# Bacillus velezensis EU07 suppresses Fusarium graminearum via transcriptomic reprogramming

**DOI:** 10.1007/s00253-026-13855-5

**Published:** 2026-05-08

**Authors:** Ömür Baysal, Catherine Jimenez-Quiros, Birsen Cevher-Keskin, Mahmut Tör

**Affiliations:** 1 Molecular Microbiology Unit, Department of Molecular Biology and Genetics, Faculty of Science, Muğla Sıtkı Koçman University, 48121 Kötekli , Muğla, Türkiye; 2 Molecular Plant and Microbial Biosciences Research Unit (MPMB-RU), University of Worcester, Henwick Grove, Worcester, WR2 6AJ UK; 3 The Scientific and Technological Research Council of Türkiye (TUBITAK), Marmara Research Centre; Life Sciences, Plant Molecular Biology and Genetics Laboratory, P.O. Box 21, 41470 Gebze Kocaeli, Türkiye

**Keywords:** *Fusarium graminearum*, Fusarium head blight, Phytopathogens, RNA-seq, Biological control agents, Molecular docking

## Abstract

*Fusarium graminearum* poses a major threat to global cereal production, necessitating sustainable control measures. This study elucidates the antagonistic mechanism of the biocontrol agent *Bacillus velezensis* EU07 against *F. graminearum* through combined transcriptomic and morphological analyses. Exposure to EU07 compromised fungal cellular integrity, causing severe hyphal distortion. RNA-seq profiling revealed that EU07 triggers a profound transcriptomic collapse rather than a standard stress response. Over 35% of the altered fungal transcriptome caused critical suppression, targeting essential metabolic hubs, secretome effectors, and master transcription factors to inhibit growth and loss of pathogenicity. In a compensatory defensive shift, the pathogen massively reallocated its transcriptional resources. This defence was characterised by a ~ 30-fold enrichment of transmembrane amino acid transporters for putative toxin efflux and an 80-fold enrichment of glutathione *S*-transferases to mitigate severe oxidative stress. Furthermore, EU07 suppressed core vulnerability nodes, including proline dehydrogenase (PRODH) and apolipophorin, disrupting the pathogen’s osmotic resilience and membrane integrity. Molecular docking analysis indicated that the *Bacillus* lipopeptide iturin A directly targets apolipophorin with high affinity (− 7.2 kcal/mol). Our findings indicated that *B. velezensis* EU07 overwhelms *F. graminearum* through simultaneous metabolic starvation and loss of virulence, revealing highly vulnerable fungal targets for next-generation RNAi-based biocontrol.

**Supplementary Information:**

The online version contains supplementary material available at 10.1007/s00253-026-13855-5.

## Highlights

- EU07 compromises fungal integrity and causes severe hyphal distortion
- The agent triggers a transcriptomic collapse, suppressing growth and virulence
- Iturin A targets apolipophorin, disrupting fungal membrane and resilience

**Supplementary Information:**

The online version contains supplementary material available at 10.1007/s00253-026-13855-5.

## Introduction

Fusarium head blight (FHB), primarily caused by *Fusarium graminearum*, is a major disease affecting cereal crops worldwide. It leads to significant yield losses and contamination of grain with mycotoxins, such as deoxynivalenol (DON), which pose serious threats to food safety and human and animal health (Wegulo 2012). Traditional approaches to managing FHB have relied heavily on chemical fungicides and resistant cultivars. However, the emergence of fungicide resistance, increasing regulatory restrictions on chemical inputs, and the limited protection conferred by resistant varieties emphasise the urgent need for sustainable alternative disease management strategies (Lee et al. 2023). Biological control agents (BCAs), particularly beneficial microorganisms, offer an environmentally friendly and sustainable strategy for managing FHB (Gao et al. 2016; Zubair et al. 2021). These agents suppress pathogens through various mechanisms, including antibiosis, competition for space and nutrients, and induction of systemic resistance in host plants (Blake et al. 2021). Compared to chemical fungicides, BCAs are generally biodegradable and less likely to induce pathogen resistance. Within this group, *Bacillus* species have shown particular promise because of their ability to form endospores, produce a wide array of secondary metabolites, and survive under a range of environmental conditions (Abdel-Aziz et al. 2017).

Species such as *Bacillus subtilis*, *Bacillus amyloliquefaciens*, *Bacillus licheniformis*, and *Bacillus pumilus* have demonstrated efficacy against various phytopathogens (Karačić et al. 2024). Their antagonistic activity is largely attributed to the production of antifungal lipopeptides, such as iturins, fengycins, and surfactins, which inhibit fungal spore germination, disrupt hyphal integrity, and interfere with fungal signalling pathways (Ongena et al. 2008). These compounds not only inhibit fungal growth but also trigger systemic resistance mechanisms in host plants (Ongena et al. 2007). Some strains also produce volatile organic compounds and enzymes capable of degrading fungal cell walls or detoxifying fungal metabolites, such as fusaric acid (Smaoui et al. 2023; Wadhwa et al. 2024).

Interestingly, recent studies have highlighted the enhanced efficacy of microbial consortia over single-strain applications (Comite et al. 2021; Nunes et al. 2024; Pérez-Moncada et al. 2024). Carefully selected bacterial–fungal consortia can broaden the spectrum of disease suppression and increase the reliability of biocontrol under varying environmental conditions. For example, arbuscular mycorrhizal fungi (AMF) have been shown to synergise with bacteria to activate plant defence pathways and modulate rhizosphere interactions (Whipps 2001; Kashyap et al. 2024; Weisany 2024). Furthermore, root-colonising strains of *Bacillus*, particularly those isolated from plant-associated niches, tend to exhibit superior biocontrol potential and rhizosphere competence compared to non-root-colonising strains. These strains are increasingly being integrated into next-generation bioformulations.

Despite these advances, the interactions between BCAs and target pathogens remain complex and occasionally contradictory. Although *Bacillus* strains generally suppress *F. graminearum* growth and mycotoxin production, certain metabolites, such as bacillomycin D, have been reported to inadvertently stimulate deoxynivalenol (DON) production in some cases (Gu et al. 2017). These observations highlight the need for mechanistic investigations that move beyond phenotypic assays to explore the molecular and transcriptional dynamics of pathogen–antagonist interactions.

Transcriptomic approaches have proven to be instrumental in unravelling these dynamics. RNA-seq analyses have revealed that pre-treatment of plants with *Bacillus* strains leads to the upregulation of jasmonic and salicylic acid pathway genes in the host, as well as pathogenesis-related proteins such as PR-1 and PR-10, upon pathogen challenge (Le Henanff et al. 2009; Rabari et al. 2023; Gebarowska et al. 2023; Gupta et al. 2024; Zhang et al. 2025). On the microbial side, transcriptomic profiling has revealed major shifts in *Bacillus* metabolic and regulatory networks during antagonistic interactions, particularly in secondary metabolite biosynthesis, redox balance, and nutrient metabolism (Medeiros et al. 2011; Wahab et al. 2023). For instance, *B. velezensis* LZN01 showed significant transcriptional changes when optimised for antifungal activity against *Fusarium oxysporum*, with hundreds of genes involved in primary and secondary metabolism being differentially expressed (Hu et al. 2024; Assena et al. 2024).

In our previous study, we identified and characterised the *B. velezensis* strain EU07, which exhibited strong antagonistic activity against *F. graminearum* K1—4 in both in vitro and in planta infection assays (Jimenez-Quiros et al. 2022). Beyond its antifungal efficacy, EU07 also promoted plant growth, suggesting its potential as a dual-function biopesticide and plant growth-promoting rhizobacterium (PGPR). Comparative genomic and proteomic analyses have placed EU07 within the *B. subtilis* species complex and highlighted its unique protein expression profile relative to that of commercial strains, indicating distinctive metabolic and biocontrol capabilities (Baysal et al. 2013; Nikolaidis et al. 2022).

In the current study, we extended this work by using RNA-seq to profile the transcriptomic changes in *F. graminearum* (K1—4) in response to EU07 treatment. Our aim was to identify differentially expressed genes (DEGs) associated with key metabolic, regulatory, and virulence pathways. By characterising these transcriptional responses, we aimed to gain mechanistic insights into the antifungal activity of EU07 and pinpoint fungal genes that may serve as candidates for RNA interference (RNAi)-based control strategies. Understanding the molecular mechanisms by which EU07 suppresses *F. graminearum* will inform the design of more targeted and effective biocontrol strategies and products.

## Materials and methods

### *F. graminearum* and *Bacillus* strains

The *F. graminearum* isolate K1—4 (*Fg*-K1—4) obtained from John Innes Centre, UK, was used in this study which was also previously described by Jimenez-Quiros et al. (2022). The fungus was cultured on potato dextrose agar (PDA) at 22–24°C and was periodically subcultured on Spezieller–Nährstoffarmer agar (SNA) according to Paredes (2024) or on 25% strength PDA to reactivate macroconidia production. The *B. velezensis* strain EU07 used in this study was previously characterised at the genome level by Baysal et al. (2024).

### Dual culture assay for transcriptomics

To determine the optimal conditions for assessing the effect of *B.* velezensis EU07 on *F. graminearum*, 50 ml of potato dextrose (PD) broth was inoculated with 100 µl of macroconidia adjusted to 10^6^ conidia/ml and incubated at 24 °C with agitation (150 rpm) for 2 days. Once the fungal cultures exhibited homogenous growth, they were treated with either sterile H₂O (control) or EU07. Briefly, bacterial broths were grown for 24 h at 28 °C (OD_600_ of 1), and 4—ml aliquots were centrifuged at 4000 rpm for 10 min to obtain a pellet. The supernatant was discarded, and the pellet was resuspended in 4 ml of sterile H₂O before adding it to the fungal cultures. Sterile H_2_O was used as a control. The bacterial–fungal interaction was allowed to proceed for 6 h. Following incubation, 2—ml samples were collected from each flask, transferred into cryogenic tubes, flash-frozen in liquid nitrogen, and stored at −  80 °C for further analysis. Three independent biological replicates per treatment were used (six flasks in total) (Van den Berge et al. 2019).

### RNA isolation and sequencing

Fungal mycelium treated with control and *Bacillus* was ground in liquid nitrogen using a mortar and pestle. Total RNA was isolated using TRIzol reagent (Invitrogen, Paisley, UK), according to the manufacturer’s instructions. RNA integrity was assessed using an Agilent (Stockport, UK) 2100 Bioanalyzer. RNA samples with RIN ≥ 7 from treated and untreated *Fg*-K1—4 were sequenced at Novogene (Cambridge, UK) on an Illumina platform (San Diego, CA, USA) with poly A-captured cDNA libraries (250–300—bp inserts) and paired-end reads (Q30 ≥ 80%). Quality control was conducted at all stages, including sample assessment, library preparation, and sequencing. The resulting raw reads were used for the subsequent analyses.

## Differential gene expression analysis

The paired-end reads for each sample were imported into the Galaxy software platform (The Galaxy Community 2024; https://usegalaxy.org/) and mapped to the *F. graminearum* reference genome (NC_026474.1) using default parameters, except for the adjustment of the maximum insert size of the paired-end library. FastQ data were first processed with optimised trimmomatic tools (adjusted parameters for MINLEN, LEADING, CROP, and HEADCROP settings) to remove adaptor sequences and low-quality bases (Chen 2023). Subsequently, RNA STAR was used to generate binary alignment map files from paired-end sequences after MultiQC analysis and then visualised using Tablet (Hutton Institute ver. 1.21.02.08; Milne et al. 2011). Raw count data from multiple samples were retrieved and merged into a single matrix using feature counts software. Differential expression analysis was performed using the *limma-voom* pipeline in R (via Sanbomics, Python 3.10) to estimate the changes in gene expression and log2 fold-change (log FC) values (Ritchie et al. 2015). The data were submitted to the iDEP platform for the visualisation of heatmaps and other gene count-based analyses (Ge et al. 2020). BLAST analyses were performed to identify the WikiPathways (WP) values corresponding to the gene integer identifiers (IDs). For gene annotation, BLASTx searches (E-value < 1e − 3) were conducted against the NCBI nr database using unigenes as query. The BLAST results were imported into Blast2 Gene Ontology (GO) for GO term assignment and functional categorisation (Conesa et al. 2005). Genes were considered differentially expressed if they exhibited a logarithmic fold change (log FC) of ≥ 2 or ≤  − 2, with a false discovery rate (FDR)-adjusted *p* value of ≤ 0.05. The protein sequences of the five genes with the lowest expression levels were retrieved from the GenBank database. BLASTp analyses were performed using the *Fusarium* taxid to determine whether these genes were conserved across other *Fusarium* species. A phylogenetic tree was constructed using the neighbour-joining method. Gene enrichment analysis was performed using ShinyGO v0.741 (Ge et al. 2020) with *F. graminearum* STRINGdb as the reference, and the results were visualised accordingly. Additonally, all key gene annotations were systematically re-verified against NCBI/FungiDB.

### Enrichment gene interaction map and network analysis

An enrichment map was generated from the RNA-seq output data using ShinyGO (Ge et al. 2020), an open-source platform for functional enrichment and network visualisation. In the enrichment map, pathways were represented as nodes, with edges representing shared genes between pathways, thereby illustrating functional relationships. To investigate gene–gene interactions both within and across pathways, selected genes were annotated using the STRING database (Szklarczyk et al. 2011). Interaction networks were constructed and visualised using Cytoscape (Shannon et al. 2003).

Based on RNA-seq data of a fungal pathogen under bacterial metabolite stress, several advanced analyses were carried out involving functional enrichment and pathway analysis. We mapped them to show their biological functions. DEGs were categorised according to related biological processes (e.g. cell wall organisation), molecular functions (e.g. transporter activity), and cellular components. Then, we visualised with colour-coded pathway maps (using tools like KEGG Mapper or Pathview) showing which steps are blocked (downregulated) or hyperactive (upregulated). In protein–protein network analysis (PPI), we determined how the proteins encoded by DEGs interact using script R code under Bioconductor ver. 3.2. (Gentleman et al. 2004). This helped identify hub genes that are central players that control the response. Top DEGs were input into the STRING database to visualise interaction networks and identified. Hub Genes using Cytoscape were identified with the most connections (nodes) using script R code. To build the integrated interaction network, the full dataset (SIF format) was utilised to establish the background topology, but the visualisation in Cytoscape was filtered to highlight the DEGs. These were attributed as potential targets for future control strategies. A network web diagram where node size represented the number of connections (degree) and expression levels were represented. A network web diagram where node size represented the number of connections (degree) and colour represents expression level (red/blue).

DEGs were categorised according to related biological processes, molecular functions, and cellular components. Pathway maps were generated to visualise downregulated and upregulated steps. PPI was conducted to determine how the proteins encoded by DEGs interact, using R scripts under Bioconductor v3.2. This facilitated the identification of hub genes central to the stress response. Top DEGs were inputted into the STRING database to construct interaction networks, and hub genes with the highest degree of connectivity were identified using Cytoscape. To identify the master regulators controlling the gene expression changes, the promoter regions of co-expressed gene clusters were analysed to find common binding motifs using Bioconductor v3.2. Fungal transcription factor (TF) regulatory networks were predicted using JASPAR 2022 (https://jaspar2022.genereg.net/), TFBSTools (https://bioconductor.org/packages/release/bioc/html/TFBSTools.html), and motifmatchr (https://bioconductor.org/packages/release/bioc/html/motifmatchr.html). Known fungal motifs were matched against extracted DNA sequences upstream of DEGs to assess TF binding probabilities. To validate these findings, Fisher’s exact test was utilised to determine significant TF enrichment (FDR < 0.05). Finally, to assess the uniqueness of the bacterial stress response, the RNA-seq profile was compared against existing datasets of the fungus under alternative stress conditions (e.g. nitrogen starvation, oxidative stress, fungicide treatment) using R scripts.

### Molecular docking-based virtual screening for protein–ligand interactions

The experimental X-ray diffraction structure of iturin A and its corresponding protein model, identified as apolipophorin based on FASTA sequence translation into amino acids using ExPASy (Berman et al. 2003), were used for docking studies. Missing residues were added with PyMOL’s builder plugin (v2.5.0), and loop regions containing these residues were refined using MODELLER (v10.1) (Webb and Sali 2016; PyMOL Molecular Graphics System, 2023: https://www.pymol.org/). The structure was further processed by removing all heteroatoms except those associated with cofactors, introducing polar hydrogen atoms as required, and assigning Kollman charges (Morris et al. 2009). A grid box (17 Å × 24 Å × 24 Å) was defined to cover the predicted active site of the modelled structure. Virtual screening was performed to assess the interactions between the iturin A ligand and target protein within a predefined grid. Docking simulations were conducted with an exhaustiveness level of 64 using AutoDock Vina (v1.1.2) (Trott and Olson 2010). Ligands with the highest binding affinity scores were selected for further evaluation using the same docking configuration.

The best docking pose of the ligand was loaded with the simulated protein structure into PyMOL, and all residues within 4 Å of the compound were visualised to identify the potential hydrophobic interactions, hydrogen bonds, and ionic interactions using Ligplot +. Predicted interactions were cross-validated using TU Dresden’s Protein–Ligand Interaction Profiler (PLIP) webserver (https://plip-tool.biotec.tu-dresden.de/plip-web/plip/index), and only interactions confirmed by both manual inspection and PLIP analysis were considered (Salentin et al. 2015).

## Results

### Optimising conditions for transcriptomic profiling of *F. graminearum* treated with *B. velezensis* EU07

**Fig. 1:**
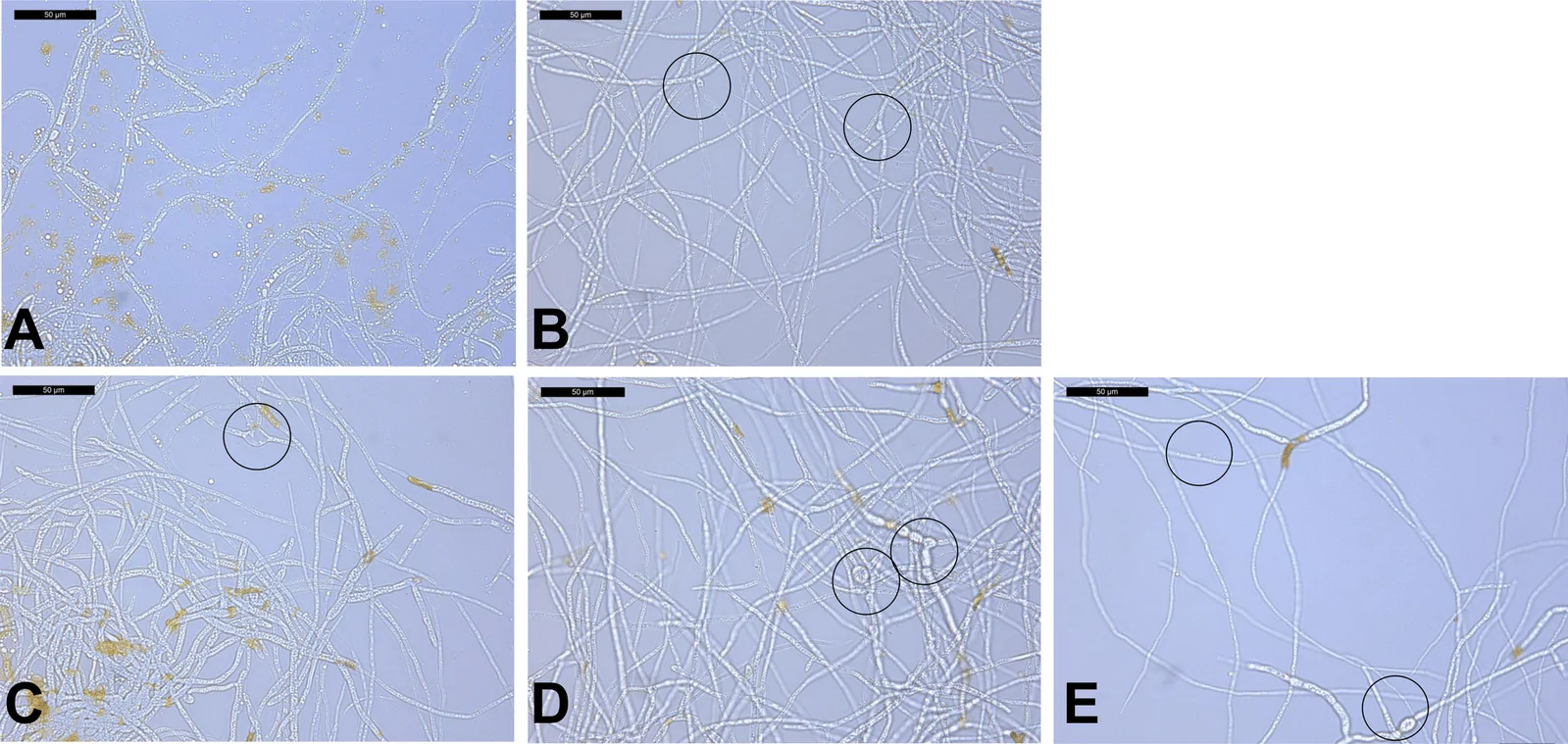
Morphological effects of *B. velezensis* EU07–derived treatments on *F. graminearum*. Cultures were treated under four different conditions: **A.** control—no treatment, **B.** LB broth only, **C.** whole EU07 culture broth, **D.** cell-free EU07 supernatant (centrifuged and 0.22-µm filtered), and **e** EU07 bacterial pellet washed and resuspended in sterile water. Scale bars = 50 µm. Circles indicate thick, rounded hyphal regions. Samples were examined using a Leica CTR5500 light microscope

We conducted a preliminary experiment to identify the treatment conditions under which *F. graminearum* K1—4 (*Fg*-K1—4) shows a reproducible physiological response to *B. velezensis* EU07, which is suitable for transcriptomic profiling. An exploratory assay was designed to test the effects of different EU07-derived treatments on fungal morphology. Five conditions were compared: (i) untreated control, (ii) LB broth only, (iii) whole EU07 culture broth, (iv) cell-free culture supernatant (centrifuged and filtered), and (v) EU07 bacterial pellet washed and resuspended in sterile water. All treatments were applied to the fungal cultures under identical conditions, each with three biological replicates. Morphological changes were assessed 48 h after treatment. In the untreated controls (Fig. 1A), the fungal hyphae appeared normal. In contrast, LB broth and all EU07-derived treatments (Fig. 1B–E) induced pronounced alterations, including localised swelling and rounded structures along the hyphae, indicative of stress or growth disruption.

Among the tested conditions, washed EU07 cells resuspended in sterile water (E) were selected for transcriptomic analysis. This condition (i) consistently elicited a clear morphological response and (ii) enabled direct bacterial–fungal interactions without confounding effects from the components of the LB broth. This part of the study established reproducible and biologically relevant conditions for downstream transcriptomic profiling, providing a robust framework for examining fungal gene expression during interactions with bacterial cells.

### DEGs in *F. graminearum* in response to *B. velezensis* EU07

RNA sequencing of the six samples generated between 12.4 and 19.0 million clean reads per library (Supplementary Table S1). The proportion of effective reads exceeded 97% in all samples, with an error rate consistently below 0.05%. Quality assessment showed that more than 94% of the bases had a Phred quality score above Q30, while the average GC content was approximately 52%. These metrics confirmed that the datasets were of high quality and suitable for downstream transcriptomic analyses.

**Fig. 2:**
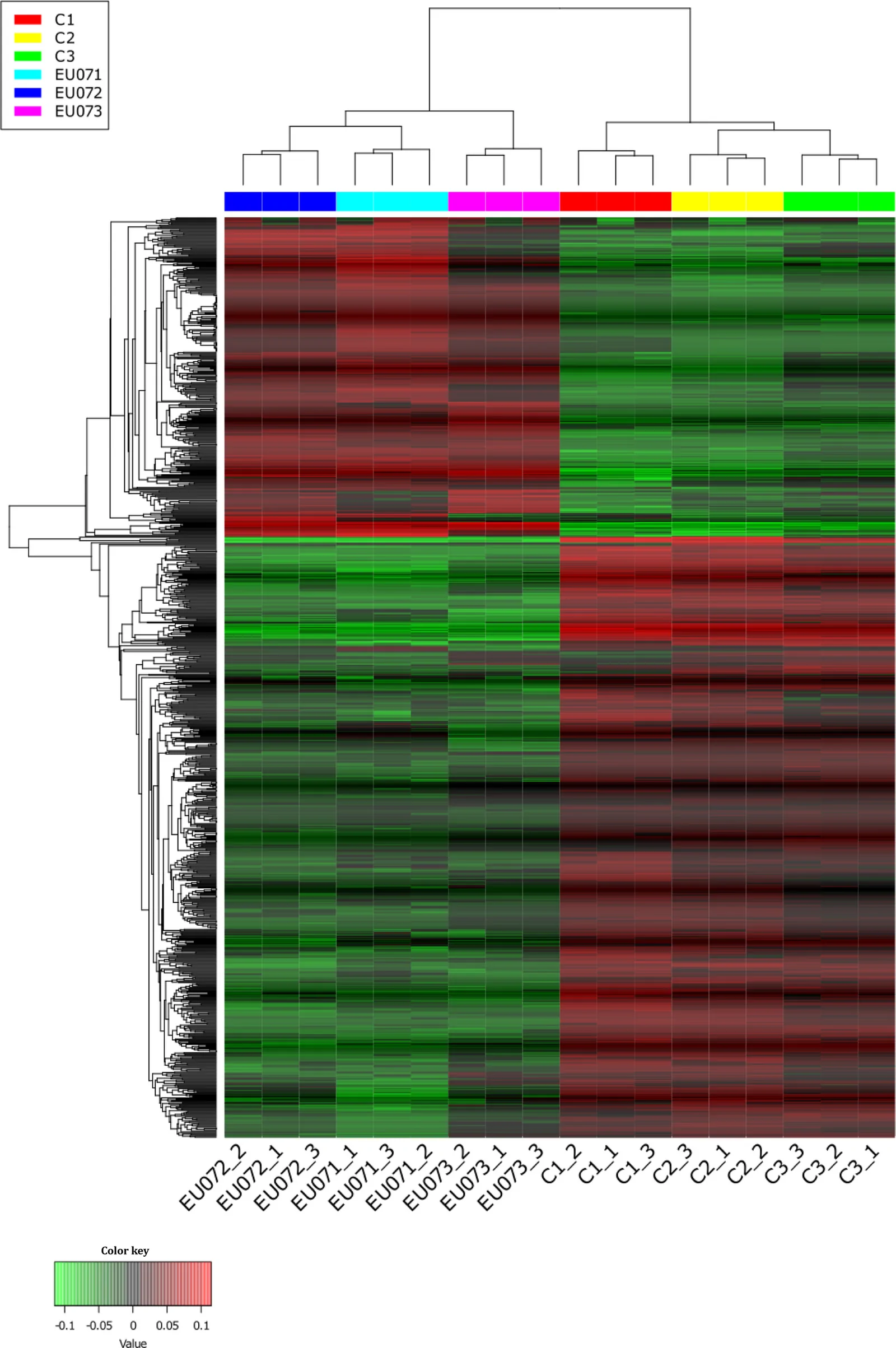
Heatmap of DEGs in *F. graminearum* treated with *B. velezensis* EU07. The heatmap shows genes significantly up- or downregulated in *F. graminearum* following exposure to metabolites secreted by *B. velezensis* EU07. Rows represent genes and columns represent biological replicates. Colour intensity reflects normalised expression levels (red: upregulated, green: downregulated). Hierarchical clustering highlights co-expression patterns. Only genes with ≥ twofold change and false discovery rate (FDR) < 0.05 are shown

**Fig. 3:**
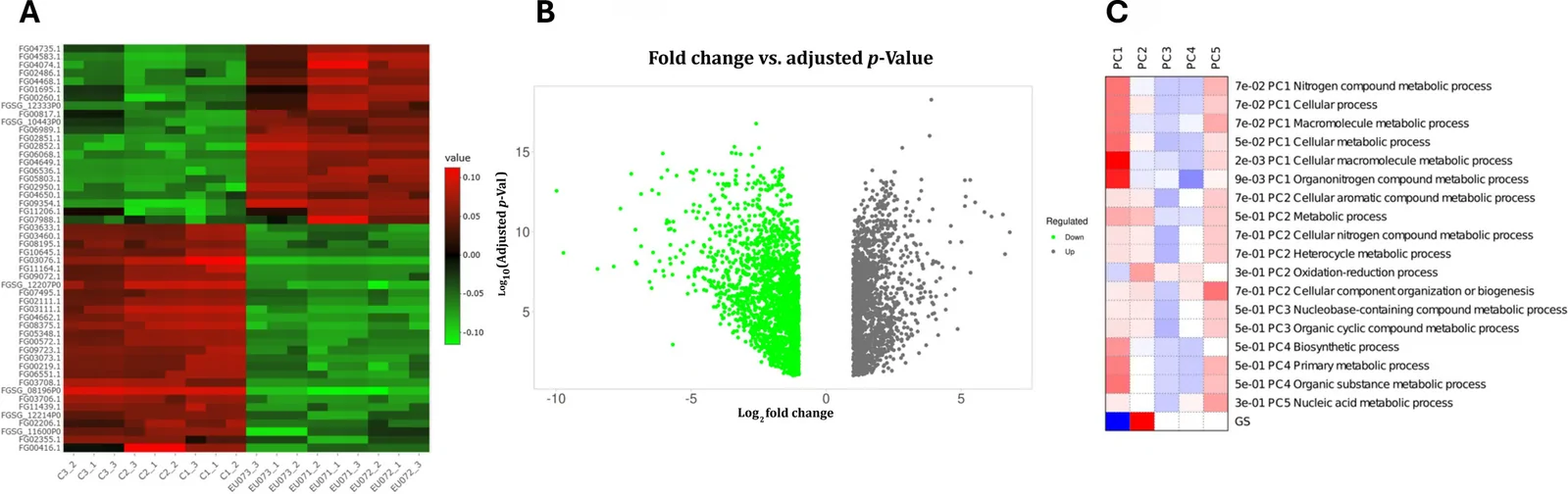
Transcriptomic responses of *F. graminearum* to metabolites of *B. velezensis* EU07. **A.** Heatmap showing the most variably expressed genes in *F. graminearum* following exposure to EU07-derived metabolites. **B.** Volcano plot summarising the number of DEGs, categorised by upregulation and downregulation according to log2 FC. **C.** Principal coordinate analysis (PCoA) displaying clustering of metabolic processes associated with DEGs in *F. graminearum* upon exposure to EU07-derived metabolites

Transcriptomic analysis revealed significant differences in the gene expression of *F. graminearum* exposed to EU07 metabolites (Supplementary Table S2). Several genes were significantly downregulated compared to the control conditions. Gene set enrichment analysis (GSEA) indicated consistent suppression of genes across several functional categories (Table 1). Heatmap analysis highlighted a clear transcriptomic shift: genes normally downregulated under control conditions were upregulated following exposure to EU07 metabolites, whereas those typically upregulated were suppressed (Fig. 2). This pattern suggests a complex regulatory response, possibly involving compensatory or stress-adaptive mechanisms that require further investigation. The 50 most strongly upregulated and downregulated genes are shown in Fig. 3A. Overall gene expression patterns and the number of DEGs, categorised by upregulation and downregulation according to log2 FC, are summarised as a volcano plot (Fig. 3B). Categorisation of DEGs by biological process, cellular component, and molecular function revealed logarithmic changes in expression (Table 2). In addition, the relationships among DEGs in the four process categories were visualised using principal coordinate analysis and presented as heatmaps (Fig. 3C).

**Table 1:** Enriched pathways among DEGs in *F. graminearum* exposed to *B. velezensis* EU07 metabolites

Direction	Adj. *p* value	nGenes	Enriched pathway/domain
Downregulated	1.2 × 10⁻^3^	5	RmlC-like cupin domain superfamily
Upregulated	4.8 × 10⁻^4^	3	Amino acid transporter, transmembrane domain
Upregulated	1.0 × 10⁻^2^	2	Amino acid permease/SLC12A domain

**Table 2:** Genes downregulated in *F. graminearum* upon exposure to *B. velezensis* EU07 products and their associated protein families

Protein domain family	EU07 vs control (fold change)	Number of genes	Adjusted *p*value
Major facilitator superfamily	− 4.3179	120	8.3e − 04
Enoyl acyl carrier protein reductase	− 4.1489	57	1.2e − 03
Short-chain dehydrogenase	− 3.9078	63	1.8e − 03
KR domain	− 3.4256	47	8.1e − 03
Sugar and other transporter	− 3.2979	52	8.1e − 03
Fungal Zn(II)_2_Cys_6_ (or C_6_ zinc) binuclear cluster domain	− 3.2788	70	8.1e − 03
Fungal specific transcription factor domain	− 3.0362	60	1.5e − 02
NADP-binding Rossmann-like domain	− 2.3874	32	8.0e − 02
Cytochrome P450	− 2.2888	35	9.1e − 02
FAD binding domain	− 2.1502	23	1.2e − 01
NADPH binding	− 2.036	23	1.4e − 01
ABC transporter	− 1.9986	23	1.4e − 01
Heterokaryon incompatibility protein HET	− 1.9432	33	1.5e − 01

#### Functional categorisation of the top DEGs and enrichment analysis

Gene expression profiling of *F. graminearum* exposed to *B. velezensis* EU07 revealed a significant transcriptional shift. The application of EU07 cells suppressed 111 genes and induced 40 genes among 3355 detected transcripts (Fig. 3B). The transcriptional response of *F. graminearum* to EU07 treatment extended beyond passive fluctuations, reflecting a coordinated adjustment of functional pathways.

Several of the most strongly induced genes encoded proteins implicated in extracellular remodelling, transcriptional regulation, transport, secondary metabolism, and detoxification. For instance, *FGSG_04649*, for an apolipoprotein which is associated with extracellular component restructuring, was upregulated 6.8-fold, suggesting reinforcement of fungal surface defences or altered interactions with the external environment. Similarly, *FGSG_09354*, which encodes an N amino acid transport system protein, was upregulated 6.6-fold, indicating a shift in nutrient acquisition or stress-induced transport mechanisms. Metabolic remodelling was also observed. *FGSG_02852* encoding maleylacetoacetate isomerase was induced 6.5-fold, potentially contributing to carbohydrate degradation and nutrient acquisition. *FGSG_04583*, which encodes a small secreted protein, showed a 5.9-fold increase, indicating the activation of secondary metabolite biosynthesis. The transporter genes *FGSG_02851* encoding fumarylacetoacetase and *FGSG_04074* (Major Facilitator Superfamily (MFS) transporter and linked to pre-rRNA-processing protein) were induced 5.5- and 5.3-fold, respectively, consistent with altered metabolite trafficking across membranes. In addition, genes involved in detoxification and redox balance, such as *FGSG_04468* (amino acid transporter transmembrane domain-containing protein) and *FGSG_06068* (*FUSOX* gene, isotrichodermin C-15 hydroxylase), were upregulated by 5.3- and 5.2-fold, respectively. Other genes are the encoded function of the gene known, including *FGSG_06536* (*FUSPO* gene, for an FAD-dependent oxidoreductase domain-containing protein) and *FGSG_05803* (*FUSSP* gene), also showed notable induction (5.1- and 4.9-fold).

In contrast, several genes were strongly repressed, pointing to targeted downregulation of transport, metabolic, and signalling pathways. *FGSG_08196* encoding aspergillopepsin-2 precursor and *FGSG_12519* encoding proline dehydrogenase likely involved in stress regulator linked to cellular transport and cell wall processes, were reduced by ~ 10- and 9.7-fold, respectively. *FGSG_03111* annotated as urea active transporter was downregulated 8.5-fold. Similarly, *FGSG_04662* (for a twin-arginine translocation (Tat) pathway signal sequence), *FGSG_12207* (*FUSCU* gene, for an uncharacterized protein), and *FGSG_08375* (for mitochondrial 2-oxoglutarate/malate carrier protein) showed reductions of 7.9-, 7.6-, and 7.2-fold, respectively, while *FGSG_11270* (*FUSLA*, for sterigmatocystin biosynthesis monooxygenase) and *FGSG_09072* (for an NADH-ubiquinone oxidoreductase-like protein) were repressed by ~ sevenfold. Downregulation extended to cell degradative enzymes (*FGSG_11164* encoding trypsin precursor linked to serine protease − sevenfold) and membrane-associated proteins (*FGSG_11439; K3VN26_FUSPC* gene encoding uracil permease, − 6.9-fold). Two additional genes, *FGSG_13802*, encoding L-amino-acid oxidase precursor which is classified as flavoenzyme catalysing oxidative deamination of L-amino acids, and *FGSG_11146*, encoding heme peroxidase and encoding essential components of the fungal antioxidant defence system that is a key mediator of hyphal chemotropism towards host, were reduced 6.8- and 6.5-fold, respectively.

We further examined five genes involving *FGSG_08196* which is required for full peroxidase activity and fungal virulence with other genes such as *FGSG_12519*, *FGSG_04649*, *FGSG_09354*, and *FGSG_02852* with the lowest expression levels and found that they were conserved across multiple *Fusarium* species (Supplementary File S1). Together, these patterns indicate that EU07 products elicit a dual strategy in *F. graminearum* involving strong induction of genes associated with defence, metabolic adaptation, and environmental interaction, accompanied by the repression of genes linked to transport, signalling, and energy metabolism. This coordinated reconfiguration highlights an adaptive transcriptional program that balances the costs of stress responses with the need to maintain cellular homeostasis.

To deduce the biological roles of the most highly responsive genes during the bacterial–fungal interaction, a functional over-representation analysis was performed on the top DEGs in *F. graminearum* exposed to *B. velezensis* EU07 metabolites. The analysis revealed a profound and targeted shift in fungal transmembrane transport, detoxification, and cell wall remodelling pathways (Table 3).

**Table 3:** Gene enrichment analysis of *F. graminearum* in response to *B. velezensis* EU07 metabolites and associated functional pathways based on RNA-seq data

Enrichment FDR	nGenes	Pathway genes	Fold enrichment	Pathway	Gene IDs
0.005894478	2	11	119.6363636	Proline metabolic process	FG03073.1, FG03076.1
0.042333332	1	11	59.81818182	Tyrosine catabolic process, and xylose isomerase-like TIM barrel	FG02852.1
0.042333332	1	11	59.81818182	Mixed, incl. ammonia transport and amino acid permease, fungi	FG03111.1
0.042333332	1	11	59.81818182	Mitochondrial carrier protein and Jlp2/Ccd25	FG08375.1
0.042333332	1	11	59.81818182	Mixed, incl. peptidase m43, pregnancy-associated plasma-A, and asexual sporulation	FG03706.1
0.054293131	1	15	43.86666667	Mixed, incl. sporocarp development involved in asexual reproduction and ccdc97-like	FG04074.1
0.054293131	1	16	41.125	Amino acid permease/SLC12A domain and ammonia transport	FG03111.1
0.008423838	2	34	38.70588235	Glutamine family amino acid biosynthetic process and ureohydrolase	FG03073.1, FG03076.1
0.054293131	1	17	38.70588235	Mostly uncharacterized, incl. peptidase m43, pregnancy-associated plasma-A, and hydrophobin	FG03706.1
0.054293131	1	17	38.70588235	Mixed, incl. sarcosine oxidase activity and threonine aldolase activity	FG06536.1
0.063125941	1	22	29.90909091	Mixed, incl. mitochondrial carrier protein and 3-hydroxyacyl-CoA dehydrogenase activity	FG08375.1
0.063125941	1	23	28.60869565	Fumarylacetoacetase-like, C-terminal, and tyrosine catabolic process	FG02852.1
0.063125941	1	24	27.41666667	Mixed, incl. velvet factor and peptidase m43, pregnancy-associated plasma-A	FG03706.1
0.063125941	1	24	27.41666667	Mixed, incl. aminotransferase class-III and pyridoxal phosphate-dependent decarboxylase	FG09723.1
0.063520185	1	25	26.32	Peptidase family A1 domain and serine carboxypeptidase	FG11164.1
0.064540527	1	28	23.5	Mixed, incl. nucleoid and mitochondrial carrier protein	FG08375.1
0.006373754	3	90	21.93333333	Glutamine family amino acid metabolic process and pyridoxal phosphate-dependent transferase	FG03073.1, FG09723.1, FG03076.1
0.025851639	2	68	19.35294118	Amino acid permease and ubiquitin protein ligase binding	FG03111.1, FG06551.1
0.006373754	3	119	16.58823529	Mixed, incl. amino acid permease and mitochondrial carrier protein	FG03111.1, FG06551.1, FG0875.1
0.086211684	1	40	16.45	Mostly uncharacterized, incl. sporocarp development involved in asexual reproduction and protein of	FG04074.1

The most significantly enriched functional categories were related to amino acid trafficking. Specifically, genes encoding transmembrane amino acid transporter proteins and associated transmembrane domains were highly overrepresented (FDR = 8.8 × 10⁻⁶), exhibiting a ~ 30-fold enrichment compared to the background genome. This robust enrichment, which captured 6 core DEGs out of a total pathway size of 27, suggests a rapid structural alteration of the fungal membrane to regulate nutrient acquisition or facilitate the efflux of toxic bacterial metabolites.

Furthermore, the transcriptomic shift highlighted a strong activation of secondary metabolism and detoxification cascades. Domains associated with fumarylacetoacetase and class Zeta glutathione *S*-transferases were the most highly enriched functional group by magnitude (80.71-fold enrichment, FDR = 6.1 × 10⁻^4^), indicating a coordinated cellular effort to mitigate EU07-induced oxidative stress and catabolize aromatic compounds (Table 3).

Finally, the analysis identified significant alterations in fungal structural components. Domains comprising glycosyl hydrolases family 16 and chitinase II, as well as the six-hairpin glycosidase-like superfamily, were significantly enriched (FDR = 6.3 × 10⁻^3^). The regulation of these specific hydrolase families points to active fungal cell wall remodelling and extracellular matrix restructuring in response to the physical stress imposed by the bacterial lipopeptides. Collectively, these enriched categories delineate a structured fungal defence programme prioritising membrane transporter modulation, ROS (reactive oxygen species) detoxification, and cell-wall reinforcement.

**Fig. 4:**
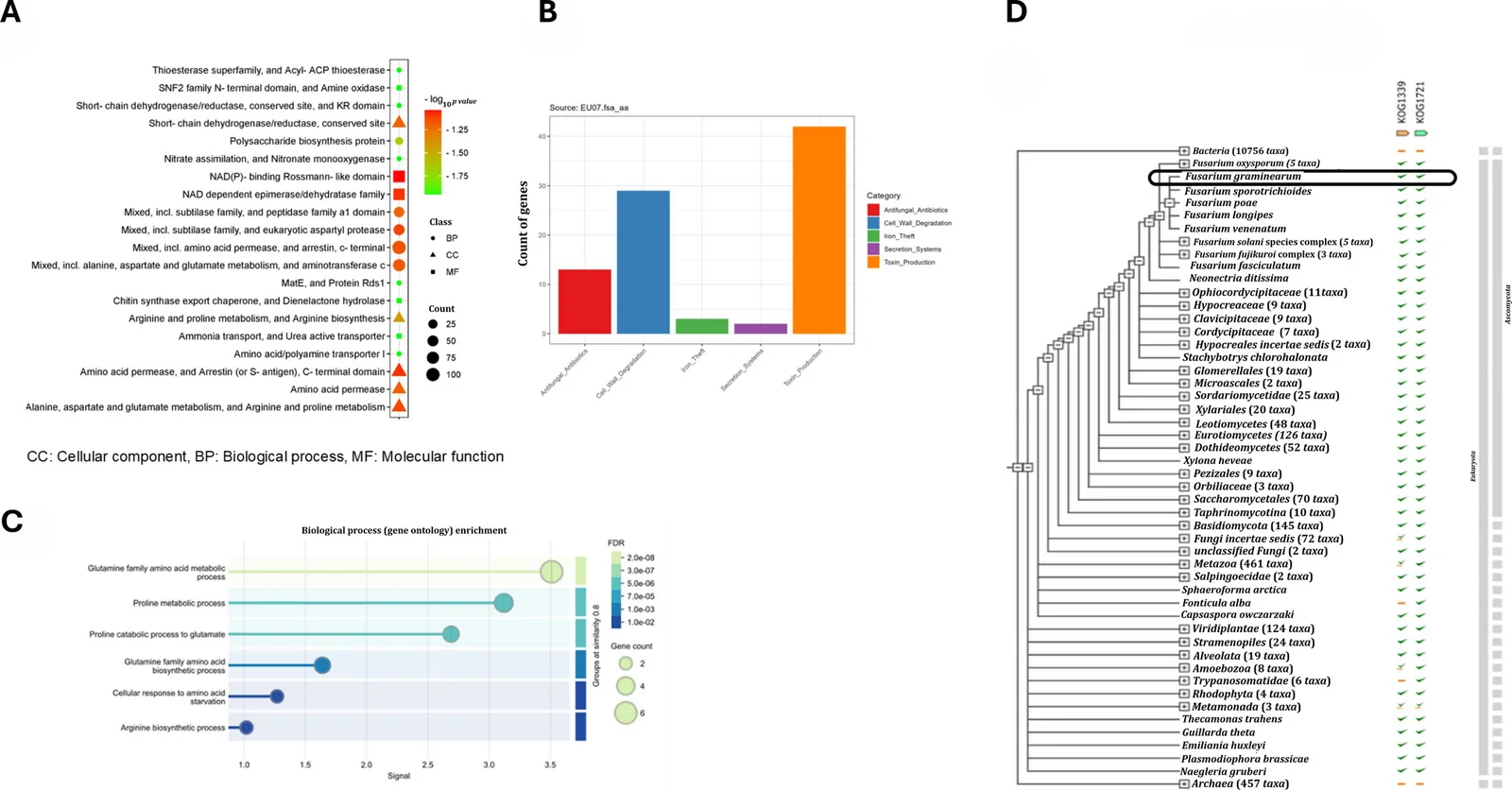
Functional categorisation of DEGs in *F. graminearum* in response to *B. velezensis* EU07 metabolites according to cellular components, biological process, and molecular function. **A.** GO classification of DEGs associated with biological process. The bar plot displays enriched GO terms, categorised by process type (e.g. metabolic process, cellular process, response to stimulus). Bars are colour coded: blue for upregulated genes, red for downregulated genes. The enrichment significance threshold was set at *p* ≤ 0.05. The number of genes contributing to each GO category is indicated on top of each bar. **B.** Categorised genes and their account in the EU07 genome according to properties. **C.** Biological process GO enrichment analysis. **D.** KOG functional classification of DEGs, derived from STRING database annotation and validated by RNA-seq expression data. Functional categories are represented by bars, with colour codes indicating expression direction: green for upregulated, orange for downregulated. Statistical significance was assessed using adjusted *p* values (Benjamini–Hochberg correction, *q* < 0.05)

DEGs were assigned to biological process (BP), molecular function (MF), and cellular component (CC) categories. Enrichment was visualised based on log2 FC (*p* value), with significant terms including membrane organisation, cellular transport, and oxidoreductase activity (Fig. 4A). These points indicate the disruption of fungal homeostasis by bacterial metabolites. To understand the diversity of stress patterns revealed by RNA expression of *F. graminearum* in response to metabolic stress induced by EU07, we categorised the inhibitory characteristics of EU07, for which we already had genomic data (Fig. 4B). To assess the functional consequences of transcriptional changes in *F. graminearum* exposed to *B. velezensis* EU07 metabolites, Gene Ontology (GO) classification and network analyses were performed (4C) and the resulting expression was confirmed on *F. graminearum* using STRING database (4D).

**Fig. 5:**
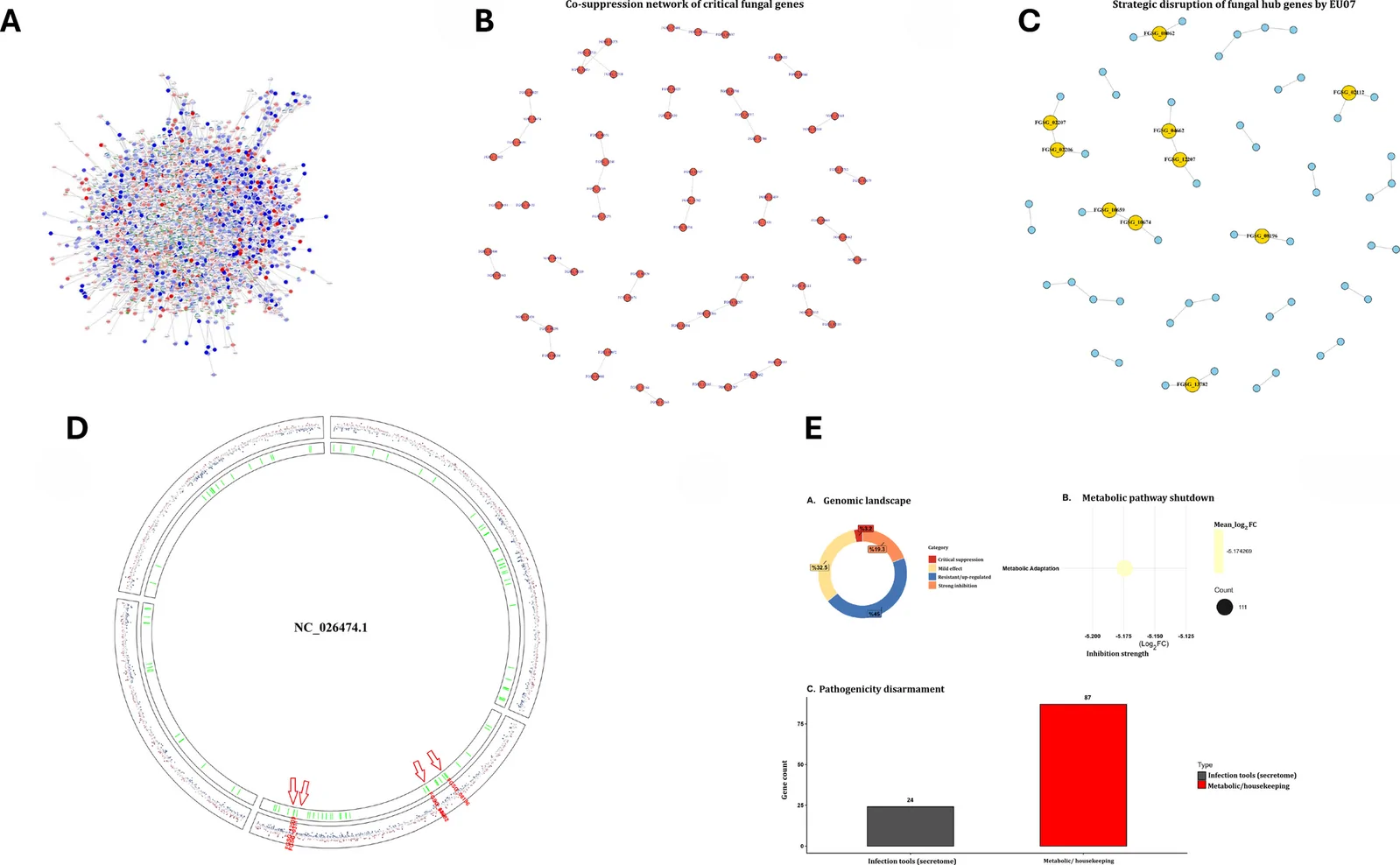
Structural impact of bacterial stress on the fungal transcriptome. **A.** Integration of RNA-seq data with the STRING database and gene–gene relationships within a network framework showing up-/downregulation in *F. graminearum*. Integrated gene interaction network constructed using RNA-seq data (SIF format) and STRING database annotations, visualised via Cytoscape. The colour changes on each node represent the specific expressed gene folding (up-/downregulated) of *F. graminearum* upon exposure to EU07 metabolites. **B.** Cytoscape analysis related to coordinated expression patterns and identified subsets of genes. **C.** Highlighted subsets of critical fungal hub genes and selective reprogramming of *F. graminearum* transcriptional networks under EU07 stress. **D.** DEGs mapped onto the fungal chromosomes. The spots (red/blue indicates up-/downregulation) represent individual genes mapped to their specific genomic loci on scafold NC_026474.1. The green bars indicate the location of BGCs. The directional red arrows show the highest-responding genes under EU07’s stress. **E.** A genome-wide description shows the alteration of the global regulatory network

GO classification further supported the functional clustering of DEGs within the biological process categories (Fig. 4C; Supplementary File Table S2). The STRING database linked DEGs to KOG1339 and KOG1721, which appeared to act cooperatively during the fungal response (Fig. 4D). Network analysis using STRING and Cytoscape identified the connections between DEGs and functional clusters. Integration of RNA-seq data with the STRING database enabled visualisation of gene–gene relationships within a network framework related to genes showing downregulation in *F. graminearum* (Fig. 5A). Cytoscape analysis also confirmed the coordinated expression patterns and identified subsets of genes that directly or indirectly interacted. Genes without connections were considered functionally unlinked under the tested conditions (Fig. 5B–C). Collectively, these analyses demonstrated that EU07 treatment induces a coordinated yet selective reprogramming of *F. graminearum* transcriptional networks.

Two complementary network analyses were performed. First, a co-expression network was generated from the most strongly downregulated genes, revealing tightly clustered nodes associated with virulence, membrane integrity, and stress responses (Fig. 5A). This focused view highlights the functional connections among repressed genes. Second, an integrated interaction network was built from the full RNA-seq dataset in SIF format (Supplementary File S2), enriched with STRING interactions, and visualised in Cytoscape (Fig. 5A). Nodes were coloured according to the fold-change direction and magnitude, enabling the visualisation of both induced and repressed genes (Fig. 5A). Highlighted subsets of critical fungal hub genes (Fig. 5B) which are functionally related DEGs and disrupted ones by EU07’ stress was filtered, unconnected nodes represented genes with no detectable interactions under the tested conditions were discarded, the connected hub genes were shown in Fig. 5C.

Together, these analyses provide both targeted and global perspectives on the transcriptional reprogramming of *F. graminearum* in response to EU07.

##### Gene enrichment and network analysis

Pathway enrichment analysis of DEGs revealed associations with virulence-related functions in *F. graminearum*. Interestingly, downregulated genes were enriched in the RmlC-like cupin domain superfamily, whereas upregulated genes were enriched in amino acid transporters and permeases (Table 4). These results suggest that EU07 metabolites modulate fungal transcriptional programs by suppressing cupin-domain proteins and enhancing transport-related processes.

**Table 4:** Functional enrichment analysis of the top 100 differentially expressed genes (DEGs) in *F. graminearum*

Enriched pathway/domain	FDR (adj.*p*value)	DEGs in pathway (nGenes)	Total genes in pathway	Fold enrichment
Amino acid transporter, transmembrane domain	8.8 × 10⁻⁶	6	26	31.04
Transmembrane amino acid transporter protein	8.8 × 10⁻⁶	6	27	29.89
Fumarylacetoacetase and glutathione *S*-transferases, class Zeta	6.1 × 10⁻^4^	3	5	80.71
Mixed, incl. fumarylacetoacetase and homogentisate 1,2-dioxygenase	6.3 × 10⁻^3^	3	11	36.69
Mixed, incl. chitinase II and glycosyl hydrolases family 16	6.3 × 10⁻^3^	4	37	14.54
Six-hairpin glycosidase-like superfamily	6.3 × 10⁻^3^	4	35	15.37

To understand the structural impact of bacterial stress on the fungal transcriptome, we constructed a global gene co-expression network (Fig. 5A–C). The topology reveals a dense, highly interconnected web of downregulated genes (blue nodes), indicating that the suppression is coordinated across multiple metabolic pathways. Filtering this global structure for the most significantly downregulated targets revealed a co-suppression network (Fig. 5C). This sparser sub-network highlights distinct clusters of genes that are simultaneously repressed, suggesting that the bacteria target specific regulatory modules essential for fungal survival.

Network centrality analysis identified specific hub genes that maintain network integrity. Prominent hubs, such as *FGSG_00062* which shows similarity to genes of *Ustilago maydis* encoding structure and function of a virally encoded fungal toxin, *FGSG_04662* encoding Tat pathway signal sequence, and *FGSG_10659* encoding asparaginase, were identified as central points of transcriptional repression (Fig. 5C). A categorisation of gene expression changes revealed that a significant portion of the affected transcriptome exhibited strong inhibition (19.3%) and critical suppression (3.2%), representing 22.5% of the total altered transcriptome. Other parts constituted resistant/upregulated parts (45%) and mild effect (32.5%). Functional analysis of the suppressed set identified a profound metabolic pathway reduction. To determine if the antagonism was localised to specific genomic regions (e.g. virulence islands), we mapped the differentially expressed genes onto the fungal chromosomes using a Circos plot. Analysis of the *B. velezensis* EU07 genome revealed a robust biosynthetic potential, specifically identifying gene clusters for the antifungal lipopeptides iturin, fengycin, and surfactin. Correspondingly, the fungal transcriptomic map (Fig. 5D) showed significant expression hotspots on scaffold NC_026474.1 that align with fungal biosynthetic gene clusters (green bars). Specifically, the high-fold-change genes highlighted by red arrows, such as *FGSG_08196* and *FGSG_02802*, encode for aspergillopepsin-2 precursor and ergosterol biosynthesis enzymes. The upregulation of these specific loci suggests a targeted fungal defence mechanism: *FGSG_02802* potentially facilitates membrane remodelling to counteract the pore-forming activity of bacterial fengycins, while the induction of *FGSG_08196* indicates an active strategy to mitigate the intracellular accumulation of bacterial metabolites. These results provide spatial evidence of a coordinated genomic response by the fungus to the metabolites of *B. velezensis* EU07 (Fig. 5D). The highlighted red zones indicate high-density regions of downregulated genes that correspond to biosynthetic gene clusters (BGCs). This suggests that the bacterial antagonist EU07 specifically targets the genomic regions responsible for secondary metabolite production, effectively silencing the fungal chemical defence system. The analysis demonstrates a genome-wide distribution of downregulated genes (inner rings labelled light green), with significant suppression events occurring across all chromosomes in *F. graminearum* (NC_026474.1). Specific high-value targets (labelled in red) are physically distributed throughout the genome, confirming that the bacterial attack is global rather than locus specific. Functional analysis of the suppressed set identified a profound metabolic pathway shutdown. The data shows a mean –log2 (*p* value) fold-change of approximately − 5.17 for metabolic adaptation genes (*n* = 111), indicating a near-total cessation of metabolic activity. Finally, we classified the downregulated genes into functional groups. While a large number of metabolic/housekeeping genes (87) were suppressed, they are consistent with starvation or growth suppression in a significant subset of infection tools (24 genes belonging to the secretome) that was also specifically targeted. This confirms that EU07 actively decreases the fungal virulence in addition to shutting down basic metabolism. Collectively, these analyses illustrate a multilayered attack strategy: EU07 induces a genome-wide collapse that shatters the global regulatory network (Fig. 5E) by breaking key hub genes, ultimately leading to a dual phenotype of metabolic starvation and loss of virulence.

**Fig. 6:**
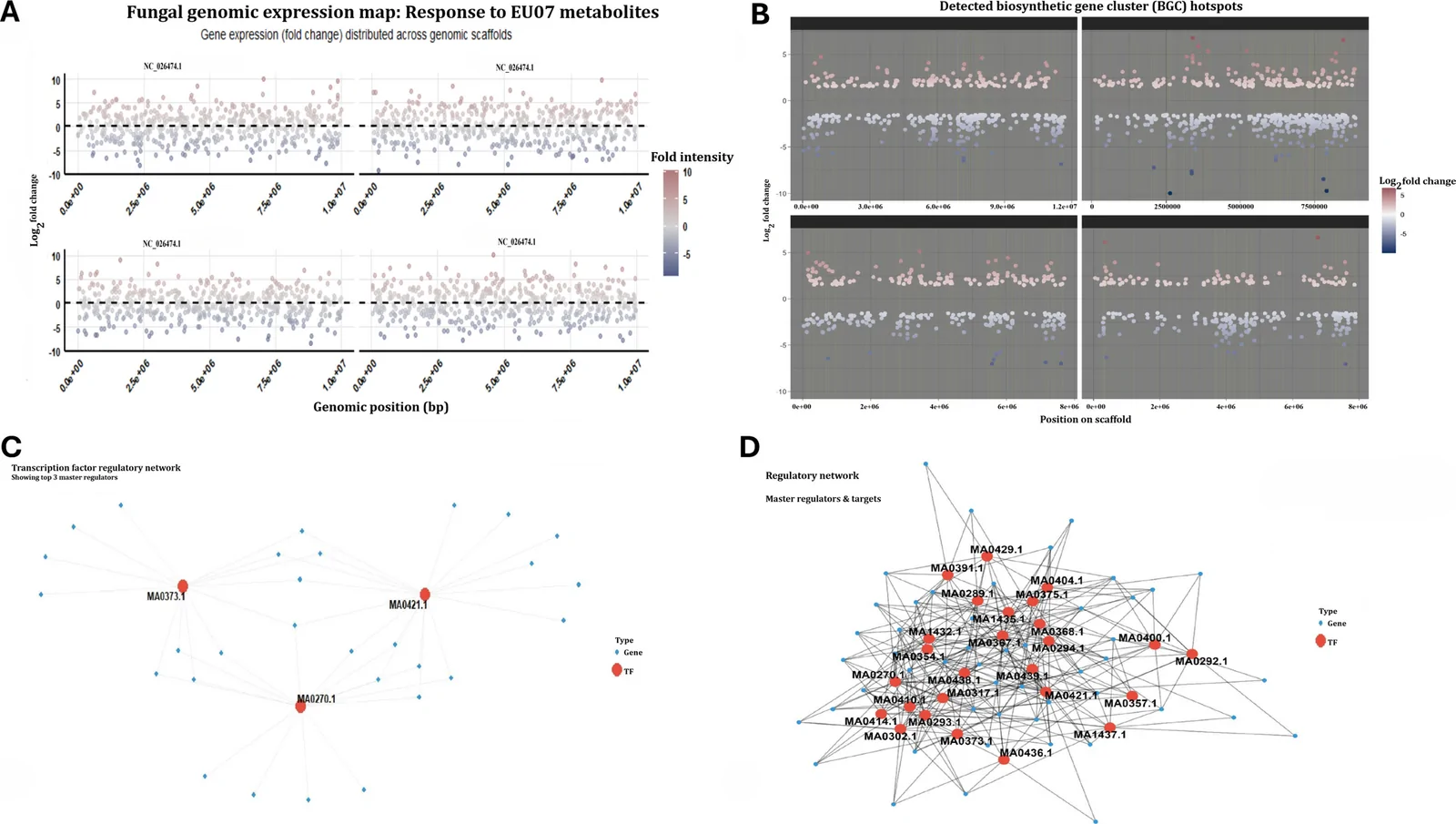
Genomic expression under stress. **A.** Spatial distribution of the bacterial-induced stress response and the fungal gene expression landscape across the major genomic scaffolds. **B.** Positional clustering analysis identified specific genomic hotspots of co-suppressed genes. **C.** The specific master transcriptional regulators (red nodes) resulting in downregulated targets. **D.** TF regulatory network related to targets in *F. graminearum*

To determine the spatial distribution of the bacterial-induced stress response, we mapped the fungal gene expression landscape across the major genomic scaffolds (Fig. 6A). The analysis reveals a pan-chromosomal suppression pattern on the whole genome of the target pathogen, where significant downregulation (blue/purple points) is distributed ubiquitously across scaffolds through NC_026474.1, rather than being localised to a single pathogenicity island. Notably, positional clustering analysis identified specific genomic hotspots of co-suppressed genes (Fig. 6B). The network illustrates a complex hierarchy where specific master regulators (red nodes) control vast arrays of downregulated targets (blue edges). We assessed the top three master regulators responsible for the observed collapse on MA0373.1, corresponding C_2_H_2_ zinc finger factors, MA0421.1 tryptophan cluster factors, and MA0270.1 AFT (Fig. 6C). Finally, to identify the drivers of this systemic failure, we reconstructed the transcription factor (TF) regulatory network (Fig. 6D). The topology around these TFs indicates that the suppression of these few key regulators propagates a cascading inhibitory signal to dozens of downstream genes. This data suggests that EU07 does not need to attack every fungal gene individually; instead, it targets the critical centres, causing a ripple effect that leads to the observed global downregulation.

**Fig. 7:**
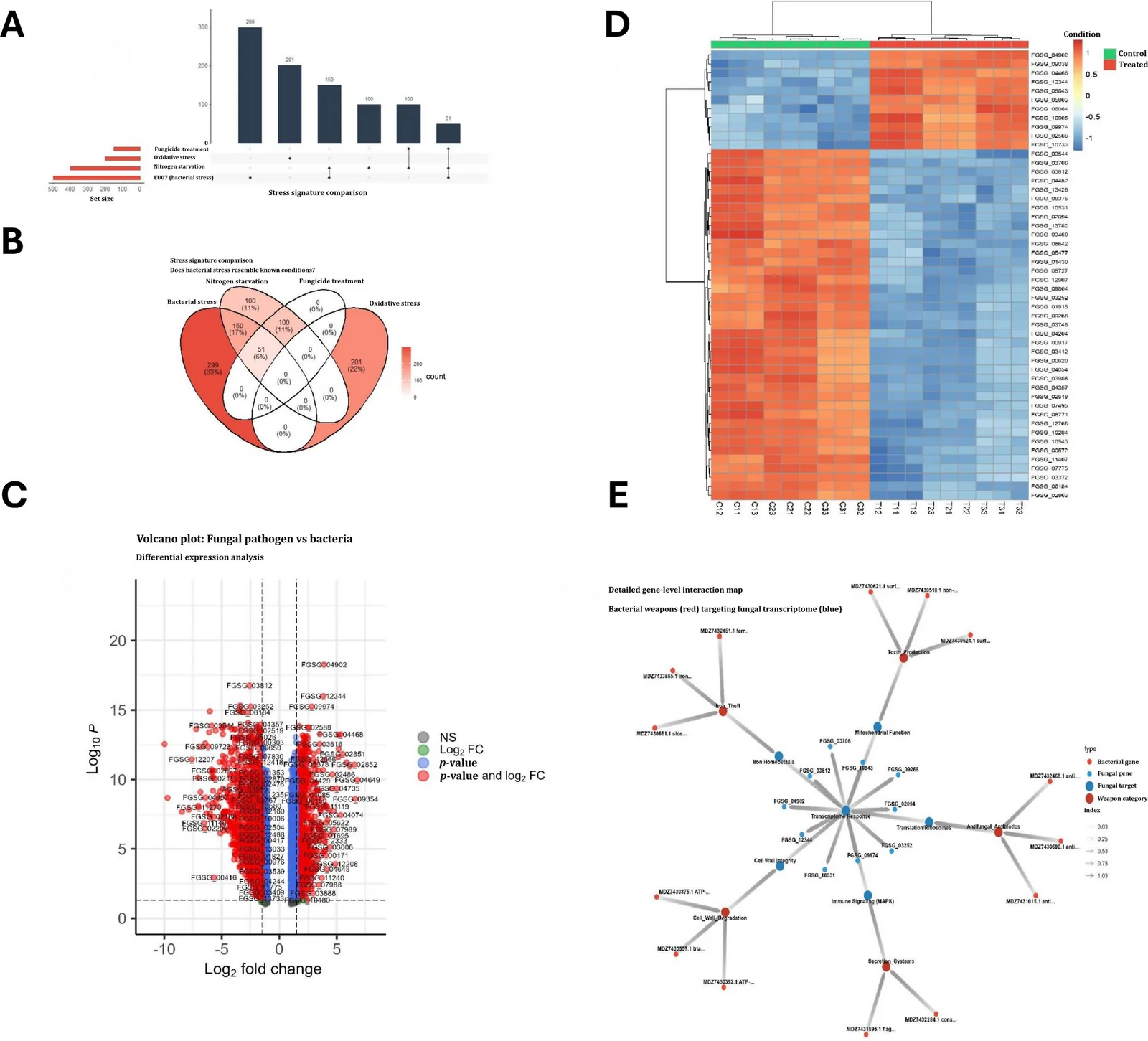
Bacterial stress signature against known stress and tested conditions. **A.** Bar plots related to known stress and comparison with EU07 stress. **B.** Venn diagram of stress signature with EU07 metabolites. **C.** Volcano plot related to transcriptional suppression in *F. graminearum*. **D.** Consistent repression and top differentially expressed genes showing a distinct switch-off pattern (blue) in treated samples compared to high expression (orange) in controls. **E.** Detailed gene-level interaction map and mechanistic link between bacterial factors and the fungal transcriptional response

To determine if this response was generic, we compared the bacterial stress signature against known stress conditions (nitrogen starvation, oxidative stress, fungicide treatment) using bar plots and Venn diagrams (Fig. 7A, B). The bacterial stress induced a unique set of 299 genes (33%) not shared with other conditions. However, a significant overlap of 150 genes (17%) with nitrogen starvation suggests that nutrient deprivation (likely iron or nitrogen competition) is a core component of the bacterial antagonism. Mapping gene expression across the fungal genome revealed that transcriptional suppression is not localised but distributed globally. Differential expression analysis revealed a profound shift in the fungal transcriptome under bacterial stress. The volcano plot (Fig. 7C) also displays a significant skew towards downregulation (left side), with numerous high-confidence targets (red points) showing log FC below − 5. This consistent repression is visualised in the heatmap (Fig. 7D), where the top differentially expressed genes show a distinct switch-off pattern (blue) in treated samples compared to high expression (orange) in controls. The fungal genomic expression map (Fig. 6A) shows widespread downregulation across all major scaffolds (NC_026474.1) that was corroborated by detailed gene-level interaction plot (Fig. 7E), which highlights that high-value targets (red labels) are physically distributed throughout the genome rather than clustered in a single pathogenicity island.

Detailed gene-level interaction map (Fig. 7E) also provides a link between bacterial virulence factors and fungal collapse. It explicitly connects specific bacterial genes (red nodes, e.g. MDZ7432451.1) to fungal pathways (blue nodes). Key interactions including bacterial siderophore genes are linked to the downregulation of fungal iron homeostasis and subsequent suppression of target genes like *FGSG_03812* encoding epoxide hydrolase. Bacterial toxin genes target mitochondrial function and cell wall integrity, leading to the collapse of genes such as for hypothetical proteins *FGSG_10543* and *FGSG_12344* encoding aromatic amino acid beta-eliminating lyase/threonine aldolase domain-containing protein functioning in metabolic pathways or as virulence factor as reported in Wang et al. (2023). Finally, we quantified the severity of the antagonism (Fig. 7E). Additionally, L-amino-acid oxidase precursor classified as flavoenzyme catalysing oxidative deamination of L-amino acids has shown downregulation. It plays a role in amino acid catabolism and nitrogen regulation and is often studied for its antibacterial properties of fungi such as *Aspergillus nidulans* or *Hebeloma cylindrosporum*, which share similar metabolic pathways with *Fusarium* (Oike and Gröger 2020). Downregulation of L-amino-acid oxidase precursor can be correlated with suppression of EU07 on target pathogen.

## Molecular docking studies

**Fig. 8:**
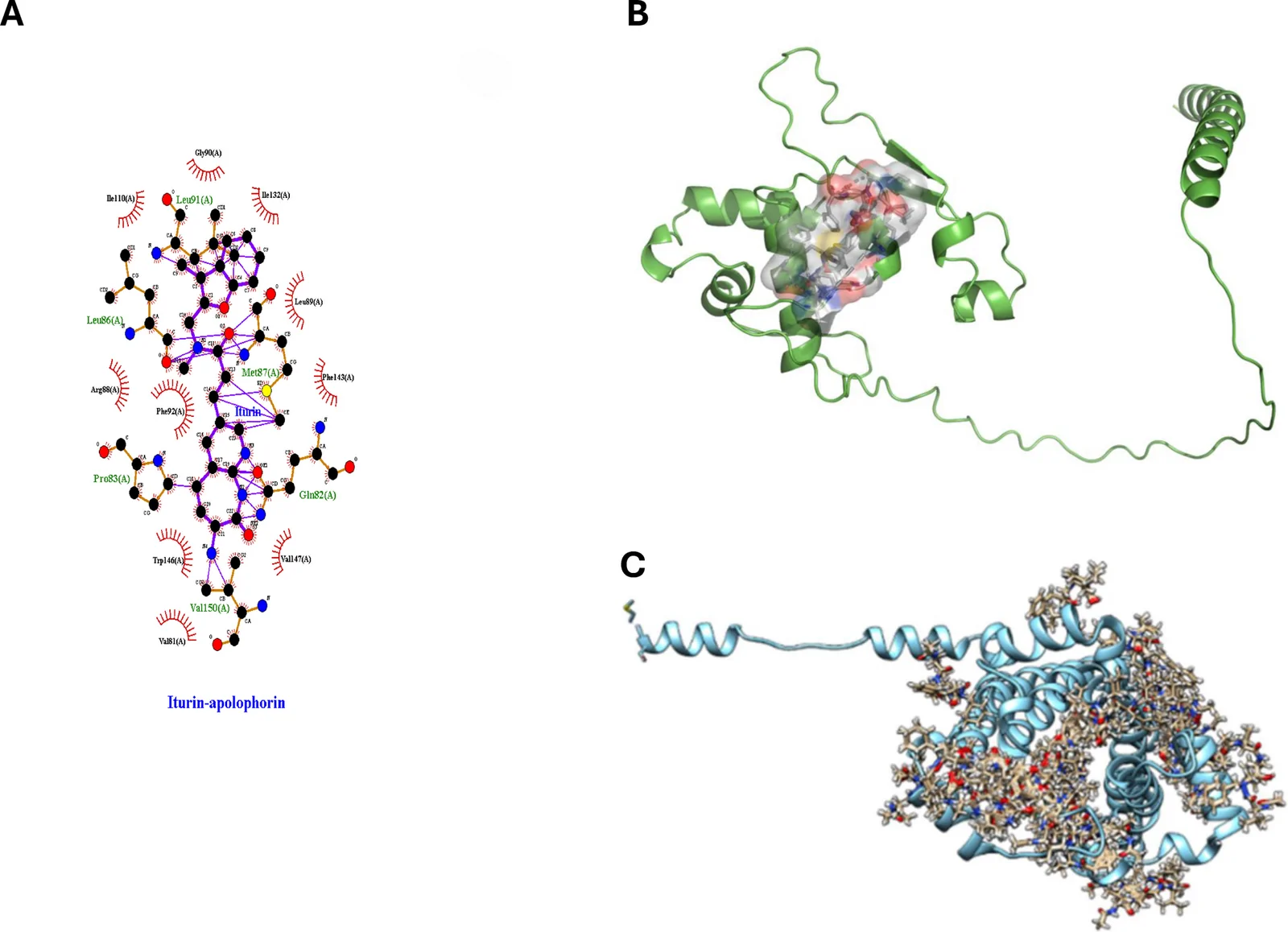
Molecular interaction between *B. velezensis* EU07–derived iturin A and *F. graminearum* apolipophorin protein. **A.** Molecular docking model shows the binding coordinates of iturin A (ligand as Unl) to the apolipophorin protein (receptor) using Ligplot +. Interaction diagram illustrates the key amino acid residues (GLU68, GLN264, ASP158, LYS60, ASP57, LYS260, TYR108, ARG112, THR204, LEU211, ASN212, ALA208, ARG207) involved in the iturin A–apolipophorin interaction. **B.** 3D visualisation of the highest possible ligand conformation. **C.** All possible ligand binding on target protein and conformations, showing the flexibility and binding hotspot regions of iturin A on the apolipophorin surface

RNA-seq analysis identified several hypothetical protein-coding genes in *F. graminearum* with altered expression in response to EU07 treatment. Among these, the apolipophorin protein, which is implicated in membrane function, was significantly upregulated. Given that EU07 produces the lipopeptide iturin A (Baysal et al. 2013), we performed molecular docking to evaluate the potential interactions between iturin A and apolipophorin. Docking simulations predicted a stable interaction with a binding energy of − 7.2 kcal/mol (Fig. 8B; Supplementary File S3) on modelled protein (Supplementary File S4). While these computional results provide a hypothesis that EU07-derived iturin A may directly target apolipophorin, subsequent genetic and biochemical validation is required to confirm this interaction in vivo.

The highest binding energy between apolipophorin (receptor) and iturin (ligand) and its coordinates on the receptor are shown in Fig. 8B and Supplementary File S3. Iturin A was predicted to interact with multiple residues of apolipophorin, including GLU68, GLN264, ASP158, LYS60, ASP57, LYS260, TYR108, ARG112, THR204, LEU211, ASN212, ALA208, and ARG207 (Fig. 5A, C). All predicted ligand binding poses on the receptor are shown in Fig. 8D. These results suggest that EU07-derived iturin A may directly target apolipophorin, potentially compromising fungal membrane–associated virulence functions.

## Discussion

Our study demonstrated that exposure of *F. graminearum* K1—4 to *B. velezensis* EU07 induced pronounced morphological and physiological changes. After 48 h of treatment, fungal cultures exhibited swollen hyphae and conglobated structures, in contrast to the untreated controls. Comparable stress responses have been reported for filamentous fungi exposed to *Bacillus* strains or their metabolites (Baysal et al. 2013; Gong et al. 2015; Patel et al. 2024). Lipopeptides such as fengycins, surfactins, and iturins are known to disrupt fungal membranes. Deleu et al. (2008) reported concentration-dependent membrane disruption by these compounds. Sampling 6 h post-treatment allowed the capture of early transcriptional responses (Schrey et al. 2005; Gu et al. 2017; He et al. 2017). RNA-seq analysis revealed a strong downregulation of genes encoding apolipophorin and proline dehydrogenase (PRODH). Apolipophorin III proteins are exchangeable apolipoproteins that play critical roles in lipid transport and membrane structure (Wang et al. 2002). PRODH catalyses the oxidation of L-proline to 1-pyrroline-5-carboxylate and regulates intracellular proline, an osmoprotectant that contributes to reactive oxygen species to (ROS) detoxification and stress tolerance (Rizzi et al. 2017; Ali et al. 2018). The coordinated downregulation of these genes indicates that EU07 metabolites compromise fungal membrane integrity and stress resilience.

To explore potential molecular interactions driving these observations, we utilised computational molecular docking. Because EU07 is known to produce the lipopeptide iturin A (Baysal et al. 2013), we modelled its interaction with the downregulated fungal apolipophorin target. Docking simulations predicted a stable interaction with a binding energy of − 7.2 kcal/mol. While these computational results provide a hypothesis that EU07-derived iturin A may physically interact with membrane-associated proteins, it is important to note that this interaction remains predictive. Subsequent genetic and biochemical validations are required to confirm this target in vivo.

The distinct overlap between the bacterial stress signature and nitrogen starvation (17% shared genes), coupled with the identification of bacterial siderophore genes targeting fungal iron homeostasis, highlights nutritional immunity as a primary weapon. The suppression of fungal mitochondrial function observed here suggests that EU07’s high-affinity siderophores likely outcompete the fungus for environmental iron, leading to the downregulation of iron-dependent respiratory chains. Transcriptomic profiling revealed a 5.3-fold induction of the MFS transporter gene *FGSG_04074* upon exposure to EU07 metabolites. In *F. graminearum* (teleomorph: *Gibberella zeae*), MFS transporters are heavily involved in membrane trafficking and detoxification. The robust upregulation of this gene likely represents a compensatory fungal defence mechanism, acting as an efflux pump to mitigate intracellular accumulation of bacterial lipopeptides. Furthermore, its association with sporocarp development suggests that EU07-induced stress may trigger protective developmental shifts within the pathogen. The functional enrichment of top differentially expressed genes reveals that *F. graminearum* shows an active, multi-faceted defence against *B. velezensis* EU07 metabolites. The most prominent signature was ~ 30-fold enrichment of transmembrane amino acid transporters. In filamentous fungi, these transporters likely act as promiscuous efflux pumps to extrude toxic bacterial lipopeptides and facilitate compensatory nutrient uptake following membrane damage. Concurrently, our analysis showed an 80-fold enrichment in domains related to fumarylacetoacetase and class Zeta glutathione *S*-transferases (GSTs). The robust induction of GSTs highlights a critical oxidative stress response, allowing the fungus to neutralise ROS induced by bacterial antagonism. The targeted enrichment of glycosyl hydrolases (family 16) and chitinase II indicates active extracellular matrix restructuring. This remodelling likely fortifies the fungal cell wall, creating a physical barrier against membrane-targeting surfactants. This massive reallocation of transcriptional resources towards membrane transport, ROS detoxification, and structural repair demonstrates that EU07 causes severe metabolic and physical stress. This defensive shift inherently suppresses the pathogen’s normal vegetative growth, underscoring the potent biocontrol efficacy of EU07.

Lipopeptides such as fengycins, surfactins, and iturins are known to disrupt fungal membranes. Deleu et al. (2008) reported concentration-dependent membrane disruption by these compounds in a 1,2-dipalmitoyl-*sn*-glycero-3-phosphatidylcholine, unilamellar vesicle model, with surfactins exhibiting up to 40-fold greater effects than fengycins. Patel et al. (2024) demonstrated that simple forms of fengycins, including agrastatin1 and plipastatin A1, induce pore formation in the fungal membrane. Baysal et al. (2013) showed that volatile organic compounds (VOCs) from *Bacillus* strains, including EU07, caused distorted, swollen, and disrupted mycelia of *F. oxysporum*, which is consistent with our observations. VOC analysis of EU07 identified a protein (ID 3835) that shares 99% identity with *S*-adenosyl-l-methionine (SAM)-dependent methyltransferases, which are typically involved in the methylation of small molecules critical for metabolism and secondary metabolite biosynthesis (Sun et al. 2020).

Gong et al. (2015) further demonstrated that purified iturin A and plipastatin A treatments caused deformation, lateral expansion, and ultrastructural damage to *F. graminearum* conidia. Our experiments with *Fg*-K1—4 treated with EU07, using both broth and bacterial pellets, produced similar morphological and ultrastructural changes. These findings guided our choice of EU07 pellet treatment for transcriptomic analysis, ensuring that the observed gene expression changes reflected direct bacterial–fungal interactions rather than medium-related effects. Sampling 6 h post-treatment allowed the capture of early transcriptional responses (Schrey et al. 2005; Gu et al. 2017; He et al. 2017).

RNA-seq analysis revealed a strong downregulation of genes encoding apolipophorin and proline dehydrogenase (PRODH). Apolipophorin III proteins are exchangeable apolipoproteins that play critical roles in lipid transport, membrane structure, and fungal virulence (Wang et al. 2002). PRODH (EC 1.5.5.2) catalyses the oxidation of L-proline to Δ^1^-pyrroline-5-carboxylate and regulates intracellular proline, an osmoprotectant that contributes to ROS detoxification, mitochondrial protection, and stress tolerance (Rizzi et al. 2017; Ali et al. 2018). Δ^1^-Pyrroline-5-carboxylate dehydrogenase, a downstream mitochondrial enzyme, participates in stress mitigation and immune defence (Liang et al. 2019). The coordinated downregulation of these genes indicates that EU07 metabolites compromise fungal membrane integrity, stress resilience, and metabolic homeostasis.

Molecular docking supported this mechanism, indicating that EU07-derived iturin binds to apolipophorin with high affinity (− 7.2 kcal/mol); these interactions suggest that EU07 metabolites may directly target membrane-associated virulence factors, which is consistent with the observed morphological and transcriptomic responses.

Beyond individual genes, RNA-seq profiling showed the coordinated upregulation of genes involved in amino acid transport, secondary metabolism, and detoxification pathways, reflecting a multilayered adaptive response to bacterial stress. Functional enrichment and network analyses confirmed that the DEGs clustered in pathways related to virulence, membrane organisation, transport, and stress responses (Figs. 4 and 6). Collectively, these global patterns highlight the fungal strategy to selectively induce protective pathways while repressing energetically costly functions, such as mitochondrial transport, serine proteases, and kinases, during bacterial challenge.

The hallmark of the interaction between bacterial antagonist EU07 and the fungal pathogen is not a typical stress response but a profound transcriptional collapse. While fungi usually respond to biotic stress by upregulating specific efflux pumps or detoxification pathways, our data reveals a switch-off phenotype where 55% of the relevant transcriptome is suppressed, including a shutdown of metabolic adaptation genes (mean log2 FC − 5.17). This aligns with metabolic paralysis as a highly effective biocontrol mechanism, where antagonists deplete the energy reserves required for the pathogen to mount a defence. Unlike fungistatic agents that merely pause growth, EU07 appears to induce a state of dormancy to the starvation-induced quiescence described in other fungi such as *Aspergillus* interactions (Nitsche et al. 2012), preventing the fungus from accessing the energy required for virulence. A critical insight from our network topology analysis is the strategic disruption of fungal hub genes (e.g. *FGSG_00062*, *FGSG_04662*). In biological networks, hub genes act as resilience anchors; their removal causes a disproportionate loss of network connectivity. Our findings indicated successful biocontrol agents often target high-degree regulatory nodes rather than peripheral effectors. By severing the communication lines between master regulators (like MA0373.1) and their downstream targets, EU07 effectively disrupts the fungal regulatory mechanism. Data depicts consistent downregulation of pathogen virulence factors. Master regulators (TFs) that the bacteria might be targeting. These are specific fungal genes (downregulated). Red node connects to many blue nodes showing that single TF controls a large portion of the fungal stress response, which is a remarkable finding.

The distinct overlap between the bacterial stress signature and nitrogen starvation (17% shared genes), coupled with the identification of bacterial siderophore genes targeting fungal iron homeostasis, highlights nutritional immunity as a primary weapon. The suppression of fungal mitochondrial function observed here suggests that EU07’s high-affinity siderophores likely outcompete the fungus for environmental iron, leading to the downregulation of iron-dependent respiratory chains. Beyond metabolic arrest, EU07 specifically targets the fungal secretome, suppressing 24 genes classified as infection tools. This pathogenicity loss is a crucial feature of effective biocontrol. Sui et al. (2026) also demonstrated the targeting of the fungal secretome profile which is specifically cell wall–degrading enzymes and effectors that are strategically more sustainable than targeting housekeeping genes, as it reduces the selective pressure for resistance development. By silencing the Biosynthetic Gene Clusters (BGCs) responsible for mycotoxin production, EU07 not only inhibits growth but potentially reduces the virulence and toxigenic potential of the fungus, addressing a key concern in agricultural mycotoxin management. In our previous study, *B. velezensis* EU07 also demonstrated superior antagonism against *F. graminearum* compared to commercial strains, significantly inhibiting fungal growth and deoxynivalenol (DON) production both in vitro and in *Brachypodium distachyon* (Jimenez-Quiros et al. 2022).

Spray-induced gene silencing (SIGS) (Bilir et al. 2022) has emerged as a promising strategy for managing *F. graminearum* and reducing FHB severity. Field trials have demonstrated that naked aqueous dsRNA sprays targeting core fungal regulatory genes, such as *CHS3b* and *MGV1*, significantly reduced both FHB incidence and DON accumulation, with two applications achieving over 90% control in some cases (Feng et al. 2025). A similar success has been reported in controlled environments: dsRNA targeting *TRI6*, a key transcriptional regulator of trichothecene biosynthesis, reduced gene expression, disease spread, and DON accumulation in wheat heads under greenhouse and growth chamber conditions (Hao et al. 2021). Together, these studies confirm the feasibility of SIGS for disease and toxin suppression.

Our findings extend this field by identifying novel candidate targets that have not yet been explored for SIGS applications. Several strongly downregulated genes, including apolipophorin and *PRODH*, were functionally linked to virulence and stress response. These genes, identified through integrated morphological, transcriptomic, and docking analyses, represent previously untested but potentially valuable targets for RNA-based silencing strategies. Importantly, the overlap between the genes suppressed by EU07 metabolites and those considered viable SIGS targets suggests complementary avenues for intervention. In line with previous proposals for engineering plants to express dsRNAs or hairpin RNAs (Morozov et al. 2019), our results provide a rational framework for prioritising the functionally critical genes. Coupling such candidate targets with advances in SIGS delivery platforms may accelerate the development of durable and environmentally sustainable RNA-based strategies for FHB and DON management.

Potential limitations include the use of broth cultures, which may not fully mimic plant–pathogen interactions, and the early time point (6 h) for transcriptomic sampling, which may not capture long-term responses. In addition, some downregulated genes remain hypothetical and require functional validation. Therefore, future studies should validate candidate targets via gene knockouts or RNAi, assess EU07 effects under plant-pathogen conditions, and evaluate the combined effects of VOCs, lipopeptides, and dsRNA strategies to maximise biocontrol efficacy. Furthermore, in this study, in addition to examining the effect of a biological agent on a target pathogen, we were able to understand how the pathogen and the biological control agent interact by using RNA-seq data to determine the pathogen’s response to the biological agent. This provides clues regarding the genes that could be targeted to neutralise the pathogen in future studies.

In summary, *B velezensis* EU07 induced profound morphological changes and transcriptional reprogramming in *F. graminearum* K1—4. The upregulation of genes linked to secondary metabolism, transport, and stress adaptation, alongside the downregulation of membrane- and metabolism-related genes, highlights a coordinated fungal defence programme which remained unclear as hypothetical protein under the stress of bacterial metabolites. Molecular docking results suggest that EU07 lipopeptides may directly target the fungal virulence proteins. Our results also provide a comprehensive view of the molecular dialogue for the interaction between a biocontrol agent and a fungal pathogen, addressing a critical gap in the current understanding of these complex biotic interactions These insights reveal the key mechanisms of bacterial–fungal interactions and identify candidate genes for targeted RNAi-based control, supporting the potential of EU07 as a sustainable biocontrol agent against FHB.

### Accession numbers

The datasets generated in this study are available under the BioProject accession number PRJNA1322080. All R Scripts and codes related to gene expression and network analysis are also available at.

https://github.com/baysalo/RNAexpression/tree/rna-expression.code.

## Supplementary Information

Below is the link to the electronic supplementary material.

ESM 1 (PDF.15.2 MB)

## Data Availability

The data supporting the findings of this study are available from the corresponding author upon reasonable request. All transcriptomic data are publicly available, as described in this paper.
