# Supplementary material for: Bacillus velezensis EU07 suppresses Fusarium graminearum via transcriptomic reprogramming
Source: Appl Microbiol Biotechnol. 2026 May 8;110:168. doi: 10.1007/s00253-026-13855-5 (PMC13234024; doi:10.1007/s00253-026-13855-5)
Supplement: Supplementary file 1 [file 253_2026_13855_MOESM1_ESM.pdf]

Supplementary Material

for "***Bacillus velezensis*** **EU07 suppresses** ***Fusarium graminearum*** **via transcriptomic reprogramming"**

Journal Name: Applied Microbiology and Biotechnology

Ömür Baysal[1,2], Catherine Jimenez-Quiros[2], Birsen Cevher-Keskin[3], and Mahmut Tör[2]

[1] Molecular Microbiology Unit, Department of Molecular Biology and Genetics, Faculty of Science, Muğla Sıtkı Koçman University, 48121 Kötekli, Muğla, Türkiye.

[2] Molecular Plant and Microbial Biosciences Research Unit (MPMB-RU), University of Worcester, Henwick Grove, Worcester, WR2 6AJ, United Kingdom.

[3] The Scientific and Technological Research Council of Türkiye (TUBITAK), Marmara Research Centre; Life Sciences, Plant Molecular Biology and Genetics Laboratory, P.O Box: 21, 41470 Gebze, Kocaeli, Türkiye

*Corresponding author:

E-mail: m.tor@worc.ac.uk

Supplementary materials

Supplementary Table S1. Summary of RNA seq sequencing reads and quality metrics.

Supplementary Table S2: DEGs obtained from the RNA seq analysis.

Supplementary File S1. Phylogenetic trees of five genes showing conservation across Fusarium species.

Supplementary File S2. Protein-protein interaction network data in SIF Format

Supplementary File S3. Molecular docking model generation details.

Supplementary File S4. Structural and sequence information for uncharacterized protein A0A2H3GNL7 from *Fusarium graminearum*

Supplementary Table 1. Summary of RNA-seq sequencing reads and quality metrics.

Sample	Raw reads	Clean reads	Raw data (bp)	Clean data (bp)	Effective (%)	Error rate (%)	Q30 (%)	GC content (%)
R1	12694917	12403487	3808475100	3721046100	97.7	0.03	94.2	51.8
R2	19517507	18985824	5855252100	5695747200	97.2	0.03	94.1	52
R3	17840027	17568959	5352008100	5270687700	98.4	0.03	94.1	52.4
R4	14149862	13838620	4244958600	4151586000	97.8	0.03	94.1	53
R5	16994840	16771164	5098452000	5031349200	98.68	0.03	94	52.7
R6	14577568	14407663	4373270400	4322298900	98.83	0.03	94.1	52.83

Supplementary Table S2: DEGs obtained from the RNA seq analysis.

Regulation	Top_Genes	Fold	FDR	Cont11	Cont12	Cont13	Cont21	Cont22	Cont23	Cont31	Cont32	Cont33
Up	FGSG_04649	6.802687015	1.07E-10	2.2688027	2.4852148	2	2	2	2	2.4052168	2.3968278	2.3974876
Up	FGSG_09354	6.627463985	2.50E-09	3.1562965	3.765011	2	2.364115	2.353841	2.3550396	3.2982451	3.3690748	3.18196
Up	FGSG_02852	6.548626357	8.28E-12	3.3996963	3.378055	3.9139052	3.2841938	2.8748957	3.6925011	3.199846	3.5358829	3.3707558
Up	FGSG_06536	6.127444527	1.12E-11	2.6910183	3.1370988	2	2.7805029	2.6377651	2.8774004	3.0942412	2.7077187	3.3707558
Up	FGSG_04583	5.86522198	5.85E-12	6.9010051	6.7326402	6.9750363	5.9540229	6.0977451	5.7002055	7.2218865	7.0571987	7.3110622
Up	FGSG_02851	5.521261696	1.45E-12	6.169847	6.1851653	6.1703363	6.2436111	5.975612	6.4361306	5.5080752	5.5947086	5.4790733
Up	FGSG_04074	5.349917417	3.60E-08	4.7070397	4.4323548	4.8733193	4.0052074	3.6887032	4.210161	4.7032988	4.4416259	4.9376179
Up	FGSG_04468	5.33803534	5.74E-14	4.0257777	3.765011	4.188785	4.6247215	4.7733906	4.3744304	4.7032988	4.262829	4.9961472
Up	FGSG_06068	5.172168673	6.27E-13	4.8775546	4.9628272	4.7932764	4.5907474	4.2902531	4.7780897	4.7387761	4.262829	5.0523944
Up	FGSG_05803	5.139732821	5.97E-14	3.7898088	2.8476671	4.3087924	3.2841938	3.4156241	3.0813961	3.0942412	3.3690748	2.7087823
Up	FGSG_02950	5.139106526	2.47E-12	4.1640552	3.378055	4.6184939	4.327482	4.5857446	3.9222766	4.666927	4.4416259	4.8129147
Up	FGSG_04735	5.012570755	4.34E-10	9.8733248	9.8359428	9.8253553	8.6194736	8.6391638	8.5097542	10.910704	10.889752	10.86821
Up	FGSG_12208	4.870279434	0.0001204	2	2	2	2	2	2	2	2	2
Up	FGSG_07989	4.75151836	3.97E-07	2	2	2	2	2	2	2	2	2
Up	FGSG_00260	4.734654513	1.82E-08	7.3697138	7.2698061	7.3831245	5.4468739	5.5313719	5.2704753	7.9980116	7.9857763	7.9445655
Up	FGSG_02486	4.620517713	4.28E-11	3.2831234	2.4852148	3.7539507	2.6546039	2.8748957	2.3550396	3.0942412	2.9633478	3.18196
Up	FGSG_04650	4.593306505	4.45E-10	3.2831234	3.765011	2.4807492	3.0034737	3.0784965	2.8774004	2.8565633	2.9633478	2.7087823
Up	FGSG_10991	4.441575576	8.90E-07	2	2	2	2	2	2	2	2	2.3974876
Up	FGSG_03006	4.431865694	7.41E-06	2	2	2	2	2	2	3.7092973	3.5358829	3.8228101
Up	FGSG_08055	4.307384449	1.62E-08	3.2831234	3.1370988	3.3684179	2.5166584	2.6377651	2.3550396	3.6359267	2.9633478	4.0607807
Up	FGSG_02949	4.223064038	8.03E-08	2.2688027	2.4852148	2	2.364115	2.6377651	2	2.2167888	2.3968278	2
Up	FGSG_11119	4.161219348	8.82E-09	2.2688027	2	2.4807492	2.1935143	2	2.3550396	2	2	2
Up	FGSG_04648	4.150557919	0.0002883	2.2688027	2.4852148	2	2.7805029	2.353841	3.0813961	2	2	2
Up	FGSG_05622	4.078754469	1.29E-07	2.4953013	3.378055	2	2.7805029	2.8748957	3.4190665	2.9802913	3.5358829	2.964684
Up	FGSG_10443	4.077845312	7.54E-09	3.1562965	3.1370988	3.1285604	3.7210377	2.8748957	4.210161	4.7387761	4.8105634	4.6029775
Up	FGSG_01695	4.072190133	1.11E-06	7.2032529	7.1285622	7.1934618	6.0446528	6.1209783	5.874995	7.895641	7.8015075	7.929627
Up	FGSG_00171	4.070159127	2.98E-05	2.2688027	2.4852148	2	2.6546039	2.353841	2.8774004	2.5718533	2.7077187	2.3974876
Up	FGSG_11206	4.007582943	1.23E-05	3.2831234	3.765011	2.4807492	2.7805029	3.0784965	2.3550396	5.8857925	5.9002223	5.9027806
Up	FGSG_09078	3.976040874	8.47E-09	2	2	2	2	2	2	2	2	2
Up	FGSG_04569	3.975693444	3.43E-06	2	2	2	2.1935143	2.353841	2.3550396	2.2167888	2.3968278	2

Up	FGSG_04828	3.969072477	6.65E-09	4.6610646	4.8868835	4.3087924	6.1878872	6.0977451	6.1937152	6.1848562	6.2272902	6.075584
Up	FGSG_02221	3.946005459	3.86E-12	5.0300296	5.2921154	4.6184939	5.1988268	5.179638	5.1395644	5.8857925	6.0176785	5.670936
Up	FGSG_06629	3.939233314	1.37E-05	2	2	2	2.6546039	3.0784965	2	2	2	2
Up	FGSG_00817	3.935983748	1.02E-08	5.7973114	5.5600358	5.9294932	7.4378284	7.3288419	7.4766159	8.5506904	8.5114961	8.5439396
Up	FGSG_12333	3.930626453	2.68E-06	3.872828	4.2010532	3.3684179	3.5190873	3.8080253	3.0813961	3.199846	2.9633478	3.3707558
Up	FGSG_04902	3.895122084	5.50E-19	7.17081	7.0952041	7.1619361	7.2815544	7.225451	7.2626125	7.2156048	7.2253567	7.1528368
Up	FGSG_11385	3.880942803	1.14E-06	2.8633281	3.378055	2	2	2	2	2.2167888	2	2.3974876
Up	FGSG_03632	3.8579462	5.82E-07	2	2	2	2.1935143	2	2.3550396	2	2	2
Up	FGSG_12344	3.834000384	9.99E-17	7.4394623	7.342684	7.8664849	7.2654925	7.225451	7.6787403	7.4530566	7.7344551	7.8760892
Up	FGSG_02229	3.827451224	2.09E-11	3.872828	3.378055	4.188785	4.7531416	4.7135134	4.7181783	5.5880596	5.5563964	5.5589053
Up	FGSG_10465	3.787605234	1.95E-05	2	2	2	2.5166584	2	2.8774004	2	2	2
Up	FGSG_06989	3.747777049	2.26E-09	3.9513288	4.3213345	3.3684179	4.4077273	4.5857446	4.1204343	5.3564544	5.4349486	5.2090653
Up	FGSG_11240	3.705350963	0.0012337	2	2	2	2.364115	2	2.6397336	3.5586239	3.5358829	3.5376798
Up	FGSG_04869	3.665309923	5.78E-07	2	2	2.4807492	2.1935143	2.353841	2	2.5718533	2.3968278	2.7087823
Up	FGSG_12406	3.619108595	1.68E-07	2	2	2	2	2	2	2.2167888	2.3968278	2
Up	FGSG_10917	3.596378505	1.28E-06	2.2688027	2	2.4807492	2.6546039	2.353841	2.8774004	2.2167888	2.3968278	2
Up	FGSG_05841	3.595343262	2.01E-08	2.4953013	2.4852148	2.4807492	2.8962916	3.4156241	2	2.7212202	3.1804273	2.7087823
Up	FGSG_11884	3.569542859	1.36E-08	4.0257777	3.378055	4.4195794	5.5913246	5.2232303	5.8190522	5.4233949	5.3920827	5.4374371
Up	FGSG_04943	3.561630075	1.10E-08	3.1562965	2.8476671	3.3684179	5.1056669	5.179638	4.9442019	4.9041484	4.9352342	4.8129147
Up	FGSG_13536	3.544609033	3.29E-07	3.2831234	3.5844678	3.1285604	2.7805029	2.8748957	3.0813961	3.199846	3.9446664	2.7087823
Up	FGSG_06084	3.529216098	5.37E-14	6.7334055	6.6198745	6.7602689	5.9805033	5.6638294	6.1715481	6.0781065	5.9304949	6.2298857
Up	FGSG_03816	3.521342893	2.76E-13	6.7334055	6.9534782	6.3766661	5.9935634	5.6951271	6.1715481	6.2600348	6.2514733	6.2052877
Up	FGSG_13959	3.507213828	2.53E-07	2.4953013	2	2.8407141	2.364115	2.353841	2.3550396	2.2167888	2.3968278	2
Up	FGSG_09290	3.501836474	1.43E-06	2.4953013	2.4852148	2.4807492	2.7805029	3.0784965	2.3550396	2.7212202	2.7077187	2.7087823
Up	FGSG_04930	3.500417878	6.75E-07	4.349408	3.9254527	4.6184939	3.2841938	3.2568854	3.260088	4.2416299	4.4416259	3.9466965
Up	FGSG_05401	3.499607315	6.09E-10	6.5437448	6.3355781	6.650969	7.1709346	7.1929315	7.0603242	8.0125782	7.904229	8.0519305
Up	FGSG_10500	3.480696764	2.93E-07	2	2	2	2.364115	2.353841	2.3550396	2	2	2
Up	FGSG_04599	3.45428482	2.58E-06	2.2688027	2.4852148	2	2	2	2	2	2	2
Up	FGSG_03797	3.449449891	3.36E-07	2	2	2	2.1935143	2.353841	2	2.2167888	2	2.3974876
Up	FGSG_02201	3.442900654	1.07E-07	3.0172362	3.378055	2.4807492	2.364115	2.6377651	2	2.2167888	2	2.3974876
Up	FGSG_06537	3.442289225	9.10E-13	4.5644712	4.6316459	4.4195794	4.7220923	4.7733906	4.5903319	5.6452659	5.5947086	5.6345505

Up	FGSG_07988	3.44012884	0.0038487	4.5136357	5.1036809	3.3684179	5.3486486	5.0403527	5.5706824	4.6296145	4.9352342	4.1665008
Up	FGSG_12281	3.425579921	5.79E-09	3.2831234	3.1370988	3.3684179	2.7805029	2.6377651	2.8774004	4.028829	3.8208439	4.1665008
Up	FGSG_11542	3.402833096	1.68E-06	2.2688027	2.4852148	2	3.1032407	2.8748957	3.260088	2	2	2
Up	FGSG_01664	3.402712539	2.17E-11	3.0172362	2.4852148	3.3684179	3.2841938	3.5586152	2.8774004	3.7092973	3.9446664	3.3707558
Up	FGSG_11533	3.391498015	0.0001721	2	2	2	2	2	2	2.2167888	2	2.3974876
Up	FGSG_12405	3.386361583	5.05E-05	2	2	2	2	2	2	2.2167888	2.3968278	2
Up	FGSG_03224	3.376838848	3.14E-07	2.4953013	2	2.8407141	2.1935143	2.353841	2	3.2982451	2.3968278	3.8228101
Up	FGSG_10386	3.375817563	3.54E-09	3.3996963	3.1370988	3.5740244	3.5895922	3.5586152	3.5622521	3.4769433	2.7077187	4.1665008
Up	FGSG_03452	3.363261509	2.07E-09	5.3528065	5.349828	5.2780294	5.0814056	4.9394155	5.1395644	6.2842483	6.4733227	5.991768
Up	FGSG_03120	3.336335396	6.83E-10	7.544488	7.2547781	7.711794	8.9443615	8.8828941	8.9186116	9.2809858	9.3663558	9.1220974
Up	FGSG_01149	3.323191217	3.45E-05	3.0172362	2.8476671	3.5740244	3.1965524	2.8748957	3.4190665	4.7032988	4.9352342	4.3572018
Up	FGSG_09240	3.308486677	5.99E-10	5.2004262	4.8067189	5.4444954	6.0446528	5.7257602	6.2582434	6.1190688	5.9891987	6.1802631
Up	FGSG_03121	3.285391374	1.42E-05	2	2	2	2.7805029	2.6377651	2.8774004	2.4052168	2.7077187	2
Up	FGSG_04789	3.253451288	5.04E-10	2.6910183	2.4852148	2.8407141	2.8962916	2.8748957	2.8774004	3.3903592	3.6853877	2.964684
Up	FGSG_03817	3.211414512	5.26E-12	5.3235909	5.2319977	5.3356788	5.8856141	5.7257602	5.9550408	5.8044166	5.7385279	5.8079414
Up	FGSG_03445	3.199610204	1.68E-10	9.0452336	8.8646883	9.1273169	9.4343613	9.4941215	9.2788996	9.6690111	9.7139873	9.5533442
Up	FGSG_13962	3.192603744	0.0002967	5.4906186	5.4053203	5.4959731	2.8962916	2.6377651	3.0813961	4.0850435	3.9446664	4.1665008
Up	FGSG_12312	3.176651524	1.31E-05	2.2688027	2	2.4807492	2	2	2	2	2	2
Up	FGSG_07744	3.176208342	2.02E-07	2.2688027	2	2.4807492	2.364115	2	2.6397336	2.2167888	2	2.3974876
Up	FGSG_03113	3.172285023	7.08E-09	3.1562965	3.1370988	3.5740244	2.8962916	2.6377651	3.0813961	3.7092973	2.9633478	4.1665008
Up	FGSG_04734	3.167876826	2.98E-06	2.8633281	3.1370988	2.4807492	2	2	2	2.2167888	2.3968278	2.3974876
Up	FGSG_08562	3.163628568	0.0002462	4.2285219	4.2010532	4.188785	4.9800625	4.8861689	4.9955781	4.7387761	4.6740912	4.7462732
Up	FGSG_05723	3.153901269	9.97E-07	5.2322171	4.5354388	5.640225	5.3887447	5.306652	5.3904893	5.1870071	5.0499834	5.2577234
Up	FGSG_02866	3.146590097	1.69E-10	3.1562965	3.1370988	3.1285604	3.1965524	3.2568854	3.0813961	2.9802913	3.1804273	2.7087823
Up	FGSG_10563	3.144917997	0.0001316	2.4953013	2.4852148	2.4807492	2.8962916	2.8748957	2.8774004	4.028829	3.8208439	4.1665008
Up	FGSG_05834	3.141329532	2.70E-06	2	2	2	3.0034737	3.0784965	2.8774004	2.2167888	2.3968278	2
Up	FGSG_08682	3.128982464	2.09E-09	7.7560934	7.7271924	7.7008668	7.4330428	7.1819267	7.5727822	8.1241013	8.0697759	8.1193104
Up	FGSG_03741	3.117328762	1.96E-11	6.310507	6.5228522	5.9664603	6.4471928	6.29449	6.5086577	6.0641898	5.9002223	6.1547968
Up	FGSG_10433	3.115034454	9.69E-12	5.0300296	5.0349724	4.9491538	5.1529987	5.2232303	4.9955781	6.0071405	5.9002223	6.0481828
Up	FGSG_04968	3.113738488	4.41E-08	2.2688027	2	2.4807492	2.1935143	2	2.3550396	2.4052168	2.3968278	2.3974876
Up	FGSG_12794	3.064774704	0.0003754	3.6078986	3.5844678	3.5740244	3.5190873	3.4156241	3.5622521	3.6359267	4.0586965	2.964684

Up	FGSG_02443	3.031131814	3.66E-09	4.0257777	4.069824	3.9139052	3.5190873	3.6887032	3.260088	4.807216	4.6740912	4.8766132
Up	FGSG_12294	3.018252715	9.68E-05	2	2	2	2	2	2	2	2	2
Up	FGSG_13411	3.016566793	0.0004925	2	2	2	2.1935143	2	2.3550396	2	2	2
Up	FGSG_01159	3.014491113	2.15E-10	6.7222549	6.7541729	6.60482	6.6339322	6.4675956	6.7066335	7.600156	7.4613623	7.6668215
Up	FGSG_06548	3.012985366	1.45E-07	4.0257777	4.4323548	3.3684179	4.1522165	4.0206066	4.210161	3.6359267	3.6853877	3.5376798
Up	FGSG_10341	3.006121917	1.38E-12	5.2004262	4.9628272	5.3356788	4.657914	4.7733906	4.4500449	4.965331	4.9352342	4.9376179
Up	FGSG_11548	2.98797536	1.58E-06	2	2	2	2.1935143	2	2.3550396	2	2	2
Up	FGSG_03225	2.982434642	1.28E-09	4.0257777	4.3213345	3.9139052	3.3668146	2.8748957	3.6925011	3.6359267	2.9633478	4.0607807
Up	FGSG_02034	2.971368396	0.0002536	2.6910183	3.5844678	2	5.3486486	5.423383	5.1845342	6.4324437	6.2272902	6.555942
Up	FGSG_07508	2.968576456	1.62E-05	6.3253218	6.153105	6.4038729	4.9800625	4.8861689	4.9955781	6.9235321	6.7011148	7.0598582
Up	FGSG_00321	2.958786496	8.02E-10	8.2275779	8.2280865	8.1416454	9.0867536	8.9164781	9.1600886	9.4172353	9.4260069	9.3411292
Up	FGSG_12666	2.957798506	3.40E-12	13.233191	13.201749	13.178941	13.26365	13.235855	13.203621	13.895239	13.857236	13.866977
Up	FGSG_11532	2.953949726	9.73E-07	2	2	2	2	2	2	2.2167888	2	2.3974876
Up	FGSG_03026	2.946684355	9.97E-12	5.4640893	5.5600358	5.2780294	6.0446528	6.1663515	5.8190522	6.4324437	6.5337304	6.2778581
Up	FGSG_03888	2.943273274	0.0157915	2	2	2	2	2	2	2.4052168	2.3968278	2.3974876
Up	FGSG_12611	2.941303697	1.43E-10	6.2182735	6.0172257	6.3206633	5.4468739	5.179638	5.6041695	6.5864309	6.6834471	6.4128878
Up	FGSG_12548	2.936781258	8.40E-09	5.9580386	5.9811576	5.85259	6.9475516	6.8889735	6.9211113	7.9112731	7.743009	8.0028193
Up	FGSG_03954	2.91815159	6.41E-07	5.3814422	5.0349724	5.5937254	4.7531416	4.1161991	5.1395644	3.8457121	4.0586965	3.5376798
Up	FGSG_06491	2.908184776	4.91E-05	2	2	2	2	2.353841	2	2	2	2
Up	FGSG_03462	2.892496314	4.52E-11	3.7898088	4.4323548	4.0578819	3.3668146	4.4455509	3.9222766	4.4698939	4.6740912	4.3572018
Up	FGSG_04830	2.887740454	9.81E-08	3.2831234	3.9254527	2	3.0034737	3.2568854	2.6397336	3.7092973	3.8208439	3.5376798
Up	FGSG_10047	2.883944998	9.80E-05	2.6910183	3.1370988	2	4.6247215	4.9394155	4.210161	3.7791165	3.9446664	3.5376798
Up	FGSG_03130	2.867687336	0.0013367	2.2688027	2.4852148	2	2.5166584	2.353841	2.6397336	2.7212202	2.3968278	2.964684
Up	FGSG_12280	2.85784468	2.91E-09	4.4062533	4.4323548	4.3087924	3.3668146	3.2568854	3.4190665	4.2902636	4.3549955	4.1665008
Up	FGSG_00093	2.844607146	0.0051124	2.2688027	2	2.4807492	2.364115	2	2.6397336	2.5718533	2	2.964684
Up	FGSG_07587	2.834683608	1.58E-05	2	2	2	2.7805029	2.353841	3.260088	2.2167888	2.3968278	2
Up	FGSG_04480	2.83141515	1.04E-07	4.5136357	4.7218364	4.188785	4.0052074	4.0206066	3.9222766	4.994976	4.9352342	4.9961472
Up	FGSG_05241	2.828592559	8.36E-06	5.2937714	5.1036809	5.3911128	5.4277566	5.5656438	5.2281446	5.0804226	4.9937494	5.1587087
Up	FGSG_05724	2.821469678	1.38E-08	9.5081899	9.4862982	9.4447256	9.2763549	9.2393369	9.2261042	8.3358337	8.3246744	8.2810547
Up	FGSG_10444	2.820950226	1.33E-05	3.5075501	3.1370988	3.7539507	5.0316238	5.0403527	4.9442019	5.7358405	5.3920827	5.9627112
Up	FGSG_09974	2.820618507	5.62E-16	9.5307355	9.6036297	9.3639055	9.3352503	9.3417718	9.2394852	9.112032	9.0827785	9.0753558

Up	FGSG_12198	2.811837014	1.27E-05	2	2	2	2	2	2	2	2	2
Up	FGSG_10992	2.802332233	3.51E-07	2.8633281	2.8476671	2.8407141	3.8981589	4.2058494	3.4190665	3.2982451	2.9633478	3.5376798
Up	FGSG_01239	2.798807088	0.000537	3.2831234	2	3.9139052	2.364115	2	2.6397336	2.4052168	2.3968278	2.3974876
Up	FGSG_01686	2.796118407	1.58E-07	4.2902308	4.2010532	4.3087924	5.5027514	5.4962661	5.4283735	5.5284909	5.2552685	5.7064263
Up	FGSG_13849	2.794918783	0.0006863	2.2688027	2	2.4807492	2	2	2	2	2	2
Up	FGSG_09061	2.789426285	0.0002316	5.167919	5.1036809	5.1553224	4.5203003	4.7733906	4.1204343	4.028829	4.262829	3.6872775
Up	FGSG_04824	2.778737032	1.54E-09	10.595658	10.610285	10.545084	11.607374	11.559464	11.610471	12.036494	11.975207	12.05826
Up	FGSG_09701	2.776756461	3.56E-07	4.4609433	4.6316459	4.188785	3.8415007	4.0206066	3.5622521	6.235408	6.3216817	6.075584
Up	FGSG_12668	2.775321534	0.0077488	2	2	2	3.444959	3.5586152	3.260088	4.028829	3.9446664	4.0607807
Up	FGSG_03662	2.76774544	3.96E-08	12.499811	12.462578	12.451383	11.848608	11.808739	11.800759	13.268285	13.239031	13.23115
Up	FGSG_03494	2.767216601	4.43E-09	3.6078986	3.5844678	3.5740244	3.7825262	3.8080253	3.6925011	4.3373111	4.1643702	4.4438634
Up	FGSG_05703	2.761824531	2.63E-05	3.9513288	4.5354388	3.7539507	3.2841938	3.5586152	3.4190665	3.4769433	3.5358829	3.3707558
Up	FGSG_09755	2.738722335	1.14E-05	2.4953013	2.8476671	2	2.1935143	2.6377651	2	2.8565633	2.3968278	3.18196
Up	FGSG_00413	2.722713301	3.25E-05	3.0172362	3.378055	2.4807492	2.1935143	2.353841	2	2	2	2
Up	FGSG_12009	2.710594949	9.54E-08	8.4054187	8.2357604	8.4978648	8.5259381	8.3750006	8.5829629	8.9225643	8.9560704	8.8198705
Up	FGSG_06001	2.710439971	3.73E-07	7.6305042	7.7380397	7.436798	8.8877757	8.8658043	8.8223008	9.4199698	9.4180164	9.3579651
Up	FGSG_02315	2.71003122	7.45E-08	7.250589	7.0610564	7.3415168	7.4425982	7.1708374	7.6062213	8.3559647	8.3584682	8.2986023
Up	FGSG_03889	2.703698953	0.0021839	2	2	2	2	2	2	2.4052168	2.3968278	2.3974876
Up	FGSG_03894	2.686700208	2.98E-06	9.3810178	9.2338408	9.441447	8.5570623	8.6471877	8.3658682	10.694549	10.664011	10.665619
Up	FGSG_07292	2.683998597	2.23E-10	9.9906539	9.9615786	9.9389876	9.3782072	9.489666	9.1657067	8.572975	8.5363144	8.5439396
Up	FGSG_01550	2.678016615	2.60E-05	2	2	2	2.1935143	2	2.3550396	2.8565633	2	3.3707558
Up	FGSG_07798	2.670585426	3.09E-06	2	2	2	2.1935143	2	2.3550396	2.5718533	2.7077187	2.3974876
Up	FGSG_04999	2.668095561	0.0001585	2	2	2	2.1935143	2.353841	2	2.7212202	2.7077187	2.7087823
Up	FGSG_20700	2.662650269	2.67E-05	2	2	2	2.1935143	2.353841	2	2	2	2
Up	FGSG_00394	2.654521748	0.0005255	2.6910183	3.1370988	2	2.1935143	2.353841	2	2	2	2
Up	FGSG_12142	2.653369886	3.78E-07	3.0172362	3.1370988	2.8407141	3.0034737	3.4156241	2.6397336	3.4769433	3.3690748	3.5376798
Up	FGSG_06124	2.645834458	3.46E-10	10.14893	10.152052	10.059369	10.188245	10.10364	10.184392	11.239985	11.216649	11.196948
Up	FGSG_00126	2.645218186	2.91E-05	2	3.1370988	2	2.1935143	2	2.3550396	2.2167888	2	2.3974876
Up	FGSG_01755	2.634507544	3.66E-06	2.8633281	2	3.3684179	3.1032407	3.2568854	2.8774004	3.4769433	3.6853877	3.18196
Up	FGSG_06587	2.633478833	9.54E-13	9.4263847	9.3829667	9.394746	8.85416	8.7808316	8.8504805	8.7507501	8.7165054	8.723558
Up	FGSG_03907	2.627506955	2.41E-06	3.0172362	2.4852148	3.3684179	3.1965524	2.8748957	3.4190665	3.0942412	2.9633478	3.18196

Up	FGSG_13851	2.622680705	0.0003877	2	2	2	2	2.6377651	2	2	2	2
Up	FGSG_11100	2.612856045	3.85E-12	4.7948149	4.8868835	4.6184939	4.7835368	4.8861689	4.5903319	4.5519634	4.0586965	4.8766132
Up	FGSG_09345	2.611994684	6.92E-07	4.0257777	4.3213345	3.5740244	4.0558932	4.2058494	3.8119585	4.1391495	3.9446664	4.265
Up	FGSG_10023	2.608584428	9.44E-05	2	2	2	2	2	2	2.2167888	2.7077187	2
Up	FGSG_13837	2.607664517	0.0001309	2	2	2	2.1935143	2	2.3550396	2.5718533	2.7077187	2.3974876
Up	FGSG_12028	2.573902694	3.77E-11	8.3485399	8.4086576	8.204887	7.7768861	7.7423879	7.7251372	8.1106233	8.0353685	8.1193104
Up	FGSG_06879	2.572096524	2.78E-05	2	2	2	2.6546039	2	3.0813961	2.9802913	3.3690748	2.3974876
Up	FGSG_08844	2.568944128	2.19E-11	9.3157495	9.3193862	9.2523278	10.052087	9.9401873	10.073702	9.9975521	10.006395	9.921236
Up	FGSG_05458	2.557944227	6.84E-11	8.0382427	8.1490155	7.8268285	8.2816438	8.2593613	8.2168902	8.7088767	8.6634198	8.6884918
Up	FGSG_07522	2.556096676	5.95E-13	7.7284952	7.7380397	7.6335064	8.0790645	8.133639	7.9304878	8.4364951	8.3860373	8.4211493
Up	FGSG_11517	2.555264003	2.62E-05	4.4609433	4.069824	4.7085304	3.444959	3.8080253	2.8774004	4.7387761	4.262829	5.0523944
Up	FGSG_04739	2.55158609	1.26E-08	4.7515947	4.7218364	4.7085304	5.4468739	5.5313719	5.2704753	5.5080752	5.1562742	5.7410644
Up	FGSG_05663	2.548465813	5.25E-06	3.7898088	3.5844678	3.9139052	3.8981589	3.9182279	3.8119585	3.4769433	3.5358829	3.3707558
Up	FGSG_02588	2.548233919	1.80E-14	9.3064464	9.3121456	9.2182494	8.9955692	8.9782109	8.9252511	8.8615131	8.9112346	8.7407766
Up	FGSG_05080	2.535196487	3.72E-06	2.2688027	2	2.4807492	3.1032407	2.353841	3.5622521	2.5718533	2.9633478	2
Up	FGSG_11453	2.534345025	5.03E-05	2	2	2	2	2	2	2.2167888	2.3968278	2
Up	FGSG_11379	2.529663876	4.96E-11	6.9966572	7.0260808	6.8813568	6.9872674	6.8477891	7.0359915	7.6889891	7.5996588	7.711174
Up	FGSG_05640	2.528770633	5.79E-11	9.9482146	9.892866	9.9226205	9.3918355	9.4140907	9.2788996	8.9011954	8.876669	8.8715923
Up	FGSG_09038	2.526027839	1.80E-14	6.7771658	6.5962238	6.8618681	7.1993948	6.9933753	7.3039581	7.104406	7.124214	7.0180995
Up	FGSG_10212	2.524893091	1.20E-06	12.839026	12.722237	12.866061	11.456962	11.522319	11.294528	13.127222	13.137255	13.049832
Up	FGSG_10942	2.518856126	1.12E-12	11.270705	11.197459	11.257384	10.849274	10.83646	10.773691	10.484634	10.468976	10.433833
Up	FGSG_00602	2.51566688	4.48E-12	7.7724024	7.8220012	7.6335064	8.3893257	8.3989584	8.2907328	8.3473715	8.4343718	8.1836833
Up	FGSG_11751	2.514541825	0.0002606	2.2688027	2.4852148	2	2	2	2	2.4052168	2.7077187	2
Up	FGSG_02827	2.512943395	2.28E-05	2	2	2	2.7805029	2.8748957	2.6397336	2.5718533	2.3968278	2.7087823
Up	FGSG_08509	2.509914131	5.71E-10	5.8799122	5.6997548	5.9664603	6.9135989	6.7617162	6.9733016	6.6622962	6.6291077	6.6317388
Up	FGSG_07795	2.507123626	0.0025391	3.9513288	4.2010532	3.5740244	2.364115	2	2.6397336	2.5718533	2.9633478	2
Up	FGSG_01440	2.4992702	3.67E-08	3.3996963	2.8476671	3.7539507	2.8962916	2.6377651	3.0813961	3.199846	3.6853877	2.3974876
Up	FGSG_06095	2.496118951	0.0006506	3.5075501	3.765011	3.1285604	2.1935143	2	2.3550396	2	2	2
Up	FGSG_13756	2.494556575	0.0091331	3.3996963	3.1370988	3.5740244	2.1935143	2.353841	2	2.2167888	2	2.3974876
Up	FGSG_12573	2.490671587	2.44E-06	3.0172362	3.1370988	2.8407141	3.444959	3.0784965	3.6925011	3.3903592	3.6853877	2.964684
Up	FGSG_12522	2.485950423	1.13E-13	5.6164066	5.4053203	5.728956	5.8856141	5.6318376	6.0308779	5.9015311	5.9601454	5.7748904

Up	FGSG_04394	2.476431996	1.07E-05	4.8367778	4.4323548	5.0898194	4.657914	4.8308815	4.3744304	5.1346989	5.1562742	5.1065306
Up	FGSG_10998	2.475161679	2.85E-07	4.5644712	4.4323548	4.6184939	4.69036	4.5857446	4.7181783	4.8402557	4.3549955	5.1587087
Up	FGSG_10213	2.47305993	5.14E-05	2.4953013	2	2.8407141	2.1935143	2	2.3550396	3.0942412	2.7077187	3.3707558
Up	FGSG_08151	2.471444711	7.18E-06	2.6910183	2	3.1285604	2.6546039	2.6377651	2.6397336	2.4052168	2.3968278	2.3974876
Up	FGSG_04429	2.464739854	1.27E-10	4.349408	4.3213345	4.3087924	4.7531416	4.8308815	4.7181783	4.2902636	3.9446664	4.5256129
Up	FGSG_07624	2.462533904	0.0010527	2	2	2	2	2	2	2.5718533	2.3968278	2.7087823
Up	FGSG_08013	2.455461878	4.60E-05	2	2	2	2.1935143	2.353841	2.3550396	2	2	2
Up	FGSG_06931	2.454976693	4.14E-11	11.070378	11.008808	11.045909	10.484389	10.459684	10.421225	10.179613	10.128939	10.165536
Up	FGSG_04754	2.448585662	6.70E-05	2.2688027	2.4852148	2	2	2	2	2.2167888	2	2.3974876
Up	FGSG_07876	2.445613518	0.001044	2	2	2	2.1935143	2	2.3550396	2.4052168	2	2.7087823
Up	FGSG_10847	2.444947805	0.0001627	2.4953013	2	2.8407141	2	2	2	2.4052168	2.3968278	2.3974876
Up	FGSG_03045	2.439664083	0.002226	2.2688027	2	2.4807492	2	2	2	2.8565633	2.7077187	2.964684
Up	FGSG_10480	2.435126334	0.0489022	2.2688027	2.4852148	2	2	2	2	2	2	2
Up	FGSG_11454	2.433337491	0.0004452	4.2902308	4.8868835	3.1285604	5.1056669	5.346621	4.7181783	4.8402557	5.1041074	4.4438634
Up	FGSG_04085	2.432693336	1.29E-09	7.8512916	7.9581125	7.6449538	7.9068273	7.9118154	7.8136811	8.8953122	8.821198	8.9025233
Up	FGSG_02669	2.430793711	1.20E-05	2.6910183	2.8476671	2.4807492	2.1935143	2	2.3550396	3.199846	3.3690748	2.964684
Up	FGSG_12530	2.429375157	1.00E-05	3.701719	3.5844678	3.7539507	4.0052074	4.0206066	3.9222766	4.2416299	4.1643702	4.265
Up	FGSG_05825	2.427296304	1.52E-06	2.4953013	2.8476671	2	2.364115	2.6377651	2	2.4052168	2.7077187	2
Up	FGSG_10905	2.416851347	7.19E-14	9.148808	9.2679217	8.9262106	8.9154885	8.7698493	8.9676783	8.7998647	8.6589066	8.8676788
Up	FGSG_04082	2.416761149	0.0002315	2.6910183	2.4852148	2.8407141	2	2	2	2.2167888	2.3968278	2
Up	FGSG_02035	2.414566664	1.88E-05	3.9513288	3.765011	4.0578819	5.2212063	4.9394155	5.3904893	5.626448	5.5170389	5.670936
Up	FGSG_11806	2.414314407	0.000654	2	2	2	2	2	2	3.0942412	2.9633478	3.18196
Up	FGSG_13172	2.41399204	0.0004819	2.8633281	2.4852148	3.1285604	3.7825262	4.2902531	2.8774004	4.807216	4.9937494	4.5256129
Up	FGSG_10442	2.408886415	0.0014839	2.2688027	2.4852148	2	2.1935143	2.353841	2	3.199846	3.5358829	2.7087823
Up	FGSG_09469	2.407241069	3.35E-05	2.8633281	2.8476671	2.8407141	3.2841938	3.6887032	2.6397336	2.4052168	2.3968278	2.3974876
Up	FGSG_05804	2.405208283	0.0001356	2.2688027	2	2.4807492	2.1935143	2	2.3550396	2.2167888	2	2.3974876
Up	FGSG_08478	2.403127178	2.56E-11	9.619055	9.5917188	9.561077	9.094351	9.0735851	9.0275257	8.8675355	8.8959742	8.7704233
Up	FGSG_01746	2.403045835	4.08E-13	6.3688748	6.4717791	6.232359	6.3077574	6.374	6.2155469	6.4645842	6.5533169	6.3692672
Up	FGSG_12868	2.402345922	9.64E-09	4.1640552	4.069824	4.188785	3.5895922	4.1161991	2.6397336	4.2416299	3.9446664	4.4438634
Up	FGSG_00738	2.400948606	7.18E-07	6.9496238	6.8169075	6.9930638	8.27631	8.2853007	8.1785015	9.1781338	9.1804804	9.1088964
Up	FGSG_07856	2.397110391	3.51E-11	10.178807	10.110918	10.16054	9.7132786	9.7029141	9.6414213	9.3812064	9.3270703	9.369081

Up	FGSG_03881	2.395218779	1.48E-06	4.2285219	4.2010532	4.188785	4.0052074	3.6887032	4.2946324	3.9093688	4.1643702	3.5376798
Up	FGSG_06757	2.39204129	1.96E-13	9.5560776	9.4338905	9.5880145	9.1995368	9.0854447	9.223413	8.9148303	8.8921338	8.8715923
Up	FGSG_04746	2.391400924	9.91E-05	2	2	2	2.8962916	2.8748957	2.8774004	3.0942412	2.7077187	3.3707558
Up	FGSG_12248	2.389358782	0.0001583	4.2902308	3.9254527	4.5224622	3.5190873	3.2568854	3.6925011	4.9041484	4.6006874	5.1065306
Up	FGSG_01828	2.3860112	2.29E-05	2.8633281	2.4852148	3.1285604	2.7805029	3.2568854	2	2.8565633	2.7077187	2.964684
Up	FGSG_03984	2.385934415	1.84E-10	9.1878364	9.2028557	9.0859202	9.5713242	9.4985633	9.5584353	9.9500989	9.9612878	9.8712943
Up	FGSG_02893	2.385779029	1.60E-10	6.7879016	6.6885859	6.8017711	6.4846757	6.374	6.5262338	6.9235321	6.8668223	6.9307712
Up	FGSG_12977	2.385367493	1.47E-10	6.0321508	5.8672052	6.1055269	5.9673238	5.9498937	5.9021734	6.7431318	7.0154424	6.3242864
Up	FGSG_11412	2.380170001	8.94E-06	3.0172362	3.1370988	2.8407141	3.1965524	2.353841	3.6925011	3.3903592	3.1804273	3.5376798
Up	FGSG_10784	2.379240774	9.13E-09	7.2112509	7.342684	7.4102108	8.0943001	8.2955472	7.9950244	8.8412549	8.9959807	8.9063435
Up	FGSG_03185	2.376103828	0.0064018	3.2831234	3.5844678	2.8407141	3.1032407	3.4156241	2.6397336	2.8565633	3.1804273	2.3974876
Up	FGSG_00210	2.375363976	0.0001552	2.2688027	2	2.4807492	2.8962916	3.5586152	2	2	2	2
Up	FGSG_03032	2.374552295	7.05E-05	4.0257777	3.378055	4.4195794	4.1980691	4.5857446	3.5622521	4.028829	3.8208439	4.1665008
Up	FGSG_11205	2.369413988	5.62E-06	12.18068	12.131373	12.144204	11.081832	11.057273	11.018432	12.912828	12.863888	12.895504
Up	FGSG_12143	2.368623467	6.63E-05	2	2	2	2.6546039	2.8748957	2.3550396	2.7212202	2.9633478	2.3974876
Up	FGSG_03571	2.368085485	0.0003104	2	2	2	2	2	2	2.2167888	2	2.3974876
Up	FGSG_08622	2.358803974	4.22E-08	5.6164066	5.7434744	5.3911128	6.466056	6.374	6.4728499	6.8844677	6.7185687	6.975096
Up	FGSG_05378	2.351648238	7.95E-12	10.922941	10.861587	10.899475	10.408408	10.411034	10.317791	10.131209	10.149997	10.044223
Up	FGSG_08272	2.348360946	4.13E-11	4.5136357	4.8067189	4.0578819	4.69036	4.4455509	4.9442019	5.0240241	5.2552685	4.7462732
Up	FGSG_03807	2.347857175	4.79E-05	2	2	2	2	2	2	2.2167888	2.3968278	2
Up	FGSG_10420	2.342415627	0.0007911	3.6078986	3.9254527	3.1285604	3.3668146	3.0784965	3.5622521	4.666927	4.5233477	4.7462732
Up	FGSG_06079	2.331870308	0.000191	3.0172362	3.378055	2.4807492	2.364115	2.353841	2.6397336	3.2982451	3.5358829	2.964684
Up	FGSG_07632	2.329681192	0.0016345	2	2	2	2	2	2	2	2	2
Up	FGSG_03689	2.327995555	5.07E-06	2.2688027	2.4852148	2	2.1935143	2	2.3550396	2.4052168	2	2.7087823
Up	FGSG_09870	2.320544622	4.34E-13	10.222989	10.215521	10.234388	9.8031793	9.8801773	9.7369752	9.7008407	9.6311615	9.7383088
Up	FGSG_04239	2.319303134	3.30E-05	2.2688027	2.4852148	2	3.1032407	3.6887032	2	2.7212202	2.7077187	2.7087823
Up	FGSG_08996	2.31924278	4.93E-09	5.2633225	5.349828	5.0898194	5.7840418	5.5991203	5.874995	5.9325022	5.7723491	6.0202512
Up	FGSG_01728	2.317869659	0.0003583	2	2	2	2	2	2	2.2167888	2.3968278	2
Up	FGSG_10368	2.317659807	5.87E-13	10.89962	11.063733	11.015363	10.41631	10.585063	10.614371	10.156024	10.453424	10.375775
Up	FGSG_03661	2.314181307	0.0019327	3.1562965	2.8476671	3.3684179	3.0034737	2.8748957	3.0813961	4.2416299	4.6740912	3.5376798
Up	FGSG_03178	2.309637063	7.66E-05	2.4953013	2.4852148	2.4807492	2.8962916	2.8748957	2.8774004	3.6359267	3.3690748	3.8228101

Up	FGSG_10010	2.309334512	9.41E-12	9.4653614	9.4137361	9.4348672	8.8964933	8.762481	8.938439	8.5852082	8.579939	8.5241867
Up	FGSG_05715	2.308819139	0.000328	3.0172362	3.1370988	2.8407141	2.1935143	2	2.3550396	3.0942412	3.3690748	2.7087823
Up	FGSG_10175	2.306027075	0.0333481	4.5136357	4.2010532	4.7085304	3.9526758	4.3699906	3.4190665	4.0850435	4.0586965	4.0607807
Up	FGSG_09181	2.303806661	4.15E-05	4.4062533	4.7218364	4.8733193	2.7805029	3.0784965	2.3550396	2.8565633	3.1804273	2.7087823
Up	FGSG_09820	2.303002409	1.34E-10	8.5854239	8.5133883	8.5716985	9.094351	9.1030531	8.9963367	9.45105	9.4627214	9.377362
Up	FGSG_05606	2.302529282	7.08E-08	3.1562965	3.378055	2.8407141	4.0558932	4.2058494	3.8119585	3.0942412	2.9633478	3.18196
Up	FGSG_05113	2.299622696	0.0027783	2	2	2	2	2	2	2.7212202	2	3.3707558
Up	FGSG_02503	2.296665995	2.01E-10	9.8440469	9.7366778	9.8650517	9.1405824	9.0616273	9.1316649	8.9417196	9.0102241	8.799473
Up	FGSG_13349	2.294868798	2.33E-05	2	2	2	2.1935143	2	2.3550396	2.4052168	2.7077187	2
Up	FGSG_04615	2.291874306	1.60E-06	2.4953013	2.4852148	2.4807492	2.1935143	2	2.3550396	2.2167888	2	2.3974876
Up	FGSG_01956	2.289236271	1.70E-12	10.457917	10.483882	10.343801	9.9103616	9.8211132	9.9109651	9.8163899	9.7673745	9.801213
Up	FGSG_03614	2.289149071	0.000683	2.6910183	2	3.1285604	2.5166584	2.6377651	2.6397336	3.2982451	3.6853877	2.7087823
Up	FGSG_20022	2.284102711	5.54E-05	3.872828	3.378055	4.188785	4.1980691	4.4455509	3.8119585	4.6296145	4.6740912	4.5256129
Up	FGSG_13574	2.283344172	8.13E-05	2	2	2	2.5166584	2	2.8774004	2.2167888	2.3968278	2
Up	FGSG_13731	2.282076769	6.37E-11	7.8816778	7.7701006	8.0238286	7.7072456	7.898432	7.6787403	8.6908756	8.7294771	8.6434254
Up	FGSG_12158	2.279696179	0.0013883	2	2	2	2.364115	2.6377651	2	2.4052168	2.7077187	2
Up	FGSG_10729	2.278836617	4.66E-06	2.6910183	3.1370988	2	2.364115	2.6377651	2	2.4052168	2.3968278	2.3974876
Up	FGSG_08335	2.270937529	1.96E-11	10.726279	10.632255	10.743155	10.25077	10.180291	10.245355	10.043494	10.03802	9.9948445
Up	FGSG_05612	2.267346294	2.90E-07	3.6078986	3.765011	3.3684179	3.3668146	3.5586152	3.0813961	4.3373111	3.9446664	4.6029775
Up	FGSG_12436	2.264394833	6.38E-05	2.6910183	2	3.1285604	3.444959	3.2568854	3.5622521	3.199846	3.1804273	3.18196
Up	FGSG_06270	2.262020632	4.92E-10	11.610898	11.612864	11.522355	10.93004	10.878817	10.893648	10.557845	10.54187	10.513635
Up	FGSG_06439	2.259792797	6.12E-05	2.4953013	2	2.8407141	3.7210377	3.4156241	3.9222766	3.970335	3.5358829	4.265
Up	FGSG_01717	2.254611383	6.32E-06	3.1562965	3.5844678	2.4807492	3.9526758	4.1161991	3.6925011	4.6296145	4.4416259	4.7462732
Up	FGSG_07725	2.254206098	0.0160319	2.4953013	2	2.8407141	2.364115	2.353841	2.3550396	2.2167888	2.3968278	2
Up	FGSG_06549	2.253378606	1.15E-06	4.5136357	4.6316459	4.3087924	4.8424709	4.7733906	4.8356119	4.965331	5.3920827	4.3572018
Up	FGSG_04000	2.252820843	8.39E-09	7.6305042	7.5902346	7.5867862	7.8894648	7.9577028	7.7251372	8.6817901	8.6222832	8.6751191
Up	FGSG_02291	2.248152688	2.58E-08	7.4394623	7.342684	7.4499101	8.3158408	8.2645866	8.2804124	8.8695373	8.8805507	8.7912326
Up	FGSG_11565	2.247218578	5.34E-05	2.6910183	3.1370988	2	2.1935143	2	2.3550396	2.5718533	2	2.964684
Up	FGSG_13602	2.246160878	0.000261	2.4953013	2.8476671	2	2.1935143	2	2.3550396	2.7212202	2.9633478	2.3974876
Up	FGSG_09866	2.245508809	2.50E-11	10.534021	10.761476	10.755089	10.089823	10.300907	10.287191	9.9117568	10.031052	10.083557
Up	FGSG_13834	2.243980369	3.68E-06	2.4953013	2.4852148	2.4807492	2.1935143	2.6377651	2	2.5718533	2.7077187	2.3974876

Up	FGSG_01154	2.238913012	2.19E-12	10.675875	10.638058	10.62818	10.25145	10.218489	10.196792	10.022056	9.9667757	10.010901
Up	FGSG_06827	2.238651484	3.77E-11	10.355189	10.305778	10.319003	9.7280054	9.673752	9.6948544	9.4814746	9.4444809	9.4524422
Up	FGSG_06554	2.238025443	0.001126	5.4640893	6.120316	4.0578819	5.0814056	4.8308815	5.2281446	4.7032988	4.4416259	4.9376179
Up	FGSG_08927	2.23707241	4.07E-08	4.7070397	4.5354388	4.7932764	5.4468739	5.346621	5.4652882	6.0216157	6.152209	5.8079414
Up	FGSG_10922	2.235769765	1.74E-09	11.158847	11.048563	11.179823	11.944831	11.950035	11.850006	12.302971	12.295395	12.244066
Up	FGSG_08561	2.232904157	8.12E-14	9.8958306	9.8409828	9.8650517	9.5982919	9.5846431	9.5238565	9.3951704	9.4392267	9.2806061
Up	FGSG_04137	2.232388562	2.76E-13	9.8568495	9.8782316	9.7478458	9.6046899	9.5443949	9.5775277	9.3092846	9.3355791	9.2143539
Up	FGSG_11105	2.229358627	0.0073733	2.4953013	2.4852148	2.4807492	2	2	2	2	2	2
Up	FGSG_05999	2.228129448	1.29E-13	9.8796117	9.8027466	9.8723747	9.7270283	9.6932588	9.6733237	9.4483738	9.4313093	9.3992151
Up	FGSG_05809	2.220140549	1.44E-06	2.8633281	2.4852148	3.1285604	3.7210377	3.5586152	3.8119585	2.8565633	2.3968278	3.18196
Up	FGSG_03736	2.218068843	4.33E-07	8.463492	8.5196883	8.3164747	7.7151516	7.7646868	7.5727822	7.3420968	7.2253567	7.3895788
Up	FGSG_07480	2.215118438	3.31E-10	11.020044	10.996439	10.958009	10.363213	10.259632	10.380737	10.21547	10.153209	10.214119
Up	FGSG_02273	2.214817015	1.70E-08	8.3006156	8.2280865	8.2875517	8.2922525	8.2435709	8.2542839	8.9643752	8.8649606	8.995118
Up	FGSG_06020	2.214554168	6.02E-10	11.859826	11.822944	11.811081	11.530212	11.472951	11.499732	10.709714	10.685249	10.667871
Up	FGSG_01569	2.213360428	7.17E-09	5.7973114	5.6546687	5.85259	5.1988268	5.4962661	4.7181783	5.3334324	5.2552685	5.3503768
Up	FGSG_04867	2.21137137	0.0004031	2.4953013	2	2.8407141	2.1935143	2.353841	2.6397336	2.2167888	2	2.3974876
Up	FGSG_04159	2.210507264	1.73E-09	5.2004262	5.510285	4.7085304	4.4077273	4.0206066	4.7181783	5.3100371	5.347904	5.2090653
Up	FGSG_11241	2.210407718	3.67E-05	2	2	2	2.1935143	2	2.3550396	2.4052168	2.7077187	2
Up	FGSG_13339	2.20895185	0.0031718	2.8633281	2	3.3684179	2	2	2	3.9093688	3.9446664	3.8228101
Up	FGSG_06019	2.203880006	8.14E-13	10.049832	10.016069	10.048636	9.8452123	9.6874344	9.9456675	9.6352872	9.6403484	9.5989078
Up	FGSG_04225	2.20358995	5.64E-08	3.3996963	3.5844678	3.1285604	3.0034737	2.8748957	3.0813961	3.5586239	3.5358829	3.5376798
Up	FGSG_12399	2.199404188	3.44E-05	2	2	2	2.5166584	2.6377651	2.3550396	2	2	2
Up	FGSG_01266	2.196753735	5.02E-06	4.0257777	3.5844678	4.3087924	5.1529987	4.9394155	5.2704753	5.1870071	4.9937494	5.3047937
Up	FGSG_08670	2.195522689	3.21E-13	10.745683	10.690654	10.717633	10.247367	10.226544	10.185775	10.156844	10.146777	10.108644
Up	FGSG_05529	2.191488699	5.25E-09	4.0965725	4.3213345	3.7539507	4.5559538	4.9394155	4.024755	5.2862562	5.1041074	5.3945636
Up	FGSG_11836	2.190428441	7.47E-07	3.6078986	3.765011	3.3684179	3.5895922	2.8748957	4.024755	3.6359267	3.3690748	3.8228101
Up	FGSG_01509	2.189964551	1.30E-10	11.113158	11.047474	11.092639	10.679347	10.58715	10.684536	10.327482	10.243391	10.343734
Up	FGSG_00798	2.188066467	3.55E-12	10.471299	10.38037	10.476262	10.113944	10.109459	10.029709	9.8369977	9.8607252	9.7447241
Up	FGSG_08722	2.187571837	2.47E-10	8.1084431	8.1326676	8.0061838	7.3693409	7.5157415	7.1194186	7.6980295	7.6090478	7.7198832
Up	FGSG_10941	2.187373731	3.55E-12	11.264954	11.273055	11.170942	10.692922	10.665285	10.632742	10.622781	10.54187	10.636016
Up	FGSG_02255	2.185479793	1.56E-07	5.2633225	5.4053203	5.0212003	4.4837433	3.9182279	4.8909283	3.9093688	4.262829	3.3707558

Up	FGSG_05818	2.180883619	3.20E-05	2.4953013	2.8476671	2	2.5166584	2.353841	2.6397336	2.4052168	2.3968278	2.3974876
Up	FGSG_02099	2.17473966	3.23E-12	10.985485	10.915139	10.969436	10.603643	10.532967	10.587415	10.642817	10.575785	10.642902
Up	FGSG_03172	2.173980448	7.40E-10	7.642384	7.7594926	7.4235657	7.3183509	7.387497	7.1537472	7.1578038	7.1372515	7.1268773
Up	FGSG_06467	2.173027546	1.47E-05	2.6910183	2.4852148	2.8407141	2.364115	2.353841	2.3550396	2.4052168	2.7077187	2
Up	FGSG_00150	2.167692116	3.41E-09	8.5574627	8.3950151	8.6246952	8.6833322	8.6107232	8.6685513	9.1404961	9.1160864	9.0989159
Up	FGSG_01871	2.164819578	5.87E-13	10.202071	10.192046	10.126238	9.8843033	9.8647765	9.8176978	9.6918179	9.6242328	9.6925848
Up	FGSG_05976	2.161706233	1.85E-05	3.0172362	2.8476671	3.1285604	2.364115	2.6377651	2	2.4052168	2.7077187	2
Up	FGSG_11413	2.158693582	0.0021361	2.4953013	2.4852148	2.4807492	2.6546039	2.353841	2.8774004	3.2982451	3.1804273	3.3707558
Up	FGSG_13963	2.156220391	0.0212843	2.6910183	2.4852148	2.8407141	2	2	2.3550396	2.2167888	2	2.3974876
Up	FGSG_10844	2.150468867	0.0004243	3.2831234	3.378055	3.1285604	2.7805029	2.353841	3.0813961	2	2	2
Up	FGSG_11128	2.149560288	3.35E-07	4.5136357	3.765011	5.0212003	5.7226193	5.9498937	5.3515835	5.9925186	5.9304949	5.991768
Up	FGSG_10786	2.148197048	9.05E-05	3.701719	3.765011	3.5740244	2.7805029	3.2568854	2	4.028829	3.6853877	4.265
Up	FGSG_11492	2.145775307	0.0003464	2	2	2	2	2	2	2.2167888	2	2.3974876
Up	FGSG_00175	2.141405628	2.91E-09	8.3521613	8.353296	8.294837	8.1063738	8.0208235	8.1160334	7.8558035	7.5902083	8.024072
Up	FGSG_03402	2.13995393	7.71E-05	3.2831234	2.8476671	3.5740244	3.2841938	3.4156241	3.0813961	3.7092973	3.6853877	3.6872775
Up	FGSG_10696	2.139946497	0.0001798	2.4953013	2.4852148	2.4807492	2.8962916	2.8748957	2.8774004	2	2	2
Up	FGSG_01798	2.138847534	7.83E-09	7.06886	6.8372271	7.1934618	7.3026957	7.2677006	7.2520883	7.0347533	7.0708531	6.9307712
Up	FGSG_13095	2.138595678	0.0006794	3.6078986	3.765011	3.3684179	2.6546039	2.6377651	2.6397336	2.8565633	2.7077187	2.964684
Up	FGSG_02485	2.135910641	3.60E-11	8.7618699	8.7936805	8.6419368	9.2260611	9.1824984	9.1824311	9.2612986	9.3635849	9.0787452
Up	FGSG_06253	2.131455063	0.0252095	2	2	2	2	2	2	2.2167888	2.3968278	2
Up	FGSG_08896	2.130682954	1.96E-11	10.751179	10.749464	10.668	10.380716	10.328613	10.350224	9.9651549	9.9631194	9.9060526
Up	FGSG_01278	2.129533948	8.85E-14	10.727674	10.719675	10.651069	10.310698	10.273954	10.259873	10.250457	10.232802	10.201737
Up	FGSG_09093	2.129083971	5.79E-10	4.7948149	4.8067189	4.7085304	4.8990917	4.8861689	4.8356119	5.5684753	5.7385279	5.3047937
Up	FGSG_10350	2.126626377	6.45E-06	2.6910183	2.8476671	2.4807492	2.364115	2.353841	2.3550396	2.9802913	3.5358829	2
Up	FGSG_07539	2.126484692	3.14E-10	8.9974717	8.7884722	9.1026217	9.430765	9.3343365	9.4405686	10.022056	10.004617	9.9731536
Up	FGSG_09315	2.12399338	0.0541692	2.8633281	2.8476671	2.8407141	2	2	2	2	2	2
Up	FGSG_01619	2.120213107	1.07E-12	6.3253218	6.1851653	6.3766661	6.6750453	6.6057619	6.6596278	6.9388664	7.0843795	6.7037513
Up	FGSG_12960	2.117721476	0.0002241	2.4953013	2	2.8407141	2.364115	2	2.6397336	2	2	2
Up	FGSG_00671	2.117395372	1.96E-12	9.6880206	9.7121323	9.5761044	9.4651598	9.3589733	9.4816879	9.2289176	9.1804804	9.211269
Up	FGSG_05546	2.113661149	2.28E-10	7.87162	7.8220012	7.8368453	7.0448635	6.8337955	7.1537472	7.9305779	7.7515126	8.0310872
Up	FGSG_04226	2.113448265	6.77E-08	4.7070397	4.8868835	4.4195794	4.1522165	4.1161991	4.1204343	4.2416299	4.3549955	4.0607807

Up	FGSG_06285	2.111024624	0.0037956	2	2	2	2.1935143	2.353841	2	2.5718533	2	2.964684
Up	FGSG_05493	2.110780229	2.18E-10	10.054285	10.040372	9.9824987	9.7807531	9.7502448	9.723706	9.2582458	9.214709	9.235766
Up	FGSG_06893	2.110026229	1.96E-13	11.057681	11.022182	11.024157	10.740664	10.652386	10.746769	10.619216	10.586519	10.592813
Up	FGSG_13025	2.109977353	0.001097	3.1562965	3.5844678	2.4807492	2	2	2	2.7212202	2.7077187	2.7087823
Up	FGSG_05613	2.109380353	0.0004625	2	2	2	3.2841938	3.5586152	2.8774004	2.9802913	2.9633478	2.964684
Up	FGSG_08597	2.108816084	1.01E-09	5.5921111	5.608128	5.4959731	5.0060735	5.2232303	4.6556708	5.5486216	5.6684096	5.3503768
Up	FGSG_04383	2.102231368	0.0004875	2.8633281	2.4852148	3.1285604	3.5190873	3.0784965	3.9222766	4.0850435	3.5358829	4.5256129
Up	FGSG_12249	2.099827159	6.79E-05	2.8633281	2	3.3684179	2.1935143	2.353841	2	2.4052168	2.3968278	2.3974876
Up	FGSG_01693	2.099432141	0.0007334	6.2955384	6.2772809	6.232359	5.8715343	6.0008798	5.636897	6.45395	6.5337304	6.3242864
Up	FGSG_08015	2.098585835	2.06E-10	8.3984303	8.2809657	8.4266952	8.2273973	8.0753633	8.2855818	7.7640849	7.6183761	7.8365939
Up	FGSG_08547	2.098269886	0.0007577	2	2	2	2.6546039	2.353841	2.8774004	3.8457121	3.9446664	3.6872775
Up	FGSG_10185	2.096119547	6.30E-06	4.7070397	4.6316459	4.7085304	4.2425091	4.2058494	4.210161	5.0240241	4.9352342	5.1587087
Up	FGSG_11922	2.094111652	3.45E-12	10.181863	10.135739	10.142482	9.8912983	9.9352807	9.7538581	9.5087042	9.4985248	9.4524422
Up	FGSG_10529	2.092055506	1.90E-05	4.6135762	5.0349724	3.9139052	5.3281747	5.2232303	5.3515835	6.3884448	6.5726412	6.1024744
Up	FGSG_05433	2.091410798	8.73E-12	10.471299	10.462784	10.39385	10.037186	9.9434491	10.041979	9.7699891	9.83495	9.6315836
Up	FGSG_11455	2.088756945	0.000652	5.1346624	4.8868835	5.2780294	5.9540229	5.8139402	6.0060392	5.9628224	6.0176785	5.8402522
Up	FGSG_10051	2.086566109	0.0002772	7.612499	7.42554	7.711794	7.4708903	7.3682091	7.485628	8.285742	8.2426005	8.2632912
Up	FGSG_10441	2.084772546	0.000133	3.1562965	3.378055	2.8407141	3.0034737	3.0784965	2.8774004	2.7212202	3.1804273	2
Up	FGSG_12209	2.084583641	0.0001024	3.0172362	3.378055	2.4807492	3.1965524	3.0784965	3.260088	3.199846	2.9633478	3.3707558
Up	FGSG_09972	2.084044734	0.0051413	4.2285219	4.2010532	4.188785	4.5907474	4.7135134	4.3744304	5.5880596	5.6684096	5.4374371
Up	FGSG_06082	2.080876413	3.80E-10	8.2510444	8.1326676	8.294837	8.4521701	8.4318447	8.3852606	8.543185	8.3360272	8.6706338
Up	FGSG_00634	2.080696428	6.21E-11	10.434194	10.383828	10.398929	9.9751668	9.8699284	9.9922575	9.5971364	9.5359244	9.5941788
Up	FGSG_08792	2.080245085	5.24E-08	8.0337397	7.8916615	8.0839393	7.5437309	7.5157415	7.485628	6.5469478	6.2514733	6.7384529
Up	FGSG_02910	2.075480985	0.0001504	4.4609433	4.3213345	4.5224622	3.0034737	3.4156241	2.3550396	4.5913113	4.6740912	4.4438634
Up	FGSG_06593	2.075071656	7.54E-09	4.0965725	4.3213345	3.7539507	3.8415007	4.2058494	3.260088	4.2902636	3.8208439	4.6029775
Up	FGSG_04455	2.074500035	3.08E-05	2.4953013	3.378055	3.1285604	2.5166584	2.8748957	2.3550396	2.9802913	2.7077187	3.18196
Up	FGSG_00247	2.07326875	0.0004936	2	2	2	2.364115	2.6377651	2	2.4052168	2	2.7087823
Up	FGSG_05701	2.071962781	5.64E-09	6.1861705	6.2772809	6.0025037	5.0814056	4.8861689	5.1845342	4.8402557	4.8742451	4.8129147
Up	FGSG_00638	2.067483232	1.59E-10	7.799181	7.832161	7.678761	8.4089556	8.2329471	8.4874957	8.4685463	8.4077195	8.4632514
Up	FGSG_01021	2.067467377	2.29E-13	10.023956	10.027167	9.9343302	9.7994657	9.7048375	9.8069525	9.6187202	9.5481786	9.6223229
Up	FGSG_00395	2.066233176	3.87E-10	10.602516	10.589473	10.531272	10.027694	9.9287125	10.040451	9.9472583	9.9241583	9.9041434

Up	FGSG_04049	2.066217242	0.0002001	2.8633281	3.1370988	2.4807492	2.1935143	2.353841	2	2.5718533	2.7077187	2.3974876
Up	FGSG_03407	2.06235245	3.11E-05	3.1562965	3.378055	2.8407141	3.2841938	2.8748957	3.5622521	2.4052168	2.3968278	2.3974876
Up	FGSG_03194	2.061878484	3.11E-06	2.6910183	2.4852148	2.8407141	2.8962916	2.6377651	3.0813961	3.3903592	3.5358829	3.18196
Up	FGSG_06289	2.061240007	1.78E-12	10.018269	9.9708045	9.9802411	9.6559041	9.533614	9.6890142	9.4735994	9.3746367	9.5037751
Up	FGSG_06130	2.060140096	1.86E-08	10.935668	10.873934	10.911374	11.266335	11.197061	11.248281	11.923246	11.941229	11.836951
Up	FGSG_07871	2.059812936	0.0020797	2.4953013	2.8476671	2	2	2	2	2.4052168	2.3968278	3.18196
Up	FGSG_11602	2.058915112	2.65E-09	9.5763453	9.4927173	9.5731114	9.7191874	9.7182296	9.6313053	10.436156	10.408425	10.397652
Up	FGSG_05662	2.05624589	0.0026167	2	2	2	2.5166584	2.6377651	2.3550396	2	2	2
Up	FGSG_04906	2.05406765	3.74E-09	8.2741352	8.2280865	8.2802294	8.5504491	8.5942161	8.4138691	9.1618901	9.2177808	9.0410167
Up	FGSG_04829	2.052384553	2.87E-07	9.0652287	9.0149575	9.0302651	9.922363	9.9401873	9.8141249	9.9166057	9.84293	9.925007
Up	FGSG_04086	2.051274926	1.38E-08	6.3688748	6.306724	6.3489364	6.3181765	6.449356	6.0793081	7.5709277	7.5614795	7.514971
Up	FGSG_04149	2.050700551	3.84E-11	9.6250545	9.6445598	9.5181483	9.4004416	9.4022865	9.3096736	8.9681168	8.9188047	8.9514135
Up	FGSG_10469	2.050013631	0.0011677	2.8633281	3.378055	2	2	2	2.3550396	2	2	2
Up	FGSG_01267	2.047965081	2.94E-11	11.528077	11.422585	11.544681	11.882791	11.839266	11.838621	12.262028	12.198043	12.259456
Up	FGSG_03813	2.046658878	6.70E-05	3.6078986	3.1370988	3.9139052	3.5190873	3.5586152	3.4190665	3.6359267	3.8208439	3.3707558
Up	FGSG_00631	2.043938539	2.47E-12	10.402236	10.329281	10.392152	10.134724	10.125344	10.05566	9.8256999	9.7757361	9.8114355
Up	FGSG_03720	2.042334713	7.37E-06	3.6078986	3.765011	3.3684179	3.0034737	3.2568854	2.6397336	4.1912995	4.262829	4.0607807
Up	FGSG_04120	2.040161554	2.98E-07	5.7541671	5.608128	5.8125445	5.0060735	4.3699906	5.3904893	5.947742	6.2026947	5.5589053
Up	FGSG_09427	2.038663802	1.66E-09	5.2004262	5.1036809	5.2179801	5.5209064	5.6638294	5.3115993	5.237485	4.6006874	5.6345505
Up	FGSG_20531	2.037662014	5.98E-07	3.1562965	3.378055	2.8407141	2.7805029	2.353841	3.0813961	2.9802913	2.9633478	2.964684
Up	FGSG_01516	2.035284703	3.41E-11	9.5016831	9.5275207	9.5025016	9.3769618	9.4093806	9.3958547	8.8913767	8.967065	8.8558744
Up	FGSG_10733	2.030761767	6.63E-14	11.293015	11.273987	11.245213	11.158849	11.152658	11.085173	10.839468	10.839785	10.785584
Up	FGSG_00845	2.027238193	1.97E-11	10.054285	9.9890811	10.033473	9.7627484	9.5699452	9.8546882	9.3671061	9.34965	9.3183711
Up	FGSG_10843	2.025847141	2.42E-11	7.3039203	7.3139731	7.20897	7.0941983	6.9551045	7.1423949	6.5569203	6.4526114	6.5943381
Up	FGSG_08895	2.02257322	4.92E-11	10.403982	10.378639	10.343801	9.9594444	9.8970987	9.9358374	9.7451269	9.816832	9.5989078
Up	FGSG_02145	2.02150306	0.0001492	2.4953013	2	2.8407141	4.0052074	3.9182279	4.024755	3.5586239	3.5358829	3.5376798
Up	FGSG_02493	2.019376429	3.07E-11	9.4108417	9.2902031	9.441447	9.1287928	9.1204492	9.0489636	8.9417196	8.876669	8.9439989
Up	FGSG_06334	2.018471595	9.01E-07	4.0257777	3.9254527	4.0578819	4.327482	3.9182279	4.5903319	4.935064	5.1562742	4.6764038
Up	FGSG_20675	2.018464986	0.003806	2.6910183	2	3.1285604	2	2	2	2	2	2
Up	FGSG_13157	2.015220117	0.0004391	2.6910183	2.8476671	2.4807492	4.1980691	4.2902531	4.024755	4.8402557	4.9352342	4.6764038
Up	FGSG_11796	2.01445429	0.0006596	2.2688027	2	2.8407141	2	2	2	2.4052168	2.7077187	2

Up	FGSG_12803	2.01423112	0.0052119	2.4953013	2.4852148	2.4807492	2	2	2	2	2	2
Up	FGSG_09471	2.012769626	7.69E-08	11.444883	11.445102	11.35906	11.123642	11.032217	11.126811	11.287925	11.230402	11.280269
Up	FGSG_06048	2.010553165	1.40E-12	11.560135	11.53181	11.502827	11.205898	11.175724	11.148253	11.785843	11.777572	11.727846
Up	FGSG_12604	2.009432276	0.0105831	2	2	2	2	2	2	2	2	2
Up	FGSG_03994	2.005805924	1.44E-06	9.0089878	8.9696574	8.9632608	8.6383062	8.7212688	8.4557453	9.3166403	9.3012384	9.2658137
Up	FGSG_01752	2.00379679	7.56E-05	3.0172362	2.4852148	3.3684179	3.0034737	2.8748957	3.0813961	2.2167888	2.3968278	2
Up	FGSG_01145	2.003727744	1.18E-05	3.9513288	4.069824	3.7539507	3.1032407	3.4156241	2.6397336	2.5718533	2.3968278	2.7087823
Up	FGSG_04709	1.999479941	1.55E-05	2.8633281	2.8476671	2.8407141	3.2841938	2.8748957	3.5622521	3.3903592	3.5358829	3.18196
Up	FGSG_07186	1.997071199	1.24E-12	10.302818	10.302128	10.217236	10.110202	10.03344	10.098881	9.8205351	9.7736502	9.801213
Up	FGSG_13403	1.994958409	8.82E-05	2.4953013	2.4852148	2.4807492	2.6546039	2.353841	3.260088	2.2167888	2	2.3974876
Up	FGSG_00032	1.993709547	1.39E-06	2.8633281	2.4852148	3.1285604	3.3668146	3.0784965	3.5622521	3.6359267	2.9633478	4.0607807
Up	FGSG_02587	1.993416444	0.0002053	2.2688027	2	2.4807492	2.6546039	2	3.0813961	2.4052168	2.3968278	2.3974876
Up	FGSG_01954	1.992924031	1.44E-06	6.0500989	6.2165286	5.7713555	5.2432439	4.8308815	5.5363994	6.247774	6.2272902	6.2052877
Up	FGSG_13979	1.98793567	2.09E-06	6.7985581	6.9534782	6.5327046	6.328521	6.29449	6.279127	6.5961351	6.2026947	6.8537291
Up	FGSG_04022	1.987894431	5.99E-12	8.0337397	7.9581125	8.0238286	8.1565887	8.1106104	8.1218255	7.9906727	7.9344186	7.9812488
Up	FGSG_09610	1.983190889	1.67E-06	8.6456852	8.668859	8.5352539	9.1924749	9.0735851	9.2207168	10.510522	10.480532	10.474286
Up	FGSG_08358	1.982426415	7.17E-09	10.095918	10.137788	9.9734469	11.031274	11.041379	10.951709	10.347729	10.393561	10.240082
Up	FGSG_10923	1.981180522	5.14E-05	2.4953013	2.8476671	2	2.364115	2.353841	2.3550396	2.2167888	2.3968278	2
Up	FGSG_06728	1.978750182	3.11E-06	3.872828	3.9254527	3.7539507	4.4077273	4.0206066	4.6556708	3.4769433	3.1804273	3.6872775
Up	FGSG_09819	1.978232381	1.64E-09	6.5437448	6.4455482	6.5571458	6.8503947	6.8337955	6.7817251	6.9690546	6.9578183	6.9156884
Up	FGSG_04471	1.977958849	1.03E-06	5.065757	4.9628272	5.0898194	5.3486486	5.4602848	5.1395644	4.3828727	3.5358829	4.8766132
Up	FGSG_05092	1.97700644	2.43E-05	3.5075501	4.069824	2.4807492	4.2425091	4.6510429	3.8119585	4.8402557	4.6006874	5.2090653
Up	FGSG_12130	1.974731146	0.0003683	2.2688027	2.4852148	2	2.5166584	2.6377651	2.3550396	2	2	2
Up	FGSG_08714	1.972084466	9.21E-11	11.467597	11.447582	11.40186	11.075321	10.993403	11.070273	10.659111	10.671872	10.578519
Up	FGSG_05989	1.970845401	0.0026022	2.2688027	2.4852148	2	2	2	2	2	2	2
Up	FGSG_00802	1.969227222	3.54E-10	10.532426	10.507847	10.471448	10.032448	10.027305	9.9489293	9.6222864	9.6055918	9.5822882
Up	FGSG_06080	1.968790256	2.90E-12	6.5181831	6.5477263	6.4038729	6.1764794	6.0257126	6.279127	6.7431318	6.7011148	6.7212064
Up	FGSG_02023	1.968573549	0.0044432	3.0172362	3.378055	2.4807492	2.6546039	3.0784965	2	2	2	2
Up	FGSG_01596	1.965705339	0.0001964	6.2955384	6.153105	6.3489364	5.4277566	5.4962661	5.2704753	7.3819108	7.5614795	7.100442
Up	FGSG_10653	1.96475638	0.0056557	2.2688027	2.4852148	2	2.364115	2	2.6397336	2.2167888	2	2.3974876
Up	FGSG_04792	1.96358381	0.0021703	2	2	2	2	2	2	2.4052168	2.7077187	2

Up	FGSG_05397	1.957966496	0.0009374	3.701719	3.378055	3.9139052	4.3681625	3.8080253	4.7181783	4.5115122	4.6006874	4.3572018
Up	FGSG_06780	1.955546813	0.001313	2.6910183	2.4852148	2.8407141	2.1935143	2	2.3550396	2	2	2
Up	FGSG_11111	1.953160391	6.36E-06	3.3996963	2.8476671	3.7539507	4.5907474	4.1161991	4.8909283	4.5913113	3.8208439	5.0523944
Up	FGSG_03299	1.950845562	0.0007729	3.3996963	2.4852148	3.9139052	3.7825262	3.0784965	4.210161	2.8565633	2.7077187	2.964684
Up	FGSG_07411	1.94989955	7.49E-10	5.899844	5.9441646	5.7713555	6.2653117	6.253029	6.1937152	6.8361499	6.9578183	6.6317388
Up	FGSG_09303	1.949555118	2.11E-08	6.1533367	5.6546687	6.4567939	7.0635623	6.9421186	7.0960705	6.8030149	6.5726412	6.945698
Up	FGSG_10820	1.948710077	3.60E-09	8.2117189	8.2585401	8.0755039	8.2301586	8.2697931	8.0985166	9.0551216	9.0589969	8.9843154
Up	FGSG_04784	1.946931508	6.41E-05	2.6910183	2	3.1285604	3.1965524	2.353841	3.8119585	3.2982451	2.3968278	3.8228101
Up	FGSG_04570	1.946526936	0.0001121	6.3971965	6.306724	6.4038729	8.7579406	8.7513576	8.680374	8.9244913	8.8452329	8.9365461
Up	FGSG_07742	1.946342412	0.0001809	2.4953013	2.4852148	2.4807492	3.2841938	3.5586152	3.0813961	2.9802913	3.1804273	2.7087823
Up	FGSG_04114	1.94480484	9.80E-06	2.4953013	2.8476671	2	3.0034737	2.6377651	3.260088	2.8565633	3.1804273	2.964684
Up	FGSG_05207	1.94244114	3.22E-06	5.3235909	5.510285	5.0212003	5.3074061	5.423383	5.0931475	5.3564544	5.2066203	5.4374371
Up	FGSG_06148	1.941696472	3.43E-05	3.9513288	4.3213345	3.3684179	3.1965524	2.6377651	3.5622521	2.7212202	2.9633478	2.3974876
Up	FGSG_04459	1.938988224	1.89E-05	3.5075501	3.9254527	2.8407141	3.3668146	3.8080253	2.6397336	4.4698939	4.262829	4.6029775
Up	FGSG_12667	1.936467706	0.0104899	2	4.069824	3.5740244	2.364115	4.1161991	3.8119585	2.5718533	4.0586965	3.6872775
Up	FGSG_04175	1.930488608	5.83E-05	3.2831234	3.1370988	3.3684179	2.7805029	2.8748957	2.8774004	2.9802913	3.1804273	2.7087823
Up	FGSG_04136	1.929477884	6.63E-11	11.057126	11.016625	11.012052	10.658486	10.584019	10.644861	10.380387	10.366136	10.328155
Up	FGSG_03590	1.92849595	1.90E-05	8.6368049	8.7514782	8.4200477	9.9776337	9.907157	9.9602881	10.811104	10.747151	10.808221
Up	FGSG_11062	1.922868873	6.42E-08	4.0257777	4.2010532	3.7539507	4.657914	4.9907667	4.1204343	4.028829	3.9446664	4.0607807
Up	FGSG_05545	1.918737891	6.28E-05	3.5075501	3.765011	3.1285604	3.9526758	3.8080253	4.024755	3.3903592	3.8208439	2.7087823
Up	FGSG_05040	1.915214526	1.54E-08	6.3831052	6.5721788	6.0719967	6.7691847	6.2739084	7.0723383	6.5961351	6.7011148	6.4128878
Up	FGSG_10554	1.913452432	1.79E-11	6.3545028	6.3355781	6.2918249	6.1992055	6.1885139	6.1261652	5.9925186	5.9891987	5.9330571
Up	FGSG_04465	1.912588483	0.0049581	2.2688027	2.4852148	2	2	2	2	2	2	2
Up	FGSG_02925	1.912332631	0.0051284	5.7321013	5.906198	5.4444954	4.0052074	3.2568854	4.4500449	4.807216	4.9352342	4.6029775
Up	FGSG_12233	1.911074594	0.0001979	2.6910183	2	3.1285604	3.7825262	3.8080253	3.6925011	4.4270393	4.5233477	4.265
Up	FGSG_11048	1.910600969	0.0199652	2	2	2	2	2	2	2.2167888	2.3968278	2
Up	FGSG_13946	1.910035396	7.80E-07	4.0257777	3.378055	4.4195794	4.0558932	3.6887032	4.2946324	4.028829	4.0586965	3.9466965
Up	FGSG_06924	1.907886613	9.77E-11	9.9695903	9.9499626	9.903685	9.6121184	9.5678332	9.5690734	9.8143128	9.7694694	9.7929826
Up	FGSG_07832	1.906665065	1.01E-08	6.8196378	6.7541729	6.8017711	7.4986383	7.489204	7.4306909	8.0798295	8.1297141	7.9593509
Up	FGSG_01504	1.906316304	2.01E-12	10.987233	11.077677	11.048066	10.742116	10.819696	10.784686	10.401295	10.441648	10.49346
Up	FGSG_13067	1.904958322	1.09E-05	2.4953013	2.4852148	2.4807492	2.5166584	2.6377651	2.3550396	2.2167888	2	2.3974876

Up	FGSG_10181	1.901779224	3.31E-12	10.394355	10.434722	10.425723	10.193206	10.249126	10.292336	9.6758911	9.8188564	9.7123585
Up	FGSG_06123	1.90170888	1.92E-12	7.112218	7.1611664	6.9930638	7.2815544	7.1024491	7.3735786	7.5208582	7.4819493	7.514971
Up	FGSG_01696	1.89822344	9.54E-08	6.5310205	6.5962238	6.3766661	7.1766717	7.23613	7.0236696	7.7981156	7.7767266	7.7542035
Up	FGSG_06083	1.890076383	0.0003289	5.6402997	5.6997548	5.4959731	4.0558932	4.3699906	3.5622521	5.107816	4.9352342	5.2090653
Up	FGSG_03554	1.887594488	4.66E-13	10.795077	10.812399	10.69165	10.264978	10.191309	10.257245	10.342694	10.385388	10.230972
Up	FGSG_12819	1.887474821	4.23E-07	4.8775546	4.7218364	4.9491538	5.1295269	4.7135134	5.3904893	4.5519634	4.4416259	4.6029775
Up	FGSG_09399	1.885898307	2.30E-08	6.2182735	5.9441646	6.3766661	6.4376679	6.1438433	6.6436126	6.7166867	6.6291077	6.7384529
Up	FGSG_00427	1.884478067	7.79E-08	4.349408	3.5844678	4.7932764	4.92659	4.8308815	4.9442019	4.5519634	4.5233477	4.5256129
Up	FGSG_03100	1.881173982	1.77E-05	2.2688027	2.8476671	2.4807492	2.364115	3.0784965	2.6397336	2.4052168	3.1804273	2.3974876
Up	FGSG_06988	1.876516275	5.46E-08	7.06886	7.2087314	6.8220828	7.3693409	7.1708374	7.4675471	8.1800181	8.2059445	8.0927348
Up	FGSG_05175	1.875815261	3.49E-10	11.619563	11.589097	11.564407	12.090602	12.04077	12.053483	12.491556	12.421648	12.494267
Up	FGSG_04451	1.874978363	3.44E-08	6.9206496	6.9157592	6.8421126	6.6505183	6.6858453	6.5262338	6.9765039	7.0012507	6.8850413
Up	FGSG_04633	1.874597197	0.007798	3.7898088	3.9254527	3.5740244	3.0034737	3.2568854	2.6397336	2	2	2
Up	FGSG_07048	1.871899656	3.94E-11	10.192003	10.178174	10.24571	9.7769812	9.716324	9.8822482	9.6341102	9.5699772	9.7867788
Up	FGSG_09083	1.870423137	0.0001899	2.4953013	2.4852148	2.4807492	3.6568115	3.4156241	3.8119585	3.9093688	3.9446664	3.8228101
Up	FGSG_11509	1.867211258	0.0001488	3.0172362	2.4852148	3.3684179	3.0034737	2.8748957	3.0813961	2.4052168	2.7077187	2
Up	FGSG_04476	1.864174684	2.13E-11	11.3794	11.329728	11.343336	10.92451	10.909162	10.85331	10.866783	10.868409	10.797975
Up	FGSG_01913	1.862027469	9.30E-05	2.6910183	2.4852148	2.8407141	2.364115	2.353841	2.3550396	2.8565633	3.1804273	2.3974876
Up	FGSG_01256	1.860131082	2.72E-08	6.1197383	6.2165286	6.3766661	6.927276	7.0669996	7.0359915	7.5310121	7.6368537	7.6395404
Up	FGSG_03522	1.858640658	0.0067419	2.8633281	3.378055	2	3.5190873	2.8748957	3.9222766	3.9093688	4.3549955	3.18196
Up	FGSG_08765	1.856641225	1.70E-05	2.4953013	2.4852148	2.4807492	2.6546039	2.8748957	2.3550396	2.2167888	2.3968278	2
Up	FGSG_08374	1.856518776	0.0034554	2	2	2	2	2	2	2.4052168	2.9633478	2.3974876
Up	FGSG_02582	1.853607806	6.80E-06	3.3996963	3.765011	2.8407141	3.7825262	4.0206066	3.4190665	4.3828727	4.1643702	4.5256129
Up	FGSG_06397	1.853238888	5.87E-06	2.4953013	2.4852148	2.4807492	3.2841938	3.4156241	3.0813961	2.9802913	2.7077187	3.18196
Up	FGSG_00915	1.852715944	2.65E-09	4.5644712	4.8067189	4.188785	4.9800625	5.0882909	4.7780897	5.4450355	5.3023297	5.5195414
Up	FGSG_03031	1.852114884	0.0010798	2.4953013	2	2.8407141	3.7210377	3.0784965	4.1204343	3.8457121	3.1804273	4.265
Up	FGSG_03778	1.849379137	1.29E-06	3.5075501	4.3213345	4.5224622	3.6568115	4.8308815	5.1845342	3.3903592	4.3549955	4.6029775
Up	FGSG_05032	1.846169993	2.73E-06	7.7615503	7.7380397	7.711794	7.3131513	7.3970452	7.1309525	8.6541853	8.6813329	8.5585788
Up	FGSG_08630	1.843426025	8.11E-08	7.4664442	7.5415319	7.2986735	8.1536827	8.133639	8.0867194	8.5531835	8.3750728	8.652552
Up	FGSG_09259	1.841662685	3.72E-07	3.7898088	3.765011	3.7539507	4.4837433	4.2902531	4.5903319	5.0524989	4.9352342	5.1065306
Up	FGSG_03780	1.840973811	0.0006245	2	2	2	2.1935143	2	2.3550396	2.8565633	3.1804273	2.3974876

Up	FGSG_02626	1.840655765	0.0250603	2.6910183	2.8476671	2.4807492	2	2	2	3.2982451	3.5358829	2.964684
Up	FGSG_02609	1.840226947	1.01E-11	6.0321508	6.0172257	5.9664603	5.7689296	5.7557564	5.7002055	5.9777469	5.3920827	6.3469521
Up	FGSG_02560	1.838725679	9.83E-05	8.3339628	8.3812423	8.1971317	9.3955301	9.3807925	9.3273266	10.057611	10.034541	10.017981
Up	FGSG_02541	1.836039347	4.23E-10	9.9529922	9.8027466	10.026925	9.4462848	9.4489346	9.3619975	9.1667824	9.1741688	9.1088964
Up	FGSG_10279	1.834961554	0.000226	3.5075501	3.378055	3.5740244	2.8962916	2.8748957	2.8774004	2.5718533	2.7077187	2.3974876
Up	FGSG_10819	1.834527695	1.36E-07	8.512861	8.3812423	8.5535913	8.0698453	7.9769307	8.0748249	8.5204318	8.4811393	8.4940407
Up	FGSG_03274	1.833580185	0.002177	2	2	2	2.364115	2.353841	2.3550396	2.5718533	2.9633478	2
Up	FGSG_11905	1.830695619	9.69E-05	3.3996963	3.5844678	3.1285604	3.2841938	3.8080253	2.3550396	3.6359267	3.3690748	3.8228101
Up	FGSG_09294	1.829610345	3.75E-12	7.7506158	7.7806311	7.6335064	7.8505153	7.7572921	7.8559952	8.2887372	8.1935159	8.3159389
Up	FGSG_09137	1.828205871	0.0002307	4.2285219	4.4323548	3.9139052	3.444959	3.5586152	3.260088	3.970335	4.262829	3.5376798
Up	FGSG_03378	1.825705046	6.95E-07	10.591068	10.551545	10.546611	11.462843	11.42825	11.412046	11.952343	11.906431	11.932305
Up	FGSG_09985	1.825150498	2.39E-06	4.6135762	5.0349724	3.9139052	3.7210377	4.1161991	3.0813961	4.2416299	3.8208439	4.5256129
Up	FGSG_01748	1.823206126	9.39E-05	2.8633281	2.4852148	3.1285604	2.8962916	2.353841	3.260088	3.6359267	3.5358829	3.6872775
Up	FGSG_12440	1.821786148	0.000151	2.2688027	2	2.4807492	2.8962916	2.353841	3.260088	2.5718533	2.7077187	2.3974876
Up	FGSG_13889	1.821644285	4.05E-08	4.7515947	4.7218364	4.7085304	4.1522165	4.2058494	4.024755	3.8457121	4.0586965	3.5376798
Up	FGSG_07629	1.821337967	0.0003713	2.2688027	2	2.4807492	3.1965524	2.8748957	3.4190665	3.199846	3.3690748	2.964684
Up	FGSG_06137	1.81866468	8.77E-09	4.9558055	4.8067189	5.0212003	5.7689296	5.8970396	5.5363994	6.0918902	5.9304949	6.1802631
Up	FGSG_13167	1.818521058	1.71E-08	3.5075501	3.1370988	3.7539507	4.0558932	4.1161991	3.9222766	3.970335	4.0586965	3.8228101
Up	FGSG_04817	1.81840303	0.0214535	2	2	2	3.1032407	3.4156241	2.8774004	3.4769433	3.3690748	3.5376798
Up	FGSG_04446	1.817389097	5.62E-06	6.4789709	7.0782312	6.4305761	7.2868688	7.5331658	7.5557675	8.0832837	8.0213724	8.1710364
Up	FGSG_05172	1.817072975	2.56E-06	3.6078986	3.9254527	3.1285604	3.0034737	3.2568854	2.6397336	3.7791165	3.3690748	4.0607807
Up	FGSG_04609	1.815054632	0.0001109	4.1640552	4.069824	4.188785	3.1965524	3.0784965	3.260088	3.8457121	3.6853877	3.9466965
Up	FGSG_10785	1.812422988	0.0004959	4.4062533	5.906198	4.7085304	4.3681625	5.179638	5.4283735	4.7387761	6.2514733	6.1288728
Up	FGSG_01546	1.811882188	0.0034823	2.2688027	2.4852148	2	2.7805029	2.353841	3.0813961	3.970335	3.9446664	3.9466965
Up	FGSG_07181	1.8110934	3.82E-12	11.15988	11.087252	11.168961	10.945664	10.900797	10.926284	10.541615	10.546764	10.504843
Up	FGSG_06522	1.808973727	3.12E-06	4.2902308	4.069824	4.4195794	4.5559538	4.3699906	4.6556708	4.5913113	4.3549955	4.7462732
Up	FGSG_02811	1.808051466	5.35E-11	9.4143102	9.4471719	9.2929149	9.0745139	9.1001333	8.9579975	9.4880046	9.4549323	9.4550527
Up	FGSG_08676	1.80690049	0.0125659	8.0337397	11.039823	10.997614	7.7347289	10.721465	10.777365	7.4742601	10.371663	10.408467
Up	FGSG_00357	1.802237525	2.70E-09	5.4906186	5.349828	5.8125445	5.7840418	5.6951271	5.9021734	6.2600348	6.2514733	6.2778581
Up	FGSG_00322	1.798420519	0.0008603	2	2	2	2.6546039	2.6377651	2.6397336	2.2167888	2.3968278	2
Up	FGSG_08302	1.79645435	0.0004959	3.0172362	2	3.5740244	2.7805029	2.353841	3.0813961	2.2167888	2	2.3974876

Up	FGSG_05031	1.796362605	0.0005338	3.3996963	3.378055	3.3684179	3.0034737	3.2568854	2.6397336	4.0850435	4.0586965	4.0607807
Up	FGSG_13352	1.795565813	6.03E-06	4.7070397	4.8067189	4.5224622	4.4837433	4.2902531	4.5903319	4.1391495	4.1643702	4.0607807
Up	FGSG_07895	1.792806272	1.12E-05	8.0825166	8.0567194	8.0238286	9.2051615	9.1714134	9.1516201	9.8194999	9.8087059	9.7638005
Up	FGSG_13723	1.792679006	1.49E-06	6.1533367	6.120316	6.1055269	7.2162053	6.9421186	7.3735786	7.895641	7.6368537	8.0588119
Up	FGSG_01006	1.79267174	2.16E-07	7.992567	7.8422497	8.0498976	8.9120534	8.8274786	8.9085948	9.4603777	9.3635849	9.4885661
Up	FGSG_10174	1.792468613	0.0002777	7.4462553	7.356828	7.4499101	6.3181765	6.4308827	6.1029268	6.7604964	6.9281194	6.4963611
Up	FGSG_11166	1.791899934	4.08E-09	5.9580386	6.0172257	5.8125445	6.7384824	6.6057619	6.7817251	6.4751405	6.2986564	6.6317388
Up	FGSG_05700	1.79140642	0.0015755	5.2937714	5.458757	5.0212003	4.657914	4.8308815	4.3744304	5.1870071	5.1562742	5.2090653
Up	FGSG_04089	1.790951504	1.01E-08	8.463492	8.2883641	8.5413923	7.2868688	7.3387849	7.1423949	8.1341278	8.07656	8.1258785
Up	FGSG_07412	1.789556781	1.02E-12	7.6482875	7.5782122	7.6449538	7.3183509	7.23613	7.3141119	7.551108	7.5122892	7.5249446
Up	FGSG_08810	1.788822389	2.44E-10	7.3553501	7.2698061	7.3555198	7.1419016	7.2146924	6.9733016	7.5003335	7.5420042	7.3895788
Up	FGSG_12306	1.774241872	3.34E-10	8.1169827	8.1244237	8.0238286	8.2162989	8.2060401	8.1390633	8.8854532	8.8571018	8.8479508
Up	FGSG_05831	1.770978688	9.77E-11	9.3231491	9.2300039	9.3288468	8.6813158	8.7136475	8.5575466	9.1957931	9.1226567	9.2019743
Up	FGSG_06572	1.77076225	6.35E-06	10.587232	10.557681	10.531272	9.2602347	9.2179474	9.2153092	10.48594	10.466396	10.439117
Up	FGSG_10245	1.769819154	5.54E-10	9.9964508	10.049109	9.8820812	9.6538489	9.5292789	9.6890142	9.3078089	9.3608087	9.211269
Up	FGSG_05916	1.769456517	1.17E-10	8.1956835	8.4554154	7.8467929	8.2356654	8.0146343	8.4043958	7.6753214	7.8258699	7.4743726
Up	FGSG_13382	1.769271353	4.64E-08	4.2902308	4.069824	4.4195794	3.7825262	3.9182279	3.8119585	4.1391495	4.3549955	4.0607807
Up	FGSG_06737	1.768007727	1.23E-08	5.3235909	5.4053203	5.1553224	5.7840418	5.6638294	5.8190522	6.443237	6.5726412	6.2298857
Up	FGSG_06619	1.765026213	3.01E-05	8.9623612	8.974252	8.8638977	8.7191544	8.6670545	8.6842935	8.572975	8.5751565	8.5041595
Up	FGSG_10518	1.763628392	0.0362205	2.4953013	2.4852148	2.4807492	2	2	2	2.2167888	2.3968278	2
Up	FGSG_09289	1.761675266	0.001783	5.2322171	5.4053203	4.9491538	5.9673238	5.9498937	5.9021734	6.354542	6.1776727	6.4552281
Up	FGSG_03998	1.759774278	5.35E-11	6.653478	6.5721788	6.650969	6.8717719	6.9024444	6.7521558	7.2467432	7.200728	7.2280252
Up	FGSG_04944	1.759664275	2.29E-11	6.3253218	6.3638664	6.2016809	6.0818165	5.5656438	6.3984523	6.247774	5.9601454	6.4342132
Up	FGSG_04982	1.759503151	2.79E-08	8.2393589	8.3103344	8.0755039	8.7464138	8.6749253	8.7418426	9.3195721	9.3635849	9.2174323
Up	FGSG_13353	1.75930248	5.18E-08	4.2902308	3.9254527	4.5224622	4.0052074	3.6887032	4.210161	4.7734018	4.9352342	4.5256129
Up	FGSG_05605	1.759166215	5.03E-10	10.89342	10.866538	10.834722	10.803195	10.738465	10.780115	10.081988	10.043225	10.054588
Up	FGSG_13647	1.756589088	0.0011122	3.5075501	3.765011	3.1285604	3.1965524	2.6377651	3.5622521	4.2902636	4.1643702	4.3572018
Up	FGSG_09231	1.755521061	2.42E-07	7.8358551	7.832161	7.7546935	8.5191804	8.3555437	8.5871558	9.256717	9.2481443	9.1988627
Up	FGSG_04933	1.754163952	1.38E-09	9.6980158	9.6871621	9.6231662	10.153756	10.086039	10.133696	10.27483	10.20211	10.280382
Up	FGSG_13673	1.752314758	5.57E-08	5.3235909	4.8868835	5.5937254	5.7226193	5.306652	5.9807654	6.410612	6.4937409	6.2540713
Up	FGSG_08199	1.746984986	1.69E-10	5.4370629	5.608128	5.1553224	5.7536574	5.9237087	5.5012819	5.8375198	5.8693008	5.7410644

Up	FGSG_09213	1.746844956	0.010978	2	2	2	2.1935143	2.353841	2	2	2	2
Up	FGSG_14038	1.746478208	0.0001927	3.1562965	3.1370988	3.1285604	4.8424709	4.7733906	4.8356119	5.4014247	5.4765776	5.2577234
Up	FGSG_06690	1.74455566	1.39E-09	5.4640893	5.8271292	4.8733193	5.484365	5.423383	5.4652882	5.9015311	6.1262878	5.5589053
Up	FGSG_03827	1.743582475	0.0401045	2	2	2	2.364115	2	2.6397336	2.7212202	3.1804273	2.7087823
Up	FGSG_06978	1.743505186	7.40E-09	7.8816778	7.7910854	7.8859117	7.810485	7.665466	7.8629285	8.5456912	8.4760172	8.5488359
Up	FGSG_10888	1.742251984	2.43E-08	7.930941	7.748806	8.0150332	7.9476643	7.898432	7.9105501	8.9922034	9.0102241	8.9063435
Up	FGSG_01926	1.741527195	3.45E-08	7.06886	7.0782312	7.5385022	7.8612426	8.0084184	7.9631169	8.2797329	8.3189643	8.4528405
Up	FGSG_06060	1.741427754	8.49E-11	9.9506054	9.9569434	9.8576913	9.7493365	9.6439882	9.7650046	10.239663	10.138696	10.271523
Up	FGSG_05035	1.741202178	2.23E-07	7.1376208	6.9719742	7.20897	7.1419016	7.0907291	7.1194186	6.8361499	6.8977964	6.7037513
Up	FGSG_07491	1.740777605	1.67E-08	8.8047878	8.7832451	8.7412711	8.2869579	8.2222444	8.2647922	7.7812006	7.6460044	7.8445799
Up	FGSG_08756	1.735774555	3.87E-09	4.349408	4.4323548	4.188785	4.657914	4.9907667	4.1204343	4.7734018	4.3549955	5.0523944
Up	FGSG_05036	1.735142505	4.54E-06	4.5644712	4.7218364	4.3087924	4.327482	3.4156241	4.8356119	4.028829	4.0586965	4.0607807
Up	FGSG_13947	1.73481136	1.48E-06	11.418786	11.31078	11.437679	12.319168	12.334279	12.213585	12.64084	12.636305	12.578382
Up	FGSG_13664	1.734336504	3.32E-10	12.2633	12.199667	12.240727	11.805056	11.722131	11.799405	11.612308	11.564875	11.593316
Up	FGSG_08841	1.732782381	6.52E-10	8.1875985	8.1651802	8.1335416	7.6215076	7.6335109	7.521124	8.2276186	8.1166081	8.2692367
Up	FGSG_07371	1.732186398	4.61E-08	4.4062533	4.6316459	4.188785	4.5559538	4.51735	5.1395644	5.0240241	5.2552685	4.7462732
Up	FGSG_06002	1.731414835	3.87E-07	6.5310205	6.5721788	6.4038729	7.153585	7.203853	7.0112415	7.9869892	7.8733942	8.0310872
Up	FGSG_01973	1.730334069	2.76E-13	9.0805911	9.0370851	9.0389684	8.7901083	8.8415333	8.6446112	9.1586193	9.1357082	9.1188085
Up	FGSG_10063	1.729571498	3.05E-07	5.6164066	5.7859081	5.4444954	4.5907474	4.51735	4.5903319	5.1870071	5.1562742	5.1587087
Up	FGSG_01255	1.729523375	3.32E-05	4.2285219	4.8868835	4.8733193	4.327482	4.9394155	5.2281446	4.4698939	5.4349486	5.2090653
Up	FGSG_02106	1.728948914	1.90E-05	5.2322171	5.2319977	5.2780294	5.7382218	6.0257126	5.5012819	6.1190688	6.0456069	6.3692672
Up	FGSG_08851	1.728736173	1.22E-10	9.998763	9.9638906	9.9482575	9.6893984	9.5314481	9.7519919	9.3854098	9.4233483	9.2776597
Up	FGSG_13153	1.728549356	1.84E-06	3.5075501	2.8476671	3.9139052	3.1032407	3.2568854	2.8774004	3.8457121	3.9446664	3.6872775
Up	FGSG_13376	1.728422408	0.0030655	2.2688027	2.4852148	2	2.1935143	2	2.3550396	3.2982451	2.9633478	3.5376798
Up	FGSG_05600	1.725423266	9.80E-09	6.5563577	6.4717791	6.5571458	5.9270474	5.8139402	5.9550408	6.6437006	6.735814	6.4759412
Up	FGSG_10154	1.725209848	0.048581	3.3996963	3.1370988	3.5740244	2	2	2	2.4052168	3.3690748	2
Up	FGSG_13654	1.724846585	0.0174192	2	2	2.4807492	2.364115	2	3.0813961	2.2167888	2.3968278	2
Up	FGSG_11753	1.724257976	0.0070516	2.2688027	2	2.4807492	2.364115	2	2.6397336	2.4052168	2	2.7087823
Up	FGSG_01705	1.72348903	3.94E-09	7.9785775	7.7271924	8.1171963	8.2273973	8.2697931	8.09263	8.1106233	8.1491527	8.0028193
Up	FGSG_04975	1.722407329	5.86E-05	6.5812576	6.5962238	6.4825439	6.3077574	6.314782	6.2155469	5.237485	5.0499834	5.3503768
Up	FGSG_12971	1.722175678	4.24E-05	4.6610646	4.5354388	4.7085304	3.444959	3.2568854	3.5622521	4.1912995	3.5358829	4.6029775

Up	FGSG_05055	1.717487315	0.0333523	4.2285219	4.069824	4.3087924	2.7805029	2	3.260088	3.3903592	3.8208439	2.7087823
Up	FGSG_12387	1.715045951	2.63E-05	4.0965725	4.2010532	3.9139052	3.444959	3.8080253	2.8774004	3.6359267	3.1804273	3.9466965
Up	FGSG_06779	1.713422722	4.54E-08	8.5353342	8.4221723	8.5596523	9.2260611	9.2233246	9.1402509	8.9848349	8.8844221	9.0164836
Up	FGSG_12434	1.7120977	0.0055258	2.2688027	2.4852148	2	3.1965524	3.4156241	3.0813961	2.4052168	2	2.7087823
Up	FGSG_05843	1.711806756	1.44E-14	12.468247	12.408437	12.44362	12.43035	12.366227	12.406545	12.494477	12.490257	12.431695
Up	FGSG_08298	1.709776079	8.91E-07	6.5310205	6.6660438	6.2918249	6.3989268	6.1438433	6.5435983	6.8279372	6.6655604	6.9156884
Up	FGSG_04138	1.70954847	0.0180899	2.6910183	3.1370988	2.4807492	2	2	2	2	2	2
Up	FGSG_04001	1.709118214	6.01E-06	3.0172362	3.378055	2.4807492	3.1032407	3.0784965	3.0813961	3.5586239	3.6853877	3.3707558
Up	FGSG_04378	1.707052192	3.03E-06	6.9966572	7.0082698	6.9005856	6.6750453	6.6701803	6.594472	7.4689883	7.3977778	7.4743726
Up	FGSG_02867	1.702049133	0.0053727	2.6910183	3.1370988	2	3.0034737	2.353841	3.4190665	2.7212202	2.9633478	2.3974876
Up	FGSG_03420	1.702021909	1.14E-06	4.0257777	3.9254527	4.0578819	4.2856211	4.6510429	3.6925011	4.5519634	4.6006874	4.5256129
Up	FGSG_06588	1.701974951	2.11E-08	4.9558055	5.1036809	4.9491538	4.9800625	5.0403527	4.9955781	5.7181748	5.347904	5.991768
Up	FGSG_04572	1.701649716	1.65E-06	6.6767699	6.8965233	6.3489364	6.8359652	6.8616483	6.7219677	6.5569203	6.152209	6.8217221
Up	FGSG_12398	1.701418121	1.32E-05	3.5075501	2.8476671	3.9139052	3.1965524	3.5586152	2.6397336	4.0850435	4.3549955	3.6872775
Up	FGSG_06390	1.700577721	1.06E-11	5.4640893	5.510285	5.3356788	5.5209064	5.346621	5.6041695	5.4873665	5.347904	5.5589053
Up	FGSG_00291	1.697383289	7.01E-13	7.14599	7.0952041	7.1297061	7.2492498	7.3387849	7.1423949	7.0907419	7.2130949	6.9307712
Up	FGSG_09512	1.695025027	9.64E-11	7.3839359	7.3708347	7.5137404	7.4986383	7.4530438	7.5812149	8.1004316	8.0900333	8.1646712
Up	FGSG_04023	1.692391446	6.51E-08	6.0321508	5.8672052	6.1055269	6.5916131	6.5554873	6.5435983	5.9015311	5.8053955	5.9330571
Up	FGSG_06524	1.692160464	3.62E-08	7.0863599	7.0436746	7.0799694	7.7230144	7.489204	7.8490285	8.3124777	8.1491527	8.4050388
Up	FGSG_01529	1.690687908	0.0625027	3.1562965	2.8476671	3.3684179	2.1935143	2	2.3550396	2.4052168	2.3968278	2.3974876
Up	FGSG_13591	1.690375693	4.61E-05	3.2831234	3.378055	3.1285604	3.1965524	2.8748957	3.4190665	4.0850435	4.3549955	3.6872775
Up	FGSG_12148	1.69013409	1.53E-09	5.2937714	5.1692654	5.3356788	4.657914	4.7733906	4.5218929	5.3791148	5.2552685	5.4374371
Up	FGSG_08372	1.690007221	5.16E-06	4.7515947	4.2010532	5.0898194	5.9405982	6.0008798	5.7902457	6.2103535	6.2272902	6.1288728
Up	FGSG_07091	1.68973427	1.60E-07	5.5673995	5.6997548	5.891554	5.2863342	5.0882909	5.6041695	5.947742	5.9002223	6.3692672
Up	FGSG_03773	1.689191793	0.000959	3.0172362	3.378055	2.4807492	3.8981589	3.0784965	4.3744304	4.1912995	4.0586965	4.265
Up	FGSG_07855	1.688483546	3.22E-07	3.6078986	3.5844678	3.5740244	4.1048586	3.9182279	4.210161	4.2902636	3.6853877	4.6764038
Up	FGSG_08891	1.686756386	0.003357	2.6910183	2.8476671	2.4807492	2	2	2	2.5718533	2.3968278	2.7087823
Up	FGSG_13004	1.684981536	2.50E-09	7.2736871	7.0782312	7.3693882	6.9067114	6.8753756	6.8531009	7.6844475	7.5711195	7.7285401
Up	FGSG_08560	1.684634318	1.48E-10	9.6069807	9.615443	9.51191	9.3467463	9.3042059	9.30459	8.9643752	8.8008584	9.0514045
Up	FGSG_08802	1.683388825	1.55E-07	6.3399861	6.306724	6.60482	6.9475516	7.1140747	6.9343369	7.083861	6.9281194	7.411249
Up	FGSG_11566	1.68266208	0.0054578	3.1562965	2.4852148	3.5740244	2.1935143	2	2.3550396	2.7212202	2.7077187	2.7087823

Up	FGSG_01747	1.682041933	2.71E-09	7.9691752	7.832161	8.0150332	8.3818951	8.2276057	8.4419212	8.739848	8.6768755	8.7364912
Up	FGSG_01635	1.681309912	9.28E-05	3.9513288	4.3213345	3.3684179	3.5895922	3.6887032	3.4190665	4.2416299	3.9446664	4.4438634
Up	FGSG_10425	1.678614549	1.86E-05	6.3399861	6.4717791	6.1055269	7.7655103	7.7572921	7.6865774	8.2797329	8.2486203	8.2453061
Up	FGSG_02654	1.676887957	0.000375	3.5075501	3.1370988	3.7539507	2.6546039	2.8748957	2.3550396	2.5718533	2.9633478	2
Up	FGSG_03238	1.675591591	2.65E-06	5.2937714	5.2319977	5.2780294	5.2432439	5.4962661	4.8356119	4.5913113	4.4416259	4.6764038
Up	FGSG_03107	1.675439462	0.0008195	3.0172362	2.8476671	3.1285604	2.364115	2.353841	2.3550396	2.2167888	2.3968278	2
Up	FGSG_11112	1.674373344	2.22E-07	4.7948149	4.3213345	5.0898194	4.69036	4.8861689	4.3744304	5.1870071	5.1041074	5.2090653
Up	FGSG_07211	1.674045376	1.91E-06	5.7321013	5.5600358	5.8125445	6.1417046	5.8139402	6.340023	6.8113703	6.8668223	6.6860823
Up	FGSG_13304	1.67282265	0.0076398	2	2	2	2.1935143	2	2.3550396	2.5718533	2.7077187	2.3974876
Up	FGSG_04097	1.672485641	0.0007677	2.6910183	2.8476671	2.4807492	3.5895922	4.1161991	3.0813961	3.5586239	4.262829	2.964684
Up	FGSG_12622	1.672240438	0.000274	3.1562965	3.765011	2	2.7805029	2.6377651	2.8774004	2.8565633	2.7077187	2.964684
Up	FGSG_06968	1.672171599	1.88E-05	3.3996963	3.1370988	3.5740244	3.444959	3.4156241	3.4190665	3.199846	2.9633478	3.3707558
Up	FGSG_00320	1.6716553	1.12E-10	8.3266186	8.2735292	8.3020857	8.5636452	8.4865337	8.5532667	8.969984	8.817153	9.0479502
Up	FGSG_12869	1.671421211	1.17E-07	6.0678264	6.120316	6.0719967	5.674773	5.8698682	5.3515835	5.9015311	6.1262878	5.5589053
Up	FGSG_06301	1.671032972	4.89E-07	5.3235909	5.5600358	5.2179801	5.8715343	5.8139402	5.9021734	6.8765263	6.7696981	6.9156884
Up	FGSG_11814	1.670235531	0.000145	5.3528065	5.510285	5.0898194	4.8133047	5.0882909	4.3744304	5.1610901	5.3023297	4.9376179
Up	FGSG_01087	1.669210821	0.0112501	2.6910183	2.4852148	3.1285604	2.364115	2.6377651	2	3.970335	3.6853877	4.1665008
Up	FGSG_01121	1.668924543	3.88E-07	4.6610646	4.8067189	5.4444954	5.5565451	5.5991203	5.5706824	5.5080752	5.2066203	5.9027806
Up	FGSG_02698	1.668476893	1.43E-06	8.4534121	8.3030481	8.5104358	8.9923198	8.9590002	8.938439	9.5898694	9.4678909	9.6407851
Up	FGSG_06614	1.668204098	1.59E-08	7.7229116	7.6139829	7.7440876	8.374426	8.3653049	8.2958654	8.7133421	8.6946233	8.6661346
Up	FGSG_09584	1.668043453	1.47E-06	4.1640552	3.378055	4.6184939	5.0060735	5.4602848	4.210161	5.2124668	4.9937494	5.3503768
Up	FGSG_12397	1.667883386	0.0006002	2.4953013	2.4852148	2.4807492	3.2841938	3.0784965	3.4190665	2.8565633	2.9633478	2.7087823
Up	FGSG_11990	1.667409628	0.0002525	8.3118172	8.0824823	8.4333122	9.7929439	9.7427755	9.7594421	10.65969	10.640167	10.61516
Up	FGSG_03309	1.666293725	2.22E-06	4.349408	4.6316459	3.9139052	3.7825262	3.4156241	4.024755	3.4769433	3.5358829	3.3707558
Up	FGSG_11122	1.665446592	0.0051927	2	2	2	2.364115	2	2.6397336	2.2167888	2	2.3974876
Up	FGSG_03738	1.662990614	4.21E-05	8.4123734	8.3673368	8.3726373	8.2948926	8.2168632	8.2855818	8.0867296	8.0900333	8.0170225
Up	FGSG_04444	1.661338522	1.88E-06	3.7898088	3.5844678	3.9139052	3.7210377	4.0206066	3.260088	4.028829	4.5233477	3.6872775
Up	FGSG_07564	1.661101439	4.23E-06	4.7948149	5.1692654	4.188785	5.9133681	5.6638294	6.0552962	6.4645842	6.59171	6.2540713
Up	FGSG_05988	1.661006674	0.0063466	2	2	2	2.5166584	2.6377651	3.0813961	2.2167888	2.3968278	2
Up	FGSG_06119	1.660128408	1.08E-05	8.7232399	8.7674491	8.6758139	8.1213252	8.1393391	8.1043792	8.3559647	8.2486203	8.4735877
Up	FGSG_08012	1.659636226	1.93E-05	4.0257777	3.9254527	4.0578819	5.1056669	4.8308815	5.2704753	3.6359267	3.9446664	3.18196

Up	FGSG_10303	1.659150973	1.60E-05	7.8614916	7.7910854	7.8467929	8.0100478	7.9184607	8.0200518	9.1684095	9.0172934	9.2448462
Up	FGSG_13278	1.658055458	0.0112706	2	2	2	2.1935143	2	2.3550396	2.2167888	2	2.3974876
Up	FGSG_05079	1.657052277	2.03E-07	7.5317704	7.3708347	7.5986089	7.2217656	7.0428732	7.3039581	8.2615538	8.2486203	8.2148237
Up	FGSG_07092	1.655551892	1.23E-08	6.9591539	7.1449564	6.9005856	6.5302	6.6383306	6.4361306	6.4645842	6.6474492	6.555942
Up	FGSG_11172	1.655051279	0.0028312	2	2	2	2.1935143	2.353841	2	2.4052168	2.3968278	2.3974876
Up	FGSG_01603	1.65418451	0.0008806	2.2688027	2	2.4807492	2.8962916	2	3.4190665	2.9802913	2.9633478	2.964684
Up	FGSG_08774	1.653120014	3.39E-08	10.574112	10.431384	10.623848	10.672258	10.634335	10.622565	11.419798	11.384597	11.3887
Up	FGSG_04375	1.648427117	6.55E-05	3.5075501	3.1370988	3.7539507	4.4077273	4.5857446	4.1204343	5.0524989	4.7439404	5.2577234
Up	FGSG_06867	1.647022709	3.97E-08	5.3235909	5.1692654	5.3911128	5.7989973	5.4602848	6.0060392	5.8044166	6.073005	5.3945636
Up	FGSG_02316	1.646922754	9.13E-09	7.1871227	7.3847067	6.9005856	7.4986383	7.6733454	7.2308067	7.8598371	7.9344186	7.7285401
Up	FGSG_01680	1.646294485	0.0143095	2.4953013	2.8476671	2	2.364115	2	2.6397336	2.5718533	2	3.18196
Up	FGSG_00569	1.645488042	0.0022557	3.7898088	4.2010532	3.1285604	4.0558932	4.0206066	4.024755	4.2902636	3.8208439	4.6029775
Up	FGSG_08695	1.643161579	2.13E-07	6.7444707	7.0082698	6.3206633	6.8858494	6.8196649	6.8669619	7.5054921	7.3977778	7.5446872
Up	FGSG_12227	1.642269199	0.008318	2.6910183	3.1370988	2	2	2	2	2.5718533	2.9633478	2
Up	FGSG_04645	1.640592313	0.0097983	3.0172362	2.8476671	3.1285604	3.5190873	3.8080253	3.0813961	2.5718533	2.7077187	2.3974876
Up	FGSG_04182	1.639677408	3.45E-07	7.0511452	6.9534782	7.063002	6.8997909	6.9290147	6.7817251	7.6097687	7.6731136	7.4743726
Up	FGSG_12684	1.633551987	0.0008405	2.4953013	2	2.8407141	3.1032407	3.0784965	3.6925011	3.2982451	3.1804273	3.3707558
Up	FGSG_06299	1.626633765	2.07E-09	5.6638035	5.8271292	5.3911128	5.5209064	5.6638294	5.2704753	5.5880596	5.7038947	5.3945636
Up	FGSG_09393	1.62487748	3.52E-09	6.1533367	6.052414	6.2016809	6.2104357	6.1885139	6.340023	6.983915	6.7185687	7.2401843
Up	FGSG_08658	1.624088624	9.76E-09	6.310507	5.9811576	6.5078422	5.8715343	6.1209783	5.4652882	6.6806551	6.7528555	6.5363539
Up	FGSG_13999	1.623914888	0.0015463	2.8633281	2.8476671	2.8407141	2.364115	2.6377651	2	2.4052168	2.3968278	2.3974876
Up	FGSG_02440	1.623186087	7.71E-10	6.9303725	6.6885859	7.063002	7.2492498	7.1819267	7.2308067	7.6753214	7.6550976	7.6303308
Up	FGSG_07462	1.622784457	0.0006862	3.1562965	3.378055	2.8407141	3.1032407	2.6377651	3.4190665	2.2167888	2	2.3974876
Up	FGSG_10261	1.622627327	0.001249	2.4953013	2.4852148	2.4807492	3.9526758	4.2058494	3.5622521	4.7734018	4.6740912	4.8766132
Up	FGSG_01161	1.62212385	9.68E-13	8.4432614	8.7514782	8.6533178	8.2922525	8.6749253	8.432631	8.3814413	8.6498375	8.4889546
Up	FGSG_11847	1.622120178	0.0018509	3.3996963	3.765011	3.3684179	2.364115	2.353841	2.3550396	3.7092973	4.0586965	3.3707558
Up	FGSG_01152	1.620128088	3.93E-10	8.0018186	7.9488049	7.9702333	8.0821245	7.9382152	8.1333402	8.512767	8.5941923	8.3555989
Up	FGSG_11833	1.619591363	0.0010539	2	2.4852148	2.4807492	3.1965524	3.2568854	3.0813961	2.8565633	2.7077187	2.964684
Up	FGSG_04048	1.61921977	0.0267858	2	2	2	3.1032407	3.5586152	2.3550396	2.7212202	3.1804273	2
Up	FGSG_04888	1.6156413	2.17E-11	6.7663496	6.6198745	6.8220828	6.5916131	6.314782	6.7521558	6.9765039	7.2130949	6.6131596
Up	FGSG_03135	1.612543371	2.29E-05	3.701719	4.069824	3.1285604	3.2841938	3.6887032	2.6397336	3.3903592	3.8208439	2.7087823

Up	FGSG_03482	1.61192278	4.22E-06	4.2902308	3.9254527	4.5224622	3.8415007	2.8748957	4.3744304	4.3828727	4.0586965	4.6029775
Up	FGSG_13538	1.61100988	7.22E-07	4.0965725	3.5844678	4.4195794	4.8133047	4.9907667	4.5218929	5.237485	5.4349486	4.9376179
Up	FGSG_09101	1.610095129	5.25E-08	8.0868701	7.832161	8.2279057	8.5438055	8.463998	8.5360188	8.8695373	8.8090287	8.8637548
Up	FGSG_06620	1.60979461	6.85E-05	6.9966572	7.0260808	6.8813568	6.2866906	6.4308827	6.0308779	5.9925186	5.9304949	5.991768
Up	FGSG_03158	1.609662463	0.043592	2.4953013	2.4852148	2.4807492	3.1965524	2.6377651	3.5622521	2.4052168	2.3968278	2.3974876
Up	FGSG_10062	1.608921051	1.92E-08	4.7515947	4.4323548	4.9491538	4.5559538	4.7135134	4.2946324	4.2416299	4.5233477	3.8228101
Up	FGSG_02529	1.6079941	3.04E-08	7.0776365	7.2547781	6.7811692	6.539135	6.374	6.6274177	6.7776545	6.3666582	7.0460725
Up	FGSG_05650	1.607594603	3.45E-07	5.5673995	5.7434744	5.3356788	5.2649499	5.6951271	4.8356119	6.4645842	6.5726412	6.4552281
Up	FGSG_02658	1.607275125	4.15E-06	10.316785	10.352407	10.192096	11.122156	11.087073	11.069524	11.341192	11.387328	11.223985
Up	FGSG_11201	1.606033456	1.78E-05	4.349408	4.7218364	3.7539507	3.9526758	4.2902531	3.4190665	3.5586239	3.6853877	3.3707558
Up	FGSG_00185	1.600771008	4.98E-07	8.3984303	8.4814692	8.2354977	9.1346997	9.1290693	9.082021	9.7986388	9.7419951	9.7950446
Up	FGSG_13392	1.599258649	1.10E-08	10.890933	10.828982	10.87538	11.043492	11.048969	10.954146	10.560326	10.544319	10.516137
Up	FGSG_06668	1.598386359	1.73E-09	10.67804	10.655327	10.615144	10.089823	10.013404	10.078177	9.8733659	9.9128305	9.7638005
Up	FGSG_06529	1.597796725	2.83E-07	7.2032529	7.1611664	7.1619361	7.8894648	7.9184607	7.7700883	8.5076343	8.5412272	8.4050388
Up	FGSG_08588	1.596607318	1.80E-06	5.5673995	5.5600358	5.4959731	5.1760947	5.306652	4.9442019	5.2620767	5.3023297	5.1587087
Up	FGSG_10765	1.596058371	1.16E-08	4.5136357	4.2010532	4.7085304	4.0558932	3.6887032	4.2946324	4.3373111	4.6006874	3.9466965
Up	FGSG_12829	1.595637089	0.0003393	3.3996963	2.8476671	3.7539507	3.3668146	3.5586152	3.0813961	3.7791165	4.1643702	3.18196
Up	FGSG_00813	1.594883625	1.08E-06	4.9933948	5.1692654	4.7932764	5.8573158	6.0257126	5.6041695	6.1719365	6.1262878	6.2052877
Up	FGSG_02144	1.594435083	5.03E-10	7.7938649	7.6716978	7.9239976	7.93418	7.9184607	8.0385416	7.9980116	8.0143231	8.0170225
Up	FGSG_10083	1.592435396	0.0004096	3.0172362	2	3.5740244	3.7825262	4.1161991	3.260088	4.0850435	3.9446664	4.1665008
Up	FGSG_05004	1.590931966	1.72E-09	5.9769267	6.1851653	6.1382955	5.8856141	6.2318429	5.9288493	6.3659317	6.3443452	6.6131596
Up	FGSG_00700	1.589340673	1.27E-05	6.8196378	7.0782312	6.4038729	6.5121623	6.5383322	6.3984523	7.4951564	7.682038	7.2033953
Up	FGSG_07156	1.589037851	0.0002813	3.3996963	2.8476671	3.7539507	3.1965524	2.6377651	3.5622521	3.9093688	3.6853877	4.0607807
Up	FGSG_06671	1.587905247	3.95E-06	4.7948149	5.0349724	4.4195794	4.6247215	4.5857446	4.5903319	5.2620767	5.4765776	4.9376179
Up	FGSG_07807	1.587569002	0.0113759	2.6910183	2	3.1285604	2.1935143	2	2.3550396	2.2167888	2	2.3974876
Up	FGSG_03567	1.58656119	8.17E-07	5.6164066	5.510285	5.640225	5.1529987	5.0403527	5.1845342	4.9041484	4.8105634	4.9376179
Up	FGSG_06378	1.583667746	3.09E-10	5.5673995	5.4053203	5.640225	5.7536574	5.9498937	5.4283735	5.6073815	5.8053955	5.3047937
Up	FGSG_02528	1.581554877	1.59E-07	5.4370629	5.0349724	5.6852725	5.6419705	5.6951271	5.5012819	6.5668244	6.59171	6.4759412
Up	FGSG_07061	1.580188284	0.0271217	4.5136357	4.2010532	4.7085304	3.2841938	3.4156241	3.0813961	3.970335	3.9446664	3.9466965
Up	FGSG_11769	1.58004202	0.0108629	2.4953013	2.4852148	2.4807492	2.1935143	2	2.3550396	2	2	2
Up	FGSG_09180	1.578757323	2.03E-08	6.2955384	6.5477263	5.891554	5.7536574	5.6638294	5.7608523	6.2962044	6.4733227	6.0202512

Up	FGSG_09362	1.57816527	7.37E-11	7.10365	7.3984466	7.3273766	7.0067227	7.0669996	7.2520883	7.1512362	7.2615289	7.3564507
Up	FGSG_00951	1.575586672	2.62E-07	6.5935482	6.153105	6.8618681	7.1182471	7.1483996	7.0236696	7.6889891	7.5996588	7.711174
Up	FGSG_06000	1.574285161	1.07E-06	8.262636	8.2509868	8.1893346	8.942679	8.9064847	8.891744	8.9848349	8.8921338	9.0093969
Up	FGSG_10162	1.57280264	3.01E-06	4.0965725	4.4323548	3.5740244	4.1980691	3.8080253	4.4500449	4.0850435	3.6853877	4.3572018
Up	FGSG_00415	1.571391287	3.95E-07	11.926321	11.87301	11.894371	12.641616	12.578052	12.617769	12.682145	12.635444	12.663517
Up	FGSG_03528	1.571028815	0.0010209	2.8633281	3.5844678	2	2.5166584	2.353841	2.6397336	2.9802913	2.7077187	3.3707558
Up	FGSG_00344	1.570956732	0.0324145	2.8633281	3.1370988	2.4807492	2	2	2	2.8565633	2.3968278	3.18196
Up	FGSG_05974	1.566268198	7.73E-09	8.0246912	8.0567194	7.9050803	8.0260259	8.1506723	7.7920493	8.0694171	7.9929661	8.0792612
Up	FGSG_12186	1.563085875	8.69E-07	4.0257777	3.765011	4.188785	3.8981589	3.6887032	4.024755	3.7092973	3.6853877	3.6872775
Up	FGSG_09564	1.562396284	6.70E-07	5.2004262	5.8271292	5.7713555	5.8573158	6.1209783	5.9807654	6.0071405	6.0998924	6.3242864
Up	FGSG_00443	1.560545447	5.70E-08	6.3399861	6.153105	6.4305761	6.6339322	6.776423	6.3792376	6.5864309	6.4526114	6.6500817
Up	FGSG_03788	1.559479227	0.0051128	2.8633281	2.4852148	3.1285604	2.1935143	2	2.3550396	3.4769433	3.5358829	3.3707558
Up	FGSG_05972	1.555503701	4.40E-06	11.263031	11.241957	11.210965	10.239851	10.218489	10.187157	10.10511	10.067266	10.088609
Up	FGSG_07201	1.5538161	2.16E-09	7.8866805	7.8014645	8.0839393	8.2816438	8.3653049	8.2168902	8.7615703	8.7121554	8.8198705
Up	FGSG_04917	1.549568698	7.44E-06	9.5162824	9.4668677	9.4803073	10.056761	10.087514	9.9341925	10.636953	10.598352	10.609313
Up	FGSG_12137	1.548486773	1.44E-08	6.653478	7.0610564	6.7602689	6.8066659	7.0669996	7.1077918	7.104406	7.3083895	7.3339349
Up	FGSG_06941	1.547063859	7.46E-08	4.5644712	4.3213345	4.7085304	4.7220923	4.5857446	4.7780897	5.0524989	5.1562742	4.8766132
Up	FGSG_00905	1.546548927	8.20E-09	5.7759005	6.1851653	5.5937254	6.1060709	6.1209783	6.1029268	6.7518403	6.8977964	6.6131596
Up	FGSG_01238	1.546176981	5.47E-09	9.2119528	9.1264838	9.2105658	8.90861	8.9590002	8.7679329	8.2976857	8.2486203	8.2869277
Up	FGSG_00579	1.545728967	0.0090497	2.4953013	2.4852148	2.4807492	3.1032407	2.6377651	3.4190665	2.4052168	2.3968278	2.3974876
Up	FGSG_11273	1.545346128	0.0004128	3.0172362	2.4852148	3.3684179	2.7805029	2.353841	3.0813961	3.4769433	3.3690748	3.5376798
Up	FGSG_00903	1.545178189	2.94E-08	5.4640893	5.5600358	5.2780294	5.1529987	4.7733906	5.3904893	5.7181748	5.8693008	5.4790733
Up	FGSG_09867	1.544641322	0.0254422	6.7334055	8.6286221	8.6304653	6.7228827	8.1730751	8.2855818	6.410612	7.6997228	7.6935963
Up	FGSG_11540	1.541335976	4.48E-06	8.1997091	8.2735292	8.1006636	8.5853741	8.6066141	8.5360188	8.7872125	8.6946233	8.8439727
Up	FGSG_08549	1.539734033	0.0004562	5.4640893	6.0172257	5.9294932	5.6419705	6.0977451	6.4361306	6.7946109	7.1882541	7.0598582
Up	FGSG_02127	1.539238249	2.00E-08	8.2236295	8.0392849	8.3092981	7.5525828	7.4346181	7.5812149	8.0448251	8.096723	7.9220993
Up	FGSG_03296	1.538032781	6.06E-11	10.641512	10.659612	10.535891	10.97106	10.930688	10.924628	10.845583	10.822737	10.802082
Up	FGSG_09639	1.537900215	6.76E-09	12.283592	12.258296	12.223651	12.485704	12.455751	12.427741	13.1339	13.119093	13.082277
Up	FGSG_20657	1.537600597	0.0094167	2.6910183	2.4852148	2.8407141	2.6546039	2	3.0813961	3.0942412	3.5358829	2.3974876
Up	FGSG_09553	1.537428226	8.07E-10	6.0500989	6.2165286	5.7713555	6.2215791	6.253029	6.1029268	6.6153499	6.8028047	6.3692672
Up	FGSG_08980	1.537376309	1.59E-07	6.6996916	6.3355781	6.9195616	6.60869	6.7013421	6.4361306	7.104406	7.249572	6.8850413

Up	FGSG_08517	1.53611957	5.10E-07	4.7515947	4.7218364	4.7085304	5.2863342	5.6318376	4.7181783	4.994976	4.9937494	4.9376179
Up	FGSG_10266	1.535714016	0.0129688	2	2	2.4807492	2	2.353841	2	2.9802913	3.3690748	2.3974876
Up	FGSG_06494	1.534806797	1.01E-10	7.7284952	7.6139829	7.7546935	7.4568136	7.387497	7.4399936	7.9906727	8.1362228	7.7542035
Up	FGSG_03279	1.534217299	0.0079504	2	2	2	2.7805029	3.0784965	2.3550396	2.7212202	2.7077187	2.7087823
Up	FGSG_02062	1.531850364	0.0020577	2.2688027	2	2.4807492	2.5166584	2.8748957	2	2.4052168	2.3968278	2.3974876
Up	FGSG_13745	1.53061955	1.59E-05	5.2633225	4.6316459	5.728956	4.7220923	4.7733906	4.5903319	5.5080752	5.4765776	5.4790733
Up	FGSG_03163	1.529639388	0.0007835	3.0172362	2	3.5740244	3.3668146	3.6887032	2.8774004	2.5718533	2.3968278	2.7087823
Up	FGSG_03125	1.529202494	3.30E-08	6.8196378	6.775389	6.7811692	6.9204536	6.9024444	6.8531009	7.2528911	7.4404773	6.9604719
Up	FGSG_04805	1.52895873	2.60E-12	12.629318	12.630254	12.592936	12.738981	12.69998	12.743544	12.756777	12.763935	12.722939
Up	FGSG_01412	1.524409685	1.31E-06	7.7669866	7.6829688	7.7652219	6.9872674	6.9157907	6.9860594	8.1072341	7.9929661	8.1518558
Up	FGSG_05047	1.524348126	2.70E-05	6.5688614	6.4717791	6.5811798	6.0065063	6.0257126	5.9021734	6.443237	6.4315984	6.3912423
Up	FGSG_10423	1.522905362	0.000525	2.8633281	2	3.3684179	3.2841938	3.0784965	3.4190665	3.199846	3.6853877	2.964684
Up	FGSG_07370	1.522800248	1.55E-07	5.2322171	5.0349724	5.3356788	5.7536574	5.9237087	5.4652882	5.9925186	5.6684096	6.2052877
Up	FGSG_11956	1.520566832	9.27E-05	2.6910183	2.8476671	2.4807492	3.1965524	2.353841	3.6925011	3.2982451	2.9633478	3.5376798
Up	FGSG_11341	1.520225518	0.0113649	2.4953013	2	2.8407141	2.364115	2.353841	2.3550396	2	2	2
Up	FGSG_07547	1.519358865	3.87E-08	6.5052304	6.4975416	6.4305761	6.9872674	6.9157907	6.9733016	7.5659982	7.6090478	7.4536368
Up	FGSG_13709	1.517696045	4.24E-07	6.3399861	6.5228522	6.0376686	5.658465	5.6638294	5.5706824	6.7691009	6.7528555	6.7212064
Up	FGSG_04353	1.517149863	7.19E-08	6.6178197	6.7962976	6.3206633	6.691168	6.449356	6.8249729	7.1643417	7.249572	7.003907
Up	FGSG_10438	1.517121808	0.0647604	2	2	2	2.5166584	2	2.8774004	3.8457121	3.5358829	4.0607807
Up	FGSG_01081	1.516843249	2.44E-11	10.526829	10.436388	10.529729	10.409626	10.345971	10.385559	10.082851	9.9867213	10.110301
Up	FGSG_04087	1.515002193	2.50E-07	9.1571127	9.0888463	9.1395078	9.1811035	9.1262017	9.1516201	9.9846804	9.988521	9.9193468
Up	FGSG_13685	1.514495388	5.70E-08	10.260086	10.261355	10.172454	11.04702	11.032982	10.972693	10.992891	10.987479	10.93144
Up	FGSG_06407	1.514204464	1.49E-09	12.000019	11.962444	11.951957	11.419045	11.375494	11.374938	11.446866	11.387328	11.439676
Up	FGSG_09389	1.514041172	0.0393384	2	2	2	3.6568115	4.0206066	3.0813961	4.7387761	4.6006874	4.8129147
Up	FGSG_13097	1.514035377	0.0011916	3.701719	3.9254527	3.3684179	2.8962916	2.6377651	3.0813961	3.199846	3.6853877	2.3974876
Up	FGSG_01062	1.513067284	5.60E-06	3.6078986	3.5844678	3.5740244	3.9526758	4.2058494	3.5622521	4.5519634	4.0586965	4.8766132
Up	FGSG_02596	1.511896334	7.14E-07	5.5166689	5.349828	5.5937254	5.5740396	5.7557564	5.3515835	5.5080752	5.5947086	5.3503768
Up	FGSG_10845	1.509602377	3.97E-10	10.964345	10.940014	10.903055	10.599912	10.577735	10.534118	10.463584	10.394918	10.46525
Up	FGSG_00227	1.509054223	6.13E-07	5.3235909	5.349828	5.2179801	4.69036	4.4455509	4.8356119	5.0804226	5.1562742	4.9961472
Up	FGSG_06983	1.508083861	0.0580964	2.4953013	2	2.8407141	2	2	2	2.5718533	2.9633478	2
Up	FGSG_13380	1.506461087	2.46E-06	6.9400302	6.9719742	6.8220828	7.821513	7.8225187	7.7327269	8.2947091	8.284221	8.2453061

Up	FGSG_03046	1.506448704	0.0541464	8.0912106	7.8819136	8.1971317	6.5302	6.5033974	6.4728499	8.0694171	8.0353685	8.0380685
Up	FGSG_09356	1.504677963	0.0100005	2.4953013	2	2.8407141	2	2.6377651	2.6397336	2.2167888	2.7077187	2.3974876
Up	FGSG_04006	1.504450762	1.15E-06	5.2004262	5.5600358	4.7085304	5.6419705	5.6951271	5.5012819	6.2600348	6.2026947	6.2540713
Up	FGSG_10190	1.504343937	3.20E-06	4.5136357	4.7218364	4.188785	4.4077273	4.7135134	3.9222766	5.3564544	5.2552685	5.3945636
Up	FGSG_00232	1.503504949	0.015361	3.2831234	3.5844678	2.8407141	2.1935143	2	2.3550396	2	2	2
Up	FGSG_10842	1.501573505	0.0007835	4.8775546	5.1692654	4.4195794	4.5907474	4.8861689	4.3744304	6.0216157	5.9304949	6.0481828
Up	FGSG_01008	1.500806833	2.70E-11	10.15205	10.057794	10.158545	9.8930418	9.8647765	9.8336671	9.7944301	9.6494771	9.865419
Up	FGSG_12272	1.498889603	9.58E-07	5.7096927	5.608128	5.728956	6.7384824	6.8053944	6.5777126	7.083861	6.9724415	7.1268773
Up	FGSG_06304	1.498481795	0.0123508	3.3996963	2.4852148	3.9139052	2.364115	2	2.6397336	3.970335	3.6853877	4.1665008
Up	FGSG_08322	1.497657261	0.0431387	3.5075501	3.765011	3.1285604	2.364115	2.6377651	2	2	2	2
Up	FGSG_06147	1.496922193	2.07E-08	7.2428067	7.2242442	7.177785	7.1766717	7.1483996	7.1194186	7.5003335	7.5022467	7.4325986
Up	FGSG_06809	1.49486591	1.65E-08	8.4158383	8.353296	8.3931471	8.6548423	8.762481	8.4419212	8.6284082	8.6315266	8.5585788
Up	FGSG_10794	1.493658004	3.85E-08	5.9769267	5.906198	5.9664603	6.6505183	6.7166741	6.4908649	7.0418719	7.0434138	6.975096
Up	FGSG_05020	1.493615582	0.0347232	2.2688027	2	2.4807492	2.6546039	2.6377651	2.6397336	2.4052168	2.3968278	2.7087823
Up	FGSG_10857	1.492856066	9.85E-09	5.5673995	5.5600358	5.4959731	5.484365	5.0882909	5.7308476	6.0216157	5.6320297	6.2778581
Up	FGSG_09693	1.491143471	0.0027016	4.1640552	4.6316459	3.7539507	3.6568115	3.6887032	3.9222766	4.7032988	5.0499834	4.3572018
Up	FGSG_07540	1.490127774	1.10E-10	7.0600298	6.9157592	7.113317	6.9067114	6.9679745	6.7521558	6.998624	6.9130376	7.0180995
Up	FGSG_05467	1.486861742	2.93E-07	7.544488	7.6139829	7.3831245	7.6832645	7.5417997	7.7327269	8.5997524	8.4862433	8.6434254
Up	FGSG_10338	1.486321821	0.0072557	2.2688027	2.4852148	2	2.364115	2.6377651	2	2.8565633	2.7077187	2.964684
Up	FGSG_07527	1.483252079	2.14E-13	9.9894918	9.8855673	10.004883	9.9215091	9.903812	9.8512059	9.9387029	9.8269256	9.9804202
Up	FGSG_04047	1.481387623	0.0198209	2.6910183	2.8476671	2.4807492	2.7805029	2.6377651	2.8774004	4.0850435	4.1643702	3.9466965
Up	FGSG_00960	1.480081511	7.40E-07	4.6135762	4.7218364	4.4195794	3.9526758	3.5586152	4.210161	3.970335	4.1643702	3.6872775
Up	FGSG_03529	1.47897119	8.03E-08	10.83574	10.776022	10.810782	10.914245	10.832947	10.907965	11.488493	11.425691	11.485085
Up	FGSG_13305	1.478509819	2.08E-05	6.1366353	5.5600358	6.4825439	4.8990917	5.179638	4.4500449	5.1610901	4.9937494	5.2577234
Up	FGSG_04912	1.477454891	1.90E-05	3.7898088	3.1370988	4.188785	3.1965524	3.0784965	3.260088	4.2902636	4.5233477	3.9466965
Up	FGSG_01491	1.477256509	8.80E-08	5.5422571	5.2319977	5.7713555	5.1529987	5.1346873	5.0931475	5.3564544	5.2066203	5.4374371
Up	FGSG_00511	1.477171788	1.09E-08	5.3235909	5.8672052	5.545677	5.5913246	5.6638294	6.2370531	5.947742	6.2272902	6.0481828
Up	FGSG_04479	1.477013431	7.37E-07	5.5921111	5.6997548	5.3911128	5.6908987	5.6638294	5.7002055	6.3772321	6.4733227	6.2052877
Up	FGSG_02103	1.476699663	0.0009668	3.6078986	3.378055	3.7539507	2.7805029	3.0784965	2.3550396	3.7092973	2.9633478	4.1665008
Up	FGSG_03768	1.474008317	0.000655	3.1562965	2.8476671	3.3684179	3.2841938	3.0784965	3.4190665	3.8457121	3.8208439	3.8228101
Up	FGSG_07291	1.473814933	5.84E-10	9.9848337	9.9592629	9.9273158	9.5482751	9.5029915	9.5062513	9.2242318	9.0794051	9.2952483

Up	FGSG_10793	1.473346019	1.60E-10	6.2340611	5.9811576	6.3766661	6.1417046	5.8970396	6.279127	6.443237	6.3443452	6.4759412
Up	FGSG_04555	1.47245446	3.02E-10	7.4050095	7.42554	7.3831245	7.3131513	7.1929315	7.4024174	7.9496278	7.8733942	8.0099383
Up	FGSG_10778	1.472016762	0.0002757	4.4062533	4.8868835	3.9139052	3.5895922	3.6887032	4.024755	4.3373111	4.8105634	4.0607807
Up	FGSG_01303	1.471411261	0.0373446	2.4953013	2	2.8407141	2.5166584	2.8748957	2	2	2	2
Up	FGSG_10791	1.47008436	8.01E-07	4.7070397	4.3213345	4.9491538	5.5388358	5.4602848	5.5363994	5.9170997	5.9304949	5.8402522
Up	FGSG_13223	1.469892177	0.0017578	4.0965725	3.765011	4.3087924	4.2425091	3.2568854	4.7780897	4.4270393	4.6006874	4.1665008
Up	FGSG_09381	1.469468201	1.59E-05	7.5819817	7.6139829	7.4629041	8.0228444	8.0084184	7.9501537	8.7935525	8.7380605	8.7829449
Up	FGSG_03660	1.469092424	0.0376386	2.8633281	2.4852148	3.1285604	2	2	2	2.4052168	2	2.7087823
Up	FGSG_09675	1.467652638	0.0017381	3.701719	4.069824	3.1285604	3.7210377	3.4156241	3.9222766	4.2416299	3.9446664	4.4438634
Up	FGSG_01981	1.465216264	0.0070713	2.8633281	3.378055	2	2.1935143	2.353841	2	2.5718533	2.3968278	2.7087823
Up	FGSG_13181	1.464035212	0.0091638	2.2688027	2.4852148	2	2.8962916	2.353841	3.260088	3.4769433	3.8208439	2.964684
Up	FGSG_05039	1.462733349	2.69E-08	8.1997091	8.2357604	8.0755039	8.4709687	8.4457125	8.4091402	8.6093677	8.5461234	8.6063304
Up	FGSG_07047	1.460356577	1.76E-05	5.7759005	6.6660438	6.7390613	5.6084049	6.5383322	6.3984523	6.1055435	6.9130376	6.7037513
Up	FGSG_13331	1.460017709	0.0254422	4.2902308	4.2010532	4.3087924	2.5166584	2.8748957	2	2.9802913	2.3968278	3.3707558
Up	FGSG_13590	1.45771996	0.0318567	2	2	2	2.8962916	3.2568854	2.3550396	2.2167888	2	2.3974876
Up	FGSG_13154	1.457324771	0.0198234	2	2	2	2.364115	2.6377651	2	2.5718533	2	2.964684
Up	FGSG_13543	1.457180294	4.16E-09	6.860895	6.8372271	6.8017711	7.4234237	7.2572536	7.4945842	7.6382282	7.6368537	7.6023441
Up	FGSG_03368	1.457070507	0.0030026	4.2902308	3.9254527	4.5224622	3.444959	3.2568854	3.5622521	3.199846	3.3690748	2.964684
Up	FGSG_04915	1.456985994	8.93E-14	11.043192	10.956361	11.042667	10.822078	10.78102	10.775529	10.882729	10.914575	10.78143
Up	FGSG_01165	1.456179065	1.12E-05	5.3814422	5.349828	5.3356788	4.2425091	4.4455509	3.9222766	4.6296145	4.5233477	4.6764038
Up	FGSG_12401	1.455953419	0.0454511	2.8633281	3.1370988	2.4807492	2	2	2	2.7212202	2.9633478	2.3974876
Up	FGSG_07569	1.455462383	1.60E-07	9.7121755	9.6416744	9.6965113	10.261608	10.300907	10.130826	10.251992	10.195893	10.241595
Up	FGSG_09040	1.453173599	4.91E-10	5.7759005	5.7859081	5.6852725	5.5027514	5.0882909	5.7608523	5.5080752	5.7385279	5.1587087
Up	FGSG_07507	1.453138467	0.0381847	3.0172362	2.8476671	3.1285604	2.364115	2.6377651	2	2.7212202	2.9633478	2.3974876
Up	FGSG_01694	1.452186646	7.74E-08	6.8910817	6.6198745	7.0458328	7.3026957	7.3387849	7.1761874	7.364982	7.4921337	7.1528368
Up	FGSG_01069	1.45188165	9.72E-07	8.0018186	8.1811658	7.7008668	8.6257784	8.5563697	8.6079392	9.2884868	9.1992511	9.3097434
Up	FGSG_05683	1.450869133	6.82E-11	7.8461644	7.8720994	7.7334032	8.2574849	8.2645866	8.1617312	8.0305821	8.0213724	7.9739864
Up	FGSG_07150	1.449538184	9.13E-09	9.6793979	9.7121323	9.5580527	9.9384925	9.9320004	9.8564262	10.503449	10.485638	10.456157
Up	FGSG_03761	1.4487857	3.34E-07	8.0516681	8.2203716	7.7652219	8.6772744	8.6591406	8.6079392	8.68634	8.5114961	8.7829449
Up	FGSG_06813	1.448561186	8.47E-07	7.1207354	7.0952041	7.063002	7.3892427	7.489204	7.187278	6.7691009	6.8028047	6.6681943
Up	FGSG_00836	1.448455912	4.50E-08	9.6793979	9.6124987	9.6603048	9.9197997	9.9021366	9.8494616	10.551625	10.458626	10.579716

Up	FGSG_03085	1.447669184	0.0009461	2.4953013	2.4852148	2.4807492	3.7825262	3.5586152	3.9222766	3.0942412	3.1804273	2.964684
Up	FGSG_12752	1.447311225	0.0001354	2.8633281	2.8476671	2.8407141	3.0034737	3.0784965	2.8774004	3.3903592	3.8208439	2.7087823
Up	FGSG_10796	1.446598066	8.17E-07	8.4768231	8.4814692	8.3931471	8.2843033	8.3006433	8.1785015	8.8574841	8.9263352	8.7148711
Up	FGSG_02600	1.445389236	1.14E-08	11.02459	10.970228	10.993142	11.401085	11.373681	11.34058	11.843505	11.817604	11.803029
Up	FGSG_07030	1.444873971	5.33E-08	6.1197383	6.1851653	6.2623983	5.674773	5.8698682	5.636897	6.1848562	5.9002223	6.5164961
Up	FGSG_06007	1.44388604	7.38E-09	5.8184092	5.608128	6.1703363	6.0939946	6.2318429	6.1261652	6.399571	6.4526114	6.555942
Up	FGSG_00595	1.443378144	1.39E-07	8.5257457	8.3950151	8.6130853	8.6486634	8.6431813	8.5829629	9.4590489	9.3635849	9.5213187
Up	FGSG_09869	1.443291377	4.12E-08	8.0427317	8.2660541	8.1171963	7.5744776	7.8916935	7.6469587	7.8106729	7.7599663	7.9445655
Up	FGSG_06465	1.442737671	5.01E-05	3.872828	3.765011	3.9139052	3.9526758	4.0206066	3.8119585	4.5913113	4.6006874	4.5256129
Up	FGSG_09317	1.441939433	2.50E-07	4.4062533	3.9254527	4.7085304	5.1760947	5.0403527	5.2281446	4.5519634	4.8742451	4.0607807
Up	FGSG_12144	1.439754841	1.15E-09	5.7973114	6.052414	5.3911128	6.1417046	6.0008798	6.1937152	5.9325022	6.073005	5.7064263
Up	FGSG_06759	1.43969294	4.98E-07	7.1207354	6.9902361	7.1619361	8.0449688	7.9641406	8.0385416	8.3095314	8.4077195	8.1324168
Up	FGSG_02649	1.437758116	1.56E-08	6.280413	6.5477263	5.85259	6.0446528	6.0501253	5.9550408	6.4324437	6.3886313	6.4128878
Up	FGSG_05416	1.437509497	7.05E-05	5.4095206	5.510285	5.2179801	5.1056669	5.0403527	5.0931475	5.2620767	5.3023297	5.1587087
Up	FGSG_04744	1.437252005	1.54E-07	7.4929307	7.5415319	7.3555198	8.2246307	8.3107819	8.0385416	8.1038368	8.123176	8.0170225
Up	FGSG_00909	1.435229039	1.88E-10	8.4019287	8.3247975	8.3931471	8.5570623	8.3457159	8.6645888	8.8453294	8.7885156	8.8399835
Up	FGSG_09881	1.435179878	1.01E-07	7.2964211	7.0436746	7.436798	6.9675462	6.8616483	6.9860594	7.3986432	7.249572	7.4743726
Up	FGSG_11771	1.435048048	0.0051588	5.8392029	5.1692654	6.232359	5.6252853	5.8698682	5.2281446	5.3334324	5.347904	5.2577234
Up	FGSG_08220	1.433927877	0.0028506	4.5136357	4.4323548	4.5224622	5.2432439	4.8308815	5.5012819	5.2124668	5.3023297	5.0523944
Up	FGSG_09004	1.432415438	8.82E-06	3.9513288	3.9254527	3.9139052	3.7210377	4.2902531	2.8774004	4.028829	4.3549955	3.5376798
Up	FGSG_06904	1.430825704	2.03E-07	5.9195042	6.306724	6.4305761	5.5913246	5.7851416	6.2155469	6.0359471	6.4102748	6.7212064
Up	FGSG_13683	1.430444164	0.0007091	7.4394623	7.4782464	7.3130965	6.0065063	6.1438433	5.7608523	6.5369058	6.3443452	6.6500817
Up	FGSG_07526	1.430324207	8.48E-08	5.8799122	5.6546687	6.0376686	6.2436111	6.2318429	6.2155469	6.4324437	6.2752577	6.5164961
Up	FGSG_00882	1.429586136	1.51E-08	6.5935482	6.5477263	6.5811798	7.2106236	7.1929315	7.1423949	7.5360624	7.5711195	7.4325986
Up	FGSG_07837	1.429582117	7.97E-08	5.5166689	5.7859081	5.0898194	5.8995578	6.1209783	5.5363994	5.2862562	5.5170389	4.9376179
Up	FGSG_07545	1.428542208	5.70E-06	4.2285219	4.3213345	4.0578819	4.5203003	4.3699906	4.5903319	5.0524989	4.9352342	5.1065306
Up	FGSG_05533	1.427962582	6.24E-09	6.280413	6.306724	6.1703363	6.7691847	6.7468578	6.7066335	6.5267935	6.6834471	6.2778581
Up	FGSG_09063	1.427375126	0.0769761	3.872828	4.2010532	3.3684179	3.0034737	3.0784965	2.8774004	3.4769433	3.6853877	3.18196
Up	FGSG_05557	1.426539331	1.58E-06	4.6610646	4.4323548	4.7932764	4.92659	4.7733906	4.9955781	4.965331	4.9352342	4.9376179
Up	FGSG_10967	1.425475922	8.86E-08	8.5884975	8.5751831	8.5166805	7.9841091	7.7572921	8.1043792	7.8678707	8.0001203	7.6486915
Up	FGSG_05712	1.42480988	0.0022072	3.0172362	2	3.5740244	4.1048586	4.1161991	4.024755	4.0850435	4.0586965	4.0607807

Up	FGSG_07554	1.424779412	6.52E-11	10.415278	10.416269	10.327908	10.226084	10.136787	10.228007	10.529005	10.522127	10.46913
Up	FGSG_07062	1.424577987	2.31E-08	6.2023113	5.6997548	6.5078422	6.2104357	5.9237087	6.3792376	6.6248622	6.4733227	6.7037513
Up	FGSG_06527	1.42343291	7.51E-09	8.4900322	8.5259608	8.7143573	9.1405824	9.2016944	9.1373946	9.0847193	9.0382958	9.1641848
Up	FGSG_08481	1.423153349	9.81E-08	5.6638035	5.1692654	5.9664603	5.4277566	5.7851416	4.8909283	5.8375198	5.7723491	5.8402522
Up	FGSG_05400	1.422106069	0.0185026	2.2688027	2	2.4807492	2.1935143	2.353841	2	3.4769433	3.3690748	3.5376798
Up	FGSG_04758	1.422007183	4.32E-08	8.0201456	7.9013439	8.0498976	8.0912658	8.0208235	8.0807844	8.4552781	8.4760172	8.3667329
Up	FGSG_09826	1.419635116	0.0727879	2	2	2	2.6546039	2.6377651	2.6397336	2.5718533	2.3968278	2.7087823
Up	FGSG_01201	1.418869916	3.70E-12	7.9501848	7.9109619	7.9050803	7.6992962	7.6967286	7.6144613	8.0234075	7.8965819	8.0792612
Up	FGSG_01455	1.415760271	0.0008131	4.0257777	4.3213345	3.5740244	3.2841938	3.5586152	2.8774004	4.3373111	4.0586965	4.5256129
Up	FGSG_06061	1.415535861	6.32E-12	10.446951	10.45623	10.350808	9.9915336	9.9450773	9.9521838	10.179613	10.140316	10.152729
Up	FGSG_13919	1.412175927	0.0072185	3.5075501	3.378055	3.5740244	3.1032407	2.8748957	3.260088	2.7212202	2.3968278	2.964684
Up	FGSG_11880	1.411782722	0.0041969	2.2688027	2.4852148	2	2.364115	2	2.6397336	2.5718533	2.3968278	2.7087823
Up	FGSG_05607	1.410391216	2.61E-08	6.1366353	5.9811576	6.2016809	6.1649807	6.0741317	6.1715481	6.6057745	6.5533169	6.5943381
Up	FGSG_10119	1.408065395	5.07E-05	6.2340611	6.306724	6.0719967	7.0385763	7.1708374	6.7962856	6.247774	6.2514733	6.1802631
Up	FGSG_08491	1.407090103	3.73E-06	7.340842	7.356828	7.2394951	7.0385763	6.9157907	7.0723383	7.8718708	7.7683708	7.9069251
Up	FGSG_00749	1.405651761	1.08E-06	4.4062533	4.9628272	4.0578819	4.6247215	4.8861689	4.5903319	5.4014247	5.5947086	5.3503768
Up	FGSG_03854	1.404699346	7.78E-05	4.2902308	3.765011	4.6184939	4.3681625	4.4455509	4.210161	5.4450355	5.5563964	5.3047937
Up	FGSG_10178	1.402919724	0.0359242	2.2688027	2	2.4807492	2	2	2	3.4769433	3.6853877	3.18196
Up	FGSG_00454	1.401935217	7.71E-10	7.9357761	7.9394368	7.8664849	7.878946	7.7572921	7.9238724	8.2338483	8.123176	8.2751578
Up	FGSG_04563	1.401721102	0.0179171	2	2	2	2.5166584	2.353841	2.6397336	2.2167888	2.3968278	2
Up	FGSG_12420	1.401386629	5.01E-05	4.1640552	3.9254527	4.3087924	5.0060735	4.8861689	5.0451875	4.2416299	3.9446664	4.4438634
Up	FGSG_13005	1.400989626	0.0003231	3.1562965	3.378055	2.8407141	4.0558932	4.2902531	3.6925011	4.2902636	4.1643702	4.3572018
Up	FGSG_07248	1.400091403	6.95E-09	6.310507	6.153105	6.4038729	6.5212093	6.4121699	6.5435983	6.4645842	6.4937409	6.3692672
Up	FGSG_05950	1.398947571	2.64E-06	7.3553501	7.3139731	7.3130965	8.238411	8.0930945	8.2958654	8.5997524	8.5412272	8.5921702
Up	FGSG_02601	1.398707162	1.01E-05	6.4522245	6.4188315	6.4038729	6.0446528	5.9498937	6.0552962	6.2600348	6.152209	6.301259
Up	FGSG_12123	1.398300519	9.21E-11	8.8844748	8.8597306	8.8241692	9.1361726	9.0942757	9.0909067	8.9903648	8.9225749	8.995118
Up	FGSG_12424	1.397887167	0.0012708	3.701719	3.765011	3.5740244	4.0558932	4.2902531	3.9222766	4.4698939	4.6006874	4.265
Up	FGSG_10148	1.397823452	2.23E-07	5.2004262	5.1036809	5.2179801	5.6084049	5.7257602	5.4283735	6.0216157	5.9891987	5.991768
Up	FGSG_20036	1.396862325	0.0781447	2	2	2	2.364115	2	2.6397336	2	2.3968278	2
Up	FGSG_20666	1.396455788	0.0024734	2.6910183	2.8476671	2.4807492	2.364115	2.6377651	2	2.2167888	2	2.3974876
Up	FGSG_11915	1.395787808	5.78E-08	10.894662	10.9211	10.82091	10.295618	10.198152	10.350224	10.279355	10.256894	10.261119

Up	FGSG_13596	1.394857763	4.62E-10	5.8799122	6.120316	5.4959731	5.4657412	5.346621	5.5012819	5.8210632	5.9304949	5.6345505
Up	FGSG_00354	1.393385483	0.0004863	3.5075501	4.069824	2.4807492	3.7210377	3.5586152	3.8119585	3.7092973	3.5358829	3.8228101
Up	FGSG_02019	1.39284759	0.0001002	7.8916659	7.8622179	7.8368453	8.742551	8.6431813	8.753082	9.8101497	9.8879776	9.656748
Up	FGSG_08122	1.392210002	6.37E-08	14.639141	14.596705	14.596295	14.573162	14.52741	14.531553	15.016359	14.992287	14.974079
Up	FGSG_08466	1.392063616	4.20E-08	6.5437448	6.3916107	6.60482	6.9872674	6.8753756	7.0112415	7.083861	7.0843795	7.0321538
Up	FGSG_09508	1.391400149	1.28E-11	7.250589	7.2994005	7.2693877	7.0385763	7.1929315	7.1194186	7.104406	7.249572	7.0460725
Up	FGSG_12550	1.390955416	0.033106	2	2	2	2.364115	2.6377651	2.8774004	2.4052168	2	2.964684
Up	FGSG_01640	1.389504571	5.69E-08	3.9513288	3.5844678	4.3087924	3.9526758	3.5586152	4.210161	3.7791165	3.8208439	3.6872775
Up	FGSG_12307	1.385908854	5.70E-09	6.5812576	6.6431438	6.4305761	6.9807236	7.0428732	6.8531009	7.2772237	7.1501722	7.3675781
Up	FGSG_00319	1.385774292	4.13E-11	6.310507	6.153105	6.4305761	6.40871	6.3932112	6.340023	6.6437006	6.61053	6.6131596
Up	FGSG_04340	1.384327471	0.0042594	2.6910183	2.8476671	2.4807492	3.0034737	3.4156241	2.6397336	2.2167888	2	2.3974876
Up	FGSG_02794	1.383420688	3.51E-08	9.0020892	8.9970078	8.9215117	9.2856758	9.3268626	9.1516201	9.6620981	9.6219158	9.6361917
Up	FGSG_02191	1.382863287	6.74E-09	6.6767699	6.306724	6.9005856	6.3181765	5.8970396	6.5777126	6.3430616	6.5726412	5.991768
Up	FGSG_04417	1.381099653	0.0240241	3.0172362	2	3.5740244	2.364115	2.353841	2.3550396	2.8565633	3.3690748	2
Up	FGSG_01548	1.377872849	3.52E-06	8.2196702	8.1490155	8.204887	9.016514	8.9525394	8.9931804	9.5492332	9.5383836	9.4936536
Up	FGSG_00750	1.377812677	6.22E-08	5.7096927	5.4053203	6.1703363	5.5740396	5.8139402	5.3904893	5.6821809	5.837702	5.6345505
Up	FGSG_02001	1.377363099	1.11E-12	9.8089013	9.8510102	9.7371893	9.9996479	9.9676804	9.9859201	9.7176077	9.7631755	9.647648
Up	FGSG_00129	1.377333104	0.0023999	2.4953013	2	2.8407141	2.364115	2.353841	2.3550396	2.5718533	2	2.964684
Up	FGSG_01881	1.376753779	9.58E-09	7.87162	7.8117694	7.8467929	8.1938425	8.1047954	8.195079	8.68634	8.6902068	8.615694
Up	FGSG_00952	1.376626202	0.0064755	3.872828	3.765011	3.9139052	3.8415007	3.9182279	3.6925011	2.7212202	2.7077187	2.7087823
Up	FGSG_04178	1.375369376	0.001007	5.3814422	5.5600358	5.0898194	5.7989973	5.6318376	5.874995	5.8375198	6.1262878	5.3945636
Up	FGSG_02499	1.373519649	1.68E-07	7.0511452	6.9534782	7.063002	7.0002667	7.1024491	6.7962856	7.9534078	7.8811649	7.9593509
Up	FGSG_08544	1.373467694	1.42E-07	6.4789709	6.3916107	6.4825439	6.379159	6.7318449	5.7902457	6.983915	6.9130376	6.9895734
Up	FGSG_01261	1.373378845	0.0016272	3.2831234	3.378055	3.1285604	2.7805029	3.0784965	2.3550396	3.7791165	3.5358829	3.9466965
Up	FGSG_06727	1.373179022	5.22E-07	7.642384	7.5902346	7.6103354	7.8824608	7.8505879	7.8279241	8.1241013	8.1362228	8.0450161
Up	FGSG_08238	1.372806976	1.66E-06	15.347149	15.296662	15.31221	15.043529	14.998608	15.001854	15.977599	15.937554	15.951952
Up	FGSG_03742	1.371573853	4.35E-05	2.6910183	2.8476671	2.8407141	2.7805029	2.8748957	2.6397336	2.5718533	2.7077187	2.3974876
Up	FGSG_10549	1.370653634	2.11E-10	12.873139	12.831041	12.854398	13.017064	13.00851	12.962895	13.000618	12.99433	12.962857
Up	FGSG_00710	1.370434035	3.95E-07	8.3914079	8.4221723	8.2728698	8.6630399	8.5900596	8.6486289	8.9530919	8.9707113	8.8676788
Up	FGSG_04655	1.369542455	0.0008924	2.8633281	2.8476671	2.8407141	2.7805029	2.8748957	2.6397336	2.5718533	2.7077187	2.3974876
Up	FGSG_06630	1.369408624	4.25E-05	6.9206496	7.0436746	6.6956874	7.9775509	7.9316604	7.9436281	8.6726471	8.6129802	8.6661346

Up	FGSG_02570	1.368371769	1.22E-08	6.6767699	6.6660438	6.60482	6.1299242	6.0741317	6.1029268	6.399571	6.5138742	6.2052877
Up	FGSG_03814	1.368020893	0.0384906	2.2688027	2	2.4807492	2.364115	2	2.6397336	2.4052168	2.3968278	2.3974876
Up	FGSG_01383	1.36731707	9.18E-06	6.6178197	6.5962238	6.5571458	5.5209064	5.2232303	5.7002055	6.0071405	5.7038947	6.2052877
Up	FGSG_03542	1.366495933	0.0063594	2.2688027	2.4852148	2	2.5166584	2	2.8774004	2.2167888	2	2.3974876
Up	FGSG_03603	1.364214026	0.0111171	2.2688027	2	2.4807492	2.364115	2	2.6397336	3.4769433	3.5358829	3.3707558
Up	FGSG_09713	1.363882637	2.71E-09	6.0853387	5.8672052	6.2016809	5.8856141	5.7257602	5.9550408	5.6638414	5.7385279	5.5195414
Up	FGSG_11118	1.359910602	0.0466024	2.2688027	2.4852148	2	2.1935143	2	2.3550396	2.4052168	2.7077187	2
Up	FGSG_01257	1.359554466	2.10E-08	6.1533367	6.3916107	5.7713555	5.9133681	5.7851416	5.9550408	6.0216157	5.9002223	6.075584
Up	FGSG_13009	1.359356002	0.0014829	3.0172362	3.1370988	2.8407141	2.5166584	2.353841	2.6397336	3.3903592	2.7077187	3.8228101
Up	FGSG_06213	1.359330966	7.51E-06	3.9513288	4.2010532	3.5740244	4.1522165	3.9182279	4.2946324	4.3373111	4.262829	4.3572018
Up	FGSG_00043	1.359068007	1.35E-05	3.872828	3.5844678	4.0578819	3.8981589	4.2058494	3.5622521	4.4270393	4.4416259	4.3572018
Up	FGSG_13016	1.357709251	0.0825292	2	2	2	2.364115	2	2.6397336	2.9802913	3.5358829	2
Up	FGSG_05181	1.356098107	3.34E-10	6.7444707	6.7962976	6.60482	6.9204536	6.9290147	6.8249729	7.104406	7.0708531	7.0735134
Up	FGSG_13933	1.355993243	8.81E-05	3.0172362	2.8476671	3.1285604	2.7805029	2.6377651	2.8774004	2.9802913	2.3968278	3.3707558
Up	FGSG_01570	1.354832668	2.20E-08	3.701719	3.5844678	3.7539507	3.5895922	3.5586152	3.5622521	3.4769433	3.5358829	3.3707558
Up	FGSG_10184	1.353702617	0.0047447	3.2831234	3.5844678	2.8407141	2.5166584	2.6377651	2.3550396	2.7212202	3.1804273	2
Up	FGSG_10595	1.35190313	0.0592224	3.2831234	3.1370988	3.3684179	3.5895922	3.9182279	3.0813961	2	2	2
Up	FGSG_11827	1.351353528	0.0058041	2.4953013	2.8476671	2	2.364115	2.6377651	2	3.0942412	2.3968278	3.5376798
Up	FGSG_13724	1.350214857	0.0553178	2	2	2	3.8415007	3.9182279	4.210161	3.6359267	3.3690748	4.1665008
Up	FGSG_01209	1.349535441	9.63E-07	3.872828	4.2010532	4.7932764	4.0052074	4.5857446	4.3744304	4.0850435	4.4416259	4.0607807
Up	FGSG_04174	1.349477204	0.0046606	4.0257777	4.5354388	3.1285604	4.92659	4.9394155	4.8356119	5.0240241	5.3920827	4.4438634
Up	FGSG_12038	1.34939463	0.0001109	4.0965725	4.4323548	3.5740244	3.8981589	3.9182279	3.8119585	4.0850435	4.0586965	4.0607807
Up	FGSG_09522	1.348529241	1.36E-07	11.161945	11.161651	11.075821	11.214639	11.137785	11.203237	11.828918	11.779652	11.811712
Up	FGSG_12576	1.348402001	0.0205701	3.701719	3.5844678	3.7539507	2.364115	2.6377651	2	2.9802913	2.9633478	2.964684
Up	FGSG_01231	1.348398417	5.78E-07	6.6996916	6.6431438	6.6735015	7.4378284	7.4621692	7.3241948	7.6288039	7.5711195	7.6303308
Up	FGSG_09640	1.347676326	1.72E-07	6.4522245	6.3355781	6.4825439	6.2653117	6.3545295	6.0793081	7.1179419	7.0708531	7.100442
Up	FGSG_05104	1.347294847	4.76E-05	4.2285219	4.3213345	4.0578819	4.7835368	4.8861689	4.5903319	4.3828727	4.6740912	3.9466965
Up	FGSG_02968	1.347292715	1.35E-06	6.8910817	6.5721788	7.0799694	5.8429557	5.9237087	5.6688985	6.9540395	6.7863464	7.0460725
Up	FGSG_03999	1.346861636	4.14E-09	7.8966342	7.9205161	7.7860511	7.5960451	7.4438603	7.65497	7.436947	7.5614795	7.2280252
Up	FGSG_13544	1.345917279	1.09E-06	4.4062533	4.5354388	4.3087924	4.2425091	4.0206066	4.3744304	4.2902636	4.1643702	4.3572018
Up	FGSG_00439	1.345741926	2.05E-09	7.4257798	7.3984466	7.3693882	7.5613806	7.5673956	7.4675471	7.7248157	7.8096741	7.5641633

Up	FGSG_04832	1.344422014	0.0067703	2.2688027	2	2.4807492	3.2841938	3.2568854	3.260088	3.199846	3.6853877	2.3974876
Up	FGSG_07930	1.343207073	3.93E-11	8.3984303	8.3103344	8.3999194	8.1710312	8.1106104	8.1447637	7.9534078	7.8177947	8.0170225
Up	FGSG_02918	1.342111108	0.0001858	3.2831234	2.8476671	3.5740244	3.9526758	3.6887032	4.1204343	4.1912995	3.6853877	4.5256129
Up	FGSG_10723	1.341212687	1.34E-08	6.665171	6.7107812	6.5327046	6.3890768	6.4675956	6.2155469	6.7518403	6.7528555	6.7037513
Up	FGSG_13550	1.341123525	0.013666	2.2688027	2.4852148	3.1285604	2.7805029	2.8748957	3.8119585	2.8565633	3.3690748	3.5376798
Up	FGSG_13350	1.339866091	1.21E-07	8.2664793	8.3030481	8.294837	8.810547	8.8450257	8.8746941	9.2242318	9.2360755	9.2448462
Up	FGSG_12171	1.339839487	0.0024723	2.4953013	2.4852148	2.4807492	2.364115	2.353841	2.3550396	2.7212202	2.3968278	2.964684
Up	FGSG_07197	1.339482893	7.22E-10	6.8404139	6.7326402	6.8618681	7.1241972	7.0669996	7.0960705	7.3819108	7.3649028	7.3339349
Up	FGSG_00040	1.338674178	0.069259	2	2	2	3.444959	3.5586152	3.260088	4.1391495	4.1643702	4.0607807
Up	FGSG_11545	1.336541314	0.0008474	3.2831234	3.765011	3.7539507	3.0034737	3.5586152	3.5622521	2.8565633	3.6853877	4.0607807
Up	FGSG_07992	1.335350807	0.0059328	3.0172362	3.378055	2.4807492	3.3668146	3.4156241	3.260088	3.8457121	3.8208439	3.8228101
Up	FGSG_05306	1.333450449	6.74E-10	8.0516681	7.9947541	8.0238286	8.366918	8.4318447	8.2060258	8.6817901	8.6129802	8.684048
Up	FGSG_06338	1.333269514	9.14E-07	4.5136357	4.3213345	4.7932764	4.5203003	4.8308815	4.210161	4.5913113	3.8208439	5.0523944
Up	FGSG_09011	1.332591163	0.0704758	2.8633281	6.052414	6.2623983	3.444959	5.4602848	5.2704753	4.1391495	6.2514733	5.5972237
Up	FGSG_13275	1.332155302	0.0063099	3.0172362	2.8476671	3.1285604	2.364115	2.353841	2.3550396	3.199846	3.1804273	3.18196
Up	FGSG_04355	1.331188925	4.59E-06	6.280413	6.153105	6.3206633	6.0571473	5.9237087	6.1029268	6.7604964	6.61053	6.8378143
Up	FGSG_08806	1.33111491	0.000403	3.5075501	3.5844678	3.3684179	3.1965524	2.8748957	3.4190665	2.7212202	2.9633478	2.3974876
Up	FGSG_11000	1.329688571	0.0603956	3.1562965	3.378055	2.8407141	2.364115	2	2.6397336	2.4052168	2.3968278	2.3974876
Up	FGSG_01727	1.32961333	0.002906	2.6910183	2.4852148	2.8407141	3.1032407	2.8748957	3.260088	3.5586239	3.3690748	3.6872775
Up	FGSG_09252	1.328326228	4.29E-10	6.9780274	6.8770274	6.9930638	6.5656126	6.5554873	6.4908649	6.908033	7.0294959	6.7037513
Up	FGSG_11848	1.328163113	1.09E-10	8.2432646	8.1811658	8.2279057	7.9239834	7.7348776	8.0138356	8.2369531	8.1100101	8.2927768
Up	FGSG_00444	1.327823652	2.36E-06	8.4364543	8.4879097	8.294837	8.1994895	8.1951353	8.1160334	8.9662472	9.0208151	8.8399835
Up	FGSG_07778	1.327548677	0.0022618	3.0172362	3.378055	2.4807492	2.364115	2.353841	2.3550396	3.2982451	3.3690748	3.18196
Up	FGSG_07174	1.327524265	6.33E-05	8.6368049	8.5133883	8.6758139	8.5810544	8.5606242	8.514165	7.9382281	7.9566533	7.852522
Up	FGSG_08174	1.326216267	4.17E-07	6.8710276	6.9719742	6.6735015	7.0573562	7.203853	6.7962856	7.1837795	7.2253567	7.0735134
Up	FGSG_13958	1.325975389	0.0010278	3.0172362	3.378055	2.4807492	2.7805029	2.6377651	2.8774004	2.4052168	2.3968278	2.3974876
Up	FGSG_08382	1.325786623	1.14E-06	4.1640552	4.5354388	3.5740244	3.7825262	3.6887032	3.8119585	4.2416299	4.4416259	4.265
Up	FGSG_05747	1.323718708	4.36E-09	4.9172105	4.8868835	4.8733193	4.657914	4.5857446	4.6556708	5.0524989	5.1562742	4.8766132
Up	FGSG_00492	1.323627701	5.64E-08	13.628023	13.581494	13.589139	13.7026	13.653235	13.664452	14.439676	14.405197	14.408275
Up	FGSG_06064	1.323441425	1.00E-08	6.9686215	6.9719742	6.9005856	7.4568136	7.387497	7.4492367	7.5560885	7.4921337	7.5928932
Up	FGSG_00867	1.322957699	0.0736273	2.8633281	3.1370988	2.4807492	2.5166584	2.353841	2.6397336	3.199846	3.5358829	2.7087823

Up	FGSG_04395	1.322822478	5.17E-07	6.8710276	6.9534782	6.6956874	7.0820218	7.1483996	6.9211113	7.7070136	7.9492797	7.3339349
Up	FGSG_01173	1.322682392	0.0001855	4.8367778	4.6316459	4.9491538	5.3074061	5.265544	5.3115993	6.235408	6.2026947	6.2052877
Up	FGSG_04270	1.322031001	3.65E-06	9.5886776	9.5827207	9.7425274	8.7444837	8.7021396	8.8329328	9.5850043	9.6126104	9.5894343
Up	FGSG_05899	1.32203038	1.22E-08	6.8091364	6.6885859	6.8421126	7.0758949	7.1596621	6.8942906	7.5758404	7.5222624	7.5641633
Up	FGSG_07214	1.321914795	8.40E-09	6.3971965	6.3355781	6.4038729	6.7150192	6.5554873	6.7817251	7.1111898	7.0434138	7.1137202
Up	FGSG_13744	1.320429869	0.0069038	6.2955384	6.4717791	6.0376686	5.658465	5.5313719	5.7002055	6.0641898	5.9601454	6.1024744
Up	FGSG_07521	1.3201695	0.0007773	3.1562965	2.8476671	3.3684179	3.2841938	3.8080253	2.3550396	3.3903592	3.5358829	3.18196
Up	FGSG_07050	1.319520608	0.0146106	3.5075501	3.765011	3.1285604	2.8962916	2.8748957	2.8774004	3.7791165	3.6853877	3.8228101
Up	FGSG_09339	1.319499939	3.86E-07	8.179468	8.1490155	8.1497038	7.7693122	7.8154152	7.762693	8.4201984	8.4448964	8.410429
Up	FGSG_03421	1.318583852	1.40E-07	5.2937714	5.2921154	5.2179801	5.368836	5.7257602	4.7780897	5.5880596	5.7038947	5.3945636
Up	FGSG_02792	1.314516918	0.0073428	4.1640552	3.765011	4.4195794	4.0558932	3.8080253	4.210161	4.3828727	4.6006874	4.0607807
Up	FGSG_08538	1.314448556	0.0325523	3.1562965	2.8476671	3.5740244	4.9800625	5.179638	4.6556708	5.5880596	5.7038947	5.3945636
Up	FGSG_01324	1.314227953	3.45E-09	8.5543222	8.5751831	8.4464558	8.5939748	8.4225248	8.6685513	9.019505	8.9959807	8.9770684
Up	FGSG_10826	1.313036399	0.0217505	2.8633281	3.1370988	2.4807492	3.5190873	3.2568854	3.6925011	2.4052168	2.7077187	2
Up	FGSG_05101	1.31180863	3.15E-08	6.1533367	6.2165286	6.0025037	6.7228827	6.6701803	6.6911344	6.9312196	6.8351685	6.9604719
Up	FGSG_09493	1.311570285	0.0017623	9.0696347	8.9924852	9.0604997	8.5415841	8.3604325	8.6243531	8.9718487	9.0172934	8.8637548
Up	FGSG_02454	1.311151274	1.41E-07	7.6658542	7.8522684	7.7008668	7.4708903	7.52448	7.7477878	8.3416141	8.45013	8.3887464
Up	FGSG_04593	1.309990466	0.0028896	3.5075501	4.069824	2.4807492	3.7825262	4.0206066	3.4190665	4.028829	4.262829	3.6872775
Up	FGSG_02756	1.308998425	1.96E-12	9.2238615	9.2414842	9.119132	9.228825	9.1347875	9.2341477	9.3923884	9.3355791	9.3828564
Up	FGSG_09215	1.308315526	7.52E-07	4.8775546	4.8067189	4.8733193	4.8990917	4.2058494	5.3115993	4.9041484	5.0499834	4.6764038
Up	FGSG_05641	1.306732664	8.65E-05	4.9558055	4.7218364	5.2179801	4.4837433	4.4455509	4.4500449	5.1346989	4.7439404	5.5195414
Up	FGSG_10214	1.305952071	0.0010518	2.8633281	3.378055	2.8407141	3.1032407	3.5586152	2.8774004	4.1391495	4.0586965	4.265
Up	FGSG_07250	1.30586626	3.09E-11	8.8231703	8.8547558	8.7034496	8.8982305	8.9031382	8.8080019	8.6795098	8.6176392	8.6795905
Up	FGSG_12174	1.302099211	1.34E-08	7.3335329	7.3284	7.2545188	7.2492498	7.225451	7.187278	7.7424007	7.7084846	7.711174
Up	FGSG_09525	1.300814071	2.39E-10	7.4731115	7.6021576	7.2394951	7.4330428	7.1929315	7.5643	7.1837795	7.2851495	7.003907
Up	FGSG_04426	1.300185592	0.0001891	2.8633281	3.1370988	2.4807492	3.8981589	3.5586152	4.1204343	3.970335	3.9446664	3.9466965
Up	FGSG_07940	1.299918142	4.97E-05	7.5189396	7.4911275	7.4629041	6.7539152	6.5033974	6.8942906	6.2721924	6.2026947	6.2778581
Up	FGSG_12754	1.299355797	9.53E-11	13.828838	13.806372	13.7655	13.842649	13.791558	13.806193	13.490729	13.429592	13.485491
Up	FGSG_11457	1.298273741	0.0001228	7.4863545	7.3284	7.6449538	8.6752495	8.5087227	8.7826325	8.489525	8.4708768	8.4423539
Up	FGSG_09615	1.298202697	1.95E-08	6.5563577	6.5721788	6.5078422	6.6750453	6.5554873	6.7670162	7.0418719	7.0843795	6.9307712
Up	FGSG_07151	1.297915529	0.0011004	6.0678264	7.6603381	7.8566725	6.0939946	7.4346181	7.5123315	6.7861576	7.6641337	7.4948146

Up	FGSG_10821	1.297786173	9.28E-06	4.2285219	4.5354388	3.7539507	4.1522165	4.3699906	3.8119585	4.8725557	4.8105634	4.8766132
Up	FGSG_07705	1.297785414	0.0207689	3.1562965	3.1370988	3.1285604	2.6546039	3.0784965	2	2.5718533	2.3968278	2.7087823
Up	FGSG_01489	1.297120412	2.49E-07	5.3235909	5.349828	5.2179801	5.0567294	4.9907667	5.0451875	5.9015311	5.5947086	6.1024744
Up	FGSG_12691	1.296584671	3.71E-08	8.2664793	8.2735292	8.1736127	8.2162989	8.2114618	8.1333402	8.6680537	8.6036168	8.6661346
Up	FGSG_13920	1.294934437	0.0017546	2.8633281	3.1370988	2.4807492	3.7825262	2.8748957	4.2946324	3.9093688	4.3549955	3.3707558
Up	FGSG_12323	1.293612884	0.0001598	3.1562965	3.378055	3.3684179	3.8981589	3.8080253	3.9222766	4.5519634	4.7439404	4.265
Up	FGSG_05284	1.293162298	4.73E-07	8.3629712	8.3812423	8.2580369	8.1213252	8.2329471	7.9038425	7.3875098	7.2615289	7.443156
Up	FGSG_09016	1.29240036	7.16E-11	9.8246274	9.7581508	9.8050899	9.4533919	9.3807925	9.4451957	9.6563119	9.5771708	9.6680429
Up	FGSG_11073	1.29197467	0.0024679	4.349408	4.2010532	4.4195794	4.1522165	4.0206066	4.210161	4.028829	4.262829	3.6872775
Up	FGSG_03976	1.291664096	5.54E-05	6.6178197	6.5228522	6.8421126	7.7881729	7.9184607	7.7175072	8.3006563	8.2059445	8.3722678
Up	FGSG_00794	1.289515881	0.0002942	3.9513288	3.9254527	3.9139052	3.3668146	3.5586152	3.0813961	4.0850435	4.262829	3.8228101
Up	FGSG_12051	1.289337857	0.0238368	3.872828	4.069824	3.5740244	3.8981589	4.1161991	3.5622521	4.3373111	4.3549955	4.265
Up	FGSG_10188	1.289179699	1.98E-08	7.7451174	7.6941525	7.7860511	7.7347289	7.6733454	7.7774459	7.1773293	6.9430453	7.4325986
Up	FGSG_02679	1.288512485	0.0037293	3.3996963	3.378055	3.3684179	2.7805029	2.6377651	3.0813961	2.4052168	2	2.7087823
Up	FGSG_09555	1.287747362	6.54E-10	8.5321451	8.4943217	8.4851833	8.8068523	8.7808316	8.7455988	8.8019627	8.9036246	8.6249972
Up	FGSG_04221	1.286870093	8.88E-08	6.3545028	6.1851653	6.5327046	6.8359652	6.5891976	6.9733016	7.1313519	7.0843795	7.1399154
Up	FGSG_04084	1.286859175	1.54E-11	8.2315156	8.1890927	8.1893346	8.2079187	8.0084184	8.3060762	8.4283697	8.2546151	8.5241867
Up	FGSG_08675	1.286621437	2.33E-09	10.177787	10.176182	10.142482	9.8307306	9.7960719	9.8424632	9.5703099	9.5110994	9.6176702
Up	FGSG_10921	1.285723109	0.0138022	3.0172362	3.1370988	2.8407141	2.7805029	2	3.260088	2.8565633	2.7077187	2.964684
Up	FGSG_01003	1.285622223	0.0008328	4.349408	4.3213345	4.3087924	4.92659	4.8308815	4.9442019	4.807216	4.5233477	4.9961472
Up	FGSG_08586	1.285201545	5.45E-09	10.303753	10.242434	10.279154	9.9291763	9.8647765	9.9059388	10.111891	10.053577	10.103661
Up	FGSG_07571	1.284899235	0.0008311	3.6078986	3.765011	3.3684179	4.1980691	4.1161991	4.210161	4.1391495	4.262829	3.9466965
Up	FGSG_00660	1.284151259	7.54E-05	5.3528065	4.8868835	5.640225	5.9805033	5.7557564	6.1029268	5.8375198	5.5170389	6.0481828
Up	FGSG_05651	1.283916044	0.0003036	4.2285219	3.1370988	4.7932764	4.0558932	4.2902531	3.6925011	4.5913113	4.4416259	4.6764038
Up	FGSG_02593	1.282041597	3.66E-05	6.6882763	6.8372271	6.4305761	5.4468739	5.306652	5.5706824	5.9325022	5.7723491	6.0202512
Up	FGSG_02059	1.280734126	0.0046913	2.8633281	2.4852148	3.1285604	2.364115	2.353841	2.3550396	3.3903592	3.3690748	3.3707558
Up	FGSG_11653	1.27857343	0.0595211	2	2	2	3.444959	3.0784965	3.6925011	2.4052168	2.7077187	2
Up	FGSG_00280	1.277936419	1.60E-07	5.7321013	5.6997548	5.6852725	6.379159	6.5033974	6.1490351	6.5668244	6.4102748	6.6500817
Up	FGSG_06008	1.276713765	2.78E-09	5.5921111	5.906198	5.4959731	5.7689296	6.1438433	5.5706824	5.8044166	5.9891987	5.9627112
Up	FGSG_00826	1.276473839	7.31E-06	4.9558055	5.0349724	4.7932764	4.5559538	4.7135134	4.2946324	5.0804226	5.3023297	4.7462732
Up	FGSG_06851	1.276076613	2.06E-08	6.1366353	6.153105	6.0376686	5.8137994	5.7557564	5.7902457	6.4645842	6.4102748	6.4552281

Up	FGSG_12848	1.275966213	0.0278625	2.6910183	2.4852148	2.8407141	2.364115	2.6377651	2	3.4769433	3.8208439	2.964684
Up	FGSG_10745	1.27391748	2.21E-07	5.5673995	5.7434744	5.2780294	5.7068462	5.5991203	5.7308476	5.6452659	5.3023297	5.8718551
Up	FGSG_06891	1.273726413	0.0090484	4.5644712	4.069824	4.8733193	6.1533896	6.374	5.7902457	7.104406	7.2968163	6.8054483
Up	FGSG_00461	1.273627292	2.95E-07	6.7110174	6.7326402	6.60482	6.7071125	6.5891976	6.7371407	7.4899606	7.4192855	7.4948146
Up	FGSG_06111	1.272891834	1.33E-05	6.605735	6.5962238	6.5327046	7.2546844	7.203853	7.220047	7.9496278	7.9418683	7.8915895
Up	FGSG_05820	1.272880984	8.43E-06	7.1207354	7.0436746	7.113317	5.9270474	5.8421752	5.9288493	6.9615665	7.0012507	6.8537291
Up	FGSG_01178	1.272126816	1.86E-05	4.5644712	4.6316459	4.5224622	3.7825262	4.1161991	4.4500449	4.8725557	5.347904	4.8766132
Up	FGSG_01502	1.271739666	3.29E-07	6.605735	6.4455482	6.6735015	6.1878872	6.1438433	6.1490351	6.0359471	5.9601454	6.0481828
Up	FGSG_13692	1.271418822	0.0040989	2.4953013	2.4852148	2.4807492	2.364115	2.6377651	2	2.5718533	2.9633478	2.7087823
Up	FGSG_11104	1.270500565	0.0647171	3.0172362	2.8476671	3.1285604	2.7805029	2.6377651	2.8774004	2.7212202	2.9633478	2.3974876
Up	FGSG_12245	1.270177704	0.0004017	3.0172362	2.8476671	3.3684179	2.7805029	2.6377651	2.8774004	2.9802913	3.3690748	2.3974876
Up	FGSG_11032	1.269526008	0.0001918	6.3253218	6.3916107	6.1703363	7.1651746	6.9807308	7.2520883	6.354542	6.59171	5.991768
Up	FGSG_01437	1.268402794	2.07E-07	7.1871227	7.1449564	7.1459111	6.7691847	6.7617162	6.6911344	7.4151838	7.4192855	7.3452367
Up	FGSG_09100	1.268280941	3.54E-09	6.2496778	5.9441646	6.4305761	6.2545022	6.4856075	5.874995	6.0359471	6.0456069	5.9627112
Up	FGSG_00684	1.268148399	2.55E-05	5.2633225	5.7434744	5.0212003	5.3281747	5.3855124	5.5363994	4.807216	5.3023297	4.5256129
Up	FGSG_11941	1.267464697	0.0003421	6.3545028	5.9811576	6.5811798	5.9673238	6.0977451	5.7308476	6.7776545	6.9430453	6.5164961
Up	FGSG_04740	1.266380126	0.0009893	3.3996963	2.4852148	3.9139052	3.1965524	3.4156241	2.8774004	3.2982451	3.6853877	2.7087823
Up	FGSG_02339	1.266234556	0.0006322	4.6610646	4.2010532	5.0212003	3.6568115	3.8080253	3.4190665	3.3903592	3.3690748	3.3707558
Up	FGSG_05702	1.266208267	0.0008673	4.5644712	4.3213345	5.0212003	3.5895922	4.0206066	3.4190665	4.7032988	4.4416259	4.8766132
Up	FGSG_01169	1.264787597	7.33E-07	7.8358551	7.9394368	7.6335064	8.5788897	8.6024932	8.4648884	8.5406746	8.4811393	8.5340969
Up	FGSG_11675	1.264502706	0.003251	4.0965725	4.6316459	3.1285604	4.6247215	4.1161991	4.9442019	4.028829	3.8208439	4.1665008
Up	FGSG_03114	1.263587654	0.0001519	3.3996963	2.4852148	3.9139052	3.1965524	2.6377651	3.5622521	3.199846	3.1804273	3.18196
Up	FGSG_00606	1.262479731	1.82E-05	4.5136357	4.6316459	4.3087924	4.2425091	4.6510429	3.5622521	4.666927	4.0586965	5.0523944
Up	FGSG_01141	1.26110245	0.0074273	6.1366353	6.306724	5.85259	8.1420001	8.0634203	8.1333402	8.6212975	8.6813329	8.4889546
Up	FGSG_01865	1.260087813	5.37E-08	8.4330387	8.4288825	8.3518317	8.2273973	8.1279162	8.2383766	8.1928659	8.1555744	8.1646712
Up	FGSG_03686	1.2576999	1.77E-05	8.0781498	7.9205161	8.1416454	8.1853305	8.0930945	8.1895743	8.5204318	8.5114961	8.4632514
Up	FGSG_02077	1.257399639	2.44E-09	12.095203	12.091752	12.012312	12.270203	12.18829	12.263889	12.619438	12.543868	12.62752
Up	FGSG_10499	1.256935612	8.43E-05	5.5921111	5.458757	5.640225	6.4753959	6.4675956	6.3984523	7.2651087	7.2130949	7.2522417
Up	FGSG_08763	1.256568643	1.24E-06	5.6638035	5.608128	5.640225	6.5030582	6.5209705	6.3984523	6.6987834	6.6655604	6.6681943
Up	FGSG_06553	1.256277642	0.0011961	8.1254721	8.0653582	8.1006636	7.9576954	7.8225187	8.0013221	8.3559647	8.3416703	8.3044043
Up	FGSG_09973	1.255743747	0.0058634	2.6910183	2.8476671	2.8407141	3.1032407	2.353841	3.5622521	2.4052168	2.3968278	2.7087823

Up	FGSG_04881	1.255528865	2.10E-08	8.3557736	8.3391171	8.2875517	8.6752495	8.6670545	8.5955051	8.1241013	8.0213724	8.1646712
Up	FGSG_01906	1.254218316	4.53E-09	6.1366353	6.2165286	6.2016809	6.548015	6.5383322	6.6436126	6.7255557	6.3886313	7.003907
Up	FGSG_01420	1.254204013	1.56E-08	5.5921111	5.5600358	5.545677	5.5209064	5.5991203	5.3515835	6.0641898	5.837702	6.2052877
Up	FGSG_05515	1.252761828	9.58E-09	8.5257457	8.6402333	8.3092981	8.9825273	8.9908774	8.8849482	9.2132388	9.1291972	9.2296806
Up	FGSG_11842	1.251312542	0.0001474	7.6600224	8.7407319	8.9120677	7.7993721	9.0220654	9.0336834	8.1173781	9.2360755	9.3495717
Up	FGSG_10180	1.24873888	1.18E-07	8.7426842	8.9228887	8.9402161	8.1242969	8.3158245	8.2276734	8.1767882	8.2665305	8.2270941
Up	FGSG_03422	1.248405161	7.52E-07	13.243952	13.196106	13.206001	13.767954	13.702317	13.745704	14.225933	14.213083	14.172365
Up	FGSG_02154	1.248398569	0.0045676	3.5075501	3.378055	3.5740244	4.327482	4.2058494	4.3744304	3.2982451	2.7077187	3.6872775
Up	FGSG_01588	1.246953251	5.66E-05	10.211073	10.194017	10.174431	11.100438	10.991828	11.126091	11.217763	11.163475	11.216311
Up	FGSG_04836	1.246558583	0.0525305	2.2688027	2.4852148	2	2.5166584	2.6377651	2.3550396	2.7212202	3.1804273	2
Up	FGSG_10316	1.246526751	3.28E-05	5.5673995	5.510285	5.545677	6.0065063	6.0501253	5.874995	6.0781065	5.8693008	6.2052877
Up	FGSG_13073	1.246379283	0.0003861	4.0257777	3.1370988	4.5224622	5.0814056	5.0403527	5.0451875	4.9041484	5.1041074	4.6029775
Up	FGSG_12392	1.245051376	0.0028797	2.4953013	2	2.8407141	2.5166584	2.353841	2.6397336	2.4052168	2.7077187	2
Up	FGSG_13730	1.245007661	8.15E-05	4.2285219	4.5354388	3.7539507	4.657914	4.9394155	4.210161	5.2124668	5.5563964	4.6764038
Up	FGSG_04571	1.244917357	3.14E-08	9.3157495	9.2028557	9.3394541	9.2561763	9.2071325	9.2180155	8.9493111	8.8882831	8.9439989
Up	FGSG_09445	1.243612377	0.0168734	3.0172362	3.5844678	2	2.364115	2.6377651	2	2.5718533	2.3968278	2.7087823
Up	FGSG_08596	1.24359175	3.71E-06	8.2741352	8.2585401	8.2126008	8.6792965	8.6749253	8.5955051	9.5163907	9.4575333	9.5088094
Up	FGSG_09851	1.24350774	1.32E-06	8.7834884	8.7024765	8.8291955	9.0867536	9.0189768	9.1600886	9.6770346	9.6472003	9.6635356
Up	FGSG_05614	1.241985264	7.02E-06	7.3481143	7.3984466	7.20897	7.188078	7.0907291	7.1982839	7.4795126	7.5902083	7.287821
Up	FGSG_11992	1.241345407	4.21E-05	5.167919	5.2319977	5.0212003	4.1980691	4.3699906	3.9222766	5.1610901	5.3023297	4.9376179
Up	FGSG_04281	1.240908183	2.35E-05	5.167919	4.9628272	5.2780294	4.6247215	4.4455509	4.7181783	5.4233949	5.0499834	5.670936
Up	FGSG_01931	1.240797874	5.59E-07	8.5480206	8.4488278	8.5596523	9.3119799	9.2286818	9.307134	9.5366898	9.5060827	9.5012514
Up	FGSG_10514	1.240792005	1.87E-07	4.4609433	4.2010532	4.9491538	4.69036	4.2902531	5.0451875	4.5519634	4.7439404	4.3572018
Up	FGSG_09390	1.240344398	0.0011597	3.872828	4.3213345	3.1285604	4.69036	4.8308815	4.4500449	5.2124668	5.1562742	5.2090653
Up	FGSG_06109	1.239511848	3.99E-05	4.9558055	5.2319977	4.5224622	5.5565451	5.423383	5.6041695	6.1976611	6.0456069	6.2778581
Up	FGSG_07432	1.238657662	2.73E-10	7.612499	7.3984466	7.7226391	7.3542322	7.23613	7.3832557	7.7640849	7.6368537	7.8204879
Up	FGSG_01350	1.238272726	2.40E-06	7.3909947	7.356828	7.3415168	7.2762202	7.1483996	7.3141119	7.3363183	7.3425624	7.2760585
Up	FGSG_01424	1.23720232	2.13E-05	8.4019287	8.3319751	8.3863428	7.4755522	7.4159539	7.4492367	8.4869193	8.3695592	8.5340969
Up	FGSG_01408	1.23668155	1.03E-08	9.6100088	9.5736661	9.561077	9.2936175	9.2152512	9.2840744	9.0249039	9.0278328	8.9551065
Up	FGSG_04456	1.236488992	0.0002966	10.894662	10.907952	10.79417	11.856672	11.799769	11.826258	12.617653	12.563922	12.604778
Up	FGSG_00374	1.23474396	2.10E-06	8.0516681	7.9394368	8.0755039	8.6833322	8.6066141	8.672503	9.2351417	9.2391022	9.1641848

Up	FGSG_00581	1.234724686	5.38E-07	7.14599	7.342684	6.8220828	6.8575557	6.6221382	6.9860594	6.908033	6.9578183	6.8054483
Up	FGSG_10413	1.234683907	0.0567952	2	2	2	3.1965524	3.4156241	2.8774004	2.5718533	2.9633478	2
Up	FGSG_07159	1.234627107	1.16E-10	6.7879016	6.7326402	6.7602689	6.60869	6.6221382	6.5435983	6.5369058	6.6474492	6.3469521
Up	FGSG_09793	1.233900189	0.0270241	2.4953013	2	2.8407141	4.4462359	4.4455509	4.3744304	4.8402557	4.8742451	4.7462732
Up	FGSG_02584	1.23364693	4.02E-08	7.2736871	7.2395919	7.2243133	6.8788278	6.7166741	6.9474424	6.9312196	6.8190773	6.975096
Up	FGSG_08230	1.233215655	0.0051312	4.349408	3.5844678	4.7932764	3.8981589	3.4156241	4.210161	4.994976	4.9937494	4.9961472
Up	FGSG_05368	1.231622965	3.12E-08	4.9558055	4.7218364	5.0898194	5.0814056	5.1346873	5.0451875	5.4663562	5.4349486	5.4374371
Up	FGSG_06078	1.231336501	5.47E-07	5.0300296	4.6316459	5.2780294	4.6247215	4.6510429	4.5218929	5.3791148	5.4349486	5.2577234
Up	FGSG_10242	1.229826233	0.0024114	4.5136357	6.052414	5.728956	5.2863342	6.4856075	6.340023	5.4663562	6.6655604	6.6131596
Up	FGSG_00726	1.229617226	2.06E-06	6.4921604	6.4455482	6.4567939	7.1651746	7.1929315	7.0482091	7.6049703	7.6183761	7.5249446
Up	FGSG_11633	1.228188687	0.0030292	5.4906186	6.4455482	7.113317	5.3074061	6.2103411	6.4728499	5.7181748	6.7863464	6.7037513
Up	FGSG_04276	1.22640137	2.31E-06	5.8184092	5.7859081	5.8125445	6.40871	6.3545295	6.4174145	6.9615665	7.0571987	6.9004463
Up	FGSG_07483	1.224912836	0.0006184	3.1562965	3.378055	2.8407141	3.1032407	3.4156241	2.6397336	2.7212202	2.7077187	2.7087823
Up	FGSG_11916	1.224716732	3.67E-07	5.065757	4.9628272	5.0898194	5.2432439	5.3855124	4.9955781	5.7875758	5.9304949	5.5589053
Up	FGSG_13728	1.223998046	1.80E-07	8.3878838	8.2280865	8.452983	8.2655829	8.2645866	8.1785015	8.2615538	8.3016973	8.1518558
Up	FGSG_04230	1.22387317	9.55E-05	6.2340611	6.120316	6.2623983	7.3542322	7.1483996	7.4584209	7.2832433	7.249572	7.2522417
Up	FGSG_01935	1.223475619	1.67E-06	7.5253693	7.4911275	7.4757821	7.1937475	7.1483996	7.1537472	7.0275995	6.9130376	7.0735134
Up	FGSG_00297	1.222683068	9.39E-08	6.2496778	6.2165286	6.2016809	6.5302	6.7318449	6.1937152	7.0418719	7.0294959	6.9895734
Up	FGSG_10201	1.221825282	5.28E-06	6.7663496	6.3638664	7.0108688	6.3890768	6.4121699	6.279127	7.083861	7.1372515	6.9604719
Up	FGSG_12194	1.221696531	1.19E-11	7.2112509	7.1119795	7.2243133	7.4330428	7.4981042	7.2730605	7.3070736	7.1756715	7.3675781
Up	FGSG_04745	1.220863914	0.0055448	3.872828	3.5844678	4.0578819	5.1056669	5.1346873	4.9955781	4.2902636	4.6006874	3.8228101
Up	FGSG_09917	1.220412552	3.18E-06	8.5605964	8.5630341	8.6533178	8.9544155	8.9908774	8.9931804	9.7030876	9.7334351	9.7297104
Up	FGSG_11895	1.218755764	0.0103786	3.1562965	3.5844678	2.4807492	4.0052074	4.1161991	3.8119585	5.237485	5.5947086	4.6764038
Up	FGSG_10128	1.216334212	0.0060489	2.4953013	2.8476671	2	2.8962916	3.2568854	2.3550396	2.7212202	2.9633478	2.3974876
Up	FGSG_06772	1.215917245	0.0016448	4.5644712	4.9628272	3.9139052	5.3281747	5.346621	5.2281446	6.0918902	6.1776727	5.9330571
Up	FGSG_10310	1.215495593	0.0028395	4.4609433	4.6316459	4.188785	3.8415007	4.1161991	3.5622521	3.7791165	3.5358829	3.9466965
Up	FGSG_09340	1.215451527	2.71E-06	8.262636	8.1651802	8.294837	8.5236891	8.3006433	8.7075894	9.2100825	9.2330424	9.1641848
Up	FGSG_09306	1.215315862	1.40E-10	7.7885291	7.8422497	7.6449538	7.8999074	7.9896083	7.7098367	8.1896647	8.1491527	8.1646712
Up	FGSG_00117	1.21367558	2.73E-07	6.7663496	6.4717791	6.9382912	7.1360243	6.9290147	7.2414868	7.02041	7.0294959	6.945698
Up	FGSG_00499	1.213474481	6.52E-10	8.2037235	8.0480285	8.2654724	7.9808337	7.8849234	7.9886991	7.919026	7.9785505	7.7877264
Up	FGSG_07963	1.213279538	2.28E-08	7.0511452	6.9347419	7.0799694	6.7228827	6.7909814	6.5607563	7.2029588	7.1372515	7.2033953

Up	FGSG_06833	1.212403855	5.51E-05	6.3971965	6.153105	6.5327046	6.7992472	6.8889735	6.6110389	7.5610519	7.4716926	7.58338
Up	FGSG_13755	1.210356541	0.0919084	2	2	2	3.1965524	2.8748957	3.4190665	4.3828727	3.8208439	4.7462732
Up	FGSG_05451	1.210221307	1.12E-08	6.5052304	6.4975416	6.4305761	6.9408247	6.7013421	7.0723383	6.8196775	6.59171	6.9604719
Up	FGSG_09727	1.21003401	3.84E-05	6.9303725	6.7541729	7.0108688	7.333838	7.3288419	7.2520883	6.8685409	6.8668223	6.8054483
Up	FGSG_12712	1.209841111	1.59E-06	5.9955706	5.906198	6.0025037	6.5302	6.314782	6.6754671	6.9158034	6.9578183	6.8054483
Up	FGSG_06644	1.209653909	1.73E-07	9.9179907	9.8831262	9.8699378	9.5214744	9.423465	9.5304035	9.8623503	9.8567896	9.8032633
Up	FGSG_01634	1.208464599	2.77E-06	7.4462553	7.356828	7.4499101	7.3693409	7.23613	7.4306909	7.6798917	7.7258501	7.5641633
Up	FGSG_02467	1.20696176	2.08E-08	6.2955384	6.3355781	6.1703363	6.1060709	6.1209783	6.0060392	5.947742	6.0176785	5.8079414
Up	FGSG_08417	1.206875326	1.24E-06	7.544488	7.6488882	7.3415168	8.1063738	8.081298	8.0446525	8.6093677	8.5214747	8.6296263
Up	FGSG_11795	1.206824833	0.0054143	2.8633281	2.4852148	3.1285604	4.3681625	4.2902531	4.3744304	4.935064	4.6740912	5.1065306
Up	FGSG_04788	1.206122767	2.31E-08	10.033009	9.9476282	10.031293	10.34167	10.314826	10.28332	10.722507	10.64588	10.731701
Up	FGSG_05003	1.205832887	1.96E-11	8.0825166	8.1077932	7.9702333	7.8030859	7.665466	7.8490285	7.8023135	7.7084846	7.8285634
Up	FGSG_13320	1.205344891	0.0105887	4.5644712	5.4053203	5.3911128	4.1048586	5.0403527	5.3515835	4.7032988	5.2066203	5.4374371
Up	FGSG_04582	1.204485323	1.79E-06	9.0407524	9.0104909	8.9859431	8.487219	8.4820546	8.4043958	8.644865	8.6768755	8.5439396
Up	FGSG_03527	1.201743439	0.0556484	2	2	2	2.5166584	2.6377651	2.3550396	2.7212202	3.1804273	2.7087823
Up	FGSG_10138	1.201522652	1.20E-07	8.6661957	8.5446165	8.6979646	8.4733014	8.4730545	8.3948599	8.4685463	8.3914885	8.4889546
Up	FGSG_12467	1.201515663	0.0474578	3.0172362	3.1370988	2.8407141	2.7805029	2.353841	3.0813961	2	2	2
Up	FGSG_06319	1.201449196	2.73E-07	6.5310205	6.4188315	6.5571458	6.4376679	6.1663515	6.594472	6.4645842	6.3443452	6.5164961
Up	FGSG_06115	1.201360486	1.79E-07	9.0362572	9.1388146	8.8342043	8.6913699	8.663103	8.6324905	8.7935525	8.7165054	8.8076666
Up	FGSG_10912	1.20060377	6.34E-05	4.7515947	4.9628272	4.4195794	3.8415007	3.8080253	3.8119585	4.1391495	4.0586965	4.1665008
Up	FGSG_11534	1.200512972	0.0212902	3.701719	4.2010532	2.8407141	3.0034737	3.2568854	2.6397336	2.8565633	2.3968278	3.18196
Up	FGSG_03079	1.200138843	2.59E-08	7.7938649	7.9394368	7.5385022	7.1477552	7.0907291	7.1194186	7.2651087	7.2851495	7.1783375
Up	FGSG_09978	1.199908322	7.42E-07	4.4062533	4.5354388	4.6184939	4.7835368	5.1346873	4.9955781	5.0240241	5.1562742	5.4374371
Up	FGSG_09479	1.198731359	0.0072318	3.6078986	2.8476671	4.0578819	3.5895922	3.5586152	3.5622521	3.7092973	2.9633478	4.1665008
Up	FGSG_01846	1.198718113	1.59E-06	5.6164066	5.349828	5.7713555	5.2212063	5.3855124	4.9442019	5.7181748	5.2066203	6.0481828
Up	FGSG_03339	1.198141741	0.0063072	2.6910183	3.1370988	2	3.0034737	3.4156241	2.3550396	3.2982451	3.1804273	3.3707558
Up	FGSG_09627	1.196912207	5.43E-05	10.675875	10.852881	10.853345	10.413883	10.567201	10.635781	10.945282	11.041355	11.11596
Up	FGSG_08971	1.196801148	7.88E-08	7.0863599	7.1771962	6.9005856	7.1002482	7.0907291	7.0236696	7.4742601	7.6368537	7.2157628
Up	FGSG_09977	1.196788558	1.62E-06	8.183539	8.0994054	8.181495	8.2601893	8.3701609	8.0446525	8.9977052	8.9412794	8.9879253
Up	FGSG_05873	1.196274421	1.64E-09	6.4249728	6.052414	6.650969	6.6587404	6.6543434	6.7219677	6.6806551	6.7185687	6.6860823
Up	FGSG_04883	1.195985854	4.78E-06	6.9400302	6.8965233	6.9005856	7.41859	7.5673956	7.1537472	7.8678707	7.7599663	7.9069251

Up	FGSG_06870	1.195006275	8.42E-07	5.065757	5.458757	4.4195794	5.0567294	4.9907667	5.0451875	5.4233949	5.4765776	5.3047937
Up	FGSG_06327	1.194377317	6.99E-05	3.7898088	3.5844678	3.9139052	4.2856211	4.3699906	4.1204343	3.8457121	3.6853877	3.9466965
Up	FGSG_11344	1.19381703	0.0741377	2	2	2	3.8981589	3.6887032	4.024755	2.5718533	2.9633478	2
Up	FGSG_07726	1.193406367	2.23E-05	4.7948149	4.6316459	5.0898194	5.3074061	4.9394155	5.6041695	4.6296145	4.9352342	4.265
Up	FGSG_07114	1.192234869	1.22E-08	10.013703	9.8609684	10.072146	10.215673	10.189936	10.153623	10.680934	10.641311	10.654305
Up	FGSG_06928	1.191702373	0.0218261	3.6078986	3.378055	3.7539507	2.5166584	2.353841	2.6397336	3.3903592	3.5358829	3.18196
Up	FGSG_06316	1.190171929	1.05E-05	5.167919	5.608128	4.4195794	5.3486486	4.9907667	5.5706824	5.4663562	5.4349486	5.4374371
Up	FGSG_10369	1.189895379	0.0299474	6.2651272	8.3030481	8.3863428	6.4184274	8.0989568	8.4279633	6.7518403	8.501448	8.4476067
Up	FGSG_09395	1.187973305	8.34E-10	6.4789709	6.4188315	6.4567939	6.7150192	6.776423	6.5607563	6.4751405	6.5726412	6.301259
Up	FGSG_13910	1.187850823	2.31E-08	8.0018186	8.1651802	7.7226391	7.8067902	7.8575207	7.662937	7.6475913	7.5711195	7.657785
Up	FGSG_07416	1.1855756	3.10E-10	7.6774476	7.7052501	7.6335064	7.7693122	7.5673956	7.8698285	7.4423369	7.0977802	7.6847264
Up	FGSG_07247	1.184999677	2.96E-06	4.5136357	4.7218364	4.3087924	4.2425091	4.4455509	3.9222766	5.0240241	4.8105634	5.1587087
Up	FGSG_06244	1.184567772	0.0212259	5.9580386	7.8014645	7.91457	5.6084049	7.4981042	7.2937323	5.2862562	6.8190773	7.0180995
Up	FGSG_06990	1.183145715	6.11E-07	4.6610646	4.8067189	4.4195794	5.4277566	5.179638	5.5706824	4.8402557	4.8742451	4.7462732
Up	FGSG_09850	1.18254031	3.49E-08	7.281305	7.1611664	7.3130965	7.5258627	7.4981042	7.4675471	7.6980295	7.7599663	7.5641633
Up	FGSG_05842	1.182144083	8.71E-07	9.5993826	9.5645544	9.5489415	10.285698	10.247808	10.237374	10.597035	10.556503	10.573723
Up	FGSG_06749	1.181765379	4.33E-11	9.1776677	9.1631643	9.106767	8.9291478	8.7587827	9.0026287	8.9866806	8.8688739	9.0340498
Up	FGSG_08866	1.181474556	3.28E-06	4.7948149	5.0349724	4.4195794	5.1056669	5.265544	4.8356119	5.6452659	5.4349486	5.7748904
Up	FGSG_00674	1.180999952	0.0517431	2.4953013	2.8476671	2	3.0034737	2.353841	3.4190665	2.5718533	2.7077187	2.3974876
Up	FGSG_10902	1.180197296	3.37E-06	4.9933948	5.1036809	4.7932764	4.1522165	4.0206066	4.210161	4.3828727	4.4416259	4.265
Up	FGSG_13953	1.179885583	0.0075924	2.6910183	2	3.1285604	2.6546039	2.8748957	2.3550396	2.4052168	2.3968278	2.3974876
Up	FGSG_00673	1.179787934	0.0088841	3.701719	4.2010532	2.8407141	4.5203003	4.8861689	3.9222766	4.028829	3.5358829	4.3572018
Up	FGSG_10197	1.179560107	0.0014712	4.2902308	4.069824	4.5224622	3.0034737	2.6377651	3.260088	3.9093688	4.0586965	3.6872775
Up	FGSG_12016	1.17915882	1.50E-08	5.0300296	5.1036809	4.8733193	5.1529987	5.0882909	5.1395644	5.1870071	5.1041074	5.2090653
Up	FGSG_06594	1.179156927	0.0265481	2.2688027	2.4852148	2.4807492	2.1935143	3.0784965	2.3550396	2.8565633	4.0586965	2.3974876
Up	FGSG_10109	1.179091551	2.50E-09	8.463492	8.2509868	8.6189019	8.7073122	8.7808316	8.5745405	8.8715365	8.8882831	8.8319719
Up	FGSG_02461	1.17809842	2.14E-10	11.477164	11.43013	11.441787	11.194279	11.108299	11.191581	11.40262	11.376373	11.363911
Up	FGSG_05539	1.177446035	3.49E-10	6.665171	6.5962238	6.650969	6.7071125	6.6057619	6.7219677	6.7518403	6.5533169	6.8694701
Up	FGSG_05413	1.177319496	9.76E-05	4.6135762	4.5354388	4.6184939	4.5559538	4.2902531	4.7181783	3.9093688	4.0586965	3.6872775
Up	FGSG_13640	1.176633881	4.02E-08	7.5758001	7.6603381	7.3967312	7.5392846	7.2883703	7.6787403	7.9980116	7.9929661	7.9593509
Up	FGSG_02623	1.176266427	3.37E-07	6.5563577	6.5477263	6.4825439	6.4753959	6.3545295	6.5086577	6.7255557	6.6655604	6.7212064

Up	FGSG_10411	1.176168006	0.0023705	2.6910183	2.4852148	2.8407141	2.7805029	3.0784965	2.3550396	2.4052168	2.3968278	2.3974876
Up	FGSG_12731	1.175722111	4.13E-11	7.8150128	7.9394368	7.5867862	7.6751814	7.6172639	7.65497	7.9869892	7.904229	8.0028193
Up	FGSG_00259	1.17558034	1.69E-08	9.2159333	9.1791721	9.1675588	9.3093709	9.2152512	9.3147394	9.6139516	9.5938177	9.5678887
Up	FGSG_07386	1.175150487	0.0023735	8.0737698	8.1408647	7.91457	9.1258303	9.0616273	9.1055957	9.8390424	9.7757361	9.8356778
Up	FGSG_09699	1.174380589	1.53E-05	8.8047878	8.8245413	8.6979646	9.4733406	9.5096084	9.3447662	10.120322	10.14355	10.030287
Up	FGSG_00776	1.173749797	3.30E-09	8.4261831	8.3175841	8.4464558	8.1093765	8.0989568	8.0385416	8.2213619	8.2182669	8.1582778
Up	FGSG_09959	1.173465416	3.98E-07	4.6135762	4.3213345	4.7932764	5.1988268	5.179638	5.1395644	4.8402557	4.9937494	4.6029775
Up	FGSG_03849	1.172806321	0.0065446	2.6910183	2.8476671	2.4807492	3.1032407	2.8748957	3.260088	2.4052168	2	2.7087823
Up	FGSG_04331	1.172283204	3.93E-07	4.6135762	4.8067189	4.3087924	4.5559538	4.51735	4.5218929	4.7032988	4.8105634	4.5256129
Up	FGSG_12909	1.171485285	0.0007338	3.7898088	4.069824	3.3684179	3.1965524	2.8748957	3.4190665	3.7092973	3.5358829	3.8228101
Up	FGSG_00336	1.170756495	9.80E-05	4.0965725	3.765011	4.3087924	4.871059	4.8861689	4.7780897	4.994976	5.1562742	4.7462732
Up	FGSG_10169	1.170552893	4.34E-08	7.8150128	7.8117694	7.7334032	7.8612426	7.9769307	7.6389028	8.2736986	8.330362	8.1454052
Up	FGSG_05052	1.169960682	1.24E-05	7.5758001	7.8622179	7.7440876	7.3026957	7.5331658	7.4119036	7.083861	7.3649028	7.0735134
Up	FGSG_13011	1.169664447	2.87E-08	5.4095206	4.9628272	5.6852725	5.5027514	5.5991203	5.3115993	5.5486216	5.0499834	5.8718551
Up	FGSG_01289	1.169526707	1.68E-05	6.7110174	7.2395919	6.4038729	7.0259191	7.203853	6.9860594	7.7597741	7.7344551	7.8604206
Up	FGSG_03162	1.169257952	0.0020618	3.872828	3.5844678	4.0578819	5.9805033	5.975612	5.9021734	5.0240241	4.9352342	5.0523944
Up	FGSG_09694	1.168351292	0.0001411	10.94169	10.903141	10.897084	10.906929	10.869416	10.85939	10.76076	10.702895	10.754132
Up	FGSG_07630	1.167767663	3.34E-05	6.8506908	6.8965233	6.7175374	6.5302	6.5891976	6.3792376	7.8838049	7.9194028	7.7794184
Up	FGSG_06177	1.166909367	2.61E-08	9.0827724	9.1591345	8.9120677	9.0806467	9.0251474	9.0550306	9.3018912	9.2360755	9.3010638
Up	FGSG_06355	1.166859414	1.72E-06	5.7096927	5.7859081	5.545677	6.2972625	6.0008798	6.4728499	6.6343121	6.5726412	6.6317388
Up	FGSG_05263	1.166300894	1.46E-09	6.9010051	6.9347419	6.7811692	6.9475516	6.9024444	6.9211113	7.2892379	7.4716926	7.0180995
Up	FGSG_04981	1.165811226	6.92E-06	6.3971965	6.2472245	6.5078422	6.8717719	6.9024444	6.7670162	7.5953256	7.6090478	7.5544581
Up	FGSG_10385	1.165571997	1.94E-06	5.5673995	5.6997548	5.3356788	6.0193342	5.6951271	6.2155469	6.354542	6.4733227	6.2052877
Up	FGSG_05522	1.165145936	0.0006079	7.3039203	7.4388982	7.063002	6.7615701	6.5554873	6.8669619	6.5668244	6.6834471	6.3692672
Up	FGSG_05488	1.164145112	3.67E-10	6.6416896	6.9157592	6.9005856	6.6001768	6.7909814	6.7962856	6.8443161	7.0571987	7.0460725
Up	FGSG_09392	1.163958302	2.66E-11	9.1735801	9.1264838	9.1395078	8.942679	8.8025486	9.0182396	9.1086461	9.0555672	9.1188085
Up	FGSG_08778	1.163584668	7.83E-06	5.167919	4.8067189	5.3911128	5.1295269	5.1346873	5.0451875	5.4873665	5.5947086	5.3047937
Up	FGSG_13404	1.163318409	1.79E-11	9.9216512	9.8584853	9.898912	9.5438428	9.4443371	9.554158	9.6736014	9.6832044	9.5965453
Up	FGSG_07896	1.162920585	1.18E-08	7.8150128	7.7052501	7.8467929	7.7578763	7.7423879	7.6865774	7.600156	7.5711195	7.5641633
Up	FGSG_10761	1.161340285	0.000135	11.346191	11.342223	11.263895	12.242435	12.225609	12.17197	12.15469	12.109441	12.133523
Up	FGSG_10822	1.160527541	5.69E-08	6.5181831	6.2472245	6.6735015	6.4376679	6.374	6.4174145	6.0781065	5.9891987	6.1024744

Up	FGSG_03750	1.160315042	1.65E-07	8.5946251	8.5872306	8.5228982	9.0465889	9.0003048	9.0057644	9.4722827	9.3440381	9.5287725
Up	FGSG_06797	1.159892244	0.0539845	2	2	2	2.1935143	2.353841	2.3550396	2.5718533	2.7077187	2.3974876
Up	FGSG_01531	1.159851193	0.0525891	2	2	2	2.7805029	2.6377651	2.8774004	2.9802913	2.7077187	3.18196
Up	FGSG_00519	1.159349911	1.95E-08	6.0139766	5.8672052	6.0719967	6.5743316	6.4856075	6.594472	6.4215692	6.2026947	6.555942
Up	FGSG_05609	1.158977079	1.02E-07	6.0853387	5.9811576	6.3206633	5.6252853	5.8421752	5.4283735	5.6452659	5.7038947	5.8079414
Up	FGSG_05386	1.158852305	0.0110108	3.0172362	3.1370988	2.8407141	3.1032407	2	3.6925011	3.4769433	3.8208439	2.964684
Up	FGSG_09396	1.15790545	7.95E-07	5.3235909	5.349828	5.2179801	5.8429557	5.5991203	5.9807654	6.1055435	5.9601454	6.1802631
Up	FGSG_00607	1.157718497	3.20E-10	6.2651272	6.2165286	6.232359	6.3989268	6.29449	6.4174145	6.5469478	6.2752577	6.7212064
Up	FGSG_01911	1.157682718	1.61E-09	6.629804	6.120316	6.9382912	6.6339322	6.4675956	6.7219677	6.6530283	6.4526114	6.7723395
Up	FGSG_07561	1.156715419	0.0006002	7.4188896	7.356828	7.3967312	8.5875291	8.6391638	8.446544	9.0050085	9.0137631	8.9290545
Up	FGSG_05369	1.156542092	0.0472068	2.4953013	2	2.8407141	4.0052074	4.0206066	3.9222766	4.5913113	4.6006874	4.5256129
Up	FGSG_00491	1.155789378	1.03E-07	10.300008	10.332863	10.178375	9.8640016	9.7742565	9.865085	9.90201	9.7398598	9.9894523
Up	FGSG_09907	1.155155157	2.64E-05	5.7541671	6.0172257	5.3356788	5.7689296	5.7851416	5.6688985	5.6821809	5.8053955	5.4790733
Up	FGSG_06834	1.154889795	0.0116603	2.6910183	2.4852148	2.8407141	2.8962916	2.6377651	3.0813961	2.4052168	2.3968278	2.3974876
Up	FGSG_05593	1.154885547	1.35E-07	8.1127192	8.0739456	8.0670189	7.6215076	7.4159539	7.7251372	7.2832433	7.3312612	7.1656435
Up	FGSG_06156	1.15448284	9.75E-11	7.7506158	7.7052501	7.7226391	7.5701251	7.4712372	7.5812149	7.7511136	7.7683708	7.6668215
Up	FGSG_07509	1.153903579	0.0009447	4.9172105	4.6316459	5.0898194	4.5559538	4.51735	4.5218929	4.965331	4.9937494	4.8766132
Up	FGSG_00176	1.153410476	7.12E-06	6.9873724	7.1611664	6.6956874	7.6506568	7.6495769	7.5643	6.4960241	6.2986564	6.6131596
Up	FGSG_12896	1.152167079	4.48E-07	6.653478	6.6660438	6.5571458	6.539135	6.29449	6.6754671	6.5267935	6.5138742	6.4963611
Up	FGSG_12132	1.151574401	0.079036	2.4953013	2.8476671	2	2.5166584	2.8748957	2	2.5718533	2	2.964684
Up	FGSG_12050	1.15123969	0.0353549	6.4522245	6.3355781	6.60482	6.2545022	5.975612	6.4908649	5.3100371	5.2066203	5.3945636
Up	FGSG_05714	1.150032821	1.52E-09	8.8387424	8.7514782	8.8391959	9.2037574	9.21255	9.1055957	9.3268756	9.230003	9.3551728
Up	FGSG_10147	1.149667272	2.32E-06	4.6610646	4.069824	5.0212003	4.5559538	4.2058494	4.8356119	4.9041484	4.9937494	4.7462732
Up	FGSG_07024	1.149626511	0.0007294	4.7070397	4.3213345	4.9491538	4.92659	5.179638	4.5218929	4.8402557	4.6006874	4.9961472
Up	FGSG_00898	1.149602015	6.32E-06	9.9167685	9.9049489	9.84534	10.641473	10.618097	10.580071	11.084934	11.076468	11.027525
Up	FGSG_01449	1.14897912	1.03E-08	7.3113806	7.3984466	7.1297061	7.6088325	7.3682091	7.740277	7.4261064	7.4716926	7.3110622
Up	FGSG_05293	1.148847606	4.85E-05	7.8564006	7.6488882	7.961104	7.0195487	6.8477891	7.0960705	6.7776545	6.8028047	6.7037513
Up	FGSG_00630	1.147503184	0.0194802	2.2688027	2.4852148	2	3.1965524	3.2568854	3.0813961	2.8565633	2.3968278	3.18196
Up	FGSG_11386	1.14741755	0.0006567	4.2285219	4.4323548	3.9139052	4.7220923	4.8861689	4.4500449	4.5115122	4.8742451	3.9466965
Up	FGSG_01478	1.147197392	5.38E-06	7.0776365	7.1119795	6.9567807	7.0067227	6.7468578	7.1537472	7.7597741	7.6909075	7.7626576
Up	FGSG_07154	1.146393832	0.0002255	7.4394623	7.2994005	7.5507256	7.2921638	7.3288419	7.1982839	7.8838049	7.8418859	7.929627

Up	FGSG_11752	1.146384936	0.042213	4.2902308	4.6316459	3.7539507	2.7805029	2.6377651	2.8774004	2.5718533	2.7077187	2.3974876
Up	FGSG_08707	1.145823071	0.0029379	2.4953013	2.4852148	2.4807492	3.3668146	3.8080253	2.6397336	3.2982451	3.5358829	2.964684
Up	FGSG_02737	1.145768924	0.0001333	5.065757	5.1692654	4.8733193	4.327482	4.3699906	4.210161	4.2902636	4.3549955	4.1665008
Up	FGSG_13397	1.145545239	0.07118	2.4953013	2.8476671	2	2.5166584	2.6377651	2.3550396	3.6359267	3.3690748	3.8228101
Up	FGSG_01103	1.145039874	8.18E-09	6.9108608	6.7962976	7.063002	7.1301229	7.1483996	7.1423949	7.436947	7.5614795	7.2522417
Up	FGSG_00323	1.144659937	4.89E-10	6.8196378	6.775389	6.7811692	7.0002667	7.1819267	6.6911344	7.1179419	7.1501722	7.0180995
Up	FGSG_05333	1.144272886	0.0828542	2.2688027	2	2.4807492	2.1935143	2	2.3550396	3.199846	3.5358829	2.7087823
Up	FGSG_04323	1.144214299	1.01E-07	7.3113806	7.19305	7.3415168	7.7269298	7.6967286	7.6708603	8.1072341	8.0561112	8.0927348
Up	FGSG_09275	1.141838149	2.59E-07	6.1026411	5.9811576	6.1382955	6.379159	6.6858453	5.874995	6.7946109	6.8190773	6.7037513
Up	FGSG_01873	1.141269968	1.03E-08	7.5253693	7.2547781	7.678761	7.9476643	7.8154152	7.9886991	8.1140046	8.1297141	8.0310872
Up	FGSG_10326	1.140782028	0.0221272	2.2688027	2.4852148	2	3.0034737	3.0784965	2.8774004	2.2167888	2	2.3974876
Up	FGSG_02547	1.14061317	8.74E-05	4.2285219	4.3213345	4.0578819	3.5895922	3.0784965	3.9222766	3.7092973	3.6853877	3.6872775
Up	FGSG_09916	1.140414118	5.91E-06	4.7070397	4.5354388	4.7932764	5.4277566	5.4962661	5.2704753	5.6638414	5.9304949	5.2577234
Up	FGSG_01290	1.140266675	9.70E-08	7.7395979	7.5538626	7.8268285	7.8251703	7.7793636	7.7847661	8.3095314	8.3132316	8.239261
Up	FGSG_03575	1.139602615	0.0004259	6.3831052	6.4188315	6.2623983	7.403991	7.3778853	7.3441517	7.2092957	7.2615289	7.0870405
Up	FGSG_06538	1.139563065	1.51E-08	6.5812576	6.5962238	6.4825439	6.7691847	6.5724409	6.8669619	6.2962044	6.4315984	6.075584
Up	FGSG_06066	1.139481114	9.52E-05	6.0321508	6.5228522	5.5937254	5.8429557	6.1438433	6.0793081	6.7691009	6.8510822	7.100442
Up	FGSG_10106	1.138995695	1.61E-05	5.7096927	5.6546687	5.6852725	5.2432439	4.9394155	5.4283735	6.235408	6.5533169	5.7410644
Up	FGSG_01281	1.138582329	2.90E-08	7.2888829	7.0610564	7.4102108	7.4801991	7.3486598	7.521124	7.4206555	7.2253567	7.5348496
Up	FGSG_08332	1.137856005	0.0001344	4.6610646	4.4323548	4.7932764	4.8990917	4.5857446	5.0931475	5.2620767	5.0499834	5.3945636
Up	FGSG_13502	1.137384398	2.20E-09	8.1423025	8.0909687	8.1089536	7.8999074	7.9832834	7.7175072	7.7769407	7.5806954	7.8915895
Up	FGSG_02901	1.136627363	6.39E-05	5.4370629	5.349828	5.4444954	5.6419705	5.8421752	5.3115993	6.1848562	5.9304949	6.3469521
Up	FGSG_06406	1.136411489	5.43E-09	6.2182735	6.4188315	5.891554	6.1417046	5.975612	6.2155469	6.2842483	6.4733227	5.991768
Up	FGSG_00369	1.136280321	3.58E-08	9.3685558	9.2261567	9.4182855	9.3365322	9.2656355	9.3197875	9.4788543	9.3412239	9.5435657
Up	FGSG_00033	1.135716847	7.85E-05	4.6135762	4.069824	4.9491538	5.2863342	5.5656438	4.8356119	5.7875758	5.4349486	6.0202512
Up	FGSG_08795	1.135668141	3.02E-06	5.3528065	5.510285	5.0898194	4.8133047	4.4455509	5.0451875	4.5519634	4.6740912	4.3572018
Up	FGSG_05709	1.135386966	7.72E-05	4.6610646	4.8868835	4.3087924	4.657914	3.8080253	5.1395644	4.807216	4.8742451	4.6764038
Up	FGSG_06057	1.134999046	2.87E-09	7.8916659	7.9300074	7.7756741	7.5303505	7.52448	7.4584209	7.8148345	7.8655814	7.7024119
Up	FGSG_05224	1.134571342	3.29E-07	7.2271151	7.1611664	7.20897	7.1937475	7.2572536	7.0359915	7.2092957	7.2375151	7.1137202
Up	FGSG_01699	1.133456132	4.34E-08	5.7321013	5.6546687	5.728956	6.1060709	5.8970396	6.2155469	6.2600348	5.837702	6.5363539
Up	FGSG_05071	1.132781863	0.0360342	2.2688027	2.4852148	2.4807492	2.5166584	2.353841	2.8774004	2.5718533	2.3968278	2.7087823

Up	FGSG_06347	1.131980126	1.74E-07	5.4370629	5.1692654	5.5937254	5.5740396	5.7257602	5.3115993	5.947742	5.7385279	6.075584
Up	FGSG_05061	1.131906694	0.0007707	3.5075501	3.1370988	3.7539507	3.2841938	3.0784965	3.4190665	3.8457121	4.3549955	2.964684
Up	FGSG_11406	1.131536846	0.0062413	2.8633281	2.4852148	3.1285604	3.7825262	3.9182279	3.5622521	4.2902636	3.9446664	4.5256129
Up	FGSG_05469	1.130322683	9.38E-08	7.6889487	7.5038947	7.8065839	7.2921638	7.225451	7.2730605	7.2711789	7.3198707	7.1656435
Up	FGSG_00464	1.128969332	9.93E-08	8.5976792	8.6169166	8.491538	8.2789793	8.0872083	8.3707408	8.414725	8.3750728	8.3887464
Up	FGSG_01609	1.127868802	7.63E-05	4.8367778	5.0349724	4.5224622	5.1760947	4.6510429	5.5012819	5.4663562	5.4349486	5.4374371
Up	FGSG_07382	1.127272811	7.14E-07	9.1044062	9.1429015	8.9769129	9.0084943	8.9231021	9.0057644	9.3018912	9.2481443	9.2894092
Up	FGSG_06258	1.126957969	7.99E-07	7.8306727	7.832161	7.7440876	8.1938425	8.133639	8.167343	8.6021623	8.5751565	8.5634257
Up	FGSG_10852	1.126583255	0.0212259	2.8633281	2.4852148	3.1285604	3.3668146	2.8748957	3.8119585	2.4052168	2.9633478	2
Up	FGSG_01339	1.125028425	4.36E-09	6.7222549	6.8572645	6.8017711	6.3691726	6.6858453	6.279127	6.8196775	7.1372515	6.7037513
Up	FGSG_13499	1.12483186	1.03E-08	6.7444707	6.8372271	6.5571458	6.7462194	6.6221382	6.7817251	7.2156048	7.249572	7.1137202
Up	FGSG_10172	1.124461613	4.93E-09	7.3697138	7.4120569	7.2394951	7.5213608	7.2883703	7.6469587	7.8718708	7.7084846	7.9593509
Up	FGSG_01335	1.124354745	0.006285	3.7898088	5.1036809	4.3087924	3.1965524	3.8080253	3.8119585	3.7791165	4.262829	3.9466965
Up	FGSG_13377	1.124277067	0.0009322	4.4609433	4.069824	4.7085304	3.7210377	3.0784965	4.1204343	4.4270393	4.5233477	4.265
Up	FGSG_00878	1.123829553	0.0014786	9.5978582	9.6124987	9.4961952	10.771795	10.789201	10.663843	11.448206	11.357	11.470967
Up	FGSG_02125	1.123764693	1.24E-06	7.112218	7.1285622	7.3967312	6.7842942	7.0059099	6.9860594	6.6897477	6.8190773	6.9004463
Up	FGSG_01618	1.122842148	1.44E-05	5.8799122	6.0172257	5.640225	5.3281747	5.7257602	4.6556708	5.1870071	4.9937494	5.3503768
Up	FGSG_02727	1.122562113	0.0012121	4.5136357	4.2010532	4.7932764	3.6568115	3.8080253	3.4190665	4.7032988	4.6740912	4.6764038
Up	FGSG_08368	1.122185696	0.014499	4.0965725	4.3213345	3.7539507	3.5895922	2.353841	4.210161	4.4270393	4.6740912	4.1665008
Up	FGSG_13239	1.121893999	4.66E-06	4.7515947	4.8067189	4.6184939	5.4277566	5.265544	5.5012819	5.0804226	4.262829	5.5589053
Up	FGSG_13412	1.121140018	3.57E-05	10.498515	10.46768	10.44386	11.140619	11.149837	11.042297	11.148555	11.075621	11.155016
Up	FGSG_12890	1.120726692	0.0005644	4.0965725	4.069824	4.6184939	4.4077273	4.5857446	4.3744304	3.9093688	4.6740912	3.5376798
Up	FGSG_09616	1.119528678	9.66E-07	7.3625498	7.3708347	7.2693877	7.0635623	7.0183365	7.0236696	7.7981156	7.6731136	7.852522
Up	FGSG_05396	1.119501901	6.72E-05	4.1640552	4.069824	4.188785	4.3681625	4.51735	4.1204343	4.8725557	4.7439404	4.9376179
Up	FGSG_08253	1.119012825	3.73E-06	4.6610646	4.5354388	4.7085304	4.92659	4.8308815	4.9442019	5.3100371	5.3023297	5.2577234
Up	FGSG_08794	1.117767204	0.035088	2.2688027	2.4852148	2	2.6546039	2.8748957	2.6397336	2.4052168	2	2.7087823
Up	FGSG_09470	1.11775519	0.007884	5.3235909	5.2319977	5.3356788	4.657914	4.6510429	4.5903319	5.1346989	4.9352342	5.2577234
Up	FGSG_07229	1.117673396	3.14E-06	6.9873724	6.9534782	6.9382912	7.4473522	7.387497	7.4213278	8.0694171	8.0213724	8.0519305
Up	FGSG_08661	1.117519648	3.54E-06	5.5921111	5.510285	5.5937254	5.8429557	5.5656438	6.0060392	6.2842483	6.3443452	6.1547968
Up	FGSG_06869	1.117248708	0.0005533	4.349408	4.3213345	4.3087924	4.4462359	4.6510429	4.1204343	5.2862562	5.3920827	5.1065306
Up	FGSG_05191	1.116963335	6.07E-07	9.2395884	9.2184314	9.1754742	9.4486578	9.3855967	9.4242559	10.038164	9.9631194	10.045956

Up	FGSG_13814	1.116611385	0.0778307	3.6078986	3.9254527	3.1285604	3.6568115	3.6887032	3.5622521	3.970335	3.6853877	4.1665008
Up	FGSG_00467	1.116225195	9.56E-06	6.9496238	7.0610564	6.7390613	7.3542322	7.2572536	7.3638362	7.7424007	7.7932944	7.6210621
Up	FGSG_09055	1.115987672	0.000735	2.8633281	2.4852148	3.1285604	3.3668146	2.6377651	3.8119585	3.5586239	3.1804273	3.8228101
Up	FGSG_07019	1.115856682	2.63E-10	8.0649697	8.0994054	7.9426701	8.1767679	8.0989568	8.167343	7.9534078	8.0629598	7.7626576
Up	FGSG_08510	1.115210685	0.0009756	5.9580386	5.8672052	5.9664603	7.0385763	6.9933753	6.9987054	7.2590128	7.3425624	7.100442
Up	FGSG_08652	1.114776008	0.0030687	3.2831234	3.5844678	3.3684179	2.7805029	2.6377651	2.8774004	2.8565633	2.3968278	3.18196
Up	FGSG_00793	1.114377163	1.64E-07	11.357046	11.448408	11.186693	10.74018	10.614009	10.782859	10.625152	10.613592	10.588064
Up	FGSG_10200	1.114341911	2.45E-05	10.618392	10.559211	10.591674	10.158837	10.128213	10.101814	10.790121	10.752456	10.761533
Up	FGSG_06139	1.113666779	8.92E-06	5.1346624	5.0349724	5.1553224	5.0316238	5.0882909	4.8909283	5.6638414	5.8693008	5.3503768
Up	FGSG_04358	1.112404321	0.0021305	4.0965725	3.765011	4.3087924	3.7210377	3.9182279	3.6925011	4.666927	4.5233477	4.8129147
Up	FGSG_07625	1.112131356	0.0044875	2.2688027	2	2.4807492	2.7805029	3.0784965	2.3550396	2.7212202	2.3968278	2.964684
Up	FGSG_09232	1.111969163	0.0003015	8.7260337	8.6631791	8.7034496	9.534937	9.5161951	9.465836	10.385992	10.328267	10.378528
Up	FGSG_04280	1.111559088	1.84E-05	5.8597011	5.906198	5.728956	6.4184274	6.5209705	6.2155469	6.8844677	6.986918	6.7037513
Up	FGSG_02937	1.111452601	3.27E-09	7.7284952	7.5902346	7.7756741	8.0821245	8.1106104	7.9631169	7.9228869	7.7767266	7.9956649
Up	FGSG_04386	1.109572324	0.0012399	4.2902308	3.378055	4.7932764	3.8981589	3.8080253	3.9222766	5.1346989	5.3023297	4.8766132
Up	FGSG_02576	1.108717824	1.12E-07	5.2633225	4.8067189	5.545677	5.1529987	4.9394155	5.2704753	5.4014247	5.2066203	5.5195414
Up	FGSG_05218	1.108591711	3.13E-06	7.2583295	7.0436746	7.3693882	6.8359652	6.8337955	6.7521558	7.2156048	7.2851495	7.0735134
Up	FGSG_01168	1.107774468	0.0002966	4.2902308	4.7218364	3.5740244	3.7210377	4.1161991	3.0813961	4.6296145	4.4416259	4.7462732
Up	FGSG_11129	1.107763512	0.0004891	9.8808658	9.9288153	9.7425274	10.72851	10.678068	10.691369	11.592447	11.576886	11.541444
Up	FGSG_09595	1.107749643	0.0007407	8.4330387	8.2280865	8.5352539	9.0197095	9.0251474	8.9252511	9.019505	9.0589969	8.9101536
Up	FGSG_00282	1.106903341	0.0001099	6.0139766	6.3638664	5.4444954	5.6084049	5.5656438	5.5706824	6.7518403	6.8190773	6.6131596
Up	FGSG_12282	1.106654501	0.0018991	2.8633281	2.8476671	2.8407141	3.1032407	3.4156241	2.6397336	3.0942412	2.9633478	3.18196
Up	FGSG_12936	1.105267534	0.0440463	2.8633281	2.8476671	2.8407141	2.7805029	3.0784965	2.3550396	2	2	2
Up	FGSG_02079	1.105216146	3.88E-07	6.8196378	6.8169075	6.7390613	7.333838	7.2146924	7.3638362	7.5610519	7.200728	7.7959868
Up	FGSG_09497	1.104711746	5.73E-07	8.8231703	8.7936805	8.7676921	9.0418817	8.9718357	9.0244369	9.4295	9.2541411	9.5262922
Up	FGSG_13569	1.104651243	0.0020099	4.9933948	4.8067189	5.0898194	5.674773	5.346621	5.874995	6.2721924	6.2986564	6.1802631
Up	FGSG_00261	1.104219227	8.49E-10	9.1022574	9.12235	8.9949171	8.6772744	8.5435304	8.7229135	8.9128904	8.7967559	8.9587901
Up	FGSG_09309	1.103487626	1.12E-06	6.0500989	5.8271292	6.1703363	6.5121623	6.4856075	6.4546071	6.7861576	6.6474492	6.8537291
Up	FGSG_09576	1.103331229	4.42E-06	6.310507	6.5228522	6.0719967	6.828696	6.8053944	6.7670162	7.104406	6.9578183	7.1783375
Up	FGSG_05070	1.10307081	0.0001081	5.3528065	5.349828	5.3356788	5.1988268	4.8308815	5.4652882	5.6073815	5.6320297	5.5195414
Up	FGSG_09238	1.10201661	2.01E-09	7.8816778	7.9765496	7.6898562	7.6003202	7.5417997	7.5727822	7.7070136	7.5711195	7.7710623

Up	FGSG_09738	1.101508353	9.30E-06	7.0150495	7.2846792	6.5811798	7.2708663	7.2780725	7.1761874	7.2651087	7.249572	7.2157628
Up	FGSG_07139	1.101488438	4.00E-09	6.7110174	6.7326402	6.60482	7.153585	7.1140747	7.1194186	7.1773293	7.0434138	7.2401843
Up	FGSG_10217	1.10125027	2.20E-05	6.4789709	6.1851653	6.6735015	6.4376679	6.3932112	6.4361306	6.6530283	6.3666582	6.8537291
Up	FGSG_08492	1.09984791	1.30E-09	8.0912106	8.1651802	7.9239976	8.1390646	8.0872083	8.1043792	8.3842444	8.4448964	8.257321
Up	FGSG_09481	1.098680174	1.16E-05	6.7222549	6.7326402	6.8421126	6.7842942	6.9551045	6.6436126	7.1446385	7.3198707	7.0870405
Up	FGSG_02126	1.098574796	5.02E-05	5.9955706	5.9441646	5.9664603	7.0002667	6.8477891	7.0603242	7.1837795	7.124214	7.1783375
Up	FGSG_08651	1.098248718	3.62E-05	5.9580386	6.120316	5.6852725	6.7615701	6.8616483	6.5607563	7.3129702	7.3198707	7.2401843
Up	FGSG_05730	1.098037184	1.14E-07	7.550805	7.4652492	7.5507256	7.3793261	7.3486598	7.3241948	7.6753214	7.5122892	7.7626576
Up	FGSG_10525	1.097659442	0.010691	3.3996963	3.1370988	3.5740244	3.1965524	3.2568854	3.260088	2.5718533	2	2.964684
Up	FGSG_05359	1.097530024	1.16E-05	4.9558055	5.1692654	4.8733193	5.658465	5.7851416	5.7002055	5.9925186	6.0998924	5.9330571
Up	FGSG_01479	1.097409005	0.0115976	3.2831234	3.5844678	2.8407141	4.1048586	3.6887032	4.3744304	5.1610901	5.1041074	5.1587087
Up	FGSG_11805	1.097282226	1.08E-05	6.5052304	6.7326402	6.1382955	7.2273045	7.2467305	7.1194186	7.5904789	7.6909075	7.411249
Up	FGSG_13131	1.096692236	8.55E-06	4.2285219	4.4323548	3.9139052	4.5907474	4.2058494	4.8356119	4.0850435	3.9446664	4.1665008
Up	FGSG_07011	1.09639115	1.28E-07	7.7284952	7.832161	7.5385022	7.0635623	7.0907291	6.9474424	7.3011528	7.1756715	7.3564507
Up	FGSG_00868	1.096267774	0.0144207	4.7070397	4.5354388	4.7932764	4.1980691	4.4455509	3.8119585	5.3791148	5.2552685	5.4374371
Up	FGSG_09666	1.095760302	2.20E-05	4.8775546	5.0349724	4.6184939	5.2432439	5.5313719	4.7780897	5.2124668	5.3920827	4.9376179
Up	FGSG_00265	1.095478525	0.0007775	3.3996963	3.765011	2.8407141	3.9526758	3.9182279	3.9222766	3.8457121	2.9633478	4.3572018
Up	FGSG_01337	1.094270891	2.27E-06	4.6135762	4.8067189	4.8733193	4.5203003	4.51735	5.0931475	5.0240241	4.9352342	5.3047937
Up	FGSG_07196	1.093585936	2.76E-10	7.4050095	7.2846792	7.436798	7.5744776	7.6495769	7.4024174	7.6193177	7.4404773	7.7198832
Up	FGSG_00482	1.093551189	0.0005624	4.0965725	4.2010532	3.9139052	3.7825262	3.4156241	4.024755	4.1391495	3.8208439	4.3572018
Up	FGSG_12849	1.093265573	0.0001775	4.6610646	4.3213345	4.8733193	4.953574	4.8861689	4.9442019	5.7181748	5.9891987	5.3047937
Up	FGSG_04806	1.092758407	0.0822007	5.4095206	7.0952041	7.3415168	5.2649499	7.3188299	7.3342078	5.5284909	7.0012507	7.2280252
Up	FGSG_07455	1.092658986	0.0007441	6.6996916	6.775389	6.5327046	6.6831292	6.5383322	6.7371407	7.1246625	7.0154424	7.1656435
Up	FGSG_09618	1.091210707	1.99E-06	7.7284952	7.6021576	7.7652219	8.3517838	8.2168632	8.3948599	8.742035	8.6724042	8.7450493
Up	FGSG_06799	1.090630892	1.43E-05	8.3521613	8.3462239	8.2728698	7.484831	7.4159539	7.6308015	7.6049703	7.5222624	7.6758017
Up	FGSG_04003	1.089870313	5.09E-08	7.606447	7.5165498	7.6563111	7.5831433	7.6090713	7.4945842	7.0347533	7.0571987	6.9604719
Up	FGSG_13098	1.089654287	0.0001954	3.7898088	3.765011	3.7539507	3.5895922	3.8080253	3.260088	4.1391495	4.3549955	3.8228101
Up	FGSG_05058	1.089597698	0.0867787	2	2	2	2.7805029	3.0784965	2.3550396	2.2167888	2.3968278	2.964684
Up	FGSG_09729	1.089595788	0.0018874	5.3235909	5.1692654	5.5937254	6.3989268	6.5383322	6.2582434	6.7077628	6.61053	6.8378143
Up	FGSG_13934	1.088994785	1.43E-09	7.3261865	7.19305	7.3967312	7.0195487	7.0059099	6.9474424	7.3930873	7.2130949	7.4948146
Up	FGSG_12974	1.088841069	3.60E-08	6.0853387	5.8672052	6.2016809	6.2326372	6.2318429	6.1490351	6.6057745	6.6655604	6.4759412

Up	FGSG_01624	1.087587735	6.16E-08	8.6278696	8.556921	8.6130853	8.598256	8.5392251	8.5703107	9.0445286	9.0066764	9.0164836
Up	FGSG_09353	1.087582248	0.0054271	2.4953013	2.8476671	2	2.7805029	3.0784965	2.3550396	2.9802913	2.9633478	2.964684
Up	FGSG_06313	1.086528935	5.28E-05	8.1169827	8.1651802	7.9793053	7.0195487	7.0789132	6.8669619	6.9235321	6.9281194	6.8537291
Up	FGSG_20590	1.085550107	0.0362111	2	2	2	2.7805029	2.353841	3.0813961	2.7212202	2.3968278	2.964684
Up	FGSG_03366	1.084319176	0.0008953	8.0649697	8.0653582	7.9883205	8.5810544	8.5262315	8.548974	8.4711854	8.3528906	8.5192059
Up	FGSG_04916	1.084097101	3.20E-05	10.577983	10.523606	10.546611	10.057539	9.9993685	10.028167	11.22247	11.193431	11.185204
Up	FGSG_12370	1.084090558	0.0014912	4.6610646	4.2010532	4.9491538	5.6419705	5.8139402	5.3515835	5.4663562	5.5563964	5.3047937
Up	FGSG_01334	1.083794286	5.98E-05	6.5437448	6.6198745	6.7811692	6.3691726	6.5209705	6.2997126	5.2620767	5.5947086	5.1065306
Up	FGSG_10528	1.083716784	4.55E-08	6.9873724	6.9902361	6.9005856	6.9741499	7.0428732	6.8107005	7.0769471	7.2253567	6.8378143
Up	FGSG_06496	1.08319098	1.88E-08	8.3485399	8.3881451	8.2202735	8.3618909	8.290433	8.3462116	8.1832408	8.1872612	8.1127123
Up	FGSG_02539	1.082004127	2.68E-05	6.1533367	6.2165286	6.0025037	6.7462194	6.7013421	6.7066335	7.1512362	7.1110577	7.1268773
Up	FGSG_00508	1.081084475	1.74E-06	8.2664793	8.2433937	8.204887	8.2601893	8.1393391	8.2907328	8.7787157	8.703416	8.7870947
Up	FGSG_05405	1.080089572	2.23E-07	5.5166689	5.2921154	5.640225	5.1760947	5.306652	4.9442019	5.0240241	5.1041074	4.8766132
Up	FGSG_14037	1.079989694	1.11E-06	6.8506908	6.7541729	6.8618681	7.0002667	7.1140747	6.7817251	7.6707365	7.6368537	7.6395404
Up	FGSG_11755	1.079957339	4.00E-06	4.2902308	3.765011	4.6184939	4.4837433	3.9182279	4.8356119	4.7032988	4.7439404	4.6029775
Up	FGSG_06694	1.079126215	3.25E-08	5.4370629	5.7434744	4.9491538	5.3074061	5.2232303	5.3115993	5.107816	4.9937494	5.1587087
Up	FGSG_11171	1.079100062	0.0039752	3.5075501	3.378055	3.5740244	3.444959	3.2568854	3.5622521	3.7791165	3.9446664	3.5376798
Up	FGSG_03282	1.07755872	4.08E-09	7.6541668	7.6257121	7.5986089	7.6629712	7.489204	7.740277	8.0554159	7.9418683	8.0994248
Up	FGSG_00353	1.077403639	6.32E-09	8.7781141	8.7244608	8.7465942	8.6152549	8.6066141	8.5360188	8.2182233	8.2486203	8.1193104
Up	FGSG_05085	1.076618238	0.0017155	6.4656596	6.5962238	6.2918249	7.6588781	7.6254103	7.6062213	7.7597741	7.8339001	7.6117334
Up	FGSG_06730	1.076451415	2.72E-08	7.9597112	7.8522684	7.9793053	8.1767679	8.1786218	8.0867194	8.3271195	8.3189643	8.2692367
Up	FGSG_07093	1.076061543	0.0021781	3.9513288	4.2010532	3.5740244	3.5895922	4.0206066	2.8774004	4.1391495	4.4416259	3.6872775
Up	FGSG_00430	1.074371753	1.28E-07	8.5946251	8.5070607	8.5954933	8.0912658	8.1450169	7.9501537	7.9112731	7.8577261	7.9069251
Up	FGSG_02090	1.073199315	7.65E-06	7.3768424	7.342684	7.3273766	7.1419016	7.0059099	7.187278	7.4951564	7.7344551	7.1268773
Up	FGSG_07126	1.072991396	0.0002117	4.2902308	3.765011	4.6184939	3.5895922	3.5586152	3.5622521	4.0850435	3.6853877	4.3572018
Up	FGSG_09687	1.072173172	1.39E-07	7.2428067	6.9902361	7.3831245	7.1993948	7.0428732	7.2626125	7.7114847	7.5711195	7.7794184
Up	FGSG_09911	1.071985605	0.0008258	5.6402997	5.9441646	5.1553224	4.4462359	3.9182279	4.7780897	5.2124668	4.9352342	5.3945636
Up	FGSG_08140	1.071079139	0.0841438	3.0172362	2.8476671	3.1285604	2.6546039	2.8748957	2.3550396	2	2	2
Up	FGSG_07301	1.070943575	0.0283517	2.8633281	3.378055	2	3.5190873	3.9182279	3.4190665	4.4270393	4.5233477	4.4438634
Up	FGSG_01534	1.070298466	5.82E-06	8.1084431	7.9394368	8.1971317	8.2896076	8.2593613	8.2383766	9.0356414	9.0895019	8.9290545
Up	FGSG_10771	1.070096721	3.12E-06	5.0300296	4.8868835	5.0898194	5.3486486	5.423383	5.1845342	5.4233949	5.4349486	5.3503768

Up	FGSG_06145	1.069365238	0.0244277	3.3996963	3.9254527	2.4807492	3.9526758	3.5586152	4.210161	4.3373111	4.7439404	3.6872775
Up	FGSG_08354	1.069225618	4.17E-12	13.31827	13.273636	13.277155	13.33513	13.32249	13.259366	13.298504	13.271544	13.259054
Up	FGSG_00690	1.068781395	2.77E-09	6.665171	6.6198745	6.628079	7.0067227	7.0789132	6.8391054	6.9690546	6.9578183	6.9156884
Up	FGSG_12837	1.067797146	0.0009782	3.1562965	2.4852148	3.5740244	3.2841938	3.6887032	2.6397336	3.199846	3.1804273	3.18196
Up	FGSG_05924	1.067253586	0.0003269	4.0257777	4.2010532	3.7539507	4.0558932	4.2058494	3.8119585	4.6296145	4.262829	4.8766132
Up	FGSG_06574	1.067208398	5.51E-06	8.1916467	8.0037709	8.2802294	7.7844204	7.8366215	7.6389028	8.2947091	8.3246744	8.2024481
Up	FGSG_05410	1.067205137	0.0002397	8.9858628	8.9228887	8.9632608	9.2079657	9.2206385	9.1055957	9.7286785	9.7505046	9.6384902
Up	FGSG_08871	1.066374759	4.75E-09	10.08399	10.172188	10.162533	9.6528202	9.7314985	9.8771207	9.4563875	9.5651614	9.629274
Up	FGSG_12711	1.065894209	0.0001145	4.7070397	4.3213345	4.9491538	4.5907474	4.7135134	4.3744304	4.5115122	4.6006874	4.3572018
Up	FGSG_01887	1.06528228	2.20E-08	8.8361586	8.9604242	8.9308942	8.7444837	8.7698493	8.8504805	8.2976857	8.4237699	8.3330697
Up	FGSG_06440	1.0648104	1.52E-05	7.4257798	7.4782464	7.2841049	8.0323679	8.0208235	7.9566499	8.5581569	8.5558662	8.4940407
Up	FGSG_10348	1.063806749	7.50E-09	8.4534121	8.5991784	8.1971317	8.1213252	8.1786218	7.9695551	8.2150779	8.1033819	8.257321
Up	FGSG_04554	1.063727818	2.82E-06	10.642252	10.571392	10.626738	11.117317	11.070759	11.076252	11.537476	11.48107	11.527869
Up	FGSG_01536	1.063662999	2.11E-05	8.0201456	8.0824823	7.8664849	7.6629712	7.7866463	7.4584209	8.5076343	8.5214747	8.4476067
Up	FGSG_00375	1.062530955	0.0790468	5.2322171	7.6716978	7.6219674	6.0571473	7.9896083	8.0748249	6.6987834	8.8292542	8.723558
Up	FGSG_06006	1.062313352	1.48E-10	7.3553501	7.2087314	7.4102108	7.4801991	7.2985951	7.5727822	7.4151838	7.2733875	7.4846298
Up	FGSG_04482	1.061888969	5.85E-09	6.7110174	6.9347419	6.3489364	6.8717719	6.7909814	6.8669619	7.0131845	7.0012507	6.9604719
Up	FGSG_10232	1.061879474	2.05E-11	10.310284	10.376905	10.430692	10.552712	10.632315	10.621543	10.530904	10.508394	10.642902
Up	FGSG_08688	1.061749937	2.36E-05	6.3253218	6.5962238	6.8017711	6.7071125	6.9290147	6.7371407	6.9912882	7.2375151	6.9156884
Up	FGSG_09776	1.059983705	1.22E-08	7.9832558	8.0824823	7.7860511	7.4615211	7.4346181	7.4024174	7.4951564	7.5122892	7.411249
Up	FGSG_00904	1.059772999	5.81E-05	6.5935482	6.4455482	6.650969	6.4753959	6.3347926	6.5262338	6.6897477	6.61053	6.7037513
Up	FGSG_03647	1.059673883	0.0049848	2.2688027	2.4852148	2	2.8962916	3.0784965	2.6397336	2.4052168	2.3968278	2.3974876
Up	FGSG_12347	1.058784456	0.0072116	4.8367778	5.2921154	4.0578819	4.8133047	4.8861689	4.6556708	5.7002901	6.0998924	5.0523944
Up	FGSG_08925	1.058094523	0.0143569	2.8633281	3.1370988	2.4807492	2.7805029	2.8748957	2.6397336	3.7092973	3.1804273	4.0607807
Up	FGSG_06293	1.057962394	0.0003321	3.9513288	4.2010532	3.5740244	3.2841938	3.4156241	3.0813961	3.199846	2.9633478	3.3707558
Up	FGSG_11025	1.05781433	0.010175	4.2902308	4.069824	4.4195794	4.5203003	4.5857446	4.4500449	3.7791165	4.1643702	3.18196
Up	FGSG_01036	1.057301664	0.0001291	4.9172105	5.0349724	4.7085304	5.4468739	5.7851416	4.8909283	5.947742	5.7038947	6.1024744
Up	FGSG_06544	1.057263215	1.65E-06	9.1404552	9.5615043	9.6715424	9.4814752	9.7650685	9.8702555	9.9763446	10.267308	10.35076
Up	FGSG_09135	1.056915597	1.13E-07	7.2112509	7.1611664	7.177785	7.2162053	6.9807308	7.3638362	7.4206555	7.4192855	7.3675781
Up	FGSG_01214	1.056879152	0.0003323	6.5688614	6.7541729	6.2623983	6.1649807	6.0257126	6.2155469	6.6343121	6.7011148	6.4963611
Up	FGSG_06792	1.05573895	0.0106408	5.6869305	5.510285	5.7713555	7.3990916	7.2572536	7.5123315	7.7769407	7.6550976	7.8682762

Up	FGSG_08415	1.054740925	8.19E-05	5.2937714	5.510285	5.2780294	5.9540229	5.9498937	6.0060392	6.7518403	6.735814	6.7723395
Up	FGSG_07671	1.054484286	0.013721	8.0516681	8.0037709	8.0150332	9.3979879	9.3927731	9.3147394	9.6139516	9.6403484	9.5262922
Up	FGSG_03789	1.054459545	0.0335903	2.6910183	3.1370988	2	3.6568115	3.5586152	3.6925011	4.5115122	4.3549955	4.6029775
Up	FGSG_10836	1.053414102	1.95E-05	5.4370629	5.7859081	5.0898194	5.3281747	5.179638	5.3904893	5.5684753	5.5563964	5.5589053
Up	FGSG_00587	1.053162206	1.08E-07	6.7771658	6.8965233	6.5571458	6.9135989	6.9290147	6.8107005	7.3592946	7.1501722	7.4948146
Up	FGSG_01287	1.052914873	1.72E-07	7.3113806	7.1771962	7.3555198	7.403991	7.2677006	7.4492367	6.8361499	6.8351685	6.7723395
Up	FGSG_11004	1.052806416	0.0710251	2.6910183	3.1370988	2	2.7805029	2.8748957	2.6397336	2.4052168	2.3968278	2.3974876
Up	FGSG_05595	1.05269528	1.42E-08	6.1197383	6.052414	6.1055269	6.032049	5.7851416	6.1715481	5.6821809	5.7723491	5.5195414
Up	FGSG_12030	1.052685537	2.92E-06	5.4640893	5.349828	5.4959731	6.2104357	6.3932112	5.9021734	5.9777469	6.3443452	5.3945636
Up	FGSG_05909	1.052652473	1.60E-06	6.860895	6.8965233	6.7390613	6.691168	6.7318449	6.5607563	6.0918902	6.1262878	5.991768
Up	FGSG_08773	1.052429628	2.28E-05	11.794784	11.737689	11.766579	11.799489	11.810527	11.698371	12.713271	12.692589	12.668305
Up	FGSG_04500	1.052325533	0.000948	5.8597011	6.120316	5.4444954	5.9540229	5.9498937	5.874995	5.9777469	5.7723491	6.1024744
Up	FGSG_12672	1.051344606	0.0005128	5.1346624	5.2319977	4.9491538	5.1529987	5.265544	4.9442019	5.107816	4.9937494	5.1587087
Up	FGSG_12908	1.050935936	0.0563855	4.8775546	5.0349724	4.6184939	3.3668146	2.6377651	3.8119585	5.2862562	5.4349486	5.0523944
Up	FGSG_09228	1.05004508	0.0004431	3.701719	3.5844678	3.7539507	3.7210377	3.9182279	3.4190665	4.2902636	4.1643702	4.3572018
Up	FGSG_05074	1.049706709	4.03E-08	5.6164066	5.458757	5.6852725	5.9935634	6.0501253	5.8472948	6.0071405	5.9002223	6.0481828
Up	FGSG_05645	1.048692979	8.18E-06	6.9591539	7.3284	6.3489364	6.9675462	6.9679745	6.8806909	7.2528911	7.124214	7.3110622
Up	FGSG_01607	1.047737899	0.0170989	2.2688027	2	2.4807492	2.1935143	2.8748957	2.8774004	2.7212202	2.9633478	2.3974876
Up	FGSG_09784	1.04586635	5.83E-05	3.9513288	3.5844678	4.188785	4.2856211	4.3699906	4.1204343	4.807216	4.9352342	4.6029775
Up	FGSG_02412	1.045311467	0.0396463	2	2	2	2.6546039	3.0784965	2.3550396	2.5718533	2.3968278	2.964684
Up	FGSG_09631	1.044618274	0.0001011	7.3697138	7.5038947	7.177785	6.9542473	7.0789132	6.8942906	7.4206555	7.4716926	7.3564507
Up	FGSG_00064	1.043227766	0.0037491	3.6078986	3.5844678	3.5740244	3.5895922	3.5586152	3.5622521	4.965331	5.1041074	4.7462732
Up	FGSG_13310	1.041541319	9.29E-06	8.5823437	8.6286221	8.4464558	9.0244894	8.9718357	8.9900172	9.6551519	9.5651614	9.6770156
Up	FGSG_00596	1.041053908	6.95E-05	8.2432646	8.3175841	8.0755039	8.366918	8.290433	8.3560734	9.112032	9.1291972	9.027049
Up	FGSG_07927	1.039457798	0.0044355	3.1562965	3.1370988	3.1285604	4.0052074	3.8080253	4.1204343	4.1912995	3.9446664	4.3572018
Up	FGSG_08757	1.039135619	0.0008531	6.3545028	6.4188315	6.2016809	7.0385763	6.8753756	7.1077918	7.0630192	7.0843795	6.975096
Up	FGSG_04090	1.038553764	3.26E-07	7.6600224	7.5782122	7.6563111	7.8288184	7.7121095	7.8559952	8.2493059	8.3189643	8.1060838
Up	FGSG_08970	1.037630246	4.32E-08	8.3593769	8.4685011	8.1497038	8.3467036	8.2060401	8.3948599	8.8635233	8.8610365	8.799473
Up	FGSG_01136	1.037411678	6.19E-06	6.1533367	5.8672052	6.3206633	6.40871	6.1885139	6.5262338	6.8605111	6.61053	7.0180995
Up	FGSG_07568	1.036914187	0.0013893	4.2285219	4.3213345	4.0578819	4.327482	3.9182279	4.5903319	5.5080752	5.5170389	5.4374371
Up	FGSG_10093	1.036598251	0.0187453	3.872828	4.2010532	3.3684179	3.5895922	3.8080253	3.260088	3.8457121	3.3690748	4.1665008

Up	FGSG_08493	1.036480953	8.03E-08	7.2349822	7.19305	7.1934618	7.6088325	7.5331658	7.5979339	7.7812006	7.6731136	7.8204879
Up	FGSG_03061	1.036314613	0.0011897	10.512337	10.499903	10.438936	11.295629	11.253996	11.252907	12.036272	12.006184	12.001799
Up	FGSG_07947	1.035513647	0.0003419	4.4062533	4.6316459	4.0578819	5.7068462	5.7257602	5.6041695	5.6638414	5.6320297	5.6345505
Up	FGSG_04279	1.035028329	1.18E-08	8.3593769	8.3391171	8.3377927	8.0790645	8.1786218	7.8835305	7.8517585	7.6997228	7.929627
Up	FGSG_00189	1.034922267	1.97E-05	7.8766576	7.8220012	7.8467929	7.2162053	7.3970452	6.9077633	6.8524363	6.59171	7.0180995
Up	FGSG_12839	1.034550773	3.97E-10	8.7176359	8.8194435	8.5166805	8.480277	8.3555437	8.514165	8.3036207	8.3805655	8.1518558
Up	FGSG_03419	1.034085276	0.0090695	3.2831234	2.8476671	3.5740244	3.7825262	3.6887032	3.8119585	3.7092973	3.9446664	3.3707558
Up	FGSG_12901	1.034033898	8.69E-07	8.9141771	8.8646883	8.8785183	8.9644	8.9296958	8.9119415	9.3840101	9.3883335	9.3155009
Up	FGSG_00423	1.033861975	0.0004309	4.9558055	4.6316459	5.1553224	4.7835368	4.7135134	4.7780897	4.1912995	4.7439404	3.18196
Up	FGSG_12183	1.033803357	9.17E-11	8.5096218	8.4355616	8.4978648	8.6320559	8.6189064	8.5575466	8.7680237	8.6813329	8.7870947
Up	FGSG_04117	1.033501046	8.59E-06	8.0382427	8.1408647	7.8467929	8.2520609	8.2114618	8.2222919	8.5631132	8.4811393	8.5778695
Up	FGSG_00136	1.033286584	1.53E-05	7.0950308	7.0082698	7.0967395	7.7540441	7.8295873	7.5812149	8.1735511	8.2365555	8.0380685
Up	FGSG_06955	1.03252537	5.35E-06	9.8933472	9.8535062	9.8478188	9.2107644	9.1461564	9.1879631	8.9187025	8.8049494	8.9624644
Up	FGSG_12862	1.032080799	0.0009871	10.437607	10.454587	10.333225	11.225404	11.173642	11.190203	12.204905	12.168183	12.176074
Up	FGSG_05434	1.031463845	1.34E-06	6.9010051	6.8572645	6.8813568	7.2546844	7.1708374	7.2834334	7.1643417	7.3312612	7.0180995
Up	FGSG_09490	1.03124708	1.94E-05	5.4095206	5.1692654	5.545677	5.5209064	5.3855124	5.5706824	6.1457448	6.3216817	5.8718551
Up	FGSG_08746	1.031136902	0.0004236	7.112218	7.0952041	7.113317	8.2329147	8.2697931	8.1043792	8.7550879	8.6452815	8.8035757
Up	FGSG_13911	1.030784974	1.62E-05	6.3253218	6.6198745	5.85259	6.60869	6.5724409	6.5607563	6.6987834	6.8668223	6.4342132
Up	FGSG_07152	1.029959393	0.001877	11.28309	11.346659	11.309567	10.716253	10.719564	10.814497	10.494397	10.509648	10.578519
Up	FGSG_12687	1.029332969	0.0273962	2.4953013	2.8476671	2.4807492	3.3668146	3.6887032	2.8774004	4.1391495	4.262829	4.9961472
Up	FGSG_05411	1.028500939	2.76E-05	7.7116792	7.8220012	7.5011983	7.7308346	7.6967286	7.6787403	8.1072341	7.9566533	8.1836833
Up	FGSG_11918	1.028044847	0.0015352	5.4095206	5.6546687	5.0212003	4.4077273	4.2058494	4.5903319	4.666927	4.7439404	4.6764038
Up	FGSG_08516	1.028029633	9.03E-06	7.2964211	7.2087314	7.2986735	7.892954	7.8295873	7.8698285	8.372999	8.4448964	8.2270941
Up	FGSG_08848	1.027758148	0.0350587	2.4953013	2	2.8407141	2.6546039	2.8748957	2.3550396	2.4052168	2	2.7087823
Up	FGSG_06256	1.026836931	2.92E-05	6.2182735	6.306724	6.0376686	6.1533896	6.0741317	6.1490351	6.8923657	6.6474492	7.0460725
Up	FGSG_02798	1.025781165	0.0002952	6.1197383	5.6997548	6.3766661	6.1180468	6.1885139	5.9550408	6.3772321	6.4526114	6.2298857
Up	FGSG_06062	1.025473326	9.35E-06	11.247559	11.179666	11.229142	10.757032	10.765439	10.658872	10.256589	10.265825	10.17981
Up	FGSG_10871	1.025176234	7.28E-05	3.872828	3.5844678	4.0578819	4.0052074	3.8080253	4.1204343	4.5913113	4.6006874	4.5256129
Up	FGSG_06362	1.025035558	2.27E-05	4.349408	4.3213345	4.3087924	4.1048586	3.8080253	4.2946324	4.2416299	4.4416259	3.9466965
Up	FGSG_08974	1.024845746	0.0020728	3.872828	3.765011	3.9139052	3.444959	2.8748957	3.8119585	3.3903592	3.5358829	3.18196
Up	FGSG_12840	1.024739728	6.29E-08	7.8202516	7.8622179	7.6898562	7.8612426	7.8505879	7.7847661	8.0867296	8.123176	7.9812488

Up	FGSG_04923	1.024196319	2.45E-09	7.5881371	7.5165498	7.5867862	7.9205685	7.8849234	7.8835305	7.7025285	7.6550976	7.6847264
Up	FGSG_08663	1.023963803	2.52E-09	11.103521	11.01328	11.106161	11.228515	11.239474	11.127531	11.498229	11.453972	11.476117
Up	FGSG_06227	1.023398009	2.03E-08	6.438663	6.3638664	6.4305761	6.5302	6.1885139	6.7371407	6.7691009	6.9578183	6.4759412
Up	FGSG_06560	1.023096119	3.69E-05	9.8311296	9.8079037	9.7689256	10.494186	10.436722	10.465171	10.971149	10.940838	10.935184
Up	FGSG_10372	1.023064501	0.0042139	5.167919	5.1036809	5.1553224	5.7382218	5.6318376	5.7608523	6.576661	6.6474492	6.4342132
Up	FGSG_02250	1.022588733	6.07E-05	5.6164066	5.349828	5.7713555	4.7835368	5.0403527	4.3744304	4.7032988	4.8105634	4.5256129
Up	FGSG_02913	1.022405236	0.0001224	4.8367778	4.5354388	5.0212003	5.4277566	5.3855124	5.3904893	4.7032988	4.3549955	4.9376179
Up	FGSG_04012	1.020787163	2.51E-05	8.9741599	8.8745529	8.9859431	9.3145841	9.2630272	9.2788996	9.3078089	9.2116306	9.3354732
Up	FGSG_09596	1.020727944	0.0012265	6.5563577	6.4975416	6.5327046	6.5743316	6.4675956	6.594472	6.5961351	6.5138742	6.6500817
Up	FGSG_00930	1.019785205	0.0020623	6.4111515	6.3638664	6.4038729	6.4753959	6.4675956	6.3984523	7.2832433	7.2733875	7.2280252
Up	FGSG_08515	1.018224253	2.96E-05	7.162584	7.1449564	7.0967395	6.7462194	6.8053944	6.6110389	7.2467432	7.4085718	6.9895734
Up	FGSG_00162	1.01762037	0.0016771	4.0257777	4.2010532	3.7539507	3.444959	3.6887032	3.0813961	4.028829	4.262829	3.6872775
Up	FGSG_05395	1.017575437	5.62E-05	6.629804	7.2242442	6.5327046	7.0511234	7.3778853	7.4024174	7.196594	7.6090478	7.7710623
Up	FGSG_05272	1.016748517	0.0009549	3.3996963	3.5844678	3.1285604	4.2425091	4.2058494	4.210161	4.5115122	4.262829	4.6764038
Up	FGSG_10005	1.016679228	3.27E-06	10.845408	10.822627	10.782557	10.933009	10.855632	10.922141	11.359113	11.325309	11.326622
Up	FGSG_11743	1.016668222	2.91E-05	5.4640893	5.608128	5.2780294	5.0060735	4.9907667	5.2704753	4.3828727	4.262829	4.6764038
Up	FGSG_01918	1.016618964	1.91E-07	5.8597011	5.906198	5.728956	5.9673238	6.1438433	5.6688985	6.1719365	6.2026947	6.075584
Up	FGSG_10321	1.016025186	4.97E-05	8.4192948	8.4355616	8.3164747	8.1478533	8.0331229	8.172933	8.6260419	8.5655436	8.6203531
Up	FGSG_08920	1.015837782	0.0004025	9.4753583	9.4471719	9.4182855	8.9594164	8.9750268	8.8539645	9.3151721	9.2330424	9.329795
Up	FGSG_13006	1.015234654	1.71E-05	7.250589	7.356828	7.0458328	7.0820218	6.9024444	7.1650109	6.7343705	6.6655604	6.7384529
Up	FGSG_09003	1.015083277	8.88E-08	7.9163379	7.7594926	7.9793053	8.0821245	7.9250757	8.1504418	8.0624335	7.9492797	8.1060838
Up	FGSG_05029	1.013908184	1.00E-08	7.5881371	7.6716978	7.436798	7.8433192	7.8011025	7.8136811	8.0901673	8.230485	7.8760892
Up	FGSG_01265	1.013390653	2.69E-06	8.8283797	8.7727337	8.7987715	8.1652715	8.1786218	8.0628316	8.2887372	8.2958954	8.2148237
Up	FGSG_12025	1.013296652	0.0002062	4.7515947	4.3213345	5.0212003	5.2212063	5.2232303	5.1395644	6.0359471	6.2272902	5.7410644
Up	FGSG_10071	1.013061808	0.0065711	3.701719	3.378055	3.9139052	3.7210377	3.4156241	3.9222766	4.1912995	4.0586965	4.265
Up	FGSG_06308	1.01286363	9.14E-08	8.5511749	8.5384247	8.4788005	8.769376	8.6391638	8.8080019	9.1553411	9.1259307	9.1188085
Up	FGSG_11615	1.012614729	0.0008952	6.2496778	6.0867644	6.3206633	7.0322616	6.9933753	6.9860594	7.2281409	7.124214	7.2641992
Up	FGSG_12808	1.01251705	3.62E-06	7.06886	7.0082698	7.0458328	7.4615211	7.6172639	7.187278	7.975882	7.8096741	8.0656606
Up	FGSG_01613	1.01201041	5.89E-07	9.8530206	9.7923768	9.8278686	9.9988386	10.005623	9.9025781	10.573895	10.575785	10.504843
Up	FGSG_02749	1.011883334	0.000204	7.3553501	7.2547781	7.3693882	7.93418	7.8849234	7.8971035	8.7332671	8.6946233	8.7061315
Up	FGSG_01638	1.011779951	2.10E-08	6.7110174	6.7541729	6.5811798	6.9675462	7.1370484	6.6754671	7.0907419	7.2968163	6.7723395

Up	FGSG_09229	1.011586773	1.61E-06	7.5381432	8.0216372	8.165687	7.188078	7.4802486	7.662937	7.2218865	7.4509576	7.6847264
Up	FGSG_10947	1.01144965	7.88E-08	7.2964211	7.1611664	7.3415168	7.41859	7.2572536	7.485628	7.7939054	7.6368537	7.8838602
Up	FGSG_06703	1.011380337	4.06E-07	7.694665	7.8819136	7.3831245	7.7032764	7.7938925	7.5123315	8.1038368	8.1166081	8.024072
Up	FGSG_01919	1.010678031	1.57E-06	6.2651272	6.2472245	6.2016809	6.8575557	6.9157907	6.7371407	6.4856201	6.2986564	6.6131596
Up	FGSG_07023	1.010522045	4.07E-08	6.2955384	6.306724	6.2016809	6.6339322	6.3545295	6.7962856	6.5369058	6.4733227	6.5363539
Up	FGSG_04214	1.010515213	0.0002879	4.5136357	4.3213345	4.6184939	4.1048586	4.51735	3.4190665	3.970335	3.6853877	4.1665008
Up	FGSG_01199	1.010364345	1.12E-06	11.308476	11.279566	11.252715	10.754636	10.739404	10.681598	10.659111	10.687467	10.565291
Up	FGSG_13439	1.010217061	0.0008309	3.9513288	4.4323548	3.1285604	4.9800625	4.5857446	5.2281446	4.935064	4.7439404	5.0523944
Up	FGSG_03429	1.010160132	0.0241263	3.1562965	3.1370988	3.1285604	3.1032407	2.6377651	3.4190665	2.4052168	2	2.7087823
Up	FGSG_07128	1.009941413	9.03E-07	7.8614916	7.8014645	7.8368453	7.6088325	7.558914	7.5727822	8.2182233	8.2182669	8.1518558
Up	FGSG_01644	1.009727885	2.08E-08	6.1026411	6.1851653	5.9294932	5.8429557	5.7257602	5.874995	5.9925186	5.9002223	6.0202512
Up	FGSG_03035	1.008822177	0.0019308	5.7321013	5.510285	5.85259	6.40871	6.5209705	6.1937152	5.5880596	5.7723491	5.3047937
Up	FGSG_11558	1.008764718	0.0976395	2	2	2	3.1032407	2.8748957	3.260088	2.5718533	2.3968278	2.7087823
Up	FGSG_01665	1.008727997	2.94E-08	7.8254716	7.6603381	7.8955278	7.9068273	7.8916935	7.8349933	7.8798378	7.9269302	7.7626576
Up	FGSG_07286	1.008452902	2.55E-07	6.7554516	6.9902361	7.1297061	7.0511234	7.1256073	7.3735786	7.1446385	7.3083895	7.3564507
Up	FGSG_00383	1.006717879	0.0001686	3.9513288	4.4323548	3.1285604	4.1048586	3.9182279	4.210161	4.2902636	4.262829	4.265
Up	FGSG_11636	1.006340956	5.34E-09	6.9496238	6.8770274	6.9382912	6.6171532	6.5209705	6.6274177	6.9312196	6.7696981	7.0180995
Up	FGSG_04780	1.006278745	0.0005582	9.2415422	9.2452908	9.1556037	8.2301586	8.1896518	8.1840485	8.8412549	8.8130966	8.8035757
Up	FGSG_06868	1.005585263	1.93E-05	7.7060301	7.5902346	7.7440876	8.2574849	8.1841473	8.2436985	8.8432936	8.7337752	8.8832693
Up	FGSG_07005	1.004753595	1.60E-06	9.4212223	9.3193862	9.4348672	9.7396796	9.6854877	9.7103137	10.255824	10.259877	10.197067
Up	FGSG_13174	1.004575333	0.0568156	3.0172362	2.4852148	3.3684179	2.364115	2.6377651	2	3.6359267	3.3690748	3.8228101
Up	FGSG_01646	1.003659569	0.0004639	5.2004262	5.4053203	4.8733193	5.1056669	5.0882909	5.0451875	5.7532926	5.7038947	5.7410644
Up	FGSG_08442	1.003533765	7.79E-08	7.2192048	7.2242442	7.1297061	7.5917573	7.3778853	7.7021252	7.7683829	7.6997228	7.7710623
Up	FGSG_13273	1.002467994	4.05E-10	9.2952028	9.2106646	9.2965486	9.3429245	9.3927731	9.1989638	9.6175296	9.5457361	9.6246436
Up	FGSG_10856	1.002024173	8.63E-10	10.49688	10.46768	10.440579	10.298252	10.23189	10.276845	10.214683	10.253904	10.105324
Up	FGSG_01122	1.001809748	3.13E-05	4.349408	5.2921154	4.5224622	4.8424709	5.0403527	4.7780897	4.7387761	4.8105634	4.9961472
Up	FGSG_03626	1.001490081	7.30E-09	7.6185257	7.529095	7.6219674	7.5348245	7.489204	7.4945842	7.541095	7.6997228	7.287821
Up	FGSG_07116	1.001342695	0.0018829	9.2415422	9.2414842	9.1556037	10.258906	10.247808	10.181622	11.102488	11.067131	11.071569
Up	FGSG_01171	1.000935696	0.009488	3.3996963	2.8476671	3.7539507	2.7805029	2.8748957	2.6397336	3.0942412	3.3690748	3.5376798
Up	FGSG_01944	1.000619547	7.46E-06	7.0422056	6.9719742	7.0284567	6.7767592	6.7468578	6.7219677	7.0489555	7.2130949	6.7889889
Down	FGSG_08395	-1.000211113	1.34E-05	8.5823437	8.5007053	8.5776841	8.5810544	8.5262315	8.548974	8.492126	8.1427022	8.7192211

Down	FGSG_12718	-1.000780859	0.0751041	2.8633281	2	3.3684179	2.5166584	2.353841	2.6397336	3.2982451	2.7077187	3.6872775
Down	FGSG_08350	-1.001971854	6.96E-06	9.0718326	9.1591345	8.8929923	8.8790051	8.8061365	8.8643665	8.1605293	8.2486203	7.9956649
Down	FGSG_07689	-1.002086149	0.0005925	4.0257777	4.2010532	3.7539507	4.0052074	3.9182279	4.024755	3.970335	4.0586965	3.8228101
Down	FGSG_13957	-1.0025813	1.08E-05	5.9195042	5.9441646	5.8125445	5.5913246	5.5991203	5.5012819	5.3334324	5.1562742	5.4374371
Down	FGSG_09451	-1.002919287	0.0003795	6.5181831	6.5477263	6.4038729	6.4938962	6.5891976	6.2997126	5.947742	6.0176785	5.8079414
Down	FGSG_03231	-1.003212965	0.0003827	6.1533367	6.052414	6.1703363	5.658465	5.5991203	5.636897	5.107816	4.8105634	5.3047937
Down	FGSG_12054	-1.003552468	0.0044817	4.7948149	5.1692654	4.188785	5.0060735	4.9907667	4.9955781	4.4698939	4.6740912	4.1665008
Down	FGSG_03890	-1.003711482	0.0815429	2.8633281	2	3.3684179	3.7210377	3.6887032	3.6925011	3.2982451	3.1804273	3.3707558
Down	FGSG_06314	-1.004115604	0.0012714	6.665171	6.8372271	6.650969	5.9270474	5.7257602	6.2370531	5.2124668	5.347904	5.2577234
Down	FGSG_00821	-1.004307883	1.69E-05	7.2428067	7.3139731	7.0799694	6.7384824	6.6858453	6.7066335	6.2721924	6.2514733	6.2298857
Down	FGSG_04332	-1.004518951	0.0003871	8.8617898	8.9370792	8.6924588	9.708336	9.6698188	9.6594531	9.9670259	9.9992719	9.865419
Down	FGSG_12760	-1.005088454	3.57E-05	11.532074	11.464009	11.513795	12.202217	12.196857	12.118657	12.670853	12.666128	12.609765
Down	FGSG_02289	-1.005387914	0.0005304	6.653478	6.3355781	6.8421126	6.4471928	6.374	6.4361306	6.0641898	6.0456069	6.0202512
Down	FGSG_12717	-1.005392814	0.0046155	5.8597011	5.7859081	5.85259	5.2649499	5.1346873	5.3115993	4.8402557	5.2066203	4.265
Down	FGSG_07500	-1.005478391	5.75E-07	8.9166249	9.076079	8.6589748	9.5647762	9.4397249	9.5984493	9.4537212	9.4045998	9.4366792
Down	FGSG_01884	-1.005630687	1.63E-05	8.7120101	8.6969277	8.6419368	9.2357116	9.2837634	9.0938565	9.130514	9.177328	9.0129446
Down	FGSG_08228	-1.005664992	0.0642347	3.9513288	3.765011	4.0578819	3.3668146	3.4156241	3.260088	3.6359267	3.5358829	3.6872775
Down	FGSG_09189	-1.005807455	0.0124109	3.2831234	2.8476671	3.5740244	3.3668146	3.5586152	3.0813961	3.0942412	2.7077187	3.3707558
Down	FGSG_02645	-1.006216189	2.17E-07	8.603768	8.6286221	8.491538	8.5236891	8.4775616	8.4830025	8.1800181	8.1555744	8.1389256
Down	FGSG_04470	-1.00654527	0.000236	5.9955706	5.906198	6.0025037	6.34899	6.0741317	6.5086577	5.4663562	5.9002223	4.7462732
Down	FGSG_05697	-1.008194812	7.46E-08	13.03777	12.993187	12.996892	13.446619	13.412773	13.393498	13.528833	13.496886	13.494401
Down	FGSG_03700	-1.009040418	0.000228	7.340842	7.342684	7.2545188	7.5303505	7.5758276	7.3928684	6.7604964	6.5533169	6.8850413
Down	FGSG_04872	-1.00942439	0.0195303	3.872828	4.4323548	2.8407141	4.5559538	4.7733906	4.2946324	4.7387761	4.5233477	4.8766132
Down	FGSG_04755	-1.009524774	2.52E-05	10.944092	10.867773	10.933714	11.230929	11.177803	11.197078	11.413295	11.408318	11.351354
Down	FGSG_00811	-1.009745666	0.0690516	3.2831234	3.5844678	3.3684179	3.1965524	3.0784965	3.4190665	3.9093688	3.8208439	4.1665008
Down	FGSG_05623	-1.010138132	4.22E-06	8.5730635	8.6402333	8.4133694	8.0132576	7.9184607	8.0200518	7.6661371	7.6997228	7.5641633
Down	FGSG_07833	-1.010676184	0.0003886	6.5812576	6.6431438	6.4305761	5.8856141	6.0741317	5.636897	5.3791148	5.1041074	5.5589053
Down	FGSG_10838	-1.011345191	0.0030468	4.8775546	6.0867644	6.1055269	6.0939946	6.5383322	6.8669619	5.3100371	6.073005	5.8718551
Down	FGSG_00166	-1.01214569	7.96E-07	7.4394623	7.5038947	7.2841049	7.0448635	7.0789132	7.0112415	6.7776545	6.59171	6.8850413
Down	FGSG_03511	-1.012547625	1.55E-05	5.2633225	4.9628272	5.4444954	5.1056669	4.7733906	5.3115993	5.107816	4.9937494	5.1587087
Down	FGSG_09983	-1.013493252	2.01E-09	12.789137	12.751974	12.779448	12.471042	12.454609	12.425109	12.417206	12.412963	12.382491

Down	FGSG_11555	-1.01408494	0.0588671	4.8775546	4.4323548	5.1553224	4.8424709	4.6510429	4.9442019	3.4769433	4.0586965	2.3974876
Down	FGSG_06918	-1.014092855	2.97E-10	9.8593964	9.8535062	9.7793511	9.8860553	9.8104344	9.8736922	9.7753377	9.6854251	9.7971037
Down	FGSG_01594	-1.014317297	6.20E-06	13.204805	13.227716	13.094052	13.075042	13.002659	13.059421	12.471431	12.380094	12.494267
Down	FGSG_05262	-1.014417457	6.06E-10	11.41273	11.346659	11.392553	11.179712	11.156177	11.115244	11.171049	11.140997	11.134806
Down	FGSG_03942	-1.014652421	0.0299501	4.0965725	3.378055	4.5224622	3.2841938	3.5586152	2.8774004	3.5586239	3.6853877	3.3707558
Down	FGSG_12700	-1.01570481	1.21E-05	5.6402997	5.458757	5.728956	5.9673238	5.8698682	5.9807654	5.8698804	5.6684096	5.991768
Down	FGSG_13246	-1.016284214	0.020029	2.8633281	2.4852148	3.1285604	3.7210377	3.5586152	3.8119585	3.2982451	3.1804273	3.5376798
Down	FGSG_08447	-1.016366085	1.72E-07	8.4330387	8.4018525	8.3795063	8.0196559	8.0989568	7.8420279	7.8758598	7.8339001	7.8604206
Down	FGSG_07279	-1.019483212	0.0001449	10.86835	10.866538	10.783852	11.319487	11.187464	11.359088	11.355546	11.292178	11.352055
Down	FGSG_05741	-1.021884479	1.28E-07	6.6882763	6.4975416	6.7811692	6.7767592	6.5724409	6.8806909	6.4751405	6.3443452	6.5363539
Down	FGSG_00769	-1.022948938	0.0001761	12.114522	12.041192	12.102258	12.733518	12.702148	12.677732	12.758534	12.759199	12.690629
Down	FGSG_01386	-1.023770557	9.67E-06	6.8910817	6.8169075	6.8813568	7.5392846	7.5331658	7.4584209	6.8113703	6.7185687	6.8378143
Down	FGSG_12557	-1.023781922	1.68E-08	9.1756253	9.0194102	9.2372812	9.296255	9.2656355	9.2394852	8.9244913	9.0066764	8.7662252
Down	FGSG_13824	-1.024167962	1.24E-06	6.0678264	6.2165286	5.8125445	5.8573158	5.9237087	5.7002055	5.8375198	5.5947086	5.991768
Down	FGSG_09742	-1.024999701	0.0001022	6.0139766	5.7859081	6.1382955	6.5568406	6.4856075	6.5435983	5.9628224	5.8693008	5.991768
Down	FGSG_08435	-1.025608314	7.91E-06	7.7615503	7.9856806	7.7860511	7.6547733	7.5758276	7.8279241	6.9540395	7.200728	6.975096
Down	FGSG_06705	-1.026280523	1.41E-06	6.2023113	6.0172257	6.2918249	6.1060709	6.0977451	6.0308779	5.7532926	5.4349486	5.9627112
Down	FGSG_11297	-1.026563327	0.0425185	4.2902308	4.7218364	3.5740244	4.1522165	3.6887032	4.4500449	3.2982451	3.5358829	2.964684
Down	FGSG_04191	-1.026743155	0.0004297	5.3814422	5.608128	5.0212003	5.9805033	5.8139402	6.0552962	6.235408	6.2986564	6.1024744
Down	FGSG_04251	-1.026984996	0.0006322	4.2285219	4.2010532	4.188785	4.8133047	4.4455509	5.0451875	3.9093688	4.0586965	3.6872775
Down	FGSG_10296	-1.027454263	4.80E-07	9.2647844	9.1510408	9.289272	9.2065643	9.1907566	9.1345326	8.9340879	8.8008584	8.998701
Down	FGSG_10741	-1.027680829	1.86E-05	8.0246912	7.8220012	8.1253921	7.4330428	7.2467305	7.521124	6.9388664	6.8510822	6.9604719
Down	FGSG_06783	-1.027886652	0.0065565	3.1562965	3.1370988	3.1285604	2.8962916	3.0784965	2.6397336	3.2982451	3.1804273	3.3707558
Down	FGSG_01307	-1.028076251	1.44E-08	8.8387424	8.9037482	8.6813836	9.16532	9.1574363	9.084989	9.1667824	9.11279	9.1545807
Down	FGSG_13759	-1.028143551	0.0005495	5.100621	5.1692654	4.9491538	4.7531416	4.3699906	4.9955781	4.2416299	3.9446664	4.5256129
Down	FGSG_13238	-1.028412956	0.006729	4.7070397	4.4323548	4.8733193	4.4837433	4.4455509	4.4500449	3.9093688	4.3549955	3.18196
Down	FGSG_02873	-1.028543343	0.0082551	4.0257777	3.5844678	4.3087924	4.7531416	4.7733906	4.6556708	3.6359267	3.5358829	3.6872775
Down	FGSG_01976	-1.028635326	0.0043939	14.520646	14.454739	14.500299	15.464173	15.424025	15.416958	15.927703	15.905587	15.88341
Down	FGSG_09400	-1.028776438	5.69E-08	8.2037235	8.3103344	7.9972798	7.8397076	7.8225187	7.7700883	7.6049703	7.4509576	7.6847264
Down	FGSG_03671	-1.028798363	0.0795198	4.0257777	3.9254527	4.0578819	4.69036	4.7733906	4.5218929	3.2982451	3.1804273	3.3707558
Down	FGSG_01850	-1.031540764	3.58E-07	8.0605495	7.8422497	8.1736127	8.4402954	8.28015	8.5053299	8.3124777	8.3132316	8.2453061

Down	FGSG_06163	-1.031590372	4.08E-05	10.77093	10.752142	10.74049	11.650787	11.642086	11.577132	11.507579	11.496961	11.462559
Down	FGSG_12302	-1.033992303	0.0009052	7.0863599	7.0610564	7.0284567	8.4259154	8.1841473	8.5575466	8.001667	7.8498277	8.0792612
Down	FGSG_06345	-1.034020664	7.69E-05	6.4522245	6.5721788	6.2623983	6.7071125	6.5724409	6.7670162	6.1719365	5.9891987	6.2778581
Down	FGSG_02320	-1.035108571	1.81E-05	10.996519	10.981683	10.925523	11.002491	10.970403	10.946823	10.835377	10.841777	10.761533
Down	FGSG_04288	-1.038101626	0.0009964	13.182985	13.096632	13.181891	13.894158	13.854854	13.845993	14.542811	14.499005	14.520561
Down	FGSG_02579	-1.038114989	2.57E-07	7.4929307	7.42554	7.4757821	7.3026957	7.1256073	7.3832557	7.6049703	7.4921337	7.6486915
Down	FGSG_03432	-1.038204311	2.61E-05	5.9389001	5.6546687	6.1055269	6.2436111	6.1209783	6.279127	6.1324685	6.073005	6.1288728
Down	FGSG_03919	-1.038299003	0.0187298	4.1640552	3.9254527	4.3087924	3.7210377	4.0206066	3.260088	2.9802913	2.9633478	2.964684
Down	FGSG_07038	-1.038708038	0.0184873	8.6007268	8.6969277	8.4066601	8.0667591	7.8849234	8.1504418	7.6193177	7.4404773	7.7198832
Down	FGSG_02275	-1.039616895	0.0301309	3.6078986	3.378055	3.7539507	3.1032407	3.0784965	3.4190665	3.2982451	2.7077187	3.6872775
Down	FGSG_20727	-1.040007758	0.0167355	4.6135762	4.8067189	4.6184939	3.8415007	3.5586152	4.024755	3.0942412	2.9633478	3.3707558
Down	FGSG_13912	-1.040776245	0.0002006	5.0300296	5.4053203	4.4195794	4.8133047	4.6510429	4.8909283	4.7734018	4.9937494	4.4438634
Down	FGSG_13803	-1.041014231	0.0130669	3.7898088	3.765011	3.7539507	3.2841938	2.353841	3.8119585	3.0942412	2.9633478	3.18196
Down	FGSG_10592	-1.041753393	0.0515663	3.872828	3.1370988	4.3087924	2.7805029	2.353841	3.0813961	2.8565633	2.7077187	2.964684
Down	FGSG_13012	-1.043568487	1.24E-07	6.7771658	6.7541729	6.7175374	7.0820218	7.0549869	7.0359915	6.9912882	6.8823924	7.0321538
Down	FGSG_09646	-1.043846204	3.08E-05	6.4921604	6.6198745	6.2623983	6.0065063	6.1438433	5.7608523	5.7358405	6.0176785	5.3047937
Down	FGSG_12761	-1.045042195	0.0058895	4.7515947	4.5354388	4.8733193	3.6568115	4.1161991	3.0813961	4.028829	3.6853877	4.3572018
Down	FGSG_01105	-1.045488505	0.0001019	5.7541671	5.6997548	5.728956	6.3387918	6.5209705	6.0308779	5.4663562	5.7038947	5.1065306
Down	FGSG_07055	-1.046562334	0.0031796	4.5136357	4.4323548	4.5224622	5.3887447	4.9907667	5.636897	4.994976	4.8742451	5.0523944
Down	FGSG_09681	-1.048781767	0.0002586	5.9769267	6.2165286	5.5937254	6.1417046	6.0741317	6.1261652	6.1976611	6.4102748	5.8718551
Down	FGSG_06241	-1.048846715	1.03E-06	5.6638035	5.8672052	5.545677	5.5027514	5.346621	5.9021734	5.5684753	5.9601454	5.2577234
Down	FGSG_01764	-1.048861123	3.01E-05	6.3545028	6.3638664	6.2623983	6.6001768	6.3545295	6.7371407	6.5569203	6.3886313	6.6500817
Down	FGSG_07843	-1.048981297	0.0140237	3.6078986	4.069824	2.8407141	3.7210377	2.8748957	4.210161	3.199846	3.1804273	3.18196
Down	FGSG_10349	-1.049342976	0.0345343	3.2831234	2.8476671	3.5740244	2.6546039	2.8748957	2.3550396	3.3903592	2.7077187	3.8228101
Down	FGSG_05277	-1.049955253	2.73E-06	9.8568495	9.8207163	9.8076387	9.5702349	9.5465415	9.5062513	9.9310552	9.8488861	9.9455731
Down	FGSG_01332	-1.051752077	0.004635	4.8367778	4.7218364	5.4959731	6.1992055	6.6543434	6.2997126	5.3100371	5.4349486	5.6345505
Down	FGSG_12838	-1.054110383	1.22E-05	6.6178197	6.775389	6.3489364	6.9807236	6.9807308	6.8942906	6.7166867	6.6474492	6.7212064
Down	FGSG_00740	-1.054582228	2.15E-05	9.9941348	9.9592629	9.94363	9.8325488	9.7742565	9.8033529	9.2809858	9.4019015	9.0753558
Down	FGSG_03897	-1.054819214	0.0016272	4.2902308	3.765011	4.7085304	4.953574	4.6510429	5.1395644	4.7387761	4.8105634	4.6029775
Down	FGSG_06360	-1.055045919	5.69E-05	5.3528065	5.349828	5.2780294	5.0567294	5.179638	4.8356119	4.9041484	4.5233477	5.1587087
Down	FGSG_07679	-1.055292606	7.54E-05	10.738091	10.697617	10.69303	11.02254	11.02915	10.926284	10.680934	10.649297	10.646332

Down	FGSG_09351	-1.055979762	0.0026177	10.414412	10.447996	10.383636	9.8279991	9.8457273	9.8265915	9.0104618	9.0066764	9.0164836
Down	FGSG_02409	-1.056038711	0.0016804	4.9558055	5.1036809	4.7085304	4.4462359	4.7135134	4.1204343	4.0850435	3.9446664	4.1665008
Down	FGSG_05309	-1.056256379	1.89E-06	8.9718078	8.974252	8.8833591	9.113919	9.0735851	9.0670887	8.6284082	8.7121554	8.4684288
Down	FGSG_01647	-1.056446105	1.38E-05	9.2724494	9.2938834	9.1635848	8.5745506	8.5175037	8.5446685	8.2244936	8.2365555	8.1454052
Down	FGSG_09049	-1.057886037	0.0003844	8.3192369	8.4221723	8.1253921	7.6588781	7.6889762	7.5385497	6.8923657	6.8028047	6.9156884
Down	FGSG_09155	-1.059087588	0.0001305	12.720224	12.691176	12.66358	13.743762	13.713704	13.686115	13.803653	13.791365	13.749213
Down	FGSG_04526	-1.059099346	0.0019827	4.8775546	4.5354388	5.1553224	3.9526758	3.4156241	4.2946324	4.028829	3.9446664	4.0607807
Down	FGSG_10012	-1.060205194	4.85E-05	8.0737698	8.1244237	8.1971317	8.3991741	8.3006433	8.5360188	8.5305887	8.6724042	8.4632514
Down	FGSG_10944	-1.060343695	6.07E-07	7.4731115	7.4521338	7.4102108	7.3892427	7.489204	7.2092065	6.8196775	6.7528555	6.8378143
Down	FGSG_11292	-1.060422205	0.002074	4.6135762	4.6316459	4.5224622	4.1048586	4.3699906	3.6925011	4.4270393	4.7439404	3.9466965
Down	FGSG_02502	-1.060787164	0.0009944	12.397283	12.358788	12.350552	13.198348	13.151461	13.157666	13.310528	13.281304	13.27373
Down	FGSG_03777	-1.061101596	8.58E-07	7.5758001	7.7380397	7.4757821	7.5348245	7.6090713	7.6787403	7.4530566	7.8418859	7.100442
Down	FGSG_12251	-1.061963357	0.0426795	2.6910183	2.8476671	2.4807492	3.2841938	3.5586152	2.8774004	2.7212202	2.7077187	2.7087823
Down	FGSG_13707	-1.062044838	0.0020019	3.5075501	2.8476671	3.9139052	4.1980691	3.9182279	4.3744304	3.7092973	3.8208439	3.5376798
Down	FGSG_02928	-1.063512054	0.0336235	4.2285219	4.8067189	5.0212003	3.6568115	4.51735	4.7181783	3.8457121	4.3549955	4.265
Down	FGSG_08127	-1.064326698	0.0003041	4.8775546	4.8868835	4.8733193	4.871059	4.8308815	4.8356119	5.1870071	5.1041074	5.2577234
Down	FGSG_01108	-1.064371029	2.55E-06	6.0853387	6.0867644	6.0025037	6.1992055	6.2318429	6.1261652	5.947742	5.7038947	6.1024744
Down	FGSG_08255	-1.065143262	1.88E-07	7.7451174	7.6716978	7.7334032	7.7502017	7.8644203	7.5298632	7.4423369	7.2253567	7.5738036
Down	FGSG_11823	-1.065952791	0.0140002	5.3528065	5.458757	5.1553224	4.0558932	4.2902531	3.6925011	4.5519634	4.6006874	4.4438634
Down	FGSG_04489	-1.066228156	1.07E-10	11.36334	11.336881	11.304162	11.276722	11.203197	11.26277	11.154725	11.111572	11.131546
Down	FGSG_09269	-1.066623148	7.37E-11	11.191051	11.142387	11.158017	11.355657	11.304971	11.318684	11.337219	11.333828	11.27806
Down	FGSG_12845	-1.067161437	0.0009408	4.1640552	4.069824	4.188785	4.7835368	4.8861689	4.5903319	4.1391495	4.1643702	4.0607807
Down	FGSG_11867	-1.067259855	0.001329	3.9513288	4.3213345	3.3684179	3.444959	3.4156241	3.4190665	3.6359267	3.3690748	3.8228101
Down	FGSG_06735	-1.068225341	6.23E-05	7.7560934	7.6716978	7.7546935	8.4686323	8.3308476	8.514165	8.8351216	8.667919	8.9252941
Down	FGSG_03839	-1.069517695	0.0745881	2.8633281	2.4852148	3.1285604	3.0034737	2.6377651	3.260088	2.9802913	2.3968278	3.3707558
Down	FGSG_08554	-1.07041268	2.15E-11	10.690967	10.630801	10.701277	10.788751	10.741279	10.778282	10.787476	10.785956	10.767846
Down	FGSG_02564	-1.070659245	1.15E-05	11.051569	10.950544	11.064143	11.406893	11.373077	11.352945	11.68771	11.657714	11.651376
Down	FGSG_06518	-1.070772262	0.0082605	3.3996963	3.1370988	3.5740244	3.2841938	3.4156241	3.0813961	3.199846	3.5358829	2.7087823
Down	FGSG_02754	-1.07160552	0.0002759	5.7321013	6.2165286	6.1703363	5.4083825	6.1885139	5.5363994	4.807216	5.3023297	4.9961472
Down	FGSG_00299	-1.071641752	0.0003453	10.902093	10.862826	10.85581	10.165344	10.150964	10.091521	9.2505855	9.3242228	9.1022504
Down	FGSG_01725	-1.071740861	0.0542787	4.4062533	4.2010532	4.5224622	3.9526758	4.1161991	3.6925011	2.7212202	2.9633478	2.3974876

Down	FGSG_08518	-1.073284462	2.19E-09	9.5763453	9.6095484	9.4545171	9.6722409	9.6539778	9.6025975	9.8059746	9.7355799	9.8093968
Down	FGSG_04911	-1.073998718	5.09E-11	12.875658	12.826283	12.839218	13.053378	13.013563	13.005616	13.057372	13.020073	13.02852
Down	FGSG_03425	-1.074459488	0.0041627	6.605735	6.6660438	6.4567939	6.2760407	6.0741317	6.3792376	5.0240241	5.2066203	4.7462732
Down	FGSG_05096	-1.074515296	9.83E-07	10.017129	9.9073534	10.037821	10.482653	10.445951	10.431755	10.912161	10.82576	10.930502
Down	FGSG_05229	-1.074971601	0.005232	5.3814422	5.4053203	5.2780294	5.674773	5.346621	5.874995	4.1912995	3.8208439	4.4438634
Down	FGSG_06127	-1.075308833	0.0168637	9.6039463	9.6329835	9.4866834	9.3518264	9.2630272	9.3521763	11.853148	11.829701	11.810693
Down	FGSG_09140	-1.075524578	7.10E-09	10.204076	10.180164	10.142482	10.524871	10.430924	10.529768	10.485287	10.423137	10.481986
Down	FGSG_09773	-1.075824479	3.16E-09	8.4432614	8.4018525	8.3999194	8.1710312	8.200598	8.0507377	7.9073809	7.9639893	7.7794184
Down	FGSG_11999	-1.076543859	0.0072187	4.0257777	3.9254527	4.0578819	4.4462359	4.7733906	3.9222766	3.7791165	3.8208439	3.6872775
Down	FGSG_08416	-1.076590244	0.0028035	4.4062533	4.7218364	3.9139052	4.4837433	4.0206066	4.7780897	3.9093688	3.9446664	3.8228101
Down	FGSG_13115	-1.077185713	0.0648194	3.7898088	4.069824	3.3684179	3.0034737	3.0784965	2.8774004	2.7212202	2	3.18196
Down	FGSG_09921	-1.0772117	1.56E-08	8.3629712	8.3881451	8.2505629	8.0512283	7.9512361	8.0628316	7.8718708	7.888894	7.7877264
Down	FGSG_03968	-1.077557628	0.0758287	3.0172362	3.1370988	2.8407141	3.3668146	3.4156241	3.260088	2.4052168	2.7077187	2
Down	FGSG_11802	-1.079998658	0.0061017	3.6078986	4.069824	2.8407141	3.1965524	2.8748957	3.4190665	3.199846	3.5358829	2.7087823
Down	FGSG_02824	-1.080130781	0.0046083	4.9172105	3.9254527	5.4444954	4.9800625	4.5857446	5.2281446	5.7532926	5.9002223	5.5195414
Down	FGSG_07672	-1.080585347	0.0065663	9.2238615	9.2604175	9.0984644	10.369488	10.343504	10.307663	10.509239	10.468976	10.483266
Down	FGSG_00485	-1.080830732	7.70E-05	5.8597011	5.9811576	5.640225	6.8646813	6.6221382	7.0112415	6.6153499	6.7185687	6.4342132
Down	FGSG_03775	-1.080880607	0.0030644	4.0257777	4.069824	3.9139052	4.6247215	4.8308815	4.2946324	3.7791165	3.3690748	4.1665008
Down	FGSG_10193	-1.081652535	0.0001967	6.8300632	6.4975416	7.0284567	6.9741499	6.6701803	7.1537472	6.0216157	6.0456069	5.9330571
Down	FGSG_11919	-1.081968218	0.0633012	2.8633281	3.1370988	2.4807492	3.0034737	3.4156241	2.3550396	2.9802913	2.7077187	3.18196
Down	FGSG_09649	-1.08441658	0.006906	4.7070397	4.4323548	4.8733193	4.1980691	4.4455509	3.8119585	3.7791165	3.6853877	3.8228101
Down	FGSG_01930	-1.085030874	7.52E-06	9.0089878	8.9370792	9.0038356	8.2628886	8.2329471	8.2060258	8.1274512	8.284221	7.8760892
Down	FGSG_07068	-1.085344279	3.34E-06	7.8614916	7.8622179	7.7756741	8.3132388	8.2382687	8.3009798	8.1106233	8.2243889	7.9145321
Down	FGSG_12458	-1.085506598	0.0006772	4.9558055	5.0349724	4.7932764	5.1056669	4.8308815	5.2704753	5.237485	5.2552685	5.1587087
Down	FGSG_03803	-1.08578269	1.69E-07	11.349365	11.318931	11.295109	11.719458	11.642588	11.708991	11.924931	11.910708	11.872536
Down	FGSG_04558	-1.087690894	6.75E-10	12.359548	12.287663	12.34486	12.1364	12.145029	12.039016	12.103516	12.075977	12.064684
Down	FGSG_03385	-1.087787213	1.58E-09	16.542644	16.507263	16.492731	16.305308	16.249175	16.273886	16.432812	16.393625	16.405737
Down	FGSG_09863	-1.087867384	0.0031457	8.5637233	8.5196883	8.7359284	9.8851795	9.9660776	9.8459666	10.317251	10.363365	10.306636
Down	FGSG_06802	-1.087879065	5.50E-06	6.0321508	5.9441646	6.0376686	6.5916131	6.6701803	6.4174145	6.399571	6.3216817	6.4128878
Down	FGSG_06647	-1.088605575	3.01E-05	9.0957916	9.0675043	9.0389684	10.006107	9.9946594	9.9292466	10.218614	10.159613	10.211034
Down	FGSG_11114	-1.089579269	5.87E-06	6.2651272	5.9811576	6.4305761	5.9935634	5.9498937	5.9550408	5.4663562	5.4765776	5.3945636

Down	FGSG_13613	-1.089720548	0.0004848	4.2285219	4.2010532	4.188785	4.1522165	3.5586152	4.5218929	4.4270393	4.5233477	4.265
Down	FGSG_02000	-1.089878338	7.26E-06	11.035329	11.021072	10.988657	10.966099	10.906658	10.959007	10.610863	10.550424	10.629098
Down	FGSG_13228	-1.089996332	0.0400693	2.6910183	2.4852148	2.8407141	3.7210377	3.8080253	3.5622521	2.8565633	2.7077187	2.964684
Down	FGSG_09223	-1.091164934	7.18E-06	11.62219	11.553464	11.604536	12.452392	12.379835	12.437063	12.593633	12.555752	12.565152
Down	FGSG_11774	-1.092378019	0.0481875	3.2831234	2	3.9139052	2.8962916	3.0784965	2.6397336	3.0942412	3.1804273	2.964684
Down	FGSG_06762	-1.092780271	3.66E-05	8.0201456	8.0392849	7.91457	8.6893647	8.6148206	8.680374	9.060389	8.9995548	9.0548506
Down	FGSG_05108	-1.092997126	2.48E-06	7.799181	7.832161	7.6898562	8.366918	8.2276057	8.4557453	7.5157543	7.4085718	7.5738036
Down	FGSG_12447	-1.093361233	6.96E-06	6.7222549	6.4717791	6.8618681	6.5829982	6.5724409	6.5086577	6.5166098	6.7011148	6.2298857
Down	FGSG_03685	-1.094024483	1.23E-06	6.9010051	6.8372271	6.8813568	7.1002482	7.0907291	7.0236696	6.9002206	6.8977964	6.8378143
Down	FGSG_11958	-1.094130749	4.21E-06	9.6115204	9.5917188	9.5489415	8.7674764	8.8061365	8.636542	8.5356404	8.5064808	8.499109
Down	FGSG_12744	-1.094287036	0.0836527	4.2902308	4.2010532	4.3087924	2.8962916	2.6377651	3.0813961	2.7212202	2.9633478	2.3974876
Down	FGSG_14016	-1.095245325	8.52E-07	10.262975	10.145957	10.291953	9.9189442	9.8987799	9.8529481	9.3783974	9.3270703	9.3635338
Down	FGSG_11280	-1.095258554	0.0006898	3.872828	3.9254527	3.7539507	3.8981589	3.6887032	4.024755	4.2902636	3.6853877	4.6764038
Down	FGSG_11843	-1.095666212	1.25E-07	8.183539	8.1571205	8.1253921	7.9137143	7.898432	7.8420279	7.541095	7.5420042	7.4743726
Down	FGSG_10971	-1.096306621	0.0077402	3.3996963	3.1370988	3.5740244	3.3668146	3.4156241	3.260088	3.9093688	4.3549955	3.18196
Down	FGSG_09208	-1.096826699	0.0158986	3.872828	4.2010532	3.5740244	3.6568115	4.2902531	3.4190665	4.0850435	4.6006874	3.8228101
Down	FGSG_12657	-1.097806685	0.0038863	6.0500989	5.8672052	6.1382955	5.8573158	5.9498937	5.6688985	5.5284909	5.5563964	5.4374371
Down	FGSG_05042	-1.098500143	0.0006856	5.167919	4.9628272	5.2780294	4.657914	4.7135134	4.5218929	4.4698939	4.6006874	4.265
Down	FGSG_09308	-1.098837315	2.23E-10	10.96789	10.855374	10.990901	11.265326	11.263815	11.179135	11.199577	11.153082	11.180479
Down	FGSG_12861	-1.099237577	9.64E-07	5.2937714	5.458757	5.0212003	5.5209064	5.3855124	5.6041695	5.6452659	5.3920827	5.8079414
Down	FGSG_06724	-1.100484568	0.000496	5.7759005	5.6997548	5.7713555	6.7842942	6.7617162	6.7219677	6.0641898	5.9891987	6.075584
Down	FGSG_08941	-1.101258334	4.49E-07	7.2736871	7.3284	7.1297061	7.1360243	6.9024444	7.2626125	6.6248622	6.7185687	6.4552281
Down	FGSG_04134	-1.101300312	7.51E-05	10.723486	10.734646	10.626738	11.35976	11.343135	11.288747	11.787961	11.705546	11.803541
Down	FGSG_13892	-1.102835718	6.11E-06	9.5932752	9.4504733	9.6432826	9.0745139	9.0883944	8.9708908	8.5024834	8.3805655	8.5537156
Down	FGSG_06635	-1.103234825	3.23E-06	6.9686215	6.5721788	7.20897	6.5212093	6.6057619	6.340023	6.3659317	6.4937409	6.1547968
Down	FGSG_04987	-1.103290433	0.0687031	2.6910183	2.4852148	2.8407141	3.6568115	3.8080253	3.5622521	2.8565633	3.1804273	2.3974876
Down	FGSG_01843	-1.104429029	8.86E-08	7.4462553	7.3139731	7.4885462	7.2921638	7.2572536	7.2414868	7.6145511	7.5806954	7.58338
Down	FGSG_03935	-1.104512188	1.83E-06	10.15205	10.035984	10.178375	10.380716	10.289417	10.38315	9.6067691	9.5359244	9.6106629
Down	FGSG_02063	-1.105025595	0.0041793	5.5673995	5.7434744	5.2780294	5.1056669	4.9907667	5.1395644	4.5913113	4.4416259	4.7462732
Down	FGSG_07960	-1.105411616	0.0033087	9.4366545	9.4862982	9.2965486	10.66258	10.661328	10.577966	11.070648	11.037013	11.03975
Down	FGSG_12104	-1.105816671	0.0133232	3.3996963	3.9254527	2.4807492	3.7825262	3.6887032	3.9222766	4.028829	3.1804273	4.5256129

Down	FGSG_12817	-1.10585213	0.0009718	6.4789709	6.3916107	6.4825439	6.1417046	6.253029	5.9288493	5.2862562	5.1562742	5.3503768
Down	FGSG_04973	-1.105954129	0.0039634	5.7096927	5.7859081	5.545677	5.4277566	5.346621	5.4283735	4.2416299	4.4416259	3.9466965
Down	FGSG_09468	-1.107504869	0.0015197	3.701719	3.1370988	4.0578819	4.2425091	4.0206066	4.3744304	3.6359267	3.8208439	3.3707558
Down	FGSG_05466	-1.108222533	0.0013896	4.0965725	4.069824	4.0578819	5.2212063	5.2232303	5.1395644	4.1391495	4.1643702	4.0607807
Down	FGSG_08749	-1.109008479	0.0001383	11.153149	11.065887	11.154016	11.633682	11.585273	11.597539	11.594566	11.560042	11.566406
Down	FGSG_10835	-1.109096086	5.30E-09	8.7176359	8.8091934	8.5352539	9.0340021	8.9782109	9.0026287	8.8814907	8.8332655	8.8676788
Down	FGSG_08188	-1.109668977	3.62E-05	5.7096927	5.608128	5.728956	5.6252853	5.6318376	5.5363994	5.4014247	5.6320297	5.0523944
Down	FGSG_07831	-1.110196291	1.89E-06	7.0058827	6.9719742	6.9567807	7.3183509	7.030657	7.485628	7.1578038	7.0977802	7.1783375
Down	FGSG_11815	-1.11184924	1.80E-05	9.9941348	9.9335416	10.020348	10.527118	10.5308	10.512235	10.239663	10.181805	10.265587
Down	FGSG_11907	-1.112099459	0.0097042	4.1640552	4.3213345	3.9139052	3.5190873	3.6887032	3.260088	3.3903592	2.9633478	3.6872775
Down	FGSG_04629	-1.112218751	0.0296462	4.7070397	4.5354388	4.7932764	4.0052074	3.9182279	4.024755	3.970335	3.8208439	4.0607807
Down	FGSG_04354	-1.112789374	1.03E-06	6.438663	6.3638664	6.4305761	6.2866906	6.374	6.1029268	6.0501376	6.1776727	5.8402522
Down	FGSG_01268	-1.113389811	3.74E-05	6.310507	6.2472245	6.2918249	6.4184274	6.449356	6.2997126	5.5880596	5.5947086	5.5195414
Down	FGSG_06225	-1.113412597	3.11E-06	6.0321508	5.7859081	6.1703363	5.6084049	5.7257602	5.3904893	6.0359471	6.2026947	5.7748904
Down	FGSG_00655	-1.113871302	1.65E-06	6.5935482	6.8169075	6.232359	6.3989268	6.374	6.340023	5.8698804	5.6320297	6.0202512
Down	FGSG_02444	-1.113973676	4.70E-06	7.3039203	7.2087314	7.3130965	6.9937817	7.2883703	6.5086577	6.7255557	6.8351685	6.5363539
Down	FGSG_01857	-1.115205725	0.0001186	7.0150495	6.775389	7.2394951	6.5121623	6.3545295	6.8107005	6.3314892	6.2272902	6.4759412
Down	FGSG_12495	-1.115405457	0.0110471	7.87162	7.9205161	7.7334032	10.140607	10.086039	10.107664	9.7109243	9.7419951	9.6106629
Down	FGSG_00770	-1.115631848	0.0004291	4.4062533	4.4323548	4.3087924	4.4077273	4.1161991	4.5903319	4.1912995	3.6853877	4.5256129
Down	FGSG_10417	-1.116186265	2.23E-09	8.3376209	8.3030481	8.2875517	8.3132388	8.3107819	8.2276734	8.1374545	8.0353685	8.1710364
Down	FGSG_12022	-1.11650422	3.61E-07	7.7831736	7.6941525	7.7860511	7.7731041	7.5503822	7.890333	7.9496278	7.8498277	7.9812488
Down	FGSG_08800	-1.116575472	1.65E-08	11.847692	11.700739	11.901547	12.041142	12.101527	11.884485	12.154485	12.196097	12.04229
Down	FGSG_04897	-1.118249123	0.0020074	4.6610646	4.9628272	4.188785	3.7825262	3.2568854	4.1204343	4.2416299	4.1643702	4.265
Down	FGSG_02309	-1.119578713	0.0017687	8.0246912	7.8522684	8.1006636	8.3080208	8.1164021	8.3996357	7.3305166	7.3198707	7.2760585
Down	FGSG_06493	-1.120564648	1.93E-08	12.154476	12.122087	12.10122	11.594574	11.570582	11.532171	11.54595	11.546668	11.478043
Down	FGSG_12402	-1.120928081	0.0011078	3.872828	3.9254527	3.7539507	3.8981589	3.8080253	3.9222766	3.3903592	3.5358829	3.18196
Down	FGSG_01213	-1.121115837	0.0005771	6.7222549	6.5721788	6.7811692	6.0939946	6.314782	5.7308476	5.6452659	5.4349486	5.7748904
Down	FGSG_02247	-1.122311572	4.24E-07	9.148808	9.1306058	9.106767	8.8123907	8.7771801	8.7605266	8.2615538	8.2546151	8.2148237
Down	FGSG_10360	-1.123952737	1.58E-05	7.1789894	7.1449564	7.1297061	7.7347289	7.7572921	7.6226545	7.5953256	7.4404773	7.6758017
Down	FGSG_05046	-1.124131611	7.52E-07	13.234295	13.174309	13.208173	12.529936	12.50377	12.468086	12.354802	12.320259	12.323356
Down	FGSG_02346	-1.124275319	3.22E-05	5.899844	5.8271292	5.891554	6.4471928	6.4856075	6.3200085	6.0781065	5.8053955	6.2540713

Down	FGSG_02322	-1.125999559	0.0003361	16.203409	16.206867	16.113699	16.823451	16.764133	16.795233	17.031235	16.983621	17.012642
Down	FGSG_07536	-1.126743336	2.83E-05	11.247073	11.262763	11.143966	10.919386	10.866841	10.884308	10.225665	10.20211	10.184537
Down	FGSG_10533	-1.126890968	0.0004258	6.4111515	6.3916107	6.3489364	6.2972625	6.449356	6.0308779	5.6073815	5.8693008	5.2090653
Down	FGSG_10491	-1.128183919	1.60E-10	8.528949	8.5322062	8.4659495	8.4756304	8.3846315	8.5097542	8.4552781	8.3750728	8.5091924
Down	FGSG_06825	-1.130820156	2.53E-07	13.7162	13.679881	13.667	13.692562	13.667606	13.629939	13.725814	13.721395	13.664059
Down	FGSG_07300	-1.130977734	0.0606124	4.2902308	4.2010532	4.3087924	4.0558932	4.2058494	3.8119585	3.7791165	4.0586965	3.3707558
Down	FGSG_07667	-1.131160625	0.0008982	5.2322171	5.1692654	5.2179801	4.8990917	4.1161991	5.3515835	4.935064	4.6006874	5.1587087
Down	FGSG_08312	-1.131600146	1.63E-07	9.2199029	9.2902031	9.0562191	8.984164	9.0220654	8.8609075	8.6284082	8.570358	8.6203531
Down	FGSG_13421	-1.131656558	2.33E-07	6.8196378	6.306724	7.1297061	6.5916131	6.253029	6.7962856	6.6057745	6.5533169	6.5943381
Down	FGSG_10840	-1.13319964	4.08E-05	6.7663496	6.5228522	6.9005856	7.5437309	7.5069498	7.4945842	6.9002206	6.986918	6.7384529
Down	FGSG_13623	-1.135301602	5.10E-07	5.167919	4.8868835	5.3356788	5.484365	5.6318376	5.2281446	5.3564544	5.2552685	5.3945636
Down	FGSG_07039	-1.135735034	8.18E-09	9.9325772	9.9452899	9.8626024	9.8939128	9.9088266	9.8336671	9.7374743	9.6876424	9.7532337
Down	FGSG_10768	-1.13671261	1.39E-08	7.6774476	7.7271924	7.5385022	7.6872892	7.52448	7.7552597	7.5560885	7.6090478	7.4325986
Down	FGSG_12830	-1.136909269	0.0198209	3.3996963	3.378055	3.3684179	4.1048586	3.9182279	4.210161	2.8565633	2.9633478	2.7087823
Down	FGSG_08452	-1.137818151	4.47E-05	12.07945	12.025229	12.049935	12.472355	12.434465	12.425694	12.427434	12.411287	12.377683
Down	FGSG_00814	-1.138381808	1.96E-08	7.3553501	7.0782312	7.5137404	7.8576757	7.7423879	7.8835305	7.5953256	7.6460044	7.4743726
Down	FGSG_08172	-1.138466621	0.0082862	7.0422056	6.7962976	7.177785	8.9223343	8.9031382	8.8539645	9.3370389	9.3468468	9.259854
Down	FGSG_09856	-1.138822894	0.0005668	6.4522245	7.7271924	7.9519165	6.8646813	7.7273281	8.0200518	7.2092957	7.8498277	8.0099383
Down	FGSG_10824	-1.138843768	0.0420898	4.9933948	4.9628272	4.9491538	4.4077273	3.8080253	4.7780897	4.7387761	4.5233477	4.8766132
Down	FGSG_13265	-1.139169585	0.0694947	3.872828	3.9254527	3.7539507	4.4077273	3.8080253	4.7780897	3.6359267	3.8208439	3.3707558
Down	FGSG_06888	-1.1397152	5.20E-06	4.7515947	4.8067189	4.6184939	4.92659	5.179638	4.5218929	4.8725557	4.8742451	4.8129147
Down	FGSG_11709	-1.139931418	0.0826342	2.8633281	2.4852148	3.1285604	3.6568115	3.9182279	3.260088	2.2167888	2.3968278	2
Down	FGSG_12268	-1.140069519	8.63E-07	9.6707233	9.5976865	9.6574817	9.3794514	9.2656355	9.4030076	8.7442187	8.6361261	8.7829449
Down	FGSG_10135	-1.140234318	1.30E-06	15.293067	15.272733	15.228016	15.532966	15.474257	15.50432	15.672105	15.629433	15.648731
Down	FGSG_05387	-1.142604092	8.34E-10	9.6310291	9.6387833	9.5489415	10.011734	9.9628666	9.973161	10.07246	9.931661	10.147896
Down	FGSG_06357	-1.142604995	0.0022571	3.7898088	4.069824	3.3684179	3.5190873	2.8748957	3.9222766	3.3903592	3.6853877	2.964684
Down	FGSG_13554	-1.142646937	0.0119726	2.8633281	2.4852148	3.1285604	3.7210377	4.1161991	3.0813961	3.0942412	2.9633478	3.18196
Down	FGSG_09168	-1.143266886	0.0067916	3.5075501	3.378055	3.5740244	3.5895922	3.2568854	3.8119585	3.0942412	2.9633478	3.18196
Down	FGSG_03555	-1.144001059	1.61E-05	7.8766576	7.7162629	7.9426701	7.4425982	7.4981042	7.2937323	6.7166867	6.7185687	6.6500817
Down	FGSG_00520	-1.146094261	0.0001769	8.528949	8.5751831	8.3931471	7.5168448	7.489204	7.4584209	6.9615665	7.0571987	6.7889889
Down	FGSG_06628	-1.146261596	0.0011939	4.7515947	4.6316459	4.8733193	5.1988268	4.9907667	5.3115993	3.9093688	3.6853877	4.0607807

Down	FGSG_08601	-1.146738726	9.22E-08	7.6482875	7.4388982	7.7546935	7.3892427	7.3387849	7.3540275	7.0275995	7.0434138	6.945698
Down	FGSG_07815	-1.148369237	0.0031883	3.9513288	4.3213345	3.3684179	3.7825262	3.5586152	3.9222766	3.970335	3.6853877	4.1665008
Down	FGSG_08309	-1.149054015	0.001224	6.1861705	6.1851653	6.1055269	5.9270474	5.7557564	6.0060392	5.8537908	5.7723491	5.8718551
Down	FGSG_01045	-1.14922064	2.27E-08	7.3039203	7.2698061	7.2545188	7.3440711	7.1596621	7.4306909	7.2281409	7.1501722	7.2401843
Down	FGSG_12898	-1.151016867	3.16E-07	9.0936299	9.0717981	9.0302651	8.6913699	8.6670545	8.6284275	8.1832408	8.123176	8.1773737
Down	FGSG_08304	-1.151087184	0.0067217	4.0965725	4.4323548	3.5740244	4.3681625	4.3699906	4.2946324	4.8725557	4.8742451	4.8129147
Down	FGSG_06915	-1.151428068	0.0001817	4.7070397	4.8868835	4.5224622	4.871059	4.2902531	5.2704753	4.807216	5.0499834	4.4438634
Down	FGSG_13970	-1.152550845	0.0177033	3.0172362	3.378055	2.4807492	3.1032407	2.8748957	3.260088	3.0942412	3.1804273	2.964684
Down	FGSG_02393	-1.15324699	0.0248053	4.2902308	3.378055	4.7932764	3.6568115	3.2568854	3.9222766	2.8565633	3.1804273	2.3974876
Down	FGSG_11835	-1.15331128	2.10E-05	9.9836668	9.9776853	9.903685	9.5713242	9.4807134	9.5754188	9.4906084	9.4653085	9.4498269
Down	FGSG_12528	-1.154825031	8.64E-06	5.8597011	5.906198	5.728956	5.5913246	5.6318376	5.4652882	5.237485	4.7439404	5.5972237
Down	FGSG_11472	-1.155638646	3.08E-06	9.3631819	9.3193862	9.3217316	9.9163748	9.8457273	9.8992097	9.5862221	9.6449199	9.4576584
Down	FGSG_05927	-1.15625733	0.0018247	7.9785775	8.0392849	7.8268285	7.5258627	7.489204	7.4766159	7.9721606	7.9269302	7.9519772
Down	FGSG_12027	-1.156415557	4.34E-09	7.3625498	7.4652492	7.1619361	7.4755522	7.558914	7.3039581	7.5610519	7.4613623	7.5928932
Down	FGSG_03851	-1.156957133	6.50E-05	11.656278	11.650669	11.575666	12.33753	12.338622	12.247426	12.208863	12.220039	12.129851
Down	FGSG_00761	-1.157249648	6.92E-06	7.544488	7.4120569	7.5867862	7.8718907	7.8575207	7.799296	7.6193177	7.743009	7.411249
Down	FGSG_03552	-1.157422023	0.0173643	2.8633281	2.4852148	3.1285604	3.2841938	3.4156241	3.0813961	2.5718533	2.3968278	2.7087823
Down	FGSG_05024	-1.157494074	1.50E-06	10.132177	10.059957	10.122148	10.301538	10.223864	10.291052	10.153562	10.112529	10.128404
Down	FGSG_08768	-1.158372079	1.68E-10	15.3436	15.307455	15.294204	15.268551	15.214127	15.235576	15.44763	15.40763	15.421218
Down	FGSG_12701	-1.158452506	0.0001657	4.8367778	4.2010532	5.2179801	4.4837433	4.51735	4.3744304	4.8725557	4.7439404	4.9376179
Down	FGSG_03606	-1.158756263	0.0642235	4.6135762	4.6316459	4.5224622	3.7210377	4.2058494	2.8774004	3.199846	3.6853877	2.3974876
Down	FGSG_09991	-1.160409892	2.29E-05	9.2278093	9.1510408	9.2182494	9.4918671	9.5096084	9.3838538	9.1271712	9.0278328	9.1577892
Down	FGSG_11098	-1.16068275	1.12E-05	12.012374	11.989363	11.949651	11.666926	11.689479	11.55322	11.191198	11.149065	11.167008
Down	FGSG_10022	-1.161683475	6.25E-08	12.792972	12.791561	12.708938	12.991456	12.957193	12.938078	12.876832	12.873902	12.8132
Down	FGSG_13530	-1.161777815	0.0007335	4.0965725	4.3213345	3.7539507	4.2425091	4.0206066	4.3744304	3.3903592	3.1804273	3.5376798
Down	FGSG_09849	-1.162671159	2.23E-11	10.023956	10.042562	9.92497	10.277045	10.223864	10.244028	10.277848	10.286453	10.204843
Down	FGSG_12342	-1.163206196	0.0302799	3.872828	3.9254527	3.7539507	3.2841938	3.2568854	3.260088	3.2982451	3.6853877	2.7087823
Down	FGSG_13826	-1.164117857	0.0948276	2.2688027	2.4852148	2	3.7210377	3.6887032	3.6925011	2.9802913	3.3690748	2.3974876
Down	FGSG_20669	-1.165519929	0.0069178	3.6078986	3.1370988	3.9139052	2.8962916	3.0784965	2.6397336	2.9802913	2.7077187	3.18196
Down	FGSG_09889	-1.167668863	4.70E-09	8.864328	8.7936805	8.8540677	8.5371311	8.4548842	8.5316745	8.2523776	8.2546151	8.1836833
Down	FGSG_02420	-1.167796474	8.75E-08	8.817942	8.804041	8.7465942	8.6609948	8.7326257	8.4964403	8.5050612	8.5412272	8.3996284

Down	FGSG_04404	-1.168332543	6.21E-05	5.2633225	5.510285	5.3911128	5.7536574	5.7557564	5.7608523	5.4014247	5.2066203	5.7410644
Down	FGSG_10064	-1.169634487	6.78E-07	8.2855435	8.2957248	8.3307216	7.9841091	7.9959056	7.9759647	7.4206555	7.3312612	7.4846298
Down	FGSG_12099	-1.169773588	5.46E-05	6.1026411	6.120316	6.0025037	6.5829982	6.5209705	6.5777126	6.3198232	6.3666582	6.2052877
Down	FGSG_11090	-1.170819429	0.0015621	6.9108608	6.9534782	6.7811692	6.4280797	6.6221382	6.1029268	5.3334324	5.1562742	5.4374371
Down	FGSG_00537	-1.172370925	1.38E-08	9.1383594	9.1751868	9.0126993	8.5169208	8.5087227	8.4372836	8.7200144	8.7550757	8.615694
Down	FGSG_12430	-1.172491563	0.0009287	5.9195042	5.6997548	6.0719967	5.4083825	5.179638	5.5363994	5.5880596	5.4765776	5.6345505
Down	FGSG_12536	-1.172957675	5.06E-05	8.1547977	7.9394368	8.2654724	7.6382363	7.6008319	7.5979339	6.8765263	7.1110577	6.5164961
Down	FGSG_00224	-1.173753107	1.94E-05	8.183539	8.1244237	8.1577176	8.0386821	8.0634203	7.9238724	7.2092957	7.3977778	6.9156884
Down	FGSG_12575	-1.177337402	1.80E-08	6.9010051	6.5228522	7.1297061	6.6001768	6.3545295	6.7371407	6.6806551	6.59171	6.7037513
Down	FGSG_11437	-1.180892316	0.000222	4.7070397	4.5354388	4.7932764	4.7531416	4.4455509	4.9442019	4.7387761	4.3549955	4.9961472
Down	FGSG_07882	-1.180979944	0.0001191	5.8392029	5.9811576	5.5937254	6.0818165	5.8970396	6.1715481	5.4233949	4.5233477	5.9330571
Down	FGSG_00722	-1.182385283	7.92E-10	10.172677	10.127513	10.132351	9.8197732	9.7521062	9.7997443	9.5910831	9.5771708	9.5386515
Down	FGSG_06592	-1.182936036	1.99E-08	9.2337109	9.301216	9.0774964	9.2936175	9.2016944	9.2969308	8.9792837	8.8688739	9.020014
Down	FGSG_04326	-1.183031315	2.98E-05	5.7973114	5.349828	6.0719967	6.5743316	6.3932112	6.6596278	6.1055435	5.9002223	6.2298857
Down	FGSG_04725	-1.183244132	0.0111403	3.2831234	3.378055	3.1285604	2.7805029	2.6377651	2.8774004	2.9802913	2.9633478	2.964684
Down	FGSG_12607	-1.18384151	2.94E-09	16.894676	16.846423	16.857304	16.910901	16.874876	16.859461	16.772505	16.743792	16.735017
Down	FGSG_01230	-1.185659636	0.0004983	7.340842	7.2547781	7.3415168	6.8788278	6.7318449	6.9343369	6.9615665	6.8028047	7.0460725
Down	FGSG_02868	-1.186601254	0.0034498	5.2004262	4.6316459	5.545677	5.0567294	4.8861689	5.1395644	4.4270393	4.1643702	4.6029775
Down	FGSG_12350	-1.18719988	0.0167707	2.4953013	2.4852148	2.4807492	3.5190873	3.2568854	3.6925011	3.3903592	3.6853877	2.964684
Down	FGSG_02130	-1.187734635	0.0095754	3.5075501	3.378055	3.5740244	3.5190873	3.5586152	3.4190665	3.4769433	3.1804273	3.6872775
Down	FGSG_12385	-1.189527525	0.0061609	3.701719	3.378055	3.9139052	2.8962916	2.8748957	2.8774004	4.028829	4.1643702	3.8228101
Down	FGSG_05839	-1.189992138	0.0005601	6.8196378	6.8372271	6.7175374	6.0571473	6.0257126	6.0060392	5.4873665	5.5170389	5.3945636
Down	FGSG_09159	-1.191121093	9.78E-05	5.1346624	5.7434744	5.545677	4.327482	5.1346873	4.6556708	4.8402557	5.3023297	4.8766132
Down	FGSG_02800	-1.191508689	0.0215712	3.7898088	4.069824	3.3684179	2.7805029	3.0784965	2.3550396	2.9802913	3.3690748	2.3974876
Down	FGSG_11435	-1.191558782	0.0073851	4.8367778	4.7218364	4.8733193	5.9133681	5.6951271	6.0308779	5.7358405	5.5563964	5.8402522
Down	FGSG_10080	-1.191675009	3.73E-07	10.168575	10.016069	10.22679	10.709343	10.645393	10.686492	10.649823	10.644739	10.588064
Down	FGSG_02321	-1.192579228	1.58E-05	15.141188	15.09218	15.104676	14.855924	14.80167	14.823035	14.191289	14.171704	14.145243
Down	FGSG_08495	-1.19275114	2.73E-08	8.6949997	8.6517518	8.6533178	8.7230802	8.6024932	8.753082	8.4283697	8.3584682	8.4317906
Down	FGSG_10099	-1.193194039	1.88E-06	7.7060301	7.7271924	7.5986089	8.0605668	8.0634203	7.9695551	7.0907419	7.1629782	6.945698
Down	FGSG_13689	-1.193205424	2.76E-07	12.947739	12.903592	12.906452	13.46219	13.446212	13.390503	13.627581	13.612847	13.575963
Down	FGSG_07572	-1.193436343	0.000238	7.6716626	7.6488882	7.6103354	8.6067805	8.6431813	8.4784952	8.4391934	8.3695592	8.4423539

Down	FGSG_02016	-1.194305	1.45E-08	9.1383594	9.1306058	9.0647677	9.4756695	9.4117376	9.4521087	9.4563875	9.5235653	9.3155009
Down	FGSG_02174	-1.194872786	0.0291921	4.0965725	4.3213345	3.7539507	4.2425091	4.7733906	3.260088	3.2982451	3.6853877	2.7087823
Down	FGSG_02833	-1.195693114	0.000123	4.6610646	4.8067189	4.4195794	4.657914	4.51735	4.7181783	4.028829	3.6853877	4.265
Down	FGSG_03635	-1.198934056	5.89E-07	7.162584	7.0952041	7.1459111	6.9675462	6.8753756	6.9733016	6.6987834	6.735814	6.5943381
Down	FGSG_11934	-1.199161391	0.0002345	6.665171	6.6431438	6.60482	6.691168	6.449356	6.8249729	6.4751405	6.4733227	6.4128878
Down	FGSG_11022	-1.199305463	2.59E-05	4.7948149	4.3213345	5.0898194	5.0814056	4.8308815	5.2281446	4.6296145	4.4416259	4.7462732
Down	FGSG_02131	-1.199318248	0.0590906	3.3996963	2.4852148	3.9139052	2.6546039	2.6377651	2.6397336	3.199846	3.5358829	2.7087823
Down	FGSG_06215	-1.199802762	0.0002892	11.747443	11.718648	11.690595	12.385848	12.33117	12.353073	12.359795	12.362507	12.290847
Down	FGSG_00311	-1.200328067	0.0407294	4.349408	4.6316459	3.9139052	3.6568115	3.8080253	3.4190665	2.7212202	2.9633478	2.3974876
Down	FGSG_02777	-1.201017507	0.0401266	2.6910183	3.1370988	2	2.6546039	2.353841	2.8774004	3.199846	2.9633478	3.3707558
Down	FGSG_09374	-1.201869262	1.10E-07	12.319875	12.306459	12.247314	12.422977	12.434465	12.321334	12.502724	12.467533	12.471563
Down	FGSG_06278	-1.202260112	0.0097983	3.2831234	3.1370988	3.3684179	3.2841938	3.5586152	2.8774004	3.4769433	3.3690748	3.5376798
Down	FGSG_02972	-1.203110314	0.000343	5.4095206	5.458757	5.2780294	5.4468739	5.265544	5.5363994	5.5684753	5.5947086	5.4790733
Down	FGSG_02031	-1.203118891	1.11E-07	7.921222	7.8220012	7.9333641	7.5392846	7.489204	7.5034852	7.5003335	7.3977778	7.5348496
Down	FGSG_10848	-1.203268134	1.23E-06	11.384272	11.32344	11.35906	11.611351	11.539665	11.595459	11.520059	11.502639	11.470967
Down	FGSG_03410	-1.203306142	0.0379824	4.0257777	4.2010532	4.3087924	2.5166584	3.2568854	3.9222766	2.7212202	2.9633478	2.964684
Down	FGSG_11278	-1.204581687	0.0273893	3.2831234	3.1370988	3.3684179	2.5166584	2.353841	2.6397336	3.199846	3.1804273	3.18196
Down	FGSG_13042	-1.204868264	0.0005633	4.5136357	4.8067189	4.0578819	4.1980691	4.2058494	4.1204343	3.7092973	3.8208439	3.5376798
Down	FGSG_11470	-1.205929912	0.0041692	3.701719	3.9254527	3.3684179	3.9526758	4.2902531	3.4190665	4.1391495	4.6006874	3.3707558
Down	FGSG_05625	-1.207038951	1.24E-06	9.6692725	9.5736661	9.6771285	9.7694079	9.716324	9.735087	10.206787	10.186516	10.160747
Down	FGSG_08528	-1.207194658	3.43E-06	8.1296981	8.1326676	8.0412601	7.5437309	7.308748	7.6708603	7.07	7.0294959	7.0460725
Down	FGSG_00377	-1.207272542	3.98E-07	8.8819718	8.8745529	8.8038869	8.7132454	8.6391638	8.6998658	8.1506853	8.123176	8.1127123
Down	FGSG_09710	-1.207725486	2.16E-06	6.5052304	6.4975416	6.4305761	7.0881229	6.9157907	7.1650109	7.083861	7.1110577	6.9895734
Down	FGSG_07615	-1.208564306	0.0112254	3.6078986	3.765011	3.3684179	4.0558932	3.8080253	4.210161	3.9093688	3.6853877	4.0607807
Down	FGSG_04493	-1.208790742	3.94E-05	6.169847	6.153105	6.1055269	6.9675462	6.8889735	6.9604299	6.9002206	7.0434138	6.6681943
Down	FGSG_03579	-1.209290743	7.59E-05	6.9966572	6.7962976	7.0967395	6.4566552	6.2739084	6.5435983	5.9170997	5.7723491	5.991768
Down	FGSG_00011	-1.209666905	0.0312581	5.4906186	5.349828	5.545677	3.5895922	3.2568854	3.8119585	3.2982451	3.3690748	3.18196
Down	FGSG_13973	-1.210344006	0.0061048	5.100621	5.4053203	4.6184939	4.1522165	3.9182279	4.2946324	4.3373111	3.6853877	4.7462732
Down	FGSG_05698	-1.210678638	0.0015179	10.942891	10.95171	10.847164	9.5918655	9.4807134	9.612916	9.8441415	9.8228966	9.7991598
Down	FGSG_11141	-1.21187181	0.0034191	5.8597011	5.9811576	5.640225	5.2649499	4.8308815	5.5363994	4.5115122	4.9937494	3.6872775
Down	FGSG_10632	-1.212287194	8.76E-09	7.8097548	7.6941525	7.8368453	7.5701251	7.5673956	7.485628	7.5003335	7.6368537	7.2760585

Down	FGSG_13676	-1.213218176	0.026523	3.9513288	4.069824	3.7539507	4.0052074	4.2058494	3.6925011	3.199846	3.5358829	2.7087823
Down	FGSG_03667	-1.213387596	4.51E-06	8.516093	8.3462239	8.5895813	8.2079187	8.2276057	8.0985166	7.5807364	7.6641337	7.4219633
Down	FGSG_05574	-1.21341833	0.0004285	5.5422571	5.510285	5.4959731	5.5565451	5.265544	5.7308476	4.8725557	4.5233477	5.1065306
Down	FGSG_13917	-1.214627029	7.47E-07	7.0241584	7.0260808	6.9382912	6.4471928	6.449356	6.3597635	6.6897477	6.4937409	6.8054483
Down	FGSG_04127	-1.214640362	1.06E-09	8.516093	8.4488278	8.4978648	8.5460234	8.4548842	8.5532667	8.1637958	8.1683327	8.0927348
Down	FGSG_14021	-1.216361824	0.0001688	6.629804	6.7326402	6.4305761	6.34899	6.2739084	6.3597635	5.9015311	5.9304949	5.8079414
Down	FGSG_04448	-1.216505605	6.88E-05	6.6996916	6.6660438	7.063002	6.1299242	6.0257126	6.4908649	5.7181748	5.3023297	6.1802631
Down	FGSG_09457	-1.216698891	1.55E-07	10.300008	10.307599	10.207619	10.534397	10.516636	10.464032	10.546003	10.50714	10.521129
Down	FGSG_03099	-1.216712707	0.012203	3.3996963	3.1370988	3.5740244	3.2841938	3.6887032	3.260088	3.8457121	3.3690748	4.265
Down	FGSG_09075	-1.217159397	0.0073098	3.2831234	3.5844678	2.8407141	3.0034737	2.8748957	3.0813961	3.0942412	3.5358829	2.3974876
Down	FGSG_12767	-1.218332451	3.69E-06	6.9873724	7.1119795	6.7602689	6.7307036	6.3545295	6.9604299	6.0641898	6.0998924	5.9627112
Down	FGSG_12112	-1.221353034	4.47E-08	9.1571127	9.1751868	9.0519257	9.8388945	9.8014746	9.788864	9.8132732	9.7483819	9.8114355
Down	FGSG_13768	-1.22151393	0.0053518	3.872828	3.9254527	3.7539507	3.5895922	3.0784965	3.9222766	2.9802913	2.3968278	3.3707558
Down	FGSG_07208	-1.221863353	6.05E-05	7.0950308	7.19305	6.9005856	7.3693409	7.2883703	7.3638362	6.9464729	6.986918	6.8378143
Down	FGSG_01883	-1.222865368	9.02E-09	9.0849505	9.1831465	8.8881838	8.7386778	8.7212688	8.6685513	8.9885239	8.9743484	8.9365461
Down	FGSG_03104	-1.223163679	0.0008885	6.1533367	6.4455482	5.6852725	7.188078	7.1596621	7.1309525	6.9002206	6.735814	6.9895734
Down	FGSG_06149	-1.223822824	0.0002845	5.8799122	5.6997548	5.9664603	5.3074061	5.5313719	4.9442019	5.0804226	4.6740912	5.3503768
Down	FGSG_10065	-1.223962519	1.16E-05	7.8097548	7.8014645	7.9239976	7.7693122	7.8644203	7.7021252	7.104406	7.1110577	7.0870405
Down	FGSG_07010	-1.224566793	0.0012209	4.5136357	4.8067189	4.0578819	4.5907474	4.9394155	4.024755	3.970335	3.1804273	4.4438634
Down	FGSG_01969	-1.225322127	9.49E-08	11.295368	11.269321	11.313159	11.889793	11.892974	11.879793	11.733495	11.749191	11.692885
Down	FGSG_07883	-1.225804343	8.16E-08	7.7116792	7.6941525	7.6449538	7.0067227	6.8337955	7.0842532	7.0630192	7.0434138	7.0180995
Down	FGSG_07985	-1.226572181	0.0218984	3.9513288	3.9254527	3.9139052	3.1032407	3.2568854	2.8774004	2.7212202	2.7077187	2.7087823
Down	FGSG_14015	-1.226684978	1.90E-05	10.069764	10.016069	10.037821	10.045048	10.004062	10.00014	9.4090004	9.4206848	9.3326369
Down	FGSG_10755	-1.227702629	0.0005025	13.62091	13.588816	13.567308	14.303772	14.248553	14.271583	14.117086	14.084156	14.083632
Down	FGSG_06830	-1.227803019	4.66E-08	14.481656	14.443704	14.434209	14.781637	14.749632	14.72596	14.75446	14.747989	14.693986
Down	FGSG_00654	-1.228459481	3.28E-06	7.992567	7.9765496	7.9239976	8.8307001	8.7250644	8.846988	8.785093	8.6724042	8.8319719
Down	FGSG_13308	-1.228569647	0.0145383	3.1562965	3.378055	2.8407141	3.7825262	3.4156241	4.1204343	3.4769433	2.9633478	3.8228101
Down	FGSG_13249	-1.229925847	0.0976395	4.0965725	3.9254527	4.188785	2.6546039	2.8748957	2.3550396	3.3903592	2.9633478	3.6872775
Down	FGSG_13049	-1.230025101	0.0009247	4.2902308	4.5354388	3.9139052	4.327482	4.5857446	3.9222766	4.2416299	4.6006874	3.6872775
Down	FGSG_04306	-1.230660259	2.01E-06	8.3737008	8.2357604	8.4200477	8.642458	8.499888	8.6921007	8.499901	8.45013	8.4838504
Down	FGSG_13295	-1.231797614	0.000525	4.0965725	3.9254527	4.188785	4.4837433	4.1161991	4.7181783	5.2124668	5.4765776	4.8129147

Down	FGSG_06975	-1.232721244	1.21E-10	9.1108336	9.0104909	9.1232303	8.9627407	8.8862879	8.9612317	8.8514196	8.6902068	8.9365461
Down	FGSG_02491	-1.233609803	9.51E-06	10.1737	10.16014	10.105672	9.4486578	9.3343365	9.472651	8.8392134	8.8727767	8.7493095
Down	FGSG_00276	-1.233977394	1.64E-06	10.499331	10.520468	10.390453	10.407188	10.363123	10.363729	10.515005	10.458626	10.504843
Down	FGSG_07304	-1.235198576	1.78E-08	9.1467243	9.3265906	9.4249411	8.7750599	9.0065557	8.9252511	8.4738197	8.4963975	8.6661346
Down	FGSG_01972	-1.236347915	8.63E-10	12.059376	12.028546	12.004558	12.228693	12.178288	12.191724	12.343504	12.32454	12.295952
Down	FGSG_13740	-1.236429843	0.0354515	2.6910183	2.4852148	2.8407141	3.2841938	3.2568854	3.260088	2.5718533	2.3968278	2.7087823
Down	FGSG_03271	-1.236479975	8.38E-06	5.9769267	6.4455482	5.8125445	5.8995578	6.1438433	6.279127	5.2620767	5.5563964	5.6345505
Down	FGSG_02624	-1.237207619	7.27E-06	9.3378353	9.2865133	9.3037887	9.5810908	9.5741599	9.5040355	9.2413391	9.1930213	9.2235695
Down	FGSG_08351	-1.237623199	2.20E-09	10.672258	10.661038	10.59905	10.464007	10.443649	10.396352	10.069851	10.034541	10.045956
Down	FGSG_13517	-1.240375033	0.0042538	5.8184092	6.052414	5.4444954	5.7068462	5.6318376	5.7308476	4.1391495	3.9446664	4.265
Down	FGSG_12145	-1.240493306	0.0552394	3.2831234	3.5844678	2.8407141	2.364115	2.353841	2.3550396	2.9802913	3.1804273	2.7087823
Down	FGSG_09761	-1.241070295	8.40E-05	5.3235909	5.2921154	5.2780294	5.1760947	4.9394155	5.3115993	5.237485	4.8742451	5.4790733
Down	FGSG_09410	-1.243234611	2.62E-09	9.6576129	9.6271603	9.6027653	9.5449521	9.5029915	9.4995936	9.9088397	9.882181	9.8712943
Down	FGSG_03272	-1.243248043	0.0586998	3.0172362	3.1370988	2.8407141	3.0034737	3.2568854	2.6397336	2.7212202	2	3.18196
Down	FGSG_13578	-1.243331709	8.53E-08	6.8300632	6.9719742	6.5811798	6.9937817	6.9024444	6.9987054	7.0131845	6.8668223	7.0870405
Down	FGSG_00338	-1.243885387	2.81E-06	14.001919	13.966124	13.952305	14.686167	14.661702	14.623474	14.805478	14.77854	14.766711
Down	FGSG_13679	-1.244519533	2.06E-10	8.2315156	8.2883641	8.0839393	8.3517838	8.2168632	8.3948599	8.0448251	8.0001203	8.024072
Down	FGSG_12368	-1.245896088	9.72E-08	8.8438959	8.9833974	8.5954933	9.2816885	9.2446352	9.2314715	9.1684095	9.0794051	9.1894874
Down	FGSG_11816	-1.247733782	1.33E-06	6.0500989	5.906198	6.1055269	5.674773	5.6318376	5.636897	5.6452659	5.3920827	5.8079414
Down	FGSG_10815	-1.248696369	0.0016447	4.8367778	4.6316459	4.9491538	5.0567294	5.2232303	4.7780897	4.5519634	4.3549955	4.6764038
Down	FGSG_07582	-1.249529633	0.0042999	7.0150495	7.0436746	6.9005856	8.6089038	8.6270435	8.5008919	9.0320711	8.9888059	9.0093969
Down	FGSG_05270	-1.250119854	0.0001981	4.9172105	4.8868835	4.8733193	4.4077273	4.0206066	4.8356119	3.8457121	3.8208439	4.265
Down	FGSG_06486	-1.251698335	5.51E-05	6.4249728	6.1851653	6.5571458	5.3887447	5.6638294	4.9442019	5.4450355	5.5170389	5.3047937
Down	FGSG_11852	-1.252869872	2.50E-07	7.7116792	7.5538626	7.7756741	8.2466164	8.2435709	8.1617312	8.2493059	8.0833124	8.3387351
Down	FGSG_00885	-1.253517267	0.0210694	3.0172362	2.8476671	3.1285604	3.1032407	2.6377651	3.4190665	2.7212202	2.3968278	2.964684
Down	FGSG_03842	-1.254054545	1.16E-05	4.7515947	4.6316459	4.7932764	4.327482	4.0206066	4.5218929	4.2416299	4.4416259	3.9466965
Down	FGSG_12070	-1.254363233	0.0361776	2.4953013	2.4852148	2.4807492	2.8962916	3.0784965	2.6397336	3.2982451	3.6853877	2.7087823
Down	FGSG_02728	-1.255704735	0.0006682	6.0500989	6.0172257	6.0025037	6.5302	6.5554873	6.4361306	6.4856201	6.1262878	6.7212064
Down	FGSG_01586	-1.25581206	0.0004326	5.5673995	5.2319977	5.7713555	5.1295269	4.9907667	5.1845342	4.4270393	4.6006874	4.1665008
Down	FGSG_04575	-1.256197244	7.35E-06	8.277948	8.2585401	8.2126008	7.6298962	7.4802486	7.6865774	7.1179419	7.124214	7.0460725
Down	FGSG_08066	-1.256418037	0.0001348	5.100621	5.1692654	5.1553224	5.2432439	5.4962661	5.2281446	4.4270393	4.3549955	4.4438634

Down	FGSG_06168	-1.256745582	2.28E-10	12.850914	12.771573	12.843261	13.245512	13.258457	13.14261	13.279433	13.27099	13.221599
Down	FGSG_13394	-1.256850978	0.0001089	7.0422056	7.0782312	6.9195616	7.6588781	7.489204	7.7327269	7.4423369	7.3649028	7.4536368
Down	FGSG_03375	-1.257403395	1.18E-05	8.1671856	8.1651802	8.0839393	7.9068273	7.8225187	7.9038425	7.1643417	7.1372515	7.1268773
Down	FGSG_02292	-1.257514949	0.059628	2.6910183	3.378055	3.7539507	2.7805029	3.8080253	3.0813961	2.2167888	2.3968278	2.7087823
Down	FGSG_06101	-1.25798033	0.0030752	3.2831234	2.8476671	3.5740244	4.4077273	4.6510429	4.024755	3.9093688	3.5358829	4.1665008
Down	FGSG_13711	-1.26038921	8.11E-07	8.3192369	8.4086576	8.1335416	7.836087	7.7348776	7.8490285	7.436947	7.2733875	7.5249446
Down	FGSG_03448	-1.260544722	1.68E-06	15.316659	15.266074	15.28154	15.809267	15.784846	15.746004	15.684951	15.662746	15.640835
Down	FGSG_01433	-1.262093104	1.44E-07	12.247345	12.180411	12.228415	12.115271	12.082421	12.060662	11.522605	11.493796	11.486362
Down	FGSG_10886	-1.263001557	1.34E-05	5.100621	4.6316459	5.3911128	4.5907474	4.6510429	4.4500449	4.3828727	4.262829	4.4438634
Down	FGSG_05717	-1.263169853	0.0123632	4.2902308	3.9254527	4.5224622	3.8981589	4.2058494	3.4190665	3.4769433	3.1804273	3.8228101
Down	FGSG_08830	-1.263220845	0.0052413	6.7334055	6.6660438	6.7175374	7.8433192	7.8295873	7.7700883	8.3502416	8.3132316	8.3216718
Down	FGSG_08235	-1.263744845	3.45E-07	7.0600298	6.5962238	7.3415168	6.7842942	6.8477891	6.6274177	6.5166098	6.4102748	6.555942
Down	FGSG_10611	-1.265366055	0.0002471	8.7148257	8.7407319	8.6072451	9.323662	9.2446352	9.3147394	8.739848	8.8292542	8.5826522
Down	FGSG_06993	-1.265871044	1.33E-05	10.51557	10.542292	10.400619	10.720677	10.704263	10.648878	10.292844	10.295204	10.223336
Down	FGSG_03230	-1.266448675	8.57E-05	7.642384	7.6716978	7.5261744	8.238411	8.2329471	8.1560975	8.1605293	8.0001203	8.2453061
Down	FGSG_04497	-1.266869396	4.77E-10	7.1207354	7.0436746	7.113317	7.3491606	7.3387849	7.2730605	7.0975901	7.0571987	7.0735134
Down	FGSG_07299	-1.267188689	1.45E-07	10.950078	10.937663	10.876595	10.484389	10.450543	10.430589	10.027446	9.9903184	9.9984282
Down	FGSG_03360	-1.268517449	6.85E-05	5.3528065	5.2921154	5.3911128	6.2104357	6.2103411	6.1261652	6.2600348	6.1776727	6.3242864
Down	FGSG_08043	-1.270448173	0.0472122	3.1562965	3.1370988	3.1285604	2.7805029	2.8748957	2.6397336	3.0942412	3.1804273	2.964684
Down	FGSG_00177	-1.270925184	0.0019987	9.6130305	9.6329835	9.5056446	9.0213046	9.0343543	8.9186116	8.9718487	8.9449914	8.9365461
Down	FGSG_02264	-1.272568945	0.0005781	4.349408	4.2010532	4.4195794	3.6568115	4.0206066	3.260088	4.4270393	4.1643702	4.6029775
Down	FGSG_05447	-1.272861488	2.88E-09	9.8427603	9.8485099	9.7504977	9.3441996	9.2233246	9.3741808	9.3004079	9.318511	9.2143539
Down	FGSG_02701	-1.274566052	9.17E-06	9.5355212	9.4927173	9.4930315	10.203078	10.097797	10.218578	10.083713	10.09593	10.003787
Down	FGSG_05929	-1.274647476	9.64E-09	10.882818	10.907952	10.770849	10.574062	10.521009	10.539537	10.349164	10.328267	10.303742
Down	FGSG_00989	-1.275014163	0.0123201	3.1562965	2.4852148	3.5740244	4.0052074	4.0206066	3.9222766	3.0942412	2.9633478	3.18196
Down	FGSG_08078	-1.275535288	2.60E-06	11.464672	11.438467	11.40523	11.64459	11.588923	11.612529	11.400891	11.319601	11.414413
Down	FGSG_05686	-1.276744911	0.0019218	5.065757	5.349828	4.6184939	5.8429557	5.8698682	5.7902457	4.2902636	3.9446664	4.5256129
Down	FGSG_12779	-1.277431115	0.0289055	2.6910183	2	3.1285604	3.0034737	2.6377651	3.260088	2.5718533	2.3968278	2.7087823
Down	FGSG_05381	-1.277759344	0.0003967	6.9010051	6.6660438	7.2693877	5.7382218	6.2318429	5.4652882	5.5880596	5.7385279	5.4790733
Down	FGSG_03369	-1.278185551	0.0002825	5.8392029	5.608128	5.9664603	5.1760947	4.7733906	5.4652882	4.8725557	4.9352342	4.7462732
Down	FGSG_03769	-1.279469234	2.02E-05	10.583386	10.548467	10.532813	10.594565	10.513347	10.587415	10.430743	10.444273	10.349358

Down	FGSG_05481	-1.279864304	0.0002329	11.487903	12.157868	12.262257	11.1574	11.873869	11.824483	10.585511	11.433639	11.420437
Down	FGSG_00406	-1.280200679	4.86E-05	11.808029	11.731596	11.797682	12.48093	12.469675	12.403576	10.814226	10.827772	10.732777
Down	FGSG_06110	-1.28032526	1.21E-06	4.9558055	4.9628272	4.8733193	5.4277566	5.5313719	5.2281446	4.7734018	4.6740912	4.8129147
Down	FGSG_03576	-1.280567863	0.0003326	5.2004262	5.5600358	4.6184939	5.0316238	4.5857446	5.3115993	4.0850435	3.9446664	4.1665008
Down	FGSG_11374	-1.281232399	1.56E-06	5.6164066	5.2921154	5.8125445	6.3181765	6.1885139	6.3597635	6.1055435	5.8053955	6.301259
Down	FGSG_09186	-1.281245612	0.0307889	3.872828	3.5844678	4.0578819	5.8715343	5.7557564	5.9021734	5.8375198	5.5170389	6.0481828
Down	FGSG_11974	-1.282409103	0.0021273	4.0965725	4.069824	4.188785	3.6568115	3.8080253	3.4190665	3.9093688	4.3549955	3.18196
Down	FGSG_13862	-1.283654739	0.0022396	4.8367778	4.8067189	4.7932764	4.7531416	4.6510429	4.7780897	3.9093688	3.3690748	4.265
Down	FGSG_04093	-1.284927023	0.0026292	3.9513288	4.069824	3.9139052	3.7210377	3.5586152	3.9222766	2.9802913	2.9633478	2.964684
Down	FGSG_01313	-1.285455035	2.98E-11	9.5576468	9.5368686	9.4930315	9.6424928	9.537936	9.6574607	9.4416615	9.3910573	9.4260741
Down	FGSG_03872	-1.285715573	0.0541498	3.5075501	3.378055	3.5740244	3.1965524	2.6377651	3.5622521	3.2982451	3.1804273	3.3707558
Down	FGSG_00045	-1.286285726	2.13E-09	8.512861	8.6169166	8.3092981	8.3262019	8.3701609	8.1895743	7.9344081	7.8096741	7.9884749
Down	FGSG_06987	-1.286831053	1.01E-07	14.81632	14.766574	14.780374	15.065679	15.020442	15.023384	14.865388	14.839761	14.824698
Down	FGSG_10776	-1.287422479	5.01E-05	5.5422571	5.7434744	5.2179801	5.7689296	5.975612	5.4283735	5.626448	5.4349486	5.7410644
Down	FGSG_03620	-1.287868692	0.0027856	7.10365	7.0082698	7.113317	7.1993948	7.1256073	7.187278	6.2842483	6.2026947	6.301259
Down	FGSG_13638	-1.287962122	2.16E-07	9.4177704	9.3479906	9.4015109	9.8613323	9.799676	9.8354306	10.357034	10.335355	10.313844
Down	FGSG_03192	-1.289012766	3.05E-09	7.606447	7.7910854	7.2986735	7.41859	7.203853	7.5298632	7.370647	7.200728	7.4640419
Down	FGSG_13625	-1.289892709	0.0037505	3.1562965	3.378055	2.8407141	2.8962916	3.2568854	2.6397336	2.5718533	2.3968278	2.7087823
Down	FGSG_02983	-1.290885308	0.0071671	2.8633281	2.4852148	3.1285604	2.8962916	3.2568854	2.3550396	3.2982451	3.3690748	3.18196
Down	FGSG_13985	-1.292352098	0.0025686	3.872828	3.5844678	4.0578819	4.5907474	4.51735	4.5903319	3.970335	4.3549955	3.3707558
Down	FGSG_03912	-1.292518966	2.37E-05	4.5644712	4.5354388	4.5224622	4.3681625	4.3699906	4.3744304	4.028829	4.1643702	3.8228101
Down	FGSG_02332	-1.293493304	0.0214432	3.1562965	2.4852148	3.5740244	2.8962916	3.4156241	2.3550396	2.9802913	2.9633478	2.964684
Down	FGSG_03118	-1.294230453	0.0159734	4.8367778	4.9628272	4.6184939	4.327482	3.9182279	4.5903319	3.4769433	3.1804273	3.6872775
Down	FGSG_13396	-1.294786704	2.90E-07	14.21678	14.167241	14.180843	14.621056	14.579245	14.57528	14.74246	14.708749	14.710398
Down	FGSG_06038	-1.296088939	0.0175019	5.4095206	5.4053203	5.3356788	3.9526758	4.2902531	3.4190665	4.3828727	4.4416259	4.265
Down	FGSG_09585	-1.297109277	1.04E-10	10.672982	10.619113	10.642528	10.785	10.808112	10.670774	10.891129	10.890715	10.824463
Down	FGSG_10545	-1.297593751	2.79E-09	8.7148257	8.7674491	8.6304653	8.2520609	8.2276057	8.2222919	8.1539741	8.0833124	8.1962202
Down	FGSG_07816	-1.298347554	0.0029793	4.9172105	4.3213345	5.2780294	4.4462359	4.2902531	4.5218929	3.970335	4.3549955	3.3707558
Down	FGSG_12627	-1.298760078	0.0568156	3.7898088	4.069824	3.3684179	3.2841938	3.0784965	3.4190665	2.4052168	2.7077187	2
Down	FGSG_00593	-1.299042566	2.20E-05	5.6638035	5.6546687	5.5937254	6.032049	5.7257602	6.2155469	5.3334324	5.3023297	5.3047937
Down	FGSG_13648	-1.299373544	0.0498842	3.3996963	2.8476671	3.7539507	3.5190873	2.8748957	3.9222766	3.7791165	3.8208439	3.6872775

Down	FGSG_00330	-1.300938718	9.22E-06	10.365976	10.343556	10.302834	10.203781	10.135361	10.184392	9.529111	9.5160987	9.4757683
Down	FGSG_00036	-1.301195286	0.0690106	3.1562965	2.8476671	3.3684179	2.7805029	2.8748957	2.6397336	3.4769433	4.0586965	2.3974876
Down	FGSG_11419	-1.301322414	0.0050811	4.2285219	3.9254527	4.4195794	4.2856211	4.51735	3.9222766	3.4769433	3.6853877	3.18196
Down	FGSG_09894	-1.301677025	2.50E-09	7.9785775	7.8819136	7.9972798	7.5917573	7.3387849	7.7327269	7.6935164	7.5806954	7.7371454
Down	FGSG_05915	-1.303507252	1.69E-07	7.0332102	7.0082698	7.0967395	6.8503947	7.1140747	6.6110389	6.354542	6.4937409	6.2298857
Down	FGSG_03277	-1.305246893	0.0034177	4.6135762	4.6316459	4.6184939	5.9270474	5.7557564	6.0060392	5.3791148	5.347904	5.3503768
Down	FGSG_12812	-1.305428201	0.0041969	3.3996963	3.5844678	3.1285604	3.3668146	3.2568854	3.4190665	2.7212202	2.7077187	2.7087823
Down	FGSG_07163	-1.305478428	0.0621192	3.701719	3.378055	3.9139052	3.0034737	3.2568854	2.6397336	2.7212202	2	3.18196
Down	FGSG_13224	-1.306803498	4.97E-05	6.2023113	6.120316	6.2016809	6.8575557	6.9679745	6.6436126	6.5864309	6.61053	6.4963611
Down	FGSG_06889	-1.306922712	0.0011965	6.3545028	6.120316	6.4825439	4.9800625	5.0403527	4.8356119	5.7002901	5.5947086	5.7410644
Down	FGSG_10868	-1.307048351	2.67E-08	10.591834	10.610285	10.521989	10.994798	10.962386	10.959816	10.910704	10.90603	10.884764
Down	FGSG_01803	-1.307765547	6.94E-06	5.8597011	5.8271292	5.8125445	5.2649499	5.346621	5.0931475	5.2124668	5.4349486	4.8766132
Down	FGSG_02791	-1.309695884	6.18E-13	10.585695	10.491914	10.591674	10.634716	10.579833	10.601993	10.50216	10.50714	10.429858
Down	FGSG_11282	-1.310108478	9.34E-06	6.8506908	6.9534782	6.650969	6.0818165	6.0501253	6.0308779	6.1719365	5.5170389	6.5752678
Down	FGSG_10979	-1.310847491	4.68E-05	5.8392029	5.906198	5.6852725	6.1764794	6.2739084	5.9807654	5.9325022	6.0456069	5.7410644
Down	FGSG_00594	-1.311576967	0.0062525	5.100621	4.9628272	5.5937254	4.1980691	3.9182279	4.6556708	4.7032988	5.2552685	4.9376179
Down	FGSG_10833	-1.31190917	1.01E-06	6.5812576	6.5477263	6.5571458	7.3389636	7.3188299	7.2834334	7.4041778	7.4192855	7.3225439
Down	FGSG_11097	-1.312871341	3.09E-07	12.99324	12.919462	12.980608	13.137047	13.099226	13.087255	13.05825	13.019413	13.03071
Down	FGSG_07704	-1.31293813	0.0414486	5.5166689	5.608128	5.3356788	4.5907474	4.51735	4.5903319	3.3903592	3.5358829	3.18196
Down	FGSG_12254	-1.313248906	0.0104984	6.0139766	5.9811576	5.9664603	5.6908987	5.8139402	5.4652882	6.2962044	6.2272902	6.301259
Down	FGSG_10602	-1.314776986	9.87E-05	4.9558055	4.8067189	5.0212003	4.871059	4.9907667	4.6556708	4.028829	3.6853877	4.265
Down	FGSG_10609	-1.316215325	9.80E-06	10.774308	10.794325	10.668	10.894798	10.796435	10.903769	10.294335	10.229762	10.292109
Down	FGSG_13678	-1.317833406	0.0020172	5.5422571	5.6997548	5.2780294	5.0060735	4.7733906	5.1395644	3.9093688	3.5358829	4.1665008
Down	FGSG_08044	-1.318302296	0.0186057	3.0172362	3.1370988	2.8407141	2.8962916	3.2568854	2.3550396	3.199846	3.1804273	3.18196
Down	FGSG_04027	-1.318482602	0.0003055	5.7096927	5.510285	5.8125445	5.8284512	5.7851416	5.7902457	5.1610901	5.3023297	4.9376179
Down	FGSG_03249	-1.318808405	6.17E-05	9.8908596	9.8001612	9.8965196	10.69692	10.627253	10.683558	11.408828	11.411676	11.341512
Down	FGSG_03638	-1.319924692	0.0002878	6.3971965	6.3355781	6.3766661	6.0939946	6.2103411	5.874995	5.4450355	5.4765776	5.3503768
Down	FGSG_06736	-1.320334289	1.08E-08	8.9835298	8.9464625	8.9355627	9.0621694	9.0735851	8.9612317	9.6139516	9.5334611	9.6269607
Down	FGSG_05919	-1.320554246	0.0069421	4.7515947	4.8868835	4.5224622	4.1048586	4.51735	3.4190665	3.8457121	3.9446664	3.6872775
Down	FGSG_04507	-1.321201578	3.34E-08	9.1674268	9.1950043	9.0519257	8.7901083	8.7326257	8.7605266	8.2767189	8.2486203	8.239261
Down	FGSG_00017	-1.322260973	0.0009082	5.3814422	5.4053203	5.2780294	4.4077273	4.51735	4.210161	5.0240241	5.3920827	4.4438634

Down	FGSG_03622	-1.32288631	0.0017993	3.701719	3.9254527	3.3684179	3.1965524	2.8748957	3.5622521	3.0942412	3.1804273	2.964684
Down	FGSG_03592	-1.323037833	0.0772853	3.6078986	3.378055	3.7539507	3.6568115	3.4156241	3.8119585	2.7212202	2	3.18196
Down	FGSG_07366	-1.324263671	5.95E-09	10.184912	10.147991	10.136412	9.9351117	9.843983	9.9374804	10.140357	10.114178	10.10033
Down	FGSG_14011	-1.327720162	2.94E-05	4.8775546	4.2010532	5.2780294	4.7531416	4.8861689	4.5218929	4.807216	4.5233477	4.9961472
Down	FGSG_08176	-1.328849701	0.0005806	3.701719	3.378055	3.9139052	3.1965524	3.2568854	3.0813961	2.9802913	2.7077187	3.18196
Down	FGSG_13003	-1.32892305	0.020663	4.6135762	4.8868835	4.188785	3.0034737	3.0784965	2.8774004	3.8457121	4.1643702	3.3707558
Down	FGSG_09833	-1.329633087	1.80E-12	11.200122	11.149514	11.164991	11.266671	11.228152	11.218183	10.989214	10.998238	10.91257
Down	FGSG_09014	-1.330048003	6.38E-08	10.794412	10.800806	10.701277	11.154495	11.113376	11.107967	11.113932	11.033531	11.127461
Down	FGSG_00381	-1.332615563	0.0654408	2.4953013	2.8476671	2	2.8962916	2.8748957	2.8774004	3.5586239	3.5358829	3.5376798
Down	FGSG_02998	-1.333361441	0.0067321	3.9513288	3.9254527	3.9139052	3.9526758	3.6887032	4.1204343	3.4769433	3.1804273	3.6872775
Down	FGSG_02752	-1.334917852	7.75E-05	5.4370629	4.6316459	5.891554	6.328521	6.253029	6.3200085	5.770536	5.8053955	5.670936
Down	FGSG_05118	-1.335398324	0.0052785	3.3996963	3.378055	3.3684179	3.7825262	2.8748957	4.2946324	3.6359267	3.8208439	3.3707558
Down	FGSG_07776	-1.335594751	6.89E-05	6.6882763	6.6198745	6.6735015	6.9741499	6.9157907	6.9474424	6.0781065	5.9304949	6.1547968
Down	FGSG_06228	-1.335855479	3.01E-05	9.6951671	9.6843607	9.6202694	10.559864	10.5221	10.510028	10.848123	10.842772	10.786621
Down	FGSG_04123	-1.339710056	2.93E-07	10.054285	9.9288153	10.0911	10.689415	10.673165	10.617449	10.395749	10.29811	10.42454
Down	FGSG_07673	-1.341889545	0.0065353	10.139531	10.131632	10.061507	11.611616	11.603945	11.531083	11.77039	11.752372	11.721897
Down	FGSG_05045	-1.342039992	3.07E-10	15.333588	15.292184	15.289312	15.250027	15.226229	15.185771	15.304887	15.280226	15.263367
Down	FGSG_01582	-1.342473641	0.0001944	5.100621	5.2319977	4.8733193	5.0567294	4.9394155	5.0931475	4.1912995	4.3549955	3.9466965
Down	FGSG_09323	-1.34482921	4.52E-08	9.6692725	9.7258202	9.5212573	9.8234349	9.7779154	9.7815646	9.7429446	9.6630628	9.7553532
Down	FGSG_03680	-1.345298964	8.34E-06	5.0300296	5.1036809	4.8733193	5.1760947	5.306652	4.9442019	5.3100371	4.9937494	5.5195414
Down	FGSG_02960	-1.346255697	0.0018839	5.6164066	5.8271292	5.2780294	5.674773	5.8698682	5.3904893	4.807216	5.3023297	3.9466965
Down	FGSG_05914	-1.34776714	8.69E-08	6.438663	6.052414	6.6735015	5.9405982	5.8698682	5.9288493	5.9925186	5.9304949	5.991768
Down	FGSG_03039	-1.350246719	0.0051653	3.7898088	3.5844678	3.9139052	3.5190873	3.4156241	3.5622521	2.9802913	3.1804273	2.7087823
Down	FGSG_05690	-1.350278344	4.01E-06	9.8695395	10.123382	10.24006	9.5021847	9.8596062	9.7218004	8.7376577	9.0861441	8.947711
Down	FGSG_10285	-1.350898121	6.39E-08	6.0853387	5.9811576	6.1055269	5.8284512	5.8698682	5.7002055	5.7358405	5.6320297	5.7748904
Down	FGSG_10507	-1.352579367	3.63E-07	9.1383594	9.1871099	9.0038356	9.1882211	9.260414	9.0182396	8.7872125	8.5655436	8.9215238
Down	FGSG_02286	-1.353248729	4.68E-07	6.7879016	6.7326402	6.7602689	6.7228827	6.7166741	6.6596278	6.5569203	6.6834471	6.3692672
Down	FGSG_03426	-1.35360108	1.27E-05	8.1127192	8.2280865	7.9050803	7.7308346	7.5069498	7.8490285	6.9002206	7.124214	6.555942
Down	FGSG_00541	-1.358195588	6.84E-05	5.167919	5.349828	4.8733193	5.0060735	5.1346873	4.7780897	4.1391495	4.0586965	4.1665008
Down	FGSG_02569	-1.358269281	4.15E-06	4.9172105	4.9628272	4.7932764	4.92659	4.7135134	5.0451875	4.5913113	4.8742451	4.1665008
Down	FGSG_00483	-1.358623253	3.08E-05	12.075896	12.020239	12.045623	12.656576	12.600954	12.624669	13.311448	13.244501	13.311313

Down	FGSG_05278	-1.359110797	8.88E-08	7.4050095	7.5165498	7.20897	7.5917573	7.4802486	7.6144613	7.551108	7.4819493	7.5544581
Down	FGSG_07399	-1.361527781	0.0005342	4.9172105	4.8067189	4.9491538	4.7220923	4.7733906	4.6556708	4.1912995	4.262829	4.0607807
Down	FGSG_13652	-1.362780655	0.0468071	2.6910183	2.4852148	2.8407141	3.0034737	3.2568854	2.6397336	3.2982451	2	3.9466965
Down	FGSG_13978	-1.363359954	1.98E-07	5.3814422	5.608128	5.0212003	5.3486486	5.2232303	5.3904893	4.965331	4.7439404	5.1065306
Down	FGSG_05193	-1.365532245	9.82E-08	5.8597011	5.6546687	5.9664603	6.1649807	6.29449	5.9288493	6.2229351	6.0176785	6.3469521
Down	FGSG_04372	-1.367111339	3.21E-08	7.7340572	7.748806	7.6335064	8.1361232	8.0146343	8.167343	7.9684295	7.9639893	7.9069251
Down	FGSG_00680	-1.367845979	7.51E-06	9.2933203	9.1950043	9.3037887	8.8772446	8.8061365	8.8609075	8.1960601	8.2243889	8.0994248
Down	FGSG_01176	-1.368797191	1.71E-05	9.3268347	9.2261567	9.3394541	9.8657783	9.8281888	9.8159125	9.6838765	9.7269816	9.5702986
Down	FGSG_20730	-1.37042281	0.0074285	3.5075501	3.5844678	3.3684179	3.3668146	2.6377651	3.8119585	2.8565633	3.1804273	2.3974876
Down	FGSG_13491	-1.371658815	0.0001266	4.1640552	4.2010532	4.0578819	3.7210377	3.5586152	3.8119585	3.7092973	4.0586965	3.18196
Down	FGSG_12750	-1.372375474	0.0112883	3.1562965	3.378055	3.5740244	3.9526758	4.1161991	4.3744304	4.4270393	5.1041074	4.5256129
Down	FGSG_11110	-1.372818315	0.000268	6.2651272	6.6198745	5.728956	6.6001768	6.5383322	6.5777126	6.5961351	5.9891987	6.975096
Down	FGSG_00277	-1.373463873	3.66E-08	11.212626	11.144427	11.194504	11.401391	11.38632	11.328109	11.656746	11.641221	11.605704
Down	FGSG_04708	-1.373830194	0.0142907	3.872828	3.765011	3.9139052	3.5190873	3.9182279	2.8774004	2.7212202	2.3968278	2.964684
Down	FGSG_10596	-1.374259746	0.0055815	2.8633281	2.8476671	2.8407141	3.1032407	2.8748957	3.260088	2.9802913	2.7077187	3.18196
Down	FGSG_02707	-1.374603133	5.30E-08	8.7288222	8.5872306	8.7781265	9.1302718	9.0343543	9.1373946	9.1536991	9.1486426	9.0922236
Down	FGSG_08440	-1.375300122	2.04E-05	5.2633225	4.9628272	5.4444954	6.1878872	6.0257126	6.4728499	5.7002901	6.073005	5.1065306
Down	FGSG_02813	-1.376763156	9.92E-08	5.899844	5.8271292	5.891554	5.7536574	5.7257602	5.7002055	5.2620767	5.2066203	5.2577234
Down	FGSG_00207	-1.377334863	3.48E-08	7.7116792	7.7162629	7.6219674	7.8397076	7.8225187	7.7700883	7.5003335	7.3977778	7.5348496
Down	FGSG_00479	-1.380468086	0.0004391	3.9513288	3.5844678	4.188785	3.8981589	3.5586152	4.1204343	3.2982451	3.5358829	2.964684
Down	FGSG_12372	-1.380661885	4.12E-06	6.5935482	6.4455482	6.650969	6.3181765	6.4675956	6.0552962	5.770536	5.5563964	5.9027806
Down	FGSG_11228	-1.380712158	3.62E-05	10.446951	10.359449	10.448767	11.013753	10.958361	10.98148	10.937669	10.897435	10.91352
Down	FGSG_11658	-1.381113772	4.42E-06	6.0321508	6.2165286	5.728956	6.691168	6.6057619	6.6911344	5.9925186	6.0176785	5.9027806
Down	FGSG_03066	-1.382332508	5.30E-06	5.6164066	5.510285	5.640225	5.5913246	5.423383	5.6688985	4.807216	4.7439404	4.8129147
Down	FGSG_01947	-1.382513856	0.0501779	9.4073648	9.4137361	9.3252936	12.753929	12.716519	12.70572	8.4364951	8.465718	8.3443783
Down	FGSG_12579	-1.38402671	0.000253	4.8367778	4.7218364	4.8733193	4.0052074	4.1161991	3.8119585	4.0850435	3.9446664	4.1665008
Down	FGSG_10913	-1.384174091	1.42E-05	8.2587825	8.1490155	8.2802294	7.5481636	7.4802486	7.5298632	6.8765263	6.7863464	6.9004463
Down	FGSG_04560	-1.38453851	4.15E-09	12.514228	12.477219	12.468799	12.795071	12.744837	12.758493	12.722299	12.692589	12.688135
Down	FGSG_08570	-1.38458067	0.0001724	3.872828	3.9254527	3.7539507	3.5190873	3.9182279	2.8774004	3.4769433	2.9633478	3.8228101
Down	FGSG_05133	-1.384859134	1.69E-11	15.15664	15.109121	15.118343	15.246707	15.197532	15.20853	15.116476	15.069291	15.097266
Down	FGSG_03212	-1.385560865	5.47E-06	8.7260337	8.6857657	8.6813836	9.1479023	9.1059671	9.1114297	9.1504097	9.1193753	9.115512

Down	FGSG_05190	-1.386641323	0.0001193	13.194252	13.156732	13.146086	14.168699	14.133134	14.116506	14.485674	14.437341	14.467572
Down	FGSG_03734	-1.388005372	3.55E-05	8.5225353	8.4619731	8.4978648	8.4941277	8.5521026	8.3412553	7.6980295	7.8096741	7.504928
Down	FGSG_13317	-1.388578684	0.0021198	5.100621	4.9628272	5.1553224	5.0814056	5.0882909	4.9955781	3.970335	3.5358829	4.265
Down	FGSG_06533	-1.391147027	8.21E-05	5.2004262	5.458757	4.7932764	4.8990917	4.7135134	5.0451875	4.4270393	4.6006874	4.265
Down	FGSG_05859	-1.391655681	0.0063872	4.4609433	4.4323548	4.4195794	3.5895922	3.8080253	3.260088	5.2620767	5.2066203	5.2577234
Down	FGSG_12316	-1.391675255	0.0133176	4.0965725	4.2010532	3.9139052	4.1980691	3.9182279	4.3744304	3.8457121	3.5358829	4.0607807
Down	FGSG_01808	-1.391709447	0.0002276	5.8597011	5.8672052	5.7713555	5.674773	5.8698682	5.5012819	4.666927	4.7439404	4.5256129
Down	FGSG_12339	-1.394349092	4.11E-05	5.2322171	4.8067189	5.4959731	4.6247215	4.3699906	4.7780897	4.3828727	3.6853877	4.8129147
Down	FGSG_12523	-1.394358697	1.97E-06	5.5422571	5.7434744	5.2179801	4.92659	4.8308815	4.9442019	4.7734018	4.9352342	4.5256129
Down	FGSG_08462	-1.395043141	1.86E-05	7.162584	7.1771962	7.063002	7.8824608	7.7720438	7.9172266	7.4742601	7.5122892	7.3675781
Down	FGSG_06093	-1.396255099	1.85E-08	7.6716626	7.5415319	7.7652219	7.4330428	7.5417997	7.2834334	6.9912882	6.8190773	7.1137202
Down	FGSG_01796	-1.396479234	0.000146	4.7070397	4.7218364	4.6184939	5.484365	5.6951271	5.1395644	4.8725557	4.262829	5.2577234
Down	FGSG_11055	-1.397028672	4.89E-07	5.6164066	5.4053203	5.728956	6.0818165	5.9237087	6.1490351	6.0918902	5.9002223	6.2052877
Down	FGSG_09786	-1.398094435	5.03E-13	13.04897	13.009227	13.003021	13.12597	13.066691	13.097579	12.977833	12.954834	12.934844
Down	FGSG_05942	-1.398259671	1.12E-06	10.3352	10.562265	10.469839	10.440355	10.5459	10.703972	10.173144	10.27912	10.340914
Down	FGSG_01443	-1.400601156	6.26E-11	11.072575	11.086192	10.971711	10.822993	10.755267	10.802827	10.561565	10.53696	10.521129
Down	FGSG_09257	-1.400631195	6.56E-05	8.8335703	8.7621451	8.8191253	7.9709627	7.8849234	7.9695551	7.1837795	7.2733875	7.0180995
Down	FGSG_03116	-1.403034623	1.51E-06	6.5563577	6.7107812	6.2918249	6.691168	6.5209705	6.7670162	5.7875758	5.8693008	5.6345505
Down	FGSG_04798	-1.40392263	0.0001461	3.701719	4.2010532	2.8407141	3.7210377	3.9182279	3.4190665	3.970335	4.0586965	3.8228101
Down	FGSG_03401	-1.404152737	7.86E-07	5.3814422	5.1692654	5.4959731	5.2432439	5.265544	5.1395644	5.2620767	5.4349486	4.9961472
Down	FGSG_10030	-1.405545452	4.54E-07	5.8799122	5.8271292	5.85259	6.0193342	6.3347926	5.5363994	5.626448	5.0499834	5.991768
Down	FGSG_10224	-1.406499481	9.83E-07	16.560192	16.519889	16.515346	16.967639	16.933848	16.913965	17.018923	16.999284	16.972351
Down	FGSG_03477	-1.407065721	7.81E-06	11.06211	11.026612	11.012052	11.200981	11.208629	11.104315	10.789592	10.717075	10.794888
Down	FGSG_02318	-1.40836786	4.96E-06	5.9580386	6.1851653	5.728956	6.34899	6.2318429	6.3984523	6.3080621	6.3216817	6.3242864
Down	FGSG_09759	-1.408378733	1.65E-05	4.7515947	4.7218364	4.7085304	4.4837433	4.2902531	4.5903319	4.5115122	4.1643702	4.7462732
Down	FGSG_01759	-1.409759633	2.48E-08	6.3971965	6.4188315	6.2918249	5.8137994	5.8970396	5.636897	6.0216157	5.9891987	5.991768
Down	FGSG_00183	-1.410311064	2.39E-06	7.6245274	7.4652492	7.6898562	7.6423884	7.5417997	7.65497	7.07	7.1110577	6.9604719
Down	FGSG_10007	-1.411075576	1.07E-10	12.000308	11.956651	11.958279	11.729982	11.705898	11.667023	11.713852	11.646929	11.713745
Down	FGSG_01989	-1.412172268	0.0293284	3.5075501	3.5844678	3.3684179	3.7210377	3.5586152	3.8119585	3.2982451	3.5358829	2.964684
Down	FGSG_05897	-1.413009734	3.28E-05	5.3814422	5.608128	5.0212003	4.5203003	4.51735	4.4500449	4.5519634	4.8742451	4.0607807
Down	FGSG_03717	-1.413452655	2.59E-06	10.299071	10.227117	10.284653	10.466936	10.408676	10.437572	9.8132732	9.724824	9.8336731

Down	FGSG_04478	-1.413954299	6.77E-13	10.900239	10.796921	10.916106	10.602578	10.582973	10.534118	10.567126	10.523369	10.54583
Down	FGSG_13212	-1.414618628	0.002568	6.2955384	5.906198	6.5327046	5.3074061	5.179638	5.3515835	4.7734018	5.0499834	4.3572018
Down	FGSG_01867	-1.414903867	4.84E-07	11.082419	11.100972	10.976249	11.271705	11.225475	11.230968	10.943383	10.877179	10.942644
Down	FGSG_09737	-1.416401947	0.0001462	7.2583295	7.1771962	7.3693882	7.6629712	7.681182	7.7021252	8.267639	8.307476	8.239261
Down	FGSG_08829	-1.418265968	1.82E-08	5.9955706	5.8672052	6.0376686	5.5913246	5.3855124	5.7002055	5.947742	5.7723491	6.0481828
Down	FGSG_03487	-1.418281108	3.49E-07	6.1197383	6.0867644	6.0719967	5.7536574	5.8698682	5.5363994	5.6821809	5.5947086	5.7410644
Down	FGSG_03936	-1.419621887	3.24E-07	11.58266	11.483478	11.5935	11.311687	11.267073	11.268656	10.572667	10.554074	10.524861
Down	FGSG_11021	-1.420476501	1.40E-06	10.397863	10.385554	10.324353	10.037974	9.9978005	9.9906758	9.2382437	9.202356	9.2081774
Down	FGSG_03117	-1.420861084	0.0689725	3.701719	3.765011	3.5740244	3.6568115	4.0206066	3.260088	2.4052168	2.7077187	2
Down	FGSG_12496	-1.421715628	0.0044543	3.7898088	4.069824	3.5740244	4.4077273	4.3699906	4.3744304	3.8457121	3.6853877	3.9466965
Down	FGSG_09198	-1.421829602	8.28E-08	13.21379	13.120403	13.219942	13.667951	13.640857	13.607281	13.588084	13.555728	13.554214
Down	FGSG_06039	-1.422915772	5.47E-09	13.726002	13.67548	13.690667	13.300654	13.262707	13.250967	12.920694	12.907583	12.868098
Down	FGSG_09814	-1.423275019	6.96E-09	12.034518	11.97568	12.007332	12.342165	12.311109	12.285982	12.362996	12.322045	12.337573
Down	FGSG_01514	-1.423887804	3.13E-06	7.8614916	7.7162629	7.91457	8.6089038	8.5175037	8.6120602	8.7956597	8.7251661	8.799473
Down	FGSG_12155	-1.427104986	0.0280085	3.6078986	4.069824	2.8407141	2.5166584	2.6377651	2.3550396	3.3903592	3.6853877	2.964684
Down	FGSG_03585	-1.427108202	6.88E-05	5.2004262	5.349828	5.640225	4.92659	5.0403527	5.2704753	4.8725557	5.3023297	4.5256129
Down	FGSG_03733	-1.427690712	1.26E-05	5.7541671	5.458757	5.9294932	5.8995578	5.4602848	6.1715481	5.2124668	5.2552685	5.1065306
Down	FGSG_06649	-1.429540594	2.37E-10	7.499477	7.5660888	7.3415168	7.5123147	7.4802486	7.4584209	7.1313519	7.2130949	6.975096
Down	FGSG_07223	-1.429962639	0.0005591	6.3971965	6.5477263	6.1703363	5.7689296	5.7257602	5.7308476	4.7734018	4.9937494	4.4438634
Down	FGSG_01376	-1.430532684	7.46E-11	9.5747963	9.5857263	9.4834989	9.8999947	9.8387374	9.8992097	9.7220462	9.6562859	9.736164
Down	FGSG_04271	-1.431161641	8.87E-09	13.7162	13.685321	13.675921	13.046149	12.978218	13.032961	13.078512	13.065325	13.034205
Down	FGSG_03675	-1.431909014	4.07E-06	6.8506908	6.9534782	6.650969	6.9204536	6.8477891	6.9077633	6.3772321	6.2752577	6.4128878
Down	FGSG_07674	-1.434321159	2.10E-09	10.505036	10.483882	10.440579	10.704883	10.621156	10.701074	10.202028	10.153209	10.184537
Down	FGSG_08831	-1.434587591	3.06E-07	6.3545028	6.306724	6.3206633	6.7384824	6.5724409	6.8107005	7.1902009	6.9578183	7.3339349
Down	FGSG_04196	-1.435301649	0.0056861	9.6517476	9.567598	9.648979	12.024337	11.981712	11.979654	12.332298	12.296485	12.301765
Down	FGSG_03580	-1.435600841	0.0008002	5.065757	5.1692654	4.8733193	5.2212063	5.306652	5.0451875	4.2902636	4.262829	4.265
Down	FGSG_01470	-1.435791317	2.64E-09	12.165839	12.115343	12.131036	12.308593	12.2597	12.26977	12.239759	12.180044	12.232701
Down	FGSG_11910	-1.436025371	0.0007368	6.3831052	6.5721788	6.0719967	4.871059	4.9394155	4.7181783	4.935064	5.2066203	4.5256129
Down	FGSG_07557	-1.436718995	1.79E-05	7.4462553	7.4911275	7.3130965	7.4137401	7.5069498	7.220047	7.1708501	7.1372515	7.1399154
Down	FGSG_06580	-1.4370195	1.58E-06	14.665031	14.623551	14.620959	13.925108	13.868753	13.894379	13.388073	13.351508	13.358264
Down	FGSG_06324	-1.437859039	1.42E-08	12.056049	12.078477	11.945607	11.800186	11.745211	11.767892	11.783721	11.726725	11.774574

Down	FGSG_11409	-1.438758318	5.39E-08	6.7879016	6.7962976	6.6956874	6.8140467	6.6701803	6.8669619	6.4215692	6.4733227	6.301259
Down	FGSG_07334	-1.438889742	5.99E-11	12.773186	12.760311	12.706891	12.397564	12.365316	12.353073	12.198748	12.185938	12.162961
Down	FGSG_10550	-1.43902782	2.51E-08	7.6774476	7.5538626	7.711794	7.5437309	7.558914	7.4584209	7.2772237	7.1756715	7.3110622
Down	FGSG_05587	-1.439171032	2.05E-05	5.100621	4.9628272	5.1553224	4.871059	4.8861689	4.8356119	4.4698939	4.9937494	3.6872775
Down	FGSG_13180	-1.439445748	0.0108076	4.1640552	4.2010532	4.0578819	3.9526758	4.2058494	3.5622521	3.2982451	2.7077187	3.6872775
Down	FGSG_12303	-1.440078495	7.77E-05	5.3235909	5.458757	5.0898194	6.5121623	6.4121699	6.5262338	6.0501376	6.2026947	5.8079414
Down	FGSG_13242	-1.441227711	0.006118	4.8367778	5.349828	3.9139052	3.7825262	3.0784965	4.210161	3.7092973	3.8208439	3.5376798
Down	FGSG_13828	-1.442507417	2.75E-05	5.5921111	5.1692654	5.85259	5.8137994	5.8698682	5.6688985	4.9041484	4.8742451	4.8766132
Down	FGSG_03329	-1.442787485	1.82E-06	6.0321508	5.7859081	6.1703363	5.5565451	5.7257602	5.3115993	5.3334324	5.5170389	5.0523944
Down	FGSG_20649	-1.444108351	0.0144217	2.4953013	2	2.8407141	3.3668146	3.4156241	3.260088	3.5586239	3.1804273	3.8228101
Down	FGSG_00669	-1.444241315	1.95E-07	9.705113	9.6183812	9.7047392	9.2398278	9.0913381	9.2943686	8.8082383	8.8727767	8.6706338
Down	FGSG_02489	-1.446379943	1.69E-11	12.733995	12.674686	12.707232	12.650791	12.595262	12.619305	12.561487	12.547535	12.509102
Down	FGSG_07130	-1.446959914	6.74E-10	15.212121	15.158152	15.180196	15.322108	15.278056	15.278868	15.102634	15.082253	15.056968
Down	FGSG_09383	-1.447858234	0.0001058	7.1376208	7.19305	6.9930638	7.1593914	7.1140747	7.1194186	6.7604964	6.7528555	6.7037513
Down	FGSG_10994	-1.44817807	0.0001473	5.6402997	5.6546687	5.545677	4.657914	4.0206066	5.0451875	4.7032988	4.8105634	4.5256129
Down	FGSG_10451	-1.448804945	0.005245	2.8633281	2.4852148	3.1285604	2.7805029	2.353841	3.0813961	3.4769433	3.5358829	3.3707558
Down	FGSG_02902	-1.450425335	1.47E-07	7.5253693	7.6373467	7.3130965	7.6465285	7.6008319	7.6062213	7.7248157	7.8418859	7.5249446
Down	FGSG_03811	-1.451273015	1.76E-07	5.6869305	5.6546687	5.640225	5.1760947	5.179638	5.1395644	4.994976	4.9937494	4.9376179
Down	FGSG_06238	-1.452050089	0.0591519	3.872828	3.765011	3.9139052	2.8962916	3.2568854	2.3550396	2.5718533	2.9633478	2
Down	FGSG_05739	-1.452457494	1.72E-05	5.7973114	5.9811576	5.4959731	5.9673238	5.8421752	6.0060392	5.4450355	5.6320297	5.1587087
Down	FGSG_05808	-1.452791455	1.14E-07	6.2023113	6.4975416	5.728956	6.0065063	6.0501253	5.874995	5.8698804	6.1262878	5.4790733
Down	FGSG_08150	-1.4552636	0.0001989	5.2633225	5.6997548	4.5224622	4.0558932	4.2058494	3.9222766	4.4270393	4.0586965	4.6764038
Down	FGSG_04502	-1.457318886	0.0082087	4.2285219	4.8067189	3.1285604	3.8981589	4.0206066	3.6925011	3.4769433	2.7077187	3.9466965
Down	FGSG_10396	-1.457698572	2.30E-10	11.247073	11.194508	11.21385	10.879489	10.809901	10.861122	10.540987	10.509648	10.506103
Down	FGSG_02899	-1.458584195	9.56E-06	5.4906186	5.7859081	5.0212003	5.8284512	5.7851416	5.7902457	5.0524989	4.9937494	5.0523944
Down	FGSG_11242	-1.459076688	0.0095754	3.2831234	3.5844678	2.8407141	3.2841938	2.353841	3.8119585	2.8565633	2.3968278	3.18196
Down	FGSG_11887	-1.459474135	0.0002108	5.4906186	5.6997548	5.1553224	4.871059	5.0403527	4.5903319	5.2862562	5.5563964	4.8766132
Down	FGSG_01458	-1.459514067	1.48E-05	4.6135762	4.3213345	4.7932764	5.1988268	5.2232303	5.0931475	4.807216	4.6740912	4.8766132
Down	FGSG_11878	-1.462980925	7.34E-06	7.7451174	7.7271924	7.678761	9.1094268	9.1489846	8.9931804	8.5305887	8.4862433	8.5192059
Down	FGSG_09373	-1.464084656	4.35E-05	14.878539	14.822984	14.851036	15.706449	15.676461	15.651473	15.722435	15.705328	15.678182
Down	FGSG_11663	-1.464673446	0.0257117	2.8633281	3.1370988	2.4807492	4.5559538	4.5857446	4.4500449	5.0804226	4.6740912	5.3503768

Down	FGSG_10612	-1.464773259	2.26E-05	7.7340572	7.7052501	7.678761	7.5168448	7.6008319	7.3441517	6.5267935	6.5138742	6.4759412
Down	FGSG_08371	-1.464974436	0.002137	4.7948149	4.5354388	4.9491538	4.5559538	4.51735	4.5218929	4.4270393	4.7439404	3.9466965
Down	FGSG_11099	-1.46587275	3.73E-06	11.666858	11.624602	11.623466	11.055997	11.038331	10.994166	10.545377	10.505884	10.518635
Down	FGSG_02911	-1.46619978	8.80E-08	9.2039587	9.076079	9.2410576	8.6711911	8.7250644	8.5229463	9.0743422	9.0794051	9.0093969
Down	FGSG_10254	-1.467108328	5.30E-09	6.2496778	6.3638664	6.0376686	6.2972625	6.3545295	6.1490351	6.1719365	6.2026947	6.075584
Down	FGSG_12602	-1.467825357	0.0247262	3.5075501	4.069824	2.4807492	3.2841938	3.0784965	3.4190665	2.5718533	2.7077187	2.3974876
Down	FGSG_10089	-1.467829394	2.01E-12	15.491185	15.43005	15.466497	15.517508	15.472776	15.474958	15.331636	15.312238	15.28517
Down	FGSG_10164	-1.467947848	4.22E-08	8.3701331	8.2433937	8.4133694	8.3027838	8.3006433	8.2222919	7.7203857	7.5902083	7.7794184
Down	FGSG_13799	-1.468843044	0.016624	3.701719	3.765011	3.5740244	3.0034737	2.6377651	3.260088	2.9802913	2.3968278	3.3707558
Down	FGSG_09440	-1.470801789	4.65E-07	9.3101748	9.076079	9.4381609	8.8433797	8.788107	8.8223008	8.0970183	8.1033819	8.024072
Down	FGSG_13486	-1.470856607	0.0091154	3.7898088	3.5844678	3.9139052	4.0558932	3.8080253	4.210161	3.2982451	3.5358829	2.964684
Down	FGSG_08107	-1.471322022	0.0006591	5.9195042	5.906198	5.85259	4.5907474	3.8080253	5.0451875	5.0524989	5.1041074	4.9376179
Down	FGSG_02379	-1.471579329	0.0039984	3.9513288	3.5844678	4.188785	3.1032407	2.8748957	3.260088	2.8565633	2.7077187	2.964684
Down	FGSG_05687	-1.472172495	0.0110821	4.4062533	4.069824	4.6184939	4.327482	4.2058494	4.3744304	3.199846	2.9633478	3.3707558
Down	FGSG_08439	-1.473326805	0.0001416	4.8367778	4.7218364	4.8733193	5.9270474	6.2318429	5.7902457	4.7387761	4.7439404	4.8766132
Down	FGSG_11462	-1.475165877	9.76E-05	6.0853387	6.3638664	5.640225	5.4277566	5.4962661	5.2704753	5.0240241	5.0499834	4.9376179
Down	FGSG_09060	-1.476073844	0.0229526	3.3996963	3.5844678	3.1285604	3.3668146	3.9182279	2.3550396	3.2982451	3.6853877	2.7087823
Down	FGSG_13416	-1.476145726	0.0308606	3.9513288	4.2010532	3.5740244	2.8962916	3.2568854	2.3550396	3.5586239	2.9633478	3.9466965
Down	FGSG_06236	-1.47628524	8.17E-10	7.1207354	7.1771962	6.9930638	6.7539152	6.7166741	6.7066335	6.7691009	6.9724415	6.4759412
Down	FGSG_01841	-1.47752898	6.52E-10	6.9966572	6.8572645	7.0458328	7.1993948	6.8196649	7.4306909	7.0975901	7.0708531	7.0598582
Down	FGSG_02452	-1.480524223	1.88E-06	6.7663496	6.7962976	6.650969	6.5121623	6.6701803	6.2370531	5.8857925	5.5563964	6.1024744
Down	FGSG_05244	-1.48083122	3.85E-06	5.065757	5.0349724	5.0212003	6.0065063	5.8698682	6.1029268	5.1610901	5.2066203	5.0523944
Down	FGSG_04442	-1.481414394	2.53E-05	4.6610646	4.8868835	4.3087924	4.4837433	4.7135134	4.1204343	4.7734018	4.3549955	5.0523944
Down	FGSG_08284	-1.482030702	1.70E-07	6.7554516	6.5962238	6.8220828	6.3890768	6.6543434	5.9550408	6.0216157	5.7723491	6.1802631
Down	FGSG_00081	-1.48369638	0.000217	5.6869305	5.608128	5.6852725	5.9935634	5.6951271	6.1715481	5.7181748	5.4349486	5.9027806
Down	FGSG_12003	-1.485046179	1.81E-10	9.9410184	9.892866	9.903685	9.7713049	9.6678482	9.7852189	9.6918179	9.6742873	9.6430764
Down	FGSG_12154	-1.486541002	3.68E-08	10.593365	10.534535	10.566311	10.413883	10.358243	10.381944	9.8153518	9.7984835	9.7659047
Down	FGSG_12384	-1.487934118	0.0001175	4.2902308	4.4323548	4.0578819	4.6247215	4.2058494	4.8909283	4.3373111	4.6006874	3.9466965
Down	FGSG_08669	-1.489284282	7.06E-11	9.6054643	9.6036297	9.5212573	9.3719696	9.3318495	9.3273266	9.0726054	9.2177808	8.8359833
Down	FGSG_13337	-1.489374017	2.82E-05	13.818485	13.783157	13.768119	14.519537	14.467213	14.484212	14.641041	14.604498	14.611195
Down	FGSG_10983	-1.489450827	1.40E-05	6.6882763	6.6660438	6.628079	7.3842929	7.5157415	7.1423949	7.6429173	7.6460044	7.5738036

Down	FGSG_07026	-1.490141946	0.0011178	3.5075501	3.9254527	2.8407141	4.2856211	4.51735	3.9222766	3.3903592	2.9633478	3.6872775
Down	FGSG_02375	-1.490437837	9.77E-06	5.167919	4.8868835	5.3356788	4.7531416	5.1346873	4.1204343	4.7734018	5.0499834	4.3572018
Down	FGSG_09684	-1.491318062	1.11E-05	7.7173064	7.7910854	7.5748659	7.4282413	7.387497	7.4399936	6.354542	6.2752577	6.3912423
Down	FGSG_08191	-1.492856605	0.0030336	3.5075501	4.069824	3.1285604	4.1522165	4.2058494	4.024755	3.199846	3.3690748	2.964684
Down	FGSG_01675	-1.493835943	5.19E-07	9.1193592	8.9650482	9.1950749	8.4592483	8.5218742	8.3212581	8.1896647	8.1935159	8.1324168
Down	FGSG_04094	-1.494962433	9.34E-06	3.7898088	3.9254527	3.5740244	3.444959	3.4156241	3.4190665	3.7791165	3.8208439	3.6872775
Down	FGSG_06233	-1.495708382	5.87E-07	7.162584	7.4388982	6.7175374	6.7071125	6.314782	6.9474424	6.3659317	6.5337304	6.1024744
Down	FGSG_11618	-1.499105386	2.05E-08	8.9068087	8.8989232	8.8291955	9.6813497	9.6479924	9.6292736	9.397947	9.3242228	9.4046271
Down	FGSG_04244	-1.500914982	0.0022605	4.2902308	3.765011	4.6184939	3.5895922	3.6887032	3.5622521	3.4769433	3.3690748	3.5376798
Down	FGSG_09669	-1.503140067	2.91E-09	6.629804	6.4975416	6.6735015	7.1766717	7.0669996	7.1982839	6.7691009	6.7863464	6.6860823
Down	FGSG_13033	-1.503824252	1.01E-05	5.5921111	5.608128	5.4959731	5.9805033	5.6951271	6.1490351	5.0240241	5.2066203	4.7462732
Down	FGSG_12098	-1.503958786	0.0215372	4.0257777	3.5844678	4.3087924	4.4077273	4.0206066	4.6556708	5.0240241	4.9937494	4.9961472
Down	FGSG_03539	-1.506954412	0.0004213	3.5075501	3.378055	3.5740244	3.9526758	4.3699906	3.260088	2.9802913	2.9633478	2.964684
Down	FGSG_00976	-1.507627117	8.63E-05	5.167919	5.2921154	4.9491538	5.0567294	5.2232303	4.7780897	5.1346989	5.5170389	4.5256129
Down	FGSG_12180	-1.510306925	1.90E-08	5.6869305	5.9441646	5.2780294	5.6419705	5.5991203	5.6041695	5.2620767	5.3920827	5.0523944
Down	FGSG_02870	-1.512133887	9.21E-11	8.4534121	8.3391171	8.4788005	8.6609948	8.663103	8.5703107	8.8755264	8.8571018	8.8319719
Down	FGSG_03336	-1.512434579	1.73E-05	11.709832	11.659255	11.674627	12.418899	12.381038	12.368989	12.819607	12.766297	12.806316
Down	FGSG_09064	-1.516675927	4.68E-06	5.9195042	6.153105	5.545677	5.8573158	5.7257602	5.9021734	5.3334324	5.6684096	4.8129147
Down	FGSG_12488	-1.517381203	9.32E-07	4.2902308	4.069824	4.4195794	4.871059	4.9907667	4.6556708	4.4270393	4.262829	4.5256129
Down	FGSG_05334	-1.517436583	0.0027745	3.7898088	3.1370988	4.3087924	3.6568115	4.0206066	3.0813961	3.199846	2.9633478	3.5376798
Down	FGSG_02504	-1.517513528	2.88E-07	12.400128	12.403761	12.328092	11.85935	11.815431	11.832894	11.231449	11.233441	11.184417
Down	FGSG_04368	-1.517628886	5.36E-09	7.1952103	7.2087314	7.0967395	7.6423884	7.8082766	7.3540275	7.7025285	7.6090478	7.7285401
Down	FGSG_10614	-1.517879189	7.04E-06	8.9166249	8.9181273	8.8291955	8.8666361	8.8760825	8.7679329	7.7292321	7.6368537	7.7542035
Down	FGSG_04057	-1.51909602	5.33E-07	6.665171	6.5228522	6.7175374	6.2972625	6.2103411	6.2997126	5.7358405	5.7723491	5.6345505
Down	FGSG_03719	-1.519627461	1.93E-06	7.992567	7.9856806	7.91457	7.3990916	7.4981042	7.1982839	7.3011528	7.2615289	7.2760585
Down	FGSG_00417	-1.521397834	2.48E-06	7.2271151	7.0952041	7.2693877	7.2815544	7.1596621	7.3141119	6.1848562	6.1262878	6.1802631
Down	FGSG_10006	-1.522415453	6.52E-08	11.397038	11.333309	11.375474	10.738242	10.718613	10.669786	10.440203	10.388117	10.425871
Down	FGSG_01950	-1.52252321	5.55E-05	13.244073	13.217095	13.185325	12.751529	12.695392	12.719871	12.958567	12.916354	12.93461
Down	FGSG_10825	-1.524238963	1.29E-07	14.879754	14.830763	14.842905	14.462118	14.427436	14.409304	13.764048	13.709987	13.751869
Down	FGSG_08233	-1.524303123	0.026034	3.2831234	3.5844678	2.8407141	2.5166584	2.6377651	2.3550396	2.2167888	2.3968278	2
Down	FGSG_09739	-1.525154497	3.22E-07	12.344401	12.295945	12.30708	13.076961	13.008316	13.057534	13.352552	13.323621	13.315449

Down	FGSG_03409	-1.526038181	0.0138201	3.7898088	3.765011	3.7539507	3.8415007	4.2902531	3.0813961	2.7212202	2.9633478	2.3974876
Down	FGSG_02861	-1.52645694	0.0568326	3.7898088	4.2010532	3.1285604	2.1935143	2.353841	2	2.7212202	3.1804273	2
Down	FGSG_00892	-1.527149327	9.82E-05	5.6869305	5.608128	5.6852725	6.5568406	6.374	6.6436126	5.626448	5.8053955	5.3503768
Down	FGSG_10319	-1.528627187	1.85E-10	8.2354426	8.1161324	8.2654724	8.5236891	8.4909989	8.4694383	8.3870422	8.2724515	8.4317906
Down	FGSG_10950	-1.5293267	7.62E-07	5.3235909	5.2319977	5.3356788	5.4083825	5.5991203	5.1845342	5.0804226	4.9352342	5.1587087
Down	FGSG_03672	-1.530208437	4.84E-08	7.5570945	7.6829688	7.3273766	6.9741499	6.9157907	6.9474424	6.8196775	6.8190773	6.7554957
Down	FGSG_03327	-1.531090936	1.45E-05	5.3814422	5.4053203	5.2780294	4.8424709	5.2232303	4.210161	4.7734018	4.6006874	4.8766132
Down	FGSG_01353	-1.531295166	3.20E-11	8.9018754	8.9323645	8.7833155	9.0559574	9.1117774	8.9085948	8.9264157	8.9375579	8.8479508
Down	FGSG_11448	-1.531913244	0.0004267	5.3814422	4.9628272	5.640225	4.9800625	5.1346873	4.7181783	4.2902636	4.6740912	3.6872775
Down	FGSG_02160	-1.532457868	5.89E-05	4.5136357	4.6316459	4.3087924	4.4837433	4.4455509	4.4500449	3.970335	3.5358829	4.265
Down	FGSG_13529	-1.533403535	1.94E-06	7.281305	7.42554	7.0284567	6.9675462	6.6858453	7.1309525	7.07	7.0843795	6.9895734
Down	FGSG_11667	-1.533904863	0.006005	2.6910183	3.1370988	2.8407141	3.1965524	3.2568854	3.5622521	2.4052168	2.3968278	2.3974876
Down	FGSG_12170	-1.534520068	5.30E-06	5.9195042	5.8672052	5.891554	6.7228827	6.7617162	6.594472	6.5063537	6.6291077	6.301259
Down	FGSG_02459	-1.53653446	6.15E-07	5.2633225	5.1692654	5.2780294	5.9673238	5.8698682	5.9807654	5.770536	5.7385279	5.7410644
Down	FGSG_02484	-1.536921517	9.87E-07	8.1753854	8.1811658	8.0839393	8.048102	7.9769307	8.0324046	7.2832433	7.1629782	7.3339349
Down	FGSG_00855	-1.537070485	1.36E-10	10.755288	10.746781	10.677785	11.126979	11.14276	11.020759	10.858743	10.771396	10.87797
Down	FGSG_03326	-1.539087685	1.78E-07	5.167919	5.510285	4.6184939	4.7220923	4.7135134	4.6556708	5.107816	4.9937494	5.1587087
Down	FGSG_09364	-1.53925528	5.03E-05	7.0422056	7.0436746	6.9567807	6.5656126	6.314782	6.7066335	6.399571	6.2514733	6.4759412
Down	FGSG_07319	-1.540052921	3.57E-09	8.0781498	8.0037709	8.0670189	8.0132576	7.9447403	7.9950244	7.8064993	7.8811649	7.657785
Down	FGSG_00197	-1.541229751	0.000403	3.872828	4.2010532	4.0578819	4.5559538	5.0403527	4.3744304	4.5519634	4.3549955	4.8129147
Down	FGSG_02476	-1.541509193	2.18E-10	9.0892967	9.0846031	9.0082743	8.8701809	8.8862879	8.7642345	8.5680525	8.4237699	8.6388404
Down	FGSG_06090	-1.542127276	1.00E-05	7.06886	7.42554	7.1459111	6.8503947	7.0059099	7.2308067	6.235408	6.4937409	6.4759412
Down	FGSG_12169	-1.54318725	0.00303	4.0965725	3.9254527	4.188785	6.1533896	6.253029	5.9550408	5.9777469	6.0176785	5.8718551
Down	FGSG_01286	-1.544433575	1.89E-05	5.4640893	5.6997548	5.0898194	4.4462359	4.5857446	4.210161	4.7734018	4.5233477	4.9376179
Down	FGSG_12699	-1.545315753	1.82E-05	5.3235909	5.349828	5.2179801	5.4083825	5.2232303	5.5012819	5.2124668	5.0499834	5.3047937
Down	FGSG_02700	-1.546359589	4.34E-09	8.5761636	8.6051154	8.4594808	8.5055693	8.5392251	8.3804369	7.8106729	7.7767266	7.7794184
Down	FGSG_09122	-1.547753795	7.66E-07	7.4863545	7.2395919	7.6219674	7.333838	7.558914	6.9604299	6.5668244	6.6474492	6.4128878
Down	FGSG_12234	-1.548509834	0.0183993	3.2831234	3.1370988	3.3684179	2.8962916	2.8748957	2.8774004	2.4052168	2.3968278	2.3974876
Down	FGSG_02392	-1.548676494	9.89E-06	5.3814422	5.608128	5.0212003	5.2649499	4.9907667	5.4283735	4.4698939	4.5233477	4.6029775
Down	FGSG_03201	-1.548896025	4.81E-06	5.065757	4.7218364	5.2780294	4.5907474	4.6510429	4.6556708	4.935064	4.8742451	4.9961472
Down	FGSG_08256	-1.549086994	1.58E-06	5.6869305	5.5600358	5.728956	6.2436111	6.4308827	5.9288493	6.6057745	6.6474492	6.4963611

Down	FGSG_00963	-1.551273789	0.0113649	3.2831234	3.378055	3.1285604	3.2841938	2.6377651	3.6925011	3.2982451	3.3690748	3.18196
Down	FGSG_03655	-1.552232981	3.67E-07	7.10365	7.0610564	7.0967395	6.82139	6.8337955	6.7219677	6.3772321	6.2272902	6.4963611
Down	FGSG_01901	-1.553869973	5.08E-11	7.544488	7.42554	7.5986089	7.3990916	7.387497	7.3241948	7.0907419	7.0434138	7.0735134
Down	FGSG_08877	-1.554729571	1.69E-10	6.5181831	6.4717791	6.4825439	6.5121623	6.6221382	6.2997126	6.5864309	6.4937409	6.6131596
Down	FGSG_04317	-1.555910748	0.0031642	3.6078986	3.1370988	3.9139052	3.1965524	3.0784965	3.260088	4.1912995	3.9446664	4.4438634
Down	FGSG_13733	-1.556257244	0.0365612	2.6910183	2.4852148	2.8407141	3.5895922	3.8080253	3.260088	2.4052168	2.7077187	2
Down	FGSG_10450	-1.556612052	0.0034571	3.5075501	3.9254527	3.5740244	3.1965524	3.4156241	3.4190665	4.0850435	4.5233477	3.9466965
Down	FGSG_01827	-1.55898461	3.37E-05	5.0300296	5.2921154	4.6184939	4.2425091	4.2902531	4.1204343	3.970335	4.0586965	3.8228101
Down	FGSG_05794	-1.560476441	0.000122	4.9558055	5.2921154	4.7085304	5.0814056	5.265544	5.2281446	5.3564544	5.4765776	5.3047937
Down	FGSG_03934	-1.56076566	1.03E-08	11.532473	11.511407	11.467803	11.220552	11.168076	11.186063	10.581243	10.617086	10.476857
Down	FGSG_06676	-1.561591046	1.18E-06	9.2952028	9.2376676	9.2672192	8.7444837	8.788107	8.6120602	8.8834733	8.8492001	8.8519181
Down	FGSG_00303	-1.561961154	2.17E-13	9.623557	9.6006612	9.561077	9.6237151	9.5971233	9.5626999	9.4970973	9.5795607	9.338304
Down	FGSG_09992	-1.562144598	2.01E-07	10.256226	10.199914	10.22679	11.142453	11.063283	11.133994	11.037645	11.038751	10.969367
Down	FGSG_08016	-1.565062718	8.32E-05	4.6135762	4.5354388	4.6184939	4.327482	4.0206066	4.5218929	3.8457121	4.1643702	3.5376798
Down	FGSG_02530	-1.565235257	7.56E-08	11.87249	11.814001	11.844977	11.634464	11.552539	11.627874	11.382783	11.361867	11.337273
Down	FGSG_09183	-1.565883432	0.0196063	3.3996963	3.378055	3.7539507	2.6546039	2.8748957	2.3550396	3.199846	2.9633478	3.3707558
Down	FGSG_11170	-1.566346609	0.0175459	4.1640552	3.5844678	4.5224622	2.364115	2.6377651	2.8774004	2.9802913	2.9633478	2.964684
Down	FGSG_02939	-1.566892359	7.22E-09	7.921222	7.5902346	8.1416454	8.1681542	8.2645866	7.9886991	8.3212806	8.4023294	8.1710364
Down	FGSG_05280	-1.56691697	7.18E-09	9.3631819	9.3337593	9.3073952	9.254821	9.2446352	9.1768778	8.7133421	8.5558662	8.7953587
Down	FGSG_11364	-1.569661417	0.0009001	3.5075501	3.5844678	3.3684179	3.0034737	3.0784965	2.8774004	3.4769433	3.3690748	3.5376798
Down	FGSG_08556	-1.570434443	9.39E-07	7.7506158	7.7052501	7.711794	8.4283221	8.3358208	8.432631	8.2244936	8.2546151	8.1324168
Down	FGSG_04661	-1.570546612	9.37E-06	5.1346624	4.8868835	5.2780294	5.0567294	4.7135134	5.2704753	4.935064	4.6740912	5.1065306
Down	FGSG_02723	-1.570667886	9.16E-07	6.4522245	6.5228522	6.2918249	6.0193342	6.0741317	5.874995	5.8375198	5.6684096	5.9330571
Down	FGSG_06613	-1.571676078	1.24E-08	6.860895	6.7962976	6.8421126	7.3990916	7.203853	7.4945842	6.6897477	6.6291077	6.6860823
Down	FGSG_12941	-1.57194736	0.00829	3.9513288	4.069824	3.7539507	3.1965524	3.2568854	3.0813961	2.5718533	2.7077187	2.3974876
Down	FGSG_13710	-1.572256751	2.73E-07	5.2633225	5.5600358	4.7932764	5.2212063	5.0882909	5.2704753	5.2620767	5.2552685	5.2090653
Down	FGSG_03915	-1.572304526	2.35E-06	7.7938649	7.7806311	7.7226391	7.6992962	7.6495769	7.662937	6.6248622	6.5138742	6.6681943
Down	FGSG_12267	-1.573707843	4.70E-06	11.553466	11.538806	11.482234	12.10797	12.031221	12.097136	12.273791	12.257089	12.223926
Down	FGSG_12745	-1.573989873	3.74E-10	17.038673	16.994363	16.997434	16.959999	16.912213	16.920263	16.989174	16.966874	16.945191
Down	FGSG_20060	-1.574378441	0.0693725	3.2831234	3.765011	3.5740244	2	2.6377651	2.3550396	2	2	2.3974876
Down	FGSG_13653	-1.575800248	9.44E-06	4.2902308	4.3213345	4.3087924	4.1980691	4.1161991	4.3744304	3.6359267	3.6853877	3.5376798

Down	FGSG_01235	-1.576781303	1.25E-09	7.8202516	7.6021576	7.9333641	7.3491606	7.3486598	7.2730605	7.2218865	7.3083895	7.0598582
Down	FGSG_08000	-1.577062752	2.93E-05	6.169847	6.153105	6.1055269	5.7226193	5.6318376	5.7308476	5.0524989	4.8742451	5.1587087
Down	FGSG_06381	-1.578863608	0.0013426	6.0139766	6.0172257	5.9294932	4.8990917	4.7135134	4.9955781	4.0850435	4.1643702	3.9466965
Down	FGSG_10707	-1.57922157	7.72E-06	5.0300296	5.1036809	4.8733193	4.3681625	4.51735	4.1204343	4.3828727	4.4416259	4.265
Down	FGSG_10103	-1.582758644	0.0011241	3.0172362	2.8476671	3.1285604	2.6546039	2.6377651	2.6397336	3.4769433	3.3690748	3.5376798
Down	FGSG_03256	-1.585631236	0.0028944	4.0257777	2.8476671	4.6184939	4.1048586	4.4455509	3.5622521	3.4769433	3.9446664	2.7087823
Down	FGSG_07889	-1.58944736	2.43E-06	5.6638035	5.5600358	5.6852725	5.0316238	5.179638	4.7780897	4.7032988	4.7439404	4.6029775
Down	FGSG_09195	-1.590063597	2.46E-10	6.3831052	6.052414	6.5811798	6.3989268	6.4856075	6.2582434	6.2600348	6.3666582	6.075584
Down	FGSG_01128	-1.590887035	0.0004925	5.2004262	5.349828	4.9491538	5.3887447	4.8308815	5.7308476	4.1912995	4.4416259	3.8228101
Down	FGSG_02842	-1.591450392	5.61E-05	4.8775546	4.6316459	5.0212003	5.2649499	5.346621	5.0931475	5.4233949	5.4765776	5.3047937
Down	FGSG_06235	-1.59225279	9.76E-05	6.0678264	5.8672052	6.1703363	5.3281747	5.1346873	5.4283735	5.0524989	4.262829	5.5589053
Down	FGSG_07489	-1.592531908	1.22E-07	10.84605	10.811116	10.795455	10.013338	10.007183	9.9308971	9.7253661	9.6608074	9.7232279
Down	FGSG_13604	-1.5934362	8.40E-05	5.2633225	5.510285	4.8733193	4.5559538	4.7135134	4.2946324	5.0804226	4.8742451	5.2090653
Down	FGSG_11300	-1.593719756	0.0019966	4.2285219	4.3213345	4.0578819	3.7825262	3.2568854	4.1204343	4.028829	3.1804273	4.5256129
Down	FGSG_04207	-1.594152728	7.01E-08	8.4933157	8.4154308	8.4851833	8.1739024	8.2060401	8.0507377	7.6475913	7.6641337	7.5641633
Down	FGSG_03600	-1.59857422	0.0064535	4.2285219	4.3213345	4.0578819	3.5895922	3.6887032	3.4190665	3.199846	2.9633478	3.3707558
Down	FGSG_07830	-1.602140181	2.23E-12	12.816757	12.763964	12.785603	13.067825	13.048441	13.000516	13.210822	13.145949	13.209475
Down	FGSG_12513	-1.604756028	1.03E-10	8.183539	8.1161324	8.165687	8.1213252	8.0453183	8.1102181	7.7380245	7.5222624	7.8682762
Down	FGSG_02519	-1.604795904	3.16E-14	11.866489	11.850695	11.797041	11.829161	11.779836	11.79125	11.721353	11.667296	11.708832
Down	FGSG_04283	-1.60493679	1.34E-07	7.4050095	7.9765496	7.711794	6.9741499	7.5157415	7.4675471	6.9002206	7.4299203	7.5348496
Down	FGSG_01772	-1.605194699	1.16E-05	8.1084431	8.1408647	8.0061838	7.6298962	7.5417997	7.6308015	7.2029588	7.3312612	7.0180995
Down	FGSG_11749	-1.605727361	3.62E-05	5.0300296	4.9628272	5.0212003	5.8429557	5.5656438	6.0060392	5.9925186	5.7723491	6.1288728
Down	FGSG_13401	-1.607733751	8.85E-05	4.7070397	4.4323548	4.8733193	4.4462359	4.51735	4.2946324	4.807216	4.7439404	4.8129147
Down	FGSG_13878	-1.607821973	6.82E-05	3.9513288	4.2010532	3.7539507	4.5203003	4.5857446	4.5218929	4.3828727	4.5233477	4.3572018
Down	FGSG_07467	-1.608880317	1.26E-08	13.180441	13.146336	13.128864	12.796352	12.752528	12.752905	12.342603	12.316682	12.302127
Down	FGSG_13750	-1.60913443	0.0032905	3.5075501	3.9254527	2.8407141	2.6546039	2.353841	2.8774004	3.0942412	3.3690748	2.7087823
Down	FGSG_00652	-1.611140919	1.23E-05	5.0300296	5.5600358	4.3087924	4.6247215	5.179638	4.2946324	5.0240241	4.8742451	5.4374371
Down	FGSG_05627	-1.611419374	2.93E-06	6.8404139	6.8965233	6.6956874	8.1966688	8.1619172	8.1447637	7.7380245	7.5996588	7.8042001
Down	FGSG_01457	-1.612803623	1.60E-05	4.4062533	4.069824	4.6184939	4.9800625	4.9394155	4.9442019	5.1870071	5.3023297	4.9961472
Down	FGSG_10537	-1.612999819	5.83E-05	3.872828	4.069824	3.5740244	3.6568115	3.8080253	3.4190665	3.3903592	3.6853877	2.964684
Down	FGSG_04974	-1.614189004	2.06E-07	6.6178197	6.5228522	6.628079	6.2653117	6.314782	6.1261652	5.947742	5.8053955	6.0202512

Down	FGSG_02211	-1.615531549	0.0010464	5.6164066	5.1692654	5.891554	5.2863342	5.3855124	5.0931475	3.9093688	3.8208439	3.9466965
Down	FGSG_09138	-1.615829084	7.71E-05	4.4062533	4.4323548	4.3087924	4.1980691	4.51735	3.6925011	3.6359267	3.3690748	3.8228101
Down	FGSG_07910	-1.616409345	1.20E-05	5.4906186	5.1692654	5.6852725	5.3281747	5.179638	5.3904893	4.4698939	4.1643702	4.6764038
Down	FGSG_12702	-1.616839991	6.86E-08	8.8769527	8.8447544	8.8241692	8.1881734	8.200598	8.0867194	7.9382281	7.9857763	7.8204879
Down	FGSG_08965	-1.618300902	3.68E-07	6.3688748	6.153105	6.5811798	6.466056	6.253029	6.5777126	5.7181748	5.5947086	5.7748904
Down	FGSG_07546	-1.620182959	3.66E-07	5.3814422	5.608128	5.0212003	5.1056669	4.9394155	5.1845342	4.8725557	5.2066203	4.3572018
Down	FGSG_02830	-1.621239718	0.0084705	3.2831234	2.8476671	3.5740244	2.6546039	2.353841	2.8774004	2.8565633	2.3968278	3.18196
Down	FGSG_06642	-1.621351338	3.70E-14	10.926589	10.84161	10.924349	10.793893	10.825011	10.670774	10.940529	10.856633	10.956528
Down	FGSG_13487	-1.623112196	5.65E-07	5.4640893	5.2921154	5.545677	5.1295269	5.0882909	5.0931475	5.626448	5.5563964	5.6345505
Down	FGSG_03207	-1.624528151	0.0014548	4.4062533	4.7218364	4.3087924	4.1048586	3.9182279	4.3744304	3.7791165	3.6853877	3.8228101
Down	FGSG_04143	-1.624734314	2.62E-10	8.3701331	8.3319751	8.3307216	8.3942583	8.200598	8.4874957	7.8106729	7.7932944	7.7626576
Down	FGSG_13311	-1.626849966	1.84E-06	4.0965725	4.2010532	3.9139052	4.327482	4.2058494	4.3744304	4.4698939	4.3549955	4.5256129
Down	FGSG_04357	-1.627238107	1.09E-14	11.114224	11.151544	10.987533	11.089456	11.024537	11.067275	10.942432	10.951026	10.86625
Down	FGSG_02678	-1.62957675	1.98E-07	6.9873724	6.9534782	6.9382912	7.0758949	7.0183365	7.0603242	6.2842483	6.0998924	6.3912423
Down	FGSG_10343	-1.630651051	2.21E-06	5.8392029	6.0172257	5.545677	5.0567294	4.8861689	5.1395644	5.0240241	5.1562742	4.8129147
Down	FGSG_03752	-1.631528577	5.79E-06	9.3756901	9.3193862	9.3464826	8.8270568	8.762481	8.8080019	8.285742	8.3640243	8.1324168
Down	FGSG_08145	-1.632725706	0.0186925	3.2831234	2.8476671	3.5740244	2.364115	2.6377651	2	3.0942412	3.5358829	2.3974876
Down	FGSG_01915	-1.6331018	2.89E-14	11.314057	11.29342	11.248969	11.399246	11.37489	11.336227	11.322314	11.328154	11.249021
Down	FGSG_12418	-1.633224633	5.68E-12	10.8234	10.794325	10.766925	10.829381	10.783753	10.787422	11.079755	11.045683	11.051872
Down	FGSG_12511	-1.634385703	3.79E-09	6.9400302	7.0952041	6.6735015	7.4520907	7.3188299	7.4945842	6.9690546	6.8028047	7.0598582
Down	FGSG_00342	-1.634485019	6.73E-10	8.2196702	8.0994054	8.2505629	8.2079187	8.1047954	8.2276734	7.7070136	7.6368537	7.711174
Down	FGSG_09999	-1.634556297	1.07E-07	7.7615503	7.7910854	7.6563111	7.742486	7.8575207	7.5643	7.6753214	7.6997228	7.6023441
Down	FGSG_12857	-1.637281274	7.75E-09	13.737124	13.698744	13.689976	13.213776	13.143683	13.196263	12.757048	12.722093	12.725914
Down	FGSG_13297	-1.637771189	7.70E-09	6.5310205	6.6198745	6.3489364	7.0881229	7.203853	6.8669619	6.7604964	6.7863464	6.6681943
Down	FGSG_13634	-1.638070026	3.47E-07	8.7399224	8.6857657	8.7089137	9.708336	9.7067583	9.621118	9.6758911	9.6288556	9.656748
Down	FGSG_06290	-1.638726156	1.25E-07	10.634838	10.537643	10.643955	10.762288	10.71002	10.726955	10.234235	10.181805	10.221804
Down	FGSG_10548	-1.639826031	0.0005959	6.9966572	7.4911275	7.5507256	7.2437947	7.8916935	7.6469587	7.3188427	7.8177947	7.6847264
Down	FGSG_06664	-1.640843539	1.98E-07	11.059897	11.065887	10.967158	11.815436	11.788477	11.754906	12.097123	12.054232	12.074054
Down	FGSG_08028	-1.640902787	8.10E-05	4.7948149	4.4323548	5.0212003	4.5559538	4.7733906	4.210161	3.7791165	3.9446664	3.5376798
Down	FGSG_09114	-1.642027991	3.29E-06	6.8810895	6.6660438	7.0108688	6.843198	6.5724409	7.0960705	6.0501376	6.152209	5.8718551
Down	FGSG_08376	-1.642567594	0.0004983	5.5422571	5.510285	5.4959731	5.0060735	5.0403527	4.8909283	3.8457121	3.9446664	3.6872775

Down	FGSG_01977	-1.643760021	4.17E-08	7.550805	7.6373467	7.4102108	7.3542322	7.4253162	7.2626125	6.9312196	7.1110577	6.6860823
Down	FGSG_04034	-1.643823006	1.04E-08	11.019475	10.956361	10.996498	11.103077	11.073739	11.044586	10.615642	10.612425	10.553158
Down	FGSG_12376	-1.644927057	2.52E-05	8.0955381	8.0824823	8.0238286	8.37692	8.5087227	8.1333402	7.8064993	7.7683708	7.7794184
Down	FGSG_06223	-1.645254564	0.0319241	2.6910183	2	3.1285604	3.2841938	2.6377651	3.8119585	4.028829	3.9446664	4.0607807
Down	FGSG_03718	-1.645416177	4.29E-08	6.8910817	6.8770274	6.8618681	6.9741499	7.1256073	6.7066335	6.443237	6.3216817	6.4963611
Down	FGSG_00455	-1.645440481	3.77E-11	12.073156	12.057521	12.00289	12.087179	12.079088	12.006513	12.088125	12.039551	12.070228
Down	FGSG_13598	-1.645843552	0.002906	3.1562965	3.1370988	3.1285604	4.4462359	4.3699906	4.4500449	5.0240241	4.8105634	5.1587087
Down	FGSG_11408	-1.648075014	3.17E-06	6.5181831	7.5415319	7.0799694	6.5829982	7.4621692	7.4024174	6.1589001	6.9724415	6.8537291
Down	FGSG_08248	-1.648516345	0.0003589	4.349408	4.2010532	4.4195794	3.8415007	3.9182279	3.6925011	3.8457121	4.0586965	3.5376798
Down	FGSG_04428	-1.64973642	9.82E-08	7.6716626	7.529095	7.7226391	7.4282413	7.3584675	7.4119036	6.7861576	6.8190773	6.6860823
Down	FGSG_03845	-1.650720751	3.67E-06	7.4797482	7.6373467	7.20897	6.9475516	7.030657	6.7670162	6.4324437	6.1776727	6.5943381
Down	FGSG_02326	-1.650967707	2.15E-08	15.177585	15.121407	15.148313	14.824493	14.794237	14.767483	14.261909	14.250498	14.206946
Down	FGSG_01000	-1.654245235	1.07E-12	10.46713	10.434722	10.414062	10.821621	10.736586	10.818069	10.482674	10.445584	10.453549
Down	FGSG_09387	-1.654711668	0.0005327	3.5075501	3.9254527	2.8407141	3.6568115	3.6887032	3.5622521	3.4769433	3.1804273	3.6872775
Down	FGSG_10126	-1.655839774	0.0330252	3.0172362	3.378055	2.4807492	2.8962916	3.4156241	2	3.0942412	2.3968278	3.5376798
Down	FGSG_08448	-1.655957244	1.59E-10	13.65878	13.61645	13.616835	14.05411	14.007673	14.013175	13.916454	13.8751	13.892119
Down	FGSG_01731	-1.656252501	1.19E-13	10.836386	10.8188	10.768234	10.719695	10.699448	10.651883	10.594012	10.569787	10.554376
Down	FGSG_06896	-1.657300608	3.96E-07	10.710848	10.725137	10.609312	9.9095006	9.7779154	9.9505575	9.264345	9.2116306	9.2508681
Down	FGSG_03101	-1.657741343	4.27E-08	7.0511452	7.0782312	6.9750363	6.3989268	6.2103411	6.5607563	6.2842483	6.3666582	6.2052877
Down	FGSG_02330	-1.658711103	4.41E-05	4.349408	4.2010532	4.7085304	4.657914	4.8861689	4.5903319	4.1912995	4.3549955	4.265
Down	FGSG_11748	-1.660664052	1.01E-05	5.5422571	5.458757	5.545677	5.1056669	5.265544	4.8356119	5.6073815	5.5170389	5.6345505
Down	FGSG_02425	-1.661579913	4.74E-06	6.0500989	6.120316	5.891554	5.658465	5.4962661	5.7308476	4.994976	5.2066203	4.6764038
Down	FGSG_05446	-1.662124641	5.33E-08	7.1789894	7.0436746	7.2243133	6.7071125	6.8053944	6.5086577	6.5668244	6.3216817	6.7212064
Down	FGSG_05331	-1.662249404	2.47E-11	8.1956835	8.0216372	8.2728698	7.6992962	7.489204	7.8279241	7.7981156	7.8177947	7.7285401
Down	FGSG_03037	-1.662602899	6.14E-05	4.349408	3.765011	4.7085304	3.6568115	3.6887032	3.5622521	3.6359267	3.6853877	3.5376798
Down	FGSG_01172	-1.66380138	3.30E-06	7.0241584	6.9347419	7.0284567	7.1241972	7.2572536	6.8806909	6.8685409	6.9130376	6.7723395
Down	FGSG_02518	-1.664543643	2.76E-13	9.8771002	9.8559979	9.8127228	9.7788684	9.7239312	9.746379	9.5112709	9.5699772	9.3801118
Down	FGSG_09013	-1.665660051	4.81E-07	6.3253218	6.052414	6.4825439	5.5388358	5.346621	5.636897	5.4014247	5.5563964	5.1587087
Down	FGSG_03349	-1.667605901	2.10E-05	6.0678264	5.9811576	6.0719967	6.0193342	5.8970396	6.0552962	4.9041484	5.1041074	4.6029775
Down	FGSG_00392	-1.667950548	0.0004874	5.5166689	5.1036809	5.7713555	5.2863342	5.0882909	5.3904893	4.7734018	3.1804273	5.4790733
Down	FGSG_13669	-1.668389523	4.29E-05	4.1640552	3.9254527	4.3087924	4.2856211	3.6887032	4.6556708	4.9041484	4.6740912	5.0523944

Down	FGSG_02819	-1.669688659	5.38E-05	6.7985581	6.6885859	6.8220828	6.0695346	6.0977451	5.9550408	5.3564544	4.8742451	5.670936
Down	FGSG_06499	-1.670308445	4.36E-09	8.7564142	8.7621451	8.6646097	8.9051585	8.7844739	8.938439	8.899237	8.8332655	8.898693
Down	FGSG_09439	-1.67148119	1.96E-12	12.957133	12.955344	12.872466	12.602059	12.555313	12.561612	12.488954	12.422314	12.489182
Down	FGSG_00295	-1.672322534	2.35E-07	6.9303725	7.1449564	6.5811798	7.4378284	7.3387849	7.4492367	7.2892379	7.249572	7.2641992
Down	FGSG_04217	-1.67396702	0.000182	4.4609433	4.3213345	4.5224622	4.0052074	3.4156241	4.3744304	3.9093688	4.1643702	3.5376798
Down	FGSG_08264	-1.674396457	0.0065199	5.2937714	5.349828	5.2780294	4.4077273	4.8308815	4.2946324	3.8457121	4.3549955	2.964684
Down	FGSG_05738	-1.675758972	5.81E-07	8.6632834	8.5446165	8.6924588	7.9643443	7.9118154	7.9304878	7.4477067	7.3425624	7.4846298
Down	FGSG_04703	-1.678194128	0.0030641	5.1346624	5.510285	4.5224622	4.0052074	4.1161991	3.8119585	3.5586239	3.8208439	3.18196
Down	FGSG_01298	-1.678734402	2.86E-07	6.9108608	6.6660438	7.0458328	7.7806582	7.7197389	7.7552597	7.656894	7.743009	7.4948146
Down	FGSG_03316	-1.681514898	0.0009253	3.6078986	3.378055	3.7539507	3.5190873	3.0784965	3.8119585	4.2416299	4.1643702	4.265
Down	FGSG_05643	-1.682066728	6.35E-09	12.837412	12.810954	12.778149	13.074274	13.043699	13.017121	12.766882	12.731795	12.736146
Down	FGSG_02004	-1.6831101	7.81E-06	12.528301	12.520663	12.449752	12.929308	12.868388	12.902349	13.038257	12.993658	13.01642
Down	FGSG_07659	-1.683982649	1.32E-06	7.7724024	7.6373467	7.8268285	7.3235317	7.1596621	7.4024174	6.5267935	6.59171	6.4128878
Down	FGSG_01733	-1.685497049	1.13E-13	11.190546	11.165673	11.129778	11.336885	11.302433	11.283589	11.139459	11.10992	11.102702
Down	FGSG_00201	-1.687455759	1.01E-08	8.3412698	8.4355616	8.1497038	8.1824819	8.1221706	8.1560975	7.733635	7.7515126	7.6486915
Down	FGSG_10281	-1.688629837	1.78E-11	12.844018	12.802582	12.799755	12.467536	12.447734	12.399111	12.171826	12.144569	12.132707
Down	FGSG_06899	-1.688729888	0.0157875	3.9513288	4.6316459	2.4807492	3.2841938	3.5586152	2.8774004	2.4052168	2.3968278	2.3974876
Down	FGSG_08968	-1.689454641	2.38E-06	5.2937714	4.8067189	5.5937254	4.8424709	4.6510429	4.9442019	4.8402557	5.0499834	4.5256129
Down	FGSG_04797	-1.691858228	0.0009441	3.701719	4.2010532	2.8407141	4.5203003	3.8080253	4.9442019	4.5913113	4.9352342	4.0607807
Down	FGSG_02150	-1.693096385	6.44E-06	6.0139766	6.3916107	5.545677	5.6419705	5.8698682	5.9021734	4.994976	5.2552685	5.0523944
Down	FGSG_07609	-1.693497309	2.80E-08	7.8044776	7.7052501	7.8167417	7.307933	7.3778853	7.1423949	6.9690546	6.7185687	7.1268773
Down	FGSG_13514	-1.69511603	9.41E-05	5.2322171	5.1036809	5.2780294	4.871059	4.9907667	4.6556708	4.3828727	3.6853877	4.8129147
Down	FGSG_04170	-1.698802289	1.59E-07	7.0332102	6.9719742	7.0108688	6.7842942	6.6858453	6.7962856	6.3314892	6.2752577	6.3242864
Down	FGSG_05073	-1.700092205	9.64E-09	7.550805	7.3708347	7.6335064	6.7767592	6.7318449	6.7371407	6.9002206	6.735814	6.9895734
Down	FGSG_03161	-1.701483532	0.004028	3.872828	4.4323548	2.8407141	3.3668146	2.8748957	3.6925011	3.199846	3.6853877	2.3974876
Down	FGSG_14002	-1.702314534	4.21E-07	4.6610646	4.5354388	4.7085304	4.7531416	4.7733906	4.6556708	4.3373111	4.0586965	4.5256129
Down	FGSG_08101	-1.702391338	5.11E-05	5.2937714	5.2319977	5.2780294	5.6908987	5.9237087	5.3115993	4.8725557	5.4349486	3.8228101
Down	FGSG_00563	-1.702851259	6.80E-09	7.8966342	7.7594926	7.9426701	7.359286	7.4621692	7.1650109	7.6429173	7.5517747	7.6668215
Down	FGSG_04028	-1.703278404	1.48E-06	5.9195042	5.8271292	5.9294932	6.1649807	6.0741317	6.1715481	5.3100371	5.2066203	5.3945636
Down	FGSG_10763	-1.70331897	2.06E-10	8.0516681	7.8916615	8.1171963	8.4639479	8.3701609	8.4694383	8.548193	8.4811393	8.5488359
Down	FGSG_01269	-1.703728813	0.0091946	2.2688027	2.4852148	2	2.364115	2.353841	2.3550396	3.4769433	3.3690748	3.5376798

Down	FGSG_13956	-1.704540046	0.0001258	5.5921111	5.2921154	5.7713555	5.368836	5.5313719	5.0931475	4.4270393	4.4416259	4.3572018
Down	FGSG_05471	-1.707590618	9.15E-08	10.9101	10.860346	10.87538	11.282387	11.267073	11.20937	10.584902	10.549205	10.555593
Down	FGSG_11775	-1.708673002	0.0057969	3.1562965	2.8476671	3.3684179	3.1032407	3.4156241	2.6397336	2.9802913	2.7077187	3.18196
Down	FGSG_11768	-1.708886491	7.11E-06	4.5136357	4.4323548	4.5224622	4.4837433	4.2902531	4.5903319	4.2902636	4.6006874	3.8228101
Down	FGSG_04050	-1.709016549	7.71E-12	12.115854	12.065617	12.147729	11.676069	11.737715	11.617662	11.620355	11.698417	11.624958
Down	FGSG_09196	-1.709150814	1.23E-08	6.665171	6.7541729	6.8813568	6.0571473	6.2318429	6.2155469	6.235408	6.2514733	6.4342132
Down	FGSG_13362	-1.709186113	5.43E-05	3.5075501	3.5844678	3.3684179	3.7210377	2.8748957	4.210161	4.0850435	3.8208439	4.265
Down	FGSG_12289	-1.71148188	0.0027458	5.2322171	4.9628272	5.3911128	4.4077273	4.2058494	4.5218929	3.7092973	3.1804273	4.0607807
Down	FGSG_01756	-1.713729259	2.17E-06	4.9558055	5.1036809	4.7085304	5.7068462	5.8421752	5.4652882	4.994976	4.8742451	5.0523944
Down	FGSG_13672	-1.715149799	2.71E-09	8.429615	8.3319751	8.491538	8.4113907	8.28015	8.4964403	8.4092308	8.2665305	8.5439396
Down	FGSG_02308	-1.718619978	6.99E-07	13.181841	13.160264	13.118124	13.418674	13.405991	13.3431	12.940588	12.890119	12.92452
Down	FGSG_03688	-1.718656177	4.03E-08	11.588429	11.548851	11.542387	12.020155	11.983297	11.96925	11.700418	11.658845	11.675641
Down	FGSG_12964	-1.719099425	0.001234	3.9513288	2.8476671	4.5224622	3.0034737	2.6377651	3.260088	3.3903592	3.8208439	2.7087823
Down	FGSG_05477	-1.719232387	3.16E-14	9.7674641	9.7581508	9.6909999	9.7445161	9.6990598	9.7045358	9.78173	9.7673745	9.7297104
Down	FGSG_02407	-1.720983807	3.21E-07	8.3521613	8.1969762	8.4133694	7.9676573	7.9832834	7.8629285	7.3305166	7.2851495	7.3452367
Down	FGSG_08727	-1.722119731	1.80E-14	10.712962	10.693443	10.646805	10.83574	10.799138	10.784686	10.827674	10.788024	10.801057
Down	FGSG_13441	-1.723382758	1.27E-06	6.438663	6.5228522	6.2623983	6.328521	6.2739084	6.2997126	5.5080752	5.8053955	5.0523944
Down	FGSG_06141	-1.724140048	2.46E-07	7.8306727	7.5538626	7.9883205	7.2708663	7.1929315	7.2626125	6.8844677	6.8028047	6.9004463
Down	FGSG_06187	-1.725189592	2.03E-06	5.3235909	5.510285	5.0212003	4.7220923	4.8861689	4.4500449	4.935064	4.5233477	5.2090653
Down	FGSG_08102	-1.725724242	2.09E-08	7.544488	7.4782464	7.7226391	7.1477552	7.308748	7.1309525	6.9235321	6.9130376	6.9895734
Down	FGSG_09031	-1.72818657	0.0003687	5.5921111	5.510285	5.5937254	4.4462359	4.4455509	4.3744304	4.935064	4.8742451	4.9376179
Down	FGSG_12555	-1.728645101	3.12E-06	13.325656	13.254169	13.310784	14.34456	14.308439	14.293238	14.269225	14.221524	14.250612
Down	FGSG_09882	-1.729768692	8.75E-06	7.2192048	7.3139731	7.0284567	6.3591166	6.2103411	6.4174145	5.8210632	5.8053955	5.7748904
Down	FGSG_09349	-1.730075573	1.41E-06	4.9558055	4.8067189	5.0898194	4.4077273	4.1161991	4.5903319	4.3373111	4.262829	4.4438634
Down	FGSG_05928	-1.730360419	1.37E-06	16.043361	15.989025	16.011797	16.548666	16.506323	16.503634	16.392386	16.360328	16.358241
Down	FGSG_13100	-1.730492857	0.0044288	3.3996963	2.4852148	3.9139052	3.3668146	3.5586152	3.0813961	3.199846	2.7077187	3.5376798
Down	FGSG_13872	-1.73298547	3.28E-05	5.2937714	5.1692654	5.3356788	5.368836	5.306652	5.3515835	4.5913113	4.4416259	4.6764038
Down	FGSG_11543	-1.735216788	0.0042365	2.6910183	3.1370988	2	3.5190873	3.0784965	3.8119585	3.2982451	3.5358829	3.18196
Down	FGSG_12380	-1.73569036	7.13E-11	10.571784	10.46768	10.587231	11.126238	11.045179	11.118869	11.201565	11.201212	11.134806
Down	FGSG_12383	-1.737782444	2.26E-06	6.1197383	6.120316	6.0376686	5.8137994	6.0257126	5.4652882	5.0524989	5.2552685	4.7462732
Down	FGSG_06186	-1.737936465	7.72E-12	8.9765081	8.9085572	8.9586812	8.5853741	8.5218742	8.5618139	8.5280562	8.6222832	8.3555989

Down	FGSG_12907	-1.738796508	5.15E-14	11.62144	11.521644	11.632838	11.831436	11.761472	11.813341	11.587894	11.570292	11.538985
Down	FGSG_05465	-1.738813622	7.56E-05	4.0257777	4.3213345	3.5740244	5.1529987	4.9907667	5.2281446	4.935064	5.0499834	4.7462732
Down	FGSG_06431	-1.739502389	0.0017124	3.5075501	3.378055	3.5740244	3.8415007	4.2058494	3.260088	3.4769433	2.7077187	3.9466965
Down	FGSG_10565	-1.739784955	0.000164	5.3235909	5.4053203	5.1553224	5.1988268	5.1346873	5.1845342	4.5115122	4.1643702	4.7462732
Down	FGSG_11347	-1.740291051	2.16E-05	6.2023113	5.9811576	6.3206633	6.3691726	6.253029	6.3984523	5.4450355	5.347904	5.4790733
Down	FGSG_09141	-1.741237409	2.83E-07	8.6338326	8.6344394	8.5475047	8.4235048	8.2593613	8.4919749	7.4583868	7.2375151	7.5928932
Down	FGSG_09280	-1.742906682	3.20E-08	9.6663664	9.6213135	9.6260572	9.2949369	9.2786072	9.223413	8.6307707	8.5751565	8.6203531
Down	FGSG_11445	-1.743422961	9.15E-11	9.2258368	9.2490874	9.119132	8.9808888	9.0034336	8.8712598	8.5204318	8.465718	8.5192059
Down	FGSG_12561	-1.743503394	0.009552	4.5136357	4.5354388	4.4195794	3.1965524	3.4156241	3.0813961	2.7212202	2.7077187	2.7087823
Down	FGSG_02251	-1.744682678	4.81E-06	5.899844	6.052414	5.640225	5.8573158	5.8970396	5.7308476	4.965331	4.8105634	5.0523944
Down	FGSG_03284	-1.74568972	1.65E-06	6.629804	6.5721788	6.60482	6.8858494	6.6221382	7.0359915	6.576661	6.4526114	6.6500817
Down	FGSG_05828	-1.745764185	3.63E-05	10.502594	10.347103	10.563298	9.9435487	9.9369181	9.8633574	9.1421531	9.1193753	9.0989159
Down	FGSG_01982	-1.746043066	2.70E-07	4.7070397	5.0349724	4.188785	4.5907474	4.51735	4.5903319	4.8402557	4.9937494	4.6029775
Down	FGSG_05871	-1.747472561	2.39E-06	6.1533367	6.0867644	6.1382955	5.6908987	5.6951271	5.6041695	5.2862562	5.4349486	5.0523944
Down	FGSG_11400	-1.748473011	2.41E-05	5.100621	4.8868835	5.2179801	4.4837433	4.0206066	4.7780897	4.0850435	4.3549955	3.6872775
Down	FGSG_11102	-1.749969225	1.18E-05	4.8367778	4.8868835	4.7085304	5.1056669	4.8308815	5.2704753	4.2902636	4.5233477	3.9466965
Down	FGSG_07826	-1.750094594	0.0155362	3.872828	4.3213345	3.1285604	3.3668146	3.2568854	3.4190665	2.4052168	2	2.7087823
Down	FGSG_03486	-1.754151883	0.0020582	2.8633281	2.4852148	3.1285604	4.0052074	3.5586152	4.2946324	2.9802913	2.7077187	3.18196
Down	FGSG_04546	-1.756039632	2.30E-08	12.051045	11.985954	12.030428	11.509903	11.502553	11.428496	10.914102	10.910784	10.850474
Down	FGSG_11624	-1.756742703	4.02E-05	4.8775546	4.7218364	4.9491538	5.8429557	5.5991203	5.9807654	5.5880596	5.5563964	5.5589053
Down	FGSG_01431	-1.758006467	2.58E-07	10.612364	10.562265	10.576809	9.596153	9.518384	9.5922046	9.9538776	9.939125	9.9022317
Down	FGSG_13298	-1.760982691	5.92E-06	8.7808037	8.646004	8.8241692	7.8824608	7.8712871	7.8065065	7.1179419	7.0977802	7.0735134
Down	FGSG_03094	-1.762445277	0.026468	3.5075501	3.9254527	2.8407141	2.364115	2.353841	2.3550396	2.8565633	2	3.3707558
Down	FGSG_12800	-1.7634448	2.30E-06	8.3339628	8.1890927	8.3863428	7.5348245	7.5842106	7.3928684	6.8443161	6.6834471	6.9307712
Down	FGSG_02506	-1.763502689	1.84E-05	7.2964211	7.2242442	7.2841049	6.7307036	6.7468578	6.6274177	5.6821809	5.6684096	5.6345505
Down	FGSG_20009	-1.763604993	0.0003927	3.6078986	3.765011	3.3684179	3.3668146	3.2568854	3.4190665	3.2982451	3.8208439	2.3974876
Down	FGSG_03589	-1.767132506	1.66E-07	6.3253218	6.2165286	6.3489364	6.2972625	6.29449	6.2155469	6.0216157	6.2752577	5.6345505
Down	FGSG_11750	-1.767286804	3.34E-10	12.622587	12.601586	12.557788	13.113031	13.050711	13.087441	12.999705	12.963552	12.969491
Down	FGSG_09365	-1.767529034	1.75E-08	8.6128533	8.5259608	8.6130853	8.3390496	8.3894229	8.195079	7.9571779	7.8258699	8.0170225
Down	FGSG_08914	-1.768604564	9.03E-09	8.4567799	8.556921	8.2580369	7.8754227	7.7498592	7.9105501	8.0624335	8.07656	7.9812488
Down	FGSG_10706	-1.768637751	0.0007426	4.0257777	3.9254527	4.0578819	3.0034737	3.2568854	2.6397336	3.4769433	3.5358829	3.3707558

Down	FGSG_02404	-1.769223031	1.66E-09	7.0600298	7.0260808	7.0108688	7.6003202	7.558914	7.5643	7.1902009	7.1756715	7.1399154
Down	FGSG_04188	-1.769233597	5.31E-05	4.6135762	4.069824	4.9491538	3.9526758	4.0206066	3.8119585	3.3903592	3.3690748	3.3707558
Down	FGSG_13156	-1.769524175	9.87E-12	8.5353342	8.5446165	8.439899	8.3416054	8.3750006	8.2168902	8.0053133	8.123176	7.8042001
Down	FGSG_10026	-1.770064413	1.74E-05	6.7444707	6.8169075	6.5811798	6.892837	6.9807308	6.7066335	5.6821809	5.6684096	5.6345505
Down	FGSG_06771	-1.770151417	7.19E-14	11.043752	10.978256	11.023061	10.783121	10.713845	10.764464	10.728586	10.725732	10.664492
Down	FGSG_00972	-1.770772974	2.08E-08	11.115823	10.993047	11.147995	10.340392	10.354573	10.236039	10.264218	10.29956	10.159147
Down	FGSG_02886	-1.771076979	0.01495	3.5075501	3.1370988	3.7539507	3.1032407	3.6887032	2.3550396	2.2167888	2.3968278	2
Down	FGSG_02119	-1.772570943	3.85E-10	8.9166249	8.9085572	8.8441702	8.9206259	8.9296958	8.8223008	8.6886096	8.6452815	8.6661346
Down	FGSG_04988	-1.772919907	5.20E-07	6.2496778	6.2165286	6.2016809	5.6419705	5.6951271	5.5363994	5.4014247	5.5170389	5.2090653
Down	FGSG_03450	-1.773049105	8.70E-05	4.8367778	5.1692654	4.7932764	5.5388358	5.8698682	5.1395644	4.5519634	4.8742451	4.6764038
Down	FGSG_09148	-1.773280477	0.0001119	4.1640552	4.069824	4.188785	3.9526758	4.1161991	3.6925011	3.6359267	3.3690748	3.8228101
Down	FGSG_02410	-1.775339531	0.0001087	5.9389001	5.7434744	6.0376686	5.2863342	5.265544	5.2281446	4.3373111	3.9446664	4.6029775
Down	FGSG_02789	-1.775891687	1.39E-10	9.1404552	9.0149575	9.1754742	8.8964933	8.7917309	8.9119415	8.9206347	8.8292542	8.9439989
Down	FGSG_11988	-1.776102429	4.33E-07	7.250589	6.8965233	7.4629041	6.6339322	6.314782	6.8249729	6.3659317	6.4937409	6.1547968
Down	FGSG_01579	-1.777103868	0.016297	3.7898088	3.9254527	3.5740244	2.6546039	2	3.0813961	3.0942412	2.9633478	3.18196
Down	FGSG_07065	-1.777282249	5.62E-05	4.6135762	4.6316459	4.5224622	5.5740396	5.8970396	5.1395644	4.9041484	4.9352342	4.8129147
Down	FGSG_12232	-1.779585948	0.0082111	4.6135762	4.8067189	4.3087924	4.8133047	4.51735	4.9955781	3.3903592	3.6853877	2.964684
Down	FGSG_03307	-1.779893893	6.78E-11	9.5763453	9.5553847	9.51191	9.1710794	9.1714134	9.082021	8.8755264	8.8412548	8.8439727
Down	FGSG_10534	-1.781503855	8.29E-05	5.2322171	5.2319977	5.1553224	4.2425091	4.0206066	4.4500449	4.028829	4.1643702	3.8228101
Down	FGSG_04939	-1.782050807	4.71E-07	7.1952103	7.0610564	7.2545188	6.9067114	6.8477891	6.8942906	6.1976611	6.073005	6.301259
Down	FGSG_00473	-1.782270061	0.0001308	4.2285219	4.5354388	3.7539507	3.6568115	3.8080253	3.4190665	4.0850435	4.262829	3.8228101
Down	FGSG_03710	-1.7855116	1.19E-13	8.683547	8.5384247	8.7359284	8.6630399	8.6944163	8.5403502	8.4472581	8.4184396	8.410429
Down	FGSG_05186	-1.787395918	6.89E-10	13.392358	13.358019	13.34101	13.764625	13.70892	13.732515	13.537752	13.487371	13.521897
Down	FGSG_05350	-1.789096727	1.75E-09	8.0201456	8.0824823	7.8664849	7.4986383	7.489204	7.4213278	7.2156048	7.1882541	7.1783375
Down	FGSG_09210	-1.791166249	2.17E-07	12.442786	12.421113	12.379108	12.819686	12.780156	12.771908	12.066473	12.016363	12.050081
Down	FGSG_00147	-1.791954632	0.0004041	4.5644712	3.9254527	4.9491538	3.2841938	3.2568854	3.260088	4.2416299	4.0586965	4.3572018
Down	FGSG_03288	-1.793570963	2.58E-06	5.167919	5.349828	4.8733193	4.6247215	4.3699906	4.7780897	4.3828727	4.3549955	4.3572018
Down	FGSG_08983	-1.793663185	2.86E-07	6.7222549	6.7107812	6.650969	7.0195487	6.9679745	6.9860594	7.1179419	7.0154424	7.1528368
Down	FGSG_02883	-1.796501753	0.0037719	3.2831234	2.8476671	3.5740244	2.7805029	2	3.260088	2.8565633	2.9633478	2.7087823
Down	FGSG_11026	-1.797857416	6.28E-06	4.5644712	4.8067189	4.188785	3.7825262	3.8080253	3.6925011	3.9093688	4.262829	3.5376798
Down	FGSG_12696	-1.799956603	3.08E-07	6.9206496	7.0610564	6.6735015	6.4846757	6.4675956	6.4546071	6.1324685	5.9304949	6.2540713

Down	FGSG_07685	-1.803635794	4.76E-07	8.6748975	8.6969277	8.5954933	7.8648007	7.8225187	7.8208202	7.4423369	7.5222624	7.2994884
Down	FGSG_03027	-1.804868714	9.03E-09	7.694665	7.7271924	7.5748659	7.3491606	7.1596621	7.4399936	6.7776545	6.7185687	6.7723395
Down	FGSG_02856	-1.811399399	0.0063094	3.2831234	2.8476671	3.5740244	2.8962916	2.353841	3.260088	2.2167888	2.3968278	2
Down	FGSG_04472	-1.81198094	0.0023821	4.2902308	4.069824	4.4195794	3.8981589	3.9182279	3.8119585	2.8565633	2.3968278	3.18196
Down	FGSG_13580	-1.812497458	7.86E-09	8.734383	8.6913575	8.6924588	8.9923198	8.9940267	8.9018781	8.9530919	8.9779764	8.85982
Down	FGSG_13805	-1.812873878	1.60E-08	5.899844	5.906198	5.8125445	5.5740396	5.5656438	5.5012819	5.1346989	4.9937494	5.2090653
Down	FGSG_08103	-1.812876057	1.26E-06	7.8512916	7.8622179	7.9239976	8.0943001	7.9447403	8.2647922	7.0275995	6.5726412	7.411249
Down	FGSG_02692	-1.814411204	1.13E-05	9.0608092	9.362083	9.3742591	9.1109258	9.4512279	9.419561	8.7088767	9.1389527	9.141675
Down	FGSG_11245	-1.815582771	0.0004928	3.701719	3.5844678	3.7539507	4.657914	4.3699906	4.8356119	4.5519634	4.8742451	4.0607807
Down	FGSG_02810	-1.817173754	9.14E-11	10.55617	10.485492	10.540495	10.908222	10.8677	10.861122	10.488547	10.513403	10.394936
Down	FGSG_01034	-1.818141684	0.001667	2.8633281	3.378055	2.4807492	3.2841938	3.5586152	2.8774004	3.2982451	3.6853877	2.7087823
Down	FGSG_11236	-1.819736461	2.98E-06	9.8285322	9.7897727	9.7819457	9.1710794	9.1658388	9.0879509	8.4119805	8.4023294	8.3555989
Down	FGSG_08632	-1.82132521	2.05E-05	5.5422571	5.349828	5.728956	5.8715343	5.7851416	5.9288493	4.9041484	4.7439404	5.0523944
Down	FGSG_07263	-1.821752912	3.54E-07	7.2112509	7.0782312	7.2545188	8.0418289	7.9705498	8.0262414	7.8189842	7.7171935	7.852522
Down	FGSG_00488	-1.821754512	1.69E-08	8.4054187	8.3103344	8.4133694	8.1739024	8.0084184	8.2436985	7.7726681	7.6909075	7.7877264
Down	FGSG_11307	-1.823433118	1.69E-08	5.9769267	5.9811576	5.891554	6.328521	6.2103411	6.3597635	6.3198232	6.2514733	6.3242864
Down	FGSG_03657	-1.823508665	0.0006814	4.4609433	4.2010532	4.6184939	4.7835368	4.8861689	4.5903319	3.199846	2.9633478	3.3707558
Down	FGSG_09650	-1.82573642	5.21E-13	12.399691	12.383179	12.331196	12.534691	12.476446	12.505396	12.575352	12.531268	12.553628
Down	FGSG_12048	-1.826456834	0.0089672	2.6910183	2.4852148	2.8407141	4.2856211	4.2058494	4.2946324	3.7791165	4.3549955	2.7087823
Down	FGSG_07296	-1.826948593	3.88E-08	6.9108608	6.5962238	7.0967395	7.1062728	7.1596621	6.9604299	7.02041	7.0571987	6.9156884
Down	FGSG_04515	-1.82924835	0.0010803	3.3996963	3.765011	2.8407141	3.0034737	3.2568854	2.6397336	2.9802913	3.1804273	2.7087823
Down	FGSG_02681	-1.829548302	0.0023227	5.6638035	5.7434744	5.4959731	5.2432439	5.346621	5.0451875	4.9041484	4.6006874	5.1065306
Down	FGSG_03476	-1.829838411	8.18E-09	10.628133	10.581966	10.590194	10.513015	10.422767	10.516638	10.14367	10.087558	10.133301
Down	FGSG_05959	-1.829974963	0.0057609	4.9933948	5.2319977	4.6184939	4.4837433	4.51735	4.4500449	3.2982451	3.1804273	3.3707558
Down	FGSG_05209	-1.831329324	1.96E-08	12.903536	12.909601	12.810596	13.368175	13.318099	13.330494	13.656202	13.64401	13.601961
Down	FGSG_05339	-1.833618968	4.46E-11	12.235384	12.211877	12.174141	12.600592	12.495738	12.615462	12.802158	12.725604	12.811673
Down	FGSG_01673	-1.834823714	0.0001461	4.2902308	3.5844678	4.7085304	3.8981589	3.8080253	3.9222766	3.7791165	2.9633478	4.265
Down	FGSG_05624	-1.836978471	8.33E-10	6.605735	6.4975416	6.628079	6.6991623	6.5554873	6.7521558	6.4960241	6.3666582	6.555942
Down	FGSG_13535	-1.838229479	3.41E-10	8.3229325	8.2357604	8.3236158	7.8540999	7.9250757	7.6865774	7.6661371	7.743009	7.514971
Down	FGSG_04937	-1.83880241	1.82E-07	6.2023113	6.0867644	6.232359	6.6505183	6.5891976	6.6274177	6.3430616	6.5533169	6.0202512
Down	FGSG_04995	-1.838839185	7.47E-11	7.8461644	8.030488	7.8467929	7.3491606	7.6575433	7.5812149	7.8598371	8.0213724	7.9145321

Down	FGSG_00080	-1.842841987	9.66E-07	5.2937714	5.458757	5.0212003	5.2863342	4.8861689	5.5363994	4.7032988	4.8742451	4.4438634
Down	FGSG_09154	-1.843010057	1.11E-08	7.9260897	7.8014645	7.961104	7.7993721	7.7273281	7.7847661	8.1703066	8.1427022	8.1324168
Down	FGSG_08532	-1.843148021	2.15E-07	9.0585944	8.9696574	9.0604997	9.016514	8.9064847	9.0428709	7.7981156	7.7599663	7.7710623
Down	FGSG_13229	-1.847374268	0.0023142	2.6910183	2.4852148	2.8407141	3.6568115	3.5586152	3.6925011	2.8565633	3.1804273	2.3974876
Down	FGSG_07165	-1.847985587	1.96E-06	11.932079	11.893167	11.885954	12.580041	12.533835	12.538522	12.384855	12.36701	12.336158
Down	FGSG_00529	-1.848629485	1.19E-13	11.13487	11.076609	11.107195	10.952787	10.877112	10.94192	11.146493	11.111572	11.115135
Down	FGSG_04260	-1.851655409	6.18E-10	11.376736	11.313502	11.360797	11.080685	11.07225	11.006741	10.382492	10.393561	10.312406
Down	FGSG_06751	-1.852477646	0.0085898	8.5448595	8.4814692	8.5228982	11.965692	11.919718	11.923972	12.285459	12.227297	12.278004
Down	FGSG_03751	-1.854140811	6.05E-09	13.388946	13.372885	13.31907	13.49104	13.438575	13.456017	13.60493	13.559667	13.584333
Down	FGSG_03932	-1.858124477	4.43E-09	10.053173	9.9981333	10.026925	10.118422	10.093399	10.05566	9.4563875	9.3440381	9.4987232
Down	FGSG_05792	-1.861686043	1.22E-08	11.470515	11.427619	11.427772	11.229895	11.193641	11.178441	10.679794	10.715989	10.573723
Down	FGSG_03727	-1.863143535	0.0258323	3.872828	4.069824	3.5740244	3.1965524	2.6377651	3.5622521	2.2167888	2.3968278	2
Down	FGSG_09071	-1.863884931	3.52E-06	4.2902308	4.4323548	4.0578819	3.7825262	3.9182279	3.5622521	3.4769433	3.1804273	3.6872775
Down	FGSG_08404	-1.867165866	5.68E-12	11.315913	11.280494	11.26575	11.612145	11.521228	11.615098	11.482292	11.44679	11.45149
Down	FGSG_12010	-1.867213202	8.36E-06	4.9558055	5.2319977	4.5224622	4.69036	4.8861689	4.3744304	4.5115122	4.6006874	4.3572018
Down	FGSG_00961	-1.868870806	3.15E-05	3.7898088	4.069824	3.3684179	3.8415007	3.8080253	3.8119585	3.9093688	4.262829	3.3707558
Down	FGSG_03639	-1.869113518	0.0003259	3.9513288	3.765011	4.0578819	5.2212063	4.7135134	5.5363994	5.4663562	5.5947086	5.2577234
Down	FGSG_11520	-1.87257869	0.0258563	4.349408	4.5354388	4.0578819	3.7210377	3.6887032	3.6925011	2.4052168	2.7077187	2
Down	FGSG_04422	-1.874127883	0.0133124	3.9513288	4.2010532	3.5740244	3.7825262	4.2058494	3.0813961	2.5718533	2.7077187	2.3974876
Down	FGSG_03285	-1.875273914	3.58E-06	6.653478	6.5228522	6.6956874	6.4753959	6.1663515	6.6596278	5.9325022	6.152209	5.5972237
Down	FGSG_01430	-1.875338246	3.76E-14	11.841264	11.783566	11.812985	11.717737	11.710691	11.635992	11.869745	11.796186	11.875949
Down	FGSG_02981	-1.87905575	3.49E-06	3.7898088	3.9254527	3.5740244	4.2425091	4.2902531	4.1204343	4.4270393	3.8208439	4.8129147
Down	FGSG_03439	-1.881071586	0.00018	6.9400302	6.6431438	7.113317	6.1649807	6.0501253	6.1937152	5.0804226	5.1041074	4.9961472
Down	FGSG_20557	-1.882280434	0.0079924	4.9172105	4.6316459	5.0898194	4.7220923	4.3699906	4.9442019	3.2982451	3.5358829	2.964684
Down	FGSG_00764	-1.88536418	0.0001677	5.0300296	4.5354388	5.3356788	4.7835368	4.0206066	5.2281446	4.994976	4.6740912	5.2090653
Down	FGSG_03443	-1.885580562	1.78E-05	6.4921604	6.052414	6.7602689	6.1878872	5.7557564	6.4546071	5.5684753	5.2552685	5.7748904
Down	FGSG_03149	-1.885686655	0.0031395	4.0257777	3.5844678	4.3087924	2.8962916	2.353841	3.260088	2.7212202	2.7077187	2.7087823
Down	FGSG_10398	-1.886129702	8.87E-05	5.1346624	5.2319977	4.9491538	5.0567294	5.0882909	4.9442019	3.8457121	3.6853877	3.9466965
Down	FGSG_05719	-1.886192323	6.19E-05	4.5644712	3.9254527	5.0212003	4.8990917	5.346621	4.210161	3.970335	4.262829	3.5376798
Down	FGSG_10440	-1.886396588	2.06E-08	6.9400302	7.0436746	6.7390613	6.6339322	6.5033974	6.6754671	6.1055435	6.0456069	6.1024744
Down	FGSG_09069	-1.887306847	0.000204	3.5075501	3.765011	3.1285604	3.7210377	4.0206066	3.260088	4.0850435	4.262829	3.8228101

Down	FGSG_01494	-1.887361548	0.0001638	4.1640552	3.9254527	4.3087924	4.8990917	4.9394155	4.7780897	3.7791165	3.5358829	3.9466965
Down	FGSG_03447	-1.889044398	0.001856	3.5075501	4.069824	2.4807492	4.2425091	4.2058494	4.210161	3.9093688	3.8208439	3.9466965
Down	FGSG_02108	-1.889382506	1.32E-08	8.6748975	8.5991784	8.6646097	8.7769495	8.7476306	8.7190977	8.2338483	8.3246744	8.0656606
Down	FGSG_09150	-1.889503012	0.0007399	3.3996963	3.1370988	3.5740244	3.3668146	3.6887032	2.8774004	2.9802913	2.7077187	3.3707558
Down	FGSG_03653	-1.890135286	0.0002925	4.2902308	4.4323548	4.0578819	3.3668146	2.8748957	3.6925011	3.5586239	3.6853877	3.3707558
Down	FGSG_07017	-1.890716794	8.11E-10	9.6009054	9.5275207	9.6260572	9.2135577	9.2179474	9.2125978	8.6541853	8.6768755	8.587419
Down	FGSG_11080	-1.891770793	8.49E-10	9.3739099	9.344446	9.3181609	9.471008	9.41644	9.4382495	8.9225643	9.0313289	8.732193
Down	FGSG_13483	-1.894874392	8.72E-05	5.5166689	5.1692654	5.728956	4.953574	4.7733906	5.0451875	4.8725557	4.6740912	4.9961472
Down	FGSG_03715	-1.898669282	2.06E-10	8.8969252	8.869629	8.8391959	9.2615849	9.1602426	9.2814893	8.7043974	8.6361261	8.7105079
Down	FGSG_04660	-1.899255994	0.0010769	4.349408	4.8868835	3.7539507	4.0558932	4.2058494	3.8119585	3.4769433	2.9633478	3.8228101
Down	FGSG_11174	-1.89977314	0.0019256	5.8392029	5.510285	6.0376686	7.5258627	7.489204	7.4766159	7.6935164	7.6997228	7.6210621
Down	FGSG_03723	-1.90146541	0.0136441	3.6078986	3.5844678	3.5740244	3.0034737	3.4156241	2.3550396	2.4052168	3.1804273	2
Down	FGSG_01792	-1.90318562	0.0097755	2	2	2	3.3668146	3.2568854	3.4190665	2.5718533	2.7077187	2.3974876
Down	FGSG_13971	-1.906858477	0.001764	3.6078986	3.9254527	3.1285604	3.1965524	3.0784965	3.260088	2.5718533	2.7077187	2.3974876
Down	FGSG_03541	-1.909054313	7.71E-06	4.4062533	4.3213345	4.4195794	4.7531416	4.9394155	4.4500449	4.3828727	4.8105634	3.6872775
Down	FGSG_01993	-1.911325158	7.65E-06	9.1086944	8.9879485	9.1395078	9.5907916	9.5867307	9.5062513	9.5641428	9.5457361	9.5188255
Down	FGSG_10495	-1.911752948	8.71E-07	5.9769267	6.052414	5.8125445	6.5568406	6.5554873	6.4728499	6.9464729	7.0434138	6.7723395
Down	FGSG_08443	-1.912099429	4.13E-10	8.9788525	8.8296212	9.0389684	9.1953038	9.1602426	9.1516201	8.5705159	8.5894568	8.4889546
Down	FGSG_08700	-1.914821263	4.86E-08	8.3701331	8.2280865	8.4200477	8.0036067	7.9705498	7.9501537	7.2092957	7.2615289	7.0870405
Down	FGSG_11014	-1.915244589	0.0001042	6.0139766	5.6997548	6.2016809	5.3887447	5.6638294	4.9442019	4.3828727	4.7439404	3.8228101
Down	FGSG_07162	-1.916982378	1.83E-10	7.550805	7.3284	7.6675797	7.9775509	7.9447403	7.9238724	7.9073809	7.9118358	7.8365939
Down	FGSG_04452	-1.917782799	1.44E-14	10.368661	10.329281	10.324353	10.076843	10.071205	9.9969924	10.036383	9.939125	10.064879
Down	FGSG_06995	-1.91953093	0.0091946	3.3996963	2	4.0578819	3.444959	3.4156241	3.4190665	2.7212202	2.7077187	2.7087823
Down	FGSG_02204	-1.920742042	1.41E-08	9.458658	9.3373303	9.489861	9.2177376	9.1461564	9.2017009	8.77231	8.7967559	8.6795905
Down	FGSG_01701	-1.921148784	5.47E-09	8.6864187	8.6574768	8.6869318	8.0003754	7.9959056	7.9436281	7.7203857	7.7515126	7.7024119
Down	FGSG_01641	-1.922679735	8.87E-11	11.910443	11.864373	11.871409	12.113402	12.029688	12.108848	12.138387	12.088617	12.12207
Down	FGSG_03952	-1.924980952	2.31E-08	7.8816778	7.9856806	7.678761	7.484831	7.489204	7.3928684	6.7691009	6.735814	6.7384529
Down	FGSG_12141	-1.925523878	0.000302	3.9513288	3.765011	4.0578819	4.0558932	4.2058494	3.8119585	3.7092973	3.9446664	3.3707558
Down	FGSG_13741	-1.926103927	0.00183	3.0172362	2.8476671	3.1285604	3.444959	3.4156241	3.4190665	3.7791165	3.9446664	3.5376798
Down	FGSG_03725	-1.926117329	8.21E-12	12.069311	12.025782	12.028791	11.982985	11.889173	11.988002	11.667429	11.611749	11.657056
Down	FGSG_02387	-1.927151947	0.0001252	4.2285219	4.069824	4.4195794	4.1048586	3.6887032	4.3744304	3.4769433	3.3690748	3.5376798

Down	FGSG_03985	-1.927320252	0.001047	4.6135762	4.6316459	4.6184939	3.444959	2.8748957	3.9222766	4.028829	3.8208439	4.1665008
Down	FGSG_10494	-1.927729245	0.0001626	4.4062533	3.9254527	4.7085304	5.5913246	5.6951271	5.3904893	5.7002901	5.6684096	5.670936
Down	FGSG_09182	-1.930207559	0.0146474	3.6078986	3.1370988	3.9139052	2.1935143	2.353841	2	2.9802913	3.1804273	2.7087823
Down	FGSG_05674	-1.930682994	1.70E-09	8.1506446	8.1731952	8.1497038	8.2922525	8.3308476	8.3412553	7.8678707	7.9118358	7.9445655
Down	FGSG_11153	-1.931688797	5.46E-06	5.1346624	5.458757	4.6184939	4.327482	4.0206066	4.5218929	5.237485	4.9352342	5.4374371
Down	FGSG_09795	-1.933563831	3.77E-06	4.8367778	4.8067189	4.7932764	5.4083825	5.265544	5.4652882	4.666927	4.7439404	4.5256129
Down	FGSG_04993	-1.934872149	1.48E-05	5.6869305	6.0172257	5.1553224	5.4277566	5.6318376	5.0931475	5.1610901	5.1562742	5.1065306
Down	FGSG_05318	-1.935936105	4.94E-06	5.9195042	5.906198	5.85259	6.7384824	6.5383322	6.8669619	6.0501376	6.1776727	5.9027806
Down	FGSG_00619	-1.938437505	1.64E-07	5.9389001	5.7434744	6.0376686	5.7840418	5.8421752	5.636897	5.3564544	5.1041074	5.5195414
Down	FGSG_01802	-1.940594083	0.0001772	5.4640893	5.6546687	5.1553224	4.953574	4.3699906	5.3115993	3.7791165	3.8208439	3.6872775
Down	FGSG_05784	-1.941027177	2.69E-09	14.010395	13.978612	13.956904	13.549001	13.498845	13.511671	13.423678	13.367328	13.413498
Down	FGSG_00005	-1.941048622	0.0086636	3.1562965	3.1370988	3.1285604	2.6546039	2.353841	2.8774004	2	2	2
Down	FGSG_09715	-1.941371619	3.90E-11	11.6723	11.643476	11.615487	11.393715	11.385121	11.313632	11.036754	11.042221	10.965711
Down	FGSG_05059	-1.941578354	1.21E-06	6.0321508	6.3916107	5.4444954	6.6255671	6.29449	6.8249729	5.9628224	5.9891987	5.9627112
Down	FGSG_10132	-1.941936455	1.59E-11	13.979631	13.922214	13.951152	14.618621	14.580686	14.569133	14.268941	14.241754	14.229897
Down	FGSG_11706	-1.947097984	1.89E-06	6.653478	6.7541729	6.4567939	6.7228827	6.776423	6.5777126	6.1719365	6.3443452	5.9027806
Down	FGSG_08420	-1.947223428	3.76E-10	8.7176359	8.7353285	8.6130853	8.2218588	8.2276057	8.1275943	8.3814413	8.5655436	8.0927348
Down	FGSG_13860	-1.947403342	0.0002669	6.3971965	6.3916107	6.3206633	6.2972625	6.5209705	5.9288493	5.107816	5.0499834	5.1065306
Down	FGSG_08967	-1.947706547	0.0004477	4.4609433	4.069824	4.7085304	3.444959	3.4156241	3.4190665	3.8457121	3.5358829	4.0607807
Down	FGSG_12149	-1.949649428	3.58E-07	10.604795	10.624969	10.49694	11.095525	11.061783	11.042297	10.794342	10.788024	10.733852
Down	FGSG_01513	-1.949680893	2.39E-07	10.874658	10.802099	10.861954	11.69618	11.607548	11.695946	12.065163	12.007959	12.05826
Down	FGSG_02325	-1.952519935	8.00E-09	14.012399	13.959622	13.980251	13.93122	13.901931	13.873182	13.359691	13.335513	13.31814
Down	FGSG_00765	-1.952565552	7.54E-09	13.042961	13.005304	12.995216	13.69131	13.653858	13.641704	13.658882	13.640581	13.610621
Down	FGSG_11971	-1.953703465	0.0048683	2.6910183	2.8476671	2.4807492	4.1048586	4.6510429	3.0813961	3.0942412	3.5358829	2.3974876
Down	FGSG_10536	-1.954302167	4.51E-06	5.8184092	5.9811576	5.545677	5.7989973	5.6951271	5.8190522	4.807216	4.9352342	4.6029775
Down	FGSG_01872	-1.955342285	1.31E-08	11.097601	11.051827	11.057734	11.740673	11.751737	11.639529	11.625103	11.564875	11.618569
Down	FGSG_12451	-1.958602659	0.0006132	3.6078986	3.5844678	3.5740244	3.2841938	3.0784965	3.4190665	3.199846	3.3690748	2.964684
Down	FGSG_03619	-1.962078948	1.12E-05	7.06886	7.1285622	6.9195616	7.4088738	7.4981042	7.220047	6.6343121	6.2026947	6.9156884
Down	FGSG_05160	-1.965720081	2.24E-09	12.767264	12.739208	12.709619	12.65465	12.612782	12.609033	12.194961	12.178074	12.146097
Down	FGSG_02277	-1.966435342	3.32E-06	5.6402997	5.6997548	5.4959731	5.8856141	5.975612	5.7002055	5.7875758	5.837702	5.670936
Down	FGSG_05705	-1.970057278	0.0028432	3.5075501	3.378055	3.5740244	4.1048586	4.2058494	3.9222766	4.0850435	4.0586965	4.0607807

Down	FGSG_09042	-1.971649333	2.44E-09	10.506661	10.416269	10.52509	10.248729	10.123907	10.288479	9.5074192	9.4523265	9.5138261
Down	FGSG_06612	-1.971848043	1.22E-08	10.849897	10.790423	10.823431	11.341361	11.349911	11.242976	11.03809	11.03004	10.979376
Down	FGSG_08352	-1.97264096	5.81E-12	10.791747	10.689258	10.805691	10.711814	10.609909	10.724102	10.221752	10.22519	10.154336
Down	FGSG_00312	-1.972889305	1.02E-05	5.5673995	5.5600358	5.4959731	4.953574	4.7733906	5.0451875	4.5913113	4.6740912	4.4438634
Down	FGSG_08405	-1.974493078	0.0023691	3.2831234	3.378055	3.1285604	4.3681625	4.3699906	4.2946324	3.2982451	2.9633478	3.5376798
Down	FGSG_10629	-1.975525992	0.0133696	3.5075501	3.5844678	3.3684179	2.5166584	2.8748957	2	2.7212202	2.3968278	2.964684
Down	FGSG_06468	-1.976177841	0.0201029	3.2831234	3.9254527	2	3.444959	2.6377651	3.9222766	2.2167888	2.3968278	2
Down	FGSG_10997	-1.9766142	2.70E-08	7.1207354	7.1449564	7.0108688	6.7539152	6.6858453	6.7371407	6.1190688	6.0456069	6.1288728
Down	FGSG_11145	-1.980561272	0.000249	4.2902308	4.5354388	3.9139052	4.953574	5.0882909	4.7181783	4.1391495	4.1643702	4.0607807
Down	FGSG_05321	-1.980598283	1.77E-10	14.862202	14.824972	14.813723	14.307903	14.265267	14.263418	13.93748	13.896224	13.912444
Down	FGSG_05371	-1.980643626	1.28E-13	10.446104	10.490311	10.311839	10.700907	10.653383	10.660862	10.593406	10.549205	10.571319
Down	FGSG_08209	-1.982440079	5.71E-08	9.7755776	9.6213135	9.8403697	10.41449	10.420428	10.322828	10.434805	10.468976	10.331
Down	FGSG_00866	-1.983625978	4.23E-12	12.539859	12.489708	12.508506	12.39218	12.347267	12.355226	12.32009	12.301199	12.276897
Down	FGSG_03745	-1.985529454	1.80E-14	12.873297	12.862361	12.798474	13.096954	13.062197	13.044633	12.924671	12.915645	12.867853
Down	FGSG_13743	-1.985662315	0.0056343	4.2285219	4.2010532	4.188785	3.0034737	3.2568854	2.6397336	3.199846	3.1804273	3.18196
Down	FGSG_13825	-1.987514162	1.04E-08	9.1446377	9.054546	9.1515965	8.8895234	8.7698493	8.9186116	8.1832408	8.2605851	8.0380685
Down	FGSG_03303	-1.988244864	5.06E-11	7.6889487	7.6603381	7.6335064	7.8288184	7.6575433	7.9105501	7.3875098	7.3759446	7.3339349
Down	FGSG_09046	-1.99283459	0.0029307	3.5075501	3.378055	3.5740244	3.0034737	2.8748957	3.0813961	2.4052168	2	2.7087823
Down	FGSG_04051	-1.994375973	5.25E-12	10.365081	10.449647	10.420737	10.440355	10.560844	10.445676	10.495693	10.606578	10.636016
Down	FGSG_12001	-1.996315368	1.54E-10	10.694538	10.648157	10.65532	10.436774	10.376458	10.4118	10.177999	10.183377	10.105324
Down	FGSG_02920	-1.997280053	0.0001948	6.5563577	6.6660438	6.3489364	6.1417046	6.0257126	6.1937152	4.7032988	4.0586965	5.1065306
Down	FGSG_12574	-1.997418159	1.51E-05	3.872828	4.069824	3.5740244	4.1048586	4.0206066	4.1204343	4.5115122	4.3549955	4.6029775
Down	FGSG_08393	-1.998926097	6.65E-09	9.4502348	9.3121456	9.4961952	10.158837	10.094866	10.13656	10.097444	10.077447	10.054588
Down	FGSG_12680	-2.000387451	0.0040507	3.2831234	3.378055	3.1285604	3.2841938	3.4156241	3.0813961	3.2982451	3.6853877	2.7087823
Down	FGSG_02695	-2.001178268	0.0001284	3.872828	3.765011	3.9139052	4.1980691	4.3699906	3.9222766	4.028829	3.8208439	4.1665008
Down	FGSG_06135	-2.001592027	4.43E-09	7.5570945	7.7162629	7.2841049	7.9273903	7.6967286	8.0507377	7.9609382	7.888894	7.9666872
Down	FGSG_00931	-2.002003631	0.0008217	4.5644712	4.9628272	3.9139052	3.5190873	3.9182279	2.8774004	3.2982451	3.3690748	3.18196
Down	FGSG_06752	-2.003796833	0.0220111	5.5673995	5.2319977	5.7713555	9.7920098	9.6479924	9.8424632	9.1797481	9.1324564	9.167372
Down	FGSG_03012	-2.004792764	1.60E-06	6.7663496	6.5721788	6.8618681	5.8573158	5.8421752	5.7902457	5.4450355	5.4349486	5.3945636
Down	FGSG_11186	-2.007041395	0.000286	3.872828	3.9254527	3.7539507	3.2841938	2.6377651	3.6925011	3.0942412	2.3968278	3.5376798
Down	FGSG_09572	-2.0073718	1.78E-13	8.6308542	8.5133883	8.6589748	8.9594164	8.9231021	8.9085948	8.8412549	8.8008584	8.815814

Down	FGSG_11310	-2.007415798	2.08E-06	8.3984303	8.3319751	8.3795063	8.5614543	8.4411047	8.5913365	8.4685463	8.4913294	8.3777816
Down	FGSG_10489	-2.008865558	0.0029768	4.6135762	4.8067189	4.3087924	3.444959	3.4156241	3.4190665	3.6359267	3.8208439	3.3707558
Down	FGSG_03298	-2.010423156	0.0002883	3.9513288	4.069824	3.7539507	3.2841938	3.4156241	3.0813961	3.2982451	3.5358829	2.964684
Down	FGSG_06300	-2.012739878	3.26E-08	8.4019287	8.2509868	8.4851833	7.6792287	7.5842106	7.7021252	7.1446385	7.1110577	7.1268773
Down	FGSG_01782	-2.012782884	4.21E-08	10.438459	10.409499	10.383636	9.9043233	9.8716416	9.8494616	9.130514	9.1518582	9.0410167
Down	FGSG_12388	-2.016462969	1.25E-07	9.0135686	8.9323645	9.0126993	9.3041389	9.2991223	9.2207168	9.90201	9.8899046	9.847648
Down	FGSG_02997	-2.017665689	0.0007438	5.4906186	5.458757	5.4444954	4.657914	4.7135134	4.5218929	3.970335	4.0586965	3.8228101
Down	FGSG_08878	-2.019265128	1.75E-05	10.46713	10.375168	10.471448	11.295959	11.265119	11.23965	11.156365	11.161083	11.085957
Down	FGSG_11162	-2.021694465	2.61E-08	7.7831736	7.6257121	7.8467929	8.4089556	8.3604325	8.3707408	8.2554428	8.0833124	8.3499995
Down	FGSG_00895	-2.024561598	0.0014748	3.9513288	3.765011	4.0578819	4.2856211	4.2902531	4.2946324	3.2982451	3.1804273	3.3707558
Down	FGSG_04525	-2.025019805	0.0080181	3.2831234	3.378055	3.1285604	2.6546039	2.6377651	2.6397336	2	2	2
Down	FGSG_06985	-2.025967523	3.03E-07	8.2587825	8.2126152	8.2202735	7.1241972	7.0789132	7.0842532	7.104406	7.1110577	7.0321538
Down	FGSG_05740	-2.027496872	0.0031598	4.7070397	4.9628272	4.3087924	6.7539152	6.776423	6.6436126	5.7358405	5.4349486	5.9330571
Down	FGSG_02668	-2.028777409	1.09E-06	5.065757	5.1036809	4.9491538	4.871059	4.6510429	4.9955781	4.6296145	4.7439404	4.4438634
Down	FGSG_02406	-2.030271383	0.0003581	3.1562965	3.5844678	2.8407141	3.8981589	4.2058494	3.4190665	3.9093688	3.3690748	4.3572018
Down	FGSG_00172	-2.03118549	6.42E-11	9.0385065	9.0194102	8.9723765	8.9923198	8.9329814	8.9644586	8.6657515	8.5847058	8.6795905
Down	FGSG_00140	-2.031857893	2.62E-10	8.6777864	8.6745166	8.5954933	8.8648604	8.6905391	8.9417172	8.1307934	8.1362228	8.0588119
Down	FGSG_02809	-2.033281523	6.46E-07	5.167919	5.0349724	5.2179801	6.0193342	5.9498937	6.0060392	5.7181748	5.5170389	5.8402522
Down	FGSG_13176	-2.033495123	9.21E-11	7.7451174	7.4911275	7.8859117	7.5303505	7.6254103	7.3342078	7.0907419	7.0154424	7.100442
Down	FGSG_10328	-2.034616794	0.0062324	3.701719	2.4852148	4.3087924	3.8981589	3.6887032	4.024755	3.3903592	3.5358829	3.18196
Down	FGSG_13375	-2.036129916	0.0218583	3.3996963	3.5844678	3.1285604	4.2425091	4.2902531	4.1204343	5.1610901	5.0499834	5.2090653
Down	FGSG_02578	-2.036672187	4.39E-09	16.016458	15.979344	15.968177	16.414785	16.371724	16.370419	16.562111	16.526624	16.531338
Down	FGSG_14001	-2.039264493	2.78E-06	7.3697138	7.4120569	7.2394951	6.8066659	6.7013421	6.8249729	5.9170997	5.9891987	5.7748904
Down	FGSG_12067	-2.040672959	5.95E-08	7.0511452	6.8965233	7.113317	6.691168	6.7909814	6.4908649	6.1457448	6.073005	6.1547968
Down	FGSG_10475	-2.042771645	0.0023424	3.6078986	4.069824	2.8407141	3.0034737	2.6377651	3.260088	3.7092973	3.6853877	3.6872775
Down	FGSG_06701	-2.043751194	3.70E-08	10.529231	10.531421	10.440579	9.4199218	9.3092715	9.4405686	9.4683253	9.4260069	9.4445822
Down	FGSG_06902	-2.044022857	1.53E-05	6.7663496	6.6660438	6.7811692	5.7689296	5.265544	6.0793081	5.1870071	5.2552685	5.0523944
Down	FGSG_03260	-2.044118481	2.28E-07	6.5812576	6.4455482	6.628079	5.9935634	6.0008798	5.9021734	5.4663562	5.4765776	5.3945636
Down	FGSG_11721	-2.044175967	0.0002758	5.5422571	5.7434744	5.2179801	4.69036	4.4455509	4.8356119	3.7092973	4.0586965	3.18196
Down	FGSG_03709	-2.046698578	4.60E-05	5.100621	5.2921154	5.2780294	4.4837433	4.3699906	4.8909283	4.4270393	4.5233477	4.265
Down	FGSG_04514	-2.049971958	0.0003113	5.2633225	5.458757	4.9491538	4.5907474	4.3699906	4.7181783	4.1912995	4.0586965	4.265

Down	FGSG_12570	-2.050306048	5.12E-07	7.2112509	6.9902361	7.3273766	7.1937475	6.8753756	7.3832557	6.1848562	6.0176785	6.2778581
Down	FGSG_00257	-2.051432307	6.14E-09	7.10365	6.8372271	7.2545188	7.3183509	7.3288419	7.220047	7.2092957	6.986918	7.3564507
Down	FGSG_00421	-2.052635446	9.60E-10	13.557525	13.506954	13.522629	13.271546	13.250743	13.204303	12.441296	12.422314	12.3941
Down	FGSG_11017	-2.053432565	3.95E-07	6.5310205	6.5962238	6.3766661	5.6252853	5.6638294	5.5012819	5.5284909	5.6684096	5.3047937
Down	FGSG_11192	-2.054171312	0.0105284	4.0965725	4.2010532	3.9139052	4.4077273	4.51735	4.210161	2.9802913	3.5358829	2
Down	FGSG_03654	-2.05419095	0.0206058	3.3996963	2.4852148	4.188785	3.1965524	3.2568854	3.0813961	2	2	2
Down	FGSG_02268	-2.058350026	0.0144566	4.2285219	4.3213345	4.188785	3.9526758	3.6887032	4.1204343	2.9802913	2	3.5376798
Down	FGSG_11079	-2.059983364	5.02E-10	11.724924	11.717278	11.64714	11.647692	11.57952	11.631431	11.352329	11.318886	11.319476
Down	FGSG_13518	-2.065482017	1.08E-07	6.860895	6.8372271	6.8017711	6.4938962	6.6543434	6.2155469	6.0216157	5.5563964	6.3242864
Down	FGSG_07932	-2.067116646	7.74E-09	10.132177	10.09413	10.08481	10.163178	10.159405	10.079666	9.4722827	9.5309935	9.3411292
Down	FGSG_03546	-2.067492996	4.42E-07	4.7948149	4.9628272	4.5224622	5.7068462	5.5313719	5.8190522	5.1870071	5.2552685	5.0523944
Down	FGSG_05898	-2.067579132	9.76E-06	8.6128533	8.5322062	8.6072451	9.8497082	9.7978751	9.8176978	10.366279	10.26879	10.394936
Down	FGSG_03367	-2.067667185	1.51E-08	9.2667045	9.3301794	9.1109005	8.9544155	8.8997838	8.9219352	8.2307368	8.330362	8.0519305
Down	FGSG_13194	-2.068301861	1.65E-05	8.2236295	8.3030481	8.0498976	7.7844204	7.7866463	7.6943723	7.3305166	7.1110577	7.4640419
Down	FGSG_01769	-2.070092924	1.04E-06	6.7110174	6.4975416	6.8220828	6.1060709	6.0008798	6.1261652	5.4014247	5.7038947	4.9376179
Down	FGSG_05822	-2.072608864	4.23E-12	7.4050095	7.2994005	7.4235657	7.8288184	7.7423879	7.8279241	7.6935164	7.6368537	7.6847264
Down	FGSG_12455	-2.073403119	9.88E-08	4.4609433	4.2010532	4.6184939	5.0060735	4.8861689	5.0451875	4.5519634	4.3549955	4.6764038
Down	FGSG_13566	-2.073819439	4.28E-08	6.4111515	6.5228522	6.2016809	6.7691847	6.8337955	6.6110389	5.8375198	5.837702	5.7748904
Down	FGSG_06517	-2.075004329	1.55E-05	5.167919	5.458757	4.7085304	4.69036	4.7135134	4.5903319	4.2902636	4.262829	4.265
Down	FGSG_02906	-2.080596196	4.99E-06	5.5921111	5.6546687	5.4444954	5.7536574	5.975612	5.3904893	5.1870071	4.9352342	5.3503768
Down	FGSG_07298	-2.081045759	1.41E-06	12.309901	12.268151	12.266432	12.41905	12.373501	12.376881	11.813923	11.782248	11.779269
Down	FGSG_02373	-2.081471391	3.04E-07	8.8257773	8.7832451	8.7833155	8.9342369	8.7844739	8.9900172	7.6753214	7.8015075	7.4640419
Down	FGSG_04543	-2.082867639	0.00041	5.100621	4.8868835	5.2179801	3.6568115	3.9182279	3.260088	3.4769433	3.5358829	3.3707558
Down	FGSG_13286	-2.083142826	5.12E-05	5.6164066	5.458757	5.6852725	4.6247215	4.51735	4.6556708	4.1912995	3.8208439	4.4438634
Down	FGSG_13089	-2.083252935	2.24E-05	7.9691752	8.5932169	8.4200477	8.9906923	9.4807134	9.472651	9.5935075	10.153209	10.200182
Down	FGSG_07925	-2.083913245	2.17E-11	15.285689	15.231949	15.253654	15.578827	15.566226	15.503107	15.366607	15.339463	15.327722
Down	FGSG_03244	-2.08405635	1.36E-08	10.030751	10.022738	9.9528702	9.9155173	9.8578786	9.8856565	9.1877929	9.1710026	9.1384305
Down	FGSG_01826	-2.086428124	7.11E-08	9.1236031	9.0803473	9.0817144	8.8142322	8.6788446	8.8574402	7.8272478	7.8577261	7.7285401
Down	FGSG_03844	-2.091625119	0.0001401	3.701719	3.765011	3.5740244	3.5895922	3.5586152	3.5622521	3.3903592	2.9633478	3.6872775
Down	FGSG_02116	-2.092177616	5.33E-09	11.769277	11.709032	11.743469	11.862468	11.807397	11.831127	11.202756	11.184823	11.154213
Down	FGSG_09173	-2.093507311	1.80E-05	4.7948149	5.2319977	4.0578819	4.8990917	4.9394155	4.8356119	4.6296145	4.3549955	4.8129147

Down	FGSG_04559	-2.094724979	2.14E-13	8.5257457	8.44221	8.5228982	8.3001582	8.2329471	8.2804124	8.0728963	7.9492797	8.1258785
Down	FGSG_05448	-2.096974459	2.95E-11	9.3083118	9.2184314	9.3109927	9.2776901	9.2914633	9.1740931	9.1221424	9.2054542	8.9624644
Down	FGSG_02329	-2.097904108	1.16E-08	12.864607	12.836729	12.809325	12.397871	12.365923	12.34412	11.633373	11.626849	11.574832
Down	FGSG_08535	-2.099531998	3.99E-09	5.5673995	5.510285	5.545677	6.1060709	6.1663515	5.9550408	5.7532926	5.4765776	5.9627112
Down	FGSG_05177	-2.100382013	2.06E-10	15.085712	15.068137	15.017572	14.964947	14.935561	14.906667	14.676206	14.650833	14.635277
Down	FGSG_13992	-2.103666735	9.23E-08	4.349408	4.3213345	4.3087924	4.8133047	4.8308815	4.7181783	4.2416299	3.9446664	4.4438634
Down	FGSG_04854	-2.107529583	0.0001602	3.2831234	2.8476671	3.5740244	3.444959	3.5586152	3.260088	2.8565633	3.1804273	2.3974876
Down	FGSG_02328	-2.108332172	9.58E-09	13.080695	13.02675	13.048716	12.480495	12.435913	12.438224	11.887141	11.845179	11.862739
Down	FGSG_03956	-2.108349992	0.0090565	4.4609433	4.4323548	4.4195794	3.8415007	4.3699906	3.0813961	2.5718533	2.7077187	2.3974876
Down	FGSG_10603	-2.108360675	1.01E-09	13.471801	13.42877	13.42972	13.437762	13.401551	13.386298	12.789129	12.699747	12.810144
Down	FGSG_10630	-2.109169707	4.12E-06	4.9558055	4.9628272	4.8733193	5.9270474	5.8698682	5.9021734	4.935064	5.0499834	4.7462732
Down	FGSG_06712	-2.10940498	0.0008254	2.4953013	2.8476671	2	3.5895922	3.4156241	3.6925011	2.9802913	3.1804273	2.7087823
Down	FGSG_10891	-2.111617514	0.0020858	5.4640893	5.349828	5.4959731	4.4077273	4.7135134	3.9222766	3.3903592	3.5358829	3.18196
Down	FGSG_20005	-2.113720831	0.000808	3.2831234	3.5844678	2.8407141	4.327482	4.51735	4.024755	3.199846	2.7077187	3.5376798
Down	FGSG_07614	-2.115822852	9.44E-05	3.3996963	3.5844678	3.1285604	3.5190873	3.8080253	3.0813961	3.199846	2.3968278	3.6872775
Down	FGSG_01107	-2.117437856	3.07E-10	8.9288021	8.974252	8.7936379	9.1243468	9.0374102	9.1287914	8.5973385	8.6036168	8.5241867
Down	FGSG_02447	-2.120561281	0.0042259	2.2688027	2.4852148	2	2.1935143	2	2.3550396	3.4769433	3.3690748	3.5376798
Down	FGSG_08424	-2.120957852	1.83E-08	6.9303725	6.7326402	7.0284567	6.8717719	6.7468578	6.9077633	7.1837795	7.124214	7.1783375
Down	FGSG_02974	-2.121567066	5.05E-09	13.566808	13.497808	13.549336	12.625976	12.579887	12.585648	13.085526	13.054198	13.050696
Down	FGSG_09740	-2.122694965	5.93E-08	6.0678264	6.2772809	5.728956	6.9542473	6.8337955	6.9860594	6.9912882	6.8668223	7.0460725
Down	FGSG_04839	-2.125234553	2.35E-05	4.7515947	4.4323548	4.9491538	4.3681625	3.9182279	4.6556708	4.2902636	4.3549955	4.1665008
Down	FGSG_11629	-2.125350054	1.01E-07	8.8126947	8.7080041	8.8291955	9.1924749	9.2206385	9.0730802	9.0031861	8.8959742	9.0410167
Down	FGSG_02987	-2.126333785	0.0003432	5.2004262	5.510285	4.7085304	4.4837433	5.0882909	3.260088	4.2902636	3.9446664	4.5256129
Down	FGSG_09524	-2.128656631	5.58E-10	8.5257457	8.4685011	8.504164	7.8288184	7.7938925	7.7774459	8.0234075	7.904229	8.0927348
Down	FGSG_11747	-2.12998978	8.62E-05	6.2651272	6.1851653	6.2623983	6.4280797	6.449356	6.3200085	6.2600348	6.59171	5.7410644
Down	FGSG_02903	-2.135632183	2.26E-09	7.1543109	6.9347419	7.2841049	7.3643222	7.3486598	7.2937323	7.0560045	6.8190773	7.2033953
Down	FGSG_02180	-2.136808762	9.09E-07	4.9172105	4.8868835	4.8733193	4.2425091	4.51735	3.9222766	4.0850435	4.1643702	3.9466965
Down	FGSG_12355	-2.137521883	5.07E-06	3.701719	3.765011	3.5740244	3.444959	3.2568854	3.5622521	3.3903592	3.3690748	3.3707558
Down	FGSG_04605	-2.137738905	8.75E-08	6.7110174	6.6885859	6.650969	6.8140467	6.8053944	6.7371407	5.8375198	5.7723491	5.8402522
Down	FGSG_12413	-2.137794364	0.0012735	3.2831234	3.765011	2.4807492	2.5166584	2.6377651	2.3550396	3.3903592	2.9633478	3.6872775
Down	FGSG_02629	-2.138919266	3.55E-10	6.8506908	6.7107812	6.9750363	6.8717719	6.6543434	7.0112415	6.5166098	6.7185687	6.2298857

Down	FGSG_03352	-2.140442829	0.002064	2.8633281	2	3.3684179	3.1032407	3.5586152	2.3550396	2.8565633	2.3968278	3.18196
Down	FGSG_12358	-2.143071131	7.15E-09	5.6869305	5.5600358	5.728956	6.1060709	6.29449	5.7902457	6.0781065	6.0176785	6.075584
Down	FGSG_05322	-2.145447037	1.61E-09	15.513137	15.466329	15.474539	14.874575	14.83542	14.826364	14.344876	14.308738	14.315166
Down	FGSG_12879	-2.145550798	1.29E-13	9.2317464	9.0675043	9.3001732	9.0714376	9.1059671	8.944988	8.7956597	8.6498375	8.8676788
Down	FGSG_07598	-2.145789775	0.0003971	3.0172362	2.4852148	3.3684179	2.7805029	2.353841	3.0813961	3.5586239	3.8208439	3.18196
Down	FGSG_03373	-2.148216081	0.0007977	2.8633281	2	3.3684179	3.3668146	3.0784965	3.5622521	2.7212202	2.3968278	2.964684
Down	FGSG_11980	-2.149096334	0.0022439	5.4370629	5.8271292	4.7932764	4.7835368	4.9394155	4.5218929	3.0942412	2.9633478	3.18196
Down	FGSG_07821	-2.149890062	2.02E-07	5.7973114	5.8271292	5.6852725	6.3387918	6.0008798	6.5435983	5.4663562	5.2066203	5.6345505
Down	FGSG_12020	-2.15080626	1.55E-08	5.5422571	5.349828	5.640225	5.0567294	4.7733906	5.3115993	5.0804226	5.3023297	4.7462732
Down	FGSG_08402	-2.15376316	0.0028716	10.577983	10.565314	10.504814	13.539293	13.515652	13.475232	9.2959491	9.2421225	9.2835464
Down	FGSG_03510	-2.154294593	4.42E-07	7.7229116	7.7910854	7.5628462	7.4330428	7.3486598	7.4306909	6.7518403	6.7528555	6.6860823
Down	FGSG_03408	-2.155784664	2.34E-07	6.2340611	6.2772809	6.1055269	5.7989973	5.8698682	5.636897	6.1190688	6.2026947	5.991768
Down	FGSG_03075	-2.156054434	1.00E-06	7.0422056	7.2395919	6.7175374	6.2760407	6.314782	6.1490351	5.8210632	5.9002223	5.670936
Down	FGSG_05185	-2.156595323	3.71E-08	6.1861705	5.7434744	6.4567939	6.3591166	6.5033974	6.1029268	5.8698804	5.837702	5.8402522
Down	FGSG_09352	-2.156975252	4.96E-07	7.4119662	7.342684	7.3967312	6.5743316	6.3545295	6.6911344	6.1457448	6.2986564	5.9027806
Down	FGSG_03578	-2.157012394	0.0019569	3.0172362	2.8476671	3.1285604	4.1980691	4.1161991	4.210161	4.028829	3.5358829	4.3572018
Down	FGSG_04440	-2.158683124	0.000462	4.349408	4.2010532	4.5224622	3.8981589	4.1161991	3.5622521	3.2982451	3.8208439	2.3974876
Down	FGSG_08687	-2.159273643	4.86E-09	9.2019533	9.2452908	9.2028411	9.1811035	9.2393369	9.0968003	8.5852082	8.6407111	8.5439396
Down	FGSG_10671	-2.161227372	1.21E-05	5.2633225	5.2319977	5.2179801	4.5203003	4.1161991	4.7780897	3.9093688	4.1643702	3.5376798
Down	FGSG_02945	-2.163652626	0.0027942	3.1562965	3.1370988	3.1285604	3.3668146	3.8080253	3.260088	2.2167888	2.3968278	2.3974876
Down	FGSG_07378	-2.164150975	3.30E-12	6.9873724	6.8965233	6.9930638	7.2273045	7.2146924	7.1761874	7.0275995	6.9430453	7.0735134
Down	FGSG_11003	-2.165024777	5.49E-06	3.872828	4.2010532	3.3684179	4.2856211	4.2902531	4.210161	3.970335	3.3690748	4.3572018
Down	FGSG_09707	-2.165131132	1.27E-07	7.5633567	7.8522684	7.5507256	7.1301229	7.387497	7.2092065	6.3430616	6.7185687	6.555942
Down	FGSG_05107	-2.168659919	2.58E-13	11.389568	11.318028	11.374615	11.133631	11.086335	11.093303	11.0527	11.060302	10.977561
Down	FGSG_07360	-2.172428719	3.73E-07	5.4370629	5.4053203	5.3911128	5.7226193	5.7257602	5.636897	5.7181748	5.9002223	5.4374371
Down	FGSG_03145	-2.172627898	7.86E-09	8.2855435	8.1571205	8.3236158	7.9543595	7.8916935	7.9304878	7.2711789	7.3977778	7.0598582
Down	FGSG_03103	-2.172640141	0.0022623	6.8300632	6.8965233	6.6735015	5.674773	5.179638	5.9807654	4.5913113	4.3549955	4.7462732
Down	FGSG_12880	-2.174250575	1.27E-09	8.4158383	8.5070607	8.2279057	7.9775509	8.0634203	7.7920493	7.551108	7.3083895	7.711174
Down	FGSG_04542	-2.177411498	0.0006791	4.4609433	4.4323548	4.4195794	3.9526758	4.2902531	3.4190665	4.0850435	3.5358829	4.4438634
Down	FGSG_07302	-2.178515452	9.09E-09	9.2970828	9.2791054	9.2296985	9.0355815	9.0343543	8.9547561	8.0448251	8.0213724	8.0170225
Down	FGSG_00153	-2.181836546	2.68E-05	5.0300296	5.349828	4.5224622	4.3681625	3.6887032	4.7780897	3.7791165	4.0586965	3.3707558

Down	FGSG_12517	-2.182892572	1.43E-08	7.8461644	7.7594926	7.8467929	6.9542473	6.9933753	6.8249729	6.8524363	6.735814	6.9004463
Down	FGSG_12168	-2.184257367	0.0016304	3.872828	4.2010532	3.3684179	2.8962916	3.2568854	2.6397336	2.5718533	2.7077187	2.3974876
Down	FGSG_04763	-2.18853136	2.61E-10	10.053173	10.024954	9.9982041	10.308087	10.267462	10.262498	9.7944301	9.7269816	9.7950446
Down	FGSG_07758	-2.18892149	0.0004707	3.1562965	3.765011	2	3.3668146	3.2568854	3.4190665	3.4769433	3.5358829	3.3707558
Down	FGSG_03767	-2.189822982	3.12E-09	7.1543109	7.342684	6.8421126	7.4520907	7.3682091	7.4492367	6.8685409	6.6474492	7.003907
Down	FGSG_04941	-2.190097006	2.34E-06	6.3545028	6.4717791	6.1382955	6.0571473	6.2318429	5.7608523	5.0240241	4.7439404	5.2090653
Down	FGSG_07497	-2.194072566	1.56E-08	8.1997091	8.1651802	8.1497038	7.6751814	7.5673956	7.6943723	6.9690546	6.9430453	6.9307712
Down	FGSG_12206	-2.194125206	7.72E-06	6.860895	6.8572645	6.7811692	7.7032764	7.5925452	7.7251372	7.2343684	7.2130949	7.1909208
Down	FGSG_12371	-2.194517384	7.94E-05	4.9172105	4.7218364	5.0212003	6.0446528	6.2739084	5.6688985	5.2124668	5.3023297	5.0523944
Down	FGSG_00790	-2.195389853	4.05E-06	6.2651272	6.2772809	6.1703363	7.6992962	7.7423879	7.5643	7.8354644	7.6909075	7.9069251
Down	FGSG_09184	-2.196159388	2.70E-06	4.2902308	4.3213345	4.188785	3.9526758	3.5586152	4.210161	4.0850435	3.6853877	4.3572018
Down	FGSG_13561	-2.196595149	1.41E-05	8.8153207	9.1015015	9.2672192	9.6924051	9.9369181	9.8856565	9.0743422	9.4653085	9.1894874
Down	FGSG_13384	-2.199139996	7.64E-06	4.2285219	3.378055	4.7085304	4.2425091	4.2058494	4.210161	3.970335	3.9446664	3.9466965
Down	FGSG_03041	-2.199334156	5.45E-05	4.6135762	4.4323548	4.7085304	4.0052074	4.2902531	3.5622521	4.3828727	4.5233477	4.1665008
Down	FGSG_02117	-2.20144852	5.01E-08	9.9434211	9.9708045	9.8278686	10.466936	10.437879	10.40825	10.002121	9.9594538	9.978607
Down	FGSG_02327	-2.201907406	9.75E-11	15.702974	15.653066	15.667325	15.461421	15.418505	15.417216	14.790698	14.758443	14.756959
Down	FGSG_12173	-2.203691838	0.0001023	5.7096927	5.458757	5.85259	7.403991	7.52448	7.1761874	7.541095	7.5517747	7.4640419
Down	FGSG_00515	-2.204082154	9.27E-11	11.550711	11.5357	11.480638	12.142092	12.075748	12.12119	12.203318	12.144569	12.195718
Down	FGSG_12516	-2.206414312	7.13E-09	7.2192048	7.2698061	7.0799694	7.2217656	7.23613	7.1309525	7.2952077	7.2851495	7.2641992
Down	FGSG_01765	-2.209208084	6.46E-07	8.3376209	8.2585401	8.3377927	9.348018	9.2152512	9.3886662	9.3755828	9.4099814	9.2717488
Down	FGSG_01476	-2.212418517	2.57E-09	9.4753583	9.376039	9.4866834	9.2629339	9.2914633	9.1431016	8.8082383	8.8252317	8.723558
Down	FGSG_11729	-2.212627736	8.93E-10	8.4867412	8.4554154	8.4333122	8.2135109	8.2060401	8.1333402	7.5953256	7.2375151	7.8285634
Down	FGSG_20548	-2.215106351	0.0007033	3.701719	4.2010532	2.8407141	2.7805029	2.6377651	2.8774004	3.7092973	3.9446664	3.3707558
Down	FGSG_07880	-2.217083778	1.07E-05	5.1346624	4.9628272	5.2179801	4.7835368	5.1346873	4.210161	4.4698939	4.4416259	4.4438634
Down	FGSG_02806	-2.223384648	3.68E-05	5.4640893	5.7434744	5.0212003	4.2856211	3.9182279	4.5218929	4.6296145	4.7439404	4.4438634
Down	FGSG_03351	-2.224175711	3.32E-07	4.0257777	4.069824	3.9139052	3.5895922	3.6887032	3.5622521	4.0850435	3.6853877	4.3572018
Down	FGSG_07046	-2.224959261	1.22E-06	4.0965725	4.2010532	3.9139052	3.7825262	3.5586152	4.024755	4.028829	3.6853877	4.265
Down	FGSG_09041	-2.233368053	6.79E-10	8.5448595	8.4554154	8.5475047	8.4779556	8.3258573	8.5360188	8.0053133	8.0833124	7.852522
Down	FGSG_01177	-2.234929389	2.35E-13	12.882091	12.841452	12.83766	12.699301	12.648696	12.665144	12.452191	12.417646	12.423721
Down	FGSG_02992	-2.235464931	1.69E-07	6.7985581	6.4975416	6.9750363	6.1299242	5.9498937	6.2155469	5.5486216	5.5947086	5.4790733
Down	FGSG_11177	-2.235599007	1.98E-08	5.3814422	5.458757	5.2179801	4.871059	4.9394155	4.7181783	4.6296145	4.6006874	4.6029775

Down	FGSG_01838	-2.237055322	1.69E-11	13.695858	13.682341	13.623902	13.46527	13.397394	13.446096	13.073853	13.034517	13.04724
Down	FGSG_07643	-2.23902685	8.59E-05	4.9558055	4.9628272	4.8733193	3.9526758	4.0206066	3.8119585	3.8457121	3.6853877	3.9466965
Down	FGSG_20723	-2.239549202	0.0003343	2.8633281	2.8476671	2.8407141	3.3668146	3.4156241	3.260088	3.6359267	3.1804273	3.9466965
Down	FGSG_08625	-2.241521037	0.0016045	3.0172362	3.378055	2.4807492	3.1965524	2.6377651	3.5622521	2.2167888	2	2.3974876
Down	FGSG_11124	-2.241569825	0.0003048	3.2831234	3.5844678	2.8407141	3.444959	3.5586152	3.260088	2.7212202	2.9633478	2.3974876
Down	FGSG_07580	-2.242086521	5.23E-06	4.9172105	4.5354388	5.1553224	5.5027514	5.306652	5.6041695	5.9170997	6.073005	5.670936
Down	FGSG_07565	-2.245880458	1.74E-09	6.5935482	6.2472245	6.8017711	6.4753959	6.6057619	6.2370531	6.3772321	6.3886313	6.301259
Down	FGSG_11500	-2.246396677	5.79E-11	7.5189396	7.19305	7.711794	7.1301229	7.1370484	7.0359915	6.8765263	7.0294959	6.6317388
Down	FGSG_08913	-2.250080425	2.46E-11	10.550664	10.540744	10.474659	10.577319	10.532967	10.535203	10.63754	10.591264	10.617493
Down	FGSG_10546	-2.253757251	3.89E-07	5.899844	5.906198	5.8125445	5.5027514	5.4602848	5.4652882	4.9041484	4.8742451	4.8766132
Down	FGSG_12716	-2.25379977	1.53E-07	5.3814422	5.510285	5.1553224	6.0818165	6.0501253	6.0308779	5.8537908	5.8053955	5.8402522
Down	FGSG_10566	-2.254753498	6.09E-09	5.3528065	5.4053203	5.2780294	4.7531416	4.5857446	4.8356119	4.7032988	4.8105634	4.6764038
Down	FGSG_02969	-2.25694119	7.82E-05	4.4062533	4.069824	4.6184939	5.1760947	5.1346873	5.1395644	4.5519634	4.9937494	3.8228101
Down	FGSG_00409	-2.258522476	1.76E-09	8.5225353	8.4221723	8.5352539	8.1390646	8.1730751	8.0138356	7.4315369	7.4085718	7.3895788
Down	FGSG_07644	-2.25973818	0.0038542	3.701719	3.5844678	3.7539507	2.1935143	2	2.3550396	2.5718533	2.3968278	2.7087823
Down	FGSG_01583	-2.260169006	0.0001503	3.3996963	2.8476671	3.7539507	2.6546039	2.8748957	2.3550396	3.0942412	2.7077187	3.3707558
Down	FGSG_13797	-2.260919048	2.01E-06	7.4530163	7.6139829	7.177785	6.9807236	6.8753756	6.9987054	6.1190688	5.9891987	6.1802631
Down	FGSG_12110	-2.261990552	3.23E-09	15.116248	15.058907	15.087671	15.724324	15.696414	15.664398	15.35645	15.348907	15.297177
Down	FGSG_12596	-2.262155855	9.78E-08	5.2633225	4.8067189	5.545677	4.953574	4.7135134	5.0931475	4.5115122	4.8742451	3.9466965
Down	FGSG_05026	-2.264383885	8.93E-14	10.102384	10.068576	10.050789	10.471028	10.422767	10.434084	10.370526	10.27027	10.401717
Down	FGSG_00235	-2.267837816	6.38E-06	5.7096927	5.6546687	5.6852725	6.1764794	6.3545295	5.874995	5.2124668	4.9352342	5.3945636
Down	FGSG_03440	-2.268025915	1.68E-06	7.7003587	7.6716978	7.6449538	8.8505755	8.8519852	8.7605266	9.1797481	9.1961396	9.0955736
Down	FGSG_07978	-2.273467315	0.006056	3.6078986	3.5844678	3.5740244	3.7210377	3.6887032	3.6925011	2.8565633	2	3.3707558
Down	FGSG_02814	-2.27462401	3.12E-09	15.501591	15.465462	15.45206	15.807073	15.759769	15.766745	15.527279	15.495009	15.493312
Down	FGSG_05294	-2.27501491	5.79E-10	13.839036	13.817281	13.786648	14.360636	14.325181	14.317967	14.347257	14.304772	14.33053
Down	FGSG_03370	-2.290909414	4.29E-07	5.9389001	5.906198	5.891554	5.5027514	5.4602848	5.5012819	5.1346989	5.347904	4.8129147
Down	FGSG_03483	-2.291082564	0.0010923	2.6910183	2.4852148	2.8407141	3.444959	3.4156241	3.5622521	2.8565633	2.7077187	2.964684
Down	FGSG_01816	-2.29176097	9.44E-05	3.9513288	4.4323548	3.7539507	4.1048586	3.9182279	4.210161	3.199846	3.3690748	2.964684
Down	FGSG_11866	-2.291827836	1.27E-05	5.5166689	5.4053203	5.545677	4.92659	4.8861689	4.8909283	4.1912995	4.4416259	3.8228101
Down	FGSG_06328	-2.293507705	5.60E-08	14.773158	14.713375	14.755672	15.352977	15.328709	15.293656	15.579042	15.564295	15.531728
Down	FGSG_12463	-2.294192429	0.0017009	3.1562965	2.8476671	3.3684179	3.1965524	3.0784965	3.260088	2.4052168	2	2.7087823

Down	FGSG_00735	-2.294377012	3.34E-10	6.6882763	6.5477263	6.7390613	7.2383189	6.9551045	7.4024174	7.1111898	7.1110577	7.0460725
Down	FGSG_02672	-2.294829883	1.69E-06	3.872828	3.5844678	4.0578819	4.327482	4.1161991	4.4500449	3.3903592	3.5358829	3.18196
Down	FGSG_04126	-2.294945881	2.79E-07	15.416052	15.356288	15.389848	15.999801	15.970465	15.941406	16.032193	16.006895	15.991263
Down	FGSG_12590	-2.29756244	0.0006274	3.2831234	3.5844678	2.8407141	3.1032407	3.4156241	2.6397336	2.5718533	2.7077187	2.3974876
Down	FGSG_00913	-2.297922352	6.29E-07	3.701719	3.9254527	3.3684179	3.7210377	3.6887032	4.024755	3.7791165	3.8208439	3.9466965
Down	FGSG_02115	-2.29914965	6.41E-10	9.5871418	9.6531815	9.4282574	9.6383411	9.6135974	9.5754188	9.2069193	9.1898963	9.167372
Down	FGSG_00050	-2.299222105	6.97E-12	8.2157	8.2660541	8.2126008	8.2107175	8.2645866	8.1333402	8.0234075	8.1683327	7.8992777
Down	FGSG_03248	-2.30266008	7.46E-08	5.7321013	5.458757	5.891554	6.40871	6.3545295	6.3792376	6.9002206	6.9281194	6.8054483
Down	FGSG_12490	-2.303477146	6.03E-06	4.4062533	4.069824	4.6184939	3.5895922	3.8080253	3.260088	3.5586239	3.5358829	3.5376798
Down	FGSG_01810	-2.304351726	5.04E-05	4.1640552	4.069824	4.188785	3.9526758	3.4156241	4.2946324	3.2982451	3.6853877	2.7087823
Down	FGSG_01557	-2.304364772	5.15E-09	7.7885291	7.8014645	7.6898562	7.6629712	7.7348776	7.4945842	6.9002206	6.8510822	6.9004463
Down	FGSG_07824	-2.304824929	5.71E-09	8.1254721	8.2203716	7.9333641	8.2601893	8.1506723	8.2804124	7.7597741	7.9492797	7.4640419
Down	FGSG_03520	-2.306670815	2.56E-11	8.7204406	8.5991784	8.7518977	9.1995368	9.1518074	9.1600886	8.8082383	8.7508407	8.799473
Down	FGSG_03154	-2.306752996	1.05E-06	5.0300296	4.8067189	5.1553224	5.368836	4.9907667	5.6041695	4.5115122	4.5233477	4.4438634
Down	FGSG_02980	-2.310051195	2.64E-07	5.3235909	5.349828	5.2179801	4.8990917	5.0882909	4.5903319	4.7734018	5.1562742	4.1665008
Down	FGSG_06188	-2.310471937	9.85E-09	14.365515	14.304465	14.340622	14.733035	14.689407	14.688947	14.722178	14.703965	14.674027
Down	FGSG_02324	-2.310474899	4.14E-11	16.442332	16.442318	16.356767	16.271203	16.202165	16.253276	15.904399	15.842682	15.9
Down	FGSG_11352	-2.311802157	0.0004096	3.872828	3.9254527	3.7539507	3.0034737	2.8748957	3.0813961	2.8565633	2.3968278	3.18196
Down	FGSG_13875	-2.312144071	1.93E-08	6.280413	6.4455482	6.0025037	6.2215791	6.374	5.9550408	5.5080752	5.4349486	5.5195414
Down	FGSG_10025	-2.313293848	1.09E-05	5.2322171	4.9628272	5.3911128	4.5203003	3.5586152	5.0451875	4.3828727	4.5233477	4.1665008
Down	FGSG_11407	-2.315704018	4.47E-14	10.0476	10.166177	10.035649	10.287688	10.289417	10.381944	10.265739	10.27912	10.30519
Down	FGSG_20602	-2.315904367	0.0001103	2.6910183	3.1370988	3.1285604	3.444959	3.0784965	3.6925011	3.199846	3.3690748	3.18196
Down	FGSG_13457	-2.319653917	0.0003299	3.1562965	3.1370988	3.1285604	3.3668146	3.4156241	3.260088	2.4052168	2.3968278	2.3974876
Down	FGSG_03072	-2.31985731	1.38E-08	9.7916691	9.7366778	9.7610567	9.7221327	9.6776745	9.6792276	9.4996847	9.4206848	9.5113199
Down	FGSG_03071	-2.327444279	0.000262	3.1562965	3.5844678	2.4807492	2.8962916	3.0784965	2.6397336	3.6359267	3.1804273	3.9466965
Down	FGSG_06217	-2.329323158	2.38E-07	5.5921111	5.6546687	5.4444954	5.674773	5.5656438	5.7002055	5.2620767	4.6740912	5.6345505
Down	FGSG_03659	-2.332189074	3.55E-09	13.702434	13.679178	13.639902	14.053282	14.047626	13.970279	13.699399	13.661741	13.671374
Down	FGSG_12159	-2.33863037	1.33E-09	6.0853387	5.9811576	6.1055269	5.8856141	5.9498937	5.7308476	6.4960241	6.4102748	6.5164961
Down	FGSG_13659	-2.339830001	3.13E-10	6.665171	6.8372271	6.3766661	6.8140467	7.0907291	6.3597635	6.2842483	6.0456069	6.4342132
Down	FGSG_07273	-2.339847965	1.04E-08	10.624394	10.575933	10.587231	11.417834	11.443305	11.301562	11.315722	11.312435	11.255025
Down	FGSG_03292	-2.341973021	0.001114	4.9172105	4.4323548	5.2179801	4.0558932	3.6887032	4.2946324	4.2902636	4.5233477	4.0607807

Down	FGSG_05159	-2.34429418	2.52E-10	14.229818	14.187167	14.187097	14.364568	14.310888	14.330761	14.226811	14.184616	14.202778
Down	FGSG_12742	-2.344495841	9.97E-12	11.48213	11.525563	11.348597	11.839143	11.721182	11.866055	11.576603	11.459821	11.624378
Down	FGSG_07610	-2.344696044	4.80E-07	6.1026411	6.153105	5.9664603	6.0065063	6.0741317	5.8472948	5.3564544	5.5947086	4.9961472
Down	FGSG_13429	-2.345974235	0.0009007	3.701719	3.1370988	4.0578819	2.6546039	2.6377651	2.6397336	2.9802913	3.1804273	2.7087823
Down	FGSG_03987	-2.350415288	7.17E-07	3.9513288	4.069824	3.7539507	4.5907474	4.4455509	4.6556708	4.1391495	4.262829	3.9466965
Down	FGSG_12102	-2.351058432	0.0013715	3.701719	4.2010532	2.8407141	3.6568115	3.5586152	3.6925011	4.0850435	3.6853877	4.3572018
Down	FGSG_02995	-2.353081133	1.33E-05	3.9513288	3.5844678	4.188785	4.2425091	4.2058494	4.210161	3.2982451	3.5358829	2.964684
Down	FGSG_00102	-2.355418424	1.02E-07	4.2902308	4.4323548	4.0578819	4.6247215	4.4455509	4.7181783	4.3828727	4.0586965	4.6029775
Down	FGSG_06460	-2.356662021	3.84E-05	4.2902308	3.5844678	4.7085304	3.2841938	3.2568854	3.4190665	4.2416299	4.4416259	3.9466965
Down	FGSG_10952	-2.357719003	6.68E-09	7.8766576	7.8522684	7.8167417	7.8397076	7.7498592	7.8420279	7.0489555	6.9281194	7.100442
Down	FGSG_04767	-2.358088776	3.41E-08	10.537206	10.514171	10.474659	11.081832	11.01061	11.066524	11.035863	10.942695	11.063039
Down	FGSG_08398	-2.360378151	7.53E-06	7.4188896	7.3139731	7.4885462	9.1551852	9.0525935	9.1685076	9.4958018	9.4985248	9.4340352
Down	FGSG_13639	-2.360855668	0.0006606	3.2831234	3.1370988	3.3684179	4.5203003	4.2058494	4.7780897	4.028829	4.262829	3.6872775
Down	FGSG_07759	-2.368673763	5.42E-05	3.7898088	3.765011	3.7539507	3.6568115	2.6377651	4.210161	3.199846	2.9633478	3.3707558
Down	FGSG_11480	-2.369400188	3.70E-10	11.591879	11.52947	11.568921	10.959874	10.897437	10.934535	10.354177	10.267308	10.373017
Down	FGSG_11218	-2.371213658	0.0007636	2.2688027	2.4852148	2	3.3668146	3.4156241	3.260088	2.4052168	2.7077187	2
Down	FGSG_01766	-2.373850434	4.10E-07	6.0853387	6.3638664	5.640225	6.6255671	6.5209705	6.6436126	6.2721924	6.3216817	6.1547968
Down	FGSG_11261	-2.373934987	2.09E-05	3.0172362	2.4852148	3.3684179	3.5895922	3.5586152	3.5622521	3.3903592	3.6853877	2.964684
Down	FGSG_01799	-2.378411011	7.62E-08	7.9453978	7.9109619	7.8955278	7.742486	7.6733454	7.7251372	7.083861	6.9281194	7.1656435
Down	FGSG_03518	-2.382756439	3.34E-08	8.5193177	8.3881451	8.5596523	9.4486578	9.4489346	9.3595485	9.5788998	9.5985389	9.4911121
Down	FGSG_11630	-2.385133777	3.48E-09	5.9955706	6.2472245	5.5937254	6.3591166	6.374	6.2582434	5.7875758	5.8693008	5.6345505
Down	FGSG_07458	-2.386850029	7.96E-11	9.2800738	9.2938834	9.1794157	9.3339673	9.2366805	9.3422877	8.5876424	8.5214747	8.587419
Down	FGSG_04579	-2.38763129	4.01E-09	6.8091364	6.5962238	6.9195616	7.3286941	7.4802486	7.0603242	7.0347533	7.0294959	6.975096
Down	FGSG_06782	-2.391452561	0.0020673	3.1562965	2	3.7539507	3.2841938	3.5586152	2.8774004	2.5718533	2.9633478	2
Down	FGSG_04794	-2.392360182	9.05E-05	3.9513288	3.1370988	4.4195794	3.7210377	3.6887032	3.6925011	3.3903592	2.9633478	3.6872775
Down	FGSG_01311	-2.39614816	2.29E-13	13.103569	13.080475	13.040884	13.153232	13.116849	13.102531	13.192547	13.146352	13.172479
Down	FGSG_13202	-2.396296883	0.0137189	3.5075501	3.378055	3.5740244	2	2	2	3.2982451	3.1804273	3.3707558
Down	FGSG_11175	-2.400104258	1.49E-06	6.1197383	6.120316	6.0376686	7.3643222	7.2883703	7.3638362	7.541095	7.4509576	7.5738036
Down	FGSG_09145	-2.401309684	4.07E-08	6.5935482	6.2472245	6.8017711	6.5302	6.4856075	6.5086577	6.2103535	6.2986564	6.0481828
Down	FGSG_04619	-2.402820511	8.80E-07	3.5075501	3.378055	3.5740244	3.6568115	3.5586152	3.6925011	4.0850435	3.6853877	4.3572018
Down	FGSG_02113	-2.403087497	2.07E-08	8.3843511	8.3743063	8.3092981	8.5526569	8.4775616	8.5403502	7.9073809	7.8965819	7.852522

Down	FGSG_04892	-2.403337989	1.08E-06	5.100621	5.0349724	5.0898194	4.8133047	4.3699906	5.0931475	4.1391495	4.3549955	3.8228101
Down	FGSG_11321	-2.405459553	0.0002578	4.6135762	4.6316459	4.5224622	4.4077273	4.4455509	4.2946324	3.0942412	3.1804273	2.964684
Down	FGSG_00115	-2.408267274	1.28E-05	3.6078986	3.5844678	3.5740244	3.5190873	3.8080253	3.0813961	3.2982451	2.9633478	3.5376798
Down	FGSG_03857	-2.409491086	1.46E-06	6.1026411	5.906198	6.2918249	5.1056669	4.9394155	5.1845342	5.0240241	4.9352342	5.1065306
Down	FGSG_02424	-2.417080148	0.0016299	4.2902308	3.5844678	4.7085304	3.2841938	2.6377651	3.6925011	3.2982451	3.5358829	2.964684
Down	FGSG_03716	-2.417601979	1.33E-10	8.0018186	7.832161	8.0755039	7.7844204	7.8505879	7.6226545	7.6522501	7.5420042	7.6935963
Down	FGSG_03986	-2.425770258	3.16E-14	12.266422	12.224948	12.222219	12.409185	12.355248	12.375367	11.906038	11.8412	11.90488
Down	FGSG_01809	-2.427431157	1.22E-08	6.9873724	7.0610564	6.8220828	6.7150192	6.5891976	6.8107005	6.1324685	5.9304949	6.2540713
Down	FGSG_05374	-2.428149826	8.85E-14	8.1630681	8.0739456	8.165687	8.2948926	8.3506382	8.1447637	7.8678707	7.904229	7.7626576
Down	FGSG_01061	-2.428563431	3.99E-08	5.9955706	5.9811576	5.9294932	6.0065063	5.6638294	6.2155469	5.3100371	5.2552685	5.3047937
Down	FGSG_05680	-2.431403472	2.54E-05	5.5921111	5.510285	5.5937254	4.5203003	4.3699906	4.5903319	4.1912995	4.262829	4.0607807
Down	FGSG_13861	-2.433592016	1.22E-07	5.9580386	5.8672052	5.9664603	5.5740396	5.7851416	5.2281446	5.2862562	5.347904	5.1587087
Down	FGSG_13883	-2.436076203	8.97E-12	6.9873724	7.1285622	6.9750363	7.0385763	7.0183365	6.9987054	7.1313519	6.9578183	7.2641992
Down	FGSG_07477	-2.436263659	2.24E-13	10.725582	10.656757	10.708115	10.599378	10.560844	10.550315	10.36486	10.388117	10.273003
Down	FGSG_13914	-2.437573456	0.0005879	3.2831234	3.1370988	3.3684179	2.7805029	3.0784965	2.3550396	3.7092973	3.3690748	3.9466965
Down	FGSG_03878	-2.438945957	8.12E-05	3.2831234	3.1370988	3.3684179	2.6546039	2.353841	2.8774004	3.2982451	3.6853877	2.7087823
Down	FGSG_01859	-2.443214511	3.55E-12	7.3553501	7.4120569	7.20897	7.2162053	7.030657	7.3039581	7.1446385	6.9130376	7.287821
Down	FGSG_00121	-2.44359116	7.84E-05	3.5075501	3.5844678	3.3684179	3.8415007	4.1161991	3.4190665	2.7212202	2.9633478	2.964684
Down	FGSG_11449	-2.449765849	2.20E-05	4.7070397	4.2010532	5.0212003	4.0558932	3.6887032	4.2946324	3.4769433	3.3690748	3.5376798
Down	FGSG_11909	-2.450552703	1.61E-06	7.2964211	7.3284	7.177785	6.7615701	6.6858453	6.7521558	5.9170997	5.7038947	6.0481828
Down	FGSG_07789	-2.45389143	0.0001029	3.3996963	3.1370988	3.5740244	2.6546039	2.6377651	2.6397336	2.9802913	2.9633478	2.964684
Down	FGSG_20636	-2.45518383	3.01E-05	3.2831234	3.1370988	3.3684179	3.1032407	3.2568854	2.8774004	2.7212202	2.3968278	2.964684
Down	FGSG_10646	-2.455977659	6.94E-07	5.5921111	5.5600358	5.545677	4.4462359	4.2058494	4.7181783	4.8402557	4.7439404	4.8766132
Down	FGSG_03643	-2.456335158	0.0004859	3.3996963	3.1370988	3.5740244	2.7805029	2	3.260088	2.8565633	2.7077187	2.964684
Down	FGSG_13680	-2.456384812	9.02E-08	5.0300296	4.6316459	5.2780294	4.92659	4.8308815	4.9442019	4.8402557	4.8742451	4.7462732
Down	FGSG_07822	-2.457673722	6.01E-06	9.5275362	9.5399712	9.4282574	10.644582	10.549116	10.650882	9.9735553	9.8841157	9.9966375
Down	FGSG_05620	-2.460859135	2.12E-10	9.4021338	9.3048684	9.411599	9.0244894	8.9296958	9.0306078	8.543185	8.5264382	8.4940407
Down	FGSG_13485	-2.461044164	0.0006136	4.2902308	4.7218364	3.5740244	3.7210377	2.8748957	4.210161	2.9802913	2.7077187	3.18196
Down	FGSG_01994	-2.463076706	2.72E-12	9.1236031	9.0194102	9.1395078	9.2191282	9.2071325	9.1431016	8.77231	8.7380605	8.7407766
Down	FGSG_02834	-2.464175	7.44E-05	4.6135762	4.8868835	4.188785	4.0558932	4.1161991	4.5903319	3.2982451	3.6853877	3.18196
Down	FGSG_13627	-2.465837216	8.68E-08	6.5181831	6.7107812	6.9567807	6.3591166	6.4308827	6.594472	5.4450355	5.4765776	5.7410644

Down	FGSG_07383	-2.467400398	5.21E-13	8.7808037	8.7299049	8.7465942	9.1624316	9.1319312	9.1055957	8.8635233	8.8090287	8.8519181
Down	FGSG_12487	-2.4697893	0.0002413	3.5075501	3.5844678	3.9139052	4.1522165	4.0206066	4.3744304	3.9093688	3.6853877	4.0607807
Down	FGSG_11306	-2.469998678	4.02E-05	5.100621	5.349828	4.7085304	5.1988268	5.265544	5.0451875	4.6296145	4.1643702	4.9376179
Down	FGSG_03399	-2.470114949	1.70E-09	7.9785775	7.9947541	7.9333641	7.4234237	7.3584675	7.4024174	7.0560045	6.986918	7.0598582
Down	FGSG_05238	-2.472274952	4.85E-05	3.6078986	3.1370988	3.9139052	2.7805029	2.6377651	2.8774004	3.7092973	3.5358829	3.8228101
Down	FGSG_14003	-2.47264557	3.30E-08	6.5935482	6.6660438	6.4305761	6.2653117	6.4121699	6.0060392	5.9925186	5.837702	6.075584
Down	FGSG_13882	-2.475300407	5.03E-10	6.665171	6.306724	6.8813568	6.9807236	7.0059099	6.8669619	6.2721924	6.3443452	6.1288728
Down	FGSG_07633	-2.476163177	0.0028644	2.2688027	2	2.8407141	3.7825262	3.6887032	3.8119585	2.2167888	2	2.3974876
Down	FGSG_08624	-2.480480878	4.09E-11	15.530021	15.455125	15.518377	14.953844	14.939191	14.880108	14.402094	14.400558	14.336825
Down	FGSG_05375	-2.481888823	4.21E-10	8.6308542	8.5133883	8.6589748	8.3517838	8.3006433	8.3162152	7.6935164	7.6276445	7.6935963
Down	FGSG_03252	-2.482513947	5.62E-16	10.369554	10.359449	10.293772	10.515847	10.486765	10.457176	10.451606	10.436383	10.400364
Down	FGSG_20502	-2.483748064	9.22E-05	4.0257777	3.9254527	4.0578819	3.444959	3.4156241	3.4190665	3.2982451	3.1804273	3.3707558
Down	FGSG_13461	-2.484988844	1.02E-12	6.7985581	6.7541729	6.7602689	7.0758949	6.9290147	7.1309525	6.9158034	6.6655604	7.0735134
Down	FGSG_07846	-2.491667983	1.00E-08	6.3253218	6.306724	6.2623983	6.0571473	5.8139402	6.1937152	5.5080752	5.7385279	5.1587087
Down	FGSG_03361	-2.493583595	2.31E-06	3.6078986	3.1370988	3.9139052	3.8981589	4.1161991	3.5622521	3.7092973	3.3690748	3.9466965
Down	FGSG_07501	-2.494942579	1.52E-06	6.5052304	6.5721788	6.3489364	6.3077574	6.5209705	5.9550408	5.1610901	5.3023297	4.9376179
Down	FGSG_03241	-2.497596036	0.0001363	3.872828	3.378055	4.188785	3.1965524	2.8748957	3.4190665	2.8565633	2.3968278	3.18196
Down	FGSG_08064	-2.498789423	3.00E-05	5.0300296	5.1692654	4.7932764	4.7220923	4.9394155	4.3744304	3.7791165	3.9446664	3.5376798
Down	FGSG_11961	-2.499473719	7.46E-11	8.6515751	8.7407319	8.4659495	9.2588832	9.2811876	9.1459467	8.8371689	8.7926417	8.815814
Down	FGSG_00067	-2.503480705	6.45E-05	4.7948149	4.8868835	4.6184939	4.4837433	4.8308815	3.9222766	5.237485	4.9937494	5.3945636
Down	FGSG_03413	-2.510614727	0.0014988	4.5644712	4.6316459	4.4195794	3.444959	3.0784965	3.6925011	2.9802913	3.3690748	2.3974876
Down	FGSG_04438	-2.513374195	2.53E-05	4.5136357	4.3213345	4.6184939	4.0558932	4.4455509	3.4190665	3.7092973	3.6853877	3.6872775
Down	FGSG_05119	-2.513721865	1.21E-06	8.2077267	8.2433937	8.0839393	8.951072	8.8658043	8.9482514	9.2521208	9.2749356	9.1609905
Down	FGSG_04874	-2.516813218	9.95E-08	10.125845	10.110918	10.055085	10.840718	10.784662	10.812708	10.177191	10.229762	10.052866
Down	FGSG_09344	-2.521320016	2.32E-09	14.346238	14.31537	14.291506	14.538807	14.499566	14.490591	13.912272	13.894546	13.863788
Down	FGSG_11070	-2.523991548	0.0003541	3.2831234	3.378055	3.1285604	2.8962916	2.353841	3.260088	2.2167888	2.3968278	2
Down	FGSG_08289	-2.52492085	2.81E-12	8.5353342	8.5932169	8.3863428	8.5918294	8.5606242	8.548974	8.4764491	8.3360272	8.5439396
Down	FGSG_11251	-2.526911434	9.49E-08	6.310507	5.7859081	6.628079	5.7068462	5.6638294	5.6688985	5.2620767	4.9937494	5.4374371
Down	FGSG_04054	-2.529639464	6.28E-14	13.602689	13.575648	13.544569	13.466515	13.414096	13.431159	13.03603	12.988272	13.017304
Down	FGSG_07665	-2.533756606	2.45E-10	8.1169827	8.1490155	7.9972798	8.2574849	8.200598	8.2276734	8.2797329	8.096723	8.3832744
Down	FGSG_07870	-2.536430867	4.70E-09	8.7994924	8.6913575	8.8191253	8.3593707	8.2955472	8.3412553	7.7380245	7.7084846	7.7024119

Down	FGSG_11572	-2.538524713	2.07E-07	7.6716626	7.42554	7.8065839	6.8717719	6.8196649	6.8391054	6.1976611	6.0176785	6.301259
Down	FGSG_00078	-2.540072516	0.001232	3.9513288	3.9254527	3.9139052	3.1965524	3.5586152	2.6397336	2.4052168	2.3968278	2.3974876
Down	FGSG_02976	-2.543663445	4.50E-10	9.6779558	9.7501358	9.51191	9.3378129	9.3392976	9.2474547	9.2021614	9.2116306	9.1384305
Down	FGSG_02118	-2.544088178	9.00E-13	9.5307355	9.4959161	9.4803073	9.833457	9.7576757	9.8265915	9.3727628	9.3552401	9.3240944
Down	FGSG_04665	-2.545689012	1.57E-05	4.0965725	4.4323548	3.5740244	4.6247215	4.2058494	4.8909283	4.2902636	4.9352342	2.964684
Down	FGSG_02741	-2.545910025	7.73E-05	3.701719	3.1370988	4.0578819	3.1032407	3.4156241	2.6397336	2.5718533	2.7077187	2.3974876
Down	FGSG_06508	-2.547768423	7.13E-10	13.789814	13.741146	13.75266	14.642138	14.607343	14.589196	15.042879	15.008674	15.010917
Down	FGSG_11936	-2.54811702	1.96E-12	10.535615	10.520468	10.466617	10.831655	10.735646	10.838438	10.974873	10.938047	10.945432
Down	FGSG_02161	-2.550959697	1.23E-08	6.2023113	6.2472245	6.0719967	5.8284512	5.6318376	5.9288493	5.3564544	5.2552685	5.3945636
Down	FGSG_04803	-2.561079005	1.43E-06	5.9955706	5.7859081	6.1382955	5.3281747	5.423383	5.1395644	4.7387761	4.6740912	4.8766132
Down	FGSG_00143	-2.568355982	1.92E-08	5.1346624	5.0349724	5.1553224	5.5209064	5.306652	5.636897	5.1870071	5.1562742	5.1587087
Down	FGSG_06136	-2.568359942	1.86E-08	7.0776365	6.8965233	7.1619361	7.7616984	7.5925452	7.8349933	8.0269992	8.0213724	7.9666872
Down	FGSG_09087	-2.568875099	5.30E-06	6.9966572	6.9157592	6.9930638	6.892837	6.9290147	6.7670162	6.0216157	5.9304949	6.0481828
Down	FGSG_20706	-2.590072063	0.0001313	3.7898088	3.378055	4.0578819	3.6568115	3.6887032	3.5622521	3.2982451	2.9633478	3.5376798
Down	FGSG_08434	-2.59083781	4.01E-10	8.1875985	8.1731952	8.1171963	8.0512283	8.1450169	7.8559952	7.5003335	7.5517747	7.3786204
Down	FGSG_03812	-2.590959415	1.72E-17	12.013232	11.988795	11.951957	11.828478	11.762858	11.806615	11.811582	11.792585	11.764086
Down	FGSG_13506	-2.591655129	1.20E-07	5.2004262	5.4053203	4.8733193	5.658465	5.6951271	5.5363994	4.7734018	4.9352342	4.5256129
Down	FGSG_03869	-2.592138923	0.0002345	4.4609433	4.069824	4.7085304	3.1965524	3.2568854	3.0813961	2.9802913	2.9633478	2.964684
Down	FGSG_07917	-2.592982192	1.01E-13	9.8970707	9.8207163	9.8869101	9.6341773	9.537936	9.6414213	9.6175296	9.5938177	9.5798982
Down	FGSG_07770	-2.594418474	0.0009426	4.2285219	4.2010532	4.188785	4.0558932	4.3699906	3.5622521	2.8565633	3.1804273	2.3974876
Down	FGSG_05324	-2.596586438	1.56E-05	5.3235909	5.1036809	5.4444954	5.3074061	5.2232303	5.3115993	4.2902636	4.1643702	4.3572018
Down	FGSG_03363	-2.599857072	3.75E-10	9.9434211	9.8510102	9.9643379	10.300224	10.225205	10.296183	10.269534	10.22824	10.246124
Down	FGSG_05615	-2.599981271	4.85E-08	11.734321	11.721383	11.661301	12.383524	12.322114	12.357375	12.01065	11.990105	11.964725
Down	FGSG_13564	-2.601237502	4.83E-10	10.070863	10.096239	10.00266	10.48381	10.415738	10.484406	9.7785374	9.729136	9.782628
Down	FGSG_12505	-2.60218251	6.30E-07	4.7070397	4.9628272	4.3087924	5.0316238	4.51735	5.3904893	4.4698939	4.6006874	4.265
Down	FGSG_05323	-2.609185371	1.59E-08	9.998763	9.9145429	9.9959709	10.596706	10.619118	10.483282	10.251992	10.234319	10.203291
Down	FGSG_10432	-2.611517548	0.0005243	4.4062533	4.3213345	4.4195794	4.0558932	3.8080253	4.210161	2.9802913	2.3968278	3.3707558
Down	FGSG_02307	-2.612861525	3.40E-10	6.6416896	6.775389	6.4038729	6.40871	6.2739084	6.4546071	6.3198232	6.2752577	6.301259
Down	FGSG_03395	-2.614769433	0.0006778	4.4609433	4.5354388	4.3087924	4.1522165	3.6887032	4.4500449	3.199846	2.3968278	3.6872775
Down	FGSG_07790	-2.615805697	0.0002602	3.7898088	3.765011	3.7539507	4.69036	4.8861689	4.3744304	4.5519634	4.262829	4.7462732
Down	FGSG_05589	-2.616066947	4.13E-11	10.400489	10.334651	10.380216	10.790623	10.788295	10.704937	10.690025	10.652706	10.661104

Down	FGSG_11522	-2.616260336	7.41E-05	2.8633281	3.1370988	2.4807492	3.444959	3.4156241	3.4190665	2.4052168	2.3968278	2.3974876
Down	FGSG_12658	-2.61643441	7.31E-08	6.8506908	6.6660438	6.9382912	6.5743316	6.449356	6.6110389	5.9170997	5.7385279	6.0202512
Down	FGSG_13417	-2.625266206	5.13E-05	3.701719	3.9254527	3.5740244	3.1965524	2.6377651	3.5622521	2.8565633	3.1804273	2.3974876
Down	FGSG_05176	-2.628183023	2.36E-09	9.473697	9.4338905	9.4282574	9.7493365	9.7502448	9.6594531	9.1170961	9.0452292	9.1220974
Down	FGSG_03173	-2.628293061	4.93E-09	9.1299458	9.1429015	9.0302651	8.9759619	8.9329814	8.9318601	7.9906727	7.9194028	8.0028193
Down	FGSG_10468	-2.629531851	7.98E-06	4.0257777	4.069824	3.9139052	4.5203003	4.7733906	4.1204343	3.7092973	3.9446664	3.3707558
Down	FGSG_03683	-2.634346447	2.26E-07	7.162584	7.1611664	7.0799694	7.93418	7.9316604	7.8490285	6.7861576	6.6474492	6.8537291
Down	FGSG_04185	-2.635837082	6.32E-06	4.6135762	4.6316459	4.5224622	4.0052074	3.6887032	4.210161	3.7791165	3.9446664	3.5376798
Down	FGSG_03563	-2.636196688	9.97E-12	9.6663664	9.6358863	9.6115439	10.326912	10.30978	10.255928	10.375465	10.368902	10.315282
Down	FGSG_03112	-2.637616138	0.0002328	4.2285219	4.4323548	4.188785	3.7210377	3.0784965	4.1204343	3.3903592	2.7077187	3.8228101
Down	FGSG_03184	-2.639524663	4.11E-06	6.5437448	6.2772809	6.6956874	6.1299242	6.0741317	6.1029268	5.2862562	5.3023297	5.2090653
Down	FGSG_20600	-2.640965448	0.0018074	4.1640552	4.4323548	3.7539507	2.364115	2.353841	2.3550396	2.4052168	2.7077187	2
Down	FGSG_12121	-2.644370945	0.0001709	4.0257777	4.069824	3.9139052	4.0558932	4.2058494	3.8119585	2.9802913	3.1804273	2.964684
Down	FGSG_02210	-2.644555739	1.12E-08	12.165324	12.106476	12.138141	12.19905	12.11026	12.198934	11.82557	11.813549	11.771436
Down	FGSG_13734	-2.651445041	0.0001206	3.5075501	3.378055	3.5740244	2.7805029	2.8748957	2.6397336	3.2982451	2.7077187	3.6872775
Down	FGSG_07885	-2.651716835	7.30E-05	4.7515947	4.5354388	4.9491538	4.0558932	4.1161991	3.9222766	3.9093688	3.5358829	4.1665008
Down	FGSG_08502	-2.652805029	3.90E-11	12.583646	12.505268	12.57883	12.91243	12.897691	12.839811	12.502724	12.507932	12.432027
Down	FGSG_13884	-2.653412849	1.79E-05	3.0172362	2.4852148	3.3684179	3.3668146	3.8080253	2.6397336	3.0942412	2.9633478	3.18196
Down	FGSG_03656	-2.654173333	4.61E-08	6.169847	6.2772809	5.9664603	6.0446528	6.1885139	5.7902457	5.3564544	5.2066203	5.4374371
Down	FGSG_03243	-2.65779489	6.98E-09	8.3914079	8.2883641	8.439899	8.238411	8.290433	8.1617312	7.942038	7.8965819	7.9371156
Down	FGSG_09813	-2.658624412	8.85E-14	9.393373	9.3967231	9.3037887	8.9693665	8.7953458	9.0459204	9.3557257	9.2571301	9.3855958
Down	FGSG_01512	-2.65975583	5.84E-10	7.8564006	7.9581125	7.6563111	7.2383189	7.1596621	7.2308067	8.1038368	8.1100101	8.0588119
Down	FGSG_02982	-2.660372223	2.39E-05	2.6910183	2.8476671	2.4807492	3.2841938	3.2568854	3.260088	2.9802913	2.7077187	3.18196
Down	FGSG_07384	-2.663680071	2.78E-06	6.438663	6.5721788	6.2016809	6.4280797	6.1438433	6.594472	5.0524989	4.8742451	5.1587087
Down	FGSG_02941	-2.66479515	4.24E-09	6.7985581	6.8770274	6.628079	6.7691847	6.6543434	6.8107005	5.9325022	5.6320297	6.1288728
Down	FGSG_03823	-2.670634402	4.34E-09	6.9873724	7.1119795	6.7602689	6.5916131	6.6543434	6.4361306	6.5267935	6.5138742	6.4963611
Down	FGSG_02671	-2.678284102	1.48E-09	10.129014	10.085662	10.08691	10.387534	10.275249	10.4118	9.8902261	9.8995014	9.8134713
Down	FGSG_04447	-2.683132064	0.0001917	3.1562965	4.4323548	4.8733193	3.5190873	4.7135134	3.9222766	4.0850435	4.4416259	4.3572018
Down	FGSG_07921	-2.68341892	1.31E-06	4.0257777	4.069824	3.9139052	3.8981589	4.0206066	3.6925011	4.5115122	4.3549955	4.6029775
Down	FGSG_07653	-2.690331443	6.35E-07	5.2633225	4.9628272	5.4444954	5.5388358	5.1346873	5.7902457	4.6296145	4.0586965	4.9961472
Down	FGSG_08283	-2.694447179	1.84E-05	4.4062533	4.2010532	4.5224622	4.1048586	4.1161991	4.024755	4.0850435	3.8208439	4.265

Down	FGSG_07303	-2.698294908	1.12E-12	10.877802	10.828982	10.840956	10.825278	10.774625	10.789243	10.154383	10.125672	10.11691
Down	FGSG_02882	-2.699016491	5.68E-09	7.7116792	7.6716978	7.6675797	7.1823862	7.0549869	7.220047	6.576661	6.6655604	6.4128878
Down	FGSG_04204	-2.702374411	8.43E-15	11.609007	11.580083	11.5523	11.517835	11.478583	11.469388	11.141945	11.123087	11.095191
Down	FGSG_04302	-2.702953391	2.10E-06	4.4609433	4.2010532	4.6184939	4.5907474	4.7733906	4.2946324	4.3373111	4.6740912	3.8228101
Down	FGSG_13479	-2.704555704	5.44E-07	4.4062533	3.9254527	4.7085304	4.2856211	4.1161991	4.3744304	4.028829	4.1643702	3.8228101
Down	FGSG_12632	-2.707162705	0.0003307	3.0172362	3.378055	3.1285604	2.8962916	3.4156241	2	2.7212202	3.1804273	2
Down	FGSG_11176	-2.708505333	6.81E-08	5.065757	5.4053203	4.5224622	5.6084049	5.423383	5.7308476	5.3100371	5.4349486	5.2090653
Down	FGSG_11155	-2.710419005	9.15E-08	5.1346624	4.8067189	5.3356788	4.4077273	4.6510429	4.024755	4.3373111	4.5233477	4.0607807
Down	FGSG_04736	-2.715842334	1.56E-05	5.2322171	5.2921154	5.0898194	5.2649499	5.6318376	4.6556708	4.4270393	3.6853877	4.8766132
Down	FGSG_08079	-2.716768937	5.76E-07	8.3155318	8.1244237	8.4066601	8.8719501	8.8380325	8.8187394	8.4764491	8.3584682	8.5241867
Down	FGSG_13974	-2.718077418	0.0004307	3.3996963	3.378055	3.3684179	3.6568115	3.6887032	3.5622521	2.8565633	3.3690748	2
Down	FGSG_03558	-2.720114452	2.19E-12	12.573599	12.548658	12.512808	12.294976	12.209443	12.291774	12.019466	11.953258	12.020866
Down	FGSG_11617	-2.722612459	1.74E-13	10.690967	10.68786	10.607851	10.272364	10.163606	10.291052	9.9127279	9.8841157	9.875198
Down	FGSG_13993	-2.724342224	1.34E-06	6.169847	6.0172257	6.232359	5.5388358	5.4602848	5.5363994	5.107816	4.8742451	5.2577234
Down	FGSG_03802	-2.726583937	1.24E-08	11.627429	11.585348	11.583868	11.650271	11.658057	11.55322	11.375054	11.328865	11.354853
Down	FGSG_07709	-2.729344617	8.70E-05	4.0965725	3.9254527	4.188785	3.7825262	3.6887032	3.8119585	2.8565633	2.9633478	2.7087823
Down	FGSG_12219	-2.730500898	0.0002402	3.701719	3.765011	3.5740244	2.6546039	2.353841	2.8774004	2.4052168	2.7077187	2
Down	FGSG_00138	-2.738826841	1.98E-07	6.2182735	6.0172257	6.3206633	5.8856141	5.9498937	5.7308476	5.5080752	5.3023297	5.6345505
Down	FGSG_02167	-2.743065518	0.0002467	4.4609433	4.5354388	4.3087924	4.4837433	4.2058494	4.6556708	3.6359267	3.6853877	3.5376798
Down	FGSG_11229	-2.74556744	8.12E-11	6.6416896	6.6431438	6.5571458	6.40871	6.253029	6.4728499	6.410612	6.4102748	6.3692672
Down	FGSG_07918	-2.747942709	8.22E-08	7.0150495	6.9157592	7.0284567	7.0758949	7.1483996	6.9077633	5.9777469	5.7038947	6.1547968
Down	FGSG_11369	-2.749894491	1.69E-06	4.2902308	4.069824	4.4195794	3.7825262	3.5586152	3.9222766	3.7092973	3.1804273	4.0607807
Down	FGSG_04852	-2.751994969	0.0001078	3.2831234	2	3.9139052	3.5190873	3.8080253	3.0813961	3.2982451	3.3690748	3.18196
Down	FGSG_06184	-2.752660586	1.51E-15	16.453293	16.400097	16.420599	16.512103	16.473294	16.463162	16.283073	16.257174	16.242762
Down	FGSG_12452	-2.762414702	0.0012049	3.701719	3.9254527	3.9139052	2.1935143	2	2.3550396	2.9802913	2.9633478	2.964684
Down	FGSG_20071	-2.764705265	0.000214	4.0257777	3.5844678	4.3087924	2.6546039	2.6377651	2.6397336	2.9802913	3.3690748	2.3974876
Down	FGSG_06815	-2.767368184	5.78E-07	6.5688614	6.4717791	6.5811798	6.4846757	6.4121699	6.4728499	5.4663562	5.3920827	5.4790733
Down	FGSG_03199	-2.769921465	4.89E-10	9.9871646	9.8831262	10.00266	9.852399	9.7258267	9.8873576	9.2914764	9.2239048	9.2923317
Down	FGSG_07028	-2.771647291	2.81E-06	3.1562965	3.1370988	3.1285604	2.8962916	3.0784965	2.6397336	2.4052168	2.3968278	2.3974876
Down	FGSG_13343	-2.774470964	2.07E-07	8.9528522	8.9370792	8.8833591	8.3339242	8.2955472	8.2855818	7.1902009	7.0294959	7.2760585
Down	FGSG_07754	-2.775699673	1.33E-05	3.5075501	3.378055	3.5740244	3.5190873	3.4156241	3.5622521	2.8565633	2.7077187	2.964684

Down	FGSG_03855	-2.778442093	4.56E-09	7.1952103	7.2547781	7.0458328	6.6171532	6.449356	6.6911344	6.1589001	6.152209	6.1024744
Down	FGSG_03069	-2.780856636	6.20E-10	10.678762	10.68085	10.590194	9.9947848	9.8818784	10.017333	9.3755828	9.2330424	9.4445822
Down	FGSG_12597	-2.785218876	2.04E-07	7.3839359	7.6373467	7.0799694	6.6171532	6.6858453	6.4546071	5.9015311	5.8053955	5.9627112
Down	FGSG_04819	-2.786742042	3.99E-05	7.2660287	7.0610564	7.3831245	6.7228827	6.374	6.9604299	5.4450355	5.6684096	5.1065306
Down	FGSG_02203	-2.787642275	2.86E-06	6.6996916	6.5962238	6.7175374	6.4376679	6.5554873	6.279127	5.1610901	4.9937494	5.2577234
Down	FGSG_10672	-2.787649484	4.89E-05	3.5075501	3.9254527	2.8407141	2.5166584	2.353841	2.6397336	3.199846	2.7077187	3.5376798
Down	FGSG_09336	-2.788963677	0.0006898	4.0965725	4.069824	4.0578819	3.5190873	4.1161991	2.3550396	2.5718533	2.3968278	2.7087823
Down	FGSG_09176	-2.789569266	0.0001588	4.5136357	3.9254527	4.8733193	3.3668146	3.4156241	3.260088	3.0942412	2.9633478	3.18196
Down	FGSG_13241	-2.792216829	3.24E-07	4.9558055	5.458757	4.0578819	4.6247215	4.7733906	4.3744304	4.965331	5.1562742	4.8129147
Down	FGSG_03174	-2.793450954	4.93E-09	12.136739	12.190318	11.993406	12.124576	12.048743	12.113216	11.0527	10.995555	11.043224
Down	FGSG_11140	-2.793764415	0.0001967	2.4953013	2	2.8407141	3.7825262	3.9182279	3.5622521	2.8565633	3.1804273	2.3974876
Down	FGSG_13243	-2.795714281	5.77E-05	2.2688027	2.4852148	2	3.1965524	3.5586152	2.6397336	3.0942412	3.3690748	2.7087823
Down	FGSG_03108	-2.797363086	5.09E-07	3.7898088	3.378055	4.0578819	3.5190873	3.6887032	3.260088	3.2982451	3.5358829	2.964684
Down	FGSG_00214	-2.80143456	2.52E-07	5.4640893	5.5600358	5.2780294	4.5203003	4.3699906	4.5903319	4.7387761	4.8105634	4.6029775
Down	FGSG_03438	-2.806124553	9.01E-06	4.7070397	4.5354388	4.7932764	4.6247215	4.7733906	4.3744304	3.4769433	3.5358829	3.3707558
Down	FGSG_12415	-2.814429881	0.0001834	4.349408	4.2010532	4.4195794	4.5907474	4.3699906	4.7181783	3.970335	4.4416259	3.18196
Down	FGSG_02220	-2.829537623	3.22E-07	7.2964211	7.1611664	7.3415168	6.3890768	6.4675956	6.2155469	5.7358405	5.7385279	5.670936
Down	FGSG_11219	-2.837995085	9.32E-07	3.1562965	2.4852148	3.5740244	3.1032407	2.6377651	3.4190665	2.8565633	2.9633478	2.7087823
Down	FGSG_05242	-2.845124367	9.97E-12	5.9955706	5.608128	6.232359	6.3077574	6.374	6.1490351	6.0216157	5.9891987	5.991768
Down	FGSG_12134	-2.847227911	7.52E-06	4.1640552	4.069824	4.188785	4.0052074	3.6887032	4.210161	3.5586239	3.5358829	3.5376798
Down	FGSG_11375	-2.848111059	1.97E-11	7.4188896	7.4120569	7.3415168	8.1361232	8.081298	8.1043792	7.5054921	7.3869025	7.5641633
Down	FGSG_10613	-2.849159197	5.78E-07	8.7316053	8.6402333	8.7359284	8.5504491	8.5900596	8.4185825	7.5003335	7.5996588	7.3225439
Down	FGSG_07600	-2.854347972	1.88E-08	4.5644712	4.7218364	4.3087924	4.9800625	5.1346873	5.0451875	5.3791148	5.4765776	5.2090653
Down	FGSG_11474	-2.859031774	3.02E-09	7.4797482	7.5415319	7.3273766	7.0820218	7.1256073	6.9474424	6.4960241	6.5138742	6.4128878
Down	FGSG_07825	-2.859462399	2.18E-05	3.701719	3.765011	3.5740244	4.1048586	3.9182279	4.210161	2.9802913	2.9633478	2.964684
Down	FGSG_07597	-2.86306385	4.56E-08	5.4906186	5.7434744	5.0898194	5.5913246	5.5656438	5.5363994	6.1324685	6.0456069	6.1547968
Down	FGSG_07551	-2.863888871	8.77E-06	4.7515947	4.4323548	4.9491538	3.8415007	3.5586152	4.024755	3.970335	4.1643702	3.8228101
Down	FGSG_13139	-2.872339893	5.30E-06	2.6910183	2.8476671	2.4807492	3.5895922	3.8080253	3.260088	3.199846	3.3690748	2.964684
Down	FGSG_13742	-2.875499791	2.00E-12	7.8097548	7.7271924	7.8065839	7.7693122	7.8154152	7.6308015	7.2832433	7.3869025	7.100442
Down	FGSG_04449	-2.875870761	6.88E-05	3.6078986	4.2010532	2.4807492	2.8962916	3.2568854	2.3550396	3.199846	2.7077187	3.5376798
Down	FGSG_10284	-2.881431078	2.85E-14	8.864328	8.8397276	8.8038869	8.7560258	8.7021396	8.7267193	8.5153264	8.3528906	8.6016258

Down	FGSG_12563	-2.883251866	9.62E-14	11.219086	11.156606	11.195478	11.148304	11.113376	11.095512	10.653313	10.587706	10.652032
Down	FGSG_03267	-2.884707998	1.15E-09	6.0139766	6.052414	5.891554	5.5209064	5.5656438	5.3904893	5.9925186	6.3666582	5.3945636
Down	FGSG_03234	-2.885232344	5.88E-09	6.2955384	6.3638664	6.1382955	7.3235317	7.3188299	7.2414868	7.0560045	7.1882541	6.8378143
Down	FGSG_05349	-2.889001375	6.74E-09	6.2340611	5.7859081	6.5078422	6.2326372	6.1209783	6.2582434	5.5684753	5.4349486	5.6345505
Down	FGSG_13939	-2.892340633	6.02E-06	3.3996963	3.5844678	3.1285604	3.1965524	3.2568854	3.0813961	2.5718533	2.3968278	2.7087823
Down	FGSG_04859	-2.893737996	1.93E-06	5.3235909	5.458757	5.0898194	5.1760947	5.179638	5.0931475	4.2416299	4.0586965	4.3572018
Down	FGSG_13926	-2.89484095	4.34E-09	6.5181831	6.5228522	6.4305761	6.2866906	6.0977451	6.3792376	6.2103535	6.152209	6.2052877
Down	FGSG_03068	-2.894985177	2.45E-10	6.7334055	6.3638664	6.9567807	6.8066659	6.8053944	6.7219677	6.1055435	6.073005	6.075584
Down	FGSG_11420	-2.900845642	1.22E-06	3.5075501	3.5844678	3.3684179	3.0034737	2.6377651	3.260088	2.7212202	2.7077187	2.7087823
Down	FGSG_05790	-2.901260899	2.71E-09	6.605735	6.7541729	6.3489364	6.7150192	6.8889735	6.4174145	6.1324685	6.2026947	5.991768
Down	FGSG_12211	-2.902022251	7.53E-09	8.8994024	8.9133502	8.7987715	8.9677129	8.8930517	8.9547561	8.1004316	8.0213724	8.1127123
Down	FGSG_03198	-2.903318019	3.49E-06	4.0257777	4.3213345	3.5740244	3.2841938	2.8748957	3.6925011	3.0942412	3.1804273	2.964684
Down	FGSG_11190	-2.904856501	3.12E-09	11.695293	11.604738	11.69817	12.422223	12.374407	12.382622	11.97274	11.962444	11.916292
Down	FGSG_05617	-2.907472368	3.97E-07	5.0300296	5.0349724	4.9491538	4.7531416	4.8308815	4.5903319	4.965331	5.0499834	4.8129147
Down	FGSG_09337	-2.908501918	4.86E-08	7.8816778	7.8220012	7.8566725	7.5744776	7.5758276	7.5034852	6.8443161	6.8668223	6.7554957
Down	FGSG_02271	-2.91086008	3.02E-10	5.6638035	5.6997548	5.545677	5.7226193	5.6951271	5.7002055	6.0641898	6.0456069	6.0202512
Down	FGSG_13196	-2.925583574	5.88E-10	7.3768424	7.5165498	7.1297061	7.7806582	7.7720438	7.7175072	7.5758404	7.4613623	7.6210621
Down	FGSG_07646	-2.928515921	1.01E-06	5.065757	4.8868835	5.1553224	4.69036	5.0403527	4.1204343	4.2902636	3.6853877	4.6764038
Down	FGSG_10601	-2.929343101	3.77E-07	4.2902308	4.5354388	3.9139052	4.0052074	3.8080253	4.1204343	4.0850435	4.1643702	3.9466965
Down	FGSG_04695	-2.937769241	6.94E-05	5.1346624	4.9628272	5.2179801	4.5203003	4.7733906	4.1204343	3.9093688	4.0586965	3.6872775
Down	FGSG_02198	-2.938324599	1.44E-08	5.5166689	5.349828	5.5937254	5.0814056	5.265544	4.7780897	4.8725557	5.2066203	4.3572018
Down	FGSG_03925	-2.938359247	1.74E-08	7.87162	7.832161	7.8268285	7.4520907	7.2677006	7.5385497	6.7255557	6.61053	6.7723395
Down	FGSG_07823	-2.939717555	1.44E-06	5.8597011	5.8672052	5.7713555	5.9405982	5.7557564	6.0308779	5.1610901	4.7439404	5.4374371
Down	FGSG_02343	-2.946975471	4.86E-08	5.8392029	5.9811576	5.5937254	6.1060709	6.3932112	5.636897	5.2862562	4.6740912	5.670936
Down	FGSG_03155	-2.949323027	2.28E-05	4.8775546	4.8067189	4.8733193	4.6247215	4.51735	4.6556708	3.4769433	3.5358829	3.3707558
Down	FGSG_04209	-2.949917196	4.20E-08	6.0853387	6.153105	5.9294932	6.3077574	6.374	6.1490351	5.2124668	5.1041074	5.2577234
Down	FGSG_13983	-2.950695593	9.64E-10	8.5031214	8.3743063	8.5413923	9.2561763	9.1880091	9.2368189	8.5973385	8.6361261	8.4889546
Down	FGSG_05440	-2.951656642	7.96E-11	8.8021425	8.7353285	8.7884859	9.1981272	9.1741926	9.1345326	8.6634457	8.5412272	8.7148711
Down	FGSG_03562	-2.954084188	2.22E-13	7.2964211	7.3284	7.177785	7.3131513	7.3486598	7.1982839	7.0131845	6.9281194	7.0321538
Down	FGSG_03220	-2.956545688	5.99E-09	5.7096927	5.5600358	5.7713555	6.5916131	6.7013421	6.3792376	6.1457448	6.2272902	5.991768
Down	FGSG_02279	-2.95875498	3.20E-07	6.2496778	6.153105	6.2623983	5.3887447	5.346621	5.3515835	5.5880596	5.5563964	5.5589053

Down	FGSG_10995	-2.959332084	6.63E-11	6.6882763	6.5721788	6.7175374	6.828696	6.7909814	6.7817251	7.0489555	7.0294959	7.003907
Down	FGSG_05025	-2.965758019	4.20E-05	5.1346624	4.9628272	5.2179801	4.69036	4.4455509	4.8356119	4.1391495	4.1643702	4.0607807
Down	FGSG_03146	-2.965930847	7.62E-07	6.2651272	6.153105	6.2918249	5.2432439	5.0882909	5.3115993	4.994976	4.8742451	5.0523944
Down	FGSG_03559	-2.977001	5.09E-10	8.2196702	8.3391171	7.9972798	7.7269298	7.6495769	7.7175072	7.2343684	6.9130376	7.443156
Down	FGSG_04658	-2.977744708	0.0007511	4.2285219	4.5354388	3.7539507	3.1965524	3.4156241	2.8774004	2.4052168	2.7077187	2
Down	FGSG_13945	-2.988368075	4.55E-08	5.3235909	5.1036809	5.4444954	4.657914	4.7733906	4.4500449	4.7734018	4.6740912	4.8129147
Down	FGSG_02965	-2.99283082	2.52E-05	4.2902308	3.765011	5.1553224	4.7531416	5.423383	4.210161	3.970335	4.0586965	4.265
Down	FGSG_07934	-2.993668409	3.55E-12	7.3188025	7.2698061	7.2841049	7.3131513	7.3778853	7.1650109	7.2343684	7.0843795	7.3110622
Down	FGSG_04019	-2.994392946	2.38E-06	3.5075501	3.765011	3.1285604	3.1032407	2.8748957	3.260088	2.9802913	2.7077187	3.18196
Down	FGSG_09950	-2.995309924	3.22E-11	15.266207	15.208132	15.239271	15.813948	15.786947	15.753515	15.707929	15.699537	15.650156
Down	FGSG_02927	-2.996463717	4.98E-07	7.7060301	7.6941525	7.6335064	7.0259191	6.9551045	7.0112415	6.1719365	5.9891987	6.2778581
Down	FGSG_11644	-3.000288139	6.30E-07	3.701719	3.5844678	3.7539507	3.5895922	3.4156241	3.6925011	3.3903592	2.9633478	3.6872775
Down	FGSG_13783	-3.00530697	1.58E-12	8.4567799	8.556921	8.5352539	8.1093765	8.1106104	8.1560975	8.3842444	8.4448964	8.3387351
Down	FGSG_04677	-3.009416168	8.75E-06	4.0965725	4.2010532	3.9139052	3.8981589	4.2902531	3.260088	4.2416299	4.6740912	3.5376798
Down	FGSG_00122	-3.015869325	1.51E-05	3.7898088	3.5844678	3.9139052	4.2856211	3.8080253	4.5903319	3.970335	3.9446664	3.9466965
Down	FGSG_10496	-3.016865673	3.54E-08	12.522889	13.313615	13.298151	13.481657	14.058479	14.079967	13.596267	14.109082	14.142614
Down	FGSG_08205	-3.019071248	1.23E-08	8.2432646	8.2126152	8.1893346	8.2628886	8.0989568	8.3312913	7.7939054	7.7344551	7.7877264
Down	FGSG_01758	-3.019145185	6.90E-06	6.0139766	5.510285	6.3206633	4.8133047	5.179638	4.210161	4.935064	4.3549955	5.3047937
Down	FGSG_07596	-3.023057915	8.53E-05	5.8597011	5.608128	6.0025037	5.0567294	4.7135134	5.2704753	6.9002206	6.7863464	6.9604719
Down	FGSG_08911	-3.028876889	3.22E-11	7.340842	7.2395919	7.3555198	7.7032764	7.7646868	7.5471843	7.1512362	7.249572	6.975096
Down	FGSG_13368	-3.030726807	6.07E-06	3.9513288	4.069824	3.7539507	3.444959	3.6887032	3.0813961	4.028829	3.8208439	4.1665008
Down	FGSG_02350	-3.03357458	0.0001471	4.2285219	3.378055	4.7085304	2.7805029	2.353841	3.0813961	3.7791165	3.8208439	3.6872775
Down	FGSG_07535	-3.051639219	1.61E-05	4.7070397	5.2319977	3.7539507	4.6247215	4.5857446	4.5903319	4.0850435	4.6740912	2.964684
Down	FGSG_01790	-3.052236179	8.73E-05	5.4640893	5.510285	5.3356788	5.1760947	5.265544	4.9955781	5.1346989	5.3920827	4.7462732
Down	FGSG_12927	-3.052619516	5.26E-05	2.2688027	2.4852148	2	3.1965524	3.0784965	3.260088	3.4769433	3.6853877	3.18196
Down	FGSG_10229	-3.054250601	5.03E-10	9.7823042	9.7447677	9.7345129	9.1154133	9.0972075	9.0489636	8.5305887	8.5214747	8.4787282
Down	FGSG_03898	-3.057985777	3.71E-06	2.6910183	2.8476671	2.4807492	3.5190873	3.5586152	3.5622521	2.5718533	2.3968278	2.7087823
Down	FGSG_12782	-3.059638725	2.99E-08	7.0422056	7.0260808	6.9750363	7.7993721	7.7044395	7.8136811	7.3762899	7.4921337	7.1783375
Down	FGSG_12244	-3.059831641	3.51E-05	6.3688748	6.3916107	6.2623983	5.2863342	5.346621	5.1395644	5.3334324	5.1562742	5.4374371
Down	FGSG_13961	-3.061321907	1.03E-06	3.2831234	2.8476671	3.5740244	3.2841938	3.5586152	2.8774004	3.0942412	2.9633478	3.18196
Down	FGSG_09111	-3.06653633	3.34E-07	6.2023113	6.2772809	6.0719967	5.7226193	5.8421752	5.5363994	4.965331	4.8742451	4.9961472

Down	FGSG_10664	-3.067629967	4.51E-06	5.065757	4.8067189	5.2179801	5.1295269	5.0403527	5.1395644	4.0850435	3.9446664	4.1665008
Down	FGSG_07579	-3.070862369	3.66E-06	5.8597011	6.052414	5.545677	5.674773	5.6951271	5.5706824	4.807216	4.5233477	4.9961472
Down	FGSG_07702	-3.074293496	3.78E-08	5.7321013	5.7859081	5.5937254	5.4468739	5.4602848	5.3515835	4.666927	4.8105634	4.4438634
Down	FGSG_07991	-3.074815642	2.36E-06	8.1254721	8.0216372	8.1416454	8.3441568	8.3308476	8.2700177	8.058929	8.0143231	8.0380685
Down	FGSG_09105	-3.082117113	8.76E-07	4.0257777	3.378055	4.4195794	3.5190873	3.8080253	3.0813961	3.5586239	3.1804273	3.8228101
Down	FGSG_01861	-3.08457899	2.72E-08	6.629804	6.6431438	6.5327046	6.2436111	6.29449	6.1029268	5.6821809	5.9304949	5.3047937
Down	FGSG_08137	-3.088010803	1.29E-08	7.340842	7.3708347	7.2243133	7.4662133	7.2467305	7.5812149	6.6715049	6.7528555	6.5164961
Down	FGSG_07666	-3.090810374	2.79E-09	8.7700148	8.79887	8.6533178	8.5745506	8.4820546	8.5787578	7.6935164	7.2968163	7.9519772
Down	FGSG_11249	-3.092259517	6.43E-10	7.112218	7.1449564	6.9930638	6.5568406	6.4675956	6.5607563	6.0918902	6.0998924	6.0202512
Down	FGSG_02267	-3.09371108	2.81E-05	6.7879016	6.6431438	6.8421126	5.9540229	6.0257126	5.7902457	4.7387761	4.6006874	4.8129147
Down	FGSG_10973	-3.098845684	1.99E-11	9.6069807	9.5275207	9.5998272	10.071464	10.018053	10.038921	9.5354294	9.4781746	9.5262922
Down	FGSG_05588	-3.100867982	5.75E-06	3.2831234	3.1370988	3.3684179	3.6568115	3.5586152	3.6925011	2.9802913	3.3690748	2.3974876
Down	FGSG_01379	-3.110086331	2.82E-06	3.7898088	3.765011	3.7539507	3.7210377	4.2058494	2.8774004	3.0942412	2.9633478	3.18196
Down	FGSG_06513	-3.114349014	1.30E-05	3.7898088	3.9254527	3.5740244	3.9526758	4.0206066	3.8119585	3.9093688	4.262829	3.3707558
Down	FGSG_08024	-3.123768132	6.82E-06	4.7948149	5.349828	4.0578819	5.6252853	5.7257602	5.4652882	4.807216	5.0499834	4.4438634
Down	FGSG_07773	-3.137564599	3.39E-07	3.7898088	3.9254527	3.5740244	3.5895922	3.6887032	3.4190665	3.4769433	2.7077187	3.9466965
Down	FGSG_05011	-3.137999518	9.62E-09	8.1423025	8.1161324	8.0839393	7.8718907	7.8849234	7.7700883	6.8361499	6.8668223	6.7554957
Down	FGSG_05682	-3.139056262	6.63E-07	7.1543109	6.9157592	7.2841049	6.5302	6.5209705	6.4546071	6.0216157	6.152209	5.8079414
Down	FGSG_11900	-3.147054432	7.30E-09	5.3814422	5.5600358	5.0898194	5.7068462	5.8698682	5.4283735	4.965331	5.3023297	4.4438634
Down	FGSG_13065	-3.147669957	3.19E-05	3.6078986	3.378055	3.7539507	3.5895922	3.5586152	3.5622521	4.2902636	3.3690748	4.8129147
Down	FGSG_03988	-3.158717796	4.62E-09	9.3756901	9.3085116	9.3569616	9.6455988	9.6559675	9.5455652	8.4229273	8.551003	8.2086492
Down	FGSG_10627	-3.160418939	4.46E-06	3.3996963	3.378055	3.3684179	4.327482	4.1161991	4.4500449	3.6359267	3.6853877	3.5376798
Down	FGSG_13632	-3.165615009	6.67E-08	5.0300296	5.1036809	4.8733193	5.1988268	4.8308815	5.4283735	4.8725557	5.1041074	4.5256129
Down	FGSG_03834	-3.168777256	1.03E-06	6.2496778	6.3638664	6.0376686	5.6908987	5.6638294	5.636897	5.3334324	5.4349486	5.1587087
Down	FGSG_03957	-3.168953016	5.23E-10	9.1776677	9.0803473	9.1872665	8.9955692	8.8310052	9.0700876	8.1004316	8.0492301	8.0860138
Down	FGSG_02305	-3.169067086	2.04E-06	5.2937714	5.510285	4.9491538	4.6247215	4.3699906	4.8356119	4.0850435	3.6853877	4.3572018
Down	FGSG_10543	-3.169647338	1.40E-15	10.911328	10.851633	10.887478	10.82345	10.758974	10.80012	10.618025	10.617086	10.55194
Down	FGSG_00026	-3.177646276	2.61E-05	4.0257777	2.8476671	4.6184939	4.5907474	4.3699906	4.7181783	3.4769433	3.3690748	3.5376798
Down	FGSG_09054	-3.185558671	4.92E-11	8.0605495	7.9947541	8.0412601	7.2437947	7.2985951	7.0960705	7.2405691	7.1110577	7.2994884
Down	FGSG_11042	-3.186842766	1.08E-07	3.2831234	3.1370988	3.3684179	2.6546039	2.8748957	2.6397336	3.0942412	3.3690748	2.7087823
Down	FGSG_05987	-3.187321073	1.60E-10	13.614422	13.560642	13.582643	13.844509	13.804397	13.797964	13.205978	13.177254	13.168707

Down	FGSG_02641	-3.198746502	1.09E-10	10.768222	10.809831	10.636806	9.9841915	9.9352807	9.9489293	9.3840101	9.4019015	9.298159
Down	FGSG_02121	-3.20012282	1.02E-06	4.349408	4.3213345	4.3087924	5.0316238	4.5857446	5.3115993	4.5913113	4.6006874	4.5256129
Down	FGSG_04527	-3.21533775	1.09E-11	11.480063	11.437635	11.466191	11.421161	11.438111	11.319314	10.784827	10.777654	10.743494
Down	FGSG_03153	-3.227824577	1.77E-07	4.7515947	5.2319977	4.0578819	4.5907474	4.7135134	4.3744304	4.2416299	4.0586965	4.4438634
Down	FGSG_13034	-3.234888641	8.35E-05	3.2831234	3.1370988	3.3684179	3.7210377	3.5586152	3.8119585	2.4052168	2.7077187	2
Down	FGSG_08396	-3.236879994	4.01E-06	6.653478	6.6660438	6.5811798	7.9375629	8.0021756	7.7920493	8.4499364	8.4130895	8.4264798
Down	FGSG_02428	-3.239930691	2.43E-08	7.3261865	7.3284	7.2394951	6.5656126	6.5554873	6.4908649	5.947742	6.0176785	5.8079414
Down	FGSG_01986	-3.242610089	1.12E-06	3.872828	3.378055	4.188785	3.2841938	3.4156241	3.0813961	3.0942412	2.9633478	3.18196
Down	FGSG_03703	-3.246524421	8.89E-07	6.8910817	6.6431438	7.063002	5.8429557	5.9498937	5.636897	5.237485	5.2552685	5.3503768
Down	FGSG_03362	-3.253527875	7.22E-06	4.5644712	5.1036809	3.9139052	4.4462359	4.51735	4.5218929	3.970335	4.4416259	3.3707558
Down	FGSG_03488	-3.259504285	0.0004108	4.4062533	4.4323548	4.3087924	2.8962916	3.2568854	2.3550396	2.5718533	2	2.964684
Down	FGSG_09029	-3.260279139	2.94E-06	4.6135762	4.8067189	4.3087924	4.6247215	4.7733906	4.3744304	3.970335	3.3690748	4.3572018
Down	FGSG_03239	-3.266601592	1.18E-05	6.1861705	6.306724	5.9664603	4.9800625	4.9394155	4.9442019	4.2902636	4.0586965	4.4438634
Down	FGSG_10318	-3.267994411	1.25E-05	2.8633281	3.378055	2	3.5190873	3.4156241	3.5622521	3.5586239	3.6853877	3.3707558
Down	FGSG_09129	-3.269565519	1.81E-09	6.665171	6.3355781	6.8618681	6.466056	6.4308827	6.4361306	5.9170997	5.9891987	5.7748904
Down	FGSG_01812	-3.27187178	3.62E-05	3.5075501	3.378055	3.5740244	3.444959	2.8748957	3.8119585	2.5718533	2	2.964684
Down	FGSG_03427	-3.272686155	3.44E-12	9.7303777	9.7871639	9.5820717	9.1796757	9.1001333	9.1713031	8.9187025	8.8332655	8.9365461
Down	FGSG_11429	-3.276031403	7.78E-07	3.5075501	2.8476671	3.9139052	4.0558932	4.2058494	3.8119585	3.2982451	3.1804273	3.3707558
Down	FGSG_12737	-3.280618127	1.96E-12	9.8593964	9.8027466	9.8303775	9.5929386	9.5007791	9.5963708	9.0462995	9.0208151	9.0058404
Down	FGSG_13526	-3.282526635	1.67E-06	5.9955706	6.0172257	5.891554	5.0814056	5.306652	4.7181783	4.5115122	4.6006874	4.3572018
Down	FGSG_10623	-3.29656124	2.21E-07	4.0965725	4.2010532	3.9139052	3.7210377	3.6887032	3.6925011	3.4769433	3.6853877	3.18196
Down	FGSG_11356	-3.299040325	0.0001601	3.0172362	2.4852148	3.3684179	4.7220923	4.0206066	5.1395644	4.6296145	4.4416259	4.7462732
Down	FGSG_04966	-3.299357354	2.29E-08	5.4906186	5.458757	5.4444954	5.5740396	5.423383	5.6688985	4.5913113	4.3549955	4.7462732
Down	FGSG_03645	-3.300919775	3.33E-06	3.701719	4.069824	3.1285604	3.6568115	3.6887032	3.5622521	3.4769433	3.6853877	3.18196
Down	FGSG_11981	-3.305043108	1.76E-06	3.6078986	3.378055	3.7539507	3.5895922	2.6377651	4.1204343	2.7212202	2.9633478	2.964684
Down	FGSG_12404	-3.30572337	3.40E-07	4.7515947	4.6316459	4.7932764	4.5907474	3.8080253	5.0451875	4.028829	3.9446664	4.1665008
Down	FGSG_12474	-3.309255255	1.01E-13	9.3396606	9.3585728	9.2334948	9.1302718	9.0942757	9.0790469	8.8913767	8.7760663	8.9365461
Down	FGSG_12475	-3.311868259	3.48E-08	6.4789709	6.3638664	6.5078422	6.9408247	6.9024444	6.8942906	7.1380105	7.200728	7.003907
Down	FGSG_08019	-3.3272068	2.60E-06	3.9513288	4.4323548	3.1285604	2.8962916	3.0784965	2.6397336	2.7212202	2.7077187	2.7087823
Down	FGSG_09804	-3.333426437	3.16E-14	8.6218818	8.3950151	8.7412711	9.1376441	9.1769665	9.0088933	8.9811365	8.9523869	8.9439989
Down	FGSG_03412	-3.335321017	1.44E-14	12.598587	12.56398	12.547531	12.269364	12.203334	12.247426	11.911635	11.872236	11.885174

Down	FGSG_07687	-3.33638259	1.06E-06	4.7948149	5.1692654	4.188785	4.4462359	4.5857446	4.210161	3.970335	3.8208439	4.0607807
Down	FGSG_09268	-3.336842311	1.26E-15	11.144821	11.145446	11.057734	11.687918	11.653583	11.634473	11.262251	11.215112	11.242991
Down	FGSG_07852	-3.340145754	5.23E-11	7.0332102	7.1119795	7.2545188	6.5656126	6.8889735	6.6596278	6.3884448	6.59171	6.5164961
Down	FGSG_03411	-3.343549545	3.13E-05	4.4609433	4.4323548	4.4195794	4.1522165	3.8080253	4.3744304	3.5586239	4.1643702	2.3974876
Down	FGSG_09053	-3.344361971	1.01E-13	11.44361	11.434304	11.366859	11.071476	11.05652	11.001253	10.696806	10.657238	10.67012
Down	FGSG_00365	-3.344853623	1.93E-10	8.4500365	8.4221723	8.3931471	9.4826336	9.41644	9.4612748	9.7440361	9.7652765	9.6544784
Down	FGSG_01771	-3.347515368	5.98E-09	11.189534	11.135225	11.16101	10.71132	10.635344	10.699138	11.32268	11.291449	11.289075
Down	FGSG_02863	-3.367175643	5.90E-14	9.8733248	9.8156049	9.84534	9.7901398	9.6757146	9.8141249	9.4172353	9.4832891	9.2835464
Down	FGSG_02492	-3.37169559	3.79E-10	6.0853387	6.2472245	5.8125445	5.7840418	5.9498937	5.5012819	5.7181748	5.9304949	5.5195414
Down	FGSG_10641	-3.374443255	2.79E-06	4.0257777	4.4323548	3.3684179	2.8962916	2.6377651	3.0813961	3.2982451	2.9633478	3.5376798
Down	FGSG_10531	-3.393903999	4.81E-16	10.487031	10.507847	10.380216	10.136933	10.06073	10.126512	10.279355	10.28499	10.206393
Down	FGSG_03547	-3.395612754	3.01E-07	4.8367778	4.3213345	5.1553224	5.8715343	5.8970396	5.7608523	5.9925186	5.9601454	5.9627112
Down	FGSG_13519	-3.400930116	3.75E-10	6.4656596	6.3638664	6.4825439	6.3181765	6.3932112	6.1490351	5.9628224	6.1262878	5.7064263
Down	FGSG_03416	-3.403232249	3.49E-07	6.9496238	7.1119795	6.6735015	6.3181765	6.2739084	6.279127	5.4233949	5.4765776	5.3047937
Down	FGSG_07575	-3.407199238	0.0001672	3.7898088	3.1370988	4.188785	4.4462359	4.3699906	4.5903319	2.9802913	3.3690748	2.3974876
Down	FGSG_10636	-3.40839928	1.08E-05	2.6910183	2.8476671	2.4807492	4.0052074	4.2902531	3.5622521	3.3903592	2.7077187	3.8228101
Down	FGSG_08226	-3.415038681	1.03E-06	3.701719	3.9254527	3.3684179	3.8415007	3.6887032	3.9222766	3.199846	3.3690748	2.964684
Down	FGSG_06511	-3.418792011	3.46E-12	8.3302954	8.3603335	8.2126008	8.4235048	8.3258573	8.432631	7.6382282	7.6183761	7.5928932
Down	FGSG_07708	-3.433187953	8.94E-09	6.2182735	6.052414	6.2918249	5.7840418	6.0008798	5.4283735	5.3100371	5.1041074	5.4374371
Down	FGSG_09279	-3.440080512	2.07E-09	5.6402997	5.4053203	5.7713555	5.7840418	5.9237087	5.5363994	4.935064	4.6740912	5.1065306
Down	FGSG_04512	-3.440613528	1.37E-09	11.109418	11.112481	11.019767	11.119924	11.007496	11.141854	10.879753	10.808545	10.883795
Down	FGSG_13080	-3.449934613	0.000205	4.0965725	4.069824	4.0578819	4.4837433	4.4455509	4.4500449	2.5718533	2.7077187	2.3974876
Down	FGSG_02964	-3.45323577	1.31E-05	4.2285219	4.3213345	4.0578819	4.327482	4.3699906	4.210161	2.9802913	2.9633478	3.5376798
Down	FGSG_00411	-3.4532903	2.13E-07	3.9513288	4.2010532	3.5740244	3.6568115	3.8080253	3.4190665	3.970335	4.1643702	3.6872775
Down	FGSG_13795	-3.45982887	2.23E-07	4.8775546	5.0349724	4.6184939	4.953574	4.7733906	5.0451875	4.2416299	4.1643702	4.265
Down	FGSG_08909	-3.466037002	2.80E-07	3.872828	4.069824	3.5740244	4.3681625	4.5857446	4.024755	4.1391495	4.4416259	3.6872775
Down	FGSG_02416	-3.468700545	4.12E-06	4.349408	3.9254527	4.6184939	4.7835368	5.2232303	4.024755	4.666927	4.6740912	4.6029775
Down	FGSG_01585	-3.479697899	1.03E-06	4.4062533	4.4323548	4.3087924	4.657914	4.8861689	4.2946324	3.9093688	3.8208439	3.9466965
Down	FGSG_13562	-3.483797643	2.14E-06	3.701719	3.378055	3.9139052	4.4077273	4.5857446	4.1204343	4.7387761	4.6006874	4.8129147
Down	FGSG_08207	-3.488097076	7.68E-11	8.0998526	7.9300074	8.1736127	8.3416054	8.5131199	8.0446525	7.5856159	7.5996588	7.504928
Down	FGSG_10599	-3.497055235	1.17E-09	6.665171	6.5721788	6.6735015	7.5392846	7.6254103	7.3540275	7.2651087	7.2733875	7.1909208

Down	FGSG_02094	-3.497781132	1.14E-15	11.600278	11.519288	11.594977	11.153041	11.14701	11.071022	11.403656	11.386645	11.354154
Down	FGSG_08633	-3.501940262	9.19E-11	9.6605367	9.5212549	9.7074714	9.6964044	9.7201326	9.5817364	8.5900726	8.5655436	8.5488359
Down	FGSG_04064	-3.52346311	2.52E-07	4.2902308	4.6316459	3.7539507	3.7825262	3.5586152	3.9222766	3.7092973	3.6853877	3.6872775
Down	FGSG_11093	-3.527482499	1.60E-08	4.5644712	4.6316459	4.5224622	4.3681625	4.5857446	4.024755	4.2416299	4.0586965	4.3572018
Down	FGSG_07683	-3.536936161	3.77E-11	9.3845586	9.3337593	9.3499841	9.3184816	9.2286818	9.3223049	8.4418866	8.4708768	8.3443783
Down	FGSG_08308	-3.549398456	3.40E-10	9.8167858	9.7740484	9.7767518	9.1169061	9.0942757	9.0610723	8.57543	8.5313848	8.5585788
Down	FGSG_07820	-3.554288116	1.58E-06	4.349408	4.5354388	4.0578819	5.2432439	5.0403527	5.3515835	4.5115122	4.8105634	4.0607807
Down	FGSG_04720	-3.561168661	2.10E-07	4.349408	4.3213345	4.3087924	4.1522165	3.9182279	4.2946324	3.6359267	3.6853877	3.5376798
Down	FGSG_12651	-3.566800902	1.56E-12	9.6721727	9.6759239	9.5820717	10.151573	10.053201	10.160674	9.436269	9.4019015	9.4046271
Down	FGSG_13169	-3.574428071	7.21E-06	5.065757	5.349828	4.6184939	5.5027514	5.1346873	5.7902457	4.8725557	4.6006874	5.0523944
Down	FGSG_04621	-3.57482681	1.32E-06	3.9513288	3.378055	4.3087924	3.2841938	3.4156241	3.0813961	2.7212202	2.3968278	2.964684
Down	FGSG_09297	-3.579207242	7.75E-09	4.5136357	4.3213345	4.6184939	4.1522165	4.2058494	4.024755	4.8725557	4.3549955	5.2090653
Down	FGSG_06234	-3.581630567	9.77E-11	9.3968837	9.344446	9.3639055	8.810547	8.8997838	8.6202671	8.2400512	8.3416703	8.0588119
Down	FGSG_06966	-3.584144957	6.01E-05	3.9513288	3.1370988	4.4195794	4.2425091	3.9182279	4.4500449	2.5718533	2.7077187	2.3974876
Down	FGSG_01561	-3.588510687	6.21E-10	9.7809614	9.6759239	9.7974163	9.1868004	9.260414	9.0151309	8.1992472	8.123176	8.2086492
Down	FGSG_07836	-3.594574887	1.01E-07	9.6310291	9.5275207	9.6461336	9.3806946	9.231353	9.4359266	7.9534078	7.7515126	8.0724769
Down	FGSG_00345	-3.598557704	1.29E-13	7.1871227	7.0260808	7.2545188	7.0573562	7.1483996	6.8669619	7.3478522	7.3759446	7.2522417
Down	FGSG_04898	-3.599017413	1.53E-05	4.2902308	4.2010532	4.3087924	3.8415007	3.0784965	4.2946324	2.8565633	2.7077187	2.964684
Down	FGSG_02411	-3.600576563	1.11E-06	4.2902308	4.4323548	4.0578819	4.6247215	3.8080253	5.0931475	4.3828727	3.6853877	4.8129147
Down	FGSG_05335	-3.601202012	2.20E-07	6.438663	6.306724	6.5078422	5.8429557	6.0008798	5.5706824	4.965331	5.1562742	4.7462732
Down	FGSG_12386	-3.601322664	3.09E-11	6.860895	6.8372271	6.8017711	6.6001768	6.6858453	6.4361306	6.399571	6.5138742	6.2052877
Down	FGSG_10542	-3.602349574	1.01E-13	8.9881921	8.8597306	9.0258937	8.8270568	8.8239435	8.7418426	8.3300301	8.2605851	8.3387351
Down	FGSG_04696	-3.607027337	4.71E-07	5.3528065	5.2921154	5.3356788	5.1760947	5.1346873	5.1395644	4.666927	4.6740912	4.6029775
Down	FGSG_00070	-3.607070191	2.83E-08	3.6078986	2.8476671	4.0578819	3.0034737	3.0784965	2.8774004	3.2982451	2.9633478	3.5376798
Down	FGSG_03278	-3.614008182	8.13E-08	7.2736871	7.42554	7.0284567	6.9475516	6.5724409	7.1761874	6.1589001	6.4102748	5.7748904
Down	FGSG_08208	-3.614057362	9.45E-12	11.376736	11.334203	11.333642	11.348685	11.330733	11.279708	10.578186	10.58533	10.503583
Down	FGSG_01712	-3.628589028	5.93E-08	6.169847	6.1851653	6.0719967	5.8856141	5.346621	6.2155469	5.107816	5.1562742	4.9961472
Down	FGSG_04903	-3.637449417	1.56E-06	8.0337397	7.8916615	8.0839393	9.6762963	9.6776745	9.5859328	10.170711	10.151604	10.123489
Down	FGSG_05692	-3.641835635	1.25E-11	5.5166689	5.8271292	5.0212003	5.6908987	5.6951271	5.6041695	5.6638414	5.8693008	5.3503768
Down	FGSG_11440	-3.645867516	9.88E-11	8.7063622	8.5507819	8.7676921	8.6173658	8.6670545	8.4784952	8.1637958	8.2121189	8.0519305
Down	FGSG_00198	-3.656852899	4.29E-08	3.0172362	3.1370988	3.7539507	3.2841938	3.8080253	3.6925011	3.199846	2.9633478	3.6872775

Down	FGSG_00119	-3.673019284	4.75E-09	3.3996963	2.8476671	3.7539507	3.1032407	3.0784965	3.260088	3.3903592	3.6853877	2.964684
Down	FGSG_05285	-3.677374217	1.01E-13	12.549354	12.596752	12.599937	12.748402	12.796031	12.771908	12.450185	12.524152	12.419384
Down	FGSG_12608	-3.682870008	3.95E-09	3.5075501	3.378055	3.5740244	3.7210377	3.5586152	3.8119585	3.2982451	3.6853877	2.964684
Down	FGSG_04518	-3.683936205	5.09E-07	4.7070397	5.1036809	4.188785	4.871059	4.6510429	4.9955781	5.0804226	5.3920827	4.6029775
Down	FGSG_01770	-3.694161419	1.79E-10	10.147889	9.9936143	10.207619	9.2949369	9.2889013	9.2125978	10.087158	10.03802	10.069997
Down	FGSG_04247	-3.701823564	2.46E-08	4.0965725	4.4323548	3.5740244	3.3668146	3.2568854	3.4190665	3.2982451	3.3690748	3.18196
Down	FGSG_08797	-3.719895958	3.25E-08	7.8866805	7.8720994	7.8167417	7.2921638	7.1819267	7.3141119	6.3198232	6.4102748	6.1547968
Down	FGSG_04155	-3.724243007	1.29E-13	10.985485	11.016625	10.903055	10.551057	10.490115	10.566332	9.9195072	9.86465	9.9362612
Down	FGSG_09348	-3.729024758	4.20E-11	6.1861705	6.3638664	5.9294932	6.1992055	5.8970396	6.3792376	5.7532926	5.6320297	5.8079414
Down	FGSG_07503	-3.731534315	6.76E-13	8.4158383	8.3319751	8.4133694	8.3467036	8.3506382	8.2752244	7.6615229	7.6550976	7.6303308
Down	FGSG_13510	-3.734343601	4.99E-06	4.4609433	4.2010532	4.6184939	3.5895922	4.1161991	2.6397336	4.0850435	3.9446664	4.1665008
Down	FGSG_07583	-3.741736989	5.64E-06	3.1562965	3.5844678	2.4807492	4.2856211	4.2058494	4.2946324	3.6359267	4.0586965	2.964684
Down	FGSG_02979	-3.742366119	7.99E-07	5.100621	5.1692654	4.9491538	4.1522165	4.0206066	4.210161	4.0850435	4.0586965	4.0607807
Down	FGSG_11196	-3.764816868	1.03E-05	5.6164066	5.458757	5.6852725	6.3691726	6.314782	6.340023	5.8698804	5.6320297	6.0202512
Down	FGSG_06512	-3.780659279	1.72E-07	5.065757	5.2921154	4.7085304	4.9800625	5.1346873	4.7181783	4.8402557	4.5233477	5.0523944
Down	FGSG_10564	-3.787128206	5.21E-06	5.6164066	5.5600358	5.5937254	4.4077273	4.2058494	4.5218929	4.807216	4.7439404	4.8129147
Down	FGSG_02944	-3.793321765	2.48E-05	6.4921604	6.1851653	6.6735015	5.3281747	5.1346873	5.4283735	4.5519634	3.9446664	4.9376179
Down	FGSG_07638	-3.798071865	2.91E-07	4.2902308	4.069824	4.4195794	4.4837433	4.8308815	3.9222766	3.7092973	4.0586965	3.18196
Down	FGSG_13407	-3.803313618	3.34E-06	4.9172105	4.4323548	5.2179801	4.3681625	3.2568854	4.9442019	3.6359267	3.8208439	3.3707558
Down	FGSG_07658	-3.80365951	2.54E-05	3.701719	3.765011	3.5740244	3.9526758	3.9182279	3.9222766	2.8565633	2	3.3707558
Down	FGSG_07502	-3.804608615	5.96E-08	3.701719	3.9254527	3.3684179	3.6568115	3.4156241	3.8119585	2.8565633	2.7077187	2.964684
Down	FGSG_02975	-3.805757745	1.01E-13	11.124319	11.114563	11.048066	10.805974	10.754339	10.770929	10.201234	10.097598	10.241595
Down	FGSG_05681	-3.806525521	1.61E-09	7.3261865	7.4521338	7.0967395	6.6669159	6.6221382	6.6274177	6.3314892	6.4733227	6.1024744
Down	FGSG_05554	-3.825732434	5.22E-13	9.2781715	9.2566506	9.2144127	9.7570157	9.7613768	9.6634297	9.1989808	9.1324564	9.1988627
Down	FGSG_06665	-3.826730359	7.31E-08	5.7973114	5.5600358	5.9294932	6.0571473	5.8421752	6.1715481	5.4873665	5.5170389	5.3945636
Down	FGSG_12349	-3.831315661	2.64E-05	3.1562965	3.378055	2.8407141	4.7531416	4.4455509	4.9442019	4.9041484	5.1041074	4.6029775
Down	FGSG_00620	-3.832458188	2.46E-14	12.40602	12.342444	12.383386	12.272883	12.235289	12.224057	11.580581	11.578082	11.516042
Down	FGSG_12444	-3.83744713	8.18E-09	5.9769267	5.9811576	5.891554	5.7689296	5.8421752	5.6041695	5.3791148	5.1041074	5.5589053
Down	FGSG_02880	-3.84232725	2.18E-13	11.953395	11.913046	11.908094	11.329178	11.335707	11.234314	10.878262	10.874261	10.815349
Down	FGSG_13263	-3.845624359	2.59E-07	3.3996963	3.765011	2.8407141	4.1980691	3.9182279	4.3744304	3.7791165	3.5358829	3.9466965
Down	FGSG_12078	-3.849524176	7.45E-07	5.1346624	5.0349724	5.1553224	4.7531416	4.3699906	4.9955781	4.0850435	3.6853877	4.3572018

Down	FGSG_09131	-3.856917306	3.12E-09	3.6078986	3.1370988	3.9139052	3.5895922	3.4156241	3.6925011	3.7092973	3.5358829	3.8228101
Down	FGSG_12768	-3.861665639	2.46E-14	10.509907	10.395865	10.534353	10.531042	10.507849	10.46631	10.145323	10.100929	10.123489
Down	FGSG_11521	-3.874249661	1.62E-05	4.4609433	4.8067189	3.9139052	4.0052074	4.2902531	3.5622521	2.8565633	2.3968278	3.18196
Down	FGSG_03856	-3.894856227	6.12E-07	7.9501848	7.9205161	7.8955278	7.7347289	7.8712871	7.485628	6.247774	6.1262878	6.301259
Down	FGSG_04958	-3.912656249	1.30E-06	7.4929307	7.5165498	7.3831245	6.6991623	6.5383322	6.7670162	5.5880596	5.6320297	5.4790733
Down	FGSG_09209	-3.913421413	5.84E-05	7.3335329	7.4782464	7.0967395	8.7807214	8.7587827	8.7343008	8.6703522	8.667919	8.6249972
Down	FGSG_08142	-3.923888491	1.72E-10	4.7070397	4.8868835	4.4195794	5.1529987	5.2232303	4.9955781	4.8402557	4.9937494	4.6029775
Down	FGSG_05771	-3.928835573	3.09E-07	4.2285219	3.765011	4.5224622	4.953574	4.7135134	5.0931475	4.7032988	4.4416259	4.8766132
Down	FGSG_08197	-3.936002106	3.94E-11	6.2955384	6.1851653	6.3206633	5.7226193	5.8421752	5.5012819	5.9325022	5.9891987	5.8079414
Down	FGSG_00806	-3.943411643	3.66E-12	9.623557	9.5947058	9.5671068	9.3262453	9.2991223	9.2658807	8.6611362	8.7337752	8.5142078
Down	FGSG_07774	-3.943870358	1.08E-10	10.939886	10.948211	10.844684	12.122348	12.051391	12.105198	11.85517	11.816591	11.827412
Down	FGSG_12926	-3.958774446	7.49E-07	4.4062533	4.5354388	4.188785	4.2425091	4.2902531	4.1204343	3.6359267	4.1643702	2.7087823
Down	FGSG_13427	-3.967257782	2.25E-06	6.3253218	6.4455482	6.1055269	5.484365	5.4602848	5.4283735	5.6073815	5.7723491	5.3503768
Down	FGSG_04431	-3.985110095	1.50E-07	5.065757	4.8067189	5.2179801	5.3074061	5.346621	5.1845342	4.5115122	4.4416259	4.5256129
Down	FGSG_01767	-3.991229027	8.28E-12	9.3305109	9.2261567	9.3464826	10.037186	10.021143	9.9651289	9.0656373	9.0172934	9.0479502
Down	FGSG_00142	-3.992790696	1.71E-09	3.3996963	2.8476671	3.7539507	3.7210377	3.4156241	3.9222766	3.3903592	3.1804273	3.5376798
Down	FGSG_00002	-3.997475288	4.08E-06	5.4640893	5.2921154	5.545677	6.34899	6.2103411	6.3984523	5.5080752	5.5947086	5.3503768
Down	FGSG_08062	-4.005326577	2.28E-07	8.0472069	7.9013439	8.1006636	7.6992962	7.9051392	7.3540275	6.3198232	6.3443452	6.2298857
Down	FGSG_04876	-4.010180336	1.01E-13	9.4653614	9.3690779	9.4739028	8.9776061	8.8623619	9.0026287	8.8371689	8.8688739	8.7364912
Down	FGSG_05752	-4.046135554	2.43E-08	4.6135762	4.3213345	4.8733193	4.4077273	4.0206066	4.6556708	4.3373111	4.262829	4.3572018
Down	FGSG_02257	-4.048069824	2.84E-09	3.3996963	3.5844678	3.1285604	3.7825262	3.0784965	4.210161	3.4769433	3.1804273	3.6872775
Down	FGSG_02278	-4.065156943	8.91E-07	5.7096927	5.608128	5.728956	5.1295269	5.2232303	4.9442019	4.2902636	3.9446664	4.5256129
Down	FGSG_10538	-4.066881501	4.93E-06	5.4370629	4.8868835	5.7713555	4.2856211	4.5857446	3.9222766	4.5115122	5.1041074	3.3707558
Down	FGSG_04657	-4.081296122	3.30E-07	5.4095206	5.1036809	5.5937254	5.0567294	5.3855124	4.5218929	4.2902636	3.9446664	4.5256129
Down	FGSG_06725	-4.088213376	3.52E-12	9.1797072	9.0972954	9.191176	8.9825273	8.981388	8.9119415	8.3701738	8.4448964	8.2751578
Down	FGSG_10325	-4.089471852	2.67E-09	3.872828	3.378055	4.188785	4.1048586	4.2902531	3.8119585	4.2902636	3.9446664	4.5256129
Down	FGSG_09494	-4.10068799	1.16E-10	8.1254721	8.1490155	8.0150332	9.0004297	8.9362595	8.9772945	9.0213069	8.9888059	8.9879253
Down	FGSG_08065	-4.11516588	6.09E-09	8.8413214	8.8397276	8.7571817	8.3441568	8.2749808	8.3262834	7.6615229	7.4192855	7.812367
Down	FGSG_00917	-4.118294635	2.46E-14	11.472596	11.407376	11.451598	11.337525	11.32824	11.25949	10.623967	10.575785	10.605793
Down	FGSG_09599	-4.138082461	9.34E-13	14.432312	14.403388	14.376043	13.779252	13.715732	13.754935	13.144272	13.082489	13.139793
Down	FGSG_08049	-4.149677974	2.85E-07	5.5166689	5.7434744	5.1553224	5.9540229	5.2232303	6.3792376	4.7032988	4.8742451	4.4438634

Down	FGSG_10674	-4.150089009	1.37E-08	4.7070397	4.2010532	5.0212003	4.8133047	4.7733906	4.7780897	4.2902636	4.5233477	3.9466965
Down	FGSG_03754	-4.15599983	1.26E-07	7.3839359	7.6021576	7.1297061	6.4566552	6.374	6.4546071	6.2229351	5.6320297	6.6131596
Down	FGSG_01580	-4.159037492	4.96E-06	5.167919	5.0349724	5.2179801	4.953574	4.8308815	4.9955781	3.6359267	3.8208439	3.3707558
Down	FGSG_08163	-4.161374874	7.63E-09	5.3528065	5.1036809	5.4959731	5.7068462	5.5991203	5.7308476	5.0524989	4.8742451	5.1587087
Down	FGSG_07608	-4.164465408	4.71E-09	4.2285219	4.3213345	4.0578819	3.3668146	3.4156241	3.260088	3.3903592	3.3690748	3.3707558
Down	FGSG_01744	-4.2007296	1.21E-12	9.5685837	9.5461564	9.5056446	9.0052739	8.9064847	9.0151309	8.742035	8.8090287	8.6016258
Down	FGSG_01823	-4.20427753	5.73E-11	7.3113806	7.342684	7.1934618	7.6670528	7.5842106	7.662937	7.0975901	6.8823924	7.2280252
Down	FGSG_11368	-4.222382974	3.86E-09	6.0853387	5.7859081	6.2623983	6.5656126	6.5209705	6.5262338	5.5284909	5.1041074	5.8079414
Down	FGSG_02820	-4.231390367	2.09E-07	7.2736871	7.342684	7.113317	7.0573562	6.9551045	7.0723383	5.7181748	5.9304949	5.3945636
Down	FGSG_03266	-4.252040328	2.70E-08	4.8775546	4.069824	5.3356788	4.4077273	4.6510429	4.024755	3.970335	4.0586965	3.8228101
Down	FGSG_12783	-4.257760604	2.39E-07	4.4609433	4.6316459	4.188785	5.1056669	5.306652	4.7780897	4.0850435	4.1643702	3.9466965
Down	FGSG_05679	-4.2597837	9.85E-10	4.0965725	4.069824	4.0578819	3.6568115	3.5586152	3.6925011	4.0850435	4.262829	3.8228101
Down	FGSG_01983	-4.260592017	4.10E-11	8.5416914	8.6745166	8.3020857	8.3390496	8.4178422	8.167343	7.7070136	7.7932944	7.5446872
Down	FGSG_01990	-4.276720109	5.21E-13	9.1236031	9.1989353	8.954087	9.1938901	9.1574363	9.1431016	8.9379088	8.9225749	8.8871407
Down	FGSG_03644	-4.286514967	1.39E-07	6.2496778	6.5721788	5.728956	6.2215791	6.1663515	6.1937152	5.1610901	5.3023297	4.9376179
Down	FGSG_08799	-4.291649733	1.90E-09	6.1861705	6.052414	6.232359	5.5209064	5.7557564	5.1395644	5.8210632	6.073005	5.4374371
Down	FGSG_11436	-4.29819987	1.11E-07	3.9513288	3.765011	4.0578819	4.5907474	4.2902531	4.7780897	4.8725557	4.9352342	4.7462732
Down	FGSG_10547	-4.308796581	2.97E-10	9.1898616	9.2261567	9.0647677	8.5767218	8.4548842	8.6079392	7.9980116	8.0001203	7.929627
Down	FGSG_13782	-4.340062165	1.68E-14	9.9601291	9.9240735	9.9108149	9.518089	9.4022865	9.543409	9.2536545	9.1160864	9.3183711
Down	FGSG_02163	-4.347541593	1.17E-07	6.1026411	5.7859081	6.2918249	5.4657412	5.4602848	5.3904893	5.107816	5.2066203	4.9376179
Down	FGSG_12094	-4.36770206	3.63E-09	5.9769267	5.8672052	6.1382955	5.3887447	5.1346873	5.7002055	5.107816	4.8742451	5.3503768
Down	FGSG_08511	-4.398410174	6.69E-12	7.5317704	7.4782464	7.5011983	7.742486	7.6495769	7.7477878	7.3592946	7.3649028	7.287821
Down	FGSG_02112	-4.431695616	4.80E-07	5.4906186	5.1036809	5.728956	5.7382218	5.8139402	5.5706824	4.7032988	4.9352342	4.3572018
Down	FGSG_03074	-4.438474452	2.03E-08	4.7070397	4.7218364	4.6184939	4.0052074	3.4156241	4.4500449	4.2416299	4.5233477	3.8228101
Down	FGSG_07911	-4.486443277	9.04E-08	6.1861705	6.3916107	5.85259	6.032049	5.8139402	6.1715481	5.0804226	4.5233477	5.4374371
Down	FGSG_11888	-4.501261422	8.29E-10	13.91392	13.836334	13.904737	13.201869	13.212049	13.101798	13.078836	13.132176	12.954349
Down	FGSG_07558	-4.512245676	1.52E-11	10.099155	10.096239	10.015946	10.128078	10.096332	10.072207	9.5764508	9.5771708	9.5088094
Down	FGSG_03001	-4.522635646	4.29E-07	5.6638035	5.6546687	5.5937254	5.0316238	4.8308815	5.1395644	4.2902636	4.3549955	4.1665008
Down	FGSG_08114	-4.528166168	6.66E-10	4.5136357	4.4323548	4.5224622	4.7531416	4.7733906	4.6556708	4.028829	4.0586965	3.9466965
Down	FGSG_03415	-4.547166138	7.26E-08	5.5422571	5.5600358	5.4444954	4.8133047	4.7733906	4.8909283	4.1912995	4.262829	4.0607807
Down	FGSG_12205	-4.572500724	1.88E-09	4.1640552	4.5354388	3.5740244	4.1980691	3.8080253	4.4500449	3.4769433	3.5358829	3.3707558

Down	FGSG_03521	-4.575488788	1.85E-08	5.2633225	5.5600358	4.7932764	5.2863342	5.6318376	4.7181783	4.666927	4.8105634	4.4438634
Down	FGSG_10454	-4.621215187	1.72E-09	7.2032529	7.1119795	7.20897	7.9102749	8.0208235	7.6943723	7.7896829	7.6909075	7.8204879
Down	FGSG_10453	-4.675665884	2.47E-12	10.404854	10.354171	10.376787	11.788057	11.759623	11.731894	11.523877	11.467584	11.51854
Down	FGSG_01830	-4.701661886	2.07E-08	5.6402997	5.458757	5.728956	5.1056669	4.7733906	5.3115993	4.5519634	4.6740912	4.3572018
Down	FGSG_01711	-4.708961935	2.44E-08	5.0300296	4.8067189	5.1553224	3.7825262	3.6887032	3.8119585	4.2416299	4.1643702	4.265
Down	FGSG_02114	-4.730970331	5.81E-12	10.535615	10.439714	10.561789	11.00047	10.9989	10.93124	9.9744857	9.882181	10.042489
Down	FGSG_07637	-4.747023471	1.85E-08	4.8775546	5.2921154	4.188785	3.9526758	4.0206066	3.8119585	3.5586239	3.6853877	3.3707558
Down	FGSG_12129	-4.793188252	1.41E-11	8.5884975	8.5322062	8.5596523	8.1739024	7.9316604	8.3060762	7.5807364	7.6276445	7.4640419
Down	FGSG_13426	-4.801653987	1.39E-14	7.8150128	7.7594926	7.7860511	7.5303505	7.5157415	7.4584209	7.5360624	7.4509576	7.5544581
Down	FGSG_08825	-4.830925711	4.79E-07	5.3528065	5.458757	5.1553224	5.1295269	5.265544	4.8909283	3.970335	3.3690748	4.3572018
Down	FGSG_03707	-4.834068475	2.27E-08	5.065757	4.6316459	5.3356788	3.8415007	3.9182279	3.6925011	4.4698939	4.262829	4.6029775
Down	FGSG_02881	-4.847489071	2.36E-08	7.4664442	7.4652492	7.3831245	6.7917901	6.7617162	6.7371407	6.1055435	6.073005	6.075584
Down	FGSG_02839	-4.857068607	6.03E-08	6.6996916	6.4717791	6.8220828	5.9540229	5.9498937	5.874995	5.8375198	5.4349486	6.1024744
Down	FGSG_09477	-4.867799093	5.43E-08	5.4906186	5.5600358	5.3356788	5.5027514	5.5313719	5.3904893	4.8725557	5.0499834	4.6029775
Down	FGSG_10659	-4.871110029	3.37E-08	5.8184092	5.6546687	5.891554	4.7531416	4.51735	4.8909283	4.7032988	4.5233477	4.8129147
Down	FGSG_03245	-4.905332577	1.98E-12	10.196038	10.190073	10.115992	10.116185	10.129645	10.012664	9.3428145	9.3242228	9.2952483
Down	FGSG_02355	-4.907348592	2.02E-07	6.7222549	6.8770274	6.4567939	7.7308346	7.5417997	7.8208202	7.8877611	7.6368537	8.0450161
Down	FGSG_03372	-4.909185586	5.47E-14	11.907062	11.914839	11.812351	12.169864	12.09274	12.159706	12.036272	11.999952	12.006256
Down	FGSG_09088	-4.918953705	4.45E-10	5.167919	4.8868835	5.3356788	5.0567294	5.0882909	4.9442019	4.5519634	4.3549955	4.6764038
Down	FGSG_07775	-4.92077326	2.14E-14	11.496928	11.432636	11.475838	11.959049	11.888327	11.941681	11.519102	11.448752	11.522279
Down	FGSG_08206	-4.925693601	5.13E-12	8.1254721	7.9205161	8.2279057	8.2246307	8.0930945	8.2647922	7.4315369	7.2375151	7.5544581
Down	FGSG_07495	-5.016332155	1.82E-14	10.915617	10.845377	10.899475	10.548294	10.513347	10.495602	9.9434622	9.882181	9.9381284
Down	FGSG_00068	-5.103051694	6.81E-08	6.4249728	6.4455482	6.3489364	5.7382218	5.9237087	5.5012819	4.965331	4.8105634	5.0523944
Down	FGSG_00218	-5.126713382	7.47E-07	7.0058827	6.7107812	7.1934618	6.3181765	6.314782	6.2370531	5.626448	5.9891987	5.0523944
Down	FGSG_12518	-5.157498698	7.30E-10	6.7444707	6.7541729	6.650969	5.8995578	5.7557564	5.9550408	5.7358405	5.7038947	5.7064263
Down	FGSG_12214	-5.194189507	5.42E-12	12.077811	12.020239	12.049935	11.816125	11.795263	11.748836	11.181158	11.155487	11.140494
Down	FGSG_00199	-5.26762738	2.36E-09	5.9195042	6.153105	5.5937254	6.2104357	6.29449	6.1029268	5.4450355	5.1041074	5.7064263
Down	FGSG_02208	-5.317071974	8.29E-09	5.2322171	5.1036809	5.3356788	4.5559538	4.51735	4.5218929	3.9093688	3.5358829	4.1665008
Down	FGSG_11600	-5.390682681	1.42E-09	9.0272245	8.9276344	9.0389684	8.6320559	8.6788446	8.4919749	8.3502416	8.2724515	8.3611766
Down	FGSG_00572	-5.538631851	3.07E-14	10.760748	10.786509	10.646805	10.644064	10.5221	10.674719	9.9800553	9.8956703	9.9966375
Down	FGSG_03314	-5.565632963	3.87E-09	5.8597011	5.6546687	5.9664603	5.5565451	5.5656438	5.4652882	4.7032988	4.5233477	4.8129147

Down	FGSG_02111	-5.617829551	8.39E-11	8.8205585	8.9464625	8.5895813	9.1243468	9.0942757	9.0700876	7.9534078	7.8096741	8.024072
Down	FGSG_02627	-5.655518243	2.34E-11	5.7541671	5.6997548	5.728956	5.7689296	5.9237087	5.5012819	5.2862562	5.2552685	5.2577234
Down	FGSG_00416	-5.67796727	0.0011359	8.7120101	8.7135105	8.6246952	11.473661	11.420078	11.439553	6.3772321	6.2272902	6.4552281
Down	FGSG_02168	-5.690968738	4.83E-08	6.3399861	6.1851653	6.4038729	5.7382218	5.423383	5.9288493	5.3564544	4.9352342	5.6345505
Down	FGSG_03073	-5.755096349	6.46E-11	7.7724024	7.7380397	7.7226391	7.2762202	7.1819267	7.2834334	6.8443161	6.8668223	6.7554957
Down	FGSG_11069	-5.761327184	1.00E-08	6.3831052	6.7541729	5.7713555	6.2326372	6.0008798	6.3597635	5.3564544	5.2552685	5.3945636
Down	FGSG_07628	-5.766360759	6.54E-10	6.2496778	6.1851653	6.232359	5.8137994	5.9237087	5.6041695	5.626448	5.7723491	5.3945636
Down	FGSG_07707	-5.783692448	4.81E-09	6.7985581	6.4975416	6.9750363	6.8646813	6.9421186	6.6911344	6.1848562	6.3666582	5.9027806
Down	FGSG_11260	-5.808072363	2.43E-11	5.4095206	5.5600358	5.1553224	4.7220923	4.3699906	4.9442019	4.807216	4.8742451	4.6764038
Down	FGSG_05348	-5.824653597	7.12E-11	8.1712913	8.030488	8.2202735	7.7993721	7.6415662	7.8629285	7.1446385	7.2253567	6.9895734
Down	FGSG_03544	-5.875886819	1.39E-14	15.498556	15.434121	15.477093	14.939891	14.912549	14.880375	14.794325	14.763378	14.759007
Down	FGSG_00219	-5.891305666	1.88E-11	8.8617898	8.7779989	8.8589911	8.4162485	8.3653049	8.3804369	7.9943468	8.0283874	7.8915895
Down	FGSG_03633	-5.915037142	2.93E-11	7.694665	7.5538626	7.7440876	7.2273045	7.1596621	7.2092065	6.8279372	6.6834471	6.9004463
Down	FGSG_11115	-6.001655721	5.77E-08	6.5181831	6.5228522	6.5078422	5.9935634	5.9498937	5.9550408	5.2124668	5.4349486	4.8766132
Down	FGSG_11269	-6.034360091	2.75E-08	6.5437448	6.4975416	6.5078422	5.7068462	5.265544	5.9807654	5.2124668	5.2552685	5.1065306
Down	FGSG_03706	-6.048395103	1.26E-15	9.0272245	9.0282747	8.9402161	8.5701983	8.463998	8.5871558	8.6976524	8.7121554	8.615694
Down	FGSG_03460	-6.095487411	4.14E-14	13.315258	13.236349	13.307186	12.141909	12.064183	12.131275	12.007017	12.016804	11.929492
Down	FGSG_02207	-6.163565871	6.91E-09	6.0500989	6.052414	5.9664603	5.3074061	5.306652	5.2281446	4.5519634	4.6006874	4.4438634
Down	FGSG_09723	-6.201743251	4.25E-13	8.5257457	8.6227812	8.3307216	8.0912658	7.9959056	8.0985166	7.8436345	7.8498277	7.7710623
Down	FGSG_06551	-6.327045177	2.79E-08	8.4054187	8.3030481	8.4266952	7.4755522	7.5157415	7.3441517	6.8605111	6.5726412	7.0460725
Down	FGSG_04957	-6.409417694	1.96E-09	6.1026411	6.0867644	6.0376686	5.6419705	5.7851416	5.3904893	4.807216	4.6006874	4.9376179
Down	FGSG_03708	-6.444744362	2.47E-13	8.3302954	8.3030481	8.3236158	9.1405824	9.1290693	9.082021	8.7658758	8.7592983	8.7061315
Down	FGSG_02206	-6.461006814	3.34E-07	6.4522245	6.5477263	6.3766661	6.60869	6.7166741	6.4908649	4.965331	4.7439404	5.2090653
Down	FGSG_11146	-6.535403936	1.36E-07	6.5688614	6.2772809	6.7602689	5.674773	5.975612	5.3515835	4.7032988	4.8105634	4.5256129
Down	FGSG_13802	-6.852678881	7.98E-10	6.8910817	6.9719742	6.9005856	6.0695346	5.975612	6.2155469	5.7181748	5.8693008	5.5589053
Down	FGSG_11439	-6.855722711	4.35E-13	7.4929307	7.2698061	7.6103354	7.2437947	7.3387849	7.0482091	7.0418719	6.9430453	7.0735134
Down	FGSG_11270	-7.010726591	1.00E-08	6.5437448	6.4975416	6.5078422	6.4471928	6.449356	6.3597635	5.2124668	5.4349486	4.8766132
Down	FGSG_11164	-7.049175963	7.79E-09	7.5189396	7.529095	7.4235657	7.2708663	7.4346181	6.9860594	6.2103535	6.152209	6.2052877
Down	FGSG_09072	-7.062506673	7.37E-11	6.8300632	6.8770274	6.6956874	7.0385763	6.8616483	7.1309525	6.247774	6.2026947	6.2298857
Down	FGSG_08375	-7.217065794	2.37E-14	12.150062	12.143662	12.070244	11.553824	11.522865	11.496949	11.030505	10.993764	11.000974
Down	FGSG_12207	-7.61623525	3.55E-12	11.967925	11.954909	11.89557	11.882133	11.864435	11.811551	10.484634	10.520884	10.378528

Down	FGSG_04662	-7.866919227	1.47E-08	8.3412698	8.4018525	8.1893346	8.2273973	8.0930945	8.2700177	7.0560045	7.1882541	6.8378143
Down	FGSG_03111	-8.468266114	2.11E-08	8.8566999	8.804041	8.8241692	8.9477206	8.8997838	8.9085948	7.5360624	7.3083895	7.6758017
Down	FGSG_12519	-9.726339756	2.07E-09	9.8995477	9.7871639	9.9226205	9.2534644	9.2260057	9.1934739	8.3981791	8.3914885	8.3443783
Down	FGSG_08196	-9.983936941	2.76E-13	11.063767	11.052914	10.988657	11.320783	11.234157	11.319314	10.688325	10.659499	10.650894

EU0711	EU0712	EU0713	EU0721	EU0722	EU0723	EU0731	EU0732	EU0733
6.3629219	6.4485311	6.1555909	6.8270804	6.6698356	6.8844113	7.1602837	7.0783871	7.1809948
7.7139978	7.5180797	7.7885525	8.2432676	8.2802004	8.1087592	8.9829459	8.955489	8.9504216
8.6252371	8.8489176	8.5496682	9.2360768	9.3662124	9.3794636	9.7522099	9.8765638	9.8039335
7.5864427	7.3936035	7.7119993	7.6677959	7.6412146	7.7429259	8.2949317	8.2581076	8.3582982
13.230016	13.177313	13.171535	12.67246	12.657516	12.593877	11.383644	11.342836	11.363858
10.63229	10.598794	10.625306	11.237163	11.22509	11.251563	12.2148	12.268046	12.143568
10.58363	10.551025	10.523213	9.6239728	9.4829516	9.6913826	8.2274073	8.2088033	8.1989157
9.9169252	9.8603926	9.8623265	9.4618202	9.4417665	9.3876817	9.1397564	9.2115874	9.0014124
9.3678858	9.2012073	9.4162269	9.2825405	9.2798198	9.1904066	10.271974	10.309686	10.171444
7.7364297	7.7216552	7.6403649	7.6920956	7.4644915	7.8056057	7.9397344	7.8712914	7.9546658
9.298723	9.2677351	9.218268	8.6177402	8.584062	8.5579336	9.6469005	9.610808	9.6227173
15.353877	15.299721	15.296472	15.043772	14.989615	15.003735	13.859257	13.844935	13.813112
3.7238255	3.5330831	3.8234137	3.3105119	3.1366761	3.4139246	6.0868657	6.0661334	6.0507505
5.0423553	4.7403036	5.2098157	4.0728969	3.5449709	4.4006546	3.396124	3.550228	3.1834889
12.381196	12.359621	12.290591	11.841642	11.789452	11.799429	10.356291	10.348617	10.303567
6.9204083	7.011299	6.7045603	6.7365211	6.5859108	6.8007223	6.2810207	6.1204673	6.3718678
6.3513412	6.3625899	6.2306822	6.603754	6.6561828	6.4546057	7.3220454	7.4618177	7.1163741
3.3106725	3.6824426	2.7091089	4.1551088	4.4101943	3.7561757	4.6747739	4.6925307	4.6052608
5.6456505	5.1003295	5.9634986	5.0916718	5.1163526	4.9811664	4.297674	4.3725921	4.1686253
5.9973125	5.8337256	6.0489734	7.0094171	7.2079771	6.6720606	6.5658347	6.8074002	6.2078724
4.5287539	4.8705531	3.9473196	4.3066864	4.2738034	4.2644835	5.3648568	5.5369839	5.2601706
3.7939455	3.8177796	3.6878577	4.1551088	3.9549305	4.2644835	4.6374231	4.6925307	4.5278714
3.6501227	3.3664558	3.8234137	4.3768933	3.9549305	4.6396102	6.2810207	6.2721744	6.2324729
5.3288274	5.4310687	5.1072743	6.5439776	6.4500929	6.6172014	6.141248	6.1728291	6.1314494
6.8726006	6.7822257	6.8545413	7.6251628	7.6412146	7.5143331	7.868986	8.0216212	7.6330084
11.390257	11.334505	11.335989	12.400633	12.343978	12.363553	9.2412799	9.2084553	9.2170683
4.7209942	4.8705531	4.4445501	6.4164385	6.5271198	6.2170221	4.3904012	4.075324	4.6052608
7.6011853	7.5378203	7.6311552	7.8236076	7.8654167	7.7170711	6.2565941	6.2959775	6.3938449
3.4903583	3.5330831	3.5382314	3.5722723	4.0414954	2.7768658	3.6422468	3.9608643	3.1834889
3.4903583	3.5330831	3.3712718	4.1145884	4.4738406	3.5372523	5.0606888	5.0128652	5.3072501

8.8619562	8.7338248	8.8763293	9.3528151	9.2020857	9.4019517	10.424348	10.351459	10.435234
8.6443435	8.5899608	8.5882516	9.4506727	9.3952677	9.4120588	9.3448426	9.3344796	9.2979636
4.9222583	5.4310687	3.9473196	3.9857156	3.6588876	4.1912539	3.5647792	2.7162254	4.0628589
10.974167	10.879696	10.956446	10.927137	10.791472	10.96277	11.536664	11.48681	11.52563
8.0689329	7.9000247	8.1201396	6.5053192	6.6698356	6.2170221	6.0729437	6.247972	5.8104792
10.921299	10.851381	10.882696	10.99498	10.972441	10.923035	11.20249	11.181288	11.163353
3.9247213	3.6824426	4.1671548	3.8929252	3.7644639	3.9462135	4.8805969	4.6189492	5.0547979
3.1053801	2.7060624	3.3712718	3.8441915	3.9549305	3.6508626	4.2489753	4.6189492	3.8247709
11.484381	11.436617	11.450368	11.115062	11.120848	11.063029	11.290467	11.3198	11.24572
8.4104693	8.2383812	8.4640832	8.294047	8.1401005	8.3465475	8.3038995	8.2940106	8.2778553
2.9906265	2.7060624	3.1824306	4.4761857	3.9549305	4.7959824	4.1985751	4.075324	4.2671646
7.5962878	7.7641735	7.2768781	7.9208146	7.9121154	7.8359532	8.6376231	8.7162536	8.4916569
3.3106725	3.3664558	3.1824306	5.4089726	5.1163526	5.5775942	5.8944734	6.1728291	5.4815611
4.2581217	4.0554482	4.357879	4.7650729	4.4738406	4.9370509	3.6422468	3.550228	3.6891623
3.1053801	2.9612664	3.1824306	3.4473206	3.421282	3.4139246	4.0921636	4.2801452	3.8247709
3.7238255	3.6824426	3.8234137	4.6278481	4.123159	4.9370509	5.0606888	4.6189492	5.3528418
5.205611	5.4726864	5.5203094	5.2866467	5.6447666	4.8445541	4.7813527	5.1756805	4.6787093
7.8144138	7.9076311	7.5937171	8.3989996	8.3665709	8.338091	9.0178829	9.0354349	8.9429878
7.8436378	7.7388132	7.8454066	7.2760325	7.3333746	7.1196804	7.9511957	7.9559037	7.8865467
6.1115561	6.6064294	6.5951447	4.9375947	5.3715813	5.3271781	6.3403369	6.4734607	6.4785512
9.6418754	9.5414831	9.6301111	9.3914868	9.3662124	9.3227321	9.9451105	9.8506802	9.9758755
9.4075665	9.3895256	9.3134611	9.6076414	9.6176099	9.5018653	10.113535	10.116019	10.050409
4.7912485	4.3515565	5.0531343	4.4438416	4.34361	4.4642052	3.7157657	3.3824986	3.9487208
4.2076351	4.1610493	4.1671548	5.3921198	5.0759055	5.5775942	5.7088847	5.65212	5.7089498
7.6011853	7.5081072	7.5842038	7.3345391	7.2630586	7.3133055	5.9715359	5.8896564	5.9943291
10.987788	10.973278	10.889471	11.093723	11.035758	11.057245	9.9403259	9.9019911	9.9182823
3.7238255	3.6824426	3.6878577	3.1593576	3.1366761	3.1302769	4.0921636	3.9608643	4.1686253
3.3106725	2.9612664	3.5382314	2.9904928	2.9701107	2.9643661	4.1463502	3.550228	4.5278714
3.9247213	4.2594452	3.3712718	3.4473206	3.8628381	2.7768658	3.8524369	3.3824986	4.1686253
4.3997744	4.6704862	3.9473196	4.2702587	4.2004465	4.2644835	4.7813527	4.5414122	5.0547979
8.1712755	8.1254995	8.1462347	8.063355	8.2071765	7.9289855	8.3594365	8.3462435	8.3968979

10.31366	10.254124	10.261957	8.2665709	8.0800152	8.3465475	5.5165681	5.4964687	5.4815611
6.1387573	5.9851925	6.1810581	6.4330113	6.3518802	6.4229682	5.7088847	5.5763924	5.7774236
4.1553175	3.9415025	4.2656663	4.0299647	3.7644639	4.1912539	4.6747739	4.8293101	4.4460942
6.0120976	5.9264999	5.9925565	6.0588757	5.9434397	6.0817401	6.206464	6.0661334	6.28045
3.6501227	3.5330831	3.6878577	4.0299647	4.4738406	3.4139246	2.7248895	3.3824986	2
2.8659503	3.1780396	2.3976902	3.3805373	3.5449709	3.1302769	4.3904012	5.123424	2.7098437
4.684541	4.5198165	4.7469892	4.7386523	4.34361	4.9811664	4.3904012	4.1813664	4.5278714
6.9125496	6.8149532	6.9012594	6.0800756	5.8505866	6.1984556	6.0012441	6.0381793	5.9053308
9.0202546	8.9407503	8.98876	9.2230325	9.2184358	9.1329025	8.064585	8.2212882	7.8231723
11.750776	11.671986	11.717852	11.812053	11.765411	11.764356	12.047575	12.038182	11.996472
7.0115184	7.0936294	6.8062596	7.0529559	7.0075186	7.0068364	5.3184099	5.72407	4.6787093
8.5385372	8.5516358	8.4112605	8.7476278	8.744272	8.6598451	9.6713609	9.5101044	9.7601898
3.4903583	3.6824426	3.1824306	4.1945222	4.34361	3.9462135	4.912217	4.6925307	5.0547979
5.3288274	5.2984878	5.2584768	5.6828387	5.9434397	5.2925838	5.2946134	5.123424	5.3970366
8.4104693	8.3261393	8.3841057	8.6411083	8.6336719	8.5542877	9.2459407	9.0909152	9.3296632
13.283648	13.29997	13.151746	12.546156	12.437982	12.557005	11.737286	11.615939	11.79186
7.7185122	7.8376847	7.4648642	7.2482078	7.1604128	7.2425732	5.2458095	5.1756805	5.2601706
2.7294717	2.3958007	2.9650943	3.630911	3.8628381	3.2790605	3.4829147	3.9608643	2.7098437
3.403311	3.3664558	3.5382314	3.8929252	3.9549305	3.7561757	4.2489753	3.7004723	4.6052608
5.7195764	5.7345728	5.5979955	6.1418678	6.0093567	6.179647	5.3648568	5.3219704	5.3970366
3.4903583	3.3664558	3.5382314	3.8929252	3.5449709	4.2644835	4.1463502	4.1813664	4.0628589
8.1613618	8.0991683	8.1135415	8.8250108	8.7116431	8.841333	5.7791646	5.6885435	5.8104792
9.1157527	9.0235898	9.0964088	8.5884498	8.5475519	8.5359188	7.0076424	7.1055801	6.8404516
5.856951	5.9264999	5.6717113	5.2682931	5.4994188	4.8915437	5.6350052	5.4964687	5.7089498
4.354085	4.0554482	4.5263082	5.7896485	5.7254286	5.7659038	6.4734725	6.7738909	6.0228156
3.9859168	3.5330831	4.2656663	4.2702587	3.6588876	4.6396102	3.9162052	3.550228	4.1686253
10.729988	10.696435	10.651733	11.704606	11.693713	11.62097	10.041195	10.061514	9.9593904
9.0018545	8.9258451	8.9669639	8.9381791	8.8318921	8.9479907	9.9044174	9.8981083	9.8503688
8.2293682	8.2800001	8.0596406	8.6446701	8.6406218	8.5542877	8.1891988	8.3116326	7.9911646
3.6501227	3.6824426	3.5382314	3.9857156	3.421282	4.334175	3.9772738	4.2801452	3.5394718
7.1110155	7.011299	7.1012589	6.7498114	6.6561828	6.7506259	4.5990795	4.9542373	4.0628589

6.3396668	6.271203	6.3020577	6.882432	6.7872233	6.8844113	7.0508988	7.0224134	7.020748
2.7294717	3.1780396	2	3.6872592	4.0414954	3.1302769	2.7248895	2.4021099	2.9660172
2.4103584	2	2.7091089	3.5111488	3.7644639	3.1302769	3.9162052	3.8365351	3.9487208
10.13692	9.9824624	10.174322	10.286475	10.266211	10.212313	9.3126228	9.278828	9.2862616
6.4631658	6.4896552	6.3250858	7.2340916	7.2357807	7.1392818	5.9102187	6.0935561	5.6370634
7.5207575	7.5572944	7.3683991	7.5849438	7.5481843	7.5292676	8.0610631	8.1447046	7.9096123
2.8659503	2.9612664	2.7091089	3.5111488	3.9549305	2.7768658	3.0991973	2.7162254	3.3724324
5.9366088	5.9851925	5.8410348	6.3912169	6.2824824	6.4229682	6.015872	6.2721744	5.6734543
7.242213	7.2573647	7.1536545	6.953078	6.8723477	6.9524728	8.2305462	8.2336661	8.1989157
9.5448058	9.4217559	9.554181	9.6293758	9.5998899	9.5648261	7.424275	7.3411675	7.445826
11.423471	11.422749	11.310975	11.539847	11.471054	11.514297	12.471477	12.38987	12.49032
15.924333	15.882014	15.855129	16.441963	16.392332	16.397602	16.730712	16.703391	16.69778
2.9906265	2.9612664	2.9650943	3.4473206	3.2859859	3.6508626	2.7248895	2.7162254	2.9660172
8.7583455	8.8370163	8.554548	8.5547713	8.525194	8.490855	9.1727666	9.0909152	9.1922014
2.5787203	2.3958007	2.7091089	5.3921198	5.2676733	5.4262461	2.4075018	2.7162254	2
9.3300359	9.2998828	9.2486961	8.5299496	8.5912544	8.3674744	8.7815338	8.7337883	8.7689328
9.5561998	9.5018304	9.4995599	9.6499019	9.6298857	9.5756572	10.223172	10.19093	10.195104
8.0075444	8.1123939	7.7718883	7.2621872	7.307406	7.1196804	6.4945145	6.2959775	6.6157808
2.7294717	3.1780396	2	3.1593576	3.421282	2.7768658	3.3037759	2.9740279	3.5394718
6.8315138	6.6250049	6.931585	6.6751515	6.6561828	6.6031543	6.515254	6.4310946	6.5389689
5.3050117	5.251441	5.2584768	5.5971992	5.2313049	5.8154814	5.3648568	5.3676132	5.4399178
5.1796526	5.1524786	5.1072743	6.0481578	5.7768019	6.2170221	6.9170326	6.7221209	7.0348031
3.1053801	3.5330831	2.3976902	4.3422168	4.4738406	4.1141074	5.4534919	5.5763924	5.2601706
6.8806794	6.8936642	6.7563059	6.3481756	6.3001473	6.3064312	6.141248	6.1468857	6.0781548
2.5787203	2.3958007	2.7091089	3.9400663	3.8628381	3.9462135	5.4318384	5.1756805	5.5997308
4.2076351	4.4381381	3.8234137	3.4473206	3.7644639	2.9643661	3.5647792	3.1926715	3.8247709
7.6445303	7.5860213	7.5937171	7.1908948	7.0706336	7.2151282	6.2930807	6.3193942	6.2078724
8.7627235	8.6859724	8.7416101	8.4689765	8.4281163	8.416494	6.5757415	6.7395848	6.3268829
12.487182	12.454848	12.407806	12.000879	11.944221	11.96373	10.903372	10.841499	10.903856
6.5055765	6.5685453	6.3250858	7.4034351	7.4085881	7.3046513	8.2147833	8.1835045	8.1863784
12.213229	12.141104	12.173747	11.889335	11.860006	11.824618	12.143946	12.13745	12.090727

2.7294717	2.9612664	2.3976902	2.5786313	2.7817736	2.30778	3.2051098	3.3824986	3.8247709
5.07088	5.251441	4.7469892	5.5971992	5.4046183	5.6881887	6.1545292	6.0096728	6.2324729
5.1262912	4.6704862	5.3953249	4.3066864	4.4738406	4.0326014	3.2051098	3.550228	2.7098437
7.083281	6.9536802	7.1012589	7.7428277	7.668673	7.7235784	8.4698033	8.4717217	8.4077393
3.403311	2.9612664	3.6878577	2.5786313	2	2.9643661	3.5647792	3.7004723	3.3724324
5.6456505	6.013667	4.9968831	7.3078306	7.1112267	7.3971067	8.29193	8.3290418	8.1926607
13.897387	13.86351	13.862418	14.005677	14.001818	13.948393	14.496734	14.499697	14.451927
7.4078966	7.3383921	7.3683991	7.4448821	7.3504308	7.4451462	7.1337051	6.9789602	7.2306851
5.6072167	5.1524786	5.872639	5.9594213	5.9877173	5.8634117	3.396124	2.9740279	3.8247709
15.913992	15.868254	15.848098	15.467916	15.420763	15.420691	14.376405	14.377693	14.314572
5.9210252	5.7345728	5.9925565	6.6958995	6.5120399	6.7758915	6.2930807	5.9509123	6.5191095
6.385808	6.3402801	6.537159	6.4493958	6.7100355	6.2534533	4.8151984	5.1756805	4.3594016
3.6501227	3.9415025	3.1824306	4.4107557	4.0414954	4.7959824	3.8524369	4.075324	3.5394718
3.5724509	3.3664558	3.6878577	3.3105119	2.7817736	3.6508626	3.7157657	3.3824986	3.9487208
12.326057	12.231383	12.308395	11.274999	11.26836	11.186517	10.19934	10.186161	10.152232
10.606948	10.491532	10.60898	11.16304	11.102717	11.130079	12.14353	12.082909	12.143162
9.6840324	9.5824532	9.6800873	11.248911	11.252569	11.150748	10.042088	10.127673	9.8895671
2.8659503	3.1780396	2.3976902	4.1945222	4.123159	4.1912539	2.5749062	2	2.9660172
12.907744	12.890171	12.812514	12.444205	12.36904	12.424735	11.153306	11.119266	11.131007
12.055351	12.034845	11.964191	11.774751	11.692877	11.761196	11.960934	11.988833	11.874778
3.6501227	3.1780396	3.9473196	3.3105119	3.6588876	2.7768658	3.4829147	3.7004723	3.1834889
3.6501227	3.6824426	3.5382314	3.1593576	3.421282	2.7768658	3.4829147	3.550228	3.3724324
3.2116748	3.3664558	2.9650943	4.2702587	3.8628381	4.5250741	2.9848891	2.7162254	3.1834889
3.6501227	3.9415025	3.1824306	2.6931321	2.5651052	2.7768658	2.7248895	2.4021099	2.9660172
3.1053801	3.3664558	2.7091089	2.7992097	2.5651052	2.9643661	4.297674	4.3725921	4.1686253
5.8238141	5.6280992	5.9634986	5.1328319	4.9914418	5.1835263	4.6374231	4.6189492	4.6052608
13.415305	13.333534	13.385361	13.138357	13.108914	13.073317	12.785152	12.798056	12.711521
3.2116748	3.5330831	2.7091089	2.9904928	2.7817736	3.1302769	3.8524369	3.8365351	3.8247709
4.5287539	4.0554482	4.8136363	5.0493029	5.1556966	4.8445541	5.5770017	5.5763924	5.5220361
11.932495	11.875257	11.883592	11.245396	11.212319	11.189458	11.5518	11.48681	11.561874
4.3069011	4.1610493	4.357879	5.3403469	5.4046183	5.1835263	5.5369952	4.9542373	5.9053308

2.4103584	2	2.7091089	3.1593576	2.5651052	3.5372523	3.3037759	3.3824986	3.1834889
7.1382268	7.0392678	7.1276945	7.2668171	7.2173046	7.2243347	6.8369191	6.9492469	6.6527056
5.5063989	5.5524845	5.3511356	6.6541008	6.5714361	6.6448917	6.8695003	6.96418	6.7063792
2.4103584	2.3958007	2.3976902	3.1593576	3.2859859	2.9643661	3.396124	3.550228	3.1834889
3.7939455	3.3664558	4.0614204	3.630911	3.8628381	3.5372523	2.7248895	2.9740279	2.3981461
11.18498	11.113895	11.145382	10.2779	10.285184	10.18192	10.261271	10.24543	10.219921
3.7238255	3.9415025	3.3712718	3.6872592	3.421282	3.8543214	3.9772738	3.7004723	4.1686253
11.940005	11.883959	11.885046	12.304837	12.240848	12.274954	12.659052	12.640227	12.617758
11.013733	10.982311	10.933216	11.201031	11.164297	11.143487	10.302123	10.280155	10.263843
10.372134	10.36464	10.266426	10.594301	10.566367	10.528862	10.75759	10.722489	10.73335
7.0692112	7.2211953	6.77315	6.5816271	6.4500929	6.6172014	5.3875313	5.0692034	5.5997308
7.9964347	7.8376847	8.038897	7.9383337	7.8831063	7.9005521	6.9780669	6.8879188	7.0065546
5.0132553	4.6704862	5.2098157	6.0800756	6.3175986	5.6881887	6.5458147	6.2233567	6.7581273
11.785075	11.747572	11.711858	11.287584	11.200021	11.279986	11.538565	11.513704	11.50306
3.9859168	3.8177796	4.0614204	4.1945222	4.2004465	4.1141074	3.9162052	3.550228	4.1686253
2.4103584	2.7060624	2.3976902	3.0773944	3.5449709	2.5613046	3.0991973	3.550228	2.7098437
9.2805199	9.0986107	9.344785	9.7342283	9.6930917	9.6813894	10.012306	9.964594	9.9993595
12.288218	12.248331	12.217093	11.608253	11.569024	11.557735	11.738113	11.725718	11.691738
9.3664483	9.3199737	9.3047989	9.546126	9.5490837	9.4478692	9.5166768	9.4316936	9.5389061
15.783911	15.778997	15.675143	15.017227	14.897878	15.038444	14.037732	13.936632	14.074613
13.586446	13.524962	13.536677	13.04887	12.981448	13.021446	13.350902	13.310639	13.331408
10.294134	10.293847	10.180649	10.702495	10.625364	10.687889	10.879075	10.805676	10.893288
2.8659503	3.1780396	2.3976902	3.1593576	2.5651052	3.5372523	3.7157657	2.7162254	4.2671646
3.3106725	2.9612664	3.5382314	4.1145884	4.0414954	4.1141074	3.7857191	3.8365351	3.6891623
8.578819	8.5564825	8.4897866	8.8437249	8.8104809	8.7833373	9.3535063	9.4073145	9.2354411
5.6456505	5.3440487	5.9338433	3.6872592	3.7644639	3.7561757	3.7857191	4.1813664	3.1834889
5.0988516	5.1524786	4.9383494	4.6563572	4.649489	4.5834785	5.0606888	4.7625408	5.2601706
3.3106725	2.9612664	3.5382314	3.9857156	4.0414954	3.8543214	3.2051098	2.7162254	3.6891623
3.2116748	2.9612664	3.3712718	5.322668	5.3715813	5.1835263	2.8607385	3.1926715	2.3981461
4.487029	4.5198165	4.357879	5.4420989	5.0342917	5.6881887	5.7267783	5.4964687	5.8744013
8.0185692	7.8691914	8.0527592	8.1258522	7.9684402	8.1814653	8.3623011	8.317459	8.3470774

7.908293	7.9302128	7.7718883	7.5049019	7.4328124	7.4992425	6.0012441	5.7587426	6.1573762
7.5564976	7.5954712	7.4012761	7.1563797	7.0706336	7.1489836	6.1278434	6.0381793	6.1573762
4.2581217	4.3515565	4.0614204	3.6872592	3.2859859	3.9462135	3.7857191	2.9740279	4.2671646
3.403311	3.5330831	3.1824306	4.3768933	3.9549305	4.6396102	4.1985751	3.9608643	4.3594016
6.4083367	6.5097858	6.1810581	6.7298298	6.7996958	6.5601733	6.9780669	6.9492469	6.9483421
2.5787203	2	2.9650943	3.4473206	3.2859859	3.5372523	3.3037759	3.550228	2.9660172
3.1053801	3.5330831	2.3976902	2.7992097	2.3100466	3.1302769	3.0991973	3.3824986	2.7098437
13.407813	13.345485	13.359105	12.618918	12.557784	12.586087	12.89593	12.865725	12.865679
3.403311	3.9415025	2.3976902	3.0773944	2.7817736	3.2790605	2.7248895	2.7162254	2.7098437
2.5787203	2	2.9650943	3.5722723	3.2859859	3.7561757	3.4829147	3.7004723	3.1834889
3.5724509	2.9612664	3.9473196	2.7992097	2.5651052	2.9643661	3.5647792	3.7004723	3.3724324
3.5724509	3.6824426	3.3712718	3.630911	4.0414954	2.9643661	3.3037759	2	3.9487208
2.7294717	3.1780396	2	4.8661672	4.7557305	4.8915437	2	2	2
5.5475752	5.8337256	5.1072743	7.2102533	6.8841084	7.4052252	8.0539932	8.0358273	8.019713
10.668905	10.632463	10.593653	10.140861	10.054855	10.131429	10.939785	10.956112	10.862096
3.7939455	3.8177796	3.6878577	4.2328874	3.8628381	4.4642052	4.0358621	4.2801452	3.6891623
6.5055765	6.271203	6.6139666	6.6398944	6.6833604	6.500798	5.0886338	4.9542373	5.1611356
3.6501227	3.3664558	3.8234137	3.3805373	3.2859859	3.4139246	3.6422468	3.550228	3.6891623
11.561789	11.494586	11.517507	10.984829	10.945334	10.930789	11.361981	11.409135	11.251749
3.2116748	2.7060624	3.5382314	3.5722723	3.8628381	3.1302769	2.8607385	2.4021099	3.1834889
6.2301152	6.013667	6.3250858	7.4448821	7.5183093	7.2695058	7.8929564	7.8633475	7.8631063
3.1053801	2.7060624	3.3712718	3.3805373	2.5651052	3.8543214	3.3037759	3.550228	2.9660172
4.7912485	4.5971173	4.9383494	5.6828387	5.4994188	5.7659038	6.7951299	6.9034969	6.6157808
2.8659503	2.9612664	2.7091089	3.8441915	3.8628381	3.7561757	4.711182	5.1756805	3.9487208
5.0423553	5.1003295	4.8773401	4.4761857	4.34361	4.5250741	3.7857191	3.1926715	4.1686253
3.7238255	4.0554482	3.1824306	3.4473206	3.2859859	3.5372523	2.5749062	2.4021099	2.7098437
12.008655	11.970027	11.936421	11.332443	11.304364	11.266123	11.251211	11.18448	11.257005
8.4160387	8.376341	8.4112605	8.9294453	8.9953352	8.8650716	8.6657226	8.5628341	8.7856528
5.9973125	5.9851925	5.9035657	5.9363573	5.7768019	5.9983304	5.9102187	5.9509123	5.8104792
10.539025	10.480099	10.48666	10.697788	10.687925	10.61451	10.222384	10.137587	10.244319
12.577238	12.512727	12.530522	11.804311	11.80268	11.71173	11.897921	11.882799	11.852632

5.7551657	5.4726864	5.9035657	5.6689139	5.5593263	5.6881887	6.9780669	6.96418	6.9483421
11.806966	11.722994	11.78063	11.412709	11.36437	11.366728	11.431482	11.394739	11.407722
4.0446218	3.9415025	4.0614204	4.3066864	4.34361	4.334175	3.4829147	3.550228	3.3724324
7.0621245	6.9239842	7.0878571	6.6541008	6.7872233	6.4068854	5.0606888	4.8931255	5.1611356
4.569306	4.6704862	4.357879	4.6278481	4.4738406	4.6936396	3.6422468	3.3824986	3.8247709
12.275062	12.169063	12.268115	12.040623	12.031742	11.954424	11.378347	11.38298	11.314304
9.5849153	9.5871976	9.4688713	8.8879732	8.9173585	8.7613687	8.6065514	8.5280819	8.623058
8.174565	8.0991683	8.139755	8.6249708	8.5138837	8.6427798	8.7556214	8.5823229	8.8546272
4.6471428	3.9415025	5.0531343	4.6278481	4.5347973	4.6396102	5.6538328	5.8580248	5.3528418
10.599625	10.657492	10.646029	10.280192	10.404309	10.288902	10.311046	10.352879	10.386742
3.9859168	4.1610493	3.6878577	6.4412268	6.4968007	6.2889872	3.7157657	4.075324	3.1834889
3.4903583	3.1780396	3.6878577	3.2369134	3.1366761	3.2790605	3.2051098	3.550228	2.7098437
5.0988516	4.990011	5.1072743	6.1113025	6.0928076	6.040638	7.1536848	7.3181044	6.9030876
15.018283	14.962448	14.962812	14.822293	14.775006	14.775289	13.287231	13.266332	13.247712
3.3106725	3.6824426	2.7091089	3.630911	3.7644639	3.4139246	4.0358621	3.9608643	4.0628589
3.1053801	3.6824426	2	2.9904928	3.1366761	2.7768658	2.5749062	2.4021099	2.7098437
8.1909008	8.058748	8.2094791	8.4115977	8.5326852	8.1767306	9.2177477	9.1958583	9.1796054
13.108872	13.082001	13.023874	12.716067	12.665418	12.672707	12.5183	12.449454	12.525585
6.7544736	6.4692397	6.9165019	6.8270804	6.7746421	6.8007223	7.092896	7.0645959	7.0896929
2.5787203	2.3958007	2.7091089	2.6931321	2.3100466	2.9643661	3.2051098	2.9740279	3.3724324
6.0838321	6.1986514	5.8410348	6.7695201	6.8120614	6.631113	4.4775267	4.5414122	4.3594016
4.756549	4.8705531	4.5263082	4.9375947	4.9017239	4.8915437	3.4829147	3.7004723	3.1834889
3.6501227	3.8177796	3.3712718	2.4542536	2.3100466	2.5613046	2.2180911	2	2.3981461
2.9906265	2.9612664	2.9650943	3.2369134	3.2859859	3.1302769	3.5647792	3.8365351	3.1834889
12.405897	12.381295	12.410164	11.833278	11.776101	11.890438	12.260046	12.242243	12.268147
3.7939455	3.3664558	4.0614204	3.8441915	3.5449709	4.0326014	4.2489753	4.4594701	3.9487208
8.367999	8.1767624	8.4846824	7.876063	7.888955	7.7808596	7.3337686	7.3181044	7.2904838
3.2116748	3.5330831	2.7091089	2.5786313	2.5651052	2.5613046	2.8607385	2	3.3724324
12.95681	12.990951	13.045916	12.576309	12.696598	12.651534	12.947748	13.033519	12.992844
6.0412208	5.7683866	6.1810581	5.8277479	5.6990392	5.8634117	3.8524369	4.1813664	3.3724324
3.7939455	3.6824426	4.0614204	4.960639	5.0759055	4.7959824	5.0886338	4.1813664	5.5997308

11.468388	11.422086	11.403131	10.983072	10.929814	10.942691	11.218782	11.229982	11.146462
3.403311	2.9612664	3.6878577	4.5690822	4.9472802	3.9462135	4.1463502	3.8365351	4.3594016
6.7278514	6.3845599	6.9165019	8.0660138	7.9684402	8.0684304	3.7157657	3.3824986	3.9487208
4.756549	4.8705531	4.8773401	5.3403469	5.0759055	5.6339509	4.746694	5.0692034	4.2671646
11.052689	10.98141	11.012493	11.128797	11.015851	11.144094	11.685331	11.659023	11.652397
4.9532328	4.9315181	4.8773401	5.4420989	5.2676733	5.5189462	5.3418203	5.2748363	5.3528418
2.7294717	3.1780396	2	3.4473206	2.5651052	3.9462135	2.9848891	2.9740279	2.9660172
11.996393	11.940669	11.940621	11.218463	11.211152	11.130691	11.400464	11.336394	11.402989
2.9906265	2.9612664	2.9650943	2.7992097	2.9701107	2.5613046	3.396124	3.550228	3.1834889
3.1053801	2.9612664	3.1824306	3.4473206	3.2859859	3.5372523	3.2051098	3.1926715	3.1834889
12.568825	12.4529	12.57142	12.039101	12.03669	11.946449	12.265029	12.332044	12.132581
3.5724509	3.3664558	3.6878577	3.9400663	4.0414954	3.7561757	5.4534919	5.4118562	5.4399178
6.8644763	6.7655792	6.8545413	6.6821007	6.5859108	6.6854555	5.0886338	5.123424	5.0547979
2.8659503	2.3958007	3.1824306	3.3105119	3.2859859	3.2790605	3.2051098	2.7162254	3.5394718
10.516518	10.488999	10.455693	10.123098	10.165003	10.052216	10.37485	10.359953	10.367435
2.9906265	3.5330831	2	3.5111488	3.8628381	3.4139246	3.0991973	3.3824986	2.7098437
3.5724509	3.1780396	3.8234137	3.630911	4.0414954	3.2790605	3.4829147	3.3824986	3.5394718
12.968702	12.940399	12.887429	12.177387	12.106433	12.155808	12.516855	12.468562	12.506485
5.8404777	6.0415904	5.4798392	5.8402274	5.5296835	6.040638	5.1429507	5.123424	5.1089463
5.0988516	5.1524786	5.0531343	4.9833211	5.0759055	5.0239729	3.9162052	4.075324	3.6891623
13.671996	13.613959	13.618469	12.975639	12.941203	12.915663	13.068651	13.019737	13.056875
5.1262912	4.8705531	5.2584768	5.4089726	5.0342917	5.6339509	4.559689	4.6189492	4.4460942
5.3050117	5.3440487	5.1594558	5.7505157	5.5883723	5.8154814	6.3050406	6.1728291	6.3718678
2.5787203	3.1780396	2	3.0773944	3.6588876	2.7768658	4.8151984	5.2748363	5.1089463
6.5769214	6.3845599	6.6508895	7.5886467	7.6202724	7.4608104	6.2442239	6.3193942	6.1050482
10.396989	10.394721	10.285629	10.662647	10.590794	10.639673	9.7145952	9.7573177	9.6110382
10.017437	10.086652	9.8244452	10.438871	10.383746	10.399713	10.755413	10.667665	10.781033
3.403311	2.7060624	3.8234137	3.3805373	3.421282	3.2790605	3.6422468	3.7004723	3.5394718
3.403311	3.9415025	2.3976902	3.4473206	3.7644639	2.9643661	3.9772738	3.8365351	4.0628589
12.735255	12.988712	12.908546	11.978826	12.225907	12.257123	12.292885	12.502524	12.501433
3.403311	3.3664558	3.3712718	3.8441915	3.9549305	3.6508626	3.3037759	3.550228	3.1834889

12.8246	12.82187	12.713454	12.262288	12.229369	12.200811	12.402008	12.378071	12.365546
12.307074	12.208472	12.293148	11.709078	11.673948	11.650298	12.078606	12.075622	12.021392
7.7230125	7.5180797	7.8050264	8.0310609	8.0385214	7.9346056	5.3875313	5.4547826	5.2601706
7.7230125	7.8691914	7.4334205	7.7494592	7.6956185	7.7105343	7.0366236	6.8721706	7.1295318
13.718022	13.714684	13.607799	14.099746	14.037059	14.067957	14.117572	14.088528	14.086364
12.04685	12.027882	11.953147	11.683984	11.643999	11.630524	11.671828	11.629469	11.653536
11.975564	11.899301	11.940155	11.475055	11.389857	11.465633	11.829445	11.794695	11.803708
2.2197205	2	2.3976902	3.741489	3.8628381	3.5372523	2.8607385	2.4021099	3.1834889
12.16505	12.123248	12.095549	11.640942	11.580815	11.60652	11.751013	11.673861	11.766289
4.7209942	4.3515565	4.9383494	4.4761857	4.2738034	4.5834785	5.0886338	5.0692034	5.0547979
9.1242835	8.9917384	9.1425104	10.088863	10.071666	10.011582	10.707782	10.734515	10.619053
13.216117	13.150917	13.169747	12.415304	12.392646	12.345473	12.437924	12.428071	12.387961
10.324065	10.243644	10.292948	10.24014	10.169815	10.215779	11.443367	11.434154	11.392794
13.748761	13.667162	13.71847	13.264163	13.245817	13.188044	13.559123	13.538028	13.520108
7.8059541	7.7808361	7.7207086	7.6920956	7.60614	7.684087	6.9092185	6.8401489	6.9183307
3.2116748	3.3664558	2.9650943	4.0728969	4.2738034	3.7561757	2.7248895	2.7162254	2.7098437
6.8887132	6.9089038	6.77315	7.141331	7.1311028	7.0592256	6.7951299	6.6500711	6.8721095
3.3106725	3.6824426	2.7091089	2.9904928	2.9701107	2.9643661	2.7248895	2.7162254	2.7098437
3.4903583	3.3664558	3.6878577	3.9857156	4.2004465	3.6508626	3.8524369	3.7004723	3.9487208
12.254614	12.219209	12.185984	11.605742	11.611206	11.528303	12.121507	12.156266	12.038931
5.1796526	5.1524786	5.1072743	5.1328319	5.4369156	4.6396102	4.6374231	4.7625408	4.4460942
2.7294717	2.7060624	2.7091089	2.8980192	2.9701107	2.7768658	3.0991973	3.1926715	2.9660172
7.2040911	7.1069057	7.1917391	7.1957589	6.9302155	7.3389609	6.015872	5.825684	6.1314494
12.711956	12.642326	12.670271	12.321344	12.261565	12.288459	12.530767	12.509221	12.493185
6.7367801	6.6793379	6.686891	6.5209071	6.4018221	6.545557	6.6050601	6.5742331	6.577886
5.9052715	5.7345728	5.9634986	5.322668	5.4369156	5.1059542	4.8151984	4.9542373	4.6052608
13.296254	13.223183	13.257652	12.447905	12.391102	12.410437	12.769052	12.767522	12.710153
12.592536	12.551782	12.521516	11.965538	11.957501	11.878825	12.246543	12.203832	12.228951
10.271271	10.216342	10.214958	9.7851395	9.7277707	9.7499307	9.4264896	9.4609414	9.3296632
13.289728	13.165266	13.300401	12.852742	12.836521	12.77434	12.829428	12.819257	12.779354
6.152168	6.0415904	6.1810581	6.3217206	6.1327901	6.4546057	6.7433306	6.7395848	6.7581273

3.7238255	3.6824426	3.6878577	4.0728969	4.123159	3.9462135	2.9848891	2.9740279	2.9660172
13.29219	13.241168	13.23161	12.532163	12.502349	12.467765	12.756177	12.733335	12.718613
9.0968054	9.0818998	9.0031099	9.2372568	9.1174347	9.2592061	10.058078	10.063249	9.9921743
3.3106725	2.9612664	3.5382314	3.8929252	4.123159	3.5372523	3.5647792	2.9740279	3.9487208
10.531348	10.44263	10.5082	10.584598	10.583599	10.490268	11.580375	11.565421	11.534931
12.325687	12.275775	12.264025	11.746023	11.698716	11.700641	12.018361	12.035091	11.940178
3.6501227	3.5330831	3.6878577	3.6872592	3.5449709	3.7561757	3.5647792	3.8365351	3.1834889
5.1796526	5.4726864	4.6771135	4.4761857	4.34361	4.5250741	3.0991973	3.3824986	2.7098437
4.2076351	4.1610493	4.1671548	3.630911	4.123159	2.7768658	2.2180911	2	2.3981461
3.6501227	3.5330831	4.0614204	3.5722723	3.5449709	3.5372523	3.5647792	3.7004723	3.3724324
7.7003693	7.6509067	7.6403649	7.5127192	7.5846785	7.3389609	7.0650342	7.0920476	6.9777419
5.1262912	5.1003295	5.0531343	5.0706429	4.9017239	5.1452616	4.559689	4.3725921	4.748599
2.4103584	2.3958007	2.3976902	2.6931321	2.9701107	2.30778	3.2051098	2.4021099	3.6891623
9.7691799	9.7420002	9.6912081	9.9035861	9.8452333	9.8677103	10.852326	10.802563	10.841254
5.4640127	5.3882147	5.4382008	5.6406543	5.7254286	5.4578126	4.0921636	4.075324	4.0628589
3.7238255	3.8177796	3.5382314	3.1593576	3.2859859	2.9643661	3.396124	3.3824986	3.3724324
8.533422	8.4820153	8.4744196	9.3175479	9.3019078	9.2389086	9.7412482	9.7138015	9.7085169
5.0132553	5.0462245	4.8773401	4.8903714	4.34361	5.2208023	3.6422468	2.7162254	4.1686253
10.776192	10.713894	10.727147	11.266933	11.192356	11.246488	11.436588	11.3906	11.422497
2.8659503	3.1780396	2.3976902	3.8441915	3.5449709	4.0326014	2	2	2
12.76788	12.7143	12.710177	12.233899	12.200265	12.174631	12.313013	12.302971	12.263306
12.695878	12.666625	12.613239	12.340412	12.299111	12.288733	12.465328	12.42605	12.444332
6.8887132	6.7144555	6.9465121	7.3033307	7.4004221	7.0998089	6.9402241	6.9189087	6.9030876
3.7238255	3.8177796	3.5382314	3.6872592	3.6588876	3.6508626	3.9162052	3.8365351	3.9487208
11.63107	11.555772	11.59534	11.396164	11.37691	11.320794	11.620708	11.584875	11.596637
4.0446218	3.6824426	4.2656663	3.5111488	3.6588876	3.2790605	2	2	2
8.6009928	8.5661273	8.5250189	8.7158125	8.6196709	8.7164018	8.735877	8.7935338	8.6137246
3.2116748	2.7060624	3.5382314	3.1593576	2.7817736	3.4139246	2.7248895	2.7162254	2.7098437
11.899578	11.824426	11.863088	11.25795	11.207061	11.216237	11.388218	11.332083	11.383213
9.887159	9.8206556	9.842513	9.449655	9.5116659	9.2858329	9.5857108	9.5378198	9.573017
6.7719527	6.7144555	6.7220159	6.0588757	5.7254286	6.2534533	5.7962123	5.7587426	5.7774236

2.4103584	2	2.7091089	3.3105119	3.421282	3.2790605	3.4829147	3.550228	3.3724324
12.038073	11.988526	11.976516	11.268088	11.252569	11.18887	11.931099	11.897946	11.903786
13.142752	13.109469	13.065919	12.646603	12.595605	12.607795	12.792073	12.760589	12.763616
3.6501227	3.6824426	3.5382314	3.1593576	2.7817736	3.4139246	3.6422468	3.3824986	3.8247709
3.9247213	4.3515565	3.1824306	3.793754	3.421282	4.0326014	4.0921636	3.9608643	4.1686253
7.7274987	7.712999	7.6311552	7.2006065	7.2540232	7.0592256	7.0149424	7.0366117	6.9483421
4.0446218	4.1610493	4.0614204	5.3403469	5.5593263	5.1452616	5.8624576	5.8896564	5.8427942
3.1053801	3.1780396	2.9650943	3.5722723	3.2859859	3.7561757	3.4829147	2.9740279	3.8247709
8.1613618	7.9959113	8.2094791	9.6805874	9.6489676	9.618186	6.6712383	6.5546339	6.7238356
10.136076	10.052752	10.107823	9.7557973	9.8631706	9.5373874	10.585633	10.688047	10.411192
3.9247213	3.6824426	4.0614204	3.8929252	3.9549305	3.7561757	3.8524369	3.9608643	3.6891623
7.242213	7.2454087	7.1276945	6.6611519	6.7362263	6.4855643	5.6907665	5.7926015	5.5220361
12.165463	12.095627	12.123731	11.676392	11.653479	11.604759	11.838216	11.779473	11.835672
7.4946757	7.4467797	7.4334205	7.9924475	8.0594175	7.8238907	6.6337947	6.6126503	6.5969578
12.393952	12.321336	12.354941	11.841254	11.806934	11.781999	12.133295	12.091435	12.114497
6.0977607	6.0689835	6.0210407	8.1930699	8.188328	8.1037793	8.7180966	8.6895454	8.6867542
10.039254	10.002136	9.9648566	11.162419	11.12333	11.107244	8.2179496	8.1315359	8.2419575
3.6501227	3.9415025	3.1824306	4.4107557	4.649489	4.0326014	4.7813527	4.8931255	4.6052608
3.8608147	3.9415025	3.6878577	4.5987644	4.7035875	4.4006546	5.5369952	5.614753	5.3970366
7.2670792	7.3717719	7.0329694	7.89712	7.9849176	7.7039678	4.8805969	4.8931255	4.8152588
10.450208	10.3674	10.443907	10.913177	10.862301	10.877806	9.9316733	9.8646752	9.9445772
12.317155	12.260634	12.262162	11.707589	11.703287	11.616607	12.042003	12.043465	11.979768
9.0652537	8.9370384	9.0795803	9.3108382	9.3365599	9.1880558	9.6999498	9.689296	9.6617333
6.2427066	6.271203	6.1032668	5.322668	5.3031474	5.2571394	5.2207741	4.7625408	5.5220361
5.7898982	5.5131371	5.9338433	5.9479354	6.1129374	5.6613247	5.6538328	5.4118562	5.8104792
3.4903583	4.3515565	3.3712718	3.8441915	4.0414954	4.1912539	4.5990795	4.6189492	4.8152588
2.9906265	2.9612664	2.9650943	3.5111488	3.7644639	3.1302769	2.5749062	2.7162254	2.3981461
7.2546997	7.2211953	7.2165815	7.0795152	7.0394212	7.069479	7.3740655	7.2466167	7.4667128
9.9575923	9.9330042	9.8701763	10.162762	10.169815	10.05995	10.583179	10.552579	10.553445
12.086899	12.049531	12.012426	11.551278	11.58262	11.423787	11.824	11.787367	11.800112
12.737064	12.658759	12.703877	11.957506	11.974447	11.844159	11.904323	11.887213	11.861038

3.6501227	3.5330831	3.6878577	3.9400663	4.123159	3.6508626	2.9848891	3.1926715	2.7098437
3.7939455	3.6824426	3.8234137	4.3066864	4.5347973	3.9462135	4.746694	4.2801452	5.0547979
3.9859168	3.9415025	3.9473196	4.3768933	4.4101943	4.2644835	4.8805969	5.1756805	4.4460942
11.866908	11.816367	11.80694	11.396956	11.370654	11.328819	11.880543	11.903767	11.795992
13.747036	13.720229	13.661512	13.597822	13.533467	13.567759	12.787552	12.757913	12.757272
2.5787203	2.7060624	2.3976902	3.3105119	3.6588876	2.7768658	3.4829147	3.3824986	3.6891623
12.014174	11.943447	11.973786	12.250211	12.178642	12.22681	11.472161	11.464009	11.420491
2.8659503	2.7060624	2.9650943	3.1593576	2.3100466	3.6508626	2.8607385	3.3824986	2
11.025622	10.992182	10.992854	10.763555	10.708742	10.752156	10.17103	10.070168	10.212212
11.055814	11.084835	10.909606	11.371926	11.287859	11.360479	12.459989	12.432775	12.428117
8.88824	8.8724296	8.7920664	8.5433682	8.4240939	8.5651979	8.7180966	8.7206573	8.6552576
11.733594	11.704008	11.652216	11.090462	11.037075	11.050143	11.131224	11.066617	11.135905
2.5787203	2.7060624	2.9650943	2.7992097	2.5651052	2.9643661	3.0991973	2.7162254	3.3724324
13.969822	13.932012	13.895967	14.011896	13.956325	13.973266	13.661298	13.649335	13.613206
4.2581217	4.0554482	4.357879	5.5523944	5.3715813	5.6339509	5.813061	5.7587426	5.8104792
12.421382	12.365156	12.366087	11.889335	11.838633	11.845651	12.005592	11.956527	11.994226
4.7209942	4.5198165	4.8136363	5.5674845	5.5593263	5.4887032	5.0886338	5.2261101	4.8789742
7.8803622	7.7042906	7.9379432	7.5088158	7.5258362	7.3971067	6.8533017	6.8401489	6.8080834
7.083281	7.1330973	6.9165019	6.9357382	6.9638538	6.8129792	7.6236703	7.5436303	7.6422183
4.5287539	4.1610493	4.7469892	4.1945222	3.6588876	4.5250741	4.297674	4.075324	4.4460942
11.648031	11.669751	11.555732	10.924209	10.909099	10.91595	11.181153	11.186073	11.155334
13.239176	13.228507	13.146301	12.916834	12.834629	12.909255	13.062389	12.99904	13.069304
11.973204	11.886848	11.948057	11.373537	11.306005	11.346318	11.729264	11.721879	11.67613
9.0846171	8.9881556	9.0693881	8.6464476	8.5176637	8.6999964	9.0341413	9.0875095	8.9166637
12.409042	12.317772	12.388123	11.752434	11.760235	11.648589	11.832547	11.798345	11.806271
4.7912485	4.3515565	5.0531343	4.4761857	4.5932826	4.2644835	5.2704178	5.1756805	5.3072501
11.583296	11.549708	11.505587	10.93261	10.881496	10.890151	10.831288	10.807747	10.796583
6.5669427	6.5296394	6.4971652	6.3912169	6.3348412	6.3575325	5.7088847	5.123424	6.0781548
2.5787203	2.9612664	2	2.8980192	2.3100466	3.2790605	2.9848891	3.1926715	2.7098437
5.856951	5.9561447	5.6353242	4.9375947	4.4738406	5.2208023	6.04469	6.3193942	5.6370634
2.7294717	2.9612664	2.3976902	3.2369134	3.2859859	3.1302769	2.7248895	2.4021099	2.9660172

2.8659503	2	3.3712718	3.0773944	2.9701107	3.1302769	2.5749062	2	2.9660172
13.998237	13.921836	13.962878	13.341022	13.317365	13.270072	12.388372	12.374582	12.342071
13.597593	13.53911	13.544976	13.259598	13.210493	13.21485	13.554747	13.524239	13.52494
2	2	2	2.6931321	2.3100466	2.9643661	3.3037759	3.3824986	3.1834889
11.786957	11.736403	11.725985	11.068752	11.049531	10.993384	9.9345633	9.9019911	9.9068647
3.5724509	3.1780396	3.8234137	3.9400663	3.6588876	4.1141074	3.9772738	3.7004723	4.1686253
4.2076351	4.1610493	4.1671548	4.5987644	4.5347973	4.5834785	4.711182	4.6189492	4.748599
5.3050117	4.9315181	5.5203094	4.5987644	4.4738406	4.6396102	4.0921636	3.7004723	4.3594016
12.27621	12.199591	12.241128	11.713537	11.673948	11.658811	12.054012	12.051353	11.996023
3.2116748	3.1780396	3.1824306	3.3105119	3.2859859	3.4139246	3.9162052	3.1926715	4.5278714
5.0423553	4.8705531	5.1072743	4.4107557	4.2738034	4.4642052	4.8482682	5.0692034	4.5278714
3.5724509	3.8177796	3.1824306	3.5111488	3.6588876	3.2790605	3.0991973	2.4021099	3.5394718
8.2760535	8.3486671	8.0800901	7.8046289	7.6412146	7.8656754	6.9780669	7.1055801	6.7749723
7.6109307	7.6141874	7.4956373	8.4487451	8.4986641	8.3037605	9.29324	9.3229406	9.2015768
9.6418754	9.5609082	9.6114999	9.9915251	9.9842168	9.9039716	10.319178	10.341486	10.235218
11.503799	11.459438	11.437217	11.414797	11.357004	11.378116	11.206879	11.138319	11.214416
12.103174	12.030062	12.07277	12.249334	12.189346	12.219923	12.892095	12.857	12.872543
3.1053801	2.7060624	3.3712718	3.1593576	3.2859859	2.9643661	3.4829147	3.9608643	2.7098437
5.0423553	4.990011	4.9968831	5.5674845	5.3031474	5.7145616	6.1143131	6.1204673	6.0507505
8.2544549	8.3542445	8.0249004	8.6676095	8.5549282	8.6834022	9.1547045	9.1044582	9.1443884
6.2427066	6.013667	6.3477521	6.6541008	6.5714361	6.6448917	7.1928328	7.1189867	7.2060539
5.3753109	5.2984878	5.5596753	5.8024604	5.8018185	5.9098007	6.2565941	6.1728291	6.4785512
3.403311	2.9612664	3.6878577	2.6931321	2.5651052	2.7768658	2.9848891	3.1926715	2.7098437
13.297292	13.236268	13.248083	12.705767	12.676661	12.641045	12.941776	12.910984	12.912817
2.2197205	2	2.3976902	3.1593576	2.9701107	3.2790605	2.5749062	2.7162254	2.3981461
12.294279	12.242704	12.234302	11.655138	11.593406	11.622277	11.966592	11.977378	11.894663
8.2821656	8.2444008	8.2094791	8.460918	8.5732057	8.2371022	8.3068764	8.2701748	8.2837523
3.8608147	2.9612664	4.357879	3.2369134	3.6588876	2.5613046	2.9848891	2.7162254	3.1834889
8.5812996	8.3981042	8.6442584	8.9250584	8.911624	8.8443218	7.0720502	7.0783871	7.0065546
2.7294717	2	3.1824306	2.8980192	2.9701107	2.7768658	4.0358621	4.5414122	3.1834889
2.4103584	2.7060624	2	3.1593576	3.1366761	3.1302769	2.5749062	2.7162254	2.3981461

5.9520259	5.8337256	5.9634986	6.882432	6.895774	6.7758915	4.746694	5.0692034	4.2671646
2.5787203	2.7060624	2.3976902	3.3105119	3.1366761	3.4139246	2.9848891	3.3824986	2.3981461
5.5063989	5.251441	5.6353242	6.1418678	5.9877173	6.1984556	5.8624576	5.4964687	6.1050482
5.6072167	5.5907872	5.5203094	4.7386523	4.9914418	4.334175	3.9162052	3.3824986	4.2671646
8.0001475	8.0101135	7.8769163	8.1536163	8.1003229	8.1137219	8.4375706	8.4293037	8.3859744
8.1446861	8.1830441	7.989303	8.5847463	8.4160154	8.6564481	8.9065068	8.8347434	8.9166637
10.775109	10.668726	10.768685	10.307974	10.242423	10.278995	10.071268	10.109318	9.9704013
4.3997744	4.0554482	4.6036804	4.1551088	4.2004465	4.0326014	4.0921636	3.8365351	4.3594016
9.795057	9.6789494	9.7979412	9.7859461	9.7228673	9.7547041	10.225534	10.2161	10.182534
3.4903583	3.3664558	3.5382314	4.4438416	4.2738034	4.5834785	4.8151984	5.123424	4.3594016
4.2581217	4.2594452	4.357879	4.0299647	3.9549305	4.0326014	3.6422468	3.550228	3.9487208
7.7408745	7.7641735	7.6031681	7.3779831	7.3333746	7.3304597	6.2317468	6.0661334	6.3268829
5.07088	4.8068978	5.2098157	4.4761857	4.2738034	4.5834785	4.1985751	4.075324	4.2671646
5.7195764	5.801426	5.5203094	5.1924505	4.9472802	5.3271781	4.8805969	4.6925307	4.9985377
4.3069011	5.8653179	5.3953249	3.4473206	4.9017239	4.4006546	3.4829147	3.9608643	4.3594016
3.9859168	3.6824426	4.1671548	3.9400663	3.9549305	3.9462135	5.2207741	5.2748363	5.1089463
12.914031	12.882472	12.833494	12.225005	12.202615	12.152798	12.569706	12.518419	12.561223
11.860029	11.718676	11.88553	11.875192	11.863351	11.792482	11.302821	11.267335	11.278575
5.6072167	5.6644705	5.4382008	5.9010549	6.1327901	5.5189462	6.2317468	6.2721744	6.1314494
5.856951	5.8653179	5.7418429	5.1123987	4.649489	5.3939735	4.4346216	4.2801452	4.5278714
7.9889804	7.7973084	8.0596406	8.2711868	8.1644158	8.2818815	8.9566504	8.8629053	8.9870265
7.6773651	7.6689217	7.5746273	8.1258522	8.0952727	8.0633091	8.1532625	8.1962094	8.0477075
2.7294717	2	3.1824306	2.5786313	2.9701107	2	2.8607385	2.4021099	3.1834889
6.546776	6.6250049	6.3477521	7.6955338	7.6754567	7.622746	4.9431589	4.9542373	4.8789742
4.9532328	5.2028082	4.5263082	5.0276426	5.2313049	4.6936396	5.1952966	5.3676132	4.9399942
2	2.3958007	2	2.5786313	2.5651052	2.5613046	3.6422468	3.8365351	3.3724324
5.0423553	5.1003295	4.8773401	5.9708164	5.8743662	5.976702	5.813061	5.825684	5.7774236
12.164016	12.087279	12.129057	11.331615	11.313635	11.254936	11.441675	11.454126	11.368028
9.5009311	9.4323413	9.458495	9.1077644	9.1199235	8.9998422	9.3375828	9.3287216	9.2862616
12.750352	12.757559	12.757235	12.344661	12.413084	12.353362	12.652631	12.721573	12.654064
3.4903583	3.8177796	2.9650943	3.1593576	3.1366761	3.4139246	3.2051098	3.3824986	2.9660172

12.033098	11.986731	12.059958	11.93532	11.952971	11.90947	11.951929	11.963046	11.962868
9.2866131	9.228795	9.2366018	8.9132945	8.9456936	8.7833373	9.2272067	9.0424877	9.3353527
9.2069273	9.1184116	9.1840388	9.2513423	9.1421317	9.2636782	8.5448491	8.55299	8.4762897
6.6258049	6.4485311	6.686891	7.3607622	7.3588839	7.2695058	5.5571371	5.6885435	5.3528418
12.398882	12.337617	12.349687	12.196472	12.150312	12.15129	12.295697	12.297455	12.23429
6.2798345	6.6250049	5.6717113	6.3217206	6.3175986	6.2353527	7.1470556	7.13227	7.1030952
8.6180065	8.4034941	8.7069648	8.4649529	8.284644	8.5432945	7.679863	7.5236079	7.76534
5.9973125	5.9561447	5.9338433	6.151914	5.9877173	6.2170221	6.7256424	6.6500711	6.7410834
3.5724509	3.6824426	3.5382314	3.6872592	3.8628381	3.4139246	3.4829147	2.9740279	3.9487208
9.6418754	9.7162436	9.4480435	9.4937682	9.4810168	9.4120588	8.9084679	8.8428459	8.9128636
13.944695	13.894584	13.883174	13.978151	13.935724	13.926174	13.732839	13.684702	13.720089
9.1595722	9.182518	9.0208489	8.7673661	8.7666809	8.6733534	7.9738488	7.9707685	7.9172196
3.4903583	3.5330831	3.3712718	4.2702587	4.2738034	4.1912539	3.3037759	3.1926715	3.3724324
12.032872	12.017814	12.045732	11.334926	11.35172	11.370879	11.678164	11.736086	11.680607
4.2076351	4.4381381	3.9473196	5.1123987	5.193996	5.0655458	4.7813527	4.2801452	5.1089463
3.6501227	3.8177796	3.3712718	3.630911	3.421282	3.7561757	4.3904012	4.6925307	3.9487208
13.228534	13.187893	13.15737	12.68581	12.623718	12.65324	12.67872	12.687673	12.608684
3.3106725	2.7060624	3.6878577	3.6872592	3.8628381	3.4139246	4.0358621	4.3725921	3.5394718
8.6443435	8.8840434	8.6848812	8.6357492	8.6816324	8.8942059	8.6634018	8.7029613	8.8346806
3.5724509	3.6824426	3.3712718	4.84155	4.7557305	4.8445541	5.761913	6.0096728	5.3970366
3.3106725	3.5330831	2.9650943	3.2369134	2.9701107	3.4139246	3.4829147	3.3824986	3.5394718
2.7294717	3.1780396	2	2.9904928	2.9701107	2.9643661	2.5749062	2.7162254	2.3981461
5.6830869	5.6999474	5.5596753	5.3403469	5.2313049	5.3609623	5.1429507	5.65212	4.2671646
4.0446218	4.0554482	3.9473196	3.9857156	4.2738034	3.5372523	4.4775267	4.2801452	4.6052608
6.7278514	6.8626932	6.4767447	6.7695201	6.8120614	6.631113	6.5658347	6.4734607	6.5969578
4.4440613	4.4381381	4.357879	4.3066864	3.5449709	4.7457183	5.2207741	5.2261101	5.1611356
5.527134	5.4310687	5.7418429	5.5824184	6.0093567	5.5485682	5.6907665	5.9509123	5.7774236
10.05183	10.030281	9.9611863	10.052456	9.9883089	10.022178	8.9414064	8.8668839	8.9541241
10.052724	10.04415	9.9482663	10.14023	10.071666	10.114198	9.3390377	9.2302387	9.3828281
5.7898982	5.8337256	5.6353242	6.1215631	5.8264086	6.2889872	6.3286672	6.3651173	6.2324729
2.8659503	2.9612664	2.7091089	3.2369134	3.1366761	3.2790605	2.8607385	2.7162254	2.9660172

2.8659503	2	3.3712718	3.9400663	3.6588876	4.1141074	3.5647792	3.8365351	3.1834889
7.6633844	7.6048597	7.6125576	7.732823	7.6481284	7.7235784	7.4886132	7.3750853	7.5375235
11.697748	11.631793	11.652785	11.50549	11.472515	11.445108	9.9451105	9.9039287	9.9277285
11.564613	11.510284	11.515632	10.919071	10.898266	10.854278	11.404638	11.436164	11.312863
3.6501227	2.9612664	4.0614204	4.4438416	4.649489	4.1141074	4.4775267	4.8931255	3.8247709
10.822535	10.834521	10.695466	10.154666	10.097152	10.117908	9.4237498	9.4423979	9.343845
2.5787203	2.7060624	2.3976902	3.4473206	3.6588876	3.1302769	3.0991973	2.4021099	3.5394718
4.5287539	4.5971173	4.357879	5.3403469	5.4994188	5.1059542	4.2489753	4.4594701	3.9487208
9.8851525	9.848586	9.8102344	9.6158302	9.6211279	9.5150543	9.6817183	9.6436453	9.6639958
5.8238141	5.8962332	5.6353242	5.7896485	5.4369156	5.9983304	4.3447825	4.1813664	4.4460942
12.863463	12.823128	12.793032	12.805693	12.742342	12.774731	13.630812	13.599545	13.602335
5.856951	5.8962332	5.7072032	5.7896485	5.8505866	5.6339509	5.2704178	5.5763924	4.8152588
3.9859168	3.6824426	4.1671548	4.1945222	4.2004465	4.1141074	4.9431589	5.3219704	4.3594016
3.1053801	3.3664558	2.7091089	3.741489	3.6588876	3.7561757	3.7157657	3.7004723	3.6891623
5.5063989	5.3440487	5.5596753	5.864867	5.9657484	5.6613247	5.941203	5.7587426	6.0507505
3.7939455	3.1780396	4.1671548	3.793754	3.6588876	3.8543214	4.5191927	4.6189492	4.3594016
7.2915241	7.0667046	7.3904001	7.3033307	7.2720378	7.2425732	7.3797314	7.408224	7.2904838
5.2311106	5.2028082	5.1594558	5.5674845	5.4685057	5.5775942	5.2207741	5.0692034	5.3072501
2.8659503	2.3958007	3.1824306	4.5690822	4.5932826	4.4642052	4.1985751	4.075324	4.2671646
9.4489042	9.506847	9.3918953	9.5575075	9.6176099	9.5206701	8.3765394	8.5330981	8.3859744
4.9835561	4.990011	5.0531343	4.6563572	4.9914418	4.2644835	4.6374231	4.8293101	4.3594016
4.9532328	4.8705531	4.9383494	5.8024604	5.8018185	5.7909055	4.559689	3.9608643	4.9399942
6.1387573	7.5669337	7.475195	6.4575188	7.6202724	7.4293101	5.1429507	6.8562486	6.8080834
3.6501227	3.8177796	3.3712718	3.8441915	3.2859859	4.1912539	4.8151984	5.0692034	4.4460942
12.7706	12.759662	12.67588	12.445992	12.430733	12.385263	12.705756	12.677232	12.684193
6.4631658	6.5097858	6.3020577	6.3996732	6.5271198	6.16059	5.3418203	5.2261101	5.3970366
11.446445	11.416098	11.364751	11.009901	10.948137	10.977137	10.758678	10.772126	10.684037
10.136076	12.682785	12.714817	9.2779609	12.095726	12.046837	9.7346309	12.114208	12.151249
7.2169106	7.2692224	7.2165815	7.7821669	7.7544665	7.8178214	7.6426454	7.6115979	7.6874061
2.8659503	3.1780396	2.3976902	2.9904928	2.5651052	3.2790605	3.2051098	2.7162254	3.5394718
3.4903583	3.6824426	3.1824306	3.2369134	3.421282	2.9643661	3.7157657	3.550228	3.8247709

4.2076351	3.9415025	4.357879	5.6548533	5.4369156	5.7909055	4.4775267	4.5414122	4.3594016
6.4738859	6.6064294	6.2060835	6.2487064	6.2281515	6.179647	5.2704178	4.9542373	5.4815611
10.18011	10.153754	10.094482	11.131973	11.063174	11.106622	11.021338	10.961703	11.01969
8.8496618	8.8607214	8.7243914	9.0034381	8.8619387	9.0447069	8.6017111	8.6205282	8.5219088
10.579913	10.51413	10.534371	10.582743	10.504854	10.566451	10.417478	10.415336	10.360477
9.0599273	9.0751609	8.9336396	9.29052	9.2276962	9.2592061	7.1733916	7.13227	7.1554928
7.7628955	7.5669337	7.9077524	8.2051811	8.1302584	8.209552	8.3157705	8.3116326	8.3470774
7.9318107	7.9887575	7.7802444	6.6821007	6.5714361	6.7506259	5.2946134	5.4547826	5.2115027
9.6394969	9.6176617	9.5517426	9.546126	9.4693534	9.5299814	9.8100056	9.7933017	9.7665207
9.4117539	9.3284991	9.3836927	9.2419673	9.1640033	9.2252164	9.0032756	9.0772439	8.866464
9.1940183	9.2073837	9.0659747	9.2939263	9.3084687	9.1880558	8.6328862	8.6485325	8.5564192
10.18338	10.155352	10.0995	10.219988	10.195999	10.149665	9.9546325	9.8844354	9.9630701
11.024255	10.994863	10.941622	10.485794	10.437611	10.439785	10.80363	10.804639	10.741943
12.536912	12.478981	12.483321	11.742911	11.672677	11.718262	11.188838	11.126751	11.190288
11.772105	11.719216	11.717852	11.042667	10.932648	11.055312	11.281415	11.220684	11.282262
9.8588119	9.8145995	9.7999974	9.4718802	9.4136418	9.4537521	9.8866199	9.8304488	9.893429
5.701447	5.8337256	5.7418429	5.6548533	5.8505866	5.6613247	5.2207741	5.4547826	4.8789742
7.63501	7.5279837	7.6311552	7.4034351	7.0910729	7.5803534	7.5401201	7.5535382	7.4667128
11.322925	11.258467	11.275948	10.822922	10.788346	10.763253	9.2161651	9.1573936	9.2139832
3.3106725	3.5330831	2.9650943	2.1678177	2.3100466	2	3.5647792	3.8365351	3.1834889
6.0838321	6.0415904	6.0210407	7.7756844	7.7089049	7.7493179	8.7136171	8.6531475	8.7132145
8.7228356	8.6726415	8.6624543	8.3496614	8.2978932	8.3080967	8.7643106	8.7029613	8.7647224
8.0331391	8.058748	7.8924167	7.7428277	7.7735609	7.6157665	7.7945884	7.8311272	7.6962762
10.659545	10.612821	10.599567	10.632471	10.601519	10.571871	10.1237	10.095821	10.096367
5.8404777	5.9851925	5.5596753	5.6828387	5.4369156	5.8154814	6.0729437	5.8580248	6.2078724
12.175759	12.090624	12.149762	12.191153	12.150921	12.137041	12.514281	12.47281	12.495093
4.6471428	4.8068978	4.4445501	5.8890928	5.8977601	5.7909055	4.2489753	4.2801452	4.1686253
10.076658	9.97705	10.064007	10.155291	10.122196	10.09425	10.478818	10.450227	10.447107
11.890354	11.871372	11.796679	12.016599	11.978224	11.960979	11.31174	11.318348	11.244208
7.3392053	7.4043967	7.1536545	7.6607773	7.6822087	7.544049	7.3453973	7.3411675	7.2904838
7.5310596	7.5475902	7.4120706	7.0475849	7.101185	6.8959802	7.3797314	7.3862163	7.3137261

2.4103584	2	2.7091089	2.3181327	2.5651052	2	3.3037759	3.3824986	3.1834889
5.6456505	5.4310687	5.7418429	6.0905599	6.1523733	5.9324476	6.2810207	6.2233567	6.28045
7.3966728	7.4363001	7.2410034	7.2806182	7.1985888	7.3046513	6.9705774	6.8074002	7.062509
2.2197205	2.3958007	2	3.5722723	3.2859859	3.7561757	3.9162052	3.8365351	3.9487208
10.081047	10.074877	9.9758117	9.8382088	9.7664092	9.8153628	9.352066	9.4263115	9.2108916
10.042859	9.9255307	10.046794	9.8319739	9.8117576	9.7594618	10.031329	10.068441	9.9314897
9.3635689	9.2882755	9.4894028	9.3473611	9.4297801	9.3033148	9.7781831	9.8566946	9.795703
11.489673	11.474881	11.391587	11.480795	11.431528	11.435726	12.013586	11.979217	11.987467
8.0258726	7.9376621	8.0036475	8.7787567	8.7149394	8.7486634	9.2977361	9.33735	9.1953333
9.6794087	9.6199786	9.6277978	9.8311926	9.8407139	9.7258238	10.3656	10.32278	10.347866
6.0555648	5.8337256	6.1555909	6.2203444	6.3687204	5.9983304	6.058886	6.1983141	5.8427942
5.8732383	6.1986514	5.30555	5.2866467	5.3715813	5.1059542	6.2191608	6.3651173	5.9943291
13.275628	13.237023	13.202565	13.787981	13.696684	13.783302	14.339085	14.271489	14.344943
13.942043	13.861921	13.910314	13.255527	13.248805	13.16731	13.516684	13.534124	13.438207
10.007327	9.9441424	9.9593476	9.6454645	9.5304961	9.6628867	9.3621184	9.3601099	9.3037792
6.5365859	6.3625899	6.6508895	6.0264801	6.1716943	5.8396456	6.3169023	5.825684	6.6708196
8.8289354	8.9031944	8.6304592	8.6693591	8.6161493	8.632443	9.1889928	9.1178753	9.1984584
10.826723	10.773388	10.768685	10.610328	10.520009	10.604849	10.594796	10.54639	10.582441
6.856306	6.7487385	6.8545413	6.9181874	6.895774	6.8491373	6.1936545	6.247972	6.0781548
5.9672799	6.9089038	6.8386263	6.0481578	7.0499006	6.8007223	5.169361	6.2959775	6.1573762
6.4523654	6.3845599	6.8062596	7.4243074	7.6342676	7.3219081	7.954996	8.1771099	8.0267627
11.299764	11.279872	11.207889	11.088501	11.041677	11.041054	11.6839	11.678961	11.628267
4.7912485	4.4381381	4.9968831	4.9375947	4.9472802	4.8445541	4.4775267	4.3725921	4.5278714
3.2116748	3.5330831	2.7091089	3.0773944	3.2859859	2.7768658	3.7857191	3.9608643	3.5394718
8.1004051	7.9302128	8.1526854	8.2479585	8.1595854	8.2416432	7.6044423	7.4723016	7.6695005
2.4103584	2	2.7091089	3.630911	3.5449709	3.6508626	3.7857191	3.3824986	4.0628589
2.7294717	2	3.1824306	3.2369134	3.1366761	3.2790605	3.0991973	3.7004723	2
2.7294717	3.1780396	2	3.4473206	3.9549305	2.5613046	3.2051098	3.1926715	3.1834889
9.3211585	9.285359	9.245682	9.7089336	9.6796582	9.6458576	10.260503	10.24543	10.215301
8.578819	8.6087481	8.4326222	7.3301219	7.3247702	7.2425732	7.0508988	6.7044429	7.2787207
5.1532186	4.990011	5.2098157	6.0155178	5.8743662	6.0613354	5.0606888	5.0128652	5.0547979

5.3522564	5.801426	4.6036804	5.1529798	4.5932826	5.4887032	3.0991973	3.3824986	2.7098437
4.487029	4.5198165	4.357879	5.5061535	5.2676733	5.6881887	4.9431589	4.8931255	4.9985377
10.092396	10.042424	10.031125	10.507474	10.459393	10.462332	11.116877	11.08893	11.084468
2.9906265	2.3958007	3.3712718	3.4473206	3.5449709	3.2790605	4.1985751	4.1813664	4.1686253
14.266116	14.219638	14.200995	13.975546	13.915989	13.940472	14.116406	14.087356	14.085204
8.4571378	8.4142138	8.3895777	8.664104	8.6849979	8.5469682	7.4674844	7.6582576	7.1809948
3.1053801	3.3664558	2.7091089	2.8980192	3.1366761	2.5613046	2.2180911	2	2.3981461
3.9859168	3.9415025	3.9473196	4.5987644	4.5347973	4.5834785	4.6747739	4.6925307	4.6052608
8.8822169	8.8209936	8.8328059	9.2419673	9.2483172	9.1426471	7.8649519	7.8311272	7.8392788
4.6087492	4.7403036	4.357879	3.8929252	3.6588876	4.0326014	2.8607385	2.7162254	2.9660172
6.1387573	6.3845599	5.7756703	5.7896485	5.9877173	5.6060478	5.2946134	5.0692034	5.4399178
6.9817831	6.9389086	7.0743297	6.8763857	6.7619501	6.9961263	6.4084399	6.5935696	6.3938449
7.6011853	7.6141874	7.475195	8.3030907	8.1302584	8.3757608	8.9940703	8.9666551	8.9797795
4.6471428	4.5198165	4.6771135	5.0276426	4.7557305	5.1835263	4.7813527	4.3725921	5.0547979
7.2975711	7.3936035	7.0878571	6.9004205	6.836479	6.8727489	7.0076424	7.0366117	6.9183307
9.0037051	8.9555032	8.9923609	8.7208828	8.6680913	8.706581	8.6399857	8.5280819	8.7044482
9.2589885	9.3056517	9.2028099	9.3208911	9.2864817	9.3607999	9.3404911	9.351617	9.3325107
7.3566872	7.3607309	7.2410034	7.7626314	7.8654167	7.5513834	8.2368034	8.2581076	8.1545502
9.3678858	9.3481981	9.2784956	9.1965847	9.1664131	9.137783	9.4643107	9.4844381	9.3828281
4.3069011	4.6704862	3.6878577	4.0299647	3.6588876	4.2644835	2.2180911	2	2.3981461
5.3288274	5.2028082	5.3511356	4.7910183	4.5932826	4.8915437	4.2489753	4.3725921	4.0628589
6.5568946	6.4896552	6.5173007	6.7365211	6.6284834	6.7506259	6.5658347	6.4310946	6.6343614
6.9204083	6.8469547	6.8858542	7.5549729	7.4878025	7.5513834	7.1668525	7.3181044	6.9483421
7.3095897	7.3717719	7.5356729	7.3907653	7.5628929	7.6773987	6.652638	6.7221209	6.9777419
4.2581217	4.5971173	3.6878577	5.5217324	5.2676733	5.6613247	5.169361	5.3676132	4.8789742
5.6456505	5.5131371	5.6717113	5.4744817	5.6447666	5.2925838	5.0606888	4.8931255	5.1611356
2.7294717	2.3958007	2.9650943	3.1593576	3.1366761	3.1302769	3.0991973	3.3824986	2.7098437
9.1208773	8.9845638	9.1425104	8.9711776	8.8649092	8.9809551	8.5797269	8.5823229	8.5169107
11.052689	10.999319	10.99465	10.604853	10.523773	10.590681	11.123224	11.156311	11.028497
8.0222255	8.1830441	7.8924167	8.3648568	8.1977831	8.4984641	8.9922222	9.0738057	8.9204538
4.1010311	4.3515565	3.6878577	3.4473206	2.9701107	3.7561757	2.9848891	3.3824986	2.3981461

9.7114679	9.7031907	9.6068094	9.9788828	9.9113783	9.9518684	10.268924	10.231611	10.24734
5.856951	5.801426	5.8410348	5.4583812	5.3031474	5.5189462	4.4775267	4.2801452	4.6052608
9.3447113	9.2438966	9.333473	9.1559811	9.0923075	9.1255506	8.7750992	8.759697	8.7305889
3.9247213	3.8177796	4.1671548	3.741489	4.0414954	3.2790605	3.6422468	3.7004723	3.5394718
6.0412208	6.2474223	5.6717113	6.5053192	6.3348412	6.5746431	7.1134449	7.145432	7.020748
3.7238255	3.5330831	3.8234137	3.1593576	3.421282	2.7768658	2.9848891	2.7162254	3.1834889
5.9973125	5.9561447	5.9338433	6.3305929	6.0928076	6.4546057	6.8203484	6.9189087	6.6527056
8.2973336	8.3261393	8.1526854	7.89712	7.8293731	7.871547	7.2496323	6.96418	7.4352681
2.4103584	2.7060624	2	3.0773944	2.9701107	3.1302769	3.2051098	2.7162254	3.5394718
3.7238255	3.3664558	4.4445501	4.7386523	4.7557305	4.7457183	4.8482682	4.8293101	5.0547979
3.403311	3.1780396	3.5382314	3.9400663	3.7644639	4.0326014	4.297674	3.9608643	4.5278714
4.5287539	4.5971173	4.357879	5.1123987	5.1163526	5.0239729	4.0358621	4.1813664	3.8247709
10.107103	10.059596	10.045062	10.157162	10.141922	10.079107	10.431867	10.423463	10.379877
7.7453057	7.5475902	7.8213144	7.6642909	7.5701914	7.6907444	6.8614237	6.9341575	6.7238356
7.6727199	7.6418141	7.5937171	7.7159928	7.7480452	7.622746	7.4886132	7.4191032	7.4974868
7.0550028	6.8626932	7.1276945	7.1312105	6.9302155	7.2334828	5.5369952	5.5369839	5.4815611
3.7238255	3.5330831	3.9473196	4.6278481	4.0414954	4.9811664	3.396124	3.8365351	2.7098437
7.1715348	7.4043967	7.3572715	6.3305929	6.3001473	6.7378252	6.9170326	6.9189087	7.1295318
11.099734	11.032746	11.055297	10.781057	10.716985	10.750564	9.9911791	9.9250713	9.9957714
10.190709	10.16649	10.102835	9.7979913	9.7146577	9.7861296	9.6609287	9.601287	9.6594672
6.2675644	6.013667	6.3920436	6.3217206	6.2095768	6.340699	6.1545292	6.2233567	6.0228156
3.4903583	3.6824426	3.1824306	3.9400663	3.1366761	4.4006546	4.5990795	4.7625408	4.3594016
11.244883	11.175838	11.204012	11.6046	11.620024	11.494457	10.752686	10.681285	10.762148
5.527134	5.4726864	5.4798392	4.9833211	4.854682	5.0239729	5.0886338	5.4964687	4.4460942
2.7294717	2.9612664	2.3976902	3.1593576	3.2859859	2.9643661	2.4075018	2.7162254	2
8.7887156	8.7295396	8.7373247	9.9475048	9.9012862	9.899668	10.851817	10.831354	10.811968
5.6072167	5.6280992	5.4798392	4.7910183	4.5347973	4.9370509	5.2946134	5.4118562	5.1089463
7.3682252	7.3936035	7.2288441	7.2054379	7.2809615	7.0280209	7.2802473	7.2708417	7.2306851
2.4103584	2.3958007	2.3976902	2.8980192	3.421282	3.5372523	3.0991973	3.550228	2.9660172
10.905053	10.888371	10.820247	10.044389	9.9828502	10.039233	9.0853168	9.0703593	9.0506623
5.4853615	5.2984878	5.5596753	5.6966305	5.2676733	5.9547445	5.8785543	5.4964687	6.1314494

10.103656	10.051035	10.045062	10.508452	10.438609	10.483574	9.2272067	9.3402147	9.0437287
3.1053801	2.3958007	3.5382314	2.3181327	2	2.5613046	2.9848891	2.4021099	3.3724324
9.4877686	9.506847	9.3532112	9.4828657	9.4674003	9.4039788	8.7921947	8.6439027	8.8703882
8.7517537	8.755062	8.7920664	7.9699096	8.1253121	8.1037793	7.8730088	7.8791918	8.0126287
2.5787203	2.3958007	2.7091089	3.3105119	2.9701107	3.5372523	2.4075018	2.4021099	2.3981461
3.5724509	3.5330831	3.5382314	3.630911	3.7644639	3.4139246	3.3037759	3.550228	2.9660172
12.891469	12.82187	12.849713	12.124482	12.03965	12.114007	12.428901	12.425375	12.372826
5.205611	5.1003295	5.2098157	5.8525998	5.8977601	5.7145616	6.1545292	6.0381793	6.2078724
7.0902647	7.1965685	6.8545413	6.8580931	6.7362263	6.8844113	7.6092734	7.6115979	7.5473614
9.0055533	8.8646347	9.0313885	9.4577766	9.4116117	9.4100431	8.823713	8.7809378	8.8062839
2.4103584	2.3958007	2.3976902	3.5111488	2.9701107	3.8543214	3.7857191	4.075324	3.3724324
6.0697677	6.1986514	5.8087227	5.864867	5.616845	5.9983304	4.3447825	4.8293101	3.5394718
8.8700947	8.8409943	8.7879285	8.8743856	8.8379517	8.8171973	8.0750997	7.8553596	8.211345
2.8659503	2.3958007	3.1824306	3.0773944	3.421282	2.5613046	2.7248895	2.9740279	2.3981461
4.1010311	3.8177796	4.2656663	5.1123987	5.0759055	5.0655458	3.0991973	2.7162254	3.3724324
8.6155882	8.5613129	8.5594113	9.3275543	9.2067761	9.3524268	8.2828873	8.2641538	8.2419575
3.8608147	4.0554482	3.5382314	3.9857156	3.1366761	4.5250741	4.4775267	4.4594701	4.4460942
7.1649344	6.9971086	7.2165815	7.3212466	7.1700519	7.3724734	6.752094	6.8879188	6.5389689
8.0075444	8.0518998	7.9748144	7.9149273	8.0697531	7.7365055	8.0433231	8.0287417	8.0819536
7.9511201	7.9151977	7.8769163	7.8546942	7.7480452	7.8656754	7.5602187	7.5236079	7.5375235
3.1053801	2.7060624	3.3712718	3.0773944	3.421282	2.5613046	3.8524369	3.8365351	3.8247709
8.9140535	8.8489176	8.868513	8.6159268	8.5768335	8.5615703	8.9959161	8.999644	8.9317648
3.7238255	3.6824426	3.6878577	3.630911	3.8628381	3.2790605	3.5647792	3.7004723	3.3724324
4.9835561	4.9315181	4.9383494	4.9833211	4.7557305	5.1059542	4.8805969	4.9542373	4.748599
9.8298967	10.095004	10.03811	10.055804	10.286292	10.260096	9.9809645	10.127673	10.02423
4.487029	3.9415025	5.2098157	4.1145884	3.7644639	4.334175	3.7157657	3.8365351	3.8247709
9.9661595	9.9716173	9.8464975	9.8552173	9.8646554	9.7499307	9.4455241	9.4921861	9.3353527
3.9859168	3.6824426	4.1671548	3.4473206	3.6588876	3.2790605	3.5647792	3.3824986	3.6891623
2.8659503	2.7060624	2.9650943	4.1551088	4.2738034	3.9462135	2.7248895	2.9740279	2.3981461
8.3737344	8.3374472	8.2994329	8.3452903	8.2934903	8.3037605	8.1697082	8.1835045	8.0954276
4.6471428	4.6704862	4.5263082	4.4438416	3.7644639	4.8445541	4.6747739	4.4594701	4.8152588

5.6456505	5.7683866	5.3953249	5.2117882	5.0342917	5.2925838	5.4958476	5.123424	5.7435929
6.0412208	6.1986514	5.7418429	6.1914136	6.3175986	5.9547445	6.2191608	6.3193942	6.0507505
9.7193726	9.6383815	9.6889908	9.9146917	9.8779498	9.8588703	10.519806	10.368397	10.600273
8.6009928	8.575708	8.51504	8.2123993	8.2395802	8.0887357	6.9248046	6.9789602	6.8080834
2.5787203	2	2.9650943	4.3422168	4.2004465	4.4006546	4.3447825	4.8293101	3.5394718
6.0697677	5.7345728	6.2548685	5.9363573	5.9657484	5.8154814	5.6724178	5.7587426	5.5220361
8.7183347	8.6636853	8.6624543	8.3075913	8.1449964	8.3674744	7.8810209	8.0216212	7.6604636
7.303593	7.1330973	7.3794415	7.2806182	7.2173046	7.364168	7.1733916	7.1842126	7.1683001
11.789641	11.731054	11.736759	12.58108	12.539774	12.528255	13.050783	13.006949	13.033878
4.756549	4.990011	4.357879	5.2117882	5.0342917	5.2925838	5.6907665	5.4964687	5.8104792
10.71029	10.677651	10.644886	10.585525	10.517179	10.56826	10.678775	10.584338	10.713827
12.067345	12.021762	12.00843	12.109901	12.078236	12.05911	12.935658	12.909297	12.908768
12.046625	11.93835	12.041828	11.582022	11.515682	11.553632	11.630543	11.668172	11.531218
9.1858911	9.0581745	9.1996983	9.4738838	9.4297801	9.4240946	9.5672086	9.5101044	9.563353
7.2732294	7.4043967	7.0047221	6.9473212	7.0499006	6.7378252	6.1143131	6.1728291	5.9943291
5.4640127	5.5131371	5.30555	5.6406543	5.5883723	5.6060478	5.761913	5.72407	5.7435929
3.9247213	4.1610493	3.5382314	5.1328319	4.9914418	5.1835263	4.9734511	4.7625408	5.1089463
6.8480892	6.7822257	6.8062596	7.1563797	7.2449308	6.9853361	7.570164	7.6210513	7.4563072
9.6033396	9.6700324	9.5121567	9.6961179	9.7584444	9.6424275	9.0269379	8.9666551	9.1605028
4.684541	4.5971173	4.6771135	4.5690822	4.7035875	4.334175	5.0031203	5.0128652	4.9399942
7.8559834	8.0030299	7.9748144	7.6572552	7.7286077	7.7039678	7.3220454	7.3411675	7.3702447
8.9548005	8.755062	9.0313885	8.6867389	8.637151	8.6427798	7.6660187	7.8229584	7.4246323
4.7912485	4.7403036	4.7469892	5.0916718	4.8060545	5.2571394	3.9772738	3.7004723	4.1686253
6.6353859	6.5685453	6.5951447	6.5741754	6.4968007	6.5601733	5.6538328	5.5369839	5.7089498
3.2116748	3.6824426	2.3976902	3.1593576	3.1366761	3.1302769	2.5749062	2	2.9660172
6.0697677	6.0415904	5.9925565	6.8763857	6.8723477	6.7883603	6.9705774	6.9189087	6.9631169
6.8644763	6.8782618	6.7392628	7.2388124	7.1700519	7.2151282	7.1992554	7.1969113	7.1425707
7.391028	7.3042218	7.3683991	7.4034351	7.5183093	7.1777034	7.274176	7.1189867	7.3591167
6.3396668	6.2474223	6.3250858	5.6548533	5.5593263	5.6613247	3.4829147	3.3824986	3.5394718
3.1053801	3.1780396	2.9650943	2.4542536	2.3100466	2.5613046	2.8607385	2	3.3724324
7.5615318	7.7725289	7.1791556	7.8516155	7.7480452	7.8597797	7.2862931	7.2094991	7.3021518

8.9432755	9.182518	8.8995273	8.5086077	8.6883556	8.6767108	8.4184302	8.5579204	8.4450552
9.0147592	9.0852575	8.8207044	8.4869454	8.4598975	8.4443381	8.4101488	8.4021449	8.3804815
9.8088823	9.7313241	9.7751281	9.9915251	9.9964584	9.8910223	10.949342	10.825233	11.006379
5.1532186	4.7403036	5.3953249	5.2682931	4.9472802	5.4578126	5.9102187	5.825684	5.935611
13.417823	13.387059	13.336618	13.909908	13.872096	13.853959	14.460725	14.427874	14.433395
3.4903583	3.3664558	3.9473196	3.3805373	3.5449709	3.5372523	3.9162052	2.9740279	4.6787093
3.4903583	2.7060624	3.9473196	3.1593576	3.421282	2.7768658	2.9848891	3.550228	2
10.09587	10.081618	9.9974755	9.3385916	9.3386982	9.2434439	9.1841441	9.233324	9.0712654
5.1532186	5.2028082	4.9968831	5.4904045	5.2676733	5.6060478	4.711182	4.6189492	4.748599
6.9046478	7.2089345	7.0468884	6.882432	7.2173046	7.1965372	7.8281285	7.9781438	8.0126287
8.4188154	8.320452	8.4058702	8.0975433	7.9629056	8.1382828	7.4621534	7.429901	7.4563072
3.7238255	3.3664558	3.9473196	3.1593576	3.1366761	3.1302769	3.9162052	3.8365351	3.9487208
12.728417	12.687211	12.660685	11.629753	11.578555	11.592813	11.748278	11.753383	11.692847
9.7339388	9.7693939	9.6462012	9.8217842	9.8407139	9.7969688	9.9142109	9.9496651	9.8740154
11.82461	11.765996	11.7728	12.031129	12.01546	11.95304	10.835933	10.894107	10.712734
8.6794998	8.9773533	8.7628484	8.4426199	8.5768335	8.6800604	7.9008588	8.0428782	8.2837523
6.191668	6.2232429	6.0489734	6.4080802	6.5568147	6.1412778	5.8624576	6.0935561	5.5220361
7.7408745	7.7641735	7.7885525	7.6033642	7.6342676	7.6366045	7.6706483	7.6304432	7.773745
10.408909	10.352151	10.3544	10.02197	9.918544	10.028762	10.430502	10.401688	10.399019
2.7294717	2.3958007	2.9650943	4.1945222	4.649489	3.4139246	3.7857191	3.9608643	3.5394718
3.7939455	3.6824426	3.8234137	4.5078205	4.5347973	4.4006546	3.8524369	4.2801452	3.1834889
7.083281	7.0802298	6.9759106	6.9357382	6.7619501	7.0068364	6.4945145	6.5146181	6.4154923
6.9590724	10.447856	10.585332	6.8395656	10.137016	9.9196423	7.0294327	10.066713	9.8992025
9.2558862	9.3142619	9.1840388	9.9991968	9.9012862	10.075296	10.745024	10.773186	10.703965
8.0112287	8.268231	8.3620077	7.9642197	8.2257819	8.5060332	6.4840318	6.7738909	6.7581273
9.6595908	9.5942851	9.6138395	9.5883513	9.4477226	9.6286251	8.9566504	8.854915	8.9942374
12.052672	11.997018	11.996825	12.264026	12.192896	12.240204	12.580201	12.558331	12.541669
14.197992	14.13063	14.153943	13.935714	13.882028	13.895074	14.209422	14.189474	14.169037
4.3997744	4.1610493	4.5263082	3.5722723	3.5449709	3.5372523	2.9848891	2.4021099	3.3724324
7.639778	7.4980653	7.6676463	7.7428277	7.5774532	7.8056057	7.8609065	8.0216212	7.6144103
8.3794472	8.320452	8.3282129	8.63037	8.612619	8.5615703	7.7295183	7.7034553	7.6962762

6.9204083	6.9389086	6.7897998	6.2393142	6.1716943	6.2170221	6.058886	5.9509123	6.1050482
2.5787203	2.3958007	2.7091089	2.7992097	2.7817736	2.9643661	3.0991973	3.3824986	2.7098437
9.404768	9.4610568	9.227464	9.0751408	9.0088267	9.0473031	9.0801062	9.0703593	9.0332654
3.1053801	2.9612664	3.1824306	3.1593576	3.5449709	2.5613046	3.4829147	4.075324	2.3981461
2.8659503	2.9612664	2.7091089	3.1593576	2.3100466	3.6508626	3.2051098	2.9740279	3.3724324
6.8644763	6.7655792	6.8545413	6.8333365	6.7362263	6.837185	5.8297152	5.65212	5.935611
3.7238255	3.9415025	3.3712718	3.6872592	3.9549305	3.2790605	4.1463502	4.075324	4.1686253
8.4679026	8.4248544	8.4004598	8.8406227	8.882605	8.6999964	8.0289724	8.164235	7.8150511
13.987347	13.945353	13.964941	14.14379	14.110153	14.130505	14.409801	14.41443	14.395773
9.49173	9.228795	9.6208355	9.102594	9.1421317	8.964567	8.6041333	8.6110713	8.5368002
8.4679026	8.4666492	8.3564299	7.9149273	7.9121154	7.8238907	6.8775319	6.9034969	6.7916228
4.2581217	3.9415025	4.4445501	4.3768933	4.5347973	4.1141074	3.6422468	3.3824986	3.9487208
7.4734654	7.5860213	7.2288441	6.958812	6.7231904	7.07966	6.8203484	6.7044429	6.8721095
3.9859168	3.5330831	4.2656663	3.9400663	4.123159	3.6508626	4.1463502	4.3725921	3.8247709
2.8659503	2	3.3712718	2.5786313	2.5651052	2.5613046	3.0991973	2.9740279	3.1834889
8.5512465	8.3653351	8.6165268	8.3187819	8.3283427	8.2141804	8.5673671	8.3575984	8.6956283
7.9814874	7.7725289	8.0664894	7.3693984	7.2079771	7.4293101	7.6426454	7.5236079	7.6962762
8.3593528	8.5418934	8.0178508	8.5698367	8.6196709	8.4205048	7.8446106	7.8870492	7.7398269
2.8659503	2.7060624	2.9650943	3.1593576	2.3100466	3.6508626	4.3904012	4.4594701	4.2671646
12.064467	12.029626	11.987383	11.60277	11.544685	11.566358	11.725098	11.707529	11.682282
11.401179	11.367974	11.322464	10.691344	10.614815	10.672836	10.600873	10.535183	10.604991
12.352438	12.308465	12.28473	12.463366	12.374262	12.45853	11.852294	11.795739	11.849157
13.450416	13.393168	13.396105	12.904538	12.874049	12.840757	12.886384	12.849983	12.862481
3.9859168	3.5330831	4.2656663	4.1551088	4.0414954	4.1912539	4.559689	4.6925307	4.3594016
4.6087492	4.8068978	4.2656663	4.5387765	4.34361	4.6396102	3.4829147	3.550228	3.3724324
5.5063989	5.7345728	5.1072743	5.3403469	5.2676733	5.3271781	5.0031203	5.2748363	4.6052608
7.391028	7.3717719	7.3003084	7.0689501	6.9302155	7.1097789	6.3169023	6.247972	6.3268829
12.473225	12.40936	12.425561	11.812845	11.829158	11.701466	12.095705	12.087605	12.043283
6.6161598	6.4896552	6.6325462	6.5286383	6.5120399	6.4546057	5.9564492	5.4547826	6.28045
2.2197205	2.3958007	2	3.741489	4.0414954	3.2790605	2.5749062	2.4021099	2.7098437
9.4529735	9.4480751	9.3504079	9.1977975	9.122408	9.1786141	8.7664748	8.6985032	8.773131

10.646574	10.639332	10.540533	9.8482835	9.8102173	9.7923334	6.452119	6.0096728	6.7410834
3.1053801	2.9612664	3.1824306	3.3105119	2.9701107	3.5372523	2.4075018	2.7162254	2.3981461
6.9514221	6.7655792	7.0189149	6.8642164	6.8485346	6.7883603	7.5145965	7.451257	7.517644
5.701447	5.9561447	5.4382008	6.1618908	6.1523733	6.179647	6.2191608	6.3424369	6.1314494
3.1053801	2.9612664	3.1824306	2.8980192	2.9701107	2.7768658	2.5749062	2.4021099	2.7098437
7.0041419	6.9971086	6.9012594	6.9357382	6.895774	6.9524728	5.5369952	5.614753	5.3970366
11.521022	11.493955	11.435896	11.076355	11.002425	11.058532	11.560557	11.562358	11.497996
8.0618448	8.138487	7.8612475	7.9001032	7.9684402	7.7300564	7.8929564	7.8473273	7.8787755
4.569306	4.7403036	4.2656663	3.741489	3.6588876	3.7561757	3.7157657	3.1926715	4.0628589
2.9906265	2.3958007	3.3712718	2.5786313	2.5651052	2.5613046	3.7857191	3.7004723	3.8247709
9.1858911	9.0581745	9.1996983	8.7326448	8.8166309	8.5432945	8.2737875	8.2581076	8.2419575
10.417264	10.408189	10.313244	10.099284	10.013959	10.08922	9.4854858	9.5303137	9.3773232
7.9777263	7.8456261	7.9964931	7.8941306	7.8594716	7.8359532	8.0820671	7.9781438	8.1220038
2.5787203	2	2.9650943	4.0728969	3.5449709	4.4006546	3.396124	3.9608643	2.3981461
7.2854516	7.2454087	7.2165815	6.8395656	6.9188261	6.6585401	7.0579838	6.9341575	7.1163741
6.0267327	6.3625899	5.4798392	5.9246856	6.2095768	5.6060478	4.3904012	4.7625408	3.9487208
8.6910308	8.5613129	8.7069648	8.1881968	7.9178478	8.3338441	8.3333958	8.1898709	8.4077393
9.4758189	9.4270583	9.4135435	9.5327332	9.4556259	9.5150543	9.0853168	9.0909152	9.0191953
3.8608147	3.5330831	4.0614204	2.9904928	3.2859859	2.5613046	2.7248895	2.7162254	2.7098437
11.573362	11.518379	11.516882	11.214574	11.189989	11.1447	11.332339	11.275589	11.327924
4.3997744	4.5971173	4.0614204	4.960639	4.7557305	5.0655458	3.0991973	2.7162254	3.3724324
5.0988516	4.990011	5.1072743	5.6118302	5.5296835	5.6060478	5.3418203	5.2748363	5.3528418
12.796719	12.726984	12.755647	12.704382	12.646554	12.668286	12.003759	11.996571	11.951799
6.6449038	6.6793379	6.4971652	7.1107541	7.1796271	6.9524728	6.1936545	6.1983141	6.1314494
4.9222583	4.6704862	5.0531343	4.7117388	4.4738406	4.8445541	4.9431589	4.6925307	5.1089463
6.5365859	6.6614533	6.3020577	6.4247487	6.5271198	6.2170221	7.1269833	6.8879188	7.2787207
6.9046478	7.0936294	7.1276945	7.2435177	7.38395	7.5068075	6.9630487	7.1714012	7.2060539
7.0478458	6.9389086	7.0468884	7.0038811	7.0182314	6.8959802	7.781807	7.8870492	7.6050206
4.7209942	4.5198165	4.8136363	4.4107557	4.7557305	3.8543214	3.7857191	3.7004723	3.8247709
3.7238255	3.8177796	3.5382314	4.9141763	4.7035875	5.0239729	4.8805969	4.8931255	4.8152588
11.153652	11.10813	11.08848	10.850557	10.809315	10.798353	10.98695	10.982022	10.93604

7.5715478	7.3042218	7.7119993	7.7023857	7.5990216	7.7300564	7.6660187	7.83925	7.4139176
8.968131	8.8449614	8.9923609	8.9294453	8.911624	8.8855272	9.0215117	8.999644	8.9870265
5.8404777	5.9561447	5.6717113	5.5824184	5.7768019	5.6613247	4.5191927	4.3725921	4.8789742
2.2197205	2	2.3976902	2.8980192	2.9701107	2.7768658	3.396124	3.3824986	3.3724324
6.7098263	6.6250049	6.686891	6.8207971	6.9748945	6.545557	6.7256424	6.5742331	6.8243582
4.487029	4.0554482	4.7469892	5.6689139	5.616845	5.6339509	6.206464	6.2959775	6.0507505
9.9623581	10.000358	9.8061483	9.8170569	9.7326575	9.8077271	8.8565904	8.890528	8.7604996
3.4903583	3.3664558	3.5382314	2.7992097	2.5651052	2.9643661	2.4075018	2	2.7098437
6.0555648	5.801426	6.1810581	4.8661672	4.649489	4.9811664	4.3447825	4.6925307	3.8247709
2.5787203	2.3958007	2.7091089	3.3805373	3.2859859	3.4139246	3.3037759	3.3824986	3.1834889
3.1053801	2.9612664	3.1824306	4.3066864	4.2004465	4.334175	3.4829147	3.7004723	3.1834889
9.5460763	9.5705235	9.4054635	9.5966501	9.5248728	9.5738576	10.350532	10.33432	10.306466
7.1110155	8.195526	8.1526854	6.8944492	8.0385214	7.8419468	7.0997782	8.0707423	8.1021177
4.1553175	4.3515565	3.8234137	4.5078205	4.4738406	4.4642052	3.2051098	3.550228	2.7098437
2.4103584	2.7060624	2	3.2369134	3.6588876	2.5613046	3.2051098	3.1926715	3.1834889
2.7294717	2.7060624	2.7091089	2.9904928	3.421282	2.30778	2.8607385	3.3824986	2
8.6956175	8.5564825	8.7200545	8.8076402	8.7949903	8.7293931	8.6161834	8.4717217	8.6911981
3.8608147	3.1780396	4.2656663	4.9833211	4.9472802	4.9370509	5.3184099	5.3676132	5.2115027
12.425357	12.390484	12.348634	12.209757	12.178343	12.14706	12.310234	12.275922	12.284783
6.1654552	6.0415904	6.1810581	6.2203444	6.1523733	6.2170221	5.4748252	5.3219704	5.5614064
3.403311	3.1780396	3.5382314	3.3105119	3.2859859	3.2790605	2.4075018	2.7162254	2
11.011895	10.985909	10.925703	11.434221	11.356477	11.417279	11.970822	11.955126	11.926108
6.8887132	6.6970036	6.9612862	6.8580931	6.8841084	6.7378252	6.9855178	6.7738909	7.1163741
3.7238255	3.5330831	3.8234137	4.2328874	3.9549305	4.4006546	2.4075018	2.4021099	2.3981461
8.7495496	8.7423572	8.6442584	8.8591367	8.7856154	8.8383381	8.0855382	8.0498948	8.0615036
9.9971444	9.9950128	9.8857489	9.9109993	9.9270961	9.7985106	10.18889	10.160459	10.157059
9.5035492	9.5118462	9.3809481	9.1941562	9.0770184	9.2137062	9.6160301	9.6273201	9.5438287
11.550439	11.48699	11.502434	11.241579	11.210568	11.178842	11.500716	11.467289	11.473696
9.4033668	9.3621059	9.333473	9.8780922	9.7472193	9.9096897	10.302869	10.250007	10.296292
8.0075444	7.981568	7.9229268	8.332097	8.3153715	8.2551811	9.0748767	9.0875095	9.0014124
11.740547	11.714887	11.653923	11.321359	11.310914	11.237991	11.26007	11.285284	11.172917

4.3069011	4.2594452	4.2656663	4.3768933	4.4738406	4.1912539	3.5647792	3.550228	3.5394718
4.2581217	4.1610493	4.2656663	4.0299647	4.0414954	3.9462135	3.9162052	4.4594701	2.9660172
10.135232	10.109918	10.048525	10.364144	10.31264	10.32144	9.2690211	9.2607983	9.2170683
13.050874	13.026536	12.96301	12.657222	12.593592	12.626579	12.720483	12.699712	12.680846
7.6819954	7.8535241	7.5937171	7.352074	7.4644915	7.3046513	6.9780669	7.0224134	6.9922202
7.6727199	7.7808361	7.8691031	7.3167885	7.4723038	7.4608104	7.2434305	7.5925031	7.2549028
10.53263	10.519104	10.471259	10.24014	10.210084	10.197196	10.02049	10.008475	9.9849531
9.4516183	9.7075548	9.7262294	9.1965847	9.355692	9.4942742	8.6209754	8.955489	8.810375
5.0132553	4.8068978	5.1072743	5.9246856	5.9207807	5.8396456	5.0031203	4.8293101	5.1089463
5.9210252	5.9851925	5.7418429	5.9479354	6.0093567	5.7909055	6.0868657	6.0935561	6.0228156
7.5361832	7.487953	7.475195	7.2621872	7.2173046	7.2151282	7.092896	7.0783871	7.0487225
9.2229027	9.1730818	9.161826	9.0672001	9.1024109	8.93403	9.352066	9.2021705	9.4314496
7.9395655	7.8923779	7.8769163	7.5625242	7.668673	7.3474123	7.2558075	7.25878	7.1935787
5.8404777	5.3882147	6.1032668	6.5591555	6.4500929	6.5746431	7.2983092	7.0783871	7.4352681
8.9140535	8.8209936	8.8956868	9.3819156	9.2573883	9.4080245	9.6702055	9.6226216	9.6571976
10.166132	10.121411	10.0995	10.010973	9.9951033	9.9323383	9.7500242	9.7400684	9.699722
8.8079069	8.755062	8.750143	8.8545304	8.849995	8.7645276	8.0433231	7.9633552	8.0683525
7.0550028	6.7822257	7.1917391	8.0035858	7.8535019	8.0530116	5.5165681	5.4964687	5.4815611
5.1262912	5.3440487	4.8136363	6.5286383	6.3175986	6.6585401	7.1992554	7.2219781	7.218422
5.1532186	5.0462245	5.2584768	4.7910183	5.0342917	4.4006546	5.0606888	5.2261101	4.8152588
7.4465063	7.6418141	8.0733058	7.1312105	7.4954899	7.6434841	6.9780669	7.2466167	7.4667128
8.6131659	8.5369974	8.5787021	8.3232339	8.2441505	8.3080967	6.9705774	7.3064331	6.4578363
7.4627421	7.4043967	7.4120706	7.2102533	7.1112267	7.2151282	7.9738488	8.1115546	7.76534
8.4517251	8.4354172	8.3564299	8.4446645	8.4677346	8.3465475	8.5423252	8.5967687	8.460757
6.6637531	6.6793379	6.537159	6.8580931	7.0394212	6.545557	7.1066277	7.0366117	7.1163741
5.3980029	5.3440487	5.3511356	5.9129186	5.6721581	6.040638	6.2565941	6.2233567	6.2324729
8.10386	7.8456261	8.2279242	7.9556425	7.7415951	8.0581695	7.5849545	7.6490456	7.4563072
2.8659503	2.7060624	2.9650943	4.6563572	4.7557305	4.4642052	6.1676892	6.247972	6.0228156
6.2045979	6.2945981	6.0210407	6.5053192	6.5859108	6.340699	5.6159286	5.4547826	5.7089498
9.2037108	9.2528823	9.0348847	9.3625807	9.3932116	9.2343589	9.9229685	9.8785357	9.9068647
4.487029	4.6704862	4.1671548	4.7910183	4.4738406	4.9811664	5.0321917	4.5414122	5.3528418

11.606011	11.547884	11.552687	12.022087	11.999667	11.949922	11.647375	11.606452	11.627687
7.8641555	7.8376847	7.7802444	7.7821669	7.668673	7.7994589	7.3453973	7.3972621	7.2306851
9.9699509	10.01099	9.9974755	10.486291	10.479883	10.512985	10.42777	10.459478	10.421927
7.2232779	7.3717719	6.931585	6.9122894	6.9073461	6.8251329	6.6432471	6.7221209	6.4989729
3.403311	3.3664558	3.3712718	3.4473206	3.421282	3.4139246	2.7248895	2.9740279	2.3981461
9.7993251	9.7942269	9.693422	9.7987908	9.6880688	9.8123134	9.0484409	9.0772439	8.9578172
3.1053801	3.3664558	2.7091089	4.0299647	4.0414954	3.9462135	2.2180911	2.4021099	2
9.369322	9.2588418	9.3671463	9.1831768	9.1784022	9.0932522	9.1711339	9.2516982	9.0227257
4.85824	4.4381381	5.1072743	5.5217324	5.5593263	5.3939735	4.3447825	4.4594701	4.1686253
11.622063	11.588083	11.544125	11.427258	11.322305	11.43523	11.641197	11.627125	11.594859
4.354085	4.3515565	4.2656663	4.5690822	4.4738406	4.6396102	3.0991973	3.3824986	2.7098437
2.5787203	2.3958007	2.7091089	3.4473206	3.421282	3.4139246	2.8607385	2.7162254	2.9660172
7.7628955	7.6778456	7.737971	7.8516155	7.8475074	7.7620174	7.237202	7.1055801	7.3137261
7.1110155	6.9971086	7.1145373	7.9296007	8.0226484	7.7300564	8.5065199	8.317459	8.6183989
9.0933335	9.0340525	9.0453226	9.0363494	8.9400709	9.0368903	8.109605	8.2274904	7.9172196
6.1786211	6.271203	6.4560311	5.9129186	5.8505866	6.2170221	6.04469	6.1983141	5.9943291
5.7551657	5.6644705	5.7418429	5.6118302	5.5296835	5.6060478	6.4628353	6.0096728	6.7581273
3.1053801	3.1780396	2.9650943	2.6931321	2.7817736	2.5613046	3.0991973	3.3824986	2.7098437
9.3447113	9.3284991	9.2547054	9.0618819	9.000747	9.0290311	9.6620915	9.6712087	9.5968974
2.8659503	2	3.3712718	3.0773944	2.9701107	3.1302769	2.4075018	2.4021099	2.3981461
5.9672799	6.0415904	5.7756703	5.9933403	5.7513439	6.1217037	5.1160479	4.8293101	5.3072501
4.487029	4.2594452	4.6036804	4.8903714	4.9472802	4.8445541	5.4958476	5.1756805	5.7089498
8.0001475	8.0030299	7.8924167	7.8941306	7.8171557	7.8947976	7.3453973	7.3181044	7.3137261
9.1579112	9.0717796	9.1327548	9.3385916	9.315	9.2681364	9.7412482	9.7444001	9.677497
8.2419661	8.1641164	8.2094791	7.5397507	7.4487389	7.5366773	6.8614237	6.7738909	6.8876817
10.115685	9.9932265	10.124327	10.319161	10.161383	10.374091	10.601479	10.592771	10.549779
4.3069011	4.2594452	4.2656663	5.8277479	5.6447666	5.9098007	5.5965964	5.4964687	5.6370634
6.9967274	6.8782618	7.0189149	7.1362796	7.1112267	7.069479	6.5757415	6.6684237	6.4154923
3.3106725	3.5330831	3.1824306	2.6931321	2.5651052	2.7768658	2	2	2
3.1053801	2.9612664	3.1824306	2.7992097	2.7817736	2.7768658	2.9848891	3.3824986	2.3981461
12.162153	12.100613	12.134363	11.966961	11.98643	11.881737	11.37516	11.377412	11.322921

7.1382268	7.0936294	7.0743297	6.8642164	6.7996958	6.837185	6.9630487	6.8879188	6.9777419
4.6471428	4.8705531	4.2656663	5.1123987	4.9472802	5.1835263	4.2489753	4.6925307	3.5394718
10.335131	10.249642	10.308919	10.17143	10.199534	10.045739	9.9555812	9.9477881	9.904953
16.470037	16.419548	16.409128	16.204718	16.162123	16.153022	15.565208	15.535713	15.534537
7.8059541	7.7973084	7.7032371	8.3884166	8.3873791	8.2950486	8.3906386	8.2880885	8.4291807
8.7406998	8.7842785	8.6624543	8.3187819	8.3326407	8.31242	8.3962399	8.4506686	8.3526987
2.9906265	3.5330831	2	2.9904928	2.3100466	3.4139246	2.9848891	2.4021099	3.3724324
5.0132553	4.6704862	5.2098157	4.960639	4.9472802	4.8915437	5.0606888	5.0692034	4.9985377
8.396451	8.3430681	8.367564	8.173478	8.3240319	7.9119928	8.2461387	8.0216212	8.3968979
7.8963888	7.7042906	7.9675151	7.6070203	7.5918679	7.5292676	7.7945884	7.7729498	7.7568857
3.6501227	4.0554482	2.9650943	2.9904928	3.1366761	2.7768658	3.396124	3.3824986	3.3724324
11.001282	10.959622	10.931342	10.64052	10.579084	10.60837	10.284109	10.243901	10.265334
7.9395655	7.9743425	7.7885525	7.5849438	7.5258362	7.5513834	7.6284375	7.3411675	7.8150511
2.7294717	2.7060624	2.7091089	3.1593576	2.7817736	3.4139246	4.1463502	4.5414122	3.5394718
10.442725	10.374279	10.399847	10.324167	10.285184	10.269021	9.9921041	10.024593	9.8992025
6.7007286	6.6614533	6.9612862	6.7095675	6.5714361	6.9413499	7.3453973	7.2219781	7.4667128
11.317727	11.305289	11.249861	11.092419	11.043645	11.074529	11.089479	11.063153	11.085312
2.9906265	3.1780396	2.7091089	3.0773944	2.7817736	3.2790605	3.0991973	3.550228	2.3981461
9.7092014	9.7313241	9.5711356	9.5489798	9.4217335	9.5774544	9.4522617	9.4477204	9.3964993
5.07088	4.9315181	5.1072743	4.6278481	4.7035875	4.4642052	3.7157657	4.1813664	2.9660172
6.9046478	6.968302	6.7220159	7.8821107	7.8772338	7.7932857	5.9564492	6.0381793	5.8104792
8.5461762	8.4972194	8.4846824	8.6919123	8.7410421	8.5432945	8.6680397	8.6805317	8.5948746
8.2102627	8.1641164	8.1526854	7.650185	7.5407733	7.6639284	7.8198163	7.5633785	7.9839383
4.684541	4.6704862	4.6036804	4.2328874	4.2738034	4.1141074	3.5647792	3.550228	3.5394718
9.5824409	9.563318	9.4919488	9.4041503	9.4095788	9.3033148	8.5992849	8.4293037	8.6956283
17.13448	17.076904	17.081161	16.938377	16.897928	16.885149	16.250439	16.238441	16.202973
3.6501227	3.8177796	3.3712718	3.3105119	2.9701107	3.5372523	3.5647792	3.1926715	3.8247709
13.981431	13.922071	13.939752	14.312753	14.264696	14.280508	14.550588	14.52163	14.533615
10.295646	10.237622	10.242434	10.250111	10.228652	10.177186	9.3988543	9.3402147	9.3964993
3.7939455	3.1780396	4.1671548	3.793754	4.0414954	3.4139246	2.9848891	3.3824986	2.7098437
9.3388591	9.2498933	9.316337	9.1880668	9.1074362	9.17387	9.0032756	8.8944313	9.0471997

8.0038507	8.0858203	7.796813	7.6143047	7.6202724	7.5143331	7.3853751	7.3064331	7.4031227
2.2197205	2.3958007	2	3.1593576	2.9701107	3.2790605	3.5647792	3.550228	3.5394718
7.7541274	7.7973084	7.5937171	7.0795152	7.3504308	6.631113	7.0366236	7.23435	6.7410834
2.5787203	2.7060624	2.3976902	3.2369134	3.2859859	3.1302769	2.5749062	2.7162254	2.3981461
3.4903583	3.8177796	2.9650943	3.1593576	2.9701107	3.2790605	3.0991973	2.9740279	3.5394718
7.0692112	7.3042218	6.6508895	7.1004161	7.2720378	6.8007223	7.2496323	7.1842126	7.2549028
2.2197205	2.3958007	2.9650943	3.5722723	3.5449709	3.5372523	2.4075018	2.7162254	2
7.2975711	7.2573647	7.2410034	7.5775092	7.4800739	7.5803534	6.9248046	6.9341575	6.8563673
3.3106725	2.9612664	3.5382314	3.8929252	3.8628381	3.9462135	3.9772738	4.2801452	3.5394718
4.7912485	4.7403036	4.7469892	5.5674845	5.6990392	5.3271781	5.4318384	5.4964687	5.3072501
4.8251331	4.7403036	4.8136363	5.3047698	5.0759055	5.4262461	5.4534919	5.8580248	4.8152588
2.9906265	2.9612664	2.9650943	2.9904928	3.5449709	2	3.0991973	3.7004723	2
8.4625302	8.2974757	8.5100245	8.1410624	8.0952727	8.0937677	8.0217431	7.9559037	8.0267627
3.4903583	3.5330831	3.3712718	3.793754	4.123159	3.2790605	3.9162052	4.075324	3.6891623
4.684541	4.7403036	4.5263082	4.5078205	4.4101943	4.5250741	4.6747739	4.6925307	4.6052608
3.6501227	2.7060624	4.1671548	3.630911	3.6588876	3.5372523	3.2051098	3.1926715	3.1834889
3.5724509	3.5330831	3.5382314	2.8980192	2.9701107	2.7768658	4.0358621	4.075324	3.9487208
3.1053801	3.1780396	2.9650943	3.4473206	3.7644639	2.9643661	2.9848891	2.9740279	2.9660172
3.5724509	4.0554482	3.9473196	3.4473206	3.2859859	3.8543214	4.3447825	4.9542373	4.1686253
5.2561672	5.3882147	5.4382008	5.3578118	5.4046183	5.8634117	4.912217	5.2748363	5.3528418
4.9532328	4.8705531	4.9383494	5.7102916	5.4994188	5.8154814	6.8369191	7.0080739	6.577886
5.0423553	4.9315181	5.0531343	5.6689139	5.4046183	5.8154814	4.559689	4.8293101	4.1686253
13.056902	12.989384	13.012836	12.722717	12.674758	12.677323	12.299252	12.288213	12.249812
4.3997744	4.1610493	4.5263082	3.3805373	3.7644639	2.7768658	3.396124	3.7004723	2.9660172
8.3242403	8.1123939	8.4326222	8.4220127	8.3153715	8.4364373	8.9045429	8.772479	8.9688401
8.0582877	7.981568	8.0249004	7.9670675	8.0065987	7.8299345	7.5849545	7.6397744	7.4667128
5.7195764	5.4310687	5.872639	6.0695146	5.9657484	6.0817401	5.0606888	4.7625408	5.2601706
7.7139978	7.7388132	7.5746273	7.8267465	7.7089049	7.8479156	7.8281285	7.7123274	7.8787755
9.0545812	8.9988775	8.9995358	9.1116301	9.0923075	9.0368903	8.5673671	8.5430783	8.5318535
5.07088	4.9315181	5.1072743	5.5971992	5.2313049	5.8154814	5.6907665	5.4118562	5.8744013
9.1529168	9.0165722	9.1745609	8.872868	8.8619387	8.789553	8.5245336	8.5027362	8.4865526

3.403311	3.1780396	3.5382314	4.0299647	3.421282	4.4006546	3.4829147	3.7004723	3.1834889
9.5549382	9.4558782	9.5419475	9.2641342	9.3322736	9.0932522	9.534565	9.4818462	9.5265254
4.9835561	4.7403036	5.1594558	4.4761857	4.0414954	4.7457183	5.2207741	5.3219704	5.0547979
8.260659	8.2974757	8.1267078	7.6642909	7.702277	7.544049	7.6236703	7.6582576	7.5860558
2.8659503	3.1780396	2.9650943	3.793754	4.2004465	3.7561757	4.2489753	4.4594701	4.8789742
9.8801242	9.9478361	9.7834656	10.064473	10.085739	10.062519	10.241958	10.327118	10.229119
2.7294717	2.7060624	2.7091089	3.741489	3.7644639	3.6508626	2.9848891	2.9740279	2.9660172
8.2973336	8.1767624	8.305235	8.3075913	8.3707567	8.1479912	8.5648824	8.5579204	8.5118952
3.403311	3.1780396	3.5382314	3.4473206	3.5449709	3.4139246	4.7813527	4.6189492	4.8789742
3.9247213	4.0554482	3.9473196	4.8165054	4.7557305	5.3609623	3.9162052	4.1813664	4.2671646
5.205611	5.2984878	4.9968831	4.4761857	4.5347973	4.334175	3.4829147	3.3824986	3.5394718
9.6978153	9.6130166	9.6711286	9.6212637	9.6385906	9.5075325	9.6039788	9.5699024	9.5826166
5.9366088	5.9264999	6.0763755	5.5523944	5.6721581	5.4887032	5.4748252	5.65212	5.2115027
4.1010311	6.6970036	7.0329694	4.7650729	6.6561828	7.069479	4.746694	6.4941862	6.6708196
4.0446218	4.0554482	3.9473196	2.9904928	2.9701107	2.9643661	3.9162052	4.2801452	3.3724324
8.0475634	7.8923779	8.0868427	7.7159928	7.6342676	7.7039678	7.0508988	6.7568398	7.2428448
4.354085	4.2594452	4.357879	3.9857156	3.6588876	4.1912539	3.5647792	3.3824986	3.8247709
3.5724509	3.5330831	3.5382314	3.8929252	3.6588876	4.0326014	2.4075018	2	2.7098437
4.2076351	4.0554482	4.2656663	4.3768933	4.123159	4.5250741	3.4829147	2.7162254	3.9487208
8.2637511	8.1449372	8.2700671	8.0228732	7.90636	8.0478352	7.8810209	7.9180576	7.7821014
9.4379961	9.4558782	9.3047989	9.5404014	9.4906648	9.4961757	9.1986416	9.1703294	9.1796054
10.291864	10.292395	10.177489	9.8590551	9.8969393	9.7225789	9.2443888	9.2547379	9.1732659
3.4903583	3.5330831	3.3712718	3.8929252	4.0414954	3.6508626	3.3037759	2.9740279	3.5394718
8.9297122	8.887894	8.8606541	9.5269551	9.4337867	9.5244018	10.49261	10.508691	10.415227
8.6748615	8.6087481	8.6304592	8.4446645	8.4832823	8.3167303	7.7383658	7.6855458	7.7312213
3.5724509	3.3664558	3.6878577	3.6872592	3.6588876	3.6508626	2.9848891	3.1926715	2.7098437
5.2561672	5.4726864	4.8773401	5.0706429	5.193996	4.8445541	5.1160479	4.8293101	5.3970366
5.9672799	6.0689835	5.7418429	6.0373597	6.1523733	5.8154814	6.1006547	6.2233567	5.935611
15.318699	15.268216	15.257584	15.187729	15.154522	15.126858	15.065705	15.026904	15.044422
8.8639951	8.7252416	8.9033577	8.5337962	8.6406218	8.3210278	8.3850155	8.4076177	8.3357685
3.9859168	4.0554482	4.2656663	4.4438416	4.2738034	4.5250741	2.5749062	2	2.9660172

8.8247542	8.8646347	8.6669677	8.4323531	8.3454582	8.4245045	8.1499507	8.077625	8.1609722
6.9514221	6.9089038	6.8858542	7.0313507	6.9967257	6.9744646	6.0303529	6.1468857	5.8427942
11.141937	11.167942	11.111014	10.441433	10.470162	10.418886	10.175108	10.209848	10.137653
8.4353641	8.3981042	8.3620077	8.2526341	8.221153	8.1908884	8.5423252	8.4664872	8.5564192
8.0969419	7.9524464	8.1267078	8.0446048	8.1449964	7.8359532	7.8446106	7.8712914	7.7568857
8.1345874	8.0655638	8.1002539	7.7361656	7.7155024	7.6639284	5.7267783	5.7587426	5.6370634
4.2076351	3.8177796	4.4445501	3.6872592	3.6588876	3.6508626	4.5990795	4.5414122	4.6052608
4.7209942	4.8068978	4.5263082	4.7117388	4.854682	4.4642052	3.7157657	2.4021099	4.3594016
9.8464906	9.8226686	9.7999974	9.3787111	9.3973209	9.3732692	8.9025764	8.9060782	8.9014029
6.8480892	6.8626932	6.7220159	6.2673093	6.0928076	6.340699	6.7256424	6.7907429	6.5969578
5.9973125	5.6999474	6.1555909	5.6966305	5.7254286	5.5775942	4.2489753	3.3824986	4.748599
4.5287539	4.6704862	4.2656663	6.1009685	6.3348412	5.7659038	6.5757415	6.7221209	6.3495506
10.196384	10.071495	10.207231	10.032884	10.003215	9.9725041	9.6976835	9.678018	9.6571976
3.8608147	3.9415025	3.6878577	4.1551088	4.123159	4.1141074	2.8607385	2.9740279	2.7098437
7.9200997	7.7302597	7.989303	7.6886492	7.7544665	7.5218197	7.954996	7.7558875	8.0751691
11.365284	11.264404	11.353595	10.043041	9.9773707	10.015565	8.9162861	8.8589157	8.9128636
9.3606837	9.408414	9.5221554	8.9796631	9.000747	9.1880558	8.8545573	8.9176318	9.0506623
4.0446218	3.6824426	4.2656663	5.2308701	4.854682	5.4578126	4.9431589	4.2801452	5.3528418
10.429018	10.318308	10.42671	10.562173	10.57818	10.449631	10.605111	10.614231	10.535022
6.0697677	5.8653179	6.1555909	5.7102916	5.5296835	5.7909055	6.351913	6.1204673	6.4989729
6.3278972	6.2232429	6.3700678	6.2673093	6.3348412	6.2889872	5.2704178	5.4964687	5.1089463
4.6087492	4.5198165	4.6036804	4.5987644	4.649489	4.5834785	3.8524369	3.9608643	3.6891623
10.30393	10.200948	10.294407	9.9732282	9.9027322	9.9490945	9.8615312	9.8506802	9.8161919
8.97946	8.9444527	8.9071778	8.6177402	8.4078915	8.7164018	8.4402844	8.1962094	8.5948746
8.7183347	8.6410485	8.689325	8.4789869	8.4638214	8.4003385	8.5821862	8.8184005	8.2297903
4.569306	4.5971173	4.4445501	4.5387765	4.5932826	4.4006546	4.5191927	4.3725921	4.6052608
8.6372082	8.599385	8.5642582	8.0201336	7.8232773	8.1087592	7.48336	7.3638677	7.5375235
15.01668	14.976021	14.945792	14.725444	14.704366	14.651928	15.147275	15.102351	15.131967
8.7757781	8.7801408	8.689325	9.5901996	9.6052288	9.4904636	10.048327	10.059778	10.010071
8.3360397	8.2741276	8.2877582	7.9090159	7.9629056	7.8117265	7.7206163	7.8147431	7.5571327
7.7672597	8.7423572	8.7243914	7.2851894	9.1664131	9.2389086	7.5849545	8.3688647	8.2175197

5.1796526	5.0462245	5.2098157	5.5217324	5.6447666	5.2925838	5.7962123	5.7587426	5.7774236
2.7294717	2.9612664	2.3976902	3.8441915	3.7644639	3.9462135	3.7157657	3.1926715	4.0628589
6.6064498	6.5492235	6.5567476	6.7365211	6.7619501	6.6172014	6.4084399	6.2959775	6.4578363
9.8194278	9.6922224	9.8325033	9.8327547	9.8163684	9.7547041	9.2034419	9.0875095	9.2535828
4.9532328	4.990011	4.8136363	4.1945222	4.2738034	4.0326014	4.1463502	4.2801452	3.9487208
5.0988516	5.1524786	4.9383494	4.5987644	4.7557305	4.334175	5.2458095	5.0692034	5.3528418
9.1242835	9.095284	9.0453226	8.9147703	8.8379517	8.8999628	9.4959579	9.3685532	9.5560623
11.086262	11.057744	11.002707	10.877674	10.814702	10.846846	10.671865	10.602348	10.679571
4.3069011	4.4381381	4.0614204	5.322668	5.3715813	5.1835263	6.1143131	6.1728291	5.9943291
8.7406998	8.7079204	8.6937552	8.6970672	8.584062	8.7486634	9.1025509	9.1245373	9.0471997
4.9835561	4.8068978	5.0531343	5.0493029	5.2676733	4.6936396	4.297674	4.6925307	3.6891623
6.0412208	5.7683866	6.1810581	5.5824184	5.1163526	5.8634117	3.7157657	3.3824986	3.9487208
8.5960947	8.5467728	8.6118526	8.6535361	8.6917055	8.5542877	9.0991205	9.0634417	9.0915784
3.7238255	3.9415025	3.3712718	3.3105119	3.421282	3.1302769	3.5647792	3.1926715	3.8247709
9.7843312	9.7610204	9.6956326	9.8051705	9.8024914	9.7128001	10.238844	10.223876	10.193539
8.2419661	8.1830441	8.1970501	8.006357	8.0279588	7.9346056	7.7339488	7.6855458	7.7568857
9.6106439	9.5316717	9.5783412	9.350636	9.3429655	9.2636782	9.5734024	9.6060553	9.4784857
11.199181	11.182907	11.208663	10.779842	10.81547	10.733739	11.253142	11.284541	11.215959
3.1053801	2.9612664	3.1824306	3.2369134	3.1366761	3.2790605	4.4346216	4.1813664	4.6052608
5.1262912	5.1524786	4.9968831	5.6828387	5.7254286	5.5485682	6.752094	6.5742331	6.8563673
11.702313	11.63466	11.659029	11.346727	11.321765	11.277224	10.977639	10.974666	10.920038
4.2581217	4.3515565	4.0614204	5.5217324	5.5296835	5.4262461	5.2946134	4.9542373	5.5614064
6.3971163	6.3845599	6.3020577	6.7231073	6.7996958	6.545557	7.6331891	7.7299096	7.4667128
5.527134	5.5131371	5.4382008	5.6118302	5.4369156	5.6881887	4.746694	5.0692034	4.2671646
7.6957977	7.5860213	7.6944214	7.1004161	7.0603043	7.0488988	6.7167164	6.8721706	6.4785512
4.1010311	4.1610493	3.9473196	3.3105119	3.421282	3.1302769	3.2051098	3.1926715	3.3724324
3.7238255	4.0554482	3.1824306	3.0773944	3.2859859	2.7768658	2.5749062	2.7162254	2.3981461
7.3566872	7.3717719	7.2288441	7.3078306	7.1311028	7.3807314	7.6426454	7.7034553	7.517644
6.9046478	7.1840957	7.1917391	6.8081477	7.0808895	6.9961263	6.7077347	7.0080739	6.9631169
5.6456505	5.7683866	5.3953249	6.3996732	6.3348412	6.3741717	5.7791646	5.7587426	5.7435929
7.1449502	7.1460169	7.0329694	7.2806182	7.121199	7.3389609	7.5197375	7.3972621	7.576479

2.8659503	3.1780396	2.3976902	3.5111488	3.2859859	3.6508626	4.0358621	3.8365351	4.1686253
7.2670792	7.3270917	7.0878571	6.5591555	6.4500929	6.5746431	6.6050601	6.631482	6.5191095
7.2915241	7.2809834	7.1917391	7.7193746	7.6550091	7.6907444	6.3286672	6.247972	6.3495506
8.3622406	8.3261393	8.2877582	8.2456149	8.1498758	8.24617	7.896913	8.0000463	7.7225641
8.8163551	8.6815424	8.8368172	8.4689765	8.4281163	8.416494	8.1762344	8.2520359	8.0337782
7.8228241	7.9000247	7.6495161	8.0871098	8.0697531	8.0110706	7.5502044	7.5535382	7.4974868
5.527134	5.8962332	6.1555909	5.2117882	5.3031474	5.6060478	5.5571371	5.7926015	5.7435929
7.9200997	7.8135948	7.9153595	7.2897461	7.3672878	7.1097789	7.1403958	7.2094991	7.0065546
2.7294717	2.9612664	2.3976902	3.1593576	3.1366761	3.1302769	3.396124	3.3824986	3.3724324
2.7294717	3.1780396	2	4.5690822	4.8060545	4.1912539	3.7857191	4.075324	3.3724324
3.9247213	3.3664558	4.2656663	3.3805373	3.2859859	3.5372523	3.8524369	3.3824986	4.1686253
7.1041317	7.0253511	7.0743297	7.8078094	7.6956185	7.8238907	8.539797	8.5967687	8.4184998
8.6887319	8.5271552	8.7330265	8.2895039	8.3110217	8.1719803	7.9625667	7.7898127	8.0615036
7.6206107	7.5279837	7.6031681	7.1563797	7.0603043	7.1586205	7.274176	7.2466167	7.2428448
5.8893438	6.0689835	5.7072032	6.1113025	6.3348412	6.1984556	6.885519	6.9935905	6.9030876
7.9739553	7.9000247	7.9379432	7.9699096	8.0279588	7.8117265	6.6050601	6.5146181	6.6343614
3.6501227	3.6824426	3.5382314	4.0299647	3.7644639	4.1912539	4.6374231	4.4594701	4.748599
4.85824	4.5971173	4.9968831	4.3768933	4.4101943	4.2644835	5.3648568	5.2261101	5.4399178
5.9052715	6.0415904	5.9634986	5.1728501	5.4994188	5.1835263	4.746694	4.9542373	4.5278714
9.9727879	9.9292723	9.9049813	9.2813969	9.2044328	9.2636782	9.3332094	9.3344796	9.2714993
4.487029	4.1610493	4.6771135	5.2497029	5.3031474	5.1059542	6.1143131	6.0096728	6.1573762
3.9247213	4.0554482	3.6878577	4.1945222	4.4101943	3.8543214	4.0358621	3.7004723	4.2671646
5.5876089	5.4726864	5.5979955	5.7102916	5.2676733	5.976702	5.169361	4.9542373	5.3072501
8.9471274	8.9953124	8.7796168	9.449655	9.4277727	9.3774017	8.1532625	8.1706868	8.0751691
9.9228054	9.8721033	9.8642929	9.4808745	9.4217335	9.4478692	9.0323438	8.98141	9.0227257
9.8791165	9.806485	9.842513	9.8194225	9.7900432	9.7547041	8.6703531	8.7337883	8.5466431
13.350978	13.27225	13.318264	13.547815	13.543235	13.457407	13.687161	13.709423	13.603367
7.793171	7.6599423	7.8131934	7.7626314	7.8048339	7.622746	7.3626665	7.13227	7.5076006
7.6680597	7.712999	7.5057508	7.3607622	7.0808895	7.5143331	7.4406305	7.2828033	7.527618
9.9803261	9.9311395	9.918293	9.9482255	9.891123	9.9111157	8.0785876	8.0707423	8.0267627
3.4903583	2.7060624	3.9473196	3.8441915	3.9549305	3.6508626	3.0991973	2.7162254	3.3724324

9.3635689	9.3085275	9.3076921	9.4394383	9.4437546	9.3397753	9.9441549	9.9345802	9.893429
7.6254264	7.6048597	7.6311552	7.5996989	7.735116	7.4451462	7.7732228	7.8712914	7.7138546
6.8231542	6.8782618	6.6508895	6.958812	7.0499006	6.763314	6.8286576	6.631482	6.9483421
10.165306	10.127938	10.091128	10.145896	10.079359	10.117908	9.9948756	9.964594	9.9649064
9.3864448	10.349361	10.348793	9.0444612	9.9691123	9.9379454	9.1906054	10.073615	10.02423
9.9671083	10.002136	10.031125	9.445577	9.4771395	9.5112983	9.3200089	9.4661961	9.2714993
14.835283	14.790233	14.768799	14.774427	14.726786	14.727729	15.197864	15.163775	15.171715
4.0446218	4.3515565	3.5382314	4.6563572	4.2738034	4.8915437	5.3648568	5.1756805	5.4815611
11.480732	11.359679	11.500539	11.953204	11.87002	11.948881	12.664285	12.657844	12.61805
2.4103584	2	2.7091089	3.6872592	3.6588876	3.6508626	3.0991973	3.1926715	2.9660172
6.4083367	6.2945981	6.4136896	7.1107541	7.0182314	7.1097789	7.6188873	7.451257	7.7138546
6.0838321	6.2945981	5.7072032	5.9010549	5.9657484	5.7404611	5.2458095	5.2748363	5.1611356
2.7294717	2.9612664	2.3976902	3.0773944	2.9701107	3.1302769	3.0991973	3.1926715	2.9660172
6.191668	5.9561447	6.3020577	5.3921198	5.3715813	5.3271781	5.7791646	6.0096728	5.4399178
9.969004	9.8246789	9.9974755	10.395664	10.392006	10.306347	10.713963	10.595171	10.766366
3.5724509	3.9415025	2.9650943	2.6931321	3.1366761	2.5613046	3.0991973	2.9740279	3.1834889
10.006404	9.9496794	9.9519695	9.934469	9.894034	9.8823244	10.094711	10.004869	10.12128
10.656019	10.668726	10.570954	10.498152	10.448544	10.510165	9.9650346	10.001254	9.904953
9.1276818	9.1085451	9.0348847	8.5125116	8.5912544	8.3295846	7.8810209	7.8473273	7.8552075
5.9366088	6.013667	5.8410348	6.0905599	6.0928076	6.16059	5.4748252	5.3676132	5.5220361
6.5055765	6.6433442	6.2306822	6.2765215	6.3348412	6.1217037	5.6350052	5.65212	5.5614064
10.487055	10.459547	10.402556	10.22832	10.19009	10.172438	10.240402	10.184568	10.235218
5.7551657	5.9264999	5.5203094	5.6966305	5.6721581	5.7145616	5.4534919	5.825684	5.3072501
6.2045979	6.1986514	6.1032668	5.6966305	5.4685057	5.8154814	5.0606888	5.0128652	5.0547979
6.718867	6.8310427	6.4767447	7.0204256	6.895774	7.0488988	6.363397	6.2233567	6.4368197
8.8721221	8.7338248	8.8956868	8.7326448	8.7474946	8.6220315	8.6209754	8.6485325	8.5318535
8.9925657	8.9407503	8.9336396	8.6339583	8.584062	8.5903385	7.8770204	7.6674111	8.0126287
9.6677841	9.6338028	9.5902713	9.4527059	9.4055044	9.4060031	8.72479	8.6623334	8.7262649
10.78375	10.73756	10.718477	10.48331	10.384781	10.48549	10.188083	10.17176	10.147389
12.624458	12.545397	12.592036	12.925827	12.899441	12.85842	13.346298	13.311734	13.320693
9.751667	9.7270314	9.6643728	9.8644109	9.7993894	9.8350286	9.894557	9.9154994	9.8161919

8.5936393	8.4301455	8.6396733	8.1027319	8.1103706	8.0003921	7.6613742	7.6304432	7.6330084
2.9906265	2.3958007	3.3712718	2.8980192	3.2859859	2.30778	3.4829147	3.8365351	2.9660172
7.7585181	7.9376621	7.422785	7.8485302	7.9005816	7.6973713	7.7903405	7.8064807	7.7225641
4.8251331	4.6704862	4.9383494	4.6843137	4.649489	4.6396102	4.8151984	4.7625408	4.8152588
8.6058744	8.6226798	8.4744196	8.006357	7.9629056	7.9568694	7.9204275	7.9180576	7.8865467
4.85824	4.6704862	4.9968831	5.0276426	4.7035875	5.2208023	6.3860942	6.3874467	6.3268829
6.191668	6.3176199	6.0210407	6.3827108	6.3348412	6.500798	6.1676892	6.0935561	6.2566609
6.0697677	5.7345728	6.2548685	5.9708164	6.0306763	5.8154814	6.2191608	6.2721744	6.1050482
6.4631658	7.7388132	7.6218864	6.2580378	7.5258362	7.4608104	6.015872	6.7738909	7.0896929
8.3737344	8.2858489	8.3508305	8.3276723	8.3326407	8.2279771	8.089001	7.7644439	8.3013003
7.4300856	8.1057963	8.2156537	6.9473212	7.7608595	7.9896339	5.8461792	6.8721706	6.7063792
7.5051651	7.6141874	7.3460574	7.5849438	7.5333241	7.6773987	7.5502044	7.2828033	7.7986703
3.5724509	3.3664558	3.6878577	4.1551088	4.123159	4.1141074	3.396124	3.550228	3.1834889
6.484527	6.427521	6.4350157	6.6541008	6.6561828	6.5601733	6.2565941	6.3193942	6.1314494
9.8740671	9.816621	9.8244452	9.5687999	9.4217335	9.6146894	8.9490485	8.9777354	8.8585836
7.7003693	7.6689217	7.6218864	7.984037	8.0437739	7.8238907	8.72479	8.4873123	8.8743017
8.7865674	8.6546731	8.8044095	8.5585524	8.698382	8.3167303	7.8322667	7.7299096	7.8709622
7.6957977	7.8054746	7.4544589	7.7461473	7.7415951	7.6571458	7.8239784	7.894864	7.6874061
7.4787973	7.3496047	7.4956373	7.7626314	7.6822087	7.7493179	8.3906386	8.3405324	8.3804815
8.5960947	8.5172455	8.5642582	8.4074106	8.3497056	8.3716236	8.4129146	8.4021449	8.3638761
4.756549	4.9315181	4.4445501	5.322668	5.193996	5.3609623	6.4413226	6.5146181	6.3038532
10.340999	10.404162	10.376614	9.9388273	9.9608064	10.026132	10.40364	10.389294	10.475724
4.684541	4.7403036	4.5263082	5.5971992	5.3031474	5.7659038	4.8151984	4.6925307	4.8789742
3.403311	3.5330831	3.1824306	3.630911	3.9549305	3.1302769	2.8607385	2.7162254	2.9660172
5.9366088	5.6644705	6.0763755	6.2203444	6.2464901	6.1018603	7.1733916	7.2946666	6.9777419
3.9859168	4.3515565	4.357879	4.8903714	4.9914418	4.8915437	5.7962123	5.9509123	5.6734543
10.042859	10.003911	10.006407	9.9654168	9.9732474	9.9337421	9.4495705	9.3908299	9.4887331
9.2037108	9.2073837	9.0863353	9.047155	9.0168615	8.9836684	9.083582	9.0909152	9.0156562
7.7453057	7.8535241	7.5057508	8.1410624	7.9903685	8.1908884	8.4724571	8.4559607	8.4291807
9.4338842	9.3228211	9.4322229	9.1745791	9.1615894	9.0932522	8.9298667	8.8428459	8.9541241
8.3389745	8.3147421	8.2521564	8.2219678	8.1595854	8.195577	7.7988238	7.8064807	7.7398269

8.3419034	8.1123939	8.4536722	8.3165507	8.284644	8.2551811	7.5197375	7.3411675	7.6330084
3.3106725	3.5330831	2.9650943	3.5722723	3.6588876	3.4139246	4.297674	3.7004723	4.6787093
7.7139978	7.4777693	7.8213144	7.891135	7.7986333	7.88902	8.0855382	7.9484135	8.1545502
7.5103813	7.4980653	7.4120706	8.2267283	8.2487063	8.1087592	8.8299348	8.8225036	8.777317
7.4410535	7.2333528	7.5257676	7.5127192	7.6202724	7.2959448	7.9358937	7.8473273	7.9693763
11.39061	11.306728	11.362666	10.873506	10.911974	10.736155	10.49261	10.480404	10.444477
9.1545835	9.0059815	9.1871843	8.7039119	8.5732057	8.7390604	8.0610631	7.9408842	8.1154055
6.9359985	6.9089038	6.8545413	7.2388124	7.2898303	7.0897696	7.4990628	7.5731522	7.3591167
9.4379961	9.3949476	9.3699172	9.077778	9.0641525	8.9971591	9.2002434	9.1044582	9.2323951
5.0132553	5.1524786	4.7469892	4.9141763	4.9017239	4.8445541	5.0886338	5.0128652	5.1089463
11.591318	11.573812	11.496107	11.300342	11.203545	11.300806	11.648549	11.641131	11.596045
9.1512481	9.0683903	9.1229327	8.9654927	9.0537765	8.7708248	8.9829459	8.9666551	8.9392565
5.8069558	7.120061	7.0878571	5.4089726	6.3518802	6.6720606	4.297674	6.247972	5.7774236
10.477099	10.412205	10.432023	9.6266769	9.5692572	9.5935301	9.4841714	9.4018405	9.503969
2.8659503	2	3.3712718	3.3105119	2.7817736	3.6508626	2.8607385	2.4021099	3.1834889
10.10193	10.052752	10.039851	9.7182599	9.6455169	9.7013071	9.2066332	9.0669047	9.2833212
3.1053801	3.3664558	2.7091089	3.2369134	2.9701107	3.4139246	2.5749062	2.7162254	2.3981461
7.8101901	7.7042906	7.8050264	7.8267465	7.8831063	7.6706793	7.1134449	7.0080739	7.1683001
10.35121	10.289487	10.301681	10.101877	10.045721	10.063802	9.4802211	9.4423979	9.4577698
5.527134	5.8653179	4.9383494	5.2497029	4.9914418	5.3939735	4.8151984	4.8293101	4.748599
3.3106725	3.8177796	2.3976902	4.2328874	3.6588876	4.5834785	4.2489753	4.075324	4.3594016
8.7822613	8.599385	8.8448067	8.3884166	8.2890739	8.3921924	8.3906386	8.2398154	8.4762897
5.6830869	5.9264999	6.3250858	5.7102916	5.9207807	6.1018603	5.8785543	6.0935561	6.0507505
5.0988516	5.1003295	4.9968831	4.7650729	4.34361	5.0239729	3.6422468	3.1926715	3.9487208
6.7719527	6.8149532	6.6139666	6.7163533	6.5714361	6.763314	6.2191608	5.9805917	6.3718678
3.3106725	3.5330831	2.9650943	3.9400663	4.123159	3.6508626	3.6422468	3.8365351	3.3724324
12.261204	12.29584	12.37439	12.317165	12.499246	12.380381	10.960255	11.236915	11.033756
8.6910308	8.5852255	8.6848812	8.3098364	8.1153683	8.3962712	8.0361656	7.9257066	8.0819536
9.9822045	9.9459904	9.9068905	9.5622234	9.5229935	9.5094166	9.3022183	9.2818112	9.2625689
7.5665485	7.6234552	7.4956373	7.7789293	7.8831063	7.622746	7.7689114	7.7981708	7.7821014
8.9081374	8.8209936	8.8841035	8.582891	8.4078915	8.6564481	8.1399696	8.0216212	8.1926607

6.2920011	6.0958662	6.4136896	6.0264801	6.2645985	5.6613247	6.4194843	6.5935696	6.1573762
5.07088	4.9315181	5.1072743	5.2866467	5.4369156	5.0239729	4.4346216	4.3725921	4.4460942
3.2116748	3.1780396	3.1824306	3.3805373	2.7817736	3.7561757	3.0991973	3.1926715	2.9660172
5.7551657	5.5907872	5.872639	5.7767218	6.0928076	5.4578126	6.4194843	6.4524332	6.3938449
11.527147	11.490793	11.451676	11.332443	11.269481	11.300806	11.450451	11.434824	11.405695
4.684541	4.1610493	4.9968831	3.741489	3.421282	3.9462135	3.3037759	3.7004723	2.7098437
5.9973125	5.6280992	6.2060835	6.5286383	6.7362263	6.179647	6.6242799	6.6865457	6.4989729
7.321509	9.6610599	9.6393274	7.1210186	9.5783345	9.5539132	7.4886132	9.5820543	9.3296632
7.8803622	7.9302128	7.7119993	7.4772031	7.38395	7.4763063	7.6613742	7.7211453	7.5375235
9.2165338	9.1635834	9.1586247	9.0144921	9.0380713	8.8942059	8.5698475	8.4612336	8.6137246
8.7539543	8.7209307	8.6804236	8.664104	8.7116431	8.5285053	8.8257899	8.7809378	8.830658
5.5063989	5.4726864	5.5203094	5.7102916	5.5296835	5.8634117	5.4958476	5.5763924	5.3528418
6.6449038	8.5709256	8.8247495	6.5286383	8.4480614	8.2506825	6.6050601	8.4506686	8.2297903
6.1786211	6.0415904	6.2060835	6.0044717	5.9657484	5.9547445	5.9564492	5.72407	6.1050482
8.9355406	8.9628234	8.7920664	8.6267728	8.5659224	8.5938945	8.3038995	8.1577542	8.3804815
11.245274	11.163978	11.226355	11.260854	11.208816	11.221994	11.344704	11.273343	11.35828
10.328502	10.202494	10.340341	9.9971086	10.028604	9.8691784	10.127915	10.1941	9.9957714
6.2798345	6.271203	6.1810581	6.3996732	6.1523733	6.5307911	6.015872	6.0661334	5.9053308
2.7294717	3.1780396	2	3.5111488	3.5449709	3.4139246	3.7857191	3.8365351	3.6891623
5.701447	5.7345728	5.5596753	5.4744817	5.3377701	5.5189462	5.2207741	5.1756805	5.2115027
2.7294717	2.9612664	2.3976902	3.0773944	3.1366761	2.9643661	3.5647792	3.550228	3.5394718
4.1010311	3.8177796	4.2656663	5.4256309	5.7254286	4.9370509	5.4748252	5.5763924	5.3072501
4.2076351	4.1610493	4.1671548	4.6563572	4.4738406	4.7457183	4.6747739	4.8293101	4.4460942
6.0838321	6.148174	5.9035657	6.1618908	6.1129374	6.1217037	6.3169023	6.6126503	5.8744013
2.7294717	2.3958007	2.9650943	3.2369134	2.9701107	3.5372523	3.396124	3.550228	3.8247709
10.049145	10.005684	10.001055	9.6039869	9.5490837	9.6146894	9.7543923	9.6982553	9.7749189
12.714359	12.688315	12.628939	12.462735	12.414606	12.417227	12.264072	12.233025	12.23467
7.9927124	7.9670807	7.9077524	7.5886467	7.6202724	7.4608104	7.8568498	7.9408842	7.7050922
5.2311106	4.990011	5.3511356	4.960639	5.0342917	4.7959824	5.8785543	5.8896564	5.8104792
9.1157527	9.0513236	9.0693881	8.7624568	8.6578515	8.7708248	8.5648824	8.5330981	8.5368002
8.0331391	7.9670807	7.989303	7.7756844	7.6132235	7.8359532	7.2309465	7.0645959	7.3252084

2.8659503	2.7060624	2.9650943	3.1593576	3.1366761	3.1302769	3.6422468	3.9608643	3.1834889
8.9297122	8.9031944	8.8448067	8.8606689	8.849995	8.7770946	9.0124225	8.98141	8.9834076
10.731105	10.63819	10.71194	10.613057	10.635836	10.493127	10.150462	10.150701	10.08965
9.4159293	9.3786202	9.3419653	10.125649	10.108475	10.048333	10.869543	10.779532	10.896178
10.53647	10.555873	10.401202	10.656927	10.563627	10.654223	10.562778	10.480404	10.582441
9.6215315	9.5512284	9.5807352	9.4114864	9.382887	9.3461149	9.0466612	8.970358	9.0610007
6.152168	5.8962332	6.278656	5.7896485	5.8018185	5.6881887	5.8785543	6.0096728	5.6734543
3.2116748	2.9612664	3.3712718	3.3105119	3.421282	3.1302769	3.4829147	3.9608643	2.7098437
5.8732383	5.6999474	5.9338433	5.6548533	5.8743662	5.2925838	5.3648568	5.3219704	5.3528418
4.9222583	4.7403036	4.9968831	4.3066864	4.123159	4.4006546	3.9772738	4.2801452	3.5394718
5.6265616	5.801426	5.30555	6.0588757	6.3001473	5.6613247	5.3418203	5.3676132	5.2601706
9.4352561	9.4270583	9.3306311	8.9711776	8.9624313	8.8855272	8.8626723	8.8468801	8.8185225
8.6794998	8.7252416	8.7157044	8.9425263	8.9202171	9.0051934	7.8405077	7.9484135	7.8709622
6.4631658	6.427521	6.3920436	6.6398944	6.5859108	6.6031543	6.4628353	6.3193942	6.5389689
8.3908053	8.4406697	8.4326222	8.5717088	8.584062	8.632443	7.8850104	7.9103677	7.924787
5.7726365	5.7683866	5.6717113	6.2107651	6.2095768	6.1217037	6.1143131	6.1728291	5.9943291
12.694303	12.659322	12.6182	12.2302	12.167858	12.199936	11.024506	10.9866	11.002808
8.2575603	8.3032542	8.0935639	8.2665709	8.0385214	8.3798863	8.007175	7.9781438	7.9766757
10.644203	10.540055	10.638005	10.241905	10.218237	10.171248	9.9024507	9.8785357	9.8661762
7.4946757	7.5378203	7.3347554	7.352074	7.2630586	7.3474123	7.0720502	6.8879188	7.1809948
8.174565	7.9743425	8.2759883	8.1207463	7.9349098	8.2141804	8.1130106	8.1962094	7.9983548
8.2791128	8.2800001	8.1907952	7.8485302	7.8475074	7.7808596	8.064585	8.0912928	7.9983548
7.2670792	7.4571837	6.9165019	7.1210186	7.1604128	6.9853361	6.8203484	6.5742331	6.9777419
8.2388269	8.1577516	8.2094791	8.5623237	8.5364163	8.4946646	7.0076424	6.8721706	7.076165
7.9395655	8.038105	8.0178508	7.8941306	8.0800152	7.9623821	7.7161446	7.8229584	7.7568857
10.32924	10.231574	10.338927	10.149035	10.181776	10.037928	10.055425	10.147434	9.9163857
6.5669427	6.4896552	6.537159	6.5053192	6.1129374	6.7378252	5.813061	5.72407	5.8427942
10.86642	10.849408	10.770783	10.795548	10.698787	10.796039	10.790397	10.814973	10.703965
8.8393358	8.6591862	8.8995273	8.4928856	8.3066588	8.5724257	9.1464191	9.1278568	9.1116094
12.37227	12.314915	12.318112	12.997044	12.922613	12.977203	13.679357	13.627223	13.671016
7.7585181	7.7302597	7.6766266	7.2435177	7.1409391	7.2516067	7.2558075	7.3862163	7.0487225

10.181746	10.174394	10.077631	10.070444	10.065223	9.9806764	10.175923	10.163697	10.127852
2.2197205	2	2.3976902	2.7992097	2.5651052	2.9643661	3.2051098	3.1926715	3.1834889
2.8659503	3.1780396	2.3976902	3.630911	3.5449709	3.6508626	2.7248895	2.9740279	2.3981461
7.4841095	7.4675132	7.3904001	7.4772031	7.2079771	7.622746	7.3220454	7.329682	7.2549028
6.8967025	7.0936294	7.2410034	6.8081477	6.9638538	6.9301405	6.525513	6.5742331	6.7749723
3.5724509	3.3664558	3.6878577	3.8929252	3.421282	4.1912539	3.9162052	3.7004723	4.0628589
6.7007286	6.8782618	6.3700678	6.756411	6.6561828	6.763314	7.0508988	6.9789602	7.062509
7.4300856	7.2692224	7.475195	7.4970421	7.4800739	7.4213264	7.5094372	7.408224	7.5473614
7.7408745	7.5572944	7.8213144	7.7560603	7.6342676	7.7870861	7.6092734	7.5731522	7.5955693
8.9869635	8.9628234	8.8995273	9.3701306	9.415669	9.2252164	9.9846872	9.9459086	9.9630701
3.7939455	3.8177796	3.6878577	4.5387765	4.4101943	4.5834785	5.0606888	4.8931255	5.1611356
11.516817	11.424076	11.497375	11.092093	11.072841	11.016754	10.746669	10.772126	10.659301
7.303593	7.2926494	7.2042138	6.8763857	6.7491456	6.9074571	6.1545292	6.1728291	6.0781548
2.8659503	2.9612664	2.7091089	3.6872592	3.2859859	3.9462135	3.396124	2.4021099	3.9487208
8.9120842	8.8993845	8.8125799	8.4909083	8.5214337	8.3633132	8.8525214	8.8101591	8.8346806
9.0055533	9.0024339	8.8956868	8.6553028	8.5804523	8.6358968	8.6749688	8.6850456	8.6043304
6.152168	6.2232429	5.9634986	6.2765215	6.4968007	5.9098007	4.9431589	5.0692034	4.748599
8.0001475	7.8054746	8.0733058	8.055349	7.9349098	8.0786187	8.3421284	8.3856007	8.2480029
8.10386	7.9524464	8.139755	7.5886467	7.5183093	7.5659411	7.237202	7.2708417	7.1425707
2.4103584	2	2.7091089	2.9904928	2.7817736	3.1302769	3.7157657	3.1926715	4.0628589
8.2102627	8.2201704	8.1718661	7.4891392	7.5481843	7.4685791	5.4318384	5.1756805	5.6370634
10.20123	10.211741	10.075935	10.120542	10.056155	10.090479	10.327997	10.250007	10.343638
5.4203434	5.4310687	5.30555	5.7372319	5.8505866	5.5189462	5.8785543	5.9509123	5.7435929
6.3396668	6.013667	6.5173007	6.0695146	6.2824824	5.7145616	5.0886338	5.4118562	4.6052608
11.717898	11.683662	11.641936	11.535777	11.463725	11.513358	11.662852	11.586686	11.678371
8.7951411	8.6410485	8.8328059	8.2314732	8.16923	8.2002504	8.5821862	8.4821341	8.6183989
8.6725368	8.6364782	8.5977384	8.4240868	8.391505	8.3633132	7.7250742	7.5134916	7.8552075
3.7939455	2.7060624	4.357879	3.3805373	3.421282	3.2790605	3.0991973	2.7162254	3.3724324
5.1262912	5.2028082	4.9383494	5.0916718	5.1163526	4.9811664	6.2442239	6.0381793	6.3718678
8.8100236	8.8047911	8.702575	7.9812226	7.9235576	7.9457804	8.29193	8.2274904	8.2954747
9.2972148	9.2408889	9.2873168	8.7722588	8.7247833	8.7645276	7.7903405	7.8229584	7.7138546

4.3997744	4.4381381	4.2656663	4.0299647	3.8628381	4.1141074	2.9848891	2.7162254	3.1834889
3.9859168	4.1610493	3.6878577	3.5111488	3.1366761	3.7561757	3.7857191	3.7004723	3.8247709
5.1796526	4.7403036	5.4798392	5.322668	5.4046183	5.1452616	6.04469	6.1468857	5.8744013
2.5787203	2.3958007	2.7091089	3.793754	3.5449709	3.9462135	4.0921636	4.1813664	3.9487208
8.4381038	8.3981042	8.4058702	8.2572947	8.1547388	8.2862839	8.0680984	8.0144653	8.0887063
8.1210115	8.0030299	8.1267078	7.9090159	7.8354433	7.88902	8.1299189	8.0498948	8.1480994
2.4103584	2.7060624	2	3.1593576	3.1366761	3.2790605	3.396124	3.1926715	3.5394718
8.716079	8.755062	8.5594113	8.7689989	8.8012065	8.6393424	8.9240621	8.8668839	8.9204538
7.5412887	7.315702	7.6403649	7.5204943	7.5333241	7.4213264	7.3797314	7.23435	7.4563072
8.9832166	8.9481456	8.9223582	8.9612142	8.942885	8.8884259	8.8767646	8.8101591	8.8820969
2.7294717	2.3958007	2.9650943	2.8980192	2.7817736	2.9643661	3.3037759	3.7004723	2.7098437
4.8906042	5.1524786	4.4445501	4.5690822	4.4738406	4.5834785	4.6374231	4.075324	5.0547979
6.2675644	6.0415904	6.3700678	6.4575188	6.4018221	6.4229682	6.1676892	6.247972	6.0228156
9.298723	9.128211	9.3532112	9.0390584	8.9568736	9.0264018	8.7707934	8.6895454	8.7898027
7.3682252	7.1965685	7.422785	8.1955003	8.0800152	8.2141804	8.7046161	8.5330981	8.8021812
7.5463762	7.487953	7.4956373	7.4407906	7.3504308	7.4372499	7.8487019	7.7558875	7.8787755
7.321509	7.5765091	7.5455105	7.6215525	7.7286077	7.8538599	6.652638	6.9935905	6.9922202
6.7719527	6.7655792	6.6690026	6.970212	6.9858514	6.8609914	6.4304448	6.4524332	6.3495506
8.561334	8.599385	8.4166306	8.1079018	8.1302584	7.9896339	8.7446856	8.6940312	8.7349001
5.8238141	5.6644705	5.872639	6.481617	6.2281515	6.6172014	5.4534919	5.3676132	5.4815611
9.2589885	9.0649931	9.3306311	8.8999445	8.8469936	8.8591735	8.8667128	8.7337883	8.9317648
6.217413	6.1222572	6.2060835	6.9473212	6.836479	6.9744646	7.1928328	7.25878	7.062509
7.1041317	7.0253511	7.0743297	7.2575425	7.0499006	7.3558145	7.4021751	7.4723016	7.2668609
10.852111	10.773388	10.819233	10.476831	10.378559	10.478774	10.082603	10.040532	10.071016
6.0412208	6.148174	5.8087227	6.4080802	6.4500929	6.2713297	6.2442239	6.0661334	6.3495506
5.8404777	5.8653179	5.7072032	5.6548533	5.6447666	5.5775942	6.1143131	6.2233567	5.935611
6.1654552	5.801426	6.3700678	5.4089726	5.4369156	5.2925838	5.4318384	5.123424	5.6370634
8.9355406	8.9031944	8.8567085	8.9367272	8.9762326	8.7988268	8.5599001	8.5919695	8.4659531
8.520554	8.5320847	8.395029	8.4988015	8.525194	8.3757608	7.7945884	7.6020821	7.9096123
7.0972149	7.0253511	7.0606743	7.058307	6.7746421	7.2151282	7.0859808	6.9935905	7.1163741
3.6501227	3.3664558	3.8234137	3.1593576	3.1366761	3.2790605	2.5749062	2.9740279	2

6.9125496	7.0253511	6.6690026	6.5515865	6.6423996	6.3575325	6.5953533	6.631482	6.4989729
3.9859168	3.8177796	4.0614204	4.4761857	3.7644639	4.8915437	4.5191927	4.3725921	4.6052608
3.9859168	3.9415025	3.9473196	4.5078205	4.7557305	4.1141074	4.9431589	4.9542373	4.8789742
8.8057871	8.8007118	8.6981719	8.4689765	8.4361274	8.4245045	8.1399696	8.2761708	7.9472539
9.8475214	9.8246789	9.7604205	9.5537236	9.4964227	9.5169287	9.076622	9.0354349	9.0575628
6.3160308	6.2474223	6.278656	6.481617	6.4180925	6.4546057	5.6538328	5.9206094	5.2601706
10.543484	10.45566	10.519474	10.408815	10.343848	10.379252	9.6655745	9.601287	9.6730107
9.4461849	9.397651	9.3864321	9.4587885	9.4055044	9.4180893	8.9259996	8.9060782	8.8937115
3.7238255	4.2594452	2.7091089	3.5111488	3.2859859	3.7561757	3.2051098	3.3824986	3.1834889
7.7364297	7.8691914	7.8454066	7.6537244	7.702277	7.7683256	7.6140883	7.6765069	7.806884
7.9043358	7.6778456	8.0036475	8.0256076	7.9121154	8.0426401	7.9281813	7.9633552	7.831248
8.7714396	8.7423572	8.689325	8.5528771	8.5912544	8.416494	8.6161834	8.5774753	8.5948746
4.756549	5.5907872	5.8410348	4.1945222	4.9472802	5.2571394	3.8524369	4.6925307	4.8152588
5.205611	5.4726864	4.7469892	5.3047698	4.7035875	5.6613247	4.6747739	4.2801452	4.9399942
11.834002	11.798578	11.757539	11.969271	11.854785	11.986905	11.21046	11.174081	11.186358
8.3708695	8.3653351	8.4219809	8.0283368	8.1449964	8.0937677	7.356933	7.6855458	7.6422183
6.6258049	6.5685453	6.5951447	6.603754	6.5568147	6.5601733	6.0303529	6.1204673	5.8744013
5.0132553	5.1003295	4.8136363	5.6548533	5.4046183	5.7909055	4.7813527	5.0692034	4.3594016
5.3753109	5.6280992	5.0531343	4.6278481	5.0759055	4.334175	4.3447825	4.7625408	3.6891623
6.0555648	5.9561447	6.0489734	5.9129186	5.8505866	5.8867927	6.2688591	6.1728291	6.3038532
11.351544	11.275469	11.316012	12.159293	12.090025	12.133686	12.465994	12.439136	12.432437
4.8251331	4.0554482	5.3511356	5.0493029	4.9472802	5.2925838	5.6350052	5.65212	5.6734543
8.8100236	8.8088588	8.6981719	8.4136868	8.3749303	8.35914	8.1399696	8.1249062	8.0954276
5.0988516	5.2028082	4.8773401	5.3047698	5.4046183	5.1059542	5.8944734	6.0661334	5.6370634
6.3396668	6.2474223	6.3250858	5.864867	5.8505866	5.7909055	5.6907665	5.7926015	5.5220361
3.1053801	2.7060624	3.3712718	3.3805373	3.7644639	2.7768658	2.4075018	2.4021099	2.3981461
6.6064498	6.4896552	6.6139666	6.5209071	6.4813989	6.470168	4.8151984	4.6925307	4.8789742
8.7583455	8.6815424	8.7243914	8.4157728	8.3665709	8.3716236	8.516841	8.4293037	8.54173
7.083281	7.2211953	6.8062596	6.5515865	6.4968007	6.5307911	7.2120155	7.0783871	7.2787207
5.205611	5.251441	5.1594558	5.5217324	5.8018185	5.1059542	6.2442239	6.3424369	6.0781548
10.718202	10.615146	10.70866	10.356563	10.238993	10.376158	10.829737	10.823187	10.775812

3.1053801	2.9612664	3.1824306	5.5674845	5.7513439	5.2571394	5.1952966	5.123424	5.2115027
8.7605362	8.5804746	8.8207044	8.5299496	8.4440944	8.5210534	7.916535	7.8633475	7.9096123
4.1010311	3.9415025	4.1671548	3.8441915	3.7644639	3.8543214	3.9772738	3.7004723	4.1686253
9.1727917	9.1085451	9.1262142	8.896961	8.9144941	8.7926509	9.2826942	9.2698413	9.2384806
7.1516424	6.8469547	7.3118824	7.917874	7.9629056	7.7746062	8.3906386	8.2999086	8.4184998
2.9906265	3.6824426	3.3712718	3.5722723	3.6588876	3.6508626	3.7857191	4.3725921	3.9487208
11.766393	11.637522	11.78375	11.980237	11.998315	11.871885	12.159881	12.173635	12.087781
12.107483	12.038747	12.064669	11.721569	11.699964	11.649017	10.91366	10.871512	10.895215
6.4414835	6.271203	6.4971652	6.4493958	6.2095768	6.6031543	5.8785543	5.65212	6.0228156
5.527134	5.6999474	5.2098157	5.2682931	4.34361	5.8154814	4.5191927	4.3725921	4.6052608
2.9906265	3.6824426	2.9650943	2.9904928	2.9701107	3.2790605	2.9848891	2.9740279	2.9660172
10.54412	10.467288	10.509457	10.456203	10.423577	10.396663	10.815698	10.73669	10.832229
7.6680597	7.7388132	7.475195	7.5435713	7.4878025	7.5068075	7.0859808	6.7568398	7.3021518
9.0968054	9.0375233	9.0453226	9.0618819	9.0380713	8.994471	8.7225623	8.7162536	8.6733398
5.6072167	5.5131371	5.5979955	5.3921198	4.9472802	5.6613247	4.9431589	5.1756805	4.6052608
6.4738859	6.4896552	6.3477521	6.2107651	6.1129374	6.2170221	6.058886	5.9206094	6.1314494
8.4132567	8.291674	8.4219809	8.3670146	8.3066588	8.3338441	7.5947313	7.5236079	7.6050206
4.9532328	5.1524786	4.6036804	5.0493029	4.854682	5.1452616	5.0886338	5.2748363	4.8789742
11.671504	11.586307	11.644799	11.650053	11.58982	11.615732	12.022442	11.976918	12.007205
9.0563654	9.0024339	8.9995358	9.7995898	9.8117576	9.6913826	10.772203	10.771065	10.712734
7.391028	7.5180797	7.1276945	6.6821007	6.6833604	6.5889691	7.3279189	7.1584751	7.4246323
4.1010311	3.9415025	4.1671548	3.741489	3.5449709	3.8543214	3.396124	2.7162254	3.8247709
2.7294717	2.3958007	2.9650943	2.9904928	2.9701107	2.9643661	2.8607385	2.7162254	2.9660172
8.1480367	8.2201704	7.9528049	8.4323531	8.4559629	8.31242	8.2523289	8.2880885	8.1545502
9.8639149	9.7589194	9.8583855	10.045063	9.9869461	10.008921	10.524289	10.511235	10.477011
6.9590724	6.7822257	7.0189149	7.141331	7.1507088	7.0384976	5.8461792	6.0096728	5.6370634
10.14197	10.066408	10.107823	9.8272799	9.8255462	9.7355149	9.8401141	9.8844354	9.7474441
7.7497233	7.7808361	7.6031681	7.4325725	7.3419279	7.4293101	7.2862931	7.5134916	6.9483421
7.958772	7.8535241	7.9528049	8.006357	7.9178478	8.0269417	7.3740655	7.3064331	7.4352681
6.6823593	6.6250049	6.6325462	6.7027497	6.6833604	6.6448917	5.6907665	5.72407	5.5997308
9.0055533	9.0200853	8.8763293	8.7376565	8.6917055	8.6900628	8.5372643	8.55299	8.460757

8.5911798	8.5072672	8.5642582	8.4157728	8.2757432	8.4561087	7.6236703	7.5336538	7.6513698
8.1107451	7.9670807	8.139755	7.9727462	8.0065987	7.8419468	8.0855382	8.1115546	7.9983548
7.924014	7.8376847	7.9001049	7.7853972	7.7415951	7.7556816	6.9013619	6.9789602	6.7581273
9.3736218	9.3565589	9.2784956	9.3186632	9.2975171	9.2502202	9.0341413	9.0634417	8.9429878
8.174565	8.0991683	8.2156537	8.2688807	8.1253121	8.384	7.3395946	7.2708417	7.4770439
7.6011853	7.7302597	7.3347554	7.6430799	7.6956185	7.4916375	8.0959017	8.1706868	7.9546658
7.7185122	7.6234552	7.7032371	7.6251628	7.6342676	7.5292676	7.8030468	7.7898127	7.7568857
8.6514436	8.5946806	8.5977384	8.8452735	8.8166309	8.7802193	8.1794864	8.1315359	8.1673659
3.8608147	3.6824426	3.9473196	3.741489	3.8628381	3.5372523	3.3037759	3.3824986	3.1834889
6.7098263	6.6793379	6.8858542	6.2011218	6.3348412	6.3741717	6.6712383	6.7044429	6.8243582
5.3753109	5.3440487	5.30555	4.5387765	4.8060545	4.1141074	5.4098549	5.72407	4.9399942
8.1312054	8.195526	7.9453932	8.3030907	8.2622881	8.2506825	7.9887555	8.0358273	7.8787755
5.4203434	5.1003295	5.5979955	5.2497029	4.9914418	5.3939735	4.9734511	5.0692034	4.8152588
8.2419661	8.1641164	8.2094791	8.2572947	8.0542218	8.3507572	8.6423445	8.6577478	8.5709604
6.0838321	5.9264999	6.2306822	6.2673093	6.1327901	6.3064312	4.5990795	4.1813664	4.8789742
6.1654552	6.271203	6.129666	6.3481756	6.3687204	6.2353527	5.6538328	5.9206094	5.2601706
4.1553175	3.9415025	4.2656663	4.5387765	4.5932826	4.4006546	4.8805969	4.7625408	4.9399942
5.3753109	5.8653179	5.4798392	5.7505157	5.7513439	5.9098007	5.8461792	6.0381793	6.1050482
8.4435678	8.4248544	8.3508305	8.6177402	8.4986641	8.6393424	8.6185814	8.5871543	8.5901234
4.487029	4.9315181	3.6878577	4.84155	4.8060545	4.8445541	5.2704178	5.2261101	5.3528418
6.152168	5.5524845	6.4971652	5.7636782	5.5296835	5.8867927	6.4084399	6.2959775	6.4578363
6.0120976	8.2201704	8.3895777	5.5971992	8.4281163	8.3674744	5.941203	8.7977081	8.7604996
8.4891943	8.3653351	8.499941	8.1410624	8.1547388	8.0321935	6.9170326	6.7907429	6.9777419
9.332983	9.3199737	9.2335623	9.2652915	9.1199235	9.311977	9.3390377	9.2240484	9.3883121
9.335924	9.4003493	9.2244052	8.7409879	8.7116431	8.7358452	8.4046013	8.4559607	8.3749676
8.6538026	8.5613129	8.653385	8.1930699	8.1003229	8.195577	8.4564604	8.3911366	8.4865526
4.6087492	4.2594452	4.8136363	5.0276426	4.854682	5.1059542	4.297674	4.2801452	4.2671646
2.5787203	2.3958007	2.9650943	2.7992097	2.9701107	2.5613046	3.3037759	2	3.9487208
6.4738859	6.4896552	6.3700678	7.3389429	7.2357807	7.3807314	7.7775213	7.7898127	7.7904097
8.3183043	8.376341	8.139755	8.1207463	8.0226484	8.1235965	8.3536902	8.2520359	8.3914465
7.3796716	7.5081072	7.1145373	7.352074	7.4644915	7.1295144	7.2246637	7.2466167	7.1425707

10.089785	10.018035	10.050253	9.7640079	9.7568462	9.6763667	9.5011656	9.4818462	9.4603756
3.1053801	2.7060624	3.3712718	3.3105119	3.421282	3.1302769	3.4829147	3.1926715	3.6891623
8.7317953	8.4820153	8.8487849	8.2123993	8.1449964	8.1861846	8.1061914	8.2150593	7.924787
2.8659503	2.9612664	2.7091089	2.7992097	3.1366761	2.30778	3.0991973	3.1926715	2.9660172
8.5487136	8.376341	8.6024585	9.3701306	9.3535787	9.2924135	10.295394	10.178178	10.347866
12.050211	12.008117	11.9806	11.426999	11.403711	11.356297	11.460512	11.450154	11.411093
5.6072167	5.6999474	5.3953249	6.2107651	6.1523733	6.179647	6.932535	6.8238675	6.9777419
7.0692112	7.4043967	7.1536545	6.8642164	6.9858514	7.0384976	7.0508988	7.1969113	7.1163741
8.4104693	8.2974757	8.4112605	7.7918363	7.77987	7.7105343	7.8730088	7.8473273	7.8392788
9.2941937	9.2588418	9.218268	8.996486	8.9144941	8.9836684	9.6713609	9.6413244	9.6412093
8.0865021	8.0655638	7.9964931	7.2714321	7.2449308	7.2058626	7.7860801	7.8064807	7.7050922
9.9041018	9.8545013	9.842513	9.3765707	9.355692	9.3033148	9.0887801	9.1606385	8.9504216
6.2920011	6.5685453	5.8087227	6.0905599	6.0928076	5.9983304	6.1936545	6.0381793	6.28045
8.3936309	8.3653351	8.3110139	8.1610965	8.1547388	8.0735335	8.0035099	8.0144653	7.9323149
5.3522564	5.251441	5.3511356	5.4420989	5.193996	5.5775942	5.3418203	5.3676132	5.2601706
6.4194705	6.4062003	6.3250858	6.0905599	6.0306763	6.0613354	6.1936545	6.1728291	6.1573762
3.8608147	4.1610493	3.3712718	4.2702587	3.9549305	4.4642052	5.1429507	4.5414122	5.5220361
8.9258134	8.8993845	8.8408175	8.7689989	8.8043046	8.6358968	8.7225623	8.8468801	8.5219088
9.705795	9.5824532	9.7153763	9.5678622	9.5002486	9.5410762	9.3806033	9.3990957	9.3008744
7.7759486	7.7473162	7.6944214	8.1207463	8.1740283	7.9678738	9.1093873	9.1958583	8.9541241
9.0147592	9.1184116	8.7796168	9.4198251	9.3745738	9.3711984	9.0887801	8.890528	9.2077934
5.0132553	5.0462245	4.8773401	4.8661672	4.7557305	4.8915437	4.0921636	3.7004723	4.3594016
9.4434604	9.3509904	9.424247	9.2182598	9.2596472	9.0780979	8.9528545	8.890528	8.9541241
8.7205869	8.7423572	8.5834848	8.4829716	8.4119592	8.460011	7.7945884	7.9633552	7.5375235
4.5287539	4.0554482	4.8136363	4.7117388	4.649489	4.6936396	5.1429507	5.0692034	5.1611356
8.260659	8.2262662	8.1845131	8.2549663	8.284644	8.1285085	8.6470505	8.5330981	8.6956283
6.2920011	6.2232429	6.2548685	6.1317512	6.1129374	6.0817401	5.4098549	5.3219704	5.4399178
3.2116748	3.1780396	3.3712718	2.9904928	2.5651052	3.2790605	2.4075018	2.4021099	2.3981461
3.9247213	3.9415025	3.8234137	4.3066864	4.7557305	4.1141074	4.559689	4.5414122	4.9399942
9.7993251	9.7942269	9.7000435	9.2973246	9.2975171	9.2206233	9.3763585	9.2516982	9.4393962
5.9210252	5.8962332	5.8410348	6.2298604	5.8743662	6.4388737	6.5658347	6.5347647	6.5389689

5.0988516	5.2028082	4.8773401	5.1529798	4.854682	5.3271781	3.7157657	3.550228	3.8247709
14.269965	14.218492	14.209915	14.291078	14.236087	14.252306	14.429567	14.376647	14.421994
7.9927124	8.0723476	7.7885525	7.89712	7.8654167	7.8359532	7.7645872	7.6020821	7.8552075
4.0446218	3.3664558	4.4445501	3.8441915	3.5449709	4.0326014	3.7857191	3.7004723	3.8247709
5.6265616	5.5131371	5.6353242	4.6563572	4.649489	4.5834785	5.1160479	5.2748363	4.8789742
9.5624911	9.5439256	9.4688713	9.0987039	9.0795778	9.0237678	8.6041333	8.4976132	8.6461308
10.709157	10.635902	10.670959	10.735437	10.64882	10.726468	9.5141032	9.3852929	9.5754229
10.896612	10.958707	10.847342	10.811876	10.780501	10.883629	10.715084	10.703703	10.771622
5.9366088	5.8962332	5.872639	5.1328319	4.9472802	5.2208023	5.5770017	6.0661334	4.748599
9.7961252	9.8446289	9.8760357	9.6952595	9.6914193	9.8046615	9.4223779	9.3769473	9.618057
8.8639951	8.9481456	8.653385	9.2146698	9.1902926	9.1474948	8.969858	8.955489	8.924234
9.4529735	9.3481981	9.4454187	9.3153148	9.2505903	9.2858329	9.0032756	8.8866142	9.0575628
12.144019	12.11629	12.059958	11.828001	11.808478	11.75287	12.337238	12.323745	12.290286
9.5100738	9.4967963	9.4215786	9.0578804	9.0872291	8.9507666	8.6113754	8.5480427	8.6137246
6.7719527	8.9664696	9.0897009	6.603754	8.7856154	8.9114081	7.4132674	9.4870254	9.4046402
8.4215867	8.3318043	8.4004598	8.2963133	8.1595854	8.3338441	8.5270888	8.5774753	8.4131296
8.0185692	7.9376621	7.989303	7.8485302	7.8048339	7.7994589	7.6890193	7.6490456	7.6695005
11.514222	11.577393	11.496107	11.452879	11.453401	11.489693	11.517518	11.51877	11.597229
7.3508834	7.5572944	7.1536545	7.6106671	7.6754567	7.6773987	8.3536902	8.3632425	8.3300807
8.8205608	8.755062	8.7754429	8.6013376	8.5512448	8.5579336	8.4671446	8.4612336	8.4131296
8.1876484	8.320452	7.9153595	7.5737775	7.4723038	7.5803534	6.9402241	6.9341575	6.8876817
2.9906265	2.9612664	2.9650943	2.8980192	2.7817736	2.9643661	3.0991973	2.7162254	3.3724324
5.07088	5.1524786	4.9383494	6.339411	6.4500929	6.1217037	6.5855807	6.8074002	6.2566609
3.4903583	3.6824426	3.1824306	4.3422168	4.34361	4.2644835	3.3037759	3.3824986	3.1834889
3.9859168	3.9415025	3.9473196	4.2702587	3.7644639	4.5834785	4.297674	4.1813664	4.3594016
4.569306	4.5971173	4.4445501	4.5987644	4.4738406	4.6936396	6.0868657	5.7926015	6.28045
6.2301152	6.5097858	5.7418429	6.6611519	6.8120614	6.3906213	6.2688591	6.3874467	6.0781548
10.526207	10.942137	10.875889	10.396721	10.767861	10.741775	10.754868	11.020035	10.994741
8.5487136	8.6410485	8.3282129	8.3452903	8.3454582	8.2596656	7.9204275	7.8229584	7.9620398
7.9739553	7.8135948	8.0178508	7.6143047	7.6956185	7.4293101	6.6712383	6.9034969	6.3268829
7.391028	7.4151098	7.2650187	7.8851251	7.979446	7.7235784	8.6564168	8.6805317	8.5948746

6.9666825	6.9971086	6.8702827	6.9415413	7.0808895	6.7758915	7.12023	7.0224134	7.2668609
9.0898533	8.9031944	9.1554162	9.8705075	9.8587071	9.7892348	11.125755	11.123429	11.067476
4.2076351	4.1610493	4.1671548	4.0728969	3.8628381	4.1912539	4.6747739	5.0692034	4.0628589
6.6543592	6.8310427	6.3477521	6.618319	6.5714361	6.5889691	5.8785543	6.0935561	5.6370634
8.1480367	8.2140486	7.9601788	7.8821107	8.0119684	7.6366045	7.9850433	7.9484135	7.9620398
7.8963888	7.8296993	7.8612475	8.2572947	8.2024874	8.218794	8.3392234	8.2025201	8.4077393
2.8659503	2	3.3712718	3.3805373	3.2859859	3.4139246	3.4829147	4.075324	2.3981461
6.8062881	6.6970036	6.8062596	6.9871448	7.0288652	6.8491373	6.885519	6.8401489	6.8721095
6.9967274	7.1069057	6.7563059	6.8017811	6.7491456	6.763314	6.752094	6.5935696	6.8404516
7.8353483	7.6955294	7.8612475	7.4448821	7.375643	7.4213264	7.2619564	7.2708417	7.1935787
13.477886	13.431597	13.412492	12.970326	12.879934	12.964725	12.844872	12.809471	12.820249
6.3278972	5.9264999	6.5567476	6.6327384	6.4500929	6.711878	7.7645872	7.9180576	7.527618
6.5868315	6.5492235	6.5173007	6.2856753	6.0723931	6.3906213	5.2946134	5.4547826	5.0547979
4.9532328	4.8068978	4.9968831	6.1215631	6.0306763	6.1217037	4.4346216	4.5414122	4.2671646
4.7209942	4.6704862	4.6771135	5.0276426	4.4101943	5.3939735	4.4346216	3.9608643	4.748599
6.8887132	7.011299	6.6325462	6.7163533	6.7619501	6.5746431	6.9402241	6.7221209	7.076165
7.8641555	7.712999	7.9001049	8.4629368	8.3997215	8.4324707	7.7206163	7.5436303	7.8231723
2.9906265	3.3664558	2.3976902	2.8980192	2.9701107	2.7768658	2.9848891	2.7162254	3.3724324
5.2561672	5.1003295	5.30555	5.3750678	5.4046183	5.2571394	5.0031203	5.2261101	4.6787093
2.7294717	2.7060624	3.3712718	2.5786313	2.5651052	2.5613046	2.8607385	3.1926715	3.1834889
8.7092907	8.8047911	8.539859	8.4426199	8.4480614	8.4245045	7.5145965	7.6304432	7.3591167
5.1796526	5.3440487	4.8773401	4.4761857	4.7035875	4.1141074	5.0321917	5.0128652	4.9985377
10.267425	10.194744	10.228761	9.9359232	9.8542298	9.9224733	10.015039	9.9683022	10.002939
9.8689999	9.8701581	9.7540711	9.8051705	9.7488283	9.7673566	8.9903717	8.9923781	8.9355155
4.0446218	3.5330831	4.357879	4.7650729	4.7557305	4.6936396	5.0031203	5.0128652	4.9399942
7.0902647	7.2692224	6.77315	7.7950451	7.7480452	7.7493179	8.5270888	8.5280819	8.4659531
9.1395131	9.1151303	9.0522396	8.9191885	8.7824769	8.9563026	8.6137814	8.6669046	8.4967431
9.5972243	9.4687902	9.6114999	9.5413571	9.5323656	9.4557077	9.3592534	9.4073145	9.2475609
7.7585181	7.6955294	7.7119993	7.4366874	7.4487389	7.3304597	7.0997782	6.9789602	7.1554928
5.5677309	5.2984878	5.7072032	5.1924505	5.1163526	5.1835263	5.9864665	5.8896564	6.0228156
5.5876089	5.7683866	5.2584768	4.3768933	4.2004465	4.4642052	3.7157657	3.550228	3.8247709

8.367999	8.2079008	8.4112605	8.7124223	8.698382	8.632443	8.4698033	8.445357	8.4397831
12.021956	12.005018	11.926103	11.893458	11.857771	11.835549	12.855585	12.832287	12.819234
6.0838321	5.8653179	6.1810581	6.2487064	6.2095768	6.1984556	6.3169023	6.4734607	6.0781548
9.2774636	9.2348547	9.2121046	8.9569231	9.0195298	8.7926509	8.969858	8.8787544	9.0014124
8.6034357	8.5369974	8.5594113	8.2195816	8.16923	8.1767306	7.9511957	8.0144653	7.8231723
9.5048565	9.4584698	9.4401549	9.3829822	9.3932116	9.2770118	9.5192459	9.5024524	9.4759124
4.6471428	4.5198165	4.7469892	4.84155	5.1556966	4.334175	3.5647792	3.3824986	3.6891623
10.368548	10.318308	10.307474	10.172047	10.181776	10.068922	9.6527622	9.5428023	9.6975148
5.7898982	5.5907872	5.9035657	5.3047698	5.2313049	5.2925838	5.2704178	5.3676132	5.1089463
9.5486138	9.521793	9.4662842	9.657855	9.5564522	9.6628867	9.6279817	9.5941047	9.6016264
9.6560651	9.6036812	9.5973823	9.3914868	9.3849578	9.3054853	8.735877	8.8142857	8.5901234
8.4435678	8.4195439	8.3564299	8.6570674	8.5475519	8.6699881	8.8586205	8.8347434	8.822579
10.61605	10.511637	10.607807	10.028119	10.049643	9.9096897	10.287127	10.281646	10.232172
12.25985	12.183233	12.224767	12.227827	12.207596	12.155507	12.295884	12.280776	12.250942
8.48655	8.5172455	8.3620077	8.0660138	8.085119	8.0269417	7.6613742	7.7034553	7.6422183
6.3396668	6.4692397	6.0763755	6.6327384	6.8120614	6.3236669	6.9170326	6.9492469	6.8243582
8.9336004	8.81696	8.9411118	9.07646	9.0433254	9.0473031	9.0484409	9.0977026	8.9504216
7.9739553	7.8376847	7.9964931	7.448962	7.375643	7.4293101	7.0650342	7.1969113	6.8563673
12.068451	12.065344	12.106805	12.564841	12.673911	12.567895	10.786137	10.883345	10.831222
3.4903583	3.9415025	4.1671548	4.0728969	4.4738406	4.0326014	4.0921636	4.5414122	4.4460942
9.3476286	9.1699226	9.4054635	8.8343982	8.8469936	8.7261563	8.2707415	8.445357	8.0055094
5.856951	5.9851925	5.6717113	6.1317512	6.1716943	5.9983304	4.9734511	4.8931255	4.9985377
8.7384788	8.7338248	8.6304592	8.7689989	8.6440842	8.7957421	8.9810834	9.0354349	8.8625292
2.8659503	3.1780396	2.3976902	2.7992097	2.3100466	3.1302769	3.3037759	2.7162254	3.6891623
7.5665485	7.5180797	7.5057508	7.6033642	7.4566367	7.6503311	6.9013619	6.9492469	6.7916228
7.7453057	7.7725289	7.6031681	7.2482078	7.2898303	7.1097789	6.4084399	6.4310946	6.3495506
11.522314	11.448399	11.484648	11.556251	11.533993	11.484434	12.086856	12.055717	12.057982
4.8906042	4.9315181	4.7469892	5.1924505	5.3715813	4.9811664	4.8151984	4.5414122	4.9985377
4.85824	4.5971173	4.9968831	4.8661672	4.8060545	4.8915437	5.4534919	5.3676132	5.4815611
4.8906042	4.7403036	4.9383494	4.0728969	3.7644639	4.2644835	3.9162052	3.9608643	3.8247709
9.1826274	9.0919496	9.161826	8.9103385	8.8796707	8.8473044	8.5599001	8.5027362	8.5564192

8.716079	8.7801408	8.5299826	8.5357157	8.4871431	8.4984641	8.8667128	8.9517476	8.7305889
12.366169	12.306668	12.314161	12.260984	12.197322	12.230242	12.101931	12.074762	12.069122
7.63501	7.6234552	7.5455105	7.4159947	7.38395	7.3558145	7.5145965	7.3064331	7.6422183
11.169813	11.131054	11.096867	11.405642	11.400132	11.315957	11.617115	11.599296	11.574558
6.9967274	6.9239842	6.9612862	7.3167885	7.1891391	7.3474123	5.9564492	5.9509123	5.9053308
5.5475752	5.4726864	5.5203094	5.9933403	5.9877173	5.9098007	6.058886	6.0381793	6.0228156
5.527134	5.3440487	5.5979955	5.9594213	5.5593263	6.1984556	6.2191608	5.9509123	6.3938449
9.6406866	9.6677945	9.4970273	10.085591	10.039162	10.037928	10.764645	10.698131	10.769522
8.5179666	8.5613129	8.3620077	7.1312105	6.8841084	7.2695058	6.9630487	7.0366117	6.8243582
8.0795001	8.058748	7.989303	8.1511142	8.1498758	8.0581695	6.7608045	6.7907429	6.6708196
8.396451	8.2262662	8.4484384	8.1486079	8.1449964	8.0633091	7.4132674	7.4723016	7.2904838
4.756549	4.9315181	4.4445501	4.7910183	4.4101943	5.0239729	4.1985751	4.6189492	3.5394718
8.1210115	8.5320847	8.5690889	7.7691726	8.4755294	8.3757608	7.4460414	8.0428782	8.019713
5.0423553	4.6704862	5.2584768	5.0276426	5.1163526	4.8445541	4.559689	4.2801452	4.748599
12.361487	12.272465	12.338414	12.12161	12.095094	12.053632	11.534443	11.51877	11.490366
5.8069558	5.7683866	6.1555909	5.6118302	5.6721581	5.6881887	5.9864665	6.0381793	5.9943291
6.6730862	6.7822257	6.4767447	6.9871448	6.8723477	7.0068364	7.1066277	7.145432	7.0065546
10.000855	10.01099	9.8799289	9.2348958	9.3408334	9.0184851	8.821633	8.890528	8.6911981
10.958032	10.909361	10.895252	10.255945	10.221717	10.196027	9.4016418	9.4609414	9.2774224
8.2637511	8.1577516	8.2581514	8.202767	8.1547388	8.1576348	7.3965968	7.4827099	7.2428448
9.3750522	9.3454004	9.2931677	8.773886	8.7149394	8.7390604	8.7772472	8.7206573	8.773131
8.7406998	8.7122702	8.6669677	8.7689989	8.7050278	8.7486634	8.8747599	8.8468801	8.8546272
9.7778572	9.7334657	9.7110121	9.3264459	9.2619026	9.2967839	9.0105977	9.0176505	8.9467095
6.4950901	6.5097858	6.3700678	6.1418678	5.9877173	6.1984556	6.1006547	5.9805917	6.1573762
4.756549	4.3515565	4.9968831	5.2497029	5.5883723	4.6936396	3.9162052	3.9608643	3.8247709
9.985954	10.025045	9.8284799	9.6857828	9.5546136	9.7176978	9.6748217	9.6343392	9.6549244
8.2293682	8.2503954	8.0935639	8.1053192	8.0594175	8.0581695	7.0294327	7.0506717	6.9483421
8.5738449	8.5369974	8.499941	8.5202878	8.4832823	8.4639027	8.2584926	8.1962094	8.2600181
11.269319	11.201591	11.22559	10.892354	10.778927	10.908116	11.125755	11.090632	11.100427
8.9355406	8.9370384	8.8247495	8.6850103	8.6578515	8.6185442	9.2857152	9.3229406	9.1859172
7.8763276	7.9887575	7.6311552	7.9030802	7.888955	7.8238907	7.8030468	7.7898127	7.7568857

8.2388269	8.7295396	8.7458829	8.0008093	8.5024841	8.416494	8.4987304	8.890528	8.7219279
8.3123437	8.3261393	8.1845131	8.6267728	8.562267	8.6009804	8.4018196	8.3856007	8.3582982
8.9120842	8.8330273	8.8802216	8.9540552	8.8855333	8.9284077	8.4591389	8.5078411	8.3470774
7.1649344	7.1715141	7.0606743	7.6143047	7.5990216	7.5366773	7.6706483	7.7558875	7.517644
7.6254264	7.7641735	7.3460574	7.2293553	7.1796271	7.2151282	7.4406305	7.5236079	7.2904838
4.7912485	4.6704862	4.8136363	5.2308701	5.3377701	5.0239729	4.8482682	5.2261101	4.2671646
12.06136	12.035279	11.975151	12.039271	12.009767	11.975364	11.488969	11.485517	11.431822
5.3980029	5.2984878	5.3953249	5.5971992	5.2313049	5.8154814	5.4098549	5.4547826	5.3072501
3.7238255	4.1610493	2.9650943	3.2369134	3.1366761	3.2790605	3.2051098	2.9740279	3.3724324
9.2085328	9.0751609	9.227464	8.7259354	8.744272	8.6115443	8.589539	8.5480427	8.5709604
7.1110155	7.0667046	7.0468884	6.7889632	6.5120399	6.9413499	6.7866248	6.7568398	6.7581273
6.1252208	6.148174	5.9925565	7.516612	7.5107428	7.4293101	6.8119911	6.9034969	6.6527056
3.2116748	2.7060624	3.5382314	3.3105119	3.5449709	2.9643661	2.4075018	2.4021099	2.3981461
9.1174629	9.0581745	9.0659747	8.820294	8.6646861	8.8738738	8.5116897	8.4612336	8.5018115
8.3033564	8.3318043	8.5642582	7.8046289	7.9573498	8.0786187	7.8030468	7.8633475	7.9323149
4.5287539	4.5971173	4.357879	5.0706429	5.0342917	5.0239729	5.0321917	5.123424	4.9985377
7.6866109	7.6234552	7.6403649	7.8821107	7.8171557	7.8538599	7.6935758	7.5535382	7.76534
9.2324038	9.1540221	9.1996983	9.4250124	9.4034629	9.3524268	10.472866	10.389294	10.494915
9.1259837	9.0270858	9.1130433	9.1770408	9.1879223	9.0704606	9.3635487	9.4045801	9.2595798
10.677625	10.623253	10.628781	10.642303	10.623611	10.57727	10.935464	10.955178	10.860124
3.3106725	3.3664558	3.1824306	3.1593576	2.7817736	3.4139246	3.8524369	3.9608643	3.6891623
6.5159872	6.4485311	6.4767447	6.5591555	6.5420437	6.4855643	5.6350052	5.1756805	5.935611
8.6818134	8.6726415	8.5787021	8.3777552	8.3497056	8.31242	8.3536902	8.2581076	8.3859744
10.365672	10.302528	10.317557	10.319161	10.267334	10.276785	10.404335	10.427509	10.319442
11.519406	11.504652	11.421277	11.154008	11.083081	11.130079	11.17016	11.231525	11.044216
5.4640127	5.8962332	6.3700678	5.2866467	5.8018185	5.6339509	5.4098549	5.5369839	5.935611
8.3765936	8.4406697	8.1907952	8.3798938	8.1930633	8.460011	8.7446856	8.772479	8.6552576
11.160304	11.090694	11.118448	11.052085	10.995665	11.014102	11.219572	11.190842	11.187932
3.3106725	3.5330831	2.9650943	3.6872592	3.421282	3.8543214	4.3447825	4.5414122	4.0628589
8.2544549	8.2323365	8.1655008	8.0008093	7.979446	7.9346056	7.3683772	7.1714012	7.4873015
7.0188574	6.8469547	7.0743297	7.4325725	7.5555573	7.2058626	8.0785876	8.0844751	8.0126287

2.7294717	2.9612664	2.3976902	2.1678177	2.3100466	2	2.4075018	2.4021099	2.3981461
7.8682243	7.8216695	7.8050264	7.4284459	7.5555573	7.187151	7.6890193	7.6304432	7.6874061
2.9906265	2.7060624	3.1824306	3.1593576	3.5449709	2.7768658	3.7157657	3.550228	3.8247709
4.354085	4.5198165	4.0614204	4.6843137	4.8060545	4.4642052	4.9431589	4.8931255	4.9399942
4.6471428	4.8068978	4.357879	5.6548533	5.8018185	5.3939735	5.7088847	6.0935561	5.1089463
4.354085	4.5198165	4.0614204	4.5987644	4.649489	4.4642052	5.0886338	5.2748363	4.8152588
3.2116748	3.3664558	2.9650943	4.0299647	3.6588876	4.334175	4.559689	3.9608643	4.9399942
3.2116748	3.3664558	2.9650943	2.4542536	2	2.7768658	2.7248895	2.4021099	2.9660172
5.205611	4.7403036	5.5596753	4.9833211	5.0759055	5.0239729	4.711182	4.6925307	4.748599
5.7898982	5.3882147	6.0489734	5.5523944	5.4994188	5.5189462	5.8785543	5.8896564	5.8104792
8.0475634	7.9000247	8.0800901	8.3053427	8.266787	8.2506825	9.0413088	8.9923781	9.0297607
10.954688	10.894126	10.903882	10.939514	10.883694	10.900957	11.323547	11.241519	11.342829
4.6087492	4.3515565	4.7469892	5.6966305	5.8505866	5.4262461	5.8624576	6.1468857	5.4399178
3.7238255	3.9415025	3.3712718	4.960639	4.34361	5.3271781	4.3904012	4.2801452	4.4460942
8.3063584	8.1830441	8.3282129	8.3713204	8.2395802	8.4043944	8.0959017	8.0844751	8.0477075
7.4734654	7.382729	7.4544589	8.006357	8.0065987	7.9119928	8.4564604	8.3519322	8.4967431
2.2197205	2	2.3976902	3.2369134	3.2859859	3.1302769	3.9162052	4.075324	3.6891623
2.7294717	3.1780396	2	2.8980192	3.1366761	2.5613046	2.5749062	2.4021099	2.7098437
7.0902647	7.011299	7.0606743	7.3607622	7.4487389	7.1681935	7.7161446	7.7123274	7.678481
4.7912485	4.8068978	4.6771135	4.9375947	4.854682	4.9370509	5.1160479	5.2748363	4.8789742
12.371733	12.307027	12.324911	11.996356	11.960628	11.93773	12.447735	12.420308	12.415751
5.701447	5.5524845	5.7418429	6.0044717	5.8505866	6.0613354	6.885519	6.9341575	6.7749723
3.1053801	3.1780396	3.1824306	4.2702587	4.4101943	4.0326014	3.2051098	2.9740279	3.3724324
9.526901	9.5341308	9.4054635	10.346756	10.296229	10.303092	10.524928	10.486882	10.50252
2.5787203	2.3958007	2.7091089	3.9400663	4.123159	3.8543214	2.4075018	2	2.7098437
6.8967025	6.7822257	6.9012594	7.2388124	7.2986449	7.07966	6.932535	6.9789602	6.8243582
4.9532328	4.8068978	4.9968831	4.7117388	4.4101943	4.9370509	5.2207741	5.3676132	4.9985377
4.569306	5.801426	4.8773401	4.6278481	5.3377701	5.0655458	4.4346216	5.0692034	5.5997308
5.9973125	5.9264999	5.9634986	6.0264801	6.2824824	5.6613247	6.1676892	6.3874467	5.8744013
4.1010311	4.0554482	4.0614204	4.5078205	4.8060545	4.0326014	4.0921636	4.3725921	3.6891623
11.680787	11.718135	11.63216	11.272124	11.241183	11.310024	11.456496	11.410498	11.520645

3.3106725	2.7060624	3.6878577	3.4473206	3.2859859	3.5372523	3.7857191	3.1926715	4.1686253
8.641969	8.5418934	8.6350736	8.6936326	8.7050278	8.5867737	8.9940703	8.9887313	8.9392565
11.440992	11.374164	11.396352	11.919419	11.882533	11.861965	12.184166	12.148932	12.158889
10.422807	10.353544	10.380741	10.10705	10.020634	10.098012	10.031329	10.063249	9.9389828
2.5787203	2	2.9650943	2.9904928	2.9701107	2.9643661	3.4829147	3.8365351	2.9660172
4.487029	4.1610493	4.6771135	4.84155	4.8060545	4.7959824	5.2946134	5.0692034	5.4399178
2.8659503	2.9612664	2.7091089	2.6931321	2.7817736	2.5613046	2.8607385	2	3.3724324
7.0621245	7.1330973	6.8702827	6.8144862	6.7619501	6.7758915	7.3102261	7.451257	7.0896929
9.5486138	9.3813543	9.5973823	9.9424493	9.9910305	9.7938802	10.832838	10.863569	10.7398
5.4203434	5.4726864	5.2584768	5.6548533	5.3377701	5.8396456	5.813061	5.8896564	5.6734543
10.944126	10.922561	10.853295	11.250373	11.208816	11.198245	12.170747	12.118386	12.162094
5.856951	5.5907872	5.9925565	6.339411	6.3348412	6.2534533	5.941203	5.7926015	6.0228156
8.2419661	8.2383812	8.1332462	7.7853972	7.702277	7.7746062	8.1762344	7.9633552	8.3128815
4.8251331	4.8068978	4.7469892	4.6843137	4.649489	4.6396102	5.3184099	5.4547826	5.1089463
4.8251331	4.9315181	4.6036804	5.7238246	5.4994188	5.8396456	4.9431589	5.0128652	4.8152588
6.0555648	6.3625899	6.1032668	6.5439776	6.7491456	6.6854555	6.4628353	6.6684237	6.577886
5.2311106	5.1524786	5.2098157	4.8165054	4.4738406	5.0239729	5.0606888	5.0692034	4.9985377
2.4103584	2.3958007	2.3976902	3.8929252	3.8628381	3.8543214	3.2051098	3.1926715	3.1834889
4.684541	4.4381381	4.8136363	4.5690822	4.4738406	4.5834785	5.5369952	5.7587426	5.2115027
3.3106725	3.3664558	3.1824306	3.5111488	3.2859859	3.6508626	3.7157657	3.550228	3.8247709
7.6157788	7.6048597	7.5157939	8.2502982	8.2165092	8.1908884	8.3068764	8.3405324	8.211345
6.2675644	6.2474223	6.1810581	6.3481756	6.2824824	6.3236669	6.5855807	6.4524332	6.6527056
2.8659503	2.9612664	2.7091089	2.5786313	2.5651052	2.5613046	2.4075018	2.7162254	2
7.8763276	7.6867146	7.9453932	7.8851251	7.9684402	7.6973713	8.2084295	8.2520359	8.1021177
4.1010311	4.0554482	4.0614204	3.5722723	3.1366761	3.8543214	3.8524369	4.075324	3.5394718
2.9906265	2.7060624	3.1824306	4.0299647	4.123159	3.8543214	3.4829147	3.3824986	3.5394718
3.1053801	3.1780396	2.9650943	3.793754	4.34361	2.7768658	3.3037759	3.1926715	3.3724324
13.724208	13.667442	13.669406	13.749939	13.687485	13.717995	15.168834	15.137616	15.13971
6.718867	6.5492235	6.7897998	6.8580931	6.6698356	6.9413499	6.8614237	6.9034969	6.7581273
2.9906265	2	3.5382314	2.9904928	2.5651052	3.2790605	4.3904012	4.5414122	4.1686253
7.6011853	7.5180797	7.5746273	6.9240614	7.0288652	6.711878	7.12023	7.1714012	7.0065546

9.9364335	9.9367265	9.8365155	9.9950173	9.9635804	9.9601581	10.73456	10.682415	10.735503
6.4083367	6.0958662	6.5760739	6.6398944	6.6144317	6.5746431	7.356933	7.2946666	7.3591167
5.1532186	5.2984878	4.8773401	6.0155178	5.8743662	6.0613354	5.0321917	5.0128652	4.9985377
9.2543325	9.2135337	9.1840388	9.8796044	9.7978359	9.8662407	10.43799	10.347194	10.465383
12.421209	12.364811	12.366087	12.431117	12.355904	12.411193	13.47544	13.450264	13.440354
6.0412208	6.1736335	5.7756703	6.5515865	6.4500929	6.5601733	6.6050601	6.5546339	6.5969578
4.6471428	4.2594452	4.8773401	5.5523944	5.4046183	5.6060478	5.0606888	4.8931255	5.1611356
2.7294717	2.3958007	2.9650943	2.8980192	2.5651052	3.1302769	3.3037759	2.4021099	3.8247709
8.0475634	8.1123939	7.8612475	7.4851714	7.5846785	7.2783728	5.4958476	5.3676132	5.5614064
2.9906265	2	3.5382314	2.8980192	2.9701107	2.7768658	2.7248895	2	3.1834889
3.2116748	2.9612664	3.3712718	3.2369134	3.1366761	3.2790605	2.8607385	2.4021099	3.1834889
3.9247213	4.0554482	3.6878577	4.3768933	4.4738406	4.1912539	3.4829147	3.3824986	3.5394718
2.8659503	2.3958007	3.1824306	2.5786313	2.5651052	2.5613046	2.7248895	2.7162254	2.7098437
2.2197205	2	2.3976902	2.5786313	2.7817736	2.30778	2.9848891	2.9740279	2.9660172
6.1115561	5.5907872	6.4136896	5.9246856	5.7513439	5.9983304	5.7267783	5.4964687	5.8744013
4.9835561	4.7403036	5.1072743	5.3047698	5.4046183	5.1059542	4.7813527	4.6189492	4.8789742
3.6501227	3.5330831	3.6878577	3.1593576	3.1366761	3.1302769	3.0991973	2.7162254	3.5394718
4.5287539	4.5198165	4.4445501	5.0493029	4.8060545	5.1835263	4.9734511	5.123424	4.748599
4.569306	4.5971173	4.4445501	3.3805373	3.5449709	3.1302769	4.5191927	4.6189492	4.3594016
4.4440613	4.1610493	4.6771135	5.7372319	5.8743662	5.4887032	4.9734511	5.0692034	4.8152588
4.569306	5.1003295	4.4445501	4.4761857	4.7035875	4.7959824	4.4775267	4.6189492	4.6787093
5.8069558	5.9851925	5.4798392	5.7636782	5.8505866	5.5775942	4.8151984	4.8931255	4.6787093
3.1053801	3.3664558	2.7091089	2.4542536	2.3100466	2.5613046	3.0991973	3.3824986	2.7098437
2.7294717	2.7060624	2.7091089	2.3181327	2.3100466	2.30778	2.7248895	3.1926715	2
9.0184251	8.9518291	8.9742659	8.1027319	8.0748932	8.0374263	8.9585446	8.955489	8.9014029
4.4440613	4.5971173	4.6036804	4.1551088	3.8628381	4.7457183	5.2207741	5.5369839	5.1611356
5.5475752	5.6644705	5.30555	5.3578118	5.3031474	5.3271781	6.3403369	6.1983141	6.4154923
8.1613618	8.0101135	8.1970501	8.472989	8.4078915	8.4443381	9.1629426	9.0806739	9.1827648
4.2581217	3.6824426	4.6036804	3.3105119	3.5449709	2.9643661	4.0358621	4.075324	3.9487208
4.354085	4.0554482	4.5263082	4.1945222	4.123159	4.1912539	3.9772738	4.4594701	3.1834889
9.2181286	9.2165989	9.1064125	9.4104407	9.2641545	9.4557077	10.491956	10.467361	10.457579

7.9043358	8.0241771	7.7207086	8.5357157	8.5176637	8.6009804	9.5089421	9.5252879	9.4784857
3.2116748	3.5330831	2.7091089	3.6872592	3.9549305	3.2790605	4.0358621	3.9608643	4.0628589
7.3682252	7.2809834	7.3460574	7.8298787	7.841488	7.7235784	8.2211091	8.0072738	8.3582982
7.7364297	7.712999	7.6495161	7.6782602	7.668673	7.5946231	7.3626665	6.9034969	7.6604636
6.1115561	5.9851925	6.129666	6.7695201	6.6423996	6.8007223	6.6712383	6.4524332	6.8080834
12.006349	11.954964	11.946665	12.118731	12.080154	12.062963	12.787685	12.747431	12.768092
3.3106725	3.1780396	3.3712718	3.3105119	3.5449709	3.1302769	3.6422468	2.9740279	4.0628589
6.6730862	6.8626932	6.6508895	7.058307	7.1891391	7.07966	7.9511957	8.0072738	8.0477075
5.9366088	6.0415904	5.7756703	6.2393142	6.1523733	6.2353527	6.2565941	6.4310946	5.9943291
3.6501227	3.6824426	3.5382314	3.9400663	4.34361	3.2790605	2.9848891	2.7162254	3.1834889
11.033795	10.9651	10.99465	11.945102	11.894211	11.902318	12.572646	12.557408	12.528692
6.0555648	5.8337256	6.2548685	6.5209071	6.5271198	6.6987273	6.7256424	6.8074002	6.7916228
2.2197205	2.3958007	2	2.3181327	2.5651052	2	3.0991973	2.9740279	3.1834889
3.3106725	3.5330831	2.9650943	3.0773944	2.7817736	3.2790605	2.8607385	2.9740279	2.7098437
2.4103584	3.6824426	3.8234137	3.630911	4.2738034	4.5834785	2.5749062	4.1813664	3.6891623
3.9859168	3.3664558	4.357879	4.5690822	4.9017239	4.1912539	3.5647792	3.8365351	3.3724324
5.07088	5.1003295	4.9383494	5.3578118	5.0759055	5.5189462	4.711182	4.2801452	4.9985377
6.2301152	6.0415904	6.3020577	6.566685	6.6284834	6.4068854	6.8614237	6.7738909	6.8876817
4.1553175	4.1610493	4.0614204	4.1145884	4.4101943	3.6508626	2.8607385	2.7162254	3.1834889
9.9746761	9.9348666	9.9030695	10.227133	10.154116	10.205356	10.237285	10.303828	10.104719
10.178473	10.171238	10.072537	10.010283	9.9171137	10.010252	10.333846	10.309686	10.302115
3.6501227	4.0554482	2.9650943	3.8929252	3.9549305	3.7561757	3.0991973	3.1926715	2.9660172
3.403311	3.5330831	3.1824306	2.7992097	2.5651052	2.9643661	2.7248895	2.7162254	2.7098437
6.8967025	6.7986822	6.8858542	7.2388124	7.1604128	7.2243347	7.6331891	7.5828601	7.6237393
3.2116748	3.5330831	2.7091089	2.5786313	2.7817736	2.30778	2	2	2
9.524325	9.4402297	9.5517426	9.5957304	9.6964306	9.4942742	9.7467394	9.7658657	9.7237803
9.8831433	9.8643067	9.7896872	10.065137	10.060049	9.9752333	10.820392	10.775304	10.804809
2.9906265	2.7060624	3.1824306	2.5786313	2.5651052	2.5613046	2.5749062	3.3824986	2
4.3997744	4.990011	4.5263082	4.2328874	4.7035875	4.6396102	4.297674	5.0128652	4.5278714
9.2820456	9.231828	9.2335623	8.809228	8.7730201	8.7518502	8.8586205	8.6669046	8.9724958
2.5787203	2.7060624	2.3976902	3.3805373	3.1366761	3.5372523	2.7248895	2.7162254	2.7098437

8.658509	8.6681703	8.5349292	8.3030907	8.085119	8.4124721	8.7424885	8.7029613	8.7219279
12.034457	11.977274	11.9806	11.769255	11.710324	11.734462	11.792754	11.771536	11.753598
4.3997744	4.5971173	4.0614204	5.1123987	5.0759055	5.0655458	5.1952966	5.4547826	4.8789742
9.4214777	9.359335	9.3726829	9.2158675	9.1950214	9.1426471	9.3988543	9.1990178	9.5215433
3.7939455	3.8177796	3.6878577	4.3422168	4.2738034	4.334175	4.1463502	3.8365351	4.3594016
9.4651132	9.3621059	9.4558892	8.8343982	8.9624313	8.5903385	9.141425	9.1606385	9.0610007
9.2165338	9.2498933	9.0659747	9.0672001	8.9817163	9.0576412	9.5534879	9.5303137	9.5165439
7.0478458	7.1069057	6.8702827	7.0689501	7.0394212	7.0068364	7.0579838	7.0506717	7.0065546
2.8659503	3.1780396	2.3976902	2.9904928	3.1366761	3.1302769	4.1463502	4.2801452	3.9487208
3.2116748	2.9612664	3.3712718	3.8441915	3.8628381	3.7561757	3.3037759	2.4021099	3.8247709
2.2197205	2.3958007	2	2.7992097	2.5651052	2.9643661	2.5749062	2.7162254	2.3981461
7.0188574	6.968302	6.9612862	6.9357382	6.5568147	7.1586205	7.0222058	6.7221209	7.218422
2.2197205	2	2.3976902	2.8980192	2.5651052	3.1302769	2.2180911	2	2.3981461
2.4103584	2.3958007	2.3976902	2.5786313	2.5651052	2.5613046	3.0991973	2.9740279	3.1834889
3.9859168	3.3664558	4.357879	3.8929252	4.0414954	3.6508626	4.9431589	4.4594701	5.2601706
8.051147	8.1189616	7.8612475	8.6780752	8.5732057	8.6867364	9.999483	10.054554	9.8798669
5.2311106	4.7403036	5.5203094	5.5523944	5.6447666	5.3609623	5.4318384	5.4547826	5.3528418
3.4903583	3.9415025	2.7091089	3.630911	3.7644639	3.4139246	2.9848891	3.1926715	2.7098437
5.0132553	5.0462245	4.8773401	5.5674845	5.616845	5.4262461	6.0012441	5.9509123	5.9943291
2	2	2	2.6931321	2.7817736	2.5613046	2.9848891	2.4021099	3.3724324
3.1053801	3.3664558	2.7091089	3.9857156	3.8628381	4.0326014	3.0991973	2.4021099	3.5394718
7.4245703	7.4467797	7.2886408	7.532079	7.2079771	7.7170711	7.0508988	7.25878	6.7410834
6.5055765	6.4062003	6.4971652	6.9815225	6.9073461	6.9635106	7.4621534	7.3750853	7.4873015
3.403311	3.5330831	3.1824306	4.3066864	3.9549305	4.5250741	4.7813527	4.6925307	4.8152588
10.32924	10.292395	10.255978	10.388778	10.321317	10.362672	10.851307	10.841499	10.800702
11.305402	11.273999	11.224825	11.032508	10.962075	11.008118	10.836963	10.796318	10.81706
15.589236	15.514979	15.551762	15.096334	15.058861	15.039463	15.16466	15.146644	15.122281
7.9814874	8.0858203	7.8612475	8.2619403	8.2257819	8.3295846	9.1825242	9.1926918	9.2015768
5.5475752	5.6644705	5.30555	5.322668	5.3377701	5.2208023	5.0606888	4.3725921	5.4815611
8.533422	8.4666492	8.4897866	8.4548444	8.4038123	8.4124721	8.9337235	8.7935338	9.0049865
4.684541	4.6704862	4.6036804	5.0493029	4.854682	5.1452616	4.7813527	4.7625408	4.748599

3.1053801	3.3664558	2.7091089	3.6872592	3.8628381	3.4139246	3.4829147	3.8365351	2.9660172
9.1225814	9.1151303	9.161826	9.7599085	9.6947621	9.7985106	10.238844	10.236232	10.235218
2.4103584	2.3958007	2.3976902	3.1593576	3.421282	2.7768658	2.2180911	2	2.3981461
11.144036	11.0722	11.104374	10.783078	10.733331	10.739369	11.29197	11.234608	11.288141
2.5787203	2.9612664	2	2.7992097	2.5651052	2.9643661	2.2180911	2.4021099	2
7.0902647	6.968302	7.1012589	7.4651674	7.5107428	7.3219081	7.8446106	7.7644439	7.8631063
6.8062881	6.5685453	7.0189149	6.7365211	6.3518802	7.0280209	6.7780693	6.8562486	6.688709
4.9532328	5.1003295	4.6771135	5.7636782	5.8018185	5.6339509	5.813061	5.5369839	5.9943291
5.3522564	5.3882147	5.2098157	6.171799	6.0093567	6.2353527	6.058886	6.0096728	6.0507505
7.9279176	7.8135948	7.9304545	7.7853972	7.668673	7.8056057	7.7689114	7.7981708	7.678481
2.4103584	2.3958007	2.3976902	3.3105119	3.2859859	3.2790605	2.2180911	2.4021099	2
8.6490808	8.6410485	8.5447719	8.4849599	8.4598975	8.416494	8.9959161	8.9960156	8.9392565
3.2116748	3.6824426	2.3976902	3.1593576	3.1366761	3.1302769	3.396124	3.550228	3.1834889
6.8726006	7.1460169	6.3920436	6.6751515	6.5420437	6.711878	6.752094	6.4524332	6.9483421
2.5787203	2.7060624	2.3976902	2.6931321	2.3100466	2.9643661	3.4829147	3.550228	3.3724324
2.8659503	2.9612664	2.7091089	2.7992097	2.3100466	3.1302769	4.1463502	4.3725921	4.0628589
3.7939455	4.2594452	2.9650943	5.322668	5.3031474	5.2571394	5.2704178	5.4118562	5.0547979
3.2116748	3.1780396	3.1824306	3.8441915	3.7644639	3.8543214	4.4346216	4.3725921	4.4460942
9.9431995	9.900971	9.8740852	9.875821	9.8482384	9.8092575	10.153773	10.144159	10.103053
4.2076351	4.1610493	4.1671548	4.6278481	4.5932826	4.5834785	4.559689	4.5414122	4.5278714
5.3753109	5.5524845	5.0531343	4.7117388	4.0414954	5.1059542	5.6159286	5.8896564	5.2115027
5.8069558	5.5131371	5.9634986	5.9129186	5.6447666	6.0613354	5.9864665	5.7926015	6.1050482
9.7417217	9.6474957	9.7240653	9.9725198	9.9594174	9.8924668	10.685653	10.693658	10.617886
7.7541274	7.6048597	7.7885525	8.1283984	8.0800152	8.0836861	7.8156421	7.7034553	7.8631063
5.205611	4.8705531	5.3953249	5.6118302	5.6447666	5.4887032	5.7088847	5.3219704	5.9652688
2.4103584	2.7060624	2.3976902	2.1678177	2	2.30778	3.3037759	3.3824986	3.3724324
6.0412208	6.0415904	5.9338433	6.6327384	6.5120399	6.6585401	6.2688591	6.0661334	6.3938449
8.6794998	8.5467728	8.6981719	8.6901899	8.584062	8.6999964	9.314103	9.1735454	9.3855727
3.2116748	3.3664558	2.9650943	4.5987644	4.2738034	4.7959824	4.5191927	4.7625408	4.1686253
8.7429174	8.6726415	8.702575	8.8713487	8.8708319	8.7770946	10.089534	10.015661	10.101384
3.3106725	2.7060624	3.6878577	2.6931321	3.2859859	2.5613046	2.8607385	2.4021099	3.3724324

5.205611	4.990011	5.30555	4.2702587	4.0414954	4.4006546	5.3648568	5.4964687	5.1611356
3.9859168	3.5330831	4.2656663	3.8929252	3.5449709	4.1141074	4.559689	4.5414122	4.5278714
3.4903583	3.3664558	3.5382314	2.8980192	3.1366761	2.5613046	2.9848891	3.1926715	2.7098437
3.3106725	3.3664558	3.1824306	3.793754	3.8628381	3.6508626	3.7157657	3.550228	3.8247709
9.5549382	9.506847	9.4970273	10.316372	10.233257	10.309595	11.017709	11.013768	10.961097
7.5514458	7.4675132	7.5257676	7.6359397	7.5481843	7.6296919	8.007175	8.0707423	7.8787755
3.9247213	3.9415025	3.8234137	4.7650729	4.649489	4.7959824	4.912217	4.6925307	5.0547979
5.6830869	5.801426	5.4382008	5.9129186	5.8743662	5.8634117	6.5757415	6.4094357	6.6708196
8.5049594	8.5613129	8.4326222	9.0458087	9.0034453	9.1007701	9.6828646	9.7444001	9.6480831
2.5787203	2.3958007	2.7091089	3.5722723	2.9701107	3.9462135	2.7248895	2.4021099	2.9660172
2.5787203	2.9612664	2	3.8929252	3.8628381	3.8543214	3.9772738	3.3824986	4.3594016
5.2807962	5.1524786	5.30555	4.7910183	4.5347973	4.9370509	5.5165681	5.3676132	5.5997308
5.0423553	5.251441	4.6771135	4.6843137	4.5347973	4.7457183	5.4534919	5.5369839	5.3072501
5.0988516	4.990011	5.1072743	4.4761857	4.5932826	4.2644835	4.9431589	4.7625408	5.0547979
5.1262912	4.8705531	5.2584768	5.1123987	5.1163526	5.0239729	5.3184099	5.3219704	5.3072501
5.5063989	5.5907872	5.30555	5.8890928	6.0516855	5.6060478	6.2317468	5.9805917	6.3938449
4.85824	4.9315181	5.1594558	5.3047698	5.4994188	5.3271781	6.206464	6.4094357	6.2078724
7.5207575	7.3936035	7.5356729	8.0844897	8.0902048	7.9842245	8.6703531	8.6252335	8.6552576
3.9859168	3.3664558	4.357879	3.1593576	3.421282	2.7768658	3.396124	3.1926715	3.5394718
7.2169106	7.3383921	6.9612862	7.2714321	7.2540232	7.1965372	6.8369191	6.7221209	6.8876817
6.8231542	6.8149532	6.7220159	6.2393142	5.9877173	6.3741717	7.0650342	7.1189867	6.9631169
10.822011	10.785824	10.746467	10.548929	10.442591	10.559193	11.151651	11.005671	11.227478
3.4903583	3.8177796	2.9650943	3.1593576	3.2859859	2.9643661	3.4829147	2.7162254	3.9487208
5.8732383	5.8337256	5.8087227	6.6110549	6.6144317	6.5158725	7.7689114	7.7558875	7.7225641
10.709157	10.642755	10.664203	10.692635	10.614815	10.675356	10.376976	10.403059	10.288981
2.8659503	2.9612664	2.7091089	2.8980192	3.2859859	2.30778	3.3037759	3.1926715	3.3724324
5.4203434	5.3882147	5.3511356	5.3578118	5.2676733	5.3609623	4.297674	4.1813664	4.3594016
7.4734654	7.5081072	7.4012761	7.4611331	7.5333241	7.3304597	7.7689114	7.6490456	7.8392788
6.152168	6.0958662	6.1032668	5.9708164	6.0093567	5.8396456	7.0437789	7.0645959	6.9631169
11.8366	11.790373	11.771228	11.289861	11.299981	11.184159	11.459843	11.464009	11.394839
4.5287539	4.7403036	4.1671548	5.3921198	4.9914418	5.6339509	5.2704178	5.2261101	5.2601706

14.865269	14.77433	14.84434	15.303891	15.286455	15.22658	16.334176	16.327524	16.280522
9.9822045	9.9478361	9.9049813	9.0240954	8.9789771	8.9755131	9.8544274	9.8263683	9.8222822
4.569306	4.3515565	4.6771135	4.7386523	4.4101943	4.9370509	5.7962123	5.7926015	5.7435929
7.2169106	7.2089345	7.1145373	7.2006065	7.1891391	7.1196804	7.5849545	7.5828601	7.527618
13.076707	12.998762	13.043315	12.180918	12.134398	12.139172	12.312828	12.271427	12.293942
2.2197205	2	2.3976902	3.9400663	3.9549305	3.8543214	4.0358621	3.3824986	4.4460942
4.6087492	4.7403036	4.357879	3.3805373	3.2859859	3.4139246	4.1985751	4.3725921	3.9487208
7.3966728	7.3383921	7.3460574	7.9412329	7.8772338	7.9233435	7.9473854	7.9633552	7.8709622
5.5063989	5.6280992	5.2584768	5.7505157	5.6447666	5.7659038	5.2946134	5.2748363	5.2601706
5.4640127	5.6280992	5.1594558	5.9363573	5.9877173	5.7909055	6.452119	6.3193942	6.5191095
4.354085	4.2594452	4.357879	4.1945222	4.2004465	4.1141074	4.4346216	3.9608643	4.748599
8.7930025	8.6546731	8.8487849	8.8939712	8.8913722	8.8709457	8.2949317	8.2025201	8.3914465
6.4523654	6.2474223	6.537159	6.2011218	6.0093567	6.2889872	6.8286576	6.8074002	6.7916228
2.5787203	2.9612664	2	2.7992097	2.5651052	2.9643661	3.0991973	3.1926715	2.9660172
10.550464	10.453064	10.539303	10.955734	10.902609	10.91737	11.889506	11.851015	11.870376
6.4950901	6.2945981	6.5760739	6.3305929	6.2095768	6.3575325	6.5356996	6.5742331	6.4368197
7.0972149	7.0253511	7.0743297	7.5775092	7.5990216	7.4608104	7.12023	7.145432	7.0348031
5.4640127	6.6970036	6.6139666	6.0373597	6.8723477	6.9074571	5.7791646	6.6126503	6.5389689
4.6087492	4.0554482	4.9383494	4.3422168	4.4738406	4.1141074	2.7248895	3.1926715	2
2.2197205	2	2.3976902	3.4473206	3.2859859	3.5372523	4.4346216	4.6189492	4.1686253
3.6501227	3.8177796	3.3712718	4.1551088	4.0414954	4.1912539	3.7857191	3.9608643	3.5394718
2.4103584	2	2.7091089	2.4542536	2.5651052	2.30778	2.2180911	2	2.3981461
8.0112287	7.8456261	8.0733058	8.278083	8.3581629	8.0937677	7.9281813	7.8791918	7.9172196
13.873233	13.81137	13.823655	14.216492	14.186416	14.152827	14.816745	14.803429	14.7701
8.7250807	8.7466046	8.5882516	8.6499963	8.6301844	8.5760261	8.7729479	8.7468008	8.7391983
2.8659503	2.9612664	2.7091089	2.5786313	2.7817736	2.30778	3.0991973	3.1926715	2.9660172
2.5787203	2.7060624	2.3976902	2.8980192	2.7817736	2.9643661	2.4075018	2	2.7098437
2.5787203	2.9612664	2	2.9904928	3.421282	2.30778	2.8607385	2.9740279	2.7098437
6.2427066	6.1736335	6.2060835	5.9246856	5.8018185	5.9547445	6.3860942	6.3651173	6.3495506
6.4194705	6.3176199	6.4136896	6.4247487	6.4341814	6.3236669	6.5953533	6.7044429	6.4154923
3.4903583	3.5330831	3.3712718	3.8441915	3.7644639	3.8543214	3.6422468	3.550228	3.6891623

6.304066	6.3625899	6.1810581	6.3038105	6.0723931	6.4229682	5.925794	5.8580248	5.935611
2.5787203	2.7060624	2.3976902	3.741489	3.2859859	4.0326014	2.9848891	2.9740279	2.9660172
3.9859168	3.3664558	4.4445501	4.960639	4.7035875	5.1059542	5.813061	5.65212	5.9053308
6.1115561	6.1222572	5.9925565	6.4164385	6.5714361	6.1412778	5.8297152	5.614753	5.9943291
7.3392053	7.3607309	7.2042138	7.4570875	7.4723038	7.3474123	7.5898512	7.4406185	7.6695005
2.7294717	2.7060624	2.7091089	3.793754	3.9549305	3.5372523	4.2489753	4.6189492	3.6891623
4.1010311	4.0554482	4.0614204	4.0299647	4.4101943	3.4139246	3.396124	2.9740279	3.6891623
2.9906265	2.7060624	3.1824306	2.1678177	2.3100466	2	2.7248895	2.4021099	2.9660172
2.7294717	2.3958007	2.9650943	3.0773944	2.5651052	3.4139246	2.5749062	2.4021099	2.7098437
9.2574382	9.0818998	9.3134611	8.2267283	8.188328	8.1767306	7.9663372	7.9707685	7.9019646
4.4440613	4.7403036	3.9473196	4.4761857	4.0414954	4.7457183	4.559689	4.4594701	4.6052608
7.9122393	7.7302597	7.9748144	8.450781	8.4716373	8.3338441	8.9006072	8.8944313	8.8466816
5.4423433	5.4310687	5.3511356	6.8763857	6.8604902	6.8007223	7.599595	7.5828601	7.5571327
6.430519	6.1736335	6.5567476	6.3996732	6.4180925	6.2889872	6.04469	5.9206094	6.1050482
10.272039	10.246646	10.186948	10.710164	10.609511	10.714267	11.578528	11.543217	11.553356
6.0412208	6.2232429	5.7072032	6.3568872	6.4180925	6.1984556	7.1536848	7.25878	6.9777419
2.7294717	3.1780396	2	2.4542536	2.3100466	2.5613046	2.2180911	2.4021099	2
8.4652189	8.3818127	8.4379136	8.9583549	8.882605	8.9396305	9.5434265	9.5353221	9.4912836
14.158979	14.107183	14.099368	14.321819	14.285768	14.263653	13.940091	13.939233	13.880329
3.3106725	3.1780396	3.3712718	3.8929252	4.123159	3.5372523	4.0921636	3.3824986	4.5278714
2.4103584	2	2.7091089	3.0773944	3.1366761	2.9643661	3.5647792	2.9740279	4.1686253
7.4519385	7.3717719	7.422785	7.9784026	7.8354433	8.0216707	8.8132832	8.8060207	8.7604996
9.845459	9.8044492	9.7751281	10.515764	10.476002	10.461359	10.870046	10.833389	10.846244
11.386361	11.381693	11.277425	11.493442	11.437526	11.454918	12.128465	12.102023	12.095345
3.1053801	2.9612664	3.1824306	2.9904928	2.5651052	3.2790605	3.2051098	3.1926715	3.1834889
8.9336004	8.9591679	8.7920664	8.9740116	8.9512944	8.9028327	9.0359365	9.0068736	9.0049865
2.2197205	2.3958007	2	2.9904928	2.3100466	3.4139246	3.4829147	3.1926715	3.6891623
2.5787203	2	2.9650943	2.1678177	2	2.30778	2.8607385	3.1926715	2.3981461
2.4103584	2	2.7091089	2.6931321	2.3100466	2.9643661	2.5749062	2.4021099	2.7098437
7.4410535	7.487953	7.2768781	7.3950009	7.3504308	7.3474123	7.1863815	7.1584751	7.1554928
7.1246853	7.120061	7.0189149	7.3345391	7.2265722	7.3474123	7.9126319	8.0144653	7.7398269

4.7209942	4.8705531	4.7469892	4.7386523	4.9914418	4.6936396	3.7857191	3.7004723	3.8247709
6.484527	6.3402801	6.5760739	6.7629804	6.6561828	6.8251329	6.8695003	6.8238675	6.8876817
4.6087492	4.2594452	4.8136363	5.2117882	4.8060545	5.4578126	5.8785543	5.9206094	5.7774236
4.9222583	4.7403036	4.9968831	4.6843137	4.4738406	4.7959824	5.6724178	5.825684	5.4399178
7.3682252	7.2926494	7.3347554	7.628764	7.5628929	7.6017054	7.7161446	7.7123274	7.6604636
3.9859168	4.3515565	3.3712718	4.2702587	3.8628381	4.5250741	5.5571371	5.72407	5.3072501
6.0697677	5.9561447	6.0763755	6.3827108	6.3853662	6.2889872	6.5757415	6.5742331	6.5191095
6.217413	6.1222572	6.2060835	6.5890405	6.6002417	6.4855643	7.0076424	7.0920476	6.8563673
5.7726365	5.6999474	5.7418429	5.3578118	5.3031474	5.3271781	5.5369952	5.3219704	5.6734543
3.1053801	3.1780396	2.9650943	4.2328874	4.123159	4.2644835	3.8524369	3.8365351	3.8247709
4.569306	4.1610493	4.8136363	4.4761857	4.0414954	4.8445541	4.8805969	4.8293101	5.0547979
8.5461762	8.4354172	8.5447719	8.6177402	8.6301844	8.5098029	8.72479	8.6805317	8.708838
7.5864427	7.4675132	7.5937171	7.9896494	8.0697531	7.8056057	8.2492371	8.2088033	8.2297903
4.8251331	4.9315181	4.6036804	4.7910183	4.7035875	4.7959824	5.5369952	5.2748363	5.7089498
2.4103584	2.3958007	2.3976902	2.7992097	2	3.2790605	2.4075018	2	2.7098437
15.426273	15.382354	15.358562	15.450566	15.398065	15.408816	15.980206	15.937155	15.962805
5.9823744	5.8337256	6.0210407	6.6751515	6.8485346	6.3741717	4.8482682	4.8931255	4.748599
4.569306	4.5198165	4.5263082	3.9400663	3.7644639	4.0326014	3.3037759	2.4021099	3.8247709
2.8659503	2.9612664	2.7091089	2.4542536	2.3100466	2.5613046	2.4075018	2.7162254	2
2.8659503	3.3664558	2	3.1593576	2.9701107	3.2790605	2.7248895	3.1926715	2
2.5787203	2.7060624	2.3976902	2.9904928	3.1366761	2.7768658	2.7248895	2.9740279	2.3981461
4.5287539	4.5198165	4.4445501	4.8661672	4.2004465	5.2571394	5.5571371	5.65212	5.3970366
3.6501227	4.1610493	3.6878577	3.8929252	3.9549305	3.8543214	4.0921636	3.7004723	4.6787093
2.7294717	2.7060624	2.7091089	2.5786313	2.5651052	2.5613046	2.2180911	2	2.3981461
3.7939455	3.6824426	3.8234137	3.9400663	4.0414954	3.7561757	5.813061	5.5763924	5.9652688
8.9548005	9.0581745	8.7200545	9.169643	9.1688189	9.0755566	9.6782741	9.6825398	9.6133816
13.068	13.002985	13.021673	13.305462	13.235813	13.281005	14.072038	14.045624	14.038121
6.9817831	6.9089038	6.9465121	7.4772031	7.5107428	7.3474123	7.6890193	7.6304432	7.6874061
6.2675644	6.2474223	6.1810581	6.4164385	6.2095768	6.5307911	6.5458147	6.3424369	6.6708196
11.883084	11.832441	11.822195	11.939676	11.869281	11.915167	12.463161	12.428744	12.438066
6.217413	5.9264999	6.3700678	7.5282277	7.4328124	7.5292676	7.30428	7.408224	7.1295318

8.4243527	8.4666492	8.2641216	8.1410624	7.9684402	8.209552	7.7945884	7.6945284	7.831248
3.1053801	3.1780396	2.9650943	2.8980192	3.421282	2	3.396124	2.9740279	3.6891623
3.3106725	3.5330831	2.9650943	3.4473206	3.421282	3.4139246	3.7857191	3.1926715	4.1686253
6.2798345	5.8337256	6.537159	5.4256309	5.4046183	5.3609623	5.5965964	5.4964687	5.6370634
4.684541	4.990011	4.1671548	5.322668	5.3715813	5.1835263	6.3860942	6.5935696	6.0781548
3.6501227	3.6824426	3.5382314	3.5722723	3.6588876	3.4139246	4.297674	4.2801452	4.2671646
2	2	2	2.5786313	2.7817736	2.30778	3.2051098	3.550228	2.7098437
10.223634	10.164905	10.174322	10.644083	10.652263	10.539966	11.854076	11.820568	11.827116
2.5787203	2.3958007	2.7091089	3.2369134	3.421282	2.9643661	2.5749062	2	2.9660172
2.7294717	3.1780396	2	2.1678177	2	2.30778	2.4075018	2	2.7098437
10.80937	10.74501	10.762372	11.013005	11.014514	10.91595	11.650894	11.64055	11.600777
2.2197205	2	2.3976902	2.7992097	2.9701107	2.5613046	3.3037759	3.7004723	2.7098437
4.6471428	4.8068978	4.357879	3.630911	3.7644639	3.4139246	5.169361	4.5414122	5.5614064
6.1115561	6.2945981	5.8087227	6.3741542	6.4658308	6.179647	6.8035851	6.5742331	6.9631169
9.8821377	9.8426462	9.8102344	9.980293	9.9524524	9.9139635	10.880575	10.907689	10.791418
2.2197205	2.3958007	2.3976902	2.3181327	2.7817736	2.7768658	3.0991973	3.1926715	2.9660172
2.9906265	3.1780396	2.7091089	2.3181327	2	2.5613046	2.2180911	2	2.3981461
3.1053801	2.3958007	3.5382314	3.3805373	3.6588876	2.9643661	3.3037759	3.1926715	3.3724324
3.7238255	4.0554482	3.1824306	2.6931321	2.7817736	2.5613046	3.0991973	3.550228	2.3981461
9.1826274	9.182518	9.0693881	8.2123993	8.0226484	8.2950486	8.5065199	8.3800436	8.5661296
6.1387573	5.9561447	6.2060835	6.4974617	6.6002417	6.2889872	6.3747902	6.4734607	6.2078724
7.0762632	7.0253511	7.0189149	7.2054379	7.0603043	7.2516067	7.7383658	7.6210513	7.7904097
5.2561672	5.251441	5.1594558	5.8024604	5.8977601	5.6339509	6.015872	6.0096728	6.0228156
2.4103584	2	2.7091089	3.3105119	3.2859859	3.2790605	3.7157657	3.7004723	3.6891623
5.4423433	5.0462245	5.6717113	5.1123987	4.7557305	5.3271781	6.0729437	6.0935561	5.9943291
4.684541	4.7403036	4.5263082	5.6118302	5.6721581	5.4578126	5.4098549	5.3676132	5.3970366
2.9906265	2.9612664	2.9650943	3.3105119	3.2859859	3.2790605	3.0991973	3.550228	2.3981461
4.0446218	3.9415025	4.0614204	3.3805373	3.8628381	2.5613046	3.4829147	2.4021099	4.0628589
9.9060821	9.9066758	9.7917552	8.5679621	8.6196709	8.416494	8.1366271	8.2336661	7.9693763
4.1553175	4.1610493	4.0614204	3.9857156	4.5347973	2.9643661	4.2489753	4.075324	4.3594016
6.2301152	6.0689835	6.278656	6.6110549	6.8723477	6.179647	6.3286672	6.1468857	6.4368197

3.6501227	2.7060624	4.1671548	2.7992097	3.2859859	2	2.8607385	3.1926715	2.3981461
6.5263233	6.5492235	6.3920436	6.7298298	6.7100355	6.6585401	7.3102261	7.2708417	7.2904838
5.0132553	4.9315181	4.9968831	3.9400663	3.5449709	4.1912539	3.8524369	3.3824986	4.2671646
5.9520259	5.7345728	6.0763755	5.3750678	5.5593263	5.0655458	5.2207741	5.0692034	5.3072501
7.0692112	7.2926494	6.6690026	7.1513809	7.0075186	7.1965372	7.2802473	7.25878	7.2428448
4.7209942	4.4381381	4.8773401	4.6843137	4.8060545	4.4642052	6.04469	5.9509123	6.0781548
4.6471428	4.8068978	4.9968831	5.3047698	5.2313049	5.5189462	4.912217	5.2748363	4.6787093
8.9850913	9.0886074	8.750143	8.889475	8.8227548	8.8621256	9.7157156	9.6712087	9.699722
2.5787203	3.1780396	2.3976902	3.0773944	3.7644639	2	2.9848891	3.1926715	2.7098437
2.4103584	2.3958007	2.3976902	2.8980192	2.9701107	2.7768658	2.2180911	2	2.3981461
5.4853615	5.5907872	5.2584768	5.2308701	4.9472802	5.3939735	5.4534919	5.3676132	5.4815611
8.4785877	8.4921691	8.3508305	8.312078	8.2165092	8.31242	8.2211091	8.2336661	8.1480994
2.7294717	2.7060624	2.7091089	2.6931321	2.3100466	2.9643661	2.7248895	2.9740279	2.3981461
5.0988516	4.8068978	5.2584768	6.0695146	6.0093567	6.040638	6.5953533	6.4094357	6.7063792
7.3392053	7.1965685	7.3683991	7.7193746	7.7672242	7.5731652	7.9511957	7.8553596	7.9839383
4.756549	5.1003295	4.1671548	5.9129186	5.4685057	6.179647	6.04469	6.3193942	5.6734543
3.5724509	3.5330831	3.5382314	4.6278481	4.4738406	4.6936396	4.5990795	4.6189492	4.5278714
5.9672799	5.9264999	5.9925565	6.0695146	6.0516855	6.0817401	7.0076424	6.8562486	7.1163741
3.1053801	2.9612664	3.1824306	3.1593576	3.1366761	3.1302769	3.8524369	3.9608643	3.8247709
10.182563	10.207125	10.143886	10.097336	10.130859	10.130205	10.74886	10.731245	10.785196
6.0267327	6.013667	5.9338433	6.0264801	5.8264086	6.1217037	5.9864665	5.7587426	6.1314494
2.5787203	2.9612664	2	2.5786313	2.7817736	2.30778	2.8607385	2	3.3724324
7.9964347	7.9227247	7.9601788	8.4426199	8.284644	8.4984641	9.2705468	9.2877591	9.1922014
11.86894	11.807246	11.819157	12.591268	12.545108	12.544196	13.724097	13.715228	13.673264
13.194506	13.135615	13.142053	13.119843	13.079328	13.066294	13.846974	13.798953	13.834236
6.9514221	6.6433442	7.1145373	7.3256911	7.3588839	7.1965372	7.575111	7.6397744	7.445826
2.2197205	2.3958007	2	3.1593576	3.2859859	2.9643661	3.0991973	3.3824986	2.7098437
2	2	2	2.7992097	2.9701107	2.5613046	3.5647792	3.550228	3.5394718
3.7238255	3.3664558	3.9473196	3.5722723	2.9701107	3.9462135	2.8607385	2.4021099	3.1834889
6.8480892	6.8310427	6.77315	6.970212	7.0808895	6.7506259	7.9320426	7.9103677	7.9019646
3.9247213	4.1610493	3.5382314	3.2369134	3.1366761	3.5372523	3.6422468	3.7004723	3.5394718

7.9318107	7.9670807	7.7802444	7.6537244	7.6822087	7.5292676	7.5197375	7.3750853	7.5955693
8.4598365	8.4406697	8.4004598	7.9527721	7.9461734	7.8773949	8.1924218	8.1315359	8.2175197
9.8961535	9.8721033	9.8081928	8.9352737	8.8913722	8.8913188	8.7380842	8.6623334	8.7520168
7.6011853	7.7890958	7.7634834	7.3907653	7.5481843	7.5586806	7.6188873	7.8229584	7.6695005
10.725509	10.63819	10.700978	10.81662	10.797703	10.741775	11.214825	11.191635	11.177675
2	2	2	2.8980192	2.9701107	2.7768658	2.4075018	2	2.7098437
4.85824	4.5198165	5.1594558	4.3422168	4.649489	4.2644835	4.8805969	5.3219704	4.5278714
8.5911798	8.5661273	8.5049916	8.3734686	8.1401005	8.490855	7.3511767	7.4618177	7.1683001
8.9490495	8.9295859	8.8606541	9.0817248	9.0693126	9.0025202	9.3126228	9.2271469	9.3353527
3.8608147	3.9415025	3.6878577	3.9857156	3.8628381	4.0326014	4.4346216	4.4594701	4.3594016
2	2	2	2.9904928	2.5651052	3.2790605	2.2180911	2	2.3981461
4.8251331	4.8068978	4.7469892	4.1551088	4.2004465	4.0326014	3.4829147	3.7004723	3.1834889
8.4918338	8.4248544	8.4484384	8.0844897	8.0173183	8.0581695	8.6772711	8.6110713	8.6867542
2.8659503	2.3958007	3.1824306	2.5786313	2	2.9643661	2	2	2
5.4853615	5.5907872	5.2584768	5.5061535	5.5593263	5.3609623	6.1807303	6.247972	6.0507505
12.914154	12.846566	12.870163	12.973074	12.910984	12.941188	13.698611	13.65366	13.684591
6.7367801	6.6433442	6.7220159	6.9758783	6.9638538	6.8959802	7.0859808	7.1055801	7.0065546
7.4519385	7.5081072	7.2768781	7.7789293	7.702277	7.7620174	8.2052422	8.2088033	8.1416196
4.487029	4.1610493	4.6771135	4.3422168	4.34361	4.2644835	5.0886338	5.3676132	4.6787093
2.8659503	2.7060624	2.9650943	4.2702587	4.34361	4.1141074	4.2489753	4.2801452	4.1686253
6.3971163	6.1222572	6.537159	6.756411	6.6284834	6.7883603	7.7602499	7.7981708	7.6604636
3.5724509	3.6824426	3.3712718	3.2369134	3.7644639	3.1302769	3.396124	3.7004723	2.9660172
4.684541	4.3515565	4.8773401	4.3768933	4.4101943	4.2644835	4.5990795	4.8293101	4.2671646
6.8726006	6.8626932	6.77315	6.9927452	6.7362263	7.1392818	6.5356996	6.8562486	6.0507505
2	2	2	2.7992097	2.9701107	2.5613046	2.5749062	2.4021099	2.7098437
3.403311	3.3664558	3.3712718	3.4473206	3.1366761	3.6508626	3.4829147	3.8365351	2.9660172
2.7294717	2	3.1824306	2.6931321	2.7817736	2.5613046	2	2	2
4.1553175	4.3515565	3.8234137	5.2682931	5.3031474	5.1452616	6.0868657	6.1728291	5.935611
3.6501227	3.5330831	3.6878577	4.3768933	4.5347973	4.1141074	3.7157657	4.075324	3.1834889
6.4083367	6.2474223	6.4560311	6.0695146	5.9434397	6.1018603	6.7256424	6.631482	6.7581273
3.403311	3.3664558	4.4445501	3.630911	4.0414954	4.4006546	3.8524369	4.075324	4.1686253

11.93468	11.894046	11.863579	11.708866	11.603223	11.717447	11.79355	11.703642	11.820032
5.2311106	5.1003295	5.2584768	6.3305929	6.1327901	6.4388737	6.8119911	6.7395848	6.8243582
5.9823744	6.013667	5.8410348	6.5286383	6.2824824	6.6585401	6.885519	6.6865457	7.0065546
2	2	2	2.3181327	2.3100466	2.7768658	2.8607385	3.1926715	2.3981461
2.7294717	2.9612664	2.3976902	2.9904928	3.1366761	2.7768658	3.396124	3.1926715	3.5394718
6.7544736	6.7822257	6.6139666	6.7431816	6.6561828	6.7378252	6.1936545	6.2959775	6.0228156
13.759959	13.734466	13.673057	14.276946	14.204924	14.254063	14.820525	14.794383	14.786871
10.824107	10.796105	10.740056	10.215205	10.145591	10.190166	10.895977	10.855583	10.876801
3.4903583	3.6824426	3.1824306	3.6872592	3.9549305	3.2790605	3.7157657	3.550228	3.8247709
3.403311	2.9612664	3.6878577	3.5111488	2.9701107	3.8543214	2.4075018	2.7162254	2
5.1796526	5.251441	4.9968831	6.8333365	7.0706336	6.4388737	7.1403958	7.0645959	7.1554928
5.4853615	5.1524786	5.6717113	5.2308701	5.1163526	5.2571394	5.9102187	5.9509123	5.8104792
6.6730862	6.6614533	6.5760739	7.6989638	7.668673	7.6366045	8.5091071	8.492472	8.4659531
8.553775	8.4406697	8.554548	9.0790948	8.9926217	9.0704606	9.9489269	9.9534118	9.8837548
7.0041419	6.968302	6.931585	5.8770308	5.6990392	5.9547445	7.4351993	7.0920476	7.6604636
5.9672799	5.8653179	5.9634986	5.6966305	5.616845	5.6881887	6.141248	6.1468857	6.0781548
9.0828675	8.9737345	9.0795803	8.9852926	9.0770184	8.7864485	9.4495705	9.3769473	9.4603756
4.2581217	4.1610493	4.2656663	5.1123987	4.7557305	5.3271781	4.9431589	4.5414122	5.2115027
2.4103584	2.3958007	2.3976902	2	2.3100466	2	3.4829147	3.8365351	2.9660172
9.1123262	9.0059815	9.1064125	7.9149273	7.7155024	8.0057412	6.6432471	6.7221209	6.4989729
3.403311	3.8177796	2.7091089	2.8980192	2.9701107	2.7768658	3.396124	3.3824986	3.3724324
8.1004051	8.0030299	8.0868427	8.0283368	7.8171557	8.1334039	8.4073777	8.4346747	8.3186374
8.0724639	7.7890958	8.2156537	8.549081	8.4361274	8.5651979	9.2505864	9.1377697	9.2979636
9.6442501	9.5043409	9.6688802	8.9497427	8.911624	8.8942059	9.2412799	9.2115874	9.2108916
2.4103584	2.3958007	2.3976902	3.0773944	2.7817736	3.2790605	2.7248895	3.1926715	2
9.6179114	9.5753072	9.5517426	10.057809	10.008597	10.012911	10.846712	10.89118	10.738727
3.9859168	4.4381381	3.1824306	3.8929252	3.7644639	3.9462135	4.0921636	4.075324	4.0628589
2	2	2	2.5786313	2.9701107	2	2.4075018	2.4021099	2.3981461
4.354085	4.5971173	4.357879	5.1328319	4.9914418	5.5775942	4.8151984	4.8931255	4.8789742
3.7939455	3.5330831	3.9473196	4.6278481	4.8060545	4.334175	3.9772738	4.4594701	3.1834889
8.4188154	8.3598005	8.367564	9.2536765	9.2184358	9.1950967	9.9460656	9.9019911	9.9296103

9.9120066	11.154419	11.049259	9.1708786	10.383746	10.413865	9.2240606	10.422112	10.456274
10.350483	10.324004	10.264938	10.16152	10.103454	10.126527	10.586245	10.423463	10.676213
3.8608147	3.9415025	3.6878577	4.0299647	4.123159	3.9462135	3.9162052	3.8365351	3.9487208
3.403311	3.3664558	3.3712718	3.8441915	3.9549305	3.6508626	3.7157657	3.7004723	3.6891623
4.9222583	5.1003295	4.6036804	4.7910183	5.0342917	4.4006546	4.746694	4.6189492	4.8152588
3.1053801	2.9612664	3.1824306	4.3066864	4.0414954	4.4642052	5.0321917	5.0128652	4.9985377
3.3106725	3.8177796	2.3976902	3.3105119	2.7817736	3.6508626	2.5749062	2.7162254	2.3981461
3.3106725	3.5330831	2.9650943	3.1593576	2.5651052	3.5372523	4.1463502	4.1813664	4.0628589
2.4103584	2.3958007	2.3976902	2.7992097	2.5651052	2.9643661	3.0991973	3.1926715	3.1834889
8.0295104	8.0171624	7.9304545	8.1857541	8.1401005	8.1382828	8.4348518	8.3462435	8.460757
2	2	2	2.6931321	2.5651052	2.7768658	3.8524369	3.9608643	3.6891623
6.8887132	6.7487385	6.9165019	6.9758783	7.0499006	6.8007223	6.9478725	6.7221209	7.0896929
13.271218	13.210939	13.220331	13.327444	13.278488	13.282107	14.115875	14.092568	14.079283
3.7939455	3.6824426	3.8234137	4.4438416	4.5932826	4.1912539	5.1952966	5.1756805	5.1611356
4.2581217	4.4381381	4.0614204	6.2107651	6.1129374	6.2170221	6.3050406	5.65212	6.7063792
8.7517537	8.6903888	8.702575	8.460918	8.2890739	8.5285053	8.4239249	8.4021449	8.3859744
5.9823744	5.8962332	5.9634986	6.2947714	6.2281515	6.2713297	6.1936545	5.7587426	6.4785512
2.2197205	2	2.3976902	2.4542536	2.3100466	2.5613046	2.4075018	2	2.7098437
2.4103584	2.7060624	2	2.6931321	2.5651052	2.7768658	2.2180911	2	2.3981461
2.9906265	3.1780396	2.7091089	3.9400663	3.421282	4.2644835	2.7248895	2.7162254	2.7098437
3.3106725	3.1780396	3.3712718	3.0773944	3.2859859	2.7768658	3.7157657	3.3824986	3.9487208
2	2	2	2.6931321	2.5651052	2.7768658	2.7248895	2.7162254	2.7098437
2.8659503	2	3.3712718	3.3105119	3.6588876	2.7768658	3.8524369	4.075324	3.5394718
12.878669	12.844833	12.800497	12.947257	12.909006	12.891124	13.697122	13.660839	13.673123
3.5724509	4.2594452	2	3.8929252	3.7644639	3.9462135	3.396124	3.7004723	2.9660172
9.6770912	9.5729174	9.6688802	9.4384127	9.3620134	9.4200938	9.1857621	9.1278568	9.1827648
6.8887132	6.7487385	6.9903883	7.0475849	6.7872233	7.2243347	7.1928328	7.2094991	7.1163741
3.1053801	3.3664558	2.7091089	3.9400663	3.2859859	4.334175	3.3037759	3.8365351	2.3981461
2.8659503	3.1780396	2.3976902	2.4542536	2.7817736	2	2.2180911	2	2.3981461
4.684541	4.9315181	4.2656663	4.84155	4.8060545	4.7959824	3.8524369	3.9608643	3.8247709
3.9247213	4.4381381	2.9650943	2.9904928	3.1366761	2.7768658	2	2	2.3981461

8.1312054	8.0925097	8.0596406	8.6049987	8.5214337	8.5974418	9.3214816	9.1377697	9.4287909
2.8659503	2.7060624	2.9650943	2	2	2	3.2051098	3.3824986	2.9660172
2.7294717	3.1780396	2	3.2369134	3.2859859	3.1302769	3.4829147	3.1926715	3.6891623
6.7098263	6.5492235	6.7563059	6.339411	6.4341814	6.1412778	6.2442239	6.3193942	6.1050482
5.6072167	5.6280992	5.6353242	5.2117882	5.1556966	5.4887032	5.4958476	5.8580248	5.2115027
3.403311	3.1780396	3.5382314	4.0299647	4.0414954	3.9462135	5.2946134	5.4547826	5.1089463
2.7294717	2.9612664	2.3976902	2.6931321	2.9701107	2.30778	2.2180911	2.4021099	2
2	2	2	2.7992097	2.3100466	3.1302769	2.7248895	3.1926715	2.3981461
4.7912485	4.7403036	4.7469892	4.9833211	5.1163526	4.7457183	6.1807303	6.0935561	6.2078724
4.569306	4.0554482	4.8773401	4.9375947	4.7557305	5.0239729	3.7857191	3.1926715	4.1686253
9.1208773	9.144397	9.1064125	9.3495452	9.2931131	9.3938147	9.8876145	9.8606904	9.9296103
4.3069011	4.2594452	4.2656663	4.3768933	3.9549305	4.6396102	4.0921636	3.3824986	4.5278714
9.3678858	9.397651	9.2244052	9.0790948	8.9817163	9.0806347	9.1954325	9.163876	9.1668985
5.2311106	4.8705531	5.4382008	5.2497029	5.1163526	5.2925838	4.6747739	4.7625408	4.5278714
4.0446218	3.5330831	4.357879	5.3578118	5.3031474	5.3271781	4.8151984	4.9542373	4.6052608
3.9247213	4.9315181	5.1594558	2.9904928	4.2738034	3.7561757	2.8607385	2.7162254	4.0628589
6.0555648	6.0958662	5.9035657	5.6406543	5.8743662	5.2571394	5.813061	5.65212	5.9053308
11.152818	11.086512	11.107698	11.713325	11.643134	11.688635	12.218559	12.206974	12.169678
3.2116748	2	3.8234137	4.1945222	4.123159	4.1912539	3.0991973	2.9740279	3.1834889
6.0555648	6.271203	5.6717113	5.1529798	5.1163526	5.1059542	3.3037759	3.7004723	2.7098437
3.7238255	3.9415025	3.3712718	3.5111488	3.2859859	3.6508626	3.396124	3.1926715	3.5394718
8.6034357	8.5467728	8.5496682	9.3007149	9.2323041	9.274798	9.9574769	9.9097256	9.9445772
3.6501227	3.6824426	3.5382314	3.741489	3.1366761	4.1141074	3.6422468	3.7004723	3.5394718
2	2	2	2.7992097	2.5651052	2.9643661	2.7248895	2.9740279	2.3981461
3.4903583	3.1780396	3.6878577	4.7117388	4.5932826	4.7457183	4.912217	5.0128652	4.748599
9.7080668	9.6222919	9.6823184	9.314197	9.3472201	9.1950967	8.8586205	8.7809378	8.8743017
3.9247213	3.9415025	3.8234137	4.7650729	4.9017239	4.5250741	5.5165681	5.72407	5.2601706
7.7139978	7.7473162	7.5649869	7.9812226	7.7861516	8.0684304	7.8770204	7.7729498	7.9172196
2.4103584	2.3958007	2.3976902	3.793754	3.6588876	3.8543214	3.5647792	3.550228	3.5394718
7.3155617	7.3717719	7.1407329	7.3564246	7.2986449	7.3304597	7.4780875	7.3064331	7.576479
4.1010311	4.1610493	3.9473196	3.1593576	3.421282	2.7768658	4.297674	4.3725921	4.1686253

2.5787203	2.3958007	2.7091089	2.8980192	3.1366761	2.5613046	2.4075018	2.7162254	2
2	2	2	3.1593576	3.2859859	2.9643661	2.8607385	3.1926715	2.3981461
9.1157527	8.9701066	9.1457477	8.3030907	8.3454582	8.1624325	8.740288	8.6345983	8.7814909
3.7238255	3.8177796	3.5382314	3.3105119	3.421282	3.1302769	4.1985751	4.6189492	3.5394718
2.8659503	2.9612664	2.7091089	2.4542536	2.5651052	2.30778	2.4075018	2.4021099	2.3981461
2.4103584	2.7060624	2	3.3105119	3.1366761	3.4139246	2.9848891	3.1926715	2.7098437
9.7735251	9.7462486	9.6889908	9.6771133	9.6437884	9.6164387	9.864565	9.9939961	9.6571976
9.2835697	9.285359	9.1713877	9.4977124	9.4337867	9.4673863	10.132956	10.075335	10.12949
2.2197205	2	2.3976902	3.2369134	3.5449709	2.9643661	2	2	2
2.4103584	2.7060624	2	3.630911	3.8628381	3.2790605	2.9848891	3.3824986	2.3981461
4.2076351	4.1610493	4.1671548	4.5078205	4.5347973	4.4006546	5.1160479	5.0128652	5.1611356
2.8659503	2.9612664	2.7091089	2.5786313	2	2.9643661	3.2051098	3.550228	2.7098437
4.684541	4.5198165	4.7469892	5.0916718	4.9914418	5.1835263	6.1006547	6.0096728	6.1314494
8.5936393	8.5172455	8.5594113	8.7242532	8.6849979	8.6733534	9.6327347	9.6982553	9.503969
8.5663513	8.5072672	8.5200381	8.9396297	8.849995	8.93403	9.4616419	9.3629299	9.4989082
8.6155882	8.6180508	8.499941	9.658736	9.5873551	9.6372671	11.055368	11.008375	11.041608
14.132635	14.092763	14.060768	13.510787	13.466231	13.461306	14.047832	14.010538	14.024648
3.9859168	4.1610493	3.6878577	3.5722723	3.5449709	3.5372523	3.5647792	4.075324	2.7098437
8.822659	8.8007118	8.7330265	8.401107	8.3539404	8.35914	7.8322667	7.8064807	7.7986703
3.4903583	3.6824426	3.1824306	4.1945222	4.2738034	4.0326014	4.3447825	3.8365351	4.6787093
3.3106725	3.1780396	3.3712718	4.3066864	4.649489	3.8543214	4.8151984	4.8931255	4.748599
4.7912485	5.1524786	4.1671548	4.9141763	4.854682	4.8915437	4.7813527	4.8931255	4.6052608
2.4103584	2.3958007	2.3976902	3.2369134	3.1366761	3.2790605	2.4075018	2	2.7098437
7.8963888	8.2323365	8.2400912	7.5996989	7.9903685	8.0374263	8.3246101	8.5967687	8.700045
4.5287539	4.7403036	4.1671548	4.3768933	4.34361	4.334175	5.0031203	5.2261101	4.6787093
7.1382268	6.9089038	7.2410034	8.1130533	7.8713373	8.2371022	7.6567148	7.7211453	7.527618
5.1796526	4.990011	5.2584768	5.9010549	6.0516855	5.6339509	4.9431589	5.123424	4.6787093
6.1115561	6.148174	5.9634986	5.9363573	5.9657484	5.8154814	6.5855807	6.6865457	6.4154923
3.5724509	3.3664558	3.6878577	3.5722723	3.8628381	3.1302769	3.7857191	3.8365351	3.6891623
3.4903583	3.3664558	3.5382314	3.4473206	3.421282	3.4139246	4.1463502	4.1813664	4.0628589
10.860815	10.794055	10.816189	11.012316	10.975193	10.955186	11.922148	11.873436	11.909995

6.718867	6.7986822	6.537159	6.0044717	5.9877173	5.9324476	5.761913	5.4964687	5.935611
2.8659503	2.9612664	2.7091089	3.630911	3.7644639	3.4139246	4.2489753	3.3824986	4.748599
2	2	2	2.5786313	2	2.9643661	2.7248895	2.7162254	2.7098437
3.8608147	3.8177796	3.8234137	4.1945222	4.34361	3.9462135	3.9772738	3.8365351	4.0628589
4.6087492	4.7403036	4.4445501	5.0706429	5.0759055	4.9811664	4.6374231	4.8931255	4.2671646
6.1654552	5.8962332	6.3020577	6.5890405	6.4500929	6.631113	6.9478725	6.8721706	6.9631169
6.8967025	6.9827772	6.686891	7.3389429	7.307406	7.2783728	7.9204275	8.0844751	7.6695005
7.4946757	7.315702	7.5552816	8.1334774	7.979446	8.1861846	9.0233227	8.955489	9.0297607
2.4103584	2.7060624	2	2.7992097	2.5651052	2.9643661	2.4075018	2	2.7098437
3.1053801	2.9612664	3.1824306	2.7992097	3.1366761	2.30778	2.9848891	3.1926715	2.7098437
2.4103584	2.7060624	3.6878577	3.5111488	3.5449709	4.2644835	2.4075018	2.7162254	3.5394718
4.1010311	4.1610493	3.9473196	5.5523944	5.4369156	5.5775942	5.761913	5.65212	5.8104792
10.500224	10.420204	10.468677	9.6194548	9.5323656	9.6111843	9.8696071	9.8726118	9.8059838
2.2197205	2	2.3976902	2.8980192	3.2859859	2.30778	2.7248895	2.7162254	2.7098437
2.7294717	2	3.1824306	2.5786313	2.9701107	2	2.2180911	2.4021099	2
7.1715348	7.3496047	6.8386263	7.6886492	7.7155024	7.5659411	7.9281813	7.9707685	7.8231723
4.3069011	4.2594452	4.2656663	4.5690822	4.2738034	4.7959824	4.5191927	4.4594701	4.5278714
4.2581217	4.2594452	4.1671548	4.3422168	4.4738406	4.1141074	4.5191927	4.2801452	4.6787093
5.9366088	5.8337256	5.9338433	6.1618908	6.2095768	6.0196392	6.8119911	6.9034969	6.6527056
2.7294717	2.9612664	2.3976902	3.1593576	2.9701107	3.2790605	2.5749062	2.4021099	2.7098437
4.6471428	4.6704862	4.5263082	4.7910183	4.5932826	4.8915437	5.3418203	4.5414122	5.8104792
8.7429174	8.7035574	8.6714669	9.0605493	9.1049258	8.9284077	10.299136	10.289078	10.251859
4.7912485	4.5971173	4.8773401	5.3047698	5.2676733	5.2571394	4.6747739	4.075324	5.0547979
4.1553175	3.6824426	4.4445501	4.2328874	4.2004465	4.1912539	3.9162052	4.075324	3.6891623
8.6467141	8.5467728	8.6396733	8.5183477	8.584062	8.3507572	9.1380859	9.1573936	9.0644304
3.5724509	3.3664558	3.6878577	3.3805373	3.6588876	2.9643661	2.8607385	3.1926715	2.3981461
6.5365859	6.4896552	6.4971652	6.0588757	5.9877173	6.040638	5.8624576	5.9206094	5.7435929
11.091492	10.996647	11.079198	10.942772	10.89027	10.906687	11.672116	11.627711	11.659218
2.5787203	2.9612664	2.3976902	2.7992097	2.7817736	2.7768658	2.7248895	2.7162254	2.7098437
13.851176	13.806546	13.784601	13.436192	13.397248	13.380741	13.906509	13.892549	13.860244
6.856306	6.7144555	6.8858542	7.6359397	7.5628929	7.6157665	8.2768272	8.317459	8.1737313

11.984728	11.945296	11.912388	12.208254	12.139314	12.182328	13.317251	13.275901	13.29811
6.1252208	6.1986514	5.9338433	6.7431816	6.8604902	6.5158725	7.6188873	7.6582576	7.517644
2.9906265	3.1780396	2.7091089	4.0728969	4.2738034	3.7561757	3.7157657	2.9740279	4.1686253
3.8608147	4.1610493	3.3712718	4.0728969	4.0414954	4.0326014	3.0991973	2.9740279	3.1834889
3.2116748	2.7060624	3.5382314	3.3805373	3.6588876	3.2790605	3.4829147	2.4021099	4.0628589
2.2197205	2.3958007	2	3.4473206	3.2859859	3.5372523	4.0358621	4.1813664	3.8247709
4.354085	3.9415025	4.7469892	3.630911	3.6588876	3.6508626	4.559689	4.2801452	4.748599
3.5724509	3.3664558	3.6878577	3.4473206	3.5449709	3.2790605	3.6422468	3.550228	3.6891623
3.9247213	3.9415025	3.8234137	3.6872592	3.421282	3.8543214	3.9772738	3.8365351	4.0628589
5.3753109	5.4726864	5.1594558	6.1914136	5.9657484	6.3064312	6.8203484	7.0224134	6.5191095
5.7551657	5.801426	5.5979955	6.0695146	6.1907599	6.040638	6.015872	5.8896564	6.0781548
3.6501227	2.9612664	4.0614204	4.3066864	4.5347973	3.9462135	3.5647792	3.550228	3.5394718
5.0423553	5.1524786	4.8136363	4.5078205	4.7035875	4.1912539	4.3904012	4.075324	4.6787093
11.730524	11.667513	11.682624	11.41088	11.43952	11.284945	11.650894	11.636477	11.605493
8.0934704	8.3598005	8.5787021	8.7980765	9.139681	9.1904066	9.4058129	9.6226216	9.6503671
9.5910829	9.5118462	9.5590456	9.2594957	9.3040981	9.1156894	9.2552172	9.1926918	9.2565844
6.6730862	6.7316988	6.5173007	6.171799	6.2645985	5.976702	6.8775319	7.0080739	6.6708196
4.9835561	4.9315181	4.9383494	4.9141763	4.5932826	5.1059542	5.1160479	4.7625408	5.3528418
3.1053801	2.9612664	3.1824306	2.7992097	2.5651052	2.9643661	2.7248895	2.9740279	2.3981461
3.8608147	3.9415025	3.6878577	4.5078205	4.7035875	4.1912539	3.7157657	3.550228	3.8247709
4.6471428	4.3515565	4.8136363	4.5690822	4.4738406	4.5834785	4.297674	4.4594701	4.1686253
14.878725	14.831868	14.814203	15.245679	15.191226	15.205815	16.029387	16.006674	15.992224
8.7714396	8.7035574	8.7287154	9.6392291	9.6455169	9.539233	10.265102	10.223876	10.24583
4.1553175	4.1610493	4.0614204	5.1728501	5.1163526	5.1452616	5.3184099	5.3219704	5.2601706
3.1053801	2.7060624	3.3712718	3.3805373	3.1366761	3.5372523	3.9772738	4.1813664	3.6891623
4.6471428	4.7403036	4.4445501	4.7650729	4.4101943	4.9811664	4.7813527	4.9542373	4.5278714
5.4423433	5.2028082	5.5596753	5.9821223	6.0093567	5.9324476	6.6712383	6.4734607	6.7916228
10.689746	10.698629	10.567337	10.222969	10.171015	10.180738	10.140484	10.130985	10.08965
2	2	2	3.1593576	3.421282	2.7768658	3.5647792	3.550228	3.5394718
3.7939455	4.0554482	3.3712718	3.3805373	3.421282	3.2790605	3.7157657	3.3824986	3.9487208
8.0258726	7.8691914	8.0664894	8.9367272	8.9953352	8.7770946	9.2287772	9.2115874	9.1859172

9.2866131	9.2196576	9.2426616	9.1396666	9.1807882	8.9998422	9.2536753	9.1767541	9.2685286
4.6471428	4.4381381	4.7469892	4.5987644	4.7557305	4.334175	3.2051098	3.3824986	2.9660172
9.0599273	9.0165722	8.9923609	9.5818636	9.5801431	9.4904636	10.254348	10.201996	10.24583
6.4083367	6.6614533	6.3020577	7.0529559	7.0075186	7.1097789	5.3184099	5.4964687	5.3072501
4.4440613	4.1610493	4.6036804	4.4107557	4.2004465	4.5250741	4.711182	4.7625408	4.6052608
4.4440613	4.6704862	4.0614204	4.1945222	4.0414954	4.2644835	5.0321917	5.0692034	4.9399942
9.5230352	9.4967963	9.4375157	9.4343028	9.315	9.4576607	10.099011	10.029926	10.106384
8.396451	8.320452	8.3620077	8.055349	8.0279588	7.9896339	8.8340679	8.8787544	8.7262649
2	2	2	3.0773944	3.421282	2.5613046	2.5749062	2.7162254	2.3981461
2.4103584	2.7060624	2	3.2369134	3.2859859	3.1302769	3.8524369	3.8365351	3.8247709
11.633164	11.580371	11.574472	11.860143	11.770568	11.854572	12.539968	12.517154	12.502381
12.088208	12.035279	12.029613	11.825845	11.783185	11.774967	11.604468	11.540729	11.60667
10.522984	10.44001	10.496836	10.556039	10.467233	10.549154	11.223516	11.173278	11.213644
6.4631658	6.2945981	6.5173007	7.0742423	7.0394212	7.0174675	7.3965968	7.1714012	7.5375235
2.9906265	3.1780396	2.7091089	2.1678177	2.3100466	2	2.2180911	2	3.1834889
3.4903583	3.5330831	3.5382314	3.3805373	3.8628381	3.5372523	4.6374231	5.3219704	4.0628589
3.9247213	3.8177796	3.9473196	4.1551088	4.0414954	4.1912539	4.9431589	5.2748363	4.4460942
5.8069558	5.7345728	5.7756703	6.0800756	5.8977601	6.16059	5.941203	5.8580248	5.9652688
3.8608147	3.6824426	3.9473196	4.6278481	4.8060545	4.334175	4.3904012	4.6189492	4.0628589
8.1480367	7.9959113	8.1970501	8.094942	8.0800152	8.0374263	8.539797	8.5628341	8.460757
12.11135	12.110946	12.054371	11.46174	11.41794	11.460286	11.780492	11.773127	11.746142
5.3288274	5.1003295	5.4382008	4.7117388	4.5347973	4.8445541	6.0303529	6.0096728	5.9943291
8.7627235	8.6226798	8.7879285	8.8468205	8.840972	8.7582029	9.3578188	9.2053163	9.4393962
5.1796526	5.3440487	4.8773401	5.5523944	5.4685057	5.5485682	5.3184099	5.2261101	5.3970366
9.3447113	9.1793794	9.3918953	9.6131058	9.5692572	9.563013	10.565887	10.533933	10.537492
3.3106725	2.9612664	3.5382314	4.0299647	4.0414954	3.9462135	3.9772738	4.6925307	2.3981461
11.010975	10.9375	10.972943	10.247186	10.212418	10.187814	10.980439	10.9866	10.913397
4.4440613	4.5971173	4.1671548	3.630911	3.5449709	3.6508626	4.1985751	3.8365351	4.4460942
5.1796526	5.5524845	4.5263082	5.8890928	6.0723931	5.5775942	6.752094	6.8401489	6.5969578
12.851823	12.791615	12.800497	12.532644	12.495657	12.475497	12.125096	12.111276	12.078482
10.003632	9.9236563	9.9721692	10.277326	10.265087	10.194856	10.888048	10.936369	10.775812

4.9532328	5.251441	4.4445501	5.4583812	4.9914418	5.7404611	5.4318384	5.4547826	5.3528418
11.149897	11.088186	11.158263	10.820168	10.780501	10.819769	10.881573	10.878426	10.868977
5.5876089	5.6999474	5.3953249	6.2487064	6.0306763	6.3906213	6.2930807	5.9805917	6.5389689
3.403311	3.5330831	3.1824306	3.5722723	3.2859859	3.7561757	3.7857191	4.075324	3.6891623
2.9906265	3.5330831	2	3.3805373	2.9701107	3.6508626	2.5749062	2	2.9660172
4.4440613	4.5198165	4.2656663	4.5078205	4.9017239	3.8543214	5.1429507	5.5763924	4.4460942
2.5787203	2.9612664	2	3.3105119	3.1366761	3.4139246	3.3037759	3.3824986	3.1834889
4.0446218	3.3664558	4.4445501	4.3768933	4.34361	4.334175	4.0921636	3.3824986	4.5278714
3.9247213	4.0554482	3.6878577	4.5387765	4.8060545	4.1141074	4.4346216	4.3725921	4.4460942
2.8659503	2.7060624	2.9650943	2.3181327	2.3100466	2.30778	2.2180911	2.4021099	2
7.3796716	7.0667046	7.5552816	7.8141494	7.8354433	7.6973713	8.0959017	8.0707423	8.0615036
10.944126	10.891252	10.885603	11.069415	11.022517	11.023362	11.430118	11.393361	11.407047
13.433869	13.372532	13.38382	13.587334	13.544278	13.53657	14.105601	14.061225	14.089314
4.8906042	3.6824426	5.4798392	5.6263142	5.6990392	5.4578126	6.4628353	6.5146181	6.3495506
3.6501227	3.5330831	3.6878577	4.0728969	4.123159	3.9462135	3.396124	2.9740279	3.6891623
2.2197205	2	2.3976902	2.4542536	2	2.7768658	2.5749062	2.7162254	2.3981461
5.5876089	5.4310687	5.6353242	6.2487064	6.1129374	6.3064312	6.6804494	6.7221209	6.577886
3.9247213	3.6824426	4.0614204	3.9857156	3.6588876	4.1912539	4.0921636	3.9608643	4.1686253
2	2	2	3.0773944	2.7817736	3.2790605	2.2180911	2.4021099	2
4.3069011	4.5198165	3.9473196	5.005652	4.854682	5.0655458	3.8524369	4.1813664	3.3724324
4.3069011	4.2594452	4.2656663	4.8165054	4.854682	4.6936396	4.7813527	4.3725921	5.0547979
3.6501227	3.6824426	3.5382314	3.3105119	3.2859859	3.2790605	3.2051098	2.7162254	3.5394718
2.8659503	2.7060624	2.9650943	2.5786313	2.9701107	2	3.4829147	3.8365351	2.9660172
9.5320394	9.5800752	9.3671463	9.3040972	9.2323041	9.2814291	9.3096577	9.2577713	9.3008744
3.7238255	3.3664558	3.9473196	4.5387765	4.5347973	4.4642052	4.1463502	4.1813664	4.0628589
2.2197205	2.3958007	2	2.3181327	2	2.5613046	2.8607385	2.7162254	2.9660172
4.3997744	4.2594452	4.4445501	3.2369134	2.7817736	3.5372523	4.4346216	4.075324	4.6787093
3.2116748	3.1780396	3.1824306	3.630911	3.7644639	3.4139246	4.2489753	4.1813664	4.2671646
7.1516424	7.1460169	7.0468884	6.6398944	6.4180925	6.763314	7.1992554	7.1714012	7.1809948
13.21162	13.162488	13.152752	13.737067	13.685386	13.701402	14.786539	14.760566	14.758633
2.5787203	2.7060624	2.3976902	3.3805373	3.1366761	3.5372523	3.396124	3.7004723	2.9660172

5.3288274	5.5524845	4.9383494	5.7636782	5.5883723	5.8396456	6.3286672	6.4941862	6.0781548
3.5724509	3.6824426	3.3712718	4.1145884	4.123159	4.0326014	2.8607385	2	3.3724324
9.0811158	9.0305734	9.0208489	9.4738838	9.3344183	9.5131776	10.17103	10.158837	10.126212
7.839499	7.6141874	7.9379432	7.6642909	7.6889292	7.544049	6.9170326	6.7568398	7.0065546
5.07088	4.7403036	5.2584768	4.8661672	4.854682	4.7959824	4.6374231	4.075324	4.9985377
2	2	2	3.0773944	3.5449709	2.30778	2.4075018	2.4021099	2.3981461
13.759	13.73179	13.673898	13.896247	13.838395	13.859694	14.114761	14.088847	14.08066
6.2427066	6.3176199	6.0489734	6.7365211	6.6698356	6.72491	6.932535	7.0506717	6.7410834
2	2	2	2.7992097	2.7817736	2.7768658	2.8607385	2.4021099	3.1834889
7.303593	7.4257438	7.0468884	7.2246035	7.1604128	7.1965372	7.1928328	6.9492469	7.3812875
2.4103584	2.3958007	2.3976902	3.8441915	3.5449709	4.0326014	2.4075018	2.7162254	2
4.2581217	4.5971173	3.6878577	3.741489	3.6588876	3.7561757	3.6422468	4.075324	2.9660172
2.2197205	2	2.3976902	2.4542536	2.5651052	2.30778	2.9848891	3.1926715	2.7098437
2.4103584	2	2.7091089	3.630911	3.7644639	3.4139246	3.0991973	3.550228	2.3981461
3.6501227	3.6824426	3.5382314	4.1551088	4.4738406	3.7561757	4.0358621	4.1813664	3.8247709
4.6087492	4.3515565	4.7469892	3.8929252	2.9701107	4.4006546	4.2489753	4.6189492	3.6891623
2	2	2	3.1593576	3.421282	2.7768658	2.7248895	2.7162254	2.7098437
3.4903583	2.9612664	3.8234137	2.1678177	2.3100466	2	2.7248895	3.1926715	2
5.2807962	5.2028082	5.2584768	5.4904045	5.6447666	5.2208023	5.4958476	5.2261101	5.6734543
5.701447	5.5907872	5.7072032	5.8024604	5.6721581	5.8396456	5.3648568	5.4118562	5.2601706
5.2807962	5.2028082	5.2584768	4.5387765	4.5932826	4.4006546	5.1160479	4.9542373	5.2115027
4.0446218	3.9415025	4.0614204	4.1551088	4.123159	4.1141074	4.1463502	4.1813664	4.0628589
3.6501227	3.6824426	3.5382314	3.0773944	2.5651052	3.4139246	3.7857191	3.8365351	3.6891623
5.1796526	5.1003295	5.1594558	4.9375947	4.7035875	5.0655458	4.711182	4.6925307	4.6787093
3.4903583	3.5330831	3.3712718	4.4438416	4.123159	4.6396102	5.5165681	5.3676132	5.5997308
8.6702082	8.6591862	8.5690889	8.1536163	8.1644158	8.0478352	7.9924583	8.0072738	7.9172196
8.3822951	8.4406697	8.203278	8.7343173	8.769854	8.6009804	9.1059731	9.0247906	9.1248105
2.7294717	2.9612664	2.3976902	3.4473206	3.5449709	3.2790605	3.7857191	3.9608643	3.5394718
7.793171	7.7557695	7.7207086	7.9208146	7.8048339	7.9402038	7.7295183	7.5436303	7.8392788
12.104683	12.034845	12.062958	12.562724	12.535819	12.495597	13.66797	13.6657	13.609836
5.2807962	5.3882147	5.0531343	5.5523944	5.4369156	5.5775942	6.5356996	6.4941862	6.5191095

2.5787203	2.3958007	2.7091089	3.1593576	3.1366761	3.1302769	2.5749062	2.7162254	2.3981461
3.6501227	3.9415025	3.1824306	3.793754	4.2004465	3.1302769	3.4829147	3.3824986	3.5394718
5.8732383	5.7345728	5.9634986	5.3578118	5.5296835	5.2571394	5.7791646	5.7926015	5.8104792
2.7294717	2	3.6878577	2.5786313	2.7817736	2.5613046	2.9848891	2.4021099	3.5394718
6.9514221	6.968302	6.8386263	6.6681687	6.7231904	6.5307911	7.5849545	7.451257	7.6962762
2.8659503	2.9612664	2.7091089	2.5786313	2.7817736	2.30778	2.8607385	2.9740279	2.7098437
5.5063989	5.3440487	5.5596753	5.3750678	5.3377701	5.3271781	4.8482682	4.6925307	4.9985377
7.8763276	8.0030299	7.6125576	7.4891392	7.3419279	7.5366773	8.0468886	8.164235	7.8552075
3.1053801	3.1780396	2.9650943	3.3105119	2.3100466	3.8543214	2.2180911	2.4021099	2.3981461
5.527134	5.3440487	5.5979955	5.2117882	5.3031474	5.0239729	5.4748252	5.5763924	5.3528418
4.3069011	4.5971173	3.8234137	3.8441915	3.7644639	3.8543214	4.5191927	4.1813664	4.748599
2.4103584	2.7060624	2	3.2369134	3.1366761	3.2790605	5.0606888	4.8293101	5.2115027
2.7294717	2.9612664	2.3976902	2.6931321	2.7817736	2.5613046	2.4075018	2.4021099	2.3981461
4.0446218	3.3664558	4.4445501	3.0773944	2.9701107	3.5372523	4.5191927	4.2801452	4.6787093
4.1553175	4.2594452	3.9473196	4.2702587	4.123159	4.334175	4.0358621	4.075324	3.9487208
6.9892746	7.0667046	6.7897998	7.2054379	6.9415156	7.3474123	7.1799012	7.0224134	7.2668609
10.305431	10.213276	10.285629	10.379723	10.318069	10.346953	11.553681	11.557442	11.489091
3.9247213	4.2594452	3.3712718	4.6563572	4.7035875	4.5250741	4.297674	4.3725921	4.1686253
3.4903583	3.3664558	3.5382314	3.2369134	2.9701107	3.4139246	3.396124	3.550228	3.1834889
2.2197205	2.3958007	2	3.1593576	3.1366761	3.1302769	2.7248895	2.4021099	2.9660172
10.102793	10.091669	10.151958	9.9453403	9.980113	9.9281187	10.649759	10.646991	10.641041
5.8732383	5.6999474	5.9338433	5.8770308	6.0306763	5.6060478	6.2810207	6.1468857	6.3495506
6.430519	6.4485311	6.3020577	7.1513809	7.0706336	7.1392818	7.2680791	7.3638677	7.1030952
4.487029	4.5971173	4.2656663	4.8165054	4.5347973	4.9811664	5.0321917	5.0692034	4.9399942
5.4203434	5.6280992	5.0531343	6.603754	6.6833604	6.4229682	6.015872	5.8580248	6.1050482
5.6072167	5.8337256	5.2098157	5.3578118	5.4046183	5.2208023	5.2458095	5.3219704	5.1611356
9.5460763	9.4739228	9.5071312	9.4219022	9.4237494	9.3248736	8.8927034	8.9777354	8.7391983
12.5682	12.511168	12.513707	10.902803	10.855596	10.85576	10.751594	10.790046	10.6502
12.588688	12.534691	12.53145	12.641599	12.593816	12.594987	13.139348	13.116066	13.103256
2	2	2	2.5786313	2	2.9643661	2	2	2
11.05626	10.96145	11.03885	11.201635	11.155832	11.154967	11.768394	11.711407	11.764709

2.9906265	3.1780396	2.7091089	2.1678177	2.3100466	2	2.8607385	2.9740279	2.7098437
2	2	2	2.3181327	2.5651052	2	2.2180911	2.4021099	2
4.1010311	3.5330831	4.5263082	4.2702587	4.0414954	4.4006546	5.4318384	5.2748363	5.5220361
6.5263233	6.2945981	6.6325462	6.9063671	6.7996958	6.9188434	7.0579838	6.9935905	7.062509
4.2581217	4.6704862	3.5382314	3.630911	3.8628381	3.2790605	4.1463502	3.7004723	4.4460942
5.9366088	5.9264999	5.8410348	5.4904045	5.3031474	5.5775942	5.3875313	4.9542373	5.6734543
3.8608147	3.5330831	4.0614204	3.3105119	3.421282	3.1302769	4.0358621	3.3824986	4.4460942
7.4946757	7.4151098	7.4648642	7.0689501	7.0808895	6.9635106	7.6426454	7.5633785	7.678481
2.9906265	2.7060624	3.1824306	3.6872592	3.6588876	3.6508626	4.1463502	3.7004723	4.4460942
3.7238255	4.0554482	3.1824306	2.7992097	2.5651052	2.9643661	3.2051098	3.1926715	3.1834889
4.9222583	4.8068978	4.9383494	5.5523944	5.3377701	5.6881887	6.3403369	6.7395848	5.7089498
2	2	2	2.4542536	2.5651052	2.30778	2.4075018	2.4021099	2.3981461
5.07088	5.4310687	4.4445501	5.3578118	4.9017239	5.6339509	4.3904012	4.5414122	4.1686253
4.3997744	4.0554482	4.6036804	4.5078205	4.7557305	4.1141074	3.9772738	4.1813664	3.6891623
5.8069558	5.7683866	5.7418429	6.2393142	6.1129374	6.2713297	6.8203484	6.5742331	6.9922202
9.0402273	8.8646347	9.0964088	9.4343028	9.382887	9.3917733	9.4998654	9.3316035	9.60634
3.7238255	3.5330831	3.8234137	3.630911	3.8628381	3.2790605	3.7157657	3.550228	3.8247709
5.7195764	5.3440487	5.9338433	5.6406543	5.2313049	5.8867927	4.1463502	4.3725921	3.8247709
6.0555648	5.9264999	6.0763755	6.5209071	5.8977601	6.8844113	6.6986968	6.631482	6.7063792
3.7939455	3.6824426	3.9473196	3.5722723	3.2859859	3.7561757	2.5749062	2.7162254	2.3981461
7.5259178	7.4777693	7.4648642	7.0475849	6.9188261	7.07966	7.2496323	7.451257	6.9483421
4.5287539	5.251441	4.6771135	5.1123987	5.2313049	5.3609623	5.8944734	6.0935561	6.4578363
4.354085	4.5971173	3.9473196	4.0728969	4.123159	3.9462135	3.8524369	3.9608643	3.6891623
3.4903583	3.8177796	2.9650943	3.5111488	3.421282	3.5372523	3.7857191	4.075324	3.3724324
3.403311	2.9612664	3.6878577	4.0299647	3.2859859	4.4642052	4.6747739	4.2801452	4.9399942
6.6637531	6.6433442	6.5760739	6.7695201	6.5271198	6.8959802	6.7433306	6.7907429	6.6343614
5.6456505	5.3440487	5.8410348	5.2308701	5.3031474	5.0655458	5.941203	5.9805917	5.8427942
2.9906265	2.9612664	2.9650943	2	2	2	2.2180911	2.4021099	2
3.9247213	3.8177796	3.9473196	3.4473206	2.9701107	3.7561757	3.9162052	3.8365351	4.0628589
3.2116748	2.9612664	3.3712718	4.0299647	3.7644639	4.1912539	3.5647792	2.9740279	3.9487208
4.354085	4.4381381	4.1671548	4.84155	4.5932826	5.0239729	4.912217	5.0128652	4.9399942

2	2	2	2.5786313	2.7817736	2.30778	3.396124	3.3824986	3.3724324
5.2311106	5.2984878	5.0531343	4.84155	4.4738406	5.0655458	5.7962123	5.7926015	5.7435929
5.8732383	6.0415904	5.6353242	5.8277479	5.7768019	5.7909055	5.6724178	5.5763924	5.7089498
4.85824	4.8068978	4.8136363	4.9375947	4.7557305	5.0239729	5.3418203	5.3219704	5.3072501
2.2197205	2.3958007	2	2.9904928	2.9701107	2.9643661	3.0991973	3.3824986	2.7098437
2.2197205	2.3958007	2	2.8980192	3.421282	2	2	2	2
2.2197205	2.3958007	2	2.8980192	2.5651052	3.1302769	3.3037759	3.7004723	3.3724324
2.9906265	2.9612664	2.9650943	3.0773944	2.9701107	3.1302769	3.5647792	3.3824986	3.6891623
2.8659503	2.7060624	3.6878577	3.8929252	4.34361	4.1912539	4.3904012	4.6189492	4.6052608
9.3017346	9.1918929	9.298995	9.4597998	9.3662124	9.4576607	9.7401474	9.782811	9.6366084
7.5361832	7.5669337	7.3904001	7.9556425	7.9461734	7.871547	6.5855807	6.6500711	6.4578363
7.839499	7.6955294	7.8691031	7.9924475	7.8713373	8.0163804	8.0959017	8.1115546	8.019713
8.9120842	8.8129151	8.8995273	9.0103568	8.9624313	8.964567	9.673669	9.6666512	9.620389
2.9906265	2.7060624	3.1824306	2.7992097	2.9701107	2.5613046	3.7157657	3.8365351	3.5394718
10.469754	10.43212	10.395774	10.188612	10.138244	10.147247	9.3806033	9.2907239	9.4073437
2	2	2	3.2369134	3.421282	2.9643661	2.2180911	2.4021099	2
2.2197205	2	2.3976902	2.3181327	2	2.5613046	2.8607385	3.1926715	2.3981461
6.745654	6.7144555	6.6690026	6.7760303	6.7996958	6.6854555	6.0868657	6.1204673	6.0507505
7.8763276	7.8054746	7.8454066	7.5049019	7.4954899	7.4213264	7.1269833	7.0366117	7.1554928
2.4103584	2.7060624	2	2.3181327	2.3100466	2.30778	3.0991973	3.550228	2.3981461
6.0412208	5.7345728	6.2060835	6.3996732	6.3175986	6.3906213	7.2680791	7.3638677	7.1030952
3.6501227	3.6824426	3.5382314	3.3105119	3.5449709	2.9643661	4.4346216	3.550228	4.9399942
4.7912485	5.1003295	4.2656663	4.1145884	3.8628381	4.2644835	5.1160479	4.8931255	5.2601706
5.6830869	5.6999474	5.5596753	5.322668	5.2676733	5.2925838	5.3648568	5.5369839	5.1089463
2.5787203	2.3958007	2.9650943	2.5786313	2.9701107	2	2.2180911	2.4021099	2
3.8608147	3.9415025	3.6878577	3.5722723	3.7644639	3.2790605	4.3447825	3.8365351	4.6787093
5.5876089	5.4726864	5.5979955	5.6828387	5.6447666	5.6339509	6.1545292	6.1204673	6.1314494
9.9100344	9.8145995	9.8973191	9.924248	9.9341843	9.8184058	11.212049	11.212891	11.150501
15.439215	15.37898	15.38842	14.923783	14.890881	14.862958	15.729803	15.701218	15.698404
2	2	2	2	2	2.30778	2	2	2
3.1053801	3.3664558	2.7091089	2.8980192	2.5651052	3.1302769	2.9848891	3.1926715	2.7098437

5.8404777	5.8337256	5.7418429	5.6966305	5.7513439	5.5485682	6.1278434	5.9805917	6.2324729
4.487029	4.2594452	4.6036804	3.630911	3.421282	3.7561757	4.6747739	4.4594701	4.8152588
3.4903583	3.1780396	3.6878577	3.3105119	3.2859859	3.2790605	4.1985751	4.5414122	3.6891623
2.9906265	2.7060624	3.1824306	3.6872592	3.8628381	3.4139246	3.3037759	3.1926715	3.3724324
2.4103584	2.3958007	2.3976902	2.3181327	2	2.5613046	2.4075018	2.7162254	2
2.4103584	2.3958007	2.3976902	3.3805373	2.9701107	3.6508626	2.5749062	2.4021099	2.7098437
3.4903583	3.3664558	3.5382314	3.9400663	4.123159	3.6508626	3.7857191	3.8365351	3.6891623
4.684541	4.4381381	4.8136363	4.84155	4.7035875	4.8915437	4.9734511	4.7625408	5.1089463
3.3106725	3.5330831	2.9650943	3.3105119	3.5449709	2.9643661	4.3447825	4.6925307	3.8247709
3.1053801	2.7060624	3.3712718	4.1551088	3.9549305	4.2644835	4.3904012	3.7004723	4.8152588
3.5724509	3.8177796	3.1824306	4.5690822	4.4101943	4.6396102	3.8524369	3.8365351	3.8247709
8.4759239	8.3818127	8.4588871	8.2895039	8.3066588	8.1814653	8.9084679	8.8826897	8.8743017
3.4903583	2.3958007	4.0614204	3.793754	4.123159	3.2790605	3.8524369	4.2801452	3.1834889
3.2116748	2.7060624	3.5382314	2.5786313	2	2.9643661	3.3037759	3.1926715	3.3724324
6.2427066	6.1736335	6.2060835	6.9240614	6.895774	6.8609914	6.2442239	6.2721744	6.1573762
2.2197205	2.3958007	2	3.6872592	3.6588876	3.6508626	2.4075018	2.4021099	2.3981461
11.533886	11.469109	11.492933	11.227398	11.175107	11.191806	11.356963	11.338545	11.315744
6.4414835	6.0689835	6.6508895	6.2487064	6.3518802	6.040638	6.525513	6.5347647	6.4578363
10.113116	10.032022	10.082707	10.035599	10.032572	9.9435307	10.296143	10.237769	10.293372
5.5063989	6.4062003	6.0489734	5.5674845	6.3518802	6.0196392	5.1429507	5.8896564	5.8104792
5.1262912	5.0462245	5.1072743	6.3038105	6.5120399	6.0196392	6.652638	6.6500711	6.5969578
3.6501227	3.3664558	3.8234137	4.1145884	3.9549305	4.1912539	4.912217	4.6925307	5.0547979
2.7294717	2.3958007	2.9650943	3.5722723	3.8628381	3.1302769	4.0358621	3.7004723	4.2671646
2.5787203	2.7060624	2.7091089	3.0773944	3.1366761	3.1302769	3.6422468	3.550228	4.3594016
11.394495	11.304569	11.372371	11.260274	11.241183	11.184749	10.682218	10.66424	10.639892
2.4103584	2	2.7091089	2.3181327	2.5651052	2	2.4075018	2.7162254	2
3.1053801	3.3664558	2.9650943	3.6872592	3.7644639	3.8543214	3.7157657	3.7004723	4.1686253
5.9366088	5.8653179	5.9035657	6.1317512	6.0723931	6.1018603	5.925794	5.6885435	6.0781548
3.403311	3.5330831	3.1824306	3.3805373	3.7644639	2.7768658	4.0358621	4.1813664	3.8247709
2.4103584	2.3958007	2.3976902	2.5786313	2.5651052	2.5613046	3.0991973	3.3824986	2.7098437
4.3069011	4.5971173	3.8234137	4.7650729	4.7557305	4.7959824	5.1429507	4.9542373	5.2601706

3.3106725	3.5330831	2.9650943	3.3805373	3.421282	3.2790605	4.0921636	3.7004723	4.3594016
2.7294717	2.9612664	2.7091089	3.1593576	3.5449709	2.5613046	2.9848891	3.3824986	2.3981461
3.7238255	3.9415025	3.3712718	3.8929252	4.0414954	3.6508626	3.4829147	3.550228	3.3724324
7.0406532	7.0936294	6.8702827	6.7231073	6.7619501	6.5889691	6.2930807	6.2959775	6.2324729
4.2581217	4.2594452	4.1671548	4.7386523	4.7557305	4.6396102	4.9734511	5.123424	4.8152588
3.9859168	3.9415025	3.9473196	3.4473206	3.7644639	3.2790605	3.7157657	3.550228	3.8247709
2	2	2	2.1678177	2.5651052	2	2.8607385	3.1926715	2.3981461
9.3447113	9.3313298	9.245682	9.1905057	9.1997348	9.0856949	9.1093873	9.0806739	9.0780682
4.354085	4.6704862	3.8234137	4.1145884	3.8628381	4.2644835	3.4829147	3.550228	3.3724324
3.6501227	2.9612664	4.0614204	2.6931321	3.1366761	2	3.2051098	2.7162254	3.5394718
6.5159872	6.3625899	6.5567476	6.4164385	6.3518802	6.3906213	6.7256424	6.6865457	6.7063792
3.403311	2.9612664	3.6878577	2.7992097	2.7817736	2.7768658	3.3037759	2.9740279	3.5394718
9.3736218	9.2795082	9.356009	9.314197	9.3084687	9.2275075	9.4223779	9.4127678	9.3717972
4.8906042	4.3515565	5.2098157	5.4089726	5.4369156	5.2925838	5.2946134	5.2261101	5.3072501
3.6501227	3.1780396	3.9473196	3.9400663	3.7644639	4.0326014	4.0358621	4.3725921	3.5394718
7.9356933	8.0171624	7.7293656	7.1158955	7.0394212	7.0998089	6.4413226	6.4524332	6.3718678
2	2	2	2.1678177	2.3100466	2	2.5749062	2.4021099	2.7098437
9.6299432	9.5776932	9.5711356	9.5451735	9.4868133	9.5094166	9.8058029	9.7049385	9.8443961
9.4956805	9.5560764	9.3134611	8.9782523	8.9679677	8.8942059	9.2161651	9.2147127	9.1572943
5.4423433	5.6644705	5.0531343	5.5971992	5.5883723	5.5189462	5.5369952	5.825684	5.1089463
6.484527	6.1736335	6.6508895	6.171799	6.2095768	6.040638	6.5953533	6.8721706	6.1828452
6.5055765	6.5685453	6.4136896	6.3655465	6.3853662	6.3741717	5.2946134	5.3676132	5.2601706
11.84514	11.814851	11.763347	11.509891	11.452414	11.473376	11.282171	11.290477	11.214416
4.9222583	5.1003295	4.6036804	5.322668	5.4369156	5.1059542	5.3875313	5.4964687	5.2115027
7.5259178	7.4777693	7.4648642	7.4118203	7.3922096	7.3474123	8.1859686	8.2025201	8.1087769
8.3123437	8.195526	8.3167697	8.7459707	8.6849979	8.7131356	9.5153906	9.4766483	9.4938296
4.354085	6.2232429	6.2306822	5.0493029	6.7872233	7.1097789	4.5191927	7.0783871	7.1425707
9.6227362	9.6130166	9.5196622	9.8674624	9.8646554	9.7752084	10.396671	10.444914	10.284577
2.8659503	2.9612664	2.7091089	2.9904928	2.9701107	2.9643661	3.6422468	3.1926715	3.9487208
4.6471428	4.4381381	4.9383494	4.7117388	4.649489	4.6936396	5.6907665	5.8580248	5.5220361
3.5724509	3.1780396	3.8234137	3.5722723	3.6588876	3.4139246	3.0991973	3.3824986	2.9660172

5.1796526	4.990011	5.3511356	5.6966305	5.5593263	5.8396456	6.0303529	6.2959775	5.7435929
8.6979053	8.796621	8.4744196	9.3701306	9.3301257	9.3184397	9.5893829	9.6036731	9.5140377
6.430519	6.013667	6.6690026	7.3736971	7.3161143	7.3389609	5.4318384	5.2748363	5.5220361
2.2197205	2.3958007	2	2.1678177	2.3100466	2	3.4829147	3.550228	3.3724324
4.85824	4.9315181	4.6771135	5.005652	5.1163526	4.7959824	5.7088847	5.4964687	5.8427942
10.137762	10.01099	10.150347	10.251864	10.244706	10.164089	10.764103	10.661952	10.80173
2.8659503	2.7060624	2.9650943	3.5111488	3.421282	3.5372523	2.7248895	2.7162254	2.7098437
4.4440613	5.2984878	4.8773401	5.2682931	5.8505866	5.5189462	5.4958476	5.8896564	5.3072501
2.2197205	2.3958007	2.3976902	3.4473206	3.421282	3.5372523	2.9848891	2.7162254	3.1834889
5.4203434	5.0462245	5.6353242	5.5371449	5.5296835	5.4578126	6.058886	5.7587426	6.2566609
4.756549	4.9315181	4.4445501	5.3047698	5.3031474	5.2208023	5.925794	5.825684	5.9652688
12.747179	12.723487	12.658703	12.918214	12.867028	12.876052	13.481958	13.462805	13.440684
8.9160201	8.9370384	8.7796168	8.7916652	8.9287594	8.5359188	8.9642124	8.9022064	8.9651752
2.7294717	3.1780396	2	2.8980192	2.9701107	2.7768658	2.4075018	2	2.7098437
2	2	2	3.0773944	2.7817736	3.2790605	2	2	2
11.944107	11.908334	11.867996	12.010056	11.952622	11.974683	12.537278	12.505717	12.508375
8.9413456	8.9628234	8.8044095	8.8954669	8.8073961	8.8884259	9.2018435	9.2240484	9.118225
8.248224	8.2323365	8.1591073	8.3648568	8.284644	8.3507572	8.1565667	7.9559037	8.2778553
5.1796526	5.1003295	5.1594558	5.0493029	4.9914418	5.0239729	4.711182	5.0692034	4.2671646
2.8659503	3.1780396	2.3976902	2.8980192	3.421282	3.1302769	3.7857191	3.3824986	4.5278714
3.4903583	3.6824426	3.1824306	3.741489	4.0414954	3.2790605	4.7813527	4.8931255	4.6052608
3.7939455	4.0554482	3.5382314	4.0728969	4.4101943	3.5372523	4.3447825	4.3725921	4.2671646
5.3753109	5.5131371	5.1072743	4.7117388	4.649489	4.6936396	5.5770017	5.614753	5.4815611
6.2920011	6.1736335	6.3020577	6.2393142	6.1716943	6.2170221	6.1278434	6.1468857	6.0507505
2.4103584	2.3958007	2.3976902	2.7992097	2.7817736	2.7768658	3.2051098	3.3824986	2.9660172
4.569306	4.3515565	4.6771135	5.322668	5.4046183	5.1452616	6.3050406	6.3874467	6.1573762
8.0969419	8.0925097	7.989303	7.876063	7.7735609	7.8832191	8.0785876	7.9633552	8.1285721
4.354085	4.2594452	4.357879	4.2328874	4.34361	4.0326014	4.0358621	3.8365351	4.1686253
3.7939455	3.6824426	3.8234137	4.2702587	3.9549305	4.4642052	4.3447825	4.2801452	4.4460942
3.3106725	3.3664558	3.1824306	3.741489	3.421282	3.9462135	3.9772738	4.1813664	3.6891623
3.1053801	2.9612664	3.1824306	2.9904928	2.9701107	2.9643661	3.6422468	4.1813664	2.7098437

4.684541	4.5971173	4.6771135	3.9857156	4.123159	3.7561757	4.912217	5.0692034	4.6787093
6.7632396	6.7986822	6.6139666	6.970212	6.8841084	6.9635106	7.7515361	7.6490456	7.7904097
11.158644	11.062863	11.142954	10.879186	10.860813	10.805271	10.835933	10.843519	10.767419
5.0988516	5.3882147	4.6036804	6.0588757	6.0723931	5.9547445	5.5571371	5.8580248	5.1089463
3.3106725	3.6824426	2.7091089	3.2369134	3.421282	2.9643661	2.4075018	2.4021099	2.3981461
3.3106725	3.1780396	3.6878577	3.0773944	2	3.7561757	3.4829147	4.075324	3.3724324
6.745654	6.5876117	6.7897998	6.1618908	6.3518802	5.8396456	6.1278434	6.3874467	5.7435929
2.5787203	2.9612664	2	3.4473206	3.6588876	3.1302769	3.0991973	2.7162254	3.3724324
5.7898982	5.5907872	5.872639	5.4089726	5.3715813	5.3609623	6.2565941	6.3193942	6.1314494
2.4103584	2.3958007	2.3976902	2.4542536	2.5651052	2.30778	3.6422468	3.7004723	3.5394718
10.78375	10.678763	10.776016	10.951426	10.851858	10.954495	11.732036	11.743679	11.659218
10.550464	10.532695	10.455693	10.595221	10.598846	10.495981	12.129306	12.096103	12.102869
5.1532186	5.1003295	5.1072743	5.6548533	5.6447666	5.5775942	5.7962123	6.0096728	5.4815611
9.698958	9.5800752	9.7044409	9.4041503	9.3429655	9.3711984	9.3535063	9.3572845	9.289196
6.0697677	6.013667	6.0210407	6.2011218	5.9657484	6.3236669	6.9092185	6.8238675	6.9334144
10.929597	10.911254	10.835363	10.691775	10.625364	10.663559	10.638579	10.608302	10.608519
2.2197205	2	2.3976902	2.6931321	3.1366761	2	2.2180911	2.4021099	2
3.6501227	3.5330831	3.6878577	3.8929252	4.2004465	3.4139246	3.2051098	3.550228	2.7098437
2.5787203	2.7060624	2.3976902	2.9904928	3.1366761	2.7768658	3.6422468	3.8365351	3.3724324
3.4903583	4.0554482	3.9473196	3.741489	4.4738406	3.8543214	4.5990795	4.8293101	4.3594016
5.4423433	5.2028082	5.5596753	5.5371449	5.5593263	5.4262461	6.058886	6.1468857	5.9053308
2.9906265	2.9612664	2.9650943	3.3805373	2.7817736	3.7561757	4.0921636	3.7004723	4.3594016
4.5287539	4.8705531	4.0614204	5.3578118	5.1556966	5.4578126	5.2946134	5.123424	5.3970366
5.205611	5.1003295	5.2098157	5.3047698	5.3031474	5.2208023	5.6724178	5.614753	5.6734543
2.9906265	3.1780396	2.7091089	2.1678177	2	2.30778	2.5749062	2.4021099	2.7098437
3.1053801	3.1780396	2.9650943	3.3105119	3.2859859	3.2790605	3.4829147	2.7162254	3.9487208
3.4903583	3.1780396	3.6878577	3.630911	3.5449709	3.6508626	4.297674	4.1813664	4.3594016
6.2301152	6.1222572	6.2306822	5.4583812	5.3377701	5.4887032	6.1807303	6.4310946	5.8104792
3.7238255	3.5330831	3.8234137	4.3768933	4.2004465	4.4642052	4.6747739	4.6925307	4.6052608
6.8231542	6.9536802	6.5567476	6.6611519	6.3853662	6.8129792	6.4413226	6.2959775	6.5191095
2	2	2	2.1678177	2.3100466	2	2.2180911	2.4021099	2

3.5724509	2.9612664	3.9473196	3.2369134	3.2859859	3.1302769	4.4346216	4.7625408	4.0628589
8.5812996	8.5418934	8.5100245	9.1868459	9.1199235	9.1595433	9.7357359	9.7444001	9.6662548
2	2	2	2.7992097	3.2859859	2	2.7248895	2	3.1834889
3.3106725	2.3958007	3.8234137	2.9904928	2.5651052	3.2790605	3.396124	3.3824986	3.3724324
9.9878251	10.049317	9.9538176	10.242492	10.075518	10.381311	9.9073625	9.8586939	9.9831421
4.3069011	5.251441	4.2656663	4.6843137	4.9914418	4.9370509	4.8482682	4.8931255	4.9399942
2.5787203	2.7060624	2.3976902	2.8980192	2.7817736	2.9643661	2.5749062	2.4021099	2.7098437
3.4903583	3.5330831	3.3712718	3.5111488	3.5449709	3.4139246	2.5749062	2	2.9660172
3.4903583	2.9612664	3.8234137	3.793754	3.6588876	3.8543214	4.0921636	3.9608643	4.1686253
6.1252208	6.0689835	6.3020577	6.7027497	6.8604902	6.4855643	7.1470556	7.0920476	7.2787207
10.80407	10.736493	10.760261	11.162729	11.08881	11.141666	12.2508	12.229166	12.212042
9.493048	9.5292083	9.3391401	9.8644109	9.8376931	9.7969688	10.637397	10.592771	10.621383
2.2197205	2	2.3976902	2.5786313	2.5651052	2.5613046	2.5749062	2.4021099	2.7098437
8.164674	7.9670807	8.2400912	7.8546942	7.90636	7.7039678	8.0035099	7.9257066	8.019713
5.6072167	5.4310687	5.6717113	6.2673093	6.3348412	6.1018603	6.5855807	6.4094357	6.688709
9.1562483	9.144397	9.0556856	8.8984535	8.7824769	8.917097	9.0069413	9.0495063	8.9128636
4.569306	4.0554482	4.8773401	4.4107557	4.2738034	4.4642052	4.5191927	3.9608643	4.8789742
5.2311106	5.3882147	4.9383494	5.4904045	5.2313049	5.6339509	6.1545292	6.1204673	6.1314494
3.6501227	3.3664558	3.8234137	3.793754	3.9549305	3.5372523	3.2051098	3.550228	2.7098437
4.9532328	4.990011	5.5203094	5.3921198	5.616845	5.4262461	5.925794	5.9509123	6.2078724
3.1053801	2.7060624	3.3712718	4.5078205	4.4738406	4.4642052	2.9848891	2.9740279	2.9660172
11.52779	11.445136	11.498641	12.169219	12.123588	12.120802	12.881525	12.860247	12.843142
4.354085	4.3515565	4.2656663	4.8165054	4.5932826	4.9370509	5.2207741	5.4118562	4.9399942
2.9906265	2.9612664	2.9650943	3.0773944	2.9701107	3.1302769	3.6422468	3.550228	3.8247709
13.780074	13.715903	13.732667	14.500531	14.443984	14.462571	15.340081	15.329983	15.29001
2	2	2	2.8980192	2.3100466	3.2790605	2.7248895	2.9740279	2.3981461
3.1053801	3.1780396	2.9650943	3.4473206	3.2859859	3.6508626	4.5191927	3.8365351	4.9399942
2.7294717	3.1780396	2	2.3181327	2.3100466	2.30778	2.2180911	2.4021099	2
9.0705604	8.9845638	9.0453226	9.1547327	9.1174347	9.0982685	9.311141	9.248652	9.3124589
4.0446218	3.8177796	4.1671548	4.1945222	4.2738034	4.1141074	3.9772738	4.2801452	3.5394718
7.0041419	7.1840957	6.6690026	6.756411	6.7100355	6.711878	7.0508988	7.0224134	7.020748

10.041058	10.000358	9.9721692	9.7582654	9.7584444	9.6645786	9.8655748	9.9077959	9.7601898
3.3106725	3.5330831	2.9650943	3.3105119	2.9701107	3.5372523	3.6422468	2.7162254	4.1686253
2.8659503	2.3958007	3.1824306	2.8980192	2	3.4139246	2.4075018	2	2.7098437
2.8659503	2.3958007	3.1824306	3.630911	3.421282	3.7561757	4.3904012	4.1813664	4.6052608
3.7238255	2.9612664	4.1671548	4.7386523	4.7557305	4.6396102	4.8151984	4.6925307	4.8789742
6.3744103	6.2474223	6.3920436	6.1618908	6.1523733	6.0817401	6.7256424	6.8238675	6.5585587
7.1041317	7.0802298	7.0189149	7.3607622	7.4487389	7.1681935	7.7945884	7.8712914	7.6513698
7.0762632	6.8936642	7.1536545	7.0367824	7.101185	6.8727489	7.2983092	7.2466167	7.2904838
2.9906265	2.3958007	3.3712718	2.5786313	2.9701107	2	2.2180911	2	2.3981461
3.9859168	4.3515565	3.3712718	4.1145884	4.5932826	3.2790605	4.0921636	4.1813664	3.9487208
4.2581217	3.9415025	4.4445501	4.9833211	5.0342917	4.8445541	5.8944734	5.8896564	5.8427942
8.5461762	8.4921691	8.4897866	8.6411083	8.6509843	8.5359188	7.0003051	6.96418	6.9777419
2.9906265	2.9612664	2.9650943	3.3805373	3.5449709	3.1302769	3.6422468	3.8365351	3.3724324
4.2076351	3.6824426	4.5263082	4.5690822	4.5347973	4.5250741	3.5647792	3.3824986	3.6891623
3.1053801	2.9612664	3.1824306	3.1593576	2.7817736	3.4139246	3.396124	3.8365351	2.7098437
3.7238255	3.9415025	3.3712718	2.9904928	2.7817736	3.1302769	3.5647792	3.9608643	2.9660172
2.2197205	2.3958007	2	2.3181327	2	2.5613046	2.5749062	2	2.9660172
2.2197205	2.3958007	2	2.5786313	2.3100466	2.9643661	2.2180911	2.4021099	2.9660172
9.5022407	9.4003493	9.4919488	9.4937682	9.4713039	9.4220956	10.062488	10.008475	10.055588
3.7939455	3.9415025	3.5382314	3.3805373	3.5449709	3.1302769	4.8482682	4.8931255	4.748599
8.9101121	8.7925186	8.9223582	8.0766004	8.0332498	8.0269417	7.7775213	7.6210513	7.8631063
6.5365859	6.5492235	6.4136896	6.0264801	6.1716943	5.7659038	5.8944734	5.8896564	5.8427942
2	2	2	2	2	2	2.8607385	2.9740279	2.7098437
5.8404777	5.9264999	5.6353242	5.5824184	5.616845	5.4578126	6.04469	5.5369839	6.3718678
5.1796526	5.251441	4.9968831	4.5690822	4.7557305	4.2644835	4.912217	4.7625408	4.9985377
2.2197205	2	2.3976902	2.7992097	2.9701107	2.5613046	2.4075018	2.4021099	2.3981461
3.9859168	3.9415025	3.9473196	4.5387765	4.649489	4.334175	5.1952966	4.8293101	5.4399178
10.93688	10.875823	10.886571	10.8241	10.718628	10.832619	11.503638	11.499039	11.447667
6.5769214	6.4692397	6.5760739	6.9473212	6.9302155	6.8727489	5.9715359	5.72407	6.1573762
6.9204083	6.7487385	6.9759106	6.2107651	6.1716943	6.16059	5.925794	5.72407	6.0507505
2.2197205	2	2.3976902	2.5786313	2.7817736	2.30778	2.8607385	2.4021099	3.1834889

5.5677309	5.801426	5.1594558	5.3047698	5.4994188	4.9811664	5.8624576	5.8896564	5.7774236
2.5787203	2.3958007	2.7091089	2.8980192	2.9701107	2.7768658	2.8607385	2.9740279	2.7098437
6.4194705	6.3176199	6.4136896	6.481617	6.5568147	6.3064312	6.614702	6.6126503	6.5585587
4.1010311	4.2594452	3.8234137	4.9375947	4.649489	5.1059542	5.2458095	5.3219704	5.1089463
8.9021969	8.8330273	8.8606541	9.0390584	9.0061385	8.9782366	9.1563559	9.0875095	9.1637042
8.8184595	8.8290272	8.6937552	9.1320742	9.0301541	9.137783	8.3038995	8.1706868	8.3694325
2	2	2	2.3181327	2.3100466	2.30778	2.2180911	2.4021099	2
6.6915732	6.5097858	6.7563059	6.9063671	6.9748945	6.7378252	7.5502044	7.6304432	7.4031227
3.8608147	3.8177796	3.9473196	3.8929252	3.9549305	3.7561757	4.711182	4.5414122	4.8789742
2.8659503	2.7060624	2.9650943	3.630911	4.123159	3.4139246	4.3447825	4.2801452	4.3594016
2.5787203	2	2.9650943	2.7992097	3.1366761	2.30778	3.2051098	3.1926715	3.1834889
3.9859168	4.0554482	3.8234137	3.5722723	3.6588876	3.4139246	3.396124	3.550228	3.1834889
7.6727199	7.7557695	7.4648642	6.9063671	7.0075186	6.6987273	6.9855178	6.8721706	7.0348031
4.569306	4.2594452	4.7469892	4.960639	4.854682	4.9811664	5.5369952	5.4118562	5.5997308
2.2197205	2.3958007	2	2.7992097	2.7817736	2.7768658	2	2	2
3.1053801	3.5330831	2.3976902	3.9400663	3.5449709	4.1912539	3.8524369	3.9608643	3.6891623
2.2197205	2	2.3976902	3.1593576	3.1366761	3.1302769	4.0358621	4.075324	3.9487208
7.5864427	7.6326639	7.422785	7.2006065	7.1985888	7.1097789	7.4077319	7.1842126	7.5473614
3.403311	3.5330831	3.1824306	2.6931321	2.5651052	2.7768658	3.4829147	3.7004723	3.1834889
4.684541	4.0554482	5.0531343	5.0493029	4.9472802	5.0655458	5.4098549	5.4118562	5.5220361
2.5787203	2	2.9650943	3.1593576	3.421282	2.7768658	2.8607385	3.1926715	2.3981461
6.7806135	6.6793379	6.77315	6.9004205	6.8485346	6.8609914	6.6712383	6.5546339	6.7238356
11.33547	11.305289	11.253617	11.640273	11.580815	11.605199	12.189014	12.160324	12.157284
5.6072167	5.6644705	5.4382008	5.7238246	5.7254286	5.6339509	6.04469	6.1204673	5.935611
9.9518524	9.848586	9.9426935	10.58784	10.566367	10.514862	11.253913	11.217572	11.230534
2.4103584	2.3958007	2.3976902	2.8980192	2.7817736	2.9643661	3.0991973	2.7162254	3.3724324
3.6501227	3.5330831	3.6878577	2.8980192	2.7817736	2.9643661	3.4829147	3.1926715	3.6891623
4.8251331	4.6704862	4.8773401	4.8661672	4.4738406	5.1059542	6.0868657	6.2233567	5.8744013
2	2	2.3976902	2.3181327	2.3100466	2.30778	2.2180911	2	2.3981461
2.5787203	2.7060624	2.9650943	2.8980192	2.5651052	3.1302769	2.8607385	3.1926715	2.7098437
4.487029	4.3515565	4.5263082	4.4761857	4.5932826	4.2644835	5.4958476	5.3219704	5.5997308

6.5365859	6.6970036	6.2306822	5.7896485	5.3715813	6.040638	6.2810207	6.4734607	6.0781548
5.6830869	5.7345728	5.5979955	5.1529798	5.4369156	5.1835263	5.4958476	5.4118562	5.6370634
2	2	2	2.1678177	2	2.30778	2.2180911	2	2.3981461
2.4103584	2.3958007	2.3976902	2.6931321	2.5651052	2.7768658	2.7248895	3.1926715	2
6.7544736	6.5492235	6.8545413	6.6958995	6.6284834	6.6720606	7.7558996	7.6020821	7.8392788
3.9859168	3.9415025	3.9473196	3.9400663	3.9549305	3.8543214	3.8524369	4.075324	3.5394718
5.2807962	5.3440487	5.7072032	5.7102916	5.8264086	5.7909055	6.3403369	6.5546339	6.3495506
5.6830869	7.4151098	7.0468884	6.5591555	7.9292449	7.8479156	7.3220454	8.3575984	8.5268897
2.5787203	2.9612664	2	3.4473206	2.9701107	3.7561757	3.3037759	2.9740279	3.5394718
8.5231368	8.575708	8.3508305	8.664104	8.5138837	8.7131356	9.1776537	9.0772439	9.2139832
2.4103584	2.3958007	2.3976902	2.7992097	2.3100466	3.1302769	2	2	2
6.9282245	6.7986822	6.9465121	6.9122894	6.7996958	6.9301405	8.0361656	8.0144653	7.9983548
3.2116748	3.3664558	2.9650943	3.8929252	3.9549305	3.7561757	4.5990795	4.7625408	4.4460942
5.3753109	5.1524786	5.4798392	5.8024604	6.0516855	5.3939735	6.5558595	6.4310946	6.6157808
5.7551657	5.4310687	5.9338433	6.3305929	6.4180925	6.1412778	6.7866248	6.5935696	6.9030876
4.0446218	3.8177796	4.1671548	4.5387765	4.2738034	4.6936396	5.0031203	5.0128652	4.9399942
2.8659503	3.1780396	2.3976902	2.7992097	2.7817736	2.7768658	2.9848891	2.9740279	2.9660172
10.922278	10.911254	10.828328	10.420809	10.379598	10.37719	10.519164	10.555047	10.428596
2.5787203	2.9612664	2	2.3181327	2.5651052	2	3.0991973	3.3824986	2.7098437
4.6087492	4.3515565	4.7469892	5.7636782	5.6990392	5.7404611	5.2704178	5.4547826	4.9985377
2	2	2	2.3181327	2.3100466	2.30778	2.8607385	2.4021099	3.1834889
2.4103584	2.3958007	2.3976902	4.2328874	4.5932826	3.7561757	4.9431589	4.7625408	5.0547979
7.9511201	7.8376847	7.9528049	8.6763361	8.7215094	8.5322168	9.0287421	9.0634417	8.9317648
2.2197205	2.3958007	2	3.5111488	3.1366761	3.9462135	3.3037759	3.550228	3.1834889
11.37604	11.283531	11.356392	10.921275	10.909818	10.837877	11.962587	11.929675	11.935035
10.869975	10.778582	10.850322	10.332473	10.309373	10.261214	10.766808	10.767877	10.705064
2.4103584	2.3958007	2.3976902	3.3105119	2.9701107	3.5372523	2.5749062	2.7162254	2.3981461
5.2561672	5.4726864	4.8773401	4.6278481	4.7557305	4.4006546	4.7813527	4.1813664	5.1611356
6.1786211	6.2474223	5.9925565	5.8151596	5.616845	5.9098007	6.351913	6.3424369	6.3038532
4.6087492	4.3515565	4.7469892	5.2497029	4.9914418	5.3939735	4.0921636	4.2801452	3.8247709
5.6456505	5.8337256	5.9925565	5.864867	6.3348412	6.2170221	5.7088847	6.1468857	5.935611

3.5724509	3.1780396	3.8234137	3.3805373	2.7817736	3.7561757	3.8524369	3.8365351	3.8247709
6.8231542	6.7316988	6.8062596	5.9129186	5.6721581	6.040638	5.6724178	5.4964687	5.7774236
6.8147458	6.8469547	6.6690026	6.7027497	6.7996958	6.545557	6.752094	6.8721706	6.5585587
2	2	2	2.3181327	2.3100466	2.30778	2.7248895	2.4021099	2.9660172
9.6118577	9.5487982	9.5638937	10.276179	10.294027	10.161695	11.300956	11.283053	11.259251
9.3256041	9.3425972	9.19658	8.9177172	8.8012065	8.9396305	9.3007258	9.2547379	9.289196
9.1106099	8.9881556	9.1262142	8.88195	8.7949903	8.8884259	9.14808	9.1990178	9.0437287
8.3822951	8.3981042	8.2521564	8.8864697	8.7793315	8.8999628	9.7952424	9.7487189	9.7811856
12.127353	12.037881	12.104727	10.926405	10.920565	10.837127	11.69644	11.654427	11.677811
7.4894023	7.5572944	7.3003084	8.0871098	8.0800152	8.0003921	8.3565662	8.5027362	8.1285721
8.8700947	8.8007118	8.8287833	8.9852926	8.8649092	9.0078615	9.8005323	9.6459625	9.8837548
2.7294717	2.9612664	2.3976902	2	2	2	2.2180911	2.4021099	2
2.7294717	2.3958007	2.9650943	2.5786313	2.5651052	2.5613046	2.7248895	2.7162254	2.7098437
9.3476286	9.3142619	9.2784956	9.4041503	9.4237494	9.3011411	9.8935673	9.9664493	9.7665207
3.5724509	3.6824426	3.3712718	3.3805373	2.3100466	3.9462135	3.0991973	3.3824986	2.7098437
2.5787203	2.3958007	2.7091089	2.3181327	2.3100466	2.5613046	3.3037759	3.3824986	3.1834889
2.8659503	2.7060624	2.9650943	3.5111488	3.7644639	3.1302769	3.9162052	3.9608643	3.8247709
2.7294717	2	3.1824306	2.4542536	2.7817736	2	2.2180911	2.7162254	2
2.4103584	2.7060624	2	3.1593576	3.2859859	2.9643661	2	2	2
3.7939455	3.3664558	4.0614204	5.1529798	5.4046183	4.7457183	4.7813527	4.8293101	4.6787093
10.103656	10.030281	10.065717	9.6797196	9.6016717	9.6628867	9.8635544	9.8486697	9.8182249
2.7294717	2.3958007	2.9650943	3.1593576	2.9701107	3.2790605	2.7248895	2.7162254	2.7098437
3.7939455	3.1780396	4.1671548	4.3422168	4.5347973	4.0326014	4.8482682	4.8293101	4.8152588
3.5724509	3.6824426	3.3712718	2.3181327	2.5651052	2	3.7157657	4.2801452	2.7098437
3.403311	3.1780396	3.5382314	3.8441915	3.9549305	3.6508626	3.2051098	3.9608643	2
3.6501227	3.6824426	3.5382314	4.1145884	3.9549305	4.1912539	5.3184099	5.1756805	5.3970366
2.2197205	2	2.3976902	2.5786313	2.9701107	2	2.2180911	2.4021099	2
3.2116748	3.5330831	2.7091089	3.1593576	2.5651052	3.5372523	3.396124	3.1926715	3.5394718
3.1053801	2.9612664	3.1824306	2.7992097	2.9701107	2.5613046	3.4829147	3.9608643	2.7098437
4.4440613	4.2594452	4.5263082	4.8903714	4.5347973	5.1059542	4.9431589	5.1756805	4.6052608
2.5787203	2	2.9650943	2.9904928	3.1366761	2.7768658	2.5749062	2	2.9660172

2.4103584	2.3958007	2.3976902	3.3805373	2.9701107	3.6508626	3.2051098	3.3824986	2.9660172
2.2197205	2	2.3976902	3.5111488	3.7644639	3.1302769	2.5749062	2.7162254	2.3981461
6.0120976	5.9851925	5.9338433	6.7889632	6.7872233	6.6987273	7.1536848	7.2219781	7.020748
2.4103584	2.7060624	2	2.1678177	2.5651052	2	2.8607385	2	3.3724324
2.2197205	2	2.3976902	2.6931321	2.7817736	2.5613046	2.9848891	2.7162254	3.1834889
7.1449502	6.9827772	7.2288441	7.1513809	6.952728	7.2959448	7.4132674	7.3862163	7.4352681
6.9437309	7.2089345	6.4767447	7.4284459	7.307406	7.4529996	7.6426454	7.4827099	7.7312213
2.7294717	2.9612664	2.3976902	3.6872592	4.0414954	3.1302769	4.4775267	3.9608643	4.8152588
6.6449038	6.6433442	6.537159	7.1055944	6.9748945	7.1489836	7.3395946	7.2094991	7.4031227
2.2197205	2.3958007	2	3.5111488	3.7644639	3.1302769	2.5749062	2.7162254	2.7098437
3.7238255	3.8177796	3.5382314	5.8024604	5.8743662	5.6339509	6.141248	6.0661334	6.1573762
2.5787203	2.9612664	2	2	2	2	2.4075018	2.7162254	2
2	2	2	2.1678177	2.3100466	2	2	2	2
2	2	2	2.5786313	2.7817736	2.30778	2.4075018	2.7162254	2
2.8659503	2.9612664	2.7091089	2.7992097	2.7817736	2.7768658	3.7857191	4.075324	3.3724324
6.430519	6.2474223	6.4971652	7.4366874	7.3672878	7.4132983	8.5870923	8.6063195	8.5068621
4.1553175	3.6824426	4.4445501	4.7910183	4.7035875	4.7959824	5.1952966	5.0128652	5.3072501
6.8726006	6.8310427	6.8062596	6.6751515	6.7231904	6.5307911	7.424275	7.5134916	7.2668609
5.701447	5.7345728	5.5979955	5.9010549	5.8743662	5.8396456	6.2317468	6.3874467	5.9943291
3.7238255	3.3664558	3.9473196	3.630911	3.5449709	3.6508626	3.396124	3.3824986	3.3724324
5.6072167	5.3440487	5.7418429	6.1618908	6.2824824	5.9324476	5.941203	6.0661334	5.7435929
8.2699154	8.1830441	8.2461364	8.1808561	8.1547388	8.1137219	8.1466313	8.1771099	8.054622
2.4103584	2.7060624	2	2	2	2	2.8607385	2.7162254	2.9660172
6.6543592	6.8149532	6.3477521	7.1663257	7.1891391	7.0488988	7.7860801	7.7729498	7.7398269
5.8732383	5.6280992	6.4136896	6.2107651	6.3001473	6.3064312	6.3403369	6.4734607	6.2078724
9.7961252	9.7270314	9.7540711	9.8743048	9.8646554	9.7892348	10.564644	10.518841	10.549779
5.3288274	5.2984878	5.2584768	5.4583812	5.4046183	5.4262461	5.6350052	5.6885435	5.5220361
2.7294717	2	3.1824306	2.5786313	2.9701107	2	3.3037759	3.3824986	3.1834889
2	2	2	2.4542536	3.2859859	2.30778	3.0991973	2.7162254	3.3724324
9.6299432	9.6013379	9.5468534	9.9080386	9.8557238	9.8691784	10.248167	10.262142	10.173033
2.9906265	2.7060624	3.1824306	2.4542536	2.7817736	2	2.9848891	2.9740279	2.9660172

2.2197205	2	2.3976902	3.2369134	3.2859859	3.1302769	2.8607385	3.1926715	2.7098437
3.5724509	3.3664558	3.6878577	3.0773944	2.9701107	3.1302769	4.5191927	4.1813664	4.748599
2.4103584	2.3958007	2.3976902	2	2.3100466	2	2	2	2
5.6456505	5.6644705	5.872639	6.1113025	6.1907599	5.9983304	6.7608045	6.8721706	6.7410834
3.5724509	3.6824426	3.3712718	3.2369134	3.1366761	3.2790605	3.2051098	3.550228	2.7098437
3.7939455	4.0554482	3.5382314	3.3805373	3.6588876	2.9643661	3.3037759	2.4021099	3.8247709
2.8659503	2.9612664	2.7091089	4.3768933	4.5347973	4.1141074	4.0358621	4.075324	3.9487208
3.5724509	3.5330831	3.5382314	4.8661672	4.7557305	4.8915437	4.9431589	4.6925307	5.1611356
3.9247213	3.3664558	4.2656663	4.0728969	4.2004465	3.8543214	4.0921636	2.9740279	4.6787093
3.1053801	3.1780396	2.9650943	3.0773944	2.9701107	3.1302769	3.396124	3.7004723	2.9660172
12.121186	12.083926	12.046599	11.784672	11.809249	11.662626	11.098097	11.07524	11.060623
2	2	2	2	2	2	2	2	2
9.1344544	9.0818998	9.0761909	9.6883736	9.6351149	9.6475696	9.3037092	9.3430738	9.2015768
3.7238255	3.8177796	3.6878577	4.7117388	4.9914418	4.2644835	4.8151984	4.2801452	5.1611356
12.070218	12.006347	12.023027	12.336839	12.283377	12.296119	12.463828	12.444803	12.423117
3.9247213	4.2594452	3.3712718	4.6563572	4.7035875	4.5250741	5.5571371	5.4118562	5.6370634
6.6353859	6.6433442	6.5173007	6.7365211	6.952728	6.3741717	6.0303529	6.1204673	5.8744013
3.403311	2.7060624	3.8234137	4.0299647	3.6588876	4.2644835	5.3418203	5.614753	4.9399942
2.9906265	2.9612664	2.9650943	2.6931321	2	3.1302769	2.7248895	2	3.1834889
8.6818134	8.4301455	8.8003068	8.1001399	8.0332498	8.0735335	9.7489301	9.7530247	9.6842005
9.4502619	9.3397886	9.4480435	9.030916	8.9624313	9.0051934	10.149633	10.178178	10.062465
11.286903	11.242015	11.220226	11.637148	11.567201	11.612668	12.357838	12.31212	12.343836
10.926674	10.908413	10.832352	11.523497	11.464704	11.487783	11.918503	11.865007	11.910948
2	2	2	2.6931321	2.5651052	2.7768658	2.4075018	2.4021099	2.3981461
3.5724509	3.1780396	3.8234137	3.6872592	4.0414954	3.1302769	4.1985751	3.8365351	4.4460942
8.9413456	8.755062	9.0066752	9.4557504	9.3808132	9.4360309	10.045657	10.029926	10.00115
2	2	2	2.4542536	2.5651052	2.30778	3.0991973	3.3824986	2.7098437
4.0446218	4.1610493	3.8234137	5.3921198	5.4994188	5.1835263	6.0012441	5.9805917	5.9652688
10.01285	9.9824624	9.931483	10.523039	10.510556	10.443732	11.02541	11.023603	10.968438
3.2116748	3.3664558	3.1824306	4.0299647	4.4101943	3.4139246	5.0031203	4.5414122	5.3072501
2	2	2	3.4473206	3.421282	3.4139246	3.0991973	2.9740279	3.5394718

7.8353483	7.7725289	7.9453932	7.8881332	7.9629056	7.8117265	8.4046013	8.5078411	8.341434
8.525715	8.5899608	8.339566	8.9088582	8.8288528	8.8942059	9.7357359	9.7400684	9.6707622
8.7972766	8.6272939	8.8487849	8.3098364	8.3066588	8.218794	8.5747957	8.6669046	8.4131296
2.8659503	2.3958007	3.1824306	3.5722723	3.6588876	3.4139246	3.9162052	3.8365351	3.9487208
2	2	2.3976902	3.2369134	3.2859859	3.1302769	2.5749062	2.4021099	2.7098437
2	2	2	2	2	2	2.9848891	2	3.5394718
2	2	2	2	2	2	2.5749062	2.7162254	2.3981461
4.569306	4.2594452	4.7469892	4.8165054	4.9017239	4.6396102	4.9431589	5.1756805	4.6052608
2.4103584	2.7060624	2	3.2369134	3.2859859	3.1302769	3.7857191	3.550228	3.9487208
12.571635	12.51148	12.520581	12.35421	12.302128	12.312129	12.080782	12.075192	12.026236
8.3794472	8.2858489	8.3620077	8.5471792	8.5948372	8.4003385	8.7380842	8.5330981	8.8625292
7.4627421	7.4151098	7.4120706	8.3605316	8.4321274	8.2002504	8.7113721	8.7337883	8.6277022
10.12081	10.056178	10.081017	10.238373	10.152901	10.243212	10.779723	10.743198	10.762148
10.785903	10.718226	10.742196	10.924941	10.895363	10.860196	11.065071	11.017352	11.052011
3.1053801	3.1780396	2.9650943	2	2	2	2.7248895	2.7162254	2.7098437
6.4194705	6.2232429	6.4971652	6.5741754	6.4968007	6.5601733	7.2120155	7.2946666	7.076165
5.527134	5.1524786	5.7418429	5.5061535	5.5296835	5.3939735	6.0303529	6.1468857	5.8427942
2	2	2	2.1678177	2	2.30778	2.4075018	2.4021099	2.3981461
8.1312054	8.2858489	8.3452093	8.1511142	8.3197082	8.2279771	8.8381891	8.854915	8.9467095
8.5638448	8.4820153	8.5349292	8.7073221	8.7215094	8.5974418	7.9008588	7.8311272	7.9096123
4.0446218	4.0554482	3.9473196	3.8441915	3.9549305	3.6508626	4.0358621	4.075324	3.9487208
2.5787203	2.7060624	2.3976902	2.8980192	3.2859859	2.30778	3.3037759	3.8365351	2.3981461
7.818625	7.7473162	7.8131934	8.3409059	8.3283427	8.2818815	7.3797314	7.3525623	7.3591167
2.4103584	2.7060624	2	2	2	2	3.4829147	2.7162254	3.9487208
2.2197205	2.3958007	2	3.4473206	3.1366761	3.6508626	2.8607385	3.1926715	2.3981461
6.484527	6.3176199	6.537159	5.3750678	5.3031474	5.3609623	5.7088847	5.72407	5.6370634
2.4103584	2.7060624	2	2.3181327	2.3100466	2.30778	3.2051098	2.9740279	3.3724324
5.9520259	5.9851925	5.8087227	5.4420989	5.1556966	5.6060478	7.3740655	7.4406185	7.2428448
3.9247213	3.6824426	4.0614204	4.3768933	4.4738406	4.1912539	4.1985751	4.5414122	3.6891623
2.5787203	2.7060624	2.3976902	2.3181327	2.3100466	2.30778	2.2180911	2	2.3981461
6.7719527	6.8936642	6.5173007	6.6541008	6.6284834	6.5889691	6.9478725	6.9189087	6.9183307

5.7898982	5.9264999	5.5203094	7.6070203	7.5481843	7.5731652	5.8785543	5.65212	6.0228156
3.2116748	3.3664558	2.9650943	3.1593576	3.2859859	2.9643661	2	2	2
2.2197205	2	2.3976902	3.0773944	2.5651052	3.4139246	2.2180911	2.4021099	2
5.856951	5.6644705	5.9338433	5.5523944	5.616845	5.5189462	5.7962123	5.8896564	5.6370634
7.5962878	7.5475902	7.5356729	7.5088158	7.5183093	7.4052252	8.2274073	8.2701748	8.1220038
6.6258049	6.3845599	6.7392628	7.5625242	7.6342676	7.3889423	7.8889888	7.8791918	7.8392788
2.5787203	2.9612664	2	3.3105119	3.421282	3.1302769	3.9772738	4.5414122	2.9660172
7.8963888	7.7890958	7.8924167	8.6867389	8.5138837	8.75503	10.14465	10.173367	10.053864
5.5475752	5.801426	5.1072743	6.6751515	6.6284834	6.631113	6.141248	6.0935561	6.1314494
2.2197205	2.3958007	2	3.2369134	2	3.8543214	3.0991973	3.550228	2.3981461
2	2	2	2	2	2	2	2	2
5.5475752	5.3882147	5.5979955	5.7636782	5.9657484	5.4262461	5.1429507	5.4964687	4.6052608
3.1053801	3.3664558	2.7091089	5.4256309	5.5593263	5.1835263	3.4829147	3.8365351	2.9660172
3.1053801	2.3958007	3.5382314	2.9904928	2.9701107	2.9643661	3.9162052	3.9608643	3.8247709
2.2197205	2.3958007	2	2.9904928	2.9701107	2.9643661	2.5749062	2.9740279	2
6.4194705	6.427521	6.3020577	6.8333365	6.8243218	6.7506259	7.2862931	7.2094991	7.3021518
6.3971163	6.4062003	6.278656	6.3127933	6.0723931	6.4388737	6.8614237	6.7738909	6.8876817
3.4903583	3.6824426	3.1824306	3.741489	3.8628381	3.5372523	4.5191927	4.075324	4.8152588
5.527134	5.2984878	5.6353242	5.2866467	5.2676733	5.2208023	5.5770017	5.2748363	5.7774236
2	2	2	2.5786313	2	2.9643661	3.7157657	3.550228	3.8247709
2	2	2	2.8980192	3.1366761	2.5613046	5.0031203	4.7625408	5.1611356
13.835481	13.750152	13.809436	13.995689	13.952839	13.944482	14.877765	14.839497	14.855541
4.2581217	4.1610493	4.2656663	4.7910183	4.4738406	4.9811664	5.169361	5.123424	5.2115027
4.3997744	3.6824426	4.8136363	4.7117388	4.4738406	4.8445541	5.0321917	4.6925307	5.2601706
2	2	2	2.8980192	3.421282	2	2.7248895	2.4021099	2.9660172
8.248224	8.2623103	8.1201396	7.4325725	7.2809615	7.4839923	7.5145965	7.2708417	7.6695005
4.0446218	4.0554482	3.9473196	4.0299647	4.4101943	3.4139246	4.0921636	4.4594701	3.5394718
3.8608147	3.9415025	3.6878577	4.0299647	4.123159	3.8543214	4.559689	4.4594701	4.6052608
2.9906265	2.9612664	2.9650943	3.2369134	3.2859859	3.1302769	2.8607385	2.9740279	2.7098437
2.7294717	2.7060624	2.7091089	2.9904928	2.3100466	3.4139246	4.0921636	3.8365351	4.3594016
2.4103584	2.7060624	2	3.3105119	3.1366761	3.4139246	3.9772738	3.8365351	4.0628589

4.5287539	4.1610493	4.7469892	4.7386523	4.7557305	4.6396102	5.4534919	5.5369839	5.3072501
4.756549	4.990011	4.357879	5.8151596	5.6721581	5.8634117	5.0031203	4.8931255	5.0547979
10.776733	10.709549	10.73254	11.004714	10.979996	10.935001	11.173018	11.154684	11.131824
3.6501227	3.6824426	3.5382314	4.3422168	4.4738406	4.1141074	4.0358621	3.7004723	4.2671646
2.8659503	2.9612664	2.7091089	2.3181327	2	2.5613046	2.7248895	3.1926715	2
2	2	2	2.1678177	2.3100466	2	2	2	2
2	2.3958007	2	2.3181327	2.3100466	2.30778	3.4829147	3.1926715	3.6891623
9.072325	8.9295859	9.1130433	9.2548422	9.2114513	9.2113931	10.065127	10.042292	10.029504
4.4440613	4.5971173	4.1671548	4.5387765	4.854682	4.0326014	4.6747739	3.7004723	5.2115027
7.3624677	7.3042218	7.3118824	7.6251628	7.6342676	7.5218197	8.4777502	8.5871543	8.2954747
3.1053801	3.1780396	3.1824306	3.5722723	3.421282	3.6508626	3.8524369	3.8365351	3.9487208
7.242213	7.3383921	7.0189149	6.9983239	6.8120614	7.07966	8.3479208	8.2641538	8.3694325
6.3971163	6.1736335	6.4971652	6.5591555	6.6002417	6.4388737	7.2434305	7.3525623	7.062509
7.1382268	7.0936294	7.0743297	5.3403469	5.2313049	5.3609623	4.8482682	4.2801452	5.2115027
4.2581217	4.3515565	4.0614204	4.0728969	4.2004465	3.8543214	4.1463502	4.1813664	4.0628589
5.7726365	5.801426	5.6353242	5.3578118	5.2313049	5.3939735	5.7267783	5.72407	5.6734543
2.8659503	2.9612664	2.7091089	3.3105119	3.2859859	3.2790605	3.2051098	3.550228	2.7098437
4.3069011	4.0554482	4.4445501	4.1945222	3.9549305	4.334175	4.8151984	4.8293101	4.748599
2.5787203	2.7060624	2.3976902	3.0773944	3.2859859	2.7768658	3.9162052	3.7004723	4.0628589
3.8608147	4.2594452	3.1824306	3.0773944	3.2859859	2.7768658	4.5990795	4.7625408	4.3594016
9.1327642	9.1508208	8.9995358	9.9568468	9.9099409	9.9096897	11.057137	11.063153	10.99024
6.0838321	6.2232429	5.8087227	6.7231073	6.8120614	6.5307911	6.3747902	6.7907429	5.7089498
2.5787203	2.9612664	2	2.6931321	2.5651052	2.7768658	2.8607385	2.7162254	2.9660172
2.8659503	2.7060624	2.9650943	3.5722723	3.6588876	3.4139246	3.0991973	3.550228	2.3981461
6.191668	6.6793379	6.8702827	6.4330113	6.7746421	6.9301405	7.7602499	8.3057825	8.3243704
12.917839	12.894723	12.828466	13.190384	13.136217	13.150806	13.701229	13.652652	13.688903
7.1382268	6.968302	7.1917391	7.4034351	7.4085881	7.3046513	8.2492371	8.1962094	8.2419575
6.430519	6.427521	6.3250858	6.171799	5.9877173	6.2534533	6.8934622	6.8238675	6.9030876
3.2116748	3.5330831	2.7091089	2.3181327	2	2.5613046	2.2180911	2	2.3981461
8.8962319	8.7801408	8.8995273	9.4678646	9.457595	9.3835785	10.044765	9.9627363	10.064179
2.8659503	3.3664558	2	3.5722723	3.5449709	3.5372523	3.3037759	3.550228	2.9660172

6.255189	6.0689835	6.3250858	6.0481578	6.0306763	5.976702	6.3169023	6.3424369	6.2324729
6.8147458	6.6433442	6.8702827	6.9063671	6.7231904	6.9853361	7.637925	7.5033038	7.7050922
9.8057036	9.7962772	9.7022439	10.037632	10.007253	9.9806764	10.605111	10.621314	10.533785
4.3069011	4.5198165	3.9473196	3.741489	3.6588876	3.7561757	3.9162052	4.1813664	3.5394718
12.399234	12.310976	12.375424	12.547108	12.552957	12.445304	13.313186	13.283921	13.282181
2.8659503	2.9612664	2.7091089	3.0773944	3.421282	2.5613046	2.9848891	2.9740279	2.9660172
2.4103584	2.3958007	2.3976902	2.4542536	2.3100466	2.5613046	2	2	2
10.044658	10.030281	9.9464111	10.096686	10.099676	9.998226	10.830254	10.762548	10.836247
2	2	2	2.4542536	2.5651052	2.30778	3.0991973	2.9740279	3.1834889
10.632888	10.531465	10.621823	11.010247	10.962075	10.964831	11.568951	11.522558	11.554576
3.2116748	3.5330831	2.7091089	3.5111488	3.1366761	3.7561757	4.0358621	3.7004723	4.2671646
2.2197205	2	2.3976902	2.1678177	2	2.30778	2.2180911	2	2.3981461
3.3106725	2	3.9473196	2.6931321	2.5651052	2.7768658	3.0991973	3.550228	2.3981461
2.7294717	2.9612664	2.3976902	2.6931321	2	3.1302769	2.2180911	2.4021099	2
2.2197205	2.3958007	2	2.4542536	2	2.7768658	2.4075018	2.4021099	2.3981461
6.3513412	6.427521	6.1555909	6.618319	6.6002417	6.545557	7.2983092	7.1584751	7.3702447
2	2	2	2	2	2	2	2	2
4.9532328	4.4381381	5.30555	4.3768933	4.2004465	4.4642052	5.7088847	5.7587426	5.5997308
10.644203	10.615146	10.563711	10.591996	10.555376	10.538121	11.517839	11.480981	11.496092
4.756549	4.6704862	4.7469892	4.7117388	4.9472802	4.334175	4.5990795	4.1813664	4.8789742
3.9859168	4.0554482	3.8234137	2.5786313	2.5651052	2.5613046	2.5749062	2.4021099	2.7098437
6.1786211	5.9851925	6.2548685	6.5890405	6.6833604	6.3906213	7.8568498	7.9026367	7.7483815
3.2116748	2	3.8234137	3.2369134	3.421282	2.9643661	2.8607385	2.4021099	3.3724324
6.5055765	6.4485311	6.4560311	5.8277479	5.5593263	5.9983304	5.6907665	5.5763924	5.7435929
2.9906265	3.1780396	2.7091089	4.6563572	4.7035875	4.5250741	5.6538328	5.5763924	5.6734543
4.487029	4.3515565	4.5263082	5.3578118	5.2313049	5.3939735	5.5165681	5.1756805	5.7435929
2.8659503	2.7060624	3.6878577	2.6931321	2.5651052	2.7768658	2.9848891	2.7162254	3.1834889
2.2197205	2	2.3976902	2.6931321	2.7817736	2.5613046	2.4075018	2	2.7098437
4.5287539	4.7403036	4.1671548	4.5078205	3.7644639	4.9370509	4.4346216	4.6189492	4.1686253
2	2	2	2.1678177	2.3100466	2	2.5749062	2	2.9660172
4.5287539	4.4381381	4.5263082	4.7117388	4.34361	5.1059542	4.9431589	4.2801452	5.3528418

2	2	2	2.5786313	2	2.9643661	2	2	2
3.7238255	3.5330831	3.8234137	4.3768933	4.4738406	4.1912539	4.0358621	4.4594701	3.3724324
12.994407	12.941789	12.935451	12.786375	12.732211	12.746053	12.358197	12.29598	12.359975
6.7098263	6.6064294	6.7045603	6.9063671	6.8723477	6.8491373	6.9630487	7.0224134	6.8404516
2	2	2	2.3181327	2.3100466	2.30778	2.4075018	2	2.7098437
2	2	2	2.3181327	2.5651052	2	2.2180911	2	2.3981461
2.8659503	2.9612664	2.7091089	2.7992097	2.7817736	2.7768658	2.8607385	2.4021099	3.1834889
3.9247213	3.5330831	4.1671548	3.630911	3.2859859	3.9462135	4.4346216	4.5414122	4.2671646
3.2116748	3.1780396	3.1824306	3.630911	3.7644639	3.4139246	3.4829147	3.8365351	2.9660172
8.6841233	8.7380973	8.5100245	8.62136	8.6406218	8.5098029	9.4841714	9.4870254	9.4261274
4.569306	4.6704862	4.357879	4.9833211	5.0342917	4.8445541	6.1278434	6.0381793	6.1573762
3.3106725	3.3664558	3.1824306	4.7386523	4.5347973	4.8445541	4.1985751	4.3725921	3.9487208
4.756549	4.9315181	4.4445501	3.8441915	3.5449709	4.0326014	4.6747739	4.075324	5.0547979
4.2076351	4.5971173	3.5382314	3.8441915	4.2004465	3.2790605	4.6747739	4.5414122	4.748599
4.3069011	4.5198165	3.9473196	4.5690822	4.8060545	4.1912539	5.1160479	5.2261101	4.9399942
2	2	2	3.1593576	3.421282	2.7768658	2.8607385	2.4021099	3.1834889
2.2197205	2.3958007	2	2.7992097	3.1366761	2.5613046	2.5749062	2.4021099	2.7098437
7.4465063	7.5378203	7.3683991	6.6110549	6.6561828	6.8959802	6.2810207	6.6684237	5.9053308
3.1053801	3.1780396	2.9650943	2.7992097	2.5651052	2.9643661	2.9848891	2.7162254	3.1834889
2	2	2	2.5786313	2.7817736	2.30778	2	2	2
4.8251331	4.5198165	4.9968831	5.0706429	4.9017239	5.1452616	5.1429507	4.6925307	5.4399178
2.4103584	2.3958007	2.3976902	2.9904928	2.3100466	3.4139246	2.7248895	2.9740279	2.3981461
4.3997744	5.1003295	4.357879	4.9833211	5.3715813	5.3939735	5.0886338	5.1756805	5.7089498
9.1661971	9.0919496	9.1294882	8.62136	8.5024841	8.6427798	9.14808	9.141059	9.0949363
4.1010311	4.1610493	3.9473196	4.0728969	3.8628381	4.1912539	3.2051098	2.4021099	3.8247709
5.3522564	5.1524786	5.4382008	5.6406543	5.7254286	5.4578126	6.0729437	6.2721744	5.7774236
3.1053801	3.6824426	2	4.3066864	4.4738406	4.1141074	4.1985751	4.2801452	4.0628589
5.7195764	5.2028082	6.0210407	6.0905599	6.0306763	6.0613354	5.6159286	5.614753	5.5614064
2.9906265	2.3958007	3.3712718	2.1678177	2	2.30778	3.7857191	4.1813664	3.1834889
6.484527	6.5097858	6.3477521	6.5741754	6.6967596	6.4388737	6.6804494	6.8562486	6.5389689
2.9906265	2.9612664	2.9650943	2.7992097	3.1366761	2.30778	2.7248895	2.7162254	2.7098437

5.3980029	5.5907872	5.0531343	4.7386523	4.5932826	4.7959824	5.1952966	5.1756805	5.1611356
2	2	2	2.1678177	2.3100466	2	2.7248895	3.1926715	2
7.321509	7.1588218	7.3904001	7.7950451	7.7480452	7.7493179	8.3565662	8.3290418	8.3243704
2.5787203	2.3958007	2.7091089	2.1678177	2	2.30778	2.2180911	2	2.3981461
4.487029	4.3515565	4.5263082	5.3578118	5.1556966	5.4578126	5.2458095	5.0128652	5.3970366
4.0446218	4.3515565	3.5382314	3.8929252	3.8628381	3.8543214	3.6422468	3.550228	3.6891623
5.3980029	5.4726864	5.2098157	5.2682931	5.193996	5.2571394	5.7088847	5.6885435	5.6734543
4.0446218	4.1610493	3.8234137	5.1328319	5.1163526	5.0655458	6.3973103	6.2959775	6.4368197
2.8659503	2.3958007	3.1824306	3.4473206	3.421282	3.4139246	4.7813527	5.2261101	4.0628589
5.0988516	4.990011	5.1072743	4.9141763	5.0759055	4.6396102	5.5571371	5.8580248	5.1089463
2.5787203	2	2.9650943	2.8980192	3.1366761	2.5613046	2.7248895	2.9740279	2.3981461
5.1262912	7.8535241	7.5842038	6.1113025	7.888955	8.0374263	6.6986968	8.1447046	7.8942763
2.5787203	2.9612664	2	2.6931321	2.9701107	2.30778	3.0991973	3.3824986	2.7098437
2.2197205	2	2.3976902	3.2369134	3.2859859	3.1302769	3.2051098	3.3824986	2.9660172
7.0261593	7.0253511	6.9165019	8.0419061	7.9573498	8.0321935	8.6185814	8.567731	8.6090351
12.805631	12.740091	12.759878	13.008406	12.946997	12.97567	13.371842	13.342776	13.340808
4.0446218	4.1610493	3.8234137	4.84155	4.649489	4.9370509	5.6159286	5.7926015	5.3528418
9.4624243	9.4506808	9.3615884	9.5327332	9.5286241	9.4419622	10.132117	10.173367	10.027748
4.6471428	4.990011	4.0614204	4.8903714	4.8060545	4.8915437	5.941203	5.4964687	6.2324729
6.0412208	6.1222572	5.8410348	6.7298298	6.8841084	6.4546057	7.6613742	7.6855458	7.576479
7.3095897	7.0667046	7.422785	7.2575425	7.1409391	7.2783728	6.2442239	6.3193942	6.1050482
5.9366088	5.9264999	5.8410348	6.0373597	6.2824824	5.6339509	5.6907665	5.7926015	5.5220361
2.2197205	2	2.3976902	2.1678177	2.3100466	2	2.8607385	3.3824986	2
3.5724509	3.3664558	3.6878577	3.2369134	3.1366761	3.2790605	2.7248895	2	3.1834889
2.8659503	2.9612664	2.7091089	3.793754	3.421282	4.0326014	2.5749062	2.7162254	2.3981461
2.5787203	2.9612664	2.3976902	2.3181327	2.3100466	2.5613046	2.7248895	2.7162254	2.7098437
2.4103584	2	2.7091089	2.8980192	2.7817736	2.9643661	2.5749062	2.7162254	2.3981461
5.8732383	5.7345728	5.9035657	5.7505157	5.8264086	5.5775942	6.7694627	6.6865457	6.7916228
10.086296	10.04415	10.074237	10.538916	10.433616	10.562827	10.522369	10.451552	10.54733
3.8608147	3.8177796	3.8234137	4.1945222	4.4101943	4.0326014	4.1985751	4.3725921	3.9487208
3.403311	3.3664558	3.5382314	3.0773944	3.1366761	2.9643661	3.0991973	2.7162254	3.3724324

10.770768	10.658619	10.769735	11.104108	11.067694	11.046255	11.489951	11.491327	11.429829
2.5787203	2.3958007	2.7091089	2.6931321	3.1366761	2	3.3037759	3.7004723	2.7098437
2	2	2	2.3181327	2.3100466	2.30778	2.9848891	2	3.5394718
2.2197205	2	2.3976902	2	2	2	2	2	2
2	2	2	2.3181327	2.5651052	2	2.5749062	2	2.9660172
2.8659503	2.9612664	2.7091089	3.793754	3.421282	4.0326014	4.1463502	4.075324	4.1686253
4.2076351	4.6704862	3.3712718	4.3422168	4.649489	3.8543214	4.7813527	4.3725921	5.0547979
5.1262912	5.1524786	4.9968831	5.005652	4.9914418	4.9370509	4.8482682	4.5414122	5.0547979
7.9852388	7.8216695	8.0319157	8.0499869	7.888955	8.1087592	8.8667128	8.8589157	8.8144545
3.6501227	3.6824426	3.5382314	3.3805373	3.6588876	2.9643661	3.7857191	2.7162254	4.3594016
3.2116748	2.9612664	3.3712718	3.9400663	4.2004465	3.6508626	4.3447825	4.2801452	4.3594016
3.3106725	3.5330831	2.9650943	2.9904928	2.9701107	3.1302769	3.2051098	3.1926715	3.1834889
2.5787203	2	2.9650943	2.9904928	3.1366761	2.7768658	3.9162052	4.075324	3.6891623
5.9210252	6.1222572	5.5596753	5.6406543	5.5296835	5.6613247	5.8297152	5.65212	5.935611
2	2.3958007	2	2	2	2	2	2	2
2.2197205	2.3958007	2	2.3181327	2.5651052	2	2	2	2
4.2581217	3.1780396	4.8136363	4.84155	4.9914418	4.5834785	5.1160479	5.1756805	4.9985377
12.463677	12.433931	12.381616	13.083481	13.050263	13.022436	13.691794	13.680611	13.642616
3.1053801	3.1780396	2.9650943	3.1593576	2.9701107	3.2790605	3.0991973	2.9740279	3.1834889
8.1379615	8.1123939	8.0527592	8.094942	8.1302584	7.9623821	7.7945884	7.5925031	7.9172196
3.3106725	2.7060624	3.6878577	3.6872592	4.2004465	2.7768658	4.559689	4.7625408	4.2671646
5.527134	5.6280992	5.30555	6.5286383	6.6284834	6.3236669	6.8934622	6.7907429	6.9334144
2.2197205	2	2.3976902	2	2	2	3.2051098	3.550228	2.7098437
12.693587	12.651699	12.624015	13.133295	13.085071	13.087576	14.014077	13.997214	13.970607
11.608749	11.555772	11.561804	11.462749	11.421979	11.421788	12.475782	12.504121	12.392744
3.2116748	3.6824426	2.3976902	3.6872592	3.7644639	3.5372523	3.9772738	4.1813664	3.6891623
2.9906265	2.9612664	2.9650943	2	2	2	2	2	2
2.9906265	2.9612664	2.9650943	2.1678177	2	2.30778	2.5749062	2.4021099	2.7098437
3.4903583	3.6824426	3.1824306	2.7992097	2.3100466	3.1302769	3.2051098	2.9740279	3.3724324
12.468542	12.448996	12.395961	12.394036	12.337309	12.375224	13.774161	13.721284	13.774762
2	2	2	2	2	2	2.5749062	2.9740279	2

4.5287539	4.4381381	4.5263082	4.7117388	4.7557305	4.5834785	5.2704178	5.3676132	5.1089463
2.5787203	2.7060624	2.3976902	2.5786313	2.3100466	2.7768658	2.4075018	2.4021099	2.3981461
12.657601	12.627145	12.575927	13.271237	13.217192	13.231212	14.45943	14.427706	14.43074
2	2	2	2	2	2	2.7248895	2.4021099	2.9660172
2.5787203	2.3958007	2.7091089	2.4542536	2.7817736	2	2.5749062	2.4021099	2.7098437
6.5159872	6.6970036	6.1810581	7.3564246	7.2809615	7.3389609	7.599595	7.8712914	7.2060539
6.3396668	6.1736335	6.3920436	5.9129186	5.8977601	5.8634117	5.3875313	5.4547826	5.3970366
4.2581217	4.3515565	4.0614204	4.2702587	4.34361	4.1141074	4.1985751	4.075324	4.2671646
2.4103584	2.7060624	2	2.4542536	2.5651052	2.30778	2.7248895	2.9740279	2.3981461
2.8659503	2.3958007	3.1824306	2.3181327	2.3100466	2.30778	2.4075018	2	2.7098437
5.7195764	5.9851925	5.2584768	5.0493029	4.7557305	5.2208023	4.912217	4.8931255	4.8789742
5.1532186	4.990011	5.2098157	5.6406543	5.4685057	5.7145616	6.5356996	6.4310946	6.577886
6.3629219	6.4062003	6.2060835	6.3741542	6.1327901	6.500798	7.0790323	6.9789602	7.1163741
3.6501227	3.8177796	3.3712718	2.6931321	2.5651052	2.7768658	3.396124	3.3824986	3.3724324
2.8659503	3.1780396	2.7091089	3.3805373	3.8628381	2.5613046	3.3037759	3.1926715	3.3724324
11.578648	11.5491	11.496107	12.106676	12.056638	12.062963	13.033702	13.008301	12.998682
13.547648	13.383143	13.594385	13.617327	13.640047	13.498658	14.357013	14.350868	14.303004
2.7294717	2.9612664	2.3976902	2.1678177	2.3100466	2	2	2	2
3.7238255	3.9415025	3.3712718	4.1551088	4.5347973	3.5372523	3.9162052	3.8365351	4.0628589
3.1053801	3.3664558	2.7091089	2.6931321	2.5651052	2.7768658	3.3037759	2.4021099	3.8247709
7.6254264	7.487953	7.8374205	7.8267465	7.8354433	7.9062838	8.1164081	8.1706868	8.054622
2	2	2	2.5786313	2	2.9643661	2.4075018	2.7162254	2
2	2	2	2.4542536	2.3100466	2.5613046	2	2	2
6.4414835	6.4692397	6.3020577	7.4201571	7.4247827	7.3219081	8.1027696	8.1115546	8.0337782
2	2	2	2.3181327	2	2.5613046	2.5749062	2	2.9660172
4.1553175	4.1610493	4.0614204	3.3805373	2.5651052	3.8543214	3.3037759	3.3824986	3.1834889
10.784289	10.705191	10.751788	11.353	11.272281	11.33839	12.155139	12.110437	12.139508
3.8608147	3.9415025	3.6878577	3.9857156	3.5449709	4.2644835	4.3447825	4.6189492	3.9487208
4.569306	4.6704862	4.6036804	4.3066864	4.5347973	4.0326014	4.2489753	4.2801452	4.1686253
8.2668366	8.3318043	8.0800901	8.450781	8.2757432	8.5247841	9.4907314	9.489608	9.4314496
2	2	2	3.630911	3.421282	3.8543214	3.3037759	3.1926715	3.5394718

11.422434	11.351337	11.382697	11.649388	11.584874	11.619663	12.548483	12.496116	12.539822
8.9199454	8.7338248	8.9959528	9.083038	9.1567495	8.9056968	9.7123517	9.7027142	9.6617333
3.2116748	2.7060624	3.5382314	3.793754	3.421282	4.0326014	4.4346216	4.5414122	4.2671646
2	2	2	2	2	2.5613046	2.7248895	3.1926715	2
2.7294717	3.1780396	2	2.7992097	2.7817736	2.7768658	2.7248895	2.7162254	2.7098437
3.7939455	4.0554482	3.3712718	2.4542536	2	2.7768658	2	2	2
2.2197205	2	2.3976902	2.6931321	2.5651052	2.7768658	2.5749062	2.4021099	2.7098437
2.4103584	2.3958007	2.3976902	3.0773944	2.9701107	3.1302769	2.9848891	3.1926715	2.7098437
2.8659503	2.7060624	2.9650943	2.5786313	2.7817736	2.30778	2.2180911	2	2.3981461
4.9835561	4.8705531	4.9968831	4.9833211	4.9472802	4.9811664	5.941203	5.72407	6.0781548
7.8682243	7.7216552	7.9001049	8.156114	8.085119	8.1334039	9.4373972	9.4609414	9.3522877
5.9520259	5.801426	5.9925565	6.4247487	6.4813989	6.2713297	6.6619681	6.7907429	6.4578363
2	2	2	3.0773944	2.9701107	3.1302769	2.9848891	2.9740279	2.9660172
2.5787203	2.7060624	2.3976902	2.1678177	2	2.30778	2.4075018	2.7162254	2
8.4812467	8.4088639	8.4431856	8.3798938	8.2757432	8.388102	8.8111882	8.7337883	8.8266242
2	2	2	2	2	2	2	2	2
3.4903583	3.9415025	2.7091089	4.5078205	4.8060545	4.0326014	4.711182	5.123424	4.0628589
2.2197205	2	2.3976902	2.4542536	2.7817736	2	2.2180911	2.4021099	2
4.354085	4.3515565	4.357879	5.5824184	5.3031474	5.7404611	5.8297152	5.614753	5.9652688
6.4631658	6.3176199	6.4971652	6.4330113	6.5859108	6.16059	7.5197375	7.3862163	7.5860558
4.3997744	4.5198165	4.1671548	3.6872592	3.9549305	3.2790605	3.7857191	3.7004723	3.8247709
6.9590724	6.6250049	7.1407329	6.756411	6.6144317	6.8007223	6.2810207	6.2233567	6.28045
4.4440613	3.6824426	4.8773401	5.3047698	5.2676733	5.2571394	4.5990795	4.7625408	4.3594016
2	2	2	2	2	2	2.4075018	2	2.7098437
2	2	2	2.5786313	2.3100466	2.7768658	2.8607385	2.7162254	2.9660172
10.324065	10.302528	10.233333	10.91059	10.921278	10.803737	10.870046	10.794231	10.884583
2	2	2	2	2	2	2	2	2
4.1553175	3.9415025	4.2656663	5.005652	4.9017239	5.0239729	5.0606888	5.123424	4.9399942
3.5724509	3.5330831	3.5382314	4.0728969	4.2004465	3.8543214	5.169361	5.0692034	5.2115027
2.2197205	2	2.3976902	2.6931321	2.5651052	2.7768658	2.4075018	2.4021099	2.3981461
5.07088	5.0462245	4.9968831	5.9129186	5.6990392	6.0196392	6.7345136	6.7395848	6.6708196

2.9906265	3.3664558	2.3976902	2.6931321	2.5651052	2.7768658	3.2051098	3.3824986	2.9660172
2.4103584	2.3958007	2.3976902	2.4542536	2	2.7768658	2.9848891	2.7162254	3.1834889
2.2197205	2	2.3976902	2.6931321	3.1366761	2	2.2180911	2	2.3981461
2.8659503	2.9612664	2.9650943	3.9400663	3.421282	4.2644835	3.2051098	3.3824986	3.3724324
2	2	2	2.1678177	2.3100466	2	3.4829147	3.1926715	3.6891623
5.0132553	4.8068978	5.1072743	5.2308701	5.1556966	5.2208023	6.1143131	6.1728291	5.9943291
9.6418754	9.4942726	9.6733735	9.5846476	9.5379599	9.5373874	9.9258759	9.9250713	9.8661762
3.7939455	3.9415025	3.8234137	4.4107557	3.9549305	4.6936396	4.8151984	4.7625408	4.8152588
5.6830869	5.7345728	5.5203094	5.6263142	5.4369156	5.7145616	5.8297152	5.9206094	5.6734543
3.3106725	2.7060624	3.6878577	3.8441915	3.8628381	3.7561757	3.9162052	4.1813664	3.5394718
2.7294717	2.7060624	2.7091089	2.8980192	3.2859859	2.30778	3.5647792	4.1813664	2.3981461
3.1053801	3.3664558	2.7091089	4.1551088	4.5932826	3.4139246	3.396124	3.550228	3.1834889
4.8906042	5.0462245	4.6771135	4.9375947	5.0759055	4.9370509	4.297674	3.9608643	4.6787093
8.288252	8.2800001	8.1845131	8.2549663	8.3066588	8.1037793	7.7071594	7.7981708	7.5473614
2	2	2	2.7992097	2.7817736	2.7768658	2	2	2
2	2	2.3976902	2.3181327	2.3100466	2.30778	2	2	2
4.756549	4.8705531	4.5263082	4.8165054	5.0342917	4.4642052	5.169361	4.5414122	5.5614064
2	2.3958007	2	2.5786313	2.7817736	2.30778	2.2180911	2	2.3981461
2.7294717	2.9612664	2.3976902	2.7992097	2.5651052	2.9643661	2.2180911	2.4021099	2
3.6501227	3.1780396	3.9473196	4.5690822	4.4101943	4.6396102	4.912217	5.0692034	4.6787093
2.5787203	2.7060624	2.3976902	2	2	2	2	2	2
2	2	2	2.4542536	2.5651052	2.30778	2	2	2
3.1053801	2.7060624	3.3712718	3.2369134	3.2859859	3.1302769	2.8607385	2.4021099	3.1834889
2	2	2	2.3181327	2	2.5613046	2	2	2
3.2116748	2.7060624	3.5382314	2.6931321	2.3100466	2.9643661	3.5647792	3.1926715	3.8247709
6.217413	6.3402801	5.9634986	7.7294726	7.7861516	7.5731652	8.7446856	8.7424763	8.6867542
6.0412208	5.6999474	6.2306822	6.6890166	6.6284834	6.6585401	6.8119911	6.7395848	6.8243582
2	2	2	2.7992097	2.9701107	2.5613046	2.4075018	2	2.7098437
6.3160308	6.3625899	6.1555909	6.3655465	6.3001473	6.340699	7.0366236	6.9492469	7.062509
2.8659503	2	3.3712718	2.6931321	2.3100466	2.9643661	2.4075018	2.7162254	2
3.6501227	3.9415025	3.9473196	3.8929252	4.4101943	3.9462135	4.0358621	4.075324	4.2671646

6.1387573	6.1986514	5.9634986	6.4736289	6.4341814	6.4229682	6.7780693	6.7907429	6.7063792
2	2	2	3.1593576	2.7817736	3.4139246	2.9848891	3.550228	2
4.1010311	4.3515565	3.6878577	2.3181327	2.3100466	2.30778	3.4829147	3.7004723	3.1834889
5.527134	5.8337256	4.9968831	4.6843137	4.5347973	4.7457183	5.169361	5.3219704	4.9399942
2.4103584	2.7060624	2	2.1678177	2.3100466	2	2.2180911	2	2.3981461
3.3106725	3.3664558	3.1824306	4.1145884	4.123159	4.0326014	4.746694	4.5414122	4.8789742
4.2076351	4.2594452	4.0614204	4.4438416	4.7557305	3.9462135	4.3904012	4.6189492	4.0628589
2	2	2	2	2	2	2	2	2
12.698308	12.701503	12.581018	12.293253	12.239704	12.25263	12.29326	12.210109	12.314251
5.3522564	5.3882147	5.2098157	5.9594213	6.1129374	5.6881887	5.9564492	5.8580248	5.9943291
7.8641555	7.8846904	7.737971	7.7950451	7.8354433	7.6706793	8.1061914	8.0980784	8.0751691
2.9906265	2.3958007	3.3712718	2.4542536	2	2.7768658	2	2	2
4.756549	4.8068978	4.6036804	4.4107557	4.2738034	4.4642052	4.6374231	4.8931255	4.2671646
4.2076351	4.3515565	3.9473196	3.630911	3.6588876	3.5372523	3.4829147	3.550228	3.3724324
2.2197205	2.3958007	2	2.6931321	2.3100466	2.9643661	2.4075018	2.7162254	2
3.5724509	3.9415025	2.9650943	3.9857156	4.0414954	3.8543214	3.7857191	3.9608643	3.5394718
2.5787203	2.7060624	2.3976902	2.1678177	2.3100466	2	2	2	2
2.9906265	2.3958007	3.3712718	2.7992097	3.1366761	2.30778	2.8607385	2	3.3724324
5.9520259	6.1222572	5.6353242	6.4330113	6.4180925	6.3575325	6.8695003	6.7907429	6.8876817
2.2197205	2	2.3976902	3.2369134	3.2859859	3.1302769	3.8524369	3.7004723	3.9487208
2.9906265	2.7060624	3.1824306	2	2	2	2.4075018	2.4021099	2.3981461
2.8659503	3.1780396	2.3976902	2.1678177	2	2.30778	2.9848891	3.1926715	2.7098437
5.1532186	4.8705531	5.30555	6.3912169	6.3175986	6.3741717	7.3909969	7.145432	7.5473614
6.8480892	6.8149532	6.77315	7.863891	7.8713373	7.7683256	8.7707934	8.6485325	8.830658
11.097136	11.093198	10.987451	11.509647	11.444989	11.479637	12.460657	12.434452	12.426452
2	2	2	2	2	2	2	2	2
6.5669427	6.1736335	6.7897998	5.8890928	5.9657484	5.7145616	5.6538328	5.4964687	5.7435929
3.7238255	3.6824426	3.6878577	3.3105119	3.6588876	2.7768658	3.7857191	3.7004723	3.8247709
10.718202	10.676538	10.648313	10.601649	10.589897	10.518609	11.035765	11.033372	10.97938
5.1262912	5.2028082	4.9383494	5.5824184	5.4685057	5.6060478	6.4734725	6.4094357	6.4785512
5.5876089	5.8653179	5.1072743	5.4904045	5.1163526	5.7145616	6.3860942	6.6500711	5.9943291

4.354085	4.6704862	3.8234137	4.6843137	4.4738406	4.7959824	4.4775267	4.7625408	4.0628589
2	2	2	2.5786313	2.9701107	2	2	2	2
6.2045979	6.0958662	6.2306822	6.7431816	6.6423996	6.7506259	7.5898512	7.5535382	7.5668383
6.9125496	6.8469547	6.8702827	6.7298298	6.6833604	6.6854555	7.4351993	7.145432	7.6237393
2.8659503	2.9612664	2.7091089	2.5786313	2.5651052	2.5613046	2.5749062	2	2.9660172
2	2	2	2.1678177	2	2.30778	2.2180911	2.4021099	2
11.672667	11.604557	11.629272	11.733535	11.676909	11.696099	12.250993	12.272552	12.167686
8.0367587	8.0858203	7.8691031	7.8941306	7.8110079	7.88902	8.6540809	8.6485325	8.5996102
3.4903583	3.5330831	3.3712718	3.3105119	3.5449709	2.9643661	4.0921636	3.8365351	4.2671646
3.2116748	3.1780396	3.1824306	3.3105119	2.3100466	3.9462135	3.4829147	3.550228	3.3724324
2.7294717	2.9612664	2.3976902	3.630911	3.421282	3.7561757	3.396124	3.1926715	3.5394718
5.5063989	5.7683866	5.0531343	4.5078205	4.2738034	4.6396102	5.5965964	5.8580248	5.2115027
3.1053801	2.7060624	3.3712718	5.0493029	4.7557305	5.2208023	4.711182	4.7625408	4.6052608
2	2	2	2.1678177	2	2.30778	3.3037759	3.550228	2.9660172
5.7374808	5.6280992	5.7418429	4.9375947	5.3377701	4.2644835	5.5965964	5.4118562	5.7089498
9.3344543	9.3199737	9.2366018	9.1892868	9.0537765	9.2252164	9.2129948	9.2084553	9.1572943
3.1053801	3.1780396	2.9650943	2.8980192	3.1366761	2.5613046	3.6422468	3.8365351	3.3724324
2	2	2	2.3181327	2.3100466	2.30778	2.7248895	3.1926715	2
7.0041419	6.9239842	6.9759106	6.8081477	6.6833604	6.837185	7.5094372	7.5436303	7.4139176
2.2197205	2.3958007	2	2.9904928	2.7817736	3.1302769	2	2	2
2.5787203	2.9612664	2	3.2369134	3.6588876	2.5613046	3.5647792	3.1926715	3.8247709
7.8353483	7.712999	7.8454066	8.0035858	7.9849176	7.9457804	6.7433306	6.7907429	6.6343614
8.5862482	8.5222089	8.5447719	9.1684064	9.1664131	9.0755566	10.422976	10.361364	10.424598
6.8147458	6.6970036	6.9012594	7.5049019	7.4328124	7.4916375	8.109605	8.0498948	8.1285721
2.9906265	2.9612664	2.9650943	3.1593576	3.2859859	2.9643661	2.5749062	2	2.9660172
6.7806135	6.6793379	6.77315	7.5625242	7.5258362	7.5143331	8.5846413	8.6850456	8.4131296
2	2	2	2.6931321	2.9701107	2.30778	2.5749062	2.4021099	2.7098437
3.4903583	3.1780396	3.6878577	4.3768933	4.2004465	4.4642052	4.3447825	4.075324	4.5278714
2	2	2	2.4542536	2.5651052	2.30778	3.0991973	2.9740279	3.1834889
2	2	2	3.1593576	2.5651052	3.5372523	3.4829147	3.7004723	3.1834889
7.4999299	7.4980653	7.3904001	7.8109829	7.841488	7.684087	8.6137814	8.5628341	8.6043304

2	2	2	2	2	2	2.2180911	2	2.3981461
3.403311	3.1780396	3.5382314	4.4107557	3.6588876	4.8445541	4.4346216	4.2801452	4.5278714
2	2	2	2.3181327	2.5651052	2	2.2180911	2	2.3981461
6.0977607	5.6644705	6.3477521	6.7889632	6.4968007	6.9524728	7.570164	7.6582576	7.4139176
6.0412208	6.3402801	5.5203094	5.6966305	5.8505866	5.4262461	6.5953533	6.5546339	6.577886
2.5787203	2.3958007	2.7091089	2.4542536	2.7817736	2	2.8607385	3.3824986	2
3.9859168	4.1610493	3.6878577	4.9141763	5.1163526	4.5834785	5.5369952	5.123424	5.8104792
2.4103584	2	2.7091089	2.9904928	2.9701107	2.9643661	2.2180911	2	2.7098437
7.6011853	7.6326639	7.4544589	7.1004161	6.9302155	7.1777034	7.6981179	7.8791918	7.4352681
2	2	2	2.7992097	3.2859859	2	2.5749062	2.9740279	2.3981461
3.4903583	3.6824426	3.1824306	4.5987644	4.9914418	3.9462135	2.9848891	2.7162254	3.1834889
2	2	2	2	2	2	2	2	2
2	2	2	2.4542536	2	2.7768658	2.9848891	2.4021099	3.3724324
8.3879741	8.3872636	8.2759883	9.6569735	9.5323656	9.6830597	10.064248	10.089024	9.9776956
2	2	2	2	2	2	2.7248895	2.4021099	2.9660172
2	2	2	3.0773944	3.1366761	2.9643661	2.9848891	2.9740279	2.9660172
9.5799622	9.5609082	9.5020881	9.6831874	9.6455169	9.6389893	10.611747	10.613047	10.553445
2	2	2	2.4542536	2.5651052	2.30778	2	2	2
3.4903583	2.7060624	3.9473196	3.8929252	3.2859859	4.2644835	3.4829147	2.9740279	3.8247709
5.6830869	5.9264999	5.30555	6.3127933	6.2281515	6.4068854	4.6374231	4.7625408	4.4460942
6.4523654	6.2945981	6.4971652	6.8017811	6.7996958	6.711878	6.4194843	6.2959775	6.4785512
5.3522564	5.0462245	5.5203094	4.7117388	4.8060545	4.5250741	5.4318384	5.4118562	5.4815611
2.4103584	2.9612664	2	2	2	2	2	2	2
3.7238255	3.3664558	3.9473196	3.630911	4.0414954	2.9643661	3.7157657	4.075324	3.1834889
4.3069011	4.5198165	3.9473196	3.741489	3.8628381	3.9462135	4.0921636	3.9608643	4.1686253
3.6501227	3.1780396	4.0614204	4.5987644	4.9017239	4.1141074	4.559689	5.123424	3.5394718
6.6064498	6.4062003	6.686891	7.5243662	7.4247827	7.5292676	8.1565667	7.9257066	8.3071025
2.2197205	3.3664558	3.3712718	2.1678177	2.7817736	3.1302769	2	2.4021099	2.9660172
2.4103584	2	2.7091089	2.7992097	2.3100466	3.1302769	2.5749062	2.9740279	2
2.9906265	3.1780396	2.7091089	3.4473206	3.2859859	3.5372523	2.7248895	2.9740279	2.3981461
2	2	2	2.8980192	2.9701107	2.7768658	3.0991973	3.1926715	2.9660172

7.6866109	7.7388132	7.5157939	7.7918363	7.8475074	7.6366045	8.1794864	8.077625	8.2175197
4.9532328	4.8705531	4.9383494	4.1945222	4.34361	3.9462135	4.711182	4.8931255	4.4460942
8.7583455	8.8047911	8.5930028	8.4487451	8.3326407	8.4755154	8.8257899	8.8787544	8.7132145
2.2197205	2	2.3976902	2.9904928	3.421282	2.30778	3.0991973	3.3824986	2.7098437
3.1053801	2.3958007	3.5382314	2.5786313	2.5651052	2.5613046	2.2180911	2	2.3981461
2	2	2	2	2	2	2	2	2
3.1053801	2.3958007	3.5382314	3.4473206	3.5449709	3.2790605	3.2051098	2.4021099	3.6891623
2.5787203	2.7060624	2.3976902	2.9904928	3.1366761	2.7768658	2.5749062	2.4021099	2.7098437
3.7238255	3.5330831	3.8234137	2.6931321	2.5651052	2.7768658	2.7248895	3.1926715	2
4.3997744	4.2594452	4.4445501	6.596416	6.6144317	6.4855643	6.5757415	6.4094357	6.6708196
2.2197205	2.3958007	2	2	2	2	2.4075018	2	2.7098437
9.9537682	9.8837198	9.912603	9.030916	9.01151	8.9563026	9.6015564	9.6689318	9.4681649
7.7094693	7.6509067	7.6586097	7.4970421	7.5031365	7.3971067	7.4021751	7.4723016	7.2668609
2.8659503	2.3958007	3.1824306	3.0773944	3.1366761	2.9643661	4.3447825	4.075324	4.5278714
7.7628955	7.6778456	7.737971	8.9554898	8.8649092	8.9507666	9.6148295	9.5303137	9.6389107
2	2	2	2.3181327	2	2.5613046	2.5749062	2.4021099	2.9660172
2	2	2	2	2	2	2	2	2
3.3106725	3.1780396	3.3712718	3.3805373	2.3100466	3.9462135	4.2489753	4.2801452	4.1686253
2	2	2	2.7992097	3.2859859	2	3.5647792	3.8365351	3.1834889
3.6501227	3.9415025	3.1824306	3.8441915	3.6588876	3.9462135	4.5990795	4.3725921	4.748599
4.1553175	4.1610493	4.0614204	4.5078205	3.6588876	4.9811664	3.8524369	4.075324	3.5394718
2.2197205	2.3958007	2	2.7992097	2.5651052	2.9643661	2.2180911	2	2.3981461
2	2	2	2.3181327	2.5651052	2	2.2180911	2.4021099	2
13.473886	13.399925	13.436317	13.846999	13.805339	13.794259	13.505633	13.478004	13.473625
2	2	2	2	2	2	2	2	2
2.4103584	2.3958007	2.3976902	2	2	2	2	2	2
2.9906265	3.1780396	2.7091089	4.1145884	4.2004465	3.9462135	4.1463502	3.8365351	4.3594016
6.1786211	6.0415904	6.2060835	7.0258985	7.1311028	6.8129792	7.5552202	7.5033038	7.5473614
2	2	2	2	2	2	2	2	2
5.3522564	5.1524786	5.4382008	5.8024604	5.8505866	5.6613247	5.0606888	5.2748363	4.748599
2	2	2	2.5786313	2.5651052	2.5613046	2	2	2

3.6501227	3.5330831	3.6878577	4.2328874	4.34361	4.0326014	4.3904012	4.1813664	4.5278714
6.9967274	6.8469547	7.0329694	6.958812	6.9415156	6.8844113	7.6890193	7.5633785	7.7483815
3.7238255	3.5330831	4.0614204	4.2702587	3.8628381	4.5250741	4.1463502	3.8365351	4.3594016
3.1053801	3.5330831	2.3976902	4.3422168	4.4738406	4.1141074	4.559689	4.8293101	4.1686253
3.403311	2.9612664	3.6878577	3.5111488	3.1366761	3.7561757	4.1985751	4.5414122	3.6891623
2	2	2	2	2	2	2.2180911	2	2.3981461
2	2	2	2.4542536	2.5651052	2.30778	2	2	2
2.4103584	2.7060624	2	2	2	2	2.7248895	2.7162254	2.7098437
2.5787203	2	2.9650943	2.9904928	2.9701107	3.1302769	2.8607385	2.7162254	3.3724324
8.3622406	8.2262662	8.395029	8.7359878	8.6883556	8.6900628	9.6988171	9.7400684	9.5945271
2	2	2	2.1678177	2.3100466	2	2	2	2
2	2	2	2	2	2	2	2	2
2.4103584	2.7060624	2	2.1678177	2	2.30778	2.2180911	2.4021099	2
2.5787203	2.7060624	2.3976902	3.3805373	3.1366761	3.5372523	2.5749062	2.7162254	2.3981461
2.2197205	2	2.3976902	2.7992097	2.7817736	2.7768658	2.5749062	2.4021099	2.7098437
2	2	2	2.4542536	2.5651052	2.30778	3.9162052	3.8365351	3.9487208
3.7238255	3.8177796	3.5382314	4.1145884	3.7644639	4.334175	3.8524369	3.3824986	4.1686253
2	2	2	2.1678177	2	2.30778	2	2	2
3.8608147	3.9415025	3.6878577	3.630911	3.6588876	3.5372523	3.396124	3.3824986	3.3724324
2	2	2	2.7992097	2.9701107	2.5613046	2.5749062	2	2.9660172
5.0988516	5.1524786	4.9383494	4.6843137	4.5347973	4.7457183	5.1160479	5.2261101	4.9399942
4.2076351	4.2594452	4.0614204	6.0264801	5.9207807	6.040638	6.1143131	5.9805917	6.1828452
2.7294717	2.9612664	2.3976902	2.7992097	2.9701107	2.7768658	3.2051098	3.550228	2.7098437
3.7939455	3.9415025	3.5382314	4.4438416	4.5347973	4.2644835	4.7813527	4.8293101	4.6787093
2	2	2	2.3181327	2.5651052	2	2.5749062	2.7162254	2.3981461
2.7294717	2.7060624	2.7091089	3.9400663	4.0414954	3.7561757	3.396124	3.3824986	3.3724324
2.2197205	2.3958007	2	2.3181327	2.5651052	2	3.2051098	2.9740279	3.3724324
2	2	2	2.3181327	2.3100466	2.30778	2	2	2
4.684541	4.9315181	4.2656663	4.8165054	4.5347973	4.9811664	5.0886338	4.9542373	5.1611356
2.4103584	2.7060624	2	2	2	2	2	2	2
5.6265616	5.5524845	5.5979955	5.7372319	5.7254286	5.6613247	6.206464	6.247972	6.1050482

7.7585181	7.6326639	7.7718883	8.1536163	8.1103706	8.1037793	8.3333958	8.3116326	8.2954747
3.7939455	3.3664558	4.0614204	3.1593576	2.9701107	3.2790605	3.3037759	3.3824986	3.1834889
4.3997744	4.4381381	4.2656663	4.4761857	4.4101943	4.4642052	3.7857191	3.8365351	3.6891623
3.403311	3.1780396	3.5382314	3.1593576	3.2859859	2.9643661	4.1463502	3.9608643	4.2671646
2	2	2	2	2	2	2.2180911	2.4021099	2
2.9906265	2	3.5382314	2.7992097	2.5651052	2.9643661	2.9848891	2.4021099	3.3724324
4.1010311	4.0554482	4.0614204	3.0773944	2.7817736	3.2790605	4.4346216	4.075324	4.6787093
4.2581217	3.8177796	4.5263082	3.793754	3.7644639	3.7561757	3.7857191	3.550228	3.9487208
2	2	2	2.1678177	2.3100466	2	2	2	2
3.3106725	3.3664558	3.1824306	3.793754	3.7644639	3.7561757	4.5990795	4.6925307	4.4460942
4.85824	5.0462245	4.5263082	6.0373597	5.9657484	6.0196392	6.452119	6.3651173	6.4785512
2	2	2	2.3181327	2.3100466	2.30778	2.2180911	2.4021099	2
8.2821656	8.2503954	8.203278	9.0048245	8.9652021	8.9507666	9.9422416	9.9608762	9.8622405
2.8659503	2.7060624	2.9650943	2.4542536	2.7817736	2	3.5647792	3.8365351	3.1834889
4.4440613	4.4381381	4.357879	4.0299647	4.2738034	3.6508626	5.7267783	5.8896564	5.4815611
3.2116748	3.1780396	3.1824306	3.0773944	2.9701107	3.1302769	3.9772738	4.1813664	3.6891623
4.1010311	4.1610493	3.9473196	4.6563572	4.5932826	4.6396102	5.6538328	5.6885435	5.5614064
2.5787203	2.7060624	2.3976902	2.3181327	2.3100466	2.30778	3.3037759	3.1926715	3.3724324
2.5787203	2	2.9650943	2.5786313	2	2.9643661	2.4075018	2.7162254	2
2	2	2	3.1593576	2.5651052	3.5372523	3.0991973	3.3824986	2.7098437
2.9906265	3.1780396	2.7091089	2.7992097	3.1366761	2.30778	3.0991973	3.3824986	2.7098437
3.9247213	4.0554482	3.6878577	4.9375947	4.123159	5.3939735	4.8482682	4.8931255	4.748599
2.4103584	2.3958007	2.3976902	3.8929252	4.0414954	3.6508626	3.6422468	3.1926715	3.9487208
3.2116748	3.3664558	2.9650943	3.630911	3.6588876	3.5372523	2.9848891	2.7162254	3.1834889
2.2197205	2.3958007	2	2.3181327	2.3100466	2.30778	3.2051098	2.9740279	3.3724324
3.2116748	2.7060624	3.5382314	3.5722723	3.7644639	3.2790605	3.396124	3.1926715	3.5394718
5.0423553	4.8068978	5.1594558	6.2765215	6.4341814	5.9983304	6.2442239	6.1728291	6.2566609
5.2807962	5.2028082	5.2584768	5.9594213	6.0516855	5.7659038	6.6432471	6.6126503	6.6343614
4.7209942	4.6704862	4.6771135	4.3768933	4.649489	4.0326014	4.1985751	4.3725921	3.9487208
3.2116748	2.9612664	3.3712718	3.8441915	4.2004465	3.2790605	3.7857191	3.9608643	3.5394718
3.5724509	3.3664558	3.6878577	3.2369134	3.1366761	3.2790605	3.4829147	2	4.1686253

3.5724509	3.1780396	3.8234137	4.4107557	4.4101943	4.334175	4.4346216	4.075324	4.6787093
2	2	2	3.0773944	2.7817736	3.2790605	3.396124	2.9740279	3.8247709
2.8659503	2.9612664	2.7091089	3.3805373	3.9549305	2.30778	3.3037759	2.9740279	3.5394718
4.6087492	4.5971173	4.5263082	4.6563572	4.9017239	4.2644835	5.3418203	5.123424	5.4815611
2	2	2	2	2	2	2.2180911	2	2.3981461
2.7294717	2	3.1824306	2.9904928	2.9701107	2.9643661	2.7248895	2.9740279	2.3981461
2.2197205	2	2.3976902	2.1678177	2.3100466	2.5613046	3.5647792	2.9740279	4.2671646
3.9859168	4.1610493	3.6878577	4.4761857	4.7557305	4.1141074	4.912217	4.8931255	5.0547979
2	2	2	2	2	2	2.4075018	2.7162254	2
12.163188	12.128547	12.090098	12.21994	12.186974	12.16181	13.240996	13.209696	13.215488
3.5724509	3.8177796	3.1824306	4.8661672	4.5347973	5.0655458	4.0358621	4.075324	3.9487208
2	2	2	2.1678177	2.3100466	2	2.5749062	2.7162254	2.3981461
5.7551657	5.6644705	5.9634986	5.4089726	5.4685057	5.7145616	4.711182	4.7625408	5.1611356
2	2	2	2.6931321	2.5651052	2.7768658	2.7248895	3.1926715	2
2	2	2	2.3181327	2.5651052	2	3.3037759	3.3824986	3.1834889
9.1327642	10.517862	10.576962	9.3861773	10.941821	10.86756	10.447463	11.485517	11.530598
3.9859168	4.1610493	3.6878577	5.7896485	5.6721581	5.8154814	5.6907665	5.8580248	5.4399178
2.9906265	2.3958007	3.3712718	3.1593576	3.2859859	3.1302769	2.7248895	2	3.1834889
3.2116748	2.7060624	3.5382314	4.4761857	4.2738034	4.5834785	2.5749062	2.7162254	2.3981461
4.756549	4.7403036	4.6771135	4.1551088	3.5449709	4.5250741	4.8482682	4.6189492	4.9985377
2.4103584	2.3958007	2.3976902	2	2	2	2.7248895	2.9740279	2.3981461
2	2	2	2.1678177	2	2.30778	2.4075018	2	2.7098437
2.8659503	3.3664558	2	2.6931321	2.3100466	2.9643661	2.2180911	2	2.3981461
3.403311	3.6824426	2.9650943	4.2328874	4.2004465	4.1912539	2	2	2
2	2	2	2	2	2	2	2	2
6.6823593	6.4062003	6.8225337	5.7505157	5.8505866	5.5485682	5.925794	5.65212	6.1050482
2	2	2	2	2	2	2	2	2
3.7238255	4.1610493	2.9650943	4.5078205	4.5347973	4.4006546	5.5165681	5.4964687	5.5220361
2.2197205	2	2.3976902	3.3805373	3.1366761	3.5372523	4.559689	4.8931255	4.0628589
2	2	2	2	2	2	2.4075018	2.7162254	2
3.1053801	3.3664558	2.9650943	2.7992097	2.3100466	3.1302769	3.7157657	3.550228	3.8247709

2.5787203	2	2.9650943	2.6931321	3.1366761	2	3.0991973	2.9740279	3.1834889
2.4103584	2	2.7091089	3.741489	3.6588876	3.7561757	3.2051098	3.550228	2.7098437
2.7294717	2.9612664	2.3976902	2.9904928	2.7817736	3.1302769	3.0991973	3.3824986	2.7098437
3.5724509	4.0554482	2.7091089	5.8024604	5.6447666	5.8867927	6.4304448	6.4734607	6.3268829
2	2	2	2.4542536	2.5651052	2.30778	2.2180911	2.4021099	2
3.1053801	3.5330831	2.3976902	3.630911	3.421282	3.7561757	3.9162052	3.9608643	3.8247709
3.4903583	3.8177796	2.9650943	4.4761857	4.7035875	4.1141074	4.9734511	4.9542373	4.9399942
4.7912485	4.6704862	4.8136363	5.1728501	4.9914418	5.2571394	6.0012441	5.9206094	6.0228156
3.6501227	3.5330831	3.6878577	3.8929252	3.8628381	3.8543214	3.8524369	3.3824986	4.1686253
2.5787203	2.7060624	2.3976902	3.5722723	3.6588876	3.4139246	3.6422468	3.7004723	3.5394718
6.1252208	5.7683866	6.3250858	6.481617	6.6002417	6.2534533	7.3279189	7.2708417	7.3252084
2	2	2	2	2	2	2.4075018	2.4021099	2.3981461
2	2	2	2.1678177	2	2.30778	2.4075018	2.7162254	2
2.2197205	2.3958007	2	2	2	2	3.2051098	3.1926715	3.1834889
2.2197205	2.7060624	2	2.9904928	2.3100466	3.4139246	3.5647792	3.3824986	3.6891623
2.2197205	2.3958007	2	2.3181327	2.3100466	2.30778	2	2	2
4.6471428	4.4381381	4.7469892	4.1551088	4.123159	4.1912539	5.1429507	4.9542373	5.2601706
4.4440613	4.2594452	4.5263082	2.7992097	2.7817736	2.7768658	4.5191927	4.7625408	4.1686253
2.8659503	2.7060624	2.9650943	3.0773944	3.1366761	2.9643661	2.8607385	3.1926715	2.3981461
2	2	2	3.0773944	2.7817736	3.2790605	2	2	2
5.1796526	5.2028082	5.0531343	6.2487064	6.1327901	6.2713297	6.5658347	6.5742331	6.4989729
2.2197205	2.3958007	2	2	2	2.30778	2.7248895	2.9740279	2.3981461
2.7294717	2.9612664	2.3976902	2.6931321	3.1366761	2	3.2051098	2.7162254	3.5394718
2.4103584	2	2.7091089	3.630911	3.6588876	3.5372523	3.7857191	3.9608643	3.5394718
5.2807962	4.8068978	5.5596753	5.4904045	5.5593263	5.3271781	6.2191608	6.631482	5.5614064
2.2197205	2	2.3976902	2.6931321	2.9701107	2.30778	2.9848891	2.4021099	3.3724324
7.3566872	7.120061	7.4648642	7.5359199	7.4878025	7.4916375	7.868986	7.6304432	8.019713
2	2	2	2.6931321	2.9701107	2.30778	2.4075018	2.7162254	2
4.7912485	4.5198165	4.9383494	4.2702587	4.0414954	4.4006546	4.4346216	4.3725921	4.4460942
2	2	2	2	2	2	2	2	2
9.6770912	9.6383815	9.6044584	10.128831	10.036529	10.135094	11.135835	11.138319	11.075147

7.083281	7.2089345	6.8225337	6.5131342	6.2645985	6.6448917	6.9402241	7.0366117	6.7749723
2.8659503	2	3.3712718	2.3181327	2.5651052	2	3.0991973	3.550228	2.3981461
7.8803622	8.1057963	7.4854524	7.4772031	7.4085881	7.4839923	8.6209754	8.5430783	8.6461308
2.7294717	2.7060624	2.7091089	2.3181327	2.5651052	2	2.7248895	3.1926715	2
2	2	2	2	2	2	2	2	2
3.5724509	3.9415025	2.9650943	4.7386523	4.7557305	4.6396102	5.7267783	5.7926015	5.5997308
3.7939455	4.1610493	3.1824306	3.5111488	3.2859859	3.6508626	3.9162052	3.7004723	4.0628589
2	2	2	2	2	2	2.4075018	2.4021099	2.3981461
2.8659503	2.9612664	2.7091089	3.793754	3.9549305	3.5372523	3.0991973	3.1926715	2.9660172
2.7294717	2	3.1824306	2.6931321	2.9701107	2.9643661	2	2	2
2	2	2	2	2	2	2	2	2
2	2	2	2.8980192	2.7817736	2.9643661	2.5749062	2.7162254	2.3981461
2.7294717	3.1780396	2	2.8980192	2.7817736	2.9643661	2.7248895	2.9740279	2.3981461
2	2	2	2.1678177	2	2.30778	2	2	2
2.9906265	2.7060624	3.1824306	3.4473206	3.6588876	3.4139246	4.0921636	4.075324	4.0628589
2	2	2	2	2	2	2	2	2
6.2427066	6.1736335	6.2060835	5.4904045	5.4994188	5.4262461	6.3747902	6.3874467	6.3268829
2	2	2	2.3181327	2.5651052	2	2.2180911	2	2.3981461
6.152168	6.0958662	6.1032668	5.8770308	6.0928076	5.5485682	6.6986968	6.7738909	6.5585587
3.1053801	2.9612664	3.1824306	2.8980192	2.7817736	2.9643661	2.4075018	2	2.7098437
2	2	2	2.3181327	2	2.5613046	2.4075018	2	2.7098437
2	2	2	2.4542536	2.3100466	2.5613046	2.5749062	2.7162254	2.3981461
2.5787203	2.3958007	2.7091089	2.7992097	2.7817736	2.7768658	2.9848891	2.7162254	3.1834889
2	2	2	2	2	2	2.8607385	2.4021099	3.1834889
2	2	2	2.1678177	2	2.30778	2	2	2
2.4103584	2	2.7091089	2.3181327	2.5651052	2	2.7248895	3.1926715	2.3981461
5.4423433	5.5907872	5.1594558	6.171799	5.8264086	6.3741717	5.8785543	6.0661334	5.5997308
3.1053801	3.1780396	2.9650943	3.741489	4.0414954	3.2790605	5.1429507	5.7926015	3.9487208
2	2	2	2	2	2	2	2	2
5.6265616	5.3882147	5.7418429	5.4420989	5.4685057	5.3271781	5.8297152	5.825684	5.7774236
8.7429174	8.6591862	8.7157044	8.7004936	8.6301844	8.6834022	9.1954325	9.141059	9.1922014

2	2	2	2.6931321	2.3100466	2.9643661	2.7248895	2.7162254	2.7098437
7.908293	7.7641735	7.9379432	7.9208146	7.7608595	7.9787949	8.1399696	8.1115546	8.1087769
4.1010311	4.0554482	4.2656663	3.3805373	4.0414954	4.0326014	3.3037759	3.1926715	3.9487208
2	2	2	2.3181327	2.5651052	2	2.5749062	2.4021099	2.7098437
7.385361	7.0802298	7.5455105	7.6677959	7.702277	7.5366773	8.0397488	7.9633552	8.054622
5.8069558	5.7683866	5.7418429	5.9129186	5.7768019	5.9547445	6.0012441	6.0096728	5.935611
8.260659	8.2444008	8.1655008	8.351842	8.3454582	8.2774656	6.452119	6.0661334	6.7063792
5.9052715	5.9264999	5.7756703	6.6327384	6.5714361	6.6031543	6.4194843	6.0935561	6.6343614
3.403311	3.5330831	4.0614204	2.7992097	2.5651052	3.1302769	2.8607385	2.7162254	3.5394718
2	2	2	2	2	2	2.2180911	2	2.3981461
7.0762632	7.2454087	6.7563059	6.6611519	6.4500929	6.763314	6.9705774	6.8721706	7.0065546
2.9906265	3.1780396	2.7091089	2.5786313	2.5651052	2.5613046	3.4829147	2.9740279	3.8247709
3.2116748	3.5330831	2.7091089	3.6872592	3.5449709	3.7561757	3.3037759	3.8365351	2.3981461
3.1053801	3.1780396	2.9650943	3.0773944	2.5651052	3.4139246	3.9772738	3.7004723	4.1686253
2	2	2	2	2	2	2.8607385	3.1926715	2.3981461
2	2	2	2	2	2	2.2180911	2	2.3981461
2	2	2	2	2	2	2.5749062	2.7162254	2.3981461
4.7209942	4.6704862	4.6771135	4.9833211	4.7035875	5.1452616	4.7813527	4.3725921	5.0547979
2.5787203	2.3958007	2.7091089	3.0773944	2.7817736	3.2790605	3.4829147	3.550228	3.3724324
2.7294717	2.7060624	2.7091089	2.7992097	2.5651052	2.9643661	2.9848891	2.9740279	2.9660172
6.4194705	6.2474223	6.4767447	7.7560603	7.7286077	7.6907444	8.516841	8.4769373	8.4967431
2	2	2	2.1678177	2.3100466	2	2.2180911	2.4021099	2
2	2	2	2.1678177	2.3100466	2	2.7248895	3.1926715	2
2.2197205	2	2.3976902	2	2	2	2.5749062	2.7162254	2.3981461
2.2197205	2.3958007	2	2.4542536	2	2.7768658	3.0991973	2.7162254	3.3724324
2	2	2	2.4542536	2.7817736	2	2.5749062	2.4021099	2.7098437
2	2	2	2.8980192	2	3.4139246	2.9848891	2.7162254	3.1834889
3.2116748	2	3.8234137	2.1678177	2.3100466	2	2.2180911	2.4021099	2
2.9906265	3.3664558	2.3976902	2.3181327	2.3100466	2.30778	2	2	2
4.1010311	3.5330831	4.4445501	5.0706429	4.9017239	5.1452616	4.8151984	4.5414122	4.9985377
3.3106725	3.1780396	3.3712718	4.1551088	4.34361	3.8543214	4.4775267	4.6925307	4.1686253

8.1379615	8.0723476	8.0935639	7.5662851	7.4247827	7.6087531	7.8770204	7.6674111	8.0055094
6.3396668	6.427521	6.129666	5.4089726	5.4994188	5.2208023	5.8461792	5.6885435	5.935611
2.2197205	2	2.3976902	2	2	2	2.5749062	2.4021099	2.7098437
2.2197205	2	2.3976902	2.4542536	2	2.7768658	2.5749062	2.9740279	2
5.2561672	5.1524786	5.2584768	5.2866467	5.2313049	5.2571394	6.206464	5.7587426	6.4989729
4.9532328	4.9315181	5.3511356	6.2011218	6.3001473	6.0196392	5.6350052	5.0692034	6.0228156
2.2197205	2	2.3976902	2.3181327	2	2.5613046	3.396124	2.4021099	3.9487208
2	2	2	2.1678177	2.3100466	2	2.7248895	2.9740279	2.3981461
5.5876089	5.5131371	5.5596753	6.4164385	6.4341814	6.3064312	6.5658347	6.3193942	6.7238356
2	2	2	2.8980192	2.3100466	3.2790605	3.8524369	3.1926715	4.2671646
2	2	2	2	2	2	2	2	2
2.2197205	2	2.3976902	2.3181327	2.3100466	2.30778	2.5749062	2.7162254	2.3981461
4.9222583	4.4381381	5.2098157	5.3047698	4.854682	5.5775942	5.7267783	5.8580248	5.5220361
2	2	2	2.1678177	2.3100466	2	2	2	2
5.6265616	5.5524845	5.5979955	5.4089726	5.4685057	5.2571394	5.5165681	5.72407	5.2115027
6.4194705	6.3176199	6.4136896	5.5217324	5.6721581	5.2571394	4.4346216	4.5414122	4.2671646
3.7238255	3.5330831	3.8234137	3.793754	3.5449709	3.9462135	4.2489753	3.8365351	4.5278714
2.4103584	2.7060624	2	2	2	2	2	2	2
2	2	2	2.5786313	2.9701107	2	2.7248895	2.4021099	2.9660172
3.3106725	3.3664558	3.1824306	2.6931321	2.9701107	2.30778	2.8607385	2.9740279	2.7098437
3.7939455	3.3664558	4.0614204	2.9904928	3.1366761	2.7768658	3.8524369	4.075324	3.5394718
4.8906042	4.990011	4.6771135	5.2866467	5.4685057	4.9811664	5.3875313	5.3676132	5.3528418
2.2197205	2.3958007	2	2.4542536	2.7817736	2	3.6422468	3.8365351	3.3724324
2	2	2	2	2	2	2	2	2
2.8659503	3.1780396	2.3976902	3.793754	4.0414954	3.4139246	4.2489753	4.2801452	4.1686253
6.8147458	6.3625899	7.0743297	7.6106671	7.5407733	7.5875059	7.9663372	8.0428782	7.8231723
2.4103584	2.3958007	2.3976902	3.1593576	3.5449709	2.5613046	3.2051098	3.1926715	3.1834889
4.4440613	4.2594452	4.5263082	5.8277479	5.7254286	5.8396456	6.9170326	7.1969113	6.4989729
2.8659503	2.7060624	2.9650943	2.7992097	2.7817736	2.7768658	2.8607385	3.1926715	2.3981461
4.2581217	4.3515565	4.0614204	4.7910183	4.2738034	5.1059542	5.941203	5.825684	6.0228156
2	2.3958007	2	2	2	2	2	2	2

2	2	2	2	2	2	2	2	2
8.3908053	8.4820153	8.3339006	8.7376565	8.7116431	8.8591735	9.4360383	9.4556675	9.5314904
2	2	2	2.1678177	2.3100466	2	2	2	2
2	2	2	2.6931321	2.3100466	3.1302769	2.8607385	3.3824986	3.1834889
6.5868315	6.7655792	6.2548685	6.6398944	6.6561828	6.5307911	5.2458095	5.5763924	4.748599
2	2	2	2.1678177	2	2.30778	2	2	2
3.9247213	4.1610493	3.5382314	3.5111488	3.1366761	3.8543214	3.9772738	4.1813664	3.6891623
6.7892225	6.6793379	6.8225337	6.6751515	6.5714361	6.6987273	6.7951299	6.6500711	6.9183307
2.9906265	2.7060624	3.1824306	3.2369134	2.5651052	3.6508626	2.8607385	2.7162254	3.1834889
4.4440613	4.2594452	4.5263082	4.5987644	4.5932826	4.5250741	4.6747739	4.3725921	4.8789742
2	2	2	2.5786313	2.3100466	2.7768658	2	2	2
2.4103584	2.7060624	2	2	2	2	2	2	2
2.2197205	2.3958007	2	2.8980192	2.3100466	3.2790605	2	2	2
2	2	2	4.0299647	4.2004465	3.7561757	3.7857191	4.075324	3.3724324
2	2	2	2.5786313	2.5651052	2.5613046	3.3037759	2.7162254	3.6891623
2	2	2	2.5786313	2.9701107	2	3.396124	3.1926715	3.5394718
2.9906265	3.1780396	2.7091089	2.1678177	2	2.30778	3.3037759	3.550228	2.9660172
2.4103584	2.7060624	2	2.3181327	2	2.5613046	2	2	2
2.2197205	2	2.3976902	2.4542536	2.5651052	2.30778	2	2	2
2	2	2	2	2	2	2	2	2
2	2	2	2	2	2	2	2	2
6.7367801	6.7986822	6.5567476	6.6751515	6.6833604	6.5746431	7.2802473	7.145432	7.3479023
3.8608147	3.5330831	4.0614204	3.630911	3.421282	3.7561757	2.8607385	2.7162254	2.9660172
5.4640127	4.8068978	5.8410348	5.3047698	5.1163526	5.3939735	6.2442239	6.1204673	6.3038532
2.2197205	2	2.3976902	3.741489	3.6588876	3.7561757	2.8607385	2.4021099	3.1834889
2	2	2	2.4542536	2.5651052	2.5613046	2.2180911	2	2.3981461
8.0439708	7.9743425	8.0036475	8.0310609	7.9629056	8.0057412	8.5747957	8.5330981	8.5564192
3.1053801	2.3958007	3.5382314	2.5786313	2.9701107	2	3.0991973	2.7162254	3.3724324
7.7408745	7.6418141	7.7293656	7.141331	6.952728	7.2243347	7.6981179	7.8229584	7.5076006
2	2	2	2	2	2	2.4075018	2.4021099	2.3981461
2	2	2	2.6931321	2.9701107	2.30778	2.5749062	2	2.9660172

2.2197205	2.3958007	2	2	2	2	2	2	2
6.0555648	6.0415904	5.9634986	6.4974617	6.3175986	6.5746431	7.0437789	6.9189087	7.1030952
2	2	2	2	2	2	2.2180911	2	2.3981461
3.1053801	2.7060624	3.3712718	3.741489	3.5449709	3.8543214	4.5990795	4.3725921	4.748599
3.5724509	2.7060624	4.0614204	3.3805373	3.6588876	2.9643661	2.9848891	2.4021099	3.3724324
2.5787203	2.9612664	2	5.2497029	5.4369156	4.9370509	6.6896019	6.5742331	6.7749723
2.2197205	2	2.3976902	2.5786313	2.5651052	2.5613046	2.4075018	2.4021099	2.3981461
2.2197205	2.3958007	2	2.9904928	3.2859859	2.5613046	2	2	2
3.2116748	2.9612664	3.3712718	2.8980192	3.1366761	2.5613046	2.5749062	2.7162254	2.3981461
5.856951	5.801426	5.8087227	5.2497029	4.854682	5.4887032	4.912217	4.7625408	4.9985377
6.7892225	6.6970036	6.77315	7.9527721	7.8354433	7.9733447	8.2179496	8.1962094	8.1800687
2	2	2	2.4542536	2.3100466	2.5613046	2	2	2
3.9859168	3.9415025	3.9473196	3.0773944	3.1366761	2.9643661	2	2	2
2	2	2	2.5786313	2.3100466	2.7768658	2.9848891	2.4021099	3.5394718
4.9222583	4.990011	4.7469892	5.6406543	5.4994188	5.7145616	6.04469	6.1204673	5.9053308
2	2	2	2	2	2	2	2	2
2	2	2	2.9904928	3.1366761	2.7768658	4.1463502	4.2801452	3.9487208
3.8608147	2.9612664	4.357879	3.3105119	3.1366761	3.4139246	4.1985751	4.1813664	4.1686253
5.0988516	5.2984878	4.7469892	4.8165054	4.9472802	4.5834785	5.6724178	5.7926015	5.4815611
2.4103584	2.7060624	2	2	2	2	2.5749062	2.7162254	2.3981461
2	2	2	2	2	2	2	2	2
2.5787203	2.9612664	2	2.6931321	2	3.1302769	2.2180911	2	2.3981461
2	2	2	2.5786313	2.9701107	2	2.5749062	2	2.9660172
2.2197205	2.3958007	2	2.1678177	2	2.30778	2.9848891	3.3824986	2.3981461
4.3069011	4.4381381	4.0614204	5.4089726	5.3031474	5.4262461	4.8482682	4.9542373	4.6787093
2	2	2	2.1678177	2.3100466	2	2.2180911	2	2.3981461
4.1010311	4.1610493	3.9473196	5.3750678	4.7035875	5.7659038	4.7813527	4.8931255	4.6052608
3.403311	3.8177796	2.7091089	4.7650729	4.7035875	4.7457183	5.0606888	5.1756805	4.8789742
7.0115184	6.9239842	7.0189149	6.6958995	6.6423996	6.6720606	7.3395946	7.0783871	7.5076006
9.6897913	9.5729174	9.693422	9.0908922	9.1347669	8.9507666	10.023207	9.9756901	10.010071
2.2197205	2.3958007	2	2.4542536	2.3100466	2.7768658	2.9848891	3.3824986	2.3981461

2	2	2	2.3181327	2.5651052	2	2.5749062	2.7162254	2.3981461
3.6501227	3.1780396	4.4445501	3.2369134	3.7644639	3.5372523	2.4075018	2.4021099	2.3981461
2.5787203	2.7060624	2.3976902	2	2	2	2.4075018	2.7162254	2
2.5787203	2.3958007	2.7091089	2.1678177	2	2.30778	3.0991973	2.9740279	3.1834889
2	2	2	2	2	2	2	2	2
5.0132553	4.3515565	5.3953249	4.5987644	4.4101943	4.6936396	5.5571371	5.3219704	5.7089498
3.6501227	3.5330831	3.6878577	2.9904928	3.1366761	2.7768658	4.3904012	4.2801452	4.4460942
2.7294717	2.7060624	2.7091089	2.6931321	2.5651052	2.7768658	2.9848891	3.3824986	2.3981461
3.1053801	3.1780396	2.9650943	3.0773944	2.7817736	3.2790605	3.3037759	3.8365351	2.3981461
2	2	2	2.3181327	2	2.5613046	2.2180911	2	2.3981461
2	2	2	2.1678177	2	2.30778	2.8607385	3.3824986	2
2	2	2	2	2	2	2.2180911	2	2.3981461
3.4903583	3.6824426	3.1824306	4.5078205	4.2004465	4.6936396	4.559689	4.8293101	4.1686253
4.1010311	3.9415025	4.1671548	5.0706429	4.9914418	5.0655458	5.6159286	5.65212	5.5220361
2.7294717	2.9612664	2.3976902	2.3181327	2.5651052	2	3.3037759	3.1926715	3.3724324
2.7294717	2.7060624	2.7091089	2.7992097	3.2859859	2	2.5749062	2.4021099	2.7098437
2	2	2	2.6931321	2.7817736	2.5613046	2	2	2
3.7939455	3.1780396	4.1671548	4.6563572	4.9472802	4.1912539	5.0321917	4.8931255	5.1089463
5.6265616	5.4310687	5.7072032	5.1123987	4.8060545	5.2925838	5.2458095	4.8931255	5.4815611
2	2	2	2.9904928	3.1366761	2.7768658	2.8607385	3.1926715	2.3981461
2.8659503	2.9612664	2.7091089	2.3181327	2	2.5613046	2.4075018	2.4021099	2.3981461
2.8659503	2.9612664	2.7091089	3.793754	3.5449709	3.9462135	4.1985751	3.9608643	4.3594016
2	2	2	2.4542536	2	2.7768658	3.2051098	3.1926715	3.1834889
2	2	2	2.1678177	2.3100466	2	2	2	2.7098437
2.2197205	2.3958007	2	2.4542536	2.3100466	2.5613046	3.0991973	2.9740279	3.1834889
9.9566372	10.000358	9.7938201	9.1038883	9.0168615	9.1007701	7.4886132	7.451257	7.4873015
4.354085	4.1610493	4.4445501	6.1618908	6.2281515	5.9983304	5.8785543	5.65212	6.0228156
2	2	2	2.3181327	2	2.5613046	2.8607385	3.3824986	2
2	2	2	2.1678177	2.3100466	2	2.2180911	2.4021099	2
2	2	2	2.4542536	2.5651052	2.30778	2.2180911	2	2.3981461
2	2	2	2	2	2	2	2	2

2	2	2	2.3181327	2	2.5613046	2.7248895	2.9740279	2.3981461
3.2116748	2	3.8234137	3.8441915	3.9549305	3.6508626	3.9162052	3.1926715	4.3594016
6.304066	6.4062003	6.0763755	6.3996732	6.3518802	6.4068854	6.9705774	6.8721706	7.0065546
2	2	2	2.3181327	2.3100466	2.30778	2.5749062	2.9740279	2
2	2	2	2	2	2	2.2180911	2.4021099	2
4.9532328	5.2028082	5.4798392	5.2497029	5.5883723	5.7909055	6.6337947	6.8074002	6.8721095
2	2	2	2	2	2	2	2	2
3.4903583	3.6824426	3.1824306	3.5722723	3.5449709	3.5372523	4.0921636	3.7004723	4.4460942
3.403311	3.6824426	2.9650943	3.4473206	3.5449709	3.2790605	3.3037759	3.7004723	2.7098437
2	2	2	2.1678177	2.5651052	2	2.2180911	2.4021099	2
2.2197205	2.3958007	2	2	2	2	2	2	2
3.2116748	2.9612664	3.3712718	2.5786313	2.9701107	2	3.0991973	3.3824986	2.7098437
2	2	2	2.8980192	2.9701107	2.7768658	3.3037759	2.4021099	3.8247709
2	2	2	3.1593576	3.1366761	3.1302769	2	2	2
2	2	2	2.3181327	2	2.5613046	2.4075018	2	2.7098437
4.3069011	4.1610493	4.357879	5.1529798	4.9017239	5.2925838	5.813061	5.9805917	5.5614064
2.2197205	2.3958007	2	3.9400663	4.2004465	3.5372523	3.9772738	4.1813664	3.6891623
6.3629219	6.2232429	6.3920436	7.1107541	7.0808895	7.0488988	7.8568498	7.9408842	7.7050922
2	2	2	2.1678177	2	2.30778	2.4075018	2	2.7098437
6.0267327	5.801426	6.129666	6.9004205	6.8723477	6.837185	7.2434305	7.1969113	7.2306851
3.3106725	3.5330831	2.9650943	3.793754	3.6588876	3.8543214	3.3037759	3.1926715	3.3724324
5.2807962	5.2984878	5.2098157	5.4420989	5.5593263	5.2208023	5.813061	6.0096728	5.5220361
2.7294717	2.3958007	2.9650943	2.1678177	2.3100466	2	2.2180911	2	2.3981461
2	2	2	3.3805373	3.421282	3.2790605	2.5749062	2	3.1834889
2.2197205	2.3958007	2	2.4542536	2.7817736	2.30778	2.5749062	2.4021099	2.7098437
5.3288274	5.4310687	5.1072743	7.1463647	7.0182314	7.1777034	6.9780669	6.8238675	7.062509
2.4103584	2.3958007	2.3976902	2.6931321	2.9701107	2.30778	2	2	2
2	2	2	2	2	2	2	2	2
4.2581217	4.3515565	4.0614204	4.7386523	4.7557305	4.6396102	2.4075018	2.4021099	2.3981461
4.4440613	4.0554482	4.6771135	5.2497029	5.3377701	5.0655458	5.3875313	5.2261101	5.4815611
2	2	2	2.1678177	2.3100466	2	2.2180911	2	2.3981461

3.2116748	3.3664558	2.9650943	3.3105119	3.5449709	2.9643661	4.0921636	3.9608643	4.1686253
2	2	2	2.3181327	2.5651052	2	2.2180911	2	2.3981461
4.0446218	4.2594452	3.6878577	3.6872592	3.5449709	3.7561757	3.0991973	2.9740279	3.3724324
2	2	2	2	2	2	3.2051098	2.7162254	3.5394718
2.4103584	2.3958007	2.3976902	2.4542536	2.3100466	2.5613046	3.3037759	3.3824986	3.1834889
2	2	2	2.1678177	2.3100466	2	2.7248895	2.4021099	2.9660172
2	2	2	2.7992097	2.5651052	2.9643661	2	2	2
2	2	2	2.4542536	2.7817736	2	3.3037759	2.9740279	3.5394718
2	2	2	2	2	2	2	2	2
2.4103584	2.3958007	2.3976902	3.0773944	3.1366761	2.9643661	2.9848891	2.9740279	2.9660172
9.5574602	9.5193127	9.4868523	9.5288837	9.4357858	9.5262641	8.3765394	8.2581076	8.4291807
2.9906265	2.3958007	3.3712718	3.0773944	2.3100466	3.5372523	3.8524369	3.7004723	3.9487208
2.8659503	2.9612664	2.7091089	2.4542536	2.5651052	2.30778	2.5749062	2.9740279	2
2	2	2	2	2	2	2.8607385	2.4021099	3.1834889
2	2	2	2	2	2	2.5749062	2.7162254	2.3981461
3.2116748	2.9612664	3.3712718	3.3105119	3.5449709	2.9643661	3.396124	3.1926715	3.5394718
6.9359985	7.0253511	6.7220159	6.0044717	5.8018185	6.1018603	6.3169023	6.3874467	6.1828452
2	2	2	2	2	2	2	2	2
2.5787203	2.3958007	2.7091089	2.7992097	2.9701107	2.5613046	3.3037759	3.550228	2.9660172
2.4103584	2.7060624	2	2.4542536	2	2.7768658	3.0991973	2.9740279	3.1834889
2	2	2	2	2	2	2	2	2
2.9906265	3.1780396	2.7091089	3.0773944	3.1366761	2.9643661	3.0991973	2.9740279	3.1834889
2	2	2	2.3181327	2.5651052	2	2	2	2
2	2	2	2	2	2	2	2	2
2	2	2	2.1678177	2.3100466	2	2	2	2
2.4103584	2.7060624	2	2.3181327	2	2.5613046	2.2180911	2	2.3981461
2	2	2	2	2	2	2	2	2
2	2	2	2.1678177	2.3100466	2	2.4075018	2.4021099	2.3981461
2	2	2	2.4542536	2	2.7768658	2	2	2
4.2076351	3.9415025	4.357879	4.9375947	5.3377701	4.2644835	4.559689	5.0128652	3.8247709
3.8608147	3.9415025	3.6878577	3.8441915	3.2859859	4.1912539	4.7813527	4.4594701	4.9985377

2	2	2	2.7992097	3.1366761	2.30778	2	2	2
2.2197205	2.3958007	2	2	2	2	2.7248895	3.1926715	2
2	2.3958007	2	2	2	2	2.2180911	2	2.3981461
2	2	2	2.6931321	2.3100466	2.9643661	3.2051098	3.3824986	2.9660172

Supplementary file S1. Phylogenetic trees of five genes showing conservation across Fusarium species.

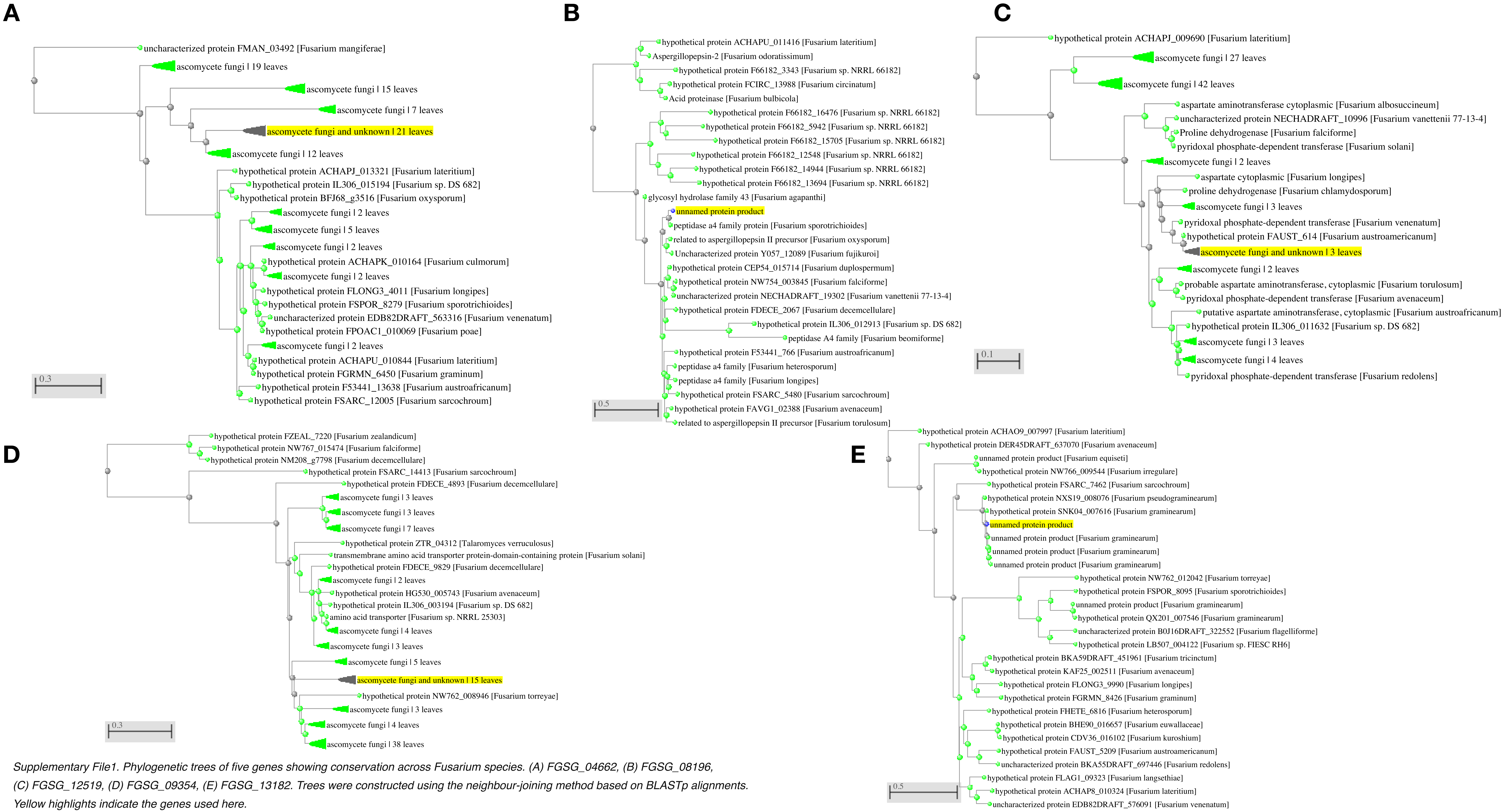

Supplementary File1. Phylogenetic trees of five genes showing conservation across Fusarium species. (A) FGSG_04662, (B) FGSG_08196, (C) FGSG_12519, (D) FGSG_09354, (E) FGSG_13182. Trees were constructed using the neighbour-joining method based on BLASTp alignments. Yellow highlights indicate the genes used here.

-Supplementary File S2. Protein Protein Interaction Network Data in SIF Format

229533.I1RB97 pp 229533.I1RIJ2
229533.I1RB97 pp 229533.I1RH76
229533.I1RB97 pp 229533.I1RL93
229533.I1RB97 pp 229533.I1RPU8
229533.I1RB97 pp 229533.I1RDB3
229533.I1RB97 pp 229533.I1SAF3
229533.I1RB97 pp 229533.I1SAI0
229533.I1RB97 pp 229533.I1RPT0
229533.I1RB97 pp 229533.I1RI58
229533.I1RB97 pp 229533.I1RNU7
229533.I1RHV5 pp 229533.I1RJU9
229533.I1RHV5 pp 229533.I1RLM3
229533.A0A0E0SGX3 pp 229533.A0A1C3YLF1
229533.A0A0E0SGX3 pp 229533.I1RTJ2
229533.A0A0E0SGX3 pp 229533.I1RYS3
229533.A0A0E0SGX3 pp 229533.I1RSI2
229533.A0A0E0SGX3 pp 229533.I1RGK9
229533.A0A0E0SGX3 pp 229533.I1RCL0
229533.A0A0E0SGX3 pp 229533.Q4I1N3
229533.I1RQC5 pp 229533.I1S7M3
229533.I1RQC5 pp 229533.I1RZ11
229533.I1RQC5 pp 229533.I1RWX8
229533.I1RQC5 pp 229533.I1S9L0
229533.I1RQC5 pp 229533.I1S0Z9
229533.I1RQC5 pp 229533.Q4IRI9
229533.I1RQC5 pp 229533.Q4I1Y1
229533.I1RQC5 pp 229533.Q4IAA0
229533.I1RQC5 pp 229533.I1RSA8
229533.I1RQC5 pp 229533.I1RYI9
229533.I1RQC5 pp 229533.I1S056
229533.I1RQC5 pp 229533.I1RWH4
229533.I1RQC5 pp 229533.I1S471
229533.I1RQC5 pp 229533.I1S7M2
229533.I1RQC5 pp 229533.Q4IB70
229533.I1RQC5 pp 229533.I1RX47
229533.I1RQC5 pp 229533.I1RZG7
229533.I1RQC5 pp 229533.I1RS75
229533.I1RQC5 pp 229533.Q4IPB3
229533.I1RQC5 pp 229533.I1S2Y5
229533.I1RQC5 pp 229533.V6QXG1
229533.I1RQC5 pp 229533.I1RX81
229533.I1RQC5 pp 229533.I1RX51
229533.I1RQC5 pp 229533.I1RSA4
229533.I1RQC5 pp 229533.I1RT47
229533.I1RQC5 pp 229533.I1RYJ9
229533.I1RQC5 pp 229533.I1RQR3
229533.I1RQC5 pp 229533.I1RZP8
229533.I1RQC5 pp 229533.I1S247
229533.I1RQC5 pp 229533.I1RSD4
229533.I1RQC5 pp 229533.I1RRL0
229533.I1RQC5 pp 229533.I1S0B1
229533.I1RQC5 pp 229533.I1RS28
229533.I1RQC5 pp 229533.Q4IBK5
229533.I1RQC5 pp 229533.I1RZQ5
229533.I1RQC5 pp 229533.I1RT91
229533.I1RQC5 pp 229533.I1RX24
229533.I1RQC5 pp 229533.I1RWU2
229533.I1RQC5 pp 229533.I1S1X9
229533.I1RQC5 pp 229533.I1RST1
229533.I1RQC5 pp 229533.I1RT17
229533.I1RQC5 pp 229533.I1RXF2
229533.I1S5C5 pp 229533.V6RQW4
229533.I1S5C5 pp 229533.I1SAJ3
229533.I1S111 pp 229533.I1S3G4
229533.I1S111 pp 229533.I1S1W8
229533.I1S111 pp 229533.V6R223
229533.I1S111 pp 229533.I1S7R5
229533.I1S111 pp 229533.V6RJ21
229533.A0A098D7G2 pp 229533.A0A0E0RX50

229533.A0A098D7G2	pp	229533.I1RZT6
229533.A0A098D7G2	pp	229533.I1RYK0
229533.A0A098D7G2	pp	229533.V6RS91
229533.A0A098D7G2	pp	229533.I1RE86
229533.A0A098D7G2	pp	229533.I1RSI2
229533.A0A098D7G2	pp	229533.I1S1W8
229533.A0A098D7G2	pp	229533.I1RNG4
229533.A0A098D7G2	pp	229533.I1S3G4
229533.A0A098D7G2	pp	229533.A0A1C3YK76
229533.A0A098D7G2	pp	229533.I1RYS3
229533.A0A098D7G2	pp	229533.A0A0E0SGX3
229533.A0A098D7G2	pp	229533.I1RCL0
229533.A0A098D7G2	pp	229533.Q4I1N3
229533.A0A098D7G2	pp	229533.I1S5C5
229533.V6RK96	pp	229533.V6RSF3
229533.V6RK96	pp	229533.V6RM02
229533.V6RK96	pp	229533.V6RXR0
229533.V6RK96	pp	229533.V6RLM7
229533.I1RJ25	pp	229533.I1RJU9
229533.I1RJ25	pp	229533.I1RLM3
229533.I1RDU0	pp	229533.I1S1W9
229533.I1RDU0	pp	229533.I1RSF1
229533.I1RDU0	pp	229533.V6RLM7
229533.I1RDU0	pp	229533.I1REW1
229533.I1RDU0	pp	229533.I1RGX1
229533.I1RDU0	pp	229533.V6R9L8
229533.I1RDU0	pp	229533.I1RG11
229533.I1RDU0	pp	229533.I1RPK0
229533.I1RDU0	pp	229533.I1RX18
229533.I1RDU0	pp	229533.I1RQ48
229533.I1RDU0	pp	229533.V6RZD4
229533.I1RDU0	pp	229533.I1S3V2
229533.I1RDU0	pp	229533.I1S149
229533.I1RDU0	pp	229533.I1RSA5
229533.I1RDU0	pp	229533.I1RNU7
229533.I1RDU0	pp	229533.I1RSU2
229533.I1RDU0	pp	229533.V6R756
229533.I1RDU0	pp	229533.I1RMC2
229533.I1RDU0	pp	229533.I1RNG4
229533.I1RDU0	pp	229533.V6RF62
229533.I1RDU0	pp	229533.I1RLZ4
229533.I1RDU0	pp	229533.I1RYK0
229533.I1RDU0	pp	229533.V6R2Z2
229533.I1RDU0	pp	229533.I1S2C4
229533.I1RDU0	pp	229533.I1S2B8
229533.I1RDU0	pp	229533.I1S6V0
229533.I1RDU0	pp	229533.I1RK50
229533.I1RDU0	pp	229533.I1RKB2
229533.I1RDU0	pp	229533.I1RX72
229533.I1RDU0	pp	229533.V6R5D2
229533.I1RDU0	pp	229533.I1RJQ5
229533.I1RDU0	pp	229533.I1S5C5
229533.I1RDU0	pp	229533.I1RIJ6
229533.I1RDU0	pp	229533.V6RV57
229533.I1RDU0	pp	229533.I1RVR2
229533.I1RDU0	pp	229533.V6QZ48
229533.I1RDU0	pp	229533.I1RR72
229533.I1RDU0	pp	229533.I1S253
229533.I1RDU0	pp	229533.Q4HVQ9
229533.I1RDU0	pp	229533.I1RZN1
229533.I1RDU0	pp	229533.V6RCJ5
229533.I1RDU0	pp	229533.I1S3F7
229533.I1RDU0	pp	229533.I1RZV5
229533.I1RDU0	pp	229533.I1RN88
229533.I1RDU0	pp	229533.I1RU18
229533.I1RDU0	pp	229533.V6RUY9
229533.I1RDU0	pp	229533.I1RF59
229533.I1RDU0	pp	229533.I1RZ39
229533.I1RDU0	pp	229533.I1RKP1
229533.I1RDU0	pp	229533.I1RMC5
229533.I1RDU0	pp	229533.I1RGC3
229533.I1RDU0	pp	229533.I1S3G4
229533.I1RDU0	pp	229533.I1RWF5

229533.I1RDU0	pp	229533.I1RSQ4
229533.I1RDU0	pp	229533.I1S5J9
229533.I1RDU0	pp	229533.I1RE76
229533.I1RDU0	pp	229533.I1RUY6
229533.I1RDU0	pp	229533.O74638
229533.I1RDU0	pp	229533.V6RHX2
229533.I1RDU0	pp	229533.V6RPS5
229533.I1RDU0	pp	229533.I1S8H9
229533.I1RDU0	pp	229533.I1RQZ6
229533.I1RDU0	pp	229533.I1RUY5
229533.I1RDU0	pp	229533.I1RQE0
229533.I1RDU0	pp	229533.I1RXH2
229533.I1RDU0	pp	229533.I1RK03
229533.I1RDU0	pp	229533.I1RDV8
229533.I1RDU0	pp	229533.I1RI40
229533.I1RDU0	pp	229533.I1S8D3
229533.I1RDU0	pp	229533.I1RWD2
229533.I1RDU0	pp	229533.I1RH82
229533.I1RDU0	pp	229533.I1RUQ3
229533.I1RDU0	pp	229533.V6QUE6
229533.I1RDU0	pp	229533.I1RUY4
229533.I1RDU0	pp	229533.I1S1J7
229533.I1RDU0	pp	229533.V6RWB3
229533.I1RDU0	pp	229533.I1RKE1
229533.I1RDU0	pp	229533.I1RY12
229533.I1RDU0	pp	229533.I1RVV3
229533.I1RDU0	pp	229533.I1RYL8
229533.I1RDU0	pp	229533.I1RFV2
229533.I1RDU0	pp	229533.V6RCC0
229533.I1RDU0	pp	229533.I1RZ16
229533.I1RDU0	pp	229533.I1RZK5
229533.I1RDU0	pp	229533.V6R3V4
229533.I1RDU0	pp	229533.I1S4S5
229533.I1RDU0	pp	229533.V6QVH2
229533.I1RDU0	pp	229533.I1RW54
229533.I1RDU0	pp	229533.I1RQV7
229533.I1RDU0	pp	229533.V6QU99
229533.I1RDU0	pp	229533.I1RPT0
229533.I1RDU0	pp	229533.I1S1W8
229533.I1RDU0	pp	229533.V6RAM6
229533.I1RDU0	pp	229533.V6R5T7
229533.I1RDU0	pp	229533.I1SAI4
229533.I1RDU0	pp	229533.I1RE86
229533.I1RDU0	pp	229533.I1RP86
229533.I1RDU0	pp	229533.Q4IR09
229533.I1RDU0	pp	229533.I1RNM7
229533.I1RDU0	pp	229533.I1RIX0
229533.I1RDU0	pp	229533.I1RH86
229533.I1RDU0	pp	229533.I1RQV8
229533.I1RDU0	pp	229533.I1RSJ2
229533.I1RDU0	pp	229533.I1RJC5
229533.I1RDU0	pp	229533.V6R0W1
229533.I1RDU0	pp	229533.I1RE47
229533.I1RDU0	pp	229533.I1RSB7
229533.I1RDU0	pp	229533.V6R1D2
229533.I1RDU0	pp	229533.I1RJN7
229533.I1RAQ4	pp	229533.I1RC11
229533.I1RAQ4	pp	229533.I1RY49
229533.I1RAQ4	pp	229533.I1RJN7
229533.I1RAQ4	pp	229533.I1S0B7
229533.I1RAQ4	pp	229533.I1RAR3
229533.I1RAQ4	pp	229533.I1S1W8
229533.I1RAQ4	pp	229533.I1RJX2
229533.I1RAQ4	pp	229533.I1RSB7
229533.I1RAQ4	pp	229533.I1RW54
229533.I1RAQ4	pp	229533.I1RFH9
229533.I1RAQ4	pp	229533.I1RM67
229533.I1RAQ4	pp	229533.I1S8C1
229533.I1RAQ4	pp	229533.I1RRG1
229533.I1RAQ4	pp	229533.I1RT33
229533.I1RAQ4	pp	229533.I1RFV2
229533.I1RAQ4	pp	229533.I1RGG8
229533.I1RAQ4	pp	229533.I1RGB2

220	229533.I1RAQ4	pp	229533.I1RDU0
221	229533.I1RAQ4	pp	229533.Q4IR09
222	229533.I1RAQ4	pp	229533.I1RMK4
223	229533.I1RAQ4	pp	229533.I1RKJ7
224	229533.I1RAQ4	pp	229533.I1RJY7
225	229533.I1RAQ4	pp	229533.I1RKB5
226	229533.I1RAQ4	pp	229533.I1RM26
227	229533.I1RAQ4	pp	229533.I1RRX5
228	229533.I1RAQ4	pp	229533.I1RVZ3
229	229533.I1RAQ4	pp	229533.I1RQE0
230	229533.I1RAQ4	pp	229533.I1RBE7
231	229533.I1RAQ4	pp	229533.I1RPT0
232	229533.I1RAQ4	pp	229533.I1RZ52
233	229533.I1RAQ4	pp	229533.I1RP86
234	229533.I1RAQ4	pp	229533.I1RVX9
235	229533.I1RAQ4	pp	229533.I1RAZ3
236	229533.I1RAQ4	pp	229533.I1RX89
237	229533.I1RAQ4	pp	229533.I1RY12
238	229533.I1RAQ4	pp	229533.I1RUY4
239	229533.I1RAQ4	pp	229533.I1RBE4
240	229533.I1RAQ4	pp	229533.I1RGC3
241	229533.I1RAQ4	pp	229533.I1RC48
242	229533.I1RAQ4	pp	229533.I1RRV4
243	229533.I1RAQ4	pp	229533.I1RIY2
244	229533.I1RAQ4	pp	229533.I1RIY0
245	229533.I1RAQ4	pp	229533.I1RKR5
246	229533.I1RAQ4	pp	229533.I1RSQ4
247	229533.I1RAQ4	pp	229533.I1RR44
248	229533.I1RAQ4	pp	229533.I1RJQ5
249	229533.I1RAQ4	pp	229533.I1RH82
250	229533.I1RAQ4	pp	229533.I1RUY5
251	229533.I1RAQ4	pp	229533.I1RN88
252	229533.I1RAQ4	pp	229533.O74638
253	229533.I1RAQ4	pp	229533.I1RUY6
254	229533.I1RAQ4	pp	229533.I1RKB2
255	229533.I1RAQ4	pp	229533.I1RBF3
256	229533.I1RAQ4	pp	229533.I1RUR8
257	229533.I1RAQ4	pp	229533.I1RVR2
258	229533.I1RAQ4	pp	229533.I1RYK0
259	229533.I1RAQ4	pp	229533.I1RNG4
260	229533.I1RAQ4	pp	229533.I1RC31
261	229533.I1RAQ4	pp	229533.I1S3G4
262	229533.I1RAQ4	pp	229533.I1RY62
263	229533.I1RAQ4	pp	229533.I1RZN1
264	229533.I1RAQ4	pp	229533.V6RV57
265	229533.I1RAQ4	pp	229533.I1RX72
266	229533.I1RAQ4	pp	229533.I1RWF5
267	229533.I1RAQ4	pp	229533.I1RZQ1
268	229533.I1RAQ4	pp	229533.I1RPU8
269	229533.I1RAQ4	pp	229533.Q4HXF0
270	229533.I1RAQ4	pp	229533.I1RSS4
271	229533.I1RAQ4	pp	229533.I1RND4
272	229533.I1RAQ4	pp	229533.I1S256
273	229533.I1RAQ4	pp	229533.I1RZ26
274	229533.I1RAQ4	pp	229533.I1S0T4
275	229533.I1RAQ4	pp	229533.I1RMX6
276	229533.I1RAQ4	pp	229533.V6RRM0
277	229533.I1RAQ4	pp	229533.I1RHJ2
278	229533.I1RAQ4	pp	229533.V6RLM7
279	229533.I1RAQ4	pp	229533.I1RNU7
280	229533.I1RAQ4	pp	229533.I1RCG5
281	229533.I1RAQ4	pp	229533.I1RFK3
282	229533.I1RAQ4	pp	229533.I1RGX1
283	229533.I1RAQ4	pp	229533.I1RQI4
284	229533.I1RAH8	pp	229533.I1RWX5
285	229533.I1RAH8	pp	229533.I1RFI3
286	229533.I1RAH8	pp	229533.I1RCP1
287	229533.I1RAH8	pp	229533.I1RYQ7
288	229533.I1RAH8	pp	229533.I1RHF2
289	229533.I1RAH8	pp	229533.I1RC93
290	229533.I1RAH8	pp	229533.I1RCM1
291	229533.I1RAH8	pp	229533.P0C431
292	229533.I1RAH8	pp	229533.I1RX27

229533.I1RAH8 pp 229533.I1RWL6
229533.I1RAH8 pp 229533.I1RTB7
229533.I1RZ57 pp 229533.I1RZ63
229533.I1RIT5 pp 229533.I1RLM3
229533.I1RIT5 pp 229533.I1RJU9
229533.I1RIT5 pp 229533.I1RXN6
229533.I1RIT5 pp 229533.V6RDP1
229533.I1RKE1 pp 229533.I1RNJ7
229533.I1RKE1 pp 229533.I1RP86
229533.I1RKE1 pp 229533.V6RK96
229533.I1RKE1 pp 229533.I1RZM4
229533.I1RKE1 pp 229533.I1RZQ1
229533.I1RKE1 pp 229533.I1RMX6
229533.I1RKE1 pp 229533.Q4IR09
229533.I1RKE1 pp 229533.V6RLM7
229533.I1RKE1 pp 229533.I1RKR5
229533.I1RKE1 pp 229533.I1RQE8
229533.I1RKE1 pp 229533.I1RMM8
229533.I1RKE1 pp 229533.I1RUY5
229533.I1RKE1 pp 229533.V6RV57
229533.I1RKE1 pp 229533.I1RX72
229533.I1RKE1 pp 229533.I1RW26
229533.I1RKE1 pp 229533.I1RQ75
229533.I1RKE1 pp 229533.I1RYK0
229533.I1RZ55 pp 229533.I1S268
229533.I1RW69 pp 229533.Q4I5U9
229533.I1RW69 pp 229533.I1RZQ1
229533.I1RW69 pp 229533.I1SAJ3
229533.I1RW69 pp 229533.I1RXY7
229533.I1RW69 pp 229533.Q4IPC8
229533.I1RW69 pp 229533.I1S0I3
229533.I1RW69 pp 229533.I1RZ70
229533.I1RW69 pp 229533.I1RZN1
229533.I1RW69 pp 229533.Q4I5I4
229533.I1RW69 pp 229533.I1S4B6
229533.I1RW69 pp 229533.I1S0A0
229533.I1RW69 pp 229533.V6RIV7
229533.I1RW69 pp 229533.I1S4B7
229533.I1RW69 pp 229533.V6RK96
229533.I1RW69 pp 229533.I1RX72
229533.I1RW69 pp 229533.O74638
229533.I1RW69 pp 229533.V6RHD0
229533.I1RW69 pp 229533.I1S183
229533.I1RW69 pp 229533.I1S3G4
229533.I1RW69 pp 229533.V6RG22
229533.I1RW69 pp 229533.I1S9C6
229533.I1RW69 pp 229533.I1RZ29
229533.I1RW69 pp 229533.I1S673
229533.I1RW69 pp 229533.I1RWI0
229533.I1RW69 pp 229533.V6RDH3
229533.I1RW69 pp 229533.V6QU65
229533.I1RW69 pp 229533.V6QXN0
229533.I1RW69 pp 229533.V6RXR0
229533.I1RW69 pp 229533.V6RQW4
229533.I1RW69 pp 229533.I1S5Z2
229533.I1RW69 pp 229533.V6RGB5
229533.I1RW69 pp 229533.I1RZ92
229533.I1RW69 pp 229533.I1RZM4
229533.I1RW69 pp 229533.I1RY49
229533.I1RW69 pp 229533.I1S053
229533.I1RW69 pp 229533.I1RYM6
229533.I1RW69 pp 229533.Q4IR09
229533.I1RW69 pp 229533.I1S4S4
229533.I1RW69 pp 229533.I1RXB1
229533.I1RW69 pp 229533.I1S1W8
229533.I1RCE4 pp 229533.I1RSK9
229533.I1RCE4 pp 229533.I1RZ94
229533.I1RLC9 pp 229533.I1RXN6
229533.I1RLC9 pp 229533.I1RLM3
229533.I1RLC9 pp 229533.V6RDP1
229533.I1S0V6 pp 229533.Q4HYR2
229533.I1S0V6 pp 229533.I1S8F8
229533.I1S0V6 pp 229533.V6RE46

229533.I1S0V6	pp	229533.I1S267
229533.I1S0V6	pp	229533.I1S8C1
229533.I1S0V6	pp	229533.I1S147
229533.I1S0V6	pp	229533.I1S8R2
229533.I1S0V6	pp	229533.Q4I5G1
229533.I1S0V6	pp	229533.V6RRM0
229533.I1S0V6	pp	229533.I1S3G4
229533.I1S0V6	pp	229533.I1S3T7
229533.I1S0V6	pp	229533.I1S2E9
229533.I1S0V6	pp	229533.I1S2C4
229533.I1S0V6	pp	229533.I1S1W8
229533.I1S0V6	pp	229533.I1S299
229533.I1S0V6	pp	229533.V6RV57
229533.I1S0V6	pp	229533.Q4HVQ9
229533.V6R223	pp	229533.V6RJ21
229533.V6R223	pp	229533.V6RBN2
229533.I1RDI7	pp	229533.I1RY11
229533.I1RUY5	pp	229533.I1S1J7
229533.I1RUY5	pp	229533.I1RWD2
229533.I1RUY5	pp	229533.I1RZK5
229533.I1RUY5	pp	229533.I1RVV3
229533.I1RUY5	pp	229533.I1RY12
229533.I1RUY5	pp	229533.I1RZ16
229533.I1RUY5	pp	229533.V6R3V4
229533.I1RUY5	pp	229533.I1RXH2
229533.I1RUY5	pp	229533.V6RCC0
229533.I1RUY5	pp	229533.V6RWB3
229533.I1RUY5	pp	229533.I1RW54
229533.I1RUY5	pp	229533.V6RPS5
229533.I1RUY5	pp	229533.I1S8D3
229533.I1RUY5	pp	229533.V6QUE6
229533.I1RUY5	pp	229533.I1RUY6
229533.I1RUY5	pp	229533.V6R0W1
229533.I1RUY5	pp	229533.Q4IR09
229533.I1RUY5	pp	229533.I1S1W8
229533.I1RUY5	pp	229533.I1SAI4
229533.I1RUY5	pp	229533.I1S5J9
229533.I1RUY5	pp	229533.V6RHX2
229533.I1RUY5	pp	229533.I1RYL8
229533.I1RUY5	pp	229533.I1S8H9
229533.I1RUY5	pp	229533.V6RAM6
229533.I1RUY5	pp	229533.V6R5T7
229533.I1RUY5	pp	229533.V6R1D2
229533.I1RUY5	pp	229533.V6QU99
229533.I1RUY5	pp	229533.I1S4S5
229533.I1RUY5	pp	229533.V6QVH2
229533.I1RUY5	pp	229533.I1S5C5
229533.I1RUY5	pp	229533.V6R756
229533.I1RUY5	pp	229533.I1S6V0
229533.I1RUY5	pp	229533.I1S149
229533.I1RUY5	pp	229533.I1S2B8
229533.I1RUY5	pp	229533.V6R9L8
229533.I1RUY5	pp	229533.V6R5D2
229533.I1RUY5	pp	229533.I1S2C4
229533.I1RUY5	pp	229533.I1RVR2
229533.I1RUY5	pp	229533.V6RF62
229533.I1RUY5	pp	229533.I1S1W9
229533.I1RUY5	pp	229533.I1S147
229533.I1RUY5	pp	229533.I1S3V2
229533.I1RUY5	pp	229533.I1S3G4
229533.I1RUY5	pp	229533.I1S3F7
229533.I1RUY5	pp	229533.V6RLM7
229533.I1RUY5	pp	229533.I1RX18
229533.I1RUY5	pp	229533.O74638
229533.I1RUY5	pp	229533.V6RCJ5
229533.I1RUY5	pp	229533.V6RV57
229533.I1RUY5	pp	229533.V6QZ48
229533.I1RUY5	pp	229533.V6RUY9
229533.I1RUY5	pp	229533.I1RZN1
229533.I1RUY5	pp	229533.I1RZV5
229533.I1RUY5	pp	229533.I1S148
229533.I1RUY5	pp	229533.I1S253
229533.I1RUY5	pp	229533.V6RZD4

229533.I1RUY5 pp 229533.I1RWF5
229533.I1RUY5 pp 229533.I1RZ39
229533.I1RUY5 pp 229533.I1RX72
229533.I1RUY5 pp 229533.Q4HVQ9
229533.I1RUY5 pp 229533.V6R2Z2
229533.I1RUY5 pp 229533.I1RYK0
229533.I1RWW4 pp 229533.I1RZY1
229533.I1RWW4 pp 229533.I1S2A8
229533.I1RWW4 pp 229533.I1S0W5
229533.I1RWW4 pp 229533.V6RGI1
229533.I1RWW4 pp 229533.I1S9T5
229533.I1RWW4 pp 229533.V6RIE5
229533.I1RWW4 pp 229533.Q4IEV4
229533.I1RWW4 pp 229533.I1S0C0
229533.I1RWW4 pp 229533.P0C431
229533.I1RCM5 pp 229533.I1RJN7
229533.I1RCM5 pp 229533.I1S4S4
229533.I1RCM5 pp 229533.Q4IR09
229533.I1RCM5 pp 229533.I1RJU3
229533.I1RCM5 pp 229533.I1RR24
229533.I1RCM5 pp 229533.I1RWL3
229533.I1RCM5 pp 229533.I1RMK7
229533.I1RCM5 pp 229533.I1RKI3
229533.I1RCM5 pp 229533.I1RNM7
229533.I1RCM5 pp 229533.I1RT41
229533.I1RCM5 pp 229533.V6RUT5
229533.I1RCM5 pp 229533.V6QYF0
229533.I1RCM5 pp 229533.I1S0F1
229533.I1RCM5 pp 229533.I1S1W8
229533.I1RCM5 pp 229533.I1RVI8
229533.I1RCM5 pp 229533.I1RZ16
229533.I1RCM5 pp 229533.I1RXD7
229533.I1RCM5 pp 229533.I1RRG3
229533.I1RCM5 pp 229533.I1S0V6
229533.I1RCM5 pp 229533.I1RP86
229533.I1RCM5 pp 229533.I1RKE1
229533.I1RCM5 pp 229533.I1RYL8
229533.I1RCM5 pp 229533.I1RX46
229533.I1RCM5 pp 229533.I1RCV1
229533.I1RCM5 pp 229533.I1RSQ7
229533.I1RCM5 pp 229533.I1S338
229533.I1RCM5 pp 229533.V6RTJ8
229533.I1RCM5 pp 229533.I1RUY4
229533.I1RCM5 pp 229533.V6REE9
229533.I1RCM5 pp 229533.I1RRX5
229533.I1RCM5 pp 229533.I1RPI5
229533.I1RCM5 pp 229533.I1RRY9
229533.I1RCM5 pp 229533.I1S8S9
229533.I1RCM5 pp 229533.I1RGB2
229533.I1RCM5 pp 229533.V6RCZ8
229533.I1RCM5 pp 229533.Q4HYR2
229533.I1RCM5 pp 229533.I1RDU0
229533.I1RCM5 pp 229533.I1RH82
229533.I1RCM5 pp 229533.I1S439
229533.I1RCM5 pp 229533.I1RJY7
229533.I1RCM5 pp 229533.Q4IJK2
229533.I1RCM5 pp 229533.I1RUQ3
229533.I1RCM5 pp 229533.I1REE2
229533.I1RCM5 pp 229533.V6QUE6
229533.I1RCM5 pp 229533.I1RCR5
229533.I1RCM5 pp 229533.I1S5B0
229533.I1RCM5 pp 229533.I1RZZ9
229533.I1RCM5 pp 229533.I1REK8
229533.I1RCM5 pp 229533.I1RZM8
229533.I1RCM5 pp 229533.I1RXF0
229533.I1RCM5 pp 229533.I1RUY5
229533.I1RCM5 pp 229533.I1RSQ4
229533.I1RCM5 pp 229533.I1RWM4
229533.I1RCM5 pp 229533.I1RZ39
229533.I1RCM5 pp 229533.O74638
229533.I1RCM5 pp 229533.I1S3T7
229533.I1RCM5 pp 229533.I1RS18
229533.I1RCM5 pp 229533.I1S2H4

512	229533.I1RCM5	pp	229533.I1RRT8
513	229533.I1RCM5	pp	229533.I1RR44
514	229533.I1RCM5	pp	229533.I1RN88
515	229533.I1RCM5	pp	229533.I1S3G4
516	229533.I1RCM5	pp	229533.I1S5R1
517	229533.I1RCM5	pp	229533.I1RFI4
518	229533.I1RCM5	pp	229533.I1RKI7
519	229533.I1RCM5	pp	229533.I1RGA4
520	229533.I1RCM5	pp	229533.V6RUY9
521	229533.I1RCM5	pp	229533.I1RTD5
522	229533.I1RCM5	pp	229533.I1RRT3
523	229533.I1RCM5	pp	229533.I1RFS3
524	229533.I1RCM5	pp	229533.I1RX72
525	229533.I1RCM5	pp	229533.I1RKB2
526	229533.I1RCM5	pp	229533.I1RUR8
527	229533.I1RCM5	pp	229533.I1S2B8
528	229533.I1RCM5	pp	229533.I1S8P5
529	229533.I1RCM5	pp	229533.V6R3J1
530	229533.I1RCM5	pp	229533.I1S148
531	229533.I1RCM5	pp	229533.I1RGC3
532	229533.I1RCM5	pp	229533.I1RN84
533	229533.I1RCM5	pp	229533.I1RPP3
534	229533.I1RCM5	pp	229533.V6RV57
535	229533.I1RCM5	pp	229533.I1S256
536	229533.I1RCM5	pp	229533.I1RFT1
537	229533.I1RCM5	pp	229533.I1RYK0
538	229533.I1RCM5	pp	229533.I1RRI2
539	229533.I1RCM5	pp	229533.I1S147
540	229533.I1RCM5	pp	229533.I1RWH4
541	229533.I1RCM5	pp	229533.I1RY62
542	229533.I1RCM5	pp	229533.I1RWX5
543	229533.I1RCM5	pp	229533.I1RD22
544	229533.I1RCM5	pp	229533.I1S0V7
545	229533.I1RCM5	pp	229533.I1RN67
546	229533.I1RCM5	pp	229533.I1RT03
547	229533.I1RCM5	pp	229533.I1S1C7
548	229533.I1RCM5	pp	229533.I1RTH6
549	229533.I1RCM5	pp	229533.I1RYW3
550	229533.I1RCM5	pp	229533.I1S2C4
551	229533.I1RCM5	pp	229533.V6RRM0
552	229533.I1RCM5	pp	229533.I1RVX0
553	229533.I1RCM5	pp	229533.I1RG11
554	229533.I1RCM5	pp	229533.I1RJV3
555	229533.I1RCM5	pp	229533.I1S0J1
556	229533.I1RCM5	pp	229533.I1RE01
557	229533.I1RCM5	pp	229533.I1RUG3
558	229533.I1RCM5	pp	229533.I1RQE7
559	229533.I1RCM5	pp	229533.I1RTA0
560	229533.I1RCM5	pp	229533.I1RFL4
561	229533.I1RCM5	pp	229533.I1RNC1
562	229533.I1RCM5	pp	229533.I1RGX1
563	229533.I1RCM5	pp	229533.I1RQI4
564	229533.I1RCM5	pp	229533.V6RLM7
565	229533.I1RCM5	pp	229533.I1RPK0
566	229533.I1RCM5	pp	229533.I1RCR8
567	229533.I1RCM5	pp	229533.V6RVG3
568	229533.I1RCM5	pp	229533.I1RSQ3
569	229533.I1RCM5	pp	229533.V6RMW6
570	229533.I1RCM5	pp	229533.V6R042
571	229533.I1RCM5	pp	229533.I1S1W3
572	229533.I1RFA1	pp	229533.I1RJU9
573	229533.I1RFA1	pp	229533.I1RLM3
574	229533.I1RCU2	pp	229533.I1RYX6
575	229533.I1RCU2	pp	229533.I1S8C1
576	229533.I1RCU2	pp	229533.I1RR12
577	229533.I1RCU2	pp	229533.V6QU99
578	229533.I1RCU2	pp	229533.I1RRX5
579	229533.I1RCU2	pp	229533.I1S094
580	229533.I1RCU2	pp	229533.I1RVI8
581	229533.I1RCU2	pp	229533.I1S4S5
582	229533.I1RCU2	pp	229533.I1S969
583	229533.I1RCU2	pp	229533.I1RJC5
584	229533.I1RCU2	pp	229533.I1RMW9

585	229533.I1RCU2	pp	229533.I1S6H3
586	229533.I1RCU2	pp	229533.I1RIF7
587	229533.I1RCU2	pp	229533.I1RYP5
588	229533.I1RCU2	pp	229533.I1RDN3
589	229533.I1RCU2	pp	229533.I1RLA2
590	229533.I1RCU2	pp	229533.V6QVV9
591	229533.I1RCU2	pp	229533.I1RVX9
592	229533.I1RCU2	pp	229533.I1RJS7
593	229533.I1RCU2	pp	229533.I1RY21
594	229533.I1RCU2	pp	229533.I1RCU8
595	229533.I1RCU2	pp	229533.V6RAM6
596	229533.I1RCU2	pp	229533.V6R0W1
597	229533.I1RCU2	pp	229533.I1RMK7
598	229533.I1RCU2	pp	229533.I1RGK3
599	229533.I1RCU2	pp	229533.I1RMA2
600	229533.I1RCU2	pp	229533.I1S0K1
601	229533.I1RCU2	pp	229533.I1REE2
602	229533.I1RCU2	pp	229533.I1RKB7
603	229533.I1RCU2	pp	229533.I1RMQ6
604	229533.I1RCU2	pp	229533.I1RST9
605	229533.I1RCU2	pp	229533.I1RDB1
606	229533.I1RCU2	pp	229533.I1S1W8
607	229533.I1RCU2	pp	229533.I1RUI2
608	229533.I1RCU2	pp	229533.I1RW54
609	229533.I1RCU2	pp	229533.I1S489
610	229533.I1RCU2	pp	229533.I1RPR1
611	229533.I1RCU2	pp	229533.I1RNM7
612	229533.I1RCU2	pp	229533.I1RNG5
613	229533.I1RCU2	pp	229533.I1RXE6
614	229533.I1RCU2	pp	229533.I1RUQ3
615	229533.I1RCU2	pp	229533.I1S3B2
616	229533.I1RCU2	pp	229533.I1RXV9
617	229533.I1RCU2	pp	229533.I1RNY6
618	229533.I1RCU2	pp	229533.I1RH67
619	229533.I1RCU2	pp	229533.V6RDP1
620	229533.I1RCU2	pp	229533.I1S0K2
621	229533.I1RCU2	pp	229533.I1S423
622	229533.I1RCU2	pp	229533.I1RMD3
623	229533.I1RCU2	pp	229533.Q4HYR2
624	229533.I1RCU2	pp	229533.I1RG33
625	229533.I1RCU2	pp	229533.I1S267
626	229533.I1RCU2	pp	229533.I1S8F8
627	229533.I1RCU2	pp	229533.I1RWM4
628	229533.I1RCU2	pp	229533.I1RH82
629	229533.I1RCU2	pp	229533.I1RL81
630	229533.I1RCU2	pp	229533.I1RPP5
631	229533.I1RCU2	pp	229533.I1RJT5
632	229533.I1RCU2	pp	229533.V6RV57
633	229533.I1RCU2	pp	229533.I1RYA2
634	229533.I1RCU2	pp	229533.I1RMQ7
635	229533.I1RCU2	pp	229533.I1RY62
636	229533.I1RCU2	pp	229533.I1RY12
637	229533.I1RCU2	pp	229533.V6R3J1
638	229533.I1RCU2	pp	229533.I1RJB9
639	229533.I1RCU2	pp	229533.I1S0A5
640	229533.I1RCU2	pp	229533.V6RUB1
641	229533.I1RCU2	pp	229533.I1RQA5
642	229533.I1RCU2	pp	229533.I1RZ25
643	229533.I1RCU2	pp	229533.I1RNG4
644	229533.I1RCU2	pp	229533.Q4HVQ9
645	229533.I1RCU2	pp	229533.V6R2Z2
646	229533.I1RCU2	pp	229533.V6R3V4
647	229533.I1RCU2	pp	229533.I1RCV1
648	229533.I1RCU2	pp	229533.I1RR68
649	229533.I1RCU2	pp	229533.I1S0V6
650	229533.I1RCU2	pp	229533.I1RZA7
651	229533.I1RCU2	pp	229533.I1RQJ7
652	229533.I1RCU2	pp	229533.I1S968
653	229533.I1RCU2	pp	229533.I1S299
654	229533.I1RCU2	pp	229533.I1RZV5
655	229533.I1RCU2	pp	229533.I1RWF5
656	229533.I1RCU2	pp	229533.I1RKB2
657	229533.I1RCU2	pp	229533.V6R9Z3

229533.I1RCU2	pp	229533.I1RY81
229533.I1RCU2	pp	229533.I1RMJ3
229533.I1RCU2	pp	229533.I1RX72
229533.I1RCU2	pp	229533.I1RYW9
229533.I1RCU2	pp	229533.V6RZD4
229533.I1RCU2	pp	229533.I1S3T7
229533.I1RCU2	pp	229533.I1RM26
229533.I1RCU2	pp	229533.I1RY35
229533.I1RCU2	pp	229533.I1RJY7
229533.I1RCU2	pp	229533.I1RLZ4
229533.I1RCU2	pp	229533.I1RQG7
229533.I1RCU2	pp	229533.I1SAH9
229533.I1RCU2	pp	229533.I1RH91
229533.I1RCU2	pp	229533.I1RGC3
229533.I1RCU2	pp	229533.I1RK55
229533.I1RCU2	pp	229533.I1S0V7
229533.I1RCU2	pp	229533.V6RRM0
229533.I1RCU2	pp	229533.I1S3G4
229533.I1RCU2	pp	229533.I1RCU9
229533.I1RCU2	pp	229533.I1S6P6
229533.I1RCU2	pp	229533.I1RFI4
229533.I1RCU2	pp	229533.V6R5D2
229533.I1RCU2	pp	229533.I1RQW1
229533.I1RCU2	pp	229533.I1S256
229533.I1RCU2	pp	229533.I1S147
229533.I1RCU2	pp	229533.I1RF85
229533.I1RCU2	pp	229533.I1RUR8
229533.I1RCU2	pp	229533.I1RVR2
229533.I1RCU2	pp	229533.I1RJS1
229533.I1RCU2	pp	229533.I1RN84
229533.I1RCU2	pp	229533.I1S6P5
229533.I1RCU2	pp	229533.I1RUE3
229533.I1RCU2	pp	229533.I1RHC8
229533.I1RCU2	pp	229533.I1RZ56
229533.I1RCU2	pp	229533.I1RZP8
229533.I1RCU2	pp	229533.I1RGZ3
229533.I1RCU2	pp	229533.I1RYC8
229533.I1RCU2	pp	229533.I1RKD3
229533.I1RCU2	pp	229533.I1RPV8
229533.I1RCU2	pp	229533.I1RUP2
229533.I1RCU2	pp	229533.I1S0X1
229533.I1RCU2	pp	229533.I1RN88
229533.I1RCU2	pp	229533.V6RE46
229533.I1RCU2	pp	229533.I1RTY9
229533.I1RCU2	pp	229533.I1RP12
229533.I1RCU2	pp	229533.V6RF62
229533.I1RCU2	pp	229533.I1S8R2
229533.I1RCU2	pp	229533.I1RYA0
229533.I1RCU2	pp	229533.I1RF40
229533.A0A1C3YMJ7	pp	229533.I1RJU9
229533.A0A1C3YMJ7	pp	229533.I1RLM3
229533.I1RYL8	pp	229533.V6RLM7
229533.I1RYL8	pp	229533.I1RZM4
229533.I1RYL8	pp	229533.V6RK96
229533.I1RYL8	pp	229533.V6RV57
229533.I1RYL8	pp	229533.I1RZQ1
229533.I1RYL8	pp	229533.Q4IR09
229533.I1RCQ2	pp	229533.I1RD70
229533.A0A0E0SJ79	pp	229533.I1RB75
229533.A0A0E0SJ79	pp	229533.I1RBE4
229533.A0A0E0SJ79	pp	229533.A0A1C3YID5
229533.A0A0E0SJ79	pp	229533.I1RMM8
229533.A0A0E0SJ79	pp	229533.I1RW06
229533.I1RWM4	pp	229533.I1S147
229533.I1RWM4	pp	229533.I1S1M4
229533.I1RWM4	pp	229533.I1S8F8
229533.I1RWM4	pp	229533.I1S2L1
229533.I1RWM4	pp	229533.I1SA33
229533.I1RWM4	pp	229533.V6RCC0
229533.I1RWM4	pp	229533.V6R5T7
229533.I1RWM4	pp	229533.I1RXH3
229533.I1RWM4	pp	229533.I1RWN7
229533.I1RWM4	pp	229533.I1RYP5

731	229533.I1RWM4	pp	229533.I1RY34
732	229533.I1RWM4	pp	229533.I1S8C1
733	229533.I1RWM4	pp	229533.I1RYX6
734	229533.I1RWM4	pp	229533.I1S8D2
735	229533.I1RWM4	pp	229533.I1S1W8
736	229533.I1RWM4	pp	229533.I1RYR3
737	229533.I1RWM4	pp	229533.I1S3T7
738	229533.I1RWM4	pp	229533.I1RX72
739	229533.I1RWM4	pp	229533.I1SAB4
740	229533.I1RWM4	pp	229533.I1S6H3
741	229533.I1RWM4	pp	229533.I1S048
742	229533.I1RWM4	pp	229533.I1S3G4
743	229533.I1RWM4	pp	229533.I1S223
744	229533.I1RWM4	pp	229533.I1S094
745	229533.I1RWM4	pp	229533.I1RYK0
746	229533.I1RWM4	pp	229533.V6RH92
747	229533.I1RWM4	pp	229533.I1RXB1
748	229533.I1RWM4	pp	229533.I1S2Q4
749	229533.I1RWM4	pp	229533.V6RCJ5
750	229533.I1RWM4	pp	229533.I1RZ25
751	229533.I1RWM4	pp	229533.I1S299
752	229533.I1RWM4	pp	229533.I1S969
753	229533.I1RWM4	pp	229533.I1S9T6
754	229533.I1RWM4	pp	229533.I1S0A5
755	229533.I1RWM4	pp	229533.V6QVV9
756	229533.I1RWM4	pp	229533.I1S7M8
757	229533.I1RWM4	pp	229533.I1S360
758	229533.I1RWM4	pp	229533.Q4HVQ9
759	229533.I1RWM4	pp	229533.V6RV57
760	229533.I1RWM4	pp	229533.I1S3T6
761	229533.I1RWM4	pp	229533.I1S968
762	229533.I1RWM4	pp	229533.I1RY81
763	229533.I1RWM4	pp	229533.I1S1K4
764	229533.I1RWM4	pp	229533.I1S8R2
765	229533.I1RWM4	pp	229533.V6RWB3
766	229533.I1RWM4	pp	229533.I1S0V6
767	229533.I1RWM4	pp	229533.I1RZ56
768	229533.I1RWM4	pp	229533.Q4HYR2
769	229533.I1RWM4	pp	229533.V6RK56
770	229533.I1RWM4	pp	229533.I1RZR5
771	229533.I1RWM4	pp	229533.I1S2C4
772	229533.I1RWM4	pp	229533.I1RYA0
773	229533.I1RWM4	pp	229533.I1S5G4
774	229533.I1RWM4	pp	229533.I1S6V0
775	229533.I1RWM4	pp	229533.V6RLM7
776	229533.I1RWM4	pp	229533.I1RXE6
777	229533.I1RWM4	pp	229533.V6RHX2
778	229533.I1RWM4	pp	229533.V6RE46
779	229533.I1RWM4	pp	229533.I1S267
780	229533.I1RWM4	pp	229533.I1S1K3
781	229533.I1RWM4	pp	229533.I1RYC8
782	229533.I1RWM4	pp	229533.I1S8D3
783	229533.I1RWM4	pp	229533.V6RRM0
784	229533.I1RWM4	pp	229533.I1S423
785	229533.I1RWM4	pp	229533.I1S0V7
786	229533.I1RZM4	pp	229533.V6R382
787	229533.I1RZM4	pp	229533.V6RLM7
788	229533.I1RZM4	pp	229533.Q4HXF0
789	229533.I1RZM4	pp	229533.I1S9C6
790	229533.I1RZM4	pp	229533.V6RM02
791	229533.I1RZM4	pp	229533.V6RSF3
792	229533.I1RZM4	pp	229533.I1S8R4
793	229533.I1RZM4	pp	229533.I1S9T6
794	229533.I1RZM4	pp	229533.I1S4B6
795	229533.I1RZM4	pp	229533.V6RHD0
796	229533.I1RZM4	pp	229533.I1S3G4
797	229533.I1RZM4	pp	229533.I1S673
798	229533.I1RZM4	pp	229533.O74638
799	229533.I1RZM4	pp	229533.V6RK96
800	229533.I1RZM4	pp	229533.V6R5D2
801	229533.I1RZM4	pp	229533.I1RZN1
802	229533.I1RZM4	pp	229533.I1S4B7
803	229533.I1RZM4	pp	229533.V6RXR0

804	229533.I1RZM4	pp	229533.V6QU65
805	229533.I1RZM4	pp	229533.I1S6A2
806	229533.I1RZM4	pp	229533.I1S0N3
807	229533.I1RZM4	pp	229533.I1S0A0
808	229533.I1RZM4	pp	229533.V6RDH3
809	229533.I1RZM4	pp	229533.I1S3L2
810	229533.I1RZM4	pp	229533.I1S1W8
811	229533.I1RZM4	pp	229533.I1S0Y7
812	229533.I1RZM4	pp	229533.I1S0K0
813	229533.I1RZM4	pp	229533.I1S8C1
814	229533.I1RZM4	pp	229533.I1S241
815	229533.I1RNM7	pp	229533.I1S266
816	229533.I1RNM7	pp	229533.V6RF62
817	229533.I1RNM7	pp	229533.I1S0R8
818	229533.I1RNM7	pp	229533.V6RXB3
819	229533.I1RNM7	pp	229533.I1S5U0
820	229533.I1RNM7	pp	229533.V6R1X4
821	229533.I1RNM7	pp	229533.I1S1K3
822	229533.I1RNM7	pp	229533.I1S2C4
823	229533.I1RNM7	pp	229533.I1RX18
824	229533.I1RNM7	pp	229533.I1S0Q7
825	229533.I1RNM7	pp	229533.I1S2Z7
826	229533.I1RNM7	pp	229533.I1RU95
827	229533.I1RNM7	pp	229533.V6RRM0
828	229533.I1RNM7	pp	229533.I1S3V2
829	229533.I1RNM7	pp	229533.V6R756
830	229533.I1RNM7	pp	229533.I1S147
831	229533.I1RNM7	pp	229533.I1RRH6
832	229533.I1RNM7	pp	229533.I1RVX8
833	229533.I1RNM7	pp	229533.Q4IN52
834	229533.I1RNM7	pp	229533.I1S0V7
835	229533.I1RNM7	pp	229533.I1RQC0
836	229533.I1RNM7	pp	229533.I1S3T7
837	229533.I1RNM7	pp	229533.I1S5X9
838	229533.I1RNM7	pp	229533.V6RLM7
839	229533.I1RNM7	pp	229533.I1RYA0
840	229533.I1RNM7	pp	229533.I1RPK0
841	229533.I1RNM7	pp	229533.I1S8R2
842	229533.I1RNM7	pp	229533.I1RWA3
843	229533.I1RNM7	pp	229533.V6QZ48
844	229533.I1RNM7	pp	229533.V6R9L8
845	229533.I1RNM7	pp	229533.I1S6P5
846	229533.I1RNM7	pp	229533.I1RVR2
847	229533.I1RNM7	pp	229533.I1RQ21
848	229533.I1RNM7	pp	229533.I1RZV5
849	229533.I1RNM7	pp	229533.I1RSF1
850	229533.I1RNM7	pp	229533.I1RWN7
851	229533.I1RNM7	pp	229533.I1RZH5
852	229533.I1RNM7	pp	229533.I1RYC8
853	229533.I1RNM7	pp	229533.I1RXD9
854	229533.I1RNM7	pp	229533.I1S0B8
855	229533.I1RNM7	pp	229533.I1RWF5
856	229533.I1RNM7	pp	229533.I1RW64
857	229533.I1RNM7	pp	229533.I1RVZ3
858	229533.I1RNM7	pp	229533.I1RY92
859	229533.I1RNM7	pp	229533.I1S968
860	229533.I1RNM7	pp	229533.I1RYX5
861	229533.I1RNM7	pp	229533.I1RX39
862	229533.I1RNM7	pp	229533.I1RX21
863	229533.I1RNM7	pp	229533.V6R5D2
864	229533.I1RNM7	pp	229533.I1RUR8
865	229533.I1RNM7	pp	229533.Q4HVQ9
866	229533.I1RNM7	pp	229533.I1RSP5
867	229533.I1RNM7	pp	229533.I1RSQ4
868	229533.I1RNM7	pp	229533.I1RY34
869	229533.I1RNM7	pp	229533.I1S3G4
870	229533.I1RNM7	pp	229533.I1RTZ1
871	229533.I1RNM7	pp	229533.V6R3J1
872	229533.I1RNM7	pp	229533.I1RTA1
873	229533.I1RNM7	pp	229533.I1RXW3
874	229533.I1RNM7	pp	229533.I1S8D2
875	229533.I1RNM7	pp	229533.V6RK56
876	229533.I1RNM7	pp	229533.I1RYW9

229533.I1RNM7 pp 229533.I1S1K4
229533.I1RNM7 pp 229533.V6RUB1
229533.I1RNM7 pp 229533.I1S1F3
229533.I1RNM7 pp 229533.I1S464
229533.I1RNM7 pp 229533.V6RZD4
229533.I1RNM7 pp 229533.I1S299
229533.I1RNM7 pp 229533.I1RY12
229533.I1RNM7 pp 229533.V6R2Z2
229533.I1RNM7 pp 229533.I1S3M4
229533.I1RNM7 pp 229533.I1S212
229533.I1RNM7 pp 229533.I1RZA7
229533.I1RNM7 pp 229533.I1RXV9
229533.I1RNM7 pp 229533.I1RP38
229533.I1RNM7 pp 229533.I1RUL3
229533.I1RNM7 pp 229533.I1RUQ3
229533.I1RNM7 pp 229533.V6R3V4
229533.I1RNM7 pp 229533.I1RZ43
229533.I1RNM7 pp 229533.I1RYK0
229533.I1RNM7 pp 229533.I1RS29
229533.I1RNM7 pp 229533.I1RX72
229533.I1RNM7 pp 229533.I1S2A1
229533.I1RNM7 pp 229533.I1S8S9
229533.I1RNM7 pp 229533.I1RNY6
229533.I1RNM7 pp 229533.I1S0K2
229533.I1RNM7 pp 229533.I1RZ68
229533.I1RNM7 pp 229533.Q4HYR2
229533.I1RNM7 pp 229533.I1RQJ7
229533.I1RNM7 pp 229533.I1RXH2
229533.I1RNM7 pp 229533.I1S0V6
229533.I1RNM7 pp 229533.I1RP02
229533.I1RNM7 pp 229533.I1RQE0
229533.I1RNM7 pp 229533.I1S423
229533.I1RNM7 pp 229533.I1RWD2
229533.I1RNM7 pp 229533.I1RYM5
229533.I1RNM7 pp 229533.I1RWM4
229533.I1RNM7 pp 229533.Q4I5G1
229533.I1RNM7 pp 229533.I1RPR9
229533.I1RNM7 pp 229533.I1RUY5
229533.I1RNM7 pp 229533.I1RY81
229533.I1RNM7 pp 229533.I1RXE6
229533.I1RNM7 pp 229533.I1S8F8
229533.I1RNM7 pp 229533.I1S3B2
229533.I1RNM7 pp 229533.I1RW13
229533.I1RNM7 pp 229533.I1RPR1
229533.I1RNM7 pp 229533.V6R1D2
229533.I1RNM7 pp 229533.I1S1M4
229533.I1RNM7 pp 229533.I1RVI8
229533.I1RNM7 pp 229533.I1S5J9
229533.I1RNM7 pp 229533.V6R0W1
229533.I1RNM7 pp 229533.V6QUG0
229533.I1RNM7 pp 229533.I1S8H9
229533.I1RNM7 pp 229533.I1RYN2
229533.I1RNM7 pp 229533.I1S5G4
229533.I1RNM7 pp 229533.I1S4S1
229533.I1RNM7 pp 229533.I1RYP5
229533.I1RNM7 pp 229533.I1S8E9
229533.I1RNM7 pp 229533.I1RYT8
229533.I1RNM7 pp 229533.I1RRK9
229533.I1RNM7 pp 229533.I1RYI6
229533.I1RNM7 pp 229533.V6RJP8
229533.I1RNM7 pp 229533.I1RWL3
229533.I1RNM7 pp 229533.I1RRS6
229533.I1RNM7 pp 229533.I1RYU3
229533.I1RNM7 pp 229533.I1RY21
229533.I1RNM7 pp 229533.I1S1W8
229533.I1RNM7 pp 229533.I1RW54
229533.I1RNM7 pp 229533.I1RS05
229533.I1RNM7 pp 229533.V6R7V8
229533.I1RNM7 pp 229533.I1S969
229533.I1RNM7 pp 229533.V6QVH2
229533.I1RNM7 pp 229533.I1RQE1
229533.I1RNM7 pp 229533.I1RP86
229533.I1RNM7 pp 229533.I1RRX5

229533.I1RNM7	pp	229533.I1S0U0
229533.I1RNM7	pp	229533.V6RAM6
229533.I1RNM7	pp	229533.I1S2F6
229533.I1RNM7	pp	229533.I1SAI4
229533.I1RNM7	pp	229533.I1RPN1
229533.I1RNM7	pp	229533.I1RYU6
229533.I1RNM7	pp	229533.I1S0T7
229533.I1RNM7	pp	229533.I1RYC7
229533.I1RNM7	pp	229533.I1S4S5
229533.I1RNM7	pp	229533.I1RPF2
229533.I1RNM7	pp	229533.V6QU99
229533.I1RNM7	pp	229533.I1RQU9
229533.I1RNM7	pp	229533.I1S0P0
229533.I1RNM7	pp	229533.I1RWG0
229533.I1RNM7	pp	229533.I1RYX6
229533.I1RNM7	pp	229533.I1S8C1
229533.I1RC55	pp	229533.I1RW71
229533.I1RC55	pp	229533.I1REC7
229533.I1RC55	pp	229533.I1RYL5
229533.I1RC55	pp	229533.I1RWH4
229533.I1RC55	pp	229533.I1RME4
229533.I1RC55	pp	229533.I1REC6
229533.I1RC55	pp	229533.I1RZ95
229533.A0A1C3YN26	pp	229533.I1S147
229533.A0A1C3YN26	pp	229533.V6RMW6
229533.A0A1C3YN26	pp	229533.I1RMQ5
229533.A0A1C3YN26	pp	229533.I1RND4
229533.A0A1C3YN26	pp	229533.I1RD22
229533.A0A1C3YN26	pp	229533.V6R382
229533.A0A1C3YN26	pp	229533.I1RSU2
229533.A0A1C3YN26	pp	229533.I1RQ75
229533.A0A1C3YN26	pp	229533.I1RPZ5
229533.A0A1C3YN26	pp	229533.V6RLM7
229533.A0A1C3YN26	pp	229533.V6R042
229533.A0A1C3YN26	pp	229533.I1RZQ1
229533.A0A1C3YN26	pp	229533.I1RB92
229533.A0A1C3YN26	pp	229533.I1RPK0
229533.A0A1C3YN26	pp	229533.I1RVX0
229533.A0A1C3YN26	pp	229533.I1RRN5
229533.A0A1C3YN26	pp	229533.I1RRV1
229533.A0A1C3YN26	pp	229533.I1RN84
229533.A0A1C3YN26	pp	229533.I1S064
229533.A0A1C3YN26	pp	229533.I1SAN9
229533.A0A1C3YN26	pp	229533.I1RSS4
229533.A0A1C3YN26	pp	229533.I1RCH5
229533.A0A1C3YN26	pp	229533.I1S0J1
229533.A0A1C3YN26	pp	229533.I1RAN1
229533.A0A1C3YN26	pp	229533.I1RHJ2
229533.A0A1C3YN26	pp	229533.I1RFF3
229533.A0A1C3YN26	pp	229533.I1RNC1
229533.A0A1C3YN26	pp	229533.I1RHU8
229533.A0A1C3YN26	pp	229533.I1RQE7
229533.A0A1C3YN26	pp	229533.I1RY74
229533.A0A1C3YN26	pp	229533.I1RTA0
229533.A0A1C3YN26	pp	229533.I1S3V2
229533.A0A1C3YN26	pp	229533.I1RKL0
229533.A0A1C3YN26	pp	229533.V6RRM0
229533.A0A1C3YN26	pp	229533.I1RGX1
229533.A0A1C3YN26	pp	229533.I1S332
229533.A0A1C3YN26	pp	229533.V6RVG3
229533.A0A1C3YN26	pp	229533.I1S149
229533.A0A1C3YN26	pp	229533.Q4I1Y5
229533.A0A1C3YN26	pp	229533.I1REC7
229533.A0A1C3YN26	pp	229533.I1RPP3
229533.A0A1C3YN26	pp	229533.I1S8P5
229533.A0A1C3YN26	pp	229533.I1RNU7
229533.A0A1C3YN26	pp	229533.I1RYK0
229533.A0A1C3YN26	pp	229533.I1RLZ4
229533.A0A1C3YN26	pp	229533.I1RAB4
229533.A0A1C3YN26	pp	229533.I1RPS2
229533.A0A1C3YN26	pp	229533.I1RBS0
229533.A0A1C3YN26	pp	229533.I1RQG4
229533.A0A1C3YN26	pp	229533.I1RW82

229533.A0A1C3YN26	pp	229533.I1RWQ6
229533.A0A1C3YN26	pp	229533.I1RFT1
229533.A0A1C3YN26	pp	229533.I1RA70
229533.A0A1C3YN26	pp	229533.I1RTA1
229533.A0A1C3YN26	pp	229533.I1RP28
229533.A0A1C3YN26	pp	229533.I1RC31
229533.A0A1C3YN26	pp	229533.I1RRI2
229533.A0A1C3YN26	pp	229533.I1RNG4
229533.A0A1C3YN26	pp	229533.I1RKB2
229533.A0A1C3YN26	pp	229533.I1RMJ3
229533.A0A1C3YN26	pp	229533.I1RWX5
229533.A0A1C3YN26	pp	229533.I1RVU7
229533.A0A1C3YN26	pp	229533.I1RN67
229533.A0A1C3YN26	pp	229533.I1RMG6
229533.A0A1C3YN26	pp	229533.I1RYW3
229533.A0A1C3YN26	pp	229533.I1RCG5
229533.A0A1C3YN26	pp	229533.I1RZV5
229533.A0A1C3YN26	pp	229533.I1RWS4
229533.A0A1C3YN26	pp	229533.V6RUY9
229533.A0A1C3YN26	pp	229533.I1RA62
229533.A0A1C3YN26	pp	229533.Q4I2W2
229533.A0A1C3YN26	pp	229533.I1RAW0
229533.A0A1C3YN26	pp	229533.I1RN63
229533.A0A1C3YN26	pp	229533.I1RX72
229533.A0A1C3YN26	pp	229533.I1RWF5
229533.A0A1C3YN26	pp	229533.I1RTD5
229533.A0A1C3YN26	pp	229533.I1RPS4
229533.A0A1C3YN26	pp	229533.I1RH91
229533.A0A1C3YN26	pp	229533.I1RT40
229533.A0A1C3YN26	pp	229533.I1RTG7
229533.A0A1C3YN26	pp	229533.I1RW43
229533.A0A1C3YN26	pp	229533.I1RCD4
229533.A0A1C3YN26	pp	229533.I1RC29
229533.A0A1C3YN26	pp	229533.I1RN88
229533.A0A1C3YN26	pp	229533.V6RV57
229533.A0A1C3YN26	pp	229533.I1RDE7
229533.A0A1C3YN26	pp	229533.I1RR95
229533.A0A1C3YN26	pp	229533.I1REC6
229533.A0A1C3YN26	pp	229533.I1RZ29
229533.A0A1C3YN26	pp	229533.I1RSZ7
229533.A0A1C3YN26	pp	229533.I1RZB2
229533.A0A1C3YN26	pp	229533.I1RZ52
229533.A0A1C3YN26	pp	229533.I1RGC3
229533.A0A1C3YN26	pp	229533.I1RSQ4
229533.A0A1C3YN26	pp	229533.I1RKP1
229533.A0A1C3YN26	pp	229533.I1S2H4
229533.A0A1C3YN26	pp	229533.I1RZN1
229533.A0A1C3YN26	pp	229533.I1RGA4
229533.A0A1C3YN26	pp	229533.I1RFI4
229533.A0A1C3YN26	pp	229533.I1RBY4
229533.A0A1C3YN26	pp	229533.I1RBY9
229533.A0A1C3YN26	pp	229533.I1RVY0
229533.A0A1C3YN26	pp	229533.I1S3G4
229533.A0A1C3YN26	pp	229533.O74638
229533.A0A1C3YN26	pp	229533.V6R3J1
229533.A0A1C3YN26	pp	229533.I1RK50
229533.A0A1C3YN26	pp	229533.I1RMD3
229533.A0A1C3YN26	pp	229533.I1RG14
229533.A0A1C3YN26	pp	229533.I1RXF0
229533.A0A1C3YN26	pp	229533.I1S5B0
229533.A0A1C3YN26	pp	229533.I1RRT8
229533.A0A1C3YN26	pp	229533.I1RJQ5
229533.A0A1C3YN26	pp	229533.I1RJH3
229533.A0A1C3YN26	pp	229533.I1RA90
229533.A0A1C3YN26	pp	229533.I1RA72
229533.A0A1C3YN26	pp	229533.I1RUY5
229533.A0A1C3YN26	pp	229533.I1RS18
229533.A0A1C3YN26	pp	229533.I1RUY6
229533.A0A1C3YN26	pp	229533.I1RA94
229533.A0A1C3YN26	pp	229533.I1RHR3
229533.A0A1C3YN26	pp	229533.I1RPL2
229533.A0A1C3YN26	pp	229533.I1RWM4
229533.A0A1C3YN26	pp	229533.I1S0Z3

229533.A0A1C3YN26	pp	229533.I1RH82
229533.A0A1C3YN26	pp	229533.I1RC95
229533.A0A1C3YN26	pp	229533.V6QUE6
229533.A0A1C3YN26	pp	229533.I1RJT5
229533.A0A1C3YN26	pp	229533.V6RCZ8
229533.A0A1C3YN26	pp	229533.V6RK96
229533.A0A1C3YN26	pp	229533.I1RBE4
229533.A0A1C3YN26	pp	229533.I1RJL9
229533.A0A1C3YN26	pp	229533.Q4HTT2
229533.A0A1C3YN26	pp	229533.I1RYI7
229533.A0A1C3YN26	pp	229533.I1RRV4
229533.A0A1C3YN26	pp	229533.I1RUQ3
229533.A0A1C3YN26	pp	229533.V6RPS5
229533.A0A1C3YN26	pp	229533.Q4IJK2
229533.A0A1C3YN26	pp	229533.I1RYM5
229533.A0A1C3YN26	pp	229533.I1RMG7
229533.A0A1C3YN26	pp	229533.I1S439
229533.A0A1C3YN26	pp	229533.I1RIE8
229533.A0A1C3YN26	pp	229533.I1RPT0
229533.A0A1C3YN26	pp	229533.I1RBE7
229533.A0A1C3YN26	pp	229533.V6RY56
229533.A0A1C3YN26	pp	229533.I1RAQ4
229533.A0A1C3YN26	pp	229533.I1RDU0
229533.A0A1C3YN26	pp	229533.Q4HYR2
229533.A0A1C3YN26	pp	229533.I1RGB2
229533.A0A1C3YN26	pp	229533.I1RYL5
229533.A0A1C3YN26	pp	229533.I1RQE0
229533.A0A1C3YN26	pp	229533.I1RFD4
229533.A0A1C3YN26	pp	229533.I1RCR5
229533.A0A1C3YN26	pp	229533.V6REE9
229533.A0A1C3YN26	pp	229533.I1RYW8
229533.A0A1C3YN26	pp	229533.I1RKE1
229533.A0A1C3YN26	pp	229533.I1REK8
229533.A0A1C3YN26	pp	229533.I1RZM4
229533.A0A1C3YN26	pp	229533.I1S4Q4
229533.A0A1C3YN26	pp	229533.I1RRY9
229533.A0A1C3YN26	pp	229533.I1RM26
229533.A0A1C3YN26	pp	229533.I1RIY0
229533.A0A1C3YN26	pp	229533.I1RRX5
229533.A0A1C3YN26	pp	229533.I1RUY4
229533.A0A1C3YN26	pp	229533.I1RCM5
229533.A0A1C3YN26	pp	229533.I1RYX6
229533.A0A1C3YN26	pp	229533.I1RQA5
229533.A0A1C3YN26	pp	229533.I1RPW1
229533.A0A1C3YN26	pp	229533.I1S0N3
229533.A0A1C3YN26	pp	229533.I1RAZ3
229533.A0A1C3YN26	pp	229533.I1RD52
229533.A0A1C3YN26	pp	229533.I1RT33
229533.A0A1C3YN26	pp	229533.I1RFV2
229533.A0A1C3YN26	pp	229533.I1RY12
229533.A0A1C3YN26	pp	229533.Q5I2J3
229533.A0A1C3YN26	pp	229533.I1RCV1
229533.A0A1C3YN26	pp	229533.I1RMW4
229533.A0A1C3YN26	pp	229533.I1RYU0
229533.A0A1C3YN26	pp	229533.I1RME6
229533.A0A1C3YN26	pp	229533.I1RYL8
229533.A0A1C3YN26	pp	229533.I1RZ16
229533.A0A1C3YN26	pp	229533.I1RP86
229533.A0A1C3YN26	pp	229533.I1RVX9
229533.A0A1C3YN26	pp	229533.I1RT41
229533.A0A1C3YN26	pp	229533.I1RW54
229533.A0A1C3YN26	pp	229533.I1RY49
229533.A0A1C3YN26	pp	229533.I1RYJ6
229533.A0A1C3YN26	pp	229533.I1RJN7
229533.A0A1C3YN26	pp	229533.I1RMM8
229533.A0A1C3YN26	pp	229533.I1S1W8
229533.A0A1C3YN26	pp	229533.I1RQE8
229533.A0A1C3YN26	pp	229533.I1RJU3
229533.A0A1C3YN26	pp	229533.I1RKB5
229533.A0A1C3YN26	pp	229533.I1RAM6
229533.A0A1C3YN26	pp	229533.V6RTJ8
229533.A0A1C3YN26	pp	229533.I1RQH1
229533.A0A1C3YN26	pp	229533.I1RGY2

229533.A0A1C3YN26 pp 229533.Q4HZS8
229533.A0A1C3YN26 pp 229533.I1RI75
229533.A0A1C3YN26 pp 229533.I1RX46
229533.A0A1C3YN26 pp 229533.I1R9Y4
229533.A0A1C3YN26 pp 229533.I1RNM7
229533.A0A1C3YN26 pp 229533.I1RVI8
229533.A0A1C3YN26 pp 229533.I1RC11
229533.A0A1C3YN26 pp 229533.I1S4S4
229533.A0A1C3YN26 pp 229533.Q4IR09
229533.A0A1C3YN26 pp 229533.I1RRG1
229533.A0A1C3YN26 pp 229533.I1RCU4
229533.A0A1C3YN26 pp 229533.I1RR24
229533.A0A1C3YN26 pp 229533.I1S1W5
229533.I1RZY1 pp 229533.I1S4D0
229533.I1RZY1 pp 229533.I1S024
229533.I1RZY1 pp 229533.V6RDH3
229533.I1RZY1 pp 229533.V6RY56
229533.I1RZY1 pp 229533.I1SAE8
229533.I1RZY1 pp 229533.I1S0H5
229533.I1RZY1 pp 229533.I1S036
229533.I1RZY1 pp 229533.Q4I1N3
229533.I1RZY1 pp 229533.V6QV31
229533.I1RZY1 pp 229533.I1S6P5
229533.I1RZY1 pp 229533.V6RUB1
229533.I1RZY1 pp 229533.I1S273
229533.I1RQE8 pp 229533.V6RK96
229533.I1RQE8 pp 229533.I1S0A0
229533.I1RQE8 pp 229533.I1RVY2
229533.I1RQE8 pp 229533.I1S0N3
229533.I1RQE8 pp 229533.I1RW69
229533.I1RQE8 pp 229533.V6QU65
229533.I1RQE8 pp 229533.I1RXS0
229533.I1RQE8 pp 229533.I1RRN4
229533.I1RQE8 pp 229533.I1S8C1
229533.I1RQE8 pp 229533.I1RYI7
229533.I1RQE8 pp 229533.I1RXV9
229533.I1RQE8 pp 229533.I1RUI6
229533.I1RQE8 pp 229533.I1RT09
229533.I1RQE8 pp 229533.I1S6A2
229533.I1RQE8 pp 229533.I1RYM6
229533.I1RQE8 pp 229533.I1RRC4
229533.I1RQE8 pp 229533.I1RUY4
229533.I1RQE8 pp 229533.I1RXB1
229533.I1RQE8 pp 229533.I1RQM4
229533.I1RQE8 pp 229533.V6RDH3
229533.I1RQE8 pp 229533.I1RXY7
229533.I1RQE8 pp 229533.I1RVX9
229533.I1RQE8 pp 229533.I1RYL8
229533.I1RQE8 pp 229533.I1RSL5
229533.I1RQE8 pp 229533.I1S0K0
229533.I1RQE8 pp 229533.I1RUP5
229533.I1RQE8 pp 229533.I1RZM4
229533.I1RQE8 pp 229533.I1RRJ3
229533.I1RQE8 pp 229533.I1S241
229533.I1RQE8 pp 229533.I1RTG4
229533.I1RQE8 pp 229533.I1RQP2
229533.I1RQE8 pp 229533.V6RLM7
229533.I1RQE8 pp 229533.V6R382
229533.I1RQE8 pp 229533.I1RZ16
229533.I1RQE8 pp 229533.I1RSU2
229533.I1RQE8 pp 229533.I1RYJ7
229533.I1RQE8 pp 229533.I1S1W8
229533.I1RQE8 pp 229533.I1S8R4
229533.I1RQE8 pp 229533.V6RSF3
229533.I1RQE8 pp 229533.I1S3L2
229533.I1RQE8 pp 229533.I1RVY1
229533.I1RQE8 pp 229533.I1RZ83
229533.I1RQE8 pp 229533.I1RXW3
229533.I1RQE8 pp 229533.V6RHD0
229533.I1RQE8 pp 229533.I1RSX9
229533.I1RQE8 pp 229533.I1RSS7
229533.I1RQE8 pp 229533.I1RSY1
229533.I1RQE8 pp 229533.I1S673

229533.I1RQE8	pp	229533.I1RZN1
229533.I1RQE8	pp	229533.I1RZ70
229533.I1RQE8	pp	229533.V6RM02
229533.I1RQE8	pp	229533.I1S4B7
229533.I1RQE8	pp	229533.I1RX72
229533.I1RQE8	pp	229533.I1S9T6
229533.I1RQE8	pp	229533.I1RYW3
229533.I1RQE8	pp	229533.Q4HXF0
229533.I1RQE8	pp	229533.V6R5D2
229533.I1RQE8	pp	229533.I1RUR8
229533.I1RQE8	pp	229533.I1RW10
229533.I1RQE8	pp	229533.V6RXR0
229533.I1RQE8	pp	229533.I1S0Y7
229533.I1RQE8	pp	229533.I1S3G4
229533.I1RQE8	pp	229533.O74638
229533.I1RQE8	pp	229533.I1RVR2
229533.I1RQE8	pp	229533.I1RW43
229533.I1RQE8	pp	229533.I1S9C6
229533.I1RQE8	pp	229533.I1RR29
229533.I1RQE8	pp	229533.I1S4B6
229533.I1RQE8	pp	229533.I1RSK5
229533.I1RMT5	pp	229533.I1RW38
229533.I1RMT5	pp	229533.I1RRH1
229533.I1RMT5	pp	229533.I1S0P4
229533.A0A0E0SF53	pp	229533.Q4IER8
229533.A0A0E0SF53	pp	229533.A0A1C3YK75
229533.A0A0E0SF53	pp	229533.I1RBL8
229533.A0A0E0SF53	pp	229533.Q4HTT2
229533.A0A0E0SF53	pp	229533.A0A1C3YJE9
229533.A0A0E0SF53	pp	229533.I1RJT1
229533.A0A0E0SF53	pp	229533.I1RQ28
229533.A0A0E0SF53	pp	229533.I1RM09
229533.A0A0E0SF53	pp	229533.I1RXX0
229533.A0A0E0SF53	pp	229533.I1RBC4
229533.A0A0E0SF53	pp	229533.I1RAL2
229533.A0A0E0SF53	pp	229533.I1RCL7
229533.A0A0E0SF53	pp	229533.Q4IR08
229533.A0A0E0SF53	pp	229533.A0A1C3YK94
229533.A0A0E0SF53	pp	229533.A0A1C3YN58
229533.A0A0E0SF53	pp	229533.A0A1C3YIS1
229533.A0A0E0SF53	pp	229533.I1S039
229533.A0A0E0SF53	pp	229533.Q4I5R3
229533.A0A0E0SF53	pp	229533.I1RJT5
229533.A0A0E0SF53	pp	229533.I1S788
229533.A0A0E0SF53	pp	229533.A0A1C3YM09
229533.A0A0E0SF53	pp	229533.I1S1W5
229533.A0A0E0SF53	pp	229533.A0A0E0SPD4
229533.A0A0E0SF53	pp	229533.I1RRT1
229533.A0A0E0SF53	pp	229533.I1S0D5
229533.A0A0E0SF53	pp	229533.I1RUQ6
229533.A0A0E0SF53	pp	229533.V6RSF3
229533.A0A0E0SF53	pp	229533.I1RQL4
229533.A0A0E0SF53	pp	229533.Q4IB89
229533.A0A0E0SF53	pp	229533.I1RDE6
229533.A0A0E0SF53	pp	229533.I1S1Y7
229533.A0A0E0SF53	pp	229533.Q4IJP1
229533.A0A0E0SF53	pp	229533.A0A0E0SH88
229533.A0A0E0SF53	pp	229533.I1RKP1
229533.A0A0E0SF53	pp	229533.I1RME4
229533.A0A0E0SF53	pp	229533.A0A1C3YLX5
229533.A0A0E0SF53	pp	229533.A0A1C3YKZ8
229533.A0A0E0SF53	pp	229533.I1RN27
229533.A0A0E0SF53	pp	229533.I1RW28
229533.A0A0E0SF53	pp	229533.I1RPR8
229533.A0A0E0SF53	pp	229533.A0A1C3YHV5
229533.A0A0E0SF53	pp	229533.Q4IB50
229533.A0A0E0SF53	pp	229533.Q4IMD1
229533.A0A0E0SF53	pp	229533.I1RWH4
229533.A0A0E0SF53	pp	229533.Q4IEV4
229533.A0A0E0SF53	pp	229533.I1RB73
229533.A0A0E0SF53	pp	229533.I1RVH7
229533.A0A0E0SF53	pp	229533.I1RRS7
229533.A0A0E0SF53	pp	229533.I1RRJ6

229533.A0A0E0SF53 pp 229533.A0A1C3YJ21
229533.A0A0E0SF53 pp 229533.I1RPF5
229533.A0A0E0SF53 pp 229533.A0A1C3YHH5
229533.A0A0E0SF53 pp 229533.I1RYR2
229533.A0A0E0SF53 pp 229533.I1RMG6
229533.A0A0E0SF53 pp 229533.I1RCN2
229533.A0A0E0SF53 pp 229533.I1RJZ7
229533.A0A0E0SF53 pp 229533.I1S0J7
229533.A0A0E0SF53 pp 229533.A0A1C3YL34
229533.A0A0E0SF53 pp 229533.I1RC29
229533.A0A0E0SF53 pp 229533.A0A1C3YM82
229533.A0A0E0SF53 pp 229533.I1RFW1
229533.I1S067 pp 229533.I1SAT1
229533.I1RGB2 pp 229533.I1RWX5
229533.I1RGB2 pp 229533.I1S5C5
229533.I1RGB2 pp 229533.V6RRM0
229533.I1RGB2 pp 229533.V6RV57
229533.I1RGB2 pp 229533.I1RVQ3
229533.I1RGB2 pp 229533.I1RSA3
229533.I1RGB2 pp 229533.I1RMC2
229533.I1RGB2 pp 229533.I1S149
229533.I1RGB2 pp 229533.I1RNU7
229533.I1RGB2 pp 229533.I1RQI4
229533.I1RGB2 pp 229533.I1RKJ4
229533.I1RGB2 pp 229533.I1RWF5
229533.I1RGB2 pp 229533.I1RT40
229533.I1RGB2 pp 229533.I1RRM3
229533.I1RGB2 pp 229533.I1RQ33
229533.I1RGB2 pp 229533.I1RZV5
229533.I1RGB2 pp 229533.I1RVX0
229533.I1RGB2 pp 229533.I1RTD5
229533.I1RGB2 pp 229533.V6RUY9
229533.I1RGB2 pp 229533.I1S147
229533.I1RGB2 pp 229533.I1S1W9
229533.I1RGB2 pp 229533.I1RSQ4
229533.I1RGB2 pp 229533.I1RZV3
229533.I1RGB2 pp 229533.I1S0W4
229533.I1RGB2 pp 229533.I1RGX1
229533.I1RGB2 pp 229533.I1RNG4
229533.I1RGB2 pp 229533.V6R9L8
229533.I1RGB2 pp 229533.I1S2B8
229533.I1RGB2 pp 229533.I1RZ39
229533.I1RGB2 pp 229533.I1RK50
229533.I1RGB2 pp 229533.I1RSF1
229533.I1RGB2 pp 229533.I1RX72
229533.I1RGB2 pp 229533.I1RSA5
229533.I1RGB2 pp 229533.I1RPU2
229533.I1RGB2 pp 229533.V6RLM7
229533.I1RGB2 pp 229533.I1RGC3
229533.I1RGB2 pp 229533.I1RJN9
229533.I1RGB2 pp 229533.I1RTA1
229533.I1RGB2 pp 229533.I1RSZ7
229533.I1RGB2 pp 229533.I1RYK0
229533.I1RGB2 pp 229533.I1RVZ2
229533.I1RGB2 pp 229533.I1S5R1
229533.I1RGB2 pp 229533.I1RPE0
229533.I1RGB2 pp 229533.I1S3F7
229533.I1RGB2 pp 229533.I1RN88
229533.I1RGB2 pp 229533.I1S148
229533.I1RGB2 pp 229533.I1RMC5
229533.I1RGB2 pp 229533.I1RUY4
229533.I1RGB2 pp 229533.I1RYI5
229533.I1RGB2 pp 229533.I1RRI1
229533.I1RGB2 pp 229533.I1RY12
229533.I1RGB2 pp 229533.I1RH82
229533.I1RGB2 pp 229533.I1RWR7
229533.I1RGB2 pp 229533.I1RKE1
229533.I1RGB2 pp 229533.I1RGY2
229533.I1RGB2 pp 229533.I1RZK5
229533.I1RGB2 pp 229533.I1RPT0
229533.I1RGB2 pp 229533.I1S1B2
229533.I1RGB2 pp 229533.I1RQD2
229533.I1RGB2 pp 229533.I1RNB2

229533.I1RGB2	pp	229533.I1RIX0
229533.I1RGB2	pp	229533.I1RUY5
229533.I1RGB2	pp	229533.I1RPY3
229533.I1RGB2	pp	229533.I1RP86
229533.I1RGB2	pp	229533.Q4IJK2
229533.I1RGB2	pp	229533.V6RPS5
229533.I1RGB2	pp	229533.I1RUQ3
229533.I1RGB2	pp	229533.I1RRV3
229533.I1RGB2	pp	229533.I1RZM8
229533.I1RGB2	pp	229533.I1RXD7
229533.I1RGB2	pp	229533.I1RTB3
229533.I1RGB2	pp	229533.I1RR24
229533.I1RGB2	pp	229533.V6RCZ8
229533.I1RGB2	pp	229533.I1RXD6
229533.I1RGB2	pp	229533.Q4IR09
229533.I1RGB2	pp	229533.I1RX95
229533.I1RGB2	pp	229533.I1S821
229533.I1RGB2	pp	229533.I1S4S7
229533.I1RGB2	pp	229533.I1RUY6
229533.I1RGB2	pp	229533.I1RJU3
229533.I1RGB2	pp	229533.I1RJN7
229533.I1RGB2	pp	229533.I1RQ85
229533.I1RGB2	pp	229533.I1RYL8
229533.I1RGB2	pp	229533.I1RXC4
229533.I1RGB2	pp	229533.I1RZ16
229533.I1RGB2	pp	229533.I1RSJ2
229533.I1RGB2	pp	229533.I1RR44
229533.I1RRH1	pp	229533.I1S074
229533.I1RRH1	pp	229533.I1RSL8
229533.I1S7R5	pp	229533.V6R223
229533.I1S7R5	pp	229533.V6RJ21
229533.A0A1C3YK30	pp	229533.I1RYL5
229533.A0A1C3YK30	pp	229533.I1RBC4
229533.A0A1C3YK30	pp	229533.I1RZ95
229533.A0A1C3YK30	pp	229533.I1RBS5
229533.A0A1C3YK30	pp	229533.I1RWH4
229533.A0A1C3YK30	pp	229533.I1RW71
229533.A0A1C3YK30	pp	229533.I1RME4
229533.A0A1C3YK30	pp	229533.I1REC7
229533.A0A1C3YK30	pp	229533.I1R9Y8
229533.A0A1C3YK30	pp	229533.I1REC6
229533.I1RVU1	pp	229533.I1S0K2
229533.I1RVU1	pp	229533.I1S8S9
229533.I1RVU1	pp	229533.I1S128
229533.I1RVU1	pp	229533.Q4I5G1
229533.I1RVU1	pp	229533.Q4IEV5
229533.I1RVU1	pp	229533.V6QU65
229533.I1RZ16	pp	229533.I1RZM4
229533.I1RZ16	pp	229533.I1RZQ1
229533.I1RZ16	pp	229533.V6RLM7
229533.I1RZ16	pp	229533.Q4IR09
229533.I1RZ16	pp	229533.V6RK96
229533.I1RZ16	pp	229533.V6RV57
229533.A0A1C3YJ04	pp	229533.I1REE2
229533.A0A1C3YJ04	pp	229533.I1RST9
229533.A0A1C3YJ04	pp	229533.I1RH82
229533.A0A1C3YJ04	pp	229533.I1RWM4
229533.A0A1C3YJ04	pp	229533.I1RJT5
229533.A0A1C3YJ04	pp	229533.I1RXE6
229533.A0A1C3YJ04	pp	229533.I1RNY6
229533.A0A1C3YJ04	pp	229533.A0A1C3YK58
229533.A0A1C3YJ04	pp	229533.I1RPR1
229533.A0A1C3YJ04	pp	229533.I1S267
229533.A0A1C3YJ04	pp	229533.A0A1C3YKR5
229533.A0A1C3YJ04	pp	229533.I1S8F8
229533.A0A1C3YJ04	pp	229533.I1RUQ3
229533.A0A1C3YJ04	pp	229533.I1RY12
229533.A0A1C3YJ04	pp	229533.A0A1C3YLB3
229533.A0A1C3YJ04	pp	229533.I1S094
229533.A0A1C3YJ04	pp	229533.I1RCV1
229533.A0A1C3YJ04	pp	229533.I1RUL3
229533.A0A1C3YJ04	pp	229533.I1RC11
229533.A0A1C3YJ04	pp	229533.I1RNM7

229533.A0A1C3YJ04 pp 229533.A0A1C3YKZ8
229533.A0A1C3YJ04 pp 229533.I1RP86
229533.A0A1C3YJ04 pp 229533.Q4I5G1
229533.A0A1C3YJ04 pp 229533.I1S8C1
229533.A0A1C3YJ04 pp 229533.I1RJY7
229533.A0A1C3YJ04 pp 229533.I1RW54
229533.A0A1C3YJ04 pp 229533.I1S1W8
229533.A0A1C3YJ04 pp 229533.I1S0P0
229533.A0A1C3YJ04 pp 229533.I1RYX6
229533.A0A1C3YJ04 pp 229533.I1RG33
229533.A0A1C3YJ04 pp 229533.I1S147
229533.A0A1C3YJ04 pp 229533.I1RZC3
229533.A0A1C3YJ04 pp 229533.I1RN84
229533.A0A1C3YJ04 pp 229533.I1RS90
229533.A0A1C3YJ04 pp 229533.V6RRM0
229533.A0A1C3YJ04 pp 229533.I1RCU2
229533.A0A1C3YJ04 pp 229533.I1RYC8
229533.A0A1C3YJ04 pp 229533.I1RY21
229533.A0A1C3YJ04 pp 229533.I1RPK0
229533.A0A1C3YJ04 pp 229533.I1RA62
229533.A0A1C3YJ04 pp 229533.I1RX72
229533.A0A1C3YJ04 pp 229533.A0A1C3YLB2
229533.A0A1C3YJ04 pp 229533.I1RF85
229533.A0A1C3YJ04 pp 229533.I1RKB2
229533.A0A1C3YJ04 pp 229533.I1RTH6
229533.A0A1C3YJ04 pp 229533.I1RYA0
229533.A0A1C3YJ04 pp 229533.I1S8R2
229533.A0A1C3YJ04 pp 229533.I1RMJ3
229533.A0A1C3YJ04 pp 229533.I1S2C4
229533.A0A1C3YJ04 pp 229533.I1S299
229533.A0A1C3YJ04 pp 229533.A0A1C3YJP4
229533.A0A1C3YJ04 pp 229533.I1S3G4
229533.A0A1C3YJ04 pp 229533.Q4HVQ9
229533.A0A1C3YJ04 pp 229533.I1RC34
229533.A0A1C3YJ04 pp 229533.I1RAX2
229533.A0A1C3YJ04 pp 229533.I1S3T7
229533.A0A1C3YJ04 pp 229533.V6RV57
229533.A0A1C3YJ04 pp 229533.I1RSQ4
229533.A0A1C3YJ04 pp 229533.I1RY32
229533.A0A1C3YJ04 pp 229533.I1REI3
229533.A0A1C3YJ04 pp 229533.I1RY92
229533.A0A1C3YJ04 pp 229533.I1RH91
229533.A0A1C3YJ04 pp 229533.I1RWF5
229533.A0A1C3YJ04 pp 229533.A0A1C3YLE2
229533.A0A1C3YJ04 pp 229533.I1RL81
229533.A0A1C3YJ04 pp 229533.I1RE53
229533.A0A1C3YJ04 pp 229533.A0A1C3YJI5
229533.A0A1C3YJ04 pp 229533.I1RUR8
229533.A0A1C3YJ04 pp 229533.I1RFI4
229533.A0A1C3YJ04 pp 229533.I1RA52
229533.A0A1C3YJ04 pp 229533.I1RGC3
229533.A0A1C3YJ04 pp 229533.I1RVR2
229533.I1RLF0 pp 229533.I1RYM2
229533.I1RLF0 pp 229533.I1RYF8
229533.I1RX44 pp 229533.I1RZT1
229533.I1RX44 pp 229533.I1RYE2
229533.I1RX44 pp 229533.I1S4Z7
229533.I1S0V7 pp 229533.V6RF62
229533.I1S0V7 pp 229533.I1S299
229533.I1S0V7 pp 229533.I1S8R2
229533.I1S0V7 pp 229533.V6RV57
229533.I1S0V7 pp 229533.I1S2C4
229533.I1S0V7 pp 229533.V6RE46
229533.I1S0V7 pp 229533.V6RRM0
229533.I1S0V7 pp 229533.Q4I5G1
229533.I1S0V7 pp 229533.V6RUB1
229533.I1S0V7 pp 229533.I1S3T7
229533.I1S0V7 pp 229533.I1S147
229533.I1S0V7 pp 229533.I1S6P5
229533.I1S0V7 pp 229533.V6R3V4
229533.I1S0V7 pp 229533.I1S2E9
229533.I1S0V7 pp 229533.Q4HVQ9
229533.I1S0V7 pp 229533.Q4HYR2

229533.I1S0V7 pp 229533.I1S3G4
229533.I1S0V7 pp 229533.I1S8C1
229533.I1S0V7 pp 229533.I1S1W8
229533.I1S0V7 pp 229533.I1S4S5
229533.I1S0V7 pp 229533.V6RZD4
229533.I1S0V7 pp 229533.I1S267
229533.I1S0V7 pp 229533.I1S8F8
229533.I1S0V7 pp 229533.V6QVV9
229533.I1RHW9 pp 229533.I1RWH4
229533.I1RHW9 pp 229533.I1RYL5
229533.I1RHW9 pp 229533.I1RZ95
229533.I1RHW9 pp 229533.I1RME4
229533.I1RHW9 pp 229533.I1RW71
229533.I1RCD9 pp 229533.I1S037
229533.I1RCD9 pp 229533.I1RD66
229533.I1RCD9 pp 229533.I1S1Z2
229533.A0A1C3YIZ6 pp 229533.A0A1C3YJ44
229533.A0A1C3YIZ6 pp 229533.I1RRL9
229533.A0A098DGN4 pp 229533.A0A098E0E1
229533.A0A098DGN4 pp 229533.A0A1C3YHL0
229533.A0A098DGN4 pp 229533.I1RL24
229533.A0A098DGN4 pp 229533.I1RYZ9
229533.A0A098DGN4 pp 229533.I1RFL7
229533.A0A098DGN4 pp 229533.V6QV09
229533.A0A098DGN4 pp 229533.I1R9Y7
229533.A0A098DGN4 pp 229533.V6RGI1
229533.I1RAN1 pp 229533.I1RHJ2
229533.I1RAN1 pp 229533.I1RJT5
229533.I1RAN1 pp 229533.I1RPM6
229533.I1RAN1 pp 229533.I1RWM4
229533.I1RAN1 pp 229533.I1RPU8
229533.I1RAN1 pp 229533.I1S147
229533.I1RAN1 pp 229533.I1RVP5
229533.I1RAN1 pp 229533.I1S4D0
229533.I1RAN1 pp 229533.V6RY56
229533.I1RAN1 pp 229533.I1RHG5
229533.I1RAN1 pp 229533.V6RLM7
229533.I1RAN1 pp 229533.I1RF85
229533.I1RAN1 pp 229533.I1S2C4
229533.I1RAN1 pp 229533.I1RP86
229533.I1RAN1 pp 229533.I1RR68
229533.I1RAN1 pp 229533.I1RCV1
229533.I1RAN1 pp 229533.I1RAQ4
229533.I1RAN1 pp 229533.I1RC11
229533.I1RAN1 pp 229533.I1RFI4
229533.I1RAN1 pp 229533.I1RCG5
229533.I1RAN1 pp 229533.I1RR12
229533.I1RAN1 pp 229533.I1S029
229533.I1RAN1 pp 229533.I1RW54
229533.I1RAN1 pp 229533.I1RYK0
229533.I1RAN1 pp 229533.I1RKB2
229533.I1RAN1 pp 229533.I1RN88
229533.I1RAN1 pp 229533.I1RNM7
229533.I1RAN1 pp 229533.I1RCU2
229533.I1RAN1 pp 229533.I1RX72
229533.I1RAN1 pp 229533.I1RWL3
229533.I1RA50 pp 229533.I1RSL8
229533.I1RA50 pp 229533.I1RMT5
229533.I1RA50 pp 229533.I1S074
229533.I1RA50 pp 229533.I1RK67
229533.A0A1C3YHG7 pp 229533.A0A1C3YKB7
229533.A0A1C3YHG7 pp 229533.I1S3G4
229533.A0A1C3YHG7 pp 229533.I1S6P6
229533.A0A1C3YHG7 pp 229533.I1RZV5
229533.A0A1C3YHG7 pp 229533.I1RFI4
229533.A0A1C3YHG7 pp 229533.I1RCU9
229533.A0A1C3YHG7 pp 229533.I1RR68
229533.A0A1C3YHG7 pp 229533.I1S3T7
229533.A0A1C3YHG7 pp 229533.A0A1C3YIE7
229533.A0A1C3YHG7 pp 229533.I1RA52
229533.A0A1C3YHG7 pp 229533.A0A1C3YLE2
229533.A0A1C3YHG7 pp 229533.I1SAH9
229533.A0A1C3YHG7 pp 229533.I1RK55

229533.A0A1C3YHG7	pp	229533.I1RQG7
229533.A0A1C3YHG7	pp	229533.I1RNG4
229533.A0A1C3YHG7	pp	229533.I1RGC3
229533.A0A1C3YHG7	pp	229533.A0A1C3YI20
229533.A0A1C3YHG7	pp	229533.I1S0A5
229533.A0A1C3YHG7	pp	229533.A0A1C3YN25
229533.A0A1C3YHG7	pp	229533.I1RH91
229533.A0A1C3YHG7	pp	229533.A0A1C3YJI5
229533.A0A1C3YHG7	pp	229533.V6R5D2
229533.A0A1C3YHG7	pp	229533.I1RY35
229533.A0A1C3YHG7	pp	229533.I1RN88
229533.A0A1C3YHG7	pp	229533.I1S6P5
229533.A0A1C3YHG7	pp	229533.I1RY62
229533.A0A1C3YHG7	pp	229533.I1RGZ3
229533.A0A1C3YHG7	pp	229533.I1RUR8
229533.A0A1C3YHG7	pp	229533.I1RL81
229533.A0A1C3YHG7	pp	229533.I1RF85
229533.A0A1C3YHG7	pp	229533.I1RKD3
229533.A0A1C3YHG7	pp	229533.I1RLZ4
229533.A0A1C3YHG7	pp	229533.I1RVR2
229533.A0A1C3YHG7	pp	229533.I1RZ25
229533.A0A1C3YHG7	pp	229533.A0A1C3YKW4
229533.A0A1C3YHG7	pp	229533.I1RQW1
229533.A0A1C3YHG7	pp	229533.V6RZD4
229533.A0A1C3YHG7	pp	229533.A0A1C3YHU3
229533.A0A1C3YHG7	pp	229533.A0A1C3YMD8
229533.A0A1C3YHG7	pp	229533.I1S0X1
229533.A0A1C3YHG7	pp	229533.I1RC34
229533.A0A1C3YHG7	pp	229533.I1RYW9
229533.A0A1C3YHG7	pp	229533.Q4HVQ9
229533.A0A1C3YHG7	pp	229533.A0A1C3YJ47
229533.A0A1C3YHG7	pp	229533.I1RX72
229533.A0A1C3YHG7	pp	229533.I1RZP8
229533.A0A1C3YHG7	pp	229533.V6RUB1
229533.A0A1C3YHG7	pp	229533.I1RKB2
229533.A0A1C3YHG7	pp	229533.V6R3J1
229533.A0A1C3YHG7	pp	229533.I1RMJ3
229533.A0A1C3YHG7	pp	229533.V6RF62
229533.A0A1C3YHG7	pp	229533.I1RZA7
229533.A0A1C3YHG7	pp	229533.I1RBY9
229533.A0A1C3YHG7	pp	229533.I1RBE6
229533.A0A1C3YHG7	pp	229533.V6RV57
229533.A0A1C3YHG7	pp	229533.I1RP12
229533.A0A1C3YHG7	pp	229533.I1RTY9
229533.A0A1C3YHG7	pp	229533.V6R2Z2
229533.A0A1C3YHG7	pp	229533.A0A1C3YLZ6
229533.A0A1C3YHG7	pp	229533.I1RYA0
229533.A0A1C3YHG7	pp	229533.I1S968
229533.A0A1C3YHG7	pp	229533.I1S299
229533.A0A1C3YHG7	pp	229533.A0A1C3YJP4
229533.A0A1C3YHG7	pp	229533.I1RWF5
229533.A0A1C3YHG7	pp	229533.A0A1C3YJ04
229533.A0A1C3YHG7	pp	229533.I1RUP2
229533.A0A1C3YHG7	pp	229533.I1S8R2
229533.A0A1C3YHG7	pp	229533.I1RAN1
229533.A0A1C3YHG7	pp	229533.V6RE46
229533.A0A1C3YHG7	pp	229533.I1RUE3
229533.A0A1C3YHG7	pp	229533.I1RF40
229533.A0A1C3YHG7	pp	229533.I1RY21
229533.A0A1C3YHG7	pp	229533.I1RJS1
229533.A0A1C3YHG7	pp	229533.I1RLA2
229533.A0A1C3YHG7	pp	229533.I1S6H3
229533.A0A1C3YHG7	pp	229533.I1RNM7
229533.A0A1C3YHG7	pp	229533.I1S0V7
229533.A0A1C3YHG7	pp	229533.I1S1W8
229533.A0A1C3YHG7	pp	229533.I1S256
229533.A0A1C3YHG7	pp	229533.I1RMW9
229533.A0A1C3YHG7	pp	229533.I1R9Y4
229533.A0A1C3YHG7	pp	229533.I1RVI8
229533.A0A1C3YHG7	pp	229533.I1RW54
229533.A0A1C3YHG7	pp	229533.I1RCU8
229533.A0A1C3YHG7	pp	229533.I1S147
229533.A0A1C3YHG7	pp	229533.I1RJS7

1680	229533.A0A1C3YHG7	pp	229533.I1RMA2
1681	229533.A0A1C3YHG7	pp	229533.I1S094
1682	229533.A0A1C3YHG7	pp	229533.V6RRM0
1683	229533.A0A1C3YHG7	pp	229533.I1RMQ6
1684	229533.A0A1C3YHG7	pp	229533.I1RGK3
1685	229533.A0A1C3YHG7	pp	229533.I1RAV0
1686	229533.A0A1C3YHG7	pp	229533.I1RMK7
1687	229533.A0A1C3YHG7	pp	229533.I1RHC8
1688	229533.A0A1C3YHG7	pp	229533.I1RDB1
1689	229533.A0A1C3YHG7	pp	229533.I1RVX9
1690	229533.A0A1C3YHG7	pp	229533.I1RKB7
1691	229533.A0A1C3YHG7	pp	229533.I1RN84
1692	229533.A0A1C3YHG7	pp	229533.V6R0W1
1693	229533.A0A1C3YHG7	pp	229533.I1RYC8
1694	229533.A0A1C3YHG7	pp	229533.I1R9X3
1695	229533.A0A1C3YHG7	pp	229533.I1RDN3
1696	229533.A0A1C3YHG7	pp	229533.I1RJC5
1697	229533.A0A1C3YHG7	pp	229533.V6QU99
1698	229533.A0A1C3YHG7	pp	229533.I1RPV8
1699	229533.A0A1C3YHG7	pp	229533.V6QVV9
1700	229533.A0A1C3YHG7	pp	229533.I1RYP5
1701	229533.A0A1C3YHG7	pp	229533.I1RZ56
1702	229533.A0A1C3YHG7	pp	229533.A0A1C3YIB0
1703	229533.A0A1C3YHG7	pp	229533.A0A1C3YIB4
1704	229533.A0A1C3YHG7	pp	229533.I1RYX6
1705	229533.A0A1C3YHG7	pp	229533.I1RM26
1706	229533.A0A1C3YHG7	pp	229533.I1RJY7
1707	229533.A0A1C3YHG7	pp	229533.I1RIF7
1708	229533.A0A1C3YHG7	pp	229533.I1RCU2
1709	229533.A0A1C3YHG7	pp	229533.I1S4S5
1710	229533.A0A1C3YHG7	pp	229533.I1S0K1
1711	229533.A0A1C3YHG7	pp	229533.V6R9Z3
1712	229533.A0A1C3YHG7	pp	229533.I1RCR6
1713	229533.A0A1C3YHG7	pp	229533.A0A1C3YM86
1714	229533.A0A1C3YHG7	pp	229533.I1S969
1715	229533.A0A1C3YHG7	pp	229533.V6RAM6
1716	229533.A0A1C3YHG7	pp	229533.I1RY81
1717	229533.A0A1C3YHG7	pp	229533.A0A1C3YKZ8
1718	229533.A0A1C3YHG7	pp	229533.I1RBE7
1719	229533.A0A1C3YHG7	pp	229533.I1RRX5
1720	229533.A0A1C3YHG7	pp	229533.A0A1C3YIJ7
1721	229533.A0A1C3YHG7	pp	229533.I1S8C1
1722	229533.A0A1C3YHG7	pp	229533.I1RPP5
1723	229533.A0A1C3YHG7	pp	229533.I1RR12
1724	229533.A0A1C3YHG7	pp	229533.I1RY12
1725	229533.A0A1C3YHG7	pp	229533.I1RJB9
1726	229533.A0A1C3YHG7	pp	229533.I1S0K2
1727	229533.A0A1C3YHG7	pp	229533.A0A1C3YLC2
1728	229533.A0A1C3YHG7	pp	229533.I1RG33
1729	229533.A0A1C3YHG7	pp	229533.I1S423
1730	229533.A0A1C3YHG7	pp	229533.A0A1C3YKY6
1731	229533.A0A1C3YHG7	pp	229533.I1RYA2
1732	229533.A0A1C3YHG7	pp	229533.V6RDP1
1733	229533.A0A1C3YHG7	pp	229533.Q4HYR2
1734	229533.A0A1C3YHG7	pp	229533.I1RWM4
1735	229533.A0A1C3YHG7	pp	229533.I1RXE6
1736	229533.A0A1C3YHG7	pp	229533.A0A1C3YKN5
1737	229533.A0A1C3YHG7	pp	229533.I1RAM9
1738	229533.A0A1C3YHG7	pp	229533.I1S0V6
1739	229533.A0A1C3YHG7	pp	229533.I1RQJ7
1740	229533.A0A1C3YHG7	pp	229533.I1RXV9
1741	229533.A0A1C3YHG7	pp	229533.A0A1C3YLB3
1742	229533.A0A1C3YHG7	pp	229533.A0A1C3YLB0
1743	229533.A0A1C3YHG7	pp	229533.A0A1C3YJW1
1744	229533.A0A1C3YHG7	pp	229533.A0A1C3YIJ9
1745	229533.A0A1C3YHG7	pp	229533.A0A1C3YLE3
1746	229533.A0A1C3YHG7	pp	229533.I1RNG5
1747	229533.A0A1C3YHG7	pp	229533.I1RNY6
1748	229533.A0A1C3YHG7	pp	229533.I1RCV1
1749	229533.A0A1C3YHG7	pp	229533.I1RA13
1750	229533.A0A1C3YHG7	pp	229533.V6R3V4
1751	229533.A0A1C3YHG7	pp	229533.A0A1C3YK58
1752	229533.A0A1C3YHG7	pp	229533.I1RH67

229533.A0A1C3YHG7 pp 229533.I1RUI2
229533.A0A1C3YHG7 pp 229533.I1RBE4
229533.A0A1C3YHG7 pp 229533.I1S3B2
229533.A0A1C3YHG7 pp 229533.A0A1C3YIP2
229533.A0A1C3YHG7 pp 229533.I1R9M7
229533.A0A1C3YHG7 pp 229533.I1RUQ3
229533.A0A1C3YHG7 pp 229533.I1RQA5
229533.A0A1C3YHG7 pp 229533.I1S489
229533.A0A1C3YHG7 pp 229533.I1RST9
229533.A0A1C3YHG7 pp 229533.I1REE2
229533.A0A1C3YHG7 pp 229533.I1RPR1
229533.A0A1C3YHG7 pp 229533.I1S8F8
229533.A0A1C3YHG7 pp 229533.I1RH82
229533.A0A1C3YHG7 pp 229533.A0A1C3YM54
229533.A0A1C3YHG7 pp 229533.I1RMD3
229533.A0A1C3YHG7 pp 229533.I1RMQ7
229533.A0A1C3YHG7 pp 229533.I1RJT5
229533.A0A1C3YHG7 pp 229533.A0A1C3YKR5
229533.A0A1C3YHG7 pp 229533.I1S267
229533.I1RR46 pp 229533.I1RYL5
229533.A0A1C3YKW4 pp 229533.I1S4D0
229533.A0A1C3YKW4 pp 229533.I1RPR1
229533.A0A1C3YKW4 pp 229533.I1RA13
229533.A0A1C3YKW4 pp 229533.I1S029
229533.A0A1C3YKW4 pp 229533.I1RB07
229533.A0A1C3YKW4 pp 229533.I1RXV9
229533.A0A1C3YKW4 pp 229533.A0A1C3YLC2
229533.A0A1C3YKW4 pp 229533.I1RDN3
229533.A0A1C3YKW4 pp 229533.I1RAQ4
229533.A0A1C3YKW4 pp 229533.A0A1C3YLR6
229533.A0A1C3YKW4 pp 229533.I1RJC5
229533.A0A1C3YKW4 pp 229533.I1RC11
229533.A0A1C3YKW4 pp 229533.I1S0V6
229533.A0A1C3YKW4 pp 229533.I1RCM5
229533.A0A1C3YKW4 pp 229533.A0A1C3YM86
229533.A0A1C3YKW4 pp 229533.I1RNM7
229533.A0A1C3YKW4 pp 229533.I1RJY7
229533.A0A1C3YKW4 pp 229533.I1RW54
229533.A0A1C3YKW4 pp 229533.I1RWA4
229533.A0A1C3YKW4 pp 229533.V6RH92
229533.A0A1C3YKW4 pp 229533.I1R9M7
229533.A0A1C3YKW4 pp 229533.V6RY56
229533.A0A1C3YKW4 pp 229533.V6R3V4
229533.A0A1C3YKW4 pp 229533.I1RCV1
229533.A0A1C3YKW4 pp 229533.Q4HYR2
229533.A0A1C3YKW4 pp 229533.I1RXH3
229533.A0A1C3YKW4 pp 229533.I1RSF5
229533.A0A1C3YKW4 pp 229533.I1RWL3
229533.A0A1C3YKW4 pp 229533.I1RUQ3
229533.A0A1C3YKW4 pp 229533.I1RA90
229533.A0A1C3YKW4 pp 229533.A0A1C3YN26
229533.A0A1C3YKW4 pp 229533.I1S8C1
229533.A0A1C3YKW4 pp 229533.I1RJT5
229533.A0A1C3YKW4 pp 229533.A0A1C3YL54
229533.A0A1C3YKW4 pp 229533.I1RDU5
229533.A0A1C3YKW4 pp 229533.I1RP86
229533.A0A1C3YKW4 pp 229533.I1RH82
229533.A0A1C3YKW4 pp 229533.A0A1C3YKZ8
229533.A0A1C3YKW4 pp 229533.I1RPM6
229533.A0A1C3YKW4 pp 229533.I1RQE1
229533.A0A1C3YKW4 pp 229533.I1RYX6
229533.A0A1C3YKW4 pp 229533.I1RSL2
229533.A0A1C3YKW4 pp 229533.V6RF62
229533.A0A1C3YKW4 pp 229533.I1RWM4
229533.A0A1C3YKW4 pp 229533.I1RR12
229533.A0A1C3YKW4 pp 229533.I1S4S5
229533.A0A1C3YKW4 pp 229533.I1RHG5
229533.A0A1C3YKW4 pp 229533.I1RZ70
229533.A0A1C3YKW4 pp 229533.I1RVP5
229533.A0A1C3YKW4 pp 229533.I1RXB1
229533.A0A1C3YKW4 pp 229533.I1RCU2
229533.A0A1C3YKW4 pp 229533.A0A1C3YN25
229533.A0A1C3YKW4 pp 229533.I1RNY6

229533.A0A1C3YKW4	pp	229533.I1S8D1
229533.A0A1C3YKW4	pp	229533.I1RF85
229533.A0A1C3YKW4	pp	229533.I1RYW9
229533.A0A1C3YKW4	pp	229533.I1RCG5
229533.A0A1C3YKW4	pp	229533.I1S2C4
229533.A0A1C3YKW4	pp	229533.I1RHJ2
229533.A0A1C3YKW4	pp	229533.V6RZD4
229533.A0A1C3YKW4	pp	229533.I1RH92
229533.A0A1C3YKW4	pp	229533.V6RLM7
229533.A0A1C3YKW4	pp	229533.I1S8D2
229533.A0A1C3YKW4	pp	229533.I1RKB2
229533.A0A1C3YKW4	pp	229533.I1S147
229533.A0A1C3YKW4	pp	229533.I1S0V7
229533.A0A1C3YKW4	pp	229533.I1RX72
229533.A0A1C3YKW4	pp	229533.I1RPZ5
229533.A0A1C3YKW4	pp	229533.A0A1C3YL82
229533.A0A1C3YKW4	pp	229533.I1RA62
229533.A0A1C3YKW4	pp	229533.V6RRM0
229533.A0A1C3YKW4	pp	229533.I1RPU8
229533.A0A1C3YKW4	pp	229533.I1RYK0
229533.A0A1C3YKW4	pp	229533.I1RUP6
229533.A0A1C3YKW4	pp	229533.I1RGC3
229533.A0A1C3YKW4	pp	229533.I1RZA7
229533.A0A1C3YKW4	pp	229533.I1RFI4
229533.A0A1C3YKW4	pp	229533.I1RN88
229533.A0A1C3YKW4	pp	229533.I1RBY9
229533.A0A1C3YKW4	pp	229533.I1S148
229533.A0A1C3YKW4	pp	229533.I1RWN7
229533.A0A1C3YKW4	pp	229533.I1S6P5
229533.A0A1C3YKW4	pp	229533.V6RUB1
229533.A0A1C3YKW4	pp	229533.I1RR68
229533.A0A1C3YKW4	pp	229533.V6RV57
229533.A0A1C3YLT5	pp	229533.I1RM26
229533.A0A1C3YLT5	pp	229533.I1RVX9
229533.A0A1C3YLT5	pp	229533.I1RSI4
229533.I1S247	pp	229533.I1S270
229533.I1S247	pp	229533.Q4I2D7
229533.I1S247	pp	229533.I1S2K7
229533.I1S247	pp	229533.Q4I5R9
229533.I1S247	pp	229533.Q4IPB8
229533.I1S247	pp	229533.Q4IPB3
229533.I1S247	pp	229533.Q4INZ9
229533.I1S247	pp	229533.Q4IBK5
229533.I1S247	pp	229533.Q4IPH4
229533.I1S247	pp	229533.I1S282
229533.I1S247	pp	229533.Q4HZB8
229533.I1S247	pp	229533.Q4HXF6
229533.I1S247	pp	229533.Q4I1Y1
229533.I1S247	pp	229533.V6QUV7
229533.I1S247	pp	229533.Q4ICA8
229533.I1S247	pp	229533.Q4IN00
229533.I1S247	pp	229533.Q4IE79
229533.I1RF85	pp	229533.I1S1W8
229533.I1RF85	pp	229533.I1RMA2
229533.I1RF85	pp	229533.I1RGK3
229533.I1RF85	pp	229533.I1RMK7
229533.I1RF85	pp	229533.I1RLA2
229533.I1RF85	pp	229533.I1RJS7
229533.I1RF85	pp	229533.I1RY21
229533.I1RF85	pp	229533.I1RJC5
229533.I1RF85	pp	229533.I1RNM7
229533.I1RF85	pp	229533.I1S969
229533.I1RF85	pp	229533.I1RYP5
229533.I1RF85	pp	229533.I1RKB7
229533.I1RF85	pp	229533.I1RIF7
229533.I1RF85	pp	229533.I1RW54
229533.I1RF85	pp	229533.I1RRX5
229533.I1RF85	pp	229533.I1RMQ6
229533.I1RF85	pp	229533.I1S4S5
229533.I1RF85	pp	229533.V6QVV9
229533.I1RF85	pp	229533.V6R0W1
229533.I1RF85	pp	229533.I1RR12
229533.I1RF85	pp	229533.I1S8C1

229533.I1RF85 pp 229533.I1RVI8
229533.I1RF85 pp 229533.I1RYX6
229533.I1RF85 pp 229533.V6QU99
229533.I1RF85 pp 229533.I1RMW9
229533.I1RF85 pp 229533.I1S0K1
229533.I1RF85 pp 229533.I1S094
229533.I1RF85 pp 229533.I1RVX9
229533.I1RF85 pp 229533.I1RNG5
229533.I1RF85 pp 229533.V6RAM6
229533.I1RF85 pp 229533.I1S6H3
229533.I1RF85 pp 229533.I1RMD3
229533.I1RF85 pp 229533.I1RXV9
229533.I1RF85 pp 229533.I1RUQ3
229533.I1RF85 pp 229533.I1S8F8
229533.I1RF85 pp 229533.I1RQA5
229533.I1RF85 pp 229533.I1RUI2
229533.I1RF85 pp 229533.I1S3B2
229533.I1RF85 pp 229533.V6R3V4
229533.I1RF85 pp 229533.I1RPR1
229533.I1RF85 pp 229533.I1RPP5
229533.I1RF85 pp 229533.I1RQJ7
229533.I1RF85 pp 229533.I1S489
229533.I1RF85 pp 229533.I1S0V6
229533.I1RF85 pp 229533.I1RYA2
229533.I1RF85 pp 229533.I1RNY6
229533.I1RF85 pp 229533.Q4HYR2
229533.I1RF85 pp 229533.I1RH67
229533.I1RF85 pp 229533.I1S0K2
229533.I1RF85 pp 229533.I1RY12
229533.I1RF85 pp 229533.I1RJB9
229533.I1RF85 pp 229533.I1RG33
229533.I1RF85 pp 229533.V6R9Z3
229533.I1RF85 pp 229533.I1S423
229533.I1RF85 pp 229533.V6RDP1
229533.I1RF85 pp 229533.I1RM26
229533.I1RF85 pp 229533.I1S267
229533.I1RF85 pp 229533.I1RJY7
229533.I1RF85 pp 229533.I1RMQ7
229533.I1RF85 pp 229533.I1RWM4
229533.I1RF85 pp 229533.I1RJT5
229533.I1RF85 pp 229533.I1RY81
229533.I1RF85 pp 229533.I1RXE6
229533.I1RF85 pp 229533.I1RST9
229533.I1RF85 pp 229533.I1S3T7
229533.I1RF85 pp 229533.I1RGZ3
229533.I1RF85 pp 229533.I1RVR2
229533.I1RF85 pp 229533.I1RH82
229533.I1RF85 pp 229533.I1RY35
229533.I1RF85 pp 229533.V6R2Z2
229533.I1RF85 pp 229533.I1S299
229533.I1RF85 pp 229533.I1RN88
229533.I1RF85 pp 229533.I1RKD3
229533.I1RF85 pp 229533.I1RZA7
229533.I1RF85 pp 229533.I1S6P5
229533.I1RF85 pp 229533.I1RFI4
229533.I1RF85 pp 229533.I1RWF5
229533.I1RF85 pp 229533.I1RX72
229533.I1RF85 pp 229533.I1RGC3
229533.I1RF85 pp 229533.I1RKB2
229533.I1RF85 pp 229533.I1RQG7
229533.I1RF85 pp 229533.I1S968
229533.I1RF85 pp 229533.I1RK55
229533.I1RF85 pp 229533.I1RMJ3
229533.I1RF85 pp 229533.V6RV57
229533.I1RF85 pp 229533.I1RLZ4
229533.I1RF85 pp 229533.I1RUR8
229533.I1RF85 pp 229533.V6R5D2
229533.I1RF85 pp 229533.I1RL81
229533.I1RF85 pp 229533.Q4HVQ9
229533.I1RF85 pp 229533.I1RYW9
229533.I1RF85 pp 229533.V6RZD4
229533.I1RF85 pp 229533.I1RZ25
229533.I1RF85 pp 229533.I1RH91

1972 229533.I1RF85 pp 229533.I1S6P6
1973 229533.I1RF85 pp 229533.V6R3J1
1974 229533.I1RF85 pp 229533.I1RNG4
1975 229533.I1RF85 pp 229533.I1RY62
1976 229533.I1RF85 pp 229533.I1S3G4
1977 229533.I1RF85 pp 229533.V6RUB1
1978 229533.I1RF85 pp 229533.I1S0A5
1979 229533.I1RF85 pp 229533.V6RE46
1980 229533.I1RF85 pp 229533.I1SAH9
1981 229533.I1RF85 pp 229533.I1RYA0
1982 229533.I1RF85 pp 229533.I1S0X1
1983 229533.I1RF85 pp 229533.I1RZV5
1984 229533.I1RF85 pp 229533.I1RR68
1985 229533.I1RF85 pp 229533.I1RZP8
1986 229533.I1RF85 pp 229533.I1RQW1
1987 229533.I1RF85 pp 229533.I1RJS1
1988 229533.I1RF85 pp 229533.I1S8R2
1989 229533.I1RF85 pp 229533.I1RP12
1990 229533.I1RF85 pp 229533.I1RTY9
1991 229533.I1RF85 pp 229533.I1RN84
1992 229533.I1RF85 pp 229533.V6RF62
1993 229533.I1RF85 pp 229533.I1RYC8
1994 229533.I1RF85 pp 229533.I1RUE3
1995 229533.I1RF85 pp 229533.V6RRM0
1996 229533.I1RF85 pp 229533.I1RHC8
1997 229533.I1RF85 pp 229533.I1S147
1998 229533.I1RF85 pp 229533.I1RUP2
1999 229533.I1RF85 pp 229533.I1S0V7
2000 229533.I1RF85 pp 229533.I1RZ56
2001 229533.I1RF85 pp 229533.I1S256
2002 229533.I1RF85 pp 229533.I1RPV8
2003 229533.A0A1C3YN27 pp 229533.I1RAI9
2004 229533.A0A1C3YN27 pp 229533.Q4HXF6
2005 229533.A0A1C3YN27 pp 229533.I1RRN5
2006 229533.A0A1C3YN27 pp 229533.I1S247
2007 229533.A0A1C3YN27 pp 229533.I1RPW0
2008 229533.A0A1C3YN27 pp 229533.I1RD39
2009 229533.A0A1C3YN27 pp 229533.I1RWF4
2010 229533.A0A1C3YN27 pp 229533.I1REC7
2011 229533.A0A1C3YN27 pp 229533.I1RZV5
2012 229533.A0A1C3YN27 pp 229533.I1RBV5
2013 229533.A0A1C3YN27 pp 229533.I1RX38
2014 229533.A0A1C3YN27 pp 229533.I1RQ88
2015 229533.A0A1C3YN27 pp 229533.I1RWH4
2016 229533.A0A1C3YN27 pp 229533.I1RBS5
2017 229533.A0A1C3YN27 pp 229533.I1RZP1
2018 229533.A0A1C3YN27 pp 229533.I1RLZ4
2019 229533.A0A1C3YN27 pp 229533.I1RW76
2020 229533.A0A1C3YN27 pp 229533.I1RP20
2021 229533.A0A1C3YN27 pp 229533.I1REC6
2022 229533.A0A1C3YN27 pp 229533.I1RYS6
2023 229533.A0A1C3YN27 pp 229533.I1RT28
2024 229533.A0A1C3YN27 pp 229533.I1RX87
2025 229533.A0A1C3YN27 pp 229533.Q4ICA8
2026 229533.A0A1C3YN27 pp 229533.I1RBC4
2027 229533.A0A1C3YN27 pp 229533.I1RQ12
2028 229533.A0A1C3YN27 pp 229533.I1RPU4
2029 229533.A0A1C3YN27 pp 229533.I1RA35
2030 229533.A0A1C3YN27 pp 229533.I1RZS5
2031 229533.A0A1C3YN27 pp 229533.A0A1C3YN77
2032 229533.A0A1C3YLT2 pp 229533.Q4I5Z5
2033 229533.I1RC29 pp 229533.I1RP86
2034 229533.I1RC29 pp 229533.I1RRG3
2035 229533.I1RC29 pp 229533.I1S338
2036 229533.I1RC29 pp 229533.V6RUT5
2037 229533.I1RC29 pp 229533.I1RQH2
2038 229533.I1RC29 pp 229533.I1RX46
2039 229533.I1RC29 pp 229533.I1RZ99
2040 229533.I1RC29 pp 229533.I1RYL8
2041 229533.I1RC29 pp 229533.V6RTJ8
2042 229533.I1RC29 pp 229533.I1RXD7
2043 229533.I1RC29 pp 229533.I1RSQ7
2044 229533.I1RC29 pp 229533.I1RPI5

2045 229533.I1RC29 pp 229533.I1RUQ2
2046 229533.I1RC29 pp 229533.I1RRX5
2047 229533.I1RC29 pp 229533.I1RNH1
2048 229533.I1RC29 pp 229533.I1REE2
2049 229533.I1RC29 pp 229533.I1RDA0
2050 229533.I1RC29 pp 229533.Q4HVW2
2051 229533.I1RC29 pp 229533.I1S4S4
2052 229533.I1RC29 pp 229533.I1RPU0
2053 229533.I1RC29 pp 229533.V6QUE6
2054 229533.I1RC29 pp 229533.I1RC95
2055 229533.I1RC29 pp 229533.I1RH82
2056 229533.I1RC29 pp 229533.I1RZ16
2057 229533.I1RC29 pp 229533.I1RMK3
2058 229533.I1RC29 pp 229533.I1RR24
2059 229533.I1RC29 pp 229533.I1RPW9
2060 229533.I1RC29 pp 229533.I1RWM4
2061 229533.I1RC29 pp 229533.Q4IBS2
2062 229533.I1RC29 pp 229533.Q4IFI0
2063 229533.I1RC29 pp 229533.I1RL23
2064 229533.I1RC29 pp 229533.I1RKI3
2065 229533.I1RC29 pp 229533.Q4IR09
2066 229533.I1RC29 pp 229533.I1RG51
2067 229533.I1RC29 pp 229533.I1S5B0
2068 229533.I1RC29 pp 229533.I1S9U2
2069 229533.I1RC29 pp 229533.I1RUY5
2070 229533.I1RC29 pp 229533.I1RXF0
2071 229533.I1RC29 pp 229533.I1RT05
2072 229533.I1RC29 pp 229533.I1RNH6
2073 229533.I1RC29 pp 229533.I1RTF4
2074 229533.I1RC29 pp 229533.I1RZM8
2075 229533.I1RC29 pp 229533.I1S1W8
2076 229533.I1RC29 pp 229533.Q4IJH1
2077 229533.I1RC29 pp 229533.I1RNA0
2078 229533.I1RC29 pp 229533.I1RMN7
2079 229533.I1RC29 pp 229533.V6QYF0
2080 229533.I1RC29 pp 229533.I1RJN7
2081 229533.I1RC29 pp 229533.I1RPH9
2082 229533.I1RC29 pp 229533.V6RCZ8
2083 229533.I1RC29 pp 229533.I1RMK7
2084 229533.I1RC29 pp 229533.I1RM06
2085 229533.I1RC29 pp 229533.I1RG56
2086 229533.I1RC29 pp 229533.I1RZF6
2087 229533.I1RC29 pp 229533.I1RJU3
2088 229533.I1RC29 pp 229533.I1RNH9
2089 229533.I1RC29 pp 229533.I1RRY9
2090 229533.I1RC29 pp 229533.I1S8X1
2091 229533.I1RC29 pp 229533.I1RX14
2092 229533.I1RC29 pp 229533.I1RCR5
2093 229533.I1RC29 pp 229533.I1REK8
2094 229533.I1RC29 pp 229533.I1RSR8
2095 229533.I1RC29 pp 229533.I1RKE1
2096 229533.I1RC29 pp 229533.I1RS18
2097 229533.I1RC29 pp 229533.Q4IJ56
2098 229533.I1RC29 pp 229533.I1S439
2099 229533.I1RC29 pp 229533.I1RV64
2100 229533.I1RC29 pp 229533.I1RCV1
2101 229533.I1RC29 pp 229533.I1RNK2
2102 229533.I1RC29 pp 229533.I1S2U2
2103 229533.I1RC29 pp 229533.I1RUY4
2104 229533.I1RC29 pp 229533.I1RVZ0
2105 229533.I1RC29 pp 229533.I1RQA2
2106 229533.I1RC29 pp 229533.I1RGB2
2107 229533.I1RC29 pp 229533.I1RFN1
2108 229533.I1RC29 pp 229533.Q4IJK2
2109 229533.I1RC29 pp 229533.I1RZE5
2110 229533.I1RC29 pp 229533.I1RC82
2111 229533.I1RC29 pp 229533.I1RGA4
2112 229533.I1RC29 pp 229533.I1RSC6
2113 229533.I1RC29 pp 229533.I1RFJ9
2114 229533.I1RC29 pp 229533.Q4IQW0
2115 229533.I1RC29 pp 229533.Q4HZ42
2116 229533.I1RC29 pp 229533.I1RDU0
2117 229533.I1RC29 pp 229533.I1RQ65

2118 229533.I1RC29 pp 229533.I1RN99
2119 229533.I1RC29 pp 229533.I1RJV4
2120 229533.I1RC29 pp 229533.V6RV57
2121 229533.I1RC29 pp 229533.I1RQ28
2122 229533.I1RC29 pp 229533.I1RK95
2123 229533.I1RC29 pp 229533.I1RDG0
2124 229533.I1RC29 pp 229533.I1RX72
2125 229533.I1RC29 pp 229533.I1RYK0
2126 229533.I1RC29 pp 229533.I1RFT1
2127 229533.I1RC29 pp 229533.I1RLV9
2128 229533.I1RC29 pp 229533.I1RKI7
2129 229533.I1RC29 pp 229533.I1RTD5
2130 229533.I1RC29 pp 229533.I1S8P5
2131 229533.I1RC29 pp 229533.Q4IAS1
2132 229533.I1RC29 pp 229533.V6RUY9
2133 229533.I1RC29 pp 229533.I1RPP3
2134 229533.I1RC29 pp 229533.I1RJL4
2135 229533.I1RC29 pp 229533.I1RRQ3
2136 229533.I1RC29 pp 229533.I1RS30
2137 229533.I1RC29 pp 229533.I1RN67
2138 229533.I1RC29 pp 229533.I1RRT8
2139 229533.I1RC29 pp 229533.V6R3J1
2140 229533.I1RC29 pp 229533.I1RRJ8
2141 229533.I1RC29 pp 229533.I1S5R1
2142 229533.I1RC29 pp 229533.I1RN88
2143 229533.I1RC29 pp 229533.Q4I662
2144 229533.I1RC29 pp 229533.O74638
2145 229533.I1RC29 pp 229533.Q4I327
2146 229533.I1RC29 pp 229533.I1RFS3
2147 229533.I1RC29 pp 229533.I1RGF6
2148 229533.I1RC29 pp 229533.I1RSI6
2149 229533.I1RC29 pp 229533.I1RRT3
2150 229533.I1RC29 pp 229533.I1RN84
2151 229533.I1RC29 pp 229533.I1RZS6
2152 229533.I1RC29 pp 229533.V6R042
2153 229533.I1RC29 pp 229533.I1S3G4
2154 229533.I1RC29 pp 229533.I1RSQ3
2155 229533.I1RC29 pp 229533.I1RMN6
2156 229533.I1RC29 pp 229533.I1S2H4
2157 229533.I1RC29 pp 229533.I1S3T7
2158 229533.I1RC29 pp 229533.V6RMW6
2159 229533.I1RC29 pp 229533.I1RSQ4
2160 229533.I1RC29 pp 229533.I1S147
2161 229533.I1RC29 pp 229533.I1RD22
2162 229533.I1RC29 pp 229533.I1RTG6
2163 229533.I1RC29 pp 229533.V6RVG3
2164 229533.I1RC29 pp 229533.I1S1W3
2165 229533.I1RC29 pp 229533.I1RZ39
2166 229533.I1RC29 pp 229533.I1RZZ9
2167 229533.I1RC29 pp 229533.I1RGX1
2168 229533.I1RC29 pp 229533.V6RRM0
2169 229533.I1RC29 pp 229533.I1S2B8
2170 229533.I1RC29 pp 229533.V6RGN5
2171 229533.I1RC29 pp 229533.I1RE01
2172 229533.I1RC29 pp 229533.I1RRR7
2173 229533.I1RC29 pp 229533.I1RTA0
2174 229533.I1RC29 pp 229533.I1RG11
2175 229533.I1RC29 pp 229533.I1RCL3
2176 229533.I1RC29 pp 229533.I1RFI4
2177 229533.I1RC29 pp 229533.I1RNC1
2178 229533.I1RC29 pp 229533.I1RC72
2179 229533.I1RC29 pp 229533.I1S0J1
2180 229533.I1RC29 pp 229533.I1RJV3
2181 229533.I1RC29 pp 229533.I1RPK0
2182 229533.I1RC29 pp 229533.I1RUG3
2183 229533.I1RC29 pp 229533.I1RK79
2184 229533.I1RC29 pp 229533.I1S274
2185 229533.I1RC29 pp 229533.I1RCR8
2186 229533.I1RC29 pp 229533.V6RLM7
2187 229533.I1RC29 pp 229533.I1RNU6
2188 229533.I1RC29 pp 229533.I1S1C7
2189 229533.I1RC29 pp 229533.I1RWX5
2190 229533.I1RC29 pp 229533.I1RYW3

2191 229533.I1RC29 pp 229533.I1RRI2
2192 229533.I1RC29 pp 229533.I1RTE9
2193 229533.I1RC29 pp 229533.I1RKC6
2194 229533.I1RC29 pp 229533.Q4HZK7
2195 229533.I1RC29 pp 229533.Q4HZ68
2196 229533.I1RC29 pp 229533.I1RTH6
2197 229533.I1RC29 pp 229533.I1RCH5
2198 229533.I1RC29 pp 229533.I1RFL4
2199 229533.I1RC29 pp 229533.I1RVX0
2200 229533.I1RC29 pp 229533.I1S2C4
2201 229533.I1RC29 pp 229533.I1S2E8
2202 229533.I1RC29 pp 229533.I1RT03
2203 229533.I1RMA7 pp 229533.I1RNF4
2204 229533.I1RMA7 pp 229533.I1RNG8
2205 229533.I1RMA7 pp 229533.I1S2B6
2206 229533.I1RMA7 pp 229533.I1S074
2207 229533.I1RMA7 pp 229533.I1RR95
2208 229533.I1RMA7 pp 229533.I1S248
2209 229533.I1RMA7 pp 229533.I1RPH1
2210 229533.I1RMA7 pp 229533.I1RSI3
2211 229533.I1RMA7 pp 229533.I1RW84
2212 229533.I1RMA7 pp 229533.I1RMR7
2213 229533.I1RMA7 pp 229533.I1RP75
2214 229533.I1RMA7 pp 229533.Q4IBS9
2215 229533.I1RMA7 pp 229533.I1S2K7
2216 229533.I1RMA7 pp 229533.I1RZW1
2217 229533.I1RMA7 pp 229533.I1RYB7
2218 229533.I1RMA7 pp 229533.I1RNR2
2219 229533.I1RMA7 pp 229533.I1S9Y1
2220 229533.I1RMA7 pp 229533.Q4HZS8
2221 229533.I1RMA7 pp 229533.I1RNR0
2222 229533.I1RMA7 pp 229533.I1S1A3
2223 229533.I1RMA7 pp 229533.I1RT91
2224 229533.I1RMA7 pp 229533.I1RZK4
2225 229533.I1RMA7 pp 229533.I1RQ72
2226 229533.I1RMA7 pp 229533.I1RPW4
2227 229533.I1RMA7 pp 229533.I1RXH6
2228 229533.I1RMA7 pp 229533.I1S296
2229 229533.I1RZ44 pp 229533.I1RZ94
2230 229533.A0A1C3YI27 pp 229533.I1RZM4
2231 229533.A0A1C3YI27 pp 229533.I1RJL9
2232 229533.A0A1C3YI27 pp 229533.I1RNJ7
2233 229533.A0A1C3YI27 pp 229533.I1RH82
2234 229533.A0A1C3YI27 pp 229533.I1RP86
2235 229533.A0A1C3YI27 pp 229533.I1RKR5
2236 229533.A0A1C3YI27 pp 229533.I1RUY5
2237 229533.A0A1C3YI27 pp 229533.I1RGB2
2238 229533.A0A1C3YI27 pp 229533.I1RGY2
2239 229533.A0A1C3YI27 pp 229533.I1RDU0
2240 229533.A0A1C3YI27 pp 229533.A0A1C3YN26
2241 229533.A0A1C3YI27 pp 229533.I1RKE1
2242 229533.A0A1C3YI27 pp 229533.I1RMM8
2243 229533.A0A1C3YI27 pp 229533.I1RCM5
2244 229533.A0A1C3YI27 pp 229533.A0A1C3YM97
2245 229533.A0A1C3YI27 pp 229533.I1RQE8
2246 229533.A0A1C3YI27 pp 229533.I1RX72
2247 229533.A0A1C3YI27 pp 229533.I1RYK0
2248 229533.A0A1C3YI27 pp 229533.Q4IR09
2249 229533.A0A1C3YI27 pp 229533.A0A1C3YKN4
2250 229533.A0A1C3YI27 pp 229533.V6RV57
2251 229533.A0A1C3YI27 pp 229533.V6RLM7
2252 229533.A0A1C3YI27 pp 229533.V6RK96
2253 229533.A0A1C3YI27 pp 229533.I1RZQ1
2254 229533.A0A1C3YI27 pp 229533.I1RC29
2255 229533.A0A1C3YI27 pp 229533.I1RQ75
2256 229533.A0A1C3YI27 pp 229533.I1RMX6
2257 229533.A0A098E104 pp 229533.I1RSI4
2258 229533.Q4IPB8 pp 229533.V6QXG1
2259 229533.Q4IPB8 pp 229533.Q4IRI9
2260 229533.I1RP61 pp 229533.I1RX38
2261 229533.I1RP61 pp 229533.I1RPW0
2262 229533.I1RP61 pp 229533.I1RQ88
2263 229533.I1RP61 pp 229533.I1RPU4

2264	229533.I1RA62	pp	229533.I1RPK0
2265	229533.I1RA62	pp	229533.V6RLM7
2266	229533.I1RA62	pp	229533.I1S444
2267	229533.I1RA62	pp	229533.I1RQI4
2268	229533.I1RA62	pp	229533.I1S272
2269	229533.I1RA62	pp	229533.I1RD80
2270	229533.I1RA62	pp	229533.I1RND4
2271	229533.I1RA62	pp	229533.V6R729
2272	229533.I1RA62	pp	229533.I1RZM3
2273	229533.I1RA62	pp	229533.I1RAN1
2274	229533.I1RA62	pp	229533.I1S0V7
2275	229533.I1RA62	pp	229533.Q4I1Y5
2276	229533.I1RA62	pp	229533.I1RNU7
2277	229533.I1RA62	pp	229533.I1S256
2278	229533.I1RA62	pp	229533.I1RCG5
2279	229533.I1RA62	pp	229533.I1RMX6
2280	229533.I1RA62	pp	229533.I1RM55
2281	229533.I1RA62	pp	229533.I1RCE5
2282	229533.I1RA62	pp	229533.I1S147
2283	229533.I1RA62	pp	229533.I1RJQ5
2284	229533.I1RA62	pp	229533.I1RPS2
2285	229533.I1RA62	pp	229533.V6RUY9
2286	229533.I1RA62	pp	229533.V6RUB1
2287	229533.I1RA62	pp	229533.I1RZA7
2288	229533.I1RA62	pp	229533.I1S6P5
2289	229533.I1RA62	pp	229533.I1RCF3
2290	229533.I1RA62	pp	229533.I1S3F7
2291	229533.I1RA62	pp	229533.I1RCZ8
2292	229533.I1RA62	pp	229533.I1RYK0
2293	229533.I1RA62	pp	229533.I1RC29
2294	229533.I1RA62	pp	229533.I1RE19
2295	229533.I1RA62	pp	229533.I1RX72
2296	229533.I1RA62	pp	229533.V6RV57
2297	229533.I1RA62	pp	229533.I1RRT3
2298	229533.I1RA62	pp	229533.I1RU18
2299	229533.I1RA62	pp	229533.I1RKB2
2300	229533.I1RA62	pp	229533.I1RKP3
2301	229533.I1RA62	pp	229533.I1RNG4
2302	229533.I1RA62	pp	229533.I1RUY5
2303	229533.I1RA62	pp	229533.I1RKR5
2304	229533.I1RA62	pp	229533.I1RZ78
2305	229533.I1RA62	pp	229533.I1RH82
2306	229533.I1RA62	pp	229533.I1RR44
2307	229533.I1RA62	pp	229533.I1SAC4
2308	229533.I1RA62	pp	229533.V6RUG6
2309	229533.I1RA62	pp	229533.I1RY62
2310	229533.I1RA62	pp	229533.I1RFI4
2311	229533.I1RA62	pp	229533.I1RXX0
2312	229533.I1RA62	pp	229533.I1RUY6
2313	229533.I1RA62	pp	229533.I1RC31
2314	229533.I1RA62	pp	229533.I1RZ13
2315	229533.I1RA62	pp	229533.I1RIY2
2316	229533.I1RA62	pp	229533.I1RZ29
2317	229533.I1RA62	pp	229533.I1RMC5
2318	229533.I1RA62	pp	229533.I1RUM7
2319	229533.I1RA62	pp	229533.I1RSX2
2320	229533.I1RA62	pp	229533.I1RM54
2321	229533.I1RA62	pp	229533.I1RDE4
2322	229533.I1RA62	pp	229533.I1RZF7
2323	229533.I1RA62	pp	229533.I1RBN0
2324	229533.I1RA62	pp	229533.I1RIE8
2325	229533.I1RA62	pp	229533.I1RMW4
2326	229533.I1RA62	pp	229533.I1RJ42
2327	229533.I1RA62	pp	229533.I1S0V6
2328	229533.I1RA62	pp	229533.I1RCM5
2329	229533.I1RA62	pp	229533.I1RBF4
2330	229533.I1RA62	pp	229533.I1RUQ3
2331	229533.I1RA62	pp	229533.V6RAN2
2332	229533.I1RA62	pp	229533.I1RGB2
2333	229533.I1RA62	pp	229533.I1RQE0
2334	229533.I1RA62	pp	229533.I1RAQ4
2335	229533.I1RA62	pp	229533.I1RSB7
2336	229533.I1RA62	pp	229533.I1RPT0

229533.I1RA62	pp	229533.V6R3R0
229533.I1RA62	pp	229533.I1RDJ1
229533.I1RA62	pp	229533.I1S9F1
229533.I1RA62	pp	229533.I1RP86
229533.I1RA62	pp	229533.I1RL20
229533.I1RA62	pp	229533.I1RKB5
229533.I1RA62	pp	229533.I1RMM6
229533.I1RA62	pp	229533.I1RGY2
229533.I1RA62	pp	229533.I1RZT6
229533.I1RA62	pp	229533.I1RVV3
229533.I1RA62	pp	229533.I1RC11
229533.I1RA62	pp	229533.I1RA63
229533.I1RA62	pp	229533.I1RMM8
229533.I1RA62	pp	229533.I1RK45
229533.I1RA62	pp	229533.Q4IR09
229533.I1RA62	pp	229533.I1RJ82
229533.A0A098E1E6	pp	229533.A0A1C3YLT9
229533.A0A098E1E6	pp	229533.A0A1C3YM29
229533.A0A098D7E2	pp	229533.V6RK96
229533.A0A098D7E2	pp	229533.I1RDU0
229533.A0A098D7E2	pp	229533.I1RPT0
229533.A0A098D7E2	pp	229533.I1RAQ4
229533.A0A098D7E2	pp	229533.I1RKE1
229533.A0A098D7E2	pp	229533.I1RW69
229533.A0A098D7E2	pp	229533.I1S0V6
229533.A0A098D7E2	pp	229533.I1RUY5
229533.A0A098D7E2	pp	229533.I1RCM5
229533.A0A098D7E2	pp	229533.I1RCU2
229533.A0A098D7E2	pp	229533.I1RYL8
229533.A0A098D7E2	pp	229533.I1RWM4
229533.A0A098D7E2	pp	229533.I1RM26
229533.A0A098D7E2	pp	229533.I1RZM4
229533.A0A098D7E2	pp	229533.I1RBE7
229533.A0A098D7E2	pp	229533.I1RNM7
229533.A0A098D7E2	pp	229533.A0A1C3YN26
229533.A0A098D7E2	pp	229533.I1RMM8
229533.A0A098D7E2	pp	229533.I1RQE8
229533.A0A098D7E2	pp	229533.A0A098DVZ4
229533.A0A098D7E2	pp	229533.I1RVX9
229533.A0A098D7E2	pp	229533.A0A1C3YL54
229533.A0A098D7E2	pp	229533.I1RP86
229533.A0A098D7E2	pp	229533.I1RGB2
229533.A0A098D7E2	pp	229533.A0A0E0SMR8
229533.A0A098D7E2	pp	229533.A0A098DR13
229533.A0A098D7E2	pp	229533.I1RZ16
229533.A0A098D7E2	pp	229533.A0A1C3YJ04
229533.A0A098D7E2	pp	229533.V6RLM7
229533.A0A098D7E2	pp	229533.I1RP97
229533.A0A098D7E2	pp	229533.I1S0V7
229533.A0A098D7E2	pp	229533.V6RPQ8
229533.A0A098D7E2	pp	229533.A0A1C3YLR6
229533.A0A098D7E2	pp	229533.I1RC11
229533.A0A098D7E2	pp	229533.A0A0E0RU55
229533.A0A098D7E2	pp	229533.I1RNU7
229533.A0A098D7E2	pp	229533.I1RAN1
229533.A0A098D7E2	pp	229533.V6RUY9
229533.A0A098D7E2	pp	229533.A0A1C3YK33
229533.A0A098D7E2	pp	229533.A0A1C3YHG7
229533.A0A098D7E2	pp	229533.A0A1C3YKW4
229533.A0A098D7E2	pp	229533.I1RF85
229533.A0A098D7E2	pp	229533.I1RC29
229533.A0A098D7E2	pp	229533.I1RCG5
229533.A0A098D7E2	pp	229533.I1RC31
229533.A0A098D7E2	pp	229533.A0A1C3YI27
229533.A0A098D7E2	pp	229533.I1RA62
229533.A0A098D7E2	pp	229533.I1RYK0
229533.I1RVA5	pp	229533.I1RX38
229533.I1RJT3	pp	229533.I1S8F0
229533.I1RJT3	pp	229533.Q4I375
229533.I1RJT3	pp	229533.I1RRN2
229533.I1RJT3	pp	229533.V6R3J1
229533.I1RJT3	pp	229533.V6RGN5
229533.I1RJT3	pp	229533.I1RZH5

229533.I1RJT3 pp 229533.I1RSX0
229533.I1RJT3 pp 229533.I1RXT3
229533.I1RJT3 pp 229533.I1S0Q7
229533.I1RJT3 pp 229533.Q4IB65
229533.I1RJT3 pp 229533.V6R1X4
229533.I1RJT3 pp 229533.Q4IBU4
229533.I1RJT3 pp 229533.I1RPA5
229533.I1RJT3 pp 229533.I1RXZ3
229533.I1RJT3 pp 229533.I1RPU2
229533.I1RJT3 pp 229533.Q4I7T5
229533.I1RJT3 pp 229533.I1RZS4
229533.I1RJT3 pp 229533.I1RVN1
229533.I1RJT3 pp 229533.Q4I2W2
229533.I1RJT3 pp 229533.Q4I099
229533.I1RJT3 pp 229533.I1RT38
229533.I1RJT3 pp 229533.Q4IPZ1
229533.I1RJT3 pp 229533.I1RPV3
229533.I1RJT3 pp 229533.Q4HZ95
229533.I1RJT3 pp 229533.I1RNJ5
229533.I1RJT3 pp 229533.I1S6K9
229533.I1RJT3 pp 229533.Q4IJW4
229533.I1RJT3 pp 229533.I1S088
229533.I1RJT3 pp 229533.I1RQ85
229533.I1RJT3 pp 229533.Q4IK03
229533.I1RJT3 pp 229533.Q4I6B0
229533.I1RJT3 pp 229533.I1RQR6
229533.A0A098E0U9 pp 229533.A0A1C3YK51
229533.A0A098E0U9 pp 229533.I1RWI6
229533.A0A098E0U9 pp 229533.I1RX09
229533.I1REM6 pp 229533.I1S2M5
229533.I1RNB3 pp 229533.I1RS22
229533.I1RF69 pp 229533.I1RQ88
229533.I1RF69 pp 229533.I1RPU4
229533.I1RF69 pp 229533.I1RX38
229533.I1RF69 pp 229533.I1RPW0
229533.A0A1C3YJM6 pp 229533.I1RVP2
229533.I1RXY9 pp 229533.I1RYD3
229533.I1RXY9 pp 229533.I1RYM5
229533.I1RXY9 pp 229533.V6RIE5
229533.A0A0E0RUG8 pp 229533.A0A0E0SP71
229533.I1RGL6 pp 229533.I1RM65
229533.I1RGL6 pp 229533.I1RNB3
229533.I1RLS5 pp 229533.I1RX38
229533.I1RLS5 pp 229533.I1RQ88
229533.I1RLS5 pp 229533.I1RPW0
229533.I1RLS5 pp 229533.I1RPU4
229533.I1RSF2 pp 229533.I1RTE6
229533.I1RSF2 pp 229533.I1S834
229533.I1RSF2 pp 229533.Q4IN00
229533.I1RSF2 pp 229533.Q4I5R9
229533.I1RSF2 pp 229533.V6REE9
229533.I1RSF2 pp 229533.I1S1B3
229533.I1RSF2 pp 229533.I1S7X9
229533.I1RSF2 pp 229533.I1RX09
229533.I1RSF2 pp 229533.I1RYN2
229533.I1RHK6 pp 229533.I1RVB6
229533.I1RAM2 pp 229533.I1RQ21
229533.I1RAM2 pp 229533.I1REC7
229533.I1RAM2 pp 229533.Q4I624
229533.I1RAM2 pp 229533.I1RME4
229533.I1RAM2 pp 229533.I1RBS5
229533.I1RAM2 pp 229533.I1RWH4
229533.I1RAM2 pp 229533.I1RZ95
229533.I1RAM2 pp 229533.I1RPS2
229533.I1RAM2 pp 229533.I1REC6
229533.I1RAM2 pp 229533.I1RK77
229533.I1RAM2 pp 229533.I1RYL5
229533.I1RAM2 pp 229533.I1RBC4
229533.I1RAM2 pp 229533.I1RW71
229533.I1RAM2 pp 229533.I1RXX0
229533.I1RDQ3 pp 229533.I1RVB6
229533.I1RDQ3 pp 229533.I1REM2
229533.I1RVT8 pp 229533.I1RY92

2483 229533.I1RVT8 pp 229533.I1S0C2
2484 229533.I1RVT8 pp 229533.I1S8S9
2485 229533.I1RVT8 pp 229533.I1S5R5
2486 229533.I1RVT8 pp 229533.I1S0K2
2487 229533.I1RVT8 pp 229533.I1S0P0
2488 229533.I1RVT8 pp 229533.V6QUG0
2489 229533.I1RVT8 pp 229533.Q4HVU7
2490 229533.I1RVT8 pp 229533.I1RY21
2491 229533.A0A1C3YJX2 pp 229533.A0A1C3YLG3
2492 229533.A0A1C3YJX2 pp 229533.I1RV17
2493 229533.A0A1C3YJX2 pp 229533.I1S4Z6
2494 229533.A0A1C3YJX2 pp 229533.I1RHZ4
2495 229533.A0A1C3YJX2 pp 229533.I1RL80
2496 229533.A0A1C3YJX2 pp 229533.I1RR23
2497 229533.A0A1C3YJX2 pp 229533.I1RW64
2498 229533.A0A1C3YJX2 pp 229533.I1R9D9
2499 229533.I1RIA5 pp 229533.I1RY35
2500 229533.I1RIA5 pp 229533.I1RKJ6
2501 229533.I1RIA5 pp 229533.I1RVB6
2502 229533.I1RIA5 pp 229533.V6RG22
2503 229533.I1RIA5 pp 229533.Q00909
2504 229533.I1RIA5 pp 229533.I1S219
2505 229533.I1S248 pp 229533.I1S2B6
2506 229533.I1S248 pp 229533.I1S2G7
2507 229533.I1S248 pp 229533.I1S2K7
2508 229533.I1S248 pp 229533.I1S296
2509 229533.I1S248 pp 229533.Q4IBS9
2510 229533.I1RSQ1 pp 229533.I1S282
2511 229533.I1RCH0 pp 229533.I1REM2
2512 229533.I1RCH0 pp 229533.I1RDQ3
2513 229533.I1RCH0 pp 229533.I1RVB6
2514 229533.I1RCD7 pp 229533.I1RWU9
2515 229533.A0A1C3YJM4 pp 229533.I1RQL4
2516 229533.A0A1C3YJM4 pp 229533.I1RD69
2517 229533.A0A1C3YJM4 pp 229533.I1RB73
2518 229533.A0A1C3YJM4 pp 229533.I1RCN2
2519 229533.A0A1C3YJM4 pp 229533.A0A1C3YJX2
2520 229533.A0A1C3YJM4 pp 229533.V6RPN0
2521 229533.A0A1C3YJM4 pp 229533.I1S0F0
2522 229533.A0A1C3YJM4 pp 229533.A0A1C3YMS7
2523 229533.A0A1C3YJM4 pp 229533.I1S5R5
2524 229533.I1REM3 pp 229533.I1REM6
2525 229533.I1REM3 pp 229533.I1REM5
2526 229533.I1RKJ1 pp 229533.I1RKJ2
2527 229533.I1RKJ1 pp 229533.I1RMG9
2528 229533.I1RKJ1 pp 229533.I1RKR7
2529 229533.I1RH44 pp 229533.I1RN04
2530 229533.I1RH44 pp 229533.I1RRF7
2531 229533.I1RH44 pp 229533.I1RW91
2532 229533.I1RH44 pp 229533.I1RPW8
2533 229533.I1S3A6 pp 229533.I1S7J8
2534 229533.I1REL9 pp 229533.I1RI22
2535 229533.I1REL9 pp 229533.I1SAS3
2536 229533.I1REL9 pp 229533.I1RFM2
2537 229533.I1REL9 pp 229533.I1RT23
2538 229533.I1REL9 pp 229533.I1RJG1
2539 229533.I1REL9 pp 229533.I1REM3
2540 229533.I1REL9 pp 229533.I1RF79
2541 229533.I1REL9 pp 229533.I1RY35
2542 229533.I1REL9 pp 229533.I1RR90
2543 229533.I1REL9 pp 229533.V6RPN0
2544 229533.I1REL9 pp 229533.I1RZZ3
2545 229533.I1REL9 pp 229533.I1SAS4
2546 229533.I1REL9 pp 229533.I1RNT1
2547 229533.I1REL9 pp 229533.I1REM5
2548 229533.I1REL9 pp 229533.I1RQ48
2549 229533.I1REL9 pp 229533.I1RQ76
2550 229533.I1REL9 pp 229533.V6RR38
2551 229533.I1REL9 pp 229533.I1S1Q3
2552 229533.I1REL9 pp 229533.I1REM6
2553 229533.I1REL9 pp 229533.I1RRQ1
2554 229533.I1REM0 pp 229533.I1RIA3
2555 229533.I1RX04 pp 229533.I1S2K7

2556	229533.I1RX04	pp	229533.I1RX77
2557	229533.I1RX04	pp	229533.I1RZZ9
2558	229533.I1RX04	pp	229533.I1S0L4
2559	229533.I1RX04	pp	229533.I1RXH6
2560	229533.I1RX04	pp	229533.I1S265
2561	229533.I1RX04	pp	229533.I1S0L3
2562	229533.I1RX04	pp	229533.V6RCG5
2563	229533.I1RDN0	pp	229533.I1RW91
2564	229533.I1RDN0	pp	229533.I1RPW8
2565	229533.I1RDN0	pp	229533.I1RVB6
2566	229533.I1RDN0	pp	229533.I1RRF7
2567	229533.I1RDN0	pp	229533.I1RN04
2568	229533.Q4IBK5	pp	229533.Q4IJH1
2569	229533.Q4IBK5	pp	229533.Q4IFI0
2570	229533.Q4IBK5	pp	229533.Q4IE79
2571	229533.Q4IBK5	pp	229533.V6QXG1
2572	229533.Q4IBK5	pp	229533.Q4IRI9
2573	229533.Q4IBK5	pp	229533.Q4IR18
2574	229533.Q4IBK5	pp	229533.Q4IPB8
2575	229533.Q4IBK5	pp	229533.Q4IF76
2576	229533.Q4IBK5	pp	229533.Q4IPB3
2577	229533.I1RUW5	pp	229533.I1RVB6
2578	229533.I1RUW5	pp	229533.I1RW91
2579	229533.I1RX77	pp	229533.Q4INZ9
2580	229533.I1RX77	pp	229533.I1S2K7
2581	229533.I1RX77	pp	229533.I1S296
2582	229533.I1RX77	pp	229533.I1S0X7
2583	229533.I1RX77	pp	229533.I1RXH6
2584	229533.I1RX77	pp	229533.Q4I4A4
2585	229533.I1RX77	pp	229533.I1S2B6
2586	229533.I1RX77	pp	229533.I1S286
2587	229533.I1RX77	pp	229533.I1RZK4
2588	229533.I1RX77	pp	229533.Q4IEA7
2589	229533.I1RXA1	pp	229533.I1S9H4
2590	229533.I1RCD1	pp	229533.I1RQB4
2591	229533.I1RCD1	pp	229533.I1RPA6
2592	229533.I1RCD1	pp	229533.I1S0X7
2593	229533.I1RCD1	pp	229533.I1RXH6
2594	229533.I1RCD1	pp	229533.I1RHQ8
2595	229533.I1RCD1	pp	229533.I1RV66
2596	229533.I1RCD1	pp	229533.I1RGF1
2597	229533.I1RCD1	pp	229533.I1RNF4
2598	229533.I1RCD1	pp	229533.I1RX20
2599	229533.I1RCD1	pp	229533.I1RHU7
2600	229533.I1RCD1	pp	229533.I1RX77
2601	229533.I1RCD1	pp	229533.I1RQ72
2602	229533.I1RCD1	pp	229533.I1S2K7
2603	229533.I1RCD1	pp	229533.I1RNY8
2604	229533.A0A0E0S6U6	pp	229533.I1RPT8
2605	229533.A0A0E0S6U6	pp	229533.I1S2J5
2606	229533.A0A0E0S6U6	pp	229533.I1RUW5
2607	229533.A0A0E0S6U6	pp	229533.I1S671
2608	229533.A0A0E0S6U6	pp	229533.I1RDL3
2609	229533.A0A0E0S6U6	pp	229533.I1REM2
2610	229533.A0A0E0S6U6	pp	229533.I1RDQ3
2611	229533.A0A0E0S6U6	pp	229533.I1S6X6
2612	229533.A0A0E0S6U6	pp	229533.I1S2M5
2613	229533.A0A0E0S6U6	pp	229533.A0A0E0SP71
2614	229533.A0A0E0S6U6	pp	229533.I1RGV3
2615	229533.A0A0E0S6U6	pp	229533.I1S3V0
2616	229533.A0A0E0S6U6	pp	229533.I1RYA9
2617	229533.A0A0E0S6U6	pp	229533.I1RF98
2618	229533.A0A0E0S6U6	pp	229533.I1RVB6
2619	229533.A0A0E0S6U6	pp	229533.A0A1C3YI89
2620	229533.A0A098D7D6	pp	229533.I1REM2
2621	229533.A0A098D7D6	pp	229533.I1REM6
2622	229533.A0A098D7D6	pp	229533.I1RNB3
2623	229533.A0A098D7D6	pp	229533.I1REL9
2624	229533.A0A098D7D6	pp	229533.I1REM3
2625	229533.A0A098D7D6	pp	229533.I1RGL6
2626	229533.A0A098D7D6	pp	229533.I1REM5
2627	229533.A0A0E0RUE8	pp	229533.A0A1C3YIP6
2628	229533.A0A0E0RUE8	pp	229533.I1S034

2629 229533.A0A0E0RUE8 pp 229533.I1RT41
2630 229533.A0A0E0RUE8 pp 229533.I1RTD0
2631 229533.A0A0E0RUE8 pp 229533.A0A1C3YK51
2632 229533.A0A0E0RUE8 pp 229533.Q4ICG5
2633 229533.A0A0E0RUE8 pp 229533.V6QW25
2634 229533.A0A0E0RUE8 pp 229533.I1RQA3
2635 229533.A0A0E0RUE8 pp 229533.A0A1C3YI02
2636 229533.A0A0E0RUE8 pp 229533.I1RE20
2637 229533.A0A0E0RUE8 pp 229533.A0A1C3YJH8
2638 229533.A0A0E0RUE8 pp 229533.I1RLT6
2639 229533.A0A0E0RUE8 pp 229533.I1RWT6
2640 229533.A0A0E0RUE8 pp 229533.V6RKE4
2641 229533.A0A0E0RUE8 pp 229533.I1RX09
2642 229533.A0A0E0RUE8 pp 229533.I1RXU1
2643 229533.A0A0E0RUE8 pp 229533.I1RWI6
2644 229533.A0A0E0RUE8 pp 229533.I1RE15
2645 229533.A0A0E0RUE8 pp 229533.A0A1C3YIN0
2646 229533.A0A0E0RUE8 pp 229533.I1RB41
2647 229533.A0A0E0RUE8 pp 229533.I1REN4
2648 229533.A0A0E0RUE8 pp 229533.I1RZA4
2649 229533.A0A0E0RUE8 pp 229533.I1RNR3
2650 229533.A0A0E0RUE8 pp 229533.I1RW84
2651 229533.A0A0E0RUE8 pp 229533.I1RD70
2652 229533.A0A0E0RUE8 pp 229533.I1RW32
2653 229533.A0A0E0RUE8 pp 229533.V6RQ48
2654 229533.I1REM5 pp 229533.I1REM6
2655 229533.A0A1C3YKC8 pp 229533.I1RYM2
2656 229533.A0A1C3YKC8 pp 229533.I1RYF8
2657 229533.A0A1C3YKC8 pp 229533.I1RD70
2658 229533.A0A0E0RUD7 pp 229533.A0A0E0S1T0
2659 229533.I1RT08 pp 229533.I1RX38
2660 229533.I1RFA2 pp 229533.I1RVB6
2661 229533.A0A098D7D4 pp 229533.I1RHK6
2662 229533.A0A098D7D4 pp 229533.I1RDQ3
2663 229533.A0A098D7D4 pp 229533.I1RIA5
2664 229533.A0A098D7D4 pp 229533.I1RCH0
2665 229533.A0A098D7D4 pp 229533.I1REM3
2666 229533.A0A098D7D4 pp 229533.I1RH44
2667 229533.A0A098D7D4 pp 229533.I1REL9
2668 229533.A0A098D7D4 pp 229533.I1REM0
2669 229533.A0A098D7D4 pp 229533.I1RDN0
2670 229533.A0A098D7D4 pp 229533.I1RUW5
2671 229533.A0A098D7D4 pp 229533.I1S1S9
2672 229533.A0A098D7D4 pp 229533.A0A0E0S6U6
2673 229533.A0A098D7D4 pp 229533.A0A098D7D6
2674 229533.A0A098D7D4 pp 229533.I1REM5
2675 229533.A0A098D7D4 pp 229533.I1REM2
2676 229533.A0A098D7D4 pp 229533.A0A0E0SP71
2677 229533.A0A098D7D4 pp 229533.I1RFA2
2678 229533.I1S2R2 pp 229533.I1S7J8
2679 229533.I1RQW2 pp 229533.I1RX38
2680 229533.I1RQW2 pp 229533.I1S1F9
2681 229533.I1RQW2 pp 229533.I1RTN8
2682 229533.I1RQW2 pp 229533.V6R556
2683 229533.I1RQW2 pp 229533.I1S1G1
2684 229533.I1RQW2 pp 229533.V6RM09
2685 229533.I1RQW2 pp 229533.I1S2L3
2686 229533.I1RQW2 pp 229533.I1RTW1
2687 229533.I1RQW2 pp 229533.V6QTX7
2688 229533.I1RQW2 pp 229533.I1S1G0
2689 229533.I1RQW2 pp 229533.I1RTV7
2690 229533.I1RQW2 pp 229533.I1RTN1
2691 229533.I1RQW2 pp 229533.V6REA4
2692 229533.I1RTN1 pp 229533.I1S2L3
2693 229533.I1RTN1 pp 229533.I1RTN8
2694 229533.I1RTN1 pp 229533.V6R556
2695 229533.I1RTN1 pp 229533.I1S1F9
2696 229533.I1RTN1 pp 229533.I1S1G1
2697 229533.I1RTN1 pp 229533.I1S1G0
2698 229533.I1RTN1 pp 229533.V6REA4
2699 229533.I1RTN1 pp 229533.I1RX38
2700 229533.I1RTN1 pp 229533.V6RM09
2701 229533.I1RTN1 pp 229533.V6QTX7

229533.I1RTN1	pp	229533.I1RTV7
229533.I1RTN1	pp	229533.I1RTW1
229533.A0A098D7C7	pp	229533.I1S1W8
229533.A0A098D7C7	pp	229533.I1RNG4
229533.A0A098D7C7	pp	229533.I1RY87
229533.A0A098D7C7	pp	229533.I1S3G4
229533.A0A098D7C7	pp	229533.A0A1C3YLB6
229533.A0A098D7C7	pp	229533.I1RBE7
229533.I1RAT6	pp	229533.I1RMK9
229533.I1RAT6	pp	229533.I1RAX2
229533.I1RAT6	pp	229533.I1S7A5
229533.I1RAT6	pp	229533.I1RD37
229533.I1RAT6	pp	229533.I1RC46
229533.I1RAT6	pp	229533.I1RND5
229533.I1RAT6	pp	229533.I1RS35
229533.I1RAT6	pp	229533.I1RBU2
229533.I1RAT6	pp	229533.I1RZ89
229533.I1RAT6	pp	229533.I1RTM8
229533.I1RAT6	pp	229533.I1RWP4
229533.I1RAT6	pp	229533.I1RN19
229533.I1RAT6	pp	229533.I1RY32
229533.I1RAT6	pp	229533.I1RVN4
229533.I1RAT6	pp	229533.I1RPN6
229533.I1RAT6	pp	229533.I1RJH3
229533.I1RAT6	pp	229533.I1RZC3
229533.I1RAT6	pp	229533.I1RKB3
229533.I1RAT6	pp	229533.I1RE59
229533.I1RD37	pp	229533.I1RZ25
229533.I1RD37	pp	229533.I1RKB3
229533.I1RD37	pp	229533.I1RY32
229533.I1RD37	pp	229533.I1RST9
229533.I1RD37	pp	229533.I1RZC3
229533.I1RD37	pp	229533.Q4IBU4
229533.I1RD37	pp	229533.I1RZV5
229533.I1RD37	pp	229533.I1RPN6
229533.I1RD37	pp	229533.I1RLZ4
229533.I1RD37	pp	229533.I1RJH3
229533.I1RD37	pp	229533.I1S267
229533.I1RD37	pp	229533.I1RZ89
229533.I1RD37	pp	229533.I1RMK9
229533.I1RD37	pp	229533.I1RE59
229533.I1RD37	pp	229533.I1RLT7
229533.I1RD37	pp	229533.I1RW71
229533.I1RD37	pp	229533.I1S7A5
229533.I1RD37	pp	229533.I1RJZ7
229533.I1RD37	pp	229533.I1RLA2
229533.I1RD37	pp	229533.I1RN19
229533.I1RD37	pp	229533.I1RS35
229533.I1RPD2	pp	229533.I1RQ88
229533.I1RPD2	pp	229533.I1RX38
229533.I1RPD2	pp	229533.I1RPU4
229533.I1RPD2	pp	229533.I1RPW0
229533.I1RZK8	pp	229533.I1S241
229533.I1RZK8	pp	229533.V6RDH3
229533.I1RZK8	pp	229533.V6RHD0
229533.I1RZK8	pp	229533.I1S1F5
229533.I1RZK8	pp	229533.V6QU65
229533.I1RZK8	pp	229533.I1S0I3
229533.I1RZK8	pp	229533.V6RQW4
229533.I1RZK8	pp	229533.I1S1W8
229533.I1RZK8	pp	229533.I1S3G4
229533.I1RZK8	pp	229533.V6QV31
229533.I1RZK8	pp	229533.V6RXR0
229533.I1RZK8	pp	229533.I1S147
229533.Q4IBU4	pp	229533.V6R1X4
229533.V6QXN0	pp	229533.V6RDH3
229533.A0A1C3YI22	pp	229533.I1RPU4
229533.A0A1C3YI22	pp	229533.A0A1C3YK37
229533.A0A1C3YI22	pp	229533.I1RX38
229533.A0A1C3YI22	pp	229533.A0A1C3YKJ6
229533.A0A1C3YI22	pp	229533.I1RQ88
229533.A0A1C3YI22	pp	229533.I1RBV5
229533.A0A1C3YI22	pp	229533.I1RAI9

2775	229533.A0A1C3YI22	pp	229533.I1RPW0
2776	229533.I1RPN6	pp	229533.I1RW71
2777	229533.I1RPN6	pp	229533.I1RZC3
2778	229533.I1RPN6	pp	229533.I1RZ25
2779	229533.I1RPN6	pp	229533.I1S7A5
2780	229533.I1RPN6	pp	229533.I1RZV5
2781	229533.I1RPN6	pp	229533.I1RY32
2782	229533.I1RPN6	pp	229533.I1RS35
2783	229533.I1RPN6	pp	229533.I1RZ89
2784	229533.I1RPN6	pp	229533.Q4IBU4
2785	229533.I1RPN6	pp	229533.I1RST9
2786	229533.I1RPN6	pp	229533.I1S267
2787	229533.I1S9Y0	pp	229533.V6RPN0
2788	229533.I1S9Y0	pp	229533.Q4IEK8
2789	229533.I1S9Y0	pp	229533.Q4IAS1
2790	229533.A0A0E0SGL0	pp	229533.I1RS35
2791	229533.A0A0E0SGL0	pp	229533.I1RCK2
2792	229533.A0A0E0SGL0	pp	229533.Q4IBU4
2793	229533.A0A0E0SGL0	pp	229533.I1R9Z1
2794	229533.A0A0E0SGL0	pp	229533.I1RE59
2795	229533.A0A0E0SGL0	pp	229533.I1RLZ4
2796	229533.A0A0E0SGL0	pp	229533.I1RJH3
2797	229533.A0A0E0SGL0	pp	229533.I1RN19
2798	229533.A0A0E0SGL0	pp	229533.I1RC71
2799	229533.A0A0E0SGL0	pp	229533.I1RSG0
2800	229533.A0A0E0SGL0	pp	229533.I1RAT6
2801	229533.A0A0E0SGL0	pp	229533.I1RMK9
2802	229533.A0A0E0SGL0	pp	229533.I1RD37
2803	229533.A0A0E0SGL0	pp	229533.I1S7A5
2804	229533.A0A0E0SGL0	pp	229533.I1RKB3
2805	229533.A0A0E0SGL0	pp	229533.I1RRK8
2806	229533.A0A0E0SGL0	pp	229533.I1RAX2
2807	229533.A0A0E0SGL0	pp	229533.I1RSI3
2808	229533.A0A0E0SGL0	pp	229533.I1RY32
2809	229533.A0A0E0SGL0	pp	229533.I1RRW0
2810	229533.A0A0E0SGL0	pp	229533.I1RPN6
2811	229533.A0A0E0SGL0	pp	229533.I1RZV5
2812	229533.A0A0E0SGL0	pp	229533.I1RZC3
2813	229533.A0A0E0SAL1	pp	229533.I1RBE7
2814	229533.A0A0E0SAL1	pp	229533.I1REA3
2815	229533.A0A0E0SAL1	pp	229533.V6RN69
2816	229533.A0A0E0SAL1	pp	229533.I1RWI5
2817	229533.A0A0E0SAL1	pp	229533.I1RQ29
2818	229533.A0A0E0SAL1	pp	229533.I1RC31
2819	229533.A0A0E0SAL1	pp	229533.A0A0E0SPV0
2820	229533.I1S053	pp	229533.V6RDH3
2821	229533.I1S053	pp	229533.V6QV31
2822	229533.I1RGD4	pp	229533.I1RNG8
2823	229533.I1RGD4	pp	229533.I1RK30
2824	229533.I1RGD4	pp	229533.I1S9X9
2825	229533.I1RGD4	pp	229533.I1RKA1
2826	229533.I1RGD4	pp	229533.I1S0P7
2827	229533.A0A098DGJ4	pp	229533.I1S9D4
2828	229533.A0A098DGJ4	pp	229533.I1RHG6
2829	229533.A0A098DGJ4	pp	229533.I1RZC3
2830	229533.A0A098DGJ4	pp	229533.A0A1C3YM01
2831	229533.A0A098DGJ4	pp	229533.I1RS35
2832	229533.A0A098DGJ4	pp	229533.I1RAT6
2833	229533.A0A098DGJ4	pp	229533.I1RY32
2834	229533.A0A098DGJ4	pp	229533.I1RD37
2835	229533.A0A098DGJ4	pp	229533.I1S2E2
2836	229533.A0A098DGJ4	pp	229533.Q4IBU4
2837	229533.A0A098DGJ4	pp	229533.I1R9K3
2838	229533.A0A098DGJ4	pp	229533.I1RPN6
2839	229533.A0A098DGJ4	pp	229533.I1RAX2
2840	229533.A0A098DGJ4	pp	229533.A0A0E0SGL0
2841	229533.A0A098DGJ4	pp	229533.A0A1C3YLU9
2842	229533.I1RA94	pp	229533.I1RUV4
2843	229533.I1RA94	pp	229533.I1RT77
2844	229533.I1RA94	pp	229533.I1S1W8
2845	229533.I1RA94	pp	229533.I1RZT6
2846	229533.I1RA94	pp	229533.I1RPE7
2847	229533.I1RA94	pp	229533.I1RRX5

2848	229533.I1RA94	pp	229533.I1RM67
2849	229533.I1RA94	pp	229533.I1RZM4
2850	229533.I1RA94	pp	229533.I1RRQ1
2851	229533.I1RA94	pp	229533.I1S241
2852	229533.I1RA94	pp	229533.I1S4S4
2853	229533.I1RA94	pp	229533.I1RAJ6
2854	229533.I1RA94	pp	229533.I1RCC2
2855	229533.I1RA94	pp	229533.I1RZY1
2856	229533.I1RA94	pp	229533.Q4IR09
2857	229533.I1RA94	pp	229533.I1RMH9
2858	229533.I1RA94	pp	229533.I1RQ60
2859	229533.I1RA94	pp	229533.I1RDQ2
2860	229533.I1RA94	pp	229533.I1RJF2
2861	229533.I1RA94	pp	229533.I1S0F1
2862	229533.I1RA94	pp	229533.I1RP86
2863	229533.I1RA94	pp	229533.V6RK96
2864	229533.I1RA94	pp	229533.I1RY49
2865	229533.I1RA94	pp	229533.I1RBW0
2866	229533.I1RA94	pp	229533.I1RD81
2867	229533.I1RA94	pp	229533.I1RW06
2868	229533.I1RA94	pp	229533.I1RAR3
2869	229533.I1RA94	pp	229533.I1S183
2870	229533.I1RA94	pp	229533.I1RMJ4
2871	229533.I1RA94	pp	229533.I1RC74
2872	229533.I1RA94	pp	229533.I1RUI6
2873	229533.I1RA94	pp	229533.I1S8E0
2874	229533.I1RA94	pp	229533.V6QUE6
2875	229533.I1RA94	pp	229533.I1S0B7
2876	229533.I1RA94	pp	229533.V6QWD8
2877	229533.I1RA94	pp	229533.I1RRV4
2878	229533.I1RA94	pp	229533.I1S4Q4
2879	229533.I1RA94	pp	229533.I1RJY7
2880	229533.I1RA94	pp	229533.I1RUY5
2881	229533.I1RA94	pp	229533.I1RYQ4
2882	229533.I1RA94	pp	229533.I1RCL0
2883	229533.I1RA94	pp	229533.I1RBE7
2884	229533.I1RA94	pp	229533.I1RC61
2885	229533.I1RA94	pp	229533.I1RVP5
2886	229533.I1RA94	pp	229533.I1RQE8
2887	229533.I1RA94	pp	229533.I1RMM8
2888	229533.I1RA94	pp	229533.I1RMQ6
2889	229533.I1RA94	pp	229533.I1RWH8
2890	229533.I1RA94	pp	229533.I1RCN8
2891	229533.I1RA94	pp	229533.I1RXF0
2892	229533.I1RA94	pp	229533.V6RBN2
2893	229533.I1RA94	pp	229533.I1RC48
2894	229533.I1RA94	pp	229533.I1RFV9
2895	229533.I1RA94	pp	229533.I1RBE4
2896	229533.I1RA94	pp	229533.I1RTU0
2897	229533.I1RA94	pp	229533.I1RG14
2898	229533.I1RA94	pp	229533.I1RZA7
2899	229533.I1RA94	pp	229533.I1RDU0
2900	229533.I1RA94	pp	229533.I1RC83
2901	229533.I1RA94	pp	229533.I1RZ52
2902	229533.I1RA94	pp	229533.I1RVZ3
2903	229533.I1RA94	pp	229533.I1RYP2
2904	229533.I1RA94	pp	229533.V6RUB1
2905	229533.I1RA94	pp	229533.I1RIY0
2906	229533.I1RA94	pp	229533.I1RBL7
2907	229533.I1RA94	pp	229533.I1RFS3
2908	229533.I1RA94	pp	229533.I1RFI8
2909	229533.I1RA94	pp	229533.I1RAQ4
2910	229533.I1RA94	pp	229533.I1RBF3
2911	229533.I1RA94	pp	229533.I1RVR2
2912	229533.I1RA94	pp	229533.V6RDH3
2913	229533.I1RA94	pp	229533.I1RSS7
2914	229533.I1RA94	pp	229533.I1RXG6
2915	229533.I1RA94	pp	229533.I1RCT7
2916	229533.I1RA94	pp	229533.I1RAZ3
2917	229533.I1RA94	pp	229533.I1RZ86
2918	229533.I1RA94	pp	229533.I1S6P5
2919	229533.I1RA94	pp	229533.Q4HXF0
2920	229533.I1RA94	pp	229533.I1SA27

2921 229533.I1RA94 pp 229533.I1S0N3
2922 229533.I1RA94 pp 229533.I1RPD6
2923 229533.I1RA94 pp 229533.V6RVJ3
2924 229533.I1RA94 pp 229533.O74638
2925 229533.I1RA94 pp 229533.I1RZ83
2926 229533.I1RA94 pp 229533.I1RNG4
2927 229533.I1RA94 pp 229533.I1S3X7
2928 229533.I1RA94 pp 229533.V6QV31
2929 229533.I1RA94 pp 229533.I1RCR3
2930 229533.I1RA94 pp 229533.I1RBC8
2931 229533.I1RA94 pp 229533.I1S0F3
2932 229533.I1RA94 pp 229533.I1RBW1
2933 229533.I1RA94 pp 229533.I1RSU2
2934 229533.I1RA94 pp 229533.I1RE58
2935 229533.I1RA94 pp 229533.I1RSQ4
2936 229533.I1RA94 pp 229533.V6RN69
2937 229533.I1RA94 pp 229533.I1S3G4
2938 229533.I1RA94 pp 229533.I1RDE7
2939 229533.I1RA94 pp 229533.I1RW43
2940 229533.I1RA94 pp 229533.I1RC54
2941 229533.I1RA94 pp 229533.I1RFK3
2942 229533.I1RA94 pp 229533.I1RQE7
2943 229533.I1RA94 pp 229533.I1RSK5
2944 229533.I1RA94 pp 229533.I1RIJ5
2945 229533.I1RA94 pp 229533.I1S0I3
2946 229533.I1RA94 pp 229533.I1S5R6
2947 229533.I1RA94 pp 229533.I1RXY7
2948 229533.I1RA94 pp 229533.I1RBA4
2949 229533.I1RA94 pp 229533.I1RKZ7
2950 229533.I1RA94 pp 229533.I1RCA0
2951 229533.I1RA94 pp 229533.I1RKB6
2952 229533.I1RA94 pp 229533.I1RNV4
2953 229533.I1RA94 pp 229533.I1S064
2954 229533.I1RA94 pp 229533.I1RLM5
2955 229533.I1RA94 pp 229533.I1RPK0
2956 229533.I1RA94 pp 229533.Q4I1N3
2957 229533.I1RA94 pp 229533.I1RDT5
2958 229533.I1RA94 pp 229533.I1S1Y7
2959 229533.I1RA94 pp 229533.I1RCG5
2960 229533.I1RA94 pp 229533.I1RYW3
2961 229533.I1RA94 pp 229533.V6RLM7
2962 229533.I1RA94 pp 229533.I1RHG5
2963 229533.I1S336 pp 229533.P0C431
2964 229533.I1S336 pp 229533.V6REE9
2965 229533.I1RX59 pp 229533.I1S1F5
2966 229533.I1RX59 pp 229533.I1RZK8
2967 229533.I1RX59 pp 229533.I1RY12
2968 229533.I1RX59 pp 229533.I1RYQ4
2969 229533.I1RX59 pp 229533.I1RY49
2970 229533.I1RX59 pp 229533.I1S147
2971 229533.I1RX59 pp 229533.I1S241
2972 229533.I1RX59 pp 229533.V6RDH3
2973 229533.I1RX59 pp 229533.V6RHD0
2974 229533.I1RX59 pp 229533.I1S1W8
2975 229533.I1RX59 pp 229533.V6RQW4
2976 229533.I1RX59 pp 229533.I1RXY7
2977 229533.I1RX59 pp 229533.I1S0I3
2978 229533.I1RX59 pp 229533.V6QU65
2979 229533.I1RX59 pp 229533.V6RXR0
2980 229533.I1RX59 pp 229533.I1S3G4
2981 229533.I1RX59 pp 229533.V6QV31
2982 229533.I1RC74 pp 229533.I1RNG4
2983 229533.I1RC74 pp 229533.I1RW11
2984 229533.I1RC74 pp 229533.I1RZA7
2985 229533.I1RC74 pp 229533.V6RHD0
2986 229533.I1RC74 pp 229533.I1S6P5
2987 229533.I1RC74 pp 229533.I1RFI8
2988 229533.I1RC74 pp 229533.V6RUB1
2989 229533.I1RC74 pp 229533.I1RWW7
2990 229533.I1RC74 pp 229533.I1S3G4
2991 229533.I1RC74 pp 229533.I1RWF5
2992 229533.I1RC74 pp 229533.I1S1W8
2993 229533.I1RC74 pp 229533.I1RE58

229533.I1RC74 pp 229533.I1S024
229533.I1RC74 pp 229533.V6QV31
229533.I1RC74 pp 229533.I1RGK9
229533.I1RC74 pp 229533.I1RSK5
229533.I1RC74 pp 229533.I1RWE8
229533.I1RC74 pp 229533.I1RY49
229533.I1RC74 pp 229533.V6RXR0
229533.I1RC74 pp 229533.I1RYQ4
229533.I1RC74 pp 229533.I1RXY7
229533.I1RC74 pp 229533.I1SAE8
229533.I1RC74 pp 229533.I1RNV4
229533.I1RC74 pp 229533.I1S0I3
229533.I1RC74 pp 229533.I1S147
229533.I1RC74 pp 229533.I1RE79
229533.I1RC74 pp 229533.I1S1F5
229533.I1RC74 pp 229533.I1RFK3
229533.I1RC74 pp 229533.I1RZK8
229533.I1RC74 pp 229533.I1S241
229533.I1RC74 pp 229533.I1RX59
229533.I1RC74 pp 229533.I1RKZ7
229533.I1RC74 pp 229533.I1RD21
229533.I1RC74 pp 229533.I1RZY1
229533.I1RC74 pp 229533.I1RTJ2
229533.I1RC74 pp 229533.I1RUI6
229533.I1RC74 pp 229533.I1RZH8
229533.I1RC74 pp 229533.I1RY12
229533.I1RC74 pp 229533.I1RM26
229533.I1RC74 pp 229533.V6RDH3
229533.I1RC74 pp 229533.I1RFV9
229533.I1RC74 pp 229533.V6RQW4
229533.I1RC74 pp 229533.V6QU65
229533.I1RC74 pp 229533.I1RVX9
229533.I1RC74 pp 229533.I1RCT7
229533.I1RC74 pp 229533.I1RPE5
229533.A0A1C3YL56 pp 229533.I1RYJ6
229533.A0A1C3YL56 pp 229533.I1S8E0
229533.A0A1C3YL56 pp 229533.I1RWF9
229533.A0A1C3YL56 pp 229533.I1RWS4
229533.A0A1C3YL56 pp 229533.I1S0Y7
229533.A0A1C3YL56 pp 229533.I1RJX2
229533.A0A1C3YL56 pp 229533.I1RNG4
229533.A0A1C3YL56 pp 229533.I1RTH6
229533.A0A1C3YL56 pp 229533.I1RBE4
229533.A0A1C3YL56 pp 229533.I1RK03
229533.A0A1C3YL56 pp 229533.I1RC61
229533.A0A1C3YL56 pp 229533.I1RHA2
229533.A0A1C3YL56 pp 229533.I1RAY9
229533.I1RCT5 pp 229533.I1RD70
229533.I1RCT5 pp 229533.I1S1C8
229533.A0A0E0SNT6 pp 229533.A0A1C3YL56
229533.A0A0E0SNT6 pp 229533.I1RJX2
229533.A0A0E0SID1 pp 229533.I1RC61
229533.A0A0E0SID1 pp 229533.I1RFV9
229533.A0A0E0SID1 pp 229533.V6RCZ8
229533.A0A0E0SID1 pp 229533.I1RBE4
229533.A0A0E0SID1 pp 229533.I1RPR1
229533.A0A0E0SID1 pp 229533.V6QUE6
229533.A0A0E0SID1 pp 229533.I1RRU9
229533.A0A0E0SID1 pp 229533.I1RUI6
229533.A0A0E0SID1 pp 229533.I1RWM4
229533.A0A0E0SID1 pp 229533.A0A1C3YM35
229533.A0A0E0SID1 pp 229533.I1RA94
229533.A0A0E0SID1 pp 229533.I1S5B0
229533.A0A0E0SID1 pp 229533.I1S0A0
229533.A0A0E0SID1 pp 229533.V6RK96
229533.A0A0E0SID1 pp 229533.I1RJ17
229533.A0A0E0SID1 pp 229533.A0A1C3YKV3
229533.A0A0E0SID1 pp 229533.I1RZK8
229533.A0A0E0SID1 pp 229533.I1RAG5
229533.A0A0E0SID1 pp 229533.I1RSI4
229533.A0A0E0SID1 pp 229533.I1RS18
229533.A0A0E0SID1 pp 229533.A0A1C3YK13
229533.A0A0E0SID1 pp 229533.I1RFC0

3067	229533.A0A0E0SID1	pp	229533.I1RZM4
3068	229533.A0A0E0SID1	pp	229533.I1RZM1
3069	229533.A0A0E0SID1	pp	229533.I1RA90
3070	229533.A0A0E0SID1	pp	229533.V6RDH3
3071	229533.A0A0E0SID1	pp	229533.A0A1C3YJR3
3072	229533.A0A0E0SID1	pp	229533.I1RRX5
3073	229533.A0A0E0SID1	pp	229533.I1RDQ0
3074	229533.A0A0E0SID1	pp	229533.I1S0N3
3075	229533.A0A0E0SID1	pp	229533.I1S241
3076	229533.A0A0E0SID1	pp	229533.I1RCL0
3077	229533.A0A0E0SID1	pp	229533.A0A1C3YL20
3078	229533.A0A0E0SID1	pp	229533.I1RM26
3079	229533.A0A0E0SID1	pp	229533.V6R1K4
3080	229533.A0A0E0SID1	pp	229533.I1RCT7
3081	229533.A0A0E0SID1	pp	229533.A0A1C3YLI1
3082	229533.A0A0E0SID1	pp	229533.V6QU65
3083	229533.A0A0E0SID1	pp	229533.I1RK03
3084	229533.A0A0E0SID1	pp	229533.I1R9Y4
3085	229533.A0A0E0SID1	pp	229533.I1RCR5
3086	229533.A0A0E0SID1	pp	229533.I1S0X6
3087	229533.A0A0E0SID1	pp	229533.I1RWH8
3088	229533.A0A0E0SID1	pp	229533.I1RVX9
3089	229533.A0A0E0SID1	pp	229533.I1RX59
3090	229533.A0A0E0SID1	pp	229533.A0A1C3YJJ2
3091	229533.A0A0E0SID1	pp	229533.A0A1C3YN26
3092	229533.A0A0E0SID1	pp	229533.A0A1C3YN30
3093	229533.A0A0E0SID1	pp	229533.I1RAU9
3094	229533.A0A0E0SID1	pp	229533.I1RQE8
3095	229533.A0A0E0SID1	pp	229533.I1RMM8
3096	229533.A0A0E0SID1	pp	229533.I1RRG1
3097	229533.A0A0E0SID1	pp	229533.I1RNM7
3098	229533.A0A0E0SID1	pp	229533.V6RTJ8
3099	229533.A0A0E0SID1	pp	229533.I1RC74
3100	229533.A0A0E0SID1	pp	229533.I1S1W8
3101	229533.A0A0E0SID1	pp	229533.I1RY49
3102	229533.A0A0E0SID1	pp	229533.I1S8E0
3103	229533.A0A0E0SID1	pp	229533.V6RMW6
3104	229533.A0A0E0SID1	pp	229533.I1S1Y7
3105	229533.A0A0E0SID1	pp	229533.A0A0E0SIE3
3106	229533.A0A0E0SID1	pp	229533.I1RW06
3107	229533.A0A0E0SID1	pp	229533.I1RSU2
3108	229533.A0A0E0SID1	pp	229533.I1RAV0
3109	229533.A0A0E0SID1	pp	229533.I1RG11
3110	229533.A0A0E0SID1	pp	229533.I1RGT3
3111	229533.A0A0E0SID1	pp	229533.I1RTA0
3112	229533.A0A0E0SID1	pp	229533.I1RYW3
3113	229533.A0A0E0SID1	pp	229533.I1RFL3
3114	229533.A0A0E0SID1	pp	229533.I1RWF5
3115	229533.A0A0E0SID1	pp	229533.I1RS17
3116	229533.A0A0E0SID1	pp	229533.I1RBC7
3117	229533.A0A0E0SID1	pp	229533.I1R9N7
3118	229533.A0A0E0SID1	pp	229533.I1RXJ5
3119	229533.A0A0E0SID1	pp	229533.V6RLM7
3120	229533.A0A0E0SID1	pp	229533.I1RA70
3121	229533.A0A0E0SID1	pp	229533.I1S2C4
3122	229533.A0A0E0SID1	pp	229533.I1RXG6
3123	229533.A0A0E0SID1	pp	229533.I1RVZ2
3124	229533.A0A0E0SID1	pp	229533.I1S9Z4
3125	229533.A0A0E0SID1	pp	229533.A0A1C3YMF6
3126	229533.A0A0E0SID1	pp	229533.I1RKB2
3127	229533.A0A0E0SID1	pp	229533.I1RFT1
3128	229533.A0A0E0SID1	pp	229533.I1S8R2
3129	229533.A0A0E0SID1	pp	229533.I1RXY7
3130	229533.A0A0E0SID1	pp	229533.I1RCH5
3131	229533.A0A0E0SID1	pp	229533.A0A1C3YIR8
3132	229533.A0A0E0SID1	pp	229533.I1S0I3
3133	229533.A0A0E0SID1	pp	229533.I1S299
3134	229533.A0A0E0SID1	pp	229533.I1RSQ4
3135	229533.A0A0E0SID1	pp	229533.A0A1C3YI20
3136	229533.A0A0E0SID1	pp	229533.I1S8P5
3137	229533.A0A0E0SID1	pp	229533.Q4I1N3
3138	229533.A0A0E0SID1	pp	229533.I1RYP6
3139	229533.A0A0E0SID1	pp	229533.V6R057

229533.A0A0E0SID1	pp	229533.V6RHD0
229533.A0A0E0SID1	pp	229533.I1RW43
229533.A0A0E0SID1	pp	229533.I1RN67
229533.A0A0E0SID1	pp	229533.I1RAH3
229533.A0A0E0SID1	pp	229533.I1RSK5
229533.A0A0E0SID1	pp	229533.I1RFI8
229533.A0A0E0SID1	pp	229533.I1RVR2
229533.A0A0E0SID1	pp	229533.I1S2H4
229533.A0A0E0SID1	pp	229533.I1RE58
229533.A0A0E0SID1	pp	229533.V6RXR0
229533.A0A0E0SID1	pp	229533.I1S3G4
229533.A0A0E0SID1	pp	229533.I1RVZ3
229533.A0A0E0SID1	pp	229533.I1RGC3
229533.A0A0E0SID1	pp	229533.V6QV31
229533.A0A0E0SID1	pp	229533.A0A1C3YJW4
229533.A0A1C3YJ89	pp	229533.I1RW69
229533.A0A1C3YJ89	pp	229533.A0A1C3YKY6
229533.A0A1C3YJ89	pp	229533.V6RDH3
229533.A0A1C3YJ89	pp	229533.I1RRX5
229533.A0A1C3YJ89	pp	229533.I1RC31
229533.A0A1C3YJ89	pp	229533.I1RBA6
229533.A0A1C3YJ89	pp	229533.I1RJI2
229533.A0A1C3YJ89	pp	229533.I1RFI8
229533.A0A1C3YJ89	pp	229533.V6QV31
229533.A0A1C3YJ89	pp	229533.I1R9Y4
229533.I1RCX5	pp	229533.I1RH68
229533.I1RCX5	pp	229533.I1RQN9
229533.I1RCX5	pp	229533.I1S0S6
229533.I1RCX5	pp	229533.I1RTB7
229533.I1RCX5	pp	229533.I1RTE6
229533.I1RCX5	pp	229533.V6REE9
229533.I1RCX5	pp	229533.P0C431
229533.A0A098DGH9	pp	229533.I1S182
229533.A0A098DGH9	pp	229533.A0A098E5C4
229533.I1S0I3	pp	229533.I1S3G4
229533.I1S0I3	pp	229533.I1S1W8
229533.I1S0I3	pp	229533.V6QV31
229533.I1S0I3	pp	229533.Q4I5I4
229533.I1S0I3	pp	229533.V6RDH3
229533.I1S0I3	pp	229533.V6RHD0
229533.I1S0I3	pp	229533.Q4HXF0
229533.I1S0I3	pp	229533.V6RXR0
229533.I1S0I3	pp	229533.I1S241
229533.I1S0I3	pp	229533.V6QU65
229533.I1S0I3	pp	229533.V6RN69
229533.I1RVR2	pp	229533.I1RZ70
229533.I1RVR2	pp	229533.V6R382
229533.I1RVR2	pp	229533.V6RLM7
229533.I1RVR2	pp	229533.I1RYA0
229533.I1RVR2	pp	229533.I1S0I3
229533.I1RVR2	pp	229533.I1S8R2
229533.I1RVR2	pp	229533.I1RYC8
229533.I1RVR2	pp	229533.Q4I1N3
229533.I1RVR2	pp	229533.V6RM02
229533.I1RVR2	pp	229533.I1S8R4
229533.I1RVR2	pp	229533.V6RN69
229533.I1RVR2	pp	229533.I1RXY7
229533.I1RVR2	pp	229533.I1RW10
229533.I1RVR2	pp	229533.V6RN31
229533.I1RVR2	pp	229533.I1S0V7
229533.I1RVR2	pp	229533.I1RVY1
229533.I1RVR2	pp	229533.V6RSF3
229533.I1RVR2	pp	229533.I1RXV3
229533.I1RVR2	pp	229533.I1S147
229533.I1RVR2	pp	229533.I1S3L8
229533.I1RVR2	pp	229533.Q4HXF0
229533.I1RVR2	pp	229533.I1S673
229533.I1RVR2	pp	229533.Q4HVQ9
229533.I1RVR2	pp	229533.I1RZ90
229533.I1RVR2	pp	229533.I1S4B6
229533.I1RVR2	pp	229533.I1RW43
229533.I1RVR2	pp	229533.I1S3G4
229533.I1RVR2	pp	229533.O74638

229533.I1RVR2	pp	229533.V6R3J1
229533.I1RVR2	pp	229533.I1S9C6
229533.I1RVR2	pp	229533.I1RW69
229533.I1RVR2	pp	229533.I1S299
229533.I1RVR2	pp	229533.I1RXW3
229533.I1RVR2	pp	229533.I1S8F8
229533.I1RVR2	pp	229533.I1S9T6
229533.I1RVR2	pp	229533.I1S4B7
229533.I1RVR2	pp	229533.I1S0K2
229533.I1RVR2	pp	229533.I1RXS0
229533.I1RVR2	pp	229533.I1RVY2
229533.I1RVR2	pp	229533.V6RY56
229533.I1RVR2	pp	229533.I1RXV9
229533.I1RVR2	pp	229533.I1S0N3
229533.I1RVR2	pp	229533.I1S0V6
229533.I1RVR2	pp	229533.I1S4D0
229533.I1RVR2	pp	229533.I1S1W8
229533.I1RVR2	pp	229533.I1RVV7
229533.I1RVR2	pp	229533.I1RYI7
229533.I1RVR2	pp	229533.V6RK96
229533.I1RVR2	pp	229533.I1RW54
229533.I1RVR2	pp	229533.V6R4J6
229533.I1RVR2	pp	229533.I1S241
229533.I1RVR2	pp	229533.I1RZM4
229533.I1RVR2	pp	229533.I1RYJ7
229533.I1RVR2	pp	229533.I1RWM4
229533.I1RVR2	pp	229533.I1S8C1
229533.I1RVR2	pp	229533.I1S8E9
229533.I1RVR2	pp	229533.I1S3L2
229533.I1RVR2	pp	229533.I1RX27
229533.I1RVR2	pp	229533.I1S6A2
229533.I1RVR2	pp	229533.I1RYM6
229533.I1RVR2	pp	229533.I1S0K0
229533.I1RVR2	pp	229533.I1RVX9
229533.I1RVR2	pp	229533.V6RTJ8
229533.I1RVR2	pp	229533.I1RXB1
229533.I1RCT7	pp	229533.I1RZA7
229533.I1RCT7	pp	229533.I1RW11
229533.I1RCT7	pp	229533.I1RMG6
229533.I1RCT7	pp	229533.I1RNG4
229533.I1RCT7	pp	229533.V6RUB1
229533.I1RCT7	pp	229533.I1RWW7
229533.I1RCT7	pp	229533.I1S024
229533.I1RCT7	pp	229533.I1S6P5
229533.I1RCT7	pp	229533.I1RFI8
229533.I1RCT7	pp	229533.I1RSK5
229533.I1RCT7	pp	229533.I1S241
229533.I1RCT7	pp	229533.I1S3G4
229533.I1RCT7	pp	229533.V6QV31
229533.I1RCT7	pp	229533.I1RD21
229533.I1RCT7	pp	229533.I1RE58
229533.I1RCT7	pp	229533.V6RS91
229533.I1RCT7	pp	229533.I1S1F5
229533.I1RCT7	pp	229533.I1RE79
229533.I1RCT7	pp	229533.I1RXY7
229533.I1RCT7	pp	229533.I1RZK8
229533.I1RCT7	pp	229533.I1RZH8
229533.I1RCT7	pp	229533.I1RZY1
229533.I1RCT7	pp	229533.I1RFK3
229533.I1RCT7	pp	229533.I1RVX9
229533.I1RCT7	pp	229533.I1SAE8
229533.I1RCT7	pp	229533.I1RMI1
229533.I1RCT7	pp	229533.I1S0I3
229533.I1RCT7	pp	229533.I1RTJ2
229533.I1RCT7	pp	229533.I1S1W8
229533.I1RCT7	pp	229533.I1RUI6
229533.I1RCT7	pp	229533.Q4I1N3
229533.I1RCT7	pp	229533.I1RX59
229533.I1RCT7	pp	229533.I1RGK9
229533.I1RCT7	pp	229533.I1RMG7
229533.I1RCT7	pp	229533.V6RSF3
229533.I1RCT7	pp	229533.I1RHK2
229533.I1RCT7	pp	229533.I1RM26

229533.I1RCT7	pp	229533.V6RQW4
229533.I1RCT7	pp	229533.I1RY49
229533.I1RCT7	pp	229533.I1RPW1
229533.I1RCT7	pp	229533.V6RDH3
229533.I1RAH3	pp	229533.I1RSK5
229533.I1RAH3	pp	229533.V6QV31
229533.I1RAH3	pp	229533.I1RE58
229533.I1RAH3	pp	229533.I1RFI8
229533.I1RAH3	pp	229533.I1S3G4
229533.I1RAH3	pp	229533.V6RXR0
229533.I1RAH3	pp	229533.I1RWF5
229533.I1RAH3	pp	229533.V6RHD0
229533.I1RAH3	pp	229533.I1RW11
229533.I1RAH3	pp	229533.I1S1W8
229533.I1RAH3	pp	229533.I1RHK2
229533.I1RAH3	pp	229533.I1RVX9
229533.I1RAH3	pp	229533.I1RNG4
229533.I1RAH3	pp	229533.I1RNV4
229533.I1RAH3	pp	229533.I1RD21
229533.I1RAH3	pp	229533.I1RKZ7
229533.I1RAH3	pp	229533.I1S0I3
229533.I1RAH3	pp	229533.I1S241
229533.I1RAH3	pp	229533.I1RE79
229533.I1RAH3	pp	229533.V6RDH3
229533.I1RAH3	pp	229533.I1S147
229533.I1RAH3	pp	229533.I1RFK3
229533.I1RAH3	pp	229533.I1RCT7
229533.I1RAH3	pp	229533.I1RY49
229533.I1RAH3	pp	229533.I1RXY7
229533.I1RAH3	pp	229533.V6QU65
229533.I1RAH3	pp	229533.I1RYQ4
229533.I1RAH3	pp	229533.I1RY12
229533.I1RAH3	pp	229533.I1RC74
229533.I1RAH3	pp	229533.I1RGK9
229533.I1RAH3	pp	229533.I1RTJ2
229533.I1RAH3	pp	229533.I1RUI6
229533.I1RAH3	pp	229533.I1RX59
229533.I1RAH3	pp	229533.I1RZK8
229533.I1RAH3	pp	229533.V6RQW4
229533.I1RAH3	pp	229533.I1RM26
229533.I1RAH3	pp	229533.I1S1F5
229533.I1S540	pp	229533.V6R283
229533.I1S540	pp	229533.Q4IN63
229533.I1S540	pp	229533.V6R042
229533.I1S540	pp	229533.V6RTJ8
229533.I1S540	pp	229533.Q4IJK2
229533.I1RGT3	pp	229533.I1S0A0
229533.I1RGT3	pp	229533.I1RPD6
229533.I1RGT3	pp	229533.I1RRD0
229533.I1RGT3	pp	229533.I1RN80
229533.I1RGT3	pp	229533.I1S2H3
229533.I1RGT3	pp	229533.I1S4F4
229533.I1RGT3	pp	229533.V6RDH3
229533.I1RGT3	pp	229533.V6QV31
229533.A0A1C3YLV9	pp	229533.I1RNG4
229533.A0A1C3YLV9	pp	229533.A0A1C3YLX8
229533.A0A1C3YLV9	pp	229533.I1RND2
229533.A0A1C3YLV9	pp	229533.I1RNJ7
229533.A0A1C3YLV9	pp	229533.I1RDB7
229533.A0A0E0RMB3	pp	229533.I1RX59
229533.A0A0E0RMB3	pp	229533.A0A1C3YLI1
229533.A0A0E0RMB3	pp	229533.V6RQW4
229533.A0A0E0RMB3	pp	229533.I1RM26
229533.A0A0E0RMB3	pp	229533.V6RDH3
229533.A0A0E0RMB3	pp	229533.I1RZK8
229533.A0A0E0RMB3	pp	229533.I1RGK9
229533.A0A0E0RMB3	pp	229533.I1S1F5
229533.A0A0E0RMB3	pp	229533.I1S1W8
229533.A0A0E0RMB3	pp	229533.I1RAC8
229533.A0A0E0RMB3	pp	229533.I1RC74
229533.A0A0E0RMB3	pp	229533.I1RTJ2
229533.A0A0E0RMB3	pp	229533.I1RY49
229533.A0A0E0RMB3	pp	229533.A0A1C3YM35

3359 229533.A0A0E0RMB3 pp 229533.I1RUI6
3360 229533.A0A0E0RMB3 pp 229533.I1S241
3361 229533.A0A0E0RMB3 pp 229533.I1RVX9
3362 229533.A0A0E0RMB3 pp 229533.I1S0I3
3363 229533.A0A0E0RMB3 pp 229533.I1RD21
3364 229533.A0A0E0RMB3 pp 229533.I1RAH3
3365 229533.A0A0E0RMB3 pp 229533.I1RXY7
3366 229533.A0A0E0RMB3 pp 229533.I1RSK5
3367 229533.A0A0E0RMB3 pp 229533.A0A0E0SID1
3368 229533.A0A0E0RMB3 pp 229533.I1RE79
3369 229533.A0A0E0RMB3 pp 229533.I1RFK3
3370 229533.A0A0E0RMB3 pp 229533.V6QV31
3371 229533.A0A0E0RMB3 pp 229533.I1RFI8
3372 229533.A0A0E0RMB3 pp 229533.I1S3G4
3373 229533.A0A0E0RMB3 pp 229533.I1RE58
3374 229533.A0A0E0RMB3 pp 229533.I1RNG4
3375 229533.I1RC86 pp 229533.I1RNJ5
3376 229533.I1RC86 pp 229533.Q4IPZ1
3377 229533.I1RC86 pp 229533.I1RPY8
3378 229533.I1RC86 pp 229533.I1RZS4
3379 229533.I1RC86 pp 229533.Q4HZ95
3380 229533.I1RC86 pp 229533.I1S8F0
3381 229533.I1RC86 pp 229533.Q4IB65
3382 229533.I1RC86 pp 229533.Q4I7T5
3383 229533.I1RC86 pp 229533.I1RJT3
3384 229533.I1RC86 pp 229533.Q4IR18
3385 229533.I1RC86 pp 229533.I1S5S8
3386 229533.I1RC86 pp 229533.Q4I099
3387 229533.I1RC86 pp 229533.I1RZH5
3388 229533.I1RC86 pp 229533.I1RRK8
3389 229533.I1RC86 pp 229533.I1RXE6
3390 229533.I1RC86 pp 229533.I1RT38
3391 229533.I1RC86 pp 229533.I1RMK1
3392 229533.I1RC86 pp 229533.Q4IK03
3393 229533.I1RC86 pp 229533.I1RQR6
3394 229533.I1RC86 pp 229533.Q4IJW4
3395 229533.I1RC86 pp 229533.Q4I6B0
3396 229533.I1RC86 pp 229533.I1RJV7
3397 229533.I1RC86 pp 229533.I1RN27
3398 229533.I1RC86 pp 229533.I1RFV5
3399 229533.I1RC86 pp 229533.Q4I375
3400 229533.A0A1C3YN78 pp 229533.I1RQ06
3401 229533.A0A1C3YN78 pp 229533.I1S1W8
3402 229533.A0A1C3YN78 pp 229533.I1RNM7
3403 229533.A0A1C3YN78 pp 229533.I1RHQ0
3404 229533.A0A1C3YN78 pp 229533.I1RFI8
3405 229533.A0A1C3YN78 pp 229533.I1RCV0
3406 229533.A0A1C3YN78 pp 229533.V6RJ21
3407 229533.A0A1C3YN78 pp 229533.I1RLY6
3408 229533.A0A1C3YN78 pp 229533.V6QV31
3409 229533.A0A1C3YN78 pp 229533.I1R9Y4
3410 229533.A0A1C3YN78 pp 229533.I1RJX2
3411 229533.A0A1C3YN78 pp 229533.I1RRX5
3412 229533.A0A1C3YN78 pp 229533.I1S3G4
3413 229533.A0A1C3YN78 pp 229533.I1RVX0
3414 229533.A0A1C3YN78 pp 229533.A0A1C3YN81
3415 229533.A0A1C3YN78 pp 229533.V6RDH3
3416 229533.A0A1C3YN78 pp 229533.I1RNS2
3417 229533.A0A1C3YN78 pp 229533.I1RH67
3418 229533.A0A1C3YN78 pp 229533.I1RWM4
3419 229533.I1RGF3 pp 229533.I1RGF4
3420 229533.I1RGF3 pp 229533.I1S6V3
3421 229533.I1RAD8 pp 229533.I1RIH0
3422 229533.I1RAD8 pp 229533.I1RK47
3423 229533.I1RAD8 pp 229533.V6QVH8
3424 229533.I1RAD8 pp 229533.Q4HTS9
3425 229533.I1RAD8 pp 229533.I1RVJ6
3426 229533.I1RAD8 pp 229533.I1REI0
3427 229533.I1RAD8 pp 229533.I1RW06
3428 229533.I1RAD8 pp 229533.I1S3F7
3429 229533.I1RAD8 pp 229533.I1S0J8
3430 229533.I1RAD8 pp 229533.I1RE49
3431 229533.I1RAD8 pp 229533.I1RKJ7

229533.A0A098E0S0	pp	229533.I1RNB8
229533.A0A098E0S0	pp	229533.I1RG90
229533.A0A098E0S0	pp	229533.I1RT35
229533.I1RGF2	pp	229533.I1S193
229533.I1RGF2	pp	229533.I1RRU9
229533.I1RGF2	pp	229533.I1S6V3
229533.I1RGF2	pp	229533.I1RGF4
229533.I1RGF2	pp	229533.I1RGF3
229533.I1RGF2	pp	229533.I1RZM1
229533.I1RGF2	pp	229533.Q4IAA0
229533.I1RGF2	pp	229533.I1S0L8
229533.I1RGF2	pp	229533.I1RY40
229533.I1RGF2	pp	229533.Q4ICM0
229533.I1RXM6	pp	229533.I1S0J8
229533.I1S5Z3	pp	229533.I1S987
229533.I1S5Z3	pp	229533.Q4INS6
229533.I1S5Z3	pp	229533.Q4IJK2
229533.I1S5Z3	pp	229533.I1S998
229533.I1RGE9	pp	229533.I1RGF2
229533.I1RGE9	pp	229533.I1RGF4
229533.I1RGE9	pp	229533.I1RGF3
229533.I1RGE9	pp	229533.I1S6V3
229533.I1RGE9	pp	229533.I1RGF1
229533.I1RGE9	pp	229533.I1RGF0
229533.I1RGE9	pp	229533.I1RT98
229533.I1RMD6	pp	229533.I1RW69
229533.I1RMD6	pp	229533.V6QV31
229533.I1RMD6	pp	229533.I1RRX5
229533.I1RMD6	pp	229533.V6RDH3
229533.I1RGF4	pp	229533.I1S6V3
229533.I1RGF4	pp	229533.I1RXX1
229533.I1RGF4	pp	229533.I1RNK1
229533.I1RGF4	pp	229533.I1RN89
229533.I1RGF4	pp	229533.I1RYJ8
229533.I1RGF4	pp	229533.I1RQ76
229533.I1RGF4	pp	229533.I1RY25
229533.I1RGF4	pp	229533.I1RR68
229533.I1RGF4	pp	229533.I1RTE7
229533.A0A098E0R6	pp	229533.I1RJU3
229533.A0A098E0R6	pp	229533.I1RS17
229533.A0A098E0R6	pp	229533.A0A1C3YKV3
229533.A0A098E0R6	pp	229533.V6RIV9
229533.A0A098E0R6	pp	229533.I1RCH5
229533.A0A098E0R6	pp	229533.I1REK8
229533.I1RCX4	pp	229533.I1RH68
229533.I1RCX4	pp	229533.I1S0S6
229533.I1RCX4	pp	229533.V6REE9
229533.I1RCX4	pp	229533.I1RTB7
229533.I1RCX4	pp	229533.I1RTE6
229533.I1RCX4	pp	229533.I1RQN9
229533.I1RCX4	pp	229533.P0C431
229533.A0A1C3YMG8	pp	229533.I1RE46
229533.A0A1C3YMG8	pp	229533.I1RMS4
229533.A0A1C3YMG8	pp	229533.V6R382
229533.A0A1C3YMG8	pp	229533.Q4IEA5
229533.A0A1C3YMG8	pp	229533.I1RKQ0
229533.A0A1C3YMG8	pp	229533.I1RSI4
229533.A0A1C3YMG8	pp	229533.I1RBD7
229533.A0A1C3YMG8	pp	229533.Q4IQM6
229533.A0A1C3YMG8	pp	229533.I1RN57
229533.A0A1C3YMG8	pp	229533.I1RFN0
229533.A0A1C3YMG8	pp	229533.I1RGF4
229533.A0A1C3YMG8	pp	229533.I1S209
229533.A0A1C3YMG8	pp	229533.I1RC54
229533.A0A1C3YMG8	pp	229533.Q4IPC8
229533.A0A1C3YMG8	pp	229533.I1RYR2
229533.A0A1C3YMG8	pp	229533.I1RPQ1
229533.A0A1C3YMG8	pp	229533.I1RAH4
229533.A0A1C3YMG8	pp	229533.I1RGF2
229533.A0A1C3YMG8	pp	229533.I1RGE1
229533.A0A1C3YMG8	pp	229533.I1RSA7
229533.A0A1C3YMG8	pp	229533.I1S6V3
229533.A0A1C3YMG8	pp	229533.I1RRS7

3505 229533.A0A1C3YMG8 pp 229533.I1RN55
3506 229533.A0A1C3YMG8 pp 229533.I1RCA5
3507 229533.A0A1C3YMG8 pp 229533.I1RGE9
3508 229533.A0A1C3YMG8 pp 229533.I1RJY5
3509 229533.A0A1C3YMG8 pp 229533.I1RKI2
3510 229533.A0A1C3YMG8 pp 229533.I1RGE7
3511 229533.A0A1C3YMG8 pp 229533.Q4IP51
3512 229533.A0A1C3YMG8 pp 229533.I1S283
3513 229533.A0A1C3YMG8 pp 229533.I1S201
3514 229533.A0A1C3YMG8 pp 229533.I1RMH6
3515 229533.A0A1C3YMG8 pp 229533.A0A1C3YMM0
3516 229533.A0A1C3YMG8 pp 229533.I1RYX9
3517 229533.A0A1C3YMG8 pp 229533.I1RUG1
3518 229533.A0A1C3YMG8 pp 229533.I1RGF3
3519 229533.A0A1C3YMG8 pp 229533.I1RGF1
3520 229533.A0A1C3YMG8 pp 229533.I1S461
3521 229533.A0A1C3YMG8 pp 229533.I1RZP8
3522 229533.A0A1C3YMG8 pp 229533.I1RFJ2
3523 229533.A0A1C3YMG8 pp 229533.I1RGE8
3524 229533.A0A1C3YMG8 pp 229533.I1RGF0
3525 229533.A0A1C3YMG8 pp 229533.I1RFX8
3526 229533.A0A1C3YMG8 pp 229533.I1RQJ4
3527 229533.A0A1C3YMG8 pp 229533.I1RSP7
3528 229533.A0A1C3YMG8 pp 229533.V6RLW4
3529 229533.A0A1C3YMG8 pp 229533.A0A1C3YMH3
3530 229533.A0A1C3YN82 pp 229533.I1S8W6
3531 229533.A0A1C3YN82 pp 229533.I1RDY1
3532 229533.A0A1C3YN82 pp 229533.I1S4Z5
3533 229533.A0A098E0M6 pp 229533.A0A1C3YN82
3534 229533.I1RYP6 pp 229533.V6RDH3
3535 229533.I1RYP6 pp 229533.I1S2H3
3536 229533.I1RYP6 pp 229533.I1S0A0
3537 229533.I1RYP6 pp 229533.V6QV31
3538 229533.I1RYP6 pp 229533.I1S4F4
3539 229533.I1RGE8 pp 229533.I1RGE9
3540 229533.I1RGE8 pp 229533.I1S6V3
3541 229533.I1RGE8 pp 229533.I1RGF4
3542 229533.I1RGE8 pp 229533.I1RGF0
3543 229533.I1RGE8 pp 229533.I1RGF3
3544 229533.I1RGE8 pp 229533.I1RGF2
3545 229533.I1RGE8 pp 229533.I1RGF1
3546 229533.A0A1C3YIP4 pp 229533.I1RZJ6
3547 229533.A0A1C3YIP4 pp 229533.I1RRM7
3548 229533.I1S058 pp 229533.Q4IQT8
3549 229533.I1RK81 pp 229533.V6R223
3550 229533.I1RK81 pp 229533.I1S4Q4
3551 229533.I1RK81 pp 229533.I1S9U1
3552 229533.I1RK81 pp 229533.I1RPI3
3553 229533.I1RK81 pp 229533.I1RY49
3554 229533.I1RK81 pp 229533.I1S7A2
3555 229533.I1RJA2 pp 229533.I1S1Q6
3556 229533.I1RJA2 pp 229533.I1RJQ9
3557 229533.I1RJA2 pp 229533.I1RLN6
3558 229533.I1RJA2 pp 229533.I1RUB4
3559 229533.I1RJA2 pp 229533.I1S8P4
3560 229533.A0A1C3YMH3 pp 229533.I1S6V3
3561 229533.A0A1C3YMH3 pp 229533.I1S4T3
3562 229533.A0A1C3YMH3 pp 229533.I1RGE9
3563 229533.A0A1C3YMH3 pp 229533.I1RGF4
3564 229533.A0A1C3YMH3 pp 229533.I1R9X8
3565 229533.A0A1C3YMH3 pp 229533.I1RGE7
3566 229533.A0A1C3YMH3 pp 229533.I1S8W6
3567 229533.A0A1C3YMH3 pp 229533.I1RGF3
3568 229533.A0A1C3YMH3 pp 229533.I1RGF2
3569 229533.A0A1C3YMH3 pp 229533.I1RGE1
3570 229533.A0A1C3YMH3 pp 229533.A0A1C3YMM0
3571 229533.A0A1C3YMH3 pp 229533.I1RRH5
3572 229533.A0A1C3YMH3 pp 229533.I1RGF0
3573 229533.A0A1C3YMH3 pp 229533.I1RGE8
3574 229533.A0A1C3YMH3 pp 229533.I1RGF1
3575 229533.I1RE58 pp 229533.I1RZY1
3576 229533.I1RE58 pp 229533.I1RZH8
3577 229533.I1RE58 pp 229533.I1S1W8

3578 229533.I1RE58 pp 229533.I1RVX9
3579 229533.I1RE58 pp 229533.I1SAE8
3580 229533.I1RE58 pp 229533.I1RGK9
3581 229533.I1RE58 pp 229533.I1RM26
3582 229533.I1RE58 pp 229533.I1RX59
3583 229533.I1RE58 pp 229533.I1RY49
3584 229533.I1RE58 pp 229533.I1RPE5
3585 229533.I1RE58 pp 229533.V6RQW4
3586 229533.I1RE58 pp 229533.I1RTJ2
3587 229533.I1RE58 pp 229533.I1RFV9
3588 229533.I1RE58 pp 229533.I1RFI8
3589 229533.I1RE58 pp 229533.I1S241
3590 229533.I1RE58 pp 229533.I1RUI6
3591 229533.I1RE58 pp 229533.I1RZA7
3592 229533.I1RE58 pp 229533.V6RDH3
3593 229533.I1RE58 pp 229533.V6RXR0
3594 229533.I1RE58 pp 229533.I1S6P5
3595 229533.I1RE58 pp 229533.V6QU65
3596 229533.I1RE58 pp 229533.I1S1F5
3597 229533.I1RE58 pp 229533.I1RNG4
3598 229533.I1RE58 pp 229533.V6RHD0
3599 229533.I1RE58 pp 229533.I1RSK5
3600 229533.I1RE58 pp 229533.I1RW11
3601 229533.I1RE58 pp 229533.V6QV31
3602 229533.I1RE58 pp 229533.I1S3G4
3603 229533.I1RE58 pp 229533.I1RZK8
3604 229533.I1RE58 pp 229533.I1RXY7
3605 229533.I1RE58 pp 229533.I1RE79
3606 229533.I1RE58 pp 229533.I1S024
3607 229533.I1RE58 pp 229533.I1RFK3
3608 229533.I1RE58 pp 229533.V6RUB1
3609 229533.I1RE58 pp 229533.Q4I1N3
3610 229533.I1RE58 pp 229533.I1RWW7
3611 229533.I1RE58 pp 229533.I1S0I3
3612 229533.I1RGE7 pp 229533.I1RGF0
3613 229533.I1RGE7 pp 229533.I1S6V3
3614 229533.I1RGE7 pp 229533.I1RGF1
3615 229533.I1RGE7 pp 229533.I1RWS7
3616 229533.I1RGE7 pp 229533.Q4I0J6
3617 229533.I1RGE7 pp 229533.I1RGF4
3618 229533.I1RGE7 pp 229533.I1RGF3
3619 229533.I1RGE7 pp 229533.I1RGF2
3620 229533.I1RGE7 pp 229533.I1RGE9
3621 229533.I1RGE7 pp 229533.I1RGE8
3622 229533.I1RCQ5 pp 229533.I1RUB4
3623 229533.I1RCQ5 pp 229533.Q4I1Y1
3624 229533.I1RCQ5 pp 229533.I1RK12
3625 229533.I1RCQ5 pp 229533.I1RMN2
3626 229533.I1RCQ5 pp 229533.I1RFH2
3627 229533.I1RCQ5 pp 229533.I1S8P4
3628 229533.I1RCQ5 pp 229533.I1RIZ1
3629 229533.I1RCQ5 pp 229533.I1RJQ9
3630 229533.I1RCQ5 pp 229533.I1RH98
3631 229533.I1RCQ5 pp 229533.I1RRQ2
3632 229533.I1RCQ5 pp 229533.I1RJA2
3633 229533.I1S2Q4 pp 229533.I1S3G4
3634 229533.I1S2Q4 pp 229533.I1S3T7
3635 229533.I1S2Q4 pp 229533.I1S6M5
3636 229533.I1S2Q4 pp 229533.I1S8C1
3637 229533.I1S2Q4 pp 229533.I1S8R2
3638 229533.I1S2Q4 pp 229533.I1S599
3639 229533.I1S2Q4 pp 229533.I1SAF0
3640 229533.I1S2Q4 pp 229533.Q4HVQ9
3641 229533.A0A1C3YMM0 pp 229533.I1RGF0
3642 229533.A0A1C3YMM0 pp 229533.I1RGF4
3643 229533.A0A1C3YMM0 pp 229533.I1RGE9
3644 229533.A0A1C3YMM0 pp 229533.I1RGE7
3645 229533.A0A1C3YMM0 pp 229533.I1RGF1
3646 229533.A0A1C3YMM0 pp 229533.I1S6V3
3647 229533.A0A1C3YMM0 pp 229533.I1RGF2
3648 229533.A0A1C3YMM0 pp 229533.I1RGE8
3649 229533.A0A1C3YMM0 pp 229533.I1RGF3
3650 229533.A0A1C3YMM0 pp 229533.I1RGE1

3651 229533.I1RH98 pp 229533.I1S8P4
3652 229533.I1RH98 pp 229533.Q4I1Y1
3653 229533.I1RH98 pp 229533.I1RJQ9
3654 229533.I1RH98 pp 229533.I1RJA2
3655 229533.I1RH98 pp 229533.I1RUB4
3656 229533.I1RH98 pp 229533.I1RIZ1
3657 229533.I1RGF7 pp 229533.I1S0S6
3658 229533.I1RGF7 pp 229533.I1RH68
3659 229533.I1RGF7 pp 229533.I1RQN9
3660 229533.I1RGF7 pp 229533.I1RX09
3661 229533.I1RGF7 pp 229533.P0C431
3662 229533.I1RGF7 pp 229533.I1RTB7
3663 229533.I1RTY0 pp 229533.I1RW54
3664 229533.I1RTY0 pp 229533.I1S1W8
3665 229533.I1RTY0 pp 229533.I1S599
3666 229533.I1RTY0 pp 229533.I1S2C4
3667 229533.I1RTY0 pp 229533.I1SAF0
3668 229533.I1RTY0 pp 229533.I1RY35
3669 229533.I1RTY0 pp 229533.I1RWL3
3670 229533.I1RTY0 pp 229533.I1RYA2
3671 229533.I1RTY0 pp 229533.I1RVB2
3672 229533.I1RTY0 pp 229533.I1RUS5
3673 229533.I1RTY0 pp 229533.I1S267
3674 229533.I1RTY0 pp 229533.I1S3G4
3675 229533.I1RTY0 pp 229533.I1S6M5
3676 229533.I1RTY0 pp 229533.I1RYQ4
3677 229533.I1RTY0 pp 229533.I1RYK1
3678 229533.I1RTY0 pp 229533.Q4HVQ9
3679 229533.I1RTY0 pp 229533.I1RYW9
3680 229533.I1RTY0 pp 229533.I1RYX6
3681 229533.I1RTY0 pp 229533.I1RYA8
3682 229533.I1RGF0 pp 229533.I1RGF4
3683 229533.I1RGF0 pp 229533.I1RGF2
3684 229533.I1RGF0 pp 229533.I1RX03
3685 229533.I1RGF0 pp 229533.I1S6V3
3686 229533.I1RGF0 pp 229533.I1RGF3
3687 229533.I1RGF0 pp 229533.I1RGF1
3688 229533.A0A098DG97 pp 229533.I1RGF1
3689 229533.A0A098DG97 pp 229533.I1RGE1
3690 229533.A0A098DG97 pp 229533.I1RGF3
3691 229533.A0A098DG97 pp 229533.I1RGF2
3692 229533.A0A098DG97 pp 229533.I1RGE9
3693 229533.A0A098DG97 pp 229533.I1RGF4
3694 229533.A0A098DG97 pp 229533.I1S6V3
3695 229533.A0A098DG97 pp 229533.A0A1C3YMG8
3696 229533.A0A098DG97 pp 229533.I1RGE8
3697 229533.A0A098DG97 pp 229533.A0A1C3YMH3
3698 229533.A0A098DG97 pp 229533.I1RGE7
3699 229533.A0A098DG97 pp 229533.A0A1C3YMM0
3700 229533.A0A098DG97 pp 229533.I1RGF0
3701 229533.A0A1C3YJI5 pp 229533.I1RD67
3702 229533.A0A1C3YJI5 pp 229533.I1RD52
3703 229533.A0A1C3YJI5 pp 229533.I1RYX6
3704 229533.A0A1C3YJI5 pp 229533.I1RBE7
3705 229533.A0A1C3YJI5 pp 229533.I1RCU2
3706 229533.A0A1C3YJI5 pp 229533.Q4IJK2
3707 229533.A0A1C3YJI5 pp 229533.I1RPN1
3708 229533.A0A1C3YJI5 pp 229533.I1RG33
3709 229533.A0A1C3YJI5 pp 229533.A0A1C3YN04
3710 229533.A0A1C3YJI5 pp 229533.Q4HYR2
3711 229533.A0A1C3YJI5 pp 229533.I1RDU0
3712 229533.A0A1C3YJI5 pp 229533.I1S4G3
3713 229533.A0A1C3YJI5 pp 229533.I1RUQ3
3714 229533.A0A1C3YJI5 pp 229533.I1SAF0
3715 229533.A0A1C3YJI5 pp 229533.I1RNM7
3716 229533.A0A1C3YJI5 pp 229533.I1RMK7
3717 229533.A0A1C3YJI5 pp 229533.I1RJS7
3718 229533.A0A1C3YJI5 pp 229533.I1RY21
3719 229533.A0A1C3YJI5 pp 229533.I1S4S7
3720 229533.A0A1C3YJI5 pp 229533.I1RA28
3721 229533.A0A1C3YJI5 pp 229533.I1RVB2
3722 229533.A0A1C3YJI5 pp 229533.I1RYA2
3723 229533.A0A1C3YJI5 pp 229533.I1RJU3

3724	229533.A0A1C3YJI5	pp	229533.I1RMQ6
3725	229533.A0A1C3YJI5	pp	229533.I1S1B8
3726	229533.A0A1C3YJI5	pp	229533.I1RWR7
3727	229533.A0A1C3YJI5	pp	229533.I1RP86
3728	229533.A0A1C3YJI5	pp	229533.I1RCR6
3729	229533.A0A1C3YJI5	pp	229533.I1S0V6
3730	229533.A0A1C3YJI5	pp	229533.I1RAM9
3731	229533.A0A1C3YJI5	pp	229533.I1RSZ7
3732	229533.A0A1C3YJI5	pp	229533.I1RXD7
3733	229533.A0A1C3YJI5	pp	229533.I1RJJ7
3734	229533.A0A1C3YJI5	pp	229533.I1RVR2
3735	229533.A0A1C3YJI5	pp	229533.I1S8S9
3736	229533.A0A1C3YJI5	pp	229533.A0A1C3YLB3
3737	229533.A0A1C3YJI5	pp	229533.I1RTY0
3738	229533.A0A1C3YJI5	pp	229533.I1RA11
3739	229533.A0A1C3YJI5	pp	229533.I1RJY7
3740	229533.A0A1C3YJI5	pp	229533.I1S5R1
3741	229533.A0A1C3YJI5	pp	229533.I1RJB0
3742	229533.A0A1C3YJI5	pp	229533.I1RBU8
3743	229533.A0A1C3YJI5	pp	229533.A0A1C3YJW1
3744	229533.A0A1C3YJI5	pp	229533.I1RAV5
3745	229533.A0A1C3YJI5	pp	229533.I1R9M7
3746	229533.A0A1C3YJI5	pp	229533.I1RZK5
3747	229533.A0A1C3YJI5	pp	229533.I1RQ85
3748	229533.A0A1C3YJI5	pp	229533.I1RPE0
3749	229533.A0A1C3YJI5	pp	229533.I1S8F8
3750	229533.A0A1C3YJI5	pp	229533.I1RG66
3751	229533.A0A1C3YJI5	pp	229533.A0A1C3YK34
3752	229533.A0A1C3YJI5	pp	229533.A0A1C3YKR5
3753	229533.A0A1C3YJI5	pp	229533.I1S2Q4
3754	229533.A0A1C3YJI5	pp	229533.A0A1C3YJN3
3755	229533.A0A1C3YJI5	pp	229533.I1REE2
3756	229533.A0A1C3YJI5	pp	229533.I1RVZ2
3757	229533.A0A1C3YJI5	pp	229533.I1RA62
3758	229533.A0A1C3YJI5	pp	229533.I1RKP1
3759	229533.A0A1C3YJI5	pp	229533.I1RWM4
3760	229533.A0A1C3YJI5	pp	229533.I1RRI1
3761	229533.A0A1C3YJI5	pp	229533.I1S3T7
3762	229533.A0A1C3YJI5	pp	229533.I1RJN9
3763	229533.A0A1C3YJI5	pp	229533.I1S423
3764	229533.A0A1C3YJI5	pp	229533.A0A1C3YJV6
3765	229533.A0A1C3YJI5	pp	229533.I1RKB2
3766	229533.A0A1C3YJI5	pp	229533.I1RTA1
3767	229533.A0A1C3YJI5	pp	229533.I1RSQ4
3768	229533.A0A1C3YJI5	pp	229533.I1RZ39
3769	229533.A0A1C3YJI5	pp	229533.I1RUY5
3770	229533.A0A1C3YJI5	pp	229533.I1S299
3771	229533.A0A1C3YJI5	pp	229533.I1RZM8
3772	229533.A0A1C3YJI5	pp	229533.I1RDF7
3773	229533.A0A1C3YJI5	pp	229533.I1RRF5
3774	229533.A0A1C3YJI5	pp	229533.I1RNG4
3775	229533.A0A1C3YJI5	pp	229533.I1RNY6
3776	229533.A0A1C3YJI5	pp	229533.I1RYI5
3777	229533.A0A1C3YJI5	pp	229533.I1S0W4
3778	229533.A0A1C3YJI5	pp	229533.I1RPR1
3779	229533.A0A1C3YJI5	pp	229533.A0A1C3YLK8
3780	229533.A0A1C3YJI5	pp	229533.I1S2B8
3781	229533.A0A1C3YJI5	pp	229533.Q4HVQ9
3782	229533.A0A1C3YJI5	pp	229533.I1RUR8
3783	229533.A0A1C3YJI5	pp	229533.V6R3J1
3784	229533.A0A1C3YJI5	pp	229533.I1R9M0
3785	229533.A0A1C3YJI5	pp	229533.I1RL81
3786	229533.A0A1C3YJI5	pp	229533.I1RSA5
3787	229533.A0A1C3YJI5	pp	229533.I1RFI4
3788	229533.A0A1C3YJI5	pp	229533.I1RGA4
3789	229533.A0A1C3YJI5	pp	229533.I1RYW9
3790	229533.A0A1C3YJI5	pp	229533.I1RA52
3791	229533.A0A1C3YJI5	pp	229533.I1RBE6
3792	229533.A0A1C3YJI5	pp	229533.I1RPU2
3793	229533.A0A1C3YJI5	pp	229533.I1RT40
3794	229533.A0A1C3YJI5	pp	229533.A0A1C3YJZ1
3795	229533.A0A1C3YJI5	pp	229533.A0A1C3YJI6
3796	229533.A0A1C3YJI5	pp	229533.I1RQ33

```
229533.A0A1C3YJI5   pp  229533.V6RAC9
229533.A0A1C3YJI5   pp  229533.I1RSF1
229533.A0A1C3YJI5   pp  229533.I1RN70
229533.A0A1C3YJI5   pp  229533.A0A1C3YLB6
229533.A0A1C3YJI5   pp  229533.I1RYA0
229533.A0A1C3YJI5   pp  229533.V6QVH8
229533.A0A1C3YJI5   pp  229533.I1S8R2
229533.A0A1C3YJI5   pp  229533.V6RLM7
229533.A0A1C3YJI5   pp  229533.I1R9W4
229533.A0A1C3YJI5   pp  229533.I1S0V7
229533.A0A1C3YJI5   pp  229533.I1RE89
229533.A0A1C3YJI5   pp  229533.I1RCH5
229533.A0A1C3YJI5   pp  229533.V6RRM0
229533.A0A1C3YJI5   pp  229533.I1RWZ3
229533.A0A1C3YJI5   pp  229533.V6RGI1
229533.A0A1C3YJI5   pp  229533.I1RF85
229533.A0A1C3YJI5   pp  229533.I1RAW0
229533.A0A1C3YJI5   pp  229533.I1RYC8
229533.A0A1C3YJI5   pp  229533.I1RR05
229533.A0A1C3YJI5   pp  229533.I1S5R6
229533.A0A1C3YJI5   pp  229533.I1S1W9
229533.A0A1C3YJI5   pp  229533.I1RZV3
229533.A0A1C3YJI5   pp  229533.V6RN69
229533.A0A1C3YJI5   pp  229533.A0A1C3YJK5
229533.A0A1C3YJI5   pp  229533.I1S599
229533.A0A1C3YJI5   pp  229533.I1RHC8
229533.A0A1C3YJI5   pp  229533.A0A1C3YMF1
229533.A0A1C3YJI5   pp  229533.I1RRM3
229533.A0A1C3YJI5   pp  229533.I1RKZ2
229533.A0A098DRV4   pp  229533.I1RJU7
229533.A0A098DRV4   pp  229533.A0A098DUU3
229533.A0A098DRV4   pp  229533.A0A1C3YID5
229533.A0A098DRV4   pp  229533.A0A0E0SG28
229533.A0A098DRV4   pp  229533.I1RY14
229533.I1R994   pp  229533.I1RUB4
229533.I1R994   pp  229533.I1S8P4
229533.I1R994   pp  229533.I1S1D3
229533.I1R994   pp  229533.Q4I1Y1
229533.I1R994   pp  229533.I1RMP0
229533.I1R994   pp  229533.I1RJQ9
229533.I1R994   pp  229533.I1RIZ1
229533.I1R994   pp  229533.I1RCQ5
229533.I1R994   pp  229533.I1RJA2
229533.I1R994   pp  229533.I1RH98
229533.A0A098DG56   pp  229533.A0A098DRV4
229533.I1S1B8   pp  229533.I1S3G4
229533.I1S1B8   pp  229533.I1S267
229533.I1S1B8   pp  229533.I1S6M5
229533.I1S1B8   pp  229533.I1S1W8
229533.I1S1B8   pp  229533.I1SAF0
229533.I1S1B8   pp  229533.I1S2C4
229533.I1S1B8   pp  229533.Q4HVQ9
229533.I1S1B8   pp  229533.I1S599
229533.I1RIZ1   pp  229533.I1RJA2
229533.I1RIZ1   pp  229533.Q4I1Y1
229533.I1RIZ1   pp  229533.I1S8P4
229533.I1RIZ1   pp  229533.I1RJQ9
229533.I1RIZ1   pp  229533.I1RUB4
229533.I1RJJ7   pp  229533.I1RVB2
229533.I1RJJ7   pp  229533.I1S3G4
229533.I1RJJ7   pp  229533.I1RYK1
229533.I1RJJ7   pp  229533.I1RWL3
229533.I1RJJ7   pp  229533.I1RYQ4
229533.I1RJJ7   pp  229533.I1RYA8
229533.I1RJJ7   pp  229533.I1RPE5
229533.I1RJJ7   pp  229533.I1RQ60
229533.I1RJJ7   pp  229533.I1SAF0
229533.I1RJJ7   pp  229533.Q4HVQ9
229533.I1RJJ7   pp  229533.I1RW54
229533.I1RJJ7   pp  229533.I1RUS5
229533.I1RJJ7   pp  229533.I1S1W8
229533.I1RJJ7   pp  229533.I1RQE1
229533.I1RJJ7   pp  229533.I1S6M5
```

229533.I1RJJ7	pp	229533.I1RY35
229533.I1RJJ7	pp	229533.I1RYX6
229533.I1RJJ7	pp	229533.I1RYW9
229533.I1RJJ7	pp	229533.I1RKZ2
229533.I1RJJ7	pp	229533.I1RNV4
229533.I1RJJ7	pp	229533.I1RKZ7
229533.I1RJJ7	pp	229533.I1RNG4
229533.I1RJJ7	pp	229533.I1S599
229533.I1RJJ7	pp	229533.I1RRF5
229533.I1RJJ7	pp	229533.I1RNY6
229533.I1RJJ7	pp	229533.I1S267
229533.I1RJJ7	pp	229533.I1S2C4
229533.I1RJJ7	pp	229533.I1RYA2
229533.I1RJQ9	pp	229533.I1S8P4
229533.I1RJQ9	pp	229533.Q4I1Y1
229533.I1RJQ9	pp	229533.I1RUB4
229533.A0A098E0H0	pp	229533.I1RJA2
229533.A0A098E0H0	pp	229533.A0A0E0SAE0
229533.A0A098E0H0	pp	229533.I1RCQ5
229533.A0A098E0H0	pp	229533.I1RH98
229533.A0A098E0H0	pp	229533.I1RUB4
229533.A0A098E0H0	pp	229533.Q4I1Y1
229533.A0A098E0H0	pp	229533.I1S8P4
229533.A0A098E0H0	pp	229533.I1R994
229533.A0A098E0H0	pp	229533.I1RIZ1
229533.A0A098E0H0	pp	229533.I1RJQ9
229533.I1RJW8	pp	229533.Q4I5I4
229533.I1RJW8	pp	229533.I1RXU0
229533.I1RJW8	pp	229533.V6QUE6
229533.I1RJW8	pp	229533.I1RVZ4
229533.I1RJW8	pp	229533.I1RK71
229533.I1RJW8	pp	229533.I1S276
229533.I1RJW8	pp	229533.I1RSH4
229533.I1RJW8	pp	229533.I1RK79
229533.I1RJW8	pp	229533.I1RZB5
229533.I1RJW8	pp	229533.I1RT35
229533.I1RJW8	pp	229533.I1S3D7
229533.I1RJW8	pp	229533.I1RR25
229533.I1RJW8	pp	229533.I1RTC2
229533.I1RJW8	pp	229533.I1RNB8
229533.I1RJW8	pp	229533.I1RRT0
229533.I1RJW8	pp	229533.I1RRG3
229533.I1RA02	pp	229533.I1RN51
229533.I1RA02	pp	229533.I1RW26
229533.I1RA02	pp	229533.I1REF2
229533.I1RA02	pp	229533.I1RTD7
229533.I1RA02	pp	229533.I1RW01
229533.I1RA02	pp	229533.I1RT45
229533.I1RA02	pp	229533.I1RY11
229533.I1RA02	pp	229533.I1RM49
229533.I1RA02	pp	229533.I1RKF9
229533.I1RA02	pp	229533.I1RZ89
229533.I1RQE1	pp	229533.I1S8D1
229533.I1RQE1	pp	229533.I1S099
229533.I1RQE1	pp	229533.I1S0I3
229533.I1RQE1	pp	229533.I1S599
229533.I1RQE1	pp	229533.I1RZ70
229533.I1RQE1	pp	229533.I1S147
229533.I1RQE1	pp	229533.V6RRM0
229533.I1RQE1	pp	229533.I1S4G3
229533.I1RQE1	pp	229533.I1RSU2
229533.I1RQE1	pp	229533.V6RQW4
229533.I1RQE1	pp	229533.V6QU65
229533.I1RQE1	pp	229533.I1S332
229533.I1RQE1	pp	229533.I1S8D2
229533.I1RQE1	pp	229533.I1RRF5
229533.I1RQE1	pp	229533.I1SAJ3
229533.I1RQE1	pp	229533.I1RYA2
229533.I1RQE1	pp	229533.I1S148
229533.I1RQE1	pp	229533.V6RSW9
229533.I1RQE1	pp	229533.I1RWZ3
229533.I1RQE1	pp	229533.I1RVX9
229533.I1RQE1	pp	229533.I1S1W8

3943	229533.I1RQE1	pp	229533.I1RYX6
3944	229533.I1RQE1	pp	229533.I1RYW9
3945	229533.I1RQE1	pp	229533.I1RWN7
3946	229533.I1RQE1	pp	229533.I1S3G4
3947	229533.I1RQE1	pp	229533.V6RHD0
3948	229533.I1RQE1	pp	229533.I1RTY0
3949	229533.I1RQE1	pp	229533.I1S2Q4
3950	229533.I1RQE1	pp	229533.V6RH92
3951	229533.I1RQE1	pp	229533.I1S1B8
3952	229533.I1RQE1	pp	229533.I1SAF0
3953	229533.I1RQE1	pp	229533.I1S6M5
3954	229533.I1RQE1	pp	229533.I1RYK1
3955	229533.I1RQE1	pp	229533.I1RUS5
3956	229533.I1RQE1	pp	229533.I1RYA8
3957	229533.I1RQE1	pp	229533.V6RXR0
3958	229533.I1RQE1	pp	229533.I1RW43
3959	229533.I1RQE1	pp	229533.I1RYQ4
3960	229533.I1RQE1	pp	229533.I1RY49
3961	229533.I1RQE1	pp	229533.I1RVB2
3962	229533.A0A1C3YLX8	pp	229533.A0A1C3YM09
3963	229533.A0A1C3YLX8	pp	229533.I1RND2
3964	229533.A0A1C3YLX8	pp	229533.V6QVM5
3965	229533.A0A1C3YLX8	pp	229533.I1RDB7
3966	229533.A0A1C3YLX8	pp	229533.I1S2I4
3967	229533.A0A1C3YLX8	pp	229533.I1RJZ7
3968	229533.A0A1C3YLX8	pp	229533.I1RJT5
3969	229533.A0A1C3YLX8	pp	229533.I1RM09
3970	229533.A0A1C3YLX8	pp	229533.I1RPB1
3971	229533.A0A1C3YLX8	pp	229533.I1RIY8
3972	229533.A0A1C3YLX8	pp	229533.Q4IMD1
3973	229533.A0A1C3YLX8	pp	229533.I1RQ28
3974	229533.A0A1C3YLX8	pp	229533.I1RPB2
3975	229533.A0A1C3YLX8	pp	229533.I1RQL4
3976	229533.A0A1C3YLX8	pp	229533.I1RDE6
3977	229533.A0A1C3YLX8	pp	229533.I1RRT1
3978	229533.A0A1C3YLX8	pp	229533.I1RNJ7
3979	229533.A0A1C3YKL7	pp	229533.I1RWZ3
3980	229533.A0A1C3YKL7	pp	229533.I1RPW1
3981	229533.A0A1C3YKL7	pp	229533.I1RYA2
3982	229533.A0A1C3YKL7	pp	229533.I1RSB7
3983	229533.A0A1C3YKL7	pp	229533.V6RY56
3984	229533.A0A1C3YKL7	pp	229533.I1RCG5
3985	229533.A0A1C3YKL7	pp	229533.I1RYA8
3986	229533.A0A1C3YKL7	pp	229533.I1RBE7
3987	229533.A0A1C3YKL7	pp	229533.I1RNV4
3988	229533.A0A1C3YKL7	pp	229533.I1RCV1
3989	229533.A0A1C3YKL7	pp	229533.I1RJB0
3990	229533.A0A1C3YKL7	pp	229533.I1RYA0
3991	229533.A0A1C3YKL7	pp	229533.I1RYK1
3992	229533.A0A1C3YKL7	pp	229533.I1RG33
3993	229533.A0A1C3YKL7	pp	229533.V6QU65
3994	229533.A0A1C3YKL7	pp	229533.I1S3G4
3995	229533.A0A1C3YKL7	pp	229533.I1RY74
3996	229533.A0A1C3YKL7	pp	229533.I1RM26
3997	229533.A0A1C3YKL7	pp	229533.I1S2Q4
3998	229533.A0A1C3YKL7	pp	229533.I1S4D0
3999	229533.A0A1C3YKL7	pp	229533.I1S0I3
4000	229533.A0A1C3YKL7	pp	229533.I1S4G3
4001	229533.A0A1C3YKL7	pp	229533.I1RAV0
4002	229533.A0A1C3YKL7	pp	229533.I1S8F8
4003	229533.A0A1C3YKL7	pp	229533.I1RYC8
4004	229533.A0A1C3YKL7	pp	229533.I1RVB2
4005	229533.A0A1C3YKL7	pp	229533.I1RQ60
4006	229533.A0A1C3YKL7	pp	229533.I1RNY6
4007	229533.A0A1C3YKL7	pp	229533.I1S4S1
4008	229533.A0A1C3YKL7	pp	229533.A0A1C3YMF1
4009	229533.A0A1C3YKL7	pp	229533.I1RYQ4
4010	229533.A0A1C3YKL7	pp	229533.I1S599
4011	229533.A0A1C3YKL7	pp	229533.I1RHC8
4012	229533.A0A1C3YKL7	pp	229533.I1RKZ7
4013	229533.A0A1C3YKL7	pp	229533.I1RDE7
4014	229533.A0A1C3YKL7	pp	229533.I1RGG7
4015	229533.A0A1C3YKL7	pp	229533.I1RW43

4016	229533.A0A1C3YKL7	pp	229533.I1S099
4017	229533.A0A1C3YKL7	pp	229533.I1RVV7
4018	229533.A0A1C3YKL7	pp	229533.I1RG66
4019	229533.A0A1C3YKL7	pp	229533.A0A1C3YKX1
4020	229533.A0A1C3YKL7	pp	229533.I1RH91
4021	229533.A0A1C3YKL7	pp	229533.I1RY49
4022	229533.A0A1C3YKL7	pp	229533.I1S1B8
4023	229533.A0A1C3YKL7	pp	229533.I1RKZ2
4024	229533.A0A1C3YKL7	pp	229533.I1RB38
4025	229533.A0A1C3YKL7	pp	229533.I1RAR3
4026	229533.A0A1C3YKL7	pp	229533.I1RL49
4027	229533.A0A1C3YKL7	pp	229533.I1S1W8
4028	229533.A0A1C3YKL7	pp	229533.I1RJJ7
4029	229533.A0A1C3YKL7	pp	229533.V6RXR0
4030	229533.A0A1C3YKL7	pp	229533.I1RVX9
4031	229533.A0A1C3YKL7	pp	229533.I1RQE1
4032	229533.A0A1C3YKL7	pp	229533.Q4HXF0
4033	229533.A0A1C3YKL7	pp	229533.I1RBY9
4034	229533.A0A1C3YKL7	pp	229533.I1RVV3
4035	229533.A0A1C3YKL7	pp	229533.I1RUS5
4036	229533.A0A1C3YKL7	pp	229533.V6QUG0
4037	229533.A0A1C3YKL7	pp	229533.I1RP86
4038	229533.A0A1C3YKL7	pp	229533.I1RDQ2
4039	229533.A0A1C3YKL7	pp	229533.I1RCR6
4040	229533.A0A1C3YKL7	pp	229533.I1RTY0
4041	229533.A0A1C3YKL7	pp	229533.I1RPI4
4042	229533.A0A1C3YKL7	pp	229533.I1RNM7
4043	229533.A0A1C3YKL7	pp	229533.I1SAF0
4044	229533.A0A1C3YKL7	pp	229533.I1RU18
4045	229533.A0A1C3YKL7	pp	229533.V6RHD0
4046	229533.A0A1C3YKL7	pp	229533.I1RL81
4047	229533.A0A1C3YKL7	pp	229533.I1RNG4
4048	229533.A0A1C3YKL7	pp	229533.I1RD67
4049	229533.A0A1C3YKL7	pp	229533.I1RYW9
4050	229533.A0A1C3YKL7	pp	229533.I1S6M5
4051	229533.A0A1C3YKL7	pp	229533.I1RYX6
4052	229533.A0A1C3YKL7	pp	229533.I1R9B6
4053	229533.A0A1C3YKL7	pp	229533.A0A1C3YN04
4054	229533.A0A1C3YKL7	pp	229533.I1RRF5
4055	229533.A0A098E3P8	pp	229533.I1RT51
4056	229533.I1S2P7	pp	229533.I1S6T1
4057	229533.I1RD15	pp	229533.I1RYZ9
4058	229533.I1RD15	pp	229533.I1S2E9
4059	229533.I1RD15	pp	229533.V6QV09
4060	229533.I1RD15	pp	229533.I1S2G7
4061	229533.I1RD15	pp	229533.I1RZB5
4062	229533.I1RD15	pp	229533.I1RFL7
4063	229533.I1RD15	pp	229533.I1RYY5
4064	229533.I1RD15	pp	229533.I1RQA3
4065	229533.I1RD15	pp	229533.I1RRN5
4066	229533.I1RD15	pp	229533.I1S0E8
4067	229533.I1RD15	pp	229533.I1RW90
4068	229533.I1RD15	pp	229533.I1RNN1
4069	229533.I1RD15	pp	229533.Q4ICA8
4070	229533.I1RD15	pp	229533.I1RYH9
4071	229533.I1RD15	pp	229533.Q4IJM4
4072	229533.I1RD15	pp	229533.I1RL24
4073	229533.I1RD15	pp	229533.I1RQ13
4074	229533.I1RD15	pp	229533.I1RPA6
4075	229533.I1RD15	pp	229533.I1S1W4
4076	229533.I1RD15	pp	229533.I1RY44
4077	229533.I1RD15	pp	229533.I1RMQ7
4078	229533.I1RD15	pp	229533.I1RUF8
4079	229533.I1RD15	pp	229533.I1RWT6
4080	229533.I1RUB9	pp	229533.I1S2P7
4081	229533.I1RUB9	pp	229533.I1S6T1
4082	229533.I1REN8	pp	229533.I1RX46
4083	229533.I1REN8	pp	229533.I1RGA4
4084	229533.I1REN8	pp	229533.I1RT04
4085	229533.I1REN8	pp	229533.I1S540
4086	229533.I1REN8	pp	229533.I1S0J1
4087	229533.I1REN8	pp	229533.I1S0R7
4088	229533.I1REN8	pp	229533.V6R042

4089	229533.I1REN8	pp	229533.I1RWJ3
4090	229533.I1REN8	pp	229533.I1RK01
4091	229533.I1REN8	pp	229533.I1RR00
4092	229533.I1REN8	pp	229533.I1RZZ1
4093	229533.I1REN8	pp	229533.I1RW25
4094	229533.I1RBY9	pp	229533.I1RG66
4095	229533.I1RBY9	pp	229533.I1RSG0
4096	229533.I1RBY9	pp	229533.I1S099
4097	229533.I1RBY9	pp	229533.I1RZK5
4098	229533.I1RBY9	pp	229533.V6QU65
4099	229533.I1RBY9	pp	229533.I1RST9
4100	229533.I1RBY9	pp	229533.I1S097
4101	229533.I1RBY9	pp	229533.I1RIM6
4102	229533.I1RBY9	pp	229533.I1S423
4103	229533.I1RBY9	pp	229533.I1RIE8
4104	229533.I1RBY9	pp	229533.I1RJT5
4105	229533.I1RBY9	pp	229533.I1RWM4
4106	229533.I1RBY9	pp	229533.I1RY12
4107	229533.I1RBY9	pp	229533.I1RNY6
4108	229533.I1RBY9	pp	229533.I1RPW1
4109	229533.I1RBY9	pp	229533.I1S267
4110	229533.I1RBY9	pp	229533.I1RRK5
4111	229533.I1RBY9	pp	229533.Q4HYR2
4112	229533.I1RBY9	pp	229533.I1RYQ4
4113	229533.I1RBY9	pp	229533.I1S3M4
4114	229533.I1RBY9	pp	229533.I1RJY7
4115	229533.I1RBY9	pp	229533.I1RYA2
4116	229533.I1RBY9	pp	229533.I1RM26
4117	229533.I1RBY9	pp	229533.I1RQE1
4118	229533.I1RBY9	pp	229533.I1RE72
4119	229533.I1RBY9	pp	229533.I1RY49
4120	229533.I1RBY9	pp	229533.I1RCR6
4121	229533.I1RBY9	pp	229533.I1RMM8
4122	229533.I1RBY9	pp	229533.I1S048
4123	229533.I1RBY9	pp	229533.I1RP86
4124	229533.I1RBY9	pp	229533.I1RDQ2
4125	229533.I1RBY9	pp	229533.I1RXD7
4126	229533.I1RBY9	pp	229533.I1RCU2
4127	229533.I1RBY9	pp	229533.V6RH92
4128	229533.I1RBY9	pp	229533.I1RLA2
4129	229533.I1RBY9	pp	229533.I1RVT2
4130	229533.I1RBY9	pp	229533.I1S2F6
4131	229533.I1RBY9	pp	229533.I1RDU5
4132	229533.I1RBY9	pp	229533.I1RVX9
4133	229533.I1RBY9	pp	229533.I1RPN4
4134	229533.I1RBY9	pp	229533.I1RVB2
4135	229533.I1RBY9	pp	229533.I1RYK1
4136	229533.I1RBY9	pp	229533.I1S8C1
4137	229533.I1RBY9	pp	229533.I1RR12
4138	229533.I1RBY9	pp	229533.I1RD67
4139	229533.I1RBY9	pp	229533.I1RY16
4140	229533.I1RBY9	pp	229533.I1RNM7
4141	229533.I1RBY9	pp	229533.I1RKZ8
4142	229533.I1RBY9	pp	229533.I1RPI4
4143	229533.I1RBY9	pp	229533.I1RYX6
4144	229533.I1RBY9	pp	229533.I1S1W8
4145	229533.I1RBY9	pp	229533.I1RQ60
4146	229533.I1RBY9	pp	229533.I1RJS7
4147	229533.I1RBY9	pp	229533.I1S3K8
4148	229533.I1RBY9	pp	229533.I1RN52
4149	229533.I1RBY9	pp	229533.I1RFH9
4150	229533.I1RBY9	pp	229533.I1RE79
4151	229533.I1RBY9	pp	229533.I1SAF0
4152	229533.I1RBY9	pp	229533.V6RXE7
4153	229533.I1RBY9	pp	229533.I1RH92
4154	229533.I1RBY9	pp	229533.I1RL76
4155	229533.I1RBY9	pp	229533.I1RKZ7
4156	229533.I1RBY9	pp	229533.I1S599
4157	229533.I1RBY9	pp	229533.I1RZ70
4158	229533.I1RBY9	pp	229533.I1RHC8
4159	229533.I1RBY9	pp	229533.I1RNK9
4160	229533.I1RBY9	pp	229533.I1RDH9
4161	229533.I1RBY9	pp	229533.I1RZC3

229533.I1RBY9	pp	229533.I1RN84
229533.I1RBY9	pp	229533.I1S8D1
229533.I1RBY9	pp	229533.I1RGG7
229533.I1RBY9	pp	229533.I1S332
229533.I1RBY9	pp	229533.V6RSW9
229533.I1RBY9	pp	229533.I1RKZ2
229533.I1RBY9	pp	229533.I1S0I3
229533.I1RBY9	pp	229533.I1RE84
229533.I1RBY9	pp	229533.I1REW3
229533.I1RBY9	pp	229533.I1S147
229533.I1RBY9	pp	229533.I1RCG5
229533.I1RBY9	pp	229533.I1S2Z7
229533.I1RBY9	pp	229533.V6RLM7
229533.I1RBY9	pp	229533.I1RNV4
229533.I1RBY9	pp	229533.I1RM41
229533.I1RBY9	pp	229533.Q4I1Y5
229533.I1RBY9	pp	229533.I1RF85
229533.I1RBY9	pp	229533.I1RZ25
229533.I1RBY9	pp	229533.I1RY32
229533.I1RBY9	pp	229533.I1RR45
229533.I1RBY9	pp	229533.I1S148
229533.I1RBY9	pp	229533.I1RYW9
229533.I1RBY9	pp	229533.I1RWF5
229533.I1RBY9	pp	229533.I1RC71
229533.I1RBY9	pp	229533.I1S1L2
229533.I1RBY9	pp	229533.I1S6M5
229533.I1RBY9	pp	229533.I1RZ29
229533.I1RBY9	pp	229533.I1RZA8
229533.I1RBY9	pp	229533.I1RSP5
229533.I1RBY9	pp	229533.I1RDE7
229533.I1RBY9	pp	229533.I1S8D2
229533.I1RBY9	pp	229533.I1S694
229533.I1RBY9	pp	229533.V6RUY9
229533.I1RBY9	pp	229533.I1RMJ3
229533.I1RBY9	pp	229533.I1RUR8
229533.I1RBY9	pp	229533.I1RW43
229533.I1RBY9	pp	229533.V6RXR0
229533.I1RBY9	pp	229533.V6RHD0
229533.I1RBY9	pp	229533.I1RRF5
229533.I1RBY9	pp	229533.I1RUS5
229533.I1RBY9	pp	229533.I1RZT0
229533.I1RBY9	pp	229533.I1RNG4
229533.I1RBY9	pp	229533.I1RYA8
229533.I1RBY9	pp	229533.I1RGC3
229533.I1RBY9	pp	229533.I1RJB0
229533.I1RBY9	pp	229533.I1S3G4
229533.I1RBY9	pp	229533.I1RI11
229533.I1RBY9	pp	229533.I1S1F3
229533.I1RBY9	pp	229533.I1RNB4
229533.I1RBY9	pp	229533.I1RWN7
229533.I1RBY9	pp	229533.I1RN88
229533.A0A1C3YK47	pp	229533.I1S147
229533.A0A1C3YK47	pp	229533.I1RFI4
229533.I1RYX6	pp	229533.Q4HXF0
229533.I1RYX6	pp	229533.I1S148
229533.I1RYX6	pp	229533.I1S318
229533.I1RYX6	pp	229533.V6R5P0
229533.I1RYX6	pp	229533.I1S1B8
229533.I1RYX6	pp	229533.I1S0V7
229533.I1RYX6	pp	229533.I1S6M5
229533.I1RYX6	pp	229533.I1S8D2
229533.I1RYX6	pp	229533.Q4HVQ9
229533.I1RYX6	pp	229533.I1S147
229533.I1RYX6	pp	229533.I1S423
229533.I1RYX6	pp	229533.V6RHD0
229533.I1RYX6	pp	229533.Q4HVU7
229533.I1RYX6	pp	229533.V6RPQ8
229533.I1RYX6	pp	229533.I1S2Q4
229533.I1RYX6	pp	229533.I1S1W8
229533.I1RYX6	pp	229533.I1S064
229533.I1RYX6	pp	229533.V6RH92
229533.I1RYX6	pp	229533.I1S3G4
229533.I1RYX6	pp	229533.I1RZ29

229533.I1RYX6 pp 229533.V6RRM0
229533.I1RYX6 pp 229533.I1S0A0
229533.I1RYX6 pp 229533.I1RZ52
229533.I1RYX6 pp 229533.Q4HYR2
229533.I1RYX6 pp 229533.I1SAF0
229533.I1RYX6 pp 229533.V6RLM7
229533.I1RYX6 pp 229533.V6RBN2
229533.I1RYX6 pp 229533.I1S8D1
229533.I1RYX6 pp 229533.I1S599
229533.I1RYX6 pp 229533.I1S8F8
229533.I1RYX6 pp 229533.I1S0I3
229533.I1RYX6 pp 229533.V6RQW4
229533.I1RYX6 pp 229533.I1S0V6
229533.I1RYX6 pp 229533.V6QU65
229533.I1RYX6 pp 229533.I1S099
229533.I1RYX6 pp 229533.I1RZ70
229533.I1RYX6 pp 229533.I1S4G3
229533.I1RYX6 pp 229533.V6RKK5
229533.A0A1C3YN04 pp 229533.I1S3G4
229533.A0A1C3YN04 pp 229533.I1S2Q4
229533.A0A1C3YN04 pp 229533.I1RDQ2
229533.A0A1C3YN04 pp 229533.I1RWL3
229533.A0A1C3YN04 pp 229533.I1RVB2
229533.A0A1C3YN04 pp 229533.I1S3T7
229533.A0A1C3YN04 pp 229533.I1RYA8
229533.A0A1C3YN04 pp 229533.I1S6M5
229533.A0A1C3YN04 pp 229533.Q4HVQ9
229533.A0A1C3YN04 pp 229533.I1RJB0
229533.A0A1C3YN04 pp 229533.I1RYK1
229533.A0A1C3YN04 pp 229533.I1RQE1
229533.A0A1C3YN04 pp 229533.I1RR68
229533.A0A1C3YN04 pp 229533.I1RYQ4
229533.A0A1C3YN04 pp 229533.I1RCR6
229533.A0A1C3YN04 pp 229533.I1RVI8
229533.A0A1C3YN04 pp 229533.I1RQ60
229533.A0A1C3YN04 pp 229533.I1RD67
229533.A0A1C3YN04 pp 229533.I1RYX6
229533.A0A1C3YN04 pp 229533.I1RUS5
229533.A0A1C3YN04 pp 229533.I1SAF0
229533.A0A1C3YN04 pp 229533.I1REE2
229533.A0A1C3YN04 pp 229533.I1RKZ2
229533.A0A1C3YN04 pp 229533.I1RYW9
229533.A0A1C3YN04 pp 229533.I1RNY6
229533.A0A1C3YN04 pp 229533.I1RPE5
229533.A0A1C3YN04 pp 229533.I1RDN3
229533.A0A1C3YN04 pp 229533.I1RNG4
229533.A0A1C3YN04 pp 229533.I1S1W8
229533.A0A1C3YN04 pp 229533.I1RKZ7
229533.A0A1C3YN04 pp 229533.I1RHC8
229533.A0A1C3YN04 pp 229533.I1S8C1
229533.A0A1C3YN04 pp 229533.I1RW54
229533.A0A1C3YN04 pp 229533.I1RG66
229533.A0A1C3YN04 pp 229533.I1S299
229533.A0A1C3YN04 pp 229533.I1RDV8
229533.A0A1C3YN04 pp 229533.I1S599
229533.A0A1C3YN04 pp 229533.I1RMA2
229533.A0A1C3YN04 pp 229533.I1RY35
229533.A0A1C3YN04 pp 229533.I1RRF5
229533.A0A1C3YN04 pp 229533.I1S267
229533.A0A1C3YN04 pp 229533.I1R9B6
229533.A0A1C3YN04 pp 229533.I1RWM4
229533.A0A1C3YN04 pp 229533.I1RYA2
229533.A0A1C3YN04 pp 229533.I1RAG3
229533.A0A1C3YN04 pp 229533.I1S2C4
229533.A0A1C3YN04 pp 229533.I1RJY7
229533.A0A1C3YN04 pp 229533.I1RNV4
229533.A0A1C3YN04 pp 229533.I1RY81
229533.A0A1C3YN04 pp 229533.I1RA13
229533.A0A1C3YN04 pp 229533.I1RBE7
229533.A0A1C3YN04 pp 229533.I1S8R2
229533.I1RCD2 pp 229533.I1RUB9
229533.I1RCD2 pp 229533.I1S2P7
229533.I1RCD2 pp 229533.I1S6T1

229533.I1RCD2 pp 229533.I1RZ18
229533.I1RNZ9 pp 229533.I1RZT1
229533.I1RNZ9 pp 229533.I1RYZ9
229533.I1RNZ9 pp 229533.V6QV09
229533.A0A1C3YI08 pp 229533.I1RVB2
229533.A0A1C3YI08 pp 229533.I1RQ60
229533.A0A1C3YI08 pp 229533.I1RYQ4
229533.A0A1C3YI08 pp 229533.A0A1C3YJI5
229533.A0A1C3YI08 pp 229533.I1RUS5
229533.A0A1C3YI08 pp 229533.I1RPE5
229533.A0A1C3YI08 pp 229533.I1SAF0
229533.A0A1C3YI08 pp 229533.I1RY35
229533.A0A1C3YI08 pp 229533.I1RW54
229533.A0A1C3YI08 pp 229533.I1RQE1
229533.A0A1C3YI08 pp 229533.I1S1W8
229533.A0A1C3YI08 pp 229533.Q4HVQ9
229533.A0A1C3YI08 pp 229533.I1RDQ2
229533.A0A1C3YI08 pp 229533.I1RNG4
229533.A0A1C3YI08 pp 229533.I1RD67
229533.A0A1C3YI08 pp 229533.I1RYA8
229533.A0A1C3YI08 pp 229533.I1RYX6
229533.A0A1C3YI08 pp 229533.I1RYK1
229533.A0A1C3YI08 pp 229533.I1RYW9
229533.A0A1C3YI08 pp 229533.I1RJB0
229533.A0A1C3YI08 pp 229533.I1R9B6
229533.A0A1C3YI08 pp 229533.I1RRF5
229533.A0A1C3YI08 pp 229533.A0A1C3YKL7
229533.A0A1C3YI08 pp 229533.I1S3G4
229533.A0A1C3YI08 pp 229533.I1S6M5
229533.A0A1C3YI08 pp 229533.I1RYA2
229533.A0A1C3YI08 pp 229533.I1RNV4
229533.A0A1C3YI08 pp 229533.I1RWL3
229533.A0A1C3YI08 pp 229533.I1S2C4
229533.A0A1C3YI08 pp 229533.I1RCR6
229533.A0A1C3YI08 pp 229533.I1RNY6
229533.A0A1C3YI08 pp 229533.I1RBE7
229533.A0A1C3YI08 pp 229533.I1RKZ7
229533.A0A1C3YI08 pp 229533.I1RHC8
229533.A0A1C3YI08 pp 229533.I1RKZ2
229533.A0A1C3YI08 pp 229533.A0A1C3YKX1
229533.A0A1C3YI08 pp 229533.I1S599
229533.A0A1C3YI08 pp 229533.I1RG66
229533.A0A1C3YI08 pp 229533.I1S267
229533.I1RS43 pp 229533.I1RVZ4
229533.I1RS43 pp 229533.V6QUE6
229533.I1RS43 pp 229533.I1RXU0
229533.I1RS43 pp 229533.I1RTC2
229533.I1RS43 pp 229533.I1RZ11
229533.I1RS43 pp 229533.Q4I5I4
229533.I1RS43 pp 229533.I1S3D7
229533.I1RS43 pp 229533.I1RT35
229533.I1RS43 pp 229533.I1S276
229533.I1RS43 pp 229533.I1RZB5
229533.I1RS43 pp 229533.I1RSH4
229533.I1RDB7 pp 229533.Q4HTS9
229533.I1RDB7 pp 229533.I1RK47
229533.I1RDB7 pp 229533.I1RTU1
229533.I1RDB7 pp 229533.I1RKJ7
229533.I1RDB7 pp 229533.I1RNJ7
229533.I1RDB7 pp 229533.I1RYU0
229533.I1RDB7 pp 229533.I1RND2
229533.I1RDB7 pp 229533.V6QT89
229533.I1S4G3 pp 229533.Q4HVQ9
229533.I1S4G3 pp 229533.I1SAF0
229533.I1S4G3 pp 229533.I1S6M5
229533.I1S4G3 pp 229533.I1S599
229533.I1RWZ3 pp 229533.I1S1W8
229533.I1RWZ3 pp 229533.I1RY35
229533.I1RWZ3 pp 229533.I1S2C4
229533.I1RWZ3 pp 229533.I1RYX6
229533.I1RWZ3 pp 229533.I1S3G4
229533.I1RWZ3 pp 229533.I1SAF0
229533.I1RWZ3 pp 229533.I1RYQ4

4381 229533.I1RWZ3 pp 229533.I1RYW9
4382 229533.I1RWZ3 pp 229533.I1S6M5
4383 229533.I1RWZ3 pp 229533.I1S267
4384 229533.I1RWZ3 pp 229533.Q4HVQ9
4385 229533.I1RWZ3 pp 229533.I1RYA2
4386 229533.I1RWZ3 pp 229533.I1RYK1
4387 229533.I1RWZ3 pp 229533.I1RYA8
4388 229533.I1RWZ3 pp 229533.I1S599
4389 229533.I1RZY9 pp 229533.V6QV09
4390 229533.A0A098D8K2 pp 229533.I1RBE7
4391 229533.A0A098D8K2 pp 229533.I1RYA0
4392 229533.A0A098D8K2 pp 229533.I1S0I3
4393 229533.A0A098D8K2 pp 229533.I1RY74
4394 229533.A0A098D8K2 pp 229533.I1RG33
4395 229533.A0A098D8K2 pp 229533.V6QU65
4396 229533.A0A098D8K2 pp 229533.I1RUQ3
4397 229533.A0A098D8K2 pp 229533.I1S8F8
4398 229533.A0A098D8K2 pp 229533.I1S4G3
4399 229533.A0A098D8K2 pp 229533.I1RM26
4400 229533.A0A098D8K2 pp 229533.I1RPW1
4401 229533.A0A098D8K2 pp 229533.I1RYC8
4402 229533.A0A098D8K2 pp 229533.A0A098DEV0
4403 229533.A0A098D8K2 pp 229533.I1S599
4404 229533.A0A098D8K2 pp 229533.A0A098DHF2
4405 229533.A0A098D8K2 pp 229533.A0A1C3YI08
4406 229533.A0A098D8K2 pp 229533.I1RYA2
4407 229533.A0A098D8K2 pp 229533.I1RWZ3
4408 229533.A0A098D8K2 pp 229533.I1S8S9
4409 229533.A0A098D8K2 pp 229533.I1RKZ2
4410 229533.A0A098D8K2 pp 229533.I1RGG7
4411 229533.A0A098D8K2 pp 229533.A0A1C3YKX1
4412 229533.A0A098D8K2 pp 229533.I1RCG5
4413 229533.A0A098D8K2 pp 229533.I1S099
4414 229533.A0A098D8K2 pp 229533.I1RL49
4415 229533.A0A098D8K2 pp 229533.I1RG66
4416 229533.A0A098D8K2 pp 229533.I1RB38
4417 229533.A0A098D8K2 pp 229533.A0A098DUS9
4418 229533.A0A098D8K2 pp 229533.I1RNV4
4419 229533.A0A098D8K2 pp 229533.V6RXR0
4420 229533.A0A098D8K2 pp 229533.I1RPI4
4421 229533.A0A098D8K2 pp 229533.I1RCV1
4422 229533.A0A098D8K2 pp 229533.I1RJJ7
4423 229533.A0A098D8K2 pp 229533.V6QUG0
4424 229533.A0A098D8K2 pp 229533.I1RAV0
4425 229533.A0A098D8K2 pp 229533.I1S1W8
4426 229533.A0A098D8K2 pp 229533.I1RVV3
4427 229533.A0A098D8K2 pp 229533.I1RSB7
4428 229533.A0A098D8K2 pp 229533.I1RNY6
4429 229533.A0A098D8K2 pp 229533.I1RUS5
4430 229533.A0A098D8K2 pp 229533.I1RKZ7
4431 229533.A0A098D8K2 pp 229533.A0A098DYP8
4432 229533.A0A098D8K2 pp 229533.I1RYA8
4433 229533.A0A098D8K2 pp 229533.I1RYK1
4434 229533.A0A098D8K2 pp 229533.I1RHC8
4435 229533.A0A098D8K2 pp 229533.A0A1C3YMF1
4436 229533.A0A098D8K2 pp 229533.I1SAF0
4437 229533.A0A098D8K2 pp 229533.I1S4S1
4438 229533.A0A098D8K2 pp 229533.I1RNM7
4439 229533.A0A098D8K2 pp 229533.I1RJB0
4440 229533.A0A098D8K2 pp 229533.I1RQ60
4441 229533.A0A098D8K2 pp 229533.I1S2Q4
4442 229533.A0A098D8K2 pp 229533.I1RU18
4443 229533.A0A098D8K2 pp 229533.I1S3G4
4444 229533.A0A098D8K2 pp 229533.A0A1C3YJW4
4445 229533.A0A098D8K2 pp 229533.I1RVB2
4446 229533.A0A098D8K2 pp 229533.I1RYX6
4447 229533.A0A098D8K2 pp 229533.I1RW43
4448 229533.A0A098D8K2 pp 229533.I1RH91
4449 229533.A0A098D8K2 pp 229533.I1RYQ4
4450 229533.A0A098D8K2 pp 229533.I1RDE7
4451 229533.A0A098D8K2 pp 229533.I1RY49
4452 229533.A0A098D8K2 pp 229533.I1RDQ2
4453 229533.A0A098D8K2 pp 229533.A0A1C3YJI5

4454	229533.A0A098D8K2	pp	229533.I1RAR3
4455	229533.A0A098D8K2	pp	229533.I1R9B6
4456	229533.A0A098D8K2	pp	229533.A0A1C3YN04
4457	229533.A0A098D8K2	pp	229533.I1S6M5
4458	229533.A0A098D8K2	pp	229533.I1RPN4
4459	229533.A0A098D8K2	pp	229533.I1RNG4
4460	229533.A0A098D8K2	pp	229533.A0A1C3YKL7
4461	229533.A0A098D8K2	pp	229533.V6RHD0
4462	229533.A0A098D8K2	pp	229533.I1RRF5
4463	229533.A0A098D8K2	pp	229533.A0A0E0SMT9
4464	229533.A0A098D8K2	pp	229533.I1RP86
4465	229533.A0A098D8K2	pp	229533.Q4HVQ9
4466	229533.A0A098D8K2	pp	229533.I1RCR6
4467	229533.A0A098D8K2	pp	229533.I1RD67
4468	229533.A0A098D8K2	pp	229533.I1RL81
4469	229533.A0A098D8K2	pp	229533.I1RVX9
4470	229533.A0A098D8K2	pp	229533.Q4HXF0
4471	229533.A0A098D8K2	pp	229533.I1RBY9
4472	229533.A0A098D8K2	pp	229533.A0A098DVZ4
4473	229533.A0A098D8K2	pp	229533.I1RYW9
4474	229533.A0A098D8K2	pp	229533.I1S1B8
4475	229533.A0A098D8K2	pp	229533.I1RQE1
4476	229533.A0A098D8K2	pp	229533.I1RRK7
4477	229533.A0A098D8K2	pp	229533.I1R9W4
4478	229533.A0A098D8K2	pp	229533.I1RTY0
4479	229533.A0A098D8K2	pp	229533.A0A098DDV4
4480	229533.A0A098DUS9	pp	229533.A0A1C3YLB3
4481	229533.A0A098DUS9	pp	229533.I1RNV4
4482	229533.A0A098DUS9	pp	229533.V6QU65
4483	229533.A0A098DUS9	pp	229533.I1RVI8
4484	229533.A0A098DUS9	pp	229533.A0A1C3YJI5
4485	229533.A0A098DUS9	pp	229533.A0A1C3YJH4
4486	229533.A0A098DUS9	pp	229533.I1S2C4
4487	229533.A0A098DUS9	pp	229533.I1RY49
4488	229533.A0A098DUS9	pp	229533.I1RQ87
4489	229533.A0A098DUS9	pp	229533.I1RYQ4
4490	229533.A0A098DUS9	pp	229533.A0A1C3YLC2
4491	229533.A0A098DUS9	pp	229533.I1RJB0
4492	229533.A0A098DUS9	pp	229533.I1RY81
4493	229533.A0A098DUS9	pp	229533.I1RYA2
4494	229533.A0A098DUS9	pp	229533.I1RYK1
4495	229533.A0A098DUS9	pp	229533.A0A1C3YKW4
4496	229533.A0A098DUS9	pp	229533.I1S8R2
4497	229533.A0A098DUS9	pp	229533.I1RJY7
4498	229533.A0A098DUS9	pp	229533.I1RBE7
4499	229533.A0A098DUS9	pp	229533.V6RH92
4500	229533.A0A098DUS9	pp	229533.I1RHC8
4501	229533.A0A098DUS9	pp	229533.I1RKZ7
4502	229533.A0A098DUS9	pp	229533.I1RZ70
4503	229533.A0A098DUS9	pp	229533.I1RYA8
4504	229533.A0A098DUS9	pp	229533.I1RUQ3
4505	229533.A0A098DUS9	pp	229533.I1RA13
4506	229533.A0A098DUS9	pp	229533.I1RNY6
4507	229533.A0A098DUS9	pp	229533.A0A1C3YKX1
4508	229533.A0A098DUS9	pp	229533.I1S3T7
4509	229533.A0A098DUS9	pp	229533.I1RKZ2
4510	229533.A0A098DUS9	pp	229533.I1S147
4511	229533.A0A098DUS9	pp	229533.A0A1C3YL54
4512	229533.A0A098DUS9	pp	229533.I1RDU5
4513	229533.A0A098DUS9	pp	229533.I1RVB2
4514	229533.A0A098DUS9	pp	229533.I1RQE1
4515	229533.A0A098DUS9	pp	229533.I1RCR6
4516	229533.A0A098DUS9	pp	229533.I1REE2
4517	229533.A0A098DUS9	pp	229533.I1RWL3
4518	229533.A0A098DUS9	pp	229533.I1RWM4
4519	229533.A0A098DUS9	pp	229533.I1RR68
4520	229533.A0A098DUS9	pp	229533.I1S148
4521	229533.A0A098DUS9	pp	229533.I1S3G4
4522	229533.A0A098DUS9	pp	229533.I1RBY9
4523	229533.A0A098DUS9	pp	229533.I1RAG3
4524	229533.A0A098DUS9	pp	229533.I1RH92
4525	229533.A0A098DUS9	pp	229533.I1S2Q4
4526	229533.A0A098DUS9	pp	229533.I1RN88

229533.A0A098DUS9	pp	229533.I1S267
229533.A0A098DUS9	pp	229533.I1S6M5
229533.A0A098DUS9	pp	229533.I1RWN7
229533.A0A098DUS9	pp	229533.A0A1C3YKL7
229533.A0A098DUS9	pp	229533.I1S599
229533.A0A098DUS9	pp	229533.I1RY35
229533.A0A098DUS9	pp	229533.I1RDV8
229533.A0A098DUS9	pp	229533.I1RMA2
229533.A0A098DUS9	pp	229533.I1S8D1
229533.A0A098DUS9	pp	229533.I1S1W8
229533.A0A098DUS9	pp	229533.I1RDQ2
229533.A0A098DUS9	pp	229533.I1S099
229533.A0A098DUS9	pp	229533.I1RW54
229533.A0A098DUS9	pp	229533.I1RB07
229533.A0A098DUS9	pp	229533.I1RG66
229533.A0A098DUS9	pp	229533.I1RDN3
229533.A0A098DUS9	pp	229533.I1R9B6
229533.A0A098DUS9	pp	229533.I1RQ60
229533.A0A098DUS9	pp	229533.I1RPE5
229533.A0A098DUS9	pp	229533.I1S8D2
229533.A0A098DUS9	pp	229533.I1SAF0
229533.A0A098DUS9	pp	229533.A0A0E0RNI4
229533.A0A098DUS9	pp	229533.I1RRF5
229533.A0A098DUS9	pp	229533.I1RGC3
229533.A0A098DUS9	pp	229533.I1S299
229533.A0A098DUS9	pp	229533.A0A1C3YN04
229533.A0A098DUS9	pp	229533.A0A098DYP8
229533.A0A098DUS9	pp	229533.I1S8C1
229533.A0A098DUS9	pp	229533.I1RNG4
229533.A0A098DUS9	pp	229533.I1RYW9
229533.A0A098DUS9	pp	229533.I1RUS5
229533.A0A098DUS9	pp	229533.I1RYX6
229533.A0A098DUS9	pp	229533.I1RD67
229533.A0A098DG62	pp	229533.I1RJW8
229533.A0A098DG62	pp	229533.I1RB36
229533.A0A098DG62	pp	229533.I1RXE5
229533.A0A098DG62	pp	229533.A0A0E0SQJ6
229533.A0A098DG62	pp	229533.A0A1C3YN58
229533.A0A098DG62	pp	229533.I1RDC9
229533.A0A098DG62	pp	229533.A0A1C3YHH5
229533.A0A098DG62	pp	229533.A0A1C3YK75
229533.A0A098DG62	pp	229533.I1RVY0
229533.A0A098DG62	pp	229533.A0A1C3YJ12
229533.A0A098DG62	pp	229533.I1RCN2
229533.A0A098DG62	pp	229533.I1RMA5
229533.A0A098DG62	pp	229533.A0A1C3YK01
229533.A0A098DG62	pp	229533.I1RS43
229533.A0A098DG62	pp	229533.A0A1C3YJZ2
229533.A0A098DG62	pp	229533.A0A0E0SPD4
229533.A0A098DG62	pp	229533.A0A1C3YL16
229533.A0A098DG62	pp	229533.A0A1C3YMF9
229533.A0A098DG62	pp	229533.I1R9Q0
229533.A0A098DG62	pp	229533.I1RGW0
229533.A0A098DG62	pp	229533.I1RKR6
229533.A0A098DG62	pp	229533.I1RB73
229533.A0A098DG62	pp	229533.V6QV38
229533.I1RA81	pp	229533.P0C431
229533.I1RA81	pp	229533.I1RAH8
229533.I1RA81	pp	229533.I1RN81
229533.I1RA81	pp	229533.I1RKG1
229533.I1RA81	pp	229533.I1RJR9
229533.I1RA81	pp	229533.I1RWL6
229533.I1RA81	pp	229533.I1RTB7
229533.I1RA81	pp	229533.I1REI2
229533.I1RA81	pp	229533.I1RAB6
229533.I1RA81	pp	229533.I1RQN9
229533.I1RA81	pp	229533.V6RR76
229533.I1RA81	pp	229533.I1RQL4
229533.I1RA81	pp	229533.I1S0S6
229533.I1RAV0	pp	229533.I1RJY7
229533.I1RAV0	pp	229533.I1RQE0
229533.I1RAV0	pp	229533.I1REE2
229533.I1RAV0	pp	229533.V6RWB3

4600	229533.I1RAV0	pp	229533.I1RIB5
4601	229533.I1RAV0	pp	229533.I1RBE7
4602	229533.I1RAV0	pp	229533.I1S8D3
4603	229533.I1RAV0	pp	229533.I1RPR1
4604	229533.I1RAV0	pp	229533.I1RNY6
4605	229533.I1RAV0	pp	229533.I1RG66
4606	229533.I1RAV0	pp	229533.I1RQA3
4607	229533.I1RAV0	pp	229533.I1RDQ2
4608	229533.I1RAV0	pp	229533.I1RRS6
4609	229533.I1RAV0	pp	229533.I1RWT6
4610	229533.I1RAV0	pp	229533.I1RWL3
4611	229533.I1RAV0	pp	229533.I1S267
4612	229533.I1RAV0	pp	229533.I1RYK1
4613	229533.I1RAV0	pp	229533.I1RT50
4614	229533.I1RAV0	pp	229533.I1RVB2
4615	229533.I1RAV0	pp	229533.I1RCR6
4616	229533.I1RAV0	pp	229533.I1RQE1
4617	229533.I1RAV0	pp	229533.I1RY12
4618	229533.I1RAV0	pp	229533.I1RYA2
4619	229533.I1RAV0	pp	229533.I1RQ60
4620	229533.I1RAV0	pp	229533.I1RJR8
4621	229533.I1RAV0	pp	229533.I1S8C1
4622	229533.I1RAV0	pp	229533.I1RYQ4
4623	229533.I1RAV0	pp	229533.I1RQJ7
4624	229533.I1RAV0	pp	229533.I1RXX1
4625	229533.I1RAV0	pp	229533.I1RYX6
4626	229533.I1RAV0	pp	229533.I1RD67
4627	229533.I1RAV0	pp	229533.I1RCU2
4628	229533.I1RAV0	pp	229533.I1S224
4629	229533.I1RAV0	pp	229533.I1RY81
4630	229533.I1RAV0	pp	229533.V6RCC0
4631	229533.I1RAV0	pp	229533.I1RHC8
4632	229533.I1RAV0	pp	229533.I1RKZ7
4633	229533.I1RAV0	pp	229533.I1RYP5
4634	229533.I1RAV0	pp	229533.I1RKZ2
4635	229533.I1RAV0	pp	229533.I1RAX2
4636	229533.I1RAV0	pp	229533.I1S6M5
4637	229533.I1RAV0	pp	229533.I1RZC3
4638	229533.I1RAV0	pp	229533.I1SAF0
4639	229533.I1RAV0	pp	229533.I1S968
4640	229533.I1RAV0	pp	229533.I1RPE5
4641	229533.I1RAV0	pp	229533.V6R5T7
4642	229533.I1RAV0	pp	229533.I1RY32
4643	229533.I1RAV0	pp	229533.I1RY44
4644	229533.I1RAV0	pp	229533.I1S599
4645	229533.I1RAV0	pp	229533.I1RMA2
4646	229533.I1RAV0	pp	229533.I1S2E9
4647	229533.I1RAV0	pp	229533.I1RPV8
4648	229533.I1RAV0	pp	229533.I1RNG4
4649	229533.I1RAV0	pp	229533.I1RQV7
4650	229533.I1RAV0	pp	229533.I1RF24
4651	229533.I1RAV0	pp	229533.I1RJS1
4652	229533.I1RAV0	pp	229533.I1S1W8
4653	229533.I1RAV0	pp	229533.I1RF85
4654	229533.I1RAV0	pp	229533.I1RSA5
4655	229533.I1RAV0	pp	229533.I1RNV4
4656	229533.I1RAV0	pp	229533.I1RYW9
4657	229533.I1RAV0	pp	229533.I1S5D0
4658	229533.I1RAV0	pp	229533.I1RRF5
4659	229533.I1RAV0	pp	229533.I1S6V0
4660	229533.I1RAV0	pp	229533.I1RW54
4661	229533.I1RAV0	pp	229533.I1RRW0
4662	229533.I1RAV0	pp	229533.I1S299
4663	229533.I1RAV0	pp	229533.I1RJB0
4664	229533.I1RAV0	pp	229533.I1S2C4
4665	229533.I1RAV0	pp	229533.I1RH91
4666	229533.I1RAV0	pp	229533.I1RIJ6
4667	229533.I1RAV0	pp	229533.I1RUR8
4668	229533.I1RAV0	pp	229533.I1SAB4
4669	229533.I1RAV0	pp	229533.I1RB05
4670	229533.I1RAV0	pp	229533.I1S8R2
4671	229533.I1RAV0	pp	229533.I1RUS5
4672	229533.I1RAV0	pp	229533.I1RK55

4673	229533.I1RAV0	pp	229533.I1RYA8
4674	229533.I1RAV0	pp	229533.I1S3G4
4675	229533.I1RAV0	pp	229533.I1RY35
4676	229533.I1RAV0	pp	229533.I1S3T7
4677	229533.I1RAV0	pp	229533.I1RFW0
4678	229533.I1RAV0	pp	229533.I1RC71
4679	229533.I1RAV0	pp	229533.V6RCJ5
4680	229533.I1RUD3	pp	229533.I1RVV7
4681	229533.I1RUD3	pp	229533.I1S569
4682	229533.I1R9D6	pp	229533.V6QWB1
4683	229533.I1R9D6	pp	229533.I1RIA5
4684	229533.I1R9D6	pp	229533.I1RSW5
4685	229533.I1R9D6	pp	229533.Q4I2M7
4686	229533.I1R9D6	pp	229533.I1RN54
4687	229533.I1R9D6	pp	229533.I1RY81
4688	229533.I1R9D6	pp	229533.I1RY35
4689	229533.I1R9D6	pp	229533.I1S050
4690	229533.I1R9D6	pp	229533.I1RCS1
4691	229533.I1RG66	pp	229533.I1RPI4
4692	229533.I1RG66	pp	229533.I1SAF0
4693	229533.I1RG66	pp	229533.I1RVV3
4694	229533.I1RG66	pp	229533.I1RUS5
4695	229533.I1RG66	pp	229533.V6QUG0
4696	229533.I1RG66	pp	229533.I1S1W8
4697	229533.I1RG66	pp	229533.I1RJJ7
4698	229533.I1RG66	pp	229533.V6RXR0
4699	229533.I1RG66	pp	229533.I1RL49
4700	229533.I1RG66	pp	229533.I1S4S1
4701	229533.I1RG66	pp	229533.I1RW43
4702	229533.I1RG66	pp	229533.I1RY49
4703	229533.I1RG66	pp	229533.I1RU18
4704	229533.I1RG66	pp	229533.I1RNM7
4705	229533.I1RG66	pp	229533.I1S2Q4
4706	229533.I1RG66	pp	229533.I1RVB2
4707	229533.I1RG66	pp	229533.I1RTY0
4708	229533.I1RG66	pp	229533.I1RH91
4709	229533.I1RG66	pp	229533.I1S3G4
4710	229533.I1RG66	pp	229533.I1RYQ4
4711	229533.I1RG66	pp	229533.I1RP86
4712	229533.I1RG66	pp	229533.I1RQE1
4713	229533.I1RG66	pp	229533.I1RPN4
4714	229533.I1RG66	pp	229533.I1RQ60
4715	229533.I1RG66	pp	229533.I1S1B8
4716	229533.I1RG66	pp	229533.I1RYK1
4717	229533.I1RG66	pp	229533.I1RYX6
4718	229533.I1RG66	pp	229533.I1RJB0
4719	229533.I1RG66	pp	229533.I1RYA8
4720	229533.I1RG66	pp	229533.I1RYW9
4721	229533.I1RG66	pp	229533.I1RSB7
4722	229533.I1RG66	pp	229533.Q4HXF0
4723	229533.I1RG66	pp	229533.I1S4G3
4724	229533.I1RG66	pp	229533.I1RRF5
4725	229533.I1RG66	pp	229533.I1RL81
4726	229533.I1RG66	pp	229533.I1RUQ3
4727	229533.I1RG66	pp	229533.I1S0I3
4728	229533.I1RG66	pp	229533.V6RHD0
4729	229533.I1RG66	pp	229533.I1RM26
4730	229533.I1RG66	pp	229533.I1RNG4
4731	229533.I1RG66	pp	229533.V6QU65
4732	229533.I1RG66	pp	229533.I1S6M5
4733	229533.I1RG66	pp	229533.I1RYA0
4734	229533.I1RG66	pp	229533.I1RY74
4735	229533.I1RG66	pp	229533.Q4HVQ9
4736	229533.I1RG66	pp	229533.I1S8S9
4737	229533.I1RG66	pp	229533.I1RVX9
4738	229533.I1RG66	pp	229533.I1RNV4
4739	229533.I1RG66	pp	229533.V6RY56
4740	229533.I1RG66	pp	229533.I1RYA2
4741	229533.I1RG66	pp	229533.I1S8F8
4742	229533.I1RG66	pp	229533.I1RWZ3
4743	229533.I1RG66	pp	229533.I1RPW1
4744	229533.I1RG66	pp	229533.I1RYC8
4745	229533.I1RG66	pp	229533.I1RGG7

229533.I1RG66 pp 229533.I1S099
229533.I1RG66 pp 229533.I1RHC8
229533.I1RG66 pp 229533.I1RKZ2
229533.I1RG66 pp 229533.I1RVV7
229533.I1RG66 pp 229533.I1S599
229533.I1RG66 pp 229533.I1RKZ7
229533.I1RG66 pp 229533.I1S4D0
229533.I1RG66 pp 229533.I1RNY6
229533.I1RPC2 pp 229533.I1S4R1
229533.I1RPC2 pp 229533.I1S098
229533.I1RPC2 pp 229533.I1RUF8
229533.I1RPC2 pp 229533.Q4I7L0
229533.I1RPC2 pp 229533.I1RYZ9
229533.I1RPC2 pp 229533.V6RGI1
229533.I1RPC2 pp 229533.I1RQ13
229533.I1RPC2 pp 229533.I1RY84
229533.I1RPC2 pp 229533.I1S9S6
229533.I1RPC2 pp 229533.I1RS56
229533.I1RPC2 pp 229533.I1RSL8
229533.I1RPC2 pp 229533.Q4IJM4
229533.I1RPC2 pp 229533.V6QV09
229533.I1RPC2 pp 229533.I1RT62
229533.A0A098E1W1 pp 229533.I1RXB5
229533.A0A098E1W1 pp 229533.A0A1C3YKT9
229533.A0A098E1W1 pp 229533.I1RH68
229533.A0A098E1W1 pp 229533.I1RJR9
229533.A0A098E1W1 pp 229533.I1RWL6
229533.A0A098E1W1 pp 229533.I1RD70
229533.A0A098E1W1 pp 229533.A0A1C3YJG7
229533.A0A098E1W1 pp 229533.I1RWA9
229533.A0A098E1W1 pp 229533.I1RQN9
229533.A0A098E1W1 pp 229533.A0A0E0SGT0
229533.A0A098E1W1 pp 229533.I1RLX6
229533.A0A098E1W1 pp 229533.I1S0S6
229533.A0A098E1W1 pp 229533.P0C431
229533.A0A098E1W1 pp 229533.A0A1C3YLH6
229533.A0A098E1W1 pp 229533.I1S549
229533.A0A098E1W1 pp 229533.A0A1C3YIZ8
229533.A0A098E1W1 pp 229533.I1RA39
229533.A0A098E1W1 pp 229533.I1RWM0
229533.A0A098DEV0 pp 229533.I1RHC8
229533.A0A098DEV0 pp 229533.I1RKZ7
229533.A0A098DEV0 pp 229533.I1RG66
229533.A0A098DEV0 pp 229533.I1S1W8
229533.A0A098DEV0 pp 229533.I1S599
229533.A0A098DEV0 pp 229533.I1RW54
229533.A0A098DEV0 pp 229533.I1S267
229533.A0A098DEV0 pp 229533.I1RY35
229533.A0A098DEV0 pp 229533.I1S3G4
229533.A0A098DEV0 pp 229533.I1RDQ2
229533.A0A098DEV0 pp 229533.I1S6M5
229533.A0A098DEV0 pp 229533.I1RVB2
229533.A0A098DEV0 pp 229533.I1RWL3
229533.A0A098DEV0 pp 229533.A0A1C3YKL7
229533.A0A098DEV0 pp 229533.I1RYA8
229533.A0A098DEV0 pp 229533.I1RJB0
229533.A0A098DEV0 pp 229533.I1RYK1
229533.A0A098DEV0 pp 229533.I1RYA2
229533.A0A098DEV0 pp 229533.Q4HVQ9
229533.A0A098DEV0 pp 229533.I1RYQ4
229533.A0A098DEV0 pp 229533.A0A1C3YJI5
229533.A0A098DEV0 pp 229533.I1RQE1
229533.A0A098DEV0 pp 229533.I1S2C4
229533.A0A098DEV0 pp 229533.I1RNV4
229533.A0A098DEV0 pp 229533.I1RBE7
229533.A0A098DEV0 pp 229533.I1RCR6
229533.A0A098DEV0 pp 229533.I1RQ60
229533.A0A098DEV0 pp 229533.I1RD67
229533.A0A098DEV0 pp 229533.A0A1C3YKX1
229533.A0A098DEV0 pp 229533.I1RKZ2
229533.A0A098DEV0 pp 229533.I1RNY6
229533.A0A098DEV0 pp 229533.I1RUS5
229533.A0A098DEV0 pp 229533.I1RYW9

229533.A0A098DEV0 pp 229533.I1RRF5
229533.A0A098DEV0 pp 229533.I1SAF0
229533.A0A098DEV0 pp 229533.I1R9B6
229533.A0A098DEV0 pp 229533.I1RYX6
229533.A0A098DEV0 pp 229533.I1RPE5
229533.A0A098DEV0 pp 229533.A0A098DYP8
229533.A0A098DEV0 pp 229533.I1RNG4
229533.A0A1C3YJF5 pp 229533.I1S096
229533.A0A1C3YJF5 pp 229533.P0C431
229533.A0A1C3YJF5 pp 229533.I1RWL6
229533.A0A1C3YJF5 pp 229533.I1RQL4
229533.A0A1C3YJF5 pp 229533.I1RJR9
229533.A0A1C3YJF5 pp 229533.I1RTB7
229533.A0A1C3YJF5 pp 229533.I1RKG1
229533.A0A1C3YJF5 pp 229533.A0A1C3YLN8
229533.A0A1C3YJF5 pp 229533.I1RA81
229533.A0A098D6W3 pp 229533.I1S1W8
229533.A0A098D6W3 pp 229533.I1S2Q4
229533.A0A098D6W3 pp 229533.I1RTY0
229533.A0A098D6W3 pp 229533.I1S3G4
229533.A0A098D6W3 pp 229533.A0A1C3YJI5
229533.A0A098D6W3 pp 229533.I1S1B8
229533.A0A098D6W3 pp 229533.I1RH91
229533.A0A098D6W3 pp 229533.I1RJJ7
229533.A0A098D6W3 pp 229533.I1RQE1
229533.A0A098D6W3 pp 229533.A0A1C3YKL7
229533.A0A098D6W3 pp 229533.A0A098E3P8
229533.A0A098D6W3 pp 229533.I1RBY9
229533.A0A098D6W3 pp 229533.I1RYX6
229533.A0A098D6W3 pp 229533.I1RNG4
229533.A0A098D6W3 pp 229533.A0A1C3YN04
229533.A0A098D6W3 pp 229533.A0A1C3YI08
229533.A0A098D6W3 pp 229533.I1RBE7
229533.A0A098D6W3 pp 229533.Q4HVQ9
229533.A0A098D6W3 pp 229533.I1S4G3
229533.A0A098D6W3 pp 229533.I1RWZ3
229533.A0A098D6W3 pp 229533.A0A098D8K2
229533.A0A098D6W3 pp 229533.A0A098DUS9
229533.A0A098D6W3 pp 229533.A0A1C3YMF1
229533.A0A098D6W3 pp 229533.I1RAV0
229533.A0A098D6W3 pp 229533.I1RG66
229533.A0A098D6W3 pp 229533.A0A098DEV0
229533.A0A1C3YL03 pp 229533.I1RN17
229533.A0A1C3YL03 pp 229533.I1RVY0
229533.I1S4R1 pp 229533.V6RGI1
229533.I1S4R1 pp 229533.V6QV09
229533.A0A1C3YL00 pp 229533.I1RSR8
229533.I1RKG1 pp 229533.I1RPL7
229533.I1RKG1 pp 229533.I1RWL6
229533.I1RKG1 pp 229533.P0C431
229533.I1RKG1 pp 229533.I1RN81
229533.I1RKG1 pp 229533.I1RTB7
229533.I1RKG1 pp 229533.I1RQL4
229533.I1S8H5 pp 229533.I1SAH9
229533.A0A0E0RTV6 pp 229533.I1RFC6
229533.A0A0E0RTV6 pp 229533.I1RWY4
229533.I1RVL7 pp 229533.I1RVT5
229533.I1RVL7 pp 229533.I1SAJ8
229533.I1RVL7 pp 229533.I1RWV9
229533.I1RVL7 pp 229533.I1S166
229533.I1RVL7 pp 229533.I1S5E5
229533.Q00909 pp 229533.V6RG22
229533.Q00909 pp 229533.V6RPN0
229533.Q00909 pp 229533.Q4HZS8
229533.I1S096 pp 229533.I1S1V5
229533.I1S096 pp 229533.Q4HYC1
229533.I1S096 pp 229533.I1S1C8
229533.I1S096 pp 229533.P0C431
229533.I1S096 pp 229533.Q5I2J3
229533.A0A098D7J4 pp 229533.I1SAJ8
229533.A0A098D7J4 pp 229533.I1RUB2
229533.A0A098D7J4 pp 229533.A0A098DEU5
229533.A0A098D7J4 pp 229533.I1S5E5

229533.A0A098D7J4 pp 229533.A0A098DC45
229533.A0A098D7J4 pp 229533.I1RVT5
229533.A0A098D7J4 pp 229533.I1RL11
229533.A0A098D7J4 pp 229533.A0A098DQH7
229533.A0A098D7J4 pp 229533.I1RDS5
229533.A0A098D7J4 pp 229533.I1RHS7
229533.A0A098D7J4 pp 229533.A0A098D8A0
229533.A0A098D7J4 pp 229533.I1RWV9
229533.A0A098D7J4 pp 229533.A0A1C3YJK1
229533.A0A098D7J4 pp 229533.I1RLA7
229533.A0A098D7J4 pp 229533.I1RVD8
229533.A0A098D7J4 pp 229533.I1RPZ7
229533.A0A098D7J4 pp 229533.I1S166
229533.A0A098D7J4 pp 229533.I1REX6
229533.A0A098D7J4 pp 229533.A0A098DC24
229533.A0A098D7J4 pp 229533.I1RF58
229533.A0A098D7J4 pp 229533.I1RJE8
229533.I1S9S6 pp 229533.V6RGI1
229533.I1S9S6 pp 229533.V6QV09
229533.A0A098D8A0 pp 229533.A0A1C3YIP3
229533.A0A098D8A0 pp 229533.A0A098E2M9
229533.A0A098D8A0 pp 229533.A0A098E3B9
229533.A0A098D8A0 pp 229533.I1RPR1
229533.A0A098D8A0 pp 229533.A0A1C3YK32
229533.A0A098D8A0 pp 229533.I1RHK0
229533.A0A098D8A0 pp 229533.I1RIV2
229533.A0A098D8A0 pp 229533.I1RVL7
229533.A0A098D8A0 pp 229533.I1RFC3
229533.A0A098D8A0 pp 229533.I1RQX6
229533.A0A098D8A0 pp 229533.I1S8H5
229533.A0A098D8A0 pp 229533.A0A0E0RTV6
229533.A0A098D8A0 pp 229533.Q00909
229533.A0A098D8A0 pp 229533.I1S9Z4
229533.A0A098D8A0 pp 229533.A0A1C3YMP1
229533.A0A098D8A0 pp 229533.I1RVM4
229533.A0A098D8A0 pp 229533.I1S5U5
229533.A0A098D8A0 pp 229533.I1RFC6
229533.A0A098D8A0 pp 229533.I1RQ48
229533.A0A098D8A0 pp 229533.I1RK69
229533.A0A098D8A0 pp 229533.A0A1C3YKA2
229533.A0A098D8A0 pp 229533.Q4HZS8
229533.A0A098D8A0 pp 229533.I1S2J4
229533.A0A098D8A0 pp 229533.I1S3K7
229533.A0A098D8A0 pp 229533.I1RF49
229533.A0A098D8A0 pp 229533.A0A098E0Z2
229533.A0A098D8A0 pp 229533.I1S1L0
229533.A0A098D8A0 pp 229533.A0A1C3YJ35
229533.I1S1T5 pp 229533.I1S1W8
229533.I1S1T5 pp 229533.I1S3G4
229533.I1S1T5 pp 229533.I1SAH9
229533.A0A1C3YJC3 pp 229533.I1RRW0
229533.A0A1C3YJC3 pp 229533.V6RGI1
229533.A0A1C3YJC3 pp 229533.I1RC17
229533.A0A1C3YJC3 pp 229533.I1RNY8
229533.A0A1C3YJC3 pp 229533.I1RMQ7
229533.A0A1C3YJC3 pp 229533.I1RY44
229533.A0A1C3YJC3 pp 229533.I1S9U1
229533.A0A1C3YJC3 pp 229533.I1RWT6
229533.A0A1C3YJC3 pp 229533.I1S1W4
229533.A0A1C3YJC3 pp 229533.I1RYY5
229533.A0A1C3YJC3 pp 229533.A0A1C3YJW1
229533.A0A1C3YJC3 pp 229533.I1RHB0
229533.A0A1C3YJC3 pp 229533.I1RGC6
229533.A0A1C3YJC3 pp 229533.I1RQA3
229533.A0A1C3YJC3 pp 229533.I1RYZ9
229533.A0A1C3YJC3 pp 229533.A0A1C3YJT5
229533.A0A1C3YJC3 pp 229533.I1S0E8
229533.A0A1C3YJC3 pp 229533.I1RRN5
229533.A0A1C3YJC3 pp 229533.V6QV09
229533.A0A1C3YJC3 pp 229533.I1RFL7
229533.A0A1C3YJC3 pp 229533.Q4IJM4
229533.A0A1C3YJC3 pp 229533.I1RT62
229533.A0A1C3YJC3 pp 229533.I1RK75

4965 229533.A0A1C3YJC3 pp 229533.I1RYZ0
4966 229533.A0A1C3YJC3 pp 229533.I1R9N1
4967 229533.A0A1C3YJC3 pp 229533.I1RB41
4968 229533.A0A1C3YJC3 pp 229533.I1RD15
4969 229533.A0A1C3YJC3 pp 229533.I1REX8
4970 229533.A0A1C3YJC3 pp 229533.I1R9Y7
4971 229533.A0A1C3YJC3 pp 229533.I1RZ63
4972 229533.A0A1C3YJC3 pp 229533.I1S098
4973 229533.A0A1C3YJC3 pp 229533.I1RL24
4974 229533.A0A1C3YJC3 pp 229533.A0A1C3YJH8
4975 229533.A0A1C3YJC3 pp 229533.I1S2E9
4976 229533.A0A1C3YJC3 pp 229533.I1RPC2
4977 229533.A0A1C3YJC3 pp 229533.A0A1C3YK57
4978 229533.A0A1C3YJC3 pp 229533.I1RAT7
4979 229533.A0A1C3YJC3 pp 229533.A0A1C3YL67
4980 229533.A0A1C3YJC3 pp 229533.I1RRT2
4981 229533.A0A1C3YJC3 pp 229533.I1S224
4982 229533.A0A1C3YJC3 pp 229533.I1S0J7
4983 229533.A0A1C3YJC3 pp 229533.I1RN89
4984 229533.A0A1C3YJC3 pp 229533.I1RM49
4985 229533.I1S2J4 pp 229533.I1S3G4
4986 229533.I1RNX8 pp 229533.I1S147
4987 229533.Q4HZS8 pp 229533.Q4I7L0
4988 229533.Q4HZS8 pp 229533.Q4IPI4
4989 229533.Q4HZS8 pp 229533.V6QUV7
4990 229533.Q4HZS8 pp 229533.Q5I2J3
4991 229533.Q4HZS8 pp 229533.V6RPN0
4992 229533.Q4HZS8 pp 229533.V6QX21
4993 229533.Q4HZS8 pp 229533.Q4I9B7
4994 229533.Q4HZS8 pp 229533.Q4I963
4995 229533.Q4HZS8 pp 229533.Q4ICA8
4996 229533.Q4HZS8 pp 229533.Q4I0J6
4997 229533.Q4HZS8 pp 229533.Q4IER8
4998 229533.Q4HZS8 pp 229533.V6REE9
4999 229533.Q4HZS8 pp 229533.Q4IBX0
5000 229533.I1RD85 pp 229533.I1RFL7
5001 229533.I1RD85 pp 229533.V6QV09
5002 229533.I1RD85 pp 229533.V6RGI1
5003 229533.I1RD85 pp 229533.I1RYZ9
5004 229533.I1RD85 pp 229533.I1RL24
5005 229533.I1RHK0 pp 229533.I1RHS7
5006 229533.I1RHK0 pp 229533.I1RJE8
5007 229533.I1RHK0 pp 229533.I1RLA7
5008 229533.I1RHK0 pp 229533.I1RPZ7
5009 229533.I1RHK0 pp 229533.I1S166
5010 229533.I1RHK0 pp 229533.I1RL11
5011 229533.I1RHK0 pp 229533.I1RVD8
5012 229533.I1RHK0 pp 229533.I1RWV9
5013 229533.I1RHK0 pp 229533.I1S5E5
5014 229533.I1RHK0 pp 229533.I1RI12
5015 229533.I1RHK0 pp 229533.I1RVT5
5016 229533.I1RHK0 pp 229533.I1RUB2
5017 229533.I1RHK0 pp 229533.I1SAJ8
5018 229533.I1RIV2 pp 229533.I1S1W8
5019 229533.I1RIV2 pp 229533.I1RVD8
5020 229533.I1RIV2 pp 229533.I1RWV9
5021 229533.I1RIV2 pp 229533.I1RKD3
5022 229533.I1RIV2 pp 229533.I1RY87
5023 229533.I1RIV2 pp 229533.I1RW06
5024 229533.I1RIV2 pp 229533.I1S3G4
5025 229533.I1RIV2 pp 229533.I1SAJ8
5026 229533.I1RIV2 pp 229533.I1RUB2
5027 229533.I1RIV2 pp 229533.I1RMG9
5028 229533.I1RIV2 pp 229533.I1SAH9
5029 229533.I1RIV2 pp 229533.I1RL11
5030 229533.I1RIV2 pp 229533.I1RWW4
5031 229533.I1RIV2 pp 229533.I1RN13
5032 229533.I1RIV2 pp 229533.I1RNG4
5033 229533.I1RIV2 pp 229533.I1S0K1
5034 229533.I1RIV2 pp 229533.I1RLA7
5035 229533.I1RIV2 pp 229533.I1S0X1
5036 229533.I1RIV2 pp 229533.I1RJE8
5037 229533.I1RIV2 pp 229533.I1RP12

5038 229533.I1RIV2 pp 229533.I1RTY9
5039 229533.I1RIV2 pp 229533.P0C431
5040 229533.I1RIV2 pp 229533.I1RUE3
5041 229533.I1RIV2 pp 229533.I1RQW1
5042 229533.I1RIV2 pp 229533.I1S166
5043 229533.I1RRQ9 pp 229533.I1RYR9
5044 229533.I1RRQ9 pp 229533.I1RYZ9
5045 229533.I1RRQ9 pp 229533.I1RS64
5046 229533.I1RRQ9 pp 229533.I1RZT1
5047 229533.I1RRQ9 pp 229533.V6QV09
5048 229533.I1RLB5 pp 229533.I1RUL3
5049 229533.I1RLB5 pp 229533.I1RMG4
5050 229533.I1RLB5 pp 229533.I1S984
5051 229533.I1RLB5 pp 229533.I1RVW7
5052 229533.I1RLB5 pp 229533.I1RNA1
5053 229533.I1RLB5 pp 229533.I1S574
5054 229533.I1RGD1 pp 229533.I1RYZ9
5055 229533.I1RGD1 pp 229533.I1S0J7
5056 229533.I1RGD1 pp 229533.I1S098
5057 229533.I1RGD1 pp 229533.V6RGI1
5058 229533.I1RGD1 pp 229533.V6QV09
5059 229533.I1RGD1 pp 229533.I1RK75
5060 229533.I1RGD1 pp 229533.I1RL24
5061 229533.I1RGD1 pp 229533.I1RS56
5062 229533.I1RGD1 pp 229533.I1RPC2
5063 229533.I1RGD1 pp 229533.I1RY84
5064 229533.I1RVD9 pp 229533.I1RY87
5065 229533.I1RVD9 pp 229533.I1S3G4
5066 229533.I1RVD9 pp 229533.I1S1W8
5067 229533.I1RVD9 pp 229533.I1RWV9
5068 229533.I1RVD9 pp 229533.I1S166
5069 229533.I1RW36 pp 229533.I1RYZ9
5070 229533.I1RW36 pp 229533.I1RXT9
5071 229533.I1RW36 pp 229533.V6QV09
5072 229533.I1RW36 pp 229533.I1S287
5073 229533.I1RW36 pp 229533.I1RYS9
5074 229533.I1RG95 pp 229533.I1RMG4
5075 229533.I1RG95 pp 229533.I1RVW7
5076 229533.I1RG95 pp 229533.I1RPE5
5077 229533.I1RG95 pp 229533.V6QTX7
5078 229533.I1RG95 pp 229533.I1RLB5
5079 229533.I1RG95 pp 229533.V6R556
5080 229533.I1RG95 pp 229533.V6RM09
5081 229533.I1RG95 pp 229533.I1S9T8
5082 229533.I1RG95 pp 229533.V6REA4
5083 229533.I1RG95 pp 229533.I1RZT6
5084 229533.I1RFC3 pp 229533.I1S1W8
5085 229533.I1RFC3 pp 229533.I1RY87
5086 229533.I1RFC3 pp 229533.I1RVD8
5087 229533.I1RFC3 pp 229533.I1S3G4
5088 229533.I1RFC3 pp 229533.I1RL35
5089 229533.I1RFC3 pp 229533.I1RL11
5090 229533.I1RFC3 pp 229533.I1RNG4
5091 229533.I1RFC3 pp 229533.I1RUB2
5092 229533.I1RFC3 pp 229533.I1RHS7
5093 229533.I1RFC3 pp 229533.I1RWV9
5094 229533.I1RFC3 pp 229533.I1RFQ4
5095 229533.I1RFC3 pp 229533.I1RLA7
5096 229533.I1RFC3 pp 229533.I1S166
5097 229533.I1RFC3 pp 229533.I1S3G0
5098 229533.I1RFC3 pp 229533.I1S478
5099 229533.A0A1C3YIP3 pp 229533.I1S5D0
5100 229533.A0A1C3YIP3 pp 229533.I1RL11
5101 229533.A0A1C3YIP3 pp 229533.I1RVT5
5102 229533.A0A1C3YIP3 pp 229533.I1RQV7
5103 229533.A0A1C3YIP3 pp 229533.I1RMA2
5104 229533.A0A1C3YIP3 pp 229533.V6R5T7
5105 229533.A0A1C3YIP3 pp 229533.I1RK02
5106 229533.A0A1C3YIP3 pp 229533.I1R9F2
5107 229533.A0A1C3YIP3 pp 229533.I1S969
5108 229533.A0A1C3YIP3 pp 229533.I1S5E5
5109 229533.A0A1C3YIP3 pp 229533.I1RSU2
5110 229533.A0A1C3YIP3 pp 229533.I1RST9

229533.A0A1C3YIP3 pp 229533.I1RRS6
229533.A0A1C3YIP3 pp 229533.I1RPZ7
229533.A0A1C3YIP3 pp 229533.I1RIB5
229533.A0A1C3YIP3 pp 229533.I1SAT2
229533.A0A1C3YIP3 pp 229533.I1RVD8
229533.A0A1C3YIP3 pp 229533.I1RUB2
229533.A0A1C3YIP3 pp 229533.I1SAJ8
229533.A0A1C3YIP3 pp 229533.I1RQV8
229533.A0A1C3YIP3 pp 229533.V6RCJ5
229533.A0A1C3YIP3 pp 229533.I1S8D3
229533.A0A1C3YIP3 pp 229533.I1RWV9
229533.A0A1C3YIP3 pp 229533.I1RK55
229533.A0A1C3YIP3 pp 229533.I1RJE8
229533.A0A1C3YIP3 pp 229533.I1S968
229533.A0A1C3YIP3 pp 229533.I1RF58
229533.A0A1C3YIP3 pp 229533.I1RYP5
229533.A0A1C3YIP3 pp 229533.I1REX6
229533.A0A1C3YIP3 pp 229533.I1RSA5
229533.A0A1C3YIP3 pp 229533.I1RH83
229533.A0A1C3YIP3 pp 229533.V6RHX2
229533.A0A1C3YIP3 pp 229533.A0A1C3YMQ9
229533.A0A1C3YIP3 pp 229533.V6RCC0
229533.A0A1C3YIP3 pp 229533.I1S9Z4
229533.A0A1C3YIP3 pp 229533.I1RIJ6
229533.A0A1C3YIP3 pp 229533.I1S6V0
229533.A0A1C3YIP3 pp 229533.I1RDS5
229533.A0A1C3YIP3 pp 229533.I1RLA7
229533.A0A1C3YIP3 pp 229533.I1RY81
229533.A0A1C3YIP3 pp 229533.A0A1C3YJK1
229533.A0A1C3YIP3 pp 229533.A0A1C3YLB6
229533.A0A1C3YIP3 pp 229533.I1S166
229533.A0A1C3YIP3 pp 229533.I1RHS7
229533.A0A1C3YIP3 pp 229533.I1S659
229533.A0A1C3YIP3 pp 229533.I1RB05
229533.A0A1C3YIP3 pp 229533.V6RWB3
229533.I1SA48 pp 229533.Q4IR09
229533.I1RVM4 pp 229533.I1S166
229533.I1RVM4 pp 229533.I1S5E5
229533.I1RVM4 pp 229533.I1RW19
229533.I1RVM4 pp 229533.I1RWV9
229533.I1RVM4 pp 229533.I1SAJ8
229533.I1RVM4 pp 229533.I1RVT5
229533.I1RVW7 pp 229533.I1S574
229533.I1RVW7 pp 229533.I1S984
229533.A0A098E2M9 pp 229533.I1RDS5
229533.A0A098E2M9 pp 229533.I1RVD8
229533.A0A098E2M9 pp 229533.I1RPZ7
229533.A0A098E2M9 pp 229533.I1RWV9
229533.A0A098E2M9 pp 229533.I1S5E5
229533.A0A098E2M9 pp 229533.I1RL11
229533.A0A098E2M9 pp 229533.I1RVT5
229533.A0A098E2M9 pp 229533.I1SAJ8
229533.A0A098E2M9 pp 229533.I1RUB2
229533.A0A098E2M9 pp 229533.I1RHS7
229533.A0A098E2M9 pp 229533.A0A1C3YJK1
229533.A0A098E2M9 pp 229533.I1RLA7
229533.A0A098E2M9 pp 229533.I1REX6
229533.A0A098E2M9 pp 229533.I1RF58
229533.A0A098E2M9 pp 229533.I1S166
229533.A0A098E2M9 pp 229533.I1RJE8
229533.A0A098E3B9 pp 229533.I1RWV9
229533.A0A098E3B9 pp 229533.I1SAJ8
229533.A0A098E3B9 pp 229533.I1RUB2
229533.A0A098E3B9 pp 229533.I1RVD8
229533.A0A098E3B9 pp 229533.I1RPZ7
229533.A0A098E3B9 pp 229533.I1S166
229533.A0A098E3B9 pp 229533.I1S5E5
229533.A0A098E3B9 pp 229533.I1REX6
229533.A0A098E3B9 pp 229533.I1RF58
229533.A0A098E3B9 pp 229533.I1RJE8
229533.A0A098E3B9 pp 229533.I1RL11
229533.A0A098E3B9 pp 229533.I1RVT5
229533.A0A098E3B9 pp 229533.I1RHS7

5184 229533.A0A098E3B9 pp 229533.A0A1C3YJK1
5185 229533.A0A098E3B9 pp 229533.I1RLA7
5186 229533.A0A098E3B9 pp 229533.I1RDS5
5187 229533.I1RKC2 pp 229533.I1RYZ9
5188 229533.I1RKC2 pp 229533.I1RL24
5189 229533.I1RKC2 pp 229533.V6QV09
5190 229533.I1RKC2 pp 229533.I1RKL6
5191 229533.I1RKC2 pp 229533.V6RGI1
5192 229533.I1RA96 pp 229533.I1S368
5193 229533.I1RA96 pp 229533.I1RN66
5194 229533.I1RA96 pp 229533.I1RAZ1
5195 229533.I1RA96 pp 229533.I1RC58
5196 229533.I1RA96 pp 229533.I1RNR9
5197 229533.I1RA96 pp 229533.I1RQ61
5198 229533.I1RA96 pp 229533.I1RMG4
5199 229533.I1RA96 pp 229533.I1RE86
5200 229533.A0A1C3YKA2 pp 229533.I1RHS7
5201 229533.A0A1C3YKA2 pp 229533.I1RUB2
5202 229533.A0A1C3YKA2 pp 229533.I1RLA7
5203 229533.A0A1C3YKA2 pp 229533.I1S166
5204 229533.A0A1C3YKA2 pp 229533.I1SAJ8
5205 229533.A0A1C3YKA2 pp 229533.I1REX6
5206 229533.A0A1C3YKA2 pp 229533.I1RF58
5207 229533.A0A1C3YKA2 pp 229533.I1RJE8
5208 229533.A0A1C3YKA2 pp 229533.I1RDS5
5209 229533.A0A1C3YKA2 pp 229533.I1RWV9
5210 229533.A0A1C3YKA2 pp 229533.I1S5E5
5211 229533.A0A1C3YKA2 pp 229533.I1RL11
5212 229533.A0A1C3YKA2 pp 229533.I1RVD8
5213 229533.A0A1C3YKA2 pp 229533.I1RPZ7
5214 229533.A0A1C3YKA2 pp 229533.I1RVT5
5215 229533.I1RC59 pp 229533.I1S147
5216 229533.I1RC59 pp 229533.I1RFI4
5217 229533.I1RBU2 pp 229533.I1RPW2
5218 229533.I1RBU2 pp 229533.I1RE63
5219 229533.I1RBU2 pp 229533.I1RVX9
5220 229533.I1RBU2 pp 229533.I1RQE0
5221 229533.I1RBU2 pp 229533.I1RFS5
5222 229533.I1RPR1 pp 229533.I1RY12
5223 229533.I1RPR1 pp 229533.V6R3V4
5224 229533.I1RPR1 pp 229533.I1S0V6
5225 229533.I1RPR1 pp 229533.I1S0K2
5226 229533.I1RPR1 pp 229533.I1RY21
5227 229533.I1RPR1 pp 229533.I1RXS0
5228 229533.I1RPR1 pp 229533.I1RXV9
5229 229533.I1RPR1 pp 229533.I1RUQ3
5230 229533.I1RPR1 pp 229533.I1S3L2
5231 229533.I1RPR1 pp 229533.I1RY81
5232 229533.I1RPR1 pp 229533.I1RVD8
5233 229533.I1RPR1 pp 229533.I1S489
5234 229533.I1RPR1 pp 229533.I1RQP2
5235 229533.I1RPR1 pp 229533.I1RWV9
5236 229533.I1RPR1 pp 229533.I1S3T7
5237 229533.I1RPR1 pp 229533.I1RYP5
5238 229533.I1RPR1 pp 229533.I1SAJ8
5239 229533.I1RPR1 pp 229533.I1RY35
5240 229533.I1RPR1 pp 229533.I1RXE6
5241 229533.I1RPR1 pp 229533.I1S6P5
5242 229533.I1RPR1 pp 229533.I1RVX9
5243 229533.I1RPR1 pp 229533.I1S166
5244 229533.I1RPR1 pp 229533.I1S8F8
5245 229533.I1RPR1 pp 229533.I1RVR2
5246 229533.I1RPR1 pp 229533.I1S4S5
5247 229533.I1RPR1 pp 229533.I1S0K0
5248 229533.I1RPR1 pp 229533.V6R5D2
5249 229533.I1RPR1 pp 229533.I1RUR8
5250 229533.I1RPR1 pp 229533.I1RRX5
5251 229533.I1RPR1 pp 229533.I1S8R4
5252 229533.I1RPR1 pp 229533.I1S0V7
5253 229533.I1RPR1 pp 229533.I1S968
5254 229533.I1RPR1 pp 229533.I1RWF5
5255 229533.I1RPR1 pp 229533.I1S147
5256 229533.I1RPR1 pp 229533.I1S8C1

5257 229533.I1RPR1 pp 229533.I1RUB2
5258 229533.I1RPR1 pp 229533.I1RR68
5259 229533.I1RPR1 pp 229533.I1RYA0
5260 229533.I1RPR1 pp 229533.V6RRM0
5261 229533.I1RPR1 pp 229533.Q4HVQ9
5262 229533.I1RPR1 pp 229533.V6RF62
5263 229533.I1RPR1 pp 229533.V6RLM7
5264 229533.I1RPR1 pp 229533.I1S0N4
5265 229533.I1RPR1 pp 229533.I1S5E5
5266 229533.I1RPR1 pp 229533.V6R3J1
5267 229533.I1RPR1 pp 229533.I1RYC8
5268 229533.I1RPR1 pp 229533.I1RZA7
5269 229533.I1RPR1 pp 229533.V6RUB1
5270 229533.I1RPR1 pp 229533.I1S1F3
5271 229533.I1RPR1 pp 229533.V6RZD4
5272 229533.I1RPR1 pp 229533.I1RQ48
5273 229533.I1S3K7 pp 229533.I1S5P3
5274 229533.I1RY38 pp 229533.I1S1X2
5275 229533.I1RBT6 pp 229533.V6RCG5
5276 229533.I1RBT6 pp 229533.I1RSQ3
5277 229533.I1RBT6 pp 229533.I1RRX5
5278 229533.I1RBT6 pp 229533.I1RS05
5279 229533.I1RBT6 pp 229533.I1RPY8
5280 229533.I1RBT6 pp 229533.I1S464
5281 229533.I1RBT6 pp 229533.I1RK39
5282 229533.I1RBT6 pp 229533.I1RFS5
5283 229533.I1RBT6 pp 229533.I1S8F0
5284 229533.I1RBT6 pp 229533.I1RE66
5285 229533.I1RBT6 pp 229533.I1RQR6
5286 229533.I1RBT6 pp 229533.I1RJB3
5287 229533.I1RBT6 pp 229533.I1S0P0
5288 229533.I1RBT6 pp 229533.I1RXE6
5289 229533.I1RBT6 pp 229533.I1RWF4
5290 229533.I1RBT6 pp 229533.I1RYC8
5291 229533.I1RBT6 pp 229533.I1S8F8
5292 229533.I1RBT6 pp 229533.I1RW13
5293 229533.I1RBT6 pp 229533.I1RE65
5294 229533.I1RBT6 pp 229533.I1RI95
5295 229533.I1RBT6 pp 229533.I1RX39
5296 229533.I1RBT6 pp 229533.I1S1F3
5297 229533.I1RBT6 pp 229533.I1RYC7
5298 229533.I1RBT6 pp 229533.I1RWG0
5299 229533.I1RBT6 pp 229533.I1RP02
5300 229533.I1RBT6 pp 229533.I1S0R8
5301 229533.I1RBT6 pp 229533.I1S0K2
5302 229533.I1RBT6 pp 229533.V6RJP8
5303 229533.I1RBT6 pp 229533.I1RG33
5304 229533.I1RBT6 pp 229533.I1S0U0
5305 229533.I1RBT6 pp 229533.I1RS29
5306 229533.I1RBT6 pp 229533.I1S5X9
5307 229533.I1RBT6 pp 229533.Q4IJW4
5308 229533.I1RBT6 pp 229533.I1RL81
5309 229533.I1RBT6 pp 229533.I1RZ68
5310 229533.I1RBT6 pp 229533.I1RN18
5311 229533.I1RBT6 pp 229533.I1RGJ4
5312 229533.I1RBT6 pp 229533.I1S0T7
5313 229533.I1RBT6 pp 229533.I1RNM7
5314 229533.I1RBT6 pp 229533.I1RYA0
5315 229533.I1RBT6 pp 229533.I1RXD9
5316 229533.I1RBT6 pp 229533.I1RWA3
5317 229533.I1RBT6 pp 229533.I1RMK7
5318 229533.I1RBT6 pp 229533.I1S266
5319 229533.I1RBT6 pp 229533.I1S0Q7
5320 229533.I1RBT6 pp 229533.I1RP38
5321 229533.I1RBT6 pp 229533.I1RKB7
5322 229533.I1RBT6 pp 229533.I1RMQ6
5323 229533.I1RBT6 pp 229533.I1RRK9
5324 229533.I1RBT6 pp 229533.I1RLN1
5325 229533.I1RBT6 pp 229533.I1RYT8
5326 229533.I1RBT6 pp 229533.I1RG62
5327 229533.I1RBT6 pp 229533.I1S5U0
5328 229533.I1RBT6 pp 229533.I1RY92
5329 229533.I1RBT6 pp 229533.I1REF0

5330 229533.I1RBT6 pp 229533.I1RZ43
5331 229533.I1RBT6 pp 229533.I1S212
5332 229533.I1RBT6 pp 229533.I1S8S9
5333 229533.I1RBT6 pp 229533.I1S5C6
5334 229533.I1RBT6 pp 229533.I1RY21
5335 229533.I1RBT6 pp 229533.I1RFJ6
5336 229533.I1RBT6 pp 229533.I1RU95
5337 229533.I1RBT6 pp 229533.I1RGI8
5338 229533.I1RBT6 pp 229533.I1RPR9
5339 229533.I1RF49 pp 229533.I1SAH9
5340 229533.I1RF49 pp 229533.I1RIF7
5341 229533.I1RF49 pp 229533.I1RNG4
5342 229533.I1RF49 pp 229533.I1RL11
5343 229533.I1RF49 pp 229533.I1RY87
5344 229533.I1RF49 pp 229533.I1S0K1
5345 229533.I1RF49 pp 229533.I1S3G4
5346 229533.I1RF49 pp 229533.I1RGK3
5347 229533.I1RF49 pp 229533.I1S1W8
5348 229533.I1RF49 pp 229533.I1SAJ8
5349 229533.I1RF49 pp 229533.I1RVD8
5350 229533.I1RF49 pp 229533.I1RKD3
5351 229533.I1RF49 pp 229533.I1RWV9
5352 229533.I1RF49 pp 229533.I1RUB2
5353 229533.I1RF49 pp 229533.I1RJE8
5354 229533.I1RF49 pp 229533.I1RF58
5355 229533.I1RF49 pp 229533.I1RP12
5356 229533.I1RF49 pp 229533.I1S166
5357 229533.I1RF49 pp 229533.I1RUE3
5358 229533.I1RF49 pp 229533.I1RLA7
5359 229533.I1RF49 pp 229533.I1RHS7
5360 229533.I1RF49 pp 229533.I1RTY9
5361 229533.I1RF49 pp 229533.I1RQW1
5362 229533.I1RF49 pp 229533.I1S0X1
5363 229533.I1RBC6 pp 229533.I1RC27
5364 229533.I1RBC6 pp 229533.I1REF5
5365 229533.I1RBC6 pp 229533.I1RNI4
5366 229533.I1RC78 pp 229533.I1RS02
5367 229533.A0A098DG39 pp 229533.I1S0K2
5368 229533.A0A098DG39 pp 229533.I1RS29
5369 229533.A0A098DG39 pp 229533.I1RS05
5370 229533.A0A098DG39 pp 229533.I1RE66
5371 229533.A0A098DG39 pp 229533.I1S0P0
5372 229533.A0A098DG39 pp 229533.I1RXD9
5373 229533.A0A098DG39 pp 229533.I1S1F3
5374 229533.A0A098DG39 pp 229533.I1S464
5375 229533.A0A098DG39 pp 229533.I1RY21
5376 229533.A0A098DG39 pp 229533.I1RKB7
5377 229533.A0A098DG39 pp 229533.I1RMQ6
5378 229533.A0A098DG39 pp 229533.I1RY92
5379 229533.A0A098DG39 pp 229533.I1RA52
5380 229533.A0A098DG39 pp 229533.A0A0E0SAR4
5381 229533.A0A098DG39 pp 229533.I1RYT8
5382 229533.A0A098DG39 pp 229533.I1RMK7
5383 229533.A0A098DG39 pp 229533.I1RNM7
5384 229533.A0A098DG39 pp 229533.I1RWA3
5385 229533.A0A098DG39 pp 229533.I1RE65
5386 229533.A0A098DG39 pp 229533.I1S0Q7
5387 229533.A0A098DG39 pp 229533.A0A1C3YJQ2
5388 229533.A0A098DG39 pp 229533.I1RP02
5389 229533.A0A098DG39 pp 229533.A0A0E0RPV7
5390 229533.A0A098DG39 pp 229533.V6RJP8
5391 229533.A0A098DG39 pp 229533.A0A1C3YLB0
5392 229533.A0A098DG39 pp 229533.I1RAM9
5393 229533.A0A098DG39 pp 229533.A0A1C3YJU7
5394 229533.A0A098DG39 pp 229533.A0A1C3YLB6
5395 229533.A0A098DG39 pp 229533.I1RFJ6
5396 229533.A0A098DG39 pp 229533.A0A0E0S8Z0
5397 229533.A0A098DG39 pp 229533.I1RBT6
5398 229533.A0A098DG39 pp 229533.I1S0R8
5399 229533.A0A098DG39 pp 229533.I1S5X9
5400 229533.I1S1L0 pp 229533.I1SAJ8
5401 229533.I1S1L0 pp 229533.I1S1W8
5402 229533.I1S1L0 pp 229533.I1S3G4

5403 229533.I1RI54 pp 229533.I1RYZ9
5404 229533.I1RI54 pp 229533.V6QV09
5405 229533.I1RI54 pp 229533.I1RL24
5406 229533.I1RI54 pp 229533.I1RZT1
5407 229533.I1RBC5 pp 229533.I1REF5
5408 229533.I1RBC5 pp 229533.I1RC27
5409 229533.I1RBC5 pp 229533.I1RNI4
5410 229533.I1RBC5 pp 229533.I1RBC6
5411 229533.I1RBH7 pp 229533.I1S034
5412 229533.I1RBH7 pp 229533.I1RYY6
5413 229533.I1RBH7 pp 229533.I1S0E3
5414 229533.I1RBH7 pp 229533.V6QW25
5415 229533.I1RBH7 pp 229533.I1RT41
5416 229533.I1RBH7 pp 229533.I1RIC2
5417 229533.I1RBH7 pp 229533.I1RCU8
5418 229533.I1RBH7 pp 229533.I1S0Z5
5419 229533.I1RBH7 pp 229533.I1S1V3
5420 229533.I1RBH7 pp 229533.I1RL09
5421 229533.I1RBH7 pp 229533.I1RIS0
5422 229533.I1RBH7 pp 229533.I1RZ91
5423 229533.A0A1C3YJ35 pp 229533.I1S1W8
5424 229533.A0A1C3YJ35 pp 229533.I1RDS5
5425 229533.A0A1C3YJ35 pp 229533.I1RY87
5426 229533.A0A1C3YJ35 pp 229533.I1RWV9
5427 229533.A0A1C3YJ35 pp 229533.I1RVD8
5428 229533.A0A1C3YJ35 pp 229533.I1RNG4
5429 229533.A0A1C3YJ35 pp 229533.I1S3G4
5430 229533.A0A1C3YJ35 pp 229533.I1RL11
5431 229533.A0A1C3YJ35 pp 229533.I1RF58
5432 229533.A0A1C3YJ35 pp 229533.I1SAJ8
5433 229533.A0A1C3YJ35 pp 229533.I1RHS7
5434 229533.A0A1C3YJ35 pp 229533.I1REX6
5435 229533.A0A1C3YJ35 pp 229533.I1RLA7
5436 229533.A0A1C3YJ35 pp 229533.A0A1C3YLB6
5437 229533.A0A1C3YJ35 pp 229533.I1S166
5438 229533.A0A1C3YJ35 pp 229533.I1RBE7
5439 229533.A0A1C3YJ35 pp 229533.I1RUB2
5440 229533.A0A1C3YJ35 pp 229533.I1RJE8
5441 229533.I1RAZ2 pp 229533.I1RQ44
5442 229533.I1RAZ2 pp 229533.I1RNG4
5443 229533.I1RAZ2 pp 229533.I1RS82
5444 229533.I1RAZ2 pp 229533.I1RSR2
5445 229533.I1RAZ2 pp 229533.I1RY38
5446 229533.A0A1C3YMP1 pp 229533.I1RUE3
5447 229533.A0A1C3YMP1 pp 229533.I1RTY9
5448 229533.A0A1C3YMP1 pp 229533.I1RP12
5449 229533.A0A1C3YMP1 pp 229533.I1RJE8
5450 229533.A0A1C3YMP1 pp 229533.I1RL13
5451 229533.A0A1C3YMP1 pp 229533.I1S0X1
5452 229533.A0A1C3YMP1 pp 229533.I1RF58
5453 229533.A0A1C3YMP1 pp 229533.I1RBE7
5454 229533.A0A1C3YMP1 pp 229533.I1R9X3
5455 229533.A0A1C3YMP1 pp 229533.I1RY87
5456 229533.A0A1C3YMP1 pp 229533.I1RGK3
5457 229533.A0A1C3YMP1 pp 229533.I1RQW1
5458 229533.A0A1C3YMP1 pp 229533.I1S1W8
5459 229533.A0A1C3YMP1 pp 229533.I1RNG4
5460 229533.A0A1C3YMP1 pp 229533.I1S5P3
5461 229533.A0A1C3YMP1 pp 229533.I1SAH9
5462 229533.A0A1C3YMP1 pp 229533.I1RIF7
5463 229533.A0A1C3YMP1 pp 229533.I1S3G4
5464 229533.A0A1C3YMP1 pp 229533.I1S0K1
5465 229533.I1RIC2 pp 229533.I1S0Z5
5466 229533.I1RIC2 pp 229533.I1RL09
5467 229533.I1RIC2 pp 229533.I1RIS0
5468 229533.I1RNW0 pp 229533.I1S686
5469 229533.I1RNW0 pp 229533.I1RNW2
5470 229533.I1RNW0 pp 229533.I1S019
5471 229533.A0A098DG35 pp 229533.I1RBH7
5472 229533.A0A098DG35 pp 229533.I1RR07
5473 229533.A0A098DG35 pp 229533.I1RIC2
5474 229533.A0A098DG35 pp 229533.I1RW37
5475 229533.A0A1C3YLJ3 pp 229533.I1RFI4

229533.A0A1C3YLJ3	pp	229533.I1S147
229533.I1RFC6	pp	229533.I1RL06
229533.I1RBJ8	pp	229533.I1S0Y7
229533.I1RBJ8	pp	229533.I1RYJ6
229533.I1RBJ8	pp	229533.I1RC61
229533.I1RBJ8	pp	229533.I1RK03
229533.I1RBJ8	pp	229533.I1S8E0
229533.I1RBJ8	pp	229533.I1RWF9
229533.I1RBJ8	pp	229533.I1RNG4
229533.I1RBJ8	pp	229533.I1RWS4
229533.A0A098DG32	pp	229533.I1RA52
229533.I1S5U5	pp	229533.I1SAJ8
229533.I1RNR9	pp	229533.I1RQ61
229533.I1RNR9	pp	229533.I1S368
229533.I1RK69	pp	229533.I1RWV9
229533.I1RK69	pp	229533.I1S166
229533.I1RK69	pp	229533.I1RLA7
229533.I1RK69	pp	229533.I1RPZ7
229533.I1RK69	pp	229533.I1RUB2
229533.I1RK69	pp	229533.I1SAJ8
229533.I1RK69	pp	229533.I1RVD8
229533.I1RK69	pp	229533.I1S5E5
229533.I1RK69	pp	229533.I1RVT5
229533.I1RK69	pp	229533.I1RL11
229533.I1RDH3	pp	229533.Q4HTT2
229533.I1RDH3	pp	229533.Q4I8B6
229533.I1RDH3	pp	229533.I1RYV3
229533.I1RDH3	pp	229533.I1RIG5
229533.I1RPW3	pp	229533.I1RYL5
229533.A0A098E0Z2	pp	229533.I1S0K0
229533.A0A098E0Z2	pp	229533.I1RVX9
229533.A0A098E0Z2	pp	229533.I1RL11
229533.A0A098E0Z2	pp	229533.I1SAJ8
229533.A0A098E0Z2	pp	229533.A0A0E0RU85
229533.A0A098E0Z2	pp	229533.I1S8C1
229533.A0A098E0Z2	pp	229533.I1RCU2
229533.A0A098E0Z2	pp	229533.A0A0E0RNI4
229533.A0A098E0Z2	pp	229533.I1RVD8
229533.A0A098E0Z2	pp	229533.I1RY21
229533.A0A098E0Z2	pp	229533.I1RJE8
229533.A0A098E0Z2	pp	229533.I1RC48
229533.A0A098E0Z2	pp	229533.I1RRX5
229533.A0A098E0Z2	pp	229533.I1RPR1
229533.A0A098E0Z2	pp	229533.A0A1C3YJW9
229533.A0A098E0Z2	pp	229533.I1S3L2
229533.A0A098E0Z2	pp	229533.I1S0K2
229533.A0A098E0Z2	pp	229533.I1R9Y4
229533.A0A098E0Z2	pp	229533.I1RBE4
229533.A0A098E0Z2	pp	229533.I1S8F8
229533.A0A098E0Z2	pp	229533.I1RG33
229533.A0A098E0Z2	pp	229533.I1RF58
229533.A0A098E0Z2	pp	229533.A0A0E0SAR4
229533.A0A098E0Z2	pp	229533.I1RDS5
229533.A0A098E0Z2	pp	229533.I1RNM7
229533.A0A098E0Z2	pp	229533.I1RXS0
229533.A0A098E0Z2	pp	229533.I1RMK7
229533.A0A098E0Z2	pp	229533.I1RQP2
229533.A0A098E0Z2	pp	229533.I1RHZ5
229533.A0A098E0Z2	pp	229533.I1REX6
229533.A0A098E0Z2	pp	229533.I1S166
229533.A0A098E0Z2	pp	229533.A0A1C3YKR5
229533.A0A098E0Z2	pp	229533.I1RY12
229533.A0A098E0Z2	pp	229533.I1RUQ3
229533.A0A098E0Z2	pp	229533.A0A1C3YLC2
229533.A0A098E0Z2	pp	229533.A0A1C3YLB3
229533.A0A098E0Z2	pp	229533.I1REE2
229533.A0A098E0Z2	pp	229533.I1RXE6
229533.A0A098E0Z2	pp	229533.V6R3J1
229533.A0A098E0Z2	pp	229533.A0A1C3YIJ9
229533.A0A098E0Z2	pp	229533.I1RVR2
229533.A0A098E0Z2	pp	229533.I1S0V6
229533.A0A098E0Z2	pp	229533.I1RLA7
229533.A0A098E0Z2	pp	229533.I1S5E5

229533.A0A098E0Z2 pp 229533.I1RC34
229533.A0A098E0Z2 pp 229533.I1RM26
229533.A0A098E0Z2 pp 229533.I1RJY7
229533.A0A098E0Z2 pp 229533.I1RKB2
229533.A0A098E0Z2 pp 229533.I1RMJ3
229533.A0A098E0Z2 pp 229533.A0A1C3YJD4
229533.A0A098E0Z2 pp 229533.I1S0V7
229533.A0A098E0Z2 pp 229533.I1S147
229533.A0A098E0Z2 pp 229533.I1S0N4
229533.A0A098E0Z2 pp 229533.Q4HVQ9
229533.A0A098E0Z2 pp 229533.V6RRM0
229533.A0A098E0Z2 pp 229533.A0A1C3YJP4
229533.A0A098E0Z2 pp 229533.A0A098E2F0
229533.A0A098E0Z2 pp 229533.I1S3T7
229533.A0A098E0Z2 pp 229533.I1RWF5
229533.A0A098E0Z2 pp 229533.A0A1C3YI20
229533.A0A098E0Z2 pp 229533.I1RUB2
229533.A0A098E0Z2 pp 229533.I1RWV9
229533.A0A098E0Z2 pp 229533.A0A1C3YLE2
229533.A0A098E0Z2 pp 229533.A0A1C3YJI5
229533.A0A098E0Z2 pp 229533.I1RR68
229533.A0A098E0Z2 pp 229533.V6R5D2
229533.A0A098E0Z2 pp 229533.I1RN84
229533.A0A098E0Z2 pp 229533.I1RNG4
229533.A0A098E0Z2 pp 229533.I1S8R4
229533.A0A098E0Z2 pp 229533.I1RUR8
229533.A0A098E0Z2 pp 229533.I1RQ48
229533.A0A098E0Z2 pp 229533.I1RGC3
229533.A0A098E0Z2 pp 229533.I1RFI4
229533.A0A098E0Z2 pp 229533.I1RYC8
229533.A0A098E0Z2 pp 229533.A0A0E0SID1
229533.A0A098E0Z2 pp 229533.I1S1F3
229533.A0A098E0Z2 pp 229533.I1RB38
229533.A0A098E0Z2 pp 229533.A0A098E3M9
229533.A0A098E0Z2 pp 229533.A0A1C3YJ04
229533.A0A098E0Z2 pp 229533.I1RE52
229533.A0A098E0Z2 pp 229533.A0A1C3YHG7
229533.A0A098E0Z2 pp 229533.I1RA52
229533.A0A098E0Z2 pp 229533.I1RL81
229533.A0A098E0Z2 pp 229533.A0A1C3YHU3
229533.A0A098E0Z2 pp 229533.I1RF85
229533.A0A098E0Z2 pp 229533.A0A1C3YKW4
229533.A0A098E0Z2 pp 229533.I1RYA0
229533.A0A098E0Z2 pp 229533.I1RHS7
229533.I1S423 pp 229533.I1S6H3
229533.I1S423 pp 229533.Q4HVQ9
229533.A0A1C3YL97 pp 229533.I1S409
229533.A0A1C3YL97 pp 229533.I1RVB5
229533.I1RE32 pp 229533.I1RZ25
229533.I1RSQ0 pp 229533.I1RX77
229533.A0A1C3YJW8 pp 229533.I1RDH0
229533.A0A1C3YJW8 pp 229533.V6RGI1
229533.A0A1C3YJW8 pp 229533.I1S0J3
229533.A0A1C3YJW8 pp 229533.A0A1C3YMC9
229533.A0A1C3YJW8 pp 229533.I1RL24
229533.A0A1C3YJW8 pp 229533.I1RYZ9
229533.A0A1C3YJW8 pp 229533.I1RY29
229533.A0A1C3YJW8 pp 229533.I1RWI8
229533.A0A1C3YJW8 pp 229533.I1RFL7
229533.A0A1C3YJW8 pp 229533.V6QV09
229533.A0A1C3YJW8 pp 229533.I1RR25
229533.A0A1C3YJW8 pp 229533.I1RMS2
229533.A0A1C3YJW8 pp 229533.I1RFQ5
229533.A0A1C3YJW8 pp 229533.I1R9Y7
229533.A0A1C3YJW8 pp 229533.I1RRN5
229533.I1RMH5 pp 229533.I1RT70
229533.I1RMH5 pp 229533.Q4IB70
229533.I1RMH5 pp 229533.I1RYB1
229533.I1RMH5 pp 229533.I1RZ59
229533.I1RMH5 pp 229533.I1RPY7
229533.I1RMH5 pp 229533.I1RMQ7
229533.I1RMH5 pp 229533.I1RZG7
229533.I1RMH5 pp 229533.I1RZD2

229533.I1RMH5 pp 229533.I1S2Z9
229533.I1RMH5 pp 229533.I1RSD4
229533.I1RMH5 pp 229533.Q4IF76
229533.I1RMH5 pp 229533.I1S246
229533.I1RMH5 pp 229533.I1RQC5
229533.I1RMH5 pp 229533.Q4IJH1
229533.I1RMH5 pp 229533.I1RTS2
229533.I1RMH5 pp 229533.I1RN09
229533.I1RMH5 pp 229533.I1RYJ9
229533.I1RMH5 pp 229533.I1S0N7
229533.I1RMH5 pp 229533.I1S7X9
229533.I1RMH5 pp 229533.I1S9Y0
229533.I1RMH5 pp 229533.Q4I5W5
229533.I1RAD3 pp 229533.I1RJT5
229533.I1RAD3 pp 229533.I1RQ28
229533.I1RAD3 pp 229533.I1RM09
229533.I1RAD3 pp 229533.I1RQL4
229533.I1RAD3 pp 229533.I1RDE6
229533.I1RAD3 pp 229533.Q4IMD1
229533.I1RAD3 pp 229533.I1RRT1
229533.I1RAD3 pp 229533.I1RJZ7
229533.I1RAD3 pp 229533.I1RB73
229533.A0A1C3YIW6 pp 229533.I1RCH5
229533.A0A1C3YIW6 pp 229533.A0A1C3YM29
229533.A0A1C3YIW6 pp 229533.I1RFL7
229533.A0A1C3YIW6 pp 229533.I1S098
229533.A0A1C3YIW6 pp 229533.I1RPC2
229533.A0A1C3YIW6 pp 229533.I1RC17
229533.A0A1C3YIW6 pp 229533.I1RWT6
229533.A0A1C3YIW6 pp 229533.A0A1C3YKV3
229533.A0A1C3YIW6 pp 229533.I1RQA3
229533.A0A1C3YIW6 pp 229533.V6RGI1
229533.A0A1C3YIW6 pp 229533.V6QV09
229533.A0A1C3YIW6 pp 229533.I1RYZ9
229533.A0A1C3YIW6 pp 229533.I1RL24
229533.I1RU89 pp 229533.I1S7X9
229533.I1RU89 pp 229533.I1RZ94
229533.I1RU89 pp 229533.I1RWL9
229533.I1S0S0 pp 229533.Q4HXF0
229533.A0A1C3YI24 pp 229533.I1S224
229533.A0A1C3YI24 pp 229533.A0A1C3YLT9
229533.A0A1C3YI24 pp 229533.A0A1C3YM29
229533.I1RLB1 pp 229533.I1RYZ9
229533.I1RLB1 pp 229533.I1RZT1
229533.I1RLB1 pp 229533.V6QV09
229533.I1RMK6 pp 229533.I1RVA8
229533.I1RMK6 pp 229533.I1RRV1
229533.I1RMK6 pp 229533.I1RYP2
229533.A0A1C3YN68 pp 229533.I1S7X9
229533.A0A1C3YN68 pp 229533.I1RWL9
229533.A0A1C3YN68 pp 229533.I1RSW9
229533.A0A1C3YN68 pp 229533.I1RZ94
229533.A0A1C3YN68 pp 229533.I1RJS2
229533.A0A1C3YJE8 pp 229533.I1RFL7
229533.A0A1C3YJE8 pp 229533.I1RL24
229533.A0A1C3YJE8 pp 229533.V6QV09
229533.A0A1C3YJE8 pp 229533.I1RZT1
229533.A0A1C3YJE8 pp 229533.I1RYZ9
229533.I1RH64 pp 229533.I1RM67
229533.I1RH64 pp 229533.I1RWD4
229533.I1RH64 pp 229533.Q4HXF0
229533.I1RC64 pp 229533.I1RM67
229533.I1RC64 pp 229533.Q4HXF0
229533.I1RC64 pp 229533.I1RWD4
229533.Q4IEV5 pp 229533.Q4IFI0
229533.Q4IEV5 pp 229533.Q4IJ56
229533.Q4IEV5 pp 229533.Q4IPI1
229533.Q4IEV5 pp 229533.Q4IJH1
229533.Q4IEV5 pp 229533.Q4IQK7
229533.Q4IEV5 pp 229533.V6QYF0
229533.Q4IEV5 pp 229533.V6R227
229533.Q4IEV5 pp 229533.Q4IF76
229533.Q4IEV5 pp 229533.V6RGY3

229533.Q4IEV5	pp	229533.Q4IN63
229533.Q4IEV5	pp	229533.V6RGN5
229533.Q4IEV5	pp	229533.Q4IR18
229533.Q4IEV5	pp	229533.Q4IQW0
229533.I1RNF5	pp	229533.Q4HWB3
229533.I1RNF5	pp	229533.I1S0K6
229533.I1RNF5	pp	229533.I1RVZ0
229533.I1RNF5	pp	229533.I1RT45
229533.I1RNF5	pp	229533.I1RNF7
229533.I1RNF5	pp	229533.Q4I9U7
229533.I1RNF5	pp	229533.V6RLW4
229533.I1RNF5	pp	229533.I1S9U2
229533.I1RNF5	pp	229533.V6R2A1
229533.I1RNF5	pp	229533.I1RQ28
229533.I1RNF5	pp	229533.I1S1B6
229533.I1RNF5	pp	229533.I1RTD7
229533.I1RNF5	pp	229533.I1RQ65
229533.I1RNF5	pp	229533.I1RSD8
229533.I1RNF5	pp	229533.I1RZF6
229533.I1RNF5	pp	229533.I1RW26
229533.I1RNF5	pp	229533.I1RNH6
229533.I1RNF5	pp	229533.Q4I8X0
229533.I1RNF5	pp	229533.I1RW71
229533.I1RE57	pp	229533.I1RN72
229533.I1RE57	pp	229533.I1S9X9
229533.I1RE57	pp	229533.I1RYH6
229533.I1RE57	pp	229533.V6RG22
229533.I1RE57	pp	229533.I1RKB7
229533.I1SAE3	pp	229533.Q4HXF0
229533.A0A1C3YLS3	pp	229533.I1RT17
229533.A0A1C3YLS3	pp	229533.I1RWM6
229533.A0A1C3YLS3	pp	229533.I1S3W0
229533.A0A1C3YLS3	pp	229533.I1RII7
229533.A0A1C3YLS3	pp	229533.I1RAH9
229533.A0A1C3YLS3	pp	229533.I1RWH7
229533.A0A1C3YLS3	pp	229533.I1S9V9
229533.A0A1C3YLS3	pp	229533.I1RF02
229533.A0A1C3YLS3	pp	229533.I1R9J7
229533.A0A1C3YLS3	pp	229533.I1S802
229533.A0A1C3YLS3	pp	229533.Q4IB50
229533.A0A1C3YLS3	pp	229533.I1S2F2
229533.A0A1C3YLS3	pp	229533.Q4I877
229533.A0A1C3YLS3	pp	229533.I1RZZ7
229533.A0A1C3YLS3	pp	229533.I1RSD4
229533.A0A1C3YLS3	pp	229533.I1RWI0
229533.A0A1C3YLS3	pp	229533.I1RWB3
229533.A0A1C3YLS3	pp	229533.I1RYX3
229533.A0A1C3YLS3	pp	229533.I1RUG4
229533.A0A1C3YLS3	pp	229533.I1RMB7
229533.A0A1C3YLS3	pp	229533.I1S3L3
229533.A0A1C3YLS3	pp	229533.A0A1C3YMB5
229533.A0A1C3YLS3	pp	229533.I1RSL6
229533.A0A1C3YLS3	pp	229533.I1RX64
229533.A0A1C3YLS3	pp	229533.I1S3Z3
229533.A0A1C3YLS3	pp	229533.I1RJ33
229533.A0A1C3YLS3	pp	229533.I1RMX3
229533.A0A1C3YLS3	pp	229533.I1RN07
229533.A0A1C3YLS3	pp	229533.I1S0N5
229533.A0A1C3YLS3	pp	229533.I1RE37
229533.A0A1C3YLS3	pp	229533.A0A1C3YM29
229533.I1RS64	pp	229533.I1RSR3
229533.I1RS64	pp	229533.I1RY98
229533.I1RS64	pp	229533.P0C431
229533.I1RS64	pp	229533.I1S0S6
229533.I1RS64	pp	229533.I1S7X9
229533.I1RS64	pp	229533.V6QYF0
229533.I1RS64	pp	229533.I1RTB7
229533.I1RS64	pp	229533.I1RWN2
229533.A0A1C3YMY8	pp	229533.I1RZT1
229533.A0A1C3YMY8	pp	229533.I1RL24
229533.A0A1C3YMY8	pp	229533.I1RFL7
229533.A0A1C3YMY8	pp	229533.V6QV09
229533.A0A1C3YMY8	pp	229533.I1RYZ9

229533.A0A098DG29	pp	229533.I1S0S0
229533.A0A098DG29	pp	229533.Q4IAA0
229533.A0A098DG29	pp	229533.I1RRU9
229533.A0A098DG29	pp	229533.I1RMK6
229533.A0A098DG29	pp	229533.I1RH64
229533.A0A098DG29	pp	229533.I1RC64
229533.A0A098DG29	pp	229533.A0A1C3YM36
229533.A0A098DG29	pp	229533.I1RUN3
229533.A0A098DG29	pp	229533.Q4IEV5
229533.A0A098DG29	pp	229533.A0A1C3YL15
229533.A0A098DG29	pp	229533.I1RB53
229533.A0A098DG29	pp	229533.I1R9X2
229533.A0A098DG29	pp	229533.I1RFT6
229533.A0A098DG29	pp	229533.I1SAE3
229533.A0A098DG29	pp	229533.I1RNI4
229533.A0A098DG29	pp	229533.A0A1C3YLS3
229533.A0A098DG29	pp	229533.I1S0D4
229533.A0A098DG29	pp	229533.I1REF5
229533.V6QUV7	pp	229533.V6R042
229533.V6QUV7	pp	229533.V6QX21
229533.A0A1C3YK36	pp	229533.I1RRI2
229533.A0A1C3YK36	pp	229533.I1RZW8
229533.A0A1C3YK36	pp	229533.I1S2C0
229533.A0A1C3YK36	pp	229533.I1RC27
229533.A0A1C3YK36	pp	229533.I1RMN6
229533.A0A1C3YK36	pp	229533.I1S5R6
229533.A0A1C3YK36	pp	229533.I1RTE9
229533.A0A1C3YK36	pp	229533.V6RGN5
229533.A0A1C3YK36	pp	229533.I1RKB6
229533.A0A1C3YK36	pp	229533.I1RQS8
229533.A0A1C3YK36	pp	229533.I1RE01
229533.A0A1C3YK36	pp	229533.I1RD64
229533.A0A1C3YK36	pp	229533.I1RCL3
229533.A0A1C3YK36	pp	229533.I1RAW0
229533.A0A1C3YK36	pp	229533.I1RFL4
229533.A0A1C3YK36	pp	229533.I1RC72
229533.A0A1C3YK36	pp	229533.A0A1C3YLB6
229533.A0A1C3YK36	pp	229533.I1RRI1
229533.A0A1C3YK36	pp	229533.I1RPK0
229533.A0A1C3YK36	pp	229533.I1RCR9
229533.A0A1C3YK36	pp	229533.I1RSD8
229533.A0A1C3YK36	pp	229533.I1RCH5
229533.A0A1C3YK36	pp	229533.I1S2E8
229533.A0A1C3YK36	pp	229533.I1RSZ7
229533.A0A1C3YK36	pp	229533.I1S9Z4
229533.A0A1C3YK36	pp	229533.I1RBG9
229533.A0A1C3YK36	pp	229533.I1R9X7
229533.A0A1C3YK36	pp	229533.I1RQE7
229533.A0A1C3YK36	pp	229533.Q4I8X0
229533.A0A1C3YK36	pp	229533.I1RZS6
229533.A0A1C3YK36	pp	229533.I1RVX0
229533.A0A1C3YK36	pp	229533.I1RSS4
229533.A0A1C3YK36	pp	229533.I1R9M0
229533.A0A1C3YK36	pp	229533.V6RIV9
229533.A0A1C3YK36	pp	229533.I1RRM5
229533.A0A1C3YK36	pp	229533.I1RB92
229533.A0A1C3YK36	pp	229533.I1RPY1
229533.A0A1C3YK36	pp	229533.I1RKC6
229533.A0A1C3YK36	pp	229533.I1S2B8
229533.A0A1C3YK36	pp	229533.I1RQ33
229533.A0A1C3YK36	pp	229533.I1RD17
229533.A0A1C3YK36	pp	229533.I1RUG3
229533.A0A1C3YK36	pp	229533.I1RC33
229533.A0A1C3YK36	pp	229533.I1RZV3
229533.A0A1C3YK36	pp	229533.I1RM96
229533.A0A1C3YK36	pp	229533.I1RBY4
229533.A0A1C3YK36	pp	229533.I1RZZ9
229533.A0A1C3YK36	pp	229533.I1RSI6
229533.A0A1C3YK36	pp	229533.I1RT40
229533.A0A1C3YK36	pp	229533.I1RFH8
229533.A0A1C3YK36	pp	229533.Q4IR18
229533.A0A1C3YK36	pp	229533.A0A1C3YLF5
229533.A0A1C3YK36	pp	229533.I1RZ39

229533.A0A1C3YK36 pp 229533.V6R283
229533.A0A1C3YK36 pp 229533.I1RKJ6
229533.A0A1C3YK36 pp 229533.I1S5R1
229533.A0A1C3YK36 pp 229533.Q4HXF0
229533.A0A1C3YK36 pp 229533.I1RTA1
229533.A0A1C3YK36 pp 229533.I1RJW6
229533.A0A1C3YK36 pp 229533.I1RRT8
229533.A0A1C3YK36 pp 229533.A0A1C3YLK8
229533.A0A1C3YK36 pp 229533.I1S2M6
229533.A0A1C3YK36 pp 229533.I1RRT2
229533.A0A1C3YK36 pp 229533.I1RAZ8
229533.A0A1C3YK36 pp 229533.I1RYI5
229533.A0A1C3YK36 pp 229533.I1RJL4
229533.A0A1C3YK36 pp 229533.I1RF19
229533.A0A1C3YK36 pp 229533.I1RFJ9
229533.A0A1C3YK36 pp 229533.I1RRJ8
229533.A0A1C3YK36 pp 229533.I1RFN1
229533.A0A1C3YK36 pp 229533.I1REK8
229533.A0A1C3YK36 pp 229533.I1RQ31
229533.A0A1C3YK36 pp 229533.I1RJ74
229533.A0A1C3YK36 pp 229533.I1RZM8
229533.A0A1C3YK36 pp 229533.I1RRP3
229533.A0A1C3YK36 pp 229533.I1RZB2
229533.A0A1C3YK36 pp 229533.A0A1C3YN63
229533.A0A1C3YK36 pp 229533.I1RRQ3
229533.A0A1C3YK36 pp 229533.V6RCZ8
229533.A0A1C3YK36 pp 229533.V6RCG5
229533.A0A1C3YK36 pp 229533.I1RN63
229533.A0A1C3YK36 pp 229533.I1RMD5
229533.A0A1C3YK36 pp 229533.I1RTD9
229533.A0A1C3YK36 pp 229533.I1RCG4
229533.A0A1C3YK36 pp 229533.I1RWK3
229533.A0A1C3YK36 pp 229533.I1RQA2
229533.A0A1C3YK36 pp 229533.I1S0F1
229533.A0A1C3YK36 pp 229533.I1RT80
229533.A0A1C3YK36 pp 229533.I1RQD2
229533.A0A1C3YK36 pp 229533.I1S5B0
229533.A0A1C3YK36 pp 229533.I1RTF4
229533.A0A1C3YK36 pp 229533.I1RPM6
229533.A0A1C3YK36 pp 229533.Q4IJK2
229533.A0A1C3YK36 pp 229533.A0A1C3YN30
229533.A0A1C3YK36 pp 229533.I1RJU3
229533.A0A1C3YK36 pp 229533.I1RB27
229533.A0A1C3YK36 pp 229533.A0A1C3YKV3
229533.A0A1C3YK36 pp 229533.I1RZF6
229533.A0A1C3YK36 pp 229533.I1RY63
229533.A0A1C3YK36 pp 229533.I1R9V7
229533.A0A1C3YK36 pp 229533.I1RMK3
229533.A0A1C3YK36 pp 229533.I1RDA0
229533.A0A1C3YK36 pp 229533.A0A1C3YKB0
229533.A0A1C3YK36 pp 229533.I1RN99
229533.A0A1C3YK36 pp 229533.V6R227
229533.A0A1C3YK36 pp 229533.I1RR24
229533.A0A1C3YK36 pp 229533.I1RNA0
229533.A0A1C3YK36 pp 229533.I1RT05
229533.A0A1C3YK36 pp 229533.I1RBT1
229533.A0A1C3YK36 pp 229533.I1RM06
229533.A0A1C3YK36 pp 229533.I1RAC2
229533.A0A1C3YK36 pp 229533.I1RRS8
229533.A0A1C3YK36 pp 229533.A0A1C3YMW0
229533.A0A1C3YK36 pp 229533.I1RUQ2
229533.A0A1C3YK36 pp 229533.I1RBC9
229533.A0A1C3YK36 pp 229533.V6QW22
229533.A0A1C3YK36 pp 229533.I1RW71
229533.A0A1C3YK36 pp 229533.I1RWD4
229533.A0A1C3YK36 pp 229533.I1RVQ0
229533.A0A1C3YK36 pp 229533.I1RT33
229533.A0A1C3YK36 pp 229533.I1RD52
229533.A0A1C3YK36 pp 229533.A0A1C3YLL3
229533.A0A1C3YK36 pp 229533.I1S0L3
229533.I1RY84 pp 229533.V6RGI1
229533.I1RY84 pp 229533.I1RYZ9
229533.I1RY84 pp 229533.I1S098

5914	229533.I1RY84	pp	229533.I1S0M6
5915	229533.I1RY84	pp	229533.V6QV09
5916	229533.I1RLU2	pp	229533.I1RRC6
5917	229533.I1RLU2	pp	229533.I1RY50
5918	229533.I1RLU2	pp	229533.I1S4Z7
5919	229533.I1RLU2	pp	229533.I1S7B8
5920	229533.I1RLU2	pp	229533.Q4IK03
5921	229533.I1RLU2	pp	229533.I1RYB8
5922	229533.I1RLU2	pp	229533.I1S9T5
5923	229533.I1RLU2	pp	229533.I1RZI8
5924	229533.I1RLU2	pp	229533.I1RSQ2
5925	229533.I1RLU2	pp	229533.I1S8Z2
5926	229533.I1RLU2	pp	229533.I1S123
5927	229533.I1RLU2	pp	229533.I1RQ38
5928	229533.I1RLU2	pp	229533.I1RY40
5929	229533.I1RLU2	pp	229533.V6QX21
5930	229533.I1RLU2	pp	229533.I1S0Z6
5931	229533.I1RLU2	pp	229533.I1RNG4
5932	229533.I1RLU2	pp	229533.I1RZK1
5933	229533.I1RLU2	pp	229533.I1RT75
5934	229533.I1RLU2	pp	229533.I1RP25
5935	229533.I1RLU2	pp	229533.I1RYL5
5936	229533.I1RLU2	pp	229533.I1RSS1
5937	229533.I1RLU2	pp	229533.I1S235
5938	229533.I1RLU2	pp	229533.V6QUV7
5939	229533.I1RLU2	pp	229533.I1RNP8
5940	229533.I1RLU2	pp	229533.I1RXF0
5941	229533.A0A1C3YLR8	pp	229533.I1S0S6
5942	229533.A0A1C3YLR8	pp	229533.I1RTB7
5943	229533.A0A1C3YLR8	pp	229533.I1RH68
5944	229533.A0A1C3YLR8	pp	229533.P0C431
5945	229533.A0A1C3YLR8	pp	229533.I1RX09
5946	229533.A0A1C3YLR8	pp	229533.I1RQN9
5947	229533.I1RYS4	pp	229533.I1S2C4
5948	229533.I1RYS4	pp	229533.Q4I5I4
5949	229533.I1RYS4	pp	229533.Q4I5W5
5950	229533.I1RYS4	pp	229533.I1RZB5
5951	229533.A0A098DGQ1	pp	229533.I1RA62
5952	229533.A0A098DGQ1	pp	229533.A0A1C3YMN0
5953	229533.A0A098DGQ1	pp	229533.I1RWE8
5954	229533.A0A098DGQ1	pp	229533.A0A1C3YHQ1
5955	229533.A0A098DGQ1	pp	229533.I1RMJ3
5956	229533.A0A098DGQ1	pp	229533.A0A1C3YJ21
5957	229533.A0A098DGQ1	pp	229533.V6R3U5
5958	229533.A0A098DGQ1	pp	229533.I1RBZ6
5959	229533.A0A098DGQ1	pp	229533.I1RZ60
5960	229533.A0A098DGQ1	pp	229533.I1RSF1
5961	229533.A0A098DGQ1	pp	229533.I1RKM3
5962	229533.A0A098DGQ1	pp	229533.I1RIU0
5963	229533.A0A098DGQ1	pp	229533.I1S024
5964	229533.A0A098DGQ1	pp	229533.I1S1F3
5965	229533.A0A098DGQ1	pp	229533.A0A1C3YL34
5966	229533.A0A098DGQ1	pp	229533.I1S268
5967	229533.A0A098DGQ1	pp	229533.A0A1C3YKK8
5968	229533.A0A098DGQ1	pp	229533.I1S9T5
5969	229533.A0A098DGQ1	pp	229533.I1S111
5970	229533.A0A098DGQ1	pp	229533.I1RKP1
5971	229533.A0A098DGQ1	pp	229533.A0A098DJ03
5972	229533.A0A098DGQ1	pp	229533.I1RUI0
5973	229533.A0A098DGQ1	pp	229533.I1RUE2
5974	229533.A0A098DGQ1	pp	229533.A0A098DRX7
5975	229533.A0A098DGQ1	pp	229533.I1RN52
5976	229533.A0A098DGQ1	pp	229533.I1RAH8
5977	229533.A0A098DGQ1	pp	229533.I1RR45
5978	229533.A0A098DGQ1	pp	229533.I1RT41
5979	229533.A0A098DGQ1	pp	229533.I1S123
5980	229533.A0A098DGQ1	pp	229533.I1RQ30
5981	229533.A0A098DGQ1	pp	229533.V6R223
5982	229533.A0A098DGQ1	pp	229533.I1REW3
5983	229533.A0A098DGQ1	pp	229533.I1RWW4
5984	229533.A0A098DGQ1	pp	229533.I1RYU3
5985	229533.A0A098DGQ1	pp	229533.I1RA32
5986	229533.A0A098DGQ1	pp	229533.I1RQC0

5987	229533.A0A098DGQ1	pp	229533.I1RMQ6
5988	229533.A0A098DGQ1	pp	229533.V6RJ21
5989	229533.A0A098DGQ1	pp	229533.Q5I2J3
5990	229533.A0A098DGQ1	pp	229533.I1RJD3
5991	229533.A0A098DGQ1	pp	229533.V6RUT5
5992	229533.A0A098DGQ1	pp	229533.I1S267
5993	229533.A0A098DGQ1	pp	229533.I1RA72
5994	229533.A0A098DGQ1	pp	229533.I1S3M2
5995	229533.A0A098DGQ1	pp	229533.I1RZY1
5996	229533.A0A098DGQ1	pp	229533.I1RWP4
5997	229533.A0A098DGQ1	pp	229533.I1RRK5
5998	229533.A0A098DGQ1	pp	229533.Q4IK03
5999	229533.A0A098DGQ1	pp	229533.A0A0E0SF53
6000	229533.A0A098DGQ1	pp	229533.I1S0Z3
6001	229533.A0A098DGQ1	pp	229533.I1RQJ0
6002	229533.A0A098DGQ1	pp	229533.A0A1C3YJZ0
6003	229533.A0A098DGQ1	pp	229533.I1RBU8
6004	229533.A0A098DGQ1	pp	229533.I1S7R5
6005	229533.A0A098DGQ1	pp	229533.I1RCM5
6006	229533.A0A098DGQ1	pp	229533.A0A1C3YKF3
6007	229533.A0A098DGQ1	pp	229533.I1RTD3
6008	229533.I1RQZ0	pp	229533.I1RZT1
6009	229533.I1RQZ0	pp	229533.V6QV09
6010	229533.I1RQZ0	pp	229533.I1RYZ9
6011	229533.I1RAS7	pp	229533.I1RNG4
6012	229533.A0A0E0SEH5	pp	229533.A0A1C3YHQ1
6013	229533.A0A0E0SEH5	pp	229533.A0A1C3YKM4
6014	229533.A0A0E0SEH5	pp	229533.A0A1C3YLL3
6015	229533.A0A0E0SEH5	pp	229533.I1S1F3
6016	229533.A0A0E0SEH5	pp	229533.I1S8Y1
6017	229533.A0A0E0SEH5	pp	229533.I1S0J8
6018	229533.A0A0E0SEH5	pp	229533.I1RMQ6
6019	229533.A0A0E0SEH5	pp	229533.I1RX17
6020	229533.A0A0E0SEH5	pp	229533.I1RUK7
6021	229533.A0A0E0SEH5	pp	229533.A0A1C3YK94
6022	229533.A0A0E0SEH5	pp	229533.I1S8Y0
6023	229533.A0A0E0SEH5	pp	229533.I1RSQ2
6024	229533.A0A0E0SEH5	pp	229533.I1RQ38
6025	229533.A0A0E0SEH5	pp	229533.A0A1C3YKP5
6026	229533.A0A0E0SEH5	pp	229533.I1RAM9
6027	229533.A0A0E0SEH5	pp	229533.I1SAJ3
6028	229533.A0A0E0SEH5	pp	229533.V6QX21
6029	229533.A0A0E0SEH5	pp	229533.I1S8Z2
6030	229533.A0A0E0SEH5	pp	229533.I1S3G4
6031	229533.A0A0E0SEH5	pp	229533.I1RWT0
6032	229533.A0A0E0SEH5	pp	229533.I1S2C4
6033	229533.A0A0E0SEH5	pp	229533.I1S212
6034	229533.A0A0E0SEH5	pp	229533.I1RJD3
6035	229533.A0A0E0SEH5	pp	229533.I1RPA4
6036	229533.A0A0E0SEH5	pp	229533.I1S1W8
6037	229533.A0A0E0SEH5	pp	229533.I1RFJ6
6038	229533.A0A0E0SEH5	pp	229533.I1RS41
6039	229533.A0A0E0SEH5	pp	229533.I1S4Z7
6040	229533.A0A0E0SEH5	pp	229533.I1S0K2
6041	229533.A0A0E0SEH5	pp	229533.I1RMK7
6042	229533.A0A0E0SEH5	pp	229533.I1RYB8
6043	229533.A0A0E0SEH5	pp	229533.I1S9T5
6044	229533.A0A0E0SEH5	pp	229533.I1RT75
6045	229533.A0A0E0SEH5	pp	229533.A0A1C3YMC1
6046	229533.A0A0E0SEH5	pp	229533.I1S123
6047	229533.A0A0E0SEH5	pp	229533.I1RBE6
6048	229533.A0A0E0SEH5	pp	229533.Q4I5G1
6049	229533.A0A0E0SEH5	pp	229533.I1S277
6050	229533.A0A0E0SEH5	pp	229533.I1S268
6051	229533.A0A0E0SEH5	pp	229533.I1RS01
6052	229533.A0A0E0SEH5	pp	229533.A0A1C3YKG0
6053	229533.I1RS56	pp	229533.I1RUD3
6054	229533.I1RS56	pp	229533.I1RZT1
6055	229533.I1RS56	pp	229533.I1S569
6056	229533.I1RS56	pp	229533.Q4IJM4
6057	229533.I1RS56	pp	229533.V6RGI1
6058	229533.I1RS56	pp	229533.V6QV09
6059	229533.I1RS56	pp	229533.I1RSJ0

6060	229533.I1RS56	pp	229533.I1RYZ9
6061	229533.I1RAS4	pp	229533.V6QVH8
6062	229533.I1RKL9	pp	229533.V6RGI1
6063	229533.I1RKL9	pp	229533.I1S2D2
6064	229533.I1RKL9	pp	229533.I1S2J5
6065	229533.I1RKL9	pp	229533.V6RH92
6066	229533.I1RKL9	pp	229533.I1S2L3
6067	229533.I1RKL9	pp	229533.I1RYZ9
6068	229533.I1RKL9	pp	229533.I1RL24
6069	229533.I1RKL9	pp	229533.V6QV09
6070	229533.I1RD03	pp	229533.I1RX53
6071	229533.I1RD03	pp	229533.I1RMN6
6072	229533.I1RD03	pp	229533.I1RYH4
6073	229533.I1RD03	pp	229533.I1RNC1
6074	229533.I1RD03	pp	229533.Q4IN63
6075	229533.I1RD03	pp	229533.I1RD22
6076	229533.I1RD03	pp	229533.I1RT93
6077	229533.I1RD03	pp	229533.I1RNY2
6078	229533.I1RD03	pp	229533.I1S060
6079	229533.I1RD03	pp	229533.V6RMW6
6080	229533.I1RD03	pp	229533.I1RZE5
6081	229533.I1RD03	pp	229533.V6R042
6082	229533.I1RD03	pp	229533.I1S0J1
6083	229533.I1RD03	pp	229533.Q4IJK2
6084	229533.I1RD03	pp	229533.I1RZZ1
6085	229533.I1RD03	pp	229533.I1RN67
6086	229533.I1RD03	pp	229533.I1REN8
6087	229533.I1RD03	pp	229533.I1RWJ3
6088	229533.I1RD03	pp	229533.I1RJZ6
6089	229533.I1RD03	pp	229533.I1RQ65
6090	229533.I1RD03	pp	229533.I1RLV9
6091	229533.I1RD03	pp	229533.I1RK01
6092	229533.I1RD03	pp	229533.I1RX46
6093	229533.I1RD03	pp	229533.I1RFM0
6094	229533.I1RD03	pp	229533.I1RRI2
6095	229533.I1RD03	pp	229533.I1S540
6096	229533.I1RD03	pp	229533.I1RWH5
6097	229533.I1RD03	pp	229533.I1RGA4
6098	229533.I1RD03	pp	229533.Q4HZK7
6099	229533.I1RD03	pp	229533.V6R283
6100	229533.I1RD03	pp	229533.I1RR00
6101	229533.I1RD03	pp	229533.V6RTJ8
6102	229533.I1RD03	pp	229533.I1RVL1
6103	229533.I1RD03	pp	229533.I1RTH6
6104	229533.I1RD03	pp	229533.I1RWF7
6105	229533.I1RD03	pp	229533.I1RZB5
6106	229533.I1RD03	pp	229533.I1RT04
6107	229533.I1RD03	pp	229533.I1RQC9
6108	229533.I1RD03	pp	229533.I1RM67
6109	229533.I1RD03	pp	229533.I1S0R7
6110	229533.I1RD03	pp	229533.I1RND6
6111	229533.I1RD03	pp	229533.I1S6U8
6112	229533.I1RD03	pp	229533.I1S8E2
6113	229533.I1RD03	pp	229533.I1S2B8
6114	229533.I1RD03	pp	229533.I1RW25
6115	229533.I1RD03	pp	229533.I1RR73
6116	229533.I1RD03	pp	229533.I1RNA0
6117	229533.I1RAP3	pp	229533.I1RJD3
6118	229533.I1RAP3	pp	229533.I1RBN0
6119	229533.I1RAP3	pp	229533.I1RMQ6
6120	229533.I1RAP3	pp	229533.I1RN52
6121	229533.I1RAP3	pp	229533.I1RRK5
6122	229533.I1RAP3	pp	229533.I1RYU3
6123	229533.I1RAP3	pp	229533.I1S267
6124	229533.I1RAP3	pp	229533.I1RRF6
6125	229533.I1RAP3	pp	229533.I1RBU8
6126	229533.I1RAP3	pp	229533.I1RFD5
6127	229533.I1RAP3	pp	229533.I1RIU0
6128	229533.I1RAP3	pp	229533.I1S1F3
6129	229533.I1RAP3	pp	229533.I1RKP1
6130	229533.I1RAP3	pp	229533.I1RZ17
6131	229533.I1RAP3	pp	229533.I1RR45
6132	229533.I1RAP3	pp	229533.I1S268

229533.I1RAP3	pp	229533.I1RQC0
229533.I1RAP3	pp	229533.I1RD96
229533.I1RAP3	pp	229533.I1S0B4
229533.I1RAP3	pp	229533.I1RX03
229533.I1RAP3	pp	229533.I1REW3
229533.I1RAP3	pp	229533.I1RZ60
229533.I1RAP3	pp	229533.I1RMA0
229533.I1RAP3	pp	229533.I1S9T5
229533.I1RAP3	pp	229533.I1S123
229533.I1RAP3	pp	229533.I1RSF1
229533.I1RAP3	pp	229533.I1RLL8
229533.I1RWJ3	pp	229533.I1RX46
229533.I1RWJ3	pp	229533.Q4IN63
229533.I1RWJ3	pp	229533.V6RTJ8
229533.I1RWJ3	pp	229533.I1S0R7
229533.I1RWJ3	pp	229533.I1S0J1
229533.I1RWJ3	pp	229533.V6R042
229533.I1RWJ3	pp	229533.I1S7A2
229533.I1RWJ3	pp	229533.I1RZE5
229533.I1RWJ3	pp	229533.V6R283
229533.I1RWJ3	pp	229533.I1RZZ1
229533.I1RWJ3	pp	229533.I1RX53
229533.I1RWJ3	pp	229533.Q4IJK2
229533.I1RWJ3	pp	229533.I1RYH4
229533.I1RAT7	pp	229533.I1RRG3
229533.I1RAT7	pp	229533.I1RYH9
229533.I1RAT7	pp	229533.I1RPU3
229533.I1RAT7	pp	229533.V6QV09
229533.I1RAT7	pp	229533.Q4IPI4
229533.I1RAT7	pp	229533.I1RFL7
229533.I1RAT7	pp	229533.I1RRX5
229533.I1RAT7	pp	229533.I1RB74
229533.I1RAT7	pp	229533.I1RVL8
229533.I1RAT7	pp	229533.I1RPI2
229533.I1RAT7	pp	229533.I1RPM7
229533.I1RAT7	pp	229533.Q4I7L0
229533.I1RAT7	pp	229533.I1RL24
229533.I1RAT7	pp	229533.I1RSK1
229533.I1RAT7	pp	229533.I1RPC2
229533.I1RAT7	pp	229533.I1RHB0
229533.I1RAT7	pp	229533.I1RQ13
229533.I1RAT7	pp	229533.I1RC24
229533.I1RAT7	pp	229533.I1RUF8
229533.I1RAT7	pp	229533.I1S0J3
229533.I1RAT7	pp	229533.I1RYY5
229533.I1RAT7	pp	229533.I1RYZ9
229533.I1RAT7	pp	229533.I1RDB1
229533.I1RAT7	pp	229533.I1RZB5
229533.I1RAT7	pp	229533.I1RP27
229533.I1RAT7	pp	229533.V6QUE6
229533.I1RAT7	pp	229533.I1RJL2
229533.I1RAT7	pp	229533.I1RFQ5
229533.I1RAT7	pp	229533.I1S098
229533.I1RAT7	pp	229533.V6RGI1
229533.I1RAT7	pp	229533.I1RB41
229533.I1RAT7	pp	229533.I1RD15
229533.I1RAT7	pp	229533.I1S2E9
229533.I1RAT7	pp	229533.I1S2G7
229533.I1RAT7	pp	229533.I1RUG4
229533.I1RAT7	pp	229533.I1RC17
229533.I1RAT7	pp	229533.I1RB75
229533.I1RAT7	pp	229533.I1REN3
229533.I1RAT7	pp	229533.I1RG11
229533.I1RAT7	pp	229533.I1RCB7
229533.I1RAT7	pp	229533.I1RXU0
229533.I1RAT7	pp	229533.I1RQA3
229533.I1RAT7	pp	229533.I1RQ26
229533.I1RAT7	pp	229533.V6RKY7
229533.I1RAT7	pp	229533.I1S0E8
229533.I1RAT7	pp	229533.I1RRN5
229533.I1RAT7	pp	229533.Q4ICA8
229533.I1RAT7	pp	229533.I1RY44
229533.I1RAT7	pp	229533.Q4IJM4

229533.I1RAT7	pp	229533.I1S1W4
229533.I1RAT7	pp	229533.I1RBJ1
229533.I1RAT7	pp	229533.I1RWT6
229533.I1RAT7	pp	229533.I1RMQ7
229533.A0A098DFV5	pp	229533.I1RTB7
229533.A0A098DFV5	pp	229533.P0C431
229533.A0A098DFV5	pp	229533.I1RTE6
229533.A0A098DFV5	pp	229533.V6REE9
229533.A0A098DFV5	pp	229533.I1RH68
229533.A0A098DFV5	pp	229533.I1S0S6
229533.A0A098DFV5	pp	229533.I1RQN9
229533.A0A098DFV5	pp	229533.I1RCD4
229533.I1RG59	pp	229533.I1RKA9
229533.I1RG59	pp	229533.I1S8F2
229533.I1RG59	pp	229533.I1RPN9
229533.I1RG59	pp	229533.I1SAF7
229533.I1RG59	pp	229533.I1RQ64
229533.I1RG59	pp	229533.I1RS35
229533.I1RG59	pp	229533.I1RT74
229533.A0A098DFX7	pp	229533.I1RG59
229533.I1RGM0	pp	229533.I1RTF1
229533.I1RGM0	pp	229533.I1RMU3
229533.I1RN07	pp	229533.V6QWX8
229533.I1RN07	pp	229533.V6RDZ4
229533.I1RN07	pp	229533.I1RZB5
229533.I1RN07	pp	229533.Q4IB50
229533.I1RN07	pp	229533.I1S3W0
229533.I1RN07	pp	229533.I1RWI0
229533.I1RN07	pp	229533.I1RZP2
229533.I1RN07	pp	229533.Q4IJ11
229533.I1RN07	pp	229533.I1RR25
229533.I1RN07	pp	229533.I1RRG3
229533.I1RN07	pp	229533.I1S3L3
229533.I1RN07	pp	229533.V6QUE6
229533.I1RN07	pp	229533.I1RRT0
229533.A0A0E0SMX5	pp	229533.I1RGM0
229533.A0A0E0SMX5	pp	229533.I1RZ14
229533.A0A0E0SMX5	pp	229533.I1RMU3
229533.A0A0E0SMX5	pp	229533.I1RUI0
229533.I1S0W0	pp	229533.I1SAT3
229533.A0A1C3YLY5	pp	229533.I1RX38
229533.A0A1C3YLY5	pp	229533.I1RQ88
229533.A0A1C3YLY5	pp	229533.I1RBV5
229533.A0A1C3YLY5	pp	229533.I1RPW0
229533.A0A1C3YLY5	pp	229533.I1RPU4
229533.A0A1C3YLY5	pp	229533.I1RAI9
229533.A0A098DFR3	pp	229533.I1RAI9
229533.A0A098DFR3	pp	229533.I1RPW0
229533.A0A098DFR3	pp	229533.I1RX38
229533.A0A098DFR3	pp	229533.I1RQ88
229533.A0A098DFR3	pp	229533.I1RPU4
229533.A0A098DFR3	pp	229533.A0A1C3YKJ6
229533.A0A098DFR3	pp	229533.A0A0E0SNZ9
229533.A0A098DFR3	pp	229533.A0A1C3YK37
229533.A0A098DFR3	pp	229533.I1RBV5
229533.A0A098DFR3	pp	229533.A0A1C3YLY5
229533.A0A1C3YKZ6	pp	229533.I1SAT3
229533.A0A1C3YKZ6	pp	229533.A0A1C3YN48
229533.A0A1C3YKZ6	pp	229533.I1RZM2
229533.A0A1C3YKZ6	pp	229533.I1S0C0
229533.A0A1C3YKZ6	pp	229533.I1S0W0
229533.A0A1C3YKZ6	pp	229533.I1RCM9
229533.A0A1C3YKZ6	pp	229533.A0A1C3YMF9
229533.I1RVP5	pp	229533.I1RW54
229533.I1RVP5	pp	229533.I1RYJ6
229533.I1RVP5	pp	229533.I1RWL3
229533.I1RVP5	pp	229533.I1RW71
229533.I1RVP5	pp	229533.V6RLM7
229533.I1RVP5	pp	229533.V6RN69
229533.I1RVP5	pp	229533.V6QT89
229533.I1RVP5	pp	229533.I1S9Z4
229533.I1RVP5	pp	229533.I1RZN1
229533.I1RVP5	pp	229533.I1RY74

6279 229533.I1RVP5 pp 229533.I1S2C4
6280 229533.I1RVP5 pp 229533.I1RWS4
6281 229533.I1RVP5 pp 229533.I1RWW7
6282 229533.A0A1C3YJK4 pp 229533.I1RJ14
6283 229533.A0A1C3YJK4 pp 229533.I1RND4
6284 229533.A0A1C3YJK4 pp 229533.V6RF62
6285 229533.A0A1C3YJK4 pp 229533.V6R3V4
6286 229533.A0A1C3YJK4 pp 229533.I1RIJ2
6287 229533.A0A1C3YJK4 pp 229533.I1REH0
6288 229533.A0A1C3YJK4 pp 229533.A0A1C3YLS8
6289 229533.A0A1C3YJK4 pp 229533.I1RAN9
6290 229533.A0A1C3YJK4 pp 229533.I1S4S5
6291 229533.A0A1C3YJK4 pp 229533.I1S308
6292 229533.A0A1C3YJK4 pp 229533.I1RAR9
6293 229533.A0A1C3YJK4 pp 229533.I1RJC5
6294 229533.I1RCY5 pp 229533.Q4I8S2
6295 229533.I1RCY5 pp 229533.I1RYI6
6296 229533.I1RCY5 pp 229533.I1RTF4
6297 229533.I1RCY5 pp 229533.I1RK72
6298 229533.I1RCY5 pp 229533.I1RW71
6299 229533.I1RCY5 pp 229533.I1RK95
6300 229533.I1RCY5 pp 229533.I1RE00
6301 229533.I1RCY5 pp 229533.I1RDG0
6302 229533.I1RCY5 pp 229533.I1RGC6
6303 229533.I1RCY5 pp 229533.I1RWX5
6304 229533.I1RCY5 pp 229533.I1RJT9
6305 229533.I1RCY5 pp 229533.I1RSD8
6306 229533.I1RSF5 pp 229533.I1RVP5
6307 229533.I1RSF5 pp 229533.I1S2C4
6308 229533.I1RSF5 pp 229533.I1RWL3
6309 229533.A0A1C3YKY7 pp 229533.I1RNG4
6310 229533.I1RK01 pp 229533.I1RKL3
6311 229533.I1RK01 pp 229533.I1S0R7
6312 229533.I1RK01 pp 229533.I1RT93
6313 229533.I1RK01 pp 229533.I1S540
6314 229533.I1RK01 pp 229533.I1RYH4
6315 229533.I1RK01 pp 229533.I1RX53
6316 229533.I1RK01 pp 229533.I1S7A2
6317 229533.I1RK01 pp 229533.I1RQ28
6318 229533.I1RK01 pp 229533.I1RTG6
6319 229533.I1RK01 pp 229533.I1RZZ1
6320 229533.I1RK01 pp 229533.I1RK81
6321 229533.I1RK01 pp 229533.I1RMQ0
6322 229533.I1RK01 pp 229533.I1RWJ3
6323 229533.I1RK01 pp 229533.I1RT80
6324 229533.I1RK01 pp 229533.I1RVL1
6325 229533.I1RB35 pp 229533.I1RR68
6326 229533.I1RB35 pp 229533.I1RNG8
6327 229533.I1RB35 pp 229533.I1S7M8
6328 229533.I1RB35 pp 229533.Q4ICM0
6329 229533.I1RB35 pp 229533.I1RMP2
6330 229533.I1RB35 pp 229533.I1RE19
6331 229533.I1RB35 pp 229533.I1RCF0
6332 229533.I1RB35 pp 229533.I1RSF5
6333 229533.I1RB35 pp 229533.I1RUK7
6334 229533.I1RB35 pp 229533.I1S4F4
6335 229533.I1RB35 pp 229533.I1RSL5
6336 229533.I1RB35 pp 229533.I1RZU6
6337 229533.I1RB35 pp 229533.I1RMG2
6338 229533.I1RB35 pp 229533.I1RKJ5
6339 229533.I1RB35 pp 229533.I1RVP5
6340 229533.I1RB35 pp 229533.I1S2X5
6341 229533.I1RB35 pp 229533.I1RWL3
6342 229533.I1RB35 pp 229533.Q4INI2
6343 229533.I1RB35 pp 229533.I1RR74
6344 229533.I1RB35 pp 229533.I1S2C4
6345 229533.I1RB35 pp 229533.I1SA33
6346 229533.I1RB35 pp 229533.I1RXT9
6347 229533.I1RB35 pp 229533.I1RN28
6348 229533.I1RB35 pp 229533.I1S0S8
6349 229533.I1RB35 pp 229533.I1RPK0
6350 229533.I1RB35 pp 229533.I1S2H3
6351 229533.I1RB35 pp 229533.I1RWD5

6352 229533.A0A098DFR1 pp 229533.I1RVP5
6353 229533.A0A098DFR1 pp 229533.I1RN28
6354 229533.A0A098DFR1 pp 229533.I1RWL3
6355 229533.A0A098DFR1 pp 229533.I1RSF5
6356 229533.A0A098DFR1 pp 229533.A0A1C3YLB6
6357 229533.A0A098DFR1 pp 229533.I1RR68
6358 229533.A0A098DFR1 pp 229533.I1S2C4
6359 229533.A0A098DFR1 pp 229533.I1RNG4
6360 229533.A0A098DFR1 pp 229533.I1RB35
6361 229533.I1RTB8 pp 229533.V6RLM7
6362 229533.I1RTB8 pp 229533.I1RYX4
6363 229533.I1RTB8 pp 229533.I1RUR5
6364 229533.I1RTB8 pp 229533.I1RVA8
6365 229533.I1RTB8 pp 229533.Q4I4A4
6366 229533.I1RTB8 pp 229533.I1RZF7
6367 229533.I1RG82 pp 229533.I1S0N8
6368 229533.A0A098E5C4 pp 229533.I1RCG6
6369 229533.A0A098E5C4 pp 229533.I1RZ42
6370 229533.A0A098E5C4 pp 229533.I1RX17
6371 229533.A0A098E5C4 pp 229533.I1RRF7
6372 229533.A0A098E5C4 pp 229533.I1RW91
6373 229533.A0A098E5C4 pp 229533.A0A1C3YM21
6374 229533.A0A098E5C4 pp 229533.A0A0E0SFV6
6375 229533.A0A098E5C4 pp 229533.I1RPW8
6376 229533.A0A098E5C4 pp 229533.I1RXF0
6377 229533.A0A098E5C4 pp 229533.A0A1C3YKV6
6378 229533.A0A098E5C4 pp 229533.I1REK9
6379 229533.A0A098E5C4 pp 229533.I1S182
6380 229533.A0A098E5C4 pp 229533.I1RE30
6381 229533.A0A098E5C4 pp 229533.A0A1C3YJC8
6382 229533.A0A098E5C4 pp 229533.I1RQ42
6383 229533.A0A098E5C4 pp 229533.A0A1C3YK82
6384 229533.A0A098E5C4 pp 229533.I1S523
6385 229533.A0A098E5C4 pp 229533.I1RW80
6386 229533.A0A098E5C4 pp 229533.I1RN04
6387 229533.A0A098E5C4 pp 229533.I1RV75
6388 229533.A0A098E5C4 pp 229533.I1RGS6
6389 229533.A0A098DRP4 pp 229533.A0A1C3YLS3
6390 229533.A0A098DFP2 pp 229533.A0A098E5C4
6391 229533.A0A098DFP2 pp 229533.I1S182
6392 229533.I1RFL0 pp 229533.I1RG82
6393 229533.I1RFL0 pp 229533.I1RQA9
6394 229533.I1RFL0 pp 229533.I1RPU7
6395 229533.I1RFL0 pp 229533.I1S0N8
6396 229533.A0A1C3YJ90 pp 229533.I1SAP6
6397 229533.A0A1C3YJ90 pp 229533.A0A1C3YKS0
6398 229533.A0A1C3YJ90 pp 229533.I1RBJ1
6399 229533.A0A1C3YJ90 pp 229533.I1RQA3
6400 229533.A0A1C3YJ90 pp 229533.I1S2E9
6401 229533.A0A1C3YJ90 pp 229533.I1RWT6
6402 229533.A0A1C3YJ90 pp 229533.I1RRI3
6403 229533.A0A1C3YJ90 pp 229533.Q4I1H6
6404 229533.A0A1C3YJ90 pp 229533.I1RY44
6405 229533.A0A1C3YJ90 pp 229533.A0A1C3YK00
6406 229533.A0A1C3YJ90 pp 229533.I1RNB8
6407 229533.A0A1C3YJ90 pp 229533.I1RB74
6408 229533.A0A1C3YJ90 pp 229533.I1RA84
6409 229533.A0A1C3YJ90 pp 229533.I1RFZ3
6410 229533.A0A1C3YJ90 pp 229533.I1RGF1
6411 229533.A0A1C3YJ90 pp 229533.I1RBW1
6412 229533.A0A1C3YJ90 pp 229533.I1RBW0
6413 229533.A0A1C3YJ90 pp 229533.I1S091
6414 229533.A0A1C3YJ90 pp 229533.I1RX70
6415 229533.I1RG99 pp 229533.Q4I1H6
6416 229533.I1RG99 pp 229533.I1RJT0
6417 229533.I1RG99 pp 229533.I1RLT6
6418 229533.I1RG99 pp 229533.I1RYB7
6419 229533.I1RG99 pp 229533.I1RW84
6420 229533.I1RG99 pp 229533.I1RS69
6421 229533.I1RG99 pp 229533.I1RPK5
6422 229533.I1RG99 pp 229533.I1RMV6
6423 229533.I1RG99 pp 229533.I1RMR7
6424 229533.I1RG99 pp 229533.I1RLH5

```
229533.I1RG99   pp  229533.I1RXF5
229533.I1RG99   pp  229533.I1RKR9
229533.I1RG99   pp  229533.I1RSZ1
229533.I1RG99   pp  229533.V6QW25
229533.I1RG99   pp  229533.I1RTG2
229533.I1RG99   pp  229533.I1S1D0
229533.I1RG99   pp  229533.Q4I6S5
229533.I1RG99   pp  229533.I1RMF2
229533.I1RG99   pp  229533.I1RTQ9
229533.I1RG99   pp  229533.I1RNA3
229533.I1RG99   pp  229533.I1S278
229533.I1RG99   pp  229533.I1RJR8
229533.I1RG99   pp  229533.I1RM09
229533.I1RG99   pp  229533.I1S8F9
229533.I1RG99   pp  229533.I1S5R2
229533.I1RG99   pp  229533.I1RYY6
229533.I1RG99   pp  229533.I1S9X9
229533.I1RG99   pp  229533.I1RMN3
229533.I1RG99   pp  229533.I1RXT2
229533.I1RG99   pp  229533.I1RX21
229533.I1RG99   pp  229533.I1RRX3
229533.I1RN23   pp  229533.I1RX77
229533.Q4IQT8   pp  229533.V6QUE6
229533.Q4IQT8   pp  229533.V6RBN2
229533.Q4IQT8   pp  229533.V6RGY3
229533.A0A0E0SMU9   pp  229533.I1RA52
229533.A0A1C3YJQ7   pp  229533.I1RKB9
229533.A0A1C3YJQ7   pp  229533.I1RFV7
229533.A0A1C3YJQ7   pp  229533.I1RN23
229533.A0A1C3YJQ7   pp  229533.I1RG48
229533.A0A1C3YJQ7   pp  229533.V6RRF6
229533.A0A1C3YJQ7   pp  229533.Q4I785
229533.A0A1C3YJQ7   pp  229533.I1RCP6
229533.A0A1C3YJQ7   pp  229533.I1RMN3
229533.A0A1C3YJQ7   pp  229533.I1RC10
229533.A0A1C3YJQ7   pp  229533.I1RG50
229533.A0A1C3YL59   pp  229533.I1RW71
229533.A0A1C3YL59   pp  229533.I1RC27
229533.A0A1C3YL59   pp  229533.I1RJZ7
229533.I1RWD1   pp  229533.I1S027
229533.I1S0G1   pp  229533.Q4I1H6
229533.I1S0G1   pp  229533.I1S8F9
229533.I1S0G1   pp  229533.I1S5R2
229533.I1RG50   pp  229533.I1RT41
229533.I1RG50   pp  229533.V6QUV7
229533.I1RRI3   pp  229533.I1RSI0
229533.I1RRI3   pp  229533.I1RRU9
229533.I1RRI3   pp  229533.I1RZM1
229533.I1RXF4   pp  229533.I1S027
229533.I1RKB9   pp  229533.I1RN23
229533.I1S034   pp  229533.Q4ILI9
229533.I1S034   pp  229533.V6QW25
229533.I1S034   pp  229533.I1S0Z3
229533.I1S034   pp  229533.Q4I1H6
229533.I1S034   pp  229533.I1S0X0
229533.I1S034   pp  229533.I1S5R2
229533.I1S034   pp  229533.I1S8F9
229533.I1S034   pp  229533.I1S0G1
229533.I1S034   pp  229533.Q4HYZ2
229533.I1S034   pp  229533.I1S0N8
229533.I1RC10   pp  229533.I1RG48
229533.I1RC10   pp  229533.I1RCP6
229533.I1RC10   pp  229533.I1RG50
229533.I1RC10   pp  229533.I1RFV7
229533.I1RC10   pp  229533.I1RKB9
229533.I1S5R2   pp  229533.Q4I1H6
229533.I1S5R2   pp  229533.I1S8F9
229533.I1S489   pp  229533.I1S9Z4
229533.I1S489   pp  229533.I1S8C1
229533.I1RG48   pp  229533.I1RMM6
229533.I1RG48   pp  229533.I1RG49
229533.I1RG48   pp  229533.I1RNM4
229533.I1RG48   pp  229533.I1RN23
```

6498 229533.I1RG48 pp 229533.I1RKB9
6499 229533.I1RRL4 pp 229533.I1RS21
6500 229533.I1RRL4 pp 229533.I1S027
6501 229533.I1RRL4 pp 229533.I1RWD1
6502 229533.A0A1C3YLQ0 pp 229533.I1S5R2
6503 229533.A0A1C3YLQ0 pp 229533.I1REC4
6504 229533.A0A1C3YLQ0 pp 229533.I1RMR5
6505 229533.A0A1C3YLQ0 pp 229533.I1RXT2
6506 229533.A0A1C3YLQ0 pp 229533.I1S8F9
6507 229533.A0A1C3YLQ0 pp 229533.I1RYY6
6508 229533.A0A1C3YLQ0 pp 229533.I1S034
6509 229533.A0A1C3YLQ0 pp 229533.I1RFU6
6510 229533.A0A1C3YLQ0 pp 229533.I1RD16
6511 229533.A0A1C3YLQ0 pp 229533.I1S0G1
6512 229533.A0A1C3YLQ0 pp 229533.Q4I1H6
6513 229533.I1RAH0 pp 229533.I1RSM8
6514 229533.I1RAH0 pp 229533.I1RJV4
6515 229533.I1RAH0 pp 229533.I1S4Z7
6516 229533.I1RAH0 pp 229533.I1RPM4
6517 229533.I1RAH0 pp 229533.I1RRM5
6518 229533.I1RAH0 pp 229533.I1RZ13
6519 229533.I1RAH0 pp 229533.I1RZ34
6520 229533.I1RAH0 pp 229533.I1RZZ9
6521 229533.I1RAH0 pp 229533.I1RRT8
6522 229533.I1RAH0 pp 229533.I1RZM0
6523 229533.I1RAH0 pp 229533.I1RE01
6524 229533.I1RAH0 pp 229533.I1S0L4
6525 229533.I1RAH0 pp 229533.I1RAK7
6526 229533.I1RAH0 pp 229533.I1RS17
6527 229533.I1RAH0 pp 229533.I1RNU6
6528 229533.I1RAH0 pp 229533.I1RLU2
6529 229533.I1RAH0 pp 229533.I1RBC9
6530 229533.I1RAH0 pp 229533.V6RIV9
6531 229533.I1RAH0 pp 229533.Q4HZE0
6532 229533.I1RAH0 pp 229533.I1RX25
6533 229533.I1RAH0 pp 229533.V6QW22
6534 229533.I1RAH0 pp 229533.I1RW25
6535 229533.I1RAH0 pp 229533.I1RSQ7
6536 229533.I1RAH0 pp 229533.I1RBT1
6537 229533.I1RAH0 pp 229533.I1RY40
6538 229533.I1RAH0 pp 229533.I1RUG3
6539 229533.I1RAH0 pp 229533.I1RQ75
6540 229533.I1RAH0 pp 229533.I1RWJ8
6541 229533.I1RAH0 pp 229533.I1RJL9
6542 229533.I1RAH0 pp 229533.I1RVQ0
6543 229533.I1RAH0 pp 229533.I1RXF0
6544 229533.I1RAH0 pp 229533.I1RNM3
6545 229533.I1RAH0 pp 229533.I1S0Z8
6546 229533.I1RAH0 pp 229533.I1RD52
6547 229533.I1RAH0 pp 229533.V6RCG5
6548 229533.I1RAH0 pp 229533.I1S0L3
6549 229533.I1RAH0 pp 229533.I1RUP0
6550 229533.I1RAH0 pp 229533.I1RBS3
6551 229533.I1RAH0 pp 229533.I1S5B0
6552 229533.I1RAH0 pp 229533.V6RPQ8
6553 229533.I1RAH0 pp 229533.I1RN18
6554 229533.I1RAH0 pp 229533.Q4IJT0
6555 229533.I1RAH0 pp 229533.I1RW99
6556 229533.I1RAH0 pp 229533.I1RMM6
6557 229533.I1RAH0 pp 229533.I1SAM9
6558 229533.I1RAH0 pp 229533.I1REK8
6559 229533.I1RAH0 pp 229533.I1RYL5
6560 229533.I1RAH0 pp 229533.I1RQE7
6561 229533.I1RAH0 pp 229533.V6QUV7
6562 229533.I1RAH0 pp 229533.I1RB92
6563 229533.I1RAH0 pp 229533.I1RD17
6564 229533.I1RAH0 pp 229533.I1RCG4
6565 229533.I1RAH0 pp 229533.I1RZW8
6566 229533.I1RAH0 pp 229533.I1RD39
6567 229533.I1RAH0 pp 229533.V6QX21
6568 229533.I1RAH0 pp 229533.I1RT00
6569 229533.I1RAH0 pp 229533.I1RWK3
6570 229533.I1RAH0 pp 229533.V6RRF6

6571	229533.I1RAH0	pp	229533.I1RJW6
6572	229533.I1RAH0	pp	229533.I1S0F6
6573	229533.I1RAH0	pp	229533.I1RBS6
6574	229533.I1RW64	pp	229533.V6RWB3
6575	229533.I1RW64	pp	229533.I1S489
6576	229533.I1RW64	pp	229533.I1S2F7
6577	229533.I1RW64	pp	229533.V6R3V4
6578	229533.I1RW64	pp	229533.I1S8D3
6579	229533.I1RW64	pp	229533.I1S8H9
6580	229533.I1RW64	pp	229533.V6QVH2
6581	229533.I1RW64	pp	229533.I1S4S5
6582	229533.I1RW64	pp	229533.I1S9Z4
6583	229533.I1RW64	pp	229533.V6RF62
6584	229533.I1RW64	pp	229533.I1S0Q7
6585	229533.I1RW64	pp	229533.V6R5T7
6586	229533.I1RW64	pp	229533.I1RZ38
6587	229533.I1RW64	pp	229533.I1RWL8
6588	229533.I1RW64	pp	229533.V6RN69
6589	229533.I1RW64	pp	229533.V6RZD4
6590	229533.I1RFV7	pp	229533.I1RT41
6591	229533.I1RFV7	pp	229533.V6QUV7
6592	229533.I1S8F9	pp	229533.Q4I1H6
6593	229533.I1RCP6	pp	229533.I1RCP7
6594	229533.I1RCP6	pp	229533.I1RKB9
6595	229533.I1RCP6	pp	229533.I1RG50
6596	229533.I1RCP6	pp	229533.I1RFV7
6597	229533.I1RCP6	pp	229533.I1RG48
6598	229533.I1RCP6	pp	229533.I1RN23
6599	229533.I1RF17	pp	229533.I1RZ25
6600	229533.A0A0E0SA52	pp	229533.I1S268
6601	229533.A0A0E0SA52	pp	229533.A0A0E0SMR8
6602	229533.A0A0E0SA52	pp	229533.I1RN23
6603	229533.A0A0E0SA52	pp	229533.A0A1C3YJQ7
6604	229533.A0A0E0SA52	pp	229533.I1RG50
6605	229533.A0A0E0SA52	pp	229533.I1RKB9
6606	229533.A0A0E0SA52	pp	229533.I1RC10
6607	229533.A0A0E0SA52	pp	229533.I1RQ41
6608	229533.A0A0E0SA52	pp	229533.I1RG48
6609	229533.A0A0E0SA52	pp	229533.I1RFV7
6610	229533.A0A0E0SA52	pp	229533.I1RCP6
6611	229533.I1RUP9	pp	229533.Q4HZB8
6612	229533.I1RUP9	pp	229533.I1RVY0
6613	229533.I1RUP9	pp	229533.Q4IMD1
6614	229533.I1RUP9	pp	229533.I1RXU0
6615	229533.I1RUP9	pp	229533.Q4IJH1
6616	229533.I1RUP9	pp	229533.Q4IF76
6617	229533.I1RUP9	pp	229533.Q4ILH3
6618	229533.I1RUP9	pp	229533.V6RCG5
6619	229533.I1RUP9	pp	229533.I1RZB5
6620	229533.I1RUP9	pp	229533.V6QUE6
6621	229533.I1REC4	pp	229533.I1S034
6622	229533.I1REC4	pp	229533.I1S0Y1
6623	229533.I1REC4	pp	229533.I1S5R2
6624	229533.I1REC4	pp	229533.I1RYY6
6625	229533.I1REC4	pp	229533.I1RSP4
6626	229533.I1REC4	pp	229533.I1RRQ1
6627	229533.I1REC4	pp	229533.I1REN4
6628	229533.I1REC4	pp	229533.I1RXT2
6629	229533.I1REC4	pp	229533.I1RTF0
6630	229533.I1REC4	pp	229533.I1RXU1
6631	229533.I1REC4	pp	229533.I1RYU1
6632	229533.I1REC4	pp	229533.V6RQ48
6633	229533.I1REC4	pp	229533.I1RG99
6634	229533.I1REC4	pp	229533.I1RMF2
6635	229533.I1REC4	pp	229533.V6R9X9
6636	229533.I1REC4	pp	229533.V6RKE4
6637	229533.I1REC4	pp	229533.I1RMR5
6638	229533.I1REC4	pp	229533.I1S8F9
6639	229533.I1REC4	pp	229533.I1REK1
6640	229533.I1REC4	pp	229533.I1RWU9
6641	229533.I1REC4	pp	229533.I1REK3
6642	229533.I1REC4	pp	229533.I1RXF5
6643	229533.I1REC4	pp	229533.I1S0G1

6644	229533.I1REC4	pp	229533.I1RIG4
6645	229533.I1REC4	pp	229533.Q4I1H6
6646	229533.I1REC4	pp	229533.I1RZA4
6647	229533.I1REC4	pp	229533.I1RW84
6648	229533.I1REC4	pp	229533.I1RPK5
6649	229533.I1REC4	pp	229533.I1RFU6
6650	229533.I1RJM0	pp	229533.I1RXU3
6651	229533.I1RJM0	pp	229533.I1S489
6652	229533.I1RJM0	pp	229533.I1RSU2
6653	229533.I1RJM0	pp	229533.I1RMW8
6654	229533.I1RJM0	pp	229533.I1RMW9
6655	229533.I1RSX7	pp	229533.I1S0S5
6656	229533.I1RSX7	pp	229533.I1RWT6
6657	229533.I1R9A6	pp	229533.I1RSU2
6658	229533.I1R9A6	pp	229533.I1RCT9
6659	229533.I1R9A6	pp	229533.I1RJM0
6660	229533.I1R9A6	pp	229533.I1RXU3
6661	229533.I1R9A6	pp	229533.I1RMW9
6662	229533.I1R9A6	pp	229533.I1RMW8
6663	229533.I1R9A6	pp	229533.I1S489
6664	229533.I1R9A6	pp	229533.I1RW64
6665	229533.I1R9A6	pp	229533.I1RHU8
6666	229533.I1R9A6	pp	229533.I1RWL8
6667	229533.I1R9A6	pp	229533.I1RQ48
6668	229533.I1R9A6	pp	229533.I1S9Z4
6669	229533.I1R9A6	pp	229533.I1RJ39
6670	229533.I1R9A6	pp	229533.I1RW82
6671	229533.I1R9A6	pp	229533.I1RR68
6672	229533.I1RMK1	pp	229533.I1S0K5
6673	229533.I1RMK1	pp	229533.Q4IBU4
6674	229533.I1RMK1	pp	229533.I1RMK2
6675	229533.I1RMK1	pp	229533.Q4ILI9
6676	229533.I1RMK1	pp	229533.Q4I877
6677	229533.I1RMK1	pp	229533.I1RMN3
6678	229533.I1RMK1	pp	229533.V6QW25
6679	229533.I1RMK1	pp	229533.I1S287
6680	229533.I1RMK1	pp	229533.I1RS69
6681	229533.I1RMK1	pp	229533.I1RWP9
6682	229533.I1RMK1	pp	229533.I1RPW0
6683	229533.I1RMK1	pp	229533.I1RQ32
6684	229533.I1RMK1	pp	229533.I1RQ70
6685	229533.I1RMK1	pp	229533.I1S1W5
6686	229533.I1RMK1	pp	229533.I1RML5
6687	229533.A0A1C3YMT4	pp	229533.I1RJ39
6688	229533.A0A1C3YMT4	pp	229533.I1RJM0
6689	229533.A0A1C3YMT4	pp	229533.I1RSU2
6690	229533.A0A1C3YMT4	pp	229533.I1S489
6691	229533.A0A1C3YMT4	pp	229533.I1R9A6
6692	229533.A0A1C3YMT4	pp	229533.I1RXU3
6693	229533.A0A1C3YMT4	pp	229533.I1RZ25
6694	229533.A0A1C3YMT4	pp	229533.I1RMW8
6695	229533.A0A1C3YMT4	pp	229533.I1RMW9
6696	229533.A0A098DFH0	pp	229533.I1S270
6697	229533.A0A098DFH0	pp	229533.I1RN81
6698	229533.A0A098DFH0	pp	229533.A0A1C3YIM8
6699	229533.A0A098DFH0	pp	229533.I1RA34
6700	229533.A0A098DFH0	pp	229533.I1RT41
6701	229533.A0A098DFH0	pp	229533.I1S275
6702	229533.A0A098DFH0	pp	229533.A0A1C3YM61
6703	229533.A0A098DFH0	pp	229533.I1RMP2
6704	229533.A0A098DFH0	pp	229533.I1RRF9
6705	229533.A0A098DFH0	pp	229533.V6RR76
6706	229533.A0A098DFH0	pp	229533.I1RXB8
6707	229533.A0A098DFH0	pp	229533.I1RZL3
6708	229533.A0A098DFH0	pp	229533.I1S0Z3
6709	229533.A0A098DFH0	pp	229533.A0A1C3YKT9
6710	229533.A0A098DFH0	pp	229533.I1RHU0
6711	229533.A0A098DFH0	pp	229533.A0A1C3YIQ1
6712	229533.A0A098DFH0	pp	229533.I1RSG9
6713	229533.A0A098DFH0	pp	229533.A0A098DTU2
6714	229533.A0A098DFH0	pp	229533.Q4I963
6715	229533.A0A098DFH0	pp	229533.I1S045
6716	229533.A0A098DFH0	pp	229533.I1RSL8

229533.A0A098DFH0	pp	229533.I1RE19
229533.A0A098DFH0	pp	229533.I1RKR9
229533.A0A098DFH0	pp	229533.A0A1C3YL06
229533.A0A098DFH0	pp	229533.I1RTA3
229533.A0A098DFH0	pp	229533.I1RA31
229533.A0A098DFH0	pp	229533.I1RTE0
229533.A0A098DFH0	pp	229533.I1RSZ6
229533.A0A098DFH0	pp	229533.I1RTG5
229533.A0A098DFH0	pp	229533.I1RD10
229533.I1RC52	pp	229533.I1S278
229533.I1RC52	pp	229533.V6R9X9
229533.I1RC52	pp	229533.I1RG99
229533.I1RC52	pp	229533.I1REK3
229533.I1RC52	pp	229533.I1RMF2
229533.I1RC52	pp	229533.I1RMR7
229533.I1RC52	pp	229533.I1RFK0
229533.I1RC52	pp	229533.I1RXT2
229533.I1RC52	pp	229533.I1RFV4
229533.I1RC52	pp	229533.I1RD63
229533.I1RC52	pp	229533.I1RNB8
229533.I1RC52	pp	229533.I1RYY6
229533.I1RC52	pp	229533.I1S8F9
229533.I1RC52	pp	229533.I1RS69
229533.I1RC52	pp	229533.Q4I1H6
229533.I1RC52	pp	229533.I1RLH5
229533.I1RC52	pp	229533.I1RG69
229533.I1RC52	pp	229533.I1RFU6
229533.I1RC52	pp	229533.I1RE18
229533.I1RC52	pp	229533.I1RW84
229533.I1RC52	pp	229533.I1RRX3
229533.I1RC52	pp	229533.I1S5R2
229533.I1RC52	pp	229533.I1RX87
229533.I1RC52	pp	229533.I1REC4
229533.A0A0E0SLL1	pp	229533.I1RMW9
229533.A0A0E0SLL1	pp	229533.I1RXU3
229533.A0A0E0SLL1	pp	229533.I1S489
229533.A0A0E0SLL1	pp	229533.I1RJ39
229533.A0A0E0SLL1	pp	229533.I1R9A6
229533.A0A0E0SLL1	pp	229533.I1RMW8
229533.A0A0E0SLL1	pp	229533.I1RSU2
229533.A0A0E0SLL1	pp	229533.A0A1C3YMT4
229533.A0A0E0SLL1	pp	229533.I1RZ25
229533.A0A0E0SLL1	pp	229533.I1RJM0
229533.I1RYY6	pp	229533.I1S0G1
229533.I1RYY6	pp	229533.Q4I1H6
229533.I1RYY6	pp	229533.I1S034
229533.I1RYY6	pp	229533.I1RZ91
229533.I1RYY6	pp	229533.I1S5R2
229533.I1RYY6	pp	229533.I1S8F9
229533.A0A1C3YK97	pp	229533.I1RBE7
229533.A0A1C3YK97	pp	229533.I1RPW1
229533.A0A1C3YK97	pp	229533.V6RQW4
229533.A0A1C3YK97	pp	229533.I1RZ29
229533.A0A1C3YK97	pp	229533.I1RNG4
229533.A0A1C3YK97	pp	229533.I1RNQ1
229533.I1RK49	pp	229533.I1S0R5
229533.I1RK49	pp	229533.I1RL16
229533.I1RK49	pp	229533.I1RPX7
229533.I1RK49	pp	229533.I1RM94
229533.I1RK49	pp	229533.I1RZT6
229533.I1RK49	pp	229533.I1RPE5
229533.I1RK49	pp	229533.V6RG04
229533.I1RK49	pp	229533.I1RQM4
229533.I1RK49	pp	229533.I1S1K6
229533.I1RK49	pp	229533.I1S8X0
229533.I1RK49	pp	229533.I1RRC4
229533.I1RK49	pp	229533.I1RXX1
229533.I1RK49	pp	229533.I1RZN1
229533.I1RK49	pp	229533.I1RQM9
229533.I1RK49	pp	229533.I1RMG4
229533.I1RK49	pp	229533.I1RVS1
229533.I1RK49	pp	229533.V6RLM7
229533.I1RK49	pp	229533.I1RWN0

229533.I1RK49	pp	229533.I1RTP2
229533.I1RK49	pp	229533.I1S2B0
229533.I1RK49	pp	229533.I1RRN4
229533.I1RM42	pp	229533.I1RZB5
229533.I1RM42	pp	229533.I1RWL3
229533.I1RM42	pp	229533.I1RRG3
229533.I1RM42	pp	229533.I1RRT0
229533.I1RM42	pp	229533.I1S2C4
229533.I1RM42	pp	229533.I1RR25
229533.I1RM42	pp	229533.Q4I5I4
229533.I1RM42	pp	229533.Q4I5W5
229533.I1RJ39	pp	229533.I1RJM0
229533.I1RJ39	pp	229533.I1RMW8
229533.I1RJ39	pp	229533.I1S489
229533.I1RJ39	pp	229533.I1RSU2
229533.I1RJ39	pp	229533.I1RXU3
229533.I1RJ39	pp	229533.I1RMW9
229533.I1RFU6	pp	229533.I1RXT2
229533.I1RFU6	pp	229533.I1S8F9
229533.I1RFU6	pp	229533.I1RG99
229533.I1RFU6	pp	229533.I1RYY6
229533.I1RFU6	pp	229533.I1S5R2
229533.I1RFU6	pp	229533.I1RMR5
229533.I1RFU6	pp	229533.I1S034
229533.I1RFU6	pp	229533.I1S0G1
229533.I1RFU6	pp	229533.Q4I1H6
229533.I1RMW9	pp	229533.I1RR68
229533.I1RMW9	pp	229533.I1RTY9
229533.I1RMW9	pp	229533.I1RW82
229533.I1RMW9	pp	229533.I1S8C1
229533.I1RMW9	pp	229533.I1RWL8
229533.I1RMW9	pp	229533.I1RPR1
229533.I1RMW9	pp	229533.I1RSU2
229533.I1RMW9	pp	229533.I1RW64
229533.I1RMW9	pp	229533.I1S489
229533.I1RMW9	pp	229533.I1RQ48
229533.I1RMW9	pp	229533.I1RXU3
229533.I1RMW9	pp	229533.I1S9Z4
229533.I1RM32	pp	229533.I1S0R5
229533.I1RM32	pp	229533.I1S2B0
229533.A0A098DFG6	pp	229533.I1RCN6
229533.A0A098DFG6	pp	229533.I1RK49
229533.A0A098DFG6	pp	229533.I1RFY0
229533.A0A098DFG6	pp	229533.A0A0E0RQT8
229533.A0A098DFG6	pp	229533.I1RW69
229533.A0A098DFG6	pp	229533.I1RDZ5
229533.Q4I1H6	pp	229533.Q4I6S5
229533.I1RP58	pp	229533.I1RUP5
229533.I1RP58	pp	229533.I1S5S8
229533.I1RMW8	pp	229533.I1RR68
229533.I1RMW8	pp	229533.I1RWL8
229533.I1RMW8	pp	229533.I1RSU2
229533.I1RMW8	pp	229533.I1S9Z4
229533.I1RMW8	pp	229533.I1RMW9
229533.I1RMW8	pp	229533.I1RQ48
229533.I1RMW8	pp	229533.I1S489
229533.I1RMW8	pp	229533.I1RW64
229533.I1RMW8	pp	229533.I1RW82
229533.I1RMW8	pp	229533.I1RXU3
229533.A0A098DFD5	pp	229533.I1RBE7
229533.A0A098E0C6	pp	229533.I1S489
229533.A0A098E0C6	pp	229533.I1RW64
229533.A0A098E0C6	pp	229533.I1RZ25
229533.A0A098E0C6	pp	229533.I1RF17
229533.A0A098E0C6	pp	229533.I1RSU2
229533.A0A098E0C6	pp	229533.I1RJM0
229533.A0A098E0C6	pp	229533.I1R9A6
229533.A0A098E0C6	pp	229533.A0A1C3YMT4
229533.A0A098E0C6	pp	229533.A0A0E0SLL1
229533.A0A098E0C6	pp	229533.I1S9Z4
229533.A0A098E0C6	pp	229533.I1RJ39
229533.A0A098E0C6	pp	229533.I1RMW9
229533.A0A098E0C6	pp	229533.I1RMW8

6863 229533.A0A098E0C6 pp 229533.I1RXU3
6864 229533.I1RA45 pp 229533.I1RAU2
6865 229533.I1RA45 pp 229533.I1RT75
6866 229533.I1RA45 pp 229533.I1RFV4
6867 229533.I1RA45 pp 229533.I1RLU2
6868 229533.I1RA45 pp 229533.I1S277
6869 229533.I1RA45 pp 229533.I1S0Z6
6870 229533.I1RA45 pp 229533.I1RJV7
6871 229533.I1RA45 pp 229533.I1RT00
6872 229533.I1RA45 pp 229533.I1RFL6
6873 229533.I1RA45 pp 229533.I1RPA4
6874 229533.I1RA45 pp 229533.V6RXW9
6875 229533.I1RA45 pp 229533.I1RXF0
6876 229533.I1RA45 pp 229533.Q4HY20
6877 229533.I1RNQ1 pp 229533.V6RQW4
6878 229533.I1RNQ1 pp 229533.I1RZ29
6879 229533.I1RNQ1 pp 229533.I1RZG9
6880 229533.I1RNQ1 pp 229533.I1RPW1
6881 229533.A0A098E033 pp 229533.I1RPD6
6882 229533.A0A098E033 pp 229533.I1RNG4
6883 229533.I1RIU0 pp 229533.I1RRD0
6884 229533.I1RIU0 pp 229533.I1RY58
6885 229533.I1RIU0 pp 229533.I1RZT1
6886 229533.I1RIU0 pp 229533.I1RNM7
6887 229533.I1RIU0 pp 229533.I1RSF1
6888 229533.I1RIU0 pp 229533.I1RNC1
6889 229533.I1RIU0 pp 229533.I1RKP1
6890 229533.I1RNP4 pp 229533.I1S6P5
6891 229533.I1RNP4 pp 229533.I1RVL8
6892 229533.I1RNP4 pp 229533.I1RZ29
6893 229533.I1RNP4 pp 229533.I1S3X8
6894 229533.I1RNP4 pp 229533.I1RZA7
6895 229533.I1RNP4 pp 229533.I1RR25
6896 229533.I1RNP4 pp 229533.I1S3N0
6897 229533.I1RNP4 pp 229533.I1S276
6898 229533.I1RNP4 pp 229533.I1RPM7
6899 229533.I1RNP4 pp 229533.V6RUB1
6900 229533.I1RNP4 pp 229533.I1RWF3
6901 229533.I1RNP4 pp 229533.Q4I5W5
6902 229533.I1RNP4 pp 229533.I1RSZ3
6903 229533.I1RNP4 pp 229533.Q4HYB8
6904 229533.I1RNP4 pp 229533.I1RVY0
6905 229533.I1RNP4 pp 229533.Q4IPC8
6906 229533.I1RNP4 pp 229533.I1RUG0
6907 229533.I1RNP4 pp 229533.V6QWX8
6908 229533.I1RNP4 pp 229533.I1RZB5
6909 229533.I1RNP4 pp 229533.I1RXU0
6910 229533.I1RNP4 pp 229533.I1RRT0
6911 229533.I1RNP4 pp 229533.I1RRG3
6912 229533.I1RNP4 pp 229533.V6QWD8
6913 229533.I1RNP4 pp 229533.I1S2V3
6914 229533.I1RNP4 pp 229533.I1RTC2
6915 229533.I1RNP4 pp 229533.V6QUE6
6916 229533.I1RNP4 pp 229533.Q4I5I4
6917 229533.I1RTE3 pp 229533.I1RVR8
6918 229533.I1RZ60 pp 229533.I1S150
6919 229533.I1RZ60 pp 229533.I1RZS5
6920 229533.I1RZ60 pp 229533.I1S830
6921 229533.I1RZ60 pp 229533.I1S0Z3
6922 229533.I1RZ60 pp 229533.V6R227
6923 229533.I1RZ60 pp 229533.I1RZV1
6924 229533.I1RZ60 pp 229533.V6RY56
6925 229533.I1RZ60 pp 229533.I1RZB4
6926 229533.I1RZ60 pp 229533.Q4HYZ2
6927 229533.I1RZ60 pp 229533.Q4ILI9
6928 229533.I1RZ60 pp 229533.Q5I2J3
6929 229533.I1RZ60 pp 229533.I1S2B6
6930 229533.I1RZ60 pp 229533.I1S287
6931 229533.I1RZ60 pp 229533.I1S278
6932 229533.I1RZ60 pp 229533.Q4IPI4
6933 229533.I1RZ60 pp 229533.I1SAM5
6934 229533.I1RHB0 pp 229533.I1S0E8
6935 229533.I1RHB0 pp 229533.I1RQA3

6936 229533.I1RHB0 pp 229533.I1RRN5
6937 229533.I1RHB0 pp 229533.I1RL24
6938 229533.I1RHB0 pp 229533.I1RPC2
6939 229533.I1RHB0 pp 229533.I1RYZ9
6940 229533.I1RHB0 pp 229533.I1RMQ7
6941 229533.I1RHB0 pp 229533.Q4IJM4
6942 229533.I1RHB0 pp 229533.I1S7X9
6943 229533.I1RHB0 pp 229533.I1RZ63
6944 229533.I1RHB0 pp 229533.I1S098
6945 229533.I1RHB0 pp 229533.I1RK75
6946 229533.I1RHB0 pp 229533.V6QV09
6947 229533.I1RHB0 pp 229533.I1RT62
6948 229533.I1RHB0 pp 229533.I1RY44
6949 229533.I1RHB0 pp 229533.I1S2E9
6950 229533.I1RHB0 pp 229533.I1RWT6
6951 229533.I1S3X8 pp 229533.V6RUB1
6952 229533.I1S3X8 pp 229533.V6QWD8
6953 229533.I1S3X8 pp 229533.I1S6P5
6954 229533.I1S123 pp 229533.I1S8Z2
6955 229533.I1S123 pp 229533.I1S3G4
6956 229533.I1S123 pp 229533.I1S1W8
6957 229533.I1S123 pp 229533.V6QUV7
6958 229533.I1S123 pp 229533.V6RKY8
6959 229533.I1RLQ5 pp 229533.I1S3X8
6960 229533.I1RLQ5 pp 229533.I1RZ29
6961 229533.I1RLQ5 pp 229533.I1S3N0
6962 229533.I1RLQ5 pp 229533.V6RUB1
6963 229533.I1RLQ5 pp 229533.I1S6P5
6964 229533.I1RLQ5 pp 229533.I1RMU5
6965 229533.I1RLQ5 pp 229533.V6QWD8
6966 229533.I1RLQ5 pp 229533.I1RZA7
6967 229533.I1RLQ5 pp 229533.I1RNP4
6968 229533.I1RLQ5 pp 229533.I1S2V3
6969 229533.I1S9T5 pp 229533.V6QUV7
6970 229533.I1S9T5 pp 229533.V6RKY8
6971 229533.A0A1C3YLW6 pp 229533.I1RCB7
6972 229533.A0A1C3YLW6 pp 229533.I1RXU0
6973 229533.A0A1C3YLW6 pp 229533.I1RPM7
6974 229533.A0A1C3YLW6 pp 229533.I1RAR2
6975 229533.A0A1C3YLW6 pp 229533.I1S276
6976 229533.A0A1C3YLW6 pp 229533.I1RK71
6977 229533.A0A1C3YLW6 pp 229533.A0A1C3YN55
6978 229533.A0A1C3YLW6 pp 229533.I1RWF3
6979 229533.A0A1C3YLW6 pp 229533.Q4HYB8
6980 229533.A0A1C3YLW6 pp 229533.V6QUE6
6981 229533.A0A1C3YLW6 pp 229533.I1RE14
6982 229533.A0A1C3YLW6 pp 229533.I1RK79
6983 229533.A0A1C3YLW6 pp 229533.I1RZ29
6984 229533.A0A1C3YLW6 pp 229533.I1REA5
6985 229533.A0A1C3YLW6 pp 229533.I1RR25
6986 229533.A0A1C3YLW6 pp 229533.I1RRT0
6987 229533.A0A1C3YLW6 pp 229533.I1RC24
6988 229533.A0A1C3YLW6 pp 229533.Q4IPC8
6989 229533.A0A1C3YLW6 pp 229533.I1RC15
6990 229533.A0A1C3YLW6 pp 229533.I1RVY0
6991 229533.A0A1C3YLW6 pp 229533.I1RVL8
6992 229533.A0A1C3YLW6 pp 229533.I1RKU0
6993 229533.A0A1C3YLW6 pp 229533.I1RG11
6994 229533.A0A1C3YLW6 pp 229533.I1RE30
6995 229533.A0A1C3YLW6 pp 229533.I1S3N0
6996 229533.A0A1C3YLW6 pp 229533.I1RIA1
6997 229533.A0A1C3YLW6 pp 229533.I1S3X8
6998 229533.A0A1C3YLW6 pp 229533.I1S6P5
6999 229533.A0A1C3YLW6 pp 229533.Q4I5W5
7000 229533.A0A1C3YLW6 pp 229533.V6QWX8
7001 229533.A0A1C3YLW6 pp 229533.I1RZA7
7002 229533.A0A1C3YLW6 pp 229533.I1RJL2
7003 229533.A0A1C3YLW6 pp 229533.I1RZB5
7004 229533.A0A1C3YLW6 pp 229533.V6QWD8
7005 229533.A0A1C3YLW6 pp 229533.I1RUG0
7006 229533.A0A1C3YLW6 pp 229533.V6RUB1
7007 229533.A0A1C3YLW6 pp 229533.I1RMU5
7008 229533.A0A1C3YLW6 pp 229533.I1RJ03

229533.A0A1C3YLW6	pp	229533.Q4I5I4
229533.A0A1C3YLW6	pp	229533.I1RSZ3
229533.A0A1C3YLW6	pp	229533.I1RLQ5
229533.A0A1C3YLW6	pp	229533.I1RNP4
229533.A0A1C3YLW6	pp	229533.I1S2V3
229533.A0A1C3YLW6	pp	229533.I1RKB1
229533.A0A1C3YLW6	pp	229533.I1RRG3
229533.A0A1C3YLW6	pp	229533.I1RHT4
229533.A0A1C3YLW6	pp	229533.I1RTC2
229533.I1RN26	pp	229533.I1RRI3
229533.I1RN26	pp	229533.I1RP27
229533.I1RN26	pp	229533.I1RRF8
229533.I1RN26	pp	229533.I1RN61
229533.I1RN26	pp	229533.I1RSV2
229533.I1RT94	pp	229533.I1RZG3
229533.I1RT94	pp	229533.I1S247
229533.I1RT94	pp	229533.V6REE9
229533.I1RT94	pp	229533.Q4HXF6
229533.I1RT94	pp	229533.I1RYS6
229533.A0A098DRX7	pp	229533.A0A1C3YIM8
229533.A0A098DRX7	pp	229533.I1RI75
229533.A0A098DRX7	pp	229533.Q4HZS8
229533.A0A098DRX7	pp	229533.I1S270
229533.A0A098DRX7	pp	229533.I1RWB3
229533.A0A098DRX7	pp	229533.Q5I2J3
229533.A0A098DRX7	pp	229533.I1RAP3
229533.A0A098DRX7	pp	229533.A0A1C3YIF4
229533.A0A098DRX7	pp	229533.I1RQP5
229533.A0A098DRX7	pp	229533.I1RT65
229533.A0A098DRX7	pp	229533.Q4I963
229533.A0A098DRX7	pp	229533.I1RT41
229533.A0A098DRX7	pp	229533.A0A1C3YIQ1
229533.A0A098DRX7	pp	229533.A0A098DZN5
229533.A0A098DRX7	pp	229533.A0A098DTU2
229533.A0A098DRX7	pp	229533.I1RW71
229533.A0A098DRX7	pp	229533.I1RJZ7
229533.A0A098DRX7	pp	229533.Q4INZ9
229533.A0A098DRX7	pp	229533.Q4IPI4
229533.A0A098DRX7	pp	229533.I1RKR9
229533.A0A098DRX7	pp	229533.I1RJK8
229533.A0A098DRX7	pp	229533.A0A098E1H5
229533.A0A098DRX7	pp	229533.I1RA72
229533.A0A098DRX7	pp	229533.I1S0Z3
229533.A0A098DRX7	pp	229533.I1RUK7
229533.A0A098DRX7	pp	229533.I1S7X9
229533.A0A098DRX7	pp	229533.A0A1C3YL06
229533.A0A098DRX7	pp	229533.I1RKI1
229533.A0A098DRX7	pp	229533.I1RRF9
229533.A0A098DRX7	pp	229533.I1RZL3
229533.A0A098DRX7	pp	229533.I1RFT2
229533.A0A098DRX7	pp	229533.A0A0E0SMG8
229533.A0A098DRX7	pp	229533.I1RAH9
229533.A0A098DRX7	pp	229533.A0A1C3YM61
229533.A0A098DRX7	pp	229533.I1RMP2
229533.A0A098DRX7	pp	229533.I1RTE0
229533.A0A098DRX7	pp	229533.I1RRF0
229533.A0A098DRX7	pp	229533.I1RDE8
229533.A0A098DRX7	pp	229533.I1RPK0
229533.A0A098DRX7	pp	229533.I1RZV1
229533.A0A098DRX7	pp	229533.I1RZ60
229533.A0A098DRX7	pp	229533.I1RE19
229533.A0A098DRX7	pp	229533.I1RR95
229533.A0A098DRX7	pp	229533.I1RQX3
229533.A0A098DRX7	pp	229533.I1RCJ9
229533.A0A098DRX7	pp	229533.I1RTA3
229533.A0A098DRX7	pp	229533.I1RBX2
229533.A0A098DRX7	pp	229533.I1RST4
229533.A0A098DRX7	pp	229533.I1RSZ6
229533.Q4HXF6	pp	229533.Q4IE79
229533.Q4HXF6	pp	229533.Q4IB70
229533.Q4HXF6	pp	229533.V6QXG1
229533.Q4HXF6	pp	229533.Q4INZ9
229533.Q4HXF6	pp	229533.Q4IRI9

7082	229533.Q4HXF6	pp	229533.V6RCZ8
7083	229533.Q4HXF6	pp	229533.Q4I1Y1
7084	229533.Q4HXF6	pp	229533.Q4IPB8
7085	229533.Q4HXF6	pp	229533.Q4IN00
7086	229533.Q4HXF6	pp	229533.Q4IF76
7087	229533.Q4HXF6	pp	229533.Q4IJH1
7088	229533.Q4HXF6	pp	229533.Q4I624
7089	229533.Q4HXF6	pp	229533.Q4HZB8
7090	229533.Q4HXF6	pp	229533.Q4IBK5
7091	229533.I1RQC0	pp	229533.I1RSF1
7092	229533.I1RQC0	pp	229533.I1RZT1
7093	229533.I1RQC0	pp	229533.I1RY58
7094	229533.I1RQC0	pp	229533.I1RRD0
7095	229533.I1S3N0	pp	229533.V6QWX8
7096	229533.I1S3N0	pp	229533.V6QWD8
7097	229533.I1S3N0	pp	229533.V6QUE6
7098	229533.I1S3N0	pp	229533.I1S6P5
7099	229533.I1S3N0	pp	229533.I1S3X8
7100	229533.I1S3N0	pp	229533.Q4I5I4
7101	229533.I1S3N0	pp	229533.V6RUB1
7102	229533.I1S3N0	pp	229533.Q4I5W5
7103	229533.I1S3N0	pp	229533.Q4IPC8
7104	229533.I1S3N0	pp	229533.Q4HYB8
7105	229533.I1RZF2	pp	229533.I1S7I5
7106	229533.I1RHT4	pp	229533.I1RKU0
7107	229533.I1RHT4	pp	229533.I1S2V3
7108	229533.I1RHT4	pp	229533.I1RJ03
7109	229533.I1RHT4	pp	229533.I1S3N0
7110	229533.I1RHT4	pp	229533.V6RUB1
7111	229533.I1RHT4	pp	229533.I1RZA7
7112	229533.I1RHT4	pp	229533.I1RZ29
7113	229533.I1RHT4	pp	229533.I1RIA1
7114	229533.I1RHT4	pp	229533.V6QWD8
7115	229533.I1RHT4	pp	229533.I1S6P5
7116	229533.I1RHT4	pp	229533.I1S3X8
7117	229533.I1RHT4	pp	229533.I1RMU5
7118	229533.I1RHT4	pp	229533.I1RLQ5
7119	229533.I1RHT4	pp	229533.I1RNP4
7120	229533.I1RCB9	pp	229533.I1REC7
7121	229533.I1RCB9	pp	229533.I1S247
7122	229533.I1RCB9	pp	229533.I1RYS6
7123	229533.I1RCB9	pp	229533.I1RPI2
7124	229533.I1RCB9	pp	229533.I1S238
7125	229533.I1RCB9	pp	229533.I1RNC0
7126	229533.I1RCB9	pp	229533.I1RT94
7127	229533.I1RCB9	pp	229533.I1S3W8
7128	229533.I1RCB9	pp	229533.I1REC6
7129	229533.I1S2V3	pp	229533.I1S6P5
7130	229533.I1S2V3	pp	229533.I1S3X8
7131	229533.I1S2V3	pp	229533.V6QWD8
7132	229533.I1S2V3	pp	229533.V6RUB1
7133	229533.I1S2V3	pp	229533.I1S3N0
7134	229533.I1RTD8	pp	229533.V6QUV7
7135	229533.I1RTD8	pp	229533.I1RUP0
7136	229533.I1RJD3	pp	229533.I1S3G4
7137	229533.I1RJD3	pp	229533.I1RN84
7138	229533.I1RJD3	pp	229533.I1RNG4
7139	229533.I1RJD3	pp	229533.V6RKY8
7140	229533.I1RJD3	pp	229533.I1RWW4
7141	229533.I1RJD3	pp	229533.V6QUV7
7142	229533.I1RJD3	pp	229533.I1RMJ3
7143	229533.I1RJD3	pp	229533.I1RYP2
7144	229533.I1RJD3	pp	229533.I1S1W8
7145	229533.I1RJD3	pp	229533.I1RZT1
7146	229533.I1RJD3	pp	229533.I1S8Z2
7147	229533.I1RJD3	pp	229533.I1RLU2
7148	229533.I1RJD3	pp	229533.I1RN46
7149	229533.I1RJ03	pp	229533.V6RUB1
7150	229533.I1RJ03	pp	229533.I1S3N0
7151	229533.I1RJ03	pp	229533.I1S2V3
7152	229533.I1RJ03	pp	229533.V6QWD8
7153	229533.I1RJ03	pp	229533.I1RZA7
7154	229533.I1RJ03	pp	229533.I1RZ29

7155 229533.I1RJ03 pp 229533.I1RNP4
7156 229533.I1RJ03 pp 229533.I1RLQ5
7157 229533.I1RJ03 pp 229533.I1RMU5
7158 229533.I1RJ03 pp 229533.I1RKU0
7159 229533.I1RJ03 pp 229533.I1S3X8
7160 229533.I1RJ03 pp 229533.I1S6P5
7161 229533.I1RYU3 pp 229533.I1RZT1
7162 229533.I1RYH4 pp 229533.I1S540
7163 229533.A0A0E0S8J8 pp 229533.A0A1C3YLW6
7164 229533.A0A0E0S8J8 pp 229533.I1S3X8
7165 229533.A0A0E0S8J8 pp 229533.I1S6P5
7166 229533.A0A0E0S8J8 pp 229533.I1RZ29
7167 229533.A0A0E0S8J8 pp 229533.I1RZA7
7168 229533.A0A0E0S8J8 pp 229533.I1S3N0
7169 229533.A0A0E0S8J8 pp 229533.V6RUB1
7170 229533.A0A0E0S8J8 pp 229533.I1RJ03
7171 229533.A0A0E0S8J8 pp 229533.I1RKU0
7172 229533.A0A0E0S8J8 pp 229533.I1REA5
7173 229533.A0A0E0S8J8 pp 229533.A0A1C3YK58
7174 229533.A0A0E0S8J8 pp 229533.I1RLQ5
7175 229533.A0A0E0S8J8 pp 229533.I1RMU5
7176 229533.A0A0E0S8J8 pp 229533.I1RNP4
7177 229533.A0A0E0S8J8 pp 229533.I1RIA1
7178 229533.A0A0E0S8J8 pp 229533.V6QWD8
7179 229533.A0A0E0S8J8 pp 229533.I1RHT4
7180 229533.A0A0E0S8J8 pp 229533.I1S2V3
7181 229533.I1REA5 pp 229533.I1RJ03
7182 229533.I1REA5 pp 229533.V6QWD8
7183 229533.I1REA5 pp 229533.I1RKU0
7184 229533.I1REA5 pp 229533.I1S6P5
7185 229533.I1REA5 pp 229533.I1RIA1
7186 229533.I1REA5 pp 229533.I1RZ29
7187 229533.I1REA5 pp 229533.I1RLQ5
7188 229533.I1REA5 pp 229533.I1RNP4
7189 229533.I1REA5 pp 229533.I1S3X8
7190 229533.I1REA5 pp 229533.I1S2V3
7191 229533.I1REA5 pp 229533.I1RMU5
7192 229533.I1REA5 pp 229533.I1S3N0
7193 229533.I1REA5 pp 229533.V6RUB1
7194 229533.I1REA5 pp 229533.I1RZA7
7195 229533.I1REA5 pp 229533.I1RHT4
7196 229533.I1RKU0 pp 229533.I1S2V3
7197 229533.I1RKU0 pp 229533.I1RNP4
7198 229533.I1RKU0 pp 229533.I1RMU5
7199 229533.I1RKU0 pp 229533.V6QWD8
7200 229533.I1RKU0 pp 229533.V6RUB1
7201 229533.I1RKU0 pp 229533.I1RLQ5
7202 229533.I1RKU0 pp 229533.I1RZ29
7203 229533.I1RKU0 pp 229533.I1RZA7
7204 229533.I1RKU0 pp 229533.I1S3N0
7205 229533.I1RKU0 pp 229533.I1S3X8
7206 229533.I1RKU0 pp 229533.I1S6P5
7207 229533.A0A098DFX9 pp 229533.V6QUV7
7208 229533.A0A098DFX9 pp 229533.V6RKY8
7209 229533.A0A098DFX9 pp 229533.I1RZT1
7210 229533.A0A098DFX9 pp 229533.I1RLU2
7211 229533.A0A098DFX9 pp 229533.I1RBA4
7212 229533.A0A098DFX9 pp 229533.A0A098DGQ1
7213 229533.A0A098DFX9 pp 229533.I1S3G4
7214 229533.A0A098DFX9 pp 229533.A0A1C3YLE2
7215 229533.A0A098DFX9 pp 229533.A0A1C3YLL1
7216 229533.A0A098DFX9 pp 229533.I1RN84
7217 229533.A0A098DFX9 pp 229533.I1RYP2
7218 229533.A0A098DFX9 pp 229533.A0A0E0SEH5
7219 229533.A0A098DFX9 pp 229533.A0A098DJ03
7220 229533.A0A098DFX9 pp 229533.I1RBF3
7221 229533.A0A098DFX9 pp 229533.I1RA32
7222 229533.A0A098DFX9 pp 229533.I1RMJ3
7223 229533.A0A098DFX9 pp 229533.A0A1C3YK11
7224 229533.A0A098DFX9 pp 229533.I1RNG4
7225 229533.A0A098DFX9 pp 229533.A0A1C3YKF3
7226 229533.A0A098DFX9 pp 229533.I1RAP3
7227 229533.A0A098DFX9 pp 229533.I1RN46

229533.A0A098DFX9 pp 229533.I1S8Z2
229533.A0A098DFX9 pp 229533.I1RC11
229533.A0A098DFX9 pp 229533.A0A1C3YJL7
229533.A0A098DFX9 pp 229533.I1S1W8
229533.A0A098DFC7 pp 229533.I1RKP1
229533.A0A098DFC7 pp 229533.A0A098DJ03
229533.A0A098DFC7 pp 229533.I1S1F3
229533.A0A098DFC7 pp 229533.I1RIU0
229533.A0A098DFC7 pp 229533.A0A1C3YJ21
229533.A0A098DFC7 pp 229533.I1RZ60
229533.A0A098DFC7 pp 229533.I1S268
229533.A0A098DFC7 pp 229533.I1S123
229533.A0A098DFC7 pp 229533.I1S9T5
229533.A0A098DFC7 pp 229533.I1RR45
229533.A0A098DFC7 pp 229533.A0A098DRX7
229533.A0A098DFC7 pp 229533.I1RQC0
229533.A0A098DFC7 pp 229533.I1RSF1
229533.A0A098DFC7 pp 229533.A0A1C3YMN0
229533.A0A098DFC7 pp 229533.I1REW3
229533.A0A098DFC7 pp 229533.A0A1C3YKK8
229533.A0A098DFC7 pp 229533.I1RMQ6
229533.A0A098DFC7 pp 229533.I1RBU8
229533.A0A098DFC7 pp 229533.A0A1C3YKF3
229533.A0A098DFC7 pp 229533.I1RJD3
229533.A0A098DFC7 pp 229533.I1RYU3
229533.A0A098DFC7 pp 229533.I1RN52
229533.A0A098DFC7 pp 229533.I1S267
229533.A0A098DFC7 pp 229533.I1RRK5
229533.A0A098DFC7 pp 229533.A0A098DFX9
229533.I1RZG3 pp 229533.Q4HXF6
229533.I1RZG3 pp 229533.V6QUE6
229533.I1RZG3 pp 229533.Q4IJ56
229533.I1RZG3 pp 229533.I1S9U2
229533.I1RZG3 pp 229533.I1S247
229533.I1RZG3 pp 229533.O74638
229533.A0A098E091 pp 229533.I1RNP4
229533.A0A098E091 pp 229533.I1RMU5
229533.A0A098E091 pp 229533.I1S6P5
229533.A0A098E091 pp 229533.I1S3X8
229533.A0A098E091 pp 229533.I1RLQ5
229533.A0A098E091 pp 229533.A0A1C3YLW6
229533.A0A098E091 pp 229533.V6QWD8
229533.A0A098E091 pp 229533.I1RIA1
229533.A0A098E091 pp 229533.I1RZ29
229533.A0A098E091 pp 229533.I1S3N0
229533.A0A098E091 pp 229533.I1RHT4
229533.A0A098E091 pp 229533.I1RZA7
229533.A0A098E091 pp 229533.V6RUB1
229533.A0A098E091 pp 229533.I1S2V3
229533.A0A098E091 pp 229533.A0A098E320
229533.A0A098E091 pp 229533.I1RJ03
229533.A0A098E091 pp 229533.A0A0E0S8J8
229533.A0A098E091 pp 229533.I1REA5
229533.A0A098E091 pp 229533.I1RKU0
229533.A0A098E091 pp 229533.A0A1C3YK58
229533.A0A098DRP2 pp 229533.I1RW16
229533.A0A098DRP2 pp 229533.I1RHM4
229533.A0A098DRP2 pp 229533.V6RR76
229533.A0A098DRP2 pp 229533.I1RWT6
229533.A0A098DRP2 pp 229533.I1REC7
229533.A0A098DRP2 pp 229533.I1RHB0
229533.A0A098DRP2 pp 229533.A0A0E0RVF4
229533.A0A098DRP2 pp 229533.A0A098DRS5
229533.A0A098DRP2 pp 229533.I1RBL8
229533.A0A098DRP2 pp 229533.I1RQA3
229533.A0A098DRP2 pp 229533.I1RT94
229533.A0A098DRP2 pp 229533.Q4HXF6
229533.A0A098DRP2 pp 229533.I1S247
229533.A0A098DRP2 pp 229533.I1RYS6
229533.A0A098DRP2 pp 229533.I1RCB9
229533.A0A098DRP2 pp 229533.I1RLX6
229533.A0A098DRP2 pp 229533.I1RPI2
229533.A0A098DRP2 pp 229533.I1RJT9

229533.A0A098DRP2 pp 229533.I1RRT6
229533.A0A098DRP2 pp 229533.A0A1C3YJI0
229533.A0A098DRP2 pp 229533.I1REC6
229533.A0A098DRP2 pp 229533.I1S7X9
229533.A0A098DRP2 pp 229533.I1RB59
229533.A0A098DRP2 pp 229533.I1RN81
229533.A0A098DRP2 pp 229533.I1RTC2
229533.A0A098DRP2 pp 229533.I1RN31
229533.A0A098DRP2 pp 229533.I1RAY2
229533.A0A098DRP2 pp 229533.I1RZG3
229533.I1S0R2 pp 229533.I1S4S8
229533.I1S0R2 pp 229533.V6RCG5
229533.I1S0R2 pp 229533.V6QW22
229533.I1RGB6 pp 229533.I1RS17
229533.I1RGB6 pp 229533.I1S0L3
229533.I1RGB6 pp 229533.I1S0R2
229533.I1RGB6 pp 229533.I1RP20
229533.I1RGB6 pp 229533.I1RWU2
229533.I1RGB6 pp 229533.I1RUC9
229533.I1RGB6 pp 229533.I1RJV4
229533.I1RGB6 pp 229533.I1S0L4
229533.I1RGB6 pp 229533.V6RCG5
229533.I1RGB6 pp 229533.I1RQN0
229533.I1RGB6 pp 229533.I1RZZ9
229533.I1RGB6 pp 229533.I1RSI0
229533.I1RGB6 pp 229533.I1RUG3
229533.I1RGB6 pp 229533.I1RJ18
229533.I1RGB6 pp 229533.I1RZM1
229533.I1RGB6 pp 229533.I1RNV0
229533.I1RGB6 pp 229533.I1S4S8
229533.I1RGB6 pp 229533.I1RVZ9
229533.I1RGB6 pp 229533.I1RN18
229533.I1RGB6 pp 229533.I1RQE7
229533.I1RJC7 pp 229533.I1RPU8
229533.I1RJC7 pp 229533.Q4HXF0
229533.I1RJC7 pp 229533.I1RS17
229533.I1RJC7 pp 229533.I1RZ20
229533.I1RJC7 pp 229533.I1RYU5
229533.I1RJC7 pp 229533.V6R057
229533.I1RJC7 pp 229533.V6R1K4
229533.I1RS66 pp 229533.I1RZL5
229533.I1RS66 pp 229533.I1RT35
229533.I1RS66 pp 229533.V6QUE6
229533.I1RS66 pp 229533.I1RZB5
229533.A0A098DRM5 pp 229533.I1RB36
229533.A0A098DRM5 pp 229533.I1RZG7
229533.A0A098DRM5 pp 229533.I1S0R2
229533.A0A098DRM5 pp 229533.I1RGB6
229533.A0A098DRM5 pp 229533.I1RKR6
229533.I1S076 pp 229533.Q4HXF0
229533.I1S076 pp 229533.V6R1K4
229533.I1S076 pp 229533.V6R057
229533.I1S076 pp 229533.I1S2C4
229533.I1RAX8 pp 229533.I1S1Z8
229533.I1RAX8 pp 229533.I1RFZ2
229533.I1RAX8 pp 229533.I1RC35
229533.I1RAX8 pp 229533.Q4IBX0
229533.I1RAX8 pp 229533.Q4IMM0
229533.I1RAX8 pp 229533.I1RQ80
229533.I1RAX8 pp 229533.I1S1C8
229533.I1RAX8 pp 229533.Q4I0J6
229533.I1RAX8 pp 229533.I1RCM6
229533.I1RJE5 pp 229533.Q4I0J6
229533.I1RJE5 pp 229533.I1RXJ5
229533.I1RP69 pp 229533.I1S029
229533.I1RP69 pp 229533.V6RQW4
229533.I1RP69 pp 229533.V6RRH2
229533.I1RP69 pp 229533.I1RQG7
229533.I1RP69 pp 229533.I1RZ52
229533.I1RP69 pp 229533.V6RY56
229533.I1RP69 pp 229533.I1S1W8
229533.I1RP69 pp 229533.I1S4Q4
229533.I1RP69 pp 229533.I1RPI5

7374 229533.I1RP69 pp 229533.I1S3G4
7375 229533.I1RP69 pp 229533.I1S338
7376 229533.I1RP69 pp 229533.I1RSQ4
7377 229533.I1RP69 pp 229533.Q4HXF0
7378 229533.I1RP69 pp 229533.I1RYI7
7379 229533.I1RP69 pp 229533.I1SAJ3
7380 229533.I1RP69 pp 229533.I1RUP2
7381 229533.I1RP69 pp 229533.I1S5B0
7382 229533.I1RP69 pp 229533.I1RRV4
7383 229533.I1RP69 pp 229533.I1S2C4
7384 229533.I1RP69 pp 229533.V6RCZ8
7385 229533.I1RP69 pp 229533.I1S4D0
7386 229533.I1RP69 pp 229533.I1RPU8
7387 229533.I1RVJ1 pp 229533.Q4HYB8
7388 229533.I1RVJ1 pp 229533.Q4I5W5
7389 229533.I1RVJ1 pp 229533.Q4IJ11
7390 229533.I1RVJ1 pp 229533.I1RZB5
7391 229533.I1RVJ1 pp 229533.V6QWX8
7392 229533.I1RVJ1 pp 229533.Q4HYQ4
7393 229533.I1RVJ1 pp 229533.Q4IJU0
7394 229533.I1RVJ1 pp 229533.Q4I5I4
7395 229533.I1RVJ1 pp 229533.I1RZU4
7396 229533.A0A0E0RTT2 pp 229533.A0A1C3YL16
7397 229533.I1RAN7 pp 229533.I1RH68
7398 229533.I1RT00 pp 229533.I1RXT7
7399 229533.I1RT00 pp 229533.I1RWZ9
7400 229533.I1RT00 pp 229533.V6QUV7
7401 229533.I1RT00 pp 229533.V6RXW9
7402 229533.I1RT00 pp 229533.I1RX87
7403 229533.I1RT00 pp 229533.V6QU20
7404 229533.I1RXR5 pp 229533.I1RZ20
7405 229533.I1RXR5 pp 229533.Q4HXF0
7406 229533.I1RXR5 pp 229533.V6R1K4
7407 229533.I1RXR5 pp 229533.V6R057
7408 229533.I1RXR5 pp 229533.I1RYU5
7409 229533.I1S1Z9 pp 229533.P0C431
7410 229533.I1S1Z9 pp 229533.V6RXE7
7411 229533.I1S1Z9 pp 229533.I1S2B6
7412 229533.I1REF6 pp 229533.I1RT52
7413 229533.I1S0S2 pp 229533.V6RG04
7414 229533.I1S0S2 pp 229533.V6RSM7
7415 229533.A0A098DF99 pp 229533.I1SAT2
7416 229533.A0A098DF99 pp 229533.I1RF85
7417 229533.A0A098DF99 pp 229533.I1RPV8
7418 229533.A0A098DF99 pp 229533.I1RH83
7419 229533.A0A098DF99 pp 229533.A0A0E0RSI1
7420 229533.A0A098DF99 pp 229533.I1RE52
7421 229533.A0A098DF99 pp 229533.A0A1C3YLB6
7422 229533.A0A098DF99 pp 229533.I1RZ56
7423 229533.A0A098DF99 pp 229533.I1RJS1
7424 229533.A0A098DF99 pp 229533.A0A1C3YHG7
7425 229533.A0A098DF99 pp 229533.I1RNG4
7426 229533.A0A098DF99 pp 229533.I1S0A5
7427 229533.A0A098DF99 pp 229533.I1RW80
7428 229533.A0A098DF99 pp 229533.I1RN04
7429 229533.A0A098DF99 pp 229533.A0A1C3YHX6
7430 229533.A0A098DF99 pp 229533.I1RCG6
7431 229533.A0A098DF99 pp 229533.I1RJE5
7432 229533.A0A098DF99 pp 229533.A0A098E1Y1
7433 229533.A0A098DF99 pp 229533.I1S659
7434 229533.A0A098DF99 pp 229533.I1RW91
7435 229533.A0A098DF99 pp 229533.I1S267
7436 229533.A0A098DF99 pp 229533.I1RA13
7437 229533.A0A098DF99 pp 229533.I1S6H3
7438 229533.A0A098DF99 pp 229533.I1S094
7439 229533.A0A098DF99 pp 229533.I1RIB5
7440 229533.A0A098DF99 pp 229533.I1RDN3
7441 229533.A0A098DF99 pp 229533.I1S5D0
7442 229533.A0A098DF99 pp 229533.I1RCU7
7443 229533.A0A098DF99 pp 229533.I1RST9
7444 229533.A0A098DF99 pp 229533.I1RQV7
7445 229533.A0A098DF99 pp 229533.I1RPW8
7446 229533.A0A098DF99 pp 229533.I1R9F2

7447 229533.A0A098DF99 pp 229533.I1RRS6
7448 229533.A0A098DF99 pp 229533.I1RCU2
7449 229533.I1RBC0 pp 229533.I1RTB8
7450 229533.I1RBC0 pp 229533.I1RY98
7451 229533.I1RBC0 pp 229533.I1RZF7
7452 229533.I1RBC0 pp 229533.I1RJT1
7453 229533.I1RBC0 pp 229533.I1S2B0
7454 229533.I1RBC0 pp 229533.I1RZ84
7455 229533.I1RBC0 pp 229533.I1RM10
7456 229533.I1RBC0 pp 229533.I1RGD5
7457 229533.I1RBC0 pp 229533.Q5I2J3
7458 229533.I1RBC0 pp 229533.I1RJP3
7459 229533.I1RRL9 pp 229533.I1RZG3
7460 229533.I1RRL9 pp 229533.I1RW25
7461 229533.I1RRL9 pp 229533.I1RX25
7462 229533.I1RRL9 pp 229533.I1RRM5
7463 229533.I1RRL9 pp 229533.I1RT28
7464 229533.I1RRL9 pp 229533.I1RZZ9
7465 229533.I1RRL9 pp 229533.I1RTC7
7466 229533.I1RRL9 pp 229533.I1RT40
7467 229533.I1RRL9 pp 229533.I1S0L4
7468 229533.I1RRL9 pp 229533.I1RS17
7469 229533.I1RRL9 pp 229533.I1RWJ8
7470 229533.I1RRL9 pp 229533.I1RZW8
7471 229533.I1RRL9 pp 229533.I1RW46
7472 229533.I1RRL9 pp 229533.I1S5R1
7473 229533.I1RRL9 pp 229533.I1RSQ7
7474 229533.I1RRL9 pp 229533.V6RIV9
7475 229533.I1RRL9 pp 229533.V6QW22
7476 229533.I1RRL9 pp 229533.I1S0L3
7477 229533.I1RRL9 pp 229533.I1RWK3
7478 229533.I1RRL9 pp 229533.V6RCG5
7479 229533.I1RRL9 pp 229533.I1RVQ0
7480 229533.I1RRL9 pp 229533.I1RZM8
7481 229533.I1RRL9 pp 229533.I1RW99
7482 229533.I1RRL9 pp 229533.I1RUG3
7483 229533.I1RRL9 pp 229533.I1RXF0
7484 229533.A0A1C3YI43 pp 229533.V6RG79
7485 229533.A0A1C3YI43 pp 229533.A0A1C3YII1
7486 229533.A0A1C3YI75 pp 229533.I1S230
7487 229533.A0A1C3YI75 pp 229533.I1REK1
7488 229533.I1RL12 pp 229533.I1RZ02
7489 229533.I1RL12 pp 229533.I1RZH4
7490 229533.I1RL12 pp 229533.I1RMT4
7491 229533.I1RL12 pp 229533.I1RN65
7492 229533.I1RFM0 pp 229533.I1S540
7493 229533.I1RFM0 pp 229533.I1RK01
7494 229533.I1RFM0 pp 229533.I1RWJ3
7495 229533.I1RFM0 pp 229533.I1RYH4
7496 229533.I1RFM0 pp 229533.I1RT93
7497 229533.I1RFM0 pp 229533.I1RX53
7498 229533.I1RFM0 pp 229533.I1RVL1
7499 229533.I1RLP4 pp 229533.I1SAS4
7500 229533.I1RLP4 pp 229533.I1RTN1
7501 229533.I1RLP4 pp 229533.I1S1G0
7502 229533.I1RLP4 pp 229533.I1RQW2
7503 229533.I1RLP4 pp 229533.I1S1G1
7504 229533.I1RLP4 pp 229533.I1RTV7
7505 229533.I1RLP4 pp 229533.I1RTW1
7506 229533.I1RLP4 pp 229533.I1S1F9
7507 229533.I1RJR0 pp 229533.Q4IBU4
7508 229533.I1RJR0 pp 229533.I1RJT1
7509 229533.I1R9G4 pp 229533.I1RS17
7510 229533.I1R9G4 pp 229533.I1RCB4
7511 229533.I1R9G4 pp 229533.V6R057
7512 229533.I1R9G4 pp 229533.I1RPU8
7513 229533.I1R9G4 pp 229533.I1RZ20
7514 229533.I1R9G4 pp 229533.V6R1K4
7515 229533.I1R9G4 pp 229533.I1RYU5
7516 229533.I1R9G4 pp 229533.Q4HXF0
7517 229533.I1RDG9 pp 229533.I1RRM9
7518 229533.I1RDG9 pp 229533.I1RMT4
7519 229533.I1RDG9 pp 229533.I1RMB3

229533.I1RDG9 pp 229533.I1RP28
229533.I1RDG9 pp 229533.I1RZ02
229533.I1RDG9 pp 229533.I1RSF2
229533.I1RDG9 pp 229533.I1RNY7
229533.I1RDG9 pp 229533.I1RJZ7
229533.I1RDG9 pp 229533.I1RJS2
229533.I1RDG9 pp 229533.I1RXZ4
229533.I1RDG9 pp 229533.I1RPP7
229533.I1RDG9 pp 229533.I1RQG4
229533.I1RDG9 pp 229533.I1RN65
229533.I1RDG9 pp 229533.I1RQH1
229533.I1RDG9 pp 229533.I1RMQ5
229533.I1RDG9 pp 229533.I1RDH0
229533.I1RDG9 pp 229533.I1RFS6
229533.I1RDG9 pp 229533.I1RSE9
229533.I1RDG9 pp 229533.I1RW71
229533.I1RDG9 pp 229533.I1RZH4
229533.I1RDG9 pp 229533.I1RPL2
229533.A0A0E0RS12 pp 229533.I1RJQ3
229533.A0A0E0RS12 pp 229533.A0A1C3YJI7
229533.A0A0E0RS12 pp 229533.A0A0E0RVF4
229533.A0A0E0RS12 pp 229533.I1R9X8
229533.A0A0E0RS12 pp 229533.I1RA73
229533.A0A0E0RS12 pp 229533.A0A1C3YMH3
229533.A0A098DR83 pp 229533.A0A1C3YJI7
229533.A0A098DR83 pp 229533.A0A0E0RS12
229533.A0A098DR83 pp 229533.I1RJQ3
229533.A0A0E0S9Y6 pp 229533.I1RT35
229533.A0A0E0S9Y6 pp 229533.I1RZM1
229533.A0A0E0S9Y6 pp 229533.I1RZH4
229533.A0A0E0S9Y6 pp 229533.I1RQ81
229533.A0A0E0S9Y6 pp 229533.Q4IAA0
229533.A0A0E0S9Y6 pp 229533.Q4HVW2
229533.A0A0E0S9Y6 pp 229533.I1RW71
229533.A0A0E0S9Y6 pp 229533.I1RRU9
229533.A0A0E0S9Y6 pp 229533.I1RN61
229533.A0A0E0S9Y6 pp 229533.I1RFT2
229533.A0A0E0S9Y6 pp 229533.I1RZS7
229533.A0A0E0S9Y6 pp 229533.I1RNB8
229533.A0A0E0S9Y6 pp 229533.V6RTJ8
229533.A0A0E0S9Y6 pp 229533.I1S6F1
229533.A0A0E0S9Y6 pp 229533.I1RE00
229533.A0A0E0S9Y6 pp 229533.A0A1C3YIA8
229533.A0A0E0S9Y6 pp 229533.I1RSM2
229533.A0A0E0S9Y6 pp 229533.I1RCI8
229533.A0A0E0S9Y6 pp 229533.I1S0N4
229533.A0A0E0S9Y6 pp 229533.I1RWD7
229533.A0A0E0S9Y6 pp 229533.A0A1C3YMI5
229533.A0A0E0S9Y6 pp 229533.I1RN65
229533.A0A0E0S9Y6 pp 229533.I1RRV1
229533.A0A0E0S9Y6 pp 229533.I1RFQ7
229533.A0A0E0S9Y6 pp 229533.I1RB53
229533.A0A0E0S9Y6 pp 229533.Q4HZ68
229533.A0A0E0S9Y6 pp 229533.I1R9X2
229533.A0A0E0S9Y6 pp 229533.I1RCM3
229533.A0A0E0S9Y6 pp 229533.I1RTH6
229533.A0A0E0S9Y6 pp 229533.I1RAU7
229533.A0A0E0S9Y6 pp 229533.I1RPU3
229533.A0A0E0S9Y6 pp 229533.I1RN67
229533.A0A0E0S9Y6 pp 229533.A0A0E0SF67
229533.A0A0E0S9Y6 pp 229533.I1S0X5
229533.A0A0E0S9Y6 pp 229533.I1RSV2
229533.A0A0E0S9Y6 pp 229533.I1RM82
229533.A0A0E0S9Y6 pp 229533.Q4IAS1
229533.A0A0E0S9Y6 pp 229533.I1RNN1
229533.A0A0E0S9Y6 pp 229533.I1RP27
229533.A0A0E0S9Y6 pp 229533.I1RCJ7
229533.A0A0E0S9Y6 pp 229533.I1RJZ7
229533.A0A0E0S6V8 pp 229533.A0A1C3YL54
229533.A0A0E0S6V8 pp 229533.I1RP86
229533.A0A0E0S6V8 pp 229533.A0A0E0SFN0
229533.A0A0E0S6V8 pp 229533.I1RXD7
229533.A0A0E0S6V8 pp 229533.I1RX46

7593	229533.A0A0E0S6V8	pp	229533.I1RDE6
7594	229533.A0A0E0S6V8	pp	229533.I1RCU2
7595	229533.A0A0E0S6V8	pp	229533.I1S4S7
7596	229533.A0A0E0S6V8	pp	229533.A0A1C3YK13
7597	229533.A0A0E0S6V8	pp	229533.I1RRX5
7598	229533.A0A0E0S6V8	pp	229533.I1RCM5
7599	229533.A0A0E0S6V8	pp	229533.I1RY12
7600	229533.A0A0E0S6V8	pp	229533.I1RJU3
7601	229533.A0A0E0S6V8	pp	229533.A0A1C3YN30
7602	229533.A0A0E0S6V8	pp	229533.I1S1J7
7603	229533.A0A0E0S6V8	pp	229533.Q4IJK2
7604	229533.A0A0E0S6V8	pp	229533.I1RSQ7
7605	229533.A0A0E0S6V8	pp	229533.I1S8C1
7606	229533.A0A0E0S6V8	pp	229533.A0A1C3YHI6
7607	229533.A0A0E0S6V8	pp	229533.I1RR24
7608	229533.A0A0E0S6V8	pp	229533.I1RD52
7609	229533.A0A0E0S6V8	pp	229533.V6R1K4
7610	229533.A0A0E0S6V8	pp	229533.I1RXV9
7611	229533.A0A0E0S6V8	pp	229533.I1RMG7
7612	229533.A0A0E0S6V8	pp	229533.I1S9F1
7613	229533.A0A0E0S6V8	pp	229533.I1RJY7
7614	229533.A0A0E0S6V8	pp	229533.I1S4Q4
7615	229533.A0A0E0S6V8	pp	229533.I1R9Y4
7616	229533.A0A0E0S6V8	pp	229533.A0A0E0SMR8
7617	229533.A0A0E0S6V8	pp	229533.A0A1C3YLE2
7618	229533.A0A0E0S6V8	pp	229533.I1S1W8
7619	229533.A0A0E0S6V8	pp	229533.I1RXW3
7620	229533.A0A0E0S6V8	pp	229533.A0A1C3YJW9
7621	229533.A0A0E0S6V8	pp	229533.I1RBE4
7622	229533.A0A0E0S6V8	pp	229533.A0A1C3YKR5
7623	229533.A0A0E0S6V8	pp	229533.I1RCR5
7624	229533.A0A0E0S6V8	pp	229533.V6R3J1
7625	229533.A0A0E0S6V8	pp	229533.A0A1C3YKV3
7626	229533.A0A0E0S6V8	pp	229533.I1REK8
7627	229533.A0A0E0S6V8	pp	229533.A0A1C3YJR3
7628	229533.A0A0E0S6V8	pp	229533.I1RBE7
7629	229533.A0A0E0S6V8	pp	229533.I1RWF5
7630	229533.A0A0E0S6V8	pp	229533.A0A1C3YLA6
7631	229533.A0A0E0S6V8	pp	229533.I1RA62
7632	229533.A0A0E0S6V8	pp	229533.I1RKB2
7633	229533.A0A0E0S6V8	pp	229533.I1RMJ3
7634	229533.A0A0E0S6V8	pp	229533.I1RUY6
7635	229533.A0A0E0S6V8	pp	229533.I1RFT1
7636	229533.A0A0E0S6V8	pp	229533.I1RAQ4
7637	229533.A0A0E0S6V8	pp	229533.Q4HXF0
7638	229533.A0A0E0S6V8	pp	229533.A0A1C3YHS4
7639	229533.A0A0E0S6V8	pp	229533.I1RYK0
7640	229533.A0A0E0S6V8	pp	229533.I1RSQ4
7641	229533.A0A0E0S6V8	pp	229533.V6RUY9
7642	229533.A0A0E0S6V8	pp	229533.I1RPP3
7643	229533.A0A0E0S6V8	pp	229533.A0A1C3YI20
7644	229533.A0A0E0S6V8	pp	229533.I1RMG6
7645	229533.A0A0E0S6V8	pp	229533.I1RZ39
7646	229533.A0A0E0S6V8	pp	229533.I1RNG4
7647	229533.A0A0E0S6V8	pp	229533.A0A1C3YKM9
7648	229533.A0A0E0S6V8	pp	229533.I1RRI2
7649	229533.A0A0E0S6V8	pp	229533.A0A1C3YJ56
7650	229533.A0A0E0S6V8	pp	229533.I1RRT8
7651	229533.A0A0E0S6V8	pp	229533.I1RN67
7652	229533.A0A0E0S6V8	pp	229533.I1RJV3
7653	229533.A0A0E0S6V8	pp	229533.A0A1C3YKY0
7654	229533.A0A0E0S6V8	pp	229533.I1RF85
7655	229533.A0A0E0S6V8	pp	229533.I1RY62
7656	229533.A0A0E0S6V8	pp	229533.O74638
7657	229533.A0A0E0S6V8	pp	229533.I1RNC1
7658	229533.A0A0E0S6V8	pp	229533.I1RM55
7659	229533.A0A0E0S6V8	pp	229533.A0A1C3YHU3
7660	229533.A0A0E0S6V8	pp	229533.I1RQ30
7661	229533.A0A0E0S6V8	pp	229533.I1S3G4
7662	229533.A0A0E0S6V8	pp	229533.A0A1C3YK33
7663	229533.A0A0E0S6V8	pp	229533.I1RN84
7664	229533.A0A0E0S6V8	pp	229533.I1RMC5
7665	229533.A0A0E0S6V8	pp	229533.I1S256

7666 229533.A0A0E0S6V8 pp 229533.I1RQE7
7667 229533.A0A0E0S6V8 pp 229533.A0A0E0SC46
7668 229533.A0A0E0S6V8 pp 229533.V6QVH8
7669 229533.A0A0E0S6V8 pp 229533.I1RSZ7
7670 229533.A0A0E0S6V8 pp 229533.I1S5R6
7671 229533.A0A0E0S6V8 pp 229533.I1RL04
7672 229533.A0A0E0S6V8 pp 229533.A0A1C3YLB6
7673 229533.A0A0E0S6V8 pp 229533.I1S0J1
7674 229533.A0A0E0S6V8 pp 229533.I1RCH5
7675 229533.A0A0E0S6V8 pp 229533.I1RVX0
7676 229533.A0A0E0S6V8 pp 229533.I1S3F7
7677 229533.A0A0E0S6V8 pp 229533.A0A1C3YJD4
7678 229533.A0A0E0S6V8 pp 229533.V6RM02
7679 229533.A0A0E0S6V8 pp 229533.I1RS17
7680 229533.A0A0E0S6V8 pp 229533.I1RUR8
7681 229533.A0A0E0S6V8 pp 229533.I1RPK0
7682 229533.A0A0E0S6V8 pp 229533.V6RRH2
7683 229533.A0A0E0S6V8 pp 229533.I1RE76
7684 229533.A0A0E0S6V8 pp 229533.I1RM54
7685 229533.A0A0E0S6V8 pp 229533.A0A1C3YHG7
7686 229533.A0A0E0S6V8 pp 229533.I1RYW3
7687 229533.A0A0E0S6V8 pp 229533.V6R057
7688 229533.A0A0E0S6V8 pp 229533.I1RGC3
7689 229533.A0A0E0S6V8 pp 229533.A0A1C3YMA1
7690 229533.I1RMU8 pp 229533.I1S7X9
7691 229533.I1RMU8 pp 229533.Q4IQT8
7692 229533.I1RMU8 pp 229533.V6RB81
7693 229533.I1RMU8 pp 229533.I1RNQ6
7694 229533.I1RMU8 pp 229533.I1RRT0
7695 229533.I1S6F1 pp 229533.Q4IAA0
7696 229533.A0A098DHK5 pp 229533.I1RB53
7697 229533.A0A098DHK5 pp 229533.I1RBZ8
7698 229533.A0A098DHK5 pp 229533.I1RE71
7699 229533.A0A098DHK5 pp 229533.I1RPS0
7700 229533.A0A098DHK5 pp 229533.I1S0N7
7701 229533.A0A098DHK5 pp 229533.I1RYJ9
7702 229533.A0A098DHK5 pp 229533.A0A098DPW7
7703 229533.A0A098DHK5 pp 229533.I1RXU7
7704 229533.A0A098DHK5 pp 229533.A0A1C3YII7
7705 229533.A0A098DHK5 pp 229533.A0A1C3YL25
7706 229533.A0A098DHK5 pp 229533.I1S471
7707 229533.A0A098DHK5 pp 229533.I1RAU7
7708 229533.A0A098DHK5 pp 229533.I1RN58
7709 229533.A0A098DHK5 pp 229533.I1RT35
7710 229533.A0A098DHK5 pp 229533.A0A0E0SF67
7711 229533.A0A098DHK5 pp 229533.A0A1C3YL15
7712 229533.A0A098DHK5 pp 229533.I1RLU5
7713 229533.A0A098DHK5 pp 229533.I1RQK2
7714 229533.A0A098DHK5 pp 229533.I1RWD7
7715 229533.A0A098DHK5 pp 229533.I1S246
7716 229533.A0A098DHK5 pp 229533.I1RZH4
7717 229533.A0A098DHK5 pp 229533.A0A0E0S9Y6
7718 229533.A0A098DHK5 pp 229533.I1RAS2
7719 229533.A0A098DHK5 pp 229533.Q4IAA0
7720 229533.A0A098DHK5 pp 229533.I1RNB8
7721 229533.A0A098DHK5 pp 229533.A0A1C3YM82
7722 229533.A0A098DHK5 pp 229533.I1RIS3
7723 229533.A0A098DHK5 pp 229533.I1RN48
7724 229533.A0A098DHK5 pp 229533.I1RS20
7725 229533.A0A098DHK5 pp 229533.A0A1C3YM64
7726 229533.A0A098DHK5 pp 229533.I1RN65
7727 229533.I1RVL1 pp 229533.I1RWJ3
7728 229533.I1RYK6 pp 229533.I1S2E9
7729 229533.I1RNY7 pp 229533.I1RPL2
7730 229533.I1RNY7 pp 229533.I1RZH4
7731 229533.I1RNY7 pp 229533.I1RRM9
7732 229533.I1RNY7 pp 229533.I1S1V1
7733 229533.I1RNY7 pp 229533.I1RZQ6
7734 229533.I1RNY7 pp 229533.I1RP28
7735 229533.I1RNY7 pp 229533.I1RQG4
7736 229533.I1RNY7 pp 229533.I1RQH1
7737 229533.I1RPV2 pp 229533.I1RTF5
7738 229533.I1RPV2 pp 229533.I1S444

229533.I1RXU7	pp	229533.Q4IAA0
229533.I1RXU7	pp	229533.V6RTJ8
229533.I1RXU7	pp	229533.Q4HZ68
229533.I1RXU7	pp	229533.Q4HVW2
229533.I1RXU7	pp	229533.I1RZH4
229533.I1RXU7	pp	229533.I1S0X5
229533.I1RXU7	pp	229533.I1RZS7
229533.I1RXU7	pp	229533.Q4IAS1
229533.I1RXU7	pp	229533.I1RZM1
229533.I1RXU7	pp	229533.I1S6F1
229533.I1RXU7	pp	229533.I1S0N4
229533.I1RTP4	pp	229533.I1S574
229533.I1RTP4	pp	229533.I1S984
229533.A0A1C3YIB2	pp	229533.I1RD73
229533.I1RY62	pp	229533.I1S8C1
229533.I1RY62	pp	229533.I1S256
229533.I1RY62	pp	229533.I1S5R6
229533.I1RY62	pp	229533.I1S338
229533.I1RY62	pp	229533.V6R1K4
229533.I1RY62	pp	229533.Q4IJK2
229533.I1RY62	pp	229533.I1S1J7
229533.I1RY62	pp	229533.V6RM02
229533.I1RY62	pp	229533.I1S4Q4
229533.I1RY62	pp	229533.I1S9F1
229533.I1RY62	pp	229533.V6QVH8
229533.I1RY62	pp	229533.V6RRH2
229533.I1RY62	pp	229533.I1S0J1
229533.I1RY62	pp	229533.V6R057
229533.I1RY62	pp	229533.I1S1W8
229533.I1RY62	pp	229533.I1S4S7
229533.I1RY62	pp	229533.Q4HXF0
229533.I1RY62	pp	229533.V6R3J1
229533.I1RY62	pp	229533.I1RYK0
229533.I1RY62	pp	229533.I1S3F7
229533.I1RY62	pp	229533.V6RUY9
229533.I1RY62	pp	229533.I1RYW3
229533.I1RY62	pp	229533.I1RZ39
229533.I1RY62	pp	229533.O74638
229533.I1RY62	pp	229533.I1S3G4
229533.I1S6H1	pp	229533.V6RAM6
229533.I1S6H1	pp	229533.V6R756
229533.I1S6H1	pp	229533.V6R1K4
229533.I1S6H1	pp	229533.V6QU99
229533.I1S6H1	pp	229533.Q4HXF0
229533.I1S6H1	pp	229533.V6R5D2
229533.I1S6H1	pp	229533.V6R057
229533.I1S6H1	pp	229533.V6R2Z2
229533.I1S6H1	pp	229533.I1SAI4
229533.I1S6H1	pp	229533.V6R0W1
229533.I1RMB3	pp	229533.I1RQG4
229533.I1RMB3	pp	229533.I1RZH4
229533.I1RMB3	pp	229533.I1RPL2
229533.I1RMB3	pp	229533.I1RQH1
229533.I1RMB3	pp	229533.I1RN65
229533.I1RMB3	pp	229533.I1RRM9
229533.I1RMB3	pp	229533.I1RP28
229533.I1RMB3	pp	229533.I1RNY7
229533.I1RMB3	pp	229533.I1RMQ5
229533.I1RYR6	pp	229533.I1RZ20
229533.I1RYR6	pp	229533.Q4HXF0
229533.I1RYR6	pp	229533.I1RYU5
229533.I1RYR6	pp	229533.V6R1K4
229533.I1RYR6	pp	229533.V6R057
229533.I1RYR6	pp	229533.I1S2C4
229533.A0A0E0SE71	pp	229533.I1S0Y2
229533.A0A0E0SE71	pp	229533.A0A1C3YII1
229533.A0A0E0SE71	pp	229533.I1RLE2
229533.A0A0E0SE71	pp	229533.I1RPI4
229533.A0A0E0SE71	pp	229533.I1RBW6
229533.A0A0E0SE71	pp	229533.V6RG79
229533.I1RD00	pp	229533.I1S0R2
229533.I1RD00	pp	229533.I1RGB6
229533.I1S256	pp	229533.V6R1K4

7812	229533.I1S256	pp	229533.I1S4Q4
7813	229533.I1S256	pp	229533.I1S4S7
7814	229533.I1S256	pp	229533.I1S8C1
7815	229533.I1S256	pp	229533.I1S9F1
7816	229533.I1S256	pp	229533.V6RUY9
7817	229533.I1S256	pp	229533.V6RRH2
7818	229533.I1S256	pp	229533.I1S338
7819	229533.I1S256	pp	229533.Q4IJK2
7820	229533.I1S256	pp	229533.I1S3F7
7821	229533.I1S256	pp	229533.V6QVH8
7822	229533.I1S256	pp	229533.Q4HXF0
7823	229533.I1S256	pp	229533.V6R057
7824	229533.I1S256	pp	229533.I1S5R6
7825	229533.I1S256	pp	229533.V6R3J1
7826	229533.I1S256	pp	229533.O74638
7827	229533.I1S256	pp	229533.V6RM02
7828	229533.I1S256	pp	229533.I1S3G4
7829	229533.I1RE42	pp	229533.I1S2J6
7830	229533.I1RE42	pp	229533.Q4I7L0
7831	229533.I1RE42	pp	229533.I1RYV4
7832	229533.I1RE42	pp	229533.I1RXV9
7833	229533.I1RCZ4	pp	229533.I1RWA5
7834	229533.I1RCZ4	pp	229533.I1RWA9
7835	229533.I1RSB8	pp	229533.I1RZH4
7836	229533.A0A1C3YMN3	pp	229533.I1RE06
7837	229533.A0A1C3YMN3	pp	229533.I1RE42
7838	229533.I1S5G3	pp	229533.V6R057
7839	229533.I1S5G3	pp	229533.V6R1K4
7840	229533.I1S5G3	pp	229533.Q4HXF0
7841	229533.I1RBW4	pp	229533.V6QUE6
7842	229533.I1RBW4	pp	229533.I1RC24
7843	229533.I1RBW4	pp	229533.I1RT35
7844	229533.I1RBW4	pp	229533.I1RG11
7845	229533.I1RBW4	pp	229533.I1RFQ1
7846	229533.I1RBW4	pp	229533.I1RK71
7847	229533.I1RBW4	pp	229533.I1RZL5
7848	229533.I1RBW4	pp	229533.I1RNB8
7849	229533.I1RBW4	pp	229533.I1RK79
7850	229533.I1RBW4	pp	229533.I1REF5
7851	229533.I1RBW4	pp	229533.I1RJL2
7852	229533.I1RBW4	pp	229533.I1RZB5
7853	229533.I1RBW4	pp	229533.I1RR25
7854	229533.I1RBW4	pp	229533.I1RE31
7855	229533.I1RBW4	pp	229533.I1RN03
7856	229533.I1RBW4	pp	229533.I1RRG3
7857	229533.I1RBW4	pp	229533.I1RQZ2
7858	229533.I1RE06	pp	229533.I1RE42
7859	229533.A0A098DRD7	pp	229533.I1RE42
7860	229533.A0A098DRD7	pp	229533.A0A1C3YMN3
7861	229533.A0A098DRD7	pp	229533.I1RE06
7862	229533.A0A098DRD7	pp	229533.A0A1C3YHW3
7863	229533.I1S2C5	pp	229533.Q4I0C5
7864	229533.I1S2C5	pp	229533.V6R057
7865	229533.I1S2C5	pp	229533.V6R1K4
7866	229533.I1S2C5	pp	229533.Q4HXF0
7867	229533.I1S328	pp	229533.I1S574
7868	229533.I1S328	pp	229533.I1S984
7869	229533.I1RMT4	pp	229533.I1S0A4
7870	229533.I1RMT4	pp	229533.I1S7X9
7871	229533.I1RMT4	pp	229533.I1RP28
7872	229533.I1RMT4	pp	229533.I1RZG0
7873	229533.I1RMT4	pp	229533.Q4IPC8
7874	229533.I1RMT4	pp	229533.Q4ICG8
7875	229533.I1RMT4	pp	229533.I1RP04
7876	229533.I1RMT4	pp	229533.I1RTK5
7877	229533.I1RMT4	pp	229533.Q4INW6
7878	229533.I1RMT4	pp	229533.Q4IER0
7879	229533.I1RMT4	pp	229533.I1RZ94
7880	229533.I1RMT4	pp	229533.I1RX64
7881	229533.I1RMT4	pp	229533.I1RN49
7882	229533.I1RMT4	pp	229533.Q4I0M9
7883	229533.I1RMT4	pp	229533.I1RT41
7884	229533.I1RMT4	pp	229533.I1RXZ4

7885	229533.I1RMT4	pp	229533.I1RN65
7886	229533.I1RMT4	pp	229533.I1S0Z3
7887	229533.I1RMT4	pp	229533.V6REE9
7888	229533.I1RMT4	pp	229533.I1RWG5
7889	229533.I1RMT4	pp	229533.I1RZH4
7890	229533.I1RMT4	pp	229533.I1RPU4
7891	229533.I1RMT4	pp	229533.I1RS52
7892	229533.I1RMT4	pp	229533.Q4I5I4
7893	229533.I1RMT4	pp	229533.I1RQ73
7894	229533.I1RP21	pp	229533.I1RRG3
7895	229533.I1RP21	pp	229533.Q4I5I4
7896	229533.I1RP21	pp	229533.I1RZB5
7897	229533.I1RP21	pp	229533.I1RRT0
7898	229533.I1RP21	pp	229533.I1RR25
7899	229533.I1RP21	pp	229533.Q4I5W5
7900	229533.I1RP21	pp	229533.I1RWL3
7901	229533.I1RP21	pp	229533.I1S2C4
7902	229533.I1RH65	pp	229533.I1RVA9
7903	229533.I1RH65	pp	229533.I1RU48
7904	229533.I1RH65	pp	229533.I1S3U8
7905	229533.I1RH65	pp	229533.I1RIE1
7906	229533.I1RYW6	pp	229533.I1RYX6
7907	229533.I1RYW6	pp	229533.Q4HXF0
7908	229533.I1RYW6	pp	229533.I1RZ20
7909	229533.I1RYW6	pp	229533.I1S9D4
7910	229533.I1RYW6	pp	229533.I1RZ52
7911	229533.I1RYW6	pp	229533.I1S2E2
7912	229533.I1RYW6	pp	229533.V6RSF3
7913	229533.I1RHT3	pp	229533.V6R1K4
7914	229533.I1RHT3	pp	229533.V6RAM6
7915	229533.I1RHT3	pp	229533.V6QU99
7916	229533.I1RHT3	pp	229533.V6R756
7917	229533.I1RHT3	pp	229533.I1RYU5
7918	229533.I1RHT3	pp	229533.I1SAI4
7919	229533.I1RHT3	pp	229533.V6R0W1
7920	229533.I1RHT3	pp	229533.I1S2C4
7921	229533.I1RHT3	pp	229533.I1RQX9
7922	229533.I1RHT3	pp	229533.I1RS17
7923	229533.I1RHT3	pp	229533.I1RZ20
7924	229533.I1RHT3	pp	229533.I1RPU8
7925	229533.I1RHT3	pp	229533.V6R2Z2
7926	229533.I1RHT3	pp	229533.Q4HXF0
7927	229533.I1RHT3	pp	229533.V6R5D2
7928	229533.I1RHT3	pp	229533.V6R057
7929	229533.A0A1C3YHT1	pp	229533.A0A1C3YN30
7930	229533.A0A1C3YHT1	pp	229533.V6R057
7931	229533.A0A1C3YHT1	pp	229533.I1RYU5
7932	229533.A0A1C3YHT1	pp	229533.A0A1C3YKJ8
7933	229533.A0A1C3YHT1	pp	229533.I1RCB4
7934	229533.A0A1C3YHT1	pp	229533.I1RPU8
7935	229533.A0A1C3YHT1	pp	229533.I1RS17
7936	229533.A0A1C3YHT1	pp	229533.V6R1K4
7937	229533.A0A1C3YHT1	pp	229533.Q4HXF0
7938	229533.A0A1C3YHT1	pp	229533.I1RZ20
7939	229533.I1RGG8	pp	229533.I1RUY6
7940	229533.I1RGG8	pp	229533.I1RZ25
7941	229533.I1RGG8	pp	229533.I1RX89
7942	229533.A0A1C3YMA8	pp	229533.Q4HXF0
7943	229533.A0A1C3YMA8	pp	229533.I1RCB4
7944	229533.A0A1C3YMA8	pp	229533.I1RZ20
7945	229533.A0A1C3YMA8	pp	229533.V6R057
7946	229533.A0A1C3YMA8	pp	229533.I1RPU8
7947	229533.A0A1C3YMA8	pp	229533.I1RYU5
7948	229533.A0A1C3YMA8	pp	229533.I1RS17
7949	229533.A0A1C3YMA8	pp	229533.V6R1K4
7950	229533.A0A1C3YMA8	pp	229533.A0A1C3YN30
7951	229533.A0A1C3YKH8	pp	229533.I1RQS9
7952	229533.A0A1C3YKH8	pp	229533.A0A1C3YKS6
7953	229533.A0A1C3YKH8	pp	229533.A0A1C3YKM8
7954	229533.A0A1C3YKH8	pp	229533.V6R1K4
7955	229533.A0A1C3YKH8	pp	229533.I1RPN4
7956	229533.A0A1C3YKH8	pp	229533.V6RBN2
7957	229533.A0A1C3YKH8	pp	229533.I1RWU3

7958 229533.A0A1C3YKH8 pp 229533.I1RB39
7959 229533.A0A1C3YKH8 pp 229533.V6RTJ8
7960 229533.A0A1C3YKH8 pp 229533.V6QV38
7961 229533.A0A1C3YKH8 pp 229533.I1RAZ3
7962 229533.A0A1C3YKH8 pp 229533.I1RB36
7963 229533.A0A1C3YKH8 pp 229533.I1S0B7
7964 229533.A0A1C3YKH8 pp 229533.I1RM35
7965 229533.A0A1C3YKH8 pp 229533.I1S4Q4
7966 229533.A0A1C3YKH8 pp 229533.I1RNF5
7967 229533.A0A1C3YKH8 pp 229533.I1RCL4
7968 229533.A0A1C3YKH8 pp 229533.I1RA90
7969 229533.A0A1C3YKH8 pp 229533.I1RF82
7970 229533.A0A1C3YKH8 pp 229533.A0A1C3YN30
7971 229533.A0A1C3YKH8 pp 229533.Q4HXF0
7972 229533.A0A1C3YKH8 pp 229533.I1RKR6
7973 229533.A0A1C3YKH8 pp 229533.A0A1C3YKU8
7974 229533.A0A1C3YKH8 pp 229533.I1RMQ4
7975 229533.A0A1C3YKH8 pp 229533.I1RBI1
7976 229533.A0A1C3YKH8 pp 229533.V6R057
7977 229533.A0A1C3YKH8 pp 229533.O74638
7978 229533.A0A1C3YKH8 pp 229533.I1RVL8
7979 229533.A0A1C3YKH8 pp 229533.I1RRV4
7980 229533.A0A1C3YKH8 pp 229533.I1RZZ9
7981 229533.A0A1C3YKH8 pp 229533.V6QUE6
7982 229533.A0A1C3YKH8 pp 229533.I1S216
7983 229533.A0A1C3YKH8 pp 229533.V6RRH2
7984 229533.A0A1C3YKH8 pp 229533.I1RSQ4
7985 229533.A0A1C3YKH8 pp 229533.A0A1C3YKY0
7986 229533.A0A1C3YKH8 pp 229533.I1RTD3
7987 229533.A0A1C3YKH8 pp 229533.I1S2C4
7988 229533.A0A1C3YKH8 pp 229533.I1RN67
7989 229533.A0A1C3YKH8 pp 229533.I1RX22
7990 229533.A0A1C3YKH8 pp 229533.I1RXF0
7991 229533.A0A1C3YKH8 pp 229533.I1RB44
7992 229533.A0A1C3YKH8 pp 229533.I1RCW6
7993 229533.A0A1C3YKH8 pp 229533.I1RWE8
7994 229533.A0A1C3YKH8 pp 229533.I1RZM8
7995 229533.A0A1C3YKH8 pp 229533.A0A1C3YLB6
7996 229533.A0A1C3YKH8 pp 229533.I1RE01
7997 229533.I1RBA5 pp 229533.I1RY84
7998 229533.I1RBA5 pp 229533.I1RYD8
7999 229533.I1RBA5 pp 229533.V6RGI1
8000 229533.I1RL78 pp 229533.I1SAS1
8001 229533.I1RL78 pp 229533.I1RVE0
8002 229533.I1RL78 pp 229533.I1SAA1
8003 229533.I1RL78 pp 229533.I1RS80
8004 229533.I1RL78 pp 229533.I1RNX0
8005 229533.I1RL78 pp 229533.I1S341
8006 229533.I1RFK7 pp 229533.I1RKI3
8007 229533.I1RFK7 pp 229533.I1RKI7
8008 229533.I1RFK7 pp 229533.I1RZ52
8009 229533.I1RFK7 pp 229533.I1RQG7
8010 229533.I1RFK7 pp 229533.I1S029
8011 229533.I1RFK7 pp 229533.I1RSQ4
8012 229533.I1RFK7 pp 229533.Q4HXF0
8013 229533.I1RFK7 pp 229533.I1S338
8014 229533.I1RFK7 pp 229533.I1RPI5
8015 229533.I1RFK7 pp 229533.I1S3G4
8016 229533.I1RFK7 pp 229533.V6RCZ8
8017 229533.I1RFK7 pp 229533.I1RNG4
8018 229533.I1RFK7 pp 229533.I1S1W8
8019 229533.I1RFK7 pp 229533.I1RN67
8020 229533.I1RFK7 pp 229533.I1RPU8
8021 229533.I1RFK7 pp 229533.I1RJT5
8022 229533.I1RFK7 pp 229533.I1S4D0
8023 229533.I1RFK7 pp 229533.I1SAJ3
8024 229533.I1RFK7 pp 229533.I1S2C4
8025 229533.I1RFK7 pp 229533.I1S5B0
8026 229533.I1RFK7 pp 229533.V6RY56
8027 229533.I1RFK7 pp 229533.I1RRV4
8028 229533.I1RFK7 pp 229533.I1RYI7
8029 229533.I1RFK7 pp 229533.I1RIY0
8030 229533.I1RFK7 pp 229533.I1RM55

8031	229533.I1RFK7	pp	229533.I1S4Q4
8032	229533.I1RFK7	pp	229533.V6RRH2
8033	229533.I1RFK7	pp	229533.I1RUP2
8034	229533.I1RFK7	pp	229533.V6RQW4
8035	229533.I1RVE0	pp	229533.I1SAS1
8036	229533.I1RVE0	pp	229533.I1S341
8037	229533.I1RVE0	pp	229533.I1SAA1
8038	229533.A0A1C3YK70	pp	229533.A0A1C3YLS3
8039	229533.I1SAA1	pp	229533.I1SAS1
8040	229533.I1S341	pp	229533.I1SAS1
8041	229533.I1S341	pp	229533.I1SAA1
8042	229533.I1RCW7	pp	229533.I1RMP2
8043	229533.I1RCW7	pp	229533.I1RCY0
8044	229533.I1RCW7	pp	229533.I1RZ02
8045	229533.I1RCW7	pp	229533.I1RE19
8046	229533.I1RCW7	pp	229533.I1RRF9
8047	229533.I1RCW7	pp	229533.I1RMT4
8048	229533.I1RCW7	pp	229533.I1RZH4
8049	229533.I1RCW7	pp	229533.I1RTA3
8050	229533.I1RCW7	pp	229533.I1RXM6
8051	229533.I1RCW7	pp	229533.I1RHI1
8052	229533.I1RJP5	pp	229533.I1RNX0
8053	229533.I1RJP5	pp	229533.I1RS80
8054	229533.I1RJP5	pp	229533.I1SAS1
8055	229533.I1RJP5	pp	229533.I1RVE0
8056	229533.I1RJP5	pp	229533.I1RL78
8057	229533.I1RJP5	pp	229533.I1S341
8058	229533.I1RJP5	pp	229533.I1SAA1
8059	229533.A0A1C3YLQ9	pp	229533.I1RUJ7
8060	229533.A0A1C3YJK9	pp	229533.I1RBD6
8061	229533.A0A1C3YJK9	pp	229533.I1RZH4
8062	229533.A0A1C3YJK9	pp	229533.I1RUP2
8063	229533.A0A1C3YJK9	pp	229533.I1RN65
8064	229533.A0A1C3YJK9	pp	229533.I1RZ02
8065	229533.A0A1C3YJK9	pp	229533.I1RHI1
8066	229533.A0A1C3YJK9	pp	229533.I1RD38
8067	229533.A0A1C3YJK9	pp	229533.I1RMT4
8068	229533.A0A1C3YJK9	pp	229533.I1RCY0
8069	229533.A0A1C3YJK9	pp	229533.I1REN4
8070	229533.A0A1C3YJK9	pp	229533.A0A1C3YL99
8071	229533.A0A1C3YJK9	pp	229533.I1RBT5
8072	229533.A0A1C3YJK9	pp	229533.I1RWF5
8073	229533.I1RYE6	pp	229533.V6R057
8074	229533.I1RYE6	pp	229533.I1RZ20
8075	229533.I1RYE6	pp	229533.I1RYU5
8076	229533.I1RYE6	pp	229533.V6R1K4
8077	229533.I1RYE6	pp	229533.Q4HXF0
8078	229533.Q4ICG8	pp	229533.Q4IQC1
8079	229533.Q4ICG8	pp	229533.Q4INW6
8080	229533.I1REK4	pp	229533.V6RTJ8
8081	229533.I1REK4	pp	229533.I1RPN4
8082	229533.I1REK4	pp	229533.I1RZB2
8083	229533.I1REK4	pp	229533.I1RWF9
8084	229533.I1REK4	pp	229533.I1RW26
8085	229533.I1REK4	pp	229533.I1RZ39
8086	229533.I1REK4	pp	229533.I1RN67
8087	229533.I1REK4	pp	229533.I1RZZ9
8088	229533.I1REK4	pp	229533.I1S3S3
8089	229533.I1REK4	pp	229533.Q4HXF0
8090	229533.I1REK4	pp	229533.I1RGA6
8091	229533.I1REK4	pp	229533.I1S2H4
8092	229533.I1REK4	pp	229533.I1RPU8
8093	229533.I1REK4	pp	229533.I1S4S4
8094	229533.I1REK4	pp	229533.I1RL04
8095	229533.I1REK4	pp	229533.I1RPR4
8096	229533.I1REK4	pp	229533.I1RM67
8097	229533.I1REK4	pp	229533.V6RBN2
8098	229533.I1REK4	pp	229533.V6QUE6
8099	229533.I1REK4	pp	229533.I1RS18
8100	229533.I1RNW8	pp	229533.I1RWE1
8101	229533.I1RNW8	pp	229533.I1RY44
8102	229533.I1RNW8	pp	229533.V6RVG3
8103	229533.I1RNW8	pp	229533.I1S1W4

8104 229533.I1RNW8 pp 229533.I1RT36
8105 229533.I1RNW8 pp 229533.I1S0M2
8106 229533.I1RNW8 pp 229533.I1S0N8
8107 229533.I1RNW8 pp 229533.I1S757
8108 229533.I1RNW8 pp 229533.I1RZE4
8109 229533.I1RNW8 pp 229533.V6QV09
8110 229533.I1RNW8 pp 229533.I1RZA4
8111 229533.I1RNW8 pp 229533.I1RRY3
8112 229533.I1RNW8 pp 229533.I1S7X9
8113 229533.I1RNW8 pp 229533.I1RQ26
8114 229533.I1RNW8 pp 229533.I1SAT1
8115 229533.I1RNW8 pp 229533.V6RKE4
8116 229533.I1RNW8 pp 229533.I1RZH4
8117 229533.I1RNW8 pp 229533.I1S2E9
8118 229533.I1RNW8 pp 229533.I1RYZ9
8119 229533.I1RNW8 pp 229533.I1RWT6
8120 229533.I1RNW8 pp 229533.Q4IR18
8121 229533.I1RNW8 pp 229533.I1RRK8
8122 229533.I1RNW8 pp 229533.Q4ICG5
8123 229533.I1RNW8 pp 229533.I1RQA3
8124 229533.I1RNW8 pp 229533.I1RTD0
8125 229533.I1RNW8 pp 229533.I1S439
8126 229533.I1RNW8 pp 229533.I1RWY1
8127 229533.I1RNW8 pp 229533.I1RQA9
8128 229533.I1RNW8 pp 229533.I1RPU7
8129 229533.I1RNW8 pp 229533.I1RXU1
8130 229533.I1RNW8 pp 229533.I1RR94
8131 229533.I1RNW8 pp 229533.I1RUN8
8132 229533.I1RNW8 pp 229533.V6RQ48
8133 229533.A0A1C3YKJ8 pp 229533.I1S216
8134 229533.A0A1C3YKJ8 pp 229533.V6R057
8135 229533.A0A1C3YKJ8 pp 229533.I1RX22
8136 229533.A0A1C3YKJ8 pp 229533.I1RZ20
8137 229533.A0A1C3YKJ8 pp 229533.I1RS17
8138 229533.A0A1C3YKJ8 pp 229533.I1RPU8
8139 229533.A0A1C3YKJ8 pp 229533.I1RCB4
8140 229533.A0A1C3YKJ8 pp 229533.I1RQ05
8141 229533.A0A1C3YKJ8 pp 229533.Q4HXF0
8142 229533.A0A1C3YKJ8 pp 229533.V6R1K4
8143 229533.A0A1C3YKJ8 pp 229533.I1RZE5
8144 229533.A0A1C3YKJ8 pp 229533.I1RYU5
8145 229533.A0A1C3YKJ8 pp 229533.I1RYV4
8146 229533.A0A1C3YKJ8 pp 229533.A0A1C3YN30
8147 229533.A0A1C3YKJ8 pp 229533.I1RCL4
8148 229533.I1RTK5 pp 229533.Q4IA62
8149 229533.I1RTK5 pp 229533.I1RZ02
8150 229533.I1RTK5 pp 229533.V6RGI1
8151 229533.I1RTK5 pp 229533.I1RZH4
8152 229533.I1RHD1 pp 229533.V6R1K4
8153 229533.I1RHD1 pp 229533.I1RS17
8154 229533.I1RHD1 pp 229533.I1RYU5
8155 229533.I1RHD1 pp 229533.I1RPU8
8156 229533.I1RHD1 pp 229533.Q4HXF0
8157 229533.I1RHD1 pp 229533.I1RZ20
8158 229533.I1RHD1 pp 229533.V6R057
8159 229533.A0A0E0SBC0 pp 229533.I1RDG9
8160 229533.A0A0E0SBC0 pp 229533.A0A1C3YLE6
8161 229533.A0A0E0SBC0 pp 229533.I1RD70
8162 229533.A0A0E0SBC0 pp 229533.A0A1C3YI80
8163 229533.A0A0E0SBC0 pp 229533.I1RZH4
8164 229533.A0A0E0SBC0 pp 229533.I1RBW7
8165 229533.A0A0E0SBC0 pp 229533.I1RPL2
8166 229533.A0A0E0SBC0 pp 229533.I1S1V1
8167 229533.A0A0E0SBC0 pp 229533.A0A1C3YI45
8168 229533.A0A0E0SBC0 pp 229533.I1RMQ5
8169 229533.A0A0E0SBC0 pp 229533.I1RAM6
8170 229533.A0A0E0SBC0 pp 229533.I1RRM9
8171 229533.A0A0E0SBC0 pp 229533.I1RMB3
8172 229533.A0A0E0SBC0 pp 229533.I1RN65
8173 229533.A0A0E0SBC0 pp 229533.A0A1C3YLF4
8174 229533.A0A0E0SBC0 pp 229533.I1RQG4
8175 229533.A0A0E0SBC0 pp 229533.I1RAU1
8176 229533.A0A0E0SBC0 pp 229533.I1RP28

8177 229533.A0A0E0SBC0 pp 229533.I1RQH1
8178 229533.V6RG79 pp 229533.V6RLM7
8179 229533.A0A0E0SF67 pp 229533.I1RXU7
8180 229533.A0A0E0SF67 pp 229533.I1RLU5
8181 229533.A0A0E0SF67 pp 229533.I1S788
8182 229533.A0A0E0SF67 pp 229533.I1RN58
8183 229533.A0A0E0SF67 pp 229533.I1RT35
8184 229533.A0A0E0SF67 pp 229533.I1RB53
8185 229533.A0A0E0SF67 pp 229533.I1S6F1
8186 229533.A0A0E0SF67 pp 229533.I1RZH4
8187 229533.A0A0E0SF67 pp 229533.Q4IAA0
8188 229533.A0A0E0SF67 pp 229533.I1RN65
8189 229533.A0A0E0SF67 pp 229533.I1RRJ6
8190 229533.A0A0E0SF67 pp 229533.A0A1C3YII7
8191 229533.A0A0E0SF67 pp 229533.A0A1C3YL15
8192 229533.A0A0E0SF67 pp 229533.A0A1C3YM82
8193 229533.A0A0E0SF67 pp 229533.I1S471
8194 229533.A0A0E0SF67 pp 229533.I1RAS2
8195 229533.A0A0E0SF67 pp 229533.I1RS20
8196 229533.A0A0E0SF67 pp 229533.I1RZS7
8197 229533.A0A0E0SF67 pp 229533.I1RNB8
8198 229533.A0A0E0SF67 pp 229533.I1RPS0
8199 229533.A0A0E0SF67 pp 229533.I1RE71
8200 229533.A0A0E0SF67 pp 229533.I1RBN9
8201 229533.A0A0E0SF67 pp 229533.I1REK3
8202 229533.I1RNL5 pp 229533.I1RRX5
8203 229533.I1RNL5 pp 229533.I1RX00
8204 229533.I1RNL5 pp 229533.I1RS50
8205 229533.I1RNL5 pp 229533.V6R3J1
8206 229533.Q4IER0 pp 229533.V6QWZ7
8207 229533.Q4I061 pp 229533.Q4IJ79
8208 229533.I1RTJ7 pp 229533.V6R2Z2
8209 229533.I1RTJ7 pp 229533.I1RZ20
8210 229533.I1RTJ7 pp 229533.V6QU99
8211 229533.I1RTJ7 pp 229533.V6R0W1
8212 229533.I1RTJ7 pp 229533.V6R1K4
8213 229533.I1RTJ7 pp 229533.V6RAM6
8214 229533.I1RTJ7 pp 229533.I1SAI4
8215 229533.I1RTJ7 pp 229533.V6R5D2
8216 229533.I1RTJ7 pp 229533.Q4HXF0
8217 229533.I1RTJ7 pp 229533.V6R756
8218 229533.I1RTJ7 pp 229533.I1RYU5
8219 229533.I1RTJ7 pp 229533.V6R057
8220 229533.A0A098DPW7 pp 229533.I1RAS2
8221 229533.A0A098DPW7 pp 229533.I1RN18
8222 229533.A0A098DPW7 pp 229533.I1RZH4
8223 229533.A0A098DPW7 pp 229533.V6R042
8224 229533.A0A098DPW7 pp 229533.A0A1C3YKB0
8225 229533.A0A098DPW7 pp 229533.I1RQE7
8226 229533.A0A098DPW7 pp 229533.I1S471
8227 229533.A0A098DPW7 pp 229533.I1RT35
8228 229533.A0A098DPW7 pp 229533.I1RD22
8229 229533.A0A098DPW7 pp 229533.I1RPM4
8230 229533.A0A098DPW7 pp 229533.I1RD17
8231 229533.A0A098DPW7 pp 229533.I1RAU7
8232 229533.A0A098DPW7 pp 229533.I1RN58
8233 229533.A0A098DPW7 pp 229533.I1RA70
8234 229533.A0A098DPW7 pp 229533.I1RTH6
8235 229533.A0A098DPW7 pp 229533.I1RPS0
8236 229533.A0A098DPW7 pp 229533.I1RRJ6
8237 229533.A0A098DPW7 pp 229533.I1RBY4
8238 229533.A0A098DPW7 pp 229533.I1S5B0
8239 229533.A0A098DPW7 pp 229533.A0A1C3YKM9
8240 229533.A0A098DPW7 pp 229533.I1RR73
8241 229533.A0A098DPW7 pp 229533.I1RE71
8242 229533.A0A098DPW7 pp 229533.I1RW99
8243 229533.A0A098DPW7 pp 229533.A0A098DXE2
8244 229533.A0A098DPW7 pp 229533.I1RWN6
8245 229533.A0A098DPW7 pp 229533.I1RS20
8246 229533.A0A098DPW7 pp 229533.A0A1C3YM82
8247 229533.A0A098DPW7 pp 229533.I1RWK3
8248 229533.A0A098DPW7 pp 229533.Q4HZ42
8249 229533.A0A098DPW7 pp 229533.I1RJV3

8250 229533.A0A098DPW7 pp 229533.I1RNB8
8251 229533.A0A098DPW7 pp 229533.I1RPP4
8252 229533.A0A098DPW7 pp 229533.I1RW25
8253 229533.A0A098DPW7 pp 229533.A0A1C3YM64
8254 229533.A0A098DPW7 pp 229533.A0A1C3YKC0
8255 229533.A0A098DPW7 pp 229533.I1RE01
8256 229533.A0A098DPW7 pp 229533.I1RBT1
8257 229533.A0A098DPW7 pp 229533.A0A1C3YL15
8258 229533.A0A098DPW7 pp 229533.A0A0E0SF67
8259 229533.A0A098DPW7 pp 229533.I1RBS6
8260 229533.A0A098DPW7 pp 229533.A0A1C3YII7
8261 229533.A0A098DPW7 pp 229533.I1RN65
8262 229533.A0A098DPW7 pp 229533.I1RZS7
8263 229533.A0A098DPW7 pp 229533.V6RIV9
8264 229533.A0A098DPW7 pp 229533.I1S0X6
8265 229533.A0A098DPW7 pp 229533.V6RMW6
8266 229533.A0A098DPW7 pp 229533.A0A0E0S9Y6
8267 229533.A0A098DPW7 pp 229533.I1RWD7
8268 229533.A0A098DPW7 pp 229533.I1S788
8269 229533.A0A098DPW7 pp 229533.I1S6F1
8270 229533.A0A098DPW7 pp 229533.I1RLU5
8271 229533.A0A098DPW7 pp 229533.I1RSI0
8272 229533.A0A098DPW7 pp 229533.I1S0L3
8273 229533.A0A098DPW7 pp 229533.I1RB53
8274 229533.A0A098DPW7 pp 229533.I1RVQ0
8275 229533.A0A098DPW7 pp 229533.Q4IAA0
8276 229533.A0A098DPW7 pp 229533.I1RQC9
8277 229533.A0A098DPW7 pp 229533.I1RXU7
8278 229533.A0A098DPW7 pp 229533.V6RTJ8
8279 229533.I1RGQ9 pp 229533.Q4I5I4
8280 229533.I1RGQ9 pp 229533.I1RZH4
8281 229533.I1RGQ9 pp 229533.I1RQ73
8282 229533.I1RGQ9 pp 229533.I1RMT4
8283 229533.I1RGQ9 pp 229533.Q4I0M9
8284 229533.I1RGQ9 pp 229533.I1RZG0
8285 229533.I1RGQ9 pp 229533.I1RZ94
8286 229533.I1RGQ9 pp 229533.I1RHI1
8287 229533.I1RGQ9 pp 229533.I1RZ02
8288 229533.I1RGQ9 pp 229533.Q4INW6
8289 229533.I1RGQ9 pp 229533.I1S0A4
8290 229533.I1RX89 pp 229533.I1S9Z4
8291 229533.I1RX89 pp 229533.I1RZ25
8292 229533.I1RK15 pp 229533.I1RN67
8293 229533.I1RK15 pp 229533.I1RSZ3
8294 229533.I1RK15 pp 229533.I1RX22
8295 229533.I1RK15 pp 229533.I1RZZ9
8296 229533.I1RK15 pp 229533.I1RSQ4
8297 229533.I1RK15 pp 229533.Q4HXF0
8298 229533.I1RK15 pp 229533.I1RZM8
8299 229533.I1RK15 pp 229533.I1S8G4
8300 229533.I1RK15 pp 229533.O74638
8301 229533.I1RK15 pp 229533.V6R2A1
8302 229533.I1RK15 pp 229533.I1RMI8
8303 229533.I1RK15 pp 229533.I1RXF0
8304 229533.I1RK15 pp 229533.V6RGY0
8305 229533.I1RK15 pp 229533.I1RWE8
8306 229533.I1RK15 pp 229533.I1RRV4
8307 229533.I1RK15 pp 229533.I1RTD3
8308 229533.I1RK15 pp 229533.V6RRH2
8309 229533.I1RK15 pp 229533.V6RTJ8
8310 229533.I1RK15 pp 229533.V6QUE6
8311 229533.I1RK15 pp 229533.I1S216
8312 229533.I1RK15 pp 229533.I1RUQ6
8313 229533.I1RK15 pp 229533.V6R057
8314 229533.I1RK15 pp 229533.I1S2C4
8315 229533.I1RK15 pp 229533.I1S0B7
8316 229533.I1RK15 pp 229533.V6RBN2
8317 229533.I1RK15 pp 229533.I1RY63
8318 229533.I1RK15 pp 229533.I1RWU3
8319 229533.I1RK15 pp 229533.I1RPW4
8320 229533.I1RK15 pp 229533.I1RKR6
8321 229533.I1RK15 pp 229533.I1RPN4
8322 229533.I1RK15 pp 229533.I1RMQ4

229533.I1RK15	pp	229533.I1RQS9
229533.I1RK15	pp	229533.I1RMA5
229533.I1RK15	pp	229533.I1RM35
229533.I1RK15	pp	229533.I1S4Q4
229533.I1RK15	pp	229533.V6QV38
229533.I1RK15	pp	229533.I1RNF5
229533.I1RK15	pp	229533.V6R1K4
229533.Q4I5G1	pp	229533.Q4IN52
229533.Q4I5G1	pp	229533.V6RN69
229533.Q4I5G1	pp	229533.V6RVJ3
229533.I1RCR2	pp	229533.I1RRX5
229533.I1RCR2	pp	229533.V6R3J1
229533.I1RCR2	pp	229533.I1RW71
229533.I1RN66	pp	229533.I1RWV4
229533.I1RN66	pp	229533.I1RNR9
229533.I1RN66	pp	229533.I1RPD3
229533.I1RN66	pp	229533.I1S368
229533.I1RN66	pp	229533.I1RSD1
229533.I1RN66	pp	229533.I1RQ61
229533.I1RN66	pp	229533.I1RTG5
229533.I1RN66	pp	229533.I1RWQ1
229533.I1RN66	pp	229533.V6QW25
229533.A0A0E0RSG8	pp	229533.I1S4Q4
229533.A0A0E0RSG8	pp	229533.I1REL7
229533.A0A0E0RSG8	pp	229533.I1RM35
229533.A0A0E0RSG8	pp	229533.A0A1C3YHS4
229533.A0A0E0RSG8	pp	229533.A0A1C3YK68
229533.A0A0E0RSG8	pp	229533.I1RAZ3
229533.A0A0E0RSG8	pp	229533.V6RBN2
229533.A0A0E0RSG8	pp	229533.I1RMQ4
229533.A0A0E0RSG8	pp	229533.A0A1C3YN30
229533.A0A0E0RSG8	pp	229533.I1RCL4
229533.A0A0E0RSG8	pp	229533.I1RQ81
229533.A0A0E0RSG8	pp	229533.I1RPN4
229533.A0A0E0RSG8	pp	229533.V6RRH2
229533.A0A0E0RSG8	pp	229533.A0A0E0S413
229533.A0A0E0RSG8	pp	229533.I1RAR1
229533.A0A0E0RSG8	pp	229533.Q4INS6
229533.A0A0E0RSG8	pp	229533.V6R057
229533.A0A0E0RSG8	pp	229533.V6R1K4
229533.A0A0E0RSG8	pp	229533.I1RRV4
229533.A0A0E0RSG8	pp	229533.I1S1P8
229533.A0A0E0RSG8	pp	229533.I1RCW6
229533.A0A0E0RSG8	pp	229533.I1RYI7
229533.A0A0E0RSG8	pp	229533.I1S2C4
229533.A0A0E0RSG8	pp	229533.I1RRT1
229533.A0A0E0RSG8	pp	229533.A0A1C3YKY0
229533.A0A0E0RSG8	pp	229533.V6QT89
229533.A0A0E0RSG8	pp	229533.I1S084
229533.A0A0E0RSG8	pp	229533.I1S0B7
229533.A0A0E0RSG8	pp	229533.I1RP99
229533.A0A0E0RSG8	pp	229533.A0A0E0SAC4
229533.A0A0E0RSG8	pp	229533.I1RKY2
229533.A0A0E0RSG8	pp	229533.I1RWE8
229533.A0A0E0RSG8	pp	229533.I1RY29
229533.A0A0E0RSG8	pp	229533.I1RPU8
229533.A0A0E0RSG8	pp	229533.A0A1C3YKU8
229533.A0A0E0RSG8	pp	229533.I1RC05
229533.A0A0E0RSG8	pp	229533.I1RTA9
229533.A0A0E0RSG8	pp	229533.I1RWI8
229533.A0A1C3YLF4	pp	229533.I1RW71
229533.A0A1C3YLF4	pp	229533.I1RMB3
229533.A0A1C3YLF4	pp	229533.I1RMT4
229533.A0A1C3YLF4	pp	229533.I1RNN3
229533.A0A1C3YLF4	pp	229533.I1RA39
229533.A0A1C3YLF4	pp	229533.I1RXZ4
229533.A0A1C3YLF4	pp	229533.I1RNY7
229533.A0A1C3YLF4	pp	229533.I1RSM8
229533.A0A1C3YLF4	pp	229533.I1RDG9
229533.A0A1C3YLF4	pp	229533.I1RJZ7
229533.A0A1C3YLF4	pp	229533.I1RB84
229533.A0A1C3YLF4	pp	229533.I1RZ02
229533.A0A1C3YLF4	pp	229533.I1RZH4

229533.A0A1C3YLF4 pp 229533.I1RN65
229533.A0A1C3YLF4 pp 229533.I1RFS6
229533.I1REX0 pp 229533.I1RYU5
229533.I1REX0 pp 229533.I1RZ20
229533.I1REX0 pp 229533.Q4HXF0
229533.I1REX0 pp 229533.V6R057
229533.I1REX0 pp 229533.I1RS17
229533.I1REX0 pp 229533.V6R1K4
229533.I1REX0 pp 229533.I1RPU8
229533.I1RLV0 pp 229533.I1RWX5
229533.I1RA39 pp 229533.I1RRM9
229533.I1RA39 pp 229533.I1S1V1
229533.I1RA39 pp 229533.I1RMB3
229533.I1RA39 pp 229533.I1RZH4
229533.I1RA39 pp 229533.I1RD70
229533.I1RA39 pp 229533.I1RZQ6
229533.I1RA39 pp 229533.I1RBW7
229533.I1RA39 pp 229533.I1RDG9
229533.I1RP04 pp 229533.I1RZ94
229533.I1RP04 pp 229533.I1RZH4
229533.I1RP04 pp 229533.Q4I0M9
229533.I1RP04 pp 229533.I1RZ02
229533.I1RP04 pp 229533.Q4I5I4
229533.I1RP04 pp 229533.Q4INW6
229533.I1RP04 pp 229533.I1S0A4
229533.I1RP04 pp 229533.I1RQ73
229533.I1RP04 pp 229533.I1RZG0
229533.I1REC0 pp 229533.I1RQE5
229533.I1REC0 pp 229533.I1RZ87
229533.I1REC0 pp 229533.I1S2I4
229533.I1REC0 pp 229533.I1RLR8
229533.A0A1C3YLP8 pp 229533.I1RP96
229533.A0A1C3YLP8 pp 229533.I1RC98
229533.A0A1C3YLP8 pp 229533.I1RML9
229533.A0A1C3YLP8 pp 229533.V6QV38
229533.A0A1C3YLP8 pp 229533.Q4HTT2
229533.A0A1C3YLP8 pp 229533.I1S951
229533.A0A1C3YLP8 pp 229533.I1S276
229533.A0A1C3YLP8 pp 229533.I1RXU0
229533.A0A1C3YLP8 pp 229533.Q4I7S1
229533.A0A1C3YLP8 pp 229533.I1RK79
229533.A0A1C3YLP8 pp 229533.I1RW91
229533.A0A1C3YLP8 pp 229533.Q4I7X1
229533.A0A1C3YLP8 pp 229533.V6QWX8
229533.A0A1C3YLP8 pp 229533.I1RCB7
229533.A0A1C3YLP8 pp 229533.I1R9Q0
229533.A0A1C3YLP8 pp 229533.I1RWF3
229533.A0A1C3YLP8 pp 229533.I1RC24
229533.A0A1C3YLP8 pp 229533.I1RZB5
229533.A0A1C3YLP8 pp 229533.I1RE30
229533.A0A1C3YLP8 pp 229533.A0A1C3YN55
229533.A0A1C3YLP8 pp 229533.I1RXE5
229533.A0A1C3YLP8 pp 229533.I1RW90
229533.A0A1C3YLP8 pp 229533.I1RBJ1
229533.A0A1C3YLP8 pp 229533.I1RCG6
229533.A0A1C3YLP8 pp 229533.I1RPW8
229533.A0A1C3YLP8 pp 229533.I1RJL2
229533.A0A1C3YLP8 pp 229533.I1RBR5
229533.A0A1C3YLP8 pp 229533.V6QUE6
229533.A0A1C3YLP8 pp 229533.I1RAR2
229533.A0A1C3YLP8 pp 229533.I1RVY0
229533.A0A1C3YLP8 pp 229533.I1RRM8
229533.A0A1C3YLP8 pp 229533.I1RRT0
229533.A0A1C3YLP8 pp 229533.I1RDC9
229533.A0A1C3YLP8 pp 229533.I1RZM2
229533.A0A1C3YLP8 pp 229533.I1RDG8
229533.A0A1C3YLP8 pp 229533.I1RA84
229533.A0A1C3YLP8 pp 229533.I1S382
229533.A0A1C3YLP8 pp 229533.Q4IQX3
229533.A0A1C3YLP8 pp 229533.I1RRG3
229533.A0A1C3YLP8 pp 229533.I1SAN0
229533.A0A1C3YLP8 pp 229533.I1RW80
229533.A0A1C3YLP8 pp 229533.I1RAW7

8469 229533.A0A1C3YLP8 pp 229533.I1RTC2
8470 229533.A0A1C3YLP8 pp 229533.I1RBC7
8471 229533.A0A1C3YLP8 pp 229533.I1RC15
8472 229533.A0A1C3YLP8 pp 229533.I1REN3
8473 229533.A0A1C3YLP8 pp 229533.I1RFU3
8474 229533.A0A1C3YLP8 pp 229533.I1RW28
8475 229533.A0A1C3YLP8 pp 229533.I1RUG4
8476 229533.A0A1C3YLP8 pp 229533.I1RZU9
8477 229533.A0A1C3YLP8 pp 229533.I1S1V5
8478 229533.A0A1C3YLP8 pp 229533.I1RRF7
8479 229533.A0A1C3YLP8 pp 229533.I1RQ42
8480 229533.A0A1C3YLP8 pp 229533.I1RKB1
8481 229533.A0A1C3YLP8 pp 229533.I1RR25
8482 229533.A0A1C3YLP8 pp 229533.I1RG11
8483 229533.A0A1C3YLP8 pp 229533.I1RPM7
8484 229533.A0A1C3YLP8 pp 229533.I1RK71
8485 229533.A0A1C3YLP8 pp 229533.I1RMV1
8486 229533.A0A1C3YLP8 pp 229533.I1R976
8487 229533.A0A1C3YLP8 pp 229533.I1RSZ3
8488 229533.A0A1C3YLP8 pp 229533.I1RVL8
8489 229533.A0A1C3YLP8 pp 229533.I1RR61
8490 229533.A0A1C3YLP8 pp 229533.I1RBZ7
8491 229533.A0A1C3YLP8 pp 229533.A0A1C3YMF9
8492 229533.A0A1C3YLP8 pp 229533.I1RN04
8493 229533.I1RZM2 pp 229533.Q4HTT2
8494 229533.I1RZM2 pp 229533.I1RZU9
8495 229533.I1RZM2 pp 229533.I1SAT3
8496 229533.I1RZM2 pp 229533.I1S382
8497 229533.I1RZM2 pp 229533.I1S1V5
8498 229533.I1RZM2 pp 229533.I1S276
8499 229533.I1RZM2 pp 229533.I1S0W0
8500 229533.I1RZM2 pp 229533.V6QUE6
8501 229533.I1RZM2 pp 229533.V6QWX8
8502 229533.I1RZM2 pp 229533.Q4I5W5
8503 229533.I1RZM2 pp 229533.Q4I7S1
8504 229533.I1RZM2 pp 229533.Q4I7X1
8505 229533.I1RZM2 pp 229533.I1S0C0
8506 229533.I1RZM2 pp 229533.I1S951
8507 229533.I1RZM2 pp 229533.V6QV38
8508 229533.I1RBK6 pp 229533.Q4ILI9
8509 229533.I1RBK6 pp 229533.I1RYW8
8510 229533.I1RBK6 pp 229533.I1RKG4
8511 229533.I1RBK6 pp 229533.I1RTG5
8512 229533.I1RBK6 pp 229533.V6RR76
8513 229533.I1RBK6 pp 229533.I1RW76
8514 229533.I1RBK6 pp 229533.I1RMP2
8515 229533.I1RBK6 pp 229533.Q4HYZ2
8516 229533.I1RBK6 pp 229533.I1RN81
8517 229533.I1RBK6 pp 229533.I1RT41
8518 229533.A0A1C3YJ14 pp 229533.I1RJT1
8519 229533.A0A1C3YJ14 pp 229533.A0A1C3YKY6
8520 229533.A0A1C3YJ14 pp 229533.A0A1C3YK11
8521 229533.A0A1C3YJ14 pp 229533.I1RSP4
8522 229533.A0A1C3YJ14 pp 229533.I1RTF0
8523 229533.A0A1C3YJ14 pp 229533.I1RTG7
8524 229533.A0A1C3YJ14 pp 229533.I1RCK2
8525 229533.A0A1C3YJ14 pp 229533.I1RQ82
8526 229533.A0A1C3YJ14 pp 229533.I1RFV6
8527 229533.A0A1C3YJ14 pp 229533.I1RY72
8528 229533.A0A1C3YJ14 pp 229533.I1RXF0
8529 229533.A0A1C3YJ14 pp 229533.I1RX17
8530 229533.A0A1C3YJ14 pp 229533.I1RRS8
8531 229533.A0A1C3YJ14 pp 229533.A0A1C3YLR6
8532 229533.A0A1C3YJ14 pp 229533.I1R9Y8
8533 229533.A0A1C3YJ14 pp 229533.I1S7L6
8534 229533.A0A1C3YJ14 pp 229533.I1RKB6
8535 229533.A0A1C3YJ14 pp 229533.I1RAD4
8536 229533.A0A1C3YJ14 pp 229533.I1RP97
8537 229533.A0A1C3YJ14 pp 229533.V6RN44
8538 229533.A0A1C3YJ14 pp 229533.I1RZH5
8539 229533.A0A1C3YJ14 pp 229533.I1RBA4
8540 229533.A0A1C3YJ14 pp 229533.I1RC18
8541 229533.A0A1C3YJ14 pp 229533.I1RBS5

229533.A0A1C3YJ14	pp	229533.I1RS50
229533.A0A1C3YJ14	pp	229533.V6R949
229533.A0A1C3YJ14	pp	229533.I1RNG4
229533.A0A1C3YJ14	pp	229533.I1RCF9
229533.A0A1C3YJ14	pp	229533.V6RM66
229533.A0A1C3YM09	pp	229533.Q4IJP1
229533.A0A1C3YM09	pp	229533.I1RRT1
229533.A0A1C3YM09	pp	229533.I1RDE6
229533.A0A1C3YM09	pp	229533.I1RJT5
229533.A0A1C3YM09	pp	229533.I1RQL4
229533.A0A1C3YM09	pp	229533.Q4HTT2
229533.A0A1C3YM09	pp	229533.I1RDN3
229533.A0A1C3YM09	pp	229533.I1RC00
229533.A0A1C3YM09	pp	229533.A0A1C3YN58
229533.A0A1C3YM09	pp	229533.I1RA13
229533.A0A1C3YM09	pp	229533.I1RQ28
229533.A0A1C3YM09	pp	229533.I1RBU8
229533.A0A1C3YM09	pp	229533.I1S8S9
229533.A0A1C3YM09	pp	229533.I1RKP1
229533.A0A1C3YM09	pp	229533.I1RB73
229533.A0A1C3YM09	pp	229533.I1RSF1
229533.A0A1C3YM09	pp	229533.I1S0A5
229533.A0A1C3YM09	pp	229533.I1RAD3
229533.A0A1C3YM09	pp	229533.I1RD23
229533.A0A1C3YM09	pp	229533.I1RND2
229533.A0A1C3YM09	pp	229533.I1RJZ7
229533.A0A1C3YM09	pp	229533.I1RCN2
229533.I1RV18	pp	229533.I1S2C4
229533.I1RV18	pp	229533.V6RAM6
229533.I1RV18	pp	229533.I1RV19
229533.I1RV18	pp	229533.V6R057
229533.I1RV18	pp	229533.V6R1K4
229533.I1RV18	pp	229533.V6R756
229533.I1RV18	pp	229533.I1RZ20
229533.I1RV18	pp	229533.Q4HXF0
229533.I1RV18	pp	229533.V6R0W1
229533.I1RV18	pp	229533.I1RYU5
229533.I1RV18	pp	229533.V6R2Z2
229533.I1RV18	pp	229533.V6R5D2
229533.I1RV18	pp	229533.V6QU99
229533.I1RV18	pp	229533.I1SAI4
229533.I1S3K6	pp	229533.I1S4Q4
229533.I1S3K6	pp	229533.V6RQW4
229533.I1S3K6	pp	229533.V6RY56
229533.I1S3K6	pp	229533.I1SAJ3
229533.I1S3K6	pp	229533.I1S5B0
229533.I1S3K6	pp	229533.I1S4D0
229533.I1S3K6	pp	229533.V6RCZ8
229533.I1S3K6	pp	229533.Q4HXF0
229533.I1S3K6	pp	229533.V6RRH2
229533.I1RCY4	pp	229533.I1RZ25
229533.I1RCY4	pp	229533.I1RXF5
229533.I1S0D7	pp	229533.I1S0Y1
229533.I1RKY2	pp	229533.I1S2C4
229533.I1RKY2	pp	229533.V6RRH2
229533.I1RKY2	pp	229533.V6R057
229533.I1RKY2	pp	229533.I1RP99
229533.I1RKY2	pp	229533.V6RBN2
229533.I1RKY2	pp	229533.I1RPU8
229533.I1RKY2	pp	229533.I1RRT1
229533.I1RKY2	pp	229533.I1RWE8
229533.I1RKY2	pp	229533.V6QT89
229533.I1RKY2	pp	229533.I1S4Q4
229533.I1RKY2	pp	229533.I1RM35
229533.I1RKY2	pp	229533.I1RY29
229533.I1RKY2	pp	229533.I1S0B7
229533.I1RKY2	pp	229533.Q4INS6
229533.I1RKY2	pp	229533.I1RWI8
229533.I1RKY2	pp	229533.I1S084
229533.I1RKY2	pp	229533.I1RPN4
229533.I1RKY2	pp	229533.I1RRV4
229533.I1RKY2	pp	229533.I1RYI7
229533.I1RKY2	pp	229533.V6R1K4

229533.I1RKY2 pp 229533.I1RTA9
229533.I1RKY2 pp 229533.I1S1P8
229533.I1RKY2 pp 229533.I1RMQ4
229533.I1RKY2 pp 229533.I1RQ81
229533.I1RVR0 pp 229533.I1S1W8
229533.I1RVR0 pp 229533.Q4HXF0
229533.I1RVR0 pp 229533.I1RZ52
229533.I1RVR0 pp 229533.I1S029
229533.I1RVR0 pp 229533.V6RQW4
229533.I1RVR0 pp 229533.I1S338
229533.I1RVR0 pp 229533.V6RRH2
229533.I1RVR0 pp 229533.I1S3G4
229533.I1RVR0 pp 229533.I1S4Q4
229533.I1RVR0 pp 229533.I1S2C4
229533.I1RVR0 pp 229533.I1SAJ3
229533.I1RVR0 pp 229533.V6RY56
229533.I1RVR0 pp 229533.I1S4D0
229533.I1RVR0 pp 229533.V6RCZ8
229533.I1RVR0 pp 229533.I1S5B0
229533.I1RVR0 pp 229533.I1RYI7
229533.I1RP96 pp 229533.I1RSZ3
229533.I1RP96 pp 229533.I1S0B6
229533.I1RP96 pp 229533.I1S560
229533.I1RP96 pp 229533.I1S1V5
229533.I1RP96 pp 229533.I1RQA8
229533.I1RP96 pp 229533.I1S951
229533.I1RP96 pp 229533.I1RRM8
229533.I1RP96 pp 229533.Q4I5W5
229533.I1RP96 pp 229533.Q4IQX3
229533.I1RP96 pp 229533.I1RVL8
229533.I1RP96 pp 229533.I1RQ42
229533.I1RP96 pp 229533.I1RVY0
229533.I1RP96 pp 229533.I1RW80
229533.I1RP96 pp 229533.I1RRG3
229533.I1RP96 pp 229533.I1RRT0
229533.I1RP96 pp 229533.I1RZM2
229533.I1RP96 pp 229533.I1RXE5
229533.I1RP96 pp 229533.I1S382
229533.I1RP96 pp 229533.I1RWF3
229533.I1RP96 pp 229533.I1RTC2
229533.I1RP96 pp 229533.Q4I7X1
229533.I1RP96 pp 229533.I1RZB5
229533.I1RP96 pp 229533.I1RXU0
229533.I1RP96 pp 229533.I1RPW8
229533.I1RP96 pp 229533.V6QWX8
229533.I1RP96 pp 229533.Q4HTT2
229533.I1RP96 pp 229533.V6QUE6
229533.I1RP96 pp 229533.I1RW91
229533.I1RP96 pp 229533.I1RPM7
229533.I1RP96 pp 229533.I1RW90
229533.I1RP96 pp 229533.I1RRF7
229533.I1RP96 pp 229533.I1S276
229533.I1RP96 pp 229533.I1RR25
229533.I1RP96 pp 229533.V6QV38
229533.I1RP96 pp 229533.I1RR61
229533.I1RP96 pp 229533.I1RUG4
229533.A0A0E0RTX4 pp 229533.I1RAI9
229533.A0A0E0RTX4 pp 229533.I1RPW0
229533.A0A0E0RTX4 pp 229533.I1RQ88
229533.A0A0E0RTX4 pp 229533.I1RX38
229533.A0A0E0RTX4 pp 229533.A0A1C3YKJ6
229533.A0A0E0RTX4 pp 229533.I1RBV5
229533.A0A0E0RTX4 pp 229533.I1RPU4
229533.A0A0E0RTX4 pp 229533.A0A1C3YK37
229533.A0A0E0RTX4 pp 229533.A0A0E0SNZ9
229533.I1RTL1 pp 229533.I1RWQ0
229533.I1RML9 pp 229533.I1RTC2
229533.I1RML9 pp 229533.I1S951
229533.I1RML9 pp 229533.I1RPW8
229533.I1RML9 pp 229533.I1S1V5
229533.I1RML9 pp 229533.I1RRG3
229533.I1RML9 pp 229533.I1RZM2
229533.I1RML9 pp 229533.Q4IQX3

8688	229533.I1RML9	pp	229533.I1RW90
8689	229533.I1RML9	pp	229533.V6QWX8
8690	229533.I1RML9	pp	229533.I1RW91
8691	229533.I1RML9	pp	229533.Q4I5W5
8692	229533.I1RML9	pp	229533.I1RWF3
8693	229533.I1RML9	pp	229533.I1RW80
8694	229533.I1RML9	pp	229533.I1RRM8
8695	229533.I1RML9	pp	229533.I1RP96
8696	229533.I1RML9	pp	229533.I1RXE5
8697	229533.I1RML9	pp	229533.I1RRT0
8698	229533.I1RML9	pp	229533.I1S382
8699	229533.I1RML9	pp	229533.I1RSZ3
8700	229533.I1RML9	pp	229533.I1RR61
8701	229533.I1RML9	pp	229533.I1RRF7
8702	229533.I1RML9	pp	229533.I1RMV1
8703	229533.I1RML9	pp	229533.Q4I7S1
8704	229533.I1RML9	pp	229533.V6QUE6
8705	229533.I1RML9	pp	229533.V6QV38
8706	229533.I1RML9	pp	229533.I1RVY0
8707	229533.I1RML9	pp	229533.I1RQ42
8708	229533.I1RML9	pp	229533.I1RVL8
8709	229533.I1RML9	pp	229533.I1S276
8710	229533.I1RML9	pp	229533.I1RN04
8711	229533.I1RML9	pp	229533.Q4I7X1
8712	229533.I1RML9	pp	229533.I1RXU0
8713	229533.I1RML9	pp	229533.I1RPM7
8714	229533.I1RML9	pp	229533.I1RZU9
8715	229533.I1RML9	pp	229533.I1RWU3
8716	229533.I1RML9	pp	229533.I1RR25
8717	229533.I1RML9	pp	229533.I1RZB5
8718	229533.I1RML9	pp	229533.I1RUG4
8719	229533.I1RJK0	pp	229533.V6R756
8720	229533.I1RJK0	pp	229533.I1RS17
8721	229533.I1RJK0	pp	229533.V6R057
8722	229533.I1RJK0	pp	229533.I1RPU8
8723	229533.I1RJK0	pp	229533.V6R5D2
8724	229533.I1RJK0	pp	229533.I1SAI4
8725	229533.I1RJK0	pp	229533.I1RYU5
8726	229533.I1RJK0	pp	229533.V6R2Z2
8727	229533.I1RJK0	pp	229533.I1RQX9
8728	229533.I1RJK0	pp	229533.V6R0W1
8729	229533.I1RJK0	pp	229533.I1RZ20
8730	229533.I1RJK0	pp	229533.V6R1K4
8731	229533.I1RJK0	pp	229533.Q4HXF0
8732	229533.I1RJK0	pp	229533.V6RAM6
8733	229533.I1RJK0	pp	229533.V6QU99
8734	229533.I1RJQ4	pp	229533.I1RNV7
8735	229533.I1RS21	pp	229533.I1RWD1
8736	229533.I1RS21	pp	229533.I1RXF4
8737	229533.I1RM30	pp	229533.I1S1W8
8738	229533.I1RM30	pp	229533.I1S029
8739	229533.I1RM30	pp	229533.V6RY56
8740	229533.I1RM30	pp	229533.Q4HXF0
8741	229533.I1RM30	pp	229533.I1RZ52
8742	229533.I1RM30	pp	229533.I1S3G4
8743	229533.I1RM30	pp	229533.I1RQG7
8744	229533.I1RM30	pp	229533.V6RQW4
8745	229533.I1RM30	pp	229533.I1RN67
8746	229533.I1RM30	pp	229533.I1S4Q4
8747	229533.I1RM30	pp	229533.I1RNG4
8748	229533.I1RM30	pp	229533.I1RSQ4
8749	229533.I1RM30	pp	229533.I1S338
8750	229533.I1RM30	pp	229533.V6RRH2
8751	229533.I1RM30	pp	229533.I1RPI5
8752	229533.I1RM30	pp	229533.I1SAJ3
8753	229533.I1RM30	pp	229533.I1S2C4
8754	229533.I1RM30	pp	229533.I1RM55
8755	229533.I1RM30	pp	229533.I1S5B0
8756	229533.I1RM30	pp	229533.I1RUP2
8757	229533.I1RM30	pp	229533.I1RPU8
8758	229533.I1RM30	pp	229533.I1RYI7
8759	229533.I1RM30	pp	229533.I1RRV4
8760	229533.I1RM30	pp	229533.I1S4D0

8761 229533.I1RM30 pp 229533.V6RCZ8
8762 229533.I1RAR0 pp 229533.I1RS21
8763 229533.I1RAR0 pp 229533.I1RMF9
8764 229533.I1RAR0 pp 229533.I1RGD2
8765 229533.I1RAR0 pp 229533.I1S027
8766 229533.I1RGU4 pp 229533.I1RQL4
8767 229533.I1RGU4 pp 229533.I1RMP2
8768 229533.A0A098E051 pp 229533.I1RMR5
8769 229533.V6R2A1 pp 229533.V6RTJ8
8770 229533.V6R2A1 pp 229533.V6RRH2
8771 229533.V6R2A1 pp 229533.V6RBN2
8772 229533.A0A098DF88 pp 229533.I1SA77
8773 229533.A0A098DKM1 pp 229533.Q4HZE0
8774 229533.A0A098DKM1 pp 229533.A0A1C3YLH6
8775 229533.A0A098DKM1 pp 229533.I1RD10
8776 229533.A0A098DKM1 pp 229533.I1RRW2
8777 229533.A0A098DKM1 pp 229533.V6RR76
8778 229533.A0A098DKM1 pp 229533.I1RA72
8779 229533.A0A098DKM1 pp 229533.I1RBD6
8780 229533.A0A098DKM1 pp 229533.I1RWA9
8781 229533.A0A098DKM1 pp 229533.A0A1C3YJJ1
8782 229533.A0A098DKM1 pp 229533.A0A1C3YLS7
8783 229533.A0A098DKM1 pp 229533.I1RJS2
8784 229533.A0A098DKM1 pp 229533.V6RB81
8785 229533.A0A098DKM1 pp 229533.I1RRF9
8786 229533.A0A098DKM1 pp 229533.A0A1C3YL99
8787 229533.A0A098DKM1 pp 229533.A0A1C3YM95
8788 229533.A0A098DKM1 pp 229533.I1RNG6
8789 229533.A0A098DKM1 pp 229533.I1S7X9
8790 229533.A0A098DKM1 pp 229533.I1RWI6
8791 229533.A0A098DKM1 pp 229533.I1S0X0
8792 229533.A0A098DKM1 pp 229533.I1RC46
8793 229533.A0A098DKM1 pp 229533.Q4ILI9
8794 229533.A0A098DKM1 pp 229533.I1RCN6
8795 229533.A0A098DKM1 pp 229533.I1RKR9
8796 229533.A0A098DKM1 pp 229533.A0A0E0SGH2
8797 229533.A0A098DKM1 pp 229533.I1RYZ7
8798 229533.A0A098DKM1 pp 229533.A0A1C3YMQ5
8799 229533.A0A098DKM1 pp 229533.I1S1B1
8800 229533.A0A098DKM1 pp 229533.I1RMF2
8801 229533.A0A098DKM1 pp 229533.A0A0E0RWX7
8802 229533.A0A098DKM1 pp 229533.I1S0Z3
8803 229533.A0A098DKM1 pp 229533.I1RAS5
8804 229533.A0A098DKM1 pp 229533.I1RNF9
8805 229533.A0A098DKM1 pp 229533.I1RWA5
8806 229533.A0A098DKM1 pp 229533.A0A1C3YKT9
8807 229533.A0A098DKM1 pp 229533.I1REZ0
8808 229533.A0A098DKM1 pp 229533.Q4HYZ2
8809 229533.A0A098DKM1 pp 229533.A0A0E0RUL1
8810 229533.A0A098DKM1 pp 229533.I1S4X2
8811 229533.A0A098DKM1 pp 229533.I1RT41
8812 229533.A0A098DKM1 pp 229533.A0A1C3YK51
8813 229533.A0A098DKM1 pp 229533.A0A1C3YI55
8814 229533.A0A098DKM1 pp 229533.I1RQP5
8815 229533.A0A098DKM1 pp 229533.I1RMR5
8816 229533.A0A098DKM1 pp 229533.A0A0E0S0U6
8817 229533.A0A098DKM1 pp 229533.A0A1C3YIM8
8818 229533.A0A098DKM1 pp 229533.I1S0S6
8819 229533.A0A098DKM1 pp 229533.I1RN81
8820 229533.A0A098DKM1 pp 229533.I1RA34
8821 229533.A0A098DKM1 pp 229533.I1S270
8822 229533.A0A098DKM1 pp 229533.I1RPP0
8823 229533.A0A098DKM1 pp 229533.I1RZX9
8824 229533.I1RWF2 pp 229533.I1S6D1
8825 229533.I1RBA8 pp 229533.I1S0W9
8826 229533.A0A0E0S0U6 pp 229533.I1RJS2
8827 229533.A0A0E0S0U6 pp 229533.A0A1C3YLL6
8828 229533.A0A0E0S0U6 pp 229533.A0A1C3YID2
8829 229533.A0A0E0S0U6 pp 229533.I1S254
8830 229533.A0A0E0S0U6 pp 229533.V6R476
8831 229533.A0A0E0S0U6 pp 229533.I1RPM8
8832 229533.A0A0E0S0U6 pp 229533.V6QUU6
8833 229533.A0A0E0S0U6 pp 229533.I1RYZ7

8834	229533.A0A0E0S0U6	pp	229533.Q4IJ79
8835	229533.A0A0E0S0U6	pp	229533.I1RN81
8836	229533.A0A0E0S0U6	pp	229533.I1RZX9
8837	229533.A0A0E0S0U6	pp	229533.A0A1C3YHZ2
8838	229533.A0A0E0S0U6	pp	229533.V6RR76
8839	229533.A0A0E0S0U6	pp	229533.I1RNF9
8840	229533.A0A1C3YLL6	pp	229533.I1RWA5
8841	229533.A0A1C3YLL6	pp	229533.I1RWI6
8842	229533.A0A1C3YLL6	pp	229533.I1RAS5
8843	229533.A0A1C3YLL6	pp	229533.I1RRF9
8844	229533.A0A1C3YLL6	pp	229533.Q4ILI9
8845	229533.A0A1C3YLL6	pp	229533.I1RQP5
8846	229533.A0A1C3YLL6	pp	229533.I1RPP0
8847	229533.A0A1C3YLL6	pp	229533.I1RWA9
8848	229533.A0A1C3YLL6	pp	229533.I1S270
8849	229533.A0A1C3YLL6	pp	229533.I1RA34
8850	229533.A0A1C3YLL6	pp	229533.I1RMR5
8851	229533.A0A1C3YLL6	pp	229533.I1RN81
8852	229533.A0A1C3YLL6	pp	229533.I1RD10
8853	229533.A0A1C3YLL6	pp	229533.I1RRW2
8854	229533.A0A1C3YLL6	pp	229533.V6RB81
8855	229533.A0A1C3YLL6	pp	229533.I1S0X0
8856	229533.A0A1C3YLL6	pp	229533.A0A1C3YLS7
8857	229533.A0A1C3YLL6	pp	229533.I1RCN6
8858	229533.A0A1C3YLL6	pp	229533.V6RR76
8859	229533.A0A1C3YLL6	pp	229533.I1S7X9
8860	229533.A0A1C3YLL6	pp	229533.A0A1C3YMQ5
8861	229533.A0A1C3YLL6	pp	229533.I1RKR9
8862	229533.A0A1C3YLL6	pp	229533.I1RC46
8863	229533.A0A1C3YLL6	pp	229533.Q4HZE0
8864	229533.A0A1C3YLL6	pp	229533.I1REZ0
8865	229533.A0A1C3YLL6	pp	229533.I1RMF2
8866	229533.A0A1C3YLL6	pp	229533.A0A1C3YM95
8867	229533.A0A1C3YLL6	pp	229533.I1RJS2
8868	229533.A0A098E030	pp	229533.I1RJZ7
8869	229533.A0A098E030	pp	229533.I1RN31
8870	229533.A0A098E030	pp	229533.I1RRJ6
8871	229533.A0A098E030	pp	229533.A0A1C3YII1
8872	229533.A0A098E030	pp	229533.I1RWJ1
8873	229533.A0A098E030	pp	229533.A0A1C3YN36
8874	229533.A0A098E030	pp	229533.A0A0E0RQT8
8875	229533.A0A098E030	pp	229533.I1RW71
8876	229533.A0A098E030	pp	229533.I1RW16
8877	229533.I1S254	pp	229533.V6RB81
8878	229533.I1S254	pp	229533.I1S270
8879	229533.I1S254	pp	229533.Q4HZE0
8880	229533.I1S254	pp	229533.Q4HYZ2
8881	229533.I1S254	pp	229533.I1S7X9
8882	229533.I1S254	pp	229533.V6RR76
8883	229533.I1S254	pp	229533.Q4ILI9
8884	229533.I1RA21	pp	229533.I1RUP5
8885	229533.I1RA21	pp	229533.I1RJS0
8886	229533.I1RA21	pp	229533.I1RFK2
8887	229533.I1RL93	pp	229533.I1RNU7
8888	229533.I1RL93	pp	229533.I1SAF3
8889	229533.I1RL93	pp	229533.I1RPU8
8890	229533.I1RL93	pp	229533.I1SAI0
8891	229533.I1RL93	pp	229533.I1RPT0
8892	229533.V6R476	pp	229533.V6RR76
8893	229533.V6R476	pp	229533.V6RB81
8894	229533.A0A1C3YID2	pp	229533.I1S0X0
8895	229533.A0A1C3YID2	pp	229533.Q4ILI9
8896	229533.A0A1C3YID2	pp	229533.I1RAS5
8897	229533.A0A1C3YID2	pp	229533.I1RF38
8898	229533.A0A1C3YID2	pp	229533.I1RWI6
8899	229533.A0A1C3YID2	pp	229533.A0A1C3YMQ9
8900	229533.A0A1C3YID2	pp	229533.I1RGI2
8901	229533.A0A1C3YID2	pp	229533.I1RRF9
8902	229533.A0A1C3YID2	pp	229533.V6RR76
8903	229533.A0A1C3YID2	pp	229533.V6RHZ8
8904	229533.A0A1C3YID2	pp	229533.I1RWA5
8905	229533.A0A1C3YID2	pp	229533.I1RWA9
8906	229533.A0A1C3YID2	pp	229533.A0A1C3YK51

8907	229533.A0A1C3YID2	pp	229533.I1RQP5
8908	229533.A0A1C3YID2	pp	229533.I1RZX9
8909	229533.A0A1C3YID2	pp	229533.A0A1C3YKT9
8910	229533.A0A1C3YID2	pp	229533.I1RQ70
8911	229533.A0A1C3YID2	pp	229533.Q4HYZ2
8912	229533.A0A1C3YID2	pp	229533.I1S270
8913	229533.A0A1C3YID2	pp	229533.I1RA34
8914	229533.A0A1C3YID2	pp	229533.I1RMR5
8915	229533.A0A1C3YID2	pp	229533.I1RPP0
8916	229533.A0A1C3YID2	pp	229533.A0A1C3YIM8
8917	229533.A0A1C3YID2	pp	229533.I1RN81
8918	229533.A0A1C3YID2	pp	229533.Q4HZE0
8919	229533.A0A1C3YID2	pp	229533.A0A1C3YLH6
8920	229533.A0A1C3YID2	pp	229533.I1REJ3
8921	229533.A0A1C3YID2	pp	229533.I1RNV4
8922	229533.A0A1C3YID2	pp	229533.A0A1C3YJJ1
8923	229533.A0A1C3YID2	pp	229533.I1RD10
8924	229533.A0A1C3YID2	pp	229533.I1RRW2
8925	229533.A0A1C3YID2	pp	229533.I1RPH3
8926	229533.A0A1C3YID2	pp	229533.A0A1C3YM95
8927	229533.A0A1C3YID2	pp	229533.I1RNG6
8928	229533.A0A1C3YID2	pp	229533.V6R476
8929	229533.A0A1C3YID2	pp	229533.I1RJS2
8930	229533.A0A1C3YID2	pp	229533.V6RB81
8931	229533.A0A1C3YID2	pp	229533.A0A1C3YLS7
8932	229533.A0A1C3YID2	pp	229533.I1RE49
8933	229533.A0A1C3YID2	pp	229533.I1S7X9
8934	229533.A0A1C3YID2	pp	229533.A0A1C3YMQ5
8935	229533.A0A1C3YID2	pp	229533.I1RKR9
8936	229533.A0A1C3YID2	pp	229533.I1RC46
8937	229533.A0A1C3YID2	pp	229533.I1RCN6
8938	229533.A0A1C3YID2	pp	229533.I1REZ0
8939	229533.A0A1C3YID2	pp	229533.I1RMF2
8940	229533.I1RKB6	pp	229533.I1S0R9
8941	229533.I1RKB6	pp	229533.I1RSY2
8942	229533.I1RKB6	pp	229533.I1S9Z4
8943	229533.I1RKB6	pp	229533.I1RSS4
8944	229533.I1RKB6	pp	229533.V6RLM7
8945	229533.I1RKB6	pp	229533.I1RTN3
8946	229533.I1RKB6	pp	229533.I1RZ86
8947	229533.I1RKB6	pp	229533.V6QT89
8948	229533.I1RKB6	pp	229533.I1RSX9
8949	229533.I1RKB6	pp	229533.I1RZ39
8950	229533.I1RKB6	pp	229533.I1RL04
8951	229533.I1RKB6	pp	229533.I1S3G4
8952	229533.I1RKB6	pp	229533.O74638
8953	229533.I1RKB6	pp	229533.I1RZ95
8954	229533.I1RKB6	pp	229533.I1S9Y1
8955	229533.I1RKB6	pp	229533.I1RWI5
8956	229533.I1RKB6	pp	229533.V6R7V8
8957	229533.I1RKB6	pp	229533.I1S1W8
8958	229533.I1RKB6	pp	229533.I1RYP2
8959	229533.I1RKB6	pp	229533.I1RSQ4
8960	229533.I1RKB6	pp	229533.V6R3J1
8961	229533.I1RKB6	pp	229533.I1RZB2
8962	229533.I1RKB6	pp	229533.I1RRS8
8963	229533.I1RKB6	pp	229533.V6RM66
8964	229533.I1RKB6	pp	229533.I1RNM7
8965	229533.I1RKB6	pp	229533.I1S2A1
8966	229533.I1RKB6	pp	229533.I1S7L6
8967	229533.I1RKB6	pp	229533.I1RPD6
8968	229533.I1RKB6	pp	229533.Q4HXF0
8969	229533.I1RKB6	pp	229533.I1S217
8970	229533.I1RKB6	pp	229533.I1RWF5
8971	229533.I1RKB6	pp	229533.I1RY49
8972	229533.I1RKB6	pp	229533.I1RQA5
8973	229533.I1RKB6	pp	229533.I1RMW4
8974	229533.I1RKB6	pp	229533.I1S4S4
8975	229533.I1RKB6	pp	229533.I1RM67
8976	229533.I1RKB6	pp	229533.I1RXF0
8977	229533.I1RKB6	pp	229533.I1RRG3
8978	229533.I1RKB6	pp	229533.I1RRX5
8979	229533.I1RKB6	pp	229533.I1RRV4

8980 229533.I1RKB6 pp 229533.I1RT33
8981 229533.I1RKB6 pp 229533.I1RP86
8982 229533.I1RKB6 pp 229533.I1RPN4
8983 229533.I1RKB6 pp 229533.I1RQE1
8984 229533.I1RT28 pp 229533.I1RWT6
8985 229533.I1RT28 pp 229533.I1S7L1
8986 229533.I1RT28 pp 229533.I1RY44
8987 229533.I1RT28 pp 229533.I1S2E9
8988 229533.I1RT28 pp 229533.I1RZP1
8989 229533.V6QUU6 pp 229533.V6RB81
8990 229533.V6QUU6 pp 229533.V6RR76
8991 229533.I1RA22 pp 229533.I1RDY1
8992 229533.I1RA22 pp 229533.I1RRH5
8993 229533.I1RA22 pp 229533.I1RBD7
8994 229533.I1RA22 pp 229533.I1RCY6
8995 229533.A0A1C3YK67 pp 229533.I1RM12
8996 229533.A0A1C3YK67 pp 229533.I1S074
8997 229533.I1S770 pp 229533.I1S9D4
8998 229533.I1S770 pp 229533.V6RQW4
8999 229533.I1S770 pp 229533.O74638
9000 229533.I1S069 pp 229533.I1S3M2
9001 229533.I1S069 pp 229533.I1S2I7
9002 229533.I1S069 pp 229533.I1S303
9003 229533.I1RI58 pp 229533.V6RB81
9004 229533.I1RI58 pp 229533.I1SAI0
9005 229533.I1RI58 pp 229533.I1RUA7
9006 229533.I1RI58 pp 229533.I1RL93
9007 229533.I1RI58 pp 229533.I1RIJ2
9008 229533.A0A1C3YHZ2 pp 229533.Q4I6S5
9009 229533.A0A1C3YHZ2 pp 229533.I1RCE7
9010 229533.A0A1C3YHZ2 pp 229533.I1RJS2
9011 229533.A0A1C3YHZ2 pp 229533.I1RC46
9012 229533.A0A1C3YHZ2 pp 229533.V6RB81
9013 229533.A0A1C3YHZ2 pp 229533.A0A1C3YMQ5
9014 229533.A0A1C3YHZ2 pp 229533.A0A1C3YM95
9015 229533.A0A1C3YHZ2 pp 229533.A0A1C3YLS7
9016 229533.A0A1C3YHZ2 pp 229533.I1RW76
9017 229533.A0A1C3YHZ2 pp 229533.I1RKR9
9018 229533.A0A1C3YHZ2 pp 229533.I1RMF2
9019 229533.A0A1C3YHZ2 pp 229533.I1RD10
9020 229533.A0A1C3YHZ2 pp 229533.I1S7X9
9021 229533.A0A1C3YHZ2 pp 229533.I1RTG5
9022 229533.A0A1C3YHZ2 pp 229533.I1RA34
9023 229533.A0A1C3YHZ2 pp 229533.A0A1C3YIM8
9024 229533.A0A1C3YHZ2 pp 229533.I1RMR5
9025 229533.A0A1C3YHZ2 pp 229533.I1RN81
9026 229533.A0A1C3YHZ2 pp 229533.I1S270
9027 229533.A0A1C3YHZ2 pp 229533.I1REZ0
9028 229533.A0A1C3YHZ2 pp 229533.I1RMJ1
9029 229533.A0A1C3YHZ2 pp 229533.I1RRW2
9030 229533.A0A1C3YHZ2 pp 229533.A0A1C3YJJ1
9031 229533.A0A1C3YHZ2 pp 229533.Q4HZE0
9032 229533.A0A1C3YHZ2 pp 229533.I1RCN6
9033 229533.A0A1C3YHZ2 pp 229533.A0A1C3YLH6
9034 229533.A0A1C3YHZ2 pp 229533.I1RA25
9035 229533.A0A1C3YHZ2 pp 229533.V6QUU6
9036 229533.A0A1C3YHZ2 pp 229533.I1RT41
9037 229533.A0A1C3YHZ2 pp 229533.A0A1C3YI55
9038 229533.A0A1C3YHZ2 pp 229533.I1RPP0
9039 229533.A0A1C3YHZ2 pp 229533.A0A1C3YK51
9040 229533.A0A1C3YHZ2 pp 229533.A0A1C3YKT9
9041 229533.A0A1C3YHZ2 pp 229533.I1RQP5
9042 229533.A0A1C3YHZ2 pp 229533.Q4HYZ2
9043 229533.A0A1C3YHZ2 pp 229533.I1S0Z3
9044 229533.A0A1C3YHZ2 pp 229533.I1RYW8
9045 229533.A0A1C3YHZ2 pp 229533.I1RAS5
9046 229533.A0A1C3YHZ2 pp 229533.I1RWA5
9047 229533.A0A1C3YHZ2 pp 229533.I1RRF9
9048 229533.A0A1C3YHZ2 pp 229533.I1RWI6
9049 229533.A0A1C3YHZ2 pp 229533.I1S1B1
9050 229533.A0A1C3YHZ2 pp 229533.Q4ILI9
9051 229533.A0A1C3YHZ2 pp 229533.V6RR76
9052 229533.A0A1C3YHZ2 pp 229533.I1S0X0

229533.A0A1C3YHZ2 pp 229533.I1RWA9
229533.A0A098DF85 pp 229533.V6RR76
229533.A0A098DF85 pp 229533.A0A098DKM1
229533.A0A098DF85 pp 229533.I1RNF9
229533.A0A098DF85 pp 229533.A0A0E0S0U6
229533.A0A098DF85 pp 229533.A0A1C3YLL6
229533.A0A098DF85 pp 229533.I1S254
229533.A0A098DF85 pp 229533.V6R476
229533.A0A098DF85 pp 229533.A0A1C3YID2
229533.A0A098DF85 pp 229533.V6QUU6
229533.A0A098DF85 pp 229533.I1RJS2
229533.A0A098DF85 pp 229533.I1RPM8
229533.A0A098DF85 pp 229533.I1RYZ7
229533.A0A098DF85 pp 229533.Q4IJ79
229533.A0A098DF85 pp 229533.A0A1C3YHZ2
229533.A0A098DF85 pp 229533.I1RN81
229533.A0A098DF85 pp 229533.I1RZX9
229533.I1RQ53 pp 229533.I1RZU5
229533.I1RYE4 pp 229533.I1RYF8
229533.I1RYE4 pp 229533.I1RZW7
229533.I1RYE4 pp 229533.I1S549
229533.I1RYE4 pp 229533.Q4HWB3
229533.I1RYE4 pp 229533.I1S287
229533.I1RYE4 pp 229533.Q4I9U7
229533.I1RYE4 pp 229533.I1RYM2
229533.A0A1C3YJE9 pp 229533.I1RXE5
229533.A0A1C3YJE9 pp 229533.A0A1C3YK75
229533.A0A1C3YJE9 pp 229533.I1RSI1
229533.A0A1C3YJE9 pp 229533.I1RWX8
229533.A0A1C3YJE9 pp 229533.I1RSH4
229533.A0A1C3YJE9 pp 229533.I1RYX3
229533.A0A1C3YJE9 pp 229533.I1RDC9
229533.A0A1C3YJE9 pp 229533.I1RQE9
229533.A0A1C3YJE9 pp 229533.I1RZU9
229533.A0A1C3YJE9 pp 229533.I1RGE1
229533.A0A1C3YJE9 pp 229533.I1RMA5
229533.A0A1C3YJE9 pp 229533.A0A1C3YL16
229533.A0A1C3YJE9 pp 229533.I1RWU3
229533.A0A1C3YJE9 pp 229533.A0A1C3YN58
229533.A0A1C3YJE9 pp 229533.V6QV38
229533.A0A1C3YJE9 pp 229533.I1R9W6
229533.A0A1C3YJE9 pp 229533.I1S1V2
229533.A0A1C3YJE9 pp 229533.A0A1C3YK01
229533.A0A1C3YJE9 pp 229533.I1RS43
229533.A0A1C3YJE9 pp 229533.I1RKR6
229533.A0A1C3YJE9 pp 229533.I1RB36
229533.A0A1C3YJE9 pp 229533.I1RI51
229533.A0A1C3YJE9 pp 229533.I1RY99
229533.A0A1C3YJE9 pp 229533.I1RB73
229533.A0A1C3YJE9 pp 229533.A0A1C3YLP1
229533.A0A1C3YJE9 pp 229533.I1RGW0
229533.A0A1C3YJE9 pp 229533.I1RUQ6
229533.A0A1C3YJE9 pp 229533.I1RJW8
229533.A0A1C3YJE9 pp 229533.I1RCN2
229533.A0A1C3YJE9 pp 229533.I1R9Q0
229533.A0A1C3YJE9 pp 229533.I1RVY0
229533.A0A1C3YJE9 pp 229533.I1RZV1
229533.A0A1C3YJE9 pp 229533.I1RHP9
229533.A0A1C3YJE9 pp 229533.A0A1C3YMF9
229533.A0A1C3YJE9 pp 229533.I1RPY4
229533.A0A1C3YJE9 pp 229533.A0A1C3YJZ2
229533.I1RCA6 pp 229533.I1RKL6
229533.I1RCA6 pp 229533.I1RQ53
229533.I1RB96 pp 229533.I1S069
229533.I1RB96 pp 229533.I1RJD9
229533.I1RB96 pp 229533.I1S303
229533.I1RB96 pp 229533.I1S2I7
229533.I1RB96 pp 229533.I1RR60
229533.I1RB96 pp 229533.I1RVC7
229533.I1RSV9 pp 229533.I1S1F3
229533.I1RSV9 pp 229533.I1RWC2
229533.I1RSV9 pp 229533.I1RX17
229533.I1RB94 pp 229533.I1RRN5

229533.I1RB94 pp 229533.V6QY25
229533.I1RB94 pp 229533.I1RT28
229533.I1RP37 pp 229533.Q4I1Y1
229533.A0A1C3YKY2 pp 229533.I1RSV9
229533.I1RJN0 pp 229533.I1RNG4
229533.I1RWQ6 pp 229533.I1S4S7
229533.I1RWQ6 pp 229533.I1S0F5
229533.A0A1C3YK64 pp 229533.I1RNN3
229533.A0A1C3YK60 pp 229533.I1RT00
229533.I1RA16 pp 229533.I1RNC2
229533.I1RA16 pp 229533.I1RCV3
229533.I1RA16 pp 229533.I1RYP0
229533.I1RLT2 pp 229533.I1RP37
229533.I1RLT2 pp 229533.Q4I1Y1
229533.A0A098E000 pp 229533.I1RJQ3
229533.A0A098E000 pp 229533.I1RP37
229533.A0A098E000 pp 229533.A0A1C3YLL4
229533.A0A098E000 pp 229533.A0A0E0SHE3
229533.A0A098E000 pp 229533.I1RLT2
229533.A0A098E000 pp 229533.Q4I1Y1
229533.A0A098DR37 pp 229533.A0A1C3YLB6
229533.A0A098DR37 pp 229533.I1RNG4
229533.A0A1C3YIJ2 pp 229533.I1RKR6
229533.A0A1C3YIJ2 pp 229533.A0A1C3YK01
229533.A0A1C3YIJ2 pp 229533.V6QV38
229533.A0A1C3YIJ2 pp 229533.I1RZU9
229533.A0A1C3YIJ2 pp 229533.A0A1C3YJE9
229533.A0A1C3YIJ2 pp 229533.I1RQE9
229533.A0A1C3YIJ2 pp 229533.A0A1C3YJ12
229533.A0A1C3YIJ2 pp 229533.I1RVY0
229533.A0A1C3YIJ2 pp 229533.I1R9Q0
229533.A0A1C3YIJ2 pp 229533.A0A1C3YJZ2
229533.A0A1C3YIJ2 pp 229533.I1RGW0
229533.A0A1C3YIJ2 pp 229533.I1RI51
229533.A0A1C3YIJ2 pp 229533.I1RHP9
229533.A0A1C3YIJ2 pp 229533.I1R9W6
229533.A0A1C3YIJ2 pp 229533.I1RXE5
229533.A0A1C3YIJ2 pp 229533.I1RDC9
229533.A0A1C3YIJ2 pp 229533.I1RGE1
229533.A0A1C3YIJ2 pp 229533.I1RMA5
229533.A0A1C3YIJ2 pp 229533.I1RUQ6
229533.A0A1C3YIJ2 pp 229533.I1RB36
229533.A0A098DZZ2 pp 229533.I1RPK5
229533.A0A098DZZ2 pp 229533.I1RG99
229533.A0A098DZZ2 pp 229533.I1RR68
229533.A0A098DZZ2 pp 229533.I1S0P7
229533.I1S6Y8 pp 229533.Q4I327
229533.I1S6Y8 pp 229533.V6RMW6
229533.I1S6Y8 pp 229533.Q4HXF0
229533.I1S6Y8 pp 229533.V6QUE6
229533.I1S6Y8 pp 229533.V6R042
229533.I1S6Y8 pp 229533.V6RCG5
229533.I1S6Y8 pp 229533.Q4I624
229533.I1S6Y8 pp 229533.V6R057
229533.I1S6Y8 pp 229533.Q4IJK2
229533.I1S6Y8 pp 229533.V6RUT5
229533.I1S6Y8 pp 229533.V6RCZ8
229533.I1S6Y8 pp 229533.V6QW22
229533.I1S6Y8 pp 229533.V6RTJ8
229533.I1S6Y8 pp 229533.V6RIV9
229533.I1S6Y8 pp 229533.V6R227
229533.I1S6Y8 pp 229533.I1S887
229533.I1RAW4 pp 229533.I1RMG6
229533.I1RAW4 pp 229533.I1S0E2
229533.I1RAW4 pp 229533.Q4IEV4
229533.I1RAW4 pp 229533.I1RFW1
229533.I1RAW4 pp 229533.Q4I5R3
229533.I1RAW4 pp 229533.Q4IER8
229533.I1RAW4 pp 229533.I1S537
229533.I1RAW4 pp 229533.I1S0T2
229533.I1RAW4 pp 229533.I1RTE9
229533.I1RAW4 pp 229533.I1RD84
229533.I1RAW4 pp 229533.I1RB11

229533.I1RAW4 pp 229533.I1RR51
229533.I1RAW4 pp 229533.Q4HZS8
229533.I1RAW4 pp 229533.I1RT41
229533.I1RAW4 pp 229533.I1RIV2
229533.I1REC5 pp 229533.I1RPE0
229533.I1REC5 pp 229533.I1S5R1
229533.I1REC5 pp 229533.I1RXC2
229533.I1REC5 pp 229533.I1S4S7
229533.I1REC5 pp 229533.I1RPP3
229533.I1REC5 pp 229533.I1RYI5
229533.I1REC5 pp 229533.I1RTA1
229533.I1REC5 pp 229533.I1S0X7
229533.I1REC5 pp 229533.I1S438
229533.I1REC5 pp 229533.I1RSZ7
229533.I1REC5 pp 229533.I1RJN9
229533.I1REC5 pp 229533.I1RX95
229533.I1REC5 pp 229533.I1RVZ2
229533.I1REC5 pp 229533.I1S9I5
229533.I1REC5 pp 229533.I1RYR0
229533.I1REC5 pp 229533.I1RKJ5
229533.I1REC5 pp 229533.I1S2B8
229533.I1REC5 pp 229533.I1RMQ6
229533.I1REC5 pp 229533.I1RSA5
229533.I1REC5 pp 229533.I1S0W4
229533.I1REC5 pp 229533.I1RXD6
229533.I1REC5 pp 229533.I1RZ39
229533.I1REC5 pp 229533.I1RRI1
229533.I1REC5 pp 229533.I1RTB3
229533.I1REC5 pp 229533.I1RJU3
229533.I1REC5 pp 229533.V6RTJ8
229533.I1REC5 pp 229533.I1RT40
229533.I1REC5 pp 229533.I1RRM3
229533.I1REC5 pp 229533.I1RR24
229533.I1REC5 pp 229533.I1RXD7
229533.I1REC5 pp 229533.I1RGA4
229533.I1REC5 pp 229533.I1RX46
229533.I1REC5 pp 229533.I1RPY3
229533.I1REC5 pp 229533.I1RTD5
229533.I1REC5 pp 229533.V6RMW6
229533.I1REC5 pp 229533.I1RNB2
229533.I1REC5 pp 229533.I1RSQ3
229533.I1REC5 pp 229533.I1RUF7
229533.I1REC5 pp 229533.V6R042
229533.I1REC5 pp 229533.I1RZK5
229533.I1REC5 pp 229533.I1RVX0
229533.I1REC5 pp 229533.I1RGB2
229533.I1REC5 pp 229533.V6RCZ8
229533.I1REC5 pp 229533.Q4IJK2
229533.I1REC5 pp 229533.I1RQE7
229533.I1REC5 pp 229533.I1S437
229533.I1REC5 pp 229533.I1S821
229533.I1REC5 pp 229533.I1RQ33
229533.I1REC5 pp 229533.I1RRY9
229533.I1REC5 pp 229533.I1REK8
229533.I1REC5 pp 229533.I1RWR7
229533.I1REC5 pp 229533.I1RNC1
229533.I1REC5 pp 229533.I1RKJ4
229533.I1REC5 pp 229533.I1RUG3
229533.I1REC5 pp 229533.I1RZM8
229533.I1REC5 pp 229533.I1RVQ3
229533.I1REC5 pp 229533.V6R9Z0
229533.I1REC5 pp 229533.I1RNY8
229533.I1REC5 pp 229533.I1S5R6
229533.I1REC5 pp 229533.I1RXC4
229533.I1REC5 pp 229533.I1RQD2
229533.I1REC5 pp 229533.I1S1B2
229533.I1REC5 pp 229533.I1RSF1
229533.I1REC5 pp 229533.I1RPU2
229533.I1REC5 pp 229533.I1RRV3
229533.I1REC5 pp 229533.I1RM37
229533.I1REC5 pp 229533.I1RQ85
229533.I1REC5 pp 229533.I1RZV3
229533.I1REC5 pp 229533.I1S1W9

9272 229533.A0A098DZV3 pp 229533.A0A0E0RW49
9273 229533.A0A098DZV3 pp 229533.I1S3C9
9274 229533.A0A098DZV3 pp 229533.I1RZ94
9275 229533.A0A098DZV3 pp 229533.Q4I7L0
9276 229533.A0A1C3YM42 pp 229533.I1RE19
9277 229533.A0A1C3YM42 pp 229533.I1RB11
9278 229533.A0A1C3YM42 pp 229533.I1RMP2
9279 229533.A0A1C3YM42 pp 229533.I1RQL4
9280 229533.I1RAB4 pp 229533.V6RTJ8
9281 229533.I1RAB4 pp 229533.I1RBD5
9282 229533.I1RAB4 pp 229533.I1S887
9283 229533.I1RAB4 pp 229533.I1RRX5
9284 229533.I1RAB4 pp 229533.I1S6K9
9285 229533.I1RAB4 pp 229533.I1RZM4
9286 229533.I1RAB4 pp 229533.V6RK96
9287 229533.I1RAB4 pp 229533.I1S1J7
9288 229533.I1RAB4 pp 229533.I1RCM5
9289 229533.I1RAB4 pp 229533.I1RW71
9290 229533.I1RAB4 pp 229533.I1RRY9
9291 229533.I1RAB4 pp 229533.Q4IJK2
9292 229533.I1RAB4 pp 229533.I1S0P0
9293 229533.I1RAB4 pp 229533.I1RBT1
9294 229533.I1RAB4 pp 229533.I1RNC1
9295 229533.I1RAB4 pp 229533.I1RRN2
9296 229533.I1RAB4 pp 229533.I1RQE8
9297 229533.I1RAB4 pp 229533.I1REK8
9298 229533.I1RAB4 pp 229533.I1RJU3
9299 229533.I1RAB4 pp 229533.I1S5R6
9300 229533.I1RAB4 pp 229533.V6RUT5
9301 229533.I1RAB4 pp 229533.I1RTA0
9302 229533.I1RAB4 pp 229533.I1RMV0
9303 229533.I1RAB4 pp 229533.I1RSX0
9304 229533.I1RAB4 pp 229533.I1RP86
9305 229533.I1RAB4 pp 229533.I1S4S4
9306 229533.I1RAB4 pp 229533.I1RTA1
9307 229533.I1RAB4 pp 229533.I1RRI2
9308 229533.I1RAB4 pp 229533.I1RPK0
9309 229533.I1RAB4 pp 229533.I1S1W3
9310 229533.I1RAB4 pp 229533.I1S1W9
9311 229533.I1RAB4 pp 229533.I1RWX5
9312 229533.I1RAB4 pp 229533.I1S8P5
9313 229533.I1RAB4 pp 229533.I1RNG4
9314 229533.I1RAB4 pp 229533.I1RXU9
9315 229533.I1RAB4 pp 229533.I1RPV3
9316 229533.I1RAB4 pp 229533.I1RN67
9317 229533.I1RAB4 pp 229533.I1RN88
9318 229533.I1RAB4 pp 229533.I1RZZ9
9319 229533.I1RAB4 pp 229533.I1RY92
9320 229533.I1RAB4 pp 229533.V6QVH8
9321 229533.I1RAB4 pp 229533.I1RC29
9322 229533.I1RAB4 pp 229533.I1RFL4
9323 229533.I1RAB4 pp 229533.I1RIH0
9324 229533.I1RAB4 pp 229533.I1RE76
9325 229533.I1RAB4 pp 229533.I1RJZ7
9326 229533.I1RAB4 pp 229533.I1RZ39
9327 229533.I1RAB4 pp 229533.I1RRT8
9328 229533.I1RAB4 pp 229533.I1RXR1
9329 229533.I1RAB4 pp 229533.Q4I7L0
9330 229533.I1RAB4 pp 229533.I1RTD5
9331 229533.I1RGU7 pp 229533.I1RZM8
9332 229533.I1RGU7 pp 229533.Q4IJK2
9333 229533.I1RGU7 pp 229533.I1RQ85
9334 229533.I1RGU7 pp 229533.I1S4S7
9335 229533.I1RGU7 pp 229533.V6RCZ8
9336 229533.I1RGU7 pp 229533.I1RZK5
9337 229533.I1RGU7 pp 229533.V6REE9
9338 229533.I1RGU7 pp 229533.I1RQD2
9339 229533.I1RGU7 pp 229533.I1S5B0
9340 229533.I1RGU7 pp 229533.I1RXD7
9341 229533.I1RGU7 pp 229533.I1RL20
9342 229533.I1RGU7 pp 229533.I1RWR7
9343 229533.I1RGU7 pp 229533.V6RTJ8
9344 229533.I1RGU7 pp 229533.I1RJU3

229533.I1RGU7 pp 229533.I1S438
229533.I1RGU7 pp 229533.I1RXC4
229533.I1RGU7 pp 229533.I1RQE7
229533.I1RGU7 pp 229533.I1S4S4
229533.I1RGU7 pp 229533.I1RVX0
229533.I1RGU7 pp 229533.I1RQ33
229533.I1RGU7 pp 229533.V6RMW6
229533.I1RGU7 pp 229533.I1RRM3
229533.I1RGU7 pp 229533.I1RJN9
229533.I1RGU7 pp 229533.I1S437
229533.I1RGU7 pp 229533.V6R042
229533.I1RGU7 pp 229533.I1RPK0
229533.I1RGU7 pp 229533.I1RTA1
229533.I1RGU7 pp 229533.I1S1W9
229533.I1RGU7 pp 229533.I1RPP3
229533.I1RGU7 pp 229533.I1RZV3
229533.I1RGU7 pp 229533.I1RYI5
229533.I1RGU7 pp 229533.I1RSQ3
229533.I1RGU7 pp 229533.I1S0W4
229533.I1RGU7 pp 229533.I1S5R6
229533.I1RGU7 pp 229533.I1S444
229533.I1RGU7 pp 229533.I1RSA5
229533.I1RGU7 pp 229533.I1RPU2
229533.I1RGU7 pp 229533.I1RNC1
229533.I1RGU7 pp 229533.I1RUG3
229533.I1RGU7 pp 229533.I1RT40
229533.I1RGU7 pp 229533.I1S2B8
229533.I1RGU7 pp 229533.I1RZ39
229533.I1RGU7 pp 229533.I1RSZ7
229533.I1RGU7 pp 229533.I1S5R1
229533.I1RGU7 pp 229533.I1RXC2
229533.I1RGU7 pp 229533.I1RYR0
229533.I1RGU7 pp 229533.I1RPE0
229533.I1RGU7 pp 229533.I1RRI1
229533.I1RGU7 pp 229533.I1RVZ2
229533.I1RWE0 pp 229533.I1RYZ0
229533.A0A098DEY3 pp 229533.I1RB11
229533.A0A098DEY3 pp 229533.V6RGI1
229533.A0A098DEY3 pp 229533.I1RAW4
229533.A0A098DEY3 pp 229533.I1RUP1
229533.A0A098DEY3 pp 229533.A0A1C3YM42
229533.A0A098DEY3 pp 229533.I1RTP1
229533.A0A098DEW5 pp 229533.I1SAT1
229533.A0A098DEW5 pp 229533.A0A1C3YLT9
229533.A0A1C3YIP1 pp 229533.I1RL13
229533.I1RTN3 pp 229533.I1S4S7
229533.I1RTN3 pp 229533.I1S2C1
229533.I1RTN3 pp 229533.I1S4S4
229533.I1RTN3 pp 229533.I1RYI5
229533.I1RTN3 pp 229533.I1RY63
229533.I1RTN3 pp 229533.I1RY57
229533.I1RTN3 pp 229533.I1RVX0
229533.I1RTN3 pp 229533.I1RW61
229533.I1RTN3 pp 229533.I1RZ95
229533.I1RTN3 pp 229533.I1S0R9
229533.I1RGB0 pp 229533.I1RW61
229533.I1RGB0 pp 229533.I1S4S7
229533.I1RGB0 pp 229533.I1RSZ7
229533.I1RGB0 pp 229533.I1RY63
229533.I1RGB0 pp 229533.I1S4S4
229533.I1RGB0 pp 229533.I1RTA1
229533.I1RGB0 pp 229533.I1RP97
229533.I1RGB0 pp 229533.I1S2C1
229533.I1RGB0 pp 229533.I1RYI5
229533.I1RGB0 pp 229533.I1S0R9
229533.I1RGB0 pp 229533.I1RJU3
229533.I1RGB0 pp 229533.I1RTE9
229533.I1RGB0 pp 229533.I1RY57
229533.I1RGB0 pp 229533.I1RSQ4
229533.I1RGB0 pp 229533.I1RZ95
229533.I1RGB0 pp 229533.I1RKB6
229533.I1REV8 pp 229533.I1S4S4
229533.I1REV8 pp 229533.I1S2C1

9418	229533.I1REV8	pp	229533.I1S4S7
9419	229533.I1REV8	pp	229533.I1RJU3
9420	229533.I1REV8	pp	229533.I1RVX0
9421	229533.I1REV8	pp	229533.I1RSQ4
9422	229533.I1REV8	pp	229533.I1RYI5
9423	229533.I1REV8	pp	229533.I1RY63
9424	229533.I1REV8	pp	229533.I1RKB6
9425	229533.I1REV8	pp	229533.I1RTA1
9426	229533.I1REV8	pp	229533.I1RTE9
9427	229533.I1REV8	pp	229533.I1RSZ7
9428	229533.I1REV8	pp	229533.I1S0R9
9429	229533.I1REV8	pp	229533.I1RW61
9430	229533.I1REV8	pp	229533.I1RZ95
9431	229533.I1REV8	pp	229533.I1RGA4
9432	229533.I1REV8	pp	229533.I1RP97
9433	229533.I1REV8	pp	229533.I1RY57
9434	229533.I1RWL3	pp	229533.I1S147
9435	229533.I1RWL3	pp	229533.I1S7M8
9436	229533.I1RWL3	pp	229533.Q4I5W5
9437	229533.I1RWL3	pp	229533.I1S0V7
9438	229533.I1RWL3	pp	229533.V6RCJ5
9439	229533.I1RWL3	pp	229533.Q4I7T5
9440	229533.I1RWL3	pp	229533.I1S9Z4
9441	229533.I1RWL3	pp	229533.I1RZ38
9442	229533.I1RWL3	pp	229533.I1SAB4
9443	229533.I1RWL3	pp	229533.I1RZU4
9444	229533.I1RWL3	pp	229533.I1S2Q4
9445	229533.I1RWL3	pp	229533.I1S6V0
9446	229533.I1RWL3	pp	229533.I1S2C4
9447	229533.I1RWL3	pp	229533.I1S223
9448	229533.I1RWL3	pp	229533.I1S0V6
9449	229533.I1RWL3	pp	229533.I1RWZ3
9450	229533.I1RWL3	pp	229533.I1S210
9451	229533.I1RWL3	pp	229533.I1S1B8
9452	229533.I1RWL3	pp	229533.I1S8C1
9453	229533.I1RWL3	pp	229533.I1S4D0
9454	229533.I1RWL3	pp	229533.I1S3T6
9455	229533.I1RWL3	pp	229533.V6R5T7
9456	229533.I1RWL3	pp	229533.I1S4G3
9457	229533.I1RWL3	pp	229533.I1RY12
9458	229533.I1RWL3	pp	229533.V6RCC0
9459	229533.I1RWL3	pp	229533.Q4I5I4
9460	229533.I1RWL3	pp	229533.I1S029
9461	229533.I1RWL3	pp	229533.V6RWB3
9462	229533.I1RWL3	pp	229533.I1S8D3
9463	229533.I1RWL3	pp	229533.I1S2F7
9464	229533.I1RWL3	pp	229533.I1RYM5
9465	229533.I1RWL3	pp	229533.V6QUE6
9466	229533.I1RWL3	pp	229533.I1RYS4
9467	229533.I1RWL3	pp	229533.V6RY56
9468	229533.I1RWL3	pp	229533.V6RHX2
9469	229533.I1RWL3	pp	229533.I1RZB5
9470	229533.I1RWL3	pp	229533.I1RY81
9471	229533.A0A1C3YJY9	pp	229533.I1RT01
9472	229533.A0A1C3YJY9	pp	229533.I1RW23
9473	229533.A0A1C3YJY9	pp	229533.I1RAX8
9474	229533.A0A1C3YJY9	pp	229533.I1RAL2
9475	229533.A0A1C3YJY9	pp	229533.I1RQ88
9476	229533.A0A1C3YJY9	pp	229533.I1RRB0
9477	229533.A0A1C3YJY9	pp	229533.I1RJK6
9478	229533.A0A1C3YJY9	pp	229533.A0A1C3YKW5
9479	229533.A0A1C3YJY9	pp	229533.I1RMP0
9480	229533.A0A1C3YJY9	pp	229533.I1RZR6
9481	229533.A0A1C3YJY9	pp	229533.I1RS41
9482	229533.A0A098DZS9	pp	229533.A0A1C3YLB6
9483	229533.A0A098DZS9	pp	229533.A0A1C3YJU7
9484	229533.A0A098DZS9	pp	229533.I1RWA3
9485	229533.A0A098DZS9	pp	229533.A0A1C3YJQ2
9486	229533.A0A098DZS9	pp	229533.I1S0Q7
9487	229533.A0A098DZS9	pp	229533.I1RA52
9488	229533.A0A098DZS9	pp	229533.I1RY92
9489	229533.A0A098DZS9	pp	229533.I1RE65
9490	229533.A0A098DZS9	pp	229533.V6RJP8

9491 229533.A0A098DZS9 pp 229533.I1S5X9
9492 229533.A0A098DZS9 pp 229533.I1RP02
9493 229533.A0A098DZS9 pp 229533.A0A0E0RPV7
9494 229533.A0A098DZS9 pp 229533.I1S0R8
9495 229533.A0A098DZS9 pp 229533.I1RS29
9496 229533.A0A098DZS9 pp 229533.I1RNM7
9497 229533.A0A098DZS9 pp 229533.I1RBT6
9498 229533.A0A098DZS9 pp 229533.I1S0K2
9499 229533.A0A098DZS9 pp 229533.A0A0E0S8Z0
9500 229533.A0A098DZS9 pp 229533.I1RYT8
9501 229533.A0A098DZS9 pp 229533.I1RFJ6
9502 229533.A0A098DZS9 pp 229533.A0A1C3YLB0
9503 229533.A0A098DZS9 pp 229533.I1S464
9504 229533.A0A098DZS9 pp 229533.I1RAM9
9505 229533.A0A098DZS9 pp 229533.A0A0E0SAR4
9506 229533.A0A098DZS9 pp 229533.I1RKB7
9507 229533.A0A098DZS9 pp 229533.I1RE66
9508 229533.A0A098DZS9 pp 229533.I1S1F3
9509 229533.A0A098DZS9 pp 229533.I1RMQ6
9510 229533.A0A098DZS9 pp 229533.I1S0P0
9511 229533.A0A098DZS9 pp 229533.I1RY21
9512 229533.A0A098DZS9 pp 229533.I1RXD9
9513 229533.A0A098DZS9 pp 229533.I1RS05
9514 229533.A0A098DZS9 pp 229533.I1RMK7
9515 229533.I1RCZ0 pp 229533.I1S1V2
9516 229533.I1RCZ0 pp 229533.Q4IPI4
9517 229533.I1RCZ0 pp 229533.I1RE51
9518 229533.I1RCZ0 pp 229533.I1S7I5
9519 229533.I1RUQ2 pp 229533.Q4I8P5
9520 229533.I1RUQ2 pp 229533.V6RIV9
9521 229533.I1RUQ2 pp 229533.I1S0J1
9522 229533.I1RUQ2 pp 229533.Q4IJ56
9523 229533.I1RUQ2 pp 229533.I1RX14
9524 229533.I1RUQ2 pp 229533.I1SA32
9525 229533.I1RUQ2 pp 229533.I1RW46
9526 229533.I1RUQ2 pp 229533.V6RCZ8
9527 229533.I1RUQ2 pp 229533.V6RCG5
9528 229533.I1RUQ2 pp 229533.I1S9U1
9529 229533.I1RUQ2 pp 229533.V6R057
9530 229533.I1RUQ2 pp 229533.V6R227
9531 229533.I1RUQ2 pp 229533.I1S296
9532 229533.I1RUQ2 pp 229533.I1RYB9
9533 229533.I1RUQ2 pp 229533.Q4IPZ1
9534 229533.I1RUQ2 pp 229533.I1RZF6
9535 229533.I1RUQ2 pp 229533.Q4IN63
9536 229533.I1RUQ2 pp 229533.Q4I5Z5
9537 229533.I1RUQ2 pp 229533.I1RZM8
9538 229533.I1RUQ2 pp 229533.Q4IEK8
9539 229533.I1RUQ2 pp 229533.Q4HZB8
9540 229533.I1RUQ2 pp 229533.I1RW99
9541 229533.I1RUQ2 pp 229533.I1S5B0
9542 229533.I1RUQ2 pp 229533.V6QW22
9543 229533.I1RUQ2 pp 229533.I1S1J6
9544 229533.I1RUQ2 pp 229533.Q4I830
9545 229533.I1RUQ2 pp 229533.V6RGN5
9546 229533.I1RUQ2 pp 229533.Q4HZK7
9547 229533.I1RUQ2 pp 229533.I1SA33
9548 229533.I1RUQ2 pp 229533.I1S8R3
9549 229533.I1RUQ2 pp 229533.I1RZB5
9550 229533.I1RUQ2 pp 229533.I1RZZ1
9551 229533.I1RUQ2 pp 229533.I1S2M6
9552 229533.I1RUQ2 pp 229533.I1RZW8
9553 229533.I1RUQ2 pp 229533.V6R283
9554 229533.I1RUQ2 pp 229533.I1RZK6
9555 229533.I1RUQ2 pp 229533.Q4I662
9556 229533.I1RUQ2 pp 229533.I1S0L3
9557 229533.I1RUQ2 pp 229533.V6R1K4
9558 229533.I1RUQ2 pp 229533.I1RW61
9559 229533.I1RUQ2 pp 229533.I1S274
9560 229533.I1RUQ2 pp 229533.I1RW25
9561 229533.I1RUQ2 pp 229533.I1RVQ0
9562 229533.I1RUQ2 pp 229533.I1S0F6
9563 229533.I1RUQ2 pp 229533.Q4IFI0

9564	229533.I1RUQ2	pp	229533.I1RWD4
9565	229533.I1RUQ2	pp	229533.I1S5R1
9566	229533.I1RUQ2	pp	229533.I1RXD7
9567	229533.I1RUQ2	pp	229533.I1S2H4
9568	229533.I1RUQ2	pp	229533.Q4HVW2
9569	229533.I1RUQ2	pp	229533.I1RWJ8
9570	229533.I1RUQ2	pp	229533.I1S0R7
9571	229533.I1RUQ2	pp	229533.Q4IJH1
9572	229533.I1RUQ2	pp	229533.Q4IQW0
9573	229533.I1RUQ2	pp	229533.Q4IF76
9574	229533.I1RUQ2	pp	229533.I1RX46
9575	229533.I1RUQ2	pp	229533.I1S1B6
9576	229533.I1RUQ2	pp	229533.I1S9U2
9577	229533.I1RUQ2	pp	229533.I1RZZ9
9578	229533.I1RUQ2	pp	229533.I1RZS6
9579	229533.I1RUQ2	pp	229533.V6QYF0
9580	229533.A0A0E0S7B3	pp	229533.I1RPU0
9581	229533.A0A0E0S7B3	pp	229533.I1RKJ6
9582	229533.A0A0E0S7B3	pp	229533.A0A1C3YIN1
9583	229533.A0A0E0S7B3	pp	229533.I1RWJ8
9584	229533.A0A0E0S7B3	pp	229533.I1RM67
9585	229533.A0A0E0S7B3	pp	229533.Q4HVW2
9586	229533.A0A0E0S7B3	pp	229533.I1S5R1
9587	229533.A0A0E0S7B3	pp	229533.I1RMK3
9588	229533.A0A0E0S7B3	pp	229533.I1RDA0
9589	229533.A0A0E0S7B3	pp	229533.I1RRT8
9590	229533.A0A0E0S7B3	pp	229533.I1S253
9591	229533.A0A0E0S7B3	pp	229533.I1S2H4
9592	229533.A0A0E0S7B3	pp	229533.I1RBN1
9593	229533.A0A0E0S7B3	pp	229533.I1RD64
9594	229533.A0A0E0S7B3	pp	229533.I1RRM5
9595	229533.A0A0E0S7B3	pp	229533.I1RR24
9596	229533.A0A0E0S7B3	pp	229533.I1RJZ7
9597	229533.A0A0E0S7B3	pp	229533.I1S9U2
9598	229533.A0A0E0S7B3	pp	229533.I1RPW9
9599	229533.A0A0E0S7B3	pp	229533.A0A1C3YIR8
9600	229533.A0A0E0S7B3	pp	229533.I1RT05
9601	229533.A0A0E0S7B3	pp	229533.Q4IFI0
9602	229533.A0A0E0S7B3	pp	229533.Q4IF76
9603	229533.A0A0E0S7B3	pp	229533.I1RW25
9604	229533.A0A0E0S7B3	pp	229533.I1RBY4
9605	229533.A0A0E0S7B3	pp	229533.I1RZZ9
9606	229533.A0A0E0S7B3	pp	229533.I1RWI5
9607	229533.A0A0E0S7B3	pp	229533.I1RAC2
9608	229533.A0A0E0S7B3	pp	229533.A0A1C3YIS7
9609	229533.A0A0E0S7B3	pp	229533.I1RBT1
9610	229533.A0A0E0S7B3	pp	229533.I1RQM6
9611	229533.A0A0E0S7B3	pp	229533.I1RZG3
9612	229533.A0A0E0S7B3	pp	229533.I1RC32
9613	229533.A0A0E0S7B3	pp	229533.I1S8E2
9614	229533.A0A0E0S7B3	pp	229533.I1RW61
9615	229533.A0A0E0S7B3	pp	229533.A0A1C3YN30
9616	229533.A0A0E0S7B3	pp	229533.I1RCS1
9617	229533.A0A0E0S7B3	pp	229533.I1RFS3
9618	229533.A0A0E0S7B3	pp	229533.Q4I662
9619	229533.A0A0E0S7B3	pp	229533.I1RJU3
9620	229533.A0A0E0S7B3	pp	229533.I1RPM4
9621	229533.A0A0E0S7B3	pp	229533.I1RNH1
9622	229533.A0A0E0S7B3	pp	229533.A0A1C3YN63
9623	229533.A0A0E0S7B3	pp	229533.I1RT80
9624	229533.A0A0E0S7B3	pp	229533.I1RNV6
9625	229533.A0A0E0S7B3	pp	229533.I1S6Y8
9626	229533.A0A0E0S7B3	pp	229533.Q4IJH1
9627	229533.A0A0E0S7B3	pp	229533.I1S6F0
9628	229533.A0A0E0S7B3	pp	229533.I1RG51
9629	229533.A0A0E0S7B3	pp	229533.I1RRQ3
9630	229533.A0A0E0S7B3	pp	229533.I1RWJ4
9631	229533.A0A0E0S7B3	pp	229533.I1RTF4
9632	229533.A0A0E0S7B3	pp	229533.I1RBG9
9633	229533.A0A0E0S7B3	pp	229533.I1RFG0
9634	229533.A0A0E0S7B3	pp	229533.I1RND6
9635	229533.A0A0E0S7B3	pp	229533.Q4HZ42
9636	229533.A0A0E0S7B3	pp	229533.V6QYF0

9637	229533.A0A0E0S7B3	pp	229533.I1RT33
9638	229533.A0A0E0S7B3	pp	229533.I1RKQ5
9639	229533.A0A0E0S7B3	pp	229533.I1RRQ6
9640	229533.A0A0E0S7B3	pp	229533.I1RJW6
9641	229533.A0A0E0S7B3	pp	229533.I1RC29
9642	229533.A0A0E0S7B3	pp	229533.A0A1C3YLK8
9643	229533.A0A0E0S7B3	pp	229533.I1RLV9
9644	229533.A0A0E0S7B3	pp	229533.I1RNA0
9645	229533.A0A0E0S7B3	pp	229533.I1RS47
9646	229533.A0A0E0S7B3	pp	229533.I1RD52
9647	229533.A0A0E0S7B3	pp	229533.Q4IAS1
9648	229533.A0A0E0S7B3	pp	229533.Q4IQK7
9649	229533.A0A0E0S7B3	pp	229533.Q4IPI1
9650	229533.A0A0E0S7B3	pp	229533.I1S0D4
9651	229533.A0A0E0S7B3	pp	229533.I1RX24
9652	229533.A0A0E0S7B3	pp	229533.I1S0L3
9653	229533.A0A0E0S7B3	pp	229533.A0A1C3YMW0
9654	229533.A0A0E0S7B3	pp	229533.I1RJL4
9655	229533.A0A0E0S7B3	pp	229533.I1RZS6
9656	229533.A0A0E0S7B3	pp	229533.I1RQS5
9657	229533.A0A0E0S7B3	pp	229533.I1RQ65
9658	229533.A0A0E0S7B3	pp	229533.I1RM06
9659	229533.A0A0E0S7B3	pp	229533.Q4IEV5
9660	229533.A0A0E0S7B3	pp	229533.I1RCL3
9661	229533.A0A0E0S7B3	pp	229533.V6R1K4
9662	229533.A0A0E0S7B3	pp	229533.I1RRT2
9663	229533.A0A0E0S7B3	pp	229533.I1S274
9664	229533.A0A0E0S7B3	pp	229533.I1RBL7
9665	229533.A0A0E0S7B3	pp	229533.I1RE87
9666	229533.A0A0E0S7B3	pp	229533.I1S0T2
9667	229533.A0A0E0S7B3	pp	229533.I1S1U7
9668	229533.A0A0E0S7B3	pp	229533.I1RC03
9669	229533.A0A0E0S7B3	pp	229533.I1RC82
9670	229533.A0A0E0S7B3	pp	229533.I1RSR3
9671	229533.A0A0E0S7B3	pp	229533.I1RFN1
9672	229533.A0A0E0S7B3	pp	229533.I1S1B6
9673	229533.A0A0E0S7B3	pp	229533.V6R283
9674	229533.A0A0E0S7B3	pp	229533.V6RGY3
9675	229533.A0A0E0S7B3	pp	229533.I1RN63
9676	229533.A0A0E0S7B3	pp	229533.I1RBS6
9677	229533.A0A0E0S7B3	pp	229533.I1RJY2
9678	229533.A0A0E0S7B3	pp	229533.I1RJV4
9679	229533.A0A0E0S7B3	pp	229533.Q4IQW0
9680	229533.A0A0E0S7B3	pp	229533.I1RQH2
9681	229533.A0A0E0S7B3	pp	229533.I1RFW5
9682	229533.A0A0E0S7B3	pp	229533.I1RTD9
9683	229533.A0A0E0S7B3	pp	229533.A0A0E0SGM4
9684	229533.A0A0E0S7B3	pp	229533.I1RRG3
9685	229533.A0A0E0S7B3	pp	229533.I1RSR8
9686	229533.A0A0E0S7B3	pp	229533.I1RR00
9687	229533.A0A0E0S7B3	pp	229533.I1RAF8
9688	229533.A0A0E0S7B3	pp	229533.I1RT40
9689	229533.A0A0E0S7B3	pp	229533.I1RMD5
9690	229533.A0A0E0S7B3	pp	229533.A0A1C3YHX6
9691	229533.A0A0E0S7B3	pp	229533.I1RT98
9692	229533.A0A0E0S7B3	pp	229533.I1RKL3
9693	229533.A0A0E0S7B3	pp	229533.I1RK79
9694	229533.A0A0E0S7B3	pp	229533.I1RBC9
9695	229533.A0A0E0S7B3	pp	229533.I1RW71
9696	229533.A0A0E0S7B3	pp	229533.Q4INZ9
9697	229533.A0A0E0S7B3	pp	229533.I1RNH9
9698	229533.A0A0E0S7B3	pp	229533.I1RT04
9699	229533.A0A0E0S7B3	pp	229533.I1RSX1
9700	229533.A0A0E0S7B3	pp	229533.I1RZW8
9701	229533.A0A0E0S7B3	pp	229533.A0A0E0SJN2
9702	229533.A0A0E0S7B3	pp	229533.Q4I830
9703	229533.A0A0E0S7B3	pp	229533.I1RSQ7
9704	229533.A0A0E0S7B3	pp	229533.I1RTI6
9705	229533.A0A0E0S7B3	pp	229533.I1S0R7
9706	229533.A0A0E0S7B3	pp	229533.I1RRI2
9707	229533.A0A0E0S7B3	pp	229533.A0A0E0SFN0
9708	229533.A0A0E0S7B3	pp	229533.A0A1C3YLF5
9709	229533.A0A0E0S7B3	pp	229533.I1RXD7

9710	229533.A0A0E0S7B3	pp	229533.I1RRJ8
9711	229533.A0A0E0S7B3	pp	229533.V6QW22
9712	229533.A0A0E0S7B3	pp	229533.Q4HZK7
9713	229533.A0A0E0S7B3	pp	229533.A0A0E0SFM9
9714	229533.A0A0E0S7B3	pp	229533.I1RRR5
9715	229533.A0A0E0S7B3	pp	229533.I1RUQ2
9716	229533.A0A0E0S7B3	pp	229533.I1S8R3
9717	229533.A0A0E0S7B3	pp	229533.I1RCG4
9718	229533.A0A0E0S7B3	pp	229533.A0A1C3YIP5
9719	229533.A0A0E0S7B3	pp	229533.I1RRP3
9720	229533.A0A0E0S7B3	pp	229533.I1RQJ5
9721	229533.A0A0E0S7B3	pp	229533.I1RZ99
9722	229533.A0A0E0S7B3	pp	229533.I1RZB5
9723	229533.A0A0E0S7B3	pp	229533.I1S558
9724	229533.A0A0E0S7B3	pp	229533.I1RS30
9725	229533.A0A0E0S7B3	pp	229533.I1S2C0
9726	229533.A0A0E0S7B3	pp	229533.Q4HZ47
9727	229533.A0A0E0S7B3	pp	229533.I1RPJ3
9728	229533.A0A0E0S7B3	pp	229533.A0A1C3YK72
9729	229533.A0A0E0S7B3	pp	229533.I1RAN5
9730	229533.A0A0E0S7B3	pp	229533.I1RCN5
9731	229533.A0A0E0S7B3	pp	229533.I1RTE9
9732	229533.A0A0E0S7B3	pp	229533.I1RQA2
9733	229533.A0A0E0S7B3	pp	229533.I1RVQ0
9734	229533.A0A0E0S7B3	pp	229533.I1RNF5
9735	229533.A0A0E0S7B3	pp	229533.I1RLP2
9736	229533.A0A0E0S7B3	pp	229533.I1RJY3
9737	229533.A0A0E0S7B3	pp	229533.I1RJV3
9738	229533.A0A0E0S7B3	pp	229533.I1RF19
9739	229533.A0A0E0S7B3	pp	229533.I1RE00
9740	229533.A0A0E0S7B3	pp	229533.A0A1C3YIJ3
9741	229533.A0A0E0S7B3	pp	229533.I1RSI0
9742	229533.A0A0E0S7B3	pp	229533.I1RC27
9743	229533.A0A0E0S7B3	pp	229533.I1RW99
9744	229533.A0A0E0S7B3	pp	229533.Q4I5Z5
9745	229533.A0A0E0S7B3	pp	229533.V6RGN5
9746	229533.A0A0E0S7B3	pp	229533.I1RFJ9
9747	229533.A0A0E0S7B3	pp	229533.I1RZE5
9748	229533.A0A0E0S7B3	pp	229533.I1S1J6
9749	229533.A0A0E0S7B3	pp	229533.I1RKC6
9750	229533.A0A0E0S7B3	pp	229533.I1RD17
9751	229533.A0A0E0S7B3	pp	229533.Q4IN63
9752	229533.A0A0E0S7B3	pp	229533.I1RAZ8
9753	229533.A0A0E0S7B3	pp	229533.I1RZM8
9754	229533.A0A0E0S7B3	pp	229533.I1REK8
9755	229533.A0A0E0S7B3	pp	229533.V6R057
9756	229533.A0A0E0S7B3	pp	229533.I1RVZ0
9757	229533.A0A0E0S7B3	pp	229533.I1S296
9758	229533.A0A0E0S7B3	pp	229533.I1RNK2
9759	229533.A0A0E0S7B3	pp	229533.A0A1C3YJG0
9760	229533.A0A0E0S7B3	pp	229533.I1RQ31
9761	229533.A0A0E0S7B3	pp	229533.I1RUG3
9762	229533.A0A0E0S7B3	pp	229533.Q4HZ68
9763	229533.A0A0E0S7B3	pp	229533.I1S0M3
9764	229533.A0A0E0S7B3	pp	229533.I1RF08
9765	229533.A0A0E0S7B3	pp	229533.I1RZF6
9766	229533.A0A0E0S7B3	pp	229533.I1RC06
9767	229533.A0A0E0S7B3	pp	229533.A0A1C3YKW9
9768	229533.A0A0E0S7B3	pp	229533.I1RMC0
9769	229533.A0A0E0S7B3	pp	229533.I1RCY5
9770	229533.A0A0E0S7B3	pp	229533.A0A1C3YKB0
9771	229533.A0A0E0S7B3	pp	229533.A0A1C3YLK9
9772	229533.A0A0E0S7B3	pp	229533.I1RKQ9
9773	229533.A0A0E0S7B3	pp	229533.I1RNH6
9774	229533.A0A0E0S7B3	pp	229533.I1RRR7
9775	229533.A0A0E0S7B3	pp	229533.I1S021
9776	229533.A0A0E0S7B3	pp	229533.I1RAU7
9777	229533.A0A0E0S7B3	pp	229533.A0A1C3YKR0
9778	229533.A0A0E0S7B3	pp	229533.I1RMQ0
9779	229533.A0A0E0S7B3	pp	229533.V6R227
9780	229533.A0A0E0S7B3	pp	229533.I1RFL4
9781	229533.A0A0E0S7B3	pp	229533.I1RSD8
9782	229533.A0A0E0S7B3	pp	229533.I1RMN7

9783 229533.A0A0E0S7B3 pp 229533.I1RI82
9784 229533.A0A0E0S7B3 pp 229533.I1RUQ9
9785 229533.A0A0E0S7B3 pp 229533.Q4I327
9786 229533.A0A0E0S7B3 pp 229533.I1RJZ6
9787 229533.A0A0E0S7B3 pp 229533.I1RX14
9788 229533.A0A0E0S7B3 pp 229533.I1S2E8
9789 229533.A0A0E0S7B3 pp 229533.I1RE01
9790 229533.A0A0E0S7B3 pp 229533.I1RQE7
9791 229533.A0A0E0S7B3 pp 229533.I1RNY2
9792 229533.A0A0E0S7B3 pp 229533.I1RSI6
9793 229533.A0A0E0S7B3 pp 229533.I1RW46
9794 229533.A0A0E0S7B3 pp 229533.A0A1C3YLS9
9795 229533.A0A0E0S7B3 pp 229533.I1RZZ1
9796 229533.A0A0E0S7B3 pp 229533.I1S060
9797 229533.A0A0E0S7B3 pp 229533.I1RZK6
9798 229533.A0A0E0S7B3 pp 229533.I1RX08
9799 229533.A0A0E0S7B3 pp 229533.V6RCG5
9800 229533.A0A0E0S7B3 pp 229533.I1S0J1
9801 229533.A0A0E0S7B3 pp 229533.I1RC72
9802 229533.A0A0E0S7B3 pp 229533.I1RR51
9803 229533.A0A0E0S7B3 pp 229533.I1RA48
9804 229533.A0A0E0S7B3 pp 229533.I1RSC6
9805 229533.A0A0E0S7B3 pp 229533.I1RQ81
9806 229533.A0A0E0S7B3 pp 229533.I1RSS4
9807 229533.A0A0E0S7B3 pp 229533.I1RPY1
9808 229533.A0A0E0S7B3 pp 229533.I1RNU6
9809 229533.A0A0E0S7B3 pp 229533.A0A1C3YK36
9810 229533.A0A0E0S7B3 pp 229533.I1S419
9811 229533.A0A0E0S7B3 pp 229533.I1S1X9
9812 229533.A0A0E0S7B3 pp 229533.I1RWB5
9813 229533.A0A0E0S7B3 pp 229533.I1RY63
9814 229533.A0A0E0S7B3 pp 229533.I1RS17
9815 229533.A0A0E0S7B3 pp 229533.I1RG56
9816 229533.A0A0E0S7B3 pp 229533.A0A1C3YKV3
9817 229533.A0A0E0S7B3 pp 229533.I1S4T3
9818 229533.A0A0E0S7B3 pp 229533.I1R9V7
9819 229533.A0A0E0S7B3 pp 229533.I1RDG0
9820 229533.A0A0E0S7B3 pp 229533.I1S0M9
9821 229533.A0A0E0S7B3 pp 229533.I1RB92
9822 229533.A0A0E0S7B3 pp 229533.I1RK95
9823 229533.A0A0E0S7B3 pp 229533.I1RQ28
9824 229533.A0A0E0S7B3 pp 229533.V6RIV9
9825 229533.A0A0E0S7B3 pp 229533.Q4IJ56
9826 229533.A0A0E0S7B3 pp 229533.I1RBI8
9827 229533.A0A0E0S7B3 pp 229533.I1RN99
9828 229533.A0A0E0S7B3 pp 229533.I1RS18
9829 229533.A0A0E0S7B3 pp 229533.V6QUE6
9830 229533.A0A0E0S7B3 pp 229533.I1R9X7
9831 229533.A0A0E0S7B3 pp 229533.I1RC33
9832 229533.A0A0E0S7B3 pp 229533.I1RB27
9833 229533.A0A0E0S7B3 pp 229533.I1RMN6
9834 229533.A0A0E0S7B3 pp 229533.Q4IBS2
9835 229533.A0A0E0S7B3 pp 229533.I1RM96
9836 229533.A0A0E0S7B3 pp 229533.Q4IEK8
9837 229533.A0A0E0S7B3 pp 229533.A0A1C3YJR3
9838 229533.A0A0E0S7B3 pp 229533.Q4I8X0
9839 229533.A0A0E0S7B3 pp 229533.Q4IAA0
9840 229533.I1RDA0 pp 229533.I1S6F0
9841 229533.I1RDA0 pp 229533.I1RFG0
9842 229533.I1RDA0 pp 229533.I1RNH1
9843 229533.I1RDA0 pp 229533.I1RRQ3
9844 229533.I1RDA0 pp 229533.I1RQ65
9845 229533.I1RDA0 pp 229533.I1RWD4
9846 229533.I1RDA0 pp 229533.I1RS47
9847 229533.I1RDA0 pp 229533.I1S0L3
9848 229533.I1RDA0 pp 229533.I1RRH5
9849 229533.I1RDA0 pp 229533.I1RZH6
9850 229533.I1RDA0 pp 229533.I1RW25
9851 229533.I1RDA0 pp 229533.Q4HZ42
9852 229533.I1RDA0 pp 229533.I1RFW5
9853 229533.I1RDA0 pp 229533.V6QW22
9854 229533.I1RDA0 pp 229533.V6R283
9855 229533.I1RDA0 pp 229533.I1RN63

229533.I1RDA0	pp	229533.I1RL23
229533.I1RDA0	pp	229533.Q4IFI0
229533.I1RDA0	pp	229533.Q4I830
229533.I1RDA0	pp	229533.I1RSX1
229533.I1RDA0	pp	229533.I1RZ99
229533.I1RDA0	pp	229533.I1RKL3
229533.I1RDA0	pp	229533.I1RWF7
229533.I1RDA0	pp	229533.I1RQJ5
229533.I1RDA0	pp	229533.I1RJZ7
229533.I1RDA0	pp	229533.I1RG51
229533.I1RDA0	pp	229533.I1RR00
229533.I1RDA0	pp	229533.I1RW71
229533.I1RDA0	pp	229533.I1RWJ4
229533.I1RDA0	pp	229533.I1RPP3
229533.I1RDA0	pp	229533.I1RS19
229533.I1RDA0	pp	229533.I1RRJ8
229533.I1RDA0	pp	229533.I1S0T2
229533.I1RDA0	pp	229533.I1RQS5
229533.I1RDA0	pp	229533.I1S9Y1
229533.I1RDA0	pp	229533.I1RFS3
229533.I1RDA0	pp	229533.I1RRT2
229533.I1RDA0	pp	229533.I1RPU0
229533.I1RDA0	pp	229533.Q4IPI1
229533.I1RDA0	pp	229533.Q4I662
229533.I1RDA0	pp	229533.I1RJL4
229533.I1RDA0	pp	229533.I1S6Y8
229533.I1RDA0	pp	229533.I1RPW9
229533.I1RDA0	pp	229533.I1RKJ6
229533.I1RDA0	pp	229533.I1RLV9
229533.I1RDA0	pp	229533.I1RPM4
229533.I1RDA0	pp	229533.I1RW61
229533.I1RDA0	pp	229533.Q4IAS1
229533.I1RDA0	pp	229533.I1S9U2
229533.I1RDA0	pp	229533.I1RM06
229533.I1RDA0	pp	229533.I1RKQ5
229533.I1RDA0	pp	229533.I1RGF6
229533.I1RDA0	pp	229533.I1RZZ9
229533.I1RDA0	pp	229533.Q4IQW0
229533.I1RDA0	pp	229533.Q4IF76
229533.I1RDA0	pp	229533.I1RZS6
229533.I1RDA0	pp	229533.I1RT05
229533.I1RDA0	pp	229533.I1RJV4
229533.I1RDA0	pp	229533.I1S1B6
229533.I1RDA0	pp	229533.I1RX24
229533.I1RDA0	pp	229533.I1RFN1
229533.I1RDA0	pp	229533.I1RTG3
229533.I1RDA0	pp	229533.I1S253
229533.I1RDA0	pp	229533.I1S6U8
229533.I1RDA0	pp	229533.Q4IQK7
229533.I1RDA0	pp	229533.I1RNA0
229533.I1RDA0	pp	229533.I1RUQ2
229533.I1RDA0	pp	229533.I1RZJ6
229533.I1RDA0	pp	229533.Q4HVW2
229533.I1RDA0	pp	229533.I1S0R7
229533.I1RDA0	pp	229533.I1RRT8
229533.I1RDA0	pp	229533.V6QYF0
229533.I1RDA0	pp	229533.I1RMK3
229533.I1RDA0	pp	229533.I1RND6
229533.I1RDA0	pp	229533.Q4IJH1
229533.I1RDA0	pp	229533.Q4I327
229533.I1RDA0	pp	229533.I1RTF4
229533.I1RDA0	pp	229533.I1RM67
229533.I1RDA0	pp	229533.I1S021
229533.I1RDA0	pp	229533.I1RNY2
229533.I1RDA0	pp	229533.I1S4T3
229533.I1RDA0	pp	229533.I1RT80
229533.I1RDA0	pp	229533.I1RNV6
229533.I1RDA0	pp	229533.I1RG56
229533.I1RDA0	pp	229533.I1S419
229533.I1RDA0	pp	229533.I1RNH6
229533.I1RDA0	pp	229533.I1RPY1
229533.I1RDA0	pp	229533.I1RMN7
229533.I1RDA0	pp	229533.I1RM96

9929	229533.I1RDA0	pp	229533.I1RMC0
9930	229533.I1RDA0	pp	229533.V6R227
9931	229533.I1RDA0	pp	229533.Q4IEK8
9932	229533.I1RDA0	pp	229533.I1RRR7
9933	229533.I1RDA0	pp	229533.I1RUG3
9934	229533.I1RDA0	pp	229533.I1S2E8
9935	229533.I1RDA0	pp	229533.I1RQ31
9936	229533.I1RDA0	pp	229533.I1RZF6
9937	229533.I1RDA0	pp	229533.I1RQE7
9938	229533.I1RDA0	pp	229533.I1S9U1
9939	229533.I1RDA0	pp	229533.Q4IN63
9940	229533.I1RDA0	pp	229533.I1RR51
9941	229533.I1RDA0	pp	229533.I1RFL4
9942	229533.I1RDA0	pp	229533.I1RSI6
9943	229533.I1RDA0	pp	229533.V6RCG5
9944	229533.I1RDA0	pp	229533.I1S0J1
9945	229533.I1RDA0	pp	229533.I1S2U2
9946	229533.I1RDA0	pp	229533.I1S060
9947	229533.I1RDA0	pp	229533.I1RSD8
9948	229533.I1RDA0	pp	229533.I1RZE5
9949	229533.I1RDA0	pp	229533.I1RN99
9950	229533.I1RDA0	pp	229533.I1RK95
9951	229533.I1RDA0	pp	229533.I1RX14
9952	229533.I1RDA0	pp	229533.I1RE00
9953	229533.I1RDA0	pp	229533.I1RQ28
9954	229533.I1RDA0	pp	229533.I1RDG0
9955	229533.I1RDA0	pp	229533.I1RS18
9956	229533.I1RDA0	pp	229533.I1S0M3
9957	229533.I1RDA0	pp	229533.I1RFJ9
9958	229533.I1RDA0	pp	229533.I1RNK2
9959	229533.I1RDA0	pp	229533.Q4IJ56
9960	229533.I1RDA0	pp	229533.I1RF19
9961	229533.I1RDA0	pp	229533.I1S0M9
9962	229533.I1RDA0	pp	229533.I1RY63
9963	229533.I1RDA0	pp	229533.I1S1X9
9964	229533.I1RDA0	pp	229533.I1RNU6
9965	229533.I1RDA0	pp	229533.I1RVZ0
9966	229533.I1RDA0	pp	229533.I1RS17
9967	229533.I1RDA0	pp	229533.I1RKC6
9968	229533.I1RDA0	pp	229533.I1RSC6
9969	229533.I1RDA0	pp	229533.I1RQA2
9970	229533.I1RDA0	pp	229533.I1S1J6
9971	229533.I1RDA0	pp	229533.I1RLP2
9972	229533.I1RDA0	pp	229533.Q4I5Z5
9973	229533.I1RDA0	pp	229533.Q4IBS2
9974	229533.I1RDA0	pp	229533.I1S1U7
9975	229533.I1RDA0	pp	229533.I1S558
9976	229533.I1RDA0	pp	229533.I1RMN6
9977	229533.I1RDA0	pp	229533.V6RGN5
9978	229533.I1RDA0	pp	229533.Q4HZ68
9979	229533.I1RDA0	pp	229533.I1RKQ9
9980	229533.I1RDA0	pp	229533.I1S5B0
9981	229533.I1RDA0	pp	229533.I1RSR3
9982	229533.I1RDA0	pp	229533.I1RG11
9983	229533.I1RDA0	pp	229533.V6RGY3
9984	229533.I1RDA0	pp	229533.I1S296
9985	229533.I1RDA0	pp	229533.I1S274
9986	229533.I1RDA0	pp	229533.I1RW99
9987	229533.I1RDA0	pp	229533.I1RF08
9988	229533.I1RDA0	pp	229533.I1RTD9
9989	229533.I1RDA0	pp	229533.I1RRI2
9990	229533.I1RDA0	pp	229533.I1S2C0
9991	229533.I1RDA0	pp	229533.I1RPJ3
9992	229533.I1RDA0	pp	229533.I1RTE9
9993	229533.I1RPE5	pp	229533.I1RY35
9994	229533.I1RPE5	pp	229533.Q4HXF0
9995	229533.I1RPE5	pp	229533.I1RUP5
9996	229533.I1RPE5	pp	229533.I1S2Q4
9997	229533.I1RPE5	pp	229533.I1RQM9
9998	229533.I1RPE5	pp	229533.I1S968
9999	229533.I1RPE5	pp	229533.I1S0I3
10000	229533.I1RPE5	pp	229533.I1RWZ3
10001	229533.I1RPE5	pp	229533.V6QXG1

10002	229533.I1RPE5	pp	229533.V6RR38
10003	229533.I1RPE5	pp	229533.I1RZD0
10004	229533.I1RPE5	pp	229533.I1RRV1
10005	229533.I1RPE5	pp	229533.I1RQ76
10006	229533.I1RPE5	pp	229533.I1S969
10007	229533.I1RPE5	pp	229533.I1RYP5
10008	229533.I1RPE5	pp	229533.I1S130
10009	229533.I1RPE5	pp	229533.I1RRA5
10010	229533.I1RPE5	pp	229533.V6RLM7
10011	229533.I1RPE5	pp	229533.I1RXX1
10012	229533.I1RPE5	pp	229533.I1RT23
10013	229533.I1RPE5	pp	229533.I1S5R5
10014	229533.I1RPE5	pp	229533.I1RYN9
10015	229533.I1RPE5	pp	229533.I1S1B8
10016	229533.I1RPE5	pp	229533.I1S4G3
10017	229533.I1RPE5	pp	229533.I1S7L6
10018	229533.I1RPE5	pp	229533.I1S0K2
10019	229533.I1RPE5	pp	229533.I1RZZ3
10020	229533.I1RPE5	pp	229533.V6RG22
10021	229533.I1RPE5	pp	229533.V6RPN0
10022	229533.I1RPE5	pp	229533.I1RTY0
10023	229533.I1RPE5	pp	229533.I1RZ92
10024	229533.I1RPE5	pp	229533.I1RY81
10025	229533.I1RPE5	pp	229533.I1RUK0
10026	229533.I1RQ31	pp	229533.Q4IJH1
10027	229533.I1RQ31	pp	229533.I1RQM6
10028	229533.I1RQ31	pp	229533.I1RT05
10029	229533.I1RQ31	pp	229533.I1S9U2
10030	229533.I1RQ31	pp	229533.I1RTF4
10031	229533.I1RQ31	pp	229533.I1RR24
10032	229533.I1RQ31	pp	229533.Q4IPI1
10033	229533.I1RQ31	pp	229533.I1RT80
10034	229533.I1RQ31	pp	229533.V6QYF0
10035	229533.I1RQ31	pp	229533.I1S6U8
10036	229533.I1RQ31	pp	229533.Q4IQK7
10037	229533.I1RQ31	pp	229533.I1RR00
10038	229533.I1RQ31	pp	229533.I1RW71
10039	229533.I1RQ31	pp	229533.I1RRQ6
10040	229533.I1RQ31	pp	229533.Q4HVW2
10041	229533.I1RQ31	pp	229533.I1RQJ5
10042	229533.I1RQ31	pp	229533.Q4IFI0
10043	229533.I1RQ31	pp	229533.V6QW22
10044	229533.I1RQ31	pp	229533.I1RZ99
10045	229533.I1RQ31	pp	229533.I1RZJ6
10046	229533.I1RQ31	pp	229533.I1RW25
10047	229533.I1RQ31	pp	229533.I1RWJ8
10048	229533.I1RQ31	pp	229533.I1RSQ7
10049	229533.I1RQ31	pp	229533.I1RZM8
10050	229533.I1RQ31	pp	229533.I1S021
10051	229533.I1RQ31	pp	229533.Q4INZ9
10052	229533.I1RQ31	pp	229533.I1RWD4
10053	229533.I1RQ31	pp	229533.V6RCZ8
10054	229533.I1RQ31	pp	229533.I1S6F0
10055	229533.I1RQ31	pp	229533.I1RTI6
10056	229533.I1RQ31	pp	229533.I1S0R7
10057	229533.I1RQ31	pp	229533.I1RVQ0
10058	229533.I1RQ31	pp	229533.I1RRH5
10059	229533.I1RQ31	pp	229533.I1RUQ2
10060	229533.I1RQ31	pp	229533.I1RR51
10061	229533.I1RQ31	pp	229533.V6R1K4
10062	229533.I1RQ31	pp	229533.V6RCG5
10063	229533.I1RQ31	pp	229533.I1RT33
10064	229533.I1RQ31	pp	229533.I1S854
10065	229533.I1RQ31	pp	229533.I1RXD7
10066	229533.I1RQ31	pp	229533.I1S0L3
10067	229533.I1RQ31	pp	229533.I1S0T2
10068	229533.I1RQ31	pp	229533.V6RSM7
10069	229533.I1RQ31	pp	229533.I1RRU9
10070	229533.I1RQ31	pp	229533.I1RTD9
10071	229533.I1RQ31	pp	229533.V6QUE6
10072	229533.I1RQ31	pp	229533.I1RSR3
10073	229533.I1RQ31	pp	229533.I1RQA2
10074	229533.I1RQ31	pp	229533.Q4IBS2

229533.I1RQ31	pp	229533.I1S0C1
229533.I1RQ31	pp	229533.I1RZB5
229533.I1RQ31	pp	229533.Q4IP34
229533.I1RQ31	pp	229533.I1S2U2
229533.I1RQ31	pp	229533.I1RZZ9
229533.I1RQ31	pp	229533.Q4IF76
229533.I1RQ31	pp	229533.I1RZK6
229533.I1RQ31	pp	229533.Q4I7F9
229533.I1RQ31	pp	229533.I1S5B0
229533.I1RQ31	pp	229533.I1RZZ1
229533.I1RQ31	pp	229533.I1S253
229533.I1RQ31	pp	229533.I1S044
229533.I1RQ31	pp	229533.I1RU69
229533.I1RQ31	pp	229533.I1RY63
229533.I1RQ31	pp	229533.I1RW99
229533.I1RQ31	pp	229533.I1RX14
229533.I1RQ31	pp	229533.I1S9Y1
229533.I1RQ31	pp	229533.I1RSC6
229533.I1RQ31	pp	229533.I1SA32
229533.I1RQ31	pp	229533.I1RVZ0
229533.I1RQ31	pp	229533.I1S5R1
229533.I1RQ31	pp	229533.I1RRT8
229533.I1RQ31	pp	229533.Q4I5Z5
229533.I1RQ31	pp	229533.I1RZE5
229533.I1RQ31	pp	229533.O74638
229533.I1RQ31	pp	229533.Q4IJ56
229533.I1RQ31	pp	229533.I1RWD7
229533.I1RQ31	pp	229533.I1RSI0
229533.I1RQ31	pp	229533.I1RQ81
229533.I1RQ31	pp	229533.I1RS20
229533.I1RQ31	pp	229533.I1RQS8
229533.I1RQ31	pp	229533.I1RZS6
229533.I1RQ31	pp	229533.I1S2H4
229533.I1RQ31	pp	229533.Q4IAA0
229533.I1RQ31	pp	229533.I1RW61
229533.I1RQ31	pp	229533.I1RRM5
229533.I1RQ31	pp	229533.I1RS18
229533.I1RQ31	pp	229533.I1RYT5
229533.I1RQ31	pp	229533.Q4I8P5
229533.I1RQ31	pp	229533.I1RYG3
229533.I1RQ31	pp	229533.I1S419
229533.I1RQ31	pp	229533.Q4IMJ1
229533.I1RQ31	pp	229533.I1S6Y8
229533.I1RQ31	pp	229533.I1RWJ4
229533.I1RQ31	pp	229533.I1RT40
229533.I1RQ31	pp	229533.Q4I662
229533.I1RQ31	pp	229533.Q4IEK8
229533.I1RQ31	pp	229533.I1RT04
229533.I1RQ31	pp	229533.I1RZF6
229533.I1RQ31	pp	229533.Q4HXF0
229533.I1RQ31	pp	229533.I1RR72
229533.I1RQ31	pp	229533.I1S9U1
229533.I1RQ31	pp	229533.I1RX24
229533.I1RQ31	pp	229533.V6R227
229533.I1RQ31	pp	229533.Q4IAS1
229533.I1RQ31	pp	229533.I1RTE9
229533.I1RQ31	pp	229533.V6R283
229533.I1RQ31	pp	229533.I1RRZ0
229533.I1RQ31	pp	229533.I1RRT2
229533.I1RQ31	pp	229533.I1RQS5
229533.I1RQ31	pp	229533.Q4HW67
229533.I1RQ31	pp	229533.I1S1B6
229533.I1RQ31	pp	229533.I1S2C0
229533.I1RQ31	pp	229533.Q4I830
229533.I1RQ31	pp	229533.I1S558
229533.I1RQ31	pp	229533.I1S2M6
229533.I1RQ31	pp	229533.I1RUG3
229533.I1RQ31	pp	229533.I1RSK1
229533.I1RQ31	pp	229533.Q4IQW0
229533.I1RQ31	pp	229533.I1S855
229533.I1RQ31	pp	229533.I1RSX1
229533.I1RQ31	pp	229533.I1S274
229533.I1RQ31	pp	229533.Q4IN63

```
229533.I1RQ31   pp  229533.I1RSV2
229533.I1RQ31   pp  229533.I1S1U7
229533.I1RQ31   pp  229533.I1RWF7
229533.I1RQ31   pp  229533.Q4I327
229533.I1RQ31   pp  229533.I1RRP3
229533.I1RQ31   pp  229533.I1RRJ8
229533.I1RQ31   pp  229533.I1RSI6
229533.I1RQ31   pp  229533.V6RGY3
229533.I1RQ31   pp  229533.V6RGY0
229533.I1RQ31   pp  229533.I1S0F6
229533.I1RQ31   pp  229533.I1RSD8
229533.I1RQ31   pp  229533.I1RS30
229533.I1RQ31   pp  229533.I1RQE7
229533.I1RQ31   pp  229533.I1RRV1
229533.I1RQ31   pp  229533.I1S2E8
229533.I1RQ31   pp  229533.I1RRQ3
229533.I1RQ31   pp  229533.I1SA33
229533.I1RQ31   pp  229533.V6RGN5
229533.I1RQ31   pp  229533.Q4HZ42
229533.I1RQ31   pp  229533.I1RS47
229533.I1RQ31   pp  229533.I1RSS4
229533.I1RQ31   pp  229533.I1S1X9
229533.I1RQ31   pp  229533.I1RW46
229533.I1RQ31   pp  229533.Q4IEV5
229533.I1RQ31   pp  229533.I1S060
229533.I1RQ31   pp  229533.I1RQ65
229533.I1RQ31   pp  229533.I1S0J1
229533.I1RQ31   pp  229533.I1S247
229533.I1RQ31   pp  229533.I1RRI2
229533.I1RQ31   pp  229533.I1S1J6
229533.I1RQ31   pp  229533.I1RWD3
229533.I1RQ31   pp  229533.Q4HZ68
229533.I1RQ31   pp  229533.I1RS17
229533.I1RQ31   pp  229533.I1S296
229533.I1RQ31   pp  229533.I1RZW8
229533.I1RQ31   pp  229533.Q4I7K4
229533.I1RQ31   pp  229533.V6RIV9
229533.I1RQ31   pp  229533.Q4HZK7
229533.I1RQ31   pp  229533.I1RS64
229533.I1RQ31   pp  229533.I1S4T3
229533.I1RQ31   pp  229533.V6R057
229533.I1RQ31   pp  229533.I1RRR7
229533.I1RQ31   pp  229533.I1RQ72
229533.I1RQ31   pp  229533.I1S8R3
229533.I1RAQ0   pp  229533.I1RRQ9
229533.I1RKF0   pp  229533.V6RKY7
229533.I1RKF0   pp  229533.I1RRJ7
229533.A0A098DZS4   pp  229533.I1RW54
229533.A0A098DZS4   pp  229533.I1RL81
229533.A0A098DZS4   pp  229533.I1RCU2
229533.A0A098DZS4   pp  229533.I1RY16
229533.A0A098DZS4   pp  229533.I1RNM7
229533.A0A098DZS4   pp  229533.I1S8F8
229533.A0A098DZS4   pp  229533.I1RI11
229533.A0A098DZS4   pp  229533.I1S694
229533.A0A098DZS4   pp  229533.I1RZA8
229533.A0A098DZS4   pp  229533.I1RYC8
229533.A0A098DZS4   pp  229533.A0A1C3YHG7
229533.A0A098DZS4   pp  229533.A0A0E0SH57
229533.A0A098DZS4   pp  229533.I1RYM5
229533.A0A098DZS4   pp  229533.I1RST9
229533.A0A098DZS4   pp  229533.I1RG33
229533.A0A098DZS4   pp  229533.I1RYA0
229533.A0A098DZS4   pp  229533.I1RF85
229533.A0A0E0S972   pp  229533.A0A1C3YI65
229533.A0A0E0S972   pp  229533.I1RRE2
229533.A0A0E0S972   pp  229533.I1RCM9
229533.A0A0E0S972   pp  229533.A0A1C3YKH1
229533.A0A1C3YLP7   pp  229533.I1RKF0
229533.A0A1C3YLP7   pp  229533.I1RRJ7
229533.I1RW61   pp  229533.I1RWR7
229533.I1RW61   pp  229533.I1RZF6
229533.I1RW61   pp  229533.I1RY63
```

229533.I1RW61 pp 229533.V6R227
229533.I1RW61 pp 229533.I1S217
229533.I1RW61 pp 229533.V6RCZ8
229533.I1RW61 pp 229533.V6RCG5
229533.I1RW61 pp 229533.I1RW71
229533.I1RW61 pp 229533.V6QYF0
229533.I1RW61 pp 229533.I1S0L3
229533.I1RW61 pp 229533.I1RZM8
229533.I1RW61 pp 229533.Q4IFI0
229533.I1RW61 pp 229533.I1RZZ9
229533.I1RW61 pp 229533.I1RWD4
229533.I1RW61 pp 229533.I1S5B0
229533.I1RW61 pp 229533.I1RXD7
229533.I1RW61 pp 229533.I1S0F1
229533.I1RW61 pp 229533.I1RYJ6
229533.I1RW61 pp 229533.I1S5R1
229533.I1RW61 pp 229533.V6QW22
229533.I1RW61 pp 229533.Q4HXF6
229533.I1RW61 pp 229533.I1S4S7
229533.I1RW61 pp 229533.I1S2M6
229533.I1RW61 pp 229533.V6R283
229533.I1RW61 pp 229533.V6RIV9
229533.I1RW61 pp 229533.Q4IPH4
229533.I1RW61 pp 229533.I1RWK3
229533.I1RW61 pp 229533.I1S2E8
229533.I1RW61 pp 229533.Q4HXF0
229533.I1RW61 pp 229533.I1RWS4
229533.I1RW61 pp 229533.V6RGN5
229533.I1RW61 pp 229533.Q4IR18
229533.I1RW61 pp 229533.I1S2C0
229533.I1RW61 pp 229533.I1RZB2
229533.I1RW61 pp 229533.I1RZW8
229533.I1RW61 pp 229533.I1RZS6
229533.A0A098D6S5 pp 229533.I1RUQ2
229533.A0A098D6S5 pp 229533.A0A098DVS9
229533.A0A098D6S5 pp 229533.I1RWD4
229533.A0A098D6S5 pp 229533.A0A0E0S7B3
229533.A0A098D6S5 pp 229533.I1RT80
229533.A0A098D6S5 pp 229533.I1RNV6
229533.A0A098D6S5 pp 229533.I1RDA0
229533.A0A098D6S5 pp 229533.V6QYF0
229533.A0A098D6S5 pp 229533.I1RQ31
229533.A0A098D6S5 pp 229533.I1RC82
229533.A0A098D6S5 pp 229533.I1RRT2
229533.A0A098D6S5 pp 229533.I1RS47
229533.A0A098D6S5 pp 229533.I1RRJ8
229533.A0A098D6S5 pp 229533.I1RFS3
229533.A0A098D6S5 pp 229533.I1RAN5
229533.A0A098D6S5 pp 229533.I1RRI2
229533.A0A1C3YMF2 pp 229533.I1S1D3
229533.A0A1C3YMF2 pp 229533.I1RMP0
229533.I1S4Q7 pp 229533.I1S8W6
229533.A0A098DY67 pp 229533.I1RWT1
229533.A0A098DY67 pp 229533.A0A0E0SMM4
229533.A0A098DY67 pp 229533.A0A1C3YI23
229533.A0A098DY67 pp 229533.A0A0E0S495
229533.A0A098DY67 pp 229533.I1RCB7
229533.A0A098DY67 pp 229533.I1R9R7
229533.A0A098DY67 pp 229533.I1RBC7
229533.A0A098DY67 pp 229533.A0A1C3YJR3
229533.A0A098DY67 pp 229533.I1RRV5
229533.A0A098DY67 pp 229533.I1RE14
229533.A0A098DY67 pp 229533.A0A1C3YII4
229533.A0A098DY67 pp 229533.I1RMT6
229533.A0A098DY67 pp 229533.Q4IPC8
229533.A0A098DY67 pp 229533.I1RUY5
229533.A0A098DY67 pp 229533.I1S5Z2
229533.A0A098DY67 pp 229533.I1RG11
229533.A0A098DY67 pp 229533.Q4IPA4
229533.A0A098DY67 pp 229533.A0A1C3YIU5
229533.A0A098DY67 pp 229533.A0A0E0SEI2
229533.A0A098DY67 pp 229533.I1S999
229533.A0A098DY67 pp 229533.I1RA38

229533.A0A098DY67	pp	229533.I1RDU0
229533.A0A098DY67	pp	229533.V6QUE6
229533.A0A098DY67	pp	229533.Q4IPN9
229533.A0A098DY67	pp	229533.I1S214
229533.A0A098DY67	pp	229533.Q4I5W5
229533.A0A098DY67	pp	229533.I1RC24
229533.A0A098DY67	pp	229533.I1RZB5
229533.A0A098DY67	pp	229533.Q4I5I4
229533.A0A098DY67	pp	229533.A0A0E0RTC6
229533.A0A098DY67	pp	229533.A0A1C3YMU9
229533.A0A098DY67	pp	229533.I1RJL2
229533.A0A098DY67	pp	229533.I1RW90
229533.A0A098DY67	pp	229533.I1RRV6
229533.A0A098DY67	pp	229533.I1RR25
229533.A0A098DY67	pp	229533.I1RK79
229533.A0A098DY67	pp	229533.I1RRT0
229533.A0A098DY67	pp	229533.A0A1C3YI81
229533.A0A098DY67	pp	229533.A0A1C3YIR8
229533.A0A098DY67	pp	229533.V6QWX8
229533.A0A098DY67	pp	229533.A0A1C3YHH7
229533.A0A098DY67	pp	229533.Q4IR09
229533.A0A098DY67	pp	229533.I1RCA8
229533.A0A098DY67	pp	229533.A0A1C3YMV0
229533.A0A098DY67	pp	229533.I1RPK5
229533.A0A098DY67	pp	229533.I1RRG3
229533.A0A098DY67	pp	229533.A0A1C3YIJ6
229533.I1RAP5	pp	229533.I1RZ25
229533.A0A0E0SA95	pp	229533.I1S3V2
229533.A0A0E0SA95	pp	229533.I1RYI6
229533.A0A0E0SA95	pp	229533.I1RA72
229533.A0A0E0SA95	pp	229533.A0A1C3YII7
229533.A0A0E0SA95	pp	229533.I1RIS3
229533.A0A0E0SA95	pp	229533.I1RN48
229533.A0A0E0SA95	pp	229533.I1RNE7
229533.I1RAP9	pp	229533.I1RMT5
229533.I1RYX5	pp	229533.I1SAZ0
229533.I1RYX5	pp	229533.V6RUT5
229533.I1RYX5	pp	229533.I1S0L8
229533.I1RYX5	pp	229533.I1S0P0
229533.I1RYX5	pp	229533.I1S422
229533.I1RYX5	pp	229533.Q4ICG5
229533.I1RYX5	pp	229533.I1S2P3
229533.I1RYX5	pp	229533.V6R3J1
229533.I1RYX5	pp	229533.I1S0B0
229533.I1RYX5	pp	229533.I1S2B9
229533.I1RYX5	pp	229533.I1S8F8
229533.I1RYX5	pp	229533.I1S1Y9
229533.I1RYX5	pp	229533.I1S7W5
229533.I1RYX5	pp	229533.I1SAK7
229533.I1RYX5	pp	229533.V6RJP8
229533.I1RYX5	pp	229533.I1S7C4
229533.I1RYX5	pp	229533.I1S9G7
229533.I1RYX5	pp	229533.I1S1W3
229533.I1RYX5	pp	229533.I1S887
229533.I1RRH8	pp	229533.V6R227
229533.I1RRH8	pp	229533.I1RSM3
229533.I1RRH8	pp	229533.I1RWD4
229533.I1RRH8	pp	229533.I1RTF4
229533.I1RRH8	pp	229533.I1S4Q7
229533.I1RRH8	pp	229533.Q4IR18
229533.I1RRH8	pp	229533.I1RZ18
229533.I1RRH8	pp	229533.I1RRT2
229533.I1RRH8	pp	229533.I1RSL8
229533.I1RG12	pp	229533.I1RSZ7
229533.I1RG12	pp	229533.I1S2B9
229533.I1RG12	pp	229533.I1RS90
229533.I1RG12	pp	229533.I1RWP9
229533.I1RG12	pp	229533.I1RY12
229533.I1RG12	pp	229533.I1RWB0
229533.I1RG12	pp	229533.I1RWC9
229533.I1RG12	pp	229533.I1RYX5
229533.I1RG12	pp	229533.I1RTG9
229533.I1RG12	pp	229533.I1RN72

10367	229533.I1RG12	pp	229533.I1RPR9
10368	229533.I1RG12	pp	229533.I1S7W5
10369	229533.I1RG12	pp	229533.I1RMV0
10370	229533.I1RG12	pp	229533.Q4I0K3
10371	229533.I1RG12	pp	229533.I1RLP1
10372	229533.I1RPV3	pp	229533.V6RRH2
10373	229533.I1RPV3	pp	229533.I1S0J1
10374	229533.I1RPV3	pp	229533.I1RXC4
10375	229533.I1RPV3	pp	229533.I1RQ33
10376	229533.I1RPV3	pp	229533.Q4I624
10377	229533.I1RPV3	pp	229533.I1RS18
10378	229533.I1RPV3	pp	229533.I1RQE7
10379	229533.I1RPV3	pp	229533.I1RRI2
10380	229533.I1RPV3	pp	229533.Q4IJK2
10381	229533.I1RPV3	pp	229533.I1RZV3
10382	229533.I1RPV3	pp	229533.I1S1W3
10383	229533.I1RPV3	pp	229533.I1RVX0
10384	229533.I1RPV3	pp	229533.I1RT03
10385	229533.I1RPV3	pp	229533.I1S1W9
10386	229533.I1RPV3	pp	229533.I1S1C7
10387	229533.I1RPV3	pp	229533.I1RQ85
10388	229533.I1RPV3	pp	229533.I1RXR1
10389	229533.I1RPV3	pp	229533.I1RYW3
10390	229533.I1RPV3	pp	229533.I1RUG3
10391	229533.I1RPV3	pp	229533.I1S437
10392	229533.I1RPV3	pp	229533.I1RZZ9
10393	229533.I1RPV3	pp	229533.I1RSQ4
10394	229533.I1RPV3	pp	229533.I1RXF0
10395	229533.I1RPV3	pp	229533.I1RZM8
10396	229533.I1RPV3	pp	229533.I1RWI5
10397	229533.I1RPV3	pp	229533.I1RZK5
10398	229533.I1RPV3	pp	229533.V6RMW6
10399	229533.I1RPV3	pp	229533.I1S887
10400	229533.I1RPV3	pp	229533.I1RXC2
10401	229533.I1RPV3	pp	229533.I1RWR7
10402	229533.I1RPV3	pp	229533.I1RZ39
10403	229533.I1RPV3	pp	229533.V6R042
10404	229533.I1RPV3	pp	229533.I1RRY9
10405	229533.I1RPV3	pp	229533.I1RRM3
10406	229533.I1RPV3	pp	229533.I1RXU9
10407	229533.I1RPV3	pp	229533.I1RRT8
10408	229533.I1RPV3	pp	229533.I1RSQ3
10409	229533.I1RPV3	pp	229533.I1S4S4
10410	229533.I1RPV3	pp	229533.I1S5R1
10411	229533.I1RPV3	pp	229533.I1RRX5
10412	229533.I1RPV3	pp	229533.V6RCZ8
10413	229533.I1RPV3	pp	229533.I1RSQ7
10414	229533.I1RPV3	pp	229533.V6QUE6
10415	229533.I1RPV3	pp	229533.I1S9Y1
10416	229533.I1RPV3	pp	229533.I1RRI1
10417	229533.I1RPV3	pp	229533.I1S5B0
10418	229533.I1RPV3	pp	229533.I1S4S7
10419	229533.I1RPV3	pp	229533.I1RTD5
10420	229533.I1RPV3	pp	229533.I1RXD6
10421	229533.I1RPV3	pp	229533.I1S0L4
10422	229533.I1RPV3	pp	229533.I1S2H4
10423	229533.I1RPV3	pp	229533.I1RR24
10424	229533.I1RPV3	pp	229533.I1RT40
10425	229533.I1RPV3	pp	229533.I1S5R6
10426	229533.I1RPV3	pp	229533.I1RQD2
10427	229533.I1RPV3	pp	229533.V6RUT5
10428	229533.I1RPV3	pp	229533.I1RTA1
10429	229533.I1RPV3	pp	229533.I1RSZ7
10430	229533.I1RPV3	pp	229533.I1S438
10431	229533.I1RPV3	pp	229533.I1RRG3
10432	229533.I1RPV3	pp	229533.I1RVZ2
10433	229533.I1RPV3	pp	229533.I1S2B8
10434	229533.I1RPV3	pp	229533.I1RXD7
10435	229533.I1RPV3	pp	229533.I1S277
10436	229533.I1RPV3	pp	229533.I1RYR0
10437	229533.I1RPV3	pp	229533.I1RX46
10438	229533.I1RPV3	pp	229533.V6R3J1
10439	229533.I1RPV3	pp	229533.I1RYI5

229533.I1RPV3 pp 229533.V6RTJ8
229533.I1RGP5 pp 229533.I1S6P5
229533.I1RGP5 pp 229533.I1S202
229533.I1RGP5 pp 229533.V6RV57
229533.I1RGP5 pp 229533.I1RZA7
229533.A0A0E0SPS2 pp 229533.I1S887
229533.A0A0E0SPS2 pp 229533.I1RZK5
229533.A0A0E0SPS2 pp 229533.I1RAM9
229533.A0A0E0SPS2 pp 229533.A0A1C3YHX6
229533.A0A0E0SPS2 pp 229533.I1RPR9
229533.A0A0E0SPS2 pp 229533.I1S9G7
229533.A0A0E0SPS2 pp 229533.I1RRY9
229533.A0A0E0SPS2 pp 229533.I1RWC9
229533.A0A0E0SPS2 pp 229533.I1RWB0
229533.A0A0E0SPS2 pp 229533.A0A1C3YKY6
229533.A0A0E0SPS2 pp 229533.A0A1C3YKV3
229533.A0A0E0SPS2 pp 229533.I1RS18
229533.A0A0E0SPS2 pp 229533.Q4IJK2
229533.A0A0E0SPS2 pp 229533.A0A1C3YK34
229533.A0A0E0SPS2 pp 229533.A0A1C3YKB0
229533.A0A0E0SPS2 pp 229533.I1S0K2
229533.A0A0E0SPS2 pp 229533.I1R9Z5
229533.A0A0E0SPS2 pp 229533.A0A1C3YJ83
229533.A0A0E0SPS2 pp 229533.I1REK8
229533.A0A0E0SPS2 pp 229533.I1RC49
229533.A0A0E0SPS2 pp 229533.A0A1C3YJW9
229533.A0A0E0SPS2 pp 229533.V6RCZ8
229533.A0A0E0SPS2 pp 229533.I1RJU3
229533.A0A0E0SPS2 pp 229533.I1RCR5
229533.A0A0E0SPS2 pp 229533.I1RFJ5
229533.A0A0E0SPS2 pp 229533.I1RMQ6
229533.A0A0E0SPS2 pp 229533.A0A1C3YLB0
229533.A0A0E0SPS2 pp 229533.I1RXF0
229533.A0A0E0SPS2 pp 229533.I1R9Y4
229533.A0A0E0SPS2 pp 229533.I1RBT1
229533.A0A0E0SPS2 pp 229533.A0A1C3YHX8
229533.A0A0E0SPS2 pp 229533.I1RZM8
229533.A0A0E0SPS2 pp 229533.I1RXR1
229533.A0A0E0SPS2 pp 229533.I1S5B0
229533.A0A0E0SPS2 pp 229533.I1RR24
229533.A0A0E0SPS2 pp 229533.I1RPA7
229533.A0A0E0SPS2 pp 229533.I1RVX0
229533.A0A0E0SPS2 pp 229533.I1S4S4
229533.A0A0E0SPS2 pp 229533.I1RT03
229533.A0A0E0SPS2 pp 229533.I1S1Y9
229533.A0A0E0SPS2 pp 229533.I1RB92
229533.A0A0E0SPS2 pp 229533.I1RN72
229533.A0A0E0SPS2 pp 229533.I1RFL4
229533.A0A0E0SPS2 pp 229533.A0A1C3YK13
229533.A0A0E0SPS2 pp 229533.I1S4S7
229533.A0A0E0SPS2 pp 229533.V6QUE6
229533.A0A0E0SPS2 pp 229533.I1S0R8
229533.A0A0E0SPS2 pp 229533.I1RAI4
229533.A0A0E0SPS2 pp 229533.I1RPA4
229533.A0A0E0SPS2 pp 229533.I1RRX5
229533.A0A0E0SPS2 pp 229533.I1RQE7
229533.A0A0E0SPS2 pp 229533.I1RCH5
229533.A0A0E0SPS2 pp 229533.I1RSQ7
229533.A0A0E0SPS2 pp 229533.I1RS90
229533.A0A0E0SPS2 pp 229533.I1S2B9
229533.A0A0E0SPS2 pp 229533.Q4I624
229533.A0A0E0SPS2 pp 229533.I1RTG6
229533.A0A0E0SPS2 pp 229533.I1RYH7
229533.A0A0E0SPS2 pp 229533.A0A1C3YLH7
229533.A0A0E0SPS2 pp 229533.I1RBD5
229533.A0A0E0SPS2 pp 229533.I1RA70
229533.A0A0E0SPS2 pp 229533.I1RCK9
229533.A0A0E0SPS2 pp 229533.I1RA85
229533.A0A0E0SPS2 pp 229533.I1RAE4
229533.A0A0E0SPS2 pp 229533.I1RD22
229533.A0A0E0SPS2 pp 229533.I1RYW3
229533.A0A0E0SPS2 pp 229533.I1S147
229533.A0A0E0SPS2 pp 229533.A0A1C3YJD4

10513	229533.A0A0E0SPS2	pp	229533.I1RD52
10514	229533.A0A0E0SPS2	pp	229533.A0A1C3YIP5
10515	229533.A0A0E0SPS2	pp	229533.A0A1C3YI04
10516	229533.A0A0E0SPS2	pp	229533.I1S0P0
10517	229533.A0A0E0SPS2	pp	229533.I1S1C7
10518	229533.A0A0E0SPS2	pp	229533.I1RXD7
10519	229533.A0A0E0SPS2	pp	229533.I1S277
10520	229533.A0A0E0SPS2	pp	229533.I1RXU9
10521	229533.A0A0E0SPS2	pp	229533.I1RFI4
10522	229533.A0A0E0SPS2	pp	229533.I1RAW0
10523	229533.A0A0E0SPS2	pp	229533.I1RA52
10524	229533.A0A0E0SPS2	pp	229533.I1RAW3
10525	229533.A0A0E0SPS2	pp	229533.I1RLP1
10526	229533.A0A0E0SPS2	pp	229533.I1RJV3
10527	229533.A0A0E0SPS2	pp	229533.V6RUT5
10528	229533.A0A0E0SPS2	pp	229533.I1RYX5
10529	229533.A0A0E0SPS2	pp	229533.I1RG12
10530	229533.A0A0E0SPS2	pp	229533.I1S2B8
10531	229533.A0A0E0SPS2	pp	229533.I1RMV0
10532	229533.A0A0E0SPS2	pp	229533.I1RAU9
10533	229533.A0A0E0SPS2	pp	229533.A0A1C3YLC1
10534	229533.A0A0E0SPS2	pp	229533.I1RAX6
10535	229533.A0A0E0SPS2	pp	229533.A0A1C3YLB6
10536	229533.A0A0E0SPS2	pp	229533.I1RA49
10537	229533.A0A0E0SPS2	pp	229533.I1R9M0
10538	229533.A0A0E0SPS2	pp	229533.I1RCG2
10539	229533.A0A0E0SPS2	pp	229533.I1S0B0
10540	229533.A0A0E0SPS2	pp	229533.I1RE01
10541	229533.A0A0E0SPS2	pp	229533.I1RX46
10542	229533.A0A0E0SPS2	pp	229533.I1RBY4
10543	229533.A0A0E0SPS2	pp	229533.I1S0J1
10544	229533.A0A0E0SPS2	pp	229533.I1RY92
10545	229533.A0A0E0SPS2	pp	229533.A0A1C3YM82
10546	229533.A0A0E0SPS2	pp	229533.I1RPP3
10547	229533.A0A0E0SPS2	pp	229533.A0A1C3YJP4
10548	229533.A0A0E0SPS2	pp	229533.I1RYI5
10549	229533.A0A0E0SPS2	pp	229533.I1RZ39
10550	229533.A0A0E0SPS2	pp	229533.A0A1C3YKM9
10551	229533.A0A0E0SPS2	pp	229533.A0A1C3YIR8
10552	229533.A0A0E0SPS2	pp	229533.I1RSQ4
10553	229533.A0A0E0SPS2	pp	229533.Q4HZB8
10554	229533.A0A0E0SPS2	pp	229533.I1RZZ9
10555	229533.A0A0E0SPS2	pp	229533.I1S1F3
10556	229533.A0A0E0SPS2	pp	229533.I1S1W3
10557	229533.A0A0E0SPS2	pp	229533.I1RN67
10558	229533.A0A0E0SPS2	pp	229533.I1RNG4
10559	229533.A0A0E0SPS2	pp	229533.A0A1C3YI20
10560	229533.A0A0E0SPS2	pp	229533.I1RC66
10561	229533.A0A0E0SPS2	pp	229533.A0A1C3YIN0
10562	229533.A0A0E0SPS2	pp	229533.I1S2H4
10563	229533.A0A0E0SPS2	pp	229533.V6R042
10564	229533.A0A0E0SPS2	pp	229533.I1RTG9
10565	229533.A0A0E0SPS2	pp	229533.A0A1C3YM60
10566	229533.A0A0E0SPS2	pp	229533.I1RT40
10567	229533.A0A0E0SPS2	pp	229533.I1RSQ3
10568	229533.A0A0E0SPS2	pp	229533.I1RCR8
10569	229533.A0A0E0SPS2	pp	229533.I1RTD5
10570	229533.A0A0E0SPS2	pp	229533.I1S0L4
10571	229533.A0A0E0SPS2	pp	229533.I1RA11
10572	229533.A0A0E0SPS2	pp	229533.I1S5R1
10573	229533.A0A0E0SPS2	pp	229533.I1RBA6
10574	229533.A0A0E0SPS2	pp	229533.I1RGA4
10575	229533.A0A0E0SPS2	pp	229533.I1S0L8
10576	229533.A0A0E0SPS2	pp	229533.I1RUG3
10577	229533.A0A0E0SPS2	pp	229533.A0A1C3YLK8
10578	229533.A0A0E0SPS2	pp	229533.V6R3J1
10579	229533.A0A0E0SPS2	pp	229533.A0A1C3YI53
10580	229533.A0A0E0SPS2	pp	229533.I1RFT1
10581	229533.A0A0E0SPS2	pp	229533.I1RNC1
10582	229533.A0A0E0SPS2	pp	229533.A0A0E0SPV0
10583	229533.A0A098DZQ8	pp	229533.I1RTB7
10584	229533.A0A098DZQ8	pp	229533.A0A0E0S972
10585	229533.A0A098DZQ8	pp	229533.I1RH68

10586 229533.A0A098DZQ8 pp 229533.I1S0S6
10587 229533.A0A098DZQ8 pp 229533.I1RX09
10588 229533.A0A098DZQ8 pp 229533.I1S4Q7
10589 229533.A0A098DZQ8 pp 229533.I1RRH8
10590 229533.A0A098DZQ8 pp 229533.I1RS64
10591 229533.A0A098DZQ8 pp 229533.P0C431
10592 229533.A0A098DZQ8 pp 229533.I1RQN9
10593 229533.I1RC49 pp 229533.V6R3J1
10594 229533.I1RC49 pp 229533.I1S0L8
10595 229533.I1RC49 pp 229533.Q4I7L0
10596 229533.I1RC49 pp 229533.I1RJV7
10597 229533.I1RC49 pp 229533.I1RLP1
10598 229533.I1RC49 pp 229533.I1RY40
10599 229533.I1RC49 pp 229533.I1RPA7
10600 229533.I1RC49 pp 229533.I1RD52
10601 229533.I1RC49 pp 229533.I1RMV0
10602 229533.I1RC49 pp 229533.I1RTG9
10603 229533.I1RC49 pp 229533.I1RN72
10604 229533.I1RC49 pp 229533.I1RRX5
10605 229533.I1RC49 pp 229533.I1S7W5
10606 229533.I1RC49 pp 229533.I1RYX5
10607 229533.I1RC49 pp 229533.I1RME4
10608 229533.I1RC49 pp 229533.I1RCK9
10609 229533.I1RC49 pp 229533.I1RQE7
10610 229533.I1RC49 pp 229533.I1RQ49
10611 229533.I1RC49 pp 229533.I1RS90
10612 229533.I1RC49 pp 229533.I1RWC9
10613 229533.I1RC49 pp 229533.I1S0B0
10614 229533.I1RC49 pp 229533.I1RPR9
10615 229533.I1RC49 pp 229533.I1RG12
10616 229533.I1RC49 pp 229533.I1S2B9
10617 229533.I1RC49 pp 229533.I1S1Y9
10618 229533.I1RC49 pp 229533.I1RWB0
10619 229533.I1RC49 pp 229533.I1RPU5
10620 229533.I1RSA5 pp 229533.I1RZX3
10621 229533.I1RSA5 pp 229533.I1RT03
10622 229533.I1RSA5 pp 229533.I1RVX0
10623 229533.I1RSA5 pp 229533.I1S2C4
10624 229533.I1RSA5 pp 229533.I1S6V0
10625 229533.I1RSA5 pp 229533.I1RUF7
10626 229533.I1RSA5 pp 229533.I1S1C7
10627 229533.I1RSA5 pp 229533.I1S0F8
10628 229533.I1RSA5 pp 229533.I1S9Z4
10629 229533.I1RSA5 pp 229533.I1S2B8
10630 229533.I1RSA5 pp 229533.I1RVQ3
10631 229533.I1RSA5 pp 229533.I1RZ56
10632 229533.I1RSA5 pp 229533.I1RSF1
10633 229533.I1RSA5 pp 229533.I1S064
10634 229533.I1RSA5 pp 229533.I1RZV3
10635 229533.I1RSA5 pp 229533.V6RCJ5
10636 229533.I1RSA5 pp 229533.I1S1W9
10637 229533.I1RSA5 pp 229533.I1S5R6
10638 229533.I1RSA5 pp 229533.I1S694
10639 229533.I1RSA5 pp 229533.I1RVF8
10640 229533.I1RSA5 pp 229533.I1RZ39
10641 229533.I1RSA5 pp 229533.I1RY35
10642 229533.I1RSA5 pp 229533.I1S3G4
10643 229533.I1RSA5 pp 229533.I1SAT2
10644 229533.I1RSA5 pp 229533.I1RVZ2
10645 229533.I1RSA5 pp 229533.I1RYI5
10646 229533.I1RSA5 pp 229533.I1RSZ7
10647 229533.I1RSA5 pp 229533.I1RZ38
10648 229533.I1RSA5 pp 229533.I1SAB4
10649 229533.I1RSA5 pp 229533.I1S968
10650 229533.I1RSA5 pp 229533.I1S5R1
10651 229533.I1RSA5 pp 229533.I1RZA8
10652 229533.I1RSA5 pp 229533.I1S0F7
10653 229533.I1RSA5 pp 229533.I1RXC2
10654 229533.I1RSA5 pp 229533.I1RTA1
10655 229533.I1RSA5 pp 229533.I1RUQ3
10656 229533.I1RSA5 pp 229533.I1RTD5
10657 229533.I1RSA5 pp 229533.I1RT40
10658 229533.I1RSA5 pp 229533.I1S0W4

10659	229533.I1RSA5	pp	229533.I1S0A5
10660	229533.I1RSA5	pp	229533.I1RZ25
10661	229533.I1RSA5	pp	229533.I1RSX0
10662	229533.I1RSA5	pp	229533.I1S2F7
10663	229533.I1RSA5	pp	229533.V6RWB3
10664	229533.I1RSA5	pp	229533.I1RZM8
10665	229533.I1RSA5	pp	229533.Q4IJK2
10666	229533.I1RSA5	pp	229533.Q4HVQ9
10667	229533.I1RSA5	pp	229533.I1RW55
10668	229533.I1RSA5	pp	229533.I1S659
10669	229533.I1RSA5	pp	229533.I1RUY5
10670	229533.I1RSA5	pp	229533.I1S4I7
10671	229533.I1RSA5	pp	229533.I1S8D3
10672	229533.I1RSA5	pp	229533.I1RWM4
10673	229533.I1RSA5	pp	229533.I1RWR7
10674	229533.I1RSA5	pp	229533.I1RY81
10675	229533.I1RSA5	pp	229533.I1S3T6
10676	229533.I1RSA5	pp	229533.I1RST9
10677	229533.I1RSA5	pp	229533.I1RZK5
10678	229533.I1RSA5	pp	229533.I1S1B2
10679	229533.I1RSA5	pp	229533.I1S267
10680	229533.I1RSA5	pp	229533.V6R9Z0
10681	229533.I1RSA5	pp	229533.I1RXC4
10682	229533.I1RSA5	pp	229533.I1S210
10683	229533.I1RSA5	pp	229533.I1RW54
10684	229533.I1RSA5	pp	229533.I1S821
10685	229533.I1RSA5	pp	229533.V6R5T7
10686	229533.I1RSA5	pp	229533.I1S1W8
10687	229533.I1RSA5	pp	229533.I1S9I5
10688	229533.I1RSA5	pp	229533.I1RYP5
10689	229533.I1RSA5	pp	229533.I1RY16
10690	229533.I1RSA5	pp	229533.V6RCZ8
10691	229533.I1RSA5	pp	229533.I1S5D0
10692	229533.I1RSA5	pp	229533.I1RX46
10693	229533.I1RSA5	pp	229533.V6RCC0
10694	229533.I1RSA5	pp	229533.I1RWL3
10695	229533.I1RSA5	pp	229533.I1S094
10696	229533.I1RSA5	pp	229533.I1S6H3
10697	229533.I1RSA5	pp	229533.I1S223
10698	229533.I1RSA5	pp	229533.I1RY86
10699	229533.I1RSA5	pp	229533.I1S048
10700	229533.I1RSA5	pp	229533.I1S969
10701	229533.I1RSA5	pp	229533.I1S999
10702	229533.I1RSA5	pp	229533.I1RXD6
10703	229533.I1RSA5	pp	229533.I1S4S7
10704	229533.I1RSA5	pp	229533.I1RXD7
10705	229533.I1RSA5	pp	229533.I1RYU6
10706	229533.I1RSA5	pp	229533.I1RX95
10707	229533.I1RSA5	pp	229533.I1RTB3
10708	229533.I1RX68	pp	229533.I1S559
10709	229533.I1RX68	pp	229533.Q4IPI4
10710	229533.I1RX68	pp	229533.I1RZD6
10711	229533.I1RX68	pp	229533.I1RXS3
10712	229533.I1RX68	pp	229533.I1S0Z3
10713	229533.I1RX68	pp	229533.V6RR76
10714	229533.I1RX68	pp	229533.I1S270
10715	229533.I1RX68	pp	229533.Q4INI2
10716	229533.I1S0F7	pp	229533.I1S4S7
10717	229533.I1S0F7	pp	229533.I1S5R6
10718	229533.I1S0F7	pp	229533.I1S1W9
10719	229533.I1S0F7	pp	229533.I1S5R1
10720	229533.I1S0F7	pp	229533.I1S2B8
10721	229533.I1S0F7	pp	229533.V6RMW6
10722	229533.I1RWC9	pp	229533.I1RY40
10723	229533.I1RWC9	pp	229533.I1RZV4
10724	229533.I1RWC9	pp	229533.I1S095
10725	229533.I1RWC9	pp	229533.I1RXR1
10726	229533.I1RWC9	pp	229533.I1S0B0
10727	229533.I1RWC9	pp	229533.V6RUT5
10728	229533.I1RWC9	pp	229533.Q4I2J8
10729	229533.I1RWC9	pp	229533.I1RXU9
10730	229533.I1RWC9	pp	229533.I1RYX5
10731	229533.I1RWC9	pp	229533.I1S0K2

10732 229533.I1RWC9 pp 229533.I1RZD9
10733 229533.I1RWC9 pp 229533.I1S9V9
10734 229533.I1RWC9 pp 229533.I1S4P6
10735 229533.I1RWC9 pp 229533.I1S1W3
10736 229533.I1RWC9 pp 229533.I1RWN8
10737 229533.I1RWC9 pp 229533.I1S9G7
10738 229533.I1RWC9 pp 229533.I1S7W5
10739 229533.I1RWC9 pp 229533.I1S887
10740 229533.I1RWC9 pp 229533.V6R3J1
10741 229533.I1RWC9 pp 229533.I1S1Y9
10742 229533.I1RWC9 pp 229533.I1S2B9
10743 229533.I1RCG2 pp 229533.I1RPR9
10744 229533.I1RCG2 pp 229533.I1RLP1
10745 229533.I1RCG2 pp 229533.I1RWB0
10746 229533.I1RCG2 pp 229533.I1RMV0
10747 229533.I1RCG2 pp 229533.I1S7W5
10748 229533.I1RCG2 pp 229533.I1S2B9
10749 229533.I1RCG2 pp 229533.I1RYX5
10750 229533.I1S559 pp 229533.Q4INI2
10751 229533.I1S559 pp 229533.Q4IPI4
10752 229533.I1S559 pp 229533.Q4ICG5
10753 229533.I1S559 pp 229533.V6RR76
10754 229533.I1S559 pp 229533.Q4HZE0
10755 229533.I1S559 pp 229533.I1S8E0
10756 229533.I1S559 pp 229533.I1S8X0
10757 229533.I1RCK9 pp 229533.I1RS90
10758 229533.I1RCK9 pp 229533.I1S1W3
10759 229533.I1RCK9 pp 229533.I1S1Y9
10760 229533.I1RCK9 pp 229533.I1S2B9
10761 229533.I1RCK9 pp 229533.I1RY40
10762 229533.I1RCK9 pp 229533.I1RKJ2
10763 229533.I1RCK9 pp 229533.I1RLP1
10764 229533.I1RCK9 pp 229533.I1RJV7
10765 229533.I1RCK9 pp 229533.I1RPS0
10766 229533.I1RCK9 pp 229533.I1RQ82
10767 229533.I1RCK9 pp 229533.I1S0L8
10768 229533.I1RCK9 pp 229533.V6RUT5
10769 229533.I1RCK9 pp 229533.I1RWC9
10770 229533.I1RCK9 pp 229533.I1RJH8
10771 229533.I1RCK9 pp 229533.I1S9G7
10772 229533.I1RCK9 pp 229533.I1RPR9
10773 229533.I1RCK9 pp 229533.V6R3J1
10774 229533.I1RCK9 pp 229533.I1RG12
10775 229533.I1RCK9 pp 229533.I1RRX5
10776 229533.I1RCK9 pp 229533.I1RWB0
10777 229533.I1RCK9 pp 229533.I1RZD9
10778 229533.I1RCK9 pp 229533.I1RMV0
10779 229533.I1RCK9 pp 229533.I1S095
10780 229533.I1RCK9 pp 229533.I1RYX5
10781 229533.I1RCK9 pp 229533.I1RYM3
10782 229533.I1RCK9 pp 229533.I1RZV4
10783 229533.I1RCK9 pp 229533.I1RPA7
10784 229533.I1RCK9 pp 229533.I1S0B0
10785 229533.I1RCK9 pp 229533.I1S7W5
10786 229533.I1RCK9 pp 229533.I1RTG9
10787 229533.I1RCK9 pp 229533.I1RZD1
10788 229533.I1RCK9 pp 229533.Q4IJW4
10789 229533.I1RCK9 pp 229533.I1RWG1
10790 229533.I1RCK9 pp 229533.I1RSB0
10791 229533.I1RCK9 pp 229533.I1RN72
10792 229533.I1RCK9 pp 229533.I1RJH7
10793 229533.I1RUQ1 pp 229533.I1RZF5
10794 229533.I1RUQ1 pp 229533.I1RYI1
10795 229533.I1RUQ1 pp 229533.I1S438
10796 229533.I1RUQ1 pp 229533.I1S437
10797 229533.I1RUQ1 pp 229533.I1RYR0
10798 229533.I1RUQ1 pp 229533.I1S1D3
10799 229533.I1RUQ1 pp 229533.Q4I0J6
10800 229533.I1RK57 pp 229533.I1RWX5
10801 229533.I1RBA6 pp 229533.I1RTG9
10802 229533.I1RBA6 pp 229533.I1S0B0
10803 229533.I1RBA6 pp 229533.I1RMF2
10804 229533.I1RBA6 pp 229533.I1RYX5

10805	229533.I1RBA6	pp	229533.I1RJ35
10806	229533.I1RBA6	pp	229533.I1S1W3
10807	229533.I1RBA6	pp	229533.I1S0L8
10808	229533.I1RBA6	pp	229533.I1S2P3
10809	229533.I1RBA6	pp	229533.I1S2B9
10810	229533.I1RBA6	pp	229533.I1RPS0
10811	229533.I1RBA6	pp	229533.I1RS90
10812	229533.I1RBA6	pp	229533.I1RMV0
10813	229533.I1RBA6	pp	229533.I1S7C4
10814	229533.I1RBA6	pp	229533.I1RYU1
10815	229533.I1RBA6	pp	229533.I1RCG2
10816	229533.I1RBA6	pp	229533.I1RND8
10817	229533.I1RBA6	pp	229533.I1RCK9
10818	229533.I1RBA6	pp	229533.I1S7W5
10819	229533.I1RBA6	pp	229533.V6R3J1
10820	229533.I1RBA6	pp	229533.I1S1Y9
10821	229533.I1RBA6	pp	229533.I1RW84
10822	229533.I1RBA6	pp	229533.I1RFY0
10823	229533.I1RBA6	pp	229533.I1RG12
10824	229533.I1RBA6	pp	229533.I1RWG1
10825	229533.I1RBA6	pp	229533.I1RY40
10826	229533.I1RBA6	pp	229533.V6RIV7
10827	229533.I1RBA6	pp	229533.I1RYM3
10828	229533.I1RBA6	pp	229533.I1RMD6
10829	229533.I1RBA6	pp	229533.I1S999
10830	229533.I1RBA6	pp	229533.I1RFK0
10831	229533.I1RBA6	pp	229533.I1RCT0
10832	229533.I1RBA6	pp	229533.I1RP86
10833	229533.I1RBA6	pp	229533.I1S053
10834	229533.I1RBA6	pp	229533.I1RC49
10835	229533.I1RBA6	pp	229533.I1S8S9
10836	229533.I1RBA6	pp	229533.I1SAZ0
10837	229533.I1RBA6	pp	229533.I1S9G7
10838	229533.I1RBA6	pp	229533.I1S422
10839	229533.I1RBA6	pp	229533.I1RPR9
10840	229533.I1RBA6	pp	229533.I1RPA7
10841	229533.I1RBA6	pp	229533.V6RUT5
10842	229533.I1RBA6	pp	229533.I1RCU4
10843	229533.I1RBA6	pp	229533.I1RH75
10844	229533.I1RBA6	pp	229533.I1S1W5
10845	229533.I1RBA6	pp	229533.I1RWB0
10846	229533.I1RBA6	pp	229533.I1RWC9
10847	229533.I1RBA6	pp	229533.I1RLP1
10848	229533.I1RBA6	pp	229533.I1RN72
10849	229533.I1RBA6	pp	229533.I1SAK7
10850	229533.I1RBA6	pp	229533.I1RQ96
10851	229533.I1RBA6	pp	229533.V6QXN0
10852	229533.I1RBA6	pp	229533.I1RKJ2
10853	229533.I1RBA6	pp	229533.I1S278
10854	229533.I1RBA6	pp	229533.I1RCH4
10855	229533.I1RB25	pp	229533.I1RXE5
10856	229533.I1RB25	pp	229533.I1RZB5
10857	229533.I1RB25	pp	229533.I1RQP3
10858	229533.I1RB25	pp	229533.I1REN3
10859	229533.I1RB25	pp	229533.Q4ICA8
10860	229533.I1RB25	pp	229533.Q4I948
10861	229533.I1RB25	pp	229533.I1RK95
10862	229533.I1RB25	pp	229533.I1S0F0
10863	229533.I1RB25	pp	229533.I1RC15
10864	229533.I1RB25	pp	229533.I1RM67
10865	229533.I1RB25	pp	229533.I1RNH1
10866	229533.I1RB25	pp	229533.I1RWF3
10867	229533.I1RB25	pp	229533.Q4IFH8
10868	229533.I1RB25	pp	229533.I1RP96
10869	229533.I1RB25	pp	229533.I1RFQ0
10870	229533.I1RB25	pp	229533.I1RKB1
10871	229533.I1RB25	pp	229533.Q4I7L0
10872	229533.I1RB25	pp	229533.I1RYT8
10873	229533.I1RB25	pp	229533.Q4HYB8
10874	229533.I1RB25	pp	229533.I1RZX0
10875	229533.I1RB25	pp	229533.I1RRF0
10876	229533.I1RB25	pp	229533.I1RC24
10877	229533.I1RB25	pp	229533.I1RB73

229533.I1RB25	pp	229533.I1S276
229533.I1RB25	pp	229533.I1RDH0
229533.I1RB25	pp	229533.I1RPM7
229533.I1RB25	pp	229533.Q4IL82
229533.I1RB25	pp	229533.I1RK71
229533.I1RB25	pp	229533.Q4IMD1
229533.I1RB25	pp	229533.I1RSG8
229533.I1RB25	pp	229533.V6RAC9
229533.I1RB25	pp	229533.Q4IPI4
229533.I1RB25	pp	229533.I1RK79
229533.I1RB25	pp	229533.I1RSZ3
229533.I1RB25	pp	229533.I1RCN2
229533.I1RB25	pp	229533.I1RS71
229533.I1RB25	pp	229533.I1RVY0
229533.I1RB25	pp	229533.I1RVL8
229533.I1RB25	pp	229533.I1RQP1
229533.I1RMV0	pp	229533.I1RRY9
229533.I1RMV0	pp	229533.I1RWC9
229533.I1RMV0	pp	229533.I1RWB0
229533.I1RMV0	pp	229533.I1S887
229533.I1RMV0	pp	229533.I1S9G7
229533.I1RMV0	pp	229533.I1RPP5
229533.I1RMV0	pp	229533.I1S0K2
229533.I1RMV0	pp	229533.I1RZK5
229533.I1RMV0	pp	229533.V6QUE6
229533.I1RMV0	pp	229533.I1S1Y9
229533.I1RMV0	pp	229533.Q4IJK2
229533.I1RMV0	pp	229533.I1RRX5
229533.I1RMV0	pp	229533.I1S5B0
229533.I1RMV0	pp	229533.I1RZM8
229533.I1RMV0	pp	229533.I1RXF0
229533.I1RMV0	pp	229533.I1RSQ7
229533.I1RMV0	pp	229533.I1RXU9
229533.I1RMV0	pp	229533.V6RCZ8
229533.I1RMV0	pp	229533.I1S4S7
229533.I1RMV0	pp	229533.I1RPS0
229533.I1RMV0	pp	229533.I1RUG3
229533.I1RMV0	pp	229533.I1RYW3
229533.I1RMV0	pp	229533.V6R042
229533.I1RMV0	pp	229533.I1RS18
229533.I1RMV0	pp	229533.I1S1C7
229533.I1RMV0	pp	229533.I1RYM3
229533.I1RMV0	pp	229533.I1RSQ3
229533.I1RMV0	pp	229533.I1S1W3
229533.I1RMV0	pp	229533.I1S4S4
229533.I1RMV0	pp	229533.I1S7W5
229533.I1RMV0	pp	229533.I1S0J1
229533.I1RMV0	pp	229533.I1RYH7
229533.I1RMV0	pp	229533.I1RR24
229533.I1RMV0	pp	229533.Q4I624
229533.I1RMV0	pp	229533.Q4HYC1
229533.I1RMV0	pp	229533.I1S5R1
229533.I1RMV0	pp	229533.I1RQE7
229533.I1RMV0	pp	229533.I1S0R8
229533.I1RMV0	pp	229533.I1SAK7
229533.I1RMV0	pp	229533.I1RPA7
229533.I1RMV0	pp	229533.I1RXR1
229533.I1RMV0	pp	229533.I1S0L4
229533.I1RMV0	pp	229533.I1RPA4
229533.I1RMV0	pp	229533.I1RX46
229533.I1RMV0	pp	229533.I1S2H4
229533.I1RMV0	pp	229533.I1RTG9
229533.I1RMV0	pp	229533.I1RXD7
229533.I1RMV0	pp	229533.I1RVX0
229533.I1RMV0	pp	229533.I1RZZ9
229533.I1RMV0	pp	229533.I1RSQ4
229533.I1RMV0	pp	229533.I1RT03
229533.I1RMV0	pp	229533.I1S147
229533.I1RMV0	pp	229533.V6RUT5
229533.I1RMV0	pp	229533.I1RS90
229533.I1RMV0	pp	229533.I1RZ39
229533.I1RMV0	pp	229533.I1S999
229533.I1RMV0	pp	229533.I1RTG6

10951	229533.I1RMV0	pp	229533.I1RWG1
10952	229533.I1RMV0	pp	229533.I1S0B0
10953	229533.I1RMV0	pp	229533.I1RYX5
10954	229533.I1RMV0	pp	229533.V6R3J1
10955	229533.I1RMV0	pp	229533.I1S0L8
10956	229533.I1RMV0	pp	229533.I1S277
10957	229533.I1RMV0	pp	229533.I1S2B9
10958	229533.I1RMV0	pp	229533.I1RT40
10959	229533.I1RMV0	pp	229533.I1RTD5
10960	229533.I1RMV0	pp	229533.I1RNC1
10961	229533.I1RMV0	pp	229533.I1RNG4
10962	229533.I1RMV0	pp	229533.I1RN67
10963	229533.I1RMV0	pp	229533.I1RPP3
10964	229533.I1RMV0	pp	229533.I1RYI5
10965	229533.I1RMV0	pp	229533.I1S1F3
10966	229533.I1RXS3	pp	229533.Q4INI2
10967	229533.I1RXS3	pp	229533.I1RZD6
10968	229533.I1RXS3	pp	229533.I1S0Z3
10969	229533.I1RXS3	pp	229533.I1S270
10970	229533.I1RXS3	pp	229533.Q4IPI4
10971	229533.I1RXS3	pp	229533.I1S559
10972	229533.I1RXS3	pp	229533.Q4HZE0
10973	229533.I1RSG0	pp	229533.I1RZT1
10974	229533.I1RSG0	pp	229533.I1S1W5
10975	229533.I1RSG0	pp	229533.I1S267
10976	229533.I1RSG0	pp	229533.I1S0M4
10977	229533.I1RSG0	pp	229533.I1S969
10978	229533.I1RSG0	pp	229533.I1RZX3
10979	229533.I1RSG0	pp	229533.I1RZC3
10980	229533.I1RSG0	pp	229533.V6RHX2
10981	229533.I1RSG0	pp	229533.I1RZ95
10982	229533.I1RSG0	pp	229533.I1S1D7
10983	229533.I1RSG0	pp	229533.I1RY44
10984	229533.I1RSG0	pp	229533.I1S224
10985	229533.I1RSG0	pp	229533.I1S9T6
10986	229533.I1RSG0	pp	229533.I1RST9
10987	229533.I1RSG0	pp	229533.I1RYP5
10988	229533.I1RSG0	pp	229533.I1RSP5
10989	229533.I1RSG0	pp	229533.Q4IPX8
10990	229533.I1RSG0	pp	229533.I1RTM8
10991	229533.I1RSG0	pp	229533.I1RZ25
10992	229533.I1RSG0	pp	229533.I1SAB4
10993	229533.I1RSG0	pp	229533.I1RWC1
10994	229533.I1RSG0	pp	229533.I1RSI3
10995	229533.I1RSG0	pp	229533.I1S968
10996	229533.I1RSG0	pp	229533.I1S2E9
10997	229533.I1RSG0	pp	229533.I1RY32
10998	229533.I1RAU9	pp	229533.I1S422
10999	229533.I1RAU9	pp	229533.I1RXG2
11000	229533.I1RAU9	pp	229533.I1RWG1
11001	229533.I1RAU9	pp	229533.I1RNM7
11002	229533.I1RAU9	pp	229533.I1RY40
11003	229533.I1RAU9	pp	229533.I1RQ96
11004	229533.I1RAU9	pp	229533.Q5I2J3
11005	229533.I1RAU9	pp	229533.Q4HZS8
11006	229533.I1RAU9	pp	229533.I1S9G7
11007	229533.I1RAU9	pp	229533.I1RRX5
11008	229533.I1RAU9	pp	229533.I1RCK9
11009	229533.I1RAU9	pp	229533.I1RPA7
11010	229533.I1RAU9	pp	229533.I1RWC9
11011	229533.I1RAU9	pp	229533.I1RN72
11012	229533.I1RAU9	pp	229533.I1RPR9
11013	229533.I1RAU9	pp	229533.I1RWB0
11014	229533.I1RAU9	pp	229533.I1RC49
11015	229533.I1RAU9	pp	229533.I1RVX9
11016	229533.I1RAU9	pp	229533.I1SAZ0
11017	229533.I1RAU9	pp	229533.I1S1Y9
11018	229533.I1RAU9	pp	229533.I1RXB5
11019	229533.I1RAU9	pp	229533.I1RXF0
11020	229533.I1RAU9	pp	229533.I1RCM5
11021	229533.I1RAU9	pp	229533.I1RYL5
11022	229533.I1RAU9	pp	229533.I1RP86
11023	229533.I1RAU9	pp	229533.V6RUT5

11024	229533.I1RAU9	pp	229533.I1RYX5
11025	229533.I1RAU9	pp	229533.I1RLP1
11026	229533.I1RAU9	pp	229533.I1S0B0
11027	229533.I1RAU9	pp	229533.V6RXW9
11028	229533.I1RAU9	pp	229533.I1S8F8
11029	229533.I1RAU9	pp	229533.I1S4Z7
11030	229533.I1RAU9	pp	229533.I1RCR5
11031	229533.I1RAU9	pp	229533.I1RC29
11032	229533.I1RAU9	pp	229533.I1RM26
11033	229533.I1RAU9	pp	229533.I1RKJ2
11034	229533.I1RAU9	pp	229533.I1RVR2
11035	229533.I1RAU9	pp	229533.I1RYM3
11036	229533.I1RAU9	pp	229533.I1RCT0
11037	229533.I1RAU9	pp	229533.I1RFT1
11038	229533.I1RAU9	pp	229533.I1RTG9
11039	229533.I1RAU9	pp	229533.I1RJT5
11040	229533.I1RAU9	pp	229533.I1RMJ3
11041	229533.I1RAU9	pp	229533.I1RX72
11042	229533.I1RAU9	pp	229533.I1S9V9
11043	229533.I1RAU9	pp	229533.I1RKE7
11044	229533.I1RAU9	pp	229533.I1RR95
11045	229533.I1RAU9	pp	229533.I1RBA6
11046	229533.I1RAU9	pp	229533.I1RBA4
11047	229533.I1RAU9	pp	229533.I1RJT8
11048	229533.I1RAU9	pp	229533.V6QX21
11049	229533.I1RAU9	pp	229533.I1S0L8
11050	229533.I1RAU9	pp	229533.I1RG12
11051	229533.I1RAU9	pp	229533.I1RYW3
11052	229533.I1RAU9	pp	229533.I1S2P3
11053	229533.I1RAU9	pp	229533.I1RJ35
11054	229533.I1RAU9	pp	229533.V6R3J1
11055	229533.I1RAU9	pp	229533.I1RGB3
11056	229533.I1RAU9	pp	229533.I1RB40
11057	229533.I1RAU9	pp	229533.I1RN84
11058	229533.I1RAU9	pp	229533.I1RMQ5
11059	229533.I1RAU9	pp	229533.I1S1W3
11060	229533.I1RAU9	pp	229533.I1RAV0
11061	229533.I1RAU9	pp	229533.I1RMV0
11062	229533.I1RAU9	pp	229533.I1S2B9
11063	229533.I1RAU9	pp	229533.I1S7W5
11064	229533.I1RAU9	pp	229533.I1RQ49
11065	229533.I1RAU9	pp	229533.I1RS90
11066	229533.I1RLE7	pp	229533.I1RN57
11067	229533.I1RLE7	pp	229533.I1S2F2
11068	229533.I1RLE7	pp	229533.I1RSJ4
11069	229533.I1RLE7	pp	229533.I1RMX3
11070	229533.I1RLE7	pp	229533.I1RSC7
11071	229533.I1RLE7	pp	229533.I1S1D3
11072	229533.I1RLE7	pp	229533.I1RSI4
11073	229533.I1RLE7	pp	229533.I1RNE4
11074	229533.I1RLE7	pp	229533.I1S1C8
11075	229533.I1RLE7	pp	229533.Q4IQM6
11076	229533.I1RLE7	pp	229533.I1RWM6
11077	229533.I1RLE7	pp	229533.I1RMB7
11078	229533.I1RLE7	pp	229533.I1RN55
11079	229533.I1RLE7	pp	229533.I1RRK8
11080	229533.I1RLE7	pp	229533.V6RLW4
11081	229533.I1RS90	pp	229533.I1S5R1
11082	229533.I1RS90	pp	229533.I1RTG9
11083	229533.I1RS90	pp	229533.I1S1W3
11084	229533.I1RS90	pp	229533.I1RUG3
11085	229533.I1RS90	pp	229533.I1S7W5
11086	229533.I1RS90	pp	229533.I1RYX5
11087	229533.I1RS90	pp	229533.I1S0B0
11088	229533.I1RS90	pp	229533.I1RXU9
11089	229533.I1RS90	pp	229533.V6R3J1
11090	229533.I1RS90	pp	229533.I1S2P3
11091	229533.I1RS90	pp	229533.I1S0V7
11092	229533.I1RS90	pp	229533.I1RWX5
11093	229533.I1RS90	pp	229533.I1S0L8
11094	229533.I1RS90	pp	229533.I1RYW3
11095	229533.I1RS90	pp	229533.I1S2B9
11096	229533.I1RS90	pp	229533.I1RXR1

11097 229533.I1RS90 pp 229533.I1RTH6
11098 229533.I1RS90 pp 229533.I1S422
11099 229533.I1RS90 pp 229533.V6RTJ8
11100 229533.I1RS90 pp 229533.I1RWB0
11101 229533.I1RS90 pp 229533.V6RUT5
11102 229533.I1RS90 pp 229533.I1RT41
11103 229533.I1RS90 pp 229533.I1RWC9
11104 229533.I1RS90 pp 229533.I1SAZ0
11105 229533.I1RS90 pp 229533.I1S9G7
11106 229533.I1RS90 pp 229533.V6RAN2
11107 229533.I1RS90 pp 229533.I1S8S9
11108 229533.I1RS90 pp 229533.I1S1Y9
11109 229533.I1RS90 pp 229533.I1RW71
11110 229533.I1RS90 pp 229533.I1S0V6
11111 229533.I1RS90 pp 229533.I1RWG1
11112 229533.I1RS90 pp 229533.I1S887
11113 229533.I1RS90 pp 229533.I1S0Z3
11114 229533.I1RS90 pp 229533.Q4IJK2
11115 229533.I1RS90 pp 229533.I1RYM3
11116 229533.A0A098DES8 pp 229533.I1RXS0
11117 229533.A0A098DES8 pp 229533.I1S0K0
11118 229533.A0A098DES8 pp 229533.I1RHZ5
11119 229533.A0A098DES8 pp 229533.I1RC48
11120 229533.A0A098DES8 pp 229533.I1RZC3
11121 229533.A0A098DES8 pp 229533.I1S8R4
11122 229533.A0A098DES8 pp 229533.I1RAX2
11123 229533.A0A098DES8 pp 229533.I1RY32
11124 229533.A0A098DES8 pp 229533.A0A1C3YKN4
11125 229533.A0A098DES8 pp 229533.I1RJR8
11126 229533.A0A098DES8 pp 229533.I1S267
11127 229533.A0A098DES8 pp 229533.I1RSG0
11128 229533.A0A098DES8 pp 229533.I1S3L2
11129 229533.A0A098DES8 pp 229533.I1RC71
11130 229533.A0A098DES8 pp 229533.I1RQP2
11131 229533.I1S2B9 pp 229533.I1S9G7
11132 229533.I1S2B9 pp 229533.Q4INW6
11133 229533.I1S2B9 pp 229533.V6RUT5
11134 229533.I1S2B9 pp 229533.I1S887
11135 229533.I1S2B9 pp 229533.I1SAZ0
11136 229533.I1S2B9 pp 229533.I1S7W5
11137 229533.I1S2B9 pp 229533.I1S7C4
11138 229533.I1S2B9 pp 229533.I1S2P3
11139 229533.I1S2B9 pp 229533.I1SAK7
11140 229533.I1S2B9 pp 229533.I1S422
11141 229533.I1S2B9 pp 229533.I1S999
11142 229533.I1S2B9 pp 229533.I1S9V9
11143 229533.A0A1C3YKK4 pp 229533.I1S130
11144 229533.A0A1C3YKK4 pp 229533.I1RCH8
11145 229533.A0A1C3YKK4 pp 229533.A0A1C3YM33
11146 229533.A0A1C3YKK4 pp 229533.I1RVN0
11147 229533.A0A1C3YKK4 pp 229533.I1RRN8
11148 229533.A0A1C3YKK4 pp 229533.I1RCJ0
11149 229533.A0A1C3YKK4 pp 229533.I1RRS3
11150 229533.I1RAP4 pp 229533.I1S2B9
11151 229533.I1RAP4 pp 229533.I1RYX5
11152 229533.I1RAP4 pp 229533.I1RWC9
11153 229533.I1RAP4 pp 229533.I1RCG2
11154 229533.I1RAP4 pp 229533.I1RCK9
11155 229533.I1RAP4 pp 229533.I1RMV0
11156 229533.I1RAP4 pp 229533.I1RC49
11157 229533.I1RAP4 pp 229533.I1RG12
11158 229533.I1RAP4 pp 229533.I1RBA6
11159 229533.I1RAP4 pp 229533.I1RAU9
11160 229533.I1RMI9 pp 229533.I1RS13
11161 229533.I1RCJ0 pp 229533.I1S130
11162 229533.I1RCJ0 pp 229533.V6REE9
11163 229533.I1RCJ0 pp 229533.I1S0L8
11164 229533.I1RCJ0 pp 229533.I1RDD6
11165 229533.I1RCJ0 pp 229533.I1RRN8
11166 229533.I1RCJ0 pp 229533.I1RVN0
11167 229533.A0A0E0SR03 pp 229533.I1S1D3
11168 229533.A0A0E0SR03 pp 229533.A0A1C3YK94
11169 229533.A0A0E0SR03 pp 229533.I1RMP0

11170 229533.A0A0E0SR03 pp 229533.A0A1C3YKW5
11171 229533.A0A0E0SR03 pp 229533.A0A1C3YMC1
11172 229533.A0A098D6R7 pp 229533.A0A098DY67
11173 229533.A0A098D6R7 pp 229533.I1RYX5
11174 229533.A0A098D6R7 pp 229533.I1RG12
11175 229533.A0A098D6R7 pp 229533.A0A0E0SPS2
11176 229533.A0A098D6R7 pp 229533.I1RC49
11177 229533.A0A098D6R7 pp 229533.I1RSA5
11178 229533.A0A098D6R7 pp 229533.I1RWC9
11179 229533.A0A098D6R7 pp 229533.I1RCG2
11180 229533.A0A098D6R7 pp 229533.I1RCK9
11181 229533.A0A098D6R7 pp 229533.I1RBA6
11182 229533.A0A098D6R7 pp 229533.I1RMV0
11183 229533.A0A098D6R7 pp 229533.I1RAU9
11184 229533.A0A098D6R7 pp 229533.I1RS90
11185 229533.A0A098D6R7 pp 229533.I1S2B9
11186 229533.A0A098D6R7 pp 229533.I1RG11
11187 229533.A0A098D6R7 pp 229533.I1RAP4
11188 229533.I1RG07 pp 229533.I1RRN8
11189 229533.I1RG07 pp 229533.I1RTK7
11190 229533.I1RG07 pp 229533.I1S050
11191 229533.I1RG07 pp 229533.I1RN41
11192 229533.I1RG07 pp 229533.I1RNF9
11193 229533.I1RG07 pp 229533.I1RKI0
11194 229533.I1RG07 pp 229533.I1S570
11195 229533.I1RG07 pp 229533.I1RYZ7
11196 229533.I1RG07 pp 229533.Q4IJ79
11197 229533.I1RG07 pp 229533.I1RT19
11198 229533.I1RG07 pp 229533.I1RWY3
11199 229533.I1RG07 pp 229533.I1S0N8
11200 229533.I1RG07 pp 229533.I1RZX9
11201 229533.I1RG07 pp 229533.I1RIG5
11202 229533.I1RG07 pp 229533.I1RRN0
11203 229533.I1RG07 pp 229533.I1RHR1
11204 229533.I1RG07 pp 229533.I1RPM8
11205 229533.I1RG07 pp 229533.I1RIM0
11206 229533.I1RG07 pp 229533.I1RNG4
11207 229533.I1RG07 pp 229533.I1S1B1
11208 229533.I1RG07 pp 229533.I1RJA7
11209 229533.I1RG07 pp 229533.I1S069
11210 229533.I1RG07 pp 229533.I1RSV0
11211 229533.I1RG07 pp 229533.I1RU37
11212 229533.I1RG07 pp 229533.I1RGR8
11213 229533.I1RG07 pp 229533.I1RM78
11214 229533.I1RG07 pp 229533.I1S721
11215 229533.I1RG07 pp 229533.I1RGZ0
11216 229533.I1RG07 pp 229533.I1RQN9
11217 229533.I1RG07 pp 229533.I1RMP5
11218 229533.I1RG07 pp 229533.I1RJS2
11219 229533.I1RX03 pp 229533.I1RZ17
11220 229533.A0A1C3YIU2 pp 229533.I1RDY1
11221 229533.A0A1C3YIU2 pp 229533.I1R9V4
11222 229533.A0A1C3YIU2 pp 229533.I1S8W6
11223 229533.A0A1C3YIU2 pp 229533.A0A1C3YKJ7
11224 229533.A0A1C3YIU2 pp 229533.I1RCY6
11225 229533.A0A1C3YIU2 pp 229533.I1RB17
11226 229533.A0A1C3YIU2 pp 229533.I1RRH5
11227 229533.I1RMR8 pp 229533.I1RZT1
11228 229533.I1RMR8 pp 229533.I1RUJ0
11229 229533.I1RDF6 pp 229533.I1RZ34
11230 229533.I1RDF6 pp 229533.I1RDJ1
11231 229533.I1RDF6 pp 229533.I1RXX1
11232 229533.I1RDF6 pp 229533.I1RX38
11233 229533.I1RDF6 pp 229533.I1RQ23
11234 229533.I1RDF6 pp 229533.I1RZ13
11235 229533.I1RDF6 pp 229533.Q4IJT0
11236 229533.I1RDF6 pp 229533.V6RPQ8
11237 229533.I1RDF6 pp 229533.I1RZV2
11238 229533.I1RDF6 pp 229533.I1RQ75
11239 229533.I1RDF6 pp 229533.I1RNM4
11240 229533.I1RDF6 pp 229533.I1RPX0
11241 229533.I1RDF6 pp 229533.I1RJL9
11242 229533.I1RDF6 pp 229533.I1RQ41

229533.I1RDF6 pp 229533.I1S272
229533.I1RDF6 pp 229533.I1RIN5
229533.A0A1C3YJS4 pp 229533.I1RVN0
229533.A0A1C3YJS4 pp 229533.I1S130
229533.A0A1C3YJS4 pp 229533.I1RCH8
229533.A0A1C3YJS4 pp 229533.A0A1C3YKK4
229533.A0A1C3YJS4 pp 229533.I1RRN8
229533.A0A1C3YJS4 pp 229533.I1RCJ0
229533.I1RG45 pp 229533.I1RMP0
229533.I1RG45 pp 229533.I1S1D3
229533.A0A098DEQ3 pp 229533.A0A1C3YKK4
229533.A0A098DEQ3 pp 229533.I1RCJ0
229533.A0A098DEQ3 pp 229533.I1RG07
229533.A0A098DEQ3 pp 229533.A0A1C3YJ00
229533.A0A098DEQ3 pp 229533.I1S130
229533.A0A098DEQ3 pp 229533.A0A1C3YJS4
229533.A0A098DEQ3 pp 229533.I1RCH8
229533.I1RSZ4 pp 229533.I1RY44
229533.I1RSZ4 pp 229533.I1RX64
229533.I1RSZ4 pp 229533.I1S2E9
229533.I1RJL1 pp 229533.V6RM09
229533.I1RJL1 pp 229533.V6RN16
229533.I1RJL1 pp 229533.V6REA4
229533.I1RJL1 pp 229533.V6RDP1
229533.I1RJL1 pp 229533.V6R556
229533.I1RJL1 pp 229533.V6QTX7
229533.I1RJL1 pp 229533.I1RSA3
229533.I1RGC0 pp 229533.I1S9L0
229533.V6RM09 pp 229533.V6RPS5
229533.V6RM09 pp 229533.V6RN16
229533.I1RZ13 pp 229533.I1S984
229533.I1RZ13 pp 229533.I1S272
229533.I1RZ13 pp 229533.I1RZT6
229533.I1RZ13 pp 229533.I1S4S9
229533.I1RZ13 pp 229533.Q4I073
229533.I1RZ13 pp 229533.I1S574
229533.I1RZ13 pp 229533.V6R0U9
229533.I1RZ13 pp 229533.I1RZV2
229533.I1RZ13 pp 229533.V6RPQ8
229533.I1RZ13 pp 229533.Q4I1Y5
229533.I1RZ13 pp 229533.I1RZ78
229533.I1RZ13 pp 229533.I1RZ34
229533.I1RZ13 pp 229533.Q4IJT0
229533.I1RZ13 pp 229533.I1S252
229533.V6REA4 pp 229533.V6RN16
229533.V6REA4 pp 229533.V6RPS5
229533.I1RB17 pp 229533.I1RD69
229533.I1RB17 pp 229533.I1RKK6
229533.I1RB17 pp 229533.I1RJQ3
229533.I1RB17 pp 229533.I1SAA0
229533.I1RB17 pp 229533.I1S8W6
229533.I1RB17 pp 229533.I1RKL2
229533.I1RB17 pp 229533.I1RBZ1
229533.I1RB17 pp 229533.I1RQ93
229533.I1RB17 pp 229533.I1S227
229533.I1RB17 pp 229533.I1RSJ0
229533.I1RB17 pp 229533.I1RCY6
229533.I1RB17 pp 229533.I1RQB7
229533.I1RB17 pp 229533.I1RS60
229533.I1RB17 pp 229533.I1RDY1
229533.I1RB17 pp 229533.I1RFN2
229533.I1RB17 pp 229533.I1RRH5
229533.I1RB17 pp 229533.I1RDA8
229533.I1RB17 pp 229533.I1RYZ0
229533.I1RB17 pp 229533.I1S4T3
229533.I1RB89 pp 229533.I1RRF0
229533.I1RB89 pp 229533.I1S575
229533.I1RB89 pp 229533.Q4INI2
229533.I1RB89 pp 229533.I1RQM8
229533.I1RB89 pp 229533.I1RP99
229533.I1RB89 pp 229533.I1RST4
229533.I1RB89 pp 229533.I1RWI6
229533.I1RB89 pp 229533.I1S559

11316 229533.I1RB89 pp 229533.I1S270
11317 229533.I1RB89 pp 229533.I1RXS3
11318 229533.I1RB89 pp 229533.I1RT41
11319 229533.I1RB89 pp 229533.I1RSD7
11320 229533.I1RB89 pp 229533.I1RX09
11321 229533.I1RB89 pp 229533.I1RBB0
11322 229533.I1RB89 pp 229533.Q4IPI4
11323 229533.I1RB89 pp 229533.I1RKR9
11324 229533.I1RB89 pp 229533.I1S0Z3
11325 229533.I1RB89 pp 229533.I1RKI1
11326 229533.I1RB89 pp 229533.I1RT65
11327 229533.I1S272 pp 229533.I1S984
11328 229533.I1S272 pp 229533.Q4IB63
11329 229533.I1S272 pp 229533.I1S4S9
11330 229533.I1S272 pp 229533.I1S574
11331 229533.I1S272 pp 229533.Q4IRI9
11332 229533.I1S272 pp 229533.Q4I073
11333 229533.I1S272 pp 229533.Q4IJT0
11334 229533.I1S272 pp 229533.V6RPQ8
11335 229533.I1S272 pp 229533.Q4I1Y5
11336 229533.A0A098D6I3 pp 229533.A0A1C3YIU2
11337 229533.A0A098D6I3 pp 229533.I1RDF6
11338 229533.A0A098D6I3 pp 229533.V6RPQ8
11339 229533.A0A098D6I3 pp 229533.I1S0C7
11340 229533.A0A098D6I3 pp 229533.I1RDY1
11341 229533.A0A098D6I3 pp 229533.I1RS60
11342 229533.A0A098D6I3 pp 229533.I1RZ13
11343 229533.A0A098D6I3 pp 229533.I1RB17
11344 229533.A0A098D6I3 pp 229533.A0A1C3YKJ7
11345 229533.A0A098D6I3 pp 229533.I1S272
11346 229533.V6QTX7 pp 229533.V6R756
11347 229533.V6QTX7 pp 229533.V6R2Z2
11348 229533.V6QTX7 pp 229533.V6RAM6
11349 229533.V6QTX7 pp 229533.V6R0W1
11350 229533.V6QTX7 pp 229533.V6RDP1
11351 229533.V6QTX7 pp 229533.V6R5D2
11352 229533.V6QTX7 pp 229533.V6RN16
11353 229533.V6QTX7 pp 229533.V6RPS5
11354 229533.V6QTX7 pp 229533.V6QU99
11355 229533.I1S202 pp 229533.V6RV57
11356 229533.I1S202 pp 229533.I1S6P5
11357 229533.I1RM99 pp 229533.I1RMP0
11358 229533.I1RM99 pp 229533.I1S1D3
11359 229533.I1RX69 pp 229533.I1S270
11360 229533.I1RX69 pp 229533.Q4IPI4
11361 229533.I1RX69 pp 229533.I1S559
11362 229533.I1RX69 pp 229533.Q4INI2
11363 229533.I1RX69 pp 229533.I1RZD6
11364 229533.I1RX69 pp 229533.I1S0Z3
11365 229533.I1RX69 pp 229533.I1RXS3
11366 229533.I1RX69 pp 229533.V6RR76
11367 229533.I1RXB8 pp 229533.I1RZL9
11368 229533.V6R556 pp 229533.V6R5D2
11369 229533.V6R556 pp 229533.V6R756
11370 229533.V6R556 pp 229533.V6RPS5
11371 229533.V6R556 pp 229533.V6RDP1
11372 229533.V6R556 pp 229533.V6RAM6
11373 229533.V6R556 pp 229533.V6RN16
11374 229533.I1RC34 pp 229533.I1RNY6
11375 229533.I1RC34 pp 229533.I1RZM8
11376 229533.I1RC34 pp 229533.V6RCZ8
11377 229533.I1RC34 pp 229533.I1S0V6
11378 229533.I1RC34 pp 229533.I1S821
11379 229533.I1RC34 pp 229533.I1RZK5
11380 229533.I1RC34 pp 229533.I1S8S9
11381 229533.I1RC34 pp 229533.Q4IJK2
11382 229533.I1RC34 pp 229533.I1RPR1
11383 229533.I1RC34 pp 229533.I1REE2
11384 229533.I1RC34 pp 229533.I1RG33
11385 229533.I1RC34 pp 229533.V6R9Z0
11386 229533.I1RC34 pp 229533.I1RRV3
11387 229533.I1RC34 pp 229533.I1RGB2
11388 229533.I1RC34 pp 229533.I1S1B2

11389	229533.I1RC34	pp	229533.Q4HYR2
11390	229533.I1RC34	pp	229533.I1RWM4
11391	229533.I1RC34	pp	229533.I1RYA2
11392	229533.I1RC34	pp	229533.I1S423
11393	229533.I1RC34	pp	229533.I1RDU0
11394	229533.I1RC34	pp	229533.I1RWR7
11395	229533.I1RC34	pp	229533.I1RXC4
11396	229533.I1RC34	pp	229533.I1RQD2
11397	229533.I1RC34	pp	229533.I1S8F8
11398	229533.I1RC34	pp	229533.I1RXD6
11399	229533.I1RC34	pp	229533.I1RD52
11400	229533.I1RC34	pp	229533.I1RNM7
11401	229533.I1RC34	pp	229533.I1RYX6
11402	229533.I1RC34	pp	229533.I1RUY5
11403	229533.I1RC34	pp	229533.I1RQ85
11404	229533.I1RC34	pp	229533.I1RJS7
11405	229533.I1RC34	pp	229533.I1RMK7
11406	229533.I1RC34	pp	229533.I1RNB2
11407	229533.I1RC34	pp	229533.I1S9I5
11408	229533.I1RC34	pp	229533.I1S438
11409	229533.I1RC34	pp	229533.I1RCR6
11410	229533.I1RC34	pp	229533.I1RPY3
11411	229533.I1RC34	pp	229533.I1RP86
11412	229533.I1RC34	pp	229533.I1RJU3
11413	229533.I1RC34	pp	229533.I1RYC8
11414	229533.I1RC34	pp	229533.I1RMQ6
11415	229533.I1RC34	pp	229533.I1RKJ5
11416	229533.I1RC34	pp	229533.I1S1W9
11417	229533.I1RC34	pp	229533.I1RZV3
11418	229533.I1RC34	pp	229533.I1RXD7
11419	229533.I1RC34	pp	229533.I1RR24
11420	229533.I1RC34	pp	229533.I1RY21
11421	229533.I1RC34	pp	229533.I1RX95
11422	229533.I1RC34	pp	229533.I1RCU2
11423	229533.I1RC34	pp	229533.I1RTB3
11424	229533.I1RC34	pp	229533.I1RPU2
11425	229533.I1RC34	pp	229533.I1RYA0
11426	229533.I1RC34	pp	229533.I1RM37
11427	229533.I1RC34	pp	229533.I1S4S7
11428	229533.I1RC34	pp	229533.I1RSF1
11429	229533.I1RC34	pp	229533.I1RCR8
11430	229533.I1RC34	pp	229533.I1S8R2
11431	229533.I1RC34	pp	229533.I1RUF7
11432	229533.I1RC34	pp	229533.I1RKJ4
11433	229533.I1RC34	pp	229533.I1RHC8
11434	229533.I1RC34	pp	229533.I1RCH5
11435	229533.I1RC34	pp	229533.I1RCR1
11436	229533.I1RC34	pp	229533.I1RRM3
11437	229533.I1RC34	pp	229533.I1RQ33
11438	229533.I1RC34	pp	229533.Q4IPZ1
11439	229533.I1RC34	pp	229533.I1RVX0
11440	229533.I1RC34	pp	229533.I1S3T7
11441	229533.I1RC34	pp	229533.V6RRM0
11442	229533.I1RC34	pp	229533.I1S0V7
11443	229533.I1RC34	pp	229533.I1RRI1
11444	229533.I1RC34	pp	229533.I1S437
11445	229533.I1RC34	pp	229533.V6RGI1
11446	229533.I1RC34	pp	229533.I1RVQ3
11447	229533.I1RC34	pp	229533.I1RVZ2
11448	229533.I1RC34	pp	229533.I1S5R6
11449	229533.I1RC34	pp	229533.I1RE89
11450	229533.I1RC34	pp	229533.I1RJN9
11451	229533.I1RC34	pp	229533.I1RKB2
11452	229533.I1RC34	pp	229533.I1RF85
11453	229533.I1RC34	pp	229533.I1RVR2
11454	229533.I1RC34	pp	229533.I1RSZ7
11455	229533.I1RC34	pp	229533.I1RTA1
11456	229533.I1RC34	pp	229533.I1S299
11457	229533.I1RC34	pp	229533.I1RGA4
11458	229533.I1RC34	pp	229533.V6R3J1
11459	229533.I1RC34	pp	229533.I1RYI5
11460	229533.I1RC34	pp	229533.I1S2B8
11461	229533.I1RC34	pp	229533.I1RFI4

229533.I1RC34	pp	229533.I1RUR8
229533.I1RC34	pp	229533.I1RDF7
229533.I1RC34	pp	229533.I1RSQ4
229533.I1RC34	pp	229533.I1RZ39
229533.I1RC34	pp	229533.I1S0W4
229533.I1RC34	pp	229533.I1RNG4
229533.I1RC34	pp	229533.I1S5R1
229533.I1RC34	pp	229533.I1RSA5
229533.I1RC34	pp	229533.I1RL81
229533.I1RC34	pp	229533.I1RXC2
229533.I1RC34	pp	229533.I1REC5
229533.I1RC34	pp	229533.I1RYW9
229533.I1RC34	pp	229533.I1RPE0
229533.I1RC34	pp	229533.I1RT40
229533.I1RC34	pp	229533.I1RKP1
229533.I1RC34	pp	229533.I1RTD5
229533.I1RC34	pp	229533.V6RAC9
229533.I1S2D3	pp	229533.I1S2P8
229533.I1S2D3	pp	229533.I1S724
229533.I1RMP2	pp	229533.Q4HZB8
229533.I1RMP2	pp	229533.I1RSZ6
229533.I1RMP2	pp	229533.I1RWQ0
229533.I1RMP2	pp	229533.I1RW46
229533.I1RMP2	pp	229533.V6R9T8
229533.I1RMP2	pp	229533.I1RZL9
229533.I1RMP2	pp	229533.I1RTA3
229533.I1RMP2	pp	229533.I1RPK0
229533.I1RMP2	pp	229533.I1RR95
229533.I1RMP2	pp	229533.I1RTG5
229533.I1RMP2	pp	229533.I1RTE0
229533.I1RMP2	pp	229533.I1RY58
229533.I1RMP2	pp	229533.Q4IMD1
229533.I1RMP2	pp	229533.I1RPX7
229533.I1RMP2	pp	229533.I1RPG1
229533.I1RMP2	pp	229533.I1RUP1
229533.I1RMP2	pp	229533.I1S7X9
229533.I1RMP2	pp	229533.I1RZR2
229533.I1RMP2	pp	229533.I1RS60
229533.I1RMP2	pp	229533.I1S092
229533.I1RMP2	pp	229533.I1RXB8
229533.I1RMP2	pp	229533.I1S8X0
229533.I1RMP2	pp	229533.I1RZD9
229533.I1RMP2	pp	229533.I1S045
229533.I1RMP2	pp	229533.I1RSB0
229533.I1RMP2	pp	229533.I1S4H6
229533.I1RMP2	pp	229533.I1RNG4
229533.I1RMP2	pp	229533.Q4IPI4
229533.I1RMP2	pp	229533.Q4ILI9
229533.I1RMP2	pp	229533.I1RRF9
229533.I1RMP2	pp	229533.V6RR76
229533.I1RMP2	pp	229533.I1RUK7
229533.I1RMP2	pp	229533.I1RW57
229533.I1RMP2	pp	229533.I1RW71
229533.I1RMP2	pp	229533.I1S0Z3
229533.I1RMP2	pp	229533.I1RZH4
229533.I1RMP2	pp	229533.I1S095
229533.I1RMP2	pp	229533.I1RZV4
229533.I1RMP2	pp	229533.I1RYW8
229533.I1RMP2	pp	229533.I1RZU6
229533.I1RMP2	pp	229533.Q5I2J3
229533.I1RMP2	pp	229533.Q4INZ9
229533.I1RMP2	pp	229533.I1RMS2
229533.I1RMP2	pp	229533.I1RMT4
229533.I1RMP2	pp	229533.I1S4Q2
229533.I1RMP2	pp	229533.I1S270
229533.I1RMP2	pp	229533.I1S1B3
229533.I1RMP2	pp	229533.Q4HZS8
229533.I1RMP2	pp	229533.I1RWJ8
229533.I1RMP2	pp	229533.I1RNF3
229533.I1RMP2	pp	229533.I1RTL1
229533.I1RMP2	pp	229533.I1RZ02
229533.I1RMP2	pp	229533.I1RT41
229533.I1RMP2	pp	229533.I1RN81

229533.I1RSA3	pp	229533.I1RUY6
229533.I1RSA3	pp	229533.V6RLM7
229533.I1RSA3	pp	229533.I1S8X0
229533.I1RSA3	pp	229533.V6RVJ3
229533.I1RSA3	pp	229533.I1RUY5
229533.A0A098DEL8	pp	229533.I1RJL1
229533.A0A098DEL8	pp	229533.V6RM09
229533.A0A098DEL8	pp	229533.V6REA4
229533.A0A098DEL8	pp	229533.V6RDP1
229533.A0A098DEL8	pp	229533.V6RN16
229533.A0A098DEL8	pp	229533.V6QTX7
229533.A0A098DEL8	pp	229533.A0A1C3YIZ4
229533.A0A098DEL8	pp	229533.V6R556
229533.A0A098DEL8	pp	229533.I1RSA3
229533.I1RE19	pp	229533.I1RZU6
229533.I1RE19	pp	229533.I1RSI4
229533.I1RE19	pp	229533.I1RW71
229533.I1RE19	pp	229533.I1RZV4
229533.I1RE19	pp	229533.Q5I2J3
229533.I1RE19	pp	229533.I1RHU0
229533.I1RE19	pp	229533.I1S095
229533.I1RE19	pp	229533.Q4INZ9
229533.I1RE19	pp	229533.I1S1Z9
229533.I1RE19	pp	229533.I1RN81
229533.I1RE19	pp	229533.I1RJB5
229533.I1RE19	pp	229533.Q4HZS8
229533.I1RE19	pp	229533.I1RT41
229533.I1RE19	pp	229533.I1S270
229533.I1RE19	pp	229533.I1RNF3
229533.I1RE19	pp	229533.I1RMT4
229533.I1RE19	pp	229533.I1RZ02
229533.I1RE19	pp	229533.I1RTL1
229533.I1RE19	pp	229533.I1RZH4
229533.I1RE19	pp	229533.I1S4Q2
229533.I1RE19	pp	229533.I1RI75
229533.I1RE19	pp	229533.I1S0Z3
229533.I1RE19	pp	229533.I1RUK7
229533.I1RE19	pp	229533.I1S1B3
229533.I1RE19	pp	229533.V6RR76
229533.I1RE19	pp	229533.I1RW57
229533.I1RE19	pp	229533.I1RGU4
229533.I1RE19	pp	229533.I1RFW1
229533.I1RE19	pp	229533.I1RWJ8
229533.I1RE19	pp	229533.I1RRF9
229533.I1RE19	pp	229533.P0C431
229533.I1RE19	pp	229533.I1S7X9
229533.I1RE19	pp	229533.I1RZP6
229533.I1RE19	pp	229533.I1S8X0
229533.I1RE19	pp	229533.I1S4H6
229533.I1RE19	pp	229533.I1RMP2
229533.I1RE19	pp	229533.Q4IER8
229533.I1RE19	pp	229533.I1RMS2
229533.I1RE19	pp	229533.I1RNG4
229533.I1RE19	pp	229533.I1RQP5
229533.I1RE19	pp	229533.I1RMG6
229533.I1RE19	pp	229533.I1RHI1
229533.I1RE19	pp	229533.I1RR95
229533.I1RE19	pp	229533.I1RPX7
229533.I1RE19	pp	229533.I1RJZ7
229533.I1RE19	pp	229533.I1RZD9
229533.I1RE19	pp	229533.I1RS60
229533.I1RE19	pp	229533.I1REC6
229533.I1RE19	pp	229533.I1RUP1
229533.I1RE19	pp	229533.I1RZR2
229533.I1RE19	pp	229533.Q4IPI4
229533.I1RE19	pp	229533.I1RMH0
229533.I1RE19	pp	229533.I1RW46
229533.I1RE19	pp	229533.V6R476
229533.I1RE19	pp	229533.I1RPK0
229533.I1RE19	pp	229533.I1RXB8
229533.I1RE19	pp	229533.I1RY58
229533.I1RE19	pp	229533.I1RWQ0
229533.I1RE19	pp	229533.I1RG11

11608 229533.I1RE19 pp 229533.Q4HZB8
11609 229533.I1RE19 pp 229533.I1S092
11610 229533.I1RE19 pp 229533.I1RPG1
11611 229533.I1RE19 pp 229533.I1RZL9
11612 229533.I1RE19 pp 229533.I1RIG3
11613 229533.I1RE19 pp 229533.I1RWX5
11614 229533.I1RE19 pp 229533.I1RSZ6
11615 229533.I1RE19 pp 229533.I1RTG5
11616 229533.I1RE19 pp 229533.I1REC7
11617 229533.I1RE19 pp 229533.I1RTA3
11618 229533.I1RE19 pp 229533.I1RTE0
11619 229533.I1RE19 pp 229533.V6QUV7
11620 229533.I1RE19 pp 229533.I1RSW5
11621 229533.I1RMI3 pp 229533.I1RPU3
11622 229533.I1RTG5 pp 229533.V6RR76
11623 229533.I1RTG5 pp 229533.I1S0Z3
11624 229533.I1RTG5 pp 229533.Q4ILI9
11625 229533.I1RTG5 pp 229533.I1S270
11626 229533.I1RTG5 pp 229533.I1RYW8
11627 229533.I1RTG5 pp 229533.I1S275
11628 229533.I1RTG5 pp 229533.I1RTQ9
11629 229533.I1RTG5 pp 229533.Q5I2J3
11630 229533.I1RTG5 pp 229533.I1RZL9
11631 229533.I1RTG5 pp 229533.I1RW71
11632 229533.I1RTG5 pp 229533.I1RX84
11633 229533.I1RTG5 pp 229533.Q4I2W2
11634 229533.I1RTG5 pp 229533.Q4HYZ2
11635 229533.I1RTG5 pp 229533.Q4HZS8
11636 229533.I1RTG5 pp 229533.I1S7X9
11637 229533.I1RTG5 pp 229533.I1S559
11638 229533.I1RTG5 pp 229533.I1RZL3
11639 229533.I1RTG5 pp 229533.Q4IPI4
11640 229533.I1RRK3 pp 229533.I1S1W9
11641 229533.I1RRK3 pp 229533.I1RRM3
11642 229533.I1RRK3 pp 229533.I1S5R6
11643 229533.I1RRK3 pp 229533.I1RZV3
11644 229533.I1RRK3 pp 229533.I1RUG3
11645 229533.I1RRK3 pp 229533.V6RMW6
11646 229533.I1RRK3 pp 229533.I1RZK5
11647 229533.I1RRK3 pp 229533.I1RWR7
11648 229533.I1RRK3 pp 229533.I1RZM8
11649 229533.I1RRK3 pp 229533.I1RVZ2
11650 229533.I1RRK3 pp 229533.I1RSA5
11651 229533.I1RRK3 pp 229533.I1RTA1
11652 229533.I1RRK3 pp 229533.I1RXD7
11653 229533.I1RRK3 pp 229533.I1S4S7
11654 229533.I1RRK3 pp 229533.I1S2B8
11655 229533.I1RRK3 pp 229533.I1RYI5
11656 229533.I1RRK3 pp 229533.I1RT40
11657 229533.I1RRK3 pp 229533.I1RZ39
11658 229533.I1RRK3 pp 229533.I1S5R1
11659 229533.I1RRK3 pp 229533.I1RSZ7
11660 229533.I1RD10 pp 229533.I1RKR7
11661 229533.I1RD10 pp 229533.I1S2C1
11662 229533.I1RD10 pp 229533.I1RMP2
11663 229533.I1RD10 pp 229533.I1RWG6
11664 229533.I1RD10 pp 229533.I1RWI6
11665 229533.I1RD10 pp 229533.I1RWL6
11666 229533.I1RD10 pp 229533.I1RRF9
11667 229533.I1RD10 pp 229533.I1RPU7
11668 229533.I1RD10 pp 229533.I1RTG9
11669 229533.I1RD10 pp 229533.V6RR76
11670 229533.I1RD10 pp 229533.I1RQA9
11671 229533.I1RD10 pp 229533.I1RX09
11672 229533.I1RD10 pp 229533.V6QUU6
11673 229533.I1RD10 pp 229533.I1RZL9
11674 229533.I1RD10 pp 229533.I1RE19
11675 229533.I1RD10 pp 229533.I1RZL3
11676 229533.I1RD10 pp 229533.I1S254
11677 229533.I1RD10 pp 229533.I1RRI4
11678 229533.I1RD10 pp 229533.I1RSB0
11679 229533.I1RD10 pp 229533.V6R476
11680 229533.I1RD10 pp 229533.I1RZV4

11681 229533.I1RD10 pp 229533.I1S095
11682 229533.I1RD10 pp 229533.I1S0Z3
11683 229533.I1RD10 pp 229533.I1RN81
11684 229533.I1RD10 pp 229533.I1S8F2
11685 229533.I1RD10 pp 229533.I1RRT4
11686 229533.I1RD10 pp 229533.I1RMK9
11687 229533.I1RD10 pp 229533.I1RXB5
11688 229533.I1RD10 pp 229533.I1RZD9
11689 229533.I1RD10 pp 229533.Q4I963
11690 229533.I1RD10 pp 229533.I1S045
11691 229533.I1RD10 pp 229533.Q4IPI4
11692 229533.I1RD10 pp 229533.V6REE9
11693 229533.I1RD10 pp 229533.I1RTG5
11694 229533.I1RD10 pp 229533.I1RT41
11695 229533.I1RD10 pp 229533.I1RPG1
11696 229533.I1RD10 pp 229533.I1RTE0
11697 229533.I1RD10 pp 229533.I1S0N8
11698 229533.I1S969 pp 229533.V6RHX2
11699 229533.I1S969 pp 229533.V6RWB3
11700 229533.I1S969 pp 229533.V6RCJ5
11701 229533.I1S969 pp 229533.I1SAB4
11702 229533.I1S969 pp 229533.I1SAT2
11703 229533.I1S969 pp 229533.V6RCC0
11704 229533.I1S969 pp 229533.V6R5T7
11705 229533.I1S969 pp 229533.V6QVV9
11706 229533.I1RTE0 pp 229533.I1RZT7
11707 229533.I1RTE0 pp 229533.Q4IPI4
11708 229533.I1RTE0 pp 229533.I1S270
11709 229533.I1RTE0 pp 229533.I1RZL3
11710 229533.I1RTE0 pp 229533.V6RR76
11711 229533.I1RTE0 pp 229533.I1RXB8
11712 229533.I1RTE0 pp 229533.I1S0Z3
11713 229533.I1RTE0 pp 229533.I1RWI6
11714 229533.I1RTE0 pp 229533.I1RZV1
11715 229533.I1RTE0 pp 229533.I1S8E0
11716 229533.I1RTE0 pp 229533.Q4I963
11717 229533.I1RTE0 pp 229533.V6RLM7
11718 229533.I1RTE0 pp 229533.I1RY27
11719 229533.I1RTE0 pp 229533.I1RTG5
11720 229533.I1RTE0 pp 229533.I1RZL9
11721 229533.I1RTE0 pp 229533.I1S0R9
11722 229533.I1RA31 pp 229533.I1RSG9
11723 229533.I1RA31 pp 229533.I1RYV4
11724 229533.I1RA31 pp 229533.I1S4Y4
11725 229533.I1RA31 pp 229533.I1RZL9
11726 229533.I1RA31 pp 229533.I1RFW6
11727 229533.I1RA31 pp 229533.I1RPG1
11728 229533.A0A098DEK1 pp 229533.I1RS29
11729 229533.A0A098DEK1 pp 229533.I1RXD9
11730 229533.A0A098DEK1 pp 229533.A0A1C3YM56
11731 229533.A0A098DEK1 pp 229533.I1S969
11732 229533.A0A098DEK1 pp 229533.I1RS05
11733 229533.A0A098DEK1 pp 229533.I1RA52
11734 229533.A0A098DEK1 pp 229533.I1S5X9
11735 229533.A0A098DEK1 pp 229533.I1RYT8
11736 229533.A0A098DEK1 pp 229533.V6RJP8
11737 229533.A0A1C3YIQ1 pp 229533.I1RXB8
11738 229533.A0A1C3YIQ1 pp 229533.I1RKI1
11739 229533.A0A1C3YIQ1 pp 229533.I1RT41
11740 229533.A0A1C3YIQ1 pp 229533.I1RQ70
11741 229533.A0A1C3YIQ1 pp 229533.I1S1B3
11742 229533.A0A1C3YIQ1 pp 229533.I1RKR9
11743 229533.A0A1C3YIQ1 pp 229533.I1RPG1
11744 229533.A0A1C3YIQ1 pp 229533.I1RHU0
11745 229533.A0A1C3YIQ1 pp 229533.Q4INI2
11746 229533.A0A1C3YIQ1 pp 229533.I1RE19
11747 229533.A0A1C3YIQ1 pp 229533.I1RRF9
11748 229533.A0A1C3YIQ1 pp 229533.I1RST4
11749 229533.A0A1C3YIQ1 pp 229533.I1RTE0
11750 229533.A0A1C3YIQ1 pp 229533.I1RQP5
11751 229533.A0A1C3YIQ1 pp 229533.I1RT65
11752 229533.A0A1C3YIQ1 pp 229533.I1S0Z3
11753 229533.A0A1C3YIQ1 pp 229533.I1RMP2

```
229533.A0A1C3YIQ1   pp  229533.Q4I963
229533.A0A1C3YIQ1   pp  229533.I1RZL9
229533.A0A1C3YL06   pp  229533.I1RXB8
229533.A0A1C3YL06   pp  229533.I1RJS2
229533.A0A1C3YL06   pp  229533.I1RE19
229533.A0A1C3YL06   pp  229533.I1RNG4
229533.A0A1C3YL06   pp  229533.I1RMP2
229533.A0A1C3YL06   pp  229533.I1S8X0
229533.A0A1C3YL06   pp  229533.I1RJZ7
229533.A0A1C3YL06   pp  229533.I1RPX7
229533.A0A1C3YL06   pp  229533.A0A1C3YMN5
229533.A0A1C3YL06   pp  229533.I1S7X9
229533.A0A1C3YL06   pp  229533.A0A1C3YL93
229533.A0A1C3YL06   pp  229533.I1RTE0
229533.A0A1C3YL06   pp  229533.I1RW46
229533.A0A1C3YL06   pp  229533.A0A1C3YLK5
229533.A0A1C3YL06   pp  229533.I1RCN6
229533.A0A1C3YL06   pp  229533.I1RPG1
229533.A0A1C3YL06   pp  229533.I1RY58
229533.A0A1C3YL06   pp  229533.I1RTG5
229533.A0A1C3YL06   pp  229533.I1RZL9
229533.A0A1C3YL06   pp  229533.I1RPK0
229533.A0A1C3YL06   pp  229533.Q4HZB8
229533.A0A1C3YL06   pp  229533.A0A1C3YMC9
229533.A0A1C3YL06   pp  229533.I1RNF3
229533.A0A1C3YL06   pp  229533.I1RWJ8
229533.A0A1C3YL06   pp  229533.Q5I2J3
229533.A0A1C3YL06   pp  229533.I1RHU0
229533.A0A1C3YL06   pp  229533.I1RZU6
229533.A0A1C3YL06   pp  229533.I1RMS2
229533.A0A1C3YL06   pp  229533.I1RUK7
229533.A0A1C3YL06   pp  229533.I1RA72
229533.A0A1C3YL06   pp  229533.I1RZH4
229533.I1RSX0   pp  229533.V6RTJ8
229533.I1RSX0   pp  229533.I1RVZ2
229533.I1RSX0   pp  229533.I1S2H4
229533.I1RSX0   pp  229533.I1RTA1
229533.I1RSX0   pp  229533.V6R3J1
229533.I1RSX0   pp  229533.V6RUT5
229533.I1RSX0   pp  229533.I1RXD7
229533.I1RSX0   pp  229533.I1RX46
229533.I1RSX0   pp  229533.I1S9Y1
229533.I1RSX0   pp  229533.I1S0L4
229533.I1RSX0   pp  229533.I1RT40
229533.I1RSX0   pp  229533.I1S2B8
229533.I1RSX0   pp  229533.I1S4S4
229533.I1RSX0   pp  229533.I1S5R1
229533.I1RSX0   pp  229533.I1RTD5
229533.I1RSX0   pp  229533.I1RT42
229533.I1RSX0   pp  229533.I1RXC2
229533.I1RSX0   pp  229533.I1RXD6
229533.I1RSX0   pp  229533.I1S4S7
229533.I1RSX0   pp  229533.I1RZ39
229533.I1RSX0   pp  229533.I1S438
229533.I1RSX0   pp  229533.I1RWI5
229533.I1RSX0   pp  229533.I1RYI5
229533.I1RSX0   pp  229533.I1RSZ7
229533.I1RSX0   pp  229533.I1S110
229533.I1RSX0   pp  229533.I1S5R6
229533.I1RSX0   pp  229533.I1RYR0
229533.I1RSX0   pp  229533.I1RZZ9
229533.I1RSX0   pp  229533.I1S5B0
229533.I1RSX0   pp  229533.I1S277
229533.I1RSX0   pp  229533.I1RUG3
229533.I1RSX0   pp  229533.I1S1W9
229533.I1RSX0   pp  229533.I1S887
229533.I1RSX0   pp  229533.I1RZV3
229533.I1RSX0   pp  229533.I1S1W3
229533.I1RSX0   pp  229533.V6QUE6
229533.I1RSX0   pp  229533.I1S437
229533.I1RSX0   pp  229533.V6RCZ8
229533.I1RSX0   pp  229533.I1RZK5
229533.I1RSX0   pp  229533.I1RXC4
```

11827	229533.I1RSX0	pp	229533.I1S1C7
11828	229533.I1RSX0	pp	229533.I1RYW3
11829	229533.I1RSX0	pp	229533.I1S0J1
11830	229533.I1RSX0	pp	229533.V6R042
11831	229533.I1RSX0	pp	229533.I1S064
11832	229533.I1RSX0	pp	229533.Q4I624
11833	229533.I1RSX0	pp	229533.V6RMW6
11834	229533.I1RSX0	pp	229533.I1RXU9
11835	229533.I1RSX0	pp	229533.I1RZM8
11836	229533.I1RSX0	pp	229533.I1RXF0
11837	229533.I1RSX0	pp	229533.V6RRH2
11838	229533.I1RSX0	pp	229533.I1RWR7
11839	229533.I1RSX0	pp	229533.I1RVX0
11840	229533.I1RSX0	pp	229533.I1RT03
11841	229533.I1RSX0	pp	229533.I1RXR1
11842	229533.I1RSX0	pp	229533.Q4IJK2
11843	229533.I1RSZ6	pp	229533.I1RTE0
11844	229533.I1RSZ6	pp	229533.I1S0Z3
11845	229533.I1RSZ6	pp	229533.Q4I963
11846	229533.I1RSZ6	pp	229533.I1RT65
11847	229533.I1RSZ6	pp	229533.I1S1B3
11848	229533.I1RSZ6	pp	229533.I1RZL9
11849	229533.I1RSZ6	pp	229533.Q4INI2
11850	229533.I1RSZ6	pp	229533.I1RT41
11851	229533.I1RSZ6	pp	229533.I1RXB8
11852	229533.I1RS03	pp	229533.Q4IPN9
11853	229533.I1RS03	pp	229533.I1RZV6
11854	229533.I1RS03	pp	229533.Q4IR09
11855	229533.I1RS03	pp	229533.I1RX24
11856	229533.I1RS03	pp	229533.Q4IF44
11857	229533.I1RS03	pp	229533.Q4IPA4
11858	229533.I1RS03	pp	229533.I1RWF3
11859	229533.I1RS03	pp	229533.I1S8V0
11860	229533.I1RS03	pp	229533.I1RSA4
11861	229533.I1RS03	pp	229533.I1RT35
11862	229533.I1RS03	pp	229533.I1S214
11863	229533.I1RS03	pp	229533.I1RYB1
11864	229533.I1RS03	pp	229533.I1S246
11865	229533.I1RS03	pp	229533.I1RUM5
11866	229533.I1RS03	pp	229533.I1S9M9
11867	229533.I1RS03	pp	229533.Q4IAA0
11868	229533.I1RS03	pp	229533.I1S1X9
11869	229533.I1RS03	pp	229533.I1RZL5
11870	229533.I1RS03	pp	229533.I1S471
11871	229533.I1RS03	pp	229533.I1S0N7
11872	229533.I1RS03	pp	229533.I1S5Z2
11873	229533.I1RS03	pp	229533.Q4I7S1
11874	229533.I1RS03	pp	229533.I1S8U9
11875	229533.I1RS03	pp	229533.Q4I7X1
11876	229533.I1RAG7	pp	229533.I1RXD6
11877	229533.I1RAG7	pp	229533.I1RM37
11878	229533.I1RAG7	pp	229533.I1S1W9
11879	229533.I1RAG7	pp	229533.I1RZV3
11880	229533.I1RAG7	pp	229533.I1S0W4
11881	229533.I1RAG7	pp	229533.I1RDF7
11882	229533.I1RAG7	pp	229533.I1RSA5
11883	229533.I1RAG7	pp	229533.I1RSF1
11884	229533.I1RAG7	pp	229533.I1RQ85
11885	229533.I1RAG7	pp	229533.I1RPU2
11886	229533.I1RAG7	pp	229533.I1RYI5
11887	229533.I1RAG7	pp	229533.I1RD52
11888	229533.I1RAG7	pp	229533.I1RJN9
11889	229533.I1RAG7	pp	229533.I1RTA1
11890	229533.I1RAG7	pp	229533.I1RXD7
11891	229533.I1RAG7	pp	229533.I1S4S7
11892	229533.I1RAG7	pp	229533.I1RVZ2
11893	229533.I1RAG7	pp	229533.I1S1B2
11894	229533.I1RAG7	pp	229533.I1RRM3
11895	229533.I1RAG7	pp	229533.I1RXC4
11896	229533.I1RAG7	pp	229533.I1RRV3
11897	229533.I1RAG7	pp	229533.I1RGC5
11898	229533.I1RAG7	pp	229533.I1RC34
11899	229533.I1RAG7	pp	229533.I1RX95

11900 229533.I1RAG7 pp 229533.V6RCZ8
11901 229533.I1RAG7 pp 229533.I1RB26
11902 229533.I1RAG7 pp 229533.I1S5R1
11903 229533.I1RAG7 pp 229533.I1RBR2
11904 229533.I1RAG7 pp 229533.I1S821
11905 229533.I1RAG7 pp 229533.I1RQD2
11906 229533.I1RAG7 pp 229533.I1RXC2
11907 229533.I1RAG7 pp 229533.I1S9I5
11908 229533.I1RAG7 pp 229533.I1S5R6
11909 229533.I1RAG7 pp 229533.I1RE89
11910 229533.I1RAG7 pp 229533.I1RVQ3
11911 229533.I1RAG7 pp 229533.I1RGA4
11912 229533.I1RAG7 pp 229533.I1RBR1
11913 229533.I1RAG7 pp 229533.I1S2B8
11914 229533.I1RAG7 pp 229533.I1RCR1
11915 229533.I1RAG7 pp 229533.I1RKJ4
11916 229533.I1RAG7 pp 229533.I1RQ33
11917 229533.I1RAG7 pp 229533.V6R9Z0
11918 229533.I1RAG7 pp 229533.I1RTD5
11919 229533.I1RAG7 pp 229533.I1RWR7
11920 229533.I1RAG7 pp 229533.I1RRI1
11921 229533.I1RAG7 pp 229533.I1RGB2
11922 229533.I1RAG7 pp 229533.I1RUG3
11923 229533.I1RAG7 pp 229533.I1RCH5
11924 229533.I1RAG7 pp 229533.I1RQE7
11925 229533.I1RAG7 pp 229533.I1RAW0
11926 229533.I1RAG7 pp 229533.I1RR24
11927 229533.I1RAG7 pp 229533.I1RNB2
11928 229533.I1RAG7 pp 229533.I1REC5
11929 229533.I1RAG7 pp 229533.I1RSZ7
11930 229533.I1RAG7 pp 229533.Q4IJK2
11931 229533.I1RAG7 pp 229533.I1RZM8
11932 229533.I1RAG7 pp 229533.I1RPE0
11933 229533.I1RAG7 pp 229533.I1RCR8
11934 229533.I1RAG7 pp 229533.I1RUF7
11935 229533.I1RAG7 pp 229533.I1S437
11936 229533.I1RAG7 pp 229533.I1RT40
11937 229533.I1RAG7 pp 229533.I1S438
11938 229533.I1RAG7 pp 229533.I1RTB3
11939 229533.I1RAG7 pp 229533.I1RZK5
11940 229533.I1RAG7 pp 229533.I1RZ39
11941 229533.I1RAG7 pp 229533.I1RVX0
11942 229533.I1RAG7 pp 229533.I1RJU3
11943 229533.I1RAG7 pp 229533.V6R042
11944 229533.I1RHU0 pp 229533.I1RRF9
11945 229533.I1RHU0 pp 229533.I1RZ70
11946 229533.I1RHU0 pp 229533.I1S8D1
11947 229533.I1RHU0 pp 229533.I1S444
11948 229533.I1RHU0 pp 229533.I1RSZ6
11949 229533.I1RHU0 pp 229533.V6QU65
11950 229533.I1RHU0 pp 229533.I1RZL9
11951 229533.I1RHU0 pp 229533.I1RTE0
11952 229533.I1RHU0 pp 229533.I1RTA3
11953 229533.I1RHU0 pp 229533.I1S3G4
11954 229533.I1RHU0 pp 229533.I1S1W8
11955 229533.I1RHU0 pp 229533.I1RYM5
11956 229533.I1RHU0 pp 229533.V6RXR0
11957 229533.I1RHU0 pp 229533.I1RWN7
11958 229533.I1RHU0 pp 229533.I1RMS5
11959 229533.I1RHU0 pp 229533.I1RPG1
11960 229533.I1RHU0 pp 229533.V6RHD0
11961 229533.I1RHU0 pp 229533.I1RMP2
11962 229533.I1RHU0 pp 229533.I1S8D2
11963 229533.I1RCS2 pp 229533.I1RZQ5
11964 229533.I1RCS2 pp 229533.I1RYA0
11965 229533.I1RCS2 pp 229533.Q4I7F9
11966 229533.I1RCS2 pp 229533.I1RG33
11967 229533.I1RCS2 pp 229533.I1RPL3
11968 229533.I1RCS2 pp 229533.Q4IPH4
11969 229533.I1RCS2 pp 229533.I1RFP9
11970 229533.I1RCS2 pp 229533.I1RG90
11971 229533.I1RCS2 pp 229533.I1S8V0
11972 229533.I1RCS2 pp 229533.I1RNK2

229533.I1RCS2	pp	229533.I1S0Z9
229533.I1RCS2	pp	229533.I1RFQ1
229533.I1RCS2	pp	229533.I1RYJ9
229533.I1RCS2	pp	229533.Q4IPB8
229533.I1RCS2	pp	229533.I1RSD4
229533.I1RCS2	pp	229533.V6QVD5
229533.I1RCS2	pp	229533.I1S0B1
229533.I1RCS2	pp	229533.I1S1X9
229533.I1RCS2	pp	229533.I1RQQ7
229533.I1RCS2	pp	229533.I1RQZ2
229533.I1RCS2	pp	229533.I1RQR3
229533.I1RCS2	pp	229533.I1RRR7
229533.I1RCS2	pp	229533.I1RYB1
229533.I1RCS2	pp	229533.I1RE24
229533.I1RCS2	pp	229533.I1RT91
229533.I1RCS2	pp	229533.V6RY33
229533.I1RCS2	pp	229533.I1RMU1
229533.I1RCS2	pp	229533.Q4HXF6
229533.I1RCS2	pp	229533.I1RPY7
229533.I1RCS2	pp	229533.I1RT35
229533.I1RCS2	pp	229533.Q4I1Y1
229533.I1RCS2	pp	229533.I1RG57
229533.I1RCS2	pp	229533.I1RDG0
229533.I1RCS2	pp	229533.I1RWX8
229533.I1RCS2	pp	229533.I1S9M9
229533.I1RCS2	pp	229533.I1RYW3
229533.I1RCS2	pp	229533.I1RK90
229533.I1RCS2	pp	229533.I1RTD0
229533.I1RCS2	pp	229533.V6QXG1
229533.I1RCS2	pp	229533.I1RQP8
229533.I1RCS2	pp	229533.I1S8F8
229533.I1RCS2	pp	229533.I1RE45
229533.I1RCS2	pp	229533.I1RX51
229533.I1RCS2	pp	229533.I1RDY2
229533.I1RCS2	pp	229533.I1RJG6
229533.I1RCS2	pp	229533.I1RVK5
229533.I1RCS2	pp	229533.I1RMV5
229533.I1RCS2	pp	229533.I1RSA4
229533.I1RCS2	pp	229533.I1RYC8
229533.I1RCS2	pp	229533.I1RS03
229533.I1RCS2	pp	229533.I1RND5
229533.I1RCS2	pp	229533.V6QX58
229533.I1RCS2	pp	229533.I1REX2
229533.I1RCS2	pp	229533.I1RK92
229533.I1RCS2	pp	229533.I1RPA0
229533.I1RCS2	pp	229533.I1RN60
229533.I1RCS2	pp	229533.Q4IJH1
229533.I1RCS2	pp	229533.I1S9L0
229533.I1RCS2	pp	229533.I1RPY8
229533.I1RCS2	pp	229533.I1RT70
229533.I1RCS2	pp	229533.I1S8U9
229533.I1RCS2	pp	229533.I1RN91
229533.I1RCS2	pp	229533.I1RZL5
229533.I1RCS2	pp	229533.I1RDE9
229533.I1RCS2	pp	229533.I1RMH5
229533.I1RCS2	pp	229533.I1RK23
229533.I1RCS2	pp	229533.Q4IB70
229533.I1RCS2	pp	229533.Q4IF76
229533.I1RCS2	pp	229533.Q4IE79
229533.I1RCS2	pp	229533.I1RKA7
229533.I1RCS2	pp	229533.I1S5Z2
229533.I1RCS2	pp	229533.I1S7M3
229533.I1RCS2	pp	229533.I1RSA8
229533.I1RCS2	pp	229533.I1RWW5
229533.I1RCS2	pp	229533.I1RU69
229533.I1RCS2	pp	229533.I1RM25
229533.I1RCS2	pp	229533.I1RN28
229533.I1RCS2	pp	229533.I1RNB8
229533.I1RCS2	pp	229533.I1RN59
229533.I1RCS2	pp	229533.I1S471
229533.I1RCS2	pp	229533.I1RX47
229533.I1RCS2	pp	229533.I1RPW9
229533.I1RCS2	pp	229533.I1RST1

12046	229533.I1RCS2	pp	229533.Q4IRI9
12047	229533.I1RCS2	pp	229533.Q4IAA0
12048	229533.I1RCS2	pp	229533.I1S9M0
12049	229533.I1RCS2	pp	229533.I1S056
12050	229533.I1RCS2	pp	229533.I1RK19
12051	229533.I1RCS2	pp	229533.I1S0N4
12052	229533.I1RCS2	pp	229533.I1RK72
12053	229533.I1RCS2	pp	229533.I1S044
12054	229533.I1RCS2	pp	229533.I1RUM5
12055	229533.I1RCS2	pp	229533.I1RRL0
12056	229533.I1RCS2	pp	229533.I1RPY5
12057	229533.I1RCS2	pp	229533.I1RS83
12058	229533.I1RCS2	pp	229533.I1S193
12059	229533.I1RCS2	pp	229533.I1RWU2
12060	229533.I1RCS2	pp	229533.I1RS28
12061	229533.I1RCS2	pp	229533.I1RN09
12062	229533.I1RCS2	pp	229533.I1RQ65
12063	229533.I1RCS2	pp	229533.I1RKK4
12064	229533.I1RCS2	pp	229533.I1RND9
12065	229533.I1RCS2	pp	229533.I1RJF9
12066	229533.I1RCS2	pp	229533.I1RG17
12067	229533.I1RCS2	pp	229533.I1RT47
12068	229533.I1RCS2	pp	229533.I1S4M5
12069	229533.I1RCS2	pp	229533.I1RFT1
12070	229533.I1RCS2	pp	229533.I1RME9
12071	229533.I1RCS2	pp	229533.I1RQC5
12072	229533.I1RCS2	pp	229533.I1RDD3
12073	229533.I1RCS2	pp	229533.I1RGC2
12074	229533.I1RCS2	pp	229533.I1RFG0
12075	229533.I1RCS2	pp	229533.I1RZ11
12076	229533.I1RCS2	pp	229533.I1RDC1
12077	229533.I1RCS2	pp	229533.I1S9L5
12078	229533.I1RCS2	pp	229533.I1RDX6
12079	229533.I1RCS2	pp	229533.I1RG13
12080	229533.I1RCS2	pp	229533.I1RX24
12081	229533.I1RCS2	pp	229533.I1RXF2
12082	229533.I1RCS2	pp	229533.I1S7M2
12083	229533.I1RCS2	pp	229533.I1RQB5
12084	229533.I1RCS2	pp	229533.I1RX81
12085	229533.I1RCS2	pp	229533.I1RT17
12086	229533.I1RCS2	pp	229533.I1RTS2
12087	229533.I1RCS2	pp	229533.Q4IBK5
12088	229533.I1RCS2	pp	229533.I1S246
12089	229533.I1RCS2	pp	229533.I1RZG7
12090	229533.I1RCS2	pp	229533.I1RS75
12091	229533.I1RCS2	pp	229533.Q4IPB3
12092	229533.I1RCS2	pp	229533.I1RSL9
12093	229533.I1RCS2	pp	229533.I1S1C6
12094	229533.I1RCS2	pp	229533.I1S2Y5
12095	229533.I1RCS2	pp	229533.I1RPZ3
12096	229533.I1RCS2	pp	229533.I1RYI9
12097	229533.I1RCS2	pp	229533.I1RPY4
12098	229533.I1RCS2	pp	229533.V6QXP4
12099	229533.I1RCS2	pp	229533.I1S9L6
12100	229533.I1RCS2	pp	229533.Q4I5R9
12101	229533.I1RCS2	pp	229533.I1RRW3
12102	229533.I1RCS2	pp	229533.I1RLU5
12103	229533.I1RCS2	pp	229533.I1RD56
12104	229533.I1RCS2	pp	229533.I1RL81
12105	229533.I1RY00	pp	229533.I1RY14
12106	229533.I1RA34	pp	229533.I1RRF9
12107	229533.I1RA34	pp	229533.I1RWI6
12108	229533.I1RA34	pp	229533.I1RSZ6
12109	229533.I1RA34	pp	229533.I1RX09
12110	229533.I1RA34	pp	229533.I1RZL9
12111	229533.I1RA34	pp	229533.I1RTE0
12112	229533.I1RA34	pp	229533.I1S8F2
12113	229533.I1RA34	pp	229533.I1RXB5
12114	229533.I1RA34	pp	229533.V6RR76
12115	229533.I1RA34	pp	229533.I1RSB0
12116	229533.I1RA34	pp	229533.I1RBD2
12117	229533.I1RA34	pp	229533.V6R476
12118	229533.I1RA34	pp	229533.I1RPG1

12119 229533.I1RA34 pp 229533.I1RN81
12120 229533.I1RA34 pp 229533.I1RTG9
12121 229533.I1RA34 pp 229533.V6QUU6
12122 229533.I1RA34 pp 229533.I1RZD9
12123 229533.I1RA34 pp 229533.I1RE19
12124 229533.I1RA34 pp 229533.I1RB15
12125 229533.I1RA34 pp 229533.I1S254
12126 229533.I1RA34 pp 229533.I1RMP2
12127 229533.I1RA34 pp 229533.I1S095
12128 229533.I1RA34 pp 229533.I1RZV4
12129 229533.I1RQM8 pp 229533.I1S0X0
12130 229533.I1RQM8 pp 229533.I1S270
12131 229533.I1RQM8 pp 229533.I1S559
12132 229533.I1RQM8 pp 229533.I1RXS3
12133 229533.I1RQM8 pp 229533.I1RX68
12134 229533.I1RQM8 pp 229533.I1RWI6
12135 229533.I1RQM8 pp 229533.Q4IPI4
12136 229533.I1RQM8 pp 229533.I1RT41
12137 229533.I1RQM8 pp 229533.I1RSD7
12138 229533.I1RQM8 pp 229533.I1RRF0
12139 229533.I1RQM8 pp 229533.I1RST4
12140 229533.I1RQM8 pp 229533.I1RT65
12141 229533.I1RQM8 pp 229533.I1RX09
12142 229533.I1RQM8 pp 229533.V6RR76
12143 229533.I1RQM8 pp 229533.I1RZD6
12144 229533.I1RQM8 pp 229533.I1S0Z3
12145 229533.I1RQM8 pp 229533.I1RX69
12146 229533.I1RQM8 pp 229533.Q4HZE0
12147 229533.A0A1C3YJW7 pp 229533.I1RN28
12148 229533.A0A1C3YJW7 pp 229533.I1RA99
12149 229533.A0A1C3YJW7 pp 229533.I1RT35
12150 229533.A0A1C3YJW7 pp 229533.I1S8U9
12151 229533.A0A1C3YJW7 pp 229533.Q4I7X1
12152 229533.A0A1C3YJW7 pp 229533.Q4IPC8
12153 229533.A0A1C3YJW7 pp 229533.I1RQZ2
12154 229533.A0A1C3YJW7 pp 229533.I1S9M9
12155 229533.A0A1C3YJW7 pp 229533.I1RA38
12156 229533.A0A1C3YJW7 pp 229533.I1RN17
12157 229533.A0A1C3YJW7 pp 229533.A0A1C3YL25
12158 229533.A0A1C3YJW7 pp 229533.I1S8V0
12159 229533.A0A1C3YJW7 pp 229533.I1SA47
12160 229533.A0A1C3YJW7 pp 229533.I1RAC1
12161 229533.A0A1C3YJW7 pp 229533.I1RMU1
12162 229533.A0A1C3YJW7 pp 229533.I1RZL5
12163 229533.A0A1C3YJW7 pp 229533.I1RDY2
12164 229533.A0A1C3YJW7 pp 229533.I1S214
12165 229533.A0A1C3YJW7 pp 229533.I1RND9
12166 229533.A0A1C3YJW7 pp 229533.I1RDX6
12167 229533.A0A1C3YJW7 pp 229533.I1RM23
12168 229533.A0A1C3YJW7 pp 229533.I1S5Z2
12169 229533.A0A1C3YJW7 pp 229533.I1RDD3
12170 229533.A0A1C3YJW7 pp 229533.Q4IF44
12171 229533.A0A1C3YJW7 pp 229533.I1RA46
12172 229533.A0A1C3YJW7 pp 229533.I1S0N7
12173 229533.A0A1C3YJW7 pp 229533.I1RUM5
12174 229533.A0A1C3YJW7 pp 229533.Q4IPA4
12175 229533.A0A1C3YJW7 pp 229533.I1S246
12176 229533.A0A1C3YJW7 pp 229533.A0A1C3YKM6
12177 229533.A0A1C3YJW7 pp 229533.Q4IPN9
12178 229533.A0A1C3YJW7 pp 229533.I1RK81
12179 229533.A0A1C3YJW7 pp 229533.I1RQK2
12180 229533.A0A1C3YJW7 pp 229533.I1RSD0
12181 229533.A0A1C3YJW7 pp 229533.I1RCZ3
12182 229533.A0A1C3YJW7 pp 229533.I1RBX0
12183 229533.A0A1C3YJW7 pp 229533.I1RAI1
12184 229533.A0A1C3YJW7 pp 229533.I1RE45
12185 229533.A0A1C3YJW7 pp 229533.I1RCH7
12186 229533.A0A1C3YJW7 pp 229533.A0A1C3YM64
12187 229533.A0A1C3YJW7 pp 229533.I1RS03
12188 229533.A0A1C3YJW7 pp 229533.I1RN91
12189 229533.A0A1C3YJW7 pp 229533.Q4IR09
12190 229533.A0A1C3YJW7 pp 229533.I1RZP2
12191 229533.I1RSG9 pp 229533.I1RVY0

229533.I1RSG9 pp 229533.I1RXX1
229533.I1RSG9 pp 229533.I1RZP8
229533.I1RSG9 pp 229533.I1RYV4
229533.I1RSG9 pp 229533.I1RZL9
229533.I1S204 pp 229533.Q4I5V3
229533.I1RRF9 pp 229533.I1S254
229533.I1RRF9 pp 229533.Q5I2J3
229533.I1RRF9 pp 229533.I1RSI4
229533.I1RRF9 pp 229533.Q4HZS8
229533.I1RRF9 pp 229533.I1RZT7
229533.I1RRF9 pp 229533.I1RZU6
229533.I1RRF9 pp 229533.I1S270
229533.I1RRF9 pp 229533.V6QUU6
229533.I1RRF9 pp 229533.I1RT41
229533.I1RRF9 pp 229533.I1RWJ8
229533.I1RRF9 pp 229533.V6RR76
229533.I1RRF9 pp 229533.I1S7X9
229533.I1RRF9 pp 229533.P0C431
229533.I1RRF9 pp 229533.I1RSX2
229533.I1RRF9 pp 229533.I1S8X0
229533.I1RRF9 pp 229533.I1S0Z3
229533.I1RRF9 pp 229533.I1RY27
229533.I1RRF9 pp 229533.I1RT65
229533.I1RRF9 pp 229533.I1RUK7
229533.I1RRF9 pp 229533.I1RZL9
229533.I1RRF9 pp 229533.I1RSF2
229533.I1RRF9 pp 229533.I1RW46
229533.I1RRF9 pp 229533.I1RXB8
229533.I1RRF9 pp 229533.I1RSZ6
229533.I1RRF9 pp 229533.Q4HZB8
229533.I1RRF9 pp 229533.I1RY58
229533.I1RRF9 pp 229533.I1RST4
229533.I1RRF9 pp 229533.I1RTE0
229533.I1RRF9 pp 229533.I1RTG5
229533.I1RRF9 pp 229533.V6R476
229533.I1RRF9 pp 229533.I1RTA3
229533.A0A098DTU2 pp 229533.A0A1C3YMN5
229533.A0A098DTU2 pp 229533.I1RMP2
229533.A0A098DTU2 pp 229533.I1RNG4
229533.A0A098DTU2 pp 229533.I1RCN6
229533.A0A098DTU2 pp 229533.A0A1C3YLK5
229533.A0A098DTU2 pp 229533.I1S7X9
229533.A0A098DTU2 pp 229533.I1S8X0
229533.A0A098DTU2 pp 229533.I1RYX9
229533.A0A098DTU2 pp 229533.I1RJZ7
229533.A0A098DTU2 pp 229533.A0A1C3YI39
229533.A0A098DTU2 pp 229533.I1RJS2
229533.A0A098DTU2 pp 229533.I1RE19
229533.A0A098DTU2 pp 229533.I1RPX7
229533.A0A098DTU2 pp 229533.I1RXB8
229533.A0A098DTU2 pp 229533.A0A1C3YL93
229533.A0A098DTU2 pp 229533.I1RTE0
229533.A0A098DTU2 pp 229533.I1RY58
229533.A0A098DTU2 pp 229533.I1RZ41
229533.A0A098DTU2 pp 229533.A0A0E0SNH8
229533.A0A098DTU2 pp 229533.I1RPG1
229533.A0A098DTU2 pp 229533.I1RZL9
229533.A0A098DTU2 pp 229533.I1RTG5
229533.A0A098DTU2 pp 229533.I1RW46
229533.A0A098DTU2 pp 229533.A0A0E0SIE1
229533.A0A098DTU2 pp 229533.Q4HZB8
229533.A0A098DTU2 pp 229533.I1RZU6
229533.A0A098DTU2 pp 229533.I1RPK0
229533.A0A098DTU2 pp 229533.Q5I2J3
229533.A0A098DTU2 pp 229533.A0A1C3YMC9
229533.A0A098DTU2 pp 229533.A0A1C3YKZ8
229533.A0A098DTU2 pp 229533.A0A0E0SNZ9
229533.A0A098DTU2 pp 229533.I1RNF3
229533.A0A098DTU2 pp 229533.I1RWJ8
229533.A0A098DTU2 pp 229533.I1RUK7
229533.A0A098DTU2 pp 229533.I1RYX7
229533.A0A098DTU2 pp 229533.A0A1C3YJT5
229533.A0A098DTU2 pp 229533.I1RHU0

229533.A0A098DTU2	pp	229533.I1RZH4
229533.A0A098DTU2	pp	229533.I1RMS2
229533.A0A098DTU2	pp	229533.A0A1C3YI44
229533.A0A098DTU2	pp	229533.I1RA72
229533.A0A098D6H2	pp	229533.I1RXB8
229533.A0A098D6H2	pp	229533.A0A1C3YM61
229533.A0A098D6H2	pp	229533.I1RMP2
229533.A0A098D6H2	pp	229533.I1RE19
229533.A0A098D6H2	pp	229533.I1RTG5
229533.A0A098D6H2	pp	229533.I1RD10
229533.A0A098D6H2	pp	229533.I1RTE0
229533.A0A098D6H2	pp	229533.I1RTA3
229533.A0A098D6H2	pp	229533.I1RA31
229533.A0A098D6H2	pp	229533.I1RSL8
229533.A0A098D6H2	pp	229533.A0A1C3YIQ1
229533.A0A098D6H2	pp	229533.I1RKR9
229533.A0A098D6H2	pp	229533.A0A1C3YL06
229533.A0A098D6H2	pp	229533.I1RSZ6
229533.A0A098D6H2	pp	229533.I1S270
229533.A0A098D6H2	pp	229533.I1RHU0
229533.A0A098D6H2	pp	229533.I1RA34
229533.A0A098D6H2	pp	229533.I1RSG9
229533.A0A098D6H2	pp	229533.I1RRF9
229533.A0A098D6H2	pp	229533.A0A098DTU2
229533.I1RPU1	pp	229533.Q4IER8
229533.I1RPU1	pp	229533.Q4I7L0
229533.I1RPU1	pp	229533.Q4HWE2
229533.I1RPU1	pp	229533.I1RRS7
229533.I1RPU1	pp	229533.I1S1V2
229533.I1RPU1	pp	229533.I1RWD7
229533.I1RPU1	pp	229533.Q4IPI4
229533.I1RPU1	pp	229533.I1RW71
229533.I1RPU1	pp	229533.I1S461
229533.I1RPU1	pp	229533.I1RT16
229533.A0A1C3YN21	pp	229533.I1RTE7
229533.A0A1C3YN21	pp	229533.I1RYJ8
229533.A0A1C3YN21	pp	229533.I1RY25
229533.I1RSD7	pp	229533.Q4INI2
229533.I1RSD7	pp	229533.I1RX68
229533.I1RSD7	pp	229533.I1S559
229533.I1RSD7	pp	229533.I1RT41
229533.I1RSD7	pp	229533.Q4IPI4
229533.I1RSD7	pp	229533.I1S270
229533.I1RSD7	pp	229533.I1RZD6
229533.I1RSD7	pp	229533.V6RR76
229533.I1RSD7	pp	229533.Q4HZE0
229533.I1RSD7	pp	229533.I1S0Z3
229533.I1RSD7	pp	229533.I1RX09
229533.I1RSD7	pp	229533.I1RST4
229533.I1RSD7	pp	229533.I1RWI6
229533.I1RSD7	pp	229533.I1RX69
229533.I1RSD7	pp	229533.I1RT65
229533.I1RSD7	pp	229533.I1RTG5
229533.I1RSD7	pp	229533.I1S8X0
229533.A0A1C3YM05	pp	229533.I1REK8
229533.A0A1C3YM05	pp	229533.I1S4S4
229533.A0A1C3YM05	pp	229533.I1S2C1
229533.A0A1C3YM05	pp	229533.I1RW61
229533.A0A1C3YM05	pp	229533.I1RY63
229533.A0A1C3YM05	pp	229533.I1RSZ7
229533.A0A1C3YM05	pp	229533.I1RJU3
229533.A0A1C3YM05	pp	229533.I1S4S7
229533.A0A1C3YM05	pp	229533.I1RY57
229533.A0A1C3YM05	pp	229533.I1S0R9
229533.A0A1C3YM05	pp	229533.I1RTE9
229533.A0A1C3YM05	pp	229533.I1RA11
229533.A0A1C3YM05	pp	229533.I1RCH5
229533.A0A1C3YM05	pp	229533.I1RVX0
229533.A0A1C3YM05	pp	229533.I1RC18
229533.A0A1C3YM05	pp	229533.I1RSQ4
229533.A0A1C3YM05	pp	229533.I1RKB6
229533.A0A1C3YM05	pp	229533.I1RZ95
229533.A0A1C3YM05	pp	229533.I1RGA4

229533.A0A1C3YM05 pp 229533.I1RTA1
229533.A0A1C3YM05 pp 229533.I1RP97
229533.A0A1C3YM05 pp 229533.I1RYI5
229533.I1RWP4 pp 229533.I1RWW1
229533.I1RWP4 pp 229533.I1RYD9
229533.I1RWP4 pp 229533.I1RYN4
229533.I1RWP4 pp 229533.I1RXF0
229533.I1RWP4 pp 229533.I1RYL5
229533.I1RWP4 pp 229533.I1S021
229533.I1RWP4 pp 229533.V6R0U9
229533.I1RWP4 pp 229533.I1RYQ1
229533.I1RWP4 pp 229533.Q4HWE2
229533.I1RWP4 pp 229533.I1S4Z7
229533.I1RPF2 pp 229533.I1RR90
229533.I1RPF2 pp 229533.I1RTP2
229533.I1RPF2 pp 229533.I1RY34
229533.I1RPF2 pp 229533.I1SAK7
229533.I1RPF2 pp 229533.I1RWM4
229533.I1RPF2 pp 229533.V6RR38
229533.I1RPF2 pp 229533.I1S2M5
229533.I1RBB0 pp 229533.V6RR76
229533.I1RBB0 pp 229533.I1RX69
229533.I1RBB0 pp 229533.I1RX09
229533.I1RBB0 pp 229533.I1RCJ9
229533.I1RBB0 pp 229533.I1S0Z3
229533.I1RBB0 pp 229533.Q4HZE0
229533.I1RBB0 pp 229533.I1RWI6
229533.I1RBB0 pp 229533.I1RCT2
229533.I1RBB0 pp 229533.I1RQM8
229533.I1RBB0 pp 229533.I1RST4
229533.I1RBB0 pp 229533.I1RTG5
229533.I1RBB0 pp 229533.I1RBR0
229533.I1RBB0 pp 229533.I1RSD7
229533.I1RBB0 pp 229533.I1S270
229533.I1RBB0 pp 229533.I1RRR5
229533.I1RBB0 pp 229533.I1RY40
229533.I1RBB0 pp 229533.I1RN81
229533.I1RBB0 pp 229533.I1RRF0
229533.I1RBB0 pp 229533.I1RT65
229533.I1RBB0 pp 229533.I1RKR9
229533.I1RBB0 pp 229533.Q4IPI4
229533.I1RBB0 pp 229533.I1RZD6
229533.I1RBB0 pp 229533.I1RT41
229533.I1RBB0 pp 229533.I1S559
229533.I1RBB0 pp 229533.I1RXS3
229533.I1RBB0 pp 229533.I1RX68
229533.I1RBB0 pp 229533.I1RKI1
229533.I1RBB0 pp 229533.I1RZL3
229533.I1S0F8 pp 229533.I1S5R6
229533.I1S0F8 pp 229533.V6RMW6
229533.I1S0F8 pp 229533.I1S2B8
229533.I1S0F8 pp 229533.I1S4S7
229533.I1S0F8 pp 229533.I1S1W9
229533.I1S0F8 pp 229533.I1S5R1
229533.A0A098D6D1 pp 229533.A0A1C3YKN4
229533.Q4IR09 pp 229533.V6RLM7
229533.Q4IR09 pp 229533.V6RUY9
229533.Q4IR09 pp 229533.V6RVG3
229533.Q4IR09 pp 229533.V6R0U9
229533.I1RGU3 pp 229533.I1SAT2
229533.I1RGU3 pp 229533.I1S2C4
229533.I1RGU3 pp 229533.I1RVX8
229533.I1RGU3 pp 229533.I1RH83
229533.I1RGU3 pp 229533.I1S6V0
229533.I1RGU3 pp 229533.I1RPV8
229533.I1RGU3 pp 229533.I1RJS1
229533.I1RGU3 pp 229533.I1RK55
229533.I1RGU3 pp 229533.I1RHU8
229533.I1RGU3 pp 229533.I1RZ56
229533.I1RGU3 pp 229533.I1S968
229533.I1RGU3 pp 229533.I1RZ89
229533.I1RGU3 pp 229533.I1RN84
229533.I1RGU3 pp 229533.V6RCJ5

12411 229533.I1RGU3 pp 229533.I1S0A5
12412 229533.I1RGU3 pp 229533.I1RRR9
12413 229533.I1RGU3 pp 229533.I1RWF5
12414 229533.I1RGU3 pp 229533.I1RW82
12415 229533.I1RGU3 pp 229533.I1RSA5
12416 229533.I1RGU3 pp 229533.V6RWB3
12417 229533.I1RGU3 pp 229533.I1RIJ6
12418 229533.I1RGU3 pp 229533.I1RQJ7
12419 229533.I1RGU3 pp 229533.I1RZ25
12420 229533.I1RGU3 pp 229533.I1RTF5
12421 229533.I1RGU3 pp 229533.I1RMK9
12422 229533.I1RGU3 pp 229533.I1S094
12423 229533.I1RGU3 pp 229533.I1RIB5
12424 229533.I1RGU3 pp 229533.I1RKY6
12425 229533.I1RGU3 pp 229533.I1RY12
12426 229533.I1RGU3 pp 229533.I1S659
12427 229533.I1RGU3 pp 229533.I1S048
12428 229533.I1RGU3 pp 229533.I1RLA2
12429 229533.I1RGU3 pp 229533.I1RST9
12430 229533.I1RGU3 pp 229533.I1RY81
12431 229533.I1RGU3 pp 229533.I1S8D3
12432 229533.I1RGU3 pp 229533.I1RWL3
12433 229533.I1RGU3 pp 229533.I1S6H3
12434 229533.I1RGU3 pp 229533.I1RWM4
12435 229533.I1RGU3 pp 229533.V6RCC0
12436 229533.I1RGU3 pp 229533.V6R5T7
12437 229533.I1RGU3 pp 229533.I1RPM6
12438 229533.I1RGU3 pp 229533.I1RYP5
12439 229533.I1RGU3 pp 229533.I1S5D0
12440 229533.I1RGU3 pp 229533.I1RW54
12441 229533.I1RGU3 pp 229533.I1S267
12442 229533.I1RGU3 pp 229533.I1RYU6
12443 229533.I1RGU3 pp 229533.I1RQV8
12444 229533.I1RGU3 pp 229533.I1RQV7
12445 229533.I1RGU3 pp 229533.V6RHX2
12446 229533.I1RGU3 pp 229533.I1RRS6
12447 229533.I1RGU3 pp 229533.I1S969
12448 229533.I1RGU3 pp 229533.I1RMA2
12449 229533.I1RI22 pp 229533.I1RY65
12450 229533.I1RI22 pp 229533.I1S1W6
12451 229533.I1RI22 pp 229533.I1S2C2
12452 229533.I1RI22 pp 229533.I1RNX0
12453 229533.I1RI22 pp 229533.I1RTP2
12454 229533.I1RI22 pp 229533.I1RYJ8
12455 229533.I1RI22 pp 229533.I1RZZ3
12456 229533.I1RI22 pp 229533.V6RPN0
12457 229533.I1RI22 pp 229533.I1SAS4
12458 229533.I1RI22 pp 229533.I1S2M5
12459 229533.I1RI22 pp 229533.I1S1Q3
12460 229533.I1RI22 pp 229533.I1RNT1
12461 229533.I1RI22 pp 229533.I1SAS3
12462 229533.I1RI22 pp 229533.I1RPF2
12463 229533.I1RI22 pp 229533.I1RT23
12464 229533.I1RI22 pp 229533.I1S5K2
12465 229533.I1RI22 pp 229533.I1RJR2
12466 229533.I1RI22 pp 229533.I1RIZ8
12467 229533.I1RI22 pp 229533.V6RR38
12468 229533.I1RI22 pp 229533.I1RI23
12469 229533.I1RI22 pp 229533.I1RY34
12470 229533.I1RI22 pp 229533.I1RJG1
12471 229533.I1RI22 pp 229533.I1RQ76
12472 229533.I1RI22 pp 229533.I1RY35
12473 229533.A0A1C3YKB9 pp 229533.I1RW61
12474 229533.A0A1C3YKB9 pp 229533.I1S0R9
12475 229533.A0A1C3YKB9 pp 229533.I1RTE9
12476 229533.A0A1C3YKB9 pp 229533.I1RSZ7
12477 229533.A0A1C3YKB9 pp 229533.I1RSQ4
12478 229533.A0A1C3YKB9 pp 229533.I1RZ95
12479 229533.A0A1C3YKB9 pp 229533.I1RKB6
12480 229533.A0A1C3YKB9 pp 229533.I1RY57
12481 229533.A0A1C3YKB9 pp 229533.I1RVX0
12482 229533.A0A1C3YKB9 pp 229533.I1RA11
12483 229533.A0A1C3YKB9 pp 229533.I1RCH5

229533.A0A1C3YKB9	pp	229533.I1S2C1
229533.A0A1C3YKB9	pp	229533.I1RYI5
229533.A0A1C3YKB9	pp	229533.I1RTA1
229533.A0A1C3YKB9	pp	229533.I1RP97
229533.A0A1C3YKB9	pp	229533.I1RJU3
229533.A0A1C3YKB9	pp	229533.I1RGA4
229533.A0A1C3YKB9	pp	229533.I1RC18
229533.A0A1C3YKB9	pp	229533.I1S4S4
229533.A0A1C3YKB9	pp	229533.I1RY63
229533.A0A1C3YKB9	pp	229533.I1REK8
229533.A0A1C3YKB9	pp	229533.I1S4S7
229533.I1RT23	pp	229533.I1RY35
229533.I1RT23	pp	229533.I1S5K2
229533.I1RT23	pp	229533.I1RZD0
229533.I1RT23	pp	229533.I1SAK7
229533.I1RT23	pp	229533.I1S2M5
229533.I1RT23	pp	229533.I1S130
229533.I1RT23	pp	229533.I1RZ92
229533.I1RT23	pp	229533.I1S1Q3
229533.I1RT23	pp	229533.V6RR38
229533.I1RT23	pp	229533.I1RZZ3
229533.I1RT23	pp	229533.I1S1W6
229533.I1RT23	pp	229533.I1RY65
229533.I1RT23	pp	229533.I1S2C2
229533.I1RT23	pp	229533.V6RPN0
229533.I1RT23	pp	229533.I1SAS3
229533.I1RT23	pp	229533.I1RYL5
229533.I1RT23	pp	229533.I1RY81
229533.I1RT23	pp	229533.I1RXX1
229533.I1RT23	pp	229533.I1SAS4
229533.I1RWY4	pp	229533.I1S694
229533.I1RWY4	pp	229533.I1RZA8
229533.I1RWY4	pp	229533.I1S1M4
229533.I1RWY4	pp	229533.I1RY16
229533.I1RWY4	pp	229533.I1S5G4
229533.I1RV65	pp	229533.I1RY58
229533.I1S0B6	pp	229533.I1S0R7
229533.I1S0B6	pp	229533.I1S7M2
229533.I1S0B6	pp	229533.I1S524
229533.I1S0B6	pp	229533.I1S7M3
229533.I1S0B6	pp	229533.I1S8V0
229533.I1S0B6	pp	229533.I1S5S2
229533.I1S0B6	pp	229533.I1S4Y4
229533.I1S0B6	pp	229533.Q4I7F9
229533.I1S0B6	pp	229533.Q4HZ42
229533.I1S0B6	pp	229533.V6QVD5
229533.I1S0B6	pp	229533.Q4IP34
229533.I1S0B6	pp	229533.I1S4M5
229533.I1S0B6	pp	229533.I1S246
229533.I1S0B6	pp	229533.V6RIS2
229533.I1S0B6	pp	229533.V6RY33
229533.I1S0B6	pp	229533.I1S8U9
229533.I1S0B6	pp	229533.Q4IAA0
229533.I1SAS4	pp	229533.V6RVJ3
229533.I1SAS4	pp	229533.V6RPN0
229533.I1RYQ0	pp	229533.I1S238
229533.I1RZZ3	pp	229533.I1S5K2
229533.I1RZZ3	pp	229533.V6RR38
229533.I1RZZ3	pp	229533.I1S2M5
229533.I1RZZ3	pp	229533.I1S1W6
229533.I1RZZ3	pp	229533.V6RPN0
229533.I1RZZ3	pp	229533.I1SAS4
229533.I1RZZ3	pp	229533.I1S1Q3
229533.I1RZZ3	pp	229533.I1S2C2
229533.I1RZZ3	pp	229533.I1SAS3
229533.I1RKY6	pp	229533.I1RZB7
229533.I1RKY6	pp	229533.I1RR68
229533.I1RKY6	pp	229533.V6QVH8
229533.I1RKY6	pp	229533.I1RYQ0
229533.I1RKY6	pp	229533.Q4I8S2
229533.I1RKY6	pp	229533.I1RTF5
229533.I1RKY6	pp	229533.I1RV65
229533.I1RYN4	pp	229533.I1S8P5

12557 229533.I1RYN4 pp 229533.I1RZM1
12558 229533.I1RYN4 pp 229533.Q4HW67
12559 229533.I1RY34 pp 229533.I1SAK7
12560 229533.I1RY34 pp 229533.I1S2M5
12561 229533.I1RY34 pp 229533.V6RR38
12562 229533.I1S1Q3 pp 229533.I1S2C2
12563 229533.I1S1Q3 pp 229533.V6RPN0
12564 229533.I1S1Q3 pp 229533.I1S5K2
12565 229533.I1S1Q3 pp 229533.I1S2M5
12566 229533.I1S1Q3 pp 229533.I1S1W6
12567 229533.I1S1Q3 pp 229533.V6RG22
12568 229533.I1S1Q3 pp 229533.V6RVJ3
12569 229533.A0A098DZM9 pp 229533.I1RR68
12570 229533.A0A098DZM9 pp 229533.I1RGU3
12571 229533.A0A098DZM9 pp 229533.I1RZB7
12572 229533.A0A098DZM9 pp 229533.I1R9G0
12573 229533.A0A098DZM9 pp 229533.I1RV65
12574 229533.A0A098DZM9 pp 229533.I1RYQ0
12575 229533.A0A098DZM9 pp 229533.I1RKY6
12576 229533.A0A098DZM9 pp 229533.I1RTF5
12577 229533.A0A098DZM9 pp 229533.I1RIH0
12578 229533.A0A098DZM9 pp 229533.V6QVH8
12579 229533.A0A1C3YLL7 pp 229533.I1RR90
12580 229533.A0A1C3YLL7 pp 229533.I1REM2
12581 229533.A0A1C3YLL7 pp 229533.I1RY35
12582 229533.A0A1C3YLL7 pp 229533.I1RJG1
12583 229533.A0A1C3YLL7 pp 229533.I1RY34
12584 229533.A0A1C3YLL7 pp 229533.I1RQ76
12585 229533.A0A1C3YLL7 pp 229533.I1RI22
12586 229533.A0A1C3YLL7 pp 229533.I1RF79
12587 229533.A0A1C3YLL7 pp 229533.I1RMA2
12588 229533.A0A1C3YLL7 pp 229533.V6RPN0
12589 229533.A0A1C3YLL7 pp 229533.I1SAK7
12590 229533.A0A1C3YLL7 pp 229533.I1S1Q3
12591 229533.A0A1C3YLL7 pp 229533.I1RCA0
12592 229533.A0A1C3YLL7 pp 229533.I1RRQ1
12593 229533.A0A1C3YLL7 pp 229533.I1REL9
12594 229533.A0A1C3YLL7 pp 229533.I1RXX1
12595 229533.A0A1C3YLL7 pp 229533.I1RY81
12596 229533.A0A1C3YLL7 pp 229533.I1RFP1
12597 229533.A0A1C3YLL7 pp 229533.I1RY65
12598 229533.A0A1C3YLL7 pp 229533.I1RPE5
12599 229533.A0A1C3YLL7 pp 229533.I1RNK1
12600 229533.A0A1C3YLL7 pp 229533.I1RI23
12601 229533.A0A1C3YLL7 pp 229533.I1S5K2
12602 229533.A0A1C3YLL7 pp 229533.I1RT23
12603 229533.A0A1C3YLL7 pp 229533.I1RJR2
12604 229533.A0A1C3YLL7 pp 229533.I1SAS3
12605 229533.A0A1C3YLL7 pp 229533.I1RZD0
12606 229533.A0A1C3YLL7 pp 229533.I1RBR4
12607 229533.A0A1C3YLL7 pp 229533.V6RR38
12608 229533.A0A1C3YLL7 pp 229533.I1SAS4
12609 229533.A0A1C3YLL7 pp 229533.I1S1W3
12610 229533.A0A1C3YLL7 pp 229533.I1S130
12611 229533.A0A1C3YLL7 pp 229533.I1RGC4
12612 229533.A0A1C3YLL7 pp 229533.I1RNX0
12613 229533.A0A1C3YLL7 pp 229533.I1RGK2
12614 229533.A0A1C3YLL7 pp 229533.A0A1C3YMS7
12615 229533.A0A1C3YLL7 pp 229533.I1S1W6
12616 229533.A0A1C3YLL7 pp 229533.V6RG22
12617 229533.A0A1C3YLL7 pp 229533.I1S2M5
12618 229533.A0A1C3YLL7 pp 229533.I1S2C2
12619 229533.A0A1C3YLL7 pp 229533.I1RZZ3
12620 229533.A0A1C3YLL7 pp 229533.I1RZ92
12621 229533.A0A1C3YLL7 pp 229533.I1RIZ8
12622 229533.A0A1C3YLL7 pp 229533.I1RCC2
12623 229533.A0A1C3YLL7 pp 229533.I1RFM2
12624 229533.A0A1C3YLL7 pp 229533.I1RE80
12625 229533.A0A1C3YLL7 pp 229533.V6RIE5
12626 229533.A0A1C3YLL7 pp 229533.A0A1C3YM25
12627 229533.A0A1C3YLL7 pp 229533.I1RPF2
12628 229533.A0A1C3YLL7 pp 229533.I1S5R5
12629 229533.A0A1C3YLL7 pp 229533.I1RNT1

229533.I1RMQ0 pp 229533.Q4HZ42
229533.I1RMQ0 pp 229533.I1RQ65
229533.I1RMQ0 pp 229533.I1RT33
229533.I1RMQ0 pp 229533.I1RTE9
229533.I1RMQ0 pp 229533.I1RVZ0
229533.I1RMQ0 pp 229533.I1S2C0
229533.I1RMQ0 pp 229533.I1S2E8
229533.I1RMQ0 pp 229533.I1RSD8
229533.I1RMQ0 pp 229533.I1RW71
229533.I1RMQ0 pp 229533.I1RPU0
229533.I1RMQ0 pp 229533.I1RZZ1
229533.I1RMQ0 pp 229533.I1RQA2
229533.I1RMQ0 pp 229533.I1S9U2
229533.I1RMQ0 pp 229533.I1RSS4
229533.I1RMQ0 pp 229533.I1RQM6
229533.I1RMQ0 pp 229533.I1S021
229533.I1RMQ0 pp 229533.I1RND7
229533.I1RMQ0 pp 229533.I1RNA0
229533.I1RMQ0 pp 229533.I1RW25
229533.I1RMQ0 pp 229533.I1RX14
229533.I1RMQ0 pp 229533.I1RZS6
229533.I1RMQ0 pp 229533.I1RNU6
229533.I1RMQ0 pp 229533.I1S0R7
229533.I1RMQ0 pp 229533.I1RT04
229533.I1RMQ0 pp 229533.I1RQS5
229533.I1SAS3 pp 229533.V6RVJ3
229533.I1SAS3 pp 229533.V6RPN0
229533.A0A0E0RPG8 pp 229533.I1RWR7
229533.A0A0E0RPG8 pp 229533.I1RAW0
229533.A0A0E0RPG8 pp 229533.I1RCH5
229533.A0A0E0RPG8 pp 229533.I1RQE7
229533.A0A0E0RPG8 pp 229533.I1RB92
229533.A0A0E0RPG8 pp 229533.I1RQ33
229533.A0A0E0RPG8 pp 229533.I1REK8
229533.A0A0E0RPG8 pp 229533.I1RUG3
229533.A0A0E0RPG8 pp 229533.A0A0E0SKJ9
229533.A0A0E0RPG8 pp 229533.I1RZM8
229533.A0A0E0RPG8 pp 229533.I1RRM3
229533.A0A0E0RPG8 pp 229533.V6RMW6
229533.A0A0E0RPG8 pp 229533.I1S5R6
229533.A0A0E0RPG8 pp 229533.I1RE01
229533.A0A0E0RPG8 pp 229533.A0A1C3YK34
229533.A0A0E0RPG8 pp 229533.A0A1C3YKV3
229533.A0A0E0RPG8 pp 229533.A0A1C3YJN3
229533.A0A0E0RPG8 pp 229533.I1RZV3
229533.A0A0E0RPG8 pp 229533.I1S1W9
229533.A0A0E0RPG8 pp 229533.I1R9M0
229533.A0A0E0RPG8 pp 229533.I1S2B8
229533.A0A0E0RPG8 pp 229533.A0A1C3YJI6
229533.A0A0E0RPG8 pp 229533.I1RT40
229533.A0A0E0RPG8 pp 229533.I1RXD7
229533.A0A0E0RPG8 pp 229533.I1RQ85
229533.A0A0E0RPG8 pp 229533.I1RPU2
229533.A0A0E0RPG8 pp 229533.I1RZ39
229533.A0A0E0RPG8 pp 229533.I1RJU3
229533.A0A0E0RPG8 pp 229533.A0A1C3YLK8
229533.A0A0E0RPG8 pp 229533.I1RTA1
229533.A0A0E0RPG8 pp 229533.I1S5R1
229533.A0A0E0RPG8 pp 229533.I1RA11
229533.A0A0E0RPG8 pp 229533.I1RRI1
229533.A0A0E0RPG8 pp 229533.I1RVZ2
229533.A0A0E0RPG8 pp 229533.I1RYI5
229533.A0A0E0RPG8 pp 229533.I1S4S7
229533.A0A0E0RPG8 pp 229533.I1RR24
229533.A0A0E0RPG8 pp 229533.I1RPE0
229533.A0A0E0RPG8 pp 229533.I1RSZ7
229533.A0A0E0RPG8 pp 229533.I1RSA5
229533.A0A0E0RPG8 pp 229533.I1RZK5
229533.A0A0E0RPG8 pp 229533.I1RD52
229533.I1RI23 pp 229533.I1RPF2
229533.I1RI23 pp 229533.I1SAK7
229533.I1RI23 pp 229533.I1S2M5
229533.I1RI23 pp 229533.V6RR38

229533.I1RI23 pp 229533.I1RJR2
229533.I1RI23 pp 229533.I1RWM4
229533.I1RI23 pp 229533.I1RNM7
229533.I1RI23 pp 229533.I1RY34
229533.I1RI23 pp 229533.I1RTP2
229533.I1RI23 pp 229533.I1RR90
229533.A0A1C3YK54 pp 229533.A0A1C3YM91
229533.A0A1C3YK54 pp 229533.I1S1Z9
229533.I1RFM2 pp 229533.I1S2M5
229533.I1RFM2 pp 229533.I1S130
229533.I1RFM2 pp 229533.I1RZ92
229533.I1RFM2 pp 229533.I1RPF2
229533.I1RFM2 pp 229533.I1RQ76
229533.I1RFM2 pp 229533.I1S2C2
229533.I1RFM2 pp 229533.I1RI22
229533.I1RFM2 pp 229533.I1RZZ3
229533.I1RFM2 pp 229533.V6RPN0
229533.I1RFM2 pp 229533.I1RNX0
229533.I1RFM2 pp 229533.I1S1Q3
229533.I1RFM2 pp 229533.I1RT23
229533.I1RFM2 pp 229533.I1RXX1
229533.I1RFM2 pp 229533.I1SAK7
229533.I1RFM2 pp 229533.I1RGK2
229533.I1RFM2 pp 229533.V6RG22
229533.I1RFM2 pp 229533.I1RI23
229533.I1RFM2 pp 229533.I1RY65
229533.I1RFM2 pp 229533.I1RJR2
229533.I1RFM2 pp 229533.V6RR38
229533.I1RFM2 pp 229533.I1S5K2
229533.I1RFM2 pp 229533.I1RQ48
229533.I1RFM2 pp 229533.I1RNT1
229533.I1RFM2 pp 229533.I1RRQ1
229533.I1RFM2 pp 229533.I1RGC4
229533.I1RFM2 pp 229533.I1S1W6
229533.I1RFM2 pp 229533.I1RIZ8
229533.I1RFM2 pp 229533.V6RVJ3
229533.I1RFM2 pp 229533.I1RJG1
229533.I1RFM2 pp 229533.I1RZJ8
229533.I1RFM2 pp 229533.I1RY35
229533.I1RFM2 pp 229533.I1SAS4
229533.I1RFM2 pp 229533.I1RY34
229533.I1RFM2 pp 229533.I1SAS3
229533.I1RFM2 pp 229533.I1RR90
229533.I1RFM2 pp 229533.I1RMH4
229533.I1RVF8 pp 229533.V6R042
229533.I1RVF8 pp 229533.I1RZV3
229533.I1RVF8 pp 229533.I1S5R6
229533.I1RVF8 pp 229533.I1S1W9
229533.I1RVF8 pp 229533.I1RVX0
229533.I1RVF8 pp 229533.V6RMW6
229533.I1RVF8 pp 229533.I1S444
229533.I1RVF8 pp 229533.I1RWR7
229533.I1RVF8 pp 229533.I1S0W4
229533.I1RVF8 pp 229533.I1S437
229533.I1RVF8 pp 229533.I1RXC4
229533.I1RVF8 pp 229533.I1RZ39
229533.I1RVF8 pp 229533.I1RVZ2
229533.I1RVF8 pp 229533.I1RYI5
229533.I1RVF8 pp 229533.I1RYR0
229533.I1RVF8 pp 229533.I1RZ78
229533.I1RVF8 pp 229533.V6REE9
229533.I1RVF8 pp 229533.I1S2B8
229533.I1RVF8 pp 229533.I1RZM8
229533.I1RVF8 pp 229533.I1S5B0
229533.I1RVF8 pp 229533.I1RXC2
229533.I1RVF8 pp 229533.V6RCZ8
229533.I1RVF8 pp 229533.I1RZK5
229533.I1RVF8 pp 229533.I1S4S7
229533.I1RVF8 pp 229533.I1S5R1
229533.I1RVF8 pp 229533.Q4IJK2
229533.I1RVF8 pp 229533.I1S4S4
229533.I1RVF8 pp 229533.I1S438
229533.I1RVF8 pp 229533.I1RXD7

12776 229533.I1RVF8 pp 229533.V6RTJ8
12777 229533.V6RR38 pp 229533.V6RVJ3
12778 229533.A0A1C3YLS9 pp 229533.I1RR24
12779 229533.A0A1C3YLS9 pp 229533.I1RW25
12780 229533.A0A1C3YLS9 pp 229533.I1RVQ0
12781 229533.A0A1C3YLS9 pp 229533.I1RS18
12782 229533.A0A1C3YLS9 pp 229533.V6QUE6
12783 229533.A0A1C3YLS9 pp 229533.I1RBT1
12784 229533.A0A1C3YLS9 pp 229533.I1RA85
12785 229533.A0A1C3YLS9 pp 229533.I1RJU3
12786 229533.A0A1C3YLS9 pp 229533.I1S0L3
12787 229533.A0A1C3YLS9 pp 229533.I1RD52
12788 229533.A0A1C3YLS9 pp 229533.I1RW99
12789 229533.A0A1C3YLS9 pp 229533.I1RDA0
12790 229533.A0A1C3YLS9 pp 229533.I1S5B0
12791 229533.A0A1C3YLS9 pp 229533.I1REK8
12792 229533.A0A1C3YLS9 pp 229533.I1RBD5
12793 229533.A0A1C3YLS9 pp 229533.I1RCR5
12794 229533.A0A1C3YLS9 pp 229533.I1RBE7
12795 229533.A0A1C3YLS9 pp 229533.I1S4S7
12796 229533.A0A1C3YLS9 pp 229533.I1RBC9
12797 229533.A0A1C3YLS9 pp 229533.I1RZM8
12798 229533.A0A1C3YLS9 pp 229533.I1RXF0
12799 229533.A0A1C3YLS9 pp 229533.Q4IJK2
12800 229533.A0A1C3YLS9 pp 229533.I1RQ31
12801 229533.A0A1C3YLS9 pp 229533.V6QW22
12802 229533.A0A1C3YLS9 pp 229533.I1RSQ7
12803 229533.A0A1C3YLS9 pp 229533.I1RZZ1
12804 229533.A0A1C3YLS9 pp 229533.I1S0T2
12805 229533.A0A1C3YLS9 pp 229533.V6RCZ8
12806 229533.A0A1C3YLS9 pp 229533.I1RRY9
12807 229533.A0A1C3YLS9 pp 229533.I1S1J7
12808 229533.A0A1C3YLS9 pp 229533.I1RBE4
12809 229533.A0A1C3YLS9 pp 229533.V6RCG5
12810 229533.A0A1C3YLS9 pp 229533.I1RCG4
12811 229533.A0A1C3YLS9 pp 229533.I1RX46
12812 229533.A0A1C3YLS9 pp 229533.I1RAX6
12813 229533.A0A1C3YLS9 pp 229533.V6RTJ8
12814 229533.A0A1C3YLS9 pp 229533.I1S887
12815 229533.A0A1C3YLS9 pp 229533.I1RR51
12816 229533.A0A1C3YLS9 pp 229533.I1RRM5
12817 229533.A0A1C3YLS9 pp 229533.I1S0R7
12818 229533.A0A1C3YLS9 pp 229533.I1RXD7
12819 229533.A0A1C3YLS9 pp 229533.I1RZK5
12820 229533.A0A1C3YLS9 pp 229533.I1S2B8
12821 229533.A0A1C3YLS9 pp 229533.Q4I8B6
12822 229533.A0A1C3YLS9 pp 229533.I1RBY4
12823 229533.A0A1C3YLS9 pp 229533.I1R9M0
12824 229533.A0A1C3YLS9 pp 229533.I1RE76
12825 229533.A0A1C3YLS9 pp 229533.A0A1C3YMA1
12826 229533.A0A1C3YLS9 pp 229533.I1RA11
12827 229533.A0A1C3YLS9 pp 229533.I1RPM4
12828 229533.A0A1C3YLS9 pp 229533.I1RRT8
12829 229533.A0A1C3YLS9 pp 229533.V6RUT5
12830 229533.A0A1C3YLS9 pp 229533.I1RF19
12831 229533.A0A1C3YLS9 pp 229533.I1RAZ8
12832 229533.A0A1C3YLS9 pp 229533.I1S1C7
12833 229533.A0A1C3YLS9 pp 229533.V6R227
12834 229533.A0A1C3YLS9 pp 229533.I1S5R1
12835 229533.A0A1C3YLS9 pp 229533.I1RYW3
12836 229533.A0A1C3YLS9 pp 229533.I1S2H4
12837 229533.A0A1C3YLS9 pp 229533.I1RBC7
12838 229533.A0A1C3YLS9 pp 229533.I1S0L4
12839 229533.A0A1C3YLS9 pp 229533.I1RZW8
12840 229533.A0A1C3YLS9 pp 229533.I1RRI2
12841 229533.A0A1C3YLS9 pp 229533.I1RGA4
12842 229533.A0A1C3YLS9 pp 229533.I1RWI5
12843 229533.A0A1C3YLS9 pp 229533.I1RBS6
12844 229533.A0A1C3YLS9 pp 229533.I1RT40
12845 229533.A0A1C3YLS9 pp 229533.I1RTH6
12846 229533.A0A1C3YLS9 pp 229533.I1RA70
12847 229533.A0A1C3YLS9 pp 229533.I1RSQ4
12848 229533.A0A1C3YLS9 pp 229533.I1S2C0

12849 229533.A0A1C3YLS9 pp 229533.I1RTD5
12850 229533.A0A1C3YLS9 pp 229533.I1RZZ9
12851 229533.A0A1C3YLS9 pp 229533.Q4HXF0
12852 229533.A0A1C3YLS9 pp 229533.I1RC66
12853 229533.A0A1C3YLS9 pp 229533.I1RT04
12854 229533.A0A1C3YLS9 pp 229533.I1RZ39
12855 229533.A0A1C3YLS9 pp 229533.V6RIV9
12856 229533.A0A1C3YLS9 pp 229533.I1S1W3
12857 229533.A0A1C3YLS9 pp 229533.I1RD17
12858 229533.A0A1C3YLS9 pp 229533.Q4I624
12859 229533.A0A1C3YLS9 pp 229533.I1RN67
12860 229533.A0A1C3YLS9 pp 229533.I1RNG4
12861 229533.A0A1C3YLS9 pp 229533.V6R057
12862 229533.A0A1C3YLS9 pp 229533.I1RDH3
12863 229533.A0A1C3YLS9 pp 229533.I1RC33
12864 229533.A0A1C3YLS9 pp 229533.I1RWK3
12865 229533.A0A1C3YLS9 pp 229533.I1RE01
12866 229533.A0A1C3YLS9 pp 229533.I1RN63
12867 229533.A0A1C3YLS9 pp 229533.I1S0J1
12868 229533.A0A1C3YLS9 pp 229533.I1RAU7
12869 229533.A0A1C3YLS9 pp 229533.I1RFL4
12870 229533.A0A1C3YLS9 pp 229533.I1RN18
12871 229533.A0A1C3YLS9 pp 229533.I1RFT1
12872 229533.A0A1C3YLS9 pp 229533.I1RC72
12873 229533.A0A1C3YLS9 pp 229533.I1RD22
12874 229533.A0A1C3YLS9 pp 229533.I1S5R6
12875 229533.A0A1C3YLS9 pp 229533.I1RRJ8
12876 229533.A0A1C3YLS9 pp 229533.I1RQE7
12877 229533.A0A1C3YLS9 pp 229533.I1RPP3
12878 229533.A0A1C3YLS9 pp 229533.I1RXR1
12879 229533.A0A1C3YLS9 pp 229533.I1RCH5
12880 229533.A0A1C3YLS9 pp 229533.I1RG11
12881 229533.A0A1C3YLS9 pp 229533.I1RJW6
12882 229533.A0A1C3YLS9 pp 229533.I1RNC1
12883 229533.A0A1C3YLS9 pp 229533.I1RCR8
12884 229533.A0A1C3YLS9 pp 229533.I1RVX0
12885 229533.A0A1C3YLS9 pp 229533.I1RRT2
12886 229533.A0A1C3YLS9 pp 229533.I1RUG3
12887 229533.A0A1C3YLS9 pp 229533.I1RT03
12888 229533.A0A1C3YLS9 pp 229533.Q4I327
12889 229533.A0A1C3YLS9 pp 229533.V6R042
12890 229533.A0A1C3YLS9 pp 229533.I1RJV3
12891 229533.A0A1C3YLS9 pp 229533.I1RB92
12892 229533.A0A1C3YLS9 pp 229533.I1RXU9
12893 229533.A0A1C3YLS9 pp 229533.I1RAW0
12894 229533.A0A1C3YLS9 pp 229533.V6RMW6
12895 229533.A0A1C3YLS9 pp 229533.I1RSQ3
12896 229533.I1RLF1 pp 229533.I1RY99
12897 229533.I1RF79 pp 229533.I1RGC4
12898 229533.I1RF79 pp 229533.I1S1W6
12899 229533.I1RF79 pp 229533.I1RYJ8
12900 229533.I1RF79 pp 229533.V6RPN0
12901 229533.I1RF79 pp 229533.I1RFM2
12902 229533.I1RF79 pp 229533.I1RT23
12903 229533.I1RF79 pp 229533.I1RZZ3
12904 229533.I1RF79 pp 229533.I1S2C2
12905 229533.I1RF79 pp 229533.I1RY65
12906 229533.I1RF79 pp 229533.I1SAS3
12907 229533.I1RF79 pp 229533.I1RNX0
12908 229533.I1RF79 pp 229533.I1SAS4
12909 229533.I1RF79 pp 229533.I1RIZ8
12910 229533.I1RF79 pp 229533.I1S5K2
12911 229533.I1RF79 pp 229533.I1RNT1
12912 229533.I1RF79 pp 229533.I1RPF2
12913 229533.I1RF79 pp 229533.I1RGK2
12914 229533.I1RF79 pp 229533.I1RI23
12915 229533.I1RF79 pp 229533.I1S2M5
12916 229533.I1RF79 pp 229533.I1RJR2
12917 229533.I1RF79 pp 229533.V6RR38
12918 229533.I1RF79 pp 229533.I1SAK7
12919 229533.I1RF79 pp 229533.I1S1Q3
12920 229533.I1RF79 pp 229533.I1RY34
12921 229533.I1RF79 pp 229533.I1RR90

12922	229533.I1RF79	pp	229533.I1RY35
12923	229533.I1RF79	pp	229533.I1RFP1
12924	229533.I1RF79	pp	229533.I1RQ76
12925	229533.I1RF79	pp	229533.I1RJG1
12926	229533.I1RJG1	pp	229533.I1S1W6
12927	229533.I1RJG1	pp	229533.I1RZJ8
12928	229533.I1RJG1	pp	229533.I1RQ48
12929	229533.I1RJG1	pp	229533.I1RMH4
12930	229533.I1RJG1	pp	229533.I1RNT1
12931	229533.I1RJG1	pp	229533.I1S5K2
12932	229533.I1RJG1	pp	229533.V6RVJ3
12933	229533.I1RJG1	pp	229533.I1RY35
12934	229533.I1RJG1	pp	229533.I1RR90
12935	229533.I1RJG1	pp	229533.V6RG22
12936	229533.I1RJG1	pp	229533.I1S2C2
12937	229533.I1RJG1	pp	229533.I1RT23
12938	229533.I1RJG1	pp	229533.I1RXX1
12939	229533.I1RJG1	pp	229533.I1RNX0
12940	229533.I1RJG1	pp	229533.I1RZZ3
12941	229533.I1RJG1	pp	229533.V6RPN0
12942	229533.I1RJG1	pp	229533.I1RY65
12943	229533.I1RJG1	pp	229533.I1S2M5
12944	229533.I1RJG1	pp	229533.I1S130
12945	229533.I1RJG1	pp	229533.I1RRQ1
12946	229533.I1RJG1	pp	229533.I1RZ92
12947	229533.I1RJG1	pp	229533.I1RQ76
12948	229533.I1RJG1	pp	229533.I1RJR2
12949	229533.I1RY35	pp	229533.I1RY81
12950	229533.I1RY35	pp	229533.V6RG22
12951	229533.I1RY35	pp	229533.V6RHX2
12952	229533.I1RY35	pp	229533.I1RY65
12953	229533.I1RY35	pp	229533.V6RCC0
12954	229533.I1RY35	pp	229533.V6RWB3
12955	229533.I1RY35	pp	229533.I1RYP5
12956	229533.I1RY35	pp	229533.V6RQW4
12957	229533.I1RY35	pp	229533.V6RPN0
12958	229533.I1RY35	pp	229533.I1S1Q3
12959	229533.I1RY35	pp	229533.I1RZZ3
12960	229533.I1RY35	pp	229533.I1S2C2
12961	229533.I1RY35	pp	229533.I1S4G3
12962	229533.I1RY35	pp	229533.V6R5T7
12963	229533.I1RY35	pp	229533.I1S8D3
12964	229533.I1RY35	pp	229533.V6RR38
12965	229533.I1RY35	pp	229533.I1S1B8
12966	229533.I1RY35	pp	229533.I1S5R5
12967	229533.I1RY35	pp	229533.V6RH92
12968	229533.I1RY35	pp	229533.I1S8C1
12969	229533.I1RY35	pp	229533.I1S969
12970	229533.I1RY35	pp	229533.I1S2Q4
12971	229533.I1RY35	pp	229533.I1S6V0
12972	229533.I1RY35	pp	229533.I1S130
12973	229533.I1RY35	pp	229533.I1S8R2
12974	229533.I1RY35	pp	229533.I1S3T7
12975	229533.I1RY35	pp	229533.V6RCJ5
12976	229533.I1RY35	pp	229533.I1S2M5
12977	229533.I1RY35	pp	229533.I1S5K2
12978	229533.I1RY35	pp	229533.I1RZ92
12979	229533.I1RY35	pp	229533.I1SAS4
12980	229533.I1RY35	pp	229533.I1S299
12981	229533.I1RY35	pp	229533.I1S9Z4
12982	229533.I1RY35	pp	229533.I1SAS3
12983	229533.I1RY35	pp	229533.I1S1W6
12984	229533.I1RY35	pp	229533.I1RZD0
12985	229533.I1RY35	pp	229533.I1S968
12986	229533.A0A0E0RTC6	pp	229533.I1RT35
12987	229533.A0A0E0RTC6	pp	229533.I1S214
12988	229533.A0A0E0RTC6	pp	229533.I1RQK2
12989	229533.A0A0E0RTC6	pp	229533.Q4IPA4
12990	229533.A0A0E0RTC6	pp	229533.A0A1C3YI81
12991	229533.A0A0E0RTC6	pp	229533.I1S246
12992	229533.A0A0E0RTC6	pp	229533.A0A1C3YJW7
12993	229533.A0A0E0RTC6	pp	229533.I1RN28
12994	229533.A0A0E0RTC6	pp	229533.I1S8V0

229533.A0A0E0RTC6 pp 229533.I1RA46
229533.A0A0E0RTC6 pp 229533.I1RDD3
229533.A0A0E0RTC6 pp 229533.I1RE45
229533.A0A0E0RTC6 pp 229533.A0A1C3YL25
229533.A0A0E0RTC6 pp 229533.I1RNB8
229533.A0A0E0RTC6 pp 229533.I1RA38
229533.A0A0E0RTC6 pp 229533.A0A1C3YKM6
229533.A0A0E0RTC6 pp 229533.I1S0N7
229533.A0A0E0RTC6 pp 229533.I1RQZ2
229533.A0A0E0RTC6 pp 229533.I1RD01
229533.A0A0E0RTC6 pp 229533.Q4IPC8
229533.A0A0E0RTC6 pp 229533.I1RK95
229533.A0A0E0RTC6 pp 229533.I1RMA1
229533.A0A0E0RTC6 pp 229533.Q4IF44
229533.A0A0E0RTC6 pp 229533.A0A1C3YII4
229533.A0A0E0RTC6 pp 229533.Q4IPN9
229533.A0A0E0RTC6 pp 229533.I1S8U9
229533.A0A0E0RTC6 pp 229533.A0A1C3YJV9
229533.A0A0E0RTC6 pp 229533.Q4I7X1
229533.A0A0E0RTC6 pp 229533.I1S5Z2
229533.A0A0E0RTC6 pp 229533.I1RS03
229533.A0A0E0RTC6 pp 229533.I1RWT5
229533.A0A0E0RTC6 pp 229533.I1RAZ8
229533.A0A0E0RTC6 pp 229533.Q4IMN9
229533.A0A0E0RTC6 pp 229533.Q4IR09
229533.A0A0E0RTC6 pp 229533.I1RZL5
229533.A0A0E0RTC6 pp 229533.I1R9Q0
229533.A0A0E0RTC6 pp 229533.A0A1C3YM64
229533.I1RXD6 pp 229533.I1RZ39
229533.I1RXD6 pp 229533.I1RYR0
229533.I1RXD6 pp 229533.V6R042
229533.I1RXD6 pp 229533.I1S2B8
229533.I1RXD6 pp 229533.I1S437
229533.I1RXD6 pp 229533.I1RYI5
229533.I1RXD6 pp 229533.I1S0W4
229533.I1RXD6 pp 229533.I1S5R6
229533.I1RXD6 pp 229533.I1RZK5
229533.I1RXD6 pp 229533.I1S5R1
229533.I1RXD6 pp 229533.V6RMW6
229533.I1RXD6 pp 229533.I1RZV3
229533.I1RXD6 pp 229533.I1RXD7
229533.I1RXD6 pp 229533.I1S1W9
229533.I1RXD6 pp 229533.I1S9I5
229533.I1RXD6 pp 229533.V6R9Z0
229533.I1RXD6 pp 229533.I1S821
229533.I1RXD6 pp 229533.I1S4S7
229533.I1RXD6 pp 229533.I1S438
229533.I1RXD6 pp 229533.V6RTJ8
229533.I1RXD6 pp 229533.Q4IJK2
229533.I1RXD6 pp 229533.I1S4S4
229533.I1RXD6 pp 229533.I1S6K9
229533.I1RXD6 pp 229533.I1S1B2
229533.I1RXD6 pp 229533.I1RZM8
229533.I1RXD6 pp 229533.I1S5B0
229533.I1RXD6 pp 229533.V6RCZ8
229533.A0A1C3YK52 pp 229533.A0A1C3YMF9
229533.A0A1C3YK52 pp 229533.A0A1C3YM02
229533.I1RE17 pp 229533.I1RQE7
229533.I1RE17 pp 229533.I1REK8
229533.I1RE17 pp 229533.I1RZM8
229533.I1RE17 pp 229533.I1RQ33
229533.I1RE17 pp 229533.I1S5R1
229533.I1RE17 pp 229533.I1RZ39
229533.I1RE17 pp 229533.I1RZK5
229533.I1RE17 pp 229533.I1RWR7
229533.I1RE17 pp 229533.I1RRM3
229533.I1RE17 pp 229533.I1RUG3
229533.I1RE17 pp 229533.I1RPE0
229533.I1RE17 pp 229533.V6RMW6
229533.I1RE17 pp 229533.I1RRI1
229533.I1RE17 pp 229533.I1RSA5
229533.I1RE17 pp 229533.I1RR24
229533.I1RE17 pp 229533.I1S5R6

229533.I1RE17	pp	229533.I1S1W9
229533.I1RE17	pp	229533.I1RSZ7
229533.I1RE17	pp	229533.I1RT40
229533.I1RE17	pp	229533.I1S2B8
229533.I1RE17	pp	229533.I1RXD7
229533.I1RE17	pp	229533.I1RZV3
229533.I1RE17	pp	229533.I1RQ85
229533.I1RE17	pp	229533.I1RVZ2
229533.I1RE17	pp	229533.I1RTA1
229533.I1RE17	pp	229533.I1RPU2
229533.I1RE17	pp	229533.I1S4S7
229533.I1RE17	pp	229533.I1RJU3
229533.I1RE17	pp	229533.I1RYI5
229533.I1RR90	pp	229533.I1S2C2
229533.I1RR90	pp	229533.I1RYJ8
229533.I1RR90	pp	229533.I1RY65
229533.I1RR90	pp	229533.V6RPN0
229533.I1RR90	pp	229533.I1RZZ3
229533.I1RR90	pp	229533.I1RY35
229533.I1RR90	pp	229533.I1S2M5
229533.I1RR90	pp	229533.I1S1W6
229533.I1RR90	pp	229533.I1S5K2
229533.I1RR90	pp	229533.I1RT23
229533.I1RR90	pp	229533.I1SAS4
229533.I1RR90	pp	229533.V6RR38
229533.I1RR90	pp	229533.I1SAS3
229533.I1RR90	pp	229533.I1SAK7
229533.I1RR90	pp	229533.I1RY34
229533.I1RR90	pp	229533.I1S1Q3
229533.A0A098D6B9	pp	229533.I1RI22
229533.A0A098D6B9	pp	229533.I1RT23
229533.A0A098D6B9	pp	229533.V6RPN0
229533.A0A098D6B9	pp	229533.I1SAS4
229533.A0A098D6B9	pp	229533.I1RZZ3
229533.A0A098D6B9	pp	229533.I1RQ76
229533.A0A098D6B9	pp	229533.I1S1Q3
229533.A0A098D6B9	pp	229533.A0A1C3YLL7
229533.A0A098D6B9	pp	229533.I1SAS3
229533.A0A098D6B9	pp	229533.I1RCA0
229533.A0A098D6B9	pp	229533.A0A1C3YMS7
229533.A0A098D6B9	pp	229533.I1RFM2
229533.A0A098D6B9	pp	229533.V6RR38
229533.A0A098D6B9	pp	229533.I1RF79
229533.A0A098D6B9	pp	229533.I1RJG1
229533.A0A098D6B9	pp	229533.I1RRQ1
229533.A0A098D6B9	pp	229533.I1RY35
229533.A0A098D6B9	pp	229533.A0A0E0SP71
229533.A0A098D6B9	pp	229533.I1RCC2
229533.A0A098D6B9	pp	229533.I1RQ48
229533.A0A098D6B9	pp	229533.I1RR90
229533.A0A098D6B9	pp	229533.I1RNT1
229533.A0A098DR13	pp	229533.I1RPS2
229533.A0A098DR13	pp	229533.I1RCE5
229533.A0A098DR13	pp	229533.I1RHJ8
229533.A0A098DR13	pp	229533.I1RZM3
229533.A0A098DR13	pp	229533.I1RD80
229533.A0A098DR13	pp	229533.I1RW78
229533.A0A098DR13	pp	229533.I1S8J1
229533.A0A098DR13	pp	229533.Q4I1Y5
229533.A0A098DR13	pp	229533.I1RWE4
229533.A0A098DR13	pp	229533.A0A0E0SCX4
229533.A0A098DR13	pp	229533.A0A0E0RU55
229533.A0A098DR13	pp	229533.I1RNU7
229533.A0A098DR13	pp	229533.I1S272
229533.A0A098DR13	pp	229533.I1RZ13
229533.A0A098DR13	pp	229533.V6RLM7
229533.A0A098DR13	pp	229533.A0A1C3YMK0
229533.A0A098DR13	pp	229533.A0A1C3YK33
229533.A0A098DR13	pp	229533.I1RM55
229533.A0A098DR13	pp	229533.I1SAC4
229533.A0A098DR13	pp	229533.A0A1C3YKW4
229533.A0A098DR13	pp	229533.I1RPK1
229533.A0A098DR13	pp	229533.I1RYW3

13141	229533.A0A098DR13	pp	229533.I1RCG5
13142	229533.A0A098DR13	pp	229533.I1RSZ6
13143	229533.A0A098DR13	pp	229533.I1S444
13144	229533.A0A098DR13	pp	229533.I1RVZ3
13145	229533.A0A098DR13	pp	229533.I1RAS9
13146	229533.A0A098DR13	pp	229533.A0A1C3YI20
13147	229533.A0A098DR13	pp	229533.I1RXB8
13148	229533.A0A098DR13	pp	229533.V6R3J1
13149	229533.A0A098DR13	pp	229533.I1RZ78
13150	229533.A0A098DR13	pp	229533.V6RV57
13151	229533.A0A098DR13	pp	229533.I1RPM4
13152	229533.A0A098DR13	pp	229533.I1RC31
13153	229533.A0A098DR13	pp	229533.I1RC29
13154	229533.A0A098DR13	pp	229533.V6RUG6
13155	229533.A0A098DR13	pp	229533.I1RRT3
13156	229533.A0A098DR13	pp	229533.A0A098E1Q1
13157	229533.A0A098DR13	pp	229533.A0A0E0SKE0
13158	229533.A0A098DR13	pp	229533.A0A1C3YHM9
13159	229533.A0A098DR13	pp	229533.A0A1C3YHI3
13160	229533.A0A098DR13	pp	229533.I1RKP3
13161	229533.A0A098DR13	pp	229533.A0A1C3YKK9
13162	229533.A0A098DR13	pp	229533.V6RUY9
13163	229533.A0A098DR13	pp	229533.I1RI39
13164	229533.A0A098DR13	pp	229533.I1RJQ5
13165	229533.A0A098DR13	pp	229533.I1RA62
13166	229533.A0A098DR13	pp	229533.I1RX72
13167	229533.A0A098DR13	pp	229533.I1S065
13168	229533.A0A098DR13	pp	229533.I1RBF4
13169	229533.A0A098DR13	pp	229533.A0A1C3YHZ7
13170	229533.A0A098DR13	pp	229533.I1RYK0
13171	229533.A0A098DR13	pp	229533.A0A1C3YLC2
13172	229533.A0A098DR13	pp	229533.A0A1C3YKI6
13173	229533.A0A098DR13	pp	229533.I1RQE0
13174	229533.A0A098DR13	pp	229533.A0A0E0S6M6
13175	229533.A0A098DR13	pp	229533.V6R3R0
13176	229533.A0A098DR13	pp	229533.I1RWW1
13177	229533.A0A098DR13	pp	229533.I1RPT0
13178	229533.A0A098DR13	pp	229533.I1RW06
13179	229533.A0A098DR13	pp	229533.A0A1C3YKN6
13180	229533.A0A098DR13	pp	229533.I1RYI6
13181	229533.A0A098DR13	pp	229533.I1RQJ7
13182	229533.A0A098DR13	pp	229533.A0A1C3YKA4
13183	229533.A0A098DR13	pp	229533.I1S1E0
13184	229533.A0A098DR13	pp	229533.I1RC11
13185	229533.A0A098DR13	pp	229533.A0A1C3YK51
13186	229533.A0A098DR13	pp	229533.I1RK45
13187	229533.A0A098DR13	pp	229533.I1RAQ4
13188	229533.A0A098DR13	pp	229533.A0A0E0RVC1
13189	229533.A0A098DR13	pp	229533.I1RMM6
13190	229533.A0A098DR13	pp	229533.I1RPL2
13191	229533.A0A098DR13	pp	229533.A0A1C3YM97
13192	229533.A0A098DR13	pp	229533.I1RCM5
13193	229533.A0A098DR13	pp	229533.I1RH82
13194	229533.A0A098DR13	pp	229533.I1RC61
13195	229533.A0A098DR13	pp	229533.I1RQA5
13196	229533.A0A098DR13	pp	229533.I1RJ42
13197	229533.A0A098DR13	pp	229533.A0A098E4T8
13198	229533.A0A098DR13	pp	229533.I1RXA6
13199	229533.A0A098DR13	pp	229533.A0A1C3YJZ0
13200	229533.A0A098DR13	pp	229533.I1RIE8
13201	229533.A0A098DR13	pp	229533.I1RBN0
13202	229533.A0A098DR13	pp	229533.A0A1C3YN98
13203	229533.A0A098DR13	pp	229533.A0A098DUT6
13204	229533.A0A098DR13	pp	229533.I1RDJ1
13205	229533.A0A098DR13	pp	229533.I1RSB7
13206	229533.A0A098DR13	pp	229533.A0A1C3YK58
13207	229533.A0A098DR13	pp	229533.I1RKL8
13208	229533.A0A098DR13	pp	229533.A0A0E0S546
13209	229533.A0A098DR13	pp	229533.I1RUM7
13210	229533.A0A098DR13	pp	229533.I1RDE4
13211	229533.A0A098DR13	pp	229533.I1RIZ7
13212	229533.A0A098DR13	pp	229533.A0A1C3YK11
13213	229533.A0A098DR13	pp	229533.A0A1C3YIJ7

13214 229533.A0A098DR13 pp 229533.Q4IR09
13215 229533.A0A098DR13 pp 229533.I1RHU0
13216 229533.A0A098DR13 pp 229533.A0A1C3YHV3
13217 229533.A0A098DR13 pp 229533.I1RDC7
13218 229533.A0A098DR13 pp 229533.A0A0E0SQ99
13219 229533.A0A098DR13 pp 229533.I1RJ82
13220 229533.A0A098DR13 pp 229533.A0A1C3YN26
13221 229533.A0A098DR13 pp 229533.I1S0P6
13222 229533.A0A098DR13 pp 229533.I1RME3
13223 229533.A0A098DR13 pp 229533.A0A0E0RNI4
13224 229533.A0A098DR13 pp 229533.A0A1C3YIQ1
13225 229533.A0A098DR13 pp 229533.I1RP86
13226 229533.A0A098DR13 pp 229533.I1RZT6
13227 229533.A0A098DR13 pp 229533.V6RN16
13228 229533.A0A098DR13 pp 229533.I1RGP7
13229 229533.A0A098DR13 pp 229533.A0A098DVZ4
13230 229533.A0A098DR13 pp 229533.A0A098DT85
13231 229533.A0A098DR13 pp 229533.A0A1C3YI19
13232 229533.A0A098DR13 pp 229533.A0A1C3YI71
13233 229533.A0A098DR13 pp 229533.A0A1C3YL54
13234 229533.A0A098DR13 pp 229533.I1RKB5
13235 229533.I1RV63 pp 229533.I1RWE4
13236 229533.I1RV63 pp 229533.I1S1E0
13237 229533.I1RV63 pp 229533.I1RXA6
13238 229533.I1RV63 pp 229533.I1RWW1
13239 229533.I1RV63 pp 229533.V6QUV7
13240 229533.A0A098D698 pp 229533.A0A098DR13
13241 229533.A0A098D698 pp 229533.I1RV63
13242 229533.I1RST4 pp 229533.I1RX69
13243 229533.I1RST4 pp 229533.Q4HY90
13244 229533.I1RST4 pp 229533.I1RYW3
13245 229533.I1RST4 pp 229533.I1RSZ6
13246 229533.I1RST4 pp 229533.I1RTG5
13247 229533.I1RST4 pp 229533.I1RTE0
13248 229533.I1RST4 pp 229533.I1RZV1
13249 229533.I1RST4 pp 229533.I1RT81
13250 229533.I1RST4 pp 229533.I1S0S8
13251 229533.I1RST4 pp 229533.I1S559
13252 229533.I1RST4 pp 229533.I1S248
13253 229533.I1RST4 pp 229533.I1RX68
13254 229533.I1RST4 pp 229533.I1S7W2
13255 229533.I1RST4 pp 229533.I1RXS3
13256 229533.I1RST4 pp 229533.I1RZL3
13257 229533.I1RST4 pp 229533.I1RVY0
13258 229533.I1RST4 pp 229533.I1S7X9
13259 229533.I1RST4 pp 229533.I1RWI6
13260 229533.I1RST4 pp 229533.V6RPN0
13261 229533.I1RST4 pp 229533.Q4IPI4
13262 229533.I1RST4 pp 229533.Q4I963
13263 229533.I1RST4 pp 229533.I1RW71
13264 229533.I1RST4 pp 229533.Q4ICA8
13265 229533.I1RST4 pp 229533.Q4I948
13266 229533.I1RST4 pp 229533.Q5I2J3
13267 229533.I1RST4 pp 229533.Q4INI2
13268 229533.I1RST4 pp 229533.I1S270
13269 229533.I1RST4 pp 229533.I1S1V2
13270 229533.I1RB67 pp 229533.I1RY99
13271 229533.I1RY86 pp 229533.I1S1W9
13272 229533.I1RY86 pp 229533.I1RZV3
13273 229533.I1RY86 pp 229533.V6R042
13274 229533.I1RY86 pp 229533.I1RYI5
13275 229533.I1RY86 pp 229533.I1RYR0
13276 229533.I1RY86 pp 229533.I1S2B8
13277 229533.I1RY86 pp 229533.I1S437
13278 229533.I1RY86 pp 229533.V6RCZ8
13279 229533.I1RY86 pp 229533.I1S0W4
13280 229533.I1RY86 pp 229533.I1RZ39
13281 229533.I1RY86 pp 229533.V6RMW6
13282 229533.I1RY86 pp 229533.I1S5R1
13283 229533.I1RY86 pp 229533.I1S5B0
13284 229533.I1RY86 pp 229533.I1RZK5
13285 229533.I1RY86 pp 229533.I1S4S7
13286 229533.I1RY86 pp 229533.I1S5R6

13287	229533.I1RY86	pp	229533.Q4IJK2
13288	229533.I1RY86	pp	229533.I1RZM8
13289	229533.I1RY86	pp	229533.V6RTJ8
13290	229533.I1RY86	pp	229533.I1S438
13291	229533.I1RY86	pp	229533.I1S4S4
13292	229533.V6RG22	pp	229533.V6RH92
13293	229533.V6RG22	pp	229533.V6RPN0
13294	229533.V6RG22	pp	229533.V6RR38
13295	229533.V6RG22	pp	229533.V6RIE5
13296	229533.V6RG22	pp	229533.V6RQW4
13297	229533.I1RXU0	pp	229533.I1S0J3
13298	229533.I1RXU0	pp	229533.I1S3N1
13299	229533.I1RXU0	pp	229533.V6QWX8
13300	229533.I1RXU0	pp	229533.Q4I5W5
13301	229533.I1RXU0	pp	229533.I1SAN0
13302	229533.I1RXU0	pp	229533.Q4HTT2
13303	229533.I1RXU0	pp	229533.Q4IQX3
13304	229533.I1RXU0	pp	229533.Q4HYI0
13305	229533.I1RXU0	pp	229533.I1S0R5
13306	229533.I1RXU0	pp	229533.Q4I099
13307	229533.I1RXU0	pp	229533.I1S182
13308	229533.I1RXU0	pp	229533.I1S0C6
13309	229533.I1RXU0	pp	229533.I1S2E9
13310	229533.I1RXU0	pp	229533.Q4I5U9
13311	229533.I1RXU0	pp	229533.V6QUE6
13312	229533.I1RXU0	pp	229533.I1RZM2
13313	229533.I1RXU0	pp	229533.I1S3N0
13314	229533.I1RXU0	pp	229533.I1RY44
13315	229533.I1RXU0	pp	229533.I1RZ42
13316	229533.I1RXU0	pp	229533.I1S276
13317	229533.I1RXU0	pp	229533.Q4IER8
13318	229533.I1RXU0	pp	229533.I1RZB5
13319	229533.I1RXU0	pp	229533.Q4I5I4
13320	229533.I1RXU0	pp	229533.V6QV38
13321	229533.I1RXU0	pp	229533.I1S9Y1
13322	229533.I1RXU0	pp	229533.Q4IJU0
13323	229533.I1RXU0	pp	229533.Q4IJM4
13324	229533.I1RLX4	pp	229533.I1S1V5
13325	229533.I1RLX4	pp	229533.I1RZB7
13326	229533.I1RLX4	pp	229533.I1RMY3
13327	229533.I1RLX4	pp	229533.I1RNT2
13328	229533.I1RLX4	pp	229533.I1RQ80
13329	229533.I1RLX4	pp	229533.I1S148
13330	229533.I1RLX4	pp	229533.I1RPZ0
13331	229533.I1RLX4	pp	229533.I1RMN1
13332	229533.I1RLX4	pp	229533.I1S382
13333	229533.I1RLX4	pp	229533.I1RMX3
13334	229533.I1RLX4	pp	229533.I1RPU4
13335	229533.I1RLX4	pp	229533.I1RNC0
13336	229533.I1RLX4	pp	229533.I1RXT9
13337	229533.I1RLX4	pp	229533.I1RX38
13338	229533.I1RLX4	pp	229533.Q4I7X1
13339	229533.I1RLX4	pp	229533.I1S951
13340	229533.I1RLX4	pp	229533.I1RPW0
13341	229533.I1RLX4	pp	229533.I1RRB0
13342	229533.I1RLX4	pp	229533.I1RRH3
13343	229533.I1RLX4	pp	229533.I1RRP7
13344	229533.I1RNM4	pp	229533.Q4IB63
13345	229533.I1RNM4	pp	229533.I1RUP0
13346	229533.I1RNM4	pp	229533.Q4IJT0
13347	229533.I1RNM4	pp	229533.I1RSM8
13348	229533.I1RNM4	pp	229533.V6QUV7
13349	229533.I1RNM4	pp	229533.I1RZV2
13350	229533.I1RNM4	pp	229533.I1RX83
13351	229533.I1RNM4	pp	229533.I1RZ13
13352	229533.I1RNM4	pp	229533.I1RSV4
13353	229533.I1RNM4	pp	229533.Q4I073
13354	229533.I1RNM4	pp	229533.I1RZ34
13355	229533.I1RNM4	pp	229533.I1S272
13356	229533.I1RNM4	pp	229533.I1RQ75
13357	229533.I1RNM4	pp	229533.Q4HZE0
13358	229533.I1RNM4	pp	229533.I1RZM0
13359	229533.I1RNM4	pp	229533.V6RPQ8

13360 229533.I1S286 pp 229533.V6QX21
13361 229533.I1S286 pp 229533.I1S4Z7
13362 229533.I1S286 pp 229533.V6QUV7
13363 229533.I1S286 pp 229533.I1S2E9
13364 229533.I1S286 pp 229533.I1S2K7
13365 229533.A0A0E0RW18 pp 229533.V6RG22
13366 229533.A0A0E0RW18 pp 229533.I1RXB6
13367 229533.A0A0E0RW18 pp 229533.I1RW69
13368 229533.A0A0E0RW18 pp 229533.I1RXV3
13369 229533.A0A0E0RW18 pp 229533.I1S3G4
13370 229533.A0A0E0RW18 pp 229533.A0A1C3YKL8
13371 229533.A0A0E0RW18 pp 229533.V6RPN0
13372 229533.A0A0E0RW18 pp 229533.A0A1C3YJH4
13373 229533.A0A0E0RW18 pp 229533.A0A1C3YMS7
13374 229533.A0A0E0RW18 pp 229533.I1RPK0
13375 229533.A0A0E0RW18 pp 229533.I1S1K6
13376 229533.A0A0E0RW18 pp 229533.I1RXX1
13377 229533.A0A0E0RW18 pp 229533.I1RY35
13378 229533.A0A0E0RW18 pp 229533.I1S5R5
13379 229533.A0A0E0RW18 pp 229533.I1RY11
13380 229533.A0A0E0RW18 pp 229533.Q00909
13381 229533.A0A0E0RW18 pp 229533.A0A1C3YMK0
13382 229533.A0A0E0RW18 pp 229533.A0A0E0SMW1
13383 229533.A0A0E0RW18 pp 229533.I1RZ92
13384 229533.A0A0E0RW18 pp 229533.I1S130
13385 229533.A0A0E0RW18 pp 229533.A0A0E0SP71
13386 229533.A0A0E0RW18 pp 229533.I1RD42
13387 229533.A0A0E0RW18 pp 229533.I1RDU5
13388 229533.A0A0E0RW18 pp 229533.V6RH92
13389 229533.A0A0E0RW18 pp 229533.I1RL16
13390 229533.A0A0E0RW18 pp 229533.I1R9Y2
13391 229533.A0A0E0RW18 pp 229533.I1S1W8
13392 229533.A0A0E0RW18 pp 229533.I1RCE6
13393 229533.A0A0E0RW18 pp 229533.I1RBT7
13394 229533.A0A0E0RW18 pp 229533.I1RPE5
13395 229533.I1RBF2 pp 229533.I1RJZ6
13396 229533.I1RBF2 pp 229533.I1RJS5
13397 229533.I1RBF2 pp 229533.I1S2B6
13398 229533.I1RBF2 pp 229533.I1RYW8
13399 229533.I1RBF2 pp 229533.I1S1V4
13400 229533.I1RBF2 pp 229533.I1RJS4
13401 229533.I1RBF2 pp 229533.I1RQH7
13402 229533.I1RBF2 pp 229533.I1RX87
13403 229533.I1RBF2 pp 229533.I1RMF2
13404 229533.I1RBF2 pp 229533.I1RG99
13405 229533.I1RBF2 pp 229533.I1RMJ1
13406 229533.I1RBF2 pp 229533.I1RNH7
13407 229533.I1RBF2 pp 229533.I1RBF3
13408 229533.I1RBF2 pp 229533.I1RND7
13409 229533.I1RBF2 pp 229533.I1RZE1
13410 229533.I1RBF2 pp 229533.I1RRX6
13411 229533.I1RBF2 pp 229533.I1S287
13412 229533.I1RBF2 pp 229533.I1RW76
13413 229533.I1RBF2 pp 229533.I1RFV4
13414 229533.I1RBF2 pp 229533.I1RD63
13415 229533.I1RBF2 pp 229533.I1RCH2
13416 229533.I1RBF2 pp 229533.I1RMN3
13417 229533.I1RBF2 pp 229533.I1RW58
13418 229533.I1RBF2 pp 229533.Q4HYZ2
13419 229533.I1RBF2 pp 229533.I1RFK0
13420 229533.I1RBF2 pp 229533.I1RQF7
13421 229533.I1RBF2 pp 229533.I1SAM5
13422 229533.I1RBF2 pp 229533.I1S183
13423 229533.I1RBF2 pp 229533.Q4ILI9
13424 229533.I1RBF2 pp 229533.I1RE18
13425 229533.I1RBF2 pp 229533.I1RS34
13426 229533.I1RBF2 pp 229533.Q4IN63
13427 229533.I1RBF2 pp 229533.I1RPG2
13428 229533.I1RBF2 pp 229533.I1RZK3
13429 229533.I1RBF2 pp 229533.I1S5B5
13430 229533.I1RBF2 pp 229533.I1RWZ9
13431 229533.I1RBF2 pp 229533.I1RWM7
13432 229533.I1RBF2 pp 229533.I1RCL9

229533.I1RBF2 pp 229533.I1RQF9
229533.I1RBF2 pp 229533.I1S830
229533.I1RBF2 pp 229533.I1RE03
229533.I1RBF2 pp 229533.I1RCQ0
229533.I1RBF2 pp 229533.I1RUN9
229533.I1RBF2 pp 229533.I1RCE7
229533.A0A1C3YKA6 pp 229533.I1RD70
229533.A0A1C3YKA6 pp 229533.V6REE9
229533.A0A1C3YKA6 pp 229533.I1RAL2
229533.A0A1C3YKA6 pp 229533.I1RBL4
229533.A0A1C3YKA6 pp 229533.I1RSK0
229533.A0A1C3YKA6 pp 229533.I1RCD4
229533.A0A1C3YKA6 pp 229533.I1RN31
229533.A0A1C3YKA6 pp 229533.I1RTE6
229533.A0A1C3YKA6 pp 229533.I1RU61
229533.A0A1C3YKA6 pp 229533.I1S604
229533.A0A1C3YKA6 pp 229533.I1SAP6
229533.A0A1C3YKA6 pp 229533.I1RF70
229533.A0A1C3YKA6 pp 229533.I1RW16
229533.A0A1C3YKA6 pp 229533.I1RCY2
229533.A0A1C3YKA6 pp 229533.I1RLX6
229533.A0A1C3YKA6 pp 229533.I1RQ67
229533.A0A1C3YKA6 pp 229533.I1RDB1
229533.A0A1C3YKA6 pp 229533.I1RKQ0
229533.A0A1C3YKA6 pp 229533.I1RXI6
229533.A0A1C3YKA6 pp 229533.I1RPZ0
229533.A0A1C3YKA6 pp 229533.I1RMK8
229533.A0A1C3YKA6 pp 229533.A0A1C3YLH6
229533.A0A1C3YKA6 pp 229533.I1RCA9
229533.A0A1C3YKA6 pp 229533.I1RD19
229533.A0A1C3YKA6 pp 229533.V6RKY7
229533.I1RUP0 pp 229533.I1RV63
229533.I1RUP0 pp 229533.I1S286
229533.I1RUP0 pp 229533.I1S272
229533.I1RUP0 pp 229533.Q4IEA7
229533.I1RUP0 pp 229533.V6QUV7
229533.I1RUP0 pp 229533.I1RWW1
229533.I1RUP0 pp 229533.I1RZM0
229533.I1RUP0 pp 229533.I1RYL5
229533.I1RUP0 pp 229533.I1RWE4
229533.A0A1C3YID3 pp 229533.I1RWZ9
229533.A0A1C3YID3 pp 229533.A0A1C3YN81
229533.A0A1C3YID3 pp 229533.I1RAH7
229533.A0A1C3YID3 pp 229533.I1RZK3
229533.A0A1C3YID3 pp 229533.A0A1C3YLS7
229533.A0A1C3YID3 pp 229533.I1RFV4
229533.A0A1C3YID3 pp 229533.I1RCH2
229533.A0A1C3YID3 pp 229533.I1RMJ1
229533.A0A1C3YID3 pp 229533.I1RND7
229533.A0A1C3YID3 pp 229533.A0A1C3YIH8
229533.A0A1C3YID3 pp 229533.I1SAM5
229533.A0A1C3YID3 pp 229533.I1RJZ6
229533.A0A1C3YID3 pp 229533.I1RFK0
229533.A0A1C3YID3 pp 229533.I1RMF2
229533.A0A1C3YID3 pp 229533.I1RNH7
229533.A0A1C3YID3 pp 229533.I1RCQ0
229533.A0A1C3YID3 pp 229533.I1RX87
229533.A0A1C3YID3 pp 229533.I1S830
229533.A0A1C3YID3 pp 229533.I1RRY9
229533.A0A1C3YID3 pp 229533.I1RCL9
229533.A0A1C3YID3 pp 229533.I1RQH7
229533.A0A1C3YID3 pp 229533.I1RQP7
229533.A0A1C3YID3 pp 229533.Q4IN63
229533.A0A1C3YID3 pp 229533.I1RJS5
229533.A0A1C3YID3 pp 229533.I1S287
229533.A0A1C3YID3 pp 229533.I1RBF2
229533.A0A1C3YID3 pp 229533.I1S1V4
229533.A0A1C3YID3 pp 229533.A0A1C3YL89
229533.A0A1C3YID3 pp 229533.I1RJS4
229533.A0A1C3YID3 pp 229533.I1S5B5
229533.A0A1C3YID3 pp 229533.I1RG99
229533.A0A1C3YID3 pp 229533.I1RW58
229533.A0A1C3YID3 pp 229533.I1RW76

13506 229533.A0A1C3YID3 pp 229533.A0A1C3YKU7
13507 229533.A0A1C3YID3 pp 229533.I1RE18
13508 229533.A0A1C3YID3 pp 229533.Q4ILI9
13509 229533.A0A1C3YID3 pp 229533.I1RPG2
13510 229533.A0A1C3YID3 pp 229533.I1RQF7
13511 229533.I1RKJ5 pp 229533.I1RXC2
13512 229533.I1RKJ5 pp 229533.I1RRI1
13513 229533.I1RKJ5 pp 229533.I1S5R1
13514 229533.I1RKJ5 pp 229533.I1RXC4
13515 229533.I1RKJ5 pp 229533.I1RPE0
13516 229533.I1RKJ5 pp 229533.I1RR24
13517 229533.I1RKJ5 pp 229533.I1RSF1
13518 229533.I1RKJ5 pp 229533.I1S1W9
13519 229533.I1RKJ5 pp 229533.I1RZV3
13520 229533.I1RKJ5 pp 229533.I1RPU2
13521 229533.I1RKJ5 pp 229533.I1RSL5
13522 229533.I1RKJ5 pp 229533.I1RTA1
13523 229533.I1RKJ5 pp 229533.I1RUG3
13524 229533.I1RKJ5 pp 229533.I1RQ85
13525 229533.I1RKJ5 pp 229533.I1RYI5
13526 229533.I1RKJ5 pp 229533.I1RZM8
13527 229533.I1RKJ5 pp 229533.I1RVZ2
13528 229533.I1RKJ5 pp 229533.I1S4S7
13529 229533.I1RKJ5 pp 229533.V6RMW6
13530 229533.I1RKJ5 pp 229533.V6RXB3
13531 229533.I1RKJ5 pp 229533.I1RXD6
13532 229533.I1RKJ5 pp 229533.I1RRM3
13533 229533.I1RKJ5 pp 229533.I1RSA5
13534 229533.I1RKJ5 pp 229533.I1S821
13535 229533.I1RKJ5 pp 229533.I1S0W4
13536 229533.I1RKJ5 pp 229533.I1RTD5
13537 229533.I1RKJ5 pp 229533.I1RWR7
13538 229533.I1RKJ5 pp 229533.I1RQE7
13539 229533.I1RKJ5 pp 229533.I1RT40
13540 229533.I1RKJ5 pp 229533.I1S5R6
13541 229533.I1RKJ5 pp 229533.I1RSZ7
13542 229533.I1RKJ5 pp 229533.Q4IJK2
13543 229533.I1RKJ5 pp 229533.I1RXD7
13544 229533.I1RKJ5 pp 229533.I1RVX0
13545 229533.I1RKJ5 pp 229533.I1S2B8
13546 229533.I1RKJ5 pp 229533.I1RZK5
13547 229533.I1RKJ5 pp 229533.I1RZ39
13548 229533.I1RKJ5 pp 229533.I1RQ33
13549 229533.I1RRI4 pp 229533.V6RR76
13550 229533.I1RRI4 pp 229533.I1RXT7
13551 229533.I1RRI4 pp 229533.I1RW84
13552 229533.I1RRI4 pp 229533.V6RXW9
13553 229533.I1RRI4 pp 229533.I1RTE0
13554 229533.I1RRI4 pp 229533.I1S0N8
13555 229533.I1RL16 pp 229533.V6RG22
13556 229533.I1RL16 pp 229533.I1S2B0
13557 229533.I1RL16 pp 229533.V6RH92
13558 229533.I1RL16 pp 229533.I1RPR9
13559 229533.I1RL16 pp 229533.V6RPN0
13560 229533.I1RL16 pp 229533.V6R3J1
13561 229533.I1RL16 pp 229533.I1RPM6
13562 229533.I1RL16 pp 229533.I1RW01
13563 229533.I1RL16 pp 229533.I1RMM8
13564 229533.I1RL16 pp 229533.I1S5R5
13565 229533.I1RL16 pp 229533.I1RZ92
13566 229533.I1RL16 pp 229533.I1RPZ1
13567 229533.I1RL16 pp 229533.I1RRK9
13568 229533.Q4IEA7 pp 229533.V6QUV7
13569 229533.Q4IEA7 pp 229533.V6QX21
13570 229533.A0A098DEF2 pp 229533.A0A0E0SDU6
13571 229533.A0A098DEF2 pp 229533.I1S1V4
13572 229533.A0A098DEF2 pp 229533.A0A1C3YKU7
13573 229533.A0A098DEF2 pp 229533.I1RY87
13574 229533.A0A098DEF2 pp 229533.I1S286
13575 229533.A0A098DEF2 pp 229533.I1RBF2
13576 229533.A0A098DEF2 pp 229533.A0A1C3YID3
13577 229533.A0A098DEF2 pp 229533.Q4IEA7
13578 229533.I1RRN2 pp 229533.I1S0L4

13579 229533.I1RRN2 pp 229533.I1S2H4
13580 229533.I1RRN2 pp 229533.I1RZ39
13581 229533.I1RRN2 pp 229533.I1RZZ9
13582 229533.I1RRN2 pp 229533.I1RXC2
13583 229533.I1RRN2 pp 229533.I1RSQ4
13584 229533.I1RRN2 pp 229533.I1S5R1
13585 229533.I1RRN2 pp 229533.I1RWI5
13586 229533.I1RRN2 pp 229533.I1RRT8
13587 229533.I1RRN2 pp 229533.I1S4S4
13588 229533.I1RRN2 pp 229533.I1S277
13589 229533.I1RRN2 pp 229533.I1S2B8
13590 229533.I1RRN2 pp 229533.V6R3J1
13591 229533.I1RRN2 pp 229533.I1RX46
13592 229533.I1RRN2 pp 229533.I1RSZ7
13593 229533.I1RRN2 pp 229533.I1S9Y1
13594 229533.I1RRN2 pp 229533.I1S438
13595 229533.I1RRN2 pp 229533.V6RRH2
13596 229533.I1RRN2 pp 229533.V6RUT5
13597 229533.I1RRN2 pp 229533.I1RVX0
13598 229533.I1RRN2 pp 229533.V6RTJ8
13599 229533.I1RRN2 pp 229533.I1RXD7
13600 229533.I1RRN2 pp 229533.I1RTD5
13601 229533.I1RRN2 pp 229533.I1RT03
13602 229533.I1RRN2 pp 229533.I1RTA1
13603 229533.I1RRN2 pp 229533.I1RT40
13604 229533.I1RRN2 pp 229533.I1RVZ2
13605 229533.I1RRN2 pp 229533.I1RYR0
13606 229533.I1RRN2 pp 229533.I1RXD6
13607 229533.I1RRN2 pp 229533.I1RXR1
13608 229533.I1RRN2 pp 229533.I1RRY9
13609 229533.I1RRN2 pp 229533.I1RWR7
13610 229533.I1RRN2 pp 229533.Q4IJK2
13611 229533.I1RRN2 pp 229533.I1S5B0
13612 229533.I1RRN2 pp 229533.I1RYI5
13613 229533.I1RRN2 pp 229533.I1RRX5
13614 229533.I1RRN2 pp 229533.I1RXU9
13615 229533.I1RRN2 pp 229533.I1RSQ7
13616 229533.I1RRN2 pp 229533.I1RZK5
13617 229533.I1RRN2 pp 229533.I1S4S7
13618 229533.I1RRN2 pp 229533.I1S5R6
13619 229533.I1RRN2 pp 229533.V6QUE6
13620 229533.I1RRN2 pp 229533.I1S887
13621 229533.I1RRN2 pp 229533.V6RMW6
13622 229533.I1RRN2 pp 229533.I1RZM8
13623 229533.I1RRN2 pp 229533.I1S1C7
13624 229533.I1RRN2 pp 229533.I1S1W3
13625 229533.I1RRN2 pp 229533.I1RYW3
13626 229533.I1RRN2 pp 229533.I1RZV3
13627 229533.I1RRN2 pp 229533.I1S1W9
13628 229533.I1RRN2 pp 229533.V6R042
13629 229533.I1RRN2 pp 229533.Q4I624
13630 229533.I1RRN2 pp 229533.I1RSQ3
13631 229533.I1RRN2 pp 229533.I1S437
13632 229533.I1RRN2 pp 229533.I1S0J1
13633 229533.I1RRN2 pp 229533.V6RCZ8
13634 229533.I1RRN2 pp 229533.I1RS18
13635 229533.I1RRN2 pp 229533.I1RXC4
13636 229533.I1RRN2 pp 229533.I1RUG3
13637 229533.I1RRN2 pp 229533.I1RXF0
13638 229533.I1RMA1 pp 229533.I1S208
13639 229533.I1RMA1 pp 229533.I1S0P4
13640 229533.I1RMA1 pp 229533.I1RMA2
13641 229533.I1RDX8 pp 229533.I1RVQ2
13642 229533.I1RDX8 pp 229533.Q4I0J6
13643 229533.I1RRF0 pp 229533.I1RVY0
13644 229533.I1RRF0 pp 229533.Q4IAK7
13645 229533.I1RRF0 pp 229533.Q4IL82
13646 229533.I1RRF0 pp 229533.Q4IPI4
13647 229533.I1RRF0 pp 229533.I1S7W2
13648 229533.I1RRF0 pp 229533.I1RRM8
13649 229533.I1RRF0 pp 229533.I1RX68
13650 229533.I1RRF0 pp 229533.I1S559
13651 229533.I1RRF0 pp 229533.I1RXS3

229533.I1RRF0	pp	229533.I1RUF8
229533.I1RRF0	pp	229533.I1RZV1
229533.I1RRF0	pp	229533.I1RX00
229533.I1RRF0	pp	229533.I1RYE8
229533.I1RRF0	pp	229533.Q4I7S1
229533.I1RRF0	pp	229533.I1S8E2
229533.I1RRF0	pp	229533.I1S086
229533.I1RRF0	pp	229533.I1RST4
229533.I1RRF0	pp	229533.I1RX69
229533.I1RRF0	pp	229533.I1S4P6
229533.I1RRF0	pp	229533.Q4HY90
229533.I1RRF0	pp	229533.I1RZL3
229533.I1RRF0	pp	229533.I1S0S8
229533.I1RRF0	pp	229533.I1RTG5
229533.I1RRF0	pp	229533.Q5I2J3
229533.I1RRF0	pp	229533.I1S1V2
229533.I1RRF0	pp	229533.I1RRN5
229533.I1RRF0	pp	229533.I1RT41
229533.I1RRF0	pp	229533.I1S270
229533.I1RRF0	pp	229533.Q4INI2
229533.I1RRF0	pp	229533.I1RW71
229533.I1RRF0	pp	229533.I1RTE0
229533.I1RRF0	pp	229533.Q4ICA8
229533.I1RRF0	pp	229533.Q4IQX3
229533.I1RRF0	pp	229533.I1S0Z3
229533.I1RRF0	pp	229533.Q4IJM4
229533.I1RRF0	pp	229533.Q4I948
229533.I1RRF0	pp	229533.I1RWI6
229533.I1RRF0	pp	229533.I1RT45
229533.I1RRF0	pp	229533.I1RSD7
229533.I1RRF0	pp	229533.I1RYS0
229533.I1RRF0	pp	229533.I1RT65
229533.I1RYX7	pp	229533.V6R382
229533.I1RYX7	pp	229533.I1S0T4
229533.I1RYX7	pp	229533.I1S4T6
229533.A0A1C3YIW0	pp	229533.I1RQ31
229533.A0A1C3YIW0	pp	229533.I1RRI2
229533.A0A1C3YIW0	pp	229533.V6QWZ7
229533.A0A1C3YIW0	pp	229533.Q4IQK7
229533.A0A1C3YIW0	pp	229533.I1RN64
229533.A0A1C3YIW0	pp	229533.I1RC32
229533.A0A1C3YI10	pp	229533.I1RSK0
229533.A0A1C3YI10	pp	229533.I1RZ94
229533.A0A1C3YI10	pp	229533.I1R976
229533.A0A1C3YI10	pp	229533.I1RRT1
229533.A0A1C3YI10	pp	229533.I1RCA5
229533.A0A1C3YI10	pp	229533.I1RD18
229533.A0A1C3YI10	pp	229533.I1RX38
229533.A0A1C3YI10	pp	229533.I1S951
229533.A0A1C3YI10	pp	229533.I1RST8
229533.A0A1C3YI10	pp	229533.I1S382
229533.A0A1C3YI10	pp	229533.I1RNC0
229533.A0A1C3YI10	pp	229533.I1S2F2
229533.A0A1C3YI10	pp	229533.I1RFU3
229533.A0A1C3YI10	pp	229533.I1RUG1
229533.A0A1C3YI10	pp	229533.I1RBW1
229533.A0A1C3YI10	pp	229533.A0A1C3YJH8
229533.A0A1C3YI10	pp	229533.I1RN89
229533.A0A1C3YI10	pp	229533.I1S1V5
229533.A0A1C3YI10	pp	229533.A0A1C3YI40
229533.A0A1C3YI10	pp	229533.I1RC35
229533.A0A1C3YI10	pp	229533.I1S283
229533.A0A1C3YI10	pp	229533.A0A1C3YI64
229533.A0A1C3YI10	pp	229533.A0A1C3YLR6
229533.A0A1C3YI10	pp	229533.I1RAN3
229533.A0A1C3YI10	pp	229533.I1RF70
229533.A0A1C3YI10	pp	229533.I1RN31
229533.A0A1C3YI10	pp	229533.I1RQ13
229533.A0A1C3YI10	pp	229533.I1RFR6
229533.A0A1C3YI10	pp	229533.I1RSJ4
229533.A0A1C3YI10	pp	229533.I1RFX8
229533.A0A1C3YI10	pp	229533.I1RKK7
229533.A0A1C3YI10	pp	229533.I1RDG8

229533.A0A1C3YI10 pp 229533.I1RBV5
229533.A0A1C3YI10 pp 229533.V6RLW4
229533.A0A1C3YI10 pp 229533.I1RLU1
229533.A0A1C3YI10 pp 229533.A0A1C3YJL7
229533.A0A1C3YI10 pp 229533.Q4IP51
229533.A0A1C3YI10 pp 229533.I1RKI2
229533.A0A1C3YI10 pp 229533.I1RCY2
229533.A0A1C3YI10 pp 229533.A0A1C3YM67
229533.A0A1C3YI10 pp 229533.I1RYX9
229533.A0A1C3YI10 pp 229533.I1S224
229533.A0A1C3YI10 pp 229533.I1S2E9
229533.A0A1C3YI10 pp 229533.A0A1C3YKJ6
229533.A0A1C3YI10 pp 229533.I1RYH9
229533.A0A1C3YI10 pp 229533.I1RFL7
229533.A0A1C3YI10 pp 229533.I1RKQ0
229533.A0A1C3YI10 pp 229533.I1RLX6
229533.A0A1C3YI10 pp 229533.I1RB00
229533.A0A1C3YI10 pp 229533.I1RAH4
229533.A0A1C3YI10 pp 229533.I1RBW0
229533.A0A1C3YI10 pp 229533.Q4IEA5
229533.A0A1C3YI10 pp 229533.I1RXG6
229533.A0A1C3YI10 pp 229533.A0A1C3YKW5
229533.A0A1C3YI10 pp 229533.I1RSI4
229533.A0A1C3YI10 pp 229533.I1RMB7
229533.A0A1C3YI10 pp 229533.I1RSA7
229533.A0A1C3YI10 pp 229533.Q4IQM6
229533.A0A1C3YI10 pp 229533.I1RDB1
229533.A0A1C3YI10 pp 229533.I1R9Q0
229533.A0A1C3YI10 pp 229533.I1RAW7
229533.A0A1C3YI10 pp 229533.A0A1C3YJT5
229533.A0A1C3YI10 pp 229533.I1RM36
229533.A0A1C3YI10 pp 229533.A0A1C3YMJ3
229533.A0A1C3YI10 pp 229533.I1RK32
229533.A0A1C3YI10 pp 229533.I1RYR2
229533.A0A1C3YI10 pp 229533.I1RMQ6
229533.A0A1C3YI10 pp 229533.A0A1C3YL89
229533.A0A1C3YI10 pp 229533.I1REX8
229533.A0A1C3YI10 pp 229533.A0A1C3YLS3
229533.A0A1C3YI10 pp 229533.I1RYQ1
229533.A0A1C3YI10 pp 229533.I1RLE7
229533.A0A1C3YI10 pp 229533.I1RQ92
229533.A0A1C3YI10 pp 229533.I1RFZ2
229533.A0A1C3YI10 pp 229533.I1RNG4
229533.A0A1C3YI10 pp 229533.I1RXI6
229533.A0A1C3YI10 pp 229533.I1RBL4
229533.A0A1C3YI10 pp 229533.I1S0N5
229533.A0A1C3YI10 pp 229533.I1RYZ9
229533.A0A1C3YI10 pp 229533.I1S461
229533.A0A1C3YI10 pp 229533.A0A1C3YIP6
229533.A0A1C3YI10 pp 229533.I1RQ67
229533.A0A1C3YI10 pp 229533.I1RSR9
229533.A0A1C3YI10 pp 229533.I1RN46
229533.A0A1C3YI10 pp 229533.I1RPW0
229533.A0A1C3YI10 pp 229533.I1RFJ2
229533.A0A1C3YI10 pp 229533.Q4IBX0
229533.A0A1C3YI10 pp 229533.I1RE46
229533.A0A1C3YI10 pp 229533.I1RN55
229533.A0A1C3YI10 pp 229533.A0A1C3YMF9
229533.A0A1C3YI10 pp 229533.I1RG77
229533.A0A1C3YI10 pp 229533.I1RMS4
229533.A0A1C3YI10 pp 229533.I1RB41
229533.A0A1C3YI10 pp 229533.A0A1C3YJN7
229533.A0A1C3YI10 pp 229533.I1RRN5
229533.A0A1C3YI10 pp 229533.A0A1C3YJ79
229533.A0A1C3YI10 pp 229533.I1RX64
229533.A0A1C3YI10 pp 229533.I1RUG2
229533.A0A1C3YI10 pp 229533.I1RW16
229533.A0A1C3YI10 pp 229533.I1RCA9
229533.A0A1C3YI10 pp 229533.I1RI34
229533.A0A1C3YI10 pp 229533.I1RBN9
229533.A0A1C3YI10 pp 229533.I1RAL2
229533.A0A1C3YI10 pp 229533.V6R382
229533.A0A1C3YI10 pp 229533.I1RZP8

13798 229533.A0A1C3YI10 pp 229533.I1RCL7
13799 229533.A0A1C3YI10 pp 229533.I1RD19
13800 229533.A0A1C3YI10 pp 229533.I1RQA3
13801 229533.A0A1C3YI10 pp 229533.I1S1D3
13802 229533.A0A1C3YI10 pp 229533.I1RSP7
13803 229533.A0A1C3YI10 pp 229533.I1RCM6
13804 229533.A0A1C3YI10 pp 229533.I1RUQ5
13805 229533.A0A1C3YI10 pp 229533.I1RA10
13806 229533.A0A1C3YI10 pp 229533.I1RQJ4
13807 229533.A0A1C3YI10 pp 229533.I1RMQ7
13808 229533.A0A1C3YI10 pp 229533.I1RRW0
13809 229533.A0A1C3YI10 pp 229533.I1RBI1
13810 229533.A0A1C3YI10 pp 229533.I1RAM7
13811 229533.A0A1C3YI10 pp 229533.I1RN57
13812 229533.A0A1C3YI10 pp 229533.I1S9J6
13813 229533.A0A1C3YI10 pp 229533.I1RU61
13814 229533.A0A1C3YI10 pp 229533.I1RD70
13815 229533.A0A1C3YI10 pp 229533.I1RPZ0
13816 229533.A0A1C3YI10 pp 229533.V6RGI1
13817 229533.A0A1C3YI10 pp 229533.I1RWT6
13818 229533.A0A1C3YI10 pp 229533.I1RRP7
13819 229533.A0A1C3YI10 pp 229533.I1RDX0
13820 229533.A0A1C3YI10 pp 229533.I1RY44
13821 229533.A0A1C3YI10 pp 229533.I1S9S6
13822 229533.A0A1C3YI10 pp 229533.I1RA12
13823 229533.A0A1C3YI10 pp 229533.I1RPU4
13824 229533.A0A1C3YI10 pp 229533.V6RVG3
13825 229533.A0A1C3YI10 pp 229533.I1RC17
13826 229533.A0A1C3YI10 pp 229533.I1RC54
13827 229533.A0A1C3YI10 pp 229533.Q4I0J6
13828 229533.A0A1C3YI10 pp 229533.I1S209
13829 229533.A0A1C3YI10 pp 229533.A0A1C3YIW6
13830 229533.A0A1C3YI10 pp 229533.Q4I7X1
13831 229533.A0A1C3YI10 pp 229533.I1RBM3
13832 229533.A0A1C3YI10 pp 229533.A0A1C3YL34
13833 229533.A0A1C3YI10 pp 229533.I1S0V7
13834 229533.A0A1C3YI10 pp 229533.A0A1C3YJ12
13835 229533.A0A1C3YI10 pp 229533.A0A1C3YI28
13836 229533.A0A1C3YI10 pp 229533.A0A1C3YK37
13837 229533.A0A1C3YI10 pp 229533.I1RMX3
13838 229533.A0A1C3YI10 pp 229533.I1S604
13839 229533.A0A1C3YI10 pp 229533.I1RRS7
13840 229533.A0A1C3YI10 pp 229533.I1RY73
13841 229533.A0A1C3YI10 pp 229533.I1RSC7
13842 229533.I1R9Q0 pp 229533.V6QV38
13843 229533.I1R9Q0 pp 229533.I1RJL2
13844 229533.I1R9Q0 pp 229533.I1RXE5
13845 229533.I1R9Q0 pp 229533.I1RZB5
13846 229533.I1R9Q0 pp 229533.I1RKE9
13847 229533.I1R9Q0 pp 229533.I1RWX8
13848 229533.I1R9Q0 pp 229533.V6R227
13849 229533.I1R9Q0 pp 229533.I1REN3
13850 229533.I1R9Q0 pp 229533.I1RAE0
13851 229533.I1R9Q0 pp 229533.I1RCM6
13852 229533.I1R9Q0 pp 229533.I1RKR6
13853 229533.I1R9Q0 pp 229533.I1RCK7
13854 229533.I1R9Q0 pp 229533.I1RWD4
13855 229533.I1R9Q0 pp 229533.I1RDC9
13856 229533.I1R9Q0 pp 229533.I1S5Z2
13857 229533.I1R9Q0 pp 229533.I1RRT0
13858 229533.I1R9Q0 pp 229533.I1RC15
13859 229533.I1R9Q0 pp 229533.I1RAR2
13860 229533.I1R9Q0 pp 229533.I1RQE9
13861 229533.I1R9Q0 pp 229533.I1RY40
13862 229533.I1R9Q0 pp 229533.I1RGE1
13863 229533.I1R9Q0 pp 229533.I1RPU4
13864 229533.I1R9Q0 pp 229533.I1RN31
13865 229533.I1R9Q0 pp 229533.I1RTC2
13866 229533.I1R9Q0 pp 229533.I1RMA5
13867 229533.I1R9Q0 pp 229533.I1RB36
13868 229533.I1R9Q0 pp 229533.I1RWU3
13869 229533.I1R9Q0 pp 229533.I1RRG3
13870 229533.I1R9Q0 pp 229533.I1RUQ6

13871	229533.I1R9Q0	pp	229533.I1RBZ7
13872	229533.I1R9Q0	pp	229533.I1RKB1
13873	229533.I1R9Q0	pp	229533.I1RQ80
13874	229533.I1R9Q0	pp	229533.I1RW16
13875	229533.I1R9Q0	pp	229533.I1RPW0
13876	229533.I1R9Q0	pp	229533.I1RK71
13877	229533.I1R9Q0	pp	229533.I1RLX6
13878	229533.I1R9Q0	pp	229533.I1RP96
13879	229533.I1R9Q0	pp	229533.I1RPM7
13880	229533.I1R9Q0	pp	229533.I1RG11
13881	229533.I1R9Q0	pp	229533.I1RA56
13882	229533.I1R9Q0	pp	229533.I1RML9
13883	229533.I1R9Q0	pp	229533.Q4IPC8
13884	229533.I1R9Q0	pp	229533.I1RR25
13885	229533.I1R9Q0	pp	229533.I1S276
13886	229533.I1R9Q0	pp	229533.I1RD70
13887	229533.I1R9Q0	pp	229533.I1RK79
13888	229533.I1R9Q0	pp	229533.I1RXU0
13889	229533.I1R9Q0	pp	229533.I1RCB7
13890	229533.I1R9Q0	pp	229533.I1RMV1
13891	229533.I1R9Q0	pp	229533.I1RLX4
13892	229533.I1R9Q0	pp	229533.I1RVL8
13893	229533.I1R9Q0	pp	229533.I1RWF3
13894	229533.I1R9Q0	pp	229533.I1RBJ1
13895	229533.I1R9Q0	pp	229533.I1RVY0
13896	229533.I1R9Q0	pp	229533.I1RA84
13897	229533.I1R9Q0	pp	229533.I1RYE2
13898	229533.I1R9Q0	pp	229533.I1RBR5
13899	229533.I1R9Q0	pp	229533.I1S3D7
13900	229533.I1R9Q0	pp	229533.I1RZM2
13901	229533.I1R9Q0	pp	229533.I1RX38
13902	229533.I1R9Q0	pp	229533.I1RPX5
13903	229533.I1R9Q0	pp	229533.I1RBL4
13904	229533.I1R9Q0	pp	229533.I1RC24
13905	229533.I1R9Q0	pp	229533.I1RQM0
13906	229533.I1R9Q0	pp	229533.I1RSZ3
13907	229533.I1R9Q0	pp	229533.I1RGW0
13908	229533.I1R9Q0	pp	229533.I1RE30
13909	229533.I1R9Q0	pp	229533.Q4IR18
13910	229533.I1R9Q0	pp	229533.I1RFZ2
13911	229533.I1R9Q0	pp	229533.I1RBV5
13912	229533.I1S1K6	pp	229533.I1S2B0
13913	229533.I1S1K6	pp	229533.V6RPN0
13914	229533.I1S1K6	pp	229533.I1S5R5
13915	229533.I1S1K6	pp	229533.V6R3J1
13916	229533.I1S1K6	pp	229533.V6RG22
13917	229533.I1S1K6	pp	229533.V6RH92
13918	229533.I1RRP7	pp	229533.I1RW16
13919	229533.I1RRP7	pp	229533.I1RRW0
13920	229533.I1RRP7	pp	229533.I1SAN9
13921	229533.I1RRP7	pp	229533.V6RVG3
13922	229533.I1RRP7	pp	229533.I1RS17
13923	229533.I1RRP7	pp	229533.I1S0D1
13924	229533.I1RRP7	pp	229533.I1S1D3
13925	229533.I1RRP7	pp	229533.V6RKY7
13926	229533.I1RRP7	pp	229533.I1RWE8
13927	229533.I1RRP7	pp	229533.I1RZE4
13928	229533.I1RRP7	pp	229533.I1RVZ7
13929	229533.I1RRP7	pp	229533.V6R057
13930	229533.I1RRP7	pp	229533.I1S1W4
13931	229533.I1RRP7	pp	229533.O74638
13932	229533.I1RRP7	pp	229533.I1S2E9
13933	229533.I1RRP7	pp	229533.I1S1V5
13934	229533.I1RRP7	pp	229533.I1RYR2
13935	229533.I1RRP7	pp	229533.I1RUN8
13936	229533.I1RRP7	pp	229533.I1RY44
13937	229533.I1RRP7	pp	229533.I1RWT6
13938	229533.I1RRP7	pp	229533.I1S4Z8
13939	229533.I1RRP7	pp	229533.I1RT75
13940	229533.I1RRP7	pp	229533.I1S0J7
13941	229533.I1RRP7	pp	229533.I1RX38
13942	229533.I1RRP7	pp	229533.I1S439
13943	229533.I1RRP7	pp	229533.I1RTD3

229533.I1RRP7	pp	229533.V6RBN2
229533.I1RRP7	pp	229533.V6R1K4
229533.I1RRP7	pp	229533.I1RW71
229533.I1RRP7	pp	229533.I1RRV4
229533.I1RRP7	pp	229533.I1S224
229533.I1RRP7	pp	229533.Q4IPN9
229533.I1RRP7	pp	229533.I1RYH9
229533.A0A0E0SMW1	pp	229533.I1S2B0
229533.A0A0E0SMW1	pp	229533.I1RRK9
229533.A0A0E0SMW1	pp	229533.I1RPR9
229533.A0A0E0SMW1	pp	229533.I1RCE6
229533.A0A0E0SMW1	pp	229533.I1R9Y2
229533.A0A0E0SMW1	pp	229533.A0A1C3YMK0
229533.A0A0E0SMW1	pp	229533.I1RA51
229533.A0A0E0SMW1	pp	229533.A0A1C3YJH4
229533.A0A0E0SMW1	pp	229533.I1RK49
229533.A0A0E0SMW1	pp	229533.I1RPM6
229533.A0A0E0SMW1	pp	229533.I1RW01
229533.A0A0E0SMW1	pp	229533.I1S5R5
229533.A0A0E0SMW1	pp	229533.A0A1C3YKL8
229533.A0A0E0SMW1	pp	229533.V6RG22
229533.A0A0E0SMW1	pp	229533.A0A1C3YMS7
229533.A0A0E0SMW1	pp	229533.I1S1K6
229533.A0A0E0SMW1	pp	229533.V6R3J1
229533.A0A0E0SMW1	pp	229533.V6RH92
229533.A0A0E0SMW1	pp	229533.I1RY11
229533.A0A0E0SMW1	pp	229533.I1RDU5
229533.A0A0E0SMW1	pp	229533.V6RPN0
229533.A0A0E0SMW1	pp	229533.I1RD42
229533.A0A0E0SMW1	pp	229533.I1RMM8
229533.A0A0E0SMW1	pp	229533.I1RZ92
229533.A0A0E0SMW1	pp	229533.I1RPZ1
229533.A0A0E0SMW1	pp	229533.V6RB81
229533.A0A0E0SMW1	pp	229533.A0A0E0SP71
229533.A0A0E0SMW1	pp	229533.I1RBT7
229533.A0A1C3YMS7	pp	229533.I1RKI1
229533.A0A1C3YMS7	pp	229533.I1RWA3
229533.A0A1C3YMS7	pp	229533.I1RBL4
229533.A0A1C3YMS7	pp	229533.I1RY92
229533.A0A1C3YMS7	pp	229533.I1REI3
229533.A0A1C3YMS7	pp	229533.I1RJG1
229533.A0A1C3YMS7	pp	229533.I1RVY0
229533.A0A1C3YMS7	pp	229533.I1RIQ7
229533.A0A1C3YMS7	pp	229533.I1RA52
229533.A0A1C3YMS7	pp	229533.I1S1K6
229533.A0A1C3YMS7	pp	229533.I1S3G4
229533.A0A1C3YMS7	pp	229533.I1RJR2
229533.A0A1C3YMS7	pp	229533.I1RZV5
229533.A0A1C3YMS7	pp	229533.I1RIZ8
229533.A0A1C3YMS7	pp	229533.I1RNT1
229533.A0A1C3YMS7	pp	229533.I1S8P5
229533.A0A1C3YMS7	pp	229533.I1RMJ3
229533.A0A1C3YMS7	pp	229533.I1RFS3
229533.A0A1C3YMS7	pp	229533.I1RY35
229533.A0A1C3YMS7	pp	229533.I1RUB4
229533.A0A1C3YMS7	pp	229533.I1RQ48
229533.A0A1C3YMS7	pp	229533.I1RH48
229533.A0A1C3YMS7	pp	229533.I1RL41
229533.A0A1C3YMS7	pp	229533.I1RRQ1
229533.A0A1C3YMS7	pp	229533.I1RN42
229533.A0A1C3YMS7	pp	229533.I1RNG4
229533.A0A1C3YMS7	pp	229533.I1S5K2
229533.A0A1C3YMS7	pp	229533.V6RR38
229533.A0A1C3YMS7	pp	229533.I1SAS4
229533.A0A1C3YMS7	pp	229533.I1REV3
229533.A0A1C3YMS7	pp	229533.I1RDP5
229533.A0A1C3YMS7	pp	229533.I1SAS3
229533.A0A1C3YMS7	pp	229533.I1R9Y2
229533.A0A1C3YMS7	pp	229533.I1RZD0
229533.A0A1C3YMS7	pp	229533.I1RUB5
229533.A0A1C3YMS7	pp	229533.I1REM2
229533.A0A1C3YMS7	pp	229533.I1RCE6
229533.A0A1C3YMS7	pp	229533.I1RFM2

14017 229533.A0A1C3YMS7 pp 229533.I1RQ76
14018 229533.A0A1C3YMS7 pp 229533.I1S6P7
14019 229533.A0A1C3YMS7 pp 229533.I1RCA0
14020 229533.A0A1C3YMS7 pp 229533.I1RN84
14021 229533.A0A1C3YMS7 pp 229533.I1S1W8
14022 229533.A0A1C3YMS7 pp 229533.I1RST4
14023 229533.A0A1C3YMS7 pp 229533.I1RXI8
14024 229533.A0A1C3YMS7 pp 229533.A0A1C3YMW5
14025 229533.A0A1C3YMS7 pp 229533.I1RBR4
14026 229533.A0A1C3YMS7 pp 229533.I1RGR2
14027 229533.A0A1C3YMS7 pp 229533.I1S724
14028 229533.A0A1C3YMS7 pp 229533.I1S7Q8
14029 229533.A0A1C3YMS7 pp 229533.Q4I073
14030 229533.A0A1C3YMS7 pp 229533.I1S1Q3
14031 229533.A0A1C3YMS7 pp 229533.I1RPE5
14032 229533.A0A1C3YMS7 pp 229533.I1S6S6
14033 229533.A0A1C3YMS7 pp 229533.I1RYT8
14034 229533.A0A1C3YMS7 pp 229533.I1RZ92
14035 229533.A0A1C3YMS7 pp 229533.I1S2M5
14036 229533.A0A1C3YMS7 pp 229533.I1RT41
14037 229533.A0A1C3YMS7 pp 229533.I1RH30
14038 229533.A0A1C3YMS7 pp 229533.Q5I2J3
14039 229533.A0A1C3YMS7 pp 229533.I1RCC2
14040 229533.A0A1C3YMS7 pp 229533.I1S2C2
14041 229533.A0A1C3YMS7 pp 229533.I1RCM8
14042 229533.A0A1C3YMS7 pp 229533.I1RMS5
14043 229533.A0A1C3YMS7 pp 229533.I1RIW5
14044 229533.A0A1C3YMS7 pp 229533.I1RGK2
14045 229533.A0A1C3YMS7 pp 229533.V6RG22
14046 229533.A0A1C3YMS7 pp 229533.I1RH32
14047 229533.A0A1C3YMS7 pp 229533.I1RT65
14048 229533.A0A1C3YMS7 pp 229533.I1RL16
14049 229533.A0A1C3YMS7 pp 229533.I1S0P0
14050 229533.A0A1C3YMS7 pp 229533.I1RFY1
14051 229533.A0A1C3YMS7 pp 229533.I1S130
14052 229533.A0A1C3YMS7 pp 229533.I1RA72
14053 229533.A0A1C3YMS7 pp 229533.I1RNX0
14054 229533.A0A1C3YMS7 pp 229533.I1RT23
14055 229533.A0A1C3YMS7 pp 229533.I1RUB1
14056 229533.A0A1C3YMS7 pp 229533.I1S0Z3
14057 229533.A0A1C3YMS7 pp 229533.I1RBL7
14058 229533.A0A1C3YMS7 pp 229533.I1RXX1
14059 229533.A0A1C3YMS7 pp 229533.I1RY65
14060 229533.A0A1C3YMS7 pp 229533.I1REL9
14061 229533.A0A1C3YMS7 pp 229533.I1S364
14062 229533.A0A1C3YMS7 pp 229533.I1RS29
14063 229533.A0A1C3YMS7 pp 229533.V6RXW9
14064 229533.A0A1C3YMS7 pp 229533.I1S8P4
14065 229533.A0A1C3YMS7 pp 229533.I1S5T5
14066 229533.A0A1C3YMS7 pp 229533.V6RJP8
14067 229533.A0A1C3YL96 pp 229533.A0A1C3YM29
14068 229533.A0A1C3YL96 pp 229533.I1RMW4
14069 229533.A0A1C3YL96 pp 229533.I1S0J7
14070 229533.A0A1C3YL96 pp 229533.I1RWT6
14071 229533.A0A1C3YL96 pp 229533.I1RY44
14072 229533.A0A1C3YL96 pp 229533.I1RM09
14073 229533.A0A1C3YL96 pp 229533.I1S2E9
14074 229533.A0A1C3YL96 pp 229533.I1RQA3
14075 229533.A0A1C3YL96 pp 229533.I1RAB7
14076 229533.I1RCN4 pp 229533.I1RT91
14077 229533.I1RCN4 pp 229533.V6RQR4
14078 229533.I1RCN4 pp 229533.I1RN64
14079 229533.I1RCN4 pp 229533.I1RYX7
14080 229533.I1RCN4 pp 229533.I1RQE7
14081 229533.I1RCN4 pp 229533.I1S4T6
14082 229533.I1S1D3 pp 229533.I1S2F2
14083 229533.I1S1D3 pp 229533.V6RGI1
14084 229533.I1S1D3 pp 229533.V6R382
14085 229533.I1S1D3 pp 229533.I1S2M1
14086 229533.I1S1D3 pp 229533.I1S1V5
14087 229533.I1S1D3 pp 229533.V6RLW4
14088 229533.I1S1D3 pp 229533.V6RKY7
14089 229533.I1S1D3 pp 229533.Q4IBX0

14090 229533.I1S1D3 pp 229533.V6R3J1
14091 229533.I1S1D3 pp 229533.I1S1W4
14092 229533.I1S1D3 pp 229533.I1S439
14093 229533.I1S1D3 pp 229533.V6QV09
14094 229533.I1S1D3 pp 229533.V6RVG3
14095 229533.I1S1D3 pp 229533.Q4I0J6
14096 229533.I1RBY8 pp 229533.Q4IJM4
14097 229533.I1RBY8 pp 229533.I1RD10
14098 229533.I1RBY8 pp 229533.I1RYH9
14099 229533.I1RBY8 pp 229533.I1S439
14100 229533.I1RBY8 pp 229533.I1RUQ5
14101 229533.I1RBY8 pp 229533.V6RKY7
14102 229533.I1RBY8 pp 229533.I1RC95
14103 229533.I1RB26 pp 229533.I1S306
14104 229533.I1RB26 pp 229533.I1RB27
14105 229533.I1RB26 pp 229533.I1S821
14106 229533.I1RB26 pp 229533.V6RCZ8
14107 229533.I1RB26 pp 229533.I1RFK0
14108 229533.I1RB26 pp 229533.I1RXC4
14109 229533.I1RB26 pp 229533.I1RZM8
14110 229533.I1RB26 pp 229533.I1RQD2
14111 229533.I1RB26 pp 229533.V6R9Z0
14112 229533.I1RB26 pp 229533.I1S4S7
14113 229533.I1RB26 pp 229533.I1RPS5
14114 229533.I1RB26 pp 229533.I1REG5
14115 229533.I1RB26 pp 229533.I1RX95
14116 229533.I1RB26 pp 229533.I1RQ85
14117 229533.I1RB26 pp 229533.I1RD52
14118 229533.I1RB26 pp 229533.I1S1B2
14119 229533.I1RB26 pp 229533.I1RRV3
14120 229533.I1RB26 pp 229533.I1RXD6
14121 229533.I1RB26 pp 229533.Q4IJK2
14122 229533.I1RB26 pp 229533.I1RGB2
14123 229533.I1RB26 pp 229533.I1RWR7
14124 229533.I1RB26 pp 229533.I1RZK5
14125 229533.I1RB26 pp 229533.I1RSF1
14126 229533.I1RB26 pp 229533.I1RXD7
14127 229533.I1RB26 pp 229533.I1RZV3
14128 229533.I1RB26 pp 229533.I1RTB3
14129 229533.I1RB26 pp 229533.I1RPY3
14130 229533.I1RB26 pp 229533.I1RE89
14131 229533.I1RB26 pp 229533.I1RNB2
14132 229533.I1RB26 pp 229533.I1RVQ3
14133 229533.I1RB26 pp 229533.I1RBR2
14134 229533.I1RB26 pp 229533.I1S1W9
14135 229533.I1RB26 pp 229533.I1RYI5
14136 229533.I1RB26 pp 229533.I1RSC9
14137 229533.I1RB26 pp 229533.I1RVZ2
14138 229533.I1RB26 pp 229533.I1RKJ4
14139 229533.I1RB26 pp 229533.I1RRM3
14140 229533.I1RB26 pp 229533.I1RCR8
14141 229533.I1RB26 pp 229533.I1RQ70
14142 229533.I1RB26 pp 229533.I1RJU3
14143 229533.I1RB26 pp 229533.I1RTA1
14144 229533.I1RB26 pp 229533.I1RJN9
14145 229533.I1RB26 pp 229533.I1RR24
14146 229533.I1RB26 pp 229533.I1RPU2
14147 229533.I1RB26 pp 229533.I1RUN9
14148 229533.I1RB26 pp 229533.I1S5R6
14149 229533.I1RB26 pp 229533.I1S7X9
14150 229533.I1RB26 pp 229533.V6RB81
14151 229533.I1RB26 pp 229533.I1RSA5
14152 229533.I1RB26 pp 229533.I1RND7
14153 229533.I1RB26 pp 229533.I1RQ33
14154 229533.I1RB26 pp 229533.I1RUF7
14155 229533.I1RB26 pp 229533.I1RCH5
14156 229533.I1RB26 pp 229533.I1RCH2
14157 229533.I1RB26 pp 229533.I1RCR1
14158 229533.I1RB26 pp 229533.I1S0W4
14159 229533.I1RB26 pp 229533.I1RDF7
14160 229533.I1RB26 pp 229533.I1RVX0
14161 229533.I1RB26 pp 229533.I1RTD5
14162 229533.I1RB26 pp 229533.I1S5R1

14163 229533.I1RB26 pp 229533.I1RBR1
14164 229533.I1RB26 pp 229533.I1RC46
14165 229533.I1RB26 pp 229533.I1RSZ7
14166 229533.I1RB26 pp 229533.I1RT40
14167 229533.I1RB26 pp 229533.I1RN70
14168 229533.I1RB26 pp 229533.I1RXC2
14169 229533.I1RB26 pp 229533.I1S2B8
14170 229533.I1RB26 pp 229533.I1RZ39
14171 229533.I1RB26 pp 229533.I1RGA4
14172 229533.I1RB26 pp 229533.I1RM74
14173 229533.I1RB26 pp 229533.I1RC34
14174 229533.I1RB26 pp 229533.I1REC5
14175 229533.I1RB26 pp 229533.I1RRI1
14176 229533.I1RB26 pp 229533.I1RPE0
14177 229533.I1S3K0 pp 229533.I1S694
14178 229533.A0A098DZ32 pp 229533.I1S3K0
14179 229533.A0A098DED7 pp 229533.I1RYX7
14180 229533.A0A098DED7 pp 229533.A0A1C3YIW0
14181 229533.A0A098DED7 pp 229533.I1S9F8
14182 229533.A0A098DED7 pp 229533.A0A1C3YN26
14183 229533.A0A098DED7 pp 229533.I1RDC5
14184 229533.A0A098DED7 pp 229533.A0A0E0SH57
14185 229533.A0A098DED7 pp 229533.I1RKL0
14186 229533.A0A098DED7 pp 229533.I1RCN4
14187 229533.A0A098DED7 pp 229533.I1RXZ9
14188 229533.A0A098DED7 pp 229533.I1RYM5
14189 229533.A0A098DED7 pp 229533.I1RX64
14190 229533.I1RWG6 pp 229533.I1S0E9
14191 229533.I1RWG6 pp 229533.I1S050
14192 229533.I1RWG6 pp 229533.I1RXB5
14193 229533.I1RWG6 pp 229533.I1S2B7
14194 229533.I1RWG6 pp 229533.I1S082
14195 229533.I1RWG6 pp 229533.I1S077
14196 229533.I1RWG6 pp 229533.I1S1V1
14197 229533.I1RWG6 pp 229533.I1S045
14198 229533.A0A098DZ08 pp 229533.I1RMF2
14199 229533.A0A1C3YHN3 pp 229533.I1RB00
14200 229533.A0A1C3YHN3 pp 229533.I1RAH4
14201 229533.A0A1C3YHN3 pp 229533.I1RFX8
14202 229533.A0A1C3YHN3 pp 229533.V6RLW4
14203 229533.A0A1C3YHN3 pp 229533.I1RN31
14204 229533.A0A1C3YHN3 pp 229533.I1RZ02
14205 229533.A0A1C3YHN3 pp 229533.I1RD01
14206 229533.A0A1C3YHN3 pp 229533.I1RMG6
14207 229533.A0A1C3YHN3 pp 229533.I1RSK0
14208 229533.A0A1C3YHN3 pp 229533.I1RAN3
14209 229533.A0A1C3YHN3 pp 229533.O74638
14210 229533.A0A1C3YHN3 pp 229533.I1RFZ2
14211 229533.A0A1C3YHN3 pp 229533.I1RAL9
14212 229533.A0A1C3YHN3 pp 229533.I1RUR0
14213 229533.A0A1C3YHN3 pp 229533.I1RB49
14214 229533.A0A1C3YHN3 pp 229533.I1RKQ0
14215 229533.A0A1C3YHN3 pp 229533.I1RDJ8
14216 229533.A0A1C3YHN3 pp 229533.I1RYX9
14217 229533.A0A1C3YHN3 pp 229533.A0A1C3YL40
14218 229533.A0A1C3YHN3 pp 229533.I1RCL7
14219 229533.A0A1C3YHN3 pp 229533.I1S788
14220 229533.A0A1C3YHN3 pp 229533.I1RJV8
14221 229533.A0A1C3YHN3 pp 229533.I1RUG2
14222 229533.A0A1C3YHN3 pp 229533.I1R9W7
14223 229533.A0A1C3YHN3 pp 229533.I1RG77
14224 229533.A0A1C3YHN3 pp 229533.I1RBL4
14225 229533.A0A1C3YHN3 pp 229533.A0A1C3YI10
14226 229533.A0A1C3YHN3 pp 229533.I1RK32
14227 229533.A0A1C3YHN3 pp 229533.I1S0D5
14228 229533.A0A1C3YHN3 pp 229533.I1RQ67
14229 229533.A0A1C3YHN3 pp 229533.I1RYQ1
14230 229533.A0A1C3YHN3 pp 229533.A0A1C3YIP6
14231 229533.A0A1C3YHN3 pp 229533.I1RMJ8
14232 229533.A0A1C3YHN3 pp 229533.I1RRS7
14233 229533.A0A1C3YHN3 pp 229533.I1RD70
14234 229533.A0A1C3YHN3 pp 229533.I1RQ73
14235 229533.A0A1C3YHN3 pp 229533.I1RD19

229533.A0A1C3YHN3 pp 229533.I1RBN9
229533.A0A1C3YHN3 pp 229533.A0A1C3YHQ0
229533.A0A1C3YHN3 pp 229533.Q4IR08
229533.A0A1C3YHN3 pp 229533.I1RLX6
229533.A0A1C3YHN3 pp 229533.I1S4P6
229533.A0A1C3YHN3 pp 229533.A0A1C3YIA8
229533.A0A1C3YHN3 pp 229533.A0A1C3YKA6
229533.A0A1C3YHN3 pp 229533.A0A1C3YJL7
229533.A0A1C3YHN3 pp 229533.A0A1C3YJ79
229533.A0A1C3YHN3 pp 229533.I1S461
229533.A0A1C3YHN3 pp 229533.I1RN46
229533.A0A1C3YHN3 pp 229533.I1RC54
229533.A0A1C3YHN3 pp 229533.I1RY73
229533.A0A1C3YHN3 pp 229533.I1RN57
229533.A0A1C3YHN3 pp 229533.I1RSC7
229533.A0A1C3YHN3 pp 229533.Q4IB89
229533.A0A1C3YHN3 pp 229533.Q4INS6
229533.A0A1C3YHN3 pp 229533.I1RW16
229533.I1RP94 pp 229533.Q4I0J6
229533.I1RCS1 pp 229533.I1RQE7
229533.I1RCS1 pp 229533.I1RVX0
229533.I1RCS1 pp 229533.I1RXR1
229533.I1RCS1 pp 229533.Q4I624
229533.I1RCS1 pp 229533.I1RJV3
229533.I1RCS1 pp 229533.I1RD17
229533.I1RCS1 pp 229533.I1RT03
229533.I1RCS1 pp 229533.I1RXU9
229533.I1RCS1 pp 229533.V6R057
229533.I1RCS1 pp 229533.I1RN18
229533.I1RCS1 pp 229533.I1RYW3
229533.I1RCS1 pp 229533.I1S1C7
229533.I1RCS1 pp 229533.I1S5R1
229533.I1RCS1 pp 229533.I1RD22
229533.I1RCS1 pp 229533.I1RFL4
229533.I1RCS1 pp 229533.I1RRT8
229533.I1RCS1 pp 229533.I1S5R6
229533.I1RCS1 pp 229533.I1RG11
229533.I1RCS1 pp 229533.I1RZW8
229533.I1RCS1 pp 229533.I1RRI2
229533.I1RCS1 pp 229533.I1RE33
229533.I1RCS1 pp 229533.I1RUG3
229533.I1RCS1 pp 229533.I1S2H4
229533.I1RCS1 pp 229533.I1S0L4
229533.I1RCS1 pp 229533.I1RTH6
229533.I1RCS1 pp 229533.I1RSQ4
229533.I1RCS1 pp 229533.I1S2C0
229533.I1RCS1 pp 229533.I1RNC1
229533.I1RCS1 pp 229533.V6RIV9
229533.I1RCS1 pp 229533.I1RZZ9
229533.I1RCS1 pp 229533.Q4I327
229533.I1RCS1 pp 229533.Q4I2M7
229533.I1RCS1 pp 229533.I1S1W3
229533.I1RCS1 pp 229533.I1RE01
229533.I1RCS1 pp 229533.I1RWI5
229533.I1RCS1 pp 229533.V6RMW6
229533.I1RCS1 pp 229533.I1S0J1
229533.I1RCS1 pp 229533.I1RZ39
229533.I1RCS1 pp 229533.I1RGF1
229533.I1RCS1 pp 229533.I1RRT2
229533.I1RCS1 pp 229533.V6R042
229533.I1RCS1 pp 229533.I1RSQ3
229533.I1RCS1 pp 229533.I1RN67
229533.I1RCS1 pp 229533.I1RGA4
229533.I1RCS1 pp 229533.I1RMQ5
229533.I1RCS1 pp 229533.I1RT40
229533.I1RCS1 pp 229533.I1RNG4
229533.I1RCS1 pp 229533.I1RTD5
229533.I1RCS1 pp 229533.I1RWK3
229533.I1RCS1 pp 229533.I1S2B8
229533.I1RCS1 pp 229533.I1RRM5
229533.I1RCS1 pp 229533.I1RN63
229533.I1RCS1 pp 229533.I1RCS2
229533.I1RCS1 pp 229533.I1RFT1

14309 229533.I1RCS1 pp 229533.I1RT04
14310 229533.I1RCS1 pp 229533.I1RE76
14311 229533.I1RCS1 pp 229533.Q4HXF0
14312 229533.I1RCS1 pp 229533.I1RF19
14313 229533.I1RCS1 pp 229533.I1RRJ8
14314 229533.I1RCS1 pp 229533.I1RPM4
14315 229533.I1RCS1 pp 229533.I1REK8
14316 229533.I1RCS1 pp 229533.I1RDA0
14317 229533.I1RCS1 pp 229533.I1RPP3
14318 229533.I1RCS1 pp 229533.Q4IJK2
14319 229533.I1RCS1 pp 229533.I1RVQ0
14320 229533.I1RCS1 pp 229533.V6QXP4
14321 229533.I1RCS1 pp 229533.I1RJW6
14322 229533.I1RCS1 pp 229533.I1RZZ1
14323 229533.I1RCS1 pp 229533.I1RD52
14324 229533.I1RCS1 pp 229533.I1S0L3
14325 229533.I1RCS1 pp 229533.I1RRY9
14326 229533.I1RCS1 pp 229533.Q4I061
14327 229533.I1RCS1 pp 229533.I1S1J7
14328 229533.I1RCS1 pp 229533.I1S887
14329 229533.I1RCS1 pp 229533.I1S5B0
14330 229533.I1RCS1 pp 229533.I1S4S7
14331 229533.I1RCS1 pp 229533.I1RZM8
14332 229533.I1RCS1 pp 229533.I1RZK5
14333 229533.I1RCS1 pp 229533.I1RSQ7
14334 229533.I1RCS1 pp 229533.I1RQ31
14335 229533.I1RCS1 pp 229533.I1RXF0
14336 229533.I1RCS1 pp 229533.I1RX46
14337 229533.I1RCS1 pp 229533.V6RCZ8
14338 229533.I1RCS1 pp 229533.V6QW22
14339 229533.I1RCS1 pp 229533.I1S0T2
14340 229533.I1RCS1 pp 229533.V6RTJ8
14341 229533.I1RCS1 pp 229533.I1RZF6
14342 229533.I1RCS1 pp 229533.V6R227
14343 229533.I1RCS1 pp 229533.V6RCG5
14344 229533.I1RCS1 pp 229533.I1RW25
14345 229533.I1RCS1 pp 229533.I1RS18
14346 229533.I1RCS1 pp 229533.I1RR51
14347 229533.I1RCS1 pp 229533.V6QUE6
14348 229533.I1RCS1 pp 229533.I1RXD7
14349 229533.I1RCS1 pp 229533.I1S0R7
14350 229533.I1RCS1 pp 229533.I1RR24
14351 229533.I1RCS1 pp 229533.V6RUT5
14352 229533.I1RCS1 pp 229533.I1RW99
14353 229533.I1RCS1 pp 229533.I1RJU3
14354 229533.I1S382 pp 229533.Q4IR18
14355 229533.I1S382 pp 229533.V6QV38
14356 229533.I1S382 pp 229533.V6R227
14357 229533.A0A098DDT3 pp 229533.I1RRS7
14358 229533.A0A098DDT3 pp 229533.I1RMX3
14359 229533.A0A098DDT3 pp 229533.I1RNA2
14360 229533.A0A098DDT3 pp 229533.I1RBC8
14361 229533.A0A098DDT3 pp 229533.A0A0E0SBE9
14362 229533.A0A098DDT3 pp 229533.I1RQ73
14363 229533.A0A098DDT3 pp 229533.I1S209
14364 229533.A0A098DDT3 pp 229533.I1RC54
14365 229533.A0A098DDT3 pp 229533.I1RA12
14366 229533.A0A098DDT3 pp 229533.I1S4P6
14367 229533.A0A098DDT3 pp 229533.I1RU61
14368 229533.A0A098DDT3 pp 229533.I1RQI6
14369 229533.A0A098DDT3 pp 229533.I1RPI0
14370 229533.A0A098DDT3 pp 229533.I1RZ98
14371 229533.A0A098DDT3 pp 229533.I1RW03
14372 229533.A0A098DDT3 pp 229533.I1RD19
14373 229533.A0A098DDT3 pp 229533.I1RRW0
14374 229533.A0A098DDT3 pp 229533.A0A1C3YN55
14375 229533.A0A098DDT3 pp 229533.A0A0E0RTV8
14376 229533.A0A098DDT3 pp 229533.I1RYP7
14377 229533.A0A098DDT3 pp 229533.A0A1C3YHN3
14378 229533.A0A098DDT3 pp 229533.I1RSC7
14379 229533.A0A098DDT3 pp 229533.I1RTN6
14380 229533.A0A098DDT3 pp 229533.A0A1C3YKA6
14381 229533.A0A098DDT3 pp 229533.I1RY73

14382	229533.A0A098DDT3	pp	229533.I1RPZ0
14383	229533.A0A098DDT3	pp	229533.I1RBN9
14384	229533.A0A098DDT3	pp	229533.A0A1C3YN69
14385	229533.A0A098DDT3	pp	229533.I1S604
14386	229533.A0A098DDT3	pp	229533.V6RSF3
14387	229533.A0A098DDT3	pp	229533.A0A1C3YM90
14388	229533.A0A098DDT3	pp	229533.Q4IB89
14389	229533.A0A098DDT3	pp	229533.I1RX64
14390	229533.A0A098DDT3	pp	229533.A0A0E0RMM5
14391	229533.A0A098DDT3	pp	229533.A0A1C3YJ79
14392	229533.A0A098DDT3	pp	229533.A0A0E0RW49
14393	229533.A0A098DDT3	pp	229533.I1RW16
14394	229533.A0A098DDT3	pp	229533.I1RFJ2
14395	229533.A0A098DDT3	pp	229533.I1RN46
14396	229533.A0A098DDT3	pp	229533.I1RE46
14397	229533.A0A098DDT3	pp	229533.A0A1C3YMC9
14398	229533.A0A098DDT3	pp	229533.I1S0D5
14399	229533.A0A098DDT3	pp	229533.A0A1C3YL34
14400	229533.A0A098DDT3	pp	229533.I1RQH8
14401	229533.A0A098DDT3	pp	229533.I1S461
14402	229533.A0A098DDT3	pp	229533.Q4INS6
14403	229533.A0A098DDT3	pp	229533.I1S788
14404	229533.A0A098DDT3	pp	229533.I1RMS2
14405	229533.A0A098DDT3	pp	229533.I1RSR9
14406	229533.A0A098DDT3	pp	229533.I1RMJ8
14407	229533.A0A098DDT3	pp	229533.I1RUG4
14408	229533.A0A098DDT3	pp	229533.I1RCL7
14409	229533.A0A098DDT3	pp	229533.I1RDJ8
14410	229533.A0A098DDT3	pp	229533.A0A1C3YLQ7
14411	229533.A0A098DDT3	pp	229533.I1RBL4
14412	229533.A0A098DDT3	pp	229533.I1RWG6
14413	229533.A0A098DDT3	pp	229533.I1RXI6
14414	229533.A0A098DDT3	pp	229533.A0A1C3YL40
14415	229533.A0A098DDT3	pp	229533.A0A098DXS1
14416	229533.A0A098DDT3	pp	229533.I1RMG6
14417	229533.A0A098DDT3	pp	229533.A0A098DTI0
14418	229533.A0A098DDT3	pp	229533.A0A1C3YI10
14419	229533.A0A098DDT3	pp	229533.I1RUG2
14420	229533.A0A098DDT3	pp	229533.I1RQ92
14421	229533.A0A098DDT3	pp	229533.I1RP99
14422	229533.A0A098DDT3	pp	229533.I1RRY1
14423	229533.A0A098DDT3	pp	229533.I1RYR2
14424	229533.A0A098DDT3	pp	229533.I1RJV8
14425	229533.A0A098DDT3	pp	229533.I1RQ67
14426	229533.A0A098DDT3	pp	229533.I1RB00
14427	229533.A0A098DDT3	pp	229533.I1RAH4
14428	229533.A0A098DDT3	pp	229533.A0A098E4E9
14429	229533.A0A098DDT3	pp	229533.I1RBW0
14430	229533.A0A098DDT3	pp	229533.A0A1C3YMJ3
14431	229533.A0A098DDT3	pp	229533.I1S224
14432	229533.A0A098DDT3	pp	229533.I1R9W7
14433	229533.A0A098DDT3	pp	229533.I1RE55
14434	229533.A0A098DDT3	pp	229533.I1RSI4
14435	229533.A0A098DDT3	pp	229533.I1RWM6
14436	229533.A0A098DDT3	pp	229533.I1S987
14437	229533.A0A098DDT3	pp	229533.I1RAL9
14438	229533.A0A098DDT3	pp	229533.A0A1C3YKW5
14439	229533.A0A098DDT3	pp	229533.A0A1C3YM63
14440	229533.A0A098DDT3	pp	229533.V6RLW4
14441	229533.A0A098DDT3	pp	229533.A0A1C3YN36
14442	229533.A0A098DDT3	pp	229533.I1RB49
14443	229533.A0A098DDT3	pp	229533.I1RPQ1
14444	229533.A0A098DDT3	pp	229533.I1RYQ1
14445	229533.A0A098DDT3	pp	229533.I1RJZ7
14446	229533.A0A098DDT3	pp	229533.I1RW24
14447	229533.A0A098DDT3	pp	229533.I1RFX8
14448	229533.A0A098DDT3	pp	229533.I1RKQ0
14449	229533.A0A098DDT3	pp	229533.I1S523
14450	229533.A0A098DDT3	pp	229533.I1RLX6
14451	229533.A0A098DDT3	pp	229533.A0A1C3YIA8
14452	229533.A0A098DDT3	pp	229533.I1RN61
14453	229533.A0A098DDT3	pp	229533.A0A1C3YJP1
14454	229533.A0A098DDT3	pp	229533.I1RY29

14455	229533.A0A098DDT3	pp	229533.A0A1C3YHM3
14456	229533.A0A098DDT3	pp	229533.I1RF70
14457	229533.A0A098DDT3	pp	229533.I1RS81
14458	229533.A0A098DDT3	pp	229533.I1RN31
14459	229533.A0A098DDT3	pp	229533.A0A1C3YK82
14460	229533.A0A098DDT3	pp	229533.A0A0E0SQG9
14461	229533.A0A098DDT3	pp	229533.I1RVV5
14462	229533.A0A098DDT3	pp	229533.I1RB74
14463	229533.A0A098DDT3	pp	229533.I1RKI2
14464	229533.A0A098DDT3	pp	229533.I1RRJ6
14465	229533.A0A098DDT3	pp	229533.I1RW71
14466	229533.A0A098DDT3	pp	229533.I1S998
14467	229533.A0A098DDT3	pp	229533.I1RWI8
14468	229533.A0A098DDT3	pp	229533.I1RYX9
14469	229533.A0A098DDT3	pp	229533.A0A1C3YJL7
14470	229533.A0A098DDT3	pp	229533.A0A1C3YL55
14471	229533.A0A098DDT3	pp	229533.I1RSJ1
14472	229533.A0A098DDT3	pp	229533.A0A1C3YI64
14473	229533.A0A098DDT3	pp	229533.I1RZ02
14474	229533.A0A098DDT3	pp	229533.I1RUR0
14475	229533.A0A098DDT3	pp	229533.A0A098E1Q1
14476	229533.A0A098DDT3	pp	229533.I1RVW0
14477	229533.A0A098DDT3	pp	229533.I1RHD2
14478	229533.A0A098DDT3	pp	229533.I1RZ94
14479	229533.A0A098DDT3	pp	229533.I1S0A4
14480	229533.A0A098DDT3	pp	229533.I1S283
14481	229533.A0A098DDT3	pp	229533.I1RCA5
14482	229533.A0A098DDT3	pp	229533.I1RRT1
14483	229533.A0A098DDT3	pp	229533.I1RQQ0
14484	229533.A0A098DDT3	pp	229533.I1RZ86
14485	229533.A0A098DDT3	pp	229533.I1RWK9
14486	229533.A0A098DDT3	pp	229533.I1RBW1
14487	229533.A0A098DDT3	pp	229533.I1RA40
14488	229533.A0A098DDT3	pp	229533.I1RSK0
14489	229533.A0A098DDT3	pp	229533.A0A0E0S1J9
14490	229533.A0A098DDT3	pp	229533.I1RCQ4
14491	229533.A0A098DDT3	pp	229533.I1RD01
14492	229533.A0A098DDT3	pp	229533.I1RAN3
14493	229533.I1RRE1	pp	229533.I1RYF2
14494	229533.I1RRE1	pp	229533.Q4I1T9
14495	229533.I1RPS4	pp	229533.I1RRN5
14496	229533.I1RPS4	pp	229533.I1S7L6
14497	229533.I1RPS4	pp	229533.I1S130
14498	229533.I1RPS4	pp	229533.I1RWD2
14499	229533.I1S3X2	pp	229533.I1S746
14500	229533.I1RHG1	pp	229533.I1S746
14501	229533.I1RHG1	pp	229533.I1RTU0
14502	229533.I1RHG1	pp	229533.I1RMJ4
14503	229533.I1S951	pp	229533.V6R227
14504	229533.I1S951	pp	229533.V6QV38
14505	229533.I1S951	pp	229533.Q4IR18
14506	229533.A0A0E0RSZ0	pp	229533.A0A1C3YKB6
14507	229533.A0A0E0RSZ0	pp	229533.I1RYF2
14508	229533.A0A0E0RSZ0	pp	229533.I1RRE1
14509	229533.A0A0E0RSZ0	pp	229533.I1RPS4
14510	229533.A0A0E0RSZ0	pp	229533.I1RYW8
14511	229533.A0A0E0RSZ0	pp	229533.I1RRN5
14512	229533.A0A0E0RSZ0	pp	229533.A0A1C3YN26
14513	229533.A0A1C3YI12	pp	229533.I1S9D4
14514	229533.A0A1C3YI12	pp	229533.A0A1C3YLU9
14515	229533.A0A1C3YI12	pp	229533.I1R9K3
14516	229533.A0A1C3YI12	pp	229533.I1RZ52
14517	229533.A0A1C3YI12	pp	229533.I1RNG4
14518	229533.A0A1C3YI12	pp	229533.I1RPD6
14519	229533.A0A1C3YI12	pp	229533.I1RMM8
14520	229533.A0A1C3YI12	pp	229533.I1RPW2
14521	229533.A0A1C3YI12	pp	229533.I1S3F3
14522	229533.A0A1C3YI12	pp	229533.A0A1C3YIV7
14523	229533.A0A1C3YI12	pp	229533.A0A1C3YM01
14524	229533.A0A1C3YI12	pp	229533.I1S2E2
14525	229533.A0A1C3YI12	pp	229533.I1RY74
14526	229533.A0A1C3YI12	pp	229533.I1RIY0
14527	229533.A0A1C3YI12	pp	229533.I1RU76

229533.A0A1C3YI12 pp 229533.I1RVX9
229533.A0A1C3YI12 pp 229533.I1RQE0
229533.A0A1C3YI12 pp 229533.I1RM26
229533.A0A1C3YI12 pp 229533.I1RK03
229533.I1RI34 pp 229533.I1S8G4
229533.I1RI34 pp 229533.I1RR68
229533.I1RI34 pp 229533.I1RX38
229533.I1RI34 pp 229533.I1RT75
229533.I1RI34 pp 229533.I1RQM7
229533.I1RI34 pp 229533.I1RZE4
229533.I1RI34 pp 229533.I1RX22
229533.I1RI34 pp 229533.O74638
229533.I1RI34 pp 229533.I1RW16
229533.I1RI34 pp 229533.I1S216
229533.I1RI34 pp 229533.I1S4Z8
229533.I1RI34 pp 229533.I1RL04
229533.I1RI34 pp 229533.V6R057
229533.I1RI34 pp 229533.I1RQM0
229533.I1RI34 pp 229533.I1RME6
229533.I1RI34 pp 229533.I1RS17
229533.I1RI34 pp 229533.I1RLX6
229533.I1RI34 pp 229533.I1RVZ7
229533.I1RI34 pp 229533.I1SAN9
229533.I1RI34 pp 229533.I1RPU8
229533.I1RI34 pp 229533.I1RMI8
229533.I1RI34 pp 229533.I1RWE8
229533.I1RI34 pp 229533.I1RPN4
229533.I1RI34 pp 229533.I1RW71
229533.I1RI34 pp 229533.I1RQS9
229533.I1RI34 pp 229533.I1RQ80
229533.I1RI34 pp 229533.V6R1K4
229533.I1RI34 pp 229533.V6RKY7
229533.I1RI34 pp 229533.I1S0B7
229533.I1RI34 pp 229533.I1RN31
229533.I1RI34 pp 229533.I1RYH9
229533.I1RI34 pp 229533.I1RPW0
229533.I1RI34 pp 229533.I1RTD3
229533.I1RI34 pp 229533.I1RMQ4
229533.I1RI34 pp 229533.I1RK95
229533.I1RI34 pp 229533.I1RUQ5
229533.I1RI34 pp 229533.I1RKK0
229533.I1RI34 pp 229533.I1RRV4
229533.I1RI34 pp 229533.V6RBN2
229533.I1RI34 pp 229533.I1RPU4
229533.I1RI34 pp 229533.I1RM35
229533.I1RF35 pp 229533.I1RMJ4
229533.I1RF35 pp 229533.I1RG29
229533.I1RF35 pp 229533.I1S746
229533.I1RF35 pp 229533.I1RHG1
229533.I1RF35 pp 229533.I1RTU0
229533.I1R9B5 pp 229533.I1RV21
229533.I1R9B5 pp 229533.I1RMJ4
229533.I1R9B5 pp 229533.I1S746
229533.I1R9B5 pp 229533.I1RHG1
229533.I1R9B5 pp 229533.I1RF35
229533.I1R9B5 pp 229533.I1RTU0
229533.I1R9B5 pp 229533.I1RG29
229533.I1RCV4 pp 229533.Q4IR08
229533.I1RCV4 pp 229533.I1S5Z5
229533.I1RCV4 pp 229533.Q4IF76
229533.I1RCV4 pp 229533.Q4IJH1
229533.I1RCV4 pp 229533.I1RKF3
229533.A0A1C3YMF9 pp 229533.I1RLX6
229533.A0A1C3YMF9 pp 229533.I1RPU4
229533.A0A1C3YMF9 pp 229533.I1S201
229533.A0A1C3YMF9 pp 229533.I1RP96
229533.A0A1C3YMF9 pp 229533.I1RPY5
229533.A0A1C3YMF9 pp 229533.I1RML9
229533.A0A1C3YMF9 pp 229533.I1RDC9
229533.A0A1C3YMF9 pp 229533.I1RW57
229533.A0A1C3YMF9 pp 229533.I1RRH5
229533.A0A1C3YMF9 pp 229533.I1RQ80
229533.A0A1C3YMF9 pp 229533.I1RKE9

14601 229533.A0A1C3YMF9 pp 229533.I1S285
14602 229533.A0A1C3YMF9 pp 229533.I1RXE5
14603 229533.A0A1C3YMF9 pp 229533.I1S2Y5
14604 229533.A0A1C3YMF9 pp 229533.I1RWD4
14605 229533.A0A1C3YMF9 pp 229533.A0A1C3YN65
14606 229533.A0A1C3YMF9 pp 229533.I1S1V2
14607 229533.A0A1C3YMF9 pp 229533.I1RW60
14608 229533.A0A1C3YMF9 pp 229533.V6R227
14609 229533.A0A1C3YMF9 pp 229533.I1S0Z1
14610 229533.A0A1C3YMF9 pp 229533.I1RSH4
14611 229533.A0A1C3YMF9 pp 229533.I1RN31
14612 229533.A0A1C3YMF9 pp 229533.A0A1C3YN58
14613 229533.A0A1C3YMF9 pp 229533.I1RF91
14614 229533.A0A1C3YMF9 pp 229533.I1RZM2
14615 229533.A0A1C3YMF9 pp 229533.I1RYX3
14616 229533.A0A1C3YMF9 pp 229533.V6QV38
14617 229533.A0A1C3YMF9 pp 229533.I1RB17
14618 229533.A0A1C3YMF9 pp 229533.I1RBD7
14619 229533.A0A1C3YMF9 pp 229533.I1RCK7
14620 229533.A0A1C3YMF9 pp 229533.I1RFZ2
14621 229533.A0A1C3YMF9 pp 229533.I1RCM6
14622 229533.A0A1C3YMF9 pp 229533.Q4IR18
14623 229533.A0A1C3YMF9 pp 229533.I1RDY1
14624 229533.A0A1C3YMF9 pp 229533.I1S3D7
14625 229533.A0A1C3YMF9 pp 229533.I1RB73
14626 229533.A0A1C3YMF9 pp 229533.I1RBL4
14627 229533.A0A1C3YMF9 pp 229533.I1RJS4
14628 229533.A0A1C3YMF9 pp 229533.A0A1C3YMH3
14629 229533.A0A1C3YMF9 pp 229533.I1RBV5
14630 229533.A0A1C3YMF9 pp 229533.I1RCN2
14631 229533.A0A1C3YMF9 pp 229533.I1RX38
14632 229533.A0A1C3YMF9 pp 229533.I1RBR5
14633 229533.A0A1C3YMF9 pp 229533.I1RPY4
14634 229533.A0A1C3YMF9 pp 229533.I1RA91
14635 229533.A0A1C3YMF9 pp 229533.I1RVY0
14636 229533.A0A1C3YMF9 pp 229533.I1RQM0
14637 229533.A0A1C3YMF9 pp 229533.I1RLX4
14638 229533.A0A1C3YMF9 pp 229533.I1RD62
14639 229533.A0A1C3YMF9 pp 229533.I1RW16
14640 229533.A0A1C3YMF9 pp 229533.I1RII1
14641 229533.A0A1C3YMF9 pp 229533.I1RD70
14642 229533.A0A1C3YMF9 pp 229533.I1RBM9
14643 229533.A0A1C3YMF9 pp 229533.I1RPW0
14644 229533.A0A1C3YMF9 pp 229533.I1S109
14645 229533.I1RPX8 pp 229533.Q4I0J6
14646 229533.A0A098DZ19 pp 229533.I1SAS3
14647 229533.A0A098DZ19 pp 229533.I1SAS4
14648 229533.A0A098DZ19 pp 229533.I1RCA0
14649 229533.A0A098DZ19 pp 229533.A0A1C3YMS7
14650 229533.A0A098DZ19 pp 229533.I1RFM2
14651 229533.A0A098DZ19 pp 229533.I1RJG1
14652 229533.A0A098DZ19 pp 229533.I1RF79
14653 229533.A0A098DZ19 pp 229533.I1RR90
14654 229533.A0A098DZ19 pp 229533.I1RNT1
14655 229533.A0A098DZ19 pp 229533.I1RI22
14656 229533.A0A098DZ19 pp 229533.I1RT23
14657 229533.A0A098DZ19 pp 229533.V6RPN0
14658 229533.A0A098DZ19 pp 229533.I1S1Q3
14659 229533.A0A098DZ19 pp 229533.A0A1C3YLL7
14660 229533.A0A098DZ19 pp 229533.I1RZZ3
14661 229533.A0A098DZ19 pp 229533.I1RY35
14662 229533.A0A098DZ19 pp 229533.I1RQ48
14663 229533.A0A098DZ19 pp 229533.V6RR38
14664 229533.A0A098DZ19 pp 229533.I1RRQ1
14665 229533.A0A098DZ19 pp 229533.I1RQ76
14666 229533.A0A098DZ19 pp 229533.A0A0E0SP71
14667 229533.A0A098DZ19 pp 229533.I1RCC2
14668 229533.I1RV21 pp 229533.I1S746
14669 229533.I1RTY1 pp 229533.I1SA62
14670 229533.I1RTY1 pp 229533.I1S2L9
14671 229533.I1RTY1 pp 229533.I1RVX2
14672 229533.I1RTY1 pp 229533.I1S6J8
14673 229533.I1RTY1 pp 229533.I1S3C0

14674 229533.I1RGU0 pp 229533.I1RSQ4
14675 229533.I1RGU0 pp 229533.I1RZ95
14676 229533.I1RGU0 pp 229533.I1RY57
14677 229533.I1RGU0 pp 229533.I1RW61
14678 229533.I1RGU0 pp 229533.I1RSZ7
14679 229533.I1RGU0 pp 229533.I1RTA1
14680 229533.I1RGU0 pp 229533.I1RYI5
14681 229533.I1RGU0 pp 229533.I1S2C1
14682 229533.I1RGU0 pp 229533.I1RJU3
14683 229533.I1RGU0 pp 229533.I1RP97
14684 229533.I1RGU0 pp 229533.I1S0R9
14685 229533.I1RGU0 pp 229533.I1S4S7
14686 229533.I1RGU0 pp 229533.I1RTE9
14687 229533.I1RGU0 pp 229533.I1S4S4
14688 229533.I1RGU0 pp 229533.I1RY63
14689 229533.I1RGU0 pp 229533.I1RKB6
14690 229533.A0A098DZ18 pp 229533.I1RE53
14691 229533.I1RBH0 pp 229533.I1S746
14692 229533.I1RBH0 pp 229533.I1RF35
14693 229533.I1RBH0 pp 229533.I1S3X2
14694 229533.I1RBH0 pp 229533.I1RS98
14695 229533.I1RBH0 pp 229533.I1RG29
14696 229533.I1RBH0 pp 229533.I1RV21
14697 229533.I1RBH0 pp 229533.I1RMJ4
14698 229533.I1RBH0 pp 229533.I1RTU0
14699 229533.I1RCD4 pp 229533.I1S0N8
14700 229533.I1RCD4 pp 229533.I1RCU4
14701 229533.I1RCD4 pp 229533.I1S0Z3
14702 229533.I1RCD4 pp 229533.I1RPA7
14703 229533.I1RCD4 pp 229533.I1RRQ0
14704 229533.I1RCD4 pp 229533.V6REE9
14705 229533.I1RCD4 pp 229533.I1RCM5
14706 229533.I1RCD4 pp 229533.I1RQA9
14707 229533.I1RCD4 pp 229533.I1S5K9
14708 229533.I1RCD4 pp 229533.I1RZH4
14709 229533.I1RCD4 pp 229533.I1RMT4
14710 229533.I1RCD4 pp 229533.Q4IER8
14711 229533.I1RCD4 pp 229533.I1RR94
14712 229533.I1RCD4 pp 229533.I1RN43
14713 229533.I1RCD4 pp 229533.I1RJK7
14714 229533.I1RCD4 pp 229533.I1S336
14715 229533.I1RCD4 pp 229533.I1S224
14716 229533.I1RCD4 pp 229533.I1RJX2
14717 229533.I1RCD4 pp 229533.I1RT41
14718 229533.I1RCD4 pp 229533.I1RKJ7
14719 229533.I1RCD4 pp 229533.V6QUU6
14720 229533.I1RCD4 pp 229533.I1RN65
14721 229533.I1RCD4 pp 229533.I1RZ02
14722 229533.I1RCD4 pp 229533.P0C431
14723 229533.I1RCD4 pp 229533.I1RN31
14724 229533.I1RCD4 pp 229533.I1RCX5
14725 229533.I1RCD4 pp 229533.I1RTQ9
14726 229533.I1RCD4 pp 229533.I1S095
14727 229533.I1RCD4 pp 229533.Q4HZS8
14728 229533.I1RCD4 pp 229533.I1RZV4
14729 229533.I1RCD4 pp 229533.I1RN81
14730 229533.I1RCD4 pp 229533.Q4ICM0
14731 229533.I1RCD4 pp 229533.I1RZ12
14732 229533.I1RCD4 pp 229533.I1RQL4
14733 229533.I1RCD4 pp 229533.I1RPP7
14734 229533.I1RCD4 pp 229533.I1RDX3
14735 229533.I1RCD4 pp 229533.I1RD19
14736 229533.I1RCD4 pp 229533.I1RWL6
14737 229533.I1RCD4 pp 229533.I1REC7
14738 229533.I1RCD4 pp 229533.I1RCY0
14739 229533.I1RCD4 pp 229533.I1S757
14740 229533.I1RCD4 pp 229533.I1RD10
14741 229533.I1RCD4 pp 229533.I1RD70
14742 229533.I1RCD4 pp 229533.I1RWB4
14743 229533.I1RCD4 pp 229533.I1RLX6
14744 229533.I1RCD4 pp 229533.I1RCT2
14745 229533.I1RCD4 pp 229533.I1RCX4
14746 229533.I1RCD4 pp 229533.I1S0R9

14747 229533.I1RCD4 pp 229533.Q5I2J3
14748 229533.I1RCD4 pp 229533.I1S2A8
14749 229533.I1RCD4 pp 229533.I1RT94
14750 229533.I1RCD4 pp 229533.I1RPS0
14751 229533.I1RCD4 pp 229533.I1RKR7
14752 229533.I1RCD4 pp 229533.I1RJU5
14753 229533.I1RCD4 pp 229533.I1RVF8
14754 229533.I1RCD4 pp 229533.I1RCJ0
14755 229533.I1RCD4 pp 229533.I1RW16
14756 229533.I1RCD4 pp 229533.I1RTG9
14757 229533.I1RCD4 pp 229533.I1RZL3
14758 229533.I1RCD4 pp 229533.I1S282
14759 229533.I1RCD4 pp 229533.I1RQ32
14760 229533.I1RCD4 pp 229533.I1RKR9
14761 229533.I1RCD4 pp 229533.I1RSF2
14762 229533.I1RCD4 pp 229533.I1REC6
14763 229533.I1RCD4 pp 229533.I1RGU7
14764 229533.I1RCD4 pp 229533.I1RR95
14765 229533.I1RCD4 pp 229533.I1RZD9
14766 229533.I1RCD4 pp 229533.I1S0L8
14767 229533.I1RCD4 pp 229533.I1RN36
14768 229533.I1RCD4 pp 229533.I1RFW1
14769 229533.I1RCD4 pp 229533.I1RPU7
14770 229533.I1RCD4 pp 229533.I1S7X9
14771 229533.I1RFI2 pp 229533.I1RW98
14772 229533.A0A1C3YHH5 pp 229533.I1RLX6
14773 229533.A0A1C3YHH5 pp 229533.I1RML9
14774 229533.A0A1C3YHH5 pp 229533.I1RYJ8
14775 229533.A0A1C3YHH5 pp 229533.I1RP96
14776 229533.A0A1C3YHH5 pp 229533.I1RF91
14777 229533.A0A1C3YHH5 pp 229533.I1RQ80
14778 229533.A0A1C3YHH5 pp 229533.I1RPU4
14779 229533.A0A1C3YHH5 pp 229533.I1S2Y5
14780 229533.A0A1C3YHH5 pp 229533.I1RPY5
14781 229533.A0A1C3YHH5 pp 229533.I1RDC9
14782 229533.A0A1C3YHH5 pp 229533.A0A1C3YII4
14783 229533.A0A1C3YHH5 pp 229533.A0A1C3YIP6
14784 229533.A0A1C3YHH5 pp 229533.I1S1V2
14785 229533.A0A1C3YHH5 pp 229533.I1RKE9
14786 229533.A0A1C3YHH5 pp 229533.Q4IA56
14787 229533.A0A1C3YHH5 pp 229533.I1RWD4
14788 229533.A0A1C3YHH5 pp 229533.I1RSI1
14789 229533.A0A1C3YHH5 pp 229533.I1RW60
14790 229533.A0A1C3YHH5 pp 229533.I1RXE5
14791 229533.A0A1C3YHH5 pp 229533.A0A1C3YL16
14792 229533.A0A1C3YHH5 pp 229533.I1RN31
14793 229533.A0A1C3YHH5 pp 229533.A0A1C3YIJ4
14794 229533.A0A1C3YHH5 pp 229533.I1RIS2
14795 229533.A0A1C3YHH5 pp 229533.A0A1C3YN58
14796 229533.A0A1C3YHH5 pp 229533.V6R227
14797 229533.A0A1C3YHH5 pp 229533.A0A1C3YHX6
14798 229533.A0A1C3YHH5 pp 229533.A0A1C3YJV9
14799 229533.A0A1C3YHH5 pp 229533.A0A1C3YK37
14800 229533.A0A1C3YHH5 pp 229533.A0A1C3YK75
14801 229533.A0A1C3YHH5 pp 229533.A0A1C3YKJ6
14802 229533.A0A1C3YHH5 pp 229533.Q4IR18
14803 229533.A0A1C3YHH5 pp 229533.V6QV38
14804 229533.A0A1C3YHH5 pp 229533.I1RSH4
14805 229533.A0A1C3YHH5 pp 229533.I1RCM6
14806 229533.A0A1C3YHH5 pp 229533.I1RYX3
14807 229533.A0A1C3YHH5 pp 229533.I1RJZ6
14808 229533.A0A1C3YHH5 pp 229533.A0A1C3YIE7
14809 229533.A0A1C3YHH5 pp 229533.I1RBV5
14810 229533.A0A1C3YHH5 pp 229533.I1RCK7
14811 229533.A0A1C3YHH5 pp 229533.A0A1C3YK53
14812 229533.A0A1C3YHH5 pp 229533.A0A1C3YK01
14813 229533.A0A1C3YHH5 pp 229533.A0A1C3YJE9
14814 229533.A0A1C3YHH5 pp 229533.I1RZM2
14815 229533.A0A1C3YHH5 pp 229533.I1RB73
14816 229533.A0A1C3YHH5 pp 229533.A0A1C3YI10
14817 229533.A0A1C3YHH5 pp 229533.Q4I7L0
14818 229533.A0A1C3YHH5 pp 229533.I1RVY0
14819 229533.A0A1C3YHH5 pp 229533.A0A1C3YLP8

14820 229533.A0A1C3YHH5 pp 229533.I1RRF5
14821 229533.A0A1C3YHH5 pp 229533.I1RQM0
14822 229533.A0A1C3YHH5 pp 229533.I1RX38
14823 229533.A0A1C3YHH5 pp 229533.A0A1C3YIP7
14824 229533.A0A1C3YHH5 pp 229533.I1RFZ2
14825 229533.A0A1C3YHH5 pp 229533.I1S3D7
14826 229533.A0A1C3YHH5 pp 229533.I1RPY4
14827 229533.A0A1C3YHH5 pp 229533.Q4IN63
14828 229533.A0A1C3YHH5 pp 229533.I1RA91
14829 229533.A0A1C3YHH5 pp 229533.A0A1C3YJZ2
14830 229533.A0A1C3YHH5 pp 229533.I1RBL4
14831 229533.A0A1C3YHH5 pp 229533.I1RLX4
14832 229533.A0A1C3YHH5 pp 229533.I1RD70
14833 229533.A0A1C3YHH5 pp 229533.A0A1C3YHV5
14834 229533.A0A1C3YHH5 pp 229533.I1RPW0
14835 229533.A0A1C3YHH5 pp 229533.I1RCN2
14836 229533.A0A1C3YHH5 pp 229533.I1RBR5
14837 229533.A0A1C3YHH5 pp 229533.I1RY99
14838 229533.A0A1C3YHH5 pp 229533.I1RS64
14839 229533.A0A1C3YHH5 pp 229533.A0A1C3YLP1
14840 229533.A0A1C3YHH5 pp 229533.A0A1C3YI81
14841 229533.A0A1C3YHH5 pp 229533.A0A1C3YM29
14842 229533.A0A1C3YHH5 pp 229533.A0A1C3YLG0
14843 229533.A0A1C3YHH5 pp 229533.I1RII1
14844 229533.A0A1C3YHH5 pp 229533.I1RW16
14845 229533.I1RDW4 pp 229533.I1RV18
14846 229533.I1RDW4 pp 229533.I1S5P3
14847 229533.I1RR21 pp 229533.Q4I0J6
14848 229533.I1RR21 pp 229533.V6R476
14849 229533.I1RD47 pp 229533.I1S2E9
14850 229533.I1RD47 pp 229533.I1RX64
14851 229533.I1RD47 pp 229533.I1RY44
14852 229533.I1RAN3 pp 229533.I1RWL9
14853 229533.I1RAN3 pp 229533.I1RN36
14854 229533.I1RAN3 pp 229533.I1RXI6
14855 229533.I1RAN3 pp 229533.I1RN31
14856 229533.I1RAN3 pp 229533.I1RKQ0
14857 229533.I1RAN3 pp 229533.I1RAW7
14858 229533.I1RAN3 pp 229533.I1RCY2
14859 229533.I1RAN3 pp 229533.I1RS37
14860 229533.I1RAN3 pp 229533.I1RSK0
14861 229533.I1RAN3 pp 229533.I1RSI4
14862 229533.I1RAN3 pp 229533.I1RBE6
14863 229533.I1RAN3 pp 229533.I1RNR6
14864 229533.I1RAN3 pp 229533.I1RCD4
14865 229533.I1RAN3 pp 229533.I1RUN8
14866 229533.I1RAN3 pp 229533.I1RKT7
14867 229533.I1RAN3 pp 229533.I1RN81
14868 229533.I1RAN3 pp 229533.I1RQ67
14869 229533.I1RAN3 pp 229533.I1S2E9
14870 229533.I1RAN3 pp 229533.I1RLU1
14871 229533.I1RAN3 pp 229533.I1RKK6
14872 229533.I1RAN3 pp 229533.I1RPZ0
14873 229533.I1RAN3 pp 229533.I1S1V5
14874 229533.I1RAN3 pp 229533.I1RZH4
14875 229533.I1RAN3 pp 229533.I1RWL6
14876 229533.I1RAN3 pp 229533.I1RLX6
14877 229533.I1RAN3 pp 229533.I1RFV6
14878 229533.I1RAN3 pp 229533.I1RGZ5
14879 229533.I1RAN3 pp 229533.I1RUG0
14880 229533.I1RAN3 pp 229533.I1RF70
14881 229533.I1RAN3 pp 229533.I1RK32
14882 229533.I1RAN3 pp 229533.I1RDB1
14883 229533.I1RAN3 pp 229533.I1RU61
14884 229533.I1RAN3 pp 229533.I1RCA9
14885 229533.I1RAN3 pp 229533.V6REE9
14886 229533.I1RAN3 pp 229533.I1RMK8
14887 229533.I1RAN3 pp 229533.I1RBL4
14888 229533.I1RAN3 pp 229533.I1SAP6
14889 229533.I1RAN3 pp 229533.I1RY44
14890 229533.I1RAN3 pp 229533.I1RM09
14891 229533.I1RAN3 pp 229533.I1RSJ0
14892 229533.I1RAN3 pp 229533.I1RUQ5

14893 229533.I1RAN3 pp 229533.V6RKY7
14894 229533.I1RAN3 pp 229533.V6RR76
14895 229533.I1RAN3 pp 229533.I1S604
14896 229533.I1RAN3 pp 229533.I1RSP7
14897 229533.I1RAN3 pp 229533.I1RTE6
14898 229533.I1RAN3 pp 229533.I1REL1
14899 229533.I1RAN3 pp 229533.I1RD19
14900 229533.I1RAN3 pp 229533.I1RJI0
14901 229533.I1RAN3 pp 229533.I1RW16
14902 229533.I1RAN3 pp 229533.I1RCL7
14903 229533.I1RAN3 pp 229533.I1RZE4
14904 229533.I1RAN3 pp 229533.I1RD70
14905 229533.I1RAN3 pp 229533.I1RJM9
14906 229533.I1RGA7 pp 229533.I1RTE6
14907 229533.I1RSL6 pp 229533.I1RW16
14908 229533.I1RSL6 pp 229533.I1S2F2
14909 229533.I1RSL6 pp 229533.I1RWM6
14910 229533.I1RSL6 pp 229533.I1RZP2
14911 229533.I1RSL6 pp 229533.I1RZT1
14912 229533.I1RSL6 pp 229533.I1RW03
14913 229533.I1RSL6 pp 229533.I1RX64
14914 229533.I1RSL6 pp 229533.I1RWB3
14915 229533.I1RPR9 pp 229533.V6RTJ8
14916 229533.I1RPR9 pp 229533.I1S0P0
14917 229533.I1RPR9 pp 229533.I1RSQ7
14918 229533.I1RPR9 pp 229533.V6RJP8
14919 229533.I1RPR9 pp 229533.I1S4S7
14920 229533.I1RPR9 pp 229533.I1RS05
14921 229533.I1RPR9 pp 229533.I1RX46
14922 229533.I1RPR9 pp 229533.I1RS18
14923 229533.I1RPR9 pp 229533.I1S5B0
14924 229533.I1RPR9 pp 229533.I1RRX5
14925 229533.I1RPR9 pp 229533.I1RXE6
14926 229533.I1RPR9 pp 229533.I1RXD7
14927 229533.I1RPR9 pp 229533.Q4IJK2
14928 229533.I1RPR9 pp 229533.V6QUE6
14929 229533.I1RPR9 pp 229533.I1S0K2
14930 229533.I1RPR9 pp 229533.V6RUT5
14931 229533.I1RPR9 pp 229533.I1RWC9
14932 229533.I1RPR9 pp 229533.I1RRY9
14933 229533.I1RPR9 pp 229533.I1RYT8
14934 229533.I1RPR9 pp 229533.I1RRK8
14935 229533.I1RPR9 pp 229533.Q4IA56
14936 229533.I1RPR9 pp 229533.I1S212
14937 229533.I1RPR9 pp 229533.V6RCZ8
14938 229533.I1RPR9 pp 229533.I1RWB0
14939 229533.I1RPR9 pp 229533.I1S4S4
14940 229533.I1RPR9 pp 229533.I1RR24
14941 229533.I1RPR9 pp 229533.I1RQA5
14942 229533.I1RPR9 pp 229533.I1S8P5
14943 229533.I1RPR9 pp 229533.I1RXF0
14944 229533.I1RPR9 pp 229533.I1RTA0
14945 229533.I1RPR9 pp 229533.I1RZM8
14946 229533.I1RPR9 pp 229533.I1S887
14947 229533.I1RPR9 pp 229533.I1RYI5
14948 229533.I1RPR9 pp 229533.I1RZK5
14949 229533.I1RPR9 pp 229533.I1S8S9
14950 229533.I1RPR9 pp 229533.I1RX39
14951 229533.I1RPR9 pp 229533.I1S0J1
14952 229533.I1RPR9 pp 229533.I1S0L8
14953 229533.I1RPR9 pp 229533.I1S1F3
14954 229533.I1RPR9 pp 229533.I1RS90
14955 229533.I1RPR9 pp 229533.V6R3J1
14956 229533.I1RPR9 pp 229533.I1S2B9
14957 229533.I1RPR9 pp 229533.I1S0B0
14958 229533.I1RPR9 pp 229533.I1RTG6
14959 229533.I1RPR9 pp 229533.I1S464
14960 229533.I1RPR9 pp 229533.I1RXD9
14961 229533.I1RPR9 pp 229533.I1S147
14962 229533.I1RPR9 pp 229533.I1RT40
14963 229533.I1RPR9 pp 229533.I1S2H4
14964 229533.I1RPR9 pp 229533.I1RTD5
14965 229533.I1RPR9 pp 229533.V6RAC9

14966	229533.I1RPR9	pp	229533.I1S1K6
14967	229533.I1RPR9	pp	229533.V6RMW6
14968	229533.I1RPR9	pp	229533.I1S0L4
14969	229533.I1RPR9	pp	229533.I1S277
14970	229533.I1RPR9	pp	229533.V6R042
14971	229533.I1RPR9	pp	229533.I1RRT8
14972	229533.I1RPR9	pp	229533.I1RSQ3
14973	229533.I1RPR9	pp	229533.I1S0Q7
14974	229533.I1RPR9	pp	229533.I1RYX5
14975	229533.I1RPR9	pp	229533.I1S5R1
14976	229533.I1RPR9	pp	229533.I1RXU9
14977	229533.I1RPR9	pp	229533.I1RUG3
14978	229533.I1RPR9	pp	229533.I1RQE7
14979	229533.I1RPR9	pp	229533.I1RZ39
14980	229533.I1RPR9	pp	229533.I1RY92
14981	229533.I1RPR9	pp	229533.I1RYH7
14982	229533.I1RPR9	pp	229533.I1RSQ4
14983	229533.I1RPR9	pp	229533.Q4I624
14984	229533.I1RPR9	pp	229533.I1RXR1
14985	229533.I1RPR9	pp	229533.I1RZJ3
14986	229533.I1RPR9	pp	229533.I1RZZ9
14987	229533.I1RPR9	pp	229533.I1RSU1
14988	229533.I1RPR9	pp	229533.I1RX64
14989	229533.I1RPR9	pp	229533.I1S1C7
14990	229533.I1RPR9	pp	229533.I1S1W3
14991	229533.I1RPR9	pp	229533.I1RVX0
14992	229533.I1RPR9	pp	229533.I1S5X9
14993	229533.I1RPR9	pp	229533.I1RYW3
14994	229533.I1RPR9	pp	229533.I1RT03
14995	229533.I1RPR9	pp	229533.I1S0R8
14996	229533.A0A0E0SNM6	pp	229533.I1RC95
14997	229533.A0A0E0SNM6	pp	229533.A0A1C3YN89
14998	229533.A0A0E0SNM6	pp	229533.P0C431
14999	229533.A0A0E0SNM6	pp	229533.I1RWT6
15000	229533.A0A0E0SNM6	pp	229533.V6RVG3
15001	229533.A0A0E0SNM6	pp	229533.I1RPP7
15002	229533.A0A0E0SNM6	pp	229533.I1RC22
15003	229533.A0A0E0SNM6	pp	229533.V6RGI1
15004	229533.A0A0E0SNM6	pp	229533.V6REE9
15005	229533.A0A0E0SNM6	pp	229533.I1RSB8
15006	229533.A0A0E0SNM6	pp	229533.I1RY44
15007	229533.A0A0E0SNM6	pp	229533.I1RTE6
15008	229533.A0A0E0SNM6	pp	229533.I1RT36
15009	229533.A0A0E0SNM6	pp	229533.I1S439
15010	229533.A0A0E0SNM6	pp	229533.I1RW16
15011	229533.A0A0E0SNM6	pp	229533.I1RBD6
15012	229533.A0A0E0SNM6	pp	229533.I1RXT9
15013	229533.A0A0E0SNM6	pp	229533.I1RZH4
15014	229533.A0A0E0SNM6	pp	229533.A0A1C3YIA8
15015	229533.A0A0E0SNM6	pp	229533.Q4INW6
15016	229533.A0A0E0SNM6	pp	229533.Q4I0M9
15017	229533.A0A0E0SNM6	pp	229533.Q4I5R3
15018	229533.A0A0E0SNM6	pp	229533.A0A1C3YK01
15019	229533.A0A0E0SNM6	pp	229533.I1RNW8
15020	229533.A0A0E0SNM6	pp	229533.I1RLX6
15021	229533.A0A0E0SNM6	pp	229533.Q4IQC1
15022	229533.A0A0E0SNM6	pp	229533.I1RTB7
15023	229533.A0A0E0SNM6	pp	229533.I1RN65
15024	229533.A0A0E0SNM6	pp	229533.I1RD70
15025	229533.A0A0E0SNM6	pp	229533.I1RH68
15026	229533.A0A0E0SNM6	pp	229533.I1RHI1
15027	229533.A0A0E0SNM6	pp	229533.I1RFZ2
15028	229533.A0A0E0SNM6	pp	229533.I1RQA3
15029	229533.A0A0E0SNM6	pp	229533.A0A1C3YI93
15030	229533.A0A0E0SNM6	pp	229533.A0A1C3YIP6
15031	229533.A0A0E0SNM6	pp	229533.I1RMT4
15032	229533.A0A0E0SNM6	pp	229533.I1RCY0
15033	229533.A0A0E0SNM6	pp	229533.I1RCM6
15034	229533.A0A0E0SNM6	pp	229533.I1S0S6
15035	229533.A0A0E0SNM6	pp	229533.I1RBL4
15036	229533.A0A0E0SNM6	pp	229533.I1RZ02
15037	229533.A0A0E0SNM6	pp	229533.I1S7X9
15038	229533.A0A0E0SNM6	pp	229533.I1RSF2

15039 229533.A0A0E0SNM6 pp 229533.I1RYZ9
15040 229533.A0A0E0SNM6 pp 229533.I1RQ80
15041 229533.A0A0E0SNM6 pp 229533.A0A1C3YL99
15042 229533.A0A0E0SNM6 pp 229533.I1RAW7
15043 229533.A0A0E0SNM6 pp 229533.Q4ICG8
15044 229533.A0A0E0SNM6 pp 229533.A0A1C3YMQ5
15045 229533.A0A0E0SNM6 pp 229533.I1RN31
15046 229533.A0A0E0SNM6 pp 229533.A0A1C3YHL0
15047 229533.A0A0E0SNM6 pp 229533.V6QV09
15048 229533.A0A0E0SNM6 pp 229533.I1RYH9
15049 229533.A0A0E0SNM6 pp 229533.I1RCD4
15050 229533.A0A0E0SNM6 pp 229533.I1RQN9
15051 229533.A0A0E0SNM6 pp 229533.I1S2E9
15052 229533.A0A0E0SNM6 pp 229533.I1S1V5
15053 229533.A0A0E0SNM6 pp 229533.I1RAN3
15054 229533.A0A0E0SNM6 pp 229533.I1RL24
15055 229533.A0A0E0SNM6 pp 229533.I1RZG0
15056 229533.I1RPZ1 pp 229533.I1RZN1
15057 229533.I1RPZ1 pp 229533.I1S272
15058 229533.I1RPZ1 pp 229533.I1RVJ8
15059 229533.I1RPZ1 pp 229533.I1RQ75
15060 229533.I1RPZ1 pp 229533.I1RRN4
15061 229533.I1RPZ1 pp 229533.I1RSM8
15062 229533.I1RPZ1 pp 229533.I1RZ13
15063 229533.I1RPZ1 pp 229533.V6RPQ8
15064 229533.I1RPZ1 pp 229533.Q4I1Y5
15065 229533.I1RPZ1 pp 229533.I1RST0
15066 229533.I1RPZ1 pp 229533.I1RSV4
15067 229533.I1RPZ1 pp 229533.I1S1K6
15068 229533.I1RPZ1 pp 229533.I1RSJ2
15069 229533.I1RPZ1 pp 229533.I1S0Y4
15070 229533.I1RPZ1 pp 229533.I1RRC4
15071 229533.I1RGB1 pp 229533.I1RY57
15072 229533.I1RGB1 pp 229533.I1RSZ7
15073 229533.I1RGB1 pp 229533.I1RW61
15074 229533.I1RGB1 pp 229533.I1RP97
15075 229533.I1RGB1 pp 229533.I1S0Q7
15076 229533.I1RGB1 pp 229533.I1RZ95
15077 229533.I1RGB1 pp 229533.I1S0R8
15078 229533.I1RGB1 pp 229533.I1RSQ4
15079 229533.I1RGB1 pp 229533.I1RYI5
15080 229533.I1RGB1 pp 229533.I1RTA1
15081 229533.I1RGB1 pp 229533.I1RKB6
15082 229533.I1RGB1 pp 229533.I1S0R9
15083 229533.I1RGB1 pp 229533.I1RJU3
15084 229533.I1RGB1 pp 229533.I1S4S4
15085 229533.I1RGB1 pp 229533.I1S4S7
15086 229533.I1RGB1 pp 229533.I1S2C1
15087 229533.I1RGB1 pp 229533.I1RSU1
15088 229533.I1RGB1 pp 229533.I1RTE9
15089 229533.I1RGB1 pp 229533.I1RY63
15090 229533.I1RRR9 pp 229533.I1RZ25
15091 229533.I1RRR9 pp 229533.I1S2F7
15092 229533.I1RRR9 pp 229533.I1RZ38
15093 229533.I1RRR9 pp 229533.I1RW55
15094 229533.I1RRR9 pp 229533.I1S267
15095 229533.I1RRR9 pp 229533.I1S3T6
15096 229533.I1RRR9 pp 229533.I1S2E9
15097 229533.I1RRR9 pp 229533.I1S210
15098 229533.I1RRR9 pp 229533.I1S223
15099 229533.I1RRR9 pp 229533.I1RST9
15100 229533.I1RRR9 pp 229533.I1S048
15101 229533.I1RRR9 pp 229533.I1SAB4
15102 229533.I1RRR9 pp 229533.Q4IB89
15103 229533.I1RRR9 pp 229533.I1RY44
15104 229533.I1RNK3 pp 229533.V6RKE4
15105 229533.I1RNK3 pp 229533.I1RZA4
15106 229533.I1RNK3 pp 229533.I1RS38
15107 229533.I1RNK3 pp 229533.I1RW83
15108 229533.I1RNK3 pp 229533.I1RZ91
15109 229533.I1RNK3 pp 229533.I1RW32
15110 229533.I1RNK3 pp 229533.I1RQX3
15111 229533.I1RNK3 pp 229533.I1S219

229533.I1RNK3 pp 229533.I1RPK5
229533.I1RNK3 pp 229533.I1RTD0
229533.I1RNK3 pp 229533.I1RNR3
229533.I1RNK3 pp 229533.V6RQ48
229533.I1RNK3 pp 229533.I1RXU1
229533.I1RNK3 pp 229533.I1RWP9
229533.I1RNK3 pp 229533.Q4ICG5
229533.I1RNK3 pp 229533.Q4INZ9
229533.I1REY8 pp 229533.I1S8X0
229533.I1REY8 pp 229533.I1RTP2
229533.I1REY8 pp 229533.I1RRC4
229533.A0A1C3YIA9 pp 229533.I1RB00
229533.A0A1C3YIA9 pp 229533.I1RCM6
229533.A0A1C3YIA9 pp 229533.I1RC35
229533.A0A1C3YIA9 pp 229533.Q4I0J6
229533.V6QYC5 pp 229533.V6RRF6
229533.I1RZ91 pp 229533.V6RQ48
229533.I1RZ91 pp 229533.Q4INZ9
229533.I1RZ91 pp 229533.I1RZL3
229533.I1RZ91 pp 229533.I1RZA4
229533.I1RZ91 pp 229533.I1S0X0
229533.I1RZ91 pp 229533.I1S0Z3
229533.I1RZ91 pp 229533.V6RKE4
229533.I1RZ91 pp 229533.I1S5R2
229533.I1RZ91 pp 229533.Q4ICG5
229533.I1RZ91 pp 229533.I1S034
229533.I1RZ91 pp 229533.I1S219
229533.I1RJI8 pp 229533.Q4I0J6
229533.I1RAW6 pp 229533.V6QU20
229533.I1RQG3 pp 229533.V6QU20
229533.I1RT65 pp 229533.I1S7X9
229533.I1RT65 pp 229533.I1RX69
229533.I1RT65 pp 229533.I1RZV1
229533.I1RT65 pp 229533.I1RXS3
229533.I1RT65 pp 229533.I1S0S8
229533.I1RT65 pp 229533.Q4HY90
229533.I1RT65 pp 229533.I1RTE0
229533.I1RT65 pp 229533.I1RTG5
229533.I1RT65 pp 229533.I1RX68
229533.I1RT65 pp 229533.I1S559
229533.I1RT65 pp 229533.Q4IPI4
229533.I1RT65 pp 229533.I1RVY0
229533.I1RT65 pp 229533.Q4ICA8
229533.I1RT65 pp 229533.I1RYW3
229533.I1RT65 pp 229533.I1S7W2
229533.I1RT65 pp 229533.I1RZL3
229533.I1RT65 pp 229533.Q4I963
229533.I1RT65 pp 229533.V6RPN0
229533.I1RT65 pp 229533.I1S248
229533.I1RT65 pp 229533.I1RWI6
229533.I1RT65 pp 229533.Q5I2J3
229533.I1RT65 pp 229533.I1RYM4
229533.I1RT65 pp 229533.Q4I948
229533.I1RT65 pp 229533.I1S270
229533.I1RT65 pp 229533.I1RVU0
229533.I1RT65 pp 229533.Q4INI2
229533.I1RT65 pp 229533.I1RW71
229533.I1RT65 pp 229533.I1S1V2
229533.A0A1C3YJP1 pp 229533.I1RT81
229533.A0A1C3YJP1 pp 229533.I1RN54
229533.A0A1C3YJP1 pp 229533.I1RWG6
229533.A0A1C3YJP1 pp 229533.I1RBL4
229533.A0A1C3YJP1 pp 229533.I1RBX2
229533.A0A1C3YJP1 pp 229533.I1RRH3
229533.A0A1C3YJP1 pp 229533.I1RDE9
229533.A0A1C3YJP1 pp 229533.I1RWM0
229533.A0A1C3YJP1 pp 229533.I1S045
229533.A0A1C3YJP1 pp 229533.I1S549
229533.A0A1C3YJP1 pp 229533.I1S082
229533.A0A1C3YJP1 pp 229533.I1S2B7
229533.A0A1C3YJP1 pp 229533.A0A1C3YKD5
229533.A0A1C3YJP1 pp 229533.I1RXB5
229533.A0A1C3YJP1 pp 229533.I1RAL2

15185 229533.A0A1C3YJP1 pp 229533.I1RUG2
15186 229533.A0A1C3YJP1 pp 229533.I1RT41
15187 229533.A0A1C3YJP1 pp 229533.I1S077
15188 229533.I1S7J1 pp 229533.V6QU20
15189 229533.V6RQ28 pp 229533.V6RRF6
15190 229533.I1S9Q3 pp 229533.Q4IJM4
15191 229533.I1S9Q3 pp 229533.V6RKY7
15192 229533.A0A1C3YI02 pp 229533.Q4ICG5
15193 229533.A0A1C3YI02 pp 229533.I1RQA3
15194 229533.A0A1C3YI02 pp 229533.I1RB41
15195 229533.A0A1C3YI02 pp 229533.I1RTD0
15196 229533.A0A1C3YI02 pp 229533.I1RW32
15197 229533.A0A1C3YI02 pp 229533.A0A1C3YK51
15198 229533.A0A1C3YI02 pp 229533.V6RQ48
15199 229533.A0A1C3YI02 pp 229533.I1RS38
15200 229533.A0A1C3YI02 pp 229533.I1RNR3
15201 229533.A0A1C3YI02 pp 229533.I1RW84
15202 229533.A0A1C3YI02 pp 229533.I1REN4
15203 229533.A0A1C3YI02 pp 229533.I1S635
15204 229533.A0A1C3YI02 pp 229533.I1RZA4
15205 229533.A0A1C3YI02 pp 229533.A0A1C3YIN0
15206 229533.A0A1C3YI02 pp 229533.I1RX09
15207 229533.A0A1C3YI02 pp 229533.I1RE20
15208 229533.A0A1C3YI02 pp 229533.A0A1C3YJH8
15209 229533.A0A1C3YI02 pp 229533.I1RPK5
15210 229533.A0A1C3YI02 pp 229533.I1S034
15211 229533.A0A1C3YI02 pp 229533.I1RQK5
15212 229533.A0A1C3YI02 pp 229533.I1SAM5
15213 229533.A0A1C3YI02 pp 229533.I1RWT6
15214 229533.A0A1C3YI02 pp 229533.I1RY44
15215 229533.A0A1C3YI02 pp 229533.I1SAT1
15216 229533.A0A1C3YI02 pp 229533.I1RLT6
15217 229533.A0A1C3YI02 pp 229533.V6RKE4
15218 229533.A0A1C3YI02 pp 229533.I1RZ91
15219 229533.A0A1C3YI02 pp 229533.I1REF9
15220 229533.A0A1C3YI02 pp 229533.I1S2E9
15221 229533.A0A1C3YI02 pp 229533.I1RXF5
15222 229533.A0A1C3YI02 pp 229533.I1RW76
15223 229533.A0A1C3YI02 pp 229533.I1RB46
15224 229533.A0A1C3YI02 pp 229533.I1RXU1
15225 229533.A0A1C3YI02 pp 229533.I1RE15
15226 229533.I1RNE1 pp 229533.I1RNE2
15227 229533.I1RNE1 pp 229533.Q4IFH8
15228 229533.I1RNE1 pp 229533.I1RWW4
15229 229533.I1RNE1 pp 229533.I1S0H1
15230 229533.I1S217 pp 229533.I1S4S7
15231 229533.I1S217 pp 229533.I1S4S4
15232 229533.I1S217 pp 229533.I1S2C1
15233 229533.A0A098DF94 pp 229533.A0A1C3YLP8
15234 229533.A0A098DF94 pp 229533.I1RZM2
15235 229533.A0A098DF94 pp 229533.I1RFZ2
15236 229533.A0A098DF94 pp 229533.A0A098DY35
15237 229533.A0A098DF94 pp 229533.I1RBV5
15238 229533.A0A098DF94 pp 229533.A0A1C3YJZ2
15239 229533.A0A098DF94 pp 229533.I1RQM0
15240 229533.A0A098DF94 pp 229533.I1RX38
15241 229533.A0A098DF94 pp 229533.Q4IR18
15242 229533.A0A098DF94 pp 229533.I1RW16
15243 229533.A0A098DF94 pp 229533.I1RBR5
15244 229533.A0A098DF94 pp 229533.I1RVY0
15245 229533.A0A098DF94 pp 229533.A0A1C3YKJ6
15246 229533.A0A098DF94 pp 229533.A0A1C3YI10
15247 229533.A0A098DF94 pp 229533.I1RBL4
15248 229533.A0A098DF94 pp 229533.A0A0E0SNY0
15249 229533.A0A098DF94 pp 229533.I1RPW0
15250 229533.A0A098DF94 pp 229533.I1RD70
15251 229533.A0A098DF94 pp 229533.I1RDC9
15252 229533.A0A098DF94 pp 229533.V6R227
15253 229533.A0A098DF94 pp 229533.I1RKE9
15254 229533.A0A098DF94 pp 229533.A0A1C3YI81
15255 229533.A0A098DF94 pp 229533.A0A0E0SHR3
15256 229533.A0A098DF94 pp 229533.I1RLX4
15257 229533.A0A098DF94 pp 229533.I1RQ80

229533.A0A098DF94	pp	229533.A0A1C3YIP6
229533.A0A098DF94	pp	229533.I1RWD4
229533.A0A098DF94	pp	229533.I1RT16
229533.A0A098DF94	pp	229533.I1RP96
229533.A0A098DF94	pp	229533.I1RML9
229533.A0A098DF94	pp	229533.A0A1C3YII4
229533.A0A098DF94	pp	229533.I1RLX6
229533.A0A098DF94	pp	229533.I1RXE5
229533.A0A098DF94	pp	229533.A0A1C3YIJ4
229533.A0A098DF94	pp	229533.A0A1C3YK37
229533.A0A098DF94	pp	229533.I1RPU4
229533.A0A098DF94	pp	229533.I1RCM6
229533.A0A098DF94	pp	229533.I1RN31
229533.A0A098DF94	pp	229533.A0A098DXS1
229533.A0A098DF94	pp	229533.A0A098E4R5
229533.A0A098DF94	pp	229533.A0A1C3YK01
229533.A0A098DF94	pp	229533.V6QV38
229533.A0A098DF94	pp	229533.A0A1C3YJV9
229533.I1RLW0	pp	229533.V6QU20
229533.I1RMT0	pp	229533.I1RTI7
229533.I1RGC5	pp	229533.I1S1W9
229533.I1RGC5	pp	229533.I1RZM8
229533.I1RGC5	pp	229533.I1RUG3
229533.I1RGC5	pp	229533.I1RZV3
229533.I1RGC5	pp	229533.V6RCZ8
229533.I1RGC5	pp	229533.I1RQ85
229533.I1RGC5	pp	229533.I1RSF1
229533.I1RGC5	pp	229533.V6R042
229533.I1RGC5	pp	229533.I1RPU2
229533.I1RGC5	pp	229533.I1RNY8
229533.I1RGC5	pp	229533.I1RXC4
229533.I1RGC5	pp	229533.I1RSQ3
229533.I1RGC5	pp	229533.I1S0M9
229533.I1RGC5	pp	229533.I1RRM3
229533.I1RGC5	pp	229533.V6RMW6
229533.I1RGC5	pp	229533.I1S128
229533.I1RGC5	pp	229533.I1RQD2
229533.I1RGC5	pp	229533.I1S0W4
229533.I1RGC5	pp	229533.I1RQ33
229533.I1RGC5	pp	229533.I1S5R6
229533.I1RGC5	pp	229533.I1RNC1
229533.I1RGC5	pp	229533.I1RQE7
229533.I1RGC5	pp	229533.Q4IJK2
229533.I1RGC5	pp	229533.I1RSZ7
229533.I1RGC5	pp	229533.I1RSA5
229533.I1RGC5	pp	229533.I1S0X7
229533.I1RGC5	pp	229533.I1RX95
229533.I1RGC5	pp	229533.I1RVX0
229533.I1RGC5	pp	229533.I1RRY9
229533.I1RGC5	pp	229533.I1RJN9
229533.I1RGC5	pp	229533.I1RWR7
229533.I1RGC5	pp	229533.I1S5R1
229533.I1RGC5	pp	229533.I1RTA1
229533.I1RGC5	pp	229533.I1RXC2
229533.I1RGC5	pp	229533.I1RVZ2
229533.I1RGC5	pp	229533.I1RZK5
229533.I1RGC5	pp	229533.I1RVU1
229533.I1RGC5	pp	229533.I1RYI5
229533.I1RGC5	pp	229533.I1S4S7
229533.I1RGC5	pp	229533.I1RPP3
229533.I1RGC5	pp	229533.I1RR24
229533.I1RGC5	pp	229533.I1RX46
229533.I1RGC5	pp	229533.I1RRX5
229533.I1RGC5	pp	229533.I1RPE0
229533.I1RGC5	pp	229533.V6RTJ8
229533.I1RGC5	pp	229533.Q4IN52
229533.I1RGC5	pp	229533.I1RRI1
229533.I1RGC5	pp	229533.I1RZ39
229533.I1RGC5	pp	229533.I1RT40
229533.I1RGC5	pp	229533.I1RTD5
229533.I1RGC5	pp	229533.I1RPY3
229533.I1RGC5	pp	229533.I1RMQ6
229533.I1RGC5	pp	229533.I1RXD7

15331 229533.I1RGC5 pp 229533.I1S2B8
15332 229533.I1RGC5 pp 229533.I1RJU3
15333 229533.I1RFG5 pp 229533.I1RNG4
15334 229533.A0A1C3YLN1 pp 229533.V6QYC5
15335 229533.A0A1C3YLN1 pp 229533.I1RRV9
15336 229533.A0A1C3YLN1 pp 229533.I1RAW6
15337 229533.A0A1C3YLN1 pp 229533.I1RQG3
15338 229533.A0A1C3YLN1 pp 229533.I1S7J1
15339 229533.A0A1C3YLN1 pp 229533.V6RQ28
15340 229533.A0A1C3YLN1 pp 229533.I1RLW0
15341 229533.A0A1C3YLN1 pp 229533.V6R5Q6
15342 229533.I1REK3 pp 229533.V6QW25
15343 229533.I1REK3 pp 229533.I1RWI6
15344 229533.I1REK3 pp 229533.I1S0X0
15345 229533.I1REK3 pp 229533.I1RW32
15346 229533.I1REK3 pp 229533.V6RR76
15347 229533.I1REK3 pp 229533.I1RZ91
15348 229533.I1REK3 pp 229533.I1RQX3
15349 229533.I1REK3 pp 229533.I1S0Z3
15350 229533.I1REK3 pp 229533.I1RKR7
15351 229533.I1REK3 pp 229533.I1RTF0
15352 229533.I1REK3 pp 229533.Q4I1H6
15353 229533.I1REK3 pp 229533.I1RZL3
15354 229533.I1REK3 pp 229533.I1REN4
15355 229533.I1REK3 pp 229533.I1RKR9
15356 229533.I1REK3 pp 229533.I1RSP4
15357 229533.I1REK3 pp 229533.I1RN81
15358 229533.I1REK3 pp 229533.I1RT41
15359 229533.I1REK3 pp 229533.I1RXU1
15360 229533.I1REK3 pp 229533.I1RG99
15361 229533.I1REK3 pp 229533.Q4ICG5
15362 229533.I1REK3 pp 229533.I1RS38
15363 229533.I1REK3 pp 229533.I1S034
15364 229533.I1RN46 pp 229533.I1S338
15365 229533.I1RN46 pp 229533.V6RUT5
15366 229533.I1RN46 pp 229533.I1RSI4
15367 229533.I1RN46 pp 229533.I1S236
15368 229533.I1RN46 pp 229533.I1RPI5
15369 229533.I1RN46 pp 229533.I1S1V5
15370 229533.I1RN46 pp 229533.I1RY57
15371 229533.I1RN46 pp 229533.Q4IP51
15372 229533.I1RN46 pp 229533.I1S2C1
15373 229533.I1RN46 pp 229533.Q4I7L0
15374 229533.I1RN46 pp 229533.V6RLW4
15375 229533.I1RN46 pp 229533.I1S2F2
15376 229533.I1RN46 pp 229533.I1S283
15377 229533.I1RN46 pp 229533.I1RSK0
15378 229533.I1RN46 pp 229533.Q4IR08
15379 229533.I1RN46 pp 229533.I1RZ94
15380 229533.I1RN46 pp 229533.I1S209
15381 229533.I1RN46 pp 229533.Q4IPI4
15382 229533.I1RN46 pp 229533.I1RXB6
15383 229533.I1RN46 pp 229533.I1RQ13
15384 229533.I1RN46 pp 229533.I1RSC7
15385 229533.I1RN46 pp 229533.Q4I0J6
15386 229533.I1RN46 pp 229533.I1S147
15387 229533.I1RN46 pp 229533.Q4IBX0
15388 229533.I1RN46 pp 229533.I1RN47
15389 229533.I1RN46 pp 229533.I1RYX9
15390 229533.I1RN46 pp 229533.I1RNE2
15391 229533.I1RN46 pp 229533.I1S237
15392 229533.I1RN46 pp 229533.I1S887
15393 229533.I1RN46 pp 229533.I1RN57
15394 229533.I1RN46 pp 229533.I1RTG6
15395 229533.I1RN46 pp 229533.I1RYR2
15396 229533.I1RN46 pp 229533.I1S1D3
15397 229533.I1RN46 pp 229533.V6R382
15398 229533.I1RN46 pp 229533.I1S0J8
15399 229533.I1RN46 pp 229533.I1RXM6
15400 229533.I1RN46 pp 229533.I1RSJ4
15401 229533.I1RN46 pp 229533.I1RW16
15402 229533.I1RN46 pp 229533.I1RN55
15403 229533.I1RN46 pp 229533.I1RPK0

15404	229533.I1RN46	pp	229533.I1RQJ4
15405	229533.I1RN46	pp	229533.I1RXU9
15406	229533.I1RN46	pp	229533.I1S1W3
15407	229533.I1RN46	pp	229533.I1S0R9
15408	229533.I1RN46	pp	229533.I1RXR1
15409	229533.I1RN46	pp	229533.I1S123
15410	229533.I1RN46	pp	229533.I1S020
15411	229533.I1RN46	pp	229533.I1S9T5
15412	229533.I1REN3	pp	229533.I1RPW8
15413	229533.I1REN3	pp	229533.I1S2G7
15414	229533.I1REN3	pp	229533.I1S5Z5
15415	229533.I1REN3	pp	229533.I1RZM2
15416	229533.I1REN3	pp	229533.I1RRG3
15417	229533.I1REN3	pp	229533.I1RTC2
15418	229533.I1REN3	pp	229533.I1RFL7
15419	229533.I1REN3	pp	229533.I1RRM8
15420	229533.I1REN3	pp	229533.I1RUF8
15421	229533.I1REN3	pp	229533.I1RW80
15422	229533.I1REN3	pp	229533.Q4IQX3
15423	229533.I1REN3	pp	229533.I1RKB1
15424	229533.I1REN3	pp	229533.Q4I5W5
15425	229533.I1REN3	pp	229533.I1RLX6
15426	229533.I1REN3	pp	229533.I1RSZ3
15427	229533.I1REN3	pp	229533.I1RT99
15428	229533.I1REN3	pp	229533.I1RPF5
15429	229533.I1REN3	pp	229533.I1S9H8
15430	229533.I1REN3	pp	229533.I1RWF3
15431	229533.I1REN3	pp	229533.I1RKI2
15432	229533.I1REN3	pp	229533.I1S2E9
15433	229533.I1REN3	pp	229533.I1RZ19
15434	229533.I1REN3	pp	229533.I1RKR8
15435	229533.I1REN3	pp	229533.I1RWT6
15436	229533.I1REN3	pp	229533.I1RY29
15437	229533.I1REN3	pp	229533.I1RML9
15438	229533.I1REN3	pp	229533.I1RQ50
15439	229533.I1REN3	pp	229533.I1RUP9
15440	229533.I1REN3	pp	229533.I1RN31
15441	229533.I1REN3	pp	229533.I1RQ42
15442	229533.I1REN3	pp	229533.I1RP96
15443	229533.I1REN3	pp	229533.I1RMQ7
15444	229533.I1REN3	pp	229533.I1S0R5
15445	229533.I1REN3	pp	229533.Q4I099
15446	229533.I1REN3	pp	229533.I1RVL8
15447	229533.I1REN3	pp	229533.Q4HYB8
15448	229533.I1REN3	pp	229533.I1RRT0
15449	229533.I1REN3	pp	229533.I1RN04
15450	229533.I1REN3	pp	229533.I1RVY0
15451	229533.I1REN3	pp	229533.I1RW16
15452	229533.I1REN3	pp	229533.I1SAD2
15453	229533.I1REN3	pp	229533.I1RR61
15454	229533.I1REN3	pp	229533.I1RUG4
15455	229533.I1REN3	pp	229533.I1RLU1
15456	229533.I1REN3	pp	229533.I1RNB8
15457	229533.I1REN3	pp	229533.Q4I5I4
15458	229533.I1REN3	pp	229533.I1RVZ4
15459	229533.I1REN3	pp	229533.I1S0E8
15460	229533.I1REN3	pp	229533.I1RQA3
15461	229533.I1REN3	pp	229533.I1RMV1
15462	229533.I1REN3	pp	229533.I1S9Y1
15463	229533.I1REN3	pp	229533.V6QUE6
15464	229533.I1REN3	pp	229533.V6QWX8
15465	229533.I1REN3	pp	229533.I1RW91
15466	229533.I1REN3	pp	229533.I1RWG8
15467	229533.I1REN3	pp	229533.I1RG11
15468	229533.I1REN3	pp	229533.I1RW90
15469	229533.I1REN3	pp	229533.I1S209
15470	229533.I1REN3	pp	229533.I1RK95
15471	229533.I1REN3	pp	229533.Q4IPC8
15472	229533.I1REN3	pp	229533.I1RRN5
15473	229533.I1REN3	pp	229533.I1S0J3
15474	229533.I1REN3	pp	229533.I1RUG0
15475	229533.I1REN3	pp	229533.Q4IJM4
15476	229533.I1REN3	pp	229533.I1RK71

229533.I1REN3	pp	229533.I1RT36
229533.I1REN3	pp	229533.I1RRF7
229533.I1REN3	pp	229533.I1RPM7
229533.I1REN3	pp	229533.I1RXE5
229533.I1REN3	pp	229533.I1RMS2
229533.I1REN3	pp	229533.I1RY44
229533.I1REN3	pp	229533.I1RXU0
229533.I1REN3	pp	229533.Q4I5U9
229533.I1REN3	pp	229533.I1S276
229533.I1REN3	pp	229533.Q4HTT2
229533.I1REN3	pp	229533.V6QV38
229533.I1REN3	pp	229533.Q4HYI0
229533.I1REN3	pp	229533.I1RK79
229533.I1REN3	pp	229533.Q4IJU0
229533.I1REN3	pp	229533.I1RYY5
229533.I1REN3	pp	229533.I1RK90
229533.I1REN3	pp	229533.I1RR25
229533.I1REN3	pp	229533.I1RFQ5
229533.I1REN3	pp	229533.I1RKF3
229533.I1REN3	pp	229533.I1RJL2
229533.I1REN3	pp	229533.I1RZB5
229533.I1S6K9	pp	229533.V6QUE6
229533.I1S6K9	pp	229533.V6RMW6
229533.I1S6K9	pp	229533.V6RUT5
229533.I1S6K9	pp	229533.V6RCZ8
229533.I1S6K9	pp	229533.I1S9Y1
229533.I1S6K9	pp	229533.V6R3J1
229533.I1S6K9	pp	229533.V6R042
229533.I1S6K9	pp	229533.V6RTJ8
229533.I1S6K9	pp	229533.I1S887
229533.I1S6K9	pp	229533.Q4I624
229533.I1S6K9	pp	229533.Q4IJK2
229533.I1S6K9	pp	229533.V6RRH2
229533.I1RE37	pp	229533.I1RIP9
229533.I1RE37	pp	229533.I1RW16
229533.I1RE37	pp	229533.I1RN57
229533.I1RE37	pp	229533.I1RSJ4
229533.I1RE37	pp	229533.I1RFZ2
229533.I1RE37	pp	229533.I1RWX7
229533.I1RE37	pp	229533.I1RW03
229533.I1RE37	pp	229533.I1RV78
229533.I1RE37	pp	229533.I1RZT1
229533.I1RE37	pp	229533.I1RX64
229533.I1RE37	pp	229533.I1S2F2
229533.I1RE37	pp	229533.I1RZ29
229533.I1RE37	pp	229533.I1RSL6
229533.I1RE37	pp	229533.I1RPP7
229533.I1RE37	pp	229533.V6RLW4
229533.I1RE37	pp	229533.I1RTN6
229533.I1RE37	pp	229533.I1RFN0
229533.I1RE37	pp	229533.I1S2E8
229533.I1RE37	pp	229533.I1RWB3
229533.I1RE37	pp	229533.I1REK6
229533.I1RE37	pp	229533.I1RN65
229533.I1RE37	pp	229533.I1RSA7
229533.I1RE37	pp	229533.I1RF02
229533.I1RE37	pp	229533.I1RUG1
229533.I1RE37	pp	229533.I1RN55
229533.I1RE37	pp	229533.I1S1C8
229533.I1RE37	pp	229533.I1RUG2
229533.I1RE37	pp	229533.Q4IR08
229533.I1RE37	pp	229533.I1RYV1
229533.I1RE37	pp	229533.I1RPU4
229533.I1RE37	pp	229533.I1RLE7
229533.I1RE37	pp	229533.Q4I0J6
229533.I1RE37	pp	229533.I1RWM6
229533.I1RE37	pp	229533.I1RMB7
229533.I1RE37	pp	229533.I1RFX8
229533.I1RE37	pp	229533.I1RYH6
229533.I1RE37	pp	229533.I1RE46
229533.I1RE37	pp	229533.I1RFJ2
229533.I1RE37	pp	229533.I1RN31
229533.I1RE37	pp	229533.I1RRT1

```
229533.I1RE37	pp	229533.I1RZZ1
229533.A0A1C3YHT4	pp	229533.I1RS38
229533.A0A1C3YHT4	pp	229533.I1RTG7
229533.A0A1C3YHT4	pp	229533.A0A1C3YKS6
229533.A0A1C3YHT4	pp	229533.Q4I8X0
229533.I1RK02	pp	229533.I1RRC4
229533.I1RK02	pp	229533.I1RSG0
229533.I1RK02	pp	229533.I1RSU2
229533.I1RK02	pp	229533.I1RZC3
229533.I1RK02	pp	229533.I1RM41
229533.I1RK02	pp	229533.I1S267
229533.I1RK02	pp	229533.I1RRN4
229533.I1RK02	pp	229533.I1RWN0
229533.I1RK02	pp	229533.I1RW69
229533.I1RK02	pp	229533.I1S9Z4
229533.I1RK02	pp	229533.I1RY32
229533.I1RK02	pp	229533.I1RR68
229533.A0A0E0SDE1	pp	229533.I1RBD2
229533.A0A0E0SDE1	pp	229533.A0A1C3YIP6
229533.A0A0E0SDE1	pp	229533.I1RLE7
229533.A0A0E0SDE1	pp	229533.I1RUG2
229533.A0A0E0SDE1	pp	229533.A0A1C3YN89
229533.A0A0E0SDE1	pp	229533.I1RN55
229533.A0A0E0SDE1	pp	229533.I1RCQ4
229533.A0A0E0SDE1	pp	229533.I1RBE4
229533.A0A0E0SDE1	pp	229533.I1RRT1
229533.A0A0E0SDE1	pp	229533.I1RN65
229533.A0A0E0SDE1	pp	229533.Q4IR08
229533.A0A0E0SDE1	pp	229533.I1RWB3
229533.A0A0E0SDE1	pp	229533.I1RN31
229533.A0A0E0SDE1	pp	229533.I1RPU4
229533.A0A0E0SDE1	pp	229533.I1RZZ1
229533.A0A0E0SDE1	pp	229533.I1RFJ2
229533.A0A0E0SDE1	pp	229533.A0A1C3YLR6
229533.A0A0E0SDE1	pp	229533.I1S1C8
229533.A0A0E0SDE1	pp	229533.I1RYH6
229533.A0A0E0SDE1	pp	229533.I1RWM6
229533.A0A0E0SDE1	pp	229533.I1RE46
229533.A0A0E0SDE1	pp	229533.I1RYV1
229533.A0A0E0SDE1	pp	229533.I1RMB7
229533.A0A0E0SDE1	pp	229533.Q4I0J6
229533.A0A0E0SDE1	pp	229533.I1RFX8
229533.A0A0E0SDE1	pp	229533.I1RFN0
229533.A0A0E0SDE1	pp	229533.I1RSL6
229533.A0A0E0SDE1	pp	229533.I1RW16
229533.A0A0E0SDE1	pp	229533.I1RB73
229533.A0A0E0SDE1	pp	229533.A0A1C3YLQ7
229533.A0A0E0SDE1	pp	229533.I1RZ29
229533.A0A0E0SDE1	pp	229533.I1RN57
229533.A0A0E0SDE1	pp	229533.A0A1C3YJI7
229533.A0A0E0SDE1	pp	229533.I1RTN6
229533.A0A0E0SDE1	pp	229533.A0A1C3YLS3
229533.A0A0E0SDE1	pp	229533.I1RPP7
229533.A0A0E0SDE1	pp	229533.I1RX64
229533.A0A0E0SDE1	pp	229533.A0A1C3YK80
229533.A0A0E0SDE1	pp	229533.I1S2F2
229533.A0A0E0SDE1	pp	229533.I1RV78
229533.A0A0E0SDE1	pp	229533.I1RW03
229533.A0A0E0SDE1	pp	229533.I1RD70
229533.A0A0E0SDE1	pp	229533.I1RZT1
229533.A0A0E0SDE1	pp	229533.I1RSJ4
229533.A0A0E0SDE1	pp	229533.I1REK6
229533.A0A0E0SDE1	pp	229533.I1S2E8
229533.A0A0E0SDE1	pp	229533.A0A1C3YI39
229533.A0A0E0SDE1	pp	229533.I1RUG1
229533.A0A0E0SDE1	pp	229533.I1RSA7
229533.A0A0E0SDE1	pp	229533.I1RIP9
229533.A0A0E0SDE1	pp	229533.I1RF02
229533.A0A0E0SDE1	pp	229533.I1RKL2
229533.A0A0E0SDE1	pp	229533.I1RCN2
229533.A0A0E0SDE1	pp	229533.V6RLW4
229533.A0A0E0SDE1	pp	229533.I1RAH9
229533.A0A0E0SDE1	pp	229533.A0A0E0SLZ3
```

229533.A0A0E0SDE1	pp	229533.I1RWX7
229533.A0A0E0SDE1	pp	229533.I1RFZ2
229533.A0A098DQF9	pp	229533.I1SAN9
229533.A0A098DQF9	pp	229533.I1RD70
229533.A0A098DQF9	pp	229533.I1RZE4
229533.A0A098DQF9	pp	229533.I1RPU8
229533.A0A098DQF9	pp	229533.I1RPW0
229533.A0A098DQF9	pp	229533.I1RCD8
229533.A0A098DQF9	pp	229533.V6R057
229533.A0A098DQF9	pp	229533.I1RBM3
229533.A0A098DQF9	pp	229533.I1R9Y9
229533.A0A098DQF9	pp	229533.I1RS17
229533.A0A098DQF9	pp	229533.I1RW16
229533.A0A098DQF9	pp	229533.V6RKY7
229533.A0A098DQF9	pp	229533.I1S216
229533.A0A098DQF9	pp	229533.A0A1C3YKJ6
229533.A0A098DQF9	pp	229533.I1RR68
229533.A0A098DQF9	pp	229533.I1RMI8
229533.A0A098DQF9	pp	229533.I1RBV5
229533.A0A098DQF9	pp	229533.I1RT75
229533.A0A098DQF9	pp	229533.A0A1C3YIS9
229533.A0A098DQF9	pp	229533.I1RQM7
229533.A0A098DQF9	pp	229533.I1RWE8
229533.A0A098DQF9	pp	229533.I1RVZ7
229533.A0A098DQF9	pp	229533.A0A1C3YKU8
229533.A0A098DQF9	pp	229533.I1RB44
229533.A0A098DQF9	pp	229533.I1RX22
229533.A0A098DQF9	pp	229533.A0A0E0SLZ3
229533.A0A098DQF9	pp	229533.A0A098E0A5
229533.A0A098DQF9	pp	229533.I1RQM0
229533.A0A098DQF9	pp	229533.I1S4Z8
229533.A0A098DQF9	pp	229533.A0A1C3YK37
229533.A0A098DQF9	pp	229533.A0A1C3YKN4
229533.A0A098DQF9	pp	229533.I1RFZ2
229533.A0A098DQF9	pp	229533.I1RTD3
229533.A0A098DQF9	pp	229533.A0A0E0SNY0
229533.A0A098DQF9	pp	229533.I1S8G4
229533.A0A098DQF9	pp	229533.I1RRV4
229533.A0A098DQF9	pp	229533.I1RKK0
229533.A0A098DQF9	pp	229533.I1RBL4
229533.A0A098DQF9	pp	229533.A0A1C3YI10
229533.A0A098DQF9	pp	229533.O74638
229533.A0A098DQF9	pp	229533.I1RPU4
229533.A0A098DQF9	pp	229533.I1RMQ4
229533.A0A098DQF9	pp	229533.I1RX38
229533.A0A098DQF9	pp	229533.I1RF82
229533.A0A098DQF9	pp	229533.I1RC22
229533.A0A098DQF9	pp	229533.I1RL04
229533.A0A098DQF9	pp	229533.I1RUQ5
229533.A0A098DQF9	pp	229533.I1RQ80
229533.A0A098DQF9	pp	229533.I1RAZ3
229533.A0A098DQF9	pp	229533.I1RW71
229533.A0A098DQF9	pp	229533.A0A1C3YK68
229533.A0A098DQF9	pp	229533.I1RPN4
229533.A0A098DQF9	pp	229533.A0A1C3YLC2
229533.A0A098DQF9	pp	229533.I1RCM6
229533.A0A098DQF9	pp	229533.A0A1C3YIP6
229533.A0A098DQF9	pp	229533.V6R1K4
229533.A0A098DQF9	pp	229533.V6RBN2
229533.A0A098DQF9	pp	229533.I1RK95
229533.A0A098DQF9	pp	229533.A0A098DY09
229533.A0A098DQF9	pp	229533.A0A1C3YKS6
229533.A0A098DQF9	pp	229533.I1RQS9
229533.A0A098DQF9	pp	229533.I1RM35
229533.A0A098DQF9	pp	229533.A0A098DR54
229533.A0A098DQF9	pp	229533.A0A1C3YN30
229533.A0A098DQF9	pp	229533.A0A098DXS1
229533.A0A098DQF9	pp	229533.I1RCL4
229533.A0A098DQF9	pp	229533.I1RLX6
229533.A0A098DQF9	pp	229533.I1RFQ7
229533.A0A098DQF9	pp	229533.I1RME6
229533.A0A098DQF9	pp	229533.I1S0B7
229533.A0A098DQF9	pp	229533.A0A0E0RNI4

15696	229533.A0A098DQF9	pp	229533.A0A0E0SAC4
15697	229533.A0A098DQF9	pp	229533.I1RYH9
15698	229533.A0A098DQF9	pp	229533.I1RB39
15699	229533.A0A098DQF9	pp	229533.I1RN31
15700	229533.A0A0E0SA06	pp	229533.I1RJZ7
15701	229533.A0A0E0SA06	pp	229533.I1RW71
15702	229533.I1RFJ2	pp	229533.I1RSI4
15703	229533.I1RFJ2	pp	229533.Q4IP51
15704	229533.I1RFJ2	pp	229533.I1RFL7
15705	229533.I1RFJ2	pp	229533.I1RYX9
15706	229533.I1RFJ2	pp	229533.I1RFZ2
15707	229533.I1RFJ2	pp	229533.I1RYR2
15708	229533.I1RFJ2	pp	229533.I1RQ92
15709	229533.I1RFJ2	pp	229533.I1RWI8
15710	229533.I1RFJ2	pp	229533.I1RKI2
15711	229533.I1RFJ2	pp	229533.I1RLX6
15712	229533.I1RFJ2	pp	229533.I1S237
15713	229533.I1RFJ2	pp	229533.I1RY29
15714	229533.I1RFJ2	pp	229533.I1RHR0
15715	229533.I1RFJ2	pp	229533.I1S2E9
15716	229533.I1RFJ2	pp	229533.I1RQ67
15717	229533.I1RFJ2	pp	229533.V6RGI1
15718	229533.I1RFJ2	pp	229533.Q4IEA5
15719	229533.I1RFJ2	pp	229533.Q4IQM6
15720	229533.I1RFJ2	pp	229533.I1RPU4
15721	229533.I1RFJ2	pp	229533.I1RM36
15722	229533.I1RFJ2	pp	229533.I1S2F2
15723	229533.I1RFJ2	pp	229533.I1S236
15724	229533.I1RFJ2	pp	229533.I1S209
15725	229533.I1RFJ2	pp	229533.I1RYQ1
15726	229533.I1RFJ2	pp	229533.Q4IR08
15727	229533.I1RFJ2	pp	229533.Q4HYB8
15728	229533.I1RFJ2	pp	229533.I1RWM6
15729	229533.I1RFJ2	pp	229533.I1RPQ1
15730	229533.I1RFJ2	pp	229533.I1RPZ0
15731	229533.I1RFJ2	pp	229533.I1RQA3
15732	229533.I1RFJ2	pp	229533.I1RN57
15733	229533.I1RFJ2	pp	229533.I1RRT1
15734	229533.I1RFJ2	pp	229533.I1RST8
15735	229533.I1RFJ2	pp	229533.I1RWB3
15736	229533.I1RFJ2	pp	229533.I1RN55
15737	229533.I1RFJ2	pp	229533.I1RW16
15738	229533.I1RFJ2	pp	229533.I1RN65
15739	229533.I1RFJ2	pp	229533.V6R382
15740	229533.I1RFJ2	pp	229533.I1RQ13
15741	229533.I1RFJ2	pp	229533.I1RKK7
15742	229533.I1RFJ2	pp	229533.I1RZ86
15743	229533.I1RFJ2	pp	229533.I1RG77
15744	229533.I1RFJ2	pp	229533.I1RRN5
15745	229533.I1RFJ2	pp	229533.I1S020
15746	229533.I1RFJ2	pp	229533.I1RZ94
15747	229533.I1RFJ2	pp	229533.I1RY44
15748	229533.I1RFJ2	pp	229533.I1RUG2
15749	229533.I1RFJ2	pp	229533.Q4I0J6
15750	229533.I1RFJ2	pp	229533.I1RWT6
15751	229533.I1RFJ2	pp	229533.I1RN99
15752	229533.I1RFJ2	pp	229533.V6RLW4
15753	229533.I1RFJ2	pp	229533.I1RFX8
15754	229533.I1RFJ2	pp	229533.I1S604
15755	229533.I1RFJ2	pp	229533.Q4IBX0
15756	229533.I1RFJ2	pp	229533.I1RYH9
15757	229533.I1RFJ2	pp	229533.I1RY73
15758	229533.I1RFJ2	pp	229533.I1RMB7
15759	229533.I1RFJ2	pp	229533.Q4IJU0
15760	229533.I1RFJ2	pp	229533.I1RSA7
15761	229533.I1RFJ2	pp	229533.I1RUG4
15762	229533.I1RFJ2	pp	229533.I1S461
15763	229533.I1RFJ2	pp	229533.I1RSC7
15764	229533.I1RFJ2	pp	229533.I1RUG1
15765	229533.I1RFJ2	pp	229533.I1RZ98
15766	229533.I1RFJ2	pp	229533.I1RN46
15767	229533.I1RFJ2	pp	229533.Q4IC11
15768	229533.I1RFJ2	pp	229533.I1RSP7

15769	229533.I1RFJ2	pp	229533.I1RQI6
15770	229533.I1RFJ2	pp	229533.I1RLE7
15771	229533.I1RFJ2	pp	229533.Q4INS6
15772	229533.I1RFJ2	pp	229533.I1RN31
15773	229533.I1RFJ2	pp	229533.I1S283
15774	229533.I1RFJ2	pp	229533.I1RSL6
15775	229533.I1RFJ2	pp	229533.I1RQJ4
15776	229533.I1RFJ2	pp	229533.I1RMX3
15777	229533.I1RFJ2	pp	229533.I1RX64
15778	229533.I1RFJ2	pp	229533.I1RRS7
15779	229533.I1RFJ2	pp	229533.I1RFR6
15780	229533.I1RFJ2	pp	229533.I1RZP8
15781	229533.I1RFJ2	pp	229533.I1RSR9
15782	229533.I1RFJ2	pp	229533.I1RTN6
15783	229533.I1RKK7	pp	229533.V6RLW4
15784	229533.I1RKK7	pp	229533.I1RSI4
15785	229533.I1RKK7	pp	229533.I1RPZ0
15786	229533.I1RKK7	pp	229533.I1S604
15787	229533.I1RKK7	pp	229533.I1RQ92
15788	229533.I1RKK7	pp	229533.I1RLX6
15789	229533.I1S604	pp	229533.I1S6Y6
15790	229533.I1S604	pp	229533.V6R382
15791	229533.I1S604	pp	229533.Q4IQM6
15792	229533.I1S604	pp	229533.Q4I6A4
15793	229533.I1S604	pp	229533.Q4I7L0
15794	229533.I1S604	pp	229533.Q4INS6
15795	229533.I1S604	pp	229533.I1S605
15796	229533.I1S604	pp	229533.V6RLW4
15797	229533.I1S604	pp	229533.Q4ICG8
15798	229533.I1RN73	pp	229533.V6R042
15799	229533.I1S419	pp	229533.V6R227
15800	229533.I1S419	pp	229533.V6RCG5
15801	229533.I1S419	pp	229533.V6RIV9
15802	229533.I1S419	pp	229533.V6RUT5
15803	229533.I1S419	pp	229533.I1S4S7
15804	229533.I1S419	pp	229533.V6RCZ8
15805	229533.I1S419	pp	229533.V6QW22
15806	229533.I1S419	pp	229533.I1S5B0
15807	229533.I1S419	pp	229533.Q4I327
15808	229533.I1S419	pp	229533.V6R042
15809	229533.I1S419	pp	229533.V6QUE6
15810	229533.I1S419	pp	229533.V6RTJ8
15811	229533.I1S419	pp	229533.V6RMW6
15812	229533.I1S419	pp	229533.I1S887
15813	229533.I1S419	pp	229533.Q4HXF0
15814	229533.I1S419	pp	229533.Q4IJK2
15815	229533.I1S419	pp	229533.I1S5R1
15816	229533.I1S419	pp	229533.I1S5R6
15817	229533.I1S419	pp	229533.V6R057
15818	229533.I1S419	pp	229533.Q4I624
15819	229533.I1RTD9	pp	229533.I1S9U2
15820	229533.I1RTD9	pp	229533.I1RZW8
15821	229533.I1RTD9	pp	229533.I1RVZ0
15822	229533.I1RTD9	pp	229533.I1S2G9
15823	229533.I1RTD9	pp	229533.I1S2E8
15824	229533.I1RTD9	pp	229533.Q4HVW2
15825	229533.I1RTD9	pp	229533.I1RTE9
15826	229533.I1RTD9	pp	229533.I1RW61
15827	229533.I1RTD9	pp	229533.V6RGN5
15828	229533.I1RTD9	pp	229533.I1RTF4
15829	229533.I1RTD9	pp	229533.V6RCG5
15830	229533.I1RTD9	pp	229533.I1S8E2
15831	229533.I1RTD9	pp	229533.Q4HWE2
15832	229533.I1RTD9	pp	229533.I1RUQ2
15833	229533.I1RTD9	pp	229533.I1RZS6
15834	229533.I1RTD9	pp	229533.I1S0R7
15835	229533.I1RTD9	pp	229533.I1RW25
15836	229533.I1RTD9	pp	229533.I1RX08
15837	229533.I1RTD9	pp	229533.V6QYF0
15838	229533.I1RTD9	pp	229533.I1RZF6
15839	229533.I1RTD9	pp	229533.I1RVQ0
15840	229533.I1RTD9	pp	229533.I1S0J1
15841	229533.I1RTD9	pp	229533.I1RY63

15842	229533.I1RTD9	pp	229533.Q4IPI1
15843	229533.I1RTD9	pp	229533.I1RX14
15844	229533.I1RTD9	pp	229533.V6QW22
15845	229533.I1RTD9	pp	229533.Q4IBS2
15846	229533.I1RTD9	pp	229533.I1S6F0
15847	229533.I1RTD9	pp	229533.I1RW99
15848	229533.I1RTD9	pp	229533.I1S0M9
15849	229533.I1RTD9	pp	229533.Q4IEK8
15850	229533.I1RTD9	pp	229533.Q4IQW0
15851	229533.I1RTD9	pp	229533.I1S0L3
15852	229533.I1RTD9	pp	229533.Q4HZ42
15853	229533.I1RTD9	pp	229533.V6R227
15854	229533.I1RTD9	pp	229533.Q4IJ56
15855	229533.I1RJI7	pp	229533.Q4I0J6
15856	229533.I1RCY2	pp	229533.I1RPZ0
15857	229533.I1RCY2	pp	229533.V6RLW4
15858	229533.I1RCY2	pp	229533.I1RFJ2
15859	229533.I1RCY2	pp	229533.I1RQ05
15860	229533.I1RCY2	pp	229533.I1RSI4
15861	229533.I1RCY2	pp	229533.I1RFX8
15862	229533.I1RCY2	pp	229533.I1RNK1
15863	229533.I1RCY2	pp	229533.Q4I6A4
15864	229533.I1RCY2	pp	229533.I1RQ92
15865	229533.I1RCY2	pp	229533.I1RKK7
15866	229533.I1RCY2	pp	229533.Q4I7L0
15867	229533.I1RCY2	pp	229533.I1RE46
15868	229533.I1RCY2	pp	229533.I1RN99
15869	229533.I1RCY2	pp	229533.I1S604
15870	229533.I1RAH9	pp	229533.I1RPF5
15871	229533.I1RAH9	pp	229533.I1RUG1
15872	229533.I1RAH9	pp	229533.I1RF02
15873	229533.I1RAH9	pp	229533.I1RLX6
15874	229533.I1RAH9	pp	229533.I1RN31
15875	229533.I1RAH9	pp	229533.I1RYV1
15876	229533.I1RAH9	pp	229533.I1RWM6
15877	229533.I1RAH9	pp	229533.I1S523
15878	229533.I1RAH9	pp	229533.I1RCN2
15879	229533.I1RAH9	pp	229533.I1RBW0
15880	229533.I1RAH9	pp	229533.I1RMB7
15881	229533.I1RAH9	pp	229533.I1RR95
15882	229533.I1RAH9	pp	229533.I1RPF1
15883	229533.I1RAH9	pp	229533.I1RSA7
15884	229533.I1RAH9	pp	229533.I1RCQ4
15885	229533.I1RAH9	pp	229533.I1RWX7
15886	229533.I1RAH9	pp	229533.I1RB73
15887	229533.I1RAH9	pp	229533.Q4HZS8
15888	229533.I1RAH9	pp	229533.I1RSI4
15889	229533.I1RAH9	pp	229533.I1RBL4
15890	229533.I1RAH9	pp	229533.I1RNN1
15891	229533.I1RAH9	pp	229533.I1RSC7
15892	229533.I1RAH9	pp	229533.I1S2F2
15893	229533.I1RAH9	pp	229533.I1RS37
15894	229533.I1RAH9	pp	229533.I1RWB3
15895	229533.I1RAH9	pp	229533.I1RFN0
15896	229533.I1RAH9	pp	229533.I1RC54
15897	229533.I1RAH9	pp	229533.I1RBW1
15898	229533.I1RAH9	pp	229533.I1RSL6
15899	229533.I1RAH9	pp	229533.I1RC01
15900	229533.I1RAH9	pp	229533.I1S283
15901	229533.I1RAH9	pp	229533.I1RUG4
15902	229533.I1RAH9	pp	229533.I1RFJ2
15903	229533.I1RAH9	pp	229533.I1RE37
15904	229533.I1RAH9	pp	229533.I1RRS7
15905	229533.I1RAH9	pp	229533.I1RMX3
15906	229533.I1RAH9	pp	229533.I1RSJ4
15907	229533.I1RAH9	pp	229533.I1RN55
15908	229533.I1RAH9	pp	229533.I1RW16
15909	229533.I1RAH9	pp	229533.I1RX64
15910	229533.I1RAH9	pp	229533.I1RZK9
15911	229533.I1RAH9	pp	229533.I1RAL2
15912	229533.I1RAH9	pp	229533.I1RN57
15913	229533.I1RAH9	pp	229533.I1RCM6
15914	229533.I1RAH9	pp	229533.I1RDJ8

229533.I1RAH9 pp 229533.I1RY58
229533.I1RAH9 pp 229533.I1RW03
229533.I1RAH9 pp 229533.I1RTN6
229533.I1RAH9 pp 229533.Q4INS6
229533.I1RAH9 pp 229533.I1RZT1
229533.I1RAH9 pp 229533.I1RD70
229533.I1RBU3 pp 229533.I1RBU5
229533.A0A1C3YMQ9 pp 229533.I1RRS6
229533.A0A1C3YMQ9 pp 229533.I1RYU6
229533.A0A1C3YMQ9 pp 229533.I1RQV8
229533.A0A1C3YMQ9 pp 229533.I1R9F2
229533.A0A1C3YMQ9 pp 229533.V6R5T7
229533.A0A1C3YMQ9 pp 229533.I1RQV7
229533.A0A1C3YMQ9 pp 229533.I1S5D0
229533.A0A1C3YMQ9 pp 229533.I1RQ70
229533.A0A1C3YMQ9 pp 229533.I1S210
229533.A0A1C3YMQ9 pp 229533.I1S659
229533.A0A1C3YMQ9 pp 229533.V6RCC0
229533.A0A1C3YMQ9 pp 229533.V6RHX2
229533.A0A1C3YMQ9 pp 229533.V6RHZ8
229533.A0A1C3YMQ9 pp 229533.I1RIB5
229533.A0A1C3YMQ9 pp 229533.I1RF38
229533.A0A1C3YMQ9 pp 229533.I1RZ38
229533.A0A1C3YMQ9 pp 229533.I1S8D3
229533.A0A1C3YMQ9 pp 229533.I1RGI2
229533.A0A1C3YMQ9 pp 229533.I1RSA5
229533.A0A1C3YMQ9 pp 229533.V6RWB3
229533.A0A1C3YMQ9 pp 229533.I1RPH3
229533.A0A1C3YMQ9 pp 229533.I1SAT2
229533.A0A1C3YMQ9 pp 229533.I1RIJ6
229533.A0A1C3YMQ9 pp 229533.I1RCL1
229533.A0A1C3YMQ9 pp 229533.I1RNV4
229533.A0A1C3YMQ9 pp 229533.I1RB05
229533.A0A1C3YMQ9 pp 229533.V6RCJ5
229533.A0A1C3YMQ9 pp 229533.I1REJ3
229533.A0A1C3YMQ9 pp 229533.I1RE52
229533.A0A1C3YMQ9 pp 229533.I1S6V0
229533.A0A1C3YMQ9 pp 229533.I1RH83
229533.I1RB49 pp 229533.I1RQ67
229533.I1RB49 pp 229533.I1RLX6
229533.I1RB49 pp 229533.I1SAP6
229533.I1RB49 pp 229533.I1RKQ0
229533.I1RB49 pp 229533.I1RU61
229533.I1RB49 pp 229533.I1RPZ0
229533.I1RB49 pp 229533.I1RXI6
229533.I1RB49 pp 229533.I1RDB1
229533.I1RB49 pp 229533.I1RW16
229533.I1RB49 pp 229533.I1RBL4
229533.I1RB49 pp 229533.I1RSK0
229533.I1RB49 pp 229533.I1RCY2
229533.I1RB49 pp 229533.I1RCD4
229533.I1RB49 pp 229533.I1RMK8
229533.I1RB49 pp 229533.V6RKY7
229533.I1RB49 pp 229533.I1S604
229533.I1RB49 pp 229533.I1RN31
229533.I1RB49 pp 229533.I1RF70
229533.I1RB49 pp 229533.V6REE9
229533.I1RB49 pp 229533.I1RCA9
229533.I1RB49 pp 229533.I1RD19
229533.I1RB49 pp 229533.I1RTE6
229533.I1RB49 pp 229533.I1RD70
229533.I1RYP9 pp 229533.Q4I0J6
229533.I1RNT9 pp 229533.I1S438
229533.I1RNT9 pp 229533.Q4IJK2
229533.I1RNT9 pp 229533.I1RZK5
229533.I1RNT9 pp 229533.V6RTJ8
229533.I1RNT9 pp 229533.I1RXD7
229533.I1RNT9 pp 229533.I1S5B0
229533.I1RNT9 pp 229533.I1RWR7
229533.I1RNT9 pp 229533.I1S4S4
229533.I1RNT9 pp 229533.I1S4S7
229533.I1RNT9 pp 229533.I1RZM8
229533.I1RNT9 pp 229533.I1RQ33

15988 229533.I1RNT9 pp 229533.I1RQD2
15989 229533.I1RNT9 pp 229533.V6RCZ8
15990 229533.I1RNT9 pp 229533.I1RQE7
15991 229533.I1RNT9 pp 229533.I1S2B8
15992 229533.I1RNT9 pp 229533.I1RVX0
15993 229533.I1RNT9 pp 229533.I1RZ39
15994 229533.I1RNT9 pp 229533.I1RXC4
15995 229533.I1RNT9 pp 229533.I1S5R6
15996 229533.I1RNT9 pp 229533.I1RQ85
15997 229533.I1RNT9 pp 229533.I1RT40
15998 229533.I1RNT9 pp 229533.I1RSZ7
15999 229533.I1RNT9 pp 229533.I1RXC2
16000 229533.I1RNT9 pp 229533.I1S0W4
16001 229533.I1RNT9 pp 229533.I1RUG3
16002 229533.I1RNT9 pp 229533.I1RRI1
16003 229533.I1RNT9 pp 229533.I1S5R1
16004 229533.I1RNT9 pp 229533.I1RRM3
16005 229533.I1RNT9 pp 229533.I1RVZ2
16006 229533.I1RNT9 pp 229533.I1RSA5
16007 229533.I1RNT9 pp 229533.I1RPE0
16008 229533.I1RNT9 pp 229533.I1RTA1
16009 229533.I1RNT9 pp 229533.V6RMW6
16010 229533.I1RNT9 pp 229533.V6R042
16011 229533.I1RNT9 pp 229533.I1RYR0
16012 229533.I1RNT9 pp 229533.I1RSQ3
16013 229533.I1RNT9 pp 229533.I1RZV3
16014 229533.I1RNT9 pp 229533.I1RPP3
16015 229533.I1RNT9 pp 229533.I1S437
16016 229533.I1RNT9 pp 229533.I1S1W9
16017 229533.I1RNT9 pp 229533.I1RYI5
16018 229533.I1RNT9 pp 229533.I1RPK0
16019 229533.I1RNT9 pp 229533.I1RPU2
16020 229533.I1RCI8 pp 229533.I1RFT6
16021 229533.I1RCI8 pp 229533.I1S1X9
16022 229533.I1RCI8 pp 229533.I1RQS5
16023 229533.I1RCI8 pp 229533.I1RN58
16024 229533.I1RCI8 pp 229533.I1RYB1
16025 229533.I1RCI8 pp 229533.I1RSS4
16026 229533.I1RCI8 pp 229533.I1RXU7
16027 229533.I1RCI8 pp 229533.I1RX24
16028 229533.I1RCI8 pp 229533.I1RW71
16029 229533.I1RCI8 pp 229533.I1RT33
16030 229533.I1RCI8 pp 229533.I1RE00
16031 229533.I1RCI8 pp 229533.I1RPU0
16032 229533.I1RCI8 pp 229533.I1RQJ5
16033 229533.I1RWB3 pp 229533.I1RYV1
16034 229533.I1RWB3 pp 229533.I1RWM6
16035 229533.I1RWB3 pp 229533.Q4INS6
16036 229533.I1RWB3 pp 229533.I1S2F2
16037 229533.I1RWB3 pp 229533.I1S523
16038 229533.I1RWB3 pp 229533.I1RZT1
16039 229533.I1RWB3 pp 229533.I1S283
16040 229533.I1RWB3 pp 229533.I1RY58
16041 229533.I1RWB3 pp 229533.I1RX64
16042 229533.A0A1C3YJZ1 pp 229533.I1RBE7
16043 229533.A0A1C3YJZ1 pp 229533.V6RSF3
16044 229533.A0A1C3YJZ1 pp 229533.I1RY87
16045 229533.A0A1C3YJZ1 pp 229533.I1RJE8
16046 229533.A0A1C3YJZ1 pp 229533.A0A1C3YLB6
16047 229533.A0A1C3YJZ1 pp 229533.I1RZ29
16048 229533.A0A1C3YJZ1 pp 229533.I1RPW1
16049 229533.A0A1C3YJZ1 pp 229533.V6QWD8
16050 229533.A0A1C3YJZ1 pp 229533.I1S1W8
16051 229533.A0A1C3YJZ1 pp 229533.Q4HVQ9
16052 229533.A0A1C3YJZ1 pp 229533.I1S3G4
16053 229533.A0A1C3YJZ1 pp 229533.I1RNG4
16054 229533.A0A1C3YJZ1 pp 229533.I1S2D5
16055 229533.A0A1C3YJZ1 pp 229533.I1RMG6
16056 229533.A0A1C3YJZ1 pp 229533.I1RA28
16057 229533.I1S3T6 pp 229533.I1S968
16058 229533.I1S3T6 pp 229533.I1S6H3
16059 229533.I1S3T6 pp 229533.I1S5D0
16060 229533.I1S3T6 pp 229533.I1SAT2

16061 229533.I1S3T6 pp 229533.I1S6V0
16062 229533.I1S3T6 pp 229533.V6R5T7
16063 229533.I1S3T6 pp 229533.V6RCJ5
16064 229533.I1S3T6 pp 229533.I1S659
16065 229533.I1S3T6 pp 229533.V6RHX2
16066 229533.I1S3T6 pp 229533.I1S8D3
16067 229533.I1S3T6 pp 229533.V6RWB3
16068 229533.I1S3T6 pp 229533.V6RCC0
16069 229533.I1S3T6 pp 229533.I1S969
16070 229533.I1RYD9 pp 229533.V6RXE7
16071 229533.I1RYD9 pp 229533.V6R0U9
16072 229533.I1RYD9 pp 229533.I1S0V4
16073 229533.A0A098DYT2 pp 229533.I1S3L2
16074 229533.A0A098DYT2 pp 229533.I1RQP2
16075 229533.A0A098DYT2 pp 229533.I1RXS0
16076 229533.A0A098DYT2 pp 229533.I1RHZ5
16077 229533.A0A098DYT2 pp 229533.I1S0K0
16078 229533.A0A098DYT2 pp 229533.I1S8R4
16079 229533.A0A098DYT2 pp 229533.I1RC48
16080 229533.A0A1C3YKN5 pp 229533.I1S5D0
16081 229533.A0A1C3YKN5 pp 229533.I1RRS6
16082 229533.A0A1C3YKN5 pp 229533.I1R9F2
16083 229533.A0A1C3YKN5 pp 229533.I1S6H3
16084 229533.A0A1C3YKN5 pp 229533.I1RDN3
16085 229533.A0A1C3YKN5 pp 229533.I1RCU2
16086 229533.A0A1C3YKN5 pp 229533.I1RPU4
16087 229533.A0A1C3YKN5 pp 229533.I1S094
16088 229533.A0A1C3YKN5 pp 229533.I1RNG4
16089 229533.A0A1C3YKN5 pp 229533.I1RST9
16090 229533.A0A1C3YKN5 pp 229533.I1S659
16091 229533.A0A1C3YKN5 pp 229533.I1S0A5
16092 229533.A0A1C3YKN5 pp 229533.I1RIB5
16093 229533.A0A1C3YKN5 pp 229533.I1RA13
16094 229533.A0A1C3YKN5 pp 229533.I1RYK0
16095 229533.A0A1C3YKN5 pp 229533.I1RQM0
16096 229533.A0A1C3YKN5 pp 229533.I1RZ56
16097 229533.A0A1C3YKN5 pp 229533.I1SAT2
16098 229533.A0A1C3YKN5 pp 229533.I1RE52
16099 229533.A0A1C3YKN5 pp 229533.I1RPV8
16100 229533.A0A1C3YKN5 pp 229533.I1RZ91
16101 229533.A0A1C3YKN5 pp 229533.A0A1C3YLB6
16102 229533.A0A1C3YKN5 pp 229533.Q4HY90
16103 229533.A0A1C3YKN5 pp 229533.I1RH83
16104 229533.A0A1C3YKN5 pp 229533.I1RJS1
16105 229533.A0A1C3YKN5 pp 229533.I1RR61
16106 229533.A0A1C3YKN5 pp 229533.I1RF85
16107 229533.A0A1C3YKN5 pp 229533.I1RCH5
16108 229533.A0A098DZN5 pp 229533.A0A1C3YLC6
16109 229533.A0A098DZN5 pp 229533.I1RFS3
16110 229533.A0A098DZN5 pp 229533.I1RX68
16111 229533.A0A098DZN5 pp 229533.I1RQP1
16112 229533.A0A098DZN5 pp 229533.A0A1C3YM61
16113 229533.A0A098DZN5 pp 229533.Q4HTT1
16114 229533.A0A098DZN5 pp 229533.I1S559
16115 229533.A0A098DZN5 pp 229533.I1S7W2
16116 229533.A0A098DZN5 pp 229533.I1RNG4
16117 229533.A0A098DZN5 pp 229533.I1RB25
16118 229533.A0A098DZN5 pp 229533.I1RXS3
16119 229533.A0A098DZN5 pp 229533.I1RBL4
16120 229533.A0A098DZN5 pp 229533.I1RVY0
16121 229533.A0A098DZN5 pp 229533.I1RFW1
16122 229533.A0A098DZN5 pp 229533.I1RJZ7
16123 229533.A0A098DZN5 pp 229533.I1RE49
16124 229533.A0A098DZN5 pp 229533.I1RKI1
16125 229533.A0A098DZN5 pp 229533.I1RC90
16126 229533.A0A098DZN5 pp 229533.I1S248
16127 229533.A0A098DZN5 pp 229533.I1S7X9
16128 229533.A0A098DZN5 pp 229533.A0A1C3YLK5
16129 229533.A0A098DZN5 pp 229533.I1RZL3
16130 229533.A0A098DZN5 pp 229533.A0A0E0S6D3
16131 229533.A0A098DZN5 pp 229533.I1RKR9
16132 229533.A0A098DZN5 pp 229533.I1RQG4
16133 229533.A0A098DZN5 pp 229533.Q4IPI4

16134 229533.A0A098DZN5 pp 229533.I1RCF0
16135 229533.A0A098DZN5 pp 229533.I1RSZ6
16136 229533.A0A098DZN5 pp 229533.I1RTG5
16137 229533.A0A098DZN5 pp 229533.A0A1C3YLA2
16138 229533.A0A098DZN5 pp 229533.I1RYW3
16139 229533.A0A098DZN5 pp 229533.A0A1C3YHP3
16140 229533.A0A098DZN5 pp 229533.I1RRF0
16141 229533.A0A098DZN5 pp 229533.I1RB89
16142 229533.A0A098DZN5 pp 229533.A0A1C3YMS7
16143 229533.A0A098DZN5 pp 229533.Q4HY90
16144 229533.A0A098DZN5 pp 229533.I1RZV1
16145 229533.A0A098DZN5 pp 229533.I1RX69
16146 229533.A0A098DZN5 pp 229533.I1RTE0
16147 229533.A0A098DZN5 pp 229533.I1RYN7
16148 229533.A0A098DZN5 pp 229533.I1S0S8
16149 229533.A0A098DZN5 pp 229533.I1RW71
16150 229533.A0A098DZN5 pp 229533.I1RT16
16151 229533.A0A098DZN5 pp 229533.Q5I2J3
16152 229533.A0A098DZN5 pp 229533.I1S1V2
16153 229533.A0A098DZN5 pp 229533.I1RR74
16154 229533.A0A098DZN5 pp 229533.Q4IQX3
16155 229533.A0A098DZN5 pp 229533.I1RA72
16156 229533.A0A098DZN5 pp 229533.I1RAN8
16157 229533.A0A098DZN5 pp 229533.Q4INI2
16158 229533.A0A098DZN5 pp 229533.A0A1C3YLL9
16159 229533.A0A098DZN5 pp 229533.V6RPN0
16160 229533.A0A098DZN5 pp 229533.A0A1C3YIQ1
16161 229533.A0A098DZN5 pp 229533.A0A1C3YI55
16162 229533.A0A098DZN5 pp 229533.I1RQF7
16163 229533.A0A098DZN5 pp 229533.I1S270
16164 229533.A0A098DZN5 pp 229533.Q4ICA8
16165 229533.A0A098DZN5 pp 229533.I1RBL7
16166 229533.A0A098DZN5 pp 229533.I1RMG2
16167 229533.A0A098DZN5 pp 229533.I1RAS5
16168 229533.A0A098DZN5 pp 229533.I1RRF9
16169 229533.A0A098DZN5 pp 229533.Q4I948
16170 229533.A0A098DZN5 pp 229533.I1RQP5
16171 229533.A0A098DZN5 pp 229533.A0A1C3YI44
16172 229533.A0A098DZN5 pp 229533.I1RQM8
16173 229533.A0A098DZN5 pp 229533.I1RQP3
16174 229533.A0A098DZN5 pp 229533.I1RWI6
16175 229533.A0A098DZN5 pp 229533.Q4I963
16176 229533.A0A098DZN5 pp 229533.I1RE51
16177 229533.A0A098DZN5 pp 229533.I1RPU1
16178 229533.A0A098DZN5 pp 229533.I1RSD7
16179 229533.A0A098DZN5 pp 229533.Q4IER8
16180 229533.A0A098DZN5 pp 229533.I1RBR0
16181 229533.A0A098DZN5 pp 229533.I1RC80
16182 229533.A0A098DZN5 pp 229533.I1RBB0
16183 229533.A0A1C3YMZ1 pp 229533.I1RKR7
16184 229533.I1RFU3 pp 229533.V6QV38
16185 229533.I1RFU3 pp 229533.I1RT16
16186 229533.I1RFU3 pp 229533.I1RML9
16187 229533.I1RFU3 pp 229533.I1RPU4
16188 229533.I1RFU3 pp 229533.I1RXE5
16189 229533.I1RFU3 pp 229533.I1RP96
16190 229533.I1RFU3 pp 229533.I1RQ80
16191 229533.I1RFU3 pp 229533.I1RLX6
16192 229533.I1RFU3 pp 229533.I1RN31
16193 229533.I1RFU3 pp 229533.V6R227
16194 229533.I1RFU3 pp 229533.I1RWD4
16195 229533.I1RFU3 pp 229533.I1RQM0
16196 229533.I1RFU3 pp 229533.I1RW16
16197 229533.I1RFU3 pp 229533.I1RKE9
16198 229533.I1RFU3 pp 229533.I1RX38
16199 229533.I1RFU3 pp 229533.I1RPW0
16200 229533.I1RFU3 pp 229533.Q4IR18
16201 229533.I1RFU3 pp 229533.I1RVY0
16202 229533.I1RFU3 pp 229533.I1RLX4
16203 229533.I1RFU3 pp 229533.I1RFZ2
16204 229533.I1RFU3 pp 229533.I1RZM2
16205 229533.A0A1C3YMY4 pp 229533.I1S2Q0
16206 229533.A0A1C3YMY4 pp 229533.I1S2J3

16207 229533.I1RAL2 pp 229533.I1RBL4
16208 229533.I1RAL2 pp 229533.I1RWG6
16209 229533.I1RAL2 pp 229533.I1RDE9
16210 229533.I1RAL2 pp 229533.I1RMP0
16211 229533.I1RAL2 pp 229533.I1RCY2
16212 229533.I1RAL2 pp 229533.I1RFZ2
16213 229533.I1RAL2 pp 229533.I1RCD4
16214 229533.I1RAL2 pp 229533.I1RFR6
16215 229533.I1RAL2 pp 229533.I1S1V5
16216 229533.I1RAL2 pp 229533.I1S7X9
16217 229533.I1RAL2 pp 229533.I1RAN3
16218 229533.I1RAL2 pp 229533.I1RC35
16219 229533.I1RAL2 pp 229533.I1RB49
16220 229533.I1RAL2 pp 229533.I1RRP7
16221 229533.I1RAL2 pp 229533.I1RK32
16222 229533.I1RAL2 pp 229533.I1RDA0
16223 229533.I1RAL2 pp 229533.I1RQN9
16224 229533.I1RAL2 pp 229533.I1RB00
16225 229533.I1RAL2 pp 229533.I1RDE8
16226 229533.I1RAL2 pp 229533.I1RN31
16227 229533.I1RAL2 pp 229533.I1RBX2
16228 229533.I1RAL2 pp 229533.I1RD70
16229 229533.I1RAL2 pp 229533.I1RRH3
16230 229533.I1RAL2 pp 229533.V6QX58
16231 229533.I1RAL2 pp 229533.I1RNC0
16232 229533.I1RAL2 pp 229533.I1RBW7
16233 229533.I1RAL2 pp 229533.I1S0S6
16234 229533.I1RAL2 pp 229533.I1REX4
16235 229533.I1RAL2 pp 229533.I1RH68
16236 229533.I1RAL2 pp 229533.I1S1D3
16237 229533.I1RAL2 pp 229533.I1RLX6
16238 229533.I1RAL2 pp 229533.I1RTB7
16239 229533.I1RAL2 pp 229533.I1RW16
16240 229533.I1RAL2 pp 229533.I1RAW7
16241 229533.I1RAL2 pp 229533.I1RCM6
16242 229533.I1RAL2 pp 229533.I1S2B7
16243 229533.I1RAL2 pp 229533.V6REE9
16244 229533.I1RAL2 pp 229533.I1RUG2
16245 229533.I1RAL2 pp 229533.I1RJK8
16246 229533.I1RAL2 pp 229533.I1RPU4
16247 229533.I1RAL2 pp 229533.I1RXB5
16248 229533.I1RAL2 pp 229533.I1S082
16249 229533.I1RAL2 pp 229533.P0C431
16250 229533.I1RAL2 pp 229533.Q4IBX0
16251 229533.I1RAL2 pp 229533.Q4I0J6
16252 229533.I1RAL2 pp 229533.I1RCJ5
16253 229533.I1RIM7 pp 229533.I1S2C4
16254 229533.I1RIM7 pp 229533.I1S6V0
16255 229533.I1RIM7 pp 229533.I1RJS1
16256 229533.I1RIM7 pp 229533.I1RVX8
16257 229533.I1RIM7 pp 229533.I1RXJ2
16258 229533.I1RIM7 pp 229533.I1SAT2
16259 229533.I1RIM7 pp 229533.I1RN84
16260 229533.I1RIM7 pp 229533.I1RMK9
16261 229533.I1RIM7 pp 229533.I1RZ25
16262 229533.I1RIM7 pp 229533.I1RZ89
16263 229533.I1RIM7 pp 229533.I1RPV8
16264 229533.I1RIM7 pp 229533.I1RZ56
16265 229533.I1RIM7 pp 229533.I1S0A5
16266 229533.I1RIM7 pp 229533.I1S968
16267 229533.I1RIM7 pp 229533.I1RK55
16268 229533.I1RIM7 pp 229533.V6RCJ5
16269 229533.I1RIM7 pp 229533.I1RW82
16270 229533.I1RIM7 pp 229533.I1RXJ0
16271 229533.I1RIM7 pp 229533.I1RRR9
16272 229533.I1RIM7 pp 229533.I1RY12
16273 229533.I1RIM7 pp 229533.I1RSA5
16274 229533.I1RIM7 pp 229533.I1S6H3
16275 229533.I1RIM7 pp 229533.I1RWF5
16276 229533.I1RIM7 pp 229533.I1S8H9
16277 229533.I1RIM7 pp 229533.I1RQJ7
16278 229533.I1RIM7 pp 229533.V6RWB3
16279 229533.I1RIM7 pp 229533.I1S267

16280 229533.I1RIM7 pp 229533.V6R5T7
16281 229533.I1RIM7 pp 229533.I1RY81
16282 229533.I1RIM7 pp 229533.I1S659
16283 229533.I1RIM7 pp 229533.I1RMA2
16284 229533.I1RIM7 pp 229533.I1S8D3
16285 229533.I1RIM7 pp 229533.I1RQV7
16286 229533.I1RIM7 pp 229533.V6RHX2
16287 229533.I1RIM7 pp 229533.I1S5D0
16288 229533.I1RIM7 pp 229533.I1RST9
16289 229533.I1RIM7 pp 229533.I1S094
16290 229533.I1RIM7 pp 229533.I1S048
16291 229533.I1RIM7 pp 229533.I1RW54
16292 229533.I1RIM7 pp 229533.I1RWL3
16293 229533.I1RIM7 pp 229533.I1RPM6
16294 229533.I1RIM7 pp 229533.I1RLA2
16295 229533.I1RIM7 pp 229533.I1RWM4
16296 229533.I1RIM7 pp 229533.I1RYP5
16297 229533.I1RIM7 pp 229533.V6RCC0
16298 229533.I1RIM7 pp 229533.I1S969
16299 229533.I1RIM7 pp 229533.I1RYU6
16300 229533.I1RIM7 pp 229533.I1RRS6
16301 229533.I1RIM7 pp 229533.I1RQV8
16302 229533.I1RCE5 pp 229533.I1RZ78
16303 229533.I1RCE5 pp 229533.I1RPS2
16304 229533.I1RCE5 pp 229533.I1RUM7
16305 229533.I1RCE5 pp 229533.I1RQE0
16306 229533.I1RCE5 pp 229533.I1RKB5
16307 229533.I1RCE5 pp 229533.I1RW06
16308 229533.I1RD44 pp 229533.I1RM07
16309 229533.I1RHJ8 pp 229533.I1RRT3
16310 229533.I1RHJ8 pp 229533.I1RZ78
16311 229533.I1RHJ8 pp 229533.V6RAN2
16312 229533.I1RHJ8 pp 229533.I1RKB5
16313 229533.I1RHJ8 pp 229533.I1RSN7
16314 229533.I1RHJ8 pp 229533.I1RSP7
16315 229533.I1RHJ8 pp 229533.I1RNU7
16316 229533.V6RPN0 pp 229533.V6RR38
16317 229533.V6RPN0 pp 229533.V6RXW9
16318 229533.I1RR05 pp 229533.I1RZ29
16319 229533.I1RR05 pp 229533.V6RSF3
16320 229533.I1RR05 pp 229533.I1S2D5
16321 229533.I1RR05 pp 229533.I1S1W8
16322 229533.I1RR05 pp 229533.I1S3G4
16323 229533.I1RR05 pp 229533.I1S1T5
16324 229533.I1RR05 pp 229533.Q4HVQ9
16325 229533.I1RR05 pp 229533.I1RY87
16326 229533.I1RS37 pp 229533.I1RVZ4
16327 229533.I1RS37 pp 229533.I1RY98
16328 229533.I1RS37 pp 229533.I1RZH4
16329 229533.I1RS37 pp 229533.Q4I0M9
16330 229533.I1RS37 pp 229533.I1RW16
16331 229533.I1RS37 pp 229533.Q4I7L0
16332 229533.I1RS37 pp 229533.I1RTN6
16333 229533.I1RS37 pp 229533.I1RZR5
16334 229533.I1RQE5 pp 229533.I1RZU5
16335 229533.I1RQE5 pp 229533.I1S2I4
16336 229533.I1RQE5 pp 229533.I1RS71
16337 229533.I1S2F7 pp 229533.I1S968
16338 229533.I1S2F7 pp 229533.V6RCJ5
16339 229533.I1S2F7 pp 229533.I1S6V0
16340 229533.I1S2F7 pp 229533.V6RHX2
16341 229533.I1S2F7 pp 229533.V6R5T7
16342 229533.I1S2F7 pp 229533.I1SAT2
16343 229533.I1S2F7 pp 229533.I1S969
16344 229533.I1S2F7 pp 229533.I1S8D3
16345 229533.I1S2F7 pp 229533.I1S659
16346 229533.I1S2F7 pp 229533.I1S5D0
16347 229533.I1S2F7 pp 229533.V6RCC0
16348 229533.I1S2F7 pp 229533.I1S6H3
16349 229533.I1S2F7 pp 229533.I1S3T6
16350 229533.I1S2F7 pp 229533.V6RWB3
16351 229533.I1RLR8 pp 229533.I1RQE5
16352 229533.I1RLR8 pp 229533.I1RS71

16353	229533.I1RE46	pp	229533.I1S237
16354	229533.I1RE46	pp	229533.I1RLX6
16355	229533.I1RE46	pp	229533.I1RYR2
16356	229533.I1RE46	pp	229533.I1RKI2
16357	229533.I1RE46	pp	229533.I1RSN7
16358	229533.I1RE46	pp	229533.I1RFZ2
16359	229533.I1RE46	pp	229533.I1RQ67
16360	229533.I1RE46	pp	229533.I1RQ92
16361	229533.I1RE46	pp	229533.I1RM36
16362	229533.I1RE46	pp	229533.Q4IEA5
16363	229533.I1RE46	pp	229533.Q4IP51
16364	229533.I1RE46	pp	229533.I1RSI4
16365	229533.I1RE46	pp	229533.I1S236
16366	229533.I1RE46	pp	229533.Q4IQM6
16367	229533.I1RE46	pp	229533.I1RYX9
16368	229533.I1RE46	pp	229533.I1RHR0
16369	229533.I1RE46	pp	229533.I1RYQ1
16370	229533.I1RE46	pp	229533.I1RFL7
16371	229533.I1RE46	pp	229533.I1S020
16372	229533.I1RE46	pp	229533.I1S1V5
16373	229533.I1RE46	pp	229533.I1RPQ1
16374	229533.I1RE46	pp	229533.Q4I0J6
16375	229533.I1RE46	pp	229533.V6RLW4
16376	229533.I1RE46	pp	229533.I1S283
16377	229533.I1RE46	pp	229533.I1RFX8
16378	229533.I1RE46	pp	229533.I1RSJ4
16379	229533.I1RE46	pp	229533.I1RRL0
16380	229533.I1RE46	pp	229533.I1RFR6
16381	229533.I1RE46	pp	229533.I1RSA7
16382	229533.I1RE46	pp	229533.I1S2F2
16383	229533.I1RE46	pp	229533.I1RPZ0
16384	229533.I1RE46	pp	229533.I1S1D3
16385	229533.I1RE46	pp	229533.I1RYH9
16386	229533.I1RE46	pp	229533.I1RMB7
16387	229533.I1RE46	pp	229533.I1RN57
16388	229533.I1RE46	pp	229533.Q4I7L0
16389	229533.I1RE46	pp	229533.I1RUG1
16390	229533.I1RE46	pp	229533.I1RLE7
16391	229533.I1RE46	pp	229533.I1RW16
16392	229533.I1RE46	pp	229533.V6R382
16393	229533.I1RE46	pp	229533.Q4IJT0
16394	229533.I1RE46	pp	229533.Q4IC11
16395	229533.I1RE46	pp	229533.I1RMS4
16396	229533.I1RE46	pp	229533.I1RF70
16397	229533.I1RE46	pp	229533.I1RG77
16398	229533.I1RE46	pp	229533.I1RN65
16399	229533.I1RE46	pp	229533.I1RQ13
16400	229533.I1RE46	pp	229533.I1RN31
16401	229533.I1RE46	pp	229533.I1RKK7
16402	229533.I1RE46	pp	229533.I1RSR9
16403	229533.I1RE46	pp	229533.I1RSP7
16404	229533.I1RE46	pp	229533.I1RN55
16405	229533.I1RE46	pp	229533.I1RZ94
16406	229533.I1RE46	pp	229533.I1RUG2
16407	229533.I1RE46	pp	229533.I1S9J6
16408	229533.I1RE46	pp	229533.I1RNE4
16409	229533.I1RE46	pp	229533.I1RMX3
16410	229533.I1RE46	pp	229533.I1RRS7
16411	229533.I1RE46	pp	229533.V6RGI1
16412	229533.I1RE46	pp	229533.I1RQJ4
16413	229533.I1RE46	pp	229533.I1RZP8
16414	229533.I1RE46	pp	229533.I1RFN0
16415	229533.I1RE46	pp	229533.I1RTN6
16416	229533.I1RE46	pp	229533.I1RPU4
16417	229533.I1RE46	pp	229533.Q4IR08
16418	229533.I1RE46	pp	229533.I1RFJ2
16419	229533.I1RE46	pp	229533.I1S209
16420	229533.I1RE46	pp	229533.Q4IBX0
16421	229533.I1RE46	pp	229533.I1S0N5
16422	229533.I1RE46	pp	229533.I1RN46
16423	229533.I1RE46	pp	229533.I1RY73
16424	229533.I1RE46	pp	229533.I1S604
16425	229533.I1RE46	pp	229533.I1RQJ0

229533.I1RE46 pp 229533.I1S461
229533.I1RE46 pp 229533.I1RSC7
229533.A0A0E0S9T3 pp 229533.I1RQE5
229533.A0A0E0S9T3 pp 229533.I1RLR8
229533.A0A1C3YJL7 pp 229533.I1S236
229533.A0A1C3YJL7 pp 229533.I1RPI5
229533.A0A1C3YJL7 pp 229533.I1RJX2
229533.A0A1C3YJL7 pp 229533.I1S283
229533.A0A1C3YJL7 pp 229533.I1RPQ1
229533.A0A1C3YJL7 pp 229533.I1RVX9
229533.A0A1C3YJL7 pp 229533.I1S338
229533.A0A1C3YJL7 pp 229533.I1S8Z2
229533.A0A1C3YJL7 pp 229533.I1S3S3
229533.A0A1C3YJL7 pp 229533.I1RSQ2
229533.A0A1C3YJL7 pp 229533.A0A1C3YLD3
229533.A0A1C3YJL7 pp 229533.I1S9F1
229533.A0A1C3YJL7 pp 229533.I1RKI2
229533.A0A1C3YJL7 pp 229533.I1RK32
229533.A0A1C3YJL7 pp 229533.I1RKI3
229533.A0A1C3YJL7 pp 229533.A0A1C3YN26
229533.A0A1C3YJL7 pp 229533.V6RUT5
229533.A0A1C3YJL7 pp 229533.A0A1C3YLR6
229533.A0A1C3YJL7 pp 229533.I1RNT2
229533.A0A1C3YJL7 pp 229533.I1RYJ6
229533.A0A1C3YJL7 pp 229533.I1RFX8
229533.A0A1C3YJL7 pp 229533.A0A1C3YL54
229533.A0A1C3YJL7 pp 229533.I1RCQ4
229533.A0A1C3YJL7 pp 229533.I1RYR1
229533.A0A1C3YJL7 pp 229533.I1RAH4
229533.A0A1C3YJL7 pp 229533.I1RBW0
229533.A0A1C3YJL7 pp 229533.I1RA85
229533.A0A1C3YJL7 pp 229533.I1RA63
229533.A0A1C3YJL7 pp 229533.A0A1C3YM63
229533.A0A1C3YJL7 pp 229533.I1RB00
229533.A0A1C3YJL7 pp 229533.I1RA94
229533.A0A1C3YJL7 pp 229533.I1RMB7
229533.A0A1C3YJL7 pp 229533.I1RBD5
229533.A0A1C3YJL7 pp 229533.I1S209
229533.A0A1C3YJL7 pp 229533.I1RQ13
229533.A0A1C3YJL7 pp 229533.I1RSI4
229533.A0A1C3YJL7 pp 229533.I1RBE4
229533.A0A1C3YJL7 pp 229533.I1RZ94
229533.A0A1C3YJL7 pp 229533.I1RU61
229533.A0A1C3YJL7 pp 229533.I1RJD3
229533.A0A1C3YJL7 pp 229533.I1RSK0
229533.A0A1C3YJL7 pp 229533.I1RKJ7
229533.A0A1C3YJL7 pp 229533.I1RA90
229533.A0A1C3YJL7 pp 229533.I1RG14
229533.A0A1C3YJL7 pp 229533.I1RG77
229533.A0A1C3YJL7 pp 229533.Q4I0J6
229533.A0A1C3YJL7 pp 229533.I1RA12
229533.A0A1C3YJL7 pp 229533.A0A1C3YLP6
229533.A0A1C3YJL7 pp 229533.I1RBH9
229533.A0A1C3YJL7 pp 229533.I1RXV9
229533.A0A1C3YJL7 pp 229533.I1RCV1
229533.A0A1C3YJL7 pp 229533.I1S887
229533.A0A1C3YJL7 pp 229533.I1RN55
229533.A0A1C3YJL7 pp 229533.I1RXM6
229533.A0A1C3YJL7 pp 229533.I1RE46
229533.A0A1C3YJL7 pp 229533.I1RW69
229533.A0A1C3YJL7 pp 229533.I1RCL7
229533.A0A1C3YJL7 pp 229533.I1RVZ9
229533.A0A1C3YJL7 pp 229533.I1RXI6
229533.A0A1C3YJL7 pp 229533.A0A1C3YN47
229533.A0A1C3YJL7 pp 229533.I1RBE7
229533.A0A1C3YJL7 pp 229533.A0A1C3YLA6
229533.A0A1C3YJL7 pp 229533.I1RN46
229533.A0A1C3YJL7 pp 229533.I1RJU6
229533.A0A1C3YJL7 pp 229533.I1RC67
229533.A0A1C3YJL7 pp 229533.I1RXW3
229533.A0A1C3YJL7 pp 229533.I1RYX9
229533.A0A1C3YJL7 pp 229533.I1RBC1
229533.A0A1C3YJL7 pp 229533.V6R3J1

16499	229533.A0A1C3YJL7	pp	229533.I1RM26
16500	229533.A0A1C3YJL7	pp	229533.Q4IBX0
16501	229533.A0A1C3YJL7	pp	229533.I1RAH9
16502	229533.A0A1C3YJL7	pp	229533.I1RSS7
16503	229533.A0A1C3YJL7	pp	229533.I1RKQ0
16504	229533.A0A1C3YJL7	pp	229533.I1RFJ2
16505	229533.A0A1C3YJL7	pp	229533.A0A1C3YJN7
16506	229533.A0A1C3YJL7	pp	229533.I1RWS4
16507	229533.A0A1C3YJL7	pp	229533.V6RQW4
16508	229533.A0A1C3YJL7	pp	229533.I1RBD7
16509	229533.A0A1C3YJL7	pp	229533.Q4IMD1
16510	229533.A0A1C3YJL7	pp	229533.I1RBW1
16511	229533.A0A1C3YJL7	pp	229533.I1S2F2
16512	229533.A0A1C3YJL7	pp	229533.I1RK03
16513	229533.A0A1C3YJL7	pp	229533.Q4HXF0
16514	229533.A0A1C3YJL7	pp	229533.I1RXB2
16515	229533.A0A1C3YJL7	pp	229533.I1RXV3
16516	229533.A0A1C3YJL7	pp	229533.I1RT75
16517	229533.A0A1C3YJL7	pp	229533.Q4I7L0
16518	229533.A0A1C3YJL7	pp	229533.I1RC14
16519	229533.A0A1C3YJL7	pp	229533.I1RSQ4
16520	229533.A0A1C3YJL7	pp	229533.I1RZZ9
16521	229533.A0A1C3YJL7	pp	229533.I1RQ38
16522	229533.A0A1C3YJL7	pp	229533.O74638
16523	229533.A0A1C3YJL7	pp	229533.I1S237
16524	229533.A0A1C3YJL7	pp	229533.I1RBL4
16525	229533.A0A1C3YJL7	pp	229533.I1RGA6
16526	229533.A0A1C3YJL7	pp	229533.I1RUG1
16527	229533.A0A1C3YJL7	pp	229533.I1RXA9
16528	229533.A0A1C3YJL7	pp	229533.I1RKI7
16529	229533.A0A1C3YJL7	pp	229533.I1RTG7
16530	229533.A0A1C3YJL7	pp	229533.I1RFZ2
16531	229533.A0A1C3YJL7	pp	229533.I1RA52
16532	229533.A0A1C3YJL7	pp	229533.Q4IPI4
16533	229533.A0A1C3YJL7	pp	229533.I1RBS5
16534	229533.A0A1C3YJL7	pp	229533.I1RSC7
16535	229533.A0A1C3YJL7	pp	229533.I1RDX0
16536	229533.A0A1C3YJL7	pp	229533.I1RKB6
16537	229533.A0A1C3YJL7	pp	229533.V6QT89
16538	229533.A0A1C3YJL7	pp	229533.V6RLW4
16539	229533.A0A1C3YJL7	pp	229533.I1R9Y8
16540	229533.A0A1C3YJL7	pp	229533.I1RSR9
16541	229533.A0A1C3YJL7	pp	229533.I1RSA7
16542	229533.A0A1C3YJL7	pp	229533.I1RX64
16543	229533.A0A1C3YJL7	pp	229533.I1RXY7
16544	229533.A0A1C3YJL7	pp	229533.I1RSL5
16545	229533.A0A1C3YJL7	pp	229533.I1S4Z7
16546	229533.A0A1C3YJL7	pp	229533.I1RGZ3
16547	229533.A0A1C3YJL7	pp	229533.V6QX21
16548	229533.A0A1C3YJL7	pp	229533.I1S020
16549	229533.A0A1C3YJL7	pp	229533.I1RTN6
16550	229533.A0A1C3YJL7	pp	229533.I1RYB8
16551	229533.A0A1C3YJL7	pp	229533.I1S1W3
16552	229533.A0A1C3YJL7	pp	229533.I1RSP7
16553	229533.A0A1C3YJL7	pp	229533.I1RBF3
16554	229533.A0A1C3YJL7	pp	229533.I1RPK0
16555	229533.A0A1C3YJL7	pp	229533.I1RCG5
16556	229533.A0A1C3YJL7	pp	229533.I1RZ86
16557	229533.A0A1C3YJL7	pp	229533.I1RD19
16558	229533.A0A1C3YJL7	pp	229533.V6R382
16559	229533.A0A1C3YJL7	pp	229533.I1RCA5
16560	229533.A0A1C3YJL7	pp	229533.I1RQJ4
16561	229533.A0A1C3YJL7	pp	229533.I1S9Z4
16562	229533.A0A1C3YJL7	pp	229533.I1RTG6
16563	229533.A0A1C3YJL7	pp	229533.I1RC54
16564	229533.A0A1C3YJL7	pp	229533.I1S296
16565	229533.A0A1C3YJL7	pp	229533.V6RN69
16566	229533.A0A1C3YJL7	pp	229533.I1S0T4
16567	229533.A0A1C3YJL7	pp	229533.I1RMS4
16568	229533.A0A1C3YJL7	pp	229533.A0A1C3YLC1
16569	229533.A0A1C3YJL7	pp	229533.I1RN57
16570	229533.A0A1C3YJL7	pp	229533.I1RXR1
16571	229533.A0A1C3YJL7	pp	229533.I1RSU2

16572 229533.A0A1C3YJL7 pp 229533.I1RA10
16573 229533.A0A1C3YJL7 pp 229533.I1RXU9
16574 229533.A0A1C3YJL7 pp 229533.I1RSY2
16575 229533.A0A1C3YJL7 pp 229533.I1S9T5
16576 229533.A0A1C3YJL7 pp 229533.I1S123
16577 229533.A0A1C3YLZ6 pp 229533.I1RST9
16578 229533.A0A1C3YLZ6 pp 229533.I1RBU8
16579 229533.A0A1C3YLZ6 pp 229533.I1RZK5
16580 229533.A0A1C3YLZ6 pp 229533.I1S8D3
16581 229533.A0A1C3YLZ6 pp 229533.I1RIB5
16582 229533.A0A1C3YLZ6 pp 229533.I1S8S9
16583 229533.A0A1C3YLZ6 pp 229533.I1S267
16584 229533.A0A1C3YLZ6 pp 229533.V6RCZ8
16585 229533.A0A1C3YLZ6 pp 229533.V6RWB3
16586 229533.A0A1C3YLZ6 pp 229533.I1S659
16587 229533.A0A1C3YLZ6 pp 229533.I1RY81
16588 229533.A0A1C3YLZ6 pp 229533.I1RD52
16589 229533.A0A1C3YLZ6 pp 229533.I1RRS6
16590 229533.A0A1C3YLZ6 pp 229533.I1RA13
16591 229533.A0A1C3YLZ6 pp 229533.I1R9F2
16592 229533.A0A1C3YLZ6 pp 229533.I1RJU3
16593 229533.A0A1C3YLZ6 pp 229533.I1S5D0
16594 229533.A0A1C3YLZ6 pp 229533.I1RCU2
16595 229533.A0A1C3YLZ6 pp 229533.I1S6H3
16596 229533.A0A1C3YLZ6 pp 229533.I1S094
16597 229533.A0A1C3YLZ6 pp 229533.I1RDN3
16598 229533.A0A1C3YLZ6 pp 229533.I1RMA2
16599 229533.A0A1C3YLZ6 pp 229533.V6RCC0
16600 229533.A0A1C3YLZ6 pp 229533.I1RE79
16601 229533.A0A1C3YLZ6 pp 229533.I1RZ56
16602 229533.A0A1C3YLZ6 pp 229533.I1RQV7
16603 229533.A0A1C3YLZ6 pp 229533.V6R5T7
16604 229533.A0A1C3YLZ6 pp 229533.I1RSF1
16605 229533.A0A1C3YLZ6 pp 229533.I1RH83
16606 229533.A0A1C3YLZ6 pp 229533.I1RPV8
16607 229533.A0A1C3YLZ6 pp 229533.I1RJS1
16608 229533.A0A1C3YLZ6 pp 229533.I1RFJ6
16609 229533.A0A1C3YLZ6 pp 229533.I1S6V0
16610 229533.A0A1C3YLZ6 pp 229533.I1SAT2
16611 229533.A0A1C3YLZ6 pp 229533.I1RKG3
16612 229533.A0A1C3YLZ6 pp 229533.I1RE52
16613 229533.A0A1C3YLZ6 pp 229533.I1RIV2
16614 229533.A0A1C3YLZ6 pp 229533.I1RRM3
16615 229533.A0A1C3YLZ6 pp 229533.I1RF85
16616 229533.A0A1C3YLZ6 pp 229533.I1S1T5
16617 229533.A0A1C3YLZ6 pp 229533.I1RQ33
16618 229533.A0A1C3YLZ6 pp 229533.Q4HXF6
16619 229533.A0A1C3YLZ6 pp 229533.I1RB05
16620 229533.A0A1C3YLZ6 pp 229533.I1RCH5
16621 229533.A0A1C3YLZ6 pp 229533.I1RBE6
16622 229533.A0A1C3YLZ6 pp 229533.I1RZ38
16623 229533.A0A1C3YLZ6 pp 229533.I1RKP1
16624 229533.A0A1C3YLZ6 pp 229533.I1RKG2
16625 229533.A0A1C3YLZ6 pp 229533.I1RRI1
16626 229533.A0A1C3YLZ6 pp 229533.A0A1C3YM61
16627 229533.A0A1C3YLZ6 pp 229533.I1RNG4
16628 229533.A0A1C3YLZ6 pp 229533.I1S5R1
16629 229533.A0A1C3YLZ6 pp 229533.I1S0A5
16630 229533.A0A1C3YLZ6 pp 229533.I1RDE7
16631 229533.A0A1C3YLZ6 pp 229533.V6RCJ5
16632 229533.A0A1C3YLZ6 pp 229533.I1RF49
16633 229533.A0A1C3YLZ6 pp 229533.I1RWP9
16634 229533.A0A1C3YLZ6 pp 229533.I1RIJ6
16635 229533.I1RWE4 pp 229533.V6QUV7
16636 229533.I1RWE4 pp 229533.I1RZM0
16637 229533.I1RWE4 pp 229533.I1S4Z7
16638 229533.I1RWE4 pp 229533.I1RWP4
16639 229533.A0A0E0SCX4 pp 229533.I1RV63
16640 229533.A0A0E0SCX4 pp 229533.I1RLP4
16641 229533.A0A0E0SCX4 pp 229533.I1RTW1
16642 229533.A0A0E0SCX4 pp 229533.I1RIB9
16643 229533.A0A0E0SCX4 pp 229533.I1RHQ4
16644 229533.A0A0E0SCX4 pp 229533.I1REZ6

16645 229533.A0A0E0SCX4 pp 229533.I1RLE3
16646 229533.A0A0E0SCX4 pp 229533.I1SAS4
16647 229533.A0A0E0SCX4 pp 229533.I1RLN1
16648 229533.I1RPZ0 pp 229533.I1RSA7
16649 229533.I1RPZ0 pp 229533.I1RY73
16650 229533.I1RPZ0 pp 229533.I1RRT1
16651 229533.I1RPZ0 pp 229533.I1RZG7
16652 229533.I1RPZ0 pp 229533.I1S461
16653 229533.I1RPZ0 pp 229533.I1RZ94
16654 229533.I1RPZ0 pp 229533.V6RLW4
16655 229533.I1RPZ0 pp 229533.I1S283
16656 229533.I1RPZ0 pp 229533.Q4I6A4
16657 229533.I1RPZ0 pp 229533.I1RSP7
16658 229533.I1RPZ0 pp 229533.I1RYR2
16659 229533.I1RPZ0 pp 229533.I1RUG2
16660 229533.I1RPZ0 pp 229533.I1RSI4
16661 229533.I1RPZ0 pp 229533.I1RQJ4
16662 229533.I1RPZ0 pp 229533.I1RQ92
16663 229533.I1RPZ0 pp 229533.I1RYX9
16664 229533.I1RPZ0 pp 229533.I1RTN6
16665 229533.I1RPZ0 pp 229533.I1S604
16666 229533.I1RPZ0 pp 229533.I1RW16
16667 229533.I1RPZ0 pp 229533.I1RZP8
16668 229533.I1RHJ0 pp 229533.I1RW82
16669 229533.I1RHJ0 pp 229533.I1RK55
16670 229533.I1RHJ0 pp 229533.I1S0A5
16671 229533.I1RHJ0 pp 229533.I1RUR8
16672 229533.I1RHJ0 pp 229533.I1RSA5
16673 229533.I1RHJ0 pp 229533.I1RWF5
16674 229533.I1RHJ0 pp 229533.I1RIJ6
16675 229533.I1RHJ0 pp 229533.I1RRR9
16676 229533.I1RHJ0 pp 229533.V6RCJ5
16677 229533.I1RHJ0 pp 229533.V6R5T7
16678 229533.I1RHJ0 pp 229533.I1SAT2
16679 229533.I1RHJ0 pp 229533.I1RMK9
16680 229533.I1RHJ0 pp 229533.I1S968
16681 229533.I1RHJ0 pp 229533.I1RW54
16682 229533.I1RHJ0 pp 229533.I1RHU8
16683 229533.I1RHJ0 pp 229533.I1S6V0
16684 229533.I1RHJ0 pp 229533.I1RZ25
16685 229533.I1RHJ0 pp 229533.I1S2C4
16686 229533.I1RHJ0 pp 229533.I1S094
16687 229533.I1RHJ0 pp 229533.I1S5D0
16688 229533.I1RHJ0 pp 229533.I1RJS1
16689 229533.I1RHJ0 pp 229533.I1RMA2
16690 229533.I1RHJ0 pp 229533.I1RQV7
16691 229533.I1RHJ0 pp 229533.I1S048
16692 229533.I1RHJ0 pp 229533.I1RLA2
16693 229533.I1RHJ0 pp 229533.V6RCC0
16694 229533.I1RHJ0 pp 229533.I1RPV8
16695 229533.I1RHJ0 pp 229533.I1S6H3
16696 229533.I1RHJ0 pp 229533.I1RYP5
16697 229533.I1RHJ0 pp 229533.I1RWL3
16698 229533.I1RHJ0 pp 229533.I1RZ56
16699 229533.I1RHJ0 pp 229533.I1RRS6
16700 229533.I1RHJ0 pp 229533.I1RZ89
16701 229533.I1RHJ0 pp 229533.I1RYU6
16702 229533.I1RHJ0 pp 229533.I1S969
16703 229533.I1RHJ0 pp 229533.I1RQV8
16704 229533.I1RHJ0 pp 229533.V6RWB3
16705 229533.I1RHJ0 pp 229533.I1RY81
16706 229533.I1RHJ0 pp 229533.I1S659
16707 229533.I1RHJ0 pp 229533.V6RHX2
16708 229533.I1RHJ0 pp 229533.I1S267
16709 229533.I1RHJ0 pp 229533.I1RST9
16710 229533.I1RHJ0 pp 229533.I1RY12
16711 229533.I1RHJ0 pp 229533.I1RIB5
16712 229533.I1RHJ0 pp 229533.I1RPM6
16713 229533.I1RHJ0 pp 229533.I1S8D3
16714 229533.A0A098DYK2 pp 229533.Q4HTT2
16715 229533.A0A098DYK2 pp 229533.Q4I7L0
16716 229533.A0A098DYK2 pp 229533.I1RYV3
16717 229533.A0A098DYK2 pp 229533.I1RBJ0

229533.A0A098DYK2	pp	229533.I1S4Z5
229533.A0A098DYK2	pp	229533.Q4I5R3
229533.I1RLX6	pp	229533.I1S1V5
229533.I1RLX6	pp	229533.I1S951
229533.I1RLX6	pp	229533.V6RLW4
229533.I1RLX6	pp	229533.I1RX38
229533.I1RLX6	pp	229533.I1RWG6
229533.I1RLX6	pp	229533.I1RSA7
229533.I1RLX6	pp	229533.I1RSC7
229533.I1RLX6	pp	229533.I1RQ67
229533.I1RLX6	pp	229533.I1S382
229533.I1RLX6	pp	229533.I1S4P6
229533.I1RLX6	pp	229533.I1S2A8
229533.I1RLX6	pp	229533.I1RXU0
229533.I1RLX6	pp	229533.I1RMP0
229533.I1RLX6	pp	229533.I1RQN9
229533.I1RLX6	pp	229533.I1RZ25
229533.I1RLX6	pp	229533.Q4IMD1
229533.I1RLX6	pp	229533.V6RVG3
229533.I1RLX6	pp	229533.I1RYR2
229533.I1RLX6	pp	229533.I1S7X9
229533.I1RLX6	pp	229533.I1S1W4
229533.I1RLX6	pp	229533.Q4IB89
229533.I1RLX6	pp	229533.I1RX64
229533.I1RLX6	pp	229533.I1RYX9
229533.I1RLX6	pp	229533.I1RZP8
229533.I1RLX6	pp	229533.I1RRP7
229533.I1RLX6	pp	229533.I1RTE6
229533.I1RLX6	pp	229533.I1S1D3
229533.I1RLX6	pp	229533.I1RSP7
229533.I1RLX6	pp	229533.I1RZE4
229533.I1RLX6	pp	229533.I1RQJ4
229533.I1RLX6	pp	229533.I1RN57
229533.I1RLX6	pp	229533.I1RWQ0
229533.I1RLX6	pp	229533.I1S219
229533.I1RLX6	pp	229533.I1RYH9
229533.I1RLX6	pp	229533.I1RMB7
229533.I1RLX6	pp	229533.I1RPW0
229533.I1RLX6	pp	229533.V6REE9
229533.I1RLX6	pp	229533.V6RGI1
229533.I1RLX6	pp	229533.I1RW16
229533.I1RLX6	pp	229533.I1RN31
229533.I1RLX6	pp	229533.I1RNC0
229533.I1RLX6	pp	229533.I1S4P4
229533.I1RLX6	pp	229533.I1RN08
229533.I1RLX6	pp	229533.I1S1C8
229533.I1RLX6	pp	229533.I1RZ94
229533.I1RLX6	pp	229533.I1RPU4
229533.I1RLX6	pp	229533.Q4IBX0
229533.I1RLX6	pp	229533.I1RRB0
229533.I1RLX6	pp	229533.I1S604
229533.I1RLX6	pp	229533.I1S439
229533.I1RLX6	pp	229533.I1S0S6
229533.I1RLX6	pp	229533.I1S050
229533.I1RLX6	pp	229533.I1RWL6
229533.I1RLX6	pp	229533.P0C431
229533.I1RLX6	pp	229533.I1RY73
229533.I1RLX6	pp	229533.Q4I7X1
229533.I1RLX6	pp	229533.I1RQ92
229533.I1RLX6	pp	229533.I1RN65
229533.I1RLX6	pp	229533.I1RQ80
229533.I1RLX6	pp	229533.I1RWB3
229533.I1RLX6	pp	229533.I1RSI4
229533.I1RLX6	pp	229533.I1S0P4
229533.I1RLX6	pp	229533.I1RN55
229533.I1RLX6	pp	229533.I1RUG2
229533.I1RLX6	pp	229533.Q4I0J6
229533.I1RLX6	pp	229533.Q4I877
229533.I1RLX6	pp	229533.I1RPZ0
229533.I1RLX6	pp	229533.I1RTC2
229533.A0A098DY81	pp	229533.I1RJZ7
229533.A0A098DY81	pp	229533.I1RW71
229533.A0A0E0S9I2	pp	229533.I1RE13

16791	229533.A0A0E0S9I2	pp	229533.I1RPK5
16792	229533.I1RBI1	pp	229533.I1RQM0
16793	229533.I1RBI1	pp	229533.I1S216
16794	229533.I1RBI1	pp	229533.V6R057
16795	229533.I1RBI1	pp	229533.I1RCD8
16796	229533.I1RBI1	pp	229533.V6RRH2
16797	229533.I1RBI1	pp	229533.I1RZ52
16798	229533.I1RBI1	pp	229533.V6R2A1
16799	229533.I1RBI1	pp	229533.I1S4Z8
16800	229533.I1RBI1	pp	229533.I1RC00
16801	229533.I1RBI1	pp	229533.I1RX38
16802	229533.I1RBI1	pp	229533.I1RL04
16803	229533.I1RBI1	pp	229533.I1RPW0
16804	229533.I1RBI1	pp	229533.O74638
16805	229533.I1RBI1	pp	229533.I1RBL4
16806	229533.I1RBI1	pp	229533.I1S8G4
16807	229533.I1RBI1	pp	229533.I1RZE4
16808	229533.I1RBI1	pp	229533.V6RLM7
16809	229533.I1RBI1	pp	229533.I1RFZ2
16810	229533.I1RBI1	pp	229533.I1RD70
16811	229533.I1RBI1	pp	229533.I1RCW6
16812	229533.I1RBI1	pp	229533.I1RS17
16813	229533.I1RBI1	pp	229533.I1RMI8
16814	229533.I1RBI1	pp	229533.I1S5C4
16815	229533.I1RBI1	pp	229533.I1RMG6
16816	229533.I1RBI1	pp	229533.I1RX22
16817	229533.I1RBI1	pp	229533.I1RBM3
16818	229533.I1RBI1	pp	229533.I1RW16
16819	229533.I1RBI1	pp	229533.I1RWE8
16820	229533.I1RBI1	pp	229533.I1SAN9
16821	229533.I1RBI1	pp	229533.I1RR68
16822	229533.I1RBI1	pp	229533.I1RQM7
16823	229533.I1RBI1	pp	229533.I1RVZ7
16824	229533.I1RBI1	pp	229533.I1RPU8
16825	229533.I1RBI1	pp	229533.I1RBV5
16826	229533.I1RBI1	pp	229533.V6RKY7
16827	229533.I1RBI1	pp	229533.I1RT75
16828	229533.I1RBI1	pp	229533.I1RN31
16829	229533.I1RBI1	pp	229533.I1RYH9
16830	229533.I1RBI1	pp	229533.I1RNG8
16831	229533.I1RBI1	pp	229533.I1RME6
16832	229533.I1RBI1	pp	229533.I1RUQ5
16833	229533.I1RBI1	pp	229533.I1RCL4
16834	229533.I1RBI1	pp	229533.I1S0B7
16835	229533.I1RBI1	pp	229533.I1RFQ7
16836	229533.I1RBI1	pp	229533.V6RUT5
16837	229533.I1RBI1	pp	229533.I1RMM8
16838	229533.I1RBI1	pp	229533.I1RC22
16839	229533.I1RBI1	pp	229533.I1RK15
16840	229533.I1RBI1	pp	229533.I1RLX6
16841	229533.I1RBI1	pp	229533.I1RM35
16842	229533.I1RBI1	pp	229533.I1RF82
16843	229533.I1RBI1	pp	229533.I1S4Q4
16844	229533.I1RBI1	pp	229533.I1RQS9
16845	229533.I1RBI1	pp	229533.V6R1K4
16846	229533.I1RBI1	pp	229533.I1RMQ4
16847	229533.I1RBI1	pp	229533.I1RKK0
16848	229533.I1RBI1	pp	229533.I1RK95
16849	229533.I1RBI1	pp	229533.I1RQ92
16850	229533.I1RBI1	pp	229533.I1RPU4
16851	229533.I1RBI1	pp	229533.V6RBN2
16852	229533.I1RBI1	pp	229533.I1RCM6
16853	229533.I1RBI1	pp	229533.I1RW71
16854	229533.I1RBI1	pp	229533.I1RRV4
16855	229533.I1RBI1	pp	229533.I1RTD3
16856	229533.I1RBI1	pp	229533.I1RRY9
16857	229533.I1RBI1	pp	229533.I1RPN4
16858	229533.I1RBI1	pp	229533.I1S1D0
16859	229533.I1RBI1	pp	229533.I1RQ80
16860	229533.Q4I5Z5	pp	229533.V6QYF0
16861	229533.Q4I5Z5	pp	229533.Q4IQK7
16862	229533.Q4I5Z5	pp	229533.V6RCG5
16863	229533.Q4I5Z5	pp	229533.V6QW22

16864	229533.Q4I5Z5	pp	229533.Q4IJH1
16865	229533.Q4I5Z5	pp	229533.Q4IFI0
16866	229533.Q4I5Z5	pp	229533.Q4IJ56
16867	229533.Q4I5Z5	pp	229533.Q4IAS1
16868	229533.Q4I5Z5	pp	229533.Q4IBS2
16869	229533.Q4I5Z5	pp	229533.Q4IN63
16870	229533.Q4I5Z5	pp	229533.Q4IEV5
16871	229533.Q4I5Z5	pp	229533.Q4IQW0
16872	229533.I1RMR3	pp	229533.I1RST9
16873	229533.I1RMR3	pp	229533.I1RQV7
16874	229533.I1RMR3	pp	229533.I1S267
16875	229533.I1RMR3	pp	229533.I1RZ25
16876	229533.I1RAH4	pp	229533.I1RE46
16877	229533.I1RAH4	pp	229533.V6R382
16878	229533.I1RAH4	pp	229533.I1RB27
16879	229533.I1RAH4	pp	229533.I1RU61
16880	229533.I1RAH4	pp	229533.I1RQI6
16881	229533.I1RAH4	pp	229533.I1RW16
16882	229533.I1RAH4	pp	229533.I1RMS4
16883	229533.I1RAH4	pp	229533.I1RY73
16884	229533.I1RAH4	pp	229533.I1RSC7
16885	229533.I1RAH4	pp	229533.Q4HYB8
16886	229533.I1RAH4	pp	229533.I1S1D3
16887	229533.I1RAH4	pp	229533.V6RGI1
16888	229533.I1RAH4	pp	229533.I1RC54
16889	229533.I1RAH4	pp	229533.Q4IR08
16890	229533.I1RAH4	pp	229533.I1RFN0
16891	229533.I1RAH4	pp	229533.I1RN57
16892	229533.I1RAH4	pp	229533.I1RPZ0
16893	229533.I1RAH4	pp	229533.I1S020
16894	229533.I1RAH4	pp	229533.I1RWT6
16895	229533.I1RAH4	pp	229533.I1S209
16896	229533.I1RAH4	pp	229533.I1RBC8
16897	229533.I1RAH4	pp	229533.Q4I0J6
16898	229533.I1RAH4	pp	229533.I1RAX3
16899	229533.I1RAH4	pp	229533.I1RMX3
16900	229533.I1RAH4	pp	229533.I1RQ92
16901	229533.I1RAH4	pp	229533.I1RFZ2
16902	229533.I1RAH4	pp	229533.I1RRS7
16903	229533.I1RAH4	pp	229533.I1RY44
16904	229533.I1RAH4	pp	229533.I1RG77
16905	229533.I1RAH4	pp	229533.I1RN55
16906	229533.I1RAH4	pp	229533.I1RXI6
16907	229533.I1RAH4	pp	229533.I1S604
16908	229533.I1RAH4	pp	229533.I1RUG2
16909	229533.I1RAH4	pp	229533.I1RQA3
16910	229533.I1RAH4	pp	229533.I1S237
16911	229533.I1RAH4	pp	229533.I1RCL7
16912	229533.I1RAH4	pp	229533.I1RCM6
16913	229533.I1RAH4	pp	229533.I1RBN9
16914	229533.I1RAH4	pp	229533.I1RX64
16915	229533.I1RAH4	pp	229533.I1S2E9
16916	229533.I1RAH4	pp	229533.I1RCA9
16917	229533.I1RAH4	pp	229533.I1RN46
16918	229533.I1RAH4	pp	229533.I1RTN6
16919	229533.I1RAH4	pp	229533.I1RQJ0
16920	229533.I1RAH4	pp	229533.I1RKI2
16921	229533.I1RAH4	pp	229533.I1RZP8
16922	229533.I1RAH4	pp	229533.I1S461
16923	229533.I1RAH4	pp	229533.I1RLX6
16924	229533.I1RAH4	pp	229533.I1RSP7
16925	229533.I1RAH4	pp	229533.I1RM36
16926	229533.I1RAH4	pp	229533.I1RBC1
16927	229533.I1RAH4	pp	229533.Q4IJU0
16928	229533.I1RAH4	pp	229533.I1RD19
16929	229533.I1RAH4	pp	229533.I1S236
16930	229533.I1RAH4	pp	229533.I1RQJ4
16931	229533.I1RAH4	pp	229533.I1RD70
16932	229533.I1RAH4	pp	229533.I1RFJ2
16933	229533.I1RAH4	pp	229533.I1RHR0
16934	229533.I1RAH4	pp	229533.Q4IBX0
16935	229533.I1RAH4	pp	229533.I1RUG1
16936	229533.I1RAH4	pp	229533.I1RDX0

16937 229533.I1RAH4 pp 229533.I1RSR9
16938 229533.I1RAH4 pp 229533.I1RBH9
16939 229533.I1RAH4 pp 229533.Q4IQM6
16940 229533.I1RAH4 pp 229533.I1S0D5
16941 229533.I1RAH4 pp 229533.Q4IC11
16942 229533.I1RAH4 pp 229533.Q4IP51
16943 229533.I1RAH4 pp 229533.I1RYQ1
16944 229533.I1RAH4 pp 229533.I1RS37
16945 229533.I1RAH4 pp 229533.I1RAH9
16946 229533.I1RAH4 pp 229533.I1RPQ1
16947 229533.I1RAH4 pp 229533.I1RYX9
16948 229533.I1RAH4 pp 229533.I1RF70
16949 229533.I1RAH4 pp 229533.I1RQ67
16950 229533.I1RAH4 pp 229533.I1RN31
16951 229533.I1RAH4 pp 229533.V6RLW4
16952 229533.I1RAH4 pp 229533.I1RFL7
16953 229533.I1RAH4 pp 229533.Q4IEA5
16954 229533.I1RAH4 pp 229533.I1RKQ0
16955 229533.I1RAH4 pp 229533.Q4I5R9
16956 229533.I1RAH4 pp 229533.I1RAW7
16957 229533.I1RAH4 pp 229533.I1RFX8
16958 229533.I1RAH4 pp 229533.I1RCY2
16959 229533.I1RAH4 pp 229533.I1RSA7
16960 229533.I1RAH4 pp 229533.I1RVA7
16961 229533.I1RAH4 pp 229533.I1RBD7
16962 229533.I1RAH4 pp 229533.I1RB00
16963 229533.I1RAH4 pp 229533.I1RSI4
16964 229533.I1RAH4 pp 229533.I1RQ13
16965 229533.I1RAH4 pp 229533.I1RYH9
16966 229533.I1RAH4 pp 229533.I1RYR2
16967 229533.I1RAH4 pp 229533.I1RKK7
16968 229533.I1RAH4 pp 229533.I1RBW0
16969 229533.I1RAH4 pp 229533.I1RMB7
16970 229533.I1RAH4 pp 229533.I1RC35
16971 229533.I1RAH4 pp 229533.I1RBL4
16972 229533.I1RAH4 pp 229533.I1RLE7
16973 229533.I1RAH4 pp 229533.I1S2F2
16974 229533.I1RAH4 pp 229533.I1RZ94
16975 229533.I1RAH4 pp 229533.I1RSK0
16976 229533.I1RAH4 pp 229533.I1RD18
16977 229533.I1RAH4 pp 229533.I1RZ86
16978 229533.I1RAH4 pp 229533.I1RBW1
16979 229533.I1RAH4 pp 229533.I1RCA5
16980 229533.I1RAH4 pp 229533.I1S283
16981 229533.I1RAH4 pp 229533.I1S1V5
16982 229533.I1RAH4 pp 229533.I1RST8
16983 229533.I1RAH4 pp 229533.I1RCQ4
16984 229533.I1RAH4 pp 229533.I1RSJ4
16985 229533.I1RAH4 pp 229533.I1RFR6
16986 229533.A0A1C3YKQ9 pp 229533.I1RJF0
16987 229533.A0A1C3YKQ9 pp 229533.I1S0Y1
16988 229533.I1RPK1 pp 229533.I1RZM0
16989 229533.I1RPK1 pp 229533.I1S3L8
16990 229533.I1RPK1 pp 229533.V6QU20
16991 229533.I1RPK1 pp 229533.I1RSK3
16992 229533.I1RPK1 pp 229533.I1RV63
16993 229533.I1RPK1 pp 229533.I1RWP4
16994 229533.I1RPK1 pp 229533.I1S4Z7
16995 229533.I1RPK1 pp 229533.V6QUV7
16996 229533.I1RPK1 pp 229533.I1RUP0
16997 229533.I1RNJ7 pp 229533.I1RY84
16998 229533.I1RNJ7 pp 229533.I1RRL5
16999 229533.I1RNJ7 pp 229533.V6QT89
17000 229533.I1RNJ7 pp 229533.I1RQX9
17001 229533.I1RNJ7 pp 229533.I1RWK4
17002 229533.I1RNJ7 pp 229533.I1RYU0
17003 229533.I1RNJ7 pp 229533.I1RZ16
17004 229533.I1RNJ7 pp 229533.I1RWQ6
17005 229533.I1RNJ7 pp 229533.I1RSE2
17006 229533.I1RNJ7 pp 229533.I1RS03
17007 229533.I1RNJ7 pp 229533.I1S334
17008 229533.I1RNJ7 pp 229533.I1RVU7
17009 229533.I1RNJ7 pp 229533.Q4HTS9

229533.I1RNJ7 pp 229533.I1S3H8
229533.I1RNJ7 pp 229533.I1RTU1
229533.I1RNJ7 pp 229533.I1S6H4
229533.I1RNJ7 pp 229533.I1RZ92
229533.I1RNJ7 pp 229533.I1SB04
229533.I1RNJ7 pp 229533.I1RSM8
229533.I1RNJ7 pp 229533.I1RP53
229533.I1RNJ7 pp 229533.I1RXV3
229533.I1RNJ7 pp 229533.I1RYL8
229533.I1RNJ7 pp 229533.I1RYP2
229533.I1RNJ7 pp 229533.I1RPQ2
229533.A0A1C3YJD6 pp 229533.I1S0Y1
229533.A0A1C3YJD6 pp 229533.I1RC58
229533.A0A1C3YJD6 pp 229533.I1RQ61
229533.A0A1C3YJD6 pp 229533.I1RAZ1
229533.A0A1C3YJD6 pp 229533.I1RPK5
229533.A0A1C3YJD6 pp 229533.I1RZP8
229533.A0A1C3YIA8 pp 229533.I1RD70
229533.A0A1C3YIA8 pp 229533.I1RBX2
229533.A0A1C3YIA8 pp 229533.V6R042
229533.A0A1C3YIA8 pp 229533.I1RHI1
229533.A0A1C3YIA8 pp 229533.I1RDE8
229533.A0A1C3YIA8 pp 229533.I1RD23
229533.A0A1C3YIA8 pp 229533.I1RY09
229533.A0A1C3YIA8 pp 229533.I1RWN6
229533.A0A1C3YIA8 pp 229533.V6RGI1
229533.A0A1C3YIA8 pp 229533.I1RZH4
229533.A0A1C3YIA8 pp 229533.I1RCJ5
229533.A0A1C3YIA8 pp 229533.I1RRH3
229533.A0A1C3YIA8 pp 229533.I1RE01
229533.A0A1C3YIA8 pp 229533.I1RNB8
229533.A0A1C3YIA8 pp 229533.I1RBW7
229533.A0A1C3YIA8 pp 229533.I1RSB8
229533.A0A1C3YIA8 pp 229533.I1RN58
229533.A0A1C3YIA8 pp 229533.I1RU61
229533.A0A1C3YIA8 pp 229533.I1RD19
229533.A0A1C3YIA8 pp 229533.I1RW16
229533.A0A1C3YIA8 pp 229533.I1RZW8
229533.A0A1C3YIA8 pp 229533.I1RL12
229533.A0A1C3YIA8 pp 229533.I1RBD6
229533.A0A1C3YIA8 pp 229533.I1RCG4
229533.A0A1C3YIA8 pp 229533.V6RMW6
229533.A0A1C3YIA8 pp 229533.I1RMH5
229533.A0A1C3YIA8 pp 229533.I1R975
229533.A0A1C3YIA8 pp 229533.I1RTH6
229533.A0A1C3YIA8 pp 229533.I1RAI8
229533.A0A1C3YIA8 pp 229533.Q4IQC1
229533.A0A1C3YIA8 pp 229533.I1RCP6
229533.A0A1C3YIA8 pp 229533.I1RXB5
229533.A0A1C3YIA8 pp 229533.I1RJK8
229533.A0A1C3YIA8 pp 229533.I1RN65
229533.A0A1C3YIA8 pp 229533.I1RAU7
229533.A0A1C3YIA8 pp 229533.I1RB53
229533.A0A1C3YIA8 pp 229533.I1RN18
229533.A0A1C3YIA8 pp 229533.I1RB92
229533.A0A1C3YIA8 pp 229533.I1RN31
229533.A0A1C3YIA8 pp 229533.I1RAL2
229533.A0A1C3YIA8 pp 229533.Q4IER0
229533.A0A1C3YIA8 pp 229533.I1RQE7
229533.A0A1C3YIA8 pp 229533.I1RL24
229533.A0A1C3YIA8 pp 229533.I1S1V2
229533.A0A1C3YIA8 pp 229533.A0A1C3YIZ2
229533.A0A1C3YIA8 pp 229533.Q4ICG8
229533.A0A1C3YIA8 pp 229533.I1RAS2
229533.A0A1C3YIA8 pp 229533.I1RAN3
229533.A0A1C3YIA8 pp 229533.I1RSK0
229533.A0A1C3YIA8 pp 229533.I1RJV4
229533.A0A1C3YIA8 pp 229533.A0A1C3YIP6
229533.A0A1C3YIA8 pp 229533.I1RN36
229533.A0A1C3YIA8 pp 229533.I1RYZ9
229533.A0A1C3YIA8 pp 229533.I1RN27
229533.A0A1C3YIA8 pp 229533.I1RWK3
229533.A0A1C3YIA8 pp 229533.I1RAW7

17083	229533.A0A1C3YIA8	pp	229533.A0A1C3YL99
17084	229533.A0A1C3YIA8	pp	229533.V6QV09
17085	229533.A0A1C3YIA8	pp	229533.I1RXU7
17086	229533.A0A1C3YIA8	pp	229533.I1RLX6
17087	229533.A0A1C3YIA8	pp	229533.I1RAH9
17088	229533.A0A1C3YIA8	pp	229533.I1RCP7
17089	229533.A0A1C3YIA8	pp	229533.I1RBC9
17090	229533.A0A1C3YIA8	pp	229533.A0A1C3YIP5
17091	229533.A0A1C3YIA8	pp	229533.I1RXI6
17092	229533.A0A1C3YIA8	pp	229533.I1S4R1
17093	229533.A0A1C3YIA8	pp	229533.I1S2B7
17094	229533.A0A1C3YIA8	pp	229533.I1RKQ0
17095	229533.A0A1C3YIA8	pp	229533.I1RCY0
17096	229533.A0A1C3YIA8	pp	229533.I1RWG6
17097	229533.A0A1C3YIA8	pp	229533.I1RMT4
17098	229533.A0A1C3YIA8	pp	229533.I1S1V5
17099	229533.A0A1C3YIA8	pp	229533.A0A1C3YKC0
17100	229533.A0A1C3YIA8	pp	229533.I1RZG0
17101	229533.A0A1C3YIA8	pp	229533.I1RBL4
17102	229533.A0A1C3YIA8	pp	229533.I1RK32
17103	229533.A0A1C3YIA8	pp	229533.A0A1C3YKM9
17104	229533.A0A1C3YIA8	pp	229533.I1RJZ7
17105	229533.A0A1C3YIA8	pp	229533.I1S0Z2
17106	229533.A0A1C3YIA8	pp	229533.I1RW25
17107	229533.A0A1C3YIA8	pp	229533.A0A1C3YKB5
17108	229533.A0A1C3YIA8	pp	229533.I1RZ02
17109	229533.A0A1C3YIA8	pp	229533.I1RBY4
17110	229533.A0A1C3YIA8	pp	229533.I1RS37
17111	229533.A0A098DSW9	pp	229533.I1RJ33
17112	229533.A0A098DSW9	pp	229533.A0A0E0SDE1
17113	229533.A0A098DSW9	pp	229533.I1RE37
17114	229533.A0A098DSW9	pp	229533.I1S461
17115	229533.A0A098DSW9	pp	229533.A0A1C3YLR6
17116	229533.A0A098DSW9	pp	229533.I1RWB3
17117	229533.A0A098DSW9	pp	229533.I1RWM6
17118	229533.A0A098DSW9	pp	229533.I1RLX6
17119	229533.A0A098DSW9	pp	229533.I1RCM6
17120	229533.A0A098DSW9	pp	229533.I1RG77
17121	229533.A0A098DSW9	pp	229533.I1RN55
17122	229533.A0A098DSW9	pp	229533.A0A1C3YIP6
17123	229533.A0A098DSW9	pp	229533.A0A1C3YK82
17124	229533.A0A098DSW9	pp	229533.I1RS41
17125	229533.A0A098DSW9	pp	229533.I1RMX3
17126	229533.A0A098DSW9	pp	229533.I1RC54
17127	229533.A0A098DSW9	pp	229533.A0A1C3YK75
17128	229533.A0A098DSW9	pp	229533.I1RD70
17129	229533.A0A098DSW9	pp	229533.I1RAU8
17130	229533.A0A098DSW9	pp	229533.A0A1C3YKP6
17131	229533.A0A098DSW9	pp	229533.I1RX64
17132	229533.A0A098DSW9	pp	229533.I1RFN0
17133	229533.A0A098DSW9	pp	229533.I1S0N5
17134	229533.A0A098DSW9	pp	229533.I1S283
17135	229533.A0A098DSW9	pp	229533.I1RN57
17136	229533.A0A098DSW9	pp	229533.I1RCU7
17137	229533.A0A098DSW9	pp	229533.I1RMS4
17138	229533.A0A098DSW9	pp	229533.A0A098DXS1
17139	229533.A0A098DSW9	pp	229533.I1RMB7
17140	229533.A0A098DSW9	pp	229533.A0A1C3YN58
17141	229533.A0A098DSW9	pp	229533.Q4HY90
17142	229533.A0A098DSW9	pp	229533.I1RW16
17143	229533.A0A098DSW9	pp	229533.A0A098DTF7
17144	229533.A0A098DSW9	pp	229533.I1RA10
17145	229533.A0A098DSW9	pp	229533.I1RSL6
17146	229533.A0A098DSW9	pp	229533.I1RN31
17147	229533.A0A098DSW9	pp	229533.I1S2F2
17148	229533.A0A098DSW9	pp	229533.A0A1C3YLS3
17149	229533.A0A098DSW9	pp	229533.I1RBL4
17150	229533.A0A098DSW9	pp	229533.A0A1C3YLQ7
17151	229533.A0A098DSW9	pp	229533.I1RB73
17152	229533.A0A098DSW9	pp	229533.I1RCN2
17153	229533.A0A098DSW9	pp	229533.I1RUG1
17154	229533.A0A098DSW9	pp	229533.I1RK96
17155	229533.A0A098DSW9	pp	229533.I1RF02

17156 229533.A0A098DSW9 pp 229533.I1RP28
17157 229533.A0A098DSW9 pp 229533.A0A0E0SLZ3
17158 229533.A0A098DSW9 pp 229533.I1RAH9
17159 229533.I1S0Y4 pp 229533.I1S2B0
17160 229533.I1RVZ3 pp 229533.I1RXY7
17161 229533.I1RVZ3 pp 229533.V6RLM7
17162 229533.I1RVZ3 pp 229533.V6RKY7
17163 229533.I1RVZ3 pp 229533.I1S0V7
17164 229533.I1RVZ3 pp 229533.I1S147
17165 229533.I1RVZ3 pp 229533.V6RRM0
17166 229533.I1RVZ3 pp 229533.I1RZ29
17167 229533.I1RVZ3 pp 229533.I1S238
17168 229533.I1RVZ3 pp 229533.I1RZT0
17169 229533.I1RVZ3 pp 229533.I1RYW9
17170 229533.I1RVZ3 pp 229533.I1S148
17171 229533.I1RVZ3 pp 229533.V6RUB1
17172 229533.I1RVZ3 pp 229533.I1S6P5
17173 229533.I1RVZ3 pp 229533.I1RZA7
17174 229533.I1RVZ3 pp 229533.I1RXE6
17175 229533.I1RVZ3 pp 229533.V6RUY9
17176 229533.I1RVZ3 pp 229533.I1S8F8
17177 229533.I1RVZ3 pp 229533.I1S3K8
17178 229533.I1RVZ3 pp 229533.I1RXX1
17179 229533.I1RVZ3 pp 229533.I1RY49
17180 229533.I1RVZ3 pp 229533.V6RH92
17181 229533.I1RVZ3 pp 229533.I1S273
17182 229533.I1RVZ3 pp 229533.I1S241
17183 229533.I1RQ92 pp 229533.I1S604
17184 229533.I1RQ92 pp 229533.Q4IQM6
17185 229533.I1RQ92 pp 229533.I1RY73
17186 229533.I1RQ92 pp 229533.I1S2E9
17187 229533.I1RQ92 pp 229533.I1RSI4
17188 229533.I1RQ92 pp 229533.Q4I6A4
17189 229533.I1RQ92 pp 229533.I1RSL8
17190 229533.I1RQ92 pp 229533.I1RYX9
17191 229533.I1RQ92 pp 229533.I1RYJ8
17192 229533.I1RQ92 pp 229533.Q4I7L0
17193 229533.I1RQ92 pp 229533.I1RW16
17194 229533.I1RQ92 pp 229533.I1RYR2
17195 229533.I1RQ92 pp 229533.I1RZ94
17196 229533.I1RQ92 pp 229533.I1RY44
17197 229533.I1RQ92 pp 229533.I1RSP7
17198 229533.I1RQ92 pp 229533.V6RLW4
17199 229533.I1RQ92 pp 229533.I1RWT6
17200 229533.I1RQ92 pp 229533.I1RZP8
17201 229533.I1RQ92 pp 229533.I1RSA7
17202 229533.I1RQ92 pp 229533.I1RQJ4
17203 229533.I1RQ92 pp 229533.I1RUG2
17204 229533.I1RQ92 pp 229533.I1RQA3
17205 229533.I1S6E5 pp 229533.I1S7S9
17206 229533.I1S6E5 pp 229533.I1S8E2
17207 229533.I1S6E5 pp 229533.Q4IR18
17208 229533.A0A0E0S9Q2 pp 229533.A0A1C3YK51
17209 229533.A0A0E0S9Q2 pp 229533.A0A1C3YKQ9
17210 229533.A0A0E0S9Q2 pp 229533.A0A1C3YJD6
17211 229533.A0A0E0S9Q2 pp 229533.I1S0Y1
17212 229533.A0A0E0S9Q2 pp 229533.I1RAZ1
17213 229533.A0A0E0S9Q2 pp 229533.I1S0Y4
17214 229533.A0A1C3YMW9 pp 229533.I1RC52
17215 229533.A0A1C3YMW9 pp 229533.I1RF62
17216 229533.A0A1C3YMW9 pp 229533.I1RD63
17217 229533.A0A1C3YMW9 pp 229533.I1RLH5
17218 229533.A0A1C3YMW9 pp 229533.I1RG99
17219 229533.A0A1C3YMW9 pp 229533.I1RF64
17220 229533.A0A0E0S9J5 pp 229533.I1S1V3
17221 229533.A0A0E0S9J5 pp 229533.A0A1C3YMQ9
17222 229533.I1S223 pp 229533.I1SAT2
17223 229533.I1S223 pp 229533.I1S2C4
17224 229533.I1S223 pp 229533.I1S659
17225 229533.I1S223 pp 229533.I1S6V0
17226 229533.I1S223 pp 229533.I1S3T6
17227 229533.I1S223 pp 229533.I1S2F7
17228 229533.I1S223 pp 229533.I1S8D3

229533.I1S223	pp	229533.V6RCJ5
229533.I1S223	pp	229533.V6RWB3
229533.I1S223	pp	229533.I1S968
229533.I1S223	pp	229533.V6R5T7
229533.I1S223	pp	229533.I1S267
229533.I1S223	pp	229533.I1S5D0
229533.I1S223	pp	229533.I1S6H3
229533.I1S223	pp	229533.V6RHX2
229533.I1S223	pp	229533.V6RCC0
229533.I1S223	pp	229533.I1S969
229533.I1RTC2	pp	229533.Q4I099
229533.I1RTC2	pp	229533.I1RZM2
229533.I1RTC2	pp	229533.I1RVL8
229533.I1RTC2	pp	229533.I1S2E9
229533.I1RTC2	pp	229533.I1S5Z5
229533.I1RTC2	pp	229533.I1RX63
229533.I1RTC2	pp	229533.Q4IQX3
229533.I1RTC2	pp	229533.Q4I5W5
229533.I1RTC2	pp	229533.I1S3N0
229533.I1RTC2	pp	229533.I1RYV4
229533.I1RTC2	pp	229533.I1RW16
229533.I1RTC2	pp	229533.I1RUF8
229533.I1RTC2	pp	229533.I1RVY0
229533.I1RTC2	pp	229533.Q4HYB8
229533.I1RTC2	pp	229533.I1S0R5
229533.I1RTC2	pp	229533.I1SAD2
229533.I1RTC2	pp	229533.I1S0J3
229533.I1RTC2	pp	229533.I1RW80
229533.I1RTC2	pp	229533.I1RWT6
229533.I1RTC2	pp	229533.I1RUG0
229533.I1RTC2	pp	229533.I1RUP9
229533.I1RTC2	pp	229533.Q4HYI0
229533.I1RTC2	pp	229533.I1RTL1
229533.I1RTC2	pp	229533.Q4HZB8
229533.I1RTC2	pp	229533.I1RW90
229533.I1RTC2	pp	229533.I1RWF3
229533.I1RTC2	pp	229533.I1RXU0
229533.I1RTC2	pp	229533.I1RW91
229533.I1RTC2	pp	229533.I1S3N1
229533.I1RTC2	pp	229533.I1RY44
229533.I1RTC2	pp	229533.Q4IPC8
229533.I1RTC2	pp	229533.I1RUG4
229533.I1RTC2	pp	229533.Q4I5U9
229533.I1RTC2	pp	229533.V6QWX8
229533.I1RTC2	pp	229533.Q4IJU0
229533.I1RTC2	pp	229533.I1S0C6
229533.I1RTC2	pp	229533.I1RXE5
229533.I1RTC2	pp	229533.I1RZB5
229533.I1RTC2	pp	229533.V6QUE6
229533.I1RTC2	pp	229533.V6QV38
229533.I1RTC2	pp	229533.Q4I5I4
229533.I1RTC2	pp	229533.I1RVZ4
229533.I1RTC2	pp	229533.I1S5Z2
229533.I1RTC2	pp	229533.I1S276
229533.I1RDG8	pp	229533.I1RT16
229533.I1RDG8	pp	229533.I1RP96
229533.I1RDG8	pp	229533.I1RPU4
229533.I1RDG8	pp	229533.I1RQ80
229533.I1RDG8	pp	229533.I1RLX6
229533.I1RDG8	pp	229533.V6R227
229533.I1RDG8	pp	229533.I1RML9
229533.I1RDG8	pp	229533.I1RXE5
229533.I1RDG8	pp	229533.I1RWD4
229533.I1RDG8	pp	229533.I1RKE9
229533.I1RDG8	pp	229533.I1RN31
229533.I1RDG8	pp	229533.I1RZM2
229533.I1RDG8	pp	229533.Q4IR18
229533.I1RDG8	pp	229533.V6QV38
229533.I1RDG8	pp	229533.I1RX38
229533.I1RDG8	pp	229533.I1RQM0
229533.I1RDG8	pp	229533.I1RFZ2
229533.I1RDG8	pp	229533.I1RLX4
229533.I1RDG8	pp	229533.I1RVY0

229533.I1RDG8	pp	229533.I1RW16
229533.I1RDG8	pp	229533.I1RPW0
229533.I1RKF3	pp	229533.I1RW91
229533.I1RKF3	pp	229533.Q4IJH1
229533.I1RKF3	pp	229533.V6QWX8
229533.I1RKF3	pp	229533.I1S0S4
229533.I1RKF3	pp	229533.I1RR61
229533.I1RKF3	pp	229533.I1S182
229533.I1RKF3	pp	229533.I1RPW8
229533.I1RKF3	pp	229533.I1RRF7
229533.I1RKF3	pp	229533.I1S9H8
229533.I1RKF3	pp	229533.I1RQ42
229533.I1RKF3	pp	229533.I1RN04
229533.I1RKF3	pp	229533.I1RTC2
229533.I1RKF3	pp	229533.I1RW80
229533.I1RKF3	pp	229533.Q4IR08
229533.I1RKF3	pp	229533.I1RSW9
229533.I1RKF3	pp	229533.I1RLI4
229533.I1RKF3	pp	229533.I1S0C6
229533.I1RKF3	pp	229533.V6RGU1
229533.I1RKF3	pp	229533.I1RWG8
229533.I1RKF3	pp	229533.I1RKR8
229533.I1RKF3	pp	229533.I1RN43
229533.I1RKF3	pp	229533.I1RZ42
229533.I1RKF3	pp	229533.I1S282
229533.A0A1C3YKZ4	pp	229533.I1RQV7
229533.A0A1C3YKZ4	pp	229533.I1RLA2
229533.A0A1C3YKZ4	pp	229533.I1S267
229533.A0A1C3YKZ4	pp	229533.I1RST9
229533.A0A1C3YKZ4	pp	229533.I1RZ25
229533.I1RRN4	pp	229533.I1RSX9
229533.I1RRN4	pp	229533.I1RZM4
229533.I1RRN4	pp	229533.I1S927
229533.I1RRN4	pp	229533.I1S2B0
229533.I1RRN4	pp	229533.I1RRQ7
229533.I1RRN4	pp	229533.I1RW71
229533.I1RRN4	pp	229533.V6RK96
229533.I1RRN4	pp	229533.I1S8X0
229533.I1RRN4	pp	229533.I1RZN1
229533.I1RRN4	pp	229533.I1RRR9
229533.I1RRN4	pp	229533.I1RW82
229533.I1RRN4	pp	229533.I1RSU2
229533.I1RRN4	pp	229533.I1RSM8
229533.I1RVF2	pp	229533.I1S267
229533.I1RVF2	pp	229533.I1RZ25
229533.I1SAB4	pp	229533.I1SAT2
229533.I1SAB4	pp	229533.V6RWB3
229533.I1SAB4	pp	229533.V6R5T7
229533.I1SAB4	pp	229533.V6RHX2
229533.I1SAB4	pp	229533.V6RCC0
229533.I1SAB4	pp	229533.V6RCJ5
229533.A0A1C3YIS2	pp	229533.I1RMQ7
229533.A0A1C3YIS2	pp	229533.A0A1C3YJT5
229533.A0A1C3YIS2	pp	229533.I1RFL7
229533.A0A1C3YIS2	pp	229533.I1R9N1
229533.A0A1C3YIS2	pp	229533.I1RUF8
229533.A0A1C3YIS2	pp	229533.I1RAT7
229533.A0A1C3YIS2	pp	229533.I1RPC2
229533.A0A1C3YIS2	pp	229533.I1S0E8
229533.A0A1C3YIS2	pp	229533.I1RQ13
229533.A0A1C3YIS2	pp	229533.A0A1C3YL67
229533.A0A1C3YIS2	pp	229533.Q4IJM4
229533.A0A1C3YIS2	pp	229533.I1RD15
229533.A0A1C3YIS2	pp	229533.I1RYY5
229533.A0A1C3YIS2	pp	229533.A0A1C3YMB6
229533.A0A0E0S9H3	pp	229533.I1RBA6
229533.A0A0E0S9H3	pp	229533.I1S2B9
229533.A0A0E0S9H3	pp	229533.I1RYX5
229533.A0A0E0S9H3	pp	229533.I1RQK4
229533.A0A0E0S9H3	pp	229533.I1RY63
229533.A0A0E0S9H3	pp	229533.I1R9Y2
229533.I1RFX8	pp	229533.I1RSA7
229533.I1RFX8	pp	229533.V6RLW4

17375	229533.I1RFX8	pp	229533.I1S1V5
17376	229533.I1RFX8	pp	229533.I1RYH9
17377	229533.I1RFX8	pp	229533.I1S283
17378	229533.I1RFX8	pp	229533.I1RMB7
17379	229533.I1RFX8	pp	229533.I1RUG1
17380	229533.I1RFX8	pp	229533.I1RM36
17381	229533.I1RFX8	pp	229533.I1S236
17382	229533.I1RFX8	pp	229533.I1RSJ4
17383	229533.I1RFX8	pp	229533.I1RHR0
17384	229533.I1RFX8	pp	229533.I1S2F2
17385	229533.I1RFX8	pp	229533.Q4IC11
17386	229533.I1RFX8	pp	229533.Q4IQM6
17387	229533.I1RFX8	pp	229533.I1RRT1
17388	229533.I1RFX8	pp	229533.I1RN31
17389	229533.I1RFX8	pp	229533.I1RQ13
17390	229533.I1RFX8	pp	229533.I1RPQ1
17391	229533.I1RFX8	pp	229533.I1S461
17392	229533.I1RFX8	pp	229533.I1RLE7
17393	229533.I1RFX8	pp	229533.I1RLX6
17394	229533.I1RFX8	pp	229533.I1RWI8
17395	229533.I1RFX8	pp	229533.I1RZ94
17396	229533.I1RFX8	pp	229533.I1RKK7
17397	229533.I1RFX8	pp	229533.I1RSJ1
17398	229533.I1RFX8	pp	229533.I1RQA3
17399	229533.I1RFX8	pp	229533.I1S237
17400	229533.I1RFX8	pp	229533.I1S2E8
17401	229533.I1RFX8	pp	229533.I1RSK0
17402	229533.I1RFX8	pp	229533.I1RQ67
17403	229533.I1RFX8	pp	229533.I1RG77
17404	229533.I1RFX8	pp	229533.I1RZ86
17405	229533.I1RFX8	pp	229533.I1S2E9
17406	229533.I1RFX8	pp	229533.I1RN55
17407	229533.I1RFX8	pp	229533.I1RMG7
17408	229533.I1RFX8	pp	229533.I1RN65
17409	229533.I1RFX8	pp	229533.I1RKI2
17410	229533.I1RFX8	pp	229533.I1RMG6
17411	229533.I1RFX8	pp	229533.I1RSR9
17412	229533.I1RFX8	pp	229533.I1RKQ0
17413	229533.I1RFX8	pp	229533.I1RXI6
17414	229533.I1RFX8	pp	229533.I1RUG2
17415	229533.I1RFX8	pp	229533.I1RMJ8
17416	229533.I1RFX8	pp	229533.I1RMX3
17417	229533.I1RFX8	pp	229533.I1RRS7
17418	229533.I1RFX8	pp	229533.I1RSN7
17419	229533.I1RFX8	pp	229533.I1RSP7
17420	229533.I1RFX8	pp	229533.I1RRP7
17421	229533.I1RFX8	pp	229533.I1RYR2
17422	229533.I1RFX8	pp	229533.I1RYR1
17423	229533.I1RFX8	pp	229533.I1RQ92
17424	229533.I1RFX8	pp	229533.V6RGI1
17425	229533.I1RFX8	pp	229533.I1RNE4
17426	229533.I1RFX8	pp	229533.I1RFZ2
17427	229533.I1RFX8	pp	229533.I1RPU4
17428	229533.I1RFX8	pp	229533.I1RQJ4
17429	229533.I1RFX8	pp	229533.I1RX64
17430	229533.I1RFX8	pp	229533.Q4IR08
17431	229533.I1RFX8	pp	229533.I1RSI4
17432	229533.I1RFX8	pp	229533.Q4IEA5
17433	229533.I1RFX8	pp	229533.Q4IER8
17434	229533.I1RFX8	pp	229533.I1S209
17435	229533.I1RFX8	pp	229533.Q4IP51
17436	229533.I1RFX8	pp	229533.I1RZP8
17437	229533.I1RFX8	pp	229533.I1RTN6
17438	229533.I1RFX8	pp	229533.I1S604
17439	229533.I1RFX8	pp	229533.I1RYX9
17440	229533.I1RFX8	pp	229533.Q4IJU0
17441	229533.I1RFX8	pp	229533.I1RZ98
17442	229533.I1RFX8	pp	229533.I1RQI6
17443	229533.I1RFX8	pp	229533.I1RPS2
17444	229533.I1RFX8	pp	229533.I1RU61
17445	229533.I1RFX8	pp	229533.I1RQJ0
17446	229533.I1RFX8	pp	229533.I1RN46
17447	229533.I1RFX8	pp	229533.I1S020

17448 229533.I1RFX8 pp 229533.I1RY73
17449 229533.I1RFX8 pp 229533.I1RSC7
17450 229533.I1RFX8 pp 229533.I1RY44
17451 229533.I1RFX8 pp 229533.I1RWT6
17452 229533.I1RFX8 pp 229533.Q4I0J6
17453 229533.I1RFX8 pp 229533.Q4HYB8
17454 229533.I1RFX8 pp 229533.I1S1D3
17455 229533.I1RFX8 pp 229533.V6R382
17456 229533.I1RFX8 pp 229533.I1RN57
17457 229533.I1RFX8 pp 229533.I1RPZ0
17458 229533.I1RFX8 pp 229533.I1RW16
17459 229533.A0A1C3YI50 pp 229533.I1RM67
17460 229533.A0A1C3YI50 pp 229533.I1RIS3
17461 229533.A0A1C3YI50 pp 229533.A0A1C3YLK2
17462 229533.A0A1C3YI50 pp 229533.I1S0F1
17463 229533.A0A1C3YI50 pp 229533.I1RVZ0
17464 229533.A0A1C3YI50 pp 229533.I1RJ17
17465 229533.A0A1C3YI50 pp 229533.I1RZV6
17466 229533.A0A1C3YI50 pp 229533.I1RDG0
17467 229533.A0A1C3YI50 pp 229533.I1R9V7
17468 229533.A0A1C3YI50 pp 229533.I1RFC0
17469 229533.A0A1C3YI50 pp 229533.I1RN48
17470 229533.A0A1C3YI50 pp 229533.I1RA48
17471 229533.A0A1C3YI50 pp 229533.A0A1C3YL10
17472 229533.A0A1C3YI50 pp 229533.I1RWH5
17473 229533.A0A1C3YI50 pp 229533.I1RBL7
17474 229533.A0A1C3YI50 pp 229533.I1RK83
17475 229533.A0A1C3YI50 pp 229533.I1RND6
17476 229533.A0A1C3YI50 pp 229533.I1RKJ6
17477 229533.A0A1C3YI50 pp 229533.I1S044
17478 229533.A0A1C3YI50 pp 229533.I1RGC2
17479 229533.A0A1C3YI50 pp 229533.A0A1C3YN57
17480 229533.A0A1C3YI50 pp 229533.I1RTG7
17481 229533.A0A1C3YI50 pp 229533.I1S8E2
17482 229533.A0A1C3YI50 pp 229533.I1RYB9
17483 229533.A0A1C3YI50 pp 229533.A0A1C3YKB0
17484 229533.A0A1C3YI50 pp 229533.I1S7S9
17485 229533.A0A1C3YI50 pp 229533.A0A1C3YII7
17486 229533.A0A1C3YI50 pp 229533.I1RFS3
17487 229533.A0A1C3YI50 pp 229533.A0A1C3YMK1
17488 229533.A0A1C3YI50 pp 229533.Q4IR18
17489 229533.A0A1C3YI50 pp 229533.I1RFL3
17490 229533.A0A1C3YI50 pp 229533.A0A1C3YL15
17491 229533.A0A1C3YI50 pp 229533.I1RRQ3
17492 229533.A0A1C3YI50 pp 229533.I1RM96
17493 229533.A0A1C3YI50 pp 229533.I1RS17
17494 229533.A0A1C3YI50 pp 229533.A0A1C3YKK5
17495 229533.A0A1C3YI50 pp 229533.I1RXF6
17496 229533.A0A1C3YI50 pp 229533.I1RWB5
17497 229533.A0A1C3YI50 pp 229533.I1RDY5
17498 229533.A0A1C3YI50 pp 229533.I1RBZ3
17499 229533.A0A1C3YI50 pp 229533.I1RE01
17500 229533.A0A1C3YHM9 pp 229533.I1RXV3
17501 229533.A0A1C3YHM9 pp 229533.O74638
17502 229533.A0A1C3YHM9 pp 229533.Q4I1Y5
17503 229533.A0A1C3YHM9 pp 229533.I1RNG1
17504 229533.A0A1C3YHM9 pp 229533.I1RA62
17505 229533.A0A1C3YHM9 pp 229533.I1S9F1
17506 229533.A0A1C3YHM9 pp 229533.A0A1C3YHZ7
17507 229533.A0A1C3YHM9 pp 229533.A0A1C3YL15
17508 229533.A0A1C3YHM9 pp 229533.I1S5S2
17509 229533.A0A1C3YHM9 pp 229533.I1S3V2
17510 229533.A0A1C3YHM9 pp 229533.I1RMW7
17511 229533.A0A1C3YHM9 pp 229533.I1RZ78
17512 229533.A0A1C3YHM9 pp 229533.I1RSJ2
17513 229533.A0A1C3YHM9 pp 229533.I1RZ40
17514 229533.A0A1C3YHM9 pp 229533.I1RNE7
17515 229533.A0A1C3YHM9 pp 229533.A0A1C3YII7
17516 229533.A0A1C3YHM9 pp 229533.A0A1C3YJL7
17517 229533.A0A1C3YHM9 pp 229533.I1RYI6
17518 229533.A0A1C3YHM9 pp 229533.I1RIX0
17519 229533.I1RN27 pp 229533.I1RW16
17520 229533.I1RN27 pp 229533.I1RWM0

229533.I1RN27 pp 229533.I1RZT1
229533.I1RN27 pp 229533.I1RT36
229533.I1RN27 pp 229533.I1RW28
229533.I1RN27 pp 229533.I1RY98
229533.I1RN27 pp 229533.I1RSU7
229533.I1RN27 pp 229533.I1S0Z2
229533.I1RN27 pp 229533.Q4IEV4
229533.I1RN27 pp 229533.I1RPR8
229533.I1RN27 pp 229533.I1RS37
229533.I1RN27 pp 229533.I1RYV4
229533.I1RN27 pp 229533.I1RN31
229533.I1RN27 pp 229533.I1RQG9
229533.A0A0E0RWP4 pp 229533.I1RLA2
229533.A0A0E0RWP4 pp 229533.I1S048
229533.A0A0E0RWP4 pp 229533.I1S6H3
229533.A0A0E0RWP4 pp 229533.I1R9F2
229533.A0A0E0RWP4 pp 229533.I1RQV8
229533.A0A0E0RWP4 pp 229533.I1RWL3
229533.A0A0E0RWP4 pp 229533.I1RRS6
229533.A0A0E0RWP4 pp 229533.V6R5T7
229533.A0A0E0RWP4 pp 229533.I1RYU6
229533.A0A0E0RWP4 pp 229533.I1S969
229533.A0A0E0RWP4 pp 229533.I1RYP5
229533.A0A0E0RWP4 pp 229533.A0A1C3YIJ7
229533.A0A0E0RWP4 pp 229533.A0A1C3YMC4
229533.A0A0E0RWP4 pp 229533.I1RIB5
229533.A0A0E0RWP4 pp 229533.I1RW54
229533.A0A0E0RWP4 pp 229533.I1S094
229533.A0A0E0RWP4 pp 229533.I1S5D0
229533.A0A0E0RWP4 pp 229533.I1RST9
229533.A0A0E0RWP4 pp 229533.I1S8D3
229533.A0A0E0RWP4 pp 229533.I1RMA2
229533.A0A0E0RWP4 pp 229533.V6RWB3
229533.A0A0E0RWP4 pp 229533.I1RA13
229533.A0A0E0RWP4 pp 229533.I1RQV7
229533.A0A0E0RWP4 pp 229533.A0A1C3YIB8
229533.A0A0E0RWP4 pp 229533.I1RDN3
229533.A0A0E0RWP4 pp 229533.V6RCC0
229533.A0A0E0RWP4 pp 229533.I1RWM4
229533.A0A0E0RWP4 pp 229533.I1S659
229533.A0A0E0RWP4 pp 229533.I1RPM6
229533.A0A0E0RWP4 pp 229533.I1RCT9
229533.A0A0E0RWP4 pp 229533.I1RY81
229533.A0A0E0RWP4 pp 229533.I1S267
229533.A0A0E0RWP4 pp 229533.A0A0E0S530
229533.A0A0E0RWP4 pp 229533.V6RHX2
229533.A0A0E0RWP4 pp 229533.I1RRR9
229533.A0A0E0RWP4 pp 229533.I1RQJ7
229533.A0A0E0RWP4 pp 229533.I1RZ25
229533.A0A0E0RWP4 pp 229533.I1RWF5
229533.A0A0E0RWP4 pp 229533.I1RMK9
229533.A0A0E0RWP4 pp 229533.I1RW82
229533.A0A0E0RWP4 pp 229533.I1RY12
229533.A0A0E0RWP4 pp 229533.I1S968
229533.A0A0E0RWP4 pp 229533.I1RPV8
229533.A0A0E0RWP4 pp 229533.A0A1C3YLE2
229533.A0A0E0RWP4 pp 229533.I1RZ56
229533.A0A0E0RWP4 pp 229533.I1S0A5
229533.A0A0E0RWP4 pp 229533.I1RIJ6
229533.A0A0E0RWP4 pp 229533.I1RSA5
229533.A0A0E0RWP4 pp 229533.I1RC46
229533.A0A0E0RWP4 pp 229533.V6RCJ5
229533.A0A0E0RWP4 pp 229533.I1RZ89
229533.A0A0E0RWP4 pp 229533.I1S6V0
229533.A0A0E0RWP4 pp 229533.I1RK55
229533.A0A0E0RWP4 pp 229533.I1S2C4
229533.A0A0E0RWP4 pp 229533.I1RN84
229533.A0A0E0RWP4 pp 229533.I1RH83
229533.A0A0E0RWP4 pp 229533.I1RJS1
229533.A0A0E0RWP4 pp 229533.I1SAT2
229533.A0A0E0RWP4 pp 229533.A0A1C3YLB6
229533.A0A0E0RWP4 pp 229533.I1RVX8
229533.A0A0E0RWP4 pp 229533.I1RE52

229533.A0A0E0RWP4 pp 229533.A0A1C3YHU3
229533.A0A0E0RWP4 pp 229533.I1RB05
229533.A0A0E0RWP4 pp 229533.I1RHU8
229533.A0A1C3YMD6 pp 229533.I1S267
229533.A0A1C3YMD6 pp 229533.I1RST9
229533.A0A1C3YMD6 pp 229533.I1RQV7
229533.A0A1C3YMD6 pp 229533.I1RZ25
229533.A0A1C3YMD6 pp 229533.I1RLA2
229533.I1R976 pp 229533.I1RDC9
229533.I1R976 pp 229533.V6R227
229533.I1R976 pp 229533.I1RT16
229533.I1R976 pp 229533.I1RP96
229533.I1R976 pp 229533.I1RQ80
229533.I1R976 pp 229533.I1RWD4
229533.I1R976 pp 229533.I1RML9
229533.I1R976 pp 229533.I1RKE9
229533.I1R976 pp 229533.I1RLX6
229533.I1R976 pp 229533.I1RPU4
229533.I1R976 pp 229533.I1RXE5
229533.I1R976 pp 229533.I1RN31
229533.I1R976 pp 229533.I1RZM2
229533.I1R976 pp 229533.I1RQM0
229533.I1R976 pp 229533.I1RCM6
229533.I1R976 pp 229533.V6QV38
229533.I1R976 pp 229533.I1RFZ2
229533.I1R976 pp 229533.I1RBV5
229533.I1R976 pp 229533.I1RBR5
229533.I1R976 pp 229533.Q4IR18
229533.I1R976 pp 229533.I1RVY0
229533.I1R976 pp 229533.I1RBL4
229533.I1R976 pp 229533.I1RW16
229533.I1R976 pp 229533.I1RLX4
229533.I1R976 pp 229533.I1RPW0
229533.I1R976 pp 229533.I1RD70
229533.I1R976 pp 229533.I1RX38
229533.I1RKD3 pp 229533.I1S267
229533.I1RKD3 pp 229533.I1RST9
229533.I1RKD3 pp 229533.V6RCZ8
229533.I1RKD3 pp 229533.I1RZK5
229533.I1RKD3 pp 229533.I1S8D3
229533.I1RKD3 pp 229533.I1S659
229533.I1RKD3 pp 229533.V6RWB3
229533.I1RKD3 pp 229533.I1S8S9
229533.I1RKD3 pp 229533.I1S094
229533.I1RKD3 pp 229533.I1RY81
229533.I1RKD3 pp 229533.I1RN13
229533.I1RKD3 pp 229533.I1S6H3
229533.I1RKD3 pp 229533.V6R5T7
229533.I1RKD3 pp 229533.I1RRM3
229533.I1RKD3 pp 229533.I1S5D0
229533.I1RKD3 pp 229533.I1RRS6
229533.I1RKD3 pp 229533.I1SAT2
229533.I1RKD3 pp 229533.I1RZ56
229533.I1RKD3 pp 229533.I1RMA2
229533.I1RKD3 pp 229533.I1RKG3
229533.I1RKD3 pp 229533.I1RQV7
229533.I1RKD3 pp 229533.I1S1T5
229533.I1RKD3 pp 229533.I1RSF1
229533.I1RKD3 pp 229533.I1RPV8
229533.I1RKD3 pp 229533.V6RCJ5
229533.I1RKD3 pp 229533.I1S6V0
229533.I1RKD3 pp 229533.V6RCC0
229533.I1RKD3 pp 229533.I1S5R1
229533.I1RKD3 pp 229533.Q4HXF6
229533.I1RKD3 pp 229533.I1S0A5
229533.I1RKD3 pp 229533.I1RQ33
229533.I1RKD3 pp 229533.I1RNG4
229533.I1RKD3 pp 229533.I1RKP1
229533.I1RKD3 pp 229533.I1RRI1
229533.I1RKD3 pp 229533.I1RKG2
229533.I1RKD3 pp 229533.I1RZ38
229533.I1RKD3 pp 229533.I1RMH4
229533.A0A098DEC1 pp 229533.A0A1C3YIP6

17667	229533.A0A098DEC1	pp	229533.I1RD70
17668	229533.A0A098DTS6	pp	229533.I1RX38
17669	229533.A0A098DTS6	pp	229533.A0A0E0SNY0
17670	229533.A0A098DTS6	pp	229533.I1RBL4
17671	229533.A0A098DTS6	pp	229533.A0A1C3YI10
17672	229533.A0A098DTS6	pp	229533.I1RVY0
17673	229533.A0A098DTS6	pp	229533.I1RBR5
17674	229533.A0A098DTS6	pp	229533.A0A1C3YJZ2
17675	229533.A0A098DTS6	pp	229533.A0A1C3YKJ6
17676	229533.A0A098DTS6	pp	229533.I1RQM0
17677	229533.A0A098DTS6	pp	229533.A0A1C3YII4
17678	229533.A0A098DTS6	pp	229533.Q4IR18
17679	229533.A0A098DTS6	pp	229533.I1RFZ2
17680	229533.A0A098DTS6	pp	229533.I1RZM2
17681	229533.A0A098DTS6	pp	229533.I1RLX6
17682	229533.A0A098DTS6	pp	229533.I1RBV5
17683	229533.A0A098DTS6	pp	229533.I1RML9
17684	229533.A0A098DTS6	pp	229533.A0A1C3YLP8
17685	229533.A0A098DTS6	pp	229533.A0A098DY35
17686	229533.A0A098DTS6	pp	229533.A0A1C3YI81
17687	229533.A0A098DTS6	pp	229533.I1RW16
17688	229533.A0A098DTS6	pp	229533.I1RD70
17689	229533.A0A098DTS6	pp	229533.I1RP96
17690	229533.A0A098DTS6	pp	229533.I1RPW0
17691	229533.A0A098DTS6	pp	229533.I1RLX4
17692	229533.A0A098DTS6	pp	229533.I1RT16
17693	229533.A0A098DTS6	pp	229533.A0A0E0SHR3
17694	229533.A0A098DTS6	pp	229533.I1RCM6
17695	229533.A0A098DTS6	pp	229533.A0A098E4R5
17696	229533.A0A098DTS6	pp	229533.A0A098DXS1
17697	229533.A0A098DTS6	pp	229533.I1RWD4
17698	229533.A0A098DTS6	pp	229533.I1RN31
17699	229533.A0A098DTS6	pp	229533.A0A1C3YIP6
17700	229533.A0A098DTS6	pp	229533.I1RPU4
17701	229533.A0A098DTS6	pp	229533.A0A098DUU3
17702	229533.A0A098DTS6	pp	229533.A0A1C3YJV9
17703	229533.A0A098DTS6	pp	229533.V6QV38
17704	229533.A0A098DTS6	pp	229533.A0A1C3YK37
17705	229533.A0A098DTS6	pp	229533.A0A1C3YIJ4
17706	229533.A0A098DTS6	pp	229533.I1RQ80
17707	229533.A0A098DTS6	pp	229533.I1RXE5
17708	229533.A0A098DTS6	pp	229533.A0A1C3YK01
17709	229533.A0A098DTS6	pp	229533.I1RKE9
17710	229533.A0A098DTS6	pp	229533.V6R227
17711	229533.A0A098DTS6	pp	229533.I1RDC9
17712	229533.I1RRH0	pp	229533.I1RWI6
17713	229533.I1S210	pp	229533.I1S6V0
17714	229533.I1S210	pp	229533.V6RHX2
17715	229533.I1S210	pp	229533.I1S969
17716	229533.I1S210	pp	229533.V6RCJ5
17717	229533.I1S210	pp	229533.I1S2C4
17718	229533.I1S210	pp	229533.I1SAT2
17719	229533.I1S210	pp	229533.I1S968
17720	229533.I1S210	pp	229533.I1S267
17721	229533.I1S210	pp	229533.I1S8D3
17722	229533.I1S210	pp	229533.I1S6H3
17723	229533.I1S210	pp	229533.V6R5T7
17724	229533.I1S210	pp	229533.V6RCC0
17725	229533.I1S210	pp	229533.I1S5D0
17726	229533.I1S210	pp	229533.I1S659
17727	229533.I1S210	pp	229533.V6RWB3
17728	229533.I1S5Z5	pp	229533.Q4IJH1
17729	229533.I1S5Z5	pp	229533.I1S9H8
17730	229533.I1S5Z5	pp	229533.V6QWX8
17731	229533.I1S5Z5	pp	229533.Q4IR08
17732	229533.I1S5Z5	pp	229533.V6RGU1
17733	229533.I1SAK2	pp	229533.V6RN31
17734	229533.A0A1C3YMW3	pp	229533.I1RV18
17735	229533.A0A1C3YMW3	pp	229533.I1RTJ7
17736	229533.A0A1C3YMW3	pp	229533.I1RPM6
17737	229533.A0A1C3YMW3	pp	229533.I1S0T1
17738	229533.A0A1C3YMW3	pp	229533.I1RJE7
17739	229533.A0A1C3YMW3	pp	229533.I1RJK0

229533.A0A1C3YMW3	pp	229533.I1RJ72
229533.A0A1C3YMW3	pp	229533.I1RY96
229533.A0A1C3YMW3	pp	229533.I1S6H1
229533.A0A1C3YMW3	pp	229533.I1RNW1
229533.A0A1C3YMW3	pp	229533.I1RTU5
229533.A0A1C3YMW3	pp	229533.I1RHT3
229533.A0A1C3YMW3	pp	229533.I1SAJ0
229533.A0A1C3YMW3	pp	229533.I1RZV5
229533.I1RX72	pp	229533.V6R382
229533.I1RX72	pp	229533.V6RLM7
229533.I1RX72	pp	229533.I1S0V7
229533.I1RX72	pp	229533.V6RUY9
229533.I1RX72	pp	229533.I1S0V6
229533.I1RX72	pp	229533.V6RPQ8
229533.I1RX72	pp	229533.I1RYK0
229533.I1RX72	pp	229533.I1RYL8
229533.I1RX72	pp	229533.V6RK96
229533.I1RX72	pp	229533.I1RZM4
229533.I1RX72	pp	229533.I1RZ16
229533.A0A1C3YHN8	pp	229533.V6R5T7
229533.A0A1C3YHN8	pp	229533.I1RMA2
229533.A0A1C3YHN8	pp	229533.I1RQV7
229533.A0A1C3YHN8	pp	229533.I1RW54
229533.A0A1C3YHN8	pp	229533.I1RRS6
229533.A0A1C3YHN8	pp	229533.I1S5D0
229533.A0A1C3YHN8	pp	229533.I1RDN3
229533.A0A1C3YHN8	pp	229533.I1S969
229533.A0A1C3YHN8	pp	229533.I1S048
229533.A0A1C3YHN8	pp	229533.I1R9I3
229533.A0A1C3YHN8	pp	229533.I1RYU6
229533.A0A1C3YHN8	pp	229533.V6RCC0
229533.A0A1C3YHN8	pp	229533.I1RWL3
229533.A0A1C3YHN8	pp	229533.I1RYP5
229533.A0A1C3YHN8	pp	229533.I1RQV8
229533.A0A1C3YHN8	pp	229533.I1S6H3
229533.A0A1C3YHN8	pp	229533.I1R9F2
229533.A0A1C3YHN8	pp	229533.I1S8D3
229533.A0A1C3YHN8	pp	229533.I1S094
229533.A0A1C3YHN8	pp	229533.I1S267
229533.A0A1C3YHN8	pp	229533.I1RLA2
229533.A0A1C3YHN8	pp	229533.A0A1C3YKR5
229533.A0A1C3YHN8	pp	229533.I1RIB5
229533.A0A1C3YHN8	pp	229533.I1RST9
229533.A0A1C3YHN8	pp	229533.I1RW82
229533.A0A1C3YHN8	pp	229533.V6RHX2
229533.A0A1C3YHN8	pp	229533.I1S0A5
229533.A0A1C3YHN8	pp	229533.I1RCT9
229533.A0A1C3YHN8	pp	229533.A0A1C3YMC4
229533.A0A1C3YHN8	pp	229533.I1RSA5
229533.A0A1C3YHN8	pp	229533.I1RRR9
229533.A0A1C3YHN8	pp	229533.I1RPM6
229533.A0A1C3YHN8	pp	229533.V6RWB3
229533.A0A1C3YHN8	pp	229533.I1S659
229533.A0A1C3YHN8	pp	229533.A0A1C3YIB8
229533.A0A1C3YHN8	pp	229533.I1RWF5
229533.A0A1C3YHN8	pp	229533.I1RY81
229533.A0A1C3YHN8	pp	229533.I1S968
229533.A0A1C3YHN8	pp	229533.I1RA13
229533.A0A1C3YHN8	pp	229533.I1RC46
229533.A0A1C3YHN8	pp	229533.I1RY12
229533.A0A1C3YHN8	pp	229533.I1RUR8
229533.A0A1C3YHN8	pp	229533.I1RIJ6
229533.A0A1C3YHN8	pp	229533.I1RZ25
229533.A0A1C3YHN8	pp	229533.I1RMK9
229533.A0A1C3YHN8	pp	229533.A0A1C3YLB6
229533.A0A1C3YHN8	pp	229533.I1RK55
229533.A0A1C3YHN8	pp	229533.I1RE52
229533.A0A1C3YHN8	pp	229533.V6RCJ5
229533.A0A1C3YHN8	pp	229533.I1RPV8
229533.A0A1C3YHN8	pp	229533.I1RZ89
229533.A0A1C3YHN8	pp	229533.I1SAT2
229533.A0A1C3YHN8	pp	229533.I1RZ56
229533.A0A1C3YHN8	pp	229533.I1RHU8

229533.A0A1C3YHN8 pp 229533.I1S6V0
229533.A0A1C3YHN8 pp 229533.I1RB05
229533.A0A1C3YHN8 pp 229533.I1S2C4
229533.A0A1C3YHN8 pp 229533.I1RJS1
229533.A0A1C3YHN8 pp 229533.I1RH83
229533.A0A1C3YHN8 pp 229533.A0A1C3YHU3
229533.I1S065 pp 229533.V6RAN2
229533.A0A0E0SMM4 pp 229533.I1RSA5
229533.A0A0E0SMM4 pp 229533.I1RBA6
229533.A0A0E0SMM4 pp 229533.I1RMV0
229533.A0A0E0SMM4 pp 229533.I1S2B9
229533.A0A0E0SMM4 pp 229533.I1RG11
229533.A0A098D628 pp 229533.A0A0E0SP71
229533.A0A098D628 pp 229533.I1RLM3
229533.A0A098D628 pp 229533.I1RJU9
229533.A0A1C3YHZ7 pp 229533.I1RCM5
229533.A0A1C3YHZ7 pp 229533.I1RPS2
229533.A0A1C3YHZ7 pp 229533.I1RBF4
229533.A0A1C3YHZ7 pp 229533.I1RMU9
229533.A0A1C3YHZ7 pp 229533.I1RNU7
229533.A0A1C3YHZ7 pp 229533.Q4I061
229533.A0A1C3YHZ7 pp 229533.Q4I1Y5
229533.A0A1C3YHZ7 pp 229533.V6R729
229533.A0A1C3YHZ7 pp 229533.I1RIE8
229533.A0A1C3YHZ7 pp 229533.I1RCG5
229533.A0A1C3YHZ7 pp 229533.I1RPK0
229533.A0A1C3YHZ7 pp 229533.V6RLM7
229533.A0A1C3YHZ7 pp 229533.I1RJQ5
229533.A0A1C3YHZ7 pp 229533.I1RNA1
229533.A0A1C3YHZ7 pp 229533.V6RAN2
229533.A0A1C3YHZ7 pp 229533.I1RDE4
229533.A0A1C3YHZ7 pp 229533.I1RPT0
229533.A0A1C3YHZ7 pp 229533.I1RUM7
229533.A0A1C3YHZ7 pp 229533.I1RKR9
229533.A0A1C3YHZ7 pp 229533.I1RP86
229533.A0A1C3YHZ7 pp 229533.A0A1C3YJQ7
229533.A0A1C3YHZ7 pp 229533.I1RC31
229533.A0A1C3YHZ7 pp 229533.I1RZ41
229533.A0A1C3YHZ7 pp 229533.I1RZ78
229533.A0A1C3YHZ7 pp 229533.I1RA62
229533.A0A1C3YHZ7 pp 229533.I1RRT3
229533.A0A1C3YHZ7 pp 229533.I1RK44
229533.A0A1C3YHZ7 pp 229533.I1RKP3
229533.A0A1C3YHZ7 pp 229533.I1RS57
229533.A0A1C3YHZ7 pp 229533.I1RDJ1
229533.A0A1C3YHZ7 pp 229533.I1RUP6
229533.A0A1C3YHZ7 pp 229533.I1RKB5
229533.A0A1C3YHZ7 pp 229533.I1RYK0
229533.A0A0E0S495 pp 229533.I1RSA5
229533.A0A0E0S495 pp 229533.I1RBA6
229533.A0A0E0S495 pp 229533.I1RMV0
229533.A0A0E0S495 pp 229533.I1RG11
229533.A0A0E0S495 pp 229533.I1S2B9
229533.I1RVB5 pp 229533.I1RYV3
229533.I1RVB5 pp 229533.I1RVB6
229533.I1RVB5 pp 229533.I1RVC0
229533.I1RVB5 pp 229533.I1S409
229533.I1RVB5 pp 229533.Q4HTT2
229533.A0A098DG04 pp 229533.A0A098DW84
229533.A0A098DG04 pp 229533.I1S409
229533.A0A098DG04 pp 229533.I1RZG7
229533.A0A098DG04 pp 229533.A0A1C3YL97
229533.A0A098DG04 pp 229533.A0A098DMM9
229533.A0A098DG04 pp 229533.I1RVB5
229533.A0A098DG04 pp 229533.I1RMH5
229533.A0A1C3YKI6 pp 229533.V6QUV7
229533.A0A1C3YKI6 pp 229533.I1RV63
229533.I1RFD1 pp 229533.Q4IJM4
229533.I1RFD1 pp 229533.I1RYH9
229533.I1RFD1 pp 229533.V6RKY7
229533.I1RFD1 pp 229533.I1RUQ5
229533.I1RFD1 pp 229533.I1S439
229533.I1S4I7 pp 229533.I1S4I8

17886 229533.I1RWC3 pp 229533.I1SAK2
17887 229533.I1RWC3 pp 229533.I1RWS7
17888 229533.I1RRV5 pp 229533.I1RSA5
17889 229533.A0A0E0S6M6 pp 229533.I1RYK0
17890 229533.A0A0E0S6M6 pp 229533.A0A1C3YKN4
17891 229533.A0A0E0S6M6 pp 229533.I1RC31
17892 229533.A0A0E0S6M6 pp 229533.I1RFW0
17893 229533.A0A0E0S6M6 pp 229533.I1RZQ1
17894 229533.A0A0E0S6M6 pp 229533.V6RAN2
17895 229533.A0A0E0S6M6 pp 229533.I1RBE7
17896 229533.A0A0E0S6M6 pp 229533.I1RRV1
17897 229533.A0A0E0S6M6 pp 229533.I1RAH6
17898 229533.A0A0E0S6M6 pp 229533.I1RER6
17899 229533.A0A0E0S6M6 pp 229533.I1RP86
17900 229533.A0A098DW84 pp 229533.I1S409
17901 229533.A0A098DW84 pp 229533.I1RVB5
17902 229533.A0A098DW84 pp 229533.I1RCG8
17903 229533.A0A098DW84 pp 229533.I1SA47
17904 229533.A0A098DW84 pp 229533.A0A1C3YL97
17905 229533.I1S000 pp 229533.I1S267
17906 229533.A0A1C3YJI2 pp 229533.I1RG07
17907 229533.I1RB84 pp 229533.I1S1V1
17908 229533.I1RB84 pp 229533.I1RWQ0
17909 229533.I1RB84 pp 229533.I1RDG9
17910 229533.I1RB84 pp 229533.I1RZH4
17911 229533.I1RB84 pp 229533.I1S5L1
17912 229533.I1RB84 pp 229533.I1REK7
17913 229533.I1RB84 pp 229533.I1RRM9
17914 229533.I1RB84 pp 229533.I1RTC2
17915 229533.I1RB84 pp 229533.I1RWL6
17916 229533.I1RB84 pp 229533.I1RWG6
17917 229533.I1RB84 pp 229533.I1RNG8
17918 229533.I1RB84 pp 229533.I1RMB3
17919 229533.I1RB84 pp 229533.I1RN31
17920 229533.I1RB84 pp 229533.I1S9H8
17921 229533.I1RB84 pp 229533.I1S4P4
17922 229533.I1RMT6 pp 229533.V6QU99
17923 229533.I1RMT6 pp 229533.V6RAM6
17924 229533.I1RMT6 pp 229533.V6RWB3
17925 229533.I1RMT6 pp 229533.I1SAI4
17926 229533.I1RMT6 pp 229533.V6R756
17927 229533.I1RMT6 pp 229533.V6R5D2
17928 229533.I1RMT6 pp 229533.V6R2Z2
17929 229533.I1RMT6 pp 229533.I1RRM1
17930 229533.I1RMT6 pp 229533.I1RZ38
17931 229533.I1RMT6 pp 229533.V6R0W1
17932 229533.I1RMT6 pp 229533.I1RQZ6
17933 229533.I1RMT6 pp 229533.I1S8D3
17934 229533.I1RMT6 pp 229533.I1RSA5
17935 229533.I1RMT6 pp 229533.I1RWL3
17936 229533.I1RMT6 pp 229533.I1RYQ4
17937 229533.I1RMT6 pp 229533.I1S0A5
17938 229533.I1RMT6 pp 229533.I1RNH4
17939 229533.A0A098DE56 pp 229533.I1SAK2
17940 229533.A0A098DE56 pp 229533.I1RKR7
17941 229533.A0A098DE56 pp 229533.A0A1C3YK51
17942 229533.A0A098DE56 pp 229533.A0A098DMK7
17943 229533.A0A098DE56 pp 229533.I1RIF9
17944 229533.A0A098DE56 pp 229533.I1RJW0
17945 229533.A0A098DE56 pp 229533.I1S1V6
17946 229533.A0A098DE56 pp 229533.I1RBM4
17947 229533.A0A098DE56 pp 229533.I1RHG0
17948 229533.A0A098DE56 pp 229533.A0A1C3YID4
17949 229533.A0A098DE56 pp 229533.I1RXP4
17950 229533.A0A098DE56 pp 229533.I1RWC3
17951 229533.A0A098DE56 pp 229533.A0A098E413
17952 229533.A0A098DE56 pp 229533.I1S2E7
17953 229533.A0A098DE56 pp 229533.I1RVV2
17954 229533.I1RWW1 pp 229533.I1S4Z7
17955 229533.I1RWW1 pp 229533.I1RZM0
17956 229533.I1RWW1 pp 229533.V6QUV7
17957 229533.I1RQS1 pp 229533.I1S5L1
17958 229533.I1RQS1 pp 229533.I1S9H8

17959 229533.A0A098D624 pp 229533.I1RVB5
17960 229533.A0A098D624 pp 229533.A0A098DG04
17961 229533.A0A098D624 pp 229533.I1S409
17962 229533.A0A098D624 pp 229533.A0A098DW84
17963 229533.A0A098D624 pp 229533.I1RB84
17964 229533.A0A098D624 pp 229533.I1RQS1
17965 229533.A0A1C3YK46 pp 229533.I1S4Z5
17966 229533.A0A1C3YK46 pp 229533.Q4I7L0
17967 229533.A0A1C3YK46 pp 229533.I1RBJ0
17968 229533.A0A1C3YK46 pp 229533.Q4HTT2
17969 229533.A0A1C3YK46 pp 229533.I1RYV3
17970 229533.A0A1C3YKN6 pp 229533.I1RV63
17971 229533.I1RXB5 pp 229533.V6RB81
17972 229533.I1RXB5 pp 229533.I1S082
17973 229533.I1RXB5 pp 229533.I1S2B7
17974 229533.I1RXB5 pp 229533.I1S7X9
17975 229533.I1RXB5 pp 229533.I1S4P4
17976 229533.I1RXB5 pp 229533.V6QX58
17977 229533.I1RXB5 pp 229533.I1S077
17978 229533.I1RXB5 pp 229533.I1RZH4
17979 229533.I1RKR8 pp 229533.I1RNK3
17980 229533.I1RKR8 pp 229533.I1RQW1
17981 229533.I1RKR8 pp 229533.I1SAH9
17982 229533.I1RKR8 pp 229533.V6QV38
17983 229533.I1RKR8 pp 229533.I1RW91
17984 229533.I1RKR8 pp 229533.I1RLI4
17985 229533.I1RKR8 pp 229533.I1RW80
17986 229533.I1RKR8 pp 229533.I1S0X1
17987 229533.I1RKR8 pp 229533.V6QWX8
17988 229533.I1RKR8 pp 229533.I1S0K1
17989 229533.I1RKR8 pp 229533.I1S050
17990 229533.I1RKR8 pp 229533.I1RQ88
17991 229533.I1RKR8 pp 229533.I1RP12
17992 229533.I1RKR8 pp 229533.I1RUG0
17993 229533.I1RKR8 pp 229533.I1RVY0
17994 229533.I1RKR8 pp 229533.I1RTY9
17995 229533.I1RKR8 pp 229533.I1RQ50
17996 229533.I1RKR8 pp 229533.I1RQ42
17997 229533.I1RKR8 pp 229533.I1S5Z5
17998 229533.I1RKR8 pp 229533.I1RPW8
17999 229533.I1RKR8 pp 229533.I1RN04
18000 229533.I1RKR8 pp 229533.I1RWG8
18001 229533.I1RKR8 pp 229533.I1RUE3
18002 229533.I1RKR8 pp 229533.I1RTC2
18003 229533.I1RKR8 pp 229533.I1RR61
18004 229533.I1RKR8 pp 229533.I1RN43
18005 229533.I1RKR8 pp 229533.I1RZP6
18006 229533.I1RKR8 pp 229533.Q4HZB8
18007 229533.I1RKR8 pp 229533.I1RRF7
18008 229533.I1RKR8 pp 229533.Q4I5I4
18009 229533.I1RKR8 pp 229533.Q4IPC8
18010 229533.I1RKR8 pp 229533.I1RNQ6
18011 229533.I1S8H1 pp 229533.V6RLW2
18012 229533.I1RYI6 pp 229533.I1S289
18013 229533.I1RYI6 pp 229533.I1S444
18014 229533.I1RYI6 pp 229533.I1RZ40
18015 229533.I1RYI6 pp 229533.I1S524
18016 229533.I1RYI6 pp 229533.I1S290
18017 229533.I1RYI6 pp 229533.Q4HW67
18018 229533.I1RYI6 pp 229533.V6RIS2
18019 229533.I1RYI6 pp 229533.I1RZ21
18020 229533.I1RYI6 pp 229533.I1S5S2
18021 229533.I1RYI6 pp 229533.V6RJP8
18022 229533.I1RYI6 pp 229533.I1S0P0
18023 229533.I1RYI6 pp 229533.I1S9F1
18024 229533.I1RYI6 pp 229533.I1S8S9
18025 229533.A0A098DG50 pp 229533.I1RA81
18026 229533.A0A098DG50 pp 229533.A0A1C3YIZ8
18027 229533.A0A098DG50 pp 229533.A0A098E1W1
18028 229533.A0A098DG50 pp 229533.A0A1C3YJF5
18029 229533.A0A098DG50 pp 229533.I1RKG1
18030 229533.A0A098DG50 pp 229533.A0A1C3YLN8
18031 229533.A0A098DG50 pp 229533.I1RJR9

18032	229533.A0A098DG50	pp	229533.I1RTB7
18033	229533.A0A098DG50	pp	229533.A0A098DVZ4
18034	229533.A0A098DG50	pp	229533.I1RQL4
18035	229533.A0A098DG50	pp	229533.I1RWL6
18036	229533.A0A098DG50	pp	229533.I1S096
18037	229533.A0A098DG50	pp	229533.P0C431
18038	229533.A0A0E0SEI2	pp	229533.I1RAP4
18039	229533.A0A0E0SEI2	pp	229533.I1S2B9
18040	229533.A0A0E0SEI2	pp	229533.I1RWC9
18041	229533.A0A0E0SEI2	pp	229533.I1RS90
18042	229533.A0A0E0SEI2	pp	229533.I1RG12
18043	229533.A0A0E0SEI2	pp	229533.I1RG11
18044	229533.A0A0E0SEI2	pp	229533.I1RBA6
18045	229533.A0A0E0SEI2	pp	229533.I1RAU9
18046	229533.A0A0E0SEI2	pp	229533.I1RMV0
18047	229533.A0A0E0SEI2	pp	229533.I1RCK9
18048	229533.A0A0E0SEI2	pp	229533.I1RCG2
18049	229533.A0A0E0SEI2	pp	229533.I1RSA5
18050	229533.A0A0E0SEI2	pp	229533.I1RC49
18051	229533.A0A0E0SEI2	pp	229533.A0A0E0SPS2
18052	229533.A0A0E0SEI2	pp	229533.I1RYX5
18053	229533.A0A0E0SGT0	pp	229533.I1RWL6
18054	229533.A0A0E0SGT0	pp	229533.I1RLX6
18055	229533.A0A0E0SGT0	pp	229533.I1S0S6
18056	229533.A0A0E0SGT0	pp	229533.I1RN31
18057	229533.A0A0E0SGT0	pp	229533.I1RAA8
18058	229533.A0A0E0SGT0	pp	229533.I1RY98
18059	229533.A0A0E0SGT0	pp	229533.I1RW16
18060	229533.A0A0E0SGT0	pp	229533.A0A1C3YLN8
18061	229533.A0A0E0SGT0	pp	229533.A0A1C3YLH6
18062	229533.A0A0E0SGT0	pp	229533.I1RJK3
18063	229533.A0A0E0SGT0	pp	229533.I1RML4
18064	229533.A0A0E0SGT0	pp	229533.I1S5K9
18065	229533.A0A0E0SGT0	pp	229533.I1RQN9
18066	229533.A0A0E0SGT0	pp	229533.I1RTE6
18067	229533.A0A0E0SGT0	pp	229533.I1RCD4
18068	229533.A0A0E0SGT0	pp	229533.I1RMH2
18069	229533.A0A0E0SGT0	pp	229533.I1RWA9
18070	229533.A0A0E0SGT0	pp	229533.I1RZD9
18071	229533.A0A0E0SGT0	pp	229533.I1RD10
18072	229533.A0A0E0SGT0	pp	229533.A0A1C3YIZ8
18073	229533.A0A0E0SGT0	pp	229533.A0A1C3YMQ5
18074	229533.A0A0E0SGT0	pp	229533.I1RRQ0
18075	229533.A0A0E0SGT0	pp	229533.I1RNM8
18076	229533.A0A0E0SGT0	pp	229533.P0C431
18077	229533.A0A0E0SGT0	pp	229533.I1RA81
18078	229533.A0A0E0SGT0	pp	229533.I1RJS9
18079	229533.A0A0E0SGT0	pp	229533.I1S095
18080	229533.A0A0E0SGT0	pp	229533.I1S0K9
18081	229533.A0A0E0SGT0	pp	229533.I1RZV4
18082	229533.A0A0E0SGT0	pp	229533.I1S207
18083	229533.A0A0E0SGT0	pp	229533.V6R476
18084	229533.A0A0E0SGT0	pp	229533.I1RTB7
18085	229533.A0A0E0SGT0	pp	229533.A0A1C3YMD9
18086	229533.A0A0E0SGT0	pp	229533.I1RH68
18087	229533.A0A0E0SGT0	pp	229533.V6REE9
18088	229533.A0A0E0SGT0	pp	229533.I1RT70
18089	229533.A0A0E0SGT0	pp	229533.I1RIE2
18090	229533.A0A0E0RSI1	pp	229533.A0A1C3YMQ9
18091	229533.A0A0E0RSI1	pp	229533.I1R9X3
18092	229533.A0A0E0RSI1	pp	229533.I1RPV8
18093	229533.A0A0E0RSI1	pp	229533.V6RHX2
18094	229533.A0A0E0RSI1	pp	229533.A0A0E0RZF3
18095	229533.A0A0E0RSI1	pp	229533.I1S3T6
18096	229533.A0A0E0RSI1	pp	229533.I1RZ56
18097	229533.A0A0E0RSI1	pp	229533.A0A1C3YKN5
18098	229533.A0A0E0RSI1	pp	229533.I1RST9
18099	229533.A0A0E0RSI1	pp	229533.I1RQW1
18100	229533.A0A0E0RSI1	pp	229533.I1S0X1
18101	229533.A0A0E0RSI1	pp	229533.I1S8D3
18102	229533.A0A0E0RSI1	pp	229533.I1RE52
18103	229533.A0A0E0RSI1	pp	229533.I1RAV0
18104	229533.A0A0E0RSI1	pp	229533.I1S6V0

229533.A0A0E0RSI1 pp 229533.I1S267
229533.A0A0E0RSI1 pp 229533.I1RW55
229533.A0A0E0RSI1 pp 229533.A0A0E0S530
229533.A0A0E0RSI1 pp 229533.I1RIM7
229533.A0A0E0RSI1 pp 229533.I1RY81
229533.A0A0E0RSI1 pp 229533.A0A1C3YIP3
229533.A0A0E0RSI1 pp 229533.I1RNG5
229533.A0A0E0RSI1 pp 229533.I1RP12
229533.A0A0E0RSI1 pp 229533.I1RA13
229533.A0A0E0RSI1 pp 229533.I1RTY9
229533.A0A0E0RSI1 pp 229533.I1RUE3
229533.A0A0E0RSI1 pp 229533.I1S2F7
229533.A0A0E0RSI1 pp 229533.I1RJS1
229533.A0A0E0RSI1 pp 229533.A0A1C3YLZ6
229533.A0A0E0RSI1 pp 229533.I1RB05
229533.A0A0E0RSI1 pp 229533.I1RHJ0
229533.A0A0E0RSI1 pp 229533.V6RWB3
229533.A0A0E0RSI1 pp 229533.A0A1C3YIB4
229533.A0A0E0RSI1 pp 229533.I1RSA5
229533.A0A0E0RSI1 pp 229533.I1RZ25
229533.A0A0E0RSI1 pp 229533.I1RIF7
229533.A0A0E0RSI1 pp 229533.I1RQV8
229533.A0A0E0RSI1 pp 229533.V6RCJ5
229533.A0A0E0RSI1 pp 229533.I1RFB0
229533.A0A0E0RSI1 pp 229533.I1RHT5
229533.A0A0E0RSI1 pp 229533.I1RZ38
229533.A0A0E0RSI1 pp 229533.I1S968
229533.A0A0E0RSI1 pp 229533.I1S969
229533.A0A0E0RSI1 pp 229533.I1RGU3
229533.A0A0E0RSI1 pp 229533.V6RCC0
229533.A0A0E0RSI1 pp 229533.I1RIJ6
229533.A0A0E0RSI1 pp 229533.I1RYU6
229533.A0A0E0RSI1 pp 229533.A0A0E0SA25
229533.A0A0E0RSI1 pp 229533.I1RDN3
229533.A0A0E0RSI1 pp 229533.I1RK55
229533.A0A0E0RSI1 pp 229533.I1S223
229533.A0A0E0RSI1 pp 229533.I1S0K1
229533.A0A0E0RSI1 pp 229533.I1S048
229533.A0A0E0RSI1 pp 229533.I1RF24
229533.A0A0E0RSI1 pp 229533.I1RYP5
229533.A0A0E0RSI1 pp 229533.V6R5T7
229533.A0A0E0RSI1 pp 229533.I1SAB4
229533.A0A0E0RSI1 pp 229533.I1SAT1
229533.A0A0E0RSI1 pp 229533.I1RE53
229533.A0A0E0RSI1 pp 229533.I1RLA2
229533.A0A0E0RSI1 pp 229533.A0A0E0RWP4
229533.A0A0E0RSI1 pp 229533.I1S6H3
229533.A0A0E0RSI1 pp 229533.I1S5D0
229533.A0A0E0RSI1 pp 229533.I1S0A5
229533.A0A0E0RSI1 pp 229533.I1RKD3
229533.A0A0E0RSI1 pp 229533.I1S210
229533.A0A0E0RSI1 pp 229533.I1SAH9
229533.A0A0E0RSI1 pp 229533.A0A1C3YHN8
229533.A0A0E0RSI1 pp 229533.I1RQV7
229533.A0A0E0RSI1 pp 229533.I1RMA2
229533.A0A0E0RSI1 pp 229533.I1S094
229533.A0A0E0RSI1 pp 229533.I1RGK3
229533.A0A1C3YKA4 pp 229533.I1RRT3
229533.A0A1C3YKA4 pp 229533.I1RZ78
229533.A0A1C3YKA4 pp 229533.A0A1C3YMJ3
229533.A0A1C3YKA4 pp 229533.I1RKB5
229533.A0A1C3YKA4 pp 229533.I1RSN7
229533.A0A1C3YKA4 pp 229533.I1RSP7
229533.I1RWA9 pp 229533.V6RQ48
229533.I1RWA9 pp 229533.I1RY98
229533.I1RWA9 pp 229533.V6R476
229533.I1RWA9 pp 229533.I1S0S6
229533.I1RWA9 pp 229533.V6QUU6
229533.I1RWA9 pp 229533.V6RKE4
229533.I1RWA9 pp 229533.Q4ICG5
229533.I1RWA9 pp 229533.I1S254
229533.I1RWA9 pp 229533.I1S207
229533.I1RWA9 pp 229533.Q4IPI4

```
229533.I1RWA9   pp  229533.V6RR76
229533.I1RWA9   pp  229533.I1RWT7
229533.I1RWA9   pp  229533.I1RWI6
229533.I1RWA9   pp  229533.I1S1B1
229533.I1RWA9   pp  229533.I1RWK0
229533.I1RWA9   pp  229533.I1RZA4
229533.I1RH83   pp  229533.I1RST9
229533.I1RH83   pp  229533.I1RJS1
229533.I1RH83   pp  229533.I1RPV8
229533.I1RH83   pp  229533.I1S8D3
229533.I1RH83   pp  229533.I1RW55
229533.I1RH83   pp  229533.I1RUE3
229533.I1RH83   pp  229533.I1S969
229533.I1RH83   pp  229533.I1RZ56
229533.I1RH83   pp  229533.V6RHX2
229533.I1RH83   pp  229533.I1S267
229533.I1RH83   pp  229533.I1RIM7
229533.I1RH83   pp  229533.I1S0X1
229533.I1RH83   pp  229533.I1S223
229533.I1RH83   pp  229533.I1S6V0
229533.I1RH83   pp  229533.I1RZ38
229533.I1RH83   pp  229533.I1RQW1
229533.I1RH83   pp  229533.I1RIF7
229533.I1RH83   pp  229533.I1RTY9
229533.I1RH83   pp  229533.I1S3T6
229533.I1RH83   pp  229533.I1RP12
229533.I1RH83   pp  229533.I1RHT5
229533.I1RH83   pp  229533.I1RY81
229533.I1RH83   pp  229533.I1RNG5
229533.I1RH83   pp  229533.V6RWB3
229533.I1RH83   pp  229533.I1S6H3
229533.I1RH83   pp  229533.I1RZ25
229533.I1RH83   pp  229533.I1RHJ0
229533.I1RH83   pp  229533.I1S2F7
229533.I1RH83   pp  229533.I1S0K1
229533.I1RH83   pp  229533.I1SAH9
229533.I1RH83   pp  229533.I1RQV8
229533.I1RH83   pp  229533.I1S048
229533.I1RH83   pp  229533.I1RYP5
229533.I1RH83   pp  229533.I1RIJ6
229533.I1RH83   pp  229533.I1RLA2
229533.I1RH83   pp  229533.I1RK55
229533.I1RH83   pp  229533.I1S968
229533.I1RH83   pp  229533.I1SAB4
229533.I1RH83   pp  229533.I1RYU6
229533.I1RH83   pp  229533.V6RCJ5
229533.I1RH83   pp  229533.V6RCC0
229533.I1RH83   pp  229533.I1RSA5
229533.I1RH83   pp  229533.I1S210
229533.I1RH83   pp  229533.I1RKD3
229533.I1RH83   pp  229533.I1S0A5
229533.I1RH83   pp  229533.I1S094
229533.I1RH83   pp  229533.V6R5T7
229533.I1RH83   pp  229533.I1RMA2
229533.I1RH83   pp  229533.I1RQV7
229533.A0A0E0SLE9   pp  229533.I1RYI9
229533.A0A0E0SLE9   pp  229533.I1R9Y2
229533.I1S2I9   pp  229533.V6RLW2
229533.I1RA38   pp  229533.I1RDJ8
229533.I1RA38   pp  229533.I1RP99
229533.I1RA38   pp  229533.I1RDH0
229533.I1RA38   pp  229533.I1RK79
229533.I1RA38   pp  229533.I1RPS0
229533.I1RA38   pp  229533.I1RFM0
229533.I1RA38   pp  229533.I1S0D5
229533.I1RA38   pp  229533.I1RMJ8
229533.I1RA38   pp  229533.Q4IB89
229533.I1RA38   pp  229533.Q4IPC8
229533.I1RA38   pp  229533.I1RC54
229533.I1RA38   pp  229533.I1RSD0
229533.I1RA38   pp  229533.I1RNB8
229533.I1RA38   pp  229533.I1S214
229533.I1RA38   pp  229533.I1S4P6
```

18251 229533.I1RA38 pp 229533.I1RSC7
18252 229533.I1RA38 pp 229533.I1S004
18253 229533.I1RA38 pp 229533.I1RS03
18254 229533.I1RA38 pp 229533.Q4IPA4
18255 229533.I1RA38 pp 229533.I1RRJ6
18256 229533.I1RA38 pp 229533.I1RQI6
18257 229533.I1RA38 pp 229533.I1RE45
18258 229533.I1RA38 pp 229533.Q4IF44
18259 229533.I1RA38 pp 229533.I1RWI8
18260 229533.I1RA38 pp 229533.I1RC24
18261 229533.I1RA38 pp 229533.I1RY29
18262 229533.I1RA38 pp 229533.I1RZP2
18263 229533.I1RA38 pp 229533.I1RW24
18264 229533.I1RA38 pp 229533.I1RE23
18265 229533.I1RA38 pp 229533.I1RRT0
18266 229533.I1RA38 pp 229533.I1RN17
18267 229533.I1RA38 pp 229533.I1RN28
18268 229533.I1RA38 pp 229533.I1RZB5
18269 229533.I1RA38 pp 229533.I1RT35
18270 229533.I1RA38 pp 229533.I1RJL2
18271 229533.I1RA38 pp 229533.Q4INS6
18272 229533.I1RA38 pp 229533.I1RWJ3
18273 229533.I1RA38 pp 229533.I1S5Z2
18274 229533.I1RA38 pp 229533.Q4I5I4
18275 229533.I1RA38 pp 229533.Q4IPN9
18276 229533.I1RA38 pp 229533.I1RAT5
18277 229533.I1RA38 pp 229533.Q4IR09
18278 229533.I1RA38 pp 229533.I1S788
18279 229533.I1RA38 pp 229533.I1RRG3
18280 229533.I1RA38 pp 229533.I1RN49
18281 229533.I1S0S6 pp 229533.I1S6S9
18282 229533.I1S0S6 pp 229533.I1S7X9
18283 229533.I1S0S6 pp 229533.V6QUU6
18284 229533.I1S0S6 pp 229533.I1S282
18285 229533.I1S0S6 pp 229533.I1S1B6
18286 229533.I1S0S6 pp 229533.V6R476
18287 229533.I1S0S6 pp 229533.I1S2A8
18288 229533.I1S0S6 pp 229533.I1S336
18289 229533.I1S0S6 pp 229533.P0C431
18290 229533.I1S0S6 pp 229533.V6RR76
18291 229533.I1S0S6 pp 229533.I1S1V3
18292 229533.I1S0S6 pp 229533.I1S0W5
18293 229533.I1S0S6 pp 229533.Q4ICG5
18294 229533.I1RWL6 pp 229533.I1S0W5
18295 229533.I1RWL6 pp 229533.V6REE9
18296 229533.I1RWL6 pp 229533.I1RZD9
18297 229533.I1RWL6 pp 229533.I1S095
18298 229533.I1RWL6 pp 229533.I1RZV4
18299 229533.I1RWL6 pp 229533.I1S0S6
18300 229533.I1RWL6 pp 229533.I1S0C0
18301 229533.I1RWL6 pp 229533.P0C431
18302 229533.I1RWL6 pp 229533.I1S096
18303 229533.I1RWL6 pp 229533.I1S5K9
18304 229533.I1RJR9 pp 229533.I1RQN9
18305 229533.I1RJR9 pp 229533.I1S1B1
18306 229533.I1RJR9 pp 229533.I1RJS2
18307 229533.I1RJR9 pp 229533.V6RR76
18308 229533.I1RJR9 pp 229533.I1S096
18309 229533.I1RJR9 pp 229533.I1RTB7
18310 229533.I1RJR9 pp 229533.P0C431
18311 229533.I1RJR9 pp 229533.I1RN81
18312 229533.I1RJR9 pp 229533.I1RWL6
18313 229533.I1RJR9 pp 229533.I1RKG1
18314 229533.I1RJR9 pp 229533.I1RT19
18315 229533.A0A1C3YI06 pp 229533.A0A1C3YJ86
18316 229533.I1RMM6 pp 229533.I1RNM4
18317 229533.I1RMM6 pp 229533.I1S4Z7
18318 229533.I1RMM6 pp 229533.I1RUP0
18319 229533.I1RMM6 pp 229533.I1SAM9
18320 229533.I1RMM6 pp 229533.I1RSW4
18321 229533.I1RMM6 pp 229533.I1RZ78
18322 229533.I1RMM6 pp 229533.I1RZM0
18323 229533.I1RMM6 pp 229533.I1RVN2

18324 229533.I1RMM6 pp 229533.I1RPK1
18325 229533.I1RMM6 pp 229533.I1RYL5
18326 229533.I1RMM6 pp 229533.I1RQ75
18327 229533.I1RMM6 pp 229533.I1RZ34
18328 229533.I1RMM6 pp 229533.V6QUV7
18329 229533.I1RMM6 pp 229533.I1RXF0
18330 229533.I1RMM6 pp 229533.Q4I1Y5
18331 229533.I1RMM6 pp 229533.I1S9S4
18332 229533.I1RMM6 pp 229533.V6QU20
18333 229533.I1RMM6 pp 229533.Q4IJT0
18334 229533.I1RH68 pp 229533.I1RJV3
18335 229533.I1RH68 pp 229533.I1RTA3
18336 229533.I1RH68 pp 229533.I1RW41
18337 229533.I1RH68 pp 229533.I1RMH2
18338 229533.I1RH68 pp 229533.I1S0K9
18339 229533.I1RH68 pp 229533.I1RNH3
18340 229533.I1RH68 pp 229533.I1S6S9
18341 229533.I1RH68 pp 229533.I1S282
18342 229533.I1RH68 pp 229533.I1RS64
18343 229533.I1RH68 pp 229533.I1RYZ7
18344 229533.I1RH68 pp 229533.I1RRN0
18345 229533.I1RH68 pp 229533.I1RJS9
18346 229533.I1RH68 pp 229533.I1S2A8
18347 229533.I1RH68 pp 229533.I1RUE4
18348 229533.I1RH68 pp 229533.I1RN65
18349 229533.I1RH68 pp 229533.V6R476
18350 229533.I1RH68 pp 229533.I1RNF9
18351 229533.I1RH68 pp 229533.I1S7X9
18352 229533.I1RH68 pp 229533.I1RXF7
18353 229533.I1RH68 pp 229533.I1RZM1
18354 229533.I1RH68 pp 229533.I1S336
18355 229533.I1RH68 pp 229533.I1RJS2
18356 229533.I1RH68 pp 229533.I1RUA4
18357 229533.I1RH68 pp 229533.I1S0W5
18358 229533.I1RH68 pp 229533.I1RZH4
18359 229533.I1RH68 pp 229533.I1RNQ5
18360 229533.I1RH68 pp 229533.I1RM09
18361 229533.I1RH68 pp 229533.I1S1B6
18362 229533.I1RH68 pp 229533.V6RR76
18363 229533.I1RH68 pp 229533.I1RN81
18364 229533.I1RH68 pp 229533.I1RZX9
18365 229533.I1RH68 pp 229533.I1RXD5
18366 229533.I1RH68 pp 229533.I1RY98
18367 229533.I1RH68 pp 229533.Q4ICG5
18368 229533.I1RH68 pp 229533.I1S0C0
18369 229533.I1RH68 pp 229533.I1RMR5
18370 229533.I1RH68 pp 229533.I1RWL6
18371 229533.I1RH68 pp 229533.I1RQL4
18372 229533.I1RH68 pp 229533.I1RKA0
18373 229533.A0A1C3YJI1 pp 229533.I1RQ30
18374 229533.A0A1C3YJI1 pp 229533.O74638
18375 229533.A0A1C3YJI1 pp 229533.I1S770
18376 229533.A0A1C3YJI1 pp 229533.I1RZ52
18377 229533.A0A1C3YJI1 pp 229533.I1RTI7
18378 229533.A0A1C3YJI1 pp 229533.I1RWF4
18379 229533.A0A1C3YJI1 pp 229533.I1RCB4
18380 229533.A0A1C3YJI1 pp 229533.I1RQ48
18381 229533.A0A1C3YJI1 pp 229533.A0A1C3YM01
18382 229533.A0A1C3YJI1 pp 229533.A0A1C3YLU9
18383 229533.A0A1C3YJI1 pp 229533.I1RE53
18384 229533.A0A1C3YJI1 pp 229533.I1R9K3
18385 229533.A0A1C3YJI1 pp 229533.I1RMG6
18386 229533.A0A1C3YJI1 pp 229533.I1RIY0
18387 229533.A0A1C3YJI1 pp 229533.I1RZ20
18388 229533.A0A1C3YJI1 pp 229533.I1S2E2
18389 229533.A0A1C3YJI1 pp 229533.V6RQW4
18390 229533.A0A1C3YJI1 pp 229533.I1RYU5
18391 229533.A0A1C3YJI1 pp 229533.I1RMG7
18392 229533.A0A1C3YJI1 pp 229533.I1S9D4
18393 229533.I1RWM0 pp 229533.I1S0Z2
18394 229533.I1RWM0 pp 229533.I1RY98
18395 229533.I1RWM0 pp 229533.I1RZS6
18396 229533.I1RP83 pp 229533.I1S3I4

229533.I1RP83	pp	229533.I1RPN8
229533.I1RP83	pp	229533.I1RQW1
229533.I1RP83	pp	229533.I1S2N3
229533.A0A098E2F6	pp	229533.I1RUE3
229533.A0A098E2F6	pp	229533.I1RBE7
229533.A0A098E2F6	pp	229533.A0A0E0RZF3
229533.A0A098E2F6	pp	229533.I1RQW1
229533.A0A098E2F6	pp	229533.I1S0X1
229533.A0A098E2F6	pp	229533.I1RTY9
229533.A0A098E2F6	pp	229533.I1S1W8
229533.A0A098E2F6	pp	229533.I1RP12
229533.A0A098E2F6	pp	229533.I1R9X3
229533.A0A098E2F6	pp	229533.A0A1C3YLB6
229533.A0A098E2F6	pp	229533.I1RGK3
229533.A0A098E2F6	pp	229533.I1RJE8
229533.A0A098E2F6	pp	229533.I1S3G4
229533.A0A098E2F6	pp	229533.I1RL13
229533.A0A098E2F6	pp	229533.I1SAH9
229533.A0A098E2F6	pp	229533.I1RY87
229533.A0A098E2F6	pp	229533.I1RNG4
229533.A0A098E2F6	pp	229533.I1RIF7
229533.A0A098E2F6	pp	229533.I1S5P3
229533.A0A098E2F6	pp	229533.I1S0K1
229533.A0A1C3YIZ8	pp	229533.I1S1B1
229533.A0A1C3YIZ8	pp	229533.I1RZM1
229533.A0A1C3YIZ8	pp	229533.P0C431
229533.A0A1C3YIZ8	pp	229533.I1RZP6
229533.A0A1C3YIZ8	pp	229533.V6REE9
229533.A0A1C3YIZ8	pp	229533.I1S050
229533.A0A1C3YIZ8	pp	229533.I1RT70
229533.A0A1C3YIZ8	pp	229533.A0A1C3YMD9
229533.A0A1C3YIZ8	pp	229533.I1S095
229533.A0A1C3YIZ8	pp	229533.I1RZV4
229533.A0A1C3YIZ8	pp	229533.I1RJR9
229533.A0A1C3YIZ8	pp	229533.I1RH68
229533.A0A1C3YIZ8	pp	229533.I1RRQ0
229533.A0A1C3YIZ8	pp	229533.I1RTB7
229533.A0A1C3YIZ8	pp	229533.I1S0S6
229533.A0A1C3YIZ8	pp	229533.I1RLX6
229533.A0A1C3YIZ8	pp	229533.I1RJM9
229533.A0A1C3YIZ8	pp	229533.I1RT19
229533.A0A1C3YIZ8	pp	229533.I1RWL6
229533.A0A1C3YIZ8	pp	229533.I1RD70
229533.A0A1C3YIZ8	pp	229533.I1RN81
229533.A0A1C3YIZ8	pp	229533.I1RQN9
229533.A0A1C3YIZ8	pp	229533.I1RG07
229533.A0A1C3YIZ8	pp	229533.I1RN31
229533.A0A1C3YIZ8	pp	229533.I1RJS2
229533.A0A1C3YIZ8	pp	229533.I1S5K9
229533.A0A1C3YIZ8	pp	229533.I1RZD9
229533.A0A1C3YIZ8	pp	229533.I1RRN0
229533.A0A1C3YIZ8	pp	229533.I1RW16
229533.A0A1C3YIZ8	pp	229533.A0A1C3YKS2
229533.A0A1C3YIZ8	pp	229533.A0A1C3YLN8
229533.A0A1C3YIZ8	pp	229533.I1RD10
229533.A0A1C3YIZ8	pp	229533.I1RCD4
229533.A0A1C3YIZ8	pp	229533.I1RTE6
229533.A0A1C3YIZ8	pp	229533.A0A1C3YMA1
229533.A0A1C3YIZ8	pp	229533.I1RA81
229533.A0A1C3YIZ8	pp	229533.I1RYZ7
229533.A0A1C3YIZ8	pp	229533.A0A1C3YMQ5
229533.I1RRV6	pp	229533.I1RSA5
229533.A0A098D5W0	pp	229533.A0A1C3YJG7
229533.A0A098D5W0	pp	229533.I1RXB5
229533.A0A098D5W0	pp	229533.A0A098DG50
229533.A0A098D5W0	pp	229533.A0A0E0SGT0
229533.A0A098D5W0	pp	229533.I1RWA9
229533.A0A098D5W0	pp	229533.I1S0S6
229533.A0A098D5W0	pp	229533.I1RWL6
229533.A0A098D5W0	pp	229533.I1RJR9
229533.A0A098D5W0	pp	229533.I1RH68
229533.A0A098D5W0	pp	229533.I1RWM0
229533.A0A098D5W0	pp	229533.A0A1C3YLH6

18470 229533.A0A098D5W0 pp 229533.A0A1C3YIZ8
18471 229533.A0A098D5W0 pp 229533.I1S549
18472 229533.I1SAT2 pp 229533.V6RCJ5
18473 229533.I1SAT2 pp 229533.V6RWB3
18474 229533.I1SAT2 pp 229533.V6R5T7
18475 229533.I1SAT2 pp 229533.V6RCC0
18476 229533.I1SAT2 pp 229533.V6RHX2
18477 229533.I1RJ82 pp 229533.I1RUM7
18478 229533.I1RJ82 pp 229533.I1SAC4
18479 229533.I1RJ82 pp 229533.I1RVW8
18480 229533.I1RJ82 pp 229533.I1RPS2
18481 229533.I1RJ82 pp 229533.I1RZ78
18482 229533.I1RJ82 pp 229533.I1RQE0
18483 229533.I1RJ82 pp 229533.V6RUY9
18484 229533.I1RJ82 pp 229533.V6RUG6
18485 229533.I1RJ82 pp 229533.I1RKB5
18486 229533.I1RJ82 pp 229533.I1S308
18487 229533.I1RXA6 pp 229533.V6QUV7
18488 229533.I1RMC5 pp 229533.I1RW06
18489 229533.I1RMC5 pp 229533.I1RUY5
18490 229533.I1RMC5 pp 229533.I1RUY6
18491 229533.I1RMC5 pp 229533.I1RP86
18492 229533.I1RMC5 pp 229533.I1RWS4
18493 229533.I1RMC5 pp 229533.I1RYJ6
18494 229533.I1RMC5 pp 229533.I1SAJ3
18495 229533.I1RMC5 pp 229533.V6QVM5
18496 229533.I1RMC5 pp 229533.I1RVW8
18497 229533.I1RMC5 pp 229533.I1S256
18498 229533.I1RMC5 pp 229533.I1RZ78
18499 229533.I1RMC5 pp 229533.I1RY62
18500 229533.I1RIB5 pp 229533.I1S0X1
18501 229533.I1RIB5 pp 229533.I1RTY9
18502 229533.I1RIB5 pp 229533.I1RQW1
18503 229533.I1RIB5 pp 229533.I1S3T6
18504 229533.I1RIB5 pp 229533.I1RUE3
18505 229533.I1RIB5 pp 229533.I1RP12
18506 229533.I1RIB5 pp 229533.I1S2F7
18507 229533.I1RIB5 pp 229533.I1RY81
18508 229533.I1RIB5 pp 229533.I1S8D3
18509 229533.I1RIB5 pp 229533.I1RNG5
18510 229533.I1RIB5 pp 229533.I1RST9
18511 229533.I1RIB5 pp 229533.I1RJS1
18512 229533.I1RIB5 pp 229533.I1RZ25
18513 229533.I1RIB5 pp 229533.I1S6V0
18514 229533.I1RIB5 pp 229533.I1RZ56
18515 229533.I1RIB5 pp 229533.I1RW55
18516 229533.I1RIB5 pp 229533.V6RWB3
18517 229533.I1RIB5 pp 229533.V6RHX2
18518 229533.I1RIB5 pp 229533.I1S0K1
18519 229533.I1RIB5 pp 229533.I1RIM7
18520 229533.I1RIB5 pp 229533.I1RYU6
18521 229533.I1RIB5 pp 229533.I1S267
18522 229533.I1RIB5 pp 229533.I1SAH9
18523 229533.I1RIB5 pp 229533.I1S968
18524 229533.I1RIB5 pp 229533.I1RPV8
18525 229533.I1RIB5 pp 229533.I1RYP5
18526 229533.I1RIB5 pp 229533.I1RQV8
18527 229533.I1RIB5 pp 229533.I1RK55
18528 229533.I1RIB5 pp 229533.I1RSA5
18529 229533.I1RIB5 pp 229533.I1S6H3
18530 229533.I1RIB5 pp 229533.I1RIJ6
18531 229533.I1RIB5 pp 229533.V6RCC0
18532 229533.I1RIB5 pp 229533.I1RLA2
18533 229533.I1RIB5 pp 229533.I1SAB4
18534 229533.I1RIB5 pp 229533.I1SAT1
18535 229533.I1RIB5 pp 229533.I1S0A5
18536 229533.I1RIB5 pp 229533.I1RQV7
18537 229533.I1RIB5 pp 229533.I1RMA2
18538 229533.I1RIB5 pp 229533.I1S223
18539 229533.I1RIB5 pp 229533.I1S048
18540 229533.I1RIB5 pp 229533.I1S210
18541 229533.I1RIB5 pp 229533.I1S969
18542 229533.I1RIB5 pp 229533.I1RKD3

18543 229533.I1RIB5 pp 229533.V6RCJ5
18544 229533.I1RIB5 pp 229533.I1RZ38
18545 229533.I1RIB5 pp 229533.V6R5T7
18546 229533.I1RIB5 pp 229533.I1S094
18547 229533.I1RIB5 pp 229533.I1RIF7
18548 229533.I1REH0 pp 229533.I1RHL0
18549 229533.I1REH0 pp 229533.V6RUG6
18550 229533.I1REH0 pp 229533.I1RHM8
18551 229533.I1REH0 pp 229533.I1RNG4
18552 229533.I1REH0 pp 229533.I1RVW8
18553 229533.I1REH0 pp 229533.I1RF73
18554 229533.I1REH0 pp 229533.I1RI39
18555 229533.I1REH0 pp 229533.I1RXM0
18556 229533.I1REH0 pp 229533.I1S308
18557 229533.I1REH0 pp 229533.I1RJ82
18558 229533.I1REH0 pp 229533.I1SAK2
18559 229533.I1REH0 pp 229533.I1S2N3
18560 229533.I1REH0 pp 229533.I1RPT0
18561 229533.I1REH0 pp 229533.I1SAC4
18562 229533.I1RP30 pp 229533.I1RPH7
18563 229533.A0A1C3YN98 pp 229533.I1RBE7
18564 229533.A0A1C3YN98 pp 229533.V6RAN2
18565 229533.A0A1C3YN98 pp 229533.I1RC31
18566 229533.A0A1C3YN98 pp 229533.I1RP86
18567 229533.A0A1C3YN98 pp 229533.I1RAH6
18568 229533.A0A1C3YN98 pp 229533.I1RYK0
18569 229533.A0A1C3YN98 pp 229533.I1RZQ1
18570 229533.A0A098DUT6 pp 229533.I1RV63
18571 229533.I1RCA8 pp 229533.I1RG11
18572 229533.I1RCA8 pp 229533.I1RW28
18573 229533.I1RCA8 pp 229533.I1RCA9
18574 229533.I1RCA8 pp 229533.I1RSA5
18575 229533.I1RCA8 pp 229533.I1RSU7
18576 229533.I1S659 pp 229533.I1SAB4
18577 229533.I1S659 pp 229533.V6RCJ5
18578 229533.I1S659 pp 229533.V6RCC0
18579 229533.I1S659 pp 229533.I1S6H3
18580 229533.I1S659 pp 229533.I1SAH9
18581 229533.I1S659 pp 229533.I1S8D3
18582 229533.I1S659 pp 229533.I1S6V0
18583 229533.I1S659 pp 229533.V6R5T7
18584 229533.I1S659 pp 229533.I1S968
18585 229533.I1S659 pp 229533.V6RWB3
18586 229533.I1S659 pp 229533.I1S969
18587 229533.I1S659 pp 229533.V6RHX2
18588 229533.I1SAC4 pp 229533.V6RUG6
18589 229533.I1SAC4 pp 229533.V6RUY9
18590 229533.A0A1C3YMV0 pp 229533.I1RMV0
18591 229533.A0A1C3YMV0 pp 229533.I1RSA5
18592 229533.A0A1C3YMV0 pp 229533.I1RBA6
18593 229533.A0A1C3YMV0 pp 229533.I1S2B9
18594 229533.A0A1C3YMV0 pp 229533.I1RG11
18595 229533.I1RE47 pp 229533.I1RUY5
18596 229533.I1RE47 pp 229533.I1RFJ1
18597 229533.I1RE47 pp 229533.I1RYJ6
18598 229533.I1RE47 pp 229533.I1RGB2
18599 229533.I1RE47 pp 229533.I1RVW8
18600 229533.I1RE47 pp 229533.I1RW06
18601 229533.I1RE47 pp 229533.I1RHM8
18602 229533.I1RE47 pp 229533.I1SAJ3
18603 229533.I1RE47 pp 229533.I1RP86
18604 229533.I1RE47 pp 229533.I1RWS4
18605 229533.I1RE47 pp 229533.I1RF73
18606 229533.I1RE47 pp 229533.I1RZ78
18607 229533.I1RKL8 pp 229533.I1RV63
18608 229533.A0A098DRH0 pp 229533.I1RP86
18609 229533.A0A098DRH0 pp 229533.A0A0E0S6V8
18610 229533.A0A098DRH0 pp 229533.I1RYJ6
18611 229533.A0A098DRH0 pp 229533.I1RDU0
18612 229533.A0A098DRH0 pp 229533.I1RFJ1
18613 229533.A0A098DRH0 pp 229533.I1RW06
18614 229533.A0A098DRH0 pp 229533.A0A1C3YHV3
18615 229533.A0A098DRH0 pp 229533.I1RC61

18616 229533.A0A098DRH0 pp 229533.I1RM54
18617 229533.A0A098DRH0 pp 229533.I1RGB2
18618 229533.A0A098DRH0 pp 229533.I1RA62
18619 229533.A0A098DRH0 pp 229533.I1RUY5
18620 229533.A0A098DRH0 pp 229533.I1RUY6
18621 229533.A0A098DRH0 pp 229533.V6QVM5
18622 229533.A0A098DRH0 pp 229533.I1RF73
18623 229533.A0A098DRH0 pp 229533.I1RWS4
18624 229533.A0A098DRH0 pp 229533.I1RAF9
18625 229533.A0A098DRH0 pp 229533.I1RZ78
18626 229533.A0A098DRH0 pp 229533.I1SAJ3
18627 229533.A0A098DRH0 pp 229533.A0A1C3YJ56
18628 229533.A0A098DRH0 pp 229533.I1RY62
18629 229533.A0A098DRH0 pp 229533.I1RCP7
18630 229533.A0A098DRH0 pp 229533.I1RVW8
18631 229533.A0A098DRH0 pp 229533.I1RM55
18632 229533.A0A098DRH0 pp 229533.I1RHM8
18633 229533.A0A098DRH0 pp 229533.A0A0E0RU55
18634 229533.A0A098DRH0 pp 229533.I1S256
18635 229533.A0A1C3YIJ6 pp 229533.I1RSA5
18636 229533.A0A1C3YIJ6 pp 229533.I1RMV0
18637 229533.A0A1C3YIJ6 pp 229533.I1RBA6
18638 229533.A0A1C3YIJ6 pp 229533.I1RG11
18639 229533.A0A1C3YIJ6 pp 229533.I1S2B9
18640 229533.A0A0E0S9F1 pp 229533.I1RTE6
18641 229533.I1R9F2 pp 229533.I1S6H3
18642 229533.I1R9F2 pp 229533.I1SAB4
18643 229533.I1R9F2 pp 229533.I1SAH9
18644 229533.I1R9F2 pp 229533.I1RMT6
18645 229533.I1R9F2 pp 229533.V6R0W1
18646 229533.I1R9F2 pp 229533.I1S094
18647 229533.I1R9F2 pp 229533.I1S968
18648 229533.I1R9F2 pp 229533.V6RCJ5
18649 229533.I1R9F2 pp 229533.I1RK55
18650 229533.I1R9F2 pp 229533.I1RYU6
18651 229533.I1R9F2 pp 229533.I1RYP5
18652 229533.I1R9F2 pp 229533.I1S0K1
18653 229533.I1R9F2 pp 229533.V6RCC0
18654 229533.I1R9F2 pp 229533.V6R5T7
18655 229533.I1R9F2 pp 229533.I1RIJ6
18656 229533.I1R9F2 pp 229533.V6RAM6
18657 229533.I1R9F2 pp 229533.I1RF24
18658 229533.I1R9F2 pp 229533.V6R5D2
18659 229533.I1R9F2 pp 229533.I1RY35
18660 229533.I1R9F2 pp 229533.I1RE53
18661 229533.I1R9F2 pp 229533.I1SAI4
18662 229533.I1R9F2 pp 229533.I1RDN3
18663 229533.I1R9F2 pp 229533.I1S0A5
18664 229533.I1R9F2 pp 229533.I1RKD3
18665 229533.I1R9F2 pp 229533.I1S210
18666 229533.I1R9F2 pp 229533.I1RSA5
18667 229533.I1R9F2 pp 229533.I1RIF7
18668 229533.I1R9F2 pp 229533.I1RUE3
18669 229533.I1R9F2 pp 229533.I1RQV7
18670 229533.I1R9F2 pp 229533.I1RFB0
18671 229533.I1R9F2 pp 229533.I1RJS1
18672 229533.I1R9F2 pp 229533.I1RHT5
18673 229533.I1R9F2 pp 229533.I1RMA2
18674 229533.I1R9F2 pp 229533.I1RRM1
18675 229533.I1R9F2 pp 229533.I1RGK3
18676 229533.I1R9F2 pp 229533.I1RZ38
18677 229533.I1R9F2 pp 229533.I1S3T6
18678 229533.I1R9F2 pp 229533.V6QU99
18679 229533.I1R9F2 pp 229533.I1RQW1
18680 229533.I1R9F2 pp 229533.I1S969
18681 229533.I1R9F2 pp 229533.I1RQV8
18682 229533.I1R9F2 pp 229533.V6R756
18683 229533.I1R9F2 pp 229533.V6R2Z2
18684 229533.I1R9F2 pp 229533.I1RP12
18685 229533.I1R9F2 pp 229533.I1RQJ7
18686 229533.I1R9F2 pp 229533.I1RTY9
18687 229533.I1R9F2 pp 229533.I1RRS6
18688 229533.I1R9F2 pp 229533.I1S0X1

```
229533.I1R9F2   pp  229533.I1S6V0
229533.I1R9F2   pp  229533.I1RGU3
229533.I1R9F2   pp  229533.I1RB05
229533.I1R9F2   pp  229533.V6RWB3
229533.I1R9F2   pp  229533.I1RNG5
229533.I1R9F2   pp  229533.I1S223
229533.I1R9F2   pp  229533.I1RA13
229533.I1R9F2   pp  229533.I1RY81
229533.I1R9F2   pp  229533.I1S2F7
229533.I1R9F2   pp  229533.I1RW55
229533.I1R9F2   pp  229533.I1RHJ0
229533.I1R9F2   pp  229533.I1RNH4
229533.I1R9F2   pp  229533.I1RDV8
229533.I1R9F2   pp  229533.I1R9X3
229533.I1R9F2   pp  229533.I1RPV8
229533.I1R9F2   pp  229533.I1RST9
229533.I1R9F2   pp  229533.I1S8D3
229533.I1R9F2   pp  229533.V6RHX2
229533.I1R9F2   pp  229533.I1RAG3
229533.I1R9F2   pp  229533.I1RZ56
229533.I1R9F2   pp  229533.I1S267
229533.I1R9F2   pp  229533.I1RE52
229533.I1R9F2   pp  229533.I1RIM7
229533.A0A0E0S546   pp  229533.I1RP86
229533.A0A0E0S546   pp  229533.I1RYK0
229533.A0A0E0S546   pp  229533.I1RC31
229533.A0A0E0S546   pp  229533.A0A1C3YKN4
229533.A0A0E0S546   pp  229533.V6RAN2
229533.A0A0E0S546   pp  229533.I1RZQ1
229533.A0A0E0S546   pp  229533.I1RAH6
229533.A0A0E0S546   pp  229533.I1RBE7
229533.I1RYJ1   pp  229533.I1S6W3
229533.I1RYJ1   pp  229533.I1S320
229533.I1RYJ1   pp  229533.I1S407
229533.I1RYJ1   pp  229533.V6R5G9
229533.I1RYJ1   pp  229533.I1S4A4
229533.I1RRS6   pp  229533.I1RZ25
229533.I1RRS6   pp  229533.I1S0K1
229533.I1RRS6   pp  229533.V6RCJ5
229533.I1RRS6   pp  229533.I1S094
229533.I1RRS6   pp  229533.I1RSA5
229533.I1RRS6   pp  229533.I1S223
229533.I1RRS6   pp  229533.I1RZ38
229533.I1RRS6   pp  229533.I1SAB4
229533.I1RRS6   pp  229533.I1S969
229533.I1RRS6   pp  229533.I1RYU6
229533.I1RRS6   pp  229533.I1S0A5
229533.I1RRS6   pp  229533.I1S048
229533.I1RRS6   pp  229533.I1S968
229533.I1RRS6   pp  229533.I1S210
229533.I1RRS6   pp  229533.I1RZ56
229533.I1RRS6   pp  229533.I1S6V0
229533.I1RRS6   pp  229533.I1SAH9
229533.I1RRS6   pp  229533.I1SAK7
229533.I1RRS6   pp  229533.I1RYP5
229533.I1RRS6   pp  229533.V6RCC0
229533.I1RRS6   pp  229533.I1S6H3
229533.I1RRS6   pp  229533.V6R5T7
229533.I1RRS6   pp  229533.I1RY35
229533.I1RRS6   pp  229533.I1RW55
229533.I1RRS6   pp  229533.I1S8D3
229533.I1RRS6   pp  229533.I1S0X1
229533.I1RRS6   pp  229533.I1RST9
229533.I1RRS6   pp  229533.I1S2F7
229533.I1RRS6   pp  229533.I1RUE3
229533.I1RRS6   pp  229533.I1S267
229533.I1RRS6   pp  229533.I1S3T6
229533.I1RRS6   pp  229533.I1RZX3
229533.I1RRS6   pp  229533.I1RY81
229533.I1RRS6   pp  229533.V6RHX2
229533.I1RRS6   pp  229533.V6RWB3
229533.I1RRS6   pp  229533.I1RTY9
229533.I1RRS6   pp  229533.I1RT23
```

18762 229533.I1R9D3 pp 229533.V6RUG6
18763 229533.I1R9D3 pp 229533.I1RHL0
18764 229533.I1R9D3 pp 229533.I1RF73
18765 229533.I1R9D3 pp 229533.I1RI39
18766 229533.I1R9D3 pp 229533.I1RXM0
18767 229533.I1R9D3 pp 229533.V6QV09
18768 229533.I1R9D3 pp 229533.I1RFL7
18769 229533.I1R9D3 pp 229533.I1S2N3
18770 229533.I1R9D3 pp 229533.I1RYZ9
18771 229533.I1R9D3 pp 229533.I1S308
18772 229533.I1R9D3 pp 229533.I1REH0
18773 229533.I1R9D3 pp 229533.I1SAK2
18774 229533.I1R9D3 pp 229533.I1RL24
18775 229533.I1R9D3 pp 229533.I1SAC4
18776 229533.I1R9D3 pp 229533.I1R9F8
18777 229533.I1R9D3 pp 229533.I1RDT7
18778 229533.I1R9D3 pp 229533.I1RDW1
18779 229533.I1R9D3 pp 229533.I1RJ82
18780 229533.I1R9D3 pp 229533.I1RHM8
18781 229533.I1R9D3 pp 229533.V6RGI1
18782 229533.I1R9D3 pp 229533.I1RVW8
18783 229533.I1R9D3 pp 229533.I1R9Y7
18784 229533.I1R9D3 pp 229533.I1RPT0
18785 229533.A0A1C3YLF7 pp 229533.I1RNG2
18786 229533.A0A1C3YLF7 pp 229533.I1RQC3
18787 229533.A0A1C3YLF7 pp 229533.I1RPP7
18788 229533.A0A1C3YLF7 pp 229533.Q4I7L0
18789 229533.A0A1C3YLF7 pp 229533.I1S4Z5
18790 229533.A0A1C3YLF7 pp 229533.A0A1C3YMQ5
18791 229533.A0A1C3YLF7 pp 229533.I1RBJ0
18792 229533.A0A1C3YLF7 pp 229533.I1R9Y7
18793 229533.A0A1C3YLF7 pp 229533.Q4HTT2
18794 229533.A0A1C3YLF7 pp 229533.I1RJU1
18795 229533.A0A1C3YLF7 pp 229533.A0A1C3YN89
18796 229533.A0A1C3YLF7 pp 229533.I1RYV3
18797 229533.I1RPT0 pp 229533.I1RX72
18798 229533.I1RPT0 pp 229533.I1RZ13
18799 229533.I1RPT0 pp 229533.I1RU18
18800 229533.I1RPT0 pp 229533.V6RUY9
18801 229533.I1RPT0 pp 229533.I1SAI0
18802 229533.I1RPT0 pp 229533.I1RSY7
18803 229533.I1RPT0 pp 229533.V6RV57
18804 229533.I1RPT0 pp 229533.I1SA27
18805 229533.I1RPT0 pp 229533.I1RZN1
18806 229533.I1RPT0 pp 229533.I1RT54
18807 229533.I1RPT0 pp 229533.I1S147
18808 229533.I1RPT0 pp 229533.I1RZ78
18809 229533.I1RPT0 pp 229533.I1RRT3
18810 229533.I1RPT0 pp 229533.I1S272
18811 229533.I1RPT0 pp 229533.I1RQ75
18812 229533.I1RPT0 pp 229533.I1RPW1
18813 229533.I1RPT0 pp 229533.I1S308
18814 229533.I1RPT0 pp 229533.I1RYK0
18815 229533.I1RPT0 pp 229533.V6RLM7
18816 229533.I1RPT0 pp 229533.I1RVW8
18817 229533.I1RPT0 pp 229533.I1S574
18818 229533.I1RPT0 pp 229533.I1RXM0
18819 229533.I1RPT0 pp 229533.I1S2N3
18820 229533.I1RPT0 pp 229533.I1S984
18821 229533.I1RPT0 pp 229533.I1S4D0
18822 229533.I1RPT0 pp 229533.I1RZT6
18823 229533.I1RPT0 pp 229533.V6RAN2
18824 229533.I1RPT0 pp 229533.I1RUY5
18825 229533.I1RPT0 pp 229533.I1RSB7
18826 229533.I1RPT0 pp 229533.I1S183
18827 229533.I1RPT0 pp 229533.I1RRV4
18828 229533.I1RPT0 pp 229533.I1RVV3
18829 229533.I1RPT0 pp 229533.I1RUY6
18830 229533.A0A0E0S977 pp 229533.I1RBF4
18831 229533.A0A0E0S977 pp 229533.A0A1C3YIG2
18832 229533.A0A0E0S977 pp 229533.I1RBY9
18833 229533.A0A0E0S977 pp 229533.A0A1C3YLR6
18834 229533.A0A0E0S977 pp 229533.I1RK44

18835	229533.A0A0E0RSS5	pp	229533.I1RYU5
18836	229533.A0A0E0RSS5	pp	229533.I1RV18
18837	229533.A0A0E0RSS5	pp	229533.A0A1C3YN30
18838	229533.A0A0E0RSS5	pp	229533.I1RS17
18839	229533.A0A0E0RSS5	pp	229533.I1S5P3
18840	229533.A0A0E0RSS5	pp	229533.I1RDW4
18841	229533.A0A0E0RSS5	pp	229533.I1RPU8
18842	229533.A0A0E0RSS5	pp	229533.I1S2C4
18843	229533.A0A0E0RSS5	pp	229533.V6R1K4
18844	229533.A0A0E0RSS5	pp	229533.I1RZ20
18845	229533.A0A0E0RSS5	pp	229533.I1RCB4
18846	229533.A0A0E0RSS5	pp	229533.V6R057
18847	229533.A0A0E0RSS5	pp	229533.Q4HXF0
18848	229533.I1RRE2	pp	229533.V6QYF0
18849	229533.I1RRE2	pp	229533.I1S201
18850	229533.I1RRE2	pp	229533.I1RWD4
18851	229533.A0A098DY52	pp	229533.I1RJS0
18852	229533.A0A098DY52	pp	229533.I1RRG1
18853	229533.A0A098DY52	pp	229533.I1RD35
18854	229533.A0A098DY52	pp	229533.I1RUP5
18855	229533.A0A098DY52	pp	229533.I1RNG4
18856	229533.A0A098DY52	pp	229533.I1RSX9
18857	229533.A0A098DY52	pp	229533.I1RND4
18858	229533.A0A098DY52	pp	229533.I1RNU7
18859	229533.A0A098DY52	pp	229533.V6RAG6
18860	229533.I1RCM9	pp	229533.I1SAT3
18861	229533.I1RCM9	pp	229533.I1S0W0
18862	229533.I1RCM9	pp	229533.I1RZM2
18863	229533.I1RCM9	pp	229533.I1S0C0
18864	229533.I1RMC2	pp	229533.I1RW00
18865	229533.I1RMC2	pp	229533.I1SAJ3
18866	229533.I1RMC2	pp	229533.I1RZ78
18867	229533.I1RMC2	pp	229533.I1RVW8
18868	229533.I1RMC2	pp	229533.I1RW06
18869	229533.I1RMC2	pp	229533.I1RYJ6
18870	229533.I1RMC2	pp	229533.I1RUY5
18871	229533.I1RMC2	pp	229533.I1RWS4
18872	229533.I1RMC2	pp	229533.I1RP86
18873	229533.I1RMC2	pp	229533.I1RZB5
18874	229533.I1S5D0	pp	229533.I1S969
18875	229533.I1S5D0	pp	229533.V6RHX2
18876	229533.I1S5D0	pp	229533.I1S8D3
18877	229533.I1S5D0	pp	229533.V6RWB3
18878	229533.I1S5D0	pp	229533.I1S6V0
18879	229533.I1S5D0	pp	229533.I1SAH9
18880	229533.I1S5D0	pp	229533.V6RCC0
18881	229533.I1S5D0	pp	229533.I1SAB4
18882	229533.I1S5D0	pp	229533.I1S6H3
18883	229533.I1S5D0	pp	229533.I1S968
18884	229533.I1S5D0	pp	229533.V6R5T7
18885	229533.I1S5D0	pp	229533.V6RCJ5
18886	229533.A0A098DEB5	pp	229533.I1RKR8
18887	229533.A0A098DEB5	pp	229533.I1RF85
18888	229533.A0A098DEB5	pp	229533.I1RJS1
18889	229533.A0A098DEB5	pp	229533.A0A0E0RSI1
18890	229533.A0A098DEB5	pp	229533.I1RH83
18891	229533.A0A098DEB5	pp	229533.I1RZ56
18892	229533.A0A098DEB5	pp	229533.I1RRF7
18893	229533.A0A098DEB5	pp	229533.I1RPV8
18894	229533.A0A098DEB5	pp	229533.I1RIV2
18895	229533.A0A098DEB5	pp	229533.I1RE52
18896	229533.A0A098DEB5	pp	229533.A0A1C3YLB6
18897	229533.A0A098DEB5	pp	229533.I1RW80
18898	229533.A0A098DEB5	pp	229533.I1S1T5
18899	229533.A0A098DEB5	pp	229533.I1S8H5
18900	229533.A0A098DEB5	pp	229533.A0A098E2F6
18901	229533.A0A098DEB5	pp	229533.I1S0A5
18902	229533.A0A098DEB5	pp	229533.I1RNG4
18903	229533.A0A098DEB5	pp	229533.I1SAT2
18904	229533.A0A098DEB5	pp	229533.A0A1C3YMP1
18905	229533.A0A098DEB5	pp	229533.I1RN04
18906	229533.A0A098DEB5	pp	229533.I1RF49
18907	229533.A0A098DEB5	pp	229533.I1RIB5

18908 229533.A0A098DEB5 pp 229533.A0A1C3YHG7
18909 229533.A0A098DEB5 pp 229533.I1RST9
18910 229533.A0A098DEB5 pp 229533.I1RCG6
18911 229533.A0A098DEB5 pp 229533.I1S659
18912 229533.A0A098DEB5 pp 229533.I1S267
18913 229533.A0A098DEB5 pp 229533.A0A098E1Y1
18914 229533.A0A098DEB5 pp 229533.I1RA13
18915 229533.A0A098DEB5 pp 229533.A0A1C3YHX6
18916 229533.A0A098DEB5 pp 229533.I1R9F2
18917 229533.A0A098DEB5 pp 229533.I1RRS6
18918 229533.A0A098DEB5 pp 229533.I1RCU2
18919 229533.A0A098DEB5 pp 229533.I1S6H3
18920 229533.A0A098DEB5 pp 229533.I1RPW8
18921 229533.A0A098DEB5 pp 229533.I1RCU7
18922 229533.A0A098DEB5 pp 229533.I1S094
18923 229533.A0A098DEB5 pp 229533.I1RDN3
18924 229533.A0A098DEB5 pp 229533.I1RW91
18925 229533.A0A098DEB5 pp 229533.I1RQV7
18926 229533.A0A098DEB5 pp 229533.I1S5D0
18927 229533.I1RNU7 pp 229533.I1RVV3
18928 229533.I1RNU7 pp 229533.I1RP86
18929 229533.I1RNU7 pp 229533.I1RUY6
18930 229533.I1RNU7 pp 229533.I1RSB7
18931 229533.I1RNU7 pp 229533.I1RPT0
18932 229533.I1RNU7 pp 229533.I1S984
18933 229533.I1RNU7 pp 229533.I1RZT6
18934 229533.I1RNU7 pp 229533.I1RT54
18935 229533.I1RNU7 pp 229533.V6RAN2
18936 229533.I1RNU7 pp 229533.I1S574
18937 229533.I1RNU7 pp 229533.I1RRV4
18938 229533.I1RNU7 pp 229533.I1RPW1
18939 229533.I1RNU7 pp 229533.I1RU18
18940 229533.I1RNU7 pp 229533.I1RUY5
18941 229533.I1RNU7 pp 229533.I1RZ78
18942 229533.I1RNU7 pp 229533.I1S147
18943 229533.I1RNU7 pp 229533.I1RRT3
18944 229533.I1RNU7 pp 229533.I1RSY7
18945 229533.I1RNU7 pp 229533.I1RQ75
18946 229533.I1RNU7 pp 229533.I1S183
18947 229533.I1RNU7 pp 229533.I1SAI0
18948 229533.I1RNU7 pp 229533.I1RZ13
18949 229533.I1RNU7 pp 229533.V6RUY9
18950 229533.I1RNU7 pp 229533.V6RV57
18951 229533.I1RNU7 pp 229533.I1S272
18952 229533.I1RNU7 pp 229533.I1RX72
18953 229533.I1RNU7 pp 229533.I1RZN1
18954 229533.I1RNU7 pp 229533.V6RLM7
18955 229533.I1RNU7 pp 229533.I1RVW8
18956 229533.I1RNU7 pp 229533.I1RYK0
18957 229533.I1RNU7 pp 229533.V6RDZ4
18958 229533.I1RN52 pp 229533.I1RZ25
18959 229533.I1RN52 pp 229533.I1RY32
18960 229533.I1RN52 pp 229533.I1S5H2
18961 229533.I1RN52 pp 229533.I1RYP2
18962 229533.I1RN52 pp 229533.I1S6D7
18963 229533.I1RN52 pp 229533.I1S048
18964 229533.I1RN52 pp 229533.I1RSF1
18965 229533.I1RN52 pp 229533.I1S267
18966 229533.I1RN52 pp 229533.I1RRD0
18967 229533.I1RN52 pp 229533.I1RST9
18968 229533.I1RME3 pp 229533.I1RZQ1
18969 229533.I1RME3 pp 229533.I1RYK0
18970 229533.I1RME3 pp 229533.V6RAN2
18971 229533.I1RME3 pp 229533.I1RP86
18972 229533.A0A098DUH5 pp 229533.I1RWS4
18973 229533.A0A098DUH5 pp 229533.I1RF73
18974 229533.A0A098DUH5 pp 229533.I1RVW8
18975 229533.A0A098DUH5 pp 229533.I1SAJ3
18976 229533.A0A098DUH5 pp 229533.A0A1C3YII3
18977 229533.A0A098DUH5 pp 229533.I1RHM8
18978 229533.A0A098DUH5 pp 229533.I1RCP7
18979 229533.A0A098DUH5 pp 229533.I1RUY5
18980 229533.A0A098DUH5 pp 229533.I1RAF9

229533.A0A098DUH5	pp	229533.I1RGB2
229533.A0A098DUH5	pp	229533.I1RFJ1
229533.A0A098DUH5	pp	229533.I1RZ78
229533.A0A098DUH5	pp	229533.A0A1C3YHV3
229533.A0A098DUH5	pp	229533.I1RC61
229533.A0A098DUH5	pp	229533.I1RP86
229533.A0A098DUH5	pp	229533.I1RDU0
229533.A0A098DUH5	pp	229533.I1RYJ6
229533.A0A098DUH5	pp	229533.I1RW06
229533.I1RLV3	pp	229533.I1S3I4
229533.I1RLV3	pp	229533.Q4HW67
229533.I1RLV3	pp	229533.I1RQP1
229533.I1RLV3	pp	229533.Q4HYQ4
229533.I1RLV3	pp	229533.I1RRG3
229533.I1RLV3	pp	229533.I1RRT0
229533.I1RLV3	pp	229533.Q4IMD1
229533.I1RLV3	pp	229533.Q4I5W5
229533.I1RLV3	pp	229533.I1RZR6
229533.I1RLV3	pp	229533.Q4ICA8
229533.I1RLV3	pp	229533.Q4IJU0
229533.I1RLV3	pp	229533.I1RQW1
229533.I1RLV3	pp	229533.I1RP83
229533.I1RLV3	pp	229533.Q4HYB8
229533.I1RLV3	pp	229533.Q4I5I4
229533.I1RLV3	pp	229533.I1RPN8
229533.I1RLV3	pp	229533.I1RZB5
229533.I1S8Z2	pp	229533.I1S9T5
229533.I1S8Z2	pp	229533.V6RKY8
229533.I1S8Z2	pp	229533.V6RJP8
229533.I1S8Z2	pp	229533.V6QUV7
229533.I1S8Z2	pp	229533.V6RCZ8
229533.I1RN42	pp	229533.I1RSC0
229533.I1RN42	pp	229533.V6RJP8
229533.I1RN42	pp	229533.I1S0P0
229533.I1RN42	pp	229533.I1S1N7
229533.I1RN42	pp	229533.I1RY92
229533.I1RN42	pp	229533.I1RRX5
229533.I1RN42	pp	229533.I1RYT8
229533.I1RN42	pp	229533.I1RVT8
229533.I1RN42	pp	229533.V6RPN0
229533.I1RN42	pp	229533.I1S8S9
229533.I1RN42	pp	229533.I1RWA3
229533.I1RN42	pp	229533.I1RP02
229533.I1RN42	pp	229533.Q4I174
229533.I1RN42	pp	229533.I1S0K2
229533.I1RN42	pp	229533.Q4HZ34
229533.I1RN42	pp	229533.I1S5X9
229533.I1RN42	pp	229533.I1RS29
229533.I1RN42	pp	229533.I1S5R5
229533.I1S3F7	pp	229533.I1SAJ3
229533.I1S3F7	pp	229533.V6QVM5
229533.I1RGP7	pp	229533.I1RZM0
229533.I1RGP7	pp	229533.I1RWP4
229533.I1RGP7	pp	229533.I1RV63
229533.I1RGP7	pp	229533.I1S4Z7
229533.I1RGP7	pp	229533.I1RUP0
229533.I1RGP7	pp	229533.V6QUV7
229533.A0A098DY22	pp	229533.I1RWI6
229533.A0A098DY22	pp	229533.I1RPE5
229533.I1RMU2	pp	229533.I1RYJ1
229533.I1RMU2	pp	229533.I1RVW8
229533.I1RMU2	pp	229533.V6R5G9
229533.I1RMU2	pp	229533.I1S320
229533.I1RMU2	pp	229533.I1RXT4
229533.I1RMU2	pp	229533.I1RWF6
229533.I1RMU2	pp	229533.I1S407
229533.I1RMU2	pp	229533.I1RWE3
229533.I1RMU2	pp	229533.I1S7I5
229533.I1RMU2	pp	229533.Q4IPI4
229533.I1RMU2	pp	229533.I1S6W3
229533.I1RMU2	pp	229533.I1RT54
229533.I1RMU2	pp	229533.I1RYC1
229533.I1RMU2	pp	229533.I1RSY7

19054 229533.I1RMU2 pp 229533.I1S1V2
19055 229533.I1RMU2 pp 229533.I1RUP6
19056 229533.I1RMU2 pp 229533.I1RR89
19057 229533.I1RMU2 pp 229533.I1S4A4
19058 229533.I1RNP9 pp 229533.I1RT01
19059 229533.I1RNP9 pp 229533.P0C431
19060 229533.I1RNP9 pp 229533.I1RW23
19061 229533.I1RNP9 pp 229533.I1RS41
19062 229533.I1RNP9 pp 229533.I1RZR6
19063 229533.I1RNP9 pp 229533.I1RQ88
19064 229533.I1S1M2 pp 229533.V6RLM7
19065 229533.I1RVW8 pp 229533.I1S4A4
19066 229533.I1RVW8 pp 229533.I1RYC1
19067 229533.I1RVW8 pp 229533.I1SAC4
19068 229533.I1RVW8 pp 229533.I1S308
19069 229533.I1RVW8 pp 229533.V6RUG6
19070 229533.I1RVW8 pp 229533.I1S6W3
19071 229533.I1RVW8 pp 229533.I1S3F7
19072 229533.I1RVW8 pp 229533.I1RWE3
19073 229533.I1RVW8 pp 229533.I1RWF6
19074 229533.I1RVW8 pp 229533.I1S407
19075 229533.I1RVW8 pp 229533.I1RXT4
19076 229533.I1RVW8 pp 229533.I1S320
19077 229533.I1RVW8 pp 229533.V6R5G9
19078 229533.I1RVW8 pp 229533.I1RYJ1
19079 229533.I1RRK5 pp 229533.I1S444
19080 229533.I1RRK5 pp 229533.I1RSF1
19081 229533.I1RRK5 pp 229533.I1S5H2
19082 229533.I1RRK5 pp 229533.I1RZC3
19083 229533.I1RRK5 pp 229533.I1RSE0
19084 229533.I1RRK5 pp 229533.I1RZ25
19085 229533.I1RRK5 pp 229533.I1RY32
19086 229533.I1RRK5 pp 229533.I1RYP2
19087 229533.I1RRK5 pp 229533.I1RST9
19088 229533.I1RRK5 pp 229533.I1S267
19089 229533.I1RRK5 pp 229533.I1S048
19090 229533.I1RRK5 pp 229533.I1S6D7
19091 229533.I1RGB5 pp 229533.Q4HZ95
19092 229533.I1RGB5 pp 229533.I1S0M2
19093 229533.I1RGB5 pp 229533.I1S0Q7
19094 229533.I1RGB5 pp 229533.Q4I6B0
19095 229533.I1RGB5 pp 229533.Q4IB65
19096 229533.I1RGB5 pp 229533.I1RZV3
19097 229533.I1RGB5 pp 229533.V6R7V8
19098 229533.I1RGB5 pp 229533.I1RQF2
19099 229533.I1RGB5 pp 229533.I1RSY2
19100 229533.I1RZ87 pp 229533.Q4I7S1
19101 229533.I1RZ87 pp 229533.Q4I650
19102 229533.I1RZ87 pp 229533.Q4HY90
19103 229533.I1RZ87 pp 229533.I1RZU5
19104 229533.I1RZ87 pp 229533.Q4IMD1
19105 229533.I1RZ87 pp 229533.Q4IAK7
19106 229533.I1RZ87 pp 229533.Q4IER8
19107 229533.I1RZ87 pp 229533.Q4ICA8
19108 229533.I1RZ87 pp 229533.Q4I948
19109 229533.I1S308 pp 229533.I1SAK2
19110 229533.I1S308 pp 229533.I1SAC4
19111 229533.I1S308 pp 229533.V6RUG6
19112 229533.A0A1C3YKK8 pp 229533.I1RTP5
19113 229533.A0A1C3YKK8 pp 229533.I1REU9
19114 229533.A0A1C3YKK8 pp 229533.I1S267
19115 229533.A0A1C3YKK8 pp 229533.I1RLM3
19116 229533.A0A1C3YKK8 pp 229533.I1RRK5
19117 229533.A0A1C3YKK8 pp 229533.I1RYQ4
19118 229533.A0A1C3YKK8 pp 229533.I1S048
19119 229533.A0A1C3YKK8 pp 229533.I1RTJ2
19120 229533.A0A1C3YKK8 pp 229533.I1RJU9
19121 229533.A0A1C3YKK8 pp 229533.I1RLA2
19122 229533.A0A1C3YKK8 pp 229533.I1S6D7
19123 229533.A0A1C3YKK8 pp 229533.I1RGU6
19124 229533.A0A1C3YKK8 pp 229533.I1RRD0
19125 229533.A0A1C3YKK8 pp 229533.I1RQQ0
19126 229533.A0A1C3YKK8 pp 229533.I1RH75

229533.A0A1C3YKK8	pp	229533.V6QVV9
229533.A0A1C3YKK8	pp	229533.I1RF24
229533.A0A1C3YKK8	pp	229533.I1RE48
229533.A0A1C3YKK8	pp	229533.I1RST9
229533.A0A1C3YKK8	pp	229533.I1RBF3
229533.A0A1C3YKK8	pp	229533.I1RY32
229533.A0A1C3YKK8	pp	229533.A0A1C3YLL1
229533.A0A1C3YKK8	pp	229533.I1RG71
229533.A0A1C3YKK8	pp	229533.I1RAP3
229533.A0A1C3YKK8	pp	229533.I1RH91
229533.A0A1C3YKK8	pp	229533.I1RGG6
229533.A0A1C3YKK8	pp	229533.I1RBU8
229533.A0A1C3YKK8	pp	229533.I1RAX2
229533.A0A1C3YKK8	pp	229533.I1RC71
229533.A0A1C3YKK8	pp	229533.I1RBY9
229533.A0A1C3YKK8	pp	229533.I1RZ25
229533.A0A1C3YKK8	pp	229533.I1RKP1
229533.A0A1C3YKK8	pp	229533.I1RYP2
229533.A0A1C3YKK8	pp	229533.I1S5H2
229533.A0A1C3YKK8	pp	229533.I1RCS6
229533.A0A1C3YKK8	pp	229533.I1REW3
229533.A0A1C3YKK8	pp	229533.I1R9K2
229533.A0A1C3YKK8	pp	229533.I1RSE0
229533.A0A1C3YKK8	pp	229533.I1RR45
229533.A0A1C3YKK8	pp	229533.I1RSF1
229533.A0A1C3YKK8	pp	229533.I1RLW5
229533.A0A1C3YKK8	pp	229533.A0A1C3YMN0
229533.A0A1C3YKK8	pp	229533.I1S444
229533.A0A1C3YKK8	pp	229533.I1RZC3
229533.A0A1C3YKK8	pp	229533.I1RGQ2
229533.A0A1C3YKK8	pp	229533.I1RE79
229533.A0A098DMQ4	pp	229533.Q4IMJ1
229533.A0A098DMQ4	pp	229533.I1RRT8
229533.A0A098DMQ4	pp	229533.I1RAF9
229533.A0A098DMQ4	pp	229533.I1RMJ3
229533.A0A098DMQ4	pp	229533.I1RWS4
229533.A0A098DMQ4	pp	229533.I1RF73
229533.A0A098DMQ4	pp	229533.I1RA62
229533.A0A098DMQ4	pp	229533.I1RZ78
229533.A0A098DMQ4	pp	229533.A0A098E2A4
229533.A0A098DMQ4	pp	229533.V6QVH8
229533.A0A098DMQ4	pp	229533.O74638
229533.A0A098DMQ4	pp	229533.A0A0E0S6X1
229533.A0A098DMQ4	pp	229533.I1RVW8
229533.A0A098DMQ4	pp	229533.I1S0H7
229533.A0A098DMQ4	pp	229533.I1S0J8
229533.A0A098DMQ4	pp	229533.I1RXW3
229533.A0A098DMQ4	pp	229533.I1RIH0
229533.A0A098DMQ4	pp	229533.A0A1C3YJ56
229533.A0A098DMQ4	pp	229533.I1RZT1
229533.A0A098DMQ4	pp	229533.I1RSS7
229533.A0A098DMQ4	pp	229533.I1S1W3
229533.A0A098DMQ4	pp	229533.A0A1C3YLA6
229533.A0A098DMQ4	pp	229533.A0A0E0SID1
229533.A0A098DMQ4	pp	229533.I1RCZ8
229533.A0A098DMQ4	pp	229533.I1RHM8
229533.A0A098DMQ4	pp	229533.I1RCP7
229533.A0A098DMQ4	pp	229533.Q4HXF0
229533.A0A098DMQ4	pp	229533.I1SAJ3
229533.A0A098DMQ4	pp	229533.V6RLM7
229533.A0A098DMQ4	pp	229533.I1RCG5
229533.A0A098DMQ4	pp	229533.I1S0R4
229533.A0A098DMQ4	pp	229533.I1RTG6
229533.A0A098DMQ4	pp	229533.I1RPK0
229533.A0A098DMQ4	pp	229533.A0A1C3YI05
229533.A0A098DMQ4	pp	229533.I1RNA2
229533.A0A098DMQ4	pp	229533.I1S0R3
229533.A0A098DMQ4	pp	229533.I1RW06
229533.A0A098DMQ4	pp	229533.A0A098E4D4
229533.A0A098DMQ4	pp	229533.A0A1C3YN26
229533.A0A098DMQ4	pp	229533.I1RY49
229533.A0A098DMQ4	pp	229533.I1RN09
229533.A0A098DMQ4	pp	229533.I1RGB2

19200	229533.A0A098DMQ4	pp	229533.I1RS10
19201	229533.A0A098DMQ4	pp	229533.I1RJT2
19202	229533.A0A098DMQ4	pp	229533.I1RFJ1
19203	229533.A0A098DMQ4	pp	229533.I1RYJ6
19204	229533.A0A098DMQ4	pp	229533.A0A1C3YHV3
19205	229533.A0A098DMQ4	pp	229533.I1RK47
19206	229533.A0A098DMQ4	pp	229533.I1RDU0
19207	229533.A0A098DMQ4	pp	229533.V6RUT5
19208	229533.A0A098DMQ4	pp	229533.I1RXV9
19209	229533.A0A098DMQ4	pp	229533.A0A1C3YLP6
19210	229533.A0A098DMQ4	pp	229533.I1RP86
19211	229533.A0A098DMQ4	pp	229533.I1RPI0
19212	229533.A0A098DMQ4	pp	229533.I1RTU1
19213	229533.A0A098DMQ4	pp	229533.I1RFB4
19214	229533.A0A098DMQ4	pp	229533.A0A098DP84
19215	229533.A0A098DMQ4	pp	229533.I1RUY5
19216	229533.A0A098DMQ4	pp	229533.I1S0N3
19217	229533.A0A098DMQ4	pp	229533.I1RA94
19218	229533.A0A098DMQ4	pp	229533.I1RGU6
19219	229533.A0A098DMQ4	pp	229533.I1RC61
19220	229533.A0A098DMQ4	pp	229533.I1RBE4
19221	229533.A0A1C3YMN0	pp	229533.I1RBU8
19222	229533.A0A1C3YMN0	pp	229533.I1RLA2
19223	229533.A0A1C3YMN0	pp	229533.I1S048
19224	229533.A0A1C3YMN0	pp	229533.I1RG71
19225	229533.A0A1C3YMN0	pp	229533.I1RGG6
19226	229533.A0A1C3YMN0	pp	229533.I1RBF3
19227	229533.A0A1C3YMN0	pp	229533.I1RAX2
19228	229533.A0A1C3YMN0	pp	229533.I1RAP3
19229	229533.A0A1C3YMN0	pp	229533.I1RE48
19230	229533.A0A1C3YMN0	pp	229533.I1RGU6
19231	229533.A0A1C3YMN0	pp	229533.I1RRD0
19232	229533.A0A1C3YMN0	pp	229533.I1RBY9
19233	229533.A0A1C3YMN0	pp	229533.I1RST9
19234	229533.A0A1C3YMN0	pp	229533.V6QVV9
19235	229533.A0A1C3YMN0	pp	229533.I1S6D7
19236	229533.A0A1C3YMN0	pp	229533.I1RZ25
19237	229533.A0A1C3YMN0	pp	229533.I1RJU9
19238	229533.A0A1C3YMN0	pp	229533.I1RY32
19239	229533.A0A1C3YMN0	pp	229533.I1RF24
19240	229533.A0A1C3YMN0	pp	229533.I1RLM3
19241	229533.A0A1C3YMN0	pp	229533.I1S5H2
19242	229533.A0A1C3YMN0	pp	229533.I1RRK5
19243	229533.A0A1C3YMN0	pp	229533.I1S267
19244	229533.A0A1C3YMN0	pp	229533.I1RYP2
19245	229533.A0A1C3YMN0	pp	229533.I1RCS6
19246	229533.A0A1C3YMN0	pp	229533.I1RMJ3
19247	229533.A0A1C3YMN0	pp	229533.I1RKP1
19248	229533.A0A1C3YMN0	pp	229533.I1S444
19249	229533.A0A1C3YMN0	pp	229533.I1RSE0
19250	229533.A0A1C3YMN0	pp	229533.I1RH91
19251	229533.A0A1C3YMN0	pp	229533.I1RLW5
19252	229533.A0A1C3YMN0	pp	229533.I1RC71
19253	229533.A0A1C3YMN0	pp	229533.I1RR45
19254	229533.A0A1C3YMN0	pp	229533.I1RZC3
19255	229533.A0A1C3YMN0	pp	229533.I1RE79
19256	229533.A0A1C3YMN0	pp	229533.I1R9K2
19257	229533.A0A1C3YMN0	pp	229533.I1REW3
19258	229533.A0A1C3YMN0	pp	229533.I1RSF1
19259	229533.I1RNW4	pp	229533.I1S1F3
19260	229533.I1REW3	pp	229533.I1RGG6
19261	229533.I1REW3	pp	229533.I1RR45
19262	229533.I1REW3	pp	229533.I1RST9
19263	229533.I1REW3	pp	229533.I1RRD0
19264	229533.I1REW3	pp	229533.I1RGU6
19265	229533.I1REW3	pp	229533.I1S5H2
19266	229533.I1REW3	pp	229533.I1RH91
19267	229533.I1REW3	pp	229533.I1S444
19268	229533.I1REW3	pp	229533.I1RJU9
19269	229533.I1REW3	pp	229533.I1RRK5
19270	229533.I1REW3	pp	229533.I1RLW5
19271	229533.I1REW3	pp	229533.I1RG71
19272	229533.I1REW3	pp	229533.I1RZ25

229533.I1REW3	pp	229533.I1RZC3
229533.I1REW3	pp	229533.I1RSF1
229533.I1REW3	pp	229533.I1RLM3
229533.I1REW3	pp	229533.I1RY32
229533.I1REW3	pp	229533.I1RKP1
229533.I1REW3	pp	229533.I1RSE0
229533.I1REW3	pp	229533.Q4I5R3
229533.I1REW3	pp	229533.I1S267
229533.I1REW3	pp	229533.I1RYP2
229533.I1REW3	pp	229533.I1RLA2
229533.I1REW3	pp	229533.I1S6D7
229533.I1REW3	pp	229533.I1S048
229533.I1RR45	pp	229533.I1S048
229533.I1RR45	pp	229533.I1RZC3
229533.I1RR45	pp	229533.I1RSF1
229533.I1RR45	pp	229533.I1S267
229533.I1RR45	pp	229533.I1S6D7
229533.I1RR45	pp	229533.I1S444
229533.I1RR45	pp	229533.I1RRK5
229533.I1RR45	pp	229533.I1RYP2
229533.I1RR45	pp	229533.I1RY32
229533.I1RR45	pp	229533.I1RST9
229533.I1RR45	pp	229533.I1RSE0
229533.I1RR45	pp	229533.I1S5H2
229533.I1RR45	pp	229533.I1RZ25
229533.I1RR45	pp	229533.I1RRD0
229533.A0A1C3YKM3	pp	229533.I1RYC1
229533.A0A1C3YKM3	pp	229533.I1S6W3
229533.A0A1C3YKM3	pp	229533.I1RMU2
229533.A0A1C3YKM3	pp	229533.I1RWE3
229533.A0A1C3YKM3	pp	229533.I1RLU4
229533.A0A1C3YKM3	pp	229533.I1RJ55
229533.A0A1C3YKM3	pp	229533.I1RXT4
229533.A0A1C3YKM3	pp	229533.I1S407
229533.A0A1C3YKM3	pp	229533.A0A1C3YMT0
229533.A0A1C3YKM3	pp	229533.I1RH94
229533.A0A1C3YKM3	pp	229533.V6R5G9
229533.A0A1C3YKM3	pp	229533.I1S320
229533.A0A1C3YKM3	pp	229533.I1R9L9
229533.A0A1C3YKM3	pp	229533.I1RI39
229533.A0A1C3YKM3	pp	229533.I1RYJ1
229533.A0A1C3YKM3	pp	229533.I1RF73
229533.A0A1C3YKM3	pp	229533.I1RUP6
229533.A0A1C3YKM3	pp	229533.I1RR89
229533.A0A1C3YKM3	pp	229533.I1S4A4
229533.A0A1C3YKM3	pp	229533.I1RSY7
229533.A0A1C3YKM3	pp	229533.A0A1C3YMT3
229533.A0A1C3YKM3	pp	229533.I1RT54
229533.A0A1C3YKM3	pp	229533.I1RLY2
229533.A0A1C3YKM3	pp	229533.I1RHM8
229533.A0A1C3YKM3	pp	229533.I1RB60
229533.A0A1C3YKM3	pp	229533.I1RWF6
229533.A0A1C3YKM3	pp	229533.I1RFY7
229533.A0A1C3YKM3	pp	229533.I1RVW8
229533.I1RWF6	pp	229533.I1S4A4
229533.I1RWF6	pp	229533.I1S6W3
229533.I1RWF6	pp	229533.I1RYC1
229533.I1RWF6	pp	229533.I1RXT4
229533.I1RWF6	pp	229533.V6R5G9
229533.I1RWF6	pp	229533.I1S320
229533.I1RWF6	pp	229533.I1RYJ1
229533.I1RWF6	pp	229533.I1S407
229533.A0A098DE24	pp	229533.A0A1C3YI88
229533.A0A098DE24	pp	229533.I1RW06
229533.A0A098DE24	pp	229533.V6QVM5
229533.A0A098DE24	pp	229533.V6RN31
229533.I1RK26	pp	229533.I1RMX6
229533.I1RK26	pp	229533.I1RSX9
229533.I1RK26	pp	229533.I1RND4
229533.I1RK26	pp	229533.I1RUP5
229533.I1RK26	pp	229533.I1RRG1
229533.I1RK26	pp	229533.I1RNG4
229533.I1S1K7	pp	229533.I1S1L0

19346 229533.A0A098DQY6 pp 229533.A0A0E0SQJ6
19347 229533.A0A098DQY6 pp 229533.I1RXE5
19348 229533.A0A098DQY6 pp 229533.I1RWX8
19349 229533.A0A098DQY6 pp 229533.I1RMA5
19350 229533.A0A098DQY6 pp 229533.V6QV38
19351 229533.A0A098DQY6 pp 229533.I1RDC9
19352 229533.A0A098DQY6 pp 229533.I1RGW0
19353 229533.A0A098DQY6 pp 229533.A0A1C3YK01
19354 229533.A0A098DQY6 pp 229533.I1RWU3
19355 229533.A0A098DQY6 pp 229533.I1RKR6
19356 229533.A0A098DQY6 pp 229533.A0A1C3YJZ2
19357 229533.A0A098DQY6 pp 229533.I1RB36
19358 229533.A0A1C3YKS9 pp 229533.I1RTA6
19359 229533.A0A1C3YKS9 pp 229533.I1RMP2
19360 229533.A0A1C3YKS9 pp 229533.I1RZP7
19361 229533.A0A1C3YKS9 pp 229533.A0A1C3YM54
19362 229533.A0A1C3YKS9 pp 229533.I1RLX6
19363 229533.A0A1C3YKS9 pp 229533.A0A1C3YL99
19364 229533.A0A1C3YKS9 pp 229533.I1RZP6
19365 229533.A0A1C3YKS9 pp 229533.I1RE19
19366 229533.A0A1C3YKS9 pp 229533.I1RKK7
19367 229533.I1RU26 pp 229533.I1S1F3
19368 229533.I1RJU6 pp 229533.I1RZ63
19369 229533.I1RJU6 pp 229533.I1RNG4
19370 229533.I1RJU6 pp 229533.I1RVX9
19371 229533.I1RJU6 pp 229533.I1RZ21
19372 229533.I1RJU6 pp 229533.I1RXV3
19373 229533.I1RJU6 pp 229533.I1RMM8
19374 229533.I1RJU6 pp 229533.I1RSX7
19375 229533.I1RJU6 pp 229533.I1S253
19376 229533.I1RJU6 pp 229533.I1RR72
19377 229533.I1RJU6 pp 229533.I1S0J7
19378 229533.I1RJU6 pp 229533.Q4IF76
19379 229533.I1RJU6 pp 229533.I1RM49
19380 229533.I1RJU6 pp 229533.I1RSX8
19381 229533.I1RJU6 pp 229533.I1RN57
19382 229533.I1RJU6 pp 229533.I1S0S5
19383 229533.I1RJU6 pp 229533.I1RN77
19384 229533.I1RJU6 pp 229533.I1RMR8
19385 229533.I1RJU6 pp 229533.I1RQU4
19386 229533.I1RJU6 pp 229533.I1S3V2
19387 229533.I1RJU6 pp 229533.I1RM26
19388 229533.I1RJU6 pp 229533.I1RK75
19389 229533.I1RPR7 pp 229533.I1RYT5
19390 229533.I1RPR7 pp 229533.I1RX70
19391 229533.I1RPR7 pp 229533.I1RT61
19392 229533.I1S3S6 pp 229533.I1S4P6
19393 229533.I1RC14 pp 229533.I1S0S5
19394 229533.I1RC14 pp 229533.I1S3V2
19395 229533.I1RC14 pp 229533.I1RMM8
19396 229533.I1RC14 pp 229533.I1RE79
19397 229533.I1RC14 pp 229533.I1RSX7
19398 229533.I1RC14 pp 229533.I1RC61
19399 229533.I1RC14 pp 229533.I1RK75
19400 229533.I1RC14 pp 229533.I1RZ63
19401 229533.I1RC14 pp 229533.I1S253
19402 229533.I1RC14 pp 229533.I1RM26
19403 229533.I1RC14 pp 229533.I1RXV3
19404 229533.I1RC14 pp 229533.I1S0J7
19405 229533.I1RC14 pp 229533.I1RFJ1
19406 229533.I1RC14 pp 229533.I1RQU4
19407 229533.I1RC14 pp 229533.I1RNG4
19408 229533.I1RC14 pp 229533.I1RFH9
19409 229533.I1RC14 pp 229533.I1RN77
19410 229533.I1RC14 pp 229533.I1RHY5
19411 229533.I1RC14 pp 229533.I1RHV1
19412 229533.I1RC14 pp 229533.I1RJU6
19413 229533.I1RC14 pp 229533.I1RZ21
19414 229533.I1RC14 pp 229533.I1RR72
19415 229533.I1RC14 pp 229533.I1RM49
19416 229533.I1RC14 pp 229533.I1RVX9
19417 229533.I1RR60 pp 229533.I1RVC7
19418 229533.I1RR60 pp 229533.I1S069

229533.I1RR60 pp 229533.I1S2I7
229533.I1RR60 pp 229533.I1S303
229533.I1RR60 pp 229533.I1S4P6
229533.I1RR60 pp 229533.I1S3M2
229533.I1RR60 pp 229533.I1RRT1
229533.A0A098DQR8 pp 229533.I1RJU6
229533.A0A098DQR8 pp 229533.I1RS05
229533.A0A098DQR8 pp 229533.I1RC14
229533.V6RUG6 pp 229533.V6RUY9
229533.I1RJH0 pp 229533.V6R042
229533.A0A0E0SDM6 pp 229533.I1RRU8
229533.A0A0E0SDM6 pp 229533.I1RBR5
229533.A0A0E0SDM6 pp 229533.Q4IMD1
229533.A0A0E0SDM6 pp 229533.Q4HY90
229533.I1RHM8 pp 229533.I1RR89
229533.I1RHM8 pp 229533.I1RP86
229533.I1RHM8 pp 229533.I1RNG4
229533.I1RHM8 pp 229533.I1RYC1
229533.I1RHM8 pp 229533.V6RUG6
229533.I1RHM8 pp 229533.I1RI39
229533.I1RHM8 pp 229533.I1RJ82
229533.I1RHM8 pp 229533.I1S4A4
229533.I1RHM8 pp 229533.I1RMC5
229533.I1RHM8 pp 229533.I1RT54
229533.I1RHM8 pp 229533.I1SAC4
229533.I1RHM8 pp 229533.I1RXT4
229533.I1RHM8 pp 229533.I1RLY2
229533.I1RHM8 pp 229533.I1S3F7
229533.I1RHM8 pp 229533.I1RYJ1
229533.I1RHM8 pp 229533.I1RSY7
229533.I1RHM8 pp 229533.I1RPT0
229533.I1RHM8 pp 229533.I1RLU4
229533.I1RHM8 pp 229533.I1RNU7
229533.I1RHM8 pp 229533.I1S6W3
229533.I1RHM8 pp 229533.I1RMU2
229533.I1RHM8 pp 229533.I1RWE3
229533.I1RHM8 pp 229533.V6R5G9
229533.I1RHM8 pp 229533.I1S308
229533.I1RHM8 pp 229533.I1RWF6
229533.I1RHM8 pp 229533.I1S320
229533.I1RHM8 pp 229533.I1S407
229533.I1RHM8 pp 229533.I1RMC2
229533.I1RHM8 pp 229533.I1RVW8
229533.I1RHM8 pp 229533.I1RJ55
229533.I1RWA4 pp 229533.I1RXD9
229533.I1RWA4 pp 229533.I1S464
229533.I1RYC1 pp 229533.I1S320
229533.I1RYC1 pp 229533.V6R5G9
229533.I1RYC1 pp 229533.I1S407
229533.I1RYC1 pp 229533.I1S6W3
229533.I1RYC1 pp 229533.I1S1V2
229533.I1RYC1 pp 229533.I1RYJ1
229533.I1RYC1 pp 229533.I1S4A4
229533.I1RYC1 pp 229533.I1S7I5
229533.I1RYC1 pp 229533.Q4IPI4
229533.I1SAT1 pp 229533.V6RKE4
229533.I1SAT1 pp 229533.Q4ICG5
229533.I1SAT1 pp 229533.V6RQ48
229533.I1RN21 pp 229533.I1RND4
229533.I1RN21 pp 229533.I1RNG4
229533.I1RN21 pp 229533.I1RRG1
229533.I1RN21 pp 229533.I1RUP5
229533.I1RN21 pp 229533.I1RSX9
229533.I1RF73 pp 229533.I1RJ82
229533.I1RF73 pp 229533.I1S6W3
229533.I1RF73 pp 229533.I1RWE3
229533.I1RF73 pp 229533.I1RT54
229533.I1RF73 pp 229533.I1S3F7
229533.I1RF73 pp 229533.I1RLU4
229533.I1RF73 pp 229533.I1RJX7
229533.I1RF73 pp 229533.I1RSY7
229533.I1RF73 pp 229533.I1SAC4
229533.I1RF73 pp 229533.I1RWF6

19492 229533.I1RF73 pp 229533.I1RP86
19493 229533.I1RF73 pp 229533.I1RR89
19494 229533.I1RF73 pp 229533.I1RZP6
19495 229533.I1RF73 pp 229533.I1RFY7
19496 229533.I1RF73 pp 229533.I1RMU2
19497 229533.I1RF73 pp 229533.I1S308
19498 229533.I1RF73 pp 229533.V6R5G9
19499 229533.I1RF73 pp 229533.I1RJ55
19500 229533.I1RF73 pp 229533.I1RI39
19501 229533.I1RF73 pp 229533.I1RGY2
19502 229533.I1RF73 pp 229533.I1S407
19503 229533.I1RF73 pp 229533.I1RHM8
19504 229533.I1RF73 pp 229533.V6RUG6
19505 229533.I1RF73 pp 229533.I1S4A4
19506 229533.I1RF73 pp 229533.I1RPT0
19507 229533.I1RF73 pp 229533.I1RNG4
19508 229533.I1RF73 pp 229533.I1RVW8
19509 229533.I1RF73 pp 229533.I1RYJ1
19510 229533.I1RF73 pp 229533.I1RH94
19511 229533.I1RF73 pp 229533.I1RMG6
19512 229533.I1RF73 pp 229533.I1S320
19513 229533.I1RF73 pp 229533.I1RYC1
19514 229533.I1RF73 pp 229533.I1RMC2
19515 229533.I1RF73 pp 229533.I1RXT4
19516 229533.I1RF73 pp 229533.I1RNU7
19517 229533.I1RF73 pp 229533.I1RLY2
19518 229533.I1RF73 pp 229533.I1RMC5
19519 229533.A0A1C3YKY6 pp 229533.I1RA35
19520 229533.A0A1C3YKY6 pp 229533.Q4ICG5
19521 229533.A0A1C3YKY6 pp 229533.I1RG62
19522 229533.A0A1C3YKY6 pp 229533.I1RYT8
19523 229533.A0A1C3YKY6 pp 229533.I1S7L6
19524 229533.A0A1C3YKY6 pp 229533.I1RW54
19525 229533.A0A1C3YKY6 pp 229533.I1RAI4
19526 229533.A0A1C3YKY6 pp 229533.I1S1W8
19527 229533.A0A1C3YKY6 pp 229533.I1RMK7
19528 229533.A0A1C3YKY6 pp 229533.I1RNM7
19529 229533.A0A1C3YKY6 pp 229533.I1RK95
19530 229533.A0A1C3YKY6 pp 229533.I1RR24
19531 229533.A0A1C3YKY6 pp 229533.I1RS29
19532 229533.A0A1C3YKY6 pp 229533.V6QXN0
19533 229533.A0A1C3YKY6 pp 229533.I1RAX6
19534 229533.A0A1C3YKY6 pp 229533.I1RY21
19535 229533.A0A1C3YKY6 pp 229533.I1S8Z2
19536 229533.A0A1C3YKY6 pp 229533.I1RAU9
19537 229533.A0A1C3YKY6 pp 229533.Q4IJK2
19538 229533.A0A1C3YKY6 pp 229533.I1RKB7
19539 229533.A0A1C3YKY6 pp 229533.V6RTJ8
19540 229533.A0A1C3YKY6 pp 229533.I1RPP5
19541 229533.A0A1C3YKY6 pp 229533.I1RA49
19542 229533.A0A1C3YKY6 pp 229533.I1RMQ6
19543 229533.A0A1C3YKY6 pp 229533.V6RBN2
19544 229533.A0A1C3YKY6 pp 229533.I1RAR3
19545 229533.A0A1C3YKY6 pp 229533.I1R9Y4
19546 229533.A0A1C3YKY6 pp 229533.I1RBT1
19547 229533.A0A1C3YKY6 pp 229533.I1S854
19548 229533.A0A1C3YKY6 pp 229533.I1RG33
19549 229533.A0A1C3YKY6 pp 229533.A0A1C3YN26
19550 229533.A0A1C3YKY6 pp 229533.I1S0K2
19551 229533.A0A1C3YKY6 pp 229533.I1RMC8
19552 229533.A0A1C3YKY6 pp 229533.I1RD52
19553 229533.A0A1C3YKY6 pp 229533.I1RA85
19554 229533.A0A1C3YKY6 pp 229533.I1RZY1
19555 229533.A0A1C3YKY6 pp 229533.I1RE66
19556 229533.A0A1C3YKY6 pp 229533.I1RPR9
19557 229533.A0A1C3YKY6 pp 229533.I1RWC9
19558 229533.A0A1C3YKY6 pp 229533.I1RX46
19559 229533.A0A1C3YKY6 pp 229533.I1S8C1
19560 229533.A0A1C3YKY6 pp 229533.I1RT16
19561 229533.A0A1C3YKY6 pp 229533.I1RRY9
19562 229533.A0A1C3YKY6 pp 229533.I1S0P0
19563 229533.A0A1C3YKY6 pp 229533.I1S053
19564 229533.A0A1C3YKY6 pp 229533.I1RWB0

229533.A0A1C3YKY6	pp	229533.I1RS05
229533.A0A1C3YKY6	pp	229533.I1S4S7
229533.A0A1C3YKY6	pp	229533.I1RRX5
229533.A0A1C3YKY6	pp	229533.I1S212
229533.A0A1C3YKY6	pp	229533.A0A1C3YLB0
229533.A0A1C3YKY6	pp	229533.I1RP86
229533.A0A1C3YKY6	pp	229533.I1RZK5
229533.A0A1C3YKY6	pp	229533.I1RSQ7
229533.A0A1C3YKY6	pp	229533.I1RBD5
229533.A0A1C3YKY6	pp	229533.V6RUT5
229533.A0A1C3YKY6	pp	229533.I1RCU2
229533.A0A1C3YKY6	pp	229533.I1S0C1
229533.A0A1C3YKY6	pp	229533.I1RQA5
229533.A0A1C3YKY6	pp	229533.I1RXD7
229533.A0A1C3YKY6	pp	229533.I1RXF0
229533.A0A1C3YKY6	pp	229533.I1RLP1
229533.A0A1C3YKY6	pp	229533.I1S887
229533.A0A1C3YKY6	pp	229533.I1RZM8
229533.A0A1C3YKY6	pp	229533.I1RCR5
229533.A0A1C3YKY6	pp	229533.I1S8S9
229533.A0A1C3YKY6	pp	229533.I1RFI4
229533.A0A1C3YKY6	pp	229533.I1RA52
229533.A0A1C3YKY6	pp	229533.Q4I5G1
229533.A0A1C3YKY6	pp	229533.I1RYX5
229533.A0A1C3YKY6	pp	229533.I1RAA2
229533.A0A1C3YKY6	pp	229533.I1RJY7
229533.A0A1C3YKY6	pp	229533.I1RAM9
229533.A0A1C3YKY6	pp	229533.I1RFJ5
229533.A0A1C3YKY6	pp	229533.I1RGC3
229533.A0A1C3YKY6	pp	229533.I1RAW3
229533.A0A1C3YKY6	pp	229533.I1RPT0
229533.A0A1C3YKY6	pp	229533.I1RAV5
229533.A0A1C3YKY6	pp	229533.A0A1C3YLB3
229533.A0A1C3YKY6	pp	229533.I1RAQ4
229533.A0A1C3YKY6	pp	229533.V6RJP8
229533.A0A1C3YKY6	pp	229533.I1RUR8
229533.A0A1C3YKY6	pp	229533.I1R9M0
229533.A0A1C3YKY6	pp	229533.I1S8F8
229533.A0A1C3YKY6	pp	229533.I1RMR9
229533.A0A1C3YKY6	pp	229533.I1R9Z5
229533.A0A1C3YKY6	pp	229533.I1S2B8
229533.A0A1C3YKY6	pp	229533.I1RUY6
229533.A0A1C3YKY6	pp	229533.I1RS18
229533.A0A1C3YKY6	pp	229533.I1RM91
229533.A0A1C3YKY6	pp	229533.I1RWA3
229533.A0A1C3YKY6	pp	229533.I1RBY4
229533.A0A1C3YKY6	pp	229533.I1S5R1
229533.A0A1C3YKY6	pp	229533.I1RMR7
229533.A0A1C3YKY6	pp	229533.I1RZ39
229533.A0A1C3YKY6	pp	229533.I1RA11
229533.A0A1C3YKY6	pp	229533.I1RN88
229533.A0A1C3YKY6	pp	229533.I1RP02
229533.A0A1C3YKY6	pp	229533.I1RY92
229533.A0A1C3YKY6	pp	229533.I1RPV3
229533.A0A1C3YKY6	pp	229533.I1RXE6
229533.A0A1C3YKY6	pp	229533.I1RWI5
229533.A0A1C3YKY6	pp	229533.I1S5B0
229533.A0A1C3YKY6	pp	229533.I1S3G4
229533.A0A1C3YKY6	pp	229533.I1S2H4
229533.A0A1C3YKY6	pp	229533.I1REE2
229533.A0A1C3YKY6	pp	229533.I1RZZ9
229533.A0A1C3YKY6	pp	229533.I1RSQ4
229533.A0A1C3YKY6	pp	229533.I1S0L4
229533.A0A1C3YKY6	pp	229533.V6QUE6
229533.A0A1C3YKY6	pp	229533.I1RRI1
229533.A0A1C3YKY6	pp	229533.I1RA29
229533.A0A1C3YKY6	pp	229533.I1RX21
229533.A0A1C3YKY6	pp	229533.V6RCZ8
229533.A0A1C3YKY6	pp	229533.I1RX00
229533.A0A1C3YKY6	pp	229533.I1RC66
229533.A0A1C3YKY6	pp	229533.I1S3T7
229533.A0A1C3YKY6	pp	229533.I1S6K9
229533.A0A1C3YKY6	pp	229533.I1RAE3

19638	229533.A0A1C3YKY6	pp	229533.I1S9Y1
19639	229533.A0A1C3YKY6	pp	229533.A0A1C3YN57
19640	229533.A0A1C3YKY6	pp	229533.I1RL81
19641	229533.A0A1C3YKY6	pp	229533.I1RPP3
19642	229533.A0A1C3YKY6	pp	229533.I1RCG5
19643	229533.A0A1C3YKY6	pp	229533.I1RYW9
19644	229533.A0A1C3YKY6	pp	229533.I1RYW3
19645	229533.A0A1C3YKY6	pp	229533.I1RQG4
19646	229533.A0A1C3YKY6	pp	229533.I1S1F3
19647	229533.A0A1C3YKY6	pp	229533.I1RP88
19648	229533.A0A1C3YKY6	pp	229533.A0A1C3YN78
19649	229533.A0A1C3YKY6	pp	229533.I1RN67
19650	229533.A0A1C3YKY6	pp	229533.I1RTA1
19651	229533.A0A1C3YKY6	pp	229533.I1S1C7
19652	229533.A0A1C3YKY6	pp	229533.I1RNG4
19653	229533.A0A1C3YKY6	pp	229533.I1RAE2
19654	229533.A0A1C3YKY6	pp	229533.I1RX39
19655	229533.A0A1C3YKY6	pp	229533.I1RBE6
19656	229533.A0A1C3YKY6	pp	229533.I1RC18
19657	229533.A0A1C3YKY6	pp	229533.I1RYC8
19658	229533.A0A1C3YKY6	pp	229533.I1S464
19659	229533.A0A1C3YKY6	pp	229533.I1RC67
19660	229533.A0A1C3YKY6	pp	229533.I1RBC7
19661	229533.A0A1C3YKY6	pp	229533.A0A1C3YLK8
19662	229533.A0A1C3YKY6	pp	229533.I1RGA4
19663	229533.A0A1C3YKY6	pp	229533.I1S1W3
19664	229533.A0A1C3YKY6	pp	229533.I1RSX0
19665	229533.A0A1C3YKY6	pp	229533.I1RYK0
19666	229533.A0A1C3YKY6	pp	229533.I1RFT1
19667	229533.A0A1C3YKY6	pp	229533.I1RYI5
19668	229533.A0A1C3YKY6	pp	229533.V6R3J1
19669	229533.A0A1C3YKY6	pp	229533.A0A1C3YM60
19670	229533.A0A1C3YKY6	pp	229533.V6RLM7
19671	229533.A0A1C3YKY6	pp	229533.A0A1C3YLE2
19672	229533.A0A1C3YKY6	pp	229533.I1RPK0
19673	229533.A0A1C3YKY6	pp	229533.I1RMD6
19674	229533.A0A1C3YKY6	pp	229533.I1RYH7
19675	229533.A0A1C3YKY6	pp	229533.I1RXD9
19676	229533.A0A1C3YKY6	pp	229533.I1RT40
19677	229533.A0A1C3YKY6	pp	229533.I1RWF5
19678	229533.A0A1C3YKY6	pp	229533.I1S9Z4
19679	229533.A0A1C3YKY6	pp	229533.I1RVX0
19680	229533.A0A1C3YKY6	pp	229533.I1RMV0
19681	229533.A0A1C3YKY6	pp	229533.I1RTD5
19682	229533.A0A1C3YKY6	pp	229533.I1RT03
19683	229533.A0A1C3YKY6	pp	229533.I1RWD3
19684	229533.A0A1C3YKY6	pp	229533.I1RBT6
19685	229533.A0A1C3YKY6	pp	229533.I1RAE5
19686	229533.A0A1C3YKY6	pp	229533.Q4I624
19687	229533.A0A1C3YKY6	pp	229533.I1RXR1
19688	229533.A0A1C3YKY6	pp	229533.Q4I7K4
19689	229533.A0A1C3YKY6	pp	229533.I1RE01
19690	229533.A0A1C3YKY6	pp	229533.V6RIV7
19691	229533.A0A1C3YKY6	pp	229533.I1RNU7
19692	229533.A0A1C3YKY6	pp	229533.I1RNK6
19693	229533.A0A1C3YKY6	pp	229533.I1RB92
19694	229533.A0A1C3YKY6	pp	229533.I1RB38
19695	229533.A0A1C3YKY6	pp	229533.I1S147
19696	229533.A0A1C3YKY6	pp	229533.A0A1C3YLC1
19697	229533.A0A1C3YKY6	pp	229533.A0A1C3YLB6
19698	229533.A0A1C3YKY6	pp	229533.I1RFL4
19699	229533.A0A1C3YKY6	pp	229533.I1S5X9
19700	229533.A0A1C3YKY6	pp	229533.I1RCR2
19701	229533.A0A1C3YKY6	pp	229533.I1RRN2
19702	229533.A0A1C3YKY6	pp	229533.I1RYA0
19703	229533.A0A1C3YKY6	pp	229533.I1RD22
19704	229533.A0A1C3YKY6	pp	229533.I1S7W5
19705	229533.A0A1C3YKY6	pp	229533.I1RN84
19706	229533.A0A1C3YKY6	pp	229533.I1RAE4
19707	229533.A0A1C3YKY6	pp	229533.I1RCH5
19708	229533.A0A1C3YKY6	pp	229533.I1RQE7
19709	229533.A0A1C3YKY6	pp	229533.I1S0R8
19710	229533.A0A1C3YKY6	pp	229533.I1S0J1

19711 229533.A0A1C3YKY6 pp 229533.I1S0Q7
19712 229533.A0A1C3YKY6 pp 229533.I1RNL5
19713 229533.A0A1C3YKY6 pp 229533.I1RE65
19714 229533.A0A1C3YKY6 pp 229533.V6R042
19715 229533.A0A1C3YKY6 pp 229533.I1RXU9
19716 229533.A0A1C3YKY6 pp 229533.I1RBS5
19717 229533.A0A1C3YKY6 pp 229533.I1RSQ3
19718 229533.A0A1C3YKY6 pp 229533.I1S5R6
19719 229533.A0A1C3YKY6 pp 229533.V6RMW6
19720 229533.A0A1C3YKY6 pp 229533.I1RF85
19721 229533.A0A1C3YKY6 pp 229533.I1RFJ6
19722 229533.A0A1C3YKY6 pp 229533.I1RAW0
19723 229533.A0A1C3YKY6 pp 229533.V6RPQ8
19724 229533.A0A1C3YKY6 pp 229533.I1RJV3
19725 229533.A0A1C3YKY6 pp 229533.I1RS90
19726 229533.A0A1C3YKY6 pp 229533.I1S855
19727 229533.A0A1C3YKY6 pp 229533.I1RNC1
19728 229533.A0A1C3YKY6 pp 229533.I1RG11
19729 229533.A0A1C3YKY6 pp 229533.I1RA70
19730 229533.A0A1C3YKY6 pp 229533.I1RUG3
19731 229533.A0A1C3YKY6 pp 229533.I1RWT0
19732 229533.A0A1C3YKY6 pp 229533.V6RN69
19733 229533.A0A1C3YKY6 pp 229533.I1RFS5
19734 229533.A0A1C3YKY6 pp 229533.I1RCR8
19735 229533.I1RM91 pp 229533.I1RW71
19736 229533.I1RM91 pp 229533.I1RRX5
19737 229533.I1RM91 pp 229533.V6R3J1
19738 229533.I1RDT7 pp 229533.I1RL24
19739 229533.I1RDT7 pp 229533.I1SAC4
19740 229533.I1RDT7 pp 229533.I1REH0
19741 229533.I1RDT7 pp 229533.I1RJ82
19742 229533.I1RDT7 pp 229533.I1RDW1
19743 229533.I1RDT7 pp 229533.I1SAK2
19744 229533.I1RDT7 pp 229533.I1RXM0
19745 229533.I1RDT7 pp 229533.V6QV09
19746 229533.I1RDT7 pp 229533.I1RF73
19747 229533.I1RDT7 pp 229533.I1RFL7
19748 229533.I1RDT7 pp 229533.I1RI39
19749 229533.I1RDT7 pp 229533.V6RUG6
19750 229533.I1RDT7 pp 229533.I1RHM8
19751 229533.I1RDT7 pp 229533.V6RGI1
19752 229533.I1RDT7 pp 229533.I1S2N3
19753 229533.I1RDT7 pp 229533.I1S308
19754 229533.I1RDT7 pp 229533.I1RHL0
19755 229533.I1RDT7 pp 229533.I1RVW8
19756 229533.I1RDT7 pp 229533.I1RYZ9
19757 229533.I1RDT7 pp 229533.I1RPT0
19758 229533.I1RAB7 pp 229533.I1RFZ7
19759 229533.I1RAB7 pp 229533.Q4IAA0
19760 229533.I1RAB7 pp 229533.I1RDK2
19761 229533.I1RAB7 pp 229533.I1RR08
19762 229533.I1RAB7 pp 229533.I1RQC4
19763 229533.I1RAB7 pp 229533.I1RMW4
19764 229533.I1RAB7 pp 229533.I1RQC3
19765 229533.I1RAB7 pp 229533.Q4IBS9
19766 229533.I1RN64 pp 229533.I1RWX5
19767 229533.I1RN64 pp 229533.I1RYW3
19768 229533.I1RN64 pp 229533.I1RTD7
19769 229533.I1RN64 pp 229533.Q4I1M9
19770 229533.I1RN64 pp 229533.I1RVM1
19771 229533.I1RN64 pp 229533.Q4I2X5
19772 229533.I1RN64 pp 229533.I1RVY4
19773 229533.I1RN64 pp 229533.I1S4T6
19774 229533.I1RN64 pp 229533.I1RNG1
19775 229533.I1RN64 pp 229533.V6RQR4
19776 229533.I1RI39 pp 229533.I1RYC1
19777 229533.I1RI39 pp 229533.I1S320
19778 229533.I1RI39 pp 229533.I1RJ82
19779 229533.I1RI39 pp 229533.I1RXT4
19780 229533.I1RI39 pp 229533.I1RWE3
19781 229533.I1RI39 pp 229533.I1RMM6
19782 229533.I1RI39 pp 229533.I1S6W3
19783 229533.I1RI39 pp 229533.I1RR89

229533.I1RI39 pp 229533.V6RUY9
229533.I1RI39 pp 229533.I1RJ42
229533.I1RI39 pp 229533.V6R5G9
229533.I1RI39 pp 229533.I1RLU4
229533.I1RI39 pp 229533.V6RUG6
229533.I1RI39 pp 229533.I1S4A4
229533.I1RI39 pp 229533.I1RN16
229533.I1RI39 pp 229533.I1RJ55
229533.I1RI39 pp 229533.I1RV63
229533.I1RI39 pp 229533.I1RLY2
229533.I1RI39 pp 229533.I1RT54
229533.I1RI39 pp 229533.I1S407
229533.I1RI39 pp 229533.I1RSY7
229533.I1RI39 pp 229533.I1S308
229533.I1RI39 pp 229533.V6RDH3
229533.I1RI39 pp 229533.I1RYJ1
229533.I1RI39 pp 229533.I1RMU2
229533.I1RI39 pp 229533.I1RWF6
229533.I1RI39 pp 229533.I1RVW8
229533.I1RI39 pp 229533.I1SAC4
229533.A0A098D5S8 pp 229533.I1RJ82
229533.A0A098D5S8 pp 229533.I1RMC5
229533.A0A098D5S8 pp 229533.I1RLY2
229533.A0A098D5S8 pp 229533.I1REH0
229533.A0A098D5S8 pp 229533.A0A1C3YMT3
229533.A0A098D5S8 pp 229533.I1RSY7
229533.A0A098D5S8 pp 229533.I1SAC4
229533.A0A098D5S8 pp 229533.I1RE47
229533.A0A098D5S8 pp 229533.A0A098DRH0
229533.A0A098D5S8 pp 229533.I1RYJ1
229533.A0A098D5S8 pp 229533.I1RLU4
229533.A0A098D5S8 pp 229533.I1R9D3
229533.A0A098D5S8 pp 229533.I1RPT0
229533.A0A098D5S8 pp 229533.A0A1C3YM97
229533.A0A098D5S8 pp 229533.I1RWE3
229533.A0A098D5S8 pp 229533.A0A0E0RSS5
229533.A0A098D5S8 pp 229533.I1RMC2
229533.A0A098D5S8 pp 229533.I1RNU7
229533.A0A098D5S8 pp 229533.I1S6W3
229533.A0A098D5S8 pp 229533.A0A098DUH5
229533.A0A098D5S8 pp 229533.I1S3F7
229533.A0A098D5S8 pp 229533.I1R9L9
229533.A0A098D5S8 pp 229533.I1S320
229533.A0A098D5S8 pp 229533.I1RGY2
229533.A0A098D5S8 pp 229533.I1RMU2
229533.A0A098D5S8 pp 229533.I1RVW8
229533.A0A098D5S8 pp 229533.I1RH94
229533.A0A098D5S8 pp 229533.I1S308
229533.A0A098D5S8 pp 229533.A0A098DMQ4
229533.A0A098D5S8 pp 229533.A0A098DIZ0
229533.A0A098D5S8 pp 229533.V6R5G9
229533.A0A098D5S8 pp 229533.I1RP86
229533.A0A098D5S8 pp 229533.A0A1C3YKM3
229533.A0A098D5S8 pp 229533.I1RWF6
229533.A0A098D5S8 pp 229533.I1RR89
229533.A0A098D5S8 pp 229533.I1RFY7
229533.A0A098D5S8 pp 229533.I1RNG4
229533.A0A098D5S8 pp 229533.I1RB60
229533.A0A098D5S8 pp 229533.A0A1C3YHP2
229533.A0A098D5S8 pp 229533.A0A098DC95
229533.A0A098D5S8 pp 229533.A0A1C3YMT0
229533.A0A098D5S8 pp 229533.I1S407
229533.A0A098D5S8 pp 229533.V6RUG6
229533.A0A098D5S8 pp 229533.I1RJ55
229533.A0A098D5S8 pp 229533.I1S4A4
229533.A0A098D5S8 pp 229533.I1RHM8
229533.A0A098D5S8 pp 229533.I1RXT4
229533.A0A098D5S8 pp 229533.I1RYC1
229533.A0A098D5S8 pp 229533.I1RF73
229533.A0A098D5S8 pp 229533.A0A098DJ33
229533.A0A098D5S8 pp 229533.I1RDT7
229533.A0A098D5S8 pp 229533.I1RI39
229533.A0A098D5S8 pp 229533.I1RT54

19857 229533.I1R995 pp 229533.I1S1F3
19858 229533.A0A098DE29 pp 229533.I1RNG4
19859 229533.A0A098DE29 pp 229533.I1RSW5
19860 229533.A0A098DE29 pp 229533.I1RCA7
19861 229533.A0A098DE29 pp 229533.I1S3S6
19862 229533.A0A098DE29 pp 229533.I1RR60
19863 229533.A0A098DE29 pp 229533.A0A1C3YHL0
19864 229533.A0A098DE29 pp 229533.I1S0E4
19865 229533.A0A098DE29 pp 229533.I1RVU9
19866 229533.A0A098DE29 pp 229533.V6RGI1
19867 229533.A0A098DE29 pp 229533.I1RN81
19868 229533.A0A098DE29 pp 229533.Q4I2M7
19869 229533.A0A098DE29 pp 229533.I1SAT1
19870 229533.A0A098DE29 pp 229533.I1RM91
19871 229533.A0A098DE29 pp 229533.I1RW71
19872 229533.A0A098DE29 pp 229533.V6RR76
19873 229533.A0A098DE29 pp 229533.A0A098E1H5
19874 229533.A0A098DE29 pp 229533.Q4I1J3
19875 229533.A0A098DE29 pp 229533.I1RSI0
19876 229533.I1RMF8 pp 229533.I1RNG4
19877 229533.A0A1C3YM21 pp 229533.I1RUX6
19878 229533.A0A1C3YM21 pp 229533.I1RAN8
19879 229533.A0A1C3YM21 pp 229533.I1RYV4
19880 229533.A0A1C3YM21 pp 229533.I1RG94
19881 229533.A0A1C3YM21 pp 229533.I1RE42
19882 229533.A0A1C3YM21 pp 229533.I1RN61
19883 229533.A0A1C3YM21 pp 229533.V6QV38
19884 229533.A0A1C3YM21 pp 229533.I1RQ42
19885 229533.A0A1C3YM21 pp 229533.I1S2R0
19886 229533.A0A1C3YM21 pp 229533.I1RXD5
19887 229533.A0A1C3YM21 pp 229533.I1RYX6
19888 229533.A0A1C3YM21 pp 229533.I1RN31
19889 229533.A0A1C3YM21 pp 229533.I1RQE9
19890 229533.A0A1C3YM21 pp 229533.V6R6F6
19891 229533.A0A1C3YM21 pp 229533.A0A1C3YN58
19892 229533.A0A1C3YM21 pp 229533.I1S0F5
19893 229533.A0A1C3YM21 pp 229533.I1RTB7
19894 229533.A0A1C3YM21 pp 229533.I1RH68
19895 229533.A0A1C3YM21 pp 229533.I1S9I5
19896 229533.A0A1C3YM21 pp 229533.I1S0C0
19897 229533.A0A1C3YM21 pp 229533.I1S0W5
19898 229533.A0A1C3YM21 pp 229533.I1S0S6
19899 229533.A0A1C3YM21 pp 229533.I1RUC6
19900 229533.A0A1C3YM21 pp 229533.I1RNG8
19901 229533.A0A1C3YM21 pp 229533.I1RME6
19902 229533.A0A1C3YM21 pp 229533.I1RQ81
19903 229533.A0A1C3YM21 pp 229533.I1RWG5
19904 229533.A0A1C3YM21 pp 229533.P0C431
19905 229533.A0A1C3YM21 pp 229533.I1RBE7
19906 229533.A0A1C3YM21 pp 229533.I1RZ29
19907 229533.A0A1C3YM21 pp 229533.I1RQ93
19908 229533.A0A1C3YM21 pp 229533.I1RUA4
19909 229533.A0A1C3YM21 pp 229533.I1S490
19910 229533.A0A1C3YM21 pp 229533.I1SAA0
19911 229533.A0A1C3YM21 pp 229533.I1S1B2
19912 229533.A0A1C3YM21 pp 229533.I1RPH6
19913 229533.A0A1C3YM21 pp 229533.I1RW80
19914 229533.A0A1C3YM21 pp 229533.I1RYE2
19915 229533.A0A1C3YM21 pp 229533.I1RMU4
19916 229533.A0A1C3YM21 pp 229533.I1RQN9
19917 229533.A0A1C3YM21 pp 229533.I1RCN2
19918 229533.A0A1C3YM21 pp 229533.I1RUF7
19919 229533.A0A1C3YM21 pp 229533.I1R9H9
19920 229533.A0A1C3YM21 pp 229533.I1RVY0
19921 229533.A0A1C3YM21 pp 229533.I1RNR2
19922 229533.A0A1C3YM21 pp 229533.A0A1C3YMN9
19923 229533.A0A1C3YM21 pp 229533.I1RJZ7
19924 229533.A0A1C3YM21 pp 229533.I1RMI1
19925 229533.A0A1C3YM21 pp 229533.I1RNG4
19926 229533.A0A1C3YM21 pp 229533.I1RB73
19927 229533.A0A1C3YM21 pp 229533.I1RU32
19928 229533.A0A1C3YM21 pp 229533.I1RWM0
19929 229533.A0A1C3YM21 pp 229533.I1RPG1

19930	229533.A0A1C3YM21	pp	229533.I1RWJ1
19931	229533.A0A1C3YM21	pp	229533.I1RXG9
19932	229533.A0A1C3YM21	pp	229533.A0A1C3YMK7
19933	229533.A0A1C3YM21	pp	229533.I1RAI5
19934	229533.A0A1C3YM21	pp	229533.I1SAN9
19935	229533.A0A1C3YM21	pp	229533.I1RUE4
19936	229533.I1RRU8	pp	229533.I1RZ87
19937	229533.I1RRU8	pp	229533.I1S9T2
19938	229533.I1RRU8	pp	229533.I1RU02
19939	229533.I1RRU8	pp	229533.Q4IER8
19940	229533.A0A1C3YKV8	pp	229533.V6QUV7
19941	229533.I1RAH6	pp	229533.I1RYG3
19942	229533.I1RAH6	pp	229533.I1RZM4
19943	229533.I1RAH6	pp	229533.I1SA55
19944	229533.I1RAH6	pp	229533.V6RN16
19945	229533.I1RAH6	pp	229533.I1RQE8
19946	229533.I1RAH6	pp	229533.I1RWS4
19947	229533.I1RAH6	pp	229533.I1S253
19948	229533.I1RAH6	pp	229533.I1RT05
19949	229533.I1RAH6	pp	229533.I1RW26
19950	229533.I1RAH6	pp	229533.I1RFW5
19951	229533.I1RAH6	pp	229533.I1RYJ6
19952	229533.I1RAH6	pp	229533.I1REF2
19953	229533.I1RAH6	pp	229533.I1RRG3
19954	229533.I1RAH6	pp	229533.I1RME3
19955	229533.I1RAH6	pp	229533.Q4HYC1
19956	229533.I1RAH6	pp	229533.I1RZB2
19957	229533.I1RAH6	pp	229533.V6RK96
19958	229533.I1RAH6	pp	229533.I1RR72
19959	229533.I1RAH6	pp	229533.I1RP31
19960	229533.I1RAH6	pp	229533.I1RM67
19961	229533.I1RAH6	pp	229533.I1S065
19962	229533.I1RAH6	pp	229533.I1S4P6
19963	229533.I1RAH6	pp	229533.Q4ID21
19964	229533.I1RAH6	pp	229533.I1RC48
19965	229533.I1RAH6	pp	229533.I1S5R6
19966	229533.I1RAH6	pp	229533.Q4I5V2
19967	229533.I1RAH6	pp	229533.I1RT09
19968	229533.I1RAH6	pp	229533.I1S835
19969	229533.I1RAH6	pp	229533.V6QWZ7
19970	229533.A0A098DY35	pp	229533.I1S276
19971	229533.A0A098DY35	pp	229533.A0A1C3YHH5
19972	229533.A0A098DY35	pp	229533.I1RZ87
19973	229533.A0A098DY35	pp	229533.V6QUE6
19974	229533.A0A098DY35	pp	229533.I1RAR2
19975	229533.A0A098DY35	pp	229533.V6QV38
19976	229533.A0A098DY35	pp	229533.Q4I7S1
19977	229533.A0A098DY35	pp	229533.I1RK71
19978	229533.A0A098DY35	pp	229533.A0A1C3YJ12
19979	229533.A0A098DY35	pp	229533.A0A1C3YI23
19980	229533.A0A098DY35	pp	229533.I1RPM7
19981	229533.A0A098DY35	pp	229533.A0A0E0RN05
19982	229533.A0A098DY35	pp	229533.I1RXU0
19983	229533.A0A098DY35	pp	229533.I1RZU9
19984	229533.A0A098DY35	pp	229533.A0A098E1Y1
19985	229533.A0A098DY35	pp	229533.I1RJL2
19986	229533.A0A098DY35	pp	229533.Q4I7X1
19987	229533.A0A098DY35	pp	229533.I1RCB7
19988	229533.A0A098DY35	pp	229533.I1RZB5
19989	229533.A0A098DY35	pp	229533.V6QWX8
19990	229533.A0A098DY35	pp	229533.I1RK79
19991	229533.A0A098DY35	pp	229533.Q4HTT2
19992	229533.A0A098DY35	pp	229533.A0A0E0RS85
19993	229533.A0A098DY35	pp	229533.A0A1C3YHX6
19994	229533.A0A098DY35	pp	229533.A0A0E0SDM6
19995	229533.A0A098DY35	pp	229533.I1RR25
19996	229533.A0A098DY35	pp	229533.I1RW91
19997	229533.A0A098DY35	pp	229533.I1RRN6
19998	229533.A0A098DY35	pp	229533.A0A1C3YJR3
19999	229533.A0A098DY35	pp	229533.A0A1C3YIR8
20000	229533.A0A098DY35	pp	229533.I1RBJ1
20001	229533.A0A098DY35	pp	229533.I1RQ42
20002	229533.A0A098DY35	pp	229533.A0A0E0S6D3

20003 229533.A0A098DY35 pp 229533.I1S382
20004 229533.A0A098DY35 pp 229533.I1REN3
20005 229533.A0A098DY35 pp 229533.I1RXE5
20006 229533.A0A098DY35 pp 229533.I1RUG4
20007 229533.A0A098DY35 pp 229533.I1RCG6
20008 229533.A0A098DY35 pp 229533.I1RWF3
20009 229533.A0A098DY35 pp 229533.A0A1C3YMF9
20010 229533.A0A098DY35 pp 229533.I1RMV1
20011 229533.A0A098DY35 pp 229533.I1RFU3
20012 229533.A0A098DY35 pp 229533.I1RBR5
20013 229533.A0A098DY35 pp 229533.A0A1C3YN55
20014 229533.A0A098DY35 pp 229533.I1RVY0
20015 229533.A0A098DY35 pp 229533.I1RC15
20016 229533.A0A098DY35 pp 229533.I1RDG8
20017 229533.A0A098DY35 pp 229533.I1RRT0
20018 229533.A0A098DY35 pp 229533.I1RA84
20019 229533.A0A098DY35 pp 229533.Q4IR08
20020 229533.A0A098DY35 pp 229533.I1RG11
20021 229533.A0A098DY35 pp 229533.A0A1C3YJZ2
20022 229533.A0A098DY35 pp 229533.I1RBZ7
20023 229533.A0A098DY35 pp 229533.I1R976
20024 229533.A0A098DY35 pp 229533.I1RVL8
20025 229533.A0A098DY35 pp 229533.I1RDC9
20026 229533.A0A098DY35 pp 229533.I1RKB1
20027 229533.A0A098DY35 pp 229533.I1RC98
20028 229533.A0A098DY35 pp 229533.I1RKL2
20029 229533.A0A098DY35 pp 229533.I1RBC7
20030 229533.A0A098DY35 pp 229533.I1S951
20031 229533.A0A098DY35 pp 229533.Q4IMD1
20032 229533.A0A098DY35 pp 229533.I1S1V5
20033 229533.A0A098DY35 pp 229533.Q4IQX3
20034 229533.A0A098DY35 pp 229533.I1RW90
20035 229533.A0A098DY35 pp 229533.I1RRM8
20036 229533.A0A098DY35 pp 229533.I1R9Q0
20037 229533.A0A098DY35 pp 229533.I1RC24
20038 229533.A0A098DY35 pp 229533.I1RW80
20039 229533.A0A098DY35 pp 229533.I1RSZ3
20040 229533.A0A098DY35 pp 229533.A0A0E0SQJ6
20041 229533.A0A098DY35 pp 229533.I1RE30
20042 229533.A0A098DY35 pp 229533.A0A1C3YLP8
20043 229533.A0A098DY35 pp 229533.I1RP96
20044 229533.A0A098DY35 pp 229533.I1RTC2
20045 229533.A0A098DY35 pp 229533.I1RML9
20046 229533.A0A098DY35 pp 229533.I1RZM2
20047 229533.A0A098DY35 pp 229533.I1RAW7
20048 229533.A0A098DY35 pp 229533.I1RRG3
20049 229533.Q4HVQ9 pp 229533.V6R3J1
20050 229533.Q4HVQ9 pp 229533.Q4IJK2
20051 229533.Q4HVQ9 pp 229533.Q4I1N3
20052 229533.Q4HVQ9 pp 229533.Q4HYR2
20053 229533.Q4HVQ9 pp 229533.V6RAC9
20054 229533.Q4HVQ9 pp 229533.V6RGI1
20055 229533.Q4HVQ9 pp 229533.V6RRM0
20056 229533.A0A098D5S2 pp 229533.A0A1C3YM21
20057 229533.A0A098D5S2 pp 229533.I1RA28
20058 229533.A0A098D5S2 pp 229533.I1S2D5
20059 229533.A0A098D5S2 pp 229533.Q4HVQ9
20060 229533.I1RMF3 pp 229533.I1RYW4
20061 229533.I1RBR5 pp 229533.I1RJL2
20062 229533.I1RBR5 pp 229533.I1REN3
20063 229533.I1RBR5 pp 229533.I1RR25
20064 229533.I1RBR5 pp 229533.I1RUG4
20065 229533.I1RBR5 pp 229533.V6QUE6
20066 229533.I1RBR5 pp 229533.I1RMV1
20067 229533.I1RBR5 pp 229533.Q4I7X1
20068 229533.I1RBR5 pp 229533.I1RG11
20069 229533.I1RBR5 pp 229533.I1RCB7
20070 229533.I1RBR5 pp 229533.Q4I7S1
20071 229533.I1RBR5 pp 229533.I1RZU9
20072 229533.I1RBR5 pp 229533.I1RXU0
20073 229533.I1RBR5 pp 229533.I1RPM7
20074 229533.I1RBR5 pp 229533.I1S0P4
20075 229533.I1RBR5 pp 229533.I1RCG6

229533.I1RBR5 pp 229533.I1RWF3
229533.I1RBR5 pp 229533.I1RK71
229533.I1RBR5 pp 229533.V6QV38
229533.I1RBR5 pp 229533.I1RDC9
229533.I1RBR5 pp 229533.I1RXE5
229533.I1RBR5 pp 229533.I1RRT0
229533.I1RBR5 pp 229533.I1RZ87
229533.I1RBR5 pp 229533.I1S276
229533.I1RBR5 pp 229533.Q4I8X0
229533.I1RBR5 pp 229533.I1S382
229533.I1RBR5 pp 229533.I1RK79
229533.I1RBR5 pp 229533.I1RBZ7
229533.I1RBR5 pp 229533.I1RVL8
229533.I1RBR5 pp 229533.I1RC15
229533.I1RBR5 pp 229533.I1RN04
229533.I1RBR5 pp 229533.I1S951
229533.I1RBR5 pp 229533.I1S1V5
229533.I1RBR5 pp 229533.I1RZB5
229533.I1RBR5 pp 229533.I1RKB1
229533.I1RBR5 pp 229533.V6QWX8
229533.I1RBR5 pp 229533.I1RKL2
229533.I1RBR5 pp 229533.I1RML9
229533.I1RBR5 pp 229533.I1RSZ3
229533.I1RBR5 pp 229533.I1RW90
229533.I1RBR5 pp 229533.I1RP96
229533.I1RBR5 pp 229533.I1RC24
229533.I1RBR5 pp 229533.I1RE30
229533.I1RBR5 pp 229533.I1RRG3
229533.I1RBR5 pp 229533.I1RDG8
229533.I1RBR5 pp 229533.I1RFU3
229533.I1RBR5 pp 229533.I1RVY0
229533.I1RBR5 pp 229533.I1RZM2
229533.I1RBR5 pp 229533.I1RTC2
229533.A0A098D5I5 pp 229533.I1RNG4
229533.I1RA35 pp 229533.I1RTD7
229533.I1RA35 pp 229533.I1S1Y7
229533.I1RA35 pp 229533.I1RWS4
229533.I1RA35 pp 229533.V6R3J1
229533.I1RA35 pp 229533.I1RC31
229533.I1RA35 pp 229533.V6RVJ3
229533.I1RA35 pp 229533.I1RL04
229533.I1RA35 pp 229533.I1RNY2
229533.I1RA35 pp 229533.Q4IPI4
229533.I1RA35 pp 229533.I1RAH6
229533.I1RA35 pp 229533.I1RS20
229533.I1RA35 pp 229533.I1RCM7
229533.I1RA35 pp 229533.I1RMR7
229533.I1RA35 pp 229533.I1RW90
229533.I1RA35 pp 229533.I1RQ86
229533.I1RA35 pp 229533.I1RWD7
229533.I1RA35 pp 229533.I1RA94
229533.I1RA35 pp 229533.I1S0F1
229533.I1RA35 pp 229533.I1RM67
229533.I1RA35 pp 229533.I1RAM9
229533.I1RA35 pp 229533.I1RMW4
229533.I1RA35 pp 229533.I1RPR9
229533.I1RA35 pp 229533.I1RRX5
229533.I1RA35 pp 229533.I1RYJ6
229533.A0A098DE23 pp 229533.I1RRU8
229533.A0A098DE23 pp 229533.A0A098DY35
229533.A0A098DE23 pp 229533.Q4IMD1
229533.A0A098DE23 pp 229533.I1RBR5
229533.A0A098DE23 pp 229533.Q4HY90
229533.I1RE72 pp 229533.Q4IJ79
229533.I1RE72 pp 229533.I1RUP6
229533.I1RE72 pp 229533.I1RST4
229533.I1RE72 pp 229533.I1RJL9
229533.I1RE72 pp 229533.I1RJW1
229533.I1RE72 pp 229533.I1RK44
229533.I1RE72 pp 229533.Q4HZS8
229533.I1RE72 pp 229533.I1RR39
229533.I1RSX3 pp 229533.I1RSX4
229533.A0A0E0RZN1 pp 229533.A0A1C3YL07

20149 229533.A0A0E0RZN1 pp 229533.I1RM96
20150 229533.A0A0E0RZN1 pp 229533.I1RPY1
20151 229533.A0A0E0RZN1 pp 229533.A0A0E0SJN2
20152 229533.A0A0E0RZN1 pp 229533.I1RMN6
20153 229533.A0A0E0RZN1 pp 229533.I1RNU6
20154 229533.A0A0E0RZN1 pp 229533.I1RN04
20155 229533.A0A0E0RZN1 pp 229533.I1RZ42
20156 229533.A0A0E0RZN1 pp 229533.I1RCG6
20157 229533.A0A0E0RZN1 pp 229533.I1RX14
20158 229533.A0A0E0RZN1 pp 229533.I1RZS6
20159 229533.A0A0E0RZN1 pp 229533.I1RM67
20160 229533.A0A0E0RZN1 pp 229533.I1RW80
20161 229533.A0A0E0RZN1 pp 229533.I1RPU0
20162 229533.A0A0E0RZN1 pp 229533.I1RJL4
20163 229533.A0A0E0RZN1 pp 229533.I1S9U2
20164 229533.A0A0E0RZN1 pp 229533.I1RVZ0
20165 229533.A0A0E0RZN1 pp 229533.I1RLV9
20166 229533.A0A0E0RZN1 pp 229533.I1RQE0
20167 229533.A0A0E0RZN1 pp 229533.I1RW91
20168 229533.A0A0E0RZN1 pp 229533.I1RA48
20169 229533.A0A0E0RZN1 pp 229533.I1RSC6
20170 229533.A0A0E0RZN1 pp 229533.I1RMK3
20171 229533.A0A0E0RZN1 pp 229533.Q4HVW2
20172 229533.A0A0E0RZN1 pp 229533.I1RNH6
20173 229533.A0A0E0RZN1 pp 229533.I1S182
20174 229533.A0A0E0RZN1 pp 229533.I1RQ42
20175 229533.A0A0E0RZN1 pp 229533.V6QYF0
20176 229533.A0A0E0RZN1 pp 229533.I1RPW8
20177 229533.I1RBZ7 pp 229533.Q4I7S1
20178 229533.I1RBZ7 pp 229533.I1RZM2
20179 229533.I1RBZ7 pp 229533.V6QV38
20180 229533.I1RBZ7 pp 229533.I1RDC9
20181 229533.I1RBZ7 pp 229533.I1RZU9
20182 229533.I1RBZ7 pp 229533.I1RVY0
20183 229533.I1RBZ7 pp 229533.I1RML9
20184 229533.I1RBZ7 pp 229533.I1RXE5
20185 229533.I1RBZ7 pp 229533.I1RP96
20186 229533.Q4IP34 pp 229533.V6QVD5
20187 229533.Q4IP34 pp 229533.V6RY33
20188 229533.Q4IP34 pp 229533.V6R227
20189 229533.Q4IP34 pp 229533.V6RIS2
20190 229533.Q4IP34 pp 229533.Q4IPN9
20191 229533.Q4IP34 pp 229533.Q4IPI1
20192 229533.Q4IP34 pp 229533.Q4IRI9
20193 229533.I1RCR1 pp 229533.I1RQD2
20194 229533.I1RCR1 pp 229533.I1S5R6
20195 229533.I1RCR1 pp 229533.I1RR24
20196 229533.I1RCR1 pp 229533.I1RGA4
20197 229533.I1RCR1 pp 229533.I1REC5
20198 229533.I1RCR1 pp 229533.I1RPE0
20199 229533.I1RCR1 pp 229533.I1RWR7
20200 229533.I1RCR1 pp 229533.I1RRI1
20201 229533.I1RCR1 pp 229533.I1S5R1
20202 229533.I1RCR1 pp 229533.I1RE89
20203 229533.I1RCR1 pp 229533.I1S1B2
20204 229533.I1RCR1 pp 229533.I1RZ39
20205 229533.I1RCR1 pp 229533.I1RRV3
20206 229533.I1RCR1 pp 229533.I1RVQ3
20207 229533.I1RCR1 pp 229533.I1RJU3
20208 229533.I1RCR1 pp 229533.I1S821
20209 229533.I1RCR1 pp 229533.I1RXD6
20210 229533.I1RCR1 pp 229533.I1RZV3
20211 229533.I1RCR1 pp 229533.V6R9Z0
20212 229533.I1RCR1 pp 229533.I1S0W4
20213 229533.I1RCR1 pp 229533.I1RZK5
20214 229533.I1RCR1 pp 229533.I1RKJ4
20215 229533.I1RCR1 pp 229533.I1RTB3
20216 229533.I1RCR1 pp 229533.I1S1W9
20217 229533.I1RCR1 pp 229533.I1RTD5
20218 229533.I1RCR1 pp 229533.I1RNB2
20219 229533.I1RCR1 pp 229533.I1RXD7
20220 229533.I1RCR1 pp 229533.I1RZM8
20221 229533.I1RCR1 pp 229533.I1RDF7

229533.I1RCR1	pp	229533.I1RCR8
229533.I1RCR1	pp	229533.I1S2B8
229533.I1RCR1	pp	229533.V6RCZ8
229533.I1RCR1	pp	229533.I1RRM3
229533.I1RCR1	pp	229533.I1RYI5
229533.I1RCR1	pp	229533.I1RT40
229533.I1RCR1	pp	229533.I1RSA5
229533.I1RCR1	pp	229533.I1RSF1
229533.I1RCR1	pp	229533.I1RVX0
229533.I1RCR1	pp	229533.I1S4S7
229533.I1RCR1	pp	229533.I1RGB2
229533.I1RCR1	pp	229533.I1RQ33
229533.I1RCR1	pp	229533.I1RJN9
229533.I1RCR1	pp	229533.I1RQ85
229533.I1RCR1	pp	229533.I1RTA1
229533.I1RCR1	pp	229533.I1RPU2
229533.I1RCR1	pp	229533.I1RD52
229533.I1RCR1	pp	229533.I1RVZ2
229533.I1RCR1	pp	229533.I1RX95
229533.I1RCR1	pp	229533.I1RXC4
229533.I1RCR1	pp	229533.Q4IJK2
229533.I1RCR1	pp	229533.I1RSZ7
229533.A0A0E0RQ52	pp	229533.I1RBF4
229533.A0A0E0RQ52	pp	229533.I1RK44
229533.A0A0E0RQ52	pp	229533.A0A1C3YIG2
229533.A0A0E0RQ52	pp	229533.I1RST4
229533.A0A0E0RQ52	pp	229533.I1RJL9
229533.A0A0E0RQ52	pp	229533.Q4IJ79
229533.A0A0E0RQ52	pp	229533.Q4HZS8
229533.A0A0E0RQ52	pp	229533.A0A1C3YLR6
229533.A0A0E0RQ52	pp	229533.I1RED5
229533.A0A0E0RQ52	pp	229533.O74638
229533.A0A0E0RQ52	pp	229533.I1RCR5
229533.A0A0E0RQ52	pp	229533.I1RT41
229533.A0A0E0RQ52	pp	229533.A0A1C3YKU0
229533.A0A0E0RQ52	pp	229533.A0A1C3YK13
229533.A0A0E0RQ52	pp	229533.I1RUP6
229533.A0A0E0RQ52	pp	229533.I1RR39
229533.A0A0E0RQ52	pp	229533.I1RCF9
229533.A0A0E0RQ52	pp	229533.I1RJW1
229533.A0A0E0RQ52	pp	229533.I1RC31
229533.A0A0E0RQ52	pp	229533.I1RBY9
229533.I1RPP2	pp	229533.I1RQ14
229533.I1RC98	pp	229533.I1RDH0
229533.I1RC98	pp	229533.I1S276
229533.I1RC98	pp	229533.I1RPM7
229533.I1RC98	pp	229533.I1RRF0
229533.I1RC98	pp	229533.I1RK79
229533.I1RC98	pp	229533.I1RSG8
229533.I1RC98	pp	229533.Q4HYB8
229533.I1RC98	pp	229533.I1RWF3
229533.I1RC98	pp	229533.I1RR25
229533.I1RC98	pp	229533.Q4IL82
229533.I1RC98	pp	229533.I1RCB7
229533.I1RC98	pp	229533.I1RG11
229533.I1RC98	pp	229533.I1RXU0
229533.I1RC98	pp	229533.Q4IJU0
229533.I1RC98	pp	229533.I1RK95
229533.I1RC98	pp	229533.I1RE30
229533.I1RC98	pp	229533.I1RUF8
229533.I1RC98	pp	229533.Q4I948
229533.I1RC98	pp	229533.Q4I5W5
229533.I1RC98	pp	229533.Q4IPI4
229533.I1RC98	pp	229533.I1RVY0
229533.I1RC98	pp	229533.I1RCN2
229533.I1RC98	pp	229533.I1RJL2
229533.I1RC98	pp	229533.I1RRM8
229533.I1RC98	pp	229533.Q4IMD1
229533.I1RC98	pp	229533.I1RVL8
229533.I1RC98	pp	229533.I1RXE5
229533.I1RC98	pp	229533.I1RZB5
229533.I1RC98	pp	229533.V6RAC9
229533.I1RC98	pp	229533.Q4ICA8

20295	229533.I1RC98	pp	229533.I1RNH1
20296	229533.I1RC98	pp	229533.Q4I7L0
20297	229533.I1RC98	pp	229533.I1RSZ3
20298	229533.I1RC98	pp	229533.I1S0F0
20299	229533.I1RC98	pp	229533.I1RTC2
20300	229533.I1RC98	pp	229533.I1RQP1
20301	229533.I1RC98	pp	229533.I1REN3
20302	229533.I1RC98	pp	229533.I1RMQ7
20303	229533.I1RC98	pp	229533.I1RUP7
20304	229533.I1RC98	pp	229533.I1RRG3
20305	229533.I1RC98	pp	229533.I1RRT0
20306	229533.I1RC98	pp	229533.I1RKB1
20307	229533.I1RC98	pp	229533.V6QUE6
20308	229533.I1RC98	pp	229533.I1RQP3
20309	229533.I1RC98	pp	229533.Q4HTT2
20310	229533.I1RC98	pp	229533.Q4IQX3
20311	229533.I1RC98	pp	229533.I1RML9
20312	229533.I1RC98	pp	229533.I1RP96
20313	229533.I1RC98	pp	229533.I1RFQ0
20314	229533.I1RC98	pp	229533.I1RM67
20315	229533.I1RC98	pp	229533.I1RYT8
20316	229533.I1RBF4	pp	229533.I1RNU7
20317	229533.I1RBF4	pp	229533.I1RX90
20318	229533.I1RBF4	pp	229533.I1RE72
20319	229533.I1RBF4	pp	229533.I1RE44
20320	229533.I1RBF4	pp	229533.V6R729
20321	229533.I1RBF4	pp	229533.I1RJQ5
20322	229533.I1RBF4	pp	229533.V6RDH3
20323	229533.I1RBF4	pp	229533.I1RQ14
20324	229533.I1RBF4	pp	229533.I1RKP3
20325	229533.I1RBF4	pp	229533.I1RZW8
20326	229533.I1RBF4	pp	229533.I1RPT0
20327	229533.I1RBF4	pp	229533.Q4IAA0
20328	229533.I1RBF4	pp	229533.I1RK44
20329	229533.I1RBF4	pp	229533.I1RDE4
20330	229533.I1RBF4	pp	229533.Q4IEV5
20331	229533.I1RBF4	pp	229533.I1RR39
20332	229533.I1RBF4	pp	229533.I1RE84
20333	229533.I1RBF4	pp	229533.I1RC31
20334	229533.I1RBF4	pp	229533.I1RQ48
20335	229533.I1RBF4	pp	229533.I1S097
20336	229533.I1RBF4	pp	229533.V6RAN2
20337	229533.I1RBF4	pp	229533.I1S1L2
20338	229533.I1RBF4	pp	229533.I1RP86
20339	229533.I1RBF4	pp	229533.I1RKB5
20340	229533.I1RBF4	pp	229533.I1RTF2
20341	229533.I1RBF4	pp	229533.I1RZ13
20342	229533.I1RBF4	pp	229533.I1RDJ1
20343	229533.I1RBF4	pp	229533.I1RKX2
20344	229533.I1RBF4	pp	229533.I1RFZ8
20345	229533.I1RBF4	pp	229533.I1RIX1
20346	229533.I1RBF4	pp	229533.I1RJW1
20347	229533.I1RBF4	pp	229533.I1RZN3
20348	229533.I1RZX4	pp	229533.I1S071
20349	229533.I1RZX4	pp	229533.V6R5G9
20350	229533.I1RZX4	pp	229533.I1S2L3
20351	229533.I1RKL2	pp	229533.I1RRH5
20352	229533.I1RKL2	pp	229533.Q4I650
20353	229533.I1RKL2	pp	229533.Q4I948
20354	229533.I1RKL2	pp	229533.Q4IER8
20355	229533.I1RKL2	pp	229533.Q4HY90
20356	229533.I1RKL2	pp	229533.I1RRU8
20357	229533.I1RKL2	pp	229533.Q4ICA8
20358	229533.I1RKL2	pp	229533.Q4IAK7
20359	229533.I1RKL2	pp	229533.Q4IMD1
20360	229533.I1RKL2	pp	229533.Q4IEV4
20361	229533.I1RKL2	pp	229533.Q4I7S1
20362	229533.I1RFM6	pp	229533.I1RW69
20363	229533.I1RFM6	pp	229533.I1S0A0
20364	229533.I1RFM6	pp	229533.I1RXB6
20365	229533.I1RFM6	pp	229533.I1RYI7
20366	229533.I1RFM6	pp	229533.V6RK96
20367	229533.I1RFM6	pp	229533.I1RM26

20368	229533.I1RFM6	pp	229533.I1RVY2
20369	229533.I1RFM6	pp	229533.I1S0N3
20370	229533.I1RFM6	pp	229533.I1RXV9
20371	229533.I1RFM6	pp	229533.I1RMW4
20372	229533.I1RFM6	pp	229533.V6RQW4
20373	229533.I1RFM6	pp	229533.I1RYX6
20374	229533.I1RFM6	pp	229533.I1RQ14
20375	229533.I1RFM6	pp	229533.I1RK95
20376	229533.I1RFM6	pp	229533.I1RZM4
20377	229533.I1RFM6	pp	229533.I1RMG7
20378	229533.I1RFM6	pp	229533.I1RXB1
20379	229533.I1RFM6	pp	229533.I1RYU5
20380	229533.I1RFM6	pp	229533.I1RZQ1
20381	229533.I1RFM6	pp	229533.V6RM02
20382	229533.I1RFM6	pp	229533.I1RVX9
20383	229533.I1RFM6	pp	229533.I1RIH0
20384	229533.I1RFM6	pp	229533.I1RYJ7
20385	229533.I1RFM6	pp	229533.I1RMM8
20386	229533.I1RFM6	pp	229533.I1RZ20
20387	229533.I1RFM6	pp	229533.I1RQE8
20388	229533.I1RFM6	pp	229533.I1S0I3
20389	229533.I1RFM6	pp	229533.I1RPW2
20390	229533.I1RFM6	pp	229533.I1RNG4
20391	229533.I1RFM6	pp	229533.I1RSL5
20392	229533.I1RFM6	pp	229533.I1RMG6
20393	229533.I1RFM6	pp	229533.V6QVH8
20394	229533.I1RFM6	pp	229533.I1RZP1
20395	229533.I1RFM6	pp	229533.I1RPK0
20396	229533.I1RFM6	pp	229533.I1RQ30
20397	229533.I1RFM6	pp	229533.V6RSF3
20398	229533.I1RFM6	pp	229533.I1RXW3
20399	229533.I1RFM6	pp	229533.Q4HXF0
20400	229533.I1RFM6	pp	229533.I1RL76
20401	229533.I1RFM6	pp	229533.I1RR68
20402	229533.I1RFM6	pp	229533.I1RFW3
20403	229533.I1RFM6	pp	229533.I1RTI7
20404	229533.I1RFM6	pp	229533.I1RVR2
20405	229533.I1RFM6	pp	229533.I1RRT8
20406	229533.I1RFM6	pp	229533.O74638
20407	229533.I1RFM6	pp	229533.I1RZ29
20408	229533.I1RQJ3	pp	229533.I1RS05
20409	229533.I1RQJ3	pp	229533.I1RY92
20410	229533.I1RQJ3	pp	229533.I1S458
20411	229533.I1RQJ3	pp	229533.I1S0P0
20412	229533.I1RQJ3	pp	229533.I1RWA3
20413	229533.I1RQJ3	pp	229533.I1S5X9
20414	229533.I1RQJ3	pp	229533.I1RXD9
20415	229533.I1RQJ3	pp	229533.I1S464
20416	229533.I1RQJ3	pp	229533.I1RS29
20417	229533.I1RQJ3	pp	229533.I1S8S9
20418	229533.I1RQJ3	pp	229533.I1RYQ1
20419	229533.I1RQJ3	pp	229533.I1S0K2
20420	229533.I1RQJ3	pp	229533.I1S0R1
20421	229533.I1RQJ3	pp	229533.I1RYT8
20422	229533.I1RQJ3	pp	229533.I1RY21
20423	229533.I1RQJ3	pp	229533.V6RJP8
20424	229533.I1RQJ3	pp	229533.I1S5C6
20425	229533.I1RVQ3	pp	229533.I1RXD6
20426	229533.I1RVQ3	pp	229533.Q4IJK2
20427	229533.I1RVQ3	pp	229533.I1RXC4
20428	229533.I1RVQ3	pp	229533.I1RX95
20429	229533.I1RVQ3	pp	229533.I1S0W4
20430	229533.I1RVQ3	pp	229533.I1RZV3
20431	229533.I1RVQ3	pp	229533.I1RYI5
20432	229533.I1RVQ3	pp	229533.I1S2B8
20433	229533.I1RVQ3	pp	229533.I1S1W9
20434	229533.I1RVQ3	pp	229533.I1RVX0
20435	229533.I1RVQ3	pp	229533.I1RZM8
20436	229533.I1RVQ3	pp	229533.I1S4S7
20437	229533.I1RVQ3	pp	229533.I1RVZ2
20438	229533.I1RVQ3	pp	229533.I1S5R1
20439	229533.I1RVQ3	pp	229533.I1RWR7
20440	229533.I1RVQ3	pp	229533.I1RZK5

20441 229533.I1RVQ3 pp 229533.V6R9Z0
20442 229533.I1RVQ3 pp 229533.I1S5R6
20443 229533.I1RVQ3 pp 229533.I1RXD7
20444 229533.I1RVQ3 pp 229533.I1S1B2
20445 229533.I1RVQ3 pp 229533.V6RCZ8
20446 229533.I1RVQ3 pp 229533.I1RZ39
20447 229533.I1RVQ3 pp 229533.I1S821
20448 229533.A0A1C3YM97 pp 229533.I1RKR5
20449 229533.A0A1C3YM97 pp 229533.I1RUY5
20450 229533.A0A1C3YM97 pp 229533.I1RHQ0
20451 229533.A0A1C3YM97 pp 229533.I1RUM7
20452 229533.A0A1C3YM97 pp 229533.I1RND4
20453 229533.A0A1C3YM97 pp 229533.V6QU63
20454 229533.A0A1C3YM97 pp 229533.I1RQI4
20455 229533.A0A1C3YM97 pp 229533.V6RLM7
20456 229533.A0A1C3YM97 pp 229533.I1RL92
20457 229533.A0A1C3YM97 pp 229533.I1RR44
20458 229533.A0A1C3YM97 pp 229533.I1RKE1
20459 229533.A0A1C3YM97 pp 229533.I1RMX6
20460 229533.A0A1C3YM97 pp 229533.I1RHM8
20461 229533.A0A1C3YM97 pp 229533.I1RPT0
20462 229533.A0A1C3YM97 pp 229533.I1RGB2
20463 229533.A0A1C3YM97 pp 229533.I1RGY2
20464 229533.A0A1C3YM97 pp 229533.I0LNZ1
20465 229533.A0A1C3YM97 pp 229533.I1RCG5
20466 229533.A0A1C3YM97 pp 229533.I1RX90
20467 229533.A0A1C3YM97 pp 229533.I1RIX0
20468 229533.A0A1C3YM97 pp 229533.I1RPS2
20469 229533.A0A1C3YM97 pp 229533.I1RP86
20470 229533.A0A1C3YM97 pp 229533.I1RHL0
20471 229533.A0A1C3YM97 pp 229533.I1RKX2
20472 229533.A0A1C3YM97 pp 229533.I1RVW8
20473 229533.A0A1C3YM97 pp 229533.V6RV57
20474 229533.A0A1C3YM97 pp 229533.I1RSJ2
20475 229533.A0A1C3YM97 pp 229533.I1RF48
20476 229533.A0A1C3YM97 pp 229533.A0A1C3YN26
20477 229533.A0A1C3YM97 pp 229533.I1RCZ8
20478 229533.A0A1C3YM97 pp 229533.I1RVV3
20479 229533.A0A1C3YM97 pp 229533.I1RC31
20480 229533.A0A1C3YM97 pp 229533.V6RW66
20481 229533.A0A1C3YM97 pp 229533.I1RA62
20482 229533.A0A1C3YM97 pp 229533.I1RF73
20483 229533.A0A1C3YM97 pp 229533.I1RYK0
20484 229533.A0A1C3YM97 pp 229533.I1RI39
20485 229533.A0A1C3YM97 pp 229533.I1S2R5
20486 229533.A0A1C3YM97 pp 229533.I1RDW1
20487 229533.A0A1C3YM97 pp 229533.V6RK40
20488 229533.A0A1C3YM97 pp 229533.I1RU18
20489 229533.A0A1C3YM97 pp 229533.I1R9F8
20490 229533.A0A1C3YM97 pp 229533.I1RZ78
20491 229533.A0A1C3YM97 pp 229533.I1RRT3
20492 229533.A0A098DQM7 pp 229533.A0A1C3YKY6
20493 229533.A0A098DQM7 pp 229533.I1RRX5
20494 229533.A0A098DQM7 pp 229533.I1RYJ6
20495 229533.A0A098DQM7 pp 229533.I1RN64
20496 229533.A0A098DQM7 pp 229533.I1RA94
20497 229533.A0A098DQM7 pp 229533.I1RQ86
20498 229533.A0A098DQM7 pp 229533.A0A1C3YJG0
20499 229533.A0A098DQM7 pp 229533.I1RC32
20500 229533.A0A098DQM7 pp 229533.I1RAH6
20501 229533.A0A098DQM7 pp 229533.A0A1C3YI30
20502 229533.A0A098DQM7 pp 229533.A0A098DR54
20503 229533.A0A098DQM7 pp 229533.I1RWD7
20504 229533.A0A098DQM7 pp 229533.I1RA35
20505 229533.A0A098DQM7 pp 229533.I1RCM7
20506 229533.A0A098DQM7 pp 229533.I1RTD7
20507 229533.A0A098DQM7 pp 229533.I1S0F1
20508 229533.A0A098DQM7 pp 229533.A0A1C3YMK0
20509 229533.A0A098DQM7 pp 229533.I1R9Y4
20510 229533.A0A098DQM7 pp 229533.V6QWZ7
20511 229533.A0A098DQM7 pp 229533.I1RWS4
20512 229533.A0A098DQM7 pp 229533.I1RMW4
20513 229533.A0A098DQM7 pp 229533.I1RM67

20514 229533.A0A098DQM7 pp 229533.I1RC31
20515 229533.A0A098DQM7 pp 229533.A0A1C3YJI4
20516 229533.A0A098DQM7 pp 229533.V6RVJ3
20517 229533.A0A098DQM7 pp 229533.I1RL04
20518 229533.A0A098DQM7 pp 229533.A0A1C3YIW0
20519 229533.A0A098DQM7 pp 229533.A0A1C3YI20
20520 229533.A0A098DQM7 pp 229533.I1RS20
20521 229533.I1RR13 pp 229533.I1RRF0
20522 229533.I1RR13 pp 229533.Q4ICA8
20523 229533.I1RR13 pp 229533.I1RT41
20524 229533.I1RR13 pp 229533.I1S0Z3
20525 229533.I1RR13 pp 229533.Q4IPI4
20526 229533.I1RGK4 pp 229533.I1S1F3
20527 229533.A0A0E0S413 pp 229533.I1RKY2
20528 229533.A0A0E0S413 pp 229533.A0A0E0SMR8
20529 229533.A0A0E0S413 pp 229533.A0A1C3YK37
20530 229533.A0A0E0S413 pp 229533.Q4HXF0
20531 229533.A0A0E0S413 pp 229533.I1RX22
20532 229533.A0A0E0S413 pp 229533.I1RPU8
20533 229533.A0A0E0S413 pp 229533.I1RE53
20534 229533.A0A0E0S413 pp 229533.I1RS17
20535 229533.A0A0E0S413 pp 229533.V6R1K4
20536 229533.A0A0E0S413 pp 229533.I1RMI8
20537 229533.A0A0E0S413 pp 229533.I1RCV1
20538 229533.A0A0E0S413 pp 229533.I1RQ14
20539 229533.A0A0E0S413 pp 229533.I1RM55
20540 229533.A0A0E0S413 pp 229533.V6RQW4
20541 229533.A0A0E0S413 pp 229533.I1S8G4
20542 229533.A0A0E0S413 pp 229533.I1RQS9
20543 229533.A0A0E0S413 pp 229533.I1S216
20544 229533.A0A0E0S413 pp 229533.A0A1C3YN30
20545 229533.A0A0E0S413 pp 229533.V6R057
20546 229533.A0A0E0S413 pp 229533.O74638
20547 229533.V6R9Z0 pp 229533.V6RCZ8
20548 229533.Q4IQX3 pp 229533.V6RAC9
20549 229533.Q4IQX3 pp 229533.V6QUE6
20550 229533.I1RRM8 pp 229533.I1RWF3
20551 229533.I1RRM8 pp 229533.Q4HYB8
20552 229533.I1RRM8 pp 229533.Q4I7L0
20553 229533.I1RRM8 pp 229533.I1S0N5
20554 229533.I1RRM8 pp 229533.I1RXU0
20555 229533.I1RRM8 pp 229533.I1S0F0
20556 229533.I1RRM8 pp 229533.I1RSG8
20557 229533.I1RRM8 pp 229533.I1RRT0
20558 229533.I1RRM8 pp 229533.I1S276
20559 229533.I1RRM8 pp 229533.Q4IMD1
20560 229533.I1RRM8 pp 229533.I1S0Z3
20561 229533.I1RRM8 pp 229533.Q4ICA8
20562 229533.I1RRM8 pp 229533.Q4I5W5
20563 229533.I1RRM8 pp 229533.I1RVY0
20564 229533.I1RRM8 pp 229533.I1RVL8
20565 229533.I1RRM8 pp 229533.I1RSZ3
20566 229533.I1RRM8 pp 229533.Q4HTT2
20567 229533.I1RRM8 pp 229533.V6RAC9
20568 229533.I1RRM8 pp 229533.I1RXE5
20569 229533.I1RRM8 pp 229533.Q4IPI4
20570 229533.I1RRM8 pp 229533.V6QUE6
20571 229533.I1RRM8 pp 229533.I1RZB5
20572 229533.I1RRM8 pp 229533.Q4I948
20573 229533.I1RRM8 pp 229533.Q4IL82
20574 229533.I1RRM8 pp 229533.I1RYT8
20575 229533.I1RRM8 pp 229533.Q4IJU0
20576 229533.I1RRM8 pp 229533.I1RTC2
20577 229533.I1RRM8 pp 229533.I1RT41
20578 229533.A0A098DT85 pp 229533.I1RN54
20579 229533.A0A098DT85 pp 229533.I1RJQ5
20580 229533.A0A098DT85 pp 229533.I1RT40
20581 229533.A0A098DT85 pp 229533.I1RWF5
20582 229533.A0A098DT85 pp 229533.V6RV57
20583 229533.A0A098DT85 pp 229533.I1RYK0
20584 229533.A0A098DT85 pp 229533.I1RA62
20585 229533.A0A098DT85 pp 229533.I1RKB2
20586 229533.A0A098DT85 pp 229533.A0A1C3YJ56

20587	229533.A0A098DT85	pp	229533.V6RLM7
20588	229533.A0A098DT85	pp	229533.I1RNG4
20589	229533.A0A098DT85	pp	229533.A0A1C3YLB6
20590	229533.A0A098DT85	pp	229533.I1RPD6
20591	229533.A0A098DT85	pp	229533.I1RC29
20592	229533.A0A098DT85	pp	229533.I1RHJ2
20593	229533.A0A098DT85	pp	229533.I1RU18
20594	229533.A0A098DT85	pp	229533.I1RC31
20595	229533.A0A098DT85	pp	229533.I1S4A5
20596	229533.A0A098DT85	pp	229533.I1RDP6
20597	229533.A0A098DT85	pp	229533.I1RGC3
20598	229533.A0A098DT85	pp	229533.A0A0E0SH88
20599	229533.A0A098DT85	pp	229533.I1RNU7
20600	229533.A0A098DT85	pp	229533.I1RGX1
20601	229533.A0A098DT85	pp	229533.I1RXN7
20602	229533.A0A098DT85	pp	229533.A0A0E0RU55
20603	229533.A0A098DT85	pp	229533.I1RBY4
20604	229533.A0A098DT85	pp	229533.I1RDE7
20605	229533.A0A098DT85	pp	229533.A0A0E0SAG8
20606	229533.A0A098DT85	pp	229533.V6QT89
20607	229533.A0A098DT85	pp	229533.I1RH76
20608	229533.A0A098DT85	pp	229533.I1S3H6
20609	229533.A0A098DT85	pp	229533.I1RQI4
20610	229533.A0A098DT85	pp	229533.A0A098E375
20611	229533.A0A098DT85	pp	229533.I1RND4
20612	229533.A0A098DT85	pp	229533.I1RAE2
20613	229533.A0A098DT85	pp	229533.I1S2C4
20614	229533.A0A098DT85	pp	229533.I1RBT2
20615	229533.A0A098DT85	pp	229533.A0A0E0SBC4
20616	229533.A0A098DT85	pp	229533.I1RP86
20617	229533.A0A098DT85	pp	229533.I1RMX6
20618	229533.A0A098DT85	pp	229533.V6RN31
20619	229533.A0A098DT85	pp	229533.I1RIX0
20620	229533.A0A098DT85	pp	229533.I1RYW3
20621	229533.A0A098DT85	pp	229533.I1RCG5
20622	229533.A0A098DT85	pp	229533.I1RZM4
20623	229533.A0A098DT85	pp	229533.A0A0E0RNI4
20624	229533.A0A098DT85	pp	229533.I1RQ14
20625	229533.A0A098DT85	pp	229533.I1RYU6
20626	229533.A0A098DT85	pp	229533.A0A1C3YN26
20627	229533.A0A098DT85	pp	229533.A0A1C3YKW4
20628	229533.A0A098DT85	pp	229533.V6RTJ8
20629	229533.A0A098DT85	pp	229533.I1RVV3
20630	229533.A0A098DT85	pp	229533.I1RSB7
20631	229533.A0A098DT85	pp	229533.I1RM78
20632	229533.A0A098DT85	pp	229533.I1RSJ2
20633	229533.A0A098DT85	pp	229533.I1RC11
20634	229533.A0A098DT85	pp	229533.I1RVX9
20635	229533.A0A098DT85	pp	229533.I1RQE8
20636	229533.A0A098DT85	pp	229533.I1RR44
20637	229533.A0A098DT85	pp	229533.A0A098DVZ4
20638	229533.A0A098DT85	pp	229533.A0A1C3YK34
20639	229533.A0A098DT85	pp	229533.V6RK96
20640	229533.A0A098DT85	pp	229533.I1RJN7
20641	229533.A0A098DT85	pp	229533.I1RWM4
20642	229533.A0A098DT85	pp	229533.I1S325
20643	229533.A0A098DT85	pp	229533.I1RUY5
20644	229533.A0A098DT85	pp	229533.I1RUY6
20645	229533.A0A098DT85	pp	229533.I1RKR5
20646	229533.A0A098DT85	pp	229533.A0A1C3YJB8
20647	229533.A0A098DT85	pp	229533.I1RCM5
20648	229533.A0A098DT85	pp	229533.I1RLI4
20649	229533.A0A098DT85	pp	229533.I1RH82
20650	229533.A0A098DT85	pp	229533.I1RAQ4
20651	229533.A0A098DT85	pp	229533.I1RDU0
20652	229533.A0A098DT85	pp	229533.I1RDB3
20653	229533.A0A098DT85	pp	229533.I1RGB2
20654	229533.A0A098DT85	pp	229533.I1RPT0
20655	229533.A0A098DT85	pp	229533.I1RQE0
20656	229533.A0A098DT85	pp	229533.I1RY12
20657	229533.A0A098DT85	pp	229533.I1RM26
20658	229533.A0A098DT85	pp	229533.A0A1C3YLC2
20659	229533.A0A098DT85	pp	229533.I1RUY4

20660 229533.A0A098DT85 pp 229533.I1RZ35
20661 229533.I1RVV3 pp 229533.V6RV57
20662 229533.I1RVV3 pp 229533.I1RX90
20663 229533.I1RVV3 pp 229533.I1RYK0
20664 229533.A0A1C3YLL0 pp 229533.I1RUP8
20665 229533.A0A0E0S134 pp 229533.I1RC31
20666 229533.A0A0E0S134 pp 229533.I1RZZ9
20667 229533.A0A0E0S134 pp 229533.I1RSQ4
20668 229533.A0A0E0S134 pp 229533.A0A1C3YIR8
20669 229533.A0A0E0S134 pp 229533.I1RC66
20670 229533.A0A0E0S134 pp 229533.I1RVN1
20671 229533.A0A0E0S134 pp 229533.I1S9Y1
20672 229533.A0A0E0S134 pp 229533.I1S5R1
20673 229533.A0A0E0S134 pp 229533.I1RRT8
20674 229533.A0A0E0S134 pp 229533.I1RA11
20675 229533.A0A0E0S134 pp 229533.I1S2H4
20676 229533.A0A0E0S134 pp 229533.A0A1C3YJI6
20677 229533.A0A0E0S134 pp 229533.I1S0L4
20678 229533.A0A0E0S134 pp 229533.I1RZ95
20679 229533.A0A0E0S134 pp 229533.I1RT40
20680 229533.A0A0E0S134 pp 229533.I1RC14
20681 229533.A0A0E0S134 pp 229533.I1RMG3
20682 229533.A0A0E0S134 pp 229533.A0A1C3YK08
20683 229533.A0A0E0S134 pp 229533.A0A1C3YLK8
20684 229533.A0A0E0S134 pp 229533.A0A1C3YJK3
20685 229533.A0A0E0S134 pp 229533.I1RBW1
20686 229533.A0A0E0S134 pp 229533.I1RSZ7
20687 229533.A0A0E0S134 pp 229533.V6RMW6
20688 229533.A0A0E0S134 pp 229533.I1RJW6
20689 229533.A0A0E0S134 pp 229533.I1RBS6
20690 229533.A0A0E0S134 pp 229533.A0A1C3YIS9
20691 229533.A0A0E0S134 pp 229533.I1S5R6
20692 229533.A0A0E0S134 pp 229533.I1RQ23
20693 229533.A0A0E0S134 pp 229533.I1RL04
20694 229533.A0A0E0S134 pp 229533.I1RZB2
20695 229533.A0A0E0S134 pp 229533.I1RPM4
20696 229533.A0A0E0S134 pp 229533.I1RW46
20697 229533.A0A0E0S134 pp 229533.I1RJV4
20698 229533.A0A0E0S134 pp 229533.I1RE01
20699 229533.A0A0E0S134 pp 229533.I1RFS3
20700 229533.A0A0E0S134 pp 229533.I1RWK3
20701 229533.A0A0E0S134 pp 229533.I1RBF3
20702 229533.A0A0E0S134 pp 229533.I1S2B8
20703 229533.A0A0E0S134 pp 229533.I1RS17
20704 229533.A0A0E0S134 pp 229533.I1S1W9
20705 229533.A0A0E0S134 pp 229533.I1RVZ2
20706 229533.A0A0E0S134 pp 229533.V6RIV9
20707 229533.A0A0E0S134 pp 229533.I1RRM5
20708 229533.A0A0E0S134 pp 229533.I1RPP3
20709 229533.A0A0E0S134 pp 229533.I1RBY4
20710 229533.A0A0E0S134 pp 229533.I1S277
20711 229533.A0A0E0S134 pp 229533.I1RYI5
20712 229533.A0A0E0S134 pp 229533.I1RWE8
20713 229533.A0A0E0S134 pp 229533.I1R9Y8
20714 229533.A0A0E0S134 pp 229533.I1RN67
20715 229533.A0A0E0S134 pp 229533.A0A1C3YIS7
20716 229533.A0A0E0S134 pp 229533.I1RE27
20717 229533.A0A0E0S134 pp 229533.I1RRI2
20718 229533.A0A0E0S134 pp 229533.I1RJU6
20719 229533.A0A0E0S134 pp 229533.I1RZW8
20720 229533.A0A0E0S134 pp 229533.I1RUG3
20721 229533.A0A0E0S134 pp 229533.I1RKB6
20722 229533.A0A0E0S134 pp 229533.I1RR24
20723 229533.A0A0E0S134 pp 229533.I1RTC7
20724 229533.A0A0E0S134 pp 229533.Q4I5V2
20725 229533.A0A0E0S134 pp 229533.I1RJV3
20726 229533.A0A0E0S134 pp 229533.I1S7A8
20727 229533.A0A0E0S134 pp 229533.I1RPA4
20728 229533.A0A0E0S134 pp 229533.A0A1C3YKY0
20729 229533.A0A0E0S134 pp 229533.I1S0B7
20730 229533.A0A0E0S134 pp 229533.I1RN18
20731 229533.A0A0E0S134 pp 229533.V6RTJ8
20732 229533.A0A0E0S134 pp 229533.I1RFL4

20733	229533.A0A0E0S134	pp	229533.I1RBW0
20734	229533.A0A0E0S134	pp	229533.I1RCH5
20735	229533.A0A0E0S134	pp	229533.A0A1C3YM63
20736	229533.A0A0E0S134	pp	229533.I1RRG3
20737	229533.A0A0E0S134	pp	229533.I1S7L6
20738	229533.A0A0E0S134	pp	229533.I1RQE7
20739	229533.A0A0E0S134	pp	229533.I1RX25
20740	229533.A0A0E0S134	pp	229533.I1RBC9
20741	229533.A0A0E0S134	pp	229533.I1RVX0
20742	229533.A0A0E0S134	pp	229533.I1RPN4
20743	229533.A0A0E0S134	pp	229533.I1RB92
20744	229533.A0A0E0S134	pp	229533.I1RW25
20745	229533.A0A0E0S134	pp	229533.I1RSQ7
20746	229533.A0A0E0S134	pp	229533.V6QW22
20747	229533.A0A0E0S134	pp	229533.A0A1C3YLR6
20748	229533.A0A0E0S134	pp	229533.I1RZM8
20749	229533.A0A0E0S134	pp	229533.V6RRH2
20750	229533.A0A0E0S134	pp	229533.I1RNL7
20751	229533.A0A0E0S134	pp	229533.I1RXF0
20752	229533.A0A0E0S134	pp	229533.I1RD17
20753	229533.A0A0E0S134	pp	229533.A0A1C3YIP5
20754	229533.A0A0E0S134	pp	229533.V6RCZ8
20755	229533.A0A0E0S134	pp	229533.Q4I624
20756	229533.A0A0E0S134	pp	229533.I1S235
20757	229533.A0A0E0S134	pp	229533.I1RVQ0
20758	229533.A0A0E0S134	pp	229533.I1RRC6
20759	229533.A0A0E0S134	pp	229533.I1RD52
20760	229533.A0A0E0S134	pp	229533.A0A0E0SFN0
20761	229533.A0A0E0S134	pp	229533.A0A0E0SLM3
20762	229533.A0A0E0S134	pp	229533.I1RQ14
20763	229533.A0A0E0S134	pp	229533.V6R1K4
20764	229533.A0A0E0S134	pp	229533.V6RCG5
20765	229533.A0A0E0S134	pp	229533.I1RXD7
20766	229533.A0A0E0S134	pp	229533.A0A1C3YKB0
20767	229533.A0A0E0S134	pp	229533.V6QUE6
20768	229533.A0A0E0S134	pp	229533.I1RG14
20769	229533.A0A0E0S134	pp	229533.I1RQ41
20770	229533.A0A0E0S134	pp	229533.A0A1C3YHX6
20771	229533.A0A0E0S134	pp	229533.A0A1C3YHS4
20772	229533.A0A0E0S134	pp	229533.I1S0L3
20773	229533.A0A0E0S134	pp	229533.A0A0E0SBE9
20774	229533.A0A0E0S134	pp	229533.I1RRV4
20775	229533.A0A0E0S134	pp	229533.I1RAZ3
20776	229533.A0A0E0S134	pp	229533.I1RCG4
20777	229533.A0A0E0S134	pp	229533.A0A1C3YK68
20778	229533.A0A0E0S134	pp	229533.A0A1C3YI30
20779	229533.A0A0E0S134	pp	229533.I1S5B0
20780	229533.A0A0E0S134	pp	229533.I1RBT1
20781	229533.A0A0E0S134	pp	229533.A0A1C3YJ14
20782	229533.A0A0E0S134	pp	229533.I1RJU3
20783	229533.A0A0E0S134	pp	229533.I1RBL7
20784	229533.A0A0E0S134	pp	229533.I1RWJ8
20785	229533.A0A0E0S134	pp	229533.I1RW99
20786	229533.A0A0E0S134	pp	229533.I1RXX1
20787	229533.A0A0E0S134	pp	229533.I1S835
20788	229533.A0A0E0S134	pp	229533.A0A1C3YK11
20789	229533.A0A0E0S134	pp	229533.I1RM67
20790	229533.A0A0E0S134	pp	229533.I1RVZ9
20791	229533.A0A0E0S134	pp	229533.I1RZJ5
20792	229533.A0A0E0S134	pp	229533.I1RK95
20793	229533.A0A0E0S134	pp	229533.A0A1C3YK34
20794	229533.A0A0E0S134	pp	229533.A0A1C3YKV3
20795	229533.A0A0E0S134	pp	229533.A0A1C3YIK4
20796	229533.A0A0E0S134	pp	229533.I1RS18
20797	229533.A0A0E0S134	pp	229533.I1RMG8
20798	229533.A0A0E0S134	pp	229533.I1S4Q4
20799	229533.A0A0E0S134	pp	229533.I1RCN8
20800	229533.A0A0E0S134	pp	229533.I1RM35
20801	229533.A0A0E0S134	pp	229533.I1RC16
20802	229533.A0A0E0S134	pp	229533.I1REK8
20803	229533.I1RUP8	pp	229533.I1S5B5
20804	229533.I1RUP8	pp	229533.I1RW24
20805	229533.I1RTH7	pp	229533.I1S1F3

20806 229533.A0A1C3YLZ8 pp 229533.I1RNG4
20807 229533.A0A1C3YLZ8 pp 229533.I1RBD7
20808 229533.A0A1C3YLZ8 pp 229533.I1S0T4
20809 229533.A0A1C3YLZ8 pp 229533.I1RWF9
20810 229533.A0A1C3YLZ8 pp 229533.I1S5I8
20811 229533.A0A1C3YLZ8 pp 229533.I1RSS7
20812 229533.A0A1C3YLZ8 pp 229533.A0A1C3YMC1
20813 229533.A0A1C3YLZ8 pp 229533.I1RHG5
20814 229533.A0A1C3YLZ8 pp 229533.I1S236
20815 229533.A0A1C3YLZ8 pp 229533.I1S1W8
20816 229533.A0A1C3YLZ8 pp 229533.I1RWW7
20817 229533.A0A1C3YLZ8 pp 229533.I1S237
20818 229533.A0A1C3YLZ8 pp 229533.I1RBE3
20819 229533.A0A1C3YLZ8 pp 229533.I1S3G4
20820 229533.A0A1C3YLZ8 pp 229533.I1RVP5
20821 229533.A0A1C3YLZ8 pp 229533.I1RBH9
20822 229533.A0A1C3YLZ8 pp 229533.I1RBD6
20823 229533.A0A1C3YLZ8 pp 229533.I1RHI1
20824 229533.A0A1C3YLX0 pp 229533.I1RBR1
20825 229533.A0A1C3YLX0 pp 229533.I1RVX0
20826 229533.A0A1C3YLX0 pp 229533.I1RCH5
20827 229533.A0A1C3YLX0 pp 229533.Q4IJK2
20828 229533.A0A1C3YLX0 pp 229533.I1RGB2
20829 229533.A0A1C3YLX0 pp 229533.I1RQD2
20830 229533.A0A1C3YLX0 pp 229533.I1RRV3
20831 229533.A0A1C3YLX0 pp 229533.I1RB26
20832 229533.A0A1C3YLX0 pp 229533.I1S1B2
20833 229533.A0A1C3YLX0 pp 229533.I1S821
20834 229533.A0A1C3YLX0 pp 229533.I1RRM3
20835 229533.A0A1C3YLX0 pp 229533.V6RCZ8
20836 229533.A0A1C3YLX0 pp 229533.I1RCR8
20837 229533.A0A1C3YLX0 pp 229533.I1RZM8
20838 229533.A0A1C3YLX0 pp 229533.I1RPU2
20839 229533.A0A1C3YLX0 pp 229533.I1RKJ4
20840 229533.A0A1C3YLX0 pp 229533.I1RQ85
20841 229533.A0A1C3YLX0 pp 229533.I1RSF1
20842 229533.A0A1C3YLX0 pp 229533.I1S1W9
20843 229533.A0A1C3YLX0 pp 229533.I1RAG7
20844 229533.A0A1C3YLX0 pp 229533.I1RZV3
20845 229533.A0A1C3YLX0 pp 229533.I1RM37
20846 229533.A0A1C3YLX0 pp 229533.I1RPE0
20847 229533.A0A1C3YLX0 pp 229533.I1RXC4
20848 229533.A0A1C3YLX0 pp 229533.I1RR24
20849 229533.A0A1C3YLX0 pp 229533.I1REC5
20850 229533.A0A1C3YLX0 pp 229533.I1RA11
20851 229533.A0A1C3YLX0 pp 229533.I1RRI1
20852 229533.A0A1C3YLX0 pp 229533.I1S5R1
20853 229533.A0A1C3YLX0 pp 229533.I1RJU3
20854 229533.A0A1C3YLX0 pp 229533.I1S2B8
20855 229533.A0A1C3YLX0 pp 229533.I1RZ39
20856 229533.A0A1C3YLX0 pp 229533.I1R9M0
20857 229533.A0A1C3YLX0 pp 229533.I1RSZ7
20858 229533.A0A1C3YLX0 pp 229533.I1RC34
20859 229533.A0A1C3YLX0 pp 229533.I1RGA4
20860 229533.A0A1C3YLX0 pp 229533.I1RXD7
20861 229533.A0A1C3YLX0 pp 229533.I1RTB3
20862 229533.A0A1C3YLX0 pp 229533.I1RNB2
20863 229533.A0A1C3YLX0 pp 229533.I1RT40
20864 229533.A0A1C3YLX0 pp 229533.I1RTD5
20865 229533.A0A1C3YLX0 pp 229533.I1RAW0
20866 229533.A0A1C3YLX0 pp 229533.I1RSA5
20867 229533.A0A1C3YLX0 pp 229533.I1RD52
20868 229533.A0A1C3YLX0 pp 229533.I1RWR7
20869 229533.A0A1C3YLX0 pp 229533.I1RDF7
20870 229533.A0A1C3YLX0 pp 229533.I1S0W4
20871 229533.A0A1C3YLX0 pp 229533.I1RE89
20872 229533.A0A1C3YLX0 pp 229533.I1RVQ3
20873 229533.A0A1C3YLX0 pp 229533.I1RXD6
20874 229533.A0A1C3YLX0 pp 229533.I1S4S7
20875 229533.A0A1C3YLX0 pp 229533.I1RZK5
20876 229533.A0A1C3YLX0 pp 229533.I1RQ33
20877 229533.A0A1C3YLX0 pp 229533.I1RYI5
20878 229533.A0A1C3YLX0 pp 229533.I1RX95

20879 229533.A0A1C3YLX0 pp 229533.I1RCR1
20880 229533.A0A1C3YLX0 pp 229533.I1RVZ2
20881 229533.A0A1C3YLX0 pp 229533.I1RJN9
20882 229533.A0A1C3YLX0 pp 229533.I1RTA1
20883 229533.I1RKR5 pp 229533.I1RMX6
20884 229533.I1RKR5 pp 229533.I1RNU7
20885 229533.I1RKR5 pp 229533.I1RPT0
20886 229533.I1RKR5 pp 229533.V6RLM7
20887 229533.I1RKR5 pp 229533.I1RU18
20888 229533.I1RKR5 pp 229533.I1RND4
20889 229533.I1RKR5 pp 229533.I1RSJ2
20890 229533.I1RKR5 pp 229533.I1RSV4
20891 229533.I1RKR5 pp 229533.Q4I1Y5
20892 229533.I1RKR5 pp 229533.I1RVV3
20893 229533.I1RKR5 pp 229533.V6RV57
20894 229533.I1RKR5 pp 229533.I1RYK0
20895 229533.I1RKR5 pp 229533.I1RP86
20896 229533.I1REU7 pp 229533.I1RX27
20897 229533.I1REU7 pp 229533.I1S8A6
20898 229533.A0A0E0SGH7 pp 229533.I1RMQ6
20899 229533.A0A0E0SGH7 pp 229533.I1S1F3
20900 229533.A0A0E0SGH7 pp 229533.I1RAM9
20901 229533.I1RWD3 pp 229533.I1S471
20902 229533.I1RWD3 pp 229533.V6RIS2
20903 229533.I1RWD3 pp 229533.Q4IJH1
20904 229533.I1RWD3 pp 229533.Q4IJ56
20905 229533.I1RWD3 pp 229533.V6R3J1
20906 229533.I1RWD3 pp 229533.V6RY33
20907 229533.I1RWD3 pp 229533.Q4IPI1
20908 229533.I1RWD3 pp 229533.I1S087
20909 229533.I1RWD3 pp 229533.V6R227
20910 229533.I1RWD3 pp 229533.I1S246
20911 229533.I1RWD3 pp 229533.I1RZ40
20912 229533.I1RWD3 pp 229533.Q4HW67
20913 229533.I1RWD3 pp 229533.I1S2C0
20914 229533.I1RWD3 pp 229533.I1RZG7
20915 229533.I1RWD3 pp 229533.Q4IF76
20916 229533.I1RWD3 pp 229533.Q4HZ68
20917 229533.I1RX27 pp 229533.I1RZ41
20918 229533.I1RX27 pp 229533.Q4I785
20919 229533.I1RX27 pp 229533.V6R3U5
20920 229533.I1S084 pp 229533.V6RQW4
20921 229533.I1S084 pp 229533.I1S8G4
20922 229533.I1S084 pp 229533.I1S3K8
20923 229533.I1S084 pp 229533.I1S6P5
20924 229533.I1S084 pp 229533.O74638
20925 229533.I1S084 pp 229533.I1S1P8
20926 229533.I1S084 pp 229533.V6RUB1
20927 229533.I1S084 pp 229533.Q4HXF0
20928 229533.I1S084 pp 229533.V6R057
20929 229533.I1S084 pp 229533.V6R1K4
20930 229533.I1S084 pp 229533.I1S216
20931 229533.I1RIM5 pp 229533.I1RRG1
20932 229533.I1RIM5 pp 229533.I1S694
20933 229533.I1RIM5 pp 229533.I1RZA8
20934 229533.I1RIM5 pp 229533.I1RY16
20935 229533.I1RC61 pp 229533.I1RSS7
20936 229533.I1RC61 pp 229533.I1S3G4
20937 229533.I1RC61 pp 229533.I1RPA7
20938 229533.I1RC61 pp 229533.I1RW00
20939 229533.I1RC61 pp 229533.Q4IR09
20940 229533.I1RC61 pp 229533.I1RP97
20941 229533.I1RC61 pp 229533.I1RMC5
20942 229533.I1RC61 pp 229533.V6RTJ8
20943 229533.I1RC61 pp 229533.I1RJU6
20944 229533.I1RC61 pp 229533.I1RZ83
20945 229533.I1RC61 pp 229533.I1RYJ6
20946 229533.I1RC61 pp 229533.I1RIX0
20947 229533.I1RC61 pp 229533.I1RJU3
20948 229533.I1RC61 pp 229533.V6RXE7
20949 229533.I1RC61 pp 229533.I1RR19
20950 229533.I1RC61 pp 229533.I1RWS4
20951 229533.I1RC61 pp 229533.I1RSJ2

20952 229533.I1RC61 pp 229533.I1RWF9
20953 229533.I1RC61 pp 229533.I1RW43
20954 229533.I1RC61 pp 229533.I1S064
20955 229533.I1RC61 pp 229533.I1RDE7
20956 229533.I1RC61 pp 229533.I1RPK0
20957 229533.I1RC61 pp 229533.I1RZ39
20958 229533.I1RC61 pp 229533.I1RXB6
20959 229533.I1RC61 pp 229533.I1RZ21
20960 229533.I1RC61 pp 229533.I1RMM8
20961 229533.I1RC61 pp 229533.I1S1W8
20962 229533.I1RC61 pp 229533.I1RQY0
20963 229533.I1RC61 pp 229533.I1S8E0
20964 229533.I1RC61 pp 229533.I1S3F7
20965 229533.I1RC61 pp 229533.I1RKZ8
20966 229533.I1RC61 pp 229533.I1S0Y7
20967 229533.I1RC61 pp 229533.I1RUP2
20968 229533.I1RC61 pp 229533.I1RW69
20969 229533.I1RC61 pp 229533.I1RSU2
20970 229533.I1RC61 pp 229533.I1RMK7
20971 229533.I1RC61 pp 229533.I1RE47
20972 229533.I1RC61 pp 229533.I1RD22
20973 229533.I1RC61 pp 229533.I1RZ52
20974 229533.I1RC61 pp 229533.I1RQG7
20975 229533.I1RC61 pp 229533.I1RYU0
20976 229533.I1RC61 pp 229533.I1RPW1
20977 229533.I1RC61 pp 229533.I1S5R6
20978 229533.I1RC61 pp 229533.I1S3V2
20979 229533.I1RC61 pp 229533.I1RSP1
20980 229533.I1RC61 pp 229533.I1RM55
20981 229533.I1RC61 pp 229533.I1RCN8
20982 229533.I1RC61 pp 229533.I1RIY0
20983 229533.I1RC61 pp 229533.I1RCG5
20984 229533.I1RC61 pp 229533.I1RZC4
20985 229533.I1RC61 pp 229533.I1S4Q4
20986 229533.I1RC61 pp 229533.I1RMC2
20987 229533.I1RC61 pp 229533.I1SAJ3
20988 229533.I1RC61 pp 229533.I1REK8
20989 229533.I1RC61 pp 229533.I1RMW4
20990 229533.I1RC61 pp 229533.I1RK03
20991 229533.I1RC61 pp 229533.V6RM02
20992 229533.I1S9I5 pp 229533.Q4IJK2
20993 229533.A0A098DQJ2 pp 229533.I1RX27
20994 229533.A0A098DQJ2 pp 229533.I1RNG4
20995 229533.A0A098DQJ2 pp 229533.I1RAU2
20996 229533.I1RHM4 pp 229533.I1S0M9
20997 229533.I1RHM4 pp 229533.I1RSI1
20998 229533.I1RHM4 pp 229533.Q4IJU0
20999 229533.I1RHM4 pp 229533.I1S050
21000 229533.I1RHM4 pp 229533.I1RZJ6
21001 229533.I1RHM4 pp 229533.I1RM09
21002 229533.I1RHM4 pp 229533.I1RQJ5
21003 229533.I1RHM4 pp 229533.I1RT98
21004 229533.I1RHM4 pp 229533.I1RZR6
21005 229533.I1RHM4 pp 229533.I1RNU6
21006 229533.I1RHM4 pp 229533.Q4HZ47
21007 229533.I1RHM4 pp 229533.I1S0J1
21008 229533.I1RHM4 pp 229533.Q4IAS1
21009 229533.I1RHM4 pp 229533.I1RJS3
21010 229533.I1RHM4 pp 229533.Q5I2J3
21011 229533.I1RHM4 pp 229533.I1RLV9
21012 229533.I1RHM4 pp 229533.I1RQ65
21013 229533.I1RHM4 pp 229533.Q4HYB8
21014 229533.I1RHM4 pp 229533.I1RSJ4
21015 229533.I1RHM4 pp 229533.I1RWE1
21016 229533.I1RHM4 pp 229533.I1RZP8
21017 229533.I1RHM4 pp 229533.I1RLE7
21018 229533.I1RHM4 pp 229533.I1S9U2
21019 229533.I1RHM4 pp 229533.I1RKJ6
21020 229533.I1RHM4 pp 229533.I1RS41
21021 229533.I1RHM4 pp 229533.I1RMB7
21022 229533.I1RHM4 pp 229533.I1RNW8
21023 229533.I1RHM4 pp 229533.I1RUG1
21024 229533.I1RPL7 pp 229533.I1RS35

229533.I1RFF8	pp	229533.I1RMX6
229533.I1RFF8	pp	229533.I1RND4
229533.I1RFF8	pp	229533.I1RSX9
229533.I1RFF8	pp	229533.I1RUP5
229533.I1RFF8	pp	229533.I1RNG4
229533.I1RFF8	pp	229533.I1RJS0
229533.I1RFF8	pp	229533.I1RRG1
229533.I1REA3	pp	229533.I1RMH2
229533.I1REA3	pp	229533.I1RX64
229533.I1REA3	pp	229533.I1RF60
229533.I1REA3	pp	229533.I1S0K9
229533.I1REA3	pp	229533.I1RQ14
229533.I1RCJ6	pp	229533.I1RCJ7
229533.I1S1A0	pp	229533.I1S1F3
229533.A0A098DDY0	pp	229533.I1RCG5
229533.A0A098DDY0	pp	229533.I1RYW3
229533.A0A098DDY0	pp	229533.V6RN31
229533.A0A098DDY0	pp	229533.I1S2C4
229533.A0A098DDY0	pp	229533.A0A1C3YKW4
229533.A0A098DDY0	pp	229533.I1RM55
229533.A0A098DDY0	pp	229533.I1RMX6
229533.A0A098DDY0	pp	229533.A0A0E0SBC4
229533.A0A098DDY0	pp	229533.A0A0E0S2K9
229533.A0A098DDY0	pp	229533.I1RBT2
229533.A0A098DDY0	pp	229533.I1RNU7
229533.A0A098DDY0	pp	229533.I1RHJ2
229533.A0A098DDY0	pp	229533.A0A0E0SH88
229533.A0A098DDY0	pp	229533.I1RQI4
229533.A0A098DDY0	pp	229533.I1RPS2
229533.A0A098DDY0	pp	229533.I1RRJ3
229533.A0A098DDY0	pp	229533.A0A1C3YLB6
229533.A0A098DDY0	pp	229533.I1S149
229533.A0A098DDY0	pp	229533.A0A098E375
229533.A0A098DDY0	pp	229533.A0A1C3YHP2
229533.A0A098DDY0	pp	229533.I1S147
229533.A0A098DDY0	pp	229533.I1RND4
229533.A0A098DDY0	pp	229533.I1S4A5
229533.A0A098DDY0	pp	229533.I1S3H6
229533.A0A098DDY0	pp	229533.I1RXN7
229533.A0A098DDY0	pp	229533.V6RLM7
229533.A0A098DDY0	pp	229533.I1RAE2
229533.A0A098DDY0	pp	229533.A0A0E0RU55
229533.A0A098DDY0	pp	229533.A0A0E0SAG8
229533.A0A098DDY0	pp	229533.I1RGX1
229533.A0A098DDY0	pp	229533.I1RDE7
229533.A0A098DDY0	pp	229533.I1RBY4
229533.A0A098DDY0	pp	229533.I1RGC3
229533.A0A098DDY0	pp	229533.I1RDP6
229533.A0A098DDY0	pp	229533.I1RWF5
229533.A0A098DDY0	pp	229533.A0A1C3YI53
229533.A0A098DDY0	pp	229533.I1RJQ5
229533.A0A098DDY0	pp	229533.A0A098DJ03
229533.A0A098DDY0	pp	229533.I1RN54
229533.A0A098DDY0	pp	229533.V6QT89
229533.A0A098DDY0	pp	229533.I1RAS9
229533.A0A098DDY0	pp	229533.I1RN88
229533.A0A098DDY0	pp	229533.I1RCZ8
229533.A0A098DDY0	pp	229533.I1RKB2
229533.A0A098DDY0	pp	229533.I1RT40
229533.A0A098DDY0	pp	229533.I1RA62
229533.A0A098DDY0	pp	229533.I1S148
229533.A0A098DDY0	pp	229533.I1RU18
229533.A0A098DDY0	pp	229533.I1RYK0
229533.A0A098DDY0	pp	229533.A0A098DXD6
229533.A0A098DDY0	pp	229533.I1RC29
229533.A0A098DDY0	pp	229533.I1RFT1
229533.A0A098DDY0	pp	229533.V6RV57
229533.A0A098DDY0	pp	229533.I1RM54
229533.A0A098DDY0	pp	229533.I1RC31
229533.A0A098DDY0	pp	229533.I1RGB2
229533.A0A098DDY0	pp	229533.I1RPD6
229533.A0A098DDY0	pp	229533.I1RPT0
229533.A0A098DDY0	pp	229533.I1RNG4

21098 229533.A0A098DDY0 pp 229533.I1RAQ4
21099 229533.A0A098DDY0 pp 229533.A0A1C3YJ56
21100 229533.A0A098DDY0 pp 229533.I1RUQ3
21101 229533.A0A098DDY0 pp 229533.I1RDU0
21102 229533.A0A098DDY0 pp 229533.I1RLI4
21103 229533.A0A098DDY0 pp 229533.I1RCM5
21104 229533.A0A098DDY0 pp 229533.A0A1C3YM97
21105 229533.A0A098DDY0 pp 229533.V6RPS5
21106 229533.A0A098DDY0 pp 229533.A0A1C3YLC2
21107 229533.A0A098DDY0 pp 229533.I1RUY4
21108 229533.A0A098DDY0 pp 229533.I1RY12
21109 229533.A0A098DDY0 pp 229533.I1RZ35
21110 229533.A0A098DDY0 pp 229533.Q4HTT2
21111 229533.A0A098DDY0 pp 229533.A0A1C3YJB8
21112 229533.A0A098DDY0 pp 229533.I1RH82
21113 229533.A0A098DDY0 pp 229533.I1RM26
21114 229533.A0A098DDY0 pp 229533.I1RQE0
21115 229533.A0A098DDY0 pp 229533.I1RT41
21116 229533.A0A098DDY0 pp 229533.I1RKR5
21117 229533.A0A098DDY0 pp 229533.I1RC11
21118 229533.A0A098DDY0 pp 229533.I1RVV3
21119 229533.A0A098DDY0 pp 229533.V6RK96
21120 229533.A0A098DDY0 pp 229533.I1RUY5
21121 229533.A0A098DDY0 pp 229533.A0A1C3YK34
21122 229533.A0A098DDY0 pp 229533.I1RYU6
21123 229533.A0A098DDY0 pp 229533.I1RWM4
21124 229533.A0A098DDY0 pp 229533.A0A1C3YL54
21125 229533.A0A098DDY0 pp 229533.I1RIX0
21126 229533.A0A098DDY0 pp 229533.A0A1C3YIK4
21127 229533.A0A098DDY0 pp 229533.I1RR44
21128 229533.A0A098DDY0 pp 229533.I1S325
21129 229533.A0A098DDY0 pp 229533.A0A098E4T8
21130 229533.A0A098DDY0 pp 229533.I1RUM7
21131 229533.A0A098DDY0 pp 229533.I1RJN7
21132 229533.A0A098DDY0 pp 229533.I1RUY6
21133 229533.A0A098DDY0 pp 229533.I1RP86
21134 229533.A0A098DDY0 pp 229533.I1RQE8
21135 229533.A0A098DDY0 pp 229533.A0A098DVZ4
21136 229533.A0A098DDY0 pp 229533.A0A1C3YN26
21137 229533.A0A098DDY0 pp 229533.I1RSJ2
21138 229533.A0A098DDY0 pp 229533.I1RGY2
21139 229533.A0A098DDY0 pp 229533.A0A0E0RNI4
21140 229533.A0A098DDY0 pp 229533.I1RZM4
21141 229533.A0A098DDY0 pp 229533.I1RM78
21142 229533.A0A098DDY0 pp 229533.I1RVX9
21143 229533.A0A098DDY0 pp 229533.I1RSB7
21144 229533.A0A098DDY0 pp 229533.A0A098DR13
21145 229533.A0A098DDY0 pp 229533.V6RTJ8
21146 229533.A0A098DDY0 pp 229533.I1RQ14
21147 229533.I1RSB7 pp 229533.I1RZ13
21148 229533.I1RSB7 pp 229533.I1RVR2
21149 229533.I1RSB7 pp 229533.I1RYK0
21150 229533.I1RSB7 pp 229533.I1RTG2
21151 229533.I1RSB7 pp 229533.V6RLM7
21152 229533.I1RSB7 pp 229533.I1RUY5
21153 229533.I1RSB7 pp 229533.I1RXB6
21154 229533.Q4I7K4 pp 229533.Q4IJ56
21155 229533.Q4I7K4 pp 229533.V6R227
21156 229533.Q4I7K4 pp 229533.V6RIS2
21157 229533.Q4I7K4 pp 229533.V6R3J1
21158 229533.Q4I7K4 pp 229533.Q4IPI1
21159 229533.Q4I7K4 pp 229533.V6RY33
21160 229533.Q4I7K4 pp 229533.Q4IF76
21161 229533.Q4I7K4 pp 229533.Q4IJH1
21162 229533.I1RKG0 pp 229533.I1RKG3
21163 229533.I1RGY2 pp 229533.V6RK40
21164 229533.I1RGY2 pp 229533.I1RU18
21165 229533.I1RGY2 pp 229533.I1RR44
21166 229533.I1RGY2 pp 229533.V6QU63
21167 229533.I1RGY2 pp 229533.I1RHM8
21168 229533.I1RGY2 pp 229533.I1RQI4
21169 229533.I1RGY2 pp 229533.I1RL92
21170 229533.I1RGY2 pp 229533.I1RKR5

229533.I1RGY2 pp 229533.I1RVW8
229533.I1RGY2 pp 229533.I1RUY5
229533.I1RGY2 pp 229533.I1RMX6
229533.I1RGY2 pp 229533.I1RYK0
229533.I1RGY2 pp 229533.I1RX90
229533.I1RGY2 pp 229533.I1RKE1
229533.I1RGY2 pp 229533.I1RPT0
229533.I1RGY2 pp 229533.I1S2R5
229533.I1RGY2 pp 229533.I1RHL0
229533.I1RGY2 pp 229533.I1RP86
229533.I1RGY2 pp 229533.I1RIX0
229533.I1RGY2 pp 229533.I1RHQ0
229533.I1RGY2 pp 229533.I1RI39
229533.I1RGY2 pp 229533.V6RV57
229533.I1RGY2 pp 229533.I1RVV3
229533.I1RGY2 pp 229533.I1RSJ2
229533.I1RGY2 pp 229533.I1RKX2
229533.I1RGY2 pp 229533.V6RW66
229533.I1RK30 pp 229533.I1RQ14
229533.A0A1C3YKI8 pp 229533.I1RRT3
229533.A0A1C3YKI8 pp 229533.I1RK45
229533.A0A1C3YKI8 pp 229533.I1RDJ1
229533.A0A1C3YKI8 pp 229533.I1RZ13
229533.A0A1C3YKI8 pp 229533.I1S9V9
229533.A0A1C3YKI8 pp 229533.I1RKP3
229533.A0A1C3YKI8 pp 229533.I1RPA7
229533.A0A1C3YKI8 pp 229533.I1RB40
229533.A0A1C3YKI8 pp 229533.A0A1C3YL99
229533.A0A1C3YKI8 pp 229533.I1RHI1
229533.A0A1C3YKI8 pp 229533.I1S9G7
229533.A0A1C3YKI8 pp 229533.I1S272
229533.A0A1C3YKI8 pp 229533.I1RBD6
229533.A0A1C3YKI8 pp 229533.I1RDE4
229533.A0A1C3YKI8 pp 229533.I1RBN0
229533.I1RRV3 pp 229533.I1S821
229533.I1RRV3 pp 229533.V6RCZ8
229533.I1RRV3 pp 229533.I1RZM8
229533.I1RRV3 pp 229533.I1S1W9
229533.I1RRV3 pp 229533.I1RTD5
229533.I1RRV3 pp 229533.I1RZV3
229533.I1RRV3 pp 229533.I1RSF1
229533.I1RRV3 pp 229533.I1RT40
229533.I1RRV3 pp 229533.I1RXD7
229533.I1RRV3 pp 229533.I1RXC4
229533.I1RRV3 pp 229533.I1RTB3
229533.I1RRV3 pp 229533.I1RVQ3
229533.I1RRV3 pp 229533.I1RXD6
229533.I1RRV3 pp 229533.V6R9Z0
229533.I1RRV3 pp 229533.I1S0W4
229533.I1RRV3 pp 229533.I1S1B2
229533.I1RRV3 pp 229533.I1RZK5
229533.I1RRV3 pp 229533.I1RSA5
229533.I1RRV3 pp 229533.I1RWR7
229533.I1RRV3 pp 229533.I1RVZ2
229533.I1RRV3 pp 229533.Q4IJK2
229533.I1RRV3 pp 229533.I1RTA1
229533.I1RRV3 pp 229533.I1RVX0
229533.I1RRV3 pp 229533.I1RX95
229533.I1RRV3 pp 229533.I1S5R1
229533.I1RRV3 pp 229533.I1S4S7
229533.I1RRV3 pp 229533.I1RYI5
229533.I1RRV3 pp 229533.I1RSZ7
229533.I1RRV3 pp 229533.I1S2B8
229533.I1RRV3 pp 229533.I1RZ39
229533.I1RWS7 pp 229533.I1S192
229533.I1RWS7 pp 229533.I1S4T3
229533.I1RWS7 pp 229533.I1RWS8
229533.I1RWS7 pp 229533.I1RX09
229533.I1RWS7 pp 229533.I1S034
229533.I1RIN3 pp 229533.I1RMP5
229533.I1RIN3 pp 229533.I1RPT0
229533.I1RIN3 pp 229533.I1SA27
229533.I1RIN3 pp 229533.I1RJA7

21244	229533.I1RGA1	pp	229533.V6RPN0
21245	229533.I1RGA1	pp	229533.I1S0Y1
21246	229533.I1RGA1	pp	229533.I1RWF4
21247	229533.I1RGA1	pp	229533.I1RMW4
21248	229533.I1RGA1	pp	229533.I1RNK1
21249	229533.I1RGA1	pp	229533.I1RPY0
21250	229533.I1RZT4	pp	229533.I1S1F3
21251	229533.I1RZT4	pp	229533.V6RLW2
21252	229533.I1RSG2	pp	229533.I1RZK6
21253	229533.I1RSG2	pp	229533.I1RX46
21254	229533.I1RSG2	pp	229533.V6R042
21255	229533.I1RSG2	pp	229533.I1RSR8
21256	229533.I1RMM8	pp	229533.I1RZM4
21257	229533.I1RMM8	pp	229533.I1S1W8
21258	229533.I1RMM8	pp	229533.I1RW54
21259	229533.I1RMM8	pp	229533.I1RYL8
21260	229533.I1RMM8	pp	229533.I1RRL5
21261	229533.I1RMM8	pp	229533.I1RYX6
21262	229533.I1RMM8	pp	229533.I1RTG4
21263	229533.I1RMM8	pp	229533.I1RQ14
21264	229533.I1RMM8	pp	229533.I1S334
21265	229533.I1RMM8	pp	229533.I1RYJ7
21266	229533.I1RMM8	pp	229533.I1S3K8
21267	229533.I1RMM8	pp	229533.I1RZ16
21268	229533.I1RMM8	pp	229533.I1RYM6
21269	229533.I1RMM8	pp	229533.I1S0A0
21270	229533.I1RMM8	pp	229533.Q4I2D7
21271	229533.I1RMM8	pp	229533.I1RQ44
21272	229533.I1RMM8	pp	229533.V6RK96
21273	229533.I1RMM8	pp	229533.I1RQE1
21274	229533.I1RMM8	pp	229533.I1RQE8
21275	229533.I1RMM8	pp	229533.I1RP86
21276	229533.I1RMM8	pp	229533.I1RVM5
21277	229533.I1RMM8	pp	229533.I1RMQ4
21278	229533.I1RMM8	pp	229533.I1S8E0
21279	229533.I1RMM8	pp	229533.I1RY49
21280	229533.I1RMM8	pp	229533.I1RVX9
21281	229533.I1RMM8	pp	229533.I1S2M0
21282	229533.I1RMM8	pp	229533.I1RRC4
21283	229533.I1RMM8	pp	229533.I1S4S4
21284	229533.I1RMM8	pp	229533.I1RXB6
21285	229533.I1RMM8	pp	229533.I1RRN4
21286	229533.I1RMM8	pp	229533.I1RW69
21287	229533.I1RMM8	pp	229533.I1RWX7
21288	229533.I1RMM8	pp	229533.I1RYI7
21289	229533.I1RMM8	pp	229533.I1S4Q4
21290	229533.I1RMM8	pp	229533.I1RWF9
21291	229533.I1RMM8	pp	229533.V6RQW4
21292	229533.I1RMM8	pp	229533.I1RX72
21293	229533.I1RMM8	pp	229533.I1S9Y1
21294	229533.I1RMM8	pp	229533.I1RQE0
21295	229533.I1RMM8	pp	229533.I1RWM4
21296	229533.I1RMM8	pp	229533.I1S0N3
21297	229533.I1RMM8	pp	229533.I1S4B7
21298	229533.I1RMM8	pp	229533.I1RYU0
21299	229533.I1RMM8	pp	229533.I1S3G4
21300	229533.I1RMM8	pp	229533.I1S3H8
21301	229533.I1RMM8	pp	229533.I1RPD6
21302	229533.I1RMM8	pp	229533.I1RNG4
21303	229533.I1RMM8	pp	229533.I1S1K6
21304	229533.I1RMM8	pp	229533.I1RNB4
21305	229533.I1RMM8	pp	229533.I1RZN1
21306	229533.I1RMM8	pp	229533.V6QV31
21307	229533.I1RMM8	pp	229533.I1RZ21
21308	229533.I1RMM8	pp	229533.I1S9C6
21309	229533.I1RMM8	pp	229533.I1RZQ1
21310	229533.I1RMM8	pp	229533.I1RVR2
21311	229533.I1RMM8	pp	229533.I1RP97
21312	229533.I1RMM8	pp	229533.I1RPZ5
21313	229533.I1RMM8	pp	229533.I1RVZ3
21314	229533.I1RMM8	pp	229533.I1RSQ4
21315	229533.I1RMM8	pp	229533.I1RNB8
21316	229533.I1RMM8	pp	229533.I1RRJ3

21317	229533.I1RMM8	pp	229533.I1RZ29
21318	229533.I1RMM8	pp	229533.I1RSU2
21319	229533.I1RMM8	pp	229533.V6RLM7
21320	229533.I1RMM8	pp	229533.V6RPQ8
21321	229533.I1RMM8	pp	229533.I1RND4
21322	229533.I1RMM8	pp	229533.V6QT89
21323	229533.I1RMM8	pp	229533.I1S4B6
21324	229533.I1RMM8	pp	229533.I1RZR5
21325	229533.I1RMM8	pp	229533.I1S3V2
21326	229533.I1RMM8	pp	229533.I1RXY7
21327	229533.I1RMM8	pp	229533.I1RW43
21328	229533.I1RMM8	pp	229533.I1RZT0
21329	229533.I1RMM8	pp	229533.I1S5R6
21330	229533.I1RMM8	pp	229533.V6RSF3
21331	229533.I1RMM8	pp	229533.I1RSL5
21332	229533.I1RMM8	pp	229533.V6RN69
21333	229533.I1RMM8	pp	229533.I1S0I3
21334	229533.I1RMM8	pp	229533.I1RZ70
21335	229533.I1RMM8	pp	229533.I1RT76
21336	229533.I1RMM8	pp	229533.V6RM02
21337	229533.I1S1P8	pp	229533.I1S8G4
21338	229533.I1S1P8	pp	229533.V6R1K4
21339	229533.I1S1P8	pp	229533.I1S3K8
21340	229533.I1S1P8	pp	229533.V6R057
21341	229533.I1S1P8	pp	229533.I1S6P5
21342	229533.I1S1P8	pp	229533.V6RUB1
21343	229533.I1S1P8	pp	229533.Q4HXF0
21344	229533.I1S1P8	pp	229533.V6RQW4
21345	229533.I1S1P8	pp	229533.I1S216
21346	229533.I1S1P8	pp	229533.O74638
21347	229533.I1S0C1	pp	229533.V6RIS2
21348	229533.I1S0C1	pp	229533.V6R227
21349	229533.I1S0C1	pp	229533.V6RY33
21350	229533.I1S0C1	pp	229533.I1S471
21351	229533.I1S0C1	pp	229533.V6R3J1
21352	229533.I1S0C1	pp	229533.I1S2C0
21353	229533.I1RP86	pp	229533.I1RYA0
21354	229533.I1RP86	pp	229533.I1RRI2
21355	229533.I1RP86	pp	229533.I1S274
21356	229533.I1RP86	pp	229533.I1S5C5
21357	229533.I1RP86	pp	229533.I1RYW3
21358	229533.I1RP86	pp	229533.V6RRM0
21359	229533.I1RP86	pp	229533.I1RSU2
21360	229533.I1RP86	pp	229533.I1RVW8
21361	229533.I1RP86	pp	229533.V6RRH2
21362	229533.I1RP86	pp	229533.I1RPS2
21363	229533.I1RP86	pp	229533.I1RX90
21364	229533.I1RP86	pp	229533.I1S149
21365	229533.I1RP86	pp	229533.Q4I1Y5
21366	229533.I1RP86	pp	229533.I1RW43
21367	229533.I1RP86	pp	229533.I1RTA0
21368	229533.I1RP86	pp	229533.I1S2B9
21369	229533.I1RP86	pp	229533.O74638
21370	229533.I1RP86	pp	229533.I1RQI4
21371	229533.I1RP86	pp	229533.V6R382
21372	229533.I1RP86	pp	229533.I1S272
21373	229533.I1RP86	pp	229533.I1S0V7
21374	229533.I1RP86	pp	229533.I1S256
21375	229533.I1RP86	pp	229533.V6RLM7
21376	229533.I1RP86	pp	229533.I1RRT8
21377	229533.I1RP86	pp	229533.I1RPK0
21378	229533.I1RP86	pp	229533.I1S147
21379	229533.I1RP86	pp	229533.I1S065
21380	229533.I1RP86	pp	229533.I1RWF5
21381	229533.I1RP86	pp	229533.I1S9Y1
21382	229533.I1RP86	pp	229533.I1RSF1
21383	229533.I1RP86	pp	229533.I1RYC8
21384	229533.I1RP86	pp	229533.V6RUY9
21385	229533.I1RP86	pp	229533.I1RZ78
21386	229533.I1RP86	pp	229533.I1RZ29
21387	229533.I1RP86	pp	229533.I1S3G4
21388	229533.I1RP86	pp	229533.I1RSQ4
21389	229533.I1RP86	pp	229533.I1RR68

21390 229533.I1RP86 pp 229533.I1RSS7
21391 229533.I1RP86 pp 229533.I1S6P5
21392 229533.I1RP86 pp 229533.I1RU18
21393 229533.I1RP86 pp 229533.V6RUB1
21394 229533.I1RP86 pp 229533.I1RSZ7
21395 229533.I1RP86 pp 229533.V6R3J1
21396 229533.I1RP86 pp 229533.I1RZN1
21397 229533.I1RP86 pp 229533.I1S3F7
21398 229533.I1RP86 pp 229533.I1S148
21399 229533.I1RP86 pp 229533.I1RTA1
21400 229533.I1RP86 pp 229533.I1RRT3
21401 229533.I1RP86 pp 229533.I1RVZ3
21402 229533.I1RP86 pp 229533.I1RX72
21403 229533.I1RP86 pp 229533.I1RZ13
21404 229533.I1RP86 pp 229533.I1S8P5
21405 229533.I1RP86 pp 229533.Q4HVQ9
21406 229533.I1RP86 pp 229533.I1RYK0
21407 229533.I1RP86 pp 229533.I1RQ48
21408 229533.I1RP86 pp 229533.V6RV57
21409 229533.I1RP86 pp 229533.I1RZA7
21410 229533.I1RP86 pp 229533.I1RRD0
21411 229533.I1RP86 pp 229533.I1S1F3
21412 229533.I1RP86 pp 229533.I1RPT0
21413 229533.I1RP86 pp 229533.I1RST9
21414 229533.I1RP86 pp 229533.V6QUE6
21415 229533.I1RP86 pp 229533.I1S0V6
21416 229533.I1RP86 pp 229533.I1RY62
21417 229533.I1RP86 pp 229533.I1RQA5
21418 229533.I1RP86 pp 229533.I1RRV4
21419 229533.I1RP86 pp 229533.I1RPD6
21420 229533.I1RP86 pp 229533.I1S0N3
21421 229533.I1RP86 pp 229533.I1RW26
21422 229533.I1RP86 pp 229533.I1RXE6
21423 229533.I1RP86 pp 229533.I1RY12
21424 229533.I1RP86 pp 229533.I1RUY5
21425 229533.I1RP86 pp 229533.I1RQE0
21426 229533.I1RP86 pp 229533.I1RUY4
21427 229533.I1RP86 pp 229533.I1S4Q4
21428 229533.I1RP86 pp 229533.I1RUQ3
21429 229533.I1RP86 pp 229533.I1RWM4
21430 229533.I1RP86 pp 229533.I1S0A0
21431 229533.I1RP86 pp 229533.I1RR44
21432 229533.I1RP86 pp 229533.V6RK96
21433 229533.I1RP86 pp 229533.I1RY49
21434 229533.I1RP86 pp 229533.I1S8F8
21435 229533.I1RP86 pp 229533.V6RPS5
21436 229533.I1RP86 pp 229533.I1RPW2
21437 229533.I1RP86 pp 229533.I1RUM7
21438 229533.I1RP86 pp 229533.I1RVI8
21439 229533.I1RP86 pp 229533.I1RQE8
21440 229533.I1RP86 pp 229533.I1RUY6
21441 229533.I1RP86 pp 229533.I1S1W8
21442 229533.I1RP86 pp 229533.I1RSB7
21443 229533.I1RP86 pp 229533.I1RSJ2
21444 229533.I1RP86 pp 229533.I1RZ16
21445 229533.I1RP86 pp 229533.Q4IR09
21446 229533.I1RP86 pp 229533.I1RRX5
21447 229533.I1RP86 pp 229533.I1RW06
21448 229533.I1RP86 pp 229533.I1RRG3
21449 229533.I1RP86 pp 229533.I1RYL8
21450 229533.I1RP86 pp 229533.I1RZM4
21451 229533.I1RP86 pp 229533.I1RVV3
21452 229533.I1RP86 pp 229533.I1RQE1
21453 229533.I1RP86 pp 229533.V6RN16
21454 229533.I1RP86 pp 229533.I1RVX9
21455 229533.I1RP86 pp 229533.I1RQ14
21456 229533.I1RC94 pp 229533.I1RME6
21457 229533.I1RC94 pp 229533.I1RP27
21458 229533.I1RC94 pp 229533.I1RLU5
21459 229533.I1RC94 pp 229533.I1RSV2
21460 229533.I1RC94 pp 229533.I1SAN9
21461 229533.I1RWN3 pp 229533.V6RLW2
21462 229533.I1RWN3 pp 229533.I1S1F3

21463	229533.I1RA03	pp	229533.I1S984
21464	229533.I1RA03	pp	229533.I1S574
21465	229533.I1RA03	pp	229533.I1RNA1
21466	229533.I1RCG5	pp	229533.I1S9U6
21467	229533.I1RCG5	pp	229533.I1S0V7
21468	229533.I1RCG5	pp	229533.I1S0S5
21469	229533.I1RCG5	pp	229533.I1RN84
21470	229533.I1RCG5	pp	229533.I1RND4
21471	229533.I1RCG5	pp	229533.V6RLM7
21472	229533.I1RCG5	pp	229533.I1RHJ2
21473	229533.I1RCG5	pp	229533.I1RE52
21474	229533.I1RCG5	pp	229533.I1S147
21475	229533.I1RCG5	pp	229533.V6RRM0
21476	229533.I1RCG5	pp	229533.I1RGX1
21477	229533.I1RCG5	pp	229533.I1RSU2
21478	229533.I1RCG5	pp	229533.I1RKB2
21479	229533.I1RCG5	pp	229533.I1S149
21480	229533.I1RCG5	pp	229533.I1RQI4
21481	229533.I1RCG5	pp	229533.I1RMJ3
21482	229533.I1RCG5	pp	229533.I1RWF5
21483	229533.I1RCG5	pp	229533.I1RNU7
21484	229533.I1RCG5	pp	229533.I1RCZ8
21485	229533.I1RCG5	pp	229533.I1S5S8
21486	229533.I1RCG5	pp	229533.I1RYW3
21487	229533.I1RCG5	pp	229533.I1RYK0
21488	229533.I1RCG5	pp	229533.I1RX72
21489	229533.I1RCG5	pp	229533.I1RKB6
21490	229533.I1RCG5	pp	229533.I1RMX6
21491	229533.I1RCG5	pp	229533.Q4HXF0
21492	229533.I1RCG5	pp	229533.I1S024
21493	229533.I1RCG5	pp	229533.I1RWF9
21494	229533.I1RCG5	pp	229533.V6RUY9
21495	229533.I1RCG5	pp	229533.I1RY35
21496	229533.I1RCG5	pp	229533.I1RPD6
21497	229533.I1RCG5	pp	229533.I1RK96
21498	229533.I1RCG5	pp	229533.I1RNB4
21499	229533.I1RCG5	pp	229533.I1RR68
21500	229533.I1RCG5	pp	229533.I1RNG4
21501	229533.I1RCG5	pp	229533.I1SA27
21502	229533.I1RCG5	pp	229533.I1RP97
21503	229533.I1RCG5	pp	229533.I1RTG2
21504	229533.I1RCG5	pp	229533.I1S1F3
21505	229533.I1RCG5	pp	229533.I1S148
21506	229533.I1RCG5	pp	229533.I1RIY2
21507	229533.I1RCG5	pp	229533.I1RGC3
21508	229533.I1RCG5	pp	229533.I1RYW9
21509	229533.I1RCG5	pp	229533.V6RV57
21510	229533.I1RCG5	pp	229533.I1RFI4
21511	229533.I1RCG5	pp	229533.I1RZ29
21512	229533.I1RCG5	pp	229533.I1RG66
21513	229533.I1RCG5	pp	229533.I1RN88
21514	229533.I1RCG5	pp	229533.I1RMD3
21515	229533.I1RCG5	pp	229533.I1RUY6
21516	229533.I1RCG5	pp	229533.I1RU18
21517	229533.I1RCG5	pp	229533.I1RVN1
21518	229533.I1RCG5	pp	229533.I1RUK7
21519	229533.I1RCG5	pp	229533.I1RDE7
21520	229533.I1RCG5	pp	229533.I1RQM9
21521	229533.I1RCG5	pp	229533.I1S3G4
21522	229533.I1RCG5	pp	229533.I1RH91
21523	229533.I1RCG5	pp	229533.I1REI3
21524	229533.I1RCG5	pp	229533.I1S0F3
21525	229533.I1RCG5	pp	229533.V6RPS5
21526	229533.I1RCG5	pp	229533.I1RUY5
21527	229533.I1RCG5	pp	229533.I1RJT5
21528	229533.I1RCG5	pp	229533.I1RNY6
21529	229533.I1RCG5	pp	229533.I1RR44
21530	229533.I1RCG5	pp	229533.I1RKR5
21531	229533.I1RCG5	pp	229533.V6RBN2
21532	229533.I1RCG5	pp	229533.I1RWM4
21533	229533.I1RCG5	pp	229533.I1RH82
21534	229533.I1RCG5	pp	229533.I1RPT0
21535	229533.I1RCG5	pp	229533.I1RRD0

21536 229533.I1RCG5 pp 229533.I1RCG6
21537 229533.I1RCG5 pp 229533.I1RGB2
21538 229533.I1RCG5 pp 229533.I1S0V6
21539 229533.I1RCG5 pp 229533.I1RCV1
21540 229533.I1RCG5 pp 229533.I1RDU0
21541 229533.I1RCG5 pp 229533.I1RCM5
21542 229533.I1RCG5 pp 229533.I1RM26
21543 229533.I1RCG5 pp 229533.I1RCL0
21544 229533.I1RCG5 pp 229533.I1RMW4
21545 229533.I1RCG5 pp 229533.I1RYM5
21546 229533.I1RCG5 pp 229533.I1RY12
21547 229533.I1RCG5 pp 229533.I1RRX5
21548 229533.I1RCG5 pp 229533.I1RQE0
21549 229533.I1RCG5 pp 229533.I1RUY4
21550 229533.I1RCG5 pp 229533.I1RQ14
21551 229533.I1RCG5 pp 229533.I1RMK4
21552 229533.I1RCG5 pp 229533.I1REK1
21553 229533.I1RCG5 pp 229533.I1RP86
21554 229533.I1RCG5 pp 229533.I1RKB5
21555 229533.I1RCG5 pp 229533.I1RGY2
21556 229533.I1RCG5 pp 229533.I1RY16
21557 229533.I1RCG5 pp 229533.I1RVX9
21558 229533.I1RCG5 pp 229533.I1S1W8
21559 229533.I1RCG5 pp 229533.Q4INI2
21560 229533.I1RCG5 pp 229533.I1RNM7
21561 229533.I1RCG5 pp 229533.I1RCM7
21562 229533.I1RCG5 pp 229533.I1RVV3
21563 229533.I1RCG5 pp 229533.I1RSB7
21564 229533.I1RCG5 pp 229533.I1RJN7
21565 229533.I1RCG5 pp 229533.I1RMM8
21566 229533.I1RCG5 pp 229533.Q4IR09
21567 229533.A0A1C3YJ95 pp 229533.I1RRG1
21568 229533.A0A1C3YJ95 pp 229533.I1RJS0
21569 229533.A0A1C3YJ95 pp 229533.I1RNG4
21570 229533.A0A1C3YJ95 pp 229533.I1RMX6
21571 229533.A0A1C3YJ95 pp 229533.I1RSX9
21572 229533.A0A1C3YJ95 pp 229533.I1RUP5
21573 229533.A0A1C3YJ95 pp 229533.I1RND4
21574 229533.I1S2C4 pp 229533.I1S4G3
21575 229533.I1S2C4 pp 229533.V6RCC0
21576 229533.I1S2C4 pp 229533.V6R6H5
21577 229533.I1S2C4 pp 229533.I1S3K8
21578 229533.I1S2C4 pp 229533.V6RY56
21579 229533.I1S2C4 pp 229533.I1S8C1
21580 229533.I1S2C4 pp 229533.V6RWB3
21581 229533.I1S2C4 pp 229533.I1S969
21582 229533.I1S2C4 pp 229533.V6R1K4
21583 229533.I1S2C4 pp 229533.V6R5T7
21584 229533.I1S2C4 pp 229533.I1S2F7
21585 229533.I1S2C4 pp 229533.I1S3T6
21586 229533.I1S2C4 pp 229533.I1S2E2
21587 229533.I1S2C4 pp 229533.Q4I8P5
21588 229533.I1S2C4 pp 229533.Q4I5I4
21589 229533.I1S2C4 pp 229533.V6RHX2
21590 229533.I1S2C4 pp 229533.V6QUE6
21591 229533.I1S2C4 pp 229533.I1SA32
21592 229533.I1S2C4 pp 229533.I1S8D3
21593 229533.I1S2C4 pp 229533.I1S2X5
21594 229533.I1S2C4 pp 229533.I1S3K6
21595 229533.I1S2C4 pp 229533.I1S4D0
21596 229533.I1S2C4 pp 229533.I1SAB4
21597 229533.I1S2C4 pp 229533.I1S8G4
21598 229533.I1S2C4 pp 229533.I1S2Q4
21599 229533.I1S2C4 pp 229533.V6QVL9
21600 229533.I1S2C4 pp 229533.I1S3G4
21601 229533.I1S2C4 pp 229533.I1S968
21602 229533.I1S2C4 pp 229533.V6RCJ5
21603 229533.I1S2C4 pp 229533.V6QZS3
21604 229533.I1S2C4 pp 229533.I1S5G3
21605 229533.I1S2C4 pp 229533.Q4HXF0
21606 229533.I1S2C4 pp 229533.V6R057
21607 229533.I1S2C4 pp 229533.Q4I5W5
21608 229533.I1S2C4 pp 229533.Q4I7T5

21609 229533.I1S2C4 pp 229533.I1S4F4
21610 229533.I1S2C4 pp 229533.V6R2A1
21611 229533.I1S2C4 pp 229533.I1S6V0
21612 229533.I1S2C4 pp 229533.V6QT89
21613 229533.I1S2C4 pp 229533.I1S2C5
21614 229533.I1S2C4 pp 229533.V6RRH2
21615 229533.I1S2C4 pp 229533.I1S7M8
21616 229533.I1S2C4 pp 229533.I1S2H3
21617 229533.I1S2C4 pp 229533.I1SA33
21618 229533.I1S2C4 pp 229533.I1S9Z4
21619 229533.I1S2C4 pp 229533.I1S6H1
21620 229533.I1RFB7 pp 229533.I1RHE5
21621 229533.I1RFB7 pp 229533.I1RHY7
21622 229533.I1RFB7 pp 229533.I1S2L3
21623 229533.A0A1C3YL07 pp 229533.I1RMN6
21624 229533.A0A1C3YL07 pp 229533.I1RRR7
21625 229533.A0A1C3YL07 pp 229533.I1S0Q7
21626 229533.A0A1C3YL07 pp 229533.I1RSI6
21627 229533.A0A1C3YL07 pp 229533.Q4HZ68
21628 229533.A0A1C3YL07 pp 229533.I1RSD8
21629 229533.A0A1C3YL07 pp 229533.I1S1X9
21630 229533.A0A1C3YL07 pp 229533.I1RM96
21631 229533.A0A1C3YL07 pp 229533.I1RPY1
21632 229533.A0A1C3YL07 pp 229533.I1RNU6
21633 229533.A0A1C3YL07 pp 229533.I1R9X7
21634 229533.A0A1C3YL07 pp 229533.I1RC72
21635 229533.A0A1C3YL07 pp 229533.V6RGN5
21636 229533.A0A1C3YL07 pp 229533.I1RCL3
21637 229533.A0A1C3YL07 pp 229533.I1S274
21638 229533.A0A1C3YL07 pp 229533.I1RC03
21639 229533.A0A1C3YL07 pp 229533.I1RTE9
21640 229533.A0A1C3YL07 pp 229533.I1S1B6
21641 229533.A0A1C3YL07 pp 229533.I1RC27
21642 229533.A0A1C3YL07 pp 229533.I1RZF6
21643 229533.A0A1C3YL07 pp 229533.I1RPJ3
21644 229533.A0A1C3YL07 pp 229533.I1RRI2
21645 229533.A0A1C3YL07 pp 229533.I1RC82
21646 229533.A0A1C3YL07 pp 229533.I1RG56
21647 229533.A0A1C3YL07 pp 229533.I1RRT2
21648 229533.A0A1C3YL07 pp 229533.I1RJL4
21649 229533.A0A1C3YL07 pp 229533.I1RB27
21650 229533.A0A1C3YL07 pp 229533.I1S0M9
21651 229533.A0A1C3YL07 pp 229533.I1RLV9
21652 229533.A0A1C3YL07 pp 229533.I1RAF8
21653 229533.A0A1C3YL07 pp 229533.I1RQ65
21654 229533.A0A1C3YL07 pp 229533.Q4IJ56
21655 229533.A0A1C3YL07 pp 229533.I1RRQ3
21656 229533.A0A1C3YL07 pp 229533.I1RRP3
21657 229533.A0A1C3YL07 pp 229533.I1RQK4
21658 229533.A0A1C3YL07 pp 229533.I1RX14
21659 229533.A0A1C3YL07 pp 229533.I1RR72
21660 229533.A0A1C3YL07 pp 229533.I1RS30
21661 229533.A0A1C3YL07 pp 229533.I1RNH6
21662 229533.A0A1C3YL07 pp 229533.I1RZS6
21663 229533.A0A1C3YL07 pp 229533.I1RRJ8
21664 229533.A0A1C3YL07 pp 229533.I1RQ31
21665 229533.A0A1C3YL07 pp 229533.I1RKL3
21666 229533.A0A1C3YL07 pp 229533.I1RX24
21667 229533.A0A1C3YL07 pp 229533.I1RZE5
21668 229533.A0A1C3YL07 pp 229533.I1RNA0
21669 229533.A0A1C3YL07 pp 229533.I1RBG9
21670 229533.A0A1C3YL07 pp 229533.I1RG51
21671 229533.A0A1C3YL07 pp 229533.I1RK03
21672 229533.A0A1C3YL07 pp 229533.Q4I662
21673 229533.A0A1C3YL07 pp 229533.I1RVZ0
21674 229533.A0A1C3YL07 pp 229533.I1RD64
21675 229533.A0A1C3YL07 pp 229533.I1RNK2
21676 229533.A0A1C3YL07 pp 229533.I1RAZ8
21677 229533.A0A1C3YL07 pp 229533.I1RKJ6
21678 229533.A0A1C3YL07 pp 229533.I1RDG0
21679 229533.A0A1C3YL07 pp 229533.I1RK95
21680 229533.A0A1C3YL07 pp 229533.I1S253
21681 229533.A0A1C3YL07 pp 229533.I1S9U2

21682 229533.A0A1C3YL07 pp 229533.I1RM67
21683 229533.A0A1C3YL07 pp 229533.I1RBD2
21684 229533.A0A1C3YL07 pp 229533.I1RQ28
21685 229533.A0A1C3YL07 pp 229533.I1RPU0
21686 229533.A0A1C3YL07 pp 229533.I1RNH1
21687 229533.A0A1C3YL07 pp 229533.I1R9V7
21688 229533.A0A1C3YL07 pp 229533.I1RSR3
21689 229533.A0A1C3YL07 pp 229533.I1RA48
21690 229533.A0A1C3YL07 pp 229533.I1RMK3
21691 229533.A0A1C3YL07 pp 229533.I1RZ99
21692 229533.A0A1C3YL07 pp 229533.V6RK56
21693 229533.A0A1C3YL07 pp 229533.I1RDA0
21694 229533.A0A1C3YL07 pp 229533.I1RT80
21695 229533.A0A1C3YL07 pp 229533.I1RW71
21696 229533.A0A1C3YL07 pp 229533.I1RAC2
21697 229533.A0A1C3YL07 pp 229533.I1RNV6
21698 229533.A0A1C3YL07 pp 229533.I1RQA2
21699 229533.A0A1C3YL07 pp 229533.I1RTF4
21700 229533.A0A1C3YL07 pp 229533.I1RT05
21701 229533.A0A1C3YL07 pp 229533.I1RM06
21702 229533.A0A1C3YL07 pp 229533.V6QYF0
21703 229533.I1S635 pp 229533.V6RKE4
21704 229533.I1S635 pp 229533.V6RQ48
21705 229533.I1S635 pp 229533.Q4ICG5
21706 229533.I1RAR1 pp 229533.V6RUB1
21707 229533.I1RAR1 pp 229533.Q4HXF0
21708 229533.I1RAR1 pp 229533.I1S084
21709 229533.I1RAR1 pp 229533.I1RZA7
21710 229533.I1RAR1 pp 229533.I1RE53
21711 229533.I1RAR1 pp 229533.I1RX22
21712 229533.I1RAR1 pp 229533.I1S1P8
21713 229533.I1RAR1 pp 229533.V6R1K4
21714 229533.I1RAR1 pp 229533.I1RQ14
21715 229533.I1RAR1 pp 229533.I1RZ20
21716 229533.I1RAR1 pp 229533.I1S8G4
21717 229533.I1RAR1 pp 229533.I1RQS9
21718 229533.I1RAR1 pp 229533.I1RZT0
21719 229533.I1RAR1 pp 229533.I1RV23
21720 229533.I1RAR1 pp 229533.O74638
21721 229533.I1RAR1 pp 229533.I1RD80
21722 229533.I1RAR1 pp 229533.I1RS17
21723 229533.I1RAR1 pp 229533.I1RDJ7
21724 229533.I1RAR1 pp 229533.I1RQX9
21725 229533.I1RAR1 pp 229533.I1RMI8
21726 229533.I1RAR1 pp 229533.I1RCV1
21727 229533.I1RAR1 pp 229533.I1RKY2
21728 229533.I1RAR1 pp 229533.I1S6P5
21729 229533.I1RAR1 pp 229533.I1RM55
21730 229533.I1RAR1 pp 229533.I1S3K8
21731 229533.I1RAR1 pp 229533.V6RQW4
21732 229533.I1RAR1 pp 229533.I1S216
21733 229533.I1RAR1 pp 229533.I1RPU8
21734 229533.I1RAR1 pp 229533.V6R057
21735 229533.I1RPH6 pp 229533.I1RZK5
21736 229533.I1RPH6 pp 229533.I1RWR7
21737 229533.I1RPH6 pp 229533.I1RRM3
21738 229533.I1RPH6 pp 229533.I1S5R6
21739 229533.I1RPH6 pp 229533.I1RPU2
21740 229533.I1RPH6 pp 229533.I1RZM8
21741 229533.I1RPH6 pp 229533.I1S2B8
21742 229533.I1RPH6 pp 229533.I1RSZ7
21743 229533.I1RPH6 pp 229533.I1RQ85
21744 229533.I1RPH6 pp 229533.I1RVZ2
21745 229533.I1RPH6 pp 229533.I1S1W9
21746 229533.I1RPH6 pp 229533.I1RZV3
21747 229533.I1RPH6 pp 229533.I1RZ39
21748 229533.I1RPH6 pp 229533.I1RT40
21749 229533.I1RPH6 pp 229533.I1RYI5
21750 229533.I1RPH6 pp 229533.I1S4S7
21751 229533.I1RPH6 pp 229533.I1S5R1
21752 229533.I1RX90 pp 229533.V6RRM0
21753 229533.I1RKJ4 pp 229533.I1RSZ7
21754 229533.I1RKJ4 pp 229533.I1RVZ2

21755 229533.I1RKJ4 pp 229533.I1RTA1
21756 229533.I1RKJ4 pp 229533.I1RX95
21757 229533.I1RKJ4 pp 229533.I1RXC4
21758 229533.I1RKJ4 pp 229533.I1RYI5
21759 229533.I1RKJ4 pp 229533.Q4IJK2
21760 229533.I1RKJ4 pp 229533.I1RM37
21761 229533.I1RKJ4 pp 229533.I1RZV3
21762 229533.I1RKJ4 pp 229533.I1RNB2
21763 229533.I1RKJ4 pp 229533.I1RXD6
21764 229533.I1RKJ4 pp 229533.I1RZM8
21765 229533.I1RKJ4 pp 229533.I1RVX0
21766 229533.I1RKJ4 pp 229533.I1RT40
21767 229533.I1RKJ4 pp 229533.I1S1W9
21768 229533.I1RKJ4 pp 229533.I1S4S7
21769 229533.I1RKJ4 pp 229533.I1RRV3
21770 229533.I1RKJ4 pp 229533.I1RZK5
21771 229533.I1RKJ4 pp 229533.I1S0W4
21772 229533.I1RKJ4 pp 229533.I1S2B8
21773 229533.I1RKJ4 pp 229533.I1RTB3
21774 229533.I1RKJ4 pp 229533.V6R9Z0
21775 229533.I1RKJ4 pp 229533.I1RRM3
21776 229533.I1RKJ4 pp 229533.I1RVQ3
21777 229533.I1RKJ4 pp 229533.I1RPU2
21778 229533.I1RKJ4 pp 229533.I1RXD7
21779 229533.I1RKJ4 pp 229533.I1RSF1
21780 229533.I1RKJ4 pp 229533.I1RWR7
21781 229533.I1RKJ4 pp 229533.I1RSA5
21782 229533.I1RKJ4 pp 229533.I1RQD2
21783 229533.I1RKJ4 pp 229533.V6RCZ8
21784 229533.I1RKJ4 pp 229533.I1RRI1
21785 229533.I1RKJ4 pp 229533.I1RZ39
21786 229533.I1RKJ4 pp 229533.I1RQ85
21787 229533.I1RKJ4 pp 229533.I1RQ33
21788 229533.I1RKJ4 pp 229533.I1RR24
21789 229533.I1RKJ4 pp 229533.I1RTD5
21790 229533.I1RKJ4 pp 229533.I1RPE0
21791 229533.I1RKJ4 pp 229533.I1S5R6
21792 229533.I1RKJ4 pp 229533.I1S821
21793 229533.I1RKJ4 pp 229533.I1S1B2
21794 229533.I1RKJ4 pp 229533.I1S5R1
21795 229533.I1RQG5 pp 229533.Q4HTT1
21796 229533.I1RQ75 pp 229533.Q4IJT0
21797 229533.I1RQ75 pp 229533.Q4I1Y5
21798 229533.I1RQ75 pp 229533.I1S569
21799 229533.I1RQ75 pp 229533.V6RPQ8
21800 229533.I1RQ75 pp 229533.I1RVJ8
21801 229533.I1RQ75 pp 229533.I1RZ29
21802 229533.I1RQ75 pp 229533.I1S272
21803 229533.I1RQ75 pp 229533.I1RZ13
21804 229533.I1RQ75 pp 229533.I1RST0
21805 229533.I1RQ75 pp 229533.I1RRT3
21806 229533.I1RQ75 pp 229533.I1S352
21807 229533.I1RQ75 pp 229533.I1RZ34
21808 229533.I1RQ75 pp 229533.Q4HZE0
21809 229533.I1RQ75 pp 229533.I1RSS1
21810 229533.I1RQ75 pp 229533.I1RZM0
21811 229533.I1RQ75 pp 229533.I1S183
21812 229533.I1RQ75 pp 229533.I1RZV2
21813 229533.I1RQ75 pp 229533.I1RSM8
21814 229533.I1RQ75 pp 229533.I1RU29
21815 229533.I1RQ75 pp 229533.I1RV63
21816 229533.I1RQ75 pp 229533.I1RZ16
21817 229533.I1RQ75 pp 229533.I1RRG3
21818 229533.I1RQ75 pp 229533.I1RW07
21819 229533.I1RQ75 pp 229533.I1RSV4
21820 229533.I1RQ75 pp 229533.V6R4J1
21821 229533.I1RQ75 pp 229533.I1RYL8
21822 229533.I1RQ75 pp 229533.I1RUP0
21823 229533.A0A0E0RU55 pp 229533.I1RNM7
21824 229533.A0A0E0RU55 pp 229533.I1RW54
21825 229533.A0A0E0RU55 pp 229533.A0A1C3YLD3
21826 229533.A0A0E0RU55 pp 229533.I1RSB7
21827 229533.A0A0E0RU55 pp 229533.I1RJX2

21828	229533.A0A0E0RU55	pp	229533.I1RCL4
21829	229533.A0A0E0RU55	pp	229533.Q4IR09
21830	229533.A0A0E0RU55	pp	229533.I1S8C1
21831	229533.A0A0E0RU55	pp	229533.I1RKJ7
21832	229533.A0A0E0RU55	pp	229533.A0A1C3YN26
21833	229533.A0A0E0RU55	pp	229533.I1RQ14
21834	229533.A0A0E0RU55	pp	229533.I1RY49
21835	229533.A0A0E0RU55	pp	229533.I1RFV2
21836	229533.A0A0E0RU55	pp	229533.I1RT33
21837	229533.A0A0E0RU55	pp	229533.I1RMM8
21838	229533.A0A0E0RU55	pp	229533.I1RJN7
21839	229533.A0A0E0RU55	pp	229533.I1RMQ6
21840	229533.A0A0E0RU55	pp	229533.A0A1C3YLC3
21841	229533.A0A0E0RU55	pp	229533.I1RKB5
21842	229533.A0A0E0RU55	pp	229533.I1S8F8
21843	229533.A0A0E0RU55	pp	229533.I1RH82
21844	229533.A0A0E0RU55	pp	229533.I1RCN8
21845	229533.A0A0E0RU55	pp	229533.I1RUY5
21846	229533.A0A0E0RU55	pp	229533.I1RUY6
21847	229533.A0A0E0RU55	pp	229533.I1RP86
21848	229533.A0A0E0RU55	pp	229533.A0A1C3YJR3
21849	229533.A0A0E0RU55	pp	229533.I1RWM4
21850	229533.A0A0E0RU55	pp	229533.I1RA90
21851	229533.A0A0E0RU55	pp	229533.Q4I5G1
21852	229533.A0A0E0RU55	pp	229533.A0A0E0S6V8
21853	229533.A0A0E0RU55	pp	229533.A0A1C3YHV8
21854	229533.A0A0E0RU55	pp	229533.I1RGG8
21855	229533.A0A0E0RU55	pp	229533.I1RMQ4
21856	229533.A0A0E0RU55	pp	229533.I1RC11
21857	229533.A0A0E0RU55	pp	229533.I1RPT0
21858	229533.A0A0E0RU55	pp	229533.I1RYI6
21859	229533.A0A0E0RU55	pp	229533.I1RJY7
21860	229533.A0A0E0RU55	pp	229533.I1RAQ4
21861	229533.A0A0E0RU55	pp	229533.I1RR44
21862	229533.A0A0E0RU55	pp	229533.I1RCM5
21863	229533.A0A0E0RU55	pp	229533.I1RC31
21864	229533.A0A0E0RU55	pp	229533.A0A1C3YKR5
21865	229533.A0A0E0RU55	pp	229533.I1RY62
21866	229533.A0A0E0RU55	pp	229533.I1RQE0
21867	229533.A0A0E0RU55	pp	229533.I1RGX1
21868	229533.A0A0E0RU55	pp	229533.I1RBE7
21869	229533.A0A0E0RU55	pp	229533.A0A1C3YJ26
21870	229533.A0A0E0RU55	pp	229533.A0A1C3YL10
21871	229533.A0A0E0RU55	pp	229533.A0A1C3YJ56
21872	229533.A0A0E0RU55	pp	229533.A0A1C3YKN4
21873	229533.A0A0E0RU55	pp	229533.I1RS47
21874	229533.A0A0E0RU55	pp	229533.V6QT89
21875	229533.A0A0E0RU55	pp	229533.I1RKB2
21876	229533.A0A0E0RU55	pp	229533.I1RX72
21877	229533.A0A0E0RU55	pp	229533.I1RDU0
21878	229533.A0A0E0RU55	pp	229533.A0A1C3YLC2
21879	229533.A0A0E0RU55	pp	229533.I1RUY4
21880	229533.A0A0E0RU55	pp	229533.V6R382
21881	229533.A0A0E0RU55	pp	229533.I1RY12
21882	229533.A0A0E0RU55	pp	229533.I1RWF5
21883	229533.A0A0E0RU55	pp	229533.Q4HXF0
21884	229533.A0A0E0RU55	pp	229533.A0A1C3YL82
21885	229533.A0A0E0RU55	pp	229533.I1RA62
21886	229533.A0A0E0RU55	pp	229533.V6RG79
21887	229533.A0A0E0RU55	pp	229533.V6RLM7
21888	229533.A0A0E0RU55	pp	229533.I1RYK0
21889	229533.A0A0E0RU55	pp	229533.V6RV57
21890	229533.A0A0E0RU55	pp	229533.I1RM54
21891	229533.A0A0E0RU55	pp	229533.I1S256
21892	229533.A0A0E0RU55	pp	229533.I1RUR8
21893	229533.A0A0E0RU55	pp	229533.I1RND4
21894	229533.A0A0E0RU55	pp	229533.I1RX89
21895	229533.A0A0E0RU55	pp	229533.I1RZN1
21896	229533.A0A0E0RU55	pp	229533.I1RSU2
21897	229533.A0A0E0RU55	pp	229533.A0A0E0SI42
21898	229533.A0A0E0RU55	pp	229533.I1RBC7
21899	229533.A0A0E0RU55	pp	229533.I1RNU7
21900	229533.A0A0E0RU55	pp	229533.I1RGC3

21901 229533.A0A0E0RU55 pp 229533.I1RIY2
21902 229533.A0A0E0RU55 pp 229533.V6RGN5
21903 229533.A0A0E0RU55 pp 229533.I1RC29
21904 229533.A0A0E0RU55 pp 229533.I1S3F7
21905 229533.A0A0E0RU55 pp 229533.A0A0E0SH88
21906 229533.A0A0E0RU55 pp 229533.V6QVH8
21907 229533.A0A0E0RU55 pp 229533.V6RRM0
21908 229533.A0A0E0RU55 pp 229533.I1RIH0
21909 229533.A0A0E0RU55 pp 229533.I1RSS4
21910 229533.A0A0E0RU55 pp 229533.O74638
21911 229533.A0A0E0RU55 pp 229533.I1S3V2
21912 229533.A0A0E0RU55 pp 229533.I1RJQ5
21913 229533.A0A0E0RU55 pp 229533.I1RMC5
21914 229533.A0A0E0RU55 pp 229533.A0A1C3YJW4
21915 229533.A0A0E0RU55 pp 229533.A0A1C3YJK3
21916 229533.A0A0E0RU55 pp 229533.I1RZ26
21917 229533.A0A0E0RU55 pp 229533.I1RM55
21918 229533.A0A0E0RU55 pp 229533.I1RQI4
21919 229533.A0A0E0RU55 pp 229533.I1RCG5
21920 229533.A0A0E0RU55 pp 229533.A0A0E0S8Z0
21921 229533.A0A0E0RU55 pp 229533.I1RCH2
21922 229533.I1RIA3 pp 229533.I1RY35
21923 229533.I1RIA3 pp 229533.V6R5G9
21924 229533.I1RIA3 pp 229533.V6RG22
21925 229533.I1S2R0 pp 229533.I1S5R6
21926 229533.I1S2R0 pp 229533.I1S2R1
21927 229533.I1S2R0 pp 229533.I1S5R1
21928 229533.I1S2R0 pp 229533.I1S4S7
21929 229533.A0A1C3YN06 pp 229533.I1RIA3
21930 229533.A0A1C3YN06 pp 229533.I1RIA1
21931 229533.A0A1C3YN06 pp 229533.V6R5G9
21932 229533.I1RMX6 pp 229533.V6RV57
21933 229533.I1RMX6 pp 229533.I1RYK0
21934 229533.I1RMX6 pp 229533.V6RLM7
21935 229533.I1RMX6 pp 229533.I1RP86
21936 229533.I1RMX6 pp 229533.I1RU18
21937 229533.I1RMX6 pp 229533.I1RN21
21938 229533.I1RMX6 pp 229533.I1RVV3
21939 229533.I1RMX6 pp 229533.I1RSJ2
21940 229533.I1RMX6 pp 229533.Q4I1Y5
21941 229533.I1RMX6 pp 229533.I1RND4
21942 229533.I1RMX6 pp 229533.I1RNU7
21943 229533.I1RMX6 pp 229533.I1S3D8
21944 229533.I1RMX6 pp 229533.I1RPT0
21945 229533.A0A1C3YMU6 pp 229533.I1RIA1
21946 229533.A0A1C3YMU6 pp 229533.I1RIA3
21947 229533.A0A1C3YMU6 pp 229533.A0A1C3YN06
21948 229533.I1S9Z4 pp 229533.V6R5T7
21949 229533.I1S9Z4 pp 229533.I1SAJ8
21950 229533.I1S9Z4 pp 229533.V6RCC0
21951 229533.I1S9Z4 pp 229533.V6QUE6
21952 229533.I1S9Z4 pp 229533.Q4IJK2
21953 229533.I1S9Z4 pp 229533.V6RCJ5
21954 229533.I1S9Z4 pp 229533.V6RHX2
21955 229533.I1S9Z4 pp 229533.V6RWB3
21956 229533.I1S9Z4 pp 229533.V6RTJ8
21957 229533.I1S9Z4 pp 229533.V6RLM7
21958 229533.I1S9Z4 pp 229533.V6RMW6
21959 229533.I1S9Z4 pp 229533.V6R042
21960 229533.I1RCD8 pp 229533.I1RQ85
21961 229533.I1RCD8 pp 229533.I1RS18
21962 229533.I1RCD8 pp 229533.I1RRV4
21963 229533.I1RCD8 pp 229533.I1RMQ4
21964 229533.I1RCD8 pp 229533.I1S5B0
21965 229533.I1RCD8 pp 229533.I1RJU3
21966 229533.I1RCD8 pp 229533.I1RQD2
21967 229533.I1RCD8 pp 229533.I1RZV3
21968 229533.I1RCD8 pp 229533.I1RPU2
21969 229533.I1RCD8 pp 229533.I1RWE8
21970 229533.I1RCD8 pp 229533.I1RE01
21971 229533.I1RCD8 pp 229533.I1RZM8
21972 229533.I1RCD8 pp 229533.V6RCZ8
21973 229533.I1RCD8 pp 229533.I1RKZ4

21974	229533.I1RCD8	pp	229533.I1REL7
21975	229533.I1RCD8	pp	229533.Q4I073
21976	229533.I1RCD8	pp	229533.I1RPK0
21977	229533.I1RCD8	pp	229533.I1RM35
21978	229533.I1RCD8	pp	229533.I1REK8
21979	229533.I1RCD8	pp	229533.I1RD22
21980	229533.I1RCD8	pp	229533.I1S5R6
21981	229533.I1RCD8	pp	229533.I1RCR5
21982	229533.I1RCD8	pp	229533.V6R1K4
21983	229533.I1RCD8	pp	229533.V6RBN2
21984	229533.I1RCD8	pp	229533.I1RUG3
21985	229533.I1RCD8	pp	229533.V6R042
21986	229533.I1RCD8	pp	229533.Q4I061
21987	229533.I1RCD8	pp	229533.Q4IJK2
21988	229533.I1RCD8	pp	229533.I1RD52
21989	229533.I1RCD8	pp	229533.I1RSQ3
21990	229533.I1RCD8	pp	229533.V6RMW6
21991	229533.I1RCD8	pp	229533.I1S4S7
21992	229533.I1RCD8	pp	229533.I1RWR7
21993	229533.I1RCD8	pp	229533.I1RPN4
21994	229533.I1RCD8	pp	229533.I1RRM3
21995	229533.I1RCD8	pp	229533.I1RE89
21996	229533.I1RCD8	pp	229533.I1RRP7
21997	229533.I1RCD8	pp	229533.V6RTJ8
21998	229533.I1RCD8	pp	229533.I1RYW3
21999	229533.I1RCD8	pp	229533.I1RCW6
22000	229533.I1RCD8	pp	229533.I1SAN9
22001	229533.I1RCD8	pp	229533.I1RME6
22002	229533.I1RCD8	pp	229533.I1RXD7
22003	229533.I1RCD8	pp	229533.I1RNG4
22004	229533.I1RCD8	pp	229533.I1S1W8
22005	229533.I1RCD8	pp	229533.V6RRH2
22006	229533.I1RCD8	pp	229533.I1RQ33
22007	229533.I1RCD8	pp	229533.I1RJN9
22008	229533.I1RCD8	pp	229533.I1RTA1
22009	229533.I1RCD8	pp	229533.I1RCL4
22010	229533.I1RCD8	pp	229533.I1RVZ2
22011	229533.I1RCD8	pp	229533.I1S4S4
22012	229533.I1RCD8	pp	229533.I1RYI5
22013	229533.I1RCD8	pp	229533.V6R057
22014	229533.I1RCD8	pp	229533.I1RQE7
22015	229533.I1RCD8	pp	229533.I1RCH5
22016	229533.I1RCD8	pp	229533.I1RPP3
22017	229533.I1RCD8	pp	229533.I1RGA4
22018	229533.I1RCD8	pp	229533.I1RT40
22019	229533.I1RCD8	pp	229533.I1RI34
22020	229533.I1RCD8	pp	229533.Q4HXF0
22021	229533.I1RCD8	pp	229533.I1RVX0
22022	229533.I1RCD8	pp	229533.I1S2B8
22023	229533.I1RCD8	pp	229533.I1RJU5
22024	229533.I1RCD8	pp	229533.I1RCN2
22025	229533.I1RCD8	pp	229533.I1RSZ7
22026	229533.I1RCD8	pp	229533.I1S5R1
22027	229533.I1RCD8	pp	229533.I1RL04
22028	229533.I1RCD8	pp	229533.I1S3G4
22029	229533.I1RCD8	pp	229533.I1RRI1
22030	229533.I1RCD8	pp	229533.I1RSQ4
22031	229533.I1RCD8	pp	229533.I1S2H4
22032	229533.I1RCD8	pp	229533.I1RPE0
22033	229533.I1RCD8	pp	229533.I1RZ39
22034	229533.A0A098DLA7	pp	229533.I1S0N3
22035	229533.A0A098DLA7	pp	229533.A0A098DVI8
22036	229533.A0A098DLA7	pp	229533.I1RMW4
22037	229533.A0A098DLA7	pp	229533.I1RYI7
22038	229533.A0A098DLA7	pp	229533.I1RQ14
22039	229533.A0A098DLA7	pp	229533.I1RBE4
22040	229533.A0A098DLA7	pp	229533.I1RA90
22041	229533.A0A098DLA7	pp	229533.I1RYX6
22042	229533.A0A098DLA7	pp	229533.I1RVY2
22043	229533.A0A098DLA7	pp	229533.I1RBE7
22044	229533.A0A098DLA7	pp	229533.I1RCN8
22045	229533.A0A098DLA7	pp	229533.I1RXB6
22046	229533.A0A098DLA7	pp	229533.I1RZM4

22047 229533.A0A098DLA7 pp 229533.I1RW69
22048 229533.A0A098DLA7 pp 229533.I1RM26
22049 229533.A0A098DLA7 pp 229533.V6RQW4
22050 229533.A0A098DLA7 pp 229533.I1S0A0
22051 229533.A0A098DLA7 pp 229533.I1RXB1
22052 229533.A0A098DLA7 pp 229533.V6RK96
22053 229533.A0A098DLA7 pp 229533.I1RYJ7
22054 229533.A0A098DLA7 pp 229533.I1RXV9
22055 229533.A0A098DLA7 pp 229533.I1RMM8
22056 229533.A0A098DLA7 pp 229533.I1RMG7
22057 229533.A0A098DLA7 pp 229533.I1RQE8
22058 229533.A0A098DLA7 pp 229533.I1RK95
22059 229533.A0A098DLA7 pp 229533.I1RPW2
22060 229533.A0A098DLA7 pp 229533.I1RVX9
22061 229533.A0A098DLA7 pp 229533.A0A1C3YJJ2
22062 229533.A0A098DLA7 pp 229533.I1RCB4
22063 229533.A0A098DLA7 pp 229533.A0A0E0SH88
22064 229533.A0A098DLA7 pp 229533.A0A098E2A4
22065 229533.A0A098DLA7 pp 229533.V6RM02
22066 229533.A0A098DLA7 pp 229533.I1RYU5
22067 229533.A0A098DLA7 pp 229533.A0A1C3YKN4
22068 229533.A0A098DLA7 pp 229533.I1RIH0
22069 229533.A0A098DLA7 pp 229533.V6QVH8
22070 229533.A0A098DLA7 pp 229533.I1RZQ1
22071 229533.A0A098DLA7 pp 229533.I1RNG4
22072 229533.A0A098DLA7 pp 229533.A0A1C3YI05
22073 229533.A0A098DLA7 pp 229533.I1RSL5
22074 229533.A0A098DLA7 pp 229533.I1RMG6
22075 229533.A0A098DLA7 pp 229533.A0A098DNS0
22076 229533.A0A098DLA7 pp 229533.A0A1C3YLA6
22077 229533.A0A098DLA7 pp 229533.I1RXW3
22078 229533.A0A098DLA7 pp 229533.I1RPK0
22079 229533.A0A098DLA7 pp 229533.I1RZP1
22080 229533.A0A098DLA7 pp 229533.I1RL76
22081 229533.A0A098DLA7 pp 229533.I1RR68
22082 229533.A0A098DLA7 pp 229533.I1RFW3
22083 229533.A0A098DLA7 pp 229533.Q4HXF0
22084 229533.A0A098DLA7 pp 229533.I1RQ30
22085 229533.A0A098DLA7 pp 229533.V6RSF3
22086 229533.A0A098DLA7 pp 229533.O74638
22087 229533.A0A098DLA7 pp 229533.I1RRT8
22088 229533.A0A098DLA7 pp 229533.I1RVR2
22089 229533.A0A098DLA7 pp 229533.A0A0E0SMR8
22090 229533.A0A098DLA7 pp 229533.I1RZ29
22091 229533.A0A098DLA7 pp 229533.I1RZ20
22092 229533.A0A098DLA7 pp 229533.I1RTI7
22093 229533.I1RIG3 pp 229533.I1RYQ0
22094 229533.I1RIG3 pp 229533.I1RZB7
22095 229533.I1RIG3 pp 229533.I1RKR7
22096 229533.A0A1C3YNC6 pp 229533.I1RJS0
22097 229533.A0A1C3YNC6 pp 229533.I1RRG1
22098 229533.A0A1C3YNC6 pp 229533.I1RNG4
22099 229533.A0A1C3YNC6 pp 229533.I1RSX9
22100 229533.A0A1C3YNC6 pp 229533.I1RUP5
22101 229533.A0A1C3YNC6 pp 229533.I1RMX6
22102 229533.A0A1C3YNC6 pp 229533.I1RND4
22103 229533.A0A1C3YJJ0 pp 229533.I1S1W9
22104 229533.A0A1C3YJJ0 pp 229533.I1RMB2
22105 229533.A0A1C3YJJ0 pp 229533.I1RCS1
22106 229533.A0A1C3YJJ0 pp 229533.I1RSQ3
22107 229533.A0A1C3YJJ0 pp 229533.I1R9M0
22108 229533.A0A1C3YJJ0 pp 229533.I1RVZ2
22109 229533.I1S855 pp 229533.V6RIS2
22110 229533.I1S855 pp 229533.Q4IF76
22111 229533.I1S855 pp 229533.V6R3J1
22112 229533.I1S855 pp 229533.V6RY33
22113 229533.I1S855 pp 229533.Q4IJH1
22114 229533.I1S855 pp 229533.V6R227
22115 229533.I1S855 pp 229533.Q4IPI1
22116 229533.I1S855 pp 229533.Q4HW67
22117 229533.I1S855 pp 229533.Q4IEK8
22118 229533.I1S855 pp 229533.Q4HZ68
22119 229533.I1R9Z7 pp 229533.I1RPR9

22120 229533.I1R9Z7 pp 229533.I1RCH5
22121 229533.A0A1C3YMU5 pp 229533.I1REM2
22122 229533.A0A1C3YMU5 pp 229533.I1RPW8
22123 229533.A0A1C3YMU5 pp 229533.I1RW91
22124 229533.A0A1C3YMU5 pp 229533.I1RVB6
22125 229533.A0A1C3YMU5 pp 229533.I1RCG6
22126 229533.A0A1C3YMU5 pp 229533.I1RRF7
22127 229533.A0A1C3YMU5 pp 229533.I1RN04
22128 229533.I1REF9 pp 229533.I1RWT6
22129 229533.I1REF9 pp 229533.I1RWI6
22130 229533.I1REF9 pp 229533.I1RLT6
22131 229533.I1REF9 pp 229533.I1RTD0
22132 229533.I1REF9 pp 229533.Q4ICG5
22133 229533.I1REF9 pp 229533.I1RX09
22134 229533.I1REF9 pp 229533.I1RQA3
22135 229533.I1REF9 pp 229533.I1RZA4
22136 229533.I1REF9 pp 229533.I1RXU1
22137 229533.I1REF9 pp 229533.I1RWA5
22138 229533.I1REF9 pp 229533.V6RKE4
22139 229533.I1REF9 pp 229533.I1RW84
22140 229533.I1REF9 pp 229533.I1REN4
22141 229533.I1REF9 pp 229533.I1RNR3
22142 229533.I1REF9 pp 229533.I1S208
22143 229533.I1REF9 pp 229533.I1RZX0
22144 229533.I1REF9 pp 229533.I1RW32
22145 229533.I1REF9 pp 229533.V6RQ48
22146 229533.I1S064 pp 229533.V6RR38
22147 229533.I1S064 pp 229533.I1S8E0
22148 229533.I1S064 pp 229533.I1S0F5
22149 229533.I1S064 pp 229533.I1S4S4
22150 229533.I1S064 pp 229533.Q4I5G1
22151 229533.I1S064 pp 229533.V6RQW4
22152 229533.I1S064 pp 229533.V6RN69
22153 229533.I1S064 pp 229533.I1S4S7
22154 229533.I1S064 pp 229533.I1S1W9
22155 229533.I1S064 pp 229533.I1S1W8
22156 229533.I1S064 pp 229533.I1S2M8
22157 229533.I1S064 pp 229533.V6R1K4
22158 229533.I1S064 pp 229533.I1S0C2
22159 229533.I1S064 pp 229533.I1S3G4
22160 229533.I1S064 pp 229533.I1S5L5
22161 229533.I1S064 pp 229533.I1S132
22162 229533.I1S064 pp 229533.I1S9Z4
22163 229533.I1S064 pp 229533.O74638
22164 229533.I1S064 pp 229533.I1S1F3
22165 229533.I1S064 pp 229533.V6RVJ3
22166 229533.A0A0E0SI42 pp 229533.I1RXF0
22167 229533.A0A0E0SI42 pp 229533.I1RRD0
22168 229533.A0A0E0SI42 pp 229533.I1RKR5
22169 229533.A0A0E0SI42 pp 229533.I1RND4
22170 229533.A0A0E0SI42 pp 229533.I1RPK0
22171 229533.A0A0E0SI42 pp 229533.V6RLM7
22172 229533.A0A0E0SI42 pp 229533.I1RMX6
22173 229533.A0A0E0SI42 pp 229533.I1RQ14
22174 229533.A0A0E0SI42 pp 229533.I1RKB6
22175 229533.A0A0E0SI42 pp 229533.A0A1C3YKV3
22176 229533.A0A0E0SI42 pp 229533.I1RC31
22177 229533.A0A0E0SI42 pp 229533.I1S0A0
22178 229533.A0A0E0SI42 pp 229533.I1RCH5
22179 229533.A0A0E0SI42 pp 229533.I1RNG4
22180 229533.A0A0E0SI42 pp 229533.I1RNU7
22181 229533.A0A0E0SI42 pp 229533.I1RUY6
22182 229533.A0A0E0SI42 pp 229533.A0A1C3YN26
22183 229533.A0A0E0SI42 pp 229533.A0A1C3YJZ0
22184 229533.A0A0E0SI42 pp 229533.I1RPT0
22185 229533.A0A0E0SI42 pp 229533.I1RM26
22186 229533.A0A0E0SI42 pp 229533.I1RP86
22187 229533.A0A0E0SI42 pp 229533.I1S3G4
22188 229533.A0A0E0SI42 pp 229533.A0A1C3YLA6
22189 229533.A0A0E0SI42 pp 229533.I1RAQ4
22190 229533.A0A0E0SI42 pp 229533.I1RJQ5
22191 229533.A0A0E0SI42 pp 229533.I1RVX9
22192 229533.A0A0E0SI42 pp 229533.I1RYK0

229533.A0A0E0SI42 pp 229533.I1S1W8
229533.A0A0E0SI42 pp 229533.I1RHQ0
229533.A0A0E0SI42 pp 229533.A0A1C3YLR6
229533.A0A0E0SI42 pp 229533.I1RW71
229533.A0A0E0SI42 pp 229533.I1R9Y8
229533.A0A0E0SI42 pp 229533.I1RJZ7
229533.I1RC81 pp 229533.I1RGC2
229533.I1RC81 pp 229533.I1RZF6
229533.I1RC81 pp 229533.I1RK83
229533.I1RC81 pp 229533.I1RJ17
229533.I1RC81 pp 229533.I1S044
229533.I1RC81 pp 229533.I1RIS3
229533.I1RC81 pp 229533.I1RVZ0
229533.I1RC81 pp 229533.I1RYB9
229533.I1RC81 pp 229533.I1RDG0
229533.I1RC81 pp 229533.I1RPU0
229533.I1RC81 pp 229533.I1REK8
229533.I1RC81 pp 229533.I1RWD4
229533.I1RC81 pp 229533.I1S0F1
229533.I1RC81 pp 229533.I1S0X6
229533.I1RC81 pp 229533.I1S0L3
229533.I1RC81 pp 229533.I1RDY5
229533.I1RC81 pp 229533.I1RWH5
229533.I1RC81 pp 229533.I1RE01
229533.I1RC81 pp 229533.I1RW25
229533.I1RC81 pp 229533.I1RT80
229533.I1RC81 pp 229533.I1RS17
229533.I1RC81 pp 229533.I1RWB5
229533.I1RC81 pp 229533.I1RQJ5
229533.I1RC81 pp 229533.I1RFC0
229533.I1RC81 pp 229533.I1RMN6
229533.I1RC81 pp 229533.I1RCL3
229533.I1RC81 pp 229533.I1RXF6
229533.I1RC81 pp 229533.V6RMW6
229533.I1RC81 pp 229533.I1RM67
229533.I1RC81 pp 229533.I1RRI2
229533.I1RC81 pp 229533.Q4IR18
229533.I1RC81 pp 229533.Q4HWE2
229533.I1RC81 pp 229533.I1RN48
229533.I1RC81 pp 229533.I1RM96
229533.I1RC81 pp 229533.I1S0L4
229533.I1RC81 pp 229533.I1RZV6
229533.I1RC81 pp 229533.I1RPS0
229533.I1RC81 pp 229533.I1RRQ3
229533.I1RC81 pp 229533.I1RQE7
229533.I1RC81 pp 229533.I1RFL3
229533.I1RC81 pp 229533.I1RQ65
229533.I1RC81 pp 229533.I1RFL4
229533.I1RC81 pp 229533.I1RKE7
229533.I1RC81 pp 229533.I1RKL3
229533.I1RC81 pp 229533.I1RD64
229533.I1RC81 pp 229533.I1RND6
229533.I1RC81 pp 229533.I1S8E2
229533.I1RC81 pp 229533.I1RSD8
229533.I1RC81 pp 229533.I1RKJ6
229533.I1RC81 pp 229533.I1RRT8
229533.I1RC81 pp 229533.I1RFS3
229533.I1RC81 pp 229533.I1RTG7
229533.I1RC81 pp 229533.I1S7S9
229533.I1RC81 pp 229533.I1RPM4
229533.V6RPQ8 pp 229533.V6RXE7
229533.A0A1C3YMU4 pp 229533.I1RNG4
229533.A0A1C3YHP2 pp 229533.I1RC31
229533.A0A1C3YHP2 pp 229533.A0A1C3YI27
229533.A0A1C3YHP2 pp 229533.I1RF73
229533.A0A1C3YHP2 pp 229533.I1RA62
229533.A0A1C3YHP2 pp 229533.I1RI39
229533.A0A1C3YHP2 pp 229533.I1RYK0
229533.A0A1C3YHP2 pp 229533.I1S2R5
229533.A0A1C3YHP2 pp 229533.I1RMX6
229533.A0A1C3YHP2 pp 229533.I1RKE1
229533.A0A1C3YHP2 pp 229533.I1RCG5
229533.A0A1C3YHP2 pp 229533.I1RX90

22266 229533.A0A1C3YHP2 pp 229533.I1RPT0
22267 229533.A0A1C3YHP2 pp 229533.A0A1C3YM97
22268 229533.A0A1C3YHP2 pp 229533.I1RVW8
22269 229533.A0A1C3YHP2 pp 229533.I1RGB2
22270 229533.A0A1C3YHP2 pp 229533.I0LNZ1
22271 229533.A0A1C3YHP2 pp 229533.Q4HWE2
22272 229533.A0A1C3YHP2 pp 229533.I1RKR5
22273 229533.A0A1C3YHP2 pp 229533.I1RQI4
22274 229533.A0A1C3YHP2 pp 229533.I1RUY5
22275 229533.A0A1C3YHP2 pp 229533.I1RL92
22276 229533.A0A1C3YHP2 pp 229533.I1RHM8
22277 229533.A0A1C3YHP2 pp 229533.V6QU63
22278 229533.A0A1C3YHP2 pp 229533.I1RSL0
22279 229533.A0A1C3YHP2 pp 229533.I1RR44
22280 229533.A0A1C3YHP2 pp 229533.I1RSJ2
22281 229533.A0A1C3YHP2 pp 229533.A0A1C3YN26
22282 229533.A0A1C3YHP2 pp 229533.I1R9F8
22283 229533.A0A1C3YHP2 pp 229533.I1RF48
22284 229533.A0A1C3YHP2 pp 229533.I1RDW1
22285 229533.A0A1C3YHP2 pp 229533.V6RK40
22286 229533.A0A1C3YHP2 pp 229533.V6RW66
22287 229533.A0A1C3YHP2 pp 229533.I1RU18
22288 229533.A0A1C3YHP2 pp 229533.I1RVV3
22289 229533.A0A1C3YHP2 pp 229533.I1RKX2
22290 229533.A0A1C3YHP2 pp 229533.I1RHQ0
22291 229533.A0A1C3YHP2 pp 229533.V6RV57
22292 229533.A0A1C3YHP2 pp 229533.I1RGY2
22293 229533.A0A1C3YHP2 pp 229533.I1RIX0
22294 229533.A0A1C3YHP2 pp 229533.A0A1C3YL54
22295 229533.A0A1C3YHP2 pp 229533.I1RCZ8
22296 229533.A0A1C3YHP2 pp 229533.A0A1C3YJ56
22297 229533.A0A1C3YHP2 pp 229533.I1RP86
22298 229533.A0A1C3YHP2 pp 229533.I1RHL0
22299 229533.I1RMF6 pp 229533.I1S1W9
22300 229533.I1RMF6 pp 229533.I1RSQ3
22301 229533.I1RMF6 pp 229533.I1RVZ2
22302 229533.I1RMI8 pp 229533.Q4INS6
22303 229533.I1RMI8 pp 229533.I1RRV4
22304 229533.I1RMI8 pp 229533.I1RRT1
22305 229533.I1RMI8 pp 229533.I1S3K8
22306 229533.I1RMI8 pp 229533.I1RTD3
22307 229533.I1RMI8 pp 229533.I1S4Q4
22308 229533.I1RMI8 pp 229533.V6R1K4
22309 229533.I1RMI8 pp 229533.V6RBN2
22310 229533.I1RMI8 pp 229533.I1S1P8
22311 229533.I1RMI8 pp 229533.V6R057
22312 229533.I1RMI8 pp 229533.I1RMQ4
22313 229533.I1RMI8 pp 229533.I1S084
22314 229533.I1RMI8 pp 229533.I1RPN4
22315 229533.I1RMI8 pp 229533.I1RMQ5
22316 229533.I1RMI8 pp 229533.V6RRH2
22317 229533.I1RMI8 pp 229533.I1RPU8
22318 229533.I1RMI8 pp 229533.I1RPP3
22319 229533.I1RMI8 pp 229533.I1RP99
22320 229533.I1RMI8 pp 229533.I1RRV1
22321 229533.I1RMI8 pp 229533.I1S2C4
22322 229533.I1RMI8 pp 229533.I1RYQ1
22323 229533.I1RMI8 pp 229533.I1RY29
22324 229533.I1RMI8 pp 229533.V6QT89
22325 229533.I1RMI8 pp 229533.I1RSD0
22326 229533.I1RMI8 pp 229533.I1RTA9
22327 229533.I1RMI8 pp 229533.I1RSC7
22328 229533.I1RMI8 pp 229533.I1RZT0
22329 229533.I1RMI8 pp 229533.I1RWE8
22330 229533.I1RMI8 pp 229533.I1RWI8
22331 229533.I1RNB2 pp 229533.I1RVQ3
22332 229533.I1RNB2 pp 229533.V6R9Z0
22333 229533.I1RNB2 pp 229533.I1RRV3
22334 229533.I1RNB2 pp 229533.I1RR24
22335 229533.I1RNB2 pp 229533.I1S1B2
22336 229533.I1RNB2 pp 229533.I1S5R1
22337 229533.I1RNB2 pp 229533.I1S5R6
22338 229533.I1RNB2 pp 229533.I1RZK5

22339 229533.I1RNB2 pp 229533.I1RQD2
22340 229533.I1RNB2 pp 229533.I1RPE0
22341 229533.I1RNB2 pp 229533.I1RTB3
22342 229533.I1RNB2 pp 229533.I1RXC4
22343 229533.I1RNB2 pp 229533.I1RSZ7
22344 229533.I1RNB2 pp 229533.I1RRM3
22345 229533.I1RNB2 pp 229533.I1RRI1
22346 229533.I1RNB2 pp 229533.I1RXD7
22347 229533.I1RNB2 pp 229533.I1RWR7
22348 229533.I1RNB2 pp 229533.I1S1W9
22349 229533.I1RNB2 pp 229533.I1RX95
22350 229533.I1RNB2 pp 229533.I1S0W4
22351 229533.I1RNB2 pp 229533.I1RZM8
22352 229533.I1RNB2 pp 229533.Q4IJK2
22353 229533.I1RNB2 pp 229533.I1RTA1
22354 229533.I1RNB2 pp 229533.I1RTD5
22355 229533.I1RNB2 pp 229533.I1RT40
22356 229533.I1RNB2 pp 229533.I1RVZ2
22357 229533.I1RNB2 pp 229533.I1S2B8
22358 229533.I1RNB2 pp 229533.I1RZ39
22359 229533.I1RNB2 pp 229533.I1RXD6
22360 229533.I1RNB2 pp 229533.V6RCZ8
22361 229533.I1RNB2 pp 229533.I1S4S7
22362 229533.I1RNB2 pp 229533.I1RZV3
22363 229533.I1RNB2 pp 229533.I1RVX0
22364 229533.I1RNB2 pp 229533.I1S821
22365 229533.I1RNB2 pp 229533.I1RSF1
22366 229533.I1RNB2 pp 229533.I1RQ33
22367 229533.I1RNB2 pp 229533.I1RPU2
22368 229533.I1RNB2 pp 229533.I1RYI5
22369 229533.I1RNB2 pp 229533.I1RQ85
22370 229533.I1RNB2 pp 229533.I1RSA5
22371 229533.I1RU18 pp 229533.V6RV57
22372 229533.I1RU18 pp 229533.I1RX90
22373 229533.I1RU18 pp 229533.I1RUY5
22374 229533.I1RU18 pp 229533.I1RVV3
22375 229533.I1RU18 pp 229533.I1RYK0
22376 229533.I1S724 pp 229533.V6RPN0
22377 229533.I1RQN6 pp 229533.I1RZU5
22378 229533.I1RQ29 pp 229533.I1RX64
22379 229533.I1RQ29 pp 229533.I1S0K9
22380 229533.A0A1C3YIS9 pp 229533.I1RFL4
22381 229533.A0A1C3YIS9 pp 229533.I1RI34
22382 229533.A0A1C3YIS9 pp 229533.V6R2A1
22383 229533.A0A1C3YIS9 pp 229533.I1S247
22384 229533.A0A1C3YIS9 pp 229533.V6RRH2
22385 229533.A0A1C3YIS9 pp 229533.I1S216
22386 229533.A0A1C3YIS9 pp 229533.I1RBI1
22387 229533.A0A1C3YIS9 pp 229533.I1S1W9
22388 229533.A0A1C3YIS9 pp 229533.I1RMQ4
22389 229533.A0A1C3YIS9 pp 229533.V6R057
22390 229533.A0A1C3YIS9 pp 229533.I1RCW6
22391 229533.A0A1C3YIS9 pp 229533.I1RWE8
22392 229533.A0A1C3YIS9 pp 229533.I1RB44
22393 229533.A0A1C3YIS9 pp 229533.I1S267
22394 229533.A0A1C3YIS9 pp 229533.I1RF82
22395 229533.A0A1C3YIS9 pp 229533.I1RZQ1
22396 229533.A0A1C3YIS9 pp 229533.I1RK15
22397 229533.A0A1C3YIS9 pp 229533.I1RXF0
22398 229533.A0A1C3YIS9 pp 229533.I1RVZ2
22399 229533.A0A1C3YIS9 pp 229533.A0A1C3YKY0
22400 229533.A0A1C3YIS9 pp 229533.Q4HXF0
22401 229533.A0A1C3YIS9 pp 229533.I1RQG4
22402 229533.A0A1C3YIS9 pp 229533.A0A1C3YKU8
22403 229533.A0A1C3YIS9 pp 229533.I1RBF3
22404 229533.A0A1C3YIS9 pp 229533.I1RTD3
22405 229533.A0A1C3YIS9 pp 229533.I1RRV4
22406 229533.A0A1C3YIS9 pp 229533.I1RVN1
22407 229533.A0A1C3YIS9 pp 229533.I1RYI5
22408 229533.A0A1C3YIS9 pp 229533.I1RC05
22409 229533.A0A1C3YIS9 pp 229533.I1RN67
22410 229533.A0A1C3YIS9 pp 229533.I1RSQ4
22411 229533.A0A1C3YIS9 pp 229533.I1RA94

22412 229533.A0A1C3YIS9 pp 229533.V6RBN2
22413 229533.A0A1C3YIS9 pp 229533.A0A1C3YJK3
22414 229533.A0A1C3YIS9 pp 229533.I1RNG4
22415 229533.A0A1C3YIS9 pp 229533.Q4IJK2
22416 229533.A0A1C3YIS9 pp 229533.I1RQA5
22417 229533.A0A1C3YIS9 pp 229533.I1S3G4
22418 229533.A0A1C3YIS9 pp 229533.I1RAZ3
22419 229533.A0A1C3YIS9 pp 229533.A0A1C3YK68
22420 229533.A0A1C3YIS9 pp 229533.A0A1C3YKH8
22421 229533.A0A1C3YIS9 pp 229533.I1S4Q4
22422 229533.A0A1C3YIS9 pp 229533.I1RRX5
22423 229533.A0A1C3YIS9 pp 229533.A0A1C3YM63
22424 229533.A0A1C3YIS9 pp 229533.I1RPN4
22425 229533.A0A1C3YIS9 pp 229533.I1REL7
22426 229533.A0A1C3YIS9 pp 229533.I1RAQ4
22427 229533.A0A1C3YIS9 pp 229533.I1RM35
22428 229533.A0A1C3YIS9 pp 229533.V6R1K4
22429 229533.A0A1C3YIS9 pp 229533.I1S0B7
22430 229533.A0A1C3YIS9 pp 229533.A0A1C3YJJ2
22431 229533.A0A1C3YIS9 pp 229533.I1S1W8
22432 229533.A0A1C3YIS9 pp 229533.I1RB39
22433 229533.A0A1C3YIS9 pp 229533.A0A1C3YLR6
22434 229533.A0A1C3YIS9 pp 229533.A0A1C3YKS6
22435 229533.A0A1C3YIS9 pp 229533.I1S0F1
22436 229533.A0A1C3YIS9 pp 229533.I1RAR3
22437 229533.A0A1C3YIS9 pp 229533.A0A1C3YN30
22438 229533.A0A1C3YIS9 pp 229533.I1RCL4
22439 229533.A0A1C3YIS9 pp 229533.I1RM67
22440 229533.A0A1C3YIS9 pp 229533.I1RE20
22441 229533.I1RW43 pp 229533.I1S1W8
22442 229533.I1RW43 pp 229533.I1RW54
22443 229533.I1RW43 pp 229533.I1S1D4
22444 229533.I1RW43 pp 229533.I1RYQ4
22445 229533.I1RW43 pp 229533.I1RY49
22446 229533.I1RW43 pp 229533.I1S2D5
22447 229533.I1RW43 pp 229533.I1RW69
22448 229533.I1RW43 pp 229533.I1S4S4
22449 229533.I1RW43 pp 229533.I1S183
22450 229533.I1RW43 pp 229533.I1RZM4
22451 229533.I1RW43 pp 229533.V6RK96
22452 229533.I1RW43 pp 229533.I1S0M4
22453 229533.I1RW43 pp 229533.I1RYX6
22454 229533.I1RW43 pp 229533.V6RRH2
22455 229533.I1RW43 pp 229533.V6RQW4
22456 229533.I1RW43 pp 229533.I1RXV9
22457 229533.I1RW43 pp 229533.I1S0A0
22458 229533.I1RW43 pp 229533.I1SAJ3
22459 229533.I1RW43 pp 229533.I1S5L5
22460 229533.I1RW43 pp 229533.I1S1F3
22461 229533.I1RW43 pp 229533.Q4I5V2
22462 229533.I1RW43 pp 229533.I1S9Z4
22463 229533.I1RW43 pp 229533.Q4HXF0
22464 229533.I1RW43 pp 229533.I1S835
22465 229533.I1RW43 pp 229533.V6R382
22466 229533.I1RW43 pp 229533.I1RZ29
22467 229533.I1RW43 pp 229533.I1S0I3
22468 229533.I1RW43 pp 229533.I1S3G4
22469 229533.I1RW43 pp 229533.I1RXW3
22470 229533.I1RW43 pp 229533.I1RXY7
22471 229533.I1RW43 pp 229533.V6RPQ8
22472 229533.I1RW43 pp 229533.V6RLM7
22473 229533.I1RQM7 pp 229533.I1RRM3
22474 229533.I1RQM7 pp 229533.V6R042
22475 229533.I1RQM7 pp 229533.I1RWE8
22476 229533.I1RQM7 pp 229533.I1RUG3
22477 229533.I1RQM7 pp 229533.V6RMW6
22478 229533.I1RQM7 pp 229533.I1RRP7
22479 229533.I1RQM7 pp 229533.I1RZV3
22480 229533.I1RQM7 pp 229533.I1S3G4
22481 229533.I1RQM7 pp 229533.I1RYW3
22482 229533.I1RQM7 pp 229533.Q4I073
22483 229533.I1RQM7 pp 229533.V6RRH2
22484 229533.I1RQM7 pp 229533.I1S2H4

22485	229533.I1RQM7	pp	229533.V6R057
22486	229533.I1RQM7	pp	229533.I1RVX0
22487	229533.I1RQM7	pp	229533.I1RRI1
22488	229533.I1RQM7	pp	229533.I1RZ39
22489	229533.I1RQM7	pp	229533.I1S5R6
22490	229533.I1RQM7	pp	229533.I1S5R1
22491	229533.I1RQM7	pp	229533.I1RSZ7
22492	229533.I1RQM7	pp	229533.I1RSQ4
22493	229533.I1RQM7	pp	229533.I1SAN9
22494	229533.I1RQM7	pp	229533.Q4IJK2
22495	229533.I1RQM7	pp	229533.I1RSQ3
22496	229533.I1RQM7	pp	229533.I1S0L8
22497	229533.I1RQM7	pp	229533.V6RBN2
22498	229533.I1RQM7	pp	229533.I1S2B8
22499	229533.I1RQM7	pp	229533.I1S1W8
22500	229533.I1RQM7	pp	229533.Q4HXF0
22501	229533.I1RQM7	pp	229533.I1S4S4
22502	229533.I1RQM7	pp	229533.I1RYI5
22503	229533.I1RQM7	pp	229533.I1RT40
22504	229533.I1RQM7	pp	229533.V6RTJ8
22505	229533.I1RQM7	pp	229533.I1RRV4
22506	229533.I1RQM7	pp	229533.I1S5B0
22507	229533.I1RQM7	pp	229533.I1RXD7
22508	229533.I1RQM7	pp	229533.I1RVZ2
22509	229533.I1RQM7	pp	229533.V6RCZ8
22510	229533.I1RQM7	pp	229533.I1RWR7
22511	229533.I1RQM7	pp	229533.I1RTA1
22512	229533.I1RQM7	pp	229533.I1RS18
22513	229533.I1RQM7	pp	229533.V6R1K4
22514	229533.I1RQM7	pp	229533.I1RZM8
22515	229533.I1RQM7	pp	229533.I1S4S7
22516	229533.I1RDK7	pp	229533.I1RXJ0
22517	229533.I1RDK7	pp	229533.I1RLV9
22518	229533.I1RDK7	pp	229533.I1RQ65
22519	229533.I1RDK7	pp	229533.I1RX46
22520	229533.I1RDK7	pp	229533.I1RTI3
22521	229533.I1RDK7	pp	229533.I1RSR8
22522	229533.I1RDK7	pp	229533.I1RZK6
22523	229533.I1RDK7	pp	229533.I1RKJ6
22524	229533.I1RDK7	pp	229533.I1RR73
22525	229533.I1RDK7	pp	229533.V6R042
22526	229533.I1RDK7	pp	229533.I1REC7
22527	229533.I1RDK7	pp	229533.I1RNH6
22528	229533.I1RDK7	pp	229533.I1RKL3
22529	229533.Q4I5V2	pp	229533.V6QWZ7
22530	229533.Q4I5V2	pp	229533.V6RBN2
22531	229533.Q4I5V2	pp	229533.Q4ID21
22532	229533.I1RPD6	pp	229533.I1S0D1
22533	229533.I1RPD6	pp	229533.V6RLM7
22534	229533.I1RPD6	pp	229533.I1S1W4
22535	229533.I1RPD6	pp	229533.V6QT89
22536	229533.I1RPD6	pp	229533.V6RVG3
22537	229533.I1RPD6	pp	229533.I1SAJ3
22538	229533.I1RPD6	pp	229533.I1RXY7
22539	229533.I1RPD6	pp	229533.I1RX64
22540	229533.I1RPD6	pp	229533.I1S0M2
22541	229533.I1RPD6	pp	229533.I1S2H3
22542	229533.I1RPD6	pp	229533.I1S2C4
22543	229533.I1RPD6	pp	229533.I1S0I3
22544	229533.I1RPD6	pp	229533.V6RRM0
22545	229533.I1RPD6	pp	229533.I1S3V2
22546	229533.I1RPD6	pp	229533.V6QVH8
22547	229533.I1RPD6	pp	229533.I1RW21
22548	229533.I1RPD6	pp	229533.O74638
22549	229533.I1RPD6	pp	229533.I1RRY3
22550	229533.I1RPD6	pp	229533.I1RYR7
22551	229533.I1RPD6	pp	229533.Q4HXF0
22552	229533.I1RPD6	pp	229533.I1S3G4
22553	229533.I1RPD6	pp	229533.I1S4F4
22554	229533.I1RPD6	pp	229533.Q4HVQ9
22555	229533.I1RPD6	pp	229533.I1RZA7
22556	229533.I1RPD6	pp	229533.I1RT75
22557	229533.I1RPD6	pp	229533.I1S6P5

22558	229533.I1RPD6	pp	229533.I1S5L5
22559	229533.I1RPD6	pp	229533.I1RUN8
22560	229533.I1RPD6	pp	229533.I1RZ20
22561	229533.I1RPD6	pp	229533.V6RUB1
22562	229533.I1RPD6	pp	229533.I1RZ29
22563	229533.I1RPD6	pp	229533.I1RVR2
22564	229533.I1RPD6	pp	229533.I1RXG6
22565	229533.I1RPD6	pp	229533.I1S207
22566	229533.I1RPD6	pp	229533.I1RW43
22567	229533.I1RPD6	pp	229533.I1S3X7
22568	229533.I1RPD6	pp	229533.I1RVZ3
22569	229533.I1RPD6	pp	229533.V6RDH3
22570	229533.I1RPD6	pp	229533.I1RRV4
22571	229533.I1RPD6	pp	229533.I1RZ52
22572	229533.I1RPD6	pp	229533.I1RYP6
22573	229533.I1RPD6	pp	229533.I1RYI7
22574	229533.I1RPD6	pp	229533.I1RUQ5
22575	229533.I1RPD6	pp	229533.I1S4D0
22576	229533.I1RPD6	pp	229533.V6RQW4
22577	229533.I1RPD6	pp	229533.I1S1L9
22578	229533.I1RPD6	pp	229533.V6QU65
22579	229533.I1RPD6	pp	229533.I1RQE0
22580	229533.I1RPD6	pp	229533.I1S0A0
22581	229533.I1RPD6	pp	229533.V6RY56
22582	229533.I1RPD6	pp	229533.I1RPW1
22583	229533.I1RPD6	pp	229533.I1RRX0
22584	229533.I1RPD6	pp	229533.I1S439
22585	229533.I1RPD6	pp	229533.I1RW69
22586	229533.I1RPD6	pp	229533.V6RBN2
22587	229533.I1RPD6	pp	229533.I1RUV4
22588	229533.I1RPD6	pp	229533.I1RYU0
22589	229533.I1RPD6	pp	229533.I1RXR3
22590	229533.I1RPD6	pp	229533.I1RQ14
22591	229533.I1RPD6	pp	229533.I1RVX9
22592	229533.I1RPD6	pp	229533.I1RQE1
22593	229533.I1RPD6	pp	229533.I1RTU0
22594	229533.I1RPD6	pp	229533.I1RQ26
22595	229533.I1RPD6	pp	229533.I1RYX6
22596	229533.I1RPD6	pp	229533.I1RPE7
22597	229533.I1RPD6	pp	229533.I1S1W8
22598	229533.I1RPD6	pp	229533.I1S2D5
22599	229533.I1RPD6	pp	229533.I1RPN4
22600	229533.I1RPD6	pp	229533.I1RPW2
22601	229533.I1RPD6	pp	229533.Q4IA56
22602	229533.I1RPD6	pp	229533.I1S029
22603	229533.I1RPD6	pp	229533.I1RYU5
22604	229533.I1RPD6	pp	229533.I1RY49
22605	229533.I1RPD6	pp	229533.I1RW06
22606	229533.I1RPD6	pp	229533.I1S8E0
22607	229533.I1RPP8	pp	229533.I1RWF4
22608	229533.I1RPP8	pp	229533.I1S0Y1
22609	229533.I1RPP8	pp	229533.I1RZS8
22610	229533.I1RVZ7	pp	229533.Q4IJK2
22611	229533.I1RVZ7	pp	229533.I1RWE8
22612	229533.I1RVZ7	pp	229533.V6RCZ8
22613	229533.I1RVZ7	pp	229533.I1SAN9
22614	229533.I1RVZ7	pp	229533.V6RBN2
22615	229533.I1RVZ7	pp	229533.I1RZV3
22616	229533.I1RVZ7	pp	229533.I1RWR7
22617	229533.I1RVZ7	pp	229533.I1S5B0
22618	229533.I1RVZ7	pp	229533.I1RZM8
22619	229533.I1RVZ7	pp	229533.V6R042
22620	229533.I1RVZ7	pp	229533.I1S1W8
22621	229533.I1RVZ7	pp	229533.I1S4S4
22622	229533.I1RVZ7	pp	229533.V6RMW6
22623	229533.I1RVZ7	pp	229533.I1RXD7
22624	229533.I1RVZ7	pp	229533.I1S5R6
22625	229533.I1RVZ7	pp	229533.I1RYM9
22626	229533.I1RVZ7	pp	229533.V6RTJ8
22627	229533.I1RVZ7	pp	229533.I1S2B8
22628	229533.I1RVZ7	pp	229533.V6R057
22629	229533.I1RVZ7	pp	229533.I1S5R1
22630	229533.I1RVZ7	pp	229533.V6RRH2

22631	229533.I1RVZ7	pp	229533.I1S4S7
22632	229533.I1RVZ7	pp	229533.I1S3G4
22633	229533.I1RVZ7	pp	229533.I1RYW3
22634	229533.I1RVZ7	pp	229533.I1RZ39
22635	229533.I1RVZ7	pp	229533.Q4HXF0
22636	229533.I1RVZ7	pp	229533.I1RYI5
22637	229533.I1RVZ7	pp	229533.Q4I073
22638	229533.I1RVZ7	pp	229533.I1S2H4
22639	229533.I1RC31	pp	229533.I1RDE4
22640	229533.I1RC31	pp	229533.I1RFV9
22641	229533.I1RC31	pp	229533.I1RK30
22642	229533.I1RC31	pp	229533.V6RAN2
22643	229533.I1RC31	pp	229533.I1RJ42
22644	229533.I1RC31	pp	229533.I1RUY5
22645	229533.I1RC31	pp	229533.I1RYM5
22646	229533.I1RC31	pp	229533.I1RIE8
22647	229533.I1RC31	pp	229533.I1S984
22648	229533.I1RC31	pp	229533.I1RKR5
22649	229533.I1RC31	pp	229533.I1RE72
22650	229533.I1RC31	pp	229533.I1RH82
22651	229533.I1RC31	pp	229533.I1RPT0
22652	229533.I1RC31	pp	229533.I1RST9
22653	229533.I1RC31	pp	229533.I1RQE0
22654	229533.I1RC31	pp	229533.V6R3R0
22655	229533.I1RC31	pp	229533.I1RK03
22656	229533.I1RC31	pp	229533.I1RM26
22657	229533.I1RC31	pp	229533.I1RPP2
22658	229533.I1RC31	pp	229533.I1RCL0
22659	229533.I1RC31	pp	229533.V6QUE6
22660	229533.I1RC31	pp	229533.I1RPW1
22661	229533.I1RC31	pp	229533.I1RJY7
22662	229533.I1RC31	pp	229533.V6RUT5
22663	229533.I1RC31	pp	229533.I1S183
22664	229533.I1RC31	pp	229533.I1RDU0
22665	229533.I1RC31	pp	229533.I1RTT4
22666	229533.I1RC31	pp	229533.I1S267
22667	229533.I1RC31	pp	229533.I1S574
22668	229533.I1RC31	pp	229533.I1RGB2
22669	229533.I1RC31	pp	229533.I1S053
22670	229533.I1RC31	pp	229533.I1RP86
22671	229533.I1RC31	pp	229533.V6QXN0
22672	229533.I1RC31	pp	229533.I1RE86
22673	229533.I1RC31	pp	229533.I1S3D9
22674	229533.I1RC31	pp	229533.I1RKB5
22675	229533.I1RC31	pp	229533.I1RJL9
22676	229533.I1RC31	pp	229533.I1RUY6
22677	229533.I1RC31	pp	229533.I1RZ92
22678	229533.I1RC31	pp	229533.I1RN46
22679	229533.I1RC31	pp	229533.I1RGY2
22680	229533.I1RC31	pp	229533.I1RNA1
22681	229533.I1RC31	pp	229533.I1RX46
22682	229533.I1RC31	pp	229533.I1RME3
22683	229533.I1RC31	pp	229533.I1RCM7
22684	229533.I1RC31	pp	229533.V6RN16
22685	229533.I1RC31	pp	229533.I1RVX9
22686	229533.I1RC31	pp	229533.V6RRM0
22687	229533.I1RC31	pp	229533.Q4IR09
22688	229533.I1RC31	pp	229533.V6RPQ8
22689	229533.I1RC31	pp	229533.I1RJ82
22690	229533.I1RC31	pp	229533.I1RRG3
22691	229533.I1RC31	pp	229533.I1RSJ2
22692	229533.I1RC31	pp	229533.I1S8C1
22693	229533.I1RC31	pp	229533.I1RQ14
22694	229533.I1RC31	pp	229533.I1S5R6
22695	229533.I1RC31	pp	229533.I1RWQ8
22696	229533.I1RC31	pp	229533.I1RDJ1
22697	229533.I1RC31	pp	229533.I1RSB7
22698	229533.I1RC31	pp	229533.I1RNM7
22699	229533.I1RC31	pp	229533.I1RND4
22700	229533.I1RC31	pp	229533.I1RQG5
22701	229533.I1RC31	pp	229533.I1REA3
22702	229533.I1RC31	pp	229533.I1RQ75
22703	229533.I1RC31	pp	229533.I1RX90

22704	229533.I1RC31	pp	229533.I1RSU2
22705	229533.I1RC31	pp	229533.I1RY87
22706	229533.I1RC31	pp	229533.I1RK45
22707	229533.I1RC31	pp	229533.I1RX64
22708	229533.I1RC31	pp	229533.I1RVV3
22709	229533.I1RC31	pp	229533.I1RW06
22710	229533.I1RC31	pp	229533.I1S272
22711	229533.I1RC31	pp	229533.I1S9Z4
22712	229533.I1RC31	pp	229533.V6R729
22713	229533.I1RC31	pp	229533.I1RRI2
22714	229533.I1RC31	pp	229533.I1RWF9
22715	229533.I1RC31	pp	229533.I1RE84
22716	229533.I1RC31	pp	229533.V6RLM7
22717	229533.I1RC31	pp	229533.I1RKP3
22718	229533.I1RC31	pp	229533.I1S5C5
22719	229533.I1RC31	pp	229533.I1RMX6
22720	229533.I1RC31	pp	229533.I1RKB2
22721	229533.I1RC31	pp	229533.I1RCG5
22722	229533.I1RC31	pp	229533.I1RZM3
22723	229533.I1RC31	pp	229533.I1RF60
22724	229533.I1RC31	pp	229533.I1RX72
22725	229533.I1RC31	pp	229533.V6RG79
22726	229533.I1RC31	pp	229533.V6RIV7
22727	229533.I1RC31	pp	229533.V6RUY9
22728	229533.I1RC31	pp	229533.Q4I1Y5
22729	229533.I1RC31	pp	229533.I1RPD6
22730	229533.I1RC31	pp	229533.I1RP97
22731	229533.I1RC31	pp	229533.I1RCE5
22732	229533.I1RC31	pp	229533.I1RNU7
22733	229533.I1RC31	pp	229533.I1RW26
22734	229533.I1RC31	pp	229533.V6RV57
22735	229533.I1RC31	pp	229533.I1RMD6
22736	229533.I1RC31	pp	229533.I1RKX2
22737	229533.I1RC31	pp	229533.I1RZ83
22738	229533.I1RC31	pp	229533.V6RUG6
22739	229533.I1RC31	pp	229533.I1RYK0
22740	229533.I1RC31	pp	229533.I1RSQ4
22741	229533.I1RC31	pp	229533.I1RQ29
22742	229533.I1RC31	pp	229533.I1RNG4
22743	229533.I1RC31	pp	229533.I1RL04
22744	229533.I1RC31	pp	229533.I1RRT8
22745	229533.I1RC31	pp	229533.I1RN67
22746	229533.I1RC31	pp	229533.I1RZ13
22747	229533.I1RC31	pp	229533.I1RU18
22748	229533.I1RC31	pp	229533.I1S065
22749	229533.I1RC31	pp	229533.I1RR73
22750	229533.I1RC31	pp	229533.I1RDE7
22751	229533.I1RC31	pp	229533.I1RJQ5
22752	229533.I1RC31	pp	229533.I1SAC4
22753	229533.I1RC31	pp	229533.I1RW43
22754	229533.I1RC31	pp	229533.I1RHH5
22755	229533.I1RC31	pp	229533.I1RRT3
22756	229533.I1RC31	pp	229533.I1RUR8
22757	229533.I1RFV3	pp	229533.I1RFV4
22758	229533.I1RFV3	pp	229533.I1S277
22759	229533.I1RFV3	pp	229533.I1RFY0
22760	229533.I1RFV3	pp	229533.I1RR72
22761	229533.I1RFV3	pp	229533.I1RM49
22762	229533.I1RFV3	pp	229533.I1RG53
22763	229533.I1RFV3	pp	229533.I1RT75
22764	229533.I1RFV3	pp	229533.I1RPA4
22765	229533.A0A1C3YKU8	pp	229533.I1S216
22766	229533.A0A1C3YKU8	pp	229533.I1RCD8
22767	229533.A0A1C3YKU8	pp	229533.V6R057
22768	229533.A0A1C3YKU8	pp	229533.I1RMI8
22769	229533.A0A1C3YKU8	pp	229533.I1RS17
22770	229533.A0A1C3YKU8	pp	229533.V6RLM7
22771	229533.A0A1C3YKU8	pp	229533.V6RRH2
22772	229533.A0A1C3YKU8	pp	229533.I1RI34
22773	229533.A0A1C3YKU8	pp	229533.I1RVX0
22774	229533.A0A1C3YKU8	pp	229533.I1RRV1
22775	229533.A0A1C3YKU8	pp	229533.I1RPU8
22776	229533.A0A1C3YKU8	pp	229533.V6R2A1

22777 229533.A0A1C3YKU8 pp 229533.I1RN63
22778 229533.A0A1C3YKU8 pp 229533.I1RQE7
22779 229533.A0A1C3YKU8 pp 229533.I1S4Z8
22780 229533.A0A1C3YKU8 pp 229533.I1RCH5
22781 229533.A0A1C3YKU8 pp 229533.I1RKY2
22782 229533.A0A1C3YKU8 pp 229533.Q4HXF0
22783 229533.A0A1C3YKU8 pp 229533.I1RQM7
22784 229533.A0A1C3YKU8 pp 229533.I1RL04
22785 229533.A0A1C3YKU8 pp 229533.I1RRP7
22786 229533.A0A1C3YKU8 pp 229533.I1REC6
22787 229533.A0A1C3YKU8 pp 229533.A0A1C3YKY0
22788 229533.A0A1C3YKU8 pp 229533.I1S8G4
22789 229533.A0A1C3YKU8 pp 229533.I1RMU9
22790 229533.A0A1C3YKU8 pp 229533.I1REC7
22791 229533.A0A1C3YKU8 pp 229533.I1RM35
22792 229533.A0A1C3YKU8 pp 229533.I1RBE7
22793 229533.A0A1C3YKU8 pp 229533.I1S4Q4
22794 229533.A0A1C3YKU8 pp 229533.I1RYW3
22795 229533.A0A1C3YKU8 pp 229533.I1RSQ4
22796 229533.A0A1C3YKU8 pp 229533.I1RAB4
22797 229533.A0A1C3YKU8 pp 229533.I1RCW6
22798 229533.A0A1C3YKU8 pp 229533.I1REL7
22799 229533.A0A1C3YKU8 pp 229533.I1RC05
22800 229533.A0A1C3YKU8 pp 229533.I1RB44
22801 229533.A0A1C3YKU8 pp 229533.V6RBN2
22802 229533.A0A1C3YKU8 pp 229533.I1RBS0
22803 229533.A0A1C3YKU8 pp 229533.I1RX22
22804 229533.A0A1C3YKU8 pp 229533.I1RWE8
22805 229533.A0A1C3YKU8 pp 229533.I1RN67
22806 229533.A0A1C3YKU8 pp 229533.I1RK15
22807 229533.A0A1C3YKU8 pp 229533.I1RAZ3
22808 229533.A0A1C3YKU8 pp 229533.I1RF82
22809 229533.A0A1C3YKU8 pp 229533.I1RNE2
22810 229533.A0A1C3YKU8 pp 229533.I1RPP3
22811 229533.A0A1C3YKU8 pp 229533.I1RQG4
22812 229533.A0A1C3YKU8 pp 229533.A0A1C3YKV3
22813 229533.A0A1C3YKU8 pp 229533.V6RK96
22814 229533.A0A1C3YKU8 pp 229533.I1RMQ4
22815 229533.A0A1C3YKU8 pp 229533.I1RBI1
22816 229533.A0A1C3YKU8 pp 229533.I1RRV4
22817 229533.A0A1C3YKU8 pp 229533.I1RTD3
22818 229533.A0A1C3YKU8 pp 229533.I1RB39
22819 229533.A0A1C3YKU8 pp 229533.I1RBE4
22820 229533.A0A1C3YKU8 pp 229533.I1S0B7
22821 229533.A0A1C3YKU8 pp 229533.I1RCL4
22822 229533.A0A1C3YKU8 pp 229533.I1RQE8
22823 229533.A0A1C3YKU8 pp 229533.A0A1C3YN30
22824 229533.A0A1C3YKU8 pp 229533.I1RMM8
22825 229533.A0A1C3YKU8 pp 229533.A0A1C3YN26
22826 229533.A0A1C3YKU8 pp 229533.I1RBT1
22827 229533.A0A1C3YKU8 pp 229533.I1RQS9
22828 229533.A0A1C3YKU8 pp 229533.V6R1K4
22829 229533.A0A1C3YKU8 pp 229533.I1RD52
22830 229533.A0A1C3YKU8 pp 229533.I1RPN4
22831 229533.A0A1C3YKU8 pp 229533.I1RZM4
22832 229533.A0A1C3YKU8 pp 229533.Q4I5R9
22833 229533.I1R9E6 pp 229533.V6R042
22834 229533.I1R9E6 pp 229533.I1RSR8
22835 229533.I1R9E6 pp 229533.I1RNH6
22836 229533.I1R9E6 pp 229533.I1RAN5
22837 229533.I1R9E6 pp 229533.I1RA48
22838 229533.I1R9E6 pp 229533.I1RAU7
22839 229533.I1R9E6 pp 229533.I1RZK6
22840 229533.I1R9E6 pp 229533.I1RX46
22841 229533.I1R9E6 pp 229533.I1RLV9
22842 229533.I1R9E6 pp 229533.I1RQ65
22843 229533.I1R9E6 pp 229533.I1RKL3
22844 229533.I1R9E6 pp 229533.I1RKJ6
22845 229533.I1R9E6 pp 229533.I1RR73
22846 229533.I1RB44 pp 229533.I1RWR7
22847 229533.I1RB44 pp 229533.V6RBN2
22848 229533.I1RB44 pp 229533.Q4I061
22849 229533.I1RB44 pp 229533.Q4IJK2

22850 229533.I1RB44 pp 229533.I1REK8
22851 229533.I1RB44 pp 229533.I1RM35
22852 229533.I1RB44 pp 229533.I1RCR5
22853 229533.I1RB44 pp 229533.I1S4Q4
22854 229533.I1RB44 pp 229533.I1REL7
22855 229533.I1RB44 pp 229533.I1RKZ4
22856 229533.I1RB44 pp 229533.I1RTD3
22857 229533.I1RB44 pp 229533.V6RCZ8
22858 229533.I1RB44 pp 229533.I1RQD2
22859 229533.I1RB44 pp 229533.I1S5B0
22860 229533.I1RB44 pp 229533.I1RZM8
22861 229533.I1RB44 pp 229533.I1RS18
22862 229533.I1RB44 pp 229533.I1RRV4
22863 229533.I1RB44 pp 229533.I1RBI1
22864 229533.I1RB44 pp 229533.I1RMQ4
22865 229533.I1RB44 pp 229533.I1RQS9
22866 229533.I1RB44 pp 229533.I1RK15
22867 229533.I1RB44 pp 229533.I1RCL4
22868 229533.I1RB44 pp 229533.I1RYW3
22869 229533.I1RB44 pp 229533.I1RCW6
22870 229533.I1RB44 pp 229533.I1S4S4
22871 229533.I1RB44 pp 229533.I1RE89
22872 229533.I1RB44 pp 229533.I1RQ85
22873 229533.I1RB44 pp 229533.I1RRP7
22874 229533.I1RB44 pp 229533.I1RF82
22875 229533.I1RB44 pp 229533.I1S0B7
22876 229533.I1RB44 pp 229533.I1S1W8
22877 229533.I1RB44 pp 229533.I1RJU3
22878 229533.I1RB44 pp 229533.V6R2A1
22879 229533.I1RB44 pp 229533.I1RE01
22880 229533.I1RB44 pp 229533.I1RVX0
22881 229533.I1RB44 pp 229533.I1RXD7
22882 229533.I1RB44 pp 229533.I1RI34
22883 229533.I1RB44 pp 229533.I1RWE8
22884 229533.I1RB44 pp 229533.I1RME6
22885 229533.I1RB44 pp 229533.V6RTJ8
22886 229533.I1RB44 pp 229533.I1RQE7
22887 229533.I1RB44 pp 229533.I1RCH5
22888 229533.I1RB44 pp 229533.I1RPU2
22889 229533.I1RB44 pp 229533.I1RPN4
22890 229533.I1RB44 pp 229533.I1RZV3
22891 229533.I1RB44 pp 229533.I1S216
22892 229533.I1RB44 pp 229533.V6R057
22893 229533.I1RB44 pp 229533.I1RZ39
22894 229533.I1RB44 pp 229533.I1S4S7
22895 229533.I1RB44 pp 229533.V6R1K4
22896 229533.I1RB44 pp 229533.I1RB73
22897 229533.I1RB44 pp 229533.I1RD52
22898 229533.I1RB44 pp 229533.I1RSQ4
22899 229533.I1RB44 pp 229533.V6RRH2
22900 229533.I1RB44 pp 229533.I1RQ33
22901 229533.I1RB44 pp 229533.I1RCD8
22902 229533.I1RB44 pp 229533.I1RPE0
22903 229533.I1RB44 pp 229533.I1RPP3
22904 229533.I1RB44 pp 229533.I1RFT1
22905 229533.I1RB44 pp 229533.I1S2H4
22906 229533.I1RB44 pp 229533.I1SAN9
22907 229533.I1RB44 pp 229533.I1S3G4
22908 229533.I1RB44 pp 229533.I1RYI5
22909 229533.I1RB44 pp 229533.I1RC00
22910 229533.I1RB44 pp 229533.I1RRM3
22911 229533.I1RB44 pp 229533.I1RRI1
22912 229533.I1RB44 pp 229533.V6RMW6
22913 229533.I1RB44 pp 229533.I1RVZ2
22914 229533.I1RB44 pp 229533.I1RJN9
22915 229533.I1RB44 pp 229533.I1RSQ3
22916 229533.I1RB44 pp 229533.I1RTA1
22917 229533.I1RB44 pp 229533.V6R042
22918 229533.I1RB44 pp 229533.I1S5R1
22919 229533.I1RB44 pp 229533.I1RC05
22920 229533.I1RB44 pp 229533.I1RNG4
22921 229533.I1RB44 pp 229533.I1RSZ7
22922 229533.I1RB44 pp 229533.I1RL04

22923 229533.I1RB44 pp 229533.I1RUG3
22924 229533.I1RB44 pp 229533.I1RX22
22925 229533.I1RB44 pp 229533.I1S2B8
22926 229533.I1RB44 pp 229533.I1S5R6
22927 229533.I1RB44 pp 229533.I1RD22
22928 229533.I1RB44 pp 229533.I1RBY4
22929 229533.I1RB44 pp 229533.I1RCN2
22930 229533.I1RB44 pp 229533.Q4I073
22931 229533.I1RB44 pp 229533.Q4HXF0
22932 229533.I1RB44 pp 229533.I1RPK0
22933 229533.I1RB44 pp 229533.I1RGA4
22934 229533.I1RB44 pp 229533.I1RT40
22935 229533.I1RKB2 pp 229533.V6R3V4
22936 229533.I1RKB2 pp 229533.I1RQJ7
22937 229533.I1RKB2 pp 229533.I1S0V6
22938 229533.I1RKB2 pp 229533.I1S8S9
22939 229533.I1RKB2 pp 229533.I1S8F8
22940 229533.I1RKB2 pp 229533.I1RMD3
22941 229533.I1RKB2 pp 229533.I1RRS8
22942 229533.I1RKB2 pp 229533.I1S8H9
22943 229533.I1RKB2 pp 229533.I1S5J9
22944 229533.I1RKB2 pp 229533.I1RS18
22945 229533.I1RKB2 pp 229533.I1RQP2
22946 229533.I1RKB2 pp 229533.I1S1W8
22947 229533.I1RKB2 pp 229533.I1S0A0
22948 229533.I1RKB2 pp 229533.Q4I5G1
22949 229533.I1RKB2 pp 229533.I1RXV9
22950 229533.I1RKB2 pp 229533.I1RNM7
22951 229533.I1RKB2 pp 229533.I1RMK7
22952 229533.I1RKB2 pp 229533.I1RUQ3
22953 229533.I1RKB2 pp 229533.V6RK96
22954 229533.I1RKB2 pp 229533.I1SAI4
22955 229533.I1RKB2 pp 229533.I1S3L2
22956 229533.I1RKB2 pp 229533.I1RXH2
22957 229533.I1RKB2 pp 229533.I1RWM4
22958 229533.I1RKB2 pp 229533.Q4IR09
22959 229533.I1RKB2 pp 229533.I1RXS0
22960 229533.I1RKB2 pp 229533.I1RXE6
22961 229533.I1RKB2 pp 229533.I1RUY5
22962 229533.I1RKB2 pp 229533.Q4HYR2
22963 229533.I1RKB2 pp 229533.I1S0K2
22964 229533.I1RKB2 pp 229533.I1RY21
22965 229533.I1RKB2 pp 229533.I1RWD2
22966 229533.I1RKB2 pp 229533.I1S5B0
22967 229533.I1RKB2 pp 229533.I1RMM8
22968 229533.I1RKB2 pp 229533.I1RYA2
22969 229533.I1RKB2 pp 229533.I1RQE8
22970 229533.I1RKB2 pp 229533.I1RRV4
22971 229533.I1RKB2 pp 229533.V6RCZ8
22972 229533.I1RKB2 pp 229533.V6R1D2
22973 229533.I1RKB2 pp 229533.V6R0W1
22974 229533.I1RKB2 pp 229533.I1RPR1
22975 229533.I1RKB2 pp 229533.I1RY12
22976 229533.I1RKB2 pp 229533.I1RNY6
22977 229533.I1RKB2 pp 229533.I1RY49
22978 229533.I1RKB2 pp 229533.I1RXZ4
22979 229533.I1RKB2 pp 229533.I1RXF0
22980 229533.I1RKB2 pp 229533.I1RPN4
22981 229533.I1RKB2 pp 229533.V6QVH2
22982 229533.I1RKB2 pp 229533.I1RVI8
22983 229533.I1RKB2 pp 229533.I1RTJ2
22984 229533.I1RKB2 pp 229533.I1RRX5
22985 229533.I1RKB2 pp 229533.I1S8C1
22986 229533.I1RKB2 pp 229533.I1RQ14
22987 229533.I1RKB2 pp 229533.I1RRJ3
22988 229533.I1RKB2 pp 229533.I1RMV0
22989 229533.I1RKB2 pp 229533.V6RF62
22990 229533.I1RKB2 pp 229533.V6RRM0
22991 229533.I1RKB2 pp 229533.I1RZM4
22992 229533.I1RKB2 pp 229533.V6RM02
22993 229533.I1RKB2 pp 229533.I1RVX0
22994 229533.I1RKB2 pp 229533.I1S4S5
22995 229533.I1RKB2 pp 229533.I1S147

22996	229533.I1RKB2	pp	229533.V6RN69
22997	229533.I1RKB2	pp	229533.I1RYA0
22998	229533.I1RKB2	pp	229533.I1S256
22999	229533.I1RKB2	pp	229533.I1RMK6
23000	229533.I1RKB2	pp	229533.I1S0V7
23001	229533.I1RKB2	pp	229533.I1S8R2
23002	229533.I1RKB2	pp	229533.V6QU99
23003	229533.I1RKB2	pp	229533.I1S8R4
23004	229533.I1RKB2	pp	229533.I1S0K0
23005	229533.I1RKB2	pp	229533.I1RVX8
23006	229533.I1RKB2	pp	229533.I1RP86
23007	229533.I1RKB2	pp	229533.V6R756
23008	229533.I1RKB2	pp	229533.I1RN84
23009	229533.I1RKB2	pp	229533.V6R9L8
23010	229533.I1RKB2	pp	229533.I1RQE1
23011	229533.I1RKB2	pp	229533.V6RAM6
23012	229533.I1RKB2	pp	229533.V6R1X4
23013	229533.I1RKB2	pp	229533.I1RKB5
23014	229533.I1RKB2	pp	229533.I1RYC8
23015	229533.I1RKB2	pp	229533.I1RX18
23016	229533.I1RKB2	pp	229533.I1S6P5
23017	229533.I1RKB2	pp	229533.I1S0B8
23018	229533.I1RKB2	pp	229533.V6RLM7
23019	229533.I1RKB2	pp	229533.I1RVR2
23020	229533.I1RKB2	pp	229533.I1RVZ3
23021	229533.I1RKB2	pp	229533.V6R5D2
23022	229533.I1RKB2	pp	229533.I1RN88
23023	229533.I1RKB2	pp	229533.I1RUR8
23024	229533.I1RKB2	pp	229533.I1S2H4
23025	229533.I1RKB2	pp	229533.V6RVJ3
23026	229533.I1RKB2	pp	229533.I1S3G4
23027	229533.I1RKB2	pp	229533.I1S3T7
23028	229533.I1RKB2	pp	229533.I1RY62
23029	229533.I1RKB2	pp	229533.V6R2Z2
23030	229533.I1RKB2	pp	229533.V6QZ48
23031	229533.I1RKB2	pp	229533.I1S299
23032	229533.I1RKB2	pp	229533.I1RSQ4
23033	229533.I1RKB2	pp	229533.I1RZA7
23034	229533.I1RKB2	pp	229533.O74638
23035	229533.I1RKB2	pp	229533.I1RYS9
23036	229533.I1RKB2	pp	229533.I1RYK0
23037	229533.I1RKB2	pp	229533.I1RSE0
23038	229533.I1RKB2	pp	229533.I1RLZ4
23039	229533.I1RKB2	pp	229533.I1RPP3
23040	229533.I1RKB2	pp	229533.I1RYW9
23041	229533.I1RKB2	pp	229533.I1RMJ3
23042	229533.I1RKB2	pp	229533.I1RL81
23043	229533.I1RKB2	pp	229533.I1RSA5
23044	229533.I1RKB2	pp	229533.V6RV57
23045	229533.I1RKB2	pp	229533.V6RUB1
23046	229533.I1RKB2	pp	229533.I1S1F3
23047	229533.I1RKB2	pp	229533.Q4HVQ9
23048	229533.I1RKB2	pp	229533.I1RR68
23049	229533.I1RKB2	pp	229533.V6R3J1
23050	229533.I1RKB2	pp	229533.V6RZD4
23051	229533.I1RKB2	pp	229533.I1RWF5
23052	229533.I1RKB2	pp	229533.I1RZV5
23053	229533.I1RTB3	pp	229533.I1RWR7
23054	229533.I1RTB3	pp	229533.I1S2B8
23055	229533.I1RTB3	pp	229533.I1RZM8
23056	229533.I1RTB3	pp	229533.I1S1W9
23057	229533.I1RTB3	pp	229533.I1RVX0
23058	229533.I1RTB3	pp	229533.I1S0W4
23059	229533.I1RTB3	pp	229533.I1S4S7
23060	229533.I1RTB3	pp	229533.I1RZV3
23061	229533.I1RTB3	pp	229533.I1RTD5
23062	229533.I1RTB3	pp	229533.I1RYI5
23063	229533.I1RTB3	pp	229533.V6R9Z0
23064	229533.I1RTB3	pp	229533.I1S821
23065	229533.I1RTB3	pp	229533.V6RCZ8
23066	229533.I1RTB3	pp	229533.I1RZ39
23067	229533.I1RTB3	pp	229533.I1RXD6
23068	229533.I1RTB3	pp	229533.I1S1B2

229533.I1RTB3 pp 229533.Q4IJK2
229533.I1RTB3 pp 229533.I1RVQ3
229533.I1RTB3 pp 229533.I1S5R1
229533.I1RTB3 pp 229533.I1RX95
229533.I1RTB3 pp 229533.I1RZK5
229533.I1RTB3 pp 229533.I1RXC4
229533.I1RTB3 pp 229533.I1RXD7
229533.I1RTB3 pp 229533.I1RVZ2
229533.I1RTB3 pp 229533.I1S5R6
229533.I1RGK5 pp 229533.I1S1F3
229533.I1RBI8 pp 229533.I1RRR7
229533.I1RBI8 pp 229533.I1RNH6
229533.I1RBI8 pp 229533.I1RMC0
229533.I1RBI8 pp 229533.I1RQ31
229533.I1RBI8 pp 229533.I1S021
229533.I1RBI8 pp 229533.I1RPJ3
229533.I1RBI8 pp 229533.I1RMD5
229533.I1RBI8 pp 229533.I1RTE9
229533.I1RBI8 pp 229533.Q4I327
229533.I1RBI8 pp 229533.I1S0R7
229533.I1RBI8 pp 229533.I1RSI6
229533.I1RBI8 pp 229533.I1RMN6
229533.I1RBI8 pp 229533.I1RUQ2
229533.I1RBI8 pp 229533.I1RC27
229533.I1RBI8 pp 229533.I1S2C0
229533.I1RBI8 pp 229533.I1RC72
229533.I1RBI8 pp 229533.I1RCN5
229533.I1RBI8 pp 229533.I1RQA2
229533.I1RBI8 pp 229533.I1RNU6
229533.I1RBI8 pp 229533.I1RKQ5
229533.I1RBI8 pp 229533.I1RM25
229533.I1RBI8 pp 229533.I1RCL3
229533.I1RBI8 pp 229533.Q4IAS1
229533.I1RBI8 pp 229533.Q4IAA0
229533.I1RBI8 pp 229533.Q4IJ56
229533.I1RBI8 pp 229533.V6RGY3
229533.I1RBI8 pp 229533.I1RJL4
229533.I1RBI8 pp 229533.Q4IPI1
229533.I1RBI8 pp 229533.I1RPY1
229533.I1RBI8 pp 229533.I1RZZ1
229533.I1RBI8 pp 229533.I1RQS5
229533.I1RBI8 pp 229533.I1S9Y0
229533.I1RBI8 pp 229533.Q4IEK8
229533.I1RBI8 pp 229533.I1RQ28
229533.I1RBI8 pp 229533.I1S1X9
229533.I1RBI8 pp 229533.I1RFN1
229533.I1RBI8 pp 229533.I1RDA0
229533.I1RBI8 pp 229533.Q4HVW2
229533.I1RBI8 pp 229533.I1RC82
229533.I1RBI8 pp 229533.Q4I5Z5
229533.I1RBI8 pp 229533.V6R283
229533.I1RBI8 pp 229533.I1RMK3
229533.I1RBI8 pp 229533.I1RFW5
229533.I1RBI8 pp 229533.Q4I830
229533.I1RBI8 pp 229533.I1RVZ0
229533.I1RBI8 pp 229533.I1RM67
229533.I1RBI8 pp 229533.I1RKJ6
229533.I1RBI8 pp 229533.I1RE00
229533.I1RBI8 pp 229533.I1RRJ8
229533.I1RBI8 pp 229533.I1RZ59
229533.I1RBI8 pp 229533.Q4HZ47
229533.I1RBI8 pp 229533.I1S9U2
229533.I1RBI8 pp 229533.I1RNA0
229533.I1RBI8 pp 229533.I1S6F0
229533.I1RBI8 pp 229533.I1RD56
229533.I1RBI8 pp 229533.I1RF08
229533.I1RBI8 pp 229533.I1RRQ6
229533.I1RBI8 pp 229533.V6QYF0
229533.I1RBI8 pp 229533.I1RT98
229533.I1RBI8 pp 229533.Q4HZ42
229533.I1RBI8 pp 229533.I1RZJ6
229533.I1RBI8 pp 229533.I1RX24
229533.I1RBI8 pp 229533.I1RZS6

23142	229533.I1RBI8	pp	229533.I1RQ65
23143	229533.I1RBI8	pp	229533.I1RQM6
23144	229533.I1RBI8	pp	229533.I1RGC9
23145	229533.I1RBI8	pp	229533.Q4IFI0
23146	229533.I1RBI8	pp	229533.I1RTF4
23147	229533.I1RBI8	pp	229533.I1RT80
23148	229533.I1RBI8	pp	229533.I1RU69
23149	229533.I1RX22	pp	229533.I1S2C4
23150	229533.I1RX22	pp	229533.I1S7B9
23151	229533.I1RX22	pp	229533.V6R057
23152	229533.I1RX22	pp	229533.I1S1P8
23153	229533.I1RX22	pp	229533.V6RRH2
23154	229533.I1RX22	pp	229533.V6QT89
23155	229533.I1RX22	pp	229533.I1S084
23156	229533.I1RX22	pp	229533.I1RZT0
23157	229533.I1RX22	pp	229533.V6R1K4
23158	229533.I1RX22	pp	229533.I1RYQ1
23159	229533.I1RX22	pp	229533.I1S216
23160	229533.I1RX22	pp	229533.Q4HZI0
23161	229533.I1RX22	pp	229533.Q4INS6
23162	229533.I1RX22	pp	229533.I1RY98
23163	229533.I1RX22	pp	229533.V6R2A1
23164	229533.I1RX22	pp	229533.I1RY29
23165	229533.I1RX22	pp	229533.I1S4Q4
23166	229533.I1RX22	pp	229533.I1S3K8
23167	229533.I1RX22	pp	229533.V6RBN2
23168	229533.I1RG92	pp	229533.I1RS07
23169	229533.I1RKX2	pp	229533.I1RVV3
23170	229533.I1RKX2	pp	229533.I1RQ14
23171	229533.I1RKX2	pp	229533.I1RP86
23172	229533.I1RKX2	pp	229533.I1RX90
23173	229533.I1RKX2	pp	229533.I1RX64
23174	229533.I1RKX2	pp	229533.V6RRM0
23175	229533.I1RKX2	pp	229533.I1RU18
23176	229533.I1S4Z8	pp	229533.I1S7W5
23177	229533.I1S4Z8	pp	229533.V6RMW6
23178	229533.I1S4Z8	pp	229533.Q4I073
23179	229533.I1S4Z8	pp	229533.V6R057
23180	229533.I1S4Z8	pp	229533.V6RCZ8
23181	229533.I1S4Z8	pp	229533.I1SAN9
23182	229533.I1S4Z8	pp	229533.I1S5B0
23183	229533.I1S4Z8	pp	229533.I1S5R6
23184	229533.I1S4Z8	pp	229533.V6R042
23185	229533.I1S4Z8	pp	229533.Q4HXF0
23186	229533.I1S4Z8	pp	229533.V6RBN2
23187	229533.I1S4Z8	pp	229533.I1S5R1
23188	229533.I1S4Z8	pp	229533.Q4IJK2
23189	229533.I1S4Z8	pp	229533.V6RRH2
23190	229533.I1S4Z8	pp	229533.V6R1K4
23191	229533.I1S4Z8	pp	229533.V6RTJ8
23192	229533.A0A0E0SMR8	pp	229533.I1RG88
23193	229533.A0A0E0SMR8	pp	229533.I1RC11
23194	229533.A0A0E0SMR8	pp	229533.A0A1C3YLR6
23195	229533.A0A0E0SMR8	pp	229533.I1S029
23196	229533.A0A0E0SMR8	pp	229533.I1RAR3
23197	229533.A0A0E0SMR8	pp	229533.I1RPW2
23198	229533.A0A0E0SMR8	pp	229533.I1RYQ4
23199	229533.A0A0E0SMR8	pp	229533.I1RHQ1
23200	229533.A0A0E0SMR8	pp	229533.I1RRG3
23201	229533.A0A0E0SMR8	pp	229533.I1RXB1
23202	229533.A0A0E0SMR8	pp	229533.I1RQE8
23203	229533.A0A0E0SMR8	pp	229533.I1RMM8
23204	229533.A0A0E0SMR8	pp	229533.I1S1P8
23205	229533.A0A0E0SMR8	pp	229533.I1RWI3
23206	229533.A0A0E0SMR8	pp	229533.I1RD56
23207	229533.A0A0E0SMR8	pp	229533.A0A1C3YKZ8
23208	229533.A0A0E0SMR8	pp	229533.I1RYJ7
23209	229533.A0A0E0SMR8	pp	229533.I1S4S4
23210	229533.A0A0E0SMR8	pp	229533.I1RP86
23211	229533.A0A0E0SMR8	pp	229533.I1RAN7
23212	229533.A0A0E0SMR8	pp	229533.I1RMK4
23213	229533.A0A0E0SMR8	pp	229533.I1RZM4
23214	229533.A0A0E0SMR8	pp	229533.A0A0E0SP71

23215	229533.A0A0E0SMR8	pp	229533.A0A1C3YJQ6
23216	229533.A0A0E0SMR8	pp	229533.I1S084
23217	229533.A0A0E0SMR8	pp	229533.A0A1C3YKY4
23218	229533.A0A0E0SMR8	pp	229533.I1RBE4
23219	229533.A0A0E0SMR8	pp	229533.A0A1C3YIK4
23220	229533.A0A0E0SMR8	pp	229533.I1RW69
23221	229533.A0A0E0SMR8	pp	229533.I1RNH6
23222	229533.A0A0E0SMR8	pp	229533.I1RC61
23223	229533.A0A0E0SMR8	pp	229533.I1RVX9
23224	229533.A0A0E0SMR8	pp	229533.I1S183
23225	229533.A0A0E0SMR8	pp	229533.I1S4D0
23226	229533.A0A0E0SMR8	pp	229533.A0A1C3YKV3
23227	229533.A0A0E0SMR8	pp	229533.V6RK96
23228	229533.A0A0E0SMR8	pp	229533.I1RYU5
23229	229533.A0A0E0SMR8	pp	229533.I1RA94
23230	229533.A0A0E0SMR8	pp	229533.I1S1W8
23231	229533.A0A0E0SMR8	pp	229533.I1RYI7
23232	229533.A0A0E0SMR8	pp	229533.I1RJT5
23233	229533.A0A0E0SMR8	pp	229533.I1RMG7
23234	229533.A0A0E0SMR8	pp	229533.I1RC74
23235	229533.A0A0E0SMR8	pp	229533.I1RA90
23236	229533.A0A0E0SMR8	pp	229533.A0A1C3YJR3
23237	229533.A0A0E0SMR8	pp	229533.I1RMG6
23238	229533.A0A0E0SMR8	pp	229533.I1RK95
23239	229533.A0A0E0SMR8	pp	229533.I1RNG4
23240	229533.A0A0E0SMR8	pp	229533.V6RQW4
23241	229533.A0A0E0SMR8	pp	229533.I1RXV9
23242	229533.A0A0E0SMR8	pp	229533.I1RE00
23243	229533.A0A0E0SMR8	pp	229533.I1RE53
23244	229533.A0A0E0SMR8	pp	229533.I1RCP7
23245	229533.A0A0E0SMR8	pp	229533.I1RVZ9
23246	229533.A0A0E0SMR8	pp	229533.Q4HXF0
23247	229533.A0A0E0SMR8	pp	229533.I1RK03
23248	229533.A0A0E0SMR8	pp	229533.I1RM26
23249	229533.A0A0E0SMR8	pp	229533.V6RY56
23250	229533.A0A0E0SMR8	pp	229533.A0A1C3YK68
23251	229533.A0A0E0SMR8	pp	229533.I1RAZ3
23252	229533.A0A0E0SMR8	pp	229533.I1S0N3
23253	229533.A0A0E0SMR8	pp	229533.I1RP97
23254	229533.A0A0E0SMR8	pp	229533.I1RR68
23255	229533.A0A0E0SMR8	pp	229533.I1RVY2
23256	229533.A0A0E0SMR8	pp	229533.I1RTA9
23257	229533.A0A0E0SMR8	pp	229533.V6RAC9
23258	229533.A0A0E0SMR8	pp	229533.I1RCV1
23259	229533.A0A0E0SMR8	pp	229533.I1RAQ4
23260	229533.A0A0E0SMR8	pp	229533.I1RPT0
23261	229533.A0A0E0SMR8	pp	229533.I1RBE7
23262	229533.A0A0E0SMR8	pp	229533.I1RIY0
23263	229533.A0A0E0SMR8	pp	229533.I1RYU0
23264	229533.A0A0E0SMR8	pp	229533.A0A0E0SMT9
23265	229533.A0A0E0SMR8	pp	229533.I1RTG7
23266	229533.A0A0E0SMR8	pp	229533.I1RZB2
23267	229533.A0A0E0SMR8	pp	229533.A0A1C3YIB2
23268	229533.A0A0E0SMR8	pp	229533.I1RGA4
23269	229533.A0A0E0SMR8	pp	229533.I1RFM6
23270	229533.A0A0E0SMR8	pp	229533.I1RW43
23271	229533.A0A0E0SMR8	pp	229533.I1RPW1
23272	229533.A0A0E0SMR8	pp	229533.I1RBC7
23273	229533.A0A0E0SMR8	pp	229533.A0A1C3YLA6
23274	229533.A0A0E0SMR8	pp	229533.I1RSQ4
23275	229533.A0A0E0SMR8	pp	229533.I1S268
23276	229533.A0A0E0SMR8	pp	229533.I1RWH9
23277	229533.A0A0E0SMR8	pp	229533.I1RFW3
23278	229533.A0A0E0SMR8	pp	229533.I1RWF9
23279	229533.A0A0E0SMR8	pp	229533.I1RE58
23280	229533.A0A0E0SMR8	pp	229533.I1RXW3
23281	229533.A0A0E0SMR8	pp	229533.A0A0E0SPV0
23282	229533.A0A0E0SMR8	pp	229533.I1RX72
23283	229533.A0A0E0SMR8	pp	229533.I1S3G4
23284	229533.A0A0E0SMR8	pp	229533.I1RZ20
23285	229533.A0A0E0SMR8	pp	229533.I1RVR2
23286	229533.A0A0E0SMR8	pp	229533.I1RRT3
23287	229533.A0A0E0SMR8	pp	229533.O74638

23288 229533.A0A0E0SMR8 pp 229533.I1RH65
23289 229533.A0A0E0SMR8 pp 229533.I1RYW6
23290 229533.A0A0E0SMR8 pp 229533.I1RPD6
23291 229533.A0A0E0SMR8 pp 229533.I1RY62
23292 229533.A0A0E0SMR8 pp 229533.I1S9Y1
23293 229533.A0A0E0SMR8 pp 229533.I1RPU8
23294 229533.A0A0E0SMR8 pp 229533.I1RN88
23295 229533.A0A0E0SMR8 pp 229533.V6R382
23296 229533.A0A0E0SMR8 pp 229533.I1RZ52
23297 229533.A0A0E0SMR8 pp 229533.V6R042
23298 229533.A0A0E0SMR8 pp 229533.I1RM25
23299 229533.A0A0E0SMR8 pp 229533.I1RBS5
23300 229533.A0A0E0SMR8 pp 229533.I1RTI7
23301 229533.A0A0E0SMR8 pp 229533.I1RPK0
23302 229533.A0A0E0SMR8 pp 229533.I1RZQ1
23303 229533.A0A0E0SMR8 pp 229533.I1RSL5
23304 229533.A0A0E0SMR8 pp 229533.A0A1C3YI05
23305 229533.A0A0E0SMR8 pp 229533.I1RG11
23306 229533.A0A0E0SMR8 pp 229533.I1S256
23307 229533.A0A0E0SMR8 pp 229533.I1S2C4
23308 229533.A0A0E0SMR8 pp 229533.I1RCB4
23309 229533.A0A0E0SMR8 pp 229533.I1RSU2
23310 229533.A0A0E0SMR8 pp 229533.V6RSF3
23311 229533.A0A0E0SMR8 pp 229533.I1RIH0
23312 229533.A0A0E0SMR8 pp 229533.I1SAJ3
23313 229533.A0A0E0SMR8 pp 229533.I1RCG5
23314 229533.A0A0E0SMR8 pp 229533.V6QVH8
23315 229533.A0A0E0SMR8 pp 229533.I1RQ30
23316 229533.A0A0E0SMR8 pp 229533.I1RCN5
23317 229533.A0A0E0SMR8 pp 229533.I1RCH5
23318 229533.A0A0E0SMR8 pp 229533.I1RNU7
23319 229533.A0A0E0SMR8 pp 229533.I1RAR1
23320 229533.I1RTA9 pp 229533.V6RQW4
23321 229533.I1RTA9 pp 229533.I1S8G4
23322 229533.I1RTA9 pp 229533.I1RX22
23323 229533.I1RTA9 pp 229533.V6R057
23324 229533.I1RTA9 pp 229533.O74638
23325 229533.I1RTA9 pp 229533.I1S216
23326 229533.I1RTA9 pp 229533.Q4HXF0
23327 229533.I1RTA9 pp 229533.V6R1K4
23328 229533.I1RQN0 pp 229533.I1S0R2
23329 229533.I1RQN0 pp 229533.I1S4S8
23330 229533.A0A098D5S0 pp 229533.I1RM26
23331 229533.A0A098D5S0 pp 229533.I1RE72
23332 229533.A0A098D5S0 pp 229533.V6RY56
23333 229533.A0A098D5S0 pp 229533.I1RTT4
23334 229533.A0A098D5S0 pp 229533.A0A0E0RQ52
23335 229533.A0A098D5S0 pp 229533.I1RPP2
23336 229533.A0A098D5S0 pp 229533.I1RBF4
23337 229533.A0A098D5S0 pp 229533.I1RFM6
23338 229533.A0A098D5S0 pp 229533.A0A1C3YM97
23339 229533.A0A098D5S0 pp 229533.I1RCV1
23340 229533.A0A098D5S0 pp 229533.A0A0E0S413
23341 229533.A0A098D5S0 pp 229533.A0A098DVZ4
23342 229533.A0A098D5S0 pp 229533.I1S3D9
23343 229533.A0A098D5S0 pp 229533.A0A098DT85
23344 229533.A0A098D5S0 pp 229533.V6QUG0
23345 229533.A0A098D5S0 pp 229533.I1RVX9
23346 229533.A0A098D5S0 pp 229533.I1RVV3
23347 229533.A0A098D5S0 pp 229533.A0A0E0S134
23348 229533.A0A098D5S0 pp 229533.A0A0E0RV94
23349 229533.A0A098D5S0 pp 229533.I1RKR5
23350 229533.A0A098D5S0 pp 229533.I1RB33
23351 229533.A0A098D5S0 pp 229533.I1S084
23352 229533.A0A098D5S0 pp 229533.I1RC61
23353 229533.A0A098D5S0 pp 229533.I1RIE8
23354 229533.A0A098D5S0 pp 229533.I1S4D0
23355 229533.A0A098D5S0 pp 229533.I1RYU5
23356 229533.A0A098D5S0 pp 229533.I1REA3
23357 229533.A0A098D5S0 pp 229533.A0A098DDY0
23358 229533.A0A098D5S0 pp 229533.A0A1C3YK11
23359 229533.A0A098D5S0 pp 229533.I1RSB7
23360 229533.A0A098D5S0 pp 229533.I1RGY2

23361	229533.A0A098D5S0	pp	229533.A0A1C3YI71
23362	229533.A0A098D5S0	pp	229533.I1RK30
23363	229533.A0A098D5S0	pp	229533.I1RBE4
23364	229533.A0A098D5S0	pp	229533.I1S029
23365	229533.A0A098D5S0	pp	229533.I1RBT1
23366	229533.A0A098D5S0	pp	229533.I1RMM8
23367	229533.A0A098D5S0	pp	229533.I1S1P8
23368	229533.A0A098D5S0	pp	229533.I1RP86
23369	229533.A0A098D5S0	pp	229533.A0A0E0SQ99
23370	229533.A0A098D5S0	pp	229533.I1RCG5
23371	229533.A0A098D5S0	pp	229533.I1S2C4
23372	229533.A0A098D5S0	pp	229533.Q4I1Y5
23373	229533.A0A098D5S0	pp	229533.I1RAR1
23374	229533.A0A098D5S0	pp	229533.I1RX90
23375	229533.A0A098D5S0	pp	229533.I1RQG5
23376	229533.A0A098D5S0	pp	229533.A0A0E0RU55
23377	229533.A0A098D5S0	pp	229533.I1RHJ2
23378	229533.A0A098D5S0	pp	229533.I1RMX6
23379	229533.A0A098D5S0	pp	229533.I1S9Z4
23380	229533.A0A098D5S0	pp	229533.A0A098DCQ7
23381	229533.A0A098D5S0	pp	229533.A0A098DLA7
23382	229533.A0A098D5S0	pp	229533.V6RLM7
23383	229533.A0A098D5S0	pp	229533.I1RCB4
23384	229533.A0A098D5S0	pp	229533.A0A0E0SI42
23385	229533.A0A098D5S0	pp	229533.V6RPQ8
23386	229533.A0A098D5S0	pp	229533.A0A1C3YHP2
23387	229533.A0A098D5S0	pp	229533.I1RPU8
23388	229533.A0A098D5S0	pp	229533.I1RU18
23389	229533.A0A098D5S0	pp	229533.A0A1C3YKK9
23390	229533.A0A098D5S0	pp	229533.I1RQ29
23391	229533.A0A098D5S0	pp	229533.I1RW43
23392	229533.A0A098D5S0	pp	229533.I1RPD6
23393	229533.A0A098D5S0	pp	229533.I1RC31
23394	229533.A0A098D5S0	pp	229533.I1RNG4
23395	229533.A0A098D5S0	pp	229533.I1RZ20
23396	229533.A0A098D5S0	pp	229533.I1RKB2
23397	229533.A0A098D5S0	pp	229533.I1RMJ3
23398	229533.A0A098D5S0	pp	229533.I1RYK0
23399	229533.A0A098D5S0	pp	229533.I1RJS2
23400	229533.A0A098D5S0	pp	229533.I1RKX2
23401	229533.A0A098D5S0	pp	229533.A0A0E0SMR8
23402	229533.A0A098D5S0	pp	229533.I1RTA9
23403	229533.A0A098D5S0	pp	229533.Q4HXF0
23404	229533.I1S6T4	pp	229533.V6RIV9
23405	229533.I1R9H9	pp	229533.I1RPU2
23406	229533.I1R9H9	pp	229533.I1RVZ2
23407	229533.I1R9H9	pp	229533.I1S5R6
23408	229533.I1R9H9	pp	229533.I1RDF7
23409	229533.I1R9H9	pp	229533.I1RQ85
23410	229533.I1R9H9	pp	229533.I1RZK5
23411	229533.I1R9H9	pp	229533.I1RT40
23412	229533.I1R9H9	pp	229533.I1S1W9
23413	229533.I1R9H9	pp	229533.I1RZV3
23414	229533.I1R9H9	pp	229533.I1RGA4
23415	229533.I1R9H9	pp	229533.I1RWR7
23416	229533.I1R9H9	pp	229533.I1RSZ7
23417	229533.I1R9H9	pp	229533.I1RAW0
23418	229533.I1R9H9	pp	229533.I1R9M0
23419	229533.I1R9H9	pp	229533.I1S4S7
23420	229533.I1R9H9	pp	229533.I1RRM3
23421	229533.I1R9H9	pp	229533.I1RZM8
23422	229533.I1R9H9	pp	229533.I1S2B8
23423	229533.I1R9H9	pp	229533.I1RYI5
23424	229533.I1R9H9	pp	229533.I1RZ39
23425	229533.I1R9H9	pp	229533.I1RPE0
23426	229533.I1R9H9	pp	229533.I1S5R1
23427	229533.I1S8G4	pp	229533.V6RRH2
23428	229533.I1S8G4	pp	229533.V6RBN2
23429	229533.I1S8G4	pp	229533.Q4INS6
23430	229533.I1S8G4	pp	229533.V6R1K4
23431	229533.I1S8G4	pp	229533.V6R057
23432	229533.I1S8G4	pp	229533.V6QT89
23433	229533.I1RK73	pp	229533.I1S9X9

23434 229533.I1RK73 pp 229533.I1RPC6
23435 229533.I1RK73 pp 229533.I1RQF7
23436 229533.I1RK73 pp 229533.I1RQ32
23437 229533.I1RK73 pp 229533.I1RSH8
23438 229533.I1RK73 pp 229533.I1RP29
23439 229533.I1RK73 pp 229533.I1RNB9
23440 229533.I1RK73 pp 229533.I1S287
23441 229533.I1RK73 pp 229533.I1RS34
23442 229533.I1RK73 pp 229533.I1S4N5
23443 229533.I1RK73 pp 229533.I1RWR8
23444 229533.I1RK73 pp 229533.I1RM71
23445 229533.I1RK73 pp 229533.I1RWB4
23446 229533.I1RK73 pp 229533.I1S7S5
23447 229533.I1RS34 pp 229533.I1S287
23448 229533.I1RS34 pp 229533.I1RYP0
23449 229533.I1RS34 pp 229533.I1RZK3
23450 229533.I1RS34 pp 229533.I1RY40
23451 229533.I1RS34 pp 229533.I1RWB4
23452 229533.I1RS34 pp 229533.I1S4N5
23453 229533.I1RS34 pp 229533.I1RTF2
23454 229533.I1RS34 pp 229533.I1RSH8
23455 229533.I1RS34 pp 229533.I1RX87
23456 229533.I1RS34 pp 229533.I1S1U6
23457 229533.I1RS34 pp 229533.I1S0V5
23458 229533.I1RS34 pp 229533.I1S7S5
23459 229533.I1RS34 pp 229533.I1RTQ9
23460 229533.I1RS34 pp 229533.I1RWR8
23461 229533.I1RS34 pp 229533.I1RTF5
23462 229533.I1RHW8 pp 229533.I1RQW2
23463 229533.I1RHW8 pp 229533.I1RME1
23464 229533.I1RHW8 pp 229533.I1S2L3
23465 229533.I1RHW8 pp 229533.I1SAK0
23466 229533.I1RHW8 pp 229533.I1RTN8
23467 229533.I1RHW8 pp 229533.I1RJ14
23468 229533.I1RHW8 pp 229533.I1RJU3
23469 229533.I1RHW8 pp 229533.I1RTN1
23470 229533.I1RHW8 pp 229533.I1RS17
23471 229533.I1RHW8 pp 229533.V6RIV9
23472 229533.I1RHW8 pp 229533.I1RUT8
23473 229533.I1RQK8 pp 229533.I1SAN9
23474 229533.I1RQK8 pp 229533.I1RRI3
23475 229533.I1RQK8 pp 229533.I1RRV1
23476 229533.I1RQK8 pp 229533.Q4IJM4
23477 229533.I1RQK8 pp 229533.I1RSV2
23478 229533.A0A0E0RR64 pp 229533.I1RGC1
23479 229533.A0A0E0RR64 pp 229533.I1RFV4
23480 229533.A0A0E0RR64 pp 229533.I1RM71
23481 229533.A0A0E0RR64 pp 229533.I1RZS5
23482 229533.A0A0E0RR64 pp 229533.A0A0E0SP22
23483 229533.A0A0E0RR64 pp 229533.I1RAH7
23484 229533.A0A0E0RR64 pp 229533.I1RQF7
23485 229533.A0A0E0RR64 pp 229533.I1S1U6
23486 229533.A0A0E0RR64 pp 229533.I1RX87
23487 229533.A0A0E0RR64 pp 229533.I1RS34
23488 229533.A0A0E0RR64 pp 229533.I1RE18
23489 229533.A0A0E0RR64 pp 229533.I1RQD6
23490 229533.A0A0E0RR64 pp 229533.I1RQN8
23491 229533.A0A0E0RR64 pp 229533.A0A1C3YKT4
23492 229533.A0A0E0RR64 pp 229533.I1RTF2
23493 229533.A0A0E0RR64 pp 229533.I1S830
23494 229533.A0A0E0RR64 pp 229533.I1S2B6
23495 229533.A0A0E0RR64 pp 229533.I1RNG2
23496 229533.A0A0E0RR64 pp 229533.I1RSW4
23497 229533.A0A0E0RR64 pp 229533.I1RQF9
23498 229533.A0A0E0RR64 pp 229533.I1RG01
23499 229533.A0A0E0RR64 pp 229533.I1RQP7
23500 229533.A0A0E0RR64 pp 229533.I1RFP5
23501 229533.A0A0E0RR64 pp 229533.I1RE44
23502 229533.A0A0E0RR64 pp 229533.I1RMN3
23503 229533.A0A0E0RR64 pp 229533.I1RSH3
23504 229533.A0A0E0RR64 pp 229533.I1S278
23505 229533.A0A0E0RR64 pp 229533.I1RN70
23506 229533.A0A0E0RR64 pp 229533.I1RAF6

229533.A0A0E0RR64	pp	229533.I1S287
229533.A0A0E0RR64	pp	229533.I1RBS3
229533.A0A0E0RR64	pp	229533.I1RY40
229533.A0A0E0RR64	pp	229533.I1RWZ9
229533.A0A0E0RR64	pp	229533.V6QXP4
229533.A0A0E0RR64	pp	229533.I1RCV3
229533.A0A0E0RR64	pp	229533.I1RCN6
229533.A0A0E0RR64	pp	229533.Q4HY20
229533.A0A0E0RR64	pp	229533.A0A0E0S3U6
229533.A0A0E0RR64	pp	229533.I1RYP0
229533.A0A0E0RR64	pp	229533.I1RNB9
229533.A0A0E0RR64	pp	229533.I1RBT0
229533.A0A0E0SMQ1	pp	229533.I1RTD5
229533.A0A0E0SMQ1	pp	229533.A0A1C3YJI6
229533.A0A0E0SMQ1	pp	229533.I1RT40
229533.A0A0E0SMQ1	pp	229533.I1RXD7
229533.A0A0E0SMQ1	pp	229533.I1RC34
229533.A0A0E0SMQ1	pp	229533.A0A1C3YLK8
229533.A0A0E0SMQ1	pp	229533.I1RGA4
229533.A0A0E0SMQ1	pp	229533.I1RTA1
229533.A0A0E0SMQ1	pp	229533.I1RJN9
229533.A0A0E0SMQ1	pp	229533.I1RVZ2
229533.A0A0E0SMQ1	pp	229533.I1RZ39
229533.A0A0E0SMQ1	pp	229533.I1RYI5
229533.A0A0E0SMQ1	pp	229533.I1RJU3
229533.A0A0E0SMQ1	pp	229533.I1RAG7
229533.A0A0E0SMQ1	pp	229533.I1RSF1
229533.A0A0E0SMQ1	pp	229533.I1S4S7
229533.A0A0E0SMQ1	pp	229533.I1S5R1
229533.A0A0E0SMQ1	pp	229533.I1RXD6
229533.A0A0E0SMQ1	pp	229533.I1RA11
229533.A0A0E0SMQ1	pp	229533.I1RQ85
229533.A0A0E0SMQ1	pp	229533.I1RRI1
229533.A0A0E0SMQ1	pp	229533.I1S0W4
229533.A0A0E0SMQ1	pp	229533.I1RPU2
229533.A0A0E0SMQ1	pp	229533.I1REC5
229533.A0A0E0SMQ1	pp	229533.I1RDF7
229533.A0A0E0SMQ1	pp	229533.I1RR24
229533.A0A0E0SMQ1	pp	229533.I1RSA5
229533.A0A0E0SMQ1	pp	229533.I1RPE0
229533.A0A0E0SMQ1	pp	229533.I1RD52
229533.A0A0E0SMQ1	pp	229533.I1RZK5
229533.A0A0E0SMQ1	pp	229533.I1RSZ7
229533.A0A0E0SMQ1	pp	229533.I1R9M0
229533.A0A0E0SMQ1	pp	229533.I1RE89
229533.A0A0E0SMQ1	pp	229533.I1S2B8
229533.A0A0E0SMQ1	pp	229533.I1RWR7
229533.A0A0E0SMQ1	pp	229533.I1RAW0
229533.A0A0E0SMQ1	pp	229533.I1RCH5
229533.A0A0E0SMQ1	pp	229533.Q4IJK2
229533.A0A0E0SMQ1	pp	229533.I1RVX0
229533.A0A0E0SMQ1	pp	229533.A0A1C3YM21
229533.A0A0E0SMQ1	pp	229533.I1RQ33
229533.A0A0E0SMQ1	pp	229533.I1RCR8
229533.A0A0E0SMQ1	pp	229533.I1RZM8
229533.A0A0E0SMQ1	pp	229533.I1RRM3
229533.A0A0E0SMQ1	pp	229533.I1S821
229533.A0A0E0SMQ1	pp	229533.A0A1C3YJV6
229533.A0A0E0SMQ1	pp	229533.I1S5R6
229533.A0A0E0SMQ1	pp	229533.A0A1C3YK34
229533.A0A0E0SMQ1	pp	229533.A0A1C3YJN3
229533.A0A0E0SMQ1	pp	229533.I1RZV3
229533.A0A0E0SMQ1	pp	229533.I1S1W9
229533.A0A1C3YKF1	pp	229533.A0A1C3YM79
229533.A0A1C3YKF1	pp	229533.I1RG11
229533.A0A1C3YKF1	pp	229533.I1RNB8
229533.A0A1C3YKF1	pp	229533.I1RCB7
229533.A0A1C3YKF1	pp	229533.I1RK92
229533.A0A1C3YKF1	pp	229533.I1RR25
229533.A0A1C3YKF1	pp	229533.I1RK79
229533.A0A1C3YKF1	pp	229533.I1RG90
229533.A0A1C3YKF1	pp	229533.I1RRU9
229533.A0A1C3YKF1	pp	229533.I1S4M5

229533.A0A1C3YKF1 pp 229533.I1RT35
229533.A0A1C3YKF1 pp 229533.I1RC03
229533.A0A1C3YKF1 pp 229533.I1RRT0
229533.A0A1C3YKF1 pp 229533.I1RC24
229533.A0A1C3YKF1 pp 229533.I1RZB5
229533.A0A1C3YKF1 pp 229533.I1RJL2
229533.A0A1C3YKF1 pp 229533.I1RE31
229533.A0A1C3YKF1 pp 229533.I1RRG3
229533.A0A1C3YKF1 pp 229533.I1S0B6
229533.I1RDN5 pp 229533.I1RPW8
229533.I1RDN5 pp 229533.I1RW91
229533.I1RDN5 pp 229533.I1RN04
229533.I1RDN5 pp 229533.I1RRF7
229533.I1RDN5 pp 229533.I1RRJ9
229533.I1RDN5 pp 229533.I1SAF7
229533.A0A098DVM8 pp 229533.I1RS34
229533.A0A098DVM8 pp 229533.A0A0E0RR64
229533.A0A098DVM8 pp 229533.I1RK73
229533.A0A098DVM8 pp 229533.A0A1C3YN54
229533.A0A098DVM8 pp 229533.I1RQC4
229533.A0A098DVM8 pp 229533.A0A0E0RU86
229533.A0A098DVM8 pp 229533.I1S7S5
229533.A0A098DVM8 pp 229533.I1S7S0
229533.A0A098DVM8 pp 229533.A0A0E0SP22
229533.A0A098DVM8 pp 229533.I1RE44
229533.A0A098DVM8 pp 229533.I1RMN3
229533.A0A098DVM8 pp 229533.I1RRC8
229533.A0A098DVM8 pp 229533.I1RWR8
229533.A0A098DVM8 pp 229533.I1RQN8
229533.A0A098DVM8 pp 229533.I1S1U6
229533.A0A098DVM8 pp 229533.I1RTQ9
229533.A0A098DVM8 pp 229533.I1RRC9
229533.A0A098DVM8 pp 229533.I1RG01
229533.A0A098DVM8 pp 229533.I1S0V5
229533.A0A098DVM8 pp 229533.I1RTF2
229533.A0A098DVM8 pp 229533.A0A1C3YKT4
229533.A0A098DVM8 pp 229533.I1S287
229533.A0A098DVM8 pp 229533.I1S278
229533.A0A098DVM8 pp 229533.I1RWZ9
229533.A0A098DVM8 pp 229533.I1RX87
229533.A0A098DVM8 pp 229533.I1RY40
229533.A0A098DVM8 pp 229533.I1S4N5
229533.A0A098DVM8 pp 229533.I1RSH8
229533.A0A098DVM8 pp 229533.I1RM71
229533.A0A098DVM8 pp 229533.I1RPC6
229533.A0A098DVM8 pp 229533.A0A0E0S643
229533.A0A098DVM8 pp 229533.I1RNB9
229533.A0A098DVM8 pp 229533.I1RQC3
229533.I1RBF8 pp 229533.I1RHD4
229533.I1RBF8 pp 229533.V6RHD3
229533.I1RBF8 pp 229533.I1RH11
229533.I1RMQ4 pp 229533.I1RSI0
229533.I1RMQ4 pp 229533.I1RWD7
229533.I1RMQ4 pp 229533.I1RWH8
229533.I1RMQ4 pp 229533.V6RBN2
229533.I1RMQ4 pp 229533.I1S4Q4
229533.I1RMQ4 pp 229533.I1RYW3
229533.I1RMQ4 pp 229533.I1RRV4
229533.I1RMQ4 pp 229533.I1RRP7
229533.I1RMQ4 pp 229533.I1RTD3
229533.I1RMQ4 pp 229533.I1S0A0
229533.I1RMQ4 pp 229533.I1S0B7
229533.I1RMQ4 pp 229533.V6R057
229533.I1RMQ4 pp 229533.I1RQ31
229533.I1RMQ4 pp 229533.I1RQS9
229533.I1RMQ4 pp 229533.I1S216
229533.I1RMQ4 pp 229533.I1S4S4
229533.I1RMQ4 pp 229533.V6RRH2
229533.I1RMQ4 pp 229533.V6R1K4
229533.I1RMQ4 pp 229533.I1RQM6
229533.I1RMQ4 pp 229533.I1RW71
229533.I1RMQ4 pp 229533.I1RPN4
229533.I1RMQ4 pp 229533.I1RPU8

229533.I1RMQ4	pp	229533.I1RRV1
229533.I1RMQ4	pp	229533.V6R2A1
229533.I1RMQ4	pp	229533.I1RQE7
229533.I1RMQ4	pp	229533.I1S2L2
229533.I1RMQ4	pp	229533.I1S0M2
229533.I1RMQ4	pp	229533.I1RMQ5
229533.I1RMQ4	pp	229533.I1S9Y1
229533.I1RMQ4	pp	229533.O74638
229533.I1RMQ4	pp	229533.I1RWE8
229533.I1RMQ4	pp	229533.V6RPQ8
229533.I1RMQ4	pp	229533.I1S8G4
229533.I1RMQ4	pp	229533.Q4HXF0
229533.I1RMQ4	pp	229533.I1RP87
229533.I1RMQ4	pp	229533.I1RS17
229533.I1RMQ4	pp	229533.I1RVZ7
229533.I1RMQ4	pp	229533.I1RS47
229533.I1RMQ4	pp	229533.I1RSQ4
229533.I1RMQ4	pp	229533.I1RTA0
229533.I1RMQ4	pp	229533.I1RS20
229533.I1RMQ4	pp	229533.I1S4Z8
229533.I1RMQ4	pp	229533.I1S8P5
229533.I1RMQ4	pp	229533.I1RQM7
229533.I1RMQ4	pp	229533.I1RX22
229533.I1RMQ4	pp	229533.I1RVZ2
229533.I1RS57	pp	229533.V6QX58
229533.I1RSH8	pp	229533.I1S4N5
229533.I1S854	pp	229533.V6RY33
229533.I1S854	pp	229533.V6R227
229533.I1S854	pp	229533.V6RIS2
229533.I1S854	pp	229533.V6R3J1
229533.I1RYB7	pp	229533.I1RZQ5
229533.I1RYB7	pp	229533.I1S1A3
229533.I1RYB7	pp	229533.I1RZH3
229533.I1RYB7	pp	229533.I1RZW1
229533.I1RYB7	pp	229533.I1S248
229533.I1RYB7	pp	229533.I1RZF7
229533.I1RYB7	pp	229533.Q4IBS9
229533.I1RYB7	pp	229533.I1S0V4
229533.I1RYB7	pp	229533.I1S074
229533.I1RPW4	pp	229533.I1RRV1
229533.I1RPW4	pp	229533.Q4IBS2
229533.I1RPW4	pp	229533.Q4IJ56
229533.I1RPW4	pp	229533.I1SAN9
229533.I1RPW4	pp	229533.I1RW71
229533.I1RPW4	pp	229533.Q4I1Y5
229533.I1RPW4	pp	229533.I1RQ81
229533.I1RPW4	pp	229533.I1S0R3
229533.I1RPW4	pp	229533.I1RVX0
229533.I1RPW4	pp	229533.I1S0N4
229533.I1RPW4	pp	229533.I1S0R4
229533.I1RPW4	pp	229533.I1RSK1
229533.I1RA00	pp	229533.I1RA98
229533.I1RA00	pp	229533.I1RAI0
229533.I1RA00	pp	229533.I1RA72
229533.I1RA00	pp	229533.I1RRC2
229533.I1RA00	pp	229533.Q5I2J3
229533.I1RA00	pp	229533.I1RFB4
229533.I1RA00	pp	229533.I1RZB7
229533.I1RA00	pp	229533.I1RIG3
229533.I1RA00	pp	229533.I1RYQ0
229533.I1RMD2	pp	229533.I1RRJ9
229533.I1RX87	pp	229533.Q4ILI9
229533.I1RX87	pp	229533.I1S4N5
229533.I1RX87	pp	229533.I1S0V5
229533.I1RX87	pp	229533.I1S278
229533.I1RX87	pp	229533.I1RXT7
229533.I1RX87	pp	229533.Q4HYZ2
229533.I1RX87	pp	229533.V6RXW9
229533.I1RX87	pp	229533.I1RZP1
229533.I1RX87	pp	229533.Q4HY20
229533.I1RX87	pp	229533.I1SAM5
229533.I1RX87	pp	229533.I1S2K7
229533.I1RX87	pp	229533.I1RXF5

23726 229533.I1RX87 pp 229533.V6QUV7
23727 229533.I1RX87 pp 229533.I1RZK4
23728 229533.I1RX87 pp 229533.I1RYP0
23729 229533.I1RX87 pp 229533.I1S0Y1
23730 229533.I1RX87 pp 229533.I1S2B6
23731 229533.I1RX87 pp 229533.I1RY40
23732 229533.I1RX87 pp 229533.I1S1U6
23733 229533.I1RX87 pp 229533.I1S296
23734 229533.I1RX87 pp 229533.V6QXP4
23735 229533.I1RX87 pp 229533.I1S051
23736 229533.I1RX87 pp 229533.I1RZS5
23737 229533.I1RX87 pp 229533.I1S1V4
23738 229533.I1RX87 pp 229533.I1RZ60
23739 229533.I1RX87 pp 229533.I1S287
23740 229533.I1RX87 pp 229533.I1RZK3
23741 229533.I1RX87 pp 229533.I1RXH6
23742 229533.I1RRJ9 pp 229533.I1S298
23743 229533.I1RZA5 pp 229533.V6QWP4
23744 229533.I1RZA5 pp 229533.I1S522
23745 229533.I1RQ64 pp 229533.I1S247
23746 229533.I1RQ64 pp 229533.I1S0A5
23747 229533.I1RQ64 pp 229533.I1RS35
23748 229533.I1RWB4 pp 229533.I1RX84
23749 229533.I1RWB4 pp 229533.I1S287
23750 229533.I1RWB4 pp 229533.I1S4N5
23751 229533.I1RWB4 pp 229533.I1RWB5
23752 229533.I1RWB4 pp 229533.I1RYU1
23753 229533.I1RWB4 pp 229533.V6REE9
23754 229533.I1RWB4 pp 229533.V6RB81
23755 229533.I1RWB4 pp 229533.I1S0K5
23756 229533.I1RWB4 pp 229533.Q4I298
23757 229533.I1RWB4 pp 229533.Q4I6S5
23758 229533.I1RWB4 pp 229533.I1S1V7
23759 229533.I1S3W8 pp 229533.V6RZD4
23760 229533.I1S3W8 pp 229533.V6RF62
23761 229533.I1S3W8 pp 229533.I1S4S5
23762 229533.I1S3W8 pp 229533.V6R3V4
23763 229533.I1RKI8 pp 229533.I1RXF0
23764 229533.I1RKI8 pp 229533.I1RZP1
23765 229533.I1RKI8 pp 229533.I1RT28
23766 229533.I1S278 pp 229533.I1S830
23767 229533.I1S278 pp 229533.Q4I6S5
23768 229533.I1S278 pp 229533.I1SAM5
23769 229533.I1S278 pp 229533.I1S296
23770 229533.I1S278 pp 229533.V6QXP4
23771 229533.I1S278 pp 229533.Q4I1H6
23772 229533.I1S278 pp 229533.I1S9X9
23773 229533.I1S278 pp 229533.I1S6S4
23774 229533.I1S278 pp 229533.I1S2K7
23775 229533.I1S278 pp 229533.Q4HY20
23776 229533.I1S278 pp 229533.I1S2B6
23777 229533.I1RCT6 pp 229533.I1RCV5
23778 229533.I1RCT6 pp 229533.I1RTC7
23779 229533.I1RCT6 pp 229533.I1RJT0
23780 229533.I1RCT6 pp 229533.V6QWP4
23781 229533.I1RCT6 pp 229533.I1RZA5
23782 229533.A0A1C3YHZ3 pp 229533.V6RZD4
23783 229533.A0A1C3YHZ3 pp 229533.I1RCB9
23784 229533.A0A1C3YHZ3 pp 229533.I1S4S5
23785 229533.A0A1C3YHZ3 pp 229533.I1S3W8
23786 229533.A0A1C3YHZ3 pp 229533.V6R3V4
23787 229533.A0A1C3YHZ3 pp 229533.V6RF62
23788 229533.I1RM71 pp 229533.I1RS34
23789 229533.I1RM71 pp 229533.I1S0V5
23790 229533.I1RM71 pp 229533.I1RSH8
23791 229533.I1RM71 pp 229533.I1RTF2
23792 229533.I1RM71 pp 229533.I1S4N5
23793 229533.I1RM71 pp 229533.I1RX87
23794 229533.I1RM71 pp 229533.I1RPC6
23795 229533.I1RM71 pp 229533.I1RTQ9
23796 229533.I1RM71 pp 229533.I1RNB9
23797 229533.I1RM71 pp 229533.I1RRC9
23798 229533.I1RM71 pp 229533.I1RWR8

23799 229533.I1RM71 pp 229533.I1RQC3
23800 229533.I1RM71 pp 229533.I1RY40
23801 229533.I1RM71 pp 229533.I1RQN8
23802 229533.I1RM71 pp 229533.I1RWZ9
23803 229533.I1RM71 pp 229533.I1RRC8
23804 229533.I1RM71 pp 229533.I1RQC4
23805 229533.I1RM71 pp 229533.I1S7S5
23806 229533.I1RM71 pp 229533.I1S287
23807 229533.I1RM71 pp 229533.I1RMM1
23808 229533.A0A098D8K3 pp 229533.I1RQC4
23809 229533.A0A098D8K3 pp 229533.A0A0E0RU86
23810 229533.A0A098D8K3 pp 229533.I1S7S5
23811 229533.A0A098D8K3 pp 229533.I1RMN3
23812 229533.A0A098D8K3 pp 229533.I1RE44
23813 229533.A0A098D8K3 pp 229533.Q4IPZ1
23814 229533.A0A098D8K3 pp 229533.I1RWR8
23815 229533.A0A098D8K3 pp 229533.I1RTQ9
23816 229533.A0A098D8K3 pp 229533.I1RX87
23817 229533.A0A098D8K3 pp 229533.I1RSH8
23818 229533.A0A098D8K3 pp 229533.I1RM71
23819 229533.A0A098D8K3 pp 229533.I1RS34
23820 229533.A0A098D8K3 pp 229533.A0A098DVM8
23821 229533.A0A098D8K3 pp 229533.A0A1C3YN54
23822 229533.A0A098D8K3 pp 229533.I1RK73
23823 229533.A0A098D8K3 pp 229533.I1RY40
23824 229533.A0A098D8K3 pp 229533.I1S4N5
23825 229533.A0A098D8K3 pp 229533.I1RPC6
23826 229533.A0A098D8K3 pp 229533.A0A0E0S643
23827 229533.A0A098D8K3 pp 229533.I1RNB9
23828 229533.A0A098D8K3 pp 229533.I1RQC3
23829 229533.A0A098D8K3 pp 229533.I1RG01
23830 229533.A0A098D8K3 pp 229533.I1RTF2
23831 229533.A0A098D8K3 pp 229533.A0A1C3YKT4
23832 229533.A0A098D8K3 pp 229533.I1S287
23833 229533.I1RQS5 pp 229533.I1S021
23834 229533.I1RQS5 pp 229533.I1RTE9
23835 229533.I1RQS5 pp 229533.I1S2C0
23836 229533.I1RQS5 pp 229533.I1S0M9
23837 229533.I1RQS5 pp 229533.Q4IJ56
23838 229533.I1RQS5 pp 229533.I1RZB5
23839 229533.I1RQS5 pp 229533.V6RGN5
23840 229533.I1RQS5 pp 229533.V6R227
23841 229533.I1RQS5 pp 229533.V6QUE6
23842 229533.I1RQS5 pp 229533.I1RRR7
23843 229533.I1RQS5 pp 229533.I1RSS4
23844 229533.I1RQS5 pp 229533.I1RX64
23845 229533.I1RQS5 pp 229533.I1S0N3
23846 229533.I1RQS5 pp 229533.I1S1X9
23847 229533.I1RQS5 pp 229533.I1RSG8
23848 229533.I1RQS5 pp 229533.I1RZF6
23849 229533.I1RQS5 pp 229533.I1RRI2
23850 229533.I1RQS5 pp 229533.I1RZZ1
23851 229533.I1RQS5 pp 229533.I1S2E8
23852 229533.I1RQS5 pp 229533.Q4IEK8
23853 229533.I1RQS5 pp 229533.V6R283
23854 229533.I1RQS5 pp 229533.I1RSR3
23855 229533.I1RQS5 pp 229533.I1RSI6
23856 229533.I1RQS5 pp 229533.I1RT33
23857 229533.I1RQS5 pp 229533.Q4I948
23858 229533.I1RQS5 pp 229533.I1RRJ8
23859 229533.I1RQS5 pp 229533.I1RSD8
23860 229533.I1RQS5 pp 229533.V6RGY3
23861 229533.I1RQS5 pp 229533.I1S6F0
23862 229533.I1RQS5 pp 229533.I1RT80
23863 229533.I1RQS5 pp 229533.I1RS47
23864 229533.I1RQS5 pp 229533.I1RTF4
23865 229533.I1RQS5 pp 229533.I1RSV2
23866 229533.I1RQS5 pp 229533.I1RRQ3
23867 229533.I1RQS5 pp 229533.Q4I830
23868 229533.I1RQS5 pp 229533.I1RW71
23869 229533.I1RQS5 pp 229533.Q4INZ9
23870 229533.I1RQS5 pp 229533.I1S0R7
23871 229533.I1RQS5 pp 229533.Q4IJH1

23872 229533.I1RQS5 pp 229533.Q4IF76
23873 229533.I1RQS5 pp 229533.I1RRT2
23874 229533.I1RQS5 pp 229533.O74638
23875 229533.I1RQS5 pp 229533.I1S0L8
23876 229533.I1RQS5 pp 229533.I1RZS6
23877 229533.I1RQS5 pp 229533.I1RX24
23878 229533.I1RQS5 pp 229533.I1S9Y1
23879 229533.I1RQS5 pp 229533.V6QYF0
23880 229533.I1RTF5 pp 229533.I1S9G7
23881 229533.I1RTF5 pp 229533.I1RYL5
23882 229533.I1RTF5 pp 229533.I1RZM0
23883 229533.I1RTF5 pp 229533.I1RXF0
23884 229533.I1RQS9 pp 229533.I1S4Q4
23885 229533.I1RQS9 pp 229533.V6R057
23886 229533.I1RQS9 pp 229533.I1S2C4
23887 229533.I1RQS9 pp 229533.V6RBN2
23888 229533.I1RQS9 pp 229533.V6RRH2
23889 229533.I1RQS9 pp 229533.Q4INS6
23890 229533.I1RQS9 pp 229533.I1RRV4
23891 229533.I1RQS9 pp 229533.V6R2A1
23892 229533.I1RQS9 pp 229533.I1S3K8
23893 229533.I1RQS9 pp 229533.I1RRV1
23894 229533.I1RQS9 pp 229533.I1RRT1
23895 229533.I1RQS9 pp 229533.I1RTD3
23896 229533.I1RQS9 pp 229533.V6QT89
23897 229533.I1RQS9 pp 229533.I1RSC7
23898 229533.I1RQS9 pp 229533.I1S084
23899 229533.I1RQS9 pp 229533.I1RWE8
23900 229533.I1RQS9 pp 229533.V6R1K4
23901 229533.I1RQS9 pp 229533.I1RSD0
23902 229533.I1RQS9 pp 229533.I1RZT0
23903 229533.I1RQS9 pp 229533.I1S1P8
23904 229533.I1RQS9 pp 229533.I1RWI8
23905 229533.I1RQS9 pp 229533.I1RTA9
23906 229533.I1RQS9 pp 229533.I1RYQ1
23907 229533.I1RQS9 pp 229533.I1RY29
23908 229533.I1RCV5 pp 229533.I1RZA5
23909 229533.I1RCV5 pp 229533.I1RCV6
23910 229533.I1RCV5 pp 229533.V6QWP4
23911 229533.I1RCV5 pp 229533.I1RTC7
23912 229533.I1RE33 pp 229533.I1RP20
23913 229533.I1RE33 pp 229533.Q4HZI0
23914 229533.I1RE33 pp 229533.I1RZ11
23915 229533.I1RE33 pp 229533.I1RGB6
23916 229533.I1RE33 pp 229533.I1S4S8
23917 229533.I1RE33 pp 229533.I1RQN0
23918 229533.A0A1C3YL39 pp 229533.I1RRQ3
23919 229533.A0A1C3YL39 pp 229533.I1RS47
23920 229533.A0A1C3YL39 pp 229533.I1RW71
23921 229533.A0A1C3YL39 pp 229533.I1RKJ6
23922 229533.A0A1C3YL39 pp 229533.I1RZ99
23923 229533.A0A1C3YL39 pp 229533.Q4IJH1
23924 229533.A0A1C3YL39 pp 229533.I1RNV6
23925 229533.A0A1C3YL39 pp 229533.I1RT80
23926 229533.A0A1C3YL39 pp 229533.I1RQJ5
23927 229533.A0A1C3YL39 pp 229533.I1RSD8
23928 229533.A0A1C3YL39 pp 229533.I1RC27
23929 229533.A0A1C3YL39 pp 229533.I1RMN6
23930 229533.A0A1C3YL39 pp 229533.V6QYF0
23931 229533.A0A1C3YL39 pp 229533.V6R283
23932 229533.A0A1C3YL39 pp 229533.I1RCL3
23933 229533.A0A1C3YL39 pp 229533.I1RQ31
23934 229533.A0A1C3YL39 pp 229533.I1RBG9
23935 229533.A0A1C3YL39 pp 229533.I1RTE9
23936 229533.A0A1C3YL39 pp 229533.I1RG51
23937 229533.A0A1C3YL39 pp 229533.Q4IAS1
23938 229533.A0A1C3YL39 pp 229533.I1RS34
23939 229533.A0A1C3YL39 pp 229533.Q4I327
23940 229533.A0A1C3YL39 pp 229533.V6R227
23941 229533.A0A1C3YL39 pp 229533.I1RRT2
23942 229533.A0A1C3YL39 pp 229533.I1RAZ8
23943 229533.A0A1C3YL39 pp 229533.Q4HZ68
23944 229533.A0A1C3YL39 pp 229533.I1RAF8

229533.A0A1C3YL39 pp 229533.I1RG56
229533.A0A1C3YL39 pp 229533.I1RNL7
229533.A0A1C3YL39 pp 229533.I1RM96
229533.A0A1C3YL39 pp 229533.I1R9V7
229533.A0A1C3YL39 pp 229533.I1RB27
229533.A0A1C3YL39 pp 229533.I1RTF5
229533.A0A1C3YL39 pp 229533.I1S060
229533.A0A1C3YL39 pp 229533.I1RPZ0
229533.I1RQ32 pp 229533.I1S1U6
229533.I1RQ32 pp 229533.I1S4N5
229533.I1RQ32 pp 229533.I1RS34
229533.I1RQ32 pp 229533.I1RTE6
229533.I1RQ32 pp 229533.V6RB81
229533.I1RQ32 pp 229533.I1S0K5
229533.I1RQ32 pp 229533.Q4I6S5
229533.I1RQ32 pp 229533.V6REE9
229533.I1RQ32 pp 229533.Q4HZS8
229533.I1RQ32 pp 229533.I1S287
229533.I1RQ32 pp 229533.I1RTQ9
229533.I1RQ32 pp 229533.I1RSJ0
229533.I1RQ32 pp 229533.I1RYU1
229533.I1RQ32 pp 229533.I1RSZ1
229533.I1RQ32 pp 229533.I1RX84
229533.I1RQ32 pp 229533.V6RR76
229533.I1RQ32 pp 229533.I1RR95
229533.I1RQ32 pp 229533.I1S2B6
229533.I1RQ32 pp 229533.I1S1V7
229533.I1RQ32 pp 229533.I1RQD6
229533.I1RQ32 pp 229533.I1RQ70
229533.I1RQ32 pp 229533.I1S034
229533.I1RQ32 pp 229533.I1RRM9
229533.I1RFA8 pp 229533.I1RFA9
229533.I1RFA8 pp 229533.I1RXQ3
229533.I1RRF7 pp 229533.I1S182
229533.I1RRF7 pp 229533.I1SAH9
229533.I1RRF7 pp 229533.I1RU81
229533.I1RRF7 pp 229533.I1S9H8
229533.I1RRF7 pp 229533.I1RW91
229533.I1RRF7 pp 229533.I1RRG3
229533.I1RRF7 pp 229533.I1RZM2
229533.I1RRF7 pp 229533.I1RSZ3
229533.I1RRF7 pp 229533.I1RSM8
229533.I1RRF7 pp 229533.V6QWX8
229533.I1RRF7 pp 229533.I1RTC2
229533.I1RRF7 pp 229533.I1S5Z5
229533.I1RRF7 pp 229533.I1RW90
229533.I1RRF7 pp 229533.I1S0K1
229533.I1RRF7 pp 229533.I1S9B5
229533.I1RRF7 pp 229533.I1RVY0
229533.I1RRF7 pp 229533.I1RUW5
229533.I1RRF7 pp 229533.I1RWF3
229533.I1RRF7 pp 229533.I1RW80
229533.I1RRF7 pp 229533.I1RV19
229533.I1RRF7 pp 229533.I1RVL8
229533.I1RRF7 pp 229533.I1RZ42
229533.I1RRF7 pp 229533.I1RRT0
229533.I1RRF7 pp 229533.I1S6R1
229533.I1RRF7 pp 229533.I1RUG4
229533.I1RRF7 pp 229533.V6RGU1
229533.I1RRF7 pp 229533.I1RWG8
229533.I1RRF7 pp 229533.I1RTY9
229533.I1RRF7 pp 229533.I1RXE5
229533.I1RRF7 pp 229533.V6QUE6
229533.I1RRF7 pp 229533.I1S0C6
229533.I1RRF7 pp 229533.Q4HTT2
229533.I1RRF7 pp 229533.Q4HYI0
229533.I1RRF7 pp 229533.I1RXU0
229533.I1RRF7 pp 229533.I1S276
229533.I1RRF7 pp 229533.I1RUE3
229533.I1RRF7 pp 229533.I1RZB5
229533.I1RRF7 pp 229533.I1S0X1
229533.I1RS69 pp 229533.Q4I6S5
229533.I1RS69 pp 229533.I1RYW4

24018 229533.I1RS69 pp 229533.Q4I1H6
24019 229533.I1RS69 pp 229533.I1S0R2
24020 229533.I1RS69 pp 229533.I1RWP9
24021 229533.I1RS69 pp 229533.I1RWR8
24022 229533.I1RG01 pp 229533.I1RWR8
24023 229533.I1RG01 pp 229533.I1RTQ9
24024 229533.I1RG01 pp 229533.I1RRC9
24025 229533.I1RG01 pp 229533.I1RQF7
24026 229533.I1RG01 pp 229533.I1RQ32
24027 229533.I1RG01 pp 229533.I1RX87
24028 229533.I1RG01 pp 229533.I1RMN3
24029 229533.I1RG01 pp 229533.I1S7S5
24030 229533.I1RG01 pp 229533.I1RRC8
24031 229533.I1RG01 pp 229533.I1RSH8
24032 229533.I1RG01 pp 229533.I1RQC4
24033 229533.I1RG01 pp 229533.I1RQC3
24034 229533.I1RG01 pp 229533.I1RYP0
24035 229533.I1RG01 pp 229533.I1RNB9
24036 229533.I1RG01 pp 229533.I1RS34
24037 229533.I1RG01 pp 229533.I1RM71
24038 229533.I1RG01 pp 229533.I1RK73
24039 229533.I1RG01 pp 229533.I1S4N5
24040 229533.I1RG01 pp 229533.I1RWB4
24041 229533.I1RG01 pp 229533.I1RJT0
24042 229533.I1RG01 pp 229533.I1RY40
24043 229533.I1RG01 pp 229533.I1S287
24044 229533.I1RG01 pp 229533.I1RMM1
24045 229533.I1RG01 pp 229533.I1RQN8
24046 229533.I1RG01 pp 229533.I1S0V5
24047 229533.I1RG01 pp 229533.I1RPC6
24048 229533.I1RG01 pp 229533.I1S1U6
24049 229533.I1RG01 pp 229533.I1RNY2
24050 229533.I1RG01 pp 229533.I1RQP7
24051 229533.I1RG01 pp 229533.I1RTF2
24052 229533.I1RG01 pp 229533.I1S278
24053 229533.I1S522 pp 229533.I1S523
24054 229533.I1S522 pp 229533.V6QWP4
24055 229533.I1S0B7 pp 229533.I1S1W9
24056 229533.I1S0B7 pp 229533.V6R2A1
24057 229533.I1S0B7 pp 229533.I1S216
24058 229533.I1S0B7 pp 229533.Q4HXF0
24059 229533.I1S0B7 pp 229533.I1S247
24060 229533.I1S0B7 pp 229533.V6RRH2
24061 229533.I1S0B7 pp 229533.V6QUE6
24062 229533.I1S0B7 pp 229533.I1S3G4
24063 229533.I1S0B7 pp 229533.I1S0F1
24064 229533.I1S0B7 pp 229533.V6R1K4
24065 229533.I1S0B7 pp 229533.V6RBN2
24066 229533.I1S0B7 pp 229533.V6R057
24067 229533.I1S0B7 pp 229533.I1S4Q4
24068 229533.I1S0B7 pp 229533.I1S1W8
24069 229533.I1RRC9 pp 229533.I1RSW4
24070 229533.I1RRC9 pp 229533.I1RYL5
24071 229533.I1RRC9 pp 229533.V6QXP4
24072 229533.I1RRC9 pp 229533.I1RX87
24073 229533.I1RRC9 pp 229533.V6QUV7
24074 229533.I1RRC9 pp 229533.I1RS34
24075 229533.I1RRC9 pp 229533.I1S287
24076 229533.I1RRC9 pp 229533.I1RWZ9
24077 229533.I1RRC9 pp 229533.I1RY40
24078 229533.I1RRC9 pp 229533.I1RTF2
24079 229533.I1RRC9 pp 229533.V6R042
24080 229533.I1RRC9 pp 229533.Q4HY20
24081 229533.A0A1C3YMP5 pp 229533.I1RNH6
24082 229533.A0A1C3YMP5 pp 229533.V6R042
24083 229533.A0A1C3YMP5 pp 229533.I1RZK6
24084 229533.A0A1C3YMP5 pp 229533.I1RAU7
24085 229533.A0A1C3YMP5 pp 229533.I1RA48
24086 229533.A0A1C3YMP5 pp 229533.I1RSR8
24087 229533.A0A1C3YMP5 pp 229533.I1RQ65
24088 229533.A0A1C3YMP5 pp 229533.I1RAN5
24089 229533.A0A1C3YMP5 pp 229533.I1RLV9
24090 229533.A0A1C3YMP5 pp 229533.I1RX46

24091 229533.A0A1C3YMP5 pp 229533.I1RKL3
24092 229533.A0A1C3YMP5 pp 229533.I1RKJ6
24093 229533.A0A1C3YMP5 pp 229533.I1RR73
24094 229533.I1RF99 pp 229533.I1RY16
24095 229533.I1RF99 pp 229533.I1S694
24096 229533.I1RF99 pp 229533.I1RRG1
24097 229533.I1RF99 pp 229533.I1RZA8
24098 229533.I1RF99 pp 229533.I1RI11
24099 229533.I1RN16 pp 229533.I1S2M8
24100 229533.I1RN16 pp 229533.V6RSF3
24101 229533.I1RN16 pp 229533.I1S132
24102 229533.I1RN16 pp 229533.V6RUY9
24103 229533.I1RN16 pp 229533.I1S2M7
24104 229533.I1S0V5 pp 229533.I1SAM5
24105 229533.I1S0V5 pp 229533.I1S0Z3
24106 229533.I1S0V5 pp 229533.I1S287
24107 229533.I1S0V5 pp 229533.Q4HY20
24108 229533.I1S0V5 pp 229533.V6QXP4
24109 229533.I1S0V5 pp 229533.I1S830
24110 229533.I1S0V5 pp 229533.I1S1U6
24111 229533.I1S0V5 pp 229533.I1S278
24112 229533.I1S0V5 pp 229533.I1S2B6
24113 229533.I1R9U9 pp 229533.I1RPA4
24114 229533.I1R9U9 pp 229533.I1RR74
24115 229533.I1R9U9 pp 229533.V6QWP4
24116 229533.I1R9U9 pp 229533.V6QUV7
24117 229533.I1R9U9 pp 229533.I1RZA5
24118 229533.I1R9U9 pp 229533.I1RCT6
24119 229533.I1R9U9 pp 229533.I1RRI2
24120 229533.I1R9U9 pp 229533.I1RXF0
24121 229533.I1R9U9 pp 229533.V6RG04
24122 229533.I1R9U9 pp 229533.I1RTC7
24123 229533.I1R9U9 pp 229533.I1RCV5
24124 229533.I1R9U9 pp 229533.I1RFW0
24125 229533.I1R9U9 pp 229533.I1RML5
24126 229533.I1R9U9 pp 229533.I1S522
24127 229533.I1RWR8 pp 229533.I1S058
24128 229533.I1RWR8 pp 229533.I1S4N5
24129 229533.I1RWR8 pp 229533.I1S7S5
24130 229533.I1RWR8 pp 229533.I1RYW4
24131 229533.I1RWR8 pp 229533.I1S287
24132 229533.I1RJG5 pp 229533.I1RNG4
24133 229533.I1RRQ7 pp 229533.I1RXS9
24134 229533.I1RRQ7 pp 229533.Q4I8Q4
24135 229533.I1RRQ7 pp 229533.I1S8H4
24136 229533.I1RRQ7 pp 229533.I1S0R1
24137 229533.I1RTF2 pp 229533.I1RY40
24138 229533.I1RTF2 pp 229533.V6QXP4
24139 229533.I1RTF2 pp 229533.I1S287
24140 229533.I1RTF2 pp 229533.I1S278
24141 229533.I1RTF2 pp 229533.I1RWZ9
24142 229533.I1RTF2 pp 229533.I1RYX9
24143 229533.I1RTF2 pp 229533.I1S4N5
24144 229533.I1RTF2 pp 229533.I1RXF0
24145 229533.I1RTF2 pp 229533.V6QUV7
24146 229533.I1RTF2 pp 229533.I1RYP0
24147 229533.I1RTF2 pp 229533.Q4HY20
24148 229533.I1RTF2 pp 229533.I1RYL5
24149 229533.I1RTF2 pp 229533.I1S2B6
24150 229533.I1RTF2 pp 229533.I1S0V5
24151 229533.I1RTF2 pp 229533.I1S1U6
24152 229533.I1RTF2 pp 229533.I1RX87
24153 229533.I1RTF2 pp 229533.I1RUN9
24154 229533.A0A1C3YMY3 pp 229533.I1RIF2
24155 229533.A0A1C3YMY3 pp 229533.I1S0B6
24156 229533.A0A1C3YMY3 pp 229533.I1RJL2
24157 229533.A0A1C3YMY3 pp 229533.I1RZB5
24158 229533.A0A1C3YMY3 pp 229533.I1RRU9
24159 229533.A0A1C3YMY3 pp 229533.I1RE31
24160 229533.A0A1C3YMY3 pp 229533.I1S2D5
24161 229533.A0A1C3YMY3 pp 229533.I1RT35
24162 229533.A0A1C3YMY3 pp 229533.I1RRT0
24163 229533.A0A1C3YMY3 pp 229533.I1RRG3

229533.A0A1C3YMY3	pp	229533.I1RC03
229533.A0A1C3YMY3	pp	229533.I1RC24
229533.A0A1C3YMY3	pp	229533.I1RT47
229533.A0A1C3YMY3	pp	229533.I1S4M5
229533.A0A1C3YMY3	pp	229533.I1RK92
229533.A0A1C3YMY3	pp	229533.I1RG11
229533.A0A1C3YMY3	pp	229533.I1RCB7
229533.A0A1C3YMY3	pp	229533.I1RK79
229533.A0A1C3YMY3	pp	229533.I1RG90
229533.A0A1C3YMY3	pp	229533.I1RNB8
229533.A0A1C3YMY3	pp	229533.I1RR25
229533.A0A098DQF5	pp	229533.I1RRQ7
229533.I1RBH5	pp	229533.I1S0Y1
229533.I1RBH5	pp	229533.I1RNT1
229533.I1RBH5	pp	229533.I1RX55
229533.I1RBH5	pp	229533.I1RPS2
229533.I1RBH5	pp	229533.V6QUV7
229533.I1RBH5	pp	229533.I1RBH7
229533.I1RBH5	pp	229533.I1RCU8
229533.I1RBH5	pp	229533.I1RTF0
229533.I1RBH5	pp	229533.Q4IBU4
229533.I1RBH5	pp	229533.I1RSP4
229533.I1RBH5	pp	229533.I1RX87
229533.I1RBH5	pp	229533.I1RVS4
229533.I1RBH5	pp	229533.I1RCK2
229533.I1RBH5	pp	229533.I1RJT1
229533.I1RQC4	pp	229533.I1S287
229533.I1RQC4	pp	229533.I1S0V5
229533.I1RQC4	pp	229533.I1RXF0
229533.I1RQC4	pp	229533.V6QXP4
229533.I1RQC4	pp	229533.I1S278
229533.I1RQC4	pp	229533.I1RZK3
229533.I1RQC4	pp	229533.I1RQP7
229533.I1RQC4	pp	229533.V6RXW9
229533.I1RQC4	pp	229533.I1RWZ9
229533.I1RQC4	pp	229533.I1RY40
229533.I1RQC4	pp	229533.I1RX87
229533.I1RQC4	pp	229533.Q4HY20
229533.I1RQC4	pp	229533.I1S4N5
229533.I1RQC4	pp	229533.V6QUV7
229533.I1RQC4	pp	229533.I1RSW4
229533.I1RQC4	pp	229533.I1RTF2
229533.I1RQC4	pp	229533.I1RYL5
229533.I1RQC4	pp	229533.I1RUN9
229533.I1RQC4	pp	229533.I1RRC8
229533.I1RQC4	pp	229533.V6R042
229533.I1RQC4	pp	229533.I1RS34
229533.I1RQC4	pp	229533.V6QW25
229533.I1RQC4	pp	229533.I1RRC9
229533.I1RQC4	pp	229533.I1S1U6
229533.I1RQC4	pp	229533.I1RQN8
229533.A0A0E0SKH4	pp	229533.I1RG48
229533.I1S8I8	pp	229533.V6R042
229533.A0A0E0RU86	pp	229533.I1RX87
229533.A0A0E0RU86	pp	229533.I1RSH8
229533.A0A0E0RU86	pp	229533.I1RWB4
229533.A0A0E0RU86	pp	229533.I1RM71
229533.A0A0E0RU86	pp	229533.I1RS34
229533.A0A0E0RU86	pp	229533.A0A1C3YN54
229533.A0A0E0RU86	pp	229533.I1RK73
229533.A0A0E0RU86	pp	229533.I1RQC4
229533.A0A0E0RU86	pp	229533.I1S7S5
229533.A0A0E0RU86	pp	229533.I1RE44
229533.A0A0E0RU86	pp	229533.I1RMN3
229533.A0A0E0RU86	pp	229533.I1RQ32
229533.A0A0E0RU86	pp	229533.I1RWR8
229533.A0A0E0RU86	pp	229533.I1RTQ9
229533.A0A0E0RU86	pp	229533.I1RG01
229533.A0A0E0RU86	pp	229533.I1RTF2
229533.A0A0E0RU86	pp	229533.A0A1C3YKT4
229533.A0A0E0RU86	pp	229533.I1RQP7
229533.A0A0E0RU86	pp	229533.I1S287
229533.A0A0E0RU86	pp	229533.I1RMM1

229533.A0A0E0RU86 pp 229533.I1RY40
229533.A0A0E0RU86 pp 229533.I1RPC6
229533.A0A0E0RU86 pp 229533.A0A0E0S643
229533.A0A0E0RU86 pp 229533.I1RQC3
229533.A0A0E0RU86 pp 229533.I1RNB9
229533.I1S835 pp 229533.V6QWZ7
229533.I1S835 pp 229533.I1S836
229533.I1S835 pp 229533.Q4IJP1
229533.I1S835 pp 229533.V6RBN2
229533.I1S835 pp 229533.O74638
229533.I1S835 pp 229533.Q4HYC1
229533.I1S835 pp 229533.Q4ID21
229533.I1S835 pp 229533.Q4I5V2
229533.I1RD02 pp 229533.Q4INI2
229533.I1RD02 pp 229533.I1RE19
229533.I1RD02 pp 229533.I1RPK0
229533.I1RD02 pp 229533.I1RR74
229533.I1RD02 pp 229533.I1RNG8
229533.I1RD02 pp 229533.I1RMP2
229533.I1RD02 pp 229533.Q4ICM0
229533.I1RD02 pp 229533.I1RZU6
229533.I1RD02 pp 229533.I1RXT9
229533.I1RD02 pp 229533.I1RUK7
229533.I1RD02 pp 229533.I1RMG2
229533.I1RD02 pp 229533.I1RWD5
229533.I1RD02 pp 229533.I1S0S8
229533.I1RPC6 pp 229533.I1RY40
229533.I1RPC6 pp 229533.I1S0V5
229533.I1RPC6 pp 229533.I1S4N5
229533.I1RPC6 pp 229533.I1RQC3
229533.I1RPC6 pp 229533.I1RQC4
229533.I1RPC6 pp 229533.I1S7S5
229533.I1RPC6 pp 229533.I1S287
229533.I1RPC6 pp 229533.I1RWR8
229533.I1RPC6 pp 229533.I1RTQ9
229533.I1RPC6 pp 229533.I1S278
229533.I1RPC6 pp 229533.I1RWB4
229533.I1RPC6 pp 229533.I1RQ32
229533.I1RPC6 pp 229533.I1RWR7
229533.I1RPC6 pp 229533.I1RQP7
229533.I1RPC6 pp 229533.I1RSH8
229533.I1RPC6 pp 229533.I1RS34
229533.I1RPC6 pp 229533.I1RX87
229533.I1RPC6 pp 229533.I1RTF2
229533.V6R729 pp 229533.V6RN31
229533.V6R729 pp 229533.V6RAN2
229533.I1RRC8 pp 229533.I1RSW4
229533.I1RRC8 pp 229533.I1RRC9
229533.I1RRC8 pp 229533.I1RX87
229533.I1RRC8 pp 229533.I1RS34
229533.I1RRC8 pp 229533.I1RYL5
229533.I1RRC8 pp 229533.Q4HY20
229533.I1RRC8 pp 229533.I1RWZ9
229533.I1RRC8 pp 229533.V6QUV7
229533.I1RRC8 pp 229533.V6R042
229533.I1RRC8 pp 229533.I1RY40
229533.I1RRC8 pp 229533.I1S287
229533.I1RRC8 pp 229533.I1RTF2
229533.I1RRC8 pp 229533.V6QXP4
229533.I1RZ21 pp 229533.V6RLM7
229533.I1S4N5 pp 229533.I1S7S5
229533.I1RNB9 pp 229533.I1RSH8
229533.I1RNB9 pp 229533.I1RQ32
229533.I1RNB9 pp 229533.I1RTQ9
229533.I1RNB9 pp 229533.I1RX87
229533.I1RNB9 pp 229533.I1S287
229533.I1RNB9 pp 229533.I1S0V5
229533.I1RNB9 pp 229533.I1RS34
229533.I1RNB9 pp 229533.I1RQC4
229533.I1RNB9 pp 229533.I1RRC8
229533.I1RNB9 pp 229533.I1RQC3
229533.I1RNB9 pp 229533.I1RY40
229533.I1RNB9 pp 229533.I1S7S5

229533.I1RNB9 pp 229533.I1RQN8
229533.I1RNB9 pp 229533.I1RRC9
229533.I1RNB9 pp 229533.I1S4N5
229533.I1RNB9 pp 229533.I1RWB4
229533.I1RNB9 pp 229533.I1RWR8
229533.I1RNB9 pp 229533.I1RPC6
229533.I1RNB9 pp 229533.I1RQP7
229533.I1RNB9 pp 229533.I1RTF2
229533.A0A1C3YMS8 pp 229533.I1RPU8
229533.A0A1C3YMS8 pp 229533.I1RNU7
229533.A0A1C3YMS8 pp 229533.I1RPT0
229533.A0A0E0SP22 pp 229533.I1RP87
229533.A0A0E0SP22 pp 229533.I1RBS3
229533.A0A0E0SP22 pp 229533.I1RSH3
229533.A0A0E0SP22 pp 229533.I1RAF6
229533.A0A0E0SP22 pp 229533.I1S287
229533.A0A0E0SP22 pp 229533.I1RG01
229533.A0A0E0SP22 pp 229533.I1S0V5
229533.A0A0E0SP22 pp 229533.A0A1C3YJK9
229533.A0A0E0SP22 pp 229533.I1RCY0
229533.A0A0E0SP22 pp 229533.I1RYP0
229533.A0A0E0SP22 pp 229533.I1RNB9
229533.A0A0E0SP22 pp 229533.I1RQC3
229533.A0A0E0SP22 pp 229533.V6QXP4
229533.A0A0E0SP22 pp 229533.I1RWZ9
229533.A0A0E0SP22 pp 229533.I1RMM1
229533.A0A0E0SP22 pp 229533.I1RY40
229533.A0A0E0SP22 pp 229533.I1RSW4
229533.A0A0E0SP22 pp 229533.I1RQD6
229533.A0A0E0SP22 pp 229533.I1RPC6
229533.A0A0E0SP22 pp 229533.I1RX87
229533.A0A0E0SP22 pp 229533.I1RJP3
229533.A0A0E0SP22 pp 229533.I1RGC1
229533.A0A0E0SP22 pp 229533.Q4HY20
229533.A0A0E0SP22 pp 229533.I1RCV3
229533.A0A0E0SP22 pp 229533.A0A1C3YII1
229533.A0A0E0SP22 pp 229533.I1S2B6
229533.A0A0E0SP22 pp 229533.I1RQP7
229533.A0A0E0SP22 pp 229533.I1RXF0
229533.A0A0E0SP22 pp 229533.I1S278
229533.A0A0E0SP22 pp 229533.I1RFP5
229533.A0A0E0SP22 pp 229533.V6QUV7
229533.A0A0E0SP22 pp 229533.A0A1C3YKT4
229533.A0A0E0SP22 pp 229533.I1RTF2
229533.A0A0E0SP22 pp 229533.I1RM71
229533.A0A0E0SP22 pp 229533.A0A1C3YJ23
229533.A0A0E0SP22 pp 229533.I1RPS3
229533.A0A0E0SP22 pp 229533.I1S1U6
229533.A0A0E0SP22 pp 229533.I1RQN8
229533.A0A0E0SP22 pp 229533.I1RRC9
229533.A0A0E0SP22 pp 229533.I1RQC4
229533.A0A0E0SP22 pp 229533.I1RMN3
229533.A0A0E0SP22 pp 229533.I1RE44
229533.A0A0E0SP22 pp 229533.I1RRC8
229533.I1RNL7 pp 229533.I1RZM0
229533.I1RNL7 pp 229533.I1S9G7
229533.I1RNL7 pp 229533.I1RZJ5
229533.I1RNL7 pp 229533.I1RX25
229533.I1RNL7 pp 229533.I1RQ82
229533.I1RNL7 pp 229533.I1RQ23
229533.I1RNL7 pp 229533.I1RRR5
229533.I1RNL7 pp 229533.I1RXF0
229533.I1RNL7 pp 229533.I1RS34
229533.I1RNL7 pp 229533.I1RQ41
229533.I1RNL7 pp 229533.Q4HY20
229533.I1RNL7 pp 229533.I1RYL5
229533.I1RQC3 pp 229533.V6QUV7
229533.I1RQC3 pp 229533.V6RXW9
229533.I1RQC3 pp 229533.I1RYL5
229533.I1RQC3 pp 229533.I1RS34
229533.I1RQC3 pp 229533.I1RTF2
229533.I1RQC3 pp 229533.Q4HY20
229533.I1RQC3 pp 229533.I1S278

229533.I1RQC3	pp	229533.I1RXF0
229533.I1RQC3	pp	229533.I1RUN9
229533.I1RQC3	pp	229533.V6QXP4
229533.I1RQC3	pp	229533.I1RX87
229533.I1RQC3	pp	229533.V6R042
229533.I1RQC3	pp	229533.I1RWZ9
229533.I1RQC3	pp	229533.I1RSW4
229533.I1RQC3	pp	229533.I1S4N5
229533.I1RQC3	pp	229533.I1RY40
229533.I1RQC3	pp	229533.I1RRC8
229533.I1RQC3	pp	229533.I1RRC9
229533.I1RQC3	pp	229533.I1S287
229533.I1RQC3	pp	229533.I1RQN8
229533.A0A098D8G7	pp	229533.I1RCV3
229533.A0A098D8G7	pp	229533.I1RY40
229533.A0A098D8G7	pp	229533.I1RMM1
229533.A0A098D8G7	pp	229533.I1RWZ9
229533.A0A098D8G7	pp	229533.I1RZK3
229533.A0A098D8G7	pp	229533.I1S4N5
229533.A0A098D8G7	pp	229533.I1RPC6
229533.A0A098D8G7	pp	229533.A0A0E0RP23
229533.A0A098D8G7	pp	229533.I1RBS3
229533.A0A098D8G7	pp	229533.I1RBT0
229533.A0A098D8G7	pp	229533.I1RP87
229533.A0A098D8G7	pp	229533.I1RXF5
229533.A0A098D8G7	pp	229533.I1SAM5
229533.A0A098D8G7	pp	229533.A0A0E0S3U6
229533.A0A098D8G7	pp	229533.I1RQP7
229533.A0A098D8G7	pp	229533.I1RSH3
229533.A0A098D8G7	pp	229533.Q4HY20
229533.A0A098D8G7	pp	229533.I1S287
229533.A0A098D8G7	pp	229533.I1RCN6
229533.A0A098D8G7	pp	229533.A0A098DRI1
229533.A0A098D8G7	pp	229533.I1RW76
229533.A0A098D8G7	pp	229533.I1RQC3
229533.A0A098D8G7	pp	229533.I1S278
229533.A0A098D8G7	pp	229533.I1RGC1
229533.A0A098D8G7	pp	229533.I1RNH7
229533.A0A098D8G7	pp	229533.I1RAH7
229533.A0A098D8G7	pp	229533.I1RNB9
229533.A0A098D8G7	pp	229533.A0A0E0SP22
229533.A0A098D8G7	pp	229533.I1RE44
229533.A0A098D8G7	pp	229533.I1RMN3
229533.A0A098D8G7	pp	229533.I1RAF6
229533.A0A098D8G7	pp	229533.I1S0V5
229533.A0A098D8G7	pp	229533.I1RYP0
229533.A0A098D8G7	pp	229533.I1RG01
229533.A0A098D8G7	pp	229533.I1RCQ0
229533.A0A098D8G7	pp	229533.I1RJP3
229533.A0A098D8G7	pp	229533.I1RRC8
229533.A0A098D8G7	pp	229533.V6RXW9
229533.A0A098D8G7	pp	229533.A0A098DLG3
229533.A0A098D8G7	pp	229533.I1RNG2
229533.A0A098D8G7	pp	229533.I1S2B6
229533.A0A098D8G7	pp	229533.I1RUN9
229533.A0A098D8G7	pp	229533.I1RTF2
229533.A0A098D8G7	pp	229533.I1S5L5
229533.A0A098D8G7	pp	229533.A0A1C3YKT4
229533.A0A098D8G7	pp	229533.A0A1C3YJ23
229533.A0A098D8G7	pp	229533.Q4INI2
229533.A0A098D8G7	pp	229533.I1RFP5
229533.A0A098D8G7	pp	229533.I1RSW4
229533.A0A098D8G7	pp	229533.I1RQN8
229533.A0A098D8G7	pp	229533.A0A1C3YKU7
229533.A0A098D8G7	pp	229533.I1RS34
229533.A0A098D8G7	pp	229533.I1RPG2
229533.A0A098D8G7	pp	229533.I1S1U6
229533.A0A098D8G7	pp	229533.I1RWR8
229533.A0A098D8G7	pp	229533.I1RE18
229533.A0A098D8G7	pp	229533.A0A0E0RR64
229533.A0A098D8G7	pp	229533.I1RQD6
229533.A0A098D8G7	pp	229533.I1RX87
229533.A0A098D8G7	pp	229533.I1RR74

229533.A0A098D8G7 pp 229533.I1RRC9
229533.A0A098D8G7 pp 229533.I1RQC4
229533.A0A098D8G7 pp 229533.A0A0E0RU86
229533.A0A098D8G7 pp 229533.I1RCB2
229533.A0A098D8G7 pp 229533.I1RQF7
229533.A0A098D8G7 pp 229533.I1S0S8
229533.A0A098D8G7 pp 229533.I1RZ84
229533.A0A098D8G7 pp 229533.I1S1V4
229533.A0A098D8G7 pp 229533.I1RZS5
229533.A0A098D8G7 pp 229533.A0A0E0SDU6
229533.I1RE44 pp 229533.I1RRC9
229533.I1RE44 pp 229533.I1S1U6
229533.I1RE44 pp 229533.I1RM71
229533.I1RE44 pp 229533.I1RQN8
229533.I1RE44 pp 229533.I1RYL5
229533.I1RE44 pp 229533.V6QUV7
229533.I1RE44 pp 229533.I1RFP5
229533.I1RE44 pp 229533.Q4I1H6
229533.I1RE44 pp 229533.I1RS34
229533.I1RE44 pp 229533.I1S2B6
229533.I1RE44 pp 229533.I1RPC6
229533.I1RE44 pp 229533.Q4HY20
229533.I1RE44 pp 229533.I1RUN9
229533.I1RE44 pp 229533.I1RZK3
229533.I1RE44 pp 229533.I1S5B0
229533.I1RE44 pp 229533.I1RMM1
229533.I1RE44 pp 229533.I1RY40
229533.I1RE44 pp 229533.I1S278
229533.I1RE44 pp 229533.V6QXP4
229533.I1RE44 pp 229533.I1RWZ9
229533.I1RE44 pp 229533.I1RQP7
229533.I1RE44 pp 229533.I1RXF0
229533.I1RE44 pp 229533.I1RNB9
229533.I1RE44 pp 229533.I1RQC3
229533.I1RE44 pp 229533.I1RRC8
229533.I1RE44 pp 229533.I1RN70
229533.I1RE44 pp 229533.I1RSH3
229533.I1RE44 pp 229533.I1RMN3
229533.I1RE44 pp 229533.I1RVA8
229533.I1RE44 pp 229533.I1RYP0
229533.I1RE44 pp 229533.I1RYX9
229533.I1RE44 pp 229533.I1S0W1
229533.I1RE44 pp 229533.I1RQC4
229533.I1RE44 pp 229533.I1RGC1
229533.I1RE44 pp 229533.I1RP87
229533.I1RE44 pp 229533.I1RZ94
229533.I1RE44 pp 229533.I1RJP3
229533.I1RE44 pp 229533.I1S4Z7
229533.I1RE44 pp 229533.I1S4N5
229533.I1RE44 pp 229533.I1S0V5
229533.I1RE44 pp 229533.I1S287
229533.I1RE44 pp 229533.I1RKJ6
229533.I1RE44 pp 229533.I1RG01
229533.I1RE44 pp 229533.I1RQD6
229533.I1RE44 pp 229533.I1RX87
229533.I1RE44 pp 229533.I1RSW4
229533.A0A098DXU9 pp 229533.I1SAF7
229533.A0A098DXU9 pp 229533.I1REI2
229533.A0A098DXU9 pp 229533.I1R9Z2
229533.I1RRH6 pp 229533.V6R1X4
229533.I1RRH6 pp 229533.I1S0Q7
229533.I1RRH6 pp 229533.I1RRW5
229533.I1RRH6 pp 229533.I1RXD9
229533.I1RRH6 pp 229533.I1RTH2
229533.I1RRH6 pp 229533.I1RVN1
229533.I1RRH6 pp 229533.I1RT38
229533.I1RRH6 pp 229533.I1S0R8
229533.A0A0E0S643 pp 229533.I1RWR8
229533.A0A0E0S643 pp 229533.I1S7S5
229533.A0A0E0S643 pp 229533.I1RS34
229533.A0A0E0S643 pp 229533.I1RK73
229533.A0A0E0S643 pp 229533.I1RM71
229533.A0A0E0S643 pp 229533.I1S4N5

24529 229533.A0A0E0S643 pp 229533.I1RPC6
24530 229533.A0A0E0S643 pp 229533.I1RNB9
24531 229533.A0A0E0S643 pp 229533.I1S287
24532 229533.A0A0E0S643 pp 229533.A0A1C3YME0
24533 229533.A0A0E0S643 pp 229533.I1RG01
24534 229533.A0A0E0S643 pp 229533.A0A1C3YKT4
24535 229533.A0A098D5I1 pp 229533.A0A1C3YN54
24536 229533.A0A098D5I1 pp 229533.I1RK73
24537 229533.A0A098D5I1 pp 229533.I1RS34
24538 229533.A0A098D5I1 pp 229533.A0A0E0RR64
24539 229533.A0A098D5I1 pp 229533.A0A098DVM8
24540 229533.A0A098D5I1 pp 229533.I1RSH8
24541 229533.A0A098D5I1 pp 229533.I1RYB7
24542 229533.A0A098D5I1 pp 229533.I1RA00
24543 229533.A0A098D5I1 pp 229533.I1RX87
24544 229533.A0A098D5I1 pp 229533.I1RZJ5
24545 229533.A0A098D5I1 pp 229533.I1S287
24546 229533.A0A098D5I1 pp 229533.I1RWB4
24547 229533.A0A098D5I1 pp 229533.I1S278
24548 229533.A0A098D5I1 pp 229533.I1RM71
24549 229533.A0A098D5I1 pp 229533.I1RQP7
24550 229533.A0A098D5I1 pp 229533.A0A098D8K3
24551 229533.A0A098D5I1 pp 229533.I1RTF5
24552 229533.A0A098D5I1 pp 229533.A0A1C3YL39
24553 229533.A0A098D5I1 pp 229533.I1RQ32
24554 229533.A0A098D5I1 pp 229533.I1RRF7
24555 229533.A0A098D5I1 pp 229533.I1RTQ9
24556 229533.A0A098D5I1 pp 229533.I1RG01
24557 229533.A0A098D5I1 pp 229533.I1RRC9
24558 229533.A0A098D5I1 pp 229533.I1S0V5
24559 229533.A0A098D5I1 pp 229533.I1RWR8
24560 229533.A0A098D5I1 pp 229533.I1RAU7
24561 229533.A0A098D5I1 pp 229533.I1RTF2
24562 229533.A0A098D5I1 pp 229533.I1RQC4
24563 229533.A0A098D5I1 pp 229533.A0A1C3YKT4
24564 229533.A0A098D5I1 pp 229533.A0A0E0RU86
24565 229533.A0A098D5I1 pp 229533.I1S7S5
24566 229533.A0A098D5I1 pp 229533.I1RPC6
24567 229533.A0A098D5I1 pp 229533.I1RRC8
24568 229533.A0A098D5I1 pp 229533.I1S4N5
24569 229533.A0A098D5I1 pp 229533.I1RNB9
24570 229533.A0A098D5I1 pp 229533.A0A0E0SP22
24571 229533.A0A098D5I1 pp 229533.I1RNL7
24572 229533.A0A098D5I1 pp 229533.I1RQC3
24573 229533.A0A098D5I1 pp 229533.A0A098D8G7
24574 229533.A0A098D5I1 pp 229533.I1RE44
24575 229533.A0A098D5I1 pp 229533.A0A0E0S643
24576 229533.A0A098D5I1 pp 229533.I1RMN3
24577 229533.A0A098D5I1 pp 229533.I1RCV3
24578 229533.A0A098D5I1 pp 229533.I1RMM1
24579 229533.A0A098D5I1 pp 229533.I1RY40
24580 229533.A0A098DWX9 pp 229533.I1RCF0
24581 229533.A0A098DWX9 pp 229533.I1S0S8
24582 229533.A0A098DWX9 pp 229533.I1S7X9
24583 229533.A0A098DWX9 pp 229533.A0A1C3YLK5
24584 229533.A0A098DWX9 pp 229533.A0A1C3YLL9
24585 229533.A0A098DWX9 pp 229533.A0A1C3YIP6
24586 229533.A0A098DWX9 pp 229533.I1RD70
24587 229533.A0A098DWX9 pp 229533.I1RB35
24588 229533.A0A098DWX9 pp 229533.A0A1C3YI44
24589 229533.A0A098DWX9 pp 229533.Q4INI2
24590 229533.A0A098DWX9 pp 229533.I1RRB0
24591 229533.A0A098DWX9 pp 229533.I1RR74
24592 229533.A0A098DWX9 pp 229533.I1RRV2
24593 229533.A0A098DWX9 pp 229533.I1RMG2
24594 229533.A0A098DWX9 pp 229533.I1RXT9
24595 229533.A0A098DWX9 pp 229533.Q4I877
24596 229533.A0A098DWX9 pp 229533.I1RD02
24597 229533.I1S4T3 pp 229533.Q4IPI1
24598 229533.I1S4T3 pp 229533.V6R283
24599 229533.I1S4T3 pp 229533.Q4INZ9
24600 229533.I1S4T3 pp 229533.Q4IBS2
24601 229533.I1S4T3 pp 229533.Q4I327

229533.I1S4T3	pp	229533.Q4IAS1
229533.I1S4T3	pp	229533.Q4IJ56
229533.I1S4T3	pp	229533.Q4HZ42
229533.I1S4T3	pp	229533.Q4IQW0
229533.I1S4T3	pp	229533.V6R227
229533.I1S4T3	pp	229533.Q4IAA0
229533.I1S4T3	pp	229533.Q4IN63
229533.I1S4T3	pp	229533.I1S8W6
229533.I1S4T3	pp	229533.Q4IJH1
229533.I1S4T3	pp	229533.Q4I5M4
229533.I1S4T3	pp	229533.Q4IEK8
229533.I1S4T3	pp	229533.Q4I830
229533.I1S4T3	pp	229533.I1S558
229533.I1S4T3	pp	229533.Q4HZ68
229533.I1S4T3	pp	229533.I1S6F0
229533.I1S4T3	pp	229533.Q4IF76
229533.I1S4T3	pp	229533.Q4HVW2
229533.I1S4T3	pp	229533.Q4IFI0
229533.I1S4T3	pp	229533.Q4I662
229533.I1S4T3	pp	229533.I1S9U2
229533.I1S4T3	pp	229533.V6RGY3
229533.I1S4T3	pp	229533.V6QYF0
229533.I1RAY2	pp	229533.I1RZJ6
229533.I1RAY2	pp	229533.I1RT98
229533.I1RAY2	pp	229533.I1RQJ5
229533.I1RAY2	pp	229533.I1S095
229533.I1RAY2	pp	229533.I1RZR6
229533.I1RAY2	pp	229533.Q4HZ47
229533.I1RAY2	pp	229533.Q4IJU0
229533.I1RAY2	pp	229533.I1RLE7
229533.I1RAY2	pp	229533.I1RM09
229533.I1RAY2	pp	229533.I1RMB7
229533.I1RAY2	pp	229533.I1RC06
229533.I1RAY2	pp	229533.I1S0M9
229533.I1RAY2	pp	229533.Q4IAS1
229533.I1RAY2	pp	229533.I1S050
229533.I1RAY2	pp	229533.I1S9U2
229533.I1RAY2	pp	229533.I1RUG1
229533.I1RAY2	pp	229533.I1RSV0
229533.I1RAY2	pp	229533.I1RSI1
229533.I1RAY2	pp	229533.I1RFN0
229533.I1RAY2	pp	229533.I1RLV9
229533.I1RAY2	pp	229533.I1RKJ6
229533.I1RAY2	pp	229533.I1RQ65
229533.I1RAY2	pp	229533.I1RJS3
229533.I1RAY2	pp	229533.Q4HYB8
229533.I1RAY2	pp	229533.I1S0J1
229533.I1RAY2	pp	229533.I1RSJ4
229533.I1RAY2	pp	229533.I1RNU6
229533.I1RAY2	pp	229533.I1RZP8
229533.I1RAY2	pp	229533.I1RS41
229533.I1RAY2	pp	229533.I1RCN5
229533.I1RAY2	pp	229533.I1RHM4
229533.I1RAY2	pp	229533.I1RWE1
229533.I1RAY2	pp	229533.I1RNW8
229533.A0A1C3YLL9	pp	229533.I1RCF0
229533.A0A1C3YLL9	pp	229533.I1RQ80
229533.A0A1C3YLL9	pp	229533.Q4INI2
229533.A0A1C3YLL9	pp	229533.I1RFZ2
229533.A0A1C3YLL9	pp	229533.I1RQ70
229533.A0A1C3YLL9	pp	229533.A0A1C3YM61
229533.A0A1C3YLL9	pp	229533.Q5I2J3
229533.A0A1C3YLL9	pp	229533.I1RR74
229533.A0A1C3YLL9	pp	229533.I1RZU6
229533.A0A1C3YLL9	pp	229533.Q4IN00
229533.A0A1C3YLL9	pp	229533.I1RT65
229533.A0A1C3YLL9	pp	229533.Q4HZS8
229533.A0A1C3YLL9	pp	229533.Q4I2D7
229533.A0A1C3YLL9	pp	229533.A0A1C3YN58
229533.A0A1C3YLL9	pp	229533.I1RMF2
229533.A0A1C3YLL9	pp	229533.I1RQF7
229533.A0A1C3YLL9	pp	229533.I1RWD5
229533.A0A1C3YLL9	pp	229533.I1S0S8

24675 229533.A0A1C3YLL9 pp 229533.Q4INZ9
24676 229533.A0A1C3YLL9 pp 229533.I1RT41
24677 229533.A0A1C3YLL9 pp 229533.I1RSH3
24678 229533.A0A1C3YLL9 pp 229533.I1RD02
24679 229533.A0A1C3YLL9 pp 229533.I1RFW0
24680 229533.A0A1C3YLL9 pp 229533.Q4I877
24681 229533.A0A1C3YLL9 pp 229533.I1RAZ9
24682 229533.A0A1C3YLL9 pp 229533.I1RYM4
24683 229533.A0A1C3YLL9 pp 229533.I1RKI1
24684 229533.A0A1C3YLL9 pp 229533.I1RR95
24685 229533.A0A1C3YLL9 pp 229533.I1RA72
24686 229533.A0A1C3YLL9 pp 229533.I1RFK0
24687 229533.A0A1C3YLL9 pp 229533.I1RBT0
24688 229533.A0A1C3YLL9 pp 229533.I1RB35
24689 229533.A0A1C3YLL9 pp 229533.Q4ICM0
24690 229533.A0A1C3YLL9 pp 229533.I1RUJ0
24691 229533.A0A1C3YLL9 pp 229533.Q4HZB8
24692 229533.A0A1C3YLL9 pp 229533.I1S0Z3
24693 229533.A0A1C3YLL9 pp 229533.I1RCM6
24694 229533.A0A1C3YLL9 pp 229533.I1RST4
24695 229533.A0A1C3YLL9 pp 229533.I1RMG2
24696 229533.A0A1C3YLL9 pp 229533.I1RXT9
24697 229533.Q4IBS2 pp 229533.Q4INZ9
24698 229533.Q4IBS2 pp 229533.Q4IFI0
24699 229533.Q4IBS2 pp 229533.Q4IPI1
24700 229533.Q4IBS2 pp 229533.V6QYF0
24701 229533.Q4IBS2 pp 229533.Q4IJ56
24702 229533.Q4IBS2 pp 229533.Q4IEV5
24703 229533.Q4IBS2 pp 229533.Q4IF76
24704 229533.Q4IBS2 pp 229533.V6R227
24705 229533.Q4IBS2 pp 229533.Q4IEK8
24706 229533.Q4IBS2 pp 229533.Q4IJH1
24707 229533.Q4IBS2 pp 229533.V6RGY3
24708 229533.Q4IBS2 pp 229533.V6RIS2
24709 229533.Q4IBS2 pp 229533.V6RY33
24710 229533.Q4IBS2 pp 229533.Q4IE79
24711 229533.Q4IBS2 pp 229533.V6R283
24712 229533.Q4IBS2 pp 229533.Q4IQW0
24713 229533.Q4IBS2 pp 229533.V6RGN5
24714 229533.I1RAB5 pp 229533.I1RJL2
24715 229533.I1RAB5 pp 229533.I1RRT0
24716 229533.I1RAB5 pp 229533.I1RT35
24717 229533.I1RAB5 pp 229533.I1RZB5
24718 229533.I1RAB5 pp 229533.I1RRU9
24719 229533.I1RAB5 pp 229533.I1RE31
24720 229533.I1RAB5 pp 229533.I1RNB8
24721 229533.I1RAB5 pp 229533.I1RRG3
24722 229533.I1RAB5 pp 229533.I1RG90
24723 229533.I1RAB5 pp 229533.I1RR25
24724 229533.I1RAB5 pp 229533.I1RK79
24725 229533.I1RAB5 pp 229533.I1RG11
24726 229533.I1RAB5 pp 229533.I1RCB7
24727 229533.I1RAB5 pp 229533.I1S0B6
24728 229533.I1RAB5 pp 229533.I1S0N7
24729 229533.I1RAB5 pp 229533.I1RK92
24730 229533.I1RAB5 pp 229533.I1RFN1
24731 229533.I1RAB5 pp 229533.I1RC03
24732 229533.I1RAB5 pp 229533.I1S4M5
24733 229533.I1RAB5 pp 229533.I1RC24
24734 229533.V6R1X4 pp 229533.V6R3J1
24735 229533.A0A098DQC9 pp 229533.Q4ICM0
24736 229533.A0A098DQC9 pp 229533.I1RST4
24737 229533.A0A098DQC9 pp 229533.I1RB35
24738 229533.A0A098DQC9 pp 229533.I1RAZ9
24739 229533.A0A098DQC9 pp 229533.I1RRF0
24740 229533.A0A098DQC9 pp 229533.I1RD02
24741 229533.A0A098DQC9 pp 229533.I1S0S8
24742 229533.A0A098DQC9 pp 229533.I1RWD5
24743 229533.A0A098DQC9 pp 229533.A0A0E0SMG8
24744 229533.A0A098DQC9 pp 229533.A0A1C3YI44
24745 229533.A0A098DQC9 pp 229533.I1RFK0
24746 229533.A0A098DQC9 pp 229533.I1RR74
24747 229533.A0A098DQC9 pp 229533.A0A1C3YLK5

24748	229533.A0A098DQC9	pp	229533.I1RCF0
24749	229533.A0A098DQC9	pp	229533.Q4INI2
24750	229533.A0A098DQC9	pp	229533.I1S270
24751	229533.A0A098DQC9	pp	229533.I1RUJ0
24752	229533.A0A098DQC9	pp	229533.I1S0Z3
24753	229533.A0A098DQC9	pp	229533.I1RB00
24754	229533.A0A098DQC9	pp	229533.I1RT41
24755	229533.A0A098DQC9	pp	229533.I1RYM4
24756	229533.A0A098DQC9	pp	229533.A0A098DZN5
24757	229533.A0A098DQC9	pp	229533.A0A1C3YIG8
24758	229533.A0A098DQC9	pp	229533.I1RT65
24759	229533.A0A098DQC9	pp	229533.Q4HZB8
24760	229533.A0A098DQC9	pp	229533.I1RKI1
24761	229533.A0A098DQC9	pp	229533.A0A1C3YIF4
24762	229533.A0A098DQC9	pp	229533.I1RMG2
24763	229533.A0A098DQC9	pp	229533.Q4I2D7
24764	229533.A0A098DQC9	pp	229533.A0A098DWX9
24765	229533.A0A098DQC9	pp	229533.A0A1C3YLL9
24766	229533.A0A098DQC9	pp	229533.Q4IPI4
24767	229533.A0A098DQC9	pp	229533.I1RKR9
24768	229533.I1RG56	pp	229533.I1RQS8
24769	229533.I1RG56	pp	229533.I1S9U2
24770	229533.I1RG56	pp	229533.I1RPW9
24771	229533.I1RG56	pp	229533.I1RGF6
24772	229533.I1RG56	pp	229533.I1RKJ6
24773	229533.I1RG56	pp	229533.I1RPU0
24774	229533.I1RG56	pp	229533.O74638
24775	229533.I1RG56	pp	229533.I1S9Y1
24776	229533.I1RG56	pp	229533.I1RM67
24777	229533.I1RG56	pp	229533.I1RJZ7
24778	229533.I1RG56	pp	229533.Q4IFI0
24779	229533.I1RG56	pp	229533.I1RRT8
24780	229533.I1RG56	pp	229533.I1RNV6
24781	229533.I1RG56	pp	229533.I1RT80
24782	229533.I1RG56	pp	229533.Q4IJH1
24783	229533.I1RG56	pp	229533.I1RL23
24784	229533.I1RG56	pp	229533.Q4IMJ1
24785	229533.I1RG56	pp	229533.V6QYF0
24786	229533.I1RG56	pp	229533.I1RPH9
24787	229533.I1RG56	pp	229533.I1RTF4
24788	229533.I1RG56	pp	229533.Q4IQK7
24789	229533.I1RG56	pp	229533.I1RNA0
24790	229533.I1RG56	pp	229533.I1RUQ2
24791	229533.I1RG56	pp	229533.Q4HXF0
24792	229533.I1RG56	pp	229533.I1RND6
24793	229533.I1RG56	pp	229533.Q4HVW2
24794	229533.I1RG56	pp	229533.I1RZS6
24795	229533.I1RG56	pp	229533.I1RX24
24796	229533.I1RG56	pp	229533.I1RZJ6
24797	229533.I1RG56	pp	229533.I1RMK3
24798	229533.I1RG56	pp	229533.I1RYT5
24799	229533.I1RG56	pp	229533.I1RM06
24800	229533.I1RG56	pp	229533.I1RT05
24801	229533.I1RG56	pp	229533.Q4INZ9
24802	229533.I1RG56	pp	229533.Q4IF76
24803	229533.I1RG56	pp	229533.Q4I662
24804	229533.I1RG56	pp	229533.I1RP27
24805	229533.I1RG56	pp	229533.Q4HZ42
24806	229533.I1RG56	pp	229533.I1RS47
24807	229533.I1RG56	pp	229533.Q4IQW0
24808	229533.I1RG56	pp	229533.I1RT33
24809	229533.I1RG56	pp	229533.I1RRH5
24810	229533.I1RG56	pp	229533.I1RJ74
24811	229533.I1RG56	pp	229533.I1RQM6
24812	229533.I1RG56	pp	229533.I1RKQ5
24813	229533.I1RG56	pp	229533.I1RQJ5
24814	229533.I1RG56	pp	229533.I1RQ65
24815	229533.I1RG56	pp	229533.I1RLV9
24816	229533.I1RG56	pp	229533.Q4IAS1
24817	229533.I1RG56	pp	229533.V6R283
24818	229533.I1RG56	pp	229533.I1RNH1
24819	229533.I1RG56	pp	229533.I1RS30
24820	229533.I1RG56	pp	229533.I1S2M6

24821 229533.I1RG56 pp 229533.I1S6F0
24822 229533.I1RG56 pp 229533.I1RRQ3
24823 229533.I1RG56 pp 229533.Q4IPI1
24824 229533.I1RG56 pp 229533.I1RQH2
24825 229533.I1RG56 pp 229533.I1RJL4
24826 229533.I1RG56 pp 229533.I1RQS5
24827 229533.I1RG56 pp 229533.I1RKL3
24828 229533.I1RG56 pp 229533.I1RRT2
24829 229533.I1RG56 pp 229533.I1S274
24830 229533.I1RG56 pp 229533.Q4I830
24831 229533.I1RG56 pp 229533.I1RW71
24832 229533.I1RG56 pp 229533.I1S1U7
24833 229533.I1RG56 pp 229533.V6RGY3
24834 229533.I1RG56 pp 229533.V6RGN5
24835 229533.I1RG56 pp 229533.I1S0R7
24836 229533.I1RG56 pp 229533.I1RSR3
24837 229533.I1RG56 pp 229533.I1RRJ8
24838 229533.I1RG56 pp 229533.V6QUE6
24839 229533.I1RG56 pp 229533.I1RZ99
24840 229533.I1RG56 pp 229533.I1RQA2
24841 229533.I1RG56 pp 229533.Q4HZK7
24842 229533.I1RG56 pp 229533.Q4HZ68
24843 229533.I1RG56 pp 229533.I1RMN6
24844 229533.I1RG56 pp 229533.I1RZB5
24845 229533.I1RG56 pp 229533.Q4IBS2
24846 229533.I1RG56 pp 229533.I1S2C0
24847 229533.I1RG56 pp 229533.I1RSH5
24848 229533.I1RG56 pp 229533.I1RKC6
24849 229533.I1RG56 pp 229533.I1RSI6
24850 229533.I1RG56 pp 229533.I1RPJ3
24851 229533.I1RG56 pp 229533.I1S1J6
24852 229533.I1RG56 pp 229533.I1RVZ0
24853 229533.I1RG56 pp 229533.Q4IN63
24854 229533.I1RG56 pp 229533.I1RMD5
24855 229533.I1RG56 pp 229533.I1RTE9
24856 229533.I1RG56 pp 229533.I1RNK2
24857 229533.I1RG56 pp 229533.I1RZE5
24858 229533.I1RG56 pp 229533.I1RRI2
24859 229533.I1RG56 pp 229533.I1RQ31
24860 229533.I1RG56 pp 229533.I1RSS4
24861 229533.I1RG56 pp 229533.I1RSC6
24862 229533.I1RG56 pp 229533.I1RNH6
24863 229533.I1RG56 pp 229533.I1RMC0
24864 229533.I1RG56 pp 229533.I1S021
24865 229533.I1RG56 pp 229533.I1RK95
24866 229533.I1RG56 pp 229533.I1RQ28
24867 229533.I1RG56 pp 229533.I1S1X9
24868 229533.I1RG56 pp 229533.Q4I327
24869 229533.I1RG56 pp 229533.I1RMN7
24870 229533.I1RG56 pp 229533.I1RNY2
24871 229533.I1RG56 pp 229533.I1RN99
24872 229533.I1RG56 pp 229533.Q4I948
24873 229533.I1RG56 pp 229533.I1S2U2
24874 229533.I1RG56 pp 229533.I1RSD8
24875 229533.I1RG56 pp 229533.I1RZF6
24876 229533.I1RG56 pp 229533.V6R227
24877 229533.I1RG56 pp 229533.I1RRR7
24878 229533.I1RG56 pp 229533.I1RPY1
24879 229533.I1RG56 pp 229533.I1RJZ6
24880 229533.I1RG56 pp 229533.I1S2E8
24881 229533.I1RG56 pp 229533.I1RQE7
24882 229533.I1RG56 pp 229533.I1RZZ1
24883 229533.I1RG56 pp 229533.I1S4T3
24884 229533.I1RG56 pp 229533.Q4IEK8
24885 229533.I1RG56 pp 229533.I1RM96
24886 229533.I1RG56 pp 229533.I1S0M9
24887 229533.I1RG56 pp 229533.I1RZK6
24888 229533.I1RG56 pp 229533.I1RNU6
24889 229533.I1RG56 pp 229533.I1RX14
24890 229533.I1RG56 pp 229533.I1S060
24891 229533.I1RG56 pp 229533.Q4IJ56
24892 229533.I1RG56 pp 229533.I1S0J1
24893 229533.A0A1C3YKV0 pp 229533.I1RBV5

24894 229533.A0A1C3YKV0 pp 229533.I1RX38
24895 229533.A0A1C3YKV0 pp 229533.I1RQ88
24896 229533.A0A1C3YKV0 pp 229533.I1RAI9
24897 229533.A0A1C3YKV0 pp 229533.I1RJ33
24898 229533.A0A1C3YKV0 pp 229533.I1RPW0
24899 229533.A0A1C3YKV0 pp 229533.I1RPU4
24900 229533.A0A098DQC2 pp 229533.A0A1C3YKV0
24901 229533.Q4IEK8 pp 229533.V6RCG5
24902 229533.Q4IEK8 pp 229533.V6R227
24903 229533.Q4IEK8 pp 229533.Q4IN63
24904 229533.Q4IEK8 pp 229533.Q4IPI1
24905 229533.Q4IEK8 pp 229533.V6QUE6
24906 229533.Q4IEK8 pp 229533.Q4IJ56
24907 229533.Q4IEK8 pp 229533.Q4INZ9
24908 229533.Q4IEK8 pp 229533.V6QW22
24909 229533.Q4IEK8 pp 229533.Q4IQK7
24910 229533.Q4IEK8 pp 229533.V6RGN5
24911 229533.Q4IEK8 pp 229533.V6R042
24912 229533.Q4IEK8 pp 229533.V6QYF0
24913 229533.Q4IEK8 pp 229533.V6RIV9
24914 229533.Q4IEK8 pp 229533.V6RGY3
24915 229533.Q4IEK8 pp 229533.Q4IJH1
24916 229533.Q4IEK8 pp 229533.V6RIS2
24917 229533.Q4IEK8 pp 229533.Q4IFI0
24918 229533.Q4IEK8 pp 229533.Q4IMJ1
24919 229533.Q4IEK8 pp 229533.Q4IF76
24920 229533.Q4IEK8 pp 229533.Q4IQW0
24921 229533.Q4IEK8 pp 229533.V6RY33
24922 229533.Q4IEK8 pp 229533.V6R283
24923 229533.A0A1C3YJN3 pp 229533.I1RPE0
24924 229533.A0A1C3YJN3 pp 229533.I1RA11
24925 229533.A0A1C3YJN3 pp 229533.I1S2H4
24926 229533.A0A1C3YJN3 pp 229533.I1REC5
24927 229533.A0A1C3YJN3 pp 229533.I1S5R1
24928 229533.A0A1C3YJN3 pp 229533.I1RR24
24929 229533.A0A1C3YJN3 pp 229533.I1RRT8
24930 229533.A0A1C3YJN3 pp 229533.I1RXC2
24931 229533.A0A1C3YJN3 pp 229533.I1RC66
24932 229533.A0A1C3YJN3 pp 229533.I1RE17
24933 229533.A0A1C3YJN3 pp 229533.I1RBT1
24934 229533.A0A1C3YJN3 pp 229533.I1S4S4
24935 229533.A0A1C3YJN3 pp 229533.I1RW54
24936 229533.A0A1C3YJN3 pp 229533.I1RWI5
24937 229533.A0A1C3YJN3 pp 229533.I1RKP1
24938 229533.A0A1C3YJN3 pp 229533.I1RSQ4
24939 229533.A0A1C3YJN3 pp 229533.I1RGU7
24940 229533.A0A1C3YJN3 pp 229533.I1RWA3
24941 229533.A0A1C3YJN3 pp 229533.I1RGB0
24942 229533.A0A1C3YJN3 pp 229533.I1S0L4
24943 229533.A0A1C3YJN3 pp 229533.I1RKJ5
24944 229533.A0A1C3YJN3 pp 229533.I1RSX0
24945 229533.A0A1C3YJN3 pp 229533.I1RY92
24946 229533.A0A1C3YJN3 pp 229533.I1RRI1
24947 229533.A0A1C3YJN3 pp 229533.I1R9H9
24948 229533.A0A1C3YJN3 pp 229533.I1RJU3
24949 229533.A0A1C3YJN3 pp 229533.I1S2B8
24950 229533.A0A1C3YJN3 pp 229533.I1RZ39
24951 229533.A0A1C3YJN3 pp 229533.I1S8F0
24952 229533.A0A1C3YJN3 pp 229533.I1RX46
24953 229533.A0A1C3YJN3 pp 229533.A0A1C3YKM9
24954 229533.A0A1C3YJN3 pp 229533.I1RRS8
24955 229533.A0A1C3YJN3 pp 229533.I1R9M0
24956 229533.A0A1C3YJN3 pp 229533.I1S4Z8
24957 229533.A0A1C3YJN3 pp 229533.I1RAD1
24958 229533.A0A1C3YJN3 pp 229533.I1S9I5
24959 229533.A0A1C3YJN3 pp 229533.A0A1C3YLK8
24960 229533.A0A1C3YJN3 pp 229533.I1RRK3
24961 229533.A0A1C3YJN3 pp 229533.I1RBY4
24962 229533.A0A1C3YJN3 pp 229533.I1RA52
24963 229533.A0A1C3YJN3 pp 229533.Q4HVQ9
24964 229533.A0A1C3YJN3 pp 229533.I1RL04
24965 229533.A0A1C3YJN3 pp 229533.I1RYT8
24966 229533.A0A1C3YJN3 pp 229533.I1RPY3

24967	229533.A0A1C3YJN3	pp	229533.I1RPV3
24968	229533.A0A1C3YJN3	pp	229533.I1RW61
24969	229533.A0A1C3YJN3	pp	229533.I1RT40
24970	229533.A0A1C3YJN3	pp	229533.I1RPX0
24971	229533.A0A1C3YJN3	pp	229533.I1RC34
24972	229533.A0A1C3YJN3	pp	229533.I1RSZ7
24973	229533.A0A1C3YJN3	pp	229533.I1RNB2
24974	229533.A0A1C3YJN3	pp	229533.I1RBR2
24975	229533.A0A1C3YJN3	pp	229533.I1RTN3
24976	229533.A0A1C3YJN3	pp	229533.A0A1C3YM05
24977	229533.A0A1C3YJN3	pp	229533.I1RXD9
24978	229533.A0A1C3YJN3	pp	229533.I1S438
24979	229533.A0A1C3YJN3	pp	229533.V6RTJ8
24980	229533.A0A1C3YJN3	pp	229533.I1RXD7
24981	229533.A0A1C3YJN3	pp	229533.I1RC97
24982	229533.A0A1C3YJN3	pp	229533.A0A1C3YLL3
24983	229533.A0A1C3YJN3	pp	229533.I1RTB3
24984	229533.A0A1C3YJN3	pp	229533.I1RY86
24985	229533.A0A1C3YJN3	pp	229533.I1RQM7
24986	229533.A0A1C3YJN3	pp	229533.I1RE66
24987	229533.A0A1C3YJN3	pp	229533.I1RS05
24988	229533.A0A1C3YJN3	pp	229533.I1RAX6
24989	229533.A0A1C3YJN3	pp	229533.I1RD52
24990	229533.A0A1C3YJN3	pp	229533.I1RGA4
24991	229533.A0A1C3YJN3	pp	229533.I1RVZ2
24992	229533.A0A1C3YJN3	pp	229533.I1S0P0
24993	229533.A0A1C3YJN3	pp	229533.I1S268
24994	229533.A0A1C3YJN3	pp	229533.I1RJN9
24995	229533.A0A1C3YJN3	pp	229533.I1RTA1
24996	229533.A0A1C3YJN3	pp	229533.I1RXD6
24997	229533.A0A1C3YJN3	pp	229533.I1RYR0
24998	229533.A0A1C3YJN3	pp	229533.I1RXF8
24999	229533.A0A1C3YJN3	pp	229533.I1RDF7
25000	229533.A0A1C3YJN3	pp	229533.I1RX95
25001	229533.A0A1C3YJN3	pp	229533.I1S0W4
25002	229533.A0A1C3YJN3	pp	229533.I1RPH6
25003	229533.A0A1C3YJN3	pp	229533.I1RQ26
25004	229533.A0A1C3YJN3	pp	229533.I1RSA5
25005	229533.A0A1C3YJN3	pp	229533.I1RTD5
25006	229533.A0A1C3YJN3	pp	229533.I1S0F7
25007	229533.A0A1C3YJN3	pp	229533.I1S2R0
25008	229533.A0A1C3YJN3	pp	229533.I1REV8
25009	229533.A0A1C3YJN3	pp	229533.I1RSQ7
25010	229533.A0A1C3YJN3	pp	229533.Q4IB65
25011	229533.A0A1C3YJN3	pp	229533.A0A1C3YKY6
25012	229533.A0A1C3YJN3	pp	229533.I1RMM9
25013	229533.A0A1C3YJN3	pp	229533.V6R9Z0
25014	229533.A0A1C3YJN3	pp	229533.I1RZK5
25015	229533.A0A1C3YJN3	pp	229533.Q4I7T5
25016	229533.A0A1C3YJN3	pp	229533.I1RYI5
25017	229533.A0A1C3YJN3	pp	229533.I1RKJ8
25018	229533.A0A1C3YJN3	pp	229533.I1RVQ3
25019	229533.A0A1C3YJN3	pp	229533.I1RE89
25020	229533.A0A1C3YJN3	pp	229533.I1RGU0
25021	229533.A0A1C3YJN3	pp	229533.I1S0F8
25022	229533.A0A1C3YJN3	pp	229533.I1RBU8
25023	229533.A0A1C3YJN3	pp	229533.I1RPP3
25024	229533.A0A1C3YJN3	pp	229533.I1S4S7
25025	229533.A0A1C3YJN3	pp	229533.I1RAW0
25026	229533.A0A1C3YJN3	pp	229533.Q4IPZ1
25027	229533.A0A1C3YJN3	pp	229533.I1RRY9
25028	229533.A0A1C3YJN3	pp	229533.I1RWR7
25029	229533.A0A1C3YJN3	pp	229533.I1RJV3
25030	229533.A0A1C3YJN3	pp	229533.I1RQD2
25031	229533.A0A1C3YJN3	pp	229533.I1RCD8
25032	229533.A0A1C3YJN3	pp	229533.I1RGB1
25033	229533.A0A1C3YJN3	pp	229533.I1RML0
25034	229533.A0A1C3YJN3	pp	229533.I1RCR1
25035	229533.A0A1C3YJN3	pp	229533.I1RRI2
25036	229533.A0A1C3YJN3	pp	229533.I1RNT9
25037	229533.A0A1C3YJN3	pp	229533.I1RVF8
25038	229533.A0A1C3YJN3	pp	229533.I1S217
25039	229533.A0A1C3YJN3	pp	229533.A0A1C3YKS0

25040 229533.A0A1C3YJN3 pp 229533.I1RKJ4
25041 229533.A0A1C3YJN3 pp 229533.I1RB44
25042 229533.A0A1C3YJN3 pp 229533.I1S437
25043 229533.A0A1C3YJN3 pp 229533.I1RBR1
25044 229533.A0A1C3YJN3 pp 229533.I1S1C7
25045 229533.A0A1C3YJN3 pp 229533.I1RCR8
25046 229533.A0A1C3YJN3 pp 229533.I1RNC1
25047 229533.A0A1C3YJN3 pp 229533.I1RZM8
25048 229533.A0A1C3YJN3 pp 229533.I1RQ33
25049 229533.A0A1C3YJN3 pp 229533.I1S8Y1
25050 229533.A0A1C3YJN3 pp 229533.I1REK8
25051 229533.A0A1C3YJN3 pp 229533.I1RG57
25052 229533.A0A1C3YJN3 pp 229533.Q4IJK2
25053 229533.A0A1C3YJN3 pp 229533.Q4I624
25054 229533.A0A1C3YJN3 pp 229533.A0A1C3YLX0
25055 229533.A0A1C3YJN3 pp 229533.I1RFL4
25056 229533.A0A1C3YJN3 pp 229533.I1RUG3
25057 229533.A0A1C3YJN3 pp 229533.I1RB92
25058 229533.A0A1C3YJN3 pp 229533.I1RUQ3
25059 229533.A0A1C3YJN3 pp 229533.I1RQE7
25060 229533.A0A1C3YJN3 pp 229533.I1RGB2
25061 229533.A0A1C3YJN3 pp 229533.I1RUF7
25062 229533.A0A1C3YJN3 pp 229533.I1RCH5
25063 229533.A0A1C3YJN3 pp 229533.I1RSQ3
25064 229533.A0A1C3YJN3 pp 229533.V6R042
25065 229533.A0A1C3YJN3 pp 229533.I1RRM3
25066 229533.A0A1C3YJN3 pp 229533.I1RT03
25067 229533.A0A1C3YJN3 pp 229533.V6RMW6
25068 229533.A0A1C3YJN3 pp 229533.I1RVX0
25069 229533.A0A1C3YJN3 pp 229533.I1RSY2
25070 229533.A0A1C3YJN3 pp 229533.I1S1B2
25071 229533.A0A1C3YJN3 pp 229533.I1S1W9
25072 229533.A0A1C3YJN3 pp 229533.I1RQ85
25073 229533.A0A1C3YJN3 pp 229533.I1RD22
25074 229533.A0A1C3YJN3 pp 229533.I1RVZ7
25075 229533.A0A1C3YJN3 pp 229533.I1S821
25076 229533.A0A1C3YJN3 pp 229533.I1S6K9
25077 229533.A0A1C3YJN3 pp 229533.I1RRN2
25078 229533.A0A1C3YJN3 pp 229533.I1RRV3
25079 229533.A0A1C3YJN3 pp 229533.I1RPU2
25080 229533.A0A1C3YJN3 pp 229533.A0A1C3YKB0
25081 229533.A0A1C3YJN3 pp 229533.V6RCZ8
25082 229533.A0A1C3YJN3 pp 229533.I1RSF1
25083 229533.A0A1C3YJN3 pp 229533.I1RG11
25084 229533.A0A1C3YJN3 pp 229533.A0A1C3YKB9
25085 229533.A0A1C3YJN3 pp 229533.I1RB26
25086 229533.A0A1C3YJN3 pp 229533.I1S5B0
25087 229533.A0A1C3YJN3 pp 229533.A0A1C3YJV6
25088 229533.A0A1C3YJN3 pp 229533.I1RGC5
25089 229533.A0A1C3YJN3 pp 229533.I1RZV3
25090 229533.A0A1C3YJN3 pp 229533.A0A1C3YK34
25091 229533.A0A1C3YJN3 pp 229533.I1S0J1
25092 229533.A0A1C3YJN3 pp 229533.A0A1C3YLB6
25093 229533.A0A1C3YJN3 pp 229533.I1S016
25094 229533.A0A1C3YJN3 pp 229533.I1RXC4
25095 229533.A0A1C3YJN3 pp 229533.I1S5R6
25096 229533.A0A1C3YJN3 pp 229533.I1RE65
25097 229533.A0A1C3YJN3 pp 229533.A0A1C3YK36
25098 229533.A0A1C3YJN3 pp 229533.I1RAG7
25099 229533.A0A1C3YJN3 pp 229533.I1RE01
25100 229533.A0A1C3YJN3 pp 229533.I1S8Y0
25101 229533.A0A1C3YJN3 pp 229533.I1RS18
25102 229533.A0A1C3YJN3 pp 229533.I1RY11
25103 229533.A0A1C3YJN3 pp 229533.I1RPK0
25104 229533.A0A1C3YJN3 pp 229533.A0A1C3YKV3
25105 229533.I1RT38 pp 229533.I1RVN1
25106 229533.I1RT38 pp 229533.I1RUG0
25107 229533.I1RT38 pp 229533.I1S277
25108 229533.I1RT38 pp 229533.Q4HZ95
25109 229533.I1RT38 pp 229533.Q4IJW4
25110 229533.I1RT38 pp 229533.V6RM66
25111 229533.I1RT38 pp 229533.Q4IPZ1
25112 229533.I1RT38 pp 229533.Q4IB65

25113	229533.I1RT38	pp	229533.Q4IK03
25114	229533.I1RT38	pp	229533.I1S0R5
25115	229533.I1RT38	pp	229533.I1RZI8
25116	229533.I1RT38	pp	229533.Q4I7T5
25117	229533.I1RT38	pp	229533.Q4I099
25118	229533.I1RT38	pp	229533.I1RZH5
25119	229533.I1RT38	pp	229533.Q4I6B0
25120	229533.I1RT38	pp	229533.I1S8F0
25121	229533.I1RT38	pp	229533.I1RT99
25122	229533.I1RT38	pp	229533.I1S0R9
25123	229533.I1RT38	pp	229533.I1S088
25124	229533.I1RT38	pp	229533.Q4IBU4
25125	229533.I1RT38	pp	229533.Q4I2W2
25126	229533.I1RT38	pp	229533.V6R1X4
25127	229533.I1RT38	pp	229533.I1RZS4
25128	229533.I1RT38	pp	229533.Q4I375
25129	229533.I1RCL3	pp	229533.I1RQ28
25130	229533.I1RCL3	pp	229533.I1RN99
25131	229533.I1RCL3	pp	229533.I1RK95
25132	229533.I1RCL3	pp	229533.I1RDG0
25133	229533.I1RCL3	pp	229533.I1S2E8
25134	229533.I1RCL3	pp	229533.I1RY63
25135	229533.I1RCL3	pp	229533.I1RSD8
25136	229533.I1RCL3	pp	229533.I1S2U2
25137	229533.I1RCL3	pp	229533.I1RMQ0
25138	229533.I1RCL3	pp	229533.I1S1X9
25139	229533.I1RCL3	pp	229533.I1RKQ9
25140	229533.I1RCL3	pp	229533.I1RCY5
25141	229533.I1RCL3	pp	229533.I1RSS4
25142	229533.I1RCL3	pp	229533.I1RF08
25143	229533.I1RCL3	pp	229533.I1RSC6
25144	229533.I1RCL3	pp	229533.V6RGY3
25145	229533.I1RCL3	pp	229533.I1RZZ1
25146	229533.I1RCL3	pp	229533.I1S296
25147	229533.I1RCL3	pp	229533.Q4HZ68
25148	229533.I1RCL3	pp	229533.I1RG56
25149	229533.I1RCL3	pp	229533.I1S4T3
25150	229533.I1RCL3	pp	229533.I1RSR3
25151	229533.I1RCL3	pp	229533.I1RPY1
25152	229533.I1RCL3	pp	229533.I1RSI6
25153	229533.I1RCL3	pp	229533.I1RZE5
25154	229533.I1RCL3	pp	229533.I1S274
25155	229533.I1RCL3	pp	229533.I1RSH5
25156	229533.I1RCL3	pp	229533.I1RFJ9
25157	229533.I1RCL3	pp	229533.I1RSI0
25158	229533.I1RCL3	pp	229533.I1RGC6
25159	229533.I1RCL3	pp	229533.I1RNY2
25160	229533.I1RCL3	pp	229533.I1RF19
25161	229533.I1RCL3	pp	229533.I1RE00
25162	229533.I1RCL3	pp	229533.I1RQA2
25163	229533.I1RCL3	pp	229533.Q4IAA0
25164	229533.I1RCL3	pp	229533.Q4I327
25165	229533.I1RCL3	pp	229533.I1RCN5
25166	229533.I1RCL3	pp	229533.I1RMN7
25167	229533.I1RCL3	pp	229533.I1S0M3
25168	229533.I1RCL3	pp	229533.Q4IJ56
25169	229533.I1RCL3	pp	229533.I1RNK2
25170	229533.I1RCL3	pp	229533.I1S021
25171	229533.I1RCL3	pp	229533.I1RNU6
25172	229533.I1RCL3	pp	229533.I1RNH6
25173	229533.I1RCL3	pp	229533.I1RVZ0
25174	229533.I1RCL3	pp	229533.I1RMC0
25175	229533.I1RCL3	pp	229533.I1RKC6
25176	229533.I1RCL3	pp	229533.I1RX14
25177	229533.I1RCL3	pp	229533.I1S060
25178	229533.I1RCL3	pp	229533.I1S1J6
25179	229533.I1RCL3	pp	229533.I1RQ31
25180	229533.I1RCL3	pp	229533.I1RUQ9
25181	229533.I1RCL3	pp	229533.I1RTE9
25182	229533.I1RCL3	pp	229533.I1RRR7
25183	229533.I1RCL3	pp	229533.I1RPJ3
25184	229533.I1RCL3	pp	229533.I1S044
25185	229533.I1RCL3	pp	229533.V6R227

25186	229533.I1RCL3	pp	229533.V6RGN5
25187	229533.I1RCL3	pp	229533.I1S2C0
25188	229533.I1RCL3	pp	229533.Q4IBS2
25189	229533.I1RCL3	pp	229533.I1RG11
25190	229533.I1RCL3	pp	229533.I1RRI2
25191	229533.I1RCL3	pp	229533.I1RZF6
25192	229533.I1RCL3	pp	229533.I1RMN6
25193	229533.I1RCL3	pp	229533.Q4IEK8
25194	229533.I1RCL3	pp	229533.I1RMD5
25195	229533.I1RCL3	pp	229533.I1RM96
25196	229533.I1RCL3	pp	229533.I1RSM3
25197	229533.I1RCL3	pp	229533.V6QUE6
25198	229533.I1RCL3	pp	229533.I1REX4
25199	229533.I1RCL3	pp	229533.I1RPW9
25200	229533.I1RCL3	pp	229533.I1RGC2
25201	229533.I1RCL3	pp	229533.I1RST1
25202	229533.I1RCL3	pp	229533.I1S9U2
25203	229533.I1RCL3	pp	229533.I1RZS6
25204	229533.I1RCL3	pp	229533.Q4I662
25205	229533.I1RCL3	pp	229533.I1RM06
25206	229533.I1RCL3	pp	229533.I1RX24
25207	229533.I1RCL3	pp	229533.I1RFS3
25208	229533.I1RCL3	pp	229533.I1RD64
25209	229533.I1RCL3	pp	229533.I1RGF6
25210	229533.I1RCL3	pp	229533.I1RQS8
25211	229533.I1RCL3	pp	229533.I1RNA0
25212	229533.I1RCL3	pp	229533.Q4IQK7
25213	229533.I1RCL3	pp	229533.I1RRT8
25214	229533.I1RCL3	pp	229533.I1RKF9
25215	229533.I1RCL3	pp	229533.I1RW61
25216	229533.I1RCL3	pp	229533.I1S7S9
25217	229533.I1RCL3	pp	229533.V6QYF0
25218	229533.I1RCL3	pp	229533.O74638
25219	229533.I1RCL3	pp	229533.I1RL23
25220	229533.I1RCL3	pp	229533.I1RG51
25221	229533.I1RCL3	pp	229533.I1RM67
25222	229533.I1RCL3	pp	229533.I1RJZ7
25223	229533.I1RCL3	pp	229533.I1S9Y1
25224	229533.I1RCL3	pp	229533.Q4IFI0
25225	229533.I1RCL3	pp	229533.I1RKJ6
25226	229533.I1RCL3	pp	229533.I1RRH5
25227	229533.I1RCL3	pp	229533.I1RPU0
25228	229533.I1RCL3	pp	229533.I1RDA0
25229	229533.I1RCL3	pp	229533.I1RS47
25230	229533.I1RCL3	pp	229533.I1RT33
25231	229533.I1RCL3	pp	229533.I1RZ99
25232	229533.I1RCL3	pp	229533.I1RQJ5
25233	229533.I1RCL3	pp	229533.I1S253
25234	229533.I1RCL3	pp	229533.Q4HZ42
25235	229533.I1RCL3	pp	229533.I1RRJ8
25236	229533.I1RCL3	pp	229533.Q4I830
25237	229533.I1RCL3	pp	229533.I1RMK3
25238	229533.I1RCL3	pp	229533.I1RRQ3
25239	229533.I1RCL3	pp	229533.I1RFG0
25240	229533.I1RCL3	pp	229533.I1RTF4
25241	229533.I1RCL3	pp	229533.Q4HVW2
25242	229533.I1RCL3	pp	229533.I1S6F0
25243	229533.I1RCL3	pp	229533.I1RSX1
25244	229533.I1RCL3	pp	229533.I1RW71
25245	229533.I1RCL3	pp	229533.I1RNH1
25246	229533.I1RCL3	pp	229533.I1RKL3
25247	229533.I1RCL3	pp	229533.Q4IJH1
25248	229533.I1RCL3	pp	229533.I1RWD4
25249	229533.I1RCL3	pp	229533.Q4HZ47
25250	229533.I1RCL3	pp	229533.I1RNV6
25251	229533.I1RCL3	pp	229533.I1RT80
25252	229533.I1RCL3	pp	229533.I1RFW5
25253	229533.I1RCL3	pp	229533.I1RR00
25254	229533.I1RCL3	pp	229533.I1RRP3
25255	229533.I1RCL3	pp	229533.I1S2M6
25256	229533.I1RCL3	pp	229533.I1RFH8
25257	229533.I1RCL3	pp	229533.I1S0F1
25258	229533.I1RCL3	pp	229533.I1S0R7

25259	229533.I1RCL3	pp	229533.Q4INZ9
25260	229533.I1RCL3	pp	229533.I1RQM6
25261	229533.I1RCL3	pp	229533.V6R283
25262	229533.I1RCL3	pp	229533.Q4IEV5
25263	229533.I1RCL3	pp	229533.I1RZJ6
25264	229533.I1RCL3	pp	229533.I1RJ74
25265	229533.I1RCL3	pp	229533.Q4IF76
25266	229533.I1RCL3	pp	229533.I1RQ65
25267	229533.I1RCL3	pp	229533.Q4IQW0
25268	229533.I1RCL3	pp	229533.I1RJL4
25269	229533.I1RCL3	pp	229533.I1RT05
25270	229533.I1RCL3	pp	229533.Q4IAS1
25271	229533.I1RCL3	pp	229533.Q4IPI1
25272	229533.I1RCL3	pp	229533.I1RRT2
25273	229533.I1RCL3	pp	229533.I1RFN1
25274	229533.I1RCL3	pp	229533.I1RE87
25275	229533.I1RCL3	pp	229533.I1RKQ5
25276	229533.I1RCL3	pp	229533.I1RQS5
25277	229533.I1RCL3	pp	229533.I1RLV9
25278	229533.I1RDP5	pp	229533.I1S5R5
25279	229533.I1RDP5	pp	229533.V6RPN0
25280	229533.I1RDP5	pp	229533.I1RQL4
25281	229533.I1RDP5	pp	229533.I1S0F0
25282	229533.I1S1C9	pp	229533.V6RIV9
25283	229533.I1RC27	pp	229533.I1S1X9
25284	229533.I1RC27	pp	229533.I1RSC6
25285	229533.I1RC27	pp	229533.I1RSS4
25286	229533.I1RC27	pp	229533.I1RY63
25287	229533.I1RC27	pp	229533.I1RSI0
25288	229533.I1RC27	pp	229533.I1RFJ9
25289	229533.I1RC27	pp	229533.I1S0M3
25290	229533.I1RC27	pp	229533.I1RK95
25291	229533.I1RC27	pp	229533.I1RF19
25292	229533.I1RC27	pp	229533.I1RQ28
25293	229533.I1RC27	pp	229533.I1RDG0
25294	229533.I1RC27	pp	229533.I1RN99
25295	229533.I1RC27	pp	229533.Q4HZK7
25296	229533.I1RC27	pp	229533.I1RZE5
25297	229533.I1RC27	pp	229533.I1RSD8
25298	229533.I1RC27	pp	229533.I1S2U2
25299	229533.I1RC27	pp	229533.I1S2E8
25300	229533.I1RC27	pp	229533.I1RPJ3
25301	229533.I1RC27	pp	229533.I1RTE9
25302	229533.I1RC27	pp	229533.I1RJZ6
25303	229533.I1RC27	pp	229533.I1RTD9
25304	229533.I1RC27	pp	229533.I1S2C0
25305	229533.I1RC27	pp	229533.I1RNH9
25306	229533.I1RC27	pp	229533.I1RZK6
25307	229533.I1RC27	pp	229533.I1RRI2
25308	229533.I1RC27	pp	229533.I1S296
25309	229533.I1RC27	pp	229533.I1RC72
25310	229533.I1RC27	pp	229533.Q4HZ68
25311	229533.I1RC27	pp	229533.I1RZF6
25312	229533.I1RC27	pp	229533.I1S0J1
25313	229533.I1RC27	pp	229533.I1S274
25314	229533.I1RC27	pp	229533.I1RF08
25315	229533.I1RC27	pp	229533.I1S1U7
25316	229533.I1RC27	pp	229533.I1RRR7
25317	229533.I1RC27	pp	229533.I1RM35
25318	229533.I1RC27	pp	229533.I1RKC6
25319	229533.I1RC27	pp	229533.I1RG56
25320	229533.I1RC27	pp	229533.V6R227
25321	229533.I1RC27	pp	229533.I1RNK2
25322	229533.I1RC27	pp	229533.I1RSR3
25323	229533.I1RC27	pp	229533.I1RVZ0
25324	229533.I1RC27	pp	229533.V6RGY3
25325	229533.I1RC27	pp	229533.I1RPY1
25326	229533.I1RC27	pp	229533.I1RE00
25327	229533.I1RC27	pp	229533.I1S558
25328	229533.I1RC27	pp	229533.I1S4T3
25329	229533.I1RC27	pp	229533.I1S4Q4
25330	229533.I1RC27	pp	229533.I1RM96
25331	229533.I1RC27	pp	229533.I1RQA2

25332 229533.I1RC27 pp 229533.Q4IEK8
25333 229533.I1RC27 pp 229533.I1RCN5
25334 229533.I1RC27 pp 229533.I1RC33
25335 229533.I1RC27 pp 229533.I1RSI6
25336 229533.I1RC27 pp 229533.I1RZS6
25337 229533.I1RC27 pp 229533.I1RGC6
25338 229533.I1RC27 pp 229533.I1RCL3
25339 229533.I1RC27 pp 229533.I1RC32
25340 229533.I1RC27 pp 229533.I1RM06
25341 229533.I1RC27 pp 229533.I1RNU6
25342 229533.I1RC27 pp 229533.I1RQ81
25343 229533.I1RC27 pp 229533.I1RW61
25344 229533.I1RC27 pp 229533.Q4IN63
25345 229533.I1RC27 pp 229533.I1RFS3
25346 229533.I1RC27 pp 229533.Q4IJ56
25347 229533.I1RC27 pp 229533.Q4I662
25348 229533.I1RC27 pp 229533.Q4IAA0
25349 229533.I1RC27 pp 229533.I1RMC0
25350 229533.I1RC27 pp 229533.I1RQ31
25351 229533.I1RC27 pp 229533.I1S021
25352 229533.I1RC27 pp 229533.I1RG51
25353 229533.I1RC27 pp 229533.I1S060
25354 229533.I1RC27 pp 229533.I1RNH6
25355 229533.I1RC27 pp 229533.I1RM67
25356 229533.I1RC27 pp 229533.I1RX14
25357 229533.I1RC27 pp 229533.I1RWJ4
25358 229533.I1RC27 pp 229533.I1RJZ7
25359 229533.I1RC27 pp 229533.Q4I327
25360 229533.I1RC27 pp 229533.I1RMN7
25361 229533.I1RC27 pp 229533.Q4IFI0
25362 229533.I1RC27 pp 229533.I1RRT8
25363 229533.I1RC27 pp 229533.I1RL23
25364 229533.I1RC27 pp 229533.Q4I830
25365 229533.I1RC27 pp 229533.V6RGN5
25366 229533.I1RC27 pp 229533.I1RKL3
25367 229533.I1RC27 pp 229533.I1RW71
25368 229533.I1RC27 pp 229533.I1RMN6
25369 229533.I1RC27 pp 229533.I1RRJ8
25370 229533.I1RC27 pp 229533.Q4IJH1
25371 229533.I1RC27 pp 229533.Q4IBS2
25372 229533.I1RC27 pp 229533.V6QUE6
25373 229533.I1RC27 pp 229533.I1RTF4
25374 229533.I1RC27 pp 229533.I1RT80
25375 229533.I1RC27 pp 229533.I1RQJ5
25376 229533.I1RC27 pp 229533.I1RZ99
25377 229533.I1RC27 pp 229533.I1RNV6
25378 229533.I1RC27 pp 229533.I1RNA0
25379 229533.I1RC27 pp 229533.I1RRQ6
25380 229533.I1RC27 pp 229533.I1RDA0
25381 229533.I1RC27 pp 229533.I1RC29
25382 229533.I1RC27 pp 229533.Q4HVW2
25383 229533.I1RC27 pp 229533.I1RN63
25384 229533.I1RC27 pp 229533.V6R283
25385 229533.I1RC27 pp 229533.I1RMK3
25386 229533.I1RC27 pp 229533.V6QYF0
25387 229533.I1RC27 pp 229533.Q4HZ42
25388 229533.I1RC27 pp 229533.I1RKF9
25389 229533.I1RC27 pp 229533.I1RFW5
25390 229533.I1RC27 pp 229533.I1RT33
25391 229533.I1RC27 pp 229533.I1RX24
25392 229533.I1RC27 pp 229533.I1RT05
25393 229533.I1RC27 pp 229533.I1RT98
25394 229533.I1RC27 pp 229533.Q4IF76
25395 229533.I1RC27 pp 229533.I1RS47
25396 229533.I1RC27 pp 229533.I1RRH5
25397 229533.I1RC27 pp 229533.I1RQM6
25398 229533.I1RC27 pp 229533.I1RD64
25399 229533.I1RC27 pp 229533.I1RGF6
25400 229533.I1RC27 pp 229533.I1RZJ6
25401 229533.I1RC27 pp 229533.I1RC82
25402 229533.I1RC27 pp 229533.I1RFN1
25403 229533.I1RC27 pp 229533.I1S9U2
25404 229533.I1RC27 pp 229533.Q4IQW0

25405	229533.I1RC27	pp	229533.Q4IEV5
25406	229533.I1RC27	pp	229533.I1RPW9
25407	229533.I1RC27	pp	229533.I1RRP3
25408	229533.I1RC27	pp	229533.I1RQ65
25409	229533.I1RC27	pp	229533.I1RKQ5
25410	229533.I1RC27	pp	229533.I1RS30
25411	229533.I1RC27	pp	229533.I1RKJ6
25412	229533.I1RC27	pp	229533.I1RPU0
25413	229533.I1RC27	pp	229533.I1RLV9
25414	229533.I1RC27	pp	229533.I1RJL4
25415	229533.I1RC27	pp	229533.I1RNH1
25416	229533.I1RC27	pp	229533.Q4IAS1
25417	229533.I1RC27	pp	229533.I1S9Y1
25418	229533.I1RC27	pp	229533.I1S6F0
25419	229533.I1RC27	pp	229533.I1RQS5
25420	229533.I1RC27	pp	229533.I1RRQ3
25421	229533.I1RC27	pp	229533.Q4IPI1
25422	229533.I1RC27	pp	229533.I1S0R7
25423	229533.I1RC27	pp	229533.I1RRT2
25424	229533.I1RC27	pp	229533.I1RFG0
25425	229533.V6RGN5	pp	229533.V6RXB3
25426	229533.V6RGN5	pp	229533.V6RGY3
25427	229533.V6RGN5	pp	229533.V6RIV9
25428	229533.I1S274	pp	229533.V6QWZ7
25429	229533.I1S274	pp	229533.Q4IEK8
25430	229533.I1S274	pp	229533.Q4IJH1
25431	229533.I1S274	pp	229533.Q4IQK7
25432	229533.I1S274	pp	229533.Q4IBS2
25433	229533.I1S274	pp	229533.Q4IJ56
25434	229533.I1S274	pp	229533.V6R227
25435	229533.I1S274	pp	229533.Q4IPI1
25436	229533.I1S274	pp	229533.V6QYF0
25437	229533.I1S274	pp	229533.Q4HZ68
25438	229533.I1S274	pp	229533.I1S9U1
25439	229533.I1S274	pp	229533.I1S558
25440	229533.I1S274	pp	229533.Q4IAS1
25441	229533.I1S274	pp	229533.O74638
25442	229533.I1S274	pp	229533.Q4IFI0
25443	229533.I1S274	pp	229533.I1S2L2
25444	229533.I1S274	pp	229533.V6RRH2
25445	229533.I1S274	pp	229533.V6R283
25446	229533.I1S274	pp	229533.V6RGN5
25447	229533.I1S274	pp	229533.Q4HZ42
25448	229533.I1S274	pp	229533.Q4I662
25449	229533.I1S274	pp	229533.Q4I327
25450	229533.I1S274	pp	229533.Q4HXF0
25451	229533.I1RD38	pp	229533.Q4HYQ4
25452	229533.I1RD38	pp	229533.I1RUG0
25453	229533.I1RD38	pp	229533.I1RRG3
25454	229533.I1RD38	pp	229533.I1RKB1
25455	229533.I1RD38	pp	229533.I1RRT0
25456	229533.I1RD38	pp	229533.I1RQR6
25457	229533.I1RD38	pp	229533.I1RT35
25458	229533.I1RD38	pp	229533.V6QUE6
25459	229533.I1RD38	pp	229533.Q4I5W5
25460	229533.I1RD38	pp	229533.I1RWF5
25461	229533.I1RD38	pp	229533.Q4IJ11
25462	229533.I1RD38	pp	229533.Q4I5I4
25463	229533.I1RD38	pp	229533.I1S9K9
25464	229533.I1RD38	pp	229533.I1RTC2
25465	229533.I1RD38	pp	229533.I1RVL8
25466	229533.I1RD38	pp	229533.I1REN4
25467	229533.I1RD38	pp	229533.I1REN3
25468	229533.I1RD38	pp	229533.I1RZB5
25469	229533.I1RD38	pp	229533.I1RT38
25470	229533.I1RD38	pp	229533.I1RD39
25471	229533.I1RD38	pp	229533.I1RK79
25472	229533.I1RD38	pp	229533.Q4I5U9
25473	229533.I1RD38	pp	229533.I1RK71
25474	229533.I1RD38	pp	229533.I1RNB8
25475	229533.I1RD38	pp	229533.I1RJL2
25476	229533.I1RD38	pp	229533.I1RG11
25477	229533.I1RD38	pp	229533.Q4HYB8

25478	229533.I1RD38	pp	229533.Q4I375
25479	229533.I1RD38	pp	229533.I1RNJ5
25480	229533.I1RD38	pp	229533.Q4HZ95
25481	229533.I1RD38	pp	229533.I1RXU0
25482	229533.I1RDV8	pp	229533.I1RK55
25483	229533.I1RDV8	pp	229533.I1RYP5
25484	229533.I1RDV8	pp	229533.I1SAI4
25485	229533.I1RDV8	pp	229533.V6RAM6
25486	229533.I1RDV8	pp	229533.V6R5D2
25487	229533.I1RDV8	pp	229533.I1S968
25488	229533.I1RDV8	pp	229533.I1RR68
25489	229533.I1RDV8	pp	229533.I1RMT6
25490	229533.I1RDV8	pp	229533.V6QU99
25491	229533.I1RDV8	pp	229533.V6R756
25492	229533.I1RDV8	pp	229533.I1RF16
25493	229533.I1RDV8	pp	229533.I1S9T6
25494	229533.I1RDV8	pp	229533.V6R0W1
25495	229533.I1RDV8	pp	229533.I1RZM4
25496	229533.I1RDV8	pp	229533.I1RQE8
25497	229533.I1RDV8	pp	229533.I1RRM1
25498	229533.I1RDV8	pp	229533.V6RE46
25499	229533.I1RDV8	pp	229533.I1RVI8
25500	229533.I1RDV8	pp	229533.I1RNH4
25501	229533.I1RDV8	pp	229533.V6R2Z2
25502	229533.I1RDV8	pp	229533.I1RT05
25503	229533.I1RDV8	pp	229533.I1RSA5
25504	229533.I1RDV8	pp	229533.I1S2Q4
25505	229533.I1RDV8	pp	229533.I1RY81
25506	229533.I1RDV8	pp	229533.I1RHL9
25507	229533.I1RDV8	pp	229533.I1S0A5
25508	229533.I1RDV8	pp	229533.I1RJY7
25509	229533.I1RDV8	pp	229533.I1RUY5
25510	229533.I1RDV8	pp	229533.I1RMA2
25511	229533.I1RDV8	pp	229533.V6RK96
25512	229533.I1RDV8	pp	229533.I1RWM4
25513	229533.I1RDV8	pp	229533.I1S8C1
25514	229533.I1RDV8	pp	229533.I1RF24
25515	229533.I1RUQ9	pp	229533.Q4IAS1
25516	229533.I1RUQ9	pp	229533.Q4IPI1
25517	229533.I1RUQ9	pp	229533.Q4IJ56
25518	229533.I1RUQ9	pp	229533.Q4IQW0
25519	229533.I1RUQ9	pp	229533.I1S558
25520	229533.I1RUQ9	pp	229533.Q4IAA0
25521	229533.I1RUQ9	pp	229533.Q4I5Z5
25522	229533.I1RUQ9	pp	229533.Q4IN63
25523	229533.I1RUQ9	pp	229533.Q4IJH1
25524	229533.I1RUQ9	pp	229533.Q4IFI0
25525	229533.I1RUQ9	pp	229533.I1RWD4
25526	229533.I1RUQ9	pp	229533.Q4IQ72
25527	229533.I1RUQ9	pp	229533.I1S0M3
25528	229533.I1RUQ9	pp	229533.Q4IEK8
25529	229533.I1RUQ9	pp	229533.Q4IEV5
25530	229533.I1RUQ9	pp	229533.Q4HZ68
25531	229533.I1RAV6	pp	229533.Q4IFI0
25532	229533.I1RAV6	pp	229533.I1RVY0
25533	229533.I1RAV6	pp	229533.I1RJZ7
25534	229533.I1RAV6	pp	229533.I1RB71
25535	229533.I1RAV6	pp	229533.I1RBG9
25536	229533.I1RAV6	pp	229533.Q4HVW2
25537	229533.I1RAV6	pp	229533.I1RBN1
25538	229533.I1RAV6	pp	229533.I1RC29
25539	229533.I1RAV6	pp	229533.I1RM67
25540	229533.I1RAV6	pp	229533.I1RW90
25541	229533.I1RAV6	pp	229533.V6R283
25542	229533.I1RAV6	pp	229533.I1RT80
25543	229533.I1RAV6	pp	229533.I1S9Y1
25544	229533.I1RAV6	pp	229533.Q4IEV5
25545	229533.I1RAV6	pp	229533.Q4IQ72
25546	229533.I1RAV6	pp	229533.I1RC24
25547	229533.I1RAV6	pp	229533.I1RBC7
25548	229533.I1RAV6	pp	229533.V6QWX8
25549	229533.I1RAV6	pp	229533.I1RW71
25550	229533.I1RAV6	pp	229533.Q4I830

25551	229533.I1RAV6	pp	229533.I1RNH1
25552	229533.I1RAV6	pp	229533.I1RE30
25553	229533.I1RAV6	pp	229533.I1RKQ5
25554	229533.I1RAV6	pp	229533.I1RS30
25555	229533.I1RAV6	pp	229533.I1RN66
25556	229533.I1RAV6	pp	229533.I1RRG3
25557	229533.I1RAV6	pp	229533.I1RK95
25558	229533.I1RAV6	pp	229533.I1RN17
25559	229533.I1RAV6	pp	229533.I1RTE9
25560	229533.I1RAV6	pp	229533.I1RR25
25561	229533.I1RAV6	pp	229533.I1RZB5
25562	229533.I1RAV6	pp	229533.Q4I8X0
25563	229533.I1RAV6	pp	229533.I1RSL8
25564	229533.I1RAV6	pp	229533.I1RF08
25565	229533.I1RAV6	pp	229533.I1RTG5
25566	229533.I1RAV6	pp	229533.I1RK79
25567	229533.I1RAV6	pp	229533.V6RGY3
25568	229533.I1RAV6	pp	229533.I1RJY3
25569	229533.I1RAV6	pp	229533.I1RJY2
25570	229533.I1RAV6	pp	229533.I1RSD1
25571	229533.I1RAV6	pp	229533.I1RCL3
25572	229533.I1RAV6	pp	229533.I1RJL2
25573	229533.I1RAV6	pp	229533.I1S255
25574	229533.I1RAV6	pp	229533.I1S9U1
25575	229533.I1RAV6	pp	229533.I1RRN6
25576	229533.I1RAV6	pp	229533.V6R227
25577	229533.I1RAV6	pp	229533.I1RG11
25578	229533.I1RAV6	pp	229533.I1RJI3
25579	229533.I1RAV6	pp	229533.V6QUE6
25580	229533.I1RAG3	pp	229533.I1RHL9
25581	229533.I1RAG3	pp	229533.I1RMA2
25582	229533.I1RAG3	pp	229533.I1SAI4
25583	229533.I1RAG3	pp	229533.I1RK55
25584	229533.I1RAG3	pp	229533.I1RYP5
25585	229533.I1RAG3	pp	229533.I1RDN3
25586	229533.I1RAG3	pp	229533.I1RSA5
25587	229533.I1RAG3	pp	229533.I1S0A5
25588	229533.I1RAG3	pp	229533.I1RF24
25589	229533.I1RAG3	pp	229533.V6R5D2
25590	229533.I1RAG3	pp	229533.I1RQE8
25591	229533.I1RAG3	pp	229533.V6R0W1
25592	229533.I1RAG3	pp	229533.V6RK96
25593	229533.I1RAG3	pp	229533.I1S8C1
25594	229533.I1RAG3	pp	229533.I1RDV8
25595	229533.I1RAG3	pp	229533.V6RAM6
25596	229533.I1RAG3	pp	229533.I1RR68
25597	229533.I1RAG3	pp	229533.I1S968
25598	229533.I1RAG3	pp	229533.I1RMT6
25599	229533.I1RAG3	pp	229533.I1RF16
25600	229533.I1RAG3	pp	229533.V6QU99
25601	229533.I1RAG3	pp	229533.I1S9T6
25602	229533.I1RAG3	pp	229533.I1RDU0
25603	229533.I1RAG3	pp	229533.I1RNH4
25604	229533.I1RAG3	pp	229533.V6RE46
25605	229533.I1RAG3	pp	229533.I1RJY7
25606	229533.I1RAG3	pp	229533.I1RY81
25607	229533.I1RAG3	pp	229533.V6R756
25608	229533.I1RAG3	pp	229533.I1RRM1
25609	229533.I1RAG3	pp	229533.I1RT05
25610	229533.I1RAG3	pp	229533.I1RVI8
25611	229533.I1RAG3	pp	229533.V6R2Z2
25612	229533.I1RAG3	pp	229533.I1S2Q4
25613	229533.I1RAG3	pp	229533.I1RZM4
25614	229533.I1RAG3	pp	229533.I1RWM4
25615	229533.I1RAG3	pp	229533.I1RUY5
25616	229533.I1RC72	pp	229533.I1RDA0
25617	229533.I1RC72	pp	229533.I1RPU0
25618	229533.I1RC72	pp	229533.Q4HVW2
25619	229533.I1RC72	pp	229533.I1S6U8
25620	229533.I1RC72	pp	229533.I1RMK3
25621	229533.I1RC72	pp	229533.I1S0L3
25622	229533.I1RC72	pp	229533.I1S0D4
25623	229533.I1RC72	pp	229533.V6R1K4

25624	229533.I1RC72	pp	229533.I1RD52
25625	229533.I1RC72	pp	229533.Q4IPI1
25626	229533.I1RC72	pp	229533.Q4IJH1
25627	229533.I1RC72	pp	229533.I1RTF4
25628	229533.I1RC72	pp	229533.I1RKQ5
25629	229533.I1RC72	pp	229533.I1RT33
25630	229533.I1RC72	pp	229533.I1RUQ2
25631	229533.I1RC72	pp	229533.I1RRH5
25632	229533.I1RC72	pp	229533.I1RNV6
25633	229533.I1RC72	pp	229533.I1S6F0
25634	229533.I1RC72	pp	229533.I1RJU3
25635	229533.I1RC72	pp	229533.I1S0R7
25636	229533.I1RC72	pp	229533.I1RT80
25637	229533.I1RC72	pp	229533.I1RTI6
25638	229533.I1RC72	pp	229533.I1RVQ0
25639	229533.I1RC72	pp	229533.I1RNH1
25640	229533.I1RC72	pp	229533.Q4HZ47
25641	229533.I1RC72	pp	229533.I1REF2
25642	229533.I1RC72	pp	229533.Q4INZ9
25643	229533.I1RC72	pp	229533.I1S0F1
25644	229533.I1RC72	pp	229533.I1RQM6
25645	229533.I1RC72	pp	229533.I1RWD4
25646	229533.I1RC72	pp	229533.I1RZJ6
25647	229533.I1RC72	pp	229533.I1RT05
25648	229533.I1RC72	pp	229533.V6R227
25649	229533.I1RC72	pp	229533.I1RQJ5
25650	229533.I1RC72	pp	229533.I1RZ99
25651	229533.I1RC72	pp	229533.I1RT98
25652	229533.I1RC72	pp	229533.I1S255
25653	229533.I1RC72	pp	229533.V6QW22
25654	229533.I1RC72	pp	229533.I1RL23
25655	229533.I1RC72	pp	229533.I1RW25
25656	229533.I1RC72	pp	229533.Q4IEK8
25657	229533.I1RC72	pp	229533.I1RZF6
25658	229533.I1RC72	pp	229533.I1RW71
25659	229533.I1RC72	pp	229533.I1RSQ7
25660	229533.I1RC72	pp	229533.Q4IFI0
25661	229533.I1RC72	pp	229533.I1RR00
25662	229533.I1RC72	pp	229533.Q4ICA8
25663	229533.I1RC72	pp	229533.I1RM06
25664	229533.I1RC72	pp	229533.I1S419
25665	229533.I1RC72	pp	229533.Q4IQK7
25666	229533.I1RC72	pp	229533.I1RFW5
25667	229533.I1RC72	pp	229533.I1RRG3
25668	229533.I1RC72	pp	229533.I1RNA0
25669	229533.I1RC72	pp	229533.I1S0T2
25670	229533.I1RC72	pp	229533.I1RG56
25671	229533.I1RC72	pp	229533.I1RCD1
25672	229533.I1RC72	pp	229533.V6QYF0
25673	229533.I1RC72	pp	229533.Q4IAA0
25674	229533.I1RC72	pp	229533.I1RX46
25675	229533.I1RC72	pp	229533.I1RG51
25676	229533.I1RC72	pp	229533.Q4IJ56
25677	229533.I1RC72	pp	229533.I1RPW9
25678	229533.I1RC72	pp	229533.I1RR51
25679	229533.I1RC72	pp	229533.I1RCS1
25680	229533.I1RC72	pp	229533.I1S0M9
25681	229533.I1RC72	pp	229533.V6RCG5
25682	229533.I1RC72	pp	229533.I1RM67
25683	229533.I1RC72	pp	229533.I1RQ81
25684	229533.I1RC72	pp	229533.I1S9U2
25685	229533.I1RC72	pp	229533.I1S5B0
25686	229533.I1RC72	pp	229533.V6RCZ8
25687	229533.I1RC72	pp	229533.I1RX14
25688	229533.I1RC72	pp	229533.I1RX08
25689	229533.I1RC72	pp	229533.I1RWJ8
25690	229533.I1RC72	pp	229533.I1RSH5
25691	229533.I1RC72	pp	229533.I1RD03
25692	229533.I1RC72	pp	229533.I1S044
25693	229533.I1RC72	pp	229533.I1RQA2
25694	229533.I1RC72	pp	229533.I1RI82
25695	229533.I1RC72	pp	229533.I1RV64
25696	229533.I1RC72	pp	229533.I1RW99

25697 229533.I1RC72 pp 229533.I1RJL2
25698 229533.I1RC72 pp 229533.Q4IBS2
25699 229533.I1RC72 pp 229533.I1RMN7
25700 229533.I1RC72 pp 229533.I1RK83
25701 229533.I1RC72 pp 229533.V6QUE6
25702 229533.I1RC72 pp 229533.I1RNH6
25703 229533.I1RC72 pp 229533.I1RZB5
25704 229533.I1RC72 pp 229533.I1S021
25705 229533.I1RC72 pp 229533.I1RGC2
25706 229533.I1RC72 pp 229533.I1RYB9
25707 229533.I1RC72 pp 229533.I1RCG4
25708 229533.I1RC72 pp 229533.I1RNH9
25709 229533.I1RC72 pp 229533.I1RJV4
25710 229533.I1RC72 pp 229533.I1RMC0
25711 229533.I1RC72 pp 229533.I1RQ31
25712 229533.I1RC72 pp 229533.I1S1B6
25713 229533.I1RC72 pp 229533.I1RSR8
25714 229533.I1RC72 pp 229533.I1RMD5
25715 229533.I1RC72 pp 229533.I1RZM8
25716 229533.I1RC72 pp 229533.I1RKQ9
25717 229533.I1RC72 pp 229533.I1RC82
25718 229533.I1RC72 pp 229533.I1RFN1
25719 229533.I1RC72 pp 229533.I1RE87
25720 229533.I1RC72 pp 229533.I1RTD9
25721 229533.I1RC72 pp 229533.I1REK8
25722 229533.I1RC72 pp 229533.I1RCY5
25723 229533.I1RC72 pp 229533.I1RF08
25724 229533.I1RC72 pp 229533.I1RQS5
25725 229533.I1RC72 pp 229533.Q4IEV5
25726 229533.I1RC72 pp 229533.I1RRT2
25727 229533.I1RC72 pp 229533.I1RZE5
25728 229533.I1RC72 pp 229533.I1RJL4
25729 229533.I1RC72 pp 229533.Q4IAS1
25730 229533.I1RC72 pp 229533.I1RQ65
25731 229533.I1RC72 pp 229533.I1RFJ9
25732 229533.I1RC72 pp 229533.I1RLV9
25733 229533.I1RC72 pp 229533.I1RSI0
25734 229533.I1RC72 pp 229533.I1RS47
25735 229533.I1RC72 pp 229533.Q4HZ42
25736 229533.I1RC72 pp 229533.I1RE00
25737 229533.I1RC72 pp 229533.I1RRZ0
25738 229533.I1RC72 pp 229533.I1RF19
25739 229533.I1RC72 pp 229533.I1RNK2
25740 229533.I1RC72 pp 229533.I1RJW6
25741 229533.I1RC72 pp 229533.I1RVZ0
25742 229533.I1RC72 pp 229533.I1RFG0
25743 229533.I1RC72 pp 229533.Q4I5Z5
25744 229533.I1RC72 pp 229533.I1RRQ3
25745 229533.I1RC72 pp 229533.I1RT04
25746 229533.I1RC72 pp 229533.Q4HXF0
25747 229533.I1RC72 pp 229533.I1RS30
25748 229533.I1RC72 pp 229533.I1RT40
25749 229533.I1RC72 pp 229533.I1RN99
25750 229533.I1RC72 pp 229533.I1RRP3
25751 229533.I1RC72 pp 229533.I1RWF7
25752 229533.I1RC72 pp 229533.I1RQ28
25753 229533.I1RC72 pp 229533.I1RJZ7
25754 229533.I1RC72 pp 229533.I1RK95
25755 229533.I1RC72 pp 229533.I1RRJ8
25756 229533.I1RC72 pp 229533.I1RDG0
25757 229533.I1RC72 pp 229533.I1RY63
25758 229533.I1RC72 pp 229533.I1RRM5
25759 229533.I1RC72 pp 229533.I1RSC6
25760 229533.I1RC72 pp 229533.Q4I830
25761 229533.I1RC72 pp 229533.I1RSX1
25762 229533.I1RC72 pp 229533.I1RZK6
25763 229533.I1RC72 pp 229533.I1RR72
25764 229533.I1RC72 pp 229533.I1RKL3
25765 229533.I1RC72 pp 229533.I1RX24
25766 229533.I1RC72 pp 229533.I1RZS6
25767 229533.I1RC72 pp 229533.I1S2M6
25768 229533.I1RC72 pp 229533.I1RZZ1
25769 229533.I1RC72 pp 229533.I1RKF9

25770	229533.I1RC72	pp	229533.I1RJZ6
25771	229533.I1RC72	pp	229533.I1S2U2
25772	229533.I1RC72	pp	229533.I1RWJ4
25773	229533.I1RC72	pp	229533.I1S6Y8
25774	229533.I1RC72	pp	229533.I1RN63
25775	229533.I1RC72	pp	229533.V6R283
25776	229533.I1RC72	pp	229533.I1RJY2
25777	229533.I1RC72	pp	229533.I1RPM4
25778	229533.I1RC72	pp	229533.I1RSR3
25779	229533.I1RC72	pp	229533.I1RJ74
25780	229533.I1RC72	pp	229533.I1RYT5
25781	229533.I1RC72	pp	229533.Q4IQW0
25782	229533.I1RC72	pp	229533.Q4I662
25783	229533.I1RC72	pp	229533.I1RD64
25784	229533.I1RC72	pp	229533.I1RGF6
25785	229533.I1RC72	pp	229533.I1RW61
25786	229533.I1RC72	pp	229533.I1RFS3
25787	229533.I1RC72	pp	229533.I1RS17
25788	229533.I1RC72	pp	229533.I1RWB5
25789	229533.I1RC72	pp	229533.I1RS20
25790	229533.I1RC72	pp	229533.I1RQS8
25791	229533.I1RC72	pp	229533.I1RQE7
25792	229533.I1RC72	pp	229533.I1RCH5
25793	229533.I1RC72	pp	229533.I1S2E8
25794	229533.I1RC72	pp	229533.I1RNU6
25795	229533.I1RC72	pp	229533.I1RRT8
25796	229533.I1RC72	pp	229533.I1S0J1
25797	229533.I1RC72	pp	229533.O74638
25798	229533.I1RC72	pp	229533.I1S5R1
25799	229533.I1RC72	pp	229533.I1S060
25800	229533.I1RC72	pp	229533.I1RSD8
25801	229533.I1RC72	pp	229533.I1RW46
25802	229533.I1RC72	pp	229533.I1RFL4
25803	229533.I1RC72	pp	229533.V6RGY3
25804	229533.I1RC72	pp	229533.I1RE01
25805	229533.I1RC72	pp	229533.I1RKJ6
25806	229533.I1RC72	pp	229533.I1S9Y1
25807	229533.I1RC72	pp	229533.V6RGN5
25808	229533.I1RC72	pp	229533.I1S1U7
25809	229533.I1RC72	pp	229533.I1RMN6
25810	229533.I1RC72	pp	229533.I1S274
25811	229533.I1RC72	pp	229533.I1S253
25812	229533.I1RC72	pp	229533.I1RGC6
25813	229533.I1RC72	pp	229533.I1RSI6
25814	229533.I1RC72	pp	229533.I1S8E2
25815	229533.I1RC72	pp	229533.I1RJV3
25816	229533.I1RC72	pp	229533.I1RCL3
25817	229533.I1RC72	pp	229533.I1RNN1
25818	229533.I1RC72	pp	229533.I1RCN5
25819	229533.I1RC72	pp	229533.I1RFH8
25820	229533.I1RC72	pp	229533.I1RFT6
25821	229533.I1RC72	pp	229533.I1S558
25822	229533.I1RC72	pp	229533.V6RXB3
25823	229533.I1RC72	pp	229533.I1RJY3
25824	229533.I1RC72	pp	229533.I1RNY2
25825	229533.I1RC72	pp	229533.Q4HZI0
25826	229533.I1RC72	pp	229533.I1RTE9
25827	229533.I1RC72	pp	229533.I1RPJ3
25828	229533.I1RC72	pp	229533.I1RZZ9
25829	229533.I1RC72	pp	229533.Q4I327
25830	229533.I1RC72	pp	229533.I1RWI5
25831	229533.I1RC72	pp	229533.I1S2C0
25832	229533.I1RC72	pp	229533.Q4IF76
25833	229533.I1RC72	pp	229533.I1RRR7
25834	229533.I1RC72	pp	229533.I1RUG3
25835	229533.I1RC72	pp	229533.I1S8R3
25836	229533.I1RC72	pp	229533.I1RM96
25837	229533.I1RC72	pp	229533.Q4HZK7
25838	229533.I1RC72	pp	229533.Q4IN63
25839	229533.I1RC72	pp	229533.I1S296
25840	229533.I1RC72	pp	229533.Q4HZ68
25841	229533.I1RC72	pp	229533.V6RIV9
25842	229533.I1RC72	pp	229533.I1RRI2

25843	229533.I1RC72	pp	229533.I1RZW8
25844	229533.I1RC72	pp	229533.I1S4T3
25845	229533.I1RC72	pp	229533.I1RD17
25846	229533.I1RC72	pp	229533.V6R057
25847	229533.I1RC72	pp	229533.Q4IN00
25848	229533.I1RC72	pp	229533.Q4HZB8
25849	229533.I1RC72	pp	229533.I1S0M3
25850	229533.I1RC72	pp	229533.I1RML0
25851	229533.I1RC72	pp	229533.I1RK79
25852	229533.I1RC72	pp	229533.Q4I8X0
25853	229533.I1RC72	pp	229533.I1RTD7
25854	229533.I1RC72	pp	229533.I1RPY1
25855	229533.I1RC72	pp	229533.I1RKC6
25856	229533.I1RC72	pp	229533.I1S1J6
25857	229533.I1RC72	pp	229533.I1S1X9
25858	229533.I1RC72	pp	229533.I1RSS4
25859	229533.I1RXI8	pp	229533.I1S0F0
25860	229533.I1RXI8	pp	229533.I1S5R5
25861	229533.I1RXI8	pp	229533.V6RPN0
25862	229533.I1RIW5	pp	229533.I1S4F4
25863	229533.I1RIW5	pp	229533.V6R756
25864	229533.I1RIW5	pp	229533.I1S2H3
25865	229533.I1RIW5	pp	229533.V6R2Z2
25866	229533.I1RIW5	pp	229533.I1S0F0
25867	229533.I1RIW5	pp	229533.I1SAI4
25868	229533.I1RIW5	pp	229533.I1RPD6
25869	229533.I1RIW5	pp	229533.V6R5D2
25870	229533.I1RIW5	pp	229533.V6RPN0
25871	229533.I1RIW5	pp	229533.V6RAM6
25872	229533.I1RIW5	pp	229533.V6QU99
25873	229533.I1RIW5	pp	229533.V6R0W1
25874	229533.I1RIW5	pp	229533.I1S5R5
25875	229533.I1RIW5	pp	229533.I1RQL4
25876	229533.Q4IJ56	pp	229533.V6R283
25877	229533.Q4IJ56	pp	229533.V6RGY3
25878	229533.Q4IJ56	pp	229533.V6RIS2
25879	229533.Q4IJ56	pp	229533.V6RGN5
25880	229533.Q4IJ56	pp	229533.Q4IQW0
25881	229533.Q4IJ56	pp	229533.Q4IN63
25882	229533.Q4IJ56	pp	229533.V6QYF0
25883	229533.Q4IJ56	pp	229533.Q4IJH1
25884	229533.Q4IJ56	pp	229533.Q4IQ72
25885	229533.Q4IJ56	pp	229533.Q4IQT8
25886	229533.Q4IJ56	pp	229533.V6QUE6
25887	229533.Q4IJ56	pp	229533.V6R227
25888	229533.Q4IJ56	pp	229533.V6RY33
25889	229533.Q4IJ56	pp	229533.Q4IQK7
25890	229533.Q4IJ56	pp	229533.Q4IPI1
25891	229533.Q4IJ56	pp	229533.Q4INZ9
25892	229533.I1RCT3	pp	229533.I1RJQ5
25893	229533.A0A1C3YHF4	pp	229533.I1RKJ7
25894	229533.A0A1C3YHF4	pp	229533.I1RV06
25895	229533.A0A1C3YHF4	pp	229533.I1RJX2
25896	229533.A0A1C3YHF4	pp	229533.A0A1C3YLD3
25897	229533.A0A1C3YHF4	pp	229533.I1RC11
25898	229533.A0A1C3YHF4	pp	229533.I1RMU5
25899	229533.A0A1C3YHF4	pp	229533.V6R0H4
25900	229533.A0A1C3YHF4	pp	229533.I1RHL9
25901	229533.A0A1C3YHF4	pp	229533.I1RCG5
25902	229533.A0A1C3YHF4	pp	229533.A0A1C3YJM7
25903	229533.A0A1C3YHF4	pp	229533.I1RCU8
25904	229533.A0A1C3YHF4	pp	229533.A0A1C3YJL9
25905	229533.A0A1C3YHF4	pp	229533.I1RA94
25906	229533.A0A1C3YHF4	pp	229533.I1RF16
25907	229533.A0A1C3YHF4	pp	229533.I1S405
25908	229533.A0A1C3YHF4	pp	229533.I1RMS5
25909	229533.A0A1C3YHF4	pp	229533.I1S0H2
25910	229533.A0A1C3YHF4	pp	229533.I1REE6
25911	229533.A0A1C3YHF4	pp	229533.A0A1C3YKH5
25912	229533.A0A1C3YHF4	pp	229533.A0A1C3YJ26
25913	229533.A0A1C3YHF4	pp	229533.I1S269
25914	229533.I1RGR2	pp	229533.I1S0F0
25915	229533.I1RGR2	pp	229533.I1RQL4

25916	229533.I1RGR2	pp	229533.V6RPN0
25917	229533.I1RGR2	pp	229533.I1S5R5
25918	229533.I1RMC0	pp	229533.I1RWJ4
25919	229533.I1RMC0	pp	229533.Q4I662
25920	229533.I1RMC0	pp	229533.I1RPM4
25921	229533.I1RMC0	pp	229533.I1RZS6
25922	229533.I1RMC0	pp	229533.Q4I830
25923	229533.I1RMC0	pp	229533.I1RX24
25924	229533.I1RMC0	pp	229533.I1RRJ8
25925	229533.I1RMC0	pp	229533.I1S0X5
25926	229533.I1RMC0	pp	229533.I1RRQ3
25927	229533.I1RMC0	pp	229533.I1RR72
25928	229533.I1RMC0	pp	229533.Q4IF76
25929	229533.I1RMC0	pp	229533.Q4HZ42
25930	229533.I1RMC0	pp	229533.I1S8E2
25931	229533.I1RMC0	pp	229533.I1RZW8
25932	229533.I1RMC0	pp	229533.V6R283
25933	229533.I1RMC0	pp	229533.I1RS47
25934	229533.I1RMC0	pp	229533.I1RRI2
25935	229533.I1RMC0	pp	229533.I1RN63
25936	229533.I1RMC0	pp	229533.I1RQ65
25937	229533.I1RMC0	pp	229533.I1S2C0
25938	229533.I1RMC0	pp	229533.I1RZ40
25939	229533.I1RMC0	pp	229533.I1RTE9
25940	229533.I1RMC0	pp	229533.V6RAC9
25941	229533.I1RMC0	pp	229533.Q4IAS1
25942	229533.I1RMC0	pp	229533.I1S558
25943	229533.I1RMC0	pp	229533.I1RRT2
25944	229533.I1RMC0	pp	229533.I1RQS5
25945	229533.I1RMC0	pp	229533.Q4IQW0
25946	229533.I1RMC0	pp	229533.I1S855
25947	229533.I1RMC0	pp	229533.Q4HXF0
25948	229533.I1RMC0	pp	229533.I1RXF6
25949	229533.I1RMC0	pp	229533.I1RPY1
25950	229533.I1RMC0	pp	229533.V6RIV9
25951	229533.I1RMC0	pp	229533.I1RSD8
25952	229533.I1RMC0	pp	229533.V6RGY3
25953	229533.I1RMC0	pp	229533.I1S4T3
25954	229533.I1RMC0	pp	229533.I1S2E8
25955	229533.I1RMC0	pp	229533.I1RQE7
25956	229533.I1RMC0	pp	229533.I1RW46
25957	229533.I1RMC0	pp	229533.I1S060
25958	229533.I1RMC0	pp	229533.I1S1X9
25959	229533.I1RMC0	pp	229533.I1RSS4
25960	229533.I1RMC0	pp	229533.I1RSD4
25961	229533.I1RMC0	pp	229533.I1S0J1
25962	229533.I1RMC0	pp	229533.Q4HZ68
25963	229533.I1RMC0	pp	229533.V6RY33
25964	229533.I1RMC0	pp	229533.I1RNU6
25965	229533.I1RMC0	pp	229533.I1RUG3
25966	229533.I1RMC0	pp	229533.Q4I327
25967	229533.I1RMC0	pp	229533.I1RR24
25968	229533.I1RMC0	pp	229533.I1RNA0
25969	229533.I1RMC0	pp	229533.I1RSI6
25970	229533.I1RMC0	pp	229533.I1RRQ6
25971	229533.I1RMC0	pp	229533.I1S9U2
25972	229533.I1RMC0	pp	229533.I1RPW9
25973	229533.I1RMC0	pp	229533.V6QYF0
25974	229533.I1RMC0	pp	229533.Q4IFI0
25975	229533.I1RMC0	pp	229533.I1RW71
25976	229533.I1RMC0	pp	229533.I1RMN6
25977	229533.I1RMC0	pp	229533.V6QW22
25978	229533.I1RMC0	pp	229533.I1RW25
25979	229533.I1RMC0	pp	229533.I1S471
25980	229533.I1RMC0	pp	229533.I1RSQ7
25981	229533.I1RMC0	pp	229533.V6RIS2
25982	229533.I1RMC0	pp	229533.V6RGN5
25983	229533.I1RMC0	pp	229533.Q4IJH1
25984	229533.I1RMC0	pp	229533.I1RQJ5
25985	229533.I1RMC0	pp	229533.Q4HZ47
25986	229533.I1RMC0	pp	229533.I1RT80
25987	229533.I1RMC0	pp	229533.I1RTF4
25988	229533.I1RMC0	pp	229533.I1RNH1

229533.I1RMC0	pp	229533.I1S0R7
229533.I1RMC0	pp	229533.Q4IPI1
229533.I1RMC0	pp	229533.I1RMK3
229533.I1RMC0	pp	229533.I1RVQ0
229533.I1RMC0	pp	229533.I1RZK6
229533.I1RMC0	pp	229533.I1RT33
229533.I1RMC0	pp	229533.Q4HVW2
229533.I1RMC0	pp	229533.I1RWJ8
229533.I1RMC0	pp	229533.I1RPU0
229533.I1RMC0	pp	229533.Q4INZ9
229533.I1RMC0	pp	229533.I1RZZ1
229533.I1RMC0	pp	229533.I1S0L3
229533.I1RMC0	pp	229533.I1RT98
229533.I1RMC0	pp	229533.Q4IAA0
229533.I1RMC0	pp	229533.I1RN99
229533.I1RMC0	pp	229533.I1RSM2
229533.I1RMC0	pp	229533.I1RNK2
229533.I1RMC0	pp	229533.V6R227
229533.I1RMC0	pp	229533.I1RVZ0
229533.I1RMC0	pp	229533.I1RZF6
229533.I1RMC0	pp	229533.I1RTD9
229533.I1RMC0	pp	229533.I1RSI0
229533.I1RMC0	pp	229533.I1RSR8
229533.I1RMC0	pp	229533.I1RZB5
229533.I1RMC0	pp	229533.I1RMD5
229533.I1RMC0	pp	229533.I1RNH6
229533.I1RMC0	pp	229533.I1S021
229533.I1RMC0	pp	229533.I1RQ31
229533.I1RMC0	pp	229533.V6RCG5
229533.I1RMC0	pp	229533.I1RSR3
229533.I1RMC0	pp	229533.I1RQA2
229533.I1RMC0	pp	229533.Q4IJ56
229533.I1RMC0	pp	229533.V6QUE6
229533.I1RMC0	pp	229533.Q4IBS2
229533.I1RMC0	pp	229533.I1RW99
229533.I1RQG9	pp	229533.I1S1V5
229533.I1RQG9	pp	229533.I1RS37
229533.I1RQG9	pp	229533.I1RWS7
229533.I1RQG9	pp	229533.I1S1V1
229533.I1RQG9	pp	229533.I1RWC3
229533.I1REC9	pp	229533.I1RY34
229533.I1REC9	pp	229533.I1RQM9
229533.I1REC9	pp	229533.I1RPF2
229533.I1REC9	pp	229533.I1RI23
229533.I1REC9	pp	229533.I1RI22
229533.I1RMN7	pp	229533.I1RRQ3
229533.I1RMN7	pp	229533.I1RNH1
229533.I1RMN7	pp	229533.I1S4T6
229533.I1RMN7	pp	229533.I1S0R7
229533.I1RMN7	pp	229533.I1RQ65
229533.I1RMN7	pp	229533.Q4HZ42
229533.I1RMN7	pp	229533.Q4IEV5
229533.I1RMN7	pp	229533.I1RUQ2
229533.I1RMN7	pp	229533.I1RT80
229533.I1RMN7	pp	229533.I1RT05
229533.I1RMN7	pp	229533.Q4IPI1
229533.I1RMN7	pp	229533.Q4IQW0
229533.I1RMN7	pp	229533.I1RNV6
229533.I1RMN7	pp	229533.I1RRT2
229533.I1RMN7	pp	229533.I1RRJ8
229533.I1RMN7	pp	229533.Q4IJH1
229533.I1RMN7	pp	229533.I1RND6
229533.I1RMN7	pp	229533.I1RWF7
229533.I1RMN7	pp	229533.I1RTF4
229533.I1RMN7	pp	229533.I1RNA0
229533.I1RMN7	pp	229533.I1S6U8
229533.I1RMN7	pp	229533.I1RZ99
229533.I1RMN7	pp	229533.I1RN64
229533.I1RMN7	pp	229533.I1RPU0
229533.I1RMN7	pp	229533.I1RZG3
229533.I1RMN7	pp	229533.I1S9U2
229533.I1RMN7	pp	229533.I1RZS6
229533.I1RMN7	pp	229533.V6QYF0

26062 229533.I1RMN7 pp 229533.I1RQ31
26063 229533.I1RMN7 pp 229533.Q4I662
26064 229533.I1RMN7 pp 229533.I1RZF6
26065 229533.I1RMN7 pp 229533.I1RYB9
26066 229533.I1RMN7 pp 229533.I1RTD7
26067 229533.I1RMN7 pp 229533.I1S021
26068 229533.I1RMN7 pp 229533.I1RRR7
26069 229533.I1RMN7 pp 229533.I1RNY2
26070 229533.I1RMN7 pp 229533.I1RYW3
26071 229533.I1RMN7 pp 229533.Q4I327
26072 229533.I1RMN7 pp 229533.I1RSI6
26073 229533.I1RMN7 pp 229533.I1RNH9
26074 229533.I1RMN7 pp 229533.Q4HZK7
26075 229533.I1RMN7 pp 229533.V6QUE6
26076 229533.I1RMN7 pp 229533.I1RRI2
26077 229533.I1RMN7 pp 229533.I1S060
26078 229533.I1RMN7 pp 229533.I1RTE9
26079 229533.I1RMN7 pp 229533.Q4IBS2
26080 229533.I1RMN7 pp 229533.I1RNU6
26081 229533.I1RMN7 pp 229533.I1RPJ3
26082 229533.I1RMN7 pp 229533.I1RQA2
26083 229533.I1RMN7 pp 229533.Q4IJ56
26084 229533.I1RMN7 pp 229533.I1RV64
26085 229533.I1RMN7 pp 229533.I1RQ28
26086 229533.I1RMN7 pp 229533.I1S2E8
26087 229533.I1RMN7 pp 229533.V6RGY3
26088 229533.I1RMN7 pp 229533.I1RNK2
26089 229533.I1RMN7 pp 229533.I1RVZ0
26090 229533.A0A1C3YIY7 pp 229533.I1RA48
26091 229533.A0A1C3YIY7 pp 229533.I1RZK6
26092 229533.A0A1C3YIY7 pp 229533.I1RSR8
26093 229533.A0A1C3YIY7 pp 229533.I1RAN5
26094 229533.A0A1C3YIY7 pp 229533.V6R042
26095 229533.A0A1C3YIY7 pp 229533.I1RNH6
26096 229533.A0A1C3YIY7 pp 229533.I1RAU7
26097 229533.A0A1C3YIY7 pp 229533.I1RKJ6
26098 229533.A0A1C3YIY7 pp 229533.I1RR73
26099 229533.A0A1C3YIY7 pp 229533.I1RLV9
26100 229533.A0A1C3YIY7 pp 229533.I1RX46
26101 229533.A0A1C3YIY7 pp 229533.I1RQ65
26102 229533.A0A1C3YIY7 pp 229533.I1RKL3
26103 229533.I1RSI6 pp 229533.V6R227
26104 229533.I1RSI6 pp 229533.I1S4T3
26105 229533.I1RSI6 pp 229533.V6RIV9
26106 229533.I1RSI6 pp 229533.I1S9U1
26107 229533.I1RSI6 pp 229533.I1RZF6
26108 229533.I1RSI6 pp 229533.V6RY33
26109 229533.I1RSI6 pp 229533.I1RX14
26110 229533.I1RSI6 pp 229533.I1S0J1
26111 229533.I1RSI6 pp 229533.Q4IBS2
26112 229533.I1RSI6 pp 229533.I1S0M9
26113 229533.I1RSI6 pp 229533.Q4IJ56
26114 229533.I1RSI6 pp 229533.V6RGN5
26115 229533.I1RSI6 pp 229533.I1RUG3
26116 229533.I1RSI6 pp 229533.V6QUE6
26117 229533.I1RSI6 pp 229533.I1S5B0
26118 229533.I1RSI6 pp 229533.Q4IN63
26119 229533.I1RSI6 pp 229533.I1RX08
26120 229533.I1RSI6 pp 229533.I1RW99
26121 229533.I1RSI6 pp 229533.V6RCG5
26122 229533.I1RSI6 pp 229533.I1S1U7
26123 229533.I1RSI6 pp 229533.Q4IEK8
26124 229533.I1RSI6 pp 229533.I1RZE5
26125 229533.I1RSI6 pp 229533.I1RSR3
26126 229533.I1RSI6 pp 229533.I1S021
26127 229533.I1RSI6 pp 229533.I1S1J6
26128 229533.I1RSI6 pp 229533.Q4HZ42
26129 229533.I1RSI6 pp 229533.I1RVZ0
26130 229533.I1RSI6 pp 229533.I1S274
26131 229533.I1RSI6 pp 229533.I1RT33
26132 229533.I1RSI6 pp 229533.V6RGY3
26133 229533.I1RSI6 pp 229533.I1S2M6
26134 229533.I1RSI6 pp 229533.Q4I5Z5

26135 229533.I1RSI6 pp 229533.I1RZK6
26136 229533.I1RSI6 pp 229533.I1RWH5
26137 229533.I1RSI6 pp 229533.Q4HZ68
26138 229533.I1RSI6 pp 229533.I1RYW3
26139 229533.I1RSI6 pp 229533.V6R283
26140 229533.I1RSI6 pp 229533.I1RTD7
26141 229533.I1RSI6 pp 229533.I1RZ99
26142 229533.I1RSI6 pp 229533.I1RZZ1
26143 229533.I1RSI6 pp 229533.I1RWF7
26144 229533.I1RSI6 pp 229533.I1RTD9
26145 229533.I1RSI6 pp 229533.I1S2E8
26146 229533.I1RSI6 pp 229533.I1S2U2
26147 229533.I1RSI6 pp 229533.I1S2C0
26148 229533.I1RSI6 pp 229533.I1RTE9
26149 229533.I1RSI6 pp 229533.I1S1X9
26150 229533.I1RSI6 pp 229533.V6QW22
26151 229533.I1RSI6 pp 229533.I1RY63
26152 229533.I1RSI6 pp 229533.I1RSQ7
26153 229533.I1RSI6 pp 229533.I1RZJ3
26154 229533.I1RSI6 pp 229533.Q4I830
26155 229533.I1RSI6 pp 229533.I1RZB5
26156 229533.I1RSI6 pp 229533.I1RSS4
26157 229533.I1RSI6 pp 229533.I1RT40
26158 229533.I1RSI6 pp 229533.Q4HZK7
26159 229533.I1RSI6 pp 229533.I1S6F0
26160 229533.I1RSI6 pp 229533.I1RUQ2
26161 229533.I1RSI6 pp 229533.I1RW71
26162 229533.I1RSI6 pp 229533.I1RT04
26163 229533.I1RSI6 pp 229533.I1RVQ0
26164 229533.I1RSI6 pp 229533.I1RX46
26165 229533.I1RSI6 pp 229533.I1RTI6
26166 229533.I1RSI6 pp 229533.Q4IPI1
26167 229533.I1RSI6 pp 229533.Q4IAS1
26168 229533.I1RSI6 pp 229533.Q4HZ47
26169 229533.I1RSI6 pp 229533.I1S0R7
26170 229533.I1RSI6 pp 229533.V6RTJ8
26171 229533.I1RSI6 pp 229533.I1RW25
26172 229533.I1RSI6 pp 229533.Q4IEV5
26173 229533.I1RSI6 pp 229533.I1S0L3
26174 229533.I1RSI6 pp 229533.Q4IFI0
26175 229533.I1RSI6 pp 229533.Q4I662
26176 229533.I1RSI6 pp 229533.I1S9U2
26177 229533.I1RSI6 pp 229533.Q4IQW0
26178 229533.I1RSI6 pp 229533.I1RZZ9
26179 229533.I1RSI6 pp 229533.I1RW61
26180 229533.I1RSI6 pp 229533.I1S1B6
26181 229533.I1RSI6 pp 229533.I1RT05
26182 229533.I1RSI6 pp 229533.I1RT98
26183 229533.I1RSI6 pp 229533.I1RX24
26184 229533.I1RSI6 pp 229533.I1RZS6
26185 229533.I1RSI6 pp 229533.Q4IF76
26186 229533.I1RSI6 pp 229533.I1S6U8
26187 229533.I1RSI6 pp 229533.Q4IQK7
26188 229533.I1RSI6 pp 229533.Q4INZ9
26189 229533.I1RSI6 pp 229533.Q4HVW2
26190 229533.I1RSI6 pp 229533.I1S253
26191 229533.I1RSI6 pp 229533.I1RW90
26192 229533.I1RSI6 pp 229533.I1S5R1
26193 229533.I1RSI6 pp 229533.V6QYF0
26194 229533.I1RSI6 pp 229533.Q4IJH1
26195 229533.I1RSI6 pp 229533.I1S9Y1
26196 229533.I1RSI6 pp 229533.I1RWJ8
26197 229533.I1RSI6 pp 229533.I1RT80
26198 229533.I1RSI6 pp 229533.I1RTF4
26199 229533.I1RSI6 pp 229533.I1S8E2
26200 229533.A0A098DQ92 pp 229533.V6R756
26201 229533.A0A098DQ92 pp 229533.I1RRM1
26202 229533.A0A098DQ92 pp 229533.I1RHU0
26203 229533.A0A098DQ92 pp 229533.I1RZM4
26204 229533.A0A098DQ92 pp 229533.V6R2Z2
26205 229533.A0A098DQ92 pp 229533.A0A1C3YKN4
26206 229533.A0A098DQ92 pp 229533.V6RAM6
26207 229533.A0A098DQ92 pp 229533.I1S9T6

26208	229533.A0A098DQ92	pp	229533.A0A098E525
26209	229533.A0A098DQ92	pp	229533.A0A0E0SH57
26210	229533.A0A098DQ92	pp	229533.I1RK44
26211	229533.A0A098DQ92	pp	229533.V6QU99
26212	229533.A0A098DQ92	pp	229533.V6RK96
26213	229533.A0A098DQ92	pp	229533.I1SAI4
26214	229533.A0A098DQ92	pp	229533.I1RDV8
26215	229533.A0A098DQ92	pp	229533.A0A1C3YIE0
26216	229533.A0A098DQ92	pp	229533.I1RAG3
26217	229533.A0A098DQ92	pp	229533.I1RMU5
26218	229533.A0A098DQ92	pp	229533.I1RIW5
26219	229533.A0A098DQ92	pp	229533.A0A1C3YHF4
26220	229533.A0A098DQ92	pp	229533.I1RNH4
26221	229533.A0A098DQ92	pp	229533.V6R5D2
26222	229533.A0A098DQ92	pp	229533.A0A1C3YIR5
26223	229533.A0A098DQ92	pp	229533.I1RYM5
26224	229533.A0A098DQ92	pp	229533.V6R0W1
26225	229533.A0A098DQ92	pp	229533.A0A1C3YIP2
26226	229533.A0A098DQ92	pp	229533.A0A1C3YKB7
26227	229533.A0A098DQ92	pp	229533.I1RQE8
26228	229533.I1RTE9	pp	229533.Q4IAS1
26229	229533.I1RTE9	pp	229533.Q4HZ42
26230	229533.I1RTE9	pp	229533.Q4IEV5
26231	229533.I1RTE9	pp	229533.Q4HXF0
26232	229533.I1RTE9	pp	229533.I1RWF7
26233	229533.I1RTE9	pp	229533.I1RW26
26234	229533.I1RTE9	pp	229533.Q4I830
26235	229533.I1RTE9	pp	229533.I1RZB2
26236	229533.I1RTE9	pp	229533.Q4IQW0
26237	229533.I1RTE9	pp	229533.Q4I662
26238	229533.I1RTE9	pp	229533.I1RWS4
26239	229533.I1RTE9	pp	229533.Q4I8X0
26240	229533.I1RTE9	pp	229533.I1RW61
26241	229533.I1RTE9	pp	229533.I1S0J1
26242	229533.I1RTE9	pp	229533.Q4HW67
26243	229533.I1RTE9	pp	229533.I1RW46
26244	229533.I1RTE9	pp	229533.V6R283
26245	229533.I1RTE9	pp	229533.V6R042
26246	229533.I1RTE9	pp	229533.I1S060
26247	229533.I1RTE9	pp	229533.V6RGN5
26248	229533.I1RTE9	pp	229533.I1S289
26249	229533.I1RTE9	pp	229533.I1S5R1
26250	229533.I1RTE9	pp	229533.V6RGY3
26251	229533.I1RTE9	pp	229533.Q4I327
26252	229533.I1RTE9	pp	229533.Q4I5M4
26253	229533.I1RTE9	pp	229533.I1S274
26254	229533.I1RTE9	pp	229533.I1S253
26255	229533.I1RTE9	pp	229533.I1RVF8
26256	229533.I1RTE9	pp	229533.I1S558
26257	229533.I1RTE9	pp	229533.Q4IF76
26258	229533.I1RTE9	pp	229533.I1S2C0
26259	229533.I1RTE9	pp	229533.I1RUG3
26260	229533.I1RTE9	pp	229533.I1RZS6
26261	229533.I1RTE9	pp	229533.I1S8R3
26262	229533.I1RTE9	pp	229533.I1RTN3
26263	229533.I1RTE9	pp	229533.Q4IN63
26264	229533.I1RTE9	pp	229533.I1RWJ4
26265	229533.I1RTE9	pp	229533.I1RX24
26266	229533.I1RTE9	pp	229533.I1RUP5
26267	229533.I1RTE9	pp	229533.I1RUQ9
26268	229533.I1RTE9	pp	229533.I1S0M3
26269	229533.I1RTE9	pp	229533.I1S4T3
26270	229533.I1RTE9	pp	229533.Q4HZK7
26271	229533.I1RTE9	pp	229533.V6RIV9
26272	229533.I1RTE9	pp	229533.I1RZW8
26273	229533.I1RTE9	pp	229533.I1S1X9
26274	229533.I1RTE9	pp	229533.V6RN31
26275	229533.I1RTE9	pp	229533.I1S6F0
26276	229533.I1RTE9	pp	229533.I1S2E8
26277	229533.I1RTE9	pp	229533.I1S296
26278	229533.I1RTE9	pp	229533.I1RX46
26279	229533.I1RTE9	pp	229533.I1RVQ0
26280	229533.I1RTE9	pp	229533.Q4HZ47

26281	229533.I1RTE9	pp	229533.Q4IPI1
26282	229533.I1RTE9	pp	229533.V6QW22
26283	229533.I1RTE9	pp	229533.I1RZ99
26284	229533.I1RTE9	pp	229533.Q4HZ68
26285	229533.I1RTE9	pp	229533.V6R057
26286	229533.I1RTE9	pp	229533.I1S0R7
26287	229533.I1RTE9	pp	229533.I1RUQ2
26288	229533.I1RTE9	pp	229533.I1RZG3
26289	229533.I1RTE9	pp	229533.I1RTI6
26290	229533.I1RTE9	pp	229533.I1RW25
26291	229533.I1RTE9	pp	229533.I1RYJ6
26292	229533.I1RTE9	pp	229533.I1RW71
26293	229533.I1RTE9	pp	229533.I1RWJ8
26294	229533.I1RTE9	pp	229533.I1S9U2
26295	229533.I1RTE9	pp	229533.Q4INZ9
26296	229533.I1RTE9	pp	229533.I1S6U8
26297	229533.I1RTE9	pp	229533.Q4IFI0
26298	229533.I1RTE9	pp	229533.Q4IQK7
26299	229533.I1RTE9	pp	229533.I1S0L3
26300	229533.I1RTE9	pp	229533.I1RZJ6
26301	229533.I1RTE9	pp	229533.V6QYF0
26302	229533.I1RTE9	pp	229533.Q4HVW2
26303	229533.I1RTE9	pp	229533.I1S0D4
26304	229533.I1RTE9	pp	229533.I1RW99
26305	229533.I1RTE9	pp	229533.I1RTF4
26306	229533.I1RTE9	pp	229533.I1RZF6
26307	229533.I1RTE9	pp	229533.Q4IJH1
26308	229533.I1RTE9	pp	229533.V6R227
26309	229533.I1RTE9	pp	229533.I1S0F1
26310	229533.I1RTE9	pp	229533.Q4IEK8
26311	229533.I1RTE9	pp	229533.Q4IBS2
26312	229533.I1RTE9	pp	229533.I1RZE5
26313	229533.I1RTE9	pp	229533.V6RCG5
26314	229533.I1RTE9	pp	229533.Q4IJ56
26315	229533.I1RTE9	pp	229533.I1S021
26316	229533.I1RTE9	pp	229533.Q4IAA0
26317	229533.I1RTE9	pp	229533.I1RZM8
26318	229533.I1RTE9	pp	229533.I1S217
26319	229533.I1RTE9	pp	229533.I1RVZ0
26320	229533.I1RTE9	pp	229533.I1S0M9
26321	229533.I1RTE9	pp	229533.Q4I5Z5
26322	229533.I1RTE9	pp	229533.I1RZZ1
26323	229533.I1RTE9	pp	229533.I1RZK6
26324	229533.I1RTE9	pp	229533.I1RX14
26325	229533.I1RTE9	pp	229533.I1RWH8
26326	229533.I1RTE9	pp	229533.I1RZB5
26327	229533.I1RYB5	pp	229533.V6RIV9
26328	229533.I1RC76	pp	229533.I1RWD0
26329	229533.I1R9X7	pp	229533.Q4HZ42
26330	229533.I1R9X7	pp	229533.I1RQ65
26331	229533.I1R9X7	pp	229533.I1S0L3
26332	229533.I1R9X7	pp	229533.V6R1K4
26333	229533.I1R9X7	pp	229533.I1RWD4
26334	229533.I1R9X7	pp	229533.I1RS30
26335	229533.I1R9X7	pp	229533.Q4HZ47
26336	229533.I1R9X7	pp	229533.I1RRP3
26337	229533.I1R9X7	pp	229533.I1RFG0
26338	229533.I1R9X7	pp	229533.I1RRQ3
26339	229533.I1R9X7	pp	229533.I1RVQ0
26340	229533.I1R9X7	pp	229533.I1S6F0
26341	229533.I1R9X7	pp	229533.I1RNH1
26342	229533.I1R9X7	pp	229533.I1RBC9
26343	229533.I1R9X7	pp	229533.I1RKL3
26344	229533.I1R9X7	pp	229533.I1RW71
26345	229533.I1R9X7	pp	229533.Q4I830
26346	229533.I1R9X7	pp	229533.I1RSX1
26347	229533.I1R9X7	pp	229533.I1RQJ5
26348	229533.I1R9X7	pp	229533.I1RWF7
26349	229533.I1R9X7	pp	229533.I1RZ99
26350	229533.I1R9X7	pp	229533.I1RSQ7
26351	229533.I1R9X7	pp	229533.V6QW22
26352	229533.I1R9X7	pp	229533.I1RC82
26353	229533.I1R9X7	pp	229533.I1RRJ8

229533.I1R9X7	pp	229533.I1RN63
229533.I1R9X7	pp	229533.V6R283
229533.I1R9X7	pp	229533.I1RC03
229533.I1R9X7	pp	229533.I1RFN1
229533.I1R9X7	pp	229533.I1RBC7
229533.I1R9X7	pp	229533.I1RS47
229533.I1R9X7	pp	229533.Q4IQW0
229533.I1R9X7	pp	229533.I1RFW5
229533.I1R9X7	pp	229533.I1RD52
229533.I1R9X7	pp	229533.I1RBS6
229533.I1R9X7	pp	229533.I1RP27
229533.I1R9X7	pp	229533.I1S1B6
229533.I1R9X7	pp	229533.I1RJV4
229533.I1R9X7	pp	229533.Q4IAS1
229533.I1R9X7	pp	229533.I1RRH5
229533.I1R9X7	pp	229533.I1RT33
229533.I1R9X7	pp	229533.I1RL23
229533.I1R9X7	pp	229533.I1RLV9
229533.I1R9X7	pp	229533.I1RJZ7
229533.I1R9X7	pp	229533.I1RRT8
229533.I1R9X7	pp	229533.Q4I5R9
229533.I1R9X7	pp	229533.I1RFQ7
229533.I1R9X7	pp	229533.I1RS03
229533.I1R9X7	pp	229533.I1RM67
229533.I1R9X7	pp	229533.Q4IFI0
229533.I1R9X7	pp	229533.I1RNV6
229533.I1R9X7	pp	229533.I1RKQ5
229533.I1R9X7	pp	229533.I1RJW6
229533.I1R9X7	pp	229533.I1RE87
229533.I1R9X7	pp	229533.I1RW25
229533.I1R9X7	pp	229533.I1RAC2
229533.I1R9X7	pp	229533.I1RT80
229533.I1R9X7	pp	229533.I1RX24
229533.I1R9X7	pp	229533.I1RTF4
229533.I1R9X7	pp	229533.I1RRT2
229533.I1R9X7	pp	229533.I1RQS5
229533.I1R9X7	pp	229533.Q4IPI1
229533.I1R9X7	pp	229533.Q4IJH1
229533.I1R9X7	pp	229533.I1RKF9
229533.I1R9X7	pp	229533.V6QYF0
229533.I1R9X7	pp	229533.I1RJU3
229533.I1R9X7	pp	229533.I1RMK3
229533.I1R9X7	pp	229533.I1RJL4
229533.I1R9X7	pp	229533.I1RRQ6
229533.I1R9X7	pp	229533.I1S0R7
229533.I1R9X7	pp	229533.I1RBG9
229533.I1R9X7	pp	229533.I1RTI6
229533.I1R9X7	pp	229533.I1RC29
229533.I1R9X7	pp	229533.Q4HVW2
229533.I1R9X7	pp	229533.I1RDA0
229533.I1R9X7	pp	229533.I1RNA0
229533.I1R9X7	pp	229533.Q4IQK7
229533.I1R9X7	pp	229533.I1RT04
229533.I1R9X7	pp	229533.I1S253
229533.I1R9X7	pp	229533.I1RUQ2
229533.I1R9X7	pp	229533.I1RBN1
229533.I1R9X7	pp	229533.I1RXD7
229533.I1R9X7	pp	229533.I1RR72
229533.I1R9X7	pp	229533.I1RB71
229533.I1R9X7	pp	229533.I1S6U8
229533.I1R9X7	pp	229533.I1RM06
229533.I1R9X7	pp	229533.I1RBT1
229533.I1R9X7	pp	229533.Q4INZ9
229533.I1R9X7	pp	229533.I1RZJ6
229533.I1R9X7	pp	229533.I1RZS6
229533.I1R9X7	pp	229533.I1RW61
229533.I1R9X7	pp	229533.Q4HZI0
229533.I1R9X7	pp	229533.I1RAF8
229533.I1R9X7	pp	229533.I1REF2
229533.I1R9X7	pp	229533.Q4IF76
229533.I1R9X7	pp	229533.I1RC32
229533.I1R9X7	pp	229533.I1RWJ4
229533.I1R9X7	pp	229533.I1RT05

26427	229533.I1R9X7	pp	229533.I1RPW4
26428	229533.I1R9X7	pp	229533.I1RG51
26429	229533.I1R9X7	pp	229533.I1RMC0
26430	229533.I1R9X7	pp	229533.I1RPM4
26431	229533.I1R9X7	pp	229533.I1S0F1
26432	229533.I1R9X7	pp	229533.Q4I662
26433	229533.I1R9X7	pp	229533.I1RQM6
26434	229533.I1R9X7	pp	229533.I1RFS3
26435	229533.I1R9X7	pp	229533.I1RZF6
26436	229533.I1R9X7	pp	229533.I1REX4
26437	229533.I1R9X7	pp	229533.I1RN58
26438	229533.I1R9X7	pp	229533.I1RGF6
26439	229533.I1R9X7	pp	229533.I1RZM8
26440	229533.I1R9X7	pp	229533.I1RR24
26441	229533.I1R9X7	pp	229533.I1S9U1
26442	229533.I1R9X7	pp	229533.I1RI82
26443	229533.I1R9X7	pp	229533.I1RPW9
26444	229533.I1R9X7	pp	229533.I1RD64
26445	229533.I1R9X7	pp	229533.I1RRR7
26446	229533.I1R9X7	pp	229533.I1S9U2
26447	229533.I1R9X7	pp	229533.Q4I327
26448	229533.I1R9X7	pp	229533.I1RNY2
26449	229533.I1R9X7	pp	229533.V6R227
26450	229533.I1R9X7	pp	229533.I1RMN7
26451	229533.I1R9X7	pp	229533.I1S4T3
26452	229533.I1R9X7	pp	229533.I1RWJ8
26453	229533.I1R9X7	pp	229533.I1S9Y1
26454	229533.I1R9X7	pp	229533.I1RNH6
26455	229533.I1R9X7	pp	229533.I1S021
26456	229533.I1R9X7	pp	229533.I1RBI8
26457	229533.I1R9X7	pp	229533.I1RKJ6
26458	229533.I1R9X7	pp	229533.V6RIV9
26459	229533.I1R9X7	pp	229533.O74638
26460	229533.I1R9X7	pp	229533.I1RPU0
26461	229533.I1R9X7	pp	229533.V6R057
26462	229533.I1R9X7	pp	229533.Q4HZB8
26463	229533.I1R9X7	pp	229533.I1RC06
26464	229533.I1R9X7	pp	229533.I1RCY5
26465	229533.I1R9X7	pp	229533.I1RPY1
26466	229533.I1R9X7	pp	229533.Q4IJ56
26467	229533.I1R9X7	pp	229533.I1RG56
26468	229533.I1R9X7	pp	229533.I1RF08
26469	229533.I1R9X7	pp	229533.I1S296
26470	229533.I1R9X7	pp	229533.I1RC33
26471	229533.I1R9X7	pp	229533.Q4HZ68
26472	229533.I1R9X7	pp	229533.I1RS17
26473	229533.I1R9X7	pp	229533.Q4IEK8
26474	229533.I1R9X7	pp	229533.I1REK8
26475	229533.I1R9X7	pp	229533.I1RM96
26476	229533.I1R9X7	pp	229533.I1RNU6
26477	229533.I1R9X7	pp	229533.Q4IAA0
26478	229533.I1R9X7	pp	229533.I1RD17
26479	229533.I1R9X7	pp	229533.I1RB27
26480	229533.I1R9X7	pp	229533.I1RKQ9
26481	229533.I1R9X7	pp	229533.Q4ICA8
26482	229533.I1R9X7	pp	229533.I1RS18
26483	229533.I1R9X7	pp	229533.I1RVZ0
26484	229533.I1R9X7	pp	229533.I1RX14
26485	229533.I1R9X7	pp	229533.I1RNK2
26486	229533.I1R9X7	pp	229533.I1S0M9
26487	229533.I1R9X7	pp	229533.I1S1J6
26488	229533.I1R9X7	pp	229533.I1RAZ8
26489	229533.I1R9X7	pp	229533.I1RW46
26490	229533.I1R9X7	pp	229533.I1RQE7
26491	229533.I1R9X7	pp	229533.I1RKC6
26492	229533.I1R9X7	pp	229533.I1S060
26493	229533.I1R9X7	pp	229533.I1S2E8
26494	229533.I1R9X7	pp	229533.I1RZE5
26495	229533.I1R9X7	pp	229533.I1RF19
26496	229533.I1R9X7	pp	229533.I1RBL7
26497	229533.I1R9X7	pp	229533.I1RE01
26498	229533.I1R9X7	pp	229533.I1S1D3
26499	229533.I1R9X7	pp	229533.I1S0J1

26500 229533.I1R9X7 pp 229533.I1S274
26501 229533.I1R9X7 pp 229533.I1S0M3
26502 229533.I1R9X7 pp 229533.V6RGY3
26503 229533.I1R9X7 pp 229533.I1RE00
26504 229533.I1R9X7 pp 229533.I1RFJ9
26505 229533.I1R9X7 pp 229533.I1RC72
26506 229533.I1R9X7 pp 229533.I1RC27
26507 229533.I1R9X7 pp 229533.V6RGN5
26508 229533.I1R9X7 pp 229533.I1RSR3
26509 229533.I1R9X7 pp 229533.I1RY63
26510 229533.I1R9X7 pp 229533.I1S1X9
26511 229533.I1R9X7 pp 229533.I1RW99
26512 229533.I1R9X7 pp 229533.I1S1U7
26513 229533.I1R9X7 pp 229533.I1RQA2
26514 229533.I1R9X7 pp 229533.I1RA48
26515 229533.I1R9X7 pp 229533.I1RG11
26516 229533.I1R9X7 pp 229533.Q4HWE2
26517 229533.I1R9X7 pp 229533.I1RCN5
26518 229533.I1R9X7 pp 229533.I1RFS5
26519 229533.I1R9X7 pp 229533.I1S558
26520 229533.I1R9X7 pp 229533.I1RGC2
26521 229533.I1R9X7 pp 229533.I1RSS4
26522 229533.I1R9X7 pp 229533.V6QUE6
26523 229533.I1R9X7 pp 229533.I1RSC6
26524 229533.I1R9X7 pp 229533.I1RAN5
26525 229533.I1R9X7 pp 229533.I1RCL3
26526 229533.I1R9X7 pp 229533.I1RB53
26527 229533.I1R9X7 pp 229533.I1RMN6
26528 229533.I1R9X7 pp 229533.I1RN99
26529 229533.I1R9X7 pp 229533.I1RJV3
26530 229533.I1R9X7 pp 229533.Q4IBS2
26531 229533.I1R9X7 pp 229533.I1RDG0
26532 229533.I1R9X7 pp 229533.I1RAS2
26533 229533.I1R9X7 pp 229533.I1RSG8
26534 229533.I1R9X7 pp 229533.I1RX08
26535 229533.I1R9X7 pp 229533.I1RK95
26536 229533.I1R9X7 pp 229533.I1RQ28
26537 229533.I1R9X7 pp 229533.I1S8R3
26538 229533.I1R9X7 pp 229533.I1RB92
26539 229533.I1R9X7 pp 229533.I1RCG4
26540 229533.I1R9X7 pp 229533.I1RSI6
26541 229533.I1R9X7 pp 229533.V6RCG5
26542 229533.I1R9X7 pp 229533.I1RMQ0
26543 229533.I1R9X7 pp 229533.Q4HZK7
26544 229533.I1R9X7 pp 229533.I1RSD8
26545 229533.I1R9X7 pp 229533.I1RFL4
26546 229533.I1R9X7 pp 229533.I1S2U2
26547 229533.I1R9X7 pp 229533.I1RZB5
26548 229533.I1R9X7 pp 229533.I1RQ31
26549 229533.I1R9X7 pp 229533.I1RRI2
26550 229533.I1R9X7 pp 229533.I1RBD2
26551 229533.I1R9X7 pp 229533.I1RZW8
26552 229533.I1R9X7 pp 229533.I1RAN0
26553 229533.I1R9X7 pp 229533.Q4I948
26554 229533.I1R9X7 pp 229533.I1S2C0
26555 229533.I1R9X7 pp 229533.Q4IN00
26556 229533.I1R9X7 pp 229533.I1RZZ1
26557 229533.I1R9X7 pp 229533.I1RTE9
26558 229533.I1R9X7 pp 229533.I1RPJ3
26559 229533.I1R9X7 pp 229533.I1RTD9
26560 229533.I1R9X7 pp 229533.I1RMD5
26561 229533.I1RFJ3 pp 229533.I1S0E2
26562 229533.I1RFJ3 pp 229533.I1RR94
26563 229533.I1RPG6 pp 229533.I1RZK6
26564 229533.I1RPG6 pp 229533.I1RSR8
26565 229533.I1RPG6 pp 229533.V6R042
26566 229533.I1RPG6 pp 229533.I1RX46
26567 229533.I1RPG6 pp 229533.I1RR73
26568 229533.I1RPG6 pp 229533.I1RQ65
26569 229533.A0A0E0S8V8 pp 229533.A0A1C3YI53
26570 229533.Q4IB65 pp 229533.Q4IPZ1
26571 229533.Q4IB65 pp 229533.V6RM66
26572 229533.Q4IB65 pp 229533.Q4IJW4

26573	229533.Q4IB65	pp	229533.V6R7V8
26574	229533.Q4IB65	pp	229533.Q4IK03
26575	229533.I1S0R5	pp	229533.Q4IJ11
26576	229533.I1S0R5	pp	229533.V6QUE6
26577	229533.I1S0R5	pp	229533.Q4HYB8
26578	229533.I1S0R5	pp	229533.Q4I5U9
26579	229533.I1S0R5	pp	229533.Q4HYQ4
26580	229533.I1S0R5	pp	229533.Q4I375
26581	229533.I1S0R5	pp	229533.Q4I5W5
26582	229533.I1S0R5	pp	229533.Q4I5I4
26583	229533.I1S0R5	pp	229533.Q4HZ95
26584	229533.I1RKC6	pp	229533.I1RY63
26585	229533.I1RKC6	pp	229533.I1S1X9
26586	229533.I1RKC6	pp	229533.I1RSS4
26587	229533.I1RKC6	pp	229533.I1RSC6
26588	229533.I1RKC6	pp	229533.I1S2C0
26589	229533.I1RKC6	pp	229533.I1RTE9
26590	229533.I1RKC6	pp	229533.I1RZK6
26591	229533.I1RKC6	pp	229533.I1RZM8
26592	229533.I1RKC6	pp	229533.I1RZZ1
26593	229533.I1RKC6	pp	229533.I1RZB5
26594	229533.I1RKC6	pp	229533.Q4HZK7
26595	229533.I1RKC6	pp	229533.I1S2E8
26596	229533.I1RKC6	pp	229533.I1RQE7
26597	229533.I1RKC6	pp	229533.I1S8R3
26598	229533.I1RKC6	pp	229533.Q4IN63
26599	229533.I1RKC6	pp	229533.I1S296
26600	229533.I1RKC6	pp	229533.V6RGN5
26601	229533.I1RKC6	pp	229533.I1RSD8
26602	229533.I1RKC6	pp	229533.I1RW99
26603	229533.I1RKC6	pp	229533.I1RRI2
26604	229533.I1RKC6	pp	229533.Q4IBS2
26605	229533.I1RKC6	pp	229533.Q4HZ68
26606	229533.I1RKC6	pp	229533.I1RZW8
26607	229533.I1RKC6	pp	229533.I1RMN6
26608	229533.I1RKC6	pp	229533.I1RNH9
26609	229533.I1RKC6	pp	229533.I1RZE5
26610	229533.I1RKC6	pp	229533.I1RNK2
26611	229533.I1RKC6	pp	229533.I1RSI0
26612	229533.I1RKC6	pp	229533.V6QUE6
26613	229533.I1RKC6	pp	229533.I1RSR8
26614	229533.I1RKC6	pp	229533.I1RMD5
26615	229533.I1RKC6	pp	229533.V6RCG5
26616	229533.I1RKC6	pp	229533.I1RVZ0
26617	229533.I1RKC6	pp	229533.I1RTD9
26618	229533.I1RKC6	pp	229533.I1RX08
26619	229533.I1RKC6	pp	229533.Q4IAA0
26620	229533.I1RKC6	pp	229533.Q4IJ56
26621	229533.I1RKC6	pp	229533.I1RSR3
26622	229533.I1RKC6	pp	229533.I1S1U7
26623	229533.I1RKC6	pp	229533.I1S0M9
26624	229533.I1RKC6	pp	229533.I1RSI6
26625	229533.I1RKC6	pp	229533.V6RGY3
26626	229533.I1RKC6	pp	229533.I1RNU6
26627	229533.I1RKC6	pp	229533.Q4I327
26628	229533.I1RKC6	pp	229533.I1RNY2
26629	229533.I1RKC6	pp	229533.I1RQ81
26630	229533.I1RKC6	pp	229533.I1S274
26631	229533.I1RKC6	pp	229533.I1S0J1
26632	229533.I1RKC6	pp	229533.I1RQA2
26633	229533.I1RKC6	pp	229533.I1RNH6
26634	229533.I1RKC6	pp	229533.I1S021
26635	229533.I1RKC6	pp	229533.I1S558
26636	229533.I1RKC6	pp	229533.I1RN99
26637	229533.I1RKC6	pp	229533.I1RMN7
26638	229533.I1RKC6	pp	229533.I1RW46
26639	229533.I1RKC6	pp	229533.I1RRR7
26640	229533.I1RKC6	pp	229533.I1RQ28
26641	229533.I1RKC6	pp	229533.I1RX14
26642	229533.I1RKC6	pp	229533.Q4HVW2
26643	229533.I1RKC6	pp	229533.I1S253
26644	229533.I1RKC6	pp	229533.V6R227
26645	229533.I1RKC6	pp	229533.I1RQ31

26646	229533.I1RKC6	pp	229533.I1RMC0
26647	229533.I1RKC6	pp	229533.I1RMK3
26648	229533.I1RKC6	pp	229533.I1RZF6
26649	229533.I1RKC6	pp	229533.I1RUG3
26650	229533.I1RKC6	pp	229533.I1RND6
26651	229533.I1RKC6	pp	229533.I1RM96
26652	229533.I1RKC6	pp	229533.Q4IAS1
26653	229533.I1RKC6	pp	229533.I1RLV9
26654	229533.I1RKC6	pp	229533.Q4IJH1
26655	229533.I1RKC6	pp	229533.I1RQJ5
26656	229533.I1RKC6	pp	229533.I1RKQ5
26657	229533.I1RKC6	pp	229533.Q4IEK8
26658	229533.I1RKC6	pp	229533.V6RIV9
26659	229533.I1RKC6	pp	229533.I1RTF4
26660	229533.I1RKC6	pp	229533.I1RZ99
26661	229533.I1RKC6	pp	229533.I1RNV6
26662	229533.I1RKC6	pp	229533.I1RT80
26663	229533.I1RKC6	pp	229533.I1S8E2
26664	229533.I1RKC6	pp	229533.I1RRJ8
26665	229533.I1RKC6	pp	229533.I1S4T3
26666	229533.I1RKC6	pp	229533.I1RQM6
26667	229533.I1RKC6	pp	229533.I1RPY1
26668	229533.I1RKC6	pp	229533.I1RSQ7
26669	229533.I1RKC6	pp	229533.Q4I662
26670	229533.I1RKC6	pp	229533.V6QW22
26671	229533.I1RKC6	pp	229533.Q4IF76
26672	229533.I1RKC6	pp	229533.I1RPM4
26673	229533.I1RKC6	pp	229533.I1RT05
26674	229533.I1RKC6	pp	229533.Q4I830
26675	229533.I1RKC6	pp	229533.I1S9U2
26676	229533.I1RKC6	pp	229533.I1RKL3
26677	229533.I1RKC6	pp	229533.I1RW61
26678	229533.I1RKC6	pp	229533.I1S2L2
26679	229533.I1RKC6	pp	229533.I1RW25
26680	229533.I1RKC6	pp	229533.I1RR24
26681	229533.I1RKC6	pp	229533.I1RW71
26682	229533.I1RKC6	pp	229533.I1RRT8
26683	229533.I1RKC6	pp	229533.I1S5R1
26684	229533.I1RKC6	pp	229533.I1RRG3
26685	229533.I1RKC6	pp	229533.Q4IFI0
26686	229533.I1RKC6	pp	229533.I1RTA1
26687	229533.I1RKC6	pp	229533.I1RZV6
26688	229533.I1RKC6	pp	229533.I1RXD7
26689	229533.I1RKC6	pp	229533.I1RM67
26690	229533.I1RKC6	pp	229533.V6R283
26691	229533.I1RKC6	pp	229533.I1RN63
26692	229533.I1RKC6	pp	229533.I1RWJ8
26693	229533.I1RKC6	pp	229533.I1RQ65
26694	229533.I1RKC6	pp	229533.I1RUQ2
26695	229533.I1RKC6	pp	229533.I1RKJ6
26696	229533.I1RKC6	pp	229533.Q4HXF0
26697	229533.I1RKC6	pp	229533.I1RPU0
26698	229533.I1RKC6	pp	229533.I1RT04
26699	229533.I1RKC6	pp	229533.I1S0L3
26700	229533.I1RKC6	pp	229533.I1S0R7
26701	229533.I1RKC6	pp	229533.I1RM06
26702	229533.I1RKC6	pp	229533.I1RS47
26703	229533.I1RKC6	pp	229533.I1RT98
26704	229533.I1RKC6	pp	229533.Q4HZ42
26705	229533.I1RKC6	pp	229533.I1RZS6
26706	229533.I1RKC6	pp	229533.I1RX24
26707	229533.I1RKC6	pp	229533.I1RT33
26708	229533.I1RKC6	pp	229533.Q4IQK7
26709	229533.I1RKC6	pp	229533.I1RZJ6
26710	229533.I1RKC6	pp	229533.I1RNA0
26711	229533.I1RKC6	pp	229533.I1RRQ3
26712	229533.I1RKC6	pp	229533.Q4INZ9
26713	229533.I1RKC6	pp	229533.I1S6F0
26714	229533.I1RKC6	pp	229533.V6QYF0
26715	229533.I1RKC6	pp	229533.I1RNH1
26716	229533.I1RKC6	pp	229533.I1RVQ0
26717	229533.I1RKC6	pp	229533.I1RRQ6
26718	229533.I1RKC6	pp	229533.Q4IQW0

26719	229533.I1RKC6	pp	229533.I1RKF9
26720	229533.I1RKC6	pp	229533.Q4HZ47
26721	229533.I1RKC6	pp	229533.I1RQS5
26722	229533.I1RKC6	pp	229533.I1RWJ4
26723	229533.I1RKC6	pp	229533.I1RRT2
26724	229533.I1RKC6	pp	229533.I1RS30
26725	229533.I1RKC6	pp	229533.I1RX46
26726	229533.I1RKC6	pp	229533.I1RRP3
26727	229533.I1RKC6	pp	229533.Q4IPI1
26728	229533.A0A098DQ47	pp	229533.I1RPY8
26729	229533.A0A098DQ47	pp	229533.I1RB38
26730	229533.A0A098DQ47	pp	229533.I1RFJ3
26731	229533.A0A098DQ47	pp	229533.Q4IPZ1
26732	229533.A0A098DQ47	pp	229533.Q4IB65
26733	229533.A0A098DQ47	pp	229533.I1RR94
26734	229533.A0A098DQ47	pp	229533.I1RB86
26735	229533.A0A0E0S814	pp	229533.A0A1C3YM61
26736	229533.A0A0E0S814	pp	229533.A0A1C3YJC0
26737	229533.A0A0E0S814	pp	229533.I1RTA6
26738	229533.A0A0E0S814	pp	229533.I1S5U9
26739	229533.A0A1C3YK72	pp	229533.I1RX53
26740	229533.A0A1C3YK72	pp	229533.I1RYH4
26741	229533.A0A1C3YK72	pp	229533.Q4IBS2
26742	229533.A0A1C3YK72	pp	229533.V6RGN5
26743	229533.A0A1C3YK72	pp	229533.I1RMN6
26744	229533.A0A1C3YK72	pp	229533.I1RC27
26745	229533.A0A1C3YK72	pp	229533.A0A1C3YL07
26746	229533.A0A1C3YK72	pp	229533.I1RNY2
26747	229533.A0A1C3YK72	pp	229533.I1RRR7
26748	229533.A0A1C3YK72	pp	229533.A0A1C3YKW9
26749	229533.A0A1C3YK72	pp	229533.Q4I327
26750	229533.A0A1C3YK72	pp	229533.I1S021
26751	229533.A0A1C3YK72	pp	229533.I1RZF6
26752	229533.A0A1C3YK72	pp	229533.I1S9U1
26753	229533.A0A1C3YK72	pp	229533.I1RC72
26754	229533.A0A1C3YK72	pp	229533.I1S0J1
26755	229533.A0A1C3YK72	pp	229533.I1RNH6
26756	229533.A0A1C3YK72	pp	229533.I1RQ31
26757	229533.A0A1C3YK72	pp	229533.I1S060
26758	229533.A0A1C3YK72	pp	229533.I1RX14
26759	229533.A0A1C3YK72	pp	229533.I1RSI6
26760	229533.A0A1C3YK72	pp	229533.Q4IEK8
26761	229533.A0A1C3YK72	pp	229533.I1RB27
26762	229533.A0A1C3YK72	pp	229533.I1S2E8
26763	229533.A0A1C3YK72	pp	229533.I1R9X7
26764	229533.A0A1C3YK72	pp	229533.I1RM96
26765	229533.A0A1C3YK72	pp	229533.I1RBI8
26766	229533.A0A1C3YK72	pp	229533.I1S4T3
26767	229533.A0A1C3YK72	pp	229533.Q4IJ56
26768	229533.A0A1C3YK72	pp	229533.I1RSD8
26769	229533.A0A1C3YK72	pp	229533.I1S0M9
26770	229533.A0A1C3YK72	pp	229533.I1RG56
26771	229533.A0A1C3YK72	pp	229533.I1RMQ0
26772	229533.A0A1C3YK72	pp	229533.I1RNU6
26773	229533.A0A1C3YK72	pp	229533.I1RPY1
26774	229533.A0A1C3YK72	pp	229533.I1RCL3
26775	229533.A0A1C3YK72	pp	229533.I1RPS3
26776	229533.A0A1C3YK72	pp	229533.V6R227
26777	229533.A0A1C3YK72	pp	229533.I1RD03
26778	229533.A0A1C3YK72	pp	229533.I1RQ28
26779	229533.A0A1C3YK72	pp	229533.I1RN99
26780	229533.A0A1C3YK72	pp	229533.I1RWJ3
26781	229533.A0A1C3YK72	pp	229533.I1RFW5
26782	229533.A0A1C3YK72	pp	229533.I1RK95
26783	229533.A0A1C3YK72	pp	229533.V6R283
26784	229533.A0A1C3YK72	pp	229533.I1RQA2
26785	229533.A0A1C3YK72	pp	229533.I1RAN5
26786	229533.A0A1C3YK72	pp	229533.I1R9V7
26787	229533.A0A1C3YK72	pp	229533.I1RDG0
26788	229533.A0A1C3YK72	pp	229533.I1RFM0
26789	229533.A0A1C3YK72	pp	229533.I1S1X9
26790	229533.A0A1C3YK72	pp	229533.I1RC03
26791	229533.A0A1C3YK72	pp	229533.I1RSR3

26792	229533.A0A1C3YK72	pp	229533.I1RBL7
26793	229533.A0A1C3YK72	pp	229533.I1RSC6
26794	229533.A0A1C3YK72	pp	229533.I1RSS4
26795	229533.A0A1C3YK72	pp	229533.I1RZ99
26796	229533.A0A1C3YK72	pp	229533.I1S274
26797	229533.A0A1C3YK72	pp	229533.I1RRJ8
26798	229533.A0A1C3YK72	pp	229533.I1RQJ5
26799	229533.A0A1C3YK72	pp	229533.Q4I830
26800	229533.A0A1C3YK72	pp	229533.I1RA48
26801	229533.A0A1C3YK72	pp	229533.I1RJL4
26802	229533.A0A1C3YK72	pp	229533.I1RE87
26803	229533.A0A1C3YK72	pp	229533.A0A1C3YMW0
26804	229533.A0A1C3YK72	pp	229533.I1RK01
26805	229533.A0A1C3YK72	pp	229533.I1RRI2
26806	229533.A0A1C3YK72	pp	229533.Q4IPI1
26807	229533.A0A1C3YK72	pp	229533.Q4IAS1
26808	229533.A0A1C3YK72	pp	229533.I1RW71
26809	229533.A0A1C3YK72	pp	229533.I1RKL3
26810	229533.A0A1C3YK72	pp	229533.I1RMD5
26811	229533.A0A1C3YK72	pp	229533.I1RSR8
26812	229533.A0A1C3YK72	pp	229533.I1RR00
26813	229533.A0A1C3YK72	pp	229533.I1RTD9
26814	229533.A0A1C3YK72	pp	229533.I1RLV9
26815	229533.A0A1C3YK72	pp	229533.I1RKQ5
26816	229533.A0A1C3YK72	pp	229533.Q4IQW0
26817	229533.A0A1C3YK72	pp	229533.I1RVL1
26818	229533.A0A1C3YK72	pp	229533.I1RTE9
26819	229533.A0A1C3YK72	pp	229533.I1RM67
26820	229533.A0A1C3YK72	pp	229533.I1RKJ6
26821	229533.A0A1C3YK72	pp	229533.I1RPJ3
26822	229533.A0A1C3YK72	pp	229533.I1RPU0
26823	229533.A0A1C3YK72	pp	229533.I1S540
26824	229533.A0A1C3YK72	pp	229533.I1RFN1
26825	229533.A0A1C3YK72	pp	229533.I1S2C0
26826	229533.A0A1C3YK72	pp	229533.I1RC82
26827	229533.A0A1C3YK72	pp	229533.I1RPW9
26828	229533.A0A1C3YK72	pp	229533.I1RZE5
26829	229533.A0A1C3YK72	pp	229533.I1S9U2
26830	229533.A0A1C3YK72	pp	229533.I1RD64
26831	229533.A0A1C3YK72	pp	229533.Q4IF76
26832	229533.A0A1C3YK72	pp	229533.I1RUQ2
26833	229533.A0A1C3YK72	pp	229533.I1RAF8
26834	229533.A0A1C3YK72	pp	229533.I1S0R7
26835	229533.A0A1C3YK72	pp	229533.I1RFJ9
26836	229533.A0A1C3YK72	pp	229533.I1RNK2
26837	229533.A0A1C3YK72	pp	229533.I1RRQ3
26838	229533.A0A1C3YK72	pp	229533.I1RNH1
26839	229533.A0A1C3YK72	pp	229533.I1RDA0
26840	229533.A0A1C3YK72	pp	229533.I1RVZ0
26841	229533.A0A1C3YK72	pp	229533.I1RMK3
26842	229533.A0A1C3YK72	pp	229533.Q4HVW2
26843	229533.A0A1C3YK72	pp	229533.I1RTF4
26844	229533.A0A1C3YK72	pp	229533.I1RS30
26845	229533.A0A1C3YK72	pp	229533.Q4HZ47
26846	229533.A0A1C3YK72	pp	229533.I1RAZ8
26847	229533.A0A1C3YK72	pp	229533.I1RQ65
26848	229533.A0A1C3YK72	pp	229533.Q4I5Z5
26849	229533.A0A1C3YK72	pp	229533.Q4IJH1
26850	229533.A0A1C3YK72	pp	229533.I1RC06
26851	229533.A0A1C3YK72	pp	229533.I1RT80
26852	229533.A0A1C3YK72	pp	229533.I1RAC2
26853	229533.A0A1C3YK72	pp	229533.I1RF08
26854	229533.A0A1C3YK72	pp	229533.Q4IFI0
26855	229533.A0A1C3YK72	pp	229533.Q4HZ42
26856	229533.A0A1C3YK72	pp	229533.I1RM06
26857	229533.A0A1C3YK72	pp	229533.Q4HZ68
26858	229533.A0A1C3YK72	pp	229533.I1RG51
26859	229533.A0A1C3YK72	pp	229533.I1RZS6
26860	229533.A0A1C3YK72	pp	229533.I1RX24
26861	229533.A0A1C3YK72	pp	229533.I1RFS3
26862	229533.A0A1C3YK72	pp	229533.I1RNA0
26863	229533.A0A1C3YK72	pp	229533.I1RC29
26864	229533.A0A1C3YK72	pp	229533.V6QYF0

229533.I1S0D8 pp 229533.Q4IA56
229533.I1RT99 pp 229533.Q4I5I4
229533.I1RT99 pp 229533.I1RXU0
229533.I1RT99 pp 229533.V6QUE6
229533.I1RT99 pp 229533.Q4HYB8
229533.I1RT99 pp 229533.Q4I375
229533.I1RT99 pp 229533.Q4I5U9
229533.I1RT99 pp 229533.I1RZB5
229533.I1RT99 pp 229533.I1RTC2
229533.I1RT99 pp 229533.Q4HYQ4
229533.I1RT99 pp 229533.Q4HZ95
229533.I1RT99 pp 229533.I1RVL8
229533.I1RT99 pp 229533.Q4I5W5
229533.I1RT99 pp 229533.Q4IJ11
229533.A0A098DQ43 pp 229533.I1RYX5
229533.A0A098DQ43 pp 229533.I1RBA6
229533.A0A098DQ43 pp 229533.I1S2B9
229533.I1RRP3 pp 229533.I1RSS4
229533.I1RRP3 pp 229533.I1RSD8
229533.I1RRP3 pp 229533.I1S2E8
229533.I1RRP3 pp 229533.I1RSI6
229533.I1RRP3 pp 229533.I1S274
229533.I1RRP3 pp 229533.V6RGN5
229533.I1RRP3 pp 229533.I1RTE9
229533.I1RRP3 pp 229533.I1RRR7
229533.I1RRP3 pp 229533.I1RUG3
229533.I1RRP3 pp 229533.V6RIV9
229533.I1RRP3 pp 229533.I1S2C0
229533.I1RRP3 pp 229533.I1S0K9
229533.I1RRP3 pp 229533.I1RZW8
229533.I1RRP3 pp 229533.Q4I8X0
229533.I1RRP3 pp 229533.I1RZM8
229533.I1RRP3 pp 229533.I1RRT2
229533.I1RRP3 pp 229533.V6RCG5
229533.I1RRP3 pp 229533.I1RRQ3
229533.I1RRP3 pp 229533.I1RRT8
229533.I1RRP3 pp 229533.I1S4T6
229533.I1RRP3 pp 229533.V6R227
229533.I1RRP3 pp 229533.I1S5R1
229533.I1RRP3 pp 229533.I1RW61
229533.I1RRP3 pp 229533.I1RZS6
229533.I1RRP3 pp 229533.I1RZF6
229533.I1RRP3 pp 229533.V6QYF0
229533.I1RRP3 pp 229533.V6R283
229533.I1RRP3 pp 229533.Q4IJ56
229533.I1RRP3 pp 229533.I1RSR3
229533.I1RRP3 pp 229533.I1RSX2
229533.I1RRP3 pp 229533.I1RRQ6
229533.I1RRP3 pp 229533.I1RX14
229533.I1RRP3 pp 229533.I1RTD9
229533.I1RRP3 pp 229533.I1S0L3
229533.I1RRP3 pp 229533.I1RVZ0
229533.I1RRP3 pp 229533.I1RT80
229533.I1RRP3 pp 229533.I1RY63
229533.I1RRP3 pp 229533.I1RTF4
229533.I1RRP3 pp 229533.I1RVQ0
229533.I1RRP3 pp 229533.I1RT33
229533.I1RRP3 pp 229533.I1S0F1
229533.I1RRP3 pp 229533.V6QW22
229533.I1RRP3 pp 229533.V6RBN2
229533.I1RRP3 pp 229533.I1S2L2
229533.I1RRP3 pp 229533.I1RWH8
229533.I1RRP3 pp 229533.I1RT05
229533.A0A0E0S6M0 pp 229533.I1RD64
229533.A0A0E0S6M0 pp 229533.I1S9Y1
229533.A0A0E0S6M0 pp 229533.I1RKJ6
229533.A0A0E0S6M0 pp 229533.I1RPU0
229533.A0A0E0S6M0 pp 229533.I1RMK3
229533.A0A0E0S6M0 pp 229533.I1RDA0
229533.A0A0E0S6M0 pp 229533.I1RNV6
229533.A0A0E0S6M0 pp 229533.I1RT80
229533.A0A0E0S6M0 pp 229533.Q4IJH1
229533.A0A0E0S6M0 pp 229533.A0A0E0SC46

26938 229533.A0A0E0S6M0 pp 229533.I1RPW9
26939 229533.A0A0E0S6M0 pp 229533.I1RAC2
26940 229533.A0A0E0S6M0 pp 229533.I1RZS6
26941 229533.A0A0E0S6M0 pp 229533.I1RX24
26942 229533.A0A0E0S6M0 pp 229533.I1S9U2
26943 229533.A0A0E0S6M0 pp 229533.I1RRT2
26944 229533.A0A0E0S6M0 pp 229533.I1RBL7
26945 229533.A0A0E0S6M0 pp 229533.V6QYF0
26946 229533.A0A0E0S6M0 pp 229533.I1RCL3
26947 229533.A0A0E0S6M0 pp 229533.Q4IPI1
26948 229533.A0A0E0S6M0 pp 229533.I1RQA2
26949 229533.A0A0E0S6M0 pp 229533.I1RBG9
26950 229533.A0A0E0S6M0 pp 229533.I1RAN5
26951 229533.A0A0E0S6M0 pp 229533.I1RG51
26952 229533.A0A0E0S6M0 pp 229533.I1RFS3
26953 229533.A0A0E0S6M0 pp 229533.I1REX4
26954 229533.A0A0E0S6M0 pp 229533.Q4IAS1
26955 229533.A0A0E0S6M0 pp 229533.I1S2C0
26956 229533.A0A0E0S6M0 pp 229533.A0A1C3YLF5
26957 229533.A0A0E0S6M0 pp 229533.I1RTI6
26958 229533.A0A0E0S6M0 pp 229533.A0A1C3YL39
26959 229533.A0A0E0S6M0 pp 229533.I1RC82
26960 229533.A0A0E0S6M0 pp 229533.I1RBC7
26961 229533.A0A0E0S6M0 pp 229533.Q4IBS2
26962 229533.A0A0E0S6M0 pp 229533.I1RTE9
26963 229533.A0A0E0S6M0 pp 229533.I1RF08
26964 229533.A0A0E0S6M0 pp 229533.I1RMN6
26965 229533.A0A0E0S6M0 pp 229533.Q4I327
26966 229533.A0A0E0S6M0 pp 229533.I1RAF8
26967 229533.A0A0E0S6M0 pp 229533.I1RS47
26968 229533.A0A0E0S6M0 pp 229533.I1S296
26969 229533.A0A0E0S6M0 pp 229533.Q4HZ42
26970 229533.A0A0E0S6M0 pp 229533.I1S021
26971 229533.A0A0E0S6M0 pp 229533.Q4HZ68
26972 229533.A0A0E0S6M0 pp 229533.I1RQ31
26973 229533.A0A0E0S6M0 pp 229533.I1RRQ3
26974 229533.A0A0E0S6M0 pp 229533.I1RNH1
26975 229533.A0A0E0S6M0 pp 229533.I1RZE5
26976 229533.A0A0E0S6M0 pp 229533.I1RQJ5
26977 229533.A0A0E0S6M0 pp 229533.A0A0E0SFM9
26978 229533.A0A0E0S6M0 pp 229533.I1RW71
26979 229533.A0A0E0S6M0 pp 229533.I1RRR7
26980 229533.A0A0E0S6M0 pp 229533.I1RVZ0
26981 229533.A0A0E0S6M0 pp 229533.V6R227
26982 229533.A0A0E0S6M0 pp 229533.I1RKL3
26983 229533.A0A0E0S6M0 pp 229533.I1RAZ8
26984 229533.A0A0E0S6M0 pp 229533.Q4I830
26985 229533.A0A0E0S6M0 pp 229533.I1RN99
26986 229533.A0A0E0S6M0 pp 229533.I1RZF6
26987 229533.A0A0E0S6M0 pp 229533.I1RDG0
26988 229533.A0A0E0S6M0 pp 229533.I1RFT1
26989 229533.A0A0E0S6M0 pp 229533.I1RK95
26990 229533.A0A0E0S6M0 pp 229533.A0A1C3YJR3
26991 229533.A0A0E0S6M0 pp 229533.Q4IEK8
26992 229533.A0A0E0S6M0 pp 229533.I1RC03
26993 229533.A0A0E0S6M0 pp 229533.I1S1X9
26994 229533.A0A0E0S6M0 pp 229533.I1R9V7
26995 229533.A0A0E0S6M0 pp 229533.V6R283
26996 229533.A0A0E0S6M0 pp 229533.I1RM96
26997 229533.A0A0E0S6M0 pp 229533.I1RA48
26998 229533.A0A0E0S6M0 pp 229533.I1S4T3
26999 229533.A0A0E0S6M0 pp 229533.I1RSD8
27000 229533.A0A0E0S6M0 pp 229533.A0A1C3YL10
27001 229533.A0A0E0S6M0 pp 229533.I1RG56
27002 229533.A0A0E0S6M0 pp 229533.I1RG11
27003 229533.A0A0E0S6M0 pp 229533.I1RB27
27004 229533.A0A0E0S6M0 pp 229533.I1R9X7
27005 229533.A0A0E0S6M0 pp 229533.I1RC72
27006 229533.A0A0E0S6M0 pp 229533.I1S060
27007 229533.A0A0E0S6M0 pp 229533.I1RC27
27008 229533.I1RFY1 pp 229533.I1RZV5
27009 229533.I1RKF9 pp 229533.I1RSD8
27010 229533.I1RKF9 pp 229533.I1RRI2

27011	229533.I1RKF9	pp	229533.I1S1F3
27012	229533.I1RKF9	pp	229533.I1S296
27013	229533.I1RKF9	pp	229533.V6RGY3
27014	229533.I1RKF9	pp	229533.I1RRQ3
27015	229533.I1RKF9	pp	229533.V6RLW4
27016	229533.I1RKF9	pp	229533.I1RMN6
27017	229533.I1RKF9	pp	229533.I1RW26
27018	229533.I1RKF9	pp	229533.I1RNU6
27019	229533.I1RKF9	pp	229533.I1S0V4
27020	229533.I1RKF9	pp	229533.Q4I8X0
27021	229533.I1RKF9	pp	229533.I1S253
27022	229533.I1RKF9	pp	229533.I1RQ28
27023	229533.I1RKF9	pp	229533.Q4I327
27024	229533.I1RKF9	pp	229533.I1RN99
27025	229533.I1RKF9	pp	229533.I1RM96
27026	229533.I1RKF9	pp	229533.Q4I9U7
27027	229533.I1RKF9	pp	229533.V6RBN2
27028	229533.I1RKF9	pp	229533.I1RZR2
27029	229533.I1RKF9	pp	229533.I1S0K6
27030	229533.I1RKF9	pp	229533.I1RRT8
27031	229533.I1RKF9	pp	229533.Q4IJ56
27032	229533.I1RKF9	pp	229533.I1RQA2
27033	229533.I1RKF9	pp	229533.I1RNF5
27034	229533.I1RKF9	pp	229533.Q4HWB3
27035	229533.I1RKF9	pp	229533.Q4IBS2
27036	229533.I1RKF9	pp	229533.I1RT45
27037	229533.I1RKF9	pp	229533.I1RN51
27038	229533.I1RKF9	pp	229533.I1RM06
27039	229533.I1RKF9	pp	229533.I1RW71
27040	229533.I1RKF9	pp	229533.I1RQJ5
27041	229533.I1RKF9	pp	229533.I1RT05
27042	229533.I1RKF9	pp	229533.I1RM49
27043	229533.I1RKF9	pp	229533.I1RZJ6
27044	229533.I1RKF9	pp	229533.I1RMN5
27045	229533.Q4HZ68	pp	229533.V6QYF0
27046	229533.Q4HZ68	pp	229533.Q4IJH1
27047	229533.Q4HZ68	pp	229533.Q4IPI1
27048	229533.Q4HZ68	pp	229533.Q4INZ9
27049	229533.Q4HZ68	pp	229533.Q4IFI0
27050	229533.Q4HZ68	pp	229533.Q4IBS2
27051	229533.Q4HZ68	pp	229533.Q4IAA0
27052	229533.Q4HZ68	pp	229533.V6R227
27053	229533.Q4HZ68	pp	229533.Q4I5M4
27054	229533.Q4HZ68	pp	229533.Q4I830
27055	229533.Q4HZ68	pp	229533.Q4IF76
27056	229533.Q4HZ68	pp	229533.Q4IJ56
27057	229533.Q4HZ68	pp	229533.Q4IAS1
27058	229533.Q4HZ68	pp	229533.V6R283
27059	229533.Q4HZ68	pp	229533.Q4IQW0
27060	229533.Q4HZ68	pp	229533.Q4I327
27061	229533.Q4HZ68	pp	229533.Q4I662
27062	229533.Q4HZ68	pp	229533.Q4IEK8
27063	229533.Q4HZ68	pp	229533.Q4IQ72
27064	229533.Q4HZ68	pp	229533.V6RIS2
27065	229533.Q4HZ68	pp	229533.Q4IEV5
27066	229533.Q4HZ68	pp	229533.Q4IN63
27067	229533.Q4HZ68	pp	229533.V6RGY3
27068	229533.Q4HZ68	pp	229533.V6RGN5
27069	229533.Q4HZ68	pp	229533.Q4I7K4
27070	229533.Q4HZ68	pp	229533.V6RY33
27071	229533.I1RD31	pp	229533.I1RWD4
27072	229533.I1RD31	pp	229533.I1RTF4
27073	229533.I1RD31	pp	229533.I1RRT2
27074	229533.I1RD31	pp	229533.I1RSL8
27075	229533.I1RD31	pp	229533.Q4IR18
27076	229533.I1RD31	pp	229533.V6R227
27077	229533.I1RD31	pp	229533.I1RSM3
27078	229533.Q4IAS1	pp	229533.Q4INZ9
27079	229533.Q4IAS1	pp	229533.V6RG22
27080	229533.Q4IAS1	pp	229533.Q4IPI1
27081	229533.Q4IAS1	pp	229533.Q4IJ56
27082	229533.Q4IAS1	pp	229533.V6QYF0
27083	229533.Q4IAS1	pp	229533.Q4IEK8

27084 229533.Q4IAS1 pp 229533.Q4IPN9
27085 229533.Q4IAS1 pp 229533.V6R227
27086 229533.Q4IAS1 pp 229533.Q4IBS2
27087 229533.Q4IAS1 pp 229533.Q4IPB3
27088 229533.Q4IAS1 pp 229533.Q4IJH1
27089 229533.Q4IAS1 pp 229533.V6QV38
27090 229533.Q4IAS1 pp 229533.Q4IFI0
27091 229533.Q4IAS1 pp 229533.Q4IN63
27092 229533.Q4IAS1 pp 229533.V6RGN5
27093 229533.Q4IAS1 pp 229533.V6RGY3
27094 229533.Q4IAS1 pp 229533.V6R283
27095 229533.Q4IAS1 pp 229533.V6RY33
27096 229533.Q4IAS1 pp 229533.V6RIS2
27097 229533.Q4IAS1 pp 229533.Q4IR09
27098 229533.Q4IAS1 pp 229533.Q4IEV5
27099 229533.Q4IAS1 pp 229533.Q4IF76
27100 229533.Q4IAS1 pp 229533.Q4IQW0
27101 229533.I1S6F0 pp 229533.Q4HZ68
27102 229533.I1S6F0 pp 229533.V6RIS2
27103 229533.I1S6F0 pp 229533.V6RY33
27104 229533.I1S6F0 pp 229533.V6RGN5
27105 229533.I1S6F0 pp 229533.I1S855
27106 229533.I1S6F0 pp 229533.Q4I327
27107 229533.I1S6F0 pp 229533.V6RGY3
27108 229533.I1S6F0 pp 229533.V6RIV9
27109 229533.I1S6F0 pp 229533.Q4IF76
27110 229533.I1S6F0 pp 229533.Q4IAS1
27111 229533.I1S6F0 pp 229533.Q4I662
27112 229533.I1S6F0 pp 229533.Q4HZ42
27113 229533.I1S6F0 pp 229533.I1S8E2
27114 229533.I1S6F0 pp 229533.Q4IAA0
27115 229533.I1S6F0 pp 229533.Q4IJ56
27116 229533.I1S6F0 pp 229533.V6RAC9
27117 229533.I1S6F0 pp 229533.Q4I830
27118 229533.I1S6F0 pp 229533.Q4HXF0
27119 229533.I1S6F0 pp 229533.V6R283
27120 229533.I1S6F0 pp 229533.V6QYF0
27121 229533.I1S6F0 pp 229533.Q4IBS2
27122 229533.I1S6F0 pp 229533.V6R227
27123 229533.I1S6F0 pp 229533.Q4IQW0
27124 229533.I1S6F0 pp 229533.Q4IFI0
27125 229533.I1S6F0 pp 229533.Q4HVW2
27126 229533.I1S6F0 pp 229533.V6RCG5
27127 229533.I1S6F0 pp 229533.V6QUE6
27128 229533.I1S6F0 pp 229533.I1S9U2
27129 229533.I1S6F0 pp 229533.Q4IJH1
27130 229533.I1S6F0 pp 229533.V6QW22
27131 229533.I1S6F0 pp 229533.Q4HZ47
27132 229533.I1S6F0 pp 229533.Q4IPI1
27133 229533.I1S6F0 pp 229533.Q4INZ9
27134 229533.A0A0E0RNT7 pp 229533.Q4IFI0
27135 229533.A0A0E0RNT7 pp 229533.I1RN51
27136 229533.A0A0E0RNT7 pp 229533.I1RKC6
27137 229533.A0A0E0RNT7 pp 229533.I1RAZ8
27138 229533.A0A0E0RNT7 pp 229533.I1RML0
27139 229533.A0A0E0RNT7 pp 229533.I1RAC2
27140 229533.A0A0E0RNT7 pp 229533.A0A1C3YHT9
27141 229533.A0A0E0RNT7 pp 229533.I1RF19
27142 229533.A0A0E0RNT7 pp 229533.I1RFJ9
27143 229533.A0A0E0RNT7 pp 229533.Q4HZ68
27144 229533.A0A0E0RNT7 pp 229533.I1S296
27145 229533.A0A0E0RNT7 pp 229533.I1RF08
27146 229533.A0A0E0RNT7 pp 229533.I1RC06
27147 229533.A0A0E0RNT7 pp 229533.I1RSD8
27148 229533.A0A0E0RNT7 pp 229533.I1RG51
27149 229533.A0A0E0RNT7 pp 229533.I1RWJ4
27150 229533.A0A0E0RNT7 pp 229533.I1RFS3
27151 229533.A0A0E0RNT7 pp 229533.Q4I662
27152 229533.A0A0E0RNT7 pp 229533.A0A1C3YK72
27153 229533.A0A0E0RNT7 pp 229533.I1RPY1
27154 229533.A0A0E0RNT7 pp 229533.I1RW26
27155 229533.A0A0E0RNT7 pp 229533.I1RJZ6
27156 229533.A0A0E0RNT7 pp 229533.I1S1X9

27157	229533.A0A0E0RNT7	pp	229533.I1RG56
27158	229533.A0A0E0RNT7	pp	229533.A0A1C3YN63
27159	229533.A0A0E0RNT7	pp	229533.I1RC33
27160	229533.A0A0E0RNT7	pp	229533.A0A0E0S6M0
27161	229533.A0A0E0RNT7	pp	229533.I1S6F0
27162	229533.A0A0E0RNT7	pp	229533.I1RY63
27163	229533.A0A0E0RNT7	pp	229533.I1RVZ0
27164	229533.A0A0E0RNT7	pp	229533.I1RX24
27165	229533.A0A0E0RNT7	pp	229533.I1RM96
27166	229533.A0A0E0RNT7	pp	229533.I1RQA2
27167	229533.A0A0E0RNT7	pp	229533.I1RRQ3
27168	229533.A0A0E0RNT7	pp	229533.I1R9V7
27169	229533.A0A0E0RNT7	pp	229533.I1RBG9
27170	229533.A0A0E0RNT7	pp	229533.I1S558
27171	229533.A0A0E0RNT7	pp	229533.V6QYF0
27172	229533.A0A0E0RNT7	pp	229533.I1RFG0
27173	229533.A0A0E0RNT7	pp	229533.A0A0E0RP78
27174	229533.A0A0E0RNT7	pp	229533.Q4IEK8
27175	229533.A0A0E0RNT7	pp	229533.I1RPU0
27176	229533.A0A0E0RNT7	pp	229533.I1RA48
27177	229533.A0A0E0RNT7	pp	229533.I1RAN5
27178	229533.A0A0E0RNT7	pp	229533.I1RS47
27179	229533.A0A0E0RNT7	pp	229533.I1RNA0
27180	229533.A0A0E0RNT7	pp	229533.I1RKJ6
27181	229533.A0A0E0RNT7	pp	229533.I1RSS4
27182	229533.A0A0E0RNT7	pp	229533.I1RCL3
27183	229533.A0A0E0RNT7	pp	229533.I1RZF6
27184	229533.A0A0E0RNT7	pp	229533.I1RM06
27185	229533.A0A0E0RNT7	pp	229533.I1RN99
27186	229533.A0A0E0RNT7	pp	229533.Q4HZ42
27187	229533.A0A0E0RNT7	pp	229533.Q4I5M4
27188	229533.A0A0E0RNT7	pp	229533.I1RBL7
27189	229533.A0A0E0RNT7	pp	229533.I1RT33
27190	229533.A0A0E0RNT7	pp	229533.I1RR72
27191	229533.A0A0E0RNT7	pp	229533.I1RD64
27192	229533.A0A0E0RNT7	pp	229533.I1RDG0
27193	229533.A0A0E0RNT7	pp	229533.I1RPW9
27194	229533.A0A0E0RNT7	pp	229533.I1RZS6
27195	229533.A0A0E0RNT7	pp	229533.I1RSM3
27196	229533.A0A0E0RNT7	pp	229533.A0A1C3YJR3
27197	229533.A0A0E0RNT7	pp	229533.I1RQ65
27198	229533.A0A0E0RNT7	pp	229533.I1RK95
27199	229533.A0A0E0RNT7	pp	229533.I1RQ28
27200	229533.A0A0E0RNT7	pp	229533.I1S9U2
27201	229533.A0A0E0RNT7	pp	229533.I1RCN5
27202	229533.A0A0E0RNT7	pp	229533.V6R227
27203	229533.A0A0E0RNT7	pp	229533.I1RSR3
27204	229533.A0A0E0RNT7	pp	229533.I1RRR7
27205	229533.A0A0E0RNT7	pp	229533.I1RX14
27206	229533.A0A0E0RNT7	pp	229533.Q4IAS1
27207	229533.A0A0E0RNT7	pp	229533.V6RGY3
27208	229533.A0A0E0RNT7	pp	229533.I1RMN6
27209	229533.A0A0E0RNT7	pp	229533.I1RMD5
27210	229533.A0A0E0RNT7	pp	229533.I1S060
27211	229533.A0A0E0RNT7	pp	229533.I1RRT2
27212	229533.A0A0E0RNT7	pp	229533.Q4IF76
27213	229533.A0A0E0RNT7	pp	229533.Q4IPI1
27214	229533.A0A0E0RNT7	pp	229533.A0A1C3YMW0
27215	229533.A0A0E0RNT7	pp	229533.Q4IBS2
27216	229533.A0A0E0RNT7	pp	229533.I1RC03
27217	229533.A0A0E0RNT7	pp	229533.V6R283
27218	229533.A0A0E0RNT7	pp	229533.I1RRI2
27219	229533.A0A0E0RNT7	pp	229533.A0A0E0SJN2
27220	229533.A0A0E0RNT7	pp	229533.A0A0E0S621
27221	229533.A0A0E0RNT7	pp	229533.A0A0E0S7B3
27222	229533.A0A0E0RNT7	pp	229533.I1RC72
27223	229533.A0A0E0RNT7	pp	229533.I1RFW5
27224	229533.A0A0E0RNT7	pp	229533.I1RT80
27225	229533.A0A0E0RNT7	pp	229533.I1RJL4
27226	229533.A0A0E0RNT7	pp	229533.I1RC27
27227	229533.A0A0E0RNT7	pp	229533.I1RC82
27228	229533.A0A0E0RNT7	pp	229533.I1RKL3
27229	229533.A0A0E0RNT7	pp	229533.Q4IJ56

229533.A0A0E0RNT7	pp	229533.Q4IJH1
229533.A0A0E0RNT7	pp	229533.Q4I830
229533.A0A0E0RNT7	pp	229533.I1RR00
229533.A0A0E0RNT7	pp	229533.I1RNU6
229533.A0A0E0RNT7	pp	229533.I1RBC7
229533.A0A0E0RNT7	pp	229533.I1RFN1
229533.A0A0E0RNT7	pp	229533.Q4IQW0
229533.A0A0E0RNT7	pp	229533.A0A1C3YJG0
229533.A0A0E0RNT7	pp	229533.I1RT98
229533.A0A0E0RNT7	pp	229533.I1RQ31
229533.A0A0E0RNT7	pp	229533.I1RTF4
229533.A0A0E0RNT7	pp	229533.I1RZJ6
229533.A0A0E0RNT7	pp	229533.Q4HVW2
229533.A0A0E0RNT7	pp	229533.Q4INZ9
229533.A0A0E0RNT7	pp	229533.Q4IAA0
229533.A0A0E0RNT7	pp	229533.I1RMK3
229533.A0A0E0RNT7	pp	229533.I1RDA0
229533.A0A0E0RNT7	pp	229533.I1RQJ5
229533.A0A0E0RNT7	pp	229533.I1RBN1
229533.A0A0E0RNT7	pp	229533.I1RZ99
229533.A0A0E0RNT7	pp	229533.I1S2C0
229533.A0A0E0RNT7	pp	229533.I1RMC0
229533.A0A0E0RNT7	pp	229533.Q4IN63
229533.A0A0E0RNT7	pp	229533.I1RRJ8
229533.A0A0E0RNT7	pp	229533.I1RTE9
229533.A0A0E0RNT7	pp	229533.I1R9X7
229533.A0A0E0RNT7	pp	229533.A0A1C3YIN1
229533.A0A0E0RNT7	pp	229533.A0A0E0SFM9
229533.A0A0E0RNT7	pp	229533.I1RB27
229533.A0A0E0RNT7	pp	229533.I1RAF8
229533.A0A0E0RNT7	pp	229533.Q4I327
229533.A0A0E0RNT7	pp	229533.I1REF2
229533.A0A0E0RNT7	pp	229533.I1RNH6
229533.A0A0E0RNT7	pp	229533.I1S021
229533.A0A0E0RNT7	pp	229533.A0A1C3YLF5
229533.A0A0E0RNT7	pp	229533.I1RSI6
229533.I1RWT7	pp	229533.V6R227
229533.I1RWT7	pp	229533.I1RY98
229533.I1RWT7	pp	229533.Q4IR18
229533.A0A1C3YHQ4	pp	229533.I1RB50
229533.A0A1C3YHQ4	pp	229533.I1RCI4
229533.A0A1C3YHQ4	pp	229533.I1RCD6
229533.A0A1C3YHQ4	pp	229533.I1RZG7
229533.A0A1C3YHQ4	pp	229533.I1RCQ6
229533.A0A1C3YHQ4	pp	229533.I1RX81
229533.A0A1C3YHQ4	pp	229533.A0A1C3YL11
229533.A0A1C3YHQ4	pp	229533.I1S0B6
229533.A0A1C3YHQ4	pp	229533.I1RTF4
229533.A0A1C3YHQ4	pp	229533.I1RD52
229533.A0A1C3YHQ4	pp	229533.I1RAZ8
229533.A0A1C3YHQ4	pp	229533.Q4IPN9
229533.A0A1C3YHQ4	pp	229533.I1RPY7
229533.A0A1C3YHQ4	pp	229533.I1RG17
229533.A0A1C3YHQ4	pp	229533.I1RQ31
229533.A0A1C3YHQ4	pp	229533.I1RLU5
229533.A0A1C3YHQ4	pp	229533.I1RW71
229533.A0A1C3YHQ4	pp	229533.I1S7M2
229533.A0A1C3YHQ4	pp	229533.I1RK19
229533.A0A1C3YHQ4	pp	229533.I1RSA8
229533.A0A1C3YHQ4	pp	229533.I1RT35
229533.A0A1C3YHQ4	pp	229533.I1S7M3
229533.A0A1C3YHQ4	pp	229533.I1RPA0
229533.A0A1C3YHQ4	pp	229533.I1RA36
229533.A0A1C3YHQ4	pp	229533.I1RWX8
229533.A0A1C3YHQ4	pp	229533.I1RAC1
229533.A0A1C3YHQ4	pp	229533.A0A1C3YJ42
229533.A0A1C3YHQ4	pp	229533.A0A1C3YJW0
229533.A0A1C3YHQ4	pp	229533.I1RMV5
229533.A0A1C3YHQ4	pp	229533.V6R227
229533.A0A1C3YHQ4	pp	229533.I1RJZ7
229533.A0A1C3YHQ4	pp	229533.I1RTS2
229533.A0A1C3YHQ4	pp	229533.I1RE71
229533.A0A1C3YHQ4	pp	229533.I1S2C0

229533.A0A1C3YHQ4	pp	229533.I1RST1
229533.A0A1C3YHQ4	pp	229533.I1RG90
229533.A0A1C3YHQ4	pp	229533.I1RX24
229533.A0A1C3YHQ4	pp	229533.I1RBV3
229533.A0A1C3YHQ4	pp	229533.A0A1C3YI62
229533.A0A1C3YHQ4	pp	229533.A0A1C3YHW5
229533.A0A1C3YHQ4	pp	229533.A0A1C3YKM6
229533.A0A1C3YHQ4	pp	229533.V6QVD5
229533.A0A1C3YHQ4	pp	229533.I1RXF2
229533.A0A1C3YHQ4	pp	229533.I1S0B1
229533.A0A1C3YHQ4	pp	229533.I1RZ40
229533.A0A1C3YHQ4	pp	229533.I1RMY3
229533.A0A1C3YHQ4	pp	229533.I1RX47
229533.A0A1C3YHQ4	pp	229533.I1RQ65
229533.A0A1C3YHQ4	pp	229533.I1RIR9
229533.A0A1C3YHQ4	pp	229533.A0A1C3YNC3
229533.A0A1C3YHQ4	pp	229533.I1RAU7
229533.A0A1C3YHQ4	pp	229533.I1RQE7
229533.A0A1C3YHQ4	pp	229533.I1S4M5
229533.A0A1C3YHQ4	pp	229533.I1S1X9
229533.A0A1C3YHQ4	pp	229533.I1RCA7
229533.A0A1C3YHQ4	pp	229533.I1RVT9
229533.A0A1C3YHQ4	pp	229533.I1RC84
229533.A0A1C3YHQ4	pp	229533.I1RSA4
229533.A0A1C3YHQ4	pp	229533.I1RT47
229533.A0A1C3YHQ4	pp	229533.I1RK92
229533.A0A1C3YHQ4	pp	229533.I1RN09
229533.A0A1C3YHQ4	pp	229533.I1S471
229533.A0A1C3YHQ4	pp	229533.I1RBH8
229533.A0A1C3YHQ4	pp	229533.V6RY33
229533.A0A1C3YHQ4	pp	229533.I1RFQ1
229533.A0A1C3YHQ4	pp	229533.I1RSD4
229533.A0A1C3YHQ4	pp	229533.V6RIS2
229533.A0A1C3YHQ4	pp	229533.I1RNB8
229533.A0A1C3YHQ4	pp	229533.I1RSH9
229533.I1RF08	pp	229533.I1RX24
229533.I1RF08	pp	229533.I1RFS3
229533.I1RF08	pp	229533.I1RJZ7
229533.I1RF08	pp	229533.I1RRQ3
229533.I1RF08	pp	229533.I1RK96
229533.I1RF08	pp	229533.I1RZS6
229533.I1RF08	pp	229533.I1RKJ6
229533.I1RF08	pp	229533.Q4IF76
229533.I1RF08	pp	229533.I1RFG0
229533.I1RF08	pp	229533.I1RZ40
229533.I1RF08	pp	229533.I1RS47
229533.I1RF08	pp	229533.I1RRJ8
229533.I1RF08	pp	229533.I1RKL3
229533.I1RF08	pp	229533.I1RQ65
229533.I1RF08	pp	229533.Q4HZ42
229533.I1RF08	pp	229533.Q4HZ68
229533.I1RF08	pp	229533.I1S296
229533.I1RF08	pp	229533.I1RFN1
229533.I1RF08	pp	229533.V6R283
229533.I1RF08	pp	229533.I1RKC6
229533.I1RF08	pp	229533.V6RGY3
229533.I1RF08	pp	229533.I1S1X9
229533.I1RF08	pp	229533.I1S2C0
229533.I1RF08	pp	229533.Q4IQW0
229533.I1RF08	pp	229533.I1RPJ3
229533.I1RF08	pp	229533.Q4HWE2
229533.I1RF08	pp	229533.Q4IAS1
229533.I1RF08	pp	229533.I1RLV9
229533.I1RF08	pp	229533.I1RTE9
229533.I1RF08	pp	229533.I1RRT2
229533.I1RF08	pp	229533.I1RQS5
229533.I1RF08	pp	229533.I1RSS4
229533.I1RF08	pp	229533.I1RJL4
229533.I1RF08	pp	229533.I1RRI2
229533.I1RF08	pp	229533.I1RSD8
229533.I1RF08	pp	229533.V6RY33
229533.I1RF08	pp	229533.I1RQE7
229533.I1RF08	pp	229533.I1S0J1

229533.I1RF08 pp 229533.I1S060
229533.I1RF08 pp 229533.I1S2E8
229533.I1RF08 pp 229533.I1S4T3
229533.I1RF08 pp 229533.I1RSD4
229533.I1RF08 pp 229533.I1RRR7
229533.I1RF08 pp 229533.I1S471
229533.I1RF08 pp 229533.Q4IFI0
229533.I1RF08 pp 229533.I1RM96
229533.I1RF08 pp 229533.I1RPY1
229533.I1RF08 pp 229533.V6QYF0
229533.I1RF08 pp 229533.V6RGN5
229533.I1RF08 pp 229533.I1RNA0
229533.I1RF08 pp 229533.I1RM25
229533.I1RF08 pp 229533.I1RNU6
229533.I1RF08 pp 229533.V6RIS2
229533.I1RF08 pp 229533.I1RG51
229533.I1RF08 pp 229533.I1RM06
229533.I1RF08 pp 229533.I1RT05
229533.I1RF08 pp 229533.I1RMN6
229533.I1RF08 pp 229533.I1RPW9
229533.I1RF08 pp 229533.I1RQM6
229533.I1RF08 pp 229533.I1RT33
229533.I1RF08 pp 229533.I1RSI6
229533.I1RF08 pp 229533.I1RM67
229533.I1RF08 pp 229533.I1S9U2
229533.I1RF08 pp 229533.I1RLU5
229533.I1RF08 pp 229533.Q4I327
229533.I1RF08 pp 229533.I1RT80
229533.I1RF08 pp 229533.Q4HZ47
229533.I1RF08 pp 229533.I1S6F0
229533.I1RF08 pp 229533.Q4IPI1
229533.I1RF08 pp 229533.I1RW71
229533.I1RF08 pp 229533.Q4IJH1
229533.I1RF08 pp 229533.I1RNH1
229533.I1RF08 pp 229533.I1RTF4
229533.I1RF08 pp 229533.I1RZJ6
229533.I1RF08 pp 229533.I1RUQ2
229533.I1RF08 pp 229533.I1RQJ5
229533.I1RF08 pp 229533.I1RMK3
229533.I1RF08 pp 229533.Q4HVW2
229533.I1RF08 pp 229533.I1RKQ5
229533.I1RF08 pp 229533.Q4INZ9
229533.I1RF08 pp 229533.V6R227
229533.I1RF08 pp 229533.I1RK95
229533.I1RF08 pp 229533.I1RT98
229533.I1RF08 pp 229533.I1RVZ0
229533.I1RF08 pp 229533.I1RG56
229533.I1RF08 pp 229533.Q4IEK8
229533.I1RF08 pp 229533.I1RQ28
229533.I1RF08 pp 229533.I1S0Z9
229533.I1RF08 pp 229533.Q4IJ56
229533.I1RF08 pp 229533.I1RF19
229533.I1RF08 pp 229533.I1RZZ1
229533.I1RF08 pp 229533.I1RN99
229533.I1RF08 pp 229533.I1RSR3
229533.I1RF08 pp 229533.I1RZB5
229533.I1RF08 pp 229533.I1RQA2
229533.I1RF08 pp 229533.V6QUE6
229533.I1RF08 pp 229533.I1RMD5
229533.I1RF08 pp 229533.I1RQ31
229533.I1RF08 pp 229533.Q4IBS2
229533.I1RF08 pp 229533.I1RMC0
229533.I1RF08 pp 229533.I1RZF6
229533.I1RF08 pp 229533.I1RNH6
229533.I1RF08 pp 229533.I1S021
229533.I1RJH5 pp 229533.I1RQ80
229533.I1RJH5 pp 229533.I1S1V5
229533.I1RJH5 pp 229533.Q4IBX0
229533.I1RJH5 pp 229533.I1RQ16
229533.I1RJH5 pp 229533.I1RRB0
229533.I1RJH5 pp 229533.I1S1C8
229533.I1RJH5 pp 229533.I1RXT9
229533.I1RJH5 pp 229533.Q4I0J6

27449	229533.I1S364	pp	229533.V6RPN0
27450	229533.I1S364	pp	229533.I1S5R5
27451	229533.I1RJL4	pp	229533.V6RCG5
27452	229533.I1RJL4	pp	229533.I1RMC0
27453	229533.I1RJL4	pp	229533.I1RQ31
27454	229533.I1RJL4	pp	229533.I1RY63
27455	229533.I1RJL4	pp	229533.I1RZE5
27456	229533.I1RJL4	pp	229533.I1RZM8
27457	229533.I1RJL4	pp	229533.I1RSC6
27458	229533.I1RJL4	pp	229533.I1RNH6
27459	229533.I1RJL4	pp	229533.I1S021
27460	229533.I1RJL4	pp	229533.I1RW99
27461	229533.I1RJL4	pp	229533.I1RN99
27462	229533.I1RJL4	pp	229533.I1RQ28
27463	229533.I1RJL4	pp	229533.I1RMN7
27464	229533.I1RJL4	pp	229533.V6QUE6
27465	229533.I1RJL4	pp	229533.I1RK95
27466	229533.I1RJL4	pp	229533.I1S2U2
27467	229533.I1RJL4	pp	229533.I1RZF6
27468	229533.I1RJL4	pp	229533.I1S9U1
27469	229533.I1RJL4	pp	229533.Q4IBS2
27470	229533.I1RJL4	pp	229533.V6R227
27471	229533.I1RJL4	pp	229533.I1RX08
27472	229533.I1RJL4	pp	229533.I1RZZ1
27473	229533.I1RJL4	pp	229533.I1RR24
27474	229533.I1RJL4	pp	229533.I1RTE4
27475	229533.I1RJL4	pp	229533.I1RKQ5
27476	229533.I1RJL4	pp	229533.Q4IPI1
27477	229533.I1RJL4	pp	229533.I1S9U2
27478	229533.I1RJL4	pp	229533.I1RXD7
27479	229533.I1RJL4	pp	229533.Q4IEK8
27480	229533.I1RJL4	pp	229533.I1S0R7
27481	229533.I1RJL4	pp	229533.I1RPU0
27482	229533.I1RJL4	pp	229533.Q4I0J6
27483	229533.I1RJL4	pp	229533.I1RWJ8
27484	229533.I1RJL4	pp	229533.I1RUQ2
27485	229533.I1RJL4	pp	229533.I1RM67
27486	229533.I1RJL4	pp	229533.Q4INZ9
27487	229533.I1RJL4	pp	229533.I1S0M9
27488	229533.I1RJL4	pp	229533.I1RT98
27489	229533.I1RJL4	pp	229533.I1RNV6
27490	229533.I1RJL4	pp	229533.Q4IJ56
27491	229533.I1RJL4	pp	229533.I1RT80
27492	229533.I1RJL4	pp	229533.I1RX14
27493	229533.I1RJL4	pp	229533.I1RTF4
27494	229533.I1RJL4	pp	229533.I1RSM3
27495	229533.I1RJL4	pp	229533.Q4IJH1
27496	229533.I1RJL4	pp	229533.I1RZJ6
27497	229533.I1RJL4	pp	229533.I1RPH9
27498	229533.I1RJL4	pp	229533.I1RSR3
27499	229533.I1RJL4	pp	229533.I1S0L3
27500	229533.I1RJL4	pp	229533.Q4HZ47
27501	229533.I1RJL4	pp	229533.I1RJU3
27502	229533.I1RJL4	pp	229533.Q4HVW2
27503	229533.I1RJL4	pp	229533.I1RQA2
27504	229533.I1RJL4	pp	229533.I1S6F0
27505	229533.I1RJL4	pp	229533.I1RMK3
27506	229533.I1RJL4	pp	229533.I1RNF5
27507	229533.I1RJL4	pp	229533.I1RZB5
27508	229533.I1RJL4	pp	229533.I1S6U8
27509	229533.I1RJL4	pp	229533.I1RVQ0
27510	229533.I1RJL4	pp	229533.I1RNH1
27511	229533.I1RJL4	pp	229533.I1RMD5
27512	229533.I1RJL4	pp	229533.I1RW90
27513	229533.I1RJL4	pp	229533.I1RW71
27514	229533.I1RJL4	pp	229533.I1RSR8
27515	229533.I1RJL4	pp	229533.I1RTD9
27516	229533.I1RJL4	pp	229533.I1RQM6
27517	229533.I1RJL4	pp	229533.I1RT05
27518	229533.I1RJL4	pp	229533.I1RZ99
27519	229533.I1RJL4	pp	229533.I1RR00
27520	229533.I1RJL4	pp	229533.I1RVZ0
27521	229533.I1RJL4	pp	229533.I1RNK2

27522 229533.I1RJL4 pp 229533.Q4IFI0
27523 229533.I1RJL4 pp 229533.I1RL23
27524 229533.I1RJL4 pp 229533.I1RSQ7
27525 229533.I1RJL4 pp 229533.I1RQJ5
27526 229533.I1RJL4 pp 229533.I1RRI2
27527 229533.I1RJL4 pp 229533.V6RGY0
27528 229533.I1RJL4 pp 229533.V6QW22
27529 229533.I1RJL4 pp 229533.V6QYF0
27530 229533.I1RJL4 pp 229533.I1RSI6
27531 229533.I1RJL4 pp 229533.I1RW25
27532 229533.I1RJL4 pp 229533.I1RNY2
27533 229533.I1RJL4 pp 229533.I1RNA0
27534 229533.I1RJL4 pp 229533.Q4HZ68
27535 229533.I1RJL4 pp 229533.I1RM06
27536 229533.I1RJL4 pp 229533.V6RGN5
27537 229533.I1RJL4 pp 229533.I1RKC6
27538 229533.I1RJL4 pp 229533.I1RMN6
27539 229533.I1RJL4 pp 229533.Q4I327
27540 229533.I1RJL4 pp 229533.Q4I830
27541 229533.I1RJL4 pp 229533.I1RRR7
27542 229533.I1RJL4 pp 229533.I1S1X9
27543 229533.I1RJL4 pp 229533.I1S1J6
27544 229533.I1RJL4 pp 229533.I1RS64
27545 229533.I1RJL4 pp 229533.I1S4T3
27546 229533.I1RJL4 pp 229533.I1RRJ8
27547 229533.I1RJL4 pp 229533.I1RWF7
27548 229533.I1RJL4 pp 229533.I1RSS4
27549 229533.I1RJL4 pp 229533.I1RN63
27550 229533.I1RJL4 pp 229533.I1S2E8
27551 229533.I1RJL4 pp 229533.I1RSD8
27552 229533.I1RJL4 pp 229533.V6RIV9
27553 229533.I1RJL4 pp 229533.I1RPY1
27554 229533.I1RJL4 pp 229533.I1RM96
27555 229533.I1RJL4 pp 229533.V6R283
27556 229533.I1RJL4 pp 229533.I1RQE7
27557 229533.I1RJL4 pp 229533.I1S9Y1
27558 229533.I1RJL4 pp 229533.I1RKJ6
27559 229533.I1RJL4 pp 229533.I1RNU6
27560 229533.I1RJL4 pp 229533.I1S1B6
27561 229533.I1RJL4 pp 229533.O74638
27562 229533.I1RJL4 pp 229533.Q4IQW0
27563 229533.I1RJL4 pp 229533.I1RW46
27564 229533.I1RJL4 pp 229533.I1RJV4
27565 229533.I1RJL4 pp 229533.I1S0J1
27566 229533.I1RJL4 pp 229533.I1S274
27567 229533.I1RJL4 pp 229533.I1RLV9
27568 229533.I1RJL4 pp 229533.I1RRT8
27569 229533.I1RJL4 pp 229533.Q4IAS1
27570 229533.I1RJL4 pp 229533.V6RGY3
27571 229533.I1RJL4 pp 229533.I1S8E2
27572 229533.I1RJL4 pp 229533.I1RRT2
27573 229533.I1RJL4 pp 229533.I1RJW6
27574 229533.I1RJL4 pp 229533.Q4IF76
27575 229533.I1RJL4 pp 229533.I1S253
27576 229533.I1RJL4 pp 229533.I1S1U7
27577 229533.I1RJL4 pp 229533.I1S558
27578 229533.I1RJL4 pp 229533.I1RT04
27579 229533.I1RJL4 pp 229533.I1RQS5
27580 229533.I1RJL4 pp 229533.I1RS47
27581 229533.I1RJL4 pp 229533.I1RJV3
27582 229533.I1RJL4 pp 229533.I1RX24
27583 229533.I1RJL4 pp 229533.I1RJZ7
27584 229533.I1RJL4 pp 229533.I1S8R3
27585 229533.I1RJL4 pp 229533.I1RQ65
27586 229533.I1RJL4 pp 229533.I1RR72
27587 229533.I1RJL4 pp 229533.Q4HZ42
27588 229533.I1RJL4 pp 229533.I1RZS6
27589 229533.I1RJL4 pp 229533.Q4HZK7
27590 229533.I1RJL4 pp 229533.I1RS30
27591 229533.I1RJL4 pp 229533.Q4IEV5
27592 229533.I1RJL4 pp 229533.I1S2C0
27593 229533.I1RJL4 pp 229533.I1RTE9
27594 229533.I1RJL4 pp 229533.I1RW61

229533.I1RJL4 pp 229533.I1RYT5
229533.I1RJL4 pp 229533.I1RK79
229533.I1RJL4 pp 229533.I1RRP3
229533.I1RJL4 pp 229533.Q4I662
229533.I1RJL4 pp 229533.I1RRQ3
229533.I1RJL4 pp 229533.I1RWJ4
229533.I1RJL4 pp 229533.I1RPM4
229533.I1RJL4 pp 229533.I1RKL3
229533.I1RQQ8 pp 229533.I1RT75
229533.I1RQQ8 pp 229533.I1RRM9
229533.I1RQQ8 pp 229533.I1RVZ0
229533.I1S5T5 pp 229533.V6RPN0
229533.A0A1C3YN63 pp 229533.I1RQ65
229533.A0A1C3YN63 pp 229533.I1RT33
229533.A0A1C3YN63 pp 229533.I1S0L3
229533.A0A1C3YN63 pp 229533.Q4HZ47
229533.A0A1C3YN63 pp 229533.I1RS30
229533.A0A1C3YN63 pp 229533.I1RRP3
229533.A0A1C3YN63 pp 229533.I1S6F0
229533.A0A1C3YN63 pp 229533.I1RRQ3
229533.A0A1C3YN63 pp 229533.I1RNA0
229533.A0A1C3YN63 pp 229533.I1RFG0
229533.A0A1C3YN63 pp 229533.I1RNH1
229533.A0A1C3YN63 pp 229533.Q4IQK7
229533.A0A1C3YN63 pp 229533.I1RC29
229533.A0A1C3YN63 pp 229533.I1RVQ0
229533.A0A1C3YN63 pp 229533.I1RBG9
229533.A0A1C3YN63 pp 229533.Q4I830
229533.A0A1C3YN63 pp 229533.V6QYF0
229533.A0A1C3YN63 pp 229533.I1RRQ6
229533.A0A1C3YN63 pp 229533.I1S2L2
229533.A0A1C3YN63 pp 229533.I1RKL3
229533.A0A1C3YN63 pp 229533.I1RBC9
229533.A0A1C3YN63 pp 229533.I1RKF9
229533.A0A1C3YN63 pp 229533.I1RM06
229533.A0A1C3YN63 pp 229533.I1RW71
229533.A0A1C3YN63 pp 229533.I1RB71
229533.A0A1C3YN63 pp 229533.I1RD52
229533.A0A1C3YN63 pp 229533.I1RZS6
229533.A0A1C3YN63 pp 229533.I1RS47
229533.A0A1C3YN63 pp 229533.Q4HZ42
229533.A0A1C3YN63 pp 229533.I1RZ99
229533.A0A1C3YN63 pp 229533.I1RW61
229533.A0A1C3YN63 pp 229533.I1RQJ5
229533.A0A1C3YN63 pp 229533.I1RRJ8
229533.A0A1C3YN63 pp 229533.I1RC32
229533.A0A1C3YN63 pp 229533.I1RG51
229533.A0A1C3YN63 pp 229533.I1RWJ4
229533.A0A1C3YN63 pp 229533.I1RKQ5
229533.A0A1C3YN63 pp 229533.I1RJW6
229533.A0A1C3YN63 pp 229533.V6QW22
229533.A0A1C3YN63 pp 229533.I1RQS5
229533.A0A1C3YN63 pp 229533.Q4I662
229533.A0A1C3YN63 pp 229533.I1RFS3
229533.A0A1C3YN63 pp 229533.I1RRT2
229533.A0A1C3YN63 pp 229533.I1RSQ7
229533.A0A1C3YN63 pp 229533.I1RN63
229533.A0A1C3YN63 pp 229533.I1RE87
229533.A0A1C3YN63 pp 229533.I1RPM4
229533.A0A1C3YN63 pp 229533.I1RR24
229533.A0A1C3YN63 pp 229533.V6R283
229533.A0A1C3YN63 pp 229533.I1RX46
229533.A0A1C3YN63 pp 229533.I1RJL4
229533.A0A1C3YN63 pp 229533.I1RD64
229533.A0A1C3YN63 pp 229533.I1S9U2
229533.A0A1C3YN63 pp 229533.I1RC03
229533.A0A1C3YN63 pp 229533.Q4IPI1
229533.A0A1C3YN63 pp 229533.I1RTA1
229533.A0A1C3YN63 pp 229533.I1RZV6
229533.A0A1C3YN63 pp 229533.Q4HXF0
229533.A0A1C3YN63 pp 229533.I1RRG3
229533.A0A1C3YN63 pp 229533.I1RFW5
229533.A0A1C3YN63 pp 229533.I1RT04

27668	229533.A0A1C3YN63	pp	229533.I1RWJ8
27669	229533.A0A1C3YN63	pp	229533.I1RBS6
27670	229533.A0A1C3YN63	pp	229533.I1RFN1
27671	229533.A0A1C3YN63	pp	229533.I1RPU0
27672	229533.A0A1C3YN63	pp	229533.I1S0R7
27673	229533.A0A1C3YN63	pp	229533.I1RC82
27674	229533.A0A1C3YN63	pp	229533.I1RKJ6
27675	229533.A0A1C3YN63	pp	229533.I1RXD7
27676	229533.A0A1C3YN63	pp	229533.I1S5R1
27677	229533.A0A1C3YN63	pp	229533.Q4IQW0
27678	229533.A0A1C3YN63	pp	229533.I1RUQ2
27679	229533.A0A1C3YN63	pp	229533.I1RT98
27680	229533.A0A1C3YN63	pp	229533.I1RJV4
27681	229533.A0A1C3YN63	pp	229533.I1RRT8
27682	229533.A0A1C3YN63	pp	229533.I1RLV9
27683	229533.A0A1C3YN63	pp	229533.Q4IJH1
27684	229533.A0A1C3YN63	pp	229533.I1RM67
27685	229533.A0A1C3YN63	pp	229533.I1RZJ6
27686	229533.A0A1C3YN63	pp	229533.Q4IAS1
27687	229533.A0A1C3YN63	pp	229533.I1RAF8
27688	229533.A0A1C3YN63	pp	229533.Q4INZ9
27689	229533.A0A1C3YN63	pp	229533.I1RTF4
27690	229533.A0A1C3YN63	pp	229533.Q4IBS2
27691	229533.A0A1C3YN63	pp	229533.I1RX08
27692	229533.A0A1C3YN63	pp	229533.I1RT80
27693	229533.A0A1C3YN63	pp	229533.I1RJZ7
27694	229533.A0A1C3YN63	pp	229533.I1RNV6
27695	229533.A0A1C3YN63	pp	229533.I1RSI6
27696	229533.A0A1C3YN63	pp	229533.I1RJU3
27697	229533.A0A1C3YN63	pp	229533.I1S8E2
27698	229533.A0A1C3YN63	pp	229533.I1RBN1
27699	229533.A0A1C3YN63	pp	229533.I1S253
27700	229533.A0A1C3YN63	pp	229533.I1RDA0
27701	229533.A0A1C3YN63	pp	229533.Q4IFI0
27702	229533.A0A1C3YN63	pp	229533.I1RAC2
27703	229533.A0A1C3YN63	pp	229533.V6RCG5
27704	229533.A0A1C3YN63	pp	229533.I1RW25
27705	229533.A0A1C3YN63	pp	229533.I1RX24
27706	229533.A0A1C3YN63	pp	229533.I1RMC0
27707	229533.A0A1C3YN63	pp	229533.I1RQM6
27708	229533.A0A1C3YN63	pp	229533.Q4HVW2
27709	229533.A0A1C3YN63	pp	229533.I1RQ31
27710	229533.A0A1C3YN63	pp	229533.I1RZF6
27711	229533.A0A1C3YN63	pp	229533.I1RUG3
27712	229533.A0A1C3YN63	pp	229533.I1RMK3
27713	229533.A0A1C3YN63	pp	229533.I1RND6
27714	229533.A0A1C3YN63	pp	229533.I1RRR7
27715	229533.A0A1C3YN63	pp	229533.V6R227
27716	229533.A0A1C3YN63	pp	229533.I1RZM8
27717	229533.A0A1C3YN63	pp	229533.I1RBT1
27718	229533.A0A1C3YN63	pp	229533.I1RT05
27719	229533.A0A1C3YN63	pp	229533.Q4IF76
27720	229533.A0A1C3YN63	pp	229533.Q4IN63
27721	229533.A0A1C3YN63	pp	229533.V6RIV9
27722	229533.A0A1C3YN63	pp	229533.Q4I327
27723	229533.A0A1C3YN63	pp	229533.I1RB92
27724	229533.A0A1C3YN63	pp	229533.I1S4T3
27725	229533.A0A1C3YN63	pp	229533.I1RK95
27726	229533.A0A1C3YN63	pp	229533.I1RNY2
27727	229533.A0A1C3YN63	pp	229533.I1RG56
27728	229533.A0A1C3YN63	pp	229533.I1RQ28
27729	229533.A0A1C3YN63	pp	229533.I1RI82
27730	229533.A0A1C3YN63	pp	229533.I1RPY1
27731	229533.A0A1C3YN63	pp	229533.I1RFL4
27732	229533.A0A1C3YN63	pp	229533.I1RDG0
27733	229533.A0A1C3YN63	pp	229533.I1S021
27734	229533.A0A1C3YN63	pp	229533.I1RSD8
27735	229533.A0A1C3YN63	pp	229533.I1RNH6
27736	229533.A0A1C3YN63	pp	229533.I1RM96
27737	229533.A0A1C3YN63	pp	229533.Q4IEK8
27738	229533.A0A1C3YN63	pp	229533.I1RZK6
27739	229533.A0A1C3YN63	pp	229533.I1RZZ1
27740	229533.A0A1C3YN63	pp	229533.I1RMN7

229533.A0A1C3YN63	pp	229533.I1RC33
229533.A0A1C3YN63	pp	229533.Q4IJ56
229533.A0A1C3YN63	pp	229533.I1S0M9
229533.A0A1C3YN63	pp	229533.I1S296
229533.A0A1C3YN63	pp	229533.I1S2E8
229533.A0A1C3YN63	pp	229533.Q4HZ68
229533.A0A1C3YN63	pp	229533.I1RC06
229533.A0A1C3YN63	pp	229533.I1RQE7
229533.A0A1C3YN63	pp	229533.I1RNU6
229533.A0A1C3YN63	pp	229533.I1RJZ6
229533.A0A1C3YN63	pp	229533.I1RBL7
229533.A0A1C3YN63	pp	229533.I1RF08
229533.A0A1C3YN63	pp	229533.I1RQ81
229533.A0A1C3YN63	pp	229533.I1RD17
229533.A0A1C3YN63	pp	229533.I1S274
229533.A0A1C3YN63	pp	229533.Q4IAA0
229533.A0A1C3YN63	pp	229533.I1REK8
229533.A0A1C3YN63	pp	229533.I1RNK2
229533.A0A1C3YN63	pp	229533.I1R9X7
229533.A0A1C3YN63	pp	229533.I1S1U7
229533.A0A1C3YN63	pp	229533.I1RB27
229533.A0A1C3YN63	pp	229533.I1RVZ0
229533.A0A1C3YN63	pp	229533.I1RSR3
229533.A0A1C3YN63	pp	229533.I1RE01
229533.A0A1C3YN63	pp	229533.I1RAZ8
229533.A0A1C3YN63	pp	229533.V6RGY3
229533.A0A1C3YN63	pp	229533.I1RX14
229533.A0A1C3YN63	pp	229533.I1RW46
229533.A0A1C3YN63	pp	229533.I1RZE5
229533.A0A1C3YN63	pp	229533.I1RE00
229533.A0A1C3YN63	pp	229533.I1S0J1
229533.A0A1C3YN63	pp	229533.I1RC72
229533.A0A1C3YN63	pp	229533.I1RQA2
229533.A0A1C3YN63	pp	229533.I1RAN5
229533.A0A1C3YN63	pp	229533.I1S558
229533.A0A1C3YN63	pp	229533.I1RSI0
229533.A0A1C3YN63	pp	229533.I1RFJ9
229533.A0A1C3YN63	pp	229533.I1RC27
229533.A0A1C3YN63	pp	229533.I1RCL3
229533.A0A1C3YN63	pp	229533.I1RF19
229533.A0A1C3YN63	pp	229533.V6RGN5
229533.A0A1C3YN63	pp	229533.I1R9V7
229533.A0A1C3YN63	pp	229533.I1RJV3
229533.A0A1C3YN63	pp	229533.I1RW99
229533.A0A1C3YN63	pp	229533.I1RZB5
229533.A0A1C3YN63	pp	229533.I1S1X9
229533.A0A1C3YN63	pp	229533.I1RMN6
229533.A0A1C3YN63	pp	229533.Q4HZK7
229533.A0A1C3YN63	pp	229533.I1RY63
229533.A0A1C3YN63	pp	229533.V6QUE6
229533.A0A1C3YN63	pp	229533.I1RCG4
229533.A0A1C3YN63	pp	229533.I1RSC6
229533.A0A1C3YN63	pp	229533.I1S8R3
229533.A0A1C3YN63	pp	229533.I1RSS4
229533.A0A1C3YN63	pp	229533.I1RA48
229533.A0A1C3YN63	pp	229533.I1RTD9
229533.A0A1C3YN63	pp	229533.I1RRI2
229533.A0A1C3YN63	pp	229533.I1RTE9
229533.A0A1C3YN63	pp	229533.I1S2C0
229533.A0A1C3YN63	pp	229533.I1RN99
229533.A0A1C3YN63	pp	229533.I1RNH9
229533.A0A1C3YN63	pp	229533.I1RZW8
229533.A0A1C3YN63	pp	229533.I1RMD5
229533.A0A1C3YN63	pp	229533.I1RK79
229533.A0A1C3YN63	pp	229533.I1RSR8
229533.A0A098DPR9	pp	229533.I1S7S9
229533.A0A098DPR9	pp	229533.I1RBY4
229533.A0A098DPR9	pp	229533.I1RFS3
229533.A0A098DPR9	pp	229533.I1RPM4
229533.A0A098DPR9	pp	229533.I1RKJ6
229533.A0A098DPR9	pp	229533.I1RZV6
229533.A0A098DPR9	pp	229533.I1RN48
229533.A0A098DPR9	pp	229533.I1RRT8

27814	229533.A0A098DPR9	pp	229533.I1RND6
27815	229533.A0A098DPR9	pp	229533.I1RD64
27816	229533.A0A098DPR9	pp	229533.I1RRQ3
27817	229533.A0A098DPR9	pp	229533.I1S0L4
27818	229533.A0A098DPR9	pp	229533.A0A1C3YL15
27819	229533.A0A098DPR9	pp	229533.I1RQ65
27820	229533.A0A098DPR9	pp	229533.I1RKE7
27821	229533.A0A098DPR9	pp	229533.A0A1C3YKK5
27822	229533.A0A098DPR9	pp	229533.I1RTG7
27823	229533.A0A098DPR9	pp	229533.I1RKL3
27824	229533.A0A098DPR9	pp	229533.A0A0E0SPV0
27825	229533.A0A098DPR9	pp	229533.A0A1C3YII7
27826	229533.A0A098DPR9	pp	229533.I1RAF8
27827	229533.A0A098DPR9	pp	229533.I1RFL3
27828	229533.A0A098DPR9	pp	229533.I1RCN0
27829	229533.A0A098DPR9	pp	229533.I1RFL4
27830	229533.A0A098DPR9	pp	229533.I1S8E2
27831	229533.A0A098DPR9	pp	229533.I1RSD8
27832	229533.A0A098DPR9	pp	229533.A0A1C3YMK1
27833	229533.A0A098DPR9	pp	229533.I1RDY5
27834	229533.A0A098DPR9	pp	229533.Q4IR18
27835	229533.A0A098DPR9	pp	229533.I1RBW7
27836	229533.A0A098DPR9	pp	229533.I1RQE7
27837	229533.A0A098DPR9	pp	229533.I1RS17
27838	229533.A0A098DPR9	pp	229533.I1S0F1
27839	229533.A0A098DPR9	pp	229533.Q4HWE2
27840	229533.A0A098DPR9	pp	229533.I1RWB5
27841	229533.A0A098DPR9	pp	229533.I1RCL3
27842	229533.A0A098DPR9	pp	229533.I1RT80
27843	229533.A0A098DPR9	pp	229533.I1RMN6
27844	229533.A0A098DPR9	pp	229533.A0A1C3YIP5
27845	229533.A0A098DPR9	pp	229533.I1RWD4
27846	229533.A0A098DPR9	pp	229533.I1RBZ3
27847	229533.A0A098DPR9	pp	229533.I1RXF6
27848	229533.A0A098DPR9	pp	229533.V6RMW6
27849	229533.A0A098DPR9	pp	229533.I1RPS0
27850	229533.A0A098DPR9	pp	229533.I1S0L3
27851	229533.A0A098DPR9	pp	229533.I1RW25
27852	229533.A0A098DPR9	pp	229533.I1RWH5
27853	229533.A0A098DPR9	pp	229533.I1RPU0
27854	229533.A0A098DPR9	pp	229533.I1RRI2
27855	229533.A0A098DPR9	pp	229533.I1RFC0
27856	229533.A0A098DPR9	pp	229533.I1RIS3
27857	229533.A0A098DPR9	pp	229533.I1RQJ5
27858	229533.A0A098DPR9	pp	229533.I1RM96
27859	229533.A0A098DPR9	pp	229533.I1RM67
27860	229533.A0A098DPR9	pp	229533.I1RE01
27861	229533.A0A098DPR9	pp	229533.I1S0X6
27862	229533.A0A098DPR9	pp	229533.I1REK8
27863	229533.A0A098DPR9	pp	229533.A0A0E0S6Y2
27864	229533.A0A098DPR9	pp	229533.I1RVZ0
27865	229533.A0A098DPR9	pp	229533.A0A1C3YL10
27866	229533.A0A098DPR9	pp	229533.I1RA48
27867	229533.A0A098DPR9	pp	229533.I1R9V7
27868	229533.A0A098DPR9	pp	229533.I1RDG0
27869	229533.A0A098DPR9	pp	229533.I1RAN5
27870	229533.A0A098DPR9	pp	229533.I1RBT1
27871	229533.A0A098DPR9	pp	229533.I1RBL7
27872	229533.A0A098DPR9	pp	229533.A0A1C3YKV3
27873	229533.A0A098DPR9	pp	229533.A0A1C3YI80
27874	229533.A0A098DPR9	pp	229533.A0A1C3YLK2
27875	229533.A0A098DPR9	pp	229533.A0A0E0RWW0
27876	229533.A0A098DPR9	pp	229533.I1RK83
27877	229533.A0A098DPR9	pp	229533.A0A1C3YJR3
27878	229533.A0A098DPR9	pp	229533.I1RB27
27879	229533.A0A098DPR9	pp	229533.I1RZF6
27880	229533.A0A098DPR9	pp	229533.I1RGC2
27881	229533.A0A098DPR9	pp	229533.A0A1C3YKB0
27882	229533.A0A098DPR9	pp	229533.I1RJ17
27883	229533.A0A098DPR9	pp	229533.I1S044
27884	229533.A0A098DPR9	pp	229533.A0A1C3YJZ9
27885	229533.A0A098DPR9	pp	229533.I1RYB9
27886	229533.A0A098DPR9	pp	229533.A0A1C3YN57

229533.A0A1C3YMS0 pp 229533.I1RZP1
229533.A0A1C3YMS0 pp 229533.A0A1C3YN77
229533.I1RFN1 pp 229533.I1RFW5
229533.I1RFN1 pp 229533.I1RQ65
229533.I1RFN1 pp 229533.V6R283
229533.I1RFN1 pp 229533.Q4IEV5
229533.I1RFN1 pp 229533.Q4HZ42
229533.I1RFN1 pp 229533.I1RT33
229533.I1RFN1 pp 229533.I1RS47
229533.I1RFN1 pp 229533.I1RRQ3
229533.I1RFN1 pp 229533.I1S6F0
229533.I1RFN1 pp 229533.I1RQJ5
229533.I1RFN1 pp 229533.I1RZ99
229533.I1RFN1 pp 229533.I1RNH1
229533.I1RFN1 pp 229533.I1RRJ8
229533.I1RFN1 pp 229533.I1RRP3
229533.I1RFN1 pp 229533.Q4HZ47
229533.I1RFN1 pp 229533.I1RUQ2
229533.I1RFN1 pp 229533.I1RSX1
229533.I1RFN1 pp 229533.I1RTI6
229533.I1RFN1 pp 229533.I1RML9
229533.I1RFN1 pp 229533.Q4I830
229533.I1RFN1 pp 229533.I1RRZ0
229533.I1RFN1 pp 229533.I1S0R7
229533.I1RFN1 pp 229533.I1RKL3
229533.I1RFN1 pp 229533.I1RT98
229533.I1RFN1 pp 229533.I1RW71
229533.I1RFN1 pp 229533.I1RR00
229533.I1RFN1 pp 229533.I1RZJ6
229533.I1RFN1 pp 229533.I1RLV9
229533.I1RFN1 pp 229533.I1RT05
229533.I1RFN1 pp 229533.Q4INZ9
229533.I1RFN1 pp 229533.Q4IAS1
229533.I1RFN1 pp 229533.I1RKQ5
229533.I1RFN1 pp 229533.Q4IQW0
229533.I1RFN1 pp 229533.I1RZS6
229533.I1RFN1 pp 229533.I1RM06
229533.I1RFN1 pp 229533.Q4IF76
229533.I1RFN1 pp 229533.I1RQS5
229533.I1RFN1 pp 229533.I1RRT2
229533.I1RFN1 pp 229533.I1RPW9
229533.I1RFN1 pp 229533.I1S9U2
229533.I1RFN1 pp 229533.I1RX24
229533.I1RFN1 pp 229533.I1RJL4
229533.I1RFN1 pp 229533.Q4IQK7
229533.I1RFN1 pp 229533.I1RGF6
229533.I1RFN1 pp 229533.I1RRT8
229533.I1RFN1 pp 229533.I1RNA0
229533.I1RFN1 pp 229533.Q4IPI1
229533.I1RFN1 pp 229533.V6QYF0
229533.I1RFN1 pp 229533.I1RM67
229533.I1RFN1 pp 229533.I1RSI6
229533.I1RFN1 pp 229533.I1RX08
229533.I1RFN1 pp 229533.I1S9Y1
229533.I1RFN1 pp 229533.I1RFS3
229533.I1RFN1 pp 229533.I1RPU0
229533.I1RFN1 pp 229533.I1RG51
229533.I1RFN1 pp 229533.I1RSH5
229533.I1RFN1 pp 229533.Q4I662
229533.I1RFN1 pp 229533.I1RW61
229533.I1RFN1 pp 229533.I1RKJ6
229533.I1RFN1 pp 229533.Q4I327
229533.I1RFN1 pp 229533.I1RZG3
229533.I1RFN1 pp 229533.I1S021
229533.I1RFN1 pp 229533.Q4IAA0
229533.I1RFN1 pp 229533.I1RNH6
229533.I1RFN1 pp 229533.Q4IJ56
229533.I1RFN1 pp 229533.I1RL23
229533.I1RFN1 pp 229533.I1RMN7
229533.I1RFN1 pp 229533.I1S0M9
229533.I1RFN1 pp 229533.Q4IFI0
229533.I1RFN1 pp 229533.I1RRX0
229533.I1RFN1 pp 229533.I1RNU6

27960 229533.I1RFN1 pp 229533.I1RQ31
27961 229533.I1RFN1 pp 229533.I1RMC0
27962 229533.I1RFN1 pp 229533.I1S253
27963 229533.I1RFN1 pp 229533.I1RJZ7
27964 229533.I1RFN1 pp 229533.I1S0J1
27965 229533.I1RFN1 pp 229533.V6RGN5
27966 229533.I1RFN1 pp 229533.I1S060
27967 229533.I1RFN1 pp 229533.I1RFZ0
27968 229533.I1RFN1 pp 229533.I1RX14
27969 229533.I1RFN1 pp 229533.Q4HVW2
27970 229533.I1RFN1 pp 229533.Q4IBS2
27971 229533.I1RFN1 pp 229533.I1RMK3
27972 229533.I1RFN1 pp 229533.Q4IJH1
27973 229533.I1RFN1 pp 229533.V6R227
27974 229533.I1RFN1 pp 229533.V6QUE6
27975 229533.I1RFN1 pp 229533.I1RMN6
27976 229533.I1RFN1 pp 229533.I1RRR7
27977 229533.I1RFN1 pp 229533.I1RTF4
27978 229533.I1RFN1 pp 229533.I1RNV6
27979 229533.I1RFN1 pp 229533.I1RT80
27980 229533.I1RFN1 pp 229533.I1RWP4
27981 229533.I1RFN1 pp 229533.I1RK95
27982 229533.I1RFN1 pp 229533.I1RZF6
27983 229533.I1RFN1 pp 229533.I1RN99
27984 229533.I1RFN1 pp 229533.I1RQM6
27985 229533.I1RFN1 pp 229533.I1RQ28
27986 229533.I1RFN1 pp 229533.I1S1X9
27987 229533.I1RFN1 pp 229533.I1RM96
27988 229533.I1RFN1 pp 229533.I1S0M3
27989 229533.I1RFN1 pp 229533.I1RY63
27990 229533.I1RFN1 pp 229533.I1RSC6
27991 229533.I1RFN1 pp 229533.Q4IEK8
27992 229533.I1RFN1 pp 229533.I1RVZ0
27993 229533.I1RFN1 pp 229533.I1RNK2
27994 229533.I1RFN1 pp 229533.I1RKC6
27995 229533.I1RFN1 pp 229533.I1S4T3
27996 229533.I1RFN1 pp 229533.I1RG56
27997 229533.I1RFN1 pp 229533.I1RSS4
27998 229533.I1RFN1 pp 229533.I1RZK6
27999 229533.I1RFN1 pp 229533.Q4I5Z5
28000 229533.I1RFN1 pp 229533.I1S2E8
28001 229533.I1RFN1 pp 229533.I1RPY1
28002 229533.I1RFN1 pp 229533.I1RSD8
28003 229533.I1RFN1 pp 229533.I1RJY3
28004 229533.I1RFN1 pp 229533.I1RTE9
28005 229533.I1RFN1 pp 229533.I1S2C0
28006 229533.I1RFN1 pp 229533.I1RRI2
28007 229533.I1RFN1 pp 229533.I1RMQ0
28008 229533.I1RFN1 pp 229533.I1RKQ9
28009 229533.I1RFN1 pp 229533.I1S2U2
28010 229533.I1RFN1 pp 229533.I1RTD9
28011 229533.I1RFN1 pp 229533.Q4HZ68
28012 229533.I1RFN1 pp 229533.I1RMD5
28013 229533.I1RFN1 pp 229533.I1RZE5
28014 229533.I1RFN1 pp 229533.I1RJY2
28015 229533.I1RFN1 pp 229533.I1S296
28016 229533.I1RFN1 pp 229533.V6RGY3
28017 229533.I1RFN1 pp 229533.I1S1U7
28018 229533.I1RFN1 pp 229533.I1RSR3
28019 229533.I1RFN1 pp 229533.I1RQA2
28020 229533.I1S190 pp 229533.V6RIV9
28021 229533.I1RRH5 pp 229533.I1RRQ3
28022 229533.I1RRH5 pp 229533.Q4HZ68
28023 229533.I1RRH5 pp 229533.Q4HY90
28024 229533.I1RRH5 pp 229533.I1RRI2
28025 229533.I1RRH5 pp 229533.Q4HZ42
28026 229533.I1RRH5 pp 229533.I1RSD8
28027 229533.I1RRH5 pp 229533.I1S8W6
28028 229533.I1RRH5 pp 229533.I1RX24
28029 229533.I1RRH5 pp 229533.V6R283
28030 229533.I1RRH5 pp 229533.I1S274
28031 229533.I1RRH5 pp 229533.I1S2C0
28032 229533.I1RRH5 pp 229533.I1S1X9

28033 229533.I1RRH5 pp 229533.I1S4T3
28034 229533.I1RRH5 pp 229533.I1RUQ9
28035 229533.I1RRH5 pp 229533.Q4IJ56
28036 229533.I1RRH5 pp 229533.Q4IFI0
28037 229533.I1RRH5 pp 229533.I1RXE5
28038 229533.I1RRH5 pp 229533.Q4IBS2
28039 229533.I1RRH5 pp 229533.I1RT80
28040 229533.I1RRH5 pp 229533.Q4IEK8
28041 229533.I1RRH5 pp 229533.Q4IJH1
28042 229533.I1RRH5 pp 229533.I1RZF6
28043 229533.I1RRH5 pp 229533.I1RZ99
28044 229533.I1RRH5 pp 229533.V6R227
28045 229533.I1RRH5 pp 229533.I1RW71
28046 229533.A0A1C3YMS2 pp 229533.I1S2C1
28047 229533.A0A1C3YMS2 pp 229533.I1RYH6
28048 229533.A0A1C3YMS2 pp 229533.I1S7X9
28049 229533.I1S1X9 pp 229533.Q4IBS2
28050 229533.I1S1X9 pp 229533.V6R227
28051 229533.I1S1X9 pp 229533.Q4IP34
28052 229533.I1S1X9 pp 229533.I1S7M3
28053 229533.I1S1X9 pp 229533.V6QUE6
28054 229533.I1S1X9 pp 229533.Q4IJ56
28055 229533.I1S1X9 pp 229533.Q4I7F9
28056 229533.I1S1X9 pp 229533.Q4HVW2
28057 229533.I1S1X9 pp 229533.Q4IJH1
28058 229533.I1S1X9 pp 229533.Q4I1Y1
28059 229533.I1S1X9 pp 229533.Q4IAA0
28060 229533.I1S1X9 pp 229533.I1S6F0
28061 229533.I1S1X9 pp 229533.Q4IRI9
28062 229533.I1S1X9 pp 229533.I1S9U2
28063 229533.I1S1X9 pp 229533.Q4HZ47
28064 229533.I1S1X9 pp 229533.I1S2C0
28065 229533.I1S1X9 pp 229533.I1S296
28066 229533.I1S1X9 pp 229533.I1S274
28067 229533.I1S1X9 pp 229533.V6QYF0
28068 229533.I1S1X9 pp 229533.Q4IEK8
28069 229533.I1S1X9 pp 229533.Q4IFI0
28070 229533.I1S1X9 pp 229533.I1S7M2
28071 229533.I1S1X9 pp 229533.Q4I5R9
28072 229533.I1S1X9 pp 229533.V6RGY3
28073 229533.I1S1X9 pp 229533.Q4ICG5
28074 229533.I1S1X9 pp 229533.Q4HZ68
28075 229533.I1S1X9 pp 229533.Q4I327
28076 229533.I1S1X9 pp 229533.I1S2Y5
28077 229533.I1S1X9 pp 229533.Q4IN63
28078 229533.I1S1X9 pp 229533.I1S2E8
28079 229533.I1S1X9 pp 229533.Q4IPI1
28080 229533.I1S1X9 pp 229533.V6QXG1
28081 229533.I1S1X9 pp 229533.V6RGN5
28082 229533.I1S1X9 pp 229533.Q4IF76
28083 229533.I1S1X9 pp 229533.Q4HWE2
28084 229533.I1S1X9 pp 229533.V6R042
28085 229533.I1S1X9 pp 229533.V6R283
28086 229533.I1S1X9 pp 229533.Q4I830
28087 229533.I1S1X9 pp 229533.I1S8E2
28088 229533.I1S1X9 pp 229533.I1S9Y1
28089 229533.I1S1X9 pp 229533.I1S4M5
28090 229533.I1S1X9 pp 229533.Q4I662
28091 229533.I1S1X9 pp 229533.Q4IAS1
28092 229533.I1S1X9 pp 229533.I1S9Y0
28093 229533.I1S1X9 pp 229533.Q4HXF0
28094 229533.I1S1X9 pp 229533.O74638
28095 229533.I1S1X9 pp 229533.Q4HZ42
28096 229533.I1S1X9 pp 229533.Q4IQW0
28097 229533.I1RHR5 pp 229533.I1S2I5
28098 229533.I1RHR5 pp 229533.Q4IPX8
28099 229533.I1RS71 pp 229533.I1RWU3
28100 229533.I1RS71 pp 229533.I1RZV9
28101 229533.I1RS71 pp 229533.I1RZX0
28102 229533.I1RS71 pp 229533.I1RYD8
28103 229533.I1RC82 pp 229533.I1RWF7
28104 229533.I1RC82 pp 229533.I1RN63
28105 229533.I1RC82 pp 229533.V6R283

28106	229533.I1RC82	pp	229533.I1RWK3
28107	229533.I1RC82	pp	229533.I1RRJ8
28108	229533.I1RC82	pp	229533.I1S2M6
28109	229533.I1RC82	pp	229533.Q4I830
28110	229533.I1RC82	pp	229533.I1RS30
28111	229533.I1RC82	pp	229533.I1RRP3
28112	229533.I1RC82	pp	229533.I1RRQ3
28113	229533.I1RC82	pp	229533.I1RS47
28114	229533.I1RC82	pp	229533.I1RFG0
28115	229533.I1RC82	pp	229533.I1RSX1
28116	229533.I1RC82	pp	229533.Q4HZ42
28117	229533.I1RC82	pp	229533.I1RFN1
28118	229533.I1RC82	pp	229533.I1RKL3
28119	229533.I1RC82	pp	229533.I1S8E2
28120	229533.I1RC82	pp	229533.I1RQ65
28121	229533.I1RC82	pp	229533.I1S253
28122	229533.I1RC82	pp	229533.I1RJ74
28123	229533.I1RC82	pp	229533.Q4IQW0
28124	229533.I1RC82	pp	229533.I1RT40
28125	229533.I1RC82	pp	229533.Q4IR18
28126	229533.I1RC82	pp	229533.I1S9Y1
28127	229533.I1RC82	pp	229533.I1RJV4
28128	229533.I1RC82	pp	229533.Q4HXF0
28129	229533.I1RC82	pp	229533.I1RSZ7
28130	229533.I1RC82	pp	229533.I1RKJ6
28131	229533.I1RC82	pp	229533.I1RYT5
28132	229533.I1RC82	pp	229533.I1RRT8
28133	229533.I1RC82	pp	229533.I1RLV9
28134	229533.I1RC82	pp	229533.Q4IAS1
28135	229533.I1RC82	pp	229533.I1S5R1
28136	229533.I1RC82	pp	229533.I1RPM4
28137	229533.I1RC82	pp	229533.O74638
28138	229533.I1RC82	pp	229533.I1RWJ4
28139	229533.I1RC82	pp	229533.I1RJW6
28140	229533.I1RC82	pp	229533.Q4I662
28141	229533.I1RC82	pp	229533.I1RD64
28142	229533.I1RC82	pp	229533.I1RGF6
28143	229533.I1RC82	pp	229533.I1RVA7
28144	229533.I1RC82	pp	229533.I1RQS8
28145	229533.I1RC82	pp	229533.I1RRT2
28146	229533.I1RC82	pp	229533.I1RFS3
28147	229533.I1RC82	pp	229533.I1RE87
28148	229533.I1RC82	pp	229533.I1RX24
28149	229533.I1RC82	pp	229533.I1RZS6
28150	229533.I1RC82	pp	229533.I1RS20
28151	229533.I1RC82	pp	229533.I1RQS5
28152	229533.I1RC82	pp	229533.I1RJZ7
28153	229533.I1RC82	pp	229533.I1RRM5
28154	229533.I1RC82	pp	229533.I1RRI1
28155	229533.I1RC82	pp	229533.I1S1B6
28156	229533.I1RC82	pp	229533.I1RJL4
28157	229533.I1RC82	pp	229533.V6RIV9
28158	229533.I1RC82	pp	229533.I1S060
28159	229533.I1RC82	pp	229533.I1RW46
28160	229533.I1RC82	pp	229533.I1RYG3
28161	229533.I1RC82	pp	229533.I1RZE1
28162	229533.I1RC82	pp	229533.I1RE01
28163	229533.I1RC82	pp	229533.I1RZZ9
28164	229533.I1RC82	pp	229533.I1S4T3
28165	229533.I1RC82	pp	229533.Q4IF76
28166	229533.I1RC82	pp	229533.I1S0J1
28167	229533.I1RC82	pp	229533.I1RS64
28168	229533.I1RC82	pp	229533.I1RS17
28169	229533.I1RC82	pp	229533.I1RM96
28170	229533.I1RC82	pp	229533.I1RPY1
28171	229533.I1RC82	pp	229533.I1RNU6
28172	229533.I1RC82	pp	229533.I1RFH8
28173	229533.I1RC82	pp	229533.I1RM25
28174	229533.I1RC82	pp	229533.I1RCH5
28175	229533.I1RC82	pp	229533.I1RP31
28176	229533.I1RC82	pp	229533.I1RQE7
28177	229533.I1RC82	pp	229533.I1RPJ3
28178	229533.I1RC82	pp	229533.I1RRR7

28179	229533.I1RC82	pp	229533.I1RTE9
28180	229533.I1RC82	pp	229533.I1S2E8
28181	229533.I1RC82	pp	229533.I1S2C0
28182	229533.I1RC82	pp	229533.I1RUG3
28183	229533.I1RC82	pp	229533.I1S1X9
28184	229533.I1RC82	pp	229533.Q4IN63
28185	229533.I1RC82	pp	229533.I1RCN5
28186	229533.I1RC82	pp	229533.I1RSS4
28187	229533.I1RC82	pp	229533.I1S1J6
28188	229533.I1RC82	pp	229533.I1RNY2
28189	229533.I1RC82	pp	229533.I1RCL3
28190	229533.I1RC82	pp	229533.I1S558
28191	229533.I1RC82	pp	229533.I1RKC6
28192	229533.I1RC82	pp	229533.Q4HZ68
28193	229533.I1RC82	pp	229533.I1RMU9
28194	229533.I1RC82	pp	229533.Q4I327
28195	229533.I1RC82	pp	229533.I1S296
28196	229533.I1RC82	pp	229533.I1RLP2
28197	229533.I1RC82	pp	229533.I1RMN6
28198	229533.I1RC82	pp	229533.I1S274
28199	229533.I1RC82	pp	229533.I1RSI6
28200	229533.I1RC82	pp	229533.V6RGY3
28201	229533.I1RC82	pp	229533.I1RD17
28202	229533.I1RC82	pp	229533.V6R057
28203	229533.I1RC82	pp	229533.I1RQ33
28204	229533.I1RC82	pp	229533.I1RQ72
28205	229533.I1RC82	pp	229533.I1S1U7
28206	229533.I1RC82	pp	229533.V6RGN5
28207	229533.I1RC82	pp	229533.I1RSD8
28208	229533.I1RC82	pp	229533.I1RFL4
28209	229533.I1RC82	pp	229533.I1RW71
28210	229533.I1RC82	pp	229533.I1RFW5
28211	229533.I1RC82	pp	229533.I1RRH5
28212	229533.I1RC82	pp	229533.I1RRI2
28213	229533.I1RC82	pp	229533.I1RT33
28214	229533.I1RC82	pp	229533.I1RR00
28215	229533.I1RC82	pp	229533.I1RD56
28216	229533.I1RC82	pp	229533.I1RQJ5
28217	229533.I1RC82	pp	229533.I1S8R3
28218	229533.I1RC82	pp	229533.I1RZW8
28219	229533.I1RC82	pp	229533.I1RZ99
28220	229533.I1RC82	pp	229533.I1S0L3
28221	229533.I1RC82	pp	229533.V6QW22
28222	229533.I1RC82	pp	229533.Q4INZ9
28223	229533.I1RC82	pp	229533.Q4HZK7
28224	229533.I1RC82	pp	229533.I1RZJ6
28225	229533.I1RC82	pp	229533.I1RSQ7
28226	229533.I1RC82	pp	229533.I1REX4
28227	229533.I1RC82	pp	229533.I1S0R7
28228	229533.I1RC82	pp	229533.I1REF2
28229	229533.I1RC82	pp	229533.I1RNA0
28230	229533.I1RC82	pp	229533.Q4IQK7
28231	229533.I1RC82	pp	229533.Q4HZ47
28232	229533.I1RC82	pp	229533.I1RWD4
28233	229533.I1RC82	pp	229533.I1RKQ5
28234	229533.I1RC82	pp	229533.I1RUQ2
28235	229533.I1RC82	pp	229533.I1S6F0
28236	229533.I1RC82	pp	229533.V6QYF0
28237	229533.I1RC82	pp	229533.I1RNH1
28238	229533.I1RC82	pp	229533.I1RRQ6
28239	229533.I1RC82	pp	229533.Q4I5R9
28240	229533.I1RC82	pp	229533.I1RM06
28241	229533.I1RC82	pp	229533.I1RL23
28242	229533.I1RC82	pp	229533.Q4IPI1
28243	229533.I1RC82	pp	229533.Q4IFI0
28244	229533.I1RC82	pp	229533.I1RVQ0
28245	229533.I1RC82	pp	229533.I1RM67
28246	229533.I1RC82	pp	229533.I1RD52
28247	229533.I1RC82	pp	229533.I1RG51
28248	229533.I1RC82	pp	229533.I1RW25
28249	229533.I1RC82	pp	229533.I1RR24
28250	229533.I1RC82	pp	229533.V6RCZ8
28251	229533.I1RC82	pp	229533.V6RCG5

28252 229533.I1RC82 pp 229533.I1RT05
28253 229533.I1RC82 pp 229533.I1S0F1
28254 229533.I1RC82 pp 229533.I1RPW9
28255 229533.I1RC82 pp 229533.V6QUE6
28256 229533.I1RC82 pp 229533.I1RQM6
28257 229533.I1RC82 pp 229533.I1RX14
28258 229533.I1RC82 pp 229533.I1S9U2
28259 229533.I1RC82 pp 229533.Q4IJH1
28260 229533.I1RC82 pp 229533.I1RTF4
28261 229533.I1RC82 pp 229533.I1RYJ8
28262 229533.I1RC82 pp 229533.Q4IBS2
28263 229533.I1RC82 pp 229533.Q4IJ56
28264 229533.I1RC82 pp 229533.I1RNV6
28265 229533.I1RC82 pp 229533.I1S0M9
28266 229533.I1RC82 pp 229533.I1S5B0
28267 229533.I1RC82 pp 229533.I1RQ81
28268 229533.I1RC82 pp 229533.I1RT80
28269 229533.I1RC82 pp 229533.I1RJU3
28270 229533.I1RC82 pp 229533.I1RW99
28271 229533.I1RC82 pp 229533.I1S2U2
28272 229533.I1RC82 pp 229533.Q4IAA0
28273 229533.I1RC82 pp 229533.I1S6U8
28274 229533.I1RC82 pp 229533.I1RDA0
28275 229533.I1RC82 pp 229533.I1RZK6
28276 229533.I1RC82 pp 229533.I1RG56
28277 229533.I1RC82 pp 229533.Q4IEK8
28278 229533.I1RC82 pp 229533.I1RZZ1
28279 229533.I1RC82 pp 229533.I1RMK3
28280 229533.I1RC82 pp 229533.I1RJZ6
28281 229533.I1RC82 pp 229533.I1RXE5
28282 229533.I1RC82 pp 229533.Q4HVW2
28283 229533.I1RC82 pp 229533.I1RZF6
28284 229533.I1RC82 pp 229533.I1RY63
28285 229533.I1RC82 pp 229533.I1RWJ8
28286 229533.I1RC82 pp 229533.I1RSC6
28287 229533.I1RC82 pp 229533.I1RN99
28288 229533.I1RC82 pp 229533.I1RPU0
28289 229533.I1RC82 pp 229533.I1RQ28
28290 229533.I1RC82 pp 229533.V6R227
28291 229533.I1RC82 pp 229533.I1RK95
28292 229533.I1RC82 pp 229533.I1RMC0
28293 229533.I1RC82 pp 229533.I1RKQ9
28294 229533.I1RC82 pp 229533.I1REK8
28295 229533.I1RC82 pp 229533.I1RQ31
28296 229533.I1RC82 pp 229533.I1RNK2
28297 229533.I1RC82 pp 229533.I1RMD5
28298 229533.I1RC82 pp 229533.I1RDG0
28299 229533.I1RC82 pp 229533.I1RSR8
28300 229533.I1RC82 pp 229533.I1RZM8
28301 229533.I1RC82 pp 229533.Q4I5Z5
28302 229533.I1RC82 pp 229533.I1RVZ0
28303 229533.I1RC82 pp 229533.I1RN58
28304 229533.I1RC82 pp 229533.I1RI82
28305 229533.I1RC82 pp 229533.I1RZB5
28306 229533.I1RC82 pp 229533.I1RTD9
28307 229533.I1RC82 pp 229533.I1RZE5
28308 229533.I1RC82 pp 229533.I1RMN7
28309 229533.I1RC82 pp 229533.I1RQA2
28310 229533.I1RC82 pp 229533.I1RCG4
28311 229533.I1RC82 pp 229533.I1RNH6
28312 229533.I1RC82 pp 229533.I1RUZ8
28313 229533.I1RC82 pp 229533.I1S021
28314 229533.I1RC82 pp 229533.I1RSM3
28315 229533.I1RC82 pp 229533.I1RSI0
28316 229533.I1RC82 pp 229533.I1RFJ9
28317 229533.I1RC82 pp 229533.I1RF19
28318 229533.I1RC82 pp 229533.I1RSR3
28319 229533.I1RC82 pp 229533.I1RE00
28320 229533.I1RC82 pp 229533.I1RF08
28321 229533.A0A1C3YMR5 pp 229533.I1RHR5
28322 229533.I1RK77 pp 229533.I1RYL5
28323 229533.I1RK77 pp 229533.I1S0P4
28324 229533.I1RK77 pp 229533.I1RME4

28325	229533.I1RK77	pp	229533.I1RNT2
28326	229533.I1RK77	pp	229533.I1RQ21
28327	229533.I1RK77	pp	229533.I1RM67
28328	229533.I1RK77	pp	229533.Q4I624
28329	229533.I1RK77	pp	229533.I1RNE4
28330	229533.I1RK77	pp	229533.I1RZV9
28331	229533.I1RK77	pp	229533.V6REZ4
28332	229533.I1RK77	pp	229533.I1RYT7
28333	229533.I1RK77	pp	229533.I1RSG9
28334	229533.I1RK77	pp	229533.I1RWH4
28335	229533.A0A1C3YMR0	pp	229533.A0A1C3YN77
28336	229533.Q4IQW0	pp	229533.V6RGN5
28337	229533.Q4IQW0	pp	229533.V6RGY3
28338	229533.Q4IQW0	pp	229533.V6R283
28339	229533.Q4IQW0	pp	229533.V6R227
28340	229533.Q4IQW0	pp	229533.Q4IRI9
28341	229533.Q4IQW0	pp	229533.V6QUE6
28342	229533.Q4IQW0	pp	229533.V6QYF0
28343	229533.I1S086	pp	229533.Q4ICA8
28344	229533.I1S086	pp	229533.I1S0Z3
28345	229533.I1S086	pp	229533.Q4IPI4
28346	229533.I1S086	pp	229533.Q4I948
28347	229533.I1RA29	pp	229533.V6RY33
28348	229533.I1RA29	pp	229533.I1RJZ7
28349	229533.I1RA29	pp	229533.I1RTF4
28350	229533.I1RA29	pp	229533.I1RD52
28351	229533.I1RA29	pp	229533.I1RRX5
28352	229533.I1RA29	pp	229533.V6R3J1
28353	229533.I1RA29	pp	229533.V6RIS2
28354	229533.I1RA29	pp	229533.I1S471
28355	229533.I1RA29	pp	229533.I1RW71
28356	229533.I1RA29	pp	229533.I1RAU7
28357	229533.I1RA29	pp	229533.I1RZ40
28358	229533.I1RA29	pp	229533.I1RQE7
28359	229533.I1RA29	pp	229533.I1RQ31
28360	229533.I1RA29	pp	229533.I1RQ65
28361	229533.I1RA29	pp	229533.I1RK95
28362	229533.I1RA29	pp	229533.I1S2C0
28363	229533.I1RA29	pp	229533.I1RRV1
28364	229533.I1RA29	pp	229533.I1RZG7
28365	229533.I1RA29	pp	229533.I1RSD4
28366	229533.I1RA29	pp	229533.I1RAZ8
28367	229533.I1RA29	pp	229533.I1RB53
28368	229533.I1RA29	pp	229533.V6R227
28369	229533.I1RA29	pp	229533.I1RLU5
28370	229533.I1RA29	pp	229533.I1RN61
28371	229533.I1RSS4	pp	229533.I1S9Y1
28372	229533.I1RSS4	pp	229533.I1S253
28373	229533.I1RSS4	pp	229533.O74638
28374	229533.I1RSS4	pp	229533.I1S5R1
28375	229533.I1RSS4	pp	229533.Q4HZI0
28376	229533.I1RSS4	pp	229533.I1RWI5
28377	229533.I1RSS4	pp	229533.I1S2B8
28378	229533.I1RSS4	pp	229533.Q4IF76
28379	229533.I1RSS4	pp	229533.I1RZZ9
28380	229533.I1RSS4	pp	229533.I1RZS6
28381	229533.I1RSS4	pp	229533.Q4IQW0
28382	229533.I1RSS4	pp	229533.I1RX24
28383	229533.I1RSS4	pp	229533.I1RW61
28384	229533.I1RSS4	pp	229533.I1RZB2
28385	229533.I1RSS4	pp	229533.I1S2H4
28386	229533.I1RSS4	pp	229533.I1RSZ7
28387	229533.I1RSS4	pp	229533.Q4IAS1
28388	229533.I1RSS4	pp	229533.I1RT04
28389	229533.I1RSS4	pp	229533.Q4HXF0
28390	229533.I1RSS4	pp	229533.I1RWJ4
28391	229533.I1RSS4	pp	229533.I1RWS4
28392	229533.I1RSS4	pp	229533.I1RYI5
28393	229533.I1RSS4	pp	229533.I1RT75
28394	229533.I1RSS4	pp	229533.V6R283
28395	229533.I1RSS4	pp	229533.Q4I662
28396	229533.I1RSS4	pp	229533.I1S0M3
28397	229533.I1RSS4	pp	229533.I1RTD5

28398 229533.I1RSS4 pp 229533.I1RUG4
28399 229533.I1RSS4 pp 229533.V6RGY3
28400 229533.I1RSS4 pp 229533.I1S274
28401 229533.I1RSS4 pp 229533.I1RW26
28402 229533.I1RSS4 pp 229533.I1S1X9
28403 229533.I1RSS4 pp 229533.Q4HZ42
28404 229533.I1RSS4 pp 229533.I1S558
28405 229533.I1RSS4 pp 229533.I1RWF9
28406 229533.I1RSS4 pp 229533.I1S2E8
28407 229533.I1RSS4 pp 229533.Q4I830
28408 229533.I1RSS4 pp 229533.Q4HZK7
28409 229533.I1RSS4 pp 229533.I1RTE9
28410 229533.I1RSS4 pp 229533.I1RSV2
28411 229533.I1RSS4 pp 229533.I1RWQ8
28412 229533.I1RSS4 pp 229533.V6QT89
28413 229533.I1RSS4 pp 229533.I1S2C0
28414 229533.I1RSS4 pp 229533.I1S5R6
28415 229533.I1RSS4 pp 229533.I1S4T3
28416 229533.I1RSS4 pp 229533.V6RGN5
28417 229533.I1RSS4 pp 229533.V6RLM7
28418 229533.I1RSS4 pp 229533.Q4HZ68
28419 229533.I1RSS4 pp 229533.V6R042
28420 229533.I1RSS4 pp 229533.I1S9U2
28421 229533.I1RSS4 pp 229533.V6RMW6
28422 229533.I1RSS4 pp 229533.I1S0J1
28423 229533.I1RSS4 pp 229533.I1RUG3
28424 229533.I1RSS4 pp 229533.I1RT05
28425 229533.I1RSS4 pp 229533.Q4IN63
28426 229533.I1RSS4 pp 229533.I1S4S4
28427 229533.I1RSS4 pp 229533.I1S0F1
28428 229533.I1RSS4 pp 229533.Q4IFI0
28429 229533.I1RSS4 pp 229533.I1RTF4
28430 229533.I1RSS4 pp 229533.Q4IJH1
28431 229533.I1RSS4 pp 229533.Q4I327
28432 229533.I1RSS4 pp 229533.I1RW25
28433 229533.I1RSS4 pp 229533.Q4I948
28434 229533.I1RSS4 pp 229533.I1RT80
28435 229533.I1RSS4 pp 229533.V6QYF0
28436 229533.I1RSS4 pp 229533.I1RZK6
28437 229533.I1RSS4 pp 229533.V6RTJ8
28438 229533.I1RSS4 pp 229533.Q4HVW2
28439 229533.I1RSS4 pp 229533.I1S0R7
28440 229533.I1RSS4 pp 229533.I1RX46
28441 229533.I1RSS4 pp 229533.Q4IPI1
28442 229533.I1RSS4 pp 229533.I1RXD7
28443 229533.I1RSS4 pp 229533.I1RZZ1
28444 229533.I1RSS4 pp 229533.I1RYJ8
28445 229533.I1RSS4 pp 229533.I1RYJ6
28446 229533.I1RSS4 pp 229533.V6RBN2
28447 229533.I1RSS4 pp 229533.I1RT33
28448 229533.I1RSS4 pp 229533.Q4INZ9
28449 229533.I1RSS4 pp 229533.Q4IBS2
28450 229533.I1RSS4 pp 229533.I1S6F0
28451 229533.I1RSS4 pp 229533.V6QUE6
28452 229533.I1RSS4 pp 229533.I1RTD9
28453 229533.I1RSS4 pp 229533.I1RXF0
28454 229533.I1RSS4 pp 229533.I1RZB5
28455 229533.I1RSS4 pp 229533.I1RVZ0
28456 229533.I1RSS4 pp 229533.I1S021
28457 229533.I1RSS4 pp 229533.V6R227
28458 229533.I1RSS4 pp 229533.I1RY63
28459 229533.I1RSS4 pp 229533.I1RZF6
28460 229533.I1RSS4 pp 229533.Q4IJ56
28461 229533.I1RSS4 pp 229533.I1RT35
28462 229533.I1RSS4 pp 229533.Q4ICA8
28463 229533.I1RSS4 pp 229533.I1RXB6
28464 229533.I1RSS4 pp 229533.Q4IEK8
28465 229533.I1RSS4 pp 229533.I1RX14
28466 229533.I1RSS4 pp 229533.Q4IAA0
28467 229533.I1RSS4 pp 229533.I1S0M9
28468 229533.I1RYS6 pp 229533.I1S247
28469 229533.I1RYS6 pp 229533.Q4I624
28470 229533.I1RYS6 pp 229533.I1RZD3

28471	229533.I1RYS6	pp	229533.Q4HXF6
28472	229533.I1RYS6	pp	229533.I1S0F5
28473	229533.I1RDY7	pp	229533.I1RKL0
28474	229533.I1RDY7	pp	229533.I1RM71
28475	229533.I1RKD0	pp	229533.Q4ICA8
28476	229533.I1RKD0	pp	229533.I1RQP3
28477	229533.I1RKD0	pp	229533.I1RRF0
28478	229533.I1RKD0	pp	229533.Q4I948
28479	229533.I1RKD0	pp	229533.Q4IL82
28480	229533.I1RKD0	pp	229533.I1S086
28481	229533.Q4HZ42	pp	229533.Q4IEK8
28482	229533.Q4HZ42	pp	229533.Q4IBS2
28483	229533.Q4HZ42	pp	229533.Q4HZ47
28484	229533.Q4HZ42	pp	229533.Q4IAA0
28485	229533.Q4HZ42	pp	229533.Q4IJ56
28486	229533.Q4HZ42	pp	229533.V6R227
28487	229533.Q4HZ42	pp	229533.V6QUE6
28488	229533.Q4HZ42	pp	229533.Q4IFI0
28489	229533.Q4HZ42	pp	229533.Q4IPI1
28490	229533.Q4HZ42	pp	229533.Q4INZ9
28491	229533.Q4HZ42	pp	229533.V6QYF0
28492	229533.Q4HZ42	pp	229533.V6RIS2
28493	229533.Q4HZ42	pp	229533.Q4I830
28494	229533.Q4HZ42	pp	229533.Q4I327
28495	229533.Q4HZ42	pp	229533.V6RY33
28496	229533.Q4HZ42	pp	229533.Q4IJH1
28497	229533.Q4HZ42	pp	229533.V6R283
28498	229533.Q4HZ42	pp	229533.Q4HZ68
28499	229533.Q4HZ42	pp	229533.Q4IEV5
28500	229533.Q4HZ42	pp	229533.V6RAC9
28501	229533.Q4HZ42	pp	229533.V6RGN5
28502	229533.Q4HZ42	pp	229533.Q4IQW0
28503	229533.Q4HZ42	pp	229533.Q4I662
28504	229533.Q4HZ42	pp	229533.Q4IF76
28505	229533.Q4HZ42	pp	229533.Q4IAS1
28506	229533.Q4HZ42	pp	229533.V6RGY3
28507	229533.I1RDY8	pp	229533.I1RZ25
28508	229533.I1RDY8	pp	229533.I1RSA5
28509	229533.I1RDY8	pp	229533.I1SAF7
28510	229533.I1RDY8	pp	229533.I1RS35
28511	229533.I1RZJ6	pp	229533.V6R227
28512	229533.I1RZJ6	pp	229533.I1S1X9
28513	229533.I1RZJ6	pp	229533.I1S060
28514	229533.I1RZJ6	pp	229533.I1S296
28515	229533.I1RZJ6	pp	229533.I1S4T3
28516	229533.I1RZJ6	pp	229533.Q4HZ68
28517	229533.I1RZJ6	pp	229533.Q4IAA0
28518	229533.I1RZJ6	pp	229533.Q4I327
28519	229533.I1RZJ6	pp	229533.I1S9U2
28520	229533.I1RZJ6	pp	229533.Q4IBS2
28521	229533.I1RZJ6	pp	229533.Q4IJ56
28522	229533.I1RZJ6	pp	229533.Q4IAS1
28523	229533.I1RZJ6	pp	229533.Q4HVW2
28524	229533.I1RZJ6	pp	229533.I1S021
28525	229533.I1RZJ6	pp	229533.V6RGY3
28526	229533.I1RZJ6	pp	229533.Q4IJH1
28527	229533.I1RZJ6	pp	229533.Q4HZ47
28528	229533.I1RZJ6	pp	229533.V6QYF0
28529	229533.I1RZJ6	pp	229533.Q4HZ42
28530	229533.I1RZJ6	pp	229533.I1S9Y0
28531	229533.I1RZJ6	pp	229533.Q4IFI0
28532	229533.I1RZJ6	pp	229533.Q4IR18
28533	229533.I1RZJ6	pp	229533.V6R283
28534	229533.I1RZJ6	pp	229533.Q4I830
28535	229533.A0A1C3YHY0	pp	229533.I1RS29
28536	229533.A0A1C3YHY0	pp	229533.I1RKF5
28537	229533.A0A1C3YHY0	pp	229533.V6RJP8
28538	229533.A0A1C3YHY0	pp	229533.I1R9N8
28539	229533.A0A1C3YHY0	pp	229533.I1RZX0
28540	229533.A0A1C3YHY0	pp	229533.I1RCZ1
28541	229533.A0A1C3YHY0	pp	229533.I1RYT8
28542	229533.A0A1C3YHY0	pp	229533.I1S5X9
28543	229533.A0A1C3YHY0	pp	229533.I1RS71

28544	229533.A0A1C3YHY0	pp	229533.I1S5C6
28545	229533.A0A1C3YHY0	pp	229533.I1RWA3
28546	229533.A0A1C3YHY0	pp	229533.I1RKF4
28547	229533.A0A1C3YHY0	pp	229533.I1RE65
28548	229533.A0A1C3YHY0	pp	229533.I1RQJ3
28549	229533.A0A1C3YHY0	pp	229533.I1S458
28550	229533.A0A1C3YHY0	pp	229533.I1RB25
28551	229533.A0A1C3YHY0	pp	229533.I1S464
28552	229533.A0A1C3YHY0	pp	229533.I1RFX9
28553	229533.A0A1C3YHY0	pp	229533.I1RYQ1
28554	229533.A0A1C3YMI6	pp	229533.I1RLV9
28555	229533.A0A1C3YMI6	pp	229533.I1RSR8
28556	229533.A0A1C3YMI6	pp	229533.I1RX46
28557	229533.A0A1C3YMI6	pp	229533.I1RAN5
28558	229533.A0A1C3YMI6	pp	229533.I1RKL3
28559	229533.A0A1C3YMI6	pp	229533.I1RZK6
28560	229533.A0A1C3YMI6	pp	229533.I1RQ65
28561	229533.A0A1C3YMI6	pp	229533.I1RKJ6
28562	229533.A0A1C3YMI6	pp	229533.I1RR73
28563	229533.A0A1C3YMI6	pp	229533.I1RA48
28564	229533.A0A1C3YMI6	pp	229533.I1RAU7
28565	229533.A0A1C3YMI6	pp	229533.V6R042
28566	229533.A0A1C3YMI6	pp	229533.I1RNH6
28567	229533.I1RN99	pp	229533.Q4IAS1
28568	229533.I1RN99	pp	229533.I1RRE2
28569	229533.I1RN99	pp	229533.I1RT04
28570	229533.I1RN99	pp	229533.I1RUQ2
28571	229533.I1RN99	pp	229533.I1RXD7
28572	229533.I1RN99	pp	229533.I1S9U2
28573	229533.I1RN99	pp	229533.I1S0R7
28574	229533.I1RN99	pp	229533.I1RPW9
28575	229533.I1RN99	pp	229533.I1RW25
28576	229533.I1RN99	pp	229533.I1RTI6
28577	229533.I1RN99	pp	229533.Q4IFI0
28578	229533.I1RN99	pp	229533.I1RR24
28579	229533.I1RN99	pp	229533.I1RZJ6
28580	229533.I1RN99	pp	229533.I1S0L3
28581	229533.I1RN99	pp	229533.I1RRT8
28582	229533.I1RN99	pp	229533.I1RQ65
28583	229533.I1RN99	pp	229533.I1RZS6
28584	229533.I1RN99	pp	229533.I1S5R1
28585	229533.I1RN99	pp	229533.Q4HZ42
28586	229533.I1RN99	pp	229533.I1RPU0
28587	229533.I1RN99	pp	229533.I1RRQ6
28588	229533.I1RN99	pp	229533.V6QYF0
28589	229533.I1RN99	pp	229533.I1S9Y1
28590	229533.I1RN99	pp	229533.I1RWJ8
28591	229533.I1RN99	pp	229533.Q4IQK7
28592	229533.I1RN99	pp	229533.I1RS47
28593	229533.I1RN99	pp	229533.I1RVQ0
28594	229533.I1RN99	pp	229533.Q4HVW2
28595	229533.I1RN99	pp	229533.I1RRH5
28596	229533.I1RN99	pp	229533.I1RNH1
28597	229533.I1RN99	pp	229533.I1RNA0
28598	229533.I1RN99	pp	229533.I1S253
28599	229533.I1RN99	pp	229533.Q4I662
28600	229533.I1RN99	pp	229533.I1RX24
28601	229533.I1RN99	pp	229533.I1RRQ3
28602	229533.I1RN99	pp	229533.I1RT80
28603	229533.I1RN99	pp	229533.I1S6F0
28604	229533.I1RN99	pp	229533.I1RPM4
28605	229533.I1RN99	pp	229533.I1RNV6
28606	229533.I1RN99	pp	229533.I1RS30
28607	229533.I1RN99	pp	229533.I1RTF4
28608	229533.I1RN99	pp	229533.I1RWJ4
28609	229533.I1RN99	pp	229533.I1RW61
28610	229533.I1RN99	pp	229533.I1RRP3
28611	229533.I1RN99	pp	229533.Q4IJH1
28612	229533.I1RN99	pp	229533.I1RWD4
28613	229533.I1RN99	pp	229533.Q4IQW0
28614	229533.I1RN99	pp	229533.I1RT05
28615	229533.I1RN99	pp	229533.I1RSQ7
28616	229533.I1RN99	pp	229533.Q4IF76

28617	229533.I1RN99	pp	229533.I1RRT2
28618	229533.I1RN99	pp	229533.V6QW22
28619	229533.I1RN99	pp	229533.Q4IPI1
28620	229533.I1RN99	pp	229533.I1RRJ8
28621	229533.I1RN99	pp	229533.Q4I327
28622	229533.I1RN99	pp	229533.I1RZ99
28623	229533.I1RN99	pp	229533.I1RQS5
28624	229533.I1RN99	pp	229533.I1RZM8
28625	229533.I1RN99	pp	229533.I1S604
28626	229533.I1RN99	pp	229533.I1RQJ5
28627	229533.I1RN99	pp	229533.I1RSI0
28628	229533.I1RN99	pp	229533.I1RW71
28629	229533.I1RN99	pp	229533.I1RZE5
28630	229533.I1RN99	pp	229533.I1RVZ0
28631	229533.I1RN99	pp	229533.I1RQ31
28632	229533.I1RN99	pp	229533.I1RUG3
28633	229533.I1RN99	pp	229533.I1RQ28
28634	229533.I1RN99	pp	229533.Q4I830
28635	229533.I1RN99	pp	229533.I1RNK2
28636	229533.I1RN99	pp	229533.V6R283
28637	229533.I1RN99	pp	229533.I1RSC6
28638	229533.I1RN99	pp	229533.I1RSR3
28639	229533.I1RN99	pp	229533.I1RSS4
28640	229533.I1RN99	pp	229533.V6R227
28641	229533.I1RN99	pp	229533.V6RGY3
28642	229533.I1RN99	pp	229533.I1RRR7
28643	229533.I1RN99	pp	229533.I1RY63
28644	229533.I1RN99	pp	229533.I1S1X9
28645	229533.I1RN99	pp	229533.I1RZF6
28646	229533.I1RN99	pp	229533.I1RPY1
28647	229533.I1RN99	pp	229533.I1S274
28648	229533.I1RN99	pp	229533.Q4IEK8
28649	229533.I1RN99	pp	229533.I1RQE7
28650	229533.I1RN99	pp	229533.I1S558
28651	229533.I1RN99	pp	229533.I1S2E8
28652	229533.I1RN99	pp	229533.I1S4T3
28653	229533.I1RN99	pp	229533.I1RZZ1
28654	229533.I1RN99	pp	229533.I1RSD8
28655	229533.I1RN99	pp	229533.V6RIV9
28656	229533.I1RN99	pp	229533.I1RQA2
28657	229533.I1RN99	pp	229533.I1RTE9
28658	229533.I1RN99	pp	229533.I1RW99
28659	229533.I1RN99	pp	229533.I1S2C0
28660	229533.I1RN99	pp	229533.V6RGN5
28661	229533.I1RN99	pp	229533.Q4IAA0
28662	229533.I1RN99	pp	229533.I1RNU6
28663	229533.I1RN99	pp	229533.Q4IBS2
28664	229533.I1RN99	pp	229533.I1S8R3
28665	229533.I1RN99	pp	229533.I1S0M9
28666	229533.I1RN99	pp	229533.I1RZW8
28667	229533.I1RN99	pp	229533.I1RS17
28668	229533.I1RN99	pp	229533.V6RCG5
28669	229533.I1RN99	pp	229533.Q4IJ56
28670	229533.I1RN99	pp	229533.I1RX08
28671	229533.I1RN99	pp	229533.I1RRI2
28672	229533.I1RN99	pp	229533.I1RSI6
28673	229533.I1RN99	pp	229533.I1RPZ0
28674	229533.I1RN99	pp	229533.I1RTD9
28675	229533.I1RN99	pp	229533.I1S0J1
28676	229533.I1RN99	pp	229533.Q4HZ68
28677	229533.I1RN99	pp	229533.I1S021
28678	229533.I1RN99	pp	229533.I1S060
28679	229533.I1RN99	pp	229533.I1RX14
28680	229533.I1RN99	pp	229533.I1RNH6
28681	229533.I1RN99	pp	229533.I1RW46
28682	229533.I1RN99	pp	229533.I1S296
28683	229533.I1S129	pp	229533.I1S130
28684	229533.I1RQA2	pp	229533.I1RZ99
28685	229533.I1RQA2	pp	229533.I1RR00
28686	229533.I1RQA2	pp	229533.I1RW71
28687	229533.I1RQA2	pp	229533.I1RSQ7
28688	229533.I1RQA2	pp	229533.V6QW22
28689	229533.I1RQA2	pp	229533.I1RQJ5

28690 229533.I1RQA2 pp 229533.Q4IQK7
28691 229533.I1RQA2 pp 229533.I1RRH5
28692 229533.I1RQA2 pp 229533.I1S0L3
28693 229533.I1RQA2 pp 229533.I1RRQ6
28694 229533.I1RQA2 pp 229533.I1RT33
28695 229533.I1RQA2 pp 229533.I1S6F0
28696 229533.I1RQA2 pp 229533.V6QYF0
28697 229533.I1RQA2 pp 229533.Q4HZ47
28698 229533.I1RQA2 pp 229533.I1RVQ0
28699 229533.I1RQA2 pp 229533.Q4IFI0
28700 229533.I1RQA2 pp 229533.I1RX08
28701 229533.I1RQA2 pp 229533.Q4IJH1
28702 229533.I1RQA2 pp 229533.I1RW25
28703 229533.I1RQA2 pp 229533.I1RTF4
28704 229533.I1RQA2 pp 229533.I1S0R7
28705 229533.I1RQA2 pp 229533.I1RSH5
28706 229533.I1RQA2 pp 229533.V6RCG5
28707 229533.I1RQA2 pp 229533.I1RT80
28708 229533.I1RQA2 pp 229533.I1RZM8
28709 229533.I1RQA2 pp 229533.I1RUQ2
28710 229533.I1RQA2 pp 229533.I1S6U8
28711 229533.I1RQA2 pp 229533.I1S021
28712 229533.I1RQA2 pp 229533.I1RZJ6
28713 229533.I1RQA2 pp 229533.Q4HVW2
28714 229533.I1RQA2 pp 229533.I1RT05
28715 229533.I1RQA2 pp 229533.Q4INZ9
28716 229533.I1RQA2 pp 229533.I1RT98
28717 229533.I1RQA2 pp 229533.I1S0F1
28718 229533.I1RQA2 pp 229533.I1RW99
28719 229533.I1RQA2 pp 229533.V6QUE6
28720 229533.I1RQA2 pp 229533.I1RQM6
28721 229533.I1RQA2 pp 229533.Q4IPI1
28722 229533.I1RQA2 pp 229533.I1S9U2
28723 229533.I1RQA2 pp 229533.I1RR24
28724 229533.I1RQA2 pp 229533.I1RS18
28725 229533.I1RQA2 pp 229533.Q4IAA0
28726 229533.I1RQA2 pp 229533.Q4IBS2
28727 229533.I1RQA2 pp 229533.I1RQS5
28728 229533.I1RQA2 pp 229533.Q4IJ56
28729 229533.I1RQA2 pp 229533.I1RWJ8
28730 229533.I1RQA2 pp 229533.I1S0M9
28731 229533.I1RQA2 pp 229533.I1RX14
28732 229533.I1RQA2 pp 229533.I1S2U2
28733 229533.I1RQA2 pp 229533.I1RRT2
28734 229533.I1RQA2 pp 229533.I1RZF6
28735 229533.I1RQA2 pp 229533.I1RZK6
28736 229533.I1RQA2 pp 229533.V6R227
28737 229533.I1RQA2 pp 229533.Q4I830
28738 229533.I1RQA2 pp 229533.I1RSX1
28739 229533.I1RQA2 pp 229533.Q4IEK8
28740 229533.I1RQA2 pp 229533.I1RRJ8
28741 229533.I1RQA2 pp 229533.I1RZZ1
28742 229533.I1RQA2 pp 229533.I1RWF7
28743 229533.I1RQA2 pp 229533.I1RTD9
28744 229533.I1RQA2 pp 229533.V6RBN2
28745 229533.I1RQA2 pp 229533.I1S0N3
28746 229533.I1RQA2 pp 229533.I1RWH8
28747 229533.I1RQA2 pp 229533.V6R283
28748 229533.I1RQA2 pp 229533.I1RVZ0
28749 229533.I1RQA2 pp 229533.I1RSR3
28750 229533.I1RQA2 pp 229533.I1RS47
28751 229533.I1RQA2 pp 229533.Q4HZ42
28752 229533.I1RQA2 pp 229533.I1RSC6
28753 229533.I1RQA2 pp 229533.I1RY63
28754 229533.I1RQA2 pp 229533.I1RRP3
28755 229533.I1RQA2 pp 229533.I1RZE5
28756 229533.I1RQA2 pp 229533.I1S0K9
28757 229533.I1RQA2 pp 229533.I1S2H4
28758 229533.I1RQA2 pp 229533.I1RSI0
28759 229533.I1RQA2 pp 229533.Q4HXF0
28760 229533.I1RQA2 pp 229533.I1RW26
28761 229533.I1RQA2 pp 229533.I1RS30
28762 229533.I1RQA2 pp 229533.I1S9Y1

28763 229533.I1RQA2 pp 229533.I1RRQ3
28764 229533.I1RQA2 pp 229533.I1S253
28765 229533.I1RQA2 pp 229533.I1S5R1
28766 229533.I1RQA2 pp 229533.I1S1B6
28767 229533.I1RQA2 pp 229533.Q4IF76
28768 229533.I1RQA2 pp 229533.I1RZZ9
28769 229533.I1RQA2 pp 229533.Q4IQW0
28770 229533.I1RQA2 pp 229533.I1RRT8
28771 229533.I1RQA2 pp 229533.Q4I327
28772 229533.I1RQA2 pp 229533.Q4IAS1
28773 229533.I1RQA2 pp 229533.I1RX24
28774 229533.I1RQA2 pp 229533.O74638
28775 229533.I1RQA2 pp 229533.I1RZS6
28776 229533.I1RQA2 pp 229533.I1RR72
28777 229533.I1RQA2 pp 229533.I1RRI2
28778 229533.I1RQA2 pp 229533.I1RZW8
28779 229533.I1RQA2 pp 229533.V6RGN5
28780 229533.I1RQA2 pp 229533.I1S274
28781 229533.I1RQA2 pp 229533.I1RW61
28782 229533.I1RQA2 pp 229533.I1RS17
28783 229533.I1RQA2 pp 229533.I1S1U7
28784 229533.I1RQA2 pp 229533.Q4I8X0
28785 229533.I1RQA2 pp 229533.I1RWJ4
28786 229533.I1RQA2 pp 229533.I1S060
28787 229533.I1RQA2 pp 229533.I1RW46
28788 229533.I1RQA2 pp 229533.I1RYG3
28789 229533.I1RQA2 pp 229533.V6RGY3
28790 229533.I1RQA2 pp 229533.Q4I662
28791 229533.I1RQA2 pp 229533.I1S558
28792 229533.I1RQA2 pp 229533.I1S0J1
28793 229533.I1RQA2 pp 229533.I1RSI6
28794 229533.I1RQA2 pp 229533.I1S1X9
28795 229533.I1RQA2 pp 229533.I1RRR7
28796 229533.I1RQA2 pp 229533.I1RUG3
28797 229533.I1RQA2 pp 229533.I1RSS4
28798 229533.I1RQA2 pp 229533.V6RIV9
28799 229533.I1RQA2 pp 229533.Q4IN63
28800 229533.I1RQA2 pp 229533.I1S4T3
28801 229533.I1RQA2 pp 229533.I1RSD8
28802 229533.I1RQA2 pp 229533.Q4HZK7
28803 229533.I1RQA2 pp 229533.I1RQE7
28804 229533.I1RQA2 pp 229533.I1S296
28805 229533.I1RQA2 pp 229533.I1S8R3
28806 229533.I1RQA2 pp 229533.I1S2E8
28807 229533.I1RQA2 pp 229533.I1S2C0
28808 229533.I1RQA2 pp 229533.Q4HZ68
28809 229533.I1RQA2 pp 229533.I1RTE9
28810 229533.I1RQA2 pp 229533.V6R057
28811 229533.I1RQA2 pp 229533.I1RTD7
28812 229533.I1RQA2 pp 229533.I1S0M3
28813 229533.I1RQA2 pp 229533.I1S1J6
28814 229533.A0A0E0SGM4 pp 229533.I1RZF6
28815 229533.A0A0E0SGM4 pp 229533.A0A1C3YJR3
28816 229533.A0A0E0SGM4 pp 229533.A0A1C3YKB0
28817 229533.A0A0E0SGM4 pp 229533.V6R227
28818 229533.A0A0E0SGM4 pp 229533.A0A1C3YKW9
28819 229533.A0A0E0SGM4 pp 229533.A0A1C3YK36
28820 229533.A0A0E0SGM4 pp 229533.I1RG56
28821 229533.A0A0E0SGM4 pp 229533.Q4IEK8
28822 229533.A0A0E0SGM4 pp 229533.Q4IJ56
28823 229533.A0A0E0SGM4 pp 229533.A0A1C3YHT4
28824 229533.A0A0E0SGM4 pp 229533.I1RB27
28825 229533.A0A0E0SGM4 pp 229533.A0A1C3YI30
28826 229533.A0A0E0SGM4 pp 229533.Q4IBS2
28827 229533.A0A0E0SGM4 pp 229533.I1RMD5
28828 229533.A0A0E0SGM4 pp 229533.V6RCG5
28829 229533.A0A0E0SGM4 pp 229533.I1RMC0
28830 229533.A0A0E0SGM4 pp 229533.I1RTD9
28831 229533.A0A0E0SGM4 pp 229533.I1RD52
28832 229533.A0A0E0SGM4 pp 229533.A0A1C3YII4
28833 229533.A0A0E0SGM4 pp 229533.I1RT33
28834 229533.A0A0E0SGM4 pp 229533.I1RQ31
28835 229533.A0A0E0SGM4 pp 229533.I1REF2

28836	229533.A0A0E0SGM4	pp	229533.I1RZM8
28837	229533.A0A0E0SGM4	pp	229533.I1RAA8
28838	229533.A0A0E0SGM4	pp	229533.I1S0L3
28839	229533.A0A0E0SGM4	pp	229533.A0A1C3YIP5
28840	229533.A0A0E0SGM4	pp	229533.I1S6F0
28841	229533.A0A0E0SGM4	pp	229533.I1RF08
28842	229533.A0A0E0SGM4	pp	229533.Q4IFI0
28843	229533.A0A0E0SGM4	pp	229533.I1REK8
28844	229533.A0A0E0SGM4	pp	229533.I1RAC2
28845	229533.A0A0E0SGM4	pp	229533.A0A1C3YL25
28846	229533.A0A0E0SGM4	pp	229533.I1RNK2
28847	229533.A0A0E0SGM4	pp	229533.I1RNH1
28848	229533.A0A0E0SGM4	pp	229533.I1RNA0
28849	229533.A0A0E0SGM4	pp	229533.I1RVQ0
28850	229533.A0A0E0SGM4	pp	229533.I1RVZ0
28851	229533.A0A0E0SGM4	pp	229533.I1RRQ6
28852	229533.A0A0E0SGM4	pp	229533.I1RAZ8
28853	229533.A0A0E0SGM4	pp	229533.I1RE00
28854	229533.A0A0E0SGM4	pp	229533.I1RFJ9
28855	229533.A0A0E0SGM4	pp	229533.I1S4S7
28856	229533.A0A0E0SGM4	pp	229533.V6QYF0
28857	229533.A0A0E0SGM4	pp	229533.I1S2L2
28858	229533.A0A0E0SGM4	pp	229533.I1RF19
28859	229533.A0A0E0SGM4	pp	229533.I1RM06
28860	229533.A0A0E0SGM4	pp	229533.I1R9V7
28861	229533.A0A0E0SGM4	pp	229533.I1RC32
28862	229533.A0A0E0SGM4	pp	229533.I1RY63
28863	229533.A0A0E0SGM4	pp	229533.I1RBC9
28864	229533.A0A0E0SGM4	pp	229533.I1RG51
28865	229533.A0A0E0SGM4	pp	229533.I1RW71
28866	229533.A0A0E0SGM4	pp	229533.I1RN99
28867	229533.A0A0E0SGM4	pp	229533.I1RPW9
28868	229533.A0A0E0SGM4	pp	229533.I1RQ28
28869	229533.A0A0E0SGM4	pp	229533.I1RQJ5
28870	229533.A0A0E0SGM4	pp	229533.I1RPU0
28871	229533.A0A0E0SGM4	pp	229533.V6QW22
28872	229533.A0A0E0SGM4	pp	229533.A0A1C3YHX9
28873	229533.A0A0E0SGM4	pp	229533.I1RK95
28874	229533.A0A0E0SGM4	pp	229533.A0A1C3YMG7
28875	229533.A0A0E0SGM4	pp	229533.I1RM67
28876	229533.A0A0E0SGM4	pp	229533.A0A1C3YKR0
28877	229533.A0A0E0SGM4	pp	229533.A0A1C3YL10
28878	229533.A0A0E0SGM4	pp	229533.V6RBN2
28879	229533.A0A0E0SGM4	pp	229533.I1RTF4
28880	229533.A0A0E0SGM4	pp	229533.I1RWH8
28881	229533.A0A0E0SGM4	pp	229533.I1S246
28882	229533.A0A0E0SGM4	pp	229533.Q4IJH1
28883	229533.A0A0E0SGM4	pp	229533.A0A1C3YJJ2
28884	229533.A0A0E0SGM4	pp	229533.I1RBL7
28885	229533.A0A0E0SGM4	pp	229533.I1RT80
28886	229533.A0A0E0SGM4	pp	229533.I1RSR3
28887	229533.A0A0E0SGM4	pp	229533.I1RJU3
28888	229533.A0A0E0SGM4	pp	229533.I1RQA2
28889	229533.A0A0E0SGM4	pp	229533.A0A1C3YMW0
28890	229533.A0A0E0SGM4	pp	229533.I1RDA0
28891	229533.A0A0E0SGM4	pp	229533.I1S0R7
28892	229533.A0A0E0SGM4	pp	229533.I1RYJ6
28893	229533.A0A0E0SGM4	pp	229533.Q4HVW2
28894	229533.A0A0E0SGM4	pp	229533.I1RCG4
28895	229533.A0A0E0SGM4	pp	229533.I1RMK3
28896	229533.A0A0E0SGM4	pp	229533.Q4INZ9
28897	229533.A0A0E0SGM4	pp	229533.I1RBD2
28898	229533.A0A0E0SGM4	pp	229533.I1RBT1
28899	229533.A0A0E0SGM4	pp	229533.I1S1X9
28900	229533.A0A0E0SGM4	pp	229533.I1RT05
28901	229533.A0A0E0SGM4	pp	229533.V6RIV9
28902	229533.A0A0E0SGM4	pp	229533.I1RSS4
28903	229533.A0A0E0SGM4	pp	229533.I1S0F1
28904	229533.A0A0E0SGM4	pp	229533.I1RFL4
28905	229533.A0A0E0SGM4	pp	229533.I1RSD8
28906	229533.A0A0E0SGM4	pp	229533.I1RPY1
28907	229533.A0A0E0SGM4	pp	229533.I1RRJ8
28908	229533.A0A0E0SGM4	pp	229533.I1RM96

28909 229533.A0A0E0SGM4 pp 229533.I1S2E8
28910 229533.A0A0E0SGM4 pp 229533.I1RN63
28911 229533.A0A0E0SGM4 pp 229533.I1RQE7
28912 229533.A0A0E0SGM4 pp 229533.I1RC33
28913 229533.A0A0E0SGM4 pp 229533.I1RNU6
28914 229533.A0A0E0SGM4 pp 229533.I1RWS4
28915 229533.A0A0E0SGM4 pp 229533.I1R9X7
28916 229533.A0A0E0SGM4 pp 229533.Q4I8X0
28917 229533.A0A0E0SGM4 pp 229533.I1S274
28918 229533.A0A0E0SGM4 pp 229533.I1RJK3
28919 229533.A0A0E0SGM4 pp 229533.V6RGY3
28920 229533.A0A0E0SGM4 pp 229533.I1RE01
28921 229533.A0A0E0SGM4 pp 229533.V6R283
28922 229533.A0A0E0SGM4 pp 229533.I1RC72
28923 229533.A0A0E0SGM4 pp 229533.I1RC03
28924 229533.A0A0E0SGM4 pp 229533.I1RC27
28925 229533.A0A0E0SGM4 pp 229533.V6RGN5
28926 229533.A0A0E0SGM4 pp 229533.I1S1U7
28927 229533.A0A0E0SGM4 pp 229533.I1RVZ2
28928 229533.A0A0E0SGM4 pp 229533.A0A1C3YJR2
28929 229533.A0A0E0SGM4 pp 229533.I1S558
28930 229533.A0A0E0SGM4 pp 229533.I1RBC7
28931 229533.A0A0E0SGM4 pp 229533.I1RG11
28932 229533.A0A0E0SGM4 pp 229533.I1RFN1
28933 229533.A0A0E0SGM4 pp 229533.I1RCL3
28934 229533.A0A0E0SGM4 pp 229533.I1RMN6
28935 229533.A0A0E0SGM4 pp 229533.I1RC82
28936 229533.A0A0E0SGM4 pp 229533.I1RML4
28937 229533.A0A0E0SGM4 pp 229533.I1RSI6
28938 229533.A0A0E0SGM4 pp 229533.I1RCW1
28939 229533.A0A0E0SGM4 pp 229533.I1RPJ3
28940 229533.A0A0E0SGM4 pp 229533.Q4IQW0
28941 229533.A0A0E0SGM4 pp 229533.I1RTE9
28942 229533.A0A0E0SGM4 pp 229533.I1RZB2
28943 229533.A0A0E0SGM4 pp 229533.I1S2C0
28944 229533.A0A0E0SGM4 pp 229533.I1RRI2
28945 229533.A0A0E0SGM4 pp 229533.I1RUG3
28946 229533.A0A0E0SGM4 pp 229533.Q4IAS1
28947 229533.A0A0E0SGM4 pp 229533.A0A1C3YJG0
28948 229533.A0A0E0SGM4 pp 229533.I1RNY2
28949 229533.A0A0E0SGM4 pp 229533.I1RJW6
28950 229533.A0A0E0SGM4 pp 229533.I1RNM8
28951 229533.A0A0E0SGM4 pp 229533.Q4I327
28952 229533.A0A0E0SGM4 pp 229533.I1RRT2
28953 229533.A0A0E0SGM4 pp 229533.I1S296
28954 229533.A0A0E0SGM4 pp 229533.Q4HZ68
28955 229533.A0A0E0SGM4 pp 229533.I1RZW8
28956 229533.A0A0E0SGM4 pp 229533.I1RS47
28957 229533.A0A0E0SGM4 pp 229533.I1RJL4
28958 229533.A0A0E0SGM4 pp 229533.Q4HZ42
28959 229533.A0A0E0SGM4 pp 229533.I1RRP3
28960 229533.A0A0E0SGM4 pp 229533.I1RD17
28961 229533.A0A0E0SGM4 pp 229533.I1RMH2
28962 229533.A0A0E0SGM4 pp 229533.I1RAF8
28963 229533.A0A0E0SGM4 pp 229533.I1RKC6
28964 229533.A0A0E0SGM4 pp 229533.I1RRQ3
28965 229533.A0A0E0SGM4 pp 229533.A0A1C3YIS7
28966 229533.A0A0E0SGM4 pp 229533.A0A1C3YN63
28967 229533.A0A0E0SGM4 pp 229533.Q4I830
28968 229533.A0A0E0SGM4 pp 229533.I1RJZ7
28969 229533.A0A0E0SGM4 pp 229533.I1RFS3
28970 229533.A0A0E0SGM4 pp 229533.A0A1C3YHT9
28971 229533.A0A0E0SGM4 pp 229533.I1RKL3
28972 229533.A0A0E0SGM4 pp 229533.I1RD64
28973 229533.A0A0E0SGM4 pp 229533.I1S0K9
28974 229533.A0A0E0SGM4 pp 229533.I1RX24
28975 229533.A0A0E0SGM4 pp 229533.I1RBG9
28976 229533.A0A0E0SGM4 pp 229533.I1S9Y1
28977 229533.A0A0E0SGM4 pp 229533.I1RKF9
28978 229533.A0A0E0SGM4 pp 229533.I1RR72
28979 229533.A0A0E0SGM4 pp 229533.A0A1C3YKK9
28980 229533.A0A0E0SGM4 pp 229533.I1RKJ6
28981 229533.A0A0E0SGM4 pp 229533.I1RRT8

229533.A0A0E0SGM4	pp	229533.I1RW61
229533.A0A0E0SGM4	pp	229533.I1S5R1
229533.A0A0E0SGM4	pp	229533.I1RSZ7
229533.A0A0E0SGM4	pp	229533.A0A1C3YM23
229533.A0A0E0SGM4	pp	229533.I1RTG7
229533.A0A0E0SGM4	pp	229533.I1RWJ4
229533.A0A0E0SGM4	pp	229533.I1S253
229533.A0A0E0SGM4	pp	229533.I1RJS9
229533.A0A0E0SGM4	pp	229533.A0A0E0SN72
229533.A0A0E0SGM4	pp	229533.Q4I1M9
229533.I1RQK4	pp	229533.I1RT98
229533.I1RQK4	pp	229533.V6QWZ7
229533.I1RQK4	pp	229533.V6RTJ8
229533.I1RQK4	pp	229533.I1RVZ0
229533.I1RQK4	pp	229533.I1RWF7
229533.I1RQK4	pp	229533.V6R283
229533.I1RQK4	pp	229533.I1S6U8
229533.I1RQK4	pp	229533.I1RZK6
229533.I1RQK4	pp	229533.I1S2L2
229533.V6QYF0	pp	229533.V6R283
229533.V6QYF0	pp	229533.V6RIV9
229533.V6QYF0	pp	229533.V6RCG5
229533.V6QYF0	pp	229533.V6RAC9
229533.V6QYF0	pp	229533.V6R227
229533.V6QYF0	pp	229533.V6RGY3
229533.V6QYF0	pp	229533.V6RGN5
229533.V6QYF0	pp	229533.V6RCZ8
229533.I1REF2	pp	229533.I1RT45
229533.I1REF2	pp	229533.I1RNF5
229533.I1REF2	pp	229533.V6QWZ7
229533.I1REF2	pp	229533.I1RSI0
229533.I1REF2	pp	229533.Q4IEK8
229533.I1REF2	pp	229533.I1RSI6
229533.I1REF2	pp	229533.I1RM96
229533.I1REF2	pp	229533.Q4I6A4
229533.I1REF2	pp	229533.I1RTD7
229533.I1REF2	pp	229533.I1RNV6
229533.I1REF2	pp	229533.V6RGY3
229533.I1REF2	pp	229533.I1RRI2
229533.I1REF2	pp	229533.I1RSR3
229533.I1REF2	pp	229533.I1RT80
229533.I1REF2	pp	229533.I1RMN7
229533.I1REF2	pp	229533.I1RI51
229533.I1REF2	pp	229533.I1RQ31
229533.I1REF2	pp	229533.I1S253
229533.I1REF2	pp	229533.I1RY63
229533.I1REF2	pp	229533.I1RQA2
229533.I1REF2	pp	229533.I1S6U8
229533.I1REF2	pp	229533.I1S021
229533.I1REF2	pp	229533.I1RMK3
229533.I1REF2	pp	229533.I1RNY2
229533.I1REF2	pp	229533.I1RN99
229533.I1REF2	pp	229533.V6QYF0
229533.I1REF2	pp	229533.V6RGN5
229533.I1REF2	pp	229533.I1RSD8
229533.I1REF2	pp	229533.I1RMN6
229533.I1REF2	pp	229533.Q4HZI0
229533.I1REF2	pp	229533.I1RZS6
229533.I1REF2	pp	229533.I1RZR2
229533.I1REF2	pp	229533.I1RKF9
229533.I1REF2	pp	229533.I1S9U2
229533.I1REF2	pp	229533.I1RFY0
229533.I1REF2	pp	229533.V6QUE6
229533.I1REF2	pp	229533.I1RR72
229533.I1REF2	pp	229533.Q4IJ56
229533.I1REF2	pp	229533.I1S0K6
229533.I1REF2	pp	229533.I1RM06
229533.I1REF2	pp	229533.Q4I9U7
229533.I1REF2	pp	229533.I1RKJ6
229533.I1REF2	pp	229533.I1RP31
229533.I1REF2	pp	229533.I1RRX0
229533.I1REF2	pp	229533.I1RG56
229533.I1REF2	pp	229533.I1RPU0

29055 229533.I1REF2 pp 229533.I1RG51
29056 229533.I1REF2 pp 229533.I1S4T3
29057 229533.I1REF2 pp 229533.I1RYT5
29058 229533.I1REF2 pp 229533.Q4HWB3
29059 229533.I1REF2 pp 229533.I1RHP9
29060 229533.I1REF2 pp 229533.I1S9Y1
29061 229533.I1REF2 pp 229533.I1RJB5
29062 229533.I1REF2 pp 229533.Q4I5M4
29063 229533.I1REF2 pp 229533.I1RK80
29064 229533.I1REF2 pp 229533.I1RMN5
29065 229533.I1REF2 pp 229533.I1RM67
29066 229533.I1REF2 pp 229533.I1RYG3
29067 229533.I1REF2 pp 229533.V6R283
29068 229533.I1REF2 pp 229533.I1RM49
29069 229533.I1REF2 pp 229533.I1RFW5
29070 229533.I1REF2 pp 229533.V6RLW4
29071 229533.I1REF2 pp 229533.I1RW28
29072 229533.I1REF2 pp 229533.I1RN51
29073 229533.I1REF2 pp 229533.I1RFN1
29074 229533.I1REF2 pp 229533.I1S1B6
29075 229533.I1REF2 pp 229533.Q4IFI0
29076 229533.I1REF2 pp 229533.I1RZH6
29077 229533.I1REF2 pp 229533.I1RJL4
29078 229533.I1REF2 pp 229533.Q4IQ72
29079 229533.I1REF2 pp 229533.I1RW71
29080 229533.I1REF2 pp 229533.I1RRT2
29081 229533.I1REF2 pp 229533.Q4IJP1
29082 229533.I1REF2 pp 229533.I1RKL3
29083 229533.I1REF2 pp 229533.I1RJL3
29084 229533.I1REF2 pp 229533.I1RW26
29085 229533.I1REF2 pp 229533.I1RNH1
29086 229533.I1REF2 pp 229533.I1RZ99
29087 229533.I1REF2 pp 229533.I1RWF7
29088 229533.I1REF2 pp 229533.I1RQJ5
29089 229533.I1REF2 pp 229533.I1RRQ3
29090 229533.I1RF84 pp 229533.I1RIJ1
29091 229533.A0A1C3YJH0 pp 229533.A0A1C3YJL4
29092 229533.A0A1C3YJH0 pp 229533.I1S575
29093 229533.Q4I830 pp 229533.V6RGY3
29094 229533.Q4I830 pp 229533.Q4IQW0
29095 229533.Q4I830 pp 229533.Q4IF76
29096 229533.Q4I830 pp 229533.V6RIS2
29097 229533.Q4I830 pp 229533.V6RGN5
29098 229533.Q4I830 pp 229533.Q4IAS1
29099 229533.Q4I830 pp 229533.Q4IJH1
29100 229533.Q4I830 pp 229533.Q4IEK8
29101 229533.Q4I830 pp 229533.Q4IFI0
29102 229533.Q4I830 pp 229533.V6RY33
29103 229533.Q4I830 pp 229533.V6R283
29104 229533.Q4I830 pp 229533.V6QYF0
29105 229533.Q4I830 pp 229533.Q4IAA0
29106 229533.Q4I830 pp 229533.Q4IBS2
29107 229533.Q4I830 pp 229533.V6R227
29108 229533.Q4I830 pp 229533.V6QUE6
29109 229533.Q4I830 pp 229533.Q4IPI1
29110 229533.Q4I830 pp 229533.Q4IJ56
29111 229533.Q4I830 pp 229533.Q4INZ9
29112 229533.I1RQQ6 pp 229533.I1RRM9
29113 229533.I1RQQ6 pp 229533.I1RT75
29114 229533.I1RPE1 pp 229533.I1RPK5
29115 229533.I1RPE1 pp 229533.I1RWI6
29116 229533.I1RPH9 pp 229533.I1RYT5
29117 229533.I1RPH9 pp 229533.I1S2U2
29118 229533.I1RPH9 pp 229533.I1RZS6
29119 229533.I1RPH9 pp 229533.I1RZ99
29120 229533.I1RPH9 pp 229533.I1RQ28
29121 229533.I1RPH9 pp 229533.I1RSI6
29122 229533.I1RPH9 pp 229533.I1RVZ0
29123 229533.I1RPH9 pp 229533.I1RX14
29124 229533.I1RPH9 pp 229533.I1RTG6
29125 229533.I1RPH9 pp 229533.I1RPI0
29126 229533.A0A1C3YKJ1 pp 229533.I1RQL4
29127 229533.I1S6B4 pp 229533.V6RIV9

229533.I1RW72 pp 229533.I1RW73
229533.I1RW72 pp 229533.I1RWI6
229533.I1RW72 pp 229533.I1S2U5
229533.I1RQK6 pp 229533.I1RRH5
229533.I1RQK6 pp 229533.Q4IBR4
229533.I1RQK6 pp 229533.Q4IR08
229533.I1RQK6 pp 229533.Q4IFH8
229533.I1S799 pp 229533.I1S9R7
229533.Q4IFI0 pp 229533.V6R227
229533.Q4IFI0 pp 229533.Q4IJ56
229533.Q4IFI0 pp 229533.V6RGN5
229533.Q4IFI0 pp 229533.Q4IPI1
229533.Q4IFI0 pp 229533.V6RMW6
229533.Q4IFI0 pp 229533.V6QUE6
229533.Q4IFI0 pp 229533.V6QYF0
229533.Q4IFI0 pp 229533.V6RCZ8
229533.Q4IFI0 pp 229533.V6RGY3
229533.Q4IFI0 pp 229533.Q4IQW0
229533.Q4IFI0 pp 229533.Q4INZ9
229533.Q4IFI0 pp 229533.Q4IJH1
229533.Q4IFI0 pp 229533.V6R042
229533.Q4IFI0 pp 229533.V6R283
229533.Q4IFI0 pp 229533.V6RIS2
229533.Q4IFI0 pp 229533.Q4IQ72
229533.Q4IFI0 pp 229533.Q4IN63
229533.Q4IFI0 pp 229533.V6RY33
229533.A0A098DXN1 pp 229533.I1RBE3
229533.A0A098DXN1 pp 229533.I1S799
229533.A0A098DXN1 pp 229533.I1S9R7
229533.I1RML3 pp 229533.I1RQL4
229533.I1RML3 pp 229533.I1S549
229533.I1RAC2 pp 229533.I1RQ31
229533.I1RAC2 pp 229533.I1S021
229533.I1RAC2 pp 229533.I1RMN7
229533.I1RAC2 pp 229533.I1RNH6
229533.I1RAC2 pp 229533.Q4I327
229533.I1RAC2 pp 229533.I1RZF6
229533.I1RAC2 pp 229533.V6R227
229533.I1RAC2 pp 229533.I1RRR7
229533.I1RAC2 pp 229533.I1RG56
229533.I1RAC2 pp 229533.I1RPY1
229533.I1RAC2 pp 229533.I1RC27
229533.I1RAC2 pp 229533.V6QXG1
229533.I1RAC2 pp 229533.I1RM96
229533.I1RAC2 pp 229533.V6RGN5
229533.I1RAC2 pp 229533.I1RMN6
229533.I1RAC2 pp 229533.Q4IEK8
229533.I1RAC2 pp 229533.I1RC33
229533.I1RAC2 pp 229533.Q4HZK7
229533.I1RAC2 pp 229533.I1RNU6
229533.I1RAC2 pp 229533.V6QUE6
229533.I1RAC2 pp 229533.Q4IBS2
229533.I1RAC2 pp 229533.I1RX64
229533.I1RAC2 pp 229533.I1RTE9
229533.I1RAC2 pp 229533.Q4IJ56
229533.I1RAC2 pp 229533.I1RB27
229533.I1RAC2 pp 229533.I1RSI6
229533.I1RAC2 pp 229533.I1S060
229533.I1RAC2 pp 229533.I1S2C0
229533.I1RAC2 pp 229533.I1RJY3
229533.I1RAC2 pp 229533.I1RX14
229533.I1RAC2 pp 229533.I1RTD9
229533.I1RAC2 pp 229533.I1RMC0
229533.I1RAC2 pp 229533.I1RC72
229533.I1RAC2 pp 229533.I1RRI2
229533.I1RAC2 pp 229533.I1S0J1
229533.I1RAC2 pp 229533.I1RN99
229533.I1RAC2 pp 229533.I1S2U2
229533.I1RAC2 pp 229533.I1S274
229533.I1RAC2 pp 229533.I1RBL7
229533.I1RAC2 pp 229533.I1RSD8
229533.I1RAC2 pp 229533.I1S296
229533.I1RAC2 pp 229533.I1RJY2

229533.I1RAC2	pp	229533.Q4HZ68
229533.I1RAC2	pp	229533.I1S2E8
229533.I1RAC2	pp	229533.I1RZK6
229533.I1RAC2	pp	229533.V6RGY3
229533.I1RAC2	pp	229533.I1RF08
229533.I1RAC2	pp	229533.I1RBC7
229533.I1RAC2	pp	229533.I1RAZ8
229533.I1RAC2	pp	229533.I1RSR3
229533.I1RAC2	pp	229533.I1S1J6
229533.I1RAC2	pp	229533.I1RKC6
229533.I1RAC2	pp	229533.I1RFN1
229533.I1RAC2	pp	229533.I1RC82
229533.I1RAC2	pp	229533.I1S1B6
229533.I1RAC2	pp	229533.I1S1U7
229533.I1RAC2	pp	229533.I1RAN5
229533.I1RAC2	pp	229533.I1RNK2
229533.I1RAC2	pp	229533.Q4IQW0
229533.I1RAC2	pp	229533.I1RCN5
229533.I1RAC2	pp	229533.I1RVZ0
229533.I1RAC2	pp	229533.I1RQA2
229533.I1RAC2	pp	229533.I1RJ74
229533.I1RAC2	pp	229533.I1RE00
229533.I1RAC2	pp	229533.I1RCL3
229533.I1RAC2	pp	229533.I1RKQ5
229533.I1RAC2	pp	229533.I1RS47
229533.I1RAC2	pp	229533.I1RFJ9
229533.I1RAC2	pp	229533.I1RF19
229533.I1RAC2	pp	229533.I1RLV9
229533.I1RAC2	pp	229533.I1RZE5
229533.I1RAC2	pp	229533.Q4IAS1
229533.I1RAC2	pp	229533.Q4IEV5
229533.I1RAC2	pp	229533.I1RSC6
229533.I1RAC2	pp	229533.I1RAV6
229533.I1RAC2	pp	229533.I1RQ65
229533.I1RAC2	pp	229533.I1RJL4
229533.I1RAC2	pp	229533.Q4IPI1
229533.I1RAC2	pp	229533.Q4HWE2
229533.I1RAC2	pp	229533.I1RSS4
229533.I1RAC2	pp	229533.Q4HZ47
229533.I1RAC2	pp	229533.I1RS30
229533.I1RAC2	pp	229533.I1S1X9
229533.I1RAC2	pp	229533.I1RJT8
229533.I1RAC2	pp	229533.I1RRT2
229533.I1RAC2	pp	229533.I1RNH1
229533.I1RAC2	pp	229533.Q4HXF0
229533.I1RAC2	pp	229533.I1RQS5
229533.I1RAC2	pp	229533.I1RQ28
229533.I1RAC2	pp	229533.I1S6F0
229533.I1RAC2	pp	229533.I1RFG0
229533.I1RAC2	pp	229533.I1RK95
229533.I1RAC2	pp	229533.I1S0R7
229533.I1RAC2	pp	229533.I1RDG0
229533.I1RAC2	pp	229533.I1RTF4
229533.I1RAC2	pp	229533.I1RRQ3
229533.I1RAC2	pp	229533.I1RAF8
229533.I1RAC2	pp	229533.Q4INZ9
229533.I1RAC2	pp	229533.Q4HZ42
229533.I1RAC2	pp	229533.I1RSX1
229533.I1RAC2	pp	229533.Q4IJH1
229533.I1RAC2	pp	229533.Q4I830
229533.I1RAC2	pp	229533.I1RKL3
229533.I1RAC2	pp	229533.I1RT80
229533.I1RAC2	pp	229533.I1RT33
229533.I1RAC2	pp	229533.I1RNV6
229533.I1RAC2	pp	229533.I1RDA0
229533.I1RAC2	pp	229533.I1RW71
229533.I1RAC2	pp	229533.Q4HVW2
229533.I1RAC2	pp	229533.I1RRH5
229533.I1RAC2	pp	229533.I1RG51
229533.I1RAC2	pp	229533.I1RRJ8
229533.I1RAC2	pp	229533.I1RZ99
229533.I1RAC2	pp	229533.I1RMK3
229533.I1RAC2	pp	229533.I1RQJ5

29274 229533.I1RAC2 pp 229533.Q4IF76
29275 229533.I1RAC2 pp 229533.I1RT05
29276 229533.I1RAC2 pp 229533.V6R283
29277 229533.I1RAC2 pp 229533.I1RQM6
29278 229533.I1RAC2 pp 229533.I1RC03
29279 229533.I1RAC2 pp 229533.I1RFT6
29280 229533.I1RAC2 pp 229533.I1S2M6
29281 229533.I1RAC2 pp 229533.I1RZH6
29282 229533.I1RAC2 pp 229533.I1RFH8
29283 229533.I1RAC2 pp 229533.I1RQS8
29284 229533.I1RAC2 pp 229533.I1RJZ7
29285 229533.I1RAC2 pp 229533.I1RD64
29286 229533.I1RAC2 pp 229533.Q4IFI0
29287 229533.I1RAC2 pp 229533.I1RGF6
29288 229533.I1RAC2 pp 229533.I1RL23
29289 229533.I1RAC2 pp 229533.I1S9U2
29290 229533.I1RAC2 pp 229533.I1RNA0
29291 229533.I1RAC2 pp 229533.Q4IQK7
29292 229533.I1RAC2 pp 229533.I1RPW9
29293 229533.I1RAC2 pp 229533.I1RC29
29294 229533.I1RAC2 pp 229533.I1RPU0
29295 229533.I1RAC2 pp 229533.I1RBG9
29296 229533.I1RAC2 pp 229533.I1RKJ6
29297 229533.I1RAC2 pp 229533.I1S9Y1
29298 229533.I1RAC2 pp 229533.V6QYF0
29299 229533.I1RAC2 pp 229533.I1RX24
29300 229533.I1RAC2 pp 229533.I1RM67
29301 229533.I1RAC2 pp 229533.I1RRT8
29302 229533.I1RAC2 pp 229533.I1RZS6
29303 229533.I1RAC2 pp 229533.I1RM06
29304 229533.I1RAC2 pp 229533.I1RC32
29305 229533.I1RAC2 pp 229533.I1RW61
29306 229533.I1RAC2 pp 229533.O74638
29307 229533.I1RAC2 pp 229533.I1REX4
29308 229533.I1RAC2 pp 229533.Q4I662
29309 229533.I1RAC2 pp 229533.I1RFS3
29310 229533.I1RQM6 pp 229533.I1RWI5
29311 229533.I1RQM6 pp 229533.Q4IFI0
29312 229533.I1RQM6 pp 229533.I1RW25
29313 229533.I1RQM6 pp 229533.Q4IF76
29314 229533.I1RQM6 pp 229533.Q4I830
29315 229533.I1RQM6 pp 229533.I1RTF4
29316 229533.I1RQM6 pp 229533.I1RYG3
29317 229533.I1RQM6 pp 229533.I1RW71
29318 229533.I1RQM6 pp 229533.I1RYT5
29319 229533.I1RQM6 pp 229533.Q4IJH1
29320 229533.I1RQM6 pp 229533.I1RRJ8
29321 229533.I1RQM6 pp 229533.I1S9Y1
29322 229533.I1RQM6 pp 229533.I1RT33
29323 229533.I1RQM6 pp 229533.I1RRT2
29324 229533.I1RQM6 pp 229533.I1RT80
29325 229533.I1RQM6 pp 229533.O74638
29326 229533.I1RQM6 pp 229533.I1RQS5
29327 229533.I1RQM6 pp 229533.I1RRT8
29328 229533.I1RQM6 pp 229533.Q4INZ9
29329 229533.I1RQM6 pp 229533.V6R283
29330 229533.I1RQM6 pp 229533.Q4HXF0
29331 229533.I1RQM6 pp 229533.I1RT04
29332 229533.I1RQM6 pp 229533.V6RGY3
29333 229533.I1RQM6 pp 229533.I1S1U7
29334 229533.I1RQM6 pp 229533.I1RUG4
29335 229533.I1RQM6 pp 229533.I1RUQ2
29336 229533.I1RQM6 pp 229533.I1RRI2
29337 229533.I1RQM6 pp 229533.I1S0R7
29338 229533.I1RQM6 pp 229533.V6R227
29339 229533.I1RQM6 pp 229533.I1RSD8
29340 229533.I1RQM6 pp 229533.I1RZB5
29341 229533.I1RQM6 pp 229533.I1RX14
29342 229533.I1RQM6 pp 229533.I1RTE9
29343 229533.I1RQM6 pp 229533.I1RZK6
29344 229533.I1RQM6 pp 229533.I1S2C0
29345 229533.I1RQM6 pp 229533.I1RZZ1
29346 229533.I1RQM6 pp 229533.I1RSS4

29347 229533.I1RQM6 pp 229533.V6QUE6
29348 229533.I1RQM6 pp 229533.I1S2E8
29349 229533.I1RQM6 pp 229533.Q4IN63
29350 229533.I1RQM6 pp 229533.V6RGN5
29351 229533.I1RQM6 pp 229533.Q4IEK8
29352 229533.I1RQM6 pp 229533.I1S0N4
29353 229533.I1RQM6 pp 229533.I1S021
29354 229533.I1RQM6 pp 229533.I1RRV1
29355 229533.I1RQM6 pp 229533.I1RSI6
29356 229533.I1RRQ6 pp 229533.I1RSS4
29357 229533.I1RRQ6 pp 229533.Q4IFI0
29358 229533.I1RRQ6 pp 229533.I1RY63
29359 229533.I1RRQ6 pp 229533.V6QYF0
29360 229533.I1RRQ6 pp 229533.I1RW25
29361 229533.I1RRQ6 pp 229533.I1S0M3
29362 229533.I1RRQ6 pp 229533.Q4I5Z5
29363 229533.I1RRQ6 pp 229533.I1RTD9
29364 229533.I1RRQ6 pp 229533.Q4HZ68
29365 229533.I1RRQ6 pp 229533.I1RS18
29366 229533.I1RRQ6 pp 229533.Q4IJ56
29367 229533.I1RRQ6 pp 229533.I1RRT8
29368 229533.I1RRQ6 pp 229533.I1RTE9
29369 229533.I1RRQ6 pp 229533.I1RTF4
29370 229533.I1RRQ6 pp 229533.Q4IJH1
29371 229533.I1RRQ6 pp 229533.Q4INZ9
29372 229533.I1RRQ6 pp 229533.V6QW22
29373 229533.I1RRQ6 pp 229533.I1S2C0
29374 229533.I1RRQ6 pp 229533.I1S6F0
29375 229533.I1RRQ6 pp 229533.I1RW26
29376 229533.I1RRQ6 pp 229533.Q4IEK8
29377 229533.I1RRQ6 pp 229533.Q4HVW2
29378 229533.I1RRQ6 pp 229533.Q4I327
29379 229533.I1RRQ6 pp 229533.Q4HZ42
29380 229533.I1RRQ6 pp 229533.V6RCG5
29381 229533.I1RRQ6 pp 229533.Q4IBS2
29382 229533.I1RRQ6 pp 229533.I1RS47
29383 229533.I1RRQ6 pp 229533.Q4IAS1
29384 229533.I1RRQ6 pp 229533.Q4IEV5
29385 229533.I1RRQ6 pp 229533.Q4IPI1
29386 229533.I1RRQ6 pp 229533.I1S0L3
29387 229533.I1RRQ6 pp 229533.I1RUQ9
29388 229533.I1RRQ6 pp 229533.I1RRT2
29389 229533.I1RRQ6 pp 229533.I1RT98
29390 229533.I1RRQ6 pp 229533.V6RGN5
29391 229533.I1RRQ6 pp 229533.V6R227
29392 229533.I1RRQ6 pp 229533.Q4IQW0
29393 229533.I1RK86 pp 229533.I1RZJ9
29394 229533.I1RK86 pp 229533.I1RRE1
29395 229533.I1RK86 pp 229533.I1RN66
29396 229533.I1RK86 pp 229533.I1RM62
29397 229533.I1RK86 pp 229533.I1RKA1
29398 229533.I1RK86 pp 229533.I1RWI0
29399 229533.I1RK86 pp 229533.I1RM09
29400 229533.I1RK86 pp 229533.I1S0Y1
29401 229533.I1RK86 pp 229533.V6QW25
29402 229533.I1RK86 pp 229533.I1S0K5
29403 229533.I1RK86 pp 229533.Q4ICM9
29404 229533.I1RK86 pp 229533.I1S0L1
29405 229533.I1RK86 pp 229533.I1RXV4
29406 229533.I1RB68 pp 229533.I1RPT2
29407 229533.A0A0E0SJU2 pp 229533.I1RNG4
29408 229533.I1RND7 pp 229533.I1RQH7
29409 229533.I1RND7 pp 229533.I1S1V4
29410 229533.I1RND7 pp 229533.I1RPG2
29411 229533.I1RND7 pp 229533.I1S213
29412 229533.I1RND7 pp 229533.I1S2B6
29413 229533.I1RND7 pp 229533.Q4IN63
29414 229533.I1RND7 pp 229533.I1S5B5
29415 229533.I1RND7 pp 229533.I1RZK3
29416 229533.I1RND7 pp 229533.I1SAM5
29417 229533.I1RND7 pp 229533.I1RNH7
29418 229533.I1RND7 pp 229533.I1RQF7
29419 229533.I1RND7 pp 229533.I1RW76

29420	229533.I1RND7	pp	229533.I1RQD6
29421	229533.I1RB61	pp	229533.I1RJD9
29422	229533.I1RG51	pp	229533.I1S9U2
29423	229533.I1RG51	pp	229533.I1RPU0
29424	229533.I1RG51	pp	229533.I1S9Y1
29425	229533.I1RG51	pp	229533.I1RRT8
29426	229533.I1RG51	pp	229533.I1RKJ6
29427	229533.I1RG51	pp	229533.O74638
29428	229533.I1RG51	pp	229533.I1RM67
29429	229533.I1RG51	pp	229533.I1RT80
29430	229533.I1RG51	pp	229533.I1RNA0
29431	229533.I1RG51	pp	229533.I1RM06
29432	229533.I1RG51	pp	229533.Q4IQK7
29433	229533.I1RG51	pp	229533.I1RZS6
29434	229533.I1RG51	pp	229533.I1RNV6
29435	229533.I1RG51	pp	229533.I1RTF4
29436	229533.I1RG51	pp	229533.I1RYT5
29437	229533.I1RG51	pp	229533.I1RPH9
29438	229533.I1RG51	pp	229533.Q4IJH1
29439	229533.I1RG51	pp	229533.Q4HVW2
29440	229533.I1RG51	pp	229533.Q4I662
29441	229533.I1RG51	pp	229533.I1RND6
29442	229533.I1RG51	pp	229533.I1S1B6
29443	229533.I1RG51	pp	229533.I1RMK3
29444	229533.I1RG51	pp	229533.I1RP27
29445	229533.I1RG51	pp	229533.I1RJ74
29446	229533.I1RG51	pp	229533.Q4IQW0
29447	229533.I1RG51	pp	229533.I1RLV9
29448	229533.I1RG51	pp	229533.Q4IF76
29449	229533.I1RG51	pp	229533.I1RQM6
29450	229533.I1RG51	pp	229533.I1RT05
29451	229533.I1RG51	pp	229533.Q4IAS1
29452	229533.I1RG51	pp	229533.I1RRT2
29453	229533.I1RG51	pp	229533.I1RQS5
29454	229533.I1RG51	pp	229533.I1RKQ5
29455	229533.I1RG51	pp	229533.I1RJZ7
29456	229533.I1RG51	pp	229533.I1RL23
29457	229533.I1RG51	pp	229533.I1RS20
29458	229533.I1RG51	pp	229533.Q4IFI0
29459	229533.I1RG51	pp	229533.I1RGF6
29460	229533.I1RG51	pp	229533.I1RQS8
29461	229533.I1RG51	pp	229533.I1RJL4
29462	229533.I1RG51	pp	229533.Q4IPI1
29463	229533.I1RG51	pp	229533.I1RPW9
29464	229533.I1RG51	pp	229533.Q4IMJ1
29465	229533.I1RG51	pp	229533.I1RX24
29466	229533.I1RG51	pp	229533.I1S0R7
29467	229533.I1RG51	pp	229533.I1RSS4
29468	229533.I1RG51	pp	229533.I1RKF9
29469	229533.I1RG51	pp	229533.I1RN99
29470	229533.I1RG51	pp	229533.Q4HXF0
29471	229533.I1RG51	pp	229533.I1RQ28
29472	229533.I1RG51	pp	229533.I1RUQ2
29473	229533.I1RG51	pp	229533.Q4INZ9
29474	229533.I1RG51	pp	229533.I1RK95
29475	229533.I1RG51	pp	229533.V6QYF0
29476	229533.I1RG51	pp	229533.I1RSD8
29477	229533.I1RG51	pp	229533.I1RZJ6
29478	229533.I1RG51	pp	229533.I1RS30
29479	229533.I1RG51	pp	229533.I1S6F0
29480	229533.I1RG51	pp	229533.I1S2U2
29481	229533.I1RG51	pp	229533.I1RRQ3
29482	229533.I1RG51	pp	229533.I1RS47
29483	229533.I1RG51	pp	229533.Q4I948
29484	229533.I1RG51	pp	229533.I1RNH1
29485	229533.I1RG51	pp	229533.I1RKL3
29486	229533.I1RG51	pp	229533.I1RRH5
29487	229533.I1RG51	pp	229533.Q4HZ42
29488	229533.I1RG51	pp	229533.I1RZZ1
29489	229533.I1RG51	pp	229533.I1RT33
29490	229533.I1RG51	pp	229533.I1RZK6
29491	229533.I1RG51	pp	229533.I1RW71
29492	229533.I1RG51	pp	229533.I1RJZ6

29493 229533.I1RG51 pp 229533.I1S2E8
29494 229533.I1RG51 pp 229533.Q4I830
29495 229533.I1RG51 pp 229533.I1RQE7
29496 229533.I1RG51 pp 229533.I1RQ65
29497 229533.I1RG51 pp 229533.Q4HZK7
29498 229533.I1RG51 pp 229533.I1RQJ5
29499 229533.I1RG51 pp 229533.I1RZ99
29500 229533.I1RG51 pp 229533.V6RGN5
29501 229533.I1RG51 pp 229533.I1RZB5
29502 229533.I1RG51 pp 229533.I1RRJ8
29503 229533.I1RG51 pp 229533.V6R283
29504 229533.I1RG51 pp 229533.V6QUE6
29505 229533.I1RG51 pp 229533.I1RMN6
29506 229533.I1RG51 pp 229533.I1S2C0
29507 229533.I1RG51 pp 229533.I1S2M6
29508 229533.I1RG51 pp 229533.Q4IBS2
29509 229533.I1RG51 pp 229533.I1RPJ3
29510 229533.I1RG51 pp 229533.I1RTE9
29511 229533.I1RG51 pp 229533.I1RMD5
29512 229533.I1RG51 pp 229533.I1RQH2
29513 229533.I1RG51 pp 229533.V6RGY3
29514 229533.I1RG51 pp 229533.I1S274
29515 229533.I1RG51 pp 229533.I1RSR3
29516 229533.I1RG51 pp 229533.I1RSH5
29517 229533.I1RG51 pp 229533.I1RRI2
29518 229533.I1RG51 pp 229533.V6RGY0
29519 229533.I1RG51 pp 229533.I1RR32
29520 229533.I1RG51 pp 229533.I1RSI6
29521 229533.I1RG51 pp 229533.I1RQ31
29522 229533.I1RG51 pp 229533.I1S1U7
29523 229533.I1RG51 pp 229533.I1RMC0
29524 229533.I1RG51 pp 229533.I1RQA2
29525 229533.I1RG51 pp 229533.I1S296
29526 229533.I1RG51 pp 229533.I1RPY1
29527 229533.I1RG51 pp 229533.Q4HZ68
29528 229533.I1RG51 pp 229533.I1RVZ0
29529 229533.I1RG51 pp 229533.V6RG06
29530 229533.I1RG51 pp 229533.Q4IN63
29531 229533.I1RG51 pp 229533.I1RNK2
29532 229533.I1RG51 pp 229533.I1RM96
29533 229533.I1RG51 pp 229533.I1RNY2
29534 229533.I1RG51 pp 229533.Q4I327
29535 229533.I1RG51 pp 229533.Q4IEK8
29536 229533.I1RG51 pp 229533.I1S0M9
29537 229533.I1RG51 pp 229533.I1S1J6
29538 229533.I1RG51 pp 229533.I1RNH6
29539 229533.I1RG51 pp 229533.Q4IJ56
29540 229533.I1RG51 pp 229533.I1S021
29541 229533.I1RG51 pp 229533.I1RKC6
29542 229533.I1RG51 pp 229533.I1RMN7
29543 229533.I1RG51 pp 229533.I1RZE5
29544 229533.I1RG51 pp 229533.I1RNU6
29545 229533.I1RG51 pp 229533.I1RZF6
29546 229533.I1RG51 pp 229533.I1S1X9
29547 229533.I1RG51 pp 229533.I1RX14
29548 229533.I1RG51 pp 229533.I1RSC6
29549 229533.I1RG51 pp 229533.I1RRR7
29550 229533.I1RG51 pp 229533.V6R227
29551 229533.I1RG51 pp 229533.I1S060
29552 229533.I1RG51 pp 229533.I1S4T3
29553 229533.I1RG51 pp 229533.I1S0J1
29554 229533.I1RNV6 pp 229533.Q4IJH1
29555 229533.I1RNV6 pp 229533.I1RTF4
29556 229533.I1RNV6 pp 229533.I1RT80
29557 229533.I1RNV6 pp 229533.Q4IF76
29558 229533.I1RNV6 pp 229533.Q4HVW2
29559 229533.I1RNV6 pp 229533.I1RQM6
29560 229533.I1RNV6 pp 229533.I1RYZ7
29561 229533.I1RNV6 pp 229533.I1RZZ9
29562 229533.I1RNV6 pp 229533.V6R283
29563 229533.I1RNV6 pp 229533.I1S9U2
29564 229533.I1RNV6 pp 229533.I1RQS8
29565 229533.I1RNV6 pp 229533.I1RPU0

29566	229533.I1RNV6	pp	229533.I1RPW9
29567	229533.I1RNV6	pp	229533.I1RRT8
29568	229533.I1RNV6	pp	229533.I1S9Y1
29569	229533.I1RNV6	pp	229533.I1S2M6
29570	229533.I1RNV6	pp	229533.I1RX24
29571	229533.I1RNV6	pp	229533.Q4HZ42
29572	229533.I1RNV6	pp	229533.V6QYF0
29573	229533.I1RNV6	pp	229533.I1RS47
29574	229533.I1RNV6	pp	229533.I1RUQ2
29575	229533.I1RNV6	pp	229533.I1RRH5
29576	229533.I1RNV6	pp	229533.I1RZS6
29577	229533.I1RNV6	pp	229533.I1RZJ6
29578	229533.I1RNV6	pp	229533.I1S0L3
29579	229533.I1RNV6	pp	229533.I1S1B6
29580	229533.I1RNV6	pp	229533.I1RYT5
29581	229533.I1RNV6	pp	229533.I1RQS5
29582	229533.I1RNV6	pp	229533.I1RRT2
29583	229533.I1RNV6	pp	229533.I1S0J7
29584	229533.I1RNV6	pp	229533.I1RWD4
29585	229533.I1RNV6	pp	229533.Q4I662
29586	229533.I1RNV6	pp	229533.I1RRJ8
29587	229533.I1RNV6	pp	229533.I1RS30
29588	229533.I1RNV6	pp	229533.I1RSX1
29589	229533.I1RNV6	pp	229533.I1RRP3
29590	229533.I1RNV6	pp	229533.I1RW25
29591	229533.I1RNV6	pp	229533.I1RRQ3
29592	229533.I1RNV6	pp	229533.I1RZ99
29593	229533.I1RNV6	pp	229533.I1S0R7
29594	229533.I1RNV6	pp	229533.I1RVZ0
29595	229533.I1RNV6	pp	229533.I1RZB5
29596	229533.I1RNV6	pp	229533.I1RQJ5
29597	229533.I1RNV6	pp	229533.I1S1U7
29598	229533.I1RNV6	pp	229533.I1RSR3
29599	229533.I1RNV6	pp	229533.I1RSI6
29600	229533.I1RNV6	pp	229533.I1RQA2
29601	229533.I1RNV6	pp	229533.I1S2C0
29602	229533.I1RNV6	pp	229533.I1RUG3
29603	229533.I1RNV6	pp	229533.V6RCG5
29604	229533.I1RNV6	pp	229533.I1RTE9
29605	229533.I1RNV6	pp	229533.I1S2U2
29606	229533.I1RNV6	pp	229533.I1S1X9
29607	229533.I1RNV6	pp	229533.I1RQ31
29608	229533.I1RNV6	pp	229533.I1RSD8
29609	229533.I1RNV6	pp	229533.I1RPJ3
29610	229533.I1RNV6	pp	229533.Q4I327
29611	229533.I1RNV6	pp	229533.I1RNY2
29612	229533.I1RNV6	pp	229533.I1RRI2
29613	229533.I1RNV6	pp	229533.I1S274
29614	229533.I1RNV6	pp	229533.I1RQE7
29615	229533.I1RNV6	pp	229533.I1RW99
29616	229533.I1RNV6	pp	229533.I1RZZ1
29617	229533.I1RNV6	pp	229533.I1S2E8
29618	229533.I1RNV6	pp	229533.I1RZK6
29619	229533.I1RNV6	pp	229533.I1S5B0
29620	229533.I1RNV6	pp	229533.Q4IJ56
29621	229533.I1RNV6	pp	229533.Q4HZ68
29622	229533.I1RNV6	pp	229533.I1S060
29623	229533.I1RNV6	pp	229533.V6QUE6
29624	229533.I1RNV6	pp	229533.I1S0J1
29625	229533.I1RNV6	pp	229533.V6RGN5
29626	229533.I1RNV6	pp	229533.Q4I5Z5
29627	229533.I1RNV6	pp	229533.I1RZF6
29628	229533.I1RNV6	pp	229533.I1S296
29629	229533.I1RNV6	pp	229533.V6R227
29630	229533.I1RNV6	pp	229533.I1S193
29631	229533.I1RNV6	pp	229533.I1RS64
29632	229533.I1RNV6	pp	229533.I1RRR7
29633	229533.I1RNV6	pp	229533.Q4IBS2
29634	229533.I1RNV6	pp	229533.Q4IEK8
29635	229533.I1RNV6	pp	229533.I1RS17
29636	229533.I1RWQ1	pp	229533.Q4ILI9
29637	229533.I1RBN1	pp	229533.V6QUE6
29638	229533.I1RBN1	pp	229533.Q4IBS2

229533.I1RBN1	pp	229533.Q4IAA0
229533.I1RBN1	pp	229533.I1RSR8
229533.I1RBN1	pp	229533.I1RMD5
229533.I1RBN1	pp	229533.Q4IJ56
229533.I1RBN1	pp	229533.I1RSM3
229533.I1RBN1	pp	229533.I1S0M9
229533.I1RBN1	pp	229533.I1RSR3
229533.I1RBN1	pp	229533.I1RNH6
229533.I1RBN1	pp	229533.I1RQA2
229533.I1RBN1	pp	229533.I1RN99
229533.I1RBN1	pp	229533.I1S021
229533.I1RBN1	pp	229533.V6R227
229533.I1RBN1	pp	229533.I1RMC0
229533.I1RBN1	pp	229533.I1RQ28
229533.I1RBN1	pp	229533.I1RQ31
229533.I1RBN1	pp	229533.I1RW99
229533.I1RBN1	pp	229533.Q4IEK8
229533.I1RBN1	pp	229533.I1RDG0
229533.I1RBN1	pp	229533.I1RK95
229533.I1RBN1	pp	229533.I1RJZ6
229533.I1RBN1	pp	229533.I1RG56
229533.I1RBN1	pp	229533.I1RTI6
229533.I1RBN1	pp	229533.I1RC06
229533.I1RBN1	pp	229533.I1RVZ0
229533.I1RBN1	pp	229533.I1RT98
229533.I1RBN1	pp	229533.I1RFJ9
229533.I1RBN1	pp	229533.I1RZB5
229533.I1RBN1	pp	229533.I1RM67
229533.I1RBN1	pp	229533.I1RF19
229533.I1RBN1	pp	229533.I1RPU0
229533.I1RBN1	pp	229533.I1S0D4
229533.I1RBN1	pp	229533.Q4IPI1
229533.I1RBN1	pp	229533.I1RQJ5
229533.I1RBN1	pp	229533.I1RRH5
229533.I1RBN1	pp	229533.I1RM06
229533.I1RBN1	pp	229533.I1RD52
229533.I1RBN1	pp	229533.I1RNA0
229533.I1RBN1	pp	229533.I1RW71
229533.I1RBN1	pp	229533.I1RD56
229533.I1RBN1	pp	229533.I1RLU5
229533.I1RBN1	pp	229533.I1RR00
229533.I1RBN1	pp	229533.I1RT33
229533.I1RBN1	pp	229533.V6QYF0
229533.I1RBN1	pp	229533.I1S6F0
229533.I1RBN1	pp	229533.I1RRQ6
229533.I1RBN1	pp	229533.I1RNU6
229533.I1RBN1	pp	229533.I1RMN6
229533.I1RBN1	pp	229533.I1RG51
229533.I1RBN1	pp	229533.I1RNH1
229533.I1RBN1	pp	229533.Q4IFI0
229533.I1RBN1	pp	229533.I1RM25
229533.I1RBN1	pp	229533.I1RBW7
229533.I1RBN1	pp	229533.Q4HVW2
229533.I1RBN1	pp	229533.I1S0J1
229533.I1RBN1	pp	229533.I1RSD4
229533.I1RBN1	pp	229533.I1RN51
229533.I1RBN1	pp	229533.I1RSI6
229533.I1RBN1	pp	229533.Q4IJH1
229533.I1RBN1	pp	229533.I1RTF4
229533.I1RBN1	pp	229533.I1RC72
229533.I1RBN1	pp	229533.I1RNY2
229533.I1RBN1	pp	229533.I1RUQ9
229533.I1RBN1	pp	229533.I1S060
229533.I1RBN1	pp	229533.I1RRR7
229533.I1RBN1	pp	229533.I1RNV6
229533.I1RBN1	pp	229533.I1RT05
229533.I1RBN1	pp	229533.I1RT80
229533.I1RBN1	pp	229533.I1RPW9
229533.I1RBN1	pp	229533.Q4I327
229533.I1RBN1	pp	229533.I1RM96
229533.I1RBN1	pp	229533.I1S9U2
229533.I1RBN1	pp	229533.V6RY33
229533.I1RBN1	pp	229533.I1RC33

29712 229533.I1RBN1 pp 229533.I1S4T3
29713 229533.I1RBN1 pp 229533.Q4IN63
29714 229533.I1RBN1 pp 229533.V6RGN5
29715 229533.I1RBN1 pp 229533.I1RFQ7
29716 229533.I1RBN1 pp 229533.V6RIS2
29717 229533.I1RBN1 pp 229533.V6RGY3
29718 229533.I1RBN1 pp 229533.I1RPY1
29719 229533.I1RBN1 pp 229533.I1RTE9
29720 229533.I1RBN1 pp 229533.I1RXF6
29721 229533.I1RBN1 pp 229533.I1RPJ3
29722 229533.I1RBN1 pp 229533.I1S471
29723 229533.I1RBN1 pp 229533.I1RCL3
29724 229533.I1RBN1 pp 229533.I1S2C0
29725 229533.I1RBN1 pp 229533.I1RBZ3
29726 229533.I1RBN1 pp 229533.I1S855
29727 229533.I1RBN1 pp 229533.I1RRI2
29728 229533.I1RBN1 pp 229533.I1RZ40
29729 229533.I1RBN1 pp 229533.I1RKC6
29730 229533.I1RBN1 pp 229533.I1S558
29731 229533.I1RBN1 pp 229533.I1RCN5
29732 229533.I1RBN1 pp 229533.I1S1X9
29733 229533.I1RBN1 pp 229533.Q4IQW0
29734 229533.I1RBN1 pp 229533.V6R283
29735 229533.I1RBN1 pp 229533.I1RC82
29736 229533.I1RBN1 pp 229533.I1RSS4
29737 229533.I1RBN1 pp 229533.I1RFN1
29738 229533.I1RBN1 pp 229533.I1RRT2
29739 229533.I1RBN1 pp 229533.I1RC03
29740 229533.I1RBN1 pp 229533.I1RJL4
29741 229533.I1RBN1 pp 229533.Q4IEV5
29742 229533.I1RBN1 pp 229533.I1RQE7
29743 229533.I1RBN1 pp 229533.Q4HZ68
29744 229533.I1RBN1 pp 229533.I1RQ65
29745 229533.I1RBN1 pp 229533.I1RSD8
29746 229533.I1RBN1 pp 229533.I1RS47
29747 229533.I1RBN1 pp 229533.Q4IAS1
29748 229533.I1RBN1 pp 229533.I1S296
29749 229533.I1RBN1 pp 229533.I1RRJ8
29750 229533.I1RBN1 pp 229533.Q4HZ42
29751 229533.I1RBN1 pp 229533.I1RRQ3
29752 229533.I1RBN1 pp 229533.I1RKJ6
29753 229533.I1RBN1 pp 229533.Q4I830
29754 229533.I1RBN1 pp 229533.I1RS30
29755 229533.I1RBN1 pp 229533.I1RZS6
29756 229533.I1RBN1 pp 229533.Q4HTT1
29757 229533.I1RBN1 pp 229533.I1RKL3
29758 229533.I1RBN1 pp 229533.I1RW26
29759 229533.I1RBN1 pp 229533.I1S253
29760 229533.I1RBN1 pp 229533.I1RND6
29761 229533.I1RBN1 pp 229533.I1RX24
29762 229533.I1RBN1 pp 229533.Q4I662
29763 229533.I1RBN1 pp 229533.I1RWJ4
29764 229533.I1RBN1 pp 229533.I1RFS3
29765 229533.I1RBN1 pp 229533.I1RU69
29766 229533.I1RBN1 pp 229533.Q4IF76
29767 229533.I1RBN1 pp 229533.I1RJZ7
29768 229533.I1RBN1 pp 229533.I1RD64
29769 229533.A0A1C3YK08 pp 229533.Q4IB89
29770 229533.A0A1C3YK08 pp 229533.I1RZS8
29771 229533.A0A1C3YK08 pp 229533.I1RQ21
29772 229533.A0A1C3YK08 pp 229533.I1RC55
29773 229533.A0A1C3YK08 pp 229533.A0A1C3YKB9
29774 229533.A0A1C3YK08 pp 229533.I1RKB6
29775 229533.A0A1C3YK08 pp 229533.I1REV8
29776 229533.A0A1C3YK08 pp 229533.A0A1C3YK30
29777 229533.A0A1C3YK08 pp 229533.I1REC7
29778 229533.A0A1C3YK08 pp 229533.I1RHW9
29779 229533.A0A1C3YK08 pp 229533.I1RQG4
29780 229533.A0A1C3YK08 pp 229533.I1RMQ5
29781 229533.A0A1C3YK08 pp 229533.I1RBS5
29782 229533.A0A1C3YK08 pp 229533.I1RSY2
29783 229533.A0A1C3YK08 pp 229533.I1RP28
29784 229533.A0A1C3YK08 pp 229533.I1RZC3

29785 229533.A0A1C3YK08 pp 229533.I1RZD3
29786 229533.A0A1C3YK08 pp 229533.I1RNG4
29787 229533.A0A1C3YK08 pp 229533.I1RC71
29788 229533.A0A1C3YK08 pp 229533.I1RY32
29789 229533.A0A1C3YK08 pp 229533.I1RFS5
29790 229533.A0A1C3YK08 pp 229533.I1RSQ4
29791 229533.A0A1C3YK08 pp 229533.A0A1C3YM05
29792 229533.A0A1C3YK08 pp 229533.I1RQH1
29793 229533.A0A1C3YK08 pp 229533.I1RAX2
29794 229533.A0A1C3YK08 pp 229533.I1RPL2
29795 229533.A0A1C3YK08 pp 229533.I1S267
29796 229533.A0A1C3YK08 pp 229533.I1RGB0
29797 229533.A0A1C3YK08 pp 229533.O74638
29798 229533.A0A1C3YK08 pp 229533.I1RSG0
29799 229533.A0A1C3YK08 pp 229533.I1RGU0
29800 229533.A0A1C3YK08 pp 229533.I1RAM6
29801 229533.A0A1C3YK08 pp 229533.I1REC6
29802 229533.A0A1C3YK08 pp 229533.I1S4S4
29803 229533.A0A1C3YK08 pp 229533.I1RQA5
29804 229533.A0A1C3YK08 pp 229533.I1RGB1
29805 229533.A0A1C3YK08 pp 229533.I1RTN3
29806 229533.A0A1C3YK08 pp 229533.I1R9Y8
29807 229533.A0A1C3YK08 pp 229533.I1S217
29808 229533.A0A1C3YK08 pp 229533.I1RAM2
29809 229533.A0A1C3YK08 pp 229533.A0A1C3YK11
29810 229533.A0A1C3YK08 pp 229533.I1RJR8
29811 229533.A0A1C3YK08 pp 229533.I1RCK2
29812 229533.A0A1C3YK08 pp 229533.I1RQE0
29813 229533.A0A1C3YK08 pp 229533.I1RRV4
29814 229533.A0A1C3YK08 pp 229533.I1RSC5
29815 229533.A0A1C3YK08 pp 229533.I1RRS8
29816 229533.Q4HVW2 pp 229533.V6RGN5
29817 229533.Q4HVW2 pp 229533.Q4IF76
29818 229533.Q4HVW2 pp 229533.Q4IQW0
29819 229533.Q4HVW2 pp 229533.Q4I662
29820 229533.Q4HVW2 pp 229533.Q4HZ68
29821 229533.Q4HVW2 pp 229533.Q4I830
29822 229533.Q4HVW2 pp 229533.Q4IAS1
29823 229533.Q4HVW2 pp 229533.Q4IEK8
29824 229533.Q4HVW2 pp 229533.Q4IBS2
29825 229533.Q4HVW2 pp 229533.Q4HZ47
29826 229533.Q4HVW2 pp 229533.V6R227
29827 229533.Q4HVW2 pp 229533.Q4IPI1
29828 229533.Q4HVW2 pp 229533.Q4HZ42
29829 229533.Q4HVW2 pp 229533.Q4IN63
29830 229533.Q4HVW2 pp 229533.V6RY33
29831 229533.Q4HVW2 pp 229533.V6QYF0
29832 229533.Q4HVW2 pp 229533.Q4IJ56
29833 229533.Q4HVW2 pp 229533.V6R283
29834 229533.Q4HVW2 pp 229533.V6RG22
29835 229533.Q4HVW2 pp 229533.Q4I327
29836 229533.Q4HVW2 pp 229533.Q4IEV5
29837 229533.Q4HVW2 pp 229533.V6RIS2
29838 229533.Q4HVW2 pp 229533.Q4IFI0
29839 229533.Q4HVW2 pp 229533.Q4I5Z5
29840 229533.Q4HVW2 pp 229533.Q4IJH1
29841 229533.Q4HVW2 pp 229533.Q4I5M4
29842 229533.Q4HVW2 pp 229533.V6RCG5
29843 229533.Q4HVW2 pp 229533.Q4IAA0
29844 229533.I1RAZ1 pp 229533.I1RXX1
29845 229533.I1RAZ1 pp 229533.V6QW25
29846 229533.I1RAZ1 pp 229533.I1RMG4
29847 229533.I1RAZ1 pp 229533.Q4ICG5
29848 229533.I1RAZ1 pp 229533.I1RE15
29849 229533.I1RAZ1 pp 229533.I1RQ61
29850 229533.I1RAZ1 pp 229533.I1RM09
29851 229533.I1RAZ1 pp 229533.I1S368
29852 229533.I1RAZ1 pp 229533.I1RJS0
29853 229533.I1RAZ1 pp 229533.I1RM77
29854 229533.I1RAZ1 pp 229533.I1RPD3
29855 229533.I1RAZ1 pp 229533.I1RJ78
29856 229533.I1RAZ1 pp 229533.I1RNR9
29857 229533.I1RAZ1 pp 229533.I1RJL9

229533.I1RAZ1	pp	229533.I1RRE1
229533.I1RAZ1	pp	229533.I1RWC2
229533.I1RAZ1	pp	229533.I1S0C9
229533.I1RAZ1	pp	229533.I1REK1
229533.I1RAZ1	pp	229533.I1RUP5
229533.I1RAZ1	pp	229533.I1RN66
229533.I1RAZ1	pp	229533.I1RZJ9
229533.I1RAZ1	pp	229533.I1SAT1
229533.I1RAZ1	pp	229533.I1RC58
229533.I1RAZ1	pp	229533.I1S0Y1
229533.I1RAZ1	pp	229533.I1RBZ9
229533.I1RAZ1	pp	229533.I1RWV4
229533.I1RCQ3	pp	229533.I1RQF2
229533.I1RCQ3	pp	229533.I1S238
229533.I1RCQ3	pp	229533.I1RQH1
229533.I1RCQ3	pp	229533.I1RSI6
229533.I1RCQ3	pp	229533.I1RMQ5
229533.I1RCQ3	pp	229533.I1S8V4
229533.I1RCQ3	pp	229533.I1RSH0
229533.I1RCQ3	pp	229533.I1RP28
229533.I1RCQ3	pp	229533.I1RXD9
229533.I1RCQ3	pp	229533.I1RPL2
229533.I1RCQ3	pp	229533.I1RQG4
229533.I1RCQ3	pp	229533.I1S2G9
229533.I1RCQ3	pp	229533.I1RD39
229533.I1RCQ3	pp	229533.I1RJU3
229533.I1RCQ3	pp	229533.I1RRM9
229533.I1RCQ3	pp	229533.I1S8V3
229533.I1RX24	pp	229533.Q4HWE2
229533.I1RX24	pp	229533.I1S1U7
229533.I1RX24	pp	229533.V6RGN5
229533.I1RX24	pp	229533.V6RGY3
229533.I1RX24	pp	229533.I1S1X9
229533.I1RX24	pp	229533.I1S274
229533.I1RX24	pp	229533.I1RX47
229533.I1RX24	pp	229533.Q4HZ68
229533.I1RX24	pp	229533.I1S2C0
229533.I1RX24	pp	229533.Q4IN63
229533.I1RX24	pp	229533.I1S0B1
229533.I1RX24	pp	229533.V6R042
229533.I1RX24	pp	229533.I1S0J1
229533.I1RX24	pp	229533.Q4I662
229533.I1RX24	pp	229533.I1S296
229533.I1RX24	pp	229533.Q4I327
229533.I1RX24	pp	229533.I1S2E8
229533.I1RX24	pp	229533.Q4IAS1
229533.I1RX24	pp	229533.I1S9Y1
229533.I1RX24	pp	229533.I1RZV6
229533.I1RX24	pp	229533.I1RX51
229533.I1RX24	pp	229533.I1RZS6
229533.I1RX24	pp	229533.I1S060
229533.I1RX24	pp	229533.I1S1B6
229533.I1RX24	pp	229533.V6QXG1
229533.I1RX24	pp	229533.O74638
229533.I1RX24	pp	229533.I1S9Y0
229533.I1RX24	pp	229533.Q4IQW0
229533.I1RX24	pp	229533.Q4HXF0
229533.I1RX24	pp	229533.Q4I1Y1
229533.I1RX24	pp	229533.Q4IF76
229533.I1RX24	pp	229533.Q4HZ42
229533.I1RX24	pp	229533.I1S8E2
229533.I1RX24	pp	229533.I1RXF2
229533.I1RX24	pp	229533.I1S4M5
229533.I1RX24	pp	229533.Q4IP34
229533.I1RX24	pp	229533.V6R283
229533.I1RX24	pp	229533.I1RZ59
229533.I1RX24	pp	229533.I1RZK6
229533.I1RX24	pp	229533.I1S021
229533.I1RX24	pp	229533.I1RZE5
229533.I1RX24	pp	229533.Q4I7F9
229533.I1RX24	pp	229533.Q4I830
229533.I1RX24	pp	229533.I1RZZ1
229533.I1RX24	pp	229533.V6QUE6

29931	229533.I1RX24	pp	229533.Q4IJH1
29932	229533.I1RX24	pp	229533.I1RYB9
29933	229533.I1RX24	pp	229533.I1RZB5
29934	229533.I1RX24	pp	229533.I1S7M3
29935	229533.I1RX24	pp	229533.I1RZF6
29936	229533.I1RX24	pp	229533.Q4HVW2
29937	229533.I1RX24	pp	229533.V6R227
29938	229533.I1RX24	pp	229533.Q4IBS2
29939	229533.I1RX24	pp	229533.Q4IEK8
29940	229533.I1RX24	pp	229533.I1RZJ6
29941	229533.I1RX24	pp	229533.Q4IJ56
29942	229533.I1RX24	pp	229533.I1S1C6
29943	229533.I1RX24	pp	229533.Q4IFI0
29944	229533.I1RX24	pp	229533.Q4IPI1
29945	229533.I1RX24	pp	229533.I1S0R7
29946	229533.I1RX24	pp	229533.I1RZG7
29947	229533.I1RX24	pp	229533.I1S7M2
29948	229533.I1RX24	pp	229533.I1S0M9
29949	229533.I1RX24	pp	229533.Q4ICG5
29950	229533.I1RX24	pp	229533.Q4IRI9
29951	229533.I1RX24	pp	229533.V6QYF0
29952	229533.I1RX24	pp	229533.I1S9U2
29953	229533.I1RX24	pp	229533.I1S6F0
29954	229533.I1RX24	pp	229533.Q4HZ47
29955	229533.I1RX24	pp	229533.Q4IAA0
29956	229533.I1RX24	pp	229533.Q4I5R9
29957	229533.I1RX24	pp	229533.I1RX81
29958	229533.I1RX24	pp	229533.I1S2Y5
29959	229533.I1RX24	pp	229533.I1RZ99
29960	229533.A0A0E0RLR7	pp	229533.I1RPD3
29961	229533.A0A0E0RLR7	pp	229533.I1R9Y8
29962	229533.A0A0E0RLR7	pp	229533.A0A1C3YME0
29963	229533.A0A0E0RLR7	pp	229533.A0A0E0SLM3
29964	229533.A0A0E0RLR7	pp	229533.I1S769
29965	229533.A0A0E0RLR7	pp	229533.I1RBA6
29966	229533.A0A0E0RLR7	pp	229533.I1RZJ9
29967	229533.A0A0E0RLR7	pp	229533.I1RC58
29968	229533.A0A0E0RLR7	pp	229533.I1RN66
29969	229533.A0A0E0RLR7	pp	229533.A0A1C3YIX0
29970	229533.A0A0E0RLR7	pp	229533.Q4I7T5
29971	229533.A0A0E0RLR7	pp	229533.I1RWV4
29972	229533.A0A0E0RLR7	pp	229533.I1RKA1
29973	229533.A0A0E0RLR7	pp	229533.I1RMG4
29974	229533.A0A0E0RLR7	pp	229533.I1RMV6
29975	229533.A0A0E0RLR7	pp	229533.I1RW84
29976	229533.A0A0E0RLR7	pp	229533.I1S0L1
29977	229533.A0A0E0RLR7	pp	229533.I1R9B8
29978	229533.A0A0E0RLR7	pp	229533.I1RNJ5
29979	229533.A0A0E0RLR7	pp	229533.I1RK86
29980	229533.A0A0E0RLR7	pp	229533.I1RMQ5
29981	229533.A0A0E0RLR7	pp	229533.I1S4H6
29982	229533.A0A0E0RLR7	pp	229533.I1RQR6
29983	229533.A0A0E0RLR7	pp	229533.I1RKR7
29984	229533.A0A0E0RLR7	pp	229533.I1RK33
29985	229533.A0A0E0RLR7	pp	229533.I1RCQ7
29986	229533.A0A0E0RLR7	pp	229533.I1RDJ7
29987	229533.A0A0E0RLR7	pp	229533.I1RJ78
29988	229533.A0A0E0RLR7	pp	229533.I1RQ61
29989	229533.A0A0E0RLR7	pp	229533.Q4I375
29990	229533.A0A0E0RLR7	pp	229533.I1RAZ1
29991	229533.A0A0E0RLR7	pp	229533.I1RM77
29992	229533.A0A098D5F5	pp	229533.I1S4T3
29993	229533.A0A098D5F5	pp	229533.Q4IBS2
29994	229533.A0A098D5F5	pp	229533.I1RG56
29995	229533.A0A098D5F5	pp	229533.I1RM96
29996	229533.A0A098D5F5	pp	229533.Q4IEK8
29997	229533.A0A098D5F5	pp	229533.A0A1C3YJN3
29998	229533.A0A098D5F5	pp	229533.I1RZF6
29999	229533.A0A098D5F5	pp	229533.I1RCL3
30000	229533.A0A098D5F5	pp	229533.A0A1C3YJR3
30001	229533.A0A098D5F5	pp	229533.V6R227
30002	229533.A0A098D5F5	pp	229533.I1RC27
30003	229533.A0A098D5F5	pp	229533.I1RBL7

30004	229533.A0A098D5F5	pp	229533.V6RGN5
30005	229533.A0A098D5F5	pp	229533.I1S274
30006	229533.A0A098D5F5	pp	229533.I1RUQ9
30007	229533.A0A098D5F5	pp	229533.A0A0E0S621
30008	229533.A0A098D5F5	pp	229533.I1RC72
30009	229533.A0A098D5F5	pp	229533.V6RGY3
30010	229533.A0A098D5F5	pp	229533.Q4IJ56
30011	229533.A0A098D5F5	pp	229533.Q4IAA0
30012	229533.A0A098D5F5	pp	229533.I1RMC0
30013	229533.A0A098D5F5	pp	229533.I1RNY2
30014	229533.A0A098D5F5	pp	229533.I1RMN7
30015	229533.A0A098D5F5	pp	229533.I1RRI2
30016	229533.A0A098D5F5	pp	229533.I1RSI6
30017	229533.A0A098D5F5	pp	229533.I1RTE9
30018	229533.A0A098D5F5	pp	229533.I1R9X7
30019	229533.A0A098D5F5	pp	229533.I1RVZ0
30020	229533.A0A098D5F5	pp	229533.I1RAZ8
30021	229533.A0A098D5F5	pp	229533.I1S2C0
30022	229533.A0A098D5F5	pp	229533.I1RKC6
30023	229533.A0A098D5F5	pp	229533.I1RB27
30024	229533.A0A098D5F5	pp	229533.A0A1C3YK72
30025	229533.A0A098D5F5	pp	229533.A0A098DVS9
30026	229533.A0A098D5F5	pp	229533.I1RMN6
30027	229533.A0A098D5F5	pp	229533.I1RRP3
30028	229533.A0A098D5F5	pp	229533.A0A0E0S6M0
30029	229533.A0A098D5F5	pp	229533.Q4HZ68
30030	229533.A0A098D5F5	pp	229533.Q4IAS1
30031	229533.A0A098D5F5	pp	229533.I1S6F0
30032	229533.A0A098D5F5	pp	229533.A0A0E0RNT7
30033	229533.A0A098D5F5	pp	229533.I1RRT2
30034	229533.A0A098D5F5	pp	229533.I1RF08
30035	229533.A0A098D5F5	pp	229533.I1RRQ3
30036	229533.A0A098D5F5	pp	229533.I1RSD8
30037	229533.A0A098D5F5	pp	229533.I1RJL4
30038	229533.A0A098D5F5	pp	229533.A0A1C3YN63
30039	229533.A0A098D5F5	pp	229533.I1RBC7
30040	229533.A0A098D5F5	pp	229533.I1RFN1
30041	229533.A0A098D5F5	pp	229533.A0A1C3YL10
30042	229533.A0A098D5F5	pp	229533.I1RRH5
30043	229533.A0A098D5F5	pp	229533.I1R9V7
30044	229533.A0A098D5F5	pp	229533.I1S1X9
30045	229533.A0A098D5F5	pp	229533.I1RC82
30046	229533.A0A098D5F5	pp	229533.I1RS47
30047	229533.A0A098D5F5	pp	229533.Q4IQW0
30048	229533.A0A098D5F5	pp	229533.I1RSS4
30049	229533.A0A098D5F5	pp	229533.A0A098DE18
30050	229533.A0A098D5F5	pp	229533.Q4HZ42
30051	229533.A0A098D5F5	pp	229533.I1RZJ6
30052	229533.A0A098D5F5	pp	229533.I1RAF8
30053	229533.A0A098D5F5	pp	229533.Q4IR18
30054	229533.A0A098D5F5	pp	229533.I1RA48
30055	229533.A0A098D5F5	pp	229533.I1RN99
30056	229533.A0A098D5F5	pp	229533.I1RK95
30057	229533.A0A098D5F5	pp	229533.I1RDG0
30058	229533.A0A098D5F5	pp	229533.I1RQA2
30059	229533.A0A098D5F5	pp	229533.V6R283
30060	229533.A0A098D5F5	pp	229533.I1RAN5
30061	229533.A0A098D5F5	pp	229533.I1RC03
30062	229533.A0A098D5F5	pp	229533.A0A0E0SGM4
30063	229533.A0A098D5F5	pp	229533.V6QYF0
30064	229533.A0A098D5F5	pp	229533.I1REF2
30065	229533.A0A098D5F5	pp	229533.A0A1C3YLF5
30066	229533.A0A098D5F5	pp	229533.Q4I830
30067	229533.A0A098D5F5	pp	229533.I1S0R7
30068	229533.A0A098D5F5	pp	229533.I1RM06
30069	229533.A0A098D5F5	pp	229533.I1RKL3
30070	229533.A0A098D5F5	pp	229533.A0A0E0SQJ6
30071	229533.A0A098D5F5	pp	229533.I1S9Y1
30072	229533.A0A098D5F5	pp	229533.I1RW71
30073	229533.A0A098D5F5	pp	229533.I1RKJ6
30074	229533.A0A098D5F5	pp	229533.I1RQJ5
30075	229533.A0A098D5F5	pp	229533.I1RD64
30076	229533.A0A098D5F5	pp	229533.I1RJZ7

30077 229533.A0A098D5F5 pp 229533.A0A0E0SFM9
30078 229533.A0A098D5F5 pp 229533.Q4IFI0
30079 229533.A0A098D5F5 pp 229533.I1RPW9
30080 229533.A0A098D5F5 pp 229533.I1RAC2
30081 229533.A0A098D5F5 pp 229533.I1RQM6
30082 229533.A0A098D5F5 pp 229533.Q4IJH1
30083 229533.A0A098D5F5 pp 229533.I1RT80
30084 229533.A0A098D5F5 pp 229533.I1RG51
30085 229533.A0A098D5F5 pp 229533.I1RFS3
30086 229533.A0A098D5F5 pp 229533.I1RNV6
30087 229533.A0A098D5F5 pp 229533.I1RBN1
30088 229533.A0A098D5F5 pp 229533.Q4HVW2
30089 229533.A0A098D5F5 pp 229533.I1RX24
30090 229533.A0A098D5F5 pp 229533.I1RBG9
30091 229533.A0A098D5F5 pp 229533.A0A1C3YIN1
30092 229533.I1RK46 pp 229533.I1RWL9
30093 229533.I1S2G9 pp 229533.Q5I2J3
30094 229533.I1S2G9 pp 229533.Q4ICA8
30095 229533.I1S2G9 pp 229533.Q4I948
30096 229533.I1S2G9 pp 229533.Q4IQT8
30097 229533.I1S2G9 pp 229533.I1S8V4
30098 229533.I1S2G9 pp 229533.Q4I6B0
30099 229533.I1S2G9 pp 229533.I1S8V3
30100 229533.I1S2G9 pp 229533.Q4IPZ1
30101 229533.I1S594 pp 229533.I1S5L1
30102 229533.I1RC91 pp 229533.I1RKR7
30103 229533.I1RC91 pp 229533.I1RCS1
30104 229533.I1RC91 pp 229533.I1RRT1
30105 229533.I1RC91 pp 229533.I1RRX6
30106 229533.I1RC91 pp 229533.I1RZ63
30107 229533.I1RC91 pp 229533.I1RLX7
30108 229533.I1RC91 pp 229533.I1RYN4
30109 229533.I1RC91 pp 229533.I1S224
30110 229533.I1RC91 pp 229533.Q4I7N9
30111 229533.I1RC91 pp 229533.I1S0J7
30112 229533.I1RC91 pp 229533.I1S2E9
30113 229533.I1RC91 pp 229533.I1RQA3
30114 229533.I1RC91 pp 229533.I1REE1
30115 229533.I1RC91 pp 229533.I1RCB3
30116 229533.I1RC91 pp 229533.I1RQB4
30117 229533.I1RC91 pp 229533.I1RRN5
30118 229533.I1RC91 pp 229533.I1RKK1
30119 229533.I1RC91 pp 229533.I1RY44
30120 229533.I1RC91 pp 229533.I1RDV9
30121 229533.I1RC91 pp 229533.I1RWT6
30122 229533.I1RC91 pp 229533.I1RMB2
30123 229533.I1RC91 pp 229533.I1RRW0
30124 229533.A0A0E0SLM3 pp 229533.I1RFL4
30125 229533.A0A0E0SLM3 pp 229533.I1RCH5
30126 229533.A0A0E0SLM3 pp 229533.I1RT03
30127 229533.A0A0E0SLM3 pp 229533.I1RB92
30128 229533.A0A0E0SLM3 pp 229533.I1RNW8
30129 229533.A0A0E0SLM3 pp 229533.I1RRH2
30130 229533.A0A0E0SLM3 pp 229533.I1RE01
30131 229533.A0A0E0SLM3 pp 229533.I1RAZ1
30132 229533.A0A0E0SLM3 pp 229533.Q4I665
30133 229533.A0A0E0SLM3 pp 229533.I1RYW3
30134 229533.A0A0E0SLM3 pp 229533.I1S1C7
30135 229533.A0A0E0SLM3 pp 229533.I1RWN5
30136 229533.A0A0E0SLM3 pp 229533.I1RA70
30137 229533.A0A0E0SLM3 pp 229533.A0A1C3YLH6
30138 229533.A0A0E0SLM3 pp 229533.I1RUG3
30139 229533.A0A0E0SLM3 pp 229533.I1S8R3
30140 229533.A0A0E0SLM3 pp 229533.I1RCR8
30141 229533.A0A0E0SLM3 pp 229533.I1RMQ5
30142 229533.A0A0E0SLM3 pp 229533.V6RDZ4
30143 229533.A0A0E0SLM3 pp 229533.I1RSQ3
30144 229533.A0A0E0SLM3 pp 229533.I1RWN6
30145 229533.A0A0E0SLM3 pp 229533.I1RD22
30146 229533.A0A0E0SLM3 pp 229533.I1RAW0
30147 229533.A0A0E0SLM3 pp 229533.I1RS90
30148 229533.A0A0E0SLM3 pp 229533.I1RFT1
30149 229533.A0A0E0SLM3 pp 229533.I1RC58

30150	229533.A0A0E0SLM3	pp	229533.I1RN67
30151	229533.A0A0E0SLM3	pp	229533.I1S5R1
30152	229533.A0A0E0SLM3	pp	229533.I1RZT6
30153	229533.A0A0E0SLM3	pp	229533.I1RWV4
30154	229533.A0A0E0SLM3	pp	229533.A0A1C3YM63
30155	229533.A0A0E0SLM3	pp	229533.A0A1C3YHV3
30156	229533.A0A0E0SLM3	pp	229533.I1RRG3
30157	229533.A0A0E0SLM3	pp	229533.A0A1C3YK34
30158	229533.A0A0E0SLM3	pp	229533.A0A1C3YKV3
30159	229533.A0A0E0SLM3	pp	229533.I1RM77
30160	229533.A0A0E0SLM3	pp	229533.I1RT40
30161	229533.A0A0E0SLM3	pp	229533.A0A1C3YME0
30162	229533.A0A0E0SLM3	pp	229533.I1RZM8
30163	229533.A0A0E0SLM3	pp	229533.A0A1C3YK13
30164	229533.A0A0E0SLM3	pp	229533.A0A1C3YLK8
30165	229533.A0A0E0SLM3	pp	229533.I1REK1
30166	229533.A0A0E0SLM3	pp	229533.A0A1C3YIP5
30167	229533.A0A0E0SLM3	pp	229533.I1RAU9
30168	229533.A0A0E0SLM3	pp	229533.I1RAX6
30169	229533.A0A0E0SLM3	pp	229533.I1RRE1
30170	229533.A0A0E0SLM3	pp	229533.I1RI82
30171	229533.A0A0E0SLM3	pp	229533.I1RM09
30172	229533.A0A0E0SLM3	pp	229533.Q4I1T9
30173	229533.A0A0E0SLM3	pp	229533.A0A1C3YI23
30174	229533.A0A0E0SLM3	pp	229533.V6QW25
30175	229533.A0A0E0SLM3	pp	229533.I1RJV4
30176	229533.A0A0E0SLM3	pp	229533.V6RTJ8
30177	229533.A0A0E0SLM3	pp	229533.I1R9M0
30178	229533.A0A0E0SLM3	pp	229533.I1RBY4
30179	229533.A0A0E0SLM3	pp	229533.I1REK8
30180	229533.A0A0E0SLM3	pp	229533.I1RCR5
30181	229533.A0A0E0SLM3	pp	229533.I1RMR5
30182	229533.A0A0E0SLM3	pp	229533.I1RQ61
30183	229533.A0A0E0SLM3	pp	229533.I1RJ78
30184	229533.A0A0E0SLM3	pp	229533.A0A1C3YHX6
30185	229533.A0A0E0SLM3	pp	229533.A0A1C3YMQ5
30186	229533.A0A0E0SLM3	pp	229533.I1RK49
30187	229533.A0A0E0SLM3	pp	229533.I1RMG4
30188	229533.A0A0E0SLM3	pp	229533.I1RX25
30189	229533.A0A0E0SLM3	pp	229533.I1RZZ9
30190	229533.A0A0E0SLM3	pp	229533.I1RPD3
30191	229533.A0A0E0SLM3	pp	229533.I1RZK5
30192	229533.A0A0E0SLM3	pp	229533.A0A1C3YHX8
30193	229533.A0A0E0SLM3	pp	229533.I1RJU3
30194	229533.A0A0E0SLM3	pp	229533.I1RN72
30195	229533.A0A0E0SLM3	pp	229533.I1RR24
30196	229533.A0A1C3YLR4	pp	229533.I1RZ94
30197	229533.A0A1C3YLR4	pp	229533.I1S1W8
30198	229533.A0A1C3YLR4	pp	229533.I1RBW0
30199	229533.A0A1C3YLR4	pp	229533.I1RRT1
30200	229533.A0A1C3YLR4	pp	229533.I1RSN7
30201	229533.A0A1C3YLR4	pp	229533.I1RWT6
30202	229533.A0A1C3YLR4	pp	229533.I1RA12
30203	229533.A0A1C3YLR4	pp	229533.I1S2E9
30204	229533.A0A1C3YLR4	pp	229533.I1RN55
30205	229533.A0A1C3YLR4	pp	229533.I1RV91
30206	229533.A0A1C3YLR4	pp	229533.I1RZ86
30207	229533.A0A1C3YLR4	pp	229533.I1RG77
30208	229533.A0A1C3YLR4	pp	229533.I1RPQ1
30209	229533.A0A1C3YLR4	pp	229533.I1RQA3
30210	229533.A0A1C3YLR4	pp	229533.A0A1C3YMJ3
30211	229533.A0A1C3YLR4	pp	229533.I1RC54
30212	229533.A0A1C3YLR4	pp	229533.I1RCA5
30213	229533.A0A1C3YLR4	pp	229533.I1RBW1
30214	229533.A0A1C3YLR4	pp	229533.I1RBC8
30215	229533.A0A1C3YLR4	pp	229533.I1S3G4
30216	229533.A0A1C3YLR4	pp	229533.I1RA10
30217	229533.A0A1C3YLR4	pp	229533.I1RY58
30218	229533.A0A1C3YLR4	pp	229533.A0A1C3YN69
30219	229533.A0A1C3YLR4	pp	229533.I1RZP8
30220	229533.A0A1C3YLR4	pp	229533.I1RN57
30221	229533.A0A1C3YLR4	pp	229533.I1RY44
30222	229533.A0A1C3YLR4	pp	229533.I1RSP7

30223	229533.A0A1C3YLR4	pp	229533.I1RX64
30224	229533.A0A1C3YLR4	pp	229533.I1RSR9
30225	229533.A0A1C3YLR4	pp	229533.I1S4P6
30226	229533.A0A098DXK5	pp	229533.I1RM09
30227	229533.A0A098DXK5	pp	229533.I1RK86
30228	229533.A0A098DXK5	pp	229533.V6QW25
30229	229533.A0A098DXK5	pp	229533.I1RND7
30230	229533.A0A098DXK5	pp	229533.I1RG57
30231	229533.A0A098DXK5	pp	229533.I1RXF5
30232	229533.A0A098DXK5	pp	229533.I1RTD0
30233	229533.A0A098DXK5	pp	229533.I1RWQ1
30234	229533.A0A098DXK5	pp	229533.I1RFK0
30235	229533.A0A098DXK5	pp	229533.I1RAZ1
30236	229533.A0A098DXK5	pp	229533.A0A0E0RLR7
30237	229533.A0A098DXK5	pp	229533.I1RX09
30238	229533.A0A098DXK5	pp	229533.A0A1C3YK51
30239	229533.A0A098DXK5	pp	229533.I1RE15
30240	229533.A0A098DXK5	pp	229533.I1RE20
30241	229533.A0A098DXK5	pp	229533.I1RAS9
30242	229533.A0A098DXK5	pp	229533.I1RNR3
30243	229533.A0A098DXK5	pp	229533.I1RXU1
30244	229533.A0A098DXK5	pp	229533.A0A0E0SGM3
30245	229533.A0A098DXK5	pp	229533.A0A1C3YIR6
30246	229533.A0A098DXK5	pp	229533.A0A0E0SLM3
30247	229533.A0A098E0E1	pp	229533.I1RNW8
30248	229533.A0A098E0E1	pp	229533.I1R9Y7
30249	229533.A0A098E0E1	pp	229533.A0A1C3YM29
30250	229533.A0A098E0E1	pp	229533.I1RK75
30251	229533.A0A098E0E1	pp	229533.I1RD15
30252	229533.A0A098E0E1	pp	229533.I1RB41
30253	229533.A0A098E0E1	pp	229533.I1RRN5
30254	229533.A0A098E0E1	pp	229533.I1RZE4
30255	229533.A0A098E0E1	pp	229533.A0A1C3YHP8
30256	229533.A0A098E0E1	pp	229533.I1S0E8
30257	229533.A0A098E0E1	pp	229533.I1RNZ9
30258	229533.A0A098E0E1	pp	229533.I1RD70
30259	229533.A0A098E0E1	pp	229533.I1RZT1
30260	229533.A0A098E0E1	pp	229533.I1RGC6
30261	229533.A0A098E0E1	pp	229533.I1RRY3
30262	229533.A0A098E0E1	pp	229533.A0A1C3YIN6
30263	229533.A0A098E0E1	pp	229533.A0A098E4P3
30264	229533.A0A098E0E1	pp	229533.I1RZY9
30265	229533.A0A098E0E1	pp	229533.I1RQ72
30266	229533.A0A098E0E1	pp	229533.A0A1C3YK43
30267	229533.A0A098E0E1	pp	229533.I1S1W4
30268	229533.A0A098E0E1	pp	229533.I1RNY8
30269	229533.A0A098E0E1	pp	229533.I1R9Y9
30270	229533.A0A098E0E1	pp	229533.I1S1D3
30271	229533.A0A098E0E1	pp	229533.I1RPC2
30272	229533.A0A098E0E1	pp	229533.I1RUN8
30273	229533.A0A098E0E1	pp	229533.V6RVG3
30274	229533.A0A098E0E1	pp	229533.I1S4R1
30275	229533.A0A098E0E1	pp	229533.I1RA43
30276	229533.A0A098E0E1	pp	229533.I1RY44
30277	229533.A0A098E0E1	pp	229533.V6RGI1
30278	229533.A0A098E0E1	pp	229533.I1RNP7
30279	229533.A0A098E0E1	pp	229533.I1RLU1
30280	229533.A0A098E0E1	pp	229533.I1RAN2
30281	229533.A0A098E0E1	pp	229533.I1REX8
30282	229533.A0A098E0E1	pp	229533.I1RZ63
30283	229533.A0A098E0E1	pp	229533.I1S9S6
30284	229533.A0A098E0E1	pp	229533.I1RT36
30285	229533.A0A098E0E1	pp	229533.V6RKY7
30286	229533.A0A098E0E1	pp	229533.A0A1C3YJC3
30287	229533.A0A098E0E1	pp	229533.A0A1C3YM91
30288	229533.A0A098E0E1	pp	229533.I1RRW0
30289	229533.A0A098E0E1	pp	229533.I1RD85
30290	229533.A0A098E0E1	pp	229533.I1RW16
30291	229533.A0A098E0E1	pp	229533.I1RRQ9
30292	229533.A0A098E0E1	pp	229533.I1RB74
30293	229533.A0A098E0E1	pp	229533.I1RGD1
30294	229533.A0A098E0E1	pp	229533.I1RW36
30295	229533.A0A098E0E1	pp	229533.I1RMA4

30296	229533.A0A098E0E1	pp	229533.I1S7X9
30297	229533.A0A098E0E1	pp	229533.A0A0E0S1U8
30298	229533.A0A098E0E1	pp	229533.A0A098E2M3
30299	229533.A0A098E0E1	pp	229533.I1S0M2
30300	229533.A0A098E0E1	pp	229533.I1RNF4
30301	229533.A0A098E0E1	pp	229533.I1RKC2
30302	229533.A0A098E0E1	pp	229533.I1S098
30303	229533.A0A098E0E1	pp	229533.I1RC97
30304	229533.A0A098E0E1	pp	229533.I1RQB4
30305	229533.A0A098E0E1	pp	229533.I1S2E9
30306	229533.A0A098E0E1	pp	229533.A0A1C3YI28
30307	229533.A0A098E0E1	pp	229533.A0A1C3YHL0
30308	229533.A0A098E0E1	pp	229533.I1S0J7
30309	229533.A0A098E0E1	pp	229533.A0A0E0SNM6
30310	229533.A0A098E0E1	pp	229533.A0A1C3YJG7
30311	229533.A0A098E0E1	pp	229533.I1RI54
30312	229533.A0A098E0E1	pp	229533.I1S0M6
30313	229533.A0A098E0E1	pp	229533.I1RV91
30314	229533.A0A098E0E1	pp	229533.I1RQA3
30315	229533.A0A098E0E1	pp	229533.I1R9N1
30316	229533.A0A098E0E1	pp	229533.I1RQG4
30317	229533.A0A098E0E1	pp	229533.I1S439
30318	229533.A0A098E0E1	pp	229533.I1RFL7
30319	229533.A0A098E0E1	pp	229533.I1RB75
30320	229533.A0A098E0E1	pp	229533.I1RC95
30321	229533.A0A098E0E1	pp	229533.A0A1C3YKY3
30322	229533.A0A098E0E1	pp	229533.I1RHB0
30323	229533.A0A098E0E1	pp	229533.I1RJK7
30324	229533.A0A098E0E1	pp	229533.V6RMM8
30325	229533.A0A098E0E1	pp	229533.A0A1C3YJW8
30326	229533.A0A098E0E1	pp	229533.A0A1C3YIW6
30327	229533.A0A098E0E1	pp	229533.I1RME5
30328	229533.A0A098E0E1	pp	229533.I1RQS4
30329	229533.A0A098E0E1	pp	229533.I1RLB1
30330	229533.A0A098E0E1	pp	229533.A0A0E0RVZ1
30331	229533.A0A098E0E1	pp	229533.A0A1C3YJE8
30332	229533.A0A098E0E1	pp	229533.I1RMM9
30333	229533.A0A098E0E1	pp	229533.I1RCB3
30334	229533.A0A098E0E1	pp	229533.I1RN65
30335	229533.A0A098E0E1	pp	229533.I1RN31
30336	229533.A0A098E0E1	pp	229533.I1RN08
30337	229533.A0A098E0E1	pp	229533.I1RYY5
30338	229533.A0A098E0E1	pp	229533.Q4IJM4
30339	229533.A0A098E0E1	pp	229533.A0A0E0RVF4
30340	229533.A0A098E0E1	pp	229533.I1RC22
30341	229533.A0A098E0E1	pp	229533.A0A1C3YJH8
30342	229533.A0A098E0E1	pp	229533.I1RN89
30343	229533.A0A098E0E1	pp	229533.A0A0E0SA11
30344	229533.A0A098E0E1	pp	229533.A0A0E0RTV8
30345	229533.A0A098E0E1	pp	229533.I1RWT6
30346	229533.A0A098E0E1	pp	229533.I1RDB1
30347	229533.A0A098E0E1	pp	229533.I1S224
30348	229533.A0A098E0E1	pp	229533.A0A1C3YMY8
30349	229533.A0A098E0E1	pp	229533.A0A1C3YIP6
30350	229533.A0A098E0E1	pp	229533.I1RYH9
30351	229533.A0A098E0E1	pp	229533.I1RT62
30352	229533.A0A098E0E1	pp	229533.A0A0E0SAC4
30353	229533.A0A098E0E1	pp	229533.I1RY84
30354	229533.A0A098E0E1	pp	229533.V6RU70
30355	229533.A0A098E0E1	pp	229533.I1RZ12
30356	229533.A0A098E0E1	pp	229533.A0A1C3YIL0
30357	229533.A0A098E0E1	pp	229533.A0A1C3YKW5
30358	229533.A0A098E0E1	pp	229533.I1RAM7
30359	229533.A0A098E0E1	pp	229533.I1RZH4
30360	229533.A0A098E0E1	pp	229533.I1RQZ0
30361	229533.A0A098E0E1	pp	229533.I1R9D3
30362	229533.A0A098E0E1	pp	229533.I1RS56
30363	229533.A0A098E0E1	pp	229533.I1RDT7
30364	229533.A0A098E0E1	pp	229533.I1RC17
30365	229533.A0A098E0E1	pp	229533.I1RKL9
30366	229533.A0A098E0E1	pp	229533.I1RKK0
30367	229533.A0A098E0E1	pp	229533.I1S1W5
30368	229533.A0A098E0E1	pp	229533.A0A1C3YJV7

229533.A0A098E0E1	pp	229533.A0A1C3YKF2
229533.A0A098E0E1	pp	229533.I1RV66
229533.A0A098E0E1	pp	229533.I1RQ26
229533.A0A098E0E1	pp	229533.Q4ICM0
229533.A0A098E0E1	pp	229533.I1RAT7
229533.A0A098E0E1	pp	229533.A0A1C3YIA8
229533.I1RQH2	pp	229533.I1RZ99
229533.I1RQH2	pp	229533.I1S060
229533.I1RQH2	pp	229533.I1RZE5
229533.I1RQH2	pp	229533.Q4HZK7
229533.I1RQH2	pp	229533.I1S0N7
229533.I1RX00	pp	229533.Q4IPZ1
229533.I1RX00	pp	229533.I1RYS4
229533.I1RX00	pp	229533.I1S887
229533.I1RX00	pp	229533.I1RXR1
229533.I1RX00	pp	229533.V6RUT5
229533.I1RX00	pp	229533.I1S1W5
229533.I1RX00	pp	229533.I1RXU9
229533.I1RX00	pp	229533.I1S0J7
229533.I1RX00	pp	229533.I1RXD7
229533.I1RX00	pp	229533.I1RX50
229533.I1RX00	pp	229533.V6R3J1
229533.I1RX00	pp	229533.I1S1W3
229533.I1RX00	pp	229533.I1S039
229533.I1RJC4	pp	229533.I1RZ25
229533.A0A1C3YK00	pp	229533.I1RRH5
229533.A0A1C3YK00	pp	229533.I1RUQ6
229533.A0A1C3YK00	pp	229533.I1RFZ3
229533.A0A1C3YK00	pp	229533.I1S0N8
229533.A0A1C3YK00	pp	229533.I1RPX5
229533.A0A1C3YK00	pp	229533.I1RJQ3
229533.A0A1C3YK00	pp	229533.I1RQA9
229533.A0A1C3YK00	pp	229533.I1RX70
229533.A0A1C3YK00	pp	229533.I1S549
229533.A0A1C3YK00	pp	229533.I1RJT1
229533.A0A1C3YK00	pp	229533.I1S091
229533.A0A1C3YK00	pp	229533.I1S8W6
229533.A0A1C3YK00	pp	229533.I1RQA3
229533.A0A1C3YK00	pp	229533.A0A1C3YL52
229533.A0A1C3YK00	pp	229533.I1RPU7
229533.A0A1C3YK00	pp	229533.I1RC17
229533.A0A1C3YK00	pp	229533.I1RV91
229533.A0A1C3YK00	pp	229533.I1RM09
229533.A0A1C3YK00	pp	229533.I1RB17
229533.A0A1C3YK00	pp	229533.I1RWT6
229533.A0A1C3YK00	pp	229533.A0A1C3YK80
229533.A0A1C3YK00	pp	229533.Q4HXF0
229533.A0A1C3YK00	pp	229533.I1RMH6
229533.A0A1C3YK00	pp	229533.I1RW08
229533.A0A1C3YK00	pp	229533.I1RMJ8
229533.A0A1C3YK00	pp	229533.I1S2E9
229533.A0A1C3YK00	pp	229533.A0A1C3YM29
229533.A0A1C3YK00	pp	229533.I1RRQ4
229533.A0A1C3YK00	pp	229533.I1RB41
229533.A0A1C3YK00	pp	229533.I1RW83
229533.A0A1C3YK00	pp	229533.I1RRN5
229533.A0A1C3YK00	pp	229533.I1SAP6
229533.A0A1C3YK00	pp	229533.A0A1C3YKS0
229533.A0A1C3YK00	pp	229533.I1S4T3
229533.A0A1C3YK00	pp	229533.I1RY44
229533.I1RKI0	pp	229533.Q4IJ79
229533.I1RKI0	pp	229533.I1RYZ7
229533.I1RKI0	pp	229533.I1RNF9
229533.I1RKI0	pp	229533.I1RNW8
229533.I1RKI0	pp	229533.I1RWI1
229533.I1RKI0	pp	229533.I1RMQ5
229533.I1RKI0	pp	229533.I1RT36
229533.I1RKI0	pp	229533.I1SAT1
229533.I1RKI0	pp	229533.I1RPL2
229533.I1RKI0	pp	229533.I1RKJ7
229533.I1RKI0	pp	229533.I1RP28
229533.I1RKI0	pp	229533.I1RZX9
229533.I1RKI0	pp	229533.I1RQG4

229533.I1RKI0	pp	229533.I1RPM8
229533.I1RKI0	pp	229533.I1S7X9
229533.I1RKI0	pp	229533.I1RQH1
229533.A0A098DXI2	pp	229533.I1RX00
229533.A0A098DXI2	pp	229533.A0A1C3YMC4
229533.A0A098DXI2	pp	229533.I1S5R5
229533.I1RN89	pp	229533.I1S193
229533.I1RN89	pp	229533.I1RWT6
229533.I1RN89	pp	229533.I1RQA3
229533.I1RN89	pp	229533.V6QV09
229533.I1RN89	pp	229533.V6RGI1
229533.I1RN89	pp	229533.I1S098
229533.I1RN89	pp	229533.I1RYZ9
229533.I1RN89	pp	229533.I1RPC2
229533.I1RGM4	pp	229533.I1RQL4
229533.A0A1C3YK90	pp	229533.I1RWI6
229533.A0A1C3YK90	pp	229533.I1RPK5
229533.A0A1C3YK90	pp	229533.I1RE15
229533.A0A1C3YK90	pp	229533.I1REN4
229533.I1RSH0	pp	229533.Q5I2J3
229533.I1RSH0	pp	229533.I1RT75
229533.I1RSH0	pp	229533.I1RYI9
229533.I1RSH0	pp	229533.I1S238
229533.I1RSH0	pp	229533.I1S2G9
229533.I1RSH0	pp	229533.I1S8P5
229533.I1RSH0	pp	229533.I1RXT9
229533.I1RSH0	pp	229533.I1RZB5
229533.I1RSH0	pp	229533.Q4I948
229533.I1RSH0	pp	229533.I1RTK6
229533.I1RSH0	pp	229533.Q4ICA8
229533.I1RSH0	pp	229533.I1RTA0
229533.I1R9X8	pp	229533.I1RJQ3
229533.A0A1C3YN58	pp	229533.I1RU02
229533.A0A1C3YN58	pp	229533.I1RQ93
229533.A0A1C3YN58	pp	229533.I1RNT2
229533.A0A1C3YN58	pp	229533.I1RY98
229533.A0A1C3YN58	pp	229533.I1RJR2
229533.A0A1C3YN58	pp	229533.Q4IER8
229533.A0A1C3YN58	pp	229533.I1S2M5
229533.A0A1C3YN58	pp	229533.I1RCU7
229533.A0A1C3YN58	pp	229533.I1S2B6
229533.A0A1C3YN58	pp	229533.I1RQL4
229533.A0A1C3YN58	pp	229533.I1RPF4
229533.A0A1C3YN58	pp	229533.I1RYZ9
229533.A0A1C3YN58	pp	229533.I1RZV9
229533.A0A1C3YN58	pp	229533.Q4I061
229533.A0A1C3YN58	pp	229533.I1RAN8
229533.A0A1C3YN58	pp	229533.I1RPN4
229533.A0A1C3YN58	pp	229533.I1RFQ0
229533.A0A1C3YN58	pp	229533.I1S0F0
229533.A0A1C3YN58	pp	229533.A0A1C3YN65
229533.A0A1C3YN58	pp	229533.I1RQA3
229533.A0A1C3YN58	pp	229533.Q4ICA8
229533.A0A1C3YN58	pp	229533.I1SAA0
229533.A0A1C3YN58	pp	229533.I1RUZ8
229533.A0A1C3YN58	pp	229533.Q4HTT2
229533.A0A1C3YN58	pp	229533.I1RBL8
229533.A0A1C3YN58	pp	229533.I1RWT2
229533.A0A1C3YN58	pp	229533.I1S0P4
229533.A0A1C3YN58	pp	229533.Q4I7L0
229533.A0A1C3YN58	pp	229533.I1RWT6
229533.A0A1C3YN58	pp	229533.I1RCW8
229533.A0A1C3YN58	pp	229533.I1RCK7
229533.A0A1C3YN58	pp	229533.I1RCK4
229533.A0A1C3YN58	pp	229533.I1RPR8
229533.A0A1C3YN58	pp	229533.I1RQ88
229533.A0A1C3YN58	pp	229533.I1RAK6
229533.A0A1C3YN58	pp	229533.I1RPY4
229533.A0A1C3YN58	pp	229533.I1RVH7
229533.A0A1C3YN58	pp	229533.I1REC6
229533.A0A1C3YN58	pp	229533.I1RB73
229533.A0A1C3YN58	pp	229533.I1RV91
229533.A0A1C3YN58	pp	229533.I1SB04

30515 229533.A0A1C3YN58 pp 229533.I1S2E9
30516 229533.A0A1C3YN58 pp 229533.Q4HTT1
30517 229533.A0A1C3YN58 pp 229533.I1RHP9
30518 229533.A0A1C3YN58 pp 229533.I1RCN2
30519 229533.A0A1C3YN58 pp 229533.Q4IEV4
30520 229533.A0A1C3YN58 pp 229533.I1RBR4
30521 229533.A0A1C3YN58 pp 229533.I1RY77
30522 229533.A0A1C3YN58 pp 229533.I1RZT1
30523 229533.A0A1C3YN58 pp 229533.I1S247
30524 229533.A0A1C3YN58 pp 229533.I1R9W6
30525 229533.A0A1C3YN58 pp 229533.I1RNF4
30526 229533.A0A1C3YN58 pp 229533.Q4IMD1
30527 229533.A0A1C3YN58 pp 229533.I1RCD8
30528 229533.A0A1C3YN58 pp 229533.I1RKC6
30529 229533.A0A1C3YN58 pp 229533.V6REZ4
30530 229533.A0A1C3YN58 pp 229533.I1RQ72
30531 229533.A0A1C3YN58 pp 229533.I1RYT7
30532 229533.A0A1C3YN58 pp 229533.I1RW28
30533 229533.A0A1C3YN58 pp 229533.I1RFW1
30534 229533.A0A1C3YN58 pp 229533.I1S3D7
30535 229533.A0A1C3YN58 pp 229533.I1RSL6
30536 229533.A0A1C3YN58 pp 229533.I1RAI9
30537 229533.A0A1C3YN58 pp 229533.I1RYW3
30538 229533.A0A1C3YN58 pp 229533.I1RND2
30539 229533.A0A1C3YN58 pp 229533.I1RQI6
30540 229533.A0A1C3YN58 pp 229533.I1RY99
30541 229533.A0A1C3YN58 pp 229533.I1REC7
30542 229533.A0A1C3YN58 pp 229533.I1RB44
30543 229533.A0A1C3YN58 pp 229533.I1RY44
30544 229533.A0A1C3YN58 pp 229533.I1S282
30545 229533.I1RA73 pp 229533.I1S109
30546 229533.I1RA73 pp 229533.Q4I7L0
30547 229533.I1RA73 pp 229533.I1RPU1
30548 229533.I1RA73 pp 229533.I1RCN2
30549 229533.I1RA73 pp 229533.I1RB73
30550 229533.I1RA73 pp 229533.I1RW60
30551 229533.I1RZ94 pp 229533.I1S788
30552 229533.I1RZ94 pp 229533.I1S219
30553 229533.I1RZ94 pp 229533.I1S3J4
30554 229533.I1RZ94 pp 229533.I1S160
30555 229533.I1RZ94 pp 229533.I1S209
30556 229533.I1RZ94 pp 229533.I1SAD2
30557 229533.I1RZ94 pp 229533.I1S987
30558 229533.I1RZ94 pp 229533.Q4INS6
30559 229533.I1RZ94 pp 229533.I1S2E9
30560 229533.I1RZ94 pp 229533.I1S4Z7
30561 229533.I1RZ94 pp 229533.I1S604
30562 229533.I1RZ94 pp 229533.Q4IR08
30563 229533.I1RZ94 pp 229533.I1S461
30564 229533.I1RZ94 pp 229533.Q4IQM6
30565 229533.I1RZ94 pp 229533.I1S998
30566 229533.I1RZ94 pp 229533.I1S471
30567 229533.I1RZ94 pp 229533.I1S5G0
30568 229533.I1RZ94 pp 229533.I1S0D6
30569 229533.I1RZ94 pp 229533.I1RZ98
30570 229533.I1RZ94 pp 229533.I1S1H4
30571 229533.I1RZ94 pp 229533.V6R382
30572 229533.I1RZ94 pp 229533.I1S523
30573 229533.I1RZ94 pp 229533.V6RLW4
30574 229533.I1RZ94 pp 229533.I1S276
30575 229533.I1RZ94 pp 229533.I1S4P6
30576 229533.I1RZ94 pp 229533.I1S008
30577 229533.I1RZ94 pp 229533.I1RZP8
30578 229533.I1RZ94 pp 229533.Q4IB89
30579 229533.I1RZ94 pp 229533.V6QX21
30580 229533.I1RZ94 pp 229533.I1S0A6
30581 229533.I1RZ94 pp 229533.V6QUV7
30582 229533.I1RZ94 pp 229533.I1S016
30583 229533.I1RZ94 pp 229533.I1S0D5
30584 229533.I1RCL5 pp 229533.I1RPP4
30585 229533.I1RCL5 pp 229533.V6RIV9
30586 229533.I1RCL5 pp 229533.I1RPL2
30587 229533.I1RCL5 pp 229533.I1RLU6

30588 229533.I1RCL5 pp 229533.I1RSA4
30589 229533.I1RCL5 pp 229533.I1RN18
30590 229533.I1RCL5 pp 229533.I1RCL7
30591 229533.I1RCL5 pp 229533.I1RNQ5
30592 229533.I1RCL5 pp 229533.I1S8V4
30593 229533.I1RCL5 pp 229533.I1RD17
30594 229533.I1RCL5 pp 229533.I1RQF2
30595 229533.I1RCL5 pp 229533.I1S0M2
30596 229533.I1RCL5 pp 229533.I1RTH6
30597 229533.I1RCL5 pp 229533.I1RQA5
30598 229533.I1RCL5 pp 229533.I1S238
30599 229533.I1RCL5 pp 229533.I1RNC1
30600 229533.I1RCL5 pp 229533.V6RXB3
30601 229533.I1RCL5 pp 229533.I1RRM9
30602 229533.I1RCL5 pp 229533.I1S8V3
30603 229533.I1RCL5 pp 229533.I1S2G9
30604 229533.I1RCL5 pp 229533.I1RCQ3
30605 229533.I1RCL5 pp 229533.V6RMW6
30606 229533.I1RCL5 pp 229533.I1RYU0
30607 229533.I1RCL5 pp 229533.I1RSK1
30608 229533.I1RCL5 pp 229533.I1RQG4
30609 229533.I1RCL5 pp 229533.V6RCG5
30610 229533.I1RCL5 pp 229533.I1RRY9
30611 229533.I1RCL5 pp 229533.I1RTA0
30612 229533.I1RCL5 pp 229533.I1S8P5
30613 229533.I1RCL5 pp 229533.I1RKK0
30614 229533.I1RCL5 pp 229533.I1RMQ5
30615 229533.I1RCL5 pp 229533.I1S8F0
30616 229533.I1RCL5 pp 229533.I1RPM4
30617 229533.I1RCL5 pp 229533.I1RQH1
30618 229533.I1RCL5 pp 229533.I1RP28
30619 229533.I1RCL5 pp 229533.I1RT40
30620 229533.I1RCL5 pp 229533.I1S0L3
30621 229533.I1RCL5 pp 229533.I1S2B8
30622 229533.I1RCL5 pp 229533.I1RSH0
30623 229533.A0A0E0RV61 pp 229533.I1RCG5
30624 229533.A0A0E0RV61 pp 229533.Q4IPZ1
30625 229533.A0A0E0RV61 pp 229533.I1RQG4
30626 229533.A0A0E0RV61 pp 229533.I1RMQ5
30627 229533.A0A0E0RV61 pp 229533.I1RP28
30628 229533.A0A0E0RV61 pp 229533.I1RD80
30629 229533.A0A0E0RV61 pp 229533.A0A1C3YLH6
30630 229533.A0A0E0RV61 pp 229533.V6R398
30631 229533.A0A0E0RV61 pp 229533.I1RMH4
30632 229533.A0A0E0RV61 pp 229533.I1RGB7
30633 229533.A0A0E0RV61 pp 229533.I1RLE2
30634 229533.A0A0E0RV61 pp 229533.A0A0E0RWX7
30635 229533.A0A0E0RV61 pp 229533.I1RPL2
30636 229533.A0A0E0RV61 pp 229533.I1RAM6
30637 229533.A0A0E0RV61 pp 229533.I1RGW2
30638 229533.A0A0E0RV61 pp 229533.I1RQH1
30639 229533.A0A0E0RV61 pp 229533.I1RPQ2
30640 229533.A0A1C3YL21 pp 229533.A0A1C3YN77
30641 229533.A0A0E0RS08 pp 229533.I1RND4
30642 229533.A0A0E0RS08 pp 229533.I1RUP5
30643 229533.A0A0E0RS08 pp 229533.I1RNG4
30644 229533.A0A0E0RS08 pp 229533.I1RSX9
30645 229533.A0A0E0RS08 pp 229533.I1RRG1
30646 229533.A0A0E0RS08 pp 229533.I1RJS0
30647 229533.A0A0E0RS08 pp 229533.A0A1C3YL21
30648 229533.I1S9U1 pp 229533.I1S9U2
30649 229533.I1S9U1 pp 229533.Q4IQT8
30650 229533.I1S9U1 pp 229533.V6QUE6
30651 229533.I1S9U1 pp 229533.I1S9Y1
30652 229533.I1S9U1 pp 229533.V6QWX8
30653 229533.I1S9U1 pp 229533.V6QYF0
30654 229533.A0A0E0SAC4 pp 229533.I1RQA9
30655 229533.A0A0E0SAC4 pp 229533.A0A0E0SNY0
30656 229533.A0A0E0SAC4 pp 229533.I1RAM6
30657 229533.A0A0E0SAC4 pp 229533.I1RWJ6
30658 229533.A0A0E0SAC4 pp 229533.I1RCU8
30659 229533.A0A0E0SAC4 pp 229533.I1RCU9
30660 229533.A0A0E0SAC4 pp 229533.I1RX38

30661	229533.A0A0E0SAC4	pp	229533.I1S549
30662	229533.A0A0E0SAC4	pp	229533.I1RL24
30663	229533.A0A0E0SAC4	pp	229533.I1RD18
30664	229533.A0A0E0SAC4	pp	229533.I1RJR8
30665	229533.A0A0E0SAC4	pp	229533.I1RGF1
30666	229533.A0A0E0SAC4	pp	229533.I1RJ88
30667	229533.A0A0E0SAC4	pp	229533.I1RDB1
30668	229533.A0A0E0SAC4	pp	229533.I1RX70
30669	229533.A0A0E0SAC4	pp	229533.I1S1V5
30670	229533.A0A0E0SAC4	pp	229533.I1RKY2
30671	229533.A0A0E0SAC4	pp	229533.A0A1C3YJH8
30672	229533.A0A0E0SAC4	pp	229533.I1RZ63
30673	229533.A0A0E0SAC4	pp	229533.I1RST8
30674	229533.A0A0E0SAC4	pp	229533.I1RAN2
30675	229533.A0A0E0SAC4	pp	229533.I1RXH6
30676	229533.A0A0E0SAC4	pp	229533.I1RV66
30677	229533.A0A0E0SAC4	pp	229533.I1RN72
30678	229533.A0A0E0SAC4	pp	229533.A0A1C3YKF2
30679	229533.A0A0E0SAC4	pp	229533.I1RRM9
30680	229533.A0A0E0SAC4	pp	229533.I1RPM4
30681	229533.A0A0E0SAC4	pp	229533.A0A1C3YJV7
30682	229533.A0A0E0SAC4	pp	229533.I1RNP7
30683	229533.A0A0E0SAC4	pp	229533.I1RYH9
30684	229533.A0A0E0SAC4	pp	229533.I1RUN8
30685	229533.A0A0E0SAC4	pp	229533.I1S224
30686	229533.A0A0E0SAC4	pp	229533.I1RQM0
30687	229533.A0A0E0SAC4	pp	229533.I1RX46
30688	229533.A0A0E0SAC4	pp	229533.A0A1C3YN39
30689	229533.A0A0E0SAC4	pp	229533.I1RTI6
30690	229533.A0A0E0SAC4	pp	229533.I1RQG4
30691	229533.A0A0E0SAC4	pp	229533.I1RNC0
30692	229533.A0A0E0SAC4	pp	229533.A0A1C3YIL0
30693	229533.A0A0E0SAC4	pp	229533.I1RB74
30694	229533.A0A0E0SAC4	pp	229533.I1S2E9
30695	229533.A0A0E0SAC4	pp	229533.I1RYH6
30696	229533.A0A0E0SAC4	pp	229533.I1RV91
30697	229533.A0A0E0SAC4	pp	229533.I1RFZ3
30698	229533.A0A0E0SAC4	pp	229533.I1S7X9
30699	229533.A0A0E0SAC4	pp	229533.I1RN31
30700	229533.A0A0E0SAC4	pp	229533.A0A1C3YHL0
30701	229533.A0A0E0SAC4	pp	229533.I1RQ26
30702	229533.A0A0E0SAC4	pp	229533.I1RAW7
30703	229533.A0A0E0SAC4	pp	229533.V6QV09
30704	229533.A0A0E0SAC4	pp	229533.I1RPU7
30705	229533.A0A0E0SAC4	pp	229533.I1RLX6
30706	229533.A0A0E0SAC4	pp	229533.I1S2B8
30707	229533.A0A0E0SAC4	pp	229533.I1RYZ9
30708	229533.A0A0E0SAC4	pp	229533.A0A1C3YKW5
30709	229533.A0A0E0SAC4	pp	229533.I1RRP7
30710	229533.A0A0E0SAC4	pp	229533.I1RJH1
30711	229533.A0A0E0SAC4	pp	229533.A0A1C3YIN6
30712	229533.A0A0E0SAC4	pp	229533.V6RTJ8
30713	229533.A0A0E0SAC4	pp	229533.I1S0Z7
30714	229533.A0A0E0SAC4	pp	229533.I1RBZ9
30715	229533.A0A0E0SAC4	pp	229533.I1RP28
30716	229533.A0A0E0SAC4	pp	229533.I1RC97
30717	229533.A0A0E0SAC4	pp	229533.I1RS47
30718	229533.A0A0E0SAC4	pp	229533.I1RMM9
30719	229533.A0A0E0SAC4	pp	229533.I1S2G7
30720	229533.A0A0E0SAC4	pp	229533.I1RN08
30721	229533.A0A0E0SAC4	pp	229533.I1RPP7
30722	229533.A0A0E0SAC4	pp	229533.I1S0N8
30723	229533.A0A0E0SAC4	pp	229533.A0A1C3YJW1
30724	229533.A0A0E0SAC4	pp	229533.I1RNF4
30725	229533.A0A0E0SAC4	pp	229533.I1RRY3
30726	229533.A0A0E0SAC4	pp	229533.V6R1K4
30727	229533.A0A0E0SAC4	pp	229533.I1RQA5
30728	229533.A0A0E0SAC4	pp	229533.I1RBD2
30729	229533.A0A0E0SAC4	pp	229533.I1RSK1
30730	229533.A0A0E0SAC4	pp	229533.I1RCL5
30731	229533.A0A0E0SAC4	pp	229533.I1RHQ8
30732	229533.A0A0E0SAC4	pp	229533.I1RC22
30733	229533.A0A0E0SAC4	pp	229533.I1RUQ5

229533.A0A0E0SAC4 pp 229533.I1RMQ5
229533.A0A0E0SAC4 pp 229533.I1RRN5
229533.A0A0E0SAC4 pp 229533.A0A1C3YJF1
229533.A0A0E0SAC4 pp 229533.I1RQB4
229533.A0A0E0SAC4 pp 229533.I1S0M2
229533.A0A0E0SAC4 pp 229533.I1RYY5
229533.A0A0E0SAC4 pp 229533.Q4IJM4
229533.A0A0E0SAC4 pp 229533.I1RMA8
229533.A0A0E0SAC4 pp 229533.A0A1C3YK37
229533.A0A0E0SAC4 pp 229533.I1RN65
229533.A0A0E0SAC4 pp 229533.I1RCB3
229533.A0A0E0SAC4 pp 229533.I1S2E8
229533.A0A0E0SAC4 pp 229533.A0A1C3YI28
229533.A0A0E0SAC4 pp 229533.I1RRW0
229533.A0A0E0SAC4 pp 229533.I1RI34
229533.A0A0E0SAC4 pp 229533.I1RM09
229533.A0A0E0SAC4 pp 229533.I1RCL8
229533.A0A0E0SAC4 pp 229533.I1RNY9
229533.A0A0E0SAC4 pp 229533.I1RZZ1
229533.A0A0E0SAC4 pp 229533.I1RDZ4
229533.A0A0E0SAC4 pp 229533.I1RZE4
229533.A0A0E0SAC4 pp 229533.I1RPU4
229533.A0A0E0SAC4 pp 229533.I1RC17
229533.A0A0E0SAC4 pp 229533.I1RQA3
229533.A0A0E0SAC4 pp 229533.V6RDP1
229533.A0A0E0SAC4 pp 229533.I1RW16
229533.A0A0E0SAC4 pp 229533.I1RI71
229533.A0A0E0SAC4 pp 229533.I1RB75
229533.A0A0E0SAC4 pp 229533.I1RZH4
229533.A0A0E0SAC4 pp 229533.I1S296
229533.A0A0E0SAC4 pp 229533.V6RGI1
229533.A0A0E0SAC4 pp 229533.I1S1D3
229533.A0A0E0SAC4 pp 229533.I1RT36
229533.A0A0E0SAC4 pp 229533.I1S439
229533.A0A0E0SAC4 pp 229533.I1RA43
229533.A0A0E0SAC4 pp 229533.I1RQ72
229533.A0A0E0SAC4 pp 229533.I1RKK0
229533.A0A0E0SAC4 pp 229533.I1S2K7
229533.A0A0E0SAC4 pp 229533.Q4IR08
229533.A0A0E0SAC4 pp 229533.I1RAM7
229533.A0A0E0SAC4 pp 229533.I1RYZ0
229533.A0A0E0SAC4 pp 229533.I1R9Y9
229533.A0A0E0SAC4 pp 229533.I1RBI1
229533.A0A0E0SAC4 pp 229533.I1S0N4
229533.A0A0E0SAC4 pp 229533.I1RB41
229533.A0A0E0SAC4 pp 229533.A0A1C3YM29
229533.A0A0E0SAC4 pp 229533.I1RSL8
229533.A0A0E0SAC4 pp 229533.I1R9Y7
229533.A0A0E0SAC4 pp 229533.V6RIS2
229533.A0A0E0SAC4 pp 229533.A0A0E0SDE1
229533.A0A0E0SAC4 pp 229533.I1RE37
229533.A0A0E0SAC4 pp 229533.I1RMQ4
229533.A0A0E0SAC4 pp 229533.A0A1C3YN89
229533.A0A0E0SAC4 pp 229533.V6RMW6
229533.A0A0E0SAC4 pp 229533.I1RYI7
229533.A0A0E0SAC4 pp 229533.I1S1W4
229533.A0A0E0SAC4 pp 229533.I1RD22
229533.A0A0E0SAC4 pp 229533.A0A1C3YM91
229533.A0A0E0SAC4 pp 229533.I1RWT6
229533.A0A0E0SAC4 pp 229533.A0A1C3YHQ3
229533.A0A0E0SAC4 pp 229533.I1RC95
229533.A0A0E0SAC4 pp 229533.I1S0D1
229533.A0A0E0SAC4 pp 229533.I1RQ21
229533.A0A0E0SAC4 pp 229533.V6RKY7
229533.A0A0E0SAC4 pp 229533.V6RVG3
229533.A0A0E0SAC4 pp 229533.I1RZM1
229533.A0A0E0SAC4 pp 229533.I1RAY4
229533.A0A0E0SAC4 pp 229533.I1RY44
229533.I1S238 pp 229533.I1S2G9
229533.I1S238 pp 229533.I1S8V3
229533.I1S238 pp 229533.Q5I2J3
229533.I1S238 pp 229533.I1S8V4
229533.I1S238 pp 229533.Q4I948

30807	229533.I1S238	pp	229533.Q4ICA8
30808	229533.I1RNS3	pp	229533.I1RPK5
30809	229533.I1RNS3	pp	229533.I1RWI6
30810	229533.A0A1C3YLX3	pp	229533.I1RQL4
30811	229533.A0A0E0RRY8	pp	229533.I1RAN8
30812	229533.A0A0E0RRY8	pp	229533.I1S1V2
30813	229533.A0A0E0RRY8	pp	229533.Q4IPI4
30814	229533.A0A0E0RRY8	pp	229533.I1S7I5
30815	229533.A0A0E0RRY8	pp	229533.A0A0E0SHR3
30816	229533.A0A0E0RRY8	pp	229533.A0A1C3YLA2
30817	229533.A0A0E0RRY8	pp	229533.Q4I7X1
30818	229533.A0A0E0RRY8	pp	229533.I1RE51
30819	229533.A0A0E0SIF3	pp	229533.I1RYZ7
30820	229533.A0A0E0SIF3	pp	229533.Q4IJ79
30821	229533.A0A0E0SIF3	pp	229533.I1RPM8
30822	229533.A0A0E0SIF3	pp	229533.I1RRN0
30823	229533.A0A0E0SIF3	pp	229533.I1RD66
30824	229533.A0A0E0SIF3	pp	229533.I1RNW8
30825	229533.A0A0E0SIF3	pp	229533.I1RQN9
30826	229533.A0A0E0SIF3	pp	229533.V6RF62
30827	229533.A0A0E0SIF3	pp	229533.I1RJS2
30828	229533.A0A0E0SIF3	pp	229533.I1RS79
30829	229533.A0A0E0SIF3	pp	229533.I1S7X9
30830	229533.A0A0E0SIF3	pp	229533.I1RMQ5
30831	229533.A0A0E0SIF3	pp	229533.I1RQG4
30832	229533.A0A0E0SIF3	pp	229533.I1RT36
30833	229533.A0A0E0SIF3	pp	229533.I1RP28
30834	229533.A0A0E0SIF3	pp	229533.I1RJS3
30835	229533.A0A0E0SIF3	pp	229533.I1RIG5
30836	229533.A0A0E0SIF3	pp	229533.I1RAW2
30837	229533.A0A0E0SIF3	pp	229533.A0A1C3YLD3
30838	229533.A0A0E0SIF3	pp	229533.I1RAM6
30839	229533.A0A0E0SIF3	pp	229533.I1RJX2
30840	229533.A0A0E0SIF3	pp	229533.I1RG07
30841	229533.A0A0E0SIF3	pp	229533.I1RKJ7
30842	229533.A0A0E0SIF3	pp	229533.I1RQH1
30843	229533.A0A0E0SIF3	pp	229533.I1S4S5
30844	229533.A0A0E0SIF3	pp	229533.I1SAT1
30845	229533.A0A0E0SIF3	pp	229533.I1RZX9
30846	229533.A0A0E0SIF3	pp	229533.I1RNF9
30847	229533.A0A0E0SIF3	pp	229533.I1RJC5
30848	229533.A0A0E0SIF3	pp	229533.A0A1C3YI65
30849	229533.A0A0E0SIF3	pp	229533.V6R3V4
30850	229533.A0A0E0SIF3	pp	229533.A0A1C3YJ26
30851	229533.A0A0E0SIF3	pp	229533.I1RPL2
30852	229533.A0A1C3YMQ0	pp	229533.I1RD22
30853	229533.I1RL24	pp	229533.I1S0J7
30854	229533.I1RL24	pp	229533.I1RUN8
30855	229533.I1RL24	pp	229533.I1RPC2
30856	229533.I1RL24	pp	229533.I1S0M6
30857	229533.I1RL24	pp	229533.I1RNP7
30858	229533.I1RL24	pp	229533.I1RLU1
30859	229533.I1RL24	pp	229533.I1S4R1
30860	229533.I1RL24	pp	229533.I1S1D3
30861	229533.I1RL24	pp	229533.I1RZ63
30862	229533.I1RL24	pp	229533.I1RRN5
30863	229533.I1RL24	pp	229533.I1RNW8
30864	229533.I1RL24	pp	229533.I1RW36
30865	229533.I1RL24	pp	229533.I1RRQ9
30866	229533.I1RL24	pp	229533.I1RNZ9
30867	229533.I1RL24	pp	229533.I1RNY8
30868	229533.I1RL24	pp	229533.I1RNF4
30869	229533.I1RL24	pp	229533.I1RT36
30870	229533.I1RL24	pp	229533.I1S9S6
30871	229533.I1RL24	pp	229533.I1S0E8
30872	229533.I1RL24	pp	229533.I1RQG4
30873	229533.I1RL24	pp	229533.I1S7X9
30874	229533.I1RL24	pp	229533.I1RZT1
30875	229533.I1RL24	pp	229533.I1RZE4
30876	229533.I1RL24	pp	229533.I1S2E9
30877	229533.I1RL24	pp	229533.V6RGI1
30878	229533.I1RL24	pp	229533.I1RZY9
30879	229533.I1RL24	pp	229533.I1RRY3

229533.I1RL24	pp	229533.I1RQB4
229533.I1RL24	pp	229533.I1RV91
229533.I1RL24	pp	229533.I1S0M2
229533.I1RL24	pp	229533.I1S098
229533.I1RL24	pp	229533.I1S1W4
229533.I1RL24	pp	229533.I1RMA4
229533.I1RL24	pp	229533.I1RQ72
229533.I1RL24	pp	229533.V6RKY7
229533.I1RL24	pp	229533.I1RRW0
229533.I1RL24	pp	229533.I1RY44
229533.I1RL24	pp	229533.Q4ICM0
229533.I1RL24	pp	229533.I1RN89
229533.I1RL24	pp	229533.V6RVG3
229533.I1RL24	pp	229533.I1RN31
229533.I1RL24	pp	229533.I1S439
229533.I1RL24	pp	229533.I1RW16
229533.I1RL24	pp	229533.I1RYH9
229533.I1RL24	pp	229533.Q4IJM4
229533.I1RL24	pp	229533.I1RQS4
229533.I1RL24	pp	229533.I1S224
229533.I1RL24	pp	229533.I1RY84
229533.I1RL24	pp	229533.I1RV66
229533.I1RL24	pp	229533.I1RN65
229533.I1RL24	pp	229533.I1RQZ0
229533.I1RL24	pp	229533.I1RT62
229533.I1RL24	pp	229533.I1S1W5
229533.I1RL24	pp	229533.I1RZ12
229533.I1RL24	pp	229533.I1RQA3
229533.I1RL24	pp	229533.V6RMM8
229533.I1RL24	pp	229533.I1RQ26
229533.I1RL24	pp	229533.I1RS56
229533.I1RL24	pp	229533.I1RMM9
229533.I1RL24	pp	229533.I1RN08
229533.I1RL24	pp	229533.I1RWT6
229533.I1RL24	pp	229533.I1RLB1
229533.I1RL24	pp	229533.I1RYY5
229533.I1RL24	pp	229533.I1RZH4
229533.I1RL24	pp	229533.V6RU70
229533.I1RL24	pp	229533.I1RME5
229533.I1RCT1	pp	229533.I1S549
229533.I1RCT1	pp	229533.I1RWM0
229533.I1RCT1	pp	229533.I1RXB5
229533.I1RC63	pp	229533.I1RQL4
229533.I1RC63	pp	229533.I1RTE6
229533.I1RC63	pp	229533.I1RTB7
229533.I1RC63	pp	229533.I1RCJ5
229533.I1RC63	pp	229533.I1RNE8
229533.I1RQF2	pp	229533.I1S2G9
229533.I1RQF2	pp	229533.I1S0M2
229533.I1RQF2	pp	229533.I1S1Z9
229533.I1RQF2	pp	229533.I1RRM9
229533.I1RQF2	pp	229533.I1RSH0
229533.I1RQF2	pp	229533.I1RQH1
229533.I1RQF2	pp	229533.I1S238
229533.I1RQF2	pp	229533.I1RQG4
229533.A0A098E4R5	pp	229533.I1RV91
229533.A0A098E4R5	pp	229533.I1RCB3
229533.A0A098E4R5	pp	229533.I1RPQ1
229533.A0A098E4R5	pp	229533.I1S193
229533.A0A098E4R5	pp	229533.A0A1C3YHH5
229533.A0A098E4R5	pp	229533.A0A1C3YI40
229533.A0A098E4R5	pp	229533.A0A1C3YMF9
229533.A0A098E4R5	pp	229533.I1RQ13
229533.A0A098E4R5	pp	229533.I1RG77
229533.A0A098E4R5	pp	229533.I1R976
229533.A0A098E4R5	pp	229533.I1RQA3
229533.A0A098E4R5	pp	229533.I1RA10
229533.A0A098E4R5	pp	229533.I1RCA5
229533.A0A098E4R5	pp	229533.I1RFL7
229533.A0A098E4R5	pp	229533.I1RMQ7
229533.A0A098E4R5	pp	229533.A0A1C3YN69
229533.A0A098E4R5	pp	229533.I1RAW7
229533.A0A098E4R5	pp	229533.A0A1C3YJT5

30953 229533.A0A098E4R5 pp 229533.A0A0E0RS85
30954 229533.A0A098E4R5 pp 229533.I1S382
30955 229533.A0A098E4R5 pp 229533.I1RFU3
30956 229533.A0A098E4R5 pp 229533.I1RDG8
30957 229533.A0A098E4R5 pp 229533.I1RWT6
30958 229533.A0A098E4R5 pp 229533.A0A0E0RW49
30959 229533.A0A098E4R5 pp 229533.I1RBW0
30960 229533.A0A098E4R5 pp 229533.A0A1C3YIJ4
30961 229533.A0A098E4R5 pp 229533.I1RSN7
30962 229533.A0A098E4R5 pp 229533.I1RX64
30963 229533.A0A098E4R5 pp 229533.I1RY44
30964 229533.A0A098E4R5 pp 229533.I1R9Q0
30965 229533.A0A098E4R5 pp 229533.A0A0E0SA11
30966 229533.A0A098E4R5 pp 229533.I1RA12
30967 229533.A0A098E4R5 pp 229533.Q4I7X1
30968 229533.A0A098E4R5 pp 229533.I1RZP8
30969 229533.A0A098E4R5 pp 229533.I1RSP7
30970 229533.A0A098E4R5 pp 229533.A0A0E0SQG9
30971 229533.A0A098E4R5 pp 229533.I1RY29
30972 229533.A0A098E4R5 pp 229533.A0A1C3YJ12
30973 229533.A0A098E4R5 pp 229533.I1S951
30974 229533.A0A098E4R5 pp 229533.I1S2E9
30975 229533.A0A098E4R5 pp 229533.I1RBW1
30976 229533.A0A098E4R5 pp 229533.I1RSR9
30977 229533.A0A098E4R5 pp 229533.I1S1V5
30978 229533.I1S0Z0 pp 229533.Q4IFH8
30979 229533.I1RJT8 pp 229533.I1RYB7
30980 229533.I1RJT8 pp 229533.I1RK67
30981 229533.I1RJT8 pp 229533.I1RW84
30982 229533.I1RJT8 pp 229533.I1S248
30983 229533.I1RJT8 pp 229533.I1RMR7
30984 229533.I1RJT8 pp 229533.I1RQA3
30985 229533.I1RJT8 pp 229533.I1S074
30986 229533.I1RJT8 pp 229533.I1S2E9
30987 229533.I1RJT8 pp 229533.Q4IBS9
30988 229533.I1RJT8 pp 229533.I1RYI3
30989 229533.I1RJT8 pp 229533.Q4IEV5
30990 229533.I1RJT8 pp 229533.I1RNY2
30991 229533.I1RJT8 pp 229533.I1RY44
30992 229533.I1RJT8 pp 229533.I1RWT6
30993 229533.I1RJT8 pp 229533.I1RW38
30994 229533.I1RJT8 pp 229533.I1RLT6
30995 229533.Q4HZK7 pp 229533.Q4IN63
30996 229533.Q4HZK7 pp 229533.Q4IQW0
30997 229533.Q4HZK7 pp 229533.Q4IFI0
30998 229533.Q4HZK7 pp 229533.Q4IEK8
30999 229533.Q4HZK7 pp 229533.V6QUE6
31000 229533.Q4HZK7 pp 229533.Q4I8B6
31001 229533.Q4HZK7 pp 229533.V6RGN5
31002 229533.Q4HZK7 pp 229533.Q4IPI1
31003 229533.I1REK7 pp 229533.I1RPK5
31004 229533.I1REK7 pp 229533.I1RYL5
31005 229533.I1REK7 pp 229533.I1REK8
31006 229533.I1REK7 pp 229533.Q4I624
31007 229533.I1REK7 pp 229533.I1RMG3
31008 229533.I1REK7 pp 229533.I1RMG8
31009 229533.I1REK7 pp 229533.I1RQ21
31010 229533.I1RW38 pp 229533.I1RXF5
31011 229533.I1RW38 pp 229533.I1S074
31012 229533.I1RW38 pp 229533.Q4IJM4
31013 229533.I1RW38 pp 229533.I1RXU1
31014 229533.I1RW38 pp 229533.I1S2E9
31015 229533.I1RW38 pp 229533.I1S0N8
31016 229533.I1RW38 pp 229533.I1RX09
31017 229533.I1RW38 pp 229533.V6RR76
31018 229533.I1RW38 pp 229533.I1S248
31019 229533.I1RW38 pp 229533.I1RW84
31020 229533.I1RW38 pp 229533.I1S2G7
31021 229533.I1RW38 pp 229533.I1RY44
31022 229533.I1RW38 pp 229533.I1RW83
31023 229533.I1RW38 pp 229533.I1RWT6
31024 229533.A0A1C3YMP7 pp 229533.I1S6N5
31025 229533.A0A1C3YMP7 pp 229533.A0A1C3YMS7

229533.A0A1C3YMP7 pp 229533.V6RPN0
229533.A0A1C3YMP7 pp 229533.I1S0F0
229533.A0A1C3YMP7 pp 229533.I1RQL4
229533.A0A1C3YMP7 pp 229533.I1S5R5
229533.A0A1C3YMP7 pp 229533.I1RCN2
229533.A0A1C3YMP7 pp 229533.I1RD69
229533.A0A1C3YMP7 pp 229533.I1RB73
229533.I1RAK3 pp 229533.I1RQL4
229533.A0A1C3YIL0 pp 229533.I1RC71
229533.A0A1C3YIL0 pp 229533.I1RX38
229533.A0A1C3YIL0 pp 229533.I1RAS9
229533.A0A1C3YIL0 pp 229533.I1RLU1
229533.A0A1C3YIL0 pp 229533.I1RNP7
229533.A0A1C3YIL0 pp 229533.I1RMF5
229533.A0A1C3YIL0 pp 229533.I1S2E9
229533.A0A1C3YIL0 pp 229533.I1RUN8
229533.A0A1C3YIL0 pp 229533.A0A1C3YJI8
229533.A0A1C3YIL0 pp 229533.I1RV91
229533.A0A1C3YIL0 pp 229533.I1RBY4
229533.A0A1C3YIL0 pp 229533.I1RY32
229533.A0A1C3YIL0 pp 229533.I1RMQ5
229533.A0A1C3YIL0 pp 229533.I1RRW0
229533.A0A1C3YIL0 pp 229533.I1RZC3
229533.A0A1C3YIL0 pp 229533.I1RQB4
229533.A0A1C3YIL0 pp 229533.I1RAX2
229533.A0A1C3YIL0 pp 229533.I1S0D1
229533.A0A1C3YIL0 pp 229533.I1RSS7
229533.A0A1C3YIL0 pp 229533.I1RB74
229533.A0A1C3YIL0 pp 229533.I1R9Y9
229533.A0A1C3YIL0 pp 229533.I1RNF4
229533.A0A1C3YIL0 pp 229533.I1RFL7
229533.A0A1C3YIL0 pp 229533.I1S0M2
229533.A0A1C3YIL0 pp 229533.I1RNY8
229533.A0A1C3YIL0 pp 229533.I1S0N4
229533.A0A1C3YIL0 pp 229533.I1RSI3
229533.A0A1C3YIL0 pp 229533.I1RA43
229533.A0A1C3YIL0 pp 229533.I1RT36
229533.A0A1C3YIL0 pp 229533.I1RBS0
229533.A0A1C3YIL0 pp 229533.I1RC97
229533.A0A1C3YIL0 pp 229533.I1RPD6
229533.A0A1C3YIL0 pp 229533.I1S1W4
229533.A0A1C3YIL0 pp 229533.V6RGI1
229533.A0A1C3YIL0 pp 229533.I1RAN2
229533.A0A1C3YIL0 pp 229533.I1RY44
229533.A0A1C3YIL0 pp 229533.I1RYZ9
229533.A0A1C3YIL0 pp 229533.I1RYH9
229533.A0A1C3YIL0 pp 229533.I1RNC0
229533.A0A1C3YIL0 pp 229533.V6RVG3
229533.A0A1C3YIL0 pp 229533.I1S224
229533.A0A1C3YIL0 pp 229533.I1RS35
229533.A0A1C3YIL0 pp 229533.V6RKY7
229533.A0A1C3YIL0 pp 229533.I1RRP7
229533.A0A1C3YIL0 pp 229533.V6QV09
229533.A0A1C3YIL0 pp 229533.A0A1C3YK37
229533.A0A1C3YIL0 pp 229533.I1RCB3
229533.A0A1C3YIL0 pp 229533.I1RRY3
229533.A0A1C3YIL0 pp 229533.I1RKK0
229533.A0A1C3YIL0 pp 229533.I1RJH1
229533.A0A1C3YIL0 pp 229533.I1RDZ4
229533.A0A1C3YIL0 pp 229533.I1RC17
229533.A0A1C3YIL0 pp 229533.I1RC95
229533.A0A1C3YIL0 pp 229533.I1RAM7
229533.A0A1C3YIL0 pp 229533.I1RZE4
229533.A0A1C3YIL0 pp 229533.I1RWT6
229533.A0A1C3YIL0 pp 229533.I1RQ26
229533.A0A1C3YIL0 pp 229533.I1RQ72
229533.A0A1C3YIL0 pp 229533.I1RBZ9
229533.A0A1C3YIL0 pp 229533.I1RDB1
229533.A0A1C3YIL0 pp 229533.I1RX70
229533.A0A1C3YIL0 pp 229533.I1RN08
229533.A0A1C3YIL0 pp 229533.A0A1C3YJN3
229533.A0A1C3YIL0 pp 229533.I1RM41
229533.A0A1C3YIL0 pp 229533.I1R9Y7

31099	229533.A0A1C3YIL0	pp	229533.I1RD18
31100	229533.A0A1C3YIL0	pp	229533.I1RYY5
31101	229533.A0A1C3YIL0	pp	229533.I1RB41
31102	229533.A0A1C3YIL0	pp	229533.A0A1C3YKF2
31103	229533.A0A1C3YIL0	pp	229533.A0A1C3YJH8
31104	229533.A0A1C3YIL0	pp	229533.A0A1C3YJV7
31105	229533.A0A1C3YIL0	pp	229533.I1RSG0
31106	229533.A0A1C3YIL0	pp	229533.I1RMM9
31107	229533.A0A1C3YIL0	pp	229533.I1RV66
31108	229533.A0A1C3YIL0	pp	229533.I1RUQ5
31109	229533.A0A1C3YIL0	pp	229533.I1RQA3
31110	229533.A0A1C3YIL0	pp	229533.I1RL24
31111	229533.A0A1C3YIL0	pp	229533.A0A1C3YLP6
31112	229533.A0A1C3YIL0	pp	229533.I1RC22
31113	229533.A0A1C3YIL0	pp	229533.I1S439
31114	229533.A0A1C3YIL0	pp	229533.I1RB75
31115	229533.A0A0E0SJN2	pp	229533.I1RM96
31116	229533.A0A0E0SJN2	pp	229533.Q4IEK8
31117	229533.A0A0E0SJN2	pp	229533.I1RPY1
31118	229533.A0A0E0SJN2	pp	229533.A0A1C3YK36
31119	229533.A0A0E0SJN2	pp	229533.I1RG56
31120	229533.A0A0E0SJN2	pp	229533.I1S4T3
31121	229533.A0A0E0SJN2	pp	229533.I1RBI8
31122	229533.A0A0E0SJN2	pp	229533.I1RSI6
31123	229533.A0A0E0SJN2	pp	229533.I1RX08
31124	229533.A0A0E0SJN2	pp	229533.I1RNH6
31125	229533.A0A0E0SJN2	pp	229533.I1S021
31126	229533.A0A0E0SJN2	pp	229533.I1RNY2
31127	229533.A0A0E0SJN2	pp	229533.Q4I327
31128	229533.A0A0E0SJN2	pp	229533.I1RQ31
31129	229533.A0A0E0SJN2	pp	229533.I1RMC0
31130	229533.A0A0E0SJN2	pp	229533.I1RW99
31131	229533.A0A0E0SJN2	pp	229533.V6RGN5
31132	229533.A0A0E0SJN2	pp	229533.I1R9X7
31133	229533.A0A0E0SJN2	pp	229533.I1RB27
31134	229533.A0A0E0SJN2	pp	229533.I1RNU6
31135	229533.A0A0E0SJN2	pp	229533.I1S0M9
31136	229533.A0A0E0SJN2	pp	229533.A0A1C3YL07
31137	229533.A0A0E0SJN2	pp	229533.I1RC27
31138	229533.A0A0E0SJN2	pp	229533.Q4IBS2
31139	229533.A0A0E0SJN2	pp	229533.I1RQD8
31140	229533.A0A0E0SJN2	pp	229533.I1RMN6
31141	229533.A0A0E0SJN2	pp	229533.Q4IJ56
31142	229533.A0A0E0SJN2	pp	229533.I1S0J1
31143	229533.A0A0E0SJN2	pp	229533.I1RQ28
31144	229533.A0A0E0SJN2	pp	229533.I1RN99
31145	229533.A0A0E0SJN2	pp	229533.I1RC72
31146	229533.A0A0E0SJN2	pp	229533.I1RX14
31147	229533.A0A0E0SJN2	pp	229533.I1RA48
31148	229533.A0A0E0SJN2	pp	229533.I1RSC6
31149	229533.A0A0E0SJN2	pp	229533.I1RSS4
31150	229533.A0A0E0SJN2	pp	229533.I1RY63
31151	229533.A0A0E0SJN2	pp	229533.A0A1C3YKW9
31152	229533.A0A0E0SJN2	pp	229533.V6R227
31153	229533.A0A0E0SJN2	pp	229533.I1RRR7
31154	229533.A0A0E0SJN2	pp	229533.I1S9U1
31155	229533.A0A0E0SJN2	pp	229533.I1S1X9
31156	229533.A0A0E0SJN2	pp	229533.I1R9V7
31157	229533.A0A0E0SJN2	pp	229533.I1RR61
31158	229533.A0A0E0SJN2	pp	229533.I1RF08
31159	229533.A0A0E0SJN2	pp	229533.I1RZF6
31160	229533.A0A0E0SJN2	pp	229533.I1RC06
31161	229533.A0A0E0SJN2	pp	229533.I1RQE7
31162	229533.A0A0E0SJN2	pp	229533.I1RSD8
31163	229533.A0A0E0SJN2	pp	229533.A0A1C3YLK9
31164	229533.A0A0E0SJN2	pp	229533.Q4HZ68
31165	229533.A0A0E0SJN2	pp	229533.A0A0E0SMM8
31166	229533.A0A0E0SJN2	pp	229533.V6R283
31167	229533.A0A0E0SJN2	pp	229533.I1RN63
31168	229533.A0A0E0SJN2	pp	229533.I1S0M3
31169	229533.A0A0E0SJN2	pp	229533.I1RE00
31170	229533.A0A0E0SJN2	pp	229533.I1RQ65
31171	229533.A0A0E0SJN2	pp	229533.I1RFJ9

31172	229533.A0A0E0SJN2	pp	229533.Q4IEV5
31173	229533.A0A0E0SJN2	pp	229533.I1RBG9
31174	229533.A0A0E0SJN2	pp	229533.I1RK70
31175	229533.A0A0E0SJN2	pp	229533.V6QYF0
31176	229533.A0A0E0SJN2	pp	229533.I1RZE5
31177	229533.A0A0E0SJN2	pp	229533.I1RRQ6
31178	229533.A0A0E0SJN2	pp	229533.I1RC29
31179	229533.A0A0E0SJN2	pp	229533.I1RNA0
31180	229533.A0A0E0SJN2	pp	229533.Q4HZ42
31181	229533.A0A0E0SJN2	pp	229533.Q4I5Z5
31182	229533.A0A0E0SJN2	pp	229533.A0A1C3YN63
31183	229533.A0A0E0SJN2	pp	229533.I1RAZ8
31184	229533.A0A0E0SJN2	pp	229533.I1RX24
31185	229533.A0A0E0SJN2	pp	229533.I1S6F0
31186	229533.A0A0E0SJN2	pp	229533.I1RKC6
31187	229533.A0A0E0SJN2	pp	229533.I1RRQ3
31188	229533.A0A0E0SJN2	pp	229533.Q4I662
31189	229533.A0A0E0SJN2	pp	229533.I1RVZ0
31190	229533.A0A0E0SJN2	pp	229533.I1RFS3
31191	229533.A0A0E0SJN2	pp	229533.A0A1C3YIP5
31192	229533.A0A0E0SJN2	pp	229533.I1RS30
31193	229533.A0A0E0SJN2	pp	229533.I1RNK2
31194	229533.A0A0E0SJN2	pp	229533.A0A1C3YK72
31195	229533.A0A0E0SJN2	pp	229533.Q4HZ47
31196	229533.A0A0E0SJN2	pp	229533.I1RG51
31197	229533.A0A0E0SJN2	pp	229533.I1RW61
31198	229533.A0A0E0SJN2	pp	229533.I1S2C0
31199	229533.A0A0E0SJN2	pp	229533.A0A1C3YIS7
31200	229533.A0A0E0SJN2	pp	229533.I1RTE9
31201	229533.A0A0E0SJN2	pp	229533.I1RCG4
31202	229533.A0A0E0SJN2	pp	229533.I1RW25
31203	229533.A0A0E0SJN2	pp	229533.I1RUQ2
31204	229533.A0A0E0SJN2	pp	229533.Q4HZK7
31205	229533.A0A0E0SJN2	pp	229533.I1S0R7
31206	229533.A0A0E0SJN2	pp	229533.I1RT98
31207	229533.A0A0E0SJN2	pp	229533.I1RAC2
31208	229533.A0A0E0SJN2	pp	229533.Q4IFI0
31209	229533.A0A0E0SJN2	pp	229533.A0A1C3YIN1
31210	229533.A0A0E0SJN2	pp	229533.I1RRI2
31211	229533.A0A0E0SJN2	pp	229533.Q4HVW2
31212	229533.A0A0E0SJN2	pp	229533.Q4INZ9
31213	229533.A0A0E0SJN2	pp	229533.I1RTD9
31214	229533.A0A0E0SJN2	pp	229533.I1RZJ6
31215	229533.A0A0E0SJN2	pp	229533.I1RMK3
31216	229533.A0A0E0SJN2	pp	229533.I1RMD5
31217	229533.A0A0E0SJN2	pp	229533.I1S6U8
31218	229533.A0A0E0SJN2	pp	229533.I1RJV4
31219	229533.A0A0E0SJN2	pp	229533.I1RSR3
31220	229533.A0A0E0SJN2	pp	229533.Q4IQW0
31221	229533.A0A0E0SJN2	pp	229533.I1RDA0
31222	229533.A0A0E0SJN2	pp	229533.I1S253
31223	229533.A0A0E0SJN2	pp	229533.V6RGY3
31224	229533.A0A0E0SJN2	pp	229533.I1RBN1
31225	229533.A0A0E0SJN2	pp	229533.I1RC82
31226	229533.A0A0E0SJN2	pp	229533.I1RBC7
31227	229533.A0A0E0SJN2	pp	229533.I1S274
31228	229533.A0A0E0SJN2	pp	229533.I1RFN1
31229	229533.A0A0E0SJN2	pp	229533.I1RBL7
31230	229533.A0A0E0SJN2	pp	229533.I1RBS6
31231	229533.A0A0E0SJN2	pp	229533.I1RCL3
31232	229533.A0A0E0SJN2	pp	229533.Q4IPI1
31233	229533.A0A0E0SJN2	pp	229533.I1RQA2
31234	229533.A0A0E0SJN2	pp	229533.A0A1C3YMW0
31235	229533.A0A0E0SJN2	pp	229533.I1RRJ8
31236	229533.A0A0E0SJN2	pp	229533.I1RJL4
31237	229533.A0A0E0SJN2	pp	229533.I1RT80
31238	229533.A0A0E0SJN2	pp	229533.I1RRT2
31239	229533.A0A0E0SJN2	pp	229533.I1RTF4
31240	229533.A0A0E0SJN2	pp	229533.I1RWF7
31241	229533.A0A0E0SJN2	pp	229533.Q4IJH1
31242	229533.A0A0E0SJN2	pp	229533.I1RQJ5
31243	229533.A0A0E0SJN2	pp	229533.I1RR00
31244	229533.A0A0E0SJN2	pp	229533.I1RQS5

31245 229533.A0A0E0SJN2 pp 229533.I1RT05
31246 229533.A0A0E0SJN2 pp 229533.I1RKL3
31247 229533.A0A0E0SJN2 pp 229533.I1S9U2
31248 229533.A0A0E0SJN2 pp 229533.I1RKQ5
31249 229533.A0A0E0SJN2 pp 229533.I1RLV9
31250 229533.A0A0E0SJN2 pp 229533.I1RW71
31251 229533.A0A0E0SJN2 pp 229533.Q4IAS1
31252 229533.A0A0E0SJN2 pp 229533.I1RPW9
31253 229533.A0A0E0SJN2 pp 229533.Q4I830
31254 229533.A0A0E0SJN2 pp 229533.I1RD64
31255 229533.A0A0E0SJN2 pp 229533.I1RM67
31256 229533.A0A0E0SJN2 pp 229533.I1RZS6
31257 229533.A0A0E0SJN2 pp 229533.I1RFW5
31258 229533.A0A0E0SJN2 pp 229533.I1RM06
31259 229533.A0A0E0SJN2 pp 229533.I1RRT8
31260 229533.A0A0E0SJN2 pp 229533.I1RPU0
31261 229533.A0A0E0SJN2 pp 229533.I1RKJ6
31262 229533.I1RJA9 pp 229533.V6RN31
31263 229533.I1S9U6 pp 229533.Q4IPZ1
31264 229533.I1S9U6 pp 229533.V6R398
31265 229533.I1RFZ3 pp 229533.I1RY44
31266 229533.I1RFZ3 pp 229533.I1RNC6
31267 229533.I1RFZ3 pp 229533.I1S4N7
31268 229533.I1RFZ3 pp 229533.I1RQB4
31269 229533.I1RFZ3 pp 229533.I1RPX5
31270 229533.I1RFZ3 pp 229533.I1RWT6
31271 229533.I1RFZ3 pp 229533.I1RZ63
31272 229533.I1RFZ3 pp 229533.I1RX70
31273 229533.I1RFZ3 pp 229533.I1SAP6
31274 229533.I1RFZ3 pp 229533.I1RM09
31275 229533.I1RFZ3 pp 229533.I1RW83
31276 229533.I1RFZ3 pp 229533.I1S2G7
31277 229533.I1RFZ3 pp 229533.I1S4N8
31278 229533.I1RFZ3 pp 229533.I1RV66
31279 229533.I1RFZ3 pp 229533.I1S2E9
31280 229533.I1RFZ3 pp 229533.I1RV91
31281 229533.I1RFZ3 pp 229533.I1RZE4
31282 229533.I1RFZ3 pp 229533.I1RUQ6
31283 229533.I1RFZ3 pp 229533.I1RQA3
31284 229533.I1RFZ3 pp 229533.I1S091
31285 229533.I1RFZ3 pp 229533.I1RRQ4
31286 229533.I1RFZ3 pp 229533.I1RRN5
31287 229533.I1RX08 pp 229533.Q4IPI1
31288 229533.I1RX08 pp 229533.V6R283
31289 229533.I1RX08 pp 229533.Q4I327
31290 229533.I1RX08 pp 229533.I1S0M3
31291 229533.I1RX08 pp 229533.Q4HZ42
31292 229533.I1RX08 pp 229533.I1RZF6
31293 229533.I1RX08 pp 229533.I1S9U2
31294 229533.I1RLX7 pp 229533.I1RRM5
31295 229533.I1RLX7 pp 229533.I1RPM4
31296 229533.I1RLX7 pp 229533.I1RW25
31297 229533.I1RLX7 pp 229533.I1S0J7
31298 229533.I1RLX7 pp 229533.I1RWJ8
31299 229533.I1RLX7 pp 229533.I1S0L4
31300 229533.I1RLX7 pp 229533.I1RR24
31301 229533.I1RLX7 pp 229533.I1RRT8
31302 229533.I1RLX7 pp 229533.I1RS17
31303 229533.I1RLX7 pp 229533.I1S5B0
31304 229533.I1RLX7 pp 229533.I1RZZ9
31305 229533.I1RLX7 pp 229533.I1RWQ8
31306 229533.I1RLX7 pp 229533.I1RTK5
31307 229533.I1RLX7 pp 229533.I1RW99
31308 229533.I1RLX7 pp 229533.I1S265
31309 229533.I1RLX7 pp 229533.I1RP20
31310 229533.I1RLX7 pp 229533.I1RNF4
31311 229533.I1RLX7 pp 229533.I1RT40
31312 229533.I1RLX7 pp 229533.I1RVQ0
31313 229533.I1RLX7 pp 229533.I1S0L3
31314 229533.I1RLX7 pp 229533.I1RSQ7
31315 229533.I1RLX7 pp 229533.I1RX04
31316 229533.I1RLX7 pp 229533.V6RCG5
31317 229533.I1RLX7 pp 229533.V6QW22

229533.I1RLX7	pp	229533.I1RUG3
229533.I1RLX7	pp	229533.I1RN18
229533.I1RLX7	pp	229533.I1RQB4
229533.I1RLX7	pp	229533.I1RSI0
229533.I1RLX7	pp	229533.I1RWK3
229533.I1RLX7	pp	229533.I1RZW8
229533.I1RLX7	pp	229533.I1RQE7
229533.I1RLX7	pp	229533.V6RIV9
229533.I1RF72	pp	229533.I1SAT1
229533.I1RIG5	pp	229533.I1RRN0
229533.I1RIG5	pp	229533.I1RP28
229533.I1RIG5	pp	229533.I1RJS2
229533.I1RIG5	pp	229533.I1RYZ7
229533.I1RIG5	pp	229533.I1RPM8
229533.I1RIG5	pp	229533.I1RJS3
229533.I1RIG5	pp	229533.I1S7X9
229533.I1RIG5	pp	229533.Q4IJ79
229533.I1RIG5	pp	229533.I1RQG4
229533.I1RIG5	pp	229533.I1RMQ5
229533.I1RIG5	pp	229533.I1RT36
229533.I1RIG5	pp	229533.I1RNW8
229533.I1RIG5	pp	229533.I1RQN9
229533.I1RIG5	pp	229533.I1RJX2
229533.I1RIG5	pp	229533.I1RZX9
229533.I1RIG5	pp	229533.I1SAT1
229533.I1RIG5	pp	229533.I1RQH1
229533.I1RIG5	pp	229533.I1RPL2
229533.I1RIG5	pp	229533.I1RKJ7
229533.I1RIG5	pp	229533.I1RNF9
229533.I1RIG5	pp	229533.Q4I8B6
229533.I1RQA9	pp	229533.V6RR76
229533.I1RQA9	pp	229533.I1RX09
229533.I1RQA9	pp	229533.V6REE9
229533.I1RQA9	pp	229533.I1RWT6
229533.I1RQA9	pp	229533.I1S224
229533.I1RQA9	pp	229533.I1RRI4
229533.I1RQA9	pp	229533.I1RW38
229533.I1RQA9	pp	229533.Q4HXT6
229533.I1RQA9	pp	229533.I1RV91
229533.I1RQA9	pp	229533.I1RTE6
229533.I1RQA9	pp	229533.I1RUI0
229533.I1RQA9	pp	229533.I1RW84
229533.I1RQA9	pp	229533.I1RRW0
229533.I1RQA9	pp	229533.I1RY44
229533.I1RQA9	pp	229533.I1S2E9
229533.I1RQA9	pp	229533.I1S6N6
229533.A0A0E0RRT0	pp	229533.I1RKB2
229533.A0A0E0RRT0	pp	229533.I1RPV8
229533.A0A0E0RRT0	pp	229533.I1RNG4
229533.A0A0E0RRT0	pp	229533.I1RJS1
229533.A0A0E0RRT0	pp	229533.I1RGZ3
229533.A0A0E0RRT0	pp	229533.I1S3H8
229533.A0A0E0RRT0	pp	229533.I1RZ56
229533.A0A0E0RRT0	pp	229533.I1S0A5
229533.A0A0E0RRT0	pp	229533.A0A1C3YKN4
229533.A0A0E0RRT0	pp	229533.I1RST9
229533.A0A0E0RRT0	pp	229533.I1RZ25
229533.A0A0E0RRT0	pp	229533.I1RA13
229533.A0A0E0RRT0	pp	229533.I1RDU0
229533.A0A0E0RRT0	pp	229533.I1S094
229533.A0A0E0RRT0	pp	229533.I1RUY5
229533.A0A0E0RRT0	pp	229533.I1RRL5
229533.A0A0E0RRT0	pp	229533.I1RLA2
229533.A0A0E0RRT0	pp	229533.I1S334
229533.A0A0E0RRT0	pp	229533.I1S8F8
229533.A0A0E0RRT0	pp	229533.I1S6H3
229533.A0A0E0RRT0	pp	229533.I1RDN3
229533.A0A0E0RRT0	pp	229533.I1RNM7
229533.I1RJS3	pp	229533.I1RQG4
229533.I1RJS3	pp	229533.I1RP28
229533.I1RJS3	pp	229533.I1RPL2
229533.I1RJS3	pp	229533.I1RMQ5
229533.I1RJS3	pp	229533.I1RQQ8

229533.I1RJS3	pp	229533.Q4IR18
229533.I1RJS3	pp	229533.I1RQH1
229533.I1RJS3	pp	229533.I1RKI0
229533.I1RJS3	pp	229533.I1RRK8
229533.I1RJS3	pp	229533.I1RRM9
229533.I1RTD7	pp	229533.V6QWZ7
229533.I1RTD7	pp	229533.V6QUE6
229533.I1RTD7	pp	229533.V6RGN5
229533.I1RTD7	pp	229533.I1RY63
229533.I1RTD7	pp	229533.I1S0J1
229533.I1RTD7	pp	229533.V6R042
229533.I1RTD7	pp	229533.I1S1U7
229533.I1RTD7	pp	229533.V6RMW6
229533.I1RTD7	pp	229533.I1RZK6
229533.I1RTD7	pp	229533.I1S274
229533.I1RTD7	pp	229533.I1RZZ1
229533.I1RTD7	pp	229533.I1S558
229533.I1RTD7	pp	229533.Q4IN63
229533.I1RTD7	pp	229533.I1RZK5
229533.I1RTD7	pp	229533.I1S2E8
229533.I1RTD7	pp	229533.I1S504
229533.I1RTD7	pp	229533.I1S0R7
229533.I1RTD7	pp	229533.I1RZB5
229533.I1RTD7	pp	229533.V6RTJ8
229533.I1RTD7	pp	229533.I1RTH6
229533.I1RTD7	pp	229533.I1RX46
229533.I1RTD7	pp	229533.I1RW26
229533.I1RTD7	pp	229533.I1RYJ6
229533.I1RTD7	pp	229533.I1S503
229533.I1RTD7	pp	229533.I1S6U8
229533.I1RTD7	pp	229533.I1RWF7
229533.I1RTD7	pp	229533.I1RWD7
229533.I1RTD7	pp	229533.I1S8E2
229533.I1RTD7	pp	229533.I1RW71
229533.I1RTD7	pp	229533.V6RLW4
229533.I1RTD7	pp	229533.V6R283
229533.I1RTD7	pp	229533.I1RWS4
229533.I1RTD7	pp	229533.I1RZS6
229533.I1RTD7	pp	229533.I1RWJ4
229533.I1RQ26	pp	229533.I1RWJ6
229533.I1RQ26	pp	229533.I1RV66
229533.I1RQ26	pp	229533.V6RTJ8
229533.I1RQ26	pp	229533.I1RX70
229533.I1RQ26	pp	229533.I1S224
229533.I1RQ26	pp	229533.I1RUQ6
229533.I1RQ26	pp	229533.V6QV09
229533.I1RQ26	pp	229533.I1RYH9
229533.I1RQ26	pp	229533.I1RYZ9
229533.I1RQ26	pp	229533.I1S0M2
229533.I1RQ26	pp	229533.I1RW25
229533.I1RQ26	pp	229533.I1RRW0
229533.I1RQ26	pp	229533.I1RYZ0
229533.I1RQ26	pp	229533.I1RYY5
229533.I1RQ26	pp	229533.I1RQB4
229533.I1RQ26	pp	229533.I1S0Z7
229533.I1RQ26	pp	229533.I1RUQ5
229533.I1RQ26	pp	229533.I1S0N4
229533.I1RQ26	pp	229533.I1RRV1
229533.I1RQ26	pp	229533.I1S439
229533.I1RQ26	pp	229533.I1RZH4
229533.I1RQ26	pp	229533.I1RQA3
229533.I1RQ26	pp	229533.I1RT36
229533.I1RQ26	pp	229533.I1RQ81
229533.I1RQ26	pp	229533.Q4IJM4
229533.I1RQ26	pp	229533.I1S8P5
229533.I1RQ26	pp	229533.I1RZM1
229533.I1RQ26	pp	229533.V6RGI1
229533.I1RQ26	pp	229533.I1S1D3
229533.I1RQ26	pp	229533.I1RWT6
229533.I1RQ26	pp	229533.I1RZZ9
229533.I1RQ26	pp	229533.I1RQG4
229533.I1RQ26	pp	229533.I1RTA0
229533.I1RQ26	pp	229533.I1RW16

31464 229533.I1RQ26 pp 229533.I1RSK1
31465 229533.I1RQ26 pp 229533.I1RYW3
31466 229533.I1RQ26 pp 229533.I1S0D1
31467 229533.I1RQ26 pp 229533.I1S1V5
31468 229533.I1RQ26 pp 229533.I1RRP7
31469 229533.I1RQ26 pp 229533.I1RUN8
31470 229533.I1RQ26 pp 229533.I1RRY3
31471 229533.I1RQ26 pp 229533.V6RVG3
31472 229533.I1RQ26 pp 229533.I1S549
31473 229533.I1RQ26 pp 229533.I1RY44
31474 229533.I1RQ26 pp 229533.I1RV91
31475 229533.I1RQ26 pp 229533.I1RZE4
31476 229533.I1RQ26 pp 229533.V6RKY7
31477 229533.I1RQ26 pp 229533.I1RQ72
31478 229533.I1RQ26 pp 229533.I1RY58
31479 229533.I1RQ26 pp 229533.I1S1W4
31480 229533.I1RQ26 pp 229533.I1S2E9
31481 229533.I1RCM2 pp 229533.I1RMP0
31482 229533.I1RCM2 pp 229533.I1S549
31483 229533.I1RCM2 pp 229533.I1RQL4
31484 229533.I1RCM2 pp 229533.I1RT41
31485 229533.I1RCM2 pp 229533.V6RGI1
31486 229533.I1RCM2 pp 229533.I1RLZ6
31487 229533.I1RCM2 pp 229533.I1S0Z3
31488 229533.I1RCM2 pp 229533.V6RN31
31489 229533.I1RBW0 pp 229533.I1RQB4
31490 229533.I1RBW0 pp 229533.I1RH64
31491 229533.I1RBW0 pp 229533.I1RQI6
31492 229533.I1RBW0 pp 229533.I1RSC7
31493 229533.I1RBW0 pp 229533.I1RRW0
31494 229533.I1RBW0 pp 229533.I1RZ98
31495 229533.I1RBW0 pp 229533.Q4IB89
31496 229533.I1RBW0 pp 229533.I1RY44
31497 229533.I1RBW0 pp 229533.I1RC54
31498 229533.I1RBW0 pp 229533.I1S064
31499 229533.I1RBW0 pp 229533.I1RN57
31500 229533.I1RBW0 pp 229533.V6RLM7
31501 229533.I1RBW0 pp 229533.I1S0S0
31502 229533.I1RBW0 pp 229533.I1S1Y7
31503 229533.I1RBW0 pp 229533.I1RSP7
31504 229533.I1RBW0 pp 229533.I1RD19
31505 229533.I1RBW0 pp 229533.V6R382
31506 229533.I1RBW0 pp 229533.I1RW16
31507 229533.I1RBW0 pp 229533.I1RMS4
31508 229533.I1RBW0 pp 229533.I1S020
31509 229533.I1RBW0 pp 229533.I1RQJ4
31510 229533.I1RBW0 pp 229533.Q4IJ84
31511 229533.I1RBW0 pp 229533.I1RX64
31512 229533.I1RBW0 pp 229533.I1RZP8
31513 229533.I1RBW0 pp 229533.I1S016
31514 229533.I1RBW0 pp 229533.I1RUG4
31515 229533.I1RBW0 pp 229533.I1RYQ1
31516 229533.I1RBW0 pp 229533.I1RSR9
31517 229533.I1RBW0 pp 229533.I1RTN6
31518 229533.I1RBW0 pp 229533.I1RWS4
31519 229533.I1RBW0 pp 229533.I1RKB2
31520 229533.I1RBW0 pp 229533.I1RQ72
31521 229533.I1RBW0 pp 229533.I1S0A6
31522 229533.I1RBW0 pp 229533.I1S6Y6
31523 229533.I1RBW0 pp 229533.I1S0D5
31524 229533.I1RBW0 pp 229533.I1RYX9
31525 229533.I1RBW0 pp 229533.I1RZT1
31526 229533.I1RBW0 pp 229533.I1RV91
31527 229533.I1RBW0 pp 229533.I1RDX0
31528 229533.I1RBW0 pp 229533.I1RFL7
31529 229533.I1RBW0 pp 229533.I1RPF5
31530 229533.I1RBW0 pp 229533.I1RY58
31531 229533.I1RBW0 pp 229533.I1RDH0
31532 229533.I1RBW0 pp 229533.I1RSZ3
31533 229533.I1RBW0 pp 229533.Q4IB96
31534 229533.I1RBW0 pp 229533.I1RDJ8
31535 229533.I1RBW0 pp 229533.I1RWI8
31536 229533.I1RBW0 pp 229533.I1RRN5

31537	229533.I1RBW0	pp	229533.I1RNG4
31538	229533.I1RBW0	pp	229533.I1RSY2
31539	229533.I1RBW0	pp	229533.I1RTB2
31540	229533.I1RBW0	pp	229533.I1S2E9
31541	229533.I1RBW0	pp	229533.I1S0Q5
31542	229533.I1RBW0	pp	229533.I1RSN7
31543	229533.I1RBW0	pp	229533.I1RYR2
31544	229533.I1RBW0	pp	229533.I1RHD2
31545	229533.I1RBW0	pp	229533.I1RQ24
31546	229533.I1RBW0	pp	229533.I1S237
31547	229533.I1RBW0	pp	229533.O74638
31548	229533.I1RBW0	pp	229533.I1SAE3
31549	229533.I1RBW0	pp	229533.I1RZI4
31550	229533.I1RBW0	pp	229533.Q4IMD1
31551	229533.I1RBW0	pp	229533.I1RFZ2
31552	229533.I1RBW0	pp	229533.I1RRJ6
31553	229533.I1RBW0	pp	229533.I1S2F2
31554	229533.I1RBW0	pp	229533.I1RNF4
31555	229533.I1RBW0	pp	229533.I1RBW1
31556	229533.I1RBW0	pp	229533.I1RXF0
31557	229533.I1RBW0	pp	229533.I1RDC0
31558	229533.I1RBW0	pp	229533.Q4I7L0
31559	229533.I1RBW0	pp	229533.Q4IEV5
31560	229533.I1RBW0	pp	229533.Q4I6A4
31561	229533.I1RBW0	pp	229533.I1S604
31562	229533.I1RBW0	pp	229533.I1RSQ4
31563	229533.I1RBW0	pp	229533.I1RL04
31564	229533.I1RBW0	pp	229533.I1S004
31565	229533.I1RBW0	pp	229533.I1RCA5
31566	229533.I1RBW0	pp	229533.I1RDE7
31567	229533.I1RBW0	pp	229533.I1RQS4
31568	229533.I1RBW0	pp	229533.I1RFJ2
31569	229533.I1RBW0	pp	229533.Q4INS6
31570	229533.I1RBW0	pp	229533.I1RZ86
31571	229533.I1RBW0	pp	229533.I1RRV4
31572	229533.I1RBW0	pp	229533.I1RMX3
31573	229533.I1RBW0	pp	229533.I1RJK7
31574	229533.I1RBW0	pp	229533.V6RLW4
31575	229533.I1RBW0	pp	229533.I1RC17
31576	229533.I1RBW0	pp	229533.I1RE23
31577	229533.I1RBW0	pp	229533.I1RN46
31578	229533.I1RBW0	pp	229533.I1RYR1
31579	229533.I1RBW0	pp	229533.I1S523
31580	229533.I1RBW0	pp	229533.I1RSA7
31581	229533.I1RBW0	pp	229533.I1S461
31582	229533.I1RBW0	pp	229533.Q4IR08
31583	229533.I1RBW0	pp	229533.I1RVW0
31584	229533.I1RBW0	pp	229533.I1S209
31585	229533.I1RBW0	pp	229533.I1RYJ6
31586	229533.I1RBW0	pp	229533.I1RMQ7
31587	229533.I1RBW0	pp	229533.I1RLX6
31588	229533.I1RBW0	pp	229533.I1S0Q4
31589	229533.I1RBW0	pp	229533.I1RSJ1
31590	229533.I1RBW0	pp	229533.I1RFI6
31591	229533.I1RBW0	pp	229533.I1RPZ0
31592	229533.I1RBW0	pp	229533.I1S998
31593	229533.I1RBW0	pp	229533.I1REK4
31594	229533.I1RBW0	pp	229533.I1RWT6
31595	229533.I1RBW0	pp	229533.I1S0B2
31596	229533.I1RBW0	pp	229533.I1RCN8
31597	229533.I1RBW0	pp	229533.I1RE46
31598	229533.I1RBW0	pp	229533.I1RKI2
31599	229533.I1RBW0	pp	229533.I1RVZ9
31600	229533.I1RBW0	pp	229533.I1RKE9
31601	229533.I1RBW0	pp	229533.I1RE55
31602	229533.I1RBW0	pp	229533.I1RSI4
31603	229533.I1RBW0	pp	229533.I1RRY1
31604	229533.I1RBW0	pp	229533.I1S236
31605	229533.I1RBW0	pp	229533.I1RS81
31606	229533.I1RBW0	pp	229533.I1RQA3
31607	229533.I1RBW0	pp	229533.I1RWB3
31608	229533.I1RBW0	pp	229533.I1RM36
31609	229533.I1RBW0	pp	229533.I1RCL7

31610	229533.I1RBW0	pp	229533.I1S0D4
31611	229533.I1RBW0	pp	229533.I1RQ05
31612	229533.I1RBW0	pp	229533.I1S283
31613	229533.I1RBW0	pp	229533.I1RPQ1
31614	229533.I1RBW0	pp	229533.I1RG77
31615	229533.I1RBW0	pp	229533.I1RQL4
31616	229533.I1RBW0	pp	229533.I1RCQ4
31617	229533.I1RBW0	pp	229533.I1RN55
31618	229533.I1RBW0	pp	229533.I1S4S4
31619	229533.I1RBW0	pp	229533.I1RRT1
31620	229533.I1RBW0	pp	229533.I1RD18
31621	229533.I1RBW0	pp	229533.I1RQ80
31622	229533.I1RBW0	pp	229533.I1RV66
31623	229533.I1RBW0	pp	229533.I1RQ13
31624	229533.I1RBW0	pp	229533.I1RZ94
31625	229533.I1RBW0	pp	229533.I1RU52
31626	229533.I1RBW0	pp	229533.I1RFX8
31627	229533.I1RBW0	pp	229533.I1S987
31628	229533.I1RBW0	pp	229533.I1RC64
31629	229533.I1RBW0	pp	229533.I1RMB7
31630	229533.I1RBW0	pp	229533.I1S224
31631	229533.I1RBW0	pp	229533.I1RN31
31632	229533.I1RZ95	pp	229533.O74638
31633	229533.I1RZ95	pp	229533.I1RZD3
31634	229533.I1RZ95	pp	229533.Q4IB89
31635	229533.I1RZ95	pp	229533.I1RZC3
31636	229533.I1RZ95	pp	229533.I1RZS8
31637	229533.I1RZ95	pp	229533.I1S267
31638	229533.I1RZ95	pp	229533.I1S217
31639	229533.I1RZ95	pp	229533.I1S4S4
31640	229533.I1RYH9	pp	229533.I1RZH4
31641	229533.I1RYH9	pp	229533.V6RDP1
31642	229533.I1RYH9	pp	229533.I1RYZ9
31643	229533.I1RYH9	pp	229533.I1RYY5
31644	229533.I1RYH9	pp	229533.I1S439
31645	229533.I1RYH9	pp	229533.Q4IJM4
31646	229533.I1RYH9	pp	229533.I1S9Q3
31647	229533.I1RYH9	pp	229533.V6RVG3
31648	229533.I1RYH9	pp	229533.I1S224
31649	229533.I1RYH9	pp	229533.V6QWB1
31650	229533.I1RYH9	pp	229533.V6RKY7
31651	229533.I1RYH9	pp	229533.V6QV09
31652	229533.I1RYH9	pp	229533.I1S9X9
31653	229533.I1RYH9	pp	229533.V6RGI1
31654	229533.I1RYH9	pp	229533.I1S1D3
31655	229533.I1RYH9	pp	229533.Q4IP51
31656	229533.I1RYH9	pp	229533.I1S1W4
31657	229533.I1RYH9	pp	229533.Q4IQM6
31658	229533.I1RYH9	pp	229533.I1RZP8
31659	229533.I1RYH9	pp	229533.I1S0M2
31660	229533.I1RYH9	pp	229533.I1S0D1
31661	229533.I1RYH9	pp	229533.I1RZE4
31662	229533.I1RYH9	pp	229533.I1S549
31663	229533.I1RYH9	pp	229533.I1RYX9
31664	229533.I1RYH9	pp	229533.I1S1V5
31665	229533.I1RYH9	pp	229533.I1S2E9
31666	229533.I1RYH9	pp	229533.I1RYR2
31667	229533.I1RYH9	pp	229533.I1S2G7
31668	229533.I1RVH2	pp	229533.I1RWM0
31669	229533.I1RVH2	pp	229533.I1S549
31670	229533.I1RVH2	pp	229533.I1RXB5
31671	229533.I1RDE8	pp	229533.I1RXB5
31672	229533.I1RDE8	pp	229533.I1RUK7
31673	229533.I1RDE8	pp	229533.I1RRH3
31674	229533.I1RDE8	pp	229533.I1S0K5
31675	229533.I1RDE8	pp	229533.Q4I2W2
31676	229533.I1RDE8	pp	229533.I1RQ70
31677	229533.I1RDE8	pp	229533.I1RMF2
31678	229533.I1RDE8	pp	229533.Q4I6S5
31679	229533.I1RDE8	pp	229533.I1RKR7
31680	229533.I1RFS6	pp	229533.I1RG34
31681	229533.I1RFS6	pp	229533.I1RRJ6
31682	229533.I1RFS6	pp	229533.I1S8C1

31683	229533.I1RFS6	pp	229533.I1RV91
31684	229533.I1RFS6	pp	229533.I1RLJ8
31685	229533.I1RFS6	pp	229533.I1RJY7
31686	229533.I1RFS6	pp	229533.I1RQS4
31687	229533.I1RFS6	pp	229533.I1S788
31688	229533.I1RFS6	pp	229533.I1RJK7
31689	229533.I1RFS6	pp	229533.I1S224
31690	229533.I1RFS6	pp	229533.I1RVV5
31691	229533.I1RFS6	pp	229533.I1RQA3
31692	229533.I1RFS6	pp	229533.I1S2E9
31693	229533.I1RFS6	pp	229533.I1RWT6
31694	229533.I1RFS6	pp	229533.I1RKB2
31695	229533.I1RFS6	pp	229533.I1RY58
31696	229533.I1RFS6	pp	229533.I1RNG1
31697	229533.I1RFS6	pp	229533.I1RZT1
31698	229533.I1RFS6	pp	229533.I1RRW0
31699	229533.I1RFS6	pp	229533.I1RMJ8
31700	229533.I1RFS6	pp	229533.I1RUR8
31701	229533.I1RFS6	pp	229533.Q4IB89
31702	229533.I1RFS6	pp	229533.I1S4P6
31703	229533.I1RFS6	pp	229533.I1RY44
31704	229533.I1RTG6	pp	229533.I1RTG7
31705	229533.I1RTG6	pp	229533.Q4HZK7
31706	229533.I1RTG6	pp	229533.Q4HVW2
31707	229533.I1RTG6	pp	229533.I1RZS6
31708	229533.I1RTG6	pp	229533.I1RWB0
31709	229533.I1RTG6	pp	229533.I1RWC9
31710	229533.I1RTG6	pp	229533.I1RZZ9
31711	229533.I1RTG6	pp	229533.Q4IJH1
31712	229533.I1RTG6	pp	229533.V6QVH8
31713	229533.I1RTG6	pp	229533.V6R042
31714	229533.I1RTG6	pp	229533.I1RXV9
31715	229533.I1RTG6	pp	229533.V6RUT5
31716	229533.I1RTG6	pp	229533.I1S7W5
31717	229533.I1RTG6	pp	229533.I1S0R7
31718	229533.I1RTG6	pp	229533.I1RZE5
31719	229533.I1RTG6	pp	229533.V6R283
31720	229533.I1RTG6	pp	229533.I1S1W3
31721	229533.I1RTG6	pp	229533.I1RTI6
31722	229533.I1RTG6	pp	229533.I1RZF6
31723	229533.I1RTG6	pp	229533.Q4IN63
31724	229533.I1RTG6	pp	229533.I1RTU1
31725	229533.I1RRC2	pp	229533.Q4HZS8
31726	229533.I1RRC2	pp	229533.I1RZD9
31727	229533.I1RRC2	pp	229533.I1RZV4
31728	229533.I1RRC2	pp	229533.I1RZ43
31729	229533.I1RRC2	pp	229533.I1RYQ0
31730	229533.I1RRC2	pp	229533.I1RZB7
31731	229533.I1RRC2	pp	229533.I1S095
31732	229533.I1RTN8	pp	229533.I1RUT8
31733	229533.I1RTN8	pp	229533.V6RIV9
31734	229533.I1RTN8	pp	229533.I1SAK0
31735	229533.I1RTN8	pp	229533.I1S2L3
31736	229533.I1RSH5	pp	229533.I1RT80
31737	229533.I1RJV7	pp	229533.I1RXT9
31738	229533.I1RJV7	pp	229533.Q4I9U7
31739	229533.I1RJV7	pp	229533.Q4I5M4
31740	229533.I1RJV7	pp	229533.I1RSL8
31741	229533.I1RJV7	pp	229533.I1RYF8
31742	229533.I1RJV7	pp	229533.I1S549
31743	229533.I1RJV7	pp	229533.I1RNF5
31744	229533.I1RJV7	pp	229533.Q4IQ72
31745	229533.I1RJV7	pp	229533.I1RX96
31746	229533.I1RJV7	pp	229533.I1RYM2
31747	229533.I1RJV7	pp	229533.I1RZW7
31748	229533.I1RJV7	pp	229533.I1RYW8
31749	229533.I1RJV7	pp	229533.Q4HWB3
31750	229533.I1RJV7	pp	229533.I1RN51
31751	229533.I1RJV7	pp	229533.I1S287
31752	229533.I1RX70	pp	229533.I1SAP6
31753	229533.I1RX70	pp	229533.I1S439
31754	229533.I1RX70	pp	229533.V6RKY7
31755	229533.I1RX70	pp	229533.I1RYY5

229533.I1RX70 pp 229533.I1S0M2
229533.I1RX70 pp 229533.I1RY44
229533.I1RX70 pp 229533.V6RVG3
229533.I1RX70 pp 229533.I1S016
229533.I1RX70 pp 229533.I1S1W4
229533.I1RX70 pp 229533.V6RDP1
229533.I1RX70 pp 229533.Q4IJM4
229533.I1RX70 pp 229533.I1S091
229533.I1RX70 pp 229533.I1S2E9
229533.I1RX70 pp 229533.I1RZ63
229533.I1RX70 pp 229533.I1S0D1
229533.I1RX70 pp 229533.I1RZE4
229533.I1RX70 pp 229533.I1RYH9
229533.I1RX70 pp 229533.I1S2G7
229533.I1RX70 pp 229533.I1S7X9
229533.I1RAI0 pp 229533.Q4HZS8
229533.I1RAI0 pp 229533.I1RI75
229533.I1RAI0 pp 229533.I1RX25
229533.I1RAI0 pp 229533.Q5I2J3
229533.I1RAI0 pp 229533.I1RCQ3
229533.I1RAI0 pp 229533.I1RAM6
229533.I1RAI0 pp 229533.I1RDB9
229533.I1RAI0 pp 229533.I1RUP0
229533.I1RAI0 pp 229533.I1RSH0
229533.I1RAI0 pp 229533.I1RR95
229533.I1RAI0 pp 229533.I1S0B1
229533.I1RAI0 pp 229533.I1RG90
229533.I1RMC7 pp 229533.I1RMC8
229533.I1RKG2 pp 229533.I1S5B0
229533.I1RKG2 pp 229533.I1RRM3
229533.I1RKG2 pp 229533.I1RUG3
229533.I1RKG2 pp 229533.I1S5R1
229533.I1RKG2 pp 229533.I1RSF1
229533.I1RKG2 pp 229533.V6RCZ8
229533.I1RKG2 pp 229533.I1RQE7
229533.I1RKG2 pp 229533.I1RKP1
229533.I1RKG2 pp 229533.I1RQ33
229533.I1RKG2 pp 229533.I1RZQ6
229533.I1RKG2 pp 229533.I1RRI1
229533.I1RKG2 pp 229533.I1RZK5
229533.A0A0E0SKJ2 pp 229533.I1RZA5
229533.A0A0E0SKJ2 pp 229533.A0A1C3YN77
229533.A0A0E0SKJ2 pp 229533.I1RKI8
229533.A0A0E0SKJ2 pp 229533.I1RCT6
229533.A0A0E0SKJ2 pp 229533.I1RZP1
229533.A0A0E0SKJ2 pp 229533.I1S0Y1
229533.A0A0E0SKJ2 pp 229533.I1RX87
229533.A0A0E0SKJ2 pp 229533.I1RPK4
229533.A0A0E0SKJ2 pp 229533.I1RTC7
229533.A0A0E0SKJ2 pp 229533.I1RCV5
229533.A0A0E0SKJ2 pp 229533.I1RML5
229533.A0A0E0SKJ2 pp 229533.I1RRL9
229533.A0A0E0SKJ2 pp 229533.I1RFW0
229533.A0A0E0SKJ2 pp 229533.I1S277
229533.A0A0E0SKJ2 pp 229533.I1RS69
229533.A0A0E0SKJ2 pp 229533.I1S522
229533.A0A0E0SKJ2 pp 229533.I1RPA4
229533.A0A0E0SKJ2 pp 229533.I1RR74
229533.A0A0E0SKJ2 pp 229533.I1R9U9
229533.A0A0E0SKJ2 pp 229533.I1RXF0
229533.A0A0E0SKJ2 pp 229533.V6QWP4
229533.A0A0E0SKJ2 pp 229533.I1RC52
229533.A0A0E0SKJ2 pp 229533.I1RNB8
229533.A0A0E0SKJ2 pp 229533.I1RBH5
229533.I1RM55 pp 229533.I1S084
229533.I1RM55 pp 229533.I1RUY6
229533.I1RM55 pp 229533.I1RVR0
229533.I1RM55 pp 229533.I1RY62
229533.I1RM55 pp 229533.I1S256
229533.I1RM55 pp 229533.I1RMC5
229533.I1RM55 pp 229533.I1S3V2
229533.I1RM55 pp 229533.I1S3K6
229533.I1RM55 pp 229533.I1RZ52

31829	229533.I1RM55	pp	229533.I1RMV8
31830	229533.I1RM55	pp	229533.I1RTA9
31831	229533.I1RM55	pp	229533.I1RSX4
31832	229533.I1RM55	pp	229533.I1RP69
31833	229533.I1RM55	pp	229533.I1RP97
31834	229533.I1RM55	pp	229533.V6RY56
31835	229533.I1RM55	pp	229533.I1S4D0
31836	229533.I1RM55	pp	229533.V6RQW4
31837	229533.I1RM55	pp	229533.Q4HXF0
31838	229533.I1RM55	pp	229533.I1RMK4
31839	229533.I1RM55	pp	229533.I1S029
31840	229533.I1RM55	pp	229533.I1S3A5
31841	229533.I1RM55	pp	229533.I1S1P8
31842	229533.I1RM55	pp	229533.I1S3F7
31843	229533.I1RM55	pp	229533.I1RZ21
31844	229533.I1RNH1	pp	229533.I1S2C0
31845	229533.I1RNH1	pp	229533.I1RPJ3
31846	229533.I1RNH1	pp	229533.I1RTE9
31847	229533.I1RNH1	pp	229533.I1RRI2
31848	229533.I1RNH1	pp	229533.I1RZB5
31849	229533.I1RNH1	pp	229533.I1RSG8
31850	229533.I1RNH1	pp	229533.I1RR25
31851	229533.I1RNH1	pp	229533.I1RTD7
31852	229533.I1RNH1	pp	229533.I1S296
31853	229533.I1RNH1	pp	229533.I1S276
31854	229533.I1RNH1	pp	229533.Q4HZ68
31855	229533.I1RNH1	pp	229533.I1RZE5
31856	229533.I1RNH1	pp	229533.I1S2U2
31857	229533.I1RNH1	pp	229533.V6RGY3
31858	229533.I1RNH1	pp	229533.I1RSR3
31859	229533.I1RNH1	pp	229533.V6RGN5
31860	229533.I1RNH1	pp	229533.Q4I5I4
31861	229533.I1RNH1	pp	229533.I1S1U7
31862	229533.I1RNH1	pp	229533.I1RNK2
31863	229533.I1RNH1	pp	229533.I1S274
31864	229533.I1RNH1	pp	229533.V6QUE6
31865	229533.I1RNH1	pp	229533.I1RVZ0
31866	229533.I1RNH1	pp	229533.I1RQA2
31867	229533.I1RNH1	pp	229533.I1S1J6
31868	229533.I1RNH1	pp	229533.I1RSI6
31869	229533.I1RNH1	pp	229533.Q4IJ56
31870	229533.I1RNH1	pp	229533.I1RQ28
31871	229533.I1RNH1	pp	229533.I1S0M9
31872	229533.I1RNH1	pp	229533.I1RVJ6
31873	229533.I1RNH1	pp	229533.I1S1X9
31874	229533.I1RNH1	pp	229533.I1RNY2
31875	229533.I1RNH1	pp	229533.I1RSS4
31876	229533.I1RNH1	pp	229533.I1S2E8
31877	229533.I1RNH1	pp	229533.I1RNU6
31878	229533.I1RNH1	pp	229533.Q4I327
31879	229533.I1RNH1	pp	229533.I1RSD8
31880	229533.I1RNH1	pp	229533.I1S021
31881	229533.I1RNH1	pp	229533.I1RQ81
31882	229533.I1RNH1	pp	229533.I1RUP9
31883	229533.I1RNH1	pp	229533.I1RNH6
31884	229533.I1RNH1	pp	229533.I1SAN0
31885	229533.I1RNH1	pp	229533.I1S0J1
31886	229533.I1RNH1	pp	229533.I1RSD4
31887	229533.I1RNH1	pp	229533.I1RQ31
31888	229533.I1RNH1	pp	229533.I1S9U2
31889	229533.I1RNH1	pp	229533.I1S060
31890	229533.I1RNH1	pp	229533.Q4IFI0
31891	229533.I1RNH1	pp	229533.V6R227
31892	229533.I1RNH1	pp	229533.I1S255
31893	229533.I1RNH1	pp	229533.I1RVY0
31894	229533.I1RNH1	pp	229533.I1RRR7
31895	229533.I1RNH1	pp	229533.I1RZF6
31896	229533.I1RNH1	pp	229533.I1RS20
31897	229533.I1RNH1	pp	229533.I1RZS6
31898	229533.I1RNH1	pp	229533.I1S9U1
31899	229533.I1RNH1	pp	229533.I1RX24
31900	229533.I1RNH1	pp	229533.Q4IQK7
31901	229533.I1RNH1	pp	229533.O74638

31902 229533.I1RNH1 pp 229533.I1RRT8
31903 229533.I1RNH1 pp 229533.V6QYF0
31904 229533.I1RNH1 pp 229533.Q4IEK8
31905 229533.I1RNH1 pp 229533.I1S9Y1
31906 229533.I1RNH1 pp 229533.I1RPU0
31907 229533.I1RNH1 pp 229533.I1RPY1
31908 229533.I1RNH1 pp 229533.I1S0R7
31909 229533.I1RNH1 pp 229533.I1S6U8
31910 229533.I1RNH1 pp 229533.Q4I662
31911 229533.I1RNH1 pp 229533.I1RW90
31912 229533.I1RNH1 pp 229533.I1RV91
31913 229533.I1RNH1 pp 229533.I1RYT5
31914 229533.I1RNH1 pp 229533.I1RTF4
31915 229533.I1RNH1 pp 229533.I1RVL8
31916 229533.I1RNH1 pp 229533.I1RS47
31917 229533.I1RNH1 pp 229533.I1RQ65
31918 229533.I1RNH1 pp 229533.Q4IJH1
31919 229533.I1RNH1 pp 229533.I1RUP7
31920 229533.I1RNH1 pp 229533.I1RT33
31921 229533.I1RNH1 pp 229533.Q4IQW0
31922 229533.I1RNH1 pp 229533.I1RT80
31923 229533.I1RNH1 pp 229533.I1RNV6
31924 229533.I1RNH1 pp 229533.I1S6F0
31925 229533.I1RNH1 pp 229533.I1S268
31926 229533.I1RNH1 pp 229533.I1RRQ3
31927 229533.I1RNH1 pp 229533.V6QWX8
31928 229533.I1RNH1 pp 229533.I1S1B6
31929 229533.I1RNH1 pp 229533.I1RQM6
31930 229533.I1RNH1 pp 229533.I1RRM8
31931 229533.I1RNH1 pp 229533.I1RS30
31932 229533.I1RNH1 pp 229533.I1RRT2
31933 229533.I1RNH1 pp 229533.Q4HZI0
31934 229533.I1RNH1 pp 229533.I1RZ99
31935 229533.I1RNH1 pp 229533.I1RWF7
31936 229533.I1RNH1 pp 229533.I1RQJ5
31937 229533.I1RNH1 pp 229533.Q4IF76
31938 229533.I1RNH1 pp 229533.I1RRJ8
31939 229533.I1RNH1 pp 229533.Q4IQX3
31940 229533.I1RNH1 pp 229533.Q4IPI1
31941 229533.I1RNH1 pp 229533.Q4I830
31942 229533.I1RNH1 pp 229533.I1RQS5
31943 229533.I1RNH1 pp 229533.I1RRG3
31944 229533.I1RNH1 pp 229533.Q4IAS1
31945 229533.I1RNH1 pp 229533.I1RW71
31946 229533.I1RNH1 pp 229533.I1RUQ2
31947 229533.I1RNH1 pp 229533.V6R283
31948 229533.I1RIH8 pp 229533.I1S2E9
31949 229533.I1RIH8 pp 229533.I1RV91
31950 229533.I1RIH8 pp 229533.I1RY44
31951 229533.I1RIH8 pp 229533.I1RQA3
31952 229533.I1RIH8 pp 229533.I1RJB3
31953 229533.I1RIH8 pp 229533.I1RWT6
31954 229533.A0A0E0SJQ1 pp 229533.A0A1C3YK01
31955 229533.A0A0E0SJQ1 pp 229533.I1RXE5
31956 229533.A0A0E0SJQ1 pp 229533.Q4HY90
31957 229533.A0A0E0SJQ1 pp 229533.A0A1C3YHX4
31958 229533.A0A0E0S2K9 pp 229533.I1S269
31959 229533.A0A0E0S2K9 pp 229533.V6RM09
31960 229533.A0A0E0S2K9 pp 229533.A0A1C3YL54
31961 229533.A0A0E0S2K9 pp 229533.V6R556
31962 229533.A0A0E0S2K9 pp 229533.A0A1C3YLE2
31963 229533.A0A0E0S2K9 pp 229533.I1RGB2
31964 229533.A0A0E0S2K9 pp 229533.I1RA13
31965 229533.A0A0E0S2K9 pp 229533.I1RDU0
31966 229533.A0A0E0S2K9 pp 229533.V6RAM6
31967 229533.A0A0E0S2K9 pp 229533.A0A1C3YKH5
31968 229533.A0A0E0S2K9 pp 229533.A0A1C3YIR5
31969 229533.A0A0E0S2K9 pp 229533.I1RJY7
31970 229533.A0A0E0S2K9 pp 229533.I1RP86
31971 229533.A0A0E0S2K9 pp 229533.I1RR44
31972 229533.A0A0E0S2K9 pp 229533.I1S149
31973 229533.A0A0E0S2K9 pp 229533.I1RZV5
31974 229533.A0A0E0S2K9 pp 229533.I1RCG5

229533.A0A0E0S2K9 pp 229533.A0A1C3YIP2
229533.A0A0E0S2K9 pp 229533.I1RWE1
229533.A0A0E0S2K9 pp 229533.I1S148
229533.A0A0E0S2K9 pp 229533.I1S0H2
229533.A0A0E0S2K9 pp 229533.V6R756
229533.A0A0E0S2K9 pp 229533.I1REE6
229533.A0A0E0S2K9 pp 229533.V6R0H4
229533.A0A0E0S2K9 pp 229533.V6RPS5
229533.A0A0E0S2K9 pp 229533.V6QU99
229533.A0A0E0S2K9 pp 229533.I1RQI4
229533.A0A0E0S2K9 pp 229533.V6REA4
229533.A0A0E0S2K9 pp 229533.I1SAI4
229533.A0A0E0S2K9 pp 229533.V6R2Z2
229533.A0A0E0S2K9 pp 229533.Q4HTT2
229533.A0A0E0S2K9 pp 229533.I1RN88
229533.A0A0E0S2K9 pp 229533.I1RJS7
229533.A0A0E0S2K9 pp 229533.I1S147
229533.A0A0E0S2K9 pp 229533.I1RBY4
229533.A0A0E0S2K9 pp 229533.I1S8C1
229533.A0A0E0S2K9 pp 229533.I1RDN3
229533.A0A0E0S2K9 pp 229533.I1S405
229533.A0A0E0S2K9 pp 229533.I1RUY5
229533.A0A0E0S2K9 pp 229533.A0A1C3YJ56
229533.A0A0E0S2K9 pp 229533.I1RNG4
229533.A0A0E0S2K9 pp 229533.V6R5D2
229533.A0A0E0S2K9 pp 229533.I1RNF4
229533.A0A0E0S2K9 pp 229533.I1RGC3
229533.A0A0E0S2K9 pp 229533.I1RV06
229533.A0A0E0S2K9 pp 229533.A0A1C3YN26
229533.A0A0E0S2K9 pp 229533.A0A1C3YKN4
229533.A0A0E0S2K9 pp 229533.I1RC00
229533.A0A0E0S2K9 pp 229533.A0A1C3YKB7
229533.A0A0E0S2K9 pp 229533.V6R0W1
229533.A0A0E0S2K9 pp 229533.V6QTX7
229533.I1RZ51 pp 229533.I1S2E9
229533.I1RBT2 pp 229533.I1RCP3
229533.I1RBT2 pp 229533.I1RZ35
229533.I1RBT2 pp 229533.I1RM78
229533.I1RBT2 pp 229533.I1RN54
229533.I1RGX2 pp 229533.I1S323
229533.I1RGX2 pp 229533.I1S6S8
229533.I1RGX2 pp 229533.I1RZH9
229533.I1RGX2 pp 229533.I1RR36
229533.I1RGX2 pp 229533.I1RR22
229533.A0A098DYZ0 pp 229533.A0A0E0SA11
229533.A0A098DYZ0 pp 229533.I1RZ63
229533.A0A098DYZ0 pp 229533.I1S2E9
229533.A0A098DYZ0 pp 229533.A0A0E0SD83
229533.A0A098DYZ0 pp 229533.I1RV91
229533.A0A098DYZ0 pp 229533.I1RPS0
229533.A0A098DYZ0 pp 229533.I1RJK7
229533.A0A098DYZ0 pp 229533.A0A1C3YM29
229533.A0A098DYZ0 pp 229533.A0A1C3YHP8
229533.A0A098DYZ0 pp 229533.A0A1C3YM82
229533.A0A098DYZ0 pp 229533.I1RQA3
229533.A0A098DYZ0 pp 229533.I1RFL7
229533.A0A098DYZ0 pp 229533.I1RC17
229533.A0A098DYZ0 pp 229533.I1RWT6
229533.A0A098DYZ0 pp 229533.I1RY44
229533.A0A1C3YMP0 pp 229533.I1S323
229533.A0A1C3YMP0 pp 229533.I1RGX2
229533.A0A0E0SH88 pp 229533.V6RLM7
229533.A0A0E0SH88 pp 229533.I1S064
229533.A0A0E0SH88 pp 229533.V6QT89
229533.A0A0E0SH88 pp 229533.Q4HTS9
229533.A0A0E0SH88 pp 229533.I1S3V2
229533.A0A0E0SH88 pp 229533.I1S685
229533.A0A0E0SH88 pp 229533.I1RKX1
229533.A0A0E0SH88 pp 229533.I1RY74
229533.A0A0E0SH88 pp 229533.I1RP53
229533.A0A0E0SH88 pp 229533.I1R9U7
229533.A0A0E0SH88 pp 229533.I1RZN1
229533.A0A0E0SH88 pp 229533.I1RND2

32048 229533.A0A0E0SH88 pp 229533.I1R9B8
32049 229533.A0A0E0SH88 pp 229533.I1RZB2
32050 229533.A0A0E0SH88 pp 229533.I1RXB6
32051 229533.A0A0E0SH88 pp 229533.A0A0E0SPV0
32052 229533.A0A0E0SH88 pp 229533.I1S0A0
32053 229533.A0A0E0SH88 pp 229533.I1S684
32054 229533.A0A0E0SH88 pp 229533.I1RHG5
32055 229533.A0A0E0SH88 pp 229533.V6QVM5
32056 229533.A0A0E0SH88 pp 229533.I1RNW2
32057 229533.A0A0E0SH88 pp 229533.I1RDB7
32058 229533.A0A0E0SH88 pp 229533.I1REB0
32059 229533.A0A0E0SH88 pp 229533.I1RXX0
32060 229533.A0A0E0SH88 pp 229533.V6RVJ3
32061 229533.A0A0E0SH88 pp 229533.I1RSX2
32062 229533.A0A0E0SH88 pp 229533.A0A1C3YLB2
32063 229533.A0A0E0SH88 pp 229533.I1RHX4
32064 229533.A0A0E0SH88 pp 229533.I1RL04
32065 229533.A0A0E0SH88 pp 229533.I1RWF9
32066 229533.A0A0E0SH88 pp 229533.I1RSE0
32067 229533.A0A0E0SH88 pp 229533.I1S8F8
32068 229533.A0A0E0SH88 pp 229533.A0A1C3YK09
32069 229533.A0A0E0SH88 pp 229533.A0A1C3YJZ0
32070 229533.A0A0E0SH88 pp 229533.I1RVJ6
32071 229533.A0A0E0SH88 pp 229533.A0A1C3YL82
32072 229533.A0A0E0SH88 pp 229533.A0A1C3YJ47
32073 229533.A0A0E0SH88 pp 229533.I1RPD6
32074 229533.A0A0E0SH88 pp 229533.I1RBE4
32075 229533.A0A0E0SH88 pp 229533.I1RXV3
32076 229533.A0A0E0SH88 pp 229533.I1RVP5
32077 229533.A0A0E0SH88 pp 229533.I1RNW6
32078 229533.A0A0E0SH88 pp 229533.I1RUE7
32079 229533.A0A0E0SH88 pp 229533.I1RP97
32080 229533.A0A0E0SH88 pp 229533.I1RE14
32081 229533.A0A0E0SH88 pp 229533.I1REA8
32082 229533.A0A0E0SH88 pp 229533.I1RC61
32083 229533.A0A0E0SH88 pp 229533.I1S019
32084 229533.A0A0E0SH88 pp 229533.I1R9W4
32085 229533.A0A0E0SH88 pp 229533.I1RCR9
32086 229533.A0A0E0SH88 pp 229533.A0A1C3YLX8
32087 229533.A0A0E0SH88 pp 229533.I1RK03
32088 229533.A0A0E0SH88 pp 229533.I1RDJ7
32089 229533.A0A0E0SH88 pp 229533.I1RK47
32090 229533.A0A0E0SH88 pp 229533.I1RJ73
32091 229533.A0A0E0SH88 pp 229533.I1RYU0
32092 229533.A0A0E0SH88 pp 229533.I1RQX9
32093 229533.A0A0E0SH88 pp 229533.I1RFM6
32094 229533.A0A0E0SH88 pp 229533.I1REI0
32095 229533.A0A0E0SH88 pp 229533.A0A1C3YLV9
32096 229533.A0A0E0SH88 pp 229533.A0A1C3YLC3
32097 229533.A0A0E0SH88 pp 229533.I1RNW1
32098 229533.A0A0E0SH88 pp 229533.I1REI6
32099 229533.A0A0E0SH88 pp 229533.A0A1C3YL20
32100 229533.A0A0E0SH88 pp 229533.I1RFV2
32101 229533.A0A0E0SH88 pp 229533.I1RW06
32102 229533.A0A0E0SH88 pp 229533.A0A1C3YLR6
32103 229533.A0A0E0SH88 pp 229533.I1RNJ7
32104 229533.A0A0E0SH88 pp 229533.I1S686
32105 229533.A0A0E0SH88 pp 229533.I1RK93
32106 229533.A0A0E0SH88 pp 229533.I1S2Y3
32107 229533.A0A0E0SH88 pp 229533.I1RTU1
32108 229533.A0A0E0SH88 pp 229533.I1RMM8
32109 229533.A0A0E0SH88 pp 229533.I1RJ72
32110 229533.A0A0E0SH88 pp 229533.I1RA64
32111 229533.A0A0E0SH88 pp 229533.I1RY49
32112 229533.A0A0E0SH88 pp 229533.I1RXY4
32113 229533.A0A0E0SH88 pp 229533.I1S8E0
32114 229533.A0A0E0SH88 pp 229533.I1RKJ7
32115 229533.A0A0E0SH88 pp 229533.A0A1C3YK11
32116 229533.I1RSV8 pp 229533.I1RX74
32117 229533.I1RSI4 pp 229533.I1RUG2
32118 229533.I1RSI4 pp 229533.I1RZK6
32119 229533.I1RSI4 pp 229533.I1S604
32120 229533.I1RSI4 pp 229533.I1S461

32121	229533.I1RSI4	pp	229533.Q4IJU0
32122	229533.I1RSI4	pp	229533.I1S236
32123	229533.I1RSI4	pp	229533.I1RY73
32124	229533.I1RSI4	pp	229533.Q4IEA5
32125	229533.I1RSI4	pp	229533.I1RWM6
32126	229533.I1RSI4	pp	229533.Q4IR08
32127	229533.I1RSI4	pp	229533.I1S283
32128	229533.I1RSI4	pp	229533.Q4IP51
32129	229533.I1RSI4	pp	229533.I1S209
32130	229533.I1RSI4	pp	229533.Q4HZS8
32131	229533.I1RSI4	pp	229533.I1S0A0
32132	229533.I1RSI4	pp	229533.I1S0R9
32133	229533.I1RSI4	pp	229533.I1S0P4
32134	229533.I1RSI4	pp	229533.I1S1Y7
32135	229533.I1RSI4	pp	229533.I1RT41
32136	229533.I1RSI4	pp	229533.I1S0Z3
32137	229533.I1RSI4	pp	229533.I1RZ94
32138	229533.I1RSI4	pp	229533.I1RSP7
32139	229533.I1RSI4	pp	229533.Q4IC11
32140	229533.I1RSI4	pp	229533.I1RWM0
32141	229533.I1RSI4	pp	229533.Q4IN00
32142	229533.I1RSI4	pp	229533.I1RWT6
32143	229533.I1RSI4	pp	229533.I1RZ98
32144	229533.I1RSI4	pp	229533.I1RUG1
32145	229533.I1RSI4	pp	229533.I1RX64
32146	229533.I1RSI4	pp	229533.I1S2E9
32147	229533.I1RSI4	pp	229533.I1RV91
32148	229533.I1RSI4	pp	229533.V6R9T8
32149	229533.I1RSI4	pp	229533.I1RSR9
32150	229533.I1RSI4	pp	229533.I1RZP8
32151	229533.I1RSI4	pp	229533.V6RGI1
32152	229533.I1RSI4	pp	229533.I1S237
32153	229533.I1RSI4	pp	229533.I1RW16
32154	229533.I1RSI4	pp	229533.I1S020
32155	229533.I1RSI4	pp	229533.V6R3J1
32156	229533.I1RSI4	pp	229533.Q4IMD1
32157	229533.I1RSI4	pp	229533.I1RY44
32158	229533.I1RSI4	pp	229533.I1S0N4
32159	229533.I1RSI4	pp	229533.I1RSJ4
32160	229533.I1RSI4	pp	229533.Q4IQM6
32161	229533.I1RSI4	pp	229533.I1S1D3
32162	229533.I1RSI4	pp	229533.V6RLM7
32163	229533.I1RSI4	pp	229533.I1S2F2
32164	229533.I1RSI4	pp	229533.V6R382
32165	229533.I1RSI4	pp	229533.V6R283
32166	229533.I1RSI4	pp	229533.V6RLW4
32167	229533.I1RSI4	pp	229533.I1RYR2
32168	229533.I1RSI4	pp	229533.I1RYX9
32169	229533.I1RSI4	pp	229533.I1RSV2
32170	229533.I1RSI4	pp	229533.I1RSX1
32171	229533.I1S1X4	pp	229533.I1S4Z7
32172	229533.I1RQI4	pp	229533.I1RUY6
32173	229533.I1RQI4	pp	229533.V6RV57
32174	229533.I1RQI4	pp	229533.V6RLM7
32175	229533.I1RQI4	pp	229533.I1RR44
32176	229533.I1RQI4	pp	229533.I1S147
32177	229533.I1RQI4	pp	229533.I1S148
32178	229533.I1RQI4	pp	229533.I1RUY5
32179	229533.I1RQI4	pp	229533.I1S149
32180	229533.I1RQI4	pp	229533.V6RPS5
32181	229533.I1RQI4	pp	229533.I1RSQ4
32182	229533.I1RV66	pp	229533.I1RZT1
32183	229533.I1RV66	pp	229533.V6RKY7
32184	229533.I1RV66	pp	229533.I1S296
32185	229533.I1RV66	pp	229533.I1RZE4
32186	229533.I1RV66	pp	229533.V6RVG3
32187	229533.I1RV66	pp	229533.I1RY44
32188	229533.I1RV66	pp	229533.I1S439
32189	229533.I1RV66	pp	229533.I1RZ63
32190	229533.I1RV66	pp	229533.I1S0M2
32191	229533.I1RV66	pp	229533.I1RYH9
32192	229533.I1RV66	pp	229533.I1S0D1
32193	229533.I1RV66	pp	229533.I1RZ94

229533.I1RV66	pp	229533.I1RX77
229533.I1RV66	pp	229533.I1RXH6
229533.I1RV66	pp	229533.I1S2K7
229533.I1RV66	pp	229533.I1S1W4
229533.I1RV66	pp	229533.I1S2B6
229533.I1RV66	pp	229533.I1S2E9
229533.I1RV66	pp	229533.V6QV09
229533.I1RV66	pp	229533.I1RX64
229533.I1RV66	pp	229533.I1RX04
229533.I1RV66	pp	229533.I1RWT6
229533.I1RV66	pp	229533.I1RV91
229533.I1RV66	pp	229533.I1S265
229533.I1RV66	pp	229533.I1RYZ9
229533.I1RV66	pp	229533.I1RZK4
229533.I1RG72	pp	229533.I1RQL4
229533.I1RPS2	pp	229533.I1RUM7
229533.I1RPS2	pp	229533.I1RXX0
229533.I1RPS2	pp	229533.V6R3R0
229533.I1RPS2	pp	229533.I1RQE0
229533.I1RPS2	pp	229533.I1RSJ2
229533.I1RPS2	pp	229533.I1SAC4
229533.I1RPS2	pp	229533.I1RRT3
229533.I1RPS2	pp	229533.I1RWP9
229533.I1RPS2	pp	229533.V6R729
229533.I1RPS2	pp	229533.I1RQ99
229533.I1RPS2	pp	229533.I1RUP6
229533.I1RPS2	pp	229533.I1RZM3
229533.I1RPS2	pp	229533.V6RUG6
229533.I1RPS2	pp	229533.I1S444
229533.I1RPS2	pp	229533.Q4HTS9
229533.I1RRJ3	pp	229533.I1RSB7
229533.I1RRJ3	pp	229533.V6RK96
229533.I1RRJ3	pp	229533.I1RZM4
229533.I1RRJ3	pp	229533.I1S3V2
229533.I1RRJ3	pp	229533.I1RW06
229533.I1RRJ3	pp	229533.I1RZ21
229533.A0A098DSW2	pp	229533.A0A098E0E1
229533.A0A098DSW2	pp	229533.I1RA32
229533.A0A098DSW2	pp	229533.A0A0E0SA11
229533.A0A098DSW2	pp	229533.I1RZ63
229533.A0A098DSW2	pp	229533.I1RSM9
229533.A0A098DSW2	pp	229533.I1S0J7
229533.A0A098DSW2	pp	229533.I1S224
229533.A0A098DSW2	pp	229533.I1RL24
229533.A0A098DSW2	pp	229533.I1RQL4
229533.A0A098DSW2	pp	229533.I1RYZ9
229533.A0A098DSW2	pp	229533.I1S098
229533.A0A098DSW2	pp	229533.I1RC17
229533.A0A098DSW2	pp	229533.I1RQD1
229533.A0A098DSW2	pp	229533.I1RME5
229533.A0A098DSW2	pp	229533.A0A1C3YM91
229533.A0A098DSW2	pp	229533.I1S2E9
229533.A0A098DSW2	pp	229533.I1REE1
229533.A0A098DSW2	pp	229533.I1RRW0
229533.A0A098DSW2	pp	229533.I1RV91
229533.A0A098DSW2	pp	229533.I1RWT6
229533.A0A098DSW2	pp	229533.A0A1C3YK66
229533.A0A098DSW2	pp	229533.I1RY44
229533.A0A098DSW2	pp	229533.A0A1C3YM82
229533.A0A098DSW2	pp	229533.I1RCB9
229533.A0A098DSW2	pp	229533.A0A0E0SD83
229533.A0A098DSW2	pp	229533.I1RA36
229533.A0A098DSW2	pp	229533.I1RPS0
229533.A0A098DSW2	pp	229533.I1RJK7
229533.A0A098DSW2	pp	229533.I1RFL7
229533.A0A098DSW2	pp	229533.V6QV09
229533.A0A098DSW2	pp	229533.A0A1C3YM29
229533.A0A098DSW2	pp	229533.I1RQA3
229533.A0A098DSW2	pp	229533.A0A1C3YHP8
229533.A0A1C3YJV7	pp	229533.I1RMB2
229533.A0A1C3YJV7	pp	229533.I1RAM7
229533.A0A1C3YJV7	pp	229533.I1RWT6
229533.A0A1C3YJV7	pp	229533.Q4IEA7

32267 229533.A0A1C3YJV7 pp 229533.I1RSS1
32268 229533.A0A1C3YJV7 pp 229533.V6RR76
32269 229533.A0A1C3YJV7 pp 229533.Q4IR87
32270 229533.A0A1C3YJV7 pp 229533.I1RB75
32271 229533.A0A1C3YJV7 pp 229533.I1RC95
32272 229533.A0A1C3YJV7 pp 229533.I1RKK0
32273 229533.A0A1C3YJV7 pp 229533.I1S439
32274 229533.A0A1C3YJV7 pp 229533.I1RQA3
32275 229533.A0A1C3YJV7 pp 229533.I1RQ26
32276 229533.A0A1C3YJV7 pp 229533.I1RCB3
32277 229533.A0A1C3YJV7 pp 229533.I1RQ31
32278 229533.A0A1C3YJV7 pp 229533.I1RLX7
32279 229533.A0A1C3YJV7 pp 229533.V6QV09
32280 229533.A0A1C3YJV7 pp 229533.I1RMM9
32281 229533.A0A1C3YJV7 pp 229533.I1RBC4
32282 229533.A0A1C3YJV7 pp 229533.I1S278
32283 229533.A0A1C3YJV7 pp 229533.I1RXX1
32284 229533.A0A1C3YJV7 pp 229533.A0A1C3YK75
32285 229533.A0A1C3YJV7 pp 229533.I1RBL8
32286 229533.A0A1C3YJV7 pp 229533.I1RWC2
32287 229533.A0A1C3YJV7 pp 229533.I1RUQ5
32288 229533.A0A1C3YJV7 pp 229533.I1RC22
32289 229533.A0A1C3YJV7 pp 229533.A0A1C3YN58
32290 229533.A0A1C3YJV7 pp 229533.I1RYH6
32291 229533.A0A1C3YJV7 pp 229533.I1RJT1
32292 229533.A0A1C3YJV7 pp 229533.I1RCD1
32293 229533.A0A1C3YJV7 pp 229533.I1RYL5
32294 229533.A0A1C3YJV7 pp 229533.I1RQL4
32295 229533.A0A1C3YJV7 pp 229533.Q4I4A4
32296 229533.A0A1C3YJV7 pp 229533.I1RYZ9
32297 229533.A0A1C3YJV7 pp 229533.I1RN08
32298 229533.A0A1C3YJV7 pp 229533.I1RPA6
32299 229533.A0A1C3YJV7 pp 229533.I1RN81
32300 229533.A0A1C3YJV7 pp 229533.I1RBZ9
32301 229533.A0A1C3YJV7 pp 229533.Q4INZ9
32302 229533.A0A1C3YJV7 pp 229533.V6RKY7
32303 229533.A0A1C3YJV7 pp 229533.I1REX4
32304 229533.A0A1C3YJV7 pp 229533.I1RL24
32305 229533.A0A1C3YJV7 pp 229533.I1RY44
32306 229533.A0A1C3YJV7 pp 229533.V6RVG3
32307 229533.A0A1C3YJV7 pp 229533.I1RMS1
32308 229533.A0A1C3YJV7 pp 229533.I1RX04
32309 229533.A0A1C3YJV7 pp 229533.I1RZ94
32310 229533.A0A1C3YJV7 pp 229533.I1RV66
32311 229533.A0A1C3YJV7 pp 229533.I1RX77
32312 229533.A0A1C3YJV7 pp 229533.I1S1W4
32313 229533.A0A1C3YJV7 pp 229533.I1RUQ2
32314 229533.A0A1C3YJV7 pp 229533.I1RBW0
32315 229533.A0A1C3YJV7 pp 229533.I1RX20
32316 229533.A0A1C3YJV7 pp 229533.I1RYH9
32317 229533.A0A1C3YJV7 pp 229533.I1RBJ1
32318 229533.A0A1C3YJV7 pp 229533.I1RX87
32319 229533.A0A1C3YJV7 pp 229533.I1RA43
32320 229533.A0A1C3YJV7 pp 229533.I1R9Y9
32321 229533.A0A1C3YJV7 pp 229533.I1RNY8
32322 229533.A0A1C3YJV7 pp 229533.I1RWH4
32323 229533.A0A1C3YJV7 pp 229533.I1RDB1
32324 229533.A0A1C3YJV7 pp 229533.I1RS47
32325 229533.A0A1C3YJV7 pp 229533.I1S2C1
32326 229533.A0A1C3YJV7 pp 229533.I1RWQ8
32327 229533.A0A1C3YJV7 pp 229533.I1S265
32328 229533.A0A1C3YJV7 pp 229533.I1RA10
32329 229533.A0A1C3YJV7 pp 229533.I1RCV9
32330 229533.A0A1C3YJV7 pp 229533.I1RQB4
32331 229533.A0A1C3YJV7 pp 229533.I1RZE4
32332 229533.A0A1C3YJV7 pp 229533.I1RRN5
32333 229533.A0A1C3YJV7 pp 229533.I1S0M2
32334 229533.A0A1C3YJV7 pp 229533.I1RC97
32335 229533.A0A1C3YJV7 pp 229533.I1RZC3
32336 229533.A0A1C3YJV7 pp 229533.I1RRI3
32337 229533.A0A1C3YJV7 pp 229533.I1S247
32338 229533.A0A1C3YJV7 pp 229533.I1RB41
32339 229533.A0A1C3YJV7 pp 229533.I1S7X9

229533.A0A1C3YJV7 pp 229533.I1S286
229533.A0A1C3YJV7 pp 229533.I1RVA8
229533.A0A1C3YJV7 pp 229533.I1RB74
229533.A0A1C3YJV7 pp 229533.I1REC7
229533.A0A1C3YJV7 pp 229533.I1RRY3
229533.A0A1C3YJV7 pp 229533.I1RFL7
229533.A0A1C3YJV7 pp 229533.I1RMA7
229533.A0A1C3YJV7 pp 229533.I1RRJ8
229533.A0A1C3YJV7 pp 229533.V6RQR4
229533.A0A1C3YJV7 pp 229533.I1RFW0
229533.A0A1C3YJV7 pp 229533.I1RX64
229533.A0A1C3YJV7 pp 229533.I1RAX2
229533.A0A1C3YJV7 pp 229533.I1RAN2
229533.A0A1C3YJV7 pp 229533.A0A1C3YLF5
229533.A0A1C3YJV7 pp 229533.I1RY32
229533.A0A1C3YJV7 pp 229533.I1S2E9
229533.A0A1C3YJV7 pp 229533.I1RA84
229533.A0A1C3YJV7 pp 229533.A0A1C3YMQ5
229533.A0A1C3YJV7 pp 229533.I1RC46
229533.A0A1C3YJV7 pp 229533.I1REC6
229533.A0A1C3YJV7 pp 229533.I1RUN8
229533.A0A1C3YJV7 pp 229533.I1S0X7
229533.A0A1C3YJV7 pp 229533.I1S4Z7
229533.A0A1C3YJV7 pp 229533.I1S248
229533.A0A1C3YJV7 pp 229533.I1RNP7
229533.A0A1C3YJV7 pp 229533.I1RQD6
229533.A0A1C3YJV7 pp 229533.I1RBW1
229533.A0A1C3YJV7 pp 229533.I1RGF1
229533.A0A1C3YJV7 pp 229533.I1RZ63
229533.I1S149 pp 229533.V6QU99
229533.I1S149 pp 229533.Q4HTT2
229533.I1S149 pp 229533.V6R756
229533.I1S149 pp 229533.I1SAI4
229533.I1S149 pp 229533.V6RPS5
229533.I1S149 pp 229533.V6REA4
229533.I1S149 pp 229533.V6QTX7
229533.I1S149 pp 229533.V6R556
229533.I1S149 pp 229533.I1S405
229533.I1S149 pp 229533.V6RAM6
229533.I1S149 pp 229533.V6RM09
229533.I1S149 pp 229533.V6R5D2
229533.I1S149 pp 229533.V6R0H4
229533.I1S149 pp 229533.I1S8C1
229533.I1S149 pp 229533.V6R0W1
229533.I1S149 pp 229533.I1S269
229533.I1S149 pp 229533.V6R2Z2
229533.I1RF63 pp 229533.I1RF64
229533.A0A1C3YLE1 pp 229533.I1RJU3
229533.A0A1C3YLE1 pp 229533.I1RCH5
229533.A0A1C3YLE1 pp 229533.I1REK8
229533.A0A1C3YLE1 pp 229533.V6RIV9
229533.A0A1C3YLE1 pp 229533.I1RS17
229533.I1RB51 pp 229533.I1RCI4
229533.I1RB51 pp 229533.I1S1C6
229533.I1RB51 pp 229533.I1RRH3
229533.A0A1C3YKU6 pp 229533.I1RW73
229533.A0A1C3YKU6 pp 229533.I1RE15
229533.A0A1C3YKU6 pp 229533.I1REN4
229533.A0A1C3YKU6 pp 229533.I1RUA0
229533.A0A1C3YKU6 pp 229533.I1S2U5
229533.A0A1C3YKU6 pp 229533.I1RPK5
229533.A0A1C3YKU6 pp 229533.I1RWI6
229533.A0A1C3YKF2 pp 229533.I1RJH1
229533.A0A1C3YKF2 pp 229533.I1S2E9
229533.A0A1C3YKF2 pp 229533.A0A1C3YKW5
229533.A0A1C3YKF2 pp 229533.I1RC97
229533.A0A1C3YKF2 pp 229533.I1S2G7
229533.A0A1C3YKF2 pp 229533.I1RBZ9
229533.A0A1C3YKF2 pp 229533.I1RV91
229533.A0A1C3YKF2 pp 229533.I1RPD6
229533.A0A1C3YKF2 pp 229533.I1RMA1
229533.A0A1C3YKF2 pp 229533.I1RYZ9
229533.A0A1C3YKF2 pp 229533.I1RDB1

229533.A0A1C3YKF2 pp 229533.I1RX70
229533.A0A1C3YKF2 pp 229533.I1RLU1
229533.A0A1C3YKF2 pp 229533.I1RNP7
229533.A0A1C3YKF2 pp 229533.I1RB74
229533.A0A1C3YKF2 pp 229533.I1RAN2
229533.A0A1C3YKF2 pp 229533.I1RQ26
229533.A0A1C3YKF2 pp 229533.I1RJR8
229533.A0A1C3YKF2 pp 229533.I1RCU9
229533.A0A1C3YKF2 pp 229533.I1RV66
229533.A0A1C3YKF2 pp 229533.I1RCU8
229533.A0A1C3YKF2 pp 229533.V6QV09
229533.A0A1C3YKF2 pp 229533.I1S549
229533.A0A1C3YKF2 pp 229533.I1RL24
229533.A0A1C3YKF2 pp 229533.I1RCB3
229533.A0A1C3YKF2 pp 229533.I1RWT6
229533.A0A1C3YKF2 pp 229533.I1RUN8
229533.A0A1C3YKF2 pp 229533.I1RQB4
229533.A0A1C3YKF2 pp 229533.V6RKY7
229533.A0A1C3YKF2 pp 229533.I1S0M2
229533.A0A1C3YKF2 pp 229533.I1RST8
229533.A0A1C3YKF2 pp 229533.I1RYH9
229533.A0A1C3YKF2 pp 229533.I1RY44
229533.A0A1C3YKF2 pp 229533.I1RKK0
229533.A0A1C3YKF2 pp 229533.A0A1C3YKL8
229533.A0A1C3YKF2 pp 229533.V6RVG3
229533.A0A1C3YKF2 pp 229533.Q4IMN9
229533.A0A1C3YKF2 pp 229533.I1RRN5
229533.A0A1C3YKF2 pp 229533.V6RDP1
229533.A0A1C3YKF2 pp 229533.I1RN08
229533.A0A1C3YKF2 pp 229533.I1RC17
229533.A0A1C3YKF2 pp 229533.I1RUQ5
229533.A0A1C3YKF2 pp 229533.I1RC22
229533.A0A1C3YKF2 pp 229533.I1RNY9
229533.A0A1C3YKF2 pp 229533.I1RI71
229533.A0A1C3YKF2 pp 229533.I1RC95
229533.A0A1C3YKF2 pp 229533.I1RDZ4
229533.A0A1C3YKF2 pp 229533.I1RQA3
229533.A0A1C3YKF2 pp 229533.I1RZE4
229533.A0A1C3YKF2 pp 229533.I1S0D1
229533.A0A1C3YKF2 pp 229533.I1RYY5
229533.A0A1C3YKF2 pp 229533.I1RMA8
229533.A0A1C3YKF2 pp 229533.I1RA43
229533.A0A1C3YKF2 pp 229533.I1S439
229533.A0A1C3YKF2 pp 229533.I1RB75
229533.A0A1C3YKF2 pp 229533.I1RMM9
229533.A0A1C3YKF2 pp 229533.I1RRY3
229533.A0A1C3YKF2 pp 229533.I1R9Y9
229533.A0A1C3YKF2 pp 229533.I1S1D3
229533.A0A1C3YKF2 pp 229533.I1RN65
229533.A0A1C3YKF2 pp 229533.I1RAM7
229533.A0A1C3YKF2 pp 229533.I1RYZ0
229533.A0A1C3YKF2 pp 229533.Q4IJM4
229533.A0A1C3YKF2 pp 229533.I1RNY8
229533.A0A1C3YKF2 pp 229533.I1RB41
229533.A0A1C3YKF2 pp 229533.I1S1W4
229533.A0A1C3YKF2 pp 229533.I1RNW8
229533.I1RF54 pp 229533.I1RF59
229533.I1RF54 pp 229533.I1RF56
229533.I1RF54 pp 229533.I1RF62
229533.I1RF54 pp 229533.I1RF58
229533.I1RF54 pp 229533.I1RF61
229533.I1RF54 pp 229533.I1RF55
229533.I1RF54 pp 229533.Q4HWQ2
229533.I1RF54 pp 229533.I1RF60
229533.I1RF54 pp 229533.I1RF63
229533.I1RF54 pp 229533.I1RF64
229533.I1RF54 pp 229533.I1RVD4
229533.I1RF54 pp 229533.I1RF57
229533.A0A1C3YIK3 pp 229533.I1RF63
229533.A0A1C3YIK3 pp 229533.A0A1C3YMZ0
229533.A0A1C3YIK3 pp 229533.I1RM91
229533.A0A1C3YIK3 pp 229533.I1RF54
229533.I1RFE0 pp 229533.I1RQL4

32486	229533.I1RFE0	pp	229533.I1S5L1
32487	229533.V6QV09	pp	229533.V6RGI1
32488	229533.V6QV09	pp	229533.V6RU70
32489	229533.V6QV09	pp	229533.V6RKY7
32490	229533.V6QV09	pp	229533.V6RVG3
32491	229533.V6QV09	pp	229533.V6RMM8
32492	229533.I1S4A5	pp	229533.I1S6X4
32493	229533.I1S3H6	pp	229533.I1S6X4
32494	229533.I1S3H6	pp	229533.I1S4A5
32495	229533.I1RE66	pp	229533.I1RNH1
32496	229533.I1RE66	pp	229533.I1S0P0
32497	229533.I1RE66	pp	229533.I1RJB3
32498	229533.I1RE66	pp	229533.I1RW26
32499	229533.I1RE66	pp	229533.I1RRG3
32500	229533.I1RE66	pp	229533.I1RGJ4
32501	229533.I1RE66	pp	229533.I1S0T7
32502	229533.I1RE66	pp	229533.I1RN42
32503	229533.I1RE66	pp	229533.I1RSC0
32504	229533.I1RE66	pp	229533.I1RRX5
32505	229533.I1RE66	pp	229533.I1RS30
32506	229533.I1RE66	pp	229533.I1S464
32507	229533.I1RE66	pp	229533.I1RWG0
32508	229533.I1RE66	pp	229533.I1RKQ5
32509	229533.I1RE66	pp	229533.I1RYC7
32510	229533.I1RE66	pp	229533.I1S268
32511	229533.I1RE66	pp	229533.I1RS05
32512	229533.I1RE66	pp	229533.I1RX39
32513	229533.I1RE66	pp	229533.I1S1F3
32514	229533.I1RE66	pp	229533.I1RXD9
32515	229533.I1RE66	pp	229533.I1RMK7
32516	229533.I1RE66	pp	229533.I1RG62
32517	229533.I1RE66	pp	229533.I1RVY0
32518	229533.I1RE66	pp	229533.V6QYF0
32519	229533.I1RE66	pp	229533.I1RV91
32520	229533.I1RE66	pp	229533.I1RPY0
32521	229533.I1RE66	pp	229533.I1RYT8
32522	229533.I1RE66	pp	229533.I1RNA0
32523	229533.I1RE66	pp	229533.I1RK39
32524	229533.I1RE66	pp	229533.I1S458
32525	229533.I1RE66	pp	229533.I1RRK9
32526	229533.I1RE66	pp	229533.I1RGC3
32527	229533.I1RE66	pp	229533.I1S7I5
32528	229533.I1RE66	pp	229533.I1RNM7
32529	229533.I1RE66	pp	229533.I1RWA3
32530	229533.I1RE66	pp	229533.I1S0U0
32531	229533.I1RE66	pp	229533.I1RY21
32532	229533.I1RE66	pp	229533.I1S5C6
32533	229533.I1RE66	pp	229533.I1REF0
32534	229533.I1RE66	pp	229533.I1S1N7
32535	229533.I1RE66	pp	229533.V6QUE6
32536	229533.I1RE66	pp	229533.I1S9U2
32537	229533.I1RE66	pp	229533.I1RMK3
32538	229533.I1RE66	pp	229533.I1RFS5
32539	229533.I1RE66	pp	229533.I1RQR6
32540	229533.I1RE66	pp	229533.I1RP02
32541	229533.I1RE66	pp	229533.I1RW13
32542	229533.I1RE66	pp	229533.I1RXE6
32543	229533.I1RE66	pp	229533.I1RY92
32544	229533.I1RE66	pp	229533.I1RMQ6
32545	229533.I1RE66	pp	229533.I1RI95
32546	229533.I1RE66	pp	229533.I1RKF4
32547	229533.I1RE66	pp	229533.I1RKB7
32548	229533.I1RE66	pp	229533.I1S266
32549	229533.I1RE66	pp	229533.I1S8F8
32550	229533.I1RE66	pp	229533.I1S0R1
32551	229533.I1RE66	pp	229533.I1RNH6
32552	229533.I1RE66	pp	229533.I1RZ68
32553	229533.I1RE66	pp	229533.I1RLN1
32554	229533.I1RE66	pp	229533.I1RJI3
32555	229533.I1RE66	pp	229533.V6RJP8
32556	229533.I1RE66	pp	229533.I1RU95
32557	229533.I1RE66	pp	229533.I1S5R5
32558	229533.I1RE66	pp	229533.I1RNJ5

32559 229533.I1RE66 pp 229533.I1S0R8
32560 229533.I1RE66 pp 229533.I1RQJ3
32561 229533.I1RE66 pp 229533.I1S0K2
32562 229533.I1RE66 pp 229533.I1RGI8
32563 229533.I1RE66 pp 229533.I1S0Q7
32564 229533.I1RE66 pp 229533.I1RS29
32565 229533.I1RE66 pp 229533.I1S5X9
32566 229533.I1RE66 pp 229533.I1S8S9
32567 229533.I1RE66 pp 229533.I1RJL2
32568 229533.I1RE66 pp 229533.I1RKF5
32569 229533.I1RE66 pp 229533.I1RK79
32570 229533.I1RE66 pp 229533.I1RZ43
32571 229533.I1RE66 pp 229533.I1RK95
32572 229533.I1RE66 pp 229533.I1RSY2
32573 229533.I1RE66 pp 229533.Q4IJM4
32574 229533.I1RE66 pp 229533.I1RZB5
32575 229533.I1RE66 pp 229533.I1S212
32576 229533.I1RE66 pp 229533.I1RP38
32577 229533.I1RE66 pp 229533.I1S5U0
32578 229533.I1RE66 pp 229533.I1RSG8
32579 229533.I1RE66 pp 229533.I1RFJ6
32580 229533.I1RPL4 pp 229533.V6RIV9
32581 229533.I1RPL4 pp 229533.I1RS17
32582 229533.I1RD84 pp 229533.I1RE42
32583 229533.I1RD84 pp 229533.I1RT41
32584 229533.I1RD84 pp 229533.I1S537
32585 229533.I1RD84 pp 229533.I1S4T3
32586 229533.I1RD84 pp 229533.I1RMG6
32587 229533.I1RD84 pp 229533.I1RQN9
32588 229533.I1RD84 pp 229533.I1RPV3
32589 229533.I1RXN7 pp 229533.I1S6X4
32590 229533.I1RXN7 pp 229533.I1S325
32591 229533.I1RXN7 pp 229533.I1S694
32592 229533.I1RXN7 pp 229533.I1RZA8
32593 229533.I1RXN7 pp 229533.I1S4A5
32594 229533.I1RXN7 pp 229533.I1RY16
32595 229533.I1RXN7 pp 229533.I1S3H6
32596 229533.A0A098DX26 pp 229533.I1RBJ5
32597 229533.A0A098DX26 pp 229533.I1RBN0
32598 229533.A0A098DX26 pp 229533.A0A0E0RZM5
32599 229533.A0A098DX26 pp 229533.I1S272
32600 229533.A0A098DX26 pp 229533.I1RRQ1
32601 229533.A0A098DX26 pp 229533.I1RIL3
32602 229533.A0A098DX26 pp 229533.A0A1C3YK73
32603 229533.A0A098DX26 pp 229533.I1RCA0
32604 229533.A0A098DX26 pp 229533.I1RI03
32605 229533.A0A098DX26 pp 229533.I1RBF4
32606 229533.A0A098DX26 pp 229533.A0A0E0SPV2
32607 229533.A0A098DX26 pp 229533.I1RU29
32608 229533.A0A098DX26 pp 229533.I1RLC7
32609 229533.A0A098DX26 pp 229533.I1RI02
32610 229533.A0A098DX26 pp 229533.I1RW07
32611 229533.A0A098DX26 pp 229533.I1R9J6
32612 229533.A0A098DX26 pp 229533.I1RGL4
32613 229533.A0A098DX26 pp 229533.I1RLM7
32614 229533.A0A098DX26 pp 229533.I1S569
32615 229533.A0A098DX26 pp 229533.I1RK45
32616 229533.A0A098DX26 pp 229533.I1RZ13
32617 229533.A0A098DX26 pp 229533.Q4HZS8
32618 229533.A0A098DX26 pp 229533.I1S984
32619 229533.A0A098DX26 pp 229533.I1RRT3
32620 229533.A0A098DX26 pp 229533.I1RE72
32621 229533.A0A098DX26 pp 229533.A0A0E0RQ52
32622 229533.A0A098DX26 pp 229533.I1S574
32623 229533.A0A098DX26 pp 229533.I1R9D1
32624 229533.A0A098DX26 pp 229533.I1RNA1
32625 229533.A0A098DX26 pp 229533.A0A1C3YLZ7
32626 229533.A0A098DX26 pp 229533.I1RDJ1
32627 229533.A0A098DX26 pp 229533.I1RLN6
32628 229533.A0A098DX26 pp 229533.I1RCC2
32629 229533.A0A098DX26 pp 229533.I1RR95
32630 229533.A0A098DX26 pp 229533.I1RDE4
32631 229533.A0A098DX26 pp 229533.I1RGF5

32632	229533.A0A098DX26	pp	229533.I1RNA5
32633	229533.A0A098DX26	pp	229533.I1RKP3
32634	229533.A0A098DX26	pp	229533.I1RIL8
32635	229533.A0A098DX26	pp	229533.I1S352
32636	229533.A0A098DX26	pp	229533.I1RDU4
32637	229533.A0A098DX26	pp	229533.V6R4J1
32638	229533.A0A1C3YJR8	pp	229533.I1RFZ3
32639	229533.A0A1C3YJR8	pp	229533.A0A1C3YL18
32640	229533.A0A1C3YJR8	pp	229533.I1RQA3
32641	229533.A0A1C3YJR8	pp	229533.I1RN07
32642	229533.A0A1C3YJR8	pp	229533.I1S836
32643	229533.A0A1C3YJR8	pp	229533.I1RC17
32644	229533.A0A1C3YJR8	pp	229533.I1RWT6
32645	229533.A0A1C3YJR8	pp	229533.I1RV91
32646	229533.A0A1C3YJR8	pp	229533.I1RM09
32647	229533.A0A1C3YJR8	pp	229533.I1S2E9
32648	229533.A0A1C3YJR8	pp	229533.I1S549
32649	229533.A0A1C3YJR8	pp	229533.A0A1C3YLC6
32650	229533.A0A1C3YJR8	pp	229533.A0A1C3YLS3
32651	229533.A0A1C3YJR8	pp	229533.Q4HXF0
32652	229533.A0A1C3YJR8	pp	229533.Q4IB50
32653	229533.A0A1C3YJR8	pp	229533.I1RB41
32654	229533.A0A1C3YJR8	pp	229533.A0A1C3YM29
32655	229533.A0A1C3YJR8	pp	229533.I1RY44
32656	229533.A0A1C3YJR8	pp	229533.I1RRN5
32657	229533.A0A0E0SAG8	pp	229533.A0A1C3YLV9
32658	229533.A0A0E0SAG8	pp	229533.I1REI0
32659	229533.A0A0E0SAG8	pp	229533.I1RAD8
32660	229533.A0A0E0SAG8	pp	229533.I1RMM8
32661	229533.A0A0E0SAG8	pp	229533.I1S5Z3
32662	229533.A0A0E0SAG8	pp	229533.I1RL04
32663	229533.A0A0E0SAG8	pp	229533.I1RNJ7
32664	229533.A0A0E0SAG8	pp	229533.V6QVM5
32665	229533.A0A0E0SAG8	pp	229533.I1RKJ7
32666	229533.A0A0E0SAG8	pp	229533.I1RW54
32667	229533.A0A0E0SAG8	pp	229533.A0A1C3YIK4
32668	229533.A0A0E0SAG8	pp	229533.I1RXB6
32669	229533.A0A0E0SAG8	pp	229533.I1RZ29
32670	229533.A0A0E0SAG8	pp	229533.I1RK47
32671	229533.A0A0E0SAG8	pp	229533.I1RK03
32672	229533.A0A0E0SAG8	pp	229533.I1RXV3
32673	229533.A0A0E0SAG8	pp	229533.A0A0E0SF53
32674	229533.A0A0E0SAG8	pp	229533.I1RSX2
32675	229533.A0A0E0SAG8	pp	229533.A0A0E0SFT7
32676	229533.A0A0E0SAG8	pp	229533.I1RDE7
32677	229533.A0A0E0SAG8	pp	229533.O74638
32678	229533.A0A0E0SAG8	pp	229533.I1RXX0
32679	229533.A0A0E0SAG8	pp	229533.I1RYU0
32680	229533.A0A0E0SAG8	pp	229533.I1RC61
32681	229533.A0A0E0SAG8	pp	229533.I1RSE0
32682	229533.A0A0E0SAG8	pp	229533.I1RE13
32683	229533.A0A0E0SAG8	pp	229533.I1RCR9
32684	229533.A0A0E0SAG8	pp	229533.I1RVP5
32685	229533.A0A0E0SAG8	pp	229533.I1RWF9
32686	229533.A0A0E0SAG8	pp	229533.I1RYI7
32687	229533.A0A0E0SAG8	pp	229533.I1S064
32688	229533.A0A0E0SAG8	pp	229533.A0A1C3YLX8
32689	229533.A0A0E0SAG8	pp	229533.I1R9W4
32690	229533.A0A0E0SAG8	pp	229533.A0A0E0SMR8
32691	229533.A0A0E0SAG8	pp	229533.Q4HTS9
32692	229533.A0A0E0SAG8	pp	229533.A0A1C3YI72
32693	229533.A0A0E0SAG8	pp	229533.I1RFK3
32694	229533.A0A0E0SAG8	pp	229533.I1RP97
32695	229533.A0A0E0SAG8	pp	229533.I1R9U7
32696	229533.A0A0E0SAG8	pp	229533.I1S0I3
32697	229533.A0A0E0SAG8	pp	229533.I1RSL5
32698	229533.A0A0E0SAG8	pp	229533.Q4HXF0
32699	229533.A0A0E0SAG8	pp	229533.V6QT89
32700	229533.A0A0E0SAG8	pp	229533.I1RDB7
32701	229533.A0A0E0SAG8	pp	229533.A0A0E0SPV0
32702	229533.A0A0E0SAG8	pp	229533.I1RHG5
32703	229533.A0A0E0SAG8	pp	229533.I1RND2
32704	229533.A0A0E0SAG8	pp	229533.A0A1C3YI05

32705 229533.A0A0E0SAG8 pp 229533.I1RE79
32706 229533.A0A0E0SAG8 pp 229533.A0A0E0SH88
32707 229533.A0A0E0SAG8 pp 229533.A0A0E0SBC4
32708 229533.A0A0E0SAG8 pp 229533.A0A1C3YLB2
32709 229533.I1RWC2 pp 229533.I1RY44
32710 229533.I1RWC2 pp 229533.I1RX00
32711 229533.I1RWC2 pp 229533.I1S0Y1
32712 229533.I1RWC2 pp 229533.I1S2B6
32713 229533.I1RWC2 pp 229533.I1S037
32714 229533.I1RWC2 pp 229533.I1S2E9
32715 229533.A0A0E0SJN8 pp 229533.I1RLX4
32716 229533.A0A0E0SJN8 pp 229533.I1RSJ1
32717 229533.A0A0E0SJN8 pp 229533.I1RCW8
32718 229533.A0A0E0SJN8 pp 229533.I1RFZ2
32719 229533.A0A0E0SJN8 pp 229533.A0A0E0SQV5
32720 229533.A0A0E0SJN8 pp 229533.I1RZB7
32721 229533.A0A0E0SJN8 pp 229533.I1RFW1
32722 229533.A0A0E0SJN8 pp 229533.I1S1Z8
32723 229533.A0A0E0SJN8 pp 229533.Q4IER8
32724 229533.A0A0E0SJN8 pp 229533.I1RU02
32725 229533.A0A0E0SJN8 pp 229533.I1RPR3
32726 229533.A0A0E0SJN8 pp 229533.I1RFY2
32727 229533.A0A0E0SJN8 pp 229533.I1RMN1
32728 229533.A0A0E0SJN8 pp 229533.I1S148
32729 229533.I1RWD5 pp 229533.V6QX58
32730 229533.I1RWD5 pp 229533.I1RZU6
32731 229533.I1RWD5 pp 229533.Q5I2J3
32732 229533.I1RWD5 pp 229533.I1S0S8
32733 229533.I1RWD5 pp 229533.Q4INI2
32734 229533.I1RWD5 pp 229533.Q4HZS8
32735 229533.I1RGC3 pp 229533.I1RZA8
32736 229533.I1RGC3 pp 229533.V6RUY9
32737 229533.I1RGC3 pp 229533.I1RWN7
32738 229533.I1RGC3 pp 229533.I1RXD9
32739 229533.I1RGC3 pp 229533.I1RWF5
32740 229533.I1RGC3 pp 229533.I1RN84
32741 229533.I1RGC3 pp 229533.I1RUR8
32742 229533.I1RGC3 pp 229533.I1S8R4
32743 229533.I1RGC3 pp 229533.V6RV57
32744 229533.I1RGC3 pp 229533.I1RML4
32745 229533.I1RGC3 pp 229533.V6R3J1
32746 229533.I1RGC3 pp 229533.I1RNG4
32747 229533.I1RGC3 pp 229533.I1RY62
32748 229533.I1RGC3 pp 229533.I1S148
32749 229533.I1RGC3 pp 229533.I1RHC8
32750 229533.I1RGC3 pp 229533.I1RZ25
32751 229533.I1RGC3 pp 229533.I1RKZ7
32752 229533.I1RGC3 pp 229533.I1RZ63
32753 229533.I1RGC3 pp 229533.I1RQI4
32754 229533.I1RGC3 pp 229533.V6RRM0
32755 229533.I1RGC3 pp 229533.I1RKK5
32756 229533.I1RGC3 pp 229533.I1RYK0
32757 229533.I1RGC3 pp 229533.I1S694
32758 229533.I1RGC3 pp 229533.I1S299
32759 229533.I1RGC3 pp 229533.I1RH91
32760 229533.I1RGC3 pp 229533.I1RND4
32761 229533.I1RGC3 pp 229533.I1RSQ4
32762 229533.I1RGC3 pp 229533.I1RMJ3
32763 229533.I1RGC3 pp 229533.I1S0V7
32764 229533.I1RGC3 pp 229533.I1RKB2
32765 229533.I1RGC3 pp 229533.I1S3T7
32766 229533.I1RGC3 pp 229533.Q4I7L0
32767 229533.I1RGC3 pp 229533.I1S256
32768 229533.I1RGC3 pp 229533.I1S147
32769 229533.I1RGC3 pp 229533.I1RRM9
32770 229533.I1RGC3 pp 229533.I1RJK3
32771 229533.I1RGC3 pp 229533.I1S8D2
32772 229533.I1RGC3 pp 229533.I1RH92
32773 229533.I1RGC3 pp 229533.I1RI11
32774 229533.I1RGC3 pp 229533.I1RPK0
32775 229533.I1RGC3 pp 229533.V6RLM7
32776 229533.I1RGC3 pp 229533.I1S1F3
32777 229533.I1RGC3 pp 229533.I1RN88

32778	229533.I1RGC3	pp	229533.I1RYW9
32779	229533.I1RGC3	pp	229533.I1RGZ3
32780	229533.I1RGC3	pp	229533.I1RNL5
32781	229533.I1RGC3	pp	229533.I1S8R2
32782	229533.I1RGC3	pp	229533.I1RZ70
32783	229533.I1RGC3	pp	229533.I1RYX6
32784	229533.I1RGC3	pp	229533.I1S8D1
32785	229533.I1RGC3	pp	229533.I1S149
32786	229533.I1RGC3	pp	229533.I1S2C4
32787	229533.I1RGC3	pp	229533.I1S8C1
32788	229533.I1RGC3	pp	229533.I1RNV4
32789	229533.I1RGC3	pp	229533.I1RYQ4
32790	229533.I1RGC3	pp	229533.I1RP86
32791	229533.I1RGC3	pp	229533.I1RQE1
32792	229533.I1RGC3	pp	229533.I1RMQ6
32793	229533.I1RGC3	pp	229533.I1RQ60
32794	229533.I1RGC3	pp	229533.V6RM02
32795	229533.I1RGC3	pp	229533.I1RKB6
32796	229533.I1RGC3	pp	229533.I1RT82
32797	229533.I1RGC3	pp	229533.I1RWL3
32798	229533.I1RGC3	pp	229533.I1RY16
32799	229533.I1RGC3	pp	229533.V6RTJ8
32800	229533.I1RGC3	pp	229533.I1S3L2
32801	229533.I1RGC3	pp	229533.I1S048
32802	229533.I1RGC3	pp	229533.I1RMK7
32803	229533.I1RGC3	pp	229533.I1RY21
32804	229533.I1RGC3	pp	229533.I1RRX5
32805	229533.I1RGC3	pp	229533.I1RW54
32806	229533.I1RGC3	pp	229533.I1RHZ5
32807	229533.I1RGC3	pp	229533.V6RH92
32808	229533.I1RGC3	pp	229533.I1RH82
32809	229533.I1RGC3	pp	229533.I1RLA2
32810	229533.I1RGC3	pp	229533.Q4IA56
32811	229533.I1RGC3	pp	229533.I1RNM7
32812	229533.I1RGC3	pp	229533.I1RUQ3
32813	229533.I1RGC3	pp	229533.I1RST9
32814	229533.I1RGC3	pp	229533.I1S0K2
32815	229533.I1RGC3	pp	229533.I1RYT8
32816	229533.I1RGC3	pp	229533.I1RR44
32817	229533.I1RGC3	pp	229533.I1RPR1
32818	229533.I1RGC3	pp	229533.I1RJY7
32819	229533.I1RGC3	pp	229533.I1S267
32820	229533.I1RGC3	pp	229533.V6RPS5
32821	229533.I1RGC3	pp	229533.I1RNY6
32822	229533.I1RGC3	pp	229533.I1S8F8
32823	229533.I1RGC3	pp	229533.I1RJT5
32824	229533.I1RGC3	pp	229533.I1RUY6
32825	229533.I1RGC3	pp	229533.I1RXE6
32826	229533.I1RGC3	pp	229533.I1S0V6
32827	229533.I1RGC3	pp	229533.I1RUY5
32828	229533.I1RGC3	pp	229533.I1RYA2
32829	229533.I1RGC3	pp	229533.I1S8S9
32830	229533.I1RGC3	pp	229533.I1RY12
32831	229533.I1RYI3	pp	229533.I1S2G7
32832	229533.I1RYI3	pp	229533.I1S2E9
32833	229533.I1S0B8	pp	229533.Q4IN52
32834	229533.I1S0B8	pp	229533.V6RM66
32835	229533.I1S0B8	pp	229533.Q4I5G1
32836	229533.I1S0B8	pp	229533.I1S8F8
32837	229533.I1S0B8	pp	229533.Q4HY20
32838	229533.I1S0B8	pp	229533.I1S464
32839	229533.I1RJ77	pp	229533.I1RW71
32840	229533.I1RJ77	pp	229533.I1RJZ7
32841	229533.I1RDP6	pp	229533.I1S3H6
32842	229533.I1RDP6	pp	229533.I1RXN7
32843	229533.I1RDP6	pp	229533.I1RLI4
32844	229533.I1RDP6	pp	229533.I1RJA3
32845	229533.I1RDP6	pp	229533.I1S4A5
32846	229533.I1RDP6	pp	229533.I1RM78
32847	229533.I1RDP6	pp	229533.I1RJ84
32848	229533.I1RWF5	pp	229533.I1S0V6
32849	229533.I1RWF5	pp	229533.I1S8C1
32850	229533.I1RWF5	pp	229533.Q4I2D7

229533.I1RWF5	pp	229533.I1RX59
229533.I1RWF5	pp	229533.V6QU65
229533.I1RWF5	pp	229533.I1S048
229533.I1RWF5	pp	229533.I1RZK8
229533.I1RWF5	pp	229533.Q4IP51
229533.I1RWF5	pp	229533.I1RY16
229533.I1RWF5	pp	229533.I1S267
229533.I1RWF5	pp	229533.I1S2F7
229533.I1RWF5	pp	229533.I1RY12
229533.I1RWF5	pp	229533.I1RWL3
229533.I1RWF5	pp	229533.I1S210
229533.I1RWF5	pp	229533.I1S8R2
229533.I1RWF5	pp	229533.I1RYQ4
229533.I1RWF5	pp	229533.I1S1F3
229533.I1RWF5	pp	229533.I1S0V7
229533.I1RWF5	pp	229533.V6RHD0
229533.I1RWF5	pp	229533.I1S256
229533.I1RWF5	pp	229533.V6RM02
229533.I1RWF5	pp	229533.V6RV57
229533.I1RWF5	pp	229533.I1RZ25
229533.I1RWF5	pp	229533.I1RZ38
229533.I1RWF5	pp	229533.V6RLM7
229533.I1RWF5	pp	229533.I1S746
229533.I1RWF5	pp	229533.I1S147
229533.I1RWF5	pp	229533.V6RUY9
229533.I1RWF5	pp	229533.I1RYK0
229533.I1RWF5	pp	229533.I1RZA8
229533.I1RWF5	pp	229533.I1S299
229533.I1RWF5	pp	229533.I1S2C4
229533.I1RWF5	pp	229533.I1S694
229533.I1RWF5	pp	229533.I1RY62
229533.I1RWF5	pp	229533.I1S3T7
229533.I1RWF5	pp	229533.I1S0K9
229533.I1RWF5	pp	229533.V6RXR0
229533.A0A1C3YMW0	pp	229533.I1R9V7
229533.A0A1C3YMW0	pp	229533.I1RSC6
229533.A0A1C3YMW0	pp	229533.I1RY63
229533.A0A1C3YMW0	pp	229533.I1RSS4
229533.A0A1C3YMW0	pp	229533.I1RA48
229533.A0A1C3YMW0	pp	229533.I1RB92
229533.A0A1C3YMW0	pp	229533.I1RN99
229533.A0A1C3YMW0	pp	229533.I1RQ28
229533.A0A1C3YMW0	pp	229533.I1RK95
229533.A0A1C3YMW0	pp	229533.I1RFL4
229533.A0A1C3YMW0	pp	229533.I1RDG0
229533.A0A1C3YMW0	pp	229533.I1RSD8
229533.A0A1C3YMW0	pp	229533.I1RN18
229533.A0A1C3YMW0	pp	229533.I1RBL7
229533.A0A1C3YMW0	pp	229533.I1RCH5
229533.A0A1C3YMW0	pp	229533.I1RSR3
229533.A0A1C3YMW0	pp	229533.I1S2E8
229533.A0A1C3YMW0	pp	229533.I1S296
229533.A0A1C3YMW0	pp	229533.I1RQE7
229533.A0A1C3YMW0	pp	229533.I1RQA2
229533.A0A1C3YMW0	pp	229533.I1RAN5
229533.A0A1C3YMW0	pp	229533.I1RJV3
229533.A0A1C3YMW0	pp	229533.I1RCL3
229533.A0A1C3YMW0	pp	229533.I1RC06
229533.A0A1C3YMW0	pp	229533.I1RD17
229533.A0A1C3YMW0	pp	229533.I1RNK2
229533.A0A1C3YMW0	pp	229533.I1REK8
229533.A0A1C3YMW0	pp	229533.Q4IJ56
229533.A0A1C3YMW0	pp	229533.I1S0M9
229533.A0A1C3YMW0	pp	229533.I1RAZ8
229533.A0A1C3YMW0	pp	229533.I1RVZ0
229533.A0A1C3YMW0	pp	229533.I1S1J6
229533.A0A1C3YMW0	pp	229533.I1RNU6
229533.A0A1C3YMW0	pp	229533.I1R9X7
229533.A0A1C3YMW0	pp	229533.I1RS17
229533.A0A1C3YMW0	pp	229533.I1RKC6
229533.A0A1C3YMW0	pp	229533.Q4I5Z5
229533.A0A1C3YMW0	pp	229533.I1RB27
229533.A0A1C3YMW0	pp	229533.I1RS18

32924	229533.A0A1C3YMW0	pp	229533.I1RE01
32925	229533.A0A1C3YMW0	pp	229533.I1RW46
32926	229533.A0A1C3YMW0	pp	229533.I1S0M3
32927	229533.A0A1C3YMW0	pp	229533.I1RZE5
32928	229533.A0A1C3YMW0	pp	229533.I1RX14
32929	229533.A0A1C3YMW0	pp	229533.I1S0J1
32930	229533.A0A1C3YMW0	pp	229533.I1RF19
32931	229533.A0A1C3YMW0	pp	229533.I1RC72
32932	229533.A0A1C3YMW0	pp	229533.I1S8R3
32933	229533.A0A1C3YMW0	pp	229533.I1RCG4
32934	229533.A0A1C3YMW0	pp	229533.I1RZF6
32935	229533.A0A1C3YMW0	pp	229533.I1RTE9
32936	229533.A0A1C3YMW0	pp	229533.I1RRR7
32937	229533.A0A1C3YMW0	pp	229533.V6R227
32938	229533.A0A1C3YMW0	pp	229533.I1RMD5
32939	229533.A0A1C3YMW0	pp	229533.I1S2C0
32940	229533.A0A1C3YMW0	pp	229533.V6RIV9
32941	229533.A0A1C3YMW0	pp	229533.I1RTD9
32942	229533.A0A1C3YMW0	pp	229533.I1RZW8
32943	229533.A0A1C3YMW0	pp	229533.I1RRI2
32944	229533.A0A1C3YMW0	pp	229533.I1S4T3
32945	229533.A0A1C3YMW0	pp	229533.I1RG56
32946	229533.A0A1C3YMW0	pp	229533.I1RR24
32947	229533.A0A1C3YMW0	pp	229533.I1RPY1
32948	229533.A0A1C3YMW0	pp	229533.I1RC27
32949	229533.A0A1C3YMW0	pp	229533.V6RGN5
32950	229533.A0A1C3YMW0	pp	229533.I1RM96
32951	229533.A0A1C3YMW0	pp	229533.I1RD64
32952	229533.A0A1C3YMW0	pp	229533.Q4IEK8
32953	229533.A0A1C3YMW0	pp	229533.I1S2H4
32954	229533.A0A1C3YMW0	pp	229533.I1RPW9
32955	229533.A0A1C3YMW0	pp	229533.I1S044
32956	229533.A0A1C3YMW0	pp	229533.I1RC33
32957	229533.A0A1C3YMW0	pp	229533.V6RCZ8
32958	229533.A0A1C3YMW0	pp	229533.I1S0L4
32959	229533.A0A1C3YMW0	pp	229533.I1S5B0
32960	229533.A0A1C3YMW0	pp	229533.I1RW99
32961	229533.A0A1C3YMW0	pp	229533.I1RMN6
32962	229533.A0A1C3YMW0	pp	229533.I1S9U2
32963	229533.A0A1C3YMW0	pp	229533.I1RX08
32964	229533.A0A1C3YMW0	pp	229533.I1RSI6
32965	229533.A0A1C3YMW0	pp	229533.I1RGC2
32966	229533.A0A1C3YMW0	pp	229533.Q4IBS2
32967	229533.A0A1C3YMW0	pp	229533.I1RWJ8
32968	229533.A0A1C3YMW0	pp	229533.V6RCG5
32969	229533.A0A1C3YMW0	pp	229533.I1RPU0
32970	229533.A0A1C3YMW0	pp	229533.I1RNA0
32971	229533.A0A1C3YMW0	pp	229533.I1RQ31
32972	229533.A0A1C3YMW0	pp	229533.I1RKJ6
32973	229533.A0A1C3YMW0	pp	229533.V6QYF0
32974	229533.A0A1C3YMW0	pp	229533.I1RRT8
32975	229533.A0A1C3YMW0	pp	229533.I1RUG3
32976	229533.A0A1C3YMW0	pp	229533.I1RZM8
32977	229533.A0A1C3YMW0	pp	229533.I1RM06
32978	229533.A0A1C3YMW0	pp	229533.I1RZS6
32979	229533.A0A1C3YMW0	pp	229533.I1S5R1
32980	229533.A0A1C3YMW0	pp	229533.I1RNY2
32981	229533.A0A1C3YMW0	pp	229533.I1RM67
32982	229533.A0A1C3YMW0	pp	229533.I1RKL3
32983	229533.A0A1C3YMW0	pp	229533.Q4I327
32984	229533.A0A1C3YMW0	pp	229533.I1RW61
32985	229533.A0A1C3YMW0	pp	229533.I1RBC9
32986	229533.A0A1C3YMW0	pp	229533.I1RZ78
32987	229533.A0A1C3YMW0	pp	229533.I1RI82
32988	229533.A0A1C3YMW0	pp	229533.I1RW71
32989	229533.A0A1C3YMW0	pp	229533.I1RG51
32990	229533.A0A1C3YMW0	pp	229533.I1RR00
32991	229533.A0A1C3YMW0	pp	229533.I1RNH6
32992	229533.A0A1C3YMW0	pp	229533.I1S021
32993	229533.A0A1C3YMW0	pp	229533.I1RFS3
32994	229533.A0A1C3YMW0	pp	229533.I1RPM4
32995	229533.A0A1C3YMW0	pp	229533.I1RQJ5
32996	229533.A0A1C3YMW0	pp	229533.I1RVQ0

32997 229533.A0A1C3YMW0 pp 229533.Q4IFI0
32998 229533.A0A1C3YMW0 pp 229533.I1RRM5
32999 229533.A0A1C3YMW0 pp 229533.I1RPP3
33000 229533.A0A1C3YMW0 pp 229533.I1S0R7
33001 229533.A0A1C3YMW0 pp 229533.V6QW22
33002 229533.A0A1C3YMW0 pp 229533.I1RAC2
33003 229533.A0A1C3YMW0 pp 229533.I1RUQ2
33004 229533.A0A1C3YMW0 pp 229533.I1RRJ8
33005 229533.A0A1C3YMW0 pp 229533.I1RBY4
33006 229533.A0A1C3YMW0 pp 229533.I1RT98
33007 229533.A0A1C3YMW0 pp 229533.I1RW25
33008 229533.A0A1C3YMW0 pp 229533.I1RSQ7
33009 229533.A0A1C3YMW0 pp 229533.Q4INZ9
33010 229533.A0A1C3YMW0 pp 229533.I1RWK3
33011 229533.A0A1C3YMW0 pp 229533.I1RTF4
33012 229533.A0A1C3YMW0 pp 229533.I1RT80
33013 229533.A0A1C3YMW0 pp 229533.I1RZJ6
33014 229533.A0A1C3YMW0 pp 229533.I1RAF8
33015 229533.A0A1C3YMW0 pp 229533.I1RN63
33016 229533.A0A1C3YMW0 pp 229533.I1RT40
33017 229533.A0A1C3YMW0 pp 229533.I1RNV6
33018 229533.A0A1C3YMW0 pp 229533.V6R283
33019 229533.A0A1C3YMW0 pp 229533.I1RJU3
33020 229533.A0A1C3YMW0 pp 229533.I1RC03
33021 229533.A0A1C3YMW0 pp 229533.I1S8E2
33022 229533.A0A1C3YMW0 pp 229533.I1RFW5
33023 229533.A0A1C3YMW0 pp 229533.I1RBS6
33024 229533.A0A1C3YMW0 pp 229533.I1RBC7
33025 229533.A0A1C3YMW0 pp 229533.I1RD52
33026 229533.A0A1C3YMW0 pp 229533.I1RDA0
33027 229533.A0A1C3YMW0 pp 229533.I1RFN1
33028 229533.A0A1C3YMW0 pp 229533.I1RBN1
33029 229533.A0A1C3YMW0 pp 229533.I1RC82
33030 229533.A0A1C3YMW0 pp 229533.Q4IQW0
33031 229533.A0A1C3YMW0 pp 229533.I1RS47
33032 229533.A0A1C3YMW0 pp 229533.Q4HVW2
33033 229533.A0A1C3YMW0 pp 229533.I1RMK3
33034 229533.A0A1C3YMW0 pp 229533.Q4HZ42
33035 229533.A0A1C3YMW0 pp 229533.I1RJV4
33036 229533.A0A1C3YMW0 pp 229533.I1RLV9
33037 229533.A0A1C3YMW0 pp 229533.Q4IAS1
33038 229533.A0A1C3YMW0 pp 229533.I1RZZ9
33039 229533.A0A1C3YMW0 pp 229533.I1RBT1
33040 229533.A0A1C3YMW0 pp 229533.I1RT33
33041 229533.A0A1C3YMW0 pp 229533.I1RQ65
33042 229533.A0A1C3YMW0 pp 229533.I1RRT2
33043 229533.A0A1C3YMW0 pp 229533.I1RJW6
33044 229533.A0A1C3YMW0 pp 229533.I1S0L3
33045 229533.A0A1C3YMW0 pp 229533.I1RX46
33046 229533.A0A1C3YMW0 pp 229533.I1RJL4
33047 229533.A0A1C3YMW0 pp 229533.I1RS30
33048 229533.A0A1C3YMW0 pp 229533.I1RRQ3
33049 229533.A0A1C3YMW0 pp 229533.Q4IPI1
33050 229533.A0A1C3YMW0 pp 229533.A0A1C3YN63
33051 229533.I1RN54 pp 229533.I1RZ35
33052 229533.I1RN54 pp 229533.Q4IB89
33053 229533.I1RN54 pp 229533.I1RZQ6
33054 229533.I1RN54 pp 229533.V6QWB1
33055 229533.I1RN54 pp 229533.I1RXB5
33056 229533.I1RN54 pp 229533.I1RSW5
33057 229533.I1RN54 pp 229533.I1RWG6
33058 229533.I1RN54 pp 229533.V6RDZ4
33059 229533.I1RN54 pp 229533.I1RZT1
33060 229533.I1RN54 pp 229533.Q4I7L0
33061 229533.A0A1C3YJG4 pp 229533.Q4ICA8
33062 229533.A0A1C3YJG4 pp 229533.I1S0Z3
33063 229533.A0A1C3YJG4 pp 229533.I1RQP3
33064 229533.A0A1C3YJG4 pp 229533.I1RT41
33065 229533.A0A1C3YJG4 pp 229533.I1RQP1
33066 229533.A0A1C3YJG4 pp 229533.I1RRF0
33067 229533.A0A1C3YJG4 pp 229533.Q4IPI4
33068 229533.A0A0E0RQK8 pp 229533.A0A0E0SA11
33069 229533.A0A0E0RQK8 pp 229533.I1RBW0

33070	229533.A0A0E0RQK8	pp	229533.A0A1C3YI64
33071	229533.A0A0E0RQK8	pp	229533.A0A1C3YLR4
33072	229533.A0A0E0RQK8	pp	229533.I1RRT1
33073	229533.A0A0E0RQK8	pp	229533.I1RZ94
33074	229533.A0A0E0RQK8	pp	229533.I1RD01
33075	229533.A0A0E0RQK8	pp	229533.I1S1W8
33076	229533.A0A0E0RQK8	pp	229533.A0A1C3YI40
33077	229533.A0A0E0RQK8	pp	229533.I1RQH8
33078	229533.A0A0E0RQK8	pp	229533.I1RA12
33079	229533.A0A0E0RQK8	pp	229533.I1RWT6
33080	229533.A0A0E0RQK8	pp	229533.A0A1C3YJN7
33081	229533.A0A0E0RQK8	pp	229533.I1RQA3
33082	229533.A0A0E0RQK8	pp	229533.I1RG77
33083	229533.A0A0E0RQK8	pp	229533.I1RN55
33084	229533.A0A0E0RQK8	pp	229533.A0A1C3YL55
33085	229533.A0A0E0RQK8	pp	229533.A0A1C3YKE4
33086	229533.A0A0E0RQK8	pp	229533.I1RYQ1
33087	229533.A0A0E0RQK8	pp	229533.I1RPQ1
33088	229533.A0A0E0RQK8	pp	229533.I1RWI8
33089	229533.A0A0E0RQK8	pp	229533.I1RV91
33090	229533.A0A0E0RQK8	pp	229533.I1S2E9
33091	229533.A0A0E0RQK8	pp	229533.I1RSN7
33092	229533.A0A0E0RQK8	pp	229533.I1RY29
33093	229533.A0A0E0RQK8	pp	229533.A0A1C3YMJ3
33094	229533.A0A0E0RQK8	pp	229533.I1RBW1
33095	229533.A0A0E0RQK8	pp	229533.I1RN57
33096	229533.A0A0E0RQK8	pp	229533.I1S3G4
33097	229533.A0A0E0RQK8	pp	229533.A0A1C3YN69
33098	229533.A0A0E0RQK8	pp	229533.I1RBU5
33099	229533.A0A0E0RQK8	pp	229533.A0A1C3YLB6
33100	229533.A0A0E0RQK8	pp	229533.Q4IB89
33101	229533.A0A0E0RQK8	pp	229533.I1RCA5
33102	229533.A0A0E0RQK8	pp	229533.I1RZ86
33103	229533.A0A0E0RQK8	pp	229533.I1RSR9
33104	229533.A0A0E0RQK8	pp	229533.I1RSP7
33105	229533.A0A0E0RQK8	pp	229533.I1RSD0
33106	229533.A0A0E0RQK8	pp	229533.I1RBC8
33107	229533.A0A0E0RQK8	pp	229533.I1RZP8
33108	229533.A0A0E0RQK8	pp	229533.I1RC54
33109	229533.A0A0E0RQK8	pp	229533.A0A1C3YKS0
33110	229533.A0A0E0RQK8	pp	229533.I1RX64
33111	229533.A0A0E0RQK8	pp	229533.I1RSC7
33112	229533.A0A0E0RQK8	pp	229533.I1RBN9
33113	229533.A0A0E0RQK8	pp	229533.A0A0E0RW49
33114	229533.A0A0E0RQK8	pp	229533.I1RY44
33115	229533.A0A0E0RQK8	pp	229533.I1S0A6
33116	229533.A0A0E0RQK8	pp	229533.I1RDH0
33117	229533.A0A0E0RQK8	pp	229533.I1S4P6
33118	229533.A0A0E0RQK8	pp	229533.I1RDJ8
33119	229533.A0A0E0RQK8	pp	229533.I1RA10
33120	229533.A0A1C3YJG1	pp	229533.A0A1C3YKV0
33121	229533.A0A098DT74	pp	229533.I1RYZ0
33122	229533.A0A098DT74	pp	229533.A0A1C3YJF1
33123	229533.A0A098DT74	pp	229533.I1RN65
33124	229533.A0A098DT74	pp	229533.A0A0E0RVZ1
33125	229533.A0A098DT74	pp	229533.I1RQA3
33126	229533.A0A098DT74	pp	229533.I1RKK0
33127	229533.A0A098DT74	pp	229533.I1RC17
33128	229533.A0A098DT74	pp	229533.I1RC95
33129	229533.A0A098DT74	pp	229533.A0A1C3YK37
33130	229533.A0A098DT74	pp	229533.I1RCB3
33131	229533.A0A098DT74	pp	229533.A0A098E0E1
33132	229533.A0A098DT74	pp	229533.A0A1C3YJV7
33133	229533.A0A098DT74	pp	229533.A0A1C3YKF2
33134	229533.A0A098DT74	pp	229533.I1RMM9
33135	229533.A0A098DT74	pp	229533.I1RX70
33136	229533.A0A098DT74	pp	229533.I1RM09
33137	229533.A0A098DT74	pp	229533.I1RN08
33138	229533.A0A098DT74	pp	229533.I1REE1
33139	229533.A0A098DT74	pp	229533.A0A0E0RTV8
33140	229533.A0A098DT74	pp	229533.I1RDB1
33141	229533.A0A098DT74	pp	229533.I1RC22
33142	229533.A0A098DT74	pp	229533.A0A0E0SA11

33143	229533.A0A098DT74	pp	229533.I1RWJ6
33144	229533.A0A098DT74	pp	229533.I1RUQ5
33145	229533.A0A098DT74	pp	229533.I1RAM6
33146	229533.A0A098DT74	pp	229533.A0A0E0SD93
33147	229533.A0A098DT74	pp	229533.I1RNC0
33148	229533.A0A098DT74	pp	229533.I1RWT6
33149	229533.A0A098DT74	pp	229533.I1RYY5
33150	229533.A0A098DT74	pp	229533.I1RZH4
33151	229533.A0A098DT74	pp	229533.I1RB75
33152	229533.A0A098DT74	pp	229533.I1RAM7
33153	229533.A0A098DT74	pp	229533.I1RD18
33154	229533.A0A098DT74	pp	229533.A0A0E0SAC4
33155	229533.A0A098DT74	pp	229533.Q4IA56
33156	229533.A0A098DT74	pp	229533.I1S439
33157	229533.A0A098DT74	pp	229533.I1S224
33158	229533.A0A098DT74	pp	229533.I1RV66
33159	229533.A0A098DT74	pp	229533.I1RBZ9
33160	229533.A0A098DT74	pp	229533.A0A1C3YIL0
33161	229533.A0A098DT74	pp	229533.A0A1C3YJH8
33162	229533.A0A098DT74	pp	229533.I1RRV1
33163	229533.A0A098DT74	pp	229533.I1RXH6
33164	229533.A0A098DT74	pp	229533.I1RQ26
33165	229533.A0A098DT74	pp	229533.A0A1C3YKW5
33166	229533.A0A098DT74	pp	229533.I1RYH9
33167	229533.A0A098DT74	pp	229533.A0A1C3YI28
33168	229533.A0A098DT74	pp	229533.I1S0M2
33169	229533.A0A098DT74	pp	229533.I1RJH1
33170	229533.A0A098DT74	pp	229533.I1RQB4
33171	229533.A0A098DT74	pp	229533.I1RL24
33172	229533.A0A098DT74	pp	229533.V6QV09
33173	229533.A0A098DT74	pp	229533.I1RYZ9
33174	229533.A0A098DT74	pp	229533.I1RRW0
33175	229533.A0A098DT74	pp	229533.A0A0E0RS85
33176	229533.A0A098DT74	pp	229533.I1S1W4
33177	229533.A0A098DT74	pp	229533.I1RAW7
33178	229533.A0A098DT74	pp	229533.V6RKY7
33179	229533.A0A098DT74	pp	229533.I1RRY3
33180	229533.A0A098DT74	pp	229533.I1RJV3
33181	229533.A0A098DT74	pp	229533.I1RY44
33182	229533.A0A098DT74	pp	229533.A0A1C3YN39
33183	229533.A0A098DT74	pp	229533.A0A1C3YHL0
33184	229533.A0A098DT74	pp	229533.A0A098E4P3
33185	229533.A0A098DT74	pp	229533.I1S549
33186	229533.A0A098DT74	pp	229533.I1RAY4
33187	229533.A0A098DT74	pp	229533.I1RQ72
33188	229533.A0A098DT74	pp	229533.I1RMF5
33189	229533.A0A098DT74	pp	229533.I1RJ88
33190	229533.A0A098DT74	pp	229533.V6RVG3
33191	229533.A0A098DT74	pp	229533.I1S216
33192	229533.A0A098DT74	pp	229533.I1S0D1
33193	229533.A0A098DT74	pp	229533.I1RB41
33194	229533.A0A098DT74	pp	229533.I1RUN8
33195	229533.A0A098DT74	pp	229533.I1RNW8
33196	229533.A0A098DT74	pp	229533.I1S1D3
33197	229533.A0A098DT74	pp	229533.A0A1C3YJI8
33198	229533.A0A098DT74	pp	229533.I1RBV5
33199	229533.A0A098DT74	pp	229533.I1R9Y7
33200	229533.A0A098DT74	pp	229533.I1S2E9
33201	229533.A0A098DT74	pp	229533.I1RHQ8
33202	229533.A0A098DT74	pp	229533.I1RV91
33203	229533.A0A098DT74	pp	229533.I1RA43
33204	229533.A0A098DT74	pp	229533.I1RNY8
33205	229533.A0A098DT74	pp	229533.I1RNY9
33206	229533.A0A098DT74	pp	229533.I1R9Y9
33207	229533.A0A098DT74	pp	229533.I1RDZ4
33208	229533.A0A098DT74	pp	229533.I1RC97
33209	229533.A0A098DT74	pp	229533.I1S2K7
33210	229533.A0A098DT74	pp	229533.I1RZE4
33211	229533.A0A098DT74	pp	229533.I1RAS9
33212	229533.A0A098DT74	pp	229533.I1RCL8
33213	229533.A0A098DT74	pp	229533.I1S1V5
33214	229533.A0A098DT74	pp	229533.A0A0E0RP92
33215	229533.A0A098DT74	pp	229533.I1RNF4

33216	229533.A0A098DT74	pp	229533.I1RT36
33217	229533.A0A098DT74	pp	229533.I1RP28
33218	229533.A0A098DT74	pp	229533.I1RBS0
33219	229533.A0A098DT74	pp	229533.I1RRM9
33220	229533.A0A098DT74	pp	229533.I1RNP7
33221	229533.A0A098DT74	pp	229533.V6RGI1
33222	229533.A0A098DT74	pp	229533.I1RLU1
33223	229533.A0A098DT74	pp	229533.I1RPD6
33224	229533.A0A098DT74	pp	229533.I1RBM3
33225	229533.A0A098DT74	pp	229533.I1RB74
33226	229533.A0A098DT74	pp	229533.I1RQG4
33227	229533.A0A098DT74	pp	229533.I1RFL7
33228	229533.A0A098DT74	pp	229533.I1RAN2
33229	229533.A0A098DT74	pp	229533.I1RWI5
33230	229533.I1RAS9	pp	229533.I1RXT2
33231	229533.I1RAS9	pp	229533.I1RYU1
33232	229533.I1RAS9	pp	229533.I1RW76
33233	229533.I1RAS9	pp	229533.I1RXU1
33234	229533.I1RAS9	pp	229533.I1RG99
33235	229533.I1RAS9	pp	229533.I1RTD0
33236	229533.I1RAS9	pp	229533.I1RWP9
33237	229533.I1RAS9	pp	229533.I1RC97
33238	229533.I1RAS9	pp	229533.I1RE15
33239	229533.I1RAS9	pp	229533.V6RKE4
33240	229533.I1RAS9	pp	229533.I1REK1
33241	229533.I1RAS9	pp	229533.I1RX87
33242	229533.I1RAS9	pp	229533.I1RE20
33243	229533.I1RAS9	pp	229533.I1REC4
33244	229533.I1RAS9	pp	229533.I1RNK3
33245	229533.I1RAS9	pp	229533.I1RYY6
33246	229533.I1RAS9	pp	229533.I1RBA6
33247	229533.I1RAS9	pp	229533.V6RQ48
33248	229533.I1RAS9	pp	229533.I1S2W9
33249	229533.I1RAS9	pp	229533.Q4I1H6
33250	229533.I1RAS9	pp	229533.I1RPL2
33251	229533.I1RAS9	pp	229533.I1RD63
33252	229533.I1RAS9	pp	229533.I1RPK5
33253	229533.I1RAS9	pp	229533.I1RMM9
33254	229533.I1RAS9	pp	229533.I1RRY3
33255	229533.I1RAS9	pp	229533.I1RKK0
33256	229533.I1RAS9	pp	229533.I1RXF5
33257	229533.I1RAS9	pp	229533.I1RZA4
33258	229533.I1RAS9	pp	229533.I1RW84
33259	229533.I1RAS9	pp	229533.I1S0M2
33260	229533.I1RAS9	pp	229533.I1RYW3
33261	229533.I1RAS9	pp	229533.I1RN08
33262	229533.I1RAS9	pp	229533.I1S278
33263	229533.I1RAS9	pp	229533.I1RC52
33264	229533.I1RUK7	pp	229533.I1S0K9
33265	229533.I1RUK7	pp	229533.Q5I2J3
33266	229533.I1RUK7	pp	229533.Q4HZS8
33267	229533.I1RUK7	pp	229533.I1S7X9
33268	229533.I1RCZ8	pp	229533.I1RQI4
33269	229533.I1RCZ8	pp	229533.I1RP86
33270	229533.I1RCZ8	pp	229533.I1RR44
33271	229533.I1RCZ8	pp	229533.I1RGY2
33272	229533.I1RCZ8	pp	229533.I1RUY5
33273	229533.I1RCZ8	pp	229533.I1RGB2
33274	229533.I1RCZ8	pp	229533.I1RJQ5
33275	229533.I1RN65	pp	229533.I1RNB8
33276	229533.I1RN65	pp	229533.I1S0D1
33277	229533.I1RN65	pp	229533.I1RZS7
33278	229533.I1RN65	pp	229533.V6RKY7
33279	229533.I1RN65	pp	229533.I1RW16
33280	229533.I1RN65	pp	229533.I1S1W4
33281	229533.I1RN65	pp	229533.V6RVG3
33282	229533.I1RN65	pp	229533.I1RY44
33283	229533.I1RN65	pp	229533.I1RXT9
33284	229533.I1RN65	pp	229533.V6RXB3
33285	229533.I1RN65	pp	229533.I1S0M2
33286	229533.I1RN65	pp	229533.I1RSB8
33287	229533.I1RN65	pp	229533.I1RT36
33288	229533.I1RN65	pp	229533.Q4IER0

229533.I1RN65 pp 229533.I1RZE4
229533.I1RN65 pp 229533.V6RGI1
229533.I1RN65 pp 229533.I1RSF2
229533.I1RN65 pp 229533.Q4INW6
229533.I1RN65 pp 229533.I1RV91
229533.I1RN65 pp 229533.I1RZG0
229533.I1RN65 pp 229533.I1S2E9
229533.I1RN65 pp 229533.I1RPS0
229533.I1RN65 pp 229533.I1RTE6
229533.I1RN65 pp 229533.Q4ICG8
229533.I1RN65 pp 229533.I1RUN8
229533.I1RN65 pp 229533.I1RQN9
229533.I1RN65 pp 229533.I1S4R1
229533.I1RN65 pp 229533.I1RRY3
229533.I1RN65 pp 229533.I1RPP7
229533.I1RN65 pp 229533.I1RQ32
229533.I1RN65 pp 229533.I1S7X9
229533.I1RN65 pp 229533.Q4I0M9
229533.I1RN65 pp 229533.I1RS52
229533.I1RN65 pp 229533.V6RLW4
229533.I1RN65 pp 229533.I1RZH4
229533.I1RN65 pp 229533.P0C431
229533.I1RN65 pp 229533.I1RWT6
229533.I1RN65 pp 229533.I1RUQ5
229533.I1RN65 pp 229533.I1RY09
229533.I1RN65 pp 229533.I1S439
229533.I1RN65 pp 229533.V6REE9
229533.I1RN65 pp 229533.Q4I5I4
229533.I1RN65 pp 229533.I1RXU7
229533.I1RN65 pp 229533.I1RPU4
229533.I1RN65 pp 229533.I1RNY7
229533.I1RN65 pp 229533.I1RQA3
229533.I1RN65 pp 229533.I1RZB5
229533.I1RN65 pp 229533.I1RQC9
229533.I1RN65 pp 229533.I1RQ26
229533.I1RN65 pp 229533.Q4IQC1
229533.I1RN65 pp 229533.I1RX46
229533.I1RN65 pp 229533.I1S0S6
229533.I1RN65 pp 229533.V6QV09
229533.I1RN65 pp 229533.I1RYZ9
229533.I1RN65 pp 229533.I1RUG2
229533.I1RN65 pp 229533.I1RW71
229533.I1RN65 pp 229533.I1RYH9
229533.I1RN65 pp 229533.I1S2B7
229533.I1RN65 pp 229533.I1S6F1
229533.I1RN65 pp 229533.I1S050
229533.I1RN65 pp 229533.I1RTB7
229533.I1RN65 pp 229533.I1RZ02
229533.I1RN65 pp 229533.I1RS37
229533.I1RZU6 pp 229533.Q4HZS8
229533.I1RZU6 pp 229533.Q5I2J3
229533.I1RZU6 pp 229533.I1S755
229533.A0A1C3YJX0 pp 229533.I1RQA3
229533.A0A1C3YJX0 pp 229533.I1RQM2
229533.A0A1C3YJX0 pp 229533.I1RV91
229533.A0A1C3YJX0 pp 229533.I1S2E9
229533.A0A1C3YJX0 pp 229533.I1RC17
229533.A0A1C3YJX0 pp 229533.I1RFZ3
229533.A0A1C3YJX0 pp 229533.I1RM09
229533.A0A1C3YJX0 pp 229533.I1RWT6
229533.A0A1C3YJX0 pp 229533.I1RY44
229533.A0A1C3YJX0 pp 229533.Q4HXF0
229533.A0A1C3YJX0 pp 229533.I1RQM0
229533.A0A1C3YJX0 pp 229533.I1RB41
229533.A0A1C3YJX0 pp 229533.I1RRN5
229533.A0A1C3YJX0 pp 229533.A0A1C3YM29
229533.A0A1C3YJX0 pp 229533.I1S549
229533.I1S148 pp 229533.V6RH92
229533.I1S148 pp 229533.V6RPS5
229533.I1S148 pp 229533.I1S8D1
229533.I1S148 pp 229533.I1S8D2
229533.I1S148 pp 229533.I1S149
229533.I1RR39 pp 229533.I1RU29

33362 229533.I1RR39 pp 229533.I1S569
33363 229533.I1RR39 pp 229533.Q4HZS8
33364 229533.I1RR39 pp 229533.I1S574
33365 229533.I1RR39 pp 229533.I1RW07
33366 229533.I1RR39 pp 229533.I1RRT3
33367 229533.I1RR39 pp 229533.I1S984
33368 229533.I1RR39 pp 229533.I1RR95
33369 229533.I1RR39 pp 229533.I1RZ13
33370 229533.I1RR39 pp 229533.I1RRQ1
33371 229533.I1RR39 pp 229533.I1S272
33372 229533.I1RR39 pp 229533.I1S352
33373 229533.I1RR39 pp 229533.V6R4J1
33374 229533.A0A098DXD6 pp 229533.I1RV06
33375 229533.A0A098DXD6 pp 229533.V6QTX7
33376 229533.A0A098DXD6 pp 229533.V6R0W1
33377 229533.A0A098DXD6 pp 229533.A0A1C3YN26
33378 229533.A0A098DXD6 pp 229533.I1RGC3
33379 229533.A0A098DXD6 pp 229533.V6R5D2
33380 229533.A0A098DXD6 pp 229533.I1RDN3
33381 229533.A0A098DXD6 pp 229533.I1RBY4
33382 229533.A0A098DXD6 pp 229533.I1RJS7
33383 229533.A0A098DXD6 pp 229533.I1RN88
33384 229533.A0A098DXD6 pp 229533.I1RNF4
33385 229533.A0A098DXD6 pp 229533.I1SAI4
33386 229533.A0A098DXD6 pp 229533.A0A1C3YKN4
33387 229533.A0A098DXD6 pp 229533.I1RNG4
33388 229533.A0A098DXD6 pp 229533.A0A1C3YJ56
33389 229533.A0A098DXD6 pp 229533.A0A098E525
33390 229533.A0A098DXD6 pp 229533.V6QU99
33391 229533.A0A098DXD6 pp 229533.I1S8C1
33392 229533.A0A098DXD6 pp 229533.I1S148
33393 229533.A0A098DXD6 pp 229533.I1REE6
33394 229533.A0A098DXD6 pp 229533.V6REA4
33395 229533.A0A098DXD6 pp 229533.I1RP86
33396 229533.A0A098DXD6 pp 229533.I1RZV5
33397 229533.A0A098DXD6 pp 229533.V6R2Z2
33398 229533.A0A098DXD6 pp 229533.I1RC00
33399 229533.A0A098DXD6 pp 229533.A0A1C3YKB7
33400 229533.A0A098DXD6 pp 229533.I1RUY5
33401 229533.A0A098DXD6 pp 229533.A0A1C3YLE2
33402 229533.A0A098DXD6 pp 229533.V6RAM6
33403 229533.A0A098DXD6 pp 229533.V6R0H4
33404 229533.A0A098DXD6 pp 229533.V6R556
33405 229533.A0A098DXD6 pp 229533.I1S405
33406 229533.A0A098DXD6 pp 229533.V6R756
33407 229533.A0A098DXD6 pp 229533.A0A0E0S2K9
33408 229533.A0A098DXD6 pp 229533.I1S147
33409 229533.A0A098DXD6 pp 229533.A0A1C3YIP2
33410 229533.A0A098DXD6 pp 229533.A0A1C3YL54
33411 229533.A0A098DXD6 pp 229533.I1RCG5
33412 229533.A0A098DXD6 pp 229533.A0A0E0RQ72
33413 229533.A0A098DXD6 pp 229533.Q4HTT2
33414 229533.A0A098DXD6 pp 229533.I1S269
33415 229533.A0A098DXD6 pp 229533.I1S149
33416 229533.A0A098DXD6 pp 229533.V6RM09
33417 229533.A0A098DXD6 pp 229533.V6RPS5
33418 229533.A0A098DXD6 pp 229533.I1RJY7
33419 229533.A0A098DXD6 pp 229533.I1RA13
33420 229533.A0A098DXD6 pp 229533.A0A1C3YKH5
33421 229533.A0A098DXD6 pp 229533.A0A1C3YIR5
33422 229533.A0A098DXD6 pp 229533.I1RQI4
33423 229533.A0A098DXD6 pp 229533.I1RDU0
33424 229533.A0A098DXD6 pp 229533.I1S0H2
33425 229533.A0A098DXD6 pp 229533.I1RGB2
33426 229533.A0A098DXD6 pp 229533.I1RWE1
33427 229533.A0A098DXD6 pp 229533.I1RR44
33428 229533.A0A0E0RMM5 pp 229533.I1S0D5
33429 229533.A0A0E0RMM5 pp 229533.I1RS18
33430 229533.A0A0E0RMM5 pp 229533.I1RRN5
33431 229533.A0A0E0RMM5 pp 229533.A0A1C3YN69
33432 229533.A0A0E0RMM5 pp 229533.I1RA43
33433 229533.A0A0E0RMM5 pp 229533.I1RA38
33434 229533.A0A0E0RMM5 pp 229533.I1RP99

33435 229533.A0A0E0RMM5 pp 229533.I1RYP7
33436 229533.A0A0E0RMM5 pp 229533.I1RQA3
33437 229533.A0A0E0RMM5 pp 229533.A0A1C3YJ79
33438 229533.A0A0E0RMM5 pp 229533.I1RZ98
33439 229533.A0A0E0RMM5 pp 229533.I1RWT6
33440 229533.A0A0E0RMM5 pp 229533.I1RSC7
33441 229533.A0A0E0RMM5 pp 229533.I1RCB3
33442 229533.A0A0E0RMM5 pp 229533.I1S044
33443 229533.A0A0E0RMM5 pp 229533.A0A0E0RW49
33444 229533.A0A0E0RMM5 pp 229533.I1RBC1
33445 229533.A0A0E0RMM5 pp 229533.I1RB41
33446 229533.A0A0E0RMM5 pp 229533.I1RKJ8
33447 229533.A0A0E0RMM5 pp 229533.I1RTN6
33448 229533.A0A0E0RMM5 pp 229533.I1RY44
33449 229533.A0A0E0RMM5 pp 229533.Q4IJ84
33450 229533.A0A0E0RMM5 pp 229533.A0A1C3YK66
33451 229533.A0A0E0RMM5 pp 229533.I1RBN9
33452 229533.A0A0E0RMM5 pp 229533.I1RW03
33453 229533.A0A0E0RMM5 pp 229533.I1RC54
33454 229533.A0A0E0RMM5 pp 229533.I1RC17
33455 229533.A0A0E0RMM5 pp 229533.I1RBC8
33456 229533.A0A0E0RMM5 pp 229533.Q4IBS2
33457 229533.A0A0E0RMM5 pp 229533.A0A1C3YN55
33458 229533.A0A0E0RMM5 pp 229533.I1RQH8
33459 229533.A0A0E0RMM5 pp 229533.I1RQD1
33460 229533.A0A0E0RMM5 pp 229533.A0A1C3YHM3
33461 229533.A0A0E0RMM5 pp 229533.I1RZ63
33462 229533.A0A0E0RMM5 pp 229533.A0A0E0SA11
33463 229533.A0A0E0RMM5 pp 229533.I1RZ94
33464 229533.A0A0E0RMM5 pp 229533.Q4INS6
33465 229533.A0A0E0RMM5 pp 229533.I1RZ86
33466 229533.A0A0E0RMM5 pp 229533.I1S2E9
33467 229533.A0A0E0RMM5 pp 229533.I1RDJ1
33468 229533.A0A0E0RMM5 pp 229533.I1RSD0
33469 229533.A0A0E0RMM5 pp 229533.A0A1C3YLQ7
33470 229533.A0A0E0RMM5 pp 229533.I1RA40
33471 229533.A0A0E0RMM5 pp 229533.I1RDC0
33472 229533.A0A0E0RMM5 pp 229533.I1RSN5
33473 229533.A0A0E0RMM5 pp 229533.I1S2H4
33474 229533.A0A0E0RMM5 pp 229533.I1RPM4
33475 229533.A0A0E0RMM5 pp 229533.I1RY29
33476 229533.A0A0E0RMM5 pp 229533.I1R9N6
33477 229533.A0A0E0RMM5 pp 229533.I1RRT1
33478 229533.A0A0E0RMM5 pp 229533.I1RUR0
33479 229533.A0A0E0RMM5 pp 229533.I1RWI8
33480 229533.A0A0E0RMM5 pp 229533.A0A1C3YJH8
33481 229533.A0A0E0RMM5 pp 229533.A0A1C3YL55
33482 229533.A0A0E0RMM5 pp 229533.I1RCQ4
33483 229533.A0A0E0RMM5 pp 229533.I1RW24
33484 229533.A0A0E0RMM5 pp 229533.I1S998
33485 229533.A0A0E0RMM5 pp 229533.I1RSJ1
33486 229533.A0A0E0RMM5 pp 229533.I1RVW0
33487 229533.A0A0E0RMM5 pp 229533.A0A1C3YK82
33488 229533.A0A0E0RMM5 pp 229533.A0A1C3YK00
33489 229533.A0A0E0RMM5 pp 229533.A0A1C3YMJ3
33490 229533.A0A0E0RMM5 pp 229533.I1RAL9
33491 229533.A0A0E0RMM5 pp 229533.I1RXF8
33492 229533.A0A0E0RMM5 pp 229533.I1RW83
33493 229533.A0A0E0RMM5 pp 229533.I1RF02
33494 229533.A0A0E0RMM5 pp 229533.A0A1C3YJX0
33495 229533.A0A0E0RMM5 pp 229533.A0A1C3YNC3
33496 229533.A0A0E0RMM5 pp 229533.I1S987
33497 229533.A0A0E0RMM5 pp 229533.I1RV91
33498 229533.A0A0E0RMM5 pp 229533.I1RWM6
33499 229533.A0A0E0RMM5 pp 229533.I1RYQ1
33500 229533.A0A0E0RMM5 pp 229533.I1RRY1
33501 229533.A0A0E0RMM5 pp 229533.A0A1C3YJR8
33502 229533.I1RF19 pp 229533.I1S504
33503 229533.I1RF19 pp 229533.V6R227
33504 229533.I1RF19 pp 229533.I1SA32
33505 229533.I1RF19 pp 229533.I1S419
33506 229533.I1RF19 pp 229533.I1RRR7
33507 229533.I1RF19 pp 229533.I1RWN5

229533.I1RF19	pp	229533.I1RPY1
229533.I1RF19	pp	229533.V6RIV9
229533.I1RF19	pp	229533.I1RNQ5
229533.I1RF19	pp	229533.Q4IEK8
229533.I1RF19	pp	229533.I1S4T3
229533.I1RF19	pp	229533.I1SA33
229533.I1RF19	pp	229533.I1RSI6
229533.I1RF19	pp	229533.Q4IBS2
229533.I1RF19	pp	229533.V6RCZ8
229533.I1RF19	pp	229533.I1RMN6
229533.I1RF19	pp	229533.I1RSS4
229533.I1RF19	pp	229533.I1RSQ3
229533.I1RF19	pp	229533.V6R042
229533.I1RF19	pp	229533.Q4IJ56
229533.I1RF19	pp	229533.I1RS17
229533.I1RF19	pp	229533.I1RT03
229533.I1RF19	pp	229533.I1RVX0
229533.I1RF19	pp	229533.V6RCG5
229533.I1RF19	pp	229533.I1RS18
229533.I1RF19	pp	229533.V6RMW6
229533.I1RF19	pp	229533.I1RY63
229533.I1RF19	pp	229533.I1RN99
229533.I1RF19	pp	229533.Q4I8P5
229533.I1RF19	pp	229533.Q4IAA0
229533.I1RF19	pp	229533.I1RR51
229533.I1RF19	pp	229533.Q4IJK2
229533.I1RF19	pp	229533.I1RW46
229533.I1RF19	pp	229533.I1RFL4
229533.I1RF19	pp	229533.I1RZM8
229533.I1RF19	pp	229533.Q4IN63
229533.I1RF19	pp	229533.I1RN18
229533.I1RF19	pp	229533.I1RMC0
229533.I1RF19	pp	229533.I1S2E8
229533.I1RF19	pp	229533.I1S0J1
229533.I1RF19	pp	229533.I1RZE5
229533.I1RF19	pp	229533.I1RQE7
229533.I1RF19	pp	229533.I1RNC1
229533.I1RF19	pp	229533.I1RJZ6
229533.I1RF19	pp	229533.I1RUG3
229533.I1RF19	pp	229533.I1RQ31
229533.I1RF19	pp	229533.I1RZZ1
229533.I1RF19	pp	229533.I1RTH6
229533.I1RF19	pp	229533.I1RNH6
229533.I1RF19	pp	229533.Q4I624
229533.I1RF19	pp	229533.I1RZB5
229533.I1RF19	pp	229533.I1S8R3
229533.I1RF19	pp	229533.Q4HZ68
229533.I1RF19	pp	229533.I1RI82
229533.I1RF19	pp	229533.Q4I327
229533.I1RF19	pp	229533.I1RFU8
229533.I1RF19	pp	229533.I1RTE9
229533.I1RF19	pp	229533.I1RPJ3
229533.I1RF19	pp	229533.I1RQ72
229533.I1RF19	pp	229533.I1S2C0
229533.I1RF19	pp	229533.I1RW99
229533.I1RF19	pp	229533.I1RKC6
229533.I1RF19	pp	229533.I1S1J6
229533.I1RF19	pp	229533.V6RGN5
229533.I1RF19	pp	229533.I1RSR8
229533.I1RF19	pp	229533.Q4I5Z5
229533.I1RF19	pp	229533.I1RZK5
229533.I1RF19	pp	229533.I1RNK2
229533.I1RF19	pp	229533.I1S5B0
229533.I1RF19	pp	229533.V6QW22
229533.I1RF19	pp	229533.I1RVZ0
229533.I1RF19	pp	229533.I1RY03
229533.I1RF19	pp	229533.I1RFJ9
229533.I1RF19	pp	229533.I1RRJ8
229533.I1RF19	pp	229533.I1RTD9
229533.I1RF19	pp	229533.I1S1C7
229533.I1RF19	pp	229533.I1RV91
229533.I1RF19	pp	229533.I1RTD5
229533.I1RF19	pp	229533.I1RSQ7

229533.I1RF19	pp	229533.I1RMD5
229533.I1RF19	pp	229533.I1RFN1
229533.I1RF19	pp	229533.I1RGA4
229533.I1RF19	pp	229533.I1RPP3
229533.I1RF19	pp	229533.I1RRI2
229533.I1RF19	pp	229533.I1RZW8
229533.I1RF19	pp	229533.I1S1B6
229533.I1RF19	pp	229533.V6R283
229533.I1RF19	pp	229533.I1RSR3
229533.I1RF19	pp	229533.I1RJV4
229533.I1RF19	pp	229533.I1RWN6
229533.I1RF19	pp	229533.I1RWK3
229533.I1RF19	pp	229533.I1RRY9
229533.I1RF19	pp	229533.I1RN63
229533.I1RF19	pp	229533.Q4IQW0
229533.I1RF19	pp	229533.V6RTJ8
229533.I1RF19	pp	229533.I1S2M6
229533.I1RF19	pp	229533.I1RFW5
229533.I1RF19	pp	229533.I1RQA2
229533.I1RF19	pp	229533.I1S558
229533.I1RF19	pp	229533.Q4HZ42
229533.I1RF19	pp	229533.I1RJ74
229533.I1RF19	pp	229533.I1RJV3
229533.I1RF19	pp	229533.I1RJW6
229533.I1RF19	pp	229533.I1S503
229533.I1RF19	pp	229533.I1S1J7
229533.I1RF19	pp	229533.I1RS47
229533.I1RF19	pp	229533.I1RT33
229533.I1RF19	pp	229533.Q4I830
229533.I1RF19	pp	229533.I1RSX1
229533.I1RF19	pp	229533.I1S0L3
229533.I1RF19	pp	229533.I1RLV9
229533.I1RF19	pp	229533.I1RRZ0
229533.I1RF19	pp	229533.I1RQ65
229533.I1RF19	pp	229533.Q4IFI0
229533.I1RF19	pp	229533.Q4IAS1
229533.I1RF19	pp	229533.I1S0F6
229533.I1RF19	pp	229533.I1RJL4
229533.I1RF19	pp	229533.I1RN51
229533.I1RF19	pp	229533.Q4IPI1
229533.I1RF19	pp	229533.I1RN67
229533.I1RF19	pp	229533.I1RRM5
229533.I1RF19	pp	229533.I1S0T2
229533.I1RF19	pp	229533.I1RRP3
229533.I1RF19	pp	229533.I1S2B8
229533.I1RF19	pp	229533.I1RRT2
229533.I1RF19	pp	229533.I1RX46
229533.I1RF19	pp	229533.I1RWD4
229533.I1RF19	pp	229533.I1RW25
229533.I1RF19	pp	229533.I1RNA0
229533.I1RF19	pp	229533.I1RPU3
229533.I1RF19	pp	229533.I1RNH1
229533.I1RF19	pp	229533.I1RJU3
229533.I1RF19	pp	229533.I1RVQ0
229533.I1RF19	pp	229533.I1S6F0
229533.I1RF19	pp	229533.I1RTF4
229533.I1RF19	pp	229533.V6QYF0
229533.I1RF19	pp	229533.I1RFG0
229533.I1RF19	pp	229533.Q4IJH1
229533.I1RF19	pp	229533.I1RRQ6
229533.I1RF19	pp	229533.I1RT04
229533.I1RF19	pp	229533.I1RZS6
229533.I1RF19	pp	229533.V6RUY9
229533.I1RF19	pp	229533.I1RM06
229533.I1RF19	pp	229533.I1RZ39
229533.I1RF19	pp	229533.I1RW61
229533.I1RF19	pp	229533.Q4I662
229533.I1RF19	pp	229533.I1RNV6
229533.I1RF19	pp	229533.I1RXD7
229533.I1RF19	pp	229533.I1S0R7
229533.I1RF19	pp	229533.I1RUQ2
229533.I1RF19	pp	229533.I1S6Y8
229533.I1RF19	pp	229533.V6R3X5

33654	229533.I1RF19	pp	229533.I1RPM4
33655	229533.I1RF19	pp	229533.I1RT40
33656	229533.I1RF19	pp	229533.Q4HVW2
33657	229533.I1RF19	pp	229533.I1RWJ4
33658	229533.I1RF19	pp	229533.I1RG51
33659	229533.I1RF19	pp	229533.I1S9U2
33660	229533.I1RF19	pp	229533.I1RMK3
33661	229533.I1RF19	pp	229533.Q4IF76
33662	229533.I1RF19	pp	229533.I1S0L4
33663	229533.I1RF19	pp	229533.I1RPU0
33664	229533.I1RF19	pp	229533.I1RZZ9
33665	229533.I1RF19	pp	229533.I1RQS8
33666	229533.I1RF19	pp	229533.I1RFH8
33667	229533.I1RF19	pp	229533.I1RWJ8
33668	229533.I1RF19	pp	229533.I1RM82
33669	229533.I1RF19	pp	229533.I1S2H4
33670	229533.I1RF19	pp	229533.I1RR24
33671	229533.I1RF19	pp	229533.I1RRT8
33672	229533.I1RF19	pp	229533.I1RM67
33673	229533.I1RF19	pp	229533.I1S5R1
33674	229533.I1RM54	pp	229533.I1RMK4
33675	229533.I1RM54	pp	229533.Q4HXF0
33676	229533.I1RM54	pp	229533.I1RNG4
33677	229533.I1RM54	pp	229533.I1RY62
33678	229533.I1RM54	pp	229533.I1S3G4
33679	229533.I1RM54	pp	229533.I1RMC5
33680	229533.I1RM54	pp	229533.I1RMG6
33681	229533.I1RM54	pp	229533.I1RZ21
33682	229533.I1RM54	pp	229533.I1S1W8
33683	229533.I1RM54	pp	229533.I1S3F7
33684	229533.I1RM54	pp	229533.I1S3V2
33685	229533.I1RM54	pp	229533.I1RM55
33686	229533.I1RM54	pp	229533.I1RUY6
33687	229533.I1RM54	pp	229533.I1RMV8
33688	229533.I1RM54	pp	229533.I1S256
33689	229533.I1RM54	pp	229533.V6RQW4
33690	229533.I1RJW1	pp	229533.I1S574
33691	229533.I1RJW1	pp	229533.I1RNA1
33692	229533.I1RJW1	pp	229533.I1RR95
33693	229533.I1RJW1	pp	229533.I1S984
33694	229533.I1RJW1	pp	229533.I1S569
33695	229533.I1RJW1	pp	229533.I1RRT3
33696	229533.I1RJW1	pp	229533.I1RLN6
33697	229533.I1RJW1	pp	229533.I1RU29
33698	229533.I1RJW1	pp	229533.I1RLM7
33699	229533.I1RJW1	pp	229533.Q4HZS8
33700	229533.I1RJW1	pp	229533.I1S272
33701	229533.I1RJW1	pp	229533.I1RZ13
33702	229533.I1RJW1	pp	229533.I1S352
33703	229533.I1RJW1	pp	229533.I1RW07
33704	229533.I1RJW1	pp	229533.I1RK45
33705	229533.I1RJW1	pp	229533.I1RLC7
33706	229533.I1RJW1	pp	229533.V6R4J1
33707	229533.I1RJW1	pp	229533.I1RKP3
33708	229533.I1RJW1	pp	229533.I1RNA5
33709	229533.I1RJW1	pp	229533.I1RRQ1
33710	229533.I1RYZ9	pp	229533.I1RZ00
33711	229533.I1RYZ9	pp	229533.Q4IJM4
33712	229533.I1RYZ9	pp	229533.I1S1W5
33713	229533.I1RYZ9	pp	229533.V6RU70
33714	229533.I1RYZ9	pp	229533.I1RZH4
33715	229533.I1RYZ9	pp	229533.V6RMM8
33716	229533.I1RYZ9	pp	229533.I1S224
33717	229533.I1RYZ9	pp	229533.I1RZY9
33718	229533.I1RYZ9	pp	229533.Q4ICM0
33719	229533.I1RYZ9	pp	229533.I1S0M2
33720	229533.I1RYZ9	pp	229533.I1S439
33721	229533.I1RYZ9	pp	229533.V6RGI1
33722	229533.I1RYZ9	pp	229533.I1S1D3
33723	229533.I1RYZ9	pp	229533.I1RZ12
33724	229533.I1RYZ9	pp	229533.I1S0E8
33725	229533.I1RYZ9	pp	229533.I1S9S6
33726	229533.I1RYZ9	pp	229533.I1RZT1

33727 229533.I1RYZ9 pp 229533.V6RKY7
33728 229533.I1RYZ9 pp 229533.I1S098
33729 229533.I1RYZ9 pp 229533.Q4IEV4
33730 229533.I1RYZ9 pp 229533.I1S1W4
33731 229533.I1RYZ9 pp 229533.I1RZE4
33732 229533.I1RYZ9 pp 229533.I1S4R1
33733 229533.I1RYZ9 pp 229533.I1S0M6
33734 229533.I1RYZ9 pp 229533.I1S7X9
33735 229533.I1RYZ9 pp 229533.I1RZ63
33736 229533.I1RYZ9 pp 229533.I1S2E9
33737 229533.I1RYZ9 pp 229533.V6RVG3
33738 229533.I1RYZ9 pp 229533.I1S0J7
33739 229533.A0A1C3YIB8 pp 229533.I1RZZ9
33740 229533.A0A1C3YIB8 pp 229533.I1RE52
33741 229533.A0A1C3YIB8 pp 229533.I1RY44
33742 229533.A0A1C3YIB8 pp 229533.I1RK55
33743 229533.A0A1C3YIB8 pp 229533.I1RBY4
33744 229533.A0A1C3YIB8 pp 229533.A0A1C3YLB6
33745 229533.A0A1C3YIB8 pp 229533.I1RQM7
33746 229533.A0A1C3YIB8 pp 229533.Q4IB89
33747 229533.A0A1C3YIB8 pp 229533.I1RHJ0
33748 229533.A0A1C3YIB8 pp 229533.I1RZ25
33749 229533.A0A1C3YIB8 pp 229533.I1SAB4
33750 229533.A0A1C3YIB8 pp 229533.I1RTM8
33751 229533.A0A1C3YIB8 pp 229533.I1S2E9
33752 229533.A0A1C3YIB8 pp 229533.I1RBL4
33753 229533.A0A1C3YIB8 pp 229533.I1RQ48
33754 229533.A0A1C3YIB8 pp 229533.I1RF24
33755 229533.A0A1C3YIB8 pp 229533.I1S267
33756 229533.A0A1C3YIB8 pp 229533.I1RKQ0
33757 229533.A0A1C3YIB8 pp 229533.I1S968
33758 229533.A0A1C3YIB8 pp 229533.I1RFB0
33759 229533.A0A1C3YIB8 pp 229533.I1REI2
33760 229533.A0A1C3YIB8 pp 229533.I1RMQ7
33761 229533.A0A1C3YIB8 pp 229533.I1RRC4
33762 229533.A0A1C3YIB8 pp 229533.I1S048
33763 229533.A0A1C3YIB8 pp 229533.I1RZ38
33764 229533.A0A1C3YIB8 pp 229533.I1RE17
33765 229533.A0A1C3YIB8 pp 229533.I1RLA2
33766 229533.A0A1C3YIB8 pp 229533.I1S2F7
33767 229533.A0A1C3YIB8 pp 229533.I1S3T6
33768 229533.A0A1C3YIB8 pp 229533.A0A1C3YJX0
33769 229533.A0A1C3YIB8 pp 229533.I1RRN4
33770 229533.A0A1C3YIB8 pp 229533.I1RYP5
33771 229533.A0A1C3YIB8 pp 229533.I1RGU3
33772 229533.A0A1C3YIB8 pp 229533.I1S210
33773 229533.A0A1C3YIB8 pp 229533.I1RST9
33774 229533.A0A1C3YIB8 pp 229533.I1RW55
33775 229533.A0A1C3YIB8 pp 229533.I1S223
33776 229533.A0A1C3YIB8 pp 229533.I1RSK0
33777 229533.A0A1C3YIB8 pp 229533.I1RIM7
33778 229533.A0A1C3YIB8 pp 229533.I1S969
33779 229533.A0A1C3YIB8 pp 229533.A0A1C3YJT5
33780 229533.A0A1C3YIB8 pp 229533.I1RHT5
33781 229533.A0A098DQ15 pp 229533.I1S2B9
33782 229533.A0A098DQ15 pp 229533.I1RBA6
33783 229533.A0A098DQ15 pp 229533.I1RYX5
33784 229533.A0A1C3YJI0 pp 229533.I1RRW0
33785 229533.A0A1C3YJI0 pp 229533.I1RRW2
33786 229533.A0A1C3YJI0 pp 229533.I1RX05
33787 229533.A0A1C3YJI0 pp 229533.I1RN09
33788 229533.A0A1C3YJI0 pp 229533.A0A1C3YM91
33789 229533.A0A1C3YJI0 pp 229533.I1S2E9
33790 229533.A0A1C3YJI0 pp 229533.A0A1C3YLT9
33791 229533.A0A1C3YJI0 pp 229533.I1RMP0
33792 229533.A0A1C3YJI0 pp 229533.I1RT36
33793 229533.A0A1C3YJI0 pp 229533.I1RN36
33794 229533.A0A1C3YJI0 pp 229533.I1RP30
33795 229533.A0A1C3YJI0 pp 229533.I1S549
33796 229533.A0A1C3YJI0 pp 229533.I1RBZ8
33797 229533.A0A1C3YJI0 pp 229533.A0A1C3YMQ5
33798 229533.A0A1C3YJI0 pp 229533.V6RR76
33799 229533.A0A1C3YJI0 pp 229533.I1RDH0

33800	229533.A0A1C3YJI0	pp	229533.I1RMH5
33801	229533.A0A1C3YJI0	pp	229533.I1RNG8
33802	229533.A0A1C3YJI0	pp	229533.A0A1C3YK36
33803	229533.A0A1C3YJI0	pp	229533.Q4IB70
33804	229533.A0A1C3YJI0	pp	229533.I1RQL4
33805	229533.A0A1C3YJI0	pp	229533.A0A1C3YN26
33806	229533.A0A1C3YJI0	pp	229533.A0A1C3YLF7
33807	229533.A0A1C3YJI0	pp	229533.A0A1C3YJW0
33808	229533.A0A1C3YJI0	pp	229533.I1RX95
33809	229533.A0A1C3YJI0	pp	229533.I1S224
33810	229533.A0A1C3YJI0	pp	229533.I1RPP7
33811	229533.A0A1C3YJI0	pp	229533.I1RTN6
33812	229533.A0A1C3YJI0	pp	229533.I1RY44
33813	229533.A0A1C3YJI0	pp	229533.I1RY58
33814	229533.A0A1C3YJI0	pp	229533.I1RPH7
33815	229533.A0A1C3YJI0	pp	229533.I1RCR9
33816	229533.A0A1C3YJI0	pp	229533.A0A1C3YKB5
33817	229533.A0A1C3YJI0	pp	229533.V6QVD5
33818	229533.A0A1C3YJI0	pp	229533.I1RQP1
33819	229533.A0A1C3YJI0	pp	229533.I1RSZ3
33820	229533.A0A1C3YJI0	pp	229533.I1RSI1
33821	229533.A0A1C3YJI0	pp	229533.I1S2C1
33822	229533.A0A1C3YJI0	pp	229533.I1RW83
33823	229533.A0A1C3YJI0	pp	229533.I1RMW4
33824	229533.A0A1C3YJI0	pp	229533.I1RN81
33825	229533.A0A1C3YJI0	pp	229533.I1S9A8
33826	229533.A0A1C3YJI0	pp	229533.I1S1V3
33827	229533.A0A1C3YJI0	pp	229533.I1RDG0
33828	229533.A0A1C3YJI0	pp	229533.A0A1C3YN89
33829	229533.A0A1C3YJI0	pp	229533.I1RBU6
33830	229533.A0A1C3YJI0	pp	229533.I1RPY3
33831	229533.A0A098DQ10	pp	229533.I1RCU3
33832	229533.A0A098DQ10	pp	229533.A0A1C3YLS3
33833	229533.I1RN08	pp	229533.I1S439
33834	229533.I1RN08	pp	229533.I1RQ81
33835	229533.I1RN08	pp	229533.I1RUQ5
33836	229533.I1RN08	pp	229533.I1RYZ0
33837	229533.I1RN08	pp	229533.I1RZH4
33838	229533.I1RN08	pp	229533.Q4IJM4
33839	229533.I1RN08	pp	229533.I1RQA3
33840	229533.I1RN08	pp	229533.I1RN65
33841	229533.I1RN08	pp	229533.I1RWT6
33842	229533.I1RN08	pp	229533.I1RWJ6
33843	229533.I1RN08	pp	229533.I1RQ72
33844	229533.I1RN08	pp	229533.I1RX70
33845	229533.I1RN08	pp	229533.I1RV66
33846	229533.I1RN08	pp	229533.I1RQ26
33847	229533.I1RN08	pp	229533.V6QV09
33848	229533.I1RN08	pp	229533.I1RNW8
33849	229533.I1RN08	pp	229533.I1RNC0
33850	229533.I1RN08	pp	229533.I1RN31
33851	229533.I1RN08	pp	229533.I1S0M2
33852	229533.I1RN08	pp	229533.I1RQB4
33853	229533.I1RN08	pp	229533.I1RYH9
33854	229533.I1RN08	pp	229533.I1RVQ0
33855	229533.I1RN08	pp	229533.V6RGI1
33856	229533.I1RN08	pp	229533.I1RRW0
33857	229533.I1RN08	pp	229533.I1RRP7
33858	229533.I1RN08	pp	229533.I1RYZ9
33859	229533.I1RN08	pp	229533.I1RRY3
33860	229533.I1RN08	pp	229533.I1S224
33861	229533.I1RN08	pp	229533.I1RT36
33862	229533.I1RN08	pp	229533.I1RRN5
33863	229533.I1RN08	pp	229533.I1RY44
33864	229533.I1RN08	pp	229533.I1S0N4
33865	229533.I1RN08	pp	229533.I1RPW0
33866	229533.I1RN08	pp	229533.I1S2G9
33867	229533.I1RN08	pp	229533.I1RX38
33868	229533.I1RN08	pp	229533.I1RY58
33869	229533.I1RN08	pp	229533.I1RZE4
33870	229533.I1RN08	pp	229533.I1S0D1
33871	229533.I1RN08	pp	229533.I1S1D3
33872	229533.I1RN08	pp	229533.I1RNF4

33873 229533.I1RN08 pp 229533.I1RW16
33874 229533.I1RN08 pp 229533.I1RNP7
33875 229533.I1RN08 pp 229533.I1RPD6
33876 229533.I1RN08 pp 229533.I1S1V5
33877 229533.I1RN08 pp 229533.I1RP28
33878 229533.I1RN08 pp 229533.I1RNY8
33879 229533.I1RN08 pp 229533.I1RUN8
33880 229533.I1RN08 pp 229533.I1S549
33881 229533.I1RN08 pp 229533.I1S1W4
33882 229533.I1RN08 pp 229533.V6RVG3
33883 229533.I1RN08 pp 229533.V6RKY7
33884 229533.I1RN08 pp 229533.I1S2E9
33885 229533.I1RN08 pp 229533.I1RV91
33886 229533.I1RJQ7 pp 229533.I1S0Y2
33887 229533.I1RJQ7 pp 229533.I1RYH1
33888 229533.I1RJQ7 pp 229533.I1RLE2
33889 229533.I1RJQ7 pp 229533.I1RPQ2
33890 229533.I1RJQ7 pp 229533.I1S5S8
33891 229533.I1RJQ7 pp 229533.I1RNE9
33892 229533.I1RK70 pp 229533.Q4IAA0
33893 229533.I1RK70 pp 229533.I1RN28
33894 229533.I1RK70 pp 229533.Q4HWE2
33895 229533.I1RK70 pp 229533.I1S9U2
33896 229533.I1RK70 pp 229533.I1RRU9
33897 229533.I1RK70 pp 229533.I1RN70
33898 229533.I1R9B7 pp 229533.I1RRG8
33899 229533.I1R9B7 pp 229533.I1S8F8
33900 229533.I1R9B7 pp 229533.I1RB38
33901 229533.I1R9B7 pp 229533.I1RFV6
33902 229533.I1R9B7 pp 229533.I1RXE6
33903 229533.I1R9B7 pp 229533.I1S7L6
33904 229533.I1R9B7 pp 229533.I1RNM7
33905 229533.A0A1C3YKN8 pp 229533.I1RNE9
33906 229533.A0A1C3YKN8 pp 229533.I1RPQ2
33907 229533.A0A1C3YKN8 pp 229533.Q4IB63
33908 229533.A0A1C3YKN8 pp 229533.I1RZ17
33909 229533.A0A1C3YKN8 pp 229533.I1RJ49
33910 229533.A0A1C3YKN8 pp 229533.I1REU0
33911 229533.A0A1C3YKN8 pp 229533.I1RJS0
33912 229533.A0A1C3YKN8 pp 229533.Q4I785
33913 229533.A0A1C3YKN8 pp 229533.I1S5S8
33914 229533.A0A1C3YKN8 pp 229533.I1RBN0
33915 229533.A0A1C3YKN8 pp 229533.I1RUP5
33916 229533.A0A1C3YKN8 pp 229533.Q4I073
33917 229533.A0A1C3YKN8 pp 229533.Q4I0K3
33918 229533.A0A1C3YKN8 pp 229533.Q4ILH3
33919 229533.A0A1C3YKN8 pp 229533.I1RW78
33920 229533.A0A1C3YKN8 pp 229533.I1RZ18
33921 229533.A0A1C3YKN8 pp 229533.I1RLE2
33922 229533.A0A1C3YKN8 pp 229533.I1RJQ7
33923 229533.A0A1C3YKN8 pp 229533.I1RNK8
33924 229533.A0A1C3YKN8 pp 229533.I1RSX9
33925 229533.A0A1C3YKN8 pp 229533.I1RYH1
33926 229533.A0A1C3YKN8 pp 229533.I1RFP1
33927 229533.A0A1C3YKN8 pp 229533.I1RK45
33928 229533.A0A1C3YKN8 pp 229533.I1S0Y2
33929 229533.A0A1C3YKN8 pp 229533.I1RPI4
33930 229533.I1RXZ4 pp 229533.Q4IB89
33931 229533.I1RXZ4 pp 229533.I1RY44
33932 229533.I1RXZ4 pp 229533.I1S4P6
33933 229533.I1RXZ4 pp 229533.I1S8C1
33934 229533.I1RXZ4 pp 229533.I1S224
33935 229533.I1RXZ4 pp 229533.I1RY58
33936 229533.I1RXZ4 pp 229533.I1RZT1
33937 229533.I1RXZ4 pp 229533.I1S2E9
33938 229533.I1RXZ4 pp 229533.I1S788
33939 229533.A0A1C3YKY3 pp 229533.I1RFL7
33940 229533.A0A1C3YKY3 pp 229533.I1RAW7
33941 229533.A0A1C3YKY3 pp 229533.I1S7X9
33942 229533.A0A1C3YKY3 pp 229533.V6QV09
33943 229533.A0A1C3YKY3 pp 229533.I1S098
33944 229533.A0A1C3YKY3 pp 229533.I1RN89
33945 229533.A0A1C3YKY3 pp 229533.I1RRW0

33946 229533.A0A1C3YKY3 pp 229533.I1S2E9
33947 229533.A0A1C3YKY3 pp 229533.I1RCH7
33948 229533.A0A1C3YKY3 pp 229533.V6RGI1
33949 229533.A0A1C3YKY3 pp 229533.I1S224
33950 229533.A0A1C3YKY3 pp 229533.I1RV91
33951 229533.A0A1C3YKY3 pp 229533.I1RC17
33952 229533.A0A1C3YKY3 pp 229533.I1RYZ9
33953 229533.A0A1C3YKY3 pp 229533.I1RGF2
33954 229533.A0A1C3YKY3 pp 229533.I1REX8
33955 229533.A0A1C3YKY3 pp 229533.I1RKJ7
33956 229533.A0A1C3YKY3 pp 229533.I1RUF8
33957 229533.A0A1C3YKY3 pp 229533.I1RT36
33958 229533.A0A1C3YKY3 pp 229533.I1S0N4
33959 229533.A0A1C3YKY3 pp 229533.I1RJX2
33960 229533.A0A1C3YKY3 pp 229533.A0A1C3YLD3
33961 229533.A0A1C3YKY3 pp 229533.I1RAN3
33962 229533.A0A1C3YKY3 pp 229533.I1RL24
33963 229533.A0A1C3YKY3 pp 229533.I1RMQ7
33964 229533.A0A1C3YKY3 pp 229533.I1S193
33965 229533.A0A1C3YKY3 pp 229533.I1RY44
33966 229533.A0A1C3YKY3 pp 229533.I1RPC2
33967 229533.A0A1C3YKY3 pp 229533.I1RN65
33968 229533.A0A1C3YKY3 pp 229533.I1RME5
33969 229533.A0A1C3YKY3 pp 229533.I1RJK7
33970 229533.A0A1C3YKY3 pp 229533.A0A1C3YM91
33971 229533.A0A1C3YKY3 pp 229533.I1RQS4
33972 229533.A0A1C3YKY3 pp 229533.I1RD70
33973 229533.A0A1C3YKY3 pp 229533.I1RK75
33974 229533.I1S0Y2 pp 229533.I1S5S8
33975 229533.I1S0Y2 pp 229533.I1S2B0
33976 229533.I1RYH1 pp 229533.I1S0Y2
33977 229533.I1RYH1 pp 229533.I1S5S8
33978 229533.I1RHU7 pp 229533.I1RPA6
33979 229533.A0A1C3YLX2 pp 229533.I1S2E9
33980 229533.A0A1C3YLX2 pp 229533.I1RWT6
33981 229533.A0A1C3YLX2 pp 229533.I1RY44
33982 229533.A0A1C3YLX2 pp 229533.I1RV91
33983 229533.A0A1C3YLX2 pp 229533.I1RC17
33984 229533.A0A1C3YLX2 pp 229533.I1RQA3
33985 229533.I1RZ35 pp 229533.V6RBN2
33986 229533.I1RZ35 pp 229533.I1S2B8
33987 229533.I1RZ35 pp 229533.V6RMW6
33988 229533.I1RZ35 pp 229533.V6RTJ8
33989 229533.I1RZ35 pp 229533.I1RZM1
33990 229533.I1RFP1 pp 229533.I1RT23
33991 229533.I1RFP1 pp 229533.I1RZ92
33992 229533.I1RFP1 pp 229533.I1RJQ7
33993 229533.I1RFP1 pp 229533.I1RPQ2
33994 229533.I1RFP1 pp 229533.I1RQ48
33995 229533.I1RFP1 pp 229533.I1RJS0
33996 229533.I1RFP1 pp 229533.I1S5S8
33997 229533.I1RFP1 pp 229533.I1RZZ3
33998 229533.I1RFP1 pp 229533.I1RNE9
33999 229533.I1RFP1 pp 229533.I1RLE2
34000 229533.I1RFP1 pp 229533.I1RYH1
34001 229533.I1RFP1 pp 229533.I1RZJ8
34002 229533.I1RFP1 pp 229533.I1RNT1
34003 229533.I1RFP1 pp 229533.I1RY35
34004 229533.I1RFP1 pp 229533.I1RI22
34005 229533.I1RFP1 pp 229533.I1RUP5
34006 229533.I1RFP1 pp 229533.V6RVJ3
34007 229533.I1RFP1 pp 229533.I1RJ49
34008 229533.I1RFP1 pp 229533.I1RR90
34009 229533.I1RFP1 pp 229533.V6RNM4
34010 229533.I1RFP1 pp 229533.I1S0Y2
34011 229533.I1RFP1 pp 229533.I1RMH4
34012 229533.A0A1C3YJB8 pp 229533.I1RJA3
34013 229533.A0A1C3YJB8 pp 229533.I1S3H6
34014 229533.A0A1C3YJB8 pp 229533.I1S6X4
34015 229533.A0A1C3YJB8 pp 229533.I1RJ84
34016 229533.A0A1C3YJB8 pp 229533.I1RXN7
34017 229533.A0A1C3YJB8 pp 229533.I1S325
34018 229533.A0A1C3YJB8 pp 229533.I1S4A5

229533.A0A1C3YJB8 pp 229533.A0A1C3YLG9
229533.A0A1C3YJB8 pp 229533.I1RCP3
229533.A0A1C3YJB8 pp 229533.I1RDP6
229533.A0A1C3YJB8 pp 229533.I1RM78
229533.I1S5S8 pp 229533.V6R398
229533.I1S5S8 pp 229533.Q4IPZ1
229533.I1S9L0 pp 229533.Q4IPB8
229533.I1S9L0 pp 229533.V6QXG1
229533.I1S9L0 pp 229533.V6QVD5
229533.I1S9L0 pp 229533.Q4IBK5
229533.I1S9L0 pp 229533.Q4IPB3
229533.I1S9L0 pp 229533.Q4IE79
229533.I1S9L0 pp 229533.Q4IRI9
229533.I1S9L0 pp 229533.Q4HXF6
229533.I1S9L0 pp 229533.Q4IB70
229533.I1S9L0 pp 229533.Q4I1Y1
229533.A0A1C3YNC7 pp 229533.V6QX58
229533.I1RH82 pp 229533.I1RR44
229533.I1RH82 pp 229533.I1RUY4
229533.I1RH82 pp 229533.I1RY12
229533.I1RH82 pp 229533.I1RPT0
229533.I1RH82 pp 229533.I1RUY5
229533.I1RH82 pp 229533.I1RJT5
229533.I1RH82 pp 229533.I1RKE1
229533.I1RH82 pp 229533.I1RWM4
229533.I1RH82 pp 229533.Q4HYR2
229533.I1RH82 pp 229533.I1RYL8
229533.I1RH82 pp 229533.I1S0V6
229533.I1RH82 pp 229533.I1RIX0
229533.I1RH82 pp 229533.I1RJN7
229533.I1RH82 pp 229533.I1RP86
229533.I1RH82 pp 229533.I1RHJ2
229533.I1RH82 pp 229533.V6RLM7
229533.I1RH82 pp 229533.I1RZ16
229533.I1RH82 pp 229533.I1RSJ2
229533.I1RH82 pp 229533.I1S0V7
229533.I1RH82 pp 229533.I1S087
229533.I1RH82 pp 229533.I1S147
229533.I1RH82 pp 229533.V6RE46
229533.I1RH82 pp 229533.I1RNU7
229533.I1RH82 pp 229533.I1RQI4
229533.I1RH82 pp 229533.I1RYK0
229533.I1RH82 pp 229533.I1RRT3
229533.I1RH82 pp 229533.I1RKB2
229533.I1RH82 pp 229533.I1RWF5
229533.I1RH82 pp 229533.I1S472
229533.I1RPQ2 pp 229533.I1S9U6
229533.I1RPQ2 pp 229533.I1S2B9
229533.I1RPQ2 pp 229533.I1RRF7
229533.I1RPQ2 pp 229533.I1S0Y2
229533.I1RPQ2 pp 229533.I1RUP5
229533.I1RPQ2 pp 229533.V6RBN2
229533.I1RPQ2 pp 229533.I1RZN1
229533.I1RPQ2 pp 229533.I1RW80
229533.I1RPQ2 pp 229533.I1RQM9
229533.I1RPQ2 pp 229533.I1RYH1
229533.I1RPQ2 pp 229533.I1S5S8
229533.I1RPQ2 pp 229533.I1RW91
229533.I1RPQ2 pp 229533.I1RPW8
229533.I1RPQ2 pp 229533.V6R398
229533.A0A1C3YML9 pp 229533.A0A1C3YNC7
229533.A0A1C3YML9 pp 229533.V6QX58
229533.A0A0E0S747 pp 229533.I1RG07
229533.I1RJ49 pp 229533.I1S0Y2
229533.I1RJ49 pp 229533.I1RLE2
229533.I1RJ49 pp 229533.I1RYH1
229533.I1RJ49 pp 229533.I1S5S8
229533.I1RJ49 pp 229533.I1RNE9
229533.I1RJ49 pp 229533.I1RJQ7
229533.I1RJ49 pp 229533.I1RPI4
229533.I1RJ49 pp 229533.I1RPQ2
229533.I1RKQ5 pp 229533.Q4INZ9
229533.I1RKQ5 pp 229533.Q4IF76

34092	229533.I1RKQ5	pp	229533.I1S0R7
34093	229533.I1RKQ5	pp	229533.I1RV91
34094	229533.I1RKQ5	pp	229533.I1RQS5
34095	229533.I1RKQ5	pp	229533.I1RTI6
34096	229533.I1RKQ5	pp	229533.Q4IPI1
34097	229533.I1RKQ5	pp	229533.I1RT05
34098	229533.I1RKQ5	pp	229533.I1RUQ2
34099	229533.I1RKQ5	pp	229533.I1S268
34100	229533.I1RKQ5	pp	229533.Q4IAS1
34101	229533.I1RKQ5	pp	229533.V6QWX8
34102	229533.I1RKQ5	pp	229533.Q4IMD1
34103	229533.I1RKQ5	pp	229533.I1RLV9
34104	229533.I1RKQ5	pp	229533.Q4IQW0
34105	229533.I1RKQ5	pp	229533.I1RNV6
34106	229533.I1RKQ5	pp	229533.I1RQM6
34107	229533.I1RKQ5	pp	229533.I1RUP7
34108	229533.I1RKQ5	pp	229533.I1RRT2
34109	229533.I1RKQ5	pp	229533.I1RT80
34110	229533.I1RKQ5	pp	229533.I1RPH9
34111	229533.I1RKQ5	pp	229533.I1RL23
34112	229533.I1RKQ5	pp	229533.Q4IJH1
34113	229533.I1RKQ5	pp	229533.I1RTF4
34114	229533.I1RKQ5	pp	229533.V6R283
34115	229533.I1RKQ5	pp	229533.Q4IFI0
34116	229533.I1RKQ5	pp	229533.I1RRG3
34117	229533.I1RKQ5	pp	229533.I1RVY0
34118	229533.I1RKQ5	pp	229533.I1S8E2
34119	229533.I1RKQ5	pp	229533.I1RW71
34120	229533.I1RKQ5	pp	229533.I1SAN0
34121	229533.I1RKQ5	pp	229533.Q4I830
34122	229533.I1RKQ5	pp	229533.I1RYG3
34123	229533.I1RKQ5	pp	229533.I1RR00
34124	229533.I1RKQ5	pp	229533.I1RW90
34125	229533.I1RKQ5	pp	229533.I1RMK3
34126	229533.I1RKQ5	pp	229533.I1RZ99
34127	229533.I1RKQ5	pp	229533.I1S9Y1
34128	229533.I1RKQ5	pp	229533.I1RRJ8
34129	229533.I1RKQ5	pp	229533.I1RPU0
34130	229533.I1RKQ5	pp	229533.I1RQJ5
34131	229533.I1RKQ5	pp	229533.I1RS30
34132	229533.I1RKQ5	pp	229533.I1RRT8
34133	229533.I1RKQ5	pp	229533.I1RQ31
34134	229533.I1RKQ5	pp	229533.I1RPW9
34135	229533.I1RKQ5	pp	229533.I1RM67
34136	229533.I1RKQ5	pp	229533.Q4IN63
34137	229533.I1RKQ5	pp	229533.I1S9U2
34138	229533.I1RKQ5	pp	229533.Q4HZ47
34139	229533.I1RKQ5	pp	229533.Q4I662
34140	229533.I1RKQ5	pp	229533.Q4I327
34141	229533.I1RKQ5	pp	229533.I1RRQ3
34142	229533.I1RKQ5	pp	229533.I1RS47
34143	229533.I1RKQ5	pp	229533.I1RNH1
34144	229533.I1RKQ5	pp	229533.I1RRH5
34145	229533.I1RKQ5	pp	229533.I1RMN7
34146	229533.I1RKQ5	pp	229533.I1RX24
34147	229533.I1RKQ5	pp	229533.V6QYF0
34148	229533.I1RKQ5	pp	229533.I1RNH6
34149	229533.I1RKQ5	pp	229533.I1S021
34150	229533.I1RKQ5	pp	229533.I1RNA0
34151	229533.I1RKQ5	pp	229533.I1RT33
34152	229533.I1RKQ5	pp	229533.I1RQ65
34153	229533.I1RKQ5	pp	229533.I1RSI6
34154	229533.I1RKQ5	pp	229533.Q4IQK7
34155	229533.I1RKQ5	pp	229533.I1RM06
34156	229533.I1RKQ5	pp	229533.I1RNU6
34157	229533.I1RKQ5	pp	229533.V6QUE6
34158	229533.I1RKQ5	pp	229533.I1RMN6
34159	229533.I1RKQ5	pp	229533.I1RZS6
34160	229533.I1RKQ5	pp	229533.I1RPY1
34161	229533.I1RKQ5	pp	229533.Q4IBS2
34162	229533.I1RKQ5	pp	229533.I1S4T3
34163	229533.I1RKQ5	pp	229533.I1RSR3
34164	229533.I1RKQ5	pp	229533.I1RPM7

34165 229533.I1RKQ5 pp 229533.I1RT36
34166 229533.I1RKQ5 pp 229533.V6RGY3
34167 229533.I1RKQ5 pp 229533.Q4IEK8
34168 229533.I1RKQ5 pp 229533.V6RGN5
34169 229533.I1RKQ5 pp 229533.I1RM96
34170 229533.I1RKQ5 pp 229533.I1S056
34171 229533.I1RKQ5 pp 229533.I1RX14
34172 229533.I1RKQ5 pp 229533.I1S1U7
34173 229533.I1RKQ5 pp 229533.I1RZF6
34174 229533.I1RKQ5 pp 229533.I1RRT0
34175 229533.I1RKQ5 pp 229533.I1RP31
34176 229533.I1RKQ5 pp 229533.I1RSD8
34177 229533.I1RKQ5 pp 229533.I1RRR7
34178 229533.I1RKQ5 pp 229533.I1RMQ0
34179 229533.I1RKQ5 pp 229533.V6R227
34180 229533.I1RKQ5 pp 229533.I1S060
34181 229533.I1RKQ5 pp 229533.I1S2U2
34182 229533.I1RKQ5 pp 229533.I1RZZ1
34183 229533.I1RKQ5 pp 229533.I1RUP9
34184 229533.I1RKQ5 pp 229533.I1S0J1
34185 229533.I1RKQ5 pp 229533.I1RZK6
34186 229533.I1RKQ5 pp 229533.I1S255
34187 229533.I1RKQ5 pp 229533.Q4IJ56
34188 229533.I1RKQ5 pp 229533.I1S0M9
34189 229533.I1RKQ5 pp 229533.I1RMD5
34190 229533.I1RKQ5 pp 229533.I1S1X9
34191 229533.I1RKQ5 pp 229533.I1S2E8
34192 229533.I1RKQ5 pp 229533.I1RQ81
34193 229533.I1RKQ5 pp 229533.I1RSS4
34194 229533.I1RKQ5 pp 229533.I1RTD7
34195 229533.I1RKQ5 pp 229533.I1RSG8
34196 229533.I1RKQ5 pp 229533.I1RSC6
34197 229533.I1RKQ5 pp 229533.I1RRI2
34198 229533.I1RKQ5 pp 229533.I1RZB5
34199 229533.I1RKQ5 pp 229533.I1RN99
34200 229533.I1RKQ5 pp 229533.I1S2C0
34201 229533.I1RKQ5 pp 229533.I1RVZ0
34202 229533.I1RKQ5 pp 229533.I1RQ28
34203 229533.I1RKQ5 pp 229533.I1RZE5
34204 229533.I1RKQ5 pp 229533.I1RPJ3
34205 229533.I1RKQ5 pp 229533.I1RNK2
34206 229533.I1RKQ5 pp 229533.I1S0M3
34207 229533.I1RKQ5 pp 229533.I1RTE9
34208 229533.I1RKQ5 pp 229533.I1RUG4
34209 229533.I1RKQ5 pp 229533.I1S0N5
34210 229533.I1RKQ5 pp 229533.I1RQA2
34211 229533.I1RKQ5 pp 229533.I1RR25
34212 229533.I1RNE9 pp 229533.I1RPQ2
34213 229533.I1RNE9 pp 229533.I1S5S8
34214 229533.I1RNE9 pp 229533.I1RPI4
34215 229533.I1RNE9 pp 229533.I1S0Y2
34216 229533.I1RNE9 pp 229533.I1RYH1
34217 229533.A0A098DV93 pp 229533.I1S5S8
34218 229533.A0A098DV93 pp 229533.Q4I785
34219 229533.A0A098DV93 pp 229533.I1RK45
34220 229533.A0A098DV93 pp 229533.I1RFP1
34221 229533.A0A098DV93 pp 229533.I1RPI4
34222 229533.A0A098DV93 pp 229533.I1RNE9
34223 229533.A0A098DV93 pp 229533.Q4IB63
34224 229533.A0A098DV93 pp 229533.I1RJ49
34225 229533.A0A098DV93 pp 229533.I1RJS0
34226 229533.A0A098DV93 pp 229533.I1REU0
34227 229533.A0A098DV93 pp 229533.Q4I073
34228 229533.A0A098DV93 pp 229533.I1RPQ2
34229 229533.A0A098DV93 pp 229533.I1RW78
34230 229533.A0A098DV93 pp 229533.I1RUP5
34231 229533.A0A098DV93 pp 229533.Q4I0K3
34232 229533.A0A098DV93 pp 229533.I1RZ18
34233 229533.A0A098DV93 pp 229533.I1RBN0
34234 229533.A0A098DV93 pp 229533.Q4ILH3
34235 229533.A0A098DV93 pp 229533.I1RNK8
34236 229533.A0A098DV93 pp 229533.I1RLE2
34237 229533.A0A098DV93 pp 229533.I1RZ17

229533.A0A098DV93	pp	229533.A0A1C3YKN8
229533.A0A098DV93	pp	229533.I1RJQ7
229533.A0A098DV93	pp	229533.I1RYH1
229533.A0A098DV93	pp	229533.I1S0Y2
229533.A0A098DV93	pp	229533.I1RSX9
229533.A0A098DPX3	pp	229533.I1RJQ7
229533.A0A098DPX3	pp	229533.A0A1C3YKN8
229533.A0A098DPX3	pp	229533.I1RLE2
229533.A0A098DPX3	pp	229533.I1S0Y2
229533.A0A098DPX3	pp	229533.I1RYH1
229533.A0A098DPX3	pp	229533.I1RFP1
229533.A0A098DPX3	pp	229533.I1S5S8
229533.A0A098DPX3	pp	229533.I1RPQ2
229533.A0A098DPX3	pp	229533.I1RJ49
229533.A0A098DPX3	pp	229533.I1RNE9
229533.A0A098DPX3	pp	229533.A0A098DV93
229533.I1RA63	pp	229533.I1S0D4
229533.I1RA63	pp	229533.I1RRG3
229533.I1RA63	pp	229533.I1RC64
229533.I1RA63	pp	229533.Q4IEV5
229533.I1RA63	pp	229533.I1RSC7
229533.I1RA63	pp	229533.I1RH64
229533.I1RA63	pp	229533.I1S0S0
229533.I1RUN2	pp	229533.Q4I963
229533.I1RUN2	pp	229533.I1S0R9
229533.I1RUN2	pp	229533.I1RZK3
229533.I1RUN2	pp	229533.I1RWI6
229533.I1RUN2	pp	229533.I1S7W2
229533.I1RUN2	pp	229533.I1S0Z3
229533.I1RBC4	pp	229533.I1RFL4
229533.I1RBC4	pp	229533.Q4I7G0
229533.I1RBC4	pp	229533.I1RQE7
229533.I1RBC4	pp	229533.I1RHW9
229533.I1RBC4	pp	229533.I1RME4
229533.I1RBC4	pp	229533.I1S247
229533.I1RBC4	pp	229533.I1REB8
229533.I1RBC4	pp	229533.I1RQ72
229533.I1RBC4	pp	229533.I1RYW3
229533.I1RBC4	pp	229533.I1RZD3
229533.I1RBC4	pp	229533.I1RUN2
229533.I1RBC4	pp	229533.I1RWX5
229533.I1RBC4	pp	229533.I1RBS6
229533.I1RBC4	pp	229533.I1RZK1
229533.I1RBC4	pp	229533.I1RG57
229533.I1RBC4	pp	229533.I1RWN6
229533.I1RBC4	pp	229533.I1RY44
229533.I1RBC4	pp	229533.I1RE15
229533.I1RBC4	pp	229533.I1REC6
229533.I1RBC4	pp	229533.I1RPP3
229533.I1RBC4	pp	229533.I1S0F9
229533.I1RBC4	pp	229533.I1RKR9
229533.I1RBC4	pp	229533.I1RQ21
229533.I1RBC4	pp	229533.I1RBS5
229533.I1RBC4	pp	229533.I1RN67
229533.I1RBC4	pp	229533.I1RQG4
229533.I1RBC4	pp	229533.I1RNF4
229533.I1RBC4	pp	229533.I1RD22
229533.I1RBC4	pp	229533.I1RPL2
229533.I1RBC4	pp	229533.I1RC66
229533.I1RBC4	pp	229533.I1RK77
229533.I1RBC4	pp	229533.I1RMQ3
229533.I1RBC4	pp	229533.I1RPK0
229533.I1RBC4	pp	229533.I1RDH7
229533.I1RBC4	pp	229533.I1RZM1
229533.I1RBC4	pp	229533.Q4I624
229533.I1RBC4	pp	229533.V6QXG1
229533.I1RBC4	pp	229533.I1RD17
229533.I1RBC4	pp	229533.I1RMB1
229533.I1RBC4	pp	229533.I1RZS8
229533.I1RBC4	pp	229533.I1RZS4
229533.I1RBC4	pp	229533.I1RN58
229533.I1RBC4	pp	229533.I1RBT8
229533.I1RBC4	pp	229533.I1S821

34311	229533.I1RBC4	pp	229533.I1S3S5
34312	229533.I1RBC4	pp	229533.I1RTH6
34313	229533.I1RBC4	pp	229533.I1RPV0
34314	229533.I1RBC4	pp	229533.I1RNC1
34315	229533.I1RBC4	pp	229533.I1RU27
34316	229533.I1RBC4	pp	229533.I1RND5
34317	229533.I1RBC4	pp	229533.I1RYL5
34318	229533.I1RBC4	pp	229533.I1RC55
34319	229533.I1RBC4	pp	229533.I1RBY7
34320	229533.I1RBC4	pp	229533.I1REC7
34321	229533.I1RBC4	pp	229533.I1RMQ5
34322	229533.I1RBC4	pp	229533.I1RGC6
34323	229533.I1RBC4	pp	229533.I1RRY9
34324	229533.I1RBC4	pp	229533.V6R042
34325	229533.I1RBC4	pp	229533.V6QUV7
34326	229533.I1RBC4	pp	229533.I1REG1
34327	229533.I1RBC4	pp	229533.I1S0Z8
34328	229533.I1RBC4	pp	229533.I1RBY6
34329	229533.I1RBC4	pp	229533.I1RSQ7
34330	229533.I1RBC4	pp	229533.I1RL79
34331	229533.I1RBC4	pp	229533.I1RNG4
34332	229533.I1RBC4	pp	229533.I1RJT9
34333	229533.I1RBC4	pp	229533.I1RZG7
34334	229533.I1RBC4	pp	229533.I1RP28
34335	229533.I1RBC4	pp	229533.I1RTU8
34336	229533.I1RBC4	pp	229533.I1RG13
34337	229533.I1RBC4	pp	229533.I1RZQ5
34338	229533.I1RBC4	pp	229533.I1RZ60
34339	229533.I1RBC4	pp	229533.I1RT41
34340	229533.I1RBC4	pp	229533.Q4HXF6
34341	229533.I1RBC4	pp	229533.I1RF94
34342	229533.I1RBC4	pp	229533.I1S2E9
34343	229533.I1RBC4	pp	229533.Q5I2J3
34344	229533.I1RBC4	pp	229533.I1RBL8
34345	229533.I1RBC4	pp	229533.I1RFY0
34346	229533.I1RBC4	pp	229533.I1RQC5
34347	229533.I1RBC4	pp	229533.I1RRU9
34348	229533.I1RBC4	pp	229533.I1S503
34349	229533.I1RBC4	pp	229533.I1RG60
34350	229533.I1RBC4	pp	229533.I1RJZ7
34351	229533.I1RBC4	pp	229533.I1RRT6
34352	229533.I1RBC4	pp	229533.I1RYS6
34353	229533.I1RBC4	pp	229533.I1RT62
34354	229533.I1RBC4	pp	229533.I1RQC9
34355	229533.I1RBC4	pp	229533.I1RR73
34356	229533.I1RBC4	pp	229533.I1RZZ9
34357	229533.I1RBC4	pp	229533.I1REK8
34358	229533.I1RBC4	pp	229533.Q4I5R9
34359	229533.I1RBC4	pp	229533.I1RCM5
34360	229533.I1RBC4	pp	229533.I1RWD7
34361	229533.I1RBC4	pp	229533.I1RRT8
34362	229533.I1RBC4	pp	229533.I1RQA5
34363	229533.I1RBC4	pp	229533.I1RX46
34364	229533.I1RBC4	pp	229533.I1RRM9
34365	229533.I1RBC4	pp	229533.I1RQH1
34366	229533.I1RBC4	pp	229533.I1RPK5
34367	229533.I1RBC4	pp	229533.I1S504
34368	229533.I1RBC4	pp	229533.V6RTJ8
34369	229533.I1RBC4	pp	229533.I1RW71
34370	229533.I1RBC4	pp	229533.I1RRF8
34371	229533.I1RBC4	pp	229533.I1RQL4
34372	229533.I1RIX0	pp	229533.I1RK45
34373	229533.I1RIX0	pp	229533.I1RP86
34374	229533.I1RIX0	pp	229533.I1RKR5
34375	229533.I1RIX0	pp	229533.I1RUY5
34376	229533.I1RIX0	pp	229533.I1S8E0
34377	229533.I1RIX0	pp	229533.I1RYK0
34378	229533.I1RIX0	pp	229533.I1RZ13
34379	229533.I1RIX0	pp	229533.I1RKJ7
34380	229533.I1RIX0	pp	229533.I1S272
34381	229533.I1RIX0	pp	229533.I1RSJ2
34382	229533.I1RIX0	pp	229533.I1RKP3
34383	229533.I1RIX0	pp	229533.I1RWF9

229533.I1RIX0	pp	229533.I1RJX2
229533.I1RIX0	pp	229533.V6RLM7
229533.I1RIX0	pp	229533.V6RV57
229533.I1RIX0	pp	229533.I1RJL9
229533.I1RIX0	pp	229533.I1RRT3
229533.I1RIX0	pp	229533.I1RUM7
229533.I1RIX0	pp	229533.Q4I1Y5
229533.I1RIX0	pp	229533.I1S5R6
229533.I1RIX0	pp	229533.I1RMX6
229533.I1RIX0	pp	229533.I1RPS2
229533.I1RMS2	pp	229533.I1RTA3
229533.I1RMS2	pp	229533.I1S276
229533.I1RMS2	pp	229533.I1RYY5
229533.I1RMS2	pp	229533.I1RRN5
229533.I1RMS2	pp	229533.I1RPM7
229533.I1RMS2	pp	229533.I1RZT1
229533.I1RMS2	pp	229533.I1RQA3
229533.I1RMS2	pp	229533.Q4IJM4
229533.I1RMS2	pp	229533.I1RXU0
229533.I1RMS2	pp	229533.I1RSC7
229533.I1RMS2	pp	229533.Q4HYI0
229533.I1RMS2	pp	229533.I1RY29
229533.I1RMS2	pp	229533.I1S2E9
229533.I1RMS2	pp	229533.I1RUG4
229533.I1RMS2	pp	229533.I1RRF9
229533.I1RMS2	pp	229533.I1RTC2
229533.I1RMS2	pp	229533.I1S209
229533.I1RMS2	pp	229533.I1S004
229533.I1RMS2	pp	229533.I1RWI8
229533.I1RMS2	pp	229533.I1RZ94
229533.I1RMS2	pp	229533.I1RWT6
229533.I1RMS2	pp	229533.I1RSZ3
229533.I1RMS2	pp	229533.I1SAD2
229533.I1RMS2	pp	229533.I1RPF5
229533.I1RMS2	pp	229533.I1RY44
229533.I1RMS2	pp	229533.I1RUF8
229533.I1RMS2	pp	229533.I1RV91
229533.I1RMS2	pp	229533.I1RVL8
229533.I1RAM3	pp	229533.I1RY33
229533.I1RAM3	pp	229533.I1RIY8
229533.I1RR44	pp	229533.I1RUY5
229533.I1RR44	pp	229533.V6RV57
229533.I1RR44	pp	229533.V6RPS5
229533.I1RR44	pp	229533.I1S148
229533.I1RR44	pp	229533.I1S147
229533.I1RR44	pp	229533.I1RSQ4
229533.I1RR44	pp	229533.V6RLM7
229533.I1RR44	pp	229533.I1RUY6
229533.I1RR44	pp	229533.I1S149
229533.I1S325	pp	229533.V6RJP8
229533.I1S325	pp	229533.I1S5X9
229533.I1S325	pp	229533.I1S4A5
229533.I1S325	pp	229533.I1S3H6
229533.A0A0E0RSG3	pp	229533.I1S2E9
229533.A0A0E0RSG3	pp	229533.A0A1C3YM29
229533.A0A0E0RSG3	pp	229533.I1RY44
229533.A0A0E0RSG3	pp	229533.A0A1C3YLT9
229533.A0A0E0RSG3	pp	229533.A0A0E0SA11
229533.A0A0E0RSG3	pp	229533.V6R227
229533.A0A0E0RSG3	pp	229533.A0A1C3YHY5
229533.A0A098E4T8	pp	229533.A0A1C3YJ56
229533.A0A098E4T8	pp	229533.Q4IPA4
229533.A0A098E4T8	pp	229533.I1S0N7
229533.A0A098E4T8	pp	229533.I1RLZ4
229533.A0A098E4T8	pp	229533.I1RQK2
229533.A0A098E4T8	pp	229533.I1RKB2
229533.A0A098E4T8	pp	229533.A0A1C3YKM6
229533.A0A098E4T8	pp	229533.I1RE45
229533.A0A098E4T8	pp	229533.Q4IF44
229533.A0A098E4T8	pp	229533.I1RA62
229533.A0A098E4T8	pp	229533.I1RF73
229533.A0A098E4T8	pp	229533.I1RYK0
229533.A0A098E4T8	pp	229533.V6RUY9

34457 229533.A0A098E4T8 pp 229533.I1RZP2
34458 229533.A0A098E4T8 pp 229533.I1RC29
34459 229533.A0A098E4T8 pp 229533.Q4IPC8
34460 229533.A0A098E4T8 pp 229533.I1RZV5
34461 229533.A0A098E4T8 pp 229533.A0A1C3YI27
34462 229533.A0A098E4T8 pp 229533.I1RC31
34463 229533.A0A098E4T8 pp 229533.I1RQI4
34464 229533.A0A098E4T8 pp 229533.I1RZQ1
34465 229533.A0A098E4T8 pp 229533.A0A1C3YI20
34466 229533.A0A098E4T8 pp 229533.I1S214
34467 229533.A0A098E4T8 pp 229533.I1RS03
34468 229533.A0A098E4T8 pp 229533.I1S8U9
34469 229533.A0A098E4T8 pp 229533.I1RNB8
34470 229533.A0A098E4T8 pp 229533.V6RVG3
34471 229533.A0A098E4T8 pp 229533.A0A0E0RTC6
34472 229533.A0A098E4T8 pp 229533.A0A1C3YJJ2
34473 229533.A0A098E4T8 pp 229533.I1RN49
34474 229533.A0A098E4T8 pp 229533.V6RLM7
34475 229533.A0A098E4T8 pp 229533.A0A1C3YII4
34476 229533.A0A098E4T8 pp 229533.A0A1C3YL54
34477 229533.A0A098E4T8 pp 229533.I1RP86
34478 229533.A0A098E4T8 pp 229533.A0A1C3YI81
34479 229533.A0A098E4T8 pp 229533.I1RZ26
34480 229533.A0A098E4T8 pp 229533.A0A1C3YN26
34481 229533.A0A098E4T8 pp 229533.A0A1C3YK33
34482 229533.A0A098E4T8 pp 229533.I1RYM6
34483 229533.A0A098E4T8 pp 229533.A0A1C3YM64
34484 229533.A0A098E4T8 pp 229533.I1RCG5
34485 229533.A0A098E4T8 pp 229533.I1RZ16
34486 229533.A0A098E4T8 pp 229533.I1S8V0
34487 229533.A0A098E4T8 pp 229533.A0A1C3YJW7
34488 229533.A0A098E4T8 pp 229533.I1S246
34489 229533.A0A098E4T8 pp 229533.A0A0E0RU55
34490 229533.A0A098E4T8 pp 229533.I1RYL8
34491 229533.A0A098E4T8 pp 229533.I1RZG7
34492 229533.A0A098E4T8 pp 229533.Q4IPN9
34493 229533.A0A098E4T8 pp 229533.I1RDD3
34494 229533.A0A098E4T8 pp 229533.I1RA46
34495 229533.A0A098E4T8 pp 229533.I1RTG4
34496 229533.A0A098E4T8 pp 229533.I1RW54
34497 229533.A0A098E4T8 pp 229533.A0A1C3YJZ0
34498 229533.A0A098E4T8 pp 229533.I1RT35
34499 229533.A0A098E4T8 pp 229533.I1RA90
34500 229533.A0A098E4T8 pp 229533.I1RC95
34501 229533.A0A098E4T8 pp 229533.I1RR44
34502 229533.A0A098E4T8 pp 229533.I1RN28
34503 229533.A0A098E4T8 pp 229533.I1RA94
34504 229533.A0A098E4T8 pp 229533.I1RUY5
34505 229533.A0A098E4T8 pp 229533.I1RC61
34506 229533.A0A098E4T8 pp 229533.I1S5Z2
34507 229533.A0A098E4T8 pp 229533.A0A1C3YJV9
34508 229533.A0A098E4T8 pp 229533.I1RY12
34509 229533.A0A098E4T8 pp 229533.I1RZL5
34510 229533.A0A098E4T8 pp 229533.I1RAQ4
34511 229533.A0A098E4T8 pp 229533.I1RKE1
34512 229533.A0A098E4T8 pp 229533.I1RCM5
34513 229533.A0A098E4T8 pp 229533.A0A1C3YL25
34514 229533.A0A098E4T8 pp 229533.I1S439
34515 229533.A0A098E4T8 pp 229533.I1RGB2
34516 229533.A0A098E4T8 pp 229533.Q4I948
34517 229533.A0A098E4T8 pp 229533.I1RDU0
34518 229533.A0A098E4T8 pp 229533.I1RQZ2
34519 229533.A0A098E4T8 pp 229533.I1RA38
34520 229533.A0A0E0S6X0 pp 229533.O74638
34521 229533.I1RUM7 pp 229533.V6R729
34522 229533.I1RUM7 pp 229533.I1RZ78
34523 229533.I1RUM7 pp 229533.I1RZM3
34524 229533.I1RUM7 pp 229533.Q4HTS9
34525 229533.I1RUM7 pp 229533.I1S444
34526 229533.I1RUM7 pp 229533.I1SAC4
34527 229533.I1RUM7 pp 229533.I1RWP9
34528 229533.I1RUM7 pp 229533.V6RUG6
34529 229533.I1RUM7 pp 229533.I1RUP6

34530	229533.I1RUM7	pp	229533.V6R3R0
34531	229533.I1RMM9	pp	229533.I1RN65
34532	229533.I1RMM9	pp	229533.I1RYZ0
34533	229533.I1RMM9	pp	229533.I1S224
34534	229533.I1RMM9	pp	229533.Q4IJM4
34535	229533.I1RMM9	pp	229533.I1S439
34536	229533.I1RMM9	pp	229533.I1RYH9
34537	229533.I1RMM9	pp	229533.I1RUQ5
34538	229533.I1RMM9	pp	229533.I1RV66
34539	229533.I1RMM9	pp	229533.I1RXH6
34540	229533.I1RMM9	pp	229533.I1RNC0
34541	229533.I1RMM9	pp	229533.V6QV09
34542	229533.I1RMM9	pp	229533.I1RQ26
34543	229533.I1RMM9	pp	229533.I1RN31
34544	229533.I1RMM9	pp	229533.I1RYY5
34545	229533.I1RMM9	pp	229533.I1RX70
34546	229533.I1RMM9	pp	229533.I1RWJ6
34547	229533.I1RMM9	pp	229533.I1RN08
34548	229533.I1RMM9	pp	229533.I1RQA3
34549	229533.I1RMM9	pp	229533.I1RZH4
34550	229533.I1RMM9	pp	229533.I1RYZ9
34551	229533.I1RMM9	pp	229533.I1RQG4
34552	229533.I1RMM9	pp	229533.I1RPL2
34553	229533.I1RMM9	pp	229533.I1RWT6
34554	229533.I1RMM9	pp	229533.I1RYW3
34555	229533.I1RMM9	pp	229533.I1S8P5
34556	229533.I1RMM9	pp	229533.I1RTA0
34557	229533.I1RMM9	pp	229533.I1RUN8
34558	229533.I1RMM9	pp	229533.I1RRY3
34559	229533.I1RMM9	pp	229533.I1RPD6
34560	229533.I1RMM9	pp	229533.I1S1D3
34561	229533.I1RMM9	pp	229533.I1RNF4
34562	229533.I1RMM9	pp	229533.I1S549
34563	229533.I1RMM9	pp	229533.I1RP28
34564	229533.I1RMM9	pp	229533.I1RNY8
34565	229533.I1RMM9	pp	229533.I1RPU3
34566	229533.I1RMM9	pp	229533.I1S2K7
34567	229533.I1RMM9	pp	229533.I1S1V5
34568	229533.I1RMM9	pp	229533.I1RV91
34569	229533.I1RMM9	pp	229533.I1RNP7
34570	229533.I1RMM9	pp	229533.I1S0D1
34571	229533.I1RMM9	pp	229533.I1S2E9
34572	229533.I1RMM9	pp	229533.I1RRM9
34573	229533.I1RMM9	pp	229533.I1RQF2
34574	229533.I1RMM9	pp	229533.I1RW16
34575	229533.I1RMM9	pp	229533.V6RKY7
34576	229533.I1RMM9	pp	229533.I1RY58
34577	229533.I1RMM9	pp	229533.I1RPW0
34578	229533.I1RMM9	pp	229533.V6RGI1
34579	229533.I1RMM9	pp	229533.I1RZE4
34580	229533.I1RMM9	pp	229533.I1RY44
34581	229533.I1RMM9	pp	229533.I1RT36
34582	229533.I1RMM9	pp	229533.V6RVG3
34583	229533.I1RMM9	pp	229533.I1S0N4
34584	229533.I1RMM9	pp	229533.I1RNW8
34585	229533.I1RMM9	pp	229533.I1S0M2
34586	229533.I1RMM9	pp	229533.I1S1W4
34587	229533.I1RMM9	pp	229533.I1RQ72
34588	229533.I1RMM9	pp	229533.I1RMQ5
34589	229533.I1RMM9	pp	229533.I1RRW0
34590	229533.I1RMM9	pp	229533.I1RQB4
34591	229533.I1RUY6	pp	229533.I1RYK0
34592	229533.I1RUY6	pp	229533.I1S3F7
34593	229533.I1RUY6	pp	229533.I1S0Y7
34594	229533.I1RUY6	pp	229533.I1RVR2
34595	229533.I1RUY6	pp	229533.I1RX89
34596	229533.I1RUY6	pp	229533.I1RXS0
34597	229533.I1RUY6	pp	229533.I1RY62
34598	229533.I1RUY6	pp	229533.I1S256
34599	229533.I1RUY6	pp	229533.I1S3L2
34600	229533.I1RUY6	pp	229533.V6RM02
34601	229533.I1RUY6	pp	229533.I1S0K0
34602	229533.I1RUY6	pp	229533.I1S5R6

34603	229533.I1RUY6	pp	229533.I1RWF5
34604	229533.I1RUY6	pp	229533.V6RLM7
34605	229533.I1RUY6	pp	229533.I1RY12
34606	229533.I1RUY6	pp	229533.I1S8R4
34607	229533.I1RMF4	pp	229533.I1RT64
34608	229533.I1RMF4	pp	229533.I1RUP0
34609	229533.I1RMF4	pp	229533.I1RZM0
34610	229533.I1RMG1	pp	229533.I1S4Z7
34611	229533.I1RMG1	pp	229533.I1RZT1
34612	229533.I1RMG1	pp	229533.I1RYE2
34613	229533.I1RUQ5	pp	229533.I1S1D3
34614	229533.I1RUQ5	pp	229533.I1RZH4
34615	229533.I1RUQ5	pp	229533.I1RWT6
34616	229533.I1RUQ5	pp	229533.I1S1W4
34617	229533.I1RUQ5	pp	229533.I1S2K7
34618	229533.I1RUQ5	pp	229533.I1RX69
34619	229533.I1RUQ5	pp	229533.V6RKY7
34620	229533.I1RUQ5	pp	229533.I1RZE4
34621	229533.I1RUQ5	pp	229533.Q4IJM4
34622	229533.I1RUQ5	pp	229533.V6RVG3
34623	229533.I1RUQ5	pp	229533.I1S0M2
34624	229533.I1RUQ5	pp	229533.I1S2B6
34625	229533.I1RUQ5	pp	229533.I1RY44
34626	229533.I1RUQ5	pp	229533.I1S439
34627	229533.I1RUQ5	pp	229533.V6RDP1
34628	229533.I1RUQ5	pp	229533.I1S0D1
34629	229533.I1RUQ5	pp	229533.I1S549
34630	229533.I1RUQ5	pp	229533.I1RYY5
34631	229533.I1RUQ5	pp	229533.I1S9Q3
34632	229533.I1RUQ5	pp	229533.I1RWJ6
34633	229533.I1RUQ5	pp	229533.I1RX70
34634	229533.I1RUQ5	pp	229533.I1RX38
34635	229533.I1RUQ5	pp	229533.I1RYH9
34636	229533.I1RUQ5	pp	229533.I1S224
34637	229533.I1RUQ5	pp	229533.I1RXH6
34638	229533.I1RUQ5	pp	229533.I1S1V5
34639	229533.I1RUQ5	pp	229533.I1S2E9
34640	229533.I1RUQ5	pp	229533.I1S2G7
34641	229533.I1RUQ5	pp	229533.I1RV66
34642	229533.I1RUQ5	pp	229533.I1RV91
34643	229533.I1S439	pp	229533.I1S4P6
34644	229533.I1S439	pp	229533.V6RKY7
34645	229533.I1S439	pp	229533.V6RMW6
34646	229533.I1S439	pp	229533.V6RVG3
34647	229533.I1S439	pp	229533.Q4IJM4
34648	229533.I1S439	pp	229533.I1S9Q3
34649	229533.I1S439	pp	229533.V6RGI1
34650	229533.I1S439	pp	229533.I1S549
34651	229533.I1S439	pp	229533.V6RUY9
34652	229533.I1S439	pp	229533.Q4IR09
34653	229533.I1S439	pp	229533.V6RDP1
34654	229533.I1S439	pp	229533.V6QV09
34655	229533.I1RAM0	pp	229533.I1RAM1
34656	229533.I1RSJ2	pp	229533.V6RPQ8
34657	229533.I1RSJ2	pp	229533.I1S8E0
34658	229533.I1RSJ2	pp	229533.Q4I1Y5
34659	229533.I1RSJ2	pp	229533.I1S5R6
34660	229533.I1RSJ2	pp	229533.I1RUM7
34661	229533.I1RSJ2	pp	229533.I1S272
34662	229533.I1RSJ2	pp	229533.I1RUY5
34663	229533.I1RSJ2	pp	229533.V6RV57
34664	229533.I1RSJ2	pp	229533.Q4I073
34665	229533.I1RSJ2	pp	229533.Q4IJT0
34666	229533.I1RSJ2	pp	229533.V6RLM7
34667	229533.I1RSJ2	pp	229533.I1RZ13
34668	229533.I1RSJ2	pp	229533.I1RWF9
34669	229533.I1RSJ2	pp	229533.I1RYK0
34670	229533.I1RC22	pp	229533.I1RRW0
34671	229533.I1RC22	pp	229533.I1RQB4
34672	229533.I1RC22	pp	229533.I1S1W4
34673	229533.I1RC22	pp	229533.I1S0M2
34674	229533.I1RC22	pp	229533.I1RRY3
34675	229533.I1RC22	pp	229533.I1S0N4

34676	229533.I1RC22	pp	229533.V6RVG3
34677	229533.I1RC22	pp	229533.I1RQ72
34678	229533.I1RC22	pp	229533.I1RY44
34679	229533.I1RC22	pp	229533.V6RGI1
34680	229533.I1RC22	pp	229533.V6RKY7
34681	229533.I1RC22	pp	229533.I1RT36
34682	229533.I1RC22	pp	229533.I1RI34
34683	229533.I1RC22	pp	229533.I1S757
34684	229533.I1RC22	pp	229533.I1RQD1
34685	229533.I1RC22	pp	229533.I1RNY9
34686	229533.I1RC22	pp	229533.I1RW16
34687	229533.I1RC22	pp	229533.I1RDZ4
34688	229533.I1RC22	pp	229533.I1S0D1
34689	229533.I1RC22	pp	229533.I1S2E9
34690	229533.I1RC22	pp	229533.I1RHQ8
34691	229533.I1RC22	pp	229533.I1RRN5
34692	229533.I1RC22	pp	229533.I1RV91
34693	229533.I1RC22	pp	229533.I1RPU7
34694	229533.I1RC22	pp	229533.I1RNY8
34695	229533.I1RC22	pp	229533.I1S2K7
34696	229533.I1RC22	pp	229533.I1RPW0
34697	229533.I1RC22	pp	229533.I1RRP7
34698	229533.I1RC22	pp	229533.I1RZE4
34699	229533.I1RC22	pp	229533.I1RCL8
34700	229533.I1RC22	pp	229533.I1S2G7
34701	229533.I1RC22	pp	229533.I1RMF5
34702	229533.I1RC22	pp	229533.I1RC97
34703	229533.I1RC22	pp	229533.I1RNF4
34704	229533.I1RC22	pp	229533.I1S7X9
34705	229533.I1RC22	pp	229533.I1S549
34706	229533.I1RC22	pp	229533.I1RUN8
34707	229533.I1RC22	pp	229533.I1RPD6
34708	229533.I1RC22	pp	229533.I1RX38
34709	229533.I1RC22	pp	229533.I1RFL7
34710	229533.I1RC22	pp	229533.I1RJ88
34711	229533.I1RC22	pp	229533.I1RP28
34712	229533.I1RC22	pp	229533.I1RCU9
34713	229533.I1RC22	pp	229533.V6RDP1
34714	229533.I1RC22	pp	229533.I1RNP7
34715	229533.I1RC22	pp	229533.I1RLU1
34716	229533.I1RC22	pp	229533.I1RQG4
34717	229533.I1RC22	pp	229533.I1RRM9
34718	229533.I1RC22	pp	229533.I1S1V5
34719	229533.I1RC22	pp	229533.I1RPL2
34720	229533.I1RC22	pp	229533.I1RI71
34721	229533.I1RC22	pp	229533.I1RPU4
34722	229533.I1RC22	pp	229533.I1RMA8
34723	229533.I1RC22	pp	229533.I1RZH4
34724	229533.I1RC22	pp	229533.I1RKK0
34725	229533.I1RC22	pp	229533.I1RM82
34726	229533.I1RC22	pp	229533.I1RMM9
34727	229533.I1RC22	pp	229533.I1RQD8
34728	229533.I1RC22	pp	229533.I1S0J7
34729	229533.I1RC22	pp	229533.I1RZ63
34730	229533.I1RC22	pp	229533.I1S0N8
34731	229533.I1RC22	pp	229533.I1RC95
34732	229533.I1RC22	pp	229533.I1RM09
34733	229533.I1RC22	pp	229533.I1RWT6
34734	229533.I1RC22	pp	229533.I1RN08
34735	229533.I1RC22	pp	229533.I1RCB3
34736	229533.I1RC22	pp	229533.I1RYY5
34737	229533.I1RC22	pp	229533.I1RQS4
34738	229533.I1RC22	pp	229533.I1RN65
34739	229533.I1RC22	pp	229533.I1RR94
34740	229533.I1RC22	pp	229533.I1RV66
34741	229533.I1RC22	pp	229533.I1RUQ5
34742	229533.I1RC22	pp	229533.I1RQA3
34743	229533.I1RC22	pp	229533.Q4IJM4
34744	229533.I1RC22	pp	229533.I1S439
34745	229533.I1RC22	pp	229533.I1RDB1
34746	229533.I1RC22	pp	229533.I1RJK7
34747	229533.I1RC22	pp	229533.I1RX70
34748	229533.I1RC22	pp	229533.I1RYZ9

34749	229533.I1RC22	pp	229533.I1RWJ6
34750	229533.I1RC22	pp	229533.I1RQA9
34751	229533.I1RC22	pp	229533.I1RYI9
34752	229533.I1RC22	pp	229533.I1RN31
34753	229533.I1RC22	pp	229533.V6QV09
34754	229533.I1RC22	pp	229533.I1RFZ3
34755	229533.I1RC22	pp	229533.I1RJH1
34756	229533.I1RC22	pp	229533.I1RPA6
34757	229533.I1RC22	pp	229533.I1RQ26
34758	229533.I1RC22	pp	229533.I1RJR8
34759	229533.I1RC22	pp	229533.I1S1Z9
34760	229533.I1RC22	pp	229533.I1RW25
34761	229533.I1RC22	pp	229533.I1RNC0
34762	229533.I1RC22	pp	229533.I1RD18
34763	229533.I1RC22	pp	229533.I1S1W5
34764	229533.I1RC22	pp	229533.I1RYH9
34765	229533.I1RC22	pp	229533.I1S224
34766	229533.I1RC22	pp	229533.I1RCU8
34767	229533.I1RC22	pp	229533.I1RXH6
34768	229533.I1RC22	pp	229533.I1RL24
34769	229533.I1RC22	pp	229533.I1RST8
34770	229533.I1RF61	pp	229533.I1RF62
34771	229533.I1RF61	pp	229533.I1RF63
34772	229533.I1RF56	pp	229533.I1RF60
34773	229533.I1RF56	pp	229533.I1RF64
34774	229533.I1RF56	pp	229533.I1RF59
34775	229533.I1RF56	pp	229533.I1RF57
34776	229533.I1RF56	pp	229533.I1RF58
34777	229533.I1RF56	pp	229533.I1RF63
34778	229533.I1RF56	pp	229533.I1RF62
34779	229533.I1RF56	pp	229533.I1RF61
34780	229533.I1RM78	pp	229533.I1S3H6
34781	229533.I1RM78	pp	229533.I1S6X4
34782	229533.I1RM78	pp	229533.I1RXN7
34783	229533.I1RM78	pp	229533.I1RN54
34784	229533.I1RM78	pp	229533.I1RZ35
34785	229533.I1RM78	pp	229533.I1S4A5
34786	229533.I1RM78	pp	229533.I1S325
34787	229533.I1RHC6	pp	229533.I1S3G4
34788	229533.I1RHC6	pp	229533.I1RNG4
34789	229533.I1RHC6	pp	229533.I1S1W8
34790	229533.A0A1C3YIF4	pp	229533.I1RCT2
34791	229533.A0A1C3YIF4	pp	229533.Q4HY90
34792	229533.A0A1C3YIF4	pp	229533.I1RLX5
34793	229533.A0A1C3YIF4	pp	229533.I1RTG5
34794	229533.A0A1C3YIF4	pp	229533.I1REK3
34795	229533.A0A1C3YIF4	pp	229533.I1RZV1
34796	229533.A0A1C3YIF4	pp	229533.I1RYW3
34797	229533.A0A1C3YIF4	pp	229533.I1RVY0
34798	229533.A0A1C3YIF4	pp	229533.I1RX69
34799	229533.A0A1C3YIF4	pp	229533.I1RJZ7
34800	229533.A0A1C3YIF4	pp	229533.I1S0S8
34801	229533.A0A1C3YIF4	pp	229533.I1RRF0
34802	229533.A0A1C3YIF4	pp	229533.I1RTE0
34803	229533.A0A1C3YIF4	pp	229533.I1RSZ6
34804	229533.A0A1C3YIF4	pp	229533.I1RY44
34805	229533.A0A1C3YIF4	pp	229533.I1S7W2
34806	229533.A0A1C3YIF4	pp	229533.I1RCJ9
34807	229533.A0A1C3YIF4	pp	229533.A0A1C3YMS7
34808	229533.A0A1C3YIF4	pp	229533.I1RZ91
34809	229533.A0A1C3YIF4	pp	229533.I1RB89
34810	229533.A0A1C3YIF4	pp	229533.Q4IPI4
34811	229533.A0A1C3YIF4	pp	229533.I1S2E9
34812	229533.A0A1C3YIF4	pp	229533.I1RCP7
34813	229533.A0A1C3YIF4	pp	229533.A0A1C3YLA2
34814	229533.A0A1C3YIF4	pp	229533.I1RKR9
34815	229533.A0A1C3YIF4	pp	229533.I1RE49
34816	229533.A0A1C3YIF4	pp	229533.I1RFS3
34817	229533.A0A1C3YIF4	pp	229533.I1RX68
34818	229533.A0A1C3YIF4	pp	229533.I1RBL4
34819	229533.A0A1C3YIF4	pp	229533.I1S7X9
34820	229533.A0A1C3YIF4	pp	229533.A0A1C3YLK5
34821	229533.A0A1C3YIF4	pp	229533.I1RXS3

34822 229533.A0A1C3YIF4 pp 229533.I1RZL3
34823 229533.A0A1C3YIF4 pp 229533.I1RCF0
34824 229533.A0A1C3YIF4 pp 229533.I1S559
34825 229533.A0A1C3YIF4 pp 229533.I1RQG4
34826 229533.A0A1C3YIF4 pp 229533.I1S248
34827 229533.A0A1C3YIF4 pp 229533.Q4I948
34828 229533.A0A1C3YIF4 pp 229533.I1RNG4
34829 229533.A0A1C3YIF4 pp 229533.I1RNK3
34830 229533.A0A1C3YIF4 pp 229533.I1RQM8
34831 229533.A0A1C3YIF4 pp 229533.I1RQP1
34832 229533.A0A1C3YIF4 pp 229533.A0A1C3YM61
34833 229533.A0A1C3YIF4 pp 229533.I1RWI6
34834 229533.A0A1C3YIF4 pp 229533.V6RPN0
34835 229533.A0A1C3YIF4 pp 229533.I1RQP3
34836 229533.A0A1C3YIF4 pp 229533.I1RBR0
34837 229533.A0A1C3YIF4 pp 229533.I1RSD7
34838 229533.A0A1C3YIF4 pp 229533.I1RR74
34839 229533.A0A1C3YIF4 pp 229533.I1RBB0
34840 229533.A0A1C3YIF4 pp 229533.I1RA72
34841 229533.A0A1C3YIF4 pp 229533.A0A1C3YME3
34842 229533.A0A1C3YIF4 pp 229533.I1RQP5
34843 229533.A0A1C3YIF4 pp 229533.Q4ICA8
34844 229533.A0A1C3YIF4 pp 229533.I1RAS5
34845 229533.A0A1C3YIF4 pp 229533.I1S1V2
34846 229533.A0A1C3YIF4 pp 229533.I1S0X0
34847 229533.A0A1C3YIF4 pp 229533.I1S270
34848 229533.A0A1C3YIF4 pp 229533.Q4I963
34849 229533.A0A1C3YIF4 pp 229533.I1RBL7
34850 229533.A0A1C3YIF4 pp 229533.I1RS38
34851 229533.A0A1C3YIF4 pp 229533.Q4INI2
34852 229533.A0A1C3YIF4 pp 229533.I1RMG2
34853 229533.A0A1C3YIF4 pp 229533.V6QW25
34854 229533.A0A1C3YIF4 pp 229533.I1RAN8
34855 229533.A0A1C3YIF4 pp 229533.I1RX09
34856 229533.A0A1C3YIF4 pp 229533.I1RRF9
34857 229533.A0A1C3YIF4 pp 229533.I1RYN9
34858 229533.A0A1C3YIF4 pp 229533.I1S034
34859 229533.A0A1C3YIF4 pp 229533.I1RT41
34860 229533.A0A1C3YIF4 pp 229533.I1RE51
34861 229533.A0A1C3YIF4 pp 229533.A0A1C3YK51
34862 229533.A0A1C3YIF4 pp 229533.I1RW71
34863 229533.A0A1C3YIF4 pp 229533.Q5I2J3
34864 229533.A0A1C3YIF4 pp 229533.A0A1C3YIQ1
34865 229533.A0A1C3YIF4 pp 229533.A0A1C3YLL9
34866 229533.A0A1C3YMX3 pp 229533.I1RNJ7
34867 229533.I1RQE9 pp 229533.I1RU02
34868 229533.I1RQE9 pp 229533.I1RZU9
34869 229533.I1RQE9 pp 229533.Q4HTT2
34870 229533.I1RQE9 pp 229533.Q4IER8
34871 229533.I1RQE9 pp 229533.I1RYV3
34872 229533.I1RQE9 pp 229533.V6QV38
34873 229533.I1RQE9 pp 229533.I1RUQ6
34874 229533.I1RQE9 pp 229533.I1RXE5
34875 229533.I1RQE9 pp 229533.Q4I5R3
34876 229533.I1RQE9 pp 229533.I1RW82
34877 229533.I1RQE9 pp 229533.I1S241
34878 229533.I1RQE9 pp 229533.I1RWU3
34879 229533.I1RQE9 pp 229533.I1RSL0
34880 229533.I1RQE9 pp 229533.I1RWW4
34881 229533.I1RQE9 pp 229533.I1RY44
34882 229533.I1RQE9 pp 229533.I1S2E9
34883 229533.I1RQE9 pp 229533.I1RT36
34884 229533.I1RQE9 pp 229533.I1RVY0
34885 229533.I1RQE9 pp 229533.Q4IMD1
34886 229533.I1RQE9 pp 229533.I1RVA7
34887 229533.I1RVD4 pp 229533.Q4HWQ2
34888 229533.A0A098DNE7 pp 229533.I1RV91
34889 229533.A0A098DNE7 pp 229533.I1RCD4
34890 229533.A0A098DNE7 pp 229533.A0A1C3YKB5
34891 229533.A0A098DNE7 pp 229533.A0A1C3YK51
34892 229533.A0A098DNE7 pp 229533.I1RUI0
34893 229533.A0A098DNE7 pp 229533.I1RTE6
34894 229533.A0A098DNE7 pp 229533.I1S0K7

34895 229533.A0A098DNE7 pp 229533.I1S2E9
34896 229533.A0A098DNE7 pp 229533.A0A1C3YMB7
34897 229533.A0A098DNE7 pp 229533.I1RD16
34898 229533.A0A098DNE7 pp 229533.I1RNW8
34899 229533.A0A098DNE7 pp 229533.I1RY44
34900 229533.A0A098DNE7 pp 229533.V6REE9
34901 229533.A0A098DNE7 pp 229533.I1RC17
34902 229533.A0A098DNE7 pp 229533.A0A0E0SA11
34903 229533.A0A098DNE7 pp 229533.I1RX09
34904 229533.A0A098DNE7 pp 229533.I1RC22
34905 229533.A0A098DNE7 pp 229533.I1RQA3
34906 229533.A0A098DNE7 pp 229533.I1RWT6
34907 229533.I1RDX7 pp 229533.I1RNJ7
34908 229533.I1RDX7 pp 229533.I1S9T8
34909 229533.I1RE25 pp 229533.V6RRF6
34910 229533.I1RJT1 pp 229533.I1RY44
34911 229533.I1RJT1 pp 229533.I1RWT6
34912 229533.I1RJT1 pp 229533.Q4IBU4
34913 229533.I1RJT1 pp 229533.I1RPN1
34914 229533.I1RJT1 pp 229533.I1S9T1
34915 229533.I1RJT1 pp 229533.I1RPK5
34916 229533.I1RJT1 pp 229533.I1RX04
34917 229533.I1RJT1 pp 229533.I1RNJ5
34918 229533.I1RJT1 pp 229533.I1RRS8
34919 229533.I1RJT1 pp 229533.I1RQ72
34920 229533.I1RJT1 pp 229533.I1RRK8
34921 229533.I1RJT1 pp 229533.I1S039
34922 229533.I1RJT1 pp 229533.V6QUV7
34923 229533.I1RJT1 pp 229533.I1RQA3
34924 229533.I1RJT1 pp 229533.Q4I2W2
34925 229533.I1RJT1 pp 229533.I1S1W5
34926 229533.I1RJT1 pp 229533.I1RTF0
34927 229533.I1RJT1 pp 229533.I1RSP4
34928 229533.I1RJT1 pp 229533.I1RZS4
34929 229533.I1RJT1 pp 229533.I1RNG4
34930 229533.I1RJT1 pp 229533.V6RM66
34931 229533.I1RJT1 pp 229533.I1RNF4
34932 229533.I1RJT1 pp 229533.I1S2E9
34933 229533.I1RJT1 pp 229533.I1S0Y1
34934 229533.I1RJT1 pp 229533.I1RV91
34935 229533.I1RJT1 pp 229533.I1RPX9
34936 229533.I1RJT1 pp 229533.I1RZH5
34937 229533.I1RJT1 pp 229533.I1RT38
34938 229533.I1RZ34 pp 229533.Q4IJT0
34939 229533.I1RZ34 pp 229533.I1S272
34940 229533.I1RZ34 pp 229533.Q4I073
34941 229533.I1RZ34 pp 229533.I1RZM0
34942 229533.I1RZ34 pp 229533.V6RPQ8
34943 229533.I1RZ34 pp 229533.Q4HZE0
34944 229533.I1RZ34 pp 229533.I1RZV2
34945 229533.I1RZ34 pp 229533.Q4IB63
34946 229533.I1RZ34 pp 229533.Q4I0K3
34947 229533.I1RM23 pp 229533.I1RWM0
34948 229533.I1RM23 pp 229533.I1RVX9
34949 229533.I1RM23 pp 229533.I1RRK7
34950 229533.I1RM23 pp 229533.I1S050
34951 229533.I1RM23 pp 229533.Q4HYC1
34952 229533.I1RM23 pp 229533.I1RWV4
34953 229533.I1RM23 pp 229533.I1RM77
34954 229533.I1RM23 pp 229533.I1RPD3
34955 229533.I1RM23 pp 229533.I1RM94
34956 229533.I1RQS4 pp 229533.I1RXZ4
34957 229533.I1RQS4 pp 229533.Q4I7N9
34958 229533.I1RQS4 pp 229533.I1RWT6
34959 229533.I1RQS4 pp 229533.I1RST9
34960 229533.I1RQS4 pp 229533.I1S334
34961 229533.I1RQS4 pp 229533.I1RRX6
34962 229533.I1RQS4 pp 229533.I1S267
34963 229533.I1RQS4 pp 229533.I1RSG0
34964 229533.I1RQS4 pp 229533.I1RRL5
34965 229533.I1RQS4 pp 229533.I1RY58
34966 229533.I1RQS4 pp 229533.V6QV09
34967 229533.I1RQS4 pp 229533.I1RYZ9

34968 229533.I1RQS4 pp 229533.I1RVN2
34969 229533.I1RQS4 pp 229533.I1RY44
34970 229533.I1RQS4 pp 229533.I1RRP7
34971 229533.I1RQS4 pp 229533.V6RGI1
34972 229533.I1RQS4 pp 229533.I1S0J7
34973 229533.I1RQS4 pp 229533.I1RSI3
34974 229533.I1RQS4 pp 229533.I1RZ63
34975 229533.I1RQS4 pp 229533.I1RZC3
34976 229533.I1RQS4 pp 229533.I1RX38
34977 229533.I1RQS4 pp 229533.I1S004
34978 229533.I1RQS4 pp 229533.I1S0M2
34979 229533.I1RQS4 pp 229533.I1S549
34980 229533.I1RQS4 pp 229533.I1S3H8
34981 229533.I1RQS4 pp 229533.I1S098
34982 229533.I1RQS4 pp 229533.I1RV91
34983 229533.I1RQS4 pp 229533.I1RTB2
34984 229533.I1RQS4 pp 229533.I1S2E9
34985 229533.I1RQS4 pp 229533.I1RY32
34986 229533.I1RQS4 pp 229533.I1S7X9
34987 229533.I1RBR3 pp 229533.I1RVS5
34988 229533.A0A1C3YI65 pp 229533.I1RRX1
34989 229533.A0A1C3YI65 pp 229533.Q4I8B6
34990 229533.A0A1C3YI65 pp 229533.I1RAW2
34991 229533.A0A1C3YI65 pp 229533.A0A1C3YII1
34992 229533.A0A1C3YI65 pp 229533.I1S4J4
34993 229533.A0A1C3YI65 pp 229533.I1RD66
34994 229533.I1RYA5 pp 229533.I1S984
34995 229533.I1RYA5 pp 229533.I1S574
34996 229533.I1RFQ5 pp 229533.I1RXU0
34997 229533.I1RFQ5 pp 229533.I1RG11
34998 229533.I1RFQ5 pp 229533.I1RPM7
34999 229533.I1RFQ5 pp 229533.I1RRN5
35000 229533.I1RFQ5 pp 229533.I1S0E8
35001 229533.I1RFQ5 pp 229533.I1S276
35002 229533.I1RFQ5 pp 229533.I1S0N5
35003 229533.I1RFQ5 pp 229533.I1RQP1
35004 229533.I1RFQ5 pp 229533.I1RV91
35005 229533.I1RFQ5 pp 229533.I1RSD4
35006 229533.I1RFQ5 pp 229533.I1RR25
35007 229533.I1RFQ5 pp 229533.I1RK79
35008 229533.I1RFQ5 pp 229533.I1RK90
35009 229533.I1RFQ5 pp 229533.I1RSZ3
35010 229533.I1RFQ5 pp 229533.I1RWI8
35011 229533.I1RFQ5 pp 229533.I1RVL8
35012 229533.I1RFQ5 pp 229533.I1RY44
35013 229533.I1RFQ5 pp 229533.I1RY29
35014 229533.I1RFQ5 pp 229533.I1RS41
35015 229533.I1RFQ5 pp 229533.I1S2E9
35016 229533.I1RFQ5 pp 229533.Q4ICA8
35017 229533.I1RFQ5 pp 229533.I1RVY0
35018 229533.I1RFQ5 pp 229533.I1RWT6
35019 229533.I1RFQ5 pp 229533.Q4IMD1
35020 229533.I1RFQ5 pp 229533.I1SAN0
35021 229533.I1RFQ5 pp 229533.Q4I5W5
35022 229533.I1RFQ5 pp 229533.Q4HYI0
35023 229533.I1RFQ5 pp 229533.I1RUF8
35024 229533.I1RFQ5 pp 229533.Q4HTT2
35025 229533.I1RFQ5 pp 229533.Q4IF76
35026 229533.I1RFQ5 pp 229533.I1SAA0
35027 229533.I1RFQ5 pp 229533.Q4I7L0
35028 229533.I1RFQ5 pp 229533.V6QUE6
35029 229533.I1RFQ5 pp 229533.I1RQA3
35030 229533.I1RFQ5 pp 229533.I1S5B0
35031 229533.I1RFQ5 pp 229533.I1RMQ7
35032 229533.I1RFQ5 pp 229533.Q4IJM4
35033 229533.I1RFQ5 pp 229533.I1RRT0
35034 229533.I1RFQ5 pp 229533.I1RYY5
35035 229533.I1RFQ5 pp 229533.I1RZB5
35036 229533.I1RFQ5 pp 229533.I1RK95
35037 229533.I1RFQ5 pp 229533.Q4HYQ4
35038 229533.I1RFQ5 pp 229533.I1S0F0
35039 229533.I1RFQ5 pp 229533.I1RRG3
35040 229533.I1RFQ5 pp 229533.I1RJL2

35041	229533.I1RFQ5	pp	229533.I1RQ93
35042	229533.I1RFQ5	pp	229533.I1RMS2
35043	229533.I1RFQ5	pp	229533.V6QWX8
35044	229533.I1RFQ5	pp	229533.I1RTC2
35045	229533.I1RFQ5	pp	229533.Q4IJH1
35046	229533.I1RFQ5	pp	229533.I1RM36
35047	229533.I1RFQ5	pp	229533.I1RKB1
35048	229533.I1RV02	pp	229533.I1S574
35049	229533.I1RV02	pp	229533.I1S984
35050	229533.I1RFJ9	pp	229533.I1RM67
35051	229533.I1RFJ9	pp	229533.I1RRT8
35052	229533.I1RFJ9	pp	229533.I1S5R1
35053	229533.I1RFJ9	pp	229533.I1RKJ6
35054	229533.I1RFJ9	pp	229533.I1RPU0
35055	229533.I1RFJ9	pp	229533.I1RWJ8
35056	229533.I1RFJ9	pp	229533.I1RPW9
35057	229533.I1RFJ9	pp	229533.I1S0L4
35058	229533.I1RFJ9	pp	229533.I1S2H4
35059	229533.I1RFJ9	pp	229533.I1S9U2
35060	229533.I1RFJ9	pp	229533.I1RR24
35061	229533.I1RFJ9	pp	229533.I1RMK3
35062	229533.I1RFJ9	pp	229533.Q4HVW2
35063	229533.I1RFJ9	pp	229533.I1RZZ9
35064	229533.I1RFJ9	pp	229533.I1RW25
35065	229533.I1RFJ9	pp	229533.I1RTF4
35066	229533.I1RFJ9	pp	229533.I1RNV6
35067	229533.I1RFJ9	pp	229533.Q4IFI0
35068	229533.I1RFJ9	pp	229533.Q4IJH1
35069	229533.I1RFJ9	pp	229533.I1RT80
35070	229533.I1RFJ9	pp	229533.I1RWJ4
35071	229533.I1RFJ9	pp	229533.I1RRM5
35072	229533.I1RFJ9	pp	229533.I1RT33
35073	229533.I1RFJ9	pp	229533.I1RJU3
35074	229533.I1RFJ9	pp	229533.I1RG51
35075	229533.I1RFJ9	pp	229533.I1RZ78
35076	229533.I1RFJ9	pp	229533.I1S8E2
35077	229533.I1RFJ9	pp	229533.Q4HZ42
35078	229533.I1RFJ9	pp	229533.I1RRQ3
35079	229533.I1RFJ9	pp	229533.I1RPM4
35080	229533.I1RFJ9	pp	229533.I1RFS3
35081	229533.I1RFJ9	pp	229533.I1RFW5
35082	229533.I1RFJ9	pp	229533.I1RWK3
35083	229533.I1RFJ9	pp	229533.I1RNH1
35084	229533.I1RFJ9	pp	229533.I1RW61
35085	229533.I1RFJ9	pp	229533.I1RN63
35086	229533.I1RFJ9	pp	229533.V6R283
35087	229533.I1RFJ9	pp	229533.I1RVQ0
35088	229533.I1RFJ9	pp	229533.I1RS30
35089	229533.I1RFJ9	pp	229533.I1RM06
35090	229533.I1RFJ9	pp	229533.I1RQJ5
35091	229533.I1RFJ9	pp	229533.I1RZS6
35092	229533.I1RFJ9	pp	229533.I1RX24
35093	229533.I1RFJ9	pp	229533.I1RZ99
35094	229533.I1RFJ9	pp	229533.I1RNA0
35095	229533.I1RFJ9	pp	229533.Q4IEV5
35096	229533.I1RFJ9	pp	229533.V6QYF0
35097	229533.I1RFJ9	pp	229533.I1RQ65
35098	229533.I1RFJ9	pp	229533.V6QW22
35099	229533.I1RFJ9	pp	229533.I1RPP3
35100	229533.I1RFJ9	pp	229533.I1S0L3
35101	229533.I1RFJ9	pp	229533.I1RS47
35102	229533.I1RFJ9	pp	229533.I1S2C0
35103	229533.I1RFJ9	pp	229533.I1RTE9
35104	229533.I1RFJ9	pp	229533.I1S0M3
35105	229533.I1RFJ9	pp	229533.I1RRJ8
35106	229533.I1RFJ9	pp	229533.I1RNK2
35107	229533.I1RFJ9	pp	229533.I1RSQ7
35108	229533.I1RFJ9	pp	229533.I1S8R3
35109	229533.I1RFJ9	pp	229533.I1RSX1
35110	229533.I1RFJ9	pp	229533.I1RW71
35111	229533.I1RFJ9	pp	229533.Q4IEK8
35112	229533.I1RFJ9	pp	229533.I1RKL3
35113	229533.I1RFJ9	pp	229533.I1RVZ0

229533.I1RFJ9 pp 229533.I1RM96
229533.I1RFJ9 pp 229533.I1S1J6
229533.I1RFJ9 pp 229533.I1RR00
229533.I1RFJ9 pp 229533.V6RIV9
229533.I1RFJ9 pp 229533.I1S4T3
229533.I1RFJ9 pp 229533.I1RKC6
229533.I1RFJ9 pp 229533.Q4I5Z5
229533.I1RFJ9 pp 229533.I1RX46
229533.I1RFJ9 pp 229533.I1RG56
229533.I1RFJ9 pp 229533.I1RRT2
229533.I1RFJ9 pp 229533.I1RJL4
229533.I1RFJ9 pp 229533.I1RPY1
229533.I1RFJ9 pp 229533.Q4IPI1
229533.I1RFJ9 pp 229533.I1S296
229533.I1RFJ9 pp 229533.Q4IAS1
229533.I1RFJ9 pp 229533.I1RQE7
229533.I1RFJ9 pp 229533.V6R227
229533.I1RFJ9 pp 229533.I1RRR7
229533.I1RFJ9 pp 229533.I1S2E8
229533.I1RFJ9 pp 229533.I1RZF6
229533.I1RFJ9 pp 229533.I1RLV9
229533.I1RFJ9 pp 229533.I1RSD8
229533.I1RFJ9 pp 229533.I1RJW6
229533.I1RFJ9 pp 229533.I1RFL4
229533.I1RFJ9 pp 229533.I1S0J1
229533.I1RFJ9 pp 229533.I1RN18
229533.I1RFJ9 pp 229533.Q4IQW0
229533.I1RFJ9 pp 229533.I1RW46
229533.I1RFJ9 pp 229533.I1RJV4
229533.I1RFJ9 pp 229533.I1RN99
229533.I1RFJ9 pp 229533.I1RFN1
229533.I1RFJ9 pp 229533.I1RQ28
229533.I1RFJ9 pp 229533.Q4IAA0
229533.I1RFJ9 pp 229533.I1RX14
229533.I1RFJ9 pp 229533.I1RK95
229533.I1RFJ9 pp 229533.I1RY63
229533.I1RFJ9 pp 229533.I1RZJ6
229533.I1RFJ9 pp 229533.Q4INZ9
229533.I1RFJ9 pp 229533.I1S1X9
229533.I1RFJ9 pp 229533.I1RS18
229533.I1RFJ9 pp 229533.I1RSS4
229533.I1RFJ9 pp 229533.Q4IJ56
229533.I1RFJ9 pp 229533.I1RT98
229533.I1RFJ9 pp 229533.I1RSC6
229533.I1RFJ9 pp 229533.I1S0M9
229533.I1RFJ9 pp 229533.I1RT40
229533.I1RFJ9 pp 229533.I1RJV3
229533.I1RFJ9 pp 229533.I1RUQ2
229533.I1RFJ9 pp 229533.I1RQA2
229533.I1RFJ9 pp 229533.I1S0R7
229533.I1RFJ9 pp 229533.I1RS17
229533.I1RFJ9 pp 229533.Q4IBS2
229533.I1RFJ9 pp 229533.I1RTI6
229533.I1RFJ9 pp 229533.I1RNU6
229533.I1RFJ9 pp 229533.I1RZE5
229533.I1RFJ9 pp 229533.I1S558
229533.I1RFJ9 pp 229533.I1RMN6
229533.I1RFJ9 pp 229533.I1RSR3
229533.I1RFJ9 pp 229533.I1RNH6
229533.I1RFJ9 pp 229533.V6RGN5
229533.I1RFJ9 pp 229533.I1S5B0
229533.I1RFJ9 pp 229533.I1RGC2
229533.I1RFJ9 pp 229533.I1RQ31
229533.I1RFJ9 pp 229533.I1S021
229533.I1RFJ9 pp 229533.I1RRI2
229533.I1RFJ9 pp 229533.I1S274
229533.I1RFJ9 pp 229533.I1RUG3
229533.I1RFJ9 pp 229533.I1RZM8
229533.I1RFJ9 pp 229533.V6RCG5
229533.I1RFJ9 pp 229533.I1S044
229533.I1RFJ9 pp 229533.I1RZW8
229533.I1RFJ9 pp 229533.I1RW99
229533.I1RFJ9 pp 229533.Q4I327

229533.I1RFJ9	pp	229533.I1RMD5
229533.I1RFJ9	pp	229533.I1RNY2
229533.I1RFJ9	pp	229533.I1RSR8
229533.I1RFJ9	pp	229533.I1RX08
229533.I1RFJ9	pp	229533.V6RCZ8
229533.I1RFJ9	pp	229533.I1RTD9
229533.I1RFJ9	pp	229533.I1RI82
229533.I1RFJ9	pp	229533.I1RSI6
229533.I1RX67	pp	229533.V6RJ22
229533.I1RX67	pp	229533.I1S1Z9
229533.I1RX67	pp	229533.I1S0Y1
229533.A0A098DDX7	pp	229533.V6RRF6
229533.A0A098DDX7	pp	229533.I1RJZ7
229533.A0A098DDX7	pp	229533.A0A0E0SC46
229533.A0A098DDX7	pp	229533.I1RYW3
229533.A0A098DDX7	pp	229533.I1RE25
229533.A0A098DDX7	pp	229533.I1RZ34
229533.A0A098DDX7	pp	229533.A0A1C3YLT9
229533.A0A098DDX7	pp	229533.I1RMJ3
229533.A0A098DDX7	pp	229533.I1RFT1
229533.A0A098DDX7	pp	229533.I1RN84
229533.A0A098DDX7	pp	229533.A0A1C3YLE2
229533.A0A098DDX7	pp	229533.I1RCR5
229533.A0A098DDX7	pp	229533.A0A1C3YK13
229533.A0A098DDX7	pp	229533.I1RZ94
229533.I1RCJ4	pp	229533.I1RJD7
229533.I1RCJ4	pp	229533.I1RCV0
229533.I1S4R0	pp	229533.I1S4R1
229533.A0A098DYT7	pp	229533.V6RQ48
229533.A0A098DYT7	pp	229533.I1REN4
229533.A0A098DYT7	pp	229533.I1RNR3
229533.A0A098DYT7	pp	229533.I1RNK3
229533.A0A098DYT7	pp	229533.V6RKE4
229533.A0A098DYT7	pp	229533.I1RZ91
229533.A0A098DYT7	pp	229533.A0A0E0SB24
229533.A0A098DYT7	pp	229533.A0A0E0SNM6
229533.A0A098DYT7	pp	229533.I1S2E9
229533.A0A098DYT7	pp	229533.I1RXU1
229533.A0A098DYT7	pp	229533.I1RLX5
229533.A0A098DYT7	pp	229533.I1RXF5
229533.A0A098DYT7	pp	229533.I1RN81
229533.A0A098DYT7	pp	229533.A0A1C3YI02
229533.A0A098DYT7	pp	229533.I1RT41
229533.A0A098DYT7	pp	229533.Q4ICG5
229533.A0A098DYT7	pp	229533.I1RE15
229533.A0A098DYT7	pp	229533.I1RB41
229533.A0A098DYT7	pp	229533.I1REK3
229533.A0A098DYT7	pp	229533.A0A1C3YIN0
229533.A0A098DYT7	pp	229533.I1RW32
229533.A0A098DYT7	pp	229533.I1RTD0
229533.A0A098DYT7	pp	229533.I1RX09
229533.A0A098DYT7	pp	229533.A0A1C3YHT4
229533.A0A098DYT7	pp	229533.A0A1C3YK51
229533.A0A098DYT7	pp	229533.A0A0E0SA11
229533.A0A098DYT7	pp	229533.A0A1C3YJH8
229533.A0A098DYT7	pp	229533.I1RE20
229533.A0A098DYT7	pp	229533.I1S1W5
229533.A0A098DYT7	pp	229533.I1RW84
229533.A0A098DYT7	pp	229533.I1RZA4
229533.A0A098DYT7	pp	229533.V6RR76
229533.A0A098DYT7	pp	229533.I1RD70
229533.A0A098DYT7	pp	229533.I1RLT6
229533.A0A098DYT7	pp	229533.I1RY44
229533.A0A098DYT7	pp	229533.I1RW38
229533.A0A098DYT7	pp	229533.I1RWT6
229533.A0A098DYT7	pp	229533.A0A1C3YIP6
229533.A0A098DYT7	pp	229533.A0A1C3YHW7
229533.A0A098DYT7	pp	229533.I1RQA3
229533.A0A098DYT7	pp	229533.V6QW25
229533.A0A098DYT7	pp	229533.A0A1C3YIF4
229533.A0A098DYT7	pp	229533.I1RWI6
229533.I1RS38	pp	229533.V6RQ48
229533.I1RS38	pp	229533.V6RKE4

35260 229533.I1RS38 pp 229533.I1RW38
35261 229533.I1RS38 pp 229533.I1RXF5
35262 229533.I1RS38 pp 229533.I1RZ91
35263 229533.I1RS38 pp 229533.I1RWP7
35264 229533.I1RS38 pp 229533.I1RY44
35265 229533.I1RS38 pp 229533.I1RW32
35266 229533.I1RS38 pp 229533.I1S2E9
35267 229533.I1RS38 pp 229533.I1RW84
35268 229533.I1RS38 pp 229533.I1S1W5
35269 229533.I1RS38 pp 229533.I1RXU1
35270 229533.I1RS38 pp 229533.I1RTD0
35271 229533.I1RS38 pp 229533.I1RT41
35272 229533.I1RS38 pp 229533.I1RX09
35273 229533.I1RS38 pp 229533.I1RZA4
35274 229533.I1RS38 pp 229533.I1RWI6
35275 229533.I1RS38 pp 229533.V6RR76
35276 229533.I1RS38 pp 229533.V6QW25
35277 229533.I1RS38 pp 229533.Q4ICG5
35278 229533.I1RS38 pp 229533.I1RWT6
35279 229533.I1RM12 pp 229533.I1RMX1
35280 229533.I1RM12 pp 229533.Q4IBS9
35281 229533.I1RM12 pp 229533.I1S248
35282 229533.I1RM12 pp 229533.I1RNM5
35283 229533.I1RM12 pp 229533.I1RM81
35284 229533.I1RM12 pp 229533.I1RMA7
35285 229533.A0A1C3YKJ5 pp 229533.I1REC7
35286 229533.A0A1C3YKJ5 pp 229533.I1RXJ0
35287 229533.A0A1C3YKJ5 pp 229533.I1RTI3
35288 229533.A0A098DDX6 pp 229533.I1S4R0
35289 229533.A0A098DDX6 pp 229533.I1RPZ8
35290 229533.A0A098DDX6 pp 229533.I1RM12
35291 229533.I1RMR7 pp 229533.I1RPR9
35292 229533.I1RMR7 pp 229533.I1RYB7
35293 229533.I1RMR7 pp 229533.I1RRX5
35294 229533.I1RMR7 pp 229533.I1RW38
35295 229533.I1RMR7 pp 229533.I1RW84
35296 229533.I1RMR7 pp 229533.I1RYI3
35297 229533.I1RMR7 pp 229533.I1S248
35298 229533.I1RMR7 pp 229533.I1RY44
35299 229533.I1RMR7 pp 229533.I1RRN5
35300 229533.I1RMR7 pp 229533.I1RWT6
35301 229533.I1RMR7 pp 229533.I1S2E9
35302 229533.I1RMR7 pp 229533.I1RQA3
35303 229533.I1RMR7 pp 229533.Q4IBS9
35304 229533.I1RMR7 pp 229533.I1RV91
35305 229533.I1RMR7 pp 229533.I1S074
35306 229533.I1RMR7 pp 229533.I1S1A3
35307 229533.I1RMR7 pp 229533.V6R3J1
35308 229533.I1RV01 pp 229533.I1S574
35309 229533.I1RV01 pp 229533.I1S984
35310 229533.I1RVS1 pp 229533.I1RW69
35311 229533.I1RMB2 pp 229533.I1RQ72
35312 229533.I1RMB2 pp 229533.I1RQA3
35313 229533.I1RMB2 pp 229533.I1RRN5
35314 229533.I1RMB2 pp 229533.Q4IR87
35315 229533.I1RMB2 pp 229533.I1S0X7
35316 229533.I1RMB2 pp 229533.I1RMB7
35317 229533.I1RMB2 pp 229533.I1RXH6
35318 229533.I1RMB2 pp 229533.I1S286
35319 229533.I1RMB2 pp 229533.I1RXX1
35320 229533.I1RMB2 pp 229533.I1RNF4
35321 229533.I1RMB2 pp 229533.I1RPK4
35322 229533.I1RMB2 pp 229533.I1RWK0
35323 229533.I1RMB2 pp 229533.I1S2E9
35324 229533.I1RMB2 pp 229533.I1RY44
35325 229533.I1RMB2 pp 229533.I1RWT6
35326 229533.I1RMB2 pp 229533.I1RSS1
35327 229533.I1RMB2 pp 229533.I1RP25
35328 229533.I1RMB2 pp 229533.I1RV91
35329 229533.I1RMB2 pp 229533.I1RX77
35330 229533.I1RMB2 pp 229533.Q4IEA7
35331 229533.I1RMB2 pp 229533.I1RXF0
35332 229533.I1RMB2 pp 229533.I1S2K7

35333 229533.A0A1C3YKT9 pp 229533.I1RYH6
35334 229533.A0A1C3YKT9 pp 229533.I1RZ02
35335 229533.A0A1C3YKT9 pp 229533.I1RPA6
35336 229533.A0A1C3YKT9 pp 229533.I1RSV0
35337 229533.A0A1C3YKT9 pp 229533.Q4INI2
35338 229533.A0A1C3YKT9 pp 229533.I1RN81
35339 229533.A0A1C3YKT9 pp 229533.I1RYI3
35340 229533.A0A1C3YKT9 pp 229533.I1S1B1
35341 229533.A0A1C3YKT9 pp 229533.I1RRI4
35342 229533.A0A1C3YKT9 pp 229533.V6RR76
35343 229533.A0A1C3YKT9 pp 229533.I1RA34
35344 229533.A0A1C3YKT9 pp 229533.I1RMR5
35345 229533.A0A1C3YKT9 pp 229533.I1RW38
35346 229533.A0A1C3YKT9 pp 229533.I1RNP1
35347 229533.A0A1C3YKT9 pp 229533.V6QUU6
35348 229533.A0A1C3YKT9 pp 229533.A0A1C3YLH7
35349 229533.A0A1C3YKT9 pp 229533.I1RX09
35350 229533.A0A1C3YKT9 pp 229533.I1S2B7
35351 229533.A0A1C3YKT9 pp 229533.I1R991
35352 229533.A0A1C3YKT9 pp 229533.I1RWI6
35353 229533.A0A1C3YKT9 pp 229533.I1S254
35354 229533.A0A1C3YKT9 pp 229533.I1RB15
35355 229533.A0A1C3YKT9 pp 229533.I1S095
35356 229533.A0A1C3YKT9 pp 229533.I1RMT4
35357 229533.A0A1C3YKT9 pp 229533.I1RRF9
35358 229533.A0A1C3YKT9 pp 229533.I1RTB7
35359 229533.A0A1C3YKT9 pp 229533.I1RZV4
35360 229533.A0A1C3YKT9 pp 229533.I1RH68
35361 229533.A0A1C3YKT9 pp 229533.I1RSB0
35362 229533.A0A1C3YKT9 pp 229533.V6R476
35363 229533.A0A1C3YKT9 pp 229533.I1S0X0
35364 229533.A0A1C3YKT9 pp 229533.P0C431
35365 229533.A0A1C3YKT9 pp 229533.I1S0S6
35366 229533.A0A1C3YKT9 pp 229533.A0A1C3YMQ5
35367 229533.A0A1C3YKT9 pp 229533.A0A1C3YMD9
35368 229533.A0A1C3YKT9 pp 229533.I1RN36
35369 229533.A0A1C3YKT9 pp 229533.I1RMP2
35370 229533.A0A1C3YKT9 pp 229533.I1RZD9
35371 229533.A0A1C3YKT9 pp 229533.I1RTG9
35372 229533.A0A1C3YKT9 pp 229533.I1RHI1
35373 229533.A0A1C3YKT9 pp 229533.I1RBD2
35374 229533.A0A1C3YKT9 pp 229533.A0A1C3YLL6
35375 229533.A0A1C3YKT9 pp 229533.I1S7X9
35376 229533.A0A1C3YKT9 pp 229533.I1RCN6
35377 229533.A0A1C3YKT9 pp 229533.I1RD10
35378 229533.A0A1C3YKT9 pp 229533.I1RTE0
35379 229533.A0A1C3YKT9 pp 229533.A0A1C3YL99
35380 229533.A0A1C3YKT9 pp 229533.I1RJS2
35381 229533.A0A1C3YKT9 pp 229533.I1RW84
35382 229533.A0A1C3YKT9 pp 229533.Q4I2W2
35383 229533.A0A1C3YKT9 pp 229533.I1RQN9
35384 229533.A0A1C3YKT9 pp 229533.I1RE19
35385 229533.A0A1C3YKT9 pp 229533.I1RKI7
35386 229533.A0A1C3YKT9 pp 229533.A0A1C3YM95
35387 229533.A0A1C3YKT9 pp 229533.I1RBD6
35388 229533.A0A1C3YKT9 pp 229533.A0A1C3YLN8
35389 229533.A0A1C3YKT9 pp 229533.I1RSZ6
35390 229533.I1RLT6 pp 229533.I1S2E9
35391 229533.I1RLT6 pp 229533.I1S074
35392 229533.I1RLT6 pp 229533.I1RS38
35393 229533.I1RLT6 pp 229533.I1RW32
35394 229533.I1RLT6 pp 229533.I1RV91
35395 229533.I1RLT6 pp 229533.I1RW76
35396 229533.I1RLT6 pp 229533.I1RYB7
35397 229533.I1RLT6 pp 229533.I1RZA4
35398 229533.I1RLT6 pp 229533.I1RW38
35399 229533.I1RLT6 pp 229533.I1RX09
35400 229533.I1RLT6 pp 229533.I1RW83
35401 229533.I1RLT6 pp 229533.I1RRN5
35402 229533.I1RLT6 pp 229533.I1RWT6
35403 229533.I1RLT6 pp 229533.I1RYI3
35404 229533.I1RLT6 pp 229533.Q4I1H6
35405 229533.I1RLT6 pp 229533.I1RXF5

35406 229533.I1RLT6 pp 229533.I1RY44
35407 229533.I1RLT6 pp 229533.I1RQA3
35408 229533.I1RLT6 pp 229533.I1RZ91
35409 229533.I1RLT6 pp 229533.I1SAT1
35410 229533.I1RLT6 pp 229533.V6RKE4
35411 229533.I1RLT6 pp 229533.I1RMR7
35412 229533.I1RLT6 pp 229533.V6RQ48
35413 229533.I1RLT6 pp 229533.Q4ICG5
35414 229533.I1RLT6 pp 229533.I1S248
35415 229533.I1RLT6 pp 229533.I1S635
35416 229533.I1RLT6 pp 229533.I1RTD0
35417 229533.I1RCV6 pp 229533.I1RRJ6
35418 229533.I1RCV6 pp 229533.I1RW71
35419 229533.I1RCV6 pp 229533.I1RJZ7
35420 229533.A0A1C3YHI6 pp 229533.I1RXV3
35421 229533.A0A1C3YHI6 pp 229533.O74638
35422 229533.A0A1C3YHI6 pp 229533.A0A1C3YHM9
35423 229533.A0A1C3YHI6 pp 229533.I1RY62
35424 229533.A0A1C3YHI6 pp 229533.I1S5S2
35425 229533.A0A1C3YHI6 pp 229533.I1S3V2
35426 229533.A0A1C3YHI6 pp 229533.I1RZ40
35427 229533.A0A1C3YHI6 pp 229533.A0A1C3YII7
35428 229533.A0A1C3YHI6 pp 229533.I1S256
35429 229533.A0A1C3YHI6 pp 229533.I1RNG1
35430 229533.A0A1C3YHI6 pp 229533.I1RA62
35431 229533.A0A1C3YHI6 pp 229533.I1RMW7
35432 229533.A0A1C3YHI6 pp 229533.A0A1C3YL15
35433 229533.A0A1C3YHI6 pp 229533.I1RNE7
35434 229533.A0A1C3YHI6 pp 229533.I1S4D0
35435 229533.A0A1C3YHI6 pp 229533.I1S9F1
35436 229533.A0A1C3YHI6 pp 229533.I1RYI6
35437 229533.A0A1C3YHI6 pp 229533.A0A1C3YJL7
35438 229533.I1RWY9 pp 229533.I1S095
35439 229533.A0A0E0SJJ3 pp 229533.I1RWY9
35440 229533.A0A0E0SJJ3 pp 229533.I1S095
35441 229533.I1RMQ7 pp 229533.I1RZ94
35442 229533.I1RMQ7 pp 229533.Q4HYQ4
35443 229533.I1RMQ7 pp 229533.I1RQ13
35444 229533.I1RMQ7 pp 229533.I1RTC2
35445 229533.I1RMQ7 pp 229533.I1RT01
35446 229533.I1RMQ7 pp 229533.I1RW90
35447 229533.I1RMQ7 pp 229533.I1RPQ1
35448 229533.I1RMQ7 pp 229533.I1RMY7
35449 229533.I1RMQ7 pp 229533.I1RRG3
35450 229533.I1RMQ7 pp 229533.V6QWX8
35451 229533.I1RMQ7 pp 229533.I1RRT0
35452 229533.I1RMQ7 pp 229533.Q4IQX3
35453 229533.I1RMQ7 pp 229533.I1RN55
35454 229533.I1RMQ7 pp 229533.I1S209
35455 229533.I1RMQ7 pp 229533.I1RTI6
35456 229533.I1RMQ7 pp 229533.I1RYJ8
35457 229533.I1RMQ7 pp 229533.I1RQP3
35458 229533.I1RMQ7 pp 229533.I1RQA3
35459 229533.I1RMQ7 pp 229533.Q4IJM4
35460 229533.I1RMQ7 pp 229533.I1RWD7
35461 229533.I1RMQ7 pp 229533.I1S004
35462 229533.I1RMQ7 pp 229533.I1S549
35463 229533.I1RMQ7 pp 229533.I1S8J7
35464 229533.I1RMQ7 pp 229533.I1S193
35465 229533.I1RMQ7 pp 229533.Q4I5I4
35466 229533.I1RMQ7 pp 229533.I1SAD2
35467 229533.I1RMQ7 pp 229533.V6QUE6
35468 229533.I1RMQ7 pp 229533.I1RMS2
35469 229533.I1RMQ7 pp 229533.I1RPC2
35470 229533.I1RMQ7 pp 229533.I1RZB5
35471 229533.I1RMQ7 pp 229533.I1RYY5
35472 229533.I1RMQ7 pp 229533.Q4INS6
35473 229533.I1RMQ7 pp 229533.I1RVL8
35474 229533.I1RMQ7 pp 229533.I1S9J6
35475 229533.I1RMQ7 pp 229533.Q4I7L0
35476 229533.I1RMQ7 pp 229533.Q4HYI0
35477 229533.I1RMQ7 pp 229533.Q4HTT2
35478 229533.I1RMQ7 pp 229533.I1RZ63

229533.I1RMQ7 pp 229533.I1S0J3
229533.I1RMQ7 pp 229533.Q4IPI4
229533.I1RMQ7 pp 229533.I1RWT6
229533.I1RMQ7 pp 229533.I1RR25
229533.I1RMQ7 pp 229533.I1RVH7
229533.I1RMQ7 pp 229533.Q4ICA8
229533.I1RMQ7 pp 229533.I1S0N5
229533.I1RMQ7 pp 229533.I1RRM8
229533.I1RMQ7 pp 229533.I1RSN7
229533.I1RMQ7 pp 229533.I1RUF8
229533.I1RMQ7 pp 229533.I1S2G7
229533.I1RMQ7 pp 229533.I1RRN5
229533.I1RMQ7 pp 229533.I1SAG3
229533.I1RMQ7 pp 229533.I1RUG4
229533.I1RMQ7 pp 229533.I1RSP7
229533.I1RMQ7 pp 229533.I1RY58
229533.I1RMQ7 pp 229533.I1S1W4
229533.I1RMQ7 pp 229533.I1RY29
229533.I1RMQ7 pp 229533.I1RPS0
229533.I1RMQ7 pp 229533.I1S0E8
229533.I1RMQ7 pp 229533.I1S2E9
229533.I1RMQ7 pp 229533.I1RW23
229533.I1RMQ7 pp 229533.Q4I5U9
229533.I1RMQ7 pp 229533.I1S098
229533.I1RMQ7 pp 229533.I1S276
229533.I1RMQ7 pp 229533.I1RWI8
229533.I1RMQ7 pp 229533.I1RRF0
229533.I1RMQ7 pp 229533.I1RZT1
229533.I1RMQ7 pp 229533.I1RY44
229533.I1RMQ7 pp 229533.I1RRR9
229533.I1RMQ7 pp 229533.I1RS41
229533.I1RMQ7 pp 229533.I1RSZ3
229533.I1RMQ7 pp 229533.I1RQ88
229533.I1RMQ7 pp 229533.I1RN57
229533.I1RMQ7 pp 229533.I1RSL8
229533.I1RMQ7 pp 229533.I1RV91
229533.I1RMQ7 pp 229533.I1RPM7
229533.I1RMQ7 pp 229533.I1RSD4
229533.I1RMQ7 pp 229533.I1RUN9
229533.I1RMQ7 pp 229533.I1RSR9
229533.I1RMQ7 pp 229533.V6RGI1
229533.I1RMQ7 pp 229533.I1RZP8
229533.I1RMQ7 pp 229533.I1RPF5
229533.I1RMQ7 pp 229533.I1RSC7
229533.I1RMQ7 pp 229533.I1RXU0
229533.I1RMQ7 pp 229533.I1RQP1
229533.A0A1C3YIM8 pp 229533.V6QUU6
229533.A0A1C3YIM8 pp 229533.V6R476
229533.A0A1C3YIM8 pp 229533.A0A1C3YIQ1
229533.A0A1C3YIM8 pp 229533.I1RPP0
229533.A0A1C3YIM8 pp 229533.A0A1C3YMQ5
229533.A0A1C3YIM8 pp 229533.I1RT41
229533.A0A1C3YIM8 pp 229533.I1RN81
229533.A0A1C3YIM8 pp 229533.I1S045
229533.A0A1C3YIM8 pp 229533.I1RRF9
229533.A0A1C3YIM8 pp 229533.I1REZ0
229533.A0A1C3YIM8 pp 229533.A0A1C3YM95
229533.A0A1C3YIM8 pp 229533.A0A1C3YLL6
229533.A0A1C3YIM8 pp 229533.I1S254
229533.A0A1C3YIM8 pp 229533.I1RSZ6
229533.A0A1C3YIM8 pp 229533.I1S0Z3
229533.A0A1C3YIM8 pp 229533.V6RR76
229533.A0A1C3YIM8 pp 229533.I1RSX3
229533.A0A1C3YIM8 pp 229533.I1RTE0
229533.A0A1C3YIM8 pp 229533.Q4I963
229533.A0A1C3YIM8 pp 229533.I1RWA5
229533.A0A1C3YIM8 pp 229533.I1RDG9
229533.A0A1C3YIM8 pp 229533.I1RRW2
229533.I1S9F1 pp 229533.O74638
229533.I1RB87 pp 229533.I1RJZ7
229533.I1RB87 pp 229533.I1RRJ6
229533.I1RB87 pp 229533.I1RW71
229533.I1RNR0 pp 229533.I1S927

35552 229533.I1RNR0 pp 229533.I1RZK4
35553 229533.I1RNR0 pp 229533.I1RZ48
35554 229533.I1RNR0 pp 229533.I1S461
35555 229533.A0A0E0SJM6 pp 229533.I1RT72
35556 229533.A0A0E0SJM6 pp 229533.A0A1C3YLP4
35557 229533.A0A0E0SJM6 pp 229533.I1RNR0
35558 229533.I1RJW5 pp 229533.I1RYE2
35559 229533.I1R9N6 pp 229533.Q4IPH4
35560 229533.I1R9N6 pp 229533.I1RFP9
35561 229533.I1R9N6 pp 229533.I1RCR5
35562 229533.I1R9N6 pp 229533.I1RCI4
35563 229533.I1R9N6 pp 229533.I1S0B1
35564 229533.I1R9N6 pp 229533.I1RPM4
35565 229533.I1R9N6 pp 229533.I1RCQ6
35566 229533.I1R9N6 pp 229533.I1RYB1
35567 229533.I1R9N6 pp 229533.I1RZQ5
35568 229533.I1R9N6 pp 229533.I1RMV5
35569 229533.I1R9N6 pp 229533.I1RD56
35570 229533.I1R9N6 pp 229533.I1RRW3
35571 229533.I1R9N6 pp 229533.I1RG90
35572 229533.I1R9N6 pp 229533.I1RZG7
35573 229533.I1R9N6 pp 229533.I1RLU5
35574 229533.I1R9N6 pp 229533.I1RYJ9
35575 229533.I1R9N6 pp 229533.I1RST1
35576 229533.I1R9N6 pp 229533.I1RND5
35577 229533.I1R9N6 pp 229533.I1RME9
35578 229533.I1R9N6 pp 229533.I1RBZ8
35579 229533.I1R9N6 pp 229533.I1S0Z9
35580 229533.I1R9N6 pp 229533.I1RPA0
35581 229533.I1R9N6 pp 229533.I1RA36
35582 229533.I1R9N6 pp 229533.I1RK92
35583 229533.I1R9N6 pp 229533.I1RSA4
35584 229533.I1R9N6 pp 229533.Q4HXF6
35585 229533.I1R9N6 pp 229533.I1RWX8
35586 229533.I1R9N6 pp 229533.V6QXG1
35587 229533.I1R9N6 pp 229533.I1S212
35588 229533.I1R9N6 pp 229533.I1RK96
35589 229533.I1R9N6 pp 229533.I1RX81
35590 229533.I1R9N6 pp 229533.Q4IB70
35591 229533.I1R9N6 pp 229533.I1RCN5
35592 229533.I1R9N6 pp 229533.I1RQC5
35593 229533.I1R9N6 pp 229533.I1RX51
35594 229533.I1R9N6 pp 229533.Q4HYR2
35595 229533.I1R9N6 pp 229533.Q4IAA0
35596 229533.I1R9N6 pp 229533.Q4I1Y1
35597 229533.I1R9N6 pp 229533.I1RC84
35598 229533.I1R9N6 pp 229533.Q4IPB3
35599 229533.I1R9N6 pp 229533.I1RAC1
35600 229533.I1R9N6 pp 229533.I1RPY4
35601 229533.I1R9N6 pp 229533.I1RCA7
35602 229533.I1R9N6 pp 229533.I1S9L5
35603 229533.I1R9N6 pp 229533.I1RG57
35604 229533.I1R9N6 pp 229533.I1RB50
35605 229533.I1R9N6 pp 229533.Q4IRI9
35606 229533.I1R9N6 pp 229533.I1RQP8
35607 229533.I1R9N6 pp 229533.Q4IBS2
35608 229533.I1R9N6 pp 229533.I1RLV9
35609 229533.I1R9N6 pp 229533.I1RN03
35610 229533.I1R9N6 pp 229533.I1RN59
35611 229533.I1R9N6 pp 229533.I1RU69
35612 229533.I1R9N6 pp 229533.I1S1C6
35613 229533.I1R9N6 pp 229533.I1S4M5
35614 229533.I1R9N6 pp 229533.I1RM25
35615 229533.I1R9N6 pp 229533.I1RFT1
35616 229533.I1R9N6 pp 229533.I1RYI9
35617 229533.I1R9N6 pp 229533.I1RK79
35618 229533.I1R9N6 pp 229533.I1RK90
35619 229533.I1R9N6 pp 229533.I1RYW3
35620 229533.I1R9N6 pp 229533.I1RS75
35621 229533.I1R9N6 pp 229533.I1RSH9
35622 229533.I1R9N6 pp 229533.Q4I5R9
35623 229533.I1R9N6 pp 229533.I1RPY5
35624 229533.I1R9N6 pp 229533.I1RXF2

35625	229533.I1R9N6	pp	229533.I1RMW7
35626	229533.I1R9N6	pp	229533.Q4IPB8
35627	229533.I1R9N6	pp	229533.I1RS28
35628	229533.I1R9N6	pp	229533.I1RFQ1
35629	229533.I1R9N6	pp	229533.I1S0Q7
35630	229533.I1R9N6	pp	229533.I1RSD4
35631	229533.I1R9N6	pp	229533.I1S9L6
35632	229533.I1R9N6	pp	229533.I1RBH8
35633	229533.I1R9N6	pp	229533.I1S7M2
35634	229533.I1R9N6	pp	229533.I1S2Y5
35635	229533.I1R9N6	pp	229533.I1S7M3
35636	229533.I1R9N6	pp	229533.I1RBV3
35637	229533.I1R9N6	pp	229533.I1RA43
35638	229533.I1R9N6	pp	229533.I1RCS2
35639	229533.I1R9N6	pp	229533.I1RK72
35640	229533.I1R9N6	pp	229533.I1RRL0
35641	229533.I1R9N6	pp	229533.I1RX47
35642	229533.I1R9N6	pp	229533.Q4IBK5
35643	229533.I1R9N6	pp	229533.Q4IE79
35644	229533.I1R9N6	pp	229533.I1R9N7
35645	229533.I1R9N6	pp	229533.I1RT17
35646	229533.I1RA06	pp	229533.I1REL7
35647	229533.I1RA06	pp	229533.I1RE00
35648	229533.I1RA06	pp	229533.I1S4Q4
35649	229533.I1RA06	pp	229533.I1S207
35650	229533.I1RA06	pp	229533.I1RCL5
35651	229533.I1RA06	pp	229533.I1RW71
35652	229533.I1RA06	pp	229533.I1RIE2
35653	229533.I1RA06	pp	229533.I1S2H3
35654	229533.I1RA06	pp	229533.I1RPI0
35655	229533.I1RA06	pp	229533.I1RN61
35656	229533.I1RA06	pp	229533.I1S4F4
35657	229533.I1RA06	pp	229533.I1RC05
35658	229533.I1RA06	pp	229533.I1RYU0
35659	229533.I1RA06	pp	229533.I1RK95
35660	229533.I1RA06	pp	229533.I1RPD6
35661	229533.I1RA06	pp	229533.I1REK8
35662	229533.I1RA06	pp	229533.I1S1J7
35663	229533.I1RA06	pp	229533.I1RGC6
35664	229533.I1RA06	pp	229533.I1S0R7
35665	229533.I1RA06	pp	229533.I1RXG6
35666	229533.I1RA06	pp	229533.I1RNA2
35667	229533.I1RA06	pp	229533.I1RQ81
35668	229533.I1RA06	pp	229533.I1RJZ7
35669	229533.I1RA06	pp	229533.I1RX46
35670	229533.I1RA06	pp	229533.I1RR95
35671	229533.I1RA06	pp	229533.I1RE76
35672	229533.I1RCT9	pp	229533.I1RK55
35673	229533.I1RCT9	pp	229533.I1RFB0
35674	229533.I1RCT9	pp	229533.I1RZZ9
35675	229533.I1RCT9	pp	229533.I1SAB4
35676	229533.I1RCT9	pp	229533.I1RZ38
35677	229533.I1RCT9	pp	229533.I1RTM8
35678	229533.I1RCT9	pp	229533.I1RQ48
35679	229533.I1RCT9	pp	229533.I1RF24
35680	229533.I1RCT9	pp	229533.I1RKQ0
35681	229533.I1RCT9	pp	229533.I1S210
35682	229533.I1RCT9	pp	229533.I1S968
35683	229533.I1RCT9	pp	229533.I1RW82
35684	229533.I1RCT9	pp	229533.I1RSK0
35685	229533.I1RCT9	pp	229533.I1RTP2
35686	229533.I1RCT9	pp	229533.I1S2E9
35687	229533.I1RCT9	pp	229533.I1RZ25
35688	229533.I1RCT9	pp	229533.Q4IB89
35689	229533.I1RCT9	pp	229533.I1RYP5
35690	229533.I1RCT9	pp	229533.I1RHU8
35691	229533.I1RCT9	pp	229533.I1RE52
35692	229533.I1RCT9	pp	229533.I1RMW9
35693	229533.I1RCT9	pp	229533.I1S048
35694	229533.I1RCT9	pp	229533.I1RHJ0
35695	229533.I1RCT9	pp	229533.I1S3T6
35696	229533.I1RCT9	pp	229533.I1RY44
35697	229533.I1RCT9	pp	229533.I1RMQ7

35698 229533.I1RCT9 pp 229533.I1RLA2
35699 229533.I1RCT9 pp 229533.I1RHT5
35700 229533.I1RCT9 pp 229533.I1S267
35701 229533.I1RCT9 pp 229533.I1S969
35702 229533.I1RCT9 pp 229533.I1RFN8
35703 229533.I1RCT9 pp 229533.I1RIM7
35704 229533.I1RCT9 pp 229533.I1RGU3
35705 229533.I1RCT9 pp 229533.I1S2F7
35706 229533.I1RCT9 pp 229533.I1RW55
35707 229533.I1RCT9 pp 229533.I1S223
35708 229533.I1RCT9 pp 229533.I1RST9
35709 229533.I1RCT9 pp 229533.I1RRC4
35710 229533.I1RCT9 pp 229533.I1S489
35711 229533.I1RCT9 pp 229533.I1RMW8
35712 229533.I1RCT9 pp 229533.I1REI2
35713 229533.I1RCT9 pp 229533.I1RRN4
35714 229533.I1RCT9 pp 229533.I1RM07
35715 229533.I1RCV9 pp 229533.I1RQ72
35716 229533.I1RCV9 pp 229533.I1RQA9
35717 229533.I1RCV9 pp 229533.I1RQB4
35718 229533.I1RCV9 pp 229533.I1RV66
35719 229533.I1RCV9 pp 229533.I1S0N8
35720 229533.I1RCV9 pp 229533.I1RGF1
35721 229533.I1RCV9 pp 229533.I1S2B0
35722 229533.I1RCV9 pp 229533.I1RQ48
35723 229533.I1RCV9 pp 229533.I1RNF4
35724 229533.I1RCV9 pp 229533.I1RPU7
35725 229533.I1RW15 pp 229533.I1RW71
35726 229533.I1RW15 pp 229533.I1RWJ1
35727 229533.I1RS07 pp 229533.I1RY44
35728 229533.I1RS07 pp 229533.V6R9T8
35729 229533.I1RS07 pp 229533.I1S267
35730 229533.I1RS07 pp 229533.I1S2I7
35731 229533.I1RS07 pp 229533.I1RWT6
35732 229533.I1RS07 pp 229533.I1RZS4
35733 229533.I1RS07 pp 229533.I1RV91
35734 229533.I1RS07 pp 229533.Q4I2W2
35735 229533.I1RS07 pp 229533.I1RWQ6
35736 229533.I1RS07 pp 229533.I1S2E9
35737 229533.I1RS07 pp 229533.I1S7L6
35738 229533.I1RS07 pp 229533.Q4IA56
35739 229533.A0A0E0SJC1 pp 229533.I1RPD6
35740 229533.A0A0E0SJC1 pp 229533.I1RA06
35741 229533.A0A0E0SJC1 pp 229533.I1RWA9
35742 229533.A0A0E0SJC1 pp 229533.A0A1C3YLE9
35743 229533.A0A0E0SJC1 pp 229533.I1RIE2
35744 229533.A0A0E0SJC1 pp 229533.I1S207
35745 229533.I1RD79 pp 229533.Q4HX89
35746 229533.I1RD79 pp 229533.I1RMK3
35747 229533.I1RKG3 pp 229533.I1RSF1
35748 229533.I1RKG3 pp 229533.I1RKP1
35749 229533.I1RKG3 pp 229533.V6RCZ8
35750 229533.I1RKG3 pp 229533.I1S5B0
35751 229533.I1RKG3 pp 229533.I1RUG3
35752 229533.I1RKG3 pp 229533.I1RRM3
35753 229533.I1RKG3 pp 229533.I1RQE7
35754 229533.I1RKG3 pp 229533.I1RZK5
35755 229533.I1RKG3 pp 229533.I1RZQ6
35756 229533.I1RKG3 pp 229533.I1RQ33
35757 229533.I1RKG3 pp 229533.I1RRI1
35758 229533.I1RKG3 pp 229533.I1S5R1
35759 229533.I1RT81 pp 229533.I1RW71
35760 229533.I1RT81 pp 229533.I1RYH0
35761 229533.I1RT81 pp 229533.I1RWN4
35762 229533.I1RP74 pp 229533.I1RW76
35763 229533.I1RVB1 pp 229533.I1RYU6
35764 229533.A0A1C3YLR9 pp 229533.I1RX91
35765 229533.A0A1C3YLR9 pp 229533.I1RYE9
35766 229533.A0A1C3YLR9 pp 229533.I1S0R1
35767 229533.I1S7B8 pp 229533.V6RGB5
35768 229533.I1S7B8 pp 229533.I1S7B9
35769 229533.I1S7B8 pp 229533.Q4I5R9
35770 229533.I1RHF8 pp 229533.I1RTI3

35771	229533.I1RHF8	pp	229533.I1RXJ0
35772	229533.I1RAU5	pp	229533.I1RQ23
35773	229533.I1RAU5	pp	229533.I1S504
35774	229533.I1RAU5	pp	229533.I1SA32
35775	229533.I1RAU5	pp	229533.I1RFU8
35776	229533.I1RAU5	pp	229533.I1RCG4
35777	229533.I1RAU5	pp	229533.Q4I662
35778	229533.I1RAU5	pp	229533.I1RNQ5
35779	229533.I1RAU5	pp	229533.I1RS18
35780	229533.I1RAU5	pp	229533.I1RB27
35781	229533.I1RAU5	pp	229533.I1RFS3
35782	229533.I1RAU5	pp	229533.I1RLU6
35783	229533.I1RAU5	pp	229533.I1RPM4
35784	229533.I1RAU5	pp	229533.I1RZM1
35785	229533.I1RAU5	pp	229533.I1RBJ5
35786	229533.I1RAU5	pp	229533.Q4I8P5
35787	229533.I1RAU5	pp	229533.I1S0M6
35788	229533.I1RAU5	pp	229533.I1RZS6
35789	229533.I1RAU5	pp	229533.I1S0M9
35790	229533.I1RAU5	pp	229533.I1RRQ3
35791	229533.I1RAU5	pp	229533.I1RQ81
35792	229533.I1RAU5	pp	229533.I1RP28
35793	229533.I1RAU5	pp	229533.I1RE22
35794	229533.I1RAU5	pp	229533.I1RFT2
35795	229533.I1RAU5	pp	229533.I1RBG9
35796	229533.I1RAU5	pp	229533.I1RGC2
35797	229533.I1RAU5	pp	229533.I1RRU9
35798	229533.I1RAU5	pp	229533.I1RBS0
35799	229533.I1RAU5	pp	229533.I1RN67
35800	229533.I1RAU5	pp	229533.I1RNG4
35801	229533.I1RAU5	pp	229533.I1RRT8
35802	229533.I1RAU5	pp	229533.I1S0F6
35803	229533.I1RAU5	pp	229533.I1S5B0
35804	229533.I1RAU5	pp	229533.I1RW99
35805	229533.I1RAU5	pp	229533.I1S5R1
35806	229533.I1RAU5	pp	229533.I1RQ65
35807	229533.I1RAU5	pp	229533.I1RWL2
35808	229533.I1RAU5	pp	229533.I1RC17
35809	229533.I1RAU5	pp	229533.I1S2G7
35810	229533.I1RAU5	pp	229533.I1RI82
35811	229533.I1RAU5	pp	229533.I1RM82
35812	229533.I1RAU5	pp	229533.I1RKJ6
35813	229533.I1RAU5	pp	229533.I1RNH6
35814	229533.I1RAU5	pp	229533.I1RS47
35815	229533.I1RAU5	pp	229533.I1RWK3
35816	229533.I1RAU5	pp	229533.I1S0L4
35817	229533.I1RAU5	pp	229533.I1S021
35818	229533.I1RAU5	pp	229533.I1RZM8
35819	229533.I1RAU5	pp	229533.I1RIL3
35820	229533.I1RAU5	pp	229533.I1RC00
35821	229533.I1RAU5	pp	229533.V6R283
35822	229533.I1RAU5	pp	229533.I1RXF0
35823	229533.I1RAU5	pp	229533.I1RD64
35824	229533.I1RAU5	pp	229533.I1RC03
35825	229533.I1RAU5	pp	229533.V6RCG5
35826	229533.I1RAU5	pp	229533.I1RB74
35827	229533.I1RAU5	pp	229533.I1S2H4
35828	229533.I1RAU5	pp	229533.V6RCZ8
35829	229533.I1RAU5	pp	229533.I1S8P5
35830	229533.I1RAU5	pp	229533.I1RS20
35831	229533.I1RAU5	pp	229533.I1RPP3
35832	229533.I1RAU5	pp	229533.I1RFT6
35833	229533.I1RAU5	pp	229533.I1RC66
35834	229533.I1RAU5	pp	229533.I1RPU3
35835	229533.I1RAU5	pp	229533.I1RWF7
35836	229533.I1RAU5	pp	229533.I1RRJ8
35837	229533.I1RAU5	pp	229533.I1RNN1
35838	229533.I1RAU5	pp	229533.I1RQG4
35839	229533.I1RAU5	pp	229533.I1RBY4
35840	229533.I1RAU5	pp	229533.I1RWI5
35841	229533.I1RAU5	pp	229533.I1RE76
35842	229533.I1RAU5	pp	229533.I1RJZ7
35843	229533.I1RAU5	pp	229533.I1S352

35844	229533.I1RAU5	pp	229533.I1RZZ9
35845	229533.I1RAU5	pp	229533.I1RCJ7
35846	229533.I1RAU5	pp	229533.I1RT40
35847	229533.I1RAU5	pp	229533.I1RKL3
35848	229533.I1RAU5	pp	229533.I1RSV2
35849	229533.I1RAU5	pp	229533.I1RR73
35850	229533.I1RAU5	pp	229533.I1RFT1
35851	229533.I1RAU5	pp	229533.I1S2E9
35852	229533.I1RAU5	pp	229533.V6R3X5
35853	229533.I1RAU5	pp	229533.I1RNA5
35854	229533.I1RAU5	pp	229533.I1RT04
35855	229533.I1RAU5	pp	229533.I1RLV9
35856	229533.I1RAU5	pp	229533.I1RZ39
35857	229533.I1RAU5	pp	229533.I1RLN6
35858	229533.I1RAU5	pp	229533.I1RJW6
35859	229533.I1RAU5	pp	229533.I1RGL4
35860	229533.I1RAU5	pp	229533.I1RRI2
35861	229533.I1RAU5	pp	229533.I1RB41
35862	229533.I1RAU5	pp	229533.I1RAU7
35863	229533.I1RAU5	pp	229533.I1S569
35864	229533.I1RAU5	pp	229533.I1RZW8
35865	229533.I1RAU5	pp	229533.I1S503
35866	229533.I1RAU5	pp	229533.I1RP27
35867	229533.I1RAU5	pp	229533.I1RJV4
35868	229533.I1RAU5	pp	229533.I1S1C7
35869	229533.I1RAU5	pp	229533.V6R057
35870	229533.I1RAU5	pp	229533.I1RD17
35871	229533.I1RAU5	pp	229533.I1RBS6
35872	229533.I1RAU5	pp	229533.I1RYW3
35873	229533.I1RAU5	pp	229533.I1RGA4
35874	229533.I1RAU5	pp	229533.I1RGF5
35875	229533.I1RAU5	pp	229533.I1RTD5
35876	229533.I1RAU5	pp	229533.I1S2C0
35877	229533.I1RAU5	pp	229533.Q4I624
35878	229533.I1RAU5	pp	229533.I1RZE4
35879	229533.I1RAU5	pp	229533.I1RV91
35880	229533.I1RAU5	pp	229533.I1S0N4
35881	229533.I1RAU5	pp	229533.I1RTH6
35882	229533.I1RAU5	pp	229533.I1RCH5
35883	229533.I1RAU5	pp	229533.I1S2E8
35884	229533.I1RAU5	pp	229533.I1RQE7
35885	229533.I1RAU5	pp	229533.I1S8R3
35886	229533.I1RAU5	pp	229533.I1RNY2
35887	229533.I1RAU5	pp	229533.I1RM96
35888	229533.I1RAU5	pp	229533.I1RFL4
35889	229533.I1RAU5	pp	229533.Q4I327
35890	229533.I1RAU5	pp	229533.I1RY44
35891	229533.I1RAU5	pp	229533.I1RSD8
35892	229533.I1RAU5	pp	229533.V6RIV9
35893	229533.I1RAU5	pp	229533.I1RN18
35894	229533.I1RAU5	pp	229533.I1RB92
35895	229533.I1RAU5	pp	229533.I1RVX0
35896	229533.I1RAU5	pp	229533.I1RWN5
35897	229533.I1RAU5	pp	229533.I1RNC1
35898	229533.I1RAU5	pp	229533.I1RUG3
35899	229533.I1RAU5	pp	229533.I1RCR8
35900	229533.I1RAU5	pp	229533.I1RRN5
35901	229533.I1RAU5	pp	229533.I1RT03
35902	229533.I1RAU5	pp	229533.Q4HZB8
35903	229533.I1RAU5	pp	229533.I1RSK1
35904	229533.I1RAU5	pp	229533.I1RRR7
35905	229533.I1RAU5	pp	229533.Q4IN63
35906	229533.I1RAU5	pp	229533.I1RMQ5
35907	229533.I1RAU5	pp	229533.I1RJV3
35908	229533.I1RAU5	pp	229533.I1RCA0
35909	229533.I1RAU5	pp	229533.I1RRE5
35910	229533.I1RAU5	pp	229533.V6R042
35911	229533.I1RAU5	pp	229533.I1S1W4
35912	229533.I1RAU5	pp	229533.I1S0J1
35913	229533.I1RAU5	pp	229533.I1RSQ3
35914	229533.I1RAU5	pp	229533.I1RCM3
35915	229533.I1RAU5	pp	229533.I1RD39
35916	229533.I1RAU5	pp	229533.I1RE01

35917 229533.I1RAU5 pp 229533.V6RMW6
35918 229533.I1RAU5 pp 229533.I1RMG3
35919 229533.I1RAU5 pp 229533.I1RWN6
35920 229533.I1RAU5 pp 229533.I1RAW0
35921 229533.I1RAU5 pp 229533.I1RW46
35922 229533.I1RAU5 pp 229533.I1RWX5
35923 229533.I1RAU5 pp 229533.I1RJP4
35924 229533.I1RAU5 pp 229533.I1RRV1
35925 229533.I1RAU5 pp 229533.I1RZS7
35926 229533.I1RAU5 pp 229533.I1RTA0
35927 229533.I1RAU5 pp 229533.I1RPP4
35928 229533.I1RAU5 pp 229533.I1RS17
35929 229533.I1RAU5 pp 229533.I1RPK0
35930 229533.I1RAU5 pp 229533.I1RD22
35931 229533.I1RAU5 pp 229533.I1SA33
35932 229533.I1RAU5 pp 229533.I1RMN6
35933 229533.I1RAU5 pp 229533.I1RZJ5
35934 229533.I1RAU5 pp 229533.I1RWJ8
35935 229533.I1RAU5 pp 229533.I1RPU0
35936 229533.I1RAU5 pp 229533.I1RM67
35937 229533.I1RAU5 pp 229533.I1S9U2
35938 229533.I1RAU5 pp 229533.I1RPW9
35939 229533.I1RAU5 pp 229533.I1RW71
35940 229533.I1RAU5 pp 229533.I1RBC9
35941 229533.I1RAU5 pp 229533.I1RR24
35942 229533.I1RAU5 pp 229533.I1RR00
35943 229533.I1RAU5 pp 229533.I1RBT1
35944 229533.I1RAU5 pp 229533.I1RMC8
35945 229533.I1RAU5 pp 229533.I1RX46
35946 229533.I1RAU5 pp 229533.I1S0X6
35947 229533.I1RAU5 pp 229533.V6RTJ8
35948 229533.I1RAU5 pp 229533.I1RCC2
35949 229533.I1RAU5 pp 229533.I1S6U8
35950 229533.I1RAU5 pp 229533.I1RKB7
35951 229533.I1RAU5 pp 229533.I1RAX6
35952 229533.I1RAU5 pp 229533.I1S7A8
35953 229533.I1RAU5 pp 229533.I1RQH3
35954 229533.I1RAU5 pp 229533.I1RT80
35955 229533.I1RAU5 pp 229533.I1RIG2
35956 229533.I1RAU5 pp 229533.I1RJU3
35957 229533.I1RAU5 pp 229533.I1RXD7
35958 229533.I1RAU5 pp 229533.I1RQC9
35959 229533.I1RAU5 pp 229533.I1RW25
35960 229533.I1RAU5 pp 229533.I1RDU4
35961 229533.I1RAU5 pp 229533.V6R4J1
35962 229533.I1RAU5 pp 229533.I1RX25
35963 229533.I1RAU5 pp 229533.I1RLM7
35964 229533.I1RAU5 pp 229533.I1S0R7
35965 229533.I1RAU5 pp 229533.I1RVQ0
35966 229533.I1RAU5 pp 229533.I1RLC7
35967 229533.I1RAU5 pp 229533.I1RI02
35968 229533.I1RAU5 pp 229533.V6R1K4
35969 229533.I1RAU5 pp 229533.I1RW07
35970 229533.I1RAU5 pp 229533.I1RZE5
35971 229533.I1RAU5 pp 229533.I1S0L3
35972 229533.I1RAU5 pp 229533.I1RE00
35973 229533.I1RAU5 pp 229533.I1RM06
35974 229533.I1RAU5 pp 229533.I1S0F5
35975 229533.I1RAU5 pp 229533.V6QUW4
35976 229533.I1RAU5 pp 229533.I1RSI0
35977 229533.I1RAU5 pp 229533.I1RFQ7
35978 229533.I1RAU5 pp 229533.I1RVZ0
35979 229533.I1RAU5 pp 229533.I1RIL8
35980 229533.I1RAU5 pp 229533.I1RRQ1
35981 229533.I1RAU5 pp 229533.I1RD52
35982 229533.I1RAU5 pp 229533.Q4IJT5
35983 229533.I1RAU5 pp 229533.I1RMG8
35984 229533.I1RAU5 pp 229533.Q4I2J8
35985 229533.I1RAU5 pp 229533.I1RB75
35986 229533.I1RAU5 pp 229533.I1RWD7
35987 229533.I1RAU5 pp 229533.I1REK8
35988 229533.I1RAU5 pp 229533.I1RQA3
35989 229533.I1RAU5 pp 229533.I1RFW5

35990	229533.I1RAU5	pp	229533.I1RCR5
35991	229533.I1RAU5	pp	229533.Q4IJK2
35992	229533.I1RAU5	pp	229533.I1RG53
35993	229533.I1RAU5	pp	229533.V6QW22
35994	229533.I1RAU5	pp	229533.I1RQJ5
35995	229533.I1RAU5	pp	229533.Q4IJM4
35996	229533.I1RAU5	pp	229533.I1RZK6
35997	229533.I1RAU5	pp	229533.I1RDG0
35998	229533.I1RAU5	pp	229533.I1RZZ1
35999	229533.I1RAU5	pp	229533.I1RC20
36000	229533.I1RAU5	pp	229533.I1RN61
36001	229533.I1RAU5	pp	229533.I1RI03
36002	229533.I1RAU5	pp	229533.I1RSQ7
36003	229533.I1RAU5	pp	229533.I1RQQ5
36004	229533.I1RAU5	pp	229533.V6RBN2
36005	229533.I1RAU5	pp	229533.I1S1J7
36006	229533.I1RAU5	pp	229533.I1RJZ6
36007	229533.I1RAU5	pp	229533.I1RRY9
36008	229533.I1RAU5	pp	229533.I1RU29
36009	229533.I1RAU5	pp	229533.Q4I283
36010	229533.I1RAU5	pp	229533.I1RBL7
36011	229533.I1RAU5	pp	229533.I1RZF6
36012	229533.I1RAU5	pp	229533.I1RRM5
36013	229533.I1RAU5	pp	229533.I1RWT6
36014	229533.I1RAU5	pp	229533.I1RSR8
36015	229533.I1RAU5	pp	229533.I1RY03
36016	229533.I1RAU5	pp	229533.I1RYY5
36017	229533.I1RAU5	pp	229533.I1RZK5
36018	229533.I1RAU5	pp	229533.I1S2B8
36019	229533.I1RJ19	pp	229533.I1RK41
36020	229533.I1RJ19	pp	229533.I1S1K2
36021	229533.Q4I963	pp	229533.V6RB81
36022	229533.Q4I963	pp	229533.V6RR76
36023	229533.Q4I963	pp	229533.Q4IPI4
36024	229533.Q4I963	pp	229533.Q4INI2
36025	229533.I1RC95	pp	229533.I1RX38
36026	229533.I1RC95	pp	229533.I1RCU9
36027	229533.I1RC95	pp	229533.I1S549
36028	229533.I1RC95	pp	229533.I1RY57
36029	229533.I1RC95	pp	229533.I1S2E9
36030	229533.I1RC95	pp	229533.I1RNP7
36031	229533.I1RC95	pp	229533.I1RUN8
36032	229533.I1RC95	pp	229533.I1RJ88
36033	229533.I1RC95	pp	229533.I1S1V5
36034	229533.I1RC95	pp	229533.I1RLU1
36035	229533.I1RC95	pp	229533.I1RQM0
36036	229533.I1RC95	pp	229533.V6RUY9
36037	229533.I1RC95	pp	229533.I1RFL7
36038	229533.I1RC95	pp	229533.I1RJZ7
36039	229533.I1RC95	pp	229533.I1RV91
36040	229533.I1RC95	pp	229533.I1RFD1
36041	229533.I1RC95	pp	229533.I1S2G7
36042	229533.I1RC95	pp	229533.I1RNF4
36043	229533.I1RC95	pp	229533.I1RDZ4
36044	229533.I1RC95	pp	229533.I1RNY9
36045	229533.I1RC95	pp	229533.I1RRN5
36046	229533.I1RC95	pp	229533.Q4IQT8
36047	229533.I1RC95	pp	229533.I1RPW0
36048	229533.I1RC95	pp	229533.I1RP28
36049	229533.I1RC95	pp	229533.I1S1W4
36050	229533.I1RC95	pp	229533.I1RC97
36051	229533.I1RC95	pp	229533.I1RNW8
36052	229533.I1RC95	pp	229533.I1RPD6
36053	229533.I1RC95	pp	229533.I1RCL8
36054	229533.I1RC95	pp	229533.I1RZE4
36055	229533.I1RC95	pp	229533.I1RY44
36056	229533.I1RC95	pp	229533.I1S0R9
36057	229533.I1RC95	pp	229533.I1RY58
36058	229533.I1RC95	pp	229533.I1RPS0
36059	229533.I1RC95	pp	229533.I1RQ72
36060	229533.I1RC95	pp	229533.V6RVG3
36061	229533.I1RC95	pp	229533.V6RKY7
36062	229533.I1RC95	pp	229533.I1RRP7

36063	229533.I1RC95	pp	229533.I1S0N5
36064	229533.I1RC95	pp	229533.I1RRY3
36065	229533.I1RC95	pp	229533.I1RW16
36066	229533.I1RC95	pp	229533.I1RL24
36067	229533.I1RC95	pp	229533.I1S0D1
36068	229533.I1RC95	pp	229533.I1S4P6
36069	229533.I1RC95	pp	229533.I1RNC0
36070	229533.I1RC95	pp	229533.I1RN31
36071	229533.I1RC95	pp	229533.I1RRV1
36072	229533.I1RC95	pp	229533.I1RRW0
36073	229533.I1RC95	pp	229533.I1S1D3
36074	229533.I1RC95	pp	229533.I1S224
36075	229533.I1RC95	pp	229533.I1S0M2
36076	229533.I1RC95	pp	229533.I1RYH9
36077	229533.I1RC95	pp	229533.I1RQB4
36078	229533.I1RC95	pp	229533.I1S9Q3
36079	229533.I1RC95	pp	229533.I1RV66
36080	229533.I1RC95	pp	229533.I1RT36
36081	229533.I1RC95	pp	229533.I1RW71
36082	229533.I1RC95	pp	229533.I1RJX2
36083	229533.I1RC95	pp	229533.I1S2C1
36084	229533.I1RC95	pp	229533.I1RJH1
36085	229533.I1RC95	pp	229533.I1RDB1
36086	229533.I1RC95	pp	229533.I1RX70
36087	229533.I1RC95	pp	229533.V6RGI1
36088	229533.I1RC95	pp	229533.V6QWB1
36089	229533.I1RC95	pp	229533.V6QV09
36090	229533.I1RC95	pp	229533.I1RWJ6
36091	229533.I1RC95	pp	229533.I1RQ26
36092	229533.I1RC95	pp	229533.I1RCU8
36093	229533.I1RC95	pp	229533.I1RJR8
36094	229533.I1RC95	pp	229533.Q4IR09
36095	229533.I1RC95	pp	229533.I1RST8
36096	229533.I1RC95	pp	229533.I1S0F5
36097	229533.I1RC95	pp	229533.I1RYZ0
36098	229533.I1RC95	pp	229533.I1RD18
36099	229533.I1RC95	pp	229533.I1RCM5
36100	229533.I1RC95	pp	229533.I1RLX6
36101	229533.I1RC95	pp	229533.I1RZH4
36102	229533.I1RC95	pp	229533.I1RYZ9
36103	229533.I1RC95	pp	229533.I1RN08
36104	229533.I1RC95	pp	229533.Q4IJM4
36105	229533.I1RC95	pp	229533.I1RWT6
36106	229533.I1RC95	pp	229533.I1RN65
36107	229533.I1RC95	pp	229533.I1RKZ4
36108	229533.I1RC95	pp	229533.I1RYY5
36109	229533.I1RC95	pp	229533.I1RCL7
36110	229533.I1RC95	pp	229533.I1RUQ5
36111	229533.I1RC95	pp	229533.I1RM09
36112	229533.I1RC95	pp	229533.I1RMA8
36113	229533.I1RC95	pp	229533.I1S439
36114	229533.I1RC95	pp	229533.I1RQA3
36115	229533.I1RC95	pp	229533.I1RMM9
36116	229533.I1RC95	pp	229533.I1RCB3
36117	229533.I1RC95	pp	229533.I1S0Z7
36118	229533.I1RC95	pp	229533.I1RKK0
36119	229533.I1RC95	pp	229533.I1RI71
36120	229533.I1RC95	pp	229533.I1RQ81
36121	229533.I1RC95	pp	229533.V6RDP1
36122	229533.I1RC88	pp	229533.I1RRJ6
36123	229533.I1RC88	pp	229533.I1RW71
36124	229533.I1RC88	pp	229533.I1RJZ7
36125	229533.I1RZL3	pp	229533.I1S0X0
36126	229533.I1RZL3	pp	229533.V6RGY0
36127	229533.I1RZL3	pp	229533.I1S8E0
36128	229533.I1RZL3	pp	229533.I1S270
36129	229533.I1RZL3	pp	229533.V6REE9
36130	229533.I1RZL3	pp	229533.I1RZV1
36131	229533.I1RZL3	pp	229533.I1S0Z3
36132	229533.I1RZL3	pp	229533.I1RZR2
36133	229533.I1RZL3	pp	229533.I1S7W2
36134	229533.I1RZL3	pp	229533.Q4IPI4
36135	229533.I1RZL3	pp	229533.I1S034

36136 229533.I1RZL3 pp 229533.Q4INI2
36137 229533.I1RZL3 pp 229533.Q4I963
36138 229533.I1S605 pp 229533.Q4IB89
36139 229533.I1RFF2 pp 229533.I1S3G4
36140 229533.I1RFF2 pp 229533.I1RNW5
36141 229533.I1RFF2 pp 229533.I1S1W8
36142 229533.I1RFF2 pp 229533.I1RNG4
36143 229533.I1RFF2 pp 229533.I1RIR8
36144 229533.A0A1C3YJF4 pp 229533.I1S3K8
36145 229533.A0A1C3YJF4 pp 229533.I1RQE0
36146 229533.A0A1C3YJF4 pp 229533.I1RZT0
36147 229533.I1RM94 pp 229533.I1RT80
36148 229533.I1RM94 pp 229533.I1RMG4
36149 229533.I1RM94 pp 229533.Q4IJU0
36150 229533.I1RM94 pp 229533.I1RRK7
36151 229533.I1RMW3 pp 229533.I1RT41
36152 229533.I1RMW3 pp 229533.I1RWN4
36153 229533.I1RMW3 pp 229533.I1RT81
36154 229533.I1RMW3 pp 229533.I1RN81
36155 229533.I1RMW3 pp 229533.I1RYH0
36156 229533.I1RMW3 pp 229533.I1RPQ7
36157 229533.I1RMW3 pp 229533.I1RW71
36158 229533.I1RX09 pp 229533.I1S095
36159 229533.I1RX09 pp 229533.I1S0Z3
36160 229533.I1RX09 pp 229533.I1RZH4
36161 229533.I1RX09 pp 229533.I1RYI3
36162 229533.I1RX09 pp 229533.I1S270
36163 229533.I1RX09 pp 229533.I1S2B7
36164 229533.I1RX09 pp 229533.I1RZV4
36165 229533.I1RX09 pp 229533.I1S0N8
36166 229533.I1RX09 pp 229533.I1S0X0
36167 229533.I1RX09 pp 229533.Q4HZE0
36168 229533.I1RX09 pp 229533.Q4ICG5
36169 229533.I1RX09 pp 229533.I1RZ65
36170 229533.I1RX09 pp 229533.I1SAT1
36171 229533.I1RX09 pp 229533.I1RX69
36172 229533.I1RX09 pp 229533.I1RY44
36173 229533.I1RX09 pp 229533.I1RZ91
36174 229533.I1RX09 pp 229533.I1S5K9
36175 229533.I1RX09 pp 229533.I1S2E9
36176 229533.I1RX09 pp 229533.I1RXS3
36177 229533.I1RX09 pp 229533.I1RX68
36178 229533.I1RX09 pp 229533.I1S7X9
36179 229533.I1RX09 pp 229533.I1S6S9
36180 229533.I1RX09 pp 229533.I1S635
36181 229533.I1RX09 pp 229533.I1S757
36182 229533.I1RX09 pp 229533.I1S559
36183 229533.I1RX09 pp 229533.I1RZD9
36184 229533.I1RUA0 pp 229533.I1RW72
36185 229533.I1RE36 pp 229533.Q4I1M9
36186 229533.I1RE36 pp 229533.I1S2W9
36187 229533.Q4HTT1 pp 229533.Q4IB89
36188 229533.Q4HTT1 pp 229533.Q4IBR4
36189 229533.Q4HTT1 pp 229533.Q4I7L0
36190 229533.Q4HTT1 pp 229533.Q4IPI4
36191 229533.Q4HTT1 pp 229533.Q4I5V3
36192 229533.Q4HTT1 pp 229533.Q4HY90
36193 229533.Q4HTT1 pp 229533.Q4HYB8
36194 229533.Q4HTT1 pp 229533.Q4IJ79
36195 229533.Q4HTT1 pp 229533.Q4IEV4
36196 229533.Q4HTT1 pp 229533.Q4I5W5
36197 229533.Q4HTT1 pp 229533.Q4IAK7
36198 229533.Q4HTT1 pp 229533.Q4IL82
36199 229533.Q4HTT1 pp 229533.Q4IMD1
36200 229533.Q4HTT1 pp 229533.Q4IJU0
36201 229533.Q4HTT1 pp 229533.Q4IER8
36202 229533.Q4HTT1 pp 229533.Q4ICA8
36203 229533.Q4HTT1 pp 229533.Q4IJ11
36204 229533.Q4HTT1 pp 229533.Q4IB50
36205 229533.Q4HTT1 pp 229533.Q4IJP1
36206 229533.Q4HTT1 pp 229533.Q4I5I4
36207 229533.Q4HTT1 pp 229533.Q4I948
36208 229533.Q4HTT1 pp 229533.V6QUE6

36209 229533.Q4HTT1 pp 229533.Q4I5R3
36210 229533.Q4HTT1 pp 229533.Q4HTT2
36211 229533.Q4HTT1 pp 229533.Q4HYQ4
36212 229533.Q4HTT1 pp 229533.Q4IQX3
36213 229533.I1RS84 pp 229533.I1RXX0
36214 229533.I1RS84 pp 229533.I1RSX2
36215 229533.I1RK71 pp 229533.I1RZ42
36216 229533.I1RK71 pp 229533.V6RG22
36217 229533.I1RK71 pp 229533.I1RNB8
36218 229533.I1RK71 pp 229533.I1RQZ3
36219 229533.I1RK71 pp 229533.Q4HYB8
36220 229533.I1RK71 pp 229533.I1RRT0
36221 229533.I1RK71 pp 229533.I1RVZ4
36222 229533.I1RK71 pp 229533.I1RRF7
36223 229533.I1RK71 pp 229533.Q4IPC8
36224 229533.I1RK71 pp 229533.V6QUE6
36225 229533.I1RK71 pp 229533.I1S0C6
36226 229533.I1RK71 pp 229533.Q4I5I4
36227 229533.I1RK71 pp 229533.I1RQZ2
36228 229533.I1RK71 pp 229533.Q4I7S1
36229 229533.I1RK71 pp 229533.I1RXE5
36230 229533.I1RK71 pp 229533.I1RXU0
36231 229533.I1RK71 pp 229533.I1RR61
36232 229533.I1RK71 pp 229533.I1S276
36233 229533.I1RK71 pp 229533.I1RMV1
36234 229533.I1RK71 pp 229533.I1S3N1
36235 229533.I1RK71 pp 229533.I1RZL5
36236 229533.I1RK71 pp 229533.I1RT35
36237 229533.I1RK71 pp 229533.Q4HTT2
36238 229533.I1RK71 pp 229533.I1RWF3
36239 229533.I1RK71 pp 229533.Q4HYI0
36240 229533.I1RK71 pp 229533.I1S5Z2
36241 229533.I1RK71 pp 229533.I1RT99
36242 229533.I1RK71 pp 229533.V6QV38
36243 229533.I1RK71 pp 229533.I1RVY0
36244 229533.I1RK71 pp 229533.I1RR25
36245 229533.I1RK71 pp 229533.I1RPM7
36246 229533.I1RK71 pp 229533.I1RLR0
36247 229533.I1RK71 pp 229533.I1RUG4
36248 229533.I1RK71 pp 229533.I1S549
36249 229533.I1RK71 pp 229533.Q4IJU0
36250 229533.I1RK71 pp 229533.I1RZU9
36251 229533.I1RK71 pp 229533.I1RTC2
36252 229533.I1RK71 pp 229533.I1RN04
36253 229533.I1RK71 pp 229533.I1RM65
36254 229533.I1RK71 pp 229533.I1RYE2
36255 229533.I1RK71 pp 229533.I1RZB5
36256 229533.I1RK71 pp 229533.I1RMP0
36257 229533.I1RK71 pp 229533.I1RK79
36258 229533.I1RK71 pp 229533.I1RZM2
36259 229533.I1RK71 pp 229533.Q4I099
36260 229533.I1RK71 pp 229533.I1RRG3
36261 229533.I1RK71 pp 229533.I1RPW8
36262 229533.I1RK71 pp 229533.Q4I5W5
36263 229533.I1RK71 pp 229533.I1RKB1
36264 229533.I1RK71 pp 229533.I1RRW3
36265 229533.I1RK71 pp 229533.I1RVL8
36266 229533.I1RK71 pp 229533.I1RX63
36267 229533.I1RK71 pp 229533.I1RW80
36268 229533.I1RK71 pp 229533.I1S182
36269 229533.I1RK71 pp 229533.I1RP96
36270 229533.I1RK71 pp 229533.I1RLV1
36271 229533.I1RK71 pp 229533.I1RS66
36272 229533.I1RK71 pp 229533.I1RML9
36273 229533.I1RK71 pp 229533.I1RS43
36274 229533.I1RK71 pp 229533.I1RW90
36275 229533.I1RK71 pp 229533.I1RUG0
36276 229533.I1RK71 pp 229533.I1S3N0
36277 229533.I1RK71 pp 229533.V6QWX8
36278 229533.I1RK71 pp 229533.I1RNP4
36279 229533.I1RK71 pp 229533.I1RSZ3
36280 229533.I1RK71 pp 229533.I1RW91
36281 229533.I1RK71 pp 229533.I1S0R5

229533.I1RK71	pp	229533.I1RQ42
229533.A0A1C3YK75	pp	229533.I1S2B6
229533.A0A1C3YK75	pp	229533.A0A1C3YME9
229533.A0A1C3YK75	pp	229533.Q4IER8
229533.A0A1C3YK75	pp	229533.I1RCK4
229533.A0A1C3YK75	pp	229533.Q4I061
229533.A0A1C3YK75	pp	229533.I1RQ93
229533.A0A1C3YK75	pp	229533.Q4HTT2
229533.A0A1C3YK75	pp	229533.I1RU02
229533.A0A1C3YK75	pp	229533.I1RZV9
229533.A0A1C3YK75	pp	229533.I1RBL8
229533.A0A1C3YK75	pp	229533.I1RCW8
229533.A0A1C3YK75	pp	229533.I1RCK7
229533.A0A1C3YK75	pp	229533.I1RUZ8
229533.A0A1C3YK75	pp	229533.I1S0F0
229533.A0A1C3YK75	pp	229533.A0A1C3YL16
229533.A0A1C3YK75	pp	229533.A0A1C3YM21
229533.A0A1C3YK75	pp	229533.I1RWT6
229533.A0A1C3YK75	pp	229533.I1RQA3
229533.A0A1C3YK75	pp	229533.I1S2M5
229533.A0A1C3YK75	pp	229533.I1RPF4
229533.A0A1C3YK75	pp	229533.I1S0P4
229533.A0A1C3YK75	pp	229533.I1RCU7
229533.A0A1C3YK75	pp	229533.A0A1C3YLL9
229533.A0A1C3YK75	pp	229533.Q4ICA8
229533.A0A1C3YK75	pp	229533.I1RY98
229533.A0A1C3YK75	pp	229533.I1RJR2
229533.A0A1C3YK75	pp	229533.A0A1C3YM09
229533.A0A1C3YK75	pp	229533.I1RPN4
229533.A0A1C3YK75	pp	229533.I1RB44
229533.A0A1C3YK75	pp	229533.I1RNT2
229533.A0A1C3YK75	pp	229533.I1RZT1
229533.A0A1C3YK75	pp	229533.I1RYW3
229533.A0A1C3YK75	pp	229533.I1RFQ0
229533.A0A1C3YK75	pp	229533.I1RAN8
229533.A0A1C3YK75	pp	229533.I1REC7
229533.A0A1C3YK75	pp	229533.I1RBR4
229533.A0A1C3YK75	pp	229533.I1RND2
229533.A0A1C3YK75	pp	229533.I1RAI9
229533.A0A1C3YK75	pp	229533.V6REZ4
229533.A0A1C3YK75	pp	229533.I1RQL4
229533.A0A1C3YK75	pp	229533.A0A1C3YLP1
229533.A0A1C3YK75	pp	229533.I1RPR8
229533.A0A1C3YK75	pp	229533.I1RQ72
229533.A0A1C3YK75	pp	229533.I1RY99
229533.A0A1C3YK75	pp	229533.I1RSL6
229533.A0A1C3YK75	pp	229533.Q4I7L0
229533.A0A1C3YK75	pp	229533.A0A1C3YMF9
229533.A0A1C3YK75	pp	229533.I1RCD8
229533.A0A1C3YK75	pp	229533.I1RQI6
229533.A0A1C3YK75	pp	229533.I1S247
229533.A0A1C3YK75	pp	229533.I1S282
229533.A0A1C3YK75	pp	229533.I1SAA0
229533.A0A1C3YK75	pp	229533.I1RB73
229533.A0A1C3YK75	pp	229533.I1SB04
229533.A0A1C3YK75	pp	229533.Q4IMD1
229533.A0A1C3YK75	pp	229533.I1RYT7
229533.A0A1C3YK75	pp	229533.I1RY44
229533.A0A1C3YK75	pp	229533.A0A1C3YM02
229533.A0A1C3YK75	pp	229533.I1RAK6
229533.A0A1C3YK75	pp	229533.Q4IEV4
229533.A0A1C3YK75	pp	229533.I1RY77
229533.A0A1C3YK75	pp	229533.A0A1C3YLK5
229533.A0A1C3YK75	pp	229533.I1S2E9
229533.A0A1C3YK75	pp	229533.I1RFW1
229533.A0A1C3YK75	pp	229533.I1RV91
229533.A0A1C3YK75	pp	229533.I1RCN2
229533.A0A1C3YK75	pp	229533.I1RQ88
229533.A0A1C3YK75	pp	229533.I1REC6
229533.A0A1C3YK75	pp	229533.I1RPY4
229533.A0A1C3YK75	pp	229533.I1RW28
229533.A0A1C3YK75	pp	229533.I1RNF4
229533.A0A1C3YK75	pp	229533.I1S3D7

229533.A0A1C3YK75 pp 229533.A0A1C3YM61
229533.I1RL82 pp 229533.I1RZP8
229533.A0A0E0SJ45 pp 229533.I1RZZ3
229533.A0A0E0SJ45 pp 229533.A0A1C3YLL7
229533.A0A0E0SJ45 pp 229533.V6RPN0
229533.A0A0E0SJ45 pp 229533.I1RI22
229533.A0A0E0SJ45 pp 229533.I1RT23
229533.A0A0E0SJ45 pp 229533.I1RCC2
229533.A0A0E0SJ45 pp 229533.A0A0E0SP71
229533.A0A0E0SJ45 pp 229533.I1RFM2
229533.A0A0E0SJ45 pp 229533.I1RRQ1
229533.A0A0E0SJ45 pp 229533.A0A1C3YMS7
229533.A0A0E0SJ45 pp 229533.I1RCA0
229533.A0A0E0SJ45 pp 229533.I1SAS4
229533.A0A0E0SJ45 pp 229533.V6RR38
229533.A0A0E0SJ45 pp 229533.I1SAS3
229533.A0A0E0SJ45 pp 229533.I1S1Q3
229533.A0A0E0SJ45 pp 229533.I1RNT1
229533.A0A0E0SJ45 pp 229533.I1RQ76
229533.A0A0E0SJ45 pp 229533.I1RQ48
229533.A0A0E0SJ45 pp 229533.I1RY35
229533.A0A0E0SJ45 pp 229533.I1RR90
229533.A0A0E0SJ45 pp 229533.I1RF79
229533.A0A0E0SJ45 pp 229533.I1RJG1
229533.I1RWF3 pp 229533.V6QV38
229533.I1RWF3 pp 229533.I1S5Z2
229533.I1RWF3 pp 229533.V6QUE6
229533.I1RWF3 pp 229533.I1RZB5
229533.I1RWF3 pp 229533.I1S276
229533.I1RWF3 pp 229533.Q4I5W5
229533.I1RWF3 pp 229533.I1S0C6
229533.I1RWF3 pp 229533.Q4IQX3
229533.I1RWF3 pp 229533.Q4IPC8
229533.I1RWF3 pp 229533.Q4I5I4
229533.I1RWF3 pp 229533.Q4HYI0
229533.I1RWF3 pp 229533.Q4HTT2
229533.I1RWF3 pp 229533.I1S3N0
229533.I1RWF3 pp 229533.I1S182
229533.I1RWF3 pp 229533.I1S3N1
229533.I1RWF3 pp 229533.I1S8D2
229533.I1RWF3 pp 229533.I1RZM2
229533.I1RWF3 pp 229533.I1RX63
229533.I1RWF3 pp 229533.I1RXE5
229533.I1RWF3 pp 229533.V6QWX8
229533.I1RWF3 pp 229533.I1RXU0
229533.I1RWF3 pp 229533.I1RZ42
229533.I1RKA9 pp 229533.I1RZ29
229533.I1RKA9 pp 229533.I1RMM8
229533.I1RKA9 pp 229533.I1RQ64
229533.I1RKA9 pp 229533.I1RMD2
229533.A0A1C3YJZ9 pp 229533.I1RZB5
229533.A0A1C3YJZ9 pp 229533.I1RJL2
229533.A0A1C3YJZ9 pp 229533.I1RA70
229533.A0A1C3YJZ9 pp 229533.A0A1C3YLK2
229533.A0A1C3YJZ9 pp 229533.I1RK79
229533.A0A1C3YJZ9 pp 229533.I1RK90
229533.A0A1C3YJZ9 pp 229533.I1RWN6
229533.A0A1C3YJZ9 pp 229533.I1RPI0
229533.A0A1C3YJZ9 pp 229533.I1RS41
229533.A0A1C3YJZ9 pp 229533.I1RC81
229533.A0A1C3YJZ9 pp 229533.I1RBZ3
229533.A0A1C3YJZ9 pp 229533.I1RCL3
229533.A0A1C3YJZ9 pp 229533.I1RWB3
229533.A0A1C3YJZ9 pp 229533.I1RDG0
229533.A0A1C3YJZ9 pp 229533.Q4IJU0
229533.A0A1C3YJZ9 pp 229533.I1RFQ5
229533.A0A1C3YJZ9 pp 229533.Q4I7N9
229533.A0A1C3YJZ9 pp 229533.I1RQE7
229533.A0A1C3YJZ9 pp 229533.V6RCG5
229533.A0A1C3YJZ9 pp 229533.I1RN18
229533.A0A1C3YJZ9 pp 229533.I1RFL4
229533.A0A1C3YJZ9 pp 229533.V6RMW6
229533.A0A1C3YJZ9 pp 229533.I1RCB7

36428 229533.A0A1C3YJZ9 pp 229533.I1RR25
36429 229533.A0A1C3YJZ9 pp 229533.Q4HTT2
36430 229533.A0A1C3YJZ9 pp 229533.I1RAI9
36431 229533.A0A1C3YJZ9 pp 229533.I1R9Y7
36432 229533.A0A1C3YJZ9 pp 229533.I1S7S9
36433 229533.A0A1C3YJZ9 pp 229533.I1RY44
36434 229533.A0A1C3YJZ9 pp 229533.I1RZM1
36435 229533.A0A1C3YJZ9 pp 229533.I1RYB9
36436 229533.A0A1C3YJZ9 pp 229533.A0A1C3YN75
36437 229533.A0A1C3YJZ9 pp 229533.I1RGF2
36438 229533.A0A1C3YJZ9 pp 229533.I1RT01
36439 229533.A0A1C3YJZ9 pp 229533.I1RUG3
36440 229533.A0A1C3YJZ9 pp 229533.I1S2E9
36441 229533.A0A1C3YJZ9 pp 229533.I1RNC1
36442 229533.A0A1C3YJZ9 pp 229533.I1RC16
36443 229533.A0A1C3YJZ9 pp 229533.V6QXG1
36444 229533.A0A1C3YJZ9 pp 229533.I1S284
36445 229533.A0A1C3YJZ9 pp 229533.I1RG11
36446 229533.A0A1C3YJZ9 pp 229533.I1RD22
36447 229533.A0A1C3YJZ9 pp 229533.I1RQ88
36448 229533.A0A1C3YJZ9 pp 229533.I1RND6
36449 229533.A0A1C3YJZ9 pp 229533.I1S0L8
36450 229533.A0A1C3YJZ9 pp 229533.I1RMK3
36451 229533.A0A1C3YJZ9 pp 229533.Q4I5I4
36452 229533.A0A1C3YJZ9 pp 229533.I1RGC2
36453 229533.A0A1C3YJZ9 pp 229533.I1REX8
36454 229533.A0A1C3YJZ9 pp 229533.I1RJV4
36455 229533.A0A1C3YJZ9 pp 229533.I1RC24
36456 229533.A0A1C3YJZ9 pp 229533.I1S193
36457 229533.A0A1C3YJZ9 pp 229533.I1S8E2
36458 229533.A0A1C3YJZ9 pp 229533.I1S0J3
36459 229533.A0A1C3YJZ9 pp 229533.I1RNB8
36460 229533.A0A1C3YJZ9 pp 229533.I1RS17
36461 229533.A0A1C3YJZ9 pp 229533.V6RTJ8
36462 229533.A0A1C3YJZ9 pp 229533.Q4I5W5
36463 229533.A0A1C3YJZ9 pp 229533.V6QWX8
36464 229533.A0A1C3YJZ9 pp 229533.I1RZZ9
36465 229533.A0A1C3YJZ9 pp 229533.I1RWB5
36466 229533.A0A1C3YJZ9 pp 229533.I1RMQ7
36467 229533.A0A1C3YJZ9 pp 229533.Q4ICM0
36468 229533.A0A1C3YJZ9 pp 229533.I1RRT0
36469 229533.A0A1C3YJZ9 pp 229533.Q4I7L0
36470 229533.A0A1C3YJZ9 pp 229533.I1S0L4
36471 229533.A0A1C3YJZ9 pp 229533.I1RFL7
36472 229533.A0A1C3YJZ9 pp 229533.Q4HYB8
36473 229533.A0A1C3YJZ9 pp 229533.V6QW22
36474 229533.A0A1C3YJZ9 pp 229533.I1RPP3
36475 229533.A0A1C3YJZ9 pp 229533.I1RG53
36476 229533.A0A1C3YJZ9 pp 229533.I1RZV6
36477 229533.A0A1C3YJZ9 pp 229533.I1RWJ8
36478 229533.A0A1C3YJZ9 pp 229533.I1S504
36479 229533.A0A1C3YJZ9 pp 229533.V6QUW4
36480 229533.A0A1C3YJZ9 pp 229533.Q4HYQ4
36481 229533.A0A1C3YJZ9 pp 229533.I1RRG3
36482 229533.A0A1C3YJZ9 pp 229533.I1RCH7
36483 229533.A0A1C3YJZ9 pp 229533.I1RNR2
36484 229533.A0A1C3YJZ9 pp 229533.I1RZR6
36485 229533.A0A1C3YJZ9 pp 229533.I1RWH5
36486 229533.A0A1C3YJZ9 pp 229533.I1RQC5
36487 229533.A0A1C3YJZ9 pp 229533.I1RWK3
36488 229533.A0A1C3YJZ9 pp 229533.I1RN67
36489 229533.A0A1C3YJZ9 pp 229533.I1S0L3
36490 229533.A0A1C3YJZ9 pp 229533.I1RY40
36491 229533.A0A1C3YJZ9 pp 229533.A0A1C3YL15
36492 229533.A0A0E0SJ27 pp 229533.I1R9Z0
36493 229533.I1RMP5 pp 229533.I1RVY4
36494 229533.I1RMP5 pp 229533.I1SA27
36495 229533.I1RMP5 pp 229533.I1RN41
36496 229533.I1RMP5 pp 229533.I1RPT0
36497 229533.I1RCI0 pp 229533.I1RCV0
36498 229533.I1RCI0 pp 229533.I1RHC6
36499 229533.I1RCI0 pp 229533.I1RFF2
36500 229533.I1RDT6 pp 229533.I1RWS7

36501	229533.I1RDT6	pp	229533.I1S4T3
36502	229533.I1RR49	pp	229533.I1RW16
36503	229533.I1RR49	pp	229533.I1RW71
36504	229533.I1RR49	pp	229533.I1RRJ6
36505	229533.I1RCN7	pp	229533.I1S4S4
36506	229533.I1RCB3	pp	229533.I1RVN2
36507	229533.I1RCB3	pp	229533.I1RRY3
36508	229533.I1RCB3	pp	229533.I1RWQ8
36509	229533.I1RCB3	pp	229533.I1RKB3
36510	229533.I1RCB3	pp	229533.I1RQH8
36511	229533.I1RCB3	pp	229533.I1S286
36512	229533.I1RCB3	pp	229533.I1RS18
36513	229533.I1RCB3	pp	229533.I1RSS1
36514	229533.I1RCB3	pp	229533.Q4INS6
36515	229533.I1RCB3	pp	229533.I1RRW0
36516	229533.I1RCB3	pp	229533.I1RSR9
36517	229533.I1RCB3	pp	229533.I1S2K7
36518	229533.I1RCB3	pp	229533.I1RXF0
36519	229533.I1RCB3	pp	229533.I1RNY8
36520	229533.I1RCB3	pp	229533.I1RMM9
36521	229533.I1RCB3	pp	229533.Q4IEA7
36522	229533.I1RCB3	pp	229533.I1RHQ8
36523	229533.I1RCB3	pp	229533.I1RP99
36524	229533.I1RCB3	pp	229533.I1RRN5
36525	229533.I1RCB3	pp	229533.I1S0M2
36526	229533.I1RCB3	pp	229533.I1RWT6
36527	229533.I1RCB3	pp	229533.I1RCL8
36528	229533.I1RCB3	pp	229533.I1RQB4
36529	229533.I1RCB3	pp	229533.I1RSI3
36530	229533.I1RCB3	pp	229533.I1RYP7
36531	229533.I1RCB3	pp	229533.I1S1W4
36532	229533.I1RCB3	pp	229533.I1RZE4
36533	229533.I1RCB3	pp	229533.I1RKK1
36534	229533.I1RCB3	pp	229533.V6RVG3
36535	229533.I1RCB3	pp	229533.V6QV09
36536	229533.I1RCB3	pp	229533.I1RQA3
36537	229533.I1RCB3	pp	229533.I1RSI4
36538	229533.I1RCB3	pp	229533.I1S439
36539	229533.I1RCB3	pp	229533.I1RY44
36540	229533.I1RCB3	pp	229533.I1RKE9
36541	229533.I1RCB3	pp	229533.I1RQ72
36542	229533.I1RCB3	pp	229533.I1S296
36543	229533.I1RCB3	pp	229533.I1RQ26
36544	229533.I1RCB3	pp	229533.I1RLX7
36545	229533.I1RCB3	pp	229533.I1RJK7
36546	229533.I1RCB3	pp	229533.I1RMB2
36547	229533.I1RCB3	pp	229533.I1RQS4
36548	229533.I1RCB3	pp	229533.V6RKY7
36549	229533.I1RCB3	pp	229533.I1RQ48
36550	229533.I1RCB3	pp	229533.I1S265
36551	229533.I1RCB3	pp	229533.I1RZK4
36552	229533.I1RCB3	pp	229533.I1RNF4
36553	229533.I1RCB3	pp	229533.I1RN08
36554	229533.I1RCB3	pp	229533.I1S2E9
36555	229533.I1RCB3	pp	229533.I1RZ63
36556	229533.I1RCB3	pp	229533.I1RFW0
36557	229533.I1RCB3	pp	229533.I1RY29
36558	229533.I1RCB3	pp	229533.I1RWI8
36559	229533.I1RCB3	pp	229533.I1S2B6
36560	229533.I1RCB3	pp	229533.I1RX64
36561	229533.I1RCB3	pp	229533.I1RDB1
36562	229533.I1RCB3	pp	229533.I1RGF1
36563	229533.I1RCB3	pp	229533.I1S2H4
36564	229533.I1RCB3	pp	229533.I1RX77
36565	229533.I1RCB3	pp	229533.I1RPK4
36566	229533.I1RCB3	pp	229533.V6QUV7
36567	229533.I1RCB3	pp	229533.I1RX04
36568	229533.I1RCB3	pp	229533.I1RE59
36569	229533.I1RCB3	pp	229533.I1RML5
36570	229533.I1RCB3	pp	229533.I1RV91
36571	229533.I1RCB3	pp	229533.I1RCD1
36572	229533.I1RCB3	pp	229533.I1RPH1
36573	229533.I1RCB3	pp	229533.I1RYZ9

229533.I1RCB3	pp	229533.I1S0X7
229533.I1RCB3	pp	229533.I1RPQ1
229533.I1RCB3	pp	229533.I1RV66
229533.I1RCB3	pp	229533.Q4IQM6
229533.I1RCB3	pp	229533.I1RSD0
229533.I1RCB3	pp	229533.I1RPA6
229533.I1RCB3	pp	229533.I1RL24
229533.I1RCB3	pp	229533.I1RFL7
229533.I1RCB3	pp	229533.I1S224
229533.I1RCB3	pp	229533.I1RXH6
229533.I1RCB3	pp	229533.I1RW24
229533.I1RCB3	pp	229533.I1RYH9
229533.I1RZU7	pp	229533.V6RJ21
229533.I1RIC3	pp	229533.I1RM03
229533.I1RIC3	pp	229533.I1RIF9
229533.I1RIC3	pp	229533.I1S2L0
229533.I1S276	pp	229533.Q4INS6
229533.I1S276	pp	229533.I1S2E9
229533.I1S276	pp	229533.I1S3N1
229533.I1S276	pp	229533.Q4HYI0
229533.I1S276	pp	229533.I1S3N0
229533.I1S276	pp	229533.V6QUE6
229533.I1S276	pp	229533.Q4I5I4
229533.I1S276	pp	229533.Q4HTT2
229533.I1S276	pp	229533.Q4IQX3
229533.I1S276	pp	229533.V6QV38
229533.I1S276	pp	229533.Q4I5U9
229533.I1S276	pp	229533.V6QWX8
229533.I1S276	pp	229533.Q4IER8
229533.Q4IPC8	pp	229533.V6QWX8
229533.Q4IPC8	pp	229533.V6RDZ4
229533.Q4IPC8	pp	229533.Q4IR09
229533.Q4IPC8	pp	229533.V6RGB5
229533.Q4IPC8	pp	229533.Q4IPN9
229533.Q4IPC8	pp	229533.V6QUE6
229533.I1S2B4	pp	229533.I1S7J8
229533.A0A0E0RRE1	pp	229533.A0A1C3YII1
229533.A0A0E0RRE1	pp	229533.I1S1W3
229533.A0A0E0RRE1	pp	229533.I1RRK7
229533.A0A0E0RRE1	pp	229533.I1RM23
229533.A0A0E0RRE1	pp	229533.I1RM94
229533.I1RPM7	pp	229533.I1S3N1
229533.I1RPM7	pp	229533.I1RSC7
229533.I1RPM7	pp	229533.I1RRF7
229533.I1RPM7	pp	229533.Q4INS6
229533.I1RPM7	pp	229533.I1S209
229533.I1RPM7	pp	229533.I1RY29
229533.I1RPM7	pp	229533.Q4HYI0
229533.I1RPM7	pp	229533.Q4IQX3
229533.I1RPM7	pp	229533.I1RRT0
229533.I1RPM7	pp	229533.I1S2E9
229533.I1RPM7	pp	229533.I1RZ42
229533.I1RPM7	pp	229533.Q4HTT2
229533.I1RPM7	pp	229533.Q4I5W5
229533.I1RPM7	pp	229533.I1RQZ3
229533.I1RPM7	pp	229533.I1RXU0
229533.I1RPM7	pp	229533.V6QUE6
229533.I1RPM7	pp	229533.I1RWF3
229533.I1RPM7	pp	229533.I1S0C6
229533.I1RPM7	pp	229533.I1RY44
229533.I1RPM7	pp	229533.I1RRM8
229533.I1RPM7	pp	229533.I1RZT1
229533.I1RPM7	pp	229533.I1RUF8
229533.I1RPM7	pp	229533.I1RW80
229533.I1RPM7	pp	229533.I1RPQ1
229533.I1RPM7	pp	229533.V6QV38
229533.I1RPM7	pp	229533.I1RXE5
229533.I1RPM7	pp	229533.I1RZM2
229533.I1RPM7	pp	229533.I1RWT6
229533.I1RPM7	pp	229533.I1RQN8
229533.I1RPM7	pp	229533.I1S276
229533.I1RPM7	pp	229533.I1RYY5
229533.I1RPM7	pp	229533.I1RTC2

36647 229533.I1RPM7 pp 229533.Q4IER8
36648 229533.I1RPM7 pp 229533.I1RRG3
36649 229533.I1RPM7 pp 229533.I1RQA3
36650 229533.I1RPM7 pp 229533.I1S182
36651 229533.I1RPM7 pp 229533.I1S3N0
36652 229533.I1RPM7 pp 229533.I1RR61
36653 229533.I1RPM7 pp 229533.I1RZB5
36654 229533.I1RPM7 pp 229533.I1RRN5
36655 229533.I1RPM7 pp 229533.I1RSR9
36656 229533.I1RPM7 pp 229533.Q4IJM4
36657 229533.I1RPM7 pp 229533.I1RW90
36658 229533.I1RPM7 pp 229533.I1RX63
36659 229533.I1RPM7 pp 229533.V6QWX8
36660 229533.I1RPM7 pp 229533.I1RW91
36661 229533.I1RPM7 pp 229533.I1RPW8
36662 229533.I1RPM7 pp 229533.I1RSZ3
36663 229533.I1RPM7 pp 229533.I1RR25
36664 229533.I1RPM7 pp 229533.I1RUG4
36665 229533.I1RPM7 pp 229533.I1RWI8
36666 229533.I1RPM7 pp 229533.I1S9Y1
36667 229533.I1RPM7 pp 229533.I1RZ94
36668 229533.I1RPM7 pp 229533.I1RVL8
36669 229533.I1RPM7 pp 229533.I1S0J3
36670 229533.I1RPM7 pp 229533.I1RQ42
36671 229533.I1RPM7 pp 229533.I1RVY0
36672 229533.A0A1C3YIJ5 pp 229533.I1RNG4
36673 229533.A0A1C3YIJ5 pp 229533.I1RPD6
36674 229533.A0A1C3YIJ5 pp 229533.I1RZ52
36675 229533.A0A1C3YIJ5 pp 229533.I1RLT3
36676 229533.A0A1C3YIJ5 pp 229533.I1RY74
36677 229533.A0A1C3YIJ5 pp 229533.A0A1C3YM01
36678 229533.A0A1C3YIJ5 pp 229533.I1S3F3
36679 229533.A0A1C3YIJ5 pp 229533.I1S0H5
36680 229533.A0A1C3YIJ5 pp 229533.I1RS84
36681 229533.A0A1C3YIJ5 pp 229533.A0A1C3YLU9
36682 229533.A0A1C3YIJ5 pp 229533.I1R9K3
36683 229533.A0A1C3YIJ5 pp 229533.I1RN51
36684 229533.A0A1C3YIJ5 pp 229533.A0A1C3YJ46
36685 229533.A0A1C3YIJ5 pp 229533.I1RMM8
36686 229533.A0A1C3YIJ5 pp 229533.I1S2E2
36687 229533.A0A1C3YIJ5 pp 229533.I1RVX9
36688 229533.A0A1C3YIJ5 pp 229533.I1RPW2
36689 229533.A0A1C3YIJ5 pp 229533.I1S9D4
36690 229533.A0A1C3YIJ5 pp 229533.A0A1C3YLV0
36691 229533.A0A1C3YIJ5 pp 229533.I1RQ14
36692 229533.A0A1C3YIJ5 pp 229533.I1RU76
36693 229533.A0A1C3YIJ5 pp 229533.I1RC83
36694 229533.A0A1C3YIJ5 pp 229533.I1RIY0
36695 229533.A0A1C3YIJ5 pp 229533.I1RQE0
36696 229533.A0A1C3YIJ5 pp 229533.I1RM26
36697 229533.A0A1C3YIJ5 pp 229533.A0A1C3YIV7
36698 229533.A0A1C3YIJ5 pp 229533.I1RK03
36699 229533.A0A1C3YIJ5 pp 229533.I1RD81
36700 229533.I1RNS2 pp 229533.I1RZU7
36701 229533.I1RGZ0 pp 229533.I1RN41
36702 229533.A0A1C3YIG3 pp 229533.I1RPK4
36703 229533.A0A1C3YIG3 pp 229533.I1RNK1
36704 229533.A0A1C3YIG3 pp 229533.I1RML5
36705 229533.A0A1C3YIG3 pp 229533.A0A1C3YJH8
36706 229533.A0A1C3YIG3 pp 229533.A0A1C3YJV7
36707 229533.A0A1C3YIG3 pp 229533.I1RBT0
36708 229533.A0A1C3YIG3 pp 229533.I1RSS1
36709 229533.A0A1C3YIG3 pp 229533.I1RCF9
36710 229533.A0A1C3YIG3 pp 229533.Q4IEA7
36711 229533.A0A1C3YIG3 pp 229533.I1RTG0
36712 229533.A0A1C3YIG3 pp 229533.I1RNF4
36713 229533.A0A1C3YIG3 pp 229533.I1RWT6
36714 229533.A0A1C3YIG3 pp 229533.I1S2K7
36715 229533.A0A1C3YIG3 pp 229533.Q4I785
36716 229533.A0A1C3YIG3 pp 229533.I1RWK0
36717 229533.A0A1C3YIG3 pp 229533.I1S2E9
36718 229533.A0A1C3YIG3 pp 229533.I1RY44
36719 229533.A0A1C3YIG3 pp 229533.I1RAY4

36720 229533.A0A1C3YIG3 pp 229533.I1RP86
36721 229533.A0A1C3YIG3 pp 229533.I1RP25
36722 229533.A0A1C3YIG3 pp 229533.I1REE1
36723 229533.A0A1C3YIG3 pp 229533.I1RRW0
36724 229533.A0A1C3YIG3 pp 229533.I1RXF0
36725 229533.A0A1C3YIG3 pp 229533.I1RCB3
36726 229533.A0A1C3YIG3 pp 229533.I1RX77
36727 229533.A0A1C3YIG3 pp 229533.I1RV91
36728 229533.A0A1C3YIG3 pp 229533.I1RVN2
36729 229533.A0A1C3YIG3 pp 229533.I1RQB4
36730 229533.A0A1C3YIG3 pp 229533.I1S0X7
36731 229533.A0A1C3YIG3 pp 229533.A0A1C3YM29
36732 229533.A0A1C3YIG3 pp 229533.A0A1C3YLN4
36733 229533.A0A1C3YIG3 pp 229533.I1RTE7
36734 229533.A0A1C3YIG3 pp 229533.I1RB41
36735 229533.A0A1C3YIG3 pp 229533.I1RHQ8
36736 229533.A0A1C3YIG3 pp 229533.I1RRN5
36737 229533.A0A1C3YIG3 pp 229533.I1RC91
36738 229533.A0A1C3YIG3 pp 229533.A0A1C3YN39
36739 229533.A0A1C3YIG3 pp 229533.Q4IR87
36740 229533.A0A1C3YIG3 pp 229533.I1S224
36741 229533.A0A1C3YIG3 pp 229533.I1RXH6
36742 229533.A0A1C3YIG3 pp 229533.I1RYL5
36743 229533.A0A1C3YIG3 pp 229533.I1RQA3
36744 229533.A0A1C3YIG3 pp 229533.I1RFW0
36745 229533.A0A1C3YIG3 pp 229533.I1RQ72
36746 229533.A0A1C3YIG3 pp 229533.V6QUV7
36747 229533.A0A1C3YIG3 pp 229533.I1S286
36748 229533.A0A1C3YIG3 pp 229533.I1RXX1
36749 229533.I1RHF4 pp 229533.I1S0N8
36750 229533.I1RVL8 pp 229533.Q4IJM4
36751 229533.I1RVL8 pp 229533.V6R2A1
36752 229533.I1RVL8 pp 229533.Q4HTT1
36753 229533.I1RVL8 pp 229533.V6QUE6
36754 229533.I1RVL8 pp 229533.I1RW80
36755 229533.I1RVL8 pp 229533.Q4I099
36756 229533.I1RVL8 pp 229533.I1S0J3
36757 229533.I1RVL8 pp 229533.I1RWI8
36758 229533.I1RVL8 pp 229533.Q4IQX3
36759 229533.I1RVL8 pp 229533.I1S0C6
36760 229533.I1RVL8 pp 229533.I1RW90
36761 229533.I1RVL8 pp 229533.I1S2E9
36762 229533.I1RVL8 pp 229533.Q4HZI0
36763 229533.I1RVL8 pp 229533.I1RW91
36764 229533.I1RVL8 pp 229533.V6RAC9
36765 229533.I1RVL8 pp 229533.I1RVZ4
36766 229533.I1RVL8 pp 229533.I1RY29
36767 229533.I1RVL8 pp 229533.V6QWX8
36768 229533.I1RVL8 pp 229533.I1RX63
36769 229533.I1RVL8 pp 229533.I1S3N0
36770 229533.I1RVL8 pp 229533.I1RVY0
36771 229533.I1RVL8 pp 229533.Q4I5I4
36772 229533.I1RVL8 pp 229533.I1S0R5
36773 229533.I1RVL8 pp 229533.I1RXE5
36774 229533.I1RVL8 pp 229533.I1S182
36775 229533.I1RVL8 pp 229533.Q4INS6
36776 229533.I1RVL8 pp 229533.I1RZM2
36777 229533.I1RVL8 pp 229533.I1RY44
36778 229533.I1RVL8 pp 229533.I1RZT1
36779 229533.I1RVL8 pp 229533.V6QV38
36780 229533.I1RVL8 pp 229533.Q4HYB8
36781 229533.I1RVL8 pp 229533.Q4HYI0
36782 229533.I1RVL8 pp 229533.I1RZ42
36783 229533.I1RVL8 pp 229533.I1RZB5
36784 229533.I1RVL8 pp 229533.I1S276
36785 229533.I1RVL8 pp 229533.V6QY25
36786 229533.I1RVL8 pp 229533.Q4IER8
36787 229533.I1RVL8 pp 229533.I1S3N1
36788 229533.I1RVL8 pp 229533.I1RWT6
36789 229533.I1RVL8 pp 229533.Q4IJU0
36790 229533.I1RVL8 pp 229533.I1RXU0
36791 229533.I1RVL8 pp 229533.I1RWF3
36792 229533.I1S0E8 pp 229533.V6QV09

36793	229533.I1S0E8	pp	229533.I1S2G7
36794	229533.I1S0E8	pp	229533.V6QUE6
36795	229533.I1S0E8	pp	229533.V6QWX8
36796	229533.I1S0E8	pp	229533.V6RGI1
36797	229533.I1S0E8	pp	229533.I1S0J3
36798	229533.I1S0E8	pp	229533.I1S2E9
36799	229533.I1S0E8	pp	229533.Q4ICM0
36800	229533.I1S0E8	pp	229533.V6RKY7
36801	229533.I1S0E8	pp	229533.Q4IJM4
36802	229533.I1S0E8	pp	229533.I1S0F0
36803	229533.I1S0E8	pp	229533.Q4I7L0
36804	229533.I1S0E8	pp	229533.I1S1W4
36805	229533.I1S0E8	pp	229533.Q4ICA8
36806	229533.I1RLU1	pp	229533.Q4IMD1
36807	229533.I1RLU1	pp	229533.I1RY44
36808	229533.I1RLU1	pp	229533.I1RN08
36809	229533.I1RLU1	pp	229533.V6RVG3
36810	229533.I1RLU1	pp	229533.Q4IBX0
36811	229533.I1RLU1	pp	229533.Q4I0J6
36812	229533.I1RLU1	pp	229533.I1RWT6
36813	229533.I1RLU1	pp	229533.V6R3J1
36814	229533.I1RLU1	pp	229533.I1RN46
36815	229533.I1RLU1	pp	229533.I1RYZ9
36816	229533.I1RLU1	pp	229533.I1S0Z9
36817	229533.I1RLU1	pp	229533.I1S1W4
36818	229533.I1RLU1	pp	229533.I1S439
36819	229533.I1RLU1	pp	229533.I1RLX6
36820	229533.I1RLU1	pp	229533.I1S1D3
36821	229533.I1RLU1	pp	229533.I1RV91
36822	229533.I1RLU1	pp	229533.I1S2E9
36823	229533.I1RLU1	pp	229533.I1RQ16
36824	229533.I1RLU1	pp	229533.I1RQA3
36825	229533.I1RLU1	pp	229533.V6R382
36826	229533.I1RLU1	pp	229533.I1RW16
36827	229533.I1RLU1	pp	229533.I1RRN5
36828	229533.I1RLU1	pp	229533.I1RMM9
36829	229533.I1RLU1	pp	229533.I1RRY3
36830	229533.I1RLU1	pp	229533.I1RLU2
36831	229533.I1RLU1	pp	229533.I1S0D1
36832	229533.I1RLU1	pp	229533.I1RMA8
36833	229533.I1RLU1	pp	229533.I1S1V5
36834	229533.I1RLU1	pp	229533.I1RNP7
36835	229533.I1RLU1	pp	229533.V6RGI1
36836	229533.I1RLU1	pp	229533.V6QV09
36837	229533.I1RLU1	pp	229533.I1S0M2
36838	229533.I1RLU1	pp	229533.I1RN31
36839	229533.I1RLU1	pp	229533.I1RSI4
36840	229533.I1RLU1	pp	229533.I1RYH9
36841	229533.I1RLU1	pp	229533.I1RTC2
36842	229533.I1RLU1	pp	229533.I1RQ26
36843	229533.I1RLU1	pp	229533.I1RUN8
36844	229533.I1RE30	pp	229533.I1RQZ3
36845	229533.I1RE30	pp	229533.I1RE54
36846	229533.I1RE30	pp	229533.I1RZ42
36847	229533.I1RE30	pp	229533.I1RK71
36848	229533.I1RE30	pp	229533.I1RPM7
36849	229533.I1RE30	pp	229533.I1S5Z2
36850	229533.I1RE30	pp	229533.I1RXX1
36851	229533.I1RE30	pp	229533.I1S276
36852	229533.I1RE30	pp	229533.I1RM65
36853	229533.I1RE30	pp	229533.V6QV38
36854	229533.I1RE30	pp	229533.Q4I5I4
36855	229533.I1RE30	pp	229533.I1RJL2
36856	229533.I1RE30	pp	229533.I1S0C6
36857	229533.I1RE30	pp	229533.I1RK79
36858	229533.I1RE30	pp	229533.Q4IER8
36859	229533.I1RE30	pp	229533.V6QUE6
36860	229533.I1RE30	pp	229533.I1RG11
36861	229533.I1RE30	pp	229533.I1RZB5
36862	229533.I1RE30	pp	229533.Q4IPC8
36863	229533.I1RE30	pp	229533.I1RN17
36864	229533.I1RE30	pp	229533.I1RSZ3
36865	229533.I1RE30	pp	229533.I1RE37

36866 229533.I1RE30 pp 229533.I1RRF7
36867 229533.I1RE30 pp 229533.I1RR25
36868 229533.I1RE30 pp 229533.I1RHQ5
36869 229533.I1RE30 pp 229533.I1RGR8
36870 229533.I1RE30 pp 229533.I1RXU0
36871 229533.I1RE30 pp 229533.I1RUG4
36872 229533.I1RE30 pp 229533.I1RP96
36873 229533.I1RE30 pp 229533.I1S3N1
36874 229533.I1RE30 pp 229533.I1RR61
36875 229533.I1RE30 pp 229533.I1REN3
36876 229533.I1RE30 pp 229533.Q4HYI0
36877 229533.I1RE30 pp 229533.I1RML9
36878 229533.I1RE30 pp 229533.I1RMV1
36879 229533.I1RE30 pp 229533.Q4HTT2
36880 229533.I1RE30 pp 229533.I1RK95
36881 229533.I1RE30 pp 229533.I1S182
36882 229533.I1RE30 pp 229533.I1RS30
36883 229533.I1RE30 pp 229533.Q4IMD1
36884 229533.I1RE30 pp 229533.I1S3N0
36885 229533.I1RE30 pp 229533.Q4I5W5
36886 229533.I1RE30 pp 229533.I1RXE5
36887 229533.I1RE30 pp 229533.I1RPW8
36888 229533.I1RE30 pp 229533.I1RRT0
36889 229533.I1RE30 pp 229533.I1RWF3
36890 229533.I1RE30 pp 229533.I1RX63
36891 229533.I1RE30 pp 229533.I1RRM8
36892 229533.I1RE30 pp 229533.I1RFW1
36893 229533.I1RE30 pp 229533.Q4IQX3
36894 229533.I1RE30 pp 229533.I1RHL8
36895 229533.I1RE30 pp 229533.I1RLV1
36896 229533.I1RE30 pp 229533.I1RZM2
36897 229533.I1RE30 pp 229533.I1RW80
36898 229533.I1RE30 pp 229533.I1RRG3
36899 229533.I1RE30 pp 229533.I1RKB1
36900 229533.I1RE30 pp 229533.I1RLR0
36901 229533.I1RE30 pp 229533.I1RVY0
36902 229533.I1RE30 pp 229533.I1RTC2
36903 229533.I1RE30 pp 229533.I1RNP4
36904 229533.I1RE30 pp 229533.I1S9Y1
36905 229533.I1RE30 pp 229533.I1RQ42
36906 229533.I1RE30 pp 229533.I1RVL8
36907 229533.I1RE30 pp 229533.I1RHL7
36908 229533.I1RE30 pp 229533.I1RW91
36909 229533.I1RE30 pp 229533.V6QWX8
36910 229533.I1RE30 pp 229533.I1RYE2
36911 229533.I1RE30 pp 229533.I1RW90
36912 229533.I1RE30 pp 229533.I1RN04
36913 229533.I1RIT1 pp 229533.I1RWY3
36914 229533.A0A1C3YN48 pp 229533.I1RC91
36915 229533.A0A1C3YN48 pp 229533.I1S0C0
36916 229533.A0A1C3YN48 pp 229533.I1RZM2
36917 229533.A0A1C3YN48 pp 229533.I1RC22
36918 229533.A0A1C3YN48 pp 229533.I1SAT3
36919 229533.A0A1C3YN48 pp 229533.I1RN08
36920 229533.A0A1C3YN48 pp 229533.I1RCI2
36921 229533.A0A1C3YN48 pp 229533.I1S0W0
36922 229533.A0A1C3YN48 pp 229533.I1RCM9
36923 229533.I1S0X7 pp 229533.V6RDP1
36924 229533.I1S0X7 pp 229533.Q4IR87
36925 229533.I1S0X7 pp 229533.I1S2E9
36926 229533.I1S0X7 pp 229533.I1S2K7
36927 229533.I1S0X7 pp 229533.I1S296
36928 229533.I1S0X7 pp 229533.I1S2B6
36929 229533.I1RE14 pp 229533.Q4I5W5
36930 229533.I1RE14 pp 229533.I1RNP4
36931 229533.I1RE14 pp 229533.I1RLR0
36932 229533.I1RE14 pp 229533.I1RLV1
36933 229533.I1RE14 pp 229533.I1RHL7
36934 229533.I1RE14 pp 229533.I1S830
36935 229533.I1RE14 pp 229533.Q4I5I4
36936 229533.I1RE14 pp 229533.I1S0C6
36937 229533.I1RE14 pp 229533.Q4I613
36938 229533.I1RE14 pp 229533.I1RHQ5

36939 229533.I1RE14 pp 229533.I1RGR8
36940 229533.I1RE14 pp 229533.I1S3N0
36941 229533.I1RE14 pp 229533.V6QUE6
36942 229533.I1RE14 pp 229533.I1S214
36943 229533.I1RE14 pp 229533.Q4IPC8
36944 229533.I1RE14 pp 229533.I1RG11
36945 229533.I1RE14 pp 229533.I1RJL3
36946 229533.I1RE14 pp 229533.I1RX63
36947 229533.I1RE14 pp 229533.Q4IPA4
36948 229533.I1RE14 pp 229533.I1S3N1
36949 229533.I1RE14 pp 229533.I1RNQ6
36950 229533.I1RE14 pp 229533.I1RSC7
36951 229533.I1RE14 pp 229533.I1RRG3
36952 229533.I1RE14 pp 229533.I1RRT0
36953 229533.I1RE14 pp 229533.I1RHL8
36954 229533.I1RE14 pp 229533.I1RQZ3
36955 229533.I1RE14 pp 229533.I1RW69
36956 229533.I1RE14 pp 229533.I1RW90
36957 229533.I1RE14 pp 229533.Q4IR09
36958 229533.I1RE14 pp 229533.V6QWX8
36959 229533.I1RE14 pp 229533.I1RM65
36960 229533.I1RE14 pp 229533.I1RUG0
36961 229533.I1RE14 pp 229533.I1S5Z2
36962 229533.I1RE14 pp 229533.I1RK79
36963 229533.I1RE14 pp 229533.I1RWI0
36964 229533.I1RE14 pp 229533.I1RZB5
36965 229533.I1RE14 pp 229533.I1RR25
36966 229533.I1RE14 pp 229533.I1RJL2
36967 229533.I1RE14 pp 229533.Q4HWE2
36968 229533.I1RE14 pp 229533.I1RPL3
36969 229533.I1RJL3 pp 229533.I1RNP1
36970 229533.I1RJL3 pp 229533.I1RZT0
36971 229533.I1RJL3 pp 229533.I1RMW7
36972 229533.I1RJL3 pp 229533.I1RMU8
36973 229533.I1RJL3 pp 229533.I1S2G9
36974 229533.I1RJL3 pp 229533.I1RTF4
36975 229533.I1RJL3 pp 229533.I1RKB5
36976 229533.I1RJL3 pp 229533.I1RRG3
36977 229533.I1RJL3 pp 229533.I1RQS6
36978 229533.I1RJL3 pp 229533.V6QUE6
36979 229533.I1RJL3 pp 229533.I1RZB5
36980 229533.I1RJL3 pp 229533.Q4ICA8
36981 229533.I1RJL3 pp 229533.I1RNQ6
36982 229533.I1RJL3 pp 229533.I1RMN7
36983 229533.I1RJL3 pp 229533.Q4IQT8
36984 229533.I1RJL3 pp 229533.I1RRT0
36985 229533.A0A1C3YN32 pp 229533.I1RMM8
36986 229533.A0A1C3YN32 pp 229533.I1RZ29
36987 229533.A0A1C3YN32 pp 229533.I1RR94
36988 229533.A0A1C3YN32 pp 229533.I1RHV1
36989 229533.A0A1C3YN32 pp 229533.I1RN77
36990 229533.A0A1C3YN32 pp 229533.I1RSX7
36991 229533.A0A1C3YN32 pp 229533.I1RC14
36992 229533.A0A1C3YN32 pp 229533.I1RFH9
36993 229533.A0A1C3YN32 pp 229533.I1RP97
36994 229533.A0A1C3YN32 pp 229533.I1RPY2
36995 229533.A0A1C3YN32 pp 229533.I1RHY5
36996 229533.A0A1C3YN32 pp 229533.I1RJU6
36997 229533.A0A1C3YN32 pp 229533.I1S0S5
36998 229533.I1RC28 pp 229533.I1RW71
36999 229533.I1RC28 pp 229533.I1RNU6
37000 229533.I1RC28 pp 229533.I1RS30
37001 229533.I1RC28 pp 229533.I1RRJ8
37002 229533.I1RC28 pp 229533.Q4HZK7
37003 229533.I1RC28 pp 229533.I1S0F5
37004 229533.I1RC28 pp 229533.I1RTF4
37005 229533.I1RC28 pp 229533.I1RJZ7
37006 229533.I1RC28 pp 229533.I1RM35
37007 229533.I1RC28 pp 229533.I1RYS0
37008 229533.I1RC28 pp 229533.I1S863
37009 229533.I1RC28 pp 229533.I1RPL1
37010 229533.I1RAR2 pp 229533.I1S182
37011 229533.I1RAR2 pp 229533.I1RS30

37012	229533.I1RAR2	pp	229533.I1S3N0
37013	229533.I1RAR2	pp	229533.I1S9H8
37014	229533.I1RAR2	pp	229533.I1RE30
37015	229533.I1RAR2	pp	229533.I1RX63
37016	229533.I1RAR2	pp	229533.I1RPW8
37017	229533.I1RAR2	pp	229533.I1RB25
37018	229533.I1RAR2	pp	229533.I1RQ92
37019	229533.I1RAR2	pp	229533.I1RZT6
37020	229533.I1RAR2	pp	229533.I1RFW1
37021	229533.I1RAR2	pp	229533.Q4HYQ4
37022	229533.I1RAR2	pp	229533.I1RHL8
37023	229533.I1RAR2	pp	229533.I1RZM2
37024	229533.I1RAR2	pp	229533.I1RRG3
37025	229533.I1RAR2	pp	229533.I1RN61
37026	229533.I1RAR2	pp	229533.I1RTC2
37027	229533.I1RAR2	pp	229533.I1RLV1
37028	229533.I1RAR2	pp	229533.I1RN04
37029	229533.I1RAR2	pp	229533.Q4IQX3
37030	229533.I1RAR2	pp	229533.I1RYE2
37031	229533.I1RAR2	pp	229533.I1RRM8
37032	229533.I1RAR2	pp	229533.I1S9Y1
37033	229533.I1RAR2	pp	229533.I1RX46
37034	229533.I1RAR2	pp	229533.Q4I5W5
37035	229533.I1RAR2	pp	229533.I1RRN6
37036	229533.I1RAR2	pp	229533.I1RM82
37037	229533.I1RAR2	pp	229533.Q4IMD1
37038	229533.I1RAR2	pp	229533.I1RCW8
37039	229533.I1RAR2	pp	229533.I1RSG9
37040	229533.I1RAR2	pp	229533.I1RKR8
37041	229533.I1RAR2	pp	229533.I1RRT0
37042	229533.I1RAR2	pp	229533.I1RC98
37043	229533.I1RAR2	pp	229533.I1RW80
37044	229533.I1RAR2	pp	229533.I1RDC9
37045	229533.I1RAR2	pp	229533.I1RBC7
37046	229533.I1RAR2	pp	229533.I1RE54
37047	229533.I1RAR2	pp	229533.I1RKB1
37048	229533.I1RAR2	pp	229533.I1RD36
37049	229533.I1RAR2	pp	229533.I1RZ42
37050	229533.I1RAR2	pp	229533.I1RW91
37051	229533.I1RAR2	pp	229533.V6QWX8
37052	229533.I1RAR2	pp	229533.I1RSZ3
37053	229533.I1RAR2	pp	229533.I1RHQ5
37054	229533.I1RAR2	pp	229533.I1RQZ3
37055	229533.I1RAR2	pp	229533.I1RC15
37056	229533.I1RAR2	pp	229533.I1RUG0
37057	229533.I1RAR2	pp	229533.I1RML9
37058	229533.I1RAR2	pp	229533.I1RP96
37059	229533.I1RAR2	pp	229533.I1S0C6
37060	229533.I1RAR2	pp	229533.I1RGR8
37061	229533.I1RAR2	pp	229533.I1RC24
37062	229533.I1RAR2	pp	229533.I1RBR5
37063	229533.I1RAR2	pp	229533.I1RW90
37064	229533.I1RAR2	pp	229533.V6QUE6
37065	229533.I1RAR2	pp	229533.I1RFX8
37066	229533.I1RAR2	pp	229533.V6RIV9
37067	229533.I1RAR2	pp	229533.I1RLR0
37068	229533.I1RAR2	pp	229533.Q4I5I4
37069	229533.I1RAR2	pp	229533.I1RWF3
37070	229533.I1RAR2	pp	229533.I1RVY0
37071	229533.I1RAR2	pp	229533.I1RRF7
37072	229533.I1RAR2	pp	229533.I1RNP4
37073	229533.I1RAR2	pp	229533.I1RXE5
37074	229533.I1RAR2	pp	229533.I1RCG6
37075	229533.I1RAR2	pp	229533.I1RQ42
37076	229533.I1RAR2	pp	229533.I1S0N4
37077	229533.I1RAR2	pp	229533.I1RVL8
37078	229533.I1RAR2	pp	229533.I1RHL7
37079	229533.I1RAR2	pp	229533.Q4ICA8
37080	229533.I1RAR2	pp	229533.Q4IPC8
37081	229533.I1RAR2	pp	229533.I1RG11
37082	229533.I1RAR2	pp	229533.I1RBJ1
37083	229533.I1RAR2	pp	229533.I1RR25
37084	229533.I1RAR2	pp	229533.I1RCL7

37085 229533.I1RAR2 pp 229533.I1RMV1
37086 229533.I1RAR2 pp 229533.I1S604
37087 229533.I1RAR2 pp 229533.I1RJI3
37088 229533.I1RAR2 pp 229533.I1RR61
37089 229533.I1RAR2 pp 229533.I1S3N1
37090 229533.I1RAR2 pp 229533.I1RK95
37091 229533.I1RAR2 pp 229533.I1RZU9
37092 229533.I1RAR2 pp 229533.Q4HYI0
37093 229533.I1RAR2 pp 229533.I1S5Z2
37094 229533.I1RAR2 pp 229533.I1RS41
37095 229533.I1RAR2 pp 229533.Q4HTT2
37096 229533.I1RAR2 pp 229533.I1RPM7
37097 229533.I1RAR2 pp 229533.I1RK71
37098 229533.I1RAR2 pp 229533.I1RXU0
37099 229533.I1RAR2 pp 229533.I1S0G0
37100 229533.I1RAR2 pp 229533.Q4I7S1
37101 229533.I1RAR2 pp 229533.I1RXX1
37102 229533.I1RAR2 pp 229533.I1RCB7
37103 229533.I1RAR2 pp 229533.I1RBE7
37104 229533.I1RAR2 pp 229533.V6QV38
37105 229533.I1RAR2 pp 229533.I1REN3
37106 229533.I1RAR2 pp 229533.I1RUG4
37107 229533.I1RAR2 pp 229533.I1S276
37108 229533.I1RAR2 pp 229533.I1RNC6
37109 229533.I1RAR2 pp 229533.I1RK79
37110 229533.I1RAR2 pp 229533.I1RDZ6
37111 229533.I1RAR2 pp 229533.I1RM65
37112 229533.I1RAR2 pp 229533.I1RCA9
37113 229533.I1RAR2 pp 229533.I1RZB5
37114 229533.I1RAR2 pp 229533.Q4IER8
37115 229533.I1RAR2 pp 229533.I1RU02
37116 229533.I1RAR2 pp 229533.I1RJL2
37117 229533.I1RG76 pp 229533.I1RJZ7
37118 229533.I1RG76 pp 229533.I1RRJ6
37119 229533.I1RG76 pp 229533.I1RW71
37120 229533.I1RKK1 pp 229533.I1RVN2
37121 229533.I1RKK1 pp 229533.Q4INZ9
37122 229533.I1RKK1 pp 229533.I1RX77
37123 229533.I1RKK1 pp 229533.I1RPK4
37124 229533.I1RVZ6 pp 229533.I1S0N8
37125 229533.I1REX8 pp 229533.V6RGI1
37126 229533.I1REX8 pp 229533.I1RN89
37127 229533.I1REX8 pp 229533.I1RYZ9
37128 229533.I1REX8 pp 229533.I1RL24
37129 229533.I1REX8 pp 229533.I1S098
37130 229533.I1REX8 pp 229533.I1RPC2
37131 229533.I1REX8 pp 229533.V6QV09
37132 229533.I1REX8 pp 229533.I1RFL7
37133 229533.I1REX8 pp 229533.I1RK75
37134 229533.I1REX8 pp 229533.I1RQA3
37135 229533.I1REX8 pp 229533.I1RGF4
37136 229533.I1REX8 pp 229533.I1S193
37137 229533.I1REX8 pp 229533.I1RME5
37138 229533.I1REX8 pp 229533.I1RWT6
37139 229533.I1RM11 pp 229533.I1RX67
37140 229533.A0A098DVE3 pp 229533.I1RT80
37141 229533.A0A098DVE3 pp 229533.I1RW90
37142 229533.A0A098DVE3 pp 229533.I1RQ42
37143 229533.A0A098DVE3 pp 229533.I1RPW8
37144 229533.A0A098DVE3 pp 229533.I1RNH1
37145 229533.A0A098DVE3 pp 229533.I1RW91
37146 229533.A0A098DVE3 pp 229533.I1RZR6
37147 229533.A0A098DVE3 pp 229533.I1RTC2
37148 229533.A0A098DVE3 pp 229533.I1RB71
37149 229533.A0A098DVE3 pp 229533.I1RKB1
37150 229533.A0A098DVE3 pp 229533.I1RKQ5
37151 229533.A0A098DVE3 pp 229533.V6QWX8
37152 229533.A0A098DVE3 pp 229533.I1RAV6
37153 229533.A0A098DVE3 pp 229533.I1REN3
37154 229533.A0A098DVE3 pp 229533.I1RW71
37155 229533.A0A098DVE3 pp 229533.A0A1C3YMU9
37156 229533.A0A098DVE3 pp 229533.A0A1C3YK88
37157 229533.A0A098DVE3 pp 229533.I1RP96

37158 229533.A0A098DVE3 pp 229533.I1RN17
37159 229533.A0A098DVE3 pp 229533.I1RML9
37160 229533.A0A098DVE3 pp 229533.A0A1C3YJT5
37161 229533.A0A098DVE3 pp 229533.I1RRG3
37162 229533.A0A098DVE3 pp 229533.Q4IJU0
37163 229533.A0A098DVE3 pp 229533.Q4HYQ4
37164 229533.A0A098DVE3 pp 229533.A0A1C3YI23
37165 229533.A0A098DVE3 pp 229533.A0A098DY67
37166 229533.A0A098DVE3 pp 229533.I1RQ31
37167 229533.A0A098DVE3 pp 229533.I1RK95
37168 229533.A0A098DVE3 pp 229533.V6QV38
37169 229533.A0A098DVE3 pp 229533.I1RYB9
37170 229533.A0A098DVE3 pp 229533.I1RZK6
37171 229533.A0A098DVE3 pp 229533.I1RNH6
37172 229533.A0A098DVE3 pp 229533.I1RJY2
37173 229533.A0A098DVE3 pp 229533.A0A0E0RN05
37174 229533.A0A098DVE3 pp 229533.A0A098E1Y1
37175 229533.A0A098DVE3 pp 229533.I1RZU9
37176 229533.A0A098DVE3 pp 229533.I1RE14
37177 229533.A0A098DVE3 pp 229533.I1RJI3
37178 229533.A0A098DVE3 pp 229533.V6QUE6
37179 229533.A0A098DVE3 pp 229533.I1RAR2
37180 229533.A0A098DVE3 pp 229533.I1S9U1
37181 229533.A0A098DVE3 pp 229533.A0A1C3YJR3
37182 229533.A0A098DVE3 pp 229533.I1S5Z2
37183 229533.A0A098DVE3 pp 229533.Q4I5I4
37184 229533.A0A098DVE3 pp 229533.I1RZB5
37185 229533.A0A098DVE3 pp 229533.I1S255
37186 229533.A0A098DVE3 pp 229533.I1RVZ4
37187 229533.A0A098DVE3 pp 229533.I1RT01
37188 229533.A0A098DVE3 pp 229533.I1RC16
37189 229533.A0A098DVE3 pp 229533.I1RK83
37190 229533.A0A098DVE3 pp 229533.I1RJL2
37191 229533.A0A098DVE3 pp 229533.Q4HTT2
37192 229533.A0A098DVE3 pp 229533.I1S9Y1
37193 229533.A0A098DVE3 pp 229533.I1RC24
37194 229533.A0A098DVE3 pp 229533.I1RT35
37195 229533.A0A098DVE3 pp 229533.I1RXE5
37196 229533.A0A098DVE3 pp 229533.Q4HYI0
37197 229533.A0A098DVE3 pp 229533.I1RCG6
37198 229533.A0A098DVE3 pp 229533.A0A1C3YIR8
37199 229533.A0A098DVE3 pp 229533.I1SAN0
37200 229533.A0A098DVE3 pp 229533.I1RW80
37201 229533.A0A098DVE3 pp 229533.A0A0E0S6D3
37202 229533.A0A098DVE3 pp 229533.I1RVY0
37203 229533.A0A098DVE3 pp 229533.I1RC15
37204 229533.A0A098DVE3 pp 229533.Q4I5W5
37205 229533.A0A098DVE3 pp 229533.I1RDC9
37206 229533.A0A098DVE3 pp 229533.I1RMQ7
37207 229533.A0A098DVE3 pp 229533.I1RRT0
37208 229533.A0A098DVE3 pp 229533.I1RBR5
37209 229533.A0A098DVE3 pp 229533.A0A1C3YKX5
37210 229533.A0A098DVE3 pp 229533.I1RN04
37211 229533.A0A098DVE3 pp 229533.I1RZS6
37212 229533.A0A098DVE3 pp 229533.A0A1C3YJZ2
37213 229533.A0A098DVE3 pp 229533.I1RVL8
37214 229533.A0A098DVE3 pp 229533.Q4IMD1
37215 229533.A0A098DVE3 pp 229533.A0A0E0SHE3
37216 229533.A0A098DVE3 pp 229533.A0A098DY35
37217 229533.A0A098DVE3 pp 229533.I1RBC7
37218 229533.A0A098DVE3 pp 229533.I1RFL7
37219 229533.A0A098DVE3 pp 229533.I1RRJ8
37220 229533.A0A098DVE3 pp 229533.I1R9Y7
37221 229533.A0A098DVE3 pp 229533.A0A0E0SFV6
37222 229533.A0A098DVE3 pp 229533.Q4IJ11
37223 229533.A0A098DVE3 pp 229533.I1RMV1
37224 229533.A0A098DVE3 pp 229533.I1S0E8
37225 229533.A0A098DVE3 pp 229533.A0A1C3YLP8
37226 229533.A0A098DVE3 pp 229533.I1RV91
37227 229533.A0A098DVE3 pp 229533.I1RZM2
37228 229533.A0A098DVE3 pp 229533.I1RW23
37229 229533.A0A098DVE3 pp 229533.I1RQ88
37230 229533.A0A098DVE3 pp 229533.I1RR61

229533.A0A098DVE3 pp 229533.I1RDH0
229533.A0A098DVE3 pp 229533.I1RRF7
229533.A0A098DVE3 pp 229533.I1S276
229533.A0A098DVE3 pp 229533.I1RPM7
229533.A0A098DVE3 pp 229533.I1RK71
229533.A0A098DVE3 pp 229533.I1RSZ3
229533.A0A098DVE3 pp 229533.I1RSG8
229533.A0A098DVE3 pp 229533.I1RNQ6
229533.A0A098DVE3 pp 229533.I1RQ65
229533.A0A098DVE3 pp 229533.I1RG11
229533.A0A098DVE3 pp 229533.I1RJY3
229533.A0A098DVE3 pp 229533.I1RS30
229533.A0A098DVE3 pp 229533.I1RK79
229533.A0A098DVE3 pp 229533.I1R9N1
229533.A0A098DVE3 pp 229533.I1RRI2
229533.A0A098DVE3 pp 229533.I1RE30
229533.A0A098DVE3 pp 229533.V6RGN5
229533.A0A098DVE3 pp 229533.I1RUG4
229533.A0A098DVE3 pp 229533.I1RRQ3
229533.A0A098DVE3 pp 229533.I1RAI9
229533.A0A098DVE3 pp 229533.I1RR25
229533.A0A098DVE3 pp 229533.I1RCB7
229533.A0A098DVE3 pp 229533.I1RXU0
229533.A0A098DVE3 pp 229533.Q4I7S1
229533.A0A098DVE3 pp 229533.I1RWF3
229533.A0A098DVE3 pp 229533.A0A1C3YN55
229533.A0A098DVE3 pp 229533.I1RNB8
229533.A0A098DVE3 pp 229533.I1RPK0
229533.A0A098DVE3 pp 229533.Q4HYB8
229533.A0A098DVE3 pp 229533.I1RRN6
229533.A0A098DVE3 pp 229533.I1S0J1
229533.I1RV06 pp 229533.V6R5D2
229533.I1RV06 pp 229533.I1S149
229533.I1RV06 pp 229533.V6RPS5
229533.I1RV06 pp 229533.I1S694
229533.I1RV06 pp 229533.V6R0W1
229533.I1RV06 pp 229533.I1SAI4
229533.I1RV06 pp 229533.I1RZA8
229533.I1RV06 pp 229533.V6R756
229533.I1RV06 pp 229533.V6R2Z2
229533.I1RV06 pp 229533.V6RAM6
229533.I1RV06 pp 229533.V6QU99
229533.I1RV06 pp 229533.I1RY16
229533.A0A0E0SCW3 pp 229533.A0A0E0SG28
229533.A0A0E0SCW3 pp 229533.I1RDY1
229533.A0A0E0SCW3 pp 229533.I1RRD2
229533.A0A0E0SCW3 pp 229533.I1RWC6
229533.A0A0E0SCW3 pp 229533.A0A0E0SKT0
229533.A0A0E0SCW3 pp 229533.A0A1C3YKJ7
229533.A0A0E0SCW3 pp 229533.A0A1C3YID5
229533.A0A0E0SCW3 pp 229533.A0A0E0SFM9
229533.A0A0E0SCW3 pp 229533.I1RJS4
229533.A0A0E0SCW3 pp 229533.A0A1C3YI56
229533.A0A0E0SCW3 pp 229533.A0A1C3YMH5
229533.A0A0E0SCW3 pp 229533.A0A0E0SMG8
229533.A0A0E0SCW3 pp 229533.I1R9Z0
229533.I1RTQ0 pp 229533.I1RWY3
229533.I1RCU1 pp 229533.I1RPP5
229533.I1RCU1 pp 229533.I1RJT7
229533.I1RCU1 pp 229533.I1RZP8
229533.I1RCU1 pp 229533.I1RQR6
229533.I1RER6 pp 229533.I1RNJ7
229533.I1RUN8 pp 229533.I1RZH4
229533.I1RUN8 pp 229533.I1S1D3
229533.I1RUN8 pp 229533.V6RKY7
229533.I1RUN8 pp 229533.I1S0D1
229533.I1RUN8 pp 229533.I1S2K7
229533.I1RUN8 pp 229533.V6RGI1
229533.I1RUN8 pp 229533.I1S0M2
229533.I1RUN8 pp 229533.V6RVG3
229533.I1RUN8 pp 229533.I1RY44
229533.I1RUN8 pp 229533.V6RDP1
229533.I1RUN8 pp 229533.Q4IJM4

37304	229533.I1RUN8	pp	229533.I1RWT6
37305	229533.I1RUN8	pp	229533.I1S1W4
37306	229533.I1RUN8	pp	229533.I1RUQ5
37307	229533.I1RUN8	pp	229533.I1S2B6
37308	229533.I1RUN8	pp	229533.I1RYY5
37309	229533.I1RUN8	pp	229533.I1S2G7
37310	229533.I1RUN8	pp	229533.I1S439
37311	229533.I1RUN8	pp	229533.I1S0D4
37312	229533.I1RUN8	pp	229533.I1RZE4
37313	229533.I1RUN8	pp	229533.I1RV91
37314	229533.I1RUN8	pp	229533.V6QV09
37315	229533.I1RUN8	pp	229533.I1S2E9
37316	229533.I1RUN8	pp	229533.I1RYZ9
37317	229533.I1RUN8	pp	229533.I1RX70
37318	229533.I1RUN8	pp	229533.I1RWJ6
37319	229533.I1RUN8	pp	229533.I1RYH9
37320	229533.I1RUN8	pp	229533.I1RXH6
37321	229533.I1RUN8	pp	229533.I1S1V5
37322	229533.I1RUN8	pp	229533.I1RV66
37323	229533.I1RKB1	pp	229533.I1S3N0
37324	229533.I1RKB1	pp	229533.I1RNH1
37325	229533.I1RKB1	pp	229533.I1RTB2
37326	229533.I1RKB1	pp	229533.Q4HZ42
37327	229533.I1RKB1	pp	229533.I1RX63
37328	229533.I1RKB1	pp	229533.I1RPW8
37329	229533.I1RKB1	pp	229533.Q4HYQ4
37330	229533.I1RKB1	pp	229533.I1RZM2
37331	229533.I1RKB1	pp	229533.I1RRM8
37332	229533.I1RKB1	pp	229533.I1RTC2
37333	229533.I1RKB1	pp	229533.I1RRG3
37334	229533.I1RKB1	pp	229533.I1RW80
37335	229533.I1RKB1	pp	229533.I1RUF8
37336	229533.I1RKB1	pp	229533.Q4IQX3
37337	229533.I1RKB1	pp	229533.I1RLV1
37338	229533.I1RKB1	pp	229533.I1RTH2
37339	229533.I1RKB1	pp	229533.V6RAC9
37340	229533.I1RKB1	pp	229533.I1RSZ3
37341	229533.I1RKB1	pp	229533.I1RPQ1
37342	229533.I1RKB1	pp	229533.I1RPF5
37343	229533.I1RKB1	pp	229533.I1RY44
37344	229533.I1RKB1	pp	229533.I1S0J3
37345	229533.I1RKB1	pp	229533.I1RXE5
37346	229533.I1RKB1	pp	229533.I1RY29
37347	229533.I1RKB1	pp	229533.Q4HYI0
37348	229533.I1RKB1	pp	229533.I1RWF3
37349	229533.I1RKB1	pp	229533.I1RKI2
37350	229533.I1RKB1	pp	229533.I1RWI8
37351	229533.I1RKB1	pp	229533.I1RT35
37352	229533.I1RKB1	pp	229533.I1S2E9
37353	229533.I1RKB1	pp	229533.I1RWT6
37354	229533.I1RKB1	pp	229533.Q4INS6
37355	229533.I1RKB1	pp	229533.I1RUP9
37356	229533.I1RKB1	pp	229533.I1RP96
37357	229533.I1RKB1	pp	229533.I1S3N1
37358	229533.I1RKB1	pp	229533.Q4HTT2
37359	229533.I1RKB1	pp	229533.I1RN57
37360	229533.I1RKB1	pp	229533.I1RML9
37361	229533.I1RKB1	pp	229533.I1RRT0
37362	229533.I1RKB1	pp	229533.I1RVY0
37363	229533.I1RKB1	pp	229533.Q4IJU0
37364	229533.I1RKB1	pp	229533.I1RNP4
37365	229533.I1RKB1	pp	229533.I1RLR0
37366	229533.I1RKB1	pp	229533.I1RQ42
37367	229533.I1RKB1	pp	229533.I1RR25
37368	229533.I1RKB1	pp	229533.Q4HYB8
37369	229533.I1RKB1	pp	229533.I1RVL8
37370	229533.I1RKB1	pp	229533.I1RMQ7
37371	229533.I1RKB1	pp	229533.I1RR61
37372	229533.I1RKB1	pp	229533.I1RQZ3
37373	229533.I1RKB1	pp	229533.I1RUG4
37374	229533.I1RKB1	pp	229533.I1RN04
37375	229533.I1RKB1	pp	229533.I1RVZ4
37376	229533.I1RKB1	pp	229533.I1RZ94

37377 229533.I1RKB1 pp 229533.I1RQA3
37378 229533.I1RKB1 pp 229533.I1RMV1
37379 229533.I1RKB1 pp 229533.I1S0C6
37380 229533.I1RKB1 pp 229533.Q4IJM4
37381 229533.I1RKB1 pp 229533.Q4I5I4
37382 229533.I1RKB1 pp 229533.V6QUE6
37383 229533.I1RKB1 pp 229533.V6QWX8
37384 229533.I1RKB1 pp 229533.I1RRN5
37385 229533.I1RKB1 pp 229533.I1RW90
37386 229533.I1RKB1 pp 229533.I1RW91
37387 229533.I1RKB1 pp 229533.I1S209
37388 229533.I1RKB1 pp 229533.I1RPM7
37389 229533.I1RKB1 pp 229533.I1RS41
37390 229533.I1RKB1 pp 229533.I1RMS2
37391 229533.I1RKB1 pp 229533.I1RRF7
37392 229533.I1RKB1 pp 229533.I1RSR9
37393 229533.I1RKB1 pp 229533.I1S276
37394 229533.I1RKB1 pp 229533.I1RSC7
37395 229533.I1RKB1 pp 229533.I1RZP8
37396 229533.I1RKB1 pp 229533.V6QV38
37397 229533.I1RKB1 pp 229533.I1RXU0
37398 229533.I1RKB1 pp 229533.Q4IER8
37399 229533.I1RKB1 pp 229533.I1RM65
37400 229533.I1RKB1 pp 229533.I1RYY5
37401 229533.I1RKB1 pp 229533.I1RZB5
37402 229533.V6RE46 pp 229533.V6RV57
37403 229533.I1RYS8 pp 229533.Q4IA62
37404 229533.I1RYS8 pp 229533.Q4I1J3
37405 229533.I1RYS8 pp 229533.Q4I8B6
37406 229533.I1RYS8 pp 229533.Q4I1B1
37407 229533.I1RYS8 pp 229533.Q4IMZ7
37408 229533.A0A098DUU3 pp 229533.I1RY59
37409 229533.A0A098DUU3 pp 229533.I1RS60
37410 229533.A0A098DUU3 pp 229533.I1R9Z0
37411 229533.A0A098DUU3 pp 229533.A0A0E0SMG8
37412 229533.A0A098DUU3 pp 229533.A0A098E454
37413 229533.A0A098DUU3 pp 229533.A0A1C3YI56
37414 229533.A0A098DUU3 pp 229533.I1RRS2
37415 229533.A0A098DUU3 pp 229533.A0A1C3YMH5
37416 229533.A0A098DUU3 pp 229533.I1RKK6
37417 229533.A0A098DUU3 pp 229533.I1RJS4
37418 229533.A0A098DUU3 pp 229533.I1RLU7
37419 229533.A0A098DUU3 pp 229533.A0A098E1K1
37420 229533.A0A098DUU3 pp 229533.I1RJU7
37421 229533.A0A098DUU3 pp 229533.I1RWI1
37422 229533.A0A098DUU3 pp 229533.A0A098DVJ5
37423 229533.A0A098DUU3 pp 229533.A0A0E0SFM9
37424 229533.A0A098DUU3 pp 229533.A0A1C3YID5
37425 229533.A0A098DUU3 pp 229533.A0A1C3YKJ7
37426 229533.A0A098DUU3 pp 229533.A0A0E0SCW3
37427 229533.A0A098DUU3 pp 229533.A0A098E1H5
37428 229533.A0A098DUU3 pp 229533.I1RDY1
37429 229533.A0A098DUU3 pp 229533.A0A098E488
37430 229533.A0A098DUU3 pp 229533.I1RZ27
37431 229533.A0A098DUU3 pp 229533.A0A098DWB1
37432 229533.A0A098DUU3 pp 229533.I1RB75
37433 229533.A0A1C3YJX9 pp 229533.I1RRJ6
37434 229533.A0A1C3YJX9 pp 229533.I1RJZ7
37435 229533.A0A1C3YJX9 pp 229533.A0A1C3YN36
37436 229533.A0A1C3YJX9 pp 229533.I1RW71
37437 229533.A0A1C3YIK9 pp 229533.I1RPM4
37438 229533.A0A1C3YIK9 pp 229533.I1RJV3
37439 229533.A0A1C3YIK9 pp 229533.I1S2C0
37440 229533.A0A1C3YIK9 pp 229533.I1RA70
37441 229533.A0A1C3YIK9 pp 229533.I1RSZ7
37442 229533.A0A1C3YIK9 pp 229533.I1RFS3
37443 229533.A0A1C3YIK9 pp 229533.I1S0L4
37444 229533.A0A1C3YIK9 pp 229533.I1RB59
37445 229533.A0A1C3YIK9 pp 229533.I1RS20
37446 229533.A0A1C3YIK9 pp 229533.I1RRI2
37447 229533.A0A1C3YIK9 pp 229533.I1S1C7
37448 229533.A0A1C3YIK9 pp 229533.I1RP27
37449 229533.A0A1C3YIK9 pp 229533.I1S2H4

37450 229533.A0A1C3YIK9 pp 229533.I1RJV4
37451 229533.A0A1C3YIK9 pp 229533.I1RBS6
37452 229533.A0A1C3YIK9 pp 229533.I1RD17
37453 229533.A0A1C3YIK9 pp 229533.V6RRH2
37454 229533.A0A1C3YIK9 pp 229533.I1RD64
37455 229533.A0A1C3YIK9 pp 229533.I1RB92
37456 229533.A0A1C3YIK9 pp 229533.I1RGA4
37457 229533.A0A1C3YIK9 pp 229533.A0A1C3YLK8
37458 229533.A0A1C3YIK9 pp 229533.I1RC82
37459 229533.A0A1C3YIK9 pp 229533.O74638
37460 229533.A0A1C3YIK9 pp 229533.I1RJW6
37461 229533.A0A1C3YIK9 pp 229533.I1RT03
37462 229533.A0A1C3YIK9 pp 229533.I1RTD5
37463 229533.A0A1C3YIK9 pp 229533.I1RXC2
37464 229533.A0A1C3YIK9 pp 229533.I1RVX0
37465 229533.A0A1C3YIK9 pp 229533.A0A1C3YJI6
37466 229533.A0A1C3YIK9 pp 229533.I1RCH5
37467 229533.A0A1C3YIK9 pp 229533.I1RT40
37468 229533.A0A1C3YIK9 pp 229533.I1S5R1
37469 229533.A0A1C3YIK9 pp 229533.I1RQE7
37470 229533.A0A1C3YIK9 pp 229533.I1RS47
37471 229533.A0A1C3YIK9 pp 229533.I1RRT8
37472 229533.A0A1C3YIK9 pp 229533.I1S2E8
37473 229533.A0A1C3YIK9 pp 229533.I1RZ39
37474 229533.A0A1C3YIK9 pp 229533.I1RRQ3
37475 229533.A0A1C3YIK9 pp 229533.I1RN67
37476 229533.A0A1C3YIK9 pp 229533.I1RN18
37477 229533.A0A1C3YIK9 pp 229533.I1RFL4
37478 229533.A0A1C3YIK9 pp 229533.A0A1C3YKM9
37479 229533.A0A1C3YIK9 pp 229533.I1S5R6
37480 229533.A0A1C3YIK9 pp 229533.I1S8P5
37481 229533.A0A1C3YIK9 pp 229533.A0A1C3YIS7
37482 229533.A0A1C3YIK9 pp 229533.I1RZZ9
37483 229533.A0A1C3YIK9 pp 229533.I1RPP3
37484 229533.A0A1C3YIK9 pp 229533.I1RBY4
37485 229533.A0A1C3YIK9 pp 229533.I1R9M0
37486 229533.A0A1C3YIK9 pp 229533.I1RD22
37487 229533.A0A1C3YIK9 pp 229533.V6RMW6
37488 229533.A0A1C3YIK9 pp 229533.I1RYI5
37489 229533.A0A1C3YIK9 pp 229533.I1RTA1
37490 229533.A0A1C3YIK9 pp 229533.I1S2B8
37491 229533.A0A1C3YIK9 pp 229533.I1RSQ3
37492 229533.A0A1C3YIK9 pp 229533.I1RJZ7
37493 229533.A0A1C3YIK9 pp 229533.I1RVZ2
37494 229533.A0A1C3YIK9 pp 229533.V6R042
37495 229533.A0A1C3YIK9 pp 229533.I1RBG9
37496 229533.A0A1C3YIK9 pp 229533.I1RN63
37497 229533.A0A1C3YIK9 pp 229533.I1S1U7
37498 229533.A0A1C3YIK9 pp 229533.I1RM96
37499 229533.A0A1C3YIK9 pp 229533.I1RAW0
37500 229533.A0A1C3YIK9 pp 229533.I1RC32
37501 229533.A0A1C3YIK9 pp 229533.I1RUG3
37502 229533.A0A1C3YIK9 pp 229533.I1RRV1
37503 229533.A0A1C3YIK9 pp 229533.I1RNC1
37504 229533.A0A1C3YIK9 pp 229533.I1S1W9
37505 229533.A0A1C3YIK9 pp 229533.I1R9X7
37506 229533.A0A1C3YIK9 pp 229533.I1RCR8
37507 229533.A0A1C3YIK9 pp 229533.I1RAQ5
37508 229533.A0A1C3YIK9 pp 229533.I1RS17
37509 229533.A0A1C3YIK9 pp 229533.V6RTJ8
37510 229533.A0A1C3YIK9 pp 229533.I1RWD7
37511 229533.A0A1C3YIK9 pp 229533.I1S0J1
37512 229533.A0A1C3YIK9 pp 229533.I1RX46
37513 229533.A0A1C3YIK9 pp 229533.I1RE01
37514 229533.A0A1C3YIK9 pp 229533.I1REK8
37515 229533.A0A1C3YIK9 pp 229533.I1RSI0
37516 229533.A0A1C3YIK9 pp 229533.I1RE00
37517 229533.A0A1C3YIK9 pp 229533.I1RTA0
37518 229533.A0A1C3YIK9 pp 229533.I1RXD7
37519 229533.A0A1C3YIK9 pp 229533.I1S0R7
37520 229533.A0A1C3YIK9 pp 229533.I1RK95
37521 229533.A0A1C3YIK9 pp 229533.I1R9V7
37522 229533.A0A1C3YIK9 pp 229533.I1RR24

229533.A0A1C3YIK9	pp	229533.I1RA48
229533.A0A1C3YIK9	pp	229533.I1RFQ7
229533.A0A1C3YIK9	pp	229533.I1RZK6
229533.A0A1C3YIK9	pp	229533.I1S0L3
229533.A0A1C3YIK9	pp	229533.I1RT80
229533.A0A1C3YIK9	pp	229533.Q4IJK2
229533.A0A1C3YIK9	pp	229533.I1RJU3
229533.A0A1C3YIK9	pp	229533.I1RD52
229533.A0A1C3YIK9	pp	229533.I1RVQ0
229533.A0A1C3YIK9	pp	229533.I1RQM6
229533.A0A1C3YIK9	pp	229533.I1RNH1
229533.A0A1C3YIK9	pp	229533.I1S5B0
229533.A0A1C3YIK9	pp	229533.I1RBT1
229533.A0A1C3YIK9	pp	229533.A0A1C3YIP5
229533.A0A1C3YIK9	pp	229533.I1RAC2
229533.A0A1C3YIK9	pp	229533.I1RQJ5
229533.A0A1C3YIK9	pp	229533.I1RW99
229533.A0A1C3YIK9	pp	229533.I1RSQ7
229533.A0A1C3YIK9	pp	229533.I1RW25
229533.A0A1C3YIK9	pp	229533.Q4HVU7
229533.A0A1C3YIK9	pp	229533.V6QUE6
229533.A0A1C3YIK9	pp	229533.I1RW71
229533.A0A1C3YIK9	pp	229533.I1S4S7
229533.A0A1C3YIK9	pp	229533.V6QYF0
229533.A0A1C3YIK9	pp	229533.I1RG51
229533.A0A1C3YIK9	pp	229533.I1RRY9
229533.A0A1C3YIK9	pp	229533.I1REX4
229533.A0A1C3YIK9	pp	229533.V6RCG5
229533.A0A1C3YIK9	pp	229533.I1RQ31
229533.A0A1C3YIK9	pp	229533.I1RSR3
229533.A0A1C3YIK9	pp	229533.I1RQA2
229533.A0A1C3YIK9	pp	229533.Q4ICA8
229533.A0A1C3YIK9	pp	229533.I1RZM8
229533.A0A1C3YIK9	pp	229533.I1RG56
229533.A0A1C3YIK9	pp	229533.A0A1C3YKB0
229533.A0A1C3YIK9	pp	229533.I1RB27
229533.A0A1C3YIK9	pp	229533.A0A1C3YJR3
229533.A0A1C3YIK9	pp	229533.I1RS18
229533.A0A1C3YIK9	pp	229533.I1RQ81
229533.A0A1C3YIK9	pp	229533.A0A1C3YKV3
229533.A0A1C3YIK9	pp	229533.I1RXC4
229533.A0A1C3YIK9	pp	229533.A0A1C3YK34
229533.A0A1C3YLS8	pp	229533.I1RZP6
229533.A0A1C3YLS8	pp	229533.I1RUG1
229533.A0A1C3YLS8	pp	229533.I1S2F2
229533.A0A1C3YLS8	pp	229533.I1RJQ5
229533.A0A1C3YLS8	pp	229533.I1RAW4
229533.A0A1C3YLS8	pp	229533.I1RSW5
229533.A0A1C3YLS8	pp	229533.I1RMG6
229533.A0A1C3YLS8	pp	229533.I1RF73
229533.A0A1C3YLS8	pp	229533.I1R9D6
229533.A0A1C3YLS8	pp	229533.I1RT41
229533.A0A1C3YLS8	pp	229533.I1RHF4
229533.A0A1C3YLS8	pp	229533.I1RVZ6
229533.A0A1C3YLS8	pp	229533.I1RIJ2
229533.A0A1C3YLS8	pp	229533.I1RMB7
229533.A0A1C3YLS8	pp	229533.I1RE37
229533.A0A1C3YLS8	pp	229533.I1S050
229533.A0A1C3YLS8	pp	229533.I1S0N8
229533.I1RJS7	pp	229533.I1RWM4
229533.I1RJS7	pp	229533.I1S267
229533.I1RJS7	pp	229533.I1RPV8
229533.I1RJS7	pp	229533.I1S149
229533.I1RJS7	pp	229533.I1RXE6
229533.I1RJS7	pp	229533.V6RPS5
229533.I1RJS7	pp	229533.I1RUQ3
229533.I1RJS7	pp	229533.Q4HVQ9
229533.I1RJS7	pp	229533.I1RZ56
229533.I1RJS7	pp	229533.I1RK50
229533.I1RJS7	pp	229533.I1RYX6
229533.I1RJS7	pp	229533.I1S6H3
229533.I1RJS7	pp	229533.I1RNM7
229533.I1RJS7	pp	229533.I1S0A5

229533.I1RJS7	pp	229533.I1S094
229533.I1S2J5	pp	229533.I1S2J8
229533.I1S2J5	pp	229533.I1SAJ7
229533.I1S2J5	pp	229533.I1S2J6
229533.I1RDL3	pp	229533.I1RVB6
229533.I1RDL3	pp	229533.I1RE53
229533.I1RDL3	pp	229533.I1REM2
229533.I1RDL3	pp	229533.I1RPY0
229533.I1S7A2	pp	229533.V6R223
229533.A0A1C3YMQ5	pp	229533.I1RZH4
229533.A0A1C3YMQ5	pp	229533.I1RRQ0
229533.A0A1C3YMQ5	pp	229533.I1RX09
229533.A0A1C3YMQ5	pp	229533.I1RWI6
229533.A0A1C3YMQ5	pp	229533.A0A1C3YN89
229533.A0A1C3YMQ5	pp	229533.I1S1V3
229533.A0A1C3YMQ5	pp	229533.I1RN81
229533.A0A1C3YMQ5	pp	229533.I1RYN2
229533.A0A1C3YMQ5	pp	229533.I1RYV4
229533.A0A1C3YMQ5	pp	229533.I1RYH6
229533.A0A1C3YMQ5	pp	229533.I1RN31
229533.A0A1C3YMQ5	pp	229533.I1RPA6
229533.A0A1C3YMQ5	pp	229533.I1RDG9
229533.A0A1C3YMQ5	pp	229533.I1RNF9
229533.A0A1C3YMQ5	pp	229533.I1S2C1
229533.A0A1C3YMQ5	pp	229533.I1RZ41
229533.A0A1C3YMQ5	pp	229533.V6QUU6
229533.A0A1C3YMQ5	pp	229533.I1RY44
229533.A0A1C3YMQ5	pp	229533.V6REE9
229533.A0A1C3YMQ5	pp	229533.I1REC6
229533.A0A1C3YMQ5	pp	229533.I1RCD4
229533.A0A1C3YMQ5	pp	229533.I1S1B3
229533.A0A1C3YMQ5	pp	229533.P0C431
229533.A0A1C3YMQ5	pp	229533.I1RH68
229533.A0A1C3YMQ5	pp	229533.I1RA52
229533.A0A1C3YMQ5	pp	229533.I1RPH7
229533.A0A1C3YMQ5	pp	229533.I1RTE6
229533.A0A1C3YMQ5	pp	229533.I1RM09
229533.A0A1C3YMQ5	pp	229533.V6R476
229533.A0A1C3YMQ5	pp	229533.I1RRF9
229533.A0A1C3YMQ5	pp	229533.I1RQ72
229533.A0A1C3YMQ5	pp	229533.I1RD10
229533.A0A1C3YMQ5	pp	229533.I1S7X9
229533.A0A1C3YMQ5	pp	229533.I1RZX9
229533.A0A1C3YMQ5	pp	229533.I1RPP7
229533.A0A1C3YMQ5	pp	229533.I1S5K9
229533.A0A1C3YMQ5	pp	229533.I1RPP0
229533.A0A1C3YMQ5	pp	229533.I1RNF4
229533.A0A1C3YMQ5	pp	229533.I1RW16
229533.A0A1C3YMQ5	pp	229533.I1RN65
229533.A0A1C3YMQ5	pp	229533.I1R9K3
229533.A0A1C3YMQ5	pp	229533.I1RWL6
229533.A0A1C3YMQ5	pp	229533.I1S9D4
229533.A0A1C3YMQ5	pp	229533.I1S095
229533.A0A1C3YMQ5	pp	229533.I1S254
229533.A0A1C3YMQ5	pp	229533.V6RR76
229533.A0A1C3YMQ5	pp	229533.I1S0S6
229533.A0A1C3YMQ5	pp	229533.I1RZV4
229533.A0A1C3YMQ5	pp	229533.I1RQL4
229533.A0A1C3YMQ5	pp	229533.I1RTB7
229533.A0A1C3YMQ5	pp	229533.Q4I5R9
229533.A0A1C3YMQ5	pp	229533.I1RT36
229533.A0A1C3YMQ5	pp	229533.I1RRW2
229533.A0A1C3YMQ5	pp	229533.I1REC7
229533.A0A1C3YMQ5	pp	229533.I1S549
229533.A0A1C3YMQ5	pp	229533.I1RZD9
229533.A0A1C3YMQ5	pp	229533.I1RJV3
229533.A0A1C3YMQ5	pp	229533.I1RN36
229533.A0A1C3YMQ5	pp	229533.I1RYZ7
229533.A0A1C3YMQ5	pp	229533.I1RJS2
229533.A0A1C3YMQ5	pp	229533.I1S2E9
229533.A0A1C3YMQ5	pp	229533.I1RQN9
229533.I1RWC6	pp	229533.I1S575
229533.I1RWC6	pp	229533.I1RY14

37669	229533.I1RWC6	pp	229533.I1S1Y5
37670	229533.I1RE27	pp	229533.I1RRI2
37671	229533.I1RE27	pp	229533.I1S8R3
37672	229533.I1RE27	pp	229533.I1RZW8
37673	229533.I1RE27	pp	229533.I1REK8
37674	229533.I1RE27	pp	229533.V6R057
37675	229533.I1RE27	pp	229533.Q4I624
37676	229533.I1RE27	pp	229533.I1RQE7
37677	229533.I1RE27	pp	229533.I1RN18
37678	229533.I1RE27	pp	229533.I1RZZ1
37679	229533.I1RE27	pp	229533.I1RE87
37680	229533.I1RE27	pp	229533.I1RFL4
37681	229533.I1RE27	pp	229533.I1SA33
37682	229533.I1RE27	pp	229533.I1RJW6
37683	229533.I1RE27	pp	229533.I1S7A8
37684	229533.I1RE27	pp	229533.I1RSV2
37685	229533.I1RE27	pp	229533.I1RI82
37686	229533.I1RE27	pp	229533.I1RW99
37687	229533.I1RE27	pp	229533.I1RV91
37688	229533.I1RE27	pp	229533.I1RJV4
37689	229533.I1RE27	pp	229533.I1RUG3
37690	229533.I1RE27	pp	229533.I1RRT8
37691	229533.I1RE27	pp	229533.I1RT40
37692	229533.I1RE27	pp	229533.I1RZM8
37693	229533.I1RE27	pp	229533.I1RXF0
37694	229533.I1RE27	pp	229533.I1S5R1
37695	229533.I1RE27	pp	229533.I1RZJ5
37696	229533.I1RE27	pp	229533.I1RXD7
37697	229533.I1RE27	pp	229533.V6RCG5
37698	229533.I1RE27	pp	229533.I1RT04
37699	229533.I1RE27	pp	229533.I1RMG8
37700	229533.I1RE27	pp	229533.I1RWJ8
37701	229533.I1RE27	pp	229533.I1RVQ0
37702	229533.I1RE27	pp	229533.I1S0R7
37703	229533.I1RE27	pp	229533.I1S0L4
37704	229533.I1RE27	pp	229533.I1RRM3
37705	229533.I1RE27	pp	229533.I1S0F6
37706	229533.I1RE27	pp	229533.I1RMG3
37707	229533.I1RE27	pp	229533.V6RIV9
37708	229533.I1RE27	pp	229533.V6R1K4
37709	229533.I1RE27	pp	229533.I1RZZ9
37710	229533.I1RE27	pp	229533.I1RR24
37711	229533.I1RE27	pp	229533.I1S0L3
37712	229533.I1RE27	pp	229533.I1SA32
37713	229533.I1RE27	pp	229533.I1RW46
37714	229533.I1RE27	pp	229533.I1RJU3
37715	229533.I1RE27	pp	229533.I1RWK3
37716	229533.I1RE27	pp	229533.Q4I8P5
37717	229533.I1RE27	pp	229533.I1RRR5
37718	229533.I1RE27	pp	229533.I1RW25
37719	229533.I1RE27	pp	229533.I1RSQ7
37720	229533.I1RE27	pp	229533.I1RRM5
37721	229533.I1RE27	pp	229533.I1RS17
37722	229533.I1RE27	pp	229533.I1RQ23
37723	229533.I1RE27	pp	229533.V6QW22
37724	229533.I1RE27	pp	229533.I1RJV3
37725	229533.I1RE27	pp	229533.I1RPM4
37726	229533.I1RIA9	pp	229533.Q00909
37727	229533.I1RIA9	pp	229533.I1RY81
37728	229533.I1RIA9	pp	229533.I1RVB6
37729	229533.I1RIA9	pp	229533.I1S166
37730	229533.I1RIA9	pp	229533.I1RW91
37731	229533.I1RIA9	pp	229533.I1RXX1
37732	229533.I1RIA9	pp	229533.I1RN04
37733	229533.I1RIA9	pp	229533.V6RG22
37734	229533.I1RIA9	pp	229533.I1RKJ6
37735	229533.I1RIA9	pp	229533.I1RY35
37736	229533.I1RIA9	pp	229533.I1RPW8
37737	229533.I1RIA9	pp	229533.I1RRF7
37738	229533.I1RF32	pp	229533.I1RZ25
37739	229533.I1RAL0	pp	229533.I1RG07
37740	229533.A0A0E0RN05	pp	229533.I1RRF7
37741	229533.A0A0E0RN05	pp	229533.I1RG11

37742 229533.A0A0E0RN05 pp 229533.I1S0C6
37743 229533.A0A0E0RN05 pp 229533.V6QUE6
37744 229533.A0A0E0RN05 pp 229533.I1RAR2
37745 229533.A0A0E0RN05 pp 229533.A0A1C3YHH5
37746 229533.A0A0E0RN05 pp 229533.I1RE54
37747 229533.A0A0E0RN05 pp 229533.I1RZ42
37748 229533.A0A0E0RN05 pp 229533.Q4HYI0
37749 229533.A0A0E0RN05 pp 229533.I1RQZ3
37750 229533.A0A0E0RN05 pp 229533.I1S3N1
37751 229533.A0A0E0RN05 pp 229533.A0A1C3YI60
37752 229533.A0A0E0RN05 pp 229533.I1RDZ6
37753 229533.A0A0E0RN05 pp 229533.I1RC15
37754 229533.A0A0E0RN05 pp 229533.A0A1C3YI23
37755 229533.A0A0E0RN05 pp 229533.I1RRT0
37756 229533.A0A0E0RN05 pp 229533.I1RPM7
37757 229533.A0A0E0RN05 pp 229533.I1RK71
37758 229533.A0A0E0RN05 pp 229533.I1RDC9
37759 229533.A0A0E0RN05 pp 229533.I1RXU0
37760 229533.A0A0E0RN05 pp 229533.A0A1C3YJ12
37761 229533.A0A0E0RN05 pp 229533.I1RBJ1
37762 229533.A0A0E0RN05 pp 229533.I1S9U1
37763 229533.A0A0E0RN05 pp 229533.A0A0E0SFV6
37764 229533.A0A0E0RN05 pp 229533.A0A0E0SIV1
37765 229533.A0A0E0RN05 pp 229533.I1RMV1
37766 229533.A0A0E0RN05 pp 229533.I1RCB7
37767 229533.A0A0E0RN05 pp 229533.I1RR61
37768 229533.A0A0E0RN05 pp 229533.I1RKB1
37769 229533.A0A0E0RN05 pp 229533.I1RAE0
37770 229533.A0A0E0RN05 pp 229533.A0A1C3YJR3
37771 229533.A0A0E0RN05 pp 229533.A0A0E0SN30
37772 229533.A0A0E0RN05 pp 229533.I1RWF3
37773 229533.A0A0E0RN05 pp 229533.I1RLV1
37774 229533.A0A0E0RN05 pp 229533.A0A1C3YN55
37775 229533.A0A0E0RN05 pp 229533.I1RAP2
37776 229533.A0A0E0RN05 pp 229533.I1REN3
37777 229533.A0A0E0RN05 pp 229533.I1RUG4
37778 229533.A0A0E0RN05 pp 229533.Q4IQX3
37779 229533.A0A0E0RN05 pp 229533.I1R9C7
37780 229533.A0A0E0RN05 pp 229533.I1RXE5
37781 229533.A0A0E0RN05 pp 229533.I1RCG6
37782 229533.A0A0E0RN05 pp 229533.I1RZB5
37783 229533.A0A0E0RN05 pp 229533.I1RR25
37784 229533.A0A0E0RN05 pp 229533.I1RE23
37785 229533.A0A0E0RN05 pp 229533.I1RRM8
37786 229533.A0A0E0RN05 pp 229533.I1RHQ5
37787 229533.A0A0E0RN05 pp 229533.I1RJL2
37788 229533.A0A0E0RN05 pp 229533.I1RK79
37789 229533.A0A0E0RN05 pp 229533.I1RW80
37790 229533.A0A0E0RN05 pp 229533.I1RM65
37791 229533.A0A0E0RN05 pp 229533.A0A0E0RUJ7
37792 229533.A0A0E0RN05 pp 229533.I1RTC2
37793 229533.A0A0E0RN05 pp 229533.A0A1C3YLP8
37794 229533.A0A0E0RN05 pp 229533.I1RML9
37795 229533.A0A0E0RN05 pp 229533.V6QV38
37796 229533.A0A0E0RN05 pp 229533.I1RZM2
37797 229533.A0A0E0RN05 pp 229533.I1S276
37798 229533.A0A0E0RN05 pp 229533.I1RP96
37799 229533.A0A0E0RN05 pp 229533.I1RHL8
37800 229533.A0A0E0RN05 pp 229533.I1RGR8
37801 229533.A0A0E0RN05 pp 229533.I1RAW7
37802 229533.A0A0E0RN05 pp 229533.I1RSZ3
37803 229533.A0A0E0RN05 pp 229533.I1RRG3
37804 229533.A0A0E0RN05 pp 229533.A0A1C3YJZ2
37805 229533.A0A0E0RN05 pp 229533.I1RBC7
37806 229533.A0A0E0RN05 pp 229533.I1RX63
37807 229533.A0A0E0RN05 pp 229533.I1RN04
37808 229533.A0A0E0RN05 pp 229533.A0A1C3YLW6
37809 229533.A0A0E0RN05 pp 229533.I1RPW8
37810 229533.A0A0E0RN05 pp 229533.I1RVL8
37811 229533.A0A0E0RN05 pp 229533.I1RB25
37812 229533.A0A0E0RN05 pp 229533.I1S182
37813 229533.A0A0E0RN05 pp 229533.I1S3N0
37814 229533.A0A0E0RN05 pp 229533.I1RE30

229533.A0A0E0RN05	pp	229533.I1R9Q0
229533.A0A0E0RN05	pp	229533.I1RQ42
229533.A0A0E0RN05	pp	229533.I1RC24
229533.A0A0E0RN05	pp	229533.I1RNP4
229533.A0A0E0RN05	pp	229533.I1RW90
229533.A0A0E0RN05	pp	229533.I1RHL7
229533.A0A0E0RN05	pp	229533.A0A1C3YK91
229533.A0A0E0RN05	pp	229533.I1RBR5
229533.A0A0E0RN05	pp	229533.I1RD36
229533.A0A0E0RN05	pp	229533.A0A1C3YIR8
229533.A0A0E0RN05	pp	229533.I1RLR0
229533.A0A0E0RN05	pp	229533.I1RVY0
229533.A0A0E0RN05	pp	229533.I1RW91
229533.A0A0E0RN05	pp	229533.I1RA84
229533.A0A0E0RN05	pp	229533.V6QWX8
229533.A0A0E0RN05	pp	229533.I1RC98
229533.I1RDZ2	pp	229533.I1RGR8
229533.I1RDZ2	pp	229533.I1RG07
229533.I1RDZ2	pp	229533.I1RN41
229533.I1RDN2	pp	229533.I1RZV5
229533.I1RDN2	pp	229533.I1RFY0
229533.I1RDN2	pp	229533.I1REC7
229533.I1REE6	pp	229533.V6R5D2
229533.I1REE6	pp	229533.I1S694
229533.I1REE6	pp	229533.I1RI11
229533.I1REE6	pp	229533.I1RZA8
229533.I1REE6	pp	229533.V6R756
229533.I1REE6	pp	229533.I1S149
229533.I1REE6	pp	229533.I1SAI4
229533.I1REE6	pp	229533.V6R2Z2
229533.I1REE6	pp	229533.V6RAM6
229533.I1REE6	pp	229533.V6R0W1
229533.I1REE6	pp	229533.V6QU99
229533.I1REE6	pp	229533.I1RMS5
229533.I1REE6	pp	229533.I1RPP5
229533.I1REE6	pp	229533.I1RY16
229533.I1REE6	pp	229533.V6RPS5
229533.I1RGV3	pp	229533.I1RRF7
229533.I1RGV3	pp	229533.I1RW91
229533.I1RGV3	pp	229533.I1RPW8
229533.I1RGV3	pp	229533.I1RN04
229533.I1RAK8	pp	229533.V6QUV7
229533.I1RBR8	pp	229533.I1RJY5
229533.A0A1C3YK31	pp	229533.I1RT35
229533.A0A1C3YK31	pp	229533.I1RNB8
229533.A0A1C3YK31	pp	229533.I1RG90
229533.A0A1C3YHL0	pp	229533.I1RQS4
229533.A0A1C3YHL0	pp	229533.I1RFJ2
229533.A0A1C3YHL0	pp	229533.I1RTC1
229533.A0A1C3YHL0	pp	229533.I1RJK7
229533.A0A1C3YHL0	pp	229533.I1RB75
229533.A0A1C3YHL0	pp	229533.I1RDV9
229533.A0A1C3YHL0	pp	229533.I1S439
229533.A0A1C3YHL0	pp	229533.A0A1C3YJ26
229533.A0A1C3YHL0	pp	229533.I1RCL7
229533.A0A1C3YHL0	pp	229533.A0A1C3YKY3
229533.A0A1C3YHL0	pp	229533.I1RQA3
229533.A0A1C3YHL0	pp	229533.Q4IJM4
229533.A0A1C3YHL0	pp	229533.V6RMM8
229533.A0A1C3YHL0	pp	229533.I1RN65
229533.A0A1C3YHL0	pp	229533.I1RC22
229533.A0A1C3YHL0	pp	229533.I1RYY5
229533.A0A1C3YHL0	pp	229533.I1RUQ3
229533.A0A1C3YHL0	pp	229533.V6RU70
229533.A0A1C3YHL0	pp	229533.I1RN08
229533.A0A1C3YHL0	pp	229533.I1RMM9
229533.A0A1C3YHL0	pp	229533.I1SAY2
229533.A0A1C3YHL0	pp	229533.A0A1C3YIW6
229533.A0A1C3YHL0	pp	229533.A0A1C3YJW8
229533.A0A1C3YHL0	pp	229533.I1RC95
229533.A0A1C3YHL0	pp	229533.I1RKK0
229533.A0A1C3YHL0	pp	229533.I1RC17
229533.A0A1C3YHL0	pp	229533.I1RN31

37888	229533.A0A1C3YHL0	pp	229533.I1REE1
37889	229533.A0A1C3YHL0	pp	229533.I1R9D3
37890	229533.A0A1C3YHL0	pp	229533.I1RWT6
37891	229533.A0A1C3YHL0	pp	229533.I1RME5
37892	229533.A0A1C3YHL0	pp	229533.I1RL24
37893	229533.A0A1C3YHL0	pp	229533.I1S224
37894	229533.A0A1C3YHL0	pp	229533.I1RZH4
37895	229533.A0A1C3YHL0	pp	229533.I1RY84
37896	229533.A0A1C3YHL0	pp	229533.A0A1C3YJH8
37897	229533.A0A1C3YHL0	pp	229533.I1RMQ7
37898	229533.A0A1C3YHL0	pp	229533.I1RWW4
37899	229533.A0A1C3YHL0	pp	229533.I1RJX2
37900	229533.A0A1C3YHL0	pp	229533.A0A1C3YL77
37901	229533.A0A1C3YHL0	pp	229533.I1RAH4
37902	229533.A0A1C3YHL0	pp	229533.I1RAM7
37903	229533.A0A1C3YHL0	pp	229533.A0A1C3YLD3
37904	229533.A0A1C3YHL0	pp	229533.A0A1C3YKV3
37905	229533.A0A1C3YHL0	pp	229533.I1RE46
37906	229533.A0A1C3YHL0	pp	229533.I1RYH9
37907	229533.A0A1C3YHL0	pp	229533.I1RD18
37908	229533.A0A1C3YHL0	pp	229533.I1RN89
37909	229533.A0A1C3YHL0	pp	229533.I1RDT7
37910	229533.A0A1C3YHL0	pp	229533.A0A1C3YIP6
37911	229533.A0A1C3YHL0	pp	229533.A0A1C3YKM8
37912	229533.A0A1C3YHL0	pp	229533.I1RQS6
37913	229533.A0A1C3YHL0	pp	229533.I1RPI2
37914	229533.A0A1C3YHL0	pp	229533.I1RQ26
37915	229533.A0A1C3YHL0	pp	229533.A0A1C3YKA7
37916	229533.A0A1C3YHL0	pp	229533.I1RKJ7
37917	229533.A0A1C3YHL0	pp	229533.I1RDB1
37918	229533.A0A1C3YHL0	pp	229533.I1RZJ3
37919	229533.A0A1C3YHL0	pp	229533.I1RS56
37920	229533.A0A1C3YHL0	pp	229533.I1RKL9
37921	229533.A0A1C3YHL0	pp	229533.I1RT62
37922	229533.A0A1C3YHL0	pp	229533.A0A1C3YKW5
37923	229533.A0A1C3YHL0	pp	229533.V6QV09
37924	229533.A0A1C3YHL0	pp	229533.I1RT14
37925	229533.A0A1C3YHL0	pp	229533.I1RAT7
37926	229533.A0A1C3YHL0	pp	229533.V6RKY7
37927	229533.A0A1C3YHL0	pp	229533.I1RAW7
37928	229533.A0A1C3YHL0	pp	229533.I1RK75
37929	229533.A0A1C3YHL0	pp	229533.A0A1C3YHV3
37930	229533.A0A1C3YHL0	pp	229533.A0A1C3YM29
37931	229533.A0A1C3YHL0	pp	229533.A0A1C3YJT5
37932	229533.A0A1C3YHL0	pp	229533.A0A1C3YIL0
37933	229533.A0A1C3YHL0	pp	229533.I1RZ12
37934	229533.A0A1C3YHL0	pp	229533.I1RY44
37935	229533.A0A1C3YHL0	pp	229533.I1RB41
37936	229533.A0A1C3YHL0	pp	229533.I1R9Y7
37937	229533.A0A1C3YHL0	pp	229533.V6RVG3
37938	229533.A0A1C3YHL0	pp	229533.I1S0E8
37939	229533.A0A1C3YHL0	pp	229533.A0A1C3YHP8
37940	229533.A0A1C3YHL0	pp	229533.I1RRY1
37941	229533.A0A1C3YHL0	pp	229533.I1S1W4
37942	229533.A0A1C3YHL0	pp	229533.I1RZE4
37943	229533.A0A1C3YHL0	pp	229533.I1RMK8
37944	229533.A0A1C3YHL0	pp	229533.I1RCH5
37945	229533.A0A1C3YHL0	pp	229533.I1RA43
37946	229533.A0A1C3YHL0	pp	229533.I1RRN5
37947	229533.A0A1C3YHL0	pp	229533.I1S1D3
37948	229533.A0A1C3YHL0	pp	229533.A0A1C3YIN6
37949	229533.A0A1C3YHL0	pp	229533.I1RYZ9
37950	229533.A0A1C3YHL0	pp	229533.I1R9Y9
37951	229533.A0A1C3YHL0	pp	229533.I1RRY3
37952	229533.A0A1C3YHL0	pp	229533.A0A1C3YIA8
37953	229533.A0A1C3YHL0	pp	229533.I1RLX6
37954	229533.A0A1C3YHL0	pp	229533.I1RV48
37955	229533.A0A1C3YHL0	pp	229533.I1RGC6
37956	229533.A0A1C3YHL0	pp	229533.A0A1C3YK43
37957	229533.A0A1C3YHL0	pp	229533.I1RW16
37958	229533.A0A1C3YHL0	pp	229533.I1RPC2
37959	229533.A0A1C3YHL0	pp	229533.I1RZT1
37960	229533.A0A1C3YHL0	pp	229533.I1RUN8

37961	229533.A0A1C3YHL0	pp	229533.I1RD70
37962	229533.A0A1C3YHL0	pp	229533.A0A1C3YJC3
37963	229533.A0A1C3YHL0	pp	229533.I1RL04
37964	229533.A0A1C3YHL0	pp	229533.I1S0M6
37965	229533.A0A1C3YHL0	pp	229533.I1RT36
37966	229533.A0A1C3YHL0	pp	229533.I1S9S6
37967	229533.A0A1C3YHL0	pp	229533.I1RC97
37968	229533.A0A1C3YHL0	pp	229533.A0A1C3YI28
37969	229533.A0A1C3YHL0	pp	229533.I1S0M2
37970	229533.A0A1C3YHL0	pp	229533.I1S1V5
37971	229533.A0A1C3YHL0	pp	229533.I1RKC2
37972	229533.A0A1C3YHL0	pp	229533.I1RNP7
37973	229533.A0A1C3YHL0	pp	229533.I1RRW0
37974	229533.A0A1C3YHL0	pp	229533.I1S2G7
37975	229533.A0A1C3YHL0	pp	229533.I1RMA4
37976	229533.A0A1C3YHL0	pp	229533.I1RLU1
37977	229533.A0A1C3YHL0	pp	229533.I1S4R1
37978	229533.A0A1C3YHL0	pp	229533.I1S7X9
37979	229533.A0A1C3YHL0	pp	229533.I1RGD1
37980	229533.A0A1C3YHL0	pp	229533.A0A1C3YM91
37981	229533.A0A1C3YHL0	pp	229533.I1S0J7
37982	229533.A0A1C3YHL0	pp	229533.I1RYX9
37983	229533.A0A1C3YHL0	pp	229533.I1RVW0
37984	229533.A0A1C3YHL0	pp	229533.I1RD85
37985	229533.A0A1C3YHL0	pp	229533.I1RB74
37986	229533.A0A1C3YHL0	pp	229533.I1S1V7
37987	229533.A0A1C3YHL0	pp	229533.I1RFL7
37988	229533.A0A1C3YHL0	pp	229533.I1RB59
37989	229533.A0A1C3YHL0	pp	229533.A0A1C3YI39
37990	229533.A0A1C3YHL0	pp	229533.I1REX8
37991	229533.A0A1C3YHL0	pp	229533.I1RAN2
37992	229533.A0A1C3YHL0	pp	229533.Q4HVQ9
37993	229533.A0A1C3YHL0	pp	229533.I1RZ63
37994	229533.A0A1C3YHL0	pp	229533.I1RC34
37995	229533.A0A1C3YHL0	pp	229533.A0A1C3YJI5
37996	229533.A0A1C3YHL0	pp	229533.I1R9N1
37997	229533.A0A1C3YHL0	pp	229533.I1RTK5
37998	229533.A0A1C3YHL0	pp	229533.A0A1C3YMA5
37999	229533.A0A1C3YHL0	pp	229533.I1S098
38000	229533.A0A1C3YHL0	pp	229533.Q4IQM6
38001	229533.A0A1C3YHL0	pp	229533.I1S2E9
38002	229533.A0A1C3YHL0	pp	229533.I1RCE7
38003	229533.A0A1C3YHL0	pp	229533.I1RV91
38004	229533.I1RCJ2	pp	229533.I1RWN0
38005	229533.I1RCJ2	pp	229533.I1RTI3
38006	229533.I1RCJ2	pp	229533.I1RPA7
38007	229533.I1RCJ2	pp	229533.I1RPS0
38008	229533.I1RCJ2	pp	229533.V6RXE7
38009	229533.I1RCJ2	pp	229533.I1RN72
38010	229533.I1RCJ2	pp	229533.I1S0L8
38011	229533.I1RCJ2	pp	229533.I1RKZ8
38012	229533.I1RCJ2	pp	229533.I1RRT3
38013	229533.I1RCJ2	pp	229533.I1RDZ5
38014	229533.I1RCJ2	pp	229533.I1RNG4
38015	229533.A0A0E0RRE0	pp	229533.I1RM94
38016	229533.A0A0E0RRE0	pp	229533.A0A1C3YII1
38017	229533.A0A0E0RRE0	pp	229533.I1RM23
38018	229533.Q4INZ9	pp	229533.V6R227
38019	229533.Q4INZ9	pp	229533.V6RGY3
38020	229533.Q4INZ9	pp	229533.V6RGN5
38021	229533.Q4INZ9	pp	229533.Q4IQW0
38022	229533.Q4INZ9	pp	229533.V6R283
38023	229533.Q4INZ9	pp	229533.Q4IPB8
38024	229533.Q4INZ9	pp	229533.Q4IPH4
38025	229533.I1RF98	pp	229533.I1RVB6
38026	229533.I1RU69	pp	229533.Q4IAS1
38027	229533.I1RU69	pp	229533.Q4IBK5
38028	229533.I1RU69	pp	229533.V6RGN5
38029	229533.I1RU69	pp	229533.I1RXF2
38030	229533.I1RU69	pp	229533.I1RV91
38031	229533.I1RU69	pp	229533.I1S2E9
38032	229533.I1RU69	pp	229533.I1S056
38033	229533.I1RU69	pp	229533.Q4HZ42

38034 229533.I1RU69 pp 229533.I1S7X9
38035 229533.I1RU69 pp 229533.I1RX47
38036 229533.I1RU69 pp 229533.I1RZV6
38037 229533.I1RU69 pp 229533.V6R042
38038 229533.I1RU69 pp 229533.I1S4M5
38039 229533.I1RU69 pp 229533.I1S8E2
38040 229533.I1RU69 pp 229533.I1RWU2
38041 229533.I1RU69 pp 229533.Q4IN63
38042 229533.I1RU69 pp 229533.Q4IF76
38043 229533.I1RU69 pp 229533.I1S2C0
38044 229533.I1RU69 pp 229533.I1RZS6
38045 229533.I1RU69 pp 229533.I1S060
38046 229533.I1RU69 pp 229533.I1RY44
38047 229533.I1RU69 pp 229533.I1RX24
38048 229533.I1RU69 pp 229533.Q4IPB8
38049 229533.I1RU69 pp 229533.V6QXG1
38050 229533.I1RU69 pp 229533.Q4I662
38051 229533.I1RU69 pp 229533.I1S0B1
38052 229533.I1RU69 pp 229533.I1S2E8
38053 229533.I1RU69 pp 229533.Q4HZ68
38054 229533.I1RU69 pp 229533.I1RWB5
38055 229533.I1RU69 pp 229533.I1RX46
38056 229533.I1RU69 pp 229533.I1RX51
38057 229533.I1RU69 pp 229533.I1RYJ9
38058 229533.I1RU69 pp 229533.I1S0J1
38059 229533.I1RU69 pp 229533.Q4IFI0
38060 229533.I1RU69 pp 229533.I1S1X9
38061 229533.I1RU69 pp 229533.Q4IPI1
38062 229533.I1RU69 pp 229533.I1RW71
38063 229533.I1RU69 pp 229533.I1RX81
38064 229533.I1RU69 pp 229533.I1S1C6
38065 229533.I1RU69 pp 229533.I1S2Y5
38066 229533.I1RU69 pp 229533.I1S7M2
38067 229533.I1RU69 pp 229533.I1RZJ6
38068 229533.I1RU69 pp 229533.I1RWH5
38069 229533.I1RU69 pp 229533.V6QYF0
38070 229533.I1RU69 pp 229533.Q4IAA0
38071 229533.I1RU69 pp 229533.I1RZG7
38072 229533.I1RU69 pp 229533.I1S246
38073 229533.I1RU69 pp 229533.I1S9U2
38074 229533.I1RU69 pp 229533.Q4IBS2
38075 229533.I1RU69 pp 229533.I1S7M3
38076 229533.I1RU69 pp 229533.Q4HVW2
38077 229533.I1RU69 pp 229533.Q4IRI9
38078 229533.I1RU69 pp 229533.Q4IE79
38079 229533.I1RU69 pp 229533.I1S021
38080 229533.I1RU69 pp 229533.Q4IJ56
38081 229533.I1RU69 pp 229533.I1S0M9
38082 229533.I1RU69 pp 229533.Q4IJH1
38083 229533.I1RU69 pp 229533.I1S9L0
38084 229533.I1RU69 pp 229533.I1RWT6
38085 229533.I1RU69 pp 229533.Q4ICG5
38086 229533.I1RU69 pp 229533.I1RVZ0
38087 229533.I1RU69 pp 229533.Q4IB70
38088 229533.I1RU69 pp 229533.I1RZF6
38089 229533.I1RU69 pp 229533.I1RZK6
38090 229533.I1RU69 pp 229533.Q4I1Y1
38091 229533.I1RU69 pp 229533.Q4I7F9
38092 229533.A0A1C3YKU3 pp 229533.I1S7A2
38093 229533.A0A1C3YKU3 pp 229533.V6R223
38094 229533.A0A1C3YKU3 pp 229533.I1R9N6
38095 229533.A0A1C3YKU3 pp 229533.I1RL32
38096 229533.A0A1C3YKU3 pp 229533.A0A1C3YL10
38097 229533.A0A1C3YKU3 pp 229533.I1RK81
38098 229533.A0A1C3YKU3 pp 229533.I1S212
38099 229533.A0A1C3YKU3 pp 229533.I1RCZ3
38100 229533.A0A1C3YKU3 pp 229533.I1RJZ5
38101 229533.A0A1C3YKU3 pp 229533.I1RLV9
38102 229533.A0A1C3YKU3 pp 229533.I1RC03
38103 229533.A0A1C3YKU3 pp 229533.I1S6Y3
38104 229533.A0A1C3YKU3 pp 229533.I1RRE0
38105 229533.A0A1C3YKU3 pp 229533.I1RYW3
38106 229533.I1RME6 pp 229533.I1RYS0

38107 229533.I1RME6 pp 229533.I1RZM1
38108 229533.I1RME6 pp 229533.V6RBN2
38109 229533.I1RME6 pp 229533.I1RQD8
38110 229533.I1RME6 pp 229533.I1RRU9
38111 229533.I1RME6 pp 229533.I1RQ81
38112 229533.I1RME6 pp 229533.V6RQW4
38113 229533.I1RME6 pp 229533.I1RRV4
38114 229533.I1RME6 pp 229533.I1RVX9
38115 229533.I1RME6 pp 229533.I1RVZ7
38116 229533.I1RME6 pp 229533.I1RW71
38117 229533.I1RME6 pp 229533.I1S4Q4
38118 229533.I1RME6 pp 229533.I1RN61
38119 229533.I1RME6 pp 229533.I1RYW3
38120 229533.I1RME6 pp 229533.I1RPW4
38121 229533.I1RME6 pp 229533.I1RWX5
38122 229533.I1RME6 pp 229533.I1S0R3
38123 229533.I1RME6 pp 229533.I1RPN4
38124 229533.I1RME6 pp 229533.I1RPU8
38125 229533.I1RME6 pp 229533.I1S0R4
38126 229533.I1RME6 pp 229533.I1RQK8
38127 229533.I1RME6 pp 229533.I1RRV1
38128 229533.I1RME6 pp 229533.Q4HXF0
38129 229533.I1RME6 pp 229533.I1RRP7
38130 229533.I1RME6 pp 229533.I1RQM7
38131 229533.I1RME6 pp 229533.I1RP27
38132 229533.I1RME6 pp 229533.I1S0L8
38133 229533.I1RME6 pp 229533.V6RY33
38134 229533.I1RME6 pp 229533.V6RRH2
38135 229533.I1RME6 pp 229533.I1RZG0
38136 229533.I1RME6 pp 229533.I1RNR2
38137 229533.I1RME6 pp 229533.I1RTG7
38138 229533.I1RME6 pp 229533.I1RTG2
38139 229533.I1RME6 pp 229533.I1RN67
38140 229533.I1RME6 pp 229533.I1S4Z8
38141 229533.A0A1C3YI89 pp 229533.I1RVB6
38142 229533.A0A1C3YI89 pp 229533.I1REM2
38143 229533.I1RLP1 pp 229533.I1S147
38144 229533.I1RLP1 pp 229533.I1S1W3
38145 229533.I1RLP1 pp 229533.I1S0J1
38146 229533.I1RLP1 pp 229533.I1RMV0
38147 229533.I1RLP1 pp 229533.Q4HZB8
38148 229533.I1RLP1 pp 229533.V6R042
38149 229533.I1RLP1 pp 229533.I1RUG3
38150 229533.I1RLP1 pp 229533.I1RSQ3
38151 229533.I1RLP1 pp 229533.I1RYW3
38152 229533.I1RLP1 pp 229533.I1RNC1
38153 229533.I1RLP1 pp 229533.I1RXU9
38154 229533.I1RLP1 pp 229533.I1S1C7
38155 229533.I1RLP1 pp 229533.I1S2B9
38156 229533.I1RLP1 pp 229533.I1RS90
38157 229533.I1RLP1 pp 229533.I1RVX0
38158 229533.I1RLP1 pp 229533.I1RTD5
38159 229533.I1RLP1 pp 229533.I1RXR1
38160 229533.I1RLP1 pp 229533.I1S5R1
38161 229533.I1RLP1 pp 229533.I1RT40
38162 229533.I1RLP1 pp 229533.I1RTG6
38163 229533.I1RLP1 pp 229533.I1S0R8
38164 229533.I1RLP1 pp 229533.I1RT03
38165 229533.I1RLP1 pp 229533.I1RYX5
38166 229533.I1RLP1 pp 229533.I1RQE7
38167 229533.I1RLP1 pp 229533.I1S0L8
38168 229533.I1RLP1 pp 229533.Q4I624
38169 229533.I1RLP1 pp 229533.I1S277
38170 229533.I1RLP1 pp 229533.V6R3J1
38171 229533.I1RLP1 pp 229533.I1RYH7
38172 229533.I1RLP1 pp 229533.I1RPP3
38173 229533.I1RLP1 pp 229533.I1S2B8
38174 229533.I1RLP1 pp 229533.I1RYI5
38175 229533.I1RLP1 pp 229533.I1S0B0
38176 229533.I1RLP1 pp 229533.I1RWC9
38177 229533.I1RLP1 pp 229533.I1S1F3
38178 229533.I1RLP1 pp 229533.I1RRY9
38179 229533.I1RLP1 pp 229533.V6RCZ8

38180 229533.I1RLP1 pp 229533.I1RN67
38181 229533.I1RLP1 pp 229533.I1RPR9
38182 229533.I1RLP1 pp 229533.I1RZM8
38183 229533.I1RLP1 pp 229533.I1RNG4
38184 229533.I1RLP1 pp 229533.I1S0K2
38185 229533.I1RLP1 pp 229533.I1RY92
38186 229533.I1RLP1 pp 229533.I1S9G7
38187 229533.I1RLP1 pp 229533.I1RZ39
38188 229533.I1RLP1 pp 229533.I1RXF0
38189 229533.I1RLP1 pp 229533.V6QUE6
38190 229533.I1RLP1 pp 229533.I1RSQ4
38191 229533.I1RLP1 pp 229533.I1S5B0
38192 229533.I1RLP1 pp 229533.I1RXD7
38193 229533.I1RLP1 pp 229533.I1RZZ9
38194 229533.I1RLP1 pp 229533.Q4IJK2
38195 229533.I1RLP1 pp 229533.I1S0L4
38196 229533.I1RLP1 pp 229533.I1RS18
38197 229533.I1RLP1 pp 229533.I1S2H4
38198 229533.I1RLP1 pp 229533.V6RUT5
38199 229533.I1RLP1 pp 229533.I1S887
38200 229533.I1RLP1 pp 229533.I1RZK5
38201 229533.I1RLP1 pp 229533.I1RRX5
38202 229533.I1RLP1 pp 229533.I1RX46
38203 229533.I1RLP1 pp 229533.I1RSQ7
38204 229533.I1RLP1 pp 229533.I1S0P0
38205 229533.I1RLP1 pp 229533.I1RR24
38206 229533.I1RLP1 pp 229533.I1S4S7
38207 229533.I1RLP1 pp 229533.I1RMQ6
38208 229533.I1RLP1 pp 229533.I1RPA4
38209 229533.I1RLP1 pp 229533.I1S4S4
38210 229533.V6R0H4 pp 229533.V6RAM6
38211 229533.V6R0H4 pp 229533.V6RPS5
38212 229533.V6R0H4 pp 229533.V6R0W1
38213 229533.V6R0H4 pp 229533.V6R756
38214 229533.V6R0H4 pp 229533.V6R2Z2
38215 229533.V6R0H4 pp 229533.V6R5D2
38216 229533.A0A1C3YKU2 pp 229533.I1R9Y2
38217 229533.I1R991 pp 229533.I1RX09
38218 229533.I1R991 pp 229533.I1S0Z7
38219 229533.I1R991 pp 229533.I1S2B7
38220 229533.I1R991 pp 229533.I1RPK5
38221 229533.I1R991 pp 229533.I1RN72
38222 229533.I1R991 pp 229533.I1RD69
38223 229533.I1R991 pp 229533.I1RCK9
38224 229533.I1R991 pp 229533.I1RA34
38225 229533.I1R991 pp 229533.I1RPS0
38226 229533.I1R991 pp 229533.I1RNG8
38227 229533.I1R991 pp 229533.I1S0L8
38228 229533.I1R991 pp 229533.I1RD10
38229 229533.I1R991 pp 229533.I1RQM0
38230 229533.I1R991 pp 229533.I1S7X9
38231 229533.I1R991 pp 229533.I1RN36
38232 229533.I1R991 pp 229533.I1RC46
38233 229533.I1R991 pp 229533.V6RB81
38234 229533.I1S405 pp 229533.I1S694
38235 229533.I1S405 pp 229533.V6R756
38236 229533.I1S405 pp 229533.V6QU99
38237 229533.I1S405 pp 229533.V6RPS5
38238 229533.I1S405 pp 229533.V6R5D2
38239 229533.I1S405 pp 229533.I1SAI4
38240 229533.I1S405 pp 229533.V6R2Z2
38241 229533.I1S405 pp 229533.V6RAM6
38242 229533.I1S405 pp 229533.V6R0W1
38243 229533.I1RQZ6 pp 229533.I1RUY5
38244 229533.I1RQZ6 pp 229533.I1RSA5
38245 229533.I1RB40 pp 229533.I1RPS0
38246 229533.I1RB40 pp 229533.I1RQ82
38247 229533.I1RB40 pp 229533.I1RN72
38248 229533.I1RB40 pp 229533.I1S9V9
38249 229533.I1RB40 pp 229533.I1RJH7
38250 229533.I1RB40 pp 229533.I1RTG9
38251 229533.I1RB40 pp 229533.Q4ICG8
38252 229533.I1RB40 pp 229533.I1S9G7

38253	229533.I1RB40	pp	229533.I1S1Y9
38254	229533.I1RB40	pp	229533.I1RPA7
38255	229533.I1RB40	pp	229533.I1RYM3
38256	229533.I1RB40	pp	229533.I1RQ49
38257	229533.I1RB40	pp	229533.I1S0L8
38258	229533.I1RB40	pp	229533.I1S2B9
38259	229533.I1RB40	pp	229533.Q4HZB8
38260	229533.I1RB40	pp	229533.I1RBA6
38261	229533.I1RB40	pp	229533.I1RY40
38262	229533.I1RB40	pp	229533.I1RJH8
38263	229533.I1RB40	pp	229533.I1RWG1
38264	229533.I1S8V3	pp	229533.V6RMW6
38265	229533.I1S8V3	pp	229533.I1S8V4
38266	229533.I1S8V3	pp	229533.Q4IPZ1
38267	229533.I1S8V3	pp	229533.V6RTJ8
38268	229533.I1S8V3	pp	229533.Q4IJK2
38269	229533.I1S8V3	pp	229533.V6RCZ8
38270	229533.I1S8V3	pp	229533.V6R3X5
38271	229533.I1RSB0	pp	229533.I1S2B7
38272	229533.I1RSB0	pp	229533.I1RX09
38273	229533.I1RSB0	pp	229533.I1S0L8
38274	229533.I1RSB0	pp	229533.I1S7X9
38275	229533.I1RSB0	pp	229533.I1S0Z7
38276	229533.I1RSB0	pp	229533.V6RB81
38277	229533.I1RKD7	pp	229533.I1S0L8
38278	229533.I1RKD7	pp	229533.I1S7W5
38279	229533.I1RKD7	pp	229533.I1S4Z8
38280	229533.I1RKD7	pp	229533.I1S2D7
38281	229533.I1RW83	pp	229533.I1S549
38282	229533.I1RW83	pp	229533.I1S248
38283	229533.I1RW83	pp	229533.I1S2E9
38284	229533.I1RW83	pp	229533.I1S074
38285	229533.I1RW83	pp	229533.I1RYV4
38286	229533.I1RW83	pp	229533.Q4IBS9
38287	229533.I1RW83	pp	229533.I1RX70
38288	229533.I1RW83	pp	229533.I1RW84
38289	229533.I1RW83	pp	229533.I1RZ63
38290	229533.I1RW83	pp	229533.I1RYB7
38291	229533.I1RW83	pp	229533.I1S2G7
38292	229533.I1RW83	pp	229533.V6RDP1
38293	229533.I1RW83	pp	229533.Q4I624
38294	229533.I1RW83	pp	229533.I1S091
38295	229533.I1RW83	pp	229533.I1RWT6
38296	229533.I1RW83	pp	229533.I1RY44
38297	229533.I1RW83	pp	229533.I1RYI3
38298	229533.A0A0E0S6K6	pp	229533.A0A1C3YL21
38299	229533.I1RQS8	pp	229533.I1RRM5
38300	229533.I1RQS8	pp	229533.V6QYF0
38301	229533.I1RQS8	pp	229533.I1RW61
38302	229533.I1RQS8	pp	229533.I1RW25
38303	229533.I1RQS8	pp	229533.I1RWJ8
38304	229533.I1RQS8	pp	229533.I1RTF4
38305	229533.I1RQS8	pp	229533.I1RZS6
38306	229533.I1RQS8	pp	229533.I1RRT8
38307	229533.I1RQS8	pp	229533.I1RVQ0
38308	229533.I1RQS8	pp	229533.I1S0L3
38309	229533.I1RQS8	pp	229533.I1S5R1
38310	229533.I1RQS8	pp	229533.I1RS47
38311	229533.I1RQS8	pp	229533.I1S9U2
38312	229533.I1RQS8	pp	229533.I1RWK3
38313	229533.I1RQS8	pp	229533.I1RR24
38314	229533.I1RQS8	pp	229533.I1RW46
38315	229533.I1RQS8	pp	229533.I1RW71
38316	229533.I1RQS8	pp	229533.I1RZZ9
38317	229533.I1RQS8	pp	229533.I1RSQ7
38318	229533.I1RQS8	pp	229533.I1S0L4
38319	229533.I1RQS8	pp	229533.I1RW99
38320	229533.I1RQS8	pp	229533.V6QW22
38321	229533.I1RQS8	pp	229533.I1RS17
38322	229533.I1RQS8	pp	229533.I1RRJ8
38323	229533.I1RQS8	pp	229533.I1RUQ2
38324	229533.I1RQS8	pp	229533.I1RZM8
38325	229533.I1RQS8	pp	229533.I1RSR3

38326 229533.I1RQS8 pp 229533.I1RSD8
38327 229533.I1RQS8 pp 229533.I1RSI6
38328 229533.I1RQS8 pp 229533.I1RUG3
38329 229533.I1RQS8 pp 229533.I1RRT2
38330 229533.I1RQS8 pp 229533.I1S1B6
38331 229533.I1RQS8 pp 229533.I1RZW8
38332 229533.I1RQS8 pp 229533.I1RT40
38333 229533.I1RQS8 pp 229533.V6RCG5
38334 229533.I1RQS8 pp 229533.I1RRI2
38335 229533.I1RQS8 pp 229533.V6RIV9
38336 229533.I1S269 pp 229533.V6RAM6
38337 229533.I1S269 pp 229533.I1SAI4
38338 229533.I1S269 pp 229533.V6R0W1
38339 229533.I1S269 pp 229533.V6QU99
38340 229533.I1S269 pp 229533.V6R756
38341 229533.I1S269 pp 229533.V6R2Z2
38342 229533.I1S269 pp 229533.V6R5D2
38343 229533.I1S269 pp 229533.V6RPS5
38344 229533.I1S269 pp 229533.I1S694
38345 229533.I1RZP7 pp 229533.I1S4N8
38346 229533.I1RZP7 pp 229533.I1S1V3
38347 229533.I1RZP7 pp 229533.I1S0L8
38348 229533.I1RZP7 pp 229533.I1S4N7
38349 229533.I1RZP7 pp 229533.I1S1V1
38350 229533.A0A1C3YM76 pp 229533.I1RQ82
38351 229533.A0A1C3YM76 pp 229533.I1RQ49
38352 229533.A0A1C3YM76 pp 229533.A0A1C3YN81
38353 229533.A0A1C3YM76 pp 229533.I1RWG1
38354 229533.A0A1C3YM76 pp 229533.I1S9V9
38355 229533.A0A1C3YM76 pp 229533.I1RY40
38356 229533.A0A1C3YM76 pp 229533.I1RC49
38357 229533.A0A1C3YM76 pp 229533.I1RB40
38358 229533.A0A1C3YM76 pp 229533.I1RPS0
38359 229533.A0A1C3YM76 pp 229533.I1RMH4
38360 229533.A0A1C3YM76 pp 229533.I1RTG9
38361 229533.A0A1C3YM76 pp 229533.I1RJH8
38362 229533.A0A1C3YM76 pp 229533.I1S0L8
38363 229533.A0A1C3YM76 pp 229533.I1S9G7
38364 229533.A0A1C3YM76 pp 229533.I1S2B9
38365 229533.A0A1C3YM76 pp 229533.I1RAU9
38366 229533.A0A1C3YM76 pp 229533.I1RPA7
38367 229533.A0A1C3YM76 pp 229533.I1RN72
38368 229533.A0A1C3YM76 pp 229533.I1RS90
38369 229533.A0A1C3YM76 pp 229533.I1RBA6
38370 229533.A0A1C3YM76 pp 229533.I1RJH7
38371 229533.A0A1C3YM76 pp 229533.A0A1C3YM82
38372 229533.A0A1C3YM76 pp 229533.I1RYM3
38373 229533.A0A1C3YM76 pp 229533.I1S1Y9
38374 229533.A0A1C3YM76 pp 229533.I1SAB8
38375 229533.A0A1C3YLI0 pp 229533.I1RTJ7
38376 229533.A0A0E0S0J3 pp 229533.I1RRX5
38377 229533.A0A0E0S0J3 pp 229533.I1RD70
38378 229533.A0A0E0S0J3 pp 229533.I1REA6
38379 229533.A0A0E0S0J3 pp 229533.I1RPK9
38380 229533.A0A0E0S0J3 pp 229533.I1RD69
38381 229533.A0A0E0S0J3 pp 229533.I1S1V3
38382 229533.A0A0E0S0J3 pp 229533.I1RPS0
38383 229533.A0A0E0S0J3 pp 229533.I1RZQ6
38384 229533.A0A0E0S0J3 pp 229533.I1RJU5
38385 229533.A0A0E0S0J3 pp 229533.I1S0L8
38386 229533.A0A0E0S0J3 pp 229533.I1RAU7
38387 229533.A0A0E0S0J3 pp 229533.Q4HZB8
38388 229533.A0A0E0S0J3 pp 229533.A0A1C3YM60
38389 229533.A0A0E0S0J3 pp 229533.A0A1C3YM82
38390 229533.A0A1C3YKH5 pp 229533.V6RAM6
38391 229533.A0A1C3YKH5 pp 229533.I1SAI4
38392 229533.A0A1C3YKH5 pp 229533.V6R0W1
38393 229533.A0A1C3YKH5 pp 229533.I1S0H2
38394 229533.A0A1C3YKH5 pp 229533.I1RMS5
38395 229533.A0A1C3YKH5 pp 229533.I1RY16
38396 229533.A0A1C3YKH5 pp 229533.V6RPS5
38397 229533.A0A1C3YKH5 pp 229533.I1S694
38398 229533.A0A1C3YKH5 pp 229533.I1RI11

38399 229533.A0A1C3YKH5 pp 229533.V6QU99
38400 229533.A0A1C3YKH5 pp 229533.V6R2Z2
38401 229533.A0A1C3YKH5 pp 229533.V6R5D2
38402 229533.A0A1C3YKH5 pp 229533.I1RZA8
38403 229533.A0A1C3YKH5 pp 229533.I1S3K0
38404 229533.A0A1C3YKH5 pp 229533.I1S149
38405 229533.A0A1C3YKH5 pp 229533.V6R756
38406 229533.I1RN63 pp 229533.Q4IAA0
38407 229533.I1RN63 pp 229533.I1RS18
38408 229533.I1RN63 pp 229533.I1RSI6
38409 229533.I1RN63 pp 229533.I1RW46
38410 229533.I1RN63 pp 229533.I1RRV1
38411 229533.I1RN63 pp 229533.I1S504
38412 229533.I1RN63 pp 229533.V6RCZ8
38413 229533.I1RN63 pp 229533.I1SA32
38414 229533.I1RN63 pp 229533.I1S8V4
38415 229533.I1RN63 pp 229533.V6RCG5
38416 229533.I1RN63 pp 229533.I1RQ31
38417 229533.I1RN63 pp 229533.I1RZM8
38418 229533.I1RN63 pp 229533.I1RR51
38419 229533.I1RN63 pp 229533.I1RPY1
38420 229533.I1RN63 pp 229533.I1RUG3
38421 229533.I1RN63 pp 229533.V6R227
38422 229533.I1RN63 pp 229533.I1S419
38423 229533.I1RN63 pp 229533.I1RNC1
38424 229533.I1RN63 pp 229533.I1S5B0
38425 229533.I1RN63 pp 229533.I1RS64
38426 229533.I1RN63 pp 229533.I1RW99
38427 229533.I1RN63 pp 229533.I1RYW3
38428 229533.I1RN63 pp 229533.I1RTD9
38429 229533.I1RN63 pp 229533.V6RIV9
38430 229533.I1RN63 pp 229533.I1RZW8
38431 229533.I1RN63 pp 229533.V6RGN5
38432 229533.I1RN63 pp 229533.I1RRI2
38433 229533.I1RN63 pp 229533.I1RZM1
38434 229533.I1RN63 pp 229533.I1S0N4
38435 229533.I1RN63 pp 229533.I1RQA2
38436 229533.I1RN63 pp 229533.I1RSR3
38437 229533.I1RN63 pp 229533.I1RRV4
38438 229533.I1RN63 pp 229533.I1SA33
38439 229533.I1RN63 pp 229533.Q4IEK8
38440 229533.I1RN63 pp 229533.I1RRU9
38441 229533.I1RN63 pp 229533.I1S1J7
38442 229533.I1RN63 pp 229533.I1S8R3
38443 229533.I1RN63 pp 229533.I1RQ81
38444 229533.I1RN63 pp 229533.Q4HZK7
38445 229533.I1RN63 pp 229533.I1RSK1
38446 229533.I1RN63 pp 229533.I1RSC6
38447 229533.I1RN63 pp 229533.I1RTH6
38448 229533.I1RN63 pp 229533.I1S2C0
38449 229533.I1RN63 pp 229533.I1RY63
38450 229533.I1RN63 pp 229533.I1RS17
38451 229533.I1RN63 pp 229533.I1RZK5
38452 229533.I1RN63 pp 229533.I1RTE9
38453 229533.I1RN63 pp 229533.V6RLM7
38454 229533.I1RN63 pp 229533.I1RN99
38455 229533.I1RN63 pp 229533.I1RVX0
38456 229533.I1RN63 pp 229533.I1RY03
38457 229533.I1RN63 pp 229533.Q4IJK2
38458 229533.I1RN63 pp 229533.Q4I8P5
38459 229533.I1RN63 pp 229533.I1RQE7
38460 229533.I1RN63 pp 229533.I1S1B6
38461 229533.I1RN63 pp 229533.I1RZZ1
38462 229533.I1RN63 pp 229533.I1S0F6
38463 229533.I1RN63 pp 229533.I1RZK6
38464 229533.I1RN63 pp 229533.Q4I624
38465 229533.I1RN63 pp 229533.I1RP27
38466 229533.I1RN63 pp 229533.I1RWD4
38467 229533.I1RN63 pp 229533.V6R057
38468 229533.I1RN63 pp 229533.I1RN67
38469 229533.I1RN63 pp 229533.V6RTJ8
38470 229533.I1RN63 pp 229533.I1RVQ0
38471 229533.I1RN63 pp 229533.I1RRP3

38472 229533.I1RN63 pp 229533.I1RRZ0
38473 229533.I1RN63 pp 229533.I1S296
38474 229533.I1RN63 pp 229533.I1S503
38475 229533.I1RN63 pp 229533.I1RNV6
38476 229533.I1RN63 pp 229533.I1RQ72
38477 229533.I1RN63 pp 229533.I1S6F0
38478 229533.I1RN63 pp 229533.I1S0T2
38479 229533.I1RN63 pp 229533.I1RRT2
38480 229533.I1RN63 pp 229533.I1RZ39
38481 229533.I1RN63 pp 229533.I1RSX1
38482 229533.I1RN63 pp 229533.Q4I5Z5
38483 229533.I1RN63 pp 229533.I1RSQ7
38484 229533.I1RN63 pp 229533.I1RTF4
38485 229533.I1RN63 pp 229533.I1S1J6
38486 229533.I1RN63 pp 229533.I1RQG4
38487 229533.I1RN63 pp 229533.I1RNK2
38488 229533.I1RN63 pp 229533.I1S2A8
38489 229533.I1RN63 pp 229533.I1RSI0
38490 229533.I1RN63 pp 229533.V6R3X5
38491 229533.I1RN63 pp 229533.I1RRJ8
38492 229533.I1RN63 pp 229533.I1RZE5
38493 229533.I1RN63 pp 229533.V6QW22
38494 229533.I1RN63 pp 229533.I1S0R7
38495 229533.I1RN63 pp 229533.Q4HVW2
38496 229533.I1RN63 pp 229533.I1RWK3
38497 229533.I1RN63 pp 229533.I1RPP3
38498 229533.I1RN63 pp 229533.I1RT05
38499 229533.I1RN63 pp 229533.I1RZZ9
38500 229533.I1RN63 pp 229533.I1S2M6
38501 229533.I1RN63 pp 229533.I1RT04
38502 229533.I1RN63 pp 229533.I1RSQ4
38503 229533.I1RN63 pp 229533.I1RUQ2
38504 229533.I1RN63 pp 229533.I1RS47
38505 229533.I1RN63 pp 229533.Q4HZ42
38506 229533.I1RN63 pp 229533.I1RXD7
38507 229533.I1RN63 pp 229533.I1RNN1
38508 229533.I1RN63 pp 229533.Q4IJ11
38509 229533.I1RN63 pp 229533.I1RWI5
38510 229533.I1RN63 pp 229533.I1RT40
38511 229533.I1RN63 pp 229533.I1S0L3
38512 229533.I1RN63 pp 229533.I1RV91
38513 229533.I1RN63 pp 229533.I1S8V3
38514 229533.I1RN63 pp 229533.V6R1K4
38515 229533.I1RN63 pp 229533.I1RX04
38516 229533.I1RN63 pp 229533.I1S2H4
38517 229533.I1RN63 pp 229533.I1RS20
38518 229533.I1RN63 pp 229533.I1S4S4
38519 229533.I1RN63 pp 229533.I1RQS8
38520 229533.I1RN63 pp 229533.I1RYN4
38521 229533.I1RN63 pp 229533.I1RWJ8
38522 229533.I1RN63 pp 229533.I1RR24
38523 229533.I1RN63 pp 229533.I1RRT8
38524 229533.I1RN63 pp 229533.I1S0L4
38525 229533.I1RN63 pp 229533.I1RRM9
38526 229533.I1RN63 pp 229533.V6QYF0
38527 229533.I1RN63 pp 229533.I1S5R1
38528 229533.I1RN63 pp 229533.I1RW61
38529 229533.I1RN63 pp 229533.I1RNA0
38530 229533.I1RN63 pp 229533.I1RRQ6
38531 229533.I1RN63 pp 229533.I1RPW4
38532 229533.I1RN63 pp 229533.I1S6Y8
38533 229533.I1RN63 pp 229533.I1RPM4
38534 229533.I1RN63 pp 229533.Q4IFI0
38535 229533.I1RN63 pp 229533.I1RRM5
38536 229533.I1RN63 pp 229533.I1RPU3
38537 229533.I1RN63 pp 229533.I1RW25
38538 229533.A0A1C3YLH8 pp 229533.I1RZ70
38539 229533.I1RTG9 pp 229533.I1RZP7
38540 229533.I1RTG9 pp 229533.I1S7X9
38541 229533.I1RTG9 pp 229533.Q4HZB8
38542 229533.I1RTG9 pp 229533.I1S0L8
38543 229533.I1RTG9 pp 229533.I1S9V9
38544 229533.I1RTG9 pp 229533.I1S2B9

38545 229533.I1RTG9 pp 229533.V6RB81
38546 229533.I1RTG9 pp 229533.I1S2B7
38547 229533.I1RTG9 pp 229533.I1S0Z7
38548 229533.I1RTG9 pp 229533.I1RWC9
38549 229533.I1RTG9 pp 229533.I1RZP6
38550 229533.I1RTG9 pp 229533.V6REE9
38551 229533.I1RTG9 pp 229533.I1RX09
38552 229533.I1RBU5 pp 229533.I1RY58
38553 229533.I1RBU5 pp 229533.I1RQA3
38554 229533.I1RBU5 pp 229533.I1RSR9
38555 229533.I1RBU5 pp 229533.I1RSP7
38556 229533.I1RBU5 pp 229533.I1RZP8
38557 229533.I1RBU5 pp 229533.I1RG77
38558 229533.I1RBU5 pp 229533.I1RN55
38559 229533.I1RBU5 pp 229533.I1RCA5
38560 229533.I1RBU5 pp 229533.I1RX64
38561 229533.I1RBU5 pp 229533.I1RBW0
38562 229533.I1RBU5 pp 229533.I1RZ86
38563 229533.I1RBU5 pp 229533.I1RWT6
38564 229533.I1RBU5 pp 229533.I1S3G4
38565 229533.I1RBU5 pp 229533.I1RY44
38566 229533.I1RBU5 pp 229533.I1RBW1
38567 229533.I1RBU5 pp 229533.I1S4P6
38568 229533.I1RBU5 pp 229533.I1RRT1
38569 229533.I1RBU5 pp 229533.I1RV91
38570 229533.I1RBU5 pp 229533.I1RSN7
38571 229533.I1RBU5 pp 229533.I1RZ94
38572 229533.I1RBU5 pp 229533.I1RN57
38573 229533.I1RBU5 pp 229533.I1S2E9
38574 229533.I1RBU5 pp 229533.I1S1W8
38575 229533.I1RBU5 pp 229533.I1RC54
38576 229533.I1RBU5 pp 229533.I1RPQ1
38577 229533.I1RPA7 pp 229533.I1S9V9
38578 229533.I1RPA7 pp 229533.I1RZG0
38579 229533.I1RPA7 pp 229533.I1RPN4
38580 229533.I1RPA7 pp 229533.I1RZP7
38581 229533.I1RPA7 pp 229533.I1RTG9
38582 229533.I1RPA7 pp 229533.I1RTA6
38583 229533.I1RPA7 pp 229533.I1S0L8
38584 229533.I1RPA7 pp 229533.I1RWG1
38585 229533.I1RPA7 pp 229533.I1RY40
38586 229533.I1RPA7 pp 229533.I1RZP6
38587 229533.I1RPA7 pp 229533.I1RTE6
38588 229533.I1RPA7 pp 229533.I1S1Y9
38589 229533.I1RPA7 pp 229533.I1S2B9
38590 229533.I1RPA7 pp 229533.I1RPS0
38591 229533.I1RPA7 pp 229533.I1S9G7
38592 229533.I1RPA7 pp 229533.I1RS90
38593 229533.I1RPA7 pp 229533.V6REE9
38594 229533.I1RPA7 pp 229533.I1RYM3
38595 229533.I1RPA7 pp 229533.I1RUP2
38596 229533.I1RPA7 pp 229533.I1RQ49
38597 229533.A0A1C3YMJ3 pp 229533.I1RQJ4
38598 229533.A0A1C3YMJ3 pp 229533.I1RSP7
38599 229533.A0A1C3YMJ3 pp 229533.I1RAU8
38600 229533.A0A1C3YMJ3 pp 229533.I1RZP8
38601 229533.A0A1C3YMJ3 pp 229533.I1RTN6
38602 229533.A0A1C3YMJ3 pp 229533.Q4IJ84
38603 229533.A0A1C3YMJ3 pp 229533.I1RCA9
38604 229533.A0A1C3YMJ3 pp 229533.I1RX64
38605 229533.A0A1C3YMJ3 pp 229533.I1RBN9
38606 229533.A0A1C3YMJ3 pp 229533.I1RBC8
38607 229533.A0A1C3YMJ3 pp 229533.I1RC54
38608 229533.A0A1C3YMJ3 pp 229533.A0A1C3YN69
38609 229533.A0A1C3YMJ3 pp 229533.I1S0D5
38610 229533.A0A1C3YMJ3 pp 229533.I1RSR9
38611 229533.A0A1C3YMJ3 pp 229533.Q4IB89
38612 229533.A0A1C3YMJ3 pp 229533.I1RDX0
38613 229533.A0A1C3YMJ3 pp 229533.I1RMJ8
38614 229533.A0A1C3YMJ3 pp 229533.I1RDH0
38615 229533.A0A1C3YMJ3 pp 229533.I1S8J1
38616 229533.A0A1C3YMJ3 pp 229533.I1RMS4
38617 229533.A0A1C3YMJ3 pp 229533.I1RY58

38618	229533.A0A1C3YMJ3	pp	229533.I1RA10
38619	229533.A0A1C3YMJ3	pp	229533.I1RDJ8
38620	229533.A0A1C3YMJ3	pp	229533.I1RP99
38621	229533.A0A1C3YMJ3	pp	229533.I1RHJ8
38622	229533.A0A1C3YMJ3	pp	229533.I1S4P6
38623	229533.A0A1C3YMJ3	pp	229533.I1RUG4
38624	229533.A0A1C3YMJ3	pp	229533.I1RRJ6
38625	229533.A0A1C3YMJ3	pp	229533.A0A1C3YN55
38626	229533.A0A1C3YMJ3	pp	229533.I1S0A6
38627	229533.A0A1C3YMJ3	pp	229533.I1RBU5
38628	229533.A0A1C3YMJ3	pp	229533.I1RBW1
38629	229533.A0A1C3YMJ3	pp	229533.I1RVW0
38630	229533.A0A1C3YMJ3	pp	229533.I1RV91
38631	229533.A0A1C3YMJ3	pp	229533.I1RQI6
38632	229533.A0A1C3YMJ3	pp	229533.I1RSC7
38633	229533.A0A1C3YMJ3	pp	229533.I1S3G4
38634	229533.A0A1C3YMJ3	pp	229533.I1RCA5
38635	229533.A0A1C3YMJ3	pp	229533.I1RY29
38636	229533.A0A1C3YMJ3	pp	229533.I1RWI8
38637	229533.A0A1C3YMJ3	pp	229533.I1RRS7
38638	229533.A0A1C3YMJ3	pp	229533.I1RZ86
38639	229533.A0A1C3YMJ3	pp	229533.I1RYQ1
38640	229533.A0A1C3YMJ3	pp	229533.I1RSD0
38641	229533.A0A1C3YMJ3	pp	229533.I1RCL7
38642	229533.A0A1C3YMJ3	pp	229533.I1RUG2
38643	229533.A0A1C3YMJ3	pp	229533.I1RSA7
38644	229533.A0A1C3YMJ3	pp	229533.I1RG77
38645	229533.A0A1C3YMJ3	pp	229533.Q4IQM6
38646	229533.A0A1C3YMJ3	pp	229533.I1RA38
38647	229533.A0A1C3YMJ3	pp	229533.I1RBC1
38648	229533.A0A1C3YMJ3	pp	229533.I1RFL7
38649	229533.A0A1C3YMJ3	pp	229533.I1RFJ2
38650	229533.A0A1C3YMJ3	pp	229533.V6RLW4
38651	229533.A0A1C3YMJ3	pp	229533.I1RYX9
38652	229533.A0A1C3YMJ3	pp	229533.I1RCM7
38653	229533.A0A1C3YMJ3	pp	229533.I1RE23
38654	229533.A0A1C3YMJ3	pp	229533.I1S461
38655	229533.A0A1C3YMJ3	pp	229533.I1RTB2
38656	229533.A0A1C3YMJ3	pp	229533.I1RSN7
38657	229533.A0A1C3YMJ3	pp	229533.I1RUR0
38658	229533.A0A1C3YMJ3	pp	229533.I1RY73
38659	229533.A0A1C3YMJ3	pp	229533.I1RAT5
38660	229533.A0A1C3YMJ3	pp	229533.I1RFZ2
38661	229533.A0A1C3YMJ3	pp	229533.I1S604
38662	229533.A0A1C3YMJ3	pp	229533.I1S283
38663	229533.A0A1C3YMJ3	pp	229533.I1RYR2
38664	229533.A0A1C3YMJ3	pp	229533.Q4INS6
38665	229533.A0A1C3YMJ3	pp	229533.I1RCQ4
38666	229533.A0A1C3YMJ3	pp	229533.I1RRT1
38667	229533.A0A1C3YMJ3	pp	229533.I1RMX3
38668	229533.A0A1C3YMJ3	pp	229533.I1S209
38669	229533.A0A1C3YMJ3	pp	229533.I1RZ94
38670	229533.A0A1C3YMJ3	pp	229533.I1RSI4
38671	229533.A0A1C3YMJ3	pp	229533.I1S1W8
38672	229533.A0A1C3YMJ3	pp	229533.I1RPZ0
38673	229533.A0A1C3YMJ3	pp	229533.Q4IEA5
38674	229533.A0A1C3YMJ3	pp	229533.I1RQ13
38675	229533.A0A1C3YMJ3	pp	229533.I1RAH4
38676	229533.A0A1C3YMJ3	pp	229533.I1S0P6
38677	229533.A0A1C3YMJ3	pp	229533.I1RMQ7
38678	229533.A0A1C3YMJ3	pp	229533.I1RBW0
38679	229533.A0A1C3YMJ3	pp	229533.I1RFX8
38680	229533.A0A1C3YMJ3	pp	229533.Q4IP51
38681	229533.A0A1C3YMJ3	pp	229533.A0A1C3YN36
38682	229533.A0A1C3YMJ3	pp	229533.I1RKI2
38683	229533.A0A1C3YMJ3	pp	229533.I1RE46
38684	229533.A0A1C3YMJ3	pp	229533.I1RSJ1
38685	229533.A0A1C3YMJ3	pp	229533.I1RA12
38686	229533.A0A1C3YMJ3	pp	229533.I1RBI6
38687	229533.A0A1C3YMJ3	pp	229533.I1RQ92
38688	229533.A0A1C3YMJ3	pp	229533.I1RRY1
38689	229533.A0A1C3YMJ3	pp	229533.I1S072
38690	229533.A0A1C3YMJ3	pp	229533.I1RNE2

229533.A0A1C3YMJ3 pp 229533.A0A1C3YML3
229533.A0A1C3YMJ3 pp 229533.I1RPQ1
229533.I1RNJ2 pp 229533.Q4I948
229533.I1RNJ2 pp 229533.I1S044
229533.I1RNJ2 pp 229533.Q4ICA8
229533.I1RNJ2 pp 229533.I1RW71
229533.I1RNJ2 pp 229533.I1RPS0
229533.I1RNJ2 pp 229533.I1S0L8
229533.I1S0J3 pp 229533.Q4IF76
229533.I1S0J3 pp 229533.I1SAA0
229533.I1S0J3 pp 229533.Q4I7L0
229533.I1S0J3 pp 229533.Q4IJM4
229533.I1S0J3 pp 229533.Q4I5W5
229533.I1S0J3 pp 229533.Q4HTT2
229533.I1S0J3 pp 229533.I1S2E9
229533.I1S0J3 pp 229533.I1SAN0
229533.I1S0J3 pp 229533.Q4HYI0
229533.I1S0J3 pp 229533.V6QWX8
229533.I1S0J3 pp 229533.I1S276
229533.I1S0J3 pp 229533.Q4IMD1
229533.I1S0J3 pp 229533.I1S0N5
229533.I1S0J3 pp 229533.Q4IJH1
229533.I1S0J3 pp 229533.Q4ICA8
229533.I1S0J3 pp 229533.V6QUE6
229533.I1S0J3 pp 229533.I1S5B0
229533.I1S0J3 pp 229533.Q4HYQ4
229533.I1RRC4 pp 229533.I1RW82
229533.I1RRC4 pp 229533.I1RSM8
229533.I1RRC4 pp 229533.I1RZN1
229533.I1RRC4 pp 229533.I1RRR9
229533.I1RRC4 pp 229533.I1S2B0
229533.I1RRC4 pp 229533.I1S8X0
229533.I1RRC4 pp 229533.I1RW71
229533.I1RRC4 pp 229533.I1RTP2
229533.I1RRC4 pp 229533.I1RSX9
229533.I1RRC4 pp 229533.I1S927
229533.I1RRC4 pp 229533.I1RZM4
229533.I1RRC4 pp 229533.I1RSU2
229533.I1RRC4 pp 229533.I1RRQ7
229533.I1RRC4 pp 229533.V6RK96
229533.I1RRN0 pp 229533.V6RR76
229533.I1RRN0 pp 229533.P0C431
229533.I1RRN0 pp 229533.I1S0S6
229533.I1RRN0 pp 229533.I1RTE6
229533.I1RRN0 pp 229533.I1RW16
229533.I1RRN0 pp 229533.I1RTB7
229533.I1RRN0 pp 229533.I1S1B1
229533.I1RRN0 pp 229533.I1RT19
229533.I1RBW1 pp 229533.I1RKB2
229533.I1RBW1 pp 229533.I1RCA5
229533.I1RBW1 pp 229533.I1S6Y6
229533.I1RBW1 pp 229533.I1RYX9
229533.I1RBW1 pp 229533.I1RVW0
229533.I1RBW1 pp 229533.I1S004
229533.I1RBW1 pp 229533.I1RL04
229533.I1RBW1 pp 229533.I1RZ86
229533.I1RBW1 pp 229533.I1RFI6
229533.I1RBW1 pp 229533.I1RRJ6
229533.I1RBW1 pp 229533.I1RFL7
229533.I1RBW1 pp 229533.Q4IB96
229533.I1RBW1 pp 229533.V6RLW4
229533.I1RBW1 pp 229533.I1S998
229533.I1RBW1 pp 229533.I1RNG4
229533.I1RBW1 pp 229533.I1RTB2
229533.I1RBW1 pp 229533.I1S523
229533.I1RBW1 pp 229533.I1RDE7
229533.I1RBW1 pp 229533.I1RSQ4
229533.I1RBW1 pp 229533.I1RSN7
229533.I1RBW1 pp 229533.I1RSA7
229533.I1RBW1 pp 229533.I1RQ24
229533.I1RBW1 pp 229533.I1RZI4
229533.I1RBW1 pp 229533.I1RYR2
229533.I1RBW1 pp 229533.Q4IMD1

38764	229533.I1RBW1	pp	229533.I1RFZ2
38765	229533.I1RBW1	pp	229533.O74638
38766	229533.I1RBW1	pp	229533.I1RHD2
38767	229533.I1RBW1	pp	229533.I1RYQ1
38768	229533.I1RBW1	pp	229533.I1S2F2
38769	229533.I1RBW1	pp	229533.I1SAE3
38770	229533.I1RBW1	pp	229533.I1RY58
38771	229533.I1RBW1	pp	229533.I1RDH0
38772	229533.I1RBW1	pp	229533.I1RNF4
38773	229533.I1RBW1	pp	229533.Q4IEV5
38774	229533.I1RBW1	pp	229533.Q4I7L0
38775	229533.I1RBW1	pp	229533.I1RRN5
38776	229533.I1RBW1	pp	229533.I1RDC0
38777	229533.I1RBW1	pp	229533.I1RDJ8
38778	229533.I1RBW1	pp	229533.I1RPF5
38779	229533.I1RBW1	pp	229533.I1RSY2
38780	229533.I1RBW1	pp	229533.I1RZP8
38781	229533.I1RBW1	pp	229533.I1RV91
38782	229533.I1RBW1	pp	229533.I1RWS4
38783	229533.I1RBW1	pp	229533.I1RUG4
38784	229533.I1RBW1	pp	229533.I1RTN6
38785	229533.I1RBW1	pp	229533.I1RSZ3
38786	229533.I1RBW1	pp	229533.I1RWI8
38787	229533.I1RBW1	pp	229533.I1S0A6
38788	229533.I1RBW1	pp	229533.I1RQ72
38789	229533.I1RBW1	pp	229533.I1RSR9
38790	229533.I1RBW1	pp	229533.I1S237
38791	229533.I1RBW1	pp	229533.I1RSP7
38792	229533.I1RBW1	pp	229533.I1S1Y7
38793	229533.I1RBW1	pp	229533.I1S0D5
38794	229533.I1RBW1	pp	229533.I1RZT1
38795	229533.I1RBW1	pp	229533.I1S0Q5
38796	229533.I1RBW1	pp	229533.I1RD19
38797	229533.I1RBW1	pp	229533.I1RDX0
38798	229533.I1RBW1	pp	229533.I1S2E9
38799	229533.I1RBW1	pp	229533.Q4IB89
38800	229533.I1RBW1	pp	229533.Q4IJ84
38801	229533.I1RBW1	pp	229533.I1RQJ4
38802	229533.I1RBW1	pp	229533.I1RN57
38803	229533.I1RBW1	pp	229533.I1S0S0
38804	229533.I1RBW1	pp	229533.I1S064
38805	229533.I1RBW1	pp	229533.I1RQB4
38806	229533.I1RBW1	pp	229533.I1RX64
38807	229533.I1RBW1	pp	229533.I1RRW0
38808	229533.I1RBW1	pp	229533.I1RZ98
38809	229533.I1RBW1	pp	229533.I1RS81
38810	229533.I1RBW1	pp	229533.I1RQI6
38811	229533.I1RBW1	pp	229533.V6RLM7
38812	229533.I1RBW1	pp	229533.I1S0D4
38813	229533.I1RBW1	pp	229533.I1RH64
38814	229533.I1RBW1	pp	229533.I1RQ05
38815	229533.I1RBW1	pp	229533.I1RYJ6
38816	229533.I1RBW1	pp	229533.I1RSC7
38817	229533.I1RBW1	pp	229533.V6R382
38818	229533.I1RBW1	pp	229533.I1RC54
38819	229533.I1RBW1	pp	229533.I1RW16
38820	229533.I1RBW1	pp	229533.I1RMS4
38821	229533.I1RBW1	pp	229533.I1RRT1
38822	229533.I1RBW1	pp	229533.I1RLX6
38823	229533.I1RBW1	pp	229533.I1RCQ4
38824	229533.I1RBW1	pp	229533.I1S020
38825	229533.I1RBW1	pp	229533.I1RSJ1
38826	229533.I1RBW1	pp	229533.I1S0B2
38827	229533.I1RBW1	pp	229533.I1RQ13
38828	229533.I1RBW1	pp	229533.I1S016
38829	229533.I1RBW1	pp	229533.I1RZ94
38830	229533.I1RBW1	pp	229533.I1REK4
38831	229533.I1RBW1	pp	229533.I1RE55
38832	229533.I1RBW1	pp	229533.I1S987
38833	229533.I1RBW1	pp	229533.I1RKI2
38834	229533.I1RBW1	pp	229533.I1RFX8
38835	229533.I1RBW1	pp	229533.I1RY44
38836	229533.I1RBW1	pp	229533.I1RM36

229533.I1RBW1	pp	229533.I1RRY1
229533.I1RBW1	pp	229533.I1RC64
229533.I1RBW1	pp	229533.I1S283
229533.I1RBW1	pp	229533.I1RMB7
229533.I1RBW1	pp	229533.I1S224
229533.I1RBW1	pp	229533.I1RV66
229533.I1RBW1	pp	229533.I1S236
229533.I1RBW1	pp	229533.I1S4S4
229533.I1RBW1	pp	229533.I1RQL4
229533.I1RBW1	pp	229533.I1RPQ1
229533.I1RBW1	pp	229533.I1RD18
229533.I1RBW1	pp	229533.I1RQA3
229533.I1RBW1	pp	229533.I1RN31
229533.I1RBW1	pp	229533.I1RVZ9
229533.I1RBW1	pp	229533.Q4IR08
229533.I1RBW1	pp	229533.I1RQ80
229533.I1RBW1	pp	229533.I1RU52
229533.I1RBW1	pp	229533.I1RCL7
229533.I1RBW1	pp	229533.I1S209
229533.I1RBW1	pp	229533.I1RSI4
229533.I1RBW1	pp	229533.I1RWB3
229533.I1RBW1	pp	229533.I1RN55
229533.I1RBW1	pp	229533.I1RG77
229533.I1RBW1	pp	229533.I1S0Q4
229533.I1RBW1	pp	229533.I1RMQ7
229533.I1RBW1	pp	229533.I1RFJ2
229533.I1RBW1	pp	229533.I1RPZ0
229533.I1RBW1	pp	229533.I1RJK7
229533.I1RBW1	pp	229533.I1RQS4
229533.I1RBW1	pp	229533.I1RN46
229533.I1RBW1	pp	229533.I1RE46
229533.I1RBW1	pp	229533.I1RKE9
229533.I1RBW1	pp	229533.I1RCN8
229533.I1RBW1	pp	229533.I1RE23
229533.I1RBW1	pp	229533.I1RWT6
229533.I1RBW1	pp	229533.I1S604
229533.I1RBW1	pp	229533.I1S461
229533.I1RBW1	pp	229533.Q4I6A4
229533.I1RBW1	pp	229533.I1RXF0
229533.I1RBW1	pp	229533.I1RMX3
229533.I1RBW1	pp	229533.Q4INS6
229533.I1RBW1	pp	229533.I1RRV4
229533.I1RBW1	pp	229533.I1RC17
229533.I1RBW1	pp	229533.I1RYR1
229533.I1RD66	pp	229533.I1RTG3
229533.I1RD66	pp	229533.Q4I8B6
229533.I1S2M6	pp	229533.V6RCG5
229533.I1S2M6	pp	229533.I1S2N0
229533.I1S2M6	pp	229533.V6RIV9
229533.I1S2M6	pp	229533.V6QYF0
229533.I1S2M6	pp	229533.V6QW22
229533.I1S2M6	pp	229533.I1S9U2
229533.I1S2M6	pp	229533.I1S5R1
229533.I1S2M6	pp	229533.I1S2M7
229533.A0A0E0SDV0	pp	229533.I1RMV6
229533.A0A0E0SDV0	pp	229533.V6RNM4
229533.A0A0E0SDV0	pp	229533.I1RZQ6
229533.A0A0E0SDV0	pp	229533.A0A1C3YJC0
229533.A0A0E0SDV0	pp	229533.I1RKR8
229533.A0A0E0SDV0	pp	229533.I1S4N7
229533.A0A0E0SDV0	pp	229533.A0A1C3YLN8
229533.A0A0E0SDV0	pp	229533.I1S1V1
229533.A0A0E0SDV0	pp	229533.I1S4N8
229533.A0A0E0SDV0	pp	229533.I1RWM7
229533.A0A0E0SDV0	pp	229533.I1RR07
229533.A0A0E0SDV0	pp	229533.I1RTG9
229533.A0A0E0SDV0	pp	229533.A0A1C3YM82
229533.A0A0E0SDV0	pp	229533.A0A1C3YM61
229533.A0A0E0SDV0	pp	229533.A0A1C3YME0
229533.A0A0E0SDV0	pp	229533.I1RE19
229533.A0A0E0SDV0	pp	229533.I1S0L8
229533.A0A0E0SDV0	pp	229533.A0A1C3YM54
229533.A0A0E0SDV0	pp	229533.I1S1V3

38910 229533.A0A0E0SDV0 pp 229533.I1RPA7
38911 229533.A0A0E0SDV0 pp 229533.I1RN72
38912 229533.A0A0E0SDV0 pp 229533.I1RTA6
38913 229533.A0A0E0SDV0 pp 229533.A0A1C3YLH7
38914 229533.A0A0E0SDV0 pp 229533.I1RNG8
38915 229533.A0A1C3YJE7 pp 229533.I1RLZ4
38916 229533.A0A1C3YLH7 pp 229533.I1RN72
38917 229533.A0A1C3YLH7 pp 229533.I1RC49
38918 229533.A0A1C3YLH7 pp 229533.I1RTA6
38919 229533.A0A1C3YLH7 pp 229533.I1R9X2
38920 229533.A0A1C3YLH7 pp 229533.I1RPA7
38921 229533.A0A1C3YLH7 pp 229533.A0A1C3YM76
38922 229533.A0A1C3YLH7 pp 229533.I1RCK9
38923 229533.A0A1C3YLH7 pp 229533.I1S2B7
38924 229533.A0A1C3YLH7 pp 229533.I1RD69
38925 229533.A0A1C3YLH7 pp 229533.I1RA34
38926 229533.A0A1C3YLH7 pp 229533.V6REE9
38927 229533.A0A1C3YLH7 pp 229533.I1RWC9
38928 229533.A0A1C3YLH7 pp 229533.I1RX09
38929 229533.A0A1C3YLH7 pp 229533.I1RNG8
38930 229533.A0A1C3YLH7 pp 229533.I1S0Z7
38931 229533.A0A1C3YLH7 pp 229533.I1RAU9
38932 229533.A0A1C3YLH7 pp 229533.I1RPK5
38933 229533.A0A1C3YLH7 pp 229533.I1S9V9
38934 229533.A0A1C3YLH7 pp 229533.A0A1C3YM54
38935 229533.A0A1C3YLH7 pp 229533.I1RZP6
38936 229533.A0A1C3YLH7 pp 229533.I1REC6
38937 229533.A0A1C3YLH7 pp 229533.I1RM09
38938 229533.A0A1C3YLH7 pp 229533.I1RZP7
38939 229533.A0A1C3YLH7 pp 229533.I1RS90
38940 229533.A0A1C3YLH7 pp 229533.I1S2B9
38941 229533.A0A1C3YLH7 pp 229533.I1RCD4
38942 229533.A0A1C3YLH7 pp 229533.I1S7X9
38943 229533.A0A1C3YLH7 pp 229533.I1RC46
38944 229533.A0A1C3YLH7 pp 229533.V6RB81
38945 229533.A0A1C3YLH7 pp 229533.A0A1C3YM82
38946 229533.A0A1C3YLH7 pp 229533.I1RQM0
38947 229533.A0A1C3YLH7 pp 229533.I1RN36
38948 229533.A0A1C3YLH7 pp 229533.I1S0L8
38949 229533.A0A1C3YLH7 pp 229533.I1RB40
38950 229533.A0A1C3YLH7 pp 229533.I1RBA6
38951 229533.A0A1C3YLH7 pp 229533.I1RD10
38952 229533.A0A1C3YLH7 pp 229533.I1RG12
38953 229533.A0A1C3YLH7 pp 229533.I1RPS0
38954 229533.A0A1C3YLH7 pp 229533.I1REC7
38955 229533.A0A1C3YLH7 pp 229533.I1RMV0
38956 229533.A0A1C3YLH7 pp 229533.Q4HZB8
38957 229533.I1RGF1 pp 229533.I1RHQ8
38958 229533.I1RGF1 pp 229533.I1RZE4
38959 229533.I1RGF1 pp 229533.I1RRN5
38960 229533.I1RGF1 pp 229533.I1RGF4
38961 229533.I1RGF1 pp 229533.I1RQA3
38962 229533.I1RGF1 pp 229533.I1RV66
38963 229533.I1RGF1 pp 229533.I1S193
38964 229533.I1RGF1 pp 229533.I1RX70
38965 229533.I1RGF1 pp 229533.I1RZ63
38966 229533.I1RGF1 pp 229533.I1RPA6
38967 229533.I1RGF1 pp 229533.I1RQB4
38968 229533.I1RGF1 pp 229533.I1RN08
38969 229533.I1RGF1 pp 229533.I1RWT6
38970 229533.I1RGF1 pp 229533.I1S6V3
38971 229533.I1RGF1 pp 229533.I1S2B6
38972 229533.I1RGF1 pp 229533.I1RYH9
38973 229533.I1RGF1 pp 229533.I1RXH6
38974 229533.I1RGF1 pp 229533.I1RY44
38975 229533.I1RGF1 pp 229533.I1RGF3
38976 229533.I1RGF1 pp 229533.I1RQ72
38977 229533.I1RGF1 pp 229533.I1RGF2
38978 229533.I1RGF1 pp 229533.I1RSS1
38979 229533.I1RGF1 pp 229533.I1S2K7
38980 229533.I1RGF1 pp 229533.I1S2E9
38981 229533.I1RGF1 pp 229533.I1S0X7
38982 229533.I1RGF1 pp 229533.I1RQ88

38983 229533.I1RGF1 pp 229533.I1RNY8
38984 229533.I1RGF1 pp 229533.I1RV91
38985 229533.I1RGF1 pp 229533.I1RX77
38986 229533.I1RGF1 pp 229533.I1RX04
38987 229533.I1RGF1 pp 229533.I1RRI3
38988 229533.I1RGF1 pp 229533.I1RLX7
38989 229533.I1RGF1 pp 229533.I1RNF4
38990 229533.I1RGF1 pp 229533.I1RW83
38991 229533.A0A1C3YL64 pp 229533.I1RW76
38992 229533.A0A1C3YL64 pp 229533.A0A1C3YLL1
38993 229533.A0A1C3YL64 pp 229533.I1RC63
38994 229533.I1RC97 pp 229533.I1RMA8
38995 229533.I1RC97 pp 229533.I1RMM9
38996 229533.I1RC97 pp 229533.I1RRY3
38997 229533.I1RC97 pp 229533.I1RN65
38998 229533.I1RC97 pp 229533.I1S0Z7
38999 229533.I1RC97 pp 229533.I1RDZ4
39000 229533.I1RC97 pp 229533.I1RG57
39001 229533.I1RC97 pp 229533.I1RPW0
39002 229533.I1RC97 pp 229533.Q4IJM4
39003 229533.I1RC97 pp 229533.I1RRN5
39004 229533.I1RC97 pp 229533.I1RN08
39005 229533.I1RC97 pp 229533.I1RKK0
39006 229533.I1RC97 pp 229533.I1RT36
39007 229533.I1RC97 pp 229533.I1RYY5
39008 229533.I1RC97 pp 229533.I1RUQ5
39009 229533.I1RC97 pp 229533.I1RQA3
39010 229533.I1RC97 pp 229533.I1RZE4
39011 229533.I1RC97 pp 229533.V6RDP1
39012 229533.I1RC97 pp 229533.I1RQ72
39013 229533.I1RC97 pp 229533.I1RJR8
39014 229533.I1RC97 pp 229533.I1RMW7
39015 229533.I1RC97 pp 229533.V6RGI1
39016 229533.I1RC97 pp 229533.I1RD18
39017 229533.I1RC97 pp 229533.I1RCU9
39018 229533.I1RC97 pp 229533.I1S439
39019 229533.I1RC97 pp 229533.I1RRW0
39020 229533.I1RC97 pp 229533.I1RI71
39021 229533.I1RC97 pp 229533.I1S0D1
39022 229533.I1RC97 pp 229533.I1RLU1
39023 229533.I1RC97 pp 229533.I1RQB4
39024 229533.I1RC97 pp 229533.I1RZH4
39025 229533.I1RC97 pp 229533.I1S0M2
39026 229533.I1RC97 pp 229533.I1RNP7
39027 229533.I1RC97 pp 229533.I1S2E9
39028 229533.I1RC97 pp 229533.I1RM82
39029 229533.I1RC97 pp 229533.I1RCU8
39030 229533.I1RC97 pp 229533.I1RWT6
39031 229533.I1RC97 pp 229533.I1RV66
39032 229533.I1RC97 pp 229533.I1RV91
39033 229533.I1RC97 pp 229533.I1RUN8
39034 229533.I1RC97 pp 229533.I1S1W4
39035 229533.I1RC97 pp 229533.I1RZH5
39036 229533.I1RC97 pp 229533.I1RY44
39037 229533.I1RC97 pp 229533.V6RVG3
39038 229533.I1RC97 pp 229533.I1RST8
39039 229533.I1RC97 pp 229533.I1RYZ9
39040 229533.I1RC97 pp 229533.V6RKY7
39041 229533.I1RC97 pp 229533.I1RNC0
39042 229533.I1RC97 pp 229533.I1RL24
39043 229533.I1RC97 pp 229533.I1RFL7
39044 229533.I1RC97 pp 229533.I1RCT6
39045 229533.I1RC97 pp 229533.I1RDB1
39046 229533.I1RC97 pp 229533.I1RYH9
39047 229533.I1RC97 pp 229533.V6QV09
39048 229533.I1RC97 pp 229533.I1S224
39049 229533.I1RC97 pp 229533.I1RWJ6
39050 229533.I1RC97 pp 229533.I1RX70
39051 229533.I1RC97 pp 229533.I1RQ26
39052 229533.I1RC97 pp 229533.I1RJH1
39053 229533.I1RC97 pp 229533.I1S2G7
39054 229533.I1RC97 pp 229533.I1RNF4
39055 229533.I1RC97 pp 229533.V6RLW4

229533.I1RC97 pp 229533.I1RLU5
229533.I1S7W5 pp 229533.Q4IJK2
229533.I1S7W5 pp 229533.V6RUT5
229533.I1S7W5 pp 229533.V6RCZ8
229533.I1S7W5 pp 229533.I1S887
229533.I1S7W5 pp 229533.I1S9G7
229533.I1S7W5 pp 229533.V6R042
229533.I1S7W5 pp 229533.Q4I624
229533.I1S7W5 pp 229533.V6R3J1
229533.I1S7W5 pp 229533.V6QUE6
229533.I1S7W5 pp 229533.Q4HZB8
229533.A0A1C3YJE3 pp 229533.V6RPN0
229533.A0A1C3YJE3 pp 229533.I1RD69
229533.A0A1C3YJE3 pp 229533.A0A1C3YMS7
229533.A0A1C3YJE3 pp 229533.I1S5R5
229533.A0A1C3YJE3 pp 229533.I1RQL4
229533.I1RH86 pp 229533.I1RUU7
229533.I1RH86 pp 229533.I1RUY5
229533.I1RH86 pp 229533.I1RSA5
229533.I1RAW2 pp 229533.I1RD66
229533.I1RAW2 pp 229533.Q4I8B6
229533.A0A098E4E9 pp 229533.I1RFL7
229533.A0A098E4E9 pp 229533.I1RYX9
229533.A0A098E4E9 pp 229533.A0A1C3YMJ3
229533.A0A098E4E9 pp 229533.A0A0E0SLZ3
229533.A0A098E4E9 pp 229533.I1RZ86
229533.A0A098E4E9 pp 229533.I1RCA5
229533.A0A098E4E9 pp 229533.I1RYR2
229533.A0A098E4E9 pp 229533.I1S998
229533.A0A098E4E9 pp 229533.I1RSN7
229533.A0A098E4E9 pp 229533.I1RUG1
229533.A0A098E4E9 pp 229533.A0A1C3YJP9
229533.A0A098E4E9 pp 229533.A0A1C3YN55
229533.A0A098E4E9 pp 229533.I1RNG4
229533.A0A098E4E9 pp 229533.I1S4P6
229533.A0A098E4E9 pp 229533.I1RSA7
229533.A0A098E4E9 pp 229533.I1RY29
229533.A0A098E4E9 pp 229533.A0A0E0SQG9
229533.A0A098E4E9 pp 229533.A0A1C3YHM3
229533.A0A098E4E9 pp 229533.A0A1C3YL34
229533.A0A098E4E9 pp 229533.A0A1C3YL55
229533.A0A098E4E9 pp 229533.I1RSC7
229533.A0A098E4E9 pp 229533.V6RLW4
229533.A0A098E4E9 pp 229533.I1RZ98
229533.A0A098E4E9 pp 229533.I1RWI8
229533.A0A098E4E9 pp 229533.I1RQI6
229533.A0A098E4E9 pp 229533.A0A1C3YI10
229533.A0A098E4E9 pp 229533.I1RBU5
229533.A0A098E4E9 pp 229533.A0A1C3YLQ7
229533.A0A098E4E9 pp 229533.I1RSZ3
229533.A0A098E4E9 pp 229533.I1RBW1
229533.A0A098E4E9 pp 229533.I1S3G4
229533.A0A098E4E9 pp 229533.Q4IPC8
229533.A0A098E4E9 pp 229533.I1RV91
229533.A0A098E4E9 pp 229533.V6RGI1
229533.A0A098E4E9 pp 229533.I1S2F2
229533.A0A098E4E9 pp 229533.I1RYQ1
229533.A0A098E4E9 pp 229533.I1RW16
229533.A0A098E4E9 pp 229533.I1RC54
229533.A0A098E4E9 pp 229533.Q4IQM6
229533.A0A098E4E9 pp 229533.I1RBC8
229533.A0A098E4E9 pp 229533.I1RMS4
229533.A0A098E4E9 pp 229533.A0A1C3YKE4
229533.A0A098E4E9 pp 229533.A0A1C3YN69
229533.A0A098E4E9 pp 229533.I1RN57
229533.A0A098E4E9 pp 229533.I1RRS7
229533.A0A098E4E9 pp 229533.I1RDJ8
229533.A0A098E4E9 pp 229533.I1RA10
229533.A0A098E4E9 pp 229533.V6R3J1
229533.A0A098E4E9 pp 229533.I1RDH0
229533.A0A098E4E9 pp 229533.I1S0A6
229533.A0A098E4E9 pp 229533.Q4IB89
229533.A0A098E4E9 pp 229533.I1RFW6

39129	229533.A0A098E4E9	pp	229533.I1RMQ7
39130	229533.A0A098E4E9	pp	229533.I1RCA9
39131	229533.A0A098E4E9	pp	229533.I1RQJ4
39132	229533.A0A098E4E9	pp	229533.I1RUG4
39133	229533.A0A098E4E9	pp	229533.I1RWT6
39134	229533.A0A098E4E9	pp	229533.A0A0E0RW49
39135	229533.A0A098E4E9	pp	229533.A0A1C3YMG8
39136	229533.A0A098E4E9	pp	229533.A0A1C3YJR3
39137	229533.A0A098E4E9	pp	229533.I1R9Y7
39138	229533.A0A098E4E9	pp	229533.I1RTN6
39139	229533.A0A098E4E9	pp	229533.I1RAU8
39140	229533.A0A098E4E9	pp	229533.I1RD19
39141	229533.A0A098E4E9	pp	229533.I1RKE9
39142	229533.A0A098E4E9	pp	229533.I1RSP7
39143	229533.A0A098E4E9	pp	229533.I1RA12
39144	229533.A0A098E4E9	pp	229533.I1RZP8
39145	229533.A0A098E4E9	pp	229533.I1RAX3
39146	229533.A0A098E4E9	pp	229533.I1RDX0
39147	229533.A0A098E4E9	pp	229533.I1S276
39148	229533.A0A098E4E9	pp	229533.I1RNE2
39149	229533.A0A098E4E9	pp	229533.A0A1C3YHN3
39150	229533.A0A098E4E9	pp	229533.I1RX64
39151	229533.A0A098E4E9	pp	229533.A0A1C3YIJ4
39152	229533.A0A098E4E9	pp	229533.A0A1C3YJQ6
39153	229533.A0A098E4E9	pp	229533.I1RBN9
39154	229533.A0A098E4E9	pp	229533.I1RMJ8
39155	229533.A0A098E4E9	pp	229533.I1RQH8
39156	229533.A0A098E4E9	pp	229533.I1S0D5
39157	229533.A0A098E4E9	pp	229533.I1RPM7
39158	229533.A0A098E4E9	pp	229533.I1RD01
39159	229533.A0A098E4E9	pp	229533.I1RZ94
39160	229533.A0A098E4E9	pp	229533.Q4INS6
39161	229533.A0A098E4E9	pp	229533.A0A1C3YI40
39162	229533.A0A098E4E9	pp	229533.I1S1W8
39163	229533.A0A098E4E9	pp	229533.I1RSR9
39164	229533.A0A098E4E9	pp	229533.I1RY73
39165	229533.A0A098E4E9	pp	229533.Q4IP51
39166	229533.A0A098E4E9	pp	229533.I1RQ13
39167	229533.A0A098E4E9	pp	229533.I1S604
39168	229533.A0A098E4E9	pp	229533.I1RRT1
39169	229533.A0A098E4E9	pp	229533.I1RAL9
39170	229533.A0A098E4E9	pp	229533.I1RSI4
39171	229533.A0A098E4E9	pp	229533.I1S209
39172	229533.A0A098E4E9	pp	229533.A0A1C3YJT5
39173	229533.A0A098E4E9	pp	229533.A0A1C3YN36
39174	229533.A0A098E4E9	pp	229533.Q4IEA5
39175	229533.A0A098E4E9	pp	229533.I1RYR1
39176	229533.A0A098E4E9	pp	229533.I1RQ92
39177	229533.A0A098E4E9	pp	229533.I1RCQ4
39178	229533.A0A098E4E9	pp	229533.A0A1C3YLR6
39179	229533.A0A098E4E9	pp	229533.I1RN31
39180	229533.A0A098E4E9	pp	229533.Q4I5I4
39181	229533.A0A098E4E9	pp	229533.A0A1C3YLR4
39182	229533.A0A098E4E9	pp	229533.Q4IC11
39183	229533.A0A098E4E9	pp	229533.I1RMX3
39184	229533.A0A098E4E9	pp	229533.I1RUG2
39185	229533.A0A098E4E9	pp	229533.A0A1C3YI64
39186	229533.A0A098E4E9	pp	229533.I1RMB7
39187	229533.A0A098E4E9	pp	229533.I1RKI2
39188	229533.A0A098E4E9	pp	229533.I1RN55
39189	229533.A0A098E4E9	pp	229533.I1RSJ1
39190	229533.A0A098E4E9	pp	229533.I1RBW0
39191	229533.A0A098E4E9	pp	229533.I1RG77
39192	229533.A0A098E4E9	pp	229533.I1RAH4
39193	229533.A0A098E4E9	pp	229533.I1RFX8
39194	229533.A0A098E4E9	pp	229533.I1S987
39195	229533.A0A098E4E9	pp	229533.A0A1C3YJL7
39196	229533.A0A098E4E9	pp	229533.A0A0E0RVF4
39197	229533.A0A098E4E9	pp	229533.I1RLX6
39198	229533.A0A098E4E9	pp	229533.I1RCL7
39199	229533.A0A098E4E9	pp	229533.A0A0E0RQK8
39200	229533.A0A098E4E9	pp	229533.I1RPQ1
39201	229533.A0A098E4E9	pp	229533.A0A098E4R5

39202 229533.A0A098E4E9 pp 229533.I1RAT5
39203 229533.A0A098E4E9 pp 229533.I1S283
39204 229533.A0A098E4E9 pp 229533.I1RQA3
39205 229533.A0A098E4E9 pp 229533.I1RW69
39206 229533.A0A098E4E9 pp 229533.I1RKB1
39207 229533.A0A098E4E9 pp 229533.I1RHR0
39208 229533.A0A098E4E9 pp 229533.I1S461
39209 229533.A0A098E4E9 pp 229533.A0A1C3YML3
39210 229533.A0A098E4E9 pp 229533.I1RE23
39211 229533.A0A098E4E9 pp 229533.I1RE55
39212 229533.A0A098E4E9 pp 229533.A0A1C3YJN7
39213 229533.A0A098E4E9 pp 229533.V6RY56
39214 229533.A0A098E4E9 pp 229533.I1RBC1
39215 229533.A0A098E4E9 pp 229533.I1RE46
39216 229533.A0A098E4E9 pp 229533.I1RPZ0
39217 229533.A0A098E4E9 pp 229533.A0A1C3YJ79
39218 229533.A0A098E4E9 pp 229533.I1RFJ2
39219 229533.A0A098E4E9 pp 229533.A0A1C3YK01
39220 229533.A0A1C3YIH5 pp 229533.I1RS90
39221 229533.A0A1C3YIH5 pp 229533.I1S2B9
39222 229533.A0A1C3YIH5 pp 229533.I1S1Y9
39223 229533.A0A1C3YIH5 pp 229533.I1RWG1
39224 229533.A0A1C3YIH5 pp 229533.I1RY40
39225 229533.A0A1C3YIH5 pp 229533.A0A1C3YLW8
39226 229533.A0A1C3YIH5 pp 229533.I1RKJ2
39227 229533.A0A1C3YIH5 pp 229533.I1RTE6
39228 229533.A0A1C3YIH5 pp 229533.I1RB40
39229 229533.A0A1C3YIH5 pp 229533.I1RNG8
39230 229533.A0A1C3YIH5 pp 229533.I1RUP2
39231 229533.A0A1C3YIH5 pp 229533.V6REE9
39232 229533.A0A1C3YIH5 pp 229533.I1RPS0
39233 229533.A0A1C3YIH5 pp 229533.I1RCJ2
39234 229533.A0A1C3YIH5 pp 229533.I1RMV0
39235 229533.A0A1C3YIH5 pp 229533.I1S0L8
39236 229533.A0A1C3YIH5 pp 229533.I1RAU9
39237 229533.A0A1C3YIH5 pp 229533.I1RYM3
39238 229533.A0A1C3YIH5 pp 229533.I1S9G7
39239 229533.A0A1C3YIH5 pp 229533.I1RBA6
39240 229533.A0A1C3YIH5 pp 229533.I1RCD4
39241 229533.A0A1C3YIH5 pp 229533.I1RQ49
39242 229533.A0A1C3YIH5 pp 229533.I1REC6
39243 229533.A0A1C3YIH5 pp 229533.I1REC7
39244 229533.A0A1C3YIH5 pp 229533.A0A1C3YM54
39245 229533.A0A1C3YIH5 pp 229533.A0A1C3YM82
39246 229533.A0A1C3YIH5 pp 229533.I1RC49
39247 229533.A0A1C3YIH5 pp 229533.I1RJU5
39248 229533.A0A1C3YIH5 pp 229533.I1RN72
39249 229533.A0A1C3YIH5 pp 229533.A0A1C3YM76
39250 229533.A0A1C3YIH5 pp 229533.I1RPN4
39251 229533.A0A1C3YIH5 pp 229533.I1RCK9
39252 229533.A0A1C3YIH5 pp 229533.I1S9V9
39253 229533.I1RJB9 pp 229533.I1RZP8
39254 229533.I1RJB9 pp 229533.Q4HYR2
39255 229533.I1RJB9 pp 229533.I1RKZ8
39256 229533.A0A098DUK3 pp 229533.A0A1C3YM54
39257 229533.A0A098DUK3 pp 229533.Q4HZB8
39258 229533.A0A098DUK3 pp 229533.I1RZP6
39259 229533.A0A098DUK3 pp 229533.I1RAE2
39260 229533.A0A098DUK3 pp 229533.A0A1C3YJC0
39261 229533.A0A098DUK3 pp 229533.I1RYM3
39262 229533.A0A098DUK3 pp 229533.I1S0L8
39263 229533.A0A098DUK3 pp 229533.I1RAE4
39264 229533.A0A098DUK3 pp 229533.I1RB40
39265 229533.A0A098DUK3 pp 229533.I1RUP2
39266 229533.A0A098DUK3 pp 229533.I1REC7
39267 229533.A0A098DUK3 pp 229533.A0A1C3YI04
39268 229533.A0A098DUK3 pp 229533.I1RAE3
39269 229533.A0A098DUK3 pp 229533.I1RWG1
39270 229533.A0A098DUK3 pp 229533.I1S1Y9
39271 229533.A0A098DUK3 pp 229533.A0A0E0SDV0
39272 229533.A0A098DUK3 pp 229533.I1S095
39273 229533.A0A098DUK3 pp 229533.I1RT36
39274 229533.A0A098DUK3 pp 229533.A0A1C3YIQ2

```
229533.A0A098DUK3   pp  229533.I1RZV4
229533.A0A098DUK3   pp  229533.I1RM09
229533.A0A098DUK3   pp  229533.I1RB15
229533.A0A098DUK3   pp  229533.I1RZP7
229533.A0A098DUK3   pp  229533.A0A1C3YLH7
229533.A0A098DUK3   pp  229533.A0A1C3YMD9
229533.A0A098DUK3   pp  229533.V6REE9
229533.A0A098DUK3   pp  229533.I1S9V9
229533.A0A098DUK3   pp  229533.I1RPS0
229533.A0A098DUK3   pp  229533.A0A1C3YM82
229533.A0A098DUK3   pp  229533.A0A1C3YIH5
229533.A0A098DUK3   pp  229533.I1RN72
229533.A0A098DUK3   pp  229533.I1S0J7
229533.A0A098DUK3   pp  229533.I1RTA6
229533.A0A098DUK3   pp  229533.I1RP88
229533.A0A098DUK3   pp  229533.I1RJU5
229533.A0A098DUK3   pp  229533.I1RAU7
229533.A0A098DUK3   pp  229533.A0A0E0SF53
229533.A0A098DUK3   pp  229533.I1RTE6
229533.A0A098DUK3   pp  229533.I1RSB0
229533.A0A098DUK3   pp  229533.I1RQG7
229533.A0A098DUK3   pp  229533.I1R991
229533.A0A098DUK3   pp  229533.I1REC6
229533.A0A098DUK3   pp  229533.A0A1C3YLW8
229533.A0A098DUK3   pp  229533.I1RKJ2
229533.A0A098DUK3   pp  229533.A0A0E0SKT2
229533.A0A098DUK3   pp  229533.A0A0E0S0J3
229533.A0A098DUK3   pp  229533.I1R9X2
229533.A0A098DUK3   pp  229533.I1RPA7
229533.A0A098DUK3   pp  229533.I1RZD9
229533.A0A098DUK3   pp  229533.I1RTG9
229533.A0A098DUK3   pp  229533.I1RAE5
229533.A0A098DUK3   pp  229533.I1S039
229533.A0A098DUK3   pp  229533.A0A0E0SNY0
229533.A0A098DUK3   pp  229533.I1S1V3
229533.A0A098DUK3   pp  229533.I1RCD4
229533.A0A1C3YK17   pp  229533.I1RC17
229533.A0A1C3YK17   pp  229533.I1RWT6
229533.A0A1C3YK17   pp  229533.I1RY44
229533.A0A1C3YK17   pp  229533.I1RC22
229533.A0A1C3YK17   pp  229533.I1RJK8
229533.A0A1C3YK17   pp  229533.I1RB74
229533.A0A1C3YK17   pp  229533.I1S2E9
229533.A0A1C3YK17   pp  229533.I1RDE8
229533.A0A1C3YK17   pp  229533.I1RBX2
229533.A0A1C3YK17   pp  229533.I1RB59
229533.A0A1C3YK17   pp  229533.I1RZE4
229533.A0A1C3YK17   pp  229533.I1RQA3
229533.A0A1C3YK17   pp  229533.I1RRN5
229533.A0A1C3YK17   pp  229533.I1RB75
229533.A0A1C3YK17   pp  229533.I1RB41
229533.I1RAX0   pp  229533.I1RW76
229533.A0A1C3YIZ3   pp  229533.I1RPS0
229533.A0A1C3YIZ3   pp  229533.I1RJY3
229533.A0A1C3YIZ3   pp  229533.I1RK79
229533.A0A1C3YIZ3   pp  229533.I1RJY2
229533.A0A1C3YIZ3   pp  229533.I1RK95
229533.A0A1C3YIZ3   pp  229533.I1RRE0
229533.A0A1C3YIZ3   pp  229533.I1RJI3
229533.A0A1C3YIZ3   pp  229533.I1RG11
229533.A0A1C3YIZ3   pp  229533.V6QUE6
229533.A0A1C3YIZ3   pp  229533.Q4I5I4
229533.A0A1C3YIZ3   pp  229533.I1RR72
229533.A0A1C3YIZ3   pp  229533.I1S9U1
229533.A0A1C3YIZ3   pp  229533.I1RRN6
229533.A0A1C3YIZ3   pp  229533.A0A1C3YJR3
229533.A0A1C3YIZ3   pp  229533.I1RB71
229533.A0A1C3YIZ3   pp  229533.I1S0L8
229533.A0A1C3YIZ3   pp  229533.I1S9Y1
229533.A0A1C3YIZ3   pp  229533.I1RW90
229533.A0A1C3YIZ3   pp  229533.I1RBC7
229533.A0A1C3YIZ3   pp  229533.I1RTH2
229533.A0A1C3YIZ3   pp  229533.A0A1C3YM82
```

229533.A0A1C3YIZ3 pp 229533.I1RAV6
229533.A0A1C3YIZ3 pp 229533.I1RS30
229533.I1RNP7 pp 229533.I1RYH9
229533.I1RNP7 pp 229533.I1S224
229533.I1RNP7 pp 229533.I1RV66
229533.I1RNP7 pp 229533.I1RST8
229533.I1RNP7 pp 229533.I1RWJ6
229533.I1RNP7 pp 229533.I1RV91
229533.I1RNP7 pp 229533.I1S439
229533.I1RNP7 pp 229533.I1RX70
229533.I1RNP7 pp 229533.I1RQA3
229533.I1RNP7 pp 229533.I1S2E9
229533.I1RNP7 pp 229533.I1RQ72
229533.I1RNP7 pp 229533.Q4IJM4
229533.I1RNP7 pp 229533.I1S0Z7
229533.I1RNP7 pp 229533.I1RZE4
229533.I1RNP7 pp 229533.V6QV09
229533.I1RNP7 pp 229533.I1RVX0
229533.I1RNP7 pp 229533.I1RYY5
229533.I1RNP7 pp 229533.I1RRG3
229533.I1RNP7 pp 229533.I1RRN5
229533.I1RNP7 pp 229533.I1RQ26
229533.I1RNP7 pp 229533.I1RWT6
229533.I1RNP7 pp 229533.I1RUQ5
229533.I1RNP7 pp 229533.I1RRY3
229533.I1RNP7 pp 229533.I1RRP7
229533.I1RNP7 pp 229533.I1RNP8
229533.I1RNP7 pp 229533.I1RYZ9
229533.I1RNP7 pp 229533.V6RDP1
229533.I1RNP7 pp 229533.I1RY44
229533.I1RNP7 pp 229533.I1S2G7
229533.I1RNP7 pp 229533.I1RUN8
229533.I1RNP7 pp 229533.V6RVG3
229533.I1RNP7 pp 229533.I1S1W4
229533.I1RNP7 pp 229533.I1RPP3
229533.I1RNP7 pp 229533.V6RKY7
229533.I1RNP7 pp 229533.I1S0D1
229533.I1RNP7 pp 229533.I1RT36
229533.I1RNP7 pp 229533.I1S0M2
229533.I1RNP7 pp 229533.I1RRW0
229533.I1RNP7 pp 229533.V6RGI1
229533.I1RNP7 pp 229533.I1RQB4
229533.A0A0E0SFI7 pp 229533.I1S0L8
229533.A0A0E0SFI7 pp 229533.I1RAU9
229533.A0A0E0SFI7 pp 229533.I1RS90
229533.A0A0E0SFI7 pp 229533.I1RJH8
229533.A0A0E0SFI7 pp 229533.I1S2B9
229533.A0A0E0SFI7 pp 229533.I1SAB8
229533.A0A0E0SFI7 pp 229533.I1S1Y9
229533.A0A0E0SFI7 pp 229533.I1RB40
229533.A0A0E0SFI7 pp 229533.I1RWG1
229533.A0A0E0SFI7 pp 229533.A0A1C3YI04
229533.A0A0E0SFI7 pp 229533.I1RY40
229533.A0A0E0SFI7 pp 229533.A0A1C3YM82
229533.A0A0E0SFI7 pp 229533.A0A1C3YLH7
229533.A0A0E0SFI7 pp 229533.I1RYM3
229533.A0A0E0SFI7 pp 229533.A0A1C3YIH5
229533.A0A0E0SFI7 pp 229533.I1RQ49
229533.A0A0E0SFI7 pp 229533.A0A1C3YM76
229533.A0A0E0SFI7 pp 229533.I1RWC9
229533.A0A0E0SFI7 pp 229533.I1RPA7
229533.A0A0E0SFI7 pp 229533.I1S9V9
229533.A0A0E0SFI7 pp 229533.I1RTG9
229533.A0A0E0SFI7 pp 229533.A0A1C3YLA0
229533.A0A0E0SFI7 pp 229533.I1RPS0
229533.A0A0E0SFI7 pp 229533.I1S9G7
229533.A0A0E0SFI7 pp 229533.I1RQ82
229533.A0A0E0SFI7 pp 229533.I1RN72
229533.A0A0E0SFI7 pp 229533.A0A1C3YN81
229533.A0A0E0SFI7 pp 229533.I1RJH7
229533.A0A1C3YI04 pp 229533.I1S1Y9
229533.A0A1C3YI04 pp 229533.A0A1C3YJ42
229533.A0A1C3YI04 pp 229533.I1RYM3

39421	229533.A0A1C3YI04	pp	229533.I1S7W5
39422	229533.A0A1C3YI04	pp	229533.I1RQ49
39423	229533.A0A1C3YI04	pp	229533.I1S9G7
39424	229533.A0A1C3YI04	pp	229533.I1RWB0
39425	229533.A0A1C3YI04	pp	229533.I1RPS0
39426	229533.A0A1C3YI04	pp	229533.I1RQ82
39427	229533.A0A1C3YI04	pp	229533.I1S8A6
39428	229533.A0A1C3YI04	pp	229533.I1REU7
39429	229533.A0A1C3YI04	pp	229533.I1S2B9
39430	229533.A0A1C3YI04	pp	229533.I1RJH8
39431	229533.A0A1C3YI04	pp	229533.I1RAU9
39432	229533.A0A1C3YI04	pp	229533.I1RBA6
39433	229533.A0A1C3YI04	pp	229533.I1S0L8
39434	229533.A0A1C3YI04	pp	229533.I1RAD4
39435	229533.A0A1C3YI04	pp	229533.Q4ICG8
39436	229533.A0A1C3YI04	pp	229533.I1RB40
39437	229533.A0A1C3YI04	pp	229533.I1RLP1
39438	229533.A0A1C3YI04	pp	229533.I1RWG1
39439	229533.A0A1C3YI04	pp	229533.I1RY40
39440	229533.A0A1C3YI04	pp	229533.A0A1C3YIH5
39441	229533.A0A1C3YI04	pp	229533.A0A1C3YM82
39442	229533.A0A1C3YI04	pp	229533.A0A1C3YLH7
39443	229533.A0A1C3YI04	pp	229533.I1RPA7
39444	229533.A0A1C3YI04	pp	229533.I1RTG9
39445	229533.A0A1C3YI04	pp	229533.A0A1C3YM76
39446	229533.A0A1C3YI04	pp	229533.I1RN72
39447	229533.A0A1C3YI04	pp	229533.I1RJH7
39448	229533.A0A1C3YI04	pp	229533.A0A1C3YKI8
39449	229533.A0A1C3YI04	pp	229533.I1S9V9
39450	229533.A0A1C3YI04	pp	229533.I1RW90
39451	229533.A0A1C3YI04	pp	229533.Q4I650
39452	229533.I1RNF4	pp	229533.I1RZC3
39453	229533.I1RNF4	pp	229533.I1S0M2
39454	229533.I1RNF4	pp	229533.I1S1W4
39455	229533.I1RNF4	pp	229533.I1RNY8
39456	229533.I1RNF4	pp	229533.I1RWQ8
39457	229533.I1RNF4	pp	229533.I1RQB4
39458	229533.I1RNF4	pp	229533.V6RKY7
39459	229533.I1RNF4	pp	229533.I1RY44
39460	229533.I1RNF4	pp	229533.V6RVG3
39461	229533.I1RNF4	pp	229533.I1RRN5
39462	229533.I1RNF4	pp	229533.I1RX64
39463	229533.I1RNF4	pp	229533.I1RWH4
39464	229533.I1RNF4	pp	229533.I1S247
39465	229533.I1RNF4	pp	229533.I1RRY3
39466	229533.I1RNF4	pp	229533.I1S286
39467	229533.I1RNF4	pp	229533.I1RP28
39468	229533.I1RNF4	pp	229533.I1RZE4
39469	229533.I1RNF4	pp	229533.I1RY32
39470	229533.I1RNF4	pp	229533.I1RRJ8
39471	229533.I1RNF4	pp	229533.I1S2E9
39472	229533.I1RNF4	pp	229533.I1RVA8
39473	229533.I1RNF4	pp	229533.I1S149
39474	229533.I1RNF4	pp	229533.I1S7X9
39475	229533.I1RNF4	pp	229533.V6RQR4
39476	229533.I1RNF4	pp	229533.I1RZ63
39477	229533.I1RNF4	pp	229533.I1RRI3
39478	229533.I1RNF4	pp	229533.I1RQD6
39479	229533.I1RNF4	pp	229533.I1S0X7
39480	229533.I1RNF4	pp	229533.I1S4Z7
39481	229533.I1RNF4	pp	229533.I1RNP7
39482	229533.I1RNF4	pp	229533.V6RR76
39483	229533.I1RNF4	pp	229533.I1S265
39484	229533.I1RNF4	pp	229533.I1S248
39485	229533.I1RNF4	pp	229533.I1RUQ2
39486	229533.I1RNF4	pp	229533.I1RS47
39487	229533.I1RNF4	pp	229533.I1RUN8
39488	229533.I1RNF4	pp	229533.I1S278
39489	229533.I1RNF4	pp	229533.I1RSS1
39490	229533.I1RNF4	pp	229533.I1RP86
39491	229533.I1RNF4	pp	229533.I1RX77
39492	229533.I1RNF4	pp	229533.Q4IEA7
39493	229533.I1RNF4	pp	229533.I1RWT6

39494	229533.I1RNF4	pp	229533.I1RX04
39495	229533.I1RNF4	pp	229533.V6RPS5
39496	229533.I1RNF4	pp	229533.Q4IR87
39497	229533.I1RNF4	pp	229533.I1RQ31
39498	229533.I1RNF4	pp	229533.I1RYZ9
39499	229533.I1RNF4	pp	229533.I1RQA3
39500	229533.I1RNF4	pp	229533.Q4INZ9
39501	229533.I1RNF4	pp	229533.I1RUQ5
39502	229533.I1RNF4	pp	229533.I1S439
39503	229533.I1RNF4	pp	229533.I1RYL5
39504	229533.I1RNF4	pp	229533.I1RYH6
39505	229533.I1RNF4	pp	229533.I1RQL4
39506	229533.I1RNF4	pp	229533.I1RYH9
39507	229533.I1RNF4	pp	229533.I1RXX1
39508	229533.I1RNF4	pp	229533.I1RPQ1
39509	229533.I1RNF4	pp	229533.I1RWC2
39510	229533.I1RNF4	pp	229533.V6QV09
39511	229533.I1RNF4	pp	229533.I1RSI4
39512	229533.I1RNF4	pp	229533.I1S2C1
39513	229533.I1RNF4	pp	229533.I1RZ94
39514	229533.I1RNF4	pp	229533.Q4I4A4
39515	229533.I1RNF4	pp	229533.I1RX87
39516	229533.I1RNF4	pp	229533.I1RPA6
39517	229533.I1RNF4	pp	229533.I1RV66
39518	229533.I1RNF4	pp	229533.I1RX20
39519	229533.I1RNF4	pp	229533.I1RQ26
39520	229533.I1RWG1	pp	229533.I1S9G7
39521	229533.I1RWG1	pp	229533.I1S1Y9
39522	229533.I1RWG1	pp	229533.I1RY40
39523	229533.I1RWG1	pp	229533.I1S9V9
39524	229533.I1RWG1	pp	229533.I1S0L8
39525	229533.I1RWG1	pp	229533.I1RYX5
39526	229533.I1RWG1	pp	229533.I1S2B9
39527	229533.A0A1C3YJD5	pp	229533.I1SAT1
39528	229533.A0A1C3YJD5	pp	229533.A0A1C3YLT9
39529	229533.A0A1C3YKN4	pp	229533.I1RZC3
39530	229533.A0A1C3YKN4	pp	229533.I1S8R4
39531	229533.A0A1C3YKN4	pp	229533.I1RPZ5
39532	229533.A0A1C3YKN4	pp	229533.I1RVY1
39533	229533.A0A1C3YKN4	pp	229533.I1RZQ1
39534	229533.A0A1C3YKN4	pp	229533.I1REW1
39535	229533.A0A1C3YKN4	pp	229533.I1SA33
39536	229533.A0A1C3YKN4	pp	229533.I1RY44
39537	229533.A0A1C3YKN4	pp	229533.V6R756
39538	229533.A0A1C3YKN4	pp	229533.I1RRP7
39539	229533.A0A1C3YKN4	pp	229533.I1S147
39540	229533.A0A1C3YKN4	pp	229533.V6RRM0
39541	229533.A0A1C3YKN4	pp	229533.V6R9L8
39542	229533.A0A1C3YKN4	pp	229533.I1RFF3
39543	229533.A0A1C3YKN4	pp	229533.I1R9K2
39544	229533.A0A1C3YKN4	pp	229533.V6RN31
39545	229533.A0A1C3YKN4	pp	229533.I1RAV0
39546	229533.A0A1C3YKN4	pp	229533.I1RXY7
39547	229533.A0A1C3YKN4	pp	229533.V6RF62
39548	229533.A0A1C3YKN4	pp	229533.I1RX18
39549	229533.A0A1C3YKN4	pp	229533.I1RT76
39550	229533.A0A1C3YKN4	pp	229533.I1RUP5
39551	229533.A0A1C3YKN4	pp	229533.I1RM41
39552	229533.A0A1C3YKN4	pp	229533.I1S1D7
39553	229533.A0A1C3YKN4	pp	229533.I1S064
39554	229533.A0A1C3YKN4	pp	229533.I1RLM5
39555	229533.A0A1C3YKN4	pp	229533.I1RZ26
39556	229533.A0A1C3YKN4	pp	229533.I1S6F3
39557	229533.A0A1C3YKN4	pp	229533.V6RM02
39558	229533.A0A1C3YKN4	pp	229533.I1RSX9
39559	229533.A0A1C3YKN4	pp	229533.V6RHD0
39560	229533.A0A1C3YKN4	pp	229533.I1S149
39561	229533.A0A1C3YKN4	pp	229533.I1RZ70
39562	229533.A0A1C3YKN4	pp	229533.I1REA8
39563	229533.A0A1C3YKN4	pp	229533.I1S3G4
39564	229533.A0A1C3YKN4	pp	229533.I1RDU6
39565	229533.A0A1C3YKN4	pp	229533.I1RI34
39566	229533.A0A1C3YKN4	pp	229533.I1RBY9

39567 229533.A0A1C3YKN4 pp 229533.I1S1H2
39568 229533.A0A1C3YKN4 pp 229533.I1S9T6
39569 229533.A0A1C3YKN4 pp 229533.I1RLZ4
39570 229533.A0A1C3YKN4 pp 229533.V6RV57
39571 229533.A0A1C3YKN4 pp 229533.I1RAX2
39572 229533.A0A1C3YKN4 pp 229533.I1RCR9
39573 229533.A0A1C3YKN4 pp 229533.I1S9C6
39574 229533.A0A1C3YKN4 pp 229533.I1RC71
39575 229533.A0A1C3YKN4 pp 229533.I1S673
39576 229533.A0A1C3YKN4 pp 229533.I1RH71
39577 229533.A0A1C3YKN4 pp 229533.I1RYR3
39578 229533.A0A1C3YKN4 pp 229533.I1RV91
39579 229533.A0A1C3YKN4 pp 229533.I1RY32
39580 229533.A0A1C3YKN4 pp 229533.I1RJU4
39581 229533.A0A1C3YKN4 pp 229533.I1RUE7
39582 229533.A0A1C3YKN4 pp 229533.I1RF59
39583 229533.A0A1C3YKN4 pp 229533.V6RZD4
39584 229533.A0A1C3YKN4 pp 229533.I1RI19
39585 229533.A0A1C3YKN4 pp 229533.I1RKB2
39586 229533.A0A1C3YKN4 pp 229533.I1RZV5
39587 229533.A0A1C3YKN4 pp 229533.I1RZA8
39588 229533.A0A1C3YKN4 pp 229533.I1RH91
39589 229533.A0A1C3YKN4 pp 229533.V6R2Z2
39590 229533.A0A1C3YKN4 pp 229533.I1S694
39591 229533.A0A1C3YKN4 pp 229533.I1S4B6
39592 229533.A0A1C3YKN4 pp 229533.A0A1C3YLE2
39593 229533.A0A1C3YKN4 pp 229533.I1S4B7
39594 229533.A0A1C3YKN4 pp 229533.I1S2E9
39595 229533.A0A1C3YKN4 pp 229533.I1RC29
39596 229533.A0A1C3YKN4 pp 229533.I1RHG2
39597 229533.A0A1C3YKN4 pp 229533.I1RZT0
39598 229533.A0A1C3YKN4 pp 229533.I1RR29
39599 229533.A0A1C3YKN4 pp 229533.V6QZ48
39600 229533.A0A1C3YKN4 pp 229533.I1RK50
39601 229533.A0A1C3YKN4 pp 229533.V6R5D2
39602 229533.A0A1C3YKN4 pp 229533.I1R9N5
39603 229533.A0A1C3YKN4 pp 229533.I1RVR2
39604 229533.A0A1C3YKN4 pp 229533.I1RHM3
39605 229533.A0A1C3YKN4 pp 229533.I1S065
39606 229533.A0A1C3YKN4 pp 229533.I1RUI6
39607 229533.A0A1C3YKN4 pp 229533.I1RH82
39608 229533.A0A1C3YKN4 pp 229533.I1RI11
39609 229533.A0A1C3YKN4 pp 229533.I1RHZ5
39610 229533.A0A1C3YKN4 pp 229533.V6RXR0
39611 229533.A0A1C3YKN4 pp 229533.I1RJS0
39612 229533.A0A1C3YKN4 pp 229533.I1RMY1
39613 229533.A0A1C3YKN4 pp 229533.I1RH30
39614 229533.A0A1C3YKN4 pp 229533.I1RAG3
39615 229533.A0A1C3YKN4 pp 229533.I1RVZ3
39616 229533.A0A1C3YKN4 pp 229533.A0A1C3YM35
39617 229533.A0A1C3YKN4 pp 229533.I1RWM4
39618 229533.A0A1C3YKN4 pp 229533.I1RC48
39619 229533.A0A1C3YKN4 pp 229533.I1RHA8
39620 229533.A0A1C3YKN4 pp 229533.I1RFI4
39621 229533.A0A1C3YKN4 pp 229533.I1RDV8
39622 229533.A0A1C3YKN4 pp 229533.I1S423
39623 229533.A0A1C3YKN4 pp 229533.I1RXH2
39624 229533.A0A1C3YKN4 pp 229533.I1RWT6
39625 229533.A0A1C3YKN4 pp 229533.I1RW10
39626 229533.A0A1C3YKN4 pp 229533.I1RFM6
39627 229533.A0A1C3YKN4 pp 229533.I1RBI1
39628 229533.A0A1C3YKN4 pp 229533.I1RWD2
39629 229533.A0A1C3YKN4 pp 229533.I1S6A2
39630 229533.A0A1C3YKN4 pp 229533.I1RRM1
39631 229533.A0A1C3YKN4 pp 229533.I1RI40
39632 229533.A0A1C3YKN4 pp 229533.V6RK96
39633 229533.A0A1C3YKN4 pp 229533.I1RPM6
39634 229533.A0A1C3YKN4 pp 229533.I1S5J9
39635 229533.A0A1C3YKN4 pp 229533.I1RA94
39636 229533.A0A1C3YKN4 pp 229533.V6RPS5
39637 229533.A0A1C3YKN4 pp 229533.I1S267
39638 229533.A0A1C3YKN4 pp 229533.V6RDH3
39639 229533.A0A1C3YKN4 pp 229533.I1RKE1

39640 229533.A0A1C3YKN4 pp 229533.I1RSG0
39641 229533.A0A1C3YKN4 pp 229533.A0A1C3YKX4
39642 229533.A0A1C3YKN4 pp 229533.I1RC61
39643 229533.A0A1C3YKN4 pp 229533.I1RFD4
39644 229533.A0A1C3YKN4 pp 229533.I1S8H9
39645 229533.A0A1C3YKN4 pp 229533.I1RXS0
39646 229533.A0A1C3YKN4 pp 229533.I1S3Y8
39647 229533.A0A1C3YKN4 pp 229533.I1RW69
39648 229533.A0A1C3YKN4 pp 229533.I1RAQ4
39649 229533.A0A1C3YKN4 pp 229533.I1RCM5
39650 229533.A0A1C3YKN4 pp 229533.I1RQA3
39651 229533.A0A1C3YKN4 pp 229533.V6R3V4
39652 229533.A0A1C3YKN4 pp 229533.Q4HYR2
39653 229533.A0A1C3YKN4 pp 229533.I1RYL8
39654 229533.A0A1C3YKN4 pp 229533.I1S241
39655 229533.A0A1C3YKN4 pp 229533.I1RNH4
39656 229533.A0A1C3YKN4 pp 229533.V6QU65
39657 229533.A0A1C3YKN4 pp 229533.V6RN16
39658 229533.A0A1C3YKN4 pp 229533.I1S0K0
39659 229533.A0A1C3YKN4 pp 229533.I1R9M7
39660 229533.A0A1C3YKN4 pp 229533.I1RZM4
39661 229533.A0A1C3YKN4 pp 229533.I1RY16
39662 229533.A0A1C3YKN4 pp 229533.V6QVH2
39663 229533.A0A1C3YKN4 pp 229533.V6RH92
39664 229533.A0A1C3YKN4 pp 229533.V6RTJ8
39665 229533.A0A1C3YKN4 pp 229533.I1RJR8
39666 229533.A0A1C3YKN4 pp 229533.A0A1C3YN32
39667 229533.A0A1C3YKN4 pp 229533.V6QU99
39668 229533.A0A1C3YKN4 pp 229533.I1RF16
39669 229533.A0A1C3YKN4 pp 229533.I1S3L2
39670 229533.A0A1C3YKN4 pp 229533.I1RD42
39671 229533.A0A1C3YKN4 pp 229533.V6RAM6
39672 229533.A0A1C3YKN4 pp 229533.A0A1C3YLK1
39673 229533.A0A1C3YKN4 pp 229533.A0A1C3YN26
39674 229533.A0A1C3YKN4 pp 229533.I1RDU5
39675 229533.A0A1C3YKN4 pp 229533.I1RKA9
39676 229533.A0A1C3YKN4 pp 229533.I1RR12
39677 229533.A0A1C3YKN4 pp 229533.V6RXE7
39678 229533.A0A1C3YKN4 pp 229533.I1RME3
39679 229533.A0A1C3YKN4 pp 229533.I1R9B1
39680 229533.A0A1C3YKN4 pp 229533.I1RYM6
39681 229533.A0A1C3YKN4 pp 229533.I1RMM8
39682 229533.A0A1C3YKN4 pp 229533.I1RC39
39683 229533.A0A1C3YKN4 pp 229533.I1RQE8
39684 229533.A0A1C3YKN4 pp 229533.I1RIJ2
39685 229533.A0A1C3YKN4 pp 229533.I1RIL0
39686 229533.A0A1C3YKN4 pp 229533.I1RBT7
39687 229533.A0A1C3YKN4 pp 229533.V6R1D2
39688 229533.A0A1C3YKN4 pp 229533.I1RH32
39689 229533.A0A1C3YKN4 pp 229533.I1RAR9
39690 229533.A0A1C3YKN4 pp 229533.I1S4S5
39691 229533.A0A1C3YKN4 pp 229533.I1RFQ4
39692 229533.A0A1C3YKN4 pp 229533.I1RP47
39693 229533.A0A1C3YKN4 pp 229533.I1RY49
39694 229533.A0A1C3YKN4 pp 229533.I1RQM4
39695 229533.A0A1C3YKN4 pp 229533.I1S3K8
39696 229533.A0A1C3YKN4 pp 229533.V6R0W1
39697 229533.A0A1C3YKN4 pp 229533.I1RJC5
39698 229533.A0A1C3YKN4 pp 229533.I1RKZ8
39699 229533.A0A1C3YKN4 pp 229533.I1RZ16
39700 229533.A0A1C3YKN4 pp 229533.I1SAI4
39701 229533.A0A1C3YKN4 pp 229533.I1RJG4
39702 229533.A0A1C3YKN4 pp 229533.I1RM66
39703 229533.A0A1C3YKN4 pp 229533.A0A1C3YN98
39704 229533.A0A1C3YKN4 pp 229533.I1S1W8
39705 229533.A0A1C3YKN4 pp 229533.I1RTG4
39706 229533.A0A1C3YKN4 pp 229533.I1RQP2
39707 229533.A0A1C3YKN4 pp 229533.I1RHL9
39708 229533.A0A1C3YKN4 pp 229533.I1RJS7
39709 229533.I1RUI2 pp 229533.Q4HYR2
39710 229533.I1RUI2 pp 229533.I1RZP8
39711 229533.I1RUP2 pp 229533.I1RYM3
39712 229533.I1RUP2 pp 229533.I1RVR0

229533.I1RUP2 pp 229533.I1RWK4
229533.I1RUP2 pp 229533.I1S3K6
229533.I1RUP2 pp 229533.I1RWP4
229533.I1RUP2 pp 229533.I1S334
229533.I1RUP2 pp 229533.I1S4N7
229533.I1RUP2 pp 229533.I1RZQ1
229533.I1RUP2 pp 229533.I1S3H8
229533.I1RUP2 pp 229533.I1RWG1
229533.I1RUP2 pp 229533.Q4HXF0
229533.I1RUP2 pp 229533.V6RRM0
229533.I1RUP2 pp 229533.I1S4N8
229533.I1RUP2 pp 229533.I1RZN1
229533.A0A0E0RP92 pp 229533.I1RQ26
229533.A0A0E0RP92 pp 229533.I1RFZ3
229533.A0A0E0RP92 pp 229533.I1RJH1
229533.A0A0E0RP92 pp 229533.I1RC97
229533.A0A0E0RP92 pp 229533.I1S2G7
229533.A0A0E0RP92 pp 229533.I1RBZ9
229533.A0A0E0RP92 pp 229533.Q4IJM4
229533.A0A0E0RP92 pp 229533.I1S2E9
229533.A0A0E0RP92 pp 229533.I1R9Y9
229533.A0A0E0RP92 pp 229533.I1RAM7
229533.A0A0E0RP92 pp 229533.I1S1W4
229533.A0A0E0RP92 pp 229533.I1RRN5
229533.A0A0E0RP92 pp 229533.I1RDZ4
229533.A0A0E0RP92 pp 229533.I1RNY9
229533.A0A0E0RP92 pp 229533.A0A0E0RVZ1
229533.A0A0E0RP92 pp 229533.I1RV91
229533.A0A0E0RP92 pp 229533.I1RQA3
229533.A0A0E0RP92 pp 229533.A0A1C3YLP4
229533.A0A0E0RP92 pp 229533.I1RZE4
229533.A0A0E0RP92 pp 229533.I1RWT6
229533.A0A0E0RP92 pp 229533.I1RYI9
229533.A0A0E0RP92 pp 229533.V6RKY7
229533.A0A0E0RP92 pp 229533.I1RCL8
229533.A0A0E0RP92 pp 229533.I1RB75
229533.A0A0E0RP92 pp 229533.I1S439
229533.A0A0E0RP92 pp 229533.I1RY44
229533.A0A0E0RP92 pp 229533.V6RVG3
229533.A0A0E0RP92 pp 229533.I1RB41
229533.A0A0E0RP92 pp 229533.I1RN08
229533.A0A0E0RP92 pp 229533.I1RUQ5
229533.A0A0E0RP92 pp 229533.A0A1C3YI28
229533.A0A0E0RP92 pp 229533.I1S0M2
229533.A0A0E0RP92 pp 229533.I1RKK0
229533.A0A0E0RP92 pp 229533.I1RC22
229533.A0A0E0RP92 pp 229533.I1RYY5
229533.A0A0E0RP92 pp 229533.I1RMA8
229533.A0A0E0RP92 pp 229533.I1RC17
229533.A0A0E0RP92 pp 229533.V6RDP1
229533.A0A0E0RP92 pp 229533.I1RI71
229533.A0A0E0RP92 pp 229533.I1RMM9
229533.A0A0E0RP92 pp 229533.I1RC95
229533.A0A0E0RP92 pp 229533.I1RCU8
229533.A0A0E0RP92 pp 229533.I1RJ88
229533.A0A0E0RP92 pp 229533.A0A1C3YIN6
229533.A0A0E0RP92 pp 229533.I1RRY3
229533.A0A0E0RP92 pp 229533.I1S0D1
229533.A0A0E0RP92 pp 229533.I1RUN8
229533.A0A0E0RP92 pp 229533.I1RST8
229533.A0A0E0RP92 pp 229533.I1RA43
229533.A0A0E0RP92 pp 229533.A0A0E0SD93
229533.A0A0E0RP92 pp 229533.A0A0E0SAC4
229533.A0A0E0RP92 pp 229533.I1RYH9
229533.A0A0E0RP92 pp 229533.I1RDB1
229533.A0A0E0RP92 pp 229533.I1RX70
229533.A0A0E0RP92 pp 229533.I1RZ63
229533.A0A0E0RP92 pp 229533.A0A0E0SA11
229533.A0A0E0RP92 pp 229533.I1RAN2
229533.A0A0E0RP92 pp 229533.I1RCU9
229533.A0A0E0RP92 pp 229533.I1RJR8
229533.A0A0E0RP92 pp 229533.A0A1C3YJH8
229533.A0A0E0RP92 pp 229533.A0A1C3YKF2

39786 229533.A0A0E0RP92 pp 229533.I1RNP7
39787 229533.A0A0E0RP92 pp 229533.A0A1C3YK17
39788 229533.A0A0E0RP92 pp 229533.A0A1C3YIL0
39789 229533.A0A0E0RP92 pp 229533.I1RB74
39790 229533.I1RAE3 pp 229533.I1RPS0
39791 229533.I1RAE3 pp 229533.I1RAU7
39792 229533.I1RAE3 pp 229533.I1S0R8
39793 229533.I1RAE3 pp 229533.I1RYH7
39794 229533.I1RAE3 pp 229533.I1S0Q7
39795 229533.I1RAE3 pp 229533.I1RKB7
39796 229533.I1RAE3 pp 229533.Q4HZB8
39797 229533.I1RAE3 pp 229533.I1RPA7
39798 229533.I1RAE3 pp 229533.I1RFT6
39799 229533.I1RAE3 pp 229533.I1RRX5
39800 229533.I1RAE3 pp 229533.I1RAW3
39801 229533.I1RAE3 pp 229533.I1RJU5
39802 229533.I1RAE3 pp 229533.I1S0L8
39803 229533.I1RAE3 pp 229533.I1RPK9
39804 229533.I1RAE3 pp 229533.I1RN72
39805 229533.I1RAE3 pp 229533.I1RPR9
39806 229533.I1RAE3 pp 229533.I1RFJ5
39807 229533.I1RAE3 pp 229533.V6R3J1
39808 229533.I1RAE3 pp 229533.I1S1V3
39809 229533.I1RAE3 pp 229533.I1REA6
39810 229533.I1RAE3 pp 229533.I1RD69
39811 229533.I1RMP0 pp 229533.I1RQS4
39812 229533.I1RMP0 pp 229533.Q4IBX0
39813 229533.I1RMP0 pp 229533.I1SA77
39814 229533.I1RMP0 pp 229533.I1RY58
39815 229533.I1RMP0 pp 229533.I1RQA3
39816 229533.I1RMP0 pp 229533.I1RZT1
39817 229533.I1RMP0 pp 229533.Q4I0J6
39818 229533.I1RMP0 pp 229533.I1RRN5
39819 229533.I1RMP0 pp 229533.I1RYH0
39820 229533.I1RMP0 pp 229533.I1SAT1
39821 229533.I1RMP0 pp 229533.I1S1D3
39822 229533.I1RMP0 pp 229533.I1RY44
39823 229533.I1RMP0 pp 229533.I1S016
39824 229533.I1RMP0 pp 229533.I1RN31
39825 229533.I1RMP0 pp 229533.I1S224
39826 229533.I1RMP0 pp 229533.I1RWT6
39827 229533.I1RMP0 pp 229533.I1RRW0
39828 229533.I1RMP0 pp 229533.I1S749
39829 229533.I1RMP0 pp 229533.I1S0D1
39830 229533.I1RMP0 pp 229533.I1RMW3
39831 229533.I1RMP0 pp 229533.I1RNP9
39832 229533.I1RMP0 pp 229533.I1S4N7
39833 229533.I1RMP0 pp 229533.I1S549
39834 229533.I1RMP0 pp 229533.I1S4N8
39835 229533.I1RMP0 pp 229533.I1RW16
39836 229533.I1RMP0 pp 229533.I1S2M1
39837 229533.I1RMP0 pp 229533.I1RW83
39838 229533.I1RMP0 pp 229533.I1RSJ4
39839 229533.I1RMP0 pp 229533.I1S1V5
39840 229533.I1RMP0 pp 229533.I1RV91
39841 229533.I1RMP0 pp 229533.V6R3J1
39842 229533.I1RMP0 pp 229533.I1S7X9
39843 229533.I1RMP0 pp 229533.I1S2E9
39844 229533.I1RMP0 pp 229533.I1RT81
39845 229533.I1RSV4 pp 229533.I1RYM1
39846 229533.I1RSV4 pp 229533.I1S2B0
39847 229533.I1RSV4 pp 229533.V6RPQ8
39848 229533.I1RSV4 pp 229533.Q4IJT0
39849 229533.I1RSV4 pp 229533.V6RG22
39850 229533.I1RSV4 pp 229533.I1S272
39851 229533.I1RK23 pp 229533.I1RU69
39852 229533.I1RK23 pp 229533.I1RST1
39853 229533.I1RK23 pp 229533.I1RX47
39854 229533.I1RK23 pp 229533.I1RT17
39855 229533.I1RK23 pp 229533.Q4IBK5
39856 229533.I1RK23 pp 229533.Q4IRI9
39857 229533.I1RK23 pp 229533.I1RK24
39858 229533.I1RK23 pp 229533.I1RPL3

39859 229533.I1RK23 pp 229533.I1RPZ3
39860 229533.I1RK23 pp 229533.I1S246
39861 229533.I1RK23 pp 229533.I1RM25
39862 229533.I1RYM3 pp 229533.I1S9V9
39863 229533.I1RYM3 pp 229533.I1RYX5
39864 229533.I1RYM3 pp 229533.I1S9G7
39865 229533.I1RYM3 pp 229533.I1S0L8
39866 229533.I1RYM3 pp 229533.I1S2B9
39867 229533.I1RYM3 pp 229533.I1S1Y9
39868 229533.A0A098DMX4 pp 229533.I1S2E9
39869 229533.A0A098DMX4 pp 229533.I1RV91
39870 229533.A0A098DMX4 pp 229533.I1RFC0
39871 229533.A0A098DMX4 pp 229533.A0A1C3YJW1
39872 229533.A0A098DMX4 pp 229533.I1RQA3
39873 229533.A0A098DMX4 pp 229533.A0A1C3YHP8
39874 229533.A0A098DMX4 pp 229533.I1RC17
39875 229533.A0A098DMX4 pp 229533.I1RWT6
39876 229533.A0A098DMX4 pp 229533.I1RY44
39877 229533.A0A098DMX4 pp 229533.A0A0E0SA11
39878 229533.I1RT93 pp 229533.I1RZS5
39879 229533.I1RT93 pp 229533.I1RZP8
39880 229533.I1RT93 pp 229533.I1S540
39881 229533.I1RT93 pp 229533.I1RYH4
39882 229533.I1RT93 pp 229533.I1RWJ3
39883 229533.I1RDK3 pp 229533.I1RWL9
39884 229533.I1RDK3 pp 229533.I1RW76
39885 229533.I1RDK3 pp 229533.I1RW84
39886 229533.I1RDK3 pp 229533.I1S0R9
39887 229533.I1RDK3 pp 229533.I1S2C1
39888 229533.I1RDK3 pp 229533.I1RY57
39889 229533.I1RQG7 pp 229533.I1S334
39890 229533.I1RQG7 pp 229533.I1RZQ1
39891 229533.I1RQG7 pp 229533.I1RTA6
39892 229533.I1RQG7 pp 229533.I1S3K6
39893 229533.I1RQG7 pp 229533.I1S4N8
39894 229533.I1RQG7 pp 229533.V6RRM0
39895 229533.I1RQG7 pp 229533.I1RVR0
39896 229533.I1RQG7 pp 229533.I1RRL5
39897 229533.I1RQG7 pp 229533.I1RZN1
39898 229533.I1RQG7 pp 229533.I1RWP4
39899 229533.I1RQG7 pp 229533.Q4HXF0
39900 229533.I1RQG7 pp 229533.I1S3H8
39901 229533.I1RQG7 pp 229533.I1S4N7
39902 229533.I1RQG7 pp 229533.I1RSL5
39903 229533.I1RQG7 pp 229533.I1RSQ4
39904 229533.I1RQG7 pp 229533.I1RWK4
39905 229533.I1RQG7 pp 229533.I1RUE2
39906 229533.I1RV79 pp 229533.I1S7S9
39907 229533.I1RYS9 pp 229533.V6QV09
39908 229533.I1S0A6 pp 229533.I1S2E9
39909 229533.I1S0A6 pp 229533.I1S3G4
39910 229533.I1S0A6 pp 229533.I1S4P6
39911 229533.I1S0A6 pp 229533.I1S1W8
39912 229533.I1RTB2 pp 229533.I1RY44
39913 229533.I1RTB2 pp 229533.I1S0D5
39914 229533.I1RTB2 pp 229533.I1RY58
39915 229533.I1RTB2 pp 229533.Q4IRI9
39916 229533.I1RTB2 pp 229533.I1RV91
39917 229533.I1RTB2 pp 229533.Q4ICA8
39918 229533.I1RTB2 pp 229533.I1S224
39919 229533.I1RTB2 pp 229533.I1S2E9
39920 229533.I1RTB2 pp 229533.I1RWT6
39921 229533.I1S9T1 pp 229533.Q4IBU4
39922 229533.I1S9T1 pp 229533.I1S9T2
39923 229533.I1RB15 pp 229533.I1RC46
39924 229533.I1RB15 pp 229533.I1S7X9
39925 229533.I1RB15 pp 229533.I1RD10
39926 229533.I1RB15 pp 229533.V6RB81
39927 229533.I1RB15 pp 229533.I1RPS0
39928 229533.I1RB15 pp 229533.I1S0L8
39929 229533.I1RB15 pp 229533.I1RQM0
39930 229533.I1RB15 pp 229533.I1S0Z7
39931 229533.I1RB15 pp 229533.I1RPK5

39932 229533.I1RB15 pp 229533.I1RCK9
39933 229533.I1RB15 pp 229533.I1RN36
39934 229533.I1RB15 pp 229533.I1RX09
39935 229533.I1RB15 pp 229533.I1S2B7
39936 229533.I1RB15 pp 229533.I1RN72
39937 229533.I1RB15 pp 229533.I1RD69
39938 229533.I1RB15 pp 229533.I1RXD6
39939 229533.I1RB15 pp 229533.I1RNG8
39940 229533.A0A1C3YIQ2 pp 229533.I1RRX5
39941 229533.A0A1C3YIQ2 pp 229533.I1RA49
39942 229533.A0A1C3YIQ2 pp 229533.I1S0L8
39943 229533.A0A1C3YIQ2 pp 229533.A0A1C3YM82
39944 229533.A0A1C3YIQ2 pp 229533.I1S1V3
39945 229533.A0A1C3YIQ2 pp 229533.I1RD69
39946 229533.A0A1C3YIQ2 pp 229533.I1RJU5
39947 229533.A0A1C3YIQ2 pp 229533.A0A1C3YM60
39948 229533.A0A1C3YIQ2 pp 229533.I1RPA7
39949 229533.A0A1C3YIQ2 pp 229533.I1RFT6
39950 229533.A0A1C3YIQ2 pp 229533.Q4HZB8
39951 229533.A0A1C3YIQ2 pp 229533.I1RN72
39952 229533.A0A1C3YIQ2 pp 229533.I1RPK9
39953 229533.A0A1C3YIQ2 pp 229533.I1REA6
39954 229533.A0A1C3YIQ2 pp 229533.I1RPS0
39955 229533.A0A1C3YIQ2 pp 229533.I1RAU7
39956 229533.A0A1C3YIQ2 pp 229533.I1RFJ5
39957 229533.A0A1C3YIQ2 pp 229533.A0A1C3YKY6
39958 229533.V6RUY9 pp 229533.V6RVG3
39959 229533.I1RDS8 pp 229533.I1RZ25
39960 229533.I1RBL8 pp 229533.I1RH68
39961 229533.I1RBL8 pp 229533.I1RWW4
39962 229533.I1RBL8 pp 229533.I1RTB7
39963 229533.I1RBL8 pp 229533.I1RQL4
39964 229533.I1RBL8 pp 229533.I1RQA5
39965 229533.I1RBL8 pp 229533.I1S0S6
39966 229533.I1RBL8 pp 229533.I1RYL5
39967 229533.I1RBL8 pp 229533.V6REE9
39968 229533.I1RBL8 pp 229533.I1RRX1
39969 229533.I1RBL8 pp 229533.P0C431
39970 229533.I1RBL8 pp 229533.I1RCD4
39971 229533.I1RBL8 pp 229533.I1RVY0
39972 229533.I1RBL8 pp 229533.I1RNF4
39973 229533.I1RBL8 pp 229533.I1REC6
39974 229533.I1RBL8 pp 229533.I1RSQ1
39975 229533.I1RBL8 pp 229533.I1RNG4
39976 229533.I1RBL8 pp 229533.I1RQN9
39977 229533.I1RBL8 pp 229533.I1S0L8
39978 229533.I1RBL8 pp 229533.I1RVA7
39979 229533.I1RBL8 pp 229533.I1RTE6
39980 229533.I1RBL8 pp 229533.I1RQ72
39981 229533.I1RBL8 pp 229533.I1S247
39982 229533.I1RBL8 pp 229533.I1RPS0
39983 229533.I1RBL8 pp 229533.I1RME4
39984 229533.I1RBL8 pp 229533.I1RT36
39985 229533.I1RBL8 pp 229533.I1RWX5
39986 229533.I1RBL8 pp 229533.I1REC7
39987 229533.I1RBL8 pp 229533.I1RWH4
39988 229533.I1RWN8 pp 229533.V6R0U9
39989 229533.I1RWN8 pp 229533.I1RZX4
39990 229533.I1S757 pp 229533.V6REE9
39991 229533.I1RAE4 pp 229533.I1RPK9
39992 229533.I1RAE4 pp 229533.I1RN72
39993 229533.I1RAE4 pp 229533.I1S1V3
39994 229533.I1RAE4 pp 229533.I1RKB7
39995 229533.I1RAE4 pp 229533.I1RD69
39996 229533.I1RAE4 pp 229533.I1S0Q7
39997 229533.I1RAE4 pp 229533.I1REA6
39998 229533.I1RAE4 pp 229533.Q4HZB8
39999 229533.I1RAE4 pp 229533.I1RMV0
40000 229533.I1RAE4 pp 229533.I1RPR9
40001 229533.I1RAE4 pp 229533.I1RWB0
40002 229533.I1RAE4 pp 229533.I1RPS0
40003 229533.I1RAE4 pp 229533.I1RAU7
40004 229533.I1RAE4 pp 229533.I1RFJ5

40005 229533.I1RAE4 pp 229533.I1RLP1
40006 229533.I1RAE4 pp 229533.I1RYH7
40007 229533.I1RAE4 pp 229533.I1RRX5
40008 229533.I1RAE4 pp 229533.I1S0R8
40009 229533.I1RAE4 pp 229533.I1RPA7
40010 229533.I1RAE4 pp 229533.I1RBA6
40011 229533.I1RAE4 pp 229533.I1S0L8
40012 229533.I1RAE4 pp 229533.I1RFT6
40013 229533.I1RAE4 pp 229533.I1RJU5
40014 229533.I1RAE4 pp 229533.I1RAW3
40015 229533.I1RAE4 pp 229533.V6R3J1
40016 229533.I1RZP6 pp 229533.I1S4N7
40017 229533.I1RZP6 pp 229533.I1S4N8
40018 229533.I1RZP6 pp 229533.V6R0R5
40019 229533.I1RZP6 pp 229533.I1S0L8
40020 229533.I1RZP6 pp 229533.I1S1V1
40021 229533.I1RZP6 pp 229533.V6R645
40022 229533.I1RZP6 pp 229533.I1S0S6
40023 229533.I1RZP6 pp 229533.V6R7A6
40024 229533.I1RZP6 pp 229533.I1RZP7
40025 229533.I1RZP6 pp 229533.I1S1V3
40026 229533.I1RZP6 pp 229533.I1RZR8
40027 229533.I1RZP6 pp 229533.V6R9L0
40028 229533.I1RZP6 pp 229533.Q4IQX3
40029 229533.I1RZP6 pp 229533.V6RHD3
40030 229533.I1RKE7 pp 229533.I1RRS8
40031 229533.I1RKE7 pp 229533.I1RWH5
40032 229533.I1RKE7 pp 229533.I1RZV6
40033 229533.I1RKE7 pp 229533.I1RN48
40034 229533.I1RKE7 pp 229533.I1RQN8
40035 229533.I1RKE7 pp 229533.I1RYB9
40036 229533.I1RKE7 pp 229533.I1S7S4
40037 229533.I1RKE7 pp 229533.I1RXB5
40038 229533.A0A1C3YK15 pp 229533.I1RFN1
40039 229533.A0A1C3YK15 pp 229533.I1RC72
40040 229533.A0A1C3YK15 pp 229533.I1RCL3
40041 229533.A0A1C3YK15 pp 229533.I1RPU0
40042 229533.A0A1C3YKT6 pp 229533.I1RJZ7
40043 229533.A0A1C3YKT6 pp 229533.I1RAM1
40044 229533.A0A1C3YKT6 pp 229533.I1S0J7
40045 229533.A0A1C3YKT6 pp 229533.I1RV91
40046 229533.A0A1C3YKT6 pp 229533.I1RBP3
40047 229533.A0A1C3YKT6 pp 229533.I1RWT6
40048 229533.A0A1C3YKT6 pp 229533.I1S2E9
40049 229533.A0A1C3YKT6 pp 229533.I1RQA3
40050 229533.A0A1C3YKT6 pp 229533.I1RW71
40051 229533.A0A1C3YKT6 pp 229533.I1RC91
40052 229533.A0A1C3YKT6 pp 229533.I1RB41
40053 229533.A0A1C3YKT6 pp 229533.A0A1C3YM29
40054 229533.A0A1C3YKT6 pp 229533.I1RZ63
40055 229533.A0A1C3YKT6 pp 229533.I1RY44
40056 229533.A0A1C3YN09 pp 229533.I1RRJ7
40057 229533.A0A1C3YN09 pp 229533.I1RMW6
40058 229533.A0A1C3YN09 pp 229533.I1RWN8
40059 229533.A0A1C3YN09 pp 229533.I1RE89
40060 229533.I1RZG0 pp 229533.Q4ICG8
40061 229533.I1RZG0 pp 229533.Q4I0M9
40062 229533.I1RZG0 pp 229533.I1RZH4
40063 229533.I1RZG0 pp 229533.Q4HZB8
40064 229533.I1RZG0 pp 229533.Q4INW6
40065 229533.I1RZG0 pp 229533.I1S0L8
40066 229533.I1S282 pp 229533.V6REE9
40067 229533.I1S282 pp 229533.P0C431
40068 229533.I1S1B6 pp 229533.Q4IPI1
40069 229533.I1S1B6 pp 229533.V6QYF0
40070 229533.I1S1B6 pp 229533.Q4IPC8
40071 229533.I1S1B6 pp 229533.I1S9U2
40072 229533.I1S1B6 pp 229533.I1S9U1
40073 229533.I1S1B6 pp 229533.P0C431
40074 229533.I1S1B6 pp 229533.V6RCG5
40075 229533.I1S1B6 pp 229533.Q4IEK8
40076 229533.I1S1B6 pp 229533.V6RGN5
40077 229533.I1S1B6 pp 229533.I1S2M6

40078 229533.I1S1B6 pp 229533.I1S1X9
40079 229533.I1S1B6 pp 229533.V6R227
40080 229533.I1S1B6 pp 229533.I1S7X9
40081 229533.A0A1C3YM54 pp 229533.I1REI0
40082 229533.A0A1C3YM54 pp 229533.I1S334
40083 229533.A0A1C3YM54 pp 229533.I1RTA6
40084 229533.A0A1C3YM54 pp 229533.I1RN72
40085 229533.A0A1C3YM54 pp 229533.I1RQ44
40086 229533.A0A1C3YM54 pp 229533.I1RPA7
40087 229533.A0A1C3YM54 pp 229533.I1RFK7
40088 229533.A0A1C3YM54 pp 229533.I1RWG1
40089 229533.A0A1C3YM54 pp 229533.I1RCU2
40090 229533.A0A1C3YM54 pp 229533.I1RRL5
40091 229533.A0A1C3YM54 pp 229533.I1S3K6
40092 229533.A0A1C3YM54 pp 229533.I1RFV2
40093 229533.A0A1C3YM54 pp 229533.I1RM30
40094 229533.A0A1C3YM54 pp 229533.I1RYM3
40095 229533.A0A1C3YM54 pp 229533.I1RNG8
40096 229533.A0A1C3YM54 pp 229533.I1RVR0
40097 229533.A0A1C3YM54 pp 229533.I1RZP6
40098 229533.A0A1C3YM54 pp 229533.I1REC6
40099 229533.A0A1C3YM54 pp 229533.I1RK47
40100 229533.A0A1C3YM54 pp 229533.I1RWP4
40101 229533.A0A1C3YM54 pp 229533.I1RSQ4
40102 229533.A0A1C3YM54 pp 229533.I1RWK4
40103 229533.A0A1C3YM54 pp 229533.I1RZN1
40104 229533.A0A1C3YM54 pp 229533.I1RC61
40105 229533.A0A1C3YM54 pp 229533.I1RTG9
40106 229533.A0A1C3YM54 pp 229533.I1S4N7
40107 229533.A0A1C3YM54 pp 229533.I1R9U7
40108 229533.A0A1C3YM54 pp 229533.I1RZP7
40109 229533.A0A1C3YM54 pp 229533.I1RSL5
40110 229533.A0A1C3YM54 pp 229533.I1RP69
40111 229533.A0A1C3YM54 pp 229533.A0A1C3YM82
40112 229533.A0A1C3YM54 pp 229533.I1RPS0
40113 229533.A0A1C3YM54 pp 229533.I1RNG4
40114 229533.A0A1C3YM54 pp 229533.I1S4N8
40115 229533.A0A1C3YM54 pp 229533.I1RUE2
40116 229533.A0A1C3YM54 pp 229533.I1S3H8
40117 229533.A0A1C3YM54 pp 229533.I1RZQ1
40118 229533.A0A1C3YM54 pp 229533.I1RMV6
40119 229533.A0A1C3YM54 pp 229533.A0A1C3YME0
40120 229533.A0A1C3YM54 pp 229533.I1REC7
40121 229533.A0A1C3YM54 pp 229533.Q4HXF0
40122 229533.A0A1C3YM54 pp 229533.V6RRM0
40123 229533.A0A1C3YM54 pp 229533.I1RF85
40124 229533.I1RFL3 pp 229533.I1RN67
40125 229533.I1RFL3 pp 229533.Q4HXF0
40126 229533.I1RFL3 pp 229533.I1RXD7
40127 229533.I1RFL3 pp 229533.I1RT33
40128 229533.I1RFL3 pp 229533.Q4IEV5
40129 229533.I1RFL3 pp 229533.I1S4T6
40130 229533.I1RFL3 pp 229533.I1RPP3
40131 229533.I1RFL3 pp 229533.I1RFT1
40132 229533.I1RFL3 pp 229533.I1S9Y1
40133 229533.I1RFL3 pp 229533.I1RSQ7
40134 229533.I1RFL3 pp 229533.I1S4S4
40135 229533.I1RFL3 pp 229533.V6QUE6
40136 229533.I1RFL3 pp 229533.I1RRT8
40137 229533.I1RFL3 pp 229533.O74638
40138 229533.I1RFL3 pp 229533.I1RX46
40139 229533.I1RFL3 pp 229533.I1S5B0
40140 229533.I1RFL3 pp 229533.I1S5R1
40141 229533.I1RFL3 pp 229533.I1RG11
40142 229533.I1RFL3 pp 229533.I1RGA4
40143 229533.I1RFL3 pp 229533.I1RM67
40144 229533.I1RFL3 pp 229533.I1RT40
40145 229533.I1RFL3 pp 229533.I1RUG3
40146 229533.I1RFL3 pp 229533.I1RSQ4
40147 229533.I1RFL3 pp 229533.I1RNC1
40148 229533.I1RFL3 pp 229533.I1S6E5
40149 229533.I1RFL3 pp 229533.I1S2H4
40150 229533.I1RFL3 pp 229533.I1RXF0

40151	229533.I1RFL3	pp	229533.I1RYB9
40152	229533.I1RFL3	pp	229533.I1RZ39
40153	229533.I1RFL3	pp	229533.I1RZZ9
40154	229533.I1RFL3	pp	229533.Q4IJK2
40155	229533.I1RFL3	pp	229533.I1RFL4
40156	229533.I1RFL3	pp	229533.I1RSZ7
40157	229533.I1RFL3	pp	229533.V6RCZ8
40158	229533.I1RFL3	pp	229533.I1RQE7
40159	229533.I1RFL3	pp	229533.V6R042
40160	229533.I1RFL3	pp	229533.I1RL04
40161	229533.I1RFL3	pp	229533.I1RFS3
40162	229533.I1RFL3	pp	229533.I1RVX0
40163	229533.I1RFL3	pp	229533.I1S0J1
40164	229533.I1RFL3	pp	229533.I1RQ28
40165	229533.I1RFL3	pp	229533.I1RSQ3
40166	229533.I1RFL3	pp	229533.I1RSS4
40167	229533.I1RFL3	pp	229533.I1RS18
40168	229533.I1RFL3	pp	229533.I1RJV3
40169	229533.I1RFL3	pp	229533.I1RK95
40170	229533.I1RFL3	pp	229533.I1RRI2
40171	229533.I1RFL3	pp	229533.I1RYW3
40172	229533.I1RFL3	pp	229533.I1RK83
40173	229533.I1RN72	pp	229533.I1RS90
40174	229533.I1RN72	pp	229533.I1RRZ3
40175	229533.I1RN72	pp	229533.I1RPA7
40176	229533.I1RN72	pp	229533.I1S834
40177	229533.I1RN72	pp	229533.I1RZG0
40178	229533.I1RN72	pp	229533.I1RSB0
40179	229533.I1RN72	pp	229533.I1S9V9
40180	229533.I1RN72	pp	229533.I1S1Y9
40181	229533.I1RN72	pp	229533.I1RTG9
40182	229533.I1RN72	pp	229533.I1RZD9
40183	229533.I1RN72	pp	229533.I1RZP7
40184	229533.I1RN72	pp	229533.I1RTA6
40185	229533.I1RN72	pp	229533.I1RUP2
40186	229533.I1RN72	pp	229533.I1RZV4
40187	229533.I1RN72	pp	229533.I1RZP6
40188	229533.I1RN72	pp	229533.I1S095
40189	229533.I1RN72	pp	229533.I1RYH6
40190	229533.I1RN72	pp	229533.I1RWG1
40191	229533.I1RN72	pp	229533.I1RYM3
40192	229533.I1RN72	pp	229533.I1S0X5
40193	229533.I1RN72	pp	229533.I1RY40
40194	229533.I1RN72	pp	229533.I1RQG7
40195	229533.I1RN72	pp	229533.I1RQ49
40196	229533.I1RN72	pp	229533.I1RP88
40197	229533.I1RN72	pp	229533.I1S0B0
40198	229533.I1RN72	pp	229533.I1RSM2
40199	229533.I1RN72	pp	229533.I1RPS0
40200	229533.I1RN72	pp	229533.I1RNG8
40201	229533.I1RN72	pp	229533.I1RWC9
40202	229533.I1RN72	pp	229533.I1S9G7
40203	229533.I1RN72	pp	229533.I1S0L8
40204	229533.I1RN72	pp	229533.I1S2B9
40205	229533.I1RN72	pp	229533.I1RPN4
40206	229533.I1RWB0	pp	229533.I1S1F3
40207	229533.I1RWB0	pp	229533.I1RY92
40208	229533.I1RWB0	pp	229533.I1RZ39
40209	229533.I1RWB0	pp	229533.I1RZZ9
40210	229533.I1RWB0	pp	229533.I1S2H4
40211	229533.I1RWB0	pp	229533.I1S5R1
40212	229533.I1RWB0	pp	229533.I1S2B8
40213	229533.I1RWB0	pp	229533.I1S2B9
40214	229533.I1RWB0	pp	229533.I1S0L4
40215	229533.I1RWB0	pp	229533.Q4HZB8
40216	229533.I1RWB0	pp	229533.I1S0J1
40217	229533.I1RWB0	pp	229533.I1S0B0
40218	229533.I1RWB0	pp	229533.I1RYX5
40219	229533.I1RWB0	pp	229533.I1S277
40220	229533.I1RWB0	pp	229533.V6R3J1
40221	229533.I1RWB0	pp	229533.I1S1W3
40222	229533.I1RWB0	pp	229533.V6R042
40223	229533.I1RWB0	pp	229533.I1S147

40224	229533.I1RWB0	pp	229533.I1S0L8
40225	229533.I1RWB0	pp	229533.I1S1C7
40226	229533.I1RWB0	pp	229533.I1RXU9
40227	229533.I1RWB0	pp	229533.I1RYW3
40228	229533.I1RWB0	pp	229533.I1RZM8
40229	229533.I1RWB0	pp	229533.I1RXF0
40230	229533.I1RWB0	pp	229533.I1RYI5
40231	229533.I1RWB0	pp	229533.I1RXR1
40232	229533.I1RWB0	pp	229533.V6RUT5
40233	229533.I1RWB0	pp	229533.I1S0R8
40234	229533.I1RWB0	pp	229533.I1RXD7
40235	229533.I1RWB0	pp	229533.V6RCZ8
40236	229533.I1RWB0	pp	229533.I1RX46
40237	229533.I1RWB0	pp	229533.I1RYH7
40238	229533.I1RWB0	pp	229533.V6QUE6
40239	229533.I1RWB0	pp	229533.I1S5B0
40240	229533.I1RWB0	pp	229533.I1S4S7
40241	229533.I1RWB0	pp	229533.Q4I624
40242	229533.I1RWB0	pp	229533.I1RZK5
40243	229533.I1RWB0	pp	229533.I1S0P0
40244	229533.I1RWB0	pp	229533.I1S9G7
40245	229533.I1RWB0	pp	229533.I1S4S4
40246	229533.I1RWB0	pp	229533.I1S887
40247	229533.I1RWB0	pp	229533.I1S0K2
40248	229533.I1RWB0	pp	229533.Q4IJK2
40249	229533.I1RWB0	pp	229533.I1RWC9
40250	229533.I1RQ72	pp	229533.I1RSR3
40251	229533.I1RQ72	pp	229533.I1RX20
40252	229533.I1RQ72	pp	229533.I1RZ43
40253	229533.I1RQ72	pp	229533.I1RXX1
40254	229533.I1RQ72	pp	229533.I1RX87
40255	229533.I1RQ72	pp	229533.I1RUQ5
40256	229533.I1RQ72	pp	229533.I1RYL5
40257	229533.I1RQ72	pp	229533.I1RW99
40258	229533.I1RQ72	pp	229533.I1S439
40259	229533.I1RQ72	pp	229533.I1S2C1
40260	229533.I1RQ72	pp	229533.I1RV66
40261	229533.I1RQ72	pp	229533.Q4IR87
40262	229533.I1RQ72	pp	229533.I1RQA3
40263	229533.I1RQ72	pp	229533.I1RYH6
40264	229533.I1RQ72	pp	229533.V6RCG5
40265	229533.I1RQ72	pp	229533.I1RQL4
40266	229533.I1RQ72	pp	229533.I1S278
40267	229533.I1RQ72	pp	229533.Q4I4A4
40268	229533.I1RQ72	pp	229533.I1RWT6
40269	229533.I1RQ72	pp	229533.I1RYN2
40270	229533.I1RQ72	pp	229533.I1RWC2
40271	229533.I1RQ72	pp	229533.V6RR76
40272	229533.I1RQ72	pp	229533.I1RYZ9
40273	229533.I1RQ72	pp	229533.I1RSS1
40274	229533.I1RQ72	pp	229533.I1RYH9
40275	229533.I1RQ72	pp	229533.Q4IEA7
40276	229533.I1RQ72	pp	229533.I1RUQ2
40277	229533.I1RQ72	pp	229533.I1RZ94
40278	229533.I1RQ72	pp	229533.V6QYF0
40279	229533.I1RQ72	pp	229533.I1RX04
40280	229533.I1RQ72	pp	229533.I1RX77
40281	229533.I1RQ72	pp	229533.I1RRN5
40282	229533.I1RQ72	pp	229533.I1RT96
40283	229533.I1RQ72	pp	229533.I1S0L3
40284	229533.I1RQ72	pp	229533.V6QV09
40285	229533.I1RQ72	pp	229533.Q4INZ9
40286	229533.I1RQ72	pp	229533.I1RZE4
40287	229533.I1RQ72	pp	229533.I1RRY3
40288	229533.I1RQ72	pp	229533.I1S247
40289	229533.I1RQ72	pp	229533.I1RQE7
40290	229533.I1RQ72	pp	229533.I1S286
40291	229533.I1RQ72	pp	229533.I1RX64
40292	229533.I1RQ72	pp	229533.I1RY44
40293	229533.I1RQ72	pp	229533.I1S0R8
40294	229533.I1RQ72	pp	229533.I1RRI2
40295	229533.I1RQ72	pp	229533.I1RWH4
40296	229533.I1RQ72	pp	229533.V6RVG3

40297	229533.I1RQ72	pp	229533.I1RSU1
40298	229533.I1RQ72	pp	229533.I1S0Q7
40299	229533.I1RQ72	pp	229533.I1RS17
40300	229533.I1RQ72	pp	229533.I1S0X7
40301	229533.I1RQ72	pp	229533.V6RKY7
40302	229533.I1RQ72	pp	229533.I1RUG3
40303	229533.I1RQ72	pp	229533.I1S4Z7
40304	229533.I1RQ72	pp	229533.I1RZC3
40305	229533.I1RQ72	pp	229533.I1S248
40306	229533.I1RQ72	pp	229533.I1S0M2
40307	229533.I1RQ72	pp	229533.I1RQB4
40308	229533.I1RQ72	pp	229533.I1RZ63
40309	229533.I1RQ72	pp	229533.I1RUN8
40310	229533.I1RQ72	pp	229533.I1RQD6
40311	229533.I1RQ72	pp	229533.I1S1W4
40312	229533.I1RQ72	pp	229533.I1S1B6
40313	229533.I1RQ72	pp	229533.V6RQR4
40314	229533.I1RQ72	pp	229533.I1RZZ9
40315	229533.I1RQ72	pp	229533.I1S265
40316	229533.I1RQ72	pp	229533.I1S2E9
40317	229533.I1RQ72	pp	229533.I1RY32
40318	229533.I1RQ72	pp	229533.I1RVA8
40319	229533.I1RQ72	pp	229533.I1RRJ8
40320	229533.I1RQ72	pp	229533.I1RS47
40321	229533.I1RQ72	pp	229533.I1S7X9
40322	229533.I1RVX1	pp	229533.I1S7S9
40323	229533.I1RJS0	pp	229533.I1S3G4
40324	229533.I1RJS0	pp	229533.I1RLE2
40325	229533.I1RJS0	pp	229533.I1S0L8
40326	229533.I1RJS0	pp	229533.I1RMH4
40327	229533.I1RJS0	pp	229533.V6RHD0
40328	229533.I1RJS0	pp	229533.I1RNG4
40329	229533.I1RJS0	pp	229533.I1RQM9
40330	229533.I1RJS0	pp	229533.I1RQD6
40331	229533.I1RJS0	pp	229533.I1S5S8
40332	229533.I1RJS0	pp	229533.I1RZA8
40333	229533.I1RJS0	pp	229533.I1S694
40334	229533.I1RJS0	pp	229533.I1RQ48
40335	229533.I1RJS0	pp	229533.I1S2A8
40336	229533.I1RJS0	pp	229533.I1RUP5
40337	229533.I1RJS0	pp	229533.V6RXR0
40338	229533.I1RJS0	pp	229533.I1S064
40339	229533.I1RJS0	pp	229533.V6RAG6
40340	229533.I1RJS0	pp	229533.I1RKR9
40341	229533.I1RJS0	pp	229533.V6RIS2
40342	229533.I1RJS0	pp	229533.V6QXG1
40343	229533.I1RJS0	pp	229533.I1RN21
40344	229533.I1RJS0	pp	229533.V6RK40
40345	229533.I1RJS0	pp	229533.I1S0C9
40346	229533.I1RJS0	pp	229533.I1RP58
40347	229533.I1RJS0	pp	229533.I1RZ41
40348	229533.I1RJS0	pp	229533.I1S0Y2
40349	229533.I1RJS0	pp	229533.V6RJ29
40350	229533.I1RJS0	pp	229533.I1RK26
40351	229533.I1RJS0	pp	229533.V6RM02
40352	229533.I1RJS0	pp	229533.Q4HXT5
40353	229533.I1RJS0	pp	229533.I1RQM4
40354	229533.I1RJS0	pp	229533.I1RPI4
40355	229533.I1RJS0	pp	229533.I1RT41
40356	229533.I1RJS0	pp	229533.I1RZM4
40357	229533.I1RJS0	pp	229533.I1RPS0
40358	229533.I1RJS0	pp	229533.I1RPQ2
40359	229533.I1RJS0	pp	229533.I1RTE9
40360	229533.I1RJS0	pp	229533.V6RW66
40361	229533.I1RJS0	pp	229533.I1RYZ2
40362	229533.I1RJS0	pp	229533.I1RPE5
40363	229533.I1RJS0	pp	229533.I1RQE8
40364	229533.I1RJS0	pp	229533.I1RVX9
40365	229533.I1RJS0	pp	229533.I1S1W8
40366	229533.I1RJS0	pp	229533.I1S0B7
40367	229533.I1RJS0	pp	229533.I1RY16
40368	229533.I1RJS0	pp	229533.I1RRQ1
40369	229533.I1RJS0	pp	229533.V6QU63

40370	229533.I1RJS0	pp	229533.I1RXX1
40371	229533.I1RJS0	pp	229533.V6RK96
40372	229533.I1RJS0	pp	229533.V6R398
40373	229533.I1RJS0	pp	229533.I1RW69
40374	229533.I1RJS0	pp	229533.I1S3D8
40375	229533.I1RJS0	pp	229533.I1RQ81
40376	229533.I1RJS0	pp	229533.I1RM26
40377	229533.I1RJS0	pp	229533.I1S2B6
40378	229533.I1RJS0	pp	229533.V6RDH3
40379	229533.I1RJS0	pp	229533.V6QU65
40380	229533.I1RJ74	pp	229533.V6RIV9
40381	229533.I1RJ74	pp	229533.I1RZW8
40382	229533.I1RJ74	pp	229533.I1RRI2
40383	229533.I1RJ74	pp	229533.I1RQ28
40384	229533.I1RJ74	pp	229533.I1RS17
40385	229533.I1RJ74	pp	229533.I1RSD8
40386	229533.I1RJ74	pp	229533.I1RW46
40387	229533.I1RJ74	pp	229533.I1S9U2
40388	229533.I1RJ74	pp	229533.I1RR24
40389	229533.I1RJ74	pp	229533.I1RN18
40390	229533.I1RJ74	pp	229533.I1RW99
40391	229533.I1RJ74	pp	229533.I1S0L4
40392	229533.I1RJ74	pp	229533.I1RQE7
40393	229533.I1RJ74	pp	229533.I1RMN6
40394	229533.I1RJ74	pp	229533.I1RJQ3
40395	229533.I1RJ74	pp	229533.I1RWJ8
40396	229533.I1RJ74	pp	229533.I1RSI6
40397	229533.I1RJ74	pp	229533.I1RSR3
40398	229533.I1RJ74	pp	229533.V6RCG5
40399	229533.I1RJ74	pp	229533.I1S5R1
40400	229533.I1RJ74	pp	229533.I1RZS6
40401	229533.I1RJ74	pp	229533.I1RW61
40402	229533.I1RJ74	pp	229533.I1RZM8
40403	229533.I1RJ74	pp	229533.I1RJU3
40404	229533.I1RJ74	pp	229533.I1RRT8
40405	229533.I1RJ74	pp	229533.I1RPM4
40406	229533.I1RJ74	pp	229533.I1RQ31
40407	229533.I1RJ74	pp	229533.I1RUG3
40408	229533.I1RJ74	pp	229533.I1S1B6
40409	229533.I1RJ74	pp	229533.I1RNV6
40410	229533.I1RJ74	pp	229533.I1RTF4
40411	229533.I1RJ74	pp	229533.I1RJV4
40412	229533.I1RJ74	pp	229533.I1RNH6
40413	229533.I1RJ74	pp	229533.I1RNY2
40414	229533.I1RJ74	pp	229533.I1RRT2
40415	229533.I1RJ74	pp	229533.I1RMK3
40416	229533.I1RJ74	pp	229533.I1RJW6
40417	229533.I1RJ74	pp	229533.I1RKL3
40418	229533.I1RJ74	pp	229533.I1RUQ2
40419	229533.I1RJ74	pp	229533.V6QW22
40420	229533.I1RJ74	pp	229533.I1RZZ9
40421	229533.I1RJ74	pp	229533.I1RRJ8
40422	229533.I1RJ74	pp	229533.I1RRM5
40423	229533.I1RJ74	pp	229533.I1RS47
40424	229533.I1RJ74	pp	229533.I1RT40
40425	229533.I1RJ74	pp	229533.I1RSQ7
40426	229533.I1RJ74	pp	229533.I1RW25
40427	229533.I1RJ74	pp	229533.I1RVQ0
40428	229533.I1RJ74	pp	229533.I1S0L3
40429	229533.I1RJ74	pp	229533.I1RNA0
40430	229533.I1RJ74	pp	229533.I1RWK3
40431	229533.I1RJ74	pp	229533.I1RN63
40432	229533.I1RJ74	pp	229533.I1RW71
40433	229533.I1RJ74	pp	229533.V6QYF0
40434	229533.I1RUP5	pp	229533.V6R398
40435	229533.I1RUP5	pp	229533.V6RK96
40436	229533.I1RUP5	pp	229533.I1RXX1
40437	229533.I1RUP5	pp	229533.I1S3D8
40438	229533.I1RUP5	pp	229533.I1RYZ2
40439	229533.I1RUP5	pp	229533.I1RW69
40440	229533.I1RUP5	pp	229533.V6RDH3
40441	229533.I1RUP5	pp	229533.V6QU63
40442	229533.I1RUP5	pp	229533.V6QU65

229533.I1RUP5	pp	229533.V6RJ29
229533.I1RUP5	pp	229533.V6RAG6
229533.I1RUP5	pp	229533.I1S2B6
229533.I1RUP5	pp	229533.I1S1W8
229533.I1RUP5	pp	229533.Q4HXT5
229533.I1RUP5	pp	229533.V6RW66
229533.I1RUP5	pp	229533.V6RIS2
229533.I1RUP5	pp	229533.I1RZM4
229533.I1RUP5	pp	229533.I1RVX9
229533.I1RUP5	pp	229533.I1S0C9
229533.I1RUP5	pp	229533.I1RY16
229533.I1RUP5	pp	229533.I1S0Y2
229533.I1RUP5	pp	229533.V6QXG1
229533.I1RUP5	pp	229533.I1S3G4
229533.I1RUP5	pp	229533.I1S064
229533.I1RUP5	pp	229533.I1RZ41
229533.I1RUP5	pp	229533.V6RXR0
229533.I1RUP5	pp	229533.V6RM02
229533.I1RUP5	pp	229533.I1S694
229533.I1RUP5	pp	229533.I1RZA8
229533.I1RUP5	pp	229533.V6RK40
229533.I1RUP5	pp	229533.I1S5S8
229533.I1RUP5	pp	229533.V6RHD0
229533.I1RUP5	pp	229533.I1S0L8
229533.I1RUP5	pp	229533.I1RUP6
229533.A0A0E0RQ57	pp	229533.I1RTN6
229533.A0A0E0RQ57	pp	229533.I1RMJ8
229533.A0A0E0RQ57	pp	229533.I1S0N5
229533.A0A0E0RQ57	pp	229533.I1RSC7
229533.A0A0E0RQ57	pp	229533.A0A1C3YJ79
229533.A0A0E0RQ57	pp	229533.I1RQI6
229533.A0A0E0RQ57	pp	229533.I1RYQ1
229533.I1RAE2	pp	229533.P0C431
229533.I1RAE2	pp	229533.I1RN72
229533.I1RAE2	pp	229533.I1RZ35
229533.I1RAE2	pp	229533.I1REA6
229533.I1RAE2	pp	229533.I1RPK9
229533.I1RAE2	pp	229533.I1RFJ5
229533.I1RAE2	pp	229533.I1RCJ5
229533.I1RAE2	pp	229533.I1S1V3
229533.I1RAE2	pp	229533.I1RB84
229533.I1RAE2	pp	229533.I1RCP3
229533.I1RAE2	pp	229533.I1S0S6
229533.I1RAE2	pp	229533.I1RD69
229533.I1RAE2	pp	229533.I1RM78
229533.I1RAE2	pp	229533.Q4HZB8
229533.I1RAE2	pp	229533.I1RRX5
229533.I1RAE2	pp	229533.I1RPS0
229533.I1RAE2	pp	229533.I1RPA7
229533.I1RAE2	pp	229533.I1RZQ6
229533.I1RAE2	pp	229533.I1RD70
229533.I1RAE2	pp	229533.I1RSW5
229533.I1RAE2	pp	229533.I1RAU7
229533.I1RAE2	pp	229533.I1RBT2
229533.I1RAE2	pp	229533.I1S0L8
229533.I1RAE2	pp	229533.I1RQN9
229533.I1RAE2	pp	229533.I1RJU5
229533.I1RAE2	pp	229533.I1RFT6
229533.I1RBW7	pp	229533.I1RQG9
229533.I1RBW7	pp	229533.I1RNY7
229533.I1RBW7	pp	229533.I1RLX6
229533.I1RBW7	pp	229533.I1RD70
229533.I1RBW7	pp	229533.Q4INZ9
229533.I1RBW7	pp	229533.I1RXB5
229533.I1RBW7	pp	229533.I1S1V1
229533.I1RBW7	pp	229533.V6RR76
229533.I1RBW7	pp	229533.I1RC81
229533.I1RBW7	pp	229533.I1S060
229533.I1RBW7	pp	229533.I1RP28
229533.I1RBW7	pp	229533.I1S0E9
229533.I1RBW7	pp	229533.I1RQG4
229533.I1RBW7	pp	229533.I1RN81
229533.I1R9X6	pp	229533.I1RFV4

40516 229533.I1R9X6 pp 229533.I1RK67
40517 229533.I1R9X6 pp 229533.I1RJT8
40518 229533.I1R9X6 pp 229533.I1RQA3
40519 229533.I1R9X6 pp 229533.I1S248
40520 229533.I1R9X6 pp 229533.I1RW84
40521 229533.I1R9X6 pp 229533.I1RMR7
40522 229533.I1R9X6 pp 229533.I1RYB7
40523 229533.I1R9X6 pp 229533.I1RMA7
40524 229533.I1R9X6 pp 229533.I1RZA5
40525 229533.I1R9X6 pp 229533.I1RLT6
40526 229533.I1R9X6 pp 229533.I1RK33
40527 229533.I1R9X6 pp 229533.I1RV91
40528 229533.I1R9X6 pp 229533.I1RY44
40529 229533.I1R9X6 pp 229533.I1S4T6
40530 229533.I1R9X6 pp 229533.I1S2E9
40531 229533.I1R9X6 pp 229533.I1S074
40532 229533.I1R9X6 pp 229533.I1RWT6
40533 229533.I1R9X6 pp 229533.I1RJT0
40534 229533.I1R9X6 pp 229533.I1RYI3
40535 229533.I1R9X6 pp 229533.I1S1A3
40536 229533.I1R9X6 pp 229533.I1RW38
40537 229533.A0A0E0SAK0 pp 229533.I1RTG3
40538 229533.A0A0E0SAK0 pp 229533.I1RWL5
40539 229533.A0A0E0SAK0 pp 229533.A0A1C3YKJ5
40540 229533.A0A0E0SAK0 pp 229533.A0A1C3YKS8
40541 229533.A0A0E0SAK0 pp 229533.A0A1C3YLQ4
40542 229533.A0A0E0SAK0 pp 229533.I1RSU7
40543 229533.A0A0E0SAK0 pp 229533.A0A1C3YMF6
40544 229533.A0A0E0SAK0 pp 229533.I1RWN0
40545 229533.A0A0E0SAK0 pp 229533.I1RTI3
40546 229533.A0A0E0SAK0 pp 229533.I1RCJ2
40547 229533.A0A0E0SAK0 pp 229533.I1RDZ5
40548 229533.A0A0E0SAK0 pp 229533.A0A1C3YII1
40549 229533.A0A0E0SAK0 pp 229533.I1RPS0
40550 229533.A0A0E0SAK0 pp 229533.V6RXE7
40551 229533.A0A0E0SAK0 pp 229533.I1S0L8
40552 229533.A0A0E0SAK0 pp 229533.I1RPA7
40553 229533.A0A0E0SAK0 pp 229533.I1RHF8
40554 229533.A0A0E0SAK0 pp 229533.A0A1C3YIH5
40555 229533.A0A0E0SAK0 pp 229533.A0A1C3YM82
40556 229533.A0A0E0SAK0 pp 229533.I1RN72
40557 229533.A0A0E0SAK0 pp 229533.I1RKZ8
40558 229533.A0A0E0SAK0 pp 229533.A0A1C3YHY5
40559 229533.A0A0E0SAK0 pp 229533.I1RDK7
40560 229533.A0A0E0SAK0 pp 229533.I1RMG4
40561 229533.A0A0E0SAK0 pp 229533.I1RNG4
40562 229533.A0A1C3YIG2 pp 229533.I1RYW3
40563 229533.A0A1C3YIG2 pp 229533.I1RFT1
40564 229533.A0A1C3YIG2 pp 229533.A0A1C3YLE2
40565 229533.A0A1C3YIG2 pp 229533.I1RMJ3
40566 229533.A0A1C3YIG2 pp 229533.I1S1L2
40567 229533.A0A1C3YIG2 pp 229533.I1RN84
40568 229533.A0A1C3YIG2 pp 229533.A0A1C3YLT9
40569 229533.A0A1C3YIG2 pp 229533.I1RJZ7
40570 229533.A0A1C3YIG2 pp 229533.I1RZ94
40571 229533.A0A1C3YIG2 pp 229533.A0A1C3YMT2
40572 229533.A0A1C3YIG2 pp 229533.I1RE72
40573 229533.A0A1C3YIG2 pp 229533.I1RCR5
40574 229533.A0A1C3YIG2 pp 229533.A0A1C3YK13
40575 229533.A0A1C3YIG2 pp 229533.A0A1C3YJA9
40576 229533.A0A1C3YIG2 pp 229533.I1S097
40577 229533.A0A098DSP0 pp 229533.A0A1C3YI04
40578 229533.A0A098DSP0 pp 229533.I1RB40
40579 229533.A0A098DSP0 pp 229533.I1S1Y9
40580 229533.A0A098DSP0 pp 229533.I1RY40
40581 229533.A0A098DSP0 pp 229533.I1RCK4
40582 229533.A0A098DSP0 pp 229533.I1RWG1
40583 229533.A0A098DSP0 pp 229533.I1S2B9
40584 229533.A0A098DSP0 pp 229533.Q4I2W2
40585 229533.A0A098DSP0 pp 229533.I1RJH8
40586 229533.A0A098DSP0 pp 229533.I1S0L8
40587 229533.A0A098DSP0 pp 229533.I1RPS0
40588 229533.A0A098DSP0 pp 229533.I1RBP9

40589	229533.A0A098DSP0	pp	229533.I1RQ49
40590	229533.A0A098DSP0	pp	229533.I1S9V9
40591	229533.A0A098DSP0	pp	229533.I1RJH7
40592	229533.A0A098DSP0	pp	229533.A0A1C3YIH5
40593	229533.A0A098DSP0	pp	229533.A0A1C3YLH7
40594	229533.A0A098DSP0	pp	229533.I1RRL3
40595	229533.A0A098DSP0	pp	229533.I1RN72
40596	229533.A0A098DSP0	pp	229533.I1RYM3
40597	229533.A0A098DSP0	pp	229533.I1RPA7
40598	229533.A0A098DSP0	pp	229533.A0A1C3YM82
40599	229533.A0A098DSP0	pp	229533.I1RTG9
40600	229533.A0A098DSP0	pp	229533.A0A0E0SFI7
40601	229533.A0A098DSP0	pp	229533.A0A1C3YN81
40602	229533.A0A098DSP0	pp	229533.I1RQ82
40603	229533.A0A098DSP0	pp	229533.I1S9G7
40604	229533.A0A098DSP0	pp	229533.A0A1C3YM76
40605	229533.I1RDD9	pp	229533.I1RQ88
40606	229533.I1RDD9	pp	229533.I1S7S9
40607	229533.I1RDD9	pp	229533.I1RGC2
40608	229533.I1RDD9	pp	229533.I1RS41
40609	229533.I1RG90	pp	229533.I1RSA8
40610	229533.I1RG90	pp	229533.I1RNC1
40611	229533.I1RG90	pp	229533.V6RCG5
40612	229533.I1RG90	pp	229533.I1S8U9
40613	229533.I1RG90	pp	229533.Q4I7X1
40614	229533.I1RG90	pp	229533.I1RSA4
40615	229533.I1RG90	pp	229533.I1RND5
40616	229533.I1RG90	pp	229533.I1RQD8
40617	229533.I1RG90	pp	229533.V6QX58
40618	229533.I1RG90	pp	229533.I1RZ11
40619	229533.I1RG90	pp	229533.V6RIV9
40620	229533.I1RG90	pp	229533.I1RT35
40621	229533.I1RG90	pp	229533.I1RT47
40622	229533.I1RG90	pp	229533.I1RSD4
40623	229533.I1RG90	pp	229533.I1RTS6
40624	229533.I1RG90	pp	229533.I1S289
40625	229533.I1RG90	pp	229533.V6RGN5
40626	229533.I1RG90	pp	229533.I1RWX8
40627	229533.I1RG90	pp	229533.I1RK72
40628	229533.I1RG90	pp	229533.V6QXG1
40629	229533.I1RG90	pp	229533.I1RVT9
40630	229533.I1RG90	pp	229533.I1RW46
40631	229533.I1RG90	pp	229533.V6RIS2
40632	229533.I1RG90	pp	229533.I1RRL0
40633	229533.I1RG90	pp	229533.Q4IP34
40634	229533.I1RG90	pp	229533.I1RX51
40635	229533.I1RG90	pp	229533.I1RS83
40636	229533.I1RG90	pp	229533.I1RRT0
40637	229533.I1RG90	pp	229533.V6RY33
40638	229533.I1RG90	pp	229533.I1RX14
40639	229533.I1RG90	pp	229533.I1S471
40640	229533.I1RG90	pp	229533.I1S7M3
40641	229533.I1RG90	pp	229533.I1RSH4
40642	229533.I1RG90	pp	229533.I1RQN0
40643	229533.I1RG90	pp	229533.I1RM22
40644	229533.I1RG90	pp	229533.I1RX47
40645	229533.I1RG90	pp	229533.I1RSD1
40646	229533.I1RG90	pp	229533.Q4IE79
40647	229533.I1RG90	pp	229533.Q4IAA0
40648	229533.I1RG90	pp	229533.I1RMM5
40649	229533.I1RG90	pp	229533.I1S8V4
40650	229533.I1RG90	pp	229533.Q4IPB8
40651	229533.I1RG90	pp	229533.Q4IRI9
40652	229533.I1RG90	pp	229533.I1RVK5
40653	229533.I1RG90	pp	229533.I1S504
40654	229533.I1RG90	pp	229533.I1RQQ7
40655	229533.I1RG90	pp	229533.I1RM25
40656	229533.I1RG90	pp	229533.I1RYW3
40657	229533.I1RG90	pp	229533.I1RK79
40658	229533.I1RG90	pp	229533.I1RSH9
40659	229533.I1RG90	pp	229533.I1RNB8
40660	229533.I1RG90	pp	229533.I1RS28
40661	229533.I1RG90	pp	229533.I1RPY5

40662	229533.I1RG90	pp	229533.I1RZV9
40663	229533.I1RG90	pp	229533.I1RK90
40664	229533.I1RG90	pp	229533.Q4HXF6
40665	229533.I1RG90	pp	229533.I1RY03
40666	229533.I1RG90	pp	229533.Q4I1Y1
40667	229533.I1RG90	pp	229533.I1RVX0
40668	229533.I1RG90	pp	229533.I1S1X9
40669	229533.I1RG90	pp	229533.Q4IB70
40670	229533.I1RG90	pp	229533.I1RZK5
40671	229533.I1RG90	pp	229533.I1RS06
40672	229533.I1RG90	pp	229533.I1RZB5
40673	229533.I1RG90	pp	229533.I1RJL2
40674	229533.I1RG90	pp	229533.I1S1J7
40675	229533.I1RG90	pp	229533.I1RJS5
40676	229533.I1RG90	pp	229533.I1RQR3
40677	229533.I1RG90	pp	229533.I1RUG9
40678	229533.I1RG90	pp	229533.I1RN78
40679	229533.I1RG90	pp	229533.I1S0B1
40680	229533.I1RG90	pp	229533.I1RZL5
40681	229533.I1RG90	pp	229533.V6QVD5
40682	229533.I1RG90	pp	229533.I1RQZ2
40683	229533.I1RG90	pp	229533.I1RPA0
40684	229533.I1RG90	pp	229533.I1RYB1
40685	229533.I1RG90	pp	229533.I1RT91
40686	229533.I1RG90	pp	229533.I1RK92
40687	229533.I1RG90	pp	229533.I1S8F0
40688	229533.I1RG90	pp	229533.I1RQP8
40689	229533.I1RG90	pp	229533.I1RYT7
40690	229533.I1RG90	pp	229533.I1RPY7
40691	229533.I1RG90	pp	229533.I1RKF8
40692	229533.I1RG90	pp	229533.I1RS72
40693	229533.I1RG90	pp	229533.I1RYJ9
40694	229533.I1RG90	pp	229533.I1S0Z9
40695	229533.I1RG90	pp	229533.I1S1C6
40696	229533.I1RG90	pp	229533.I1S8V0
40697	229533.I1RG90	pp	229533.Q4IPH4
40698	229533.I1RG90	pp	229533.I1S5S2
40699	229533.I1RG90	pp	229533.I1RYI9
40700	229533.I1RG90	pp	229533.I1S8U8
40701	229533.I1RG90	pp	229533.I1RJW6
40702	229533.I1RG90	pp	229533.I1RT04
40703	229533.I1RG90	pp	229533.Q4I5R9
40704	229533.I1RG90	pp	229533.I1RRQ4
40705	229533.I1RG90	pp	229533.I1S503
40706	229533.I1RG90	pp	229533.I1RR25
40707	229533.I1RG90	pp	229533.I1RPL3
40708	229533.I1RG90	pp	229533.I1RME9
40709	229533.I1RG90	pp	229533.I1S0R7
40710	229533.I1RG90	pp	229533.I1RZZ1
40711	229533.I1RG90	pp	229533.Q4IQW0
40712	229533.I1RG90	pp	229533.I1RPZ3
40713	229533.I1RG90	pp	229533.I1S214
40714	229533.I1RG90	pp	229533.I1S2E8
40715	229533.I1RG90	pp	229533.I1RT17
40716	229533.I1RG90	pp	229533.I1RQE7
40717	229533.I1RG90	pp	229533.I1RMY3
40718	229533.I1RG90	pp	229533.Q4I7F9
40719	229533.I1RG90	pp	229533.I1RY40
40720	229533.I1RG90	pp	229533.I1S4Y4
40721	229533.I1RG90	pp	229533.I1RSL9
40722	229533.I1RG90	pp	229533.I1RHI5
40723	229533.I1RG90	pp	229533.I1RV91
40724	229533.I1RG90	pp	229533.I1RZQ5
40725	229533.I1RG90	pp	229533.I1RS30
40726	229533.I1RG90	pp	229533.Q4IJK2
40727	229533.I1RG90	pp	229533.I1RN67
40728	229533.I1RG90	pp	229533.I1RQC5
40729	229533.I1RG90	pp	229533.I1RND9
40730	229533.I1RG90	pp	229533.I1RXF2
40731	229533.I1RG90	pp	229533.I1RMV1
40732	229533.I1RG90	pp	229533.I1S0L3
40733	229533.I1RG90	pp	229533.I1S7M2
40734	229533.I1RG90	pp	229533.V6R3X5

40735 229533.I1RG90 pp 229533.I1RMW7
40736 229533.I1RG90 pp 229533.I1S9L5
40737 229533.I1RG90 pp 229533.I1RPP3
40738 229533.I1RG90 pp 229533.I1S9L6
40739 229533.I1RG90 pp 229533.I1RZ39
40740 229533.I1RG90 pp 229533.I1RWU2
40741 229533.I1RG90 pp 229533.I1RLU5
40742 229533.I1RG90 pp 229533.I1RKK4
40743 229533.I1RG90 pp 229533.I1RS75
40744 229533.I1RG90 pp 229533.I1RID3
40745 229533.I1RG90 pp 229533.I1RJH1
40746 229533.I1RG90 pp 229533.I1S4M5
40747 229533.I1RG90 pp 229533.I1RRG3
40748 229533.I1RG90 pp 229533.I1RMV5
40749 229533.I1RG90 pp 229533.I1RW25
40750 229533.I1RG90 pp 229533.I1RJF9
40751 229533.I1RG90 pp 229533.I1RS03
40752 229533.I1RG90 pp 229533.I1RWJ8
40753 229533.I1RG90 pp 229533.I1S0B6
40754 229533.I1RG90 pp 229533.I1S2Y5
40755 229533.I1RG90 pp 229533.I1RN63
40756 229533.I1RG90 pp 229533.I1S524
40757 229533.I1RG90 pp 229533.I1RJG6
40758 229533.I1RG90 pp 229533.I1RQB5
40759 229533.I1RG90 pp 229533.I1RPW9
40760 229533.I1RG90 pp 229533.Q4IBK5
40761 229533.I1RG90 pp 229533.I1RST1
40762 229533.I1RG90 pp 229533.Q4IPB3
40763 229533.I1RG90 pp 229533.I1RXJ6
40764 229533.I1RG90 pp 229533.I1S8V3
40765 229533.I1RG90 pp 229533.I1RPY4
40766 229533.I1RG90 pp 229533.I1S246
40767 229533.I1RG90 pp 229533.I1RN59
40768 229533.I1RG90 pp 229533.Q4IPN9
40769 229533.I1RG90 pp 229533.I1RU69
40770 229533.I1RG90 pp 229533.I1RZG7
40771 229533.I1RG90 pp 229533.I1RX81
40772 229533.I1RG90 pp 229533.I1RK96
40773 229533.I1RG90 pp 229533.I1RX24
40774 229533.A0A1C3YME9 pp 229533.I1REC7
40775 229533.A0A1C3YME9 pp 229533.I1RWX5
40776 229533.A0A1C3YME9 pp 229533.I1RPS0
40777 229533.A0A1C3YME9 pp 229533.I1RCD4
40778 229533.A0A1C3YME9 pp 229533.I1RTE6
40779 229533.A0A1C3YME9 pp 229533.I1RNG4
40780 229533.A0A1C3YME9 pp 229533.I1REC6
40781 229533.A0A1C3YME9 pp 229533.I1S247
40782 229533.A0A1C3YME9 pp 229533.I1S0L8
40783 229533.A0A1C3YME9 pp 229533.I1RYL5
40784 229533.A0A1C3YME9 pp 229533.I1RT36
40785 229533.A0A1C3YME9 pp 229533.I1RSQ1
40786 229533.A0A1C3YME9 pp 229533.I1RQN9
40787 229533.A0A1C3YME9 pp 229533.V6REE9
40788 229533.A0A1C3YME9 pp 229533.I1S0S6
40789 229533.A0A1C3YME9 pp 229533.A0A1C3YN58
40790 229533.A0A1C3YME9 pp 229533.P0C431
40791 229533.A0A1C3YME9 pp 229533.I1RH68
40792 229533.A0A1C3YME9 pp 229533.I1RTB7
40793 229533.A0A1C3YLW8 pp 229533.I1RJQ5
40794 229533.A0A1C3YLW8 pp 229533.A0A1C3YM82
40795 229533.A0A1C3YLW8 pp 229533.I1RBA6
40796 229533.A0A1C3YLW8 pp 229533.I1RPS0
40797 229533.A0A1C3YLW8 pp 229533.I1S0L8
40798 229533.A0A1C3YLW8 pp 229533.V6QX21
40799 229533.A0A1C3YLW8 pp 229533.I1RS90
40800 229533.A0A1C3YLW8 pp 229533.I1RSK9
40801 229533.A0A1C3YLW8 pp 229533.I1S2B9
40802 229533.A0A1C3YLW8 pp 229533.I1RN72
40803 229533.A0A1C3YLW8 pp 229533.I1RTA6
40804 229533.A0A1C3YLW8 pp 229533.I1RNG8
40805 229533.A0A1C3YLW8 pp 229533.I1RAU9
40806 229533.A0A1C3YLW8 pp 229533.I1RWC9
40807 229533.A0A1C3YLW8 pp 229533.I1RPA7

40808 229533.A0A1C3YLW8 pp 229533.I1RYM3
40809 229533.A0A1C3YLW8 pp 229533.I1RWG1
40810 229533.A0A1C3YLW8 pp 229533.I1RCK9
40811 229533.I1S002 pp 229533.I1S7S9
40812 229533.I1R9Y7 pp 229533.I1S2G9
40813 229533.I1R9Y7 pp 229533.I1RWL3
40814 229533.I1R9Y7 pp 229533.I1S4R1
40815 229533.I1R9Y7 pp 229533.I1RNP7
40816 229533.I1R9Y7 pp 229533.I1RDT7
40817 229533.I1R9Y7 pp 229533.I1RUG0
40818 229533.I1R9Y7 pp 229533.I1RC24
40819 229533.I1R9Y7 pp 229533.I1RGD1
40820 229533.I1R9Y7 pp 229533.I1S283
40821 229533.I1R9Y7 pp 229533.I1RZ63
40822 229533.I1R9Y7 pp 229533.V6QWX8
40823 229533.I1R9Y7 pp 229533.I1RA84
40824 229533.I1R9Y7 pp 229533.Q4HVW2
40825 229533.I1R9Y7 pp 229533.I1RD85
40826 229533.I1R9Y7 pp 229533.I1S0R5
40827 229533.I1R9Y7 pp 229533.I1RAN2
40828 229533.I1R9Y7 pp 229533.I1RAH4
40829 229533.I1R9Y7 pp 229533.I1RW90
40830 229533.I1R9Y7 pp 229533.V6RLW4
40831 229533.I1R9Y7 pp 229533.I1S0M6
40832 229533.I1R9Y7 pp 229533.I1RYH9
40833 229533.I1R9Y7 pp 229533.I1RDB1
40834 229533.I1R9Y7 pp 229533.I1S224
40835 229533.I1R9Y7 pp 229533.I1RUN8
40836 229533.I1R9Y7 pp 229533.Q4IQM6
40837 229533.I1R9Y7 pp 229533.I1RVJ1
40838 229533.I1R9Y7 pp 229533.I1RPC2
40839 229533.I1R9Y7 pp 229533.I1RFX8
40840 229533.I1R9Y7 pp 229533.I1RRM8
40841 229533.I1R9Y7 pp 229533.Q4I5W5
40842 229533.I1R9Y7 pp 229533.Q4I099
40843 229533.I1R9Y7 pp 229533.I1RL24
40844 229533.I1R9Y7 pp 229533.I1RV91
40845 229533.I1R9Y7 pp 229533.I1RJV8
40846 229533.I1R9Y7 pp 229533.I1RKC2
40847 229533.I1R9Y7 pp 229533.I1RVL8
40848 229533.I1R9Y7 pp 229533.Q4IAS1
40849 229533.I1R9Y7 pp 229533.I1S2G7
40850 229533.I1R9Y7 pp 229533.I1RYZ9
40851 229533.I1R9Y7 pp 229533.I1RAT7
40852 229533.I1R9Y7 pp 229533.I1RC97
40853 229533.I1R9Y7 pp 229533.I1RC18
40854 229533.I1R9Y7 pp 229533.I1S2E9
40855 229533.I1R9Y7 pp 229533.I1RZP8
40856 229533.I1R9Y7 pp 229533.I1RB74
40857 229533.I1R9Y7 pp 229533.I1RFL7
40858 229533.I1R9Y7 pp 229533.I1S2C4
40859 229533.I1R9Y7 pp 229533.I1RYX9
40860 229533.I1R9Y7 pp 229533.I1S098
40861 229533.I1R9Y7 pp 229533.I1S0V4
40862 229533.I1R9Y7 pp 229533.I1RQ88
40863 229533.I1R9Y7 pp 229533.I1RN08
40864 229533.I1R9Y7 pp 229533.I1RJL2
40865 229533.I1R9Y7 pp 229533.Q4IP51
40866 229533.I1R9Y7 pp 229533.I1RUN2
40867 229533.I1R9Y7 pp 229533.I1RZB5
40868 229533.I1R9Y7 pp 229533.I1RSI4
40869 229533.I1R9Y7 pp 229533.V6QV09
40870 229533.I1R9Y7 pp 229533.I1RB75
40871 229533.I1R9Y7 pp 229533.I1RRG3
40872 229533.I1R9Y7 pp 229533.I1RQ26
40873 229533.I1R9Y7 pp 229533.I1RYY5
40874 229533.I1R9Y7 pp 229533.I1RIR0
40875 229533.I1R9Y7 pp 229533.I1RYZ0
40876 229533.I1R9Y7 pp 229533.I1RKL9
40877 229533.I1R9Y7 pp 229533.I1RB41
40878 229533.I1R9Y7 pp 229533.I1RD15
40879 229533.I1R9Y7 pp 229533.I1S7X9
40880 229533.I1R9Y7 pp 229533.I1RD38

40881 229533.I1R9Y7 pp 229533.I1RC22
40882 229533.I1R9Y7 pp 229533.V6RMM8
40883 229533.I1R9Y7 pp 229533.I1RQS4
40884 229533.I1R9Y7 pp 229533.I1S0R9
40885 229533.I1R9Y7 pp 229533.I1RK75
40886 229533.I1R9Y7 pp 229533.I1RFJ2
40887 229533.I1R9Y7 pp 229533.I1S461
40888 229533.I1R9Y7 pp 229533.I1RHB0
40889 229533.I1R9Y7 pp 229533.I1RS41
40890 229533.I1R9Y7 pp 229533.Q4IJM4
40891 229533.I1R9Y7 pp 229533.I1RR25
40892 229533.I1R9Y7 pp 229533.I1S439
40893 229533.I1R9Y7 pp 229533.I1RRY3
40894 229533.I1R9Y7 pp 229533.I1RK71
40895 229533.I1R9Y7 pp 229533.I1RRN5
40896 229533.I1R9Y7 pp 229533.I1RMM9
40897 229533.I1R9Y7 pp 229533.I1RAI9
40898 229533.I1R9Y7 pp 229533.I1RZE4
40899 229533.I1R9Y7 pp 229533.I1RJK7
40900 229533.I1R9Y7 pp 229533.Q4HZ68
40901 229533.I1R9Y7 pp 229533.I1RQF2
40902 229533.I1R9Y7 pp 229533.I1RCS6
40903 229533.I1R9Y7 pp 229533.I1RKK0
40904 229533.I1R9Y7 pp 229533.I1RC17
40905 229533.I1R9Y7 pp 229533.V6RGI1
40906 229533.I1R9Y7 pp 229533.I1RQA3
40907 229533.I1R9Y7 pp 229533.I1REN3
40908 229533.I1R9Y7 pp 229533.Q4I5I4
40909 229533.I1R9Y7 pp 229533.I1S0E8
40910 229533.I1R9Y7 pp 229533.I1S0M2
40911 229533.I1R9Y7 pp 229533.I1RC95
40912 229533.I1R9Y7 pp 229533.I1RRS7
40913 229533.I1R9Y7 pp 229533.I1RKK2
40914 229533.I1R9Y7 pp 229533.I1RMD3
40915 229533.I1R9Y7 pp 229533.I1RXU0
40916 229533.I1R9Y7 pp 229533.I1RCB7
40917 229533.I1R9Y7 pp 229533.I1RWT6
40918 229533.I1R9Y7 pp 229533.I1RRW0
40919 229533.I1R9Y7 pp 229533.I1RDE0
40920 229533.I1R9Y7 pp 229533.I1S1W4
40921 229533.I1R9Y7 pp 229533.I1RBJ1
40922 229533.I1R9Y7 pp 229533.I1RRT0
40923 229533.I1R9Y7 pp 229533.I1RNY8
40924 229533.I1R9Y7 pp 229533.I1RY44
40925 229533.I1R9Y7 pp 229533.I1RA43
40926 229533.I1R9Y7 pp 229533.V6RVG3
40927 229533.I1R9Y7 pp 229533.I1RMQ7
40928 229533.I1R9Y7 pp 229533.I1RZM1
40929 229533.I1R9Y7 pp 229533.I1RC16
40930 229533.I1R9Y7 pp 229533.I1RT99
40931 229533.I1R9Y7 pp 229533.V6RKY7
40932 229533.I1R9Y7 pp 229533.I1RC15
40933 229533.I1R9Y7 pp 229533.V6RU70
40934 229533.I1R9Y7 pp 229533.I1RAM7
40935 229533.I1R9Y7 pp 229533.I1RE46
40936 229533.I1R9Y7 pp 229533.I1R9Y9
40937 229533.I1RXF6 pp 229533.I1S6E5
40938 229533.I1RXF6 pp 229533.Q4IBS2
40939 229533.I1RXF6 pp 229533.I1S6F0
40940 229533.I1RXF6 pp 229533.Q4IFI0
40941 229533.I1RXF6 pp 229533.Q4I662
40942 229533.I1RXF6 pp 229533.V6RAC9
40943 229533.I1RXF6 pp 229533.Q4IAA0
40944 229533.I1RXF6 pp 229533.Q4HZ42
40945 229533.I1RXF6 pp 229533.I1RYQ1
40946 229533.A0A1C3YKU0 pp 229533.I1RY98
40947 229533.A0A1C3YKU0 pp 229533.I1RIC2
40948 229533.A0A1C3YKU0 pp 229533.I1S097
40949 229533.A0A1C3YKU0 pp 229533.I1S0S6
40950 229533.A0A1C3YKU0 pp 229533.I1RE72
40951 229533.A0A1C3YKU0 pp 229533.A0A1C3YMT2
40952 229533.A0A1C3YKU0 pp 229533.A0A1C3YLH6
40953 229533.A0A1C3YKU0 pp 229533.I1RUP6

229533.Q4IN00 pp 229533.V6QX21
229533.Q4IN00 pp 229533.Q4INI2
229533.Q4IN00 pp 229533.V6R227
229533.Q4IN00 pp 229533.Q4IPB8
229533.Q4IN00 pp 229533.V6QX58
229533.I1RBI5 pp 229533.I1S1B1
229533.I1RBI5 pp 229533.I1RQP5
229533.I1RBI5 pp 229533.I1RST4
229533.I1RBI5 pp 229533.I1S0Z3
229533.I1RBI5 pp 229533.V6RR76
229533.I1RBI5 pp 229533.I1RT41
229533.I1RBI5 pp 229533.I1RNW8
229533.I1RBI5 pp 229533.Q4ILI9
229533.I1RBI5 pp 229533.I1RD10
229533.I1RBI5 pp 229533.I1RMR5
229533.I1RBI5 pp 229533.I1S2C1
229533.I1RBI5 pp 229533.I1RN81
229533.I1RBI5 pp 229533.I1RKG4
229533.I1RKJ2 pp 229533.I1RWC9
229533.I1RKJ2 pp 229533.I1RYM3
229533.I1RKJ2 pp 229533.I1RPA7
229533.I1RKJ2 pp 229533.I1S2B9
229533.I1RKJ2 pp 229533.I1RS90
229533.I1RKJ2 pp 229533.I1RWG1
229533.I1RKJ2 pp 229533.I1S0L8
229533.I1RKJ2 pp 229533.I1RPS0
229533.I1RKJ2 pp 229533.I1RTA6
229533.I1RKJ2 pp 229533.I1RN72
229533.I1RKJ2 pp 229533.I1RNG8
229533.A0A0E0RU69 pp 229533.I1RNG8
229533.A0A0E0RU69 pp 229533.I1RTA6
229533.A0A0E0RU69 pp 229533.A0A1C3YM61
229533.A0A0E0RU69 pp 229533.A0A1C3YJC0
229533.A0A0E0RU69 pp 229533.I1RUP2
229533.I1RCF9 pp 229533.I1RMB2
229533.I1RCF9 pp 229533.Q4IEA7
229533.I1RCF9 pp 229533.I1RE72
229533.I1RCF9 pp 229533.I1RSS1
229533.I1RCF9 pp 229533.I1RX77
229533.I1RCF9 pp 229533.I1RFW0
229533.I1RCF9 pp 229533.I1S286
229533.I1RCF9 pp 229533.Q4IJ79
229533.I1RCF9 pp 229533.I1RST4
229533.A0A0E0SKT2 pp 229533.I1RNG8
229533.A0A0E0SKT2 pp 229533.A0A1C3YLH7
229533.A0A0E0SKT2 pp 229533.I1RMV6
229533.A0A0E0SKT2 pp 229533.I1S4N8
229533.A0A0E0SKT2 pp 229533.A0A1C3YM54
229533.A0A0E0SKT2 pp 229533.I1RPA7
229533.A0A0E0SKT2 pp 229533.I1S1V1
229533.A0A0E0SKT2 pp 229533.I1RTA6
229533.A0A0E0SKT2 pp 229533.A0A1C3YKS9
229533.A0A0E0SKT2 pp 229533.I1S4N7
229533.A0A0E0SKT2 pp 229533.I1RN72
229533.A0A0E0SKT2 pp 229533.A0A1C3YJC0
229533.A0A0E0SKT2 pp 229533.I1RTG9
229533.A0A0E0SKT2 pp 229533.I1S0L8
229533.A0A0E0SKT2 pp 229533.A0A1C3YME0
229533.A0A0E0SKT2 pp 229533.I1S1V3
229533.A0A0E0SKT2 pp 229533.A0A1C3YM61
229533.A0A0E0SKT2 pp 229533.A0A1C3YM82
229533.I1RRZ0 pp 229533.I1RW25
229533.I1RRZ0 pp 229533.V6R3X5
229533.I1RRZ0 pp 229533.I1S2B8
229533.I1RRZ0 pp 229533.I1S8E2
229533.I1RRZ0 pp 229533.I1RS47
229533.I1RRZ0 pp 229533.I1S0L3
229533.I1RRZ0 pp 229533.I1RS30
229533.I1RRZ0 pp 229533.I1S8V3
229533.I1RRZ0 pp 229533.I1RZ39
229533.I1RRZ0 pp 229533.V6RUY9
229533.I1RRZ0 pp 229533.I1RXD7
229533.I1RRZ0 pp 229533.I1RZZ9

41027 229533.I1RRZ0 pp 229533.I1RTE9
41028 229533.I1RRZ0 pp 229533.I1RT05
41029 229533.I1RRZ0 pp 229533.I1S0R7
41030 229533.I1RRZ0 pp 229533.I1S238
41031 229533.I1RRZ0 pp 229533.I1RT04
41032 229533.I1RRZ0 pp 229533.I1RTH6
41033 229533.I1RRZ0 pp 229533.I1S0L4
41034 229533.I1RRZ0 pp 229533.I1RY03
41035 229533.I1RRZ0 pp 229533.I1RTD5
41036 229533.I1RRZ0 pp 229533.I1S2H4
41037 229533.I1RRZ0 pp 229533.I1RYW3
41038 229533.I1RRZ0 pp 229533.Q4HX89
41039 229533.I1RRZ0 pp 229533.I1RT40
41040 229533.I1RRZ0 pp 229533.I1RV91
41041 229533.I1RRZ0 pp 229533.I1S5R1
41042 229533.I1RRZ0 pp 229533.I1RSR3
41043 229533.I1RRZ0 pp 229533.V6RTJ8
41044 229533.I1RRZ0 pp 229533.I1S1C7
41045 229533.I1RRZ0 pp 229533.I1RWJ8
41046 229533.I1RRZ0 pp 229533.I1S1B6
41047 229533.I1RRZ0 pp 229533.I1S4S4
41048 229533.I1RRZ0 pp 229533.I1RSQ7
41049 229533.I1RRZ0 pp 229533.I1RWN6
41050 229533.I1RRZ0 pp 229533.I1S503
41051 229533.I1RRZ0 pp 229533.I1RS18
41052 229533.I1RRZ0 pp 229533.I1RX46
41053 229533.I1RRZ0 pp 229533.I1RUG3
41054 229533.I1RRZ0 pp 229533.I1S0J1
41055 229533.I1RRZ0 pp 229533.I1RWK3
41056 229533.I1RRZ0 pp 229533.I1RW46
41057 229533.I1RRZ0 pp 229533.I1S1C6
41058 229533.I1RRZ0 pp 229533.I1S8V4
41059 229533.I1RRZ0 pp 229533.I1RW99
41060 229533.I1RRZ0 pp 229533.I1RVX0
41061 229533.I1RRZ0 pp 229533.I1RZZ1
41062 229533.I1RRZ0 pp 229533.I1RT03
41063 229533.I1RRZ0 pp 229533.V6RGN5
41064 229533.I1RRZ0 pp 229533.I1RZK6
41065 229533.I1RRZ0 pp 229533.I1S504
41066 229533.I1RRZ0 pp 229533.I1RWN5
41067 229533.I1RRZ0 pp 229533.Q4IJK2
41068 229533.I1RRZ0 pp 229533.V6RIV9
41069 229533.I1RRZ0 pp 229533.I1RSQ3
41070 229533.I1RRZ0 pp 229533.V6R042
41071 229533.I1RRZ0 pp 229533.V6RMW6
41072 229533.I1RRZ0 pp 229533.I1RZM8
41073 229533.I1RRZ0 pp 229533.I1RYB9
41074 229533.I1RSU7 pp 229533.I1S0F0
41075 229533.I1RSU7 pp 229533.I1SB04
41076 229533.I1RSU7 pp 229533.Q4I7L0
41077 229533.I1RSU7 pp 229533.I1RWD0
41078 229533.I1RSU7 pp 229533.I1RW28
41079 229533.I1RSU7 pp 229533.Q4IB50
41080 229533.I1RSU7 pp 229533.Q4IEV4
41081 229533.I1RSU7 pp 229533.Q4IER8
41082 229533.I1RAE5 pp 229533.I1RFT6
41083 229533.I1RAE5 pp 229533.I1S0L8
41084 229533.I1RAE5 pp 229533.I1RJU5
41085 229533.I1RAE5 pp 229533.I1RQN9
41086 229533.I1RAE5 pp 229533.I1RSW5
41087 229533.I1RAE5 pp 229533.I1RPS0
41088 229533.I1RAE5 pp 229533.I1S1V3
41089 229533.I1RAE5 pp 229533.I1RD69
41090 229533.I1RAE5 pp 229533.I1S0S6
41091 229533.I1RAE5 pp 229533.Q4HZB8
41092 229533.I1RAE5 pp 229533.I1RAU7
41093 229533.I1RAE5 pp 229533.I1RPA7
41094 229533.I1RAE5 pp 229533.I1RRX5
41095 229533.I1RAE5 pp 229533.P0C431
41096 229533.I1RAE5 pp 229533.I1RFJ5
41097 229533.I1RAE5 pp 229533.I1RPK9
41098 229533.I1RAE5 pp 229533.I1RN72
41099 229533.I1RAE5 pp 229533.I1REA6

41100 229533.I1RAE5 pp 229533.I1RCJ5
41101 229533.I1RW14 pp 229533.I1S268
41102 229533.I1RA87 pp 229533.Q4IER8
41103 229533.I1RA87 pp 229533.Q4I7N9
41104 229533.I1RA87 pp 229533.Q4IMD1
41105 229533.I1RA87 pp 229533.Q4IAK7
41106 229533.I1RA87 pp 229533.I1RYV3
41107 229533.I1RA87 pp 229533.I1RMQ7
41108 229533.I1RA87 pp 229533.I1RY40
41109 229533.I1RA87 pp 229533.I1RN49
41110 229533.I1RA87 pp 229533.I1RFW1
41111 229533.I1RA87 pp 229533.Q4I5R3
41112 229533.I1RA87 pp 229533.I1RSD1
41113 229533.I1RA87 pp 229533.Q4I7L0
41114 229533.I1RA87 pp 229533.I1RBJ0
41115 229533.I1RA87 pp 229533.Q4HTT1
41116 229533.A0A0E0SID2 pp 229533.I1RJZ7
41117 229533.A0A0E0SID2 pp 229533.I1RW71
41118 229533.A0A0E0SB24 pp 229533.I1RY44
41119 229533.A0A0E0SB24 pp 229533.I1RLT6
41120 229533.A0A0E0SB24 pp 229533.V6RR76
41121 229533.A0A0E0SB24 pp 229533.I1RX09
41122 229533.A0A0E0SB24 pp 229533.I1RMR7
41123 229533.A0A0E0SB24 pp 229533.I1RMT5
41124 229533.A0A0E0SB24 pp 229533.A0A1C3YKT9
41125 229533.A0A0E0SB24 pp 229533.I1RW83
41126 229533.A0A0E0SB24 pp 229533.I1RXF5
41127 229533.A0A0E0SB24 pp 229533.I1RB41
41128 229533.A0A0E0SB24 pp 229533.I1RJT8
41129 229533.A0A0E0SB24 pp 229533.Q4IJM4
41130 229533.A0A0E0SB24 pp 229533.A0A1C3YM48
41131 229533.A0A0E0SB24 pp 229533.I1R9X6
41132 229533.A0A0E0SB24 pp 229533.I1RXU1
41133 229533.A0A0E0SB24 pp 229533.I1RQA3
41134 229533.A0A0E0SB24 pp 229533.I1RS38
41135 229533.A0A0E0SB24 pp 229533.I1S2E9
41136 229533.A0A0E0SB24 pp 229533.I1RPU7
41137 229533.A0A0E0SB24 pp 229533.I1S0N8
41138 229533.A0A0E0SB24 pp 229533.I1RW84
41139 229533.A0A0E0SB24 pp 229533.I1RV91
41140 229533.A0A0E0SB24 pp 229533.A0A1C3YK51
41141 229533.A0A0E0SB24 pp 229533.I1RN81
41142 229533.A0A0E0SB24 pp 229533.I1RW32
41143 229533.A0A0E0SB24 pp 229533.I1RWT6
41144 229533.A0A0E0SB24 pp 229533.I1S2G7
41145 229533.A0A0E0SB24 pp 229533.A0A1C3YJH8
41146 229533.A0A0E0SB24 pp 229533.I1RE20
41147 229533.A0A0E0SB24 pp 229533.I1REN4
41148 229533.A0A0E0SB24 pp 229533.I1RQA9
41149 229533.I1RTE6 pp 229533.I1S0N8
41150 229533.I1RTE6 pp 229533.I1RTQ9
41151 229533.I1RTE6 pp 229533.I1RWL6
41152 229533.I1RTE6 pp 229533.I1S336
41153 229533.I1RTE6 pp 229533.I1RZH4
41154 229533.I1RTE6 pp 229533.I1RYM4
41155 229533.I1RTE6 pp 229533.I1S2A8
41156 229533.I1RTE6 pp 229533.I1S757
41157 229533.I1RTE6 pp 229533.I1S0R9
41158 229533.I1RTE6 pp 229533.I1S0L8
41159 229533.I1RTE6 pp 229533.I1RWB4
41160 229533.I1RTE6 pp 229533.I1S7X9
41161 229533.I1RTE6 pp 229533.I1RW16
41162 229533.I1RTE6 pp 229533.I1S282
41163 229533.I1RP88 pp 229533.I1S1V3
41164 229533.I1RP88 pp 229533.I1S0S6
41165 229533.I1RP88 pp 229533.P0C431
41166 229533.I1RP88 pp 229533.I1RPA7
41167 229533.I1RP88 pp 229533.I1RPK9
41168 229533.I1RP88 pp 229533.Q4HZB8
41169 229533.I1RP88 pp 229533.I1RRX5
41170 229533.I1RP88 pp 229533.I1RSW5
41171 229533.I1RP88 pp 229533.I1S0L8
41172 229533.I1RP88 pp 229533.I1RQN9

41173 229533.I1RP88 pp 229533.I1RPS0
41174 229533.I1RP88 pp 229533.I1RZQ6
41175 229533.A0A0E0RQ26 pp 229533.I1RBD6
41176 229533.A0A0E0RQ26 pp 229533.I1RZP7
41177 229533.A0A0E0RQ26 pp 229533.A0A1C3YL99
41178 229533.A0A0E0RQ26 pp 229533.I1RZP6
41179 229533.A0A0E0RQ26 pp 229533.I1RHI1
41180 229533.A0A0E0RQ26 pp 229533.I1RTA6
41181 229533.A0A1C3YKE4 pp 229533.I1RPQ1
41182 229533.A0A1C3YKE4 pp 229533.I1RD47
41183 229533.A0A1C3YKE4 pp 229533.I1RNH1
41184 229533.A0A1C3YKE4 pp 229533.I1RSR9
41185 229533.A0A1C3YKE4 pp 229533.I1RZJ9
41186 229533.A0A1C3YKE4 pp 229533.I1RSP7
41187 229533.A0A1C3YKE4 pp 229533.I1RSG8
41188 229533.A0A1C3YKE4 pp 229533.I1RWT6
41189 229533.A0A1C3YKE4 pp 229533.I1RA12
41190 229533.A0A1C3YKE4 pp 229533.I1RZP8
41191 229533.A0A1C3YKE4 pp 229533.I1RME4
41192 229533.A0A1C3YKE4 pp 229533.I1RV66
41193 229533.A0A1C3YKE4 pp 229533.I1RSZ4
41194 229533.A0A1C3YKE4 pp 229533.I1S0A6
41195 229533.A0A1C3YKE4 pp 229533.I1RQE3
41196 229533.A0A1C3YKE4 pp 229533.I1RUG4
41197 229533.A0A1C3YKE4 pp 229533.I1RAT5
41198 229533.A0A1C3YKE4 pp 229533.I1RW90
41199 229533.A0A1C3YKE4 pp 229533.I1RYK6
41200 229533.A0A1C3YKE4 pp 229533.I1RE00
41201 229533.A0A1C3YKE4 pp 229533.I1RQA3
41202 229533.A0A1C3YKE4 pp 229533.I1RDJ8
41203 229533.A0A1C3YKE4 pp 229533.I1RA10
41204 229533.A0A1C3YKE4 pp 229533.I1RBW0
41205 229533.A0A1C3YKE4 pp 229533.A0A1C3YLR4
41206 229533.A0A1C3YKE4 pp 229533.I1RRT1
41207 229533.A0A1C3YKE4 pp 229533.I1RG77
41208 229533.A0A1C3YKE4 pp 229533.I1RN55
41209 229533.A0A1C3YKE4 pp 229533.I1RY58
41210 229533.A0A1C3YKE4 pp 229533.I1RK95
41211 229533.A0A1C3YKE4 pp 229533.I1S2E8
41212 229533.A0A1C3YKE4 pp 229533.I1S1W8
41213 229533.A0A1C3YKE4 pp 229533.I1RZ94
41214 229533.A0A1C3YKE4 pp 229533.I1RQI6
41215 229533.A0A1C3YKE4 pp 229533.I1S4P6
41216 229533.A0A1C3YKE4 pp 229533.I1RDH0
41217 229533.A0A1C3YKE4 pp 229533.I1RBC8
41218 229533.A0A1C3YKE4 pp 229533.I1RC54
41219 229533.A0A1C3YKE4 pp 229533.I1RQB4
41220 229533.A0A1C3YKE4 pp 229533.I1RY44
41221 229533.A0A1C3YKE4 pp 229533.Q4IB89
41222 229533.A0A1C3YKE4 pp 229533.I1RSC7
41223 229533.A0A1C3YKE4 pp 229533.A0A1C3YN69
41224 229533.A0A1C3YKE4 pp 229533.A0A1C3YKS0
41225 229533.A0A1C3YKE4 pp 229533.I1RN57
41226 229533.A0A1C3YKE4 pp 229533.I1RV91
41227 229533.A0A1C3YKE4 pp 229533.I1S3G4
41228 229533.A0A1C3YKE4 pp 229533.A0A1C3YL55
41229 229533.A0A1C3YKE4 pp 229533.I1RWI8
41230 229533.A0A1C3YKE4 pp 229533.I1RBW1
41231 229533.A0A1C3YKE4 pp 229533.I1S2E9
41232 229533.A0A1C3YKE4 pp 229533.I1RBU5
41233 229533.A0A1C3YKE4 pp 229533.I1RCA5
41234 229533.A0A1C3YKE4 pp 229533.I1RZ86
41235 229533.A0A1C3YKE4 pp 229533.A0A1C3YMJ3
41236 229533.A0A1C3YKE4 pp 229533.I1RSN7
41237 229533.I1RV82 pp 229533.I1S7S9
41238 229533.I1RPS0 pp 229533.Q4IB89
41239 229533.I1RPS0 pp 229533.I1S2B9
41240 229533.I1RPS0 pp 229533.Q4HWE2
41241 229533.I1RPS0 pp 229533.I1RSC7
41242 229533.I1RPS0 pp 229533.I1RZS7
41243 229533.I1RPS0 pp 229533.I1RS90
41244 229533.I1RPS0 pp 229533.I1RY44
41245 229533.I1RPS0 pp 229533.I1RUP2

41246 229533.I1RPS0 pp 229533.Q4IN00
41247 229533.I1RPS0 pp 229533.I1RWB5
41248 229533.I1RPS0 pp 229533.I1RTN0
41249 229533.I1RPS0 pp 229533.I1S4P6
41250 229533.I1RPS0 pp 229533.I1RZG0
41251 229533.I1RPS0 pp 229533.I1S282
41252 229533.I1RPS0 pp 229533.I1RZV6
41253 229533.I1RPS0 pp 229533.I1RTE6
41254 229533.I1RPS0 pp 229533.I1RSB0
41255 229533.I1RPS0 pp 229533.Q4HZB8
41256 229533.I1RPS0 pp 229533.I1RRJ6
41257 229533.I1RPS0 pp 229533.I1RTG9
41258 229533.I1RPS0 pp 229533.I1RQG7
41259 229533.I1RPS0 pp 229533.I1RUP5
41260 229533.I1RPS0 pp 229533.I1RS20
41261 229533.I1RPS0 pp 229533.I1S0X5
41262 229533.I1RPS0 pp 229533.I1RZD9
41263 229533.I1RPS0 pp 229533.V6REE9
41264 229533.I1RPS0 pp 229533.I1S9G7
41265 229533.I1RPS0 pp 229533.I1S0L8
41266 229533.I1RPS0 pp 229533.I1S0N7
41267 229533.I1RPS0 pp 229533.I1S9V9
41268 229533.I1RPS0 pp 229533.I1S2B6
41269 229533.I1RPS0 pp 229533.I1S2E9
41270 229533.I1RPS0 pp 229533.Q4IEV4
41271 229533.I1RPS0 pp 229533.I1RQA3
41272 229533.I1RPS0 pp 229533.I1S6F1
41273 229533.I1RPS0 pp 229533.Q4IAA0
41274 229533.I1RPS0 pp 229533.I1RWD7
41275 229533.I1RPS0 pp 229533.Q4I948
41276 229533.I1RPS0 pp 229533.Q4ICA8
41277 229533.I1RPS0 pp 229533.I1RY40
41278 229533.I1RPS0 pp 229533.I1RWG1
41279 229533.I1RPS0 pp 229533.I1RWT6
41280 229533.I1RPS0 pp 229533.I1RYM3
41281 229533.I1RPS0 pp 229533.I1RRT1
41282 229533.I1RPS0 pp 229533.I1S095
41283 229533.I1RPS0 pp 229533.I1S183
41284 229533.I1RPS0 pp 229533.I1RQL4
41285 229533.I1RPS0 pp 229533.I1RQ81
41286 229533.I1RPS0 pp 229533.I1RW71
41287 229533.I1RPS0 pp 229533.I1RTA6
41288 229533.I1RPS0 pp 229533.I1RZV4
41289 229533.I1RPS0 pp 229533.I1S788
41290 229533.I1RPS0 pp 229533.Q4INZ9
41291 229533.I1RPS0 pp 229533.I1RSM2
41292 229533.I1RBX3 pp 229533.I1RDJ8
41293 229533.I1RBX3 pp 229533.I1S287
41294 229533.I1RBX3 pp 229533.I1RPU8
41295 229533.A0A0E0S9G7 pp 229533.I1RT41
41296 229533.A0A0E0S9G7 pp 229533.I1RRF0
41297 229533.A0A0E0S9G7 pp 229533.I1RQP1
41298 229533.A0A0E0S9G7 pp 229533.I1RRM8
41299 229533.A0A0E0S9G7 pp 229533.Q4IPI4
41300 229533.A0A0E0S9G7 pp 229533.Q4IL82
41301 229533.A0A0E0S9G7 pp 229533.Q4ICA8
41302 229533.A0A0E0S9G7 pp 229533.I1RQP3
41303 229533.A0A0E0S9G7 pp 229533.Q4I948
41304 229533.I1S0V1 pp 229533.I1S7S9
41305 229533.I1RSG8 pp 229533.I1RVL8
41306 229533.I1RSG8 pp 229533.I1RW90
41307 229533.I1RSG8 pp 229533.I1S9Y1
41308 229533.I1RSG8 pp 229533.I1RW71
41309 229533.I1RSG8 pp 229533.V6QWX8
41310 229533.I1RSG8 pp 229533.I1RT80
41311 229533.I1RSG8 pp 229533.I1RV91
41312 229533.I1RSG8 pp 229533.I1RVY0
41313 229533.I1RSG8 pp 229533.Q4HYQ4
41314 229533.I1RSG8 pp 229533.I1RX64
41315 229533.I1RSG8 pp 229533.I1RUP7
41316 229533.I1RSG8 pp 229533.I1S268
41317 229533.I1RSG8 pp 229533.I1RZS6
41318 229533.I1RSG8 pp 229533.I1RZB5

41319 229533.I1RSG8 pp 229533.I1S0M9
41320 229533.I1RSG8 pp 229533.Q4I5I4
41321 229533.I1RSG8 pp 229533.I1RUP9
41322 229533.I1RSG8 pp 229533.V6QUE6
41323 229533.I1RSG8 pp 229533.Q4IQX3
41324 229533.I1RSG8 pp 229533.I1S021
41325 229533.I1RSZ3 pp 229533.I1RYY5
41326 229533.I1RSZ3 pp 229533.I1S9J6
41327 229533.I1RSZ3 pp 229533.Q4IER8
41328 229533.I1RSZ3 pp 229533.I1RU02
41329 229533.I1RSZ3 pp 229533.Q4HYI0
41330 229533.I1RSZ3 pp 229533.I1RZB5
41331 229533.I1RSZ3 pp 229533.Q4HTT2
41332 229533.I1RSZ3 pp 229533.Q4INS6
41333 229533.I1RSZ3 pp 229533.I1RWH8
41334 229533.I1RSZ3 pp 229533.V6QV38
41335 229533.I1RSZ3 pp 229533.Q4IJM4
41336 229533.I1RSZ3 pp 229533.V6QUE6
41337 229533.I1RSZ3 pp 229533.Q4I5I4
41338 229533.I1RSZ3 pp 229533.I1RXE5
41339 229533.I1RSZ3 pp 229533.I1S209
41340 229533.I1RSZ3 pp 229533.Q4IJU0
41341 229533.I1RSZ3 pp 229533.I1RW90
41342 229533.I1RSZ3 pp 229533.I1RTC2
41343 229533.I1RSZ3 pp 229533.I1RUR0
41344 229533.I1RSZ3 pp 229533.I1RWT6
41345 229533.I1RSZ3 pp 229533.I1RT35
41346 229533.I1RSZ3 pp 229533.V6QWX8
41347 229533.I1RSZ3 pp 229533.I1RZ94
41348 229533.I1RSZ3 pp 229533.I1S182
41349 229533.I1RSZ3 pp 229533.I1RW91
41350 229533.I1RSZ3 pp 229533.I1RTN6
41351 229533.I1RSZ3 pp 229533.I1RZ42
41352 229533.I1RSZ3 pp 229533.I1RZP8
41353 229533.I1RSZ3 pp 229533.Q4IQX3
41354 229533.I1RSZ3 pp 229533.I1SAD2
41355 229533.I1RSZ3 pp 229533.I1S276
41356 229533.I1RSZ3 pp 229533.I1S004
41357 229533.I1RSZ3 pp 229533.V6R2A1
41358 229533.I1RSZ3 pp 229533.Q4I5U9
41359 229533.I1RSZ3 pp 229533.I1S0N5
41360 229533.I1RSZ3 pp 229533.I1RVY0
41361 229533.I1RSZ3 pp 229533.I1RY44
41362 229533.I1RSZ3 pp 229533.I1RUG4
41363 229533.I1RSZ3 pp 229533.I1RWF3
41364 229533.I1RSZ3 pp 229533.I1RVW0
41365 229533.I1RSZ3 pp 229533.I1RV91
41366 229533.I1RSZ3 pp 229533.I1S0J3
41367 229533.I1RSZ3 pp 229533.O74638
41368 229533.I1RSZ3 pp 229533.I1RY29
41369 229533.I1RSZ3 pp 229533.I1RXU0
41370 229533.I1RSZ3 pp 229533.I1S2E9
41371 229533.I1RSZ3 pp 229533.I1S9Y1
41372 229533.I1RSZ3 pp 229533.I1S3N1
41373 229533.I1RSZ3 pp 229533.I1RWI8
41374 229533.I1RSZ3 pp 229533.I1S0C6
41375 229533.I1RSZ3 pp 229533.I1RW80
41376 229533.I1RSZ3 pp 229533.Q4I5W5
41377 229533.I1RSZ3 pp 229533.I1RUP9
41378 229533.I1RSZ3 pp 229533.I1RUF8
41379 229533.I1RSZ3 pp 229533.Q4HYB8
41380 229533.I1RSZ3 pp 229533.I1RVL8
41381 229533.I1RSZ3 pp 229533.I1RZM2
41382 229533.I1RSZ3 pp 229533.I1S3N0
41383 229533.I1RSZ3 pp 229533.I1RX63
41384 229533.A0A1C3YI80 pp 229533.I1S0E9
41385 229533.A0A1C3YI80 pp 229533.A0A1C3YIP6
41386 229533.A0A1C3YI80 pp 229533.I1RNY7
41387 229533.A0A1C3YI80 pp 229533.I1RLX6
41388 229533.A0A1C3YI80 pp 229533.I1RB84
41389 229533.A0A1C3YI80 pp 229533.A0A1C3YIA8
41390 229533.A0A1C3YI80 pp 229533.I1RT41
41391 229533.A0A1C3YI80 pp 229533.I1RQG9

41392	229533.A0A1C3YI80	pp	229533.V6RR76
41393	229533.A0A1C3YI80	pp	229533.I1RN81
41394	229533.A0A1C3YI80	pp	229533.I1RAL2
41395	229533.A0A1C3YI80	pp	229533.I1S050
41396	229533.A0A1C3YI80	pp	229533.I1RBN1
41397	229533.A0A1C3YI80	pp	229533.I1RAI8
41398	229533.A0A1C3YI80	pp	229533.I1RKR8
41399	229533.A0A1C3YI80	pp	229533.I1S1V1
41400	229533.A0A1C3YI80	pp	229533.I1RXB5
41401	229533.A0A1C3YI80	pp	229533.I1RWC3
41402	229533.A0A1C3YI80	pp	229533.I1S060
41403	229533.A0A1C3YI80	pp	229533.I1RP28
41404	229533.A0A1C3YI80	pp	229533.I1RAU1
41405	229533.A0A1C3YI80	pp	229533.A0A1C3YIZ2
41406	229533.A0A1C3YI80	pp	229533.I1RCN6
41407	229533.A0A1C3YI80	pp	229533.I1RC81
41408	229533.A0A1C3YI80	pp	229533.I1RZQ6
41409	229533.A0A1C3YI80	pp	229533.I1RD70
41410	229533.A0A1C3YI80	pp	229533.I1RQG4
41411	229533.A0A1C3YI80	pp	229533.A0A1C3YLE6
41412	229533.I1S1K9	pp	229533.I1S7S9
41413	229533.I1RX64	pp	229533.I1RY58
41414	229533.I1RX64	pp	229533.I1RX90
41415	229533.I1RX64	pp	229533.I1S2K7
41416	229533.I1RX64	pp	229533.I1RZP8
41417	229533.I1RX64	pp	229533.I1S2E8
41418	229533.I1RX64	pp	229533.I1S0A6
41419	229533.I1RX64	pp	229533.I1S0D5
41420	229533.I1RX64	pp	229533.Q4IN00
41421	229533.I1RX64	pp	229533.I1RYK6
41422	229533.I1RX64	pp	229533.Q4IB89
41423	229533.I1RX64	pp	229533.I1S4P6
41424	229533.I1RX64	pp	229533.I1S998
41425	229533.I1RX64	pp	229533.I1S523
41426	229533.I1RX64	pp	229533.I1RY44
41427	229533.I1RX64	pp	229533.V6RLW4
41428	229533.I1RX64	pp	229533.I1RZ86
41429	229533.I1RX64	pp	229533.I1RYQ1
41430	229533.I1RX64	pp	229533.I1RYX9
41431	229533.I1RX64	pp	229533.I1S2W9
41432	229533.I1RX64	pp	229533.Q4HZB8
41433	229533.I1RX64	pp	229533.I1S3G4
41434	229533.I1RX64	pp	229533.I1S461
41435	229533.I1RX64	pp	229533.I1S2E9
41436	229533.I1RX64	pp	229533.I1S2F2
41437	229533.I1RX64	pp	229533.I1RYR2
41438	229533.I1RX64	pp	229533.Q4I0J6
41439	229533.I1RX64	pp	229533.Q4IE79
41440	229533.I1RX64	pp	229533.I1RY73
41441	229533.I1RX64	pp	229533.I1S209
41442	229533.I1RX64	pp	229533.Q4IBX0
41443	229533.I1RX64	pp	229533.Q4IER8
41444	229533.I1RX64	pp	229533.Q4INS6
41445	229533.I1RX64	pp	229533.I1RZJ9
41446	229533.I1RX64	pp	229533.Q4IEA5
41447	229533.I1RX64	pp	229533.I1RZ94
41448	229533.I1RX64	pp	229533.I1RXH6
41449	229533.I1RX64	pp	229533.I1S987
41450	229533.I1RX64	pp	229533.I1RZ02
41451	229533.I1RX64	pp	229533.Q4INZ9
41452	229533.I1RX64	pp	229533.I1S1W8
41453	229533.I1RX64	pp	229533.I1S283
41454	229533.I1RZK9	pp	229533.I1S2E9
41455	229533.I1RZK9	pp	229533.I1RZT1
41456	229533.I1RZK9	pp	229533.I1S004
41457	229533.I1RZK9	pp	229533.Q4IB89
41458	229533.I1RZK9	pp	229533.I1S098
41459	229533.I1RZK9	pp	229533.I1S4P6
41460	229533.I1RZK9	pp	229533.I1S788
41461	229533.I1RK54	pp	229533.I1RPU8
41462	229533.I1RK54	pp	229533.I1S287
41463	229533.I1RIC5	pp	229533.I1RS41
41464	229533.I1RIC5	pp	229533.I1RQ88

41465	229533.I1RIC5	pp	229533.I1S7S9
41466	229533.I1RSP7	pp	229533.I1RYR1
41467	229533.I1RSP7	pp	229533.I1S209
41468	229533.I1RSP7	pp	229533.I1S604
41469	229533.I1RSP7	pp	229533.I1S987
41470	229533.I1RSP7	pp	229533.I1RY73
41471	229533.I1RSP7	pp	229533.I1RY80
41472	229533.I1RSP7	pp	229533.I1RZV9
41473	229533.I1RSP7	pp	229533.Q4IC11
41474	229533.I1RSP7	pp	229533.I1S0P6
41475	229533.I1RSP7	pp	229533.I1S0L3
41476	229533.I1RSP7	pp	229533.I1S1W8
41477	229533.I1RSP7	pp	229533.Q4INS6
41478	229533.I1RSP7	pp	229533.I1RZ94
41479	229533.I1RSP7	pp	229533.Q4IP51
41480	229533.I1RSP7	pp	229533.I1S461
41481	229533.I1RSP7	pp	229533.Q4IEA5
41482	229533.I1RSP7	pp	229533.I1RUG2
41483	229533.I1RSP7	pp	229533.I1RUR0
41484	229533.I1RSP7	pp	229533.Q4HZQ1
41485	229533.I1RSP7	pp	229533.I1RT03
41486	229533.I1RSP7	pp	229533.I1S283
41487	229533.I1RSP7	pp	229533.I1RYQ1
41488	229533.I1RSP7	pp	229533.I1S0D5
41489	229533.I1RSP7	pp	229533.I1S4P6
41490	229533.I1RSP7	pp	229533.I1RSR9
41491	229533.I1RSP7	pp	229533.Q4IB89
41492	229533.I1RSP7	pp	229533.I1S0C2
41493	229533.I1RSP7	pp	229533.Q4IQM6
41494	229533.I1RSP7	pp	229533.I1RW16
41495	229533.I1RSP7	pp	229533.I1S1C7
41496	229533.I1RSP7	pp	229533.I1S8J1
41497	229533.I1RSP7	pp	229533.I1RV91
41498	229533.I1RSP7	pp	229533.I1RY29
41499	229533.I1RSP7	pp	229533.I1RZ98
41500	229533.I1RSP7	pp	229533.I1RUG4
41501	229533.I1RSP7	pp	229533.I1RWI8
41502	229533.I1RSP7	pp	229533.I1RZP8
41503	229533.I1RSP7	pp	229533.V6RLW4
41504	229533.I1RSP7	pp	229533.I1S0A6
41505	229533.I1RSP7	pp	229533.I1RTN6
41506	229533.I1RSP7	pp	229533.I1S998
41507	229533.I1RSP7	pp	229533.I1RX64
41508	229533.I1RSP7	pp	229533.I1RYR2
41509	229533.I1RSP7	pp	229533.I1S3G4
41510	229533.I1RSP7	pp	229533.I1RVL8
41511	229533.I1RSP7	pp	229533.I1RYX9
41512	229533.I1RSP7	pp	229533.I1RZ86
41513	229533.A0A0E0RPW7	pp	229533.I1RJS1
41514	229533.A0A0E0RPW7	pp	229533.I1RPV8
41515	229533.A0A0E0RPW7	pp	229533.I1RZ38
41516	229533.A0A0E0RPW7	pp	229533.I1RKB2
41517	229533.A0A0E0RPW7	pp	229533.I1S0A5
41518	229533.A0A0E0RPW7	pp	229533.I1RZ56
41519	229533.A0A0E0RPW7	pp	229533.I1RGZ3
41520	229533.A0A0E0RPW7	pp	229533.I1RZ25
41521	229533.A0A0E0RPW7	pp	229533.I1RNG4
41522	229533.A0A0E0RPW7	pp	229533.I1S3H8
41523	229533.A0A0E0RPW7	pp	229533.A0A1C3YKN4
41524	229533.A0A0E0RPW7	pp	229533.I1RA13
41525	229533.A0A0E0RPW7	pp	229533.I1RDU0
41526	229533.A0A0E0RPW7	pp	229533.I1RC22
41527	229533.A0A0E0RPW7	pp	229533.I1RST9
41528	229533.A0A0E0RPW7	pp	229533.I1RUY5
41529	229533.A0A0E0RPW7	pp	229533.I1S8F8
41530	229533.A0A0E0RPW7	pp	229533.I1S094
41531	229533.A0A0E0RPW7	pp	229533.I1RLA2
41532	229533.A0A0E0RPW7	pp	229533.I1S6H3
41533	229533.A0A0E0RPW7	pp	229533.I1RNM7
41534	229533.A0A0E0RPW7	pp	229533.I1RRL5
41535	229533.A0A0E0RPW7	pp	229533.I1S334
41536	229533.A0A0E0RPW7	pp	229533.I1RDN3
41537	229533.A0A098DPM7	pp	229533.I1RFA2

41538 229533.I1RDQ0 pp 229533.I1RW71
41539 229533.I1RDQ0 pp 229533.I1RJE5
41540 229533.I1RDQ0 pp 229533.I1RJZ7
41541 229533.I1RDQ0 pp 229533.I1RXJ5
41542 229533.A0A1C3YMN6 pp 229533.I1RG62
41543 229533.A0A1C3YMN6 pp 229533.I1S212
41544 229533.A0A1C3YMN6 pp 229533.I1S0Q7
41545 229533.A0A1C3YMN6 pp 229533.I1S0R8
41546 229533.A0A1C3YMN6 pp 229533.I1RZ43
41547 229533.A0A1C3YMN6 pp 229533.I1RAM9
41548 229533.A0A1C3YMN6 pp 229533.I1RAV5
41549 229533.A0A1C3YMN6 pp 229533.I1RX39
41550 229533.A0A1C3YMN6 pp 229533.I1RKB7
41551 229533.A0A1C3YMN6 pp 229533.I1RBE6
41552 229533.A0A0E0S675 pp 229533.I1RS47
41553 229533.I1RQF6 pp 229533.Q4I9U7
41554 229533.I1RQF6 pp 229533.I1RX96
41555 229533.I1RQF6 pp 229533.I1S549
41556 229533.I1RQF6 pp 229533.I1RYF8
41557 229533.I1RQF6 pp 229533.I1RXT9
41558 229533.I1RQF6 pp 229533.V6RGY3
41559 229533.I1RQF6 pp 229533.I1RZW7
41560 229533.I1RQF6 pp 229533.I1RSL8
41561 229533.I1RQF6 pp 229533.Q4HWB3
41562 229533.I1RQF6 pp 229533.I1S287
41563 229533.I1RQF6 pp 229533.I1RYM2
41564 229533.I1RB42 pp 229533.I1RBF3
41565 229533.A0A1C3YMJ2 pp 229533.I1RFU6
41566 229533.A0A1C3YMJ2 pp 229533.I1RC63
41567 229533.I1SB04 pp 229533.Q4IR08
41568 229533.I1SB04 pp 229533.V6RK56
41569 229533.I1SB04 pp 229533.Q4HTT2
41570 229533.I1SB04 pp 229533.Q4I948
41571 229533.I1SB04 pp 229533.Q4I5R3
41572 229533.I1SB04 pp 229533.Q4IPI4
41573 229533.I1SB04 pp 229533.Q4ICA8
41574 229533.I1SB04 pp 229533.Q4IER8
41575 229533.I1SB04 pp 229533.Q4IAK7
41576 229533.I1SB04 pp 229533.Q4I5V3
41577 229533.I1SB04 pp 229533.Q4HY90
41578 229533.I1SB04 pp 229533.Q4HYQ4
41579 229533.I1SB04 pp 229533.Q4I7S1
41580 229533.I1SB04 pp 229533.Q4IB96
41581 229533.I1SB04 pp 229533.Q4IB50
41582 229533.I1SB04 pp 229533.Q4IMD1
41583 229533.I1SB04 pp 229533.Q4IEV4
41584 229533.I1SB04 pp 229533.Q4I7L0
41585 229533.A0A0E0S664 pp 229533.V6RLM7
41586 229533.A0A0E0S664 pp 229533.I1RHJ2
41587 229533.A0A0E0S664 pp 229533.I1RUY6
41588 229533.I1RK39 pp 229533.I1RKB7
41589 229533.I1RK39 pp 229533.I1RY21
41590 229533.I1RK39 pp 229533.I1RMQ6
41591 229533.I1RK39 pp 229533.I1RXD9
41592 229533.I1RK39 pp 229533.I1RWA3
41593 229533.I1RK39 pp 229533.I1S1F3
41594 229533.I1RK39 pp 229533.I1RYT8
41595 229533.I1RK39 pp 229533.I1RNM7
41596 229533.I1RK39 pp 229533.I1RMK7
41597 229533.I1RK39 pp 229533.I1S464
41598 229533.I1RK39 pp 229533.I1S5X9
41599 229533.I1RK39 pp 229533.I1S0P0
41600 229533.I1RK39 pp 229533.I1RS05
41601 229533.I1RK39 pp 229533.I1S0Q7
41602 229533.I1RK39 pp 229533.I1S0R8
41603 229533.I1RK39 pp 229533.I1RY92
41604 229533.I1RK39 pp 229533.I1S0K2
41605 229533.I1RK39 pp 229533.I1RS29
41606 229533.I1RK39 pp 229533.V6RJP8
41607 229533.I1RK39 pp 229533.I1RP02
41608 229533.I1RYW7 pp 229533.I1S208
41609 229533.I1RE40 pp 229533.I1RE65
41610 229533.I1RE40 pp 229533.I1RFJ6

41611	229533.I1RE40	pp	229533.I1S5X9
41612	229533.I1RE40	pp	229533.I1RWA3
41613	229533.I1RE40	pp	229533.I1S464
41614	229533.I1RE40	pp	229533.I1RXD9
41615	229533.I1RE40	pp	229533.I1S458
41616	229533.I1RE40	pp	229533.I1S0P0
41617	229533.I1RE40	pp	229533.I1RY92
41618	229533.I1RE40	pp	229533.V6RJP8
41619	229533.I1RE40	pp	229533.I1RKF4
41620	229533.I1RE40	pp	229533.I1RMK7
41621	229533.I1RE40	pp	229533.I1RS05
41622	229533.I1RE40	pp	229533.I1RP02
41623	229533.I1RE40	pp	229533.I1RYT8
41624	229533.I1RE40	pp	229533.I1RS29
41625	229533.I1RE40	pp	229533.I1RKF5
41626	229533.I1RE40	pp	229533.I1S0K2
41627	229533.I1RE40	pp	229533.V6QWX8
41628	229533.I1RE40	pp	229533.I1RY21
41629	229533.I1RE40	pp	229533.I1S0R1
41630	229533.I1RE40	pp	229533.I1S8S9
41631	229533.I1RE40	pp	229533.I1RYQ3
41632	229533.I1RE40	pp	229533.I1S5C6
41633	229533.I1RN13	pp	229533.I1S2M8
41634	229533.I1RN13	pp	229533.I1RYV4
41635	229533.I1RN13	pp	229533.I1RN14
41636	229533.I1RY77	pp	229533.I1S109
41637	229533.I1RY77	pp	229533.Q4IMD1
41638	229533.I1RY77	pp	229533.Q4I7L0
41639	229533.I1RY77	pp	229533.I1SB04
41640	229533.I1S0T7	pp	229533.I1S1F3
41641	229533.I1S0T7	pp	229533.I1S464
41642	229533.I1S0T7	pp	229533.I1S5X9
41643	229533.I1S0T7	pp	229533.V6RJP8
41644	229533.I1RA43	pp	229533.I1RUN8
41645	229533.I1RA43	pp	229533.I1RPM4
41646	229533.I1RA43	pp	229533.I1S1V5
41647	229533.I1RA43	pp	229533.I1RAS9
41648	229533.I1RA43	pp	229533.I1RNP7
41649	229533.I1RA43	pp	229533.I1RLU1
41650	229533.I1RA43	pp	229533.I1S1W4
41651	229533.I1RA43	pp	229533.I1RAN2
41652	229533.I1RA43	pp	229533.V6RKY7
41653	229533.I1RA43	pp	229533.I1RY44
41654	229533.I1RA43	pp	229533.I1S0D1
41655	229533.I1RA43	pp	229533.V6RVG3
41656	229533.I1RA43	pp	229533.I1RC97
41657	229533.I1RA43	pp	229533.I1RNF4
41658	229533.I1RA43	pp	229533.I1RBS0
41659	229533.I1RA43	pp	229533.I1S1D3
41660	229533.I1RA43	pp	229533.I1RPD6
41661	229533.I1RA43	pp	229533.I1RNY8
41662	229533.I1RA43	pp	229533.I1RFL7
41663	229533.I1RA43	pp	229533.I1RB74
41664	229533.I1RA43	pp	229533.I1RT36
41665	229533.I1RA43	pp	229533.I1S2E9
41666	229533.I1RA43	pp	229533.I1RV91
41667	229533.I1RA43	pp	229533.V6RGI1
41668	229533.I1RA43	pp	229533.I1RPU3
41669	229533.I1RA43	pp	229533.I1RQ72
41670	229533.I1RA43	pp	229533.I1RNW8
41671	229533.I1RA43	pp	229533.I1S0N4
41672	229533.I1RA43	pp	229533.I1RBM3
41673	229533.I1RA43	pp	229533.I1RNY9
41674	229533.I1RA43	pp	229533.I1S0M2
41675	229533.I1RA43	pp	229533.I1RMF5
41676	229533.I1RA43	pp	229533.I1RNC0
41677	229533.I1RA43	pp	229533.I1RDZ4
41678	229533.I1RA43	pp	229533.I1S224
41679	229533.I1RA43	pp	229533.I1RZE4
41680	229533.I1RA43	pp	229533.I1RQB4
41681	229533.I1RA43	pp	229533.I1RYH9
41682	229533.I1RA43	pp	229533.I1RRW0
41683	229533.I1RA43	pp	229533.I1RRY3

41684	229533.I1RA43	pp	229533.I1RBZ9
41685	229533.I1RA43	pp	229533.I1RC95
41686	229533.I1RA43	pp	229533.I1RD18
41687	229533.I1RA43	pp	229533.I1RL24
41688	229533.I1RA43	pp	229533.I1RQ26
41689	229533.I1RA43	pp	229533.I1RJH1
41690	229533.I1RA43	pp	229533.V6QV09
41691	229533.I1RA43	pp	229533.Q4IBS2
41692	229533.I1RA43	pp	229533.I1S9U2
41693	229533.I1RA43	pp	229533.I1RCB3
41694	229533.I1RA43	pp	229533.I1RV66
41695	229533.I1RA43	pp	229533.I1RM09
41696	229533.I1RA43	pp	229533.I1RAW7
41697	229533.I1RA43	pp	229533.I1RB75
41698	229533.I1RA43	pp	229533.I1RX70
41699	229533.I1RA43	pp	229533.I1RYZ9
41700	229533.I1RA43	pp	229533.I1S439
41701	229533.I1RA43	pp	229533.I1RDB1
41702	229533.I1RA43	pp	229533.I1RYZ0
41703	229533.I1RA43	pp	229533.I1RWJ6
41704	229533.I1RA43	pp	229533.I1RWT6
41705	229533.I1RA43	pp	229533.I1RN65
41706	229533.I1RA43	pp	229533.Q4IJM4
41707	229533.I1RA43	pp	229533.I1RZH4
41708	229533.I1RA43	pp	229533.I1RAM7
41709	229533.I1RA43	pp	229533.I1RKK0
41710	229533.I1RA43	pp	229533.I1RC17
41711	229533.I1RA43	pp	229533.I1RQA3
41712	229533.I1RA43	pp	229533.I1RMM9
41713	229533.I1RA43	pp	229533.I1RN08
41714	229533.I1RA43	pp	229533.I1RUQ5
41715	229533.I1RA43	pp	229533.I1RC22
41716	229533.I1RA43	pp	229533.I1RYY5
41717	229533.I1RIU9	pp	229533.V6QUV7
41718	229533.I1RGJ4	pp	229533.I1S0R8
41719	229533.I1RGJ4	pp	229533.I1S464
41720	229533.I1RGJ4	pp	229533.V6RJP8
41721	229533.I1RGJ4	pp	229533.I1S5X9
41722	229533.I1RGJ4	pp	229533.I1RP02
41723	229533.I1RGJ4	pp	229533.I1S1F3
41724	229533.I1RGJ4	pp	229533.I1S0K2
41725	229533.I1RGJ4	pp	229533.I1RXD9
41726	229533.I1RGJ4	pp	229533.I1S0Q7
41727	229533.I1RGJ4	pp	229533.I1RS29
41728	229533.I1RGJ4	pp	229533.I1S0P0
41729	229533.I1RGJ4	pp	229533.I1RWA3
41730	229533.I1RGJ4	pp	229533.I1RY92
41731	229533.I1RGJ4	pp	229533.I1RYT8
41732	229533.I1RGJ4	pp	229533.I1RMK7
41733	229533.I1RGJ4	pp	229533.I1RMQ6
41734	229533.I1RGJ4	pp	229533.I1RS05
41735	229533.I1RGJ4	pp	229533.I1RY21
41736	229533.I1RGJ4	pp	229533.I1RNM7
41737	229533.I1RGJ4	pp	229533.I1RKB7
41738	229533.A0A1C3YKP8	pp	229533.I1RY99
41739	229533.A0A1C3YKP8	pp	229533.A0A1C3YLP1
41740	229533.A0A1C3YKP8	pp	229533.I1S109
41741	229533.A0A1C3YKP8	pp	229533.I1RA73
41742	229533.A0A1C3YKP8	pp	229533.Q4HY90
41743	229533.A0A1C3YKP8	pp	229533.Q4IEV4
41744	229533.A0A1C3YKP8	pp	229533.I1RFW1
41745	229533.A0A1C3YKP8	pp	229533.I1RW28
41746	229533.A0A1C3YKP8	pp	229533.I1RB73
41747	229533.A0A1C3YKP8	pp	229533.I1SB04
41748	229533.A0A1C3YKP8	pp	229533.I1RPR8
41749	229533.A0A1C3YKP8	pp	229533.Q4I7L0
41750	229533.A0A1C3YKP8	pp	229533.I1RCN2
41751	229533.A0A1C3YKP8	pp	229533.I1RXE5
41752	229533.A0A1C3YKP8	pp	229533.A0A1C3YM68
41753	229533.A0A1C3YKP8	pp	229533.Q4IER8
41754	229533.A0A1C3YKP8	pp	229533.I1RW60
41755	229533.I1RNU0	pp	229533.I1S287
41756	229533.I1RNU0	pp	229533.I1RPU8

229533.A0A1C3YIA5 pp 229533.Q4I7L0
229533.A0A1C3YIA5 pp 229533.I1RAK6
229533.A0A1C3YIA5 pp 229533.I1RFQ0
229533.A0A1C3YIA5 pp 229533.I1RB73
229533.A0A1C3YIA5 pp 229533.I1SB04
229533.A0A1C3YIA5 pp 229533.I1RCN2
229533.A0A1C3YIA5 pp 229533.A0A1C3YN58
229533.A0A1C3YIA5 pp 229533.Q4IMD1
229533.A0A1C3YIA5 pp 229533.I1RAN8
229533.A0A1C3YIA5 pp 229533.I1S109
229533.A0A1C3YIA5 pp 229533.I1RUZ8
229533.A0A1C3YIA5 pp 229533.A0A1C3YK75
229533.I1RNA2 pp 229533.I1RZ86
229533.I1RNA2 pp 229533.I1S523
229533.I1RNA2 pp 229533.I1RUR0
229533.I1RNA2 pp 229533.I1RYQ1
229533.I1RNA2 pp 229533.I1RWM6
229533.I1RNA2 pp 229533.I1S4P6
229533.I1RNA2 pp 229533.Q4IB89
229533.I1RNA2 pp 229533.I1RWI8
229533.I1RNA2 pp 229533.I1RSL6
229533.I1RNA2 pp 229533.I1RP99
229533.I1RNA2 pp 229533.I1RX64
229533.I1RNA2 pp 229533.I1RQH8
229533.I1RNA2 pp 229533.I1RSC7
229533.I1RNA2 pp 229533.I1RZ98
229533.I1RNA2 pp 229533.Q4INS6
229533.I1RS80 pp 229533.I1S341
229533.I1RS80 pp 229533.I1RVE0
229533.I1RS80 pp 229533.I1SAA1
229533.I1RS80 pp 229533.Q4IP51
229533.I1RS80 pp 229533.I1SAS1
229533.I1RS80 pp 229533.I1RSB7
229533.A0A1C3YLP1 pp 229533.I1S0N5
229533.A0A1C3YLP1 pp 229533.I1S109
229533.A0A1C3YLP1 pp 229533.I1RUZ8
229533.A0A1C3YLP1 pp 229533.I1RSH4
229533.A0A1C3YLP1 pp 229533.I1RSI1
229533.A0A1C3YLP1 pp 229533.Q4IER8
229533.A0A1C3YLP1 pp 229533.I1RT16
229533.A0A1C3YLP1 pp 229533.I1S1Y7
229533.A0A1C3YLP1 pp 229533.Q4HTT2
229533.A0A1C3YLP1 pp 229533.I1S7M3
229533.A0A1C3YLP1 pp 229533.A0A1C3YM68
229533.A0A1C3YLP1 pp 229533.I1RLF1
229533.A0A1C3YLP1 pp 229533.I1S1U7
229533.A0A1C3YLP1 pp 229533.A0A1C3YM82
229533.A0A1C3YLP1 pp 229533.A0A1C3YLP8
229533.A0A1C3YLP1 pp 229533.Q4IMD1
229533.A0A1C3YLP1 pp 229533.I1RFW1
229533.A0A1C3YLP1 pp 229533.I1RAN8
229533.A0A1C3YLP1 pp 229533.I1S1V2
229533.A0A1C3YLP1 pp 229533.I1RC32
229533.A0A1C3YLP1 pp 229533.A0A1C3YN58
229533.A0A1C3YLP1 pp 229533.I1RFQ0
229533.A0A1C3YLP1 pp 229533.I1RB73
229533.A0A1C3YLP1 pp 229533.I1RCN2
229533.A0A1C3YLP1 pp 229533.I1SB04
229533.A0A1C3YLP1 pp 229533.I1RW60
229533.A0A1C3YLP1 pp 229533.Q4I7L0
229533.A0A1C3YLP1 pp 229533.I1RAK6
229533.A0A1C3YLP1 pp 229533.I1RB67
229533.I1RJT0 pp 229533.I1RW84
229533.I1RJT0 pp 229533.I1RZW1
229533.I1RJT0 pp 229533.I1RQA3
229533.I1RJT0 pp 229533.I1RYB7
229533.I1RJT0 pp 229533.I1RM12
229533.I1RJT0 pp 229533.I1RJT8
229533.I1RJT0 pp 229533.I1RJT2
229533.I1RJT0 pp 229533.I1RK67
229533.I1RJT0 pp 229533.I1RWM7
229533.I1RJT0 pp 229533.I1RTD0
229533.I1RJT0 pp 229533.I1RMR7

41830	229533.I1RJT0	pp	229533.I1RV91
41831	229533.I1RJT0	pp	229533.I1S074
41832	229533.I1RJT0	pp	229533.I1S2E9
41833	229533.I1RJT0	pp	229533.I1S1A3
41834	229533.I1RJT0	pp	229533.I1RY44
41835	229533.I1RJT0	pp	229533.Q4IBS9
41836	229533.I1RJT0	pp	229533.I1RPC6
41837	229533.I1RJT0	pp	229533.I1RNB9
41838	229533.I1RJT0	pp	229533.I1RLT6
41839	229533.I1RJT0	pp	229533.I1RMA7
41840	229533.I1RJT0	pp	229533.I1RZK4
41841	229533.I1RJT0	pp	229533.I1RW83
41842	229533.I1RJT0	pp	229533.I1RW38
41843	229533.I1RJT0	pp	229533.I1S248
41844	229533.I1RJT0	pp	229533.I1RWT6
41845	229533.I1RJT0	pp	229533.I1S0F5
41846	229533.I1RJT0	pp	229533.I1RS34
41847	229533.I1RJT0	pp	229533.I1S1W5
41848	229533.I1RJT0	pp	229533.I1RPH1
41849	229533.I1RJT0	pp	229533.I1S2G7
41850	229533.I1RBC8	pp	229533.I1RH70
41851	229533.I1RBC8	pp	229533.Q4IB89
41852	229533.I1RBC8	pp	229533.I1RQI6
41853	229533.I1RBC8	pp	229533.I1RZ98
41854	229533.I1RBC8	pp	229533.I1S4P6
41855	229533.I1RBC8	pp	229533.I1RC54
41856	229533.I1RBC8	pp	229533.I1RSC7
41857	229533.I1RBC8	pp	229533.I1RSR9
41858	229533.I1RBC8	pp	229533.I1RKB6
41859	229533.I1RBC8	pp	229533.I1S0D5
41860	229533.I1RBC8	pp	229533.I1RNA2
41861	229533.I1RBC8	pp	229533.I1RUG4
41862	229533.I1RBC8	pp	229533.I1S0A6
41863	229533.I1RBC8	pp	229533.I1RBN9
41864	229533.I1RBC8	pp	229533.I1RJV8
41865	229533.I1RBC8	pp	229533.I1RX64
41866	229533.I1RBC8	pp	229533.I1RWI8
41867	229533.I1RBC8	pp	229533.I1RP99
41868	229533.I1RBC8	pp	229533.I1RZP8
41869	229533.I1RBC8	pp	229533.I1RRN5
41870	229533.I1RBC8	pp	229533.I1RDH0
41871	229533.I1RBC8	pp	229533.I1RDJ8
41872	229533.I1RBC8	pp	229533.Q4IEV4
41873	229533.I1RBC8	pp	229533.I1RY29
41874	229533.I1RBC8	pp	229533.I1RSP7
41875	229533.I1RBC8	pp	229533.I1RW28
41876	229533.I1RBC8	pp	229533.I1RD19
41877	229533.I1RBC8	pp	229533.I1RQJ4
41878	229533.I1RBC8	pp	229533.I1RYQ1
41879	229533.I1RBC8	pp	229533.I1RFZ2
41880	229533.I1RBC8	pp	229533.I1RVV5
41881	229533.I1RBC8	pp	229533.I1RQ67
41882	229533.I1RBC8	pp	229533.I1RPR8
41883	229533.I1RBC8	pp	229533.I1S3G4
41884	229533.I1RBC8	pp	229533.I1RNG4
41885	229533.I1RBC8	pp	229533.I1RVW0
41886	229533.I1RBC8	pp	229533.I1RBW1
41887	229533.I1RBC8	pp	229533.I1RHD2
41888	229533.I1RBC8	pp	229533.I1RSN7
41889	229533.I1RBC8	pp	229533.I1RM26
41890	229533.I1RBC8	pp	229533.I1RF70
41891	229533.I1RBC8	pp	229533.I1RFJ2
41892	229533.I1RBC8	pp	229533.I1RRJ6
41893	229533.I1RBC8	pp	229533.I1RBU5
41894	229533.I1RBC8	pp	229533.I1S998
41895	229533.I1RBC8	pp	229533.Q4IBX0
41896	229533.I1RBC8	pp	229533.I1S209
41897	229533.I1RBC8	pp	229533.Q4INS6
41898	229533.I1RBC8	pp	229533.I1RJZ7
41899	229533.I1RBC8	pp	229533.I1RBF3
41900	229533.I1RBC8	pp	229533.I1RG77
41901	229533.I1RBC8	pp	229533.I1RQH8
41902	229533.I1RBC8	pp	229533.I1RVZ9

41903 229533.I1RBC8 pp 229533.I1RCM6
41904 229533.I1RBC8 pp 229533.I1RZ86
41905 229533.I1RBC8 pp 229533.I1RPI0
41906 229533.I1RBC8 pp 229533.I1RCA5
41907 229533.I1RBC8 pp 229533.I1RZ94
41908 229533.I1RBC8 pp 229533.Q4I0J6
41909 229533.I1RBC8 pp 229533.I1RWM6
41910 229533.I1RBC8 pp 229533.I1RBE7
41911 229533.I1RBC8 pp 229533.I1RPQ1
41912 229533.I1RBC8 pp 229533.I1RMS2
41913 229533.I1RBC8 pp 229533.I1RD01
41914 229533.I1RBC8 pp 229533.I1S788
41915 229533.I1RBC8 pp 229533.I1S283
41916 229533.I1RBC8 pp 229533.I1RRT1
41917 229533.I1RBC8 pp 229533.I1RVX9
41918 229533.I1RBC8 pp 229533.I1RUR0
41919 229533.I1RBC8 pp 229533.I1RSJ1
41920 229533.I1RBC8 pp 229533.I1RRY1
41921 229533.I1RBC8 pp 229533.I1S987
41922 229533.I1RBC8 pp 229533.I1RKI2
41923 229533.I1RBC8 pp 229533.I1S1W8
41924 229533.I1RBC8 pp 229533.I1RNC2
41925 229533.I1RBC8 pp 229533.I1RBW0
41926 229533.I1RBC8 pp 229533.Q4IP51
41927 229533.I1RY99 pp 229533.I1SB04
41928 229533.I1RY99 pp 229533.I1S0N5
41929 229533.I1RY99 pp 229533.Q4HTT2
41930 229533.I1RY99 pp 229533.Q4I7L0
41931 229533.I1RY99 pp 229533.I1S109
41932 229533.I1RY99 pp 229533.I1S1Y7
41933 229533.I1RY99 pp 229533.Q4IMD1
41934 229533.I1RY99 pp 229533.Q4IER8
41935 229533.I1RY99 pp 229533.I1S1V2
41936 229533.I1RY99 pp 229533.I1S1U7
41937 229533.I1RNY8 pp 229533.I1S0M2
41938 229533.I1RNY8 pp 229533.I1RSI3
41939 229533.I1RNY8 pp 229533.I1RYZ9
41940 229533.I1RNY8 pp 229533.I1RX77
41941 229533.I1RNY8 pp 229533.I1RV91
41942 229533.I1RNY8 pp 229533.I1RQB4
41943 229533.I1RNY8 pp 229533.I1S2E9
41944 229533.I1RNY8 pp 229533.I1S098
41945 229533.I1RNY8 pp 229533.I1RZK4
41946 229533.I1RNY8 pp 229533.I1S2B6
41947 229533.I1RNY8 pp 229533.I1RT62
41948 229533.I1RNY8 pp 229533.I1RYZ0
41949 229533.I1RNY8 pp 229533.I1S1W4
41950 229533.I1RNY8 pp 229533.I1RY44
41951 229533.I1RNY8 pp 229533.I1RWT6
41952 229533.I1RNY8 pp 229533.I1RXH6
41953 229533.I1RNY8 pp 229533.I1RQ72
41954 229533.I1RNY8 pp 229533.I1S296
41955 229533.I1RNY8 pp 229533.I1RQ26
41956 229533.I1RNY8 pp 229533.V6QV09
41957 229533.I1RNY8 pp 229533.I1S2K7
41958 229533.I1RNY8 pp 229533.I1RPA6
41959 229533.I1RNY8 pp 229533.I1RZE4
41960 229533.I1RNY8 pp 229533.I1RQA3
41961 229533.I1RNY8 pp 229533.I1RRN5
41962 229533.I1RNY8 pp 229533.I1RV66
41963 229533.I1RNY8 pp 229533.I1RPH1
41964 229533.I1RNY8 pp 229533.I1RRY3
41965 229533.I1RNY8 pp 229533.I1S0X7
41966 229533.I1RNY8 pp 229533.I1RNY9
41967 229533.A0A0E0SBE9 pp 229533.I1RJU3
41968 229533.A0A0E0SBE9 pp 229533.A0A1C3YIP5
41969 229533.A0A0E0SBE9 pp 229533.I1RAH0
41970 229533.A0A0E0SBE9 pp 229533.I1RVQ0
41971 229533.A0A0E0SBE9 pp 229533.I1RD52
41972 229533.A0A0E0SBE9 pp 229533.I1S0L3
41973 229533.A0A0E0SBE9 pp 229533.I1RQ41
41974 229533.A0A0E0SBE9 pp 229533.I1RWK3
41975 229533.A0A0E0SBE9 pp 229533.A0A1C3YM63

41976 229533.A0A0E0SBE9 pp 229533.I1RBC9
41977 229533.A0A0E0SBE9 pp 229533.I1RSQ7
41978 229533.A0A0E0SBE9 pp 229533.V6QW22
41979 229533.A0A0E0SBE9 pp 229533.I1RJW6
41980 229533.A0A0E0SBE9 pp 229533.A0A1C3YLK8
41981 229533.A0A0E0SBE9 pp 229533.I1RRM5
41982 229533.A0A0E0SBE9 pp 229533.I1RBS6
41983 229533.A0A0E0SBE9 pp 229533.I1RBY4
41984 229533.A0A0E0SBE9 pp 229533.I1RX25
41985 229533.A0A0E0SBE9 pp 229533.I1S7A8
41986 229533.A0A0E0SBE9 pp 229533.I1RW25
41987 229533.A0A0E0SBE9 pp 229533.I1RJV4
41988 229533.A0A0E0SBE9 pp 229533.A0A1C3YIS7
41989 229533.A0A0E0SBE9 pp 229533.I1RRC6
41990 229533.A0A0E0SBE9 pp 229533.I1RFL6
41991 229533.A0A0E0SBE9 pp 229533.A0A0E0SFN0
41992 229533.A0A0E0SBE9 pp 229533.I1RE27
41993 229533.A0A0E0SBE9 pp 229533.I1RNL7
41994 229533.A0A0E0SBE9 pp 229533.I1RQ23
41995 229533.A0A0E0SBE9 pp 229533.I1RRC8
41996 229533.A0A0E0SBE9 pp 229533.I1RPM4
41997 229533.A0A0E0SBE9 pp 229533.I1REK8
41998 229533.A0A0E0SBE9 pp 229533.I1RWJ8
41999 229533.A0A0E0SBE9 pp 229533.I1RD17
42000 229533.A0A0E0SBE9 pp 229533.I1S5R1
42001 229533.A0A0E0SBE9 pp 229533.I1RCG4
42002 229533.A0A0E0SBE9 pp 229533.I1RN18
42003 229533.A0A0E0SBE9 pp 229533.I1RC16
42004 229533.A0A0E0SBE9 pp 229533.I1RFL4
42005 229533.A0A0E0SBE9 pp 229533.V6RIV9
42006 229533.A0A0E0SBE9 pp 229533.I1RCH5
42007 229533.A0A0E0SBE9 pp 229533.I1RRT8
42008 229533.A0A0E0SBE9 pp 229533.I1RRC9
42009 229533.A0A0E0SBE9 pp 229533.I1RZJ5
42010 229533.A0A0E0SBE9 pp 229533.A0A1C3YKB0
42011 229533.A0A0E0SBE9 pp 229533.I1RE01
42012 229533.A0A0E0SBE9 pp 229533.I1RQE7
42013 229533.A0A0E0SBE9 pp 229533.I1RW46
42014 229533.A0A0E0SBE9 pp 229533.I1RMG8
42015 229533.A0A0E0SBE9 pp 229533.I1RR24
42016 229533.A0A0E0SBE9 pp 229533.I1RTF5
42017 229533.A0A0E0SBE9 pp 229533.A0A1C3YKV3
42018 229533.A0A0E0SBE9 pp 229533.I1RS17
42019 229533.A0A0E0SBE9 pp 229533.I1RB92
42020 229533.A0A0E0SBE9 pp 229533.A0A1C3YHX6
42021 229533.A0A0E0SBE9 pp 229533.I1S0L4
42022 229533.A0A0E0SBE9 pp 229533.I1RW99
42023 229533.A0A0E0SBE9 pp 229533.I1RRI2
42024 229533.A0A0E0SBE9 pp 229533.I1RUG3
42025 229533.A0A0E0SBE9 pp 229533.I1RZZ9
42026 229533.A0A0E0SBE9 pp 229533.I1RZW8
42027 229533.A0A0E0SBE9 pp 229533.I1RBT1
42028 229533.A0A0E0SBE9 pp 229533.I1RXF0
42029 229533.A0A0E0SBE9 pp 229533.I1RZM8
42030 229533.A0A0E0SBE9 pp 229533.I1RMG3
42031 229533.A0A0E0SBE9 pp 229533.I1RYL5
42032 229533.A0A0E0SBE9 pp 229533.V6RCG5
42033 229533.I1RW32 pp 229533.I1RY44
42034 229533.I1RW32 pp 229533.V6RQ48
42035 229533.I1RW32 pp 229533.I1RWT6
42036 229533.I1RW32 pp 229533.I1SAT1
42037 229533.I1RW32 pp 229533.I1RW38
42038 229533.I1RW32 pp 229533.I1RX09
42039 229533.I1RW32 pp 229533.V6RKE4
42040 229533.I1RW32 pp 229533.I1SAM5
42041 229533.I1RW32 pp 229533.I1RZ91
42042 229533.I1RW32 pp 229533.I1S0X0
42043 229533.I1RW32 pp 229533.I1S034
42044 229533.I1RW32 pp 229533.I1RXF5
42045 229533.I1RW32 pp 229533.I1RXU1
42046 229533.I1RW32 pp 229533.I1RW84
42047 229533.I1RW32 pp 229533.V6QW25
42048 229533.I1RW32 pp 229533.I1RW76

42049 229533.I1RW32 pp 229533.Q4ICG5
42050 229533.I1RW32 pp 229533.I1RZA4
42051 229533.I1RW32 pp 229533.I1S2E9
42052 229533.I1RW32 pp 229533.I1S635
42053 229533.I1RQ73 pp 229533.Q4I5I4
42054 229533.I1RQ73 pp 229533.I1RZ94
42055 229533.I1RQ73 pp 229533.I1RZH4
42056 229533.I1RQ73 pp 229533.Q4INW6
42057 229533.I1RQ73 pp 229533.I1RZ02
42058 229533.I1RQ73 pp 229533.Q4I0M9
42059 229533.I1RAV7 pp 229533.I1RCU1
42060 229533.I1RAV7 pp 229533.I1RPP5
42061 229533.I1RAV7 pp 229533.I1RQR6
42062 229533.I1RAV7 pp 229533.Q4IPZ1
42063 229533.I1RYE5 pp 229533.I1S0B8
42064 229533.I1RYE5 pp 229533.Q4I5G1
42065 229533.I1RYE5 pp 229533.I1S1F3
42066 229533.I1RYE5 pp 229533.I1RYT8
42067 229533.I1RYE5 pp 229533.I1S212
42068 229533.A0A1C3YM68 pp 229533.I1SB04
42069 229533.A0A1C3YM68 pp 229533.I1RB73
42070 229533.A0A1C3YM68 pp 229533.I1RZH4
42071 229533.A0A1C3YM68 pp 229533.I1RZG0
42072 229533.A0A1C3YM68 pp 229533.Q4I7L0
42073 229533.A0A1C3YM68 pp 229533.Q4I0M9
42074 229533.A0A1C3YM68 pp 229533.I1RRR9
42075 229533.A0A1C3YM68 pp 229533.I1RCN2
42076 229533.A0A1C3YM68 pp 229533.I1RS52
42077 229533.A0A1C3YM68 pp 229533.Q4IAK7
42078 229533.A0A1C3YM68 pp 229533.I1RD59
42079 229533.A0A1C3YM68 pp 229533.I1RMT4
42080 229533.A0A1C3YM68 pp 229533.I1RW60
42081 229533.A0A1C3YM68 pp 229533.Q4INW6
42082 229533.A0A1C3YM68 pp 229533.I1RCY0
42083 229533.A0A1C3YM68 pp 229533.I1S109
42084 229533.A0A1C3YM68 pp 229533.I1RY99
42085 229533.A0A1C3YM68 pp 229533.I1RA73
42086 229533.I1RYC7 pp 229533.I1S1F3
42087 229533.I1RYC7 pp 229533.I1S0Q7
42088 229533.I1RYC7 pp 229533.I1S5X9
42089 229533.I1RYC7 pp 229533.I1S464
42090 229533.I1RYC7 pp 229533.V6RJP8
42091 229533.I1RYC7 pp 229533.I1S0K2
42092 229533.I1RYC7 pp 229533.I1S0R8
42093 229533.I1RYC7 pp 229533.I1S0P0
42094 229533.I1RYC7 pp 229533.I1RYT8
42095 229533.A0A1C3YJ12 pp 229533.I1RC15
42096 229533.A0A1C3YJ12 pp 229533.I1RDC9
42097 229533.A0A1C3YJ12 pp 229533.I1RCK7
42098 229533.A0A1C3YJ12 pp 229533.I1RRT0
42099 229533.A0A1C3YJ12 pp 229533.V6R227
42100 229533.A0A1C3YJ12 pp 229533.A0A1C3YK37
42101 229533.A0A1C3YJ12 pp 229533.I1RAR2
42102 229533.A0A1C3YJ12 pp 229533.I1RPU4
42103 229533.A0A1C3YJ12 pp 229533.I1S193
42104 229533.A0A1C3YJ12 pp 229533.I1RWX8
42105 229533.A0A1C3YJ12 pp 229533.I1RKE9
42106 229533.A0A1C3YJ12 pp 229533.I1RXE5
42107 229533.A0A1C3YJ12 pp 229533.I1RZB5
42108 229533.A0A1C3YJ12 pp 229533.I1S5Z2
42109 229533.A0A1C3YJ12 pp 229533.I1RQE9
42110 229533.A0A1C3YJ12 pp 229533.I1RUQ6
42111 229533.A0A1C3YJ12 pp 229533.I1RKB1
42112 229533.A0A1C3YJ12 pp 229533.I1RJL2
42113 229533.A0A1C3YJ12 pp 229533.A0A1C3YJE9
42114 229533.A0A1C3YJ12 pp 229533.I1RB36
42115 229533.A0A1C3YJ12 pp 229533.V6QV38
42116 229533.A0A1C3YJ12 pp 229533.A0A1C3YJV9
42117 229533.A0A1C3YJ12 pp 229533.I1RTC2
42118 229533.A0A1C3YJ12 pp 229533.I1RKR6
42119 229533.A0A1C3YJ12 pp 229533.I1RCM6
42120 229533.A0A1C3YJ12 pp 229533.I1REN3
42121 229533.A0A1C3YJ12 pp 229533.I1RCB7

42122	229533.A0A1C3YJ12	pp	229533.I1RRG3
42123	229533.A0A1C3YJ12	pp	229533.I1RXU0
42124	229533.A0A1C3YJ12	pp	229533.I1RAE0
42125	229533.A0A1C3YJ12	pp	229533.I1RQ80
42126	229533.A0A1C3YJ12	pp	229533.A0A1C3YK01
42127	229533.A0A1C3YJ12	pp	229533.I1RP96
42128	229533.A0A1C3YJ12	pp	229533.I1RMA5
42129	229533.A0A1C3YJ12	pp	229533.I1RG11
42130	229533.A0A1C3YJ12	pp	229533.I1RML9
42131	229533.A0A1C3YJ12	pp	229533.I1RGE1
42132	229533.A0A1C3YJ12	pp	229533.Q4IPC8
42133	229533.A0A1C3YJ12	pp	229533.I1RWD4
42134	229533.A0A1C3YJ12	pp	229533.I1RLX6
42135	229533.A0A1C3YJ12	pp	229533.A0A1C3YL11
42136	229533.A0A1C3YJ12	pp	229533.I1REC4
42137	229533.A0A1C3YJ12	pp	229533.I1S0N4
42138	229533.A0A1C3YJ12	pp	229533.I1RWU3
42139	229533.A0A1C3YJ12	pp	229533.I1RY40
42140	229533.A0A1C3YJ12	pp	229533.I1RA56
42141	229533.A0A1C3YJ12	pp	229533.I1RK79
42142	229533.A0A1C3YJ12	pp	229533.I1RW16
42143	229533.A0A1C3YJ12	pp	229533.I1RBZ7
42144	229533.A0A1C3YJ12	pp	229533.I1RN31
42145	229533.A0A1C3YJ12	pp	229533.I1RBJ1
42146	229533.A0A1C3YJ12	pp	229533.I1RD70
42147	229533.A0A1C3YJ12	pp	229533.I1RWF3
42148	229533.A0A1C3YJ12	pp	229533.I1RLX4
42149	229533.A0A1C3YJ12	pp	229533.I1S3D7
42150	229533.A0A1C3YJ12	pp	229533.I1S276
42151	229533.A0A1C3YJ12	pp	229533.A0A1C3YN55
42152	229533.A0A1C3YJ12	pp	229533.I1RK71
42153	229533.A0A1C3YJ12	pp	229533.I1RHP9
42154	229533.A0A1C3YJ12	pp	229533.I1RBV5
42155	229533.A0A1C3YJ12	pp	229533.I1RPM7
42156	229533.A0A1C3YJ12	pp	229533.I1RFZ2
42157	229533.A0A1C3YJ12	pp	229533.I1RE30
42158	229533.A0A1C3YJ12	pp	229533.I1R9W6
42159	229533.A0A1C3YJ12	pp	229533.I1RSZ3
42160	229533.A0A1C3YJ12	pp	229533.I1RPW0
42161	229533.A0A1C3YJ12	pp	229533.I1RC24
42162	229533.A0A1C3YJ12	pp	229533.Q4IR18
42163	229533.A0A1C3YJ12	pp	229533.I1RGW0
42164	229533.A0A1C3YJ12	pp	229533.I1RMV1
42165	229533.A0A1C3YJ12	pp	229533.I1RBL4
42166	229533.A0A1C3YJ12	pp	229533.I1RI51
42167	229533.A0A1C3YJ12	pp	229533.I1RX38
42168	229533.A0A1C3YJ12	pp	229533.I1RYE2
42169	229533.A0A1C3YJ12	pp	229533.I1RRT2
42170	229533.A0A1C3YJ12	pp	229533.A0A1C3YKJ6
42171	229533.A0A1C3YJ12	pp	229533.A0A1C3YLP8
42172	229533.A0A1C3YJ12	pp	229533.I1RZM2
42173	229533.A0A1C3YJ12	pp	229533.I1RPX5
42174	229533.A0A1C3YJ12	pp	229533.I1RR25
42175	229533.A0A1C3YJ12	pp	229533.A0A1C3YJZ2
42176	229533.A0A1C3YJ12	pp	229533.I1RVL8
42177	229533.A0A1C3YJ12	pp	229533.I1RVY0
42178	229533.A0A1C3YJ12	pp	229533.I1RQM0
42179	229533.A0A1C3YJ12	pp	229533.I1RA84
42180	229533.A0A1C3YJ12	pp	229533.I1RBR5
42181	229533.A0A0E0RNL0	pp	229533.I1RY99
42182	229533.A0A0E0RNL0	pp	229533.A0A1C3YLP1
42183	229533.A0A0E0RNL0	pp	229533.A0A1C3YIA5
42184	229533.A0A0E0RNL0	pp	229533.I1SAP6
42185	229533.A0A0E0RNL0	pp	229533.I1S0H1
42186	229533.A0A0E0RNL0	pp	229533.I1RPU1
42187	229533.A0A0E0RNL0	pp	229533.A0A1C3YK75
42188	229533.A0A0E0RNL0	pp	229533.I1RYX3
42189	229533.A0A0E0RNL0	pp	229533.I1S0F0
42190	229533.A0A0E0RNL0	pp	229533.I1RUZ8
42191	229533.A0A0E0RNL0	pp	229533.I1S109
42192	229533.A0A0E0RNL0	pp	229533.I1RYN7
42193	229533.A0A0E0RNL0	pp	229533.I1S201
42194	229533.A0A0E0RNL0	pp	229533.Q4I7L0

42195 229533.A0A0E0RNL0 pp 229533.I1RAK6
42196 229533.A0A0E0RNL0 pp 229533.A0A1C3YLD0
42197 229533.A0A0E0RNL0 pp 229533.I1RPJ1
42198 229533.A0A0E0RNL0 pp 229533.I1RB25
42199 229533.A0A0E0RNL0 pp 229533.I1RRH5
42200 229533.A0A0E0RNL0 pp 229533.Q4IFH8
42201 229533.A0A0E0RNL0 pp 229533.I1RB73
42202 229533.A0A0E0RNL0 pp 229533.I1SB04
42203 229533.A0A0E0RNL0 pp 229533.I1RW60
42204 229533.A0A0E0RNL0 pp 229533.I1RY77
42205 229533.A0A0E0RNL0 pp 229533.I1RCN2
42206 229533.A0A0E0RNL0 pp 229533.I1RY90
42207 229533.A0A0E0RNL0 pp 229533.I1RFQ0
42208 229533.A0A0E0RNL0 pp 229533.A0A1C3YN58
42209 229533.I1RA12 pp 229533.I1RFX8
42210 229533.I1RA12 pp 229533.I1RLE7
42211 229533.I1RA12 pp 229533.I1S987
42212 229533.I1RA12 pp 229533.I1RAH4
42213 229533.I1RA12 pp 229533.I1RB00
42214 229533.I1RA12 pp 229533.I1RBW0
42215 229533.I1RA12 pp 229533.I1RMB7
42216 229533.I1RA12 pp 229533.I1RRT1
42217 229533.I1RA12 pp 229533.I1RD01
42218 229533.I1RA12 pp 229533.I1RQ13
42219 229533.I1RA12 pp 229533.I1RZ94
42220 229533.I1RA12 pp 229533.I1RE46
42221 229533.I1RA12 pp 229533.I1RBH9
42222 229533.I1RA12 pp 229533.I1RM36
42223 229533.I1RA12 pp 229533.Q4IBX0
42224 229533.I1RA12 pp 229533.I1RMX3
42225 229533.I1RA12 pp 229533.I1RYR1
42226 229533.I1RA12 pp 229533.I1S236
42227 229533.I1RA12 pp 229533.I1RFJ2
42228 229533.I1RA12 pp 229533.I1RKB1
42229 229533.I1RA12 pp 229533.I1S461
42230 229533.I1RA12 pp 229533.I1RBC1
42231 229533.I1RA12 pp 229533.I1S209
42232 229533.I1RA12 pp 229533.I1RPQ1
42233 229533.I1RA12 pp 229533.Q4IR08
42234 229533.I1RA12 pp 229533.I1RD47
42235 229533.I1RA12 pp 229533.I1RN46
42236 229533.I1RA12 pp 229533.I1RKI2
42237 229533.I1RA12 pp 229533.I1S604
42238 229533.I1RA12 pp 229533.I1RCL7
42239 229533.I1RA12 pp 229533.I1RQ92
42240 229533.I1RA12 pp 229533.Q4INS6
42241 229533.I1RA12 pp 229533.I1RN55
42242 229533.I1RA12 pp 229533.I1RQA3
42243 229533.I1RA12 pp 229533.Q4IEA5
42244 229533.I1RA12 pp 229533.I1RG77
42245 229533.I1RA12 pp 229533.I1RQH8
42246 229533.I1RA12 pp 229533.I1RAL9
42247 229533.I1RA12 pp 229533.I1RUG2
42248 229533.I1RA12 pp 229533.I1RSI4
42249 229533.I1RA12 pp 229533.Q4IMD1
42250 229533.I1RA12 pp 229533.I1RQ67
42251 229533.I1RA12 pp 229533.I1RKE9
42252 229533.I1RA12 pp 229533.Q4I0J6
42253 229533.I1RA12 pp 229533.I1RWT6
42254 229533.I1RA12 pp 229533.Q4IP51
42255 229533.I1RA12 pp 229533.I1RV66
42256 229533.I1RA12 pp 229533.I1RJV8
42257 229533.I1RA12 pp 229533.I1RMQ7
42258 229533.I1RA12 pp 229533.Q4IQM6
42259 229533.I1RA12 pp 229533.I1S283
42260 229533.I1RA12 pp 229533.I1RSZ4
42261 229533.I1RA12 pp 229533.I1RSZ3
42262 229533.I1RA12 pp 229533.I1RAT5
42263 229533.I1RA12 pp 229533.I1RYQ1
42264 229533.I1RA12 pp 229533.I1RY44
42265 229533.I1RA12 pp 229533.V6RLW4
42266 229533.I1RA12 pp 229533.I1RBU5
42267 229533.I1RA12 pp 229533.I1RWI8

42268	229533.I1RA12	pp	229533.I1S1D3
42269	229533.I1RA12	pp	229533.I1RSA7
42270	229533.I1RA12	pp	229533.I1RXA9
42271	229533.I1RA12	pp	229533.Q4IB89
42272	229533.I1RA12	pp	229533.Q4IPI4
42273	229533.I1RA12	pp	229533.I1RN57
42274	229533.I1RA12	pp	229533.I1RY29
42275	229533.I1RA12	pp	229533.I1S237
42276	229533.I1RA12	pp	229533.I1RMS4
42277	229533.I1RA12	pp	229533.I1S998
42278	229533.I1RA12	pp	229533.I1RUG1
42279	229533.I1RA12	pp	229533.I1S2E9
42280	229533.I1RA12	pp	229533.V6R382
42281	229533.I1RA12	pp	229533.I1RSR9
42282	229533.I1RA12	pp	229533.I1RSN7
42283	229533.I1RA12	pp	229533.I1RF70
42284	229533.I1RA12	pp	229533.I1RYR2
42285	229533.I1RA12	pp	229533.I1RC35
42286	229533.I1RA12	pp	229533.I1RCA5
42287	229533.I1RA12	pp	229533.I1S0D5
42288	229533.I1RA12	pp	229533.I1RZ86
42289	229533.I1RA12	pp	229533.I1RBD7
42290	229533.I1RA12	pp	229533.I1RFZ2
42291	229533.I1RA12	pp	229533.I1RPM7
42292	229533.I1RA12	pp	229533.I1RYX9
42293	229533.I1RA12	pp	229533.I1RRS7
42294	229533.I1RA12	pp	229533.I1RBC8
42295	229533.I1RA12	pp	229533.I1RDX0
42296	229533.I1RA12	pp	229533.I1RFL7
42297	229533.I1RA12	pp	229533.I1RHD2
42298	229533.I1RA12	pp	229533.I1RAU8
42299	229533.I1RA12	pp	229533.I1RFN0
42300	229533.I1RA12	pp	229533.I1RD19
42301	229533.I1RA12	pp	229533.I1RQB4
42302	229533.I1RA12	pp	229533.I1RC54
42303	229533.I1RA12	pp	229533.I1RSJ4
42304	229533.I1RA12	pp	229533.I1RSP7
42305	229533.I1RA12	pp	229533.I1RSC7
42306	229533.I1RA12	pp	229533.I1S2F2
42307	229533.I1RA12	pp	229533.I1RQJ4
42308	229533.I1RA12	pp	229533.I1RQI6
42309	229533.I1RA12	pp	229533.I1RX64
42310	229533.I1RA12	pp	229533.I1RXB2
42311	229533.I1RA12	pp	229533.I1RBW1
42312	229533.I1RA12	pp	229533.I1S016
42313	229533.I1RA12	pp	229533.I1S4P6
42314	229533.I1RA12	pp	229533.I1RBN9
42315	229533.I1RA12	pp	229533.I1RZP8
42316	229533.I1RA12	pp	229533.I1S0A6
42317	229533.I1RA12	pp	229533.I1RUG4
42318	229533.I1RA12	pp	229533.I1RYK6
42319	229533.I1RA12	pp	229533.I1RDJ8
42320	229533.I1RA12	pp	229533.I1RDH0
42321	229533.I1RA12	pp	229533.Q4I624
42322	229533.I1RJB3	pp	229533.V6RJP8
42323	229533.I1RJB3	pp	229533.I1S0K2
42324	229533.I1RJB3	pp	229533.I1RKB7
42325	229533.I1RJB3	pp	229533.I1RP02
42326	229533.I1RJB3	pp	229533.I1RS29
42327	229533.I1RJB3	pp	229533.I1RMQ6
42328	229533.I1RJB3	pp	229533.I1S0R8
42329	229533.I1RJB3	pp	229533.I1S0P0
42330	229533.I1RJB3	pp	229533.I1S0Q7
42331	229533.I1RJB3	pp	229533.I1RS05
42332	229533.I1RJB3	pp	229533.I1S5X9
42333	229533.I1RJB3	pp	229533.I1RZ51
42334	229533.I1RJB3	pp	229533.I1RYT8
42335	229533.I1RJB3	pp	229533.I1S1F3
42336	229533.I1RJB3	pp	229533.I1RXD9
42337	229533.I1RJB3	pp	229533.I1RMK7
42338	229533.I1RJB3	pp	229533.I1S464
42339	229533.I1RJB3	pp	229533.I1RY21
42340	229533.I1RJB3	pp	229533.I1RWA3

42341	229533.I1RJB3	pp	229533.I1RNM7
42342	229533.I1RJB3	pp	229533.I1RY92
42343	229533.I1RQF5	pp	229533.I1RZP2
42344	229533.A0A098DTF7	pp	229533.I1RRR6
42345	229533.A0A098DTF7	pp	229533.A0A1C3YL40
42346	229533.A0A098DTF7	pp	229533.I1S0N5
42347	229533.A0A098DTF7	pp	229533.I1RSP4
42348	229533.A0A098DTF7	pp	229533.I1RXE5
42349	229533.A0A098DTF7	pp	229533.A0A1C3YLP8
42350	229533.A0A098DTF7	pp	229533.I1RLX7
42351	229533.A0A098DTF7	pp	229533.I1RNC6
42352	229533.A0A098DTF7	pp	229533.A0A1C3YJQ6
42353	229533.A0A098DTF7	pp	229533.I1RAR2
42354	229533.A0A098DTF7	pp	229533.A0A1C3YJZ2
42355	229533.A0A098DTF7	pp	229533.I1RMA5
42356	229533.A0A098DTF7	pp	229533.I1RST8
42357	229533.A0A098DTF7	pp	229533.I1SAN0
42358	229533.A0A098DTF7	pp	229533.I1RRN6
42359	229533.A0A098DTF7	pp	229533.I1S0F5
42360	229533.A0A098DTF7	pp	229533.A0A1C3YLT1
42361	229533.A0A098DTF7	pp	229533.A0A0E0SA25
42362	229533.A0A098DTF7	pp	229533.I1RP96
42363	229533.A0A098DTF7	pp	229533.I1RJZ7
42364	229533.A0A098DTF7	pp	229533.I1RVY0
42365	229533.A0A098DTF7	pp	229533.I1RT16
42366	229533.A0A098DTF7	pp	229533.A0A098DY35
42367	229533.A0A098DTF7	pp	229533.A0A098E2M3
42368	229533.A0A098DTF7	pp	229533.I1RBR5
42369	229533.A0A098DTF7	pp	229533.I1RGW0
42370	229533.A0A098DTF7	pp	229533.A0A1C3YLS3
42371	229533.I1RJ10	pp	229533.I1RW98
42372	229533.I1RJ10	pp	229533.I1RY33
42373	229533.I1R9Y9	pp	229533.I1RB75
42374	229533.I1R9Y9	pp	229533.I1S439
42375	229533.I1R9Y9	pp	229533.I1RQ72
42376	229533.I1R9Y9	pp	229533.I1RY44
42377	229533.I1R9Y9	pp	229533.I1RAY4
42378	229533.I1R9Y9	pp	229533.I1RYZ0
42379	229533.I1R9Y9	pp	229533.I1RB41
42380	229533.I1R9Y9	pp	229533.I1RRN5
42381	229533.I1R9Y9	pp	229533.I1RNW8
42382	229533.I1R9Y9	pp	229533.I1S0D1
42383	229533.I1R9Y9	pp	229533.I1RW16
42384	229533.I1R9Y9	pp	229533.I1RHQ8
42385	229533.I1R9Y9	pp	229533.I1RZH4
42386	229533.I1R9Y9	pp	229533.I1RRP7
42387	229533.I1R9Y9	pp	229533.I1RN65
42388	229533.I1R9Y9	pp	229533.Q4IJM4
42389	229533.I1R9Y9	pp	229533.I1RMM9
42390	229533.I1R9Y9	pp	229533.I1S1D3
42391	229533.I1R9Y9	pp	229533.I1RSK1
42392	229533.I1R9Y9	pp	229533.I1S2K7
42393	229533.I1R9Y9	pp	229533.I1RA43
42394	229533.I1R9Y9	pp	229533.I1RI34
42395	229533.I1R9Y9	pp	229533.I1RRY3
42396	229533.I1R9Y9	pp	229533.I1RN08
42397	229533.I1R9Y9	pp	229533.I1RNY9
42398	229533.I1R9Y9	pp	229533.I1RAM7
42399	229533.I1R9Y9	pp	229533.I1RDZ4
42400	229533.I1R9Y9	pp	229533.I1RBI1
42401	229533.I1R9Y9	pp	229533.I1RQA3
42402	229533.I1R9Y9	pp	229533.I1RNY8
42403	229533.I1R9Y9	pp	229533.I1S2B6
42404	229533.I1R9Y9	pp	229533.I1RCL8
42405	229533.I1R9Y9	pp	229533.V6RDP1
42406	229533.I1R9Y9	pp	229533.I1RZE4
42407	229533.I1R9Y9	pp	229533.I1RUQ5
42408	229533.I1R9Y9	pp	229533.I1RMA8
42409	229533.I1R9Y9	pp	229533.I1RC22
42410	229533.I1R9Y9	pp	229533.I1RC17
42411	229533.I1R9Y9	pp	229533.V6RGI1
42412	229533.I1R9Y9	pp	229533.I1RI71
42413	229533.I1R9Y9	pp	229533.I1RYY5

42414	229533.I1R9Y9	pp	229533.I1RQB4
42415	229533.I1R9Y9	pp	229533.I1S0M2
42416	229533.I1R9Y9	pp	229533.I1RCB3
42417	229533.I1R9Y9	pp	229533.I1S1W4
42418	229533.I1R9Y9	pp	229533.I1RT36
42419	229533.I1R9Y9	pp	229533.I1RWT6
42420	229533.I1R9Y9	pp	229533.I1RKK0
42421	229533.I1R9Y9	pp	229533.I1RYJ8
42422	229533.I1R9Y9	pp	229533.I1S0N4
42423	229533.I1R9Y9	pp	229533.I1RRW0
42424	229533.I1R9Y9	pp	229533.I1RNC0
42425	229533.I1R9Y9	pp	229533.I1REE1
42426	229533.I1R9Y9	pp	229533.V6RKY7
42427	229533.I1R9Y9	pp	229533.I1RN31
42428	229533.I1R9Y9	pp	229533.I1RD22
42429	229533.I1R9Y9	pp	229533.I1RXH6
42430	229533.I1R9Y9	pp	229533.I1RC95
42431	229533.I1R9Y9	pp	229533.V6RVG3
42432	229533.I1R9Y9	pp	229533.I1RST8
42433	229533.I1R9Y9	pp	229533.I1RAM6
42434	229533.I1R9Y9	pp	229533.I1RUN8
42435	229533.I1R9Y9	pp	229533.I1RYH9
42436	229533.I1R9Y9	pp	229533.I1S224
42437	229533.I1R9Y9	pp	229533.I1RWJ6
42438	229533.I1R9Y9	pp	229533.I1RC97
42439	229533.I1R9Y9	pp	229533.I1RX38
42440	229533.I1R9Y9	pp	229533.I1RMF5
42441	229533.I1R9Y9	pp	229533.I1RCU8
42442	229533.I1R9Y9	pp	229533.I1S2G7
42443	229533.I1R9Y9	pp	229533.I1S549
42444	229533.I1R9Y9	pp	229533.I1RD18
42445	229533.I1R9Y9	pp	229533.I1RCU9
42446	229533.I1R9Y9	pp	229533.I1RNF4
42447	229533.I1R9Y9	pp	229533.I1RJR8
42448	229533.I1R9Y9	pp	229533.I1RL24
42449	229533.I1R9Y9	pp	229533.I1RBZ9
42450	229533.I1R9Y9	pp	229533.I1S1V5
42451	229533.I1R9Y9	pp	229533.I1RP28
42452	229533.I1R9Y9	pp	229533.I1S1V2
42453	229533.I1R9Y9	pp	229533.I1RV66
42454	229533.I1R9Y9	pp	229533.I1RFL7
42455	229533.I1R9Y9	pp	229533.I1RJ88
42456	229533.I1R9Y9	pp	229533.I1RQG4
42457	229533.I1R9Y9	pp	229533.I1RNP7
42458	229533.I1R9Y9	pp	229533.I1RB74
42459	229533.I1R9Y9	pp	229533.I1RDB1
42460	229533.I1R9Y9	pp	229533.I1S7X9
42461	229533.I1R9Y9	pp	229533.I1RX70
42462	229533.I1R9Y9	pp	229533.I1RAN2
42463	229533.I1R9Y9	pp	229533.I1RQ26
42464	229533.I1R9Y9	pp	229533.V6QV09
42465	229533.I1R9Y9	pp	229533.I1RAW7
42466	229533.I1R9Y9	pp	229533.I1RJH1
42467	229533.I1R9Y9	pp	229533.I1RYZ9
42468	229533.I1R9Y9	pp	229533.I1RBV5
42469	229533.I1R9Y9	pp	229533.V6RTJ8
42470	229533.I1R9Y9	pp	229533.I1S2E9
42471	229533.I1R9Y9	pp	229533.I1RV91
42472	229533.I1R9Y9	pp	229533.I1RLX6
42473	229533.I1R9X9	pp	229533.I1S285
42474	229533.I1RZF3	pp	229533.Q4I7S1
42475	229533.I1RZF3	pp	229533.Q4IPI4
42476	229533.I1RZF3	pp	229533.I1SB04
42477	229533.I1RZF3	pp	229533.Q4IEV4
42478	229533.I1RZF3	pp	229533.Q4HY90
42479	229533.I1RZF3	pp	229533.Q4I5V3
42480	229533.I1RZF3	pp	229533.Q4IB96
42481	229533.I1RZF3	pp	229533.Q4I948
42482	229533.I1RZF3	pp	229533.Q4ICA8
42483	229533.I1RZF3	pp	229533.Q4IAK7
42484	229533.I1RZF3	pp	229533.I1RZU9
42485	229533.I1RZF3	pp	229533.Q4IMD1
42486	229533.A0A1C3YLF2	pp	229533.I1RWX4

42487 229533.I1RPI0 pp 229533.Q4IB89
42488 229533.I1RPI0 pp 229533.I1RWI8
42489 229533.I1RPI0 pp 229533.I1RSJ1
42490 229533.I1RPI0 pp 229533.I1RZ86
42491 229533.I1RPI0 pp 229533.I1S4P6
42492 229533.I1RPI0 pp 229533.I1RYQ1
42493 229533.I1RPI0 pp 229533.I1RRJ6
42494 229533.I1RPI0 pp 229533.I1RWM6
42495 229533.I1RPI0 pp 229533.I1RZK4
42496 229533.I1RPI0 pp 229533.V6RSF3
42497 229533.I1RPI0 pp 229533.I1RWB3
42498 229533.I1RPI0 pp 229533.I1RQH8
42499 229533.I1RPI0 pp 229533.I1S523
42500 229533.I1RPI0 pp 229533.Q4INS6
42501 229533.I1RPI0 pp 229533.I1S788
42502 229533.I1RPI0 pp 229533.I1RSC7
42503 229533.I1RPI0 pp 229533.I1RX64
42504 229533.I1RPI0 pp 229533.I1RSL6
42505 229533.I1RPI0 pp 229533.I1RZ98
42506 229533.I1RPI0 pp 229533.I1RUR0
42507 229533.I1RPI0 pp 229533.I1S0D5
42508 229533.I1RPI0 pp 229533.I1RTN6
42509 229533.I1RPI0 pp 229533.I1RQC5
42510 229533.I1RPI0 pp 229533.I1S183
42511 229533.A0A1C3YLU6 pp 229533.I1RBF3
42512 229533.A0A1C3YLU6 pp 229533.I1RNG4
42513 229533.A0A1C3YLU6 pp 229533.I1RBX1
42514 229533.A0A1C3YLU6 pp 229533.Q4HXF0
42515 229533.A0A1C3YLU6 pp 229533.I1RK47
42516 229533.A0A1C3YLU6 pp 229533.I1RM30
42517 229533.A0A1C3YLU6 pp 229533.I1R9U7
42518 229533.A0A1C3YLU6 pp 229533.I1RP69
42519 229533.A0A1C3YLU6 pp 229533.I1RSL5
42520 229533.A0A1C3YLU6 pp 229533.I1S3K6
42521 229533.A0A1C3YLU6 pp 229533.I1RVR0
42522 229533.A0A1C3YLU6 pp 229533.I1RA94
42523 229533.A0A1C3YLU6 pp 229533.I1RFK7
42524 229533.A0A1C3YLU6 pp 229533.I1REI0
42525 229533.A0A1C3YLN8 pp 229533.I1RTB7
42526 229533.A0A1C3YLN8 pp 229533.I1S2G7
42527 229533.A0A1C3YLN8 pp 229533.I1RJR9
42528 229533.A0A1C3YLN8 pp 229533.I1RA81
42529 229533.A0A1C3YLN8 pp 229533.I1RKG1
42530 229533.A0A1C3YLN8 pp 229533.I1RN81
42531 229533.A0A1C3YLN8 pp 229533.I1RW83
42532 229533.A0A1C3YLN8 pp 229533.I1RQN9
42533 229533.A0A1C3YLN8 pp 229533.I1REN4
42534 229533.A0A1C3YLN8 pp 229533.I1RXU1
42535 229533.A0A1C3YLN8 pp 229533.I1RLT6
42536 229533.A0A1C3YLN8 pp 229533.I1R9X6
42537 229533.A0A1C3YLN8 pp 229533.A0A1C3YM48
42538 229533.A0A1C3YLN8 pp 229533.I1RE19
42539 229533.A0A1C3YLN8 pp 229533.I1RWL6
42540 229533.A0A1C3YLN8 pp 229533.I1RY44
42541 229533.A0A1C3YLN8 pp 229533.I1RWT6
42542 229533.A0A1C3YLN8 pp 229533.I1S1B1
42543 229533.A0A1C3YLN8 pp 229533.I1RV91
42544 229533.A0A1C3YLN8 pp 229533.I1RMR7
42545 229533.A0A1C3YLN8 pp 229533.I1RNG8
42546 229533.A0A1C3YLN8 pp 229533.I1S2E9
42547 229533.A0A1C3YLN8 pp 229533.I1RX09
42548 229533.A0A1C3YLN8 pp 229533.V6RR76
42549 229533.A0A1C3YLN8 pp 229533.I1RB41
42550 229533.A0A1C3YLN8 pp 229533.P0C431
42551 229533.A0A1C3YLN8 pp 229533.I1S096
42552 229533.A0A1C3YLN8 pp 229533.I1RQA3
42553 229533.A0A1C3YLN8 pp 229533.I1RW84
42554 229533.A0A1C3YLN8 pp 229533.I1RJT8
42555 229533.A0A1C3YLN8 pp 229533.I1RKH9
42556 229533.A0A1C3YLN8 pp 229533.I1RW32
42557 229533.I1RWG0 pp 229533.I1RY21
42558 229533.I1RWG0 pp 229533.V6RJP8
42559 229533.I1RWG0 pp 229533.I1S0K2

42560	229533.I1RWG0	pp	229533.I1S0P0
42561	229533.I1RWG0	pp	229533.I1S0Q7
42562	229533.I1RWG0	pp	229533.I1RYT8
42563	229533.I1RWG0	pp	229533.I1RY92
42564	229533.I1RWG0	pp	229533.I1S5X9
42565	229533.I1RWG0	pp	229533.I1S1F3
42566	229533.I1RWG0	pp	229533.I1S0R8
42567	229533.I1RWG0	pp	229533.I1RXD9
42568	229533.I1RWG0	pp	229533.I1S464
42569	229533.I1RW03	pp	229533.I1RWB3
42570	229533.I1RW03	pp	229533.I1S7B9
42571	229533.I1RW03	pp	229533.I1RZ86
42572	229533.I1RW03	pp	229533.I1S004
42573	229533.I1RW03	pp	229533.I1RZT1
42574	229533.I1RW03	pp	229533.I1S2F2
42575	229533.I1RW03	pp	229533.I1S0D5
42576	229533.I1RW03	pp	229533.I1RWM6
42577	229533.I1RW03	pp	229533.I1RY98
42578	229533.I1RW03	pp	229533.Q4INS6
42579	229533.I1RW03	pp	229533.I1RYV1
42580	229533.I1RW03	pp	229533.I1RX22
42581	229533.I1RW03	pp	229533.I1RWX7
42582	229533.A0A098DF98	pp	229533.I1RAM7
42583	229533.A0A098DF98	pp	229533.I1RX09
42584	229533.A0A098DF98	pp	229533.I1RMM9
42585	229533.A0A098DF98	pp	229533.A0A1C3YI43
42586	229533.A0A098DF98	pp	229533.I1RPU4
42587	229533.A0A098DF98	pp	229533.I1RL12
42588	229533.A0A098DF98	pp	229533.I1RB53
42589	229533.A0A098DF98	pp	229533.I1RKK0
42590	229533.A0A098DF98	pp	229533.I1RN08
42591	229533.A0A098DF98	pp	229533.I1RXB5
42592	229533.A0A098DF98	pp	229533.P0C431
42593	229533.A0A098DF98	pp	229533.I1RW99
42594	229533.A0A098DF98	pp	229533.I1RN58
42595	229533.A0A098DF98	pp	229533.I1RC95
42596	229533.A0A098DF98	pp	229533.I1RDG9
42597	229533.A0A098DF98	pp	229533.A0A1C3YK37
42598	229533.A0A098DF98	pp	229533.I1RUQ5
42599	229533.A0A098DF98	pp	229533.I1RC22
42600	229533.A0A098DF98	pp	229533.Q4IQC1
42601	229533.A0A098DF98	pp	229533.V6REE9
42602	229533.A0A098DF98	pp	229533.I1RN65
42603	229533.A0A098DF98	pp	229533.I1RPL2
42604	229533.A0A098DF98	pp	229533.A0A0E0S9Y6
42605	229533.A0A098DF98	pp	229533.Q4I0M9
42606	229533.A0A098DF98	pp	229533.I1S439
42607	229533.A0A098DF98	pp	229533.I1RCM6
42608	229533.A0A098DF98	pp	229533.I1S6F1
42609	229533.A0A098DF98	pp	229533.A0A1C3YIA8
42610	229533.A0A098DF98	pp	229533.I1RB84
42611	229533.A0A098DF98	pp	229533.A0A098DHK5
42612	229533.A0A098DF98	pp	229533.I1RNY7
42613	229533.A0A098DF98	pp	229533.I1S0S6
42614	229533.A0A098DF98	pp	229533.I1RYH9
42615	229533.A0A098DF98	pp	229533.I1RXU7
42616	229533.A0A098DF98	pp	229533.I1RQH1
42617	229533.A0A098DF98	pp	229533.A0A098DT74
42618	229533.A0A098DF98	pp	229533.I1RTB7
42619	229533.A0A098DF98	pp	229533.I1RYZ9
42620	229533.A0A098DF98	pp	229533.I1RH68
42621	229533.A0A098DF98	pp	229533.I1RL24
42622	229533.A0A098DF98	pp	229533.I1RN49
42623	229533.A0A098DF98	pp	229533.V6RGI1
42624	229533.A0A098DF98	pp	229533.I1SAT1
42625	229533.A0A098DF98	pp	229533.I1RMB3
42626	229533.A0A098DF98	pp	229533.A0A0E0SE71
42627	229533.A0A098DF98	pp	229533.A0A1C3YIP6
42628	229533.A0A098DF98	pp	229533.I1RQ26
42629	229533.A0A098DF98	pp	229533.I1RSB8
42630	229533.A0A098DF98	pp	229533.I1RM67
42631	229533.A0A098DF98	pp	229533.A0A098E0E1
42632	229533.A0A098DF98	pp	229533.I1RT36

229533.A0A098DF98	pp	229533.A0A098DQ41
229533.A0A098DF98	pp	229533.I1RDB1
229533.A0A098DF98	pp	229533.I1RQ80
229533.A0A098DF98	pp	229533.I1RMT4
229533.A0A098DF98	pp	229533.Q4IPC8
229533.A0A098DF98	pp	229533.I1RAW7
229533.A0A098DF98	pp	229533.I1RAM6
229533.A0A098DF98	pp	229533.V6QV09
229533.A0A098DF98	pp	229533.I1RXT9
229533.A0A098DF98	pp	229533.I1RZ02
229533.A0A098DF98	pp	229533.A0A1C3YI28
229533.A0A098DF98	pp	229533.I1RNC0
229533.A0A098DF98	pp	229533.I1RMQ5
229533.A0A098DF98	pp	229533.I1S1W4
229533.A0A098DF98	pp	229533.A0A0E0SAC4
229533.A0A098DF98	pp	229533.I1S0M2
229533.A0A098DF98	pp	229533.A0A0E0RS85
229533.A0A098DF98	pp	229533.A0A1C3YII1
229533.A0A098DF98	pp	229533.I1RRY3
229533.A0A098DF98	pp	229533.V6RVG3
229533.A0A098DF98	pp	229533.I1RAS2
229533.A0A098DF98	pp	229533.I1RTE6
229533.A0A098DF98	pp	229533.I1RQE7
229533.A0A098DF98	pp	229533.I1RCW7
229533.A0A098DF98	pp	229533.I1RNB8
229533.A0A098DF98	pp	229533.I1RBS6
229533.A0A098DF98	pp	229533.A0A1C3YJK9
229533.A0A098DF98	pp	229533.Q4INW6
229533.A0A098DF98	pp	229533.Q4ICG8
229533.A0A098DF98	pp	229533.I1RZS7
229533.A0A098DF98	pp	229533.I1RNW8
229533.A0A098DF98	pp	229533.I1RA43
229533.A0A098DF98	pp	229533.I1R9Y9
229533.A0A098DF98	pp	229533.I1RQN9
229533.A0A098DF98	pp	229533.I1RPW0
229533.A0A098DF98	pp	229533.I1RTK5
229533.A0A098DF98	pp	229533.I1RV91
229533.A0A098DF98	pp	229533.I1RD70
229533.A0A098DF98	pp	229533.A0A0E0SBC0
229533.A0A098DF98	pp	229533.A0A0E0SF67
229533.A0A098DF98	pp	229533.I1RP28
229533.A0A098DF98	pp	229533.Q4IER0
229533.A0A098DF98	pp	229533.I1RQG4
229533.A0A098DF98	pp	229533.A0A098DPW7
229533.A0A098DF98	pp	229533.I1RGQ9
229533.A0A098DF98	pp	229533.A0A0E0SNM6
229533.A0A098DF98	pp	229533.I1RC97
229533.A0A098DF98	pp	229533.I1RFZ2
229533.A0A098DF98	pp	229533.I1RFL7
229533.A0A098DF98	pp	229533.I1RPM4
229533.A0A098DF98	pp	229533.A0A0E0SLZ3
229533.A0A098DF98	pp	229533.I1S7X9
229533.A0A098DF98	pp	229533.I1RRT8
229533.A0A098DF98	pp	229533.I1RZG0
229533.A0A098DF98	pp	229533.I1RN66
229533.A0A098DF98	pp	229533.A0A098DNS0
229533.A0A098DF98	pp	229533.A0A1C3YLF4
229533.A0A098DF98	pp	229533.I1S1V5
229533.A0A098DF98	pp	229533.I1RAN2
229533.A0A098DF98	pp	229533.A0A1C3YIR8
229533.A0A098DF98	pp	229533.I1RA39
229533.A0A098DF98	pp	229533.A0A1C3YHL0
229533.A0A098DF98	pp	229533.I1RP04
229533.A0A098DF98	pp	229533.I1RRM9
229533.A0A098DF98	pp	229533.I1RCD4
229533.A0A098DX11	pp	229533.I1RWT6
229533.A0A098DX11	pp	229533.I1RQA3
229533.I1RKQ9	pp	229533.I1RQ31
229533.I1RKQ9	pp	229533.I1RM96
229533.I1RKQ9	pp	229533.I1RZF6
229533.I1RKQ9	pp	229533.I1RRI2
229533.I1RKQ9	pp	229533.I1RN99
229533.I1RKQ9	pp	229533.I1S2C0

42706 229533.I1RKQ9 pp 229533.I1RVZ0
42707 229533.I1RKQ9 pp 229533.I1S2E8
42708 229533.I1RKQ9 pp 229533.I1S1U7
42709 229533.I1RKQ9 pp 229533.I1RRJ8
42710 229533.I1RKQ9 pp 229533.Q4HZ47
42711 229533.I1RKQ9 pp 229533.I1RW71
42712 229533.I1RKQ9 pp 229533.I1RQM6
42713 229533.A0A1C3YJF7 pp 229533.I1RCM8
42714 229533.A0A1C3YJF7 pp 229533.I1RFV2
42715 229533.A0A1C3YJF7 pp 229533.I1RD79
42716 229533.A0A1C3YJF7 pp 229533.A0A1C3YLB6
42717 229533.A0A1C3YJF7 pp 229533.I1RN32
42718 229533.A0A1C3YJF7 pp 229533.A0A1C3YLR9
42719 229533.A0A1C3YJF7 pp 229533.I1RNG4
42720 229533.I1S0M2 pp 229533.I1S0N4
42721 229533.I1S0M2 pp 229533.I1S1D3
42722 229533.I1S0M2 pp 229533.V6RKY7
42723 229533.I1S0M2 pp 229533.V6RGI1
42724 229533.I1S0M2 pp 229533.I1S1V5
42725 229533.I1S0M2 pp 229533.I1S2E9
42726 229533.I1S0M2 pp 229533.V6RVG3
42727 229533.I1S0M2 pp 229533.I1S2G9
42728 229533.I1S0M2 pp 229533.I1S1W4
42729 229533.I1S0M2 pp 229533.Q4IEK8
42730 229533.I1S0M2 pp 229533.V6QV09
42731 229533.I1S0M2 pp 229533.I1S439
42732 229533.I1S0M2 pp 229533.Q4IJM4
42733 229533.I1S0M2 pp 229533.I1S224
42734 229533.I1RN32 pp 229533.I1RPY8
42735 229533.I1RN32 pp 229533.V6RG04
42736 229533.I1RN32 pp 229533.I1RNJ5
42737 229533.I1RN32 pp 229533.Q4IPZ1
42738 229533.I1RN32 pp 229533.I1S0I9
42739 229533.I1RWT5 pp 229533.I1RZV4
42740 229533.I1RWT5 pp 229533.I1S2E9
42741 229533.I1RWT5 pp 229533.I1RYJ8
42742 229533.I1RWT5 pp 229533.I1RZD9
42743 229533.I1RWT5 pp 229533.I1S095
42744 229533.I1RWT5 pp 229533.I1RWT6
42745 229533.I1RWT5 pp 229533.I1RY44
42746 229533.I1RSU1 pp 229533.I1RYI5
42747 229533.I1RSU1 pp 229533.I1S0Q7
42748 229533.I1RSU1 pp 229533.I1RYN2
42749 229533.I1RSU1 pp 229533.I1RZ43
42750 229533.I1RSU1 pp 229533.I1RSU2
42751 229533.I1RSU1 pp 229533.I1S5R1
42752 229533.I1RSU1 pp 229533.I1SB04
42753 229533.I1RSU1 pp 229533.I1S0R8
42754 229533.I1RSU1 pp 229533.I1RSV9
42755 229533.I1RSU1 pp 229533.I1RSZ7
42756 229533.I1S8V4 pp 229533.V6RCZ8
42757 229533.I1S8V4 pp 229533.Q4IJK2
42758 229533.I1S8V4 pp 229533.V6R3X5
42759 229533.I1S8V4 pp 229533.Q4IPZ1
42760 229533.I1S8V4 pp 229533.V6RMW6
42761 229533.I1S8V4 pp 229533.V6RTJ8
42762 229533.I1RY73 pp 229533.I1RYR2
42763 229533.I1RY73 pp 229533.V6RLW4
42764 229533.I1RY73 pp 229533.I1RYH9
42765 229533.I1RY73 pp 229533.I1S283
42766 229533.I1RY73 pp 229533.Q4IQM6
42767 229533.I1RY73 pp 229533.I1RZ94
42768 229533.I1RY73 pp 229533.I1RYX9
42769 229533.I1RY73 pp 229533.I1S604
42770 229533.I1RY73 pp 229533.I1S461
42771 229533.I1RY73 pp 229533.I1RZP8
42772 229533.I1RQB4 pp 229533.V6RVG3
42773 229533.I1RQB4 pp 229533.I1S1W4
42774 229533.I1RQB4 pp 229533.I1RZK4
42775 229533.I1RQB4 pp 229533.V6RKY7
42776 229533.I1RQB4 pp 229533.I1RY44
42777 229533.I1RQB4 pp 229533.I1RSS1
42778 229533.I1RQB4 pp 229533.I1S0D1

42779 229533.I1RQB4 pp 229533.I1RZT1
42780 229533.I1RQB4 pp 229533.I1RSI3
42781 229533.I1RQB4 pp 229533.I1S2K7
42782 229533.I1RQB4 pp 229533.I1S0M2
42783 229533.I1RQB4 pp 229533.I1RRY3
42784 229533.I1RQB4 pp 229533.I1RWQ8
42785 229533.I1RQB4 pp 229533.I1RYZ9
42786 229533.I1RQB4 pp 229533.I1S0D5
42787 229533.I1RQB4 pp 229533.I1RSR9
42788 229533.I1RQB4 pp 229533.Q4IQM6
42789 229533.I1RQB4 pp 229533.I1RXF0
42790 229533.I1RQB4 pp 229533.I1S2E9
42791 229533.I1RQB4 pp 229533.I1RX64
42792 229533.I1RQB4 pp 229533.I1RWT6
42793 229533.I1RQB4 pp 229533.I1RX70
42794 229533.I1RQB4 pp 229533.I1S2B6
42795 229533.I1RQB4 pp 229533.I1RXH6
42796 229533.I1RQB4 pp 229533.I1RX77
42797 229533.I1RQB4 pp 229533.I1RUQ5
42798 229533.I1RQB4 pp 229533.I1S439
42799 229533.I1RQB4 pp 229533.I1RV91
42800 229533.I1RQB4 pp 229533.I1RX04
42801 229533.I1RQB4 pp 229533.I1S296
42802 229533.I1RQB4 pp 229533.I1RYH9
42803 229533.I1RQB4 pp 229533.I1RZ94
42804 229533.I1RQB4 pp 229533.I1S265
42805 229533.I1RQB4 pp 229533.I1RRN5
42806 229533.I1RQB4 pp 229533.I1RZE4
42807 229533.I1RQB4 pp 229533.V6QV09
42808 229533.I1RQB4 pp 229533.I1RYX9
42809 229533.I1RQB4 pp 229533.I1RUN8
42810 229533.I1RQB4 pp 229533.I1S0X7
42811 229533.I1RQB4 pp 229533.I1RZ63
42812 229533.I1RQB4 pp 229533.I1RSI4
42813 229533.I1RR19 pp 229533.I1S9Z4
42814 229533.I1RR19 pp 229533.I1RW06
42815 229533.I1S0U0 pp 229533.I1S464
42816 229533.I1S0U0 pp 229533.V6RJP8
42817 229533.I1S0U0 pp 229533.I1S1F3
42818 229533.I1S0U0 pp 229533.I1S5X9
42819 229533.I1RQK5 pp 229533.I1S2E9
42820 229533.I1RQK5 pp 229533.I1S0N8
42821 229533.I1RQK5 pp 229533.I1RY44
42822 229533.A0A1C3YLB7 pp 229533.I1RS94
42823 229533.A0A1C3YLB7 pp 229533.I1RNT2
42824 229533.A0A1C3YLB7 pp 229533.I1RNG5
42825 229533.A0A1C3YLB7 pp 229533.I1S279
42826 229533.A0A1C3YI28 pp 229533.A0A1C3YJV7
42827 229533.A0A1C3YI28 pp 229533.A0A1C3YKF2
42828 229533.A0A1C3YI28 pp 229533.I1RD18
42829 229533.A0A1C3YI28 pp 229533.I1RL24
42830 229533.A0A1C3YI28 pp 229533.I1S224
42831 229533.A0A1C3YI28 pp 229533.I1RYH9
42832 229533.A0A1C3YI28 pp 229533.I1RNC0
42833 229533.A0A1C3YI28 pp 229533.I1RYZ9
42834 229533.A0A1C3YI28 pp 229533.I1RQ26
42835 229533.A0A1C3YI28 pp 229533.I1RN31
42836 229533.A0A1C3YI28 pp 229533.I1RCB3
42837 229533.A0A1C3YI28 pp 229533.I1RJH1
42838 229533.A0A1C3YI28 pp 229533.I1RMM9
42839 229533.A0A1C3YI28 pp 229533.I1RCK9
42840 229533.A0A1C3YI28 pp 229533.A0A1C3YKW5
42841 229533.A0A1C3YI28 pp 229533.V6QV09
42842 229533.A0A1C3YI28 pp 229533.A0A1C3YK37
42843 229533.A0A1C3YI28 pp 229533.I1RM09
42844 229533.A0A1C3YI28 pp 229533.I1RC95
42845 229533.A0A1C3YI28 pp 229533.I1S0Z7
42846 229533.A0A1C3YI28 pp 229533.I1RKK0
42847 229533.A0A1C3YI28 pp 229533.I1RQA3
42848 229533.A0A1C3YI28 pp 229533.I1RC17
42849 229533.A0A1C3YI28 pp 229533.I1RZH4
42850 229533.A0A1C3YI28 pp 229533.I1RAW7
42851 229533.A0A1C3YI28 pp 229533.A0A1C3YIL0

42852	229533.A0A1C3YI28	pp	229533.I1RBZ9
42853	229533.A0A1C3YI28	pp	229533.I1RN65
42854	229533.A0A1C3YI28	pp	229533.I1RWJ6
42855	229533.A0A1C3YI28	pp	229533.A0A1C3YJF1
42856	229533.A0A1C3YI28	pp	229533.I1RX70
42857	229533.A0A1C3YI28	pp	229533.I1RAM7
42858	229533.A0A1C3YI28	pp	229533.A0A1C3YJN3
42859	229533.A0A1C3YI28	pp	229533.I1RYZ0
42860	229533.A0A1C3YI28	pp	229533.I1RWT6
42861	229533.A0A1C3YI28	pp	229533.I1RB75
42862	229533.A0A1C3YI28	pp	229533.I1RC22
42863	229533.A0A1C3YI28	pp	229533.I1RUQ5
42864	229533.A0A1C3YI28	pp	229533.I1RDB1
42865	229533.A0A1C3YI28	pp	229533.I1RV66
42866	229533.A0A1C3YI28	pp	229533.I1S439
42867	229533.A0A1C3YI28	pp	229533.A0A1C3YJH8
42868	229533.A0A1C3YI28	pp	229533.I1S0B6
42869	229533.A0A1C3YI28	pp	229533.I1RYY5
42870	229533.A0A1C3YI28	pp	229533.I1RV91
42871	229533.A0A1C3YI28	pp	229533.I1RN08
42872	229533.A0A1C3YI28	pp	229533.A0A1C3YKJ6
42873	229533.A0A1C3YI28	pp	229533.I1RFL7
42874	229533.A0A1C3YI28	pp	229533.I1RBV5
42875	229533.A0A1C3YI28	pp	229533.I1S0M2
42876	229533.A0A1C3YI28	pp	229533.I1RBS0
42877	229533.A0A1C3YI28	pp	229533.I1RJT3
42878	229533.A0A1C3YI28	pp	229533.I1RPD6
42879	229533.A0A1C3YI28	pp	229533.I1RC97
42880	229533.A0A1C3YI28	pp	229533.I1RQB4
42881	229533.A0A1C3YI28	pp	229533.I1S2E9
42882	229533.A0A1C3YI28	pp	229533.I1RRW0
42883	229533.A0A1C3YI28	pp	229533.I1RZ25
42884	229533.A0A1C3YI28	pp	229533.I1RBM3
42885	229533.A0A1C3YI28	pp	229533.I1RT36
42886	229533.A0A1C3YI28	pp	229533.I1RNF4
42887	229533.A0A1C3YI28	pp	229533.I1RB74
42888	229533.A0A1C3YI28	pp	229533.I1RAN2
42889	229533.A0A1C3YI28	pp	229533.I1S1V5
42890	229533.A0A1C3YI28	pp	229533.V6RGI1
42891	229533.A0A1C3YI28	pp	229533.I1RZE4
42892	229533.A0A1C3YI28	pp	229533.I1S0N4
42893	229533.A0A1C3YI28	pp	229533.I1RNP7
42894	229533.A0A1C3YI28	pp	229533.I1RY58
42895	229533.A0A1C3YI28	pp	229533.I1RLU1
42896	229533.A0A1C3YI28	pp	229533.I1RDZ4
42897	229533.A0A1C3YI28	pp	229533.I1RA43
42898	229533.A0A1C3YI28	pp	229533.I1RB41
42899	229533.A0A1C3YI28	pp	229533.I1S1D3
42900	229533.A0A1C3YI28	pp	229533.I1RAS9
42901	229533.A0A1C3YI28	pp	229533.I1RUN8
42902	229533.A0A1C3YI28	pp	229533.I1RNW8
42903	229533.A0A1C3YI28	pp	229533.I1R9Y7
42904	229533.A0A1C3YI28	pp	229533.A0A1C3YJI8
42905	229533.A0A1C3YI28	pp	229533.I1RMF5
42906	229533.A0A1C3YI28	pp	229533.I1RNY8
42907	229533.A0A1C3YI28	pp	229533.I1RQ72
42908	229533.A0A1C3YI28	pp	229533.I1R9Y9
42909	229533.A0A1C3YI28	pp	229533.I1S549
42910	229533.A0A1C3YI28	pp	229533.I1S0D1
42911	229533.A0A1C3YI28	pp	229533.I1RW16
42912	229533.A0A1C3YI28	pp	229533.V6RKY7
42913	229533.A0A1C3YI28	pp	229533.I1RY44
42914	229533.A0A1C3YI28	pp	229533.I1RSA4
42915	229533.A0A1C3YI28	pp	229533.V6RVG3
42916	229533.A0A1C3YI28	pp	229533.I1S1W4
42917	229533.A0A1C3YI28	pp	229533.I1RRY3
42918	229533.A0A1C3YM90	pp	229533.I1RDU5
42919	229533.A0A1C3YM90	pp	229533.I1RMD1
42920	229533.A0A1C3YM90	pp	229533.I1RBT7
42921	229533.I1RI27	pp	229533.I1S0R8
42922	229533.I1RI27	pp	229533.I1RYE5
42923	229533.I1RI27	pp	229533.I1S1F3
42924	229533.I1RI27	pp	229533.I1RYN2

42925 229533.I1RI27 pp 229533.I1RX39
42926 229533.I1RI27 pp 229533.I1S212
42927 229533.I1RI27 pp 229533.I1RMQ6
42928 229533.I1RKZ8 pp 229533.I1RWF9
42929 229533.I1RKZ8 pp 229533.I1RUI2
42930 229533.I1RKZ8 pp 229533.V6RE46
42931 229533.I1RKZ8 pp 229533.I1RY21
42932 229533.I1RKZ8 pp 229533.V6RPQ8
42933 229533.I1RKZ8 pp 229533.I1RWN0
42934 229533.I1RKZ8 pp 229533.I1RMG4
42935 229533.I1RKZ8 pp 229533.I1RYD9
42936 229533.I1RNF7 pp 229533.I1S464
42937 229533.I1RNF7 pp 229533.I1RW71
42938 229533.I1RNF7 pp 229533.I1RYT8
42939 229533.I1RNF7 pp 229533.I1RY21
42940 229533.I1RNF7 pp 229533.I1S5C6
42941 229533.I1RNF7 pp 229533.V6RJP8
42942 229533.I1RNF7 pp 229533.I1RWA3
42943 229533.I1RNF7 pp 229533.I1RXD9
42944 229533.I1RNF7 pp 229533.I1RRC5
42945 229533.I1RNF7 pp 229533.I1RS05
42946 229533.I1RNF7 pp 229533.I1S5X9
42947 229533.I1RNF7 pp 229533.I1S0K2
42948 229533.I1RNF7 pp 229533.I1S8S9
42949 229533.I1RNF7 pp 229533.I1RS29
42950 229533.I1RDC5 pp 229533.I1RX70
42951 229533.I1RDC5 pp 229533.I1RQA3
42952 229533.I1RDC5 pp 229533.I1RW54
42953 229533.I1RDC5 pp 229533.I1RLX7
42954 229533.I1RDC5 pp 229533.I1S2E9
42955 229533.I1RDC5 pp 229533.I1RV91
42956 229533.I1RDC5 pp 229533.I1RWT6
42957 229533.I1RDC5 pp 229533.I1RY44
42958 229533.I1RDC5 pp 229533.I1RKL0
42959 229533.A0A1C3YJ79 pp 229533.I1RYR2
42960 229533.A0A1C3YJ79 pp 229533.I1RY29
42961 229533.A0A1C3YJ79 pp 229533.I1RKI2
42962 229533.A0A1C3YJ79 pp 229533.I1RWI8
42963 229533.A0A1C3YJ79 pp 229533.I1RVV5
42964 229533.A0A1C3YJ79 pp 229533.A0A1C3YL55
42965 229533.A0A1C3YJ79 pp 229533.I1RJV8
42966 229533.A0A1C3YJ79 pp 229533.I1RAL9
42967 229533.A0A1C3YJ79 pp 229533.I1R9W7
42968 229533.A0A1C3YJ79 pp 229533.I1RG34
42969 229533.A0A1C3YJ79 pp 229533.Q4IEA5
42970 229533.A0A1C3YJ79 pp 229533.A0A1C3YML3
42971 229533.A0A1C3YJ79 pp 229533.I1RY73
42972 229533.A0A1C3YJ79 pp 229533.I1RQH8
42973 229533.A0A1C3YJ79 pp 229533.I1RKQ0
42974 229533.A0A1C3YJ79 pp 229533.I1RYX9
42975 229533.A0A1C3YJ79 pp 229533.Q4I5R9
42976 229533.A0A1C3YJ79 pp 229533.I1RQ67
42977 229533.A0A1C3YJ79 pp 229533.Q4IP51
42978 229533.A0A1C3YJ79 pp 229533.I1RPQ1
42979 229533.A0A1C3YJ79 pp 229533.Q4INS6
42980 229533.A0A1C3YJ79 pp 229533.I1RYQ1
42981 229533.A0A1C3YJ79 pp 229533.I1RRS7
42982 229533.A0A1C3YJ79 pp 229533.I1S987
42983 229533.A0A1C3YJ79 pp 229533.I1S209
42984 229533.A0A1C3YJ79 pp 229533.I1RYV1
42985 229533.A0A1C3YJ79 pp 229533.V6RLW4
42986 229533.A0A1C3YJ79 pp 229533.I1S998
42987 229533.A0A1C3YJ79 pp 229533.I1RWM6
42988 229533.A0A1C3YJ79 pp 229533.I1RC54
42989 229533.A0A1C3YJ79 pp 229533.I1RA10
42990 229533.A0A1C3YJ79 pp 229533.Q4IQM6
42991 229533.A0A1C3YJ79 pp 229533.I1RDJ8
42992 229533.A0A1C3YJ79 pp 229533.I1RUG2
42993 229533.A0A1C3YJ79 pp 229533.A0A1C3YLR6
42994 229533.A0A1C3YJ79 pp 229533.I1RN55
42995 229533.A0A1C3YJ79 pp 229533.I1RRT1
42996 229533.A0A1C3YJ79 pp 229533.I1RF70
42997 229533.A0A1C3YJ79 pp 229533.I1RCA5

42998 229533.A0A1C3YJ79 pp 229533.I1RP99
42999 229533.A0A1C3YJ79 pp 229533.Q4IC11
43000 229533.A0A1C3YJ79 pp 229533.I1RDV4
43001 229533.A0A1C3YJ79 pp 229533.I1RG77
43002 229533.A0A1C3YJ79 pp 229533.A0A1C3YMG8
43003 229533.A0A1C3YJ79 pp 229533.I1RD01
43004 229533.A0A1C3YJ79 pp 229533.A0A1C3YM29
43005 229533.A0A1C3YJ79 pp 229533.I1RHD2
43006 229533.A0A1C3YJ79 pp 229533.I1RUR0
43007 229533.A0A1C3YJ79 pp 229533.I1RSK0
43008 229533.A0A1C3YJ79 pp 229533.I1S283
43009 229533.A0A1C3YJ79 pp 229533.I1RFJ2
43010 229533.A0A1C3YJ79 pp 229533.I1RZ94
43011 229533.A0A1C3YJ79 pp 229533.I1S461
43012 229533.A0A1C3YJ79 pp 229533.I1RAH4
43013 229533.A0A1C3YJ79 pp 229533.I1RUG4
43014 229533.A0A1C3YJ79 pp 229533.I1RN57
43015 229533.A0A1C3YJ79 pp 229533.A0A1C3YN69
43016 229533.A0A1C3YJ79 pp 229533.I1RYH9
43017 229533.A0A1C3YJ79 pp 229533.Q4IB89
43018 229533.A0A1C3YJ79 pp 229533.I1RFX8
43019 229533.A0A1C3YJ79 pp 229533.I1RW03
43020 229533.A0A1C3YJ79 pp 229533.I1RD19
43021 229533.A0A1C3YJ79 pp 229533.I1RSP7
43022 229533.A0A1C3YJ79 pp 229533.I1RZP8
43023 229533.A0A1C3YJ79 pp 229533.I1RMJ8
43024 229533.A0A1C3YJ79 pp 229533.I1RLJ8
43025 229533.A0A1C3YJ79 pp 229533.I1RTN6
43026 229533.A0A1C3YJ79 pp 229533.I1RA12
43027 229533.A0A1C3YJ79 pp 229533.I1RE46
43028 229533.A0A1C3YJ79 pp 229533.A0A1C3YN55
43029 229533.A0A1C3YJ79 pp 229533.I1S4P6
43030 229533.A0A1C3YJ79 pp 229533.I1RBN9
43031 229533.A0A1C3YJ79 pp 229533.I1RSC7
43032 229533.A0A1C3YJ79 pp 229533.I1RCA9
43033 229533.A0A1C3YJ79 pp 229533.I1S0D5
43034 229533.A0A1C3YJ79 pp 229533.I1RSR9
43035 229533.I1RZ81 pp 229533.Q4IPZ1
43036 229533.V6RKY7 pp 229533.V6RVG3
43037 229533.A0A0E0RW49 pp 229533.I1RY09
43038 229533.A0A0E0RW49 pp 229533.Q4IEK8
43039 229533.A0A0E0RW49 pp 229533.I1RKE9
43040 229533.A0A0E0RW49 pp 229533.I1RSS8
43041 229533.A0A0E0RW49 pp 229533.I1RA12
43042 229533.A0A0E0RW49 pp 229533.I1RYP7
43043 229533.A0A0E0RW49 pp 229533.I1RMQ7
43044 229533.A0A0E0RW49 pp 229533.I1RPZ0
43045 229533.A0A0E0RW49 pp 229533.I1RE46
43046 229533.A0A0E0RW49 pp 229533.A0A1C3YJW8
43047 229533.A0A0E0RW49 pp 229533.I1RMX3
43048 229533.A0A0E0RW49 pp 229533.I1S209
43049 229533.A0A0E0RW49 pp 229533.Q4IR08
43050 229533.A0A0E0RW49 pp 229533.I1RQ92
43051 229533.A0A0E0RW49 pp 229533.I1S604
43052 229533.A0A0E0RW49 pp 229533.A0A1C3YN36
43053 229533.A0A0E0RW49 pp 229533.I1RAL9
43054 229533.A0A0E0RW49 pp 229533.I1RY73
43055 229533.A0A0E0RW49 pp 229533.I1RSI4
43056 229533.A0A0E0RW49 pp 229533.A0A1C3YJT5
43057 229533.A0A0E0RW49 pp 229533.A0A1C3YK82
43058 229533.A0A0E0RW49 pp 229533.Q4INS6
43059 229533.A0A0E0RW49 pp 229533.I1RS81
43060 229533.A0A0E0RW49 pp 229533.A0A1C3YI14
43061 229533.A0A0E0RW49 pp 229533.I1RBL8
43062 229533.A0A0E0RW49 pp 229533.I1RCQ4
43063 229533.A0A0E0RW49 pp 229533.I1RE55
43064 229533.A0A0E0RW49 pp 229533.I1RQH8
43065 229533.A0A0E0RW49 pp 229533.A0A1C3YML3
43066 229533.A0A0E0RW49 pp 229533.Q4IQX3
43067 229533.A0A0E0RW49 pp 229533.I1RTL0
43068 229533.A0A0E0RW49 pp 229533.I1RM36
43069 229533.A0A0E0RW49 pp 229533.A0A1C3YI40
43070 229533.A0A0E0RW49 pp 229533.I1RQ13

43071 229533.A0A0E0RW49 pp 229533.I1RD01
43072 229533.A0A0E0RW49 pp 229533.I1RXJ1
43073 229533.A0A0E0RW49 pp 229533.I1RZ94
43074 229533.A0A0E0RW49 pp 229533.A0A1C3YJN7
43075 229533.A0A0E0RW49 pp 229533.I1RPQ1
43076 229533.A0A0E0RW49 pp 229533.I1RFJ2
43077 229533.A0A0E0RW49 pp 229533.A0A1C3YJ79
43078 229533.A0A0E0RW49 pp 229533.I1S283
43079 229533.A0A0E0RW49 pp 229533.I1RKB1
43080 229533.A0A0E0RW49 pp 229533.I1RQL4
43081 229533.A0A0E0RW49 pp 229533.I1RUR0
43082 229533.A0A0E0RW49 pp 229533.I1S461
43083 229533.A0A0E0RW49 pp 229533.I1RAT5
43084 229533.A0A0E0RW49 pp 229533.I1RLX6
43085 229533.A0A0E0RW49 pp 229533.I1RN46
43086 229533.A0A0E0RW49 pp 229533.I1RSJ1
43087 229533.A0A0E0RW49 pp 229533.A0A1C3YJV7
43088 229533.A0A0E0RW49 pp 229533.I1RE23
43089 229533.A0A0E0RW49 pp 229533.I1RC98
43090 229533.A0A0E0RW49 pp 229533.I1S788
43091 229533.A0A0E0RW49 pp 229533.I1RMT4
43092 229533.A0A0E0RW49 pp 229533.A0A1C3YJL7
43093 229533.A0A0E0RW49 pp 229533.I1RCL7
43094 229533.A0A0E0RW49 pp 229533.I1RKI2
43095 229533.A0A0E0RW49 pp 229533.I1RN55
43096 229533.A0A0E0RW49 pp 229533.I1S0B2
43097 229533.A0A0E0RW49 pp 229533.I1RG77
43098 229533.A0A0E0RW49 pp 229533.I1RFX8
43099 229533.A0A0E0RW49 pp 229533.I1S987
43100 229533.A0A0E0RW49 pp 229533.Q4HVW2
43101 229533.A0A0E0RW49 pp 229533.I1RUG2
43102 229533.A0A0E0RW49 pp 229533.A0A0E0SA11
43103 229533.A0A0E0RW49 pp 229533.A0A1C3YN55
43104 229533.A0A0E0RW49 pp 229533.I1RMS2
43105 229533.A0A0E0RW49 pp 229533.I1S4P6
43106 229533.A0A0E0RW49 pp 229533.I1RBW0
43107 229533.A0A0E0RW49 pp 229533.A0A1C3YJF3
43108 229533.A0A0E0RW49 pp 229533.I1RAH4
43109 229533.A0A0E0RW49 pp 229533.A0A1C3YME9
43110 229533.A0A0E0RW49 pp 229533.A0A1C3YLR4
43111 229533.A0A0E0RW49 pp 229533.I1RC22
43112 229533.A0A0E0RW49 pp 229533.I1RY44
43113 229533.A0A0E0RW49 pp 229533.I1S282
43114 229533.A0A0E0RW49 pp 229533.A0A1C3YI64
43115 229533.A0A0E0RW49 pp 229533.I1RZ02
43116 229533.A0A0E0RW49 pp 229533.I1S9J6
43117 229533.A0A0E0RW49 pp 229533.I1RN31
43118 229533.A0A0E0RW49 pp 229533.I1RAP2
43119 229533.A0A0E0RW49 pp 229533.I1RRT1
43120 229533.A0A0E0RW49 pp 229533.I1S5G0
43121 229533.A0A0E0RW49 pp 229533.I1RSP7
43122 229533.A0A0E0RW49 pp 229533.I1RRS7
43123 229533.A0A0E0RW49 pp 229533.Q4IB89
43124 229533.A0A0E0RW49 pp 229533.I1RQJ4
43125 229533.A0A0E0RW49 pp 229533.I1RBC8
43126 229533.A0A0E0RW49 pp 229533.I1RC54
43127 229533.A0A0E0RW49 pp 229533.I1RCA9
43128 229533.A0A0E0RW49 pp 229533.I1RBN9
43129 229533.A0A0E0RW49 pp 229533.A0A1C3YLB6
43130 229533.A0A0E0RW49 pp 229533.I1RQB4
43131 229533.A0A0E0RW49 pp 229533.A0A1C3YN69
43132 229533.A0A0E0RW49 pp 229533.I1RX64
43133 229533.A0A0E0RW49 pp 229533.Q4IA62
43134 229533.A0A0E0RW49 pp 229533.I1RSC7
43135 229533.A0A0E0RW49 pp 229533.I1RN57
43136 229533.A0A0E0RW49 pp 229533.I1S008
43137 229533.A0A0E0RW49 pp 229533.I1RQI6
43138 229533.A0A0E0RW49 pp 229533.Q4IJ84
43139 229533.A0A0E0RW49 pp 229533.A0A1C3YMC9
43140 229533.A0A0E0RW49 pp 229533.I1RW16
43141 229533.A0A0E0RW49 pp 229533.I1RZ98
43142 229533.A0A0E0RW49 pp 229533.A0A1C3YM91
43143 229533.A0A0E0RW49 pp 229533.A0A0E0SMT9

43144	229533.A0A0E0RW49	pp	229533.I1RD25
43145	229533.A0A0E0RW49	pp	229533.I1RZP8
43146	229533.A0A0E0RW49	pp	229533.I1S0A6
43147	229533.A0A0E0RW49	pp	229533.I1RTN6
43148	229533.A0A0E0RW49	pp	229533.I1RG57
43149	229533.A0A0E0RW49	pp	229533.I1RSZ3
43150	229533.A0A0E0RW49	pp	229533.I1RWI8
43151	229533.A0A0E0RW49	pp	229533.I1S0N5
43152	229533.A0A0E0RW49	pp	229533.A0A1C3YL55
43153	229533.A0A0E0RW49	pp	229533.I1RY29
43154	229533.A0A0E0RW49	pp	229533.A0A1C3YHN3
43155	229533.A0A0E0RW49	pp	229533.A0A1C3YM61
43156	229533.A0A0E0RW49	pp	229533.I1RMG6
43157	229533.A0A0E0RW49	pp	229533.I1RUG4
43158	229533.A0A0E0RW49	pp	229533.I1RSN7
43159	229533.A0A0E0RW49	pp	229533.I1RQ72
43160	229533.A0A0E0RW49	pp	229533.I1RGD5
43161	229533.A0A0E0RW49	pp	229533.I1RA10
43162	229533.A0A0E0RW49	pp	229533.A0A0E0SQG9
43163	229533.A0A0E0RW49	pp	229533.A0A1C3YM67
43164	229533.A0A0E0RW49	pp	229533.I1RDJ8
43165	229533.A0A0E0RW49	pp	229533.I1S2E9
43166	229533.A0A0E0RW49	pp	229533.I1RDH0
43167	229533.A0A0E0RW49	pp	229533.I1S523
43168	229533.A0A0E0RW49	pp	229533.I1RYR2
43169	229533.A0A0E0RW49	pp	229533.I1RY58
43170	229533.A0A0E0RW49	pp	229533.I1RNF4
43171	229533.A0A0E0RW49	pp	229533.I1RSY2
43172	229533.A0A0E0RW49	pp	229533.A0A1C3YHM3
43173	229533.A0A0E0RW49	pp	229533.V6RLW4
43174	229533.A0A0E0RW49	pp	229533.I1RB25
43175	229533.A0A0E0RW49	pp	229533.A0A1C3YMJ3
43176	229533.A0A0E0RW49	pp	229533.I1RYX9
43177	229533.A0A0E0RW49	pp	229533.I1RRN5
43178	229533.A0A0E0RW49	pp	229533.I1S6Y6
43179	229533.A0A0E0RW49	pp	229533.I1RJZ7
43180	229533.A0A0E0RW49	pp	229533.I1RSR9
43181	229533.A0A0E0RW49	pp	229533.I1RSA7
43182	229533.A0A0E0RW49	pp	229533.I1S0D5
43183	229533.A0A0E0RW49	pp	229533.I1RVW0
43184	229533.A0A0E0RW49	pp	229533.I1RPM2
43185	229533.A0A0E0RW49	pp	229533.I1RW24
43186	229533.A0A0E0RW49	pp	229533.I1R9W7
43187	229533.A0A0E0RW49	pp	229533.I1RZT1
43188	229533.A0A0E0RW49	pp	229533.Q4IAS1
43189	229533.A0A0E0RW49	pp	229533.I1RDX0
43190	229533.A0A0E0RW49	pp	229533.I1RRJ6
43191	229533.A0A0E0RW49	pp	229533.I1RMJ8
43192	229533.A0A0E0RW49	pp	229533.I1RRM8
43193	229533.A0A0E0RW49	pp	229533.I1S998
43194	229533.A0A0E0RW49	pp	229533.I1RD19
43195	229533.A0A0E0RW49	pp	229533.I1S0D6
43196	229533.A0A0E0RW49	pp	229533.I1RUF8
43197	229533.A0A0E0RW49	pp	229533.A0A1C3YKE4
43198	229533.A0A0E0RW49	pp	229533.I1RJV8
43199	229533.A0A0E0RW49	pp	229533.Q4IQM6
43200	229533.A0A0E0RW49	pp	229533.A0A1C3YI39
43201	229533.A0A0E0RW49	pp	229533.I1RCA5
43202	229533.A0A0E0RW49	pp	229533.I1RZ86
43203	229533.A0A0E0RW49	pp	229533.I1REP7
43204	229533.A0A0E0RW49	pp	229533.I1RYQ1
43205	229533.A0A0E0RW49	pp	229533.I1RPF5
43206	229533.A0A0E0RW49	pp	229533.I1RHD2
43207	229533.A0A0E0RW49	pp	229533.I1RBW1
43208	229533.A0A0E0RW49	pp	229533.I1RBU5
43209	229533.A0A0E0RW49	pp	229533.A0A1C3YJ90
43210	229533.A0A0E0RW49	pp	229533.A0A1C3YI10
43211	229533.I1RYR1	pp	229533.I1S209
43212	229533.I1RYR1	pp	229533.I1S461
43213	229533.I1RYR1	pp	229533.Q4INS6
43214	229533.I1RQY0	pp	229533.I1RW06
43215	229533.I1RQY0	pp	229533.I1S9Z4
43216	229533.A0A1C3YKS0	pp	229533.I1RSQ3

43217	229533.A0A1C3YKS0	pp	229533.I1RGA4
43218	229533.A0A1C3YKS0	pp	229533.I1RNC1
43219	229533.A0A1C3YKS0	pp	229533.A0A1C3YLK8
43220	229533.A0A1C3YKS0	pp	229533.I1S2E9
43221	229533.A0A1C3YKS0	pp	229533.I1RT40
43222	229533.A0A1C3YKS0	pp	229533.I1RTD5
43223	229533.A0A1C3YKS0	pp	229533.I1RAW0
43224	229533.A0A1C3YKS0	pp	229533.I1RY44
43225	229533.A0A1C3YKS0	pp	229533.I1RSR9
43226	229533.A0A1C3YKS0	pp	229533.I1RRI2
43227	229533.A0A1C3YKS0	pp	229533.I1RSL8
43228	229533.A0A1C3YKS0	pp	229533.I1RBW1
43229	229533.A0A1C3YKS0	pp	229533.I1S5R1
43230	229533.A0A1C3YKS0	pp	229533.I1RRQ4
43231	229533.A0A1C3YKS0	pp	229533.I1RMP0
43232	229533.A0A1C3YKS0	pp	229533.I1S2G7
43233	229533.A0A1C3YKS0	pp	229533.I1RRT8
43234	229533.A0A1C3YKS0	pp	229533.I1RZ39
43235	229533.A0A1C3YKS0	pp	229533.I1RME4
43236	229533.A0A1C3YKS0	pp	229533.I1RYI5
43237	229533.A0A1C3YKS0	pp	229533.I1RVX0
43238	229533.A0A1C3YKS0	pp	229533.I1RPP3
43239	229533.A0A1C3YKS0	pp	229533.I1RCH5
43240	229533.A0A1C3YKS0	pp	229533.I1RPX5
43241	229533.A0A1C3YKS0	pp	229533.I1RA10
43242	229533.A0A1C3YKS0	pp	229533.I1RTA1
43243	229533.A0A1C3YKS0	pp	229533.I1R9M0
43244	229533.A0A1C3YKS0	pp	229533.I1RQE7
43245	229533.A0A1C3YKS0	pp	229533.I1RRY9
43246	229533.A0A1C3YKS0	pp	229533.I1RD22
43247	229533.A0A1C3YKS0	pp	229533.I1S2B8
43248	229533.A0A1C3YKS0	pp	229533.I1RML8
43249	229533.A0A1C3YKS0	pp	229533.I1RZK5
43250	229533.A0A1C3YKS0	pp	229533.I1S549
43251	229533.A0A1C3YKS0	pp	229533.Q4IJM4
43252	229533.A0A1C3YKS0	pp	229533.I1REK8
43253	229533.A0A1C3YKS0	pp	229533.I1RYZ0
43254	229533.A0A1C3YKS0	pp	229533.I1RG77
43255	229533.A0A1C3YKS0	pp	229533.I1RSZ7
43256	229533.A0A1C3YKS0	pp	229533.I1RQA3
43257	229533.A0A1C3YKS0	pp	229533.I1S091
43258	229533.A0A1C3YKS0	pp	229533.I1RCA5
43259	229533.A0A1C3YKS0	pp	229533.Q4IJK2
43260	229533.A0A1C3YKS0	pp	229533.I1RBW0
43261	229533.A0A1C3YKS0	pp	229533.Q4I7N9
43262	229533.A0A1C3YKS0	pp	229533.I1RZ94
43263	229533.A0A1C3YKS0	pp	229533.V6RTJ8
43264	229533.A0A1C3YKS0	pp	229533.I1S5B0
43265	229533.A0A1C3YKS0	pp	229533.I1RUQ6
43266	229533.A0A1C3YKS0	pp	229533.I1RPQ1
43267	229533.A0A1C3YKS0	pp	229533.V6RCZ8
43268	229533.A0A1C3YKS0	pp	229533.I1RXD7
43269	229533.A0A1C3YKS0	pp	229533.I1RD52
43270	229533.A0A1C3YKS0	pp	229533.I1RZM8
43271	229533.A0A1C3YKS0	pp	229533.I1S4S7
43272	229533.A0A1C3YKS0	pp	229533.I1RWT6
43273	229533.A0A1C3YKS0	pp	229533.I1RW71
43274	229533.A0A1C3YKS0	pp	229533.I1RA12
43275	229533.A0A1C3YKS0	pp	229533.I1RS07
43276	229533.A0A1C3YKS0	pp	229533.I1RZJ9
43277	229533.A0A1C3YKS0	pp	229533.I1RFZ3
43278	229533.A0A1C3YKS0	pp	229533.I1RX70
43279	229533.A0A1C3YKS0	pp	229533.I1RJU3
43280	229533.A0A1C3YKG4	pp	229533.I1RW06
43281	229533.A0A1C3YKG4	pp	229533.I1RBE4
43282	229533.A0A1C3YKG4	pp	229533.I1S368
43283	229533.A0A1C3YKG4	pp	229533.I1RMM8
43284	229533.A0A1C3YKG4	pp	229533.I1RFV2
43285	229533.A0A1C3YKG4	pp	229533.I1REI2
43286	229533.A0A1C3YL34	pp	229533.I1S183
43287	229533.A0A1C3YL34	pp	229533.Q4ICA8
43288	229533.A0A1C3YL34	pp	229533.I1RQH8
43289	229533.A0A1C3YL34	pp	229533.I1RYR1

43290 229533.A0A1C3YL34 pp 229533.Q4INS6
43291 229533.A0A1C3YL34 pp 229533.V6QUE6
43292 229533.A0A1C3YL34 pp 229533.I1S209
43293 229533.A0A1C3YL34 pp 229533.I1S461
43294 229533.A0A1C3YL34 pp 229533.A0A1C3YN36
43295 229533.A0A1C3YL34 pp 229533.I1RBC1
43296 229533.A0A1C3YL34 pp 229533.I1RSI4
43297 229533.A0A1C3YL34 pp 229533.I1RTN6
43298 229533.A0A1C3YL34 pp 229533.I1RK49
43299 229533.A0A1C3YL34 pp 229533.I1RD19
43300 229533.A0A1C3YL34 pp 229533.I1RAL9
43301 229533.A0A1C3YL34 pp 229533.I1RKI2
43302 229533.A0A1C3YL34 pp 229533.I1RW71
43303 229533.A0A1C3YL34 pp 229533.I1S283
43304 229533.A0A1C3YL34 pp 229533.I1RSP7
43305 229533.A0A1C3YL34 pp 229533.I1RWM6
43306 229533.A0A1C3YL34 pp 229533.I1RW03
43307 229533.A0A1C3YL34 pp 229533.I1S1V2
43308 229533.A0A1C3YL34 pp 229533.A0A1C3YML3
43309 229533.A0A1C3YL34 pp 229533.I1RUR0
43310 229533.A0A1C3YL34 pp 229533.I1RQJ4
43311 229533.A0A1C3YL34 pp 229533.I1RRT1
43312 229533.A0A1C3YL34 pp 229533.Q4IB89
43313 229533.A0A1C3YL34 pp 229533.I1RZT1
43314 229533.A0A1C3YL34 pp 229533.A0A1C3YLR6
43315 229533.A0A1C3YL34 pp 229533.I1RMJ8
43316 229533.A0A1C3YL34 pp 229533.I1S987
43317 229533.A0A1C3YL34 pp 229533.Q4HYB8
43318 229533.A0A1C3YL34 pp 229533.I1S0D5
43319 229533.A0A1C3YL34 pp 229533.A0A1C3YN26
43320 229533.A0A1C3YL34 pp 229533.I1RAH4
43321 229533.A0A1C3YL34 pp 229533.I1RSC7
43322 229533.A0A1C3YL34 pp 229533.I1RYX9
43323 229533.A0A1C3YL34 pp 229533.I1RYR2
43324 229533.A0A1C3YL34 pp 229533.I1RQI6
43325 229533.A0A1C3YL34 pp 229533.I1RC54
43326 229533.A0A1C3YL34 pp 229533.I1S0N4
43327 229533.A0A1C3YL34 pp 229533.I1RNG4
43328 229533.A0A1C3YL34 pp 229533.I1RJV8
43329 229533.A0A1C3YL34 pp 229533.I1RZ86
43330 229533.A0A1C3YL34 pp 229533.A0A1C3YL55
43331 229533.A0A1C3YL34 pp 229533.I1RMN6
43332 229533.A0A1C3YL34 pp 229533.I1S004
43333 229533.A0A1C3YL34 pp 229533.I1RWI8
43334 229533.A0A1C3YL34 pp 229533.I1RRS7
43335 229533.A0A1C3YL34 pp 229533.I1RVW0
43336 229533.A0A1C3YL34 pp 229533.I1RP99
43337 229533.A0A1C3YL34 pp 229533.I1RY29
43338 229533.A0A1C3YL34 pp 229533.I1RRJ6
43339 229533.A0A1C3YL34 pp 229533.I1RPF5
43340 229533.A0A1C3YL34 pp 229533.I1RDJ8
43341 229533.A0A1C3YL34 pp 229533.I1RFN1
43342 229533.A0A1C3YL34 pp 229533.I1S998
43343 229533.A0A1C3YL34 pp 229533.I1RWI0
43344 229533.A0A1C3YL34 pp 229533.I1RYQ1
43345 229533.A0A1C3YL34 pp 229533.I1RP27
43346 229533.A0A1C3YL34 pp 229533.I1RJZ7
43347 229533.A0A1C3YL34 pp 229533.A0A1C3YLQ7
43348 229533.A0A1C3YL34 pp 229533.I1S7I5
43349 229533.A0A0E0RWF7 pp 229533.V6RSM7
43350 229533.A0A0E0RWF7 pp 229533.V6RG04
43351 229533.A0A0E0RWF7 pp 229533.I1RDZ5
43352 229533.A0A0E0RWF7 pp 229533.I1RN32
43353 229533.I1RXZ3 pp 229533.V6RJP8
43354 229533.I1RXZ3 pp 229533.I1RYE5
43355 229533.I1RXZ3 pp 229533.I1S0P0
43356 229533.I1RXZ3 pp 229533.I1S8F0
43357 229533.I1RXZ3 pp 229533.Q4I375
43358 229533.I1RXZ3 pp 229533.Q4IPZ1
43359 229533.I1RXZ3 pp 229533.Q4IJW4
43360 229533.I1RXZ3 pp 229533.Q4I7T5
43361 229533.I1RXZ3 pp 229533.Q4IB65
43362 229533.I1RXZ3 pp 229533.I1RZS4

229533.I1RXZ3 pp 229533.I1RY92
229533.I1RXZ3 pp 229533.I1S0R8
229533.I1RXZ3 pp 229533.Q4HZ95
229533.I1RXZ3 pp 229533.Q4I6B0
229533.I1RXZ3 pp 229533.I1S464
229533.I1RXZ3 pp 229533.I1S5X9
229533.I1RXZ3 pp 229533.I1RYT8
229533.I1RXZ3 pp 229533.Q4I099
229533.V6RGI1 pp 229533.V6RMM8
229533.V6RGI1 pp 229533.V6RKY7
229533.V6RGI1 pp 229533.V6RVG3
229533.V6RGI1 pp 229533.V6RU70
229533.V6RGI1 pp 229533.V6RLW4
229533.I1RG49 pp 229533.I1RKB9
229533.I1RQH8 pp 229533.I1RSP7
229533.I1RQH8 pp 229533.I1RW03
229533.I1RQH8 pp 229533.I1S1U7
229533.I1RQH8 pp 229533.I1S4P6
229533.I1RQH8 pp 229533.I1RUG4
229533.I1RQH8 pp 229533.I1RVT9
229533.I1RQH8 pp 229533.I1S209
229533.I1RQH8 pp 229533.Q4IB89
229533.I1RQH8 pp 229533.I1S0D5
229533.I1RQH8 pp 229533.Q4INS6
229533.I1RQH8 pp 229533.I1RUR0
229533.I1RQH8 pp 229533.I1RZ98
229533.I1RQH8 pp 229533.I1RSR9
229533.I1RQH8 pp 229533.I1RZP8
229533.I1RQH8 pp 229533.I1RSC7
229533.I1RQH8 pp 229533.I1RYP7
229533.I1RQH8 pp 229533.I1RYQ1
229533.I1RQH8 pp 229533.I1S998
229533.I1RQH8 pp 229533.I1RY29
229533.I1RQH8 pp 229533.I1RRY1
229533.I1RQH8 pp 229533.I1RVW0
229533.I1RQH8 pp 229533.I1RWI8
229533.I1RQH8 pp 229533.I1S987
229533.I1RQH8 pp 229533.I1RZ86
229533.I1RQH8 pp 229533.I1RZJ6
229533.I1RQH8 pp 229533.I1RZ94
229533.I1RQH8 pp 229533.I1RW24
229533.I1RQH8 pp 229533.I1RRT1
229533.I1RQH8 pp 229533.I1RSN7
229533.I1RQH8 pp 229533.I1RWM6
229533.I1RAT0 pp 229533.I1RRS2
229533.A0A098DPC2 pp 229533.A0A1C3YJF7
229533.A0A098DPC2 pp 229533.I1RYD3
229533.A0A098DPC2 pp 229533.I1RN32
229533.A0A098DPC2 pp 229533.A0A1C3YJJ7
229533.A0A098DPC2 pp 229533.A0A1C3YLQ4
229533.A0A098DPC2 pp 229533.I1RQM4
229533.A0A098DPC2 pp 229533.I1RDZ5
229533.A0A098DPC2 pp 229533.I1RWN0
229533.A0A098DPC2 pp 229533.A0A1C3YMF6
229533.A0A098DPC2 pp 229533.A0A1C3YLC3
229533.A0A098DPC2 pp 229533.I1RZN1
229533.A0A098DPC2 pp 229533.A0A1C3YLB7
229533.A0A098DPC2 pp 229533.I1RFV2
229533.A0A098DPC2 pp 229533.A0A0E0SIE3
229533.A0A098DPC2 pp 229533.I1RKZ8
229533.A0A098DPC2 pp 229533.I1RZ81
229533.A0A098DPC2 pp 229533.I1RFY0
229533.A0A098DPC2 pp 229533.I1RYR1
229533.A0A098DPC2 pp 229533.V6RG04
229533.A0A098DPC2 pp 229533.I1RW69
229533.A0A098DPC2 pp 229533.A0A1C3YKG4
229533.A0A098DPC2 pp 229533.A0A0E0RWF7
229533.A0A098DPC2 pp 229533.I1RK49
229533.A0A098DPC2 pp 229533.I1RMG4
229533.A0A1C3YK33 pp 229533.I1RV91
229533.A0A1C3YK33 pp 229533.I1RT40
229533.A0A1C3YK33 pp 229533.I1RTD5
229533.A0A1C3YK33 pp 229533.I1RWF5

229533.A0A1C3YK33	pp	229533.V6RUY9
229533.A0A1C3YK33	pp	229533.I1RT04
229533.A0A1C3YK33	pp	229533.I1RRZ0
229533.A0A1C3YK33	pp	229533.A0A1C3YLE2
229533.A0A1C3YK33	pp	229533.I1S503
229533.A0A1C3YK33	pp	229533.I1RJW6
229533.A0A1C3YK33	pp	229533.I1RBY9
229533.A0A1C3YK33	pp	229533.A0A1C3YLK8
229533.A0A1C3YK33	pp	229533.I1RMJ3
229533.A0A1C3YK33	pp	229533.V6R3J1
229533.A0A1C3YK33	pp	229533.I1RFT1
229533.A0A1C3YK33	pp	229533.I1RI39
229533.A0A1C3YK33	pp	229533.I1RBS6
229533.A0A1C3YK33	pp	229533.I1RGA4
229533.A0A1C3YK33	pp	229533.I1RIF9
229533.A0A1C3YK33	pp	229533.I1RPP3
229533.A0A1C3YK33	pp	229533.I1RYK0
229533.A0A1C3YK33	pp	229533.I1RJV4
229533.A0A1C3YK33	pp	229533.I1RA62
229533.A0A1C3YK33	pp	229533.I1RRT8
229533.A0A1C3YK33	pp	229533.I1RN67
229533.A0A1C3YK33	pp	229533.I1RX72
229533.A0A1C3YK33	pp	229533.I1S2H4
229533.A0A1C3YK33	pp	229533.I1RA11
229533.A0A1C3YK33	pp	229533.I1RNG4
229533.A0A1C3YK33	pp	229533.I1S3G4
229533.A0A1C3YK33	pp	229533.V6RUG6
229533.A0A1C3YK33	pp	229533.I1RWK3
229533.A0A1C3YK33	pp	229533.I1RCR8
229533.A0A1C3YK33	pp	229533.I1S0L4
229533.A0A1C3YK33	pp	229533.I1RBS0
229533.A0A1C3YK33	pp	229533.I1S0F6
229533.A0A1C3YK33	pp	229533.I1RZ78
229533.A0A1C3YK33	pp	229533.I1RN84
229533.A0A1C3YK33	pp	229533.I1RRV1
229533.A0A1C3YK33	pp	229533.I1RC31
229533.A0A1C3YK33	pp	229533.I1RN88
229533.A0A1C3YK33	pp	229533.I1RFS3
229533.A0A1C3YK33	pp	229533.I1RPM4
229533.A0A1C3YK33	pp	229533.I1RY62
229533.A0A1C3YK33	pp	229533.V6RMW6
229533.A0A1C3YK33	pp	229533.I1RSQ4
229533.A0A1C3YK33	pp	229533.I1RGZ3
229533.A0A1C3YK33	pp	229533.V6R042
229533.A0A1C3YK33	pp	229533.I1RE22
229533.A0A1C3YK33	pp	229533.I1RZZ9
229533.A0A1C3YK33	pp	229533.I1RC66
229533.A0A1C3YK33	pp	229533.I1RC29
229533.A0A1C3YK33	pp	229533.I1RSQ3
229533.A0A1C3YK33	pp	229533.I1R9M0
229533.A0A1C3YK33	pp	229533.I1RH91
229533.A0A1C3YK33	pp	229533.I1RUR8
229533.A0A1C3YK33	pp	229533.I1RRM5
229533.A0A1C3YK33	pp	229533.I1RSU2
229533.A0A1C3YK33	pp	229533.I1RPZ5
229533.A0A1C3YK33	pp	229533.I1RZ39
229533.A0A1C3YK33	pp	229533.A0A1C3YKM9
229533.A0A1C3YK33	pp	229533.I1S2B8
229533.A0A1C3YK33	pp	229533.I1SA33
229533.A0A1C3YK33	pp	229533.I1RBY4
229533.A0A1C3YK33	pp	229533.V6R3X5
229533.A0A1C3YK33	pp	229533.I1S256
229533.A0A1C3YK33	pp	229533.I1SAC4
229533.A0A1C3YK33	pp	229533.I1RVZ3
229533.A0A1C3YK33	pp	229533.I1S9Y1
229533.A0A1C3YK33	pp	229533.I1RFI4
229533.A0A1C3YK33	pp	229533.I1RD22
229533.A0A1C3YK33	pp	229533.I1S147
229533.A0A1C3YK33	pp	229533.I1S5R1
229533.A0A1C3YK33	pp	229533.I1RFF3
229533.A0A1C3YK33	pp	229533.I1RCG5
229533.A0A1C3YK33	pp	229533.I1RGC3
229533.A0A1C3YK33	pp	229533.I1RUG3

43509 229533.A0A1C3YK33 pp 229533.I1RE01
43510 229533.A0A1C3YK33 pp 229533.I1RYW3
43511 229533.A0A1C3YK33 pp 229533.I1RW46
43512 229533.A0A1C3YK33 pp 229533.A0A1C3YLB6
43513 229533.A0A1C3YK33 pp 229533.I1S0J1
43514 229533.A0A1C3YK33 pp 229533.V6RLM7
43515 229533.A0A1C3YK33 pp 229533.I1S1C7
43516 229533.A0A1C3YK33 pp 229533.I1RNC1
43517 229533.A0A1C3YK33 pp 229533.I1RRI2
43518 229533.A0A1C3YK33 pp 229533.I1RA70
43519 229533.A0A1C3YK33 pp 229533.I1RAU7
43520 229533.A0A1C3YK33 pp 229533.I1RKB5
43521 229533.A0A1C3YK33 pp 229533.I1RP86
43522 229533.A0A1C3YK33 pp 229533.V6RIV9
43523 229533.A0A1C3YK33 pp 229533.I1S8R3
43524 229533.A0A1C3YK33 pp 229533.I1S0R7
43525 229533.A0A1C3YK33 pp 229533.I1RTH6
43526 229533.A0A1C3YK33 pp 229533.I1RJV3
43527 229533.A0A1C3YK33 pp 229533.I1RXD7
43528 229533.A0A1C3YK33 pp 229533.I1S2L0
43529 229533.A0A1C3YK33 pp 229533.I1RWN5
43530 229533.A0A1C3YK33 pp 229533.I1RC33
43531 229533.A0A1C3YK33 pp 229533.I1RWN6
43532 229533.A0A1C3YK33 pp 229533.I1RAW0
43533 229533.A0A1C3YK33 pp 229533.I1S2N3
43534 229533.A0A1C3YK33 pp 229533.I1RFL4
43535 229533.A0A1C3YK33 pp 229533.V6RVG3
43536 229533.A0A1C3YK33 pp 229533.I1RCH5
43537 229533.A0A1C3YK33 pp 229533.I1RGX1
43538 229533.A0A1C3YK33 pp 229533.I1RQE7
43539 229533.A0A1C3YK33 pp 229533.I1RX46
43540 229533.A0A1C3YK33 pp 229533.I1RAX6
43541 229533.A0A1C3YK33 pp 229533.V6RTJ8
43542 229533.A0A1C3YK33 pp 229533.I1RVX0
43543 229533.A0A1C3YK33 pp 229533.I1RR12
43544 229533.A0A1C3YK33 pp 229533.I1S0L3
43545 229533.A0A1C3YK33 pp 229533.I1RT03
43546 229533.A0A1C3YK33 pp 229533.I1RBT1
43547 229533.A0A1C3YK33 pp 229533.I1RRG3
43548 229533.A0A1C3YK33 pp 229533.I1RB92
43549 229533.A0A1C3YK33 pp 229533.A0A1C3YN26
43550 229533.A0A1C3YK33 pp 229533.I1RBC9
43551 229533.A0A1C3YK33 pp 229533.I1RD17
43552 229533.A0A1C3YK33 pp 229533.I1RSQ7
43553 229533.A0A1C3YK33 pp 229533.I1S8C1
43554 229533.A0A1C3YK33 pp 229533.I1RWJ8
43555 229533.A0A1C3YK33 pp 229533.I1RJU3
43556 229533.A0A1C3YK33 pp 229533.Q4I624
43557 229533.A0A1C3YK33 pp 229533.I1RR24
43558 229533.A0A1C3YK33 pp 229533.I1RD52
43559 229533.A0A1C3YK33 pp 229533.I1RNU7
43560 229533.A0A1C3YK33 pp 229533.I1RW25
43561 229533.A0A1C3YK33 pp 229533.Q4IR09
43562 229533.A0A1C3YK33 pp 229533.I1RJ82
43563 229533.A0A1C3YK33 pp 229533.Q4I8P5
43564 229533.A0A1C3YK33 pp 229533.I1RC11
43565 229533.A0A1C3YK33 pp 229533.I1RS18
43566 229533.A0A1C3YK33 pp 229533.I1RNQ5
43567 229533.A0A1C3YK33 pp 229533.I1RZM8
43568 229533.A0A1C3YK33 pp 229533.I1S1W8
43569 229533.A0A1C3YK33 pp 229533.E6PBP1
43570 229533.A0A1C3YK33 pp 229533.I1RIC3
43571 229533.A0A1C3YK33 pp 229533.I1RI82
43572 229533.A0A1C3YK33 pp 229533.I1RJ42
43573 229533.A0A1C3YK33 pp 229533.I1RBE4
43574 229533.A0A1C3YK33 pp 229533.I1S504
43575 229533.A0A1C3YK33 pp 229533.A0A1C3YKB0
43576 229533.A0A1C3YK33 pp 229533.I1RZZ1
43577 229533.A0A1C3YK33 pp 229533.I1RY03
43578 229533.A0A1C3YK33 pp 229533.V6RCZ8
43579 229533.A0A1C3YK33 pp 229533.V6QUE6
43580 229533.A0A1C3YK33 pp 229533.I1RGB2
43581 229533.A0A1C3YK33 pp 229533.I1RPT0

43582 229533.A0A1C3YK33 pp 229533.I1RDU0
43583 229533.A0A1C3YK33 pp 229533.I1RK95
43584 229533.A0A1C3YK33 pp 229533.I1RFU8
43585 229533.A0A1C3YK33 pp 229533.I1S5B0
43586 229533.A0A1C3YK33 pp 229533.I1RC95
43587 229533.A0A1C3YK33 pp 229533.I1RCG4
43588 229533.A0A1C3YK33 pp 229533.I1RUY5
43589 229533.A0A1C3YK33 pp 229533.I1RZK5
43590 229533.A0A1C3YK33 pp 229533.I1RJY7
43591 229533.A0A1C3YK33 pp 229533.I1RCM5
43592 229533.A0A1C3YK33 pp 229533.I1RF19
43593 229533.A0A1C3YK33 pp 229533.A0A1C3YLC2
43594 229533.A0A1C3YK33 pp 229533.I1RW99
43595 229533.A0A1C3YK33 pp 229533.A0A1C3YK34
43596 229533.A0A1C3YK33 pp 229533.I1RBE7
43597 229533.A0A1C3YK33 pp 229533.I1RY12
43598 229533.A0A1C3YK33 pp 229533.I1RFD4
43599 229533.A0A1C3YK33 pp 229533.I1REK8
43600 229533.A0A1C3YK33 pp 229533.I1SA32
43601 229533.A0A1C3YK33 pp 229533.I1RCR5
43602 229533.A0A1C3YK33 pp 229533.I1RBL7
43603 229533.A0A1C3YK33 pp 229533.I1S439
43604 229533.A0A1C3YK33 pp 229533.A0A1C3YKV3
43605 229533.A0A1C3YK33 pp 229533.A0A1C3YKR5
43606 229533.A0A1C3YK33 pp 229533.I1RRY9
43607 229533.A0A1C3YK33 pp 229533.Q4IJK2
43608 229533.I1RZ68 pp 229533.I1S0K2
43609 229533.I1RZ68 pp 229533.I1S0P0
43610 229533.I1RZ68 pp 229533.I1S1F3
43611 229533.I1RZ68 pp 229533.I1S5X9
43612 229533.I1RZ68 pp 229533.I1S0R8
43613 229533.I1RZ68 pp 229533.I1S464
43614 229533.I1RZ68 pp 229533.I1S0Q7
43615 229533.I1RZ68 pp 229533.V6RJP8
43616 229533.I1RF10 pp 229533.I1RNG4
43617 229533.A0A098DPA8 pp 229533.I1S7X9
43618 229533.I1RDQ9 pp 229533.I1RMQ7
43619 229533.I1RDQ9 pp 229533.I1RPF5
43620 229533.I1RDQ9 pp 229533.I1S2E9
43621 229533.I1RDQ9 pp 229533.I1RSZ3
43622 229533.I1RDQ9 pp 229533.I1RPM7
43623 229533.I1RDQ9 pp 229533.I1RRN5
43624 229533.I1RDQ9 pp 229533.I1RFL7
43625 229533.I1RDQ9 pp 229533.I1RY58
43626 229533.I1RDQ9 pp 229533.I1RKB1
43627 229533.I1RDQ9 pp 229533.I1REE1
43628 229533.I1RDQ9 pp 229533.I1RY44
43629 229533.A0A1C3YHJ9 pp 229533.I1RFJ6
43630 229533.A0A1C3YHJ9 pp 229533.I1S0R8
43631 229533.A0A1C3YHJ9 pp 229533.I1S5X9
43632 229533.A0A1C3YHJ9 pp 229533.I1S0K2
43633 229533.A0A1C3YHJ9 pp 229533.I1RBT6
43634 229533.A0A1C3YHJ9 pp 229533.I1RS29
43635 229533.A0A1C3YHJ9 pp 229533.I1RP02
43636 229533.A0A1C3YHJ9 pp 229533.I1S0Q7
43637 229533.A0A1C3YHJ9 pp 229533.V6RJP8
43638 229533.A0A1C3YHJ9 pp 229533.A0A1C3YJQ2
43639 229533.A0A1C3YHJ9 pp 229533.I1RE65
43640 229533.A0A1C3YHJ9 pp 229533.I1RMQ6
43641 229533.A0A1C3YHJ9 pp 229533.A0A1C3YLB6
43642 229533.A0A1C3YHJ9 pp 229533.A0A1C3YJU7
43643 229533.A0A1C3YHJ9 pp 229533.I1RKB7
43644 229533.A0A1C3YHJ9 pp 229533.I1RY92
43645 229533.A0A1C3YHJ9 pp 229533.I1RY21
43646 229533.A0A1C3YHJ9 pp 229533.I1RWA3
43647 229533.A0A1C3YHJ9 pp 229533.I1RA52
43648 229533.A0A1C3YHJ9 pp 229533.I1RNM7
43649 229533.A0A1C3YHJ9 pp 229533.I1RXD9
43650 229533.A0A1C3YHJ9 pp 229533.I1RMK7
43651 229533.A0A1C3YHJ9 pp 229533.I1S464
43652 229533.A0A1C3YHJ9 pp 229533.I1RYT8
43653 229533.A0A1C3YHJ9 pp 229533.I1S1F3
43654 229533.A0A1C3YHJ9 pp 229533.I1RS05

43655	229533.A0A1C3YHJ9	pp	229533.I1RAM9
43656	229533.A0A1C3YHJ9	pp	229533.A0A1C3YLB0
43657	229533.A0A1C3YHJ9	pp	229533.I1S0P0
43658	229533.A0A1C3YHJ9	pp	229533.I1RE66
43659	229533.I1RSR9	pp	229533.V6R382
43660	229533.I1RSR9	pp	229533.I1S0D5
43661	229533.I1RSR9	pp	229533.V6RLW4
43662	229533.I1RSR9	pp	229533.I1S8P5
43663	229533.I1RSR9	pp	229533.I1RXB2
43664	229533.I1RSR9	pp	229533.I1RYX9
43665	229533.I1RSR9	pp	229533.Q4IB89
43666	229533.I1RSR9	pp	229533.I1RTA0
43667	229533.I1RSR9	pp	229533.I1S016
43668	229533.I1RSR9	pp	229533.I1RY44
43669	229533.I1RSR9	pp	229533.I1RYR2
43670	229533.I1RSR9	pp	229533.I1RXA9
43671	229533.I1RSR9	pp	229533.I1S2E9
43672	229533.I1RSR9	pp	229533.I1RZ98
43673	229533.I1RSR9	pp	229533.I1RUG4
43674	229533.I1RSR9	pp	229533.I1S4P6
43675	229533.I1RSR9	pp	229533.I1RWI8
43676	229533.I1RSR9	pp	229533.I1S0A6
43677	229533.I1RSR9	pp	229533.I1RY29
43678	229533.I1RSR9	pp	229533.I1RTN6
43679	229533.I1RSR9	pp	229533.I1RZ86
43680	229533.I1RSR9	pp	229533.I1RZP8
43681	229533.I1RSR9	pp	229533.I1RSZ3
43682	229533.I1RSR9	pp	229533.I1S998
43683	229533.I1RSR9	pp	229533.Q4INS6
43684	229533.I1RSR9	pp	229533.I1RX64
43685	229533.I1RSR9	pp	229533.I1RYQ1
43686	229533.I1RSR9	pp	229533.I1S604
43687	229533.I1RSR9	pp	229533.Q4IQM6
43688	229533.I1RSR9	pp	229533.I1RZ94
43689	229533.I1RSR9	pp	229533.I1S461
43690	229533.I1RSR9	pp	229533.I1S209
43691	229533.I1RSR9	pp	229533.I1S283
43692	229533.I1RSR9	pp	229533.I1S987
43693	229533.I1RSR9	pp	229533.I1RWT6
43694	229533.I1RSR9	pp	229533.I1RV66
43695	229533.I1RE65	pp	229533.I1RI95
43696	229533.I1RE65	pp	229533.I1RW13
43697	229533.I1RE65	pp	229533.I1RP02
43698	229533.I1RE65	pp	229533.V6QUE6
43699	229533.I1RE65	pp	229533.I1RQR6
43700	229533.I1RE65	pp	229533.I1RFS5
43701	229533.I1RE65	pp	229533.I1S5R5
43702	229533.I1RE65	pp	229533.I1S0Q7
43703	229533.I1RE65	pp	229533.I1RXE6
43704	229533.I1RE65	pp	229533.I1RQJ3
43705	229533.I1RE65	pp	229533.I1RZ68
43706	229533.I1RE65	pp	229533.I1RP38
43707	229533.I1RE65	pp	229533.V6RJP8
43708	229533.I1RE65	pp	229533.I1RJL2
43709	229533.I1RE65	pp	229533.I1RU95
43710	229533.I1RE65	pp	229533.I1RSG8
43711	229533.I1RE65	pp	229533.I1RZB5
43712	229533.I1RE65	pp	229533.I1RGI8
43713	229533.I1RE65	pp	229533.I1S8F8
43714	229533.I1RE65	pp	229533.I1RFJ6
43715	229533.I1RE65	pp	229533.I1S212
43716	229533.I1RE65	pp	229533.I1RKF4
43717	229533.I1RE65	pp	229533.I1RZ43
43718	229533.I1RE65	pp	229533.I1S5U0
43719	229533.I1RE65	pp	229533.I1RK95
43720	229533.I1RE65	pp	229533.I1RLN1
43721	229533.I1RE65	pp	229533.I1RKF5
43722	229533.I1RE65	pp	229533.Q4IJM4
43723	229533.I1RE65	pp	229533.I1RYQ1
43724	229533.I1RE65	pp	229533.I1S8S9
43725	229533.I1RE65	pp	229533.I1S268
43726	229533.I1RE65	pp	229533.I1RNJ5
43727	229533.I1RE65	pp	229533.I1S0K2

43728 229533.I1RE65 pp 229533.I1RSY2
43729 229533.I1RE65 pp 229533.I1S266
43730 229533.I1RE65 pp 229533.I1RK39
43731 229533.I1RE65 pp 229533.I1RYC7
43732 229533.I1RE65 pp 229533.I1RX39
43733 229533.I1RE65 pp 229533.I1RXD9
43734 229533.I1RE65 pp 229533.I1S0R8
43735 229533.I1RE65 pp 229533.I1RV91
43736 229533.I1RE65 pp 229533.I1S5X9
43737 229533.I1RE65 pp 229533.I1RSC0
43738 229533.I1RE65 pp 229533.I1RS29
43739 229533.I1RE65 pp 229533.I1RWG0
43740 229533.I1RE65 pp 229533.I1RKQ5
43741 229533.I1RE65 pp 229533.I1S1F3
43742 229533.I1RE65 pp 229533.I1S5C6
43743 229533.I1RE65 pp 229533.I1RR65
43744 229533.I1RE65 pp 229533.I1RP87
43745 229533.I1RE65 pp 229533.I1RS05
43746 229533.I1RE65 pp 229533.I1RJB3
43747 229533.I1RE65 pp 229533.I1RMQ6
43748 229533.I1RE65 pp 229533.I1S0P0
43749 229533.I1RE65 pp 229533.I1RKB7
43750 229533.I1RE65 pp 229533.I1S464
43751 229533.I1RE65 pp 229533.I1RGJ4
43752 229533.I1RE65 pp 229533.I1RNH1
43753 229533.I1RE65 pp 229533.I1RY92
43754 229533.I1RE65 pp 229533.I1S0T7
43755 229533.I1RE65 pp 229533.I1REF0
43756 229533.I1RE65 pp 229533.I1RN42
43757 229533.I1RE65 pp 229533.I1RQ54
43758 229533.I1RE65 pp 229533.I1RS30
43759 229533.I1RE65 pp 229533.I1RY21
43760 229533.I1RE65 pp 229533.I1S0R1
43761 229533.I1RE65 pp 229533.I1RW26
43762 229533.I1RE65 pp 229533.I1RRX5
43763 229533.I1RE65 pp 229533.I1S458
43764 229533.I1RE65 pp 229533.I1S1N7
43765 229533.I1RE65 pp 229533.I1RRK9
43766 229533.I1RE65 pp 229533.I1S0U0
43767 229533.I1RE65 pp 229533.I1RVY0
43768 229533.I1RE65 pp 229533.I1RGC3
43769 229533.I1RE65 pp 229533.I1RNM7
43770 229533.I1RE65 pp 229533.I1RYT8
43771 229533.I1RE65 pp 229533.I1RMK7
43772 229533.I1RE65 pp 229533.I1RG62
43773 229533.I1RE65 pp 229533.I1RPY0
43774 229533.I1RE65 pp 229533.I1RWA3
43775 229533.I1RE65 pp 229533.I1RNA0
43776 229533.I1REF0 pp 229533.I1RYT8
43777 229533.I1REF0 pp 229533.I1RNM7
43778 229533.I1REF0 pp 229533.I1RP02
43779 229533.I1REF0 pp 229533.I1RY21
43780 229533.I1REF0 pp 229533.I1RMK7
43781 229533.I1REF0 pp 229533.I1S0K2
43782 229533.I1REF0 pp 229533.I1RMQ6
43783 229533.I1REF0 pp 229533.I1RKB7
43784 229533.I1REF0 pp 229533.I1S0Q7
43785 229533.I1REF0 pp 229533.I1RS29
43786 229533.I1REF0 pp 229533.I1S0P0
43787 229533.I1REF0 pp 229533.I1RY92
43788 229533.I1REF0 pp 229533.I1RWA3
43789 229533.I1REF0 pp 229533.V6RJP8
43790 229533.I1REF0 pp 229533.I1RS05
43791 229533.I1REF0 pp 229533.I1S0R8
43792 229533.I1REF0 pp 229533.I1S1F3
43793 229533.I1REF0 pp 229533.I1S464
43794 229533.I1REF0 pp 229533.I1S5X9
43795 229533.I1REF0 pp 229533.I1RFJ6
43796 229533.I1REF0 pp 229533.I1RXD9
43797 229533.I1RZC4 pp 229533.I1S9Z4
43798 229533.A0A0E0S082 pp 229533.I1RS05
43799 229533.A0A0E0S082 pp 229533.I1RYT8
43800 229533.A0A0E0S082 pp 229533.I1RMK7

43801 229533.A0A0E0S082 pp 229533.I1RNM7
43802 229533.A0A0E0S082 pp 229533.I1RY21
43803 229533.A0A0E0S082 pp 229533.I1RKB7
43804 229533.A0A0E0S082 pp 229533.I1RMQ6
43805 229533.A0A0E0S082 pp 229533.A0A0E0SAR4
43806 229533.A0A0E0S082 pp 229533.I1S0P0
43807 229533.A0A0E0S082 pp 229533.I1RE66
43808 229533.A0A0E0S082 pp 229533.V6RJP8
43809 229533.A0A0E0S082 pp 229533.I1RXD9
43810 229533.A0A0E0S082 pp 229533.I1RP02
43811 229533.A0A0E0S082 pp 229533.I1RA52
43812 229533.A0A0E0S082 pp 229533.I1RS29
43813 229533.A0A0E0S082 pp 229533.I1RWA3
43814 229533.A0A0E0S082 pp 229533.I1S0K2
43815 229533.A0A0E0S082 pp 229533.I1RY92
43816 229533.A0A0E0S082 pp 229533.A0A1C3YLB0
43817 229533.A0A0E0S082 pp 229533.I1RAM9
43818 229533.A0A0E0S082 pp 229533.A0A1C3YJU7
43819 229533.A0A0E0S082 pp 229533.A0A1C3YLB6
43820 229533.A0A0E0S082 pp 229533.I1RE65
43821 229533.A0A0E0S082 pp 229533.I1S1F3
43822 229533.A0A0E0S082 pp 229533.A0A1C3YJQ2
43823 229533.A0A0E0S082 pp 229533.I1S0Q7
43824 229533.A0A0E0S082 pp 229533.I1S464
43825 229533.A0A0E0S082 pp 229533.I1RBT6
43826 229533.A0A0E0S082 pp 229533.I1S5X9
43827 229533.A0A0E0S082 pp 229533.I1S0R8
43828 229533.A0A0E0S082 pp 229533.I1RFJ6
43829 229533.A0A0E0S082 pp 229533.A0A0E0S8Z0
43830 229533.I1RTN0 pp 229533.I1RWT6
43831 229533.I1RTN0 pp 229533.I1RY44
43832 229533.I1RTN0 pp 229533.I1S2E9
43833 229533.I1RTN0 pp 229533.I1RV91
43834 229533.I1RTN0 pp 229533.I1RZ63
43835 229533.A0A1C3YLQ7 pp 229533.I1RX64
43836 229533.A0A1C3YLQ7 pp 229533.I1S0D5
43837 229533.A0A1C3YLQ7 pp 229533.I1RD70
43838 229533.A0A1C3YLQ7 pp 229533.I1RZT1
43839 229533.A0A1C3YLQ7 pp 229533.I1RY58
43840 229533.A0A1C3YLQ7 pp 229533.I1REK6
43841 229533.A0A1C3YLQ7 pp 229533.I1RSC7
43842 229533.A0A1C3YLQ7 pp 229533.I1RP99
43843 229533.A0A1C3YLQ7 pp 229533.I1RSL6
43844 229533.A0A1C3YLQ7 pp 229533.I1RFN0
43845 229533.A0A1C3YLQ7 pp 229533.I1RDV4
43846 229533.A0A1C3YLQ7 pp 229533.I1RC54
43847 229533.A0A1C3YLQ7 pp 229533.A0A1C3YN69
43848 229533.A0A1C3YLQ7 pp 229533.I1RZ86
43849 229533.A0A1C3YLQ7 pp 229533.I1RUG1
43850 229533.A0A1C3YLQ7 pp 229533.A0A1C3YLS3
43851 229533.A0A1C3YLQ7 pp 229533.I1RZ29
43852 229533.A0A1C3YLQ7 pp 229533.I1RIP9
43853 229533.A0A1C3YLQ7 pp 229533.I1RVV5
43854 229533.A0A1C3YLQ7 pp 229533.I1RSJ4
43855 229533.A0A1C3YLQ7 pp 229533.I1RW03
43856 229533.A0A1C3YLQ7 pp 229533.I1RV78
43857 229533.A0A1C3YLQ7 pp 229533.I1RE37
43858 229533.A0A1C3YLQ7 pp 229533.I1RFJ2
43859 229533.A0A1C3YLQ7 pp 229533.Q4INS6
43860 229533.A0A1C3YLQ7 pp 229533.I1RWX7
43861 229533.A0A1C3YLQ7 pp 229533.I1S2F2
43862 229533.A0A1C3YLQ7 pp 229533.I1RAH9
43863 229533.A0A1C3YLQ7 pp 229533.I1RLJ8
43864 229533.A0A1C3YLQ7 pp 229533.I1RPI0
43865 229533.A0A1C3YLQ7 pp 229533.Q4I0J6
43866 229533.A0A1C3YLQ7 pp 229533.I1RBE4
43867 229533.A0A1C3YLQ7 pp 229533.I1RWB3
43868 229533.A0A1C3YLQ7 pp 229533.I1RLE7
43869 229533.A0A1C3YLQ7 pp 229533.I1RRT1
43870 229533.A0A1C3YLQ7 pp 229533.I1RUR0
43871 229533.A0A1C3YLQ7 pp 229533.I1RMB7
43872 229533.A0A1C3YLQ7 pp 229533.I1RW06
43873 229533.A0A1C3YLQ7 pp 229533.I1RYV1

43874 229533.A0A1C3YLQ7 pp 229533.A0A1C3YLR6
43875 229533.A0A1C3YLQ7 pp 229533.I1RWM6
43876 229533.A0A1C3YLQ7 pp 229533.I1RAL9
43877 229533.A0A1C3YLQ7 pp 229533.I1RG34
43878 229533.I1RRD0 pp 229533.I1RXN5
43879 229533.I1RRD0 pp 229533.I1RYU3
43880 229533.I1RRD0 pp 229533.V6RK56
43881 229533.I1RRD0 pp 229533.I1RRK5
43882 229533.I1RRD0 pp 229533.I1RWF6
43883 229533.A0A1C3YJG6 pp 229533.I1RY98
43884 229533.A0A1C3YJG6 pp 229533.I1RWA9
43885 229533.A0A1C3YJG6 pp 229533.I1RD84
43886 229533.A0A1C3YJG6 pp 229533.I1RWT7
43887 229533.A0A1C3YJG6 pp 229533.I1RAF0
43888 229533.A0A1C3YJG6 pp 229533.I1RWQ0
43889 229533.A0A1C3YJG6 pp 229533.I1RSW5
43890 229533.I1RRY3 pp 229533.I1RT36
43891 229533.I1RRY3 pp 229533.I1S0M2
43892 229533.I1RRY3 pp 229533.I1S0D1
43893 229533.I1RRY3 pp 229533.V6RGI1
43894 229533.I1RRY3 pp 229533.I1RY44
43895 229533.I1RRY3 pp 229533.I1S1W4
43896 229533.I1RRY3 pp 229533.I1S1D3
43897 229533.I1RRY3 pp 229533.I1RZE4
43898 229533.I1RRY3 pp 229533.V6RVG3
43899 229533.I1RRY3 pp 229533.I1RS41
43900 229533.I1RRY3 pp 229533.I1S1V5
43901 229533.I1RRY3 pp 229533.I1RZ63
43902 229533.I1RRY3 pp 229533.I1S2E9
43903 229533.I1RRY3 pp 229533.I1S549
43904 229533.I1RRY3 pp 229533.V6RKY7
43905 229533.I1RRY3 pp 229533.I1S8X0
43906 229533.I1RRY3 pp 229533.I1RV91
43907 229533.I1RRY3 pp 229533.I1RZQ6
43908 229533.I1RRY3 pp 229533.I1RWT6
43909 229533.I1RRY3 pp 229533.I1RUN8
43910 229533.I1RRY3 pp 229533.V6QV09
43911 229533.I1RRY3 pp 229533.I1RYY5
43912 229533.I1RRY3 pp 229533.I1RZH4
43913 229533.I1RRY3 pp 229533.I1RY49
43914 229533.I1RRY3 pp 229533.I1RXX1
43915 229533.I1RRY3 pp 229533.I1RUQ5
43916 229533.I1RRY3 pp 229533.I1RX70
43917 229533.I1RRY3 pp 229533.I1S0Z7
43918 229533.I1RRY3 pp 229533.I1RV66
43919 229533.I1RRY3 pp 229533.I1S439
43920 229533.I1RRY3 pp 229533.I1RWJ6
43921 229533.I1RRY3 pp 229533.I1S224
43922 229533.I1RRY3 pp 229533.I1RYH9
43923 229533.I1RRY3 pp 229533.I1RYZ0
43924 229533.I1RRY3 pp 229533.I1RYZ9
43925 229533.I1RBL4 pp 229533.I1S270
43926 229533.I1RBL4 pp 229533.I1RN31
43927 229533.I1RBL4 pp 229533.I1RFU3
43928 229533.I1RBL4 pp 229533.I1RNC0
43929 229533.I1RBL4 pp 229533.I1RT41
43930 229533.I1RBL4 pp 229533.I1RFX8
43931 229533.I1RBL4 pp 229533.I1RDG8
43932 229533.I1RBL4 pp 229533.I1RD18
43933 229533.I1RBL4 pp 229533.I1S0Z3
43934 229533.I1RBL4 pp 229533.I1RNT2
43935 229533.I1RBL4 pp 229533.I1RCL7
43936 229533.I1RBL4 pp 229533.I1RCU7
43937 229533.I1RBL4 pp 229533.Q4I0J6
43938 229533.I1RBL4 pp 229533.I1RTC2
43939 229533.I1RBL4 pp 229533.I1RQA3
43940 229533.I1RBL4 pp 229533.I1REN3
43941 229533.I1RBL4 pp 229533.I1RFJ2
43942 229533.I1RBL4 pp 229533.I1RSI4
43943 229533.I1RBL4 pp 229533.I1S0P4
43944 229533.I1RBL4 pp 229533.I1RQ80
43945 229533.I1RBL4 pp 229533.I1RCM6
43946 229533.I1RBL4 pp 229533.Q4I7X1

43947 229533.I1RBL4 pp 229533.I1RLX6
43948 229533.I1RBL4 pp 229533.I1RWB3
43949 229533.I1RBL4 pp 229533.I1RCT9
43950 229533.I1RBL4 pp 229533.Q4IBX0
43951 229533.I1RBL4 pp 229533.I1S2A8
43952 229533.I1RBL4 pp 229533.I1RT65
43953 229533.I1RBL4 pp 229533.I1S1C8
43954 229533.I1RBL4 pp 229533.I1RPU4
43955 229533.I1RBL4 pp 229533.I1RBL7
43956 229533.I1RBL4 pp 229533.I1RZV9
43957 229533.I1RBL4 pp 229533.I1RWG6
43958 229533.I1RBL4 pp 229533.I1RKI1
43959 229533.I1RBL4 pp 229533.I1RFS3
43960 229533.I1RBL4 pp 229533.I1RX38
43961 229533.I1RBL4 pp 229533.Q4IPI4
43962 229533.I1RBL4 pp 229533.I1RKR9
43963 229533.I1RBL4 pp 229533.V6RPN0
43964 229533.I1RBL4 pp 229533.I1S382
43965 229533.I1RBL4 pp 229533.I1S951
43966 229533.I1RBL4 pp 229533.I1RPK5
43967 229533.I1RBL4 pp 229533.I1RFR6
43968 229533.I1RBL4 pp 229533.I1RPZ0
43969 229533.I1RBL4 pp 229533.I1RWT6
43970 229533.I1RBL4 pp 229533.I1RLU1
43971 229533.I1RBL4 pp 229533.V6RLW4
43972 229533.I1RBL4 pp 229533.I1S1V5
43973 229533.I1RBL4 pp 229533.I1RRP7
43974 229533.I1RBL4 pp 229533.I1RVY0
43975 229533.I1RBL4 pp 229533.I1RRF0
43976 229533.I1RBL4 pp 229533.I1RYX9
43977 229533.I1RBL4 pp 229533.I1RFZ2
43978 229533.I1RBL4 pp 229533.I1RBV5
43979 229533.I1RBL4 pp 229533.I1RD70
43980 229533.I1RBL4 pp 229533.I1RSL6
43981 229533.I1RBL4 pp 229533.I1RRR9
43982 229533.I1RBL4 pp 229533.I1RMP0
43983 229533.I1RBL4 pp 229533.I1RI34
43984 229533.I1RBL4 pp 229533.Q4IMD1
43985 229533.I1RBL4 pp 229533.I1RST4
43986 229533.I1RBL4 pp 229533.I1RW16
43987 229533.I1RBL4 pp 229533.I1RPW0
43988 229533.I1RBL4 pp 229533.V6REZ4
43989 229533.I1RBL4 pp 229533.I1RXU0
43990 229533.I1RBL4 pp 229533.I1S1D3
43991 229533.I1RBL4 pp 229533.I1RBM3
43992 229533.I1RBL4 pp 229533.I1RLX4
43993 229533.I1RB64 pp 229533.I1S464
43994 229533.I1RB64 pp 229533.I1S1F3
43995 229533.I1RB64 pp 229533.I1RY92
43996 229533.I1RB64 pp 229533.I1RWA3
43997 229533.I1RB64 pp 229533.I1RE65
43998 229533.I1RB64 pp 229533.I1RXD9
43999 229533.I1RB64 pp 229533.I1RBT6
44000 229533.I1RB64 pp 229533.I1S0Q7
44001 229533.I1RB64 pp 229533.I1RYT8
44002 229533.I1RB64 pp 229533.I1S0R8
44003 229533.I1RB64 pp 229533.I1RS05
44004 229533.I1RB64 pp 229533.I1S5X9
44005 229533.I1RB64 pp 229533.I1RFJ6
44006 229533.I1RB64 pp 229533.I1RNM7
44007 229533.I1RB64 pp 229533.I1S0P0
44008 229533.I1RB64 pp 229533.I1RE66
44009 229533.I1RB64 pp 229533.I1RMQ6
44010 229533.I1RB64 pp 229533.I1RMK7
44011 229533.I1RB64 pp 229533.I1RY21
44012 229533.I1RB64 pp 229533.I1RKB7
44013 229533.I1RB64 pp 229533.V6RJP8
44014 229533.I1RB64 pp 229533.I1S0K2
44015 229533.I1RB64 pp 229533.I1RP02
44016 229533.I1RB64 pp 229533.I1RS29
44017 229533.I1RT36 pp 229533.V6RGI1
44018 229533.I1RT36 pp 229533.I1S0M2
44019 229533.I1RT36 pp 229533.I1S282

229533.I1RT36 pp 229533.V6RVG3
229533.I1RT36 pp 229533.I1S0D1
229533.I1RT36 pp 229533.I1S9D4
229533.I1RT36 pp 229533.I1S549
229533.I1RT36 pp 229533.V6RKY7
229533.I1RT36 pp 229533.I1S0E4
229533.I1RT36 pp 229533.I1S1W4
229533.I1RT36 pp 229533.I1RX95
229533.I1RT36 pp 229533.I1RV91
229533.I1RT36 pp 229533.I1RW82
229533.I1RT36 pp 229533.I1S2E9
229533.I1RT36 pp 229533.I1S439
229533.I1RT36 pp 229533.I1SAT1
229533.I1RT36 pp 229533.I1RWT6
229533.I1RT36 pp 229533.I1RZE4
229533.I1RT36 pp 229533.I1RY44
229533.I1RT36 pp 229533.I1RYZ9
229533.I1RT36 pp 229533.I1S0N5
229533.I1RT36 pp 229533.I1S0K9
229533.I1RT36 pp 229533.I1RZB5
229533.I1RT36 pp 229533.V6QV09
229533.I1RT36 pp 229533.I1S241
229533.I1RT36 pp 229533.I1RUK0
229533.I1RT36 pp 229533.I1RTG3
229533.I1RT36 pp 229533.I1S050
229533.I1RT36 pp 229533.I1RUN8
229533.I1RT36 pp 229533.I1RZH4
229533.I1RT36 pp 229533.I1RVU9
229533.I1RT36 pp 229533.V6QUE6
229533.I1RT36 pp 229533.I1S0Z3
229533.I1RT36 pp 229533.I1RUQ5
229533.I1RT36 pp 229533.I1RT41
229533.I1RT36 pp 229533.I1RYH9
229533.A0A1C3YL40 pp 229533.I1RKQ0
229533.A0A1C3YL40 pp 229533.I1RXI6
229533.A0A1C3YL40 pp 229533.I1RC86
229533.A0A1C3YL40 pp 229533.I1RQM0
229533.A0A1C3YL40 pp 229533.I1RN27
229533.A0A1C3YL40 pp 229533.I1RSK0
229533.A0A1C3YL40 pp 229533.I1S193
229533.A0A1C3YL40 pp 229533.I1RC73
229533.A0A1C3YL40 pp 229533.A0A1C3YLC6
229533.A0A1C3YL40 pp 229533.I1RAP1
229533.A0A1C3YL40 pp 229533.I1RU61
229533.A0A1C3YL40 pp 229533.I1RAP2
229533.I1RF15 pp 229533.I1RNG4
229533.I1RSI3 pp 229533.I1S278
229533.I1RSI3 pp 229533.Q4IBU4
229533.I1RSI3 pp 229533.I1S224
229533.I1RSI3 pp 229533.I1RWT6
229533.I1RSI3 pp 229533.I1RY44
229533.I1RSI3 pp 229533.I1RX87
229533.I1RSI3 pp 229533.I1RSS1
229533.I1RSI3 pp 229533.I1RZC3
229533.I1RSI3 pp 229533.I1RV66
229533.I1RSI3 pp 229533.I1S248
229533.I1RSI3 pp 229533.I1S0X7
229533.I1RSI3 pp 229533.I1RX77
229533.I1RSI3 pp 229533.I1RY32
229533.I1RSI3 pp 229533.I1S2E9
229533.I1RSI3 pp 229533.I1RZ25
229533.A0A098DTI0 pp 229533.I1RSI4
229533.A0A098DTI0 pp 229533.Q4IMM0
229533.A0A098DTI0 pp 229533.I1RLX6
229533.A0A098DTI0 pp 229533.I1RYX9
229533.A0A098DTI0 pp 229533.I1RPQ1
229533.A0A098DTI0 pp 229533.I1RB49
229533.A0A098DTI0 pp 229533.V6RLW4
229533.A0A098DTI0 pp 229533.I1RQ13
229533.A0A098DTI0 pp 229533.I1RD01
229533.A0A098DTI0 pp 229533.A0A1C3YI10
229533.A0A098DTI0 pp 229533.I1RW16
229533.A0A098DTI0 pp 229533.I1RYR2

44093 229533.A0A098DTI0 pp 229533.I1RSK0
44094 229533.A0A098DTI0 pp 229533.I1RFZ2
44095 229533.A0A098DTI0 pp 229533.I1RYQ1
44096 229533.A0A098DTI0 pp 229533.Q4IB89
44097 229533.A0A098DTI0 pp 229533.I1RZ94
44098 229533.A0A098DTI0 pp 229533.A0A1C3YKA6
44099 229533.A0A098DTI0 pp 229533.A0A1C3YLN8
44100 229533.A0A098DTI0 pp 229533.I1RRT1
44101 229533.A0A098DTI0 pp 229533.I1RSC7
44102 229533.A0A098DTI0 pp 229533.I1RN31
44103 229533.A0A098DTI0 pp 229533.I1RFN0
44104 229533.A0A098DTI0 pp 229533.I1R9W7
44105 229533.A0A098DTI0 pp 229533.I1RFX8
44106 229533.A0A098DTI0 pp 229533.I1RJV8
44107 229533.A0A098DTI0 pp 229533.I1RC54
44108 229533.A0A098DTI0 pp 229533.I1RAH4
44109 229533.A0A098DTI0 pp 229533.I1RBC8
44110 229533.A0A098DTI0 pp 229533.I1RAN3
44111 229533.A0A098DTI0 pp 229533.I1RZ86
44112 229533.A0A098DTI0 pp 229533.I1RRS7
44113 229533.A0A098DTI0 pp 229533.I1RUR0
44114 229533.A0A098DTI0 pp 229533.I1RCH2
44115 229533.A0A098DTI0 pp 229533.I1RE46
44116 229533.A0A098DTI0 pp 229533.I1RCA5
44117 229533.A0A098DTI0 pp 229533.I1RCL7
44118 229533.A0A098DTI0 pp 229533.I1S461
44119 229533.A0A098DTI0 pp 229533.I1RBN9
44120 229533.A0A098DTI0 pp 229533.A0A1C3YHN3
44121 229533.A0A098DTI0 pp 229533.I1RD70
44122 229533.A0A098DTI0 pp 229533.I1RMJ8
44123 229533.A0A098DTI0 pp 229533.I1RA12
44124 229533.A0A098DTI0 pp 229533.I1RSR9
44125 229533.A0A098DTI0 pp 229533.I1RFJ2
44126 229533.A0A098DTI0 pp 229533.A0A1C3YJ79
44127 229533.A0A098DTI0 pp 229533.I1S604
44128 229533.A0A098DTI0 pp 229533.I1RY73
44129 229533.A0A098DTI0 pp 229533.I1RUG2
44130 229533.I1RE39 pp 229533.I1RY92
44131 229533.I1RE39 pp 229533.I1RR65
44132 229533.I1RE39 pp 229533.I1S458
44133 229533.I1RE39 pp 229533.I1RYQ1
44134 229533.I1RE39 pp 229533.I1S464
44135 229533.I1RE39 pp 229533.I1RXD9
44136 229533.I1RE39 pp 229533.I1RKF5
44137 229533.I1RE39 pp 229533.I1RMK7
44138 229533.I1RE39 pp 229533.I1RSC0
44139 229533.I1RE39 pp 229533.I1S0R8
44140 229533.I1RE39 pp 229533.I1S0I9
44141 229533.I1RE39 pp 229533.I1RS29
44142 229533.I1RE39 pp 229533.I1S0K2
44143 229533.I1RE39 pp 229533.I1RYT8
44144 229533.I1RE39 pp 229533.I1RFJ6
44145 229533.I1RE39 pp 229533.V6QWX8
44146 229533.I1RE39 pp 229533.I1S5X9
44147 229533.I1RE39 pp 229533.I1RQJ3
44148 229533.I1RE39 pp 229533.I1RKF4
44149 229533.I1RE39 pp 229533.V6RJP8
44150 229533.I1RE39 pp 229533.I1S8S9
44151 229533.I1RE39 pp 229533.I1S0R1
44152 229533.I1RE39 pp 229533.I1RWA3
44153 229533.I1RE39 pp 229533.I1S5C6
44154 229533.I1RE39 pp 229533.I1RE65
44155 229533.I1RE39 pp 229533.I1RY21
44156 229533.I1RE39 pp 229533.I1RP02
44157 229533.I1RE39 pp 229533.I1RS05
44158 229533.I1RE39 pp 229533.I1RYQ3
44159 229533.I1RE39 pp 229533.I1S0P0
44160 229533.I1RF03 pp 229533.I1RNG4
44161 229533.I1RP99 pp 229533.I1RPA0
44162 229533.I1RP99 pp 229533.I1S0D5
44163 229533.I1RP99 pp 229533.I1RX69
44164 229533.I1RP99 pp 229533.I1RPI0
44165 229533.I1RP99 pp 229533.Q4IB89

44166	229533.I1RP99	pp	229533.I1S216
44167	229533.I1RP99	pp	229533.I1RTN6
44168	229533.I1RP99	pp	229533.I1RUG4
44169	229533.I1RP99	pp	229533.I1RUR0
44170	229533.I1RP99	pp	229533.I1S8G4
44171	229533.I1RP99	pp	229533.I1RQS9
44172	229533.I1RP99	pp	229533.I1S209
44173	229533.I1RP99	pp	229533.Q4INS6
44174	229533.I1RP99	pp	229533.I1RQH8
44175	229533.I1RP99	pp	229533.I1S998
44176	229533.I1RP99	pp	229533.I1S987
44177	229533.I1RP99	pp	229533.I1RSC7
44178	229533.I1RP99	pp	229533.I1RSJ1
44179	229533.I1RP99	pp	229533.I1RZ98
44180	229533.I1RP99	pp	229533.I1RZ86
44181	229533.I1RP99	pp	229533.I1RYQ1
44182	229533.I1RP99	pp	229533.I1RRT1
44183	229533.I1RP99	pp	229533.I1RSD0
44184	229533.I1RP99	pp	229533.I1RX22
44185	229533.I1RP99	pp	229533.I1RWI8
44186	229533.I1RP99	pp	229533.I1RX68
44187	229533.I1RP99	pp	229533.I1RWM6
44188	229533.I1RP99	pp	229533.Q4HYZ2
44189	229533.I1RP99	pp	229533.I1RY29
44190	229533.I1RP99	pp	229533.I1RVV5
44191	229533.I1RP99	pp	229533.I1RW24
44192	229533.I1RDB9	pp	229533.I1RJH5
44193	229533.I1RMI1	pp	229533.I1RZ43
44194	229533.I1RMI1	pp	229533.V6RS91
44195	229533.I1RMI1	pp	229533.I1S0Q7
44196	229533.I1RMI1	pp	229533.I1S212
44197	229533.I1RMI1	pp	229533.I1S0R8
44198	229533.I1RMI1	pp	229533.I1RZY1
44199	229533.I1RMI1	pp	229533.I1RX39
44200	229533.I1RMI1	pp	229533.Q4I1N3
44201	229533.I1RMI1	pp	229533.I1RW11
44202	229533.I1RC33	pp	229533.I1RM06
44203	229533.I1RC33	pp	229533.I1RNA0
44204	229533.I1RC33	pp	229533.I1RE22
44205	229533.I1RC33	pp	229533.I1RZS6
44206	229533.I1RC33	pp	229533.I1RRQ6
44207	229533.I1RC33	pp	229533.V6QYF0
44208	229533.I1RC33	pp	229533.I1S6Y8
44209	229533.I1RC33	pp	229533.I1RWJ4
44210	229533.I1RC33	pp	229533.I1RG51
44211	229533.I1RC33	pp	229533.Q4I662
44212	229533.I1RC33	pp	229533.I1RE76
44213	229533.I1RC33	pp	229533.I1RPM4
44214	229533.I1RC33	pp	229533.I1RW25
44215	229533.I1RC33	pp	229533.I1S7W2
44216	229533.I1RC33	pp	229533.I1RW61
44217	229533.I1RC33	pp	229533.Q4IFI0
44218	229533.I1RC33	pp	229533.I1RCS1
44219	229533.I1RC33	pp	229533.I1S2H4
44220	229533.I1RC33	pp	229533.I1RN51
44221	229533.I1RC33	pp	229533.I1RPU3
44222	229533.I1RC33	pp	229533.I1RVQ0
44223	229533.I1RC33	pp	229533.I1S2B8
44224	229533.I1RC33	pp	229533.I1RRM5
44225	229533.I1RC33	pp	229533.I1RRT8
44226	229533.I1RC33	pp	229533.I1RWD4
44227	229533.I1RC33	pp	229533.I1RDA0
44228	229533.I1RC33	pp	229533.I1RN67
44229	229533.I1RC33	pp	229533.I1S5R1
44230	229533.I1RC33	pp	229533.I1RRP3
44231	229533.I1RC33	pp	229533.I1RM67
44232	229533.I1RC33	pp	229533.Q4HVW2
44233	229533.I1RC33	pp	229533.I1RMK3
44234	229533.I1RC33	pp	229533.I1RM82
44235	229533.I1RC33	pp	229533.I1RWJ8
44236	229533.I1RC33	pp	229533.I1RW26
44237	229533.I1RC33	pp	229533.I1S0F6
44238	229533.I1RC33	pp	229533.I1RUQ2

44239	229533.I1RC33	pp	229533.I1RPU0
44240	229533.I1RC33	pp	229533.I1RXD7
44241	229533.I1RC33	pp	229533.I1RPP3
44242	229533.I1RC33	pp	229533.V6R3X5
44243	229533.I1RC33	pp	229533.I1S0R7
44244	229533.I1RC33	pp	229533.I1RZ39
44245	229533.I1RC33	pp	229533.V6QW22
44246	229533.I1RC33	pp	229533.I1RT04
44247	229533.I1RC33	pp	229533.I1RRJ8
44248	229533.I1RC33	pp	229533.Q4IJH1
44249	229533.I1RC33	pp	229533.I1RTF4
44250	229533.I1RC33	pp	229533.I1RSQ7
44251	229533.I1RC33	pp	229533.Q4I830
44252	229533.I1RC33	pp	229533.I1RTD5
44253	229533.I1RC33	pp	229533.V6RUY9
44254	229533.I1RC33	pp	229533.I1RNV6
44255	229533.I1RC33	pp	229533.I1RV91
44256	229533.I1RC33	pp	229533.I1RC66
44257	229533.I1RC33	pp	229533.I1RJU3
44258	229533.I1RC33	pp	229533.I1RSX1
44259	229533.I1RC33	pp	229533.I1RT40
44260	229533.I1RC33	pp	229533.V6RTJ8
44261	229533.I1RC33	pp	229533.I1RFW5
44262	229533.I1RC33	pp	229533.I1RWI5
44263	229533.I1RC33	pp	229533.I1RFH8
44264	229533.I1RC33	pp	229533.I1RWK3
44265	229533.I1RC33	pp	229533.I1S2M6
44266	229533.I1RC33	pp	229533.I1RZZ9
44267	229533.I1RC33	pp	229533.Q4IF76
44268	229533.I1RC33	pp	229533.I1RN63
44269	229533.I1RC33	pp	229533.I1RJ74
44270	229533.I1RC33	pp	229533.Q4IQW0
44271	229533.I1RC33	pp	229533.I1S0L4
44272	229533.I1RC33	pp	229533.I1S1B6
44273	229533.I1RC33	pp	229533.I1RJV4
44274	229533.I1RC33	pp	229533.V6R283
44275	229533.I1RC33	pp	229533.Q4IEV5
44276	229533.I1RC33	pp	229533.I1RR24
44277	229533.I1RC33	pp	229533.I1S9U2
44278	229533.I1RC33	pp	229533.I1RQ65
44279	229533.I1RC33	pp	229533.I1RFN1
44280	229533.I1RC33	pp	229533.I1S0T2
44281	229533.I1RC33	pp	229533.I1RGA4
44282	229533.I1RC33	pp	229533.I1RC82
44283	229533.I1RC33	pp	229533.I1RQS8
44284	229533.I1RC33	pp	229533.I1S0L3
44285	229533.I1RC33	pp	229533.I1RD64
44286	229533.I1RC33	pp	229533.I1RN99
44287	229533.I1RC33	pp	229533.I1RD52
44288	229533.I1RC33	pp	229533.I1RRT2
44289	229533.I1RC33	pp	229533.I1RX46
44290	229533.I1RC33	pp	229533.I1RS47
44291	229533.I1RC33	pp	229533.I1RT33
44292	229533.I1RC33	pp	229533.I1RT03
44293	229533.I1RC33	pp	229533.I1RDG0
44294	229533.I1RC33	pp	229533.Q4IPI1
44295	229533.I1RC33	pp	229533.I1RJL4
44296	229533.I1RC33	pp	229533.I1RY63
44297	229533.I1RC33	pp	229533.I1RVX0
44298	229533.I1RC33	pp	229533.Q4HZ42
44299	229533.I1RC33	pp	229533.I1RSS4
44300	229533.I1RC33	pp	229533.Q4IAS1
44301	229533.I1RC33	pp	229533.I1RFG0
44302	229533.I1RC33	pp	229533.I1RLV9
44303	229533.I1RC33	pp	229533.I1RRQ3
44304	229533.I1RC33	pp	229533.I1RZZ1
44305	229533.I1RC33	pp	229533.I1RSC6
44306	229533.I1RC33	pp	229533.I1S6F0
44307	229533.I1RC33	pp	229533.I1RJW6
44308	229533.I1RC33	pp	229533.I1RNH1
44309	229533.I1RC33	pp	229533.I1RRZ0
44310	229533.I1RC33	pp	229533.I1RCH5
44311	229533.I1RC33	pp	229533.I1S503

44312 229533.I1RC33 pp 229533.I1S2E8
44313 229533.I1RC33 pp 229533.I1RFU8
44314 229533.I1RC33 pp 229533.I1RQE7
44315 229533.I1RC33 pp 229533.I1RS17
44316 229533.I1RC33 pp 229533.I1RJZ6
44317 229533.I1RC33 pp 229533.I1RTH6
44318 229533.I1RC33 pp 229533.I1RSD8
44319 229533.I1RC33 pp 229533.I1RZB5
44320 229533.I1RC33 pp 229533.I1S0J1
44321 229533.I1RC33 pp 229533.I1SA32
44322 229533.I1RC33 pp 229533.I1S8R3
44323 229533.I1RC33 pp 229533.I1RCG4
44324 229533.I1RC33 pp 229533.I1RN18
44325 229533.I1RC33 pp 229533.I1RFL4
44326 229533.I1RC33 pp 229533.I1RC72
44327 229533.I1RC33 pp 229533.Q4IJK2
44328 229533.I1RC33 pp 229533.I1RZW8
44329 229533.I1RC33 pp 229533.I1RRI2
44330 229533.I1RC33 pp 229533.I1RW46
44331 229533.I1RC33 pp 229533.V6R227
44332 229533.I1RC33 pp 229533.I1RQ72
44333 229533.I1RC33 pp 229533.I1RE01
44334 229533.I1RC33 pp 229533.Q4HZ68
44335 229533.I1RC33 pp 229533.I1RF08
44336 229533.I1RC33 pp 229533.I1RMD5
44337 229533.I1RC33 pp 229533.I1S1C7
44338 229533.I1RC33 pp 229533.I1RRR7
44339 229533.I1RC33 pp 229533.I1RSR8
44340 229533.I1RC33 pp 229533.Q4I624
44341 229533.I1RC33 pp 229533.I1S296
44342 229533.I1RC33 pp 229533.I1RY03
44343 229533.I1RC33 pp 229533.I1S504
44344 229533.I1RC33 pp 229533.I1RTD9
44345 229533.I1RC33 pp 229533.Q4IEK8
44346 229533.I1RC33 pp 229533.I1REK8
44347 229533.I1RC33 pp 229533.I1RZE5
44348 229533.I1RC33 pp 229533.I1RWN6
44349 229533.I1RC33 pp 229533.I1RD17
44350 229533.I1RC33 pp 229533.I1RFJ9
44351 229533.I1RC33 pp 229533.I1RNQ5
44352 229533.I1RC33 pp 229533.I1RSR3
44353 229533.I1RC33 pp 229533.I1RRY9
44354 229533.I1RC33 pp 229533.I1RNK2
44355 229533.I1RC33 pp 229533.I1RVZ0
44356 229533.I1RC33 pp 229533.I1RCL3
44357 229533.I1RC33 pp 229533.I1RWN5
44358 229533.I1RC33 pp 229533.I1RJV3
44359 229533.I1RC33 pp 229533.I1S1J6
44360 229533.I1RC33 pp 229533.I1S1J7
44361 229533.I1RC33 pp 229533.V6RIV9
44362 229533.I1RC33 pp 229533.I1RKC6
44363 229533.I1RC33 pp 229533.I1S4T3
44364 229533.I1RC33 pp 229533.I1RQA2
44365 229533.I1RC33 pp 229533.Q4I5Z5
44366 229533.I1RC33 pp 229533.I1S419
44367 229533.I1RC33 pp 229533.I1RCN5
44368 229533.I1RC33 pp 229533.I1S558
44369 229533.I1RC33 pp 229533.I1RPY1
44370 229533.I1RC33 pp 229533.I1RZK5
44371 229533.I1RC33 pp 229533.I1RTE9
44372 229533.I1RC33 pp 229533.I1RR51
44373 229533.I1RC33 pp 229533.I1RPJ3
44374 229533.I1RC33 pp 229533.I1RSQ3
44375 229533.I1RC33 pp 229533.Q4IAA0
44376 229533.I1RC33 pp 229533.Q4IN63
44377 229533.I1RC33 pp 229533.I1S2C0
44378 229533.I1RC33 pp 229533.I1RS18
44379 229533.I1RC33 pp 229533.Q4I8P5
44380 229533.I1RC33 pp 229533.V6RCG5
44381 229533.I1RC33 pp 229533.V6R042
44382 229533.I1RC33 pp 229533.V6RGN5
44383 229533.I1RC33 pp 229533.V6RMW6
44384 229533.I1RC33 pp 229533.I1RW99

229533.I1RC33 pp 229533.Q4IJ56
229533.I1RC33 pp 229533.I1S5B0
229533.I1RC33 pp 229533.I1RSI6
229533.I1RC33 pp 229533.I1RD22
229533.I1RC33 pp 229533.V6RCZ8
229533.I1RC33 pp 229533.I1RNY2
229533.I1RC33 pp 229533.Q4IBS2
229533.I1RC33 pp 229533.I1SA33
229533.I1RC33 pp 229533.Q4I327
229533.I1RC33 pp 229533.I1RMN6
229533.I1RC33 pp 229533.I1RNH6
229533.I1RC33 pp 229533.I1RMC0
229533.I1RC33 pp 229533.I1RI82
229533.I1RC33 pp 229533.I1RUG3
229533.I1RC33 pp 229533.I1RNC1
229533.I1RC33 pp 229533.I1RCR8
229533.I1RC33 pp 229533.I1RQ31
229533.I1RC33 pp 229533.I1RZM8
229533.A0A0E0S502 pp 229533.I1RYT8
229533.A0A0E0S502 pp 229533.I1RNM7
229533.A0A0E0S502 pp 229533.I1RMK7
229533.A0A0E0S502 pp 229533.I1RY21
229533.A0A0E0S502 pp 229533.I1RMQ6
229533.A0A0E0S502 pp 229533.I1RKB7
229533.A0A0E0S502 pp 229533.A0A0E0SAR4
229533.A0A0E0S502 pp 229533.A0A1C3YJU7
229533.A0A0E0S502 pp 229533.A0A1C3YLB6
229533.A0A0E0S502 pp 229533.I1RE65
229533.A0A0E0S502 pp 229533.V6RJP8
229533.A0A0E0S502 pp 229533.I1S0Q7
229533.A0A0E0S502 pp 229533.A0A1C3YJQ2
229533.A0A0E0S502 pp 229533.I1RP02
229533.A0A0E0S502 pp 229533.I1RBT6
229533.A0A0E0S502 pp 229533.I1S0K2
229533.A0A0E0S502 pp 229533.I1RS29
229533.A0A0E0S502 pp 229533.I1S0R8
229533.A0A0E0S502 pp 229533.I1RAM9
229533.A0A0E0S502 pp 229533.A0A1C3YLB0
229533.A0A0E0S502 pp 229533.I1S5X9
229533.A0A0E0S502 pp 229533.I1RE66
229533.A0A0E0S502 pp 229533.I1RFJ6
229533.A0A0E0S502 pp 229533.I1S0P0
229533.A0A0E0S502 pp 229533.A0A0E0S8Z0
229533.A0A0E0S502 pp 229533.I1RS05
229533.A0A0E0S502 pp 229533.I1S1F3
229533.A0A0E0S502 pp 229533.I1RXD9
229533.A0A0E0S502 pp 229533.I1S464
229533.A0A0E0S502 pp 229533.I1RA52
229533.A0A0E0S502 pp 229533.I1RWA3
229533.A0A0E0S502 pp 229533.I1RY92
229533.I1RQ67 pp 229533.I1RUG2
229533.I1RQ67 pp 229533.I1RSK0
229533.I1RQ67 pp 229533.I1RY73
229533.I1RQ67 pp 229533.I1S461
229533.I1RQ67 pp 229533.I1RSR9
229533.I1RQ67 pp 229533.I1RZ94
229533.I1RQ67 pp 229533.I1S604
229533.I1RQ67 pp 229533.I1RSC7
229533.I1RQ67 pp 229533.I1RRT1
229533.I1RQ67 pp 229533.Q4IMM0
229533.I1RQ67 pp 229533.I1RRS7
229533.I1RQ67 pp 229533.I1RSI4
229533.I1RQ67 pp 229533.Q4IB89
229533.I1RQ67 pp 229533.I1RZ86
229533.I1RQ67 pp 229533.I1RW16
229533.I1RQ67 pp 229533.I1RYQ1
229533.I1RQ67 pp 229533.V6RLW4
229533.I1RQ67 pp 229533.I1RYR2
229533.I1RQ67 pp 229533.I1RYX9
229533.I1RWH4 pp 229533.Q4I7G0
229533.I1RWH4 pp 229533.I1RZD3
229533.I1RWH4 pp 229533.I1RYW3
229533.I1RWH4 pp 229533.I1RWN6

44458 229533.I1RWH4 pp 229533.I1S247
44459 229533.I1RWH4 pp 229533.I1RZK1
44460 229533.I1RWH4 pp 229533.I1RYL5
44461 229533.I1RWH4 pp 229533.I1RZM1
44462 229533.I1RWH4 pp 229533.I1RY44
44463 229533.I1RWH4 pp 229533.I1S3S5
44464 229533.I1RWH4 pp 229533.I1S821
44465 229533.I1RWH4 pp 229533.I1S0F9
44466 229533.I1RWH4 pp 229533.V6QUV7
44467 229533.I1RWH4 pp 229533.I1RZS4
44468 229533.I1RWH4 pp 229533.Q4I624
44469 229533.I1RWH4 pp 229533.V6QXG1
44470 229533.I1RWH4 pp 229533.I1RZS8
44471 229533.I1RWH4 pp 229533.I1RZG7
44472 229533.I1RWH4 pp 229533.Q4HXF6
44473 229533.I1RWH4 pp 229533.I1S2E9
44474 229533.I1RWH4 pp 229533.I1RZQ5
44475 229533.I1RWH4 pp 229533.I1S0Z8
44476 229533.I1RWH4 pp 229533.I1RZ60
44477 229533.I1RWH4 pp 229533.I1S503
44478 229533.I1RWH4 pp 229533.I1RZZ9
44479 229533.I1RWH4 pp 229533.V6R042
44480 229533.I1RWH4 pp 229533.I1RX46
44481 229533.I1RWH4 pp 229533.Q5I2J3
44482 229533.I1RWH4 pp 229533.I1RYS6
44483 229533.I1RWH4 pp 229533.I1S504
44484 229533.I1RWH4 pp 229533.V6RTJ8
44485 229533.I1RRV2 pp 229533.I1RT08
44486 229533.I1RRV2 pp 229533.Q4ICM0
44487 229533.Q4I877 pp 229533.Q4ICM0
44488 229533.A0A1C3YMC9 pp 229533.A0A1C3YN55
44489 229533.A0A1C3YMC9 pp 229533.I1RMQ7
44490 229533.A0A1C3YMC9 pp 229533.I1S276
44491 229533.A0A1C3YMC9 pp 229533.I1RPM7
44492 229533.A0A1C3YMC9 pp 229533.I1RZT1
44493 229533.A0A1C3YMC9 pp 229533.I1RMS2
44494 229533.A0A1C3YMC9 pp 229533.I1RTA3
44495 229533.A0A1C3YMC9 pp 229533.I1RK90
44496 229533.A0A1C3YMC9 pp 229533.I1RYY5
44497 229533.A0A1C3YMC9 pp 229533.I1REN3
44498 229533.A0A1C3YMC9 pp 229533.I1S209
44499 229533.A0A1C3YMC9 pp 229533.I1RC17
44500 229533.A0A1C3YMC9 pp 229533.I1RUG4
44501 229533.A0A1C3YMC9 pp 229533.I1RFQ5
44502 229533.A0A1C3YMC9 pp 229533.I1RRF9
44503 229533.A0A1C3YMC9 pp 229533.I1RBC8
44504 229533.A0A1C3YMC9 pp 229533.I1RB41
44505 229533.A0A1C3YMC9 pp 229533.I1RH70
44506 229533.A0A1C3YMC9 pp 229533.I1RXU0
44507 229533.A0A1C3YMC9 pp 229533.Q4IJM4
44508 229533.A0A1C3YMC9 pp 229533.I1RRN5
44509 229533.A0A1C3YMC9 pp 229533.Q4HYI0
44510 229533.A0A1C3YMC9 pp 229533.I1RQA3
44511 229533.A0A1C3YMC9 pp 229533.I1RDH0
44512 229533.A0A1C3YMC9 pp 229533.I1RSC7
44513 229533.A0A1C3YMC9 pp 229533.I1RKB1
44514 229533.A0A1C3YMC9 pp 229533.I1RE19
44515 229533.A0A1C3YMC9 pp 229533.I1RWT6
44516 229533.A0A1C3YMC9 pp 229533.I1RY44
44517 229533.A0A1C3YMC9 pp 229533.I1RUF8
44518 229533.A0A1C3YMC9 pp 229533.I1RKI2
44519 229533.A0A1C3YMC9 pp 229533.I1RY29
44520 229533.A0A1C3YMC9 pp 229533.I1S2E9
44521 229533.A0A1C3YMC9 pp 229533.I1RWI8
44522 229533.A0A1C3YMC9 pp 229533.I1RV91
44523 229533.A0A1C3YMC9 pp 229533.I1RSZ3
44524 229533.A0A1C3YMC9 pp 229533.I1RPF5
44525 229533.A0A1C3YMC9 pp 229533.I1RMP2
44526 229533.A0A1C3YMC9 pp 229533.I1RTC2
44527 229533.A0A1C3YMC9 pp 229533.I1RFL7
44528 229533.A0A1C3YMC9 pp 229533.I1RVT2
44529 229533.A0A1C3YMC9 pp 229533.I1S004
44530 229533.A0A1C3YMC9 pp 229533.I1RZ94

44531 229533.A0A1C3YMC9 pp 229533.I1SAD2
44532 229533.A0A1C3YMC9 pp 229533.I1RVL8
44533 229533.A0A1C3YK43 pp 229533.I1RMJ8
44534 229533.A0A1C3YK43 pp 229533.I1RQA3
44535 229533.A0A1C3YK43 pp 229533.I1RCS6
44536 229533.A0A1C3YK43 pp 229533.I1RWT6
44537 229533.A0A1C3YK43 pp 229533.I1RMD3
44538 229533.A0A1C3YK43 pp 229533.I1RDJ8
44539 229533.A0A1C3YK43 pp 229533.I1RY58
44540 229533.A0A1C3YK43 pp 229533.I1RL24
44541 229533.A0A1C3YK43 pp 229533.I1RPC2
44542 229533.A0A1C3YK43 pp 229533.I1RA38
44543 229533.A0A1C3YK43 pp 229533.I1RK75
44544 229533.A0A1C3YK43 pp 229533.A0A1C3YM29
44545 229533.A0A1C3YK43 pp 229533.I1S004
44546 229533.A0A1C3YK43 pp 229533.I1R9Y7
44547 229533.A0A1C3YK43 pp 229533.I1RV91
44548 229533.A0A1C3YK43 pp 229533.I1RKK2
44549 229533.A0A1C3YK43 pp 229533.I1RDE0
44550 229533.A0A1C3YK43 pp 229533.V6RGI1
44551 229533.A0A1C3YK43 pp 229533.I1S2E9
44552 229533.A0A1C3YK43 pp 229533.I1S098
44553 229533.A0A1C3YK43 pp 229533.I1RC17
44554 229533.A0A1C3YK43 pp 229533.Q4IB89
44555 229533.A0A1C3YK43 pp 229533.I1RJV8
44556 229533.A0A1C3YK43 pp 229533.I1RRM8
44557 229533.A0A1C3YK43 pp 229533.I1S4P6
44558 229533.A0A1C3YK43 pp 229533.I1RYZ9
44559 229533.A0A1C3YK43 pp 229533.V6QV09
44560 229533.A0A1C3YK43 pp 229533.I1RY44
44561 229533.A0A1C3YK43 pp 229533.I1RFL7
44562 229533.I1RL06 pp 229533.I1RYY2
44563 229533.A0A1C3YL52 pp 229533.I1RX70
44564 229533.A0A1C3YL52 pp 229533.I1RPX5
44565 229533.A0A1C3YL52 pp 229533.I1RUQ6
44566 229533.A0A1C3YL52 pp 229533.I1RV91
44567 229533.A0A1C3YL52 pp 229533.I1S2E9
44568 229533.A0A1C3YL52 pp 229533.I1S2G7
44569 229533.A0A1C3YL52 pp 229533.I1RB74
44570 229533.A0A1C3YL52 pp 229533.I1RFZ3
44571 229533.A0A1C3YL52 pp 229533.I1RW83
44572 229533.A0A1C3YL52 pp 229533.I1RRQ4
44573 229533.A0A1C3YL52 pp 229533.I1RQA3
44574 229533.A0A1C3YL52 pp 229533.A0A1C3YM91
44575 229533.A0A1C3YL52 pp 229533.I1RC17
44576 229533.A0A1C3YL52 pp 229533.I1RWT6
44577 229533.A0A1C3YL52 pp 229533.I1RB41
44578 229533.A0A1C3YL52 pp 229533.I1RY44
44579 229533.A0A1C3YL52 pp 229533.I1RRN5
44580 229533.A0A1C3YL52 pp 229533.I1RKE9
44581 229533.I1RW13 pp 229533.I1RYT8
44582 229533.I1RW13 pp 229533.I1S464
44583 229533.I1RW13 pp 229533.I1RY92
44584 229533.I1RW13 pp 229533.I1RY21
44585 229533.I1RW13 pp 229533.I1S0P0
44586 229533.I1RW13 pp 229533.I1RXD9
44587 229533.I1RW13 pp 229533.I1S0K2
44588 229533.I1RW13 pp 229533.I1S1F3
44589 229533.I1RW13 pp 229533.I1S5X9
44590 229533.I1RW13 pp 229533.V6RJP8
44591 229533.I1RW13 pp 229533.I1S0R8
44592 229533.I1RW13 pp 229533.I1S0Q7
44593 229533.I1RW13 pp 229533.I1RWA3
44594 229533.I1RWM6 pp 229533.Q4IR08
44595 229533.I1RWM6 pp 229533.Q4INS6
44596 229533.I1RWM6 pp 229533.Q4I0J6
44597 229533.I1RWM6 pp 229533.I1S283
44598 229533.I1RWM6 pp 229533.I1S788
44599 229533.I1RWM6 pp 229533.I1S998
44600 229533.I1RWM6 pp 229533.I1S004
44601 229533.I1RWM6 pp 229533.I1RZ86
44602 229533.I1RWM6 pp 229533.I1RYQ1
44603 229533.I1RWM6 pp 229533.I1RZ98

229533.I1RWM6 pp 229533.I1RWX7
229533.I1RWM6 pp 229533.I1S987
229533.I1RWM6 pp 229533.Q4IMD1
229533.I1RWM6 pp 229533.I1RY58
229533.I1RWM6 pp 229533.I1S0D5
229533.I1RWM6 pp 229533.I1RZT1
229533.I1RWM6 pp 229533.I1RYX9
229533.I1RWM6 pp 229533.I1RYV1
229533.I1RWM6 pp 229533.I1S4P6
229533.I1RWM6 pp 229533.I1RZ29
229533.I1RWM6 pp 229533.Q4IB89
229533.I1RWM6 pp 229533.I1RX64
229533.I1RWM6 pp 229533.I1S2F2
229533.I1S266 pp 229533.I1S464
229533.I1S266 pp 229533.V6RJP8
229533.I1S266 pp 229533.I1S5X9
229533.I1S987 pp 229533.Q4INS6
229533.I1S987 pp 229533.Q4IB89
229533.I1S2H3 pp 229533.V6R9Z3
229533.I1S2H3 pp 229533.V6RY56
229533.I1S2H3 pp 229533.V6RRM0
229533.I1S2H3 pp 229533.I1S6P6
229533.I1S2H3 pp 229533.V6RN31
229533.I1S2H3 pp 229533.I1S5L5
229533.I1S2H3 pp 229533.I1S4F4
229533.I1S2H3 pp 229533.I1S4D0
229533.I1RAW9 pp 229533.I1S212
229533.I1RAW9 pp 229533.I1RYE5
229533.I1RAW9 pp 229533.I1S1F3
229533.I1RAW9 pp 229533.I1RG62
229533.I1RAW9 pp 229533.I1RBE6
229533.I1RAW9 pp 229533.I1RMQ6
229533.I1RAW9 pp 229533.I1RYN2
229533.I1RAW9 pp 229533.I1RX39
229533.I1RAW9 pp 229533.I1S0R8
229533.I1RWZ0 pp 229533.I1S570
229533.A0A1C3YJ17 pp 229533.I1RMK7
229533.A0A1C3YJ17 pp 229533.I1RPY0
229533.A0A1C3YJ17 pp 229533.I1RVR2
229533.A0A1C3YJ17 pp 229533.I1RMJ3
229533.A0A1C3YJ17 pp 229533.A0A1C3YLE2
229533.A0A1C3YJ17 pp 229533.I1RN84
229533.I1RYA0 pp 229533.I1S3T7
229533.I1RYA0 pp 229533.I1S8R2
229533.I1RYA0 pp 229533.V6RUB1
229533.I1RYA0 pp 229533.Q4I1N3
229533.I1RYA0 pp 229533.I1S6P5
229533.I1RYA0 pp 229533.I1S3G4
229533.I1RYA0 pp 229533.I1S0B8
229533.I1RYA0 pp 229533.I1S3V2
229533.I1RYA0 pp 229533.Q4IN52
229533.I1RYA0 pp 229533.Q4HVQ9
229533.I1RYA0 pp 229533.I1S299
229533.I1RYA0 pp 229533.I1S1F3
229533.I1RYA0 pp 229533.I1RZA7
229533.I1RYA0 pp 229533.V6RRM0
229533.I1RYA0 pp 229533.I1S968
229533.I1RYA0 pp 229533.I1S147
229533.I1RYA0 pp 229533.I1S1W8
229533.I1RYA0 pp 229533.Q4I5G1
229533.I1RYA0 pp 229533.I1RYP5
229533.I1RYA0 pp 229533.I1S0V7
229533.I1RYA0 pp 229533.I1S0Q7
229533.I1RYA0 pp 229533.I1RYX6
229533.I1RYA0 pp 229533.I1S3B2
229533.I1RYA0 pp 229533.V6RS91
229533.I1RYA0 pp 229533.I1RYT8
229533.I1RYA0 pp 229533.I1S0K2
229533.I1RYA0 pp 229533.V6RJP8
229533.I1RYA0 pp 229533.I1S0P0
229533.I1RYA0 pp 229533.I1S969
229533.I1RYA0 pp 229533.I1S8C1
229533.I1RYA0 pp 229533.I1S0V6

44677 229533.I1RFJ5 pp 229533.I1RPR9
44678 229533.I1RFJ5 pp 229533.I1RZ43
44679 229533.I1RFJ5 pp 229533.I1S0R8
44680 229533.I1RFJ5 pp 229533.I1RRX5
44681 229533.I1RFJ5 pp 229533.I1RG62
44682 229533.I1RFJ5 pp 229533.I1RYH7
44683 229533.I1RFJ5 pp 229533.V6R3J1
44684 229533.I1RFJ5 pp 229533.I1RP88
44685 229533.I1RFJ5 pp 229533.I1RX39
44686 229533.I1RFJ5 pp 229533.I1S0Q7
44687 229533.I1RBB8 pp 229533.V6R949
44688 229533.I1RKF2 pp 229533.I1SAP6
44689 229533.I1RKF2 pp 229533.I1RWF4
44690 229533.I1RKF2 pp 229533.I1RYD3
44691 229533.I1RKF2 pp 229533.I1RS40
44692 229533.I1RKF2 pp 229533.I1S835
44693 229533.I1RKF2 pp 229533.I1RZ15
44694 229533.I1RPQ1 pp 229533.Q4IC11
44695 229533.I1RPQ1 pp 229533.I1RZ94
44696 229533.I1RPQ1 pp 229533.I1S283
44697 229533.I1RPQ1 pp 229533.I1S987
44698 229533.I1RPQ1 pp 229533.I1RQ13
44699 229533.I1RPQ1 pp 229533.I1RWI8
44700 229533.I1RPQ1 pp 229533.I1RSZ3
44701 229533.I1RPQ1 pp 229533.I1RV66
44702 229533.I1RPQ1 pp 229533.I1RRT1
44703 229533.I1RPQ1 pp 229533.I1RQ92
44704 229533.I1RPQ1 pp 229533.I1RYQ1
44705 229533.I1RPQ1 pp 229533.I1RSI4
44706 229533.I1RPQ1 pp 229533.I1RWT6
44707 229533.I1RPQ1 pp 229533.I1RQA3
44708 229533.I1RPQ1 pp 229533.I1S998
44709 229533.I1RPQ1 pp 229533.I1RQH8
44710 229533.I1RPQ1 pp 229533.Q4IQM6
44711 229533.I1RPQ1 pp 229533.I1S604
44712 229533.I1RPQ1 pp 229533.I1RSA7
44713 229533.I1RPQ1 pp 229533.I1RQ67
44714 229533.I1RPQ1 pp 229533.I1S209
44715 229533.I1RPQ1 pp 229533.I1RQ72
44716 229533.I1RPQ1 pp 229533.I1S523
44717 229533.I1RPQ1 pp 229533.I1RUG4
44718 229533.I1RPQ1 pp 229533.V6RLW4
44719 229533.I1RPQ1 pp 229533.Q4IB89
44720 229533.I1RPQ1 pp 229533.I1S0A6
44721 229533.I1RPQ1 pp 229533.I1RZ86
44722 229533.I1RPQ1 pp 229533.I1RZP8
44723 229533.I1RPQ1 pp 229533.I1RXB2
44724 229533.I1RPQ1 pp 229533.I1RY44
44725 229533.I1RPQ1 pp 229533.I1RX64
44726 229533.I1RPQ1 pp 229533.I1RYX9
44727 229533.I1RPQ1 pp 229533.I1RSN7
44728 229533.I1RPQ1 pp 229533.I1RQJ4
44729 229533.I1RPQ1 pp 229533.I1RYR2
44730 229533.I1RPQ1 pp 229533.I1RSP7
44731 229533.I1RPQ1 pp 229533.I1S4P6
44732 229533.I1RPQ1 pp 229533.I1S276
44733 229533.I1RPQ1 pp 229533.I1S2E9
44734 229533.I1RPQ1 pp 229533.I1S016
44735 229533.I1RPQ1 pp 229533.I1S0D5
44736 229533.I1RPQ1 pp 229533.I1RRV1
44737 229533.I1RPQ1 pp 229533.I1RY29
44738 229533.I1RPQ1 pp 229533.I1RXA9
44739 229533.I1RPQ1 pp 229533.I1RSC7
44740 229533.I1RPQ1 pp 229533.I1RRS7
44741 229533.I1RPQ1 pp 229533.I1RSR9
44742 229533.I1RPQ1 pp 229533.I1RQB4
44743 229533.I1RPQ1 pp 229533.V6R382
44744 229533.I1RDL5 pp 229533.I1RZA8
44745 229533.I1RDL5 pp 229533.I1S694
44746 229533.I1RDL5 pp 229533.I1RI11
44747 229533.I1RDL5 pp 229533.I1RY16
44748 229533.I1RDL5 pp 229533.I1RRG1
44749 229533.A0A098E3X8 pp 229533.I1RYD3

229533.A0A098E3X8 pp 229533.A0A1C3YKM8
229533.I1RYQ1 pp 229533.I1RYR1
229533.I1RYQ1 pp 229533.I1RYX9
229533.I1RYQ1 pp 229533.Q4I0J6
229533.I1RYQ1 pp 229533.I1S4P6
229533.I1RYQ1 pp 229533.I1S209
229533.I1RYQ1 pp 229533.I1RZ98
229533.I1RYQ1 pp 229533.I1RZP8
229533.I1RYQ1 pp 229533.I1S6Y6
229533.I1RYQ1 pp 229533.Q4IB89
229533.I1RYQ1 pp 229533.I1S464
229533.I1RYQ1 pp 229533.Q4INS6
229533.I1RYQ1 pp 229533.V6RJP8
229533.I1RYQ1 pp 229533.I1S0D5
229533.I1RYQ1 pp 229533.I1S5C6
229533.I1RYQ1 pp 229533.I1S283
229533.I1RYQ1 pp 229533.V6QW22
229533.I1RYQ1 pp 229533.I1S5X9
229533.I1RYQ1 pp 229533.I1S5Z3
229533.I1RYQ1 pp 229533.I1S0B2
229533.I1RYQ1 pp 229533.I1S0K2
229533.I1RYQ1 pp 229533.I1S8S9
229533.I1RYQ1 pp 229533.Q4IQM6
229533.I1RYQ1 pp 229533.V6RLW4
229533.I1RYQ1 pp 229533.I1S458
229533.I1RYQ1 pp 229533.I1RYR2
229533.I1RYQ1 pp 229533.I1S788
229533.I1RYQ1 pp 229533.I1S987
229533.I1RYQ1 pp 229533.I1S0P0
229533.I1RYQ1 pp 229533.I1RYT8
229533.I1RYQ1 pp 229533.I1S998
229533.I1RYQ1 pp 229533.I1RZ94
229533.I1RYQ1 pp 229533.I1S8G4
229533.I1RYQ1 pp 229533.I1RZ86
229533.I1RZJ3 pp 229533.V6RGI1
229533.I1RZJ3 pp 229533.I1S8S9
229533.I1RZJ3 pp 229533.I1RZR5
229533.I1RZJ3 pp 229533.V6R3J1
229533.A0A098DWK6 pp 229533.I1S1V4
229533.A0A098DWK6 pp 229533.I1RZ84
229533.A0A1C3YHV5 pp 229533.A0A1C3YK75
229533.A0A1C3YHV5 pp 229533.A0A1C3YJQ6
229533.A0A1C3YHV5 pp 229533.I1RWX8
229533.A0A1C3YHV5 pp 229533.I1RB36
229533.A0A1C3YHV5 pp 229533.I1RXE5
229533.A0A1C3YHV5 pp 229533.Q4IQX3
229533.A0A1C3YHV5 pp 229533.I1RSI1
229533.A0A1C3YHV5 pp 229533.I1RB17
229533.A0A1C3YHV5 pp 229533.I1RUQ6
229533.A0A1C3YHV5 pp 229533.I1RYX3
229533.A0A1C3YHV5 pp 229533.A0A1C3YI68
229533.A0A1C3YHV5 pp 229533.I1RMA5
229533.A0A1C3YHV5 pp 229533.I1RSV0
229533.A0A1C3YHV5 pp 229533.I1RRH5
229533.A0A1C3YHV5 pp 229533.I1RGE1
229533.A0A1C3YHV5 pp 229533.A0A1C3YL16
229533.A0A1C3YHV5 pp 229533.A0A1C3YN58
229533.A0A1C3YHV5 pp 229533.I1RSH4
229533.A0A1C3YHV5 pp 229533.I1S1V2
229533.A0A1C3YHV5 pp 229533.I1RDC9
229533.A0A1C3YHV5 pp 229533.A0A1C3YK01
229533.A0A1C3YHV5 pp 229533.A0A1C3YIJ2
229533.A0A1C3YHV5 pp 229533.I1RKR6
229533.A0A1C3YHV5 pp 229533.I1RT19
229533.A0A1C3YHV5 pp 229533.I1RQE9
229533.A0A1C3YHV5 pp 229533.I1RAF0
229533.A0A1C3YHV5 pp 229533.I1RGW0
229533.A0A1C3YHV5 pp 229533.I1S8W6
229533.A0A1C3YHV5 pp 229533.V6QV38
229533.A0A1C3YHV5 pp 229533.A0A1C3YIE7
229533.A0A1C3YHV5 pp 229533.I1RHP9
229533.A0A1C3YHV5 pp 229533.I1R9Q0
229533.A0A1C3YHV5 pp 229533.I1RDY1

44823 229533.A0A1C3YHV5 pp 229533.I1RI51
44824 229533.A0A1C3YHV5 pp 229533.I1S1Y5
44825 229533.A0A1C3YHV5 pp 229533.I1RJL5
44826 229533.A0A1C3YHV5 pp 229533.I1R9W6
44827 229533.A0A1C3YHV5 pp 229533.I1RS43
44828 229533.A0A1C3YHV5 pp 229533.I1RB73
44829 229533.A0A1C3YHV5 pp 229533.I1RVY0
44830 229533.A0A1C3YHV5 pp 229533.I1RY99
44831 229533.A0A1C3YHV5 pp 229533.I1RBZ1
44832 229533.A0A1C3YHV5 pp 229533.A0A1C3YLP1
44833 229533.A0A1C3YHV5 pp 229533.A0A1C3YJ12
44834 229533.A0A1C3YHV5 pp 229533.I1RCN2
44835 229533.A0A1C3YHV5 pp 229533.I1RPY4
44836 229533.A0A1C3YHV5 pp 229533.A0A1C3YN82
44837 229533.A0A1C3YHV5 pp 229533.I1S1X2
44838 229533.A0A1C3YHV5 pp 229533.I1S4T3
44839 229533.A0A1C3YHV5 pp 229533.I1S109
44840 229533.A0A1C3YHV5 pp 229533.I1RA91
44841 229533.A0A1C3YHV5 pp 229533.A0A1C3YJZ2
44842 229533.A0A1C3YHV5 pp 229533.I1RJW8
44843 229533.A0A1C3YHV5 pp 229533.A0A1C3YMF9
44844 229533.A0A1C3YHV5 pp 229533.I1RRM7
44845 229533.A0A1C3YHV5 pp 229533.I1RDA8
44846 229533.I1RI95 pp 229533.I1RYT8
44847 229533.I1RI95 pp 229533.I1RS05
44848 229533.I1RI95 pp 229533.I1RY21
44849 229533.I1RI95 pp 229533.I1RNM7
44850 229533.I1RI95 pp 229533.I1RMK7
44851 229533.I1RI95 pp 229533.V6RJP8
44852 229533.I1RI95 pp 229533.I1RMQ6
44853 229533.I1RI95 pp 229533.I1RKB7
44854 229533.I1RI95 pp 229533.I1RP02
44855 229533.I1RI95 pp 229533.I1S0Q7
44856 229533.I1RI95 pp 229533.I1S5X9
44857 229533.I1RI95 pp 229533.I1S0K2
44858 229533.I1RI95 pp 229533.I1S0R8
44859 229533.I1RI95 pp 229533.I1S0P0
44860 229533.I1RI95 pp 229533.I1S1F3
44861 229533.I1RI95 pp 229533.I1RS29
44862 229533.I1RI95 pp 229533.I1RXD9
44863 229533.I1RI95 pp 229533.I1S464
44864 229533.I1RI95 pp 229533.I1RWA3
44865 229533.I1RI95 pp 229533.I1RY92
44866 229533.Q4I1N3 pp 229533.V6RG79
44867 229533.Q4I1N3 pp 229533.V6RS91
44868 229533.Q4I1N3 pp 229533.V6RDH3
44869 229533.I1RGW8 pp 229533.I1S3D5
44870 229533.I1RGW8 pp 229533.I1SAF3
44871 229533.I1S523 pp 229533.Q4IB89
44872 229533.I1S523 pp 229533.Q4INS6
44873 229533.I1RLN1 pp 229533.I1RS05
44874 229533.I1RLN1 pp 229533.I1RMK7
44875 229533.I1RLN1 pp 229533.I1RYT8
44876 229533.I1RLN1 pp 229533.V6RJP8
44877 229533.I1RLN1 pp 229533.I1RY21
44878 229533.I1RLN1 pp 229533.I1RNM7
44879 229533.I1RLN1 pp 229533.I1S0Q7
44880 229533.I1RLN1 pp 229533.I1RTW1
44881 229533.I1RLN1 pp 229533.I1RMQ6
44882 229533.I1RLN1 pp 229533.I1RLP4
44883 229533.I1RLN1 pp 229533.I1S5X9
44884 229533.I1RLN1 pp 229533.I1S0K2
44885 229533.I1RLN1 pp 229533.I1RS29
44886 229533.I1RLN1 pp 229533.I1RP02
44887 229533.I1RLN1 pp 229533.I1SAS4
44888 229533.I1RLN1 pp 229533.I1S0R8
44889 229533.I1RLN1 pp 229533.I1S0P0
44890 229533.I1RLN1 pp 229533.I1RXD9
44891 229533.I1RLN1 pp 229533.I1S1F3
44892 229533.I1RLN1 pp 229533.I1RY92
44893 229533.I1RLN1 pp 229533.I1RWA3
44894 229533.I1RLN1 pp 229533.I1S464
44895 229533.I1RY74 pp 229533.I1RYP2

44896	229533.I1RY74	pp	229533.I1RYK0
44897	229533.I1RY74	pp	229533.I1RZT6
44898	229533.I1RY74	pp	229533.I1RZ83
44899	229533.I1RY74	pp	229533.I1S1F5
44900	229533.I1RY74	pp	229533.I1RZ52
44901	229533.I1RY74	pp	229533.I1S3G4
44902	229533.I1RY74	pp	229533.I1RYQ4
44903	229533.I1RY74	pp	229533.Q4HXF0
44904	229533.I1RY74	pp	229533.I1SAJ3
44905	229533.I1RY74	pp	229533.I1S0T4
44906	229533.I1RY74	pp	229533.I1S9Z4
44907	229533.I1RY74	pp	229533.I1S1W8
44908	229533.I1RY74	pp	229533.I1RYU0
44909	229533.I1SAF3	pp	229533.V6RB81
44910	229533.I1SAF3	pp	229533.I1SAI0
44911	229533.A0A098DWE5	pp	229533.I1RGW8
44912	229533.A0A098DWE5	pp	229533.I1SAF3
44913	229533.A0A098DWE5	pp	229533.I1REL7
44914	229533.I1RS77	pp	229533.I1S0A0
44915	229533.I1RS77	pp	229533.I1RVX9
44916	229533.I1RPK2	pp	229533.V6R949
44917	229533.I1RS01	pp	229533.I1RWT0
44918	229533.I1RS01	pp	229533.I1S212
44919	229533.I1RS01	pp	229533.Q4I5G1
44920	229533.I1RS01	pp	229533.I1RX17
44921	229533.I1RS01	pp	229533.I1RWA3
44922	229533.I1RS01	pp	229533.I1S277
44923	229533.A0A098DKG5	pp	229533.I1RC83
44924	229533.A0A098DKG5	pp	229533.I1RQE0
44925	229533.A0A098DKG5	pp	229533.V6RY56
44926	229533.A0A098DKG5	pp	229533.I1RM26
44927	229533.A0A098DKG5	pp	229533.I1S4D0
44928	229533.A0A098DKG5	pp	229533.I1RF38
44929	229533.A0A098DKG5	pp	229533.I1RXX0
44930	229533.A0A098DKG5	pp	229533.V6R9Z3
44931	229533.A0A098DKG5	pp	229533.I1RYP6
44932	229533.A0A098DKG5	pp	229533.I1RF53
44933	229533.A0A098DKG5	pp	229533.A0A1C3YN25
44934	229533.A0A098DKG5	pp	229533.I1S6P6
44935	229533.A0A098DKG5	pp	229533.I1RSX2
44936	229533.A0A098DKG5	pp	229533.I1S2C4
44937	229533.A0A098DKG5	pp	229533.I1RD81
44938	229533.A0A098DKG5	pp	229533.A0A1C3YJ36
44939	229533.A0A098DKG5	pp	229533.I1RNG7
44940	229533.A0A098DKG5	pp	229533.V6RN31
44941	229533.A0A098DKG5	pp	229533.I1RR68
44942	229533.A0A098DKG5	pp	229533.I1RGT3
44943	229533.A0A098DKG5	pp	229533.I1RZ20
44944	229533.A0A098DKG5	pp	229533.I1S5L5
44945	229533.A0A098DKG5	pp	229533.I1S029
44946	229533.A0A098DKG5	pp	229533.I1RYU5
44947	229533.A0A098DKG5	pp	229533.I1RF40
44948	229533.A0A098DKG5	pp	229533.A0A1C3YLV0
44949	229533.A0A098DKG5	pp	229533.I1RVX9
44950	229533.A0A098DKG5	pp	229533.I1RPD6
44951	229533.A0A098DKG5	pp	229533.V6RRM0
44952	229533.A0A098DKG5	pp	229533.A0A098E2F0
44953	229533.A0A098DKG5	pp	229533.I1RCB4
44954	229533.A0A098DCQ7	pp	229533.I1RKX2
44955	229533.A0A098DCQ7	pp	229533.I1RGY2
44956	229533.A0A098DCQ7	pp	229533.I1RIY2
44957	229533.A0A098DCQ7	pp	229533.I1RP86
44958	229533.A0A098DCQ7	pp	229533.I1RQ14
44959	229533.A0A098DCQ7	pp	229533.I1RC31
44960	229533.A0A098DCQ7	pp	229533.A0A1C3YI71
44961	229533.A0A098DCQ7	pp	229533.I1S0V4
44962	229533.A0A098DCQ7	pp	229533.I1RVV3
44963	229533.A0A098DCQ7	pp	229533.A0A1C3YHV8
44964	229533.A0A098DCQ7	pp	229533.I1RYK0
44965	229533.A0A098DCQ7	pp	229533.I1RGK9
44966	229533.A0A098DCQ7	pp	229533.A0A0E0SQ99
44967	229533.A0A098DCQ7	pp	229533.I1RHQ0
44968	229533.A0A098DCQ7	pp	229533.I1RPK0

44969 229533.A0A098DCQ7 pp 229533.I1RSB7
44970 229533.A0A098DCQ7 pp 229533.V6RLM7
44971 229533.A0A098DCQ7 pp 229533.I1RU18
44972 229533.A0A098DCQ7 pp 229533.A0A1C3YKK9
44973 229533.A0A098DCQ7 pp 229533.I1RUY5
44974 229533.A0A098DCQ7 pp 229533.I1RJQ5
44975 229533.A0A098DCQ7 pp 229533.A0A0E0RMK7
44976 229533.A0A098DCQ7 pp 229533.A0A1C3YN26
44977 229533.A0A098DCQ7 pp 229533.I1RKR5
44978 229533.A0A098DCQ7 pp 229533.I1RND4
44979 229533.A0A098DCQ7 pp 229533.I1RHJ2
44980 229533.A0A098DCQ7 pp 229533.I1RMX6
44981 229533.A0A098DCQ7 pp 229533.I1RBF4
44982 229533.A0A098DCQ7 pp 229533.Q4IL82
44983 229533.A0A098DCQ7 pp 229533.A0A1C3YID4
44984 229533.A0A098DCQ7 pp 229533.A0A1C3YM97
44985 229533.A0A098DCQ7 pp 229533.A0A1C3YHP2
44986 229533.A0A098DCQ7 pp 229533.I1RTJ2
44987 229533.A0A098DCQ7 pp 229533.I1RDU0
44988 229533.A0A098DCQ7 pp 229533.I1RIE8
44989 229533.A0A098DCQ7 pp 229533.I1RX90
44990 229533.A0A098DCQ7 pp 229533.I1RPT0
44991 229533.A0A098DCQ7 pp 229533.A0A1C3YN83
44992 229533.A0A098DCQ7 pp 229533.Q4I1Y5
44993 229533.A0A098DCQ7 pp 229533.I1RNU7
44994 229533.I1RF70 pp 229533.I1RUG2
44995 229533.I1RF70 pp 229533.I1RFJ2
44996 229533.I1RF70 pp 229533.I1S461
44997 229533.I1RF70 pp 229533.I1RY73
44998 229533.I1RF70 pp 229533.I1S604
44999 229533.I1RF70 pp 229533.I1RJV8
45000 229533.I1RF70 pp 229533.I1RSC7
45001 229533.I1RF70 pp 229533.I1RRT1
45002 229533.I1RF70 pp 229533.I1RPQ1
45003 229533.I1RF70 pp 229533.I1RYQ1
45004 229533.I1RF70 pp 229533.I1RRS7
45005 229533.I1RF70 pp 229533.I1RZ94
45006 229533.I1RF70 pp 229533.Q4IB89
45007 229533.I1RF70 pp 229533.I1RSI4
45008 229533.I1RF70 pp 229533.I1RFN0
45009 229533.I1RF70 pp 229533.I1RYX9
45010 229533.I1RF70 pp 229533.I1RQ13
45011 229533.I1RF70 pp 229533.I1RZ86
45012 229533.I1RF70 pp 229533.I1RSR9
45013 229533.I1RF70 pp 229533.V6RLW4
45014 229533.I1RF70 pp 229533.I1RFZ2
45015 229533.I1RF70 pp 229533.Q4IMM0
45016 229533.I1RF70 pp 229533.I1RMJ8
45017 229533.I1RF70 pp 229533.I1RYR2
45018 229533.I1RF70 pp 229533.I1RFX8
45019 229533.I1S5H2 pp 229533.I1S8Z2
45020 229533.I1S5H2 pp 229533.V6RKY8
45021 229533.A0A1C3YK82 pp 229533.I1RZ86
45022 229533.A0A1C3YK82 pp 229533.I1S523
45023 229533.A0A1C3YK82 pp 229533.I1S998
45024 229533.A0A1C3YK82 pp 229533.I1RF02
45025 229533.A0A1C3YK82 pp 229533.I1RD73
45026 229533.A0A1C3YK82 pp 229533.I1RX64
45027 229533.A0A1C3YK82 pp 229533.A0A1C3YN26
45028 229533.A0A1C3YK82 pp 229533.I1RUR0
45029 229533.A0A1C3YK82 pp 229533.A0A1C3YL55
45030 229533.A0A1C3YK82 pp 229533.I1RP99
45031 229533.A0A1C3YK82 pp 229533.I1RWI8
45032 229533.A0A1C3YK82 pp 229533.I1RSC7
45033 229533.A0A1C3YK82 pp 229533.I1RAV0
45034 229533.A0A1C3YK82 pp 229533.I1RRT1
45035 229533.A0A1C3YK82 pp 229533.I1RD01
45036 229533.A0A1C3YK82 pp 229533.I1RZ94
45037 229533.A0A1C3YK82 pp 229533.I1RY29
45038 229533.A0A1C3YK82 pp 229533.I1RAH9
45039 229533.A0A1C3YK82 pp 229533.I1RZ98
45040 229533.A0A1C3YK82 pp 229533.I1RFX8
45041 229533.A0A1C3YK82 pp 229533.I1RQI6

45042 229533.A0A1C3YK82 pp 229533.V6RDH3
45043 229533.A0A1C3YK82 pp 229533.I1S987
45044 229533.A0A1C3YK82 pp 229533.I1RPI0
45045 229533.A0A1C3YK82 pp 229533.I1RBC8
45046 229533.A0A1C3YK82 pp 229533.I1RBW0
45047 229533.A0A1C3YK82 pp 229533.I1RSJ1
45048 229533.A0A1C3YK82 pp 229533.I1RWB3
45049 229533.A0A1C3YK82 pp 229533.I1RNA2
45050 229533.A0A1C3YK82 pp 229533.I1RC54
45051 229533.A0A1C3YK82 pp 229533.Q4IB89
45052 229533.A0A1C3YK82 pp 229533.I1RKI2
45053 229533.A0A1C3YK82 pp 229533.I1RFJ2
45054 229533.A0A1C3YK82 pp 229533.I1RBE7
45055 229533.A0A1C3YK82 pp 229533.A0A1C3YLQ7
45056 229533.A0A1C3YK82 pp 229533.I1RE55
45057 229533.A0A1C3YK82 pp 229533.I1RDC0
45058 229533.A0A1C3YK82 pp 229533.I1RWM6
45059 229533.A0A1C3YK82 pp 229533.Q4INS6
45060 229533.A0A1C3YK82 pp 229533.I1RBW1
45061 229533.A0A1C3YK82 pp 229533.I1RSI4
45062 229533.A0A1C3YK82 pp 229533.I1RAL9
45063 229533.A0A1C3YK82 pp 229533.I1RQH8
45064 229533.A0A1C3YK82 pp 229533.I1S209
45065 229533.I1RQS2 pp 229533.I1RZ43
45066 229533.I1RQS2 pp 229533.I1S212
45067 229533.I1RQS2 pp 229533.I1S0Q7
45068 229533.I1RQS2 pp 229533.I1S0R8
45069 229533.I1RQS2 pp 229533.I1RX39
45070 229533.I1RTH9 pp 229533.Q4IPI4
45071 229533.I1RTH9 pp 229533.I1S1V2
45072 229533.I1RTH9 pp 229533.Q4I7X1
45073 229533.I1RTH9 pp 229533.I1S7I5
45074 229533.V6QZS3 pp 229533.V6RY56
45075 229533.A0A1C3YHU7 pp 229533.I1RH76
45076 229533.A0A1C3YHU7 pp 229533.I1S2C4
45077 229533.A0A1C3YHU7 pp 229533.I1RDB3
45078 229533.A0A1C3YHU7 pp 229533.Q4IR87
45079 229533.A0A1C3YHU7 pp 229533.V6RY56
45080 229533.I1RGI8 pp 229533.I1RMQ6
45081 229533.I1RGI8 pp 229533.I1RKB7
45082 229533.I1RGI8 pp 229533.I1RY21
45083 229533.I1RGI8 pp 229533.I1RNM7
45084 229533.I1RGI8 pp 229533.I1RYT8
45085 229533.I1RGI8 pp 229533.I1S0P0
45086 229533.I1RGI8 pp 229533.I1RMK7
45087 229533.I1RGI8 pp 229533.I1RS05
45088 229533.I1RGI8 pp 229533.I1RS29
45089 229533.I1RGI8 pp 229533.V6RJP8
45090 229533.I1RGI8 pp 229533.I1RP02
45091 229533.I1RGI8 pp 229533.I1RY92
45092 229533.I1RGI8 pp 229533.I1S0K2
45093 229533.I1RGI8 pp 229533.I1S0R8
45094 229533.I1RGI8 pp 229533.I1S5X9
45095 229533.I1RGI8 pp 229533.I1RWA3
45096 229533.I1RGI8 pp 229533.I1S1F3
45097 229533.I1RGI8 pp 229533.I1S0Q7
45098 229533.I1RGI8 pp 229533.I1RXD9
45099 229533.I1RGI8 pp 229533.I1S464
45100 229533.I1R9U7 pp 229533.I1RW06
45101 229533.I1R9U7 pp 229533.I1RZ52
45102 229533.I1R9U7 pp 229533.I1RQG7
45103 229533.I1R9U7 pp 229533.I1S334
45104 229533.I1R9U7 pp 229533.I1RXV3
45105 229533.I1R9U7 pp 229533.Q4IMJ1
45106 229533.I1R9U7 pp 229533.I1RAD8
45107 229533.I1R9U7 pp 229533.I1S3H8
45108 229533.I1R9U7 pp 229533.I1REI3
45109 229533.I1R9U7 pp 229533.I1RFB4
45110 229533.I1R9U7 pp 229533.I1RRL5
45111 229533.I1R9U7 pp 229533.I1RAM9
45112 229533.I1R9U7 pp 229533.I1RCP7
45113 229533.I1R9U7 pp 229533.I1RJT2
45114 229533.I1R9U7 pp 229533.I1RYJ6

45115	229533.I1R9U7	pp	229533.I1S0H7
45116	229533.I1R9U7	pp	229533.V6QVM5
45117	229533.I1R9U7	pp	229533.I1RWS4
45118	229533.I1R9U7	pp	229533.I1RIY0
45119	229533.I1R9U7	pp	229533.I1RBE4
45120	229533.I1R9U7	pp	229533.I1RUP2
45121	229533.I1R9U7	pp	229533.I1RNJ7
45122	229533.I1R9U7	pp	229533.I1RZQ1
45123	229533.I1R9U7	pp	229533.I1S072
45124	229533.I1RU95	pp	229533.I1S0R8
45125	229533.I1RU95	pp	229533.I1RXD9
45126	229533.I1RU95	pp	229533.I1S5X9
45127	229533.I1RU95	pp	229533.I1S1F3
45128	229533.I1RU95	pp	229533.I1RY92
45129	229533.I1RU95	pp	229533.I1S464
45130	229533.I1RU95	pp	229533.I1S0P0
45131	229533.I1RU95	pp	229533.I1RWA3
45132	229533.I1RU95	pp	229533.I1RYT8
45133	229533.I1RU95	pp	229533.I1RY21
45134	229533.I1RU95	pp	229533.I1S0Q7
45135	229533.I1RU95	pp	229533.V6RJP8
45136	229533.I1RU95	pp	229533.I1S0K2
45137	229533.I1RYD5	pp	229533.I1RZG7
45138	229533.I1S998	pp	229533.Q4INS6
45139	229533.I1S998	pp	229533.Q4IB89
45140	229533.A0A098DP90	pp	229533.V6RLM7
45141	229533.A0A098DP90	pp	229533.A0A1C3YJI4
45142	229533.A0A098DP90	pp	229533.I1RW06
45143	229533.A0A098DP90	pp	229533.I1RFV9
45144	229533.A0A098DP90	pp	229533.I1RYK0
45145	229533.A0A098DP90	pp	229533.I1RND4
45146	229533.A0A098DP90	pp	229533.A0A098DVZ4
45147	229533.A0A098DP90	pp	229533.I1S9T8
45148	229533.I1RBA4	pp	229533.V6QX21
45149	229533.I1RBA4	pp	229533.V6RPQ8
45150	229533.I1RBA4	pp	229533.I1RC67
45151	229533.I1RBA4	pp	229533.V6R949
45152	229533.I1RBA4	pp	229533.I1RBX1
45153	229533.I1RBA4	pp	229533.I1RRV1
45154	229533.I1RBA4	pp	229533.I1RYP2
45155	229533.I1RBA4	pp	229533.I1S9T5
45156	229533.I1RBA4	pp	229533.I1RMJ3
45157	229533.I1RBA4	pp	229533.I1RN84
45158	229533.I1RBA4	pp	229533.I1S123
45159	229533.I1RBA4	pp	229533.I1RBS5
45160	229533.I1RBA4	pp	229533.I1RQ38
45161	229533.I1RBA4	pp	229533.I1RSC7
45162	229533.I1RBA4	pp	229533.I1RXY7
45163	229533.I1RBA4	pp	229533.I1RE79
45164	229533.I1RBA4	pp	229533.I1S1W7
45165	229533.I1RBA4	pp	229533.I1RY74
45166	229533.I1RBA4	pp	229533.I1RSQ4
45167	229533.I1RBA4	pp	229533.I1RZN1
45168	229533.I1RBA4	pp	229533.I1RPK0
45169	229533.I1RBA4	pp	229533.I1RSS7
45170	229533.I1RBA4	pp	229533.I1RWI5
45171	229533.I1RBA4	pp	229533.I1RKB6
45172	229533.I1RBA4	pp	229533.I1S209
45173	229533.I1RBA4	pp	229533.I1RT75
45174	229533.I1RBA4	pp	229533.I1RYM5
45175	229533.I1RBA4	pp	229533.I1S4Z7
45176	229533.I1RBA4	pp	229533.I1RBE4
45177	229533.I1RBA4	pp	229533.I1RCL0
45178	229533.I1RBA4	pp	229533.V6RQW4
45179	229533.I1RBA4	pp	229533.I1RBF3
45180	229533.I1RBA4	pp	229533.I1RJT5
45181	229533.I1RBA4	pp	229533.I1RNE2
45182	229533.I1RBA4	pp	229533.I1RMW4
45183	229533.I1RBA4	pp	229533.I1RYB8
45184	229533.I1RBA4	pp	229533.I1RJD3
45185	229533.I1RBA4	pp	229533.I1RMK6
45186	229533.I1RBA4	pp	229533.I1S8Z2
45187	229533.I1RBA4	pp	229533.I1RVZ9

45188 229533.I1RBA4 pp 229533.I1RM67
45189 229533.I1RBA4 pp 229533.I1RP86
45190 229533.I1RBA4 pp 229533.I1RFV2
45191 229533.I1RBA4 pp 229533.I1RKI2
45192 229533.I1RBA4 pp 229533.I1RSQ2
45193 229533.I1RBA4 pp 229533.I1S7L6
45194 229533.I1RBA4 pp 229533.I1RY49
45195 229533.A0A1C3YN75 pp 229533.I1RSH4
45196 229533.A0A1C3YN75 pp 229533.I1RSA4
45197 229533.A0A1C3YN75 pp 229533.I1RBH8
45198 229533.A0A1C3YN75 pp 229533.I1RRW3
45199 229533.A0A1C3YN75 pp 229533.I1S7M3
45200 229533.A0A1C3YN75 pp 229533.I1RVT9
45201 229533.A0A1C3YN75 pp 229533.I1RXF2
45202 229533.A0A1C3YN75 pp 229533.I1RE00
45203 229533.A0A1C3YN75 pp 229533.I1S0P4
45204 229533.A0A1C3YN75 pp 229533.I1S0B1
45205 229533.A0A1C3YN75 pp 229533.I1S7M2
45206 229533.A0A1C3YN75 pp 229533.I1RBV3
45207 229533.A0A1C3YN75 pp 229533.I1RG90
45208 229533.A0A1C3YN75 pp 229533.I1RKE7
45209 229533.A0A1C3YN75 pp 229533.I1RB50
45210 229533.A0A1C3YN75 pp 229533.I1RCQ6
45211 229533.A0A1C3YN75 pp 229533.I1RA36
45212 229533.A0A1C3YN75 pp 229533.I1RZL3
45213 229533.A0A1C3YN75 pp 229533.I1RGF2
45214 229533.Q4I1Y5 pp 229533.V6RAN2
45215 229533.Q4I1Y5 pp 229533.V6R4J1
45216 229533.I1S5U0 pp 229533.I1S5X9
45217 229533.I1S5U0 pp 229533.V6RJP8
45218 229533.I1RQ30 pp 229533.I1RXB1
45219 229533.I1RQ30 pp 229533.I1S9D4
45220 229533.I1RQ30 pp 229533.I1RVX9
45221 229533.I1RQ30 pp 229533.I1RYU5
45222 229533.I1RQ30 pp 229533.I1RYD3
45223 229533.I1RQ30 pp 229533.I1S1W8
45224 229533.I1RQ30 pp 229533.I1S8X1
45225 229533.I1RQ30 pp 229533.I1S887
45226 229533.I1RQ30 pp 229533.I1RVN1
45227 229533.I1RQ30 pp 229533.I1RVY2
45228 229533.I1RQ30 pp 229533.Q4I283
45229 229533.I1RQ30 pp 229533.I1S2E2
45230 229533.I1RQ30 pp 229533.O74638
45231 229533.I1RQ30 pp 229533.I1RYI7
45232 229533.I1RQ30 pp 229533.I1RYW6
45233 229533.I1RQ30 pp 229533.I1S3G4
45234 229533.I1RQ30 pp 229533.I1RTI7
45235 229533.I1RQ30 pp 229533.V6RSF3
45236 229533.I1RQ30 pp 229533.I1RZ52
45237 229533.I1RQ30 pp 229533.I1S770
45238 229533.I1RQ30 pp 229533.Q4HXF0
45239 229533.I1RQ30 pp 229533.I1RXU9
45240 229533.I1RQ30 pp 229533.V6QVH8
45241 229533.I1RQ30 pp 229533.I1RY62
45242 229533.I1RQ30 pp 229533.I1RXR1
45243 229533.I1RQ30 pp 229533.I1RSL5
45244 229533.I1RQ30 pp 229533.I1RZ20
45245 229533.I1RQ30 pp 229533.I1S256
45246 229533.I1RQ30 pp 229533.I1RS40
45247 229533.I1RSJ1 pp 229533.Q4HZ68
45248 229533.I1RSJ1 pp 229533.I1RSP7
45249 229533.I1RSJ1 pp 229533.I1RX64
45250 229533.I1RSJ1 pp 229533.I1RTN6
45251 229533.I1RSJ1 pp 229533.I1RZ98
45252 229533.I1RSJ1 pp 229533.I1S0D5
45253 229533.I1RSJ1 pp 229533.Q4IB89
45254 229533.I1RSJ1 pp 229533.I1S523
45255 229533.I1RSJ1 pp 229533.I1RZ86
45256 229533.I1RSJ1 pp 229533.I1RY29
45257 229533.I1RSJ1 pp 229533.I1S5R2
45258 229533.I1RSJ1 pp 229533.I1S4P6
45259 229533.I1RSJ1 pp 229533.Q4INS6
45260 229533.I1RSJ1 pp 229533.I1S0Q4

45261 229533.I1RSJ1 pp 229533.Q4IAS1
45262 229533.I1RSJ1 pp 229533.I1RWI8
45263 229533.I1RSJ1 pp 229533.I1S0Q5
45264 229533.I1RSJ1 pp 229533.Q4INZ9
45265 229533.I1RSJ1 pp 229533.Q4IR08
45266 229533.I1RSJ1 pp 229533.I1S209
45267 229533.I1RSJ1 pp 229533.I1S788
45268 229533.I1RSJ1 pp 229533.I1RZ94
45269 229533.I1RSJ1 pp 229533.I1RUR0
45270 229533.I1RSJ1 pp 229533.I1RWM6
45271 229533.I1RP38 pp 229533.I1S0R8
45272 229533.I1RP38 pp 229533.I1S0Q7
45273 229533.I1RP38 pp 229533.V6RJP8
45274 229533.I1RP38 pp 229533.I1S5X9
45275 229533.I1RP38 pp 229533.I1RWA3
45276 229533.I1RP38 pp 229533.I1S464
45277 229533.I1RP38 pp 229533.I1S0K2
45278 229533.I1RP38 pp 229533.I1RXD9
45279 229533.I1RP38 pp 229533.I1S1F3
45280 229533.I1RP38 pp 229533.I1RS29
45281 229533.I1RP38 pp 229533.I1S0P0
45282 229533.I1RP38 pp 229533.I1RY92
45283 229533.I1RP38 pp 229533.I1RS05
45284 229533.I1RP38 pp 229533.I1RY21
45285 229533.I1RP38 pp 229533.I1RYT8
45286 229533.I1RQH7 pp 229533.I1S1V4
45287 229533.I1RQH7 pp 229533.I1RZK3
45288 229533.I1RQH7 pp 229533.I1S8C5
45289 229533.I1RQH7 pp 229533.I1SAM5
45290 229533.I1RQH7 pp 229533.I1RW76
45291 229533.I1RQH7 pp 229533.I1S2B6
45292 229533.I1RQH7 pp 229533.Q4IN63
45293 229533.I1RQH7 pp 229533.I1S287
45294 229533.I1RQH7 pp 229533.I1S5B5
45295 229533.I1RYQ7 pp 229533.V6R3U5
45296 229533.A0A1C3YKG0 pp 229533.I1RE79
45297 229533.A0A1C3YKG0 pp 229533.I1RNG4
45298 229533.A0A1C3YKG0 pp 229533.I1RGK9
45299 229533.A0A1C3YKG0 pp 229533.I1RHK2
45300 229533.A0A1C3YKG0 pp 229533.I1RRG1
45301 229533.A0A1C3YKG0 pp 229533.A0A1C3YLB6
45302 229533.A0A1C3YKG0 pp 229533.I1RTJ2
45303 229533.A0A1C3YKG0 pp 229533.I1RYM5
45304 229533.A0A1C3YKG0 pp 229533.I1RPM6
45305 229533.A0A1C3YKG0 pp 229533.I1RFY0
45306 229533.I1RB22 pp 229533.I1RTA6
45307 229533.A0A098DP68 pp 229533.I1S268
45308 229533.I1RN82 pp 229533.I1RW76
45309 229533.I1RN82 pp 229533.I1RZ60
45310 229533.I1RN82 pp 229533.I1RXU1
45311 229533.I1RN82 pp 229533.I1RTD0
45312 229533.I1RN82 pp 229533.I1RZS5
45313 229533.I1RZ02 pp 229533.Q4ICG8
45314 229533.I1RZ02 pp 229533.I1S0A4
45315 229533.I1RZ02 pp 229533.Q4IER0
45316 229533.I1RZ02 pp 229533.I1S7X9
45317 229533.I1RZ02 pp 229533.Q4HTT1
45318 229533.I1RZ02 pp 229533.I1S0K9
45319 229533.I1RZ02 pp 229533.Q4IPC8
45320 229533.I1RZ02 pp 229533.Q4I0M9
45321 229533.I1RZ02 pp 229533.I1RZ94
45322 229533.I1RZ02 pp 229533.I1RZG0
45323 229533.I1RZ02 pp 229533.Q4INW6
45324 229533.I1RZ02 pp 229533.Q4I5I4
45325 229533.I1RZ02 pp 229533.V6REE9
45326 229533.I1RZ02 pp 229533.I1RZH4
45327 229533.I1RZ02 pp 229533.I1S0Z3
45328 229533.I1RYC8 pp 229533.V6RJP8
45329 229533.I1RYC8 pp 229533.I1S8C1
45330 229533.I1RYC8 pp 229533.I1S0P0
45331 229533.I1RYC8 pp 229533.I1S147
45332 229533.I1RYC8 pp 229533.I1S0K2
45333 229533.I1RYC8 pp 229533.I1S3B2

45334 229533.I1RYC8 pp 229533.Q4I5G1
45335 229533.I1RYC8 pp 229533.I1RYX6
45336 229533.I1RYC8 pp 229533.I1S0V7
45337 229533.I1RYC8 pp 229533.I1S3V2
45338 229533.I1RYC8 pp 229533.V6RS91
45339 229533.I1RYC8 pp 229533.I1S969
45340 229533.I1RYC8 pp 229533.I1S0V6
45341 229533.I1RYC8 pp 229533.I1RYT8
45342 229533.I1RYC8 pp 229533.I1S1F3
45343 229533.I1RYC8 pp 229533.I1S1W8
45344 229533.I1RYC8 pp 229533.I1S0Q7
45345 229533.I1RYC8 pp 229533.V6RRM0
45346 229533.I1RYC8 pp 229533.I1RYP5
45347 229533.I1RYC8 pp 229533.I1RZA7
45348 229533.I1RYC8 pp 229533.I1S8R2
45349 229533.I1RYC8 pp 229533.I1S299
45350 229533.I1RYC8 pp 229533.I1S0B8
45351 229533.I1RYC8 pp 229533.Q4I1N3
45352 229533.I1RYC8 pp 229533.V6RUB1
45353 229533.I1RYC8 pp 229533.I1S3T7
45354 229533.I1RYC8 pp 229533.I1S968
45355 229533.I1RYC8 pp 229533.I1S6P5
45356 229533.I1RYC8 pp 229533.Q4HVQ9
45357 229533.I1RYC8 pp 229533.I1S3G4
45358 229533.I1RYC8 pp 229533.Q4IN52
45359 229533.I1RHF2 pp 229533.V6R3U5
45360 229533.I1RL88 pp 229533.I1RTA6
45361 229533.A0A0E0SHE3 pp 229533.I1RVY0
45362 229533.A0A0E0SHE3 pp 229533.I1RR25
45363 229533.A0A0E0SHE3 pp 229533.I1RN17
45364 229533.A0A0E0SHE3 pp 229533.I1RJQ3
45365 229533.A0A0E0SHE3 pp 229533.I1RMP9
45366 229533.A0A0E0SHE3 pp 229533.I1S9Y1
45367 229533.A0A0E0SHE3 pp 229533.I1RK95
45368 229533.A0A0E0SHE3 pp 229533.I1RB71
45369 229533.A0A0E0SHE3 pp 229533.I1RJY2
45370 229533.A0A0E0SHE3 pp 229533.V6RFX5
45371 229533.A0A0E0SHE3 pp 229533.I1RJL2
45372 229533.A0A0E0SHE3 pp 229533.I1RW90
45373 229533.A0A0E0SHE3 pp 229533.V6QWX8
45374 229533.A0A0E0SHE3 pp 229533.I1RJY3
45375 229533.A0A0E0SHE3 pp 229533.I1RJM5
45376 229533.A0A0E0SHE3 pp 229533.A0A1C3YIR8
45377 229533.A0A0E0SHE3 pp 229533.I1RRG3
45378 229533.A0A0E0SHE3 pp 229533.I1S255
45379 229533.A0A0E0SHE3 pp 229533.I1RDC9
45380 229533.A0A0E0SHE3 pp 229533.I1S7M8
45381 229533.A0A0E0SHE3 pp 229533.I1RRN6
45382 229533.A0A0E0SHE3 pp 229533.A0A1C3YJR3
45383 229533.A0A0E0SHE3 pp 229533.A0A1C3YK88
45384 229533.A0A0E0SHE3 pp 229533.V6QUE6
45385 229533.A0A0E0SHE3 pp 229533.I1S9U1
45386 229533.A0A0E0SHE3 pp 229533.I1RAV6
45387 229533.A0A0E0SHE3 pp 229533.I1RG11
45388 229533.A0A0E0SHE3 pp 229533.I1RBC7
45389 229533.A0A0E0SHE3 pp 229533.I1RJI3
45390 229533.I1RUR0 pp 229533.I1RZ98
45391 229533.I1RUR0 pp 229533.Q4IB89
45392 229533.I1RUR0 pp 229533.Q4HY90
45393 229533.I1RUR0 pp 229533.I1S4P6
45394 229533.I1RUR0 pp 229533.Q4IMD1
45395 229533.I1RUR0 pp 229533.I1RYQ1
45396 229533.I1RUR0 pp 229533.I1S998
45397 229533.I1RUR0 pp 229533.I1S0D5
45398 229533.I1RUR0 pp 229533.I1RVW0
45399 229533.I1RUR0 pp 229533.I1RVV5
45400 229533.I1RUR0 pp 229533.I1RWI8
45401 229533.I1RUR0 pp 229533.I1RZ86
45402 229533.I1RUR0 pp 229533.I1RY29
45403 229533.I1RUR0 pp 229533.Q4IJ84
45404 229533.I1RUR0 pp 229533.I1RX64
45405 229533.I1RUR0 pp 229533.I1S209
45406 229533.I1RUR0 pp 229533.I1RVL8

45407 229533.I1RUR0 pp 229533.I1RZB5
45408 229533.I1RUR0 pp 229533.Q4ICA8
45409 229533.I1RUR0 pp 229533.Q4INS6
45410 229533.I1RUR0 pp 229533.I1RWM6
45411 229533.I1RUR0 pp 229533.Q4I948
45412 229533.I1RUR0 pp 229533.I1S283
45413 229533.I1RUR0 pp 229533.I1S788
45414 229533.I1RUR0 pp 229533.I1RZJ9
45415 229533.I1RUR0 pp 229533.I1RZ94
45416 229533.I1RUR0 pp 229533.I1S987
45417 229533.A0A1C3YLT9 pp 229533.I1RDQ9
45418 229533.A0A1C3YLT9 pp 229533.V6QUV7
45419 229533.A0A1C3YLT9 pp 229533.A0A1C3YLW9
45420 229533.A0A1C3YLT9 pp 229533.I1RGN1
45421 229533.A0A1C3YLT9 pp 229533.I1RZQ6
45422 229533.A0A1C3YLT9 pp 229533.I1RNL7
45423 229533.A0A1C3YLT9 pp 229533.I1S9G0
45424 229533.A0A1C3YLT9 pp 229533.I1S645
45425 229533.A0A1C3YLT9 pp 229533.I1RMA8
45426 229533.A0A1C3YLT9 pp 229533.I1RSI1
45427 229533.A0A1C3YLT9 pp 229533.V6RDH3
45428 229533.A0A1C3YLT9 pp 229533.I1RNN7
45429 229533.A0A1C3YLT9 pp 229533.I1RAE7
45430 229533.A0A1C3YLT9 pp 229533.I1RZ05
45431 229533.A0A1C3YLT9 pp 229533.I1S2I7
45432 229533.A0A1C3YLT9 pp 229533.I1RMW4
45433 229533.A0A1C3YLT9 pp 229533.I1S067
45434 229533.A0A1C3YLT9 pp 229533.I1RAS9
45435 229533.A0A1C3YLT9 pp 229533.I1RC66
45436 229533.A0A1C3YLT9 pp 229533.I1RR15
45437 229533.A0A1C3YLT9 pp 229533.I1S7W0
45438 229533.A0A1C3YLT9 pp 229533.I1RY72
45439 229533.A0A1C3YLT9 pp 229533.I1RT41
45440 229533.A0A1C3YLT9 pp 229533.I1RX44
45441 229533.A0A1C3YLT9 pp 229533.I1RCD9
45442 229533.A0A1C3YLT9 pp 229533.I1RCI5
45443 229533.A0A1C3YLT9 pp 229533.I1SAP1
45444 229533.A0A1C3YLT9 pp 229533.I1RI71
45445 229533.A0A1C3YLT9 pp 229533.I1RWL9
45446 229533.A0A1C3YLT9 pp 229533.I1R9Z9
45447 229533.A0A1C3YLT9 pp 229533.I1S303
45448 229533.I1RRN6 pp 229533.V6QUE6
45449 229533.I1RRN6 pp 229533.I1RS30
45450 229533.I1RRN6 pp 229533.I1S255
45451 229533.I1RRN6 pp 229533.I1RSR3
45452 229533.I1RRN6 pp 229533.I1S9U1
45453 229533.I1RRN6 pp 229533.I1S9Y1
45454 229533.I1RRN6 pp 229533.V6QWX8
45455 229533.I1RRN6 pp 229533.I1RVY0
45456 229533.I1RRN6 pp 229533.I1RW90
45457 229533.I1RRN6 pp 229533.I1RZB5
45458 229533.I1RC93 pp 229533.V6R3U5
45459 229533.I1RML8 pp 229533.I1RWI6
45460 229533.I1RML8 pp 229533.I1S0V5
45461 229533.I1RML8 pp 229533.I1RWX8
45462 229533.I1RCB4 pp 229533.I1S5L5
45463 229533.I1RCB4 pp 229533.I1S5G3
45464 229533.I1RCB4 pp 229533.I1RZ20
45465 229533.I1RCB4 pp 229533.I1RMG6
45466 229533.I1RCB4 pp 229533.I1RE63
45467 229533.I1RCB4 pp 229533.I1SAJ0
45468 229533.I1RCB4 pp 229533.I1RNG4
45469 229533.I1RCB4 pp 229533.I1RE53
45470 229533.I1RCB4 pp 229533.Q4HXF0
45471 229533.I1RCB4 pp 229533.I1S4F4
45472 229533.I1RCB4 pp 229533.I1RPD6
45473 229533.I1RCB4 pp 229533.I1REP3
45474 229533.I1RCB4 pp 229533.I1RR68
45475 229533.I1RCB4 pp 229533.I1RHT3
45476 229533.I1RCB4 pp 229533.I1RIB6
45477 229533.I1RCB4 pp 229533.I1RTI7
45478 229533.I1RCB4 pp 229533.I1RNG7
45479 229533.I1RCB4 pp 229533.I1REV2

229533.I1RCB4	pp	229533.I1S076
229533.I1RCB4	pp	229533.I1RYW6
229533.I1RCB4	pp	229533.I1RPU8
229533.I1RCB4	pp	229533.I1RXR5
229533.I1RCB4	pp	229533.I1RZ52
229533.I1RCB4	pp	229533.I1S6H1
229533.I1RCB4	pp	229533.I1RJC7
229533.I1RCB4	pp	229533.I1RQ30
229533.I1RCB4	pp	229533.I1S3G4
229533.I1RCB4	pp	229533.I1S685
229533.I1RCB4	pp	229533.I1S770
229533.I1RCB4	pp	229533.I1RS40
229533.I1RCB4	pp	229533.I1S2H3
229533.I1RCB4	pp	229533.O74638
229533.I1RCB4	pp	229533.I1RYR6
229533.I1RCB4	pp	229533.I1S8X1
229533.I1RCB4	pp	229533.I1S2C5
229533.I1RCB4	pp	229533.I1S2C4
229533.I1RCB4	pp	229533.I1RF53
229533.I1RCB4	pp	229533.I1RYD3
229533.I1RCB4	pp	229533.I1S9D4
229533.I1RCB4	pp	229533.I1RJK0
229533.I1RCB4	pp	229533.I1RJT5
229533.I1RCB4	pp	229533.V6RY56
229533.I1RCB4	pp	229533.I1RQE0
229533.I1RCB4	pp	229533.I1RJ73
229533.I1RCB4	pp	229533.I1RF38
229533.I1RCB4	pp	229533.I1RK93
229533.I1RCB4	pp	229533.I1RM26
229533.I1RCB4	pp	229533.V6RQW4
229533.I1RCB4	pp	229533.I1S1W8
229533.I1RCB4	pp	229533.I1RYU5
229533.I1RCB4	pp	229533.I1RVX9
229533.I1RCB4	pp	229533.I1S029
229533.I1RCB4	pp	229533.I1S2E2
229533.I1RCB4	pp	229533.I1RCV1
229533.I1RCB4	pp	229533.I1S4D0
229533.I1RCB4	pp	229533.I1RTJ7
229533.I1RCB4	pp	229533.I1RV18
229533.I1RCB4	pp	229533.I1RHD1
229533.I1RCB4	pp	229533.I1RPW2
229533.I1RCB4	pp	229533.I1RYE6
229533.I1RCB4	pp	229533.I1RD81
229533.I1RCB4	pp	229533.I1RMG7
229533.I1RCB4	pp	229533.I1RYI7
229533.I1RCB4	pp	229533.I1RU76
229533.I1RCB4	pp	229533.I1REX0
229533.I1RCB4	pp	229533.I1RFM6
229533.I1RCB4	pp	229533.I1RIY0
229533.I1RCB4	pp	229533.I1RN80
229533.I1RHD2	pp	229533.I1RZ94
229533.I1RHD2	pp	229533.Q4IB89
229533.I1RHD2	pp	229533.I1RZ86
229533.I1RHD2	pp	229533.I1RSC7
229533.I1RHD2	pp	229533.I1RQH8
229533.I1RHD2	pp	229533.I1RKI2
229533.I1RHD2	pp	229533.I1RP99
229533.I1RHD2	pp	229533.I1RWI8
229533.I1RHD2	pp	229533.I1RUG4
229533.I1RHD2	pp	229533.I1RRJ6
229533.I1RHD2	pp	229533.I1S5Z3
229533.I1RHD2	pp	229533.Q4INS6
229533.I1RHD2	pp	229533.I1RPQ1
229533.I1RHD2	pp	229533.I1RJV8
229533.I1RHD2	pp	229533.I1S0D5
229533.I1RHD2	pp	229533.I1S209
229533.I1RHD2	pp	229533.I1RYQ1
229533.I1RHD2	pp	229533.I1RSR9
229533.I1RHD2	pp	229533.I1S788
229533.I1RJM4	pp	229533.I1RTA6
229533.I1S0A4	pp	229533.Q4I0M9
229533.I1S0A4	pp	229533.Q4INW6
229533.I1S0A4	pp	229533.Q4I5I4

45553 229533.I1RJM5 pp 229533.I1S9Y1
45554 229533.I1RJM5 pp 229533.I1S4Q7
45555 229533.I1RJM5 pp 229533.V6RFX5
45556 229533.I1RJM5 pp 229533.I1RRN6
45557 229533.I1RJM5 pp 229533.I1RVY0
45558 229533.I1RJM5 pp 229533.I1S8W6
45559 229533.I1RJM5 pp 229533.I1RN17
45560 229533.I1RFX5 pp 229533.I1RYD3
45561 229533.I1R9U3 pp 229533.I1RTA6
45562 229533.I1RFK3 pp 229533.V6RS91
45563 229533.I1RFK3 pp 229533.I1RW11
45564 229533.I1RFK3 pp 229533.I1S767
45565 229533.I1RFK3 pp 229533.Q4HZQ1
45566 229533.I1RFK3 pp 229533.I1RYD3
45567 229533.I1RFK3 pp 229533.I1RNG4
45568 229533.I1RFK3 pp 229533.I1RZU2
45569 229533.I1RFK3 pp 229533.I1RHK2
45570 229533.I1RFK3 pp 229533.I1RZE7
45571 229533.I1RFK3 pp 229533.I1S029
45572 229533.I1RFK3 pp 229533.I1RPY0
45573 229533.I1RFK3 pp 229533.I1S6D1
45574 229533.I1RFK3 pp 229533.I1RGK9
45575 229533.I1RFK3 pp 229533.I1S1W8
45576 229533.I1RFK3 pp 229533.I1S241
45577 229533.I1RFK3 pp 229533.I1S4D0
45578 229533.I1RFK3 pp 229533.I1RXY7
45579 229533.I1RFK3 pp 229533.I1RNM7
45580 229533.I1RFK3 pp 229533.I1RTJ2
45581 229533.I1RFK3 pp 229533.I1RTT0
45582 229533.I1RFK3 pp 229533.I1RJT5
45583 229533.I1RFK3 pp 229533.I1S3G4
45584 229533.I1RFK3 pp 229533.I1RP70
45585 229533.I1RFK3 pp 229533.I1RXB6
45586 229533.I1RFK3 pp 229533.V6QV31
45587 229533.I1RFK3 pp 229533.I1RVW6
45588 229533.I1RFK3 pp 229533.I1RPW2
45589 229533.I1RFK3 pp 229533.I1RZQ1
45590 229533.I1RFK3 pp 229533.I1RZK8
45591 229533.I1RFK3 pp 229533.V6RLM7
45592 229533.I1RFK3 pp 229533.Q4I5G1
45593 229533.I1RFK3 pp 229533.V6RQW4
45594 229533.I1RFK3 pp 229533.I1RMI1
45595 229533.I1RFK3 pp 229533.I1RUI6
45596 229533.I1RFK3 pp 229533.Q4I1N3
45597 229533.I1RFK3 pp 229533.I1RX59
45598 229533.I1RFK3 pp 229533.V6RY56
45599 229533.I1RFK3 pp 229533.V6RDH3
45600 229533.I1RFK3 pp 229533.I1RMW4
45601 229533.A0A0E0RPS5 pp 229533.Q4I7L0
45602 229533.A0A0E0RPS5 pp 229533.Q4IB50
45603 229533.A0A0E0RPS5 pp 229533.I1RBJ0
45604 229533.A0A0E0RPS5 pp 229533.I1S4Z5
45605 229533.A0A0E0RPS5 pp 229533.Q4I8C9
45606 229533.A0A0E0RPS5 pp 229533.A0A1C3YIS1
45607 229533.A0A0E0RPS5 pp 229533.Q4IJP1
45608 229533.A0A0E0RPS5 pp 229533.I1RYV3
45609 229533.A0A0E0RPS5 pp 229533.Q4HTT2
45610 229533.A0A0E0RPS5 pp 229533.Q4I5R3
45611 229533.I1RCA5 pp 229533.I1RFL7
45612 229533.I1RCA5 pp 229533.I1RYQ1
45613 229533.I1RCA5 pp 229533.I1RYX9
45614 229533.I1RCA5 pp 229533.Q4IMD1
45615 229533.I1RCA5 pp 229533.Q4IQM6
45616 229533.I1RCA5 pp 229533.I1RJV8
45617 229533.I1RCA5 pp 229533.I1RQ67
45618 229533.I1RCA5 pp 229533.I1RFZ2
45619 229533.I1RCA5 pp 229533.I1RXB2
45620 229533.I1RCA5 pp 229533.I1S2E9
45621 229533.I1RCA5 pp 229533.I1S2F2
45622 229533.I1RCA5 pp 229533.I1RNF4
45623 229533.I1RCA5 pp 229533.I1RHD2
45624 229533.I1RCA5 pp 229533.I1RY44
45625 229533.I1RCA5 pp 229533.I1RXA9

45626 229533.I1RCA5 pp 229533.I1RSN7
45627 229533.I1RCA5 pp 229533.I1RYR2
45628 229533.I1RCA5 pp 229533.I1RY29
45629 229533.I1RCA5 pp 229533.I1S016
45630 229533.I1RCA5 pp 229533.I1RWI8
45631 229533.I1RCA5 pp 229533.I1S020
45632 229533.I1RCA5 pp 229533.I1RQI6
45633 229533.I1RCA5 pp 229533.I1S4P6
45634 229533.I1RCA5 pp 229533.I1RSZ3
45635 229533.I1RCA5 pp 229533.I1RSC7
45636 229533.I1RCA5 pp 229533.I1RQB4
45637 229533.I1RCA5 pp 229533.V6R382
45638 229533.I1RCA5 pp 229533.I1RF70
45639 229533.I1RCA5 pp 229533.I1RPF5
45640 229533.I1RCA5 pp 229533.I1RQ72
45641 229533.I1RCA5 pp 229533.I1RMS4
45642 229533.I1RCA5 pp 229533.I1S998
45643 229533.I1RCA5 pp 229533.I1RUG4
45644 229533.I1RCA5 pp 229533.Q4IB89
45645 229533.I1RCA5 pp 229533.I1RN57
45646 229533.I1RCA5 pp 229533.I1RG90
45647 229533.I1RCA5 pp 229533.I1S0A6
45648 229533.I1RCA5 pp 229533.I1RSA7
45649 229533.I1RCA5 pp 229533.I1S2E8
45650 229533.I1RCA5 pp 229533.I1RRS7
45651 229533.I1RCA5 pp 229533.I1RDH0
45652 229533.I1RCA5 pp 229533.I1RZ98
45653 229533.I1RCA5 pp 229533.V6RLW4
45654 229533.I1RCA5 pp 229533.I1RDX0
45655 229533.I1RCA5 pp 229533.I1RDJ8
45656 229533.I1RCA5 pp 229533.I1RZ86
45657 229533.I1RCA5 pp 229533.I1RWT6
45658 229533.I1RCA5 pp 229533.Q4IP51
45659 229533.I1RCA5 pp 229533.I1S276
45660 229533.I1RCA5 pp 229533.I1RPM7
45661 229533.I1RCA5 pp 229533.Q4I0J6
45662 229533.I1RCA5 pp 229533.I1S0D5
45663 229533.I1RCA5 pp 229533.I1RSI4
45664 229533.I1RCA5 pp 229533.I1RKE9
45665 229533.I1RCA5 pp 229533.I1RSR9
45666 229533.I1RCA5 pp 229533.I1RE46
45667 229533.I1RCA5 pp 229533.I1RPQ1
45668 229533.I1RCA5 pp 229533.I1RKB1
45669 229533.I1RCA5 pp 229533.I1RMQ7
45670 229533.I1RCA5 pp 229533.I1RHR0
45671 229533.I1RCA5 pp 229533.I1S209
45672 229533.I1RCA5 pp 229533.I1RTN6
45673 229533.I1RCA5 pp 229533.I1RKI2
45674 229533.I1RCA5 pp 229533.I1RX64
45675 229533.I1RCA5 pp 229533.I1RZP8
45676 229533.I1RCA5 pp 229533.I1RMX3
45677 229533.I1RCA5 pp 229533.I1RYR1
45678 229533.I1RCA5 pp 229533.I1RQJ4
45679 229533.I1RCA5 pp 229533.Q4IC11
45680 229533.I1RCA5 pp 229533.I1RQH8
45681 229533.I1RCA5 pp 229533.I1RSP7
45682 229533.I1RCA5 pp 229533.I1RD19
45683 229533.I1RCA5 pp 229533.I1RMB7
45684 229533.I1RCA5 pp 229533.Q4INS6
45685 229533.I1RCA5 pp 229533.I1S0L3
45686 229533.I1RCA5 pp 229533.I1RFX8
45687 229533.I1RCA5 pp 229533.I1S987
45688 229533.I1RCA5 pp 229533.I1S604
45689 229533.I1RCA5 pp 229533.I1RCB3
45690 229533.I1RCA5 pp 229533.I1RZ94
45691 229533.I1RCA5 pp 229533.I1RRT4
45692 229533.I1RCA5 pp 229533.I1RQA3
45693 229533.I1RCA5 pp 229533.I1RN46
45694 229533.I1RCA5 pp 229533.I1S461
45695 229533.I1RCA5 pp 229533.I1RD01
45696 229533.I1RCA5 pp 229533.I1RCL7
45697 229533.I1RCA5 pp 229533.I1RFJ2
45698 229533.I1RCA5 pp 229533.I1RRT1

229533.I1RCA5 pp 229533.I1RQ13
229533.I1RCA5 pp 229533.I1RCQ4
229533.I1RCA5 pp 229533.Q4IBX0
229533.I1RCA5 pp 229533.I1RG77
229533.I1RCA5 pp 229533.I1RUG2
229533.I1RCA5 pp 229533.I1S283
229533.I1RCA5 pp 229533.I1RV66
229533.I1RCA5 pp 229533.I1RCM6
229533.I1RCA5 pp 229533.I1RN55
229533.I1RZ09 pp 229533.I1RZU5
229533.I1RJI3 pp 229533.I1RZB5
229533.I1RJI3 pp 229533.I1RJY3
229533.I1RJI3 pp 229533.I1RJL2
229533.I1RJI3 pp 229533.I1RJM5
229533.I1RJI3 pp 229533.V6QWX8
229533.I1RJI3 pp 229533.I1RJY2
229533.I1RJI3 pp 229533.I1S9U1
229533.I1RJI3 pp 229533.I1RW90
229533.I1RJI3 pp 229533.I1S255
229533.I1RJI3 pp 229533.I1RRN6
229533.I1RJI3 pp 229533.V6QUE6
229533.I1RJI3 pp 229533.I1S9Y1
229533.I1RJI3 pp 229533.I1S268
229533.I1RJI3 pp 229533.I1RRG3
229533.I1RJI3 pp 229533.I1RS30
229533.I1RJI3 pp 229533.I1RYE2
229533.I1RJI3 pp 229533.I1RN17
229533.I1RJI3 pp 229533.I1RK95
229533.I1RJI3 pp 229533.I1RR25
229533.I1RJI3 pp 229533.I1RVY0
229533.I1RJI3 pp 229533.I1RK79
229533.I1S1X2 pp 229533.Q4I7L0
229533.I1S1X2 pp 229533.I1S4Z5
229533.I1S1X2 pp 229533.Q4HTT2
229533.I1RWF4 pp 229533.I1S0D7
229533.I1RWF4 pp 229533.I1S770
229533.I1RWF4 pp 229533.I1RXF0
229533.I1RWF4 pp 229533.I1RYD3
229533.I1RWF4 pp 229533.I1RZ25
229533.I1RWF4 pp 229533.I1RZE7
229533.I1RWF4 pp 229533.I1S2M5
229533.I1RWF4 pp 229533.V6RR38
229533.A0A0E0SHE5 pp 229533.I1RZ09
229533.I1RHA9 pp 229533.I1RTA6
229533.I1R9Z0 pp 229533.I1RBV3
229533.I1R9Z0 pp 229533.I1RSD4
229533.I1R9Z0 pp 229533.I1RK90
229533.I1R9Z0 pp 229533.I1RY59
229533.I1R9Z0 pp 229533.I1RG57
229533.I1R9Z0 pp 229533.I1RS28
229533.I1R9Z0 pp 229533.I1RXF2
229533.I1R9Z0 pp 229533.I1RK92
229533.I1R9Z0 pp 229533.I1S7X9
229533.I1R9Z0 pp 229533.I1RY14
229533.I1R9Z0 pp 229533.V6QXG1
229533.I1R9Z0 pp 229533.I1RSA4
229533.I1R9Z0 pp 229533.I1RA22
229533.I1R9Z0 pp 229533.I1RCS2
229533.I1R9Z0 pp 229533.I1RBD7
229533.I1R9Z0 pp 229533.I1RND5
229533.I1R9Z0 pp 229533.I1RG90
229533.I1R9Z0 pp 229533.I1RJS4
229533.I1R9Z0 pp 229533.I1RJL5
229533.I1R9Z0 pp 229533.I1RBH8
229533.I1R9Z0 pp 229533.I1S4M5
229533.I1R9Z0 pp 229533.I1S1Y5
229533.I1R9Z0 pp 229533.I1RDY1
229533.I1R9Z0 pp 229533.I1RZG7
229533.I1R9Z0 pp 229533.I1RB50
229533.I1R9Z0 pp 229533.I1RWC6
229533.I1R9Z0 pp 229533.I1RX81
229533.I1R9Z0 pp 229533.I1RCI4
229533.I1R9Z0 pp 229533.I1RA36

45772	229533.I1R9Z0	pp	229533.I1S7M3
45773	229533.I1R9Z0	pp	229533.I1S575
45774	229533.I1R9Z0	pp	229533.I1S7M2
45775	229533.I1RQQ0	pp	229533.I1RYQ4
45776	229533.I1RQQ0	pp	229533.I1RTP5
45777	229533.I1RQQ0	pp	229533.I1S4P6
45778	229533.I1RQQ0	pp	229533.I1RTJ2
45779	229533.I1RQQ0	pp	229533.Q4IB89
45780	229533.I1RQQ0	pp	229533.I1RVW0
45781	229533.I1S109	pp	229533.Q4I7L0
45782	229533.I1RN84	pp	229533.I1RNU7
45783	229533.I1RN84	pp	229533.V6QX21
45784	229533.I1RN84	pp	229533.I1RYA0
45785	229533.I1RN84	pp	229533.I1S123
45786	229533.I1RN84	pp	229533.I1S147
45787	229533.I1RN84	pp	229533.I1S8R2
45788	229533.I1RN84	pp	229533.I1S256
45789	229533.I1RN84	pp	229533.I1S0V7
45790	229533.I1RN84	pp	229533.V6RM02
45791	229533.I1RN84	pp	229533.V6R1X4
45792	229533.I1RN84	pp	229533.I1S8R4
45793	229533.I1RN84	pp	229533.V6RRM0
45794	229533.I1RN84	pp	229533.I1S3L8
45795	229533.I1RN84	pp	229533.I1RYC8
45796	229533.I1RN84	pp	229533.I1S9T5
45797	229533.I1RN84	pp	229533.V6R382
45798	229533.I1RN84	pp	229533.I1RXY7
45799	229533.I1RN84	pp	229533.I1RN88
45800	229533.I1RN84	pp	229533.I1RQ38
45801	229533.I1RN84	pp	229533.I1RQG7
45802	229533.I1RN84	pp	229533.I1S3G4
45803	229533.I1RN84	pp	229533.I1RSK5
45804	229533.I1RN84	pp	229533.I1RSQ4
45805	229533.I1RN84	pp	229533.I1S3T7
45806	229533.I1RN84	pp	229533.I1S4Z7
45807	229533.I1RN84	pp	229533.V6RKY8
45808	229533.I1RN84	pp	229533.I1RVR2
45809	229533.I1RN84	pp	229533.I1RUR8
45810	229533.I1RN84	pp	229533.I1RNB4
45811	229533.I1RN84	pp	229533.I1RYB8
45812	229533.I1RN84	pp	229533.I1RNG4
45813	229533.I1RN84	pp	229533.I1RVZ3
45814	229533.I1RN84	pp	229533.I1RY62
45815	229533.I1RN84	pp	229533.I1S673
45816	229533.I1RN84	pp	229533.I1RYK0
45817	229533.I1RN84	pp	229533.I1RY50
45818	229533.I1RN84	pp	229533.I1RUY4
45819	229533.I1RN84	pp	229533.I1RT88
45820	229533.I1RN84	pp	229533.I1RZ90
45821	229533.I1RN84	pp	229533.I1RY12
45822	229533.I1RN84	pp	229533.I1RSS7
45823	229533.I1RN84	pp	229533.I1RZ38
45824	229533.I1RN84	pp	229533.I1RR95
45825	229533.I1RN84	pp	229533.I1RYW9
45826	229533.I1RN84	pp	229533.I1RYA2
45827	229533.I1RN84	pp	229533.I1RWF5
45828	229533.I1RN84	pp	229533.I1S3T6
45829	229533.I1RN84	pp	229533.V6RUY9
45830	229533.I1RN84	pp	229533.I1S299
45831	229533.I1RN84	pp	229533.I1RT75
45832	229533.I1RN84	pp	229533.I1S8S9
45833	229533.I1RN84	pp	229533.I1RYP2
45834	229533.I1RN84	pp	229533.I1RXE6
45835	229533.I1RN84	pp	229533.V6RK96
45836	229533.I1RN84	pp	229533.I1S0V6
45837	229533.I1RN84	pp	229533.I1RPT0
45838	229533.I1RN84	pp	229533.V6RPN0
45839	229533.I1RN84	pp	229533.I1RW55
45840	229533.I1RN84	pp	229533.I1RYL5
45841	229533.I1RN84	pp	229533.I1RNY6
45842	229533.I1RN84	pp	229533.I1RVV7
45843	229533.I1RN84	pp	229533.I1RY21
45844	229533.I1RN84	pp	229533.I1RXS0

229533.I1RN84	pp	229533.I1RUI6
229533.I1RN84	pp	229533.I1RT82
229533.I1RN84	pp	229533.I1S8Z2
229533.I1RN84	pp	229533.I1RSQ2
229533.I1RN84	pp	229533.I1RPR1
229533.I1RN84	pp	229533.I1RYQ4
229533.I1RN84	pp	229533.I1S3L2
229533.I1RN84	pp	229533.I1S5R5
229533.I1RN84	pp	229533.I1RQE8
229533.I1RN84	pp	229533.I1S8F8
229533.I1RN84	pp	229533.I1RQL4
229533.I1RN84	pp	229533.Q4HZS8
229533.I1RN84	pp	229533.I1RNM7
229533.I1RN84	pp	229533.I1S1W8
229533.I1RN84	pp	229533.I1RYU6
229533.I1RN84	pp	229533.I1S034
229533.I1RN84	pp	229533.I1S0K0
229533.I1RN84	pp	229533.I1RQP2
229533.I1RN84	pp	229533.I1RP86
229533.I1RN84	pp	229533.I1S241
229533.I1RN84	pp	229533.I1S223
229533.I1RN84	pp	229533.I1S3J4
229533.I1RN84	pp	229533.I1RZM4
229533.I1RN84	pp	229533.I1RRX5
229533.I1RN84	pp	229533.I1S8C1
229533.I1RN84	pp	229533.Q5I2J3
229533.I1RZ86	pp	229533.V6R382
229533.I1RZ86	pp	229533.I1RZP8
229533.I1RZ86	pp	229533.I1S0A6
229533.I1RZ86	pp	229533.I1S0D5
229533.I1RZ86	pp	229533.I1S4P6
229533.I1RZ86	pp	229533.I1RZT1
229533.I1RZ86	pp	229533.Q4IB89
229533.I1RZ86	pp	229533.I1RZ98
229533.I1RZ86	pp	229533.I1S998
229533.I1RZ86	pp	229533.I1S523
229533.I1RZ86	pp	229533.Q4I7L0
229533.I1RZ86	pp	229533.I1S3G4
229533.I1RZ86	pp	229533.Q4INS6
229533.I1RZ86	pp	229533.Q4IBX0
229533.I1RZ86	pp	229533.I1S283
229533.I1RZ86	pp	229533.Q4IR08
229533.I1RZ86	pp	229533.Q4I0J6
229533.I1RZ86	pp	229533.I1S209
229533.I1RZ86	pp	229533.I1RZ94
229533.I1RZ86	pp	229533.I1S987
229533.I1RZ86	pp	229533.I1S5Z3
229533.I1RZ86	pp	229533.I1S1W8
229533.I1RDK1	pp	229533.I1RWI6
229533.I1RDK1	pp	229533.I1RPE5
229533.I1RN17	pp	229533.V6QWX8
229533.I1RN17	pp	229533.I1RRG3
229533.I1RN17	pp	229533.I1S214
229533.I1RN17	pp	229533.I1RVY0
229533.I1RN17	pp	229533.I1S9Y1
229533.I1RN17	pp	229533.I1RW90
229533.I1RN17	pp	229533.I1RRG4
229533.I1RN17	pp	229533.Q4IPN9
229533.I1RN17	pp	229533.I1RRN6
229533.I1RN17	pp	229533.I1S255
229533.I1RN17	pp	229533.V6QUE6
229533.I1RN17	pp	229533.I1S9U1
229533.I1RN17	pp	229533.I1RR25
229533.I1RN17	pp	229533.I1RZB5
229533.I1RN17	pp	229533.V6RFX5
229533.A0A098DP54	pp	229533.I1RVY0
229533.A0A098DP54	pp	229533.A0A0E0SHE3
229533.A0A098DP54	pp	229533.I1RRN6
229533.A0A098DP54	pp	229533.I1RR78
229533.A0A098DP54	pp	229533.V6RFX5
229533.A0A098DP54	pp	229533.I1RJM5
229533.A0A098DP54	pp	229533.A0A098DVJ5
229533.A0A098DP54	pp	229533.I1RJI3

45918	229533.A0A098DP54	pp	229533.I1R9Z0
45919	229533.A0A098DP54	pp	229533.I1RB17
45920	229533.A0A098DP54	pp	229533.I1RN17
45921	229533.A0A098DWB8	pp	229533.I1RW28
45922	229533.A0A098DWB8	pp	229533.A0A0E0RNL0
45923	229533.A0A098DWB8	pp	229533.I1RPF4
45924	229533.A0A098DWB8	pp	229533.I1RPR8
45925	229533.A0A098DWB8	pp	229533.Q4IEV4
45926	229533.A0A098DWB8	pp	229533.A0A1C3YHH5
45927	229533.A0A098DWB8	pp	229533.I1S1X2
45928	229533.A0A098DWB8	pp	229533.I1RQ19
45929	229533.A0A098DWB8	pp	229533.I1S109
45930	229533.A0A098DWB8	pp	229533.A0A1C3YHV5
45931	229533.A0A098DWB8	pp	229533.A0A1C3YMF9
45932	229533.I1RT11	pp	229533.I1RW36
45933	229533.I1RT11	pp	229533.I1RZG7
45934	229533.I1RG46	pp	229533.I1RMJ3
45935	229533.I1RG46	pp	229533.I1RVR2
45936	229533.I1RG46	pp	229533.I1RMK7
45937	229533.I1RG46	pp	229533.I1RPY0
45938	229533.I1RG46	pp	229533.I1RN84
45939	229533.I1S5L1	pp	229533.I1S7K6
45940	229533.I1RWK9	pp	229533.I1RZK1
45941	229533.I1RWK9	pp	229533.Q4I7G0
45942	229533.I1RWK9	pp	229533.I1S0F9
45943	229533.I1RWK9	pp	229533.I1RZS8
45944	229533.I1RWK9	pp	229533.I1RZD3
45945	229533.I1RWK9	pp	229533.I1S3S5
45946	229533.I1RWK9	pp	229533.I1RYL5
45947	229533.I1RWK9	pp	229533.I1S0Z8
45948	229533.I1RWK9	pp	229533.I1S821
45949	229533.I1RA65	pp	229533.I1RQP3
45950	229533.I1RA65	pp	229533.I1RRF7
45951	229533.I1RA65	pp	229533.I1RKR8
45952	229533.I1RA65	pp	229533.I1RCG6
45953	229533.I1RA65	pp	229533.Q4ICA8
45954	229533.I1RA65	pp	229533.I1RN04
45955	229533.I1RA65	pp	229533.I1RW80
45956	229533.I1RA65	pp	229533.V6QWX8
45957	229533.I1RA65	pp	229533.I1RQP1
45958	229533.I1RA65	pp	229533.I1RRF0
45959	229533.I1RA65	pp	229533.I1RW91
45960	229533.I1RA65	pp	229533.I1RKF3
45961	229533.I1RA65	pp	229533.I1RPW8
45962	229533.I1RA65	pp	229533.I1S9H8
45963	229533.I1RA65	pp	229533.I1RQ42
45964	229533.I1RA65	pp	229533.Q4IPI4
45965	229533.I1RA65	pp	229533.I1RR61
45966	229533.I1RA65	pp	229533.I1S5Z5
45967	229533.I1RRC3	pp	229533.I1S7W2
45968	229533.I1RRC3	pp	229533.I1RT41
45969	229533.I1RRC3	pp	229533.I1S0Z3
45970	229533.I1RRC3	pp	229533.I1RWI6
45971	229533.I1RRC3	pp	229533.Q4I963
45972	229533.I1RRC3	pp	229533.I1RZK3
45973	229533.A0A1C3YIU4	pp	229533.I1RKK6
45974	229533.A0A1C3YIU4	pp	229533.A0A1C3YKJ7
45975	229533.A0A1C3YIU4	pp	229533.A0A1C3YMH5
45976	229533.A0A1C3YIU4	pp	229533.I1RJS4
45977	229533.I1RDJ9	pp	229533.I1RY11
45978	229533.I1RDJ9	pp	229533.I1RZR5
45979	229533.I1RDJ9	pp	229533.I1RT96
45980	229533.I1RDJ9	pp	229533.V6RLW2
45981	229533.I1RA40	pp	229533.I1RNJ2
45982	229533.I1RA40	pp	229533.I1RWI8
45983	229533.I1RA40	pp	229533.I1S0L8
45984	229533.I1RA40	pp	229533.I1RVU7
45985	229533.I1RA40	pp	229533.I1RPS0
45986	229533.I1RA40	pp	229533.Q4INS6
45987	229533.I1RA40	pp	229533.Q4I948
45988	229533.I1RA40	pp	229533.Q4ICA8
45989	229533.I1RA40	pp	229533.Q4IB89
45990	229533.I1RA40	pp	229533.I1RD18

45991 229533.I1RA40 pp 229533.I1RN72
45992 229533.I1RA40 pp 229533.I1RJU5
45993 229533.I1RP70 pp 229533.I1S0C2
45994 229533.I1RP70 pp 229533.V6RDH3
45995 229533.I1RP70 pp 229533.V6QV31
45996 229533.I1RP70 pp 229533.Q4I1N3
45997 229533.I1RP70 pp 229533.V6RS91
45998 229533.I1RP70 pp 229533.I1RXY7
45999 229533.I1RLW5 pp 229533.V6RKY8
46000 229533.I1RLW5 pp 229533.I1RR45
46001 229533.I1RLW5 pp 229533.I1RRK5
46002 229533.I1RLW5 pp 229533.I1RT75
46003 229533.I1RLW5 pp 229533.I1RSF1
46004 229533.I1RLW5 pp 229533.I1S8Z2
46005 229533.I1RLW5 pp 229533.I1RN52
46006 229533.A0A0E0S1J9 pp 229533.I1RFN0
46007 229533.A0A0E0S1J9 pp 229533.I1RC54
46008 229533.A0A0E0S1J9 pp 229533.I1RBC8
46009 229533.A0A0E0S1J9 pp 229533.I1RRS7
46010 229533.A0A0E0S1J9 pp 229533.I1RYR2
46011 229533.A0A0E0S1J9 pp 229533.I1RFZ2
46012 229533.A0A0E0S1J9 pp 229533.Q4IMM0
46013 229533.A0A0E0S1J9 pp 229533.I1S604
46014 229533.A0A0E0S1J9 pp 229533.I1RYX9
46015 229533.A0A0E0S1J9 pp 229533.I1RB49
46016 229533.A0A0E0S1J9 pp 229533.I1RSI4
46017 229533.A0A0E0S1J9 pp 229533.I1RY73
46018 229533.A0A0E0S1J9 pp 229533.I1RSC7
46019 229533.A0A0E0S1J9 pp 229533.I1S461
46020 229533.A0A0E0S1J9 pp 229533.I1RYQ1
46021 229533.A0A0E0S1J9 pp 229533.A0A1C3YKA6
46022 229533.A0A0E0S1J9 pp 229533.I1RPQ1
46023 229533.A0A0E0S1J9 pp 229533.I1RA12
46024 229533.A0A0E0S1J9 pp 229533.I1RFJ2
46025 229533.A0A0E0S1J9 pp 229533.I1RE46
46026 229533.A0A0E0S1J9 pp 229533.A0A1C3YJ79
46027 229533.A0A0E0S1J9 pp 229533.I1RUG2
46028 229533.A0A0E0S1J9 pp 229533.I1RW16
46029 229533.A0A0E0S1J9 pp 229533.I1RCL7
46030 229533.A0A0E0S1J9 pp 229533.I1R9W7
46031 229533.A0A0E0S1J9 pp 229533.I1RHM4
46032 229533.A0A0E0S1J9 pp 229533.I1RJV8
46033 229533.A0A0E0S1J9 pp 229533.I1RD70
46034 229533.A0A0E0S1J9 pp 229533.I1RMJ8
46035 229533.A0A0E0S1J9 pp 229533.I1RSR9
46036 229533.A0A0E0S1J9 pp 229533.I1RB84
46037 229533.A0A0E0S1J9 pp 229533.I1RLX6
46038 229533.A0A0E0S1J9 pp 229533.Q4IB89
46039 229533.A0A0E0S1J9 pp 229533.I1RN31
46040 229533.A0A0E0S1J9 pp 229533.I1RFX8
46041 229533.A0A0E0S1J9 pp 229533.I1RBN9
46042 229533.A0A0E0S1J9 pp 229533.I1RSK0
46043 229533.A0A0E0S1J9 pp 229533.A0A1C3YHN3
46044 229533.A0A0E0S1J9 pp 229533.V6RLW4
46045 229533.A0A0E0S1J9 pp 229533.I1RZ86
46046 229533.A0A0E0S1J9 pp 229533.I1RAH4
46047 229533.A0A0E0S1J9 pp 229533.I1RC35
46048 229533.A0A0E0S1J9 pp 229533.I1RZ94
46049 229533.A0A0E0S1J9 pp 229533.I1RAN3
46050 229533.A0A0E0S1J9 pp 229533.I1RQ13
46051 229533.A0A0E0S1J9 pp 229533.I1RRT1
46052 229533.A0A0E0S1J9 pp 229533.I1RCA5
46053 229533.A0A0E0S1J9 pp 229533.I1RD01
46054 229533.A0A0E0S1J9 pp 229533.A0A1C3YI10
46055 229533.A0A0E0S1J9 pp 229533.I1RWG6
46056 229533.A0A1C3YJC4 pp 229533.V6RN69
46057 229533.A0A1C3YJC4 pp 229533.I1RPU2
46058 229533.A0A1C3YJC4 pp 229533.I1RTP2
46059 229533.A0A1C3YJC4 pp 229533.I1S1W9
46060 229533.A0A1C3YJC4 pp 229533.I1RAW0
46061 229533.A0A1C3YJC4 pp 229533.I1RHU8
46062 229533.A0A1C3YJC4 pp 229533.A0A1C3YJI6
46063 229533.A0A1C3YJC4 pp 229533.I1RQ33

46064 229533.A0A1C3YJC4 pp 229533.I1RAJ0
46065 229533.A0A1C3YJC4 pp 229533.I1RYP2
46066 229533.A0A1C3YJC4 pp 229533.I1RVZ2
46067 229533.A0A1C3YJC4 pp 229533.I1RYI5
46068 229533.A0A1C3YJC4 pp 229533.I1RTA1
46069 229533.A0A1C3YJC4 pp 229533.I1RW82
46070 229533.A0A1C3YJC4 pp 229533.I1RRI1
46071 229533.A0A1C3YJC4 pp 229533.I1RQM9
46072 229533.A0A1C3YJC4 pp 229533.I1S5R1
46073 229533.A0A1C3YJC4 pp 229533.I1S2B8
46074 229533.A0A1C3YJC4 pp 229533.I1RCT9
46075 229533.A0A1C3YJC4 pp 229533.V6RCZ8
46076 229533.A0A1C3YJC4 pp 229533.I1RRG7
46077 229533.A0A1C3YJC4 pp 229533.I1RFN8
46078 229533.A0A1C3YJC4 pp 229533.I1RXD7
46079 229533.A0A1C3YJC4 pp 229533.I1RED2
46080 229533.A0A1C3YJC4 pp 229533.I1S4S7
46081 229533.A0A1C3YJC4 pp 229533.I1RD52
46082 229533.A0A1C3YJC4 pp 229533.I1RJU3
46083 229533.I1RCQ4 pp 229533.I1RN57
46084 229533.I1RCQ4 pp 229533.I1RY29
46085 229533.I1RCQ4 pp 229533.I1RSR9
46086 229533.I1RCQ4 pp 229533.Q4IB89
46087 229533.I1RCQ4 pp 229533.I1RWI8
46088 229533.I1RCQ4 pp 229533.I1RZ86
46089 229533.I1RCQ4 pp 229533.I1RYQ1
46090 229533.I1RCQ4 pp 229533.I1RT74
46091 229533.I1RCQ4 pp 229533.I1RSC7
46092 229533.I1RCQ4 pp 229533.I1S998
46093 229533.I1RCQ4 pp 229533.I1RUF8
46094 229533.I1RCQ4 pp 229533.I1RZ98
46095 229533.I1RCQ4 pp 229533.I1RDJ8
46096 229533.I1RCQ4 pp 229533.I1RSA7
46097 229533.I1RCQ4 pp 229533.I1RDH0
46098 229533.I1RCQ4 pp 229533.I1RX64
46099 229533.I1RCQ4 pp 229533.V6RLW4
46100 229533.I1RCQ4 pp 229533.I1RD01
46101 229533.I1RCQ4 pp 229533.I1RQI6
46102 229533.I1RCQ4 pp 229533.I1RQJ4
46103 229533.I1RCQ4 pp 229533.I1RDC0
46104 229533.I1RCQ4 pp 229533.I1S0D5
46105 229533.I1RCQ4 pp 229533.I1RSP7
46106 229533.I1RCQ4 pp 229533.I1RZ94
46107 229533.I1RCQ4 pp 229533.I1RRT1
46108 229533.I1RCQ4 pp 229533.I1RFX8
46109 229533.I1RCQ4 pp 229533.I1RUR0
46110 229533.I1RCQ4 pp 229533.I1RKI2
46111 229533.I1RCQ4 pp 229533.I1S283
46112 229533.I1RCQ4 pp 229533.I1S987
46113 229533.I1RCQ4 pp 229533.I1RE46
46114 229533.I1RCQ4 pp 229533.I1RPZ0
46115 229533.I1RCQ4 pp 229533.I1RSJ1
46116 229533.I1RCQ4 pp 229533.I1RWM6
46117 229533.I1RCQ4 pp 229533.Q4INS6
46118 229533.I1RCQ4 pp 229533.I1S209
46119 229533.I1RCQ4 pp 229533.I1RE55
46120 229533.I1RCQ4 pp 229533.I1RE37
46121 229533.I1RCQ4 pp 229533.I1RN55
46122 229533.I1RCQ4 pp 229533.I1RWB3
46123 229533.I1RCQ4 pp 229533.I1RFJ2
46124 229533.I1RCY6 pp 229533.I1RMH6
46125 229533.I1RCY6 pp 229533.I1RDY1
46126 229533.I1RCY6 pp 229533.I1RJS4
46127 229533.I1RCY6 pp 229533.I1RFN2
46128 229533.A0A1C3YLS4 pp 229533.I1RFT1
46129 229533.A0A1C3YLS4 pp 229533.Q4I7T5
46130 229533.A0A1C3YLS4 pp 229533.I1RN63
46131 229533.A0A1C3YLS4 pp 229533.I1RPK5
46132 229533.A0A1C3YLS4 pp 229533.I1S2M6
46133 229533.A0A1C3YLS4 pp 229533.I1RQS8
46134 229533.A0A1C3YLS4 pp 229533.I1RSR3
46135 229533.A0A1C3YLS4 pp 229533.I1RGC6
46136 229533.A0A1C3YLS4 pp 229533.I1RAB6

46137	229533.A0A1C3YLS4	pp	229533.I1RJ74
46138	229533.A0A1C3YLS4	pp	229533.I1RRZ0
46139	229533.A0A1C3YLS4	pp	229533.I1RFH8
46140	229533.A0A1C3YLS4	pp	229533.I1RW60
46141	229533.A0A1C3YLS4	pp	229533.I1RQ31
46142	229533.A0A1C3YLS4	pp	229533.I1RZD3
46143	229533.A0A1C3YLS4	pp	229533.I1RS47
46144	229533.A0A1C3YLS4	pp	229533.I1RME4
46145	229533.A0A1C3YLS4	pp	229533.I1RPV0
46146	229533.A0A1C3YLS4	pp	229533.I1RT62
46147	229533.A0A1C3YLS4	pp	229533.I1RBC4
46148	229533.A0A0E0SH57	pp	229533.I1RST9
46149	229533.A0A0E0SH57	pp	229533.I1RS29
46150	229533.A0A0E0SH57	pp	229533.I1S8D3
46151	229533.A0A0E0SH57	pp	229533.V6RWB3
46152	229533.A0A0E0SH57	pp	229533.I1RYM5
46153	229533.A0A0E0SH57	pp	229533.I1RP02
46154	229533.A0A0E0SH57	pp	229533.I1S8S9
46155	229533.A0A0E0SH57	pp	229533.I1RPM6
46156	229533.A0A0E0SH57	pp	229533.V6QU65
46157	229533.A0A0E0SH57	pp	229533.I1S250
46158	229533.A0A0E0SH57	pp	229533.I1RXE6
46159	229533.A0A0E0SH57	pp	229533.I1S8F8
46160	229533.A0A0E0SH57	pp	229533.I1RNM7
46161	229533.A0A0E0SH57	pp	229533.A0A1C3YMG4
46162	229533.A0A0E0SH57	pp	229533.A0A1C3YJH4
46163	229533.A0A0E0SH57	pp	229533.V6RK56
46164	229533.A0A0E0SH57	pp	229533.I1RCN8
46165	229533.A0A0E0SH57	pp	229533.A0A1C3YK58
46166	229533.A0A0E0SH57	pp	229533.I1S1W8
46167	229533.A0A0E0SH57	pp	229533.A0A1C3YIJ9
46168	229533.A0A0E0SH57	pp	229533.I1S3B2
46169	229533.A0A0E0SH57	pp	229533.I1RW54
46170	229533.A0A0E0SH57	pp	229533.I1RG62
46171	229533.A0A0E0SH57	pp	229533.I1RVI8
46172	229533.A0A0E0SH57	pp	229533.I1RYJ6
46173	229533.A0A0E0SH57	pp	229533.I1RHL9
46174	229533.A0A0E0SH57	pp	229533.I1RS05
46175	229533.A0A0E0SH57	pp	229533.A0A1C3YN26
46176	229533.A0A0E0SH57	pp	229533.I1RWL3
46177	229533.A0A0E0SH57	pp	229533.I1RMQ6
46178	229533.A0A0E0SH57	pp	229533.V6RH92
46179	229533.A0A0E0SH57	pp	229533.I1RD42
46180	229533.A0A0E0SH57	pp	229533.I1RRG1
46181	229533.A0A0E0SH57	pp	229533.I1RDU5
46182	229533.A0A0E0SH57	pp	229533.I1RY16
46183	229533.A0A0E0SH57	pp	229533.I1RYD3
46184	229533.A0A0E0SH57	pp	229533.I1S2C4
46185	229533.A0A0E0SH57	pp	229533.I1RF42
46186	229533.A0A0E0SH57	pp	229533.I1RHU0
46187	229533.A0A0E0SH57	pp	229533.I1RF16
46188	229533.A0A0E0SH57	pp	229533.I1RBT7
46189	229533.A0A0E0SH57	pp	229533.I1RMS5
46190	229533.A0A0E0SH57	pp	229533.I1RCW1
46191	229533.A0A0E0SH57	pp	229533.I1RKL0
46192	229533.A0A0E0SH57	pp	229533.I1RYX7
46193	229533.A0A0E0SH57	pp	229533.I1RB38
46194	229533.A0A0E0SH57	pp	229533.I1RI91
46195	229533.A0A0E0SH57	pp	229533.A0A1C3YJL9
46196	229533.A0A0E0SH57	pp	229533.I1RBA4
46197	229533.A0A0E0SH57	pp	229533.A0A1C3YKY9
46198	229533.A0A0E0SH57	pp	229533.I1RE79
46199	229533.A0A0E0SH57	pp	229533.A0A1C3YKG0
46200	229533.A0A0E0SH57	pp	229533.A0A1C3YJU7
46201	229533.A0A0E0SH57	pp	229533.V6RLM7
46202	229533.A0A0E0SH57	pp	229533.A0A1C3YN83
46203	229533.A0A0E0SH57	pp	229533.A0A1C3YJC7
46204	229533.A0A0E0SH57	pp	229533.A0A1C3YJM7
46205	229533.A0A0E0SH57	pp	229533.I1RCG5
46206	229533.A0A0E0SH57	pp	229533.I1RNC1
46207	229533.A0A0E0SH57	pp	229533.I1RUQ7
46208	229533.A0A0E0SH57	pp	229533.A0A1C3YL46
46209	229533.A0A0E0SH57	pp	229533.I1RWS4

46210 229533.A0A0E0SH57 pp 229533.I1S0S5
46211 229533.A0A0E0SH57 pp 229533.A0A1C3YJA1
46212 229533.A0A0E0SH57 pp 229533.I1RPY0
46213 229533.A0A0E0SH57 pp 229533.I1RWA3
46214 229533.A0A0E0SH57 pp 229533.I1RZ38
46215 229533.A0A0E0SH57 pp 229533.I1S694
46216 229533.A0A0E0SH57 pp 229533.I1RMJ3
46217 229533.A0A0E0SH57 pp 229533.I1RF80
46218 229533.A0A0E0SH57 pp 229533.I1RXY9
46219 229533.A0A0E0SH57 pp 229533.I1S2P8
46220 229533.A0A0E0SH57 pp 229533.I1RI11
46221 229533.A0A0E0SH57 pp 229533.V6RHD0
46222 229533.A0A0E0SH57 pp 229533.V6RXR0
46223 229533.A0A0E0SH57 pp 229533.I1S3G4
46224 229533.A0A0E0SH57 pp 229533.I1RA52
46225 229533.A0A0E0SH57 pp 229533.I1RKP1
46226 229533.A0A0E0SH57 pp 229533.I1RNG4
46227 229533.A0A0E0SH57 pp 229533.I1RXD9
46228 229533.A0A0E0SH57 pp 229533.I1RC31
46229 229533.A0A0E0SH57 pp 229533.I1RLZ4
46230 229533.A0A0E0SH57 pp 229533.I1RVR7
46231 229533.A0A0E0SH57 pp 229533.I1RZA8
46232 229533.A0A0E0SH57 pp 229533.A0A1C3YJ21
46233 229533.A0A0E0SH57 pp 229533.I1RZV5
46234 229533.I1S016 pp 229533.Q4IJK2
46235 229533.I1S016 pp 229533.V6RCZ8
46236 229533.I1S016 pp 229533.I1S4S7
46237 229533.I1S016 pp 229533.I1S5B0
46238 229533.I1S016 pp 229533.I1S549
46239 229533.I1S016 pp 229533.I1S2B8
46240 229533.I1S016 pp 229533.I1S5R1
46241 229533.I1S016 pp 229533.V6RTJ8
46242 229533.A0A1C3YKY5 pp 229533.A0A1C3YM61
46243 229533.A0A1C3YKY5 pp 229533.I1R9U3
46244 229533.A0A1C3YKY5 pp 229533.I1RTA6
46245 229533.I1RQ21 pp 229533.I1S821
46246 229533.I1RQ21 pp 229533.I1RQR6
46247 229533.I1RQ21 pp 229533.I1RUQ3
46248 229533.I1RQ21 pp 229533.I1RWH4
46249 229533.I1RQ21 pp 229533.I1RYL5
46250 229533.I1RQ21 pp 229533.I1S3S5
46251 229533.I1RQ21 pp 229533.I1RZD3
46252 229533.I1RQ21 pp 229533.V6REZ4
46253 229533.I1RQ21 pp 229533.I1RY40
46254 229533.I1RQ21 pp 229533.I1S2A1
46255 229533.I1RQ21 pp 229533.Q4I7G0
46256 229533.I1RQ21 pp 229533.I1RTU8
46257 229533.I1RQ21 pp 229533.I1S247
46258 229533.I1RQ21 pp 229533.I1S0R9
46259 229533.I1RQ21 pp 229533.I1RZK1
46260 229533.I1RQ21 pp 229533.Q4I099
46261 229533.I1RQ21 pp 229533.I1RW54
46262 229533.I1RQ21 pp 229533.I1RQG4
46263 229533.I1RQ21 pp 229533.I1RZV9
46264 229533.I1RQ21 pp 229533.I1RT38
46265 229533.I1RQ21 pp 229533.Q4I7T5
46266 229533.I1RQ21 pp 229533.I1RYT7
46267 229533.I1RQ21 pp 229533.I1S0F9
46268 229533.I1RQ21 pp 229533.I1RQA5
46269 229533.I1RQ21 pp 229533.I1S0P4
46270 229533.I1RQ21 pp 229533.I1RT62
46271 229533.I1RQ21 pp 229533.I1RRT6
46272 229533.I1RQ21 pp 229533.I1RW71
46273 229533.I1RQ21 pp 229533.I1RSQ4
46274 229533.I1RQ21 pp 229533.I1RZS8
46275 229533.I1RQ21 pp 229533.I1RQL4
46276 229533.I1RQ21 pp 229533.I1RRU9
46277 229533.I1RQ21 pp 229533.V6R7V8
46278 229533.I1RQ21 pp 229533.I1RRS8
46279 229533.I1RQ21 pp 229533.I1S0Z8
46280 229533.I1RQ21 pp 229533.I1RSV9
46281 229533.I1RQ21 pp 229533.I1RQH1
46282 229533.I1RQ21 pp 229533.I1RRF8

46283 229533.I1RQ21 pp 229533.Q4I375
46284 229533.I1RQ21 pp 229533.V6QUV7
46285 229533.I1RQ21 pp 229533.I1RZ95
46286 229533.I1RQ21 pp 229533.I1RU27
46287 229533.I1RQ21 pp 229533.I1RRM9
46288 229533.I1RQ21 pp 229533.V6R3J1
46289 229533.I1RQ21 pp 229533.I1RZS4
46290 229533.A0A098DDS8 pp 229533.A0A1C3YIH7
46291 229533.A0A098DDS8 pp 229533.A0A1C3YJG7
46292 229533.A0A098DDS8 pp 229533.I1RFL7
46293 229533.A0A098DDS8 pp 229533.I1REG5
46294 229533.I1RSF1 pp 229533.I1RVZ2
46295 229533.I1RSF1 pp 229533.I1RXC2
46296 229533.I1RSF1 pp 229533.I1RTA1
46297 229533.I1RSF1 pp 229533.I1S3T7
46298 229533.I1RSF1 pp 229533.I1S5R1
46299 229533.I1RSF1 pp 229533.I1RYI5
46300 229533.I1RSF1 pp 229533.I1S2B8
46301 229533.I1RSF1 pp 229533.V6RKY8
46302 229533.I1RSF1 pp 229533.I1RZ39
46303 229533.I1RSF1 pp 229533.Q4HVQ9
46304 229533.I1RSF1 pp 229533.I1RT40
46305 229533.I1RSF1 pp 229533.I1RSZ7
46306 229533.I1RSF1 pp 229533.I1S0W4
46307 229533.I1RSF1 pp 229533.I1RTD5
46308 229533.I1RSF1 pp 229533.I1S5R6
46309 229533.I1RSF1 pp 229533.I1RUG3
46310 229533.I1RSF1 pp 229533.I1RUF7
46311 229533.I1RSF1 pp 229533.I1RVX0
46312 229533.I1RSF1 pp 229533.I1RVQ3
46313 229533.I1RSF1 pp 229533.V6RMW6
46314 229533.I1RSF1 pp 229533.I1S5H2
46315 229533.I1RSF1 pp 229533.I1S437
46316 229533.I1RSF1 pp 229533.I1S1W9
46317 229533.I1RSF1 pp 229533.I1RSQ3
46318 229533.I1RSF1 pp 229533.I1RSU1
46319 229533.I1RSF1 pp 229533.V6R042
46320 229533.I1RSF1 pp 229533.I1S6D7
46321 229533.I1RSF1 pp 229533.I1RZV3
46322 229533.I1RSF1 pp 229533.I1RXD6
46323 229533.I1RSF1 pp 229533.V6RLM7
46324 229533.I1RSF1 pp 229533.I1S9I5
46325 229533.I1RSF1 pp 229533.I1RX95
46326 229533.I1RSF1 pp 229533.I1RX46
46327 229533.I1RSF1 pp 229533.I1RYU3
46328 229533.I1RSF1 pp 229533.Q4IJK2
46329 229533.I1RSF1 pp 229533.I1S438
46330 229533.I1RSF1 pp 229533.I1S4S7
46331 229533.I1RSF1 pp 229533.I1S1B2
46332 229533.I1RSF1 pp 229533.V6RTJ8
46333 229533.I1RSF1 pp 229533.I1S5B0
46334 229533.I1RSF1 pp 229533.I1RUQ3
46335 229533.I1RSF1 pp 229533.I1RXD7
46336 229533.I1RSF1 pp 229533.I1RUY5
46337 229533.I1RSF1 pp 229533.I1RTB3
46338 229533.I1RSF1 pp 229533.V6R9Z0
46339 229533.I1RSF1 pp 229533.I1RWR7
46340 229533.I1RSF1 pp 229533.I1RZK5
46341 229533.I1RSF1 pp 229533.I1S821
46342 229533.I1RSF1 pp 229533.I1RXC4
46343 229533.I1RSF1 pp 229533.I1RZM8
46344 229533.I1RSF1 pp 229533.V6RCZ8
46345 229533.I1RRG7 pp 229533.I1RTP2
46346 229533.I1RY11 pp 229533.I1S2I9
46347 229533.I1RY11 pp 229533.I1RZR5
46348 229533.I1RY11 pp 229533.I1S8H1
46349 229533.I1RY11 pp 229533.I1S8M3
46350 229533.I1RY11 pp 229533.I1SAF8
46351 229533.I1RY11 pp 229533.I1RYW0
46352 229533.I1RY11 pp 229533.I1RZN8
46353 229533.I1RY11 pp 229533.V6QT89
46354 229533.I1RY11 pp 229533.I1RZJ3
46355 229533.I1RY11 pp 229533.I1SAV2

46356 229533.I1RY11 pp 229533.Q4IJW4
46357 229533.I1RY11 pp 229533.I1S1K6
46358 229533.I1RY11 pp 229533.Q4I5M4
46359 229533.A0A098DDK9 pp 229533.A0A0E0SP71
46360 229533.A0A098DDK9 pp 229533.I1RY11
46361 229533.A0A098DP43 pp 229533.I1RA52
46362 229533.A0A098DP43 pp 229533.A0A0E0SAR4
46363 229533.A0A098DP43 pp 229533.I1RY21
46364 229533.A0A098DP43 pp 229533.I1RW71
46365 229533.A0A098DP43 pp 229533.I1S5C6
46366 229533.A0A098DP43 pp 229533.I1S464
46367 229533.A0A098DP43 pp 229533.I1RXD9
46368 229533.A0A098DP43 pp 229533.V6RJP8
46369 229533.A0A098DP43 pp 229533.I1RS05
46370 229533.A0A098DP43 pp 229533.A0A1C3YL46
46371 229533.A0A098DP43 pp 229533.A0A0E0RPV7
46372 229533.A0A098DP43 pp 229533.I1S0K2
46373 229533.A0A098DP43 pp 229533.I1RMK7
46374 229533.A0A098DP43 pp 229533.I1S5X9
46375 229533.A0A098DP43 pp 229533.I1RWA3
46376 229533.A0A098DP43 pp 229533.I1RS29
46377 229533.A0A098DP43 pp 229533.I1RYT8
46378 229533.A0A098DP43 pp 229533.I1S8S9
46379 229533.A0A098DP43 pp 229533.I1RFJ6
46380 229533.A0A1C3YJT3 pp 229533.I1RTA6
46381 229533.A0A1C3YJT3 pp 229533.A0A1C3YM61
46382 229533.A0A1C3YJF9 pp 229533.I1RYD3
46383 229533.I1RC43 pp 229533.I1RTA6
46384 229533.I1RW68 pp 229533.I1RZH6
46385 229533.I1RW68 pp 229533.Q4I8X0
46386 229533.I1RW68 pp 229533.I1S4D0
46387 229533.I1RW68 pp 229533.I1RY63
46388 229533.I1RW68 pp 229533.Q4IER0
46389 229533.I1RW68 pp 229533.I1S274
46390 229533.I1RW68 pp 229533.V6QWZ7
46391 229533.I1RMH4 pp 229533.I1RTJ2
46392 229533.I1RMH4 pp 229533.I1S267
46393 229533.I1RMH4 pp 229533.I1RXX1
46394 229533.I1RMH4 pp 229533.I1RW54
46395 229533.I1RMH4 pp 229533.I1RST9
46396 229533.I1RMH4 pp 229533.I1RPI4
46397 229533.I1RMH4 pp 229533.I1RQ76
46398 229533.I1RMH4 pp 229533.I1RRS8
46399 229533.I1RMH4 pp 229533.I1S1Q3
46400 229533.I1RMH4 pp 229533.I1S0Y2
46401 229533.I1RMH4 pp 229533.V6RR38
46402 229533.I1RMH4 pp 229533.I1SAS4
46403 229533.I1RMH4 pp 229533.I1RSX9
46404 229533.I1RMH4 pp 229533.I1RUP5
46405 229533.I1RMH4 pp 229533.I1RYD3
46406 229533.I1RMH4 pp 229533.I1RZJ8
46407 229533.I1RMH4 pp 229533.I1SAS3
46408 229533.I1RMH4 pp 229533.I1S5S8
46409 229533.I1RMH4 pp 229533.I1RQ48
46410 229533.I1RMH4 pp 229533.I1S2A8
46411 229533.I1RMH4 pp 229533.I1S9U6
46412 229533.I1RMH4 pp 229533.V6RVJ3
46413 229533.I1RMH4 pp 229533.I1RZV5
46414 229533.I1RF06 pp 229533.I1RZ25
46415 229533.I1RCX1 pp 229533.I1RTA6
46416 229533.I1RIM3 pp 229533.I1RUP7
46417 229533.I1RIM3 pp 229533.I1RVY0
46418 229533.I1RKP5 pp 229533.I1RXB6
46419 229533.I1RKP5 pp 229533.I1S0H2
46420 229533.I1RKP5 pp 229533.I1RYD3
46421 229533.I1R9V4 pp 229533.I1RK50
46422 229533.I1R9V4 pp 229533.I1RQB7
46423 229533.I1R9V4 pp 229533.I1RSB5
46424 229533.I1R9V4 pp 229533.I1RXT8
46425 229533.I1R9U0 pp 229533.I1RTA6
46426 229533.I1RFI8 pp 229533.I1RYP6
46427 229533.I1RFI8 pp 229533.I1RFK3
46428 229533.I1RFI8 pp 229533.I1S3G4

46429	229533.I1RFI8	pp	229533.V6RIV7
46430	229533.I1RFI8	pp	229533.I1RP70
46431	229533.I1RFI8	pp	229533.I1RMD6
46432	229533.I1RFI8	pp	229533.I1RZU2
46433	229533.I1RFI8	pp	229533.I1RGT3
46434	229533.I1RFI8	pp	229533.I1RNG4
46435	229533.I1RFI8	pp	229533.I1RTJ2
46436	229533.I1RFI8	pp	229533.I1RVW6
46437	229533.I1RFI8	pp	229533.I1S0I3
46438	229533.I1RFI8	pp	229533.V6QXN0
46439	229533.I1RFI8	pp	229533.I1RW11
46440	229533.I1RFI8	pp	229533.I1S767
46441	229533.I1RFI8	pp	229533.I1RZK8
46442	229533.I1RFI8	pp	229533.I1RX59
46443	229533.I1RFI8	pp	229533.V6RS91
46444	229533.I1RFI8	pp	229533.I1RZE7
46445	229533.I1RFI8	pp	229533.I1RTT0
46446	229533.I1RFI8	pp	229533.I1S1W8
46447	229533.I1RFI8	pp	229533.I1S053
46448	229533.I1RFI8	pp	229533.I1RGK9
46449	229533.I1RFI8	pp	229533.Q4HZQ1
46450	229533.I1S1Y5	pp	229533.I1S4T3
46451	229533.I1RTI7	pp	229533.I1RXB1
46452	229533.I1RTI7	pp	229533.I1RVY2
46453	229533.I1RTI7	pp	229533.I1RYD3
46454	229533.I1RTI7	pp	229533.I1S9D4
46455	229533.I1RTI7	pp	229533.I1RZP0
46456	229533.I1RTI7	pp	229533.I1S1D1
46457	229533.I1RTI7	pp	229533.I1S8X1
46458	229533.I1RTI7	pp	229533.I1S2E2
46459	229533.I1RTI7	pp	229533.I1RYU0
46460	229533.I1RTI7	pp	229533.V6RSF3
46461	229533.I1RTI7	pp	229533.I1RYI7
46462	229533.I1RTI7	pp	229533.V6QVH8
46463	229533.I1RTI7	pp	229533.I1RYJ7
46464	229533.I1RTI7	pp	229533.I1S770
46465	229533.I1RTI7	pp	229533.I1RYU5
46466	229533.I1RTI7	pp	229533.I1RZ20
46467	229533.I1RTI7	pp	229533.I1RZ52
46468	229533.I1RTI7	pp	229533.I1RWF9
46469	229533.I1RTI7	pp	229533.Q4HXF0
46470	229533.I1RTI7	pp	229533.O74638
46471	229533.I1RTI7	pp	229533.I1S7M8
46472	229533.I1RE87	pp	229533.I1RPW4
46473	229533.I1RE87	pp	229533.I1RKQ5
46474	229533.I1RE87	pp	229533.I1RM06
46475	229533.I1RE87	pp	229533.I1RTF4
46476	229533.I1RE87	pp	229533.I1S0R7
46477	229533.I1RE87	pp	229533.I1RZJ5
46478	229533.I1RE87	pp	229533.I1RUQ2
46479	229533.I1RE87	pp	229533.I1RNH1
46480	229533.I1RE87	pp	229533.Q4IJH1
46481	229533.I1RE87	pp	229533.I1RQA2
46482	229533.I1RE87	pp	229533.I1RRG3
46483	229533.I1RE87	pp	229533.Q4IJ56
46484	229533.I1RE87	pp	229533.V6QUE6
46485	229533.I1RE87	pp	229533.I1RRE0
46486	229533.I1RE87	pp	229533.I1RQ81
46487	229533.I1RE87	pp	229533.I1RZB5
46488	229533.I1RE87	pp	229533.I1RT35
46489	229533.I1RE87	pp	229533.I1RJL2
46490	229533.I1RE87	pp	229533.I1RZF6
46491	229533.I1RE87	pp	229533.I1RX14
46492	229533.I1RE87	pp	229533.I1RK92
46493	229533.I1RE87	pp	229533.Q4IAA0
46494	229533.I1RE87	pp	229533.I1RK79
46495	229533.I1RE87	pp	229533.I1RJL4
46496	229533.I1RE87	pp	229533.Q4IF76
46497	229533.I1RE87	pp	229533.I1RRI2
46498	229533.I1RE87	pp	229533.I1RFN1
46499	229533.I1RE87	pp	229533.I1RQ23
46500	229533.I1RE87	pp	229533.Q4HZK7
46501	229533.I1RE87	pp	229533.I1RFT6

46502 229533.I1RE87 pp 229533.I1RFS3
46503 229533.I1RE87 pp 229533.V6RGN5
46504 229533.I1RE87 pp 229533.Q4IQW0
46505 229533.I1RE87 pp 229533.Q4HZ42
46506 229533.I1RE87 pp 229533.I1S2C0
46507 229533.I1RE87 pp 229533.I1RSI6
46508 229533.I1RE87 pp 229533.I1RG90
46509 229533.I1RE87 pp 229533.I1RQ65
46510 229533.I1RE87 pp 229533.I1RKC6
46511 229533.I1RE87 pp 229533.I1S2E8
46512 229533.I1RE87 pp 229533.I1RNB8
46513 229533.I1RE87 pp 229533.I1RNU6
46514 229533.I1RE87 pp 229533.I1S4M5
46515 229533.I1RT52 pp 229533.I1RYV3
46516 229533.I1RT52 pp 229533.Q4HTT2
46517 229533.I1RAJ0 pp 229533.I1RRG7
46518 229533.I1RAJ0 pp 229533.I1RCT9
46519 229533.I1RAJ0 pp 229533.I1RTP2
46520 229533.I1RAJ0 pp 229533.I1RFN8
46521 229533.I1RAJ0 pp 229533.I1RPG8
46522 229533.I1RAJ0 pp 229533.I1RHU8
46523 229533.I1RAJ0 pp 229533.I1RR68
46524 229533.I1RAJ0 pp 229533.I1RED2
46525 229533.I1RAJ0 pp 229533.I1RW82
46526 229533.I1RAJ0 pp 229533.I1RCM8
46527 229533.I1RAJ0 pp 229533.I1RQM9
46528 229533.A0A1C3YJB1 pp 229533.I1RT52
46529 229533.I1RJA3 pp 229533.I1S325
46530 229533.I1RJA3 pp 229533.I1RLI4
46531 229533.I1RJA3 pp 229533.I1RXN7
46532 229533.I1RJA3 pp 229533.I1RX74
46533 229533.I1RJA3 pp 229533.I1RM78
46534 229533.I1RJA3 pp 229533.I1S6X4
46535 229533.I1RJA3 pp 229533.I1RQC1
46536 229533.I1RJA3 pp 229533.I1RSV8
46537 229533.I1RJA3 pp 229533.I1S4A5
46538 229533.I1RJA3 pp 229533.I1S3H6
46539 229533.A0A098E3T0 pp 229533.I1RFI8
46540 229533.A0A098E3T0 pp 229533.V6QV31
46541 229533.A0A098E3T0 pp 229533.A0A0E0RX50
46542 229533.A0A098E3T0 pp 229533.V6RDH3
46543 229533.A0A098E3T0 pp 229533.V6RS91
46544 229533.A0A098E3T0 pp 229533.I1S0C2
46545 229533.A0A098E3T0 pp 229533.I1RCL0
46546 229533.A0A098E3T0 pp 229533.A0A0E0SFT7
46547 229533.A0A098E3T0 pp 229533.I1RN33
46548 229533.A0A098E3T0 pp 229533.Q4I1N3
46549 229533.A0A098E3T0 pp 229533.I1RXY7
46550 229533.A0A098E3T0 pp 229533.I1RFK3
46551 229533.I1RDX9 pp 229533.I1RZ87
46552 229533.I1RDX9 pp 229533.I1S0P5
46553 229533.I1RDX9 pp 229533.I1RZU5
46554 229533.I1RDX9 pp 229533.I1RJL5
46555 229533.A0A098DVT4 pp 229533.I1RDE7
46556 229533.A0A098DVT4 pp 229533.I1RH11
46557 229533.A0A098DVT4 pp 229533.Q00909
46558 229533.I1RB58 pp 229533.I1S0H2
46559 229533.I1RB58 pp 229533.I1RXB6
46560 229533.I1RB58 pp 229533.I1RK35
46561 229533.I1RB58 pp 229533.I1RYD3
46562 229533.I1RJ84 pp 229533.I1S325
46563 229533.I1RJ84 pp 229533.I1RJA3
46564 229533.I1RJ84 pp 229533.I1RX74
46565 229533.I1RJ84 pp 229533.I1RXN7
46566 229533.I1RJ84 pp 229533.I1RSV8
46567 229533.I1RJ84 pp 229533.I1S6X4
46568 229533.I1RJ84 pp 229533.I1RLI4
46569 229533.I1RJ84 pp 229533.I1RM78
46570 229533.I1RJ84 pp 229533.I1S4A5
46571 229533.I1RJ84 pp 229533.I1S3H6
46572 229533.I1RJ84 pp 229533.I1RQC1
46573 229533.A0A0E0RPD9 pp 229533.I1S2M5
46574 229533.A0A0E0RPD9 pp 229533.A0A0E0SP71

46575	229533.I1RZ52	pp	229533.I1S9D4
46576	229533.I1RZ52	pp	229533.I1S2E2
46577	229533.I1RZ52	pp	229533.V6RBN2
46578	229533.I1RZ52	pp	229533.I1S3K6
46579	229533.I1RZ52	pp	229533.I1S8X1
46580	229533.I1RZ52	pp	229533.O74638
46581	229533.I1RZ52	pp	229533.V6RQW4
46582	229533.I1RZ52	pp	229533.I1S2F2
46583	229533.I1RZ52	pp	229533.V6QVH8
46584	229533.I1RZ52	pp	229533.Q4HVQ9
46585	229533.I1RZ52	pp	229533.Q4HXF0
46586	229533.I1RZ52	pp	229533.I1S770
46587	229533.I1RZ52	pp	229533.V6RVJ3
46588	229533.I1RZ52	pp	229533.V6RLM7
46589	229533.A0A1C3YHT9	pp	229533.I1RTE9
46590	229533.A0A1C3YHT9	pp	229533.I1RPJ3
46591	229533.A0A1C3YHT9	pp	229533.I1S2C0
46592	229533.A0A1C3YHT9	pp	229533.I1RN41
46593	229533.A0A1C3YHT9	pp	229533.I1RRR7
46594	229533.A0A1C3YHT9	pp	229533.Q4HZ68
46595	229533.A0A1C3YHT9	pp	229533.I1S4T3
46596	229533.A0A1C3YHT9	pp	229533.I1RSS4
46597	229533.A0A1C3YHT9	pp	229533.I1RM96
46598	229533.A0A1C3YHT9	pp	229533.I1S2E8
46599	229533.A0A1C3YHT9	pp	229533.Q4I8X0
46600	229533.A0A1C3YHT9	pp	229533.I1S274
46601	229533.A0A1C3YHT9	pp	229533.I1R9X7
46602	229533.A0A1C3YHT9	pp	229533.I1RC72
46603	229533.A0A1C3YHT9	pp	229533.Q4I327
46604	229533.A0A1C3YHT9	pp	229533.I1R9V7
46605	229533.A0A1C3YHT9	pp	229533.I1S558
46606	229533.A0A1C3YHT9	pp	229533.I1RK95
46607	229533.A0A1C3YHT9	pp	229533.I1RCL3
46608	229533.A0A1C3YHT9	pp	229533.I1RZZ1
46609	229533.A0A1C3YHT9	pp	229533.I1RKJ6
46610	229533.A0A1C3YHT9	pp	229533.I1RR72
46611	229533.A0A1C3YHT9	pp	229533.I1RBL7
46612	229533.A0A1C3YHT9	pp	229533.I1RQA2
46613	229533.A0A1C3YHT9	pp	229533.O74638
46614	229533.A0A1C3YHT9	pp	229533.I1RZF6
46615	229533.A0A1C3YHT9	pp	229533.I1RWJ4
46616	229533.A0A1C3YHT9	pp	229533.I1RS47
46617	229533.A0A1C3YHT9	pp	229533.I1RFS3
46618	229533.A0A1C3YHT9	pp	229533.I1RD64
46619	229533.A0A1C3YHT9	pp	229533.A0A1C3YJR3
46620	229533.A0A1C3YHT9	pp	229533.V6R227
46621	229533.A0A1C3YHT9	pp	229533.Q4HZ42
46622	229533.A0A1C3YHT9	pp	229533.Q4IEK8
46623	229533.A0A1C3YHT9	pp	229533.I1RM82
46624	229533.A0A1C3YHT9	pp	229533.I1RRP3
46625	229533.A0A1C3YHT9	pp	229533.I1RPW9
46626	229533.A0A1C3YHT9	pp	229533.I1RRQ3
46627	229533.A0A1C3YHT9	pp	229533.I1RM67
46628	229533.A0A1C3YHT9	pp	229533.Q4IJH1
46629	229533.A0A1C3YHT9	pp	229533.Q4IJ56
46630	229533.A0A1C3YHT9	pp	229533.Q4I830
46631	229533.A0A1C3YHT9	pp	229533.I1RBC7
46632	229533.A0A1C3YHT9	pp	229533.I1RC82
46633	229533.A0A1C3YHT9	pp	229533.I1RB27
46634	229533.A0A1C3YHT9	pp	229533.Q4IBS2
46635	229533.A0A1C3YHT9	pp	229533.I1RTF4
46636	229533.A0A1C3YHT9	pp	229533.I1RNV6
46637	229533.A0A1C3YHT9	pp	229533.Q4IQW0
46638	229533.A0A1C3YHT9	pp	229533.I1RT80
46639	229533.A0A1C3YHT9	pp	229533.I1RRT2
46640	229533.A0A1C3YHT9	pp	229533.I1RQ31
46641	229533.A0A1C3YHT9	pp	229533.A0A1C3YLF5
46642	229533.A0A1C3YHT9	pp	229533.Q4HVW2
46643	229533.A0A1C3YHT9	pp	229533.I1RT05
46644	229533.A0A1C3YHT9	pp	229533.Q4IFI0
46645	229533.A0A1C3YHT9	pp	229533.Q4HXF0
46646	229533.A0A1C3YHT9	pp	229533.V6QYF0
46647	229533.A0A1C3YHT9	pp	229533.I1RF08

229533.A0A1C3YHT9 pp 229533.I1RT33
229533.A0A1C3YHT9 pp 229533.I1RAZ8
229533.A0A1C3YHT9 pp 229533.I1RN61
229533.A0A1C3YHT9 pp 229533.I1RK03
229533.A0A1C3YHT9 pp 229533.Q4IPI1
229533.I1RFN2 pp 229533.I1S0P5
229533.I1RFN2 pp 229533.I1RWH6
229533.I1RE78 pp 229533.I1RLS1
229533.I1RE78 pp 229533.I1RVA8
229533.I1RE78 pp 229533.I1RWU9
229533.A0A098DJ03 pp 229533.I1RMJ3
229533.A0A098DJ03 pp 229533.I1RMP0
229533.A0A098DJ03 pp 229533.I1RBS0
229533.A0A098DJ03 pp 229533.A0A1C3YM60
229533.A0A098DJ03 pp 229533.I1RT75
229533.A0A098DJ03 pp 229533.A0A1C3YJ21
229533.A0A098DJ03 pp 229533.A0A1C3YMA5
229533.A0A098DJ03 pp 229533.A0A1C3YIE7
229533.A0A098DJ03 pp 229533.V6R3J1
229533.A0A098DJ03 pp 229533.I1RYB8
229533.A0A098DJ03 pp 229533.I1R9Y8
229533.A0A098DJ03 pp 229533.I1RSF1
229533.A0A098DJ03 pp 229533.V6RKY8
229533.A0A098DJ03 pp 229533.I1RBF3
229533.A0A098DJ03 pp 229533.A0A1C3YMN0
229533.A0A098DJ03 pp 229533.I1S4Z7
229533.A0A098DJ03 pp 229533.A0A1C3YKK8
229533.A0A098DJ03 pp 229533.I1RKP1
229533.A0A098DJ03 pp 229533.I1RQ38
229533.A0A098DJ03 pp 229533.I1RCD4
229533.A0A098DJ03 pp 229533.I1RQL4
229533.A0A098DJ03 pp 229533.I1S020
229533.A0A098DJ03 pp 229533.A0A098DVZ4
229533.A0A098DJ03 pp 229533.I1RZY1
229533.A0A098DJ03 pp 229533.V6RLM7
229533.A0A098DJ03 pp 229533.I1S123
229533.A0A098DJ03 pp 229533.A0A0E0SID1
229533.A0A098DJ03 pp 229533.I1RQ21
229533.A0A098DJ03 pp 229533.Q4I5R9
229533.A0A098DJ03 pp 229533.I1S9T5
229533.A0A098DJ03 pp 229533.I1RGK9
229533.A0A098DJ03 pp 229533.I1RG57
229533.A0A098DJ03 pp 229533.I1RTE6
229533.A0A098DJ03 pp 229533.V6QX21
229533.A0A098DJ03 pp 229533.I1REW3
229533.A0A098DJ03 pp 229533.I1RWW4
229533.A0A098DJ03 pp 229533.I1RBU1
229533.A0A098DJ03 pp 229533.I1RCH2
229533.A0A098DJ03 pp 229533.I1RR45
229533.A0A098DJ03 pp 229533.I1RN52
229533.A0A098DJ03 pp 229533.I1RJD3
229533.A0A098DJ03 pp 229533.I1RSQ2
229533.A0A098DJ03 pp 229533.I1RMQ6
229533.A0A098DJ03 pp 229533.I1S8Z2
229533.A0A098DJ03 pp 229533.I1RP96
229533.A0A098DJ03 pp 229533.I1RD63
229533.A0A098DJ03 pp 229533.I1RA32
229533.A0A098DJ03 pp 229533.A0A1C3YN26
229533.A0A098DJ03 pp 229533.I1RRK5
229533.A0A098DJ03 pp 229533.I1RAP3
229533.A0A098DJ03 pp 229533.I1RBM1
229533.A0A098DJ03 pp 229533.I1RCM5
229533.A0A098DJ03 pp 229533.I1RBU8
229533.A0A098DJ03 pp 229533.I1RTJ2
229533.A0A098DJ03 pp 229533.V6REE9
229533.A0A098DJ03 pp 229533.A0A1C3YKF3
229533.A0A098DJ03 pp 229533.I1RBF2
229533.A0A098DDI4 pp 229533.I1RRQ1
229533.A0A098DDI4 pp 229533.I1RR90
229533.A0A098DDI4 pp 229533.V6RR38
229533.A0A098DDI4 pp 229533.I1RY35
229533.A0A098DDI4 pp 229533.I1S1Q3
229533.A0A098DDI4 pp 229533.I1RJG1

46721 229533.A0A098DDI4 pp 229533.I1RQ76
46722 229533.A0A098DDI4 pp 229533.I1RF79
46723 229533.A0A098DDI4 pp 229533.I1RCC2
46724 229533.A0A098DDI4 pp 229533.I1RNT1
46725 229533.A0A098DDI4 pp 229533.A0A0E0SP71
46726 229533.A0A098DDI4 pp 229533.I1RQ48
46727 229533.A0A098DDI4 pp 229533.I1RCA0
46728 229533.A0A098DDI4 pp 229533.I1RT23
46729 229533.A0A098DDI4 pp 229533.A0A098DVT4
46730 229533.A0A098DDI4 pp 229533.I1RI22
46731 229533.A0A098DDI4 pp 229533.I1SAS3
46732 229533.A0A098DDI4 pp 229533.I1RZZ3
46733 229533.A0A098DDI4 pp 229533.A0A1C3YLL7
46734 229533.A0A098DDI4 pp 229533.I1SAS4
46735 229533.A0A098DDI4 pp 229533.V6RPN0
46736 229533.A0A098DDI4 pp 229533.I1RFM2
46737 229533.A0A098DDI4 pp 229533.A0A1C3YMS7
46738 229533.I1RVT9 pp 229533.I1RX81
46739 229533.I1RVT9 pp 229533.V6RY33
46740 229533.I1RVT9 pp 229533.I1RWX8
46741 229533.I1RVT9 pp 229533.I1S7M2
46742 229533.I1RVT9 pp 229533.I1S7M3
46743 229533.I1RVT9 pp 229533.I1S1X9
46744 229533.I1RVT9 pp 229533.I1RXF2
46745 229533.I1RVT9 pp 229533.I1RX24
46746 229533.I1RVT9 pp 229533.I1S4M5
46747 229533.I1RVT9 pp 229533.Q4IP34
46748 229533.I1RVT9 pp 229533.I1S0B1
46749 229533.A0A1C3YIL3 pp 229533.A0A1C3YK01
46750 229533.A0A1C3YIL3 pp 229533.Q4IEV4
46751 229533.A0A1C3YIL3 pp 229533.I1RFW1
46752 229533.A0A1C3YIL3 pp 229533.I1RW28
46753 229533.A0A1C3YIL3 pp 229533.Q4IB96
46754 229533.A0A1C3YIL3 pp 229533.I1SB04
46755 229533.A0A1C3YIL3 pp 229533.Q4I5V3
46756 229533.A0A1C3YIL3 pp 229533.I1RB73
46757 229533.A0A1C3YIL3 pp 229533.I1RPR8
46758 229533.A0A1C3YIL3 pp 229533.Q4I7L0
46759 229533.A0A1C3YIL3 pp 229533.I1RCN2
46760 229533.A0A1C3YIL3 pp 229533.Q4HTT1
46761 229533.A0A1C3YIL3 pp 229533.I1RFN9
46762 229533.A0A1C3YIL3 pp 229533.I1RT41
46763 229533.A0A1C3YIL3 pp 229533.I1RXE5
46764 229533.A0A1C3YIL3 pp 229533.Q4IPI4
46765 229533.A0A1C3YIL3 pp 229533.Q4ICA8
46766 229533.A0A1C3YIL3 pp 229533.Q4I7S1
46767 229533.A0A1C3YIL3 pp 229533.Q4IER8
46768 229533.A0A1C3YIL3 pp 229533.I1S0Z3
46769 229533.A0A1C3YIL3 pp 229533.I1RZF3
46770 229533.A0A1C3YIL3 pp 229533.Q4I948
46771 229533.A0A1C3YIL3 pp 229533.I1RZT1
46772 229533.A0A1C3YIL3 pp 229533.I1RSU7
46773 229533.A0A1C3YIL3 pp 229533.Q4HY90
46774 229533.I1RB71 pp 229533.I1RWJ4
46775 229533.I1RB71 pp 229533.I1RG51
46776 229533.I1RB71 pp 229533.I1RNA0
46777 229533.I1RB71 pp 229533.I1RC29
46778 229533.I1RB71 pp 229533.V6QYF0
46779 229533.I1RB71 pp 229533.I1RVY0
46780 229533.I1RB71 pp 229533.I1RZS6
46781 229533.I1RB71 pp 229533.I1RJL4
46782 229533.I1RB71 pp 229533.V6QWX8
46783 229533.I1RB71 pp 229533.I1RT80
46784 229533.I1RB71 pp 229533.I1RDA0
46785 229533.I1RB71 pp 229533.I1S253
46786 229533.I1RB71 pp 229533.I1RC24
46787 229533.I1RB71 pp 229533.I1RC82
46788 229533.I1RB71 pp 229533.I1RW90
46789 229533.I1RB71 pp 229533.I1S9U2
46790 229533.I1RB71 pp 229533.I1RBC7
46791 229533.I1RB71 pp 229533.I1RFN1
46792 229533.I1RB71 pp 229533.I1RC03
46793 229533.I1RB71 pp 229533.I1S9Y1

229533.I1RB71 pp 229533.I1RQ28
229533.I1RB71 pp 229533.I1RRG3
229533.I1RB71 pp 229533.I1RK95
229533.I1RB71 pp 229533.I1RRJ8
229533.I1RB71 pp 229533.I1RKJ6
229533.I1RB71 pp 229533.I1RVZ0
229533.I1RB71 pp 229533.I1RPJ3
229533.I1RB71 pp 229533.I1RS30
229533.I1RB71 pp 229533.I1RNK2
229533.I1RB71 pp 229533.I1RKC6
229533.I1RB71 pp 229533.I1S558
229533.I1RB71 pp 229533.I1RE30
229533.I1RB71 pp 229533.I1RRQ3
229533.I1RB71 pp 229533.I1RZE5
229533.I1RB71 pp 229533.I1RSR3
229533.I1RB71 pp 229533.I1RNH1
229533.I1RB71 pp 229533.I1RR25
229533.I1RB71 pp 229533.I1RC72
229533.I1RB71 pp 229533.I1RJY2
229533.I1RB71 pp 229533.I1RK79
229533.I1RB71 pp 229533.I1RN17
229533.I1RB71 pp 229533.I1RRH5
229533.I1RB71 pp 229533.I1RQ65
229533.I1RB71 pp 229533.I1RNU6
229533.I1RB71 pp 229533.I1S0J1
229533.I1RB71 pp 229533.I1RKQ5
229533.I1RB71 pp 229533.I1RRI2
229533.I1RB71 pp 229533.I1RWF3
229533.I1RB71 pp 229533.I1RSG8
229533.I1RB71 pp 229533.Q4I8X0
229533.I1RB71 pp 229533.I1RZB5
229533.I1RB71 pp 229533.I1RG56
229533.I1RB71 pp 229533.Q4HZK7
229533.I1RB71 pp 229533.I1RJY3
229533.I1RB71 pp 229533.I1RRN6
229533.I1RB71 pp 229533.I1S255
229533.I1RB71 pp 229533.I1RJL2
229533.I1RB71 pp 229533.I1S9U1
229533.I1RB71 pp 229533.V6QUE6
229533.I1RB71 pp 229533.V6RGN5
229533.I1RB71 pp 229533.I1RCB7
229533.I1RB71 pp 229533.I1RP20
229533.I1RB71 pp 229533.I1RJI3
229533.I1RB71 pp 229533.I1RSI6
229533.I1RB71 pp 229533.I1RG11
229533.A0A1C3YKK9 pp 229533.A0A1C3YLZ7
229533.A0A1C3YKK9 pp 229533.I1RDJ1
229533.A0A1C3YKK9 pp 229533.I1RSJ2
229533.A0A1C3YKK9 pp 229533.I1RRQ1
229533.A0A1C3YKK9 pp 229533.V6R4J1
229533.A0A1C3YKK9 pp 229533.I1RDU4
229533.A0A1C3YKK9 pp 229533.I1RLC7
229533.A0A1C3YKK9 pp 229533.I1RQ14
229533.A0A1C3YKK9 pp 229533.I1RIL8
229533.A0A1C3YKK9 pp 229533.I1RW71
229533.A0A1C3YKK9 pp 229533.I1RN61
229533.A0A1C3YKK9 pp 229533.I1RW07
229533.A0A1C3YKK9 pp 229533.I1RK45
229533.A0A1C3YKK9 pp 229533.I1RCC2
229533.A0A1C3YKK9 pp 229533.I1RPW4
229533.A0A1C3YKK9 pp 229533.I1RLM7
229533.A0A1C3YKK9 pp 229533.I1RBE7
229533.A0A1C3YKK9 pp 229533.I1RQE0
229533.A0A1C3YKK9 pp 229533.I1RI02
229533.A0A1C3YKK9 pp 229533.I1RQL4
229533.A0A1C3YKK9 pp 229533.I1RIX0
229533.A0A1C3YKK9 pp 229533.I1S984
229533.A0A1C3YKK9 pp 229533.I1S574
229533.A0A1C3YKK9 pp 229533.I1RKR5
229533.A0A1C3YKK9 pp 229533.I1RP86
229533.A0A1C3YKK9 pp 229533.I1RME6
229533.A0A1C3YKK9 pp 229533.I1RA62
229533.A0A1C3YKK9 pp 229533.V6RAN2

46867 229533.A0A1C3YKK9 pp 229533.I1RDE4
46868 229533.A0A1C3YKK9 pp 229533.I1RI03
46869 229533.A0A1C3YKK9 pp 229533.I1RJZ7
46870 229533.A0A1C3YKK9 pp 229533.I1RKP3
46871 229533.A0A1C3YKK9 pp 229533.I1RNR2
46872 229533.A0A1C3YKK9 pp 229533.I1S352
46873 229533.A0A1C3YKK9 pp 229533.I1RBN0
46874 229533.A0A1C3YKK9 pp 229533.I1RU29
46875 229533.A0A1C3YKK9 pp 229533.I1RZ13
46876 229533.A0A1C3YKK9 pp 229533.I1RBJ5
46877 229533.A0A1C3YKK9 pp 229533.I1RLN6
46878 229533.A0A1C3YKK9 pp 229533.I1RQ81
46879 229533.A0A1C3YKK9 pp 229533.I1RRT3
46880 229533.A0A1C3YKK9 pp 229533.I1RIL3
46881 229533.A0A1C3YKK9 pp 229533.I1RCA0
46882 229533.A0A1C3YKK9 pp 229533.I1RBY9
46883 229533.A0A1C3YKK9 pp 229533.I1RMX6
46884 229533.A0A1C3YKK9 pp 229533.I1RP27
46885 229533.A0A1C3YKK9 pp 229533.I1RGF5
46886 229533.A0A1C3YKK9 pp 229533.I1RZ29
46887 229533.A0A1C3YKK9 pp 229533.I1RNA5
46888 229533.A0A1C3YKK9 pp 229533.I1RSK1
46889 229533.A0A1C3YKK9 pp 229533.I1S0R4
46890 229533.A0A1C3YKK9 pp 229533.I1RH91
46891 229533.A0A1C3YKK9 pp 229533.I1RC31
46892 229533.A0A1C3YKK9 pp 229533.I1R9J6
46893 229533.A0A1C3YKK9 pp 229533.I1S569
46894 229533.A0A1C3YKK9 pp 229533.I1RGL4
46895 229533.A0A1C3YKK9 pp 229533.I1RC72
46896 229533.A0A1C3YKK9 pp 229533.I1S272
46897 229533.A0A1C3YKK9 pp 229533.I1S0R3
46898 229533.A0A1C3YKK9 pp 229533.I1R9D1
46899 229533.A0A1C3YKK9 pp 229533.I1RNA1
46900 229533.A0A1C3YKK9 pp 229533.A0A1C3YMI5
46901 229533.A0A1C3YKK9 pp 229533.I1SAN9
46902 229533.A0A1C3YKK9 pp 229533.I1RRV1
46903 229533.I1RR72 pp 229533.I1RYJ6
46904 229533.I1RR72 pp 229533.I1RTI6
46905 229533.I1RR72 pp 229533.I1S0R7
46906 229533.I1RR72 pp 229533.I1S6F0
46907 229533.I1RR72 pp 229533.I1RT33
46908 229533.I1RR72 pp 229533.I1RRC6
46909 229533.I1RR72 pp 229533.I1RRX5
46910 229533.I1RR72 pp 229533.I1S2L2
46911 229533.I1RR72 pp 229533.I1RUY5
46912 229533.I1RR72 pp 229533.I1RW71
46913 229533.I1RR72 pp 229533.I1S9U2
46914 229533.I1RR72 pp 229533.V6QUE6
46915 229533.I1RR72 pp 229533.I1RT80
46916 229533.I1RR72 pp 229533.I1S4S7
46917 229533.I1RR72 pp 229533.Q4IQ72
46918 229533.I1RR72 pp 229533.Q4IJH1
46919 229533.I1RR72 pp 229533.I1RWS4
46920 229533.I1RR72 pp 229533.I1RW26
46921 229533.I1RR72 pp 229533.Q4IBS2
46922 229533.I1RR72 pp 229533.V6R227
46923 229533.I1RR72 pp 229533.I1S0F1
46924 229533.I1RR72 pp 229533.I1RT05
46925 229533.I1RR72 pp 229533.I1RTA1
46926 229533.I1RR72 pp 229533.I1S1W8
46927 229533.I1RR72 pp 229533.I1S3G4
46928 229533.I1RR72 pp 229533.I1RVZ2
46929 229533.I1RR72 pp 229533.Q4IEK8
46930 229533.I1RR72 pp 229533.Q4IJ56
46931 229533.I1RR72 pp 229533.I1RY63
46932 229533.I1RR72 pp 229533.V6QWZ7
46933 229533.I1RR72 pp 229533.I1S9Y1
46934 229533.I1RR72 pp 229533.I1RZZ1
46935 229533.I1RR72 pp 229533.I1RRT2
46936 229533.I1RR72 pp 229533.Q4I5M4
46937 229533.I1RR72 pp 229533.I1RZS6
46938 229533.I1RR72 pp 229533.I1RSU2
46939 229533.I1RR72 pp 229533.I1RT75

46940 229533.I1RR72 pp 229533.I1RWJ4
46941 229533.I1RR72 pp 229533.I1RWI5
46942 229533.I1RR72 pp 229533.I1S253
46943 229533.I1RR72 pp 229533.I1RTE9
46944 229533.I1RR72 pp 229533.I1S558
46945 229533.I1RR72 pp 229533.Q4I1M9
46946 229533.I1RR72 pp 229533.I1S4T3
46947 229533.I1RR72 pp 229533.I1RRI2
46948 229533.I1RR72 pp 229533.I1RSI6
46949 229533.I1RR72 pp 229533.Q4IPZ1
46950 229533.I1RR72 pp 229533.Q4HZ68
46951 229533.I1RR72 pp 229533.I1RSS4
46952 229533.I1RR72 pp 229533.Q4I8X0
46953 229533.A0A1C3YHI3 pp 229533.I1RTJ2
46954 229533.A0A1C3YHI3 pp 229533.I1RFK3
46955 229533.A0A1C3YHI3 pp 229533.I1RVM7
46956 229533.A0A1C3YHI3 pp 229533.I1RM91
46957 229533.A0A1C3YHI3 pp 229533.I1RRZ6
46958 229533.A0A1C3YHI3 pp 229533.I1RG14
46959 229533.A0A1C3YHI3 pp 229533.I1RYD3
46960 229533.A0A1C3YHI3 pp 229533.I1RPT0
46961 229533.A0A1C3YHI3 pp 229533.I1RVV7
46962 229533.A0A1C3YHI3 pp 229533.I1RMU9
46963 229533.A0A1C3YHI3 pp 229533.I1RBF3
46964 229533.A0A1C3YHI3 pp 229533.I1RFB0
46965 229533.A0A1C3YHI3 pp 229533.I1S1F3
46966 229533.A0A1C3YHI3 pp 229533.A0A1C3YJ17
46967 229533.A0A1C3YHI3 pp 229533.A0A1C3YN47
46968 229533.A0A1C3YHI3 pp 229533.I1RGK9
46969 229533.A0A1C3YHI3 pp 229533.I1RNU7
46970 229533.A0A1C3YHI3 pp 229533.I1RNM7
46971 229533.A0A1C3YHI3 pp 229533.V6RKK5
46972 229533.A0A1C3YHI3 pp 229533.A0A1C3YMZ0
46973 229533.A0A1C3YHI3 pp 229533.I1RTI7
46974 229533.A0A1C3YHI3 pp 229533.I1RGL3
46975 229533.A0A1C3YHI3 pp 229533.I1RHZ4
46976 229533.A0A1C3YHI3 pp 229533.I1RMQ6
46977 229533.A0A1C3YHI3 pp 229533.I1S049
46978 229533.A0A1C3YHI3 pp 229533.A0A1C3YLG7
46979 229533.A0A1C3YHI3 pp 229533.A0A1C3YJL2
46980 229533.A0A1C3YHI3 pp 229533.I1RM49
46981 229533.I1RMP9 pp 229533.I1RVN2
46982 229533.I1RMP9 pp 229533.Q4I785
46983 229533.I1RMP9 pp 229533.I1RQ75
46984 229533.I1RMP9 pp 229533.I1RNM4
46985 229533.I1RMP9 pp 229533.I1RZ34
46986 229533.I1RMP9 pp 229533.I1S272
46987 229533.I1RMP9 pp 229533.Q4HZE0
46988 229533.I1RMP9 pp 229533.V6QU20
46989 229533.I1RMP9 pp 229533.I1RNK8
46990 229533.I1RMP9 pp 229533.I1RNU0
46991 229533.I1RMP9 pp 229533.Q4IJT0
46992 229533.I1RBB9 pp 229533.I1RGF9
46993 229533.I1RBB9 pp 229533.I1RJW6
46994 229533.I1RBB9 pp 229533.I1RZB4
46995 229533.I1RL13 pp 229533.I1S3K7
46996 229533.I1RL13 pp 229533.I1S5P3
46997 229533.I1SA47 pp 229533.I1SA48
46998 229533.I1SA47 pp 229533.Q4IR09
46999 229533.I1SA47 pp 229533.Q4IPN9
47000 229533.I1SA47 pp 229533.Q4IQK7
47001 229533.O74638 pp 229533.V6RRM0
47002 229533.O74638 pp 229533.Q4HXF0
47003 229533.O74638 pp 229533.V6RMW6
47004 229533.O74638 pp 229533.V6R3J1
47005 229533.O74638 pp 229533.Q4IB89
47006 229533.O74638 pp 229533.V6RGN5
47007 229533.O74638 pp 229533.V6R042
47008 229533.O74638 pp 229533.Q4IF76
47009 229533.O74638 pp 229533.V6RIV9
47010 229533.O74638 pp 229533.V6RLM7
47011 229533.O74638 pp 229533.V6R283
47012 229533.O74638 pp 229533.V6R382

47013	229533.O74638	pp	229533.V6RSF3
47014	229533.O74638	pp	229533.V6RCZ8
47015	229533.O74638	pp	229533.V6QVH8
47016	229533.O74638	pp	229533.Q4I5V2
47017	229533.O74638	pp	229533.Q4IMJ1
47018	229533.O74638	pp	229533.V6R2A1
47019	229533.O74638	pp	229533.Q4I283
47020	229533.O74638	pp	229533.V6RCG5
47021	229533.O74638	pp	229533.V6RRH2
47022	229533.O74638	pp	229533.Q4IEK8
47023	229533.O74638	pp	229533.V6R1K4
47024	229533.O74638	pp	229533.V6R227
47025	229533.O74638	pp	229533.V6RUT5
47026	229533.O74638	pp	229533.V6R057
47027	229533.O74638	pp	229533.Q4I624
47028	229533.O74638	pp	229533.V6RTJ8
47029	229533.O74638	pp	229533.Q4IJH1
47030	229533.O74638	pp	229533.Q4I2J8
47031	229533.O74638	pp	229533.Q4IJ56
47032	229533.O74638	pp	229533.V6RK96
47033	229533.O74638	pp	229533.V6QUE6
47034	229533.O74638	pp	229533.V6RBN2
47035	229533.O74638	pp	229533.V6RQW4
47036	229533.O74638	pp	229533.V6RY56
47037	229533.O74638	pp	229533.Q4IJK2
47038	229533.I1S2L2	pp	229533.Q4IFI0
47039	229533.I1S2L2	pp	229533.V6QYF0
47040	229533.I1S2L2	pp	229533.V6R1K4
47041	229533.I1RBF3	pp	229533.I1RP86
47042	229533.I1RBF3	pp	229533.I1RMK6
47043	229533.I1RBF3	pp	229533.I1RQE1
47044	229533.I1RBF3	pp	229533.I1RQL4
47045	229533.I1RBF3	pp	229533.I1RDE6
47046	229533.I1RBF3	pp	229533.I1S0K0
47047	229533.I1RBF3	pp	229533.I1RM49
47048	229533.I1RBF3	pp	229533.I1RY12
47049	229533.I1RBF3	pp	229533.I1RRX5
47050	229533.I1RBF3	pp	229533.I1RK95
47051	229533.I1RBF3	pp	229533.I1S4S7
47052	229533.I1RBF3	pp	229533.I1S8C1
47053	229533.I1RBF3	pp	229533.I1RYX6
47054	229533.I1RBF3	pp	229533.I1S574
47055	229533.I1RBF3	pp	229533.I1RCM7
47056	229533.I1RBF3	pp	229533.I1RMQ6
47057	229533.I1RBF3	pp	229533.I1S6D7
47058	229533.I1RBF3	pp	229533.I1RXS0
47059	229533.I1RBF3	pp	229533.I1RGG6
47060	229533.I1RBF3	pp	229533.I1RSQ2
47061	229533.I1RBF3	pp	229533.V6QUG0
47062	229533.I1RBF3	pp	229533.I1S4S1
47063	229533.I1RBF3	pp	229533.I1RC11
47064	229533.I1RBF3	pp	229533.I1RBT1
47065	229533.I1RBF3	pp	229533.I1RVZ9
47066	229533.I1RBF3	pp	229533.I1RWW4
47067	229533.I1RBF3	pp	229533.I1RJD3
47068	229533.I1RBF3	pp	229533.I1S3L2
47069	229533.I1RBF3	pp	229533.I1RN52
47070	229533.I1RBF3	pp	229533.I1RY21
47071	229533.I1RBF3	pp	229533.I1S984
47072	229533.I1RBF3	pp	229533.I1S8Z2
47073	229533.I1RBF3	pp	229533.V6RKK5
47074	229533.I1RBF3	pp	229533.I1RC48
47075	229533.I1RBF3	pp	229533.I1S029
47076	229533.I1RBF3	pp	229533.I1RM35
47077	229533.I1RBF3	pp	229533.I1RQP2
47078	229533.I1RBF3	pp	229533.V6RY56
47079	229533.I1RBF3	pp	229533.I1S4D0
47080	229533.I1RBF3	pp	229533.I1S0B7
47081	229533.I1RBF3	pp	229533.I1RE48
47082	229533.I1RBF3	pp	229533.I1RHZ5
47083	229533.I1RBF3	pp	229533.I1RXF0
47084	229533.I1RBF3	pp	229533.I1S4Q4
47085	229533.I1RBF3	pp	229533.I1RC74

47086	229533.I1RBF3	pp	229533.Q4I5G1
47087	229533.I1RBF3	pp	229533.I1RFY0
47088	229533.I1RBF3	pp	229533.I1RRV4
47089	229533.I1RBF3	pp	229533.I1S0Q4
47090	229533.I1RBF3	pp	229533.I1RCL0
47091	229533.I1RBF3	pp	229533.I1RJY7
47092	229533.I1RBF3	pp	229533.I1RP97
47093	229533.I1RBF3	pp	229533.I1RT75
47094	229533.I1RBF3	pp	229533.I1RX58
47095	229533.I1RBF3	pp	229533.I1RRK5
47096	229533.I1RBF3	pp	229533.I1RMW4
47097	229533.I1RBF3	pp	229533.I1S4Z7
47098	229533.I1RBF3	pp	229533.I1RCV1
47099	229533.I1RBF3	pp	229533.I1S0Q5
47100	229533.I1RBF3	pp	229533.I1RR68
47101	229533.I1RBF3	pp	229533.I1RC31
47102	229533.I1RBF3	pp	229533.I1RZB2
47103	229533.I1RBF3	pp	229533.I1RPY0
47104	229533.I1RBF3	pp	229533.I1RYK0
47105	229533.I1RBF3	pp	229533.I1S8P5
47106	229533.I1RBF3	pp	229533.I1RPD6
47107	229533.I1RBF3	pp	229533.I1S6D1
47108	229533.I1RBF3	pp	229533.I1RWF9
47109	229533.I1RBF3	pp	229533.I1RG71
47110	229533.I1RBF3	pp	229533.I1RZ63
47111	229533.I1RBF3	pp	229533.I1RUR8
47112	229533.I1RBF3	pp	229533.I1RSK5
47113	229533.I1RBF3	pp	229533.V6RKY8
47114	229533.I1RBF3	pp	229533.I1RC67
47115	229533.I1RBF3	pp	229533.I1RYP2
47116	229533.I1RBF3	pp	229533.I1RSQ4
47117	229533.I1RBF3	pp	229533.I1S0J7
47118	229533.I1RBF3	pp	229533.I1RWI5
47119	229533.I1RBF3	pp	229533.I1RMJ3
47120	229533.I1RBF3	pp	229533.I1RLW5
47121	229533.I1RBF3	pp	229533.I1S1F3
47122	229533.I1RBF3	pp	229533.I1RKB2
47123	229533.I1RBF3	pp	229533.I1S9Y1
47124	229533.I1RBF3	pp	229533.I1RQ38
47125	229533.I1RBF3	pp	229533.I1RPU8
47126	229533.I1RBF3	pp	229533.I1RBS5
47127	229533.I1RBF3	pp	229533.I1RC54
47128	229533.I1RBF3	pp	229533.I1S8R4
47129	229533.I1RBF3	pp	229533.I1RYB8
47130	229533.I1RBF3	pp	229533.I1S147
47131	229533.I1RBF3	pp	229533.I1RZ86
47132	229533.I1RBF3	pp	229533.I1S0S5
47133	229533.I1RBF3	pp	229533.I1RN84
47134	229533.I1RBF3	pp	229533.I1RSU2
47135	229533.I1RBF3	pp	229533.I1RVR2
47136	229533.I1RBF3	pp	229533.I1RPK0
47137	229533.I1RBF3	pp	229533.I1RRV1
47138	229533.I1RBF3	pp	229533.V6RLM7
47139	229533.I1RBF3	pp	229533.I1RCG5
47140	229533.I1RBF3	pp	229533.I1RNA1
47141	229533.I1RBF3	pp	229533.I1RE52
47142	229533.I1RBF3	pp	229533.I1RR45
47143	229533.I1RBF3	pp	229533.I1RTA0
47144	229533.I1RBF3	pp	229533.I1RKB6
47145	229533.I1RBF3	pp	229533.I1RFK3
47146	229533.I1RBF3	pp	229533.I1RWE8
47147	229533.I1RBF3	pp	229533.I1RE79
47148	229533.I1RBF3	pp	229533.V6RRM0
47149	229533.I1RBF3	pp	229533.V6QX21
47150	229533.I1RBF3	pp	229533.I1S123
47151	229533.I1RBF3	pp	229533.I1REW3
47152	229533.I1RBF3	pp	229533.I1S9T5
47153	229533.I1RBF3	pp	229533.V6RM02
47154	229533.I1RBF3	pp	229533.I1RK75
47155	229533.I1RBF3	pp	229533.V6RRH2
47156	229533.I1RBF3	pp	229533.I1RCS6
47157	229533.I1RBF3	pp	229533.I1S5H2
47158	229533.I1RNJ6	pp	229533.I1RSK7

47159 229533.I1RNJ6 pp 229533.I1RTF4
47160 229533.I1RNJ6 pp 229533.I1RRI2
47161 229533.A0A0E0RPC5 pp 229533.I1RPM4
47162 229533.A0A0E0RPC5 pp 229533.I1RE22
47163 229533.A0A0E0RPC5 pp 229533.A0A0E0RU84
47164 229533.A0A0E0RPC5 pp 229533.A0A1C3YM60
47165 229533.A0A0E0RPC5 pp 229533.V6RMW6
47166 229533.A0A0E0RPC5 pp 229533.I1RVT9
47167 229533.A0A0E0RPC5 pp 229533.V6RIV9
47168 229533.A0A0E0RPC5 pp 229533.I1RN67
47169 229533.A0A0E0RPC5 pp 229533.I1RVQ0
47170 229533.A0A0E0RPC5 pp 229533.I1RQR6
47171 229533.A0A0E0RPC5 pp 229533.I1RDE6
47172 229533.A0A0E0RPC5 pp 229533.I1RBS6
47173 229533.A0A0E0RPC5 pp 229533.I1RW99
47174 229533.A0A0E0RPC5 pp 229533.A0A1C3YKV3
47175 229533.A0A0E0RPC5 pp 229533.I1RBC9
47176 229533.A0A098DW62 pp 229533.I1RNJ6
47177 229533.Q4ID21 pp 229533.V6QYF0
47178 229533.A0A1C3YLL1 pp 229533.Q4IPC8
47179 229533.A0A1C3YLL1 pp 229533.A0A1C3YLQ8
47180 229533.A0A1C3YLL1 pp 229533.I1RSF1
47181 229533.A0A1C3YLL1 pp 229533.I1S7J8
47182 229533.A0A1C3YLL1 pp 229533.I1S5N3
47183 229533.A0A1C3YLL1 pp 229533.I1RBF3
47184 229533.A0A1C3YLL1 pp 229533.A0A1C3YMN0
47185 229533.A0A1C3YLL1 pp 229533.A0A1C3YNB1
47186 229533.A0A1C3YLL1 pp 229533.I1S9T5
47187 229533.A0A1C3YLL1 pp 229533.I1RJ04
47188 229533.A0A1C3YLL1 pp 229533.I1R9Y8
47189 229533.A0A1C3YLL1 pp 229533.V6RKY8
47190 229533.A0A1C3YLL1 pp 229533.I1RSY1
47191 229533.A0A1C3YLL1 pp 229533.I1REW3
47192 229533.A0A1C3YLL1 pp 229533.I1RKX6
47193 229533.A0A1C3YLL1 pp 229533.I1RKP1
47194 229533.A0A1C3YLL1 pp 229533.I1RR45
47195 229533.A0A1C3YLL1 pp 229533.I1RMJ3
47196 229533.A0A1C3YLL1 pp 229533.I1RLP9
47197 229533.A0A1C3YLL1 pp 229533.I1RK24
47198 229533.A0A1C3YLL1 pp 229533.I1S2P0
47199 229533.A0A1C3YLL1 pp 229533.I1RT75
47200 229533.A0A1C3YLL1 pp 229533.I1RE24
47201 229533.A0A1C3YLL1 pp 229533.I1RRK5
47202 229533.A0A1C3YLL1 pp 229533.I1RCH9
47203 229533.A0A1C3YLL1 pp 229533.I1RKZ4
47204 229533.A0A1C3YLL1 pp 229533.I1RDU9
47205 229533.A0A1C3YLL1 pp 229533.I1S273
47206 229533.A0A1C3YLL1 pp 229533.I1RJX3
47207 229533.A0A1C3YLL1 pp 229533.I1RN52
47208 229533.A0A1C3YLL1 pp 229533.I1RBU8
47209 229533.A0A1C3YLL1 pp 229533.I1RAB6
47210 229533.A0A1C3YLL1 pp 229533.I1RA32
47211 229533.A0A1C3YLL1 pp 229533.I1S1V6
47212 229533.A0A1C3YLL1 pp 229533.I1RJD3
47213 229533.A0A1C3YLL1 pp 229533.I1S8Z2
47214 229533.I1R9Y1 pp 229533.I1RRJ8
47215 229533.I1R9Y1 pp 229533.Q4IB96
47216 229533.I1R9Y1 pp 229533.I1RFW1
47217 229533.I1R9Y1 pp 229533.I1RPR8
47218 229533.I1R9Y1 pp 229533.Q4IEV4
47219 229533.I1R9Y1 pp 229533.I1RW28
47220 229533.I1R9Y1 pp 229533.Q4I7L0
47221 229533.I1R9Y1 pp 229533.Q4IPI4
47222 229533.I1R9Y1 pp 229533.Q4I5V3
47223 229533.I1R9Y1 pp 229533.I1RCN2
47224 229533.I1R9Y1 pp 229533.I1RSU7
47225 229533.I1R9Y1 pp 229533.I1SB04
47226 229533.I1R9Y1 pp 229533.Q4I7S1
47227 229533.I1R9Y1 pp 229533.I1RZF3
47228 229533.I1R9Y1 pp 229533.I1RB73
47229 229533.I1R9Y1 pp 229533.I1S0Z3
47230 229533.I1R9Y1 pp 229533.Q4HY90
47231 229533.I1R9Y1 pp 229533.Q4ICA8

47232 229533.I1R9Y1 pp 229533.I1RXE5
47233 229533.I1R9Y1 pp 229533.I1RZT1
47234 229533.I1R9Y1 pp 229533.I1RFN9
47235 229533.I1R9Y1 pp 229533.I1RT41
47236 229533.I1R9Y1 pp 229533.Q4I948
47237 229533.I1R9Y1 pp 229533.Q4IER8
47238 229533.A0A098DDH1 pp 229533.I1RBE7
47239 229533.A0A098DDH1 pp 229533.A0A1C3YLB6
47240 229533.A0A098DDH1 pp 229533.I1RF58
47241 229533.A0A098DDH1 pp 229533.I1S166
47242 229533.A0A098DDH1 pp 229533.I1RL13
47243 229533.A0A098DDH1 pp 229533.I1RY87
47244 229533.A0A098DDH1 pp 229533.I1S3G4
47245 229533.A0A098DDH1 pp 229533.I1S1W8
47246 229533.A0A098DDH1 pp 229533.I1RNG4
47247 229533.A0A098DDH1 pp 229533.I1RL11
47248 229533.A0A098DDH1 pp 229533.I1RUB2
47249 229533.A0A098DDH1 pp 229533.I1S5P3
47250 229533.I1REV2 pp 229533.I1RZ20
47251 229533.I1REV2 pp 229533.I1RPD6
47252 229533.I1REV2 pp 229533.I1S5L5
47253 229533.I1REV2 pp 229533.I1RR68
47254 229533.I1REV2 pp 229533.I1RNG7
47255 229533.I1REV2 pp 229533.I1RXG6
47256 229533.I1REV2 pp 229533.V6RF62
47257 229533.I1REV2 pp 229533.I1S4S5
47258 229533.I1REV2 pp 229533.I1RGT3
47259 229533.I1REV2 pp 229533.I1RF40
47260 229533.I1REV2 pp 229533.I1RVX9
47261 229533.I1REV2 pp 229533.I1S6P6
47262 229533.I1REV2 pp 229533.I1RF53
47263 229533.I1REV2 pp 229533.I1S2C4
47264 229533.I1REV2 pp 229533.I1RQE0
47265 229533.I1REV2 pp 229533.I1RM26
47266 229533.I1REV2 pp 229533.V6RN31
47267 229533.I1REV2 pp 229533.V6R9Z3
47268 229533.I1REV2 pp 229533.I1RYP6
47269 229533.I1REV2 pp 229533.I1RNH4
47270 229533.I1REV2 pp 229533.I1S0R9
47271 229533.I1REV2 pp 229533.V6RRM0
47272 229533.I1REV2 pp 229533.I1RN80
47273 229533.I1REV2 pp 229533.V6R3V4
47274 229533.I1REV2 pp 229533.I1RRM1
47275 229533.I1REV2 pp 229533.I1RJC5
47276 229533.I1REV2 pp 229533.I1RLM5
47277 229533.I1REV2 pp 229533.I1RSX2
47278 229533.I1REV2 pp 229533.I1RXX0
47279 229533.I1REV2 pp 229533.I1RF38
47280 229533.I1RSW9 pp 229533.I1RU89
47281 229533.I1RSW9 pp 229533.Q4IB50
47282 229533.I1RSW9 pp 229533.Q4I5U9
47283 229533.I1RSW9 pp 229533.Q4I5I4
47284 229533.I1RSW9 pp 229533.I1RXU0
47285 229533.I1RSW9 pp 229533.I1RTC2
47286 229533.I1RSW9 pp 229533.Q4HTT2
47287 229533.I1RSW9 pp 229533.I1S5Z5
47288 229533.I1RWD0 pp 229533.Q4I948
47289 229533.I1RWD0 pp 229533.Q4IEV4
47290 229533.I1RWD0 pp 229533.Q4IB96
47291 229533.I1RWD0 pp 229533.Q4IER8
47292 229533.I1RWD0 pp 229533.I1RZF3
47293 229533.I1RWD0 pp 229533.Q4IPI4
47294 229533.I1RWD0 pp 229533.Q4ICA8
47295 229533.I1RWD0 pp 229533.I1RXE5
47296 229533.I1RWD0 pp 229533.Q4I7L0
47297 229533.I1RWD0 pp 229533.Q4HY90
47298 229533.I1RWD0 pp 229533.Q4I5V3
47299 229533.I1RWD0 pp 229533.Q4I7S1
47300 229533.I1RWD0 pp 229533.I1RZT1
47301 229533.I1RWD0 pp 229533.I1SB04
47302 229533.I1S0C0 pp 229533.I1S0W0
47303 229533.I1S0C0 pp 229533.I1S4J4
47304 229533.I1S0C0 pp 229533.I1SAT3

229533.I1S0C0 pp 229533.P0C431
229533.I1S0C0 pp 229533.Q4IMM0
229533.I1S0C0 pp 229533.Q4IBX0
229533.I1S0C0 pp 229533.I1S0S6
229533.A0A098DW43 pp 229533.I1RG99
229533.A0A098DW43 pp 229533.I1RD63
229533.A0A098DW43 pp 229533.I1RC52
229533.A0A098DDF3 pp 229533.I1S268
229533.I1RGJ3 pp 229533.I1RVR2
229533.I1RGJ3 pp 229533.I1RMK7
229533.I1RGJ3 pp 229533.I1RN84
229533.I1RGJ3 pp 229533.I1RPY0
229533.I1RGJ3 pp 229533.I1RMJ3
229533.A0A1C3YIJ0 pp 229533.I1RD80
229533.A0A1C3YIJ0 pp 229533.I1S3V2
229533.A0A1C3YIJ0 pp 229533.I1RPS2
229533.A0A1C3YIJ0 pp 229533.I1RZ21
229533.A0A1C3YIJ0 pp 229533.I1RBB9
229533.A0A1C3YIJ0 pp 229533.I1RYI6
229533.A0A1C3YIJ0 pp 229533.I1RJW6
229533.A0A1C3YIJ0 pp 229533.I1RSE0
229533.A0A1C3YIJ0 pp 229533.I1RA62
229533.A0A1C3YIJ0 pp 229533.A0A1C3YJL7
229533.A0A1C3YIJ0 pp 229533.I1RNE7
229533.A0A1C3YIJ0 pp 229533.I1RZB4
229533.A0A1C3YIJ0 pp 229533.I1RNM7
229533.A0A1C3YII9 pp 229533.I1RMR5
229533.A0A1C3YII9 pp 229533.I1RJS2
229533.I1RKP1 pp 229533.I1RR60
229533.I1RKP1 pp 229533.I1S3T7
229533.I1RKP1 pp 229533.I1RRI1
229533.I1RKP1 pp 229533.I1RPE0
229533.I1RKP1 pp 229533.I1RXC2
229533.I1RKP1 pp 229533.I1S5R1
229533.I1RKP1 pp 229533.I1RZ39
229533.I1RKP1 pp 229533.I1RT40
229533.I1RKP1 pp 229533.V6RKY8
229533.I1RKP1 pp 229533.I1RMU3
229533.I1RKP1 pp 229533.I1S2B8
229533.I1RKP1 pp 229533.I1RTD5
229533.I1RKP1 pp 229533.I1RSA5
229533.I1RKP1 pp 229533.I1RSZ7
229533.I1RKP1 pp 229533.Q4HVQ9
229533.I1RKP1 pp 229533.I1S0W4
229533.I1RKP1 pp 229533.I1RLW5
229533.I1RKP1 pp 229533.I1RYI5
229533.I1RKP1 pp 229533.I1S069
229533.I1RKP1 pp 229533.V6R042
229533.I1RKP1 pp 229533.I1RPP3
229533.I1RKP1 pp 229533.I1RSQ3
229533.I1RKP1 pp 229533.V6RMW6
229533.I1RKP1 pp 229533.I1RTA1
229533.I1RKP1 pp 229533.I1RQ33
229533.I1RKP1 pp 229533.I1RRM3
229533.I1RKP1 pp 229533.I1RVX0
229533.I1RKP1 pp 229533.I1RVZ2
229533.I1RKP1 pp 229533.I1RR45
229533.I1RKP1 pp 229533.I1RQE7
229533.I1RKP1 pp 229533.I1S5H2
229533.I1RKP1 pp 229533.I1RQC0
229533.I1RKP1 pp 229533.I1RNC1
229533.I1RKP1 pp 229533.I1RN52
229533.I1RKP1 pp 229533.I1S5R6
229533.I1RKP1 pp 229533.I1RUG3
229533.I1RKP1 pp 229533.I1RYU3
229533.I1RKP1 pp 229533.I1RSF1
229533.I1RKP1 pp 229533.I1RNM7
229533.I1RKP1 pp 229533.I1RXD7
229533.I1RKP1 pp 229533.I1RPU2
229533.I1RKP1 pp 229533.I1RPY3
229533.I1RKP1 pp 229533.V6RTJ8
229533.I1RKP1 pp 229533.I1S1W9
229533.I1RKP1 pp 229533.I1RZV3

47378	229533.I1RKP1	pp	229533.I1S6D7
47379	229533.I1RKP1	pp	229533.I1RX46
47380	229533.I1RKP1	pp	229533.I1RZY1
47381	229533.I1RKP1	pp	229533.I1S4S7
47382	229533.I1RKP1	pp	229533.I1RRX5
47383	229533.I1RKP1	pp	229533.I1RP86
47384	229533.I1RKP1	pp	229533.V6RLM7
47385	229533.I1RKP1	pp	229533.I1RX95
47386	229533.I1RKP1	pp	229533.I1RR24
47387	229533.I1RKP1	pp	229533.Q4IJK2
47388	229533.I1RKP1	pp	229533.I1RZM8
47389	229533.I1RKP1	pp	229533.I1RUQ3
47390	229533.I1RKP1	pp	229533.I1RRY9
47391	229533.I1RKP1	pp	229533.I1RVC7
47392	229533.I1RKP1	pp	229533.I1RWR7
47393	229533.I1RKP1	pp	229533.I1RUY5
47394	229533.I1RKP1	pp	229533.I1RZK5
47395	229533.I1RKP1	pp	229533.I1RQA5
47396	229533.I1RKP1	pp	229533.I1S5B0
47397	229533.I1RKP1	pp	229533.I1S3M2
47398	229533.I1RKP1	pp	229533.V6RCZ8
47399	229533.I1RKP1	pp	229533.I1RYM5
47400	229533.I1RKP1	pp	229533.I1RQ85
47401	229533.I1RKP1	pp	229533.I1RXC4
47402	229533.I1RKP1	pp	229533.I1RRK5
47403	229533.I1RG58	pp	229533.Q4IB89
47404	229533.I1RG58	pp	229533.I1RJU4
47405	229533.I1RG58	pp	229533.I1S7M8
47406	229533.I1RG58	pp	229533.I1RTJ2
47407	229533.I1RG58	pp	229533.I1RGK9
47408	229533.I1RG58	pp	229533.I1RTI7
47409	229533.I1RH11	pp	229533.I1RWM4
47410	229533.I1RH11	pp	229533.I1RHD4
47411	229533.I1RH11	pp	229533.V6RHD3
47412	229533.I1RH11	pp	229533.I1RNH4
47413	229533.I1RBH2	pp	229533.Q4HY90
47414	229533.I1RBH2	pp	229533.I1RZG7
47415	229533.I1RBH2	pp	229533.I1RXE5
47416	229533.I1R9K3	pp	229533.I1RYU5
47417	229533.I1R9K3	pp	229533.I1RX80
47418	229533.I1R9K3	pp	229533.I1REP3
47419	229533.I1R9K3	pp	229533.I1RN19
47420	229533.I1R9K3	pp	229533.I1RT36
47421	229533.I1R9K3	pp	229533.I1RCB4
47422	229533.I1R9K3	pp	229533.I1RS40
47423	229533.I1R9K3	pp	229533.I1RM68
47424	229533.I1R9K3	pp	229533.I1S770
47425	229533.I1R9K3	pp	229533.I1RQ30
47426	229533.I1R9K3	pp	229533.I1RZ20
47427	229533.I1R9K3	pp	229533.I1RM69
47428	229533.I1R9K3	pp	229533.I1RMG6
47429	229533.I1R9K3	pp	229533.I1S8X1
47430	229533.I1R9K3	pp	229533.I1RTI7
47431	229533.I1R9K3	pp	229533.I1RIB6
47432	229533.I1R9K3	pp	229533.I1RE53
47433	229533.I1R9K3	pp	229533.I1RNG4
47434	229533.I1R9K3	pp	229533.I1S7A5
47435	229533.I1R9K3	pp	229533.I1RMG7
47436	229533.I1R9K3	pp	229533.I1RZ52
47437	229533.I1R9K3	pp	229533.I1RIY0
47438	229533.I1R9K3	pp	229533.I1RI56
47439	229533.I1R9K3	pp	229533.I1RBK1
47440	229533.I1R9K3	pp	229533.I1R9Z1
47441	229533.I1R9K3	pp	229533.I1S7M8
47442	229533.I1R9K3	pp	229533.I1RYW6
47443	229533.I1R9K3	pp	229533.I1RYD3
47444	229533.I1R9K3	pp	229533.I1RJF1
47445	229533.Q4ILH3	pp	229533.V6R382
47446	229533.Q4ILH3	pp	229533.Q4IQ08
47447	229533.V6QV31	pp	229533.V6RUB1
47448	229533.V6QV31	pp	229533.V6RDH3
47449	229533.V6QV31	pp	229533.V6QXN0
47450	229533.V6QV31	pp	229533.V6RIV7

47451	229533.V6QV31	pp	229533.V6RS91
47452	229533.A0A1C3YII8	pp	229533.V6RIE5
47453	229533.A0A1C3YII8	pp	229533.I1RD80
47454	229533.A0A1C3YII8	pp	229533.I1RDW1
47455	229533.A0A1C3YII8	pp	229533.I1S2D3
47456	229533.A0A1C3YII8	pp	229533.I1S2P8
47457	229533.I1RH33	pp	229533.I1S1W4
47458	229533.I1RH33	pp	229533.I1RVC7
47459	229533.I1RH33	pp	229533.I1RHY7
47460	229533.A0A1C3YII6	pp	229533.I1RT23
47461	229533.A0A1C3YLQ1	pp	229533.I1S4S9
47462	229533.A0A1C3YLQ1	pp	229533.I1S5B1
47463	229533.A0A1C3YLQ1	pp	229533.I1RNK8
47464	229533.A0A1C3YLQ1	pp	229533.I1RP81
47465	229533.A0A1C3YLQ1	pp	229533.I1RZ17
47466	229533.A0A1C3YLQ1	pp	229533.Q4I4A4
47467	229533.A0A1C3YLQ1	pp	229533.I1RMP9
47468	229533.A0A1C3YLQ1	pp	229533.I1RK45
47469	229533.A0A1C3YLQ1	pp	229533.I1S252
47470	229533.A0A1C3YLQ1	pp	229533.Q4I785
47471	229533.A0A1C3YLQ1	pp	229533.I1RA25
47472	229533.A0A1C3YLQ1	pp	229533.I1RBN0
47473	229533.A0A1C3YLQ1	pp	229533.I1RYB9
47474	229533.A0A1C3YLQ1	pp	229533.I1RTM9
47475	229533.A0A1C3YLQ1	pp	229533.Q4IB63
47476	229533.A0A1C3YLQ1	pp	229533.I1REU0
47477	229533.A0A1C3YLQ1	pp	229533.I1RZ18
47478	229533.A0A1C3YLQ1	pp	229533.Q4I0K3
47479	229533.A0A1C3YLQ1	pp	229533.I1R9W2
47480	229533.A0A1C3YLQ1	pp	229533.I1S8P5
47481	229533.A0A1C3YLQ1	pp	229533.Q4I073
47482	229533.A0A1C3YLQ1	pp	229533.I1RTA0
47483	229533.A0A1C3YLQ1	pp	229533.A0A1C3YN83
47484	229533.A0A1C3YLQ1	pp	229533.Q4HXT5
47485	229533.I1RZU2	pp	229533.I1S0C2
47486	229533.I1RZU2	pp	229533.V6RDH3
47487	229533.I1RZU2	pp	229533.Q4I1N3
47488	229533.I1RZU2	pp	229533.V6RS91
47489	229533.I1RZU2	pp	229533.V6QV31
47490	229533.I1S0V3	pp	229533.I1S1W4
47491	229533.A0A1C3YN73	pp	229533.I1RPA0
47492	229533.A0A1C3YN73	pp	229533.V6RRF6
47493	229533.A0A1C3YN73	pp	229533.Q4I5V2
47494	229533.I1RP81	pp	229533.I1S272
47495	229533.I1RP81	pp	229533.Q4I0K3
47496	229533.I1RP81	pp	229533.Q4I073
47497	229533.I1RP81	pp	229533.I1RST0
47498	229533.I1RP81	pp	229533.Q4IB63
47499	229533.I1RP81	pp	229533.I1RZ13
47500	229533.I1RP81	pp	229533.I1S252
47501	229533.I1RP81	pp	229533.I1RRT3
47502	229533.I1RG71	pp	229533.V6RKY8
47503	229533.I1RG71	pp	229533.I1RSF1
47504	229533.I1RG71	pp	229533.I1RR45
47505	229533.I1RG71	pp	229533.I1RKP1
47506	229533.I1RG71	pp	229533.I1S8Z2
47507	229533.I1RG71	pp	229533.I1RT75
47508	229533.I1RG71	pp	229533.I1RN52
47509	229533.I1RG71	pp	229533.I1RRK5
47510	229533.A0A1C3YKZ0	pp	229533.I1RSI1
47511	229533.A0A1C3YKZ0	pp	229533.I1S255
47512	229533.A0A1C3YKZ0	pp	229533.I1S201
47513	229533.A0A1C3YKZ0	pp	229533.I1RPV5
47514	229533.A0A1C3YKZ0	pp	229533.A0A1C3YN82
47515	229533.A0A1C3YKZ0	pp	229533.I1RB53
47516	229533.A0A1C3YKZ0	pp	229533.I1RAV6
47517	229533.A0A1C3YKZ0	pp	229533.I1RZ40
47518	229533.A0A1C3YKZ0	pp	229533.Q4HW67
47519	229533.A0A1C3YKZ0	pp	229533.I1RLV3
47520	229533.A0A1C3YKZ0	pp	229533.I1RN48
47521	229533.A0A1C3YKZ0	pp	229533.I1RB71
47522	229533.A0A1C3YKZ0	pp	229533.I1RIS3
47523	229533.V6RF62	pp	229533.V6RLM7

47524 229533.V6RF62 pp 229533.V6RRM0
47525 229533.I1RLE2 pp 229533.I1S5S8
47526 229533.I1RLE2 pp 229533.I1S9U6
47527 229533.I1RLE2 pp 229533.I1RYH1
47528 229533.I1RLE2 pp 229533.I1RP58
47529 229533.I1RLE2 pp 229533.I1RPQ2
47530 229533.I1RLE2 pp 229533.I1S0Y2
47531 229533.I1RLE2 pp 229533.I1RNE9
47532 229533.I1RLE2 pp 229533.I1RW91
47533 229533.I1RLE2 pp 229533.I1RUP5
47534 229533.I1RLE2 pp 229533.I1RPI4
47535 229533.I1RLE2 pp 229533.I1RYD9
47536 229533.Q4HXF0 pp 229533.Q4INZ9
47537 229533.Q4HXF0 pp 229533.Q4IJH1
47538 229533.Q4HXF0 pp 229533.V6RQW4
47539 229533.Q4HXF0 pp 229533.V6RTJ8
47540 229533.Q4HXF0 pp 229533.V6RY56
47541 229533.Q4HXF0 pp 229533.V6RBN2
47542 229533.Q4HXF0 pp 229533.Q4HZQ1
47543 229533.Q4HXF0 pp 229533.V6QYF0
47544 229533.Q4HXF0 pp 229533.V6R1K4
47545 229533.Q4HXF0 pp 229533.V6R227
47546 229533.Q4HXF0 pp 229533.Q4I624
47547 229533.Q4HXF0 pp 229533.V6RCZ8
47548 229533.Q4HXF0 pp 229533.Q4IJK2
47549 229533.Q4HXF0 pp 229533.Q4IEK8
47550 229533.Q4HXF0 pp 229533.V6QUE6
47551 229533.Q4HXF0 pp 229533.Q4IF76
47552 229533.Q4HXF0 pp 229533.Q4IJ56
47553 229533.Q4HXF0 pp 229533.Q4HZ68
47554 229533.Q4HXF0 pp 229533.V6R057
47555 229533.Q4HXF0 pp 229533.V6RK96
47556 229533.Q4HXF0 pp 229533.Q4IQW0
47557 229533.Q4HXF0 pp 229533.Q4IMJ1
47558 229533.Q4HXF0 pp 229533.Q4HZI0
47559 229533.Q4HXF0 pp 229533.V6QVH8
47560 229533.Q4HXF0 pp 229533.V6R2A1
47561 229533.Q4HXF0 pp 229533.V6R283
47562 229533.Q4HXF0 pp 229533.V6RGN5
47563 229533.Q4HXF0 pp 229533.V6RSF3
47564 229533.Q4HXF0 pp 229533.Q4I375
47565 229533.Q4HXF0 pp 229533.Q4IEV5
47566 229533.Q4HXF0 pp 229533.V6RN69
47567 229533.Q4HXF0 pp 229533.V6R382
47568 229533.Q4HXF0 pp 229533.V6R042
47569 229533.Q4HXF0 pp 229533.Q4IN63
47570 229533.Q4HXF0 pp 229533.V6RRH2
47571 229533.I1RCI2 pp 229533.I1S193
47572 229533.I1RCI2 pp 229533.I1RIT1
47573 229533.I1RCI2 pp 229533.I1RT19
47574 229533.I1RCI2 pp 229533.I1RZP6
47575 229533.I1RCI2 pp 229533.I1S282
47576 229533.I1RCI2 pp 229533.I1RZM2
47577 229533.I1RCI2 pp 229533.I1RTQ0
47578 229533.I1RCI2 pp 229533.I1RYX3
47579 229533.I1RCI2 pp 229533.I1RWY3
47580 229533.A0A1C3YIR3 pp 229533.I1RJV4
47581 229533.A0A1C3YIR3 pp 229533.I1RXE5
47582 229533.A0A1C3YIR3 pp 229533.Q4HY90
47583 229533.A0A1C3YIR3 pp 229533.A0A1C3YK01
47584 229533.I1RYI9 pp 229533.V6RIS2
47585 229533.I1RYI9 pp 229533.I1S7M2
47586 229533.I1RYI9 pp 229533.I1S8Y1
47587 229533.I1RYI9 pp 229533.I1S7M3
47588 229533.I1RYI9 pp 229533.V6QXG1
47589 229533.I1RYI9 pp 229533.I1RZ11
47590 229533.I1RYI9 pp 229533.I1RZG7
47591 229533.I1RYI9 pp 229533.I1S0N4
47592 229533.I1RYI9 pp 229533.V6RY33
47593 229533.I1RYI9 pp 229533.I1S5S2
47594 229533.I1RYI9 pp 229533.I1RZZ1
47595 229533.I1RYI9 pp 229533.Q4IF76
47596 229533.I1RYI9 pp 229533.I1S2Y5

47597	229533.I1RYI9	pp	229533.Q4IBK5
47598	229533.I1RYI9	pp	229533.Q4IJH1
47599	229533.I1RYI9	pp	229533.I1RYJ9
47600	229533.I1RYI9	pp	229533.I1S524
47601	229533.I1RYI9	pp	229533.I1S0B1
47602	229533.I1RYI9	pp	229533.Q4IPH4
47603	229533.I1RMJ3	pp	229533.I1RY62
47604	229533.I1RMJ3	pp	229533.I1RNG4
47605	229533.I1RMJ3	pp	229533.I1RWF5
47606	229533.I1RMJ3	pp	229533.I1S2P0
47607	229533.I1RMJ3	pp	229533.I1RPD6
47608	229533.I1RMJ3	pp	229533.I1RT75
47609	229533.I1RMJ3	pp	229533.I1RYP2
47610	229533.I1RMJ3	pp	229533.I1RNB4
47611	229533.I1RMJ3	pp	229533.I1S673
47612	229533.I1RMJ3	pp	229533.I1S299
47613	229533.I1RMJ3	pp	229533.I1RZ90
47614	229533.I1RMJ3	pp	229533.I1RT88
47615	229533.I1RMJ3	pp	229533.V6RUY9
47616	229533.I1RMJ3	pp	229533.I1S3G4
47617	229533.I1RMJ3	pp	229533.I1RSS7
47618	229533.I1RMJ3	pp	229533.I1S024
47619	229533.I1RMJ3	pp	229533.I1RN88
47620	229533.I1RMJ3	pp	229533.I1S3L8
47621	229533.I1RMJ3	pp	229533.I1S3T7
47622	229533.I1RMJ3	pp	229533.I1S4Z7
47623	229533.I1RMJ3	pp	229533.V6RKY8
47624	229533.I1RMJ3	pp	229533.I1RVR2
47625	229533.I1RMJ3	pp	229533.I1RSQ4
47626	229533.I1RMJ3	pp	229533.I1RUR8
47627	229533.I1RMJ3	pp	229533.I1RYB8
47628	229533.I1RMJ3	pp	229533.I1S6P6
47629	229533.I1RMJ3	pp	229533.I1RVZ3
47630	229533.I1RMJ3	pp	229533.I1RSK5
47631	229533.I1RMJ3	pp	229533.I1RQ38
47632	229533.I1RMJ3	pp	229533.V6R9L8
47633	229533.I1RMJ3	pp	229533.I1S0V7
47634	229533.I1RMJ3	pp	229533.I1RYC8
47635	229533.I1RMJ3	pp	229533.I1RNU7
47636	229533.I1RMJ3	pp	229533.I1S256
47637	229533.I1RMJ3	pp	229533.I1S147
47638	229533.I1RMJ3	pp	229533.I1RSY1
47639	229533.I1RMJ3	pp	229533.I1S5N3
47640	229533.I1RMJ3	pp	229533.I1RYA0
47641	229533.I1RMJ3	pp	229533.V6RRM0
47642	229533.I1RMJ3	pp	229533.I1RXY7
47643	229533.I1RMJ3	pp	229533.I1S8R2
47644	229533.I1RMJ3	pp	229533.V6RLM7
47645	229533.I1RMJ3	pp	229533.I1S8R4
47646	229533.I1RMJ3	pp	229533.I1S7J8
47647	229533.I1RMJ3	pp	229533.V6RM02
47648	229533.I1RMJ3	pp	229533.I1S8C1
47649	229533.I1RMJ3	pp	229533.I1RR45
47650	229533.I1RMJ3	pp	229533.V6R382
47651	229533.I1RMJ3	pp	229533.I1S123
47652	229533.I1RMJ3	pp	229533.I1S9T5
47653	229533.I1RMJ3	pp	229533.I1RRX5
47654	229533.I1RMJ3	pp	229533.I1RWW4
47655	229533.I1RMJ3	pp	229533.V6RN31
47656	229533.I1RMJ3	pp	229533.I1S241
47657	229533.I1RMJ3	pp	229533.V6QX21
47658	229533.I1RMJ3	pp	229533.I1RZM4
47659	229533.I1RMJ3	pp	229533.I1S3J4
47660	229533.I1RMJ3	pp	229533.I1S273
47661	229533.I1RMJ3	pp	229533.I1RQL4
47662	229533.I1RMJ3	pp	229533.I1S0K0
47663	229533.I1RMJ3	pp	229533.I1RNM7
47664	229533.I1RMJ3	pp	229533.I1S1W8
47665	229533.I1RMJ3	pp	229533.I1RSB7
47666	229533.I1RMJ3	pp	229533.I1RZY1
47667	229533.I1RMJ3	pp	229533.I1RMK7
47668	229533.I1RMJ3	pp	229533.I1RQP2
47669	229533.I1RMJ3	pp	229533.I1S3L2

47670 229533.I1RMJ3 pp 229533.I1S8Z2
47671 229533.I1RMJ3 pp 229533.I1RP86
47672 229533.I1RMJ3 pp 229533.I1RQE8
47673 229533.I1RMJ3 pp 229533.I1S8F8
47674 229533.I1RMJ3 pp 229533.I1RSQ2
47675 229533.I1RMJ3 pp 229533.I1RRK5
47676 229533.I1RMJ3 pp 229533.I1RY49
47677 229533.I1RMJ3 pp 229533.I1RXE6
47678 229533.I1RMJ3 pp 229533.I1RUI6
47679 229533.I1RMJ3 pp 229533.I1RQ12
47680 229533.I1RMJ3 pp 229533.I1RVV7
47681 229533.I1RMJ3 pp 229533.V6RPN0
47682 229533.I1RMJ3 pp 229533.V6RK96
47683 229533.I1RMJ3 pp 229533.I1S5R5
47684 229533.I1RMJ3 pp 229533.I1RMY1
47685 229533.I1RMJ3 pp 229533.I1RPR1
47686 229533.I1RMJ3 pp 229533.I1RYM5
47687 229533.I1RMJ3 pp 229533.V6R9Z3
47688 229533.I1RMJ3 pp 229533.I1RY12
47689 229533.I1RMJ3 pp 229533.I1RXS0
47690 229533.I1RMJ3 pp 229533.I1RUY4
47691 229533.I1RMJ3 pp 229533.I1RPT0
47692 229533.I1RMJ3 pp 229533.I1S0V6
47693 229533.I1RME2 pp 229533.I1S253
47694 229533.I1RME2 pp 229533.I1RT05
47695 229533.I1RME2 pp 229533.I1RW68
47696 229533.I1RME2 pp 229533.I1RRE0
47697 229533.I1S290 pp 229533.I1S5S2
47698 229533.I1S290 pp 229533.O74638
47699 229533.I1S290 pp 229533.I1S3V2
47700 229533.I1S290 pp 229533.V6RG79
47701 229533.I1RZ17 pp 229533.I1S4S9
47702 229533.I1RZ17 pp 229533.Q4HXT5
47703 229533.I1RZ17 pp 229533.I1S5B1
47704 229533.I1RZ17 pp 229533.Q4ILH3
47705 229533.I1RZ17 pp 229533.Q4I0K3
47706 229533.I1RZ17 pp 229533.I1RZV2
47707 229533.I1RZ17 pp 229533.I1RZ18
47708 229533.I1RZ17 pp 229533.Q4I073
47709 229533.I1RZ17 pp 229533.I1S252
47710 229533.I1RZ17 pp 229533.Q4IB63
47711 229533.I1RZ17 pp 229533.I1S0B4
47712 229533.I1RZ17 pp 229533.Q4I785
47713 229533.A0A1C3YM01 pp 229533.I1RQ30
47714 229533.A0A1C3YM01 pp 229533.I1RS40
47715 229533.A0A1C3YM01 pp 229533.I1S770
47716 229533.A0A1C3YM01 pp 229533.I1REP3
47717 229533.A0A1C3YM01 pp 229533.I1RCB4
47718 229533.A0A1C3YM01 pp 229533.I1RT36
47719 229533.A0A1C3YM01 pp 229533.I1RM68
47720 229533.A0A1C3YM01 pp 229533.I1RZ52
47721 229533.A0A1C3YM01 pp 229533.I1RTI7
47722 229533.A0A1C3YM01 pp 229533.A0A1C3YMQ5
47723 229533.A0A1C3YM01 pp 229533.I1RI56
47724 229533.A0A1C3YM01 pp 229533.I1R9Z1
47725 229533.A0A1C3YM01 pp 229533.I1RJF1
47726 229533.A0A1C3YM01 pp 229533.I1RYW6
47727 229533.A0A1C3YM01 pp 229533.I1S8X1
47728 229533.A0A1C3YM01 pp 229533.I1RMG7
47729 229533.A0A1C3YM01 pp 229533.I1RIY0
47730 229533.A0A1C3YM01 pp 229533.I1RZ20
47731 229533.A0A1C3YM01 pp 229533.I1RBK1
47732 229533.A0A1C3YM01 pp 229533.I1RYU5
47733 229533.A0A1C3YM01 pp 229533.I1RM69
47734 229533.A0A1C3YM01 pp 229533.I1RX80
47735 229533.A0A1C3YM01 pp 229533.I1RMG6
47736 229533.A0A1C3YM01 pp 229533.I1RNG4
47737 229533.A0A1C3YM01 pp 229533.I1S7A5
47738 229533.A0A1C3YM01 pp 229533.I1RE53
47739 229533.A0A1C3YM01 pp 229533.I1R989
47740 229533.A0A1C3YM01 pp 229533.I1RN19
47741 229533.A0A1C3YM01 pp 229533.I1RIB6
47742 229533.A0A1C3YM01 pp 229533.I1RYD3

47743 229533.A0A1C3YM01 pp 229533.I1S7M8
47744 229533.I1RKX1 pp 229533.I1S984
47745 229533.I1RKX1 pp 229533.I1S574
47746 229533.I1RKX1 pp 229533.I1RNA1
47747 229533.I1RST0 pp 229533.Q4I0K3
47748 229533.I1RST0 pp 229533.Q4I073
47749 229533.I1RST0 pp 229533.I1S272
47750 229533.I1RST0 pp 229533.I1RVJ8
47751 229533.I1RST0 pp 229533.Q4I1Y5
47752 229533.I1RST0 pp 229533.I1RZ13
47753 229533.I1RST0 pp 229533.I1S4S9
47754 229533.I1RST0 pp 229533.I1S2B0
47755 229533.I1RST0 pp 229533.I1S252
47756 229533.I1RST0 pp 229533.V6RPQ8
47757 229533.I1RST0 pp 229533.I1RZ34
47758 229533.I1RST0 pp 229533.Q4IB63
47759 229533.I1RQA8 pp 229533.I1S4T3
47760 229533.I1RQA8 pp 229533.Q4HY90
47761 229533.I1RQA8 pp 229533.I1RR61
47762 229533.I1RQA8 pp 229533.I1RXE5
47763 229533.I1RQA8 pp 229533.I1RS60
47764 229533.I1RK78 pp 229533.I1S5S2
47765 229533.I1RK78 pp 229533.I1RZ40
47766 229533.I1RK78 pp 229533.I1RNE7
47767 229533.I1RK78 pp 229533.I1RYI6
47768 229533.I1RK78 pp 229533.O74638
47769 229533.I1RK78 pp 229533.I1S3V2
47770 229533.I1RK78 pp 229533.I1RMW7
47771 229533.I1RK78 pp 229533.I1RXV3
47772 229533.A0A1C3YLE2 pp 229533.V6RRM0
47773 229533.A0A1C3YLE2 pp 229533.I1RV23
47774 229533.A0A1C3YLE2 pp 229533.I1RMQ5
47775 229533.A0A1C3YLE2 pp 229533.I1S0V7
47776 229533.A0A1C3YLE2 pp 229533.I1S147
47777 229533.A0A1C3YLE2 pp 229533.I1RE79
47778 229533.A0A1C3YLE2 pp 229533.I1S256
47779 229533.A0A1C3YLE2 pp 229533.I1RHC8
47780 229533.A0A1C3YLE2 pp 229533.I1RGX1
47781 229533.A0A1C3YLE2 pp 229533.I1RML4
47782 229533.A0A1C3YLE2 pp 229533.I1RXY7
47783 229533.A0A1C3YLE2 pp 229533.I1RG46
47784 229533.A0A1C3YLE2 pp 229533.I1RYC8
47785 229533.A0A1C3YLE2 pp 229533.V6R1X4
47786 229533.A0A1C3YLE2 pp 229533.I1S8R4
47787 229533.A0A1C3YLE2 pp 229533.V6R382
47788 229533.A0A1C3YLE2 pp 229533.I1RB38
47789 229533.A0A1C3YLE2 pp 229533.A0A1C3YMS7
47790 229533.A0A1C3YLE2 pp 229533.I1RE52
47791 229533.A0A1C3YLE2 pp 229533.I1RHJ2
47792 229533.A0A1C3YLE2 pp 229533.V6QX21
47793 229533.A0A1C3YLE2 pp 229533.V6RM02
47794 229533.A0A1C3YLE2 pp 229533.I1RD80
47795 229533.A0A1C3YLE2 pp 229533.I1RCG5
47796 229533.A0A1C3YLE2 pp 229533.I1RF85
47797 229533.A0A1C3YLE2 pp 229533.I1S123
47798 229533.A0A1C3YLE2 pp 229533.V6RUY9
47799 229533.A0A1C3YLE2 pp 229533.I1RBY9
47800 229533.A0A1C3YLE2 pp 229533.I1RNU7
47801 229533.A0A1C3YLE2 pp 229533.I1RYA0
47802 229533.A0A1C3YLE2 pp 229533.I1RMH2
47803 229533.A0A1C3YLE2 pp 229533.I1S149
47804 229533.A0A1C3YLE2 pp 229533.I1RT75
47805 229533.A0A1C3YLE2 pp 229533.I1S8R2
47806 229533.A0A1C3YLE2 pp 229533.I1RGJ3
47807 229533.A0A1C3YLE2 pp 229533.I1RBE6
47808 229533.A0A1C3YLE2 pp 229533.I1RWF5
47809 229533.A0A1C3YLE2 pp 229533.I1RR95
47810 229533.A0A1C3YLE2 pp 229533.I1RL81
47811 229533.A0A1C3YLE2 pp 229533.A0A1C3YN70
47812 229533.A0A1C3YLE2 pp 229533.I1RYW9
47813 229533.A0A1C3YLE2 pp 229533.I1RBA4
47814 229533.A0A1C3YLE2 pp 229533.I1RZ90
47815 229533.A0A1C3YLE2 pp 229533.I1S9T5

47816 229533.A0A1C3YLE2 pp 229533.I1RY62
47817 229533.A0A1C3YLE2 pp 229533.I1RT88
47818 229533.A0A1C3YLE2 pp 229533.I1S673
47819 229533.A0A1C3YLE2 pp 229533.I1RSS7
47820 229533.A0A1C3YLE2 pp 229533.I1RNB4
47821 229533.A0A1C3YLE2 pp 229533.I1RVZ3
47822 229533.A0A1C3YLE2 pp 229533.I1RQG7
47823 229533.A0A1C3YLE2 pp 229533.I1RC67
47824 229533.A0A1C3YLE2 pp 229533.I1RE53
47825 229533.A0A1C3YLE2 pp 229533.I1RYP2
47826 229533.A0A1C3YLE2 pp 229533.I1RNG4
47827 229533.A0A1C3YLE2 pp 229533.I1S299
47828 229533.A0A1C3YLE2 pp 229533.I1S3L8
47829 229533.A0A1C3YLE2 pp 229533.I1S3T7
47830 229533.A0A1C3YLE2 pp 229533.I1RYK0
47831 229533.A0A1C3YLE2 pp 229533.I1RGC3
47832 229533.A0A1C3YLE2 pp 229533.I1RJK3
47833 229533.A0A1C3YLE2 pp 229533.I1RQ38
47834 229533.A0A1C3YLE2 pp 229533.I1RH91
47835 229533.A0A1C3YLE2 pp 229533.V6RKY8
47836 229533.A0A1C3YLE2 pp 229533.I1RSK5
47837 229533.A0A1C3YLE2 pp 229533.I1RFB0
47838 229533.A0A1C3YLE2 pp 229533.I1RZ38
47839 229533.A0A1C3YLE2 pp 229533.I1RUR8
47840 229533.A0A1C3YLE2 pp 229533.I1RC29
47841 229533.A0A1C3YLE2 pp 229533.I1RKB2
47842 229533.A0A1C3YLE2 pp 229533.I1RKK5
47843 229533.A0A1C3YLE2 pp 229533.I1S3G4
47844 229533.A0A1C3YLE2 pp 229533.I1REI3
47845 229533.A0A1C3YLE2 pp 229533.I1RSQ4
47846 229533.A0A1C3YLE2 pp 229533.I1S4Z7
47847 229533.A0A1C3YLE2 pp 229533.I1RFI4
47848 229533.A0A1C3YLE2 pp 229533.I1RYB8
47849 229533.A0A1C3YLE2 pp 229533.I1RN88
47850 229533.A0A1C3YLE2 pp 229533.I1S4D0
47851 229533.A0A1C3YLE2 pp 229533.A0A1C3YM35
47852 229533.A0A1C3YLE2 pp 229533.I1RY50
47853 229533.A0A1C3YLE2 pp 229533.I1RXE6
47854 229533.A0A1C3YLE2 pp 229533.I1RBF3
47855 229533.A0A1C3YLE2 pp 229533.I1REE2
47856 229533.A0A1C3YLE2 pp 229533.I1RW55
47857 229533.A0A1C3YLE2 pp 229533.I1RNY6
47858 229533.A0A1C3YLE2 pp 229533.I1RVR2
47859 229533.A0A1C3YLE2 pp 229533.V6RPS5
47860 229533.A0A1C3YLE2 pp 229533.I1RUI6
47861 229533.A0A1C3YLE2 pp 229533.I1RHZ5
47862 229533.A0A1C3YLE2 pp 229533.V6RK96
47863 229533.A0A1C3YLE2 pp 229533.I1RMY1
47864 229533.A0A1C3YLE2 pp 229533.I1RVV7
47865 229533.A0A1C3YLE2 pp 229533.I1RJT5
47866 229533.A0A1C3YLE2 pp 229533.I1S5R5
47867 229533.A0A1C3YLE2 pp 229533.A0A1C3YLP6
47868 229533.A0A1C3YLE2 pp 229533.I1RA90
47869 229533.A0A1C3YLE2 pp 229533.I1RC48
47870 229533.A0A1C3YLE2 pp 229533.I1S8F8
47871 229533.A0A1C3YLE2 pp 229533.I1S3T6
47872 229533.A0A1C3YLE2 pp 229533.I1RPR1
47873 229533.A0A1C3YLE2 pp 229533.V6RPN0
47874 229533.A0A1C3YLE2 pp 229533.I1RYA2
47875 229533.A0A1C3YLE2 pp 229533.I1RCM5
47876 229533.A0A1C3YLE2 pp 229533.I1RAM9
47877 229533.A0A1C3YLE2 pp 229533.I1RIM7
47878 229533.A0A1C3YLE2 pp 229533.I1S0V6
47879 229533.A0A1C3YLE2 pp 229533.I1RY12
47880 229533.A0A1C3YLE2 pp 229533.I1RA72
47881 229533.A0A1C3YLE2 pp 229533.I1S8S9
47882 229533.A0A1C3YLE2 pp 229533.I1RUY4
47883 229533.A0A1C3YLE2 pp 229533.I1S8C1
47884 229533.A0A1C3YLE2 pp 229533.I1RYL5
47885 229533.A0A1C3YLE2 pp 229533.I1RCV1
47886 229533.A0A1C3YLE2 pp 229533.I1S034
47887 229533.A0A1C3YLE2 pp 229533.I1RUQ3
47888 229533.A0A1C3YLE2 pp 229533.I1RZM4

47889 229533.A0A1C3YLE2 pp 229533.I1RGU3
47890 229533.A0A1C3YLE2 pp 229533.I1RPT0
47891 229533.A0A1C3YLE2 pp 229533.V6RY56
47892 229533.A0A1C3YLE2 pp 229533.I1RG33
47893 229533.A0A1C3YLE2 pp 229533.I1RXS0
47894 229533.A0A1C3YLE2 pp 229533.I1RJY7
47895 229533.A0A1C3YLE2 pp 229533.I1RAV5
47896 229533.A0A1C3YLE2 pp 229533.I1RRX5
47897 229533.A0A1C3YLE2 pp 229533.A0A1C3YLI1
47898 229533.A0A1C3YLE2 pp 229533.I1S223
47899 229533.A0A1C3YLE2 pp 229533.I1RAU9
47900 229533.A0A1C3YLE2 pp 229533.I1RDE6
47901 229533.A0A1C3YLE2 pp 229533.I1S241
47902 229533.A0A1C3YLE2 pp 229533.I1RQL4
47903 229533.A0A1C3YLE2 pp 229533.I1RP86
47904 229533.A0A1C3YLE2 pp 229533.I1S0K0
47905 229533.A0A1C3YLE2 pp 229533.I1RCU2
47906 229533.A0A1C3YLE2 pp 229533.I1RHT5
47907 229533.A0A1C3YLE2 pp 229533.I1RCR6
47908 229533.A0A1C3YLE2 pp 229533.I1RJD3
47909 229533.A0A1C3YLE2 pp 229533.I1RZJ6
47910 229533.A0A1C3YLE2 pp 229533.I1S3L2
47911 229533.A0A1C3YLE2 pp 229533.I1S3J4
47912 229533.A0A1C3YLE2 pp 229533.I1RYU6
47913 229533.A0A1C3YLE2 pp 229533.I1S8Z2
47914 229533.A0A1C3YLE2 pp 229533.I1RYQ4
47915 229533.A0A1C3YLE2 pp 229533.I1RC39
47916 229533.A0A1C3YLE2 pp 229533.I1RQE8
47917 229533.A0A1C3YLE2 pp 229533.Q5I2J3
47918 229533.A0A1C3YLE2 pp 229533.I1RT82
47919 229533.A0A1C3YLE2 pp 229533.I1RY21
47920 229533.A0A1C3YLE2 pp 229533.I1RAG9
47921 229533.A0A1C3YLE2 pp 229533.I1RSQ2
47922 229533.A0A1C3YLE2 pp 229533.I1RJN7
47923 229533.A0A1C3YLE2 pp 229533.I1RQP2
47924 229533.A0A1C3YLE2 pp 229533.I1RA32
47925 229533.A0A1C3YLE2 pp 229533.I1RMQ6
47926 229533.A0A1C3YLE2 pp 229533.I1S1W8
47927 229533.A0A1C3YLE2 pp 229533.I1R9Y4
47928 229533.A0A1C3YLE2 pp 229533.I1RMK7
47929 229533.A0A1C3YLE2 pp 229533.A0A1C3YN26
47930 229533.A0A1C3YLE2 pp 229533.I1RC11
47931 229533.A0A1C3YLE2 pp 229533.I1RNM7
47932 229533.A0A1C3YLE2 pp 229533.I1RL49
47933 229533.A0A1C3YLE2 pp 229533.I1RHK2
47934 229533.A0A1C3YLE2 pp 229533.Q4HZS8
47935 229533.I1RP53 pp 229533.I1RYY2
47936 229533.I1RP53 pp 229533.I1RXH3
47937 229533.I1RP53 pp 229533.I1RYU0
47938 229533.I1RP53 pp 229533.I1RXV3
47939 229533.I1RP53 pp 229533.I1S2C4
47940 229533.A0A1C3YJA7 pp 229533.I1RNG4
47941 229533.A0A1C3YN70 pp 229533.I1RMK7
47942 229533.A0A1C3YN70 pp 229533.I1RN84
47943 229533.A0A1C3YN70 pp 229533.I1RPY0
47944 229533.A0A1C3YN70 pp 229533.I1RMJ3
47945 229533.A0A1C3YN70 pp 229533.I1RVR2
47946 229533.I1RKK9 pp 229533.I1S3V2
47947 229533.I1RKK9 pp 229533.I1RYI6
47948 229533.I1RKK9 pp 229533.O74638
47949 229533.I1RKK9 pp 229533.I1RXV3
47950 229533.I1RKK9 pp 229533.I1RMW7
47951 229533.I1RKK9 pp 229533.I1S5S2
47952 229533.I1RKK9 pp 229533.I1RZ40
47953 229533.I1RKK9 pp 229533.I1RNE7
47954 229533.Q4IB96 pp 229533.Q4IQX3
47955 229533.Q4IB96 pp 229533.Q4ICA8
47956 229533.Q4IB96 pp 229533.V6RY33
47957 229533.Q4IB96 pp 229533.Q4IPI4
47958 229533.Q4IB96 pp 229533.Q4IEV4
47959 229533.A0A098DZL0 pp 229533.I1RXX1
47960 229533.A0A098DZL0 pp 229533.I1RST9
47961 229533.A0A098DZL0 pp 229533.A0A1C3YLP6

47962	229533.A0A098DZL0	pp	229533.I1RYM5
47963	229533.A0A098DZL0	pp	229533.I1S267
47964	229533.A0A098DZL0	pp	229533.I1RWM4
47965	229533.A0A098DZL0	pp	229533.I1RTJ2
47966	229533.A0A098DZL0	pp	229533.V6RG22
47967	229533.A0A098DZL0	pp	229533.I1RLA2
47968	229533.A0A098DZL0	pp	229533.A0A0E0RW18
47969	229533.A0A098DZL0	pp	229533.I1RCE6
47970	229533.A0A098DZL0	pp	229533.I1RGK9
47971	229533.A0A098DZL0	pp	229533.I1S130
47972	229533.A0A098DZL0	pp	229533.I1RL16
47973	229533.A0A098DZL0	pp	229533.I1S048
47974	229533.A0A098DZL0	pp	229533.I1RYD3
47975	229533.A0A098DZL0	pp	229533.I1R9Y2
47976	229533.A0A098DZL0	pp	229533.I1RZ25
47977	229533.A0A098DZL0	pp	229533.A0A0E0SH57
47978	229533.A0A098DZL0	pp	229533.A0A1C3YKL8
47979	229533.A0A098DZL0	pp	229533.I1S1K6
47980	229533.A0A098DZL0	pp	229533.I1RVZ3
47981	229533.A0A098DZL0	pp	229533.I1RSS7
47982	229533.A0A098DZL0	pp	229533.I1RY35
47983	229533.A0A098DZL0	pp	229533.A0A0E0SMW1
47984	229533.A0A098DZL0	pp	229533.A0A1C3YKN4
47985	229533.A0A098DZL0	pp	229533.I1RNG4
47986	229533.I1S252	pp	229533.Q4I0K3
47987	229533.I1S252	pp	229533.Q4I785
47988	229533.I1S252	pp	229533.Q4ILH3
47989	229533.I1S252	pp	229533.Q4IB63
47990	229533.I1S252	pp	229533.I1S272
47991	229533.I1S252	pp	229533.Q4I073
47992	229533.I1S252	pp	229533.I1S4S9
47993	229533.A0A1C3YJA3	pp	229533.Q4IAA0
47994	229533.A0A1C3YJA3	pp	229533.I1RIX1
47995	229533.A0A1C3YJA3	pp	229533.I1RN59
47996	229533.A0A1C3YJA3	pp	229533.V6RDH3
47997	229533.A0A1C3YJA3	pp	229533.I1S246
47998	229533.A0A1C3YJ21	pp	229533.I1RGA4
47999	229533.A0A1C3YJ21	pp	229533.A0A1C3YLK8
48000	229533.A0A1C3YJ21	pp	229533.I1RC34
48001	229533.A0A1C3YJ21	pp	229533.Q4HVQ9
48002	229533.A0A1C3YJ21	pp	229533.I1RKG2
48003	229533.A0A1C3YJ21	pp	229533.I1RTD5
48004	229533.A0A1C3YJ21	pp	229533.I1RT40
48005	229533.A0A1C3YJ21	pp	229533.A0A1C3YLL1
48006	229533.A0A1C3YJ21	pp	229533.I1RIU0
48007	229533.A0A1C3YJ21	pp	229533.I1RDF7
48008	229533.A0A1C3YJ21	pp	229533.I1S5R6
48009	229533.A0A1C3YJ21	pp	229533.I1RSZ7
48010	229533.A0A1C3YJ21	pp	229533.A0A1C3YJI6
48011	229533.A0A1C3YJ21	pp	229533.I1RSA5
48012	229533.A0A1C3YJ21	pp	229533.I1RA11
48013	229533.A0A1C3YJ21	pp	229533.I1S0W4
48014	229533.A0A1C3YJ21	pp	229533.I1RF24
48015	229533.A0A1C3YJ21	pp	229533.I1S0V7
48016	229533.A0A1C3YJ21	pp	229533.I1RYI5
48017	229533.A0A1C3YJ21	pp	229533.I1S5R1
48018	229533.A0A1C3YJ21	pp	229533.I1RXC2
48019	229533.A0A1C3YJ21	pp	229533.I1RD22
48020	229533.A0A1C3YJ21	pp	229533.I1RSQ3
48021	229533.A0A1C3YJ21	pp	229533.I1RPP3
48022	229533.A0A1C3YJ21	pp	229533.V6R042
48023	229533.A0A1C3YJ21	pp	229533.A0A1C3YJI5
48024	229533.A0A1C3YJ21	pp	229533.I1RVZ2
48025	229533.A0A1C3YJ21	pp	229533.I1RMU3
48026	229533.A0A1C3YJ21	pp	229533.A0A1C3YKK8
48027	229533.A0A1C3YJ21	pp	229533.I1RZ39
48028	229533.A0A1C3YJ21	pp	229533.A0A1C3YKM9
48029	229533.A0A1C3YJ21	pp	229533.I1RTA1
48030	229533.A0A1C3YJ21	pp	229533.I1RRM3
48031	229533.A0A1C3YJ21	pp	229533.I1RJN9
48032	229533.A0A1C3YJ21	pp	229533.I1R9Y8
48033	229533.A0A1C3YJ21	pp	229533.V6RMW6
48034	229533.A0A1C3YJ21	pp	229533.I1RG71

48035 229533.A0A1C3YJ21 pp 229533.I1RKP1
48036 229533.A0A1C3YJ21 pp 229533.I1RLW5
48037 229533.A0A1C3YJ21 pp 229533.V6RKY8
48038 229533.A0A1C3YJ21 pp 229533.I1S3T7
48039 229533.A0A1C3YJ21 pp 229533.I1RE79
48040 229533.A0A1C3YJ21 pp 229533.I1S2B8
48041 229533.A0A1C3YJ21 pp 229533.I1RNC1
48042 229533.A0A1C3YJ21 pp 229533.I1RRI1
48043 229533.A0A1C3YJ21 pp 229533.I1R9M0
48044 229533.A0A1C3YJ21 pp 229533.I1RUG3
48045 229533.A0A1C3YJ21 pp 229533.I1RKD3
48046 229533.A0A1C3YJ21 pp 229533.I1RPE0
48047 229533.A0A1C3YJ21 pp 229533.I1RPU2
48048 229533.A0A1C3YJ21 pp 229533.I1S1W9
48049 229533.A0A1C3YJ21 pp 229533.I1RCH5
48050 229533.A0A1C3YJ21 pp 229533.A0A1C3YLZ6
48051 229533.A0A1C3YJ21 pp 229533.I1RSF1
48052 229533.A0A1C3YJ21 pp 229533.A0A1C3YMN0
48053 229533.A0A1C3YJ21 pp 229533.I1RBZ6
48054 229533.A0A1C3YJ21 pp 229533.I1RZV3
48055 229533.A0A1C3YJ21 pp 229533.I1RKG3
48056 229533.A0A1C3YJ21 pp 229533.V6RLM7
48057 229533.A0A1C3YJ21 pp 229533.V6RTJ8
48058 229533.A0A1C3YJ21 pp 229533.I1RR45
48059 229533.A0A1C3YJ21 pp 229533.I1RQ33
48060 229533.A0A1C3YJ21 pp 229533.V6QVV9
48061 229533.A0A1C3YJ21 pp 229533.I1REW3
48062 229533.A0A1C3YJ21 pp 229533.I1RB92
48063 229533.A0A1C3YJ21 pp 229533.I1RAX6
48064 229533.A0A1C3YJ21 pp 229533.A0A1C3YM09
48065 229533.A0A1C3YJ21 pp 229533.I1RAW0
48066 229533.A0A1C3YJ21 pp 229533.I1RFJ6
48067 229533.A0A1C3YJ21 pp 229533.I1RVX0
48068 229533.A0A1C3YJ21 pp 229533.I1S5H2
48069 229533.A0A1C3YJ21 pp 229533.I1RX46
48070 229533.A0A1C3YJ21 pp 229533.I1RQC0
48071 229533.A0A1C3YJ21 pp 229533.I1RE89
48072 229533.A0A1C3YJ21 pp 229533.A0A1C3YN26
48073 229533.A0A1C3YJ21 pp 229533.I1RA32
48074 229533.A0A1C3YJ21 pp 229533.I1RQE7
48075 229533.A0A1C3YJ21 pp 229533.A0A1C3YKZ8
48076 229533.A0A1C3YJ21 pp 229533.I1RRX5
48077 229533.A0A1C3YJ21 pp 229533.I1RZY1
48078 229533.A0A1C3YJ21 pp 229533.I1S4S7
48079 229533.A0A1C3YJ21 pp 229533.I1R9Y4
48080 229533.A0A1C3YJ21 pp 229533.I1RXD7
48081 229533.A0A1C3YJ21 pp 229533.I1RYU3
48082 229533.A0A1C3YJ21 pp 229533.I1RN52
48083 229533.A0A1C3YJ21 pp 229533.I1RP86
48084 229533.A0A1C3YJ21 pp 229533.I1RE48
48085 229533.A0A1C3YJ21 pp 229533.I1RX95
48086 229533.A0A1C3YJ21 pp 229533.I1RPY3
48087 229533.A0A1C3YJ21 pp 229533.I1RNM7
48088 229533.A0A1C3YJ21 pp 229533.I1RD52
48089 229533.A0A1C3YJ21 pp 229533.I1RR24
48090 229533.A0A1C3YJ21 pp 229533.A0A1C3YK11
48091 229533.A0A1C3YJ21 pp 229533.I1RCM7
48092 229533.A0A1C3YJ21 pp 229533.I1RUY5
48093 229533.A0A1C3YJ21 pp 229533.I1RJU3
48094 229533.A0A1C3YJ21 pp 229533.A0A1C3YJV6
48095 229533.A0A1C3YJ21 pp 229533.I1S5B0
48096 229533.A0A1C3YJ21 pp 229533.I1REE2
48097 229533.A0A1C3YJ21 pp 229533.I1S6D7
48098 229533.A0A1C3YJ21 pp 229533.I1RYM5
48099 229533.A0A1C3YJ21 pp 229533.A0A1C3YKV3
48100 229533.A0A1C3YJ21 pp 229533.I1RXC4
48101 229533.A0A1C3YJ21 pp 229533.A0A1C3YK34
48102 229533.A0A1C3YJ21 pp 229533.V6RCZ8
48103 229533.A0A1C3YJ21 pp 229533.I1RRY9
48104 229533.A0A1C3YJ21 pp 229533.I1REK8
48105 229533.A0A1C3YJ21 pp 229533.I1RWR7
48106 229533.A0A1C3YJ21 pp 229533.I1RUQ3
48107 229533.A0A1C3YJ21 pp 229533.I1RZM8

229533.A0A1C3YJ21 pp 229533.I1RQ85
229533.A0A1C3YJ21 pp 229533.I1RDU0
229533.A0A1C3YJ21 pp 229533.A0A1C3YKF3
229533.A0A1C3YJ21 pp 229533.A0A1C3YKB0
229533.A0A1C3YJ21 pp 229533.I1RGG6
229533.A0A1C3YJ21 pp 229533.I1RAP3
229533.A0A1C3YJ21 pp 229533.Q4IJK2
229533.A0A1C3YJ21 pp 229533.I1RQA5
229533.A0A1C3YJ21 pp 229533.I1RZK5
229533.A0A1C3YJ21 pp 229533.I1S3M2
229533.A0A1C3YJ21 pp 229533.I1RRK5
229533.A0A1C3YJ21 pp 229533.A0A1C3YJN3
229533.A0A1C3YJ21 pp 229533.A0A1C3YLB3
229533.A0A1C3YJ21 pp 229533.A0A1C3YLB0
229533.I1R9W9 pp 229533.I1S5B1
229533.I1R9W9 pp 229533.Q4IB63
229533.I1R9W9 pp 229533.I1RNC0
229533.I1R9W9 pp 229533.I1RC36
229533.I1R9W9 pp 229533.I1REU0
229533.A0A1C3YIQ6 pp 229533.I1RYI6
229533.A0A1C3YIQ6 pp 229533.I1RXV3
229533.A0A1C3YIQ6 pp 229533.O74638
229533.A0A1C3YIQ6 pp 229533.I1S5S2
229533.A0A1C3YIQ6 pp 229533.I1RZ40
229533.A0A1C3YIQ6 pp 229533.I1RNE7
229533.A0A1C3YIQ6 pp 229533.I1S3V2
229533.A0A1C3YIQ6 pp 229533.A0A1C3YL15
229533.A0A1C3YIQ6 pp 229533.I1RMW7
229533.I1RZ25 pp 229533.V6RPC7
229533.I1RZ25 pp 229533.I1S404
229533.I1RZ25 pp 229533.I1SAB4
229533.I1RZ25 pp 229533.V6QZ48
229533.I1RZ25 pp 229533.V6R0A3
229533.I1RZ25 pp 229533.I1S1Q8
229533.I1RZ25 pp 229533.I1RZA8
229533.I1RZ25 pp 229533.V6RCJ5
229533.I1RZ25 pp 229533.V6RKR9
229533.I1RZ25 pp 229533.I1S694
229533.I1RZ25 pp 229533.I1S968
229533.I1RZ25 pp 229533.I1S5D0
229533.I1RZ25 pp 229533.I1S958
229533.I1RZ25 pp 229533.I1S2S9
229533.I1RZ25 pp 229533.V6R5T7
229533.I1RZ25 pp 229533.I1SAI4
229533.I1RZ25 pp 229533.V6RDX9
229533.I1RZ25 pp 229533.V6R378
229533.I1RZ25 pp 229533.I1RZX3
229533.I1RZ25 pp 229533.V6RD34
229533.I1RZ25 pp 229533.V6RH92
229533.I1RZ25 pp 229533.V6R2Z2
229533.I1RZ25 pp 229533.I1S0A5
229533.I1RZ25 pp 229533.V6RE46
229533.I1RZ25 pp 229533.I1S6H3
229533.I1RZ25 pp 229533.I1S6V0
229533.I1RZ25 pp 229533.I1S0V7
229533.I1RZ25 pp 229533.V6R1D2
229533.I1RZ25 pp 229533.V6R0W1
229533.I1RZ25 pp 229533.V6R756
229533.I1RZ25 pp 229533.I1RZ38
229533.I1RZ25 pp 229533.V6R9L8
229533.I1RZ25 pp 229533.I1S094
229533.I1RZ25 pp 229533.V6RCC0
229533.I1RZ25 pp 229533.I1RZ56
229533.I1RZ25 pp 229533.I1RZC3
229533.I1RZ25 pp 229533.I1S969
229533.I1RZ25 pp 229533.I1S210
229533.I1RZ25 pp 229533.V6R4J6
229533.I1RZ25 pp 229533.V6RRR4
229533.I1RZ25 pp 229533.I1S223
229533.I1RZ25 pp 229533.I1SA56
229533.I1RZ25 pp 229533.I1S2F7
229533.I1RZ25 pp 229533.V6QVV9
229533.I1RZ25 pp 229533.I1S0V6

48181	229533.I1RZ25	pp	229533.I1S4I8
48182	229533.I1RZ25	pp	229533.I1S099
48183	229533.I1RZ25	pp	229533.V6RWB3
48184	229533.I1RZ25	pp	229533.V6QU99
48185	229533.I1RZ25	pp	229533.V6RW01
48186	229533.I1RZ25	pp	229533.I1S359
48187	229533.I1RZ25	pp	229533.V6RHX2
48188	229533.I1RZ25	pp	229533.V6RAM6
48189	229533.I1RZ25	pp	229533.Q4HYR2
48190	229533.I1RZ25	pp	229533.I1S000
48191	229533.I1RZ25	pp	229533.I1S8D3
48192	229533.I1RZ25	pp	229533.I1S4I7
48193	229533.I1RZ25	pp	229533.I1S267
48194	229533.I1RZ25	pp	229533.I1S3T6
48195	229533.I1RZ25	pp	229533.I1S5J9
48196	229533.I1RCQ8	pp	229533.I1RNG4
48197	229533.Q4I785	pp	229533.Q4IB63
48198	229533.Q4I785	pp	229533.Q4ILH3
48199	229533.Q4I785	pp	229533.Q4IQ08
48200	229533.I1RW90	pp	229533.Q4I7S1
48201	229533.I1RW90	pp	229533.V6RGN5
48202	229533.I1RW90	pp	229533.I1S0E8
48203	229533.I1RW90	pp	229533.I1RXU0
48204	229533.I1RW90	pp	229533.Q4IQT8
48205	229533.I1RW90	pp	229533.I1S8A6
48206	229533.I1RW90	pp	229533.I1S276
48207	229533.I1RW90	pp	229533.Q4HYB8
48208	229533.I1RW90	pp	229533.Q4IPZ1
48209	229533.I1RW90	pp	229533.I1RWF3
48210	229533.I1RW90	pp	229533.I1RYE2
48211	229533.I1RW90	pp	229533.I1S9Y1
48212	229533.I1RW90	pp	229533.Q4IJ11
48213	229533.I1RW90	pp	229533.Q4IMD1
48214	229533.I1RW90	pp	229533.Q4I099
48215	229533.I1RW90	pp	229533.V6QV38
48216	229533.I1RW90	pp	229533.I1RZB5
48217	229533.I1RW90	pp	229533.Q4I5I4
48218	229533.I1RW90	pp	229533.V6QUE6
48219	229533.I1RW90	pp	229533.Q4I5W5
48220	229533.I1RW90	pp	229533.I1S5Z2
48221	229533.I1RW90	pp	229533.I1RZM2
48222	229533.I1RW90	pp	229533.I1RZU9
48223	229533.I1RW90	pp	229533.Q4IJU0
48224	229533.I1RW90	pp	229533.I1S9U1
48225	229533.I1RW90	pp	229533.Q4HYI0
48226	229533.I1RW90	pp	229533.Q4IJM4
48227	229533.I1RW90	pp	229533.I1S255
48228	229533.I1RW90	pp	229533.I1RXE5
48229	229533.I1RW90	pp	229533.V6QWX8
48230	229533.I1RW90	pp	229533.I1RW91
48231	229533.I1RW90	pp	229533.I1S0R7
48232	229533.I1RW90	pp	229533.Q4HTT2
48233	229533.I1RW90	pp	229533.Q4HYQ4
48234	229533.I1RW90	pp	229533.I1RZR6
48235	229533.I1RCQ9	pp	229533.I1RNG4
48236	229533.I1RMA8	pp	229533.I1RNY8
48237	229533.I1RMA8	pp	229533.V6RVG3
48238	229533.I1RMA8	pp	229533.V6RKY7
48239	229533.I1RMA8	pp	229533.I1RZE4
48240	229533.I1RMA8	pp	229533.I1S3G4
48241	229533.I1RMA8	pp	229533.I1RNP7
48242	229533.I1RMA8	pp	229533.I1RW71
48243	229533.I1RMA8	pp	229533.I1RUN8
48244	229533.I1RMA8	pp	229533.I1S439
48245	229533.I1RMA8	pp	229533.I1RUQ5
48246	229533.I1RMA8	pp	229533.I1RMP0
48247	229533.I1RMA8	pp	229533.I1S267
48248	229533.I1RMA8	pp	229533.I1RPK5
48249	229533.I1RMA8	pp	229533.I1RST9
48250	229533.I1RMA8	pp	229533.I1RYH9
48251	229533.I1RMA8	pp	229533.I1RX70
48252	229533.I1RMA8	pp	229533.I1RYZ9
48253	229533.I1RMA8	pp	229533.I1S1W8

48254	229533.I1RE84	pp	229533.I1RJL9
48255	229533.I1RE84	pp	229533.I1RK44
48256	229533.I1RE84	pp	229533.Q4IJ79
48257	229533.I1RTM9	pp	229533.I1S5B1
48258	229533.I1RTM9	pp	229533.I1RZ17
48259	229533.I1RTM9	pp	229533.I1RZ18
48260	229533.I1RTM9	pp	229533.Q4I0K3
48261	229533.I1RTM9	pp	229533.Q4I073
48262	229533.I1RTM9	pp	229533.Q4IB63
48263	229533.I1RTM9	pp	229533.Q4HXT5
48264	229533.I1RPJ3	pp	229533.I1S7M3
48265	229533.I1RPJ3	pp	229533.Q4I327
48266	229533.I1RPJ3	pp	229533.I1S021
48267	229533.I1RPJ3	pp	229533.V6RGN5
48268	229533.I1RPJ3	pp	229533.Q4I8X0
48269	229533.I1RPJ3	pp	229533.Q4IJ56
48270	229533.I1RPJ3	pp	229533.Q4IAA0
48271	229533.I1RPJ3	pp	229533.V6QUE6
48272	229533.I1RPJ3	pp	229533.I1RY63
48273	229533.I1RPJ3	pp	229533.I1RSS4
48274	229533.I1RPJ3	pp	229533.I1S060
48275	229533.I1RPJ3	pp	229533.Q4HZ68
48276	229533.I1RPJ3	pp	229533.V6R227
48277	229533.I1RPJ3	pp	229533.I1RZF6
48278	229533.I1RPJ3	pp	229533.I1RRR7
48279	229533.I1RPJ3	pp	229533.Q4I5Z5
48280	229533.I1RPJ3	pp	229533.I1RZE5
48281	229533.I1RPJ3	pp	229533.I1RVZ0
48282	229533.I1RPJ3	pp	229533.Q4IEK8
48283	229533.I1RPJ3	pp	229533.V6QWZ7
48284	229533.I1RPJ3	pp	229533.Q4IN63
48285	229533.I1RPJ3	pp	229533.I1RSI6
48286	229533.I1RPJ3	pp	229533.I1RZB5
48287	229533.I1RPJ3	pp	229533.I1RT80
48288	229533.I1RPJ3	pp	229533.Q4IJH1
48289	229533.I1RPJ3	pp	229533.I1RQ31
48290	229533.I1RPJ3	pp	229533.I1S2C0
48291	229533.I1RPJ3	pp	229533.I1RTE9
48292	229533.I1RPJ3	pp	229533.I1S274
48293	229533.I1RPJ3	pp	229533.I1RYB9
48294	229533.I1RPJ3	pp	229533.Q4HVW2
48295	229533.I1RPJ3	pp	229533.I1RTD7
48296	229533.I1RPJ3	pp	229533.I1S6U8
48297	229533.I1RPJ3	pp	229533.I1S1U7
48298	229533.I1RPJ3	pp	229533.I1RRI2
48299	229533.I1RPJ3	pp	229533.I1S253
48300	229533.I1RPJ3	pp	229533.I1RUQ2
48301	229533.I1RPJ3	pp	229533.I1S558
48302	229533.I1RPJ3	pp	229533.I1RQA2
48303	229533.I1RPJ3	pp	229533.Q4IQW0
48304	229533.I1RPJ3	pp	229533.Q4IAS1
48305	229533.I1RPJ3	pp	229533.Q4I830
48306	229533.I1RPJ3	pp	229533.I1RQM6
48307	229533.I1RPJ3	pp	229533.I1RRQ6
48308	229533.I1RPJ3	pp	229533.Q4IPI1
48309	229533.I1RPJ3	pp	229533.I1S0F1
48310	229533.I1RPJ3	pp	229533.I1RWF7
48311	229533.I1RPJ3	pp	229533.I1RRJ8
48312	229533.I1RPJ3	pp	229533.V6QYF0
48313	229533.I1RPJ3	pp	229533.V6R283
48314	229533.I1RPJ3	pp	229533.Q4I1M9
48315	229533.I1RPJ3	pp	229533.Q4HZ42
48316	229533.I1RPJ3	pp	229533.Q4IQK7
48317	229533.I1RPJ3	pp	229533.I1S9U2
48318	229533.I1RPJ3	pp	229533.I1RT33
48319	229533.I1RPJ3	pp	229533.I1RPU0
48320	229533.I1RPJ3	pp	229533.Q4I5M4
48321	229533.I1RPJ3	pp	229533.I1RZS6
48322	229533.I1RPJ3	pp	229533.I1RS47
48323	229533.I1RPJ3	pp	229533.I1RQ65
48324	229533.I1RPJ3	pp	229533.Q4I662
48325	229533.I1RPJ3	pp	229533.I1RR72
48326	229533.I1RPJ3	pp	229533.I1RW26

229533.I1RPJ3	pp	229533.Q4IEV5
229533.I1RPJ3	pp	229533.Q4IFI0
229533.I1RPJ3	pp	229533.I1RS19
229533.I1RPJ3	pp	229533.I1RWJ4
229533.I1RPJ3	pp	229533.I1RRQ3
229533.I1RAP1	pp	229533.I1RDH3
229533.I1RAP1	pp	229533.I1RKR8
229533.I1RAP1	pp	229533.I1RCG6
229533.I1RAP1	pp	229533.Q4ICA8
229533.I1RAP1	pp	229533.I1RAP2
229533.I1RAP1	pp	229533.I1RPW8
229533.I1RAP1	pp	229533.I1S9H8
229533.I1RAP1	pp	229533.I1RQP3
229533.I1RAP1	pp	229533.I1RYV4
229533.I1RAP1	pp	229533.I1RC73
229533.I1RAP1	pp	229533.Q4IJH1
229533.I1RAP1	pp	229533.I1RRF7
229533.I1RAP1	pp	229533.I1RKF3
229533.I1RAP1	pp	229533.I1S5Z5
229533.I1RAP1	pp	229533.I1RR61
229533.I1RAP1	pp	229533.I1RW80
229533.I1RAP1	pp	229533.V6QWX8
229533.I1RAP1	pp	229533.I1RRF0
229533.I1RAP1	pp	229533.Q4IAS1
229533.I1RAP1	pp	229533.I1RW91
229533.I1RAP1	pp	229533.I1RQP1
229533.I1RAP1	pp	229533.I1RT65
229533.I1RAP1	pp	229533.I1RQ42
229533.I1RAP1	pp	229533.Q4IPI4
229533.I1RAP1	pp	229533.I1RN04
229533.A0A1C3YHX2	pp	229533.I1RMS2
229533.A0A1C3YHX2	pp	229533.A0A1C3YMC9
229533.A0A1C3YHX2	pp	229533.I1RBC8
229533.I1REU0	pp	229533.I1RP81
229533.I1REU0	pp	229533.I1S5B1
229533.I1REU0	pp	229533.Q4ILH3
229533.I1REU0	pp	229533.I1RW60
229533.I1REU0	pp	229533.I1RNK8
229533.I1REU0	pp	229533.I1S4S9
229533.I1REU0	pp	229533.I1RK45
229533.I1REU0	pp	229533.I1RWM0
229533.I1REU0	pp	229533.I1S252
229533.I1REU0	pp	229533.I1RXT9
229533.I1REU0	pp	229533.I1RZ17
229533.I1REU0	pp	229533.Q4I073
229533.I1REU0	pp	229533.I1RZE1
229533.I1REU0	pp	229533.I1RTM9
229533.I1REU0	pp	229533.Q4I0K3
229533.I1REU0	pp	229533.Q4I785
229533.I1REU0	pp	229533.Q4IB63
229533.I1REU0	pp	229533.Q4HXT5
229533.I1REU0	pp	229533.I1RZ18
229533.I1RIL7	pp	229533.I1S574
229533.I1RIL7	pp	229533.I1S984
229533.I1RIL7	pp	229533.I1RNA1
229533.I1RI71	pp	229533.I1RK95
229533.I1RI71	pp	229533.I1RUQ5
229533.I1RI71	pp	229533.I1RST9
229533.I1RI71	pp	229533.I1S267
229533.I1RI71	pp	229533.I1RKK0
229533.I1RI71	pp	229533.I1RPK5
229533.I1RI71	pp	229533.I1RX70
229533.I1RI71	pp	229533.V6RKY7
229533.I1RI71	pp	229533.I1S439
229533.I1RI71	pp	229533.I1RW71
229533.I1RI71	pp	229533.I1RYH9
229533.I1RI71	pp	229533.I1S1W8
229533.I1RI71	pp	229533.V6RVG3
229533.I1RI71	pp	229533.I1RZE4
229533.I1RI71	pp	229533.I1RLU1
229533.I1RI71	pp	229533.I1RM96
229533.I1RI71	pp	229533.I1RNP7
229533.I1RI71	pp	229533.I1S3G4

48400 229533.I1RI71 pp 229533.I1RNY8
48401 229533.I1RI71 pp 229533.I1RUN8
48402 229533.I1RI71 pp 229533.I1RMP0
48403 229533.Q4IB63 pp 229533.Q4ILH3
48404 229533.A0A1C3YHX1 pp 229533.A0A1C3YM29
48405 229533.A0A1C3YHX1 pp 229533.A0A1C3YLT9
48406 229533.I1REB0 pp 229533.I1RXV3
48407 229533.I1REB0 pp 229533.I1RYU0
48408 229533.I1REB0 pp 229533.I1S2C4
48409 229533.I1REB0 pp 229533.I1RNG4
48410 229533.A0A1C3YJ98 pp 229533.A0A1C3YME3
48411 229533.A0A1C3YMC1 pp 229533.I1RS05
48412 229533.A0A1C3YMC1 pp 229533.I1RMP0
48413 229533.A0A1C3YMC1 pp 229533.I1S1F3
48414 229533.A0A1C3YMC1 pp 229533.I1RYU6
48415 229533.A0A1C3YMC1 pp 229533.I1REI0
48416 229533.A0A1C3YMC1 pp 229533.I1RSQ4
48417 229533.A0A1C3YMC1 pp 229533.I1RBE6
48418 229533.A0A1C3YMC1 pp 229533.I1RYN2
48419 229533.A0A1C3YMC1 pp 229533.I1RX39
48420 229533.A0A1C3YMC1 pp 229533.I1RXY7
48421 229533.A0A1C3YMC1 pp 229533.I1RYT8
48422 229533.A0A1C3YMC1 pp 229533.I1RE52
48423 229533.A0A1C3YMC1 pp 229533.I1S2M1
48424 229533.A0A1C3YMC1 pp 229533.I1RXD9
48425 229533.A0A1C3YMC1 pp 229533.A0A1C3YMF2
48426 229533.A0A1C3YMC1 pp 229533.I1RG62
48427 229533.A0A1C3YMC1 pp 229533.I1RA52
48428 229533.A0A1C3YMC1 pp 229533.I1RB38
48429 229533.A0A1C3YMC1 pp 229533.I1RE65
48430 229533.A0A1C3YMC1 pp 229533.I1RMK7
48431 229533.A0A1C3YMC1 pp 229533.I1RM99
48432 229533.A0A1C3YMC1 pp 229533.I1RGK9
48433 229533.A0A1C3YMC1 pp 229533.I1S6Y3
48434 229533.A0A1C3YMC1 pp 229533.I1RG45
48435 229533.A0A1C3YMC1 pp 229533.I1RNM7
48436 229533.A0A1C3YMC1 pp 229533.I1RFS5
48437 229533.A0A1C3YMC1 pp 229533.I1S0R8
48438 229533.A0A1C3YMC1 pp 229533.I1R9U7
48439 229533.A0A1C3YMC1 pp 229533.I1S0Q7
48440 229533.A0A1C3YMC1 pp 229533.I1RY21
48441 229533.A0A1C3YMC1 pp 229533.I1RXC9
48442 229533.A0A1C3YMC1 pp 229533.I1RKB7
48443 229533.A0A1C3YMC1 pp 229533.I1RBT6
48444 229533.A0A1C3YMC1 pp 229533.I1RMQ6
48445 229533.A0A1C3YMC1 pp 229533.I1RE66
48446 229533.A0A1C3YMC1 pp 229533.I1RRX5
48447 229533.A0A1C3YMC1 pp 229533.I1RP02
48448 229533.A0A1C3YMC1 pp 229533.I1RXE6
48449 229533.A0A1C3YMC1 pp 229533.I1RX17
48450 229533.A0A1C3YMC1 pp 229533.I1R9Y4
48451 229533.A0A1C3YMC1 pp 229533.I1RFY0
48452 229533.A0A1C3YMC1 pp 229533.I1S8F8
48453 229533.A0A1C3YMC1 pp 229533.I1RTJ2
48454 229533.A0A1C3YMC1 pp 229533.I1R994
48455 229533.A0A1C3YMC1 pp 229533.I1RAV5
48456 229533.A0A1C3YMC1 pp 229533.I1RK47
48457 229533.A0A1C3YMC1 pp 229533.I1S212
48458 229533.A0A1C3YMC1 pp 229533.I1S0K2
48459 229533.A0A1C3YMC1 pp 229533.I1S8S9
48460 229533.A0A1C3YMC1 pp 229533.I1RPR9
48461 229533.A0A1C3YMC1 pp 229533.I1RAM9
48462 229533.I1RNK8 pp 229533.I1RSS1
48463 229533.I1RNK8 pp 229533.I1RP81
48464 229533.I1RNK8 pp 229533.V6R382
48465 229533.I1RNK8 pp 229533.I1RTM9
48466 229533.I1RNK8 pp 229533.Q4I073
48467 229533.I1RNK8 pp 229533.I1S4S9
48468 229533.I1RNK8 pp 229533.I1RTG0
48469 229533.I1RNK8 pp 229533.Q4HXT5
48470 229533.I1RNK8 pp 229533.I1RPK0
48471 229533.I1RNK8 pp 229533.Q4I0K3
48472 229533.I1RNK8 pp 229533.Q4IB63

48473 229533.I1RNK8 pp 229533.Q4ILH3
48474 229533.I1RNK8 pp 229533.I1RZ18
48475 229533.I1RNK8 pp 229533.I1RX27
48476 229533.I1RNK8 pp 229533.I1RZ17
48477 229533.I1RNK8 pp 229533.I1S252
48478 229533.I1RNK8 pp 229533.Q4I785
48479 229533.I1RNK8 pp 229533.I1RST0
48480 229533.I1RG69 pp 229533.V6QUW4
48481 229533.I1RG69 pp 229533.I1RRX3
48482 229533.I1RG69 pp 229533.I1RZX4
48483 229533.I1RG69 pp 229533.I1RG99
48484 229533.I1RTE4 pp 229533.I1RW71
48485 229533.A0A098DE31 pp 229533.I1RN52
48486 229533.A0A098DE31 pp 229533.I1RA32
48487 229533.A0A098DE31 pp 229533.I1S8Z2
48488 229533.A0A098DE31 pp 229533.A0A1C3YJ21
48489 229533.A0A098DE31 pp 229533.I1RT75
48490 229533.A0A098DE31 pp 229533.I1RBF3
48491 229533.A0A098DE31 pp 229533.I1R9Y8
48492 229533.A0A098DE31 pp 229533.V6RKY8
48493 229533.A0A098DE31 pp 229533.I1RSF1
48494 229533.A0A098DE31 pp 229533.I1RRK5
48495 229533.A0A098DE31 pp 229533.I1RBU8
48496 229533.A0A098DE31 pp 229533.A0A1C3YKK8
48497 229533.A0A098DE31 pp 229533.I1RKP1
48498 229533.A0A098DE31 pp 229533.A0A1C3YMN0
48499 229533.A0A098DE31 pp 229533.I1REW3
48500 229533.A0A098DE31 pp 229533.I1RR45
48501 229533.I1RB91 pp 229533.I1RYI6
48502 229533.I1RB91 pp 229533.I1S5S2
48503 229533.I1RB91 pp 229533.V6QUW4
48504 229533.I1RB91 pp 229533.O74638
48505 229533.I1RB91 pp 229533.I1RZ40
48506 229533.I1RB91 pp 229533.I1RMW7
48507 229533.I1RB91 pp 229533.I1S3V2
48508 229533.I1RB91 pp 229533.I1RNE7
48509 229533.I1RB91 pp 229533.I1RXV3
48510 229533.I1RQ58 pp 229533.I1RRJ8
48511 229533.A0A1C3YLF0 pp 229533.I1RNK8
48512 229533.A0A1C3YLF0 pp 229533.I1RE20
48513 229533.A0A1C3YLF0 pp 229533.I1RQ58
48514 229533.A0A1C3YLF0 pp 229533.I1RNU6
48515 229533.I1RZ18 pp 229533.Q4IB63
48516 229533.I1RZ18 pp 229533.Q4I785
48517 229533.I1RZ18 pp 229533.I1S252
48518 229533.I1RZ18 pp 229533.I1S5B1
48519 229533.I1RZ18 pp 229533.Q4HXT5
48520 229533.I1RZ18 pp 229533.Q4ILH3
48521 229533.I1RZ18 pp 229533.I1S4S9
48522 229533.I1RZ18 pp 229533.I1S4Q7
48523 229533.I1RZ18 pp 229533.Q4I0K3
48524 229533.I1RZ18 pp 229533.Q4I073
48525 229533.A0A1C3YLU9 pp 229533.I1RCB4
48526 229533.A0A1C3YLU9 pp 229533.I1RT36
48527 229533.A0A1C3YLU9 pp 229533.I1RM68
48528 229533.A0A1C3YLU9 pp 229533.I1REP3
48529 229533.A0A1C3YLU9 pp 229533.I1RJF1
48530 229533.A0A1C3YLU9 pp 229533.I1RBK1
48531 229533.A0A1C3YLU9 pp 229533.I1S8X1
48532 229533.A0A1C3YLU9 pp 229533.I1RTI7
48533 229533.A0A1C3YLU9 pp 229533.A0A1C3YMQ5
48534 229533.A0A1C3YLU9 pp 229533.I1RZ52
48535 229533.A0A1C3YLU9 pp 229533.I1RMG7
48536 229533.A0A1C3YLU9 pp 229533.I1R989
48537 229533.A0A1C3YLU9 pp 229533.I1RI56
48538 229533.A0A1C3YLU9 pp 229533.I1RN19
48539 229533.A0A1C3YLU9 pp 229533.I1RIY0
48540 229533.A0A1C3YLU9 pp 229533.I1RYU5
48541 229533.A0A1C3YLU9 pp 229533.I1RYW6
48542 229533.A0A1C3YLU9 pp 229533.I1RX80
48543 229533.A0A1C3YLU9 pp 229533.I1R9Z1
48544 229533.A0A1C3YLU9 pp 229533.I1S7M8
48545 229533.A0A1C3YLU9 pp 229533.I1RYD3

48546 229533.A0A1C3YLU9 pp 229533.I1RIB6
48547 229533.A0A1C3YLU9 pp 229533.I1S770
48548 229533.A0A1C3YLU9 pp 229533.I1RM69
48549 229533.A0A1C3YLU9 pp 229533.I1RZ20
48550 229533.A0A1C3YLU9 pp 229533.I1RQ30
48551 229533.A0A1C3YLU9 pp 229533.I1RS40
48552 229533.A0A1C3YLU9 pp 229533.I1RNG4
48553 229533.A0A1C3YLU9 pp 229533.I1RE53
48554 229533.A0A1C3YLU9 pp 229533.I1RMG6
48555 229533.A0A1C3YLU9 pp 229533.I1S7A5
48556 229533.I1RHX4 pp 229533.I1RXV3
48557 229533.I1RHX4 pp 229533.I1RNG4
48558 229533.I1RHX4 pp 229533.I1S2C4
48559 229533.I1RHX4 pp 229533.I1RYU0
48560 229533.I1RNN3 pp 229533.I1RPZ3
48561 229533.I1RNN3 pp 229533.I1RTF5
48562 229533.A0A098DH99 pp 229533.I1RZ52
48563 229533.A0A098DH99 pp 229533.A0A098DQN8
48564 229533.A0A098DH99 pp 229533.I1RHK2
48565 229533.A0A098DH99 pp 229533.I1RRK9
48566 229533.A0A098DH99 pp 229533.I1RGK9
48567 229533.A0A098DH99 pp 229533.I1RA51
48568 229533.A0A098DH99 pp 229533.I1S6Y3
48569 229533.A0A098DH99 pp 229533.I1RAN6
48570 229533.A0A098DH99 pp 229533.A0A1C3YKL8
48571 229533.A0A098DH99 pp 229533.I1RTJ2
48572 229533.A0A098DH99 pp 229533.I1RW01
48573 229533.A0A098DH99 pp 229533.I1RZR5
48574 229533.A0A098DH99 pp 229533.I1RY11
48575 229533.A0A098DH99 pp 229533.I1S8U8
48576 229533.A0A098DH99 pp 229533.I1RT96
48577 229533.A0A098DH99 pp 229533.I1RC18
48578 229533.A0A098DH99 pp 229533.I1RE53
48579 229533.A0A098DH99 pp 229533.I1RVX8
48580 229533.A0A098DH99 pp 229533.I1RYD3
48581 229533.A0A098DH99 pp 229533.I1RZJ3
48582 229533.A0A098DH99 pp 229533.A0A1C3YIL2
48583 229533.A0A098DH99 pp 229533.I1S0R9
48584 229533.Q4I0K3 pp 229533.Q4ILH3
48585 229533.Q4I0K3 pp 229533.Q4I785
48586 229533.Q4I0K3 pp 229533.Q4IB63
48587 229533.Q4I0K3 pp 229533.Q4IJ79
48588 229533.I1S0F1 pp 229533.V6RCZ8
48589 229533.I1S0F1 pp 229533.I1S6E5
48590 229533.I1S0F1 pp 229533.V6QYF0
48591 229533.I1S0F1 pp 229533.I1S5B0
48592 229533.I1S0F1 pp 229533.I1S274
48593 229533.I1S0F1 pp 229533.I1S5R6
48594 229533.I1S0F1 pp 229533.Q4I327
48595 229533.I1S0F1 pp 229533.V6QVD5
48596 229533.I1S0F1 pp 229533.Q4IFI0
48597 229533.I1S0F1 pp 229533.Q4I8X0
48598 229533.I1S0F1 pp 229533.Q4IPI1
48599 229533.I1S0F1 pp 229533.I1S5R1
48600 229533.I1S0F1 pp 229533.I1S4S7
48601 229533.I1S0F1 pp 229533.I1S253
48602 229533.I1S0F1 pp 229533.Q4I1M9
48603 229533.I1RWF9 pp 229533.I1RXB6
48604 229533.I1RWF9 pp 229533.I1S3M4
48605 229533.I1RWF9 pp 229533.I1RXY7
48606 229533.I1RWF9 pp 229533.I1S064
48607 229533.I1RWF9 pp 229533.V6RMW6
48608 229533.I1RWF9 pp 229533.V6QWZ7
48609 229533.I1RWF9 pp 229533.I1S0S5
48610 229533.I1RWF9 pp 229533.I1S2Z7
48611 229533.I1RWF9 pp 229533.I1RYU0
48612 229533.I1RWF9 pp 229533.I1S1W8
48613 229533.I1RWF9 pp 229533.I1RZ21
48614 229533.I1RWF9 pp 229533.V6RXE7
48615 229533.I1RWF9 pp 229533.I1S3G4
48616 229533.I1RWF9 pp 229533.I1S6D1
48617 229533.I1RWF9 pp 229533.I1S3V2
48618 229533.I1RWF9 pp 229533.I1S334

229533.I1RWF9 pp 229533.I1S2B8
229533.I1RWF9 pp 229533.I1S8E9
229533.I1RWF9 pp 229533.I1RYD3
229533.I1RWF9 pp 229533.I1S8E0
229533.I1RWF9 pp 229533.I1S2F6
229533.I1RWF9 pp 229533.I1S3H8
229533.I1RWF9 pp 229533.I1RZ83
229533.I1RWF9 pp 229533.V6RS91
229533.I1S5B1 pp 229533.V6QUV7
229533.I1S5B1 pp 229533.Q4ILH3
229533.I1S5B1 pp 229533.Q4HY20
229533.I1S5B1 pp 229533.Q4IB63
229533.I1S5B1 pp 229533.Q4I073
229533.A0A098DRZ5 pp 229533.I1RMN7
229533.A0A098DRZ5 pp 229533.I1RQ31
229533.A0A098DRZ5 pp 229533.A0A1C3YJG0
229533.A0A098DRZ5 pp 229533.Q4IN63
229533.A0A098DRZ5 pp 229533.V6RGY0
229533.A0A098DRZ5 pp 229533.I1RSI6
229533.A0A098DRZ5 pp 229533.Q4IBS2
229533.A0A098DRZ5 pp 229533.I1RMN6
229533.A0A098DRZ5 pp 229533.V6QUE6
229533.A0A098DRZ5 pp 229533.V6RGN5
229533.A0A098DRZ5 pp 229533.I1RC27
229533.A0A098DRZ5 pp 229533.I1RTD7
229533.A0A098DRZ5 pp 229533.I1RMD5
229533.A0A098DRZ5 pp 229533.A0A1C3YMG4
229533.A0A098DRZ5 pp 229533.I1RBD2
229533.A0A098DRZ5 pp 229533.A0A1C3YL07
229533.A0A098DRZ5 pp 229533.I1RAH6
229533.A0A098DRZ5 pp 229533.A0A0E0S621
229533.A0A098DRZ5 pp 229533.I1R9X7
229533.A0A098DRZ5 pp 229533.I1RB27
229533.A0A098DRZ5 pp 229533.I1RPJ3
229533.A0A098DRZ5 pp 229533.I1RTE9
229533.A0A098DRZ5 pp 229533.I1S2C0
229533.A0A098DRZ5 pp 229533.Q4I8X0
229533.A0A098DRZ5 pp 229533.Q4IJ56
229533.A0A098DRZ5 pp 229533.I1RM96
229533.A0A098DRZ5 pp 229533.I1RZB5
229533.A0A098DRZ5 pp 229533.I1RRU2
229533.A0A098DRZ5 pp 229533.Q4IEK8
229533.A0A098DRZ5 pp 229533.I1RNY2
229533.A0A098DRZ5 pp 229533.I1RG56
229533.A0A098DRZ5 pp 229533.V6R227
229533.A0A098DRZ5 pp 229533.I1RRR7
229533.A0A098DRZ5 pp 229533.I1RZV9
229533.A0A098DRZ5 pp 229533.A0A1C3YN86
229533.A0A098DRZ5 pp 229533.I1RCW1
229533.A0A098DRZ5 pp 229533.I1RQA2
229533.A0A098DRZ5 pp 229533.I1S558
229533.A0A098DRZ5 pp 229533.Q4I327
229533.A0A098DRZ5 pp 229533.V6RGY3
229533.A0A098DRZ5 pp 229533.A0A1C3YJR3
229533.A0A098DRZ5 pp 229533.I1RRI2
229533.A0A098DRZ5 pp 229533.Q4HZ68
229533.A0A098DRZ5 pp 229533.I1RF08
229533.A0A098DRZ5 pp 229533.I1RRQ3
229533.A0A098DRZ5 pp 229533.I1RNH1
229533.A0A098DRZ5 pp 229533.I1S274
229533.A0A098DRZ5 pp 229533.Q4INZ9
229533.A0A098DRZ5 pp 229533.I1RRE0
229533.A0A098DRZ5 pp 229533.A0A098DVS9
229533.A0A098DRZ5 pp 229533.I1RRP3
229533.A0A098DRZ5 pp 229533.A0A1C3YIP5
229533.A0A098DRZ5 pp 229533.I1RZZ1
229533.A0A098DRZ5 pp 229533.Q4HXF0
229533.A0A098DRZ5 pp 229533.I1RWH8
229533.A0A098DRZ5 pp 229533.I1RJZ6
229533.A0A098DRZ5 pp 229533.I1RSD8
229533.A0A098DRZ5 pp 229533.I1RW26
229533.A0A098DRZ5 pp 229533.I1RS47
229533.A0A098DRZ5 pp 229533.Q4HZ42

48692	229533.A0A098DRZ5	pp	229533.I1RRT2
48693	229533.A0A098DRZ5	pp	229533.I1RJL4
48694	229533.A0A098DRZ5	pp	229533.I1RT33
48695	229533.A0A098DRZ5	pp	229533.I1S0F1
48696	229533.A0A098DRZ5	pp	229533.A0A1C3YMG7
48697	229533.A0A098DRZ5	pp	229533.I1RK95
48698	229533.A0A098DRZ5	pp	229533.I1R9V7
48699	229533.A0A098DRZ5	pp	229533.Q4IPI1
48700	229533.A0A098DRZ5	pp	229533.I1RY63
48701	229533.A0A098DRZ5	pp	229533.I1RSS4
48702	229533.A0A098DRZ5	pp	229533.Q4I1M9
48703	229533.A0A098DRZ5	pp	229533.Q4IAS1
48704	229533.A0A098DRZ5	pp	229533.I1RAV6
48705	229533.A0A098DRZ5	pp	229533.I1RT05
48706	229533.A0A098DRZ5	pp	229533.I1S6U8
48707	229533.A0A098DRZ5	pp	229533.V6QWZ7
48708	229533.A0A098DRZ5	pp	229533.Q4IQW0
48709	229533.A0A098DRZ5	pp	229533.I1RE00
48710	229533.A0A098DRZ5	pp	229533.I1RC82
48711	229533.A0A098DRZ5	pp	229533.I1RAZ8
48712	229533.A0A098DRZ5	pp	229533.A0A0E0SGM4
48713	229533.A0A098DRZ5	pp	229533.I1S253
48714	229533.A0A098DRZ5	pp	229533.I1RG51
48715	229533.A0A098DRZ5	pp	229533.I1RZH6
48716	229533.A0A098DRZ5	pp	229533.I1RS19
48717	229533.A0A098DRZ5	pp	229533.I1RWJ4
48718	229533.A0A098DRZ5	pp	229533.Q4IJH1
48719	229533.A0A098DRZ5	pp	229533.V6R283
48720	229533.A0A098DRZ5	pp	229533.I1RT80
48721	229533.A0A098DRZ5	pp	229533.I1REX4
48722	229533.A0A098DRZ5	pp	229533.I1RQJ5
48723	229533.A0A098DRZ5	pp	229533.I1RM06
48724	229533.A0A098DRZ5	pp	229533.I1RR72
48725	229533.A0A098DRZ5	pp	229533.I1RNV6
48726	229533.A0A098DRZ5	pp	229533.I1RWF7
48727	229533.A0A098DRZ5	pp	229533.I1RRT8
48728	229533.A0A098DRZ5	pp	229533.I1RM67
48729	229533.A0A098DRZ5	pp	229533.I1RKF9
48730	229533.A0A098DRZ5	pp	229533.I1RRJ8
48731	229533.A0A098DRZ5	pp	229533.A0A0E0SFM9
48732	229533.A0A098DRZ5	pp	229533.A0A1C3YHT9
48733	229533.A0A098DRZ5	pp	229533.I1RKJ6
48734	229533.A0A098DRZ5	pp	229533.Q4I7L0
48735	229533.A0A098DRZ5	pp	229533.Q4I830
48736	229533.A0A098DRZ5	pp	229533.A0A1C3YK00
48737	229533.A0A098DRZ5	pp	229533.Q4HYC1
48738	229533.A0A098DRZ5	pp	229533.I1RA35
48739	229533.A0A098DF57	pp	229533.I1RRK5
48740	229533.A0A098DF57	pp	229533.I1RA32
48741	229533.A0A098DF57	pp	229533.I1S8Z2
48742	229533.A0A098DF57	pp	229533.I1RR45
48743	229533.A0A098DF57	pp	229533.I1RN52
48744	229533.A0A098DF57	pp	229533.I1RBU8
48745	229533.A0A098DF57	pp	229533.I1REW3
48746	229533.A0A098DF57	pp	229533.I1RSF1
48747	229533.A0A098DF57	pp	229533.I1RBF3
48748	229533.A0A098DF57	pp	229533.V6RKY8
48749	229533.A0A098DF57	pp	229533.I1R9Y8
48750	229533.A0A098DF57	pp	229533.A0A1C3YJ21
48751	229533.A0A098DF57	pp	229533.I1RT75
48752	229533.A0A098DF57	pp	229533.A0A1C3YMN0
48753	229533.A0A098DF57	pp	229533.A0A1C3YKK8
48754	229533.A0A098DF57	pp	229533.I1RKP1
48755	229533.A0A1C3YK09	pp	229533.V6QT89
48756	229533.A0A1C3YK09	pp	229533.I1RNJ7
48757	229533.A0A1C3YK09	pp	229533.I1RW06
48758	229533.A0A1C3YK09	pp	229533.Q4HTS9
48759	229533.A0A1C3YK09	pp	229533.I1RBE4
48760	229533.A0A1C3YK09	pp	229533.V6QVM5
48761	229533.A0A1C3YK09	pp	229533.I1RMM8
48762	229533.A0A1C3YK09	pp	229533.I1RDB7
48763	229533.A0A1C3YK09	pp	229533.I1RYU0
48764	229533.I1RMG4	pp	229533.I1RQJ0

48765	229533.I1RMG4	pp	229533.I1RW69
48766	229533.I1RMG4	pp	229533.I1RNR9
48767	229533.I1RMG4	pp	229533.I1S4S9
48768	229533.I1RMG4	pp	229533.I1RVW7
48769	229533.I1RMG4	pp	229533.I1RRN4
48770	229533.I1RMG4	pp	229533.V6RXW9
48771	229533.I1RMG4	pp	229533.I1S368
48772	229533.I1RMG4	pp	229533.I1RRC4
48773	229533.I1RMG4	pp	229533.I1RZT6
48774	229533.I1RMG4	pp	229533.I1S2B0
48775	229533.I1RMG4	pp	229533.I1RPD3
48776	229533.I1RMG4	pp	229533.I1RPZ1
48777	229533.I1RMG4	pp	229533.I1RQM4
48778	229533.I1RMG4	pp	229533.I1RWN0
48779	229533.I1RMG4	pp	229533.I1RPQ2
48780	229533.I1RMG4	pp	229533.I1RUP5
48781	229533.I1RMG4	pp	229533.V6RG04
48782	229533.I1RMG4	pp	229533.I1S0R5
48783	229533.I1RMG4	pp	229533.I1RST0
48784	229533.I1RMG4	pp	229533.I1S252
48785	229533.I1RMG4	pp	229533.I1RN66
48786	229533.I1RMG4	pp	229533.I1RQ61
48787	229533.I1RMG4	pp	229533.I1RWV4
48788	229533.I1RMG4	pp	229533.I1RNG4
48789	229533.I1RMG4	pp	229533.I1RSM8
48790	229533.I1RMG6	pp	229533.I1S788
48791	229533.I1RMG6	pp	229533.I1RVX9
48792	229533.I1RMG6	pp	229533.I1RYU5
48793	229533.I1RMG6	pp	229533.I1RYJ7
48794	229533.I1RMG6	pp	229533.I1RYD3
48795	229533.I1RMG6	pp	229533.I1S1W8
48796	229533.I1RMG6	pp	229533.I1RWD7
48797	229533.I1RMG6	pp	229533.I1RQE0
48798	229533.I1RMG6	pp	229533.I1S9D4
48799	229533.I1RMG6	pp	229533.I1RVY2
48800	229533.I1RMG6	pp	229533.I1RMH1
48801	229533.I1RMG6	pp	229533.I1RXB1
48802	229533.I1RMG6	pp	229533.I1S8X1
48803	229533.I1RMG6	pp	229533.I1RT41
48804	229533.I1RMG6	pp	229533.I1RYX6
48805	229533.I1RMG6	pp	229533.I1S0Z3
48806	229533.I1RMG6	pp	229533.I1RPW1
48807	229533.I1RMG6	pp	229533.I1RTU1
48808	229533.I1RMG6	pp	229533.V6RQW4
48809	229533.I1RMG6	pp	229533.P0C431
48810	229533.I1RMG6	pp	229533.I1S887
48811	229533.I1RMG6	pp	229533.I1S2E2
48812	229533.I1RMG6	pp	229533.I1S0N3
48813	229533.I1RMG6	pp	229533.I1RS40
48814	229533.I1RMG6	pp	229533.Q4I1N3
48815	229533.I1RMG6	pp	229533.I1RVN1
48816	229533.I1RMG6	pp	229533.V6RSF3
48817	229533.I1RMG6	pp	229533.I1RN88
48818	229533.I1RMG6	pp	229533.I1RZ29
48819	229533.I1RMG6	pp	229533.I1RYI7
48820	229533.I1RMG6	pp	229533.I1RYW6
48821	229533.I1RMG6	pp	229533.I1RTH6
48822	229533.I1RMG6	pp	229533.I1S3G4
48823	229533.I1RMG6	pp	229533.I1RRJ6
48824	229533.I1RMG6	pp	229533.I1RQ30
48825	229533.I1RMG6	pp	229533.I1RZ52
48826	229533.I1RMG6	pp	229533.O74638
48827	229533.I1RMG6	pp	229533.I1S770
48828	229533.I1RMG6	pp	229533.I1RTI7
48829	229533.I1RMG6	pp	229533.I1RZ20
48830	229533.I1RMG6	pp	229533.I1S0D5
48831	229533.I1RMG6	pp	229533.I1RNG4
48832	229533.I1RMG6	pp	229533.I1RY62
48833	229533.I1RMG6	pp	229533.I1RSL5
48834	229533.I1RMG6	pp	229533.Q4HXF0
48835	229533.I1RMG6	pp	229533.V6R382
48836	229533.I1RMG6	pp	229533.I1RSC7
48837	229533.I1RMG6	pp	229533.I1RR05

48838	229533.I1RMG6	pp	229533.V6QVH8
48839	229533.I1RMG6	pp	229533.I1S256
48840	229533.I1S2A9	pp	229533.I1S9T8
48841	229533.I1S2A9	pp	229533.V6RLM7
48842	229533.Q4I073	pp	229533.Q4IB63
48843	229533.Q4I073	pp	229533.Q4I785
48844	229533.Q4I073	pp	229533.Q4I0K3
48845	229533.Q4I073	pp	229533.Q4ILH3
48846	229533.Q4I073	pp	229533.Q4IQ08
48847	229533.I1RA66	pp	229533.I1RZ40
48848	229533.I1RA66	pp	229533.I1S5S2
48849	229533.I1RA66	pp	229533.O74638
48850	229533.I1RA66	pp	229533.I1S3V2
48851	229533.I1RA66	pp	229533.I1RXV3
48852	229533.I1RA66	pp	229533.I1RNE7
48853	229533.I1RA66	pp	229533.I1RMW7
48854	229533.I1RA66	pp	229533.I1RYI6
48855	229533.A0A1C3YKB6	pp	229533.Q4ILH3
48856	229533.A0A1C3YKB6	pp	229533.I1RMM6
48857	229533.A0A1C3YKB6	pp	229533.I1RC36
48858	229533.A0A1C3YKB6	pp	229533.I1RRN5
48859	229533.A0A1C3YKB6	pp	229533.I1S252
48860	229533.A0A1C3YKB6	pp	229533.I1RPS4
48861	229533.A0A1C3YKB6	pp	229533.I1RTI6
48862	229533.A0A1C3YKB6	pp	229533.I1R9W9
48863	229533.A0A1C3YKB6	pp	229533.I1RNK8
48864	229533.A0A1C3YKB6	pp	229533.I1REU0
48865	229533.A0A1C3YKB6	pp	229533.A0A1C3YN83
48866	229533.A0A1C3YKB6	pp	229533.I1RBE2
48867	229533.A0A1C3YKB6	pp	229533.Q4I073
48868	229533.A0A1C3YKB6	pp	229533.A0A1C3YN26
48869	229533.A0A1C3YKB6	pp	229533.I1RBN0
48870	229533.A0A1C3YKB6	pp	229533.Q4I0K3
48871	229533.A0A1C3YKB6	pp	229533.I1RLR7
48872	229533.A0A1C3YKB6	pp	229533.I1RJL9
48873	229533.I1RAQ5	pp	229533.I1RSQ4
48874	229533.I1RAQ5	pp	229533.I1S9Y1
48875	229533.I1RAQ5	pp	229533.I1RT80
48876	229533.I1RAQ5	pp	229533.O74638
48877	229533.I1RAQ5	pp	229533.I1RS20
48878	229533.I1RAQ5	pp	229533.I1RWD7
48879	229533.I1RAQ5	pp	229533.I1S1X9
48880	229533.I1RAQ5	pp	229533.V6RR38
48881	229533.I1RAQ5	pp	229533.I1RRI2
48882	229533.I1RAQ5	pp	229533.I1RFM2
48883	229533.I1RAQ5	pp	229533.V6RVJ3
48884	229533.I1RAQ5	pp	229533.I1SAS4
48885	229533.I1RAQ5	pp	229533.I1RB27
48886	229533.I1RAQ5	pp	229533.I1RZF6
48887	229533.I1RAQ5	pp	229533.I1RX24
48888	229533.I1RAQ5	pp	229533.I1SAS3
48889	229533.I1RAQ5	pp	229533.V6R283
48890	229533.I1RAQ5	pp	229533.V6QYF0
48891	229533.I1RAQ5	pp	229533.I1RJG1
48892	229533.I1RAQ5	pp	229533.I1RJZ7
48893	229533.I1RAQ5	pp	229533.I1RW71
48894	229533.I1RAQ5	pp	229533.I1RFP1
48895	229533.I1RAQ5	pp	229533.I1S1Q3
48896	229533.I1RAQ5	pp	229533.I1RZG3
48897	229533.I1RNT1	pp	229533.I1RWF4
48898	229533.I1RNT1	pp	229533.I1S2C2
48899	229533.I1RNT1	pp	229533.I1S5K2
48900	229533.I1RNT1	pp	229533.I1RY65
48901	229533.I1RNT1	pp	229533.I1RZ92
48902	229533.I1RNT1	pp	229533.I1S5R5
48903	229533.I1RNT1	pp	229533.I1RZZ3
48904	229533.I1RNT1	pp	229533.I1SAS4
48905	229533.I1RNT1	pp	229533.V6RIE5
48906	229533.I1RNT1	pp	229533.I1S2M5
48907	229533.I1RNT1	pp	229533.V6RPN0
48908	229533.I1RNT1	pp	229533.I1RNX0
48909	229533.I1RNT1	pp	229533.I1S1W6
48910	229533.I1RNT1	pp	229533.I1SAS3

48911	229533.I1RNT1	pp	229533.I1RT23
48912	229533.I1RNT1	pp	229533.V6RG22
48913	229533.I1RNT1	pp	229533.I1RRA5
48914	229533.I1RNT1	pp	229533.I1RXX1
48915	229533.I1RNT1	pp	229533.I1RRQ1
48916	229533.I1RNT1	pp	229533.I1RQ76
48917	229533.I1RNT1	pp	229533.V6RR38
48918	229533.I1RNT1	pp	229533.I1RYD3
48919	229533.I1RNT1	pp	229533.I1S1Q3
48920	229533.I1RNT1	pp	229533.I1RR90
48921	229533.I1RMG7	pp	229533.I1RQE0
48922	229533.I1RMG7	pp	229533.I1RXB1
48923	229533.I1RMG7	pp	229533.I1S8X1
48924	229533.I1RMG7	pp	229533.I1RVY2
48925	229533.I1RMG7	pp	229533.I1S1W8
48926	229533.I1RMG7	pp	229533.I1S9D4
48927	229533.I1RMG7	pp	229533.V6RQW4
48928	229533.I1RMG7	pp	229533.I1RYU5
48929	229533.I1RMG7	pp	229533.I1RPW1
48930	229533.I1RMG7	pp	229533.I1RVX9
48931	229533.I1RMG7	pp	229533.I1S2E2
48932	229533.I1RMG7	pp	229533.I1RYD3
48933	229533.I1RMG7	pp	229533.I1RYJ7
48934	229533.I1RMG7	pp	229533.V6QVH8
48935	229533.I1RMG7	pp	229533.I1RS40
48936	229533.I1RMG7	pp	229533.I1RYI7
48937	229533.I1RMG7	pp	229533.I1RYX6
48938	229533.I1RMG7	pp	229533.I1S770
48939	229533.I1RMG7	pp	229533.I1RTI7
48940	229533.I1RMG7	pp	229533.I1RRJ6
48941	229533.I1RMG7	pp	229533.I1RZ52
48942	229533.I1RMG7	pp	229533.I1RQ30
48943	229533.I1RMG7	pp	229533.V6RSF3
48944	229533.I1RMG7	pp	229533.I1RN88
48945	229533.I1RMG7	pp	229533.I1RTH6
48946	229533.I1RMG7	pp	229533.I1RZ29
48947	229533.I1RMG7	pp	229533.I1RSL5
48948	229533.I1RMG7	pp	229533.I1RVN1
48949	229533.I1RMG7	pp	229533.I1S3G4
48950	229533.I1RMG7	pp	229533.I1RYW6
48951	229533.I1RMG7	pp	229533.I1RZ20
48952	229533.I1RMG7	pp	229533.O74638
48953	229533.I1RMG7	pp	229533.V6R382
48954	229533.I1RMG7	pp	229533.I1RY62
48955	229533.I1RMG7	pp	229533.I1S256
48956	229533.I1RMG7	pp	229533.I1RNG4
48957	229533.I1RMG7	pp	229533.Q4HXF0
48958	229533.A0A1C3YHP5	pp	229533.I1RZ17
48959	229533.A0A1C3YHP5	pp	229533.I1RNK8
48960	229533.A0A1C3YHP5	pp	229533.Q4HXT5
48961	229533.A0A1C3YHP5	pp	229533.I1RZ18
48962	229533.A0A1C3YHP5	pp	229533.Q4I0K3
48963	229533.A0A1C3YHP5	pp	229533.Q4IB63
48964	229533.A0A1C3YHP5	pp	229533.A0A1C3YLQ1
48965	229533.A0A1C3YHP5	pp	229533.I1S5B1
48966	229533.A0A1C3YHP5	pp	229533.I1RJL9
48967	229533.A0A1C3YHP5	pp	229533.I1R9W2
48968	229533.A0A1C3YHP5	pp	229533.I1REU0
48969	229533.A0A1C3YHP5	pp	229533.I1RTM9
48970	229533.I1RFB0	pp	229533.I1S094
48971	229533.I1RFB0	pp	229533.I1RQV7
48972	229533.I1RFB0	pp	229533.I1S5D0
48973	229533.I1RFB0	pp	229533.I1S049
48974	229533.I1RFB0	pp	229533.I1RMA2
48975	229533.I1RFB0	pp	229533.I1RWL3
48976	229533.I1RFB0	pp	229533.I1RW54
48977	229533.I1RFB0	pp	229533.I1RYP5
48978	229533.I1RFB0	pp	229533.V6R5T7
48979	229533.I1RFB0	pp	229533.I1S6H3
48980	229533.I1RFB0	pp	229533.V6RCC0
48981	229533.I1RFB0	pp	229533.I1RLA2
48982	229533.I1RFB0	pp	229533.I1S048
48983	229533.I1RFB0	pp	229533.I1RYU6

48984	229533.I1RFB0	pp	229533.I1RY81
48985	229533.I1RFB0	pp	229533.I1S659
48986	229533.I1RFB0	pp	229533.I1S969
48987	229533.I1RFB0	pp	229533.I1RQV8
48988	229533.I1RFB0	pp	229533.I1RQJ7
48989	229533.I1RFB0	pp	229533.I1RY12
48990	229533.I1RFB0	pp	229533.I1RRS6
48991	229533.I1RFB0	pp	229533.I1S267
48992	229533.I1RFB0	pp	229533.V6RWB3
48993	229533.I1RFB0	pp	229533.V6RHX2
48994	229533.I1RFB0	pp	229533.I1S0A5
48995	229533.I1RFB0	pp	229533.I1S8D3
48996	229533.I1RFB0	pp	229533.I1RW82
48997	229533.I1RFB0	pp	229533.I1RVV7
48998	229533.I1RFB0	pp	229533.I1RST9
48999	229533.I1RFB0	pp	229533.I1RIB5
49000	229533.I1RFB0	pp	229533.I1S968
49001	229533.I1RFB0	pp	229533.I1RIJ6
49002	229533.I1RFB0	pp	229533.I1RPM6
49003	229533.I1RFB0	pp	229533.I1RK55
49004	229533.I1RFB0	pp	229533.I1RWM4
49005	229533.I1RFB0	pp	229533.I1RRR9
49006	229533.I1RFB0	pp	229533.I1RWF5
49007	229533.I1RFB0	pp	229533.I1RMK9
49008	229533.I1RFB0	pp	229533.V6RCJ5
49009	229533.I1RFB0	pp	229533.I1RHU8
49010	229533.I1RFB0	pp	229533.I1RZ25
49011	229533.I1RFB0	pp	229533.I1RSA5
49012	229533.I1RFB0	pp	229533.I1S2C4
49013	229533.I1RFB0	pp	229533.I1RZ56
49014	229533.I1RFB0	pp	229533.I1RZ89
49015	229533.I1RFB0	pp	229533.I1RPV8
49016	229533.I1RFB0	pp	229533.I1S6V0
49017	229533.I1RFB0	pp	229533.I1RJS1
49018	229533.I1RFB0	pp	229533.I1RVX8
49019	229533.I1RFB0	pp	229533.I1RN84
49020	229533.I1RFB0	pp	229533.I1RH83
49021	229533.I1RFB0	pp	229533.I1SAT2
49022	229533.I1RZG9	pp	229533.Q4HZQ1
49023	229533.I1RZG9	pp	229533.Q4HWE2
49024	229533.I1RJL9	pp	229533.I1RNM4
49025	229533.I1RJL9	pp	229533.I1RZ29
49026	229533.I1RJL9	pp	229533.Q4I0K3
49027	229533.I1RJL9	pp	229533.I1RSM8
49028	229533.I1RJL9	pp	229533.I1RQG4
49029	229533.I1RJL9	pp	229533.I1RST0
49030	229533.I1RJL9	pp	229533.Q4I073
49031	229533.I1RJL9	pp	229533.I1RZM0
49032	229533.I1RJL9	pp	229533.I1RRT3
49033	229533.I1RJL9	pp	229533.V6RPQ8
49034	229533.I1RJL9	pp	229533.I1S252
49035	229533.I1RJL9	pp	229533.I1RKP3
49036	229533.I1RJL9	pp	229533.I1S272
49037	229533.I1RJL9	pp	229533.I1RQ75
49038	229533.I1RJL9	pp	229533.Q4HZE0
49039	229533.I1RJL9	pp	229533.I1RVJ8
49040	229533.I1RJL9	pp	229533.I1RZ13
49041	229533.I1RJL9	pp	229533.Q4I1Y5
49042	229533.I1RJL9	pp	229533.I1RZ34
49043	229533.I1RJL9	pp	229533.Q4IJT0
49044	229533.I1RJL9	pp	229533.Q4IB63
49045	229533.I1RJL9	pp	229533.I1RNU7
49046	229533.I1RJL9	pp	229533.I1RSV4
49047	229533.I1RJL9	pp	229533.I1RNK8
49048	229533.I1RJL9	pp	229533.I1RYL8
49049	229533.I1RJL9	pp	229533.I1RPZ1
49050	229533.I1RJL9	pp	229533.I1RSJ2
49051	229533.I1RJL9	pp	229533.I1RRG3
49052	229533.I1RJL9	pp	229533.I1S183
49053	229533.I1RJL9	pp	229533.I1RMP9
49054	229533.I1RJL9	pp	229533.I1RXF0
49055	229533.I1RJL9	pp	229533.I1RK45
49056	229533.I1RJL9	pp	229533.I1RRC6

49057 229533.I1RJL9 pp 229533.I1RZ16
49058 229533.I1RJL9 pp 229533.I1RZV2
49059 229533.I1RJL9 pp 229533.V6RG22
49060 229533.I1RJL9 pp 229533.I1RKE1
49061 229533.I1RJL9 pp 229533.I1RPT0
49062 229533.I1RUE7 pp 229533.I1RVZ3
49063 229533.I1RUE7 pp 229533.I1RXV3
49064 229533.I1RUE7 pp 229533.I1RYU0
49065 229533.I1RUE7 pp 229533.I1S2C4
49066 229533.I1S5X3 pp 229533.I1S9T8
49067 229533.I1S5X3 pp 229533.V6RLM7
49068 229533.I1S4F4 pp 229533.V6R9Z3
49069 229533.I1S4F4 pp 229533.V6RN31
49070 229533.I1S4F4 pp 229533.V6RRM0
49071 229533.I1S4F4 pp 229533.V6RY56
49072 229533.I1S4F4 pp 229533.I1S5L5
49073 229533.I1S4F4 pp 229533.I1S6P6
49074 229533.I1S4S9 pp 229533.Q4IB63
49075 229533.I1S4S9 pp 229533.Q4ILH3
49076 229533.I1S4S9 pp 229533.Q4I073
49077 229533.I1S4S9 pp 229533.Q4I0K3
49078 229533.I1S4S9 pp 229533.Q4I785
49079 229533.I1S4S9 pp 229533.I1S5B3
49080 229533.A0A098DD87 pp 229533.Q4ILH3
49081 229533.A0A098DD87 pp 229533.A0A1C3YLQ1
49082 229533.A0A098DD87 pp 229533.I1RP81
49083 229533.A0A098DD87 pp 229533.I1RLE2
49084 229533.A0A098DD87 pp 229533.I1S5B3
49085 229533.A0A098DD87 pp 229533.I1RRT3
49086 229533.A0A098DD87 pp 229533.I1RZ17
49087 229533.A0A098DD87 pp 229533.I1RST0
49088 229533.A0A098DD87 pp 229533.I1RDJ1
49089 229533.A0A098DD87 pp 229533.I1RK45
49090 229533.A0A098DD87 pp 229533.I1RZ13
49091 229533.A0A098DD87 pp 229533.I1S252
49092 229533.A0A098DD87 pp 229533.I1R9W9
49093 229533.A0A098DD87 pp 229533.I1RDE4
49094 229533.A0A098DD87 pp 229533.Q4I785
49095 229533.A0A098DD87 pp 229533.I1RTM9
49096 229533.A0A098DD87 pp 229533.I1REU0
49097 229533.A0A098DD87 pp 229533.Q4IB63
49098 229533.A0A098DD87 pp 229533.I1RNK8
49099 229533.A0A098DD87 pp 229533.I1RKP3
49100 229533.A0A098DD87 pp 229533.I1RZ18
49101 229533.A0A098DD87 pp 229533.I1RW91
49102 229533.A0A098DD87 pp 229533.I1S272
49103 229533.A0A098DD87 pp 229533.Q4I0K3
49104 229533.A0A098DD87 pp 229533.I1S5B1
49105 229533.A0A098DD87 pp 229533.A0A1C3YN83
49106 229533.A0A098DD87 pp 229533.I1RMG4
49107 229533.A0A098DD87 pp 229533.Q4I073
49108 229533.A0A098DD87 pp 229533.A0A1C3YKB6
49109 229533.A0A098DD87 pp 229533.I1RBN0
49110 229533.A0A098DD87 pp 229533.A0A1C3YHP5
49111 229533.A0A098DD87 pp 229533.I1RJL9
49112 229533.A0A098DD87 pp 229533.I1S4S9
49113 229533.I1RZD0 pp 229533.I1S130
49114 229533.I1RZD0 pp 229533.I1RZZ3
49115 229533.I1RZD0 pp 229533.Q4IAS1
49116 229533.I1RZD0 pp 229533.V6RPN0
49117 229533.I1RZD0 pp 229533.V6RG22
49118 229533.I1RZD0 pp 229533.V6RQW4
49119 229533.I1RZD0 pp 229533.Q4HVW2
49120 229533.I1RZD0 pp 229533.V6RR38
49121 229533.I1RZD0 pp 229533.I1S0I3
49122 229533.I1RZD0 pp 229533.I1S2B0
49123 229533.I1REA8 pp 229533.I1RYU0
49124 229533.I1REA8 pp 229533.I1S2C4
49125 229533.I1REA8 pp 229533.I1RXV3
49126 229533.I1REA8 pp 229533.I1RVZ3
49127 229533.I1REA8 pp 229533.I1RNG4
49128 229533.I1RU13 pp 229533.I1RV34
49129 229533.I1RU13 pp 229533.I1RV76

49130 229533.I1RU13 pp 229533.I1SAJ3
49131 229533.I1RU13 pp 229533.I1S1F7
49132 229533.I1RU13 pp 229533.I1S6Z3
49133 229533.I1RJ05 pp 229533.Q4I073
49134 229533.I1RJ05 pp 229533.I1RNF5
49135 229533.I1RJ05 pp 229533.I1RMH4
49136 229533.I1RCL0 pp 229533.I1RTG4
49137 229533.I1RCL0 pp 229533.I1RGK9
49138 229533.I1RCL0 pp 229533.I1RVW6
49139 229533.I1RCL0 pp 229533.I1RZY1
49140 229533.I1RCL0 pp 229533.I1RTT0
49141 229533.I1RCL0 pp 229533.I1RFV9
49142 229533.I1RCL0 pp 229533.I1S8F8
49143 229533.I1RCL0 pp 229533.I1RVX9
49144 229533.I1RCL0 pp 229533.I1RZE7
49145 229533.I1RCL0 pp 229533.I1RDZ7
49146 229533.I1RCL0 pp 229533.I1RE90
49147 229533.I1RCL0 pp 229533.V6RS91
49148 229533.I1RCL0 pp 229533.I1S1W8
49149 229533.I1RCL0 pp 229533.Q4HZQ1
49150 229533.I1RCL0 pp 229533.V6QV31
49151 229533.I1RCL0 pp 229533.I1S3G4
49152 229533.I1RCL0 pp 229533.I1S767
49153 229533.I1RCL0 pp 229533.V6RDH3
49154 229533.I1RCL0 pp 229533.I1S9C6
49155 229533.I1RCL0 pp 229533.I1RCT7
49156 229533.I1RCL0 pp 229533.I1RYM6
49157 229533.I1RCL0 pp 229533.I1RWD7
49158 229533.I1RCL0 pp 229533.I1RSQ4
49159 229533.I1RCL0 pp 229533.I1RM26
49160 229533.I1RCL0 pp 229533.I1S0J8
49161 229533.I1RCL0 pp 229533.I1S241
49162 229533.I1RCL0 pp 229533.I1RTJ2
49163 229533.I1RCL0 pp 229533.I1RG33
49164 229533.I1RCL0 pp 229533.I1RFI8
49165 229533.I1RCL0 pp 229533.I1RVN1
49166 229533.I1RCL0 pp 229533.I1RYK0
49167 229533.I1RCL0 pp 229533.I1RSI2
49168 229533.I1RCL0 pp 229533.I1S4B7
49169 229533.I1RCL0 pp 229533.I1RSC0
49170 229533.I1RCL0 pp 229533.I1RZU2
49171 229533.I1RCL0 pp 229533.I1S4B6
49172 229533.I1RCL0 pp 229533.I1RJU4
49173 229533.I1RCL0 pp 229533.I1RKB6
49174 229533.I1RCL0 pp 229533.I1RWX5
49175 229533.I1RCL0 pp 229533.I1RNG4
49176 229533.I1RCL0 pp 229533.I1RW11
49177 229533.I1RCL0 pp 229533.I1RCL1
49178 229533.I1RCL0 pp 229533.I1RSY1
49179 229533.I1RCL0 pp 229533.I1RL81
49180 229533.I1RCL0 pp 229533.Q4I1N3
49181 229533.I1RCL0 pp 229533.I1RYA0
49182 229533.I1RCL0 pp 229533.I1RZ70
49183 229533.I1RCL0 pp 229533.V6RG79
49184 229533.I1RCL0 pp 229533.I1RYS3
49185 229533.I1RCL0 pp 229533.I1RSY2
49186 229533.I1RCL0 pp 229533.I1RE79
49187 229533.I1RCL0 pp 229533.V6RPQ8
49188 229533.I1RCL0 pp 229533.I1RFK3
49189 229533.I1RCL0 pp 229533.I1RYC8
49190 229533.I1RCL0 pp 229533.I1RP70
49191 229533.I1RCL0 pp 229533.I1RRV1
49192 229533.I1R9P9 pp 229533.I1RV34
49193 229533.I1R9P9 pp 229533.I1S1F7
49194 229533.I1R9P9 pp 229533.I1REP6
49195 229533.I1R9P9 pp 229533.I1RIM9
49196 229533.I1R9P9 pp 229533.I1RJI9
49197 229533.I1R9P9 pp 229533.I1S6Z3
49198 229533.A0A0E0RSU2 pp 229533.I1RIM9
49199 229533.A0A0E0RSU2 pp 229533.I1RV76
49200 229533.A0A0E0RSU2 pp 229533.A0A1C3YIH6
49201 229533.A0A0E0RSU2 pp 229533.I1S1F7
49202 229533.A0A0E0RSU2 pp 229533.I1RJI9

49203 229533.A0A0E0RSU2 pp 229533.I1SAJ3
49204 229533.A0A0E0RSU2 pp 229533.A0A1C3YIF2
49205 229533.A0A0E0RSU2 pp 229533.A0A1C3YJ49
49206 229533.A0A0E0RSU2 pp 229533.I1REP6
49207 229533.A0A0E0RSU2 pp 229533.I1RTY1
49208 229533.A0A0E0RSU2 pp 229533.I1RV34
49209 229533.I1RVX2 pp 229533.I1SAJ3
49210 229533.I1RVX2 pp 229533.I1S1F7
49211 229533.I1R9E0 pp 229533.I1RYD3
49212 229533.I1S3C0 pp 229533.I1S6Z3
49213 229533.I1S3C0 pp 229533.I1SAJ3
49214 229533.A0A1C3YKR0 pp 229533.V6R1K4
49215 229533.A0A1C3YKR0 pp 229533.I1RW71
49216 229533.A0A1C3YKR0 pp 229533.I1RT33
49217 229533.A0A1C3YKR0 pp 229533.I1RP29
49218 229533.A0A1C3YKR0 pp 229533.I1RMK3
49219 229533.A0A1C3YKR0 pp 229533.I1RM67
49220 229533.A0A1C3YKR0 pp 229533.I1RSW4
49221 229533.A0A1C3YKR0 pp 229533.I1RT05
49222 229533.A0A1C3YKR0 pp 229533.I1RSM9
49223 229533.A0A1C3YKR0 pp 229533.I1RQ65
49224 229533.A0A1C3YKR0 pp 229533.A0A1C3YN63
49225 229533.A0A1C3YKR0 pp 229533.I1RRP3
49226 229533.A0A1C3YKR0 pp 229533.I1RS30
49227 229533.A0A1C3YKR0 pp 229533.Q4IFI0
49228 229533.A0A1C3YKR0 pp 229533.I1S253
49229 229533.A0A1C3YKR0 pp 229533.I1S0R7
49230 229533.A0A1C3YKR0 pp 229533.V6QYF0
49231 229533.A0A1C3YKR0 pp 229533.I1RNH6
49232 229533.A0A1C3YKR0 pp 229533.A0A1C3YL07
49233 229533.A0A1C3YKR0 pp 229533.I1RB27
49234 229533.A0A1C3YKR0 pp 229533.Q4I1M9
49235 229533.A0A1C3YKR0 pp 229533.I1RMQ4
49236 229533.A0A1C3YKR0 pp 229533.I1RMN6
49237 229533.A0A1C3YKR0 pp 229533.I1SA27
49238 229533.A0A1C3YKR0 pp 229533.I1RNY2
49239 229533.A0A1C3YKR0 pp 229533.I1RK72
49240 229533.A0A1C3YKR0 pp 229533.I1RSR3
49241 229533.A0A1C3YKR0 pp 229533.I1RM96
49242 229533.A0A1C3YKR0 pp 229533.I1RQK4
49243 229533.A0A1C3YKR0 pp 229533.I1RK03
49244 229533.A0A1C3YKR0 pp 229533.I1RPY1
49245 229533.A0A1C3YKR0 pp 229533.I1R9X7
49246 229533.A0A1C3YKR0 pp 229533.I1RZS6
49247 229533.A0A1C3YKR0 pp 229533.I1RR72
49248 229533.A0A1C3YKR0 pp 229533.Q4I8X0
49249 229533.A0A1C3YKR0 pp 229533.I1RBG9
49250 229533.A0A1C3YKR0 pp 229533.I1S274
49251 229533.A0A1C3YKR0 pp 229533.I1RNU6
49252 229533.A0A1C3YKR0 pp 229533.I1RPJ3
49253 229533.A0A1C3YKR0 pp 229533.I1RTD7
49254 229533.A0A1C3YKR0 pp 229533.I1RKC6
49255 229533.I1RVY8 pp 229533.I1S0P5
49256 229533.A0A098DP19 pp 229533.I1RW68
49257 229533.A0A098DP19 pp 229533.I1RFN1
49258 229533.A0A098DP19 pp 229533.I1RRT2
49259 229533.A0A098DP19 pp 229533.A0A098DVZ4
49260 229533.A0A098DP19 pp 229533.I1RJL4
49261 229533.A0A098DP19 pp 229533.I1RT75
49262 229533.A0A098DP19 pp 229533.I1REF2
49263 229533.A0A098DP19 pp 229533.I1RAF8
49264 229533.A0A098DP19 pp 229533.I1RQ65
49265 229533.A0A098DP19 pp 229533.I1RS47
49266 229533.A0A098DP19 pp 229533.I1RT33
49267 229533.A0A098DP19 pp 229533.Q4HZ42
49268 229533.A0A098DP19 pp 229533.A0A1C3YN63
49269 229533.A0A098DP19 pp 229533.I1RRQ3
49270 229533.A0A098DP19 pp 229533.I1RC05
49271 229533.A0A098DP19 pp 229533.I1RAC2
49272 229533.A0A098DP19 pp 229533.I1RRP3
49273 229533.A0A098DP19 pp 229533.I1RG56
49274 229533.A0A098DP19 pp 229533.A0A1C3YHT9
49275 229533.A0A098DP19 pp 229533.V6RBG5

49276	229533.A0A098DP19	pp	229533.A0A0E0SFM9
49277	229533.A0A098DP19	pp	229533.I1RB71
49278	229533.A0A098DP19	pp	229533.I1RR72
49279	229533.A0A098DP19	pp	229533.I1RB27
49280	229533.A0A098DP19	pp	229533.Q4I8X0
49281	229533.A0A098DP19	pp	229533.I1RW71
49282	229533.A0A098DP19	pp	229533.I1R9X7
49283	229533.A0A098DP19	pp	229533.I1RZS6
49284	229533.A0A098DP19	pp	229533.I1S2L2
49285	229533.A0A098DP19	pp	229533.V6QYF0
49286	229533.A0A098DP19	pp	229533.Q4IJ56
49287	229533.A0A098DP19	pp	229533.Q4I830
49288	229533.A0A098DP19	pp	229533.I1RPK0
49289	229533.A0A098DP19	pp	229533.A0A0E0S7B3
49290	229533.A0A098DP19	pp	229533.I1RWJ4
49291	229533.A0A098DP19	pp	229533.A0A0E0SGM4
49292	229533.A0A098DP19	pp	229533.I1RG51
49293	229533.A0A098DP19	pp	229533.I1RFS3
49294	229533.A0A098DP19	pp	229533.V6RGN5
49295	229533.A0A098DP19	pp	229533.A0A0E0S621
49296	229533.A0A098DP19	pp	229533.I1RC72
49297	229533.A0A098DP19	pp	229533.I1RBL7
49298	229533.A0A098DP19	pp	229533.A0A098DRR8
49299	229533.A0A098DP19	pp	229533.I1S274
49300	229533.A0A098DP19	pp	229533.I1RC03
49301	229533.A0A098DP19	pp	229533.I1RD64
49302	229533.A0A098DP19	pp	229533.I1RCL3
49303	229533.A0A098DP19	pp	229533.I1RQA2
49304	229533.A0A098DP19	pp	229533.I1RC27
49305	229533.A0A098DP19	pp	229533.I1RM67
49306	229533.A0A098DP19	pp	229533.A0A1C3YL07
49307	229533.A0A098DP19	pp	229533.I1RME2
49308	229533.A0A098DP19	pp	229533.I1S558
49309	229533.A0A098DP19	pp	229533.I1S9Y1
49310	229533.A0A098DP19	pp	229533.I1S2C0
49311	229533.A0A098DP19	pp	229533.I1RMN6
49312	229533.A0A098DP19	pp	229533.I1RKJ6
49313	229533.A0A098DP19	pp	229533.V6RGY0
49314	229533.A0A098DP19	pp	229533.I1S253
49315	229533.A0A098DP19	pp	229533.A0A1C3YJG0
49316	229533.A0A098DP19	pp	229533.I1RQ31
49317	229533.A0A098DP19	pp	229533.I1RDA0
49318	229533.A0A098DP19	pp	229533.I1RTE9
49319	229533.A0A098DP19	pp	229533.I1RW90
49320	229533.A0A098DP19	pp	229533.I1RPJ3
49321	229533.A0A098DP19	pp	229533.I1RNV6
49322	229533.A0A098DP19	pp	229533.V6R227
49323	229533.A0A098DP19	pp	229533.Q4HZK7
49324	229533.A0A098DP19	pp	229533.I1RT80
49325	229533.A0A098DP19	pp	229533.I1RRI2
49326	229533.A0A098DP19	pp	229533.I1RTE4
49327	229533.A0A098DP19	pp	229533.I1RTF4
49328	229533.A0A098DP19	pp	229533.I1RRR7
49329	229533.A0A098DP19	pp	229533.I1RNN3
49330	229533.A0A098DP19	pp	229533.I1S0F1
49331	229533.A0A098DP19	pp	229533.A0A098DRZ5
49332	229533.A0A098DP19	pp	229533.I1RF08
49333	229533.A0A098DP19	pp	229533.V6QWZ7
49334	229533.A0A098DP19	pp	229533.I1RVZ0
49335	229533.A0A098DP19	pp	229533.I1RZF6
49336	229533.A0A098DP19	pp	229533.I1RAQ5
49337	229533.A0A098DP19	pp	229533.Q4IEK8
49338	229533.A0A098DP19	pp	229533.I1RT05
49339	229533.A0A098DP19	pp	229533.I1RM96
49340	229533.A0A098DP19	pp	229533.I1RDG0
49341	229533.A0A098DP19	pp	229533.I1RK95
49342	229533.A0A098DP19	pp	229533.I1RQ28
49343	229533.A0A098DP19	pp	229533.I1RKC6
49344	229533.A0A098DP19	pp	229533.I1RAZ8
49345	229533.A0A098DP19	pp	229533.I1RJ05
49346	229533.A0A098DP19	pp	229533.I1RY63
49347	229533.A0A098DP19	pp	229533.I1R9V7
49348	229533.A0A098DP19	pp	229533.I1RA48

49349 229533.A0A098DP19 pp 229533.I1RSS4
49350 229533.A0A098DP19 pp 229533.I1RN99
49351 229533.A0A098DP19 pp 229533.I1RSD8
49352 229533.A0A098DP19 pp 229533.A0A1C3YKR0
49353 229533.I1RAM9 pp 229533.I1RT38
49354 229533.I1RAM9 pp 229533.I1RB64
49355 229533.I1RAM9 pp 229533.I1RY92
49356 229533.I1RAM9 pp 229533.I1RUR8
49357 229533.I1RAM9 pp 229533.I1RFI4
49358 229533.I1RAM9 pp 229533.I1RGC3
49359 229533.I1RAM9 pp 229533.I1RAW3
49360 229533.I1RAM9 pp 229533.Q4I099
49361 229533.I1RAM9 pp 229533.I1RN88
49362 229533.I1RAM9 pp 229533.I1RWA3
49363 229533.I1RAM9 pp 229533.I1RPV3
49364 229533.I1RAM9 pp 229533.I1RPY0
49365 229533.I1RAM9 pp 229533.Q4HVQ9
49366 229533.I1RAM9 pp 229533.I1RC34
49367 229533.I1RAM9 pp 229533.I1RAW9
49368 229533.I1RAM9 pp 229533.I1S3T7
49369 229533.I1RAM9 pp 229533.I1RSX0
49370 229533.I1RAM9 pp 229533.I1RPY8
49371 229533.I1RAM9 pp 229533.I1RHC8
49372 229533.I1RAM9 pp 229533.I1RSQ4
49373 229533.I1RAM9 pp 229533.V6R3J1
49374 229533.I1RAM9 pp 229533.I1RK39
49375 229533.I1RAM9 pp 229533.I1RJT3
49376 229533.I1RAM9 pp 229533.I1RN84
49377 229533.I1RAM9 pp 229533.V6R1X4
49378 229533.I1RAM9 pp 229533.I1RBE6
49379 229533.I1RAM9 pp 229533.I1RX39
49380 229533.I1RAM9 pp 229533.I1S0Q7
49381 229533.I1RAM9 pp 229533.I1RC18
49382 229533.I1RAM9 pp 229533.I1RXD9
49383 229533.I1RAM9 pp 229533.I1RYE5
49384 229533.I1RAM9 pp 229533.I1RL81
49385 229533.I1RAM9 pp 229533.I1RYW9
49386 229533.I1RAM9 pp 229533.I1RWT0
49387 229533.I1RAM9 pp 229533.I1RQJ3
49388 229533.I1RAM9 pp 229533.I1RRM3
49389 229533.I1RAM9 pp 229533.I1RSU1
49390 229533.I1RAM9 pp 229533.I1S5U0
49391 229533.I1RAM9 pp 229533.I1S1F3
49392 229533.I1RAM9 pp 229533.I1RKB2
49393 229533.I1RAM9 pp 229533.I1RYC8
49394 229533.I1RAM9 pp 229533.I1RKF4
49395 229533.I1RAM9 pp 229533.I1RLN1
49396 229533.I1RAM9 pp 229533.I1S464
49397 229533.I1RAM9 pp 229533.I1S266
49398 229533.I1RAM9 pp 229533.I1RFJ6
49399 229533.I1RAM9 pp 229533.I1RBT6
49400 229533.I1RAM9 pp 229533.I1RNJ5
49401 229533.I1RAM9 pp 229533.I1RU95
49402 229533.I1RAM9 pp 229533.I1RB38
49403 229533.I1RAM9 pp 229533.I1RHJ2
49404 229533.I1RAM9 pp 229533.I1S5X9
49405 229533.I1RAM9 pp 229533.I1S7W5
49406 229533.I1RAM9 pp 229533.I1RYA0
49407 229533.I1RAM9 pp 229533.I1RGI8
49408 229533.I1RAM9 pp 229533.I1RF85
49409 229533.I1RAM9 pp 229533.V6RN31
49410 229533.I1RAM9 pp 229533.I1RMV0
49411 229533.I1RAM9 pp 229533.I1RCM2
49412 229533.I1RAM9 pp 229533.I1RTK1
49413 229533.I1RAM9 pp 229533.I1RCH5
49414 229533.I1RAM9 pp 229533.I1RKB7
49415 229533.I1RAM9 pp 229533.I1S5C6
49416 229533.I1RAM9 pp 229533.I1S0R8
49417 229533.I1RAM9 pp 229533.I1RY21
49418 229533.I1RAM9 pp 229533.I1RMI1
49419 229533.I1RAM9 pp 229533.I1RMQ6
49420 229533.I1RAM9 pp 229533.I1RB86
49421 229533.I1RAM9 pp 229533.I1S8Z2

49422	229533.I1RAM9	pp	229533.I1RFS5
49423	229533.I1RAM9	pp	229533.I1RX17
49424	229533.I1RAM9	pp	229533.I1RRK9
49425	229533.I1RAM9	pp	229533.I1S1A0
49426	229533.I1RAM9	pp	229533.I1S0R1
49427	229533.I1RAM9	pp	229533.I1RYT8
49428	229533.I1RAM9	pp	229533.I1RRN2
49429	229533.I1RAM9	pp	229533.I1RVU1
49430	229533.I1RAM9	pp	229533.I1REF0
49431	229533.I1RAM9	pp	229533.I1RE65
49432	229533.I1RAM9	pp	229533.I1RG62
49433	229533.I1RAM9	pp	229533.I1REI0
49434	229533.I1RAM9	pp	229533.I1RYN2
49435	229533.I1RAM9	pp	229533.I1RI95
49436	229533.I1RAM9	pp	229533.I1RMW4
49437	229533.I1RAM9	pp	229533.I1RCR6
49438	229533.I1RAM9	pp	229533.I1RI27
49439	229533.I1RAM9	pp	229533.I1RP38
49440	229533.I1RAM9	pp	229533.I1RLP1
49441	229533.I1RAM9	pp	229533.I1RPA4
49442	229533.I1RAM9	pp	229533.I1RE66
49443	229533.I1RAM9	pp	229533.I1RZ43
49444	229533.I1RAM9	pp	229533.I1RQA5
49445	229533.I1RAM9	pp	229533.I1S0U0
49446	229533.I1RAM9	pp	229533.I1S8C1
49447	229533.I1RAM9	pp	229533.I1RNM7
49448	229533.I1RAM9	pp	229533.I1RMK7
49449	229533.I1RAM9	pp	229533.Q4I5G1
49450	229533.I1RAM9	pp	229533.I1RBC1
49451	229533.I1RAM9	pp	229533.I1S0P0
49452	229533.I1RAM9	pp	229533.I1RJY7
49453	229533.I1RAM9	pp	229533.I1RW01
49454	229533.I1RAM9	pp	229533.I1RYC7
49455	229533.I1RAM9	pp	229533.I1RJB3
49456	229533.I1RAM9	pp	229533.I1RAV5
49457	229533.I1RAM9	pp	229533.I1S128
49458	229533.I1RAM9	pp	229533.I1RGJ4
49459	229533.I1RAM9	pp	229533.I1RWG0
49460	229533.I1RAM9	pp	229533.I1RS29
49461	229533.I1RAM9	pp	229533.I1RCU2
49462	229533.I1RAM9	pp	229533.I1RPP5
49463	229533.I1RAM9	pp	229533.I1RS05
49464	229533.I1RAM9	pp	229533.I1S0T7
49465	229533.I1RAM9	pp	229533.I1S0K2
49466	229533.I1RAM9	pp	229533.I1RRX5
49467	229533.I1RAM9	pp	229533.I1RZ68
49468	229533.I1RAM9	pp	229533.I1S6K9
49469	229533.I1RAM9	pp	229533.I1RG33
49470	229533.I1RAM9	pp	229533.I1RK47
49471	229533.I1RAM9	pp	229533.I1RWB0
49472	229533.I1RAM9	pp	229533.I1S8F8
49473	229533.I1RAM9	pp	229533.I1RUQ3
49474	229533.I1RAM9	pp	229533.I1S2A1
49475	229533.I1RAM9	pp	229533.I1S212
49476	229533.I1RAM9	pp	229533.I1RGB1
49477	229533.I1RAM9	pp	229533.V6RJP8
49478	229533.I1RAM9	pp	229533.I1RKF5
49479	229533.I1RAM9	pp	229533.I1RQS2
49480	229533.I1RAM9	pp	229533.I1RQ85
49481	229533.I1RAM9	pp	229533.I1REE2
49482	229533.I1RAM9	pp	229533.I1RQR6
49483	229533.I1RAM9	pp	229533.I1RPR9
49484	229533.I1RAM9	pp	229533.I1RJU8
49485	229533.I1RAM9	pp	229533.I1RMR7
49486	229533.I1RAM9	pp	229533.I1RXB6
49487	229533.I1RAM9	pp	229533.I1RYA2
49488	229533.I1RAM9	pp	229533.I1RP02
49489	229533.I1RAM9	pp	229533.I1RXE6
49490	229533.I1RAM9	pp	229533.I1RW13
49491	229533.I1RAM9	pp	229533.I1S8S9
49492	229533.I1RAM9	pp	229533.I1RNY6
49493	229533.I1S6J8	pp	229533.I1S6Z3
49494	229533.I1S6J8	pp	229533.I1SAJ3

49495	229533.I1RLI4	pp	229533.I1RM78
49496	229533.I1RLI4	pp	229533.I1RW80
49497	229533.I1RLI4	pp	229533.I1S9H8
49498	229533.I1RLI4	pp	229533.I1S5Z5
49499	229533.I1RLI4	pp	229533.V6QWX8
49500	229533.I1RLI4	pp	229533.I1RQ42
49501	229533.I1RLI4	pp	229533.I1RPW8
49502	229533.I1RLI4	pp	229533.Q4IPI4
49503	229533.I1RLI4	pp	229533.I1S325
49504	229533.I1RLI4	pp	229533.I1RW91
49505	229533.I1RLI4	pp	229533.I1RN04
49506	229533.I1RLI4	pp	229533.I1RQP3
49507	229533.I1RLI4	pp	229533.I1RRF7
49508	229533.I1RLI4	pp	229533.I1RR61
49509	229533.I1RLI4	pp	229533.I1RQP1
49510	229533.I1RLI4	pp	229533.I1S3H6
49511	229533.I1RLI4	pp	229533.Q4ICA8
49512	229533.I1RLI4	pp	229533.I1S4A5
49513	229533.I1RLI4	pp	229533.I1S6X4
49514	229533.I1RLI4	pp	229533.I1RXN7
49515	229533.I1RLI4	pp	229533.I1RRF0
49516	229533.I1S2L9	pp	229533.I1SAJ3
49517	229533.I1S2L9	pp	229533.I1S6Z3
49518	229533.I1RVY7	pp	229533.I1S099
49519	229533.I1RW11	pp	229533.Q4I1N3
49520	229533.I1RW11	pp	229533.I1RZK8
49521	229533.I1RW11	pp	229533.I1RXY7
49522	229533.I1RW11	pp	229533.I1S0C2
49523	229533.I1RW11	pp	229533.I1S241
49524	229533.I1RW11	pp	229533.I1RX59
49525	229533.I1RW11	pp	229533.V6RDH3
49526	229533.I1RW11	pp	229533.V6RS91
49527	229533.I1RW11	pp	229533.V6QV31
49528	229533.I1SA62	pp	229533.I1SAJ3
49529	229533.I1RSE2	pp	229533.I1RZ40
49530	229533.I1RSE2	pp	229533.I1RY32
49531	229533.I1RSE2	pp	229533.O74638
49532	229533.I1RSE2	pp	229533.I1S5S2
49533	229533.I1RSE2	pp	229533.I1RXV3
49534	229533.I1RSE2	pp	229533.I1RZC3
49535	229533.I1RSE2	pp	229533.I1S3V2
49536	229533.I1RSE2	pp	229533.I1RYI6
49537	229533.I1RZ20	pp	229533.V6RQW4
49538	229533.I1RZ20	pp	229533.I1S084
49539	229533.I1RZ20	pp	229533.I1S9D4
49540	229533.I1RZ20	pp	229533.I1S1W8
49541	229533.I1RZ20	pp	229533.I1S2D5
49542	229533.I1RZ20	pp	229533.I1S1P8
49543	229533.I1RZ20	pp	229533.V6RY56
49544	229533.I1RZ20	pp	229533.I1S029
49545	229533.I1RZ20	pp	229533.I1S8X1
49546	229533.I1RZ20	pp	229533.I1S3K8
49547	229533.I1RZ20	pp	229533.I1RZT0
49548	229533.I1RZ20	pp	229533.I1S5G3
49549	229533.I1RZ20	pp	229533.I1S6P5
49550	229533.I1RZ20	pp	229533.I1RZA7
49551	229533.I1RZ20	pp	229533.I1S2E2
49552	229533.I1RZ20	pp	229533.I1S4D0
49553	229533.I1RZ20	pp	229533.I1RZ52
49554	229533.I1RZ20	pp	229533.O74638
49555	229533.I1RZ20	pp	229533.I1S2C4
49556	229533.I1RZ20	pp	229533.I1S5L5
49557	229533.I1RZ20	pp	229533.I1S076
49558	229533.I1RZ20	pp	229533.I1S3G4
49559	229533.I1RZ20	pp	229533.V6RUB1
49560	229533.I1RZ20	pp	229533.Q4HXF0
49561	229533.I1RZ20	pp	229533.I1S770
49562	229533.I1RZ20	pp	229533.I1S2H3
49563	229533.I1RZ20	pp	229533.Q4HXF6
49564	229533.I1RZ20	pp	229533.I1SAJ0
49565	229533.I1RZ20	pp	229533.I1S2C5
49566	229533.I1RZ20	pp	229533.I1S6H1
49567	229533.I1RZ20	pp	229533.I1S4F4

229533.I1RZ20 pp 229533.I1S685
229533.A0A0E0S9X0 pp 229533.I1SAJ3
229533.A0A0E0S9X0 pp 229533.A0A1C3YIH6
229533.A0A0E0S9X0 pp 229533.A0A1C3YJ49
229533.A0A0E0S9X0 pp 229533.I1S1F7
229533.A0A0E0S9X0 pp 229533.A0A1C3YIF2
229533.A0A0E0S9X0 pp 229533.I1RV34
229533.A0A0E0S9X0 pp 229533.I1RTY1
229533.A0A0E0S9X0 pp 229533.I1RJI9
229533.A0A0E0S9X0 pp 229533.I1RIM9
229533.A0A0E0S9X0 pp 229533.I1RV76
229533.I1S6L5 pp 229533.I1S9I6
229533.I1REI6 pp 229533.I1RNA1
229533.I1REI6 pp 229533.I1S574
229533.I1REI6 pp 229533.I1S984
229533.I1RCP1 pp 229533.P0C431
229533.I1RCP1 pp 229533.I1RG57
229533.I1RCP1 pp 229533.I1RJ26
229533.I1RCP1 pp 229533.I1RZT1
229533.I1RCP1 pp 229533.I1RIX1
229533.I1RCP1 pp 229533.I1RTA3
229533.I1RCP1 pp 229533.I1RE19
229533.I1RCP1 pp 229533.I1RMP2
229533.I1RCP1 pp 229533.I1RJZ4
229533.I1RCP1 pp 229533.I1RRF9
229533.I1RCP1 pp 229533.I1RTB7
229533.I1RCP1 pp 229533.I1RJZ0
229533.I1RCP1 pp 229533.V6R3U5
229533.A0A098E1U3 pp 229533.I1REP6
229533.A0A098E1U3 pp 229533.I1RV76
229533.A0A098E1U3 pp 229533.I1RV34
229533.A0A098E1U3 pp 229533.A0A1C3YIH6
229533.A0A098E1U3 pp 229533.I1SAJ3
229533.A0A098E1U3 pp 229533.I1S1F7
229533.A0A098E1U3 pp 229533.I1RJI9
229533.I1RIB2 pp 229533.I1S3L2
229533.I1RIB2 pp 229533.I1RQP2
229533.I1RIB2 pp 229533.I1RUK0
229533.I1RIB2 pp 229533.I1RXS0
229533.I1RIB2 pp 229533.I1S8R4
229533.I1RIB2 pp 229533.I1S0K0
229533.I1RL81 pp 229533.Q4I1N3
229533.I1RL81 pp 229533.I1S3V2
229533.I1RL81 pp 229533.I1S8R2
229533.I1RL81 pp 229533.I1S0V7
229533.I1RL81 pp 229533.I1S147
229533.I1RL81 pp 229533.V6RRM0
229533.I1RL81 pp 229533.I1RN84
229533.I1RL81 pp 229533.I1S3T7
229533.I1RL81 pp 229533.I1S0Q7
229533.I1RL81 pp 229533.I1S0B8
229533.I1RL81 pp 229533.Q4IN52
229533.I1RL81 pp 229533.I1S3G4
229533.I1RL81 pp 229533.I1S0V6
229533.I1RL81 pp 229533.I1RQJ7
229533.I1RL81 pp 229533.I1RY92
229533.I1RL81 pp 229533.I1RUR8
229533.I1RL81 pp 229533.I1RY81
229533.I1RL81 pp 229533.Q4I5G1
229533.I1RL81 pp 229533.I1RVR2
229533.I1RL81 pp 229533.V6RUB1
229533.I1RL81 pp 229533.I1RSQ4
229533.I1RL81 pp 229533.I1S6P5
229533.I1RL81 pp 229533.I1S968
229533.I1RL81 pp 229533.I1S0K2
229533.I1RL81 pp 229533.I1RUQ3
229533.I1RL81 pp 229533.Q4HVQ9
229533.I1RL81 pp 229533.I1RS29
229533.I1RL81 pp 229533.I1RXE6
229533.I1RL81 pp 229533.I1RPR1
229533.I1RL81 pp 229533.I1S1F3
229533.I1RL81 pp 229533.I1RWM4
229533.I1RL81 pp 229533.I1RP02

49641 229533.I1RL81 pp 229533.I1S299
49642 229533.I1RL81 pp 229533.I1RZA7
49643 229533.I1RL81 pp 229533.I1S3B2
49644 229533.I1RL81 pp 229533.V6RJP8
49645 229533.I1RL81 pp 229533.I1RMJ3
49646 229533.I1RL81 pp 229533.I1RMD3
49647 229533.I1RL81 pp 229533.V6RS91
49648 229533.I1RL81 pp 229533.I1RYP5
49649 229533.I1RL81 pp 229533.I1RVI8
49650 229533.I1RL81 pp 229533.I1RY21
49651 229533.I1RL81 pp 229533.I1RYT8
49652 229533.I1RL81 pp 229533.I1RMA2
49653 229533.I1RL81 pp 229533.I1RYX6
49654 229533.I1RL81 pp 229533.I1RMQ6
49655 229533.I1RL81 pp 229533.I1S1W8
49656 229533.I1RL81 pp 229533.I1RMK7
49657 229533.I1RL81 pp 229533.I1S0P0
49658 229533.I1RL81 pp 229533.I1RNM7
49659 229533.I1RL81 pp 229533.I1S8C1
49660 229533.I1RL81 pp 229533.I1S969
49661 229533.I1RL81 pp 229533.I1RP86
49662 229533.I1RL81 pp 229533.I1RRX5
49663 229533.I1RQY8 pp 229533.Q4IPZ1
49664 229533.I1RQY8 pp 229533.Q4IB65
49665 229533.V6RDH3 pp 229533.V6RS91
49666 229533.V6RDH3 pp 229533.V6RK96
49667 229533.V6RDH3 pp 229533.V6RXR0
49668 229533.V6RDH3 pp 229533.V6RIV7
49669 229533.V6RDH3 pp 229533.V6RHD0
49670 229533.I1RLE1 pp 229533.I1S3L2
49671 229533.I1RLE1 pp 229533.I1RXS0
49672 229533.I1RLE1 pp 229533.I1S0K0
49673 229533.I1RLE1 pp 229533.I1S8R4
49674 229533.I1RLE1 pp 229533.I1RQP2
49675 229533.I1RUP6 pp 229533.I1RYJ1
49676 229533.I1RUP6 pp 229533.I1RWF6
49677 229533.I1RUP6 pp 229533.I1RZX4
49678 229533.I1RUP6 pp 229533.V6R5G9
49679 229533.I1RUP6 pp 229533.V6R729
49680 229533.I1RUP6 pp 229533.I1S097
49681 229533.I1RUP6 pp 229533.I1RWA9
49682 229533.I1RUP6 pp 229533.Q4IJ79
49683 229533.I1RUP6 pp 229533.I1RYC1
49684 229533.I1RUP6 pp 229533.I1RWL9
49685 229533.I1RUP6 pp 229533.I1S8F8
49686 229533.I1RUP6 pp 229533.I1RW71
49687 229533.A0A098DP11 pp 229533.I1RTB7
49688 229533.A0A098DP11 pp 229533.I1RYV4
49689 229533.A0A098DP11 pp 229533.I1RAH8
49690 229533.A0A098DP11 pp 229533.A0A0E0SN30
49691 229533.A0A098DP11 pp 229533.I1RWG5
49692 229533.A0A098DP11 pp 229533.A0A0E0SIE1
49693 229533.A0A098DP11 pp 229533.I1RCP1
49694 229533.A0A098DP11 pp 229533.I1RTA3
49695 229533.A0A098DP11 pp 229533.I1RRF9
49696 229533.A0A098DP11 pp 229533.I1RUP6
49697 229533.A0A098DP11 pp 229533.I1RE19
49698 229533.A0A1C3YL98 pp 229533.I1RVY0
49699 229533.V6RN31 pp 229533.V6RUB1
49700 229533.A0A098DD64 pp 229533.A0A1C3YI88
49701 229533.A0A098DD64 pp 229533.V6QVM5
49702 229533.A0A098DD64 pp 229533.I1RW06
49703 229533.A0A098DD64 pp 229533.V6RN31
49704 229533.I1RQ48 pp 229533.I1S2M5
49705 229533.I1RQ48 pp 229533.I1RVT5
49706 229533.I1RQ48 pp 229533.I1RRC6
49707 229533.I1RQ48 pp 229533.I1RZT6
49708 229533.I1RQ48 pp 229533.I1RT50
49709 229533.I1RQ48 pp 229533.I1RT14
49710 229533.I1RQ48 pp 229533.I1SAJ8
49711 229533.I1RQ48 pp 229533.I1RWM4
49712 229533.I1RQ48 pp 229533.I1RST9
49713 229533.I1RQ48 pp 229533.I1RYD3

49714 229533.I1RQ48 pp 229533.I1RQ76
49715 229533.I1RQ48 pp 229533.I1RY65
49716 229533.I1RQ48 pp 229533.I1S1Q3
49717 229533.I1RQ48 pp 229533.I1S166
49718 229533.I1RQ48 pp 229533.I1RUY5
49719 229533.I1RQ48 pp 229533.V6RG22
49720 229533.I1RQ48 pp 229533.I1S048
49721 229533.I1RQ48 pp 229533.I1RXF0
49722 229533.I1RQ48 pp 229533.I1S267
49723 229533.I1RQ48 pp 229533.I1RWC1
49724 229533.I1RQ48 pp 229533.I1RVD8
49725 229533.I1RQ48 pp 229533.V6RR38
49726 229533.I1RQ48 pp 229533.I1RTJ2
49727 229533.I1RQ48 pp 229533.I1S2C2
49728 229533.I1RQ48 pp 229533.I1RXB6
49729 229533.I1RQ48 pp 229533.I1S5R5
49730 229533.I1RQ48 pp 229533.I1S8X1
49731 229533.I1RQ48 pp 229533.V6RPN0
49732 229533.I1RQ48 pp 229533.I1RY81
49733 229533.I1RQ48 pp 229533.I1RRN4
49734 229533.I1RQ48 pp 229533.I1S489
49735 229533.I1RQ48 pp 229533.I1RYL5
49736 229533.I1RQ48 pp 229533.I1RSE6
49737 229533.I1RQ48 pp 229533.I1RYK0
49738 229533.I1RQ48 pp 229533.I1RXX1
49739 229533.I1RQ48 pp 229533.I1RZ25
49740 229533.I1RQ48 pp 229533.I1RRC4
49741 229533.I1RQ48 pp 229533.I1RRR9
49742 229533.I1RQ48 pp 229533.I1RUJ5
49743 229533.I1RQ48 pp 229533.I1S5E5
49744 229533.I1RQ48 pp 229533.I1RR68
49745 229533.I1RQ48 pp 229533.I1S5K2
49746 229533.I1RQ48 pp 229533.I1RUB2
49747 229533.I1RQ48 pp 229533.V6RIE5
49748 229533.I1RQ48 pp 229533.I1RW82
49749 229533.I1RQ48 pp 229533.I1RY35
49750 229533.I1RQ48 pp 229533.I1RTM8
49751 229533.I1RQ48 pp 229533.I1RXY7
49752 229533.I1RQ48 pp 229533.V6RVJ3
49753 229533.I1RQ48 pp 229533.I1RQM9
49754 229533.I1RQ48 pp 229533.I1S770
49755 229533.I1RQ48 pp 229533.I1RZJ8
49756 229533.I1RQ48 pp 229533.I1RWV9
49757 229533.I1RQ48 pp 229533.I1SAS3
49758 229533.I1RQ48 pp 229533.I1RWF4
49759 229533.I1RQ48 pp 229533.I1RS40
49760 229533.I1RQ48 pp 229533.I1RZ89
49761 229533.I1RQ48 pp 229533.I1RSU2
49762 229533.I1RQ48 pp 229533.I1S0Y2
49763 229533.I1RQ48 pp 229533.I1SAS4
49764 229533.I1RPH7 pp 229533.I1RX05
49765 229533.I1RPH7 pp 229533.I1S9A8
49766 229533.I1RPH7 pp 229533.Q4ICG5
49767 229533.I1RPH7 pp 229533.I1RZ94
49768 229533.I1S2Y3 pp 229533.I1S574
49769 229533.I1S2Y3 pp 229533.I1S984
49770 229533.I1S9E7 pp 229533.Q4IPZ1
49771 229533.I1S9E7 pp 229533.Q4IB65
49772 229533.I1RK03 pp 229533.I1RYX6
49773 229533.I1RK03 pp 229533.V6R2Z2
49774 229533.I1RK03 pp 229533.I1RPD6
49775 229533.I1RK03 pp 229533.I1RNB4
49776 229533.I1RK03 pp 229533.I1RM69
49777 229533.I1RK03 pp 229533.I1RWW7
49778 229533.I1RK03 pp 229533.V6QU99
49779 229533.I1RK03 pp 229533.I1RMG6
49780 229533.I1RK03 pp 229533.I1RNG4
49781 229533.I1RK03 pp 229533.I1RSS7
49782 229533.I1RK03 pp 229533.I1RW00
49783 229533.I1RK03 pp 229533.I1S5I8
49784 229533.I1RK03 pp 229533.I1RZ83
49785 229533.I1RK03 pp 229533.I1S2L2
49786 229533.I1RK03 pp 229533.I1RWF9

49787 229533.I1RK03 pp 229533.I1RP97
49788 229533.I1RK03 pp 229533.I1RXB6
49789 229533.I1RK03 pp 229533.V6RAM6
49790 229533.I1RK03 pp 229533.I1SAI4
49791 229533.I1RK03 pp 229533.I1RPK0
49792 229533.I1RK03 pp 229533.I1S1W8
49793 229533.I1RK03 pp 229533.V6RLM7
49794 229533.I1RK03 pp 229533.Q4HXF0
49795 229533.I1RK03 pp 229533.I1RUY5
49796 229533.I1RK03 pp 229533.I1RZ21
49797 229533.I1RK03 pp 229533.I1RZ92
49798 229533.I1RK03 pp 229533.I1RV23
49799 229533.I1RK03 pp 229533.I1RMK4
49800 229533.I1RK03 pp 229533.I1S3G4
49801 229533.I1RK03 pp 229533.I1RZ52
49802 229533.I1RK03 pp 229533.I1RTI7
49803 229533.I1RK03 pp 229533.I1S253
49804 229533.I1RK03 pp 229533.I1S3V2
49805 229533.I1RK03 pp 229533.I1RPW2
49806 229533.I1RK03 pp 229533.V6R5D2
49807 229533.I1RK03 pp 229533.I1RXV3
49808 229533.I1RK03 pp 229533.I1RSU2
49809 229533.I1RK03 pp 229533.I1RR72
49810 229533.I1RK03 pp 229533.I1RRV4
49811 229533.I1RK03 pp 229533.I1S0T4
49812 229533.I1RK03 pp 229533.V6R0W1
49813 229533.I1RK03 pp 229533.I1RM68
49814 229533.I1RK03 pp 229533.I1RW43
49815 229533.I1RK03 pp 229533.I1RNH4
49816 229533.I1RK03 pp 229533.I1RVP5
49817 229533.I1RK03 pp 229533.I1RMG7
49818 229533.I1RK03 pp 229533.I1RXF0
49819 229533.I1RK03 pp 229533.V6R756
49820 229533.I1RK03 pp 229533.Q4HZ68
49821 229533.I1RK03 pp 229533.I1RY74
49822 229533.I1RK03 pp 229533.I1RRM1
49823 229533.I1RK03 pp 229533.I1RM55
49824 229533.I1RK03 pp 229533.I1RQ30
49825 229533.I1RK03 pp 229533.I1RY12
49826 229533.I1RK03 pp 229533.I1RYU0
49827 229533.I1RK03 pp 229533.I1SAJ3
49828 229533.I1S702 pp 229533.I1S8R4
49829 229533.I1S163 pp 229533.I1S166
49830 229533.A0A1C3YLL4 pp 229533.I1RND4
49831 229533.A0A1C3YLL4 pp 229533.I1RFV9
49832 229533.A0A1C3YLL4 pp 229533.I1RLT2
49833 229533.A0A1C3YLL4 pp 229533.V6RLM7
49834 229533.A0A1C3YLL4 pp 229533.I1RBM9
49835 229533.A0A1C3YLL4 pp 229533.I1RP37
49836 229533.A0A1C3YLL4 pp 229533.I1RW06
49837 229533.A0A1C3YLL4 pp 229533.Q4I1Y1
49838 229533.A0A1C3YLL4 pp 229533.I1S9T8
49839 229533.A0A1C3YLL4 pp 229533.I1RYK0
49840 229533.A0A1C3YLL4 pp 229533.I1RJQ3
49841 229533.I1RW57 pp 229533.Q4IB96
49842 229533.I1RT88 pp 229533.I1RVR2
49843 229533.I1RDS6 pp 229533.I1S3L2
49844 229533.I1RDS6 pp 229533.I1S8R4
49845 229533.I1RDS6 pp 229533.I1RQP2
49846 229533.I1RDS6 pp 229533.I1RXS0
49847 229533.I1RDS6 pp 229533.I1S0K0
49848 229533.I1RDS6 pp 229533.I1RHZ5
49849 229533.I1RA64 pp 229533.I1S984
49850 229533.I1RA64 pp 229533.I1S574
49851 229533.I1RA64 pp 229533.I1RNA1
49852 229533.A0A098DNY9 pp 229533.A0A1C3YJG7
49853 229533.A0A098DNY9 pp 229533.I1S549
49854 229533.A0A098DNY9 pp 229533.I1RWM0
49855 229533.A0A098DNY9 pp 229533.I1RXB5
49856 229533.I1RHZ7 pp 229533.I1RXS0
49857 229533.I1RHZ7 pp 229533.I1S8R4
49858 229533.I1RHZ7 pp 229533.I1RQP2
49859 229533.I1RHZ7 pp 229533.I1S3L2

49860	229533.I1RHZ7	pp	229533.I1S0K0
49861	229533.I1RBU8	pp	229533.I1RYI5
49862	229533.I1RBU8	pp	229533.I1RPP3
49863	229533.I1RBU8	pp	229533.I1RT40
49864	229533.I1RBU8	pp	229533.I1RTA1
49865	229533.I1RBU8	pp	229533.I1RJN9
49866	229533.I1RBU8	pp	229533.I1RTD5
49867	229533.I1RBU8	pp	229533.I1RVZ2
49868	229533.I1RBU8	pp	229533.I1S0W4
49869	229533.I1RBU8	pp	229533.I1RIU0
49870	229533.I1RBU8	pp	229533.I1RSA5
49871	229533.I1RBU8	pp	229533.I1RC34
49872	229533.I1RBU8	pp	229533.Q4HVQ9
49873	229533.I1RBU8	pp	229533.I1RGA4
49874	229533.I1RBU8	pp	229533.I1RKG2
49875	229533.I1RBU8	pp	229533.I1RDF7
49876	229533.I1RBU8	pp	229533.I1RKD3
49877	229533.I1RBU8	pp	229533.I1RG71
49878	229533.I1RBU8	pp	229533.I1RPU2
49879	229533.I1RBU8	pp	229533.I1RZ39
49880	229533.I1RBU8	pp	229533.I1RMU3
49881	229533.I1RBU8	pp	229533.I1RSZ7
49882	229533.I1RBU8	pp	229533.I1RPE0
49883	229533.I1RBU8	pp	229533.I1S1W9
49884	229533.I1RBU8	pp	229533.I1S2B8
49885	229533.I1RBU8	pp	229533.I1RZV3
49886	229533.I1RBU8	pp	229533.I1RRI1
49887	229533.I1RBU8	pp	229533.V6RKY8
49888	229533.I1RBU8	pp	229533.I1S3T7
49889	229533.I1RBU8	pp	229533.I1RLW5
49890	229533.I1RBU8	pp	229533.I1RXC2
49891	229533.I1RBU8	pp	229533.V6RMW6
49892	229533.I1RBU8	pp	229533.I1S5R1
49893	229533.I1RBU8	pp	229533.I1RRM3
49894	229533.I1RBU8	pp	229533.I1RR45
49895	229533.I1RBU8	pp	229533.V6RLM7
49896	229533.I1RBU8	pp	229533.I1RE89
49897	229533.I1RBU8	pp	229533.V6R042
49898	229533.I1RBU8	pp	229533.I1RKG3
49899	229533.I1RBU8	pp	229533.I1RQC0
49900	229533.I1RBU8	pp	229533.I1RUG3
49901	229533.I1RBU8	pp	229533.I1RSQ3
49902	229533.I1RBU8	pp	229533.I1RD22
49903	229533.I1RBU8	pp	229533.I1RVX0
49904	229533.I1RBU8	pp	229533.I1RNC1
49905	229533.I1RBU8	pp	229533.I1S4S7
49906	229533.I1RBU8	pp	229533.I1RE79
49907	229533.I1RBU8	pp	229533.I1RCH5
49908	229533.I1RBU8	pp	229533.I1S5H2
49909	229533.I1RBU8	pp	229533.I1RX95
49910	229533.I1RBU8	pp	229533.I1RQE7
49911	229533.I1RBU8	pp	229533.I1RQ33
49912	229533.I1RBU8	pp	229533.I1S6D7
49913	229533.I1RBU8	pp	229533.I1S5R6
49914	229533.I1RBU8	pp	229533.I1RD52
49915	229533.I1RBU8	pp	229533.I1RP86
49916	229533.I1RBU8	pp	229533.I1RNM7
49917	229533.I1RBU8	pp	229533.I1REW3
49918	229533.I1RBU8	pp	229533.I1RXD7
49919	229533.I1RBU8	pp	229533.I1RE48
49920	229533.I1RBU8	pp	229533.I1RN52
49921	229533.I1RBU8	pp	229533.I1RPY3
49922	229533.I1RBU8	pp	229533.I1RYU3
49923	229533.I1RBU8	pp	229533.V6RTJ8
49924	229533.I1RBU8	pp	229533.I1RCM7
49925	229533.I1RBU8	pp	229533.I1RJU3
49926	229533.I1RBU8	pp	229533.I1RX46
49927	229533.I1RBU8	pp	229533.I1RR24
49928	229533.I1RBU8	pp	229533.I1RXC4
49929	229533.I1RBU8	pp	229533.I1RZM8
49930	229533.I1RBU8	pp	229533.I1RQ85
49931	229533.I1RBU8	pp	229533.I1S5B0
49932	229533.I1RBU8	pp	229533.I1RRK5

49933 229533.I1RBU8 pp 229533.I1RUY5
49934 229533.I1RBU8 pp 229533.I1REE2
49935 229533.I1RBU8 pp 229533.V6RCZ8
49936 229533.I1RBU8 pp 229533.I1RUQ3
49937 229533.I1RBU8 pp 229533.I1RDU0
49938 229533.I1RBU8 pp 229533.I1RGG6
49939 229533.I1RBU8 pp 229533.Q4IJK2
49940 229533.I1RBU8 pp 229533.I1RWR7
49941 229533.I1RBU8 pp 229533.I1REK8
49942 229533.I1RBU8 pp 229533.I1RZK5
49943 229533.I1RBU8 pp 229533.I1RRY9
49944 229533.A0A098DD55 pp 229533.I1S166
49945 229533.A0A098DD55 pp 229533.I1S163
49946 229533.A0A098DD55 pp 229533.I1RBE7
49947 229533.A0A098DD55 pp 229533.A0A1C3YLB6
49948 229533.A0A098DD55 pp 229533.I1RNG4
49949 229533.A0A098DD55 pp 229533.I1RL11
49950 229533.A0A098DD55 pp 229533.I1S3G4
49951 229533.A0A098DD55 pp 229533.I1RY87
49952 229533.A0A098DD55 pp 229533.I1S1W8
49953 229533.I1RN80 pp 229533.I1RQE0
49954 229533.I1RN80 pp 229533.V6R9Z3
49955 229533.I1RN80 pp 229533.I1RSQ2
49956 229533.I1RN80 pp 229533.I1RRM1
49957 229533.I1RN80 pp 229533.I1RNH4
49958 229533.I1RN80 pp 229533.V6R3V4
49959 229533.I1RN80 pp 229533.I1S4S5
49960 229533.I1RN80 pp 229533.I1RYL5
49961 229533.I1RN80 pp 229533.I1RVX9
49962 229533.I1RN80 pp 229533.I1RSX2
49963 229533.I1RN80 pp 229533.I1RZ20
49964 229533.I1RN80 pp 229533.I1RXX0
49965 229533.I1RN80 pp 229533.I1RPD6
49966 229533.I1RN80 pp 229533.I1S6P6
49967 229533.I1RN80 pp 229533.V6RF62
49968 229533.I1RN80 pp 229533.I1RR68
49969 229533.I1RN80 pp 229533.I1S4Z7
49970 229533.I1RN80 pp 229533.I1S5L5
49971 229533.I1RN80 pp 229533.I1S0R9
49972 229533.I1RN80 pp 229533.I1RNG7
49973 229533.I1RN80 pp 229533.V6QUV7
49974 229533.I1RN80 pp 229533.V6RRM0
49975 229533.I1RN80 pp 229533.I1RXG6
49976 229533.I1RN80 pp 229533.I1RYP6
49977 229533.I1RN80 pp 229533.I1S2C4
49978 229533.I1RN80 pp 229533.V6RN31
49979 229533.I1RGT2 pp 229533.I1S3L2
49980 229533.I1RGT2 pp 229533.I1S0K0
49981 229533.I1RGT2 pp 229533.I1RQP2
49982 229533.I1RGT2 pp 229533.I1RXS0
49983 229533.I1RGT2 pp 229533.I1S8R4
49984 229533.I1RGT2 pp 229533.I1RHZ5
49985 229533.A0A1C3YKF3 pp 229533.I1S123
49986 229533.A0A1C3YKF3 pp 229533.I1RSY2
49987 229533.A0A1C3YKF3 pp 229533.V6RLM7
49988 229533.A0A1C3YKF3 pp 229533.I1S9T5
49989 229533.A0A1C3YKF3 pp 229533.I1RR45
49990 229533.A0A1C3YKF3 pp 229533.I1RCG5
49991 229533.A0A1C3YKF3 pp 229533.I1RBA4
49992 229533.A0A1C3YKF3 pp 229533.V6RN31
49993 229533.A0A1C3YKF3 pp 229533.I1RLU2
49994 229533.A0A1C3YKF3 pp 229533.I1RE79
49995 229533.A0A1C3YKF3 pp 229533.Q4I8S2
49996 229533.A0A1C3YKF3 pp 229533.I1RN84
49997 229533.A0A1C3YKF3 pp 229533.I1RQ38
49998 229533.A0A1C3YKF3 pp 229533.V6QX21
49999 229533.A0A1C3YKF3 pp 229533.I1REW3
50000 229533.A0A1C3YKF3 pp 229533.I1RRV1
50001 229533.A0A1C3YKF3 pp 229533.I1RKP1
50002 229533.A0A1C3YKF3 pp 229533.I1RSF1
50003 229533.A0A1C3YKF3 pp 229533.A0A1C3YMN0
50004 229533.A0A1C3YKF3 pp 229533.I1RPR8
50005 229533.A0A1C3YKF3 pp 229533.I1SB04

50006 229533.A0A1C3YKF3 pp 229533.I1S7M8
50007 229533.A0A1C3YKF3 pp 229533.I1RW28
50008 229533.A0A1C3YKF3 pp 229533.I1S064
50009 229533.A0A1C3YKF3 pp 229533.A0A1C3YKK8
50010 229533.A0A1C3YKF3 pp 229533.I1RTA0
50011 229533.A0A1C3YKF3 pp 229533.A0A1C3YLL1
50012 229533.A0A1C3YKF3 pp 229533.V6RKY8
50013 229533.A0A1C3YKF3 pp 229533.A0A1C3YN83
50014 229533.A0A1C3YKF3 pp 229533.A0A1C3YLE2
50015 229533.A0A1C3YKF3 pp 229533.I1R9Y8
50016 229533.A0A1C3YKF3 pp 229533.I1RZV5
50017 229533.A0A1C3YKF3 pp 229533.I1S8P5
50018 229533.A0A1C3YKF3 pp 229533.I1RVZ3
50019 229533.A0A1C3YKF3 pp 229533.I1RC67
50020 229533.A0A1C3YKF3 pp 229533.Q4IEV4
50021 229533.A0A1C3YKF3 pp 229533.A0A1C3YMA5
50022 229533.A0A1C3YKF3 pp 229533.I1RBF3
50023 229533.A0A1C3YKF3 pp 229533.I1RYP2
50024 229533.A0A1C3YKF3 pp 229533.I1RYB8
50025 229533.A0A1C3YKF3 pp 229533.I1S4Z7
50026 229533.A0A1C3YKF3 pp 229533.I1RT75
50027 229533.A0A1C3YKF3 pp 229533.I1RPD6
50028 229533.A0A1C3YKF3 pp 229533.I1RYL5
50029 229533.A0A1C3YKF3 pp 229533.I1RMJ3
50030 229533.A0A1C3YKF3 pp 229533.I1RQ12
50031 229533.A0A1C3YKF3 pp 229533.V6RY56
50032 229533.A0A1C3YKF3 pp 229533.I1S8Z2
50033 229533.A0A1C3YKF3 pp 229533.I1REN3
50034 229533.A0A1C3YKF3 pp 229533.V6RDH3
50035 229533.A0A1C3YKF3 pp 229533.I1RKZ4
50036 229533.A0A1C3YKF3 pp 229533.I1RAP3
50037 229533.A0A1C3YKF3 pp 229533.I1RMW4
50038 229533.A0A1C3YKF3 pp 229533.I1RPF4
50039 229533.A0A1C3YKF3 pp 229533.I1RBU8
50040 229533.A0A1C3YKF3 pp 229533.P0C431
50041 229533.A0A1C3YKF3 pp 229533.I1RRK5
50042 229533.A0A1C3YKF3 pp 229533.I1RJD3
50043 229533.A0A1C3YKF3 pp 229533.I1RYB1
50044 229533.A0A1C3YKF3 pp 229533.I1RA32
50045 229533.A0A1C3YKF3 pp 229533.I1RSQ2
50046 229533.A0A1C3YKF3 pp 229533.I1RWW4
50047 229533.A0A1C3YKF3 pp 229533.I1RN52
50048 229533.A0A1C3YKF3 pp 229533.I1RTC2
50049 229533.A0A1C3YKF3 pp 229533.A0A1C3YLR6
50050 229533.A0A1C3YKF3 pp 229533.I1RZY1
50051 229533.A0A1C3YKF3 pp 229533.I1RKM2
50052 229533.A0A1C3YKF3 pp 229533.I1RMK6
50053 229533.A0A1C3YKF3 pp 229533.I1RP86
50054 229533.I1RSR6 pp 229533.I1RYV4
50055 229533.I1RSR6 pp 229533.I1S490
50056 229533.I1RSR6 pp 229533.I1RVV7
50057 229533.I1RSR6 pp 229533.I1RWG5
50058 229533.I1RIY0 pp 229533.I1RK95
50059 229533.I1RIY0 pp 229533.I1RMG7
50060 229533.I1RIY0 pp 229533.I1RW06
50061 229533.I1RIY0 pp 229533.I1RK47
50062 229533.I1RIY0 pp 229533.I1RYU5
50063 229533.I1RIY0 pp 229533.I1S8X1
50064 229533.I1RIY0 pp 229533.I1RVX9
50065 229533.I1RIY0 pp 229533.V6RBN2
50066 229533.I1RIY0 pp 229533.I1S3K6
50067 229533.I1RIY0 pp 229533.V6RQW4
50068 229533.I1RIY0 pp 229533.I1RYD3
50069 229533.I1RIY0 pp 229533.I1RMD1
50070 229533.I1RIY0 pp 229533.I1RYI7
50071 229533.I1RIY0 pp 229533.I1RM26
50072 229533.I1RIY0 pp 229533.I1RVJ6
50073 229533.I1RIY0 pp 229533.I1RK03
50074 229533.I1RIY0 pp 229533.I1RVR0
50075 229533.I1RIY0 pp 229533.I1RRV4
50076 229533.I1RIY0 pp 229533.I1RJF1
50077 229533.I1RIY0 pp 229533.I1S9D4
50078 229533.I1RIY0 pp 229533.I1RM30

50079 229533.I1RIY0 pp 229533.I1S770
50080 229533.I1RIY0 pp 229533.I1S2E2
50081 229533.I1RIY0 pp 229533.I1RXF0
50082 229533.I1RIY0 pp 229533.I1RXV3
50083 229533.I1RIY0 pp 229533.O74638
50084 229533.I1RIY0 pp 229533.V6RVJ3
50085 229533.I1RIY0 pp 229533.I1RSQ4
50086 229533.I1RIY0 pp 229533.I1RVX8
50087 229533.I1RIY0 pp 229533.I1RSS7
50088 229533.I1RIY0 pp 229533.I1RYW6
50089 229533.I1RIY0 pp 229533.I1RM55
50090 229533.I1RIY0 pp 229533.I1RZ52
50091 229533.I1RIY0 pp 229533.I1RZ20
50092 229533.I1RIY0 pp 229533.I1RTI7
50093 229533.I1RIY0 pp 229533.V6QVH8
50094 229533.I1RIY0 pp 229533.I1RM69
50095 229533.I1RIY0 pp 229533.I1RMG6
50096 229533.I1RIY0 pp 229533.I1RY74
50097 229533.I1RIY0 pp 229533.I1RPD6
50098 229533.I1RIY0 pp 229533.I1RP69
50099 229533.I1RIY0 pp 229533.Q4HXF0
50100 229533.I1RIY0 pp 229533.I1RQ30
50101 229533.I1RIY0 pp 229533.I1RNG4
50102 229533.I1RIY0 pp 229533.I1RP97
50103 229533.I1RIY0 pp 229533.I1RM68
50104 229533.I1RIY0 pp 229533.V6RLM7
50105 229533.I1RIY0 pp 229533.I1RS40
50106 229533.I1RIY0 pp 229533.I1RSL5
50107 229533.I1RIY0 pp 229533.I1RPK0
50108 229533.A0A098DTS1 pp 229533.I1RQE8
50109 229533.A0A098DTS1 pp 229533.I1RDZ7
50110 229533.A0A098DTS1 pp 229533.I1RGK9
50111 229533.A0A098DTS1 pp 229533.V6QXN0
50112 229533.A0A098DTS1 pp 229533.I1S1W8
50113 229533.A0A098DTS1 pp 229533.I1RC74
50114 229533.A0A098DTS1 pp 229533.V6QU65
50115 229533.A0A098DTS1 pp 229533.I1RA94
50116 229533.A0A098DTS1 pp 229533.I1RJS0
50117 229533.A0A098DTS1 pp 229533.I1R9N5
50118 229533.A0A098DTS1 pp 229533.I1RVW6
50119 229533.A0A098DTS1 pp 229533.I1RCT7
50120 229533.A0A098DTS1 pp 229533.I1RQA5
50121 229533.A0A098DTS1 pp 229533.I1RZE7
50122 229533.A0A098DTS1 pp 229533.A0A1C3YJ89
50123 229533.A0A098DTS1 pp 229533.V6RDH3
50124 229533.A0A098DTS1 pp 229533.I1RBE7
50125 229533.A0A098DTS1 pp 229533.I1RTJ2
50126 229533.A0A098DTS1 pp 229533.I1RW69
50127 229533.A0A098DTS1 pp 229533.A0A1C3YJU0
50128 229533.A0A098DTS1 pp 229533.I1RZK8
50129 229533.A0A098DTS1 pp 229533.I1RCL0
50130 229533.A0A098DTS1 pp 229533.I1RX59
50131 229533.A0A098DTS1 pp 229533.Q4HZQ1
50132 229533.A0A098DTS1 pp 229533.I1RZM4
50133 229533.A0A098DTS1 pp 229533.I1S053
50134 229533.A0A098DTS1 pp 229533.I1RQM4
50135 229533.A0A098DTS1 pp 229533.I1RGT3
50136 229533.A0A098DTS1 pp 229533.V6RK96
50137 229533.A0A098DTS1 pp 229533.V6RS91
50138 229533.A0A098DTS1 pp 229533.I1RE90
50139 229533.A0A098DTS1 pp 229533.A0A0E0RX50
50140 229533.A0A098DTS1 pp 229533.I1RTT0
50141 229533.A0A098DTS1 pp 229533.I1RY49
50142 229533.A0A098DTS1 pp 229533.I1RAH3
50143 229533.A0A098DTS1 pp 229533.A0A0E0RMB3
50144 229533.A0A098DTS1 pp 229533.I1RMD6
50145 229533.A0A098DTS1 pp 229533.I1S767
50146 229533.A0A098DTS1 pp 229533.Q4I1N3
50147 229533.A0A098DTS1 pp 229533.V6RIV7
50148 229533.A0A098DTS1 pp 229533.I1S241
50149 229533.A0A098DTS1 pp 229533.I1RE79
50150 229533.A0A098DTS1 pp 229533.I1RCT2
50151 229533.A0A098DTS1 pp 229533.I1RXY7

50152 229533.A0A098DTS1 pp 229533.I1S0I3
50153 229533.A0A098DTS1 pp 229533.I1RUP5
50154 229533.A0A098DTS1 pp 229533.I1RFK3
50155 229533.A0A098DTS1 pp 229533.A0A0E0SID1
50156 229533.A0A098DTS1 pp 229533.V6RXR0
50157 229533.A0A098DTS1 pp 229533.I1RP70
50158 229533.A0A098DTS1 pp 229533.V6QV31
50159 229533.A0A098DTS1 pp 229533.I1RZU2
50160 229533.A0A098DTS1 pp 229533.I1S3G4
50161 229533.A0A098DTS1 pp 229533.I1RE58
50162 229533.A0A098DTS1 pp 229533.A0A098E3T0
50163 229533.A0A098DTS1 pp 229533.I1RYP6
50164 229533.A0A098DTS1 pp 229533.A0A1C3YKN4
50165 229533.A0A098DTS1 pp 229533.I1RW11
50166 229533.A0A098DTS1 pp 229533.I1RNG4
50167 229533.A0A098DTS1 pp 229533.V6RHD0
50168 229533.A0A098DTS1 pp 229533.A0A1C3YN78
50169 229533.A0A098DGA1 pp 229533.I1RVR2
50170 229533.A0A098DGA1 pp 229533.I1RPY0
50171 229533.A0A098DGA1 pp 229533.A0A1C3YLE2
50172 229533.A0A098DGA1 pp 229533.I1RN84
50173 229533.A0A098DGA1 pp 229533.I1RMJ3
50174 229533.A0A098DGA1 pp 229533.I1RMK7
50175 229533.A0A098DGA1 pp 229533.A0A098DPB8
50176 229533.I1S568 pp 229533.I1S8R4
50177 229533.A0A098DD42 pp 229533.A0A1C3YIN1
50178 229533.A0A098DD42 pp 229533.I1RR73
50179 229533.A0A098DD42 pp 229533.I1RKJ6
50180 229533.A0A098DD42 pp 229533.A0A098DE18
50181 229533.A0A098DD42 pp 229533.I1RSR8
50182 229533.A0A098DD42 pp 229533.I1RX46
50183 229533.A0A098DD42 pp 229533.I1RAN5
50184 229533.A0A098DD42 pp 229533.I1RLV9
50185 229533.A0A098DD42 pp 229533.I1RA48
50186 229533.A0A098DD42 pp 229533.I1RZK6
50187 229533.A0A098DD42 pp 229533.I1RKL3
50188 229533.A0A098DD42 pp 229533.I1RAU7
50189 229533.A0A098DD42 pp 229533.I1RQ65
50190 229533.A0A098DD42 pp 229533.V6R042
50191 229533.A0A098DD42 pp 229533.I1RNH6
50192 229533.I1RG33 pp 229533.I1S0K2
50193 229533.I1RG33 pp 229533.I1RS29
50194 229533.I1RG33 pp 229533.I1RUQ3
50195 229533.I1RG33 pp 229533.Q4I5G1
50196 229533.I1RG33 pp 229533.I1RY81
50197 229533.I1RG33 pp 229533.I1RG66
50198 229533.I1RG33 pp 229533.I1RQJ7
50199 229533.I1RG33 pp 229533.I1RJY7
50200 229533.I1RG33 pp 229533.I1RMD3
50201 229533.I1RG33 pp 229533.V6RJP8
50202 229533.I1RG33 pp 229533.I1S0V6
50203 229533.I1RG33 pp 229533.I1RNM7
50204 229533.I1RG33 pp 229533.I1RPR1
50205 229533.I1RG33 pp 229533.I1RXE6
50206 229533.I1RG33 pp 229533.I1S3B2
50207 229533.I1RG33 pp 229533.I1RYP5
50208 229533.I1RG33 pp 229533.I1RKB7
50209 229533.I1RG33 pp 229533.I1RMQ6
50210 229533.I1RG33 pp 229533.I1RP02
50211 229533.I1RG33 pp 229533.I1RVI8
50212 229533.I1RG33 pp 229533.I1RYT8
50213 229533.I1RG33 pp 229533.I1RWM4
50214 229533.I1RG33 pp 229533.I1RG62
50215 229533.I1RG33 pp 229533.I1RY21
50216 229533.I1RG33 pp 229533.I1RYX6
50217 229533.I1RG33 pp 229533.I1RMK7
50218 229533.I1RG33 pp 229533.I1RP86
50219 229533.I1RG33 pp 229533.I1RMA2
50220 229533.I1RG33 pp 229533.V6RS91
50221 229533.I1RG33 pp 229533.I1RRX5
50222 229533.I1RG33 pp 229533.I1S1W8
50223 229533.I1RG33 pp 229533.I1S8C1
50224 229533.I1RG33 pp 229533.Q4I1N3

229533.I1RG33 pp 229533.I1S969
229533.I1RG33 pp 229533.I1S0P0
229533.I1RG33 pp 229533.I1S8R2
229533.I1RG33 pp 229533.I1S0Q7
229533.I1RG33 pp 229533.I1RSQ4
229533.I1RG33 pp 229533.I1RY92
229533.I1RG33 pp 229533.I1RK55
229533.I1RG33 pp 229533.I1S0B8
229533.I1RG33 pp 229533.I1RUR8
229533.I1RG33 pp 229533.I1RN84
229533.I1RG33 pp 229533.Q4IN52
229533.I1RG33 pp 229533.V6RRM0
229533.I1RG33 pp 229533.I1S3T7
229533.I1RG33 pp 229533.I1S3V2
229533.I1RG33 pp 229533.I1S968
229533.I1RG33 pp 229533.I1S147
229533.I1RG33 pp 229533.I1S3G4
229533.I1RG33 pp 229533.V6RUB1
229533.I1RG33 pp 229533.Q4HVQ9
229533.I1RG33 pp 229533.I1S0V7
229533.I1RG33 pp 229533.I1RHJ2
229533.I1RG33 pp 229533.I1RVR2
229533.I1RG33 pp 229533.I1S299
229533.I1RG33 pp 229533.I1RKB2
229533.I1RG33 pp 229533.I1RZA7
229533.I1RG33 pp 229533.I1S6P5
229533.I1RG33 pp 229533.I1S1F3
229533.I1RG33 pp 229533.I1RMJ3
229533.A0A0E0SHR3 pp 229533.A0A1C3YI62
229533.A0A0E0SHR3 pp 229533.Q4IMD1
229533.A0A0E0SHR3 pp 229533.A0A1C3YLC6
229533.A0A0E0SHR3 pp 229533.I1RT16
229533.A0A0E0SHR3 pp 229533.I1S7M2
229533.A0A0E0SHR3 pp 229533.I1RXF2
229533.A0A0E0SHR3 pp 229533.I1RBV3
229533.A0A0E0SHR3 pp 229533.I1RLX6
229533.A0A0E0SHR3 pp 229533.I1S951
229533.A0A0E0SHR3 pp 229533.A0A1C3YKN4
229533.A0A0E0SHR3 pp 229533.I1RR00
229533.A0A0E0SHR3 pp 229533.Q4IQK7
229533.A0A0E0SHR3 pp 229533.I1S1V5
229533.A0A0E0SHR3 pp 229533.I1RAN3
229533.A0A0E0SHR3 pp 229533.I1RC32
229533.A0A0E0SHR3 pp 229533.I1R976
229533.A0A0E0SHR3 pp 229533.I1RE51
229533.A0A0E0SHR3 pp 229533.I1RAW7
229533.A0A0E0SHR3 pp 229533.I1RFW1
229533.A0A0E0SHR3 pp 229533.O74638
229533.A0A0E0SHR3 pp 229533.I1RSA4
229533.A0A0E0SHR3 pp 229533.I1RFQ7
229533.A0A0E0SHR3 pp 229533.I1R9Q0
229533.A0A0E0SHR3 pp 229533.A0A0E0SQV5
229533.A0A0E0SHR3 pp 229533.I1RMW7
229533.A0A0E0SHR3 pp 229533.A0A1C3YHH5
229533.A0A0E0SHR3 pp 229533.P0C431
229533.A0A0E0SHR3 pp 229533.I1RFU3
229533.A0A0E0SHR3 pp 229533.I1RW16
229533.A0A0E0SHR3 pp 229533.I1RTH9
229533.A0A0E0SHR3 pp 229533.A0A1C3YLA2
229533.A0A0E0SHR3 pp 229533.I1S382
229533.A0A0E0SHR3 pp 229533.A0A1C3YJ12
229533.A0A0E0SHR3 pp 229533.I1RPA0
229533.A0A0E0SHR3 pp 229533.Q4I7X1
229533.A0A0E0SHR3 pp 229533.Q4IPI4
229533.A0A0E0SHR3 pp 229533.A0A1C3YN75
229533.A0A0E0SHR3 pp 229533.I1S7M3
229533.A0A0E0SHR3 pp 229533.I1RA36
229533.A0A0E0SHR3 pp 229533.A0A1C3YI80
229533.A0A0E0SHR3 pp 229533.I1S1U7
229533.A0A0E0SHR3 pp 229533.I1RN31
229533.A0A0E0SHR3 pp 229533.Q4IER8
229533.A0A0E0SHR3 pp 229533.I1RCW8
229533.A0A0E0SHR3 pp 229533.I1RW06

50298 229533.A0A0E0SHR3 pp 229533.I1RWP4
50299 229533.A0A0E0SHR3 pp 229533.I1RDG8
50300 229533.A0A0E0SHR3 pp 229533.I1RU02
50301 229533.A0A0E0SHR3 pp 229533.I1RAN8
50302 229533.A0A0E0SHR3 pp 229533.A0A1C3YHP3
50303 229533.A0A0E0SHR3 pp 229533.I1SA47
50304 229533.A0A0E0SHR3 pp 229533.I1RG90
50305 229533.A0A0E0SHR3 pp 229533.I1RE00
50306 229533.A0A0E0SHR3 pp 229533.A0A1C3YMF9
50307 229533.A0A0E0SHR3 pp 229533.I1RCG8
50308 229533.I1RQ18 pp 229533.I1S3L2
50309 229533.I1RQ18 pp 229533.I1RQP2
50310 229533.I1RQ18 pp 229533.I1S0K0
50311 229533.I1RQ18 pp 229533.I1RXS0
50312 229533.I1RQ18 pp 229533.I1S8R4
50313 229533.A0A098DNW3 pp 229533.A0A0E0SAR4
50314 229533.A0A098DNW3 pp 229533.I1S5C6
50315 229533.A0A098DNW3 pp 229533.I1RY21
50316 229533.A0A098DNW3 pp 229533.A0A1C3YL46
50317 229533.A0A098DNW3 pp 229533.I1RPM4
50318 229533.A0A098DNW3 pp 229533.I1RMK7
50319 229533.A0A098DNW3 pp 229533.I1RWA3
50320 229533.A0A098DNW3 pp 229533.I1RYT8
50321 229533.A0A098DNW3 pp 229533.I1RA52
50322 229533.A0A098DNW3 pp 229533.I1RXD9
50323 229533.A0A098DNW3 pp 229533.I1RS05
50324 229533.A0A098DNW3 pp 229533.I1S1Z2
50325 229533.A0A098DNW3 pp 229533.I1RW71
50326 229533.A0A098DNW3 pp 229533.I1S464
50327 229533.A0A098DNW3 pp 229533.A0A0E0RPV7
50328 229533.A0A098DNW3 pp 229533.V6RJP8
50329 229533.A0A098DNW3 pp 229533.I1S8S9
50330 229533.A0A098DNW3 pp 229533.I1RFJ6
50331 229533.A0A098DNW3 pp 229533.I1S0K2
50332 229533.A0A098DNW3 pp 229533.I1S5X9
50333 229533.A0A098DNW3 pp 229533.I1RS29
50334 229533.I1RLR2 pp 229533.I1RYD3
50335 229533.I1S097 pp 229533.I1S098
50336 229533.I1S097 pp 229533.Q4HZS8
50337 229533.I1S097 pp 229533.I1S2L0
50338 229533.I1RW58 pp 229533.Q4IEA7
50339 229533.I1RW58 pp 229533.I1S286
50340 229533.I1RW58 pp 229533.I1S575
50341 229533.I1RW58 pp 229533.I1S1V4
50342 229533.I1RW58 pp 229533.I1RY87
50343 229533.I1RGQ1 pp 229533.I1S8R4
50344 229533.I1RGQ1 pp 229533.I1RXS0
50345 229533.I1RGQ1 pp 229533.I1RHZ5
50346 229533.I1RGQ1 pp 229533.I1RQP2
50347 229533.I1RGQ1 pp 229533.I1S3L2
50348 229533.I1RGQ1 pp 229533.I1S0K0
50349 229533.A0A0E0SMY1 pp 229533.A0A1C3YMU9
50350 229533.A0A0E0SMY1 pp 229533.Q4ICA8
50351 229533.A0A0E0SMY1 pp 229533.I1RW80
50352 229533.A0A0E0SMY1 pp 229533.I1S5Z5
50353 229533.A0A0E0SMY1 pp 229533.A0A1C3YLK8
50354 229533.A0A0E0SMY1 pp 229533.I1RN04
50355 229533.A0A0E0SMY1 pp 229533.I1S9H8
50356 229533.A0A0E0SMY1 pp 229533.I1RKR8
50357 229533.A0A0E0SMY1 pp 229533.A0A1C3YLG5
50358 229533.A0A0E0SMY1 pp 229533.I1RQ42
50359 229533.A0A0E0SMY1 pp 229533.I1RQP3
50360 229533.A0A0E0SMY1 pp 229533.I1RRF7
50361 229533.A0A0E0SMY1 pp 229533.I1RPW8
50362 229533.A0A0E0SMY1 pp 229533.I1RQP1
50363 229533.A0A0E0SMY1 pp 229533.Q4IPI4
50364 229533.A0A0E0SMY1 pp 229533.I1RR61
50365 229533.A0A0E0SMY1 pp 229533.I1RKF3
50366 229533.A0A0E0SMY1 pp 229533.V6QWX8
50367 229533.A0A0E0SMY1 pp 229533.A0A1C3YHX6
50368 229533.A0A0E0SMY1 pp 229533.I1RRF0
50369 229533.A0A0E0SMY1 pp 229533.I1RW91
50370 229533.A0A0E0SMY1 pp 229533.I1RCG6

50371	229533.A0A098D6Y5	pp	229533.I1S767
50372	229533.A0A098D6Y5	pp	229533.A0A0E0RX50
50373	229533.A0A098D6Y5	pp	229533.V6RS91
50374	229533.A0A098D6Y5	pp	229533.I1RE90
50375	229533.A0A098D6Y5	pp	229533.I1RZK8
50376	229533.A0A098D6Y5	pp	229533.Q4HZQ1
50377	229533.A0A098D6Y5	pp	229533.V6QXN0
50378	229533.A0A098D6Y5	pp	229533.I1RTJ2
50379	229533.A0A098D6Y5	pp	229533.I1RVW6
50380	229533.A0A098D6Y5	pp	229533.I1S053
50381	229533.A0A098D6Y5	pp	229533.I1RA94
50382	229533.A0A098D6Y5	pp	229533.I1RX59
50383	229533.A0A098D6Y5	pp	229533.I1RC74
50384	229533.A0A098D6Y5	pp	229533.I1RCL0
50385	229533.A0A098D6Y5	pp	229533.I1RP70
50386	229533.A0A098D6Y5	pp	229533.I1RZE7
50387	229533.A0A098D6Y5	pp	229533.A0A0E0SID1
50388	229533.A0A098D6Y5	pp	229533.I1RE79
50389	229533.A0A098D6Y5	pp	229533.I1RBE7
50390	229533.A0A098D6Y5	pp	229533.I1RGK9
50391	229533.A0A098D6Y5	pp	229533.A0A1C3YJ89
50392	229533.A0A098D6Y5	pp	229533.I1RFK3
50393	229533.A0A098D6Y5	pp	229533.I1S0I3
50394	229533.A0A098D6Y5	pp	229533.I1RCT7
50395	229533.A0A098D6Y5	pp	229533.I1RAH3
50396	229533.A0A098D6Y5	pp	229533.I1S1W8
50397	229533.A0A098D6Y5	pp	229533.I1RGT3
50398	229533.A0A098D6Y5	pp	229533.A0A0E0RMB3
50399	229533.A0A098D6Y5	pp	229533.A0A1C3YN78
50400	229533.A0A098D6Y5	pp	229533.V6RIV7
50401	229533.A0A098D6Y5	pp	229533.I1RDZ7
50402	229533.A0A098D6Y5	pp	229533.I1RMD6
50403	229533.A0A098D6Y5	pp	229533.I1RW11
50404	229533.A0A098D6Y5	pp	229533.I1RNG4
50405	229533.A0A098D6Y5	pp	229533.I1RTT0
50406	229533.A0A098D6Y5	pp	229533.I1RZU2
50407	229533.A0A098D6Y5	pp	229533.A0A098E3T0
50408	229533.A0A098D6Y5	pp	229533.I1RYP6
50409	229533.A0A098D6Y5	pp	229533.I1S3G4
50410	229533.A0A098D6Y5	pp	229533.I1RE58
50411	229533.A0A1C3YJH4	pp	229533.I1S148
50412	229533.A0A1C3YJH4	pp	229533.I1S1K6
50413	229533.A0A1C3YJH4	pp	229533.I1RBY9
50414	229533.A0A1C3YJH4	pp	229533.I1RSS7
50415	229533.A0A1C3YJH4	pp	229533.I1RZ25
50416	229533.A0A1C3YJH4	pp	229533.I1RN88
50417	229533.A0A1C3YJH4	pp	229533.I1RY35
50418	229533.A0A1C3YJH4	pp	229533.I1RWN7
50419	229533.A0A1C3YJH4	pp	229533.I1S8D2
50420	229533.A0A1C3YJH4	pp	229533.A0A1C3YM90
50421	229533.A0A1C3YJH4	pp	229533.I1RGC3
50422	229533.A0A1C3YJH4	pp	229533.I1S147
50423	229533.A0A1C3YJH4	pp	229533.I1RNG4
50424	229533.A0A1C3YJH4	pp	229533.I1RVZ3
50425	229533.A0A1C3YJH4	pp	229533.I1RH92
50426	229533.A0A1C3YJH4	pp	229533.A0A1C3YKN4
50427	229533.A0A1C3YJH4	pp	229533.I1RI19
50428	229533.A0A1C3YJH4	pp	229533.A0A1C3YKL8
50429	229533.A0A1C3YJH4	pp	229533.I1S048
50430	229533.A0A1C3YJH4	pp	229533.I1RYX6
50431	229533.A0A1C3YJH4	pp	229533.I1RDU5
50432	229533.A0A1C3YJH4	pp	229533.I1RQE1
50433	229533.A0A1C3YJH4	pp	229533.I1RYD3
50434	229533.A0A1C3YJH4	pp	229533.I1S8D1
50435	229533.A0A1C3YJH4	pp	229533.A0A1C3YKW4
50436	229533.A0A1C3YJH4	pp	229533.V6RH92
50437	229533.A0A1C3YJH4	pp	229533.I1RZ70
50438	229533.A0A1C3YJH4	pp	229533.I1RL16
50439	229533.A0A1C3YJH4	pp	229533.I1S130
50440	229533.A0A1C3YJH4	pp	229533.A0A1C3YL54
50441	229533.A0A1C3YJH4	pp	229533.I1RST9
50442	229533.A0A1C3YJH4	pp	229533.I1RLA2
50443	229533.A0A1C3YJH4	pp	229533.I1RJG4

229533.A0A1C3YJH4	pp	229533.I1R9Y2
229533.A0A1C3YJH4	pp	229533.I1RGK9
229533.A0A1C3YJH4	pp	229533.I1RWM4
229533.A0A1C3YJH4	pp	229533.I1RCE6
229533.A0A1C3YJH4	pp	229533.I1RB07
229533.A0A1C3YJH4	pp	229533.I1RTJ2
229533.A0A1C3YJH4	pp	229533.V6RG22
229533.A0A1C3YJH4	pp	229533.I1RXX1
229533.A0A1C3YJH4	pp	229533.A0A1C3YLP6
229533.A0A1C3YJH4	pp	229533.I1S267
229533.A0A1C3YJH4	pp	229533.I1RYM5
229533.A0A1C3YJH4	pp	229533.A0A1C3YLC2
229533.I1RWZ4	pp	229533.I1S3L2
229533.I1RWZ4	pp	229533.I1RXS0
229533.I1RWZ4	pp	229533.I1S0K0
229533.I1RWZ4	pp	229533.I1S8R4
229533.A0A098DD40	pp	229533.A0A1C3YLA2
229533.A0A098DD40	pp	229533.Q4I7X1
229533.A0A098DD40	pp	229533.A0A0E0SHR3
229533.A0A098DD40	pp	229533.I1RW58
229533.A0A098DD40	pp	229533.I1S7I5
229533.A0A098DD40	pp	229533.I1RE51
229533.A0A098DD40	pp	229533.I1RAN8
229533.A0A098DD40	pp	229533.I1S1V2
229533.A0A098DD40	pp	229533.Q4IPI4
229533.A0A0E0SIK8	pp	229533.A0A1C3YN55
229533.A0A0E0SIK8	pp	229533.I1RMQ7
229533.A0A0E0SIK8	pp	229533.I1RSC7
229533.A0A0E0SIK8	pp	229533.I1REN3
229533.A0A0E0SIK8	pp	229533.A0A1C3YMC9
229533.A0A0E0SIK8	pp	229533.I1RTC2
229533.A0A0E0SIK8	pp	229533.I1S209
229533.A0A0E0SIK8	pp	229533.I1RDH0
229533.A0A0E0SIK8	pp	229533.I1RRN5
229533.A0A0E0SIK8	pp	229533.I1RUF8
229533.A0A0E0SIK8	pp	229533.I1RMS2
229533.A0A0E0SIK8	pp	229533.I1RSZ3
229533.A0A0E0SIK8	pp	229533.I1RZT1
229533.A0A0E0SIK8	pp	229533.I1RKI2
229533.A0A0E0SIK8	pp	229533.A0A1C3YJA2
229533.A0A0E0SIK8	pp	229533.A0A1C3YM61
229533.A0A0E0SIK8	pp	229533.A0A1C3YJT5
229533.A0A0E0SIK8	pp	229533.I1RRJ6
229533.A0A0E0SIK8	pp	229533.A0A1C3YI39
229533.A0A0E0SIK8	pp	229533.I1RZ94
229533.A0A1C3YLC2	pp	229533.I1S3T7
229533.A0A1C3YLC2	pp	229533.I1RN84
229533.A0A1C3YLC2	pp	229533.I1RNU7
229533.A0A1C3YLC2	pp	229533.I1RN88
229533.A0A1C3YLC2	pp	229533.I1RWN7
229533.A0A1C3YLC2	pp	229533.I1RCG5
229533.A0A1C3YLC2	pp	229533.I1S147
229533.A0A1C3YLC2	pp	229533.I1S3X7
229533.A0A1C3YLC2	pp	229533.I1RPZ5
229533.A0A1C3YLC2	pp	229533.I1RJS1
229533.A0A1C3YLC2	pp	229533.I1RUR8
229533.A0A1C3YLC2	pp	229533.I1RSU2
229533.A0A1C3YLC2	pp	229533.I1RRP7
229533.A0A1C3YLC2	pp	229533.I1RF85
229533.A0A1C3YLC2	pp	229533.I1RFK3
229533.A0A1C3YLC2	pp	229533.I1RK55
229533.A0A1C3YLC2	pp	229533.I1RIJ5
229533.A0A1C3YLC2	pp	229533.I1RFI4
229533.A0A1C3YLC2	pp	229533.I1RGC3
229533.A0A1C3YLC2	pp	229533.I1RVZ3
229533.A0A1C3YLC2	pp	229533.I1S8R2
229533.A0A1C3YLC2	pp	229533.V6RRM0
229533.A0A1C3YLC2	pp	229533.I1RAN1
229533.A0A1C3YLC2	pp	229533.I1RZ70
229533.A0A1C3YLC2	pp	229533.I1RHJ2
229533.A0A1C3YLC2	pp	229533.I1RH92
229533.A0A1C3YLC2	pp	229533.I1S968
229533.A0A1C3YLC2	pp	229533.I1RYA0

50517	229533.A0A1C3YLC2	pp	229533.I1RI34
50518	229533.A0A1C3YLC2	pp	229533.V6RLM7
50519	229533.A0A1C3YLC2	pp	229533.I1RWF5
50520	229533.A0A1C3YLC2	pp	229533.A0A1C3YLE2
50521	229533.A0A1C3YLC2	pp	229533.I1RZA8
50522	229533.A0A1C3YLC2	pp	229533.I1RYC8
50523	229533.A0A1C3YLC2	pp	229533.I1RWL5
50524	229533.A0A1C3YLC2	pp	229533.I1S299
50525	229533.A0A1C3YLC2	pp	229533.V6RUY9
50526	229533.A0A1C3YLC2	pp	229533.I1RR68
50527	229533.A0A1C3YLC2	pp	229533.I1RPV8
50528	229533.A0A1C3YLC2	pp	229533.I1RC31
50529	229533.A0A1C3YLC2	pp	229533.I1S0A5
50530	229533.A0A1C3YLC2	pp	229533.I1RNG4
50531	229533.A0A1C3YLC2	pp	229533.I1S8D1
50532	229533.A0A1C3YLC2	pp	229533.I1RZV5
50533	229533.A0A1C3YLC2	pp	229533.I1RZ56
50534	229533.A0A1C3YLC2	pp	229533.I1S148
50535	229533.A0A1C3YLC2	pp	229533.I1RP97
50536	229533.A0A1C3YLC2	pp	229533.I1RL81
50537	229533.A0A1C3YLC2	pp	229533.I1RAQ4
50538	229533.A0A1C3YLC2	pp	229533.I1RPT0
50539	229533.A0A1C3YLC2	pp	229533.I1S694
50540	229533.A0A1C3YLC2	pp	229533.I1RH91
50541	229533.A0A1C3YLC2	pp	229533.I1RBY9
50542	229533.A0A1C3YLC2	pp	229533.A0A1C3YLN6
50543	229533.A0A1C3YLC2	pp	229533.I1S8D2
50544	229533.A0A1C3YLC2	pp	229533.I1RCV1
50545	229533.A0A1C3YLC2	pp	229533.I1RI11
50546	229533.A0A1C3YLC2	pp	229533.I1RCR3
50547	229533.A0A1C3YLC2	pp	229533.I1RY12
50548	229533.A0A1C3YLC2	pp	229533.I1RMJ3
50549	229533.A0A1C3YLC2	pp	229533.I1RG33
50550	229533.A0A1C3YLC2	pp	229533.V6RH92
50551	229533.A0A1C3YLC2	pp	229533.I1RTU0
50552	229533.A0A1C3YLC2	pp	229533.I1RDU0
50553	229533.A0A1C3YLC2	pp	229533.I1RKB2
50554	229533.A0A1C3YLC2	pp	229533.I1RXH3
50555	229533.A0A1C3YLC2	pp	229533.I1RA62
50556	229533.A0A1C3YLC2	pp	229533.A0A1C3YN78
50557	229533.A0A1C3YLC2	pp	229533.I1RYK0
50558	229533.A0A1C3YLC2	pp	229533.I1RY81
50559	229533.A0A1C3YLC2	pp	229533.I1RA13
50560	229533.A0A1C3YLC2	pp	229533.I1S6H3
50561	229533.A0A1C3YLC2	pp	229533.I1RJY7
50562	229533.A0A1C3YLC2	pp	229533.I1RNM7
50563	229533.A0A1C3YLC2	pp	229533.I1RQE0
50564	229533.A0A1C3YLC2	pp	229533.I1RPR1
50565	229533.A0A1C3YLC2	pp	229533.I1RCU2
50566	229533.A0A1C3YLC2	pp	229533.I1RMA2
50567	229533.A0A1C3YLC2	pp	229533.I1RXB1
50568	229533.A0A1C3YLC2	pp	229533.I1RPE7
50569	229533.A0A1C3YLC2	pp	229533.I1REE2
50570	229533.A0A1C3YLC2	pp	229533.I1RW54
50571	229533.A0A1C3YLC2	pp	229533.I1RUY5
50572	229533.A0A1C3YLC2	pp	229533.I1RB07
50573	229533.A0A1C3YLC2	pp	229533.I1RWM4
50574	229533.A0A1C3YLC2	pp	229533.I1RDN3
50575	229533.A0A1C3YLC2	pp	229533.I1S969
50576	229533.A0A1C3YLC2	pp	229533.I1RUV4
50577	229533.A0A1C3YLC2	pp	229533.I1RSL2
50578	229533.A0A1C3YLC2	pp	229533.I1RBI1
50579	229533.A0A1C3YLC2	pp	229533.I1RYP5
50580	229533.A0A1C3YLC2	pp	229533.I1RC11
50581	229533.A0A1C3YLC2	pp	229533.I1S8C1
50582	229533.A0A1C3YLC2	pp	229533.I1RMJ4
50583	229533.A0A1C3YLC2	pp	229533.I1S8F8
50584	229533.A0A1C3YLC2	pp	229533.I1RDU5
50585	229533.A0A1C3YLC2	pp	229533.I1RA90
50586	229533.A0A1C3YLC2	pp	229533.I1RYX6
50587	229533.A0A1C3YLC2	pp	229533.I1RJF2
50588	229533.A0A1C3YLC2	pp	229533.I1RQE1
50589	229533.A0A1C3YLC2	pp	229533.I1RR12

50590 229533.A0A1C3YLC2 pp 229533.I1RP86
50591 229533.A0A1C3YLC2 pp 229533.I1S094
50592 229533.A0A1C3YLC2 pp 229533.A0A1C3YN26
50593 229533.A0A1C3YLC2 pp 229533.I1RY16
50594 229533.A0A1C3YLC2 pp 229533.I1RMH9
50595 229533.I1S2R6 pp 229533.I1S6L5
50596 229533.I1S2R6 pp 229533.I1S7S9
50597 229533.I1S2R6 pp 229533.I1S9I6
50598 229533.I1RPF5 pp 229533.I1RRN5
50599 229533.I1RPF5 pp 229533.I1RRS7
50600 229533.I1RPF5 pp 229533.I1RZ94
50601 229533.I1RPF5 pp 229533.I1RRJ6
50602 229533.I1RPF5 pp 229533.I1S209
50603 229533.I1RPF5 pp 229533.I1S004
50604 229533.I1RPF5 pp 229533.I1S788
50605 229533.I1RPF5 pp 229533.I1RSC7
50606 229533.I1RPF5 pp 229533.I1RSK0
50607 229533.I1RPF5 pp 229533.I1RXU0
50608 229533.I1RPF5 pp 229533.I1RSZ3
50609 229533.I1RPF5 pp 229533.I1RUF8
50610 229533.I1RPF5 pp 229533.I1RY29
50611 229533.I1RPF5 pp 229533.I1S2E9
50612 229533.I1RPF5 pp 229533.I1S0D5
50613 229533.I1RPF5 pp 229533.Q4HTT2
50614 229533.I1RPF5 pp 229533.I1RTC2
50615 229533.I1RPF5 pp 229533.I1RZT1
50616 229533.I1RPF5 pp 229533.I1RY44
50617 229533.I1RPF5 pp 229533.I1RWI8
50618 229533.I1RPF5 pp 229533.Q4IER8
50619 229533.I1RPF5 pp 229533.I1S4P6
50620 229533.I1RPF5 pp 229533.Q4IB89
50621 229533.I1RGP8 pp 229533.I1S3L2
50622 229533.I1RGP8 pp 229533.I1RXS0
50623 229533.I1RGP8 pp 229533.I1S8R4
50624 229533.I1RGP8 pp 229533.I1RQP2
50625 229533.I1RGP8 pp 229533.I1S0K0
50626 229533.I1RGP8 pp 229533.I1RHZ5
50627 229533.V6RKK5 pp 229533.V6RLM7
50628 229533.A0A098DD30 pp 229533.A0A098DRI1
50629 229533.A0A098DD30 pp 229533.A0A1C3YL16
50630 229533.A0A0E0SFT7 pp 229533.I1RXB6
50631 229533.A0A0E0SFT7 pp 229533.I1RPK0
50632 229533.A0A0E0SFT7 pp 229533.I1S064
50633 229533.A0A0E0SFT7 pp 229533.I1RCT7
50634 229533.A0A0E0SFT7 pp 229533.I1RQ12
50635 229533.A0A0E0SFT7 pp 229533.I1RMW4
50636 229533.A0A0E0SFT7 pp 229533.A0A1C3YLB0
50637 229533.A0A0E0SFT7 pp 229533.I1RE90
50638 229533.A0A0E0SFT7 pp 229533.I1RP70
50639 229533.A0A0E0SFT7 pp 229533.I1RCN8
50640 229533.A0A0E0SFT7 pp 229533.I1RWF9
50641 229533.A0A0E0SFT7 pp 229533.I1RVW6
50642 229533.A0A0E0SFT7 pp 229533.I1RC61
50643 229533.A0A0E0SFT7 pp 229533.I1RXY7
50644 229533.A0A0E0SFT7 pp 229533.I1S767
50645 229533.A0A0E0SFT7 pp 229533.I1RBF3
50646 229533.A0A0E0SFT7 pp 229533.V6RVJ3
50647 229533.A0A0E0SFT7 pp 229533.I1RZE7
50648 229533.A0A0E0SFT7 pp 229533.I1RE79
50649 229533.A0A0E0SFT7 pp 229533.I1RFK3
50650 229533.A0A0E0SFT7 pp 229533.I1RCS6
50651 229533.A0A0E0SFT7 pp 229533.V6RKK5
50652 229533.A0A0E0SFT7 pp 229533.I1RW11
50653 229533.A0A0E0SFT7 pp 229533.I1R9W4
50654 229533.A0A0E0SFT7 pp 229533.A0A1C3YN47
50655 229533.A0A0E0SFT7 pp 229533.Q4I1N3
50656 229533.A0A0E0SFT7 pp 229533.I1RZU2
50657 229533.A0A0E0SFT7 pp 229533.I1RP97
50658 229533.A0A0E0SFT7 pp 229533.I1RMM8
50659 229533.A0A0E0SFT7 pp 229533.I1RZ29
50660 229533.A0A0E0SFT7 pp 229533.I1RCL0
50661 229533.A0A0E0SFT7 pp 229533.Q4HZQ1
50662 229533.A0A0E0SFT7 pp 229533.I1RDZ7

50663	229533.A0A0E0SFT7	pp	229533.A0A1C3YHI3
50664	229533.A0A0E0SFT7	pp	229533.V6RS91
50665	229533.A0A0E0SFT7	pp	229533.I1RTT0
50666	229533.A0A0E0SFT7	pp	229533.A0A1C3YJU0
50667	229533.A0A0E0SFT7	pp	229533.I1RCG7
50668	229533.Q4HW67	pp	229533.V6R0U9
50669	229533.Q4HW67	pp	229533.V6RIS2
50670	229533.Q4HW67	pp	229533.Q4I963
50671	229533.Q4HW67	pp	229533.V6R227
50672	229533.Q4HW67	pp	229533.Q4I7K4
50673	229533.Q4HW67	pp	229533.V6RY33
50674	229533.Q4HW67	pp	229533.Q4IPI1
50675	229533.I1RD62	pp	229533.I1RWT1
50676	229533.I1RD62	pp	229533.I1RMP0
50677	229533.I1S0K2	pp	229533.V6RKK5
50678	229533.I1S0K2	pp	229533.Q4IJM4
50679	229533.I1S0K2	pp	229533.I1S5C6
50680	229533.I1S0K2	pp	229533.I1S212
50681	229533.I1S0K2	pp	229533.Q4I5G1
50682	229533.I1S0K2	pp	229533.I1S887
50683	229533.I1S0K2	pp	229533.I1S8S9
50684	229533.I1S0K2	pp	229533.I1S0T7
50685	229533.I1S0K2	pp	229533.I1S1W8
50686	229533.I1S0K2	pp	229533.I1S8C1
50687	229533.I1S0K2	pp	229533.I1S128
50688	229533.I1S0K2	pp	229533.V6RJP8
50689	229533.I1S0K2	pp	229533.I1S0P0
50690	229533.I1S0K2	pp	229533.I1S1N7
50691	229533.I1S0K2	pp	229533.I1S8F8
50692	229533.I1S0K2	pp	229533.I1S0U0
50693	229533.I1S0K2	pp	229533.I1S7W5
50694	229533.I1S0K2	pp	229533.I1S0Q7
50695	229533.I1S0K2	pp	229533.I1S266
50696	229533.I1S0K2	pp	229533.I1S5X9
50697	229533.I1S0K2	pp	229533.V6RUT5
50698	229533.I1S0K2	pp	229533.I1S1W3
50699	229533.I1S0K2	pp	229533.I1S0R1
50700	229533.I1S0K2	pp	229533.I1S0R8
50701	229533.I1S0K2	pp	229533.V6RRM0
50702	229533.I1S0K2	pp	229533.I1S8R2
50703	229533.I1S0K2	pp	229533.I1S5U0
50704	229533.I1S0K2	pp	229533.I1S8Y1
50705	229533.I1S0K2	pp	229533.Q4HZ34
50706	229533.I1S0K2	pp	229533.I1S464
50707	229533.I1S0K2	pp	229533.I1S299
50708	229533.I1S0K2	pp	229533.Q4I174
50709	229533.I1S0K2	pp	229533.I1S458
50710	229533.I1S0K2	pp	229533.V6R3J1
50711	229533.I1S0K2	pp	229533.I1S3G4
50712	229533.I1S0K2	pp	229533.I1S1F3
50713	229533.I1S0K2	pp	229533.I1S3T7
50714	229533.I1RAV4	pp	229533.I1RQE5
50715	229533.I1RAV4	pp	229533.I1RB53
50716	229533.I1RAV4	pp	229533.I1RNE7
50717	229533.I1RAV4	pp	229533.I1RJR8
50718	229533.I1RAV4	pp	229533.V6QW25
50719	229533.I1RAV4	pp	229533.I1S1D0
50720	229533.I1RAV4	pp	229533.I1RG99
50721	229533.I1RAV4	pp	229533.I1RTG2
50722	229533.I1RAV4	pp	229533.I1RN58
50723	229533.I1RAV4	pp	229533.I1RNB8
50724	229533.I1RAV4	pp	229533.I1RM09
50725	229533.I1RAV4	pp	229533.I1RBH8
50726	229533.I1RAV4	pp	229533.Q4HW67
50727	229533.I1RAV4	pp	229533.I1RPK5
50728	229533.I1RAV4	pp	229533.I1S5B2
50729	229533.I1RAV4	pp	229533.I1RRI3
50730	229533.I1RAV4	pp	229533.I1RCK7
50731	229533.I1RHI1	pp	229533.I1S214
50732	229533.I1RHI1	pp	229533.I1RMP2
50733	229533.I1RHI1	pp	229533.I1RTK5
50734	229533.I1RHI1	pp	229533.I1RJL4
50735	229533.I1RHI1	pp	229533.I1S1V5

50736	229533.I1RHI1	pp	229533.I1RRT8
50737	229533.I1RHI1	pp	229533.I1RZG0
50738	229533.I1RHI1	pp	229533.I1RXT9
50739	229533.I1RHI1	pp	229533.I1S7X9
50740	229533.I1RHI1	pp	229533.I1RRT3
50741	229533.I1RHI1	pp	229533.I1RZP2
50742	229533.I1RHI1	pp	229533.I1RP04
50743	229533.I1RHI1	pp	229533.V6QX58
50744	229533.I1RHI1	pp	229533.Q4IPC8
50745	229533.I1RHI1	pp	229533.I1RQE7
50746	229533.I1RHI1	pp	229533.I1RPU4
50747	229533.I1RHI1	pp	229533.Q4I7X1
50748	229533.I1RHI1	pp	229533.I1RQ80
50749	229533.I1RHI1	pp	229533.I1RN49
50750	229533.I1RHI1	pp	229533.I1RW99
50751	229533.I1RHI1	pp	229533.I1RTG5
50752	229533.I1RHI1	pp	229533.Q4IPN9
50753	229533.I1RHI1	pp	229533.I1RN28
50754	229533.I1RHI1	pp	229533.I1RN65
50755	229533.I1RHI1	pp	229533.I1RM67
50756	229533.I1RHI1	pp	229533.I1RQ81
50757	229533.I1RHI1	pp	229533.I1RQD4
50758	229533.I1RNV0	pp	229533.I1RZG7
50759	229533.I1RNV0	pp	229533.I1S0R2
50760	229533.I1RSF6	pp	229533.I1S0B1
50761	229533.I1RSF6	pp	229533.I1RWA4
50762	229533.I1RYM5	pp	229533.I1S2P8
50763	229533.I1RYM5	pp	229533.I1RZV5
50764	229533.I1RYM5	pp	229533.V6RLM7
50765	229533.I1RYM5	pp	229533.V6RHD0
50766	229533.I1RYM5	pp	229533.I1RZ38
50767	229533.I1RYM5	pp	229533.I1RZA8
50768	229533.I1RYM5	pp	229533.I1S2C4
50769	229533.I1RYM5	pp	229533.I1S0S5
50770	229533.I1RYM5	pp	229533.I1S694
50771	229533.I1RYM5	pp	229533.I1S3G4
50772	229533.I1RYM5	pp	229533.V6RXR0
50773	229533.I1RYM5	pp	229533.V6RWB3
50774	229533.I1RYM5	pp	229533.I1S250
50775	229533.I1RYM5	pp	229533.I1S8F8
50776	229533.I1RYM5	pp	229533.I1RYX7
50777	229533.I1RYM5	pp	229533.I1S1W8
50778	229533.I1RYM5	pp	229533.V6RK56
50779	229533.I1RYM5	pp	229533.I1S8S9
50780	229533.I1RYM5	pp	229533.V6QU65
50781	229533.I1RYM5	pp	229533.I1S8D3
50782	229533.I1RYM5	pp	229533.V6RH92
50783	229533.I1RYM5	pp	229533.I1S3B2
50784	229533.I1REX1	pp	229533.I1RNG4
50785	229533.I1RLX1	pp	229533.I1RQP2
50786	229533.I1RLX1	pp	229533.I1RXS0
50787	229533.I1RLX1	pp	229533.I1S3L2
50788	229533.I1RLX1	pp	229533.I1S0K0
50789	229533.I1RLX1	pp	229533.I1S8R4
50790	229533.I1RGG6	pp	229533.I1RSF1
50791	229533.I1RGG6	pp	229533.I1RKP1
50792	229533.I1RGG6	pp	229533.I1RR45
50793	229533.I1RGG6	pp	229533.V6RKY8
50794	229533.I1RGG6	pp	229533.I1S8Z2
50795	229533.I1RGG6	pp	229533.I1RT75
50796	229533.I1RGG6	pp	229533.I1RN52
50797	229533.I1RGG6	pp	229533.I1RRK5
50798	229533.I1RUC9	pp	229533.I1S0R2
50799	229533.I1RUC9	pp	229533.I1RZG7
50800	229533.V6R3V4	pp	229533.V6RLM7
50801	229533.V6R3V4	pp	229533.V6RRM0
50802	229533.V6R3V4	pp	229533.V6R9Z3
50803	229533.I1RX98	pp	229533.I1S9I6
50804	229533.I1RX98	pp	229533.I1S6L5
50805	229533.I1RX98	pp	229533.I1S1J6
50806	229533.I1RX98	pp	229533.I1S9E7
50807	229533.I1RX98	pp	229533.Q4IPZ1
50808	229533.I1RX98	pp	229533.Q4IB65

50809 229533.I1RX98 pp 229533.I1S2R6
50810 229533.I1RK47 pp 229533.I1RPI5
50811 229533.I1RK47 pp 229533.I1RXV3
50812 229533.I1RK47 pp 229533.I1RWS4
50813 229533.I1RK47 pp 229533.I1S0J8
50814 229533.I1RK47 pp 229533.I1RTG7
50815 229533.I1RK47 pp 229533.I1S0H7
50816 229533.I1RK47 pp 229533.V6RUT5
50817 229533.I1RK47 pp 229533.I1S338
50818 229533.I1RK47 pp 229533.I1RZ52
50819 229533.I1RK47 pp 229533.I1RQG7
50820 229533.I1RK47 pp 229533.I1RW06
50821 229533.I1RK47 pp 229533.Q4HXF0
50822 229533.I1RK47 pp 229533.I1RTG6
50823 229533.I1RK47 pp 229533.I1RTU1
50824 229533.I1RK47 pp 229533.I1RZQ1
50825 229533.I1RK47 pp 229533.Q4IMJ1
50826 229533.I1RK47 pp 229533.V6QT89
50827 229533.I1RK47 pp 229533.I1RYJ6
50828 229533.I1RK47 pp 229533.I1S334
50829 229533.I1RK47 pp 229533.V6QVM5
50830 229533.I1RK47 pp 229533.I1RRL5
50831 229533.I1RK47 pp 229533.I1RNJ7
50832 229533.I1RK47 pp 229533.Q4HTS9
50833 229533.I1RK47 pp 229533.I1S3H8
50834 229533.I1RK47 pp 229533.I1RYL8
50835 229533.I1RK47 pp 229533.I1S1W3
50836 229533.I1RK47 pp 229533.I1RMG6
50837 229533.I1RK47 pp 229533.I1RUP2
50838 229533.I1RK47 pp 229533.I1RK54
50839 229533.I1RK47 pp 229533.I1S072
50840 229533.I1RK47 pp 229533.I1RXM6
50841 229533.I1RK47 pp 229533.I1RYU0
50842 229533.I1RK47 pp 229533.I1RPK0
50843 229533.I1RK47 pp 229533.I1RXV9
50844 229533.I1RK47 pp 229533.V6R382
50845 229533.I1RK47 pp 229533.V6QVH8
50846 229533.I1RKF8 pp 229533.I1RYI6
50847 229533.I1RKF8 pp 229533.I1RN48
50848 229533.I1RKF8 pp 229533.I1RSA4
50849 229533.I1RKF8 pp 229533.I1RZS7
50850 229533.I1RKF8 pp 229533.Q4HW67
50851 229533.I1RKF8 pp 229533.I1S5S2
50852 229533.I1RKF8 pp 229533.I1RRU9
50853 229533.I1RKF8 pp 229533.I1RZ40
50854 229533.I1RKF8 pp 229533.V6RIS2
50855 229533.I1RKF8 pp 229533.I1S524
50856 229533.I1RKF8 pp 229533.I1RNE7
50857 229533.I1RKF8 pp 229533.I1S3V2
50858 229533.I1RKF8 pp 229533.I1RMW7
50859 229533.I1RKF8 pp 229533.Q4HZ68
50860 229533.V6QVL9 pp 229533.V6RY56
50861 229533.A0A1C3YM36 pp 229533.I1RFT6
50862 229533.A0A1C3YM36 pp 229533.I1RT76
50863 229533.A0A1C3YM36 pp 229533.Q4IAA0
50864 229533.A0A1C3YM36 pp 229533.I1RRQ4
50865 229533.A0A1C3YM36 pp 229533.V6RG22
50866 229533.A0A1C3YM36 pp 229533.V6RNM4
50867 229533.A0A1C3YM36 pp 229533.I1REF5
50868 229533.A0A1C3YM36 pp 229533.I1RRR7
50869 229533.A0A1C3YM36 pp 229533.I1RRU9
50870 229533.A0A1C3YM36 pp 229533.Q4HXF0
50871 229533.A0A1C3YM36 pp 229533.A0A1C3YM86
50872 229533.A0A1C3YM36 pp 229533.I1RK03
50873 229533.A0A1C3YM36 pp 229533.I1RB53
50874 229533.A0A1C3YM36 pp 229533.I1RBW1
50875 229533.A0A1C3YM36 pp 229533.I1RUN3
50876 229533.A0A1C3YM36 pp 229533.I1RBW0
50877 229533.A0A1C3YM36 pp 229533.I1RNI4
50878 229533.A0A1C3YM36 pp 229533.I1R9X2
50879 229533.A0A1C3YHQ3 pp 229533.I1S1W8
50880 229533.A0A1C3YHQ3 pp 229533.I1S3K8
50881 229533.A0A1C3YHQ3 pp 229533.I1RY49

50882 229533.A0A1C3YHQ3 pp 229533.I1RMM8
50883 229533.A0A1C3YHQ3 pp 229533.I1RQE8
50884 229533.A0A1C3YHQ3 pp 229533.I1RZM4
50885 229533.A0A1C3YHQ3 pp 229533.A0A1C3YL54
50886 229533.A0A1C3YHQ3 pp 229533.I1RA28
50887 229533.A0A1C3YHQ3 pp 229533.I1RM26
50888 229533.A0A1C3YHQ3 pp 229533.I1RCL0
50889 229533.A0A1C3YHQ3 pp 229533.I1RBE7
50890 229533.A0A1C3YHQ3 pp 229533.V6RDH3
50891 229533.A0A1C3YHQ3 pp 229533.I1RVX9
50892 229533.A0A1C3YHQ3 pp 229533.V6RK96
50893 229533.A0A1C3YHQ3 pp 229533.I1RW69
50894 229533.A0A1C3YHQ3 pp 229533.I1RC48
50895 229533.A0A1C3YHQ3 pp 229533.I1RVR2
50896 229533.A0A1C3YHQ3 pp 229533.A0A1C3YJW4
50897 229533.A0A1C3YHQ3 pp 229533.I1S3G4
50898 229533.A0A1C3YHQ3 pp 229533.I1RZT0
50899 229533.A0A1C3YHQ3 pp 229533.I1RNG4
50900 229533.A0A1C3YHQ3 pp 229533.A0A1C3YKN4
50901 229533.A0A1C3YHQ3 pp 229533.Q4IMD1
50902 229533.A0A1C3YHQ3 pp 229533.V6RM02
50903 229533.A0A1C3YHQ3 pp 229533.I1RXY7
50904 229533.A0A1C3YHQ3 pp 229533.A0A1C3YI05
50905 229533.A0A0E0S4G6 pp 229533.V6RJP8
50906 229533.A0A0E0S4G6 pp 229533.I1RS29
50907 229533.A0A0E0S4G6 pp 229533.I1RS05
50908 229533.A0A0E0S4G6 pp 229533.I1RYT8
50909 229533.A0A0E0S4G6 pp 229533.I1S5X9
50910 229533.A0A0E0S4G6 pp 229533.A0A1C3YM56
50911 229533.A0A0E0S4G6 pp 229533.I1RA52
50912 229533.A0A0E0S4G6 pp 229533.I1RXD9
50913 229533.A0A0E0SH52 pp 229533.A0A1C3YLC1
50914 229533.A0A0E0SH52 pp 229533.I1S0J8
50915 229533.A0A0E0SH52 pp 229533.V6R382
50916 229533.A0A0E0SH52 pp 229533.I1RXB6
50917 229533.A0A0E0SH52 pp 229533.I1RPK0
50918 229533.A0A0E0SH52 pp 229533.I1RA12
50919 229533.A0A0E0SH52 pp 229533.I1S1W3
50920 229533.A0A0E0SH52 pp 229533.I1S209
50921 229533.A0A0E0SH52 pp 229533.I1RXR1
50922 229533.A0A0E0SH52 pp 229533.I1RBN9
50923 229533.A0A0E0SH52 pp 229533.I1RSR9
50924 229533.A0A0E0SH52 pp 229533.I1RK47
50925 229533.A0A0E0SH52 pp 229533.I1RBC1
50926 229533.A0A0E0SH52 pp 229533.I1RXU9
50927 229533.A0A0E0SH52 pp 229533.I1RBD5
50928 229533.A0A0E0SH52 pp 229533.I1RM26
50929 229533.A0A0E0SH52 pp 229533.I1RA85
50930 229533.A0A0E0SH52 pp 229533.V6RUT5
50931 229533.A0A0E0SH52 pp 229533.I1RBD7
50932 229533.A0A0E0SH52 pp 229533.I1RN46
50933 229533.A0A0E0SH52 pp 229533.I1S237
50934 229533.A0A0E0SH52 pp 229533.I1RVY2
50935 229533.A0A0E0SH52 pp 229533.I1RSS7
50936 229533.A0A0E0SH52 pp 229533.I1RCA5
50937 229533.A0A0E0SH52 pp 229533.A0A1C3YJL7
50938 229533.A0A0E0SH52 pp 229533.I1RTU1
50939 229533.A0A0E0SH52 pp 229533.I1S887
50940 229533.A0A0E0SH52 pp 229533.I1RVX9
50941 229533.A0A0E0SH52 pp 229533.I1RBH9
50942 229533.A0A0E0SH52 pp 229533.A0A1C3YLP6
50943 229533.A0A0E0SH52 pp 229533.I1RPQ1
50944 229533.A0A0E0SH52 pp 229533.I1S236
50945 229533.A0A0E0SH52 pp 229533.I1RXB1
50946 229533.I1RVV9 pp 229533.I1S1X2
50947 229533.I1RVV9 pp 229533.I1RYR9
50948 229533.I1RVV9 pp 229533.I1RYE8
50949 229533.I1RJA8 pp 229533.I1RVX9
50950 229533.I1RJA8 pp 229533.I1S7B8
50951 229533.I1RJA8 pp 229533.I1S0A0
50952 229533.I1RJA8 pp 229533.I1RM26
50953 229533.I1RJA8 pp 229533.V6RGB5
50954 229533.I1RJA8 pp 229533.I1RNG7

50955 229533.I1RJA8 pp 229533.I1RNG4
50956 229533.I1RV17 pp 229533.I1RV18
50957 229533.I1RV17 pp 229533.I1RV19
50958 229533.I1RV17 pp 229533.I1RV24
50959 229533.I1RZ40 pp 229533.V6RIS2
50960 229533.I1RZ40 pp 229533.Q4IP34
50961 229533.I1RZ40 pp 229533.I1S290
50962 229533.I1RZ40 pp 229533.I1S3V2
50963 229533.I1RZ40 pp 229533.Q4IJ56
50964 229533.I1RZ40 pp 229533.Q4IAA0
50965 229533.I1RZ40 pp 229533.Q4I7F9
50966 229533.I1RZ40 pp 229533.Q4IBS2
50967 229533.I1RZ40 pp 229533.Q4IEK8
50968 229533.I1RZ40 pp 229533.I1S854
50969 229533.I1RZ40 pp 229533.I1S855
50970 229533.I1RZ40 pp 229533.Q4I7K4
50971 229533.I1RZ40 pp 229533.Q4IAS1
50972 229533.I1RZ40 pp 229533.I1S289
50973 229533.I1RZ40 pp 229533.V6RNM4
50974 229533.I1RZ40 pp 229533.I1S9F1
50975 229533.I1RZ40 pp 229533.I1S6F0
50976 229533.I1RZ40 pp 229533.I1S5S2
50977 229533.I1RZ40 pp 229533.I1S0C1
50978 229533.I1RZ40 pp 229533.Q4HZ42
50979 229533.I1RZ40 pp 229533.Q4HW67
50980 229533.I1RZ40 pp 229533.I1S524
50981 229533.I1RZ40 pp 229533.Q4I662
50982 229533.I1RZ40 pp 229533.Q4I830
50983 229533.I1RZ40 pp 229533.Q4HZ68
50984 229533.I1RZ40 pp 229533.Q4IFI0
50985 229533.I1RZ40 pp 229533.Q4IJH1
50986 229533.I1RZ40 pp 229533.Q4HVW2
50987 229533.I1RZ40 pp 229533.Q4IF76
50988 229533.A0A1C3YIL8 pp 229533.I1RWW7
50989 229533.A0A1C3YIL8 pp 229533.I1RK03
50990 229533.A0A1C3YIL8 pp 229533.A0A1C3YLZ8
50991 229533.I1RMB6 pp 229533.I1RRQ2
50992 229533.I1RMB6 pp 229533.I1RYD3
50993 229533.I1RZG7 pp 229533.I1S855
50994 229533.I1RZG7 pp 229533.Q4HZ68
50995 229533.I1RZG7 pp 229533.Q4IB70
50996 229533.I1RZG7 pp 229533.I1S8V0
50997 229533.I1RZG7 pp 229533.Q4IPH4
50998 229533.I1RZG7 pp 229533.Q4IAA0
50999 229533.I1RZG7 pp 229533.I1S0Z9
51000 229533.I1RZG7 pp 229533.I1S0B1
51001 229533.I1RZG7 pp 229533.I1S0C1
51002 229533.I1RZG7 pp 229533.Q4I7K4
51003 229533.I1RZG7 pp 229533.Q4IP34
51004 229533.I1RZG7 pp 229533.V6RY33
51005 229533.I1RZG7 pp 229533.V6RIS2
51006 229533.I1RZG7 pp 229533.Q4HXF6
51007 229533.I1RZG7 pp 229533.Q4I1Y1
51008 229533.I1RZG7 pp 229533.I1S1X9
51009 229533.I1RZG7 pp 229533.I1RZQ5
51010 229533.I1RZG7 pp 229533.I1RZL5
51011 229533.I1RZG7 pp 229533.I1S056
51012 229533.I1RZG7 pp 229533.Q4I7F9
51013 229533.I1RZG7 pp 229533.Q4IPB8
51014 229533.I1RZG7 pp 229533.Q4IEK8
51015 229533.I1RZG7 pp 229533.I1S2Z9
51016 229533.I1RZG7 pp 229533.V6QX58
51017 229533.I1RZG7 pp 229533.I1S471
51018 229533.I1RZG7 pp 229533.I1S9L0
51019 229533.I1RZG7 pp 229533.Q4IE79
51020 229533.I1RZG7 pp 229533.Q4IPB3
51021 229533.I1RZG7 pp 229533.Q4IRI9
51022 229533.I1RZG7 pp 229533.I1S8U9
51023 229533.I1RZG7 pp 229533.V6QXG1
51024 229533.I1RZG7 pp 229533.I1S7M3
51025 229533.I1RZG7 pp 229533.Q4I5R9
51026 229533.I1RZG7 pp 229533.I1S9L5
51027 229533.I1RZG7 pp 229533.Q4IR09

51028	229533.I1RZG7	pp	229533.I1S524
51029	229533.I1RZG7	pp	229533.I1S9L6
51030	229533.I1RZG7	pp	229533.I1S4M5
51031	229533.I1RZG7	pp	229533.I1S2Y5
51032	229533.I1RZG7	pp	229533.I1S854
51033	229533.I1RZG7	pp	229533.I1S9M0
51034	229533.I1RZG7	pp	229533.I1S1C6
51035	229533.I1RZG7	pp	229533.I1S5S2
51036	229533.I1RZG7	pp	229533.I1S7M2
51037	229533.I1RZG7	pp	229533.Q4HVW2
51038	229533.I1RZG7	pp	229533.Q4IAS1
51039	229533.I1RZG7	pp	229533.Q4IBK5
51040	229533.I1RR51	pp	229533.Q4I061
51041	229533.I1RR51	pp	229533.I1RRY9
51042	229533.I1RR51	pp	229533.I1RZK5
51043	229533.I1RR51	pp	229533.I1S1J7
51044	229533.I1RR51	pp	229533.I1S887
51045	229533.I1RR51	pp	229533.Q4IJK2
51046	229533.I1RR51	pp	229533.I1RZZ1
51047	229533.I1RR51	pp	229533.V6QUE6
51048	229533.I1RR51	pp	229533.V6RCG5
51049	229533.I1RR51	pp	229533.I1RXX8
51050	229533.I1RR51	pp	229533.I1RXF0
51051	229533.I1RR51	pp	229533.V6RCZ8
51052	229533.I1RR51	pp	229533.I1RS18
51053	229533.I1RR51	pp	229533.V6RUT5
51054	229533.I1RR51	pp	229533.I1RW25
51055	229533.I1RR51	pp	229533.V6R227
51056	229533.I1RR51	pp	229533.I1RZM8
51057	229533.I1RR51	pp	229533.I1RXD7
51058	229533.I1RR51	pp	229533.I1S0R7
51059	229533.I1RR51	pp	229533.I1S5B0
51060	229533.I1RR51	pp	229533.I1RW99
51061	229533.I1RR51	pp	229533.V6RTJ8
51062	229533.I1RR51	pp	229533.I1RZM1
51063	229533.I1RR51	pp	229533.I1RX46
51064	229533.I1RR51	pp	229533.I1S0L3
51065	229533.I1RR51	pp	229533.I1S419
51066	229533.I1RR51	pp	229533.I1S537
51067	229533.I1RR51	pp	229533.I1RSQ7
51068	229533.I1RR51	pp	229533.I1S2C0
51069	229533.I1RR51	pp	229533.I1RVQ0
51070	229533.I1RR51	pp	229533.V6QW22
51071	229533.I1RR51	pp	229533.I1S4S7
51072	229533.I1RR51	pp	229533.I1RTH6
51073	229533.I1RR51	pp	229533.Q4I624
51074	229533.I1RR51	pp	229533.V6RIV9
51075	229533.I1RR51	pp	229533.V6RMW6
51076	229533.I1RR51	pp	229533.V6R042
51077	229533.I1RR51	pp	229533.I1RZW8
51078	229533.I1RR51	pp	229533.I1RRI2
51079	229533.I1RR51	pp	229533.I1RSQ3
51080	229533.I1RR51	pp	229533.I1RZ39
51081	229533.I1RR51	pp	229533.I1RYW3
51082	229533.I1RR51	pp	229533.I1S1C7
51083	229533.I1RR51	pp	229533.I1RSQ4
51084	229533.I1RR51	pp	229533.I1RZZ9
51085	229533.I1RR51	pp	229533.I1RXU9
51086	229533.I1RR51	pp	229533.Q4I327
51087	229533.I1RR51	pp	229533.I1S0L4
51088	229533.I1RR51	pp	229533.I1RWI5
51089	229533.I1RR51	pp	229533.I1RUG3
51090	229533.I1RR51	pp	229533.I1S0N4
51091	229533.I1RR51	pp	229533.I1RVX0
51092	229533.I1RR51	pp	229533.I1S2H4
51093	229533.I1RR51	pp	229533.I1RXR1
51094	229533.I1RR51	pp	229533.I1RT03
51095	229533.I1RR51	pp	229533.I1RT04
51096	229533.I1RR51	pp	229533.I1S5R1
51097	229533.I1RR51	pp	229533.V6RGN5
51098	229533.I1RR51	pp	229533.Q4HXF0
51099	229533.I1RR51	pp	229533.I1S5R6
51100	229533.I1RR51	pp	229533.I1S6Y8

51101 229533.I1RR51 pp 229533.I1RRT8
51102 229533.I1RR51 pp 229533.I1RTD5
51103 229533.I1RR51 pp 229533.I1S2B8
51104 229533.I1RR51 pp 229533.I1RRM5
51105 229533.I1RR51 pp 229533.I1S0J1
51106 229533.I1RR51 pp 229533.I1RRT2
51107 229533.I1RR51 pp 229533.I1RT40
51108 229533.I1RR51 pp 229533.I1RWK3
51109 229533.I1RR51 pp 229533.I1RRJ8
51110 229533.I1RR51 pp 229533.I1S1W3
51111 229533.I1RJ18 pp 229533.I1S0R2
51112 229533.I1RJ18 pp 229533.I1RKR6
51113 229533.I1RJ18 pp 229533.I1RZG7
51114 229533.I1REW4 pp 229533.I1RY33
51115 229533.I1REW4 pp 229533.I1RW98
51116 229533.I1RGN6 pp 229533.I1S8R4
51117 229533.I1RGN6 pp 229533.I1RXS0
51118 229533.I1RGN6 pp 229533.I1RHZ5
51119 229533.I1RGN6 pp 229533.I1RQP2
51120 229533.I1RGN6 pp 229533.I1S0K0
51121 229533.I1RGN6 pp 229533.I1S3L2
51122 229533.I1S3B2 pp 229533.I1S694
51123 229533.I1S3B2 pp 229533.I1S8F8
51124 229533.I1RVW6 pp 229533.I1RXY7
51125 229533.I1RVW6 pp 229533.V6QV31
51126 229533.I1RVW6 pp 229533.Q4I1N3
51127 229533.I1RVW6 pp 229533.I1S0C2
51128 229533.I1RVW6 pp 229533.I1RWF4
51129 229533.I1RVW6 pp 229533.I1RY32
51130 229533.I1RVW6 pp 229533.V6RS91
51131 229533.I1RVW6 pp 229533.V6RDH3
51132 229533.A0A1C3YJ26 pp 229533.I1RMG6
51133 229533.A0A1C3YJ26 pp 229533.I1RB73
51134 229533.A0A1C3YJ26 pp 229533.I1S2A8
51135 229533.A0A1C3YJ26 pp 229533.I1S0F3
51136 229533.A0A1C3YJ26 pp 229533.I1RTI7
51137 229533.A0A1C3YJ26 pp 229533.I1RKI0
51138 229533.A0A1C3YJ26 pp 229533.I1RCN2
51139 229533.A0A1C3YJ26 pp 229533.I1S0J8
51140 229533.A0A1C3YJ26 pp 229533.I1RQ88
51141 229533.A0A1C3YJ26 pp 229533.I1SAA0
51142 229533.A0A1C3YJ26 pp 229533.O74638
51143 229533.A0A1C3YJ26 pp 229533.I1RZA9
51144 229533.A0A1C3YJ26 pp 229533.I1RFL7
51145 229533.A0A1C3YJ26 pp 229533.I1S7X9
51146 229533.A0A1C3YJ26 pp 229533.I1SA27
51147 229533.A0A1C3YJ26 pp 229533.I1RPK0
51148 229533.A0A1C3YJ26 pp 229533.V6QVM5
51149 229533.A0A1C3YJ26 pp 229533.I1RCD4
51150 229533.A0A1C3YJ26 pp 229533.I1RT36
51151 229533.A0A1C3YJ26 pp 229533.I1RY98
51152 229533.A0A1C3YJ26 pp 229533.V6RGI1
51153 229533.A0A1C3YJ26 pp 229533.A0A1C3YKG7
51154 229533.A0A1C3YJ26 pp 229533.V6R382
51155 229533.A0A1C3YJ26 pp 229533.I1RAI9
51156 229533.A0A1C3YJ26 pp 229533.I1S2C1
51157 229533.A0A1C3YJ26 pp 229533.I1RTE6
51158 229533.A0A1C3YJ26 pp 229533.A0A1C3YLC1
51159 229533.A0A1C3YJ26 pp 229533.I1RNW8
51160 229533.A0A1C3YJ26 pp 229533.I1RIG5
51161 229533.A0A1C3YJ26 pp 229533.I1RTU1
51162 229533.A0A1C3YJ26 pp 229533.I1S9M0
51163 229533.A0A1C3YJ26 pp 229533.I1RJX2
51164 229533.A0A1C3YJ26 pp 229533.I1RBJ0
51165 229533.A0A1C3YJ26 pp 229533.A0A1C3YJL7
51166 229533.A0A1C3YJ26 pp 229533.I1RIX0
51167 229533.A0A1C3YJ26 pp 229533.I1RDB1
51168 229533.A0A1C3YJ26 pp 229533.A0A1C3YLD3
51169 229533.A0A1C3YJ26 pp 229533.I1RCP3
51170 229533.A0A1C3YJ26 pp 229533.I1RSJ2
51171 229533.A0A1C3YJ26 pp 229533.Q4ICA8
51172 229533.A0A1C3YJ26 pp 229533.I1RC95
51173 229533.A0A1C3YJ26 pp 229533.Q4I948

51174 229533.A0A1C3YJ26 pp 229533.A0A1C3YLW0
51175 229533.A0A1C3YJ26 pp 229533.I1RQ93
51176 229533.A0A1C3YJ26 pp 229533.I1RUY6
51177 229533.A0A1C3YJ26 pp 229533.A0A1C3YKY3
51178 229533.A0A1C3YJ26 pp 229533.V6REE9
51179 229533.A0A1C3YJ26 pp 229533.I1RYV3
51180 229533.A0A1C3YJ26 pp 229533.I1RAQ4
51181 229533.A0A1C3YJ26 pp 229533.I1S439
51182 229533.A0A1C3YJ26 pp 229533.I1RN65
51183 229533.A0A1C3YJ26 pp 229533.A0A1C3YK01
51184 229533.A0A1C3YJ26 pp 229533.I1RK47
51185 229533.A0A0E0SBQ2 pp 229533.I1RV19
51186 229533.A0A0E0SBQ2 pp 229533.I1S2N4
51187 229533.A0A0E0SBQ2 pp 229533.I1S5P3
51188 229533.A0A0E0SBQ2 pp 229533.A0A0E0SP71
51189 229533.A0A0E0SBQ2 pp 229533.I1REM8
51190 229533.A0A0E0SBQ2 pp 229533.I1RLM3
51191 229533.A0A0E0SBQ2 pp 229533.I1RJU9
51192 229533.A0A0E0SBQ2 pp 229533.I1RVM7
51193 229533.I1RNE7 pp 229533.I1S290
51194 229533.I1RNE7 pp 229533.V6RIS2
51195 229533.I1RNE7 pp 229533.I1RRU9
51196 229533.I1RNE7 pp 229533.I1RWD3
51197 229533.I1RNE7 pp 229533.Q4I7K4
51198 229533.I1RNE7 pp 229533.I1RZS7
51199 229533.I1RNE7 pp 229533.I1S289
51200 229533.I1RNE7 pp 229533.I1RSE2
51201 229533.I1RNE7 pp 229533.V6RJP8
51202 229533.I1RNE7 pp 229533.I1S524
51203 229533.I1RNE7 pp 229533.I1S9F1
51204 229533.I1RNE7 pp 229533.I1S5S2
51205 229533.I1RNE7 pp 229533.I1S504
51206 229533.I1RNE7 pp 229533.I1RQF9
51207 229533.I1RNE7 pp 229533.I1RNI4
51208 229533.I1RNE7 pp 229533.I1RZ40
51209 229533.I1RNE7 pp 229533.I1RQJ4
51210 229533.I1RNE7 pp 229533.I1S503
51211 229533.I1RNE7 pp 229533.Q4HW67
51212 229533.I1RIE8 pp 229533.I1RPZ1
51213 229533.I1RIE8 pp 229533.I1RJL9
51214 229533.I1RIE8 pp 229533.V6RAN2
51215 229533.I1RIE8 pp 229533.V6R4J1
51216 229533.I1RIE8 pp 229533.I1RIX0
51217 229533.I1RIE8 pp 229533.I1RQ14
51218 229533.I1RIE8 pp 229533.I1RRU9
51219 229533.I1RIE8 pp 229533.I1RXF0
51220 229533.I1RIE8 pp 229533.I1RP86
51221 229533.I1RIE8 pp 229533.I1RKR5
51222 229533.I1RIE8 pp 229533.I1RMM6
51223 229533.I1RIE8 pp 229533.I1RQH1
51224 229533.I1RIE8 pp 229533.I1RIL8
51225 229533.I1RIE8 pp 229533.I1S574
51226 229533.I1RIE8 pp 229533.I1RPW4
51227 229533.I1RIE8 pp 229533.I1RW71
51228 229533.I1RIE8 pp 229533.I1RLC7
51229 229533.I1RIE8 pp 229533.I1RLM7
51230 229533.I1RIE8 pp 229533.I1RK45
51231 229533.I1RIE8 pp 229533.I1S352
51232 229533.I1RIE8 pp 229533.I1RNA5
51233 229533.I1RIE8 pp 229533.I1S984
51234 229533.I1RIE8 pp 229533.I1RQE0
51235 229533.I1RIE8 pp 229533.I1RLN6
51236 229533.I1RIE8 pp 229533.I1S4Z7
51237 229533.I1RIE8 pp 229533.I1RW07
51238 229533.I1RIE8 pp 229533.I1RU29
51239 229533.I1RIE8 pp 229533.I1RYL5
51240 229533.I1RIE8 pp 229533.I1RRQ1
51241 229533.I1RIE8 pp 229533.I1RRT3
51242 229533.I1RIE8 pp 229533.I1RSJ2
51243 229533.I1RIE8 pp 229533.I1RJZ7
51244 229533.I1RIE8 pp 229533.I1RNR2
51245 229533.I1RIE8 pp 229533.I1RZ13
51246 229533.I1RIE8 pp 229533.I1RQ81

51247 229533.I1RIE8 pp 229533.I1RKP3
51248 229533.I1RIE8 pp 229533.I1RIL3
51249 229533.I1RIE8 pp 229533.I1RZ29
51250 229533.I1RIE8 pp 229533.I1S0R3
51251 229533.I1RIE8 pp 229533.I1S272
51252 229533.I1RIE8 pp 229533.I1RNA1
51253 229533.I1RIE8 pp 229533.I1RQ75
51254 229533.I1RIE8 pp 229533.I1S569
51255 229533.I1RIE8 pp 229533.I1RRV1
51256 229533.I1RIE8 pp 229533.I1RMX6
51257 229533.I1RIE8 pp 229533.I1S0R4
51258 229533.I1RP29 pp 229533.I1RYP1
51259 229533.I1RP29 pp 229533.I1RTQ9
51260 229533.I1RP29 pp 229533.I1RPW0
51261 229533.I1RP29 pp 229533.I1SA27
51262 229533.I1RV24 pp 229533.I1RVD9
51263 229533.I1RB11 pp 229533.I1RUP1
51264 229533.I1RB11 pp 229533.I1RVY0
51265 229533.I1RB11 pp 229533.I1RDG0
51266 229533.A0A1C3YKN2 pp 229533.V6REE9
51267 229533.A0A1C3YKN2 pp 229533.I1RE31
51268 229533.A0A1C3YKN2 pp 229533.I1RWM7
51269 229533.A0A1C3YKN2 pp 229533.I1RNI4
51270 229533.A0A1C3YKN2 pp 229533.I1RCI8
51271 229533.A0A1C3YKN2 pp 229533.I1RC27
51272 229533.A0A1C3YKN2 pp 229533.I1RNE7
51273 229533.A0A1C3YKN2 pp 229533.I1RY11
51274 229533.A0A1C3YKN2 pp 229533.I1RZR5
51275 229533.A0A1C3YKN2 pp 229533.I1R9X2
51276 229533.A0A1C3YKN2 pp 229533.I1S0R9
51277 229533.A0A1C3YKN2 pp 229533.I1RKE7
51278 229533.A0A1C3YKN2 pp 229533.A0A1C3YM29
51279 229533.A0A1C3YKN2 pp 229533.I1REF5
51280 229533.A0A1C3YKN2 pp 229533.I1RCD4
51281 229533.A0A1C3YKN2 pp 229533.I1RC46
51282 229533.A0A1C3YKN2 pp 229533.I1RZH5
51283 229533.A0A1C3YKN2 pp 229533.I1S0X5
51284 229533.A0A1C3YKN2 pp 229533.I1RJT3
51285 229533.A0A1C3YKN2 pp 229533.I1RSX0
51286 229533.A0A1C3YKN2 pp 229533.I1RRI3
51287 229533.A0A1C3YKN2 pp 229533.Q4HW67
51288 229533.A0A1C3YKN2 pp 229533.A0A1C3YL15
51289 229533.I1RV58 pp 229533.I1S1J6
51290 229533.I1RV58 pp 229533.I1S9I6
51291 229533.I1RV58 pp 229533.I1S6L5
51292 229533.I1RV58 pp 229533.I1RX98
51293 229533.I1RV58 pp 229533.I1RV79
51294 229533.I1RV58 pp 229533.I1S2R6
51295 229533.A0A1C3YKS2 pp 229533.I1RMP2
51296 229533.A0A1C3YKS2 pp 229533.I1RE19
51297 229533.A0A1C3YKS2 pp 229533.A0A1C3YMA1
51298 229533.A0A1C3YKS2 pp 229533.I1RQN9
51299 229533.A0A1C3YKS2 pp 229533.A0A1C3YLS8
51300 229533.A0A1C3YKS2 pp 229533.P0C431
51301 229533.A0A1C3YKS2 pp 229533.I1RZP6
51302 229533.A0A1C3YKS2 pp 229533.I1RRF9
51303 229533.A0A1C3YKS2 pp 229533.I1S050
51304 229533.A0A1C3YKS2 pp 229533.I1RLX6
51305 229533.A0A1C3YKS2 pp 229533.I1S0S6
51306 229533.A0A1C3YKS2 pp 229533.I1RQL4
51307 229533.I1RXB6 pp 229533.I1S8F8
51308 229533.I1RXB6 pp 229533.I1RYU0
51309 229533.I1RXB6 pp 229533.I1S835
51310 229533.I1RXB6 pp 229533.I1S3V2
51311 229533.I1RXB6 pp 229533.Q4I5G1
51312 229533.I1RXB6 pp 229533.V6R1K4
51313 229533.I1RXB6 pp 229533.I1S0H2
51314 229533.I1RXB6 pp 229533.V6RG22
51315 229533.I1RXB6 pp 229533.V6RQW4
51316 229533.I1RXB6 pp 229533.I1S064
51317 229533.I1RXB6 pp 229533.V6RLM7
51318 229533.I1RXB6 pp 229533.Q4HYQ4
51319 229533.I1RXB6 pp 229533.I1S1W9

51320	229533.I1RXB6	pp	229533.I1S2A1
51321	229533.I1RXB6	pp	229533.I1S236
51322	229533.I1RXB6	pp	229533.I1RZ84
51323	229533.I1RXB6	pp	229533.Q4I5R9
51324	229533.I1RXB6	pp	229533.V6R7V8
51325	229533.I1RXB6	pp	229533.Q4I5V2
51326	229533.I1RXB6	pp	229533.I1RZ29
51327	229533.I1RXB6	pp	229533.V6R3J1
51328	229533.I1RXB6	pp	229533.V6RVJ3
51329	229533.I1RXB6	pp	229533.I1RZH6
51330	229533.I1RXB6	pp	229533.V6RRH2
51331	229533.I1RXB6	pp	229533.I1RYK0
51332	229533.I1RXB6	pp	229533.I1S237
51333	229533.A0A1C3YJZ6	pp	229533.I1RNI4
51334	229533.A0A1C3YJZ6	pp	229533.I1RC27
51335	229533.A0A1C3YJZ6	pp	229533.I1RY11
51336	229533.A0A1C3YJZ6	pp	229533.A0A1C3YM29
51337	229533.A0A1C3YJZ6	pp	229533.I1REF5
51338	229533.A0A1C3YJZ6	pp	229533.I1RC46
51339	229533.I1RC48	pp	229533.I1RY72
51340	229533.I1RC48	pp	229533.I1RW10
51341	229533.I1RC48	pp	229533.I1RUR8
51342	229533.I1RC48	pp	229533.I1S4B6
51343	229533.I1RC48	pp	229533.I1RGC3
51344	229533.I1RC48	pp	229533.I1RIB2
51345	229533.I1RC48	pp	229533.I1RVR2
51346	229533.I1RC48	pp	229533.I1RLE1
51347	229533.I1RC48	pp	229533.I1RR29
51348	229533.I1RC48	pp	229533.I1S702
51349	229533.I1RC48	pp	229533.I1S9T6
51350	229533.I1RC48	pp	229533.I1S673
51351	229533.I1RC48	pp	229533.I1S1D7
51352	229533.I1RC48	pp	229533.I1RSS7
51353	229533.I1RC48	pp	229533.I1S9C6
51354	229533.I1RC48	pp	229533.I1S299
51355	229533.I1RC48	pp	229533.I1RDS6
51356	229533.I1RC48	pp	229533.I1RHZ7
51357	229533.I1RC48	pp	229533.I1RSK5
51358	229533.I1RC48	pp	229533.I1S4B7
51359	229533.I1RC48	pp	229533.V6RM02
51360	229533.I1RC48	pp	229533.I1RZ70
51361	229533.I1RC48	pp	229533.I1RGT2
51362	229533.I1RC48	pp	229533.I1RVY1
51363	229533.I1RC48	pp	229533.I1RKB2
51364	229533.I1RC48	pp	229533.I1RXY7
51365	229533.I1RC48	pp	229533.I1RMJ3
51366	229533.I1RC48	pp	229533.I1RN84
51367	229533.I1RC48	pp	229533.I1RW54
51368	229533.I1RC48	pp	229533.I1S568
51369	229533.I1RC48	pp	229533.I1S8R2
51370	229533.I1RC48	pp	229533.V6R382
51371	229533.I1RC48	pp	229533.I1RSB7
51372	229533.I1RC48	pp	229533.I1RTG4
51373	229533.I1RC48	pp	229533.I1RD80
51374	229533.I1RC48	pp	229533.I1RQ18
51375	229533.I1RC48	pp	229533.I1RYM6
51376	229533.I1RC48	pp	229533.I1RGQ1
51377	229533.I1RC48	pp	229533.I1RMM8
51378	229533.I1RC48	pp	229533.I1RV23
51379	229533.I1RC48	pp	229533.I1S8C1
51380	229533.I1RC48	pp	229533.I1RQE8
51381	229533.I1RC48	pp	229533.I1RWZ4
51382	229533.I1RC48	pp	229533.I1RGP8
51383	229533.I1RC48	pp	229533.I1S241
51384	229533.I1RC48	pp	229533.V6RY56
51385	229533.I1RC48	pp	229533.I1RC49
51386	229533.I1RC48	pp	229533.I1RVY2
51387	229533.I1RC48	pp	229533.I1RZM4
51388	229533.I1RC48	pp	229533.I1RUY6
51389	229533.I1RC48	pp	229533.I1RLX1
51390	229533.I1RC48	pp	229533.I1RJT5
51391	229533.I1RC48	pp	229533.I1RXB1
51392	229533.I1RC48	pp	229533.I1RUK0

51393 229533.I1RC48 pp 229533.I1RJY7
51394 229533.I1RC48 pp 229533.I1RH32
51395 229533.I1RC48 pp 229533.I1RM26
51396 229533.I1RC48 pp 229533.V6RK96
51397 229533.I1RC48 pp 229533.I1RVX9
51398 229533.I1RC48 pp 229533.I1RT09
51399 229533.I1RC48 pp 229533.I1RGN6
51400 229533.I1RC48 pp 229533.I1S0N3
51401 229533.I1RC48 pp 229533.I1RMY1
51402 229533.I1RC48 pp 229533.I1S6A2
51403 229533.I1RC48 pp 229533.I1RPR1
51404 229533.I1RC48 pp 229533.I1RCV1
51405 229533.I1RC48 pp 229533.I1RHA8
51406 229533.I1RC48 pp 229533.I1S4D0
51407 229533.A0A1C3YKY4 pp 229533.I1R9B6
51408 229533.A0A1C3YKY4 pp 229533.I1RYX6
51409 229533.A0A1C3YKY4 pp 229533.I1RAG9
51410 229533.A0A1C3YKY4 pp 229533.I1RXX1
51411 229533.A0A1C3YKY4 pp 229533.I1RAQ4
51412 229533.A0A1C3YKY4 pp 229533.I1RY49
51413 229533.A0A1C3YKY4 pp 229533.A0A1C3YLP6
51414 229533.A0A1C3YKY4 pp 229533.I1RPW1
51415 229533.A0A1C3YKY4 pp 229533.I1RPM6
51416 229533.A0A1C3YKY4 pp 229533.I1RA94
51417 229533.A0A1C3YKY4 pp 229533.I1RAR3
51418 229533.A0A1C3YKY4 pp 229533.I1RXV9
51419 229533.A0A1C3YKY4 pp 229533.V6QWD8
51420 229533.A0A1C3YKY4 pp 229533.I1RRV4
51421 229533.A0A1C3YKY4 pp 229533.I1RA13
51422 229533.A0A1C3YKY4 pp 229533.I1S0A0
51423 229533.A0A1C3YKY4 pp 229533.I1RBE7
51424 229533.A0A1C3YKY4 pp 229533.V6RQW4
51425 229533.A0A1C3YKY4 pp 229533.I1RYQ4
51426 229533.A0A1C3YKY4 pp 229533.I1RQ60
51427 229533.A0A1C3YKY4 pp 229533.I1RXX0
51428 229533.A0A1C3YKY4 pp 229533.I1RA90
51429 229533.A0A1C3YKY4 pp 229533.I1RK03
51430 229533.A0A1C3YKY4 pp 229533.I1S094
51431 229533.A0A1C3YKY4 pp 229533.I1RM26
51432 229533.A0A1C3YKY4 pp 229533.I1RSX2
51433 229533.A0A1C3YKY4 pp 229533.I1S6H3
51434 229533.A0A1C3YKY4 pp 229533.I1RDN3
51435 229533.A0A1C3YKY4 pp 229533.I1RMK4
51436 229533.A0A1C3YKY4 pp 229533.I1RDQ2
51437 229533.A0A1C3YKY4 pp 229533.I1RKZ7
51438 229533.A0A1C3YKY4 pp 229533.I1RAJ6
51439 229533.A0A1C3YKY4 pp 229533.I1RM67
51440 229533.A0A1C3YKY4 pp 229533.I1RXX8
51441 229533.A0A1C3YKY4 pp 229533.V6RLM7
51442 229533.A0A1C3YKY4 pp 229533.I1RVX9
51443 229533.A0A1C3YKY4 pp 229533.I1S1W8
51444 229533.A0A1C3YKY4 pp 229533.I1RZ56
51445 229533.A0A1C3YKY4 pp 229533.I1RIH0
51446 229533.A0A1C3YKY4 pp 229533.V6QVH8
51447 229533.A0A1C3YKY4 pp 229533.I1RAY9
51448 229533.A0A1C3YKY4 pp 229533.I1S0A5
51449 229533.A0A1C3YKY4 pp 229533.I1RPV8
51450 229533.A0A1C3YKY4 pp 229533.I1RAU7
51451 229533.A0A1C3YKY4 pp 229533.I1RPD6
51452 229533.A0A1C3YKY4 pp 229533.I1RNV4
51453 229533.A0A1C3YKY4 pp 229533.I1S0T4
51454 229533.A0A1C3YKY4 pp 229533.I1RNB4
51455 229533.A0A1C3YKY4 pp 229533.I1RCG5
51456 229533.A0A1C3YKY4 pp 229533.I1RZ83
51457 229533.A0A1C3YKY4 pp 229533.I1R9W4
51458 229533.A0A1C3YKY4 pp 229533.Q4HXF0
51459 229533.A0A1C3YKY4 pp 229533.I1RJS1
51460 229533.A0A1C3YKY4 pp 229533.I1RP97
51461 229533.A0A1C3YKY4 pp 229533.I1RNG4
51462 229533.A0A1C3YKY4 pp 229533.I1RZ29
51463 229533.A0A1C3YKY4 pp 229533.I1RDE7
51464 229533.A0A1C3YKY4 pp 229533.I1S3G4
51465 229533.A0A1C3YKY4 pp 229533.I1RXW3

51466 229533.A0A1C3YKY4 pp 229533.I1RA52
51467 229533.A0A1C3YKY4 pp 229533.I1RSS7
51468 229533.A0A1C3YKY4 pp 229533.I1S0Y7
51469 229533.I1RXE6 pp 229533.I1S8F8
51470 229533.I1RXE6 pp 229533.I1RZ43
51471 229533.I1RXE6 pp 229533.I1S8C1
51472 229533.I1RXE6 pp 229533.I1S423
51473 229533.I1RXE6 pp 229533.V6RRM0
51474 229533.I1RXE6 pp 229533.I1RYC8
51475 229533.I1RXE6 pp 229533.V6R3V4
51476 229533.I1RXE6 pp 229533.V6RJP8
51477 229533.I1RXE6 pp 229533.I1S0P0
51478 229533.I1RXE6 pp 229533.I1RYA0
51479 229533.I1RXE6 pp 229533.V6RXB3
51480 229533.I1RXE6 pp 229533.V6RLM7
51481 229533.I1RXE6 pp 229533.I1S0V7
51482 229533.I1RXE6 pp 229533.Q4IB65
51483 229533.I1RXE6 pp 229533.I1S0K2
51484 229533.I1RXE6 pp 229533.I1RYX6
51485 229533.I1RXE6 pp 229533.I1S0Q7
51486 229533.I1RXE6 pp 229533.Q4HYR2
51487 229533.I1RXE6 pp 229533.V6RF62
51488 229533.I1RXE6 pp 229533.I1S8R2
51489 229533.I1RXE6 pp 229533.I1S1F3
51490 229533.I1RXE6 pp 229533.I1S0R8
51491 229533.I1RXE6 pp 229533.Q4I5G1
51492 229533.I1RXE6 pp 229533.V6RZD4
51493 229533.I1RXE6 pp 229533.I1S4S5
51494 229533.I1RXE6 pp 229533.I1RYM5
51495 229533.I1RXE6 pp 229533.I1S299
51496 229533.I1RXE6 pp 229533.I1RY12
51497 229533.I1RXE6 pp 229533.Q4IPZ1
51498 229533.I1RXE6 pp 229533.I1S0V6
51499 229533.I1RXE6 pp 229533.V6RUB1
51500 229533.I1RXE6 pp 229533.I1S5X9
51501 229533.I1RXE6 pp 229533.I1S8S9
51502 229533.I1RXE6 pp 229533.I1S464
51503 229533.I1RXE6 pp 229533.I1RYP2
51504 229533.I1RXE6 pp 229533.I1S6P5
51505 229533.I1RXE6 pp 229533.I1RY21
51506 229533.I1RXE6 pp 229533.I1RZA7
51507 229533.I1RXE6 pp 229533.I1RYT8
51508 229533.I1RXE6 pp 229533.I1S0B8
51509 229533.I1RXE6 pp 229533.I1S3T7
51510 229533.I1RXE6 pp 229533.I1RY92
51511 229533.I1S235 pp 229533.Q4I5R9
51512 229533.I1S235 pp 229533.I1S821
51513 229533.I1S235 pp 229533.I1S3S5
51514 229533.I1S235 pp 229533.Q4I7G0
51515 229533.I1S235 pp 229533.V6QX21
51516 229533.I1S235 pp 229533.I1S4Z7
51517 229533.I1S235 pp 229533.V6QUV7
51518 229533.I1S235 pp 229533.Q4I624
51519 229533.I1S235 pp 229533.Q4IN00
51520 229533.I1RNI4 pp 229533.Q4HW67
51521 229533.I1RNI4 pp 229533.I1RZ40
51522 229533.I1RNI4 pp 229533.Q4IAA0
51523 229533.I1RNI4 pp 229533.I1RSA7
51524 229533.I1RNI4 pp 229533.Q4IJU0
51525 229533.I1RNI4 pp 229533.I1RRU9
51526 229533.I1RNI4 pp 229533.I1RQJ4
51527 229533.I1RNI4 pp 229533.V6RNM4
51528 229533.I1RVV7 pp 229533.V6R223
51529 229533.I1RVV7 pp 229533.I1S569
51530 229533.I1RVV7 pp 229533.I1S490
51531 229533.I1RVV7 pp 229533.V6RY56
51532 229533.I1RVV7 pp 229533.V6RRM0
51533 229533.I1RVV7 pp 229533.I1S049
51534 229533.I1RVV7 pp 229533.V6RJ21
51535 229533.I1RVV7 pp 229533.I1RWE8
51536 229533.I1RVV7 pp 229533.I1S111
51537 229533.I1RVV7 pp 229533.I1S4D0
51538 229533.I1RVV7 pp 229533.I1RXH3

51539 229533.I1RVV7 pp 229533.Q4HVU7
51540 229533.I1RVV7 pp 229533.I1RYV4
51541 229533.I1RVV7 pp 229533.I1S7R5
51542 229533.I1RVV7 pp 229533.I1RWG5
51543 229533.I1RVV7 pp 229533.I1S029
51544 229533.I1RB34 pp 229533.I1RFZ2
51545 229533.I1RB34 pp 229533.I1RJU1
51546 229533.I1RB34 pp 229533.I1RC01
51547 229533.I1S2E2 pp 229533.I1S770
51548 229533.I1S2E2 pp 229533.I1S8X1
51549 229533.I1S2E2 pp 229533.I1S7M8
51550 229533.I1S2E2 pp 229533.I1S7A5
51551 229533.A0A0E0SD83 pp 229533.I1R9X2
51552 229533.A0A0E0SD83 pp 229533.I1RJZ4
51553 229533.A0A0E0SD83 pp 229533.I1RC20
51554 229533.A0A0E0SD83 pp 229533.A0A0E0SQI9
51555 229533.A0A0E0SD83 pp 229533.I1RNN1
51556 229533.A0A0E0SD83 pp 229533.I1RK00
51557 229533.A0A0E0SD83 pp 229533.I1S345
51558 229533.A0A0E0SD83 pp 229533.I1RRS1
51559 229533.A0A0E0SD83 pp 229533.A0A1C3YM29
51560 229533.A0A0E0SD83 pp 229533.I1RBB7
51561 229533.A0A0E0SD83 pp 229533.I1RSY1
51562 229533.A0A0E0SD83 pp 229533.A0A1C3YMI5
51563 229533.A0A0E0SD83 pp 229533.I1RN58
51564 229533.A0A0E0SD83 pp 229533.I1RA70
51565 229533.A0A0E0SD83 pp 229533.A0A1C3YKP2
51566 229533.A0A0E0SD83 pp 229533.I1RRU9
51567 229533.A0A0E0SD83 pp 229533.I1RTN0
51568 229533.I1RYR9 pp 229533.I1S1X2
51569 229533.A0A1C3YMT2 pp 229533.I1S2L0
51570 229533.A0A1C3YMT2 pp 229533.I1RB03
51571 229533.A0A1C3YMT2 pp 229533.I1RK44
51572 229533.A0A1C3YMT2 pp 229533.I1RYW3
51573 229533.A0A1C3YMT2 pp 229533.I1RUP6
51574 229533.A0A1C3YMT2 pp 229533.I1RBY9
51575 229533.A0A1C3YMT2 pp 229533.I1RBF4
51576 229533.A0A1C3YMT2 pp 229533.I1S098
51577 229533.A0A1C3YMT2 pp 229533.Q4HZS8
51578 229533.I1RJY2 pp 229533.I1RK79
51579 229533.I1RJY2 pp 229533.V6QUE6
51580 229533.I1RJY2 pp 229533.V6RGN5
51581 229533.I1RJY2 pp 229533.I1RRN6
51582 229533.I1RJY2 pp 229533.I1RSI6
51583 229533.I1RJY2 pp 229533.I1RKQ5
51584 229533.I1RJY2 pp 229533.I1S9Y1
51585 229533.I1RJY2 pp 229533.V6QWX8
51586 229533.I1RJY2 pp 229533.I1S9U1
51587 229533.I1RJY2 pp 229533.I1RN17
51588 229533.I1RJY2 pp 229533.I1RR25
51589 229533.I1RJY2 pp 229533.I1RVY0
51590 229533.I1RJY2 pp 229533.I1RQ65
51591 229533.I1RJY2 pp 229533.I1RZB5
51592 229533.I1RJY2 pp 229533.I1RK95
51593 229533.I1RJY2 pp 229533.I1RW90
51594 229533.I1RJY2 pp 229533.I1RS30
51595 229533.I1RJY2 pp 229533.I1RMK3
51596 229533.I1RJY2 pp 229533.I1S255
51597 229533.I1RJY2 pp 229533.I1RRG3
51598 229533.I1RJY2 pp 229533.I1RM06
51599 229533.A0A1C3YMG4 pp 229533.I1S0T4
51600 229533.A0A1C3YMG4 pp 229533.Q4IMJ1
51601 229533.A0A1C3YMG4 pp 229533.I1RR72
51602 229533.A0A1C3YMG4 pp 229533.I1RWJ4
51603 229533.A0A1C3YMG4 pp 229533.I1RZI8
51604 229533.A0A1C3YMG4 pp 229533.I1S558
51605 229533.A0A1C3YMG4 pp 229533.I1S0J8
51606 229533.A0A1C3YMG4 pp 229533.I1RPJ3
51607 229533.A0A1C3YMG4 pp 229533.I1RCW1
51608 229533.A0A1C3YMG4 pp 229533.I1RTD7
51609 229533.A0A1C3YMG4 pp 229533.I1RYM5
51610 229533.A0A1C3YMG4 pp 229533.A0A1C3YMG7
51611 229533.A0A1C3YMG4 pp 229533.V6R382

229533.A0A1C3YMG4 pp 229533.I1RKB1
229533.A0A1C3YMG4 pp 229533.I1RNY2
229533.A0A1C3YMG4 pp 229533.I1RS35
229533.A0A1C3YMG4 pp 229533.I1RW06
229533.A0A1C3YMG4 pp 229533.I1RS19
229533.A0A1C3YIN3 pp 229533.A0A1C3YKS2
229533.A0A1C3YIN3 pp 229533.I1RW16
229533.A0A1C3YIN3 pp 229533.I1RWQ0
229533.A0A1C3YIN3 pp 229533.I1RKR8
229533.A0A1C3YIN3 pp 229533.Q4HZB8
229533.A0A1C3YIN3 pp 229533.I1RN31
229533.A0A1C3YIN3 pp 229533.I1RTC2
229533.I1S8F8 pp 229533.Q4I1N3
229533.I1S8F8 pp 229533.V6RF62
229533.I1S8F8 pp 229533.I1S8R2
229533.I1S8F8 pp 229533.V6R756
229533.I1S8F8 pp 229533.V6RRM0
229533.I1S8F8 pp 229533.Q4HVQ9
229533.I1S8F8 pp 229533.Q4IN52
229533.I1S8F8 pp 229533.V6RUB1
229533.I1S8F8 pp 229533.V6RXB3
229533.I1S8F8 pp 229533.V6R3J1
229533.I1S8F8 pp 229533.I1S968
229533.I1S8F8 pp 229533.V6QZ48
229533.I1S8F8 pp 229533.V6R2Z2
229533.I1S8F8 pp 229533.V6R9L8
229533.I1S8F8 pp 229533.I1S8F9
229533.I1S8F8 pp 229533.V6RZD4
229533.I1S8F8 pp 229533.I1S8S9
229533.I1S8F8 pp 229533.I1S8H9
229533.I1S8F8 pp 229533.V6RJP8
229533.I1S8F8 pp 229533.V6R3V4
229533.I1S8F8 pp 229533.V6RS91
229533.I1S8F8 pp 229533.Q4I7X1
229533.I1S8F8 pp 229533.V6QU99
229533.I1S8F8 pp 229533.V6R0W1
229533.I1S8F8 pp 229533.Q4I5G1
229533.I1S8F8 pp 229533.V6R1D2
229533.I1S8F8 pp 229533.V6QUG0
229533.I1S8F8 pp 229533.I1S969
229533.I1S8F8 pp 229533.V6RAM6
229533.I1S8F8 pp 229533.I1SAI4
229533.I1S8F8 pp 229533.V6QVH2
229533.I1S289 pp 229533.I1S5S2
229533.I1S289 pp 229533.Q4IN63
229533.I1S289 pp 229533.I1S524
229533.I1S289 pp 229533.Q4HW67
229533.I1S289 pp 229533.Q4HZ68
229533.I1S289 pp 229533.I1S3V2
229533.I1S289 pp 229533.V6RIS2
229533.I1S289 pp 229533.Q4IMD1
229533.A0A1C3YK94 pp 229533.I1RY21
229533.A0A1C3YK94 pp 229533.I1RVU1
229533.A0A1C3YK94 pp 229533.I1R9Y4
229533.A0A1C3YK94 pp 229533.I1RS05
229533.A0A1C3YK94 pp 229533.I1RKB7
229533.A0A1C3YK94 pp 229533.I1R994
229533.A0A1C3YK94 pp 229533.I1RYT8
229533.A0A1C3YK94 pp 229533.I1RMQ6
229533.A0A1C3YK94 pp 229533.I1RAV5
229533.A0A1C3YK94 pp 229533.A0A1C3YMF2
229533.A0A1C3YK94 pp 229533.I1S0K2
229533.A0A1C3YK94 pp 229533.I1RG62
229533.A0A1C3YK94 pp 229533.I1RX17
229533.A0A1C3YK94 pp 229533.I1RYN2
229533.A0A1C3YK94 pp 229533.I1REI0
229533.A0A1C3YK94 pp 229533.A0A1C3YLZ8
229533.A0A1C3YK94 pp 229533.I1RNM7
229533.A0A1C3YK94 pp 229533.I1RUQ3
229533.A0A1C3YK94 pp 229533.I1RPP5
229533.A0A1C3YK94 pp 229533.I1RE66
229533.A0A1C3YK94 pp 229533.I1RK47
229533.A0A1C3YK94 pp 229533.I1RMK7

51685 229533.A0A1C3YK94 pp 229533.Q4IK03
51686 229533.A0A1C3YK94 pp 229533.I1S039
51687 229533.A0A1C3YK94 pp 229533.I1RRX5
51688 229533.A0A1C3YK94 pp 229533.I1S8F8
51689 229533.A0A1C3YK94 pp 229533.I1RGK9
51690 229533.A0A1C3YK94 pp 229533.I1S212
51691 229533.A0A1C3YK94 pp 229533.I1RYU6
51692 229533.A0A1C3YK94 pp 229533.A0A1C3YLM6
51693 229533.A0A1C3YK94 pp 229533.I1RPR9
51694 229533.A0A1C3YK94 pp 229533.I1RJT1
51695 229533.A0A1C3YK94 pp 229533.I1S8S9
51696 229533.A0A1C3YK94 pp 229533.I1RFY0
51697 229533.A0A1C3YK94 pp 229533.I1RAG5
51698 229533.A0A1C3YK94 pp 229533.I1RXE6
51699 229533.A0A1C3YK94 pp 229533.V6R3J1
51700 229533.A0A1C3YK94 pp 229533.Q4HWQ2
51701 229533.A0A1C3YK94 pp 229533.I1RE19
51702 229533.A0A1C3YK94 pp 229533.I1RAM9
51703 229533.A0A1C3YK94 pp 229533.I1RZ43
51704 229533.A0A1C3YK94 pp 229533.I1RBE6
51705 229533.A0A1C3YK94 pp 229533.I1RX39
51706 229533.A0A1C3YK94 pp 229533.A0A1C3YLB0
51707 229533.A0A1C3YK94 pp 229533.A0A1C3YLX8
51708 229533.A0A1C3YK94 pp 229533.I1RP02
51709 229533.A0A1C3YK94 pp 229533.I1RTJ2
51710 229533.A0A1C3YK94 pp 229533.I1RWT0
51711 229533.A0A1C3YK94 pp 229533.Q4IBU4
51712 229533.A0A1C3YK94 pp 229533.I1RZH5
51713 229533.A0A1C3YK94 pp 229533.I1RMG9
51714 229533.A0A1C3YK94 pp 229533.I1RXD9
51715 229533.A0A1C3YK94 pp 229533.I1RKJ1
51716 229533.A0A1C3YK94 pp 229533.I1RA52
51717 229533.A0A1C3YK94 pp 229533.I1RWF4
51718 229533.A0A1C3YK94 pp 229533.I1RZV5
51719 229533.A0A1C3YK94 pp 229533.I1S277
51720 229533.A0A1C3YK94 pp 229533.Q4ING3
51721 229533.A0A1C3YK94 pp 229533.A0A1C3YKP5
51722 229533.A0A1C3YK94 pp 229533.A0A1C3YM60
51723 229533.A0A1C3YK94 pp 229533.I1RXY7
51724 229533.A0A1C3YK94 pp 229533.I1RM99
51725 229533.A0A1C3YK94 pp 229533.I1RBT6
51726 229533.A0A1C3YK94 pp 229533.I1RSQ4
51727 229533.A0A1C3YK94 pp 229533.A0A1C3YLB6
51728 229533.A0A1C3YK94 pp 229533.I1RB38
51729 229533.A0A1C3YK94 pp 229533.I1RC18
51730 229533.A0A1C3YK94 pp 229533.I1R9U7
51731 229533.A0A1C3YK94 pp 229533.I1S1F3
51732 229533.A0A1C3YK94 pp 229533.I1S2M1
51733 229533.A0A1C3YK94 pp 229533.I1RCM2
51734 229533.A0A1C3YK94 pp 229533.I1S0R8
51735 229533.A0A1C3YK94 pp 229533.I1RG45
51736 229533.A0A1C3YK94 pp 229533.I1RMP0
51737 229533.A0A1C3YK94 pp 229533.I1RE52
51738 229533.A0A1C3YK94 pp 229533.A0A1C3YKM4
51739 229533.A0A1C3YK94 pp 229533.I1RFS5
51740 229533.A0A1C3YK94 pp 229533.I1RZI8
51741 229533.A0A1C3YK94 pp 229533.I1S6Y3
51742 229533.A0A1C3YK94 pp 229533.I1RE65
51743 229533.A0A1C3YK94 pp 229533.I1RFJ6
51744 229533.A0A1C3YK94 pp 229533.I1S0Q7
51745 229533.I1S887 pp 229533.V6RTJ8
51746 229533.I1S887 pp 229533.V6RCZ8
51747 229533.I1S887 pp 229533.V6QUE6
51748 229533.I1S887 pp 229533.Q4I624
51749 229533.I1S887 pp 229533.V6R3J1
51750 229533.I1S887 pp 229533.Q4IJK2
51751 229533.I1S887 pp 229533.O74638
51752 229533.I1S887 pp 229533.I1S9Y1
51753 229533.I1S887 pp 229533.V6QVH8
51754 229533.I1S887 pp 229533.V6R042
51755 229533.I1RE90 pp 229533.I1RFK3
51756 229533.I1RE90 pp 229533.I1RXY7
51757 229533.I1RE90 pp 229533.V6QV31

```
229533.I1RE90   pp  229533.I1RTT0
229533.I1RE90   pp  229533.I1RG09
229533.I1RE90   pp  229533.I1RXH1
229533.I1RE90   pp  229533.V6R5D2
229533.I1RE90   pp  229533.I1RFI8
229533.I1RE90   pp  229533.V6R0W1
229533.I1RE90   pp  229533.I1SAI4
229533.I1RE90   pp  229533.V6R756
229533.I1RE90   pp  229533.V6R2Z2
229533.I1RE90   pp  229533.V6RS91
229533.I1RE90   pp  229533.V6QU99
229533.I1RE90   pp  229533.I1RLM2
229533.I1RE90   pp  229533.I1RN33
229533.I1RE90   pp  229533.Q4I1N3
229533.I1RE90   pp  229533.I1S0C2
229533.I1RE90   pp  229533.I1RIY8
229533.I1RE90   pp  229533.V6RAM6
229533.I1RE90   pp  229533.V6RDH3
229533.I1REF5   pp  229533.I1RW23
229533.I1REF5   pp  229533.V6RNM4
229533.I1REF5   pp  229533.I1RRU9
229533.I1REF5   pp  229533.Q4IAA0
229533.I1REF5   pp  229533.I1RS66
229533.I1REF5   pp  229533.I1RNI4
229533.I1REF5   pp  229533.I1S289
229533.I1REF5   pp  229533.I1RNE7
229533.I1REF5   pp  229533.I1RN48
229533.I1REF5   pp  229533.I1RT01
229533.I1REF5   pp  229533.I1RZ40
229533.I1REF5   pp  229533.I1RQ88
229533.I1REF5   pp  229533.Q4HW67
229533.I1REF5   pp  229533.I1RKF8
229533.I1S4L8   pp  229533.V6QUG0
229533.I1S4L8   pp  229533.V6R7V8
229533.I1S4L8   pp  229533.I1S4S1
229533.I1RYI8   pp  229533.I1RZT0
229533.I1RYI8   pp  229533.I1S3K8
229533.A0A1C3YJC9   pp  229533.Q4IJU0
229533.A0A1C3YJC9   pp  229533.I1RWT7
229533.A0A1C3YJC9   pp  229533.I1RB53
229533.A0A1C3YJC9   pp  229533.I1RWA9
229533.A0A1C3YJC9   pp  229533.I1REF5
229533.A0A1C3YJC9   pp  229533.I1RPP4
229533.A0A1C3YJC9   pp  229533.I1RK83
229533.A0A1C3YJC9   pp  229533.I1RNC1
229533.A0A1C3YJC9   pp  229533.V6RNM4
229533.A0A1C3YJC9   pp  229533.I1RA84
229533.A0A1C3YJC9   pp  229533.I1RYB9
229533.A0A1C3YJC9   pp  229533.V6RG06
229533.A0A1C3YJC9   pp  229533.I1RCI3
229533.A0A1C3YJC9   pp  229533.I1RBJ1
229533.A0A1C3YJC9   pp  229533.A0A1C3YKX5
229533.A0A1C3YJC9   pp  229533.V6QUW4
229533.A0A1C3YJC9   pp  229533.I1RNE7
229533.A0A1C3YJC9   pp  229533.I1RNI4
229533.A0A1C3YJC9   pp  229533.I1RZZ9
229533.A0A1C3YJC9   pp  229533.I1RAF0
229533.A0A1C3YJC9   pp  229533.I1S8E2
229533.A0A1C3YJC9   pp  229533.Q4HW67
229533.A0A1C3YJC9   pp  229533.A0A1C3YL15
229533.A0A1C3YJC9   pp  229533.I1RZ40
229533.A0A1C3YJC9   pp  229533.A0A1C3YJG6
229533.A0A1C3YKR5   pp  229533.I1S267
229533.A0A1C3YKR5   pp  229533.I1S2F7
229533.A0A1C3YKR5   pp  229533.A0A1C3YLI1
229533.A0A1C3YKR5   pp  229533.I1RAV5
229533.A0A1C3YKR5   pp  229533.I1S8F8
229533.A0A1C3YKR5   pp  229533.I1RY81
229533.A0A1C3YKR5   pp  229533.I1RA90
229533.A0A1C3YKR5   pp  229533.I1RJY7
229533.A0A1C3YKR5   pp  229533.I1RNY6
229533.A0A1C3YKR5   pp  229533.A0A1C3YLB3
229533.A0A1C3YKR5   pp  229533.A0A1C3YKX1
```

51831	229533.A0A1C3YKR5	pp	229533.I1RC48
51832	229533.A0A1C3YKR5	pp	229533.I1RAQ4
51833	229533.A0A1C3YKR5	pp	229533.I1RAM9
51834	229533.A0A1C3YKR5	pp	229533.I1RPR1
51835	229533.A0A1C3YKR5	pp	229533.I1REE2
51836	229533.A0A1C3YKR5	pp	229533.I1RHZ5
51837	229533.A0A1C3YKR5	pp	229533.I1S0V6
51838	229533.A0A1C3YKR5	pp	229533.I1RST9
51839	229533.A0A1C3YKR5	pp	229533.I1RXE6
51840	229533.A0A1C3YKR5	pp	229533.I1RCM5
51841	229533.A0A1C3YKR5	pp	229533.V6RK96
51842	229533.A0A1C3YKR5	pp	229533.I1RXZ4
51843	229533.A0A1C3YKR5	pp	229533.I1RJT5
51844	229533.A0A1C3YKR5	pp	229533.A0A1C3YKY6
51845	229533.A0A1C3YKR5	pp	229533.I1S0K2
51846	229533.A0A1C3YKR5	pp	229533.I1RY12
51847	229533.A0A1C3YKR5	pp	229533.I1RG33
51848	229533.A0A1C3YKR5	pp	229533.A0A1C3YLC2
51849	229533.A0A1C3YKR5	pp	229533.I1RXS0
51850	229533.A0A1C3YKR5	pp	229533.I1S8C1
51851	229533.A0A1C3YKR5	pp	229533.A0A1C3YKZ8
51852	229533.A0A1C3YKR5	pp	229533.I1RUY4
51853	229533.A0A1C3YKR5	pp	229533.A0A1C3YL54
51854	229533.A0A1C3YKR5	pp	229533.I1RUQ3
51855	229533.A0A1C3YKR5	pp	229533.I1RQ60
51856	229533.A0A1C3YKR5	pp	229533.I1RYA2
51857	229533.A0A1C3YKR5	pp	229533.I1S3T6
51858	229533.A0A1C3YKR5	pp	229533.A0A1C3YLP6
51859	229533.A0A1C3YKR5	pp	229533.I1R9Y4
51860	229533.A0A1C3YKR5	pp	229533.I1RCR6
51861	229533.A0A1C3YKR5	pp	229533.I1R9B6
51862	229533.A0A1C3YKR5	pp	229533.I1RDE6
51863	229533.A0A1C3YKR5	pp	229533.I1S0K0
51864	229533.A0A1C3YKR5	pp	229533.I1S8E0
51865	229533.A0A1C3YKR5	pp	229533.I1RCU2
51866	229533.A0A1C3YKR5	pp	229533.I1RFS6
51867	229533.A0A1C3YKR5	pp	229533.I1RQE8
51868	229533.A0A1C3YKR5	pp	229533.I1RA49
51869	229533.A0A1C3YKR5	pp	229533.I1RYQ4
51870	229533.A0A1C3YKR5	pp	229533.I1RZM4
51871	229533.A0A1C3YKR5	pp	229533.I1RRX5
51872	229533.A0A1C3YKR5	pp	229533.I1RKB7
51873	229533.A0A1C3YKR5	pp	229533.I1RMQ6
51874	229533.A0A1C3YKR5	pp	229533.I1RJN7
51875	229533.A0A1C3YKR5	pp	229533.I1RNM7
51876	229533.A0A1C3YKR5	pp	229533.I1RDQ2
51877	229533.A0A1C3YKR5	pp	229533.I1S210
51878	229533.A0A1C3YKR5	pp	229533.I1S223
51879	229533.A0A1C3YKR5	pp	229533.I1RY21
51880	229533.A0A1C3YKR5	pp	229533.I1RMK7
51881	229533.A0A1C3YKR5	pp	229533.I1RYC8
51882	229533.A0A1C3YKR5	pp	229533.I1RWL3
51883	229533.A0A1C3YKR5	pp	229533.I1RXY7
51884	229533.A0A1C3YKR5	pp	229533.I1RMA2
51885	229533.A0A1C3YKR5	pp	229533.I1RQP2
51886	229533.A0A1C3YKR5	pp	229533.I1S3L2
51887	229533.A0A1C3YKR5	pp	229533.A0A1C3YLB6
51888	229533.A0A1C3YKR5	pp	229533.I1RGX1
51889	229533.A0A1C3YKR5	pp	229533.I1RAI4
51890	229533.A0A1C3YKR5	pp	229533.I1RKZ7
51891	229533.A0A1C3YKR5	pp	229533.I1RYP5
51892	229533.A0A1C3YKR5	pp	229533.I1RW54
51893	229533.A0A1C3YKR5	pp	229533.I1RB38
51894	229533.A0A1C3YKR5	pp	229533.V6R382
51895	229533.A0A1C3YKR5	pp	229533.I1RE52
51896	229533.A0A1C3YKR5	pp	229533.I1RHC8
51897	229533.A0A1C3YKR5	pp	229533.I1RYW9
51898	229533.A0A1C3YKR5	pp	229533.I1RAV0
51899	229533.A0A1C3YKR5	pp	229533.I1RN84
51900	229533.A0A1C3YKR5	pp	229533.I1RLZ4
51901	229533.A0A1C3YKR5	pp	229533.I1RL81
51902	229533.A0A1C3YKR5	pp	229533.I1S8R4
51903	229533.A0A1C3YKR5	pp	229533.A0A1C3YM29

51904	229533.A0A1C3YKR5	pp	229533.I1S968
51905	229533.A0A1C3YKR5	pp	229533.I1S0V7
51906	229533.A0A1C3YKR5	pp	229533.I1RNV4
51907	229533.A0A1C3YKR5	pp	229533.I1S256
51908	229533.A0A1C3YKR5	pp	229533.I1S2C4
51909	229533.A0A1C3YKR5	pp	229533.I1RFJ6
51910	229533.A0A1C3YKR5	pp	229533.A0A1C3YMK0
51911	229533.A0A1C3YKR5	pp	229533.A0A1C3YLE2
51912	229533.A0A1C3YKR5	pp	229533.I1RWF5
51913	229533.A0A1C3YKR5	pp	229533.V6RRM0
51914	229533.A0A1C3YKR5	pp	229533.A0A1C3YM60
51915	229533.A0A1C3YKR5	pp	229533.I1RF85
51916	229533.A0A1C3YKR5	pp	229533.V6RUY9
51917	229533.A0A1C3YKR5	pp	229533.I1RZ38
51918	229533.A0A1C3YKR5	pp	229533.I1S8R2
51919	229533.A0A1C3YKR5	pp	229533.I1RMJ3
51920	229533.A0A1C3YKR5	pp	229533.I1RKB2
51921	229533.A0A1C3YKR5	pp	229533.I1RZV5
51922	229533.A0A1C3YKR5	pp	229533.I1RYA0
51923	229533.A0A1C3YKR5	pp	229533.I1RYK0
51924	229533.A0A1C3YKR5	pp	229533.V6R3J1
51925	229533.A0A1C3YKR5	pp	229533.I1RHJ0
51926	229533.A0A1C3YKR5	pp	229533.I1S299
51927	229533.A0A1C3YKR5	pp	229533.V6RM02
51928	229533.A0A1C3YKR5	pp	229533.I1RSS7
51929	229533.A0A1C3YKR5	pp	229533.I1RBY9
51930	229533.A0A1C3YKR5	pp	229533.Q4HVQ9
51931	229533.A0A1C3YKR5	pp	229533.I1RC31
51932	229533.A0A1C3YKR5	pp	229533.I1RY62
51933	229533.A0A1C3YKR5	pp	229533.I1RGC3
51934	229533.A0A1C3YKR5	pp	229533.I1RC34
51935	229533.A0A1C3YKR5	pp	229533.I1RN88
51936	229533.A0A1C3YKR5	pp	229533.I1RBE6
51937	229533.A0A1C3YKR5	pp	229533.I1RFI4
51938	229533.A0A1C3YKR5	pp	229533.I1RA52
51939	229533.A0A1C3YKR5	pp	229533.I1RY35
51940	229533.A0A1C3YKR5	pp	229533.I1RSQ4
51941	229533.A0A1C3YKR5	pp	229533.I1RVR2
51942	229533.A0A1C3YKR5	pp	229533.I1RSK5
51943	229533.A0A1C3YKR5	pp	229533.I1RBF3
51944	229533.A0A1C3YKR5	pp	229533.I1RH91
51945	229533.A0A1C3YKR5	pp	229533.I1RUR8
51946	229533.A0A1C3YKR5	pp	229533.I1S3T7
51947	229533.A0A1C3YKR5	pp	229533.I1RK55
51948	229533.I1REW2	pp	229533.I1RTA6
51949	229533.I1REW2	pp	229533.I1REW3
51950	229533.I1REW2	pp	229533.Q4I5R3
51951	229533.A0A0E0SN30	pp	229533.I1RVY0
51952	229533.A0A0E0SN30	pp	229533.I1RNN1
51953	229533.A0A0E0SN30	pp	229533.I1RN27
51954	229533.A0A0E0SN30	pp	229533.A0A1C3YN58
51955	229533.A0A0E0SN30	pp	229533.I1RWG5
51956	229533.A0A0E0SN30	pp	229533.I1RYV4
51957	229533.A0A0E0SN30	pp	229533.A0A1C3YK75
51958	229533.V6RG06	pp	229533.V6RNM4
51959	229533.A0A1C3YKY9	pp	229533.I1RFY0
51960	229533.A0A1C3YKY9	pp	229533.I1RYM5
51961	229533.A0A1C3YKY9	pp	229533.I1RTJ2
51962	229533.A0A1C3YKY9	pp	229533.I1RPM6
51963	229533.A0A1C3YKY9	pp	229533.I1RRG1
51964	229533.A0A1C3YKY9	pp	229533.A0A1C3YLB6
51965	229533.A0A1C3YKY9	pp	229533.I1RGK9
51966	229533.A0A1C3YKY9	pp	229533.I1RE79
51967	229533.A0A1C3YKY9	pp	229533.I1RNG4
51968	229533.A0A1C3YKY9	pp	229533.I1RR72
51969	229533.A0A1C3YKY9	pp	229533.I1S8D2
51970	229533.I1RLA2	pp	229533.I1RZC3
51971	229533.I1RLA2	pp	229533.I1RX18
51972	229533.I1RLA2	pp	229533.I1RZA8
51973	229533.I1RLA2	pp	229533.I1RPV8
51974	229533.I1RLA2	pp	229533.I1RZ56
51975	229533.I1RLA2	pp	229533.I1S0A5
51976	229533.I1RLA2	pp	229533.V6R9L8

51977 229533.I1RLA2 pp 229533.I1RW82
51978 229533.I1RLA2 pp 229533.I1S6V0
51979 229533.I1RLA2 pp 229533.I1RQ48
51980 229533.I1RLA2 pp 229533.I1RR45
51981 229533.I1RLA2 pp 229533.I1RZX3
51982 229533.I1RLA2 pp 229533.I1RSA5
51983 229533.I1RLA2 pp 229533.I1RY32
51984 229533.I1RLA2 pp 229533.V6R756
51985 229533.I1RLA2 pp 229533.I1RZ38
51986 229533.I1RLA2 pp 229533.I1S968
51987 229533.I1RLA2 pp 229533.I1RRR9
51988 229533.I1RLA2 pp 229533.I1RXH2
51989 229533.I1RLA2 pp 229533.I1RWM4
51990 229533.I1RLA2 pp 229533.I1RW55
51991 229533.I1RLA2 pp 229533.I1RWF5
51992 229533.I1RLA2 pp 229533.V6R2Z2
51993 229533.I1RLA2 pp 229533.V6RHX2
51994 229533.I1RLA2 pp 229533.V6QZ48
51995 229533.I1RLA2 pp 229533.V6RCJ5
51996 229533.I1RLA2 pp 229533.I1S694
51997 229533.I1RLA2 pp 229533.I1RRK5
51998 229533.I1RLA2 pp 229533.I1S5J9
51999 229533.I1RLA2 pp 229533.I1RST9
52000 229533.I1RLA2 pp 229533.I1SAB4
52001 229533.I1RLA2 pp 229533.I1RMR3
52002 229533.I1RLA2 pp 229533.I1S000
52003 229533.I1RLA2 pp 229533.I1S223
52004 229533.I1RLA2 pp 229533.I1S3T6
52005 229533.I1RLA2 pp 229533.I1S8D3
52006 229533.I1RLA2 pp 229533.I1RY12
52007 229533.I1RLA2 pp 229533.I1S4I7
52008 229533.I1RLA2 pp 229533.I1S267
52009 229533.I1RLA2 pp 229533.I1S4I8
52010 229533.I1RLA2 pp 229533.V6RH92
52011 229533.I1RLA2 pp 229533.I1RY81
52012 229533.I1RLA2 pp 229533.I1RY16
52013 229533.I1RLA2 pp 229533.I1S6H3
52014 229533.I1RLA2 pp 229533.V6QU99
52015 229533.I1RLA2 pp 229533.I1S094
52016 229533.I1RLA2 pp 229533.I1RVF2
52017 229533.I1RLA2 pp 229533.V6QVV9
52018 229533.I1RLA2 pp 229533.V6RWB3
52019 229533.I1RLA2 pp 229533.V6R0W1
52020 229533.I1RLA2 pp 229533.I1S2F7
52021 229533.I1RLA2 pp 229533.V6R1D2
52022 229533.I1RLA2 pp 229533.I1RPN6
52023 229533.I1RLA2 pp 229533.I1SAI4
52024 229533.I1RLA2 pp 229533.I1RQV8
52025 229533.I1RLA2 pp 229533.V6RCC0
52026 229533.I1RLA2 pp 229533.V6RAM6
52027 229533.I1RLA2 pp 229533.I1RMF1
52028 229533.I1RLA2 pp 229533.I1RN52
52029 229533.I1RLA2 pp 229533.I1S5D0
52030 229533.I1RLA2 pp 229533.I1RW54
52031 229533.I1RLA2 pp 229533.I1RYP5
52032 229533.I1RLA2 pp 229533.I1S969
52033 229533.I1RLA2 pp 229533.I1RRS6
52034 229533.I1RLA2 pp 229533.V6R5T7
52035 229533.I1RLA2 pp 229533.I1RQV7
52036 229533.I1RLA2 pp 229533.I1RMA2
52037 229533.I1RLA2 pp 229533.I1S210
52038 229533.A0A1C3YK79 pp 229533.I1RPV5
52039 229533.A0A1C3YK79 pp 229533.I1S289
52040 229533.A0A1C3YK79 pp 229533.Q4IEK8
52041 229533.A0A1C3YK79 pp 229533.I1S201
52042 229533.A0A1C3YK79 pp 229533.I1RSI1
52043 229533.A0A1C3YK79 pp 229533.I1RZ40
52044 229533.A0A1C3YK79 pp 229533.Q4IAS1
52045 229533.A0A1C3YK79 pp 229533.I1RIS3
52046 229533.A0A1C3YK79 pp 229533.I1S1U7
52047 229533.A0A1C3YK79 pp 229533.I1RKF8
52048 229533.A0A1C3YK79 pp 229533.I1SAM5
52049 229533.A0A1C3YK79 pp 229533.I1RB53

52050 229533.A0A1C3YK79 pp 229533.I1RLV3
52051 229533.A0A1C3YK79 pp 229533.I1RC32
52052 229533.A0A1C3YK79 pp 229533.Q4HW67
52053 229533.A0A1C3YK79 pp 229533.I1RAV4
52054 229533.A0A1C3YK79 pp 229533.I1R9X2
52055 229533.A0A1C3YK79 pp 229533.A0A1C3YL15
52056 229533.A0A1C3YK79 pp 229533.I1RNE7
52057 229533.A0A1C3YK79 pp 229533.I1RN48
52058 229533.I1RHR3 pp 229533.V6R382
52059 229533.I1RHR3 pp 229533.I1RSY2
52060 229533.I1RHR3 pp 229533.I1RNG4
52061 229533.I1RHR3 pp 229533.I1RPK0
52062 229533.I1RHR3 pp 229533.I1RNJ7
52063 229533.I1RCN8 pp 229533.I1RRG3
52064 229533.I1RCN8 pp 229533.I1RVX9
52065 229533.I1RCN8 pp 229533.I1RMQ6
52066 229533.I1RCN8 pp 229533.I1RXB6
52067 229533.I1RCN8 pp 229533.I1RRV4
52068 229533.I1RCN8 pp 229533.I1RQ12
52069 229533.I1RCN8 pp 229533.V6R1K4
52070 229533.I1RCN8 pp 229533.I1RK95
52071 229533.I1RCN8 pp 229533.I1RGZ3
52072 229533.I1RCN8 pp 229533.I1S0A0
52073 229533.I1RCN8 pp 229533.V6RVJ3
52074 229533.I1RCN8 pp 229533.V6RQW4
52075 229533.I1RCN8 pp 229533.I1RYM5
52076 229533.I1RCN8 pp 229533.I1RFM6
52077 229533.I1RCN8 pp 229533.I1RIY0
52078 229533.I1RCN8 pp 229533.Q4I5G1
52079 229533.I1RCN8 pp 229533.I1RZ52
52080 229533.I1RCN8 pp 229533.I1RYK0
52081 229533.I1RCN8 pp 229533.I1RM26
52082 229533.I1RCN8 pp 229533.I1RMJ3
52083 229533.I1RCN8 pp 229533.I1RTG7
52084 229533.I1RCN8 pp 229533.I1RZ29
52085 229533.I1RCN8 pp 229533.I1RKB2
52086 229533.I1RCN8 pp 229533.I1RSQ4
52087 229533.I1RCN8 pp 229533.I1RZH6
52088 229533.I1RCN8 pp 229533.I1RMW4
52089 229533.I1RCN8 pp 229533.I1RXY7
52090 229533.I1RCN8 pp 229533.I1S1F3
52091 229533.I1RCN8 pp 229533.I1RCV1
52092 229533.I1RCN8 pp 229533.Q4HXF0
52093 229533.I1RCN8 pp 229533.O74638
52094 229533.I1RCN8 pp 229533.V6RN69
52095 229533.I1RCN8 pp 229533.I1RRZ6
52096 229533.I1RCN8 pp 229533.I1S9Y1
52097 229533.I1RCN8 pp 229533.I1RIH0
52098 229533.I1RCN8 pp 229533.I1RY74
52099 229533.I1RCN8 pp 229533.V6RLM7
52100 229533.I1RCN8 pp 229533.I1S064
52101 229533.I1RCN8 pp 229533.V6QVH8
52102 229533.I1RCN8 pp 229533.I1RGL3
52103 229533.I1RCN8 pp 229533.I1RSX9
52104 229533.I1RCN8 pp 229533.I1RCQ1
52105 229533.I1RCN8 pp 229533.I1SAJ3
52106 229533.I1RCN8 pp 229533.I1RKB6
52107 229533.I1RCN8 pp 229533.I1RWT0
52108 229533.I1RBN4 pp 229533.I1RJ33
52109 229533.I1RBN4 pp 229533.I1SA77
52110 229533.A0A1C3YJ91 pp 229533.I1S172
52111 229533.A0A1C3YJ91 pp 229533.I1RQG9
52112 229533.I1RWH1 pp 229533.I1RY74
52113 229533.I1RWH1 pp 229533.V6RN69
52114 229533.I1RHM1 pp 229533.I1RVY0
52115 229533.A0A0E0SJ19 pp 229533.Q4IJU0
52116 229533.A0A0E0SJ19 pp 229533.I1RAS2
52117 229533.A0A0E0SJ19 pp 229533.I1RDG0
52118 229533.A0A0E0SJ19 pp 229533.I1RD39
52119 229533.A0A0E0SJ19 pp 229533.I1RBZ3
52120 229533.A0A0E0SJ19 pp 229533.I1RB53
52121 229533.A0A0E0SJ19 pp 229533.V6RNM4
52122 229533.A0A0E0SJ19 pp 229533.I1RK83

52123 229533.A0A0E0SJ19 pp 229533.I1R9X2
52124 229533.A0A0E0SJ19 pp 229533.I1RPK4
52125 229533.A0A0E0SJ19 pp 229533.I1REF5
52126 229533.A0A0E0SJ19 pp 229533.A0A1C3YJC9
52127 229533.A0A0E0SJ19 pp 229533.I1RBJ1
52128 229533.A0A0E0SJ19 pp 229533.I1RN48
52129 229533.A0A0E0SJ19 pp 229533.I1RIS3
52130 229533.A0A0E0SJ19 pp 229533.I1RT35
52131 229533.A0A0E0SJ19 pp 229533.I1RMB2
52132 229533.A0A0E0SJ19 pp 229533.I1RNI4
52133 229533.A0A0E0SJ19 pp 229533.A0A1C3YL15
52134 229533.A0A0E0SJ19 pp 229533.I1RDD3
52135 229533.A0A0E0SJ19 pp 229533.I1RWK3
52136 229533.A0A0E0SJ19 pp 229533.A0A1C3YKK5
52137 229533.A0A0E0SJ19 pp 229533.I1RNB8
52138 229533.A0A0E0SJ19 pp 229533.Q4IAA0
52139 229533.A0A0E0SJ19 pp 229533.A0A1C3YJJ0
52140 229533.A0A0E0SJ19 pp 229533.I1RKK1
52141 229533.A0A0E0SJ19 pp 229533.Q4HW67
52142 229533.A0A0E0SJ19 pp 229533.I1RA84
52143 229533.A0A0E0SJ19 pp 229533.I1RZ40
52144 229533.A0A0E0SJ19 pp 229533.I1RRU9
52145 229533.A0A0E0SJ19 pp 229533.A0A1C3YKX5
52146 229533.A0A0E0SJ19 pp 229533.I1RV65
52147 229533.A0A0E0SJ19 pp 229533.I1RN58
52148 229533.A0A0E0SJ19 pp 229533.I1RAE0
52149 229533.A0A0E0SJ19 pp 229533.I1REE1
52150 229533.A0A0E0SJ19 pp 229533.A0A1C3YIG3
52151 229533.A0A0E0SJ19 pp 229533.I1RYB9
52152 229533.I1RZD2 pp 229533.Q4IB70
52153 229533.I1RWG5 pp 229533.I1S1U7
52154 229533.I1RWG5 pp 229533.I1RYV4
52155 229533.A0A098DCZ4 pp 229533.A0A1C3YL15
52156 229533.A0A098DCZ4 pp 229533.Q4HW67
52157 229533.A0A098DCZ4 pp 229533.I1RAV4
52158 229533.A0A098DCZ4 pp 229533.I1RCA7
52159 229533.A0A098DCZ4 pp 229533.Q4IAS1
52160 229533.A0A098DCZ4 pp 229533.I1RQM0
52161 229533.A0A098DCZ4 pp 229533.A0A098DN79
52162 229533.A0A098DCZ4 pp 229533.I1RKF8
52163 229533.A0A098DCZ4 pp 229533.A0A1C3YM36
52164 229533.A0A098DCZ4 pp 229533.A0A1C3YII7
52165 229533.A0A098DCZ4 pp 229533.Q4IQM6
52166 229533.A0A098DCZ4 pp 229533.I1RZ40
52167 229533.A0A098DCZ4 pp 229533.I1RZG7
52168 229533.A0A098DCZ4 pp 229533.A0A1C3YKX5
52169 229533.A0A098DCZ4 pp 229533.Q4IJU0
52170 229533.A0A098DCZ4 pp 229533.I1RNE7
52171 229533.A0A098DCZ4 pp 229533.I1R9X2
52172 229533.A0A098DCZ4 pp 229533.A0A1C3YKN2
52173 229533.A0A098DCZ4 pp 229533.A0A1C3YJZ6
52174 229533.A0A098DCZ4 pp 229533.I1RAS2
52175 229533.A0A098DCZ4 pp 229533.A0A1C3YM29
52176 229533.A0A098DCZ4 pp 229533.A0A1C3YK01
52177 229533.A0A098DCZ4 pp 229533.I1RNI4
52178 229533.A0A098DCZ4 pp 229533.I1RZS7
52179 229533.A0A098DCZ4 pp 229533.I1RB53
52180 229533.A0A098DCZ4 pp 229533.A0A0E0SD83
52181 229533.A0A098DCZ4 pp 229533.I1RJY2
52182 229533.A0A098DCZ4 pp 229533.I1S289
52183 229533.A0A098DCZ4 pp 229533.Q4IAA0
52184 229533.A0A098DCZ4 pp 229533.I1REF5
52185 229533.A0A098DCZ4 pp 229533.A0A1C3YJC9
52186 229533.A0A098DCZ4 pp 229533.V6RNM4
52187 229533.A0A098DCZ4 pp 229533.V6RG06
52188 229533.A0A098DCZ4 pp 229533.A0A1C3YK79
52189 229533.A0A098DCZ4 pp 229533.I1RRU9
52190 229533.A0A098DCZ4 pp 229533.I1RPU4
52191 229533.A0A098DCZ4 pp 229533.I1RN58
52192 229533.A0A098DCZ4 pp 229533.A0A0E0SJ19
52193 229533.A0A1C3YJC7 pp 229533.A0A1C3YN26
52194 229533.A0A1C3YJC7 pp 229533.I1RDC5
52195 229533.A0A1C3YJC7 pp 229533.I1RYM5

52196 229533.A0A1C3YJC7 pp 229533.I1RX64
52197 229533.A0A0E0S3S1 pp 229533.I1RRQ7
52198 229533.I1RCU4 pp 229533.V6REE9
52199 229533.I1RCU4 pp 229533.V6R3J1
52200 229533.I1RCU4 pp 229533.I1RR95
52201 229533.I1RCU4 pp 229533.I1RKR5
52202 229533.I1RCU4 pp 229533.I1RMX6
52203 229533.I1RCU4 pp 229533.I1RJS5
52204 229533.I1RCU4 pp 229533.I1RT41
52205 229533.I1RCU4 pp 229533.V6QUV7
52206 229533.I1RCU4 pp 229533.I1RF57
52207 229533.A0A098DN79 pp 229533.A0A1C3YMK1
52208 229533.A0A098DN79 pp 229533.A0A1C3YL15
52209 229533.A0A098DN79 pp 229533.I1S9H8
52210 229533.A0A098DN79 pp 229533.I1R9W6
52211 229533.A0A098DN79 pp 229533.I1RG53
52212 229533.A0A098DN79 pp 229533.A0A1C3YKK5
52213 229533.A0A098DN79 pp 229533.Q4HW67
52214 229533.A0A098DN79 pp 229533.I1RLX7
52215 229533.A0A098DN79 pp 229533.I1RI51
52216 229533.A0A098DN79 pp 229533.I1RBZ3
52217 229533.A0A098DN79 pp 229533.I1RB53
52218 229533.A0A098DN79 pp 229533.I1RNI4
52219 229533.A0A098DN79 pp 229533.V6RNM4
52220 229533.A0A098DN79 pp 229533.I1RK83
52221 229533.A0A098DN79 pp 229533.A0A1C3YJC9
52222 229533.A0A098DN79 pp 229533.A0A1C3YLK2
52223 229533.A0A098DN79 pp 229533.I1S224
52224 229533.A0A098DN79 pp 229533.I1REF5
52225 229533.A0A098DN79 pp 229533.A0A1C3YKX5
52226 229533.A0A098DN79 pp 229533.A0A1C3YIX7
52227 229533.A0A098DN79 pp 229533.I1RDV9
52228 229533.A0A098DN79 pp 229533.I1RJK7
52229 229533.A0A098DN79 pp 229533.I1RSI0
52230 229533.A0A098DN79 pp 229533.A0A1C3YM29
52231 229533.A0A098DN79 pp 229533.I1RQS4
52232 229533.A0A098DN79 pp 229533.I1S044
52233 229533.A0A098DN79 pp 229533.A0A1C3YJK5
52234 229533.A0A098DN79 pp 229533.I1S4Y4
52235 229533.A0A098DN79 pp 229533.I1RA94
52236 229533.A0A098DN79 pp 229533.A0A0E0SQJ6
52237 229533.A0A098DN79 pp 229533.Q4IJU0
52238 229533.A0A098DN79 pp 229533.I1RWQ8
52239 229533.A0A098DN79 pp 229533.I1RHP9
52240 229533.A0A098DN79 pp 229533.A0A0E0SQI9
52241 229533.A0A098DN79 pp 229533.I1RRU9
52242 229533.A0A098DN79 pp 229533.I1RGC2
52243 229533.A0A098DN79 pp 229533.Q4I2J8
52244 229533.A0A098DN79 pp 229533.I1RZ40
52245 229533.A0A098DN79 pp 229533.I1RDG0
52246 229533.A0A098DN79 pp 229533.A0A1C3YHQ0
52247 229533.A0A098DN79 pp 229533.A0A1C3YM91
52248 229533.A0A098DN79 pp 229533.I1RBE4
52249 229533.A0A098DN79 pp 229533.I1REE1
52250 229533.A0A098DN79 pp 229533.I1RYB9
52251 229533.A0A098DN79 pp 229533.I1RNY9
52252 229533.A0A098DN79 pp 229533.A0A0E0RTW3
52253 229533.A0A098DN79 pp 229533.I1RRW0
52254 229533.A0A098DN79 pp 229533.A0A0E0SJ19
52255 229533.A0A098DN79 pp 229533.I1RKR8
52256 229533.A0A098DN79 pp 229533.I1RC15
52257 229533.A0A098DN79 pp 229533.Q4IAA0
52258 229533.A0A1C3YII2 pp 229533.A0A1C3YLU9
52259 229533.A0A1C3YII2 pp 229533.I1RMG6
52260 229533.A0A1C3YII2 pp 229533.I1R9K3
52261 229533.A0A1C3YII2 pp 229533.A0A1C3YM01
52262 229533.A0A1C3YII2 pp 229533.I1RCB4
52263 229533.A0A1C3YII2 pp 229533.I1RZ20
52264 229533.A0A1C3YII2 pp 229533.I1RE53
52265 229533.A0A1C3YII2 pp 229533.I1RTI7
52266 229533.A0A1C3YII2 pp 229533.I1RQ48
52267 229533.A0A1C3YII2 pp 229533.I1RZ52
52268 229533.A0A1C3YII2 pp 229533.I1RPU8

52269 229533.A0A1C3YII2 pp 229533.I1RQ30
52270 229533.A0A1C3YII2 pp 229533.I1S9D4
52271 229533.A0A1C3YII2 pp 229533.I1RYU5
52272 229533.A0A1C3YII2 pp 229533.O74638
52273 229533.A0A1C3YII2 pp 229533.I1S5S3
52274 229533.A0A1C3YII2 pp 229533.V6RQW4
52275 229533.A0A1C3YII2 pp 229533.I1S2E2
52276 229533.A0A1C3YII2 pp 229533.I1RMG7
52277 229533.A0A1C3YII2 pp 229533.I1RIY0
52278 229533.I1S250 pp 229533.V6R382
52279 229533.I1RDZ7 pp 229533.I1RJL1
52280 229533.I1RDZ7 pp 229533.V6RS91
52281 229533.I1RDZ7 pp 229533.I1RIV9
52282 229533.I1RDZ7 pp 229533.I1RG55
52283 229533.I1RDZ7 pp 229533.I1RLT7
52284 229533.I1RDZ7 pp 229533.V6RDH3
52285 229533.I1RDZ7 pp 229533.I1S0C2
52286 229533.I1RDZ7 pp 229533.V6QV31
52287 229533.I1RDZ7 pp 229533.I1S0N3
52288 229533.I1RDZ7 pp 229533.I1RSY9
52289 229533.I1RDZ7 pp 229533.I1RSY8
52290 229533.I1RDZ7 pp 229533.I1RFI8
52291 229533.I1RDZ7 pp 229533.I1RSK5
52292 229533.I1RDZ7 pp 229533.I1RHJ9
52293 229533.I1RDZ7 pp 229533.I1RXY7
52294 229533.I1RDZ7 pp 229533.Q4I1N3
52295 229533.I1RDZ7 pp 229533.I1RFK3
52296 229533.I1RDZ7 pp 229533.I1RN33
52297 229533.I1RDZ7 pp 229533.I1RG54
52298 229533.I1RDZ7 pp 229533.V6R382
52299 229533.I1RD35 pp 229533.I1S2Y4
52300 229533.I1RD35 pp 229533.V6RK40
52301 229533.I1RD35 pp 229533.V6QU63
52302 229533.I1RD35 pp 229533.V6RW66
52303 229533.I1RD35 pp 229533.V6RAG6
52304 229533.I1RD35 pp 229533.I1RGX0
52305 229533.I1RD35 pp 229533.I1RT41
52306 229533.I1RD35 pp 229533.I1RQW2
52307 229533.I1RUQ7 pp 229533.I1RYM5
52308 229533.I1RUQ7 pp 229533.I1S8D2
52309 229533.I1RJC5 pp 229533.I1S8R2
52310 229533.I1RJC5 pp 229533.I1S0V7
52311 229533.I1RJC5 pp 229533.I1RPV8
52312 229533.I1RJC5 pp 229533.I1RJS1
52313 229533.I1RJC5 pp 229533.I1RZ56
52314 229533.I1RJC5 pp 229533.I1RNG4
52315 229533.I1RJC5 pp 229533.V6RLM7
52316 229533.I1RJC5 pp 229533.I1S3T7
52317 229533.I1RJC5 pp 229533.I1S0A5
52318 229533.I1RJC5 pp 229533.I1RW64
52319 229533.I1RJC5 pp 229533.I1S6P6
52320 229533.I1RJC5 pp 229533.I1RKB2
52321 229533.I1RJC5 pp 229533.V6R9Z3
52322 229533.I1RJC5 pp 229533.I1RUY5
52323 229533.I1RJC5 pp 229533.I1RYW9
52324 229533.I1RJC5 pp 229533.I1S299
52325 229533.I1RJC5 pp 229533.I1RN80
52326 229533.I1RJC5 pp 229533.I1RNY6
52327 229533.I1RJC5 pp 229533.I1RNH4
52328 229533.I1RJC5 pp 229533.I1RST9
52329 229533.I1RJC5 pp 229533.I1RRM1
52330 229533.I1RJC5 pp 229533.I1S094
52331 229533.I1RJC5 pp 229533.I1RPR1
52332 229533.I1RJC5 pp 229533.I1S6H3
52333 229533.I1RJC5 pp 229533.I1S8F8
52334 229533.I1RJC5 pp 229533.I1RNM7
52335 229533.I1RKK5 pp 229533.V6RSM7
52336 229533.I1RKK5 pp 229533.I1RWF5
52337 229533.I1RKK5 pp 229533.I1S8C1
52338 229533.I1RKK5 pp 229533.I1RN88
52339 229533.I1RKK5 pp 229533.I1RN84
52340 229533.I1RKK5 pp 229533.V6RSF3
52341 229533.I1RZE7 pp 229533.V6QV31

52342 229533.I1RZE7 pp 229533.V6RS91
52343 229533.I1RZE7 pp 229533.V6RDH3
52344 229533.I1RZE7 pp 229533.I1S0C2
52345 229533.I1RZE7 pp 229533.Q4I1N3
52346 229533.A0A1C3YIX7 pp 229533.A0A1C3YNB1
52347 229533.A0A1C3YIX7 pp 229533.I1RY74
52348 229533.A0A1C3YIX7 pp 229533.I1RSY1
52349 229533.A0A1C3YIX7 pp 229533.I1S6P5
52350 229533.A0A1C3YIX7 pp 229533.A0A1C3YJK5
52351 229533.A0A1C3YIX7 pp 229533.I1R9K3
52352 229533.A0A1C3YIX7 pp 229533.A0A1C3YLU9
52353 229533.A0A1C3YIX7 pp 229533.Q4HZ95
52354 229533.A0A1C3YIX7 pp 229533.V6RUB1
52355 229533.A0A1C3YIX7 pp 229533.I1RZ52
52356 229533.A0A1C3YIX7 pp 229533.I1RLT3
52357 229533.A0A1C3YIX7 pp 229533.I1RK03
52358 229533.A0A1C3YIX7 pp 229533.I1RQE0
52359 229533.A0A1C3YIX7 pp 229533.A0A1C3YM01
52360 229533.A0A1C3YIX7 pp 229533.I1S0H5
52361 229533.A0A1C3YIX7 pp 229533.I1RS84
52362 229533.A0A1C3YIX7 pp 229533.I1RM26
52363 229533.A0A1C3YIX7 pp 229533.I1RTG7
52364 229533.A0A1C3YIX7 pp 229533.A0A1C3YLV0
52365 229533.A0A1C3YIX7 pp 229533.I1S273
52366 229533.A0A1C3YIX7 pp 229533.I1RC74
52367 229533.A0A1C3YIX7 pp 229533.I1RCT7
52368 229533.A0A1C3YIX7 pp 229533.I1RU76
52369 229533.A0A1C3YIX7 pp 229533.I1RIY0
52370 229533.A0A1C3YIX7 pp 229533.V6QV31
52371 229533.A0A1C3YIX7 pp 229533.I1RE58
52372 229533.A0A1C3YIX7 pp 229533.I1S3F3
52373 229533.A0A1C3YIX7 pp 229533.I1RAB6
52374 229533.A0A1C3YIX7 pp 229533.I1RC83
52375 229533.A0A1C3YIX7 pp 229533.I1RZA7
52376 229533.A0A1C3YIX7 pp 229533.I1RD81
52377 229533.A0A1C3YIX7 pp 229533.I1RN51
52378 229533.A0A1C3YIX7 pp 229533.V6RK96
52379 229533.A0A1C3YIX7 pp 229533.I1RPD6
52380 229533.A0A1C3YIX7 pp 229533.I1RPW2
52381 229533.A0A1C3YIX7 pp 229533.I1SAE8
52382 229533.A0A1C3YIX7 pp 229533.I1RNG4
52383 229533.A0A1C3YIX7 pp 229533.A0A1C3YJ46
52384 229533.A0A1C3YIX7 pp 229533.I1S2E2
52385 229533.A0A1C3YIX7 pp 229533.I1RMM8
52386 229533.A0A1C3YIX7 pp 229533.I1RBE4
52387 229533.A0A1C3YIX7 pp 229533.I1RQE8
52388 229533.A0A1C3YIX7 pp 229533.I1RRG1
52389 229533.A0A1C3YIX7 pp 229533.I1S024
52390 229533.A0A1C3YIX7 pp 229533.I1RZM4
52391 229533.A0A1C3YIX7 pp 229533.I1S9D4
52392 229533.A0A1C3YIX7 pp 229533.A0A1C3YK58
52393 229533.A0A1C3YIX7 pp 229533.I1RQ14
52394 229533.A0A1C3YIX7 pp 229533.I1RA94
52395 229533.A0A1C3YIX7 pp 229533.I1RGA0
52396 229533.A0A1C3YIX7 pp 229533.I1RZY1
52397 229533.A0A1C3YIX7 pp 229533.I1RZH8
52398 229533.A0A1C3YIX7 pp 229533.I1RVX9
52399 229533.I1RLL8 pp 229533.I1RZ17
52400 229533.I1RLL8 pp 229533.I1RMA0
52401 229533.I1RLL8 pp 229533.Q4ILH3
52402 229533.I1RLL8 pp 229533.I1RRF6
52403 229533.I1RB32 pp 229533.I1RKG1
52404 229533.A0A1C3YMC0 pp 229533.I1RSK5
52405 229533.A0A1C3YMC0 pp 229533.V6REE9
52406 229533.A0A1C3YMC0 pp 229533.I1RCD4
52407 229533.A0A1C3YMC0 pp 229533.I1RKR5
52408 229533.A0A1C3YMC0 pp 229533.V6R3J1
52409 229533.A0A1C3YMC0 pp 229533.I1RW55
52410 229533.A0A1C3YMC0 pp 229533.I1RBA6
52411 229533.A0A1C3YMC0 pp 229533.I1RCU4
52412 229533.A0A1C3YMC0 pp 229533.A0A1C3YN26
52413 229533.A0A1C3YMC0 pp 229533.I1RC18
52414 229533.A0A1C3YMC0 pp 229533.I1RC11

52415 229533.A0A1C3YMC0 pp 229533.I1RT41
52416 229533.A0A1C3YMC0 pp 229533.I1RJS5
52417 229533.A0A1C3YMC0 pp 229533.I1RMX6
52418 229533.A0A1C3YMC0 pp 229533.V6QUV7
52419 229533.I1RBM9 pp 229533.I1RRH5
52420 229533.I1RBM9 pp 229533.I1RDY1
52421 229533.I1RBM9 pp 229533.I1REC2
52422 229533.I1S0B4 pp 229533.Q4ILH3
52423 229533.I1RE48 pp 229533.I1S8Z2
52424 229533.I1RE48 pp 229533.I1RN52
52425 229533.I1RE48 pp 229533.I1RRK5
52426 229533.I1RE48 pp 229533.V6RKY8
52427 229533.I1RE48 pp 229533.I1RT75
52428 229533.I1RE48 pp 229533.I1REW3
52429 229533.I1RE48 pp 229533.I1RKP1
52430 229533.I1RE48 pp 229533.I1RR45
52431 229533.I1RE48 pp 229533.I1RSF1
52432 229533.A0A098DCZ3 pp 229533.A0A098DN79
52433 229533.A0A098DCZ3 pp 229533.I1RD35
52434 229533.A0A098DCZ3 pp 229533.I1RMJ3
52435 229533.A0A098DCZ3 pp 229533.A0A1C3YIX7
52436 229533.A0A098DCZ3 pp 229533.I1RA94
52437 229533.A0A098DCZ3 pp 229533.A0A1C3YJK5
52438 229533.A0A098DCZ3 pp 229533.I1RBE4
52439 229533.A0A1C3YLG0 pp 229533.I1RA83
52440 229533.A0A1C3YLG0 pp 229533.I1RXE5
52441 229533.A0A1C3YLG0 pp 229533.I1RYW7
52442 229533.A0A1C3YLG0 pp 229533.I1RSR2
52443 229533.A0A1C3YLG0 pp 229533.I1RZY6
52444 229533.A0A1C3YLG0 pp 229533.I1RWT1
52445 229533.A0A1C3YLG0 pp 229533.I1RYX3
52446 229533.A0A1C3YLG0 pp 229533.I1RBP9
52447 229533.A0A1C3YLG0 pp 229533.I1RS64
52448 229533.A0A1C3YLG0 pp 229533.I1RAL5
52449 229533.A0A1C3YLG0 pp 229533.I1S1X2
52450 229533.A0A1C3YLG0 pp 229533.I1RYM4
52451 229533.A0A1C3YLG0 pp 229533.Q4HY90
52452 229533.I1RYV4 pp 229533.Q4HTT2
52453 229533.I1RYV4 pp 229533.Q4IER8
52454 229533.I1RYV4 pp 229533.V6QWB1
52455 229533.I1RYV4 pp 229533.I1S490
52456 229533.I1RYV4 pp 229533.Q4IF76
52457 229533.I1RYV4 pp 229533.I1S247
52458 229533.I1RYV4 pp 229533.Q4IAS1
52459 229533.I1RYV4 pp 229533.Q4IMD1
52460 229533.I1RYV4 pp 229533.I1S1U7
52461 229533.I1RYV4 pp 229533.Q4IJH1
52462 229533.I1RFD5 pp 229533.I1RZ17
52463 229533.I1RFD5 pp 229533.I1RRF6
52464 229533.I1RFD5 pp 229533.I1RMA0
52465 229533.I1RFD5 pp 229533.I1S0B4
52466 229533.I1RFD5 pp 229533.Q4ILH3
52467 229533.I1RFD5 pp 229533.I1RLL8
52468 229533.A0A0E0SGM9 pp 229533.I1RQ76
52469 229533.A0A0E0SGM9 pp 229533.I1RC91
52470 229533.A0A0E0SGM9 pp 229533.I1RVN2
52471 229533.A0A0E0SGM9 pp 229533.I1RQ72
52472 229533.A0A0E0SGM9 pp 229533.I1REE1
52473 229533.A0A0E0SGM9 pp 229533.A0A1C3YIG3
52474 229533.A0A0E0SGM9 pp 229533.I1RX77
52475 229533.A0A0E0SGM9 pp 229533.I1RQB4
52476 229533.A0A0E0SGM9 pp 229533.I1RXF0
52477 229533.A0A0E0SGM9 pp 229533.Q4INZ9
52478 229533.A0A0E0SGM9 pp 229533.I1RCB3
52479 229533.A0A0E0SGM9 pp 229533.I1RQ41
52480 229533.A0A0E0SGM9 pp 229533.A0A0E0SJ19
52481 229533.A0A0E0SGM9 pp 229533.I1RSS1
52482 229533.A0A0E0SGM9 pp 229533.I1RNF4
52483 229533.A0A0E0SGM9 pp 229533.Q4IEA7
52484 229533.A0A0E0SGM9 pp 229533.V6RLW2
52485 229533.A0A0E0SGM9 pp 229533.I1RD39
52486 229533.A0A0E0SGM9 pp 229533.I1RXX1
52487 229533.A0A0E0SGM9 pp 229533.I1RLX7

52488 229533.A0A0E0SGM9 pp 229533.I1RPK4
52489 229533.A0A0E0SGM9 pp 229533.I1S286
52490 229533.A0A0E0SGM9 pp 229533.I1RD18
52491 229533.A0A0E0SGM9 pp 229533.A0A1C3YJV7
52492 229533.A0A0E0SGM9 pp 229533.I1S253
52493 229533.A0A0E0SGM9 pp 229533.I1RFW0
52494 229533.A0A0E0RP32 pp 229533.I1RBM9
52495 229533.A0A0E0RP32 pp 229533.A0A1C3YLG0
52496 229533.A0A0E0RP32 pp 229533.I1RD01
52497 229533.A0A0E0RP32 pp 229533.I1RAL5
52498 229533.I1RSX8 pp 229533.I1RWT6
52499 229533.I1RY50 pp 229533.I1RZJ6
52500 229533.I1RY50 pp 229533.Q5I2J3
52501 229533.I1RY50 pp 229533.Q4IAA0
52502 229533.I1RY50 pp 229533.I1RYL5
52503 229533.A0A1C3YMF0 pp 229533.I1RT96
52504 229533.A0A1C3YMF0 pp 229533.I1RZR5
52505 229533.A0A1C3YMF0 pp 229533.V6RLW2
52506 229533.A0A1C3YMF0 pp 229533.I1RY11
52507 229533.I1RWY2 pp 229533.I1S0M9
52508 229533.I1RWY2 pp 229533.I1RYD3
52509 229533.I1S216 pp 229533.I1S2C4
52510 229533.I1S216 pp 229533.V6QT89
52511 229533.I1S216 pp 229533.Q4INS6
52512 229533.I1S216 pp 229533.V6RRH2
52513 229533.I1S216 pp 229533.V6R057
52514 229533.I1S216 pp 229533.V6R2A1
52515 229533.I1S216 pp 229533.V6RBN2
52516 229533.I1S216 pp 229533.I1S4Q4
52517 229533.I1S216 pp 229533.V6R1K4
52518 229533.A0A1C3YLI1 pp 229533.I1RW54
52519 229533.A0A1C3YLI1 pp 229533.I1RQP2
52520 229533.A0A1C3YLI1 pp 229533.I1RTG4
52521 229533.A0A1C3YLI1 pp 229533.I1RC74
52522 229533.A0A1C3YLI1 pp 229533.I1RZM4
52523 229533.A0A1C3YLI1 pp 229533.I1S3L2
52524 229533.A0A1C3YLI1 pp 229533.I1RY21
52525 229533.A0A1C3YLI1 pp 229533.I1S241
52526 229533.A0A1C3YLI1 pp 229533.I1RY49
52527 229533.A0A1C3YLI1 pp 229533.I1RQE8
52528 229533.A0A1C3YLI1 pp 229533.I1S0K0
52529 229533.A0A1C3YLI1 pp 229533.I1RDZ7
52530 229533.A0A1C3YLI1 pp 229533.I1RYX6
52531 229533.A0A1C3YLI1 pp 229533.I1RVX9
52532 229533.A0A1C3YLI1 pp 229533.I1RZ92
52533 229533.A0A1C3YLI1 pp 229533.I1S0N3
52534 229533.A0A1C3YLI1 pp 229533.I1RYU6
52535 229533.A0A1C3YLI1 pp 229533.I1S8C1
52536 229533.A0A1C3YLI1 pp 229533.I1RBE7
52537 229533.A0A1C3YLI1 pp 229533.I1RUR8
52538 229533.A0A1C3YLI1 pp 229533.I1RM26
52539 229533.A0A1C3YLI1 pp 229533.I1RCT7
52540 229533.A0A1C3YLI1 pp 229533.V6RDH3
52541 229533.A0A1C3YLI1 pp 229533.I1RVR2
52542 229533.A0A1C3YLI1 pp 229533.I1RBF3
52543 229533.A0A1C3YLI1 pp 229533.I1RJY7
52544 229533.A0A1C3YLI1 pp 229533.I1RE58
52545 229533.A0A1C3YLI1 pp 229533.I1RYL5
52546 229533.A0A1C3YLI1 pp 229533.I1RA90
52547 229533.A0A1C3YLI1 pp 229533.I1RX59
52548 229533.A0A1C3YLI1 pp 229533.I1RZK8
52549 229533.A0A1C3YLI1 pp 229533.I1RDE7
52550 229533.A0A1C3YLI1 pp 229533.I1RXS0
52551 229533.A0A1C3YLI1 pp 229533.I1RSK5
52552 229533.A0A1C3YLI1 pp 229533.I1RW43
52553 229533.A0A1C3YLI1 pp 229533.A0A1C3YLP6
52554 229533.A0A1C3YLI1 pp 229533.I1RZ29
52555 229533.A0A1C3YLI1 pp 229533.A0A1C3YMC0
52556 229533.A0A1C3YLI1 pp 229533.V6RK96
52557 229533.A0A1C3YLI1 pp 229533.I1RSS7
52558 229533.A0A1C3YLI1 pp 229533.I1RA94
52559 229533.A0A1C3YLI1 pp 229533.A0A1C3YM35
52560 229533.A0A1C3YLI1 pp 229533.I1RUI6

52561 229533.A0A1C3YLI1 pp 229533.I1S673
52562 229533.A0A1C3YLI1 pp 229533.I1RNG4
52563 229533.A0A1C3YLI1 pp 229533.I1RP97
52564 229533.A0A1C3YLI1 pp 229533.I1RAH3
52565 229533.A0A1C3YLI1 pp 229533.I1RHZ5
52566 229533.A0A1C3YLI1 pp 229533.I1RYK0
52567 229533.A0A1C3YLI1 pp 229533.I1RE53
52568 229533.A0A1C3YLI1 pp 229533.I1RVZ2
52569 229533.A0A1C3YLI1 pp 229533.I1RMY1
52570 229533.A0A1C3YLI1 pp 229533.I1RC48
52571 229533.A0A1C3YLI1 pp 229533.I1RFV9
52572 229533.A0A1C3YLI1 pp 229533.V6RM02
52573 229533.A0A1C3YLI1 pp 229533.I1RE52
52574 229533.A0A1C3YLI1 pp 229533.I1RCS6
52575 229533.A0A1C3YLI1 pp 229533.I1RMJ3
52576 229533.A0A1C3YLI1 pp 229533.V6RLM7
52577 229533.A0A1C3YLI1 pp 229533.V6R382
52578 229533.A0A1C3YLI1 pp 229533.I1RN84
52579 229533.A0A1C3YLI1 pp 229533.I1S274
52580 229533.A0A1C3YLI1 pp 229533.I1S8R4
52581 229533.A0A1C3YLI1 pp 229533.I1RXY7
52582 229533.I1RQ45 pp 229533.I1RTA0
52583 229533.I1RQ45 pp 229533.I1RZM1
52584 229533.I1RQ45 pp 229533.I1S8P5
52585 229533.A0A1C3YME4 pp 229533.I1RRN5
52586 229533.A0A1C3YME4 pp 229533.I1RT28
52587 229533.I1RQ76 pp 229533.I1RY81
52588 229533.I1RQ76 pp 229533.I1S1Q3
52589 229533.I1RQ76 pp 229533.I1RXX1
52590 229533.I1RQ76 pp 229533.I1RRQ1
52591 229533.I1RQ76 pp 229533.V6RR38
52592 229533.I1RQ76 pp 229533.I1RXB6
52593 229533.I1RQ76 pp 229533.I1RT23
52594 229533.I1RQ76 pp 229533.I1SAK7
52595 229533.I1RQ76 pp 229533.I1S2M5
52596 229533.I1RQ76 pp 229533.I1S130
52597 229533.I1RQ76 pp 229533.I1RZD0
52598 229533.I1RQ76 pp 229533.V6RPN0
52599 229533.I1RQ76 pp 229533.I1RZ92
52600 229533.I1RQ76 pp 229533.I1RZZ3
52601 229533.I1RQ76 pp 229533.V6RG22
52602 229533.I1RQ76 pp 229533.I1RY65
52603 229533.I1RQ76 pp 229533.I1RVN0
52604 229533.I1RQ76 pp 229533.I1S5K2
52605 229533.I1RQ76 pp 229533.V6RVJ3
52606 229533.I1RQ76 pp 229533.I1S2C2
52607 229533.I1RQ76 pp 229533.V6RIE5
52608 229533.I1RQ76 pp 229533.I1RR90
52609 229533.I1RQ76 pp 229533.I1S0Y2
52610 229533.I1RQ76 pp 229533.I1RWF4
52611 229533.I1RQ76 pp 229533.I1RSX9
52612 229533.I1RQ76 pp 229533.I1S1W6
52613 229533.I1RQ76 pp 229533.I1SAS4
52614 229533.I1RQ76 pp 229533.I1SAS3
52615 229533.I1RQ76 pp 229533.I1RY35
52616 229533.I1RMA0 pp 229533.I1S0B4
52617 229533.I1RMA0 pp 229533.I1RZ17
52618 229533.I1RMA0 pp 229533.Q4ILH3
52619 229533.I1RMA0 pp 229533.I1RRF6
52620 229533.I1RJI2 pp 229533.I1S053
52621 229533.I1RJI2 pp 229533.V6QXN0
52622 229533.I1RJI2 pp 229533.V6RIV7
52623 229533.I1RJI2 pp 229533.I1RMD6
52624 229533.I1RTT0 pp 229533.V6RAM6
52625 229533.I1RTT0 pp 229533.V6QU99
52626 229533.I1RTT0 pp 229533.V6R756
52627 229533.I1RTT0 pp 229533.V6R2Z2
52628 229533.I1RTT0 pp 229533.V6RS91
52629 229533.I1RTT0 pp 229533.V6R5D2
52630 229533.I1RTT0 pp 229533.V6R0W1
52631 229533.I1RTT0 pp 229533.V6RDH3
52632 229533.I1RTT0 pp 229533.I1RXH1
52633 229533.I1RTT0 pp 229533.V6QV31

229533.I1RTT0 pp 229533.I1RXY7
229533.I1RTT0 pp 229533.I1SAI4
229533.I1RTT0 pp 229533.I1S0C2
229533.I1RTT0 pp 229533.Q4I1N3
229533.I1RPI5 pp 229533.V6RUT5
229533.I1RPI5 pp 229533.I1RYI7
229533.I1RPI5 pp 229533.I1S3K6
229533.I1RPI5 pp 229533.I1RVR0
229533.I1RPI5 pp 229533.I1S338
229533.I1RPI5 pp 229533.I1RTU1
229533.I1RPI5 pp 229533.I1S887
229533.I1RPI5 pp 229533.I1S1W3
229533.I1RPI5 pp 229533.I1RXR1
229533.I1RPI5 pp 229533.I1RXU9
229533.I1RPI5 pp 229533.I1RUE2
229533.I1RPI5 pp 229533.I1RPK0
229533.I1RPI5 pp 229533.V6R382
229533.I1RPI5 pp 229533.I1RRG3
229533.I1RPI5 pp 229533.V6QVH8
229533.I1RPI5 pp 229533.I1S1B3
229533.I1RPI5 pp 229533.I1RTG7
229533.I1RPI5 pp 229533.Q4HXF0
229533.I1RPI5 pp 229533.I1RZ39
229533.I1RPI5 pp 229533.I1RTA1
229533.I1RPI5 pp 229533.O74638
229533.I1RPI5 pp 229533.I1RZB2
229533.A0A1C3YJW5 pp 229533.I1RZS5
229533.A0A1C3YJW5 pp 229533.A0A1C3YJY5
229533.I1REI0 pp 229533.I1S072
229533.I1REI0 pp 229533.I1RYL8
229533.I1REI0 pp 229533.I1RUP2
229533.I1REI0 pp 229533.I1S0H7
229533.I1REI0 pp 229533.I1RPK0
229533.I1REI0 pp 229533.I1RZQ1
229533.I1REI0 pp 229533.I1S334
229533.I1REI0 pp 229533.I1RYJ6
229533.I1REI0 pp 229533.I1RIY0
229533.I1REI0 pp 229533.Q4IMJ1
229533.I1REI0 pp 229533.I1S3H8
229533.I1REI0 pp 229533.I1RW06
229533.I1REI0 pp 229533.I1RFB4
229533.I1REI0 pp 229533.I1RQG7
229533.I1REI0 pp 229533.I1RNJ7
229533.I1REI0 pp 229533.I1RZ52
229533.I1REI0 pp 229533.I1RRL5
229533.I1REI0 pp 229533.I1RWS4
229533.I1REI0 pp 229533.I1REI3
229533.I1REI0 pp 229533.I1RJT2
229533.I1REI0 pp 229533.V6QVM5
229533.I1REI0 pp 229533.I1RXV3
229533.A0A1C3YJ87 pp 229533.A0A1C3YKR1
229533.I1RC11 pp 229533.I1RRX5
229533.I1RC11 pp 229533.I1RW71
229533.I1RC11 pp 229533.I1RZM4
229533.I1RC11 pp 229533.I1S0L3
229533.I1RC11 pp 229533.I1RC12
229533.I1RC11 pp 229533.I1RVV3
229533.I1RC11 pp 229533.I1RGG8
229533.I1RC11 pp 229533.I1RKB5
229533.I1RC11 pp 229533.I1RQE8
229533.I1RC11 pp 229533.I1RJN7
229533.I1RC11 pp 229533.I1RCU4
229533.I1RC11 pp 229533.I1RJD3
229533.I1RC11 pp 229533.I1RP86
229533.I1RC11 pp 229533.I1RUY5
229533.I1RC11 pp 229533.I1RSB7
229533.I1RC11 pp 229533.I1RJT5
229533.I1RC11 pp 229533.I1RCM5
229533.I1RC11 pp 229533.V6RK96
229533.I1RC11 pp 229533.I1RWM4
229533.I1RC11 pp 229533.I1RCV1
229533.I1RC11 pp 229533.I1RMW4
229533.I1RC11 pp 229533.I1RUY6

52707 229533.I1RC11 pp 229533.I1RKR5
52708 229533.I1RC11 pp 229533.I1RH82
52709 229533.I1RC11 pp 229533.I1RPT0
52710 229533.I1RC11 pp 229533.I1RDU0
52711 229533.I1RC11 pp 229533.I1RUY4
52712 229533.I1RC11 pp 229533.I1RPP3
52713 229533.I1RC11 pp 229533.I1S0V6
52714 229533.I1RC11 pp 229533.I1RQE0
52715 229533.I1RC11 pp 229533.I1S8P5
52716 229533.I1RC11 pp 229533.I1RGB2
52717 229533.I1RC11 pp 229533.I1RYK0
52718 229533.I1RC11 pp 229533.I1RT75
52719 229533.I1RC11 pp 229533.I1RMJ3
52720 229533.I1RC11 pp 229533.V6RUY9
52721 229533.I1RC11 pp 229533.I1RN88
52722 229533.I1RC11 pp 229533.I1RIY2
52723 229533.I1RC11 pp 229533.I1RX72
52724 229533.I1RC11 pp 229533.I1RU18
52725 229533.I1RC11 pp 229533.V6RV57
52726 229533.I1RC11 pp 229533.I1RMT6
52727 229533.I1RC11 pp 229533.I1SA27
52728 229533.I1RC11 pp 229533.I1RX89
52729 229533.I1RC11 pp 229533.I1RYW9
52730 229533.I1RC11 pp 229533.I1RSS7
52731 229533.I1RC11 pp 229533.I1RJQ5
52732 229533.I1RC11 pp 229533.I1RC31
52733 229533.I1RC11 pp 229533.I1RZ78
52734 229533.I1RC11 pp 229533.I1RC29
52735 229533.I1RC11 pp 229533.I1RPK0
52736 229533.I1RC11 pp 229533.I1RJZ7
52737 229533.I1RC11 pp 229533.I1RSQ4
52738 229533.I1RC11 pp 229533.I1S0F3
52739 229533.I1RC11 pp 229533.I1RN84
52740 229533.I1RC11 pp 229533.I1REI3
52741 229533.I1RC11 pp 229533.I1S0S5
52742 229533.I1RC11 pp 229533.V6RLM7
52743 229533.I1RC11 pp 229533.I1RTA0
52744 229533.I1RC11 pp 229533.I1RHJ2
52745 229533.I1RC11 pp 229533.I1RVX0
52746 229533.I1RC11 pp 229533.V6RRM0
52747 229533.I1RC11 pp 229533.I1RXY7
52748 229533.I1RC11 pp 229533.I1RNU7
52749 229533.I1RC11 pp 229533.I1RGX1
52750 229533.I1RC11 pp 229533.I1RND4
52751 229533.I1RC11 pp 229533.V6RRH2
52752 229533.I1RC11 pp 229533.I1S0V7
52753 229533.I1RC11 pp 229533.I1RMX6
52754 229533.I1RC11 pp 229533.I1RYW3
52755 229533.I1RC11 pp 229533.I1RCG5
52756 229533.I1RC11 pp 229533.I1S9T5
52757 229533.I1RRF6 pp 229533.I1RZ17
52758 229533.I1RRF6 pp 229533.Q4ILH3
52759 229533.I1RRF6 pp 229533.I1S0B4
52760 229533.I1RRF6 pp 229533.I1RTE5
52761 229533.I1RRF6 pp 229533.I1RX03
52762 229533.I1REV4 pp 229533.I1S685
52763 229533.I1REV4 pp 229533.I1RNG4
52764 229533.A0A098DCT9 pp 229533.A0A0E0RR40
52765 229533.A0A098DCT9 pp 229533.I1RB74
52766 229533.A0A098DCT9 pp 229533.A0A0E0SGM9
52767 229533.A0A098DCT9 pp 229533.I1RLX7
52768 229533.A0A098DCT9 pp 229533.I1RX70
52769 229533.A0A098DCT9 pp 229533.I1S216
52770 229533.A0A098DCT9 pp 229533.I1RB75
52771 229533.A0A098DCT9 pp 229533.I1RJK7
52772 229533.A0A098DCT9 pp 229533.I1S224
52773 229533.A0A098DCT9 pp 229533.I1RRW0
52774 229533.A0A098DCT9 pp 229533.I1RZJ6
52775 229533.A0A098DCT9 pp 229533.I1S0J7
52776 229533.A0A098DCT9 pp 229533.A0A1C3YM29
52777 229533.A0A098DCT9 pp 229533.I1RWT6
52778 229533.A0A098DCT9 pp 229533.I1RZE4
52779 229533.A0A098DCT9 pp 229533.I1RC91

52780	229533.A0A098DCT9	pp	229533.A0A1C3YHP8
52781	229533.A0A098DCT9	pp	229533.I1RQA3
52782	229533.A0A1C3YJ84	pp	229533.I1RQE1
52783	229533.A0A1C3YJ84	pp	229533.I1RBY9
52784	229533.A0A1C3YJ84	pp	229533.I1RNG4
52785	229533.I1RHK2	pp	229533.I1RW11
52786	229533.I1RHK2	pp	229533.V6R3J1
52787	229533.I1RHK2	pp	229533.I1RMJ3
52788	229533.I1RHK2	pp	229533.I1RYI5
52789	229533.I1RHK2	pp	229533.I1RSF1
52790	229533.I1RHK2	pp	229533.I1RVR2
52791	229533.I1RHK2	pp	229533.I1RPY0
52792	229533.I1RHK2	pp	229533.I1S7J8
52793	229533.I1RHK2	pp	229533.I1RY11
52794	229533.I1RHK2	pp	229533.I1S1W9
52795	229533.I1RHK2	pp	229533.V6QV31
52796	229533.I1RHK2	pp	229533.I1RN84
52797	229533.I1RHK2	pp	229533.I1RZJ3
52798	229533.I1RHK2	pp	229533.I1RQ85
52799	229533.I1RHK2	pp	229533.I1RRM3
52800	229533.I1RHK2	pp	229533.I1RRX5
52801	229533.I1RHK2	pp	229533.I1RZK8
52802	229533.I1RHK2	pp	229533.I1RX59
52803	229533.I1RHK2	pp	229533.I1RTJ2
52804	229533.I1RHK2	pp	229533.I1RMK7
52805	229533.I1RSC5	pp	229533.Q4IR08
52806	229533.I1RSC5	pp	229533.I1RZK5
52807	229533.I1RSC5	pp	229533.I1S0S5
52808	229533.I1RRR5	pp	229533.I1RX25
52809	229533.I1RRR5	pp	229533.I1S9G7
52810	229533.I1RRR5	pp	229533.V6RIE5
52811	229533.I1RRR5	pp	229533.I1RTF5
52812	229533.I1RD96	pp	229533.I1RMA0
52813	229533.I1RD96	pp	229533.I1RFD5
52814	229533.I1RD96	pp	229533.I1RZ17
52815	229533.I1RD96	pp	229533.Q4ILH3
52816	229533.I1RD96	pp	229533.I1RRF6
52817	229533.V6R6H5	pp	229533.V6RY56
52818	229533.A0A098DCT0	pp	229533.I1RE53
52819	229533.A0A098DCT0	pp	229533.I1REM2
52820	229533.I1RDH1	pp	229533.I1RTI7
52821	229533.I1RDH1	pp	229533.I1RNC2
52822	229533.A0A1C3YJ33	pp	229533.V6RN44
52823	229533.A0A1C3YJ33	pp	229533.I1S2I7
52824	229533.A0A1C3YJ33	pp	229533.I1RFV6
52825	229533.A0A1C3YJ33	pp	229533.I1RI71
52826	229533.A0A1C3YJ33	pp	229533.I1RNN7
52827	229533.A0A1C3YJ33	pp	229533.A0A1C3YLT9
52828	229533.A0A1C3YJ33	pp	229533.Q4I7N9
52829	229533.A0A1C3YJ33	pp	229533.I1RMA8
52830	229533.I1S088	pp	229533.V6R1X4
52831	229533.I1S088	pp	229533.Q4I099
52832	229533.I1S088	pp	229533.Q4IB65
52833	229533.I1S088	pp	229533.Q4HZ95
52834	229533.I1S088	pp	229533.Q4IJW4
52835	229533.I1S088	pp	229533.I1S8F0
52836	229533.I1RJY7	pp	229533.I1RNM7
52837	229533.I1RJY7	pp	229533.I1RQP2
52838	229533.I1RJY7	pp	229533.I1RMA2
52839	229533.I1RJY7	pp	229533.I1RMK7
52840	229533.I1RJY7	pp	229533.I1RY21
52841	229533.I1RJY7	pp	229533.I1RW54
52842	229533.I1RJY7	pp	229533.I1RRG1
52843	229533.I1RJY7	pp	229533.I1RWL3
52844	229533.I1RJY7	pp	229533.I1RT82
52845	229533.I1RJY7	pp	229533.I1RMW9
52846	229533.I1RJY7	pp	229533.I1RVI8
52847	229533.I1RJY7	pp	229533.I1S3L2
52848	229533.I1RJY7	pp	229533.Q4IA56
52849	229533.I1RJY7	pp	229533.I1RY81
52850	229533.I1RJY7	pp	229533.I1RQE8
52851	229533.I1RJY7	pp	229533.I1RT09
52852	229533.I1RJY7	pp	229533.I1S8E0

52853	229533.I1RJY7	pp	229533.I1S8F8
52854	229533.I1RJY7	pp	229533.I1S0K2
52855	229533.I1RJY7	pp	229533.I1RYP5
52856	229533.I1RJY7	pp	229533.I1RXS0
52857	229533.I1RJY7	pp	229533.I1S8C1
52858	229533.I1RJY7	pp	229533.V6RK96
52859	229533.I1RJY7	pp	229533.I1RUQ3
52860	229533.I1RJY7	pp	229533.I1RRX5
52861	229533.I1RJY7	pp	229533.I1S0K0
52862	229533.I1RJY7	pp	229533.I1RQE9
52863	229533.I1RJY7	pp	229533.I1RZM4
52864	229533.I1RJY7	pp	229533.I1RY12
52865	229533.I1RJY7	pp	229533.I1RYI6
52866	229533.I1RJY7	pp	229533.I1RXE6
52867	229533.I1RJY7	pp	229533.I1RUY4
52868	229533.I1RJY7	pp	229533.I1RST9
52869	229533.I1RJY7	pp	229533.I1S0V6
52870	229533.I1RJY7	pp	229533.I1RWM4
52871	229533.I1RJY7	pp	229533.I1RYA2
52872	229533.I1RJY7	pp	229533.V6R5D2
52873	229533.I1RJY7	pp	229533.I1RP86
52874	229533.I1RJY7	pp	229533.I1RXZ4
52875	229533.I1RJY7	pp	229533.V6RPS5
52876	229533.I1RJY7	pp	229533.I1RNY6
52877	229533.I1RJY7	pp	229533.I1S489
52878	229533.I1RJY7	pp	229533.I1RPR1
52879	229533.I1RJY7	pp	229533.I1RKK5
52880	229533.I1RJY7	pp	229533.I1RSK5
52881	229533.I1RJY7	pp	229533.I1RUR8
52882	229533.I1RJY7	pp	229533.I1RY62
52883	229533.I1RJY7	pp	229533.I1S0B8
52884	229533.I1RJY7	pp	229533.I1RK55
52885	229533.I1RJY7	pp	229533.I1RW82
52886	229533.I1RJY7	pp	229533.I1RSS7
52887	229533.I1RJY7	pp	229533.I1RN88
52888	229533.I1RJY7	pp	229533.I1RY35
52889	229533.I1RJY7	pp	229533.V6R3J1
52890	229533.I1RJY7	pp	229533.I1RVA7
52891	229533.I1RJY7	pp	229533.I1RVR2
52892	229533.I1RJY7	pp	229533.I1RYW9
52893	229533.I1RJY7	pp	229533.I1RSL0
52894	229533.I1RJY7	pp	229533.I1S968
52895	229533.I1RJY7	pp	229533.I1RLZ4
52896	229533.I1RJY7	pp	229533.I1RMJ3
52897	229533.I1RJY7	pp	229533.I1RL81
52898	229533.I1RJY7	pp	229533.I1RKB2
52899	229533.I1RJY7	pp	229533.I1RWF5
52900	229533.I1RJY7	pp	229533.I1S3T7
52901	229533.I1RJY7	pp	229533.I1RYK0
52902	229533.I1RJY7	pp	229533.I1S2Q4
52903	229533.I1RJY7	pp	229533.V6RUY9
52904	229533.I1RJY7	pp	229533.I1RR68
52905	229533.I1RJY7	pp	229533.I1RQP8
52906	229533.I1RJY7	pp	229533.I1S2C4
52907	229533.I1RJY7	pp	229533.I1RZV5
52908	229533.I1RJY7	pp	229533.I1S299
52909	229533.I1RJY7	pp	229533.I1RQ21
52910	229533.I1RJY7	pp	229533.I1RYC8
52911	229533.I1RJY7	pp	229533.I1S256
52912	229533.I1RJY7	pp	229533.I1S149
52913	229533.I1RJY7	pp	229533.I1RXY7
52914	229533.I1RJY7	pp	229533.I1RYA0
52915	229533.I1RJY7	pp	229533.I1S8R2
52916	229533.I1RJY7	pp	229533.V6RN69
52917	229533.I1RJY7	pp	229533.V6RLM7
52918	229533.I1RJY7	pp	229533.V6RRM0
52919	229533.I1RJY7	pp	229533.V6R382
52920	229533.I1RJY7	pp	229533.Q4HWE2
52921	229533.I1RJY7	pp	229533.I1RMQ5
52922	229533.I1RJY7	pp	229533.I1RPK0
52923	229533.I1RJY7	pp	229533.V6RM02
52924	229533.I1RJY7	pp	229533.I1RML4
52925	229533.I1RJY7	pp	229533.I1S8R4

229533.I1RJY7 pp 229533.I1S0V7
229533.I1RJY7 pp 229533.I1RN84
229533.I1RYU5 pp 229533.I1S1W8
229533.I1RYU5 pp 229533.V6RQW4
229533.I1RYU5 pp 229533.I1S4F4
229533.I1RYU5 pp 229533.I1SAJ0
229533.I1RYU5 pp 229533.I1S9D4
229533.I1RYU5 pp 229533.I1S029
229533.I1RYU5 pp 229533.I1S5G3
229533.I1RYU5 pp 229533.I1S8X1
229533.I1RYU5 pp 229533.V6RY56
229533.I1RYU5 pp 229533.I1S4D0
229533.I1RYU5 pp 229533.I1RZ20
229533.I1RYU5 pp 229533.I1S5L5
229533.I1RYU5 pp 229533.I1S076
229533.I1RYU5 pp 229533.I1S0N8
229533.I1RYU5 pp 229533.O74638
229533.I1RYU5 pp 229533.I1S2E2
229533.I1RYU5 pp 229533.Q4HXF0
229533.I1RYU5 pp 229533.I1S3G4
229533.I1RYU5 pp 229533.I1S2C4
229533.I1RYU5 pp 229533.Q4IBD0
229533.I1RYU5 pp 229533.I1S2C5
229533.I1RYU5 pp 229533.I1RYW6
229533.I1RYU5 pp 229533.I1S2H3
229533.I1RYU5 pp 229533.I1RZ52
229533.I1RYU5 pp 229533.I1S6H1
229533.I1RYU5 pp 229533.I1S770
229533.I1RR12 pp 229533.I1RVZ3
229533.I1RR12 pp 229533.I1RZA7
229533.I1RR12 pp 229533.I1RTU0
229533.I1RR12 pp 229533.I1S3F3
229533.I1RR12 pp 229533.I1RW54
229533.I1RR12 pp 229533.I1RUV4
229533.I1RR12 pp 229533.I1S6P5
229533.I1RR12 pp 229533.I1S5L5
229533.I1RR12 pp 229533.I1RR68
229533.I1RR12 pp 229533.V6RUY9
229533.I1RR12 pp 229533.I1S3X7
229533.I1RR12 pp 229533.I1RWM4
229533.I1RR12 pp 229533.V6RRM0
229533.I1RR12 pp 229533.V6RUB1
229533.I1RIY2 pp 229533.I1RP86
229533.I1RIY2 pp 229533.I1RJ17
229533.I1RIY2 pp 229533.I1RY27
229533.I1RIY2 pp 229533.I1RRD0
229533.I1RIY2 pp 229533.I1RSB7
229533.I1RIY2 pp 229533.I1RU18
229533.I1RIY2 pp 229533.I1RVV3
229533.I1RDU5 pp 229533.I1S8D2
229533.I1RDU5 pp 229533.I1RVZ3
229533.I1RDU5 pp 229533.I1RNG4
229533.I1RDU5 pp 229533.I1RY35
229533.I1RDU5 pp 229533.I1RN88
229533.I1RDU5 pp 229533.I1RSS7
229533.I1RDU5 pp 229533.I1S148
229533.I1RDU5 pp 229533.I1RZ25
229533.I1RDU5 pp 229533.I1RWN7
229533.I1RDU5 pp 229533.I1RGC3
229533.I1RDU5 pp 229533.I1S8D1
229533.I1RDU5 pp 229533.I1S130
229533.I1RDU5 pp 229533.I1S1K6
229533.I1RDU5 pp 229533.I1RI19
229533.I1RDU5 pp 229533.I1RH92
229533.I1RDU5 pp 229533.I1S147
229533.I1RDU5 pp 229533.I1RZ70
229533.I1RDU5 pp 229533.I1RJG4
229533.I1RDU5 pp 229533.I1RYX6
229533.I1RDU5 pp 229533.I1RQE1
229533.I1RDU5 pp 229533.I1RYD3
229533.I1RDU5 pp 229533.I1RLA2
229533.I1RDU5 pp 229533.I1RGK9
229533.I1RDU5 pp 229533.I1RL16

52999 229533.I1RDU5 pp 229533.I1RST9
53000 229533.I1RDU5 pp 229533.V6RG22
53001 229533.I1RDU5 pp 229533.V6RH92
53002 229533.I1RDU5 pp 229533.I1S048
53003 229533.I1RDU5 pp 229533.I1RWM4
53004 229533.I1RDU5 pp 229533.I1RXX1
53005 229533.I1RDU5 pp 229533.I1S267
53006 229533.I1RDU5 pp 229533.I1RTJ2
53007 229533.I1RDU5 pp 229533.I1RYM5
53008 229533.I1S0Y1 pp 229533.Q4IBU4
53009 229533.I1S0Y1 pp 229533.I1S1D0
53010 229533.I1S0Y1 pp 229533.I1S0Y4
53011 229533.I1RQ14 pp 229533.Q4HXF0
53012 229533.I1RQ14 pp 229533.V6RLM7
53013 229533.I1RQ14 pp 229533.I1RW43
53014 229533.I1RQ14 pp 229533.I1RYK0
53015 229533.I1RQ14 pp 229533.I1RTA9
53016 229533.I1RQ14 pp 229533.I1S9Z4
53017 229533.I1RQ14 pp 229533.V6RPQ8
53018 229533.I1RQ14 pp 229533.I1RQ29
53019 229533.I1RQ14 pp 229533.I1RU18
53020 229533.I1RQ14 pp 229533.Q4I1Y5
53021 229533.I1RQ14 pp 229533.I1RQG5
53022 229533.I1RQ14 pp 229533.I1S1P8
53023 229533.I1RQ14 pp 229533.I1RVX9
53024 229533.I1RQ14 pp 229533.I1RVV3
53025 229533.I1RQ14 pp 229533.I1S029
53026 229533.I1RQ14 pp 229533.I1S084
53027 229533.I1RQ14 pp 229533.I1RX90
53028 229533.I1RQ14 pp 229533.I1S2C4
53029 229533.I1RQ14 pp 229533.I1S3D9
53030 229533.I1RQ14 pp 229533.I1RSB7
53031 229533.I1RQ14 pp 229533.I1RTT4
53032 229533.I1RQ14 pp 229533.I1S4D0
53033 229533.I1RQ14 pp 229533.V6RY56
53034 229533.A0A0E0S3R4 pp 229533.I1RPW8
53035 229533.A0A0E0S3R4 pp 229533.I1RN04
53036 229533.A0A0E0S3R4 pp 229533.I1RW91
53037 229533.A0A0E0S3R4 pp 229533.I1RRF7
53038 229533.A0A0E0S3R4 pp 229533.I1RCG6
53039 229533.I1RJX7 pp 229533.I1RMJ8
53040 229533.I1RJX7 pp 229533.I1RWI8
53041 229533.I1RMK4 pp 229533.I1S0T4
53042 229533.I1RMK4 pp 229533.I1S0S2
53043 229533.I1RMK4 pp 229533.V6RLM7
53044 229533.I1RMK4 pp 229533.I1RNV4
53045 229533.I1RMK4 pp 229533.I1RNB4
53046 229533.I1RMK4 pp 229533.I1S0A5
53047 229533.I1RMK4 pp 229533.I1RZ56
53048 229533.I1RMK4 pp 229533.I1RPV8
53049 229533.I1RMK4 pp 229533.I1S0Y7
53050 229533.I1RMK4 pp 229533.I1S3V2
53051 229533.I1RMK4 pp 229533.I1RPD6
53052 229533.I1RMK4 pp 229533.I1RZ83
53053 229533.I1RMK4 pp 229533.I1RNG4
53054 229533.I1RMK4 pp 229533.I1S3G4
53055 229533.I1RMK4 pp 229533.I1RZ29
53056 229533.I1RMK4 pp 229533.I1RP97
53057 229533.I1RMK4 pp 229533.I1RXW3
53058 229533.I1RMK4 pp 229533.I1RSS7
53059 229533.I1RMK4 pp 229533.Q4HXF0
53060 229533.I1RMK4 pp 229533.I1RZS3
53061 229533.I1RMK4 pp 229533.I1RPW1
53062 229533.I1RMK4 pp 229533.I1RXX1
53063 229533.I1RMK4 pp 229533.V6RQW4
53064 229533.I1RMK4 pp 229533.I1RXV9
53065 229533.I1RMK4 pp 229533.I1RSX2
53066 229533.I1RMK4 pp 229533.I1RYX6
53067 229533.I1RMK4 pp 229533.I1S0A0
53068 229533.I1RMK4 pp 229533.I1RVX9
53069 229533.I1RMK4 pp 229533.I1RXX0
53070 229533.I1RMK4 pp 229533.I1S1W8
53071 229533.I1RMK4 pp 229533.I1RPM6

53072 229533.I1RMK4 pp 229533.I1RRV4
53073 229533.I1RMK4 pp 229533.I1RY49
53074 229533.I1RMK4 pp 229533.I1RQ60
53075 229533.I1RMK4 pp 229533.I1S094
53076 229533.I1RMK4 pp 229533.I1S6H3
53077 229533.I1RMK4 pp 229533.I1RYQ4
53078 229533.I1RA25 pp 229533.I1RZ60
53079 229533.I1RA25 pp 229533.I1SAM5
53080 229533.I1RA25 pp 229533.I1RW76
53081 229533.I1RA25 pp 229533.Q4HYZ2
53082 229533.I1RA25 pp 229533.I1RWP4
53083 229533.I1RA25 pp 229533.I1RTG5
53084 229533.I1RA25 pp 229533.I1RPL3
53085 229533.I1RA25 pp 229533.I1RCN6
53086 229533.I1RA25 pp 229533.I1RZS5
53087 229533.I1RA25 pp 229533.I1RBZ7
53088 229533.I1S338 pp 229533.V6R382
53089 229533.I1S338 pp 229533.I1S887
53090 229533.I1S338 pp 229533.V6RUT5
53091 229533.I1S338 pp 229533.I1S3K6
53092 229533.I1S338 pp 229533.O74638
53093 229533.I1S338 pp 229533.Q4HXF0
53094 229533.I1S338 pp 229533.V6QVH8
53095 229533.A0A1C3YI71 pp 229533.I1RC31
53096 229533.A0A1C3YI71 pp 229533.I1RA62
53097 229533.A0A1C3YI71 pp 229533.I1S352
53098 229533.A0A1C3YI71 pp 229533.I1RKP3
53099 229533.A0A1C3YI71 pp 229533.I1RNR2
53100 229533.A0A1C3YI71 pp 229533.I1RLN6
53101 229533.A0A1C3YI71 pp 229533.I1RP27
53102 229533.A0A1C3YI71 pp 229533.I1RBY9
53103 229533.A0A1C3YI71 pp 229533.I1R9D1
53104 229533.A0A1C3YI71 pp 229533.I1S0R3
53105 229533.A0A1C3YI71 pp 229533.I1RRV1
53106 229533.A0A1C3YI71 pp 229533.I1RNA5
53107 229533.A0A1C3YI71 pp 229533.I1RGF5
53108 229533.A0A1C3YI71 pp 229533.I1RJZ7
53109 229533.A0A1C3YI71 pp 229533.I1RZ13
53110 229533.A0A1C3YI71 pp 229533.I1RQ14
53111 229533.A0A1C3YI71 pp 229533.I1RNA1
53112 229533.A0A1C3YI71 pp 229533.I1S569
53113 229533.A0A1C3YI71 pp 229533.V6R4J1
53114 229533.A0A1C3YI71 pp 229533.I1RRT3
53115 229533.A0A1C3YI71 pp 229533.I1RS20
53116 229533.A0A1C3YI71 pp 229533.I1RGL4
53117 229533.A0A1C3YI71 pp 229533.I1R9J6
53118 229533.A0A1C3YI71 pp 229533.I1RDU4
53119 229533.A0A1C3YI71 pp 229533.I1RIL3
53120 229533.A0A1C3YI71 pp 229533.A0A1C3YKK9
53121 229533.A0A1C3YI71 pp 229533.I1RW71
53122 229533.A0A1C3YI71 pp 229533.I1RH91
53123 229533.A0A1C3YI71 pp 229533.I1RN61
53124 229533.A0A1C3YI71 pp 229533.I1RIL8
53125 229533.A0A1C3YI71 pp 229533.A0A1C3YK73
53126 229533.A0A1C3YI71 pp 229533.I1RCC2
53127 229533.A0A1C3YI71 pp 229533.I1RCA0
53128 229533.A0A1C3YI71 pp 229533.I1RZ29
53129 229533.A0A1C3YI71 pp 229533.I1RMX6
53130 229533.A0A1C3YI71 pp 229533.I1S0R4
53131 229533.A0A1C3YI71 pp 229533.I1RP86
53132 229533.A0A1C3YI71 pp 229533.I1S272
53133 229533.A0A1C3YI71 pp 229533.I1RME6
53134 229533.A0A1C3YI71 pp 229533.I1RIX0
53135 229533.A0A1C3YI71 pp 229533.I1RMA7
53136 229533.A0A1C3YI71 pp 229533.V6RAN2
53137 229533.A0A1C3YI71 pp 229533.I1RC72
53138 229533.A0A1C3YI71 pp 229533.I1RW07
53139 229533.A0A1C3YI71 pp 229533.I1RRQ1
53140 229533.A0A1C3YI71 pp 229533.I1RK45
53141 229533.A0A1C3YI71 pp 229533.I1RPW4
53142 229533.A0A1C3YI71 pp 229533.I1RI02
53143 229533.A0A1C3YI71 pp 229533.I1RLC7
53144 229533.A0A1C3YI71 pp 229533.I1RDJ1

53145	229533.A0A1C3YI71	pp	229533.A0A1C3YLZ7
53146	229533.A0A1C3YI71	pp	229533.I1S021
53147	229533.A0A1C3YI71	pp	229533.I1RQE0
53148	229533.A0A1C3YI71	pp	229533.I1RBE7
53149	229533.A0A1C3YI71	pp	229533.I1RSJ2
53150	229533.A0A1C3YI71	pp	229533.I1RBN0
53151	229533.A0A1C3YI71	pp	229533.I1S984
53152	229533.A0A1C3YI71	pp	229533.I1RLM7
53153	229533.A0A1C3YI71	pp	229533.I1RBJ5
53154	229533.A0A1C3YI71	pp	229533.I1RQ81
53155	229533.A0A1C3YI71	pp	229533.I1RB14
53156	229533.A0A1C3YI71	pp	229533.I1S574
53157	229533.A0A1C3YI71	pp	229533.I1RKR5
53158	229533.A0A1C3YI71	pp	229533.I1RDE4
53159	229533.A0A1C3YI71	pp	229533.V6RBN2
53160	229533.A0A1C3YI71	pp	229533.I1R9V7
53161	229533.A0A1C3YI71	pp	229533.I1RQ31
53162	229533.A0A1C3YI71	pp	229533.I1RU29
53163	229533.A0A1C3YI71	pp	229533.I1RI03
53164	229533.I1S0V4	pp	229533.I1S4S4
53165	229533.I1S0V4	pp	229533.V6R0U9
53166	229533.A0A0E0S3L9	pp	229533.I1RHY9
53167	229533.A0A0E0S3L9	pp	229533.I1RZ25
53168	229533.I1S0T2	pp	229533.I1S419
53169	229533.I1S0T2	pp	229533.I1S887
53170	229533.I1S0T2	pp	229533.V6RCZ8
53171	229533.I1S0T2	pp	229533.Q4IJK2
53172	229533.I1S0T2	pp	229533.Q4I061
53173	229533.I1S0T2	pp	229533.V6R227
53174	229533.I1S0T2	pp	229533.V6QUE6
53175	229533.I1S0T2	pp	229533.I1S1J7
53176	229533.I1S0T2	pp	229533.V6RCG5
53177	229533.I1S0T2	pp	229533.V6RTJ8
53178	229533.I1S0T2	pp	229533.Q4I327
53179	229533.I1S0T2	pp	229533.I1S537
53180	229533.I1S0T2	pp	229533.V6RUT5
53181	229533.I1S0T2	pp	229533.I1S5B0
53182	229533.I1S0T2	pp	229533.I1S2C0
53183	229533.I1S0T2	pp	229533.V6QW22
53184	229533.I1S0T2	pp	229533.V6R042
53185	229533.I1S0T2	pp	229533.I1S1C7
53186	229533.I1S0T2	pp	229533.V6RMW6
53187	229533.I1S0T2	pp	229533.I1S4S7
53188	229533.I1S0T2	pp	229533.Q4I624
53189	229533.I1S0T2	pp	229533.V6RGN5
53190	229533.I1S0T2	pp	229533.I1S5R1
53191	229533.I1S0T2	pp	229533.I1S0Y2
53192	229533.I1S0T2	pp	229533.I1S5R6
53193	229533.I1S0T2	pp	229533.I1S6Y8
53194	229533.I1S0T2	pp	229533.I1S2B8
53195	229533.I1S0T2	pp	229533.V6RIV9
53196	229533.I1S0T2	pp	229533.I1S2H4
53197	229533.I1S0T2	pp	229533.I1S1W3
53198	229533.I1S0T2	pp	229533.Q4HXF0
53199	229533.I1RZP0	pp	229533.Q4IB89
53200	229533.I1RZP0	pp	229533.I1S7M8
53201	229533.A0A1C3YHV8	pp	229533.I1RY27
53202	229533.A0A1C3YHV8	pp	229533.I1RP86
53203	229533.A0A1C3YHV8	pp	229533.I1RJ17
53204	229533.A0A1C3YHV8	pp	229533.I1RA62
53205	229533.A0A1C3YHV8	pp	229533.I1RRD0
53206	229533.A0A1C3YHV8	pp	229533.I1RU18
53207	229533.A0A1C3YHV8	pp	229533.I1RSB7
53208	229533.A0A1C3YHV8	pp	229533.I1RC11
53209	229533.A0A1C3YHV8	pp	229533.I1RIY2
53210	229533.A0A1C3YHV8	pp	229533.I1RVV3
53211	229533.A0A1C3YHV8	pp	229533.I1RAQ4
53212	229533.A0A1C3YHV8	pp	229533.I1RCG5
53213	229533.A0A0E0S3I7	pp	229533.I1RNA1
53214	229533.A0A0E0S3I7	pp	229533.I1S984
53215	229533.A0A0E0S3I7	pp	229533.I1S574
53216	229533.I1RCY0	pp	229533.I1S0Z3
53217	229533.I1RCY0	pp	229533.Q4I7X1

53218	229533.I1RCY0	pp	229533.I1RS52
53219	229533.I1RCY0	pp	229533.I1RPU4
53220	229533.I1RCY0	pp	229533.Q4I0M9
53221	229533.I1RCY0	pp	229533.I1RZP6
53222	229533.I1RCY0	pp	229533.I1RN65
53223	229533.I1RCY0	pp	229533.Q4IQC1
53224	229533.I1RCY0	pp	229533.I1S050
53225	229533.I1RCY0	pp	229533.I1RME6
53226	229533.I1RCY0	pp	229533.I1S0R7
53227	229533.I1RCY0	pp	229533.I1RT41
53228	229533.I1RCY0	pp	229533.I1RM67
53229	229533.I1RCY0	pp	229533.V6REE9
53230	229533.I1RCY0	pp	229533.I1RN49
53231	229533.I1RCY0	pp	229533.I1RQN8
53232	229533.I1RCY0	pp	229533.Q4INW6
53233	229533.I1RCY0	pp	229533.I1RNB8
53234	229533.I1RCY0	pp	229533.I1RMP2
53235	229533.I1RCY0	pp	229533.I1RP28
53236	229533.I1RCY0	pp	229533.I1RD70
53237	229533.I1RCY0	pp	229533.Q4IPC8
53238	229533.I1RCY0	pp	229533.I1RE19
53239	229533.I1RCY0	pp	229533.I1RGQ9
53240	229533.I1RCY0	pp	229533.I1RP04
53241	229533.I1RCY0	pp	229533.I1RTK5
53242	229533.I1RCY0	pp	229533.I1S7X9
53243	229533.I1RCY0	pp	229533.Q4ICG8
53244	229533.I1RCY0	pp	229533.I1RKR7
53245	229533.I1RCY0	pp	229533.I1RZG0
53246	229533.I1S1D0	pp	229533.V6QW25
53247	229533.I1RH36	pp	229533.I1RVX9
53248	229533.I1RH36	pp	229533.I1RJA8
53249	229533.I1RH36	pp	229533.I1RM26
53250	229533.I1RH36	pp	229533.I1S7B8
53251	229533.I1RH36	pp	229533.I1S6P6
53252	229533.I1RH36	pp	229533.V6RGB5
53253	229533.I1RH36	pp	229533.I1RNG4
53254	229533.I1RH36	pp	229533.I1S0A0
53255	229533.I1RH36	pp	229533.I1RW69
53256	229533.I1RH36	pp	229533.Q4IPC8
53257	229533.I1RH36	pp	229533.I1RNG7
53258	229533.I1RPA5	pp	229533.I1RT38
53259	229533.I1RPA5	pp	229533.I1RS22
53260	229533.I1RPA5	pp	229533.I1RZW1
53261	229533.I1RPA5	pp	229533.I1RVN1
53262	229533.I1RMD9	pp	229533.I1RY27
53263	229533.A0A0E0RNI4	pp	229533.V6RRM0
53264	229533.A0A0E0RNI4	pp	229533.I1RIJ5
53265	229533.A0A0E0RNI4	pp	229533.I1RFK3
53266	229533.A0A0E0RNI4	pp	229533.A0A1C3YIT6
53267	229533.A0A0E0RNI4	pp	229533.I1RSU2
53268	229533.A0A0E0RNI4	pp	229533.I1RPZ5
53269	229533.A0A0E0RNI4	pp	229533.I1RL81
53270	229533.A0A0E0RNI4	pp	229533.I1RNG4
53271	229533.A0A0E0RNI4	pp	229533.I1S147
53272	229533.A0A0E0RNI4	pp	229533.I1RWL5
53273	229533.A0A0E0RNI4	pp	229533.I1S0A5
53274	229533.A0A0E0RNI4	pp	229533.I1RI34
53275	229533.A0A0E0RNI4	pp	229533.I1RYA0
53276	229533.A0A0E0RNI4	pp	229533.I1RC31
53277	229533.A0A0E0RNI4	pp	229533.I1RP97
53278	229533.A0A0E0RNI4	pp	229533.I1RZ70
53279	229533.A0A0E0RNI4	pp	229533.I1RZV5
53280	229533.A0A0E0RNI4	pp	229533.I1RAN1
53281	229533.A0A0E0RNI4	pp	229533.I1S8R2
53282	229533.A0A0E0RNI4	pp	229533.I1RR68
53283	229533.A0A0E0RNI4	pp	229533.V6RUY9
53284	229533.A0A0E0RNI4	pp	229533.A0A0E0RU55
53285	229533.A0A0E0RNI4	pp	229533.I1RNU7
53286	229533.A0A0E0RNI4	pp	229533.I1RCG5
53287	229533.A0A0E0RNI4	pp	229533.I1RZA8
53288	229533.A0A0E0RNI4	pp	229533.V6RLM7
53289	229533.A0A0E0RNI4	pp	229533.A0A1C3YLE2
53290	229533.A0A0E0RNI4	pp	229533.A0A1C3YKW4

53291 229533.A0A0E0RNI4 pp 229533.I1RF85
53292 229533.A0A0E0RNI4 pp 229533.I1RH92
53293 229533.A0A0E0RNI4 pp 229533.A0A1C3YK33
53294 229533.A0A0E0RNI4 pp 229533.I1RRP7
53295 229533.A0A0E0RNI4 pp 229533.I1RWF5
53296 229533.A0A0E0RNI4 pp 229533.I1RHJ2
53297 229533.A0A0E0RNI4 pp 229533.A0A1C3YLB6
53298 229533.A0A0E0RNI4 pp 229533.I1S968
53299 229533.A0A0E0RNI4 pp 229533.A0A1C3YHU3
53300 229533.A0A0E0RNI4 pp 229533.I1RJS1
53301 229533.A0A0E0RNI4 pp 229533.I1RZ56
53302 229533.A0A0E0RNI4 pp 229533.I1S299
53303 229533.A0A0E0RNI4 pp 229533.A0A1C3YJP4
53304 229533.A0A0E0RNI4 pp 229533.I1RBY9
53305 229533.A0A0E0RNI4 pp 229533.I1S148
53306 229533.A0A0E0RNI4 pp 229533.I1S8D1
53307 229533.A0A0E0RNI4 pp 229533.I1RWN7
53308 229533.A0A0E0RNI4 pp 229533.I1RYK0
53309 229533.A0A0E0RNI4 pp 229533.I1RPV8
53310 229533.A0A0E0RNI4 pp 229533.A0A1C3YN78
53311 229533.A0A0E0RNI4 pp 229533.I1RA62
53312 229533.A0A0E0RNI4 pp 229533.I1RKB2
53313 229533.A0A0E0RNI4 pp 229533.I1RN88
53314 229533.A0A0E0RNI4 pp 229533.I1RYC8
53315 229533.A0A0E0RNI4 pp 229533.A0A1C3YHG7
53316 229533.A0A0E0RNI4 pp 229533.I1RVZ3
53317 229533.A0A0E0RNI4 pp 229533.I1RFI4
53318 229533.A0A0E0RNI4 pp 229533.I1RGC3
53319 229533.A0A0E0RNI4 pp 229533.I1RK55
53320 229533.A0A0E0RNI4 pp 229533.I1RMJ3
53321 229533.A0A0E0RNI4 pp 229533.I1RCR3
53322 229533.A0A0E0RNI4 pp 229533.I1S8D2
53323 229533.A0A0E0RNI4 pp 229533.I1RN84
53324 229533.A0A0E0RNI4 pp 229533.I1RH91
53325 229533.A0A0E0RNI4 pp 229533.I1S694
53326 229533.A0A0E0RNI4 pp 229533.I1RUR8
53327 229533.A0A0E0RNI4 pp 229533.I1S969
53328 229533.A0A0E0RNI4 pp 229533.I1S3X7
53329 229533.A0A0E0RNI4 pp 229533.I1RPE7
53330 229533.A0A0E0RNI4 pp 229533.I1S3T7
53331 229533.A0A0E0RNI4 pp 229533.I1REE2
53332 229533.A0A0E0RNI4 pp 229533.I1RXB1
53333 229533.A0A0E0RNI4 pp 229533.I1RA13
53334 229533.A0A0E0RNI4 pp 229533.A0A0E0RZZ3
53335 229533.A0A0E0RNI4 pp 229533.I1RI11
53336 229533.A0A0E0RNI4 pp 229533.I1RCU2
53337 229533.A0A0E0RNI4 pp 229533.A0A1C3YKR5
53338 229533.A0A0E0RNI4 pp 229533.I1RBI1
53339 229533.A0A0E0RNI4 pp 229533.I1RDU0
53340 229533.A0A0E0RNI4 pp 229533.I1RTU0
53341 229533.A0A0E0RNI4 pp 229533.I1RR12
53342 229533.A0A0E0RNI4 pp 229533.I1RNH4
53343 229533.A0A0E0RNI4 pp 229533.I1RJF2
53344 229533.A0A0E0RNI4 pp 229533.I1RA90
53345 229533.A0A0E0RNI4 pp 229533.I1RYX6
53346 229533.A0A0E0RNI4 pp 229533.I1S8F8
53347 229533.A0A0E0RNI4 pp 229533.I1RG33
53348 229533.A0A0E0RNI4 pp 229533.I1RQE0
53349 229533.A0A0E0RNI4 pp 229533.I1S8C1
53350 229533.A0A0E0RNI4 pp 229533.I1RJY7
53351 229533.A0A0E0RNI4 pp 229533.I1RY81
53352 229533.A0A0E0RNI4 pp 229533.A0A1C3YJZ0
53353 229533.A0A0E0RNI4 pp 229533.I1RPR1
53354 229533.A0A0E0RNI4 pp 229533.I1RUV4
53355 229533.A0A0E0RNI4 pp 229533.A0A1C3YK58
53356 229533.A0A0E0RNI4 pp 229533.I1RSL2
53357 229533.A0A0E0RNI4 pp 229533.I1RCV1
53358 229533.A0A0E0RNI4 pp 229533.I1RP86
53359 229533.A0A0E0RNI4 pp 229533.A0A0E0RU85
53360 229533.A0A0E0RNI4 pp 229533.A0A1C3YJH4
53361 229533.A0A0E0RNI4 pp 229533.A0A1C3YLN6
53362 229533.A0A0E0RNI4 pp 229533.I1RPT0
53363 229533.A0A0E0RNI4 pp 229533.I1RWM4

53364 229533.A0A0E0RNI4 pp 229533.I1RQE1
53365 229533.A0A0E0RNI4 pp 229533.I1RUY5
53366 229533.A0A0E0RNI4 pp 229533.I1RDU5
53367 229533.A0A0E0RNI4 pp 229533.A0A1C3YLB3
53368 229533.A0A0E0RNI4 pp 229533.A0A1C3YL54
53369 229533.A0A0E0RNI4 pp 229533.I1RAQ4
53370 229533.A0A0E0RNI4 pp 229533.I1RRM1
53371 229533.A0A0E0RNI4 pp 229533.I1RC11
53372 229533.A0A0E0RNI4 pp 229533.I1RMH9
53373 229533.A0A0E0RNI4 pp 229533.I1RYP5
53374 229533.A0A0E0RNI4 pp 229533.A0A1C3YLC2
53375 229533.A0A0E0RNI4 pp 229533.I1RY12
53376 229533.A0A0E0RNI4 pp 229533.I1RY16
53377 229533.A0A0E0RNI4 pp 229533.A0A0E0S409
53378 229533.A0A0E0RNI4 pp 229533.I1RDN3
53379 229533.A0A0E0RNI4 pp 229533.I1RB07
53380 229533.A0A0E0RNI4 pp 229533.I1RW54
53381 229533.A0A0E0RNI4 pp 229533.I1S094
53382 229533.A0A0E0RNI4 pp 229533.I1RMA2
53383 229533.A0A0E0RNI4 pp 229533.A0A1C3YN26
53384 229533.A0A0E0RNI4 pp 229533.I1S6H3
53385 229533.A0A0E0RNI4 pp 229533.I1RNM7
53386 229533.A0A0E0RNI4 pp 229533.I1RMJ4
53387 229533.A0A0E0RNI4 pp 229533.I1RXH3
53388 229533.A0A0E0RNI4 pp 229533.V6RH92
53389 229533.I1RYM6 pp 229533.V6RM02
53390 229533.I1RYM6 pp 229533.I1RZM4
53391 229533.I1RYM6 pp 229533.I1RZ26
53392 229533.I1RYM6 pp 229533.V6RK96
53393 229533.I1RYM6 pp 229533.I1S1W8
53394 229533.I1RYM6 pp 229533.I1S3G4
53395 229533.I1S5C4 pp 229533.I1S5C5
53396 229533.I1S5C4 pp 229533.V6RB81
53397 229533.A0A1C3YKG8 pp 229533.I1RMP2
53398 229533.A0A1C3YKG8 pp 229533.I1RE19
53399 229533.A0A1C3YKG8 pp 229533.Q4I2W2
53400 229533.A0A1C3YKG8 pp 229533.I1RFV5
53401 229533.A0A1C3YKG8 pp 229533.I1RRK8
53402 229533.A0A1C3YKG8 pp 229533.I1RQL4
53403 229533.I1S3V2 pp 229533.I1S8F8
53404 229533.I1S3V2 pp 229533.Q4HXF0
53405 229533.I1S3V2 pp 229533.V6QT89
53406 229533.I1S3V2 pp 229533.V6RJP8
53407 229533.I1S3V2 pp 229533.V6RIS2
53408 229533.I1S3V2 pp 229533.I1S9F1
53409 229533.I1S3V2 pp 229533.Q4HW67
53410 229533.I1S3V2 pp 229533.V6RLM7
53411 229533.I1S3V2 pp 229533.I1S5S2
53412 229533.I1S3V2 pp 229533.I1S524
53413 229533.A0A1C3YK05 pp 229533.I1RSK3
53414 229533.A0A1C3YK05 pp 229533.I1RDH6
53415 229533.I1RD42 pp 229533.I1S2D2
53416 229533.I1RD42 pp 229533.I1RGK9
53417 229533.I1RD42 pp 229533.I1S130
53418 229533.I1RD42 pp 229533.I1RL16
53419 229533.I1RD42 pp 229533.I1S048
53420 229533.I1RD42 pp 229533.I1RYD3
53421 229533.I1RD42 pp 229533.I1RLA2
53422 229533.I1RD42 pp 229533.I1RJF2
53423 229533.I1RD42 pp 229533.I1RKL9
53424 229533.I1RD42 pp 229533.I1RWM4
53425 229533.I1RD42 pp 229533.V6RG22
53426 229533.I1RD42 pp 229533.I1RTJ2
53427 229533.I1RD42 pp 229533.I1S267
53428 229533.I1RD42 pp 229533.I1RST9
53429 229533.I1RD42 pp 229533.I1RXX1
53430 229533.I1RD42 pp 229533.I1RNG4
53431 229533.I1RD42 pp 229533.I1RYM5
53432 229533.I1RD42 pp 229533.I1RSS7
53433 229533.I1RD42 pp 229533.I1RZ25
53434 229533.I1RD42 pp 229533.I1S1K6
53435 229533.I1RD42 pp 229533.I1RVZ3
53436 229533.I1RD42 pp 229533.I1RY35

229533.I1RAZ6 pp 229533.Q4HZ95
229533.I1RAZ6 pp 229533.I1RZS4
229533.I1RAZ6 pp 229533.I1S088
229533.I1RAZ6 pp 229533.Q4IB65
229533.I1RAZ6 pp 229533.I1RPA5
229533.I1RAZ6 pp 229533.Q4IJW4
229533.I1RAZ6 pp 229533.I1RZH5
229533.I1RAZ6 pp 229533.I1RFV5
229533.I1RAZ6 pp 229533.I1RJT3
229533.I1RAZ6 pp 229533.I1S8F0
229533.I1RAZ6 pp 229533.V6R1X4
229533.I1RAZ6 pp 229533.I1RRK8
229533.I1RAZ6 pp 229533.I1S4H6
229533.I1RAZ6 pp 229533.Q4I099
229533.I1RAZ6 pp 229533.I1RT38
229533.I1RAZ6 pp 229533.I1RVN1
229533.I1S0Z6 pp 229533.V6RXW9
229533.I1S0Z6 pp 229533.I1S277
229533.I1S0Z6 pp 229533.Q4HY20
229533.I1RJQ5 pp 229533.I1RNA1
229533.I1RJQ5 pp 229533.V6R729
229533.I1RJQ5 pp 229533.I1RMX6
229533.I1RJQ5 pp 229533.I1RPU8
229533.I1RJQ5 pp 229533.I1S2C4
229533.I1RJQ5 pp 229533.I1S332
229533.I1RJQ5 pp 229533.I1RND4
229533.I1RJQ5 pp 229533.V6R382
229533.I1RJQ5 pp 229533.I1RYK0
229533.I1RJQ5 pp 229533.I1S0I3
229533.I1RJQ5 pp 229533.V6RLM7
229533.I1RJQ5 pp 229533.I1RNG4
229533.I1RJQ5 pp 229533.I1RMG6
229533.I1RJQ5 pp 229533.I1S0N9
229533.I1RJQ5 pp 229533.I1RUY6
229533.I1RJQ5 pp 229533.I1RZ39
229533.I1RJQ5 pp 229533.I1RNU7
229533.I1RJQ5 pp 229533.I1RP86
229533.I1RJQ5 pp 229533.I1RPT0
229533.I1RJQ5 pp 229533.I1RPE5
229533.I1RJQ5 pp 229533.I1RMW4
229533.I1RJQ5 pp 229533.I1RKZ8
229533.I1RJQ5 pp 229533.I1RPW1
229533.I1RJQ5 pp 229533.I1RTP4
229533.I1RJQ5 pp 229533.V6RAN2
229533.I1RJQ5 pp 229533.V6RXE7
229533.I1RJQ5 pp 229533.I1RYX6
229533.I1RJQ5 pp 229533.I1RKR5
229533.I1RJQ5 pp 229533.I1S574
229533.I1RJQ5 pp 229533.I1SAS8
229533.I1RJQ5 pp 229533.I1S984
229533.I1RJQ5 pp 229533.I1RUY5
229533.I1RJQ5 pp 229533.I1RJT5
229533.I1RJQ5 pp 229533.I1SAS7
229533.I1RJQ5 pp 229533.I1S219
229533.I1RJQ5 pp 229533.I1S328
229533.I1RJQ5 pp 229533.I1S0A0
229533.I1RJQ5 pp 229533.I1RQE0
229533.A0A0E0S390 pp 229533.I1RYC8
229533.A0A1C3YLC3 pp 229533.I1RZT6
229533.A0A1C3YLC3 pp 229533.I1RC58
229533.A0A1C3YLC3 pp 229533.I1RZM4
229533.A0A1C3YLC3 pp 229533.I1RXB1
229533.A0A1C3YLC3 pp 229533.I1RDZ5
229533.A0A1C3YLC3 pp 229533.I1RPZ1
229533.A0A1C3YLC3 pp 229533.I1RFV2
229533.A0A1C3YLC3 pp 229533.A0A1C3YLL3
229533.A0A1C3YLC3 pp 229533.I1RP86
229533.A0A1C3YLC3 pp 229533.I1RPQ2
229533.A0A1C3YLC3 pp 229533.I1RA90
229533.A0A1C3YLC3 pp 229533.I1RE86
229533.A0A1C3YLC3 pp 229533.I1RVP5
229533.A0A1C3YLC3 pp 229533.I1RVX9
229533.A0A1C3YLC3 pp 229533.I1RAR3

229533.A0A1C3YLC3 pp 229533.I1RH75
229533.A0A1C3YLC3 pp 229533.I1RRL5
229533.A0A1C3YLC3 pp 229533.I1RCH4
229533.A0A1C3YLC3 pp 229533.I1RQE8
229533.A0A1C3YLC3 pp 229533.I1RBE4
229533.A0A1C3YLC3 pp 229533.A0A1C3YMV8
229533.A0A1C3YLC3 pp 229533.I1RRN4
229533.A0A1C3YLC3 pp 229533.I1RMM8
229533.A0A1C3YLC3 pp 229533.I1S334
229533.A0A1C3YLC3 pp 229533.I1RW06
229533.A0A1C3YLC3 pp 229533.I1RUY5
229533.A0A1C3YLC3 pp 229533.I1RRC4
229533.A0A1C3YLC3 pp 229533.V6RK96
229533.A0A1C3YLC3 pp 229533.A0A1C3YN26
229533.A0A1C3YLC3 pp 229533.I1RDU0
229533.A0A1C3YLC3 pp 229533.I1RW69
229533.A0A1C3YLC3 pp 229533.I1RQE0
229533.A0A1C3YLC3 pp 229533.I1RVY2
229533.A0A1C3YLC3 pp 229533.I1RED2
229533.A0A1C3YLC3 pp 229533.I1REI2
229533.A0A1C3YLC3 pp 229533.A0A1C3YM54
229533.A0A1C3YLC3 pp 229533.I1RM26
229533.A0A1C3YLC3 pp 229533.I1RAQ4
229533.A0A1C3YLC3 pp 229533.A0A1C3YME0
229533.A0A1C3YLC3 pp 229533.I1RNG4
229533.A0A1C3YLC3 pp 229533.I1RPT0
229533.A0A1C3YLC3 pp 229533.V6R382
229533.A0A1C3YLC3 pp 229533.I1RK49
229533.A0A1C3YLC3 pp 229533.I1RD89
229533.A0A1C3YLC3 pp 229533.I1RSM8
229533.A0A1C3YLC3 pp 229533.I1S3H8
229533.A0A1C3YLC3 pp 229533.I1RBE6
229533.A0A1C3YLC3 pp 229533.I1RUP2
229533.A0A1C3YLC3 pp 229533.I1RF55
229533.A0A1C3YLC3 pp 229533.I1RND4
229533.A0A1C3YLC3 pp 229533.V6RLM7
229533.A0A1C3YLC3 pp 229533.I1RED3
229533.A0A1C3YLC3 pp 229533.I1RFS5
229533.A0A1C3YLC3 pp 229533.I1RBA4
229533.A0A1C3YLC3 pp 229533.I1REB5
229533.A0A1C3YLC3 pp 229533.I1RHG5
229533.A0A1C3YLC3 pp 229533.I1RM37
229533.A0A1C3YLC3 pp 229533.I1RWN0
229533.A0A1C3YLC3 pp 229533.I1RQG7
229533.A0A1C3YLC3 pp 229533.A0A1C3YLQ4
229533.A0A1C3YLC3 pp 229533.I1RNU7
229533.A0A1C3YLC3 pp 229533.I1S2B0
229533.A0A1C3YLC3 pp 229533.I1RZ29
229533.A0A1C3YLC3 pp 229533.I1SAV3
229533.A0A1C3YLC3 pp 229533.I1RSQ4
229533.A0A1C3YLC3 pp 229533.I1S5C5
229533.A0A1C3YLC3 pp 229533.I1RQJ4
229533.A0A1C3YLC3 pp 229533.I1S8H4
229533.A0A0E0RMK7 pp 229533.V6RN31
229533.A0A0E0S310 pp 229533.I1RNG4
229533.I1RML5 pp 229533.I1RSS1
229533.I1RML5 pp 229533.I1RQB4
229533.I1RML5 pp 229533.I1RSQ0
229533.I1RML5 pp 229533.I1S274
229533.I1RML5 pp 229533.I1RTC7
229533.I1RML5 pp 229533.I1RV66
229533.I1RML5 pp 229533.I1RX77
229533.I1RML5 pp 229533.I1S8A6
229533.I1RML5 pp 229533.Q4I4A4
229533.I1RML5 pp 229533.I1RTG0
229533.I1S130 pp 229533.V6RR38
229533.I1S130 pp 229533.I1S1Q3
229533.I1S130 pp 229533.V6RH92
229533.I1S130 pp 229533.V6QVH8
229533.I1S130 pp 229533.I1S5R5
229533.I1S130 pp 229533.I1S2M5
229533.I1S130 pp 229533.I1S968
229533.I1S130 pp 229533.I1S969

53583	229533.I1S130	pp	229533.V6RRH2
53584	229533.I1S130	pp	229533.V6RG22
53585	229533.I1S130	pp	229533.I1S183
53586	229533.I1S130	pp	229533.I1S2B0
53587	229533.I1S130	pp	229533.V6RPN0
53588	229533.A0A0E0SQ99	pp	229533.I1RRQ1
53589	229533.A0A0E0SQ99	pp	229533.I1RSJ2
53590	229533.A0A0E0SQ99	pp	229533.I1RLC7
53591	229533.A0A0E0SQ99	pp	229533.A0A1C3YLZ7
53592	229533.A0A0E0SQ99	pp	229533.I1RPW4
53593	229533.A0A0E0SQ99	pp	229533.I1RDJ1
53594	229533.A0A0E0SQ99	pp	229533.I1RCC2
53595	229533.A0A0E0SQ99	pp	229533.I1RIX0
53596	229533.A0A0E0SQ99	pp	229533.I1RP86
53597	229533.A0A0E0SQ99	pp	229533.I1RK45
53598	229533.A0A0E0SQ99	pp	229533.I1RW07
53599	229533.A0A0E0SQ99	pp	229533.I1RI02
53600	229533.A0A0E0SQ99	pp	229533.V6R4J1
53601	229533.A0A0E0SQ99	pp	229533.I1RBY9
53602	229533.A0A0E0SQ99	pp	229533.I1RDU4
53603	229533.A0A0E0SQ99	pp	229533.I1RKR5
53604	229533.A0A0E0SQ99	pp	229533.I1RGF5
53605	229533.A0A0E0SQ99	pp	229533.I1RIE8
53606	229533.A0A0E0SQ99	pp	229533.V6RAN2
53607	229533.A0A0E0SQ99	pp	229533.I1RQ14
53608	229533.A0A0E0SQ99	pp	229533.I1RNA5
53609	229533.A0A0E0SQ99	pp	229533.I1RIL8
53610	229533.A0A0E0SQ99	pp	229533.I1RC31
53611	229533.A0A0E0SQ99	pp	229533.A0A1C3YHZ7
53612	229533.A0A0E0SQ99	pp	229533.I1RW71
53613	229533.A0A0E0SQ99	pp	229533.I1RA62
53614	229533.A0A0E0SQ99	pp	229533.I1RDE4
53615	229533.A0A0E0SQ99	pp	229533.I1RI03
53616	229533.A0A0E0SQ99	pp	229533.I1RBN0
53617	229533.A0A0E0SQ99	pp	229533.I1RKP3
53618	229533.A0A0E0SQ99	pp	229533.I1RBJ5
53619	229533.A0A0E0SQ99	pp	229533.I1RU29
53620	229533.A0A0E0SQ99	pp	229533.I1RNR2
53621	229533.A0A0E0SQ99	pp	229533.I1RLM7
53622	229533.A0A0E0SQ99	pp	229533.I1RQE0
53623	229533.A0A0E0SQ99	pp	229533.I1RJL9
53624	229533.A0A0E0SQ99	pp	229533.I1RQ81
53625	229533.A0A0E0SQ99	pp	229533.I1RIL3
53626	229533.A0A0E0SQ99	pp	229533.I1S352
53627	229533.A0A0E0SQ99	pp	229533.I1RCA0
53628	229533.A0A0E0SQ99	pp	229533.I1RZ29
53629	229533.A0A0E0SQ99	pp	229533.I1RBE7
53630	229533.A0A0E0SQ99	pp	229533.I1S984
53631	229533.A0A0E0SQ99	pp	229533.I1RMX6
53632	229533.A0A0E0SQ99	pp	229533.I1S574
53633	229533.A0A0E0SQ99	pp	229533.I1RH91
53634	229533.A0A0E0SQ99	pp	229533.A0A1C3YK73
53635	229533.A0A0E0SQ99	pp	229533.I1RJZ7
53636	229533.A0A0E0SQ99	pp	229533.I1S0R4
53637	229533.A0A0E0SQ99	pp	229533.I1R9J6
53638	229533.A0A0E0SQ99	pp	229533.I1RZ13
53639	229533.A0A0E0SQ99	pp	229533.I1RGL4
53640	229533.A0A0E0SQ99	pp	229533.I1RRT3
53641	229533.A0A0E0SQ99	pp	229533.I1S569
53642	229533.A0A0E0SQ99	pp	229533.I1RLN6
53643	229533.A0A0E0SQ99	pp	229533.Q4I1Y5
53644	229533.A0A0E0SQ99	pp	229533.I1R9D1
53645	229533.A0A0E0SQ99	pp	229533.I1S0R3
53646	229533.A0A0E0SQ99	pp	229533.I1RNA1
53647	229533.A0A0E0SQ99	pp	229533.I1RRV1
53648	229533.A0A0E0SQ99	pp	229533.I1S272
53649	229533.I1RA85	pp	229533.I1REK8
53650	229533.I1RA85	pp	229533.I1RZK5
53651	229533.I1RA85	pp	229533.I1RCR5
53652	229533.I1RA85	pp	229533.I1S419
53653	229533.I1RA85	pp	229533.I1RS18
53654	229533.I1RA85	pp	229533.I1RN46
53655	229533.I1RA85	pp	229533.V6QUE6

53656	229533.I1RA85	pp	229533.Q4IJK2
53657	229533.I1RA85	pp	229533.I1S5B0
53658	229533.I1RA85	pp	229533.I1S0K2
53659	229533.I1RA85	pp	229533.I1RXV9
53660	229533.I1RA85	pp	229533.I1RZM8
53661	229533.I1RA85	pp	229533.I1RK95
53662	229533.I1RA85	pp	229533.I1S4S7
53663	229533.I1RA85	pp	229533.I1RSQ7
53664	229533.I1RA85	pp	229533.I1RXF0
53665	229533.I1RA85	pp	229533.V6RCZ8
53666	229533.I1RA85	pp	229533.I1RWB0
53667	229533.I1RA85	pp	229533.I1RRX5
53668	229533.I1RA85	pp	229533.I1RPR9
53669	229533.I1RA85	pp	229533.I1S6K9
53670	229533.I1RA85	pp	229533.I1S0T2
53671	229533.I1RA85	pp	229533.I1RWC9
53672	229533.I1RA85	pp	229533.I1RR51
53673	229533.I1RA85	pp	229533.I1RRY9
53674	229533.I1RA85	pp	229533.I1S338
53675	229533.I1RA85	pp	229533.I1RX46
53676	229533.I1RA85	pp	229533.I1RFL4
53677	229533.I1RA85	pp	229533.I1RCH5
53678	229533.I1RA85	pp	229533.I1RW06
53679	229533.I1RA85	pp	229533.I1RCS1
53680	229533.I1RA85	pp	229533.I1RKI3
53681	229533.I1RA85	pp	229533.V6RTJ8
53682	229533.I1RA85	pp	229533.I1RAX6
53683	229533.I1RA85	pp	229533.V6QVH8
53684	229533.I1RA85	pp	229533.I1RPI5
53685	229533.I1RA85	pp	229533.I1RXD7
53686	229533.I1RA85	pp	229533.I1RQE7
53687	229533.I1RA85	pp	229533.I1RR24
53688	229533.I1RA85	pp	229533.I1RVX0
53689	229533.I1RA85	pp	229533.I1RLP1
53690	229533.I1RA85	pp	229533.I1RBT1
53691	229533.I1RA85	pp	229533.I1RT03
53692	229533.I1RA85	pp	229533.Q4I624
53693	229533.I1RA85	pp	229533.I1RB92
53694	229533.I1RA85	pp	229533.I1RJU3
53695	229533.I1RA85	pp	229533.I1RD52
53696	229533.I1RA85	pp	229533.I1RIH0
53697	229533.I1RA85	pp	229533.I1S9Y1
53698	229533.I1RA85	pp	229533.I1S0J1
53699	229533.I1RA85	pp	229533.I1RJV3
53700	229533.I1RA85	pp	229533.I1RRT8
53701	229533.I1RA85	pp	229533.I1RQ30
53702	229533.I1RA85	pp	229533.I1RPK0
53703	229533.I1RA85	pp	229533.I1RAW0
53704	229533.I1RA85	pp	229533.I1RMV0
53705	229533.I1RA85	pp	229533.I1S5R1
53706	229533.I1RA85	pp	229533.I1RRN2
53707	229533.I1RA85	pp	229533.I1S1C7
53708	229533.I1RA85	pp	229533.O74638
53709	229533.I1RA85	pp	229533.I1S2B9
53710	229533.I1RA85	pp	229533.I1RD22
53711	229533.I1RA85	pp	229533.I1RYW3
53712	229533.I1RA85	pp	229533.I1RS90
53713	229533.I1RA85	pp	229533.I1S2H4
53714	229533.I1RA85	pp	229533.I1RNC1
53715	229533.I1RA85	pp	229533.I1RXB2
53716	229533.I1RA85	pp	229533.I1RRI2
53717	229533.I1RA85	pp	229533.I1RUG3
53718	229533.I1RA85	pp	229533.I1S0L4
53719	229533.I1RA85	pp	229533.I1RE01
53720	229533.I1RA85	pp	229533.I1RCR8
53721	229533.I1RA85	pp	229533.I1RWI5
53722	229533.I1RA85	pp	229533.V6R042
53723	229533.I1RA85	pp	229533.I1RSQ4
53724	229533.I1RA85	pp	229533.I1S7W5
53725	229533.I1RA85	pp	229533.I1RSQ3
53726	229533.I1RA85	pp	229533.I1RZZ9
53727	229533.I1RA85	pp	229533.I1RPP3
53728	229533.I1RA85	pp	229533.I1RBY4

53729	229533.I1RA85	pp	229533.I1RC66
53730	229533.I1RA85	pp	229533.I1RSX0
53731	229533.I1RA85	pp	229533.I1RX00
53732	229533.I1RA85	pp	229533.I1RYX5
53733	229533.I1RA85	pp	229533.V6R3J1
53734	229533.I1RA85	pp	229533.I1RPV3
53735	229533.I1RA85	pp	229533.I1S6Y8
53736	229533.I1RA85	pp	229533.I1RZ39
53737	229533.I1RA85	pp	229533.I1RN67
53738	229533.I1RA85	pp	229533.I1RXW3
53739	229533.I1RA85	pp	229533.I1RGA4
53740	229533.I1RA85	pp	229533.I1RMG6
53741	229533.I1RA85	pp	229533.I1RT40
53742	229533.I1RA85	pp	229533.I1RS47
53743	229533.I1RA85	pp	229533.I1RKI7
53744	229533.I1RA85	pp	229533.I1RAB4
53745	229533.I1RA85	pp	229533.I1RTD5
53746	229533.I1RA85	pp	229533.I1RTA1
53747	229533.I1RA85	pp	229533.I1RC67
53748	229533.I1RA85	pp	229533.I1RFT1
53749	229533.I1RA85	pp	229533.I1RXA9
53750	229533.I1RAJ9	pp	229533.I1RZR5
53751	229533.I1RBT7	pp	229533.I1RZ25
53752	229533.I1RBT7	pp	229533.I1S1K6
53753	229533.I1RBT7	pp	229533.I1RSS7
53754	229533.I1RBT7	pp	229533.I1RY35
53755	229533.I1RBT7	pp	229533.I1RNG4
53756	229533.I1RBT7	pp	229533.I1RVZ3
53757	229533.I1RBT7	pp	229533.I1RST9
53758	229533.I1RBT7	pp	229533.I1RTJ2
53759	229533.I1RBT7	pp	229533.I1RWM4
53760	229533.I1RBT7	pp	229533.V6RG22
53761	229533.I1RBT7	pp	229533.I1S267
53762	229533.I1RBT7	pp	229533.I1S130
53763	229533.I1RBT7	pp	229533.I1RXX1
53764	229533.I1RBT7	pp	229533.I1RYM5
53765	229533.I1RBT7	pp	229533.I1RYD3
53766	229533.I1RBT7	pp	229533.I1RLA2
53767	229533.I1RBT7	pp	229533.I1RL16
53768	229533.I1RBT7	pp	229533.I1RCE6
53769	229533.I1RBT7	pp	229533.I1S048
53770	229533.I1RBT7	pp	229533.I1RGK9
53771	229533.I1RFV2	pp	229533.I1RQJ4
53772	229533.I1RFV2	pp	229533.I1RHG5
53773	229533.I1RFV2	pp	229533.I1S5C5
53774	229533.I1RFV2	pp	229533.V6RLM7
53775	229533.I1RFV2	pp	229533.I1RUP2
53776	229533.I1RFV2	pp	229533.I1S8H4
53777	229533.I1RFV2	pp	229533.V6R382
53778	229533.I1RFV2	pp	229533.I1S2B0
53779	229533.I1RFV2	pp	229533.I1RZN1
53780	229533.I1RFV2	pp	229533.I1RWN0
53781	229533.I1RFV2	pp	229533.I1RNU7
53782	229533.I1RFV2	pp	229533.I1RM37
53783	229533.I1RFV2	pp	229533.I1SAV3
53784	229533.I1RFV2	pp	229533.I1RFV4
53785	229533.I1RFV2	pp	229533.I1RQG7
53786	229533.I1RFV2	pp	229533.I1RSM8
53787	229533.I1RFV2	pp	229533.I1RM26
53788	229533.I1RFV2	pp	229533.I1RRC4
53789	229533.I1RFV2	pp	229533.I1RSQ4
53790	229533.I1RFV2	pp	229533.I1RN32
53791	229533.I1RFV2	pp	229533.I1RNG4
53792	229533.I1RFV2	pp	229533.I1RND4
53793	229533.I1RFV2	pp	229533.I1RVY2
53794	229533.I1RFV2	pp	229533.I1S3H8
53795	229533.I1RFV2	pp	229533.I1RK49
53796	229533.I1RFV2	pp	229533.I1RQE0
53797	229533.I1RFV2	pp	229533.I1RZ29
53798	229533.I1RFV2	pp	229533.I1RRN4
53799	229533.I1RFV2	pp	229533.V6RK96
53800	229533.I1RFV2	pp	229533.I1RPT0
53801	229533.I1RFV2	pp	229533.I1RMG4

229533.I1RFV2 pp 229533.I1S368
229533.I1RFV2 pp 229533.I1RVP5
229533.I1RFV2 pp 229533.I1RW69
229533.I1RFV2 pp 229533.I1RUY5
229533.I1RFV2 pp 229533.I1S334
229533.I1RFV2 pp 229533.I1RW06
229533.I1RFV2 pp 229533.I1RRL5
229533.I1RFV2 pp 229533.I1RQE8
229533.I1RFV2 pp 229533.I1RMM8
229533.I1RFV2 pp 229533.I1S5P3
229533.I1RFV2 pp 229533.I1RXB1
229533.I1RFV2 pp 229533.I1RZT6
229533.I1RFV2 pp 229533.I1RZM4
229533.I1RFV2 pp 229533.I1RPZ1
229533.I1RFV2 pp 229533.I1RQM4
229533.I1RFV2 pp 229533.I1RH75
229533.I1RFV2 pp 229533.I1RP86
229533.I1RFV2 pp 229533.I1RVX9
229533.I1RFV2 pp 229533.I1RPQ2
229533.A0A1C3YID9 pp 229533.I1RUP0
229533.A0A1C3YID9 pp 229533.I1RQ38
229533.A0A1C3YID9 pp 229533.I1RPK4
229533.A0A1C3YID9 pp 229533.A0A1C3YKR7
229533.A0A1C3YID9 pp 229533.I1RFW0
229533.A0A1C3YID9 pp 229533.I1RSQ2
229533.A0A1C3YID9 pp 229533.I1RMP9
229533.A0A1C3YID9 pp 229533.I1RZ34
229533.A0A1C3YID9 pp 229533.I1RMM6
229533.A0A1C3YID9 pp 229533.I1RYB8
229533.A0A1C3YID9 pp 229533.A0A1C3YIG3
229533.A0A1C3YID9 pp 229533.I1S4Z7
229533.A0A1C3YID9 pp 229533.Q4IEA7
229533.A0A1C3YID9 pp 229533.I1RLU2
229533.A0A1C3YID9 pp 229533.I1S286
229533.A0A1C3YID9 pp 229533.I1RSS1
229533.A0A1C3YID9 pp 229533.V6QUV7
229533.A0A1C3YID9 pp 229533.I1RT00
229533.A0A1C3YID9 pp 229533.A0A1C3YIL9
229533.A0A1C3YID9 pp 229533.I1RYL5
229533.A0A1C3YID9 pp 229533.I1RZK1
229533.A0A1C3YID9 pp 229533.V6QX21
229533.I1S6D7 pp 229533.V6RKY8
229533.I1S6D7 pp 229533.I1S8Z2
229533.A0A1C3YID4 pp 229533.I1RIF9
229533.A0A1C3YID4 pp 229533.I1RXP4
229533.A0A1C3YID4 pp 229533.I1S308
229533.A0A1C3YID4 pp 229533.I1RHG0
229533.A0A1C3YID4 pp 229533.I1RWC3
229533.A0A1C3YID4 pp 229533.I1RJW0
229533.A0A1C3YID4 pp 229533.V6RN31
229533.A0A1C3YID4 pp 229533.I1RCH0
229533.A0A1C3YID4 pp 229533.I1S5Y0
229533.A0A1C3YID4 pp 229533.I1S2E7
229533.A0A1C3YID4 pp 229533.I1REH0
229533.A0A1C3YID4 pp 229533.I1RDT7
229533.A0A1C3YID4 pp 229533.I1R9D3
229533.A0A1C3YID4 pp 229533.I1SAK2
229533.A0A1C3YID4 pp 229533.I1S1V6
229533.A0A1C3YID4 pp 229533.I1RBM4
229533.I1RBE3 pp 229533.I1RWW7
229533.I1RBE3 pp 229533.I1RK03
229533.I1RBE3 pp 229533.I1S799
229533.I1RBE3 pp 229533.I1S9R7
229533.I1S767 pp 229533.V6RDH3
229533.I1S767 pp 229533.V6QV31
229533.I1S767 pp 229533.V6RS91
229533.I1S767 pp 229533.Q4I1N3
229533.A0A0E0RX50 pp 229533.A0A1C3YI72
229533.A0A0E0RX50 pp 229533.I1RVW6
229533.A0A0E0RX50 pp 229533.A0A1C3YN47
229533.A0A0E0RX50 pp 229533.I1RCL0
229533.A0A0E0RX50 pp 229533.I1RM26
229533.A0A0E0RX50 pp 229533.I1RYA0

53875 229533.A0A0E0RX50 pp 229533.I1S0S5
53876 229533.A0A0E0RX50 pp 229533.I1RG33
53877 229533.A0A0E0RX50 pp 229533.I1RJU8
53878 229533.A0A0E0RX50 pp 229533.Q4I1N3
53879 229533.A0A0E0RX50 pp 229533.A0A1C3YLU7
53880 229533.A0A0E0RX50 pp 229533.I1S8F8
53881 229533.A0A0E0RX50 pp 229533.I1RVX8
53882 229533.A0A0E0RX50 pp 229533.I1REE1
53883 229533.A0A0E0RX50 pp 229533.A0A0E0SFT7
53884 229533.A0A0E0RX50 pp 229533.V6RDH3
53885 229533.A0A0E0RX50 pp 229533.I1RYC8
53886 229533.A0A0E0RX50 pp 229533.I1RZU2
53887 229533.A0A0E0RX50 pp 229533.I1RPK0
53888 229533.A0A0E0RX50 pp 229533.I1RN77
53889 229533.A0A0E0RX50 pp 229533.I1RWX5
53890 229533.A0A0E0RX50 pp 229533.I1RHV1
53891 229533.A0A0E0RX50 pp 229533.I1RYU6
53892 229533.A0A0E0RX50 pp 229533.I1RP70
53893 229533.A0A0E0RX50 pp 229533.I1RE90
53894 229533.A0A0E0RX50 pp 229533.I1S1W8
53895 229533.A0A0E0RX50 pp 229533.I1RFK3
53896 229533.A0A0E0RX50 pp 229533.A0A1C3YJK5
53897 229533.A0A0E0RX50 pp 229533.I1RYS3
53898 229533.A0A0E0RX50 pp 229533.I1RZE7
53899 229533.A0A0E0RX50 pp 229533.I1S3G4
53900 229533.A0A0E0RX50 pp 229533.I1S6D1
53901 229533.A0A0E0RX50 pp 229533.I1RSI2
53902 229533.A0A0E0RX50 pp 229533.I1S8E9
53903 229533.A0A0E0RX50 pp 229533.I1RFI8
53904 229533.A0A0E0RX50 pp 229533.V6QV31
53905 229533.A0A0E0RX50 pp 229533.I1RTT0
53906 229533.A0A0E0RX50 pp 229533.I1RSQ4
53907 229533.A0A0E0RX50 pp 229533.I1RDZ7
53908 229533.A0A0E0RX50 pp 229533.I1RL81
53909 229533.A0A0E0RX50 pp 229533.I1S767
53910 229533.A0A0E0RX50 pp 229533.I1RE53
53911 229533.A0A0E0RX50 pp 229533.I1RNG4
53912 229533.A0A0E0RX50 pp 229533.I1RHY5
53913 229533.A0A0E0RX50 pp 229533.I1RW11
53914 229533.A0A0E0RX50 pp 229533.I1RWF9
53915 229533.A0A0E0RX50 pp 229533.I1RZP1
53916 229533.A0A0E0RX50 pp 229533.I1RUP7
53917 229533.A0A0E0RX50 pp 229533.I1RVX9
53918 229533.A0A0E0RX50 pp 229533.Q4HZQ1
53919 229533.A0A1C3YMK7 pp 229533.Q4IBX0
53920 229533.A0A1C3YMK7 pp 229533.I1RP25
53921 229533.A0A1C3YMK7 pp 229533.Q4I963
53922 229533.A0A1C3YMK7 pp 229533.I1RYL5
53923 229533.A0A1C3YMK7 pp 229533.I1RSP4
53924 229533.A0A1C3YMK7 pp 229533.V6R223
53925 229533.A0A1C3YMK7 pp 229533.I1RKA1
53926 229533.A0A1C3YMK7 pp 229533.V6QUV7
53927 229533.A0A1C3YMK7 pp 229533.Q4IMM0
53928 229533.A0A1C3YMK7 pp 229533.I1RLU2
53929 229533.A0A1C3YMK7 pp 229533.I1RWF4
53930 229533.A0A1C3YMK7 pp 229533.I1S4Z7
53931 229533.I1RAJ6 pp 229533.I1RTG7
53932 229533.I1RAJ6 pp 229533.I1RQE8
53933 229533.I1RAJ6 pp 229533.I1RYQ4
53934 229533.I1RAJ6 pp 229533.I1RDE7
53935 229533.I1RAJ6 pp 229533.I1RNG4
53936 229533.I1RAJ6 pp 229533.I1RQ60
53937 229533.I1RAJ6 pp 229533.I1RC31
53938 229533.I1RAJ6 pp 229533.I1RWF9
53939 229533.I1RAJ6 pp 229533.I1RZM4
53940 229533.I1RAJ6 pp 229533.I1RMK4
53941 229533.I1RAJ6 pp 229533.I1S0Y7
53942 229533.I1RAJ6 pp 229533.I1RK03
53943 229533.I1RAJ6 pp 229533.I1RY74
53944 229533.I1RAJ6 pp 229533.I1RDQ2
53945 229533.I1RAJ6 pp 229533.I1RL04
53946 229533.I1RAJ6 pp 229533.I1RSS7
53947 229533.I1RAJ6 pp 229533.V6RLM7

53948 229533.I1RAJ6 pp 229533.I1RAY9
53949 229533.I1RAJ6 pp 229533.I1S0N3
53950 229533.I1RAJ6 pp 229533.I1RP97
53951 229533.I1RAJ6 pp 229533.I1RPK0
53952 229533.I1RAJ6 pp 229533.I1RNV4
53953 229533.I1RAJ6 pp 229533.V6RK96
53954 229533.I1RAJ6 pp 229533.I1RBE4
53955 229533.I1RAJ6 pp 229533.I1RKZ7
53956 229533.I1RAJ6 pp 229533.I1RC61
53957 229533.I1S0N8 pp 229533.V6REE9
53958 229533.I1S0N8 pp 229533.I1S224
53959 229533.I1S0N8 pp 229533.V6RR76
53960 229533.I1S0N8 pp 229533.I1S549
53961 229533.I1S0N8 pp 229533.I1S6N6
53962 229533.I1S0N8 pp 229533.I1S2E9
53963 229533.I1S0N8 pp 229533.Q4HXT6
53964 229533.A0A1C3YHX9 pp 229533.I1RN84
53965 229533.A0A1C3YHX9 pp 229533.I1S549
53966 229533.A0A1C3YHX9 pp 229533.I1RN88
53967 229533.A0A1C3YHX9 pp 229533.I1RGC3
53968 229533.A0A1C3YHX9 pp 229533.I1RRP3
53969 229533.A0A1C3YHX9 pp 229533.A0A1C3YLE2
53970 229533.A0A1C3YHX9 pp 229533.I1RJY7
53971 229533.A0A1C3YHX9 pp 229533.I1RQA2
53972 229533.A0A1C3YHX9 pp 229533.I1S8C1
53973 229533.A0A1C3YHX9 pp 229533.A0A1C3YJJ2
53974 229533.I1RW84 pp 229533.I1RYB7
53975 229533.I1RW84 pp 229533.I1RZA4
53976 229533.I1RW84 pp 229533.I1S0N8
53977 229533.I1RW84 pp 229533.I1S1A3
53978 229533.I1RW84 pp 229533.I1RX09
53979 229533.I1RW84 pp 229533.I1RY44
53980 229533.I1RW84 pp 229533.Q4I1H6
53981 229533.I1RW84 pp 229533.V6RR76
53982 229533.I1RW84 pp 229533.I1RZ91
53983 229533.I1RW84 pp 229533.I1RWT6
53984 229533.I1RW84 pp 229533.I1SAT1
53985 229533.I1RW84 pp 229533.I1RXF5
53986 229533.I1RW84 pp 229533.V6RKE4
53987 229533.I1RW84 pp 229533.I1S074
53988 229533.I1RW84 pp 229533.Q4ICG5
53989 229533.I1RW84 pp 229533.I1RYI3
53990 229533.I1RW84 pp 229533.I1RXU1
53991 229533.I1RW84 pp 229533.I1S635
53992 229533.I1RW84 pp 229533.I1S2E9
53993 229533.I1RW84 pp 229533.Q4IBS9
53994 229533.I1RW84 pp 229533.V6RQ48
53995 229533.I1RW84 pp 229533.I1S248
53996 229533.I1RP25 pp 229533.I1S235
53997 229533.I1RP25 pp 229533.V6RN44
53998 229533.I1RP25 pp 229533.I1RQ81
53999 229533.I1RP25 pp 229533.V6R223
54000 229533.I1RP25 pp 229533.I1RYL5
54001 229533.I1RP25 pp 229533.V6QUV7
54002 229533.I1RP25 pp 229533.I1S549
54003 229533.I1RP25 pp 229533.I1RPK4
54004 229533.I1RP25 pp 229533.I1RSI0
54005 229533.I1RP25 pp 229533.I1RQL4
54006 229533.I1RDK2 pp 229533.I1RMA7
54007 229533.I1RDK2 pp 229533.I1RYB7
54008 229533.I1RDK2 pp 229533.I1RJT8
54009 229533.I1RDK2 pp 229533.I1RW84
54010 229533.I1RDK2 pp 229533.I1RWN2
54011 229533.I1RDK2 pp 229533.I1RM12
54012 229533.I1RDK2 pp 229533.I1RRR9
54013 229533.I1RDK2 pp 229533.I1RMR7
54014 229533.I1RDK2 pp 229533.I1RJT0
54015 229533.I1RDK2 pp 229533.I1S074
54016 229533.I1RDK2 pp 229533.Q4IBS9
54017 229533.I1RDK2 pp 229533.I1S1A3
54018 229533.I1RDK2 pp 229533.I1S248
54019 229533.I1RDK2 pp 229533.I1RW38
54020 229533.A0A1C3YN83 pp 229533.I1RT54

229533.A0A1C3YN83 pp 229533.I1RBF3
229533.A0A1C3YN83 pp 229533.I1RZ17
229533.A0A1C3YN83 pp 229533.I1RDJ1
229533.A0A1C3YN83 pp 229533.I1RRT3
229533.A0A1C3YN83 pp 229533.I1RST0
229533.A0A1C3YN83 pp 229533.I1RZ13
229533.A0A1C3YN83 pp 229533.I1RSY7
229533.A0A1C3YN83 pp 229533.I1RK45
229533.A0A1C3YN83 pp 229533.I1R9Y8
229533.A0A1C3YN83 pp 229533.I1RDI6
229533.A0A1C3YN83 pp 229533.I1S252
229533.A0A1C3YN83 pp 229533.I1RDE4
229533.A0A1C3YN83 pp 229533.I1RQ75
229533.A0A1C3YN83 pp 229533.I1REU0
229533.A0A1C3YN83 pp 229533.I1R9W9
229533.A0A1C3YN83 pp 229533.I1RE86
229533.A0A1C3YN83 pp 229533.I1S272
229533.A0A1C3YN83 pp 229533.Q4I785
229533.A0A1C3YN83 pp 229533.Q4IB63
229533.A0A1C3YN83 pp 229533.V6RK56
229533.A0A1C3YN83 pp 229533.I1RBN0
229533.A0A1C3YN83 pp 229533.Q4I073
229533.A0A1C3YN83 pp 229533.Q4ILH3
229533.A0A1C3YN83 pp 229533.I1RJL9
229533.A0A1C3YN83 pp 229533.Q4I0K3
229533.A0A1C3YN83 pp 229533.I1RKP3
229533.A0A1C3YN83 pp 229533.I1RP81
229533.A0A1C3YN83 pp 229533.Q4IQ08
229533.A0A1C3YN83 pp 229533.I1RBA4
229533.A0A1C3YN83 pp 229533.I1RC36
229533.A0A1C3YN83 pp 229533.I1RZ18
229533.A0A1C3YN83 pp 229533.I1RNK8
229533.A0A1C3YN83 pp 229533.I1S5C5
229533.A0A1C3YN83 pp 229533.V6RKK5
229533.A0A1C3YN83 pp 229533.I1S4S9
229533.A0A1C3YN83 pp 229533.I1RBE7
229533.A0A1C3YN83 pp 229533.I1RYM5
229533.I1RNE8 pp 229533.I1RWN2
229533.I1S8C1 pp 229533.I1S8E0
229533.I1S8C1 pp 229533.I1S8F8
229533.I1S8C1 pp 229533.V6RRM0
229533.I1S8C1 pp 229533.V6RPS5
229533.I1S8C1 pp 229533.I1S8R4
229533.I1S8C1 pp 229533.V6RUY9
229533.I1S8C1 pp 229533.V6RK96
229533.I1S8C1 pp 229533.Q4IA56
229533.I1S8C1 pp 229533.V6R3J1
229533.I1S8C1 pp 229533.V6RM02
229533.I1S8C1 pp 229533.I1S8R2
229533.I1S8C1 pp 229533.V6R382
229533.I1S8C1 pp 229533.I1S968
229533.I1S8C1 pp 229533.V6RLM7
229533.I1RCP9 pp 229533.I1RSL8
229533.I1RCP9 pp 229533.I1RSD1
229533.I1RWN2 pp 229533.Q4IBS9
229533.I1RWN2 pp 229533.I1S074
229533.A0A0E0RNW9 pp 229533.I1S2E9
229533.A0A0E0RNW9 pp 229533.I1RN27
229533.A0A0E0RNW9 pp 229533.I1RNF4
229533.A0A0E0RNW9 pp 229533.I1RTG0
229533.A0A0E0RNW9 pp 229533.I1RPK4
229533.A0A0E0RNW9 pp 229533.I1S0Z6
229533.A0A0E0RNW9 pp 229533.I1RFW0
229533.A0A0E0RNW9 pp 229533.I1RML5
229533.A0A0E0RNW9 pp 229533.I1RGF1
229533.A0A0E0RNW9 pp 229533.A0A1C3YJV7
229533.A0A0E0RNW9 pp 229533.A0A0E0SA11
229533.A0A0E0RNW9 pp 229533.I1RQB4
229533.A0A0E0RNW9 pp 229533.A0A1C3YID9
229533.A0A0E0RNW9 pp 229533.I1RY44
229533.A0A0E0RNW9 pp 229533.I1S235
229533.A0A0E0RNW9 pp 229533.A0A1C3YIG3
229533.A0A0E0RNW9 pp 229533.A0A0E0SGM9

54094	229533.A0A0E0RNW9	pp	229533.I1S286
54095	229533.A0A0E0RNW9	pp	229533.I1RCB3
54096	229533.A0A0E0RNW9	pp	229533.I1RQ72
54097	229533.A0A0E0RNW9	pp	229533.I1RXF0
54098	229533.A0A0E0RNW9	pp	229533.I1RP25
54099	229533.A0A0E0RNW9	pp	229533.I1RMB2
54100	229533.A0A0E0RNW9	pp	229533.I1RX77
54101	229533.A0A0E0RNW9	pp	229533.I1RSS1
54102	229533.A0A0E0RNW9	pp	229533.Q4IEA7
54103	229533.A0A0E0RNW9	pp	229533.I1RXX1
54104	229533.A0A0E0RNW9	pp	229533.I1RYL5
54105	229533.A0A1C3YKS6	pp	229533.I1REL7
54106	229533.A0A1C3YKS6	pp	229533.I1RM26
54107	229533.A0A1C3YKS6	pp	229533.I1S4Q4
54108	229533.A0A1C3YKS6	pp	229533.I1RE00
54109	229533.A0A1C3YKS6	pp	229533.A0A1C3YM63
54110	229533.A0A1C3YKS6	pp	229533.V6RQW4
54111	229533.A0A1C3YKS6	pp	229533.I1RM35
54112	229533.A0A1C3YKS6	pp	229533.I1RBE7
54113	229533.A0A1C3YKS6	pp	229533.I1RPN4
54114	229533.A0A1C3YKS6	pp	229533.I1RW71
54115	229533.A0A1C3YKS6	pp	229533.I1RVX9
54116	229533.A0A1C3YKS6	pp	229533.V6RBN2
54117	229533.A0A1C3YKS6	pp	229533.I1RME6
54118	229533.A0A1C3YKS6	pp	229533.A0A1C3YM21
54119	229533.A0A1C3YKS6	pp	229533.V6RRH2
54120	229533.A0A1C3YKS6	pp	229533.I1RYS0
54121	229533.A0A1C3YKS6	pp	229533.I1RB39
54122	229533.A0A1C3YKS6	pp	229533.I1RTD3
54123	229533.A0A1C3YKS6	pp	229533.I1RAZ3
54124	229533.A0A1C3YKS6	pp	229533.I1RF82
54125	229533.A0A1C3YKS6	pp	229533.I1S0B7
54126	229533.A0A1C3YKS6	pp	229533.I1RCD8
54127	229533.A0A1C3YKS6	pp	229533.V6R057
54128	229533.A0A1C3YKS6	pp	229533.I1RK15
54129	229533.A0A1C3YKS6	pp	229533.I1S216
54130	229533.A0A1C3YKS6	pp	229533.I1RCL4
54131	229533.A0A1C3YKS6	pp	229533.I1RBI1
54132	229533.A0A1C3YKS6	pp	229533.I1RMQ4
54133	229533.A0A1C3YKS6	pp	229533.A0A1C3YN26
54134	229533.A0A1C3YKS6	pp	229533.I1RQS9
54135	229533.A0A1C3YKS6	pp	229533.A0A1C3YKV3
54136	229533.A0A1C3YKS6	pp	229533.I1RQ81
54137	229533.A0A1C3YKS6	pp	229533.I1RMQ6
54138	229533.A0A1C3YKS6	pp	229533.I1RFL4
54139	229533.A0A1C3YKS6	pp	229533.I1RCH5
54140	229533.A0A1C3YKS6	pp	229533.A0A1C3YN30
54141	229533.A0A1C3YKS6	pp	229533.I1RRV4
54142	229533.A0A1C3YKS6	pp	229533.V6R2A1
54143	229533.A0A1C3YKS6	pp	229533.I1RQI6
54144	229533.A0A1C3YKS6	pp	229533.V6R1K4
54145	229533.A0A1C3YKS6	pp	229533.I1RI34
54146	229533.A0A1C3YKS6	pp	229533.I1SAN9
54147	229533.A0A1C3YKS6	pp	229533.A0A1C3YKY0
54148	229533.A0A1C3YKS6	pp	229533.I1RTG7
54149	229533.A0A1C3YKS6	pp	229533.I1RRV1
54150	229533.A0A1C3YKS6	pp	229533.I1RRP7
54151	229533.A0A1C3YKS6	pp	229533.I1RCW6
54152	229533.A0A1C3YKS6	pp	229533.I1RDE7
54153	229533.A0A1C3YKS6	pp	229533.I1RPU8
54154	229533.A0A1C3YKS6	pp	229533.I1RB44
54155	229533.A0A1C3YKS6	pp	229533.I1RN67
54156	229533.A0A1C3YKS6	pp	229533.I1RA70
54157	229533.A0A1C3YKS6	pp	229533.I1RJZ7
54158	229533.A0A1C3YKS6	pp	229533.I1RX22
54159	229533.A0A1C3YKS6	pp	229533.I1RMI8
54160	229533.A0A1C3YKS6	pp	229533.A0A1C3YKU8
54161	229533.A0A1C3YKS6	pp	229533.I1RC05
54162	229533.A0A1C3YKS6	pp	229533.I1S4Z8
54163	229533.A0A1C3YKS6	pp	229533.I1RWE8
54164	229533.A0A1C3YKS6	pp	229533.V6R3J1
54165	229533.A0A1C3YKS6	pp	229533.I1RVZ7
54166	229533.A0A1C3YKS6	pp	229533.I1S8G4

```
229533.A0A1C3YKS6	pp	229533.I1RQM7
229533.A0A1C3YKS6	pp	229533.Q4HXF0
229533.A0A1C3YKS6	pp	229533.I1RWI8
229533.I1S074	pp	229533.I1S1W5
229533.I1S074	pp	229533.Q4HZS8
229533.I1S074	pp	229533.V6R9X9
229533.I1S074	pp	229533.I1S248
229533.I1S074	pp	229533.I1S2E9
229533.I1S074	pp	229533.Q4IBS9
229533.I1S074	pp	229533.I1S1A3
229533.I1S074	pp	229533.Q5I2J3
229533.I1S3H8	pp	229533.V6R756
229533.I1S3H8	pp	229533.V6R9L8
229533.I1S3H8	pp	229533.Q4IMJ1
229533.I1S3H8	pp	229533.V6R2Z2
229533.I1S3H8	pp	229533.I1SAI4
229533.I1S3H8	pp	229533.V6R0W1
229533.I1S3H8	pp	229533.V6QZ48
229533.I1S3H8	pp	229533.I1S5J9
229533.I1S3H8	pp	229533.V6QU99
229533.I1S3H8	pp	229533.V6RAM6
229533.I1S3H8	pp	229533.I1S8E0
229533.I1S3H8	pp	229533.V6R1D2
229533.I1RY27	pp	229533.Q4HZI0
229533.I1RY27	pp	229533.I1RZ78
229533.I1RY27	pp	229533.V6R382
229533.A0A1C3YJP0	pp	229533.I1RM49
229533.A0A1C3YJP0	pp	229533.I1RNF5
229533.A0A1C3YJP0	pp	229533.I1REF2
229533.A0A1C3YJP0	pp	229533.I1RW26
229533.A0A1C3YJP0	pp	229533.I1RK80
229533.A0A1C3YJP0	pp	229533.I1RKF9
229533.A0A0E0RU58	pp	229533.I1RK44
229533.A0A0E0RU58	pp	229533.I1RUP6
229533.A0A0E0RU58	pp	229533.I1RBY9
229533.A0A0E0RU58	pp	229533.Q4IJ79
229533.A0A0E0RU58	pp	229533.A0A1C3YIG2
229533.A0A0E0RU58	pp	229533.A0A1C3YKU0
229533.A0A0E0RU58	pp	229533.I1RBF4
229533.A0A0E0RU58	pp	229533.Q4HZS8
229533.A0A0E0RU58	pp	229533.A0A1C3YLR6
229533.Q4IBS9	pp	229533.Q4IR08
229533.I1S0A5	pp	229533.V6RZD4
229533.I1S0A5	pp	229533.V6QZ48
229533.I1S0A5	pp	229533.I1S968
229533.I1S0A5	pp	229533.I1S6V0
229533.I1S0A5	pp	229533.V6R2Z2
229533.I1S0A5	pp	229533.I1SAH9
229533.I1S0A5	pp	229533.I1S969
229533.I1S0A5	pp	229533.V6RF62
229533.I1S0A5	pp	229533.I1SAB4
229533.I1S0A5	pp	229533.V6R5D2
229533.I1S0A5	pp	229533.V6RCJ5
229533.I1S0A5	pp	229533.I1SAT2
229533.I1S0A5	pp	229533.V6R756
229533.I1S0A5	pp	229533.I1S5D0
229533.I1S0A5	pp	229533.V6QVH2
229533.I1S0A5	pp	229533.I1S223
229533.I1S0A5	pp	229533.V6R9L8
229533.I1S0A5	pp	229533.V6QU99
229533.I1S0A5	pp	229533.I1S210
229533.I1S0A5	pp	229533.I1S0K1
229533.I1S0A5	pp	229533.I1S4S5
229533.I1S0A5	pp	229533.I1S0X1
229533.I1S0A5	pp	229533.V6RHX2
229533.I1S0A5	pp	229533.I1SAI4
229533.I1S0A5	pp	229533.V6R5T7
229533.I1S0A5	pp	229533.V6R0W1
229533.I1S0A5	pp	229533.V6RAM6
229533.I1S0A5	pp	229533.I1S659
229533.I1S0A5	pp	229533.I1S267
229533.I1S0A5	pp	229533.I1S5J9
229533.I1S0A5	pp	229533.V6RCC0
```

54240	229533.I1S0A5	pp	229533.V6R1D2
54241	229533.I1S0A5	pp	229533.I1S8H9
54242	229533.I1S0A5	pp	229533.I1S423
54243	229533.I1S0A5	pp	229533.V6RWB3
54244	229533.I1S0A5	pp	229533.I1S8D3
54245	229533.I1S0A5	pp	229533.V6R3V4
54246	229533.I1S0A5	pp	229533.I1S2F7
54247	229533.I1S0A5	pp	229533.Q4HYR2
54248	229533.I1S0A5	pp	229533.I1S3T6
54249	229533.I1RN51	pp	229533.I1S558
54250	229533.I1RN51	pp	229533.I1RZS6
54251	229533.I1RN51	pp	229533.Q4IJH1
54252	229533.I1RN51	pp	229533.I1RR72
54253	229533.I1RN51	pp	229533.I1RTE9
54254	229533.I1RN51	pp	229533.I1S253
54255	229533.I1RN51	pp	229533.I1RWJ4
54256	229533.I1RN51	pp	229533.I1RPJ3
54257	229533.I1RN51	pp	229533.Q4IQW0
54258	229533.I1RN51	pp	229533.I1RRT2
54259	229533.I1RN51	pp	229533.I1RTD7
54260	229533.I1RN51	pp	229533.Q4IAS1
54261	229533.I1RN51	pp	229533.Q4HZ68
54262	229533.I1RN51	pp	229533.Q4IPI1
54263	229533.I1RN51	pp	229533.Q4IF76
54264	229533.I1RN51	pp	229533.I1RT45
54265	229533.I1RN51	pp	229533.Q4IBS2
54266	229533.I1RN51	pp	229533.I1RQ31
54267	229533.I1RN51	pp	229533.I1RTI6
54268	229533.I1RN51	pp	229533.I1RTI7
54269	229533.I1RN51	pp	229533.V6RLW4
54270	229533.I1RN51	pp	229533.I1RT98
54271	229533.I1RN51	pp	229533.Q4IFI0
54272	229533.I1RN51	pp	229533.I1RY63
54273	229533.I1RN51	pp	229533.Q4IN63
54274	229533.I1RN51	pp	229533.I1S9U2
54275	229533.I1RN51	pp	229533.Q4I327
54276	229533.I1RN51	pp	229533.V6QYF0
54277	229533.I1RN51	pp	229533.Q4IEV5
54278	229533.I1RN51	pp	229533.I1RRQ6
54279	229533.I1RN51	pp	229533.I1RS47
54280	229533.I1RN51	pp	229533.I1S4T3
54281	229533.I1RN51	pp	229533.V6R227
54282	229533.I1RN51	pp	229533.Q4IJ56
54283	229533.I1RN51	pp	229533.Q4IEK8
54284	229533.I1RN51	pp	229533.I1RW26
54285	229533.I1RN51	pp	229533.I1RRJ8
54286	229533.A0A1C3YIF8	pp	229533.A0A1C3YKU0
54287	229533.A0A1C3YIF8	pp	229533.A0A1C3YIG2
54288	229533.A0A1C3YIF8	pp	229533.I1RBF4
54289	229533.A0A1C3YIF8	pp	229533.A0A1C3YLR6
54290	229533.A0A1C3YIF8	pp	229533.Q4HZS8
54291	229533.A0A1C3YIF8	pp	229533.I1RK44
54292	229533.A0A1C3YIF8	pp	229533.A0A1C3YLH6
54293	229533.A0A1C3YIF8	pp	229533.Q4IJ79
54294	229533.A0A1C3YIF8	pp	229533.I1RUP6
54295	229533.A0A1C3YIF8	pp	229533.I1RBY9
54296	229533.A0A1C3YIF8	pp	229533.I1RWL9
54297	229533.I1RNM5	pp	229533.I1S074
54298	229533.I1RNM5	pp	229533.I1RZ16
54299	229533.I1RNM5	pp	229533.Q4IBS9
54300	229533.I1RIG6	pp	229533.I1RUK0
54301	229533.I1RIG6	pp	229533.I1RT36
54302	229533.I1RPV8	pp	229533.I1S267
54303	229533.I1RPV8	pp	229533.I1S659
54304	229533.I1RPV8	pp	229533.I1S8H9
54305	229533.I1RPV8	pp	229533.I1S5J9
54306	229533.I1RPV8	pp	229533.I1RXH2
54307	229533.I1RPV8	pp	229533.I1RST9
54308	229533.I1RPV8	pp	229533.I1S8D3
54309	229533.I1RPV8	pp	229533.V6RWB3
54310	229533.I1RPV8	pp	229533.I1RY81
54311	229533.I1RPV8	pp	229533.I1RW55
54312	229533.I1RPV8	pp	229533.I1RWM4

54313	229533.I1RPV8	pp	229533.I1S423
54314	229533.I1RPV8	pp	229533.I1S2F7
54315	229533.I1RPV8	pp	229533.V6RHX2
54316	229533.I1RPV8	pp	229533.Q4HYR2
54317	229533.I1RPV8	pp	229533.I1S0K1
54318	229533.I1RPV8	pp	229533.I1S3T6
54319	229533.I1RPV8	pp	229533.I1RWD2
54320	229533.I1RPV8	pp	229533.V6QVH2
54321	229533.I1RPV8	pp	229533.I1S223
54322	229533.I1RPV8	pp	229533.V6R3V4
54323	229533.I1RPV8	pp	229533.I1RQJ7
54324	229533.I1RPV8	pp	229533.V6RCC0
54325	229533.I1RPV8	pp	229533.I1RRS6
54326	229533.I1RPV8	pp	229533.I1RYP5
54327	229533.I1RPV8	pp	229533.I1SAI4
54328	229533.I1RPV8	pp	229533.I1S4S5
54329	229533.I1RPV8	pp	229533.I1S969
54330	229533.I1RPV8	pp	229533.V6R5T7
54331	229533.I1RPV8	pp	229533.V6QU99
54332	229533.I1RPV8	pp	229533.I1S048
54333	229533.I1RPV8	pp	229533.I1RQV7
54334	229533.I1RPV8	pp	229533.I1RQV8
54335	229533.I1RPV8	pp	229533.I1S210
54336	229533.I1RPV8	pp	229533.V6R0W1
54337	229533.I1RPV8	pp	229533.V6RAM6
54338	229533.I1RPV8	pp	229533.V6R9L8
54339	229533.I1RPV8	pp	229533.I1RQ76
54340	229533.I1RPV8	pp	229533.V6R1D2
54341	229533.I1RPV8	pp	229533.I1S5D0
54342	229533.I1RPV8	pp	229533.I1SAT2
54343	229533.I1RPV8	pp	229533.I1RTY9
54344	229533.I1RPV8	pp	229533.V6RF62
54345	229533.I1RPV8	pp	229533.I1RX18
54346	229533.I1RPV8	pp	229533.V6R2Z2
54347	229533.I1RPV8	pp	229533.I1RUE3
54348	229533.I1RPV8	pp	229533.I1S6V0
54349	229533.I1RPV8	pp	229533.I1RZ25
54350	229533.I1RPV8	pp	229533.I1S0X1
54351	229533.I1RPV8	pp	229533.I1S968
54352	229533.I1RPV8	pp	229533.I1RQW1
54353	229533.I1RPV8	pp	229533.V6R756
54354	229533.I1RPV8	pp	229533.I1RSA5
54355	229533.I1RPV8	pp	229533.I1RZV5
54356	229533.I1RPV8	pp	229533.V6RZD4
54357	229533.I1RPV8	pp	229533.I1RY35
54358	229533.I1RPV8	pp	229533.V6R5D2
54359	229533.I1RPV8	pp	229533.V6QZ48
54360	229533.I1RPV8	pp	229533.I1RZ38
54361	229533.I1RPV8	pp	229533.I1SAB4
54362	229533.I1RPV8	pp	229533.V6RCJ5
54363	229533.I1RPV8	pp	229533.I1SAH9
54364	229533.I1RBD5	pp	229533.I1RBY4
54365	229533.I1RBD5	pp	229533.I1S2H4
54366	229533.I1RBD5	pp	229533.I1RC66
54367	229533.I1RBD5	pp	229533.I1RYX5
54368	229533.I1RBD5	pp	229533.I1RX00
54369	229533.I1RBD5	pp	229533.I1RPV3
54370	229533.I1RBD5	pp	229533.I1RGA4
54371	229533.I1RBD5	pp	229533.I1S6Y8
54372	229533.I1RBD5	pp	229533.I1RWI5
54373	229533.I1RBD5	pp	229533.I1RSQ4
54374	229533.I1RBD5	pp	229533.I1RRT8
54375	229533.I1RBD5	pp	229533.I1S5R1
54376	229533.I1RBD5	pp	229533.I1RTD5
54377	229533.I1RBD5	pp	229533.I1RKI7
54378	229533.I1RBD5	pp	229533.O74638
54379	229533.I1RBD5	pp	229533.I1RZZ9
54380	229533.I1RBD5	pp	229533.I1RT40
54381	229533.I1RBD5	pp	229533.I1S9Y1
54382	229533.I1RBD5	pp	229533.I1RXA9
54383	229533.I1RBD5	pp	229533.I1RZ39
54384	229533.I1RBD5	pp	229533.Q4I624
54385	229533.I1RBD5	pp	229533.I1RN67

54386 229533.I1RBD5 pp 229533.I1RS47
54387 229533.I1RBD5 pp 229533.I1RMG6
54388 229533.I1RBD5 pp 229533.I1RXB2
54389 229533.I1RBD5 pp 229533.V6QVH8
54390 229533.I1RBD5 pp 229533.I1S0L4
54391 229533.I1RBD5 pp 229533.I1RCH5
54392 229533.I1RBD5 pp 229533.I1RRI2
54393 229533.I1RBD5 pp 229533.I1RTA1
54394 229533.I1RBD5 pp 229533.I1RC67
54395 229533.I1RBD5 pp 229533.I1RPP3
54396 229533.I1RBD5 pp 229533.I1RQE7
54397 229533.I1RBD5 pp 229533.I1RYW3
54398 229533.I1RBD5 pp 229533.I1RFT1
54399 229533.I1RBD5 pp 229533.I1RFL4
54400 229533.I1RBD5 pp 229533.I1RIH0
54401 229533.I1RBD5 pp 229533.I1S1C7
54402 229533.I1RBD5 pp 229533.I1RXW3
54403 229533.I1RBD5 pp 229533.I1RSX0
54404 229533.I1RBD5 pp 229533.I1RVX0
54405 229533.I1RBD5 pp 229533.I1RJV3
54406 229533.I1RBD5 pp 229533.I1RQ30
54407 229533.I1RBD5 pp 229533.I1RT03
54408 229533.I1RBD5 pp 229533.V6R3J1
54409 229533.I1RBD5 pp 229533.I1S7W5
54410 229533.I1RBD5 pp 229533.I1RS90
54411 229533.I1RBD5 pp 229533.I1S0J1
54412 229533.I1RBD5 pp 229533.I1RJU3
54413 229533.I1RBD5 pp 229533.I1RE01
54414 229533.I1RBD5 pp 229533.I1RMV0
54415 229533.I1RBD5 pp 229533.I1RD52
54416 229533.I1RBD5 pp 229533.I1RRX5
54417 229533.I1RBD5 pp 229533.I1RNC1
54418 229533.I1RBD5 pp 229533.I1RSQ7
54419 229533.I1RBD5 pp 229533.I1RUG3
54420 229533.I1RBD5 pp 229533.I1RPK0
54421 229533.I1RBD5 pp 229533.I1RD22
54422 229533.I1RBD5 pp 229533.I1RCR8
54423 229533.I1RBD5 pp 229533.I1RRN2
54424 229533.I1RBD5 pp 229533.I1S4S7
54425 229533.I1RBD5 pp 229533.V6R042
54426 229533.I1RBD5 pp 229533.I1S0T2
54427 229533.I1RBD5 pp 229533.I1RSQ3
54428 229533.I1RBD5 pp 229533.I1S2B9
54429 229533.I1RBD5 pp 229533.I1RW06
54430 229533.I1RBD5 pp 229533.I1S338
54431 229533.I1RBD5 pp 229533.I1RN46
54432 229533.I1RBD5 pp 229533.I1REK8
54433 229533.I1RBD5 pp 229533.I1RKI3
54434 229533.I1RBD5 pp 229533.I1S0K2
54435 229533.I1RBD5 pp 229533.I1RCR5
54436 229533.I1RBD5 pp 229533.I1RX46
54437 229533.I1RBD5 pp 229533.I1RCS1
54438 229533.I1RBD5 pp 229533.V6RTJ8
54439 229533.I1RBD5 pp 229533.Q4IJK2
54440 229533.I1RBD5 pp 229533.I1RBT1
54441 229533.I1RBD5 pp 229533.I1RR24
54442 229533.I1RBD5 pp 229533.I1RXV9
54443 229533.I1RBD5 pp 229533.I1RK95
54444 229533.I1RBD5 pp 229533.I1RPI5
54445 229533.I1RBD5 pp 229533.I1RXD7
54446 229533.I1RBD5 pp 229533.V6QUE6
54447 229533.I1RBD5 pp 229533.I1RS18
54448 229533.I1RBD5 pp 229533.I1RLP1
54449 229533.I1RBD5 pp 229533.I1RWB0
54450 229533.I1RBD5 pp 229533.I1S5B0
54451 229533.I1RBD5 pp 229533.I1RWC9
54452 229533.I1RBD5 pp 229533.I1RRY9
54453 229533.I1RBD5 pp 229533.I1RPR9
54454 229533.I1RBD5 pp 229533.I1RZK5
54455 229533.I1RBD5 pp 229533.I1RZM8
54456 229533.I1RBD5 pp 229533.I1RR51
54457 229533.I1RBD5 pp 229533.I1RXF0
54458 229533.I1RBD5 pp 229533.I1S419

54459	229533.I1RBD5	pp	229533.V6RCZ8
54460	229533.I1RBD5	pp	229533.I1S6K9
54461	229533.A0A0E0SKP9	pp	229533.I1RDK2
54462	229533.A0A0E0SKP9	pp	229533.I1RA50
54463	229533.A0A0E0SKP9	pp	229533.I1RWN2
54464	229533.A0A0E0SKP9	pp	229533.I1RAB7
54465	229533.A0A0E0SKP9	pp	229533.I1S074
54466	229533.A0A0E0SKP9	pp	229533.I1RNM5
54467	229533.A0A098D586	pp	229533.I1S0N8
54468	229533.A0A098D586	pp	229533.I1RW84
54469	229533.A0A098D586	pp	229533.I1RDK2
54470	229533.A0A098D586	pp	229533.I1RNE8
54471	229533.A0A098D586	pp	229533.I1RWN2
54472	229533.A0A098D586	pp	229533.I1S074
54473	229533.A0A098D586	pp	229533.Q4IBS9
54474	229533.A0A098D586	pp	229533.I1RNM5
54475	229533.A0A098D586	pp	229533.A0A0E0SKP9
54476	229533.I1RZR2	pp	229533.I1S0X0
54477	229533.I1RZ56	pp	229533.V6RF62
54478	229533.I1RZ56	pp	229533.I1S6V0
54479	229533.I1RZ56	pp	229533.V6RCJ5
54480	229533.I1RZ56	pp	229533.I1SAH9
54481	229533.I1RZ56	pp	229533.I1S0X1
54482	229533.I1RZ56	pp	229533.V6R756
54483	229533.I1RZ56	pp	229533.V6R5D2
54484	229533.I1RZ56	pp	229533.I1SAB4
54485	229533.I1RZ56	pp	229533.I1SAT2
54486	229533.I1RZ56	pp	229533.V6QZ48
54487	229533.I1RZ56	pp	229533.V6R2Z2
54488	229533.I1RZ56	pp	229533.V6R9L8
54489	229533.I1RZ56	pp	229533.V6RZD4
54490	229533.I1RZ56	pp	229533.I1RZV5
54491	229533.I1RZ56	pp	229533.I1S2F7
54492	229533.I1RZ56	pp	229533.I1S5J9
54493	229533.I1RZ56	pp	229533.I1S968
54494	229533.I1RZ56	pp	229533.I1S659
54495	229533.I1RZ56	pp	229533.I1S3T6
54496	229533.I1RZ56	pp	229533.I1S267
54497	229533.I1RZ56	pp	229533.I1S8H9
54498	229533.I1RZ56	pp	229533.V6R3V4
54499	229533.I1RZ56	pp	229533.V6RWB3
54500	229533.I1RZ56	pp	229533.I1S8D3
54501	229533.I1RZ56	pp	229533.V6RCC0
54502	229533.I1RZ56	pp	229533.I1S210
54503	229533.I1RZ56	pp	229533.V6RHX2
54504	229533.I1RZ56	pp	229533.Q4HYR2
54505	229533.I1RZ56	pp	229533.I1S423
54506	229533.I1RZ56	pp	229533.I1S4S5
54507	229533.I1RZ56	pp	229533.I1S5D0
54508	229533.I1RZ56	pp	229533.V6R1D2
54509	229533.I1RZ56	pp	229533.I1S969
54510	229533.I1RZ56	pp	229533.V6R0W1
54511	229533.I1RZ56	pp	229533.I1S0K1
54512	229533.I1RZ56	pp	229533.V6R5T7
54513	229533.I1RZ56	pp	229533.I1SAI4
54514	229533.I1RZ56	pp	229533.V6RAM6
54515	229533.I1RZ56	pp	229533.I1S048
54516	229533.I1RZ56	pp	229533.I1S223
54517	229533.I1RZ56	pp	229533.V6QU99
54518	229533.I1RZ56	pp	229533.V6QVH2
54519	229533.I1RRE9	pp	229533.I1SAF7
54520	229533.I1RK80	pp	229533.Q4HWB3
54521	229533.I1RK80	pp	229533.I1RT45
54522	229533.I1RK80	pp	229533.V6RLW4
54523	229533.I1RK80	pp	229533.I1S0K6
54524	229533.I1RK80	pp	229533.I1S9G7
54525	229533.I1RK80	pp	229533.I1RMN5
54526	229533.I1RK80	pp	229533.I1RKF9
54527	229533.I1RK80	pp	229533.I1RNF5
54528	229533.I1RK80	pp	229533.I1RW26
54529	229533.I1RK80	pp	229533.I1RZR2
54530	229533.I1RK80	pp	229533.Q4I9U7
54531	229533.I1RK80	pp	229533.I1RM49

54532	229533.I1RG62	pp	229533.Q4I7T5
54533	229533.I1RG62	pp	229533.V6R3J1
54534	229533.I1RG62	pp	229533.I1RRX5
54535	229533.I1RG62	pp	229533.I1RL81
54536	229533.I1RG62	pp	229533.I1S0P0
54537	229533.I1RG62	pp	229533.I1RYE5
54538	229533.I1RG62	pp	229533.I1S1F3
54539	229533.I1RG62	pp	229533.I1RTL0
54540	229533.I1RG62	pp	229533.I1RX39
54541	229533.I1RG62	pp	229533.I1RI27
54542	229533.I1RG62	pp	229533.I1RYN2
54543	229533.I1RG62	pp	229533.I1RXD9
54544	229533.I1RG62	pp	229533.I1S464
54545	229533.I1RG62	pp	229533.I1RNM7
54546	229533.I1RG62	pp	229533.I1RS05
54547	229533.I1RG62	pp	229533.I1RYT8
54548	229533.I1RG62	pp	229533.I1RP02
54549	229533.I1RG62	pp	229533.I1RWA3
54550	229533.I1RG62	pp	229533.I1RPM4
54551	229533.I1RG62	pp	229533.I1RNY6
54552	229533.I1RG62	pp	229533.I1RXE6
54553	229533.I1RG62	pp	229533.I1RMK7
54554	229533.I1RG62	pp	229533.I1RY21
54555	229533.I1RG62	pp	229533.I1S0Q7
54556	229533.I1RG62	pp	229533.I1RY92
54557	229533.I1RG62	pp	229533.I1S8F8
54558	229533.I1RG62	pp	229533.I1RSQ4
54559	229533.I1RG62	pp	229533.I1RXZ3
54560	229533.I1RG62	pp	229533.I1RNJ5
54561	229533.I1RG62	pp	229533.I1RYC8
54562	229533.I1RG62	pp	229533.I1RMQ6
54563	229533.I1RG62	pp	229533.I1RNQ5
54564	229533.I1RG62	pp	229533.I1RYM5
54565	229533.I1RG62	pp	229533.V6RJP8
54566	229533.I1RG62	pp	229533.I1RKQ6
54567	229533.I1RG62	pp	229533.I1RMI1
54568	229533.I1RG62	pp	229533.I1RKB7
54569	229533.I1RG62	pp	229533.I1S5X9
54570	229533.I1RG62	pp	229533.I1RL71
54571	229533.I1RG62	pp	229533.I1RS29
54572	229533.I1RG62	pp	229533.I1S212
54573	229533.I1RG62	pp	229533.I1RPP5
54574	229533.I1RG62	pp	229533.I1RQ21
54575	229533.I1RG62	pp	229533.I1RQS2
54576	229533.I1RG62	pp	229533.I1RQR6
54577	229533.I1RG62	pp	229533.I1RYA0
54578	229533.I1RG62	pp	229533.I1RPR9
54579	229533.I1RG62	pp	229533.I1S8S9
54580	229533.I1RG62	pp	229533.I1S0K2
54581	229533.I1RG62	pp	229533.I1RZ43
54582	229533.I1RG62	pp	229533.I1S0R8
54583	229533.I1RGZ3	pp	229533.V6RAM6
54584	229533.I1RGZ3	pp	229533.I1RZV5
54585	229533.I1RGZ3	pp	229533.V6RUY9
54586	229533.I1RGZ3	pp	229533.I1RZA8
54587	229533.I1RGZ3	pp	229533.V6QU99
54588	229533.I1RGZ3	pp	229533.I1SAI4
54589	229533.I1RGZ3	pp	229533.I1RY16
54590	229533.I1RGZ3	pp	229533.I1RJC5
54591	229533.I1RGZ3	pp	229533.V6R5D2
54592	229533.I1RGZ3	pp	229533.I1S4S5
54593	229533.I1RGZ3	pp	229533.V6R0W1
54594	229533.I1RGZ3	pp	229533.I1S694
54595	229533.I1RGZ3	pp	229533.I1RQP7
54596	229533.I1RGZ3	pp	229533.V6QVH2
54597	229533.I1RGZ3	pp	229533.V6R1D2
54598	229533.I1RGZ3	pp	229533.I1RI11
54599	229533.I1RGZ3	pp	229533.I1RX18
54600	229533.I1RGZ3	pp	229533.I1S8H9
54601	229533.I1RGZ3	pp	229533.V6QZ48
54602	229533.I1RGZ3	pp	229533.I1RYK0
54603	229533.I1RGZ3	pp	229533.V6R2Z2
54604	229533.I1RGZ3	pp	229533.I1RXH2

54605	229533.I1RGZ3	pp	229533.V6RN69
54606	229533.I1RGZ3	pp	229533.I1S5J9
54607	229533.I1RGZ3	pp	229533.V6RF62
54608	229533.I1RGZ3	pp	229533.V6RLM7
54609	229533.I1RGZ3	pp	229533.V6RZD4
54610	229533.I1RGZ3	pp	229533.I1RLZ4
54611	229533.I1RGZ3	pp	229533.I1RWF5
54612	229533.I1RGZ3	pp	229533.I1RKB6
54613	229533.I1RGZ3	pp	229533.V6R3V4
54614	229533.I1RGZ3	pp	229533.I1RKB5
54615	229533.I1RGZ3	pp	229533.V6R9L8
54616	229533.I1RGZ3	pp	229533.V6R756
54617	229533.I1RGZ3	pp	229533.I1RY12
54618	229533.I1RGZ3	pp	229533.I1RI40
54619	229533.I1RGZ3	pp	229533.I1RWD2
54620	229533.A0A1C3YJU4	pp	229533.I1S472
54621	229533.A0A1C3YJU4	pp	229533.I1RM55
54622	229533.A0A1C3YJU4	pp	229533.I1RB01
54623	229533.A0A1C3YJU4	pp	229533.V6RN44
54624	229533.A0A1C3YJU4	pp	229533.I1S087
54625	229533.A0A1C3YJU4	pp	229533.A0A1C3YLD0
54626	229533.A0A1C3YJU4	pp	229533.V6RV57
54627	229533.A0A1C3YJU4	pp	229533.I1RB14
54628	229533.A0A1C3YJU4	pp	229533.I1RDY3
54629	229533.A0A1C3YJU4	pp	229533.I1RH82
54630	229533.I1R9W5	pp	229533.I1RCJ0
54631	229533.I1R9W5	pp	229533.I1RBB4
54632	229533.A0A1C3YIW9	pp	229533.I1RZM9
54633	229533.A0A1C3YIW9	pp	229533.I1RJF5
54634	229533.A0A1C3YIW9	pp	229533.I1RFJ6
54635	229533.A0A1C3YIW9	pp	229533.A0A1C3YKY6
54636	229533.A0A1C3YIW9	pp	229533.I1S212
54637	229533.A0A1C3YIW9	pp	229533.I1RQS2
54638	229533.A0A1C3YIW9	pp	229533.I1RHG0
54639	229533.A0A1C3YIW9	pp	229533.I1RPR9
54640	229533.A0A1C3YIW9	pp	229533.A0A1C3YKQ4
54641	229533.A0A1C3YIW9	pp	229533.I1RRY9
54642	229533.A0A1C3YIW9	pp	229533.I1S8S9
54643	229533.A0A1C3YIW9	pp	229533.I1RAM9
54644	229533.A0A1C3YIW9	pp	229533.I1RCG4
54645	229533.A0A1C3YIW9	pp	229533.A0A1C3YLB0
54646	229533.A0A1C3YIW9	pp	229533.I1RD17
54647	229533.A0A1C3YIW9	pp	229533.I1RFJ5
54648	229533.A0A1C3YIW9	pp	229533.I1RBT6
54649	229533.A0A1C3YIW9	pp	229533.I1RXP4
54650	229533.A0A1C3YIW9	pp	229533.I1S1G0
54651	229533.A0A1C3YIW9	pp	229533.I1RWT0
54652	229533.A0A1C3YIW9	pp	229533.I1RZ43
54653	229533.A0A1C3YIW9	pp	229533.I1RKB7
54654	229533.A0A1C3YIW9	pp	229533.Q4I5G1
54655	229533.A0A1C3YIW9	pp	229533.I1S5X9
54656	229533.A0A1C3YIW9	pp	229533.I1RKQ6
54657	229533.A0A1C3YIW9	pp	229533.A0A1C3YKM9
54658	229533.A0A1C3YIW9	pp	229533.I1S3U5
54659	229533.A0A1C3YIW9	pp	229533.I1RAV5
54660	229533.A0A1C3YIW9	pp	229533.I1RRM5
54661	229533.A0A1C3YIW9	pp	229533.I1RS29
54662	229533.A0A1C3YIW9	pp	229533.V6RIV9
54663	229533.A0A1C3YIW9	pp	229533.I1S0R8
54664	229533.A0A1C3YIW9	pp	229533.I1S0K2
54665	229533.A0A1C3YIW9	pp	229533.A0A1C3YKP5
54666	229533.A0A1C3YIW9	pp	229533.I1RYT8
54667	229533.A0A1C3YIW9	pp	229533.I1RG62
54668	229533.A0A1C3YIW9	pp	229533.I1RNQ5
54669	229533.A0A1C3YIW9	pp	229533.I1RMI1
54670	229533.A0A1C3YIW9	pp	229533.I1REZ6
54671	229533.A0A1C3YIW9	pp	229533.I1RLU6
54672	229533.A0A1C3YIW9	pp	229533.I1S2E7
54673	229533.A0A1C3YIW9	pp	229533.I1RYN2
54674	229533.A0A1C3YIW9	pp	229533.I1RA52
54675	229533.A0A1C3YIW9	pp	229533.I1RPP1
54676	229533.A0A1C3YIW9	pp	229533.I1RF87
54677	229533.A0A1C3YIW9	pp	229533.I1RL71

54678 229533.A0A1C3YIW9 pp 229533.I1RAE4
54679 229533.A0A1C3YIW9 pp 229533.I1RFS5
54680 229533.A0A1C3YIW9 pp 229533.I1RD22
54681 229533.A0A1C3YIW9 pp 229533.I1RAW3
54682 229533.A0A1C3YIW9 pp 229533.I1S504
54683 229533.A0A1C3YIW9 pp 229533.I1RNM7
54684 229533.A0A1C3YIW9 pp 229533.I1RPM4
54685 229533.A0A1C3YIW9 pp 229533.I1RAI4
54686 229533.A0A1C3YIW9 pp 229533.I1RP02
54687 229533.A0A1C3YIW9 pp 229533.I1RWA3
54688 229533.A0A1C3YIW9 pp 229533.I1RE65
54689 229533.A0A1C3YIW9 pp 229533.A0A1C3YK94
54690 229533.A0A1C3YIW9 pp 229533.I1RXE6
54691 229533.A0A1C3YIW9 pp 229533.V6RJP8
54692 229533.A0A1C3YIW9 pp 229533.I1RB38
54693 229533.A0A1C3YIW9 pp 229533.I1RTQ5
54694 229533.A0A1C3YIW9 pp 229533.A0A1C3YJU7
54695 229533.A0A1C3YIW9 pp 229533.A0A1C3YLB6
54696 229533.A0A1C3YIW9 pp 229533.I1RE01
54697 229533.A0A1C3YIW9 pp 229533.I1RMK7
54698 229533.A0A1C3YIW9 pp 229533.I1S503
54699 229533.A0A1C3YIW9 pp 229533.I1RHX8
54700 229533.A0A1C3YIW9 pp 229533.I1RJW6
54701 229533.A0A1C3YIW9 pp 229533.I1RJW0
54702 229533.A0A1C3YIW9 pp 229533.V6RCG5
54703 229533.A0A1C3YIW9 pp 229533.A0A1C3YJQ2
54704 229533.A0A1C3YIW9 pp 229533.I1S0Q7
54705 229533.A0A1C3YIW9 pp 229533.I1RUP9
54706 229533.A0A1C3YIW9 pp 229533.I1RY21
54707 229533.A0A1C3YIW9 pp 229533.I1R9Z5
54708 229533.A0A1C3YIW9 pp 229533.V6R3J1
54709 229533.A0A1C3YIW9 pp 229533.I1RS05
54710 229533.A0A1C3YIW9 pp 229533.I1RXZ3
54711 229533.A0A1C3YIW9 pp 229533.I1RDC3
54712 229533.A0A1C3YIW9 pp 229533.I1RE66
54713 229533.A0A1C3YIW9 pp 229533.I1R9Y4
54714 229533.A0A1C3YIW9 pp 229533.I1S8F0
54715 229533.A0A1C3YIW9 pp 229533.I1S0P0
54716 229533.A0A1C3YIW9 pp 229533.I1RMY6
54717 229533.A0A1C3YIW9 pp 229533.A0A1C3YMC1
54718 229533.A0A1C3YIW9 pp 229533.I1RBM4
54719 229533.A0A1C3YIW9 pp 229533.I1RTL0
54720 229533.A0A1C3YIW9 pp 229533.I1RY92
54721 229533.A0A1C3YIW9 pp 229533.I1RAE3
54722 229533.A0A1C3YIW9 pp 229533.I1S0L3
54723 229533.A0A1C3YIW9 pp 229533.A0A1C3YMN6
54724 229533.A0A1C3YIW9 pp 229533.I1RX39
54725 229533.A0A1C3YIW9 pp 229533.I1RMQ6
54726 229533.A0A1C3YIW9 pp 229533.I1S1F3
54727 229533.A0A1C3YIW9 pp 229533.I1RBE6
54728 229533.A0A1C3YIW9 pp 229533.I1S1V6
54729 229533.A0A1C3YIW9 pp 229533.I1RA49
54730 229533.A0A1C3YIW9 pp 229533.I1RJM7
54731 229533.A0A1C3YIW9 pp 229533.I1RIF9
54732 229533.A0A1C3YIW9 pp 229533.I1RWK3
54733 229533.A0A1C3YIW9 pp 229533.I1RXD9
54734 229533.A0A1C3YIW9 pp 229533.I1S464
54735 229533.A0A1C3YIW9 pp 229533.I1RTD5
54736 229533.A0A1C3YIW9 pp 229533.I1RRX5
54737 229533.A0A1C3YIW9 pp 229533.A0A1C3YLM6
54738 229533.A0A1C3YIW9 pp 229533.A0A1C3YM60
54739 229533.A0A1C3YIW9 pp 229533.I1RL35
54740 229533.I1RK82 pp 229533.I1RK83
54741 229533.I1RZN8 pp 229533.Q4IJM4
54742 229533.I1RRL5 pp 229533.I1RSX2
54743 229533.I1RRL5 pp 229533.I1RWT6
54744 229533.I1RRL5 pp 229533.I1RXX0
54745 229533.I1RRL5 pp 229533.I1S334
54746 229533.I1RRL5 pp 229533.I1RW06
54747 229533.I1RRL5 pp 229533.I1S224
54748 229533.I1RRL5 pp 229533.I1S267
54749 229533.I1RRL5 pp 229533.I1S5J9
54750 229533.I1RRL5 pp 229533.I1RXH2

54751	229533.I1RRL5	pp	229533.I1SAI4
54752	229533.I1RRL5	pp	229533.V6R1D2
54753	229533.I1RRL5	pp	229533.V6QU99
54754	229533.I1RRL5	pp	229533.I1S8E0
54755	229533.I1RRL5	pp	229533.I1RY44
54756	229533.I1RRL5	pp	229533.V6R9L8
54757	229533.I1RRL5	pp	229533.V6RAM6
54758	229533.I1RRL5	pp	229533.I1RZC3
54759	229533.I1RRL5	pp	229533.I1RUP2
54760	229533.I1RRL5	pp	229533.V6R0W1
54761	229533.I1RRL5	pp	229533.I1RX18
54762	229533.I1RRL5	pp	229533.V6R756
54763	229533.I1RRL5	pp	229533.V6QZ48
54764	229533.I1RRL5	pp	229533.Q4IMJ1
54765	229533.I1RRL5	pp	229533.I1RRW0
54766	229533.I1RRL5	pp	229533.I1RZN1
54767	229533.I1RRL5	pp	229533.V6R2Z2
54768	229533.I1RRL5	pp	229533.I1RWF9
54769	229533.I1RRL5	pp	229533.I1S2E9
54770	229533.I1RRL5	pp	229533.I1S0H7
54771	229533.I1RRL5	pp	229533.I1RY32
54772	229533.I1RAA8	pp	229533.I1S0K9
54773	229533.I1RAA8	pp	229533.I1RJS9
54774	229533.I1RAA8	pp	229533.I1S549
54775	229533.I1RAA8	pp	229533.I1RQA2
54776	229533.I1RAA8	pp	229533.I1RRP3
54777	229533.I1RAA8	pp	229533.I1RJK3
54778	229533.I1RAA8	pp	229533.I1RMH2
54779	229533.I1RAA8	pp	229533.I1RML4
54780	229533.I1RBB4	pp	229533.I1RKF7
54781	229533.I1RMN5	pp	229533.I1RW26
54782	229533.I1RMN5	pp	229533.I1RNF5
54783	229533.I1RZP4	pp	229533.I1S087
54784	229533.I1RX39	pp	229533.I1S1V6
54785	229533.I1RX39	pp	229533.I1RY21
54786	229533.I1RX39	pp	229533.I1S1F3
54787	229533.I1RX39	pp	229533.I1S3U5
54788	229533.I1RX39	pp	229533.I1RYN2
54789	229533.I1RX39	pp	229533.I1RYT8
54790	229533.I1RX39	pp	229533.I1RYE5
54791	229533.I1RX39	pp	229533.I1RY92
54792	229533.I1RX39	pp	229533.I1S0P0
54793	229533.I1RX39	pp	229533.I1S1G0
54794	229533.I1RX39	pp	229533.Q4I5G1
54795	229533.I1RX39	pp	229533.I1RZM9
54796	229533.I1RX39	pp	229533.I1RXP4
54797	229533.I1RX39	pp	229533.I1S464
54798	229533.I1RX39	pp	229533.I1RZ43
54799	229533.I1RX39	pp	229533.I1RXD9
54800	229533.I1RX39	pp	229533.I1RYH7
54801	229533.I1RX39	pp	229533.I1S212
54802	229533.I1RX39	pp	229533.I1S5X9
54803	229533.I1RX39	pp	229533.I1RXE6
54804	229533.I1RX39	pp	229533.I1S2E7
54805	229533.I1RX39	pp	229533.I1S8S9
54806	229533.I1RX39	pp	229533.V6RJP8
54807	229533.I1RX39	pp	229533.I1S0R8
54808	229533.I1RX39	pp	229533.I1S0K2
54809	229533.I1RX39	pp	229533.V6RCG5
54810	229533.I1RX39	pp	229533.I1S0Q7
54811	229533.I1RJS1	pp	229533.I1RQV7
54812	229533.I1RJS1	pp	229533.I1RMA2
54813	229533.I1RJS1	pp	229533.I1S210
54814	229533.I1RJS1	pp	229533.I1S223
54815	229533.I1RJS1	pp	229533.I1RQ76
54816	229533.I1RJS1	pp	229533.I1RJS7
54817	229533.I1RJS1	pp	229533.V6QVH2
54818	229533.I1RJS1	pp	229533.V6RAM6
54819	229533.I1RJS1	pp	229533.I1S969
54820	229533.I1RJS1	pp	229533.I1S5D0
54821	229533.I1RJS1	pp	229533.I1RRS6
54822	229533.I1RJS1	pp	229533.I1S0K1
54823	229533.I1RJS1	pp	229533.I1SAI4

54824	229533.I1RJS1	pp	229533.I1RMK4
54825	229533.I1RJS1	pp	229533.I1RQV8
54826	229533.I1RJS1	pp	229533.V6R5T7
54827	229533.I1RJS1	pp	229533.I1RLA2
54828	229533.I1RJS1	pp	229533.I1S4S5
54829	229533.I1RJS1	pp	229533.V6RCC0
54830	229533.I1RJS1	pp	229533.I1RYP5
54831	229533.I1RJS1	pp	229533.I1S048
54832	229533.I1RJS1	pp	229533.V6QU99
54833	229533.I1RJS1	pp	229533.I1RWM4
54834	229533.I1RJS1	pp	229533.V6R0W1
54835	229533.I1RJS1	pp	229533.I1S423
54836	229533.I1RJS1	pp	229533.V6R1D2
54837	229533.I1RJS1	pp	229533.I1RXH2
54838	229533.I1RJS1	pp	229533.V6RHX2
54839	229533.I1RJS1	pp	229533.I1RNG5
54840	229533.I1RJS1	pp	229533.I1S2F7
54841	229533.I1RJS1	pp	229533.I1S5J9
54842	229533.I1RJS1	pp	229533.I1S8D3
54843	229533.I1RJS1	pp	229533.I1S267
54844	229533.I1RJS1	pp	229533.Q4HYR2
54845	229533.I1RJS1	pp	229533.I1RW55
54846	229533.I1RJS1	pp	229533.I1RST9
54847	229533.I1RJS1	pp	229533.I1S3T6
54848	229533.I1RJS1	pp	229533.V6RWB3
54849	229533.I1RJS1	pp	229533.I1S8H9
54850	229533.I1RJS1	pp	229533.I1RQJ7
54851	229533.I1RJS1	pp	229533.I1S659
54852	229533.I1RJS1	pp	229533.I1RY81
54853	229533.I1RJS1	pp	229533.I1RSA5
54854	229533.I1RJS1	pp	229533.I1RWD2
54855	229533.I1RJS1	pp	229533.V6RZD4
54856	229533.I1RJS1	pp	229533.V6R3V4
54857	229533.I1RJS1	pp	229533.V6R5D2
54858	229533.I1RJS1	pp	229533.I1RY35
54859	229533.I1RJS1	pp	229533.I1RZ25
54860	229533.I1RJS1	pp	229533.I1RKD3
54861	229533.I1RJS1	pp	229533.I1RLZ4
54862	229533.I1RJS1	pp	229533.I1RZV5
54863	229533.I1RJS1	pp	229533.V6R2Z2
54864	229533.I1RJS1	pp	229533.I1S968
54865	229533.I1RJS1	pp	229533.I1SAH9
54866	229533.I1RJS1	pp	229533.I1RK55
54867	229533.I1RJS1	pp	229533.I1RZ38
54868	229533.I1RJS1	pp	229533.I1SAB4
54869	229533.I1RJS1	pp	229533.V6QZ48
54870	229533.I1RJS1	pp	229533.V6R756
54871	229533.I1RJS1	pp	229533.I1RQW1
54872	229533.I1RJS1	pp	229533.V6RCJ5
54873	229533.I1RJS1	pp	229533.I1SAT2
54874	229533.I1RJS1	pp	229533.I1RUE3
54875	229533.I1RJS1	pp	229533.I1RX18
54876	229533.I1RJS1	pp	229533.V6R9L8
54877	229533.I1RJS1	pp	229533.I1RTY9
54878	229533.I1RJS1	pp	229533.I1RPV8
54879	229533.I1RJS1	pp	229533.I1S0X1
54880	229533.I1RJS1	pp	229533.I1RP12
54881	229533.I1RJS1	pp	229533.I1S6V0
54882	229533.I1RJS1	pp	229533.V6RF62
54883	229533.I1RJB5	pp	229533.I1S0X0
54884	229533.I1RJB5	pp	229533.I1RKF9
54885	229533.I1RJB5	pp	229533.I1RJZ7
54886	229533.I1RJB5	pp	229533.I1RWI6
54887	229533.I1RJB5	pp	229533.I1RW71
54888	229533.I1RJB5	pp	229533.I1RZL3
54889	229533.I1RJB5	pp	229533.I1RMP2
54890	229533.I1RJB5	pp	229533.I1RK80
54891	229533.A0A098D8B8	pp	229533.I1RMY6
54892	229533.A0A098D8B8	pp	229533.I1RMQ6
54893	229533.A0A098D8B8	pp	229533.I1RKB7
54894	229533.A0A098D8B8	pp	229533.A0A1C3YI09
54895	229533.A0A098D8B8	pp	229533.I1RG62
54896	229533.A0A098D8B8	pp	229533.A0A098DMK7

229533.A0A098D8B8 pp 229533.I1RYN2
229533.A0A098D8B8 pp 229533.I1RTQ5
229533.A0A098D8B8 pp 229533.I1RDC3
229533.A0A098D8B8 pp 229533.I1S1F3
229533.A0A098D8B8 pp 229533.A0A1C3YIW9
229533.A0A098D8B8 pp 229533.I1RBM4
229533.A0A098D8B8 pp 229533.I1RTL0
229533.A0A098D8B8 pp 229533.I1RJF5
229533.A0A098D8B8 pp 229533.A0A1C3YLM6
229533.A0A098D8B8 pp 229533.A0A1C3YKQ4
229533.A0A098D8B8 pp 229533.I1RL35
229533.A0A098D8B8 pp 229533.A0A098E413
229533.A0A098D8B8 pp 229533.I1REZ6
229533.A0A098D8B8 pp 229533.A0A0E0RPV7
229533.A0A098D8B8 pp 229533.A0A1C3YN65
229533.A0A098D8B8 pp 229533.I1RYH7
229533.A0A098D8B8 pp 229533.I1S1V6
229533.A0A098D8B8 pp 229533.I1RL71
229533.A0A098D8B8 pp 229533.I1RPP1
229533.A0A098D8B8 pp 229533.I1RHX8
229533.A0A098D8B8 pp 229533.A0A098DPE0
229533.A0A098D8B8 pp 229533.A0A098DW31
229533.A0A098D8B8 pp 229533.I1RYE8
229533.A0A098D8B8 pp 229533.I1RXP4
229533.A0A098D8B8 pp 229533.I1S1G0
229533.A0A098D8B8 pp 229533.V6R8C4
229533.A0A098D8B8 pp 229533.I1S0R8
229533.A0A098D8B8 pp 229533.I1RN18
229533.A0A098D8B8 pp 229533.A0A098DC40
229533.A0A098D8B8 pp 229533.I1S3U5
229533.A0A098D8B8 pp 229533.A0A098DNZ7
229533.A0A098D8B8 pp 229533.I1RF87
229533.A0A098D8B8 pp 229533.A0A098DWS1
229533.A0A098D8B8 pp 229533.I1S8F0
229533.A0A098D8B8 pp 229533.I1RKQ6
229533.A0A098D8B8 pp 229533.I1S2E7
229533.A0A098D8B8 pp 229533.I1S212
229533.A0A098D8B8 pp 229533.I1RHG0
229533.A0A098D8B8 pp 229533.A0A098DI50
229533.A0A098D8B8 pp 229533.I1S0Q7
229533.A0A098D8B8 pp 229533.I1RJW0
229533.A0A098D8B8 pp 229533.I1RAM9
229533.A0A098D8B8 pp 229533.I1RIF9
229533.A0A098D8B8 pp 229533.I1RX39
229533.A0A098D8B8 pp 229533.I1RZ43
229533.A0A098D8B8 pp 229533.A0A1C3YLB0
229533.A0A098D8B8 pp 229533.A0A1C3YM43
229533.A0A098D8B8 pp 229533.I1RBE6
229533.A0A098D8B8 pp 229533.A0A1C3YII3
229533.A0A098D8B8 pp 229533.I1RAV5
229533.I1RX17 pp 229533.I1RXF0
229533.I1RX17 pp 229533.I1RX39
229533.I1RX17 pp 229533.I1S277
229533.I1RX17 pp 229533.I1RYE5
229533.I1RX17 pp 229533.I1S212
229533.I1RX17 pp 229533.I1S1F3
229533.I1RX17 pp 229533.I1S6Y3
229533.I1RX17 pp 229533.Q4I5G1
229533.I1S334 pp 229533.I1SAI4
229533.I1S334 pp 229533.V6R0W1
229533.I1S334 pp 229533.I1S5J9
229533.I1S334 pp 229533.V6R1D2
229533.I1S334 pp 229533.V6QU99
229533.I1S334 pp 229533.V6RAM6
229533.I1S334 pp 229533.V6QZ48
229533.I1S334 pp 229533.V6R9L8
229533.I1S334 pp 229533.V6R756
229533.I1S334 pp 229533.I1S8E0
229533.I1S334 pp 229533.Q4IMJ1
229533.I1S334 pp 229533.V6R2Z2
229533.A0A1C3YLD5 pp 229533.I1S1Z9
229533.A0A1C3YLD5 pp 229533.I1RX67
229533.I1RDN3 pp 229533.V6QU99

54970	229533.I1RDN3	pp	229533.I1RQV8
54971	229533.I1RDN3	pp	229533.I1RY16
54972	229533.I1RDN3	pp	229533.I1RHT5
54973	229533.I1RDN3	pp	229533.I1RIF7
54974	229533.I1RDN3	pp	229533.I1RVI8
54975	229533.I1RDN3	pp	229533.I1S4S5
54976	229533.I1RDN3	pp	229533.I1RMK4
54977	229533.I1RDN3	pp	229533.V6RAM6
54978	229533.I1RDN3	pp	229533.I1S969
54979	229533.I1RDN3	pp	229533.I1S0K1
54980	229533.I1RDN3	pp	229533.I1RRS6
54981	229533.I1RDN3	pp	229533.V6R1D2
54982	229533.I1RDN3	pp	229533.V6R0W1
54983	229533.I1RDN3	pp	229533.I1S223
54984	229533.I1RDN3	pp	229533.I1RJG4
54985	229533.I1RDN3	pp	229533.I1RLA2
54986	229533.I1RDN3	pp	229533.I1RMA2
54987	229533.I1RDN3	pp	229533.I1S5D0
54988	229533.I1RDN3	pp	229533.I1S048
54989	229533.I1RDN3	pp	229533.V6QVH2
54990	229533.I1RDN3	pp	229533.I1RQV7
54991	229533.I1RDN3	pp	229533.I1RGU3
54992	229533.I1RDN3	pp	229533.I1S8C1
54993	229533.I1RDN3	pp	229533.I1RGK3
54994	229533.I1RDN3	pp	229533.I1RST9
54995	229533.I1RDN3	pp	229533.V6R5T7
54996	229533.I1RDN3	pp	229533.I1S210
54997	229533.I1RDN3	pp	229533.I1RIB5
54998	229533.I1RDN3	pp	229533.V6RCC0
54999	229533.I1RDN3	pp	229533.I1RJS7
55000	229533.I1RDN3	pp	229533.I1SAI4
55001	229533.I1RDN3	pp	229533.I1RI40
55002	229533.I1RDN3	pp	229533.I1S8D3
55003	229533.I1RDN3	pp	229533.I1REE2
55004	229533.I1RDN3	pp	229533.I1S3T6
55005	229533.I1RDN3	pp	229533.I1RJC5
55006	229533.I1RDN3	pp	229533.I1RWM4
55007	229533.I1RDN3	pp	229533.I1S423
55008	229533.I1RDN3	pp	229533.I1RYP5
55009	229533.I1RDN3	pp	229533.Q4HYR2
55010	229533.I1RDN3	pp	229533.I1RW55
55011	229533.I1RDN3	pp	229533.V6RPS5
55012	229533.I1RDN3	pp	229533.V6RWB3
55013	229533.I1RDN3	pp	229533.I1RXH2
55014	229533.I1RDN3	pp	229533.I1S2F7
55015	229533.I1RDN3	pp	229533.I1S5J9
55016	229533.I1RDN3	pp	229533.I1RH67
55017	229533.I1RDN3	pp	229533.I1S267
55018	229533.I1RDN3	pp	229533.I1RNG5
55019	229533.I1RDN3	pp	229533.V6RHX2
55020	229533.I1RDN3	pp	229533.I1RNH4
55021	229533.I1RDN3	pp	229533.I1S8H9
55022	229533.I1RDN3	pp	229533.I1RWD2
55023	229533.I1RDN3	pp	229533.V6R3V4
55024	229533.I1RDN3	pp	229533.I1RUQ3
55025	229533.I1RDN3	pp	229533.I1RDV8
55026	229533.I1RDN3	pp	229533.I1RJY7
55027	229533.I1RDN3	pp	229533.I1RIM7
55028	229533.I1RDN3	pp	229533.I1RRM1
55029	229533.I1RDN3	pp	229533.I1RF24
55030	229533.I1RDN3	pp	229533.I1S659
55031	229533.I1RDN3	pp	229533.I1RY81
55032	229533.I1RDN3	pp	229533.I1RR68
55033	229533.I1RDN3	pp	229533.I1S0A5
55034	229533.I1RDN3	pp	229533.I1RMT6
55035	229533.I1RDN3	pp	229533.I1RZ25
55036	229533.I1RDN3	pp	229533.V6RZD4
55037	229533.I1RDN3	pp	229533.I1RLZ4
55038	229533.I1RDN3	pp	229533.I1RFB0
55039	229533.I1RDN3	pp	229533.I1RZ38
55040	229533.I1RDN3	pp	229533.I1S968
55041	229533.I1RDN3	pp	229533.I1RSA5
55042	229533.I1RDN3	pp	229533.I1RZV5

55043 229533.I1RDN3 pp 229533.I1S299
55044 229533.I1RDN3 pp 229533.I1RI19
55045 229533.I1RDN3 pp 229533.I1RF59
55046 229533.I1RDN3 pp 229533.I1S2Q4
55047 229533.I1RDN3 pp 229533.V6RCJ5
55048 229533.I1RDN3 pp 229533.V6R2Z2
55049 229533.I1RDN3 pp 229533.I1SAH9
55050 229533.I1RDN3 pp 229533.V6QZ48
55051 229533.I1RDN3 pp 229533.I1REW1
55052 229533.I1RDN3 pp 229533.I1SAB4
55053 229533.I1RDN3 pp 229533.V6R9L8
55054 229533.I1RDN3 pp 229533.I1RN88
55055 229533.I1RDN3 pp 229533.I1S3T7
55056 229533.I1RDN3 pp 229533.I1RWX5
55057 229533.I1RDN3 pp 229533.I1RQW1
55058 229533.I1RDN3 pp 229533.I1RKD3
55059 229533.I1RDN3 pp 229533.I1S0X1
55060 229533.I1RDN3 pp 229533.I1S8R2
55061 229533.I1RDN3 pp 229533.V6R5D2
55062 229533.I1RDN3 pp 229533.I1RHJ0
55063 229533.I1RDN3 pp 229533.I1S6V0
55064 229533.I1RDN3 pp 229533.I1RH83
55065 229533.I1RDN3 pp 229533.I1S149
55066 229533.I1RDN3 pp 229533.I1RIJ6
55067 229533.I1RDN3 pp 229533.V6RF62
55068 229533.I1RDN3 pp 229533.I1RK55
55069 229533.I1RDN3 pp 229533.I1RGC3
55070 229533.I1RDN3 pp 229533.I1RF85
55071 229533.I1RDN3 pp 229533.I1RX18
55072 229533.I1RDN3 pp 229533.I1RUE3
55073 229533.I1RDN3 pp 229533.I1RTY9
55074 229533.I1RDN3 pp 229533.I1RP12
55075 229533.I1RDN3 pp 229533.I1SAT2
55076 229533.I1RDN3 pp 229533.V6R756
55077 229533.I1RZ43 pp 229533.I1S212
55078 229533.I1RZ43 pp 229533.I1S0Q7
55079 229533.I1RZ43 pp 229533.I1S1F3
55080 229533.I1RZ43 pp 229533.I1S458
55081 229533.I1RZ43 pp 229533.I1S0R8
55082 229533.I1RDY3 pp 229533.I1RRB5
55083 229533.I1RDY3 pp 229533.I1RGA0
55084 229533.I1RDY3 pp 229533.I1RUI0
55085 229533.I1RDY3 pp 229533.I1S472
55086 229533.I1RDY3 pp 229533.I1RDZ4
55087 229533.I1S087 pp 229533.I1S7A3
55088 229533.I1S087 pp 229533.V6RV57
55089 229533.I1S087 pp 229533.I1S472
55090 229533.I1S087 pp 229533.Q4I7K4
55091 229533.I1RKI3 pp 229533.I1RZB2
55092 229533.I1RKI3 pp 229533.I1RZ39
55093 229533.I1RKI3 pp 229533.O74638
55094 229533.I1RKI3 pp 229533.Q4HXF0
55095 229533.I1RKI3 pp 229533.I1RTA1
55096 229533.I1RKI3 pp 229533.I1RNG4
55097 229533.I1RKI3 pp 229533.V6R382
55098 229533.I1RKI3 pp 229533.V6QVH8
55099 229533.I1RKI3 pp 229533.I1RXU9
55100 229533.I1RKI3 pp 229533.I1RPK0
55101 229533.I1RKI3 pp 229533.I1RUE2
55102 229533.I1RKI3 pp 229533.I1RXR1
55103 229533.I1RKI3 pp 229533.I1S1W3
55104 229533.I1RKI3 pp 229533.I1RP69
55105 229533.I1RKI3 pp 229533.I1RRG3
55106 229533.I1RKI3 pp 229533.V6RUT5
55107 229533.I1RKI3 pp 229533.I1S1B3
55108 229533.I1RKI3 pp 229533.I1RN46
55109 229533.I1RKI3 pp 229533.I1S887
55110 229533.I1RKI3 pp 229533.I1RM67
55111 229533.I1RKI3 pp 229533.I1RVR0
55112 229533.I1RKI3 pp 229533.I1RM30
55113 229533.I1RKI3 pp 229533.I1RYI7
55114 229533.I1RKI3 pp 229533.I1S3K6
55115 229533.A0A1C3YML7 pp 229533.I1RW06

55116 229533.A0A1C3YML7 pp 229533.I1S9T8
55117 229533.A0A1C3YML7 pp 229533.V6RLM7
55118 229533.A0A1C3YML7 pp 229533.I1RYK0
55119 229533.A0A1C3YML7 pp 229533.I1RND4
55120 229533.A0A1C3YML7 pp 229533.I1RFV9
55121 229533.I1S6H3 pp 229533.V6R2Z2
55122 229533.I1S6H3 pp 229533.V6R5D2
55123 229533.I1S6H3 pp 229533.I1SAH9
55124 229533.I1S6H3 pp 229533.V6RZD4
55125 229533.I1S6H3 pp 229533.I1S968
55126 229533.I1S6H3 pp 229533.I1SAB4
55127 229533.I1S6H3 pp 229533.I1S969
55128 229533.I1S6H3 pp 229533.V6QZ48
55129 229533.I1S6H3 pp 229533.I1SAT2
55130 229533.I1S6H3 pp 229533.I1S6V0
55131 229533.I1S6H3 pp 229533.V6QVH2
55132 229533.I1S6H3 pp 229533.V6R9L8
55133 229533.I1S6H3 pp 229533.V6R1D2
55134 229533.I1S6H3 pp 229533.V6RF62
55135 229533.I1S6H3 pp 229533.V6RCJ5
55136 229533.I1S6H3 pp 229533.V6R756
55137 229533.I1S6H3 pp 229533.V6RAM6
55138 229533.I1S6H3 pp 229533.V6R0W1
55139 229533.I1S6H3 pp 229533.V6RHX2
55140 229533.I1S6H3 pp 229533.V6QU99
55141 229533.I1S6H3 pp 229533.V6RCC0
55142 229533.I1S6H3 pp 229533.I1S8D3
55143 229533.I1S6H3 pp 229533.V6RWB3
55144 229533.I1S6H3 pp 229533.V6R5T7
55145 229533.I1S6H3 pp 229533.I1S8H9
55146 229533.I1S6H3 pp 229533.I1SAI4
55147 229533.I1S6H3 pp 229533.Q4HYR2
55148 229533.I1S6H3 pp 229533.V6R3V4
55149 229533.I1S0R8 pp 229533.I1S0T7
55150 229533.I1S0R8 pp 229533.I1S8F0
55151 229533.I1S0R8 pp 229533.I1S8S9
55152 229533.I1S0R8 pp 229533.I1S464
55153 229533.I1S0R8 pp 229533.V6R3J1
55154 229533.I1S0R8 pp 229533.I1S212
55155 229533.I1S0R8 pp 229533.I1S1F3
55156 229533.I1S0R8 pp 229533.Q4IPZ1
55157 229533.I1S0R8 pp 229533.I1S8F8
55158 229533.I1S0R8 pp 229533.I1S5C6
55159 229533.I1S0R8 pp 229533.I1S5U0
55160 229533.I1S0R8 pp 229533.I1S0U0
55161 229533.I1S0R8 pp 229533.Q4I6B0
55162 229533.I1S0R8 pp 229533.I1S7W5
55163 229533.I1S0R8 pp 229533.I1S5X9
55164 229533.I1S0R8 pp 229533.I1S266
55165 229533.I1S0R8 pp 229533.V6RJP8
55166 229533.I1RCC3 pp 229533.I1RVR8
55167 229533.I1RCC3 pp 229533.I1RT74
55168 229533.I1RSV0 pp 229533.I1RWT2
55169 229533.I1RSV0 pp 229533.Q4IJ79
55170 229533.I1RSV0 pp 229533.I1RYZ7
55171 229533.I1RSV0 pp 229533.I1RZX9
55172 229533.I1RSV0 pp 229533.I1S095
55173 229533.A0A0E0S648 pp 229533.I1RZT6
55174 229533.A0A0E0S648 pp 229533.A0A1C3YLR6
55175 229533.A0A0E0S648 pp 229533.I1RAV7
55176 229533.A0A0E0S648 pp 229533.I1RWQ8
55177 229533.A0A0E0S648 pp 229533.I1RBA4
55178 229533.A0A0E0S648 pp 229533.I1RBT2
55179 229533.A0A0E0S648 pp 229533.I1RBX1
55180 229533.A0A0E0S648 pp 229533.I1RYP2
55181 229533.A0A0E0S648 pp 229533.I1RN63
55182 229533.A0A0E0S648 pp 229533.I1RQ48
55183 229533.A0A0E0S648 pp 229533.A0A1C3YLU6
55184 229533.A0A0E0S648 pp 229533.I1RP97
55185 229533.A0A0E0S648 pp 229533.I1RJW6
55186 229533.A0A0E0S648 pp 229533.I1RK97
55187 229533.I1S094 pp 229533.I1SAI4
55188 229533.I1S094 pp 229533.I1S4S5

55189 229533.I1S094 pp 229533.V6RCC0
55190 229533.I1S094 pp 229533.V6QVH2
55191 229533.I1S094 pp 229533.V6R5T7
55192 229533.I1S094 pp 229533.I1S210
55193 229533.I1S094 pp 229533.I1S5D0
55194 229533.I1S094 pp 229533.V6QU99
55195 229533.I1S094 pp 229533.V6RWB3
55196 229533.I1S094 pp 229533.I1S223
55197 229533.I1S094 pp 229533.I1S0K1
55198 229533.I1S094 pp 229533.V6R0W1
55199 229533.I1S094 pp 229533.Q4HYR2
55200 229533.I1S094 pp 229533.I1S8H9
55201 229533.I1S094 pp 229533.I1S969
55202 229533.I1S094 pp 229533.V6R1D2
55203 229533.I1S094 pp 229533.V6RAM6
55204 229533.I1S094 pp 229533.I1S659
55205 229533.I1S094 pp 229533.I1S5J9
55206 229533.I1S094 pp 229533.V6RCJ5
55207 229533.I1S094 pp 229533.I1S2F7
55208 229533.I1S094 pp 229533.I1S267
55209 229533.I1S094 pp 229533.V6R3V4
55210 229533.I1S094 pp 229533.V6R2Z2
55211 229533.I1S094 pp 229533.I1SAB4
55212 229533.I1S094 pp 229533.I1S8D3
55213 229533.I1S094 pp 229533.I1S3T6
55214 229533.I1S094 pp 229533.V6RHX2
55215 229533.I1S094 pp 229533.I1S968
55216 229533.I1S094 pp 229533.I1SAH9
55217 229533.I1S094 pp 229533.V6QZ48
55218 229533.I1S094 pp 229533.I1S423
55219 229533.I1S094 pp 229533.V6RZD4
55220 229533.I1S094 pp 229533.I1S6V0
55221 229533.I1S094 pp 229533.V6R756
55222 229533.I1S094 pp 229533.I1SAT2
55223 229533.I1S094 pp 229533.V6R5D2
55224 229533.I1S094 pp 229533.V6RF62
55225 229533.I1S094 pp 229533.I1S0X1
55226 229533.I1S094 pp 229533.V6R9L8
55227 229533.I1S472 pp 229533.V6RV57
55228 229533.A0A0E0RPV7 pp 229533.I1RAE3
55229 229533.A0A0E0RPV7 pp 229533.A0A1C3YMN6
55230 229533.A0A0E0RPV7 pp 229533.A0A1C3YLM6
55231 229533.A0A0E0RPV7 pp 229533.I1RXD9
55232 229533.A0A0E0RPV7 pp 229533.A0A0E0RU85
55233 229533.A0A0E0RPV7 pp 229533.A0A1C3YM60
55234 229533.A0A0E0RPV7 pp 229533.I1RK39
55235 229533.A0A0E0RPV7 pp 229533.I1RE40
55236 229533.A0A0E0RPV7 pp 229533.A0A1C3YHQ1
55237 229533.A0A0E0RPV7 pp 229533.I1S0T7
55238 229533.A0A0E0RPV7 pp 229533.I1RGJ4
55239 229533.A0A0E0RPV7 pp 229533.I1RS05
55240 229533.A0A0E0RPV7 pp 229533.I1RRX5
55241 229533.A0A0E0RPV7 pp 229533.I1S8F0
55242 229533.A0A0E0RPV7 pp 229533.A0A1C3YMC1
55243 229533.A0A0E0RPV7 pp 229533.A0A1C3YIW9
55244 229533.A0A0E0RPV7 pp 229533.I1S464
55245 229533.A0A0E0RPV7 pp 229533.I1RBR2
55246 229533.A0A0E0RPV7 pp 229533.I1RYE5
55247 229533.A0A0E0RPV7 pp 229533.I1RYC7
55248 229533.A0A0E0RPV7 pp 229533.I1RJB3
55249 229533.A0A0E0RPV7 pp 229533.I1RX39
55250 229533.A0A0E0RPV7 pp 229533.I1S0P0
55251 229533.A0A0E0RPV7 pp 229533.I1RE66
55252 229533.A0A0E0RPV7 pp 229533.I1RBE6
55253 229533.A0A0E0RPV7 pp 229533.I1RDC3
55254 229533.A0A0E0RPV7 pp 229533.I1RWG0
55255 229533.A0A0E0RPV7 pp 229533.I1RTL0
55256 229533.A0A0E0RPV7 pp 229533.I1RYQ3
55257 229533.A0A0E0RPV7 pp 229533.I1S1F3
55258 229533.A0A0E0RPV7 pp 229533.I1R9Z5
55259 229533.A0A0E0RPV7 pp 229533.I1RKF4
55260 229533.A0A0E0RPV7 pp 229533.Q4HZB8
55261 229533.A0A0E0RPV7 pp 229533.A0A1C3YL46

55262 229533.A0A0E0RPV7 pp 229533.I1S8F8
55263 229533.A0A0E0RPV7 pp 229533.I1RNM7
55264 229533.A0A0E0RPV7 pp 229533.I1RSU1
55265 229533.A0A0E0RPV7 pp 229533.I1RRK9
55266 229533.A0A0E0RPV7 pp 229533.I1RRS8
55267 229533.A0A0E0RPV7 pp 229533.I1S0U0
55268 229533.A0A0E0RPV7 pp 229533.I1RAW3
55269 229533.A0A0E0RPV7 pp 229533.I1RMK7
55270 229533.A0A0E0RPV7 pp 229533.I1RYX5
55271 229533.A0A0E0RPV7 pp 229533.I1RWA3
55272 229533.A0A0E0RPV7 pp 229533.I1RI27
55273 229533.A0A0E0RPV7 pp 229533.I1RNF7
55274 229533.A0A0E0RPV7 pp 229533.I1RFI4
55275 229533.A0A0E0RPV7 pp 229533.I1RG62
55276 229533.A0A0E0RPV7 pp 229533.I1RYN2
55277 229533.A0A0E0RPV7 pp 229533.I1RA52
55278 229533.A0A0E0RPV7 pp 229533.I1RQ85
55279 229533.A0A0E0RPV7 pp 229533.I1RL71
55280 229533.A0A0E0RPV7 pp 229533.I1RYT8
55281 229533.A0A0E0RPV7 pp 229533.V6RJP8
55282 229533.A0A0E0RPV7 pp 229533.I1S458
55283 229533.A0A0E0RPV7 pp 229533.I1RCH5
55284 229533.A0A0E0RPV7 pp 229533.I1R9Y4
55285 229533.A0A0E0RPV7 pp 229533.I1RXZ3
55286 229533.A0A0E0RPV7 pp 229533.I1S5X9
55287 229533.A0A0E0RPV7 pp 229533.I1RZ68
55288 229533.A0A0E0RPV7 pp 229533.I1RKQ6
55289 229533.A0A0E0RPV7 pp 229533.A0A1C3YIJ9
55290 229533.A0A0E0RPV7 pp 229533.A0A1C3YHJ9
55291 229533.A0A0E0RPV7 pp 229533.V6RCG5
55292 229533.A0A0E0RPV7 pp 229533.I1REF0
55293 229533.A0A0E0RPV7 pp 229533.I1RB50
55294 229533.A0A0E0RPV7 pp 229533.A0A0E0S082
55295 229533.A0A0E0RPV7 pp 229533.A0A0E0SAR4
55296 229533.A0A0E0RPV7 pp 229533.I1RJU3
55297 229533.A0A0E0RPV7 pp 229533.I1RB64
55298 229533.A0A0E0RPV7 pp 229533.I1RS29
55299 229533.A0A0E0RPV7 pp 229533.I1RY92
55300 229533.A0A0E0RPV7 pp 229533.I1RKF5
55301 229533.A0A0E0RPV7 pp 229533.A0A1C3YI23
55302 229533.A0A0E0RPV7 pp 229533.I1S0Q7
55303 229533.A0A0E0RPV7 pp 229533.I1RMQ6
55304 229533.A0A0E0RPV7 pp 229533.I1RE39
55305 229533.A0A0E0RPV7 pp 229533.I1RKB7
55306 229533.A0A0E0RPV7 pp 229533.I1REZ6
55307 229533.A0A0E0RPV7 pp 229533.I1S8Z2
55308 229533.A0A0E0RPV7 pp 229533.A0A1C3YJQ2
55309 229533.A0A0E0RPV7 pp 229533.I1RAE4
55310 229533.A0A0E0RPV7 pp 229533.I1RMI1
55311 229533.A0A0E0RPV7 pp 229533.A0A0E0S502
55312 229533.A0A0E0RPV7 pp 229533.I1RQR6
55313 229533.A0A0E0RPV7 pp 229533.I1RAV5
55314 229533.A0A0E0RPV7 pp 229533.I1REE2
55315 229533.A0A0E0RPV7 pp 229533.I1RFY0
55316 229533.A0A0E0RPV7 pp 229533.I1RY21
55317 229533.A0A0E0RPV7 pp 229533.Q4IPZ1
55318 229533.A0A0E0RPV7 pp 229533.I1S5C6
55319 229533.A0A0E0RPV7 pp 229533.I1RFS5
55320 229533.A0A0E0RPV7 pp 229533.I1RW13
55321 229533.A0A0E0RPV7 pp 229533.I1S266
55322 229533.A0A0E0RPV7 pp 229533.I1RAW9
55323 229533.A0A0E0RPV7 pp 229533.I1RB38
55324 229533.A0A0E0RPV7 pp 229533.A0A1C3YK94
55325 229533.A0A0E0RPV7 pp 229533.I1RFJ5
55326 229533.A0A0E0RPV7 pp 229533.I1RXE6
55327 229533.A0A0E0RPV7 pp 229533.I1RP02
55328 229533.A0A0E0RPV7 pp 229533.A0A1C3YLB6
55329 229533.A0A0E0RPV7 pp 229533.I1RRT0
55330 229533.A0A0E0RPV7 pp 229533.I1RYH7
55331 229533.A0A0E0RPV7 pp 229533.A0A1C3YJU7
55332 229533.A0A0E0RPV7 pp 229533.I1RBR1
55333 229533.A0A0E0RPV7 pp 229533.I1RI95
55334 229533.A0A0E0RPV7 pp 229533.I1RLN1

229533.A0A0E0RPV7	pp	229533.A0A1C3YI30
229533.A0A0E0RPV7	pp	229533.I1RE65
229533.A0A0E0RPV7	pp	229533.I1S0R8
229533.A0A0E0RPV7	pp	229533.I1RS01
229533.A0A0E0RPV7	pp	229533.A0A1C3YM56
229533.A0A0E0RPV7	pp	229533.I1S0K2
229533.A0A0E0RPV7	pp	229533.I1RQ72
229533.A0A0E0RPV7	pp	229533.I1RBT6
229533.A0A0E0RPV7	pp	229533.I1RAM9
229533.A0A0E0RPV7	pp	229533.I1RQS2
229533.A0A0E0RPV7	pp	229533.I1RAA2
229533.A0A0E0RPV7	pp	229533.I1RPR9
229533.A0A0E0RPV7	pp	229533.I1RGI8
229533.A0A0E0RPV7	pp	229533.I1S8S9
229533.A0A0E0RPV7	pp	229533.I1RU95
229533.A0A0E0RPV7	pp	229533.I1RZ43
229533.A0A0E0RPV7	pp	229533.I1RGB1
229533.A0A0E0RPV7	pp	229533.A0A0E0S8Z0
229533.A0A0E0RPV7	pp	229533.I1S5U0
229533.A0A0E0RPV7	pp	229533.A0A1C3YLB0
229533.A0A0E0RPV7	pp	229533.I1RP38
229533.A0A0E0RPV7	pp	229533.A0A1C3YKY6
229533.A0A0E0RPV7	pp	229533.I1RFJ6
229533.A0A0E0RPV7	pp	229533.I1S212
229533.I1RQD5	pp	229533.I1RQE3
229533.I1RB01	pp	229533.I1RB14
229533.I1RB01	pp	229533.I1S472
229533.A0A1C3YHK3	pp	229533.I1S5S2
229533.A0A1C3YHK3	pp	229533.I1RXF2
229533.A0A1C3YHK3	pp	229533.I1S7M2
229533.A0A1C3YHK3	pp	229533.I1RN09
229533.A0A1C3YHK3	pp	229533.A0A1C3YI62
229533.A0A1C3YHK3	pp	229533.I1RT47
229533.A0A1C3YHK3	pp	229533.I1RG17
229533.A0A1C3YHK3	pp	229533.I1RYI9
229533.A0A1C3YHK3	pp	229533.I1RSA8
229533.A0A1C3YHK3	pp	229533.I1RBV3
229533.A0A1C3YHK3	pp	229533.I1S7M3
229533.A0A1C3YHK3	pp	229533.I1RX51
229533.A0A1C3YHK3	pp	229533.I1RQC5
229533.A0A1C3YHK3	pp	229533.I1RT35
229533.A0A1C3YHK3	pp	229533.I1RVR8
229533.A0A1C3YHK3	pp	229533.I1RZ11
229533.A0A1C3YHK3	pp	229533.I1RWX8
229533.A0A1C3YHK3	pp	229533.I1RQD8
229533.A0A1C3YHK3	pp	229533.I1RAD1
229533.A0A1C3YHK3	pp	229533.I1S0B6
229533.A0A1C3YHK3	pp	229533.I1RVT9
229533.A0A1C3YHK3	pp	229533.I1RPA0
229533.A0A1C3YHK3	pp	229533.V6QXG1
229533.A0A1C3YHK3	pp	229533.I1S471
229533.A0A1C3YHK3	pp	229533.I1RK92
229533.A0A1C3YHK3	pp	229533.I1RSA4
229533.A0A1C3YHK3	pp	229533.I1RBZ8
229533.A0A1C3YHK3	pp	229533.I1RZL5
229533.A0A1C3YHK3	pp	229533.V6RIS2
229533.A0A1C3YHK3	pp	229533.I1RND5
229533.A0A1C3YHK3	pp	229533.Q4IP34
229533.A0A1C3YHK3	pp	229533.I1RA36
229533.A0A1C3YHK3	pp	229533.I1RST1
229533.A0A1C3YHK3	pp	229533.I1RG13
229533.A0A1C3YHK3	pp	229533.I1RZG7
229533.A0A1C3YHK3	pp	229533.I1RS83
229533.A0A1C3YHK3	pp	229533.I1RRW3
229533.A0A1C3YHK3	pp	229533.I1RZQ5
229533.A0A1C3YHK3	pp	229533.I1RJM7
229533.A0A1C3YHK3	pp	229533.I1RLU5
229533.A0A1C3YHK3	pp	229533.Q4IBK5
229533.A0A1C3YHK3	pp	229533.I1RCC3
229533.A0A1C3YHK3	pp	229533.I1RBH8
229533.A0A1C3YHK3	pp	229533.A0A1C3YNC3
229533.A0A1C3YHK3	pp	229533.Q4I7F9
229533.A0A1C3YHK3	pp	229533.I1RCQ6

55408 229533.A0A1C3YHK3 pp 229533.I1RSD1
55409 229533.A0A1C3YHK3 pp 229533.I1RK72
55410 229533.A0A1C3YHK3 pp 229533.V6RY33
55411 229533.A0A1C3YHK3 pp 229533.A0A1C3YHQ4
55412 229533.A0A1C3YHK3 pp 229533.I1RCS2
55413 229533.A0A1C3YHK3 pp 229533.I1RZZ1
55414 229533.A0A1C3YHK3 pp 229533.I1RMW7
55415 229533.A0A1C3YHK3 pp 229533.Q4IPB3
55416 229533.A0A1C3YHK3 pp 229533.I1RPY4
55417 229533.A0A1C3YHK3 pp 229533.I1RRL0
55418 229533.A0A1C3YHK3 pp 229533.I1RSD4
55419 229533.A0A1C3YHK3 pp 229533.I1S2Y5
55420 229533.A0A1C3YHK3 pp 229533.I1RK90
55421 229533.A0A1C3YHK3 pp 229533.I1RPY5
55422 229533.A0A1C3YHK3 pp 229533.I1RS75
55423 229533.A0A1C3YHK3 pp 229533.I1RS28
55424 229533.A0A1C3YHK3 pp 229533.I1RFQ1
55425 229533.A0A1C3YHK3 pp 229533.I1RQR3
55426 229533.A0A1C3YHK3 pp 229533.I1RT91
55427 229533.A0A1C3YHK3 pp 229533.I1RK96
55428 229533.A0A1C3YHK3 pp 229533.I1RX24
55429 229533.A0A1C3YHK3 pp 229533.I1RN59
55430 229533.A0A1C3YHK3 pp 229533.I1S1X9
55431 229533.A0A1C3YHK3 pp 229533.I1RNI1
55432 229533.A0A1C3YHK3 pp 229533.I1RG57
55433 229533.A0A1C3YHK3 pp 229533.I1S4M5
55434 229533.A0A1C3YHK3 pp 229533.I1RB50
55435 229533.A0A1C3YHK3 pp 229533.I1RX81
55436 229533.A0A1C3YHK3 pp 229533.I1RJL2
55437 229533.A0A1C3YHK3 pp 229533.I1RYJ9
55438 229533.A0A1C3YHK3 pp 229533.I1S0Z9
55439 229533.A0A1C3YHK3 pp 229533.I1RMV5
55440 229533.A0A1C3YHK3 pp 229533.I1RNB8
55441 229533.A0A1C3YHK3 pp 229533.I1RWU2
55442 229533.A0A1C3YHK3 pp 229533.I1RCA7
55443 229533.A0A1C3YHK3 pp 229533.I1RFL7
55444 229533.A0A1C3YHK3 pp 229533.Q4I1Y1
55445 229533.A0A1C3YHK3 pp 229533.A0A1C3YL11
55446 229533.A0A1C3YHK3 pp 229533.I1RG90
55447 229533.A0A1C3YHK3 pp 229533.I1RC84
55448 229533.A0A1C3YHK3 pp 229533.I1RPY7
55449 229533.A0A1C3YHK3 pp 229533.I1RAE1
55450 229533.A0A1C3YHK3 pp 229533.I1S0B1
55451 229533.A0A1C3YHK3 pp 229533.I1RCI4
55452 229533.A0A1C3YHK3 pp 229533.V6QVD5
55453 229533.A0A1C3YHK3 pp 229533.I1REX2
55454 229533.A0A1C3YHK3 pp 229533.I1RCI3
55455 229533.A0A1C3YHK3 pp 229533.I1RRQ4
55456 229533.A0A1C3YHK3 pp 229533.Q4IPH4
55457 229533.A0A1C3YHK3 pp 229533.I1RFQ0
55458 229533.I1R9N7 pp 229533.I1RNL7
55459 229533.I1R9N7 pp 229533.Q4HZE0
55460 229533.I1R9N7 pp 229533.I1RJY7
55461 229533.I1R9N7 pp 229533.I1RD39
55462 229533.I1R9N7 pp 229533.I1RAK7
55463 229533.I1R9N7 pp 229533.I1RTF5
55464 229533.I1R9N7 pp 229533.I1S272
55465 229533.I1R9N7 pp 229533.I1RX83
55466 229533.I1R9N7 pp 229533.I1RYL5
55467 229533.I1R9N7 pp 229533.V6QUV7
55468 229533.I1R9N7 pp 229533.I1RUN9
55469 229533.I1R9N7 pp 229533.V6RRF6
55470 229533.I1R9N7 pp 229533.I1S4Z7
55471 229533.I1R9N7 pp 229533.I1RNM3
55472 229533.I1R9N7 pp 229533.V6RXW9
55473 229533.I1R9N7 pp 229533.I1RV63
55474 229533.I1R9N7 pp 229533.Q4HY20
55475 229533.I1R9N7 pp 229533.V6QX21
55476 229533.I1R9N7 pp 229533.I1RYB8
55477 229533.I1R9N7 pp 229533.I1RSQ2
55478 229533.I1R9N7 pp 229533.I1RD46
55479 229533.I1R9N7 pp 229533.I1RZM0
55480 229533.I1R9N7 pp 229533.I1RAH0

55481	229533.I1R9N7	pp	229533.I1RAS3
55482	229533.I1R9N7	pp	229533.I1RQ38
55483	229533.I1R9N7	pp	229533.I1R9U9
55484	229533.I1R9N7	pp	229533.I1RUP0
55485	229533.I1R9N7	pp	229533.I1S235
55486	229533.I1R9N7	pp	229533.I1RXF0
55487	229533.I1R9N7	pp	229533.I1S051
55488	229533.I1R9N7	pp	229533.I1RSM8
55489	229533.I1R9N7	pp	229533.I1S0Y1
55490	229533.I1R9N7	pp	229533.I1RX25
55491	229533.I1R9N7	pp	229533.I1SAM9
55492	229533.I1R9N7	pp	229533.I1RMM6
55493	229533.I1R9N7	pp	229533.I1RNM4
55494	229533.I1R9N7	pp	229533.I1RDD0
55495	229533.I1RA13	pp	229533.I1S0A5
55496	229533.I1RA13	pp	229533.V6R2Z2
55497	229533.I1RA13	pp	229533.I1S299
55498	229533.I1RA13	pp	229533.I1RF24
55499	229533.I1RA13	pp	229533.I1RSA5
55500	229533.I1RA13	pp	229533.V6RZD4
55501	229533.I1RA13	pp	229533.I1RK55
55502	229533.I1RA13	pp	229533.I1RZ25
55503	229533.I1RA13	pp	229533.I1RLZ4
55504	229533.I1RA13	pp	229533.I1S968
55505	229533.I1RA13	pp	229533.I1RGC3
55506	229533.I1RA13	pp	229533.I1RFB0
55507	229533.I1RA13	pp	229533.I1RN88
55508	229533.I1RA13	pp	229533.I1RMT6
55509	229533.I1RA13	pp	229533.I1RZ38
55510	229533.I1RA13	pp	229533.I1RKD3
55511	229533.I1RA13	pp	229533.I1RR68
55512	229533.I1RA13	pp	229533.I1RAC0
55513	229533.I1RA13	pp	229533.I1SAB4
55514	229533.I1RA13	pp	229533.I1S2Q4
55515	229533.I1RA13	pp	229533.I1RZV5
55516	229533.I1RA13	pp	229533.V6QZ48
55517	229533.I1RA13	pp	229533.I1RI19
55518	229533.I1RA13	pp	229533.I1RIJ6
55519	229533.I1RA13	pp	229533.I1RF59
55520	229533.I1RA13	pp	229533.V6RCJ5
55521	229533.I1RA13	pp	229533.V6R5D2
55522	229533.I1RA13	pp	229533.I1RCR9
55523	229533.I1RA13	pp	229533.I1S3T7
55524	229533.I1RA13	pp	229533.I1SAH9
55525	229533.I1RA13	pp	229533.I1SAT2
55526	229533.I1RA13	pp	229533.I1REW1
55527	229533.I1RA13	pp	229533.V6R9L8
55528	229533.I1RA13	pp	229533.I1RX18
55529	229533.I1RA13	pp	229533.V6RF62
55530	229533.I1RA13	pp	229533.I1S8R2
55531	229533.I1RA13	pp	229533.I1RB05
55532	229533.I1RA13	pp	229533.I1RHJ0
55533	229533.I1RA13	pp	229533.I1S0X1
55534	229533.I1RA13	pp	229533.I1RP12
55535	229533.I1RA13	pp	229533.I1S149
55536	229533.I1RA13	pp	229533.I1S8C1
55537	229533.I1RA13	pp	229533.I1RTY9
55538	229533.I1RA13	pp	229533.I1RUE3
55539	229533.I1RA13	pp	229533.I1RH83
55540	229533.I1RA13	pp	229533.I1RQW1
55541	229533.I1RA13	pp	229533.I1S4S5
55542	229533.I1RA13	pp	229533.I1RC39
55543	229533.I1RA13	pp	229533.V6QU99
55544	229533.I1RA13	pp	229533.I1RHT5
55545	229533.I1RA13	pp	229533.I1RMK4
55546	229533.I1RA13	pp	229533.V6R756
55547	229533.I1RA13	pp	229533.I1RF85
55548	229533.I1RA13	pp	229533.I1RCU2
55549	229533.I1RA13	pp	229533.I1RQV8
55550	229533.I1RA13	pp	229533.I1RIF7
55551	229533.I1RA13	pp	229533.I1RGU3
55552	229533.I1RA13	pp	229533.V6QVH2
55553	229533.I1RA13	pp	229533.V6RAM6

55554	229533.I1RA13	pp	229533.I1S6V0
55555	229533.I1RA13	pp	229533.I1SAI4
55556	229533.I1RA13	pp	229533.I1S223
55557	229533.I1RA13	pp	229533.I1RDN3
55558	229533.I1RA13	pp	229533.I1S0K1
55559	229533.I1RA13	pp	229533.I1RRS6
55560	229533.I1RA13	pp	229533.I1S969
55561	229533.I1RA13	pp	229533.V6RCC0
55562	229533.I1RA13	pp	229533.V6R5T7
55563	229533.I1RA13	pp	229533.I1RJS7
55564	229533.I1RA13	pp	229533.I1S210
55565	229533.I1RA13	pp	229533.I1RYP5
55566	229533.I1RA13	pp	229533.I1RY16
55567	229533.I1RA13	pp	229533.I1RJC5
55568	229533.I1RA13	pp	229533.V6R1D2
55569	229533.I1RA13	pp	229533.I1RQV7
55570	229533.I1RA13	pp	229533.I1RGK3
55571	229533.I1RA13	pp	229533.I1RJG4
55572	229533.I1RA13	pp	229533.I1RMA2
55573	229533.I1RA13	pp	229533.V6RHX2
55574	229533.I1RA13	pp	229533.I1REE2
55575	229533.I1RA13	pp	229533.I1S5D0
55576	229533.I1RA13	pp	229533.I1RST9
55577	229533.I1RA13	pp	229533.I1RDV8
55578	229533.I1RA13	pp	229533.I1S048
55579	229533.I1RA13	pp	229533.I1RIM7
55580	229533.I1RA13	pp	229533.I1RXH2
55581	229533.I1RA13	pp	229533.I1RLA2
55582	229533.I1RA13	pp	229533.I1S8H9
55583	229533.I1RA13	pp	229533.I1RWM4
55584	229533.I1RA13	pp	229533.I1RVI8
55585	229533.I1RA13	pp	229533.I1S267
55586	229533.I1RA13	pp	229533.V6R0W1
55587	229533.I1RA13	pp	229533.I1S423
55588	229533.I1RA13	pp	229533.I1S5J9
55589	229533.I1RA13	pp	229533.I1RH67
55590	229533.I1RA13	pp	229533.I1RAG3
55591	229533.I1RA13	pp	229533.Q4HYR2
55592	229533.I1RA13	pp	229533.I1S8D3
55593	229533.I1RA13	pp	229533.I1RIB5
55594	229533.I1RA13	pp	229533.V6RPS5
55595	229533.I1RA13	pp	229533.I1RUQ3
55596	229533.I1RA13	pp	229533.I1RNH4
55597	229533.I1RA13	pp	229533.I1RNG5
55598	229533.I1RA13	pp	229533.I1RW55
55599	229533.I1RA13	pp	229533.I1S659
55600	229533.I1RA13	pp	229533.I1S2F7
55601	229533.I1RA13	pp	229533.I1S3T6
55602	229533.I1RA13	pp	229533.I1RY81
55603	229533.I1RA13	pp	229533.I1RJY7
55604	229533.I1RA13	pp	229533.I1RI40
55605	229533.I1RA13	pp	229533.V6RWB3
55606	229533.I1RA13	pp	229533.I1RRM1
55607	229533.I1RA13	pp	229533.I1RWD2
55608	229533.I1RA13	pp	229533.V6R3V4
55609	229533.I1RPA4	pp	229533.I1RXD7
55610	229533.I1RPA4	pp	229533.I1S3S3
55611	229533.I1RPA4	pp	229533.V6RTJ8
55612	229533.I1RPA4	pp	229533.I1RZH5
55613	229533.I1RPA4	pp	229533.I1S1W8
55614	229533.I1RPA4	pp	229533.I1RZ39
55615	229533.I1RPA4	pp	229533.V6R3J1
55616	229533.I1RPA4	pp	229533.I1RSZ7
55617	229533.I1RPA4	pp	229533.I1RSX0
55618	229533.I1RPA4	pp	229533.I1RX17
55619	229533.I1RPA4	pp	229533.I1S2C1
55620	229533.I1RPA4	pp	229533.V6RM66
55621	229533.I1RPA4	pp	229533.I1RVN1
55622	229533.I1RPA4	pp	229533.Q4I099
55623	229533.I1RPA4	pp	229533.I1RSQ4
55624	229533.I1RPA4	pp	229533.I1RYI5
55625	229533.I1RPA4	pp	229533.I1RW61
55626	229533.I1RPA4	pp	229533.I1S4S7

55627 229533.I1RPA4 pp 229533.I1RPV3
55628 229533.I1RPA4 pp 229533.I1RT38
55629 229533.I1RPA4 pp 229533.I1S277
55630 229533.I1RPA4 pp 229533.I1RSQ7
55631 229533.I1RPA4 pp 229533.I1RPE0
55632 229533.I1RPA4 pp 229533.I1S3G4
55633 229533.I1RPA4 pp 229533.I1RTA1
55634 229533.I1RPA4 pp 229533.I1S0Z6
55635 229533.I1RPA4 pp 229533.I1RRI1
55636 229533.I1RPA4 pp 229533.I1S2B8
55637 229533.I1RPA4 pp 229533.V6RCZ8
55638 229533.I1RPA4 pp 229533.I1RVZ2
55639 229533.I1RPA4 pp 229533.I1S4S4
55640 229533.I1RPA4 pp 229533.I1S6K9
55641 229533.I1RPA4 pp 229533.I1RXC2
55642 229533.I1RPA4 pp 229533.Q4IB65
55643 229533.I1RPA4 pp 229533.I1RSU1
55644 229533.I1RPA4 pp 229533.O74638
55645 229533.I1RPA4 pp 229533.I1RY57
55646 229533.I1RPA4 pp 229533.I1RWT0
55647 229533.I1RPA4 pp 229533.I1RRM3
55648 229533.I1RPA4 pp 229533.I1RYW3
55649 229533.I1RPA4 pp 229533.I1S5R1
55650 229533.I1RPA4 pp 229533.I1RXT3
55651 229533.I1RPA4 pp 229533.I1S0R9
55652 229533.I1RPA4 pp 229533.I1RZM8
55653 229533.I1RPA4 pp 229533.I1RXF0
55654 229533.I1RPA4 pp 229533.I1RZS4
55655 229533.I1RPA4 pp 229533.I1RS50
55656 229533.I1RPA4 pp 229533.I1RRN2
55657 229533.I1RPA4 pp 229533.I1RQR6
55658 229533.I1RPA4 pp 229533.I1S9G7
55659 229533.I1RPA4 pp 229533.Q4I665
55660 229533.I1RPA4 pp 229533.I1RPR9
55661 229533.I1RPA4 pp 229533.V6RDZ4
55662 229533.I1RPA4 pp 229533.V6QWP4
55663 229533.I1RPA4 pp 229533.I1RXC4
55664 229533.I1RPA4 pp 229533.I1RWB0
55665 229533.I1RPA4 pp 229533.I1RPU2
55666 229533.I1RPA4 pp 229533.I1S7W5
55667 229533.I1RPA4 pp 229533.Q4IK03
55668 229533.I1RPA4 pp 229533.I1RVX0
55669 229533.I1RPA4 pp 229533.I1RQE7
55670 229533.I1RPA4 pp 229533.Q4IJK2
55671 229533.I1RPA4 pp 229533.I1RQ85
55672 229533.I1RPA4 pp 229533.Q4HZ95
55673 229533.I1RPA4 pp 229533.I1RQ33
55674 229533.I1RPA4 pp 229533.I1RQ82
55675 229533.I1RPA4 pp 229533.Q4IPZ1
55676 229533.A0A098D562 pp 229533.I1RKB7
55677 229533.A0A098D562 pp 229533.I1RG62
55678 229533.A0A098D562 pp 229533.A0A1C3YIW9
55679 229533.A0A098D562 pp 229533.I1RX39
55680 229533.A0A098D562 pp 229533.A0A098D8B8
55681 229533.A0A098D562 pp 229533.I1RVR8
55682 229533.A0A098D562 pp 229533.I1RZ43
55683 229533.A0A098D562 pp 229533.A0A1C3YLB0
55684 229533.A0A098D562 pp 229533.I1S0R8
55685 229533.A0A098D562 pp 229533.I1RCC3
55686 229533.A0A098D562 pp 229533.A0A0E0RPV7
55687 229533.A0A098D562 pp 229533.A0A1C3YHK3
55688 229533.A0A098DCP9 pp 229533.I1RKB2
55689 229533.A0A098DCP9 pp 229533.I1RNG4
55690 229533.A0A098DCP9 pp 229533.I1S3H8
55691 229533.A0A098DCP9 pp 229533.I1S0A5
55692 229533.A0A098DCP9 pp 229533.A0A1C3YKN4
55693 229533.A0A098DCP9 pp 229533.I1RPV8
55694 229533.A0A098DCP9 pp 229533.I1RZ25
55695 229533.A0A098DCP9 pp 229533.I1RZ56
55696 229533.A0A098DCP9 pp 229533.I1RGZ3
55697 229533.A0A098DCP9 pp 229533.I1RRL5
55698 229533.A0A098DCP9 pp 229533.I1RJS1
55699 229533.A0A098DCP9 pp 229533.I1RNM7

55700 229533.A0A098DCP9 pp 229533.I1S334
55701 229533.A0A098DCP9 pp 229533.I1RDN3
55702 229533.A0A098DCP9 pp 229533.I1RLA2
55703 229533.A0A098DCP9 pp 229533.I1S6H3
55704 229533.A0A098DCP9 pp 229533.I1S8F8
55705 229533.A0A098DCP9 pp 229533.I1S094
55706 229533.A0A098DCP9 pp 229533.I1RUY5
55707 229533.A0A098DCP9 pp 229533.I1RST9
55708 229533.A0A098DCP9 pp 229533.I1RA13
55709 229533.A0A098DCP9 pp 229533.I1RDU0
55710 229533.I1S147 pp 229533.V6RH92
55711 229533.I1S147 pp 229533.I1S8D2
55712 229533.I1S147 pp 229533.I1S1W8
55713 229533.I1S147 pp 229533.I1S299
55714 229533.I1S147 pp 229533.I1S6P5
55715 229533.I1S147 pp 229533.V6RV57
55716 229533.I1S147 pp 229533.V6RPS5
55717 229533.I1S147 pp 229533.I1S746
55718 229533.I1S147 pp 229533.V6RXR0
55719 229533.I1S147 pp 229533.I1S8F8
55720 229533.I1S147 pp 229533.V6QU65
55721 229533.I1S147 pp 229533.V6RUY9
55722 229533.I1S147 pp 229533.I1S3T7
55723 229533.I1S147 pp 229533.I1S148
55724 229533.I1S147 pp 229533.V6RUB1
55725 229533.I1S147 pp 229533.I1S8D1
55726 229533.I1S147 pp 229533.I1S149
55727 229533.I1S147 pp 229533.V6RHD0
55728 229533.I1S147 pp 229533.I1S3G4
55729 229533.I1S147 pp 229533.V6RRM0
55730 229533.I1S147 pp 229533.I1S2C4
55731 229533.I1S147 pp 229533.V6RLM7
55732 229533.I1S147 pp 229533.I1S8R2
55733 229533.I1S147 pp 229533.I1S7W5
55734 229533.I1RET4 pp 229533.I1RSX9
55735 229533.I1RET4 pp 229533.I1RNG4
55736 229533.I1RET4 pp 229533.I1RND4
55737 229533.I1RET4 pp 229533.I1RRG1
55738 229533.I1RET4 pp 229533.I1RJS0
55739 229533.I1RET4 pp 229533.I1RUP5
55740 229533.A0A1C3YJ80 pp 229533.I1RBC1
55741 229533.A0A1C3YJ80 pp 229533.I1RYR1
55742 229533.A0A1C3YJ80 pp 229533.Q4INI2
55743 229533.A0A1C3YJ80 pp 229533.I1RMK1
55744 229533.A0A0E0SH36 pp 229533.I1RBL7
55745 229533.A0A0E0SH36 pp 229533.I1RTE9
55746 229533.A0A0E0SH36 pp 229533.I1RNF5
55747 229533.A0A0E0SH36 pp 229533.A0A1C3YJR3
55748 229533.A0A0E0SH36 pp 229533.A0A1C3YN86
55749 229533.A0A0E0SH36 pp 229533.I1RB27
55750 229533.A0A0E0SH36 pp 229533.I1RCL3
55751 229533.A0A0E0SH36 pp 229533.I1R9X7
55752 229533.A0A0E0SH36 pp 229533.I1S0K6
55753 229533.A0A0E0SH36 pp 229533.I1RVZ0
55754 229533.A0A0E0SH36 pp 229533.I1S9Y1
55755 229533.A0A0E0SH36 pp 229533.I1RSD8
55756 229533.A0A0E0SH36 pp 229533.I1RDG0
55757 229533.A0A0E0SH36 pp 229533.I1RKF9
55758 229533.A0A0E0SH36 pp 229533.I1RBG9
55759 229533.A0A0E0SH36 pp 229533.I1RC82
55760 229533.A0A0E0SH36 pp 229533.Q4I9U7
55761 229533.A0A0E0SH36 pp 229533.I1RM49
55762 229533.A0A0E0SH36 pp 229533.I1RK95
55763 229533.A0A0E0SH36 pp 229533.I1RD64
55764 229533.A0A0E0SH36 pp 229533.I1RCT2
55765 229533.A0A0E0SH36 pp 229533.A0A1C3YJP0
55766 229533.A0A0E0SH36 pp 229533.I1R9V7
55767 229533.A0A0E0SH36 pp 229533.I1RQ31
55768 229533.A0A0E0SH36 pp 229533.I1RBC7
55769 229533.A0A0E0SH36 pp 229533.I1REF2
55770 229533.A0A0E0SH36 pp 229533.I1RZR2
55771 229533.A0A0E0SH36 pp 229533.I1RK80
55772 229533.A0A0E0SH36 pp 229533.I1RDA0

55773 229533.A0A0E0SH36 pp 229533.I1RAF8
55774 229533.A0A0E0SH36 pp 229533.I1RMN5
55775 229533.A0A0E0SH36 pp 229533.I1RNV6
55776 229533.A0A0E0SH36 pp 229533.I1RJB5
55777 229533.A0A0E0SH36 pp 229533.I1RSI6
55778 229533.A0A0E0SH36 pp 229533.I1RT80
55779 229533.A0A0E0SH36 pp 229533.I1RM96
55780 229533.A0A0E0SH36 pp 229533.I1RRQ3
55781 229533.A0A0E0SH36 pp 229533.I1RAC2
55782 229533.A0A0E0SH36 pp 229533.I1RW26
55783 229533.A0A0E0SH36 pp 229533.I1RG56
55784 229533.A0A0E0SH36 pp 229533.I1RJZ7
55785 229533.A0A0E0SH36 pp 229533.I1RA02
55786 229533.A0A0E0SH36 pp 229533.I1RN51
55787 229533.A0A0E0SH36 pp 229533.I1RRH5
55788 229533.A0A0E0SH36 pp 229533.V6R283
55789 229533.A0A0E0SH36 pp 229533.I1RQJ5
55790 229533.A0A0E0SH36 pp 229533.Q4HWB3
55791 229533.A0A0E0SH36 pp 229533.I1RW71
55792 229533.A0A0E0SH36 pp 229533.I1RFS3
55793 229533.A0A0E0SH36 pp 229533.I1RG51
55794 229533.I1RFI4 pp 229533.I1S0R8
55795 229533.I1RFI4 pp 229533.I1S2C4
55796 229533.I1RFI4 pp 229533.V6RLM7
55797 229533.I1RFI4 pp 229533.I1RPK0
55798 229533.I1RFI4 pp 229533.I1RYA0
55799 229533.I1RFI4 pp 229533.I1S8R2
55800 229533.I1RFI4 pp 229533.V6RF62
55801 229533.I1RFI4 pp 229533.I1S7W5
55802 229533.I1RFI4 pp 229533.I1RNU7
55803 229533.I1RFI4 pp 229533.I1RN84
55804 229533.I1RFI4 pp 229533.I1RUR8
55805 229533.I1RFI4 pp 229533.V6RRM0
55806 229533.I1RFI4 pp 229533.I1RY92
55807 229533.I1RFI4 pp 229533.I1RYC8
55808 229533.I1RFI4 pp 229533.I1S0V7
55809 229533.I1RFI4 pp 229533.I1RPZ5
55810 229533.I1RFI4 pp 229533.I1RSQ4
55811 229533.I1RFI4 pp 229533.I1RR68
55812 229533.I1RFI4 pp 229533.I1RMV0
55813 229533.I1RFI4 pp 229533.V6RUY9
55814 229533.I1RFI4 pp 229533.I1RKB2
55815 229533.I1RFI4 pp 229533.V6RV57
55816 229533.I1RFI4 pp 229533.I1RZA7
55817 229533.I1RFI4 pp 229533.I1RMJ3
55818 229533.I1RFI4 pp 229533.V6R3J1
55819 229533.I1RFI4 pp 229533.I1S3T7
55820 229533.I1RFI4 pp 229533.V6RUB1
55821 229533.I1RFI4 pp 229533.I1S3G4
55822 229533.I1RFI4 pp 229533.V6RZD4
55823 229533.I1RFI4 pp 229533.I1RYW9
55824 229533.I1RFI4 pp 229533.I1RVR2
55825 229533.I1RFI4 pp 229533.I1S6P5
55826 229533.I1RFI4 pp 229533.Q4HVQ9
55827 229533.I1RFI4 pp 229533.I1RXW3
55828 229533.I1RFI4 pp 229533.I1RN88
55829 229533.I1RFI4 pp 229533.I1RL81
55830 229533.I1RFI4 pp 229533.I1RYK0
55831 229533.I1RFI4 pp 229533.I1RGC3
55832 229533.I1RFI4 pp 229533.I1RNG4
55833 229533.I1RFI4 pp 229533.I1S299
55834 229533.I1RFI4 pp 229533.V6R3V4
55835 229533.I1RFI4 pp 229533.I1RVZ3
55836 229533.I1RFI4 pp 229533.I1S464
55837 229533.I1RFI4 pp 229533.I1RM26
55838 229533.I1RFI4 pp 229533.I1S8S9
55839 229533.I1RFI4 pp 229533.I1RJY7
55840 229533.I1RFI4 pp 229533.I1RPT0
55841 229533.I1RFI4 pp 229533.I1RPR9
55842 229533.I1RFI4 pp 229533.I1RNY6
55843 229533.I1RFI4 pp 229533.I1RPR1
55844 229533.I1RFI4 pp 229533.I1S0V6
55845 229533.I1RFI4 pp 229533.I1RY12

55846 229533.I1RFI4 pp 229533.I1RY21
55847 229533.I1RFI4 pp 229533.I1RSL2
55848 229533.I1RFI4 pp 229533.I1RWB0
55849 229533.I1RFI4 pp 229533.I1RXH3
55850 229533.I1RFI4 pp 229533.I1RUQ3
55851 229533.I1RFI4 pp 229533.I1RP02
55852 229533.I1RFI4 pp 229533.I1S423
55853 229533.I1RFI4 pp 229533.I1RXV9
55854 229533.I1RFI4 pp 229533.I1RWM4
55855 229533.I1RFI4 pp 229533.Q4HYR2
55856 229533.I1RFI4 pp 229533.I1RJS7
55857 229533.I1RFI4 pp 229533.I1RH82
55858 229533.I1RFI4 pp 229533.I1S1W8
55859 229533.I1RFI4 pp 229533.I1RW54
55860 229533.I1RFI4 pp 229533.I1RJT5
55861 229533.I1RFI4 pp 229533.I1RG33
55862 229533.I1RFI4 pp 229533.I1RP86
55863 229533.I1RFI4 pp 229533.I1RRV4
55864 229533.I1RFI4 pp 229533.I1S8F8
55865 229533.I1RFI4 pp 229533.I1RMQ6
55866 229533.I1RFI4 pp 229533.I1RYU6
55867 229533.I1RFI4 pp 229533.I1RNM7
55868 229533.I1RFI4 pp 229533.I1RJC5
55869 229533.I1RFI4 pp 229533.I1RKB7
55870 229533.I1RFI4 pp 229533.I1RYT8
55871 229533.I1RFI4 pp 229533.I1RVX9
55872 229533.I1RFI4 pp 229533.I1RG62
55873 229533.I1RFI4 pp 229533.I1RRX5
55874 229533.I1RFI4 pp 229533.I1S0P0
55875 229533.I1RFI4 pp 229533.I1RWL3
55876 229533.I1RFI4 pp 229533.I1S4S5
55877 229533.I1RFI4 pp 229533.I1RNX8
55878 229533.I1RFI4 pp 229533.I1RXB1
55879 229533.I1RFI4 pp 229533.I1RLP1
55880 229533.I1RFI4 pp 229533.I1S8C1
55881 229533.I1RXJ0 pp 229533.I1RXJ2
55882 229533.I1RKR7 pp 229533.I1RPP0
55883 229533.I1RKR7 pp 229533.I1RTQ9
55884 229533.I1RKR7 pp 229533.I1RN81
55885 229533.I1RKR7 pp 229533.I1RX87
55886 229533.I1RKR7 pp 229533.Q5I2J3
55887 229533.I1RKR7 pp 229533.Q4HYZ2
55888 229533.I1RKR7 pp 229533.I1RTC1
55889 229533.I1RKR7 pp 229533.I1RT41
55890 229533.I1RKR7 pp 229533.I1RQ70
55891 229533.I1RKR7 pp 229533.V6RR76
55892 229533.I1RKR7 pp 229533.I1RYW8
55893 229533.I1RKR7 pp 229533.V6REE9
55894 229533.I1RKR7 pp 229533.I1S0Z3
55895 229533.I1RKR7 pp 229533.I1S9S4
55896 229533.I1RKR7 pp 229533.I1RMG9
55897 229533.I1RKR7 pp 229533.I1RYP1
55898 229533.I1RKR7 pp 229533.I1RQP5
55899 229533.I1RKR7 pp 229533.I1RPK5
55900 229533.I1RKR7 pp 229533.I1RKR9
55901 229533.I1RKR7 pp 229533.Q4ILI9
55902 229533.I1RKR7 pp 229533.I1RX09
55903 229533.I1RKR7 pp 229533.I1RX76
55904 229533.I1RKR7 pp 229533.I1RMF2
55905 229533.I1RKR7 pp 229533.I1S7X9
55906 229533.I1RKR7 pp 229533.I1RWI6
55907 229533.I1RKR7 pp 229533.I1RRW2
55908 229533.I1RKR7 pp 229533.I1RND7
55909 229533.I1RKR7 pp 229533.V6RB81
55910 229533.I1RKR7 pp 229533.I1RZX6
55911 229533.I1RKR7 pp 229533.I1RZ84
55912 229533.I1RKR7 pp 229533.I1RQK5
55913 229533.I1RKR7 pp 229533.I1RTG5
55914 229533.I1RKR7 pp 229533.I1RS79
55915 229533.A0A0E0RU85 pp 229533.I1RA52
55916 229533.A0A0E0RU85 pp 229533.A0A1C3YHG7
55917 229533.A0A0E0RU85 pp 229533.I1RFI4
55918 229533.A0A0E0RU85 pp 229533.I1S3T7

55919 229533.A0A0E0RU85 pp 229533.I1RVZ3
55920 229533.A0A0E0RU85 pp 229533.I1RGC3
55921 229533.A0A0E0RU85 pp 229533.I1S464
55922 229533.A0A0E0RU85 pp 229533.I1RUR8
55923 229533.A0A0E0RU85 pp 229533.I1S299
55924 229533.A0A0E0RU85 pp 229533.I1RC29
55925 229533.A0A0E0RU85 pp 229533.I1RY92
55926 229533.A0A0E0RU85 pp 229533.I1S6P5
55927 229533.A0A0E0RU85 pp 229533.I1RZA7
55928 229533.A0A0E0RU85 pp 229533.A0A1C3YN25
55929 229533.A0A0E0RU85 pp 229533.A0A1C3YJI5
55930 229533.A0A0E0RU85 pp 229533.I1RYK0
55931 229533.A0A0E0RU85 pp 229533.I1RVR2
55932 229533.A0A0E0RU85 pp 229533.I1RSQ4
55933 229533.A0A0E0RU85 pp 229533.I1RC71
55934 229533.A0A0E0RU85 pp 229533.I1RN88
55935 229533.A0A0E0RU85 pp 229533.A0A1C3YI20
55936 229533.A0A0E0RU85 pp 229533.I1RA62
55937 229533.A0A0E0RU85 pp 229533.A0A0E0SPS2
55938 229533.A0A0E0RU85 pp 229533.A0A1C3YJ47
55939 229533.A0A0E0RU85 pp 229533.I1RKB2
55940 229533.A0A0E0RU85 pp 229533.I1RMJ3
55941 229533.A0A0E0RU85 pp 229533.I1RR68
55942 229533.A0A0E0RU85 pp 229533.A0A1C3YM60
55943 229533.A0A0E0RU85 pp 229533.I1S3G4
55944 229533.A0A0E0RU85 pp 229533.V6RUY9
55945 229533.A0A0E0RU85 pp 229533.I1RL81
55946 229533.A0A0E0RU85 pp 229533.I1RCH5
55947 229533.A0A0E0RU85 pp 229533.I1RAN1
55948 229533.A0A0E0RU85 pp 229533.I1RYW9
55949 229533.A0A0E0RU85 pp 229533.A0A1C3YLE2
55950 229533.A0A0E0RU85 pp 229533.I1RBE6
55951 229533.A0A0E0RU85 pp 229533.V6RV57
55952 229533.A0A0E0RU85 pp 229533.A0A1C3YKN4
55953 229533.A0A0E0RU85 pp 229533.V6RZD4
55954 229533.A0A0E0RU85 pp 229533.I1S8R2
55955 229533.A0A0E0RU85 pp 229533.I1RNG4
55956 229533.A0A0E0RU85 pp 229533.Q4HVQ9
55957 229533.A0A0E0RU85 pp 229533.I1S0R8
55958 229533.A0A0E0RU85 pp 229533.I1RNU7
55959 229533.A0A0E0RU85 pp 229533.I1RC34
55960 229533.A0A0E0RU85 pp 229533.V6R3J1
55961 229533.A0A0E0RU85 pp 229533.V6RUB1
55962 229533.A0A0E0RU85 pp 229533.V6RF62
55963 229533.A0A0E0RU85 pp 229533.I1RXW3
55964 229533.A0A0E0RU85 pp 229533.I1S2C4
55965 229533.A0A0E0RU85 pp 229533.I1RYA0
55966 229533.A0A0E0RU85 pp 229533.I1RCG5
55967 229533.A0A0E0RU85 pp 229533.A0A1C3YKW4
55968 229533.A0A0E0RU85 pp 229533.I1RF85
55969 229533.A0A0E0RU85 pp 229533.A0A1C3YK33
55970 229533.A0A0E0RU85 pp 229533.A0A1C3YK47
55971 229533.A0A0E0RU85 pp 229533.V6RRM0
55972 229533.A0A0E0RU85 pp 229533.V6RLM7
55973 229533.A0A0E0RU85 pp 229533.I1RPZ5
55974 229533.A0A0E0RU85 pp 229533.I1RPK0
55975 229533.A0A0E0RU85 pp 229533.I1S0V7
55976 229533.A0A0E0RU85 pp 229533.I1RE52
55977 229533.A0A0E0RU85 pp 229533.I1RMV0
55978 229533.A0A0E0RU85 pp 229533.I1RW54
55979 229533.A0A0E0RU85 pp 229533.A0A1C3YJ04
55980 229533.A0A0E0RU85 pp 229533.A0A1C3YJU7
55981 229533.A0A0E0RU85 pp 229533.A0A1C3YLB6
55982 229533.A0A0E0RU85 pp 229533.I1S1W8
55983 229533.A0A0E0RU85 pp 229533.I1RB38
55984 229533.A0A0E0RU85 pp 229533.I1RJS7
55985 229533.A0A0E0RU85 pp 229533.I1S7W5
55986 229533.A0A0E0RU85 pp 229533.I1RNM7
55987 229533.A0A0E0RU85 pp 229533.I1RYC8
55988 229533.A0A0E0RU85 pp 229533.I1RN84
55989 229533.A0A0E0RU85 pp 229533.I1RRX5
55990 229533.A0A0E0RU85 pp 229533.I1RJC5
55991 229533.A0A0E0RU85 pp 229533.I1RXB1

55992	229533.A0A0E0RU85	pp	229533.I1RYT8
55993	229533.A0A0E0RU85	pp	229533.I1RCU2
55994	229533.A0A0E0RU85	pp	229533.I1RG62
55995	229533.A0A0E0RU85	pp	229533.I1S4S5
55996	229533.A0A0E0RU85	pp	229533.I1RKB7
55997	229533.A0A0E0RU85	pp	229533.I1RMQ6
55998	229533.A0A0E0RU85	pp	229533.A0A1C3YKZ8
55999	229533.A0A0E0RU85	pp	229533.A0A0E0SAR4
56000	229533.A0A0E0RU85	pp	229533.I1S0P0
56001	229533.A0A0E0RU85	pp	229533.I1R9Y4
56002	229533.A0A0E0RU85	pp	229533.A0A1C3YN26
56003	229533.A0A0E0RU85	pp	229533.I1S8C1
56004	229533.A0A0E0RU85	pp	229533.I1RWL3
56005	229533.A0A0E0RU85	pp	229533.I1RP86
56006	229533.A0A0E0RU85	pp	229533.I1RY21
56007	229533.A0A0E0RU85	pp	229533.A0A1C3YK11
56008	229533.A0A0E0RU85	pp	229533.I1RNX8
56009	229533.A0A0E0RU85	pp	229533.I1RLP1
56010	229533.A0A0E0RU85	pp	229533.A0A1C3YL54
56011	229533.A0A0E0RU85	pp	229533.I1RYU6
56012	229533.A0A0E0RU85	pp	229533.I1RXH3
56013	229533.A0A0E0RU85	pp	229533.I1RCV1
56014	229533.A0A0E0RU85	pp	229533.A0A1C3YLB0
56015	229533.A0A0E0RU85	pp	229533.I1RCM5
56016	229533.A0A0E0RU85	pp	229533.I1R9M7
56017	229533.A0A0E0RU85	pp	229533.V6R3V4
56018	229533.A0A0E0RU85	pp	229533.I1RVX9
56019	229533.A0A0E0RU85	pp	229533.I1S8S9
56020	229533.A0A0E0RU85	pp	229533.I1RXV9
56021	229533.A0A0E0RU85	pp	229533.I1RUQ3
56022	229533.A0A0E0RU85	pp	229533.I1RAM9
56023	229533.A0A0E0RU85	pp	229533.I1S0V6
56024	229533.A0A0E0RU85	pp	229533.Q4HYR2
56025	229533.A0A0E0RU85	pp	229533.I1RPT0
56026	229533.A0A0E0RU85	pp	229533.I1RG33
56027	229533.A0A0E0RU85	pp	229533.A0A1C3YLB3
56028	229533.A0A0E0RU85	pp	229533.I1RC59
56029	229533.A0A0E0RU85	pp	229533.I1RPR9
56030	229533.A0A0E0RU85	pp	229533.I1RM26
56031	229533.A0A0E0RU85	pp	229533.A0A1C3YLC2
56032	229533.A0A0E0RU85	pp	229533.I1RWB0
56033	229533.A0A0E0RU85	pp	229533.I1RJY7
56034	229533.A0A0E0RU85	pp	229533.I1RY12
56035	229533.A0A0E0RU85	pp	229533.A0A1C3YM86
56036	229533.A0A0E0RU85	pp	229533.I1RAV5
56037	229533.A0A0E0RU85	pp	229533.A0A1C3YKY6
56038	229533.A0A0E0RU85	pp	229533.A0A1C3YKR5
56039	229533.A0A0E0RU85	pp	229533.A0A1C3YIK4
56040	229533.A0A0E0RU85	pp	229533.I1RJT5
56041	229533.A0A0E0RU85	pp	229533.I1S8F8
56042	229533.A0A0E0RU85	pp	229533.A0A1C3YLJ3
56043	229533.A0A0E0RU85	pp	229533.I1RPR1
56044	229533.A0A0E0RU85	pp	229533.A0A1C3YK58
56045	229533.A0A0E0RU85	pp	229533.I1RNY6
56046	229533.A0A0E0RU85	pp	229533.I1RSL2
56047	229533.A0A0E0RU85	pp	229533.I1RP02
56048	229533.A0A0E0RU85	pp	229533.I1S423
56049	229533.A0A0E0RU85	pp	229533.I1RWM4
56050	229533.A0A0E0RU85	pp	229533.I1REE2
56051	229533.A0A0E0RU85	pp	229533.I1RRV4
56052	229533.A0A0E0RU85	pp	229533.I1RH82
56053	229533.A0A1C3YL89	pp	229533.I1RPG2
56054	229533.A0A1C3YL89	pp	229533.I1RAH5
56055	229533.A0A1C3YL89	pp	229533.I1RCH2
56056	229533.A0A1C3YL89	pp	229533.I1RD23
56057	229533.A0A1C3YL89	pp	229533.I1S1U6
56058	229533.A0A1C3YL89	pp	229533.I1RJS5
56059	229533.A0A1C3YL89	pp	229533.Q4IN63
56060	229533.A0A1C3YL89	pp	229533.I1RND7
56061	229533.A0A1C3YL89	pp	229533.I1RZ84
56062	229533.A0A1C3YL89	pp	229533.I1RS79
56063	229533.A0A1C3YL89	pp	229533.I1S7X9
56064	229533.A0A1C3YL89	pp	229533.A0A1C3YLH6

56065 229533.A0A1C3YL89 pp 229533.I1RXG6
56066 229533.A0A1C3YL89 pp 229533.I1SAM5
56067 229533.A0A1C3YL89 pp 229533.I1RW76
56068 229533.A0A1C3YL89 pp 229533.I1RJS4
56069 229533.A0A1C3YL89 pp 229533.I1RQD6
56070 229533.A0A1C3YL89 pp 229533.I1RN70
56071 229533.A0A1C3YL89 pp 229533.I1RBF2
56072 229533.A0A1C3YL89 pp 229533.I1RZ63
56073 229533.A0A1C3YL89 pp 229533.A0A1C3YLP9
56074 229533.A0A1C3YL89 pp 229533.I1RKI7
56075 229533.A0A1C3YL89 pp 229533.I1RC46
56076 229533.A0A1C3YL89 pp 229533.I1RYP1
56077 229533.A0A1C3YL89 pp 229533.I1RGC1
56078 229533.A0A1C3YL89 pp 229533.I1RSH3
56079 229533.A0A1C3YL89 pp 229533.I1RMF2
56080 229533.A0A1C3YL89 pp 229533.I1RAH7
56081 229533.A0A1C3YL89 pp 229533.V6REE9
56082 229533.A0A1C3YL89 pp 229533.I1RP87
56083 229533.A0A1C3YL89 pp 229533.I1RCD4
56084 229533.A0A1C3YL89 pp 229533.I1RE18
56085 229533.A0A1C3YL89 pp 229533.I1RM74
56086 229533.A0A1C3YL89 pp 229533.A0A1C3YME3
56087 229533.A0A1C3YL89 pp 229533.I1RZK3
56088 229533.A0A1C3YL89 pp 229533.I1RJZ6
56089 229533.A0A1C3YL89 pp 229533.I1S5B5
56090 229533.A0A1C3YL89 pp 229533.V6RB81
56091 229533.A0A1C3YL89 pp 229533.I1RRU2
56092 229533.A0A1C3YL89 pp 229533.I1S2B6
56093 229533.A0A1C3YL89 pp 229533.I1S9S4
56094 229533.A0A1C3YL89 pp 229533.I1RUN1
56095 229533.A0A1C3YL89 pp 229533.I1RN36
56096 229533.A0A1C3YL89 pp 229533.I1RNH7
56097 229533.A0A1C3YL89 pp 229533.I1S213
56098 229533.A0A1C3YL89 pp 229533.I1RPK5
56099 229533.A0A1C3YL89 pp 229533.I1RFK0
56100 229533.A0A1C3YL89 pp 229533.I1S1V4
56101 229533.A0A1C3YL89 pp 229533.I1RA72
56102 229533.A0A1C3YL89 pp 229533.I1RKA7
56103 229533.A0A1C3YL89 pp 229533.I1RE92
56104 229533.A0A1C3YL89 pp 229533.I1RT41
56105 229533.A0A1C3YL89 pp 229533.I1RMQ6
56106 229533.A0A1C3YL89 pp 229533.I1RE20
56107 229533.A0A1C3YL89 pp 229533.I1RQH7
56108 229533.A0A1C3YL89 pp 229533.I1S287
56109 229533.A0A1C3YL89 pp 229533.I1RCQ0
56110 229533.A0A1C3YL89 pp 229533.I1RQF7
56111 229533.A0A1C3YL89 pp 229533.Q5I2J3
56112 229533.A0A1C3YL89 pp 229533.I1RP29
56113 229533.A0A1C3YL89 pp 229533.I1RJY4
56114 229533.A0A1C3YL89 pp 229533.I1RW71
56115 229533.I1RTG4 pp 229533.Q4IMD1
56116 229533.I1RTG4 pp 229533.I1S3G4
56117 229533.I1RTG4 pp 229533.I1RZT0
56118 229533.I1RTG4 pp 229533.I1RVX9
56119 229533.I1RTG4 pp 229533.Q4HYQ4
56120 229533.I1RTG4 pp 229533.I1RXY7
56121 229533.I1RTG4 pp 229533.I1RZ26
56122 229533.I1RTG4 pp 229533.I1RZM4
56123 229533.I1RTG4 pp 229533.I1RVR2
56124 229533.I1RTG4 pp 229533.I1RYM6
56125 229533.I1RTG4 pp 229533.V6RM02
56126 229533.I1RTG4 pp 229533.I1RZ52
56127 229533.I1RTG4 pp 229533.I1RY49
56128 229533.I1RTG4 pp 229533.I1S1W8
56129 229533.I1RTG4 pp 229533.V6RK96
56130 229533.I1RTG4 pp 229533.I1S3K8
56131 229533.I1RTG4 pp 229533.V6RDH3
56132 229533.I1RTG4 pp 229533.I1RW69
56133 229533.A0A098DCP6 pp 229533.A0A098DLB3
56134 229533.A0A098DCP6 pp 229533.I1RKR7
56135 229533.A0A098DCP6 pp 229533.A0A1C3YL89
56136 229533.A0A0E0SH09 pp 229533.I1RJH7
56137 229533.A0A0E0SH09 pp 229533.I1RFW9

56138 229533.A0A0E0SH09 pp 229533.I1RWC6
56139 229533.I1RAF0 pp 229533.I1RQP1
56140 229533.I1RAF0 pp 229533.I1RWT7
56141 229533.I1RAF0 pp 229533.I1RQP3
56142 229533.I1RAF0 pp 229533.Q4IQX3
56143 229533.A0A1C3YJP4 pp 229533.A0A1C3YLB3
56144 229533.A0A1C3YJP4 pp 229533.I1S8F8
56145 229533.A0A1C3YJP4 pp 229533.I1RPT0
56146 229533.A0A1C3YJP4 pp 229533.I1RXV9
56147 229533.A0A1C3YJP4 pp 229533.I1RG33
56148 229533.A0A1C3YJP4 pp 229533.A0A1C3YK58
56149 229533.A0A1C3YJP4 pp 229533.I1RPR1
56150 229533.A0A1C3YJP4 pp 229533.A0A1C3YLJ3
56151 229533.A0A1C3YJP4 pp 229533.I1S0V6
56152 229533.A0A1C3YJP4 pp 229533.I1RCM5
56153 229533.A0A1C3YJP4 pp 229533.I1RH82
56154 229533.A0A1C3YJP4 pp 229533.A0A1C3YKY6
56155 229533.A0A1C3YJP4 pp 229533.I1RC59
56156 229533.A0A1C3YJP4 pp 229533.I1RM26
56157 229533.A0A1C3YJP4 pp 229533.I1RRV4
56158 229533.A0A1C3YJP4 pp 229533.I1REE2
56159 229533.A0A1C3YJP4 pp 229533.I1RWB0
56160 229533.A0A1C3YJP4 pp 229533.I1RWM4
56161 229533.A0A1C3YJP4 pp 229533.I1RN46
56162 229533.A0A1C3YJP4 pp 229533.I1RNM7
56163 229533.A0A1C3YJP4 pp 229533.A0A1C3YLC2
56164 229533.A0A1C3YJP4 pp 229533.I1RPR9
56165 229533.A0A1C3YJP4 pp 229533.I1S1W8
56166 229533.A0A1C3YJP4 pp 229533.I1RW54
56167 229533.A0A1C3YJP4 pp 229533.I1RVX9
56168 229533.A0A1C3YJP4 pp 229533.A0A1C3YN26
56169 229533.A0A1C3YJP4 pp 229533.I1R9Y4
56170 229533.A0A1C3YJP4 pp 229533.I1RCU2
56171 229533.A0A1C3YJP4 pp 229533.I1RWL3
56172 229533.A0A1C3YJP4 pp 229533.I1S8R2
56173 229533.A0A1C3YJP4 pp 229533.I1RRX5
56174 229533.A0A1C3YJP4 pp 229533.I1RAN1
56175 229533.A0A1C3YJP4 pp 229533.I1RMQ6
56176 229533.A0A1C3YJP4 pp 229533.A0A1C3YK11
56177 229533.A0A1C3YJP4 pp 229533.A0A1C3YL54
56178 229533.A0A1C3YJP4 pp 229533.I1RLP1
56179 229533.A0A1C3YJP4 pp 229533.I1RYA0
56180 229533.A0A1C3YJP4 pp 229533.I1RNX8
56181 229533.A0A1C3YJP4 pp 229533.A0A1C3YK33
56182 229533.A0A1C3YJP4 pp 229533.I1RNU7
56183 229533.A0A1C3YJP4 pp 229533.I1RF85
56184 229533.A0A1C3YJP4 pp 229533.A0A1C3YKW4
56185 229533.A0A1C3YJP4 pp 229533.I1RP86
56186 229533.A0A1C3YJP4 pp 229533.I1S2C4
56187 229533.A0A1C3YJP4 pp 229533.I1S7W5
56188 229533.A0A1C3YJP4 pp 229533.A0A1C3YLB6
56189 229533.A0A1C3YJP4 pp 229533.I1RMV0
56190 229533.A0A1C3YJP4 pp 229533.V6RLM7
56191 229533.A0A1C3YJP4 pp 229533.A0A1C3YK47
56192 229533.A0A1C3YJP4 pp 229533.I1RN84
56193 229533.A0A1C3YJP4 pp 229533.I1RZA7
56194 229533.A0A1C3YJP4 pp 229533.I1RPK0
56195 229533.A0A1C3YJP4 pp 229533.I1S0V7
56196 229533.A0A1C3YJP4 pp 229533.I1S299
56197 229533.A0A1C3YJP4 pp 229533.I1RYC8
56198 229533.A0A1C3YJP4 pp 229533.I1RNG4
56199 229533.A0A1C3YJP4 pp 229533.V6RRM0
56200 229533.A0A1C3YJP4 pp 229533.A0A1C3YKN4
56201 229533.A0A1C3YJP4 pp 229533.I1RVR2
56202 229533.A0A1C3YJP4 pp 229533.I1RL81
56203 229533.A0A1C3YJP4 pp 229533.I1RVZ3
56204 229533.A0A1C3YJP4 pp 229533.A0A1C3YLE2
56205 229533.A0A1C3YJP4 pp 229533.I1RSQ4
56206 229533.A0A1C3YJP4 pp 229533.I1RC29
56207 229533.A0A1C3YJP4 pp 229533.I1S3T7
56208 229533.A0A1C3YJP4 pp 229533.I1S6P5
56209 229533.A0A1C3YJP4 pp 229533.V6RUY9
56210 229533.A0A1C3YJP4 pp 229533.I1S3G4

56211 229533.A0A1C3YJP4 pp 229533.I1RR68
56212 229533.A0A1C3YJP4 pp 229533.I1RMJ3
56213 229533.A0A1C3YJP4 pp 229533.V6RUB1
56214 229533.A0A1C3YJP4 pp 229533.I1RXW3
56215 229533.A0A1C3YJP4 pp 229533.I1RKB2
56216 229533.A0A1C3YJP4 pp 229533.I1RA62
56217 229533.A0A1C3YJP4 pp 229533.I1RYK0
56218 229533.A0A1C3YJP4 pp 229533.V6RV57
56219 229533.I1RA36 pp 229533.I1RZ11
56220 229533.I1RA36 pp 229533.I1RCK4
56221 229533.I1RA36 pp 229533.I1RQD8
56222 229533.I1RA36 pp 229533.I1RT47
56223 229533.I1RA36 pp 229533.I1RG17
56224 229533.I1RA36 pp 229533.I1RN09
56225 229533.I1RA36 pp 229533.Q4IRI9
56226 229533.I1RA36 pp 229533.Q4IAA0
56227 229533.I1RA36 pp 229533.I1RM25
56228 229533.I1RA36 pp 229533.I1RNB8
56229 229533.I1RA36 pp 229533.I1RSH9
56230 229533.I1RA36 pp 229533.I1RT35
56231 229533.I1RA36 pp 229533.I1RWX8
56232 229533.I1RA36 pp 229533.I1RSD4
56233 229533.I1RA36 pp 229533.I1RPY5
56234 229533.I1RA36 pp 229533.I1RVT9
56235 229533.I1RA36 pp 229533.I1RJL2
56236 229533.I1RA36 pp 229533.I1RS28
56237 229533.I1RA36 pp 229533.I1RFQ1
56238 229533.I1RA36 pp 229533.I1RG57
56239 229533.I1RA36 pp 229533.I1S289
56240 229533.I1RA36 pp 229533.I1RRT0
56241 229533.I1RA36 pp 229533.Q4I1Y1
56242 229533.I1RA36 pp 229533.I1RAC1
56243 229533.I1RA36 pp 229533.I1RRL0
56244 229533.I1RA36 pp 229533.I1RK72
56245 229533.I1RA36 pp 229533.I1RE24
56246 229533.I1RA36 pp 229533.I1RCB7
56247 229533.I1RA36 pp 229533.I1RZB5
56248 229533.I1RA36 pp 229533.Q4I7X1
56249 229533.I1RA36 pp 229533.I1S8U9
56250 229533.I1RA36 pp 229533.V6RY33
56251 229533.I1RA36 pp 229533.I1RSH4
56252 229533.I1RA36 pp 229533.I1RVK5
56253 229533.I1RA36 pp 229533.I1RX47
56254 229533.I1RA36 pp 229533.I1RQP8
56255 229533.I1RA36 pp 229533.I1RQQ7
56256 229533.I1RA36 pp 229533.I1S7M3
56257 229533.I1RA36 pp 229533.I1RYW3
56258 229533.I1RA36 pp 229533.I1RBJ1
56259 229533.I1RA36 pp 229533.I1RSA8
56260 229533.I1RA36 pp 229533.I1RBH8
56261 229533.I1RA36 pp 229533.I1RK90
56262 229533.I1RA36 pp 229533.I1RG11
56263 229533.I1RA36 pp 229533.I1RND5
56264 229533.I1RA36 pp 229533.Q4IP34
56265 229533.I1RA36 pp 229533.I1RSA4
56266 229533.I1RA36 pp 229533.I1RK79
56267 229533.I1RA36 pp 229533.I1RZL5
56268 229533.I1RA36 pp 229533.V6QX58
56269 229533.I1RA36 pp 229533.I1RS83
56270 229533.I1RA36 pp 229533.I1RBZ8
56271 229533.I1RA36 pp 229533.V6RIS2
56272 229533.I1RA36 pp 229533.I1RX51
56273 229533.I1RA36 pp 229533.I1RK92
56274 229533.I1RA36 pp 229533.I1S471
56275 229533.I1RA36 pp 229533.I1S0P4
56276 229533.I1RA36 pp 229533.V6QXG1
56277 229533.I1RA36 pp 229533.I1RPA0
56278 229533.I1RA36 pp 229533.I1RZQ5
56279 229533.I1RA36 pp 229533.I1RAD1
56280 229533.I1RA36 pp 229533.I1RKF8
56281 229533.I1RA36 pp 229533.Q4IB70
56282 229533.I1RA36 pp 229533.I1RYI9
56283 229533.I1RA36 pp 229533.I1RE31

229533.I1RA36 pp 229533.I1S1C6
229533.I1RA36 pp 229533.I1RZV9
229533.I1RA36 pp 229533.I1RR25
229533.I1RA36 pp 229533.I1S8V0
229533.I1RA36 pp 229533.I1RJS5
229533.I1RA36 pp 229533.I1RCA7
229533.I1RA36 pp 229533.I1RC84
229533.I1RA36 pp 229533.I1RCN5
229533.I1RA36 pp 229533.I1S9L5
229533.I1RA36 pp 229533.Q4IPH4
229533.I1RA36 pp 229533.I1RT91
229533.I1RA36 pp 229533.I1RQR3
229533.I1RA36 pp 229533.I1RCR5
229533.I1RA36 pp 229533.I1S4M5
229533.I1RA36 pp 229533.I1RFP9
229533.I1RA36 pp 229533.I1RRQ4
229533.I1RA36 pp 229533.I1RQZ2
229533.I1RA36 pp 229533.I1RE00
229533.I1RA36 pp 229533.I1RJF9
229533.I1RA36 pp 229533.I1RA46
229533.I1RA36 pp 229533.I1S1X9
229533.I1RA36 pp 229533.I1RFT1
229533.I1RA36 pp 229533.V6QVD5
229533.I1RA36 pp 229533.I1RYB1
229533.I1RA36 pp 229533.I1RS06
229533.I1RA36 pp 229533.I1RDD3
229533.I1RA36 pp 229533.I1RK19
229533.I1RA36 pp 229533.Q4I7F9
229533.I1RA36 pp 229533.I1RWU2
229533.I1RA36 pp 229533.I1S2Y5
229533.I1RA36 pp 229533.I1S0B1
229533.I1RA36 pp 229533.I1S0Z9
229533.I1RA36 pp 229533.I1RZZ1
229533.I1RA36 pp 229533.I1RRG3
229533.I1RA36 pp 229533.I1RG90
229533.I1RA36 pp 229533.I1S5S2
229533.I1RA36 pp 229533.I1RPL3
229533.I1RA36 pp 229533.I1RS75
229533.I1RA36 pp 229533.I1RQC5
229533.I1RA36 pp 229533.I1RND9
229533.I1RA36 pp 229533.Q4IBK5
229533.I1RA36 pp 229533.I1S7M2
229533.I1RA36 pp 229533.I1RW90
229533.I1RA36 pp 229533.I1RXF2
229533.I1RA36 pp 229533.I1S246
229533.I1RA36 pp 229533.I1RB50
229533.I1RA36 pp 229533.I1S0R7
229533.I1RA36 pp 229533.I1RQB5
229533.I1RA36 pp 229533.I1RBU6
229533.I1RA36 pp 229533.I1RCS2
229533.I1RA36 pp 229533.I1RDE9
229533.I1RA36 pp 229533.I1RAF3
229533.I1RA36 pp 229533.I1RAU1
229533.I1RA36 pp 229533.I1RPZ3
229533.I1RA36 pp 229533.I1RC24
229533.I1RA36 pp 229533.I1RLU5
229533.I1RA36 pp 229533.I1S4Y4
229533.I1RA36 pp 229533.I1RN59
229533.I1RA36 pp 229533.I1RD56
229533.I1RA36 pp 229533.I1RSL9
229533.I1RA36 pp 229533.I1S9L6
229533.I1RA36 pp 229533.I1RU69
229533.I1RA36 pp 229533.I1RT17
229533.I1RA36 pp 229533.I1RMY3
229533.I1RA36 pp 229533.I1RQM6
229533.I1RA36 pp 229533.I1RMW7
229533.I1RA36 pp 229533.I1S214
229533.I1RA36 pp 229533.I1RST1
229533.I1RA36 pp 229533.I1RCQ6
229533.I1RA36 pp 229533.I1RG13
229533.I1RA36 pp 229533.I1RME9
229533.I1RA36 pp 229533.I1RPW9
229533.I1RA36 pp 229533.I1S524

56357 229533.I1RA36 pp 229533.Q4IPB3
56358 229533.I1RA36 pp 229533.I1RCU7
56359 229533.I1RA36 pp 229533.I1RPY4
56360 229533.I1RA36 pp 229533.I1RAE1
56361 229533.I1RA36 pp 229533.I1S0B6
56362 229533.I1RA36 pp 229533.I1RTS2
56363 229533.I1RA36 pp 229533.I1RZG7
56364 229533.I1RA36 pp 229533.I1RJS4
56365 229533.I1RA36 pp 229533.Q4IPN9
56366 229533.I1RA36 pp 229533.I1RJG6
56367 229533.I1RA36 pp 229533.I1RCD6
56368 229533.I1RA36 pp 229533.I1RK96
56369 229533.I1RA36 pp 229533.I1RCI3
56370 229533.I1RA36 pp 229533.I1RCI4
56371 229533.I1RA36 pp 229533.I1RA84
56372 229533.I1RA36 pp 229533.I1RX81
56373 229533.I1RA36 pp 229533.I1RMV5
56374 229533.I1RA36 pp 229533.I1RX24
56375 229533.I1RA36 pp 229533.I1RDC1
56376 229533.I1RTK6 pp 229533.I1S8V3
56377 229533.I1RTK6 pp 229533.I1S8V4
56378 229533.I1RTK6 pp 229533.I1S0R7
56379 229533.I1RTK6 pp 229533.I1RWF5
56380 229533.A0A098D558 pp 229533.I1S147
56381 229533.A0A098D558 pp 229533.I1RFI4
56382 229533.A0A098D558 pp 229533.A0A0E0RU85
56383 229533.A0A098D558 pp 229533.I1RA49
56384 229533.A0A098D558 pp 229533.A0A1C3YJP4
56385 229533.I1RC84 pp 229533.Q4IRI9
56386 229533.I1RC84 pp 229533.I1RK72
56387 229533.I1RC84 pp 229533.I1RS28
56388 229533.I1RC84 pp 229533.I1RRL0
56389 229533.I1RC84 pp 229533.I1RFQ1
56390 229533.I1RC84 pp 229533.I1RPY5
56391 229533.I1RC84 pp 229533.I1RNI1
56392 229533.I1RC84 pp 229533.I1RSD4
56393 229533.I1RC84 pp 229533.V6RIS2
56394 229533.I1RC84 pp 229533.V6RG22
56395 229533.I1RC84 pp 229533.V6RY33
56396 229533.I1RC84 pp 229533.I1RS83
56397 229533.I1RC84 pp 229533.I1RWX8
56398 229533.I1RC84 pp 229533.Q4IP34
56399 229533.I1RC84 pp 229533.I1RT47
56400 229533.I1RC84 pp 229533.I1RN09
56401 229533.I1RC84 pp 229533.I1RG17
56402 229533.I1RC84 pp 229533.I1RT35
56403 229533.I1RC84 pp 229533.I1RVT9
56404 229533.I1RC84 pp 229533.I1RZ11
56405 229533.I1RC84 pp 229533.I1RQD8
56406 229533.I1RC84 pp 229533.V6QXG1
56407 229533.I1RC84 pp 229533.I1RX51
56408 229533.I1RC84 pp 229533.I1RSA8
56409 229533.I1RC84 pp 229533.I1RSA4
56410 229533.I1RC84 pp 229533.Q4IE79
56411 229533.I1RC84 pp 229533.I1S7M3
56412 229533.I1RC84 pp 229533.I1RND5
56413 229533.I1RC84 pp 229533.I1RSH9
56414 229533.I1RC84 pp 229533.I1RX47
56415 229533.I1RC84 pp 229533.I1RM25
56416 229533.I1RC84 pp 229533.I1RMM5
56417 229533.I1RC84 pp 229533.I1RRQ4
56418 229533.I1RC84 pp 229533.I1RNB8
56419 229533.I1RC84 pp 229533.I1RZL5
56420 229533.I1RC84 pp 229533.I1RCR5
56421 229533.I1RC84 pp 229533.I1S2Y5
56422 229533.I1RC84 pp 229533.Q4IPH4
56423 229533.I1RC84 pp 229533.I1RCN5
56424 229533.I1RC84 pp 229533.I1RG90
56425 229533.I1RC84 pp 229533.Q4IB70
56426 229533.I1RC84 pp 229533.I1S4Y4
56427 229533.I1RC84 pp 229533.I1S0Z9
56428 229533.I1RC84 pp 229533.I1RYJ9
56429 229533.I1RC84 pp 229533.I1RG57

229533.I1RC84	pp	229533.I1RYI9
229533.I1RC84	pp	229533.Q4I1Y1
229533.I1RC84	pp	229533.I1S0B1
229533.I1RC84	pp	229533.I1RK90
229533.I1RC84	pp	229533.I1RVA7
229533.I1RC84	pp	229533.I1S0R7
229533.I1RC84	pp	229533.V6QVD5
229533.I1RC84	pp	229533.I1S7M2
229533.I1RC84	pp	229533.I1RYW3
229533.I1RC84	pp	229533.I1RXF2
229533.I1RC84	pp	229533.I1S1X9
229533.I1RC84	pp	229533.I1RQP8
229533.I1RC84	pp	229533.I1RPY7
229533.I1RC84	pp	229533.I1RQR3
229533.I1RC84	pp	229533.I1RQZ2
229533.I1RC84	pp	229533.I1S9L6
229533.I1RC84	pp	229533.I1S5S2
229533.I1RC84	pp	229533.I1RCI3
229533.I1RC84	pp	229533.I1RD56
229533.I1RC84	pp	229533.I1RPE5
229533.I1RC84	pp	229533.I1RCD6
229533.I1RC84	pp	229533.I1RRW3
229533.I1RC84	pp	229533.I1RT91
229533.I1RC84	pp	229533.I1RND9
229533.I1RC84	pp	229533.I1RN99
229533.I1RC84	pp	229533.I1RLS1
229533.I1RC84	pp	229533.I1RCI4
229533.I1RC84	pp	229533.Q4HXF6
229533.I1RC84	pp	229533.I1RMV5
229533.I1RC84	pp	229533.I1RZQ5
229533.I1RC84	pp	229533.I1RQC5
229533.I1RC84	pp	229533.I1RX24
229533.I1RC84	pp	229533.I1REX2
229533.I1RC84	pp	229533.I1RME9
229533.I1RC84	pp	229533.I1RZZ1
229533.I1RC84	pp	229533.Q4I7F9
229533.I1RC84	pp	229533.I1RG13
229533.I1RC84	pp	229533.I1S130
229533.I1RC84	pp	229533.I1RX81
229533.I1RC84	pp	229533.I1RPA0
229533.I1RC84	pp	229533.I1RK96
229533.I1RC84	pp	229533.I1RK19
229533.I1RC84	pp	229533.I1RWU2
229533.I1RC84	pp	229533.I1RTS2
229533.I1RC84	pp	229533.I1RZG7
229533.I1RC84	pp	229533.I1RK92
229533.I1RC84	pp	229533.Q4IPB3
229533.I1RC84	pp	229533.I1RFT1
229533.I1RC84	pp	229533.I1S0B6
229533.I1RC84	pp	229533.I1S4M5
229533.I1RC84	pp	229533.I1S524
229533.I1RC84	pp	229533.I1RCA7
229533.I1RC84	pp	229533.I1RMW7
229533.I1RC84	pp	229533.I1RN59
229533.I1RC84	pp	229533.I1RPY4
229533.I1RC84	pp	229533.I1RCS2
229533.I1RC84	pp	229533.I1RU69
229533.I1RC84	pp	229533.I1S9L5
229533.I1RC84	pp	229533.I1RST1
229533.I1RC84	pp	229533.Q4IBK5
229533.I1RC84	pp	229533.I1RCQ6
229533.I1RC84	pp	229533.I1RS75
229533.I1RC84	pp	229533.I1RZD2
229533.I1REQ0	pp	229533.I1RNG4
229533.I1RBH8	pp	229533.I1RBV3
229533.I1RBH8	pp	229533.I1RQB5
229533.I1RBH8	pp	229533.I1S246
229533.I1RBH8	pp	229533.I1RCS2
229533.I1RBH8	pp	229533.I1RRG3
229533.I1RBH8	pp	229533.I1S0R7
229533.I1RBH8	pp	229533.I1S5S2
229533.I1RBH8	pp	229533.I1S2Y5
229533.I1RBH8	pp	229533.I1RS75

56503	229533.I1RBH8	pp	229533.I1RX51
56504	229533.I1RBH8	pp	229533.I1RXF2
56505	229533.I1RBH8	pp	229533.I1RJF9
56506	229533.I1RBH8	pp	229533.I1S4M5
56507	229533.I1RBH8	pp	229533.I1RWX8
56508	229533.I1RBH8	pp	229533.I1RT35
56509	229533.I1RBH8	pp	229533.I1S7M2
56510	229533.I1RBH8	pp	229533.I1RVT9
56511	229533.I1RBH8	pp	229533.I1S1C6
56512	229533.I1RBH8	pp	229533.I1RDD3
56513	229533.I1RBH8	pp	229533.I1RK19
56514	229533.I1RBH8	pp	229533.I1RZG7
56515	229533.I1RBH8	pp	229533.I1RFT1
56516	229533.I1RBH8	pp	229533.I1RTS2
56517	229533.I1RBH8	pp	229533.V6QXG1
56518	229533.I1RBH8	pp	229533.I1RCA7
56519	229533.I1RBH8	pp	229533.I1RWU2
56520	229533.I1RBH8	pp	229533.I1RSA4
56521	229533.I1RBH8	pp	229533.I1RCQ6
56522	229533.I1RBH8	pp	229533.I1RND5
56523	229533.I1RBH8	pp	229533.I1S9L5
56524	229533.I1RBH8	pp	229533.I1RD56
56525	229533.I1RBH8	pp	229533.Q4IPB3
56526	229533.I1RBH8	pp	229533.I1S524
56527	229533.I1RBH8	pp	229533.V6QX58
56528	229533.I1RBH8	pp	229533.I1S9L6
56529	229533.I1RBH8	pp	229533.I1RPY4
56530	229533.I1RBH8	pp	229533.I1RE24
56531	229533.I1RBH8	pp	229533.I1RQQ7
56532	229533.I1RBH8	pp	229533.I1RK96
56533	229533.I1RBH8	pp	229533.I1RCI3
56534	229533.I1RBH8	pp	229533.I1RJG6
56535	229533.I1RBH8	pp	229533.I1RQN0
56536	229533.I1RBH8	pp	229533.I1RX24
56537	229533.I1RBH8	pp	229533.I1RSH4
56538	229533.I1RBH8	pp	229533.I1RMW7
56539	229533.I1RBH8	pp	229533.I1RCD6
56540	229533.I1RBH8	pp	229533.I1RDC1
56541	229533.I1RBH8	pp	229533.I1RXZ3
56542	229533.I1RBH8	pp	229533.I1RMV5
56543	229533.I1RBH8	pp	229533.I1RX81
56544	229533.I1RBH8	pp	229533.V6RY33
56545	229533.I1RBH8	pp	229533.I1RWW5
56546	229533.I1RBH8	pp	229533.I1RSA8
56547	229533.I1RBH8	pp	229533.I1RCI4
56548	229533.I1RBH8	pp	229533.I1REX2
56549	229533.I1RBH8	pp	229533.I1RN59
56550	229533.I1RBH8	pp	229533.I1S1X9
56551	229533.I1RBH8	pp	229533.I1RX47
56552	229533.I1RBH8	pp	229533.I1RQZ2
56553	229533.I1RBH8	pp	229533.I1S7M3
56554	229533.I1RBH8	pp	229533.I1RDE9
56555	229533.I1RBH8	pp	229533.I1RU69
56556	229533.I1RBH8	pp	229533.I1S8U9
56557	229533.I1RBH8	pp	229533.I1S0Z9
56558	229533.I1RBH8	pp	229533.I1RJV2
56559	229533.I1RBH8	pp	229533.I1RG90
56560	229533.I1RBH8	pp	229533.I1S0B6
56561	229533.I1RBH8	pp	229533.I1S0B1
56562	229533.I1RBH8	pp	229533.I1RT47
56563	229533.I1RBH8	pp	229533.I1RST1
56564	229533.I1RBH8	pp	229533.I1RK72
56565	229533.I1RBH8	pp	229533.I1RCR5
56566	229533.I1RBH8	pp	229533.I1RFP9
56567	229533.I1RBH8	pp	229533.I1RN09
56568	229533.I1RBH8	pp	229533.I1RRL0
56569	229533.I1RBH8	pp	229533.I1S8V0
56570	229533.I1RBH8	pp	229533.I1RSD4
56571	229533.I1RBH8	pp	229533.I1RG17
56572	229533.I1RBH8	pp	229533.I1RK90
56573	229533.I1RBH8	pp	229533.I1RZ11
56574	229533.I1RBH8	pp	229533.I1RQD8
56575	229533.I1RBH8	pp	229533.I1RYW3

56576 229533.I1RBH8 pp 229533.I1RS28
56577 229533.I1RBH8 pp 229533.I1RF91
56578 229533.I1RBH8 pp 229533.I1RQP8
56579 229533.I1RBH8 pp 229533.I1RPY5
56580 229533.I1RBH8 pp 229533.I1RFQ1
56581 229533.I1RBH8 pp 229533.I1RVK5
56582 229533.I1RBH8 pp 229533.I1RSH9
56583 229533.I1RBH8 pp 229533.I1RM25
56584 229533.I1RBH8 pp 229533.V6RIS2
56585 229533.I1RBH8 pp 229533.I1RG57
56586 229533.I1RBH8 pp 229533.I1RNB8
56587 229533.I1RBH8 pp 229533.I1RS83
56588 229533.I1RBH8 pp 229533.Q4IP34
56589 229533.I1RBH8 pp 229533.Q4IRI9
56590 229533.I1RBH8 pp 229533.I1RZQ5
56591 229533.I1RBH8 pp 229533.Q4IAA0
56592 229533.I1RBH8 pp 229533.Q4I1Y1
56593 229533.I1RBH8 pp 229533.I1RCN5
56594 229533.I1RBH8 pp 229533.Q4IB70
56595 229533.I1RBH8 pp 229533.I1RC84
56596 229533.I1RBH8 pp 229533.Q4I7F9
56597 229533.I1RBH8 pp 229533.I1RMM5
56598 229533.I1RBH8 pp 229533.I1RK92
56599 229533.I1RBH8 pp 229533.I1RPA0
56600 229533.I1RBH8 pp 229533.I1RZZ1
56601 229533.I1RBH8 pp 229533.I1RZL5
56602 229533.I1S2N4 pp 229533.I1S5P3
56603 229533.I1RVM7 pp 229533.I1S2N4
56604 229533.I1RSA4 pp 229533.I1RTS2
56605 229533.I1RSA4 pp 229533.I1RW25
56606 229533.I1RSA4 pp 229533.I1RX25
56607 229533.I1RSA4 pp 229533.I1RUN8
56608 229533.I1RSA4 pp 229533.I1RZG7
56609 229533.I1RSA4 pp 229533.I1RX24
56610 229533.I1RSA4 pp 229533.Q4IPB3
56611 229533.I1RSA4 pp 229533.I1S524
56612 229533.I1RSA4 pp 229533.I1S9L6
56613 229533.I1RSA4 pp 229533.I1RX81
56614 229533.I1RSA4 pp 229533.I1S0B6
56615 229533.I1RSA4 pp 229533.Q4IBK5
56616 229533.I1RSA4 pp 229533.I1RWL2
56617 229533.I1RSA4 pp 229533.Q4I5R9
56618 229533.I1RSA4 pp 229533.I1S2G9
56619 229533.I1RSA4 pp 229533.I1RYI9
56620 229533.I1RSA4 pp 229533.I1S2Y5
56621 229533.I1RSA4 pp 229533.I1RST1
56622 229533.I1RSA4 pp 229533.I1S246
56623 229533.I1RSA4 pp 229533.I1RU69
56624 229533.I1RSA4 pp 229533.Q4IMD1
56625 229533.I1RSA4 pp 229533.V6QVD5
56626 229533.I1RSA4 pp 229533.I1RWU2
56627 229533.I1RSA4 pp 229533.I1RT17
56628 229533.I1RSA4 pp 229533.I1RYB1
56629 229533.I1RSA4 pp 229533.I1RSL9
56630 229533.I1RSA4 pp 229533.I1S4M5
56631 229533.I1RSA4 pp 229533.V6QW22
56632 229533.I1RSA4 pp 229533.I1S4Y4
56633 229533.I1RSA4 pp 229533.I1RT40
56634 229533.I1RSA4 pp 229533.I1S9L5
56635 229533.I1RSA4 pp 229533.Q4IPH4
56636 229533.I1RSA4 pp 229533.I1S0R7
56637 229533.I1RSA4 pp 229533.I1S8V0
56638 229533.I1RSA4 pp 229533.I1RT04
56639 229533.I1RSA4 pp 229533.I1RZQ5
56640 229533.I1RSA4 pp 229533.I1RW24
56641 229533.I1RSA4 pp 229533.Q4I948
56642 229533.I1RSA4 pp 229533.I1RXF2
56643 229533.I1RSA4 pp 229533.I1S1C6
56644 229533.I1RSA4 pp 229533.I1RZZ1
56645 229533.I1RSA4 pp 229533.I1RZL5
56646 229533.I1RSA4 pp 229533.I1S7M2
56647 229533.I1RSA4 pp 229533.I1RTD7
56648 229533.I1RSA4 pp 229533.I1RYW3

56649 229533.I1RSA4 pp 229533.Q4I7F9
56650 229533.I1RSA4 pp 229533.I1RYJ9
56651 229533.I1RSA4 pp 229533.I1S5S2
56652 229533.I1RSA4 pp 229533.I1S0B1
56653 229533.I1RSA4 pp 229533.Q4I6B0
56654 229533.I1RSA4 pp 229533.I1S0Z9
56655 229533.I1RSA4 pp 229533.I1RZB5
56656 229533.I1RSA4 pp 229533.I1RVK5
56657 229533.I1RSA4 pp 229533.V6RY33
56658 229533.I1RSA4 pp 229533.Q4I1Y1
56659 229533.I1RSA4 pp 229533.I1S1X9
56660 229533.I1RSA4 pp 229533.I1RT35
56661 229533.I1RSA4 pp 229533.I1RSH4
56662 229533.I1RSA4 pp 229533.I1RT91
56663 229533.I1RSA4 pp 229533.I1RWX8
56664 229533.I1RSA4 pp 229533.Q4HXF6
56665 229533.I1RSA4 pp 229533.I1S289
56666 229533.I1RSA4 pp 229533.V6QXG1
56667 229533.I1RSA4 pp 229533.I1RVT9
56668 229533.I1RSA4 pp 229533.Q4IAA0
56669 229533.I1RSA4 pp 229533.I1RSH9
56670 229533.I1RSA4 pp 229533.I1RSD4
56671 229533.I1RSA4 pp 229533.Q4IRI9
56672 229533.I1RSA4 pp 229533.Q4IB70
56673 229533.I1RSA4 pp 229533.I1RX51
56674 229533.I1RSA4 pp 229533.Q4IP34
56675 229533.I1RSA4 pp 229533.I1S8F8
56676 229533.I1RSA4 pp 229533.I1RZ11
56677 229533.I1RSA4 pp 229533.Q4IE79
56678 229533.I1RSA4 pp 229533.I1RT47
56679 229533.I1RSA4 pp 229533.I1RSA8
56680 229533.I1RSA4 pp 229533.V6QX58
56681 229533.I1RSA4 pp 229533.I1S7M3
56682 229533.I1RSA4 pp 229533.I1S471
56683 229533.I1RSA4 pp 229533.Q4IPB8
56684 229533.I1RSA4 pp 229533.I1S8U9
56685 229533.I1RSA4 pp 229533.I1RX47
56686 229533.I1RSA4 pp 229533.V6RIS2
56687 229533.I1REW1 pp 229533.I1S8F8
56688 229533.I1REW1 pp 229533.I1RLA2
56689 229533.I1REW1 pp 229533.I1RST9
56690 229533.I1REW1 pp 229533.I1S094
56691 229533.I1REW1 pp 229533.I1RUY5
56692 229533.I1REW1 pp 229533.I1RNM7
56693 229533.I1REW1 pp 229533.I1S6H3
56694 229533.I1REW1 pp 229533.I1RRL5
56695 229533.I1REW1 pp 229533.I1S3H8
56696 229533.I1REW1 pp 229533.I1S334
56697 229533.I1REW1 pp 229533.I1RNG4
56698 229533.I1REW1 pp 229533.I1RJS1
56699 229533.I1REW1 pp 229533.I1RPV8
56700 229533.I1REW1 pp 229533.I1RKB2
56701 229533.I1REW1 pp 229533.I1RZ25
56702 229533.I1REW1 pp 229533.I1RZ56
56703 229533.I1REW1 pp 229533.I1RZ38
56704 229533.I1REW1 pp 229533.I1S0A5
56705 229533.I1REW1 pp 229533.I1RGZ3
56706 229533.I1RML4 pp 229533.I1S549
56707 229533.I1RML4 pp 229533.I1RRP3
56708 229533.I1RML4 pp 229533.I1S8R4
56709 229533.I1RML4 pp 229533.I1RN84
56710 229533.I1RML4 pp 229533.I1S8C1
56711 229533.I1RML4 pp 229533.I1RNM8
56712 229533.I1RML4 pp 229533.I1RN88
56713 229533.I1RML4 pp 229533.I1RQA2
56714 229533.I1RML4 pp 229533.I1S3L2
56715 229533.I1RV19 pp 229533.I1S2N4
56716 229533.I1RV19 pp 229533.I1RV20
56717 229533.I1RV19 pp 229533.I1RVB6
56718 229533.I1RV19 pp 229533.I1RW91
56719 229533.I1RFQ1 pp 229533.I1RS75
56720 229533.I1RFQ1 pp 229533.I1RQB5
56721 229533.I1RFQ1 pp 229533.I1S2Y5

56722	229533.I1RFQ1	pp	229533.Q4IBK5
56723	229533.I1RFQ1	pp	229533.I1S9L5
56724	229533.I1RFQ1	pp	229533.I1RW71
56725	229533.I1RFQ1	pp	229533.I1RZJ1
56726	229533.I1RFQ1	pp	229533.I1RWU2
56727	229533.I1RFQ1	pp	229533.I1RKK4
56728	229533.I1RFQ1	pp	229533.I1S9L6
56729	229533.I1RFQ1	pp	229533.I1RFT1
56730	229533.I1RFQ1	pp	229533.I1S4M5
56731	229533.I1RFQ1	pp	229533.I1RG13
56732	229533.I1RFQ1	pp	229533.I1RLU5
56733	229533.I1RFQ1	pp	229533.I1RJF9
56734	229533.I1RFQ1	pp	229533.I1S7M2
56735	229533.I1RFQ1	pp	229533.I1RME9
56736	229533.I1RFQ1	pp	229533.I1RND9
56737	229533.I1RFQ1	pp	229533.I1RMY3
56738	229533.I1RFQ1	pp	229533.I1RXF2
56739	229533.I1RFQ1	pp	229533.I1RQC5
56740	229533.I1RFQ1	pp	229533.I1S1C6
56741	229533.I1RFQ1	pp	229533.I1S5S2
56742	229533.I1RFQ1	pp	229533.Q4IB96
56743	229533.I1RFQ1	pp	229533.I1RND5
56744	229533.I1RFQ1	pp	229533.I1RYI9
56745	229533.I1RFQ1	pp	229533.I1RSA4
56746	229533.I1RFQ1	pp	229533.V6QXG1
56747	229533.I1RFQ1	pp	229533.I1RPZ3
56748	229533.I1RFQ1	pp	229533.I1RMV5
56749	229533.I1RFQ1	pp	229533.I1RW25
56750	229533.I1RFQ1	pp	229533.I1RT17
56751	229533.I1RFQ1	pp	229533.I1S8F8
56752	229533.I1RFQ1	pp	229533.I1RPY4
56753	229533.I1RFQ1	pp	229533.I1RJG6
56754	229533.I1RFQ1	pp	229533.I1RN59
56755	229533.I1RFQ1	pp	229533.I1RN03
56756	229533.I1RFQ1	pp	229533.I1S524
56757	229533.I1RFQ1	pp	229533.I1RT35
56758	229533.I1RFQ1	pp	229533.I1RU69
56759	229533.I1RFQ1	pp	229533.I1RWX8
56760	229533.I1RFQ1	pp	229533.I1RWW5
56761	229533.I1RFQ1	pp	229533.I1RX51
56762	229533.I1RFQ1	pp	229533.Q4IPB3
56763	229533.I1RFQ1	pp	229533.V6RY33
56764	229533.I1RFQ1	pp	229533.I1RZZ9
56765	229533.I1RFQ1	pp	229533.I1RZG7
56766	229533.I1RFQ1	pp	229533.I1RX81
56767	229533.I1RFQ1	pp	229533.I1S7M3
56768	229533.I1RFQ1	pp	229533.I1RS66
56769	229533.I1RFQ1	pp	229533.I1RMW7
56770	229533.I1RFQ1	pp	229533.I1RX24
56771	229533.I1RFQ1	pp	229533.I1RSA8
56772	229533.I1RFQ1	pp	229533.I1RK96
56773	229533.I1RFQ1	pp	229533.I1RX47
56774	229533.I1RFQ1	pp	229533.I1S0B1
56775	229533.I1RFQ1	pp	229533.I1S0B6
56776	229533.I1RFQ1	pp	229533.I1RPY8
56777	229533.I1RFQ1	pp	229533.I1RFQ7
56778	229533.I1RFQ1	pp	229533.I1RJV2
56779	229533.I1RFQ1	pp	229533.I1RST1
56780	229533.I1RFQ1	pp	229533.I1S0Z9
56781	229533.I1RFQ1	pp	229533.I1RG90
56782	229533.I1RFQ1	pp	229533.I1RPY5
56783	229533.I1RFQ1	pp	229533.I1RQD8
56784	229533.I1RFQ1	pp	229533.I1RS28
56785	229533.I1RFQ1	pp	229533.I1RZ11
56786	229533.I1RFQ1	pp	229533.I1RYJ9
56787	229533.I1RFQ1	pp	229533.I1RG17
56788	229533.I1RFQ1	pp	229533.I1RSD4
56789	229533.I1RFQ1	pp	229533.Q4IPH4
56790	229533.I1RFQ1	pp	229533.I1RRL0
56791	229533.I1RFQ1	pp	229533.I1RT47
56792	229533.I1RFQ1	pp	229533.Q4IP34
56793	229533.I1RFQ1	pp	229533.I1RRQ4
56794	229533.I1RFQ1	pp	229533.I1RN09

56795	229533.I1RFQ1	pp	229533.I1RMM5
56796	229533.I1RFQ1	pp	229533.I1RS83
56797	229533.I1RFQ1	pp	229533.I1RK72
56798	229533.I1RFQ1	pp	229533.I1RQP8
56799	229533.I1RFQ1	pp	229533.V6RIS2
56800	229533.I1RFQ1	pp	229533.I1RK90
56801	229533.I1RFQ1	pp	229533.I1RYW3
56802	229533.I1RFQ1	pp	229533.Q4IAA0
56803	229533.I1RFQ1	pp	229533.Q4IRI9
56804	229533.I1RFQ1	pp	229533.I1S471
56805	229533.I1RFQ1	pp	229533.I1RG11
56806	229533.I1RFQ1	pp	229533.I1RNB8
56807	229533.I1RFQ1	pp	229533.Q4I1Y1
56808	229533.I1RFQ1	pp	229533.I1RZZ1
56809	229533.I1RFQ1	pp	229533.I1RG57
56810	229533.I1RFQ1	pp	229533.Q4IB70
56811	229533.I1RFQ1	pp	229533.I1RPL3
56812	229533.I1RFQ1	pp	229533.Q4I7F9
56813	229533.I1RFQ1	pp	229533.I1RM25
56814	229533.I1RFQ1	pp	229533.I1RT70
56815	229533.I1RFQ1	pp	229533.I1RJS5
56816	229533.I1RFQ1	pp	229533.I1S1X9
56817	229533.I1RFQ1	pp	229533.I1RK92
56818	229533.I1RFQ1	pp	229533.I1RZQ5
56819	229533.I1RFQ1	pp	229533.I1RQR3
56820	229533.I1RFQ1	pp	229533.I1RPA0
56821	229533.I1RSU2	pp	229533.V6R042
56822	229533.I1RSU2	pp	229533.I1S5R6
56823	229533.I1RSU2	pp	229533.V6RN69
56824	229533.I1RSU2	pp	229533.I1RYK0
56825	229533.I1RSU2	pp	229533.V6RUY9
56826	229533.I1RSU2	pp	229533.I1RWF5
56827	229533.I1RSU2	pp	229533.I1S0J1
56828	229533.I1RSU2	pp	229533.I1RWL8
56829	229533.I1RSU2	pp	229533.V6RRM0
56830	229533.I1RSU2	pp	229533.I1RY35
56831	229533.I1RSU2	pp	229533.I1S253
56832	229533.I1RSU2	pp	229533.I1RW43
56833	229533.I1RSU2	pp	229533.I1RSZ7
56834	229533.I1RSU2	pp	229533.I1RZJ8
56835	229533.I1RSU2	pp	229533.I1S9Z4
56836	229533.I1RSU2	pp	229533.I1RX89
56837	229533.I1RSU2	pp	229533.I1S9Y1
56838	229533.I1RSU2	pp	229533.I1RYW3
56839	229533.I1RSU2	pp	229533.I1RW64
56840	229533.I1RSU2	pp	229533.I1RYU6
56841	229533.I1RSU2	pp	229533.V6RLM7
56842	229533.I1RSU2	pp	229533.I1RSX9
56843	229533.I1RSU2	pp	229533.V6QUE6
56844	229533.I1RSU2	pp	229533.I1RX46
56845	229533.I1RSU2	pp	229533.I1S2C4
56846	229533.I1RSU2	pp	229533.V6RTJ8
56847	229533.I1RSU2	pp	229533.I1RT50
56848	229533.I1RSU2	pp	229533.I1RUY5
56849	229533.I1RSU2	pp	229533.I1RVP5
56850	229533.I1RSU2	pp	229533.I1RW54
56851	229533.I1RSU2	pp	229533.I1S489
56852	229533.I1RSU2	pp	229533.I1RXU3
56853	229533.I1RSU2	pp	229533.I1RZM4
56854	229533.I1RSU2	pp	229533.V6RK96
56855	229533.I1RSU2	pp	229533.I1RWL3
56856	229533.I1RSU2	pp	229533.I1RXB6
56857	229533.I1REM8	pp	229533.I1RV19
56858	229533.I1REM8	pp	229533.I1S2N4
56859	229533.I1REM8	pp	229533.I1RVM7
56860	229533.I1REM8	pp	229533.I1S5P3
56861	229533.I1RSD4	pp	229533.I1RWU2
56862	229533.I1RSD4	pp	229533.Q4I830
56863	229533.I1RSD4	pp	229533.I1RXF2
56864	229533.I1RSD4	pp	229533.I1S4M5
56865	229533.I1RSD4	pp	229533.I1S6F0
56866	229533.I1RSD4	pp	229533.I1S7M2
56867	229533.I1RSD4	pp	229533.I1S9L5

229533.I1RSD4	pp	229533.I1RSL9
229533.I1RSD4	pp	229533.I1S5S2
229533.I1RSD4	pp	229533.Q4HW67
229533.I1RSD4	pp	229533.Q4IAS1
229533.I1RSD4	pp	229533.I1S2Y5
229533.I1RSD4	pp	229533.Q4IBK5
229533.I1RSD4	pp	229533.I1S854
229533.I1RSD4	pp	229533.I1S9L6
229533.I1RSD4	pp	229533.Q4IMD1
229533.I1RSD4	pp	229533.I1RYI9
229533.I1RSD4	pp	229533.Q4HZ42
229533.I1RSD4	pp	229533.I1RX24
229533.I1RSD4	pp	229533.I1RZG7
229533.I1RSD4	pp	229533.Q4IE79
229533.I1RSD4	pp	229533.Q4IPB3
229533.I1RSD4	pp	229533.I1RTS2
229533.I1RSD4	pp	229533.I1RX47
229533.I1RSD4	pp	229533.I1RX81
229533.I1RSD4	pp	229533.I1S7M3
229533.I1RSD4	pp	229533.Q4I662
229533.I1RSD4	pp	229533.I1S524
229533.I1RSD4	pp	229533.I1RST1
229533.I1RSD4	pp	229533.V6RIS2
229533.I1RSD4	pp	229533.I1RT36
229533.I1RSD4	pp	229533.I1S0B6
229533.I1RSD4	pp	229533.I1RUN9
229533.I1RSD4	pp	229533.I1S9Y1
229533.I1RSD4	pp	229533.Q4IFI0
229533.I1RSD4	pp	229533.Q4IJ56
229533.I1RSD4	pp	229533.Q4IP34
229533.I1RSD4	pp	229533.I1RSH9
229533.I1RSD4	pp	229533.I1RT47
229533.I1RSD4	pp	229533.V6QUE6
229533.I1RSD4	pp	229533.I1RVY0
229533.I1RSD4	pp	229533.I1RT41
229533.I1RSD4	pp	229533.Q4IJH1
229533.I1RSD4	pp	229533.Q4I5I4
229533.I1RSD4	pp	229533.Q4IAA0
229533.I1RSD4	pp	229533.I1S0J3
229533.I1RSD4	pp	229533.I1RWD3
229533.I1RSD4	pp	229533.Q4IBS2
229533.I1RSD4	pp	229533.Q4IRI9
229533.I1RSD4	pp	229533.I1RZ11
229533.I1RSD4	pp	229533.I1RWI5
229533.I1RSD4	pp	229533.Q4I7K4
229533.I1RSD4	pp	229533.Q4HVW2
229533.I1RSD4	pp	229533.I1S1X9
229533.I1RSD4	pp	229533.Q4IF76
229533.I1RSD4	pp	229533.Q4HXF6
229533.I1RSD4	pp	229533.V6RY33
229533.I1RSD4	pp	229533.I1RT91
229533.I1RSD4	pp	229533.I1RT35
229533.I1RSD4	pp	229533.I1RU69
229533.I1RSD4	pp	229533.I1RWX8
229533.I1RSD4	pp	229533.I1RZQ5
229533.I1RSD4	pp	229533.I1RYB1
229533.I1RSD4	pp	229533.Q4I7F9
229533.I1RSD4	pp	229533.I1RZZ1
229533.I1RSD4	pp	229533.I1RVT9
229533.I1RSD4	pp	229533.Q4I1Y1
229533.I1RSD4	pp	229533.V6QXG1
229533.I1RSD4	pp	229533.Q4IER8
229533.I1RSD4	pp	229533.I1S0C1
229533.I1RSD4	pp	229533.I1RX51
229533.I1RSD4	pp	229533.Q4IEK8
229533.I1RSD4	pp	229533.I1S0N5
229533.I1RSD4	pp	229533.Q4HZ68
229533.I1RSD4	pp	229533.V6QX58
229533.I1RSD4	pp	229533.I1RYW3
229533.I1RSD4	pp	229533.Q4IPH4
229533.I1RSD4	pp	229533.I1S8V0
229533.I1RSD4	pp	229533.I1S9J6
229533.I1RSD4	pp	229533.I1RVK5

56941 229533.I1RSD4 pp 229533.I1RYJ9
56942 229533.I1RSD4 pp 229533.I1S0Z3
56943 229533.I1RSD4 pp 229533.I1RSD7
56944 229533.I1RSD4 pp 229533.I1RZL5
56945 229533.I1RSD4 pp 229533.I1S8U9
56946 229533.I1RSD4 pp 229533.Q4I2J8
56947 229533.I1RSD4 pp 229533.I1S0Z9
56948 229533.I1RSD4 pp 229533.I1S0B1
56949 229533.I1RSD4 pp 229533.I1S855
56950 229533.I1RSD4 pp 229533.Q4IB70
56951 229533.I1RSD4 pp 229533.V6QVD5
56952 229533.A0A0E0S4D3 pp 229533.I1RV17
56953 229533.A0A0E0S4D3 pp 229533.Q00909
56954 229533.A0A0E0S4D3 pp 229533.A0A1C3YI59
56955 229533.A0A0E0S4D3 pp 229533.I1REM8
56956 229533.A0A0E0S4D3 pp 229533.A0A0E0SBQ2
56957 229533.A0A0E0S4D3 pp 229533.I1RV24
56958 229533.A0A0E0S4D3 pp 229533.I1S2N4
56959 229533.A0A1C3YLF1 pp 229533.I1RUP7
56960 229533.A0A1C3YLF1 pp 229533.I1RVY0
56961 229533.A0A1C3YLF1 pp 229533.I1RE53
56962 229533.I1RQ13 pp 229533.I1S209
56963 229533.I1RQ13 pp 229533.I1RR73
56964 229533.I1RQ13 pp 229533.I1RZP8
56965 229533.I1RQ13 pp 229533.I1RYQ1
56966 229533.I1RQ13 pp 229533.I1RZ63
56967 229533.I1RQ13 pp 229533.I1RX64
56968 229533.I1RQ13 pp 229533.I1RUF8
56969 229533.I1RQ13 pp 229533.I1S0E8
56970 229533.I1RQ13 pp 229533.V6R382
56971 229533.I1RQ13 pp 229533.I1RQ67
56972 229533.I1RQ13 pp 229533.I1RQA3
56973 229533.I1RQ13 pp 229533.I1RSP7
56974 229533.I1RQ13 pp 229533.I1RYY5
56975 229533.I1RQ13 pp 229533.I1RWT6
56976 229533.I1RQ13 pp 229533.I1RYX9
56977 229533.I1RQ13 pp 229533.Q4IBX0
56978 229533.I1RQ13 pp 229533.Q4I7L0
56979 229533.I1RQ13 pp 229533.V6RLW4
56980 229533.I1RQ13 pp 229533.Q4I0J6
56981 229533.I1RQ13 pp 229533.I1S283
56982 229533.I1RQ13 pp 229533.I1RYR2
56983 229533.I1RQ13 pp 229533.I1RSR9
56984 229533.I1RQ13 pp 229533.I1S2E9
56985 229533.I1RQ13 pp 229533.I1RZ94
56986 229533.I1RQ13 pp 229533.I1RZM1
56987 229533.I1RQ13 pp 229533.I1S098
56988 229533.I1RQ13 pp 229533.Q4IJM4
56989 229533.I1RQ13 pp 229533.I1RSC7
56990 229533.I1RQ13 pp 229533.I1RY44
56991 229533.I1RQ13 pp 229533.Q4IMM0
56992 229533.I1RQ13 pp 229533.I1RSN7
56993 229533.A0A098D4Y6 pp 229533.A0A0E0SP71
56994 229533.A0A098D4Y6 pp 229533.I1S5P3
56995 229533.A0A098D4Y6 pp 229533.I1S2N4
56996 229533.A0A098D4Y6 pp 229533.I1RVM7
56997 229533.A0A098D4Y6 pp 229533.I1RJU9
56998 229533.A0A098D4Y6 pp 229533.I1RV19
56999 229533.A0A098D4Y6 pp 229533.I1REM8
57000 229533.A0A098D4Y6 pp 229533.A0A0E0S4D3
57001 229533.A0A098D4Y6 pp 229533.I1RLM3
57002 229533.Q4I5M4 pp 229533.Q4IJ56
57003 229533.Q4I5M4 pp 229533.Q4I662
57004 229533.Q4I5M4 pp 229533.Q4IFI0
57005 229533.Q4I5M4 pp 229533.Q4I9U7
57006 229533.Q4I5M4 pp 229533.Q4IQ72
57007 229533.I1RS83 pp 229533.I1RYI9
57008 229533.I1RS83 pp 229533.I1RYB1
57009 229533.I1RS83 pp 229533.Q4IPH4
57010 229533.I1RS83 pp 229533.I1S0B1
57011 229533.I1RS83 pp 229533.I1S7M2
57012 229533.I1RS83 pp 229533.I1S0R7
57013 229533.I1RS83 pp 229533.I1RT04

57014 229533.I1RS83 pp 229533.Q4I7F9
57015 229533.I1RS83 pp 229533.I1RWX8
57016 229533.I1RS83 pp 229533.I1RZZ1
57017 229533.I1RS83 pp 229533.I1RXF2
57018 229533.I1RS83 pp 229533.I1RSA4
57019 229533.I1RS83 pp 229533.V6RY33
57020 229533.I1RS83 pp 229533.Q4IB70
57021 229533.I1RS83 pp 229533.I1S5S2
57022 229533.I1RS83 pp 229533.I1RSD4
57023 229533.I1RS83 pp 229533.I1RW25
57024 229533.I1RS83 pp 229533.V6QWP4
57025 229533.I1RS83 pp 229533.I1RT47
57026 229533.I1RS83 pp 229533.I1S524
57027 229533.I1RS83 pp 229533.I1S9L6
57028 229533.I1RS83 pp 229533.I1RYW3
57029 229533.I1RS83 pp 229533.I1RX81
57030 229533.I1RS83 pp 229533.I1S0B6
57031 229533.I1RS83 pp 229533.I1S246
57032 229533.I1RS83 pp 229533.I1RZ11
57033 229533.I1RS83 pp 229533.Q4IF76
57034 229533.I1RS83 pp 229533.V6RIS2
57035 229533.I1RS83 pp 229533.I1S9L5
57036 229533.I1RS83 pp 229533.I1RSA8
57037 229533.I1RS83 pp 229533.I1S4M5
57038 229533.I1RS83 pp 229533.I1S7M3
57039 229533.I1RS83 pp 229533.Q4IJH1
57040 229533.I1RT47 pp 229533.I1S1X9
57041 229533.I1RT47 pp 229533.I1RU69
57042 229533.I1RT47 pp 229533.I1S0B6
57043 229533.I1RT47 pp 229533.I1RX24
57044 229533.I1RT47 pp 229533.I1RWU2
57045 229533.I1RT47 pp 229533.I1RW25
57046 229533.I1RT47 pp 229533.I1S4M5
57047 229533.I1RT47 pp 229533.I1RT91
57048 229533.I1RT47 pp 229533.I1RZG7
57049 229533.I1RT47 pp 229533.I1RX81
57050 229533.I1RT47 pp 229533.Q4HXF6
57051 229533.I1RT47 pp 229533.I1RZQ5
57052 229533.I1RT47 pp 229533.I1S524
57053 229533.I1RT47 pp 229533.I1S9L5
57054 229533.I1RT47 pp 229533.Q4IBK5
57055 229533.I1RT47 pp 229533.I1RZZ1
57056 229533.I1RT47 pp 229533.I1S2Y5
57057 229533.I1RT47 pp 229533.Q4IPB3
57058 229533.I1RT47 pp 229533.Q4I7F9
57059 229533.I1RT47 pp 229533.I1RT70
57060 229533.I1RT47 pp 229533.V6RIS2
57061 229533.I1RT47 pp 229533.I1S4Y4
57062 229533.I1RT47 pp 229533.I1RYI9
57063 229533.I1RT47 pp 229533.Q4IP34
57064 229533.I1RT47 pp 229533.I1RXF2
57065 229533.I1RT47 pp 229533.Q4IB70
57066 229533.I1RT47 pp 229533.I1S0R7
57067 229533.I1RT47 pp 229533.Q4I1Y1
57068 229533.I1RT47 pp 229533.Q4IE79
57069 229533.I1RT47 pp 229533.I1RZ11
57070 229533.I1RT47 pp 229533.V6RY33
57071 229533.I1RT47 pp 229533.I1S7M3
57072 229533.I1RT47 pp 229533.I1RYW3
57073 229533.I1RT47 pp 229533.I1S5S2
57074 229533.I1RT47 pp 229533.I1S7M2
57075 229533.I1RT47 pp 229533.I1RVT9
57076 229533.I1RT47 pp 229533.I1S0F5
57077 229533.I1RT47 pp 229533.I1S9L6
57078 229533.I1RT47 pp 229533.I1RWX8
57079 229533.I1RT47 pp 229533.Q4IPH4
57080 229533.I1RT47 pp 229533.I1S0B1
57081 229533.I1RT47 pp 229533.V6QXG1
57082 229533.I1RT47 pp 229533.I1S0Z9
57083 229533.I1RT47 pp 229533.Q4IAA0
57084 229533.I1RT47 pp 229533.I1RYB1
57085 229533.I1RT47 pp 229533.I1RX51
57086 229533.I1RT47 pp 229533.Q4IRI9

57087 229533.I1RT47 pp 229533.V6QVD5
57088 229533.I1SAN9 pp 229533.V6RRH2
57089 229533.I1SAN9 pp 229533.Q4HXF0
57090 229533.I1SAN9 pp 229533.V6RQW4
57091 229533.I1SAN9 pp 229533.V6RBN2
57092 229533.I1RFQ4 pp 229533.I1RWJ5
57093 229533.I1RFQ4 pp 229533.I1RY74
57094 229533.I1RFQ4 pp 229533.I1RK03
57095 229533.I1RZ11 pp 229533.Q4IPH4
57096 229533.I1RZ11 pp 229533.I1RZG7
57097 229533.I1RZ11 pp 229533.V6QXG1
57098 229533.I1RZ11 pp 229533.I1S524
57099 229533.I1RZ11 pp 229533.I1S9L6
57100 229533.I1RZ11 pp 229533.Q4I7F9
57101 229533.I1RZ11 pp 229533.I1RZZ1
57102 229533.I1RZ11 pp 229533.I1S7M3
57103 229533.I1RZ11 pp 229533.I1S246
57104 229533.I1RZ11 pp 229533.V6RIS2
57105 229533.I1RZ11 pp 229533.V6RY33
57106 229533.I1RZ11 pp 229533.Q4IB70
57107 229533.I1RZ11 pp 229533.Q4IJH1
57108 229533.I1RZ11 pp 229533.I1S5S2
57109 229533.I1RZ11 pp 229533.I1S2Y5
57110 229533.I1RZ11 pp 229533.I1S0R7
57111 229533.I1RZ11 pp 229533.Q4IF76
57112 229533.I1RZ11 pp 229533.Q4IBK5
57113 229533.I1RZ11 pp 229533.I1S4M5
57114 229533.I1RZ11 pp 229533.I1S9L5
57115 229533.I1RZ11 pp 229533.I1S7M2
57116 229533.I1RZ11 pp 229533.I1S0B1
57117 229533.A0A1C3YLI6 pp 229533.I1RZT1
57118 229533.A0A1C3YLI6 pp 229533.I1RX84
57119 229533.A0A1C3YLI6 pp 229533.I1S1W5
57120 229533.A0A1C3YLI6 pp 229533.I1RSD1
57121 229533.I1RQD8 pp 229533.Q4IPH4
57122 229533.I1RQD8 pp 229533.I1S0B1
57123 229533.I1RQD8 pp 229533.I1RSH9
57124 229533.I1RQD8 pp 229533.I1RSD4
57125 229533.I1RQD8 pp 229533.I1S8V0
57126 229533.I1RQD8 pp 229533.I1RZ11
57127 229533.I1RQD8 pp 229533.I1RZZ1
57128 229533.I1RQD8 pp 229533.I1RRQ4
57129 229533.I1RQD8 pp 229533.Q4I7F9
57130 229533.I1RQD8 pp 229533.I1RT47
57131 229533.I1RQD8 pp 229533.I1RS83
57132 229533.I1RQD8 pp 229533.V6RIS2
57133 229533.I1RQD8 pp 229533.I1S1X9
57134 229533.I1RQD8 pp 229533.I1RQR3
57135 229533.I1RQD8 pp 229533.I1RQZ2
57136 229533.I1RQD8 pp 229533.I1S9L6
57137 229533.I1RQD8 pp 229533.Q4IB70
57138 229533.I1RQD8 pp 229533.I1S7M3
57139 229533.I1RQD8 pp 229533.I1RS75
57140 229533.I1RQD8 pp 229533.I1RX47
57141 229533.I1RQD8 pp 229533.Q4HZ42
57142 229533.I1RQD8 pp 229533.I1RQP8
57143 229533.I1RQD8 pp 229533.I1RSA8
57144 229533.I1RQD8 pp 229533.I1RZL5
57145 229533.I1RQD8 pp 229533.I1S8U9
57146 229533.I1RQD8 pp 229533.I1S2Y5
57147 229533.I1RQD8 pp 229533.I1S4M5
57148 229533.I1RQD8 pp 229533.I1RW25
57149 229533.I1RQD8 pp 229533.I1S9L5
57150 229533.I1RQD8 pp 229533.I1RSA4
57151 229533.I1RQD8 pp 229533.I1RYW3
57152 229533.I1RQD8 pp 229533.I1S1C6
57153 229533.I1RQD8 pp 229533.I1RWX8
57154 229533.I1RQD8 pp 229533.I1S524
57155 229533.I1RQD8 pp 229533.I1S7A8
57156 229533.I1RQD8 pp 229533.I1RZG7
57157 229533.I1RQD8 pp 229533.I1RYI9
57158 229533.I1RQD8 pp 229533.V6RY33
57159 229533.I1RQD8 pp 229533.I1RTS2

57160 229533.I1RQD8 pp 229533.I1RT35
57161 229533.I1RQD8 pp 229533.I1RT17
57162 229533.I1RQD8 pp 229533.I1RX24
57163 229533.I1RQD8 pp 229533.I1RXF2
57164 229533.I1RQD8 pp 229533.I1S4Y4
57165 229533.I1RQD8 pp 229533.I1RX81
57166 229533.I1RQD8 pp 229533.I1S5S2
57167 229533.I1RQD8 pp 229533.I1S0B6
57168 229533.I1RQD8 pp 229533.I1S7M2
57169 229533.I1RQD8 pp 229533.I1S0R7
57170 229533.I1RQD8 pp 229533.I1RT04
57171 229533.I1RBX1 pp 229533.I1RP97
57172 229533.I1RBX1 pp 229533.I1RYP2
57173 229533.I1RBX1 pp 229533.I1RN63
57174 229533.I1RBX1 pp 229533.I1RQ48
57175 229533.I1RBX1 pp 229533.I1RWQ8
57176 229533.I1RX18 pp 229533.I1RZ38
57177 229533.I1RX18 pp 229533.I1RZ25
57178 229533.I1RX18 pp 229533.I1RZ56
57179 229533.I1RX18 pp 229533.I1S0A5
57180 229533.I1RX18 pp 229533.I1S6H3
57181 229533.I1RX18 pp 229533.I1S094
57182 229533.I1RX18 pp 229533.I1S8F8
57183 229533.I1RX18 pp 229533.I1S3H8
57184 229533.I1RX18 pp 229533.I1S334
57185 229533.Q4IPH4 pp 229533.V6RY33
57186 229533.Q4IPH4 pp 229533.V6QXG1
57187 229533.Q4IPH4 pp 229533.Q4IRI9
57188 229533.Q4IPH4 pp 229533.V6RIS2
57189 229533.Q4IPH4 pp 229533.V6RCZ8
57190 229533.I1RXU9 pp 229533.I1S0L4
57191 229533.I1RXU9 pp 229533.I1S2H4
57192 229533.I1RXU9 pp 229533.I1RZZ9
57193 229533.I1RXU9 pp 229533.O74638
57194 229533.I1RXU9 pp 229533.I1S5R1
57195 229533.I1RXU9 pp 229533.I1S9Y1
57196 229533.I1RXU9 pp 229533.V6R3J1
57197 229533.I1RXU9 pp 229533.I1RYX5
57198 229533.I1RXU9 pp 229533.I1RYW3
57199 229533.I1RXU9 pp 229533.I1RZ39
57200 229533.I1RXU9 pp 229533.I1S1C7
57201 229533.I1RXU9 pp 229533.I1S2B9
57202 229533.I1RXU9 pp 229533.V6R042
57203 229533.I1RXU9 pp 229533.I1S6Y8
57204 229533.I1RXU9 pp 229533.I1RXW3
57205 229533.I1RXU9 pp 229533.Q4I624
57206 229533.I1RXU9 pp 229533.Q4IJK2
57207 229533.I1RXU9 pp 229533.V6QVH8
57208 229533.I1RXU9 pp 229533.I1RXV9
57209 229533.I1RXU9 pp 229533.I1S0J1
57210 229533.I1RXU9 pp 229533.I1RZK5
57211 229533.I1RXU9 pp 229533.I1S0K2
57212 229533.I1RXU9 pp 229533.V6RTJ8
57213 229533.I1RXU9 pp 229533.V6QUE6
57214 229533.I1RXU9 pp 229533.I1S338
57215 229533.I1RXU9 pp 229533.I1S7W5
57216 229533.I1RXU9 pp 229533.I1S0T2
57217 229533.I1RXU9 pp 229533.I1S6K9
57218 229533.I1RXU9 pp 229533.V6RCZ8
57219 229533.I1RXU9 pp 229533.I1S5B0
57220 229533.I1RXU9 pp 229533.I1RZM8
57221 229533.I1RXU9 pp 229533.I1S4S7
57222 229533.I1RXU9 pp 229533.I1S419
57223 229533.A0A098D6U0 pp 229533.I1S8H5
57224 229533.A0A098D6U0 pp 229533.A0A0E0RTV6
57225 229533.A0A098D6U0 pp 229533.I1RVL7
57226 229533.A0A098D6U0 pp 229533.Q00909
57227 229533.A0A098D6U0 pp 229533.A0A098D7J4
57228 229533.A0A098D6U0 pp 229533.A0A098D8A0
57229 229533.A0A098D6U0 pp 229533.I1S1T5
57230 229533.A0A098D6U0 pp 229533.I1S2J4
57231 229533.A0A098D6U0 pp 229533.Q4HZS8
57232 229533.A0A098D6U0 pp 229533.I1RHK0

57233 229533.A0A098D6U0 pp 229533.I1RIV2
57234 229533.A0A098D6U0 pp 229533.I1S9Z4
57235 229533.A0A098D6U0 pp 229533.I1RVD9
57236 229533.A0A098D6U0 pp 229533.I1RFC3
57237 229533.A0A098D6U0 pp 229533.A0A1C3YIP3
57238 229533.A0A098D6U0 pp 229533.I1RVM4
57239 229533.A0A098D6U0 pp 229533.A0A098E2M9
57240 229533.A0A098D6U0 pp 229533.A0A098E3B9
57241 229533.A0A098D6U0 pp 229533.A0A1C3YKA2
57242 229533.A0A098D6U0 pp 229533.I1RPR1
57243 229533.A0A098D6U0 pp 229533.I1S3K7
57244 229533.A0A098D6U0 pp 229533.I1RF49
57245 229533.A0A098D6U0 pp 229533.I1RFQ4
57246 229533.A0A098D6U0 pp 229533.I1S1L0
57247 229533.A0A098D6U0 pp 229533.A0A1C3YJ35
57248 229533.A0A098D6U0 pp 229533.A0A1C3YMP1
57249 229533.A0A098D6U0 pp 229533.I1RQ48
57250 229533.A0A098D6U0 pp 229533.I1RFC6
57251 229533.A0A098D6U0 pp 229533.I1S5U5
57252 229533.A0A098D6U0 pp 229533.I1RK69
57253 229533.A0A098D6U0 pp 229533.A0A098E0Z2
57254 229533.I1RMS4 pp 229533.I1RRS7
57255 229533.I1RMS4 pp 229533.I1RMX3
57256 229533.I1RMS4 pp 229533.I1RSC7
57257 229533.I1RMS4 pp 229533.I1RQJ4
57258 229533.I1RMS4 pp 229533.V6R382
57259 229533.I1RMS4 pp 229533.I1RN57
57260 229533.I1RMS4 pp 229533.I1RSR9
57261 229533.I1RMS4 pp 229533.I1RSP7
57262 229533.I1RMS4 pp 229533.I1RX64
57263 229533.I1RMS4 pp 229533.I1RN55
57264 229533.I1RMS4 pp 229533.I1RZP8
57265 229533.I1RMS4 pp 229533.I1S283
57266 229533.I1RMS4 pp 229533.Q4IBX0
57267 229533.I1RMS4 pp 229533.I1RXB8
57268 229533.I1RMS4 pp 229533.I1S461
57269 229533.I1RMS4 pp 229533.I1RZ94
57270 229533.I1RMS4 pp 229533.I1RSA7
57271 229533.I1RMS4 pp 229533.I1RQ13
57272 229533.I1RMS4 pp 229533.I1RUG4
57273 229533.I1RMS4 pp 229533.Q4IQM6
57274 229533.I1RMS4 pp 229533.V6RLW4
57275 229533.I1RMS4 pp 229533.I1RPQ1
57276 229533.I1RMS4 pp 229533.Q4IP51
57277 229533.I1RMS4 pp 229533.Q4IC11
57278 229533.I1RMS4 pp 229533.I1RYR2
57279 229533.I1RMS4 pp 229533.I1RSN7
57280 229533.I1RMS4 pp 229533.I1RYX9
57281 229533.A0A0E0SGX2 pp 229533.I1RGW8
57282 229533.A0A0E0SGX2 pp 229533.I1RWT6
57283 229533.A0A0E0SGX2 pp 229533.I1RQA3
57284 229533.A0A0E0SGX2 pp 229533.I1REL7
57285 229533.A0A0E0SGX2 pp 229533.I1SAF3
57286 229533.A0A098D4K1 pp 229533.I1S3G4
57287 229533.A0A098D4K1 pp 229533.I1S1W8
57288 229533.A0A098D4K1 pp 229533.I1RFQ4
57289 229533.A0A098D4K1 pp 229533.I1RNG4
57290 229533.A0A098D4K1 pp 229533.I1RY87
57291 229533.A0A098D4K1 pp 229533.I1RBE7
57292 229533.A0A098D4K1 pp 229533.A0A1C3YLB6
57293 229533.A0A098D4K1 pp 229533.A0A098D6U0
57294 229533.A0A098D4K1 pp 229533.A0A1C3YMF1
57295 229533.I1RMW7 pp 229533.I1RW25
57296 229533.I1RMW7 pp 229533.I1RN48
57297 229533.I1RMW7 pp 229533.I1RX81
57298 229533.I1RMW7 pp 229533.I1RNE7
57299 229533.I1RMW7 pp 229533.I1S9F1
57300 229533.I1RMW7 pp 229533.I1S0B6
57301 229533.I1RMW7 pp 229533.I1RZG7
57302 229533.I1RMW7 pp 229533.I1RPY4
57303 229533.I1RMW7 pp 229533.I1S9L6
57304 229533.I1RMW7 pp 229533.I1S4M5
57305 229533.I1RMW7 pp 229533.Q4I7F9

57306 229533.I1RMW7 pp 229533.I1S524
57307 229533.I1RMW7 pp 229533.I1S2Y5
57308 229533.I1RMW7 pp 229533.I1RW71
57309 229533.I1RMW7 pp 229533.Q4HYR2
57310 229533.I1RMW7 pp 229533.I1S9L5
57311 229533.I1RMW7 pp 229533.I1RZZ1
57312 229533.I1RMW7 pp 229533.I1RS75
57313 229533.I1RMW7 pp 229533.I1RSP7
57314 229533.I1RMW7 pp 229533.I1RPA0
57315 229533.I1RMW7 pp 229533.I1RYI9
57316 229533.I1RMW7 pp 229533.Q4HW67
57317 229533.I1RMW7 pp 229533.I1S4Y4
57318 229533.I1RMW7 pp 229533.I1RYI6
57319 229533.I1RMW7 pp 229533.I1RYW3
57320 229533.I1RMW7 pp 229533.I1RSE2
57321 229533.I1RMW7 pp 229533.I1S0R7
57322 229533.I1RMW7 pp 229533.I1RQP8
57323 229533.I1RMW7 pp 229533.I1RT04
57324 229533.I1RMW7 pp 229533.V6RY33
57325 229533.I1RMW7 pp 229533.I1RPY5
57326 229533.I1RMW7 pp 229533.I1S5S2
57327 229533.I1RMW7 pp 229533.I1RS28
57328 229533.I1RMW7 pp 229533.I1S7M2
57329 229533.I1RMW7 pp 229533.V6QXG1
57330 229533.I1RMW7 pp 229533.I1S3V2
57331 229533.I1RMW7 pp 229533.I1RT35
57332 229533.I1RMW7 pp 229533.I1RXF2
57333 229533.I1RMW7 pp 229533.I1S290
57334 229533.I1RMW7 pp 229533.I1RWX8
57335 229533.I1RMW7 pp 229533.I1RS83
57336 229533.I1RMW7 pp 229533.V6RIS2
57337 229533.I1RMW7 pp 229533.I1RZ40
57338 229533.I1RMW7 pp 229533.I1RND5
57339 229533.I1RMW7 pp 229533.I1RN09
57340 229533.I1RMW7 pp 229533.I1RSA4
57341 229533.I1RMW7 pp 229533.I1RRQ4
57342 229533.I1RMW7 pp 229533.Q4IPH4
57343 229533.I1RMW7 pp 229533.I1RNB8
57344 229533.I1RMW7 pp 229533.Q4I7K4
57345 229533.I1RMW7 pp 229533.I1RYJ9
57346 229533.I1RMW7 pp 229533.I1RT47
57347 229533.I1RMW7 pp 229533.I1S0B1
57348 229533.I1RMW7 pp 229533.I1RWD3
57349 229533.I1RMW7 pp 229533.I1RQD8
57350 229533.I1RMW7 pp 229533.Q4HWE2
57351 229533.I1RMW7 pp 229533.I1RZ11
57352 229533.I1RMW7 pp 229533.I1S289
57353 229533.I1RMW7 pp 229533.Q4I6A4
57354 229533.I1RMW7 pp 229533.Q4IAA0
57355 229533.I1RMW7 pp 229533.I1RSD4
57356 229533.I1RMW7 pp 229533.I1S0Q7
57357 229533.I1RMW7 pp 229533.I1RQR3
57358 229533.I1RMW7 pp 229533.I1S7M3
57359 229533.I1RMW7 pp 229533.I1RSA8
57360 229533.I1S1D7 pp 229533.I1S8R4
57361 229533.I1S1D7 pp 229533.I1S267
57362 229533.I1S1D7 pp 229533.I1S3L2
57363 229533.I1RSE6 pp 229533.I1RTE6
57364 229533.I1RSE6 pp 229533.I1RYD8
57365 229533.I1RSA8 pp 229533.Q4IPB3
57366 229533.I1RSA8 pp 229533.I1S524
57367 229533.I1RSA8 pp 229533.I1RZG7
57368 229533.I1RSA8 pp 229533.I1RSW2
57369 229533.I1RSA8 pp 229533.I1RX81
57370 229533.I1RSA8 pp 229533.Q4IJH1
57371 229533.I1RSA8 pp 229533.I1RWW5
57372 229533.I1RSA8 pp 229533.Q4IF76
57373 229533.I1RSA8 pp 229533.I1RT91
57374 229533.I1RSA8 pp 229533.Q4IBK5
57375 229533.I1RSA8 pp 229533.I1RST1
57376 229533.I1RSA8 pp 229533.I1RYJ9
57377 229533.I1RSA8 pp 229533.I1S246
57378 229533.I1RSA8 pp 229533.Q4IPB8

57379 229533.I1RSA8 pp 229533.I1S0Z9
57380 229533.I1RSA8 pp 229533.I1S2Y5
57381 229533.I1RSA8 pp 229533.I1RZD0
57382 229533.I1RSA8 pp 229533.I1S0B1
57383 229533.I1RSA8 pp 229533.I1S9L5
57384 229533.I1RSA8 pp 229533.I1RYB1
57385 229533.I1RSA8 pp 229533.V6RY33
57386 229533.I1RSA8 pp 229533.Q4IPH4
57387 229533.I1RSA8 pp 229533.I1S9L6
57388 229533.I1RSA8 pp 229533.Q4IE79
57389 229533.I1RSA8 pp 229533.I1RYW3
57390 229533.I1RSA8 pp 229533.I1RX47
57391 229533.I1RSA8 pp 229533.I1S5S2
57392 229533.I1RSA8 pp 229533.I1RXF2
57393 229533.I1RSA8 pp 229533.I1S7M3
57394 229533.I1RSA8 pp 229533.I1RT47
57395 229533.I1RSA8 pp 229533.Q4IJ11
57396 229533.I1RSA8 pp 229533.Q4I1Y1
57397 229533.I1RSA8 pp 229533.V6RIS2
57398 229533.I1RSA8 pp 229533.Q4IB70
57399 229533.I1RSA8 pp 229533.I1RZ11
57400 229533.I1RSA8 pp 229533.I1RYI9
57401 229533.I1RSA8 pp 229533.I1S7M2
57402 229533.I1RSA8 pp 229533.Q4IP34
57403 229533.I1RSA8 pp 229533.I1RZZ1
57404 229533.I1RSA8 pp 229533.I1S9L0
57405 229533.I1RSA8 pp 229533.I1RT35
57406 229533.I1RSA8 pp 229533.Q4IAA0
57407 229533.I1RSA8 pp 229533.I1RSD4
57408 229533.I1RSA8 pp 229533.V6QXG1
57409 229533.I1RSA8 pp 229533.I1RWX8
57410 229533.I1RSA8 pp 229533.I1S1W3
57411 229533.A0A098D458 pp 229533.I1RSE6
57412 229533.I1S0B1 pp 229533.Q4IPB3
57413 229533.I1S0B1 pp 229533.I1S9L6
57414 229533.I1S0B1 pp 229533.I1S524
57415 229533.I1S0B1 pp 229533.I1S5S2
57416 229533.I1S0B1 pp 229533.I1S7M2
57417 229533.I1S0B1 pp 229533.I1S2Y5
57418 229533.I1S0B1 pp 229533.I1S1C6
57419 229533.I1S0B1 pp 229533.I1S4Y4
57420 229533.I1S0B1 pp 229533.I1S0B6
57421 229533.I1S0B1 pp 229533.I1S9L5
57422 229533.I1S0B1 pp 229533.I1S0R7
57423 229533.I1S0B1 pp 229533.V6QVD5
57424 229533.I1S0B1 pp 229533.I1S246
57425 229533.I1S0B1 pp 229533.Q4I1Y1
57426 229533.I1S0B1 pp 229533.Q4IB70
57427 229533.I1S0B1 pp 229533.Q4IP34
57428 229533.I1S0B1 pp 229533.V6RIS2
57429 229533.I1S0B1 pp 229533.I1S4M5
57430 229533.I1S0B1 pp 229533.I1S0Z9
57431 229533.I1S0B1 pp 229533.I1S7M3
57432 229533.I1S0B1 pp 229533.V6RY33
57433 229533.I1S0B1 pp 229533.Q4I7F9
57434 229533.I1S0B1 pp 229533.Q4IPH4
57435 229533.I1S0B1 pp 229533.I1S471
57436 229533.I1S0B1 pp 229533.I1S8V0
57437 229533.I1S0B1 pp 229533.I1S1X9
57438 229533.I1S0B1 pp 229533.V6QXG1
57439 229533.I1S0B1 pp 229533.Q4IAA0
57440 229533.I1S0B1 pp 229533.Q4IRI9
57441 229533.I1S0B1 pp 229533.I1S8U9
57442 229533.V6R382 pp 229533.V6RLM7
57443 229533.V6R382 pp 229533.V6RM02
57444 229533.V6R382 pp 229533.V6RLW4
57445 229533.V6R382 pp 229533.V6R3J1
57446 229533.V6R382 pp 229533.V6RQW4
57447 229533.V6R382 pp 229533.V6RK96
57448 229533.I1RPW0 pp 229533.I1S330
57449 229533.I1RPW0 pp 229533.I1RUQ5
57450 229533.I1RPW0 pp 229533.I1RRW0
57451 229533.I1RPW0 pp 229533.I1RQ80

57452	229533.I1RPW0	pp	229533.I1RZ12
57453	229533.I1RPW0	pp	229533.I1RTG0
57454	229533.I1RPW0	pp	229533.I1RQ26
57455	229533.I1RPW0	pp	229533.I1RTN1
57456	229533.I1RPW0	pp	229533.I1S439
57457	229533.I1RPW0	pp	229533.I1S0M2
57458	229533.I1RPW0	pp	229533.I1S2R1
57459	229533.I1RPW0	pp	229533.I1RRN5
57460	229533.I1RPW0	pp	229533.I1RQS4
57461	229533.I1RPW0	pp	229533.I1S1W4
57462	229533.I1RPW0	pp	229533.I1S9H9
57463	229533.I1RPW0	pp	229533.I1RWT6
57464	229533.I1RPW0	pp	229533.I1RQA3
57465	229533.I1RPW0	pp	229533.I1RXA1
57466	229533.I1RPW0	pp	229533.I1RZT1
57467	229533.I1RPW0	pp	229533.I1RRP7
57468	229533.I1RPW0	pp	229533.V6RVG3
57469	229533.I1RPW0	pp	229533.I1RQW2
57470	229533.I1RPW0	pp	229533.I1RY44
57471	229533.I1RPW0	pp	229533.I1S2E9
57472	229533.I1RPW0	pp	229533.I1RW16
57473	229533.I1RPW0	pp	229533.I1RRY3
57474	229533.I1RPW0	pp	229533.I1S1D3
57475	229533.I1RPW0	pp	229533.I1RWT1
57476	229533.I1RPW0	pp	229533.I1RS41
57477	229533.I1RPW0	pp	229533.V6R2L8
57478	229533.I1RPW0	pp	229533.I1RZG3
57479	229533.I1RPW0	pp	229533.I1RV91
57480	229533.I1RPW0	pp	229533.I1RVA5
57481	229533.I1RPW0	pp	229533.I1RZH4
57482	229533.I1RPW0	pp	229533.Q4I7X1
57483	229533.I1RPW0	pp	229533.I1RQM0
57484	229533.I1RPW0	pp	229533.I1S339
57485	229533.I1RPW0	pp	229533.I1S382
57486	229533.I1RPW0	pp	229533.I1S0J7
57487	229533.I1RPW0	pp	229533.I1SA96
57488	229533.I1RPW0	pp	229533.I1RRX6
57489	229533.I1RPW0	pp	229533.I1RT08
57490	229533.I1RPW0	pp	229533.I1RYH9
57491	229533.I1RPW0	pp	229533.I1S951
57492	229533.I1RPW0	pp	229533.I1S224
57493	229533.I1RPW0	pp	229533.I1S1V5
57494	229533.I1RPW0	pp	229533.Q4I8S2
57495	229533.I1RPW0	pp	229533.I1RX38
57496	229533.I1RPW0	pp	229533.I1RZ63
57497	229533.I1RDH6	pp	229533.I1RFJ1
57498	229533.I1RDH6	pp	229533.I1RMV8
57499	229533.I1RDH6	pp	229533.I1RM55
57500	229533.I1RDH6	pp	229533.I1RK03
57501	229533.I1RDH6	pp	229533.I1RSX8
57502	229533.I1RDH6	pp	229533.I1RM54
57503	229533.I1RDH6	pp	229533.I1RZ21
57504	229533.I1RDH6	pp	229533.I1RSX7
57505	229533.I1RDH6	pp	229533.I1RJU6
57506	229533.I1S7M3	pp	229533.Q4IAA0
57507	229533.I1S7M3	pp	229533.V6RIS2
57508	229533.I1S7M3	pp	229533.Q4ICA8
57509	229533.I1S7M3	pp	229533.Q4IP34
57510	229533.I1S7M3	pp	229533.Q4IRI9
57511	229533.I1S7M3	pp	229533.V6QXG1
57512	229533.I1S7M3	pp	229533.V6QX58
57513	229533.I1S7M3	pp	229533.Q4I7F9
57514	229533.I1S7M3	pp	229533.Q4IB70
57515	229533.I1S7M3	pp	229533.I1S8U9
57516	229533.I1S7M3	pp	229533.V6RY33
57517	229533.I1S7M3	pp	229533.Q4I1Y1
57518	229533.I1S7M3	pp	229533.I1S9L5
57519	229533.I1S7M3	pp	229533.I1S8V0
57520	229533.I1S7M3	pp	229533.Q4IPH4
57521	229533.I1S7M3	pp	229533.Q4I7X1
57522	229533.I1S7M3	pp	229533.I1S9L6
57523	229533.I1S7M3	pp	229533.Q4IPB3
57524	229533.A0A1C3YMK0	pp	229533.I1S1W8

57525 229533.A0A1C3YMK0 pp 229533.I1RA35
57526 229533.A0A1C3YMK0 pp 229533.A0A1C3YMK9
57527 229533.A0A1C3YMK0 pp 229533.I1RZ92
57528 229533.A0A1C3YMK0 pp 229533.I1RY49
57529 229533.A0A1C3YMK0 pp 229533.I1S8C1
57530 229533.A0A1C3YMK0 pp 229533.I1RQV7
57531 229533.A0A1C3YMK0 pp 229533.I1RQV8
57532 229533.A0A1C3YMK0 pp 229533.I1RPT0
57533 229533.A0A1C3YMK0 pp 229533.I1RJY7
57534 229533.A0A1C3YMK0 pp 229533.V6RQW4
57535 229533.A0A1C3YMK0 pp 229533.I1RQE0
57536 229533.A0A1C3YMK0 pp 229533.I1S130
57537 229533.A0A1C3YMK0 pp 229533.I1RL16
57538 229533.A0A1C3YMK0 pp 229533.I1RH32
57539 229533.A0A1C3YMK0 pp 229533.V6RHX2
57540 229533.A0A1C3YMK0 pp 229533.I1RUR8
57541 229533.A0A1C3YMK0 pp 229533.V6RG22
57542 229533.A0A1C3YMK0 pp 229533.V6RWB3
57543 229533.A0A1C3YMK0 pp 229533.I1RY12
57544 229533.A0A1C3YMK0 pp 229533.I1RXX1
57545 229533.A0A1C3YMK0 pp 229533.I1R9Y8
57546 229533.A0A1C3YMK0 pp 229533.I1RGC3
57547 229533.A0A1C3YMK0 pp 229533.I1RY35
57548 229533.A0A1C3YMK0 pp 229533.V6RPN0
57549 229533.A0A1C3YMK0 pp 229533.I1RUB1
57550 229533.A0A1C3YMK0 pp 229533.I1RDL0
57551 229533.A0A1C3YMK0 pp 229533.I1RLZ4
57552 229533.A0A1C3YMK0 pp 229533.I1S1K6
57553 229533.A0A1C3YMK0 pp 229533.I1S8P5
57554 229533.A0A1C3YMK0 pp 229533.I1S3G4
57555 229533.A0A1C3YMK0 pp 229533.I1S8D3
57556 229533.A0A1C3YMK0 pp 229533.I1RH30
57557 229533.A0A1C3YMK0 pp 229533.I1RWF5
57558 229533.A0A1C3YMK0 pp 229533.I1RBA6
57559 229533.A0A1C3YMK0 pp 229533.I1RZV5
57560 229533.A0A1C3YMK0 pp 229533.I1RWI5
57561 229533.A0A1C3YMK0 pp 229533.I1RZJ8
57562 229533.A0A1C3YMK0 pp 229533.I1S5R5
57563 229533.A0A1C3YMK0 pp 229533.I1RNU7
57564 229533.A0A1C3YMK0 pp 229533.I1RP97
57565 229533.A0A1C3YMK0 pp 229533.I1RUB5
57566 229533.A0A1C3YMK0 pp 229533.A0A1C3YMP8
57567 229533.A0A1C3YMK0 pp 229533.I1S6P7
57568 229533.A0A1C3YMK0 pp 229533.I1RKB6
57569 229533.A0A1C3YMK0 pp 229533.V6R729
57570 229533.A0A1C3YMK0 pp 229533.I1RBU1
57571 229533.A0A1C3YMK0 pp 229533.I1S064
57572 229533.A0A1C3YMK0 pp 229533.I1RHJ2
57573 229533.A0A1C3YMK0 pp 229533.A0A1C3YMS7
57574 229533.I1RY58 pp 229533.I1S9J6
57575 229533.I1RY58 pp 229533.I1RZK9
57576 229533.I1RY58 pp 229533.I1S004
57577 229533.I1RY58 pp 229533.I1S549
57578 229533.I1RY58 pp 229533.I1RZT1
57579 229533.I1RY58 pp 229533.I1S788
57580 229533.I1RY58 pp 229533.Q4IJ84
57581 229533.I1RY58 pp 229533.I1S0D5
57582 229533.I1RY58 pp 229533.I1RZ94
57583 229533.I1RY58 pp 229533.I1S0A6
57584 229533.I1RY58 pp 229533.I1RZ86
57585 229533.I1RY58 pp 229533.I1S0N5
57586 229533.I1RY58 pp 229533.Q4IB89
57587 229533.I1RY58 pp 229533.I1RZ63
57588 229533.I1RY58 pp 229533.I1S224
57589 229533.I1RY58 pp 229533.I1RYU3
57590 229533.I1RY58 pp 229533.I1S4P6
57591 229533.I1RY58 pp 229533.Q4HTT2
57592 229533.I1RY58 pp 229533.I1S098
57593 229533.I1RY58 pp 229533.I1RZI4
57594 229533.I1RY58 pp 229533.I1S2E9
57595 229533.I1RY58 pp 229533.I1RZ12
57596 229533.I1RY58 pp 229533.I1S0J7
57597 229533.I1RY58 pp 229533.Q4IB96

57598 229533.I1RY58 pp 229533.I1S7X9
57599 229533.A0A0E0SGV2 pp 229533.I1S182
57600 229533.A0A0E0SGV2 pp 229533.I1RCG6
57601 229533.A0A0E0SGV2 pp 229533.I1RPW8
57602 229533.A0A0E0SGV2 pp 229533.I1RQE0
57603 229533.A0A0E0SGV2 pp 229533.I1RQ42
57604 229533.A0A0E0SGV2 pp 229533.I1RZ42
57605 229533.A0A0E0SGV2 pp 229533.I1RW91
57606 229533.A0A0E0SGV2 pp 229533.I1RW80
57607 229533.A0A0E0SGV2 pp 229533.I1RN04
57608 229533.I1RVJ8 pp 229533.Q4I073
57609 229533.I1RVJ8 pp 229533.Q4I0K3
57610 229533.I1RVJ8 pp 229533.Q4IB63
57611 229533.I1RVJ8 pp 229533.V6RPQ8
57612 229533.I1RVJ8 pp 229533.I1RZ13
57613 229533.I1RVJ8 pp 229533.Q4I1Y5
57614 229533.I1RVJ8 pp 229533.I1RZ34
57615 229533.A0A1C3YNC3 pp 229533.I1RQG4
57616 229533.A0A1C3YNC3 pp 229533.I1RCA7
57617 229533.A0A1C3YNC3 pp 229533.I1S9L5
57618 229533.A0A1C3YNC3 pp 229533.I1RWU2
57619 229533.A0A1C3YNC3 pp 229533.Q4I830
57620 229533.A0A1C3YNC3 pp 229533.I1RFT1
57621 229533.A0A1C3YNC3 pp 229533.I1RKK4
57622 229533.A0A1C3YNC3 pp 229533.I1RDD3
57623 229533.A0A1C3YNC3 pp 229533.I1S4M5
57624 229533.A0A1C3YNC3 pp 229533.I1RS75
57625 229533.A0A1C3YNC3 pp 229533.I1RA46
57626 229533.A0A1C3YNC3 pp 229533.I1S2Y5
57627 229533.A0A1C3YNC3 pp 229533.I1RAU1
57628 229533.A0A1C3YNC3 pp 229533.I1RCS2
57629 229533.A0A1C3YNC3 pp 229533.Q4IAA0
57630 229533.A0A1C3YNC3 pp 229533.Q4IBK5
57631 229533.A0A1C3YNC3 pp 229533.I1RMM5
57632 229533.A0A1C3YNC3 pp 229533.I1S9L6
57633 229533.A0A1C3YNC3 pp 229533.Q4IRI9
57634 229533.A0A1C3YNC3 pp 229533.I1RD56
57635 229533.A0A1C3YNC3 pp 229533.I1RRW3
57636 229533.A0A1C3YNC3 pp 229533.I1RSH9
57637 229533.A0A1C3YNC3 pp 229533.I1RLU5
57638 229533.A0A1C3YNC3 pp 229533.I1RM25
57639 229533.A0A1C3YNC3 pp 229533.I1RNB8
57640 229533.A0A1C3YNC3 pp 229533.I1RME9
57641 229533.A0A1C3YNC3 pp 229533.I1RFQ1
57642 229533.A0A1C3YNC3 pp 229533.I1RG13
57643 229533.A0A1C3YNC3 pp 229533.I1RND9
57644 229533.A0A1C3YNC3 pp 229533.I1RS28
57645 229533.A0A1C3YNC3 pp 229533.I1RPY5
57646 229533.A0A1C3YNC3 pp 229533.I1RSD4
57647 229533.A0A1C3YNC3 pp 229533.I1RQC5
57648 229533.A0A1C3YNC3 pp 229533.I1RRL0
57649 229533.A0A1C3YNC3 pp 229533.I1RXF2
57650 229533.A0A1C3YNC3 pp 229533.I1S7M2
57651 229533.A0A1C3YNC3 pp 229533.I1RA43
57652 229533.A0A1C3YNC3 pp 229533.I1S5S2
57653 229533.A0A1C3YNC3 pp 229533.I1RK72
57654 229533.A0A1C3YNC3 pp 229533.Q4HYB8
57655 229533.A0A1C3YNC3 pp 229533.V6RY33
57656 229533.A0A1C3YNC3 pp 229533.I1R9Z0
57657 229533.A0A1C3YNC3 pp 229533.I1S0R7
57658 229533.A0A1C3YNC3 pp 229533.I1RBV3
57659 229533.A0A1C3YNC3 pp 229533.I1RSL9
57660 229533.A0A1C3YNC3 pp 229533.I1RE24
57661 229533.A0A1C3YNC3 pp 229533.I1S4Y4
57662 229533.A0A1C3YNC3 pp 229533.I1RBH8
57663 229533.A0A1C3YNC3 pp 229533.I1RQQ7
57664 229533.A0A1C3YNC3 pp 229533.I1RAD1
57665 229533.A0A1C3YNC3 pp 229533.Q4IEK8
57666 229533.A0A1C3YNC3 pp 229533.V6QX58
57667 229533.A0A1C3YNC3 pp 229533.I1RQR3
57668 229533.A0A1C3YNC3 pp 229533.I1S854
57669 229533.A0A1C3YNC3 pp 229533.I1RQZ2
57670 229533.A0A1C3YNC3 pp 229533.I1RT91

57671	229533.A0A1C3YNC3	pp	229533.I1RYI9
57672	229533.A0A1C3YNC3	pp	229533.I1RCG6
57673	229533.A0A1C3YNC3	pp	229533.I1RZZ1
57674	229533.A0A1C3YNC3	pp	229533.I1RND5
57675	229533.A0A1C3YNC3	pp	229533.I1RX24
57676	229533.A0A1C3YNC3	pp	229533.I1RDC1
57677	229533.A0A1C3YNC3	pp	229533.I1RSA4
57678	229533.A0A1C3YNC3	pp	229533.Q4I7F9
57679	229533.A0A1C3YNC3	pp	229533.I1RVT9
57680	229533.A0A1C3YNC3	pp	229533.I1RZQ5
57681	229533.A0A1C3YNC3	pp	229533.I1RX81
57682	229533.A0A1C3YNC3	pp	229533.I1RT35
57683	229533.A0A1C3YNC3	pp	229533.I1RK96
57684	229533.A0A1C3YNC3	pp	229533.Q4IPH4
57685	229533.A0A1C3YNC3	pp	229533.I1RWX8
57686	229533.A0A1C3YNC3	pp	229533.I1RPY4
57687	229533.A0A1C3YNC3	pp	229533.I1S8V0
57688	229533.A0A1C3YNC3	pp	229533.I1S524
57689	229533.A0A1C3YNC3	pp	229533.I1RWF3
57690	229533.A0A1C3YNC3	pp	229533.V6QXG1
57691	229533.A0A1C3YNC3	pp	229533.I1RF08
57692	229533.A0A1C3YNC3	pp	229533.Q4HZ68
57693	229533.A0A1C3YNC3	pp	229533.Q4IPB3
57694	229533.A0A1C3YNC3	pp	229533.I1S8F8
57695	229533.A0A1C3YNC3	pp	229533.I1R9N6
57696	229533.A0A1C3YNC3	pp	229533.I1RX51
57697	229533.A0A1C3YNC3	pp	229533.I1RPM4
57698	229533.A0A1C3YNC3	pp	229533.I1RRQ4
57699	229533.A0A1C3YNC3	pp	229533.I1RCQ6
57700	229533.A0A1C3YNC3	pp	229533.I1S0B1
57701	229533.A0A1C3YNC3	pp	229533.I1RCR5
57702	229533.A0A1C3YNC3	pp	229533.I1S8U9
57703	229533.A0A1C3YNC3	pp	229533.I1RZG7
57704	229533.A0A1C3YNC3	pp	229533.I1S7M3
57705	229533.A0A1C3YNC3	pp	229533.I1RCD6
57706	229533.A0A1C3YNC3	pp	229533.I1RYB1
57707	229533.A0A1C3YNC3	pp	229533.I1RCI3
57708	229533.A0A1C3YNC3	pp	229533.V6QVD5
57709	229533.A0A1C3YNC3	pp	229533.I1RCI4
57710	229533.A0A1C3YNC3	pp	229533.I1REX2
57711	229533.A0A1C3YNC3	pp	229533.I1RX47
57712	229533.A0A1C3YNC3	pp	229533.Q4IE79
57713	229533.A0A1C3YNC3	pp	229533.I1RW25
57714	229533.A0A1C3YNC3	pp	229533.I1RPY7
57715	229533.A0A1C3YNC3	pp	229533.I1RMV5
57716	229533.A0A1C3YNC3	pp	229533.I1RYJ9
57717	229533.A0A1C3YNC3	pp	229533.I1RG90
57718	229533.A0A1C3YNC3	pp	229533.I1RSA8
57719	229533.A0A1C3YNC3	pp	229533.Q4IFI0
57720	229533.A0A1C3YNC3	pp	229533.I1S0Z9
57721	229533.A0A1C3YNC3	pp	229533.V6RIS2
57722	229533.A0A1C3YNC3	pp	229533.I1RDE9
57723	229533.A0A1C3YNC3	pp	229533.I1RAC1
57724	229533.A0A1C3YNC3	pp	229533.Q4IP34
57725	229533.A0A1C3YNC3	pp	229533.Q4I1Y1
57726	229533.A0A1C3YNC3	pp	229533.I1RB50
57727	229533.A0A1C3YNC3	pp	229533.I1RS83
57728	229533.A0A1C3YNC3	pp	229533.I1RN59
57729	229533.A0A1C3YNC3	pp	229533.I1RG57
57730	229533.A0A1C3YNC3	pp	229533.I1RQP8
57731	229533.A0A1C3YNC3	pp	229533.I1RQD8
57732	229533.A0A1C3YNC3	pp	229533.I1RZ11
57733	229533.A0A1C3YNC3	pp	229533.I1RN03
57734	229533.A0A1C3YNC3	pp	229533.I1RU69
57735	229533.A0A1C3YNC3	pp	229533.I1RRU9
57736	229533.A0A1C3YNC3	pp	229533.I1RZ15
57737	229533.A0A1C3YNC3	pp	229533.I1RVK5
57738	229533.A0A1C3YNC3	pp	229533.I1RK90
57739	229533.A0A1C3YNC3	pp	229533.I1RMW7
57740	229533.A0A1C3YNC3	pp	229533.Q4IBS2
57741	229533.A0A1C3YNC3	pp	229533.I1RG17
57742	229533.A0A1C3YNC3	pp	229533.I1RYW3
57743	229533.A0A1C3YNC3	pp	229533.I1RBZ8

57744 229533.A0A1C3YNC3 pp 229533.I1RPW9
57745 229533.A0A1C3YNC3 pp 229533.I1RT47
57746 229533.A0A1C3YNC3 pp 229533.I1RST1
57747 229533.A0A1C3YNC3 pp 229533.I1RA36
57748 229533.A0A1C3YNC3 pp 229533.I1RN09
57749 229533.A0A1C3YNC3 pp 229533.I1RZL5
57750 229533.A0A1C3YNC3 pp 229533.I1RFQ7
57751 229533.A0A1C3YNC3 pp 229533.Q4HXF6
57752 229533.A0A1C3YNC3 pp 229533.I1RPA0
57753 229533.A0A1C3YNC3 pp 229533.I1S0B6
57754 229533.A0A1C3YNC3 pp 229533.I1S1X9
57755 229533.A0A1C3YNC3 pp 229533.I1RAE1
57756 229533.A0A1C3YNC3 pp 229533.I1RK92
57757 229533.A0A1C3YNC3 pp 229533.Q4IB70
57758 229533.A0A1C3YNC3 pp 229533.I1S855
57759 229533.A0A1C3YNC3 pp 229533.I1RC84
57760 229533.A0A1C3YNC3 pp 229533.I1RCN5
57761 229533.I1RAG6 pp 229533.I1RMN3
57762 229533.I1RGD8 pp 229533.I1S279
57763 229533.I1RCW1 pp 229533.I1RPJ3
57764 229533.I1RCW1 pp 229533.I1RTD7
57765 229533.I1RCW1 pp 229533.I1S558
57766 229533.I1RCW1 pp 229533.I1S0T4
57767 229533.I1RCW1 pp 229533.Q4IMJ1
57768 229533.I1RCW1 pp 229533.I1RW26
57769 229533.I1RCW1 pp 229533.I1RWJ4
57770 229533.I1RCW1 pp 229533.I1RZI8
57771 229533.I1RCW1 pp 229533.I1S2P8
57772 229533.I1RCW1 pp 229533.I1RWS4
57773 229533.I1RCW1 pp 229533.I1RNY2
57774 229533.I1RCW1 pp 229533.I1RW06
57775 229533.I1RCW1 pp 229533.I1S250
57776 229533.I1RCW1 pp 229533.I1RYM5
57777 229533.I1RCW1 pp 229533.V6R382
57778 229533.I1RCW1 pp 229533.I1RS19
57779 229533.I1RCW1 pp 229533.I1RY63
57780 229533.I1RCW1 pp 229533.I1RYJ6
57781 229533.I1RCW1 pp 229533.I1RI91
57782 229533.I1RCW1 pp 229533.I1RYX7
57783 229533.I1RRW0 pp 229533.I1RS35
57784 229533.I1RRW0 pp 229533.I1RRX6
57785 229533.I1RRW0 pp 229533.I1S334
57786 229533.I1RRW0 pp 229533.V6QV09
57787 229533.I1RRW0 pp 229533.I1RTG0
57788 229533.I1RRW0 pp 229533.I1RYH9
57789 229533.I1RRW0 pp 229533.I1RUQ5
57790 229533.I1RRW0 pp 229533.I1RZ12
57791 229533.I1RRW0 pp 229533.I1S004
57792 229533.I1RRW0 pp 229533.I1RSG0
57793 229533.I1RRW0 pp 229533.I1RYZ9
57794 229533.I1RRW0 pp 229533.I1RXZ4
57795 229533.I1RRW0 pp 229533.I1S549
57796 229533.I1RRW0 pp 229533.I1S439
57797 229533.I1RRW0 pp 229533.I1RWT6
57798 229533.I1RRW0 pp 229533.Q4I7N9
57799 229533.I1RRW0 pp 229533.I1S0N8
57800 229533.I1RRW0 pp 229533.I1S1V5
57801 229533.I1RRW0 pp 229533.I1S267
57802 229533.I1RRW0 pp 229533.I1RTM8
57803 229533.I1RRW0 pp 229533.I1RST9
57804 229533.I1RRW0 pp 229533.I1RX38
57805 229533.I1RRW0 pp 229533.I1RZ63
57806 229533.I1RRW0 pp 229533.I1S7X9
57807 229533.I1RRW0 pp 229533.I1RRY3
57808 229533.I1RRW0 pp 229533.I1S0J7
57809 229533.I1RRW0 pp 229533.I1S3H8
57810 229533.I1RRW0 pp 229533.I1RY32
57811 229533.I1RRW0 pp 229533.I1RW24
57812 229533.I1RRW0 pp 229533.I1RTB2
57813 229533.I1RRW0 pp 229533.I1RV91
57814 229533.I1RRW0 pp 229533.I1RZS4
57815 229533.I1RRW0 pp 229533.V6RKY7
57816 229533.I1RRW0 pp 229533.I1S2E9

229533.I1RRW0	pp	229533.I1S098
229533.I1RRW0	pp	229533.I1RW16
229533.I1RRW0	pp	229533.I1RZX3
229533.I1RRW0	pp	229533.I1RRW1
229533.I1RRW0	pp	229533.V6RVG3
229533.I1RRW0	pp	229533.V6RGI1
229533.I1RRW0	pp	229533.I1RY44
229533.I1RRW0	pp	229533.I1RY58
229533.I1RRW0	pp	229533.I1RVN2
229533.I1RRW0	pp	229533.I1S0M2
229533.I1RRW0	pp	229533.I1S1W4
229533.I1RRW0	pp	229533.I1RSI3
229533.I1RRW0	pp	229533.I1RZC3
229533.I1S4M5	pp	229533.Q4IB96
229533.I1S4M5	pp	229533.Q4IAA0
229533.I1S4M5	pp	229533.Q4IPB3
229533.I1S4M5	pp	229533.Q4IRI9
229533.I1S4M5	pp	229533.V6QX58
229533.I1S4M5	pp	229533.I1S5S2
229533.I1S4M5	pp	229533.V6QXG1
229533.I1S4M5	pp	229533.I1S524
229533.I1S4M5	pp	229533.I1S9L6
229533.I1S4M5	pp	229533.I1S9L5
229533.I1S4M5	pp	229533.V6RY33
229533.I1S4M5	pp	229533.Q4IQW0
229533.I1S4M5	pp	229533.I1S7M3
229533.I1S4M5	pp	229533.I1S7M2
229533.I1S4M5	pp	229533.V6QVD5
229533.I1S4M5	pp	229533.V6RIS2
229533.I1S4M5	pp	229533.Q4IB70
229533.I1S4M5	pp	229533.I1S4Y4
229533.I1S4M5	pp	229533.Q4I1Y1
229533.I1S4M5	pp	229533.Q4IP34
229533.I1S4M5	pp	229533.Q4I7F9
229533.A0A1C3YMJ1	pp	229533.I1RU13
229533.I1RZC3	pp	229533.I1S968
229533.I1RZC3	pp	229533.I1S969
229533.I1RZC3	pp	229533.I1S2K7
229533.I1RZC3	pp	229533.I1S7A5
229533.I1RZC3	pp	229533.I1S267
229533.I1RZC3	pp	229533.I1S2E9
229533.I1RZC3	pp	229533.I1S0M4
229533.I1RZC3	pp	229533.I1S334
229533.I1RZC3	pp	229533.I1S3H8
229533.I1RZC3	pp	229533.I1RZV5
229533.I1RZC3	pp	229533.I1RZK4
229533.I1RZC3	pp	229533.V6RHX2
229533.I1RZC3	pp	229533.I1S224
229533.I1RZC3	pp	229533.I1S9T6
229533.I1RZC3	pp	229533.Q4IBU4
229533.I1RZC3	pp	229533.Q4IPX8
229533.I1RZC3	pp	229533.I1S2B6
229533.I1RZC3	pp	229533.I1SAB4
229533.I1RZC3	pp	229533.I1RZX3
229533.I1RZC3	pp	229533.I1S0V6
229533.I1RZC3	pp	229533.I1S7M8
229533.I1RZC3	pp	229533.I1RZT1
229533.I1RZC3	pp	229533.I1S048
229533.I1RZC3	pp	229533.I1S1D7
229533.I1RZC3	pp	229533.I1S296
229533.I1RZC3	pp	229533.I1S0V7
229533.I1RCI3	pp	229533.I1RX47
229533.I1RCI3	pp	229533.I1S7M3
229533.I1RCI3	pp	229533.I1RSA8
229533.I1RCI3	pp	229533.V6RY33
229533.I1RCI3	pp	229533.I1RT47
229533.I1RCI3	pp	229533.I1RQD8
229533.I1RCI3	pp	229533.I1RZ11
229533.I1RCI3	pp	229533.I1RK90
229533.I1RCI3	pp	229533.V6RIS2
229533.I1RCI3	pp	229533.I1RQP8
229533.I1RCI3	pp	229533.I1RYW3
229533.I1RCI3	pp	229533.I1RPA0

57890 229533.I1RCI3 pp 229533.I1RS83
57891 229533.I1RCI3 pp 229533.I1RZQ5
57892 229533.I1RCI3 pp 229533.I1RK72
57893 229533.I1RCI3 pp 229533.I1RK92
57894 229533.I1RCI3 pp 229533.I1RFQ1
57895 229533.I1RCI3 pp 229533.I1RXF2
57896 229533.I1RCI3 pp 229533.I1RSD4
57897 229533.I1RCI3 pp 229533.Q4IAA0
57898 229533.I1RCI3 pp 229533.Q4I7F9
57899 229533.I1RCI3 pp 229533.I1RMM5
57900 229533.I1RCI3 pp 229533.I1RZZ1
57901 229533.I1RCI3 pp 229533.I1S7M2
57902 229533.I1RCI3 pp 229533.I1RQR3
57903 229533.I1RCI3 pp 229533.I1RWX8
57904 229533.I1RCI3 pp 229533.I1RME6
57905 229533.I1RCI3 pp 229533.I1RYI9
57906 229533.I1RCI3 pp 229533.I1S0Z9
57907 229533.I1RCI3 pp 229533.I1RQB5
57908 229533.I1RCI3 pp 229533.I1RSA4
57909 229533.I1RCI3 pp 229533.I1S4Y4
57910 229533.I1RCI3 pp 229533.I1S0B1
57911 229533.I1RCI3 pp 229533.Q4ICA8
57912 229533.I1RCI3 pp 229533.I1RG90
57913 229533.I1RCI3 pp 229533.I1RCR5
57914 229533.I1RCI3 pp 229533.I1RFP9
57915 229533.I1RCI3 pp 229533.I1RS75
57916 229533.I1RCI3 pp 229533.I1RCS2
57917 229533.I1RCI3 pp 229533.I1S0B6
57918 229533.I1RCI3 pp 229533.I1RCQ6
57919 229533.I1RCI3 pp 229533.I1S4M5
57920 229533.I1RCI3 pp 229533.I1RRQ4
57921 229533.I1RCI3 pp 229533.Q4IPB3
57922 229533.I1RCI3 pp 229533.Q4IPH4
57923 229533.I1RCI3 pp 229533.I1RKK4
57924 229533.I1RCI3 pp 229533.I1RT04
57925 229533.I1RCI3 pp 229533.I1RFT1
57926 229533.I1RCI3 pp 229533.I1S0R7
57927 229533.I1RCI3 pp 229533.I1S524
57928 229533.I1RCI3 pp 229533.I1S9L5
57929 229533.I1RCI3 pp 229533.I1RK96
57930 229533.I1RCI3 pp 229533.I1S5S2
57931 229533.I1RCI3 pp 229533.I1RG13
57932 229533.I1RCI3 pp 229533.Q4IB96
57933 229533.I1RCI3 pp 229533.I1RX81
57934 229533.I1RCI3 pp 229533.I1S9L6
57935 229533.I1RCI3 pp 229533.I1RW25
57936 229533.I1RCI3 pp 229533.I1RMV5
57937 229533.I1RCI3 pp 229533.I1REX2
57938 229533.I1RCI3 pp 229533.I1RMW7
57939 229533.I1RBQ4 pp 229533.I1RJL9
57940 229533.I1RBQ4 pp 229533.I1RPZ1
57941 229533.I1RBQ4 pp 229533.I1RQ75
57942 229533.I1RBQ4 pp 229533.I1RST0
57943 229533.I1RBQ4 pp 229533.I1RJ14
57944 229533.A0A1C3YLB2 pp 229533.I1REI0
57945 229533.A0A1C3YLB2 pp 229533.A0A1C3YLV9
57946 229533.A0A1C3YLB2 pp 229533.I1RNG4
57947 229533.A0A1C3YLB2 pp 229533.I1RVU7
57948 229533.A0A1C3YLB2 pp 229533.I1RMJ3
57949 229533.A0A1C3YLB2 pp 229533.I1RNJ7
57950 229533.A0A1C3YLB2 pp 229533.I1RK47
57951 229533.A0A1C3YLB2 pp 229533.A0A1C3YLX8
57952 229533.A0A1C3YLB2 pp 229533.A0A1C3YLE2
57953 229533.A0A1C3YLB2 pp 229533.I1RDB7
57954 229533.A0A1C3YLB2 pp 229533.I1RM26
57955 229533.A0A1C3YLB2 pp 229533.I1RP86
57956 229533.A0A1C3YLB2 pp 229533.I1RND2
57957 229533.A0A1C3YLB2 pp 229533.I1RVX9
57958 229533.A0A1C3YLB2 pp 229533.I1S0V6
57959 229533.A0A1C3YLB2 pp 229533.I1RCG5
57960 229533.A0A1C3YLB2 pp 229533.I1R9U7
57961 229533.A0A1C3YLB2 pp 229533.I1RHR3
57962 229533.A0A1C3YLB2 pp 229533.V6RPN0

229533.A0A1C3YLB2	pp	229533.A0A1C3YMS7
229533.A0A1C3YLB2	pp	229533.I1S5R5
229533.A0A1C3YLB2	pp	229533.I1RN84
229533.A0A1C3YLB2	pp	229533.I1S0V7
229533.I1RMA4	pp	229533.I1RPK4
229533.I1RMA4	pp	229533.V6RGI1
229533.I1RMA4	pp	229533.I1RX38
229533.I1RMA4	pp	229533.I1RZ63
229533.I1RMA4	pp	229533.I1S098
229533.I1RMA4	pp	229533.I1RVN2
229533.I1RMA4	pp	229533.I1S0J7
229533.I1RMA4	pp	229533.I1S549
229533.I1RMA4	pp	229533.I1S2E9
229533.I1RMA4	pp	229533.I1S004
229533.I1RMA4	pp	229533.I1RZC3
229533.I1RMA4	pp	229533.I1RV91
229533.I1RMA4	pp	229533.I1RRX6
229533.I1RMA4	pp	229533.I1RPW0
229533.I1RMA4	pp	229533.I1RRW0
229533.I1RMA4	pp	229533.I1RY44
229533.I1RMA4	pp	229533.I1S224
229533.I1RMA4	pp	229533.I1RYZ9
229533.I1RMA4	pp	229533.I1S0R1
229533.I1RMA4	pp	229533.I1RY58
229533.I1RMA4	pp	229533.I1RTG0
229533.I1RMA4	pp	229533.V6QV09
229533.I1RMA4	pp	229533.Q4I7N9
229533.I1RMA4	pp	229533.I1RPU4
229533.I1RMA4	pp	229533.I1RQA3
229533.I1RMA4	pp	229533.I1S267
229533.I1RMA4	pp	229533.I1RWT6
229533.I1RMV5	pp	229533.I1RZG7
229533.I1RMV5	pp	229533.I1S0B6
229533.I1RMV5	pp	229533.I1RN59
229533.I1RMV5	pp	229533.I1RMW7
229533.I1RMV5	pp	229533.Q4IPB3
229533.I1RMV5	pp	229533.I1RT41
229533.I1RMV5	pp	229533.I1RX81
229533.I1RMV5	pp	229533.Q4I298
229533.I1RMV5	pp	229533.I1RTF4
229533.I1RMV5	pp	229533.I1S7M2
229533.I1RMV5	pp	229533.I1S7M3
229533.I1RMV5	pp	229533.I1RSA8
229533.I1RMV5	pp	229533.Q4I1Y1
229533.I1RMV5	pp	229533.I1RYI9
229533.I1RMV5	pp	229533.Q4IB70
229533.I1RMV5	pp	229533.I1S1C6
229533.I1RMV5	pp	229533.I1RZ11
229533.I1RMV5	pp	229533.I1RPA0
229533.I1RMV5	pp	229533.I1RZZ1
229533.I1RMV5	pp	229533.I1RS75
229533.I1RMV5	pp	229533.I1RPZ3
229533.I1RMV5	pp	229533.I1RS83
229533.I1RMV5	pp	229533.I1RQD8
229533.I1RMV5	pp	229533.I1S2Y5
229533.I1RMV5	pp	229533.Q4IP34
229533.I1RMV5	pp	229533.Q4I7F9
229533.I1RMV5	pp	229533.I1RWJ0
229533.I1RMV5	pp	229533.I1RQC5
229533.I1RMV5	pp	229533.I1S0Z9
229533.I1RMV5	pp	229533.I1RPY5
229533.I1RMV5	pp	229533.I1RRW3
229533.I1RMV5	pp	229533.I1RSH4
229533.I1RMV5	pp	229533.Q4IAA0
229533.I1RMV5	pp	229533.I1RST1
229533.I1RMV5	pp	229533.I1RPY7
229533.I1RMV5	pp	229533.I1RU69
229533.I1RMV5	pp	229533.I1S0P4
229533.I1RMV5	pp	229533.I1RXF2
229533.I1RMV5	pp	229533.Q4IRI9
229533.I1RMV5	pp	229533.I1S5S2
229533.I1RMV5	pp	229533.I1RTS2
229533.I1RMV5	pp	229533.I1RT17

58036 229533.I1RMV5 pp 229533.I1RWX8
58037 229533.I1RMV5 pp 229533.I1S524
58038 229533.I1RMV5 pp 229533.I1RX24
58039 229533.I1RMV5 pp 229533.I1RPY4
58040 229533.I1RMV5 pp 229533.I1RT35
58041 229533.I1RMV5 pp 229533.I1RRL0
58042 229533.I1RMV5 pp 229533.I1S7X9
58043 229533.I1RMV5 pp 229533.Q4IBK5
58044 229533.I1RMV5 pp 229533.I1RPL3
58045 229533.I1RMV5 pp 229533.I1RZQ5
58046 229533.I1RMV5 pp 229533.I1RWU2
58047 229533.I1RMV5 pp 229533.I1RS28
58048 229533.I1RMV5 pp 229533.I1S1X9
58049 229533.I1RMV5 pp 229533.I1RNB8
58050 229533.I1RMV5 pp 229533.I1RSD4
58051 229533.I1RMV5 pp 229533.I1RQR3
58052 229533.I1RMV5 pp 229533.I1S4M5
58053 229533.I1RMV5 pp 229533.I1RT91
58054 229533.I1RMV5 pp 229533.I1RSH9
58055 229533.I1RMV5 pp 229533.I1S0B1
58056 229533.I1RMV5 pp 229533.V6QVD5
58057 229533.I1RMV5 pp 229533.I1RND9
58058 229533.I1RMV5 pp 229533.Q4I7K4
58059 229533.I1RMV5 pp 229533.I1RRQ4
58060 229533.I1RMV5 pp 229533.V6QXG1
58061 229533.I1RMV5 pp 229533.I1RWD3
58062 229533.I1RMV5 pp 229533.I1RVT9
58063 229533.I1RMV5 pp 229533.I1RX47
58064 229533.I1RMV5 pp 229533.Q4IPH4
58065 229533.I1RMV5 pp 229533.I1RT47
58066 229533.I1RMV5 pp 229533.I1RX51
58067 229533.I1RMV5 pp 229533.I1RSA4
58068 229533.I1RMV5 pp 229533.I1RND5
58069 229533.I1RMV5 pp 229533.I1RN09
58070 229533.I1RMV5 pp 229533.I1S056
58071 229533.I1RMV5 pp 229533.I1S471
58072 229533.I1RMV5 pp 229533.V6RY33
58073 229533.I1RMV5 pp 229533.V6RIS2
58074 229533.A0A0E0SQG9 pp 229533.I1RV91
58075 229533.A0A0E0SQG9 pp 229533.I1S098
58076 229533.A0A0E0SQG9 pp 229533.I1S2E9
58077 229533.A0A0E0SQG9 pp 229533.I1RBV5
58078 229533.A0A0E0SQG9 pp 229533.I1S0J7
58079 229533.A0A0E0SQG9 pp 229533.A0A1C3YHL0
58080 229533.A0A0E0SQG9 pp 229533.I1RVW0
58081 229533.A0A0E0SQG9 pp 229533.I1RSN7
58082 229533.A0A0E0SQG9 pp 229533.I1RCA5
58083 229533.A0A0E0SQG9 pp 229533.A0A1C3YI39
58084 229533.A0A0E0SQG9 pp 229533.I1RZ86
58085 229533.A0A0E0SQG9 pp 229533.I1RFW1
58086 229533.A0A0E0SQG9 pp 229533.A0A1C3YMJ3
58087 229533.A0A0E0SQG9 pp 229533.I1S7X9
58088 229533.A0A0E0SQG9 pp 229533.I1RTB2
58089 229533.A0A0E0SQG9 pp 229533.I1RBW1
58090 229533.A0A0E0SQG9 pp 229533.I1RB74
58091 229533.A0A0E0SQG9 pp 229533.I1RFL7
58092 229533.A0A0E0SQG9 pp 229533.I1S549
58093 229533.A0A0E0SQG9 pp 229533.I1RPK4
58094 229533.A0A0E0SQG9 pp 229533.I1S004
58095 229533.A0A0E0SQG9 pp 229533.A0A1C3YKE4
58096 229533.A0A0E0SQG9 pp 229533.A0A1C3YKJ6
58097 229533.A0A0E0SQG9 pp 229533.I1RX38
58098 229533.A0A0E0SQG9 pp 229533.I1RQ88
58099 229533.A0A0E0SQG9 pp 229533.I1RCD4
58100 229533.A0A0E0SQG9 pp 229533.I1RZ63
58101 229533.A0A0E0SQG9 pp 229533.V6RSF3
58102 229533.A0A0E0SQG9 pp 229533.I1RAI9
58103 229533.A0A0E0SQG9 pp 229533.I1RY44
58104 229533.A0A0E0SQG9 pp 229533.A0A1C3YHP8
58105 229533.A0A0E0SQG9 pp 229533.I1RDJ8
58106 229533.A0A0E0SQG9 pp 229533.A0A1C3YN55
58107 229533.A0A0E0SQG9 pp 229533.Q4IJ84
58108 229533.A0A0E0SQG9 pp 229533.I1RA10

58109	229533.A0A0E0SQG9	pp	229533.I1RDH0
58110	229533.A0A0E0SQG9	pp	229533.I1S1W4
58111	229533.A0A0E0SQG9	pp	229533.I1RY58
58112	229533.A0A0E0SQG9	pp	229533.I1R9Y9
58113	229533.A0A0E0SQG9	pp	229533.I1RZR6
58114	229533.A0A0E0SQG9	pp	229533.A0A1C3YN69
58115	229533.A0A0E0SQG9	pp	229533.I1RW23
58116	229533.A0A0E0SQG9	pp	229533.A0A1C3YK43
58117	229533.A0A0E0SQG9	pp	229533.I1RPW0
58118	229533.A0A0E0SQG9	pp	229533.I1RQ41
58119	229533.A0A0E0SQG9	pp	229533.I1RSH9
58120	229533.A0A0E0SQG9	pp	229533.Q4IJP1
58121	229533.A0A0E0SQG9	pp	229533.I1RBM3
58122	229533.A0A0E0SQG9	pp	229533.I1RRN5
58123	229533.A0A0E0SQG9	pp	229533.I1RBC8
58124	229533.A0A0E0SQG9	pp	229533.A0A1C3YJT5
58125	229533.A0A0E0SQG9	pp	229533.I1S0D5
58126	229533.A0A0E0SQG9	pp	229533.I1RQD1
58127	229533.A0A0E0SQG9	pp	229533.I1RS41
58128	229533.A0A0E0SQG9	pp	229533.I1RQ26
58129	229533.A0A0E0SQG9	pp	229533.I1RTG0
58130	229533.A0A0E0SQG9	pp	229533.I1RVN2
58131	229533.A0A0E0SQG9	pp	229533.A0A1C3YI31
58132	229533.A0A0E0SQG9	pp	229533.I1RSR9
58133	229533.A0A0E0SQG9	pp	229533.I1RMJ8
58134	229533.A0A0E0SQG9	pp	229533.V6RGI1
58135	229533.A0A0E0SQG9	pp	229533.I1RC54
58136	229533.A0A0E0SQG9	pp	229533.I1RW03
58137	229533.A0A0E0SQG9	pp	229533.A0A1C3YKS0
58138	229533.A0A0E0SQG9	pp	229533.I1RZ12
58139	229533.A0A0E0SQG9	pp	229533.I1RSP7
58140	229533.A0A0E0SQG9	pp	229533.I1RRW0
58141	229533.A0A0E0SQG9	pp	229533.I1RZK9
58142	229533.A0A0E0SQG9	pp	229533.I1RRY1
58143	229533.A0A0E0SQG9	pp	229533.I1RMA4
58144	229533.A0A0E0SQG9	pp	229533.I1RN49
58145	229533.A0A0E0SQG9	pp	229533.A0A1C3YM91
58146	229533.A0A0E0SQG9	pp	229533.I1RPQ1
58147	229533.A0A0E0SQG9	pp	229533.I1RX64
58148	229533.A0A0E0SQG9	pp	229533.I1RZC3
58149	229533.A0A0E0SQG9	pp	229533.I1RD18
58150	229533.A0A0E0SQG9	pp	229533.I1RKE9
58151	229533.A0A0E0SQG9	pp	229533.I1RA12
58152	229533.A0A0E0SQG9	pp	229533.I1RUQ6
58153	229533.A0A0E0SQG9	pp	229533.I1RM49
58154	229533.A0A0E0SQG9	pp	229533.A0A1C3YM29
58155	229533.A0A0E0SQG9	pp	229533.I1S096
58156	229533.A0A0E0SQG9	pp	229533.I1RAU6
58157	229533.A0A0E0SQG9	pp	229533.A0A1C3YK66
58158	229533.A0A0E0SQG9	pp	229533.A0A1C3YIJ4
58159	229533.A0A0E0SQG9	pp	229533.I1RT01
58160	229533.A0A0E0SQG9	pp	229533.I1S224
58161	229533.A0A0E0SQG9	pp	229533.I1RQ19
58162	229533.A0A0E0SQG9	pp	229533.I1RPU4
58163	229533.A0A0E0SQG9	pp	229533.I1RBW0
58164	229533.A0A0E0SQG9	pp	229533.I1REE1
58165	229533.A0A0E0SQG9	pp	229533.I1RWT6
58166	229533.A0A0E0SQG9	pp	229533.I1RRX6
58167	229533.A0A0E0SQG9	pp	229533.Q4I5R3
58168	229533.A0A0E0SQG9	pp	229533.A0A1C3YK37
58169	229533.A0A0E0SQG9	pp	229533.A0A1C3YJZ9
58170	229533.A0A0E0SQG9	pp	229533.I1RME5
58171	229533.A0A0E0SQG9	pp	229533.I1RT41
58172	229533.A0A0E0SQG9	pp	229533.Q4HTT2
58173	229533.A0A0E0SQG9	pp	229533.I1RC91
58174	229533.A0A0E0SQG9	pp	229533.I1RMQ7
58175	229533.A0A0E0SQG9	pp	229533.I1RZ94
58176	229533.A0A0E0SQG9	pp	229533.I1S091
58177	229533.A0A0E0SQG9	pp	229533.A0A1C3YI40
58178	229533.A0A0E0SQG9	pp	229533.I1RQ13
58179	229533.A0A0E0SQG9	pp	229533.I1RQS4
58180	229533.A0A0E0SQG9	pp	229533.I1RDV9
58181	229533.A0A0E0SQG9	pp	229533.I1S267

58182 229533.A0A0E0SQG9 pp 229533.I1RUR0
58183 229533.A0A0E0SQG9 pp 229533.I1RJK7
58184 229533.A0A0E0SQG9 pp 229533.I1RKH9
58185 229533.A0A0E0SQG9 pp 229533.Q4I7N9
58186 229533.A0A0E0SQG9 pp 229533.I1RQA3
58187 229533.A0A0E0SQG9 pp 229533.I1RG77
58188 229533.A0A0E0SQG9 pp 229533.V6REE9
58189 229533.A0A0E0SQG9 pp 229533.I1RAN0
58190 229533.A0A0E0SQG9 pp 229533.Q4IER8
58191 229533.Q4I7F9 pp 229533.V6R227
58192 229533.Q4I7F9 pp 229533.V6RY33
58193 229533.Q4I7F9 pp 229533.V6RIS2
58194 229533.Q4I7F9 pp 229533.Q4IRI9
58195 229533.Q4I7F9 pp 229533.V6QVD5
58196 229533.Q4I7F9 pp 229533.Q4IPI1
58197 229533.I1RRW9 pp 229533.I1RZ21
58198 229533.I1RVN2 pp 229533.I1RVN3
58199 229533.I1RVN2 pp 229533.I1S098
58200 229533.I1RVN2 pp 229533.I1RZ12
58201 229533.I1RVN2 pp 229533.I1S224
58202 229533.I1RVN2 pp 229533.I1S0J7
58203 229533.I1RVN2 pp 229533.I1S2E9
58204 229533.I1RVN2 pp 229533.I1RY44
58205 229533.I1RVN2 pp 229533.I1RZ63
58206 229533.I1RVN2 pp 229533.I1RVU1
58207 229533.I1RVN2 pp 229533.I1RY58
58208 229533.I1RVN2 pp 229533.I1RWT6
58209 229533.I1RX81 pp 229533.Q4IPH4
58210 229533.I1RX81 pp 229533.I1S0B1
58211 229533.I1RX81 pp 229533.I1RYJ9
58212 229533.I1RX81 pp 229533.V6RY33
58213 229533.I1RX81 pp 229533.I1S0Z9
58214 229533.I1RX81 pp 229533.V6QVD5
58215 229533.I1RX81 pp 229533.I1RYB1
58216 229533.I1RX81 pp 229533.I1RYW3
58217 229533.I1RX81 pp 229533.V6QXG1
58218 229533.I1RX81 pp 229533.I1S1X9
58219 229533.I1RX81 pp 229533.Q4I1H6
58220 229533.I1RX81 pp 229533.V6QX58
58221 229533.I1RX81 pp 229533.Q4HXF6
58222 229533.I1RX81 pp 229533.V6RIS2
58223 229533.I1RX81 pp 229533.I1RZQ5
58224 229533.I1RX81 pp 229533.I1RZZ1
58225 229533.I1RX81 pp 229533.Q4IRI9
58226 229533.I1RX81 pp 229533.Q4I7F9
58227 229533.I1RX81 pp 229533.I1S7M3
58228 229533.I1RX81 pp 229533.Q4I1Y1
58229 229533.I1RX81 pp 229533.Q4IE79
58230 229533.I1RX81 pp 229533.Q4IP34
58231 229533.I1RX81 pp 229533.Q4IB70
58232 229533.I1RX81 pp 229533.I1RZ11
58233 229533.I1RX81 pp 229533.I1S9L5
58234 229533.I1RX81 pp 229533.Q4IFI0
58235 229533.I1RX81 pp 229533.I1S7M2
58236 229533.I1RX81 pp 229533.I1S9L6
58237 229533.I1RX81 pp 229533.I1RXF2
58238 229533.I1RX81 pp 229533.I1S2Y5
58239 229533.I1RX81 pp 229533.I1S524
58240 229533.I1RX81 pp 229533.Q4IPB3
58241 229533.I1RX81 pp 229533.I1S4M5
58242 229533.I1RX81 pp 229533.I1S5S2
58243 229533.I1RX81 pp 229533.I1S1C6
58244 229533.I1RX81 pp 229533.I1S0B6
58245 229533.I1RX81 pp 229533.I1RYI9
58246 229533.I1RX81 pp 229533.I1S0R7
58247 229533.I1RX81 pp 229533.I1RZG7
58248 229533.I1RX81 pp 229533.Q4IBK5
58249 229533.I1RCA7 pp 229533.Q4IJM4
58250 229533.I1RCA7 pp 229533.Q4I1Y1
58251 229533.I1RCA7 pp 229533.I1S0Z9
58252 229533.I1RCA7 pp 229533.I1RYB1
58253 229533.I1RCA7 pp 229533.I1RCR5
58254 229533.I1RCA7 pp 229533.Q4IB70

58255 229533.I1RCA7 pp 229533.I1RPA0
58256 229533.I1RCA7 pp 229533.I1S7M3
58257 229533.I1RCA7 pp 229533.Q4I7F9
58258 229533.I1RCA7 pp 229533.I1RFP9
58259 229533.I1RCA7 pp 229533.I1RCN5
58260 229533.I1RCA7 pp 229533.I1RG57
58261 229533.I1RCA7 pp 229533.I1RZZ1
58262 229533.I1RCA7 pp 229533.I1RD39
58263 229533.I1RCA7 pp 229533.I1RSA8
58264 229533.I1RCA7 pp 229533.I1RK92
58265 229533.I1RCA7 pp 229533.I1RCQ6
58266 229533.I1RCA7 pp 229533.I1RJV2
58267 229533.I1RCA7 pp 229533.I1RZQ5
58268 229533.I1RCA7 pp 229533.I1RPL3
58269 229533.I1RCA7 pp 229533.Q4IPB3
58270 229533.I1RCA7 pp 229533.I1RQD8
58271 229533.I1RCA7 pp 229533.I1RZ11
58272 229533.I1RCA7 pp 229533.I1RT91
58273 229533.I1RCA7 pp 229533.I1RZG7
58274 229533.I1RCA7 pp 229533.I1RRU9
58275 229533.I1RCA7 pp 229533.I1RG17
58276 229533.I1RCA7 pp 229533.I1RQR3
58277 229533.I1RCA7 pp 229533.I1S1X9
58278 229533.I1RCA7 pp 229533.I1RX81
58279 229533.I1RCA7 pp 229533.I1RS83
58280 229533.I1RCA7 pp 229533.Q4IP34
58281 229533.I1RCA7 pp 229533.I1RCI3
58282 229533.I1RCA7 pp 229533.I1RCI4
58283 229533.I1RCA7 pp 229533.I1RRC6
58284 229533.I1RCA7 pp 229533.I1S0B1
58285 229533.I1RCA7 pp 229533.I1RG90
58286 229533.I1RCA7 pp 229533.I1RFI5
58287 229533.I1RCA7 pp 229533.I1RPY5
58288 229533.I1RCA7 pp 229533.I1S7M2
58289 229533.I1RCA7 pp 229533.I1RRQ4
58290 229533.I1RCA7 pp 229533.Q4IPH4
58291 229533.I1RCA7 pp 229533.I1RK72
58292 229533.I1RCA7 pp 229533.I1RMQ7
58293 229533.I1RCA7 pp 229533.I1RX47
58294 229533.I1RCA7 pp 229533.I1RYI9
58295 229533.I1RCA7 pp 229533.Q4IRI9
58296 229533.I1RCA7 pp 229533.I1RT47
58297 229533.I1RCA7 pp 229533.Q4IAA0
58298 229533.I1RCA7 pp 229533.I1S1C6
58299 229533.I1RCA7 pp 229533.I1RWX8
58300 229533.I1RCA7 pp 229533.I1RPZ3
58301 229533.I1RCA7 pp 229533.V6RIS2
58302 229533.I1RCA7 pp 229533.I1RN09
58303 229533.I1RCA7 pp 229533.I1S4Y4
58304 229533.I1RCA7 pp 229533.I1S2Y5
58305 229533.I1RCA7 pp 229533.I1S471
58306 229533.I1RCA7 pp 229533.I1RT35
58307 229533.I1RCA7 pp 229533.I1RS75
58308 229533.I1RCA7 pp 229533.I1RN59
58309 229533.I1RCA7 pp 229533.I1RFQ1
58310 229533.I1RCA7 pp 229533.I1RMW7
58311 229533.I1RCA7 pp 229533.I1RSD4
58312 229533.I1RCA7 pp 229533.I1RZJ1
58313 229533.I1RCA7 pp 229533.V6RY33
58314 229533.I1RCA7 pp 229533.I1S0B6
58315 229533.I1RCA7 pp 229533.I1RW71
58316 229533.I1RCA7 pp 229533.I1RS28
58317 229533.I1RCA7 pp 229533.I1RU69
58318 229533.I1RCA7 pp 229533.I1RRL0
58319 229533.I1RCA7 pp 229533.I1RKK4
58320 229533.I1RCA7 pp 229533.I1RQC5
58321 229533.I1RCA7 pp 229533.I1RSH9
58322 229533.I1RCA7 pp 229533.I1RNB8
58323 229533.I1RCA7 pp 229533.I1RQ41
58324 229533.I1RCA7 pp 229533.I1RST1
58325 229533.I1RCA7 pp 229533.I1RLU5
58326 229533.I1RCA7 pp 229533.I1RPY4
58327 229533.I1RCA7 pp 229533.I1RM25

58328	229533.I1RCA7	pp	229533.I1S524
58329	229533.I1RCA7	pp	229533.I1RK96
58330	229533.I1RCA7	pp	229533.I1RSA4
58331	229533.I1RCA7	pp	229533.I1RX51
58332	229533.I1RCA7	pp	229533.I1RND5
58333	229533.I1RCA7	pp	229533.I1RX24
58334	229533.I1RCA7	pp	229533.I1RD56
58335	229533.I1RCA7	pp	229533.V6QXG1
58336	229533.I1RCA7	pp	229533.I1REX2
58337	229533.I1RCA7	pp	229533.I1RJZ7
58338	229533.I1RCA7	pp	229533.I1RYW3
58339	229533.I1RCA7	pp	229533.I1RCS2
58340	229533.I1RCA7	pp	229533.I1RK90
58341	229533.I1RCA7	pp	229533.I1S5S2
58342	229533.I1RCA7	pp	229533.I1RFT1
58343	229533.I1RCA7	pp	229533.I1RMV5
58344	229533.I1RCA7	pp	229533.I1RWU2
58345	229533.I1RCA7	pp	229533.I1RXF2
58346	229533.I1RCA7	pp	229533.I1S4M5
58347	229533.I1RCA7	pp	229533.Q4IBK5
58348	229533.I1RCA7	pp	229533.I1RT17
58349	229533.I1RCA7	pp	229533.Q4IB96
58350	229533.I1RCA7	pp	229533.I1RG13
58351	229533.I1RCA7	pp	229533.I1RME9
58352	229533.I1RCA7	pp	229533.I1RQB5
58353	229533.I1RCA7	pp	229533.I1RND9
58354	229533.A0A1C3YM29	pp	229533.I1S549
58355	229533.A0A1C3YM29	pp	229533.I1SAA0
58356	229533.A0A1C3YM29	pp	229533.I1S0M6
58357	229533.A0A1C3YM29	pp	229533.I1RSD0
58358	229533.A0A1C3YM29	pp	229533.I1RW83
58359	229533.A0A1C3YM29	pp	229533.I1RRJ6
58360	229533.A0A1C3YM29	pp	229533.I1RPX5
58361	229533.A0A1C3YM29	pp	229533.Q4IPA4
58362	229533.A0A1C3YM29	pp	229533.I1RVW0
58363	229533.A0A1C3YM29	pp	229533.I1S7W0
58364	229533.A0A1C3YM29	pp	229533.I1RM94
58365	229533.A0A1C3YM29	pp	229533.I1RYR2
58366	229533.A0A1C3YM29	pp	229533.I1S0J7
58367	229533.A0A1C3YM29	pp	229533.I1S098
58368	229533.A0A1C3YM29	pp	229533.I1S2E9
58369	229533.A0A1C3YM29	pp	229533.I1S9L6
58370	229533.A0A1C3YM29	pp	229533.A0A1C3YM91
58371	229533.A0A1C3YM29	pp	229533.I1RUR8
58372	229533.A0A1C3YM29	pp	229533.I1RP28
58373	229533.A0A1C3YM29	pp	229533.I1RND9
58374	229533.A0A1C3YM29	pp	229533.I1S9G0
58375	229533.A0A1C3YM29	pp	229533.I1RV91
58376	229533.A0A1C3YM29	pp	229533.I1RBY9
58377	229533.A0A1C3YM29	pp	229533.I1R9X6
58378	229533.A0A1C3YM29	pp	229533.I1REX8
58379	229533.A0A1C3YM29	pp	229533.Q4IAK7
58380	229533.A0A1C3YM29	pp	229533.Q4IB50
58381	229533.A0A1C3YM29	pp	229533.V6RGY0
58382	229533.A0A1C3YM29	pp	229533.I1RCA7
58383	229533.A0A1C3YM29	pp	229533.I1RZ63
58384	229533.A0A1C3YM29	pp	229533.I1RRW0
58385	229533.A0A1C3YM29	pp	229533.I1RUM5
58386	229533.A0A1C3YM29	pp	229533.I1RMA4
58387	229533.A0A1C3YM29	pp	229533.I1RGF1
58388	229533.A0A1C3YM29	pp	229533.I1RU69
58389	229533.A0A1C3YM29	pp	229533.I1RSH9
58390	229533.A0A1C3YM29	pp	229533.I1RA56
58391	229533.A0A1C3YM29	pp	229533.I1SAP6
58392	229533.A0A1C3YM29	pp	229533.Q4I8C9
58393	229533.A0A1C3YM29	pp	229533.I1RGD1
58394	229533.A0A1C3YM29	pp	229533.I1RQB4
58395	229533.A0A1C3YM29	pp	229533.I1RPK4
58396	229533.A0A1C3YM29	pp	229533.I1RZP2
58397	229533.A0A1C3YM29	pp	229533.I1RFQ1
58398	229533.A0A1C3YM29	pp	229533.A0A1C3YN69
58399	229533.A0A1C3YM29	pp	229533.V6RGI1
58400	229533.A0A1C3YM29	pp	229533.I1RA99

229533.A0A1C3YM29 pp 229533.I1R9Y9
229533.A0A1C3YM29 pp 229533.I1RZ05
229533.A0A1C3YM29 pp 229533.I1S1V1
229533.A0A1C3YM29 pp 229533.I1S9S6
229533.A0A1C3YM29 pp 229533.I1RAV2
229533.A0A1C3YM29 pp 229533.A0A1C3YMF9
229533.A0A1C3YM29 pp 229533.I1RY44
229533.A0A1C3YM29 pp 229533.I1RRN5
229533.A0A1C3YM29 pp 229533.I1RVN2
229533.A0A1C3YM29 pp 229533.I1S645
229533.A0A1C3YM29 pp 229533.I1RY58
229533.A0A1C3YM29 pp 229533.I1RT47
229533.A0A1C3YM29 pp 229533.I1RZK9
229533.A0A1C3YM29 pp 229533.I1S109
229533.A0A1C3YM29 pp 229533.I1RCH5
229533.A0A1C3YM29 pp 229533.I1RQD1
229533.A0A1C3YM29 pp 229533.I1RK75
229533.A0A1C3YM29 pp 229533.I1RMU1
229533.A0A1C3YM29 pp 229533.I1RD15
229533.A0A1C3YM29 pp 229533.I1S9L5
229533.A0A1C3YM29 pp 229533.I1RQP8
229533.A0A1C3YM29 pp 229533.I1RB41
229533.A0A1C3YM29 pp 229533.I1RNE4
229533.A0A1C3YM29 pp 229533.I1RCP3
229533.A0A1C3YM29 pp 229533.I1RZ12
229533.A0A1C3YM29 pp 229533.A0A1C3YMZ7
229533.A0A1C3YM29 pp 229533.I1R9Z9
229533.A0A1C3YM29 pp 229533.I1RHU7
229533.A0A1C3YM29 pp 229533.I1RUQ6
229533.A0A1C3YM29 pp 229533.I1SAP1
229533.A0A1C3YM29 pp 229533.I1RD70
229533.A0A1C3YM29 pp 229533.I1RZT1
229533.A0A1C3YM29 pp 229533.I1RTN0
229533.A0A1C3YM29 pp 229533.V6QV09
229533.A0A1C3YM29 pp 229533.I1RLX7
229533.A0A1C3YM29 pp 229533.I1RYZ9
229533.A0A1C3YM29 pp 229533.I1RDQ9
229533.A0A1C3YM29 pp 229533.I1RQ26
229533.A0A1C3YM29 pp 229533.I1RM49
229533.A0A1C3YM29 pp 229533.I1RQH1
229533.A0A1C3YM29 pp 229533.I1RTG0
229533.A0A1C3YM29 pp 229533.Q4IBS2
229533.A0A1C3YM29 pp 229533.I1RFS6
229533.A0A1C3YM29 pp 229533.I1RBT0
229533.A0A1C3YM29 pp 229533.A0A1C3YMU9
229533.A0A1C3YM29 pp 229533.I1S259
229533.A0A1C3YM29 pp 229533.I1RRY1
229533.A0A1C3YM29 pp 229533.I1S8C1
229533.A0A1C3YM29 pp 229533.I1RDA0
229533.A0A1C3YM29 pp 229533.I1RPH1
229533.A0A1C3YM29 pp 229533.I1RR15
229533.A0A1C3YM29 pp 229533.I1RV66
229533.A0A1C3YM29 pp 229533.I1S270
229533.A0A1C3YM29 pp 229533.I1RN91
229533.A0A1C3YM29 pp 229533.I1S0T3
229533.A0A1C3YM29 pp 229533.I1RWT6
229533.A0A1C3YM29 pp 229533.I1RX70
229533.A0A1C3YM29 pp 229533.I1RDB1
229533.A0A1C3YM29 pp 229533.I1RC91
229533.A0A1C3YM29 pp 229533.I1RGB6
229533.A0A1C3YM29 pp 229533.I1RL24
229533.A0A1C3YM29 pp 229533.I1RNI4
229533.A0A1C3YM29 pp 229533.I1RTE4
229533.A0A1C3YM29 pp 229533.I1RMB2
229533.A0A1C3YM29 pp 229533.I1RD18
229533.A0A1C3YM29 pp 229533.I1RN89
229533.A0A1C3YM29 pp 229533.I1RUI0
229533.A0A1C3YM29 pp 229533.I1RRX6
229533.A0A1C3YM29 pp 229533.I1RX20
229533.A0A1C3YM29 pp 229533.I1RZK4
229533.A0A1C3YM29 pp 229533.I1RAE1
229533.A0A1C3YM29 pp 229533.I1S224
229533.A0A1C3YM29 pp 229533.I1RQ93

58474 229533.A0A1C3YM29 pp 229533.I1RY12
58475 229533.A0A1C3YM29 pp 229533.I1RME5
58476 229533.A0A1C3YM29 pp 229533.I1RXZ4
58477 229533.A0A1C3YM29 pp 229533.I1REE1
58478 229533.A0A1C3YM29 pp 229533.I1RDG0
58479 229533.A0A1C3YM29 pp 229533.I1RN07
58480 229533.A0A1C3YM29 pp 229533.Q4I7N9
58481 229533.A0A1C3YM29 pp 229533.Q4IE79
58482 229533.A0A1C3YM29 pp 229533.I1RAU6
58483 229533.A0A1C3YM29 pp 229533.I1RC17
58484 229533.A0A1C3YM29 pp 229533.I1RDY2
58485 229533.A0A1C3YM29 pp 229533.I1RFI5
58486 229533.A0A1C3YM29 pp 229533.I1RC22
58487 229533.A0A1C3YM29 pp 229533.I1RQA3
58488 229533.A0A1C3YM29 pp 229533.I1RDV9
58489 229533.A0A1C3YM29 pp 229533.I1RHB0
58490 229533.A0A1C3YM29 pp 229533.I1RJK7
58491 229533.A0A1C3YM29 pp 229533.I1S091
58492 229533.A0A1C3YM29 pp 229533.I1RQS4
58493 229533.A0A1C3YM29 pp 229533.I1RJY7
58494 229533.I1S5K9 pp 229533.V6REE9
58495 229533.I1RPA0 pp 229533.I1RSH9
58496 229533.I1RPA0 pp 229533.I1RSD4
58497 229533.I1RPA0 pp 229533.I1RS28
58498 229533.I1RPA0 pp 229533.I1RPY5
58499 229533.I1RPA0 pp 229533.Q4HXF6
58500 229533.I1RPA0 pp 229533.I1RSA4
58501 229533.I1RPA0 pp 229533.V6RY33
58502 229533.I1RPA0 pp 229533.I1RT35
58503 229533.I1RPA0 pp 229533.V6QX58
58504 229533.I1RPA0 pp 229533.I1RQR3
58505 229533.I1RPA0 pp 229533.I1RWX8
58506 229533.I1RPA0 pp 229533.I1S1X9
58507 229533.I1RPA0 pp 229533.I1RZZ1
58508 229533.I1RPA0 pp 229533.I1RT91
58509 229533.I1RPA0 pp 229533.I1RVT9
58510 229533.I1RPA0 pp 229533.Q4I7F9
58511 229533.I1RPA0 pp 229533.V6QXG1
58512 229533.I1RPA0 pp 229533.I1RX51
58513 229533.I1RPA0 pp 229533.I1RZQ5
58514 229533.I1RPA0 pp 229533.I1S8F8
58515 229533.I1RPA0 pp 229533.I1S7M3
58516 229533.I1RPA0 pp 229533.Q4IPH4
58517 229533.I1RPA0 pp 229533.Q4IAA0
58518 229533.I1RPA0 pp 229533.I1RX47
58519 229533.I1RPA0 pp 229533.I1RRQ4
58520 229533.I1RPA0 pp 229533.Q4IE79
58521 229533.I1RPA0 pp 229533.I1S0B1
58522 229533.I1RPA0 pp 229533.Q4IRI9
58523 229533.I1RPA0 pp 229533.I1RSA8
58524 229533.I1RPA0 pp 229533.I1S9L5
58525 229533.I1RPA0 pp 229533.V6QVD5
58526 229533.I1RPA0 pp 229533.I1RYB1
58527 229533.I1RPA0 pp 229533.I1RWU2
58528 229533.I1RPA0 pp 229533.I1S0Z9
58529 229533.I1RPA0 pp 229533.I1RYJ9
58530 229533.I1RPA0 pp 229533.V6RIS2
58531 229533.I1RPA0 pp 229533.Q4IP34
58532 229533.I1RPA0 pp 229533.I1RS75
58533 229533.I1RPA0 pp 229533.I1S4M5
58534 229533.I1RPA0 pp 229533.I1RS83
58535 229533.I1RPA0 pp 229533.I1S2Y5
58536 229533.I1RPA0 pp 229533.I1RZ11
58537 229533.I1RPA0 pp 229533.Q4IBK5
58538 229533.I1RPA0 pp 229533.Q4I1Y1
58539 229533.I1RPA0 pp 229533.I1S9L6
58540 229533.I1RPA0 pp 229533.I1RQD8
58541 229533.I1RPA0 pp 229533.I1RQP8
58542 229533.I1RPA0 pp 229533.I1RRW3
58543 229533.I1RPA0 pp 229533.I1RT47
58544 229533.I1RPA0 pp 229533.Q4IB70
58545 229533.I1RPA0 pp 229533.I1RSL9
58546 229533.I1RPA0 pp 229533.I1RYW3

58547	229533.I1RPA0	pp	229533.I1RYI9
58548	229533.I1RPA0	pp	229533.I1RXF2
58549	229533.I1RPA0	pp	229533.I1RQC5
58550	229533.I1RPA0	pp	229533.I1RPW9
58551	229533.I1RPA0	pp	229533.I1S7M2
58552	229533.I1RPA0	pp	229533.I1RX81
58553	229533.I1RPA0	pp	229533.I1RST1
58554	229533.I1RPA0	pp	229533.I1RX24
58555	229533.I1RPA0	pp	229533.I1S0B6
58556	229533.I1RPA0	pp	229533.I1RT04
58557	229533.I1RPA0	pp	229533.I1S5S2
58558	229533.I1RPA0	pp	229533.I1S0R7
58559	229533.I1RPA0	pp	229533.V6QYF0
58560	229533.I1RPA0	pp	229533.I1RPY4
58561	229533.I1RPA0	pp	229533.Q4IPB3
58562	229533.I1RPA0	pp	229533.I1S524
58563	229533.I1RPA0	pp	229533.I1RTF4
58564	229533.I1RPA0	pp	229533.I1RW25
58565	229533.I1RPA0	pp	229533.I1RZG7
58566	229533.I1RPA0	pp	229533.I1RU69
58567	229533.I1RMV8	pp	229533.I1S3V2
58568	229533.I1RMV8	pp	229533.I1RRW9
58569	229533.I1RMV8	pp	229533.I1RZ21
58570	229533.I1RBV5	pp	229533.I1RFZ2
58571	229533.I1RBV5	pp	229533.I1RQ80
58572	229533.I1RBV5	pp	229533.I1RTG0
58573	229533.I1RBV5	pp	229533.I1RP61
58574	229533.I1RBV5	pp	229533.I1RME5
58575	229533.I1RBV5	pp	229533.Q4I7X1
58576	229533.I1RBV5	pp	229533.I1RZ12
58577	229533.I1RBV5	pp	229533.I1RLX4
58578	229533.I1RBV5	pp	229533.I1RY44
58579	229533.I1RBV5	pp	229533.I1RFL7
58580	229533.I1RBV5	pp	229533.I1RD18
58581	229533.I1RBV5	pp	229533.I1S1V5
58582	229533.I1RBV5	pp	229533.V6RKY7
58583	229533.I1RBV5	pp	229533.V6RVG3
58584	229533.I1RBV5	pp	229533.I1S951
58585	229533.I1RBV5	pp	229533.I1RXA1
58586	229533.I1RBV5	pp	229533.I1RDB1
58587	229533.I1RBV5	pp	229533.I1RX38
58588	229533.I1RBV5	pp	229533.I1RWT6
58589	229533.I1RBV5	pp	229533.I1S9H9
58590	229533.I1RBV5	pp	229533.I1RW16
58591	229533.I1RBV5	pp	229533.I1RPK5
58592	229533.I1RBV5	pp	229533.I1RYH9
58593	229533.I1RBV5	pp	229533.I1S0D1
58594	229533.I1RBV5	pp	229533.I1RRX6
58595	229533.I1RBV5	pp	229533.I1S224
58596	229533.I1RBV5	pp	229533.I1RDG8
58597	229533.I1RBV5	pp	229533.I1S2R1
58598	229533.I1RBV5	pp	229533.I1RZG3
58599	229533.I1RBV5	pp	229533.I1RS41
58600	229533.I1RBV5	pp	229533.I1RRY3
58601	229533.I1RBV5	pp	229533.V6R2L8
58602	229533.I1RBV5	pp	229533.I1RN31
58603	229533.I1RBV5	pp	229533.I1RLX6
58604	229533.I1RBV5	pp	229533.I1RNC0
58605	229533.I1RBV5	pp	229533.I1RV91
58606	229533.I1RBV5	pp	229533.I1RRP7
58607	229533.I1RBV5	pp	229533.I1RQW2
58608	229533.I1RBV5	pp	229533.I1S2E9
58609	229533.I1RBV5	pp	229533.I1RF69
58610	229533.I1RBV5	pp	229533.I1RLS5
58611	229533.I1RBV5	pp	229533.I1S382
58612	229533.I1RBV5	pp	229533.I1RWT1
58613	229533.I1RBV5	pp	229533.I1RZT1
58614	229533.I1RBV5	pp	229533.I1S330
58615	229533.I1RBV5	pp	229533.I1SA96
58616	229533.I1RBV5	pp	229533.I1RQM0
58617	229533.I1RBV5	pp	229533.I1RK90
58618	229533.I1RBV5	pp	229533.I1RFU3
58619	229533.I1RBV5	pp	229533.I1RD70

58620	229533.I1RBV5	pp	229533.I1RBZ9
58621	229533.I1RBV5	pp	229533.I1RC22
58622	229533.I1RBV5	pp	229533.I1RT08
58623	229533.I1RBV5	pp	229533.I1RL04
58624	229533.I1RBV5	pp	229533.I1RVA5
58625	229533.I1RBV5	pp	229533.I1RPD2
58626	229533.I1RBV5	pp	229533.I1RC95
58627	229533.I1RBV5	pp	229533.I1S339
58628	229533.I1RBV5	pp	229533.I1RPU4
58629	229533.I1RBV5	pp	229533.I1RQS4
58630	229533.I1RBV5	pp	229533.I1S439
58631	229533.I1RBV5	pp	229533.I1RDV9
58632	229533.I1RBV5	pp	229533.I1RC17
58633	229533.I1RBV5	pp	229533.I1RJK7
58634	229533.I1RBV5	pp	229533.I1RTN1
58635	229533.I1RBV5	pp	229533.I1REE1
58636	229533.I1RBV5	pp	229533.I1RMA4
58637	229533.I1RBV5	pp	229533.I1RZE4
58638	229533.I1RBV5	pp	229533.I1RRW0
58639	229533.I1RBV5	pp	229533.I1RCM6
58640	229533.I1RBV5	pp	229533.I1RQA3
58641	229533.I1RBV5	pp	229533.I1RPW0
58642	229533.I1RBV5	pp	229533.I1RRN5
58643	229533.I1RBV5	pp	229533.I1RI34
58644	229533.I1RS75	pp	229533.V6RY33
58645	229533.I1RS75	pp	229533.I1RT91
58646	229533.I1RS75	pp	229533.I1RT35
58647	229533.I1RS75	pp	229533.I1RSA4
58648	229533.I1RS75	pp	229533.I1RZZ1
58649	229533.I1RS75	pp	229533.I1RWX8
58650	229533.I1RS75	pp	229533.I1RX51
58651	229533.I1RS75	pp	229533.I1RZQ5
58652	229533.I1RS75	pp	229533.V6QXG1
58653	229533.I1RS75	pp	229533.Q4I1Y1
58654	229533.I1RS75	pp	229533.Q4IRI9
58655	229533.I1RS75	pp	229533.Q4IB70
58656	229533.I1RS75	pp	229533.I1RYW3
58657	229533.I1RS75	pp	229533.I1RSD4
58658	229533.I1RS75	pp	229533.I1RS83
58659	229533.I1RS75	pp	229533.I1S9L6
58660	229533.I1RS75	pp	229533.V6RIS2
58661	229533.I1RS75	pp	229533.I1RT47
58662	229533.I1RS75	pp	229533.I1S9L5
58663	229533.I1RS75	pp	229533.I1RZ11
58664	229533.I1RS75	pp	229533.I1S7M3
58665	229533.I1RS75	pp	229533.I1RWU2
58666	229533.I1RS75	pp	229533.I1S2Y5
58667	229533.I1RS75	pp	229533.Q4IE79
58668	229533.I1RS75	pp	229533.I1S4M5
58669	229533.I1RS75	pp	229533.I1RX81
58670	229533.I1RS75	pp	229533.I1S0B1
58671	229533.I1RS75	pp	229533.I1RSA8
58672	229533.I1RS75	pp	229533.Q4IBK5
58673	229533.I1RS75	pp	229533.I1S524
58674	229533.I1RS75	pp	229533.I1RYI9
58675	229533.I1RS75	pp	229533.I1RYJ9
58676	229533.I1RS75	pp	229533.Q4IPB3
58677	229533.I1RS75	pp	229533.I1RZG7
58678	229533.I1RS75	pp	229533.I1RXF2
58679	229533.I1RS75	pp	229533.I1S0Z9
58680	229533.I1RS75	pp	229533.Q4HXF6
58681	229533.I1RS75	pp	229533.I1RST1
58682	229533.I1RS75	pp	229533.I1S5S2
58683	229533.I1RS75	pp	229533.I1RUG0
58684	229533.I1RS75	pp	229533.I1S7M2
58685	229533.I1RWT0	pp	229533.Q4HZ95
58686	229533.I1RWT0	pp	229533.V6RVJ3
58687	229533.I1RWT0	pp	229533.I1S277
58688	229533.I1RWT0	pp	229533.I1S212
58689	229533.I1RWT0	pp	229533.I1RZM9
58690	229533.I1RWT0	pp	229533.I1S064
58691	229533.I1RWT0	pp	229533.I1RYE5
58692	229533.I1RWT0	pp	229533.Q4I5G1

58693 229533.I1RWT0 pp 229533.I1S5R5
58694 229533.I1RWT0 pp 229533.I1RX39
58695 229533.I1RWT0 pp 229533.I1RXB6
58696 229533.I1RWT0 pp 229533.I1S1F3
58697 229533.I1RWT0 pp 229533.I1RX17
58698 229533.I1RBM5 pp 229533.I1RMT5
58699 229533.I1RBM5 pp 229533.I1RM11
58700 229533.I1RBM5 pp 229533.I1S1Z9
58701 229533.I1RBM5 pp 229533.I1RX67
58702 229533.A0A1C3YKJ6 pp 229533.I1RLX6
58703 229533.A0A1C3YKJ6 pp 229533.I1RV91
58704 229533.A0A1C3YKJ6 pp 229533.I1RQW2
58705 229533.A0A1C3YKJ6 pp 229533.I1R9R7
58706 229533.A0A1C3YKJ6 pp 229533.I1S2E9
58707 229533.A0A1C3YKJ6 pp 229533.I1RBV5
58708 229533.A0A1C3YKJ6 pp 229533.I1RXA1
58709 229533.A0A1C3YKJ6 pp 229533.I1RWT1
58710 229533.A0A1C3YKJ6 pp 229533.I1S9H9
58711 229533.A0A1C3YKJ6 pp 229533.I1S2R1
58712 229533.A0A1C3YKJ6 pp 229533.I1RAW7
58713 229533.A0A1C3YKJ6 pp 229533.A0A1C3YKW5
58714 229533.A0A1C3YKJ6 pp 229533.I1RQ80
58715 229533.A0A1C3YKJ6 pp 229533.A0A1C3YN27
58716 229533.A0A1C3YKJ6 pp 229533.I1RQ26
58717 229533.A0A1C3YKJ6 pp 229533.I1RAN2
58718 229533.A0A1C3YKJ6 pp 229533.I1RTG0
58719 229533.A0A1C3YKJ6 pp 229533.I1RB74
58720 229533.A0A1C3YKJ6 pp 229533.I1RDB1
58721 229533.A0A1C3YKJ6 pp 229533.I1R9Q0
58722 229533.A0A1C3YKJ6 pp 229533.I1S1V5
58723 229533.A0A1C3YKJ6 pp 229533.I1RZ12
58724 229533.A0A1C3YKJ6 pp 229533.I1RD18
58725 229533.A0A1C3YKJ6 pp 229533.I1S330
58726 229533.A0A1C3YKJ6 pp 229533.I1RX38
58727 229533.A0A1C3YKJ6 pp 229533.I1S951
58728 229533.A0A1C3YKJ6 pp 229533.I1RFZ2
58729 229533.A0A1C3YKJ6 pp 229533.I1S9Z3
58730 229533.A0A1C3YKJ6 pp 229533.I1RBL4
58731 229533.A0A1C3YKJ6 pp 229533.I1RT08
58732 229533.A0A1C3YKJ6 pp 229533.I1RL04
58733 229533.A0A1C3YKJ6 pp 229533.I1RMA4
58734 229533.A0A1C3YKJ6 pp 229533.I1S339
58735 229533.A0A1C3YKJ6 pp 229533.I1S0M2
58736 229533.A0A1C3YKJ6 pp 229533.I1RRY3
58737 229533.A0A1C3YKJ6 pp 229533.I1RN08
58738 229533.A0A1C3YKJ6 pp 229533.I1RB59
58739 229533.A0A1C3YKJ6 pp 229533.I1R976
58740 229533.A0A1C3YKJ6 pp 229533.Q4I7X1
58741 229533.A0A1C3YKJ6 pp 229533.I1RPD2
58742 229533.A0A1C3YKJ6 pp 229533.I1S224
58743 229533.A0A1C3YKJ6 pp 229533.I1RS41
58744 229533.A0A1C3YKJ6 pp 229533.A0A1C3YM91
58745 229533.A0A1C3YKJ6 pp 229533.I1RP61
58746 229533.A0A1C3YKJ6 pp 229533.V6R2L8
58747 229533.A0A1C3YKJ6 pp 229533.I1RRX6
58748 229533.A0A1C3YKJ6 pp 229533.I1RDG8
58749 229533.A0A1C3YKJ6 pp 229533.I1RLX4
58750 229533.A0A1C3YKJ6 pp 229533.I1S0J7
58751 229533.A0A1C3YKJ6 pp 229533.I1RME5
58752 229533.A0A1C3YKJ6 pp 229533.I1RBM3
58753 229533.A0A1C3YKJ6 pp 229533.I1RMM9
58754 229533.A0A1C3YKJ6 pp 229533.I1RRP7
58755 229533.A0A1C3YKJ6 pp 229533.I1RC95
58756 229533.A0A1C3YKJ6 pp 229533.I1RZG3
58757 229533.A0A1C3YKJ6 pp 229533.I1RN31
58758 229533.A0A1C3YKJ6 pp 229533.I1RZT1
58759 229533.A0A1C3YKJ6 pp 229533.I1RPU4
58760 229533.A0A1C3YKJ6 pp 229533.I1RLS5
58761 229533.A0A1C3YKJ6 pp 229533.I1RBI1
58762 229533.A0A1C3YKJ6 pp 229533.I1R9Y9
58763 229533.A0A1C3YKJ6 pp 229533.I1SA96
58764 229533.A0A1C3YKJ6 pp 229533.I1S382
58765 229533.A0A1C3YKJ6 pp 229533.I1RAM7

58766 229533.A0A1C3YKJ6 pp 229533.I1RF69
58767 229533.A0A1C3YKJ6 pp 229533.I1RD70
58768 229533.A0A1C3YKJ6 pp 229533.I1RCM6
58769 229533.A0A1C3YKJ6 pp 229533.I1RPK5
58770 229533.A0A1C3YKJ6 pp 229533.I1RA43
58771 229533.A0A1C3YKJ6 pp 229533.A0A1C3YLY5
58772 229533.A0A1C3YKJ6 pp 229533.I1RQA3
58773 229533.A0A1C3YKJ6 pp 229533.A0A1C3YMG7
58774 229533.A0A1C3YKJ6 pp 229533.I1RNC0
58775 229533.A0A1C3YKJ6 pp 229533.I1S1D3
58776 229533.A0A1C3YKJ6 pp 229533.I1RI34
58777 229533.A0A1C3YKJ6 pp 229533.I1RFU3
58778 229533.A0A1C3YKJ6 pp 229533.I1RPW0
58779 229533.A0A1C3YKJ6 pp 229533.I1RQM0
58780 229533.A0A1C3YKJ6 pp 229533.I1RTN1
58781 229533.A0A1C3YKJ6 pp 229533.I1RW16
58782 229533.A0A1C3YKJ6 pp 229533.I1REE1
58783 229533.A0A1C3YKJ6 pp 229533.I1RY44
58784 229533.A0A1C3YKJ6 pp 229533.A0A1C3YMF9
58785 229533.A0A1C3YKJ6 pp 229533.I1RRW0
58786 229533.A0A1C3YKJ6 pp 229533.I1RQS4
58787 229533.A0A1C3YKJ6 pp 229533.V6RVG3
58788 229533.A0A1C3YKJ6 pp 229533.I1RDV9
58789 229533.A0A1C3YKJ6 pp 229533.A0A1C3YM29
58790 229533.A0A1C3YKJ6 pp 229533.I1RWT6
58791 229533.A0A1C3YKJ6 pp 229533.I1S439
58792 229533.A0A1C3YKJ6 pp 229533.I1RJK7
58793 229533.A0A1C3YKJ6 pp 229533.I1S1W4
58794 229533.A0A1C3YKJ6 pp 229533.A0A1C3YKV0
58795 229533.A0A1C3YKJ6 pp 229533.I1RB75
58796 229533.A0A1C3YKJ6 pp 229533.I1RVA5
58797 229533.I1RBL9 pp 229533.I1RTF1
58798 229533.I1RK96 pp 229533.I1RZG7
58799 229533.I1RK96 pp 229533.Q4IPB3
58800 229533.I1RK96 pp 229533.I1S524
58801 229533.I1RK96 pp 229533.I1RQB5
58802 229533.I1RK96 pp 229533.I1RVL8
58803 229533.I1RK96 pp 229533.I1S9L5
58804 229533.I1RK96 pp 229533.I1RM25
58805 229533.I1RK96 pp 229533.I1RQJ5
58806 229533.I1RK96 pp 229533.I1RKF9
58807 229533.I1RK96 pp 229533.I1RNU6
58808 229533.I1RK96 pp 229533.I1RX81
58809 229533.I1RK96 pp 229533.I1RSA4
58810 229533.I1RK96 pp 229533.I1RKK4
58811 229533.I1RK96 pp 229533.I1RMV5
58812 229533.I1RK96 pp 229533.I1RW25
58813 229533.I1RK96 pp 229533.Q4I830
58814 229533.I1RK96 pp 229533.I1S4M5
58815 229533.I1RK96 pp 229533.I1RU69
58816 229533.I1RK96 pp 229533.I1RVT9
58817 229533.I1RK96 pp 229533.I1S9L6
58818 229533.I1RK96 pp 229533.I1RWX8
58819 229533.I1RK96 pp 229533.I1RMW7
58820 229533.I1RK96 pp 229533.V6RY33
58821 229533.I1RK96 pp 229533.Q4HZI0
58822 229533.I1RK96 pp 229533.I1RXF2
58823 229533.I1RK96 pp 229533.I1RZF6
58824 229533.I1RK96 pp 229533.I1S7M2
58825 229533.I1RK96 pp 229533.I1RS75
58826 229533.I1RK96 pp 229533.I1S0B6
58827 229533.I1RK96 pp 229533.I1S5S2
58828 229533.I1RK96 pp 229533.I1RQR3
58829 229533.I1RK96 pp 229533.I1S7M3
58830 229533.I1RK96 pp 229533.I1S0B1
58831 229533.I1RK96 pp 229533.I1S0R7
58832 229533.I1RK96 pp 229533.I1RX47
58833 229533.I1RK96 pp 229533.I1RPY7
58834 229533.I1RK96 pp 229533.I1RSA8
58835 229533.I1RK96 pp 229533.I1RYI9
58836 229533.I1RK96 pp 229533.I1RQD8
58837 229533.I1RK96 pp 229533.I1RT04
58838 229533.I1RK96 pp 229533.Q4IPH4

58839	229533.I1RK96	pp	229533.I1RZ11
58840	229533.I1RK96	pp	229533.I1S0Z9
58841	229533.I1RK96	pp	229533.I1RT47
58842	229533.I1RK96	pp	229533.I1S4Y4
58843	229533.I1RK96	pp	229533.Q4IP34
58844	229533.I1RK96	pp	229533.I1RRQ4
58845	229533.I1RK96	pp	229533.I1RZZ1
58846	229533.I1RK96	pp	229533.I1RS83
58847	229533.I1RK96	pp	229533.Q4I7F9
58848	229533.I1RK96	pp	229533.I1RQP8
58849	229533.I1RK96	pp	229533.V6RIS2
58850	229533.I1RK96	pp	229533.I1RYW3
58851	229533.I1RK96	pp	229533.I1RSD4
58852	229533.I1RK96	pp	229533.I1RPA0
58853	229533.I1RK96	pp	229533.V6QY25
58854	229533.I1RK96	pp	229533.I1RMM5
58855	229533.I1RK96	pp	229533.I1RN43
58856	229533.I1RBM3	pp	229533.I1S439
58857	229533.I1RBM3	pp	229533.I1S224
58858	229533.I1RBM3	pp	229533.I1RQS4
58859	229533.I1RBM3	pp	229533.I1RFL7
58860	229533.I1RBM3	pp	229533.I1RZ12
58861	229533.I1RBM3	pp	229533.I1RRX6
58862	229533.I1RBM3	pp	229533.I1RTG0
58863	229533.I1RBM3	pp	229533.I1RI34
58864	229533.I1RBM3	pp	229533.I1RPW0
58865	229533.I1RBM3	pp	229533.V6R9P0
58866	229533.I1RBM3	pp	229533.I1S5X9
58867	229533.I1RBM3	pp	229533.I1S1W4
58868	229533.I1RBM3	pp	229533.I1RQA3
58869	229533.I1RBM3	pp	229533.I1RFZ2
58870	229533.I1RBM3	pp	229533.I1RCM6
58871	229533.I1RBM3	pp	229533.I1RQ80
58872	229533.I1RBM3	pp	229533.I1RPU4
58873	229533.I1RBM3	pp	229533.I1RZT1
58874	229533.I1RBM3	pp	229533.I1RWT6
58875	229533.I1RBM3	pp	229533.I1RBV5
58876	229533.I1RBM3	pp	229533.I1RD18
58877	229533.I1RBM3	pp	229533.I1RY44
58878	229533.I1RBM3	pp	229533.V6RVG3
58879	229533.I1RBM3	pp	229533.I1RME5
58880	229533.I1RBM3	pp	229533.I1RX38
58881	229533.I1RBM3	pp	229533.I1RD70
58882	229533.I1RBM3	pp	229533.I1REE1
58883	229533.I1RBM3	pp	229533.I1S2E9
58884	229533.I1RBM3	pp	229533.I1RRP7
58885	229533.I1RBM3	pp	229533.I1RMA4
58886	229533.I1RBM3	pp	229533.I1S1V5
58887	229533.I1RBM3	pp	229533.I1RMM9
58888	229533.I1RBM3	pp	229533.I1RJK7
58889	229533.I1RBM3	pp	229533.I1RRW0
58890	229533.I1RBM3	pp	229533.I1RNP7
58891	229533.I1RBM3	pp	229533.I1RSJ4
58892	229533.I1RBM3	pp	229533.I1RNC0
58893	229533.I1RBM3	pp	229533.I1RDB1
58894	229533.I1RBM3	pp	229533.I1RC22
58895	229533.I1RBM3	pp	229533.I1RDV9
58896	229533.I1RK92	pp	229533.I1S0B1
58897	229533.I1RK92	pp	229533.V6QVD5
58898	229533.I1RK92	pp	229533.I1RQR3
58899	229533.I1RK92	pp	229533.I1S0Z9
58900	229533.I1RK92	pp	229533.Q4I7F9
58901	229533.I1RK92	pp	229533.I1RPA0
58902	229533.I1RK92	pp	229533.I1S1X9
58903	229533.I1RK92	pp	229533.I1RZZ1
58904	229533.I1RK92	pp	229533.V6QXG1
58905	229533.I1RK92	pp	229533.Q4IB70
58906	229533.I1RK92	pp	229533.I1RPL3
58907	229533.I1RK92	pp	229533.Q4I1Y1
58908	229533.I1RK92	pp	229533.Q4IRI9
58909	229533.I1RK92	pp	229533.I1RWX8
58910	229533.I1RK92	pp	229533.I1RVT9
58911	229533.I1RK92	pp	229533.I1RZB5

58912	229533.I1RK92	pp	229533.Q4IP34
58913	229533.I1RK92	pp	229533.I1RZQ5
58914	229533.I1RK92	pp	229533.I1RVK5
58915	229533.I1RK92	pp	229533.I1RS83
58916	229533.I1RK92	pp	229533.I1RQP8
58917	229533.I1RK92	pp	229533.V6RIS2
58918	229533.I1RK92	pp	229533.Q4IAA0
58919	229533.I1RK92	pp	229533.I1RYW3
58920	229533.I1RK92	pp	229533.I1RNB8
58921	229533.I1RK92	pp	229533.I1S471
58922	229533.I1RK92	pp	229533.I1RR25
58923	229533.I1RK92	pp	229533.I1RSH9
58924	229533.I1RK92	pp	229533.I1RM25
58925	229533.I1RK92	pp	229533.I1RZ11
58926	229533.I1RK92	pp	229533.I1RQD8
58927	229533.I1RK92	pp	229533.I1RPY7
58928	229533.I1RK92	pp	229533.I1RSD4
58929	229533.I1RK92	pp	229533.I1RRQ4
58930	229533.I1RK92	pp	229533.I1RS28
58931	229533.I1RK92	pp	229533.I1RPY5
58932	229533.I1RK92	pp	229533.I1RYB1
58933	229533.I1RK92	pp	229533.I1RX47
58934	229533.I1RK92	pp	229533.I1RT47
58935	229533.I1RK92	pp	229533.I1RMV5
58936	229533.I1RK92	pp	229533.I1RRT0
58937	229533.I1RK92	pp	229533.I1S7M3
58938	229533.I1RK92	pp	229533.I1RK96
58939	229533.I1RK92	pp	229533.I1RX14
58940	229533.I1RK92	pp	229533.I1RX81
58941	229533.I1RK92	pp	229533.I1RRL0
58942	229533.I1RK92	pp	229533.V6RY33
58943	229533.I1RK92	pp	229533.I1RX24
58944	229533.I1RK92	pp	229533.I1RQQ7
58945	229533.I1RK92	pp	229533.I1RST1
58946	229533.I1RK92	pp	229533.I1RPW9
58947	229533.I1RK92	pp	229533.V6QX58
58948	229533.I1RK92	pp	229533.I1RND5
58949	229533.I1RK92	pp	229533.I1S0B6
58950	229533.I1RK92	pp	229533.I1RPY4
58951	229533.I1RK92	pp	229533.I1RX51
58952	229533.I1RK92	pp	229533.I1RSA4
58953	229533.I1RK92	pp	229533.I1S524
58954	229533.I1RK92	pp	229533.Q4IPB3
58955	229533.I1RK92	pp	229533.I1RU69
58956	229533.I1RK92	pp	229533.I1RZG7
58957	229533.I1RK92	pp	229533.I1S9L6
58958	229533.I1RK92	pp	229533.I1RN59
58959	229533.I1RK92	pp	229533.Q4IQW0
58960	229533.I1RK92	pp	229533.I1RMW7
58961	229533.I1RK92	pp	229533.I1RME9
58962	229533.I1RK92	pp	229533.I1S4Y4
58963	229533.I1RK92	pp	229533.I1RZB7
58964	229533.I1RK92	pp	229533.I1S4M5
58965	229533.I1RK92	pp	229533.I1RSL9
58966	229533.I1RK92	pp	229533.I1S9L5
58967	229533.I1RK92	pp	229533.I1S5S2
58968	229533.I1RK92	pp	229533.I1RWU2
58969	229533.I1RK92	pp	229533.I1S1C6
58970	229533.I1RK92	pp	229533.I1S7M2
58971	229533.I1RK92	pp	229533.I1S2Y5
58972	229533.I1RK92	pp	229533.I1S0R7
58973	229533.I1RK92	pp	229533.I1RXF2
58974	229533.I1RK92	pp	229533.I1RS75
58975	229533.I1RK92	pp	229533.I1RLU5
58976	229533.I1RK92	pp	229533.I1RRW3
58977	229533.I1RAA0	pp	229533.I1RRW9
58978	229533.I1RAA0	pp	229533.I1RMV8
58979	229533.I1RAA0	pp	229533.I1RZ21
58980	229533.I1RY32	pp	229533.I1S2K7
58981	229533.I1RY32	pp	229533.I1S267
58982	229533.I1RY32	pp	229533.I1RY44
58983	229533.I1RY32	pp	229533.I1RY81
58984	229533.I1RY32	pp	229533.V6RHX2

58985 229533.I1RY32 pp 229533.Q4IBU4
58986 229533.I1RY32 pp 229533.I1S0M4
58987 229533.I1RY32 pp 229533.I1RZ89
58988 229533.I1RY32 pp 229533.I1S296
58989 229533.I1RY32 pp 229533.I1RZX3
58990 229533.I1RY32 pp 229533.I1RZK4
58991 229533.I1RY32 pp 229533.I1S1D7
58992 229533.I1RY32 pp 229533.I1S2B6
58993 229533.I1RY32 pp 229533.I1S0V7
58994 229533.I1RY32 pp 229533.I1RZE7
58995 229533.I1RY32 pp 229533.I1RZC3
58996 229533.I1RY32 pp 229533.I1S0V6
58997 229533.I1RY32 pp 229533.I1RZ95
58998 229533.I1RY32 pp 229533.I1S969
58999 229533.I1RY32 pp 229533.I1S334
59000 229533.I1RY32 pp 229533.I1S3H8
59001 229533.I1RY32 pp 229533.I1S7A5
59002 229533.I1RY32 pp 229533.I1S224
59003 229533.I1RY32 pp 229533.I1S2E9
59004 229533.I1RY32 pp 229533.I1S048
59005 229533.I1RY32 pp 229533.I1RYP5
59006 229533.I1RY32 pp 229533.I1S968
59007 229533.I1RY32 pp 229533.I1SAB4
59008 229533.I1RY32 pp 229533.I1S9T6
59009 229533.I1RY32 pp 229533.I1RZ25
59010 229533.I1S524 pp 229533.Q4IPH4
59011 229533.I1S524 pp 229533.Q4I7F9
59012 229533.I1S524 pp 229533.I1S9L6
59013 229533.I1S524 pp 229533.V6RIS2
59014 229533.I1S524 pp 229533.I1S9L5
59015 229533.I1S524 pp 229533.Q4I1Y1
59016 229533.I1S524 pp 229533.V6RY33
59017 229533.I1S524 pp 229533.V6QXG1
59018 229533.I1S524 pp 229533.I1S5S2
59019 229533.I1S524 pp 229533.Q4HW67
59020 229533.I1S524 pp 229533.I1S7M2
59021 229533.I1S524 pp 229533.I1S7M3
59022 229533.I1RRQ0 pp 229533.I1RX09
59023 229533.I1RRQ0 pp 229533.I1RRQ1
59024 229533.I1RRQ0 pp 229533.I1RZV4
59025 229533.I1RRQ0 pp 229533.V6REE9
59026 229533.I1RRQ0 pp 229533.I1S095
59027 229533.I1RRQ0 pp 229533.I1RZD9
59028 229533.I1RRQ0 pp 229533.I1RWL6
59029 229533.I1S098 pp 229533.V6QV09
59030 229533.I1S098 pp 229533.V6RGI1
59031 229533.I1S098 pp 229533.Q4IJM4
59032 229533.I1S098 pp 229533.I1S7X9
59033 229533.I1S098 pp 229533.Q4I7L0
59034 229533.I1S098 pp 229533.I1S2E9
59035 229533.I1S098 pp 229533.Q4I963
59036 229533.I1S098 pp 229533.I1S4R1
59037 229533.I1S098 pp 229533.I1S9S6
59038 229533.I1S098 pp 229533.I1S0J7
59039 229533.I1S098 pp 229533.I1S224
59040 229533.I1S098 pp 229533.I1S0M6
59041 229533.A0A1C3YM86 pp 229533.I1RCV1
59042 229533.A0A1C3YM86 pp 229533.I1RXH3
59043 229533.A0A1C3YM86 pp 229533.I1RCM5
59044 229533.A0A1C3YM86 pp 229533.V6R3V4
59045 229533.A0A1C3YM86 pp 229533.I1RDN3
59046 229533.A0A1C3YM86 pp 229533.Q4HYR2
59047 229533.A0A1C3YM86 pp 229533.I1RJC5
59048 229533.A0A1C3YM86 pp 229533.I1RXB1
59049 229533.A0A1C3YM86 pp 229533.I1RCU2
59050 229533.A0A1C3YM86 pp 229533.I1RXV9
59051 229533.A0A1C3YM86 pp 229533.I1RA13
59052 229533.A0A1C3YM86 pp 229533.I1S4S5
59053 229533.A0A1C3YM86 pp 229533.I1RJY7
59054 229533.A0A1C3YM86 pp 229533.I1S8C1
59055 229533.A0A1C3YM86 pp 229533.I1RPR1
59056 229533.A0A1C3YM86 pp 229533.I1RH82
59057 229533.A0A1C3YM86 pp 229533.I1RF85

59058 229533.A0A1C3YM86 pp 229533.I1RD70
59059 229533.A0A1C3YM86 pp 229533.I1RSL2
59060 229533.A0A1C3YM86 pp 229533.I1RWM4
59061 229533.A0A1C3YM86 pp 229533.I1RPZ5
59062 229533.A0A1C3YM86 pp 229533.V6RE46
59063 229533.A0A1C3YM86 pp 229533.A0A1C3YN25
59064 229533.A0A1C3YM86 pp 229533.I1RN88
59065 229533.A0A1C3YM86 pp 229533.I1S6P5
59066 229533.A0A1C3YM86 pp 229533.V6RF62
59067 229533.A0A1C3YM86 pp 229533.I1RJT5
59068 229533.A0A1C3YM86 pp 229533.I1RFI4
59069 229533.A0A1C3YM86 pp 229533.I1RGC3
59070 229533.A0A1C3YM86 pp 229533.I1S148
59071 229533.A0A1C3YM86 pp 229533.V6RUB1
59072 229533.A0A1C3YM86 pp 229533.V6RV57
59073 229533.A0A1C3YM86 pp 229533.I1RZA7
59074 229533.A0A1C3YM86 pp 229533.V6RZD4
59075 229533.A0A1C3YM86 pp 229533.I1RYK0
59076 229533.A0A0E0RUQ7 pp 229533.I1RZJ1
59077 229533.A0A0E0RUQ7 pp 229533.I1RCA7
59078 229533.A0A0E0RUQ7 pp 229533.I1S9L5
59079 229533.A0A0E0RUQ7 pp 229533.A0A0E0S1T0
59080 229533.A0A0E0RUQ7 pp 229533.A0A1C3YK13
59081 229533.A0A0E0RUQ7 pp 229533.I1RKK4
59082 229533.A0A0E0RUQ7 pp 229533.I1S4M5
59083 229533.A0A0E0RUQ7 pp 229533.I1RJF9
59084 229533.A0A0E0RUQ7 pp 229533.I1RFT1
59085 229533.A0A0E0RUQ7 pp 229533.I1RX81
59086 229533.A0A0E0RUQ7 pp 229533.I1RAD1
59087 229533.A0A0E0RUQ7 pp 229533.I1RK96
59088 229533.A0A0E0RUQ7 pp 229533.I1S0B6
59089 229533.A0A0E0RUQ7 pp 229533.I1RME9
59090 229533.A0A0E0RUQ7 pp 229533.A0A1C3YKM6
59091 229533.A0A0E0RUQ7 pp 229533.I1RG13
59092 229533.A0A0E0RUQ7 pp 229533.I1RQC5
59093 229533.A0A0E0RUQ7 pp 229533.I1S1C6
59094 229533.A0A0E0RUQ7 pp 229533.A0A1C3YI62
59095 229533.A0A0E0RUQ7 pp 229533.A0A1C3YNC3
59096 229533.A0A0E0RUQ7 pp 229533.I1S9L6
59097 229533.A0A0E0RUQ7 pp 229533.I1RAE1
59098 229533.A0A0E0RUQ7 pp 229533.A0A1C3YL11
59099 229533.A0A0E0RUQ7 pp 229533.I1RYI9
59100 229533.A0A0E0RUQ7 pp 229533.A0A0E0SC46
59101 229533.A0A0E0RUQ7 pp 229533.I1RST1
59102 229533.A0A0E0RUQ7 pp 229533.I1RPZ3
59103 229533.A0A0E0RUQ7 pp 229533.I1RWW5
59104 229533.A0A0E0RUQ7 pp 229533.I1RAF3
59105 229533.A0A0E0RUQ7 pp 229533.I1RLU5
59106 229533.A0A0E0RUQ7 pp 229533.I1RT17
59107 229533.A0A0E0RUQ7 pp 229533.I1RS75
59108 229533.A0A0E0RUQ7 pp 229533.I1S2Y5
59109 229533.A0A0E0RUQ7 pp 229533.I1S4Y4
59110 229533.A0A0E0RUQ7 pp 229533.I1RN59
59111 229533.A0A0E0RUQ7 pp 229533.I1RBV3
59112 229533.A0A0E0RUQ7 pp 229533.I1RBU6
59113 229533.A0A0E0RUQ7 pp 229533.I1RYZ7
59114 229533.A0A0E0RUQ7 pp 229533.I1RCS2
59115 229533.A0A0E0RUQ7 pp 229533.I1RQB5
59116 229533.A0A0E0RUQ7 pp 229533.I1RXF2
59117 229533.A0A0E0RUQ7 pp 229533.Q4IBK5
59118 229533.A0A0E0RUQ7 pp 229533.I1RMW7
59119 229533.A0A0E0RUQ7 pp 229533.I1RB50
59120 229533.A0A0E0RUQ7 pp 229533.I1S7M2
59121 229533.A0A0E0RUQ7 pp 229533.I1RSA4
59122 229533.A0A0E0RUQ7 pp 229533.I1RND5
59123 229533.A0A0E0RUQ7 pp 229533.I1RT47
59124 229533.A0A0E0RUQ7 pp 229533.I1S5S2
59125 229533.A0A0E0RUQ7 pp 229533.A0A0E0S8Y8
59126 229533.A0A0E0RUQ7 pp 229533.I1RN09
59127 229533.A0A0E0RUQ7 pp 229533.I1RT35
59128 229533.A0A0E0RUQ7 pp 229533.I1RCI4
59129 229533.A0A0E0RUQ7 pp 229533.I1RWX8
59130 229533.A0A0E0RUQ7 pp 229533.I1RG17

59131 229533.A0A0E0RUQ7 pp 229533.I1RGF2
59132 229533.A0A0E0RUQ7 pp 229533.V6RIS2
59133 229533.A0A0E0RUQ7 pp 229533.I1REX2
59134 229533.A0A0E0RUQ7 pp 229533.V6QXG1
59135 229533.A0A0E0RUQ7 pp 229533.I1RMV5
59136 229533.A0A0E0RUQ7 pp 229533.I1RS83
59137 229533.A0A0E0RUQ7 pp 229533.I1RJG6
59138 229533.A0A0E0RUQ7 pp 229533.I1S471
59139 229533.A0A0E0RUQ7 pp 229533.I1RX51
59140 229533.A0A0E0RUQ7 pp 229533.I1RCH7
59141 229533.A0A0E0RUQ7 pp 229533.I1RCI3
59142 229533.A0A0E0RUQ7 pp 229533.V6RY33
59143 229533.A0A0E0RUQ7 pp 229533.I1S524
59144 229533.A0A0E0RUQ7 pp 229533.I1RBH8
59145 229533.A0A0E0RUQ7 pp 229533.I1RPY5
59146 229533.A0A0E0RUQ7 pp 229533.I1S7M3
59147 229533.A0A0E0RUQ7 pp 229533.Q4IPB3
59148 229533.A0A0E0RUQ7 pp 229533.I1RX47
59149 229533.A0A0E0RUQ7 pp 229533.A0A1C3YHK3
59150 229533.A0A0E0RUQ7 pp 229533.I1RS28
59151 229533.A0A0E0RUQ7 pp 229533.I1RPY4
59152 229533.A0A0E0RUQ7 pp 229533.I1RFQ1
59153 229533.A0A0E0RUQ7 pp 229533.I1R9N6
59154 229533.A0A0E0RUQ7 pp 229533.I1RSD4
59155 229533.A0A0E0RUQ7 pp 229533.I1RSA8
59156 229533.A0A0E0RUQ7 pp 229533.I1RCQ6
59157 229533.A0A0E0RUQ7 pp 229533.I1RJV2
59158 229533.A0A0E0RUQ7 pp 229533.I1RK72
59159 229533.A0A0E0RUQ7 pp 229533.Q4I662
59160 229533.A0A0E0RUQ7 pp 229533.I1RRL0
59161 229533.A0A0E0RUQ7 pp 229533.Q4IAA0
59162 229533.A0A0E0RUQ7 pp 229533.I1S0B1
59163 229533.A0A0E0RUQ7 pp 229533.I1RZG7
59164 229533.A0A0E0RUQ7 pp 229533.I1RG90
59165 229533.A0A0E0RUQ7 pp 229533.I1RYJ9
59166 229533.A0A0E0RUQ7 pp 229533.I1RMM5
59167 229533.A0A0E0RUQ7 pp 229533.Q4IRI9
59168 229533.A0A0E0RUQ7 pp 229533.I1RNB8
59169 229533.A0A0E0RUQ7 pp 229533.I1S0Z9
59170 229533.A0A0E0RUQ7 pp 229533.Q4IPH4
59171 229533.A0A0E0RUQ7 pp 229533.I1RZ11
59172 229533.A0A0E0RUQ7 pp 229533.I1RQD8
59173 229533.A0A0E0RUQ7 pp 229533.I1RYM5
59174 229533.A0A0E0RUQ7 pp 229533.Q4HZ68
59175 229533.A0A0E0RUQ7 pp 229533.I1S193
59176 229533.A0A0E0RUQ7 pp 229533.I1RQP8
59177 229533.A0A0E0RUQ7 pp 229533.I1RNF9
59178 229533.A0A0E0RUQ7 pp 229533.I1RRQ4
59179 229533.A0A0E0RUQ7 pp 229533.I1RCR5
59180 229533.A0A0E0RUQ7 pp 229533.I1RPL3
59181 229533.A0A0E0RUQ7 pp 229533.I1RZZ1
59182 229533.A0A0E0RUQ7 pp 229533.Q4I7F9
59183 229533.A0A0E0RUQ7 pp 229533.I1RK90
59184 229533.A0A0E0RUQ7 pp 229533.I1RYW3
59185 229533.A0A0E0RUQ7 pp 229533.Q4I1Y1
59186 229533.A0A0E0RUQ7 pp 229533.I1RAC1
59187 229533.A0A0E0RUQ7 pp 229533.I1RZQ5
59188 229533.A0A0E0RUQ7 pp 229533.A0A1C3YIM4
59189 229533.A0A0E0RUQ7 pp 229533.I1RQR3
59190 229533.A0A0E0RUQ7 pp 229533.I1RT91
59191 229533.A0A0E0RUQ7 pp 229533.I1RG57
59192 229533.A0A0E0RUQ7 pp 229533.A0A1C3YK19
59193 229533.A0A0E0RUQ7 pp 229533.I1RJM9
59194 229533.A0A0E0RUQ7 pp 229533.I1RC84
59195 229533.A0A0E0RUQ7 pp 229533.I1RBZ8
59196 229533.A0A0E0RUQ7 pp 229533.I1RA36
59197 229533.A0A0E0RUQ7 pp 229533.I1RPA0
59198 229533.A0A0E0RUQ7 pp 229533.I1RK92
59199 229533.I1RYW2 pp 229533.I1S0N7
59200 229533.I1RYW2 pp 229533.Q4IEK8
59201 229533.I1RYW2 pp 229533.I1RYW3
59202 229533.I1RHG5 pp 229533.I1S2C4
59203 229533.I1RHG5 pp 229533.I1RYJ6

59204 229533.I1RHG5 pp 229533.I1RW71
59205 229533.I1RHG5 pp 229533.V6QT89
59206 229533.I1RHG5 pp 229533.V6RN69
59207 229533.I1RHG5 pp 229533.I1RY74
59208 229533.I1RHG5 pp 229533.I1RK03
59209 229533.I1RHG5 pp 229533.I1RJZ7
59210 229533.I1RHG5 pp 229533.V6RLM7
59211 229533.I1RHG5 pp 229533.I1RSU2
59212 229533.I1RHG5 pp 229533.I1RP97
59213 229533.I1RHG5 pp 229533.I1RR68
59214 229533.I1RHG5 pp 229533.I1RWL3
59215 229533.I1RHG5 pp 229533.I1RST9
59216 229533.I1RHG5 pp 229533.I1RPK0
59217 229533.I1RHG5 pp 229533.I1RZN1
59218 229533.I1RHG5 pp 229533.I1RSS7
59219 229533.I1RHG5 pp 229533.I1S9Z4
59220 229533.I1RHG5 pp 229533.I1RW54
59221 229533.I1RHG5 pp 229533.I1RWS4
59222 229533.I1RHG5 pp 229533.I1RNG4
59223 229533.I1RHG5 pp 229533.I1RWW7
59224 229533.A0A1C3YN69 pp 229533.I1RFI6
59225 229533.A0A1C3YN69 pp 229533.I1S998
59226 229533.A0A1C3YN69 pp 229533.I1RCA5
59227 229533.A0A1C3YN69 pp 229533.I1RBU5
59228 229533.A0A1C3YN69 pp 229533.Q4IJH1
59229 229533.A0A1C3YN69 pp 229533.I1RRT1
59230 229533.A0A1C3YN69 pp 229533.I1RC91
59231 229533.A0A1C3YN69 pp 229533.I1RQ24
59232 229533.A0A1C3YN69 pp 229533.I1RQ13
59233 229533.A0A1C3YN69 pp 229533.I1S5Z3
59234 229533.A0A1C3YN69 pp 229533.I1RUR0
59235 229533.A0A1C3YN69 pp 229533.I1S004
59236 229533.A0A1C3YN69 pp 229533.I1RZ94
59237 229533.A0A1C3YN69 pp 229533.I1RD18
59238 229533.A0A1C3YN69 pp 229533.I1RD01
59239 229533.A0A1C3YN69 pp 229533.I1S2E9
59240 229533.A0A1C3YN69 pp 229533.I1RPK4
59241 229533.A0A1C3YN69 pp 229533.I1RVV5
59242 229533.A0A1C3YN69 pp 229533.I1RBW1
59243 229533.A0A1C3YN69 pp 229533.I1RZ86
59244 229533.A0A1C3YN69 pp 229533.I1S987
59245 229533.A0A1C3YN69 pp 229533.I1RY29
59246 229533.A0A1C3YN69 pp 229533.Q4IAS1
59247 229533.A0A1C3YN69 pp 229533.I1RU52
59248 229533.A0A1C3YN69 pp 229533.I1RTG0
59249 229533.A0A1C3YN69 pp 229533.I1RRX6
59250 229533.A0A1C3YN69 pp 229533.I1S224
59251 229533.A0A1C3YN69 pp 229533.I1RAL9
59252 229533.A0A1C3YN69 pp 229533.I1RRY1
59253 229533.A0A1C3YN69 pp 229533.I1RZ12
59254 229533.A0A1C3YN69 pp 229533.I1RBW0
59255 229533.A0A1C3YN69 pp 229533.I1RWM6
59256 229533.A0A1C3YN69 pp 229533.I1RFL7
59257 229533.A0A1C3YN69 pp 229533.I1RVW0
59258 229533.A0A1C3YN69 pp 229533.Q4IB96
59259 229533.A0A1C3YN69 pp 229533.I1RPQ1
59260 229533.A0A1C3YN69 pp 229533.I1RQA3
59261 229533.A0A1C3YN69 pp 229533.I1RTB2
59262 229533.A0A1C3YN69 pp 229533.I1RSR9
59263 229533.A0A1C3YN69 pp 229533.I1RY58
59264 229533.A0A1C3YN69 pp 229533.I1RSN7
59265 229533.A0A1C3YN69 pp 229533.I1S0D5
59266 229533.A0A1C3YN69 pp 229533.I1RZI4
59267 229533.A0A1C3YN69 pp 229533.Q4I7N9
59268 229533.A0A1C3YN69 pp 229533.I1RDH0
59269 229533.A0A1C3YN69 pp 229533.I1RDJ8
59270 229533.A0A1C3YN69 pp 229533.I1RRN5
59271 229533.A0A1C3YN69 pp 229533.I1RA10
59272 229533.A0A1C3YN69 pp 229533.I1RS41
59273 229533.A0A1C3YN69 pp 229533.I1RZT1
59274 229533.A0A1C3YN69 pp 229533.I1RMJ8
59275 229533.A0A1C3YN69 pp 229533.I1RYC3
59276 229533.A0A1C3YN69 pp 229533.I1RMQ7

59277	229533.A0A1C3YN69	pp	229533.I1RG77
59278	229533.A0A1C3YN69	pp	229533.I1RPF5
59279	229533.A0A1C3YN69	pp	229533.I1RN55
59280	229533.A0A1C3YN69	pp	229533.I1RA38
59281	229533.A0A1C3YN69	pp	229533.I1RUG2
59282	229533.A0A1C3YN69	pp	229533.I1S4P6
59283	229533.A0A1C3YN69	pp	229533.I1RN57
59284	229533.A0A1C3YN69	pp	229533.I1RV91
59285	229533.A0A1C3YN69	pp	229533.I1RA12
59286	229533.A0A1C3YN69	pp	229533.I1RF02
59287	229533.A0A1C3YN69	pp	229533.I1RKE9
59288	229533.A0A1C3YN69	pp	229533.I1RY44
59289	229533.A0A1C3YN69	pp	229533.I1RQS4
59290	229533.A0A1C3YN69	pp	229533.I1RWI8
59291	229533.A0A1C3YN69	pp	229533.I1RJK7
59292	229533.A0A1C3YN69	pp	229533.I1S0A6
59293	229533.A0A1C3YN69	pp	229533.I1RWT6
59294	229533.A0A1C3YN69	pp	229533.I1RDV9
59295	229533.A0A1C3YN69	pp	229533.I1RRW0
59296	229533.A0A1C3YN69	pp	229533.I1RSP7
59297	229533.A0A1C3YN69	pp	229533.I1RD19
59298	229533.A0A1C3YN69	pp	229533.I1REE1
59299	229533.A0A1C3YN69	pp	229533.I1RMA4
59300	229533.A0A1C3YN69	pp	229533.I1RSC7
59301	229533.A0A1C3YN69	pp	229533.I1RME5
59302	229533.A0A1C3YN69	pp	229533.I1RX64
59303	229533.A0A1C3YN69	pp	229533.Q4IJ84
59304	229533.A0A1C3YN69	pp	229533.Q4INS6
59305	229533.A0A1C3YN69	pp	229533.I1RBC8
59306	229533.A0A1C3YN69	pp	229533.I1RZP8
59307	229533.A0A1C3YN69	pp	229533.I1RBN9
59308	229533.A0A1C3YN69	pp	229533.I1RC54
59309	229533.A0A1C3YN69	pp	229533.I1RQP3
59310	229533.A0A1C3YN69	pp	229533.I1S209
59311	229533.A0A1C3YI62	pp	229533.A0A1C3YI81
59312	229533.A0A1C3YI62	pp	229533.I1RK72
59313	229533.A0A1C3YI62	pp	229533.I1RRL0
59314	229533.A0A1C3YI62	pp	229533.A0A1C3YN75
59315	229533.A0A1C3YI62	pp	229533.I1RSD4
59316	229533.A0A1C3YI62	pp	229533.I1RS28
59317	229533.A0A1C3YI62	pp	229533.I1RFQ1
59318	229533.A0A1C3YI62	pp	229533.I1RPY5
59319	229533.A0A1C3YI62	pp	229533.I1S7M3
59320	229533.A0A1C3YI62	pp	229533.I1S8U9
59321	229533.A0A1C3YI62	pp	229533.I1RSH9
59322	229533.A0A1C3YI62	pp	229533.I1RM25
59323	229533.A0A1C3YI62	pp	229533.A0A1C3YJW0
59324	229533.A0A1C3YI62	pp	229533.I1RNB8
59325	229533.A0A1C3YI62	pp	229533.A0A1C3YL22
59326	229533.A0A1C3YI62	pp	229533.I1RJV2
59327	229533.A0A1C3YI62	pp	229533.Q4IRI9
59328	229533.A0A1C3YI62	pp	229533.I1RT47
59329	229533.A0A1C3YI62	pp	229533.I1RG17
59330	229533.A0A1C3YI62	pp	229533.Q4IAA0
59331	229533.A0A1C3YI62	pp	229533.I1RN09
59332	229533.A0A1C3YI62	pp	229533.I1RQD8
59333	229533.A0A1C3YI62	pp	229533.I1RMM5
59334	229533.A0A1C3YI62	pp	229533.A0A1C3YJ42
59335	229533.A0A1C3YI62	pp	229533.I1RX51
59336	229533.A0A1C3YI62	pp	229533.I1RWX8
59337	229533.A0A1C3YI62	pp	229533.I1RVT9
59338	229533.A0A1C3YI62	pp	229533.I1RK90
59339	229533.A0A1C3YI62	pp	229533.I1RQP8
59340	229533.A0A1C3YI62	pp	229533.I1RVK5
59341	229533.A0A1C3YI62	pp	229533.I1RZ11
59342	229533.A0A1C3YI62	pp	229533.I1RT35
59343	229533.A0A1C3YI62	pp	229533.I1S471
59344	229533.A0A1C3YI62	pp	229533.I1RF91
59345	229533.A0A1C3YI62	pp	229533.V6QXG1
59346	229533.A0A1C3YI62	pp	229533.V6QX58
59347	229533.A0A1C3YI62	pp	229533.V6RIS2
59348	229533.A0A1C3YI62	pp	229533.I1RSA4
59349	229533.A0A1C3YI62	pp	229533.I1RS83

59350 229533.A0A1C3YI62 pp 229533.I1RG57
59351 229533.A0A1C3YI62 pp 229533.I1RAC1
59352 229533.A0A1C3YI62 pp 229533.I1RND5
59353 229533.A0A1C3YI62 pp 229533.Q4I1Y1
59354 229533.A0A1C3YI62 pp 229533.Q4IP34
59355 229533.A0A1C3YI62 pp 229533.I1RQQ7
59356 229533.A0A1C3YI62 pp 229533.I1RC84
59357 229533.A0A1C3YI62 pp 229533.I1RE24
59358 229533.A0A1C3YI62 pp 229533.I1RZQ5
59359 229533.A0A1C3YI62 pp 229533.A0A1C3YK19
59360 229533.A0A1C3YI62 pp 229533.Q4IB70
59361 229533.A0A1C3YI62 pp 229533.I1RCN5
59362 229533.A0A1C3YI62 pp 229533.V6RY33
59363 229533.A0A1C3YI62 pp 229533.Q4I7F9
59364 229533.A0A1C3YI62 pp 229533.I1RPA0
59365 229533.A0A1C3YI62 pp 229533.I1RSH4
59366 229533.A0A1C3YI62 pp 229533.I1RPL3
59367 229533.A0A1C3YI62 pp 229533.I1RK92
59368 229533.A0A1C3YI62 pp 229533.I1RZZ1
59369 229533.A0A1C3YI62 pp 229533.I1RSA8
59370 229533.A0A1C3YI62 pp 229533.I1RX47
59371 229533.A0A1C3YI62 pp 229533.I1RBZ8
59372 229533.A0A1C3YI62 pp 229533.I1RQZ2
59373 229533.A0A1C3YI62 pp 229533.I1RA36
59374 229533.A0A1C3YI62 pp 229533.I1RG13
59375 229533.A0A1C3YI62 pp 229533.I1RZL5
59376 229533.A0A1C3YI62 pp 229533.I1RQB5
59377 229533.A0A1C3YI62 pp 229533.I1RQR3
59378 229533.A0A1C3YI62 pp 229533.I1S214
59379 229533.A0A1C3YI62 pp 229533.I1RT91
59380 229533.A0A1C3YI62 pp 229533.A0A1C3YIA6
59381 229533.A0A1C3YI62 pp 229533.I1RD56
59382 229533.A0A1C3YI62 pp 229533.A0A1C3YIM4
59383 229533.A0A1C3YI62 pp 229533.I1S246
59384 229533.A0A1C3YI62 pp 229533.I1RAF3
59385 229533.A0A1C3YI62 pp 229533.I1RLU5
59386 229533.A0A1C3YI62 pp 229533.I1S1X9
59387 229533.A0A1C3YI62 pp 229533.I1S9L6
59388 229533.A0A1C3YI62 pp 229533.I1S0Z9
59389 229533.A0A1C3YI62 pp 229533.Q4IBK5
59390 229533.A0A1C3YI62 pp 229533.I1RAU1
59391 229533.A0A1C3YI62 pp 229533.I1RCS2
59392 229533.A0A1C3YI62 pp 229533.I1RG90
59393 229533.A0A1C3YI62 pp 229533.A0A1C3YNC3
59394 229533.A0A1C3YI62 pp 229533.I1RBU6
59395 229533.A0A1C3YI62 pp 229533.I1RBV3
59396 229533.A0A1C3YI62 pp 229533.I1RRG3
59397 229533.A0A1C3YI62 pp 229533.A0A1C3YM53
59398 229533.A0A1C3YI62 pp 229533.I1S0B1
59399 229533.A0A1C3YI62 pp 229533.I1S2Y5
59400 229533.A0A1C3YI62 pp 229533.I1S0R7
59401 229533.A0A1C3YI62 pp 229533.I1RS75
59402 229533.A0A1C3YI62 pp 229533.I1S5S2
59403 229533.A0A1C3YI62 pp 229533.V6QVD5
59404 229533.A0A1C3YI62 pp 229533.I1RXF2
59405 229533.A0A1C3YI62 pp 229533.I1RCR5
59406 229533.A0A1C3YI62 pp 229533.I1RAV4
59407 229533.A0A1C3YI62 pp 229533.I1RDD3
59408 229533.A0A1C3YI62 pp 229533.I1RFT1
59409 229533.A0A1C3YI62 pp 229533.I1RFP9
59410 229533.A0A1C3YI62 pp 229533.I1RRQ4
59411 229533.A0A1C3YI62 pp 229533.I1S7M2
59412 229533.A0A1C3YI62 pp 229533.A0A1C3YL25
59413 229533.A0A1C3YI62 pp 229533.I1S4M5
59414 229533.A0A1C3YI62 pp 229533.I1R9X6
59415 229533.A0A1C3YI62 pp 229533.I1RJF9
59416 229533.A0A1C3YI62 pp 229533.I1RA46
59417 229533.A0A1C3YI62 pp 229533.Q4IPH4
59418 229533.A0A1C3YI62 pp 229533.I1RWU2
59419 229533.A0A1C3YI62 pp 229533.I1RYI9
59420 229533.A0A1C3YI62 pp 229533.I1S1C6
59421 229533.A0A1C3YI62 pp 229533.I1S8V0
59422 229533.A0A1C3YI62 pp 229533.I1RK19

59423	229533.A0A1C3YI62	pp	229533.A0A1C3YK13
59424	229533.A0A1C3YI62	pp	229533.I1RYW3
59425	229533.A0A1C3YI62	pp	229533.I1S9L5
59426	229533.A0A1C3YI62	pp	229533.I1RCA7
59427	229533.A0A1C3YI62	pp	229533.I1RQC5
59428	229533.A0A1C3YI62	pp	229533.I1R9Z0
59429	229533.A0A1C3YI62	pp	229533.A0A1C3YKM6
59430	229533.A0A1C3YI62	pp	229533.I1RAD1
59431	229533.A0A1C3YI62	pp	229533.Q4IPN9
59432	229533.A0A1C3YI62	pp	229533.I1S4Y4
59433	229533.A0A1C3YI62	pp	229533.I1RMV5
59434	229533.A0A1C3YI62	pp	229533.I1RT17
59435	229533.A0A1C3YI62	pp	229533.I1RPZ3
59436	229533.A0A1C3YI62	pp	229533.I1RCI4
59437	229533.A0A1C3YI62	pp	229533.I1RZG7
59438	229533.A0A1C3YI62	pp	229533.I1REX2
59439	229533.A0A1C3YI62	pp	229533.I1RCI3
59440	229533.A0A1C3YI62	pp	229533.I1RCD6
59441	229533.A0A1C3YI62	pp	229533.I1RJG6
59442	229533.A0A1C3YI62	pp	229533.I1RMW7
59443	229533.A0A1C3YI62	pp	229533.I1RTS2
59444	229533.A0A1C3YI62	pp	229533.I1R9N6
59445	229533.A0A1C3YI62	pp	229533.I1R9Z2
59446	229533.A0A1C3YI62	pp	229533.I1RCQ6
59447	229533.A0A1C3YI62	pp	229533.I1RPY4
59448	229533.A0A1C3YI62	pp	229533.I1RWW5
59449	229533.A0A1C3YI62	pp	229533.Q4IPB3
59450	229533.A0A1C3YI62	pp	229533.I1RN59
59451	229533.A0A1C3YI62	pp	229533.I1RU69
59452	229533.A0A1C3YI62	pp	229533.I1RK96
59453	229533.A0A1C3YI62	pp	229533.I1S524
59454	229533.A0A1C3YI62	pp	229533.I1RX24
59455	229533.A0A1C3YI62	pp	229533.I1RB50
59456	229533.A0A1C3YI62	pp	229533.A0A1C3YL11
59457	229533.A0A1C3YI62	pp	229533.I1RDE9
59458	229533.A0A1C3YI62	pp	229533.I1RAE1
59459	229533.A0A1C3YI62	pp	229533.I1RDC1
59460	229533.A0A1C3YI62	pp	229533.I1S0B6
59461	229533.A0A1C3YI62	pp	229533.I1RX81
59462	229533.A0A1C3YI62	pp	229533.I1RST1
59463	229533.I1RNM8	pp	229533.I1RRP3
59464	229533.I1RNM8	pp	229533.I1RQA2
59465	229533.I1RNM8	pp	229533.I1S549
59466	229533.I1RNM8	pp	229533.I1S0K9
59467	229533.I1RNM8	pp	229533.I1S1Z9
59468	229533.I1RNM8	pp	229533.I1RRU2
59469	229533.I1S5R5	pp	229533.I1S5T5
59470	229533.I1S5R5	pp	229533.I1S7Q8
59471	229533.I1S5R5	pp	229533.V6RXW9
59472	229533.I1S5R5	pp	229533.I1S8S9
59473	229533.I1S5R5	pp	229533.V6RG22
59474	229533.I1S5R5	pp	229533.V6RJP8
59475	229533.I1S5R5	pp	229533.I1S5R6
59476	229533.I1S5R5	pp	229533.Q5I2J3
59477	229533.I1S5R5	pp	229533.I1S724
59478	229533.I1S5R5	pp	229533.I1S6S6
59479	229533.I1RAD1	pp	229533.I1RS83
59480	229533.I1RAD1	pp	229533.I1RT47
59481	229533.I1RAD1	pp	229533.I1RPA0
59482	229533.I1RAD1	pp	229533.I1RQP8
59483	229533.I1RAD1	pp	229533.I1RYW3
59484	229533.I1RAD1	pp	229533.V6RIS2
59485	229533.I1RAD1	pp	229533.I1S3V2
59486	229533.I1RAD1	pp	229533.I1S2C0
59487	229533.I1RAD1	pp	229533.I1S238
59488	229533.I1RAD1	pp	229533.I1S7M3
59489	229533.I1RAD1	pp	229533.I1RSA8
59490	229533.I1RAD1	pp	229533.I1S0B1
59491	229533.I1RAD1	pp	229533.I1RG90
59492	229533.I1RAD1	pp	229533.I1RSA4
59493	229533.I1RAD1	pp	229533.I1RND5
59494	229533.I1RAD1	pp	229533.Q4IPH4
59495	229533.I1RAD1	pp	229533.I1RRQ4

59496 229533.I1RAD1 pp 229533.V6QXG1
59497 229533.I1RAD1 pp 229533.I1RQD8
59498 229533.I1RAD1 pp 229533.I1RT35
59499 229533.I1RAD1 pp 229533.I1RCR5
59500 229533.I1RAD1 pp 229533.I1RZ11
59501 229533.I1RAD1 pp 229533.I1RFP9
59502 229533.I1RAD1 pp 229533.I1RZZ1
59503 229533.I1RAD1 pp 229533.Q4I7F9
59504 229533.I1RAD1 pp 229533.I1RWX8
59505 229533.I1RAD1 pp 229533.I1RT04
59506 229533.I1RAD1 pp 229533.Q4HZ47
59507 229533.I1RAD1 pp 229533.I1S9L6
59508 229533.I1RAD1 pp 229533.V6RY33
59509 229533.I1RAD1 pp 229533.I1RSY2
59510 229533.I1RAD1 pp 229533.I1S8V4
59511 229533.I1RAD1 pp 229533.I1RBH8
59512 229533.I1RAD1 pp 229533.I1RQR3
59513 229533.I1RAD1 pp 229533.I1RJH1
59514 229533.I1RAD1 pp 229533.I1RS75
59515 229533.I1RAD1 pp 229533.I1RYI6
59516 229533.I1RAD1 pp 229533.I1RKF8
59517 229533.I1RAD1 pp 229533.I1RYI9
59518 229533.I1RAD1 pp 229533.I1RFQ1
59519 229533.I1RAD1 pp 229533.Q4HW67
59520 229533.I1RAD1 pp 229533.I1S7A8
59521 229533.I1RAD1 pp 229533.I1RS28
59522 229533.I1RAD1 pp 229533.I1S9L5
59523 229533.I1RAD1 pp 229533.I1RCA7
59524 229533.I1RAD1 pp 229533.I1RSD4
59525 229533.I1RAD1 pp 229533.I1S4Y4
59526 229533.I1RAD1 pp 229533.I1S289
59527 229533.I1RAD1 pp 229533.I1RKK4
59528 229533.I1RAD1 pp 229533.I1RZ40
59529 229533.I1RAD1 pp 229533.I1RK72
59530 229533.I1RAD1 pp 229533.I1RFT1
59531 229533.I1RAD1 pp 229533.I1RBV3
59532 229533.I1RAD1 pp 229533.I1S4M5
59533 229533.I1RAD1 pp 229533.I1RMM5
59534 229533.I1RAD1 pp 229533.I1RT98
59535 229533.I1RAD1 pp 229533.I1RBU6
59536 229533.I1RAD1 pp 229533.I1RNB8
59537 229533.I1RAD1 pp 229533.I1S7M2
59538 229533.I1RAD1 pp 229533.I1S0B6
59539 229533.I1RAD1 pp 229533.I1RBZ8
59540 229533.I1RAD1 pp 229533.I1RC84
59541 229533.I1RAD1 pp 229533.I1RIS3
59542 229533.I1RAD1 pp 229533.I1RAE1
59543 229533.I1RAD1 pp 229533.I1RK92
59544 229533.I1RAD1 pp 229533.I1RXF2
59545 229533.I1RAD1 pp 229533.I1S5S2
59546 229533.I1RAD1 pp 229533.I1RN48
59547 229533.I1RAD1 pp 229533.I1S8V3
59548 229533.I1RAD1 pp 229533.I1RNE7
59549 229533.I1RAD1 pp 229533.I1S0R7
59550 229533.I1RAD1 pp 229533.I1RMV5
59551 229533.I1RAD1 pp 229533.I1RMW7
59552 229533.I1RAD1 pp 229533.Q4IF76
59553 229533.I1RAD1 pp 229533.I1RCI3
59554 229533.I1RAD1 pp 229533.I1RPY4
59555 229533.I1RAD1 pp 229533.I1RB50
59556 229533.I1RAD1 pp 229533.I1S524
59557 229533.I1RAD1 pp 229533.I1S8E2
59558 229533.I1RAD1 pp 229533.I1REX2
59559 229533.I1RAD1 pp 229533.I1RZG7
59560 229533.I1RAD1 pp 229533.I1RCQ6
59561 229533.I1RAD1 pp 229533.I1RX81
59562 229533.I1RAD1 pp 229533.I1RCI4
59563 229533.I1RAD1 pp 229533.I1RK96
59564 229533.I1RAD1 pp 229533.I1RW25
59565 229533.I1RAD1 pp 229533.I1RQ13
59566 229533.A0A0E0SIE1 pp 229533.I1RRJ7
59567 229533.A0A0E0SIE1 pp 229533.I1RN65
59568 229533.A0A0E0SIE1 pp 229533.I1RB53

59569 229533.A0A0E0SIE1 pp 229533.I1RWN8
59570 229533.A0A0E0SIE1 pp 229533.I1RRF9
59571 229533.A0A0E0SIE1 pp 229533.A0A1C3YIP6
59572 229533.A0A0E0SIE1 pp 229533.V6REE9
59573 229533.A0A0E0SIE1 pp 229533.A0A1C3YN09
59574 229533.A0A0E0SIE1 pp 229533.I1RH68
59575 229533.A0A0E0SIE1 pp 229533.I1RPU4
59576 229533.A0A0E0SIE1 pp 229533.I1RTB7
59577 229533.A0A0E0SIE1 pp 229533.I1RMW6
59578 229533.A0A0E0SIE1 pp 229533.P0C431
59579 229533.A0A0E0SIE1 pp 229533.I1RAH8
59580 229533.A0A0E0SIE1 pp 229533.I1RN49
59581 229533.A0A0E0SIE1 pp 229533.Q4ICM0
59582 229533.A0A0E0SIE1 pp 229533.I1RM67
59583 229533.A0A0E0SIE1 pp 229533.I1RE89
59584 229533.A0A0E0SIE1 pp 229533.I1RD70
59585 229533.A0A0E0SIE1 pp 229533.I1S0S6
59586 229533.A0A0E0SIE1 pp 229533.A0A1C3YIA8
59587 229533.A0A0E0SIE1 pp 229533.I1RTA3
59588 229533.A0A0E0SIE1 pp 229533.Q4HZB8
59589 229533.A0A0E0SIE1 pp 229533.I1RQG9
59590 229533.A0A0E0SIE1 pp 229533.I1RCP1
59591 229533.A0A0E0SIE1 pp 229533.I1RTE6
59592 229533.A0A0E0SIE1 pp 229533.I1RS64
59593 229533.A0A0E0SIE1 pp 229533.A0A1C3YII1
59594 229533.A0A0E0SIE1 pp 229533.I1RCW7
59595 229533.A0A0E0SIE1 pp 229533.A0A1C3YLH6
59596 229533.A0A0E0SIE1 pp 229533.I1RT36
59597 229533.A0A0E0SIE1 pp 229533.Q4IPC8
59598 229533.A0A0E0SIE1 pp 229533.I1RP04
59599 229533.A0A0E0SIE1 pp 229533.A0A1C3YL06
59600 229533.A0A0E0SIE1 pp 229533.I1RGQ9
59601 229533.A0A0E0SIE1 pp 229533.I1RCD4
59602 229533.A0A0E0SIE1 pp 229533.I1RZG0
59603 229533.A0A0E0SIE1 pp 229533.I1RCM1
59604 229533.A0A0E0SIE1 pp 229533.I1RQN9
59605 229533.A0A0E0SIE1 pp 229533.I1RTK5
59606 229533.A0A0E0SIE1 pp 229533.I1S1B6
59607 229533.A0A0E0SIE1 pp 229533.A0A1C3YM61
59608 229533.A0A0E0SIE1 pp 229533.I1RMP2
59609 229533.A0A0E0SIE1 pp 229533.I1S7X9
59610 229533.A0A0E0SIE1 pp 229533.I1RE19
59611 229533.A0A0E0SIE1 pp 229533.A0A1C3YJK9
59612 229533.A0A0E0SIE1 pp 229533.A0A0E0SNM6
59613 229533.A0A1C3YIJ9 pp 229533.I1RJS7
59614 229533.A0A1C3YIJ9 pp 229533.I1RMK7
59615 229533.A0A1C3YIJ9 pp 229533.I1S6P5
59616 229533.A0A1C3YIJ9 pp 229533.I1RWA3
59617 229533.A0A1C3YIJ9 pp 229533.I1RAI4
59618 229533.A0A1C3YIJ9 pp 229533.I1RNM7
59619 229533.A0A1C3YIJ9 pp 229533.I1RMJ3
59620 229533.A0A1C3YIJ9 pp 229533.I1RAW3
59621 229533.A0A1C3YIJ9 pp 229533.I1RC86
59622 229533.A0A1C3YIJ9 pp 229533.I1RKB2
59623 229533.A0A1C3YIJ9 pp 229533.I1RGC3
59624 229533.A0A1C3YIJ9 pp 229533.V6RZD4
59625 229533.A0A1C3YIJ9 pp 229533.I1RCU2
59626 229533.A0A1C3YIJ9 pp 229533.I1RVZ3
59627 229533.A0A1C3YIJ9 pp 229533.I1RA52
59628 229533.A0A1C3YIJ9 pp 229533.I1S0B8
59629 229533.A0A1C3YIJ9 pp 229533.I1S1F3
59630 229533.A0A1C3YIJ9 pp 229533.A0A1C3YN25
59631 229533.A0A1C3YIJ9 pp 229533.Q4IB65
59632 229533.A0A1C3YIJ9 pp 229533.I1RY92
59633 229533.A0A1C3YIJ9 pp 229533.I1RYT8
59634 229533.A0A1C3YIJ9 pp 229533.I1RG62
59635 229533.A0A1C3YIJ9 pp 229533.I1RKB7
59636 229533.A0A1C3YIJ9 pp 229533.I1RYX6
59637 229533.A0A1C3YIJ9 pp 229533.I1RMQ6
59638 229533.A0A1C3YIJ9 pp 229533.I1RUR8
59639 229533.A0A1C3YIJ9 pp 229533.I1S3T7
59640 229533.A0A1C3YIJ9 pp 229533.I1R9Y4
59641 229533.A0A1C3YIJ9 pp 229533.I1RL81

59642 229533.A0A1C3YIJ9 pp 229533.I1RX39
59643 229533.A0A1C3YIJ9 pp 229533.I1RQU9
59644 229533.A0A1C3YIJ9 pp 229533.I1RBE6
59645 229533.A0A1C3YIJ9 pp 229533.I1RY21
59646 229533.A0A1C3YIJ9 pp 229533.I1RRX5
59647 229533.A0A1C3YIJ9 pp 229533.I1RYP2
59648 229533.A0A1C3YIJ9 pp 229533.V6RUB1
59649 229533.A0A1C3YIJ9 pp 229533.A0A1C3YMC1
59650 229533.A0A1C3YIJ9 pp 229533.I1RE66
59651 229533.A0A1C3YIJ9 pp 229533.I1RZA7
59652 229533.A0A1C3YIJ9 pp 229533.A0A1C3YIW9
59653 229533.A0A1C3YIJ9 pp 229533.I1S8C1
59654 229533.A0A1C3YIJ9 pp 229533.I1S8R2
59655 229533.A0A1C3YIJ9 pp 229533.I1S464
59656 229533.A0A1C3YIJ9 pp 229533.I1S0P0
59657 229533.A0A1C3YIJ9 pp 229533.I1S0R8
59658 229533.A0A1C3YIJ9 pp 229533.I1S299
59659 229533.A0A1C3YIJ9 pp 229533.A0A1C3YM60
59660 229533.A0A1C3YIJ9 pp 229533.A0A1C3YLE2
59661 229533.A0A1C3YIJ9 pp 229533.I1RG33
59662 229533.A0A1C3YIJ9 pp 229533.I1RS05
59663 229533.A0A1C3YIJ9 pp 229533.I1S4S5
59664 229533.A0A1C3YIJ9 pp 229533.I1RP86
59665 229533.A0A1C3YIJ9 pp 229533.I1S0K2
59666 229533.A0A1C3YIJ9 pp 229533.I1R9B7
59667 229533.A0A1C3YIJ9 pp 229533.I1RXD9
59668 229533.A0A1C3YIJ9 pp 229533.Q4HYR2
59669 229533.A0A1C3YIJ9 pp 229533.I1RA49
59670 229533.A0A1C3YIJ9 pp 229533.V6RF62
59671 229533.A0A1C3YIJ9 pp 229533.A0A1C3YKR5
59672 229533.A0A1C3YIJ9 pp 229533.I1RYA0
59673 229533.A0A1C3YIJ9 pp 229533.I1RJY7
59674 229533.A0A1C3YIJ9 pp 229533.I1RS29
59675 229533.A0A1C3YIJ9 pp 229533.I1RPK0
59676 229533.A0A1C3YIJ9 pp 229533.V6RLM7
59677 229533.A0A1C3YIJ9 pp 229533.Q4I5G1
59678 229533.A0A1C3YIJ9 pp 229533.I1R9M7
59679 229533.A0A1C3YIJ9 pp 229533.I1S5X9
59680 229533.A0A1C3YIJ9 pp 229533.V6R3V4
59681 229533.A0A1C3YIJ9 pp 229533.I1RE52
59682 229533.A0A1C3YIJ9 pp 229533.A0A1C3YLB6
59683 229533.A0A1C3YIJ9 pp 229533.I1RB38
59684 229533.A0A1C3YIJ9 pp 229533.I1RYM5
59685 229533.A0A1C3YIJ9 pp 229533.I1RHJ2
59686 229533.A0A1C3YIJ9 pp 229533.A0A1C3YJU7
59687 229533.A0A1C3YIJ9 pp 229533.A0A1C3YKW8
59688 229533.A0A1C3YIJ9 pp 229533.I1RZ43
59689 229533.A0A1C3YIJ9 pp 229533.I1RBT6
59690 229533.A0A1C3YIJ9 pp 229533.V6RJP8
59691 229533.A0A1C3YIJ9 pp 229533.A0A1C3YLB0
59692 229533.A0A1C3YIJ9 pp 229533.Q4IPZ1
59693 229533.A0A1C3YIJ9 pp 229533.I1RYC8
59694 229533.A0A1C3YIJ9 pp 229533.I1S8F8
59695 229533.A0A1C3YIJ9 pp 229533.I1RAV5
59696 229533.A0A1C3YIJ9 pp 229533.A0A1C3YLB3
59697 229533.A0A1C3YIJ9 pp 229533.I1RLZ6
59698 229533.A0A1C3YIJ9 pp 229533.A0A1C3YK94
59699 229533.A0A1C3YIJ9 pp 229533.I1RQJ7
59700 229533.A0A1C3YIJ9 pp 229533.I1RPR1
59701 229533.A0A1C3YIJ9 pp 229533.I1S0Q7
59702 229533.A0A1C3YIJ9 pp 229533.V6RRM0
59703 229533.A0A1C3YIJ9 pp 229533.I1S0V6
59704 229533.A0A1C3YIJ9 pp 229533.I1RAM9
59705 229533.A0A1C3YIJ9 pp 229533.I1S8S9
59706 229533.A0A1C3YIJ9 pp 229533.I1RWM4
59707 229533.A0A1C3YIJ9 pp 229533.I1RN84
59708 229533.A0A1C3YIJ9 pp 229533.V6RXB3
59709 229533.A0A1C3YIJ9 pp 229533.I1RF85
59710 229533.A0A1C3YIJ9 pp 229533.I1RPR9
59711 229533.A0A1C3YIJ9 pp 229533.I1S423
59712 229533.A0A1C3YIJ9 pp 229533.I1RP02
59713 229533.A0A1C3YIJ9 pp 229533.A0A1C3YJQ2
59714 229533.A0A1C3YIJ9 pp 229533.I1RE65

59715 229533.A0A1C3YIJ9 pp 229533.I1RPY8
59716 229533.A0A1C3YIJ9 pp 229533.A0A1C3YK58
59717 229533.A0A1C3YIJ9 pp 229533.I1RGI4
59718 229533.A0A1C3YIJ9 pp 229533.I1RFJ6
59719 229533.A0A1C3YIJ9 pp 229533.A0A1C3YKY6
59720 229533.A0A1C3YIJ9 pp 229533.I1RY12
59721 229533.A0A1C3YIJ9 pp 229533.I1S0V7
59722 229533.A0A1C3YIJ9 pp 229533.I1RQR6
59723 229533.A0A1C3YIJ9 pp 229533.I1RXE6
59724 229533.A0A1C3YIJ9 pp 229533.A0A1C3YJ04
59725 229533.A0A1C3YIJ9 pp 229533.I1REE2
59726 229533.A0A1C3YIJ9 pp 229533.I1RFS5
59727 229533.I1RXF2 pp 229533.Q4IPB3
59728 229533.I1RXF2 pp 229533.I1S5S2
59729 229533.I1RXF2 pp 229533.I1S0R7
59730 229533.I1RXF2 pp 229533.I1S8V0
59731 229533.I1RXF2 pp 229533.I1S524
59732 229533.I1RXF2 pp 229533.I1RZG7
59733 229533.I1RXF2 pp 229533.I1S0B6
59734 229533.I1RXF2 pp 229533.Q4I1Y1
59735 229533.I1RXF2 pp 229533.I1RYI9
59736 229533.I1RXF2 pp 229533.Q4IPH4
59737 229533.I1RXF2 pp 229533.I1S4Y4
59738 229533.I1RXF2 pp 229533.I1S1C6
59739 229533.I1RXF2 pp 229533.I1S9L5
59740 229533.I1RXF2 pp 229533.I1S4M5
59741 229533.I1RXF2 pp 229533.I1RYW3
59742 229533.I1RXF2 pp 229533.I1RYB1
59743 229533.I1RXF2 pp 229533.I1S0B1
59744 229533.I1RXF2 pp 229533.I1RYJ9
59745 229533.I1RXF2 pp 229533.Q4IB70
59746 229533.I1RXF2 pp 229533.Q4I7X1
59747 229533.I1RXF2 pp 229533.I1RZL5
59748 229533.I1RXF2 pp 229533.I1S2Y5
59749 229533.I1RXF2 pp 229533.I1S0Z9
59750 229533.I1RXF2 pp 229533.I1S9L6
59751 229533.I1RXF2 pp 229533.I1S8U9
59752 229533.I1RXF2 pp 229533.I1RZZ1
59753 229533.I1RXF2 pp 229533.I1S246
59754 229533.I1RXF2 pp 229533.I1S1X9
59755 229533.I1RXF2 pp 229533.Q4IRI9
59756 229533.I1RXF2 pp 229533.Q4IP34
59757 229533.I1RXF2 pp 229533.I1S7M3
59758 229533.I1RXF2 pp 229533.Q4IAA0
59759 229533.I1RXF2 pp 229533.Q4I7F9
59760 229533.I1RXF2 pp 229533.Q4ICA8
59761 229533.I1RXF2 pp 229533.I1S471
59762 229533.I1RXF2 pp 229533.I1RZ11
59763 229533.I1RXF2 pp 229533.I1RZQ5
59764 229533.I1RXF2 pp 229533.V6RIS2
59765 229533.I1RXF2 pp 229533.V6RY33
59766 229533.I1RXF2 pp 229533.V6QX58
59767 229533.I1RXF2 pp 229533.V6QXG1
59768 229533.I1RW08 pp 229533.I1S0E2
59769 229533.I1RFL7 pp 229533.I1RJL2
59770 229533.I1RFL7 pp 229533.I1RZB5
59771 229533.I1RFL7 pp 229533.I1RQA3
59772 229533.I1RFL7 pp 229533.I1RN08
59773 229533.I1RFL7 pp 229533.I1RYY5
59774 229533.I1RFL7 pp 229533.I1S461
59775 229533.I1RFL7 pp 229533.I1RJK7
59776 229533.I1RFL7 pp 229533.I1RIR0
59777 229533.I1RFL7 pp 229533.I1S439
59778 229533.I1RFL7 pp 229533.V6RMM8
59779 229533.I1RFL7 pp 229533.I1RQS4
59780 229533.I1RFL7 pp 229533.I1RHB0
59781 229533.I1RFL7 pp 229533.I1RMM9
59782 229533.I1RFL7 pp 229533.I1RYZ0
59783 229533.I1RFL7 pp 229533.Q4INS6
59784 229533.I1RFL7 pp 229533.I1RMS2
59785 229533.I1RFL7 pp 229533.I1RFQ5
59786 229533.I1RFL7 pp 229533.I1S2A1
59787 229533.I1RFL7 pp 229533.Q4IJM4

59788	229533.I1RFL7	pp	229533.I1RME5
59789	229533.I1RFL7	pp	229533.I1RLB1
59790	229533.I1RFL7	pp	229533.I1RN55
59791	229533.I1RFL7	pp	229533.I1RKK0
59792	229533.I1RFL7	pp	229533.I1RQP3
59793	229533.I1RFL7	pp	229533.I1RZH4
59794	229533.I1RFL7	pp	229533.I1RRT0
59795	229533.I1RFL7	pp	229533.I1RYZ9
59796	229533.I1RFL7	pp	229533.I1RN65
59797	229533.I1RFL7	pp	229533.I1RMQ7
59798	229533.I1RFL7	pp	229533.V6QUE6
59799	229533.I1RFL7	pp	229533.I1RWT6
59800	229533.I1RFL7	pp	229533.V6RU70
59801	229533.I1RFL7	pp	229533.I1RM49
59802	229533.I1RFL7	pp	229533.I1RKE9
59803	229533.I1RFL7	pp	229533.V6RG22
59804	229533.I1RFL7	pp	229533.I1RYI9
59805	229533.I1RFL7	pp	229533.I1S0Z3
59806	229533.I1RFL7	pp	229533.I1S2N3
59807	229533.I1RFL7	pp	229533.I1RJX2
59808	229533.I1RFL7	pp	229533.I1RZ12
59809	229533.I1RFL7	pp	229533.Q4IP51
59810	229533.I1RFL7	pp	229533.I1RT01
59811	229533.I1RFL7	pp	229533.V6R7V8
59812	229533.I1RFL7	pp	229533.I1S283
59813	229533.I1RFL7	pp	229533.I1RTC2
59814	229533.I1RFL7	pp	229533.I1RJI1
59815	229533.I1RFL7	pp	229533.I1RY84
59816	229533.I1RFL7	pp	229533.I1RZ94
59817	229533.I1RFL7	pp	229533.I1RW90
59818	229533.I1RFL7	pp	229533.V6QV09
59819	229533.I1RFL7	pp	229533.V6QWX8
59820	229533.I1RFL7	pp	229533.I1RJH1
59821	229533.I1RFL7	pp	229533.I1RQ13
59822	229533.I1RFL7	pp	229533.I1RXM0
59823	229533.I1RFL7	pp	229533.I1RKJ7
59824	229533.I1RFL7	pp	229533.I1RPI2
59825	229533.I1RFL7	pp	229533.I1RU80
59826	229533.I1RFL7	pp	229533.I1RQZ0
59827	229533.I1RFL7	pp	229533.Q4HYQ4
59828	229533.I1RFL7	pp	229533.I1RSI4
59829	229533.I1RFL7	pp	229533.I1RRG3
59830	229533.I1RFL7	pp	229533.I1S1W5
59831	229533.I1RFL7	pp	229533.I1RV66
59832	229533.I1RFL7	pp	229533.I1RL24
59833	229533.I1RFL7	pp	229533.I1RNC0
59834	229533.I1RFL7	pp	229533.I1RTG0
59835	229533.I1RFL7	pp	229533.I1RRF0
59836	229533.I1RFL7	pp	229533.I1RN89
59837	229533.I1RFL7	pp	229533.I1S224
59838	229533.I1RFL7	pp	229533.I1RQ26
59839	229533.I1RFL7	pp	229533.I1RS56
59840	229533.I1RFL7	pp	229533.I1RZT1
59841	229533.I1RFL7	pp	229533.I1RYH9
59842	229533.I1RFL7	pp	229533.I1RKL9
59843	229533.I1RFL7	pp	229533.I1RQS6
59844	229533.I1RFL7	pp	229533.I1RRY3
59845	229533.I1RFL7	pp	229533.I1RTN0
59846	229533.I1RFL7	pp	229533.I1RRX6
59847	229533.I1RFL7	pp	229533.I1RZP8
59848	229533.I1RFL7	pp	229533.I1RFX8
59849	229533.I1RFL7	pp	229533.I1RT41
59850	229533.I1RFL7	pp	229533.I1RPP7
59851	229533.I1RFL7	pp	229533.I1RKB1
59852	229533.I1RFL7	pp	229533.I1RS41
59853	229533.I1RFL7	pp	229533.I1RPQ1
59854	229533.I1RFL7	pp	229533.I1RSR9
59855	229533.I1RFL7	pp	229533.I1RZY9
59856	229533.I1RFL7	pp	229533.I1RZJ3
59857	229533.I1RFL7	pp	229533.I1RRN5
59858	229533.I1RFL7	pp	229533.I1RY58
59859	229533.I1RFL7	pp	229533.I1RMA4
59860	229533.I1RFL7	pp	229533.I1RRW0

59861 229533.I1RFL7 pp 229533.I1RK79
59862 229533.I1RFL7 pp 229533.I1S0M2
59863 229533.I1RFL7 pp 229533.I1RNZ9
59864 229533.I1RFL7 pp 229533.I1S0E8
59865 229533.I1RFL7 pp 229533.I1RSP7
59866 229533.I1RFL7 pp 229533.I1RQB4
59867 229533.I1RFL7 pp 229533.I1RQ72
59868 229533.I1RFL7 pp 229533.I1RZ63
59869 229533.I1RFL7 pp 229533.I1S0J3
59870 229533.I1RFL7 pp 229533.I1RG11
59871 229533.I1RFL7 pp 229533.I1S9S6
59872 229533.I1RFL7 pp 229533.I1RT36
59873 229533.I1RFL7 pp 229533.I1RR25
59874 229533.I1RFL7 pp 229533.I1RUG4
59875 229533.I1RFL7 pp 229533.I1RRQ9
59876 229533.I1RFL7 pp 229533.I1RGD1
59877 229533.I1RFL7 pp 229533.Q4I7L0
59878 229533.I1RFL7 pp 229533.I1RK75
59879 229533.I1RFL7 pp 229533.I1RLS9
59880 229533.I1RFL7 pp 229533.V6RGI1
59881 229533.I1RFL7 pp 229533.I1RG90
59882 229533.I1RFL7 pp 229533.I1RPK4
59883 229533.I1RFL7 pp 229533.I1S4R1
59884 229533.I1RFL7 pp 229533.I1RXU0
59885 229533.I1RFL7 pp 229533.I1RRS7
59886 229533.I1RFL7 pp 229533.I1RT94
59887 229533.I1RFL7 pp 229533.I1RNP7
59888 229533.I1RFL7 pp 229533.I1RZC3
59889 229533.I1RFL7 pp 229533.I1RNY8
59890 229533.I1RFL7 pp 229533.V6RLW4
59891 229533.I1RFL7 pp 229533.I1RI54
59892 229533.I1RFL7 pp 229533.I1RLU1
59893 229533.I1RFL7 pp 229533.I1RN57
59894 229533.I1RFL7 pp 229533.I1S0M6
59895 229533.I1RFL7 pp 229533.I1S1W4
59896 229533.I1RFL7 pp 229533.I1S1D3
59897 229533.I1RFL7 pp 229533.I1S0J7
59898 229533.I1RFL7 pp 229533.I1RY29
59899 229533.I1RFL7 pp 229533.I1RPC2
59900 229533.I1RFL7 pp 229533.I1S004
59901 229533.I1RFL7 pp 229533.I1S098
59902 229533.I1RFL7 pp 229533.I1RY44
59903 229533.I1RFL7 pp 229533.I1S2E9
59904 229533.I1RFL7 pp 229533.V6RVG3
59905 229533.I1RFL7 pp 229533.I1RUN8
59906 229533.I1RFL7 pp 229533.I1S7X9
59907 229533.I1RFL7 pp 229533.I1RWI8
59908 229533.I1RFL7 pp 229533.Q4IPI4
59909 229533.I1RFL7 pp 229533.I1RKC2
59910 229533.I1RFL7 pp 229533.I1RSZ3
59911 229533.I1RFL7 pp 229533.I1S2G7
59912 229533.I1RFL7 pp 229533.I1RNF4
59913 229533.I1RFL7 pp 229533.I1RQ88
59914 229533.I1RFL7 pp 229533.I1RSN7
59915 229533.I1RFL7 pp 229533.I1RV91
59916 229533.I1RFL7 pp 229533.I1RPF5
59917 229533.I1RFL7 pp 229533.I1RUF8
59918 229533.I1RFL7 pp 229533.Q4IQM6
59919 229533.I1RFL7 pp 229533.I1RYX9
59920 229533.I1RFL7 pp 229533.Q4IAK7
59921 229533.I1RFL7 pp 229533.I1RNZ8
59922 229533.A0A1C3YIH2 pp 229533.I1RY58
59923 229533.A0A1C3YIH2 pp 229533.I1RBW1
59924 229533.A0A1C3YIH2 pp 229533.A0A1C3YM91
59925 229533.A0A1C3YIH2 pp 229533.A0A1C3YN69
59926 229533.A0A1C3YIH2 pp 229533.I1RSC7
59927 229533.A0A1C3YIH2 pp 229533.Q4IPI4
59928 229533.A0A1C3YIH2 pp 229533.I1RQP3
59929 229533.A0A1C3YIH2 pp 229533.Q4ICA8
59930 229533.A0A1C3YIH2 pp 229533.I1RQP1
59931 229533.A0A1C3YIH2 pp 229533.I1RBW0
59932 229533.A0A1C3YIH2 pp 229533.I1RRG3
59933 229533.A0A1C3YHV2 pp 229533.Q4IR09

229533.A0A1C3YHV2 pp 229533.I1RD18
229533.A0A1C3YHV2 pp 229533.I1RZP2
229533.A0A1C3YHV2 pp 229533.Q4IPN9
229533.A0A1C3YHV2 pp 229533.I1S214
229533.A0A1C3YHV2 pp 229533.A0A1C3YIH2
229533.I1S207 pp 229533.I1S208
229533.I1RME5 pp 229533.I1S549
229533.I1RME5 pp 229533.I1S0J7
229533.I1RME5 pp 229533.I1RX38
229533.I1RME5 pp 229533.I1S2E9
229533.I1RME5 pp 229533.I1S004
229533.I1RME5 pp 229533.I1S098
229533.I1RME5 pp 229533.I1RPK4
229533.I1RME5 pp 229533.I1RQ88
229533.I1RME5 pp 229533.I1RV91
229533.I1RME5 pp 229533.I1RY44
229533.I1RME5 pp 229533.I1RPW0
229533.I1RME5 pp 229533.I1RW16
229533.I1RME5 pp 229533.I1RZ63
229533.I1RME5 pp 229533.I1RN89
229533.I1RME5 pp 229533.I1RVN2
229533.I1RME5 pp 229533.I1RY58
229533.I1RME5 pp 229533.I1RRN5
229533.I1RME5 pp 229533.I1RN31
229533.I1RME5 pp 229533.I1S224
229533.I1RME5 pp 229533.I1RS41
229533.I1RME5 pp 229533.V6RGI1
229533.I1RME5 pp 229533.I1RRX6
229533.I1RME5 pp 229533.I1RZC3
229533.I1RME5 pp 229533.I1RRW0
229533.I1RME5 pp 229533.I1RTG0
229533.I1RME5 pp 229533.I1S0R1
229533.I1RME5 pp 229533.I1RYZ9
229533.I1RME5 pp 229533.I1RQA3
229533.I1RME5 pp 229533.V6QV09
229533.I1RME5 pp 229533.Q4I7N9
229533.I1RME5 pp 229533.I1RPU4
229533.I1RME5 pp 229533.I1S267
229533.I1RME5 pp 229533.I1RWT6
229533.A0A1C3YL11 pp 229533.I1S0B6
229533.A0A1C3YL11 pp 229533.I1RST1
229533.A0A1C3YL11 pp 229533.I1RAE1
229533.A0A1C3YL11 pp 229533.I1RMW7
229533.A0A1C3YL11 pp 229533.I1RN59
229533.A0A1C3YL11 pp 229533.I1RGW0
229533.A0A1C3YL11 pp 229533.I1R9Q0
229533.A0A1C3YL11 pp 229533.I1RMV5
229533.A0A1C3YL11 pp 229533.I1RAH7
229533.A0A1C3YL11 pp 229533.I1RUG0
229533.A0A1C3YL11 pp 229533.Q4IFI0
229533.A0A1C3YL11 pp 229533.I1RB50
229533.A0A1C3YL11 pp 229533.I1REX2
229533.A0A1C3YL11 pp 229533.I1RCI4
229533.A0A1C3YL11 pp 229533.I1RND9
229533.A0A1C3YL11 pp 229533.I1RQC5
229533.A0A1C3YL11 pp 229533.I1RCI3
229533.A0A1C3YL11 pp 229533.I1RG13
229533.A0A1C3YL11 pp 229533.I1RCD6
229533.A0A1C3YL11 pp 229533.I1RTS2
229533.A0A1C3YL11 pp 229533.I1RME9
229533.A0A1C3YL11 pp 229533.I1S6F0
229533.A0A1C3YL11 pp 229533.I1RZG7
229533.A0A1C3YL11 pp 229533.I1S524
229533.A0A1C3YL11 pp 229533.Q4IPB3
229533.A0A1C3YL11 pp 229533.I1RRW3
229533.A0A1C3YL11 pp 229533.I1S9L6
229533.A0A1C3YL11 pp 229533.I1RPY4
229533.A0A1C3YL11 pp 229533.I1RCQ6
229533.A0A1C3YL11 pp 229533.I1R9N6
229533.A0A1C3YL11 pp 229533.Q4HZ42
229533.A0A1C3YL11 pp 229533.I1RD56
229533.A0A1C3YL11 pp 229533.I1RX81
229533.A0A1C3YL11 pp 229533.I1RX24

60007	229533.A0A1C3YL11	pp	229533.I1RMA5
60008	229533.A0A1C3YL11	pp	229533.A0A1C3YNC3
60009	229533.A0A1C3YL11	pp	229533.I1RK96
60010	229533.A0A1C3YL11	pp	229533.I1RM06
60011	229533.A0A1C3YL11	pp	229533.I1RZJ9
60012	229533.A0A1C3YL11	pp	229533.I1RCS2
60013	229533.A0A1C3YL11	pp	229533.I1RPU0
60014	229533.A0A1C3YL11	pp	229533.I1S0Z9
60015	229533.A0A1C3YL11	pp	229533.I1RG90
60016	229533.A0A1C3YL11	pp	229533.Q4IBK5
60017	229533.A0A1C3YL11	pp	229533.V6QVD5
60018	229533.A0A1C3YL11	pp	229533.I1RQB5
60019	229533.A0A1C3YL11	pp	229533.I1RQP8
60020	229533.A0A1C3YL11	pp	229533.I1RS75
60021	229533.A0A1C3YL11	pp	229533.Q4I1Y1
60022	229533.A0A1C3YL11	pp	229533.A0A1C3YLF4
60023	229533.A0A1C3YL11	pp	229533.I1RYB1
60024	229533.A0A1C3YL11	pp	229533.I1S0B1
60025	229533.A0A1C3YL11	pp	229533.I1S2Y5
60026	229533.A0A1C3YL11	pp	229533.I1S2C0
60027	229533.A0A1C3YL11	pp	229533.I1RAC1
60028	229533.A0A1C3YL11	pp	229533.I1RK19
60029	229533.A0A1C3YL11	pp	229533.I1RG57
60030	229533.A0A1C3YL11	pp	229533.I1RRQ4
60031	229533.A0A1C3YL11	pp	229533.I1RPY7
60032	229533.A0A1C3YL11	pp	229533.I1RWU2
60033	229533.A0A1C3YL11	pp	229533.I1RCR5
60034	229533.A0A1C3YL11	pp	229533.V6QXG1
60035	229533.A0A1C3YL11	pp	229533.Q4IPH4
60036	229533.A0A1C3YL11	pp	229533.I1S4M5
60037	229533.A0A1C3YL11	pp	229533.I1RT35
60038	229533.A0A1C3YL11	pp	229533.I1RCA7
60039	229533.A0A1C3YL11	pp	229533.I1RFT1
60040	229533.A0A1C3YL11	pp	229533.I1RZQ5
60041	229533.A0A1C3YL11	pp	229533.I1RZZ1
60042	229533.A0A1C3YL11	pp	229533.I1RVT9
60043	229533.A0A1C3YL11	pp	229533.I1RWX8
60044	229533.A0A1C3YL11	pp	229533.I1S9L5
60045	229533.A0A1C3YL11	pp	229533.I1RX51
60046	229533.A0A1C3YL11	pp	229533.I1S854
60047	229533.A0A1C3YL11	pp	229533.Q4I7F9
60048	229533.A0A1C3YL11	pp	229533.I1S1X9
60049	229533.A0A1C3YL11	pp	229533.Q4IEK8
60050	229533.A0A1C3YL11	pp	229533.I1RXE5
60051	229533.A0A1C3YL11	pp	229533.I1RYI9
60052	229533.A0A1C3YL11	pp	229533.I1RSA4
60053	229533.A0A1C3YL11	pp	229533.I1RT91
60054	229533.A0A1C3YL11	pp	229533.I1RAD1
60055	229533.A0A1C3YL11	pp	229533.I1RQR3
60056	229533.A0A1C3YL11	pp	229533.Q4HXF6
60057	229533.A0A1C3YL11	pp	229533.I1RND5
60058	229533.A0A1C3YL11	pp	229533.I1RBH8
60059	229533.A0A1C3YL11	pp	229533.I1S4Y4
60060	229533.A0A1C3YL11	pp	229533.V6RY33
60061	229533.A0A1C3YL11	pp	229533.I1RKR6
60062	229533.A0A1C3YL11	pp	229533.I1RBU6
60063	229533.A0A1C3YL11	pp	229533.I1RP27
60064	229533.A0A1C3YL11	pp	229533.I1RCN5
60065	229533.A0A1C3YL11	pp	229533.I1RBV3
60066	229533.A0A1C3YL11	pp	229533.I1RRL0
60067	229533.A0A1C3YL11	pp	229533.I1RK72
60068	229533.A0A1C3YL11	pp	229533.I1RC84
60069	229533.A0A1C3YL11	pp	229533.I1S855
60070	229533.A0A1C3YL11	pp	229533.Q4IB70
60071	229533.A0A1C3YL11	pp	229533.I1S7M2
60072	229533.A0A1C3YL11	pp	229533.I1RDC9
60073	229533.A0A1C3YL11	pp	229533.I1RMQ7
60074	229533.A0A1C3YL11	pp	229533.I1RPY5
60075	229533.A0A1C3YL11	pp	229533.I1RXF2
60076	229533.A0A1C3YL11	pp	229533.I1RS28
60077	229533.A0A1C3YL11	pp	229533.I1RK92
60078	229533.A0A1C3YL11	pp	229533.I1S5S2
60079	229533.A0A1C3YL11	pp	229533.I1RPA0

60080 229533.A0A1C3YL11 pp 229533.V6QV38
60081 229533.A0A1C3YL11 pp 229533.I1RFQ1
60082 229533.A0A1C3YL11 pp 229533.I1RNI1
60083 229533.A0A1C3YL11 pp 229533.I1RG17
60084 229533.A0A1C3YL11 pp 229533.I1RZ11
60085 229533.A0A1C3YL11 pp 229533.I1RA36
60086 229533.A0A1C3YL11 pp 229533.I1RNB8
60087 229533.A0A1C3YL11 pp 229533.I1RSD4
60088 229533.A0A1C3YL11 pp 229533.V6RGY3
60089 229533.A0A1C3YL11 pp 229533.I1RBZ8
60090 229533.A0A1C3YL11 pp 229533.I1RK90
60091 229533.A0A1C3YL11 pp 229533.I1RYW3
60092 229533.A0A1C3YL11 pp 229533.I1RM25
60093 229533.A0A1C3YL11 pp 229533.I1RSH9
60094 229533.A0A1C3YL11 pp 229533.I1RQD8
60095 229533.A0A1C3YL11 pp 229533.Q4I7K4
60096 229533.A0A1C3YL11 pp 229533.I1RWD3
60097 229533.A0A1C3YL11 pp 229533.Q4IP34
60098 229533.A0A1C3YL11 pp 229533.I1RS83
60099 229533.A0A1C3YL11 pp 229533.I1RT47
60100 229533.A0A1C3YL11 pp 229533.I1RN09
60101 229533.A0A1C3YL11 pp 229533.V6RIS2
60102 229533.A0A1C3YL11 pp 229533.I1RMC0
60103 229533.A0A1C3YL11 pp 229533.Q4IE79
60104 229533.A0A1C3YL11 pp 229533.I1RSA8
60105 229533.A0A1C3YL11 pp 229533.I1S7M3
60106 229533.I1RJF3 pp 229533.I1RXA1
60107 229533.I1RJF3 pp 229533.I1SAT1
60108 229533.I1RJF3 pp 229533.I1S9H4
60109 229533.I1RJF3 pp 229533.I1RL99
60110 229533.I1S0T4 pp 229533.I1S2P8
60111 229533.I1S0T4 pp 229533.I1S250
60112 229533.I1S7M2 pp 229533.V6RY33
60113 229533.I1S7M2 pp 229533.Q4IP34
60114 229533.I1S7M2 pp 229533.I1S9L6
60115 229533.I1S7M2 pp 229533.V6RIS2
60116 229533.I1S7M2 pp 229533.I1S9L5
60117 229533.I1S7M2 pp 229533.Q4IAA0
60118 229533.I1S7M2 pp 229533.Q4I1Y1
60119 229533.I1S7M2 pp 229533.I1S8U9
60120 229533.I1S7M2 pp 229533.Q4IRI9
60121 229533.I1S7M2 pp 229533.Q4IPB3
60122 229533.I1S7M2 pp 229533.Q4I7X1
60123 229533.I1S7M2 pp 229533.Q4ICA8
60124 229533.I1S7M2 pp 229533.Q4IB70
60125 229533.I1S7M2 pp 229533.V6QXG1
60126 229533.I1S7M2 pp 229533.V6QX58
60127 229533.I1S7M2 pp 229533.Q4IPH4
60128 229533.I1S7M2 pp 229533.Q4I7F9
60129 229533.I1S7M2 pp 229533.I1S8V0
60130 229533.I1RM45 pp 229533.I1RZ29
60131 229533.I1RM45 pp 229533.I1RRV2
60132 229533.I1REE1 pp 229533.I1S2E9
60133 229533.I1REE1 pp 229533.I1S549
60134 229533.I1REE1 pp 229533.I1RZ63
60135 229533.I1REE1 pp 229533.I1S004
60136 229533.I1REE1 pp 229533.I1S0J7
60137 229533.I1REE1 pp 229533.V6RVG3
60138 229533.I1REE1 pp 229533.I1RV91
60139 229533.I1REE1 pp 229533.I1S098
60140 229533.I1REE1 pp 229533.I1RY44
60141 229533.I1REE1 pp 229533.I1RX38
60142 229533.I1REE1 pp 229533.I1RPW0
60143 229533.I1REE1 pp 229533.I1RPK4
60144 229533.I1REE1 pp 229533.I1RPU7
60145 229533.I1REE1 pp 229533.I1RY58
60146 229533.I1REE1 pp 229533.I1RFL7
60147 229533.I1REE1 pp 229533.I1RZC3
60148 229533.I1REE1 pp 229533.I1RZT1
60149 229533.I1REE1 pp 229533.I1RMA4
60150 229533.I1REE1 pp 229533.Q4ICM0
60151 229533.I1REE1 pp 229533.I1RRW0
60152 229533.I1REE1 pp 229533.I1RRP7

60153 229533.I1REE1 pp 229533.V6RGI1
60154 229533.I1REE1 pp 229533.Q4I7N9
60155 229533.I1REE1 pp 229533.I1RQA3
60156 229533.I1REE1 pp 229533.I1RVN2
60157 229533.I1REE1 pp 229533.I1RQA9
60158 229533.I1REE1 pp 229533.I1RJK7
60159 229533.I1REE1 pp 229533.I1RZ12
60160 229533.I1REE1 pp 229533.I1RRX6
60161 229533.I1REE1 pp 229533.I1S0N8
60162 229533.I1REE1 pp 229533.I1RTG0
60163 229533.I1REE1 pp 229533.I1RQS4
60164 229533.I1REE1 pp 229533.I1S224
60165 229533.I1REE1 pp 229533.I1RQ80
60166 229533.I1REE1 pp 229533.I1S267
60167 229533.I1REE1 pp 229533.I1RUP7
60168 229533.I1REE1 pp 229533.I1RWT6
60169 229533.I1REE1 pp 229533.I1RPU4
60170 229533.I1REE1 pp 229533.I1RME5
60171 229533.A0A0E0SFP0 pp 229533.I1REV6
60172 229533.A0A0E0SFP0 pp 229533.I1RYH6
60173 229533.A0A0E0SFP0 pp 229533.I1RSK5
60174 229533.A0A0E0SFP0 pp 229533.I1RQE8
60175 229533.A0A0E0SFP0 pp 229533.I1RFB6
60176 229533.A0A0E0SFP0 pp 229533.I1S0K2
60177 229533.A0A0E0SFP0 pp 229533.A0A1C3YK08
60178 229533.A0A0E0SFP0 pp 229533.V6RBN2
60179 229533.A0A0E0SFP0 pp 229533.A0A1C3YK11
60180 229533.A0A0E0SFP0 pp 229533.I1RAJ6
60181 229533.A0A0E0SFP0 pp 229533.I1RZM4
60182 229533.A0A0E0SFP0 pp 229533.I1RZ95
60183 229533.A0A0E0SFP0 pp 229533.V6RN69
60184 229533.A0A0E0SFP0 pp 229533.A0A1C3YLI1
60185 229533.A0A0E0SFP0 pp 229533.I1RZ83
60186 229533.A0A0E0SFP0 pp 229533.I1RVY2
60187 229533.A0A0E0SFP0 pp 229533.I1RP97
60188 229533.A0A0E0SFP0 pp 229533.I1S0N3
60189 229533.A0A0E0SFP0 pp 229533.I1RAD4
60190 229533.A0A0E0SFP0 pp 229533.A0A1C3YJJ2
60191 229533.A0A0E0SFP0 pp 229533.I1R982
60192 229533.A0A0E0SFP0 pp 229533.I1RSS7
60193 229533.A0A0E0SFP0 pp 229533.A0A1C3YLP6
60194 229533.A0A0E0SFP0 pp 229533.I1RDX0
60195 229533.A0A0E0SFP0 pp 229533.I1RZE1
60196 229533.A0A0E0SFP0 pp 229533.V6RIE5
60197 229533.A0A0E0SFP0 pp 229533.V6RK96
60198 229533.A0A0E0SFP0 pp 229533.I1RK02
60199 229533.A0A0E0SFP0 pp 229533.V6R382
60200 229533.A0A0E0SFP0 pp 229533.I1S8A6
60201 229533.A0A0E0SFP0 pp 229533.I1RA90
60202 229533.A0A0E0SFP0 pp 229533.I1RXY7
60203 229533.I1RL99 pp 229533.I1S9H4
60204 229533.I1RL99 pp 229533.I1RXA1
60205 229533.I1S5S2 pp 229533.Q4IPH4
60206 229533.I1S5S2 pp 229533.Q4I7F9
60207 229533.I1S5S2 pp 229533.I1S855
60208 229533.I1S5S2 pp 229533.I1S7M3
60209 229533.I1S5S2 pp 229533.I1S9F1
60210 229533.I1S5S2 pp 229533.I1S854
60211 229533.I1S5S2 pp 229533.Q4HZ42
60212 229533.I1S5S2 pp 229533.V6RIS2
60213 229533.I1S5S2 pp 229533.I1S9L6
60214 229533.I1S5S2 pp 229533.V6RY33
60215 229533.I1S5S2 pp 229533.I1S9L5
60216 229533.I1S5S2 pp 229533.Q4HW67
60217 229533.I1S5S2 pp 229533.I1S7M2
60218 229533.I1RH14 pp 229533.I1RXA1
60219 229533.I1RH14 pp 229533.I1S9H4
60220 229533.I1RH14 pp 229533.I1RL99
60221 229533.I1RH14 pp 229533.I1RJF3
60222 229533.A0A1C3YK37 pp 229533.I1RPK5
60223 229533.A0A1C3YK37 pp 229533.I1RAM7
60224 229533.A0A1C3YK37 pp 229533.I1S1W4
60225 229533.A0A1C3YK37 pp 229533.I1RWT6

60226 229533.A0A1C3YK37 pp 229533.I1RLX4
60227 229533.A0A1C3YK37 pp 229533.V6RKY7
60228 229533.A0A1C3YK37 pp 229533.I1RME5
60229 229533.A0A1C3YK37 pp 229533.I1RY44
60230 229533.A0A1C3YK37 pp 229533.I1S339
60231 229533.A0A1C3YK37 pp 229533.V6RVG3
60232 229533.A0A1C3YK37 pp 229533.Q4I7X1
60233 229533.A0A1C3YK37 pp 229533.I1RP61
60234 229533.A0A1C3YK37 pp 229533.A0A1C3YKV0
60235 229533.A0A1C3YK37 pp 229533.I1RN08
60236 229533.A0A1C3YK37 pp 229533.A0A1C3YM91
60237 229533.A0A1C3YK37 pp 229533.I1S0M2
60238 229533.A0A1C3YK37 pp 229533.I1RC22
60239 229533.A0A1C3YK37 pp 229533.I1RVA5
60240 229533.A0A1C3YK37 pp 229533.I1RZT1
60241 229533.A0A1C3YK37 pp 229533.I1RTN1
60242 229533.A0A1C3YK37 pp 229533.I1RLS5
60243 229533.A0A1C3YK37 pp 229533.I1REE1
60244 229533.A0A1C3YK37 pp 229533.I1RF69
60245 229533.A0A1C3YK37 pp 229533.I1RRW0
60246 229533.A0A1C3YK37 pp 229533.I1RPU4
60247 229533.A0A1C3YK37 pp 229533.I1RMA4
60248 229533.A0A1C3YK37 pp 229533.I1RD70
60249 229533.A0A1C3YK37 pp 229533.I1RRP7
60250 229533.A0A1C3YK37 pp 229533.I1RBM3
60251 229533.A0A1C3YK37 pp 229533.I1RSR3
60252 229533.A0A1C3YK37 pp 229533.I1RC95
60253 229533.A0A1C3YK37 pp 229533.I1S0D1
60254 229533.A0A1C3YK37 pp 229533.I1RMM9
60255 229533.A0A1C3YK37 pp 229533.V6R2L8
60256 229533.A0A1C3YK37 pp 229533.I1RRY3
60257 229533.A0A1C3YK37 pp 229533.I1RW16
60258 229533.A0A1C3YK37 pp 229533.I1RA43
60259 229533.A0A1C3YK37 pp 229533.I1S1D3
60260 229533.A0A1C3YK37 pp 229533.I1RS41
60261 229533.A0A1C3YK37 pp 229533.I1RD18
60262 229533.A0A1C3YK37 pp 229533.I1RZH4
60263 229533.A0A1C3YK37 pp 229533.I1RPW0
60264 229533.A0A1C3YK37 pp 229533.I1S951
60265 229533.A0A1C3YK37 pp 229533.I1RBI1
60266 229533.A0A1C3YK37 pp 229533.I1RX38
60267 229533.A0A1C3YK37 pp 229533.I1S9H9
60268 229533.A0A1C3YK37 pp 229533.A0A1C3YMF9
60269 229533.A0A1C3YK37 pp 229533.I1RI34
60270 229533.A0A1C3YK37 pp 229533.I1RKB0
60271 229533.A0A1C3YK37 pp 229533.I1S084
60272 229533.A0A1C3YK37 pp 229533.I1S2R1
60273 229533.A0A1C3YK37 pp 229533.I1RQA3
60274 229533.A0A1C3YK37 pp 229533.A0A1C3YLY5
60275 229533.A0A1C3YK37 pp 229533.I1S1V5
60276 229533.A0A1C3YK37 pp 229533.I1RCM6
60277 229533.A0A1C3YK37 pp 229533.I1RAR1
60278 229533.A0A1C3YK37 pp 229533.V6QYF0
60279 229533.A0A1C3YK37 pp 229533.I1RJK7
60280 229533.A0A1C3YK37 pp 229533.I1RXA1
60281 229533.A0A1C3YK37 pp 229533.I1RDV9
60282 229533.A0A1C3YK37 pp 229533.I1S439
60283 229533.A0A1C3YK37 pp 229533.I1RPD2
60284 229533.A0A1C3YK37 pp 229533.I1RB59
60285 229533.A0A1C3YK37 pp 229533.I1RZ12
60286 229533.A0A1C3YK37 pp 229533.I1RQS4
60287 229533.A0A1C3YK37 pp 229533.I1RQ26
60288 229533.A0A1C3YK37 pp 229533.I1RZE5
60289 229533.A0A1C3YK37 pp 229533.I1R976
60290 229533.A0A1C3YK37 pp 229533.I1RTG0
60291 229533.A0A1C3YK37 pp 229533.I1RTA9
60292 229533.A0A1C3YK37 pp 229533.I1RUN8
60293 229533.A0A1C3YK37 pp 229533.A0A1C3YKW5
60294 229533.A0A1C3YK37 pp 229533.I1RT08
60295 229533.A0A1C3YK37 pp 229533.I1RBV5
60296 229533.A0A1C3YK37 pp 229533.I1RWT1
60297 229533.A0A1C3YK37 pp 229533.I1RL04
60298 229533.A0A1C3YK37 pp 229533.I1S2E9

60299	229533.A0A1C3YK37	pp	229533.I1RQW2
60300	229533.A0A1C3YK37	pp	229533.I1RAW7
60301	229533.A0A1C3YK37	pp	229533.I1RQ80
60302	229533.A0A1C3YK37	pp	229533.I1SA96
60303	229533.A0A1C3YK37	pp	229533.I1RC97
60304	229533.A0A1C3YK37	pp	229533.I1S382
60305	229533.A0A1C3YK37	pp	229533.I1RFZ2
60306	229533.A0A1C3YK37	pp	229533.I1S1P8
60307	229533.A0A1C3YK37	pp	229533.I1RNC0
60308	229533.A0A1C3YK37	pp	229533.I1R9R7
60309	229533.A0A1C3YK37	pp	229533.I1RFU3
60310	229533.A0A1C3YK37	pp	229533.I1RLX6
60311	229533.A0A1C3YK37	pp	229533.A0A1C3YKJ6
60312	229533.A0A1C3YK37	pp	229533.I1RBZ9
60313	229533.A0A1C3YK37	pp	229533.I1RQM0
60314	229533.A0A1C3YK37	pp	229533.I1RN31
60315	229533.A0A1C3YK37	pp	229533.I1S330
60316	229533.A0A1C3YK37	pp	229533.I1RDB1
60317	229533.A0A1C3YK37	pp	229533.I1RRX6
60318	229533.A0A1C3YK37	pp	229533.I1R9Q0
60319	229533.A0A1C3YK37	pp	229533.I1RZG3
60320	229533.A0A1C3YK37	pp	229533.I1RAN2
60321	229533.A0A1C3YK37	pp	229533.A0A1C3YN27
60322	229533.A0A1C3YK37	pp	229533.I1RBL4
60323	229533.A0A1C3YK37	pp	229533.I1S0J7
60324	229533.A0A1C3YK37	pp	229533.I1RDG8
60325	229533.A0A1C3YK37	pp	229533.I1S224
60326	229533.A0A1C3YK37	pp	229533.I1RYH9
60327	229533.A0A1C3YMD4	pp	229533.I1RS71
60328	229533.A0A1C3YMD4	pp	229533.I1RFX9
60329	229533.A0A1C3YMD4	pp	229533.I1RKL6
60330	229533.A0A1C3YMD4	pp	229533.Q4IFH8
60331	229533.A0A1C3YMD4	pp	229533.I1RDY5
60332	229533.A0A1C3YMD4	pp	229533.I1RZU5
60333	229533.I1RI91	pp	229533.I1RYM5
60334	229533.I1RI91	pp	229533.V6R382
60335	229533.I1RI91	pp	229533.I1RW06
60336	229533.I1RI91	pp	229533.I1S0T4
60337	229533.I1RT70	pp	229533.I1S9L0
60338	229533.I1RT70	pp	229533.I1RWL6
60339	229533.I1RT70	pp	229533.Q4I1Y1
60340	229533.I1RT70	pp	229533.I1S056
60341	229533.I1RP72	pp	229533.I1RW06
60342	229533.I1RP72	pp	229533.I1RR94
60343	229533.I1RP72	pp	229533.I1RYK0
60344	229533.I1RP72	pp	229533.V6RV57
60345	229533.I1RP72	pp	229533.I1SAF8
60346	229533.I1RP72	pp	229533.V6RLM7
60347	229533.I1RP72	pp	229533.I1S9T8
60348	229533.I1RBV3	pp	229533.I1RSD4
60349	229533.I1RBV3	pp	229533.I1RNB8
60350	229533.I1RBV3	pp	229533.I1RS28
60351	229533.I1RBV3	pp	229533.I1RFQ1
60352	229533.I1RBV3	pp	229533.I1RPY5
60353	229533.I1RBV3	pp	229533.I1RK72
60354	229533.I1RBV3	pp	229533.I1RRT0
60355	229533.I1RBV3	pp	229533.I1RRL0
60356	229533.I1RBV3	pp	229533.V6RY33
60357	229533.I1RBV3	pp	229533.I1RE24
60358	229533.I1RBV3	pp	229533.I1RQQ7
60359	229533.I1RBV3	pp	229533.I1S471
60360	229533.I1RBV3	pp	229533.I1RQD8
60361	229533.I1RBV3	pp	229533.I1RZ11
60362	229533.I1RBV3	pp	229533.I1RG17
60363	229533.I1RBV3	pp	229533.V6QX58
60364	229533.I1RBV3	pp	229533.Q4IRI9
60365	229533.I1RBV3	pp	229533.I1RSA4
60366	229533.I1RBV3	pp	229533.I1RND5
60367	229533.I1RBV3	pp	229533.I1RN09
60368	229533.I1RBV3	pp	229533.Q4IAA0
60369	229533.I1RBV3	pp	229533.I1RX51
60370	229533.I1RBV3	pp	229533.I1RQZ2
60371	229533.I1RBV3	pp	229533.I1RT47

60372 229533.I1RBV3 pp 229533.I1RM25
60373 229533.I1RBV3 pp 229533.I1RSH9
60374 229533.I1RBV3 pp 229533.I1RT35
60375 229533.I1RBV3 pp 229533.I1RVT9
60376 229533.I1RBV3 pp 229533.I1S1X9
60377 229533.I1RBV3 pp 229533.I1RQP8
60378 229533.I1RBV3 pp 229533.I1RWX8
60379 229533.I1RBV3 pp 229533.I1RS06
60380 229533.I1RBV3 pp 229533.V6QXG1
60381 229533.I1RBV3 pp 229533.Q4I7F9
60382 229533.I1RBV3 pp 229533.I1RK79
60383 229533.I1RBV3 pp 229533.I1RVK5
60384 229533.I1RBV3 pp 229533.I1RZZ1
60385 229533.I1RBV3 pp 229533.Q4I7X1
60386 229533.I1RBV3 pp 229533.I1RZQ5
60387 229533.I1RBV3 pp 229533.I1RK90
60388 229533.I1RBV3 pp 229533.I1RYW3
60389 229533.I1RBV3 pp 229533.I1S8U9
60390 229533.I1RBV3 pp 229533.I1RX47
60391 229533.I1RBV3 pp 229533.I1RR25
60392 229533.I1RBV3 pp 229533.I1RZL5
60393 229533.I1RBV3 pp 229533.I1S7M3
60394 229533.I1RBV3 pp 229533.I1RPA0
60395 229533.I1RBV3 pp 229533.Q4IPH4
60396 229533.I1RBV3 pp 229533.I1S8V0
60397 229533.I1RBV3 pp 229533.I1RK92
60398 229533.I1RBV3 pp 229533.I1RSA8
60399 229533.I1RBV3 pp 229533.Q4IB70
60400 229533.I1RBV3 pp 229533.I1RFP9
60401 229533.I1RBV3 pp 229533.V6RIS2
60402 229533.I1RBV3 pp 229533.I1S0B1
60403 229533.I1RBV3 pp 229533.I1RCR5
60404 229533.I1RBV3 pp 229533.I1RYB1
60405 229533.I1RBV3 pp 229533.I1RC84
60406 229533.I1RBV3 pp 229533.Q4IP34
60407 229533.I1RBV3 pp 229533.I1RCN5
60408 229533.I1RBV3 pp 229533.I1S9L5
60409 229533.I1RBV3 pp 229533.I1RS83
60410 229533.I1RBV3 pp 229533.I1RG90
60411 229533.I1RBV3 pp 229533.I1S0Z9
60412 229533.I1RBV3 pp 229533.I1RG57
60413 229533.I1RBV3 pp 229533.I1RCA7
60414 229533.I1RBV3 pp 229533.I1S5S2
60415 229533.I1RBV3 pp 229533.I1S214
60416 229533.I1RBV3 pp 229533.I1RJL2
60417 229533.I1RBV3 pp 229533.I1RDD3
60418 229533.I1RBV3 pp 229533.Q4I1Y1
60419 229533.I1RBV3 pp 229533.I1RFT1
60420 229533.I1RBV3 pp 229533.I1S4M5
60421 229533.I1RBV3 pp 229533.I1RJF9
60422 229533.I1RBV3 pp 229533.I1RCQ6
60423 229533.I1RBV3 pp 229533.I1RXF2
60424 229533.I1RBV3 pp 229533.I1RZB5
60425 229533.I1RBV3 pp 229533.I1RWU2
60426 229533.I1RBV3 pp 229533.I1RPY4
60427 229533.I1RBV3 pp 229533.I1S7M2
60428 229533.I1RBV3 pp 229533.I1RRG3
60429 229533.I1RBV3 pp 229533.I1RK19
60430 229533.I1RBV3 pp 229533.I1RSL9
60431 229533.I1RBV3 pp 229533.I1S0R7
60432 229533.I1RBV3 pp 229533.I1S524
60433 229533.I1RBV3 pp 229533.Q4IPB3
60434 229533.I1RBV3 pp 229533.I1RS75
60435 229533.I1RBV3 pp 229533.I1S2Y5
60436 229533.I1RBV3 pp 229533.I1RTS2
60437 229533.I1RBV3 pp 229533.I1S4Y4
60438 229533.I1RBV3 pp 229533.I1RZG7
60439 229533.I1RBV3 pp 229533.I1S1C6
60440 229533.I1RBV3 pp 229533.I1S246
60441 229533.I1RBV3 pp 229533.I1RD56
60442 229533.I1RBV3 pp 229533.I1RJG6
60443 229533.I1RBV3 pp 229533.I1RCS2
60444 229533.I1RBV3 pp 229533.I1RLU5

60445 229533.I1RBV3 pp 229533.Q4IPN9
60446 229533.I1RBV3 pp 229533.I1S9L6
60447 229533.I1RBV3 pp 229533.I1RCD6
60448 229533.I1RBV3 pp 229533.I1RCI3
60449 229533.I1RBV3 pp 229533.I1RCI4
60450 229533.I1RBV3 pp 229533.I1RDC1
60451 229533.I1RBV3 pp 229533.I1RK96
60452 229533.I1RBV3 pp 229533.I1RX24
60453 229533.I1RBV3 pp 229533.I1RMV5
60454 229533.I1RBV3 pp 229533.I1RC24
60455 229533.I1RBV3 pp 229533.I1RDE9
60456 229533.I1RBV3 pp 229533.I1RX81
60457 229533.I1RBV3 pp 229533.I1RN59
60458 229533.I1RBV3 pp 229533.I1RMW7
60459 229533.I1RBV3 pp 229533.I1RU69
60460 229533.I1RBV3 pp 229533.I1RST1
60461 229533.I1RBV3 pp 229533.I1RPW9
60462 229533.I1RBV3 pp 229533.I1S0B6
60463 229533.I1RPK4 pp 229533.I1S098
60464 229533.I1RPK4 pp 229533.I1RX77
60465 229533.I1RPK4 pp 229533.I1S224
60466 229533.I1RPK4 pp 229533.I1S0J7
60467 229533.I1RPK4 pp 229533.I1S2E9
60468 229533.I1RPK4 pp 229533.I1RV66
60469 229533.I1RPK4 pp 229533.I1RQH7
60470 229533.I1RPK4 pp 229533.I1RRX6
60471 229533.I1RPK4 pp 229533.I1RWT6
60472 229533.I1RPK4 pp 229533.I1RZ63
60473 229533.I1RPK4 pp 229533.I1RTG0
60474 229533.I1RPK4 pp 229533.I1RY44
60475 229533.I1RPK4 pp 229533.I1RZ12
60476 229533.I1RPK4 pp 229533.I1RQS4
60477 229533.I1RPK4 pp 229533.Q4IEA7
60478 229533.I1RPK4 pp 229533.I1RSS1
60479 229533.I1RPK4 pp 229533.I1RZA5
60480 229533.I1RPK4 pp 229533.I1RRN5
60481 229533.I1RPK4 pp 229533.I1RXF0
60482 229533.I1RPK4 pp 229533.I1RVN2
60483 229533.I1RPK4 pp 229533.I1RQA3
60484 229533.I1RPK4 pp 229533.I1RQB4
60485 229533.I1RPK4 pp 229533.I1S286
60486 229533.I1RPK4 pp 229533.I1RY58
60487 229533.I1RPK4 pp 229533.I1RRW0
60488 229533.I1RIE2 pp 229533.I1RPD6
60489 229533.I1RIE2 pp 229533.I1RWA9
60490 229533.I1RIE2 pp 229533.I1RX38
60491 229533.I1RIE2 pp 229533.I1S207
60492 229533.I1R9U8 pp 229533.I1RH14
60493 229533.I1R9U8 pp 229533.I1RJF3
60494 229533.I1R9U8 pp 229533.I1RXA1
60495 229533.I1R9U8 pp 229533.I1RL99
60496 229533.I1R9U8 pp 229533.I1RG10
60497 229533.I1R9U8 pp 229533.I1S9H4
60498 229533.I1RB50 pp 229533.I1RJM7
60499 229533.I1RB50 pp 229533.Q4IBK5
60500 229533.I1RB50 pp 229533.I1S246
60501 229533.I1RB50 pp 229533.I1S2Y5
60502 229533.I1RB50 pp 229533.I1RVZ2
60503 229533.I1RB50 pp 229533.I1RCS2
60504 229533.I1RB50 pp 229533.I1RJH1
60505 229533.I1RB50 pp 229533.I1RRG3
60506 229533.I1RB50 pp 229533.I1RDD3
60507 229533.I1RB50 pp 229533.I1RS75
60508 229533.I1RB50 pp 229533.I1RFT1
60509 229533.I1RB50 pp 229533.I1S4M5
60510 229533.I1RB50 pp 229533.I1RWU2
60511 229533.I1RB50 pp 229533.I1RJF9
60512 229533.I1RB50 pp 229533.I1S9L5
60513 229533.I1RB50 pp 229533.I1RK19
60514 229533.I1RB50 pp 229533.I1RPM4
60515 229533.I1RB50 pp 229533.I1RCA7
60516 229533.I1RB50 pp 229533.I1RQC5
60517 229533.I1RB50 pp 229533.I1RND9

60518	229533.I1RB50	pp	229533.I1RBV3
60519	229533.I1RB50	pp	229533.I1RBU6
60520	229533.I1RB50	pp	229533.I1RCQ6
60521	229533.I1RB50	pp	229533.I1RS30
60522	229533.I1RB50	pp	229533.I1S524
60523	229533.I1RB50	pp	229533.I1RPY4
60524	229533.I1RB50	pp	229533.I1S0R7
60525	229533.I1RB50	pp	229533.I1RT04
60526	229533.I1RB50	pp	229533.I1S214
60527	229533.I1RB50	pp	229533.I1RMY3
60528	229533.I1RB50	pp	229533.I1RSH0
60529	229533.I1RB50	pp	229533.Q4IPB3
60530	229533.I1RB50	pp	229533.I1RME9
60531	229533.I1RB50	pp	229533.I1RK96
60532	229533.I1RB50	pp	229533.I1RG13
60533	229533.I1RB50	pp	229533.I1RLU5
60534	229533.I1RB50	pp	229533.I1RD56
60535	229533.I1RB50	pp	229533.I1S5S2
60536	229533.I1RB50	pp	229533.I1S7M2
60537	229533.I1RB50	pp	229533.I1RX81
60538	229533.I1RB50	pp	229533.Q4IJW4
60539	229533.I1RB50	pp	229533.I1RDC1
60540	229533.I1RB50	pp	229533.I1RX24
60541	229533.I1RB50	pp	229533.I1S9L6
60542	229533.I1RB50	pp	229533.Q4IPN9
60543	229533.I1RB50	pp	229533.I1RXF2
60544	229533.I1RB50	pp	229533.I1RMV5
60545	229533.I1RB50	pp	229533.Q4I5R9
60546	229533.I1RB50	pp	229533.I1RDE9
60547	229533.I1RB50	pp	229533.Q4IB65
60548	229533.I1RB50	pp	229533.I1RYI9
60549	229533.I1RB50	pp	229533.I1RW25
60550	229533.I1RB50	pp	229533.I1S1C6
60551	229533.I1RB50	pp	229533.I1RX25
60552	229533.I1RB50	pp	229533.I1RCU7
60553	229533.I1RB50	pp	229533.I1REX2
60554	229533.I1RB50	pp	229533.I1S0B6
60555	229533.I1RB50	pp	229533.I1RZJ5
60556	229533.I1RB50	pp	229533.I1S8F0
60557	229533.I1RB50	pp	229533.I1RCI4
60558	229533.I1RB50	pp	229533.I1S4Y4
60559	229533.I1RB50	pp	229533.I1RML8
60560	229533.I1RB50	pp	229533.I1RST1
60561	229533.I1RB50	pp	229533.I1RCI3
60562	229533.I1RB50	pp	229533.I1RSL9
60563	229533.I1RB50	pp	229533.I1S2G9
60564	229533.I1RB50	pp	229533.I1RJG6
60565	229533.I1RB50	pp	229533.I1RCD6
60566	229533.I1RB50	pp	229533.I1RT17
60567	229533.I1RB50	pp	229533.I1RFQ0
60568	229533.I1RB50	pp	229533.I1RPW9
60569	229533.I1RB50	pp	229533.I1RMW7
60570	229533.I1RB50	pp	229533.I1RU69
60571	229533.I1RB50	pp	229533.I1RPZ3
60572	229533.I1RB50	pp	229533.I1RZS7
60573	229533.I1RB50	pp	229533.I1RRT0
60574	229533.I1RB50	pp	229533.Q4IQW0
60575	229533.I1RB50	pp	229533.I1RN59
60576	229533.I1RB50	pp	229533.I1RFT6
60577	229533.I1RB50	pp	229533.I1RRL0
60578	229533.I1RB50	pp	229533.I1RC24
60579	229533.I1RB50	pp	229533.I1RWW5
60580	229533.I1RB50	pp	229533.I1RTS2
60581	229533.I1RB50	pp	229533.I1S289
60582	229533.I1RB50	pp	229533.I1RZG7
60583	229533.I1RB50	pp	229533.I1RK72
60584	229533.I1RB50	pp	229533.I1RX51
60585	229533.I1RB50	pp	229533.I1RSD4
60586	229533.I1RB50	pp	229533.I1RQD8
60587	229533.I1RB50	pp	229533.I1RFQ1
60588	229533.I1RB50	pp	229533.I1RPY5
60589	229533.I1RB50	pp	229533.I1RRU9
60590	229533.I1RB50	pp	229533.I1RZ11

60591	229533.I1RB50	pp	229533.V6QXG1
60592	229533.I1RB50	pp	229533.I1RVT9
60593	229533.I1RB50	pp	229533.I1RS28
60594	229533.I1RB50	pp	229533.I1RT35
60595	229533.I1RB50	pp	229533.I1RNB8
60596	229533.I1RB50	pp	229533.I1RC17
60597	229533.I1RB50	pp	229533.I1S471
60598	229533.I1RB50	pp	229533.I1RS17
60599	229533.I1RB50	pp	229533.I1RF91
60600	229533.I1RB50	pp	229533.I1RWX8
60601	229533.I1RB50	pp	229533.I1RSH9
60602	229533.I1RB50	pp	229533.V6QX58
60603	229533.I1RB50	pp	229533.I1RM25
60604	229533.I1RB50	pp	229533.I1RG11
60605	229533.I1RB50	pp	229533.Q4IRI9
60606	229533.I1RB50	pp	229533.I1RSA4
60607	229533.I1RB50	pp	229533.Q4IP34
60608	229533.I1RB50	pp	229533.Q4IAA0
60609	229533.I1RB50	pp	229533.I1RMM5
60610	229533.I1RB50	pp	229533.V6RIS2
60611	229533.I1RB50	pp	229533.I1RS83
60612	229533.I1RB50	pp	229533.I1RND5
60613	229533.I1RB50	pp	229533.I1RRQ4
60614	229533.I1RB50	pp	229533.I1RZQ5
60615	229533.I1RB50	pp	229533.I1RLU6
60616	229533.I1RB50	pp	229533.I1RE31
60617	229533.I1RB50	pp	229533.I1RQQ7
60618	229533.I1RB50	pp	229533.I1S8V0
60619	229533.I1RB50	pp	229533.I1RR25
60620	229533.I1RB50	pp	229533.Q4IPB8
60621	229533.I1RB50	pp	229533.I1RBH8
60622	229533.I1RB50	pp	229533.I1RE24
60623	229533.I1RB50	pp	229533.Q4I6B0
60624	229533.I1RB50	pp	229533.Q4IPH4
60625	229533.I1RB50	pp	229533.Q4I7F9
60626	229533.I1RB50	pp	229533.I1RZZ1
60627	229533.I1RB50	pp	229533.I1RSH4
60628	229533.I1RB50	pp	229533.I1RYW3
60629	229533.I1RB50	pp	229533.I1RPL3
60630	229533.I1RB50	pp	229533.I1RK90
60631	229533.I1RB50	pp	229533.V6RY33
60632	229533.I1RB50	pp	229533.I1RK79
60633	229533.I1RB50	pp	229533.I1RSA8
60634	229533.I1RB50	pp	229533.Q4IE79
60635	229533.I1RB50	pp	229533.I1RT91
60636	229533.I1RB50	pp	229533.I1RQR3
60637	229533.I1RB50	pp	229533.I1RVK5
60638	229533.I1RB50	pp	229533.I1RX47
60639	229533.I1RB50	pp	229533.I1RQZ2
60640	229533.I1RB50	pp	229533.I1S1X9
60641	229533.I1RB50	pp	229533.I1RQP8
60642	229533.I1RB50	pp	229533.I1RS06
60643	229533.I1RB50	pp	229533.I1S7M3
60644	229533.I1RB50	pp	229533.I1RSY2
60645	229533.I1RB50	pp	229533.I1RZB5
60646	229533.I1RB50	pp	229533.I1S8U9
60647	229533.I1RB50	pp	229533.I1RE71
60648	229533.I1RB50	pp	229533.I1RG57
60649	229533.I1RB50	pp	229533.I1RJL2
60650	229533.I1RB50	pp	229533.I1S0Z9
60651	229533.I1RB50	pp	229533.Q4HXF6
60652	229533.I1RB50	pp	229533.V6RCG5
60653	229533.I1RB50	pp	229533.I1RCB7
60654	229533.I1RB50	pp	229533.I1RG17
60655	229533.I1RB50	pp	229533.Q4I1Y1
60656	229533.I1RB50	pp	229533.I1RCN5
60657	229533.I1RB50	pp	229533.I1RC84
60658	229533.I1RB50	pp	229533.I1RG90
60659	229533.I1RB50	pp	229533.I1RYJ9
60660	229533.I1RB50	pp	229533.I1RN09
60661	229533.I1RB50	pp	229533.I1RT47
60662	229533.I1RB50	pp	229533.Q4IB70
60663	229533.I1RB50	pp	229533.Q4IPZ1

60664 229533.I1RB50 pp 229533.I1RYB1
60665 229533.I1RB50 pp 229533.I1RPY7
60666 229533.I1RB50 pp 229533.I1RPA0
60667 229533.I1RB50 pp 229533.I1RZV9
60668 229533.I1RB50 pp 229533.I1RK92
60669 229533.I1RB50 pp 229533.I1S0B1
60670 229533.I1RB50 pp 229533.I1RZL5
60671 229533.I1RB50 pp 229533.V6QVD5
60672 229533.I1RB50 pp 229533.I1RFP9
60673 229533.I1RB50 pp 229533.I1RBZ8
60674 229533.I1RB50 pp 229533.I1RCR5
60675 229533.A0A1C3YLC1 pp 229533.I1RD52
60676 229533.A0A1C3YLC1 pp 229533.I1RRX5
60677 229533.A0A1C3YLC1 pp 229533.I1RPI5
60678 229533.A0A1C3YLC1 pp 229533.I1RXD7
60679 229533.A0A1C3YLC1 pp 229533.I1RAX6
60680 229533.A0A1C3YLC1 pp 229533.I1RBT1
60681 229533.A0A1C3YLC1 pp 229533.I1RSQ7
60682 229533.A0A1C3YLC1 pp 229533.I1S4S7
60683 229533.A0A1C3YLC1 pp 229533.I1R9Y4
60684 229533.A0A1C3YLC1 pp 229533.I1RLP1
60685 229533.A0A1C3YLC1 pp 229533.I1RJU3
60686 229533.A0A1C3YLC1 pp 229533.I1RW06
60687 229533.A0A1C3YLC1 pp 229533.I1RTU1
60688 229533.A0A1C3YLC1 pp 229533.I1RX46
60689 229533.A0A1C3YLC1 pp 229533.I1S419
60690 229533.A0A1C3YLC1 pp 229533.I1RS18
60691 229533.A0A1C3YLC1 pp 229533.I1S338
60692 229533.A0A1C3YLC1 pp 229533.I1RKI3
60693 229533.A0A1C3YLC1 pp 229533.I1S0T2
60694 229533.A0A1C3YLC1 pp 229533.V6RTJ8
60695 229533.A0A1C3YLC1 pp 229533.V6QUE6
60696 229533.A0A1C3YLC1 pp 229533.I1RCS1
60697 229533.A0A1C3YLC1 pp 229533.A0A1C3YLD3
60698 229533.A0A1C3YLC1 pp 229533.I1RJX2
60699 229533.A0A1C3YLC1 pp 229533.I1S5B0
60700 229533.A0A1C3YLC1 pp 229533.I1RR24
60701 229533.A0A1C3YLC1 pp 229533.I1RN46
60702 229533.A0A1C3YLC1 pp 229533.I1REK8
60703 229533.A0A1C3YLC1 pp 229533.I1RXF0
60704 229533.A0A1C3YLC1 pp 229533.I1RN67
60705 229533.A0A1C3YLC1 pp 229533.I1RZM8
60706 229533.A0A1C3YLC1 pp 229533.I1RCR5
60707 229533.A0A1C3YLC1 pp 229533.I1RMG6
60708 229533.A0A1C3YLC1 pp 229533.I1S0K2
60709 229533.A0A1C3YLC1 pp 229533.I1RR51
60710 229533.A0A1C3YLC1 pp 229533.I1RS47
60711 229533.A0A1C3YLC1 pp 229533.Q4IJK2
60712 229533.A0A1C3YLC1 pp 229533.I1RAB4
60713 229533.A0A1C3YLC1 pp 229533.V6RCZ8
60714 229533.A0A1C3YLC1 pp 229533.I1RK95
60715 229533.A0A1C3YLC1 pp 229533.I1RTA1
60716 229533.A0A1C3YLC1 pp 229533.I1RXV9
60717 229533.A0A1C3YLC1 pp 229533.I1RPP3
60718 229533.A0A1C3YLC1 pp 229533.I1RC67
60719 229533.A0A1C3YLC1 pp 229533.I1S6K9
60720 229533.A0A1C3YLC1 pp 229533.I1R9M0
60721 229533.A0A1C3YLC1 pp 229533.I1RK47
60722 229533.A0A1C3YLC1 pp 229533.I1RFT1
60723 229533.A0A1C3YLC1 pp 229533.I1RWB0
60724 229533.A0A1C3YLC1 pp 229533.I1RXW3
60725 229533.A0A1C3YLC1 pp 229533.I1RYX5
60726 229533.A0A1C3YLC1 pp 229533.I1RRY9
60727 229533.A0A1C3YLC1 pp 229533.V6R3J1
60728 229533.A0A1C3YLC1 pp 229533.I1S6Y8
60729 229533.A0A1C3YLC1 pp 229533.I1RWC9
60730 229533.A0A1C3YLC1 pp 229533.I1RPV3
60731 229533.A0A1C3YLC1 pp 229533.I1RSX0
60732 229533.A0A1C3YLC1 pp 229533.A0A1C3YLK8
60733 229533.A0A1C3YLC1 pp 229533.I1RGA4
60734 229533.A0A1C3YLC1 pp 229533.I1RPR9
60735 229533.A0A1C3YLC1 pp 229533.I1RKI7
60736 229533.A0A1C3YLC1 pp 229533.I1RXM6

60737 229533.A0A1C3YLC1 pp 229533.O74638
60738 229533.A0A1C3YLC1 pp 229533.I1RTD5
60739 229533.A0A1C3YLC1 pp 229533.I1RZK5
60740 229533.A0A1C3YLC1 pp 229533.I1S5R1
60741 229533.A0A1C3YLC1 pp 229533.I1RT40
60742 229533.A0A1C3YLC1 pp 229533.Q4I624
60743 229533.A0A1C3YLC1 pp 229533.I1RXA9
60744 229533.A0A1C3YLC1 pp 229533.I1RRT8
60745 229533.A0A1C3YLC1 pp 229533.I1RA11
60746 229533.A0A1C3YLC1 pp 229533.V6QVH8
60747 229533.A0A1C3YLC1 pp 229533.I1RCH5
60748 229533.A0A1C3YLC1 pp 229533.I1RBY4
60749 229533.A0A1C3YLC1 pp 229533.I1S7W5
60750 229533.A0A1C3YLC1 pp 229533.I1S0J1
60751 229533.A0A1C3YLC1 pp 229533.I1S9Y1
60752 229533.A0A1C3YLC1 pp 229533.I1RQE7
60753 229533.A0A1C3YLC1 pp 229533.I1RXB2
60754 229533.A0A1C3YLC1 pp 229533.I1RIH0
60755 229533.A0A1C3YLC1 pp 229533.I1RFL4
60756 229533.A0A1C3YLC1 pp 229533.I1RE01
60757 229533.A0A1C3YLC1 pp 229533.I1S0L4
60758 229533.A0A1C3YLC1 pp 229533.I1RPK0
60759 229533.A0A1C3YLC1 pp 229533.I1RMV0
60760 229533.A0A1C3YLC1 pp 229533.I1RB92
60761 229533.A0A1C3YLC1 pp 229533.I1RVX0
60762 229533.A0A1C3YLC1 pp 229533.I1RC66
60763 229533.A0A1C3YLC1 pp 229533.I1S2H4
60764 229533.A0A1C3YLC1 pp 229533.I1RT03
60765 229533.A0A1C3YLC1 pp 229533.I1RJV3
60766 229533.A0A1C3YLC1 pp 229533.I1RD22
60767 229533.A0A1C3YLC1 pp 229533.I1RRN2
60768 229533.A0A1C3YLC1 pp 229533.I1RX00
60769 229533.A0A1C3YLC1 pp 229533.I1S2B9
60770 229533.A0A1C3YLC1 pp 229533.I1S0J8
60771 229533.A0A1C3YLC1 pp 229533.I1RQ30
60772 229533.A0A1C3YLC1 pp 229533.I1RAW0
60773 229533.A0A1C3YLC1 pp 229533.I1RRI2
60774 229533.A0A1C3YLC1 pp 229533.I1RSQ4
60775 229533.A0A1C3YLC1 pp 229533.I1RS90
60776 229533.A0A1C3YLC1 pp 229533.I1RUG3
60777 229533.A0A1C3YLC1 pp 229533.I1RNC1
60778 229533.A0A1C3YLC1 pp 229533.I1RZZ9
60779 229533.A0A1C3YLC1 pp 229533.I1RYW3
60780 229533.A0A1C3YLC1 pp 229533.I1S1C7
60781 229533.A0A1C3YLC1 pp 229533.I1RCR8
60782 229533.A0A1C3YLC1 pp 229533.V6R042
60783 229533.A0A1C3YLC1 pp 229533.I1RWI5
60784 229533.A0A1C3YLC1 pp 229533.I1RSQ3
60785 229533.A0A1C3YLC1 pp 229533.A0A1C3YLS9
60786 229533.A0A1C3YLC1 pp 229533.I1RZ39
60787 229533.A0A1C3YLC1 pp 229533.I1RA70
60788 229533.I1RPU4 pp 229533.I1S2R1
60789 229533.I1RPU4 pp 229533.I1S9H9
60790 229533.I1RPU4 pp 229533.I1RZ12
60791 229533.I1RPU4 pp 229533.I1S9B2
60792 229533.I1RPU4 pp 229533.I1RW16
60793 229533.I1RPU4 pp 229533.I1RYK0
60794 229533.I1RPU4 pp 229533.I1RQ80
60795 229533.I1RPU4 pp 229533.I1RQ01
60796 229533.I1RPU4 pp 229533.I1RXA1
60797 229533.I1RPU4 pp 229533.I1RSL6
60798 229533.I1RPU4 pp 229533.I1RRQ7
60799 229533.I1RPU4 pp 229533.I1S1D3
60800 229533.I1RPU4 pp 229533.I1RPW0
60801 229533.I1RPU4 pp 229533.I1RRN5
60802 229533.I1RPU4 pp 229533.I1RT08
60803 229533.I1RPU4 pp 229533.I1RZH4
60804 229533.I1RPU4 pp 229533.I1RTG0
60805 229533.I1RPU4 pp 229533.I1RWT6
60806 229533.I1RPU4 pp 229533.I1RZE4
60807 229533.I1RPU4 pp 229533.I1RUN8
60808 229533.I1RPU4 pp 229533.I1S330
60809 229533.I1RPU4 pp 229533.I1RY44

60810 229533.I1RPU4 pp 229533.I1RZG3
60811 229533.I1RPU4 pp 229533.I1S2E9
60812 229533.I1RPU4 pp 229533.I1RQA3
60813 229533.I1RPU4 pp 229533.I1RQW2
60814 229533.I1RPU4 pp 229533.I1RQM0
60815 229533.I1RPU4 pp 229533.V6RLW4
60816 229533.I1RPU4 pp 229533.I1RZ02
60817 229533.I1RPU4 pp 229533.Q4I7X1
60818 229533.I1RPU4 pp 229533.I1RQS4
60819 229533.I1RPU4 pp 229533.I1RWT1
60820 229533.I1RPU4 pp 229533.I1SA96
60821 229533.I1RPU4 pp 229533.I1RRW0
60822 229533.I1RPU4 pp 229533.I1S382
60823 229533.I1RPU4 pp 229533.I1S604
60824 229533.I1RPU4 pp 229533.I1RTN1
60825 229533.I1RPU4 pp 229533.I1RVA5
60826 229533.I1RPU4 pp 229533.I1S224
60827 229533.I1RPU4 pp 229533.I1S339
60828 229533.I1RPU4 pp 229533.I1RRX6
60829 229533.I1RPU4 pp 229533.I1S0J7
60830 229533.I1RPU4 pp 229533.I1RRP7
60831 229533.I1RPU4 pp 229533.I1RZT1
60832 229533.I1RPU4 pp 229533.I1RX38
60833 229533.I1RPU4 pp 229533.I1S951
60834 229533.I1RPU4 pp 229533.V6R2L8
60835 229533.I1RPU4 pp 229533.I1S1V5
60836 229533.I1RPU4 pp 229533.I1RS41
60837 229533.A0A098DCM1 pp 229533.I1RA36
60838 229533.A0A098DCM1 pp 229533.I1RC84
60839 229533.A0A098DCM1 pp 229533.I1RBH8
60840 229533.A0A098DCM1 pp 229533.V6RY33
60841 229533.A0A098DCM1 pp 229533.I1RWX8
60842 229533.A0A098DCM1 pp 229533.I1RSA4
60843 229533.A0A098DCM1 pp 229533.I1RFQ1
60844 229533.A0A098DCM1 pp 229533.I1RSD4
60845 229533.A0A098DCM1 pp 229533.V6RIS2
60846 229533.A0A098DCM1 pp 229533.I1RS83
60847 229533.A0A098DCM1 pp 229533.I1RT47
60848 229533.A0A098DCM1 pp 229533.I1RZ11
60849 229533.A0A098DCM1 pp 229533.I1RQD8
60850 229533.A0A098DCM1 pp 229533.Q4IPH4
60851 229533.A0A098DCM1 pp 229533.A0A1C3YM29
60852 229533.A0A098DCM1 pp 229533.I1RG90
60853 229533.A0A098DCM1 pp 229533.I1RMW7
60854 229533.A0A098DCM1 pp 229533.I1RSA8
60855 229533.A0A098DCM1 pp 229533.I1S0B1
60856 229533.A0A098DCM1 pp 229533.I1S7M3
60857 229533.A0A098DCM1 pp 229533.I1RZZ1
60858 229533.A0A098DCM1 pp 229533.A0A1C3YNC3
60859 229533.A0A098DCM1 pp 229533.I1S4M5
60860 229533.A0A098DCM1 pp 229533.I1RCI3
60861 229533.A0A098DCM1 pp 229533.I1RMV5
60862 229533.A0A098DCM1 pp 229533.Q4I7F9
60863 229533.A0A098DCM1 pp 229533.I1RX81
60864 229533.A0A098DCM1 pp 229533.I1RCA7
60865 229533.A0A098DCM1 pp 229533.I1RPA0
60866 229533.A0A098DCM1 pp 229533.I1RS75
60867 229533.A0A098DCM1 pp 229533.I1RK96
60868 229533.A0A098DCM1 pp 229533.I1RK92
60869 229533.A0A098DCM1 pp 229533.I1S524
60870 229533.A0A098DCM1 pp 229533.A0A0E0RUQ7
60871 229533.A0A098DCM1 pp 229533.A0A1C3YI62
60872 229533.A0A098DCM1 pp 229533.I1RAD1
60873 229533.A0A098DCM1 pp 229533.I1RXF2
60874 229533.A0A098DCM1 pp 229533.I1RCQ6
60875 229533.A0A098DCM1 pp 229533.A0A0E0SC46
60876 229533.A0A098DCM1 pp 229533.A0A1C3YL11
60877 229533.A0A098DCM1 pp 229533.I1S7M2
60878 229533.A0A098DCM1 pp 229533.I1S5S2
60879 229533.A0A098DCM1 pp 229533.I1RBV3
60880 229533.A0A098DCM1 pp 229533.I1RB50
60881 229533.A0A1C3YN56 pp 229533.V6RK40
60882 229533.A0A1C3YN56 pp 229533.V6RW66

60883 229533.A0A1C3YN56 pp 229533.I1RZ25
60884 229533.A0A1C3YN56 pp 229533.V6QU63
60885 229533.I1R9A0 pp 229533.I1RVA9
60886 229533.I1R9A0 pp 229533.I1RIE1
60887 229533.I1R9A0 pp 229533.I1S3U8
60888 229533.I1R9A0 pp 229533.I1RU48
60889 229533.I1R9A0 pp 229533.I1RH65
60890 229533.I1R9A0 pp 229533.I1RGH6
60891 229533.A0A1C3YJA9 pp 229533.I1RKR7
60892 229533.A0A1C3YJA9 pp 229533.I1RE19
60893 229533.A0A1C3YJA9 pp 229533.Q4IJ79
60894 229533.A0A1C3YJA9 pp 229533.I1RBY9
60895 229533.A0A1C3YJA9 pp 229533.I1RBF4
60896 229533.A0A1C3YJA9 pp 229533.I1RUP6
60897 229533.A0A1C3YJA9 pp 229533.A0A1C3YKU0
60898 229533.A0A1C3YJA9 pp 229533.Q4I5Z5
60899 229533.A0A1C3YJA9 pp 229533.I1RK44
60900 229533.A0A1C3YJA9 pp 229533.A0A1C3YLR6
60901 229533.A0A1C3YJA9 pp 229533.Q4HZS8
60902 229533.A0A1C3YJA9 pp 229533.I1RT41
60903 229533.I1RSE4 pp 229533.I1RSK7
60904 229533.I1S9H4 pp 229533.V6RIV9
60905 229533.I1RX38 pp 229533.I1S207
60906 229533.I1RX38 pp 229533.I1S339
60907 229533.I1RX38 pp 229533.V6R2L8
60908 229533.I1RX38 pp 229533.I1S1W4
60909 229533.I1RX38 pp 229533.I1RZE4
60910 229533.I1RX38 pp 229533.Q4IJU0
60911 229533.I1RX38 pp 229533.I1RZT1
60912 229533.I1RX38 pp 229533.I1S439
60913 229533.I1RX38 pp 229533.I1S1V5
60914 229533.I1RX38 pp 229533.I1S951
60915 229533.I1RX38 pp 229533.I1S0M2
60916 229533.I1RX38 pp 229533.I1S0N5
60917 229533.I1RX38 pp 229533.I1S2E9
60918 229533.I1RX38 pp 229533.I1RXA1
60919 229533.I1RX38 pp 229533.I1RYH9
60920 229533.I1RX38 pp 229533.I1S2R1
60921 229533.I1RX38 pp 229533.I1S0D1
60922 229533.I1RX38 pp 229533.I1S224
60923 229533.I1RX38 pp 229533.Q4I7X1
60924 229533.I1RX38 pp 229533.I1RZG3
60925 229533.I1RX38 pp 229533.V6RVG3
60926 229533.I1RX38 pp 229533.I1S9H9
60927 229533.I1RX38 pp 229533.I1RX39
60928 229533.I1RX38 pp 229533.I1S0J7
60929 229533.I1RX38 pp 229533.I1RZ12
60930 229533.I1RX38 pp 229533.I1S330
60931 229533.I1RX38 pp 229533.I1SA96
60932 229533.I1RX38 pp 229533.I1RY44
60933 229533.I1RX38 pp 229533.I1S382
60934 229533.I1RK50 pp 229533.I1RUY5
60935 229533.I1RK50 pp 229533.I1RRQ2
60936 229533.I1RK50 pp 229533.I1RZV5
60937 229533.I1RK50 pp 229533.I1RNG4
60938 229533.I1RK50 pp 229533.I1RRH5
60939 229533.I1RK50 pp 229533.I1RQB7
60940 229533.I1RSE0 pp 229533.V6QT89
60941 229533.I1RSE0 pp 229533.I1S386
60942 229533.I1RSE0 pp 229533.I1RYU0
60943 229533.I1RSE0 pp 229533.I1RSL2
60944 229533.I1RSE0 pp 229533.I1RT12
60945 229533.I1RSE0 pp 229533.V6QVM5
60946 229533.I1RSE0 pp 229533.I1RXJ0
60947 229533.I1RLM0 pp 229533.V6QU63
60948 229533.I1RLM0 pp 229533.I1RZ25
60949 229533.I1RLM0 pp 229533.V6RW66
60950 229533.I1RLM0 pp 229533.V6RK40
60951 229533.A0A1C3YJ78 pp 229533.I1RJF3
60952 229533.A0A1C3YJ78 pp 229533.I1RL99
60953 229533.A0A1C3YJ78 pp 229533.I1RH14
60954 229533.A0A1C3YJ78 pp 229533.I1R9U8
60955 229533.A0A1C3YJ78 pp 229533.I1RXA1

60956 229533.A0A1C3YJ78 pp 229533.I1S9H4
60957 229533.I1RP73 pp 229533.V6RV57
60958 229533.I1RP73 pp 229533.I1RW06
60959 229533.I1RP73 pp 229533.V6RLM7
60960 229533.I1RP73 pp 229533.I1RYK0
60961 229533.I1RP73 pp 229533.I1S9T8
60962 229533.A0A1C3YJ77 pp 229533.I1S7X9
60963 229533.I1S549 pp 229533.I1S749
60964 229533.I1S549 pp 229533.Q4IER8
60965 229533.I1S549 pp 229533.I1SA77
60966 229533.I1S549 pp 229533.V6RVG3
60967 229533.I1S549 pp 229533.I1S7X9
60968 229533.I1RUB4 pp 229533.I1S8P4
60969 229533.I1RUB4 pp 229533.I1S8P5
60970 229533.I1RUB4 pp 229533.I1S6P7
60971 229533.I1RUB4 pp 229533.I1RUB5
60972 229533.I1RPI3 pp 229533.I1S7A2
60973 229533.I1RCR9 pp 229533.I1RVR7
60974 229533.I1RCR9 pp 229533.I1S0A5
60975 229533.I1RCR9 pp 229533.I1RSE0
60976 229533.I1RCR9 pp 229533.I1RG09
60977 229533.I1RCR9 pp 229533.I1RF80
60978 229533.I1RCR9 pp 229533.I1RDN3
60979 229533.I1RCR9 pp 229533.Q4HTS9
60980 229533.I1RCR9 pp 229533.V6QVM5
60981 229533.I1RCR9 pp 229533.I1RLM3
60982 229533.I1RCR9 pp 229533.I1RJE9
60983 229533.I1RCR9 pp 229533.I1RT12
60984 229533.I1RCR9 pp 229533.I1RLM2
60985 229533.I1RCR9 pp 229533.I1RJU9
60986 229533.I1RCR9 pp 229533.I1RSL2
60987 229533.I1RCR9 pp 229533.I1RYU0
60988 229533.I1REN2 pp 229533.I1SAT1
60989 229533.I1RZH5 pp 229533.Q4IBU4
60990 229533.I1RZH5 pp 229533.I1S8F8
60991 229533.I1RZH5 pp 229533.Q4IB65
60992 229533.I1RZH5 pp 229533.Q4I375
60993 229533.I1RZH5 pp 229533.I1S4H6
60994 229533.I1RZH5 pp 229533.Q4I7T5
60995 229533.I1RZH5 pp 229533.V6R1X4
60996 229533.I1RZH5 pp 229533.Q4IK03
60997 229533.I1RZH5 pp 229533.V6R3J1
60998 229533.I1RZH5 pp 229533.Q4IPZ1
60999 229533.I1RZH5 pp 229533.Q4I099
61000 229533.I1RZH5 pp 229533.Q4IJW4
61001 229533.I1RZH5 pp 229533.I1S039
61002 229533.I1RZH5 pp 229533.I1S8F0
61003 229533.I1RZH5 pp 229533.I1RZS4
61004 229533.I1RZH5 pp 229533.Q4I2W2
61005 229533.I1RZH5 pp 229533.I1S1W5
61006 229533.I1RZH5 pp 229533.Q4HZ95
61007 229533.I1RZH5 pp 229533.V6QUV7
61008 229533.I1RZH5 pp 229533.I1S088
61009 229533.I1RZH5 pp 229533.I1S0R9
61010 229533.Q4I7N9 pp 229533.V6QUV7
61011 229533.Q4I7N9 pp 229533.Q4IAK7
61012 229533.Q4I7N9 pp 229533.Q4IMD1
61013 229533.Q4I7N9 pp 229533.Q4IJP1
61014 229533.Q4I7N9 pp 229533.Q4IB50
61015 229533.I1RHH5 pp 229533.I1RQJ7
61016 229533.I1RHH5 pp 229533.I1S2M8
61017 229533.I1RHH5 pp 229533.I1S0M4
61018 229533.I1RHH5 pp 229533.I1S132
61019 229533.I1RHH5 pp 229533.I1RJU9
61020 229533.I1RHH5 pp 229533.I1RXV9
61021 229533.I1RHH5 pp 229533.I1RKD4
61022 229533.I1RHH5 pp 229533.I1S386
61023 229533.I1RHH5 pp 229533.I1RLM3
61024 229533.I1RHH5 pp 229533.I1RND4
61025 229533.I1RHH5 pp 229533.I1RIV0
61026 229533.I1RHH5 pp 229533.I1RK10
61027 229533.I1RHH5 pp 229533.I1RP86
61028 229533.I1RHH5 pp 229533.I1RT12

61029	229533.I1RHH5	pp	229533.I1RXJ0
61030	229533.I1RHH5	pp	229533.I1RNB4
61031	229533.I1RHH5	pp	229533.I1RXW3
61032	229533.I1RHH5	pp	229533.I1RJE9
61033	229533.I1RDC1	pp	229533.I1RS28
61034	229533.I1RDC1	pp	229533.I1S4M5
61035	229533.I1RDC1	pp	229533.I1RXF2
61036	229533.I1RDC1	pp	229533.I1S7X9
61037	229533.I1RDC1	pp	229533.I1RSA4
61038	229533.I1RDC1	pp	229533.I1RND5
61039	229533.I1RDC1	pp	229533.I1RG90
61040	229533.I1RDC1	pp	229533.I1RSD4
61041	229533.I1RDC1	pp	229533.I1RG57
61042	229533.I1RDC1	pp	229533.I1RK92
61043	229533.I1RDC1	pp	229533.I1RX81
61044	229533.I1RDC1	pp	229533.V6QXG1
61045	229533.I1RDC1	pp	229533.I1RK90
61046	229533.I1RDC1	pp	229533.I1S7M3
61047	229533.I1RDC1	pp	229533.I1RZG7
61048	229533.I1RDC1	pp	229533.I1S7M2
61049	229533.I1RDC1	pp	229533.I1RDK3
61050	229533.I1RMH2	pp	229533.I1RQ42
61051	229533.I1RMH2	pp	229533.I1RY12
61052	229533.I1RMH2	pp	229533.I1RMR5
61053	229533.I1RMH2	pp	229533.I1RW91
61054	229533.I1RMH2	pp	229533.I1RQA2
61055	229533.I1RMH2	pp	229533.I1RTB7
61056	229533.I1RMH2	pp	229533.I1S0S6
61057	229533.I1RMH2	pp	229533.I1S0A0
61058	229533.I1RMH2	pp	229533.I1RPS7
61059	229533.I1RMH2	pp	229533.I1S7X9
61060	229533.I1RMH2	pp	229533.I1RUK7
61061	229533.I1RMH2	pp	229533.I1S0Y1
61062	229533.I1RMH2	pp	229533.I1RN04
61063	229533.I1RMH2	pp	229533.P0C431
61064	229533.I1RMH2	pp	229533.I1RQ29
61065	229533.I1RMH2	pp	229533.I1RQN9
61066	229533.I1RMH2	pp	229533.I1S549
61067	229533.I1RMH2	pp	229533.I1RNM8
61068	229533.I1RMH2	pp	229533.I1RWF5
61069	229533.I1RMH2	pp	229533.I1RN84
61070	229533.I1RMH2	pp	229533.I1RRP3
61071	229533.I1RMH2	pp	229533.I1RT36
61072	229533.I1RMH2	pp	229533.I1RML4
61073	229533.I1RKK0	pp	229533.I1RPU7
61074	229533.I1RKK0	pp	229533.I1S2E9
61075	229533.I1RKK0	pp	229533.I1RRM9
61076	229533.I1RKK0	pp	229533.I1RZH5
61077	229533.I1RKK0	pp	229533.I1RRT8
61078	229533.I1RKK0	pp	229533.I1RNP7
61079	229533.I1RKK0	pp	229533.I1RV91
61080	229533.I1RKK0	pp	229533.I1RPX0
61081	229533.I1RKK0	pp	229533.V6RTJ8
61082	229533.I1RKK0	pp	229533.I1RZJ5
61083	229533.I1RKK0	pp	229533.I1RMF5
61084	229533.I1RKK0	pp	229533.I1RNF4
61085	229533.I1RKK0	pp	229533.I1RLU1
61086	229533.I1RKK0	pp	229533.I1RYZ9
61087	229533.I1RKK0	pp	229533.I1S2G7
61088	229533.I1RKK0	pp	229533.I1RQG4
61089	229533.I1RKK0	pp	229533.I1RL24
61090	229533.I1RKK0	pp	229533.I1RP28
61091	229533.I1RKK0	pp	229533.I1RRX5
61092	229533.I1RKK0	pp	229533.I1RPD6
61093	229533.I1RKK0	pp	229533.I1RUN8
61094	229533.I1RKK0	pp	229533.I1RST8
61095	229533.I1RKK0	pp	229533.I1RVQ0
61096	229533.I1RKK0	pp	229533.I1S224
61097	229533.I1RKK0	pp	229533.I1RNC0
61098	229533.I1RKK0	pp	229533.I1RQ26
61099	229533.I1RKK0	pp	229533.I1RQA9
61100	229533.I1RKK0	pp	229533.I1S7X9
61101	229533.I1RKK0	pp	229533.I1RX70

61102 229533.I1RKK0 pp 229533.I1RXH6
61103 229533.I1RKK0 pp 229533.I1RWJ6
61104 229533.I1RKK0 pp 229533.I1RX25
61105 229533.I1RKK0 pp 229533.V6QV09
61106 229533.I1RKK0 pp 229533.I1RYH9
61107 229533.I1RKK0 pp 229533.I1RNC1
61108 229533.I1RKK0 pp 229533.I1S1W4
61109 229533.I1RKK0 pp 229533.I1RV66
61110 229533.I1RKK0 pp 229533.I1RRV1
61111 229533.I1RKK0 pp 229533.I1S0M2
61112 229533.I1RKK0 pp 229533.I1RPW0
61113 229533.I1RKK0 pp 229533.I1RMQ5
61114 229533.I1RKK0 pp 229533.I1RWT6
61115 229533.I1RKK0 pp 229533.V6RKY7
61116 229533.I1RKK0 pp 229533.I1RRW0
61117 229533.I1RKK0 pp 229533.I1RQB4
61118 229533.I1RKK0 pp 229533.Q4IJM4
61119 229533.I1RKK0 pp 229533.I1RT36
61120 229533.I1RKK0 pp 229533.V6RGI1
61121 229533.I1RKK0 pp 229533.I1RN65
61122 229533.I1RKK0 pp 229533.I1RY44
61123 229533.I1RKK0 pp 229533.I1RRN5
61124 229533.I1RKK0 pp 229533.I1RPU4
61125 229533.I1RKK0 pp 229533.I1RZM1
61126 229533.I1RKK0 pp 229533.V6RVG3
61127 229533.I1RKK0 pp 229533.I1RNY9
61128 229533.I1RKK0 pp 229533.I1RN08
61129 229533.I1RKK0 pp 229533.V6RDP1
61130 229533.I1RKK0 pp 229533.I1RQF2
61131 229533.I1RKK0 pp 229533.I1RUQ5
61132 229533.I1RKK0 pp 229533.I1RQA3
61133 229533.I1RKK0 pp 229533.I1S0N4
61134 229533.I1RKK0 pp 229533.Q4IPC8
61135 229533.I1RKK0 pp 229533.I1RZE4
61136 229533.I1RKK0 pp 229533.I1RY58
61137 229533.I1RKK0 pp 229533.I1S0D1
61138 229533.I1RKK0 pp 229533.I1RSG0
61139 229533.I1RKK0 pp 229533.I1RMA8
61140 229533.I1RKK0 pp 229533.I1RM35
61141 229533.I1RKK0 pp 229533.I1RYY5
61142 229533.I1RKK0 pp 229533.I1S439
61143 229533.I1RKK0 pp 229533.I1RQ72
61144 229533.I1RKK0 pp 229533.I1RZH4
61145 229533.I1RKK0 pp 229533.I1RRP7
61146 229533.I1RKK0 pp 229533.I1S1D3
61147 229533.I1RKK0 pp 229533.I1RNW8
61148 229533.I1RKK0 pp 229533.I1S0N8
61149 229533.I1RKK0 pp 229533.I1RM41
61150 229533.I1RKK0 pp 229533.I1RMM9
61151 229533.I1RKK0 pp 229533.I1S2K7
61152 229533.I1RKK0 pp 229533.I1RRY3
61153 229533.I1RKK0 pp 229533.I1S0Z7
61154 229533.I1RHQ0 pp 229533.I1S267
61155 229533.I1RHQ0 pp 229533.I1RM41
61156 229533.I1RHQ0 pp 229533.I1RT12
61157 229533.I1RHQ0 pp 229533.I1RMX6
61158 229533.I1RHQ0 pp 229533.V6RY56
61159 229533.I1RHQ0 pp 229533.I1S4D0
61160 229533.I1RHQ0 pp 229533.I1RKR5
61161 229533.I1RHQ0 pp 229533.I1RZC3
61162 229533.I1RHQ0 pp 229533.I1RPT0
61163 229533.I1RHQ0 pp 229533.I1RSG0
61164 229533.I1RHQ0 pp 229533.V6RN31
61165 229533.I1RHQ0 pp 229533.V6RK40
61166 229533.I1RHQ0 pp 229533.V6RW66
61167 229533.I1RHQ0 pp 229533.I1RVN1
61168 229533.I1RHQ0 pp 229533.V6QU63
61169 229533.I1RHQ0 pp 229533.I1RXM0
61170 229533.I1RHQ0 pp 229533.I1S386
61171 229533.I1RHQ0 pp 229533.I1RXJ0
61172 229533.I1RHQ0 pp 229533.I1RY32
61173 229533.I1RHQ0 pp 229533.I1RJE9
61174 229533.A0A098DCL3 pp 229533.I1RSE0

61175 229533.A0A098DCL3 pp 229533.A0A1C3YJW1
61176 229533.A0A098DCL3 pp 229533.I1RCR9
61177 229533.A0A098DCL3 pp 229533.I1RHH5
61178 229533.A0A098DCL3 pp 229533.I1RHQ0
61179 229533.V6RRR4 pp 229533.V6RW66
61180 229533.I1RDV9 pp 229533.I1RY44
61181 229533.I1RDV9 pp 229533.I1RFL7
61182 229533.I1RDV9 pp 229533.I1RZC3
61183 229533.I1RDV9 pp 229533.I1RRW0
61184 229533.I1RDV9 pp 229533.I1RVN2
61185 229533.I1RDV9 pp 229533.I1RPW0
61186 229533.I1RDV9 pp 229533.V6RGI1
61187 229533.I1RDV9 pp 229533.I1S098
61188 229533.I1RDV9 pp 229533.I1S2E9
61189 229533.I1RDV9 pp 229533.I1RV91
61190 229533.I1RDV9 pp 229533.I1RY58
61191 229533.I1RDV9 pp 229533.I1RZT1
61192 229533.I1RDV9 pp 229533.I1S004
61193 229533.I1RDV9 pp 229533.I1S0J7
61194 229533.I1RDV9 pp 229533.I1S549
61195 229533.I1RDV9 pp 229533.I1RWT6
61196 229533.I1RDV9 pp 229533.I1RZ63
61197 229533.I1RDV9 pp 229533.I1RPK4
61198 229533.I1RDV9 pp 229533.I1RPU4
61199 229533.I1RDV9 pp 229533.Q4I7N9
61200 229533.I1RDV9 pp 229533.I1S267
61201 229533.I1RDV9 pp 229533.I1RQA3
61202 229533.I1RDV9 pp 229533.I1RX38
61203 229533.I1RDV9 pp 229533.I1RQS4
61204 229533.I1RDV9 pp 229533.I1RJK7
61205 229533.I1RDV9 pp 229533.I1RRX6
61206 229533.I1RDV9 pp 229533.I1S224
61207 229533.I1RDV9 pp 229533.I1RTG0
61208 229533.I1RAG5 pp 229533.V6RLM7
61209 229533.I1RAG5 pp 229533.Q4I1B1
61210 229533.I1RAG5 pp 229533.I1RW06
61211 229533.I1RAG5 pp 229533.I1RFV9
61212 229533.I1RAG5 pp 229533.I1RH82
61213 229533.I1RAG5 pp 229533.I1RXY7
61214 229533.I1RAG5 pp 229533.I1S9T8
61215 229533.I1RAG5 pp 229533.I1REC4
61216 229533.I1RAG5 pp 229533.I1RIH0
61217 229533.I1RAG5 pp 229533.I1RND4
61218 229533.I1RAG5 pp 229533.I1RYK0
61219 229533.I1RAG5 pp 229533.V6RV57
61220 229533.I1RAG5 pp 229533.V6QVH8
61221 229533.I1RAG5 pp 229533.I1RQK4
61222 229533.I1RVM1 pp 229533.I1RYU6
61223 229533.A0A1C3YJ86 pp 229533.I1RT38
61224 229533.A0A1C3YJ86 pp 229533.I1RZS4
61225 229533.A0A1C3YJ86 pp 229533.I1S088
61226 229533.A0A1C3YJ86 pp 229533.I1RVN1
61227 229533.A0A1C3YJ86 pp 229533.I1S1W5
61228 229533.A0A1C3YJ86 pp 229533.I1RJT1
61229 229533.A0A1C3YJ86 pp 229533.I1RC86
61230 229533.A0A1C3YJ86 pp 229533.Q4I6B0
61231 229533.A0A1C3YJ86 pp 229533.I1RZH5
61232 229533.A0A1C3YJ86 pp 229533.A0A1C3YN81
61233 229533.A0A1C3YJ86 pp 229533.I1RJT3
61234 229533.A0A1C3YJ86 pp 229533.Q4IPZ1
61235 229533.A0A1C3YJ86 pp 229533.I1S8F0
61236 229533.A0A1C3YJ86 pp 229533.I1RCK2
61237 229533.A0A1C3YJ86 pp 229533.I1RFV5
61238 229533.A0A1C3YJ86 pp 229533.I1S1D0
61239 229533.A0A1C3YJ86 pp 229533.I1RPA5
61240 229533.A0A1C3YJ86 pp 229533.I1S4H6
61241 229533.A0A1C3YJ86 pp 229533.Q4IJW4
61242 229533.A0A1C3YJ86 pp 229533.A0A1C3YKG8
61243 229533.A0A1C3YJ86 pp 229533.I1RAZ6
61244 229533.A0A1C3YJ86 pp 229533.Q4I2W2
61245 229533.A0A1C3YJ86 pp 229533.I1RRK8
61246 229533.Q4I2X5 pp 229533.Q4IN63
61247 229533.Q4I2X5 pp 229533.V6RQR4

61248	229533.I1RJK7	pp	229533.I1RQA3
61249	229533.I1RJK7	pp	229533.Q4IER8
61250	229533.I1RJK7	pp	229533.Q4I7N9
61251	229533.I1RJK7	pp	229533.I1RST9
61252	229533.I1RJK7	pp	229533.I1RXZ4
61253	229533.I1RJK7	pp	229533.I1RSG0
61254	229533.I1RJK7	pp	229533.I1RRL5
61255	229533.I1RJK7	pp	229533.Q4HZS8
61256	229533.I1RJK7	pp	229533.I1RTQ9
61257	229533.I1RJK7	pp	229533.I1RL24
61258	229533.I1RJK7	pp	229533.V6REE9
61259	229533.I1RJK7	pp	229533.I1S267
61260	229533.I1RJK7	pp	229533.I1RZ12
61261	229533.I1RJK7	pp	229533.I1S224
61262	229533.I1RJK7	pp	229533.V6QV09
61263	229533.I1RJK7	pp	229533.I1RNC0
61264	229533.I1RJK7	pp	229533.I1RRX6
61265	229533.I1RJK7	pp	229533.I1RQ80
61266	229533.I1RJK7	pp	229533.I1RZH4
61267	229533.I1RJK7	pp	229533.I1RTG0
61268	229533.I1RJK7	pp	229533.I1RWT6
61269	229533.I1RJK7	pp	229533.I1RT41
61270	229533.I1RJK7	pp	229533.I1S334
61271	229533.I1RJK7	pp	229533.I1S0Z3
61272	229533.I1RJK7	pp	229533.I1RPU4
61273	229533.I1RJK7	pp	229533.I1RYZ9
61274	229533.I1RJK7	pp	229533.Q5I2J3
61275	229533.I1RJK7	pp	229533.I1RZC3
61276	229533.I1RJK7	pp	229533.I1RPW0
61277	229533.I1RJK7	pp	229533.I1RRW0
61278	229533.I1RJK7	pp	229533.I1RY58
61279	229533.I1RJK7	pp	229533.I1RRP7
61280	229533.I1RJK7	pp	229533.I1RM41
61281	229533.I1RJK7	pp	229533.I1S0M2
61282	229533.I1RJK7	pp	229533.I1RMJ8
61283	229533.I1RJK7	pp	229533.I1RY44
61284	229533.I1RJK7	pp	229533.I1RTB2
61285	229533.I1RJK7	pp	229533.I1S3H8
61286	229533.I1RJK7	pp	229533.I1RSI3
61287	229533.I1RJK7	pp	229533.I1S0J7
61288	229533.I1RJK7	pp	229533.I1S098
61289	229533.I1RJK7	pp	229533.I1S2E9
61290	229533.I1RJK7	pp	229533.I1RVN2
61291	229533.I1RJK7	pp	229533.V6RGI1
61292	229533.I1RJK7	pp	229533.I1RZ63
61293	229533.I1RJK7	pp	229533.I1RX38
61294	229533.I1RJK7	pp	229533.I1RPK4
61295	229533.I1RJK7	pp	229533.I1RQD1
61296	229533.I1RJK7	pp	229533.I1RV91
61297	229533.I1RJK7	pp	229533.I1RY32
61298	229533.I1RJK7	pp	229533.I1S7X9
61299	229533.I1RJK7	pp	229533.I1RR95
61300	229533.I1RJK7	pp	229533.I1S004
61301	229533.I1RJK7	pp	229533.I1RMP0
61302	229533.I1RJK7	pp	229533.I1S549
61303	229533.I1RQ12	pp	229533.V6RVJ3
61304	229533.I1RQ12	pp	229533.I1RZY1
61305	229533.I1RQ12	pp	229533.I1RZE7
61306	229533.I1RQ12	pp	229533.I1RXX1
61307	229533.I1RQ12	pp	229533.I1S064
61308	229533.I1RQ12	pp	229533.I1S024
61309	229533.I1RQ12	pp	229533.I1RVW6
61310	229533.I1RQ12	pp	229533.I1RXB6
61311	229533.A0A1C3YJ70	pp	229533.I1RZ60
61312	229533.A0A1C3YJ70	pp	229533.I1REJ4
61313	229533.A0A1C3YJ70	pp	229533.V6R227
61314	229533.A0A1C3YJ70	pp	229533.I1RA52
61315	229533.A0A1C3YJ70	pp	229533.I1RSE9
61316	229533.A0A1C3YKE3	pp	229533.V6RW66
61317	229533.A0A1C3YKE3	pp	229533.V6QU63
61318	229533.A0A1C3YKE3	pp	229533.V6RK40
61319	229533.A0A1C3YKE3	pp	229533.I1RZ25
61320	229533.A0A1C3YHQ1	pp	229533.Q4IK03

61321 229533.A0A1C3YHQ1 pp 229533.I1RZI8
61322 229533.A0A1C3YHQ1 pp 229533.I1RWA3
61323 229533.A0A1C3YHQ1 pp 229533.Q4I6B0
61324 229533.A0A1C3YHQ1 pp 229533.I1S0R8
61325 229533.A0A1C3YHQ1 pp 229533.I1S0K2
61326 229533.A0A1C3YHQ1 pp 229533.Q4I099
61327 229533.A0A1C3YHQ1 pp 229533.A0A1C3YK11
61328 229533.A0A1C3YHQ1 pp 229533.A0A1C3YKM4
61329 229533.A0A1C3YHQ1 pp 229533.I1RPA4
61330 229533.A0A1C3YHQ1 pp 229533.I1S8Y0
61331 229533.A0A1C3YHQ1 pp 229533.I1RS01
61332 229533.A0A1C3YHQ1 pp 229533.I1RVZ2
61333 229533.A0A1C3YHQ1 pp 229533.I1S277
61334 229533.A0A1C3YHQ1 pp 229533.A0A1C3YJZ6
61335 229533.A0A1C3YHQ1 pp 229533.I1RKB2
61336 229533.A0A1C3YHQ1 pp 229533.A0A1C3YLB6
61337 229533.A0A1C3YHQ1 pp 229533.I1S1F3
61338 229533.A0A1C3YHQ1 pp 229533.Q4IPZ1
61339 229533.A0A1C3YHQ1 pp 229533.A0A1C3YKN2
61340 229533.A0A1C3YHQ1 pp 229533.Q4IB65
61341 229533.A0A1C3YHQ1 pp 229533.Q4I5G1
61342 229533.A0A1C3YHQ1 pp 229533.A0A1C3YLB0
61343 229533.A0A1C3YHQ1 pp 229533.I1RWT0
61344 229533.A0A1C3YHQ1 pp 229533.A0A1C3YLL3
61345 229533.A0A1C3YHQ1 pp 229533.Q4IJW4
61346 229533.A0A1C3YHQ1 pp 229533.I1RX17
61347 229533.A0A1C3YHQ1 pp 229533.I1RKB7
61348 229533.A0A1C3YHQ1 pp 229533.A0A1C3YJN3
61349 229533.A0A1C3YHQ1 pp 229533.A0A1C3YKP5
61350 229533.A0A1C3YHQ1 pp 229533.I1RX21
61351 229533.A0A1C3YHQ1 pp 229533.I1RPT2
61352 229533.A0A1C3YHQ1 pp 229533.I1S8Y1
61353 229533.A0A1C3YHQ1 pp 229533.I1RLV7
61354 229533.A0A1C3YHQ1 pp 229533.I1RX39
61355 229533.A0A1C3YHQ1 pp 229533.I1S212
61356 229533.A0A1C3YHQ1 pp 229533.I1RS50
61357 229533.A0A1C3YHQ1 pp 229533.I1RZM9
61358 229533.A0A1C3YHQ1 pp 229533.I1S268
61359 229533.A0A1C3YHQ1 pp 229533.I1RB66
61360 229533.A0A1C3YHQ1 pp 229533.I1RNP8
61361 229533.A0A1C3YHQ1 pp 229533.A0A1C3YIW9
61362 229533.I1RH70 pp 229533.I1RMS2
61363 229533.I1S004 pp 229533.Q4IB89
61364 229533.I1S004 pp 229533.I1S0D5
61365 229533.I1S004 pp 229533.I1S4P6
61366 229533.I1S004 pp 229533.I1S2E9
61367 229533.I1S004 pp 229533.I1S224
61368 229533.I1S004 pp 229533.I1S788
61369 229533.I1RE13 pp 229533.I1RSI0
61370 229533.I1RE13 pp 229533.V6QWB1
61371 229533.I1RE13 pp 229533.I1RMW4
61372 229533.I1RE13 pp 229533.I1RNP7
61373 229533.I1RE13 pp 229533.I1S4Z7
61374 229533.I1RE13 pp 229533.I1RZ94
61375 229533.I1RE13 pp 229533.Q4HVU7
61376 229533.I1RE13 pp 229533.V6RRM0
61377 229533.I1RJX2 pp 229533.I1RN65
61378 229533.I1RJX2 pp 229533.I1RK47
61379 229533.I1RJX2 pp 229533.I1RUY6
61380 229533.I1RJX2 pp 229533.I1S439
61381 229533.I1RJX2 pp 229533.Q4I948
61382 229533.I1RJX2 pp 229533.V6REE9
61383 229533.I1RJX2 pp 229533.I1RQ93
61384 229533.I1RJX2 pp 229533.Q4ICA8
61385 229533.I1RJX2 pp 229533.I1RTU1
61386 229533.I1RJX2 pp 229533.V6RGI1
61387 229533.I1RJX2 pp 229533.I1RYV3
61388 229533.I1RJX2 pp 229533.I1RY98
61389 229533.I1RJX2 pp 229533.I1S2C1
61390 229533.I1RJX2 pp 229533.I1RPK0
61391 229533.I1RJX2 pp 229533.I1RSJ2
61392 229533.I1RJX2 pp 229533.I1RT36
61393 229533.I1RJX2 pp 229533.I1RTE6

61394 229533.I1RJX2 pp 229533.I1RMG6
61395 229533.I1RJX2 pp 229533.I1S7X9
61396 229533.I1RJX2 pp 229533.I1S2A8
61397 229533.I1RJX2 pp 229533.I1RNW8
61398 229533.I1RJX2 pp 229533.O74638
61399 229533.I1RJX2 pp 229533.I1RKI0
61400 229533.I1RJX2 pp 229533.I1RQ88
61401 229533.I1RJX2 pp 229533.I1S0F3
61402 229533.I1RJX2 pp 229533.I1SAA0
61403 229533.I1RJX2 pp 229533.I1S0J8
61404 229533.I1RJX2 pp 229533.I1RTI7
61405 229533.I1RJX2 pp 229533.V6QVM5
61406 229533.I1RJX2 pp 229533.I1RZA9
61407 229533.I1RJX2 pp 229533.I1SA27
61408 229533.I1RZ83 pp 229533.V6RK96
61409 229533.I1RZ83 pp 229533.I1S0N3
61410 229533.I1RZ83 pp 229533.I1S0Y7
61411 229533.I1RZ83 pp 229533.I1S0S2
61412 229533.I1RZ83 pp 229533.I1RZS3
61413 229533.I1RZ83 pp 229533.V6RLM7
61414 229533.I1RZ83 pp 229533.I1RZM4
61415 229533.I1RZ70 pp 229533.I1S3G4
61416 229533.I1RZ70 pp 229533.I1S1W8
61417 229533.I1RZ70 pp 229533.I1S8D1
61418 229533.I1RZ70 pp 229533.V6RM02
61419 229533.I1RZ70 pp 229533.V6RK96
61420 229533.I1RZ70 pp 229533.V6RH92
61421 229533.I1RZ70 pp 229533.I1S148
61422 229533.I1RZ70 pp 229533.I1S147
61423 229533.I1RZ70 pp 229533.I1S8D2
61424 229533.I1RZ70 pp 229533.I1RZM4
61425 229533.A0A1C3YLD3 pp 229533.I1RPK0
61426 229533.A0A1C3YLD3 pp 229533.V6RGI1
61427 229533.A0A1C3YLD3 pp 229533.I1RZA9
61428 229533.A0A1C3YLD3 pp 229533.I1RCN2
61429 229533.A0A1C3YLD3 pp 229533.V6R382
61430 229533.A0A1C3YLD3 pp 229533.I1RAI9
61431 229533.A0A1C3YLD3 pp 229533.I1S2A8
61432 229533.A0A1C3YLD3 pp 229533.I1RTE6
61433 229533.A0A1C3YLD3 pp 229533.I1RT36
61434 229533.A0A1C3YLD3 pp 229533.I1RFL7
61435 229533.A0A1C3YLD3 pp 229533.I1S7X9
61436 229533.A0A1C3YLD3 pp 229533.I1S0F3
61437 229533.A0A1C3YLD3 pp 229533.I1RB73
61438 229533.A0A1C3YLD3 pp 229533.I1RNW8
61439 229533.A0A1C3YLD3 pp 229533.I1RKI0
61440 229533.A0A1C3YLD3 pp 229533.I1RIG5
61441 229533.A0A1C3YLD3 pp 229533.I1RMG6
61442 229533.A0A1C3YLD3 pp 229533.I1RQ88
61443 229533.A0A1C3YLD3 pp 229533.I1S0J8
61444 229533.A0A1C3YLD3 pp 229533.I1RC95
61445 229533.A0A1C3YLD3 pp 229533.I1RBJ0
61446 229533.A0A1C3YLD3 pp 229533.I1SAA0
61447 229533.A0A1C3YLD3 pp 229533.I1RCD4
61448 229533.A0A1C3YLD3 pp 229533.V6QVM5
61449 229533.A0A1C3YLD3 pp 229533.I1RK47
61450 229533.A0A1C3YLD3 pp 229533.I1RN65
61451 229533.A0A1C3YLD3 pp 229533.O74638
61452 229533.A0A1C3YLD3 pp 229533.I1RAF9
61453 229533.A0A1C3YLD3 pp 229533.I1RUY6
61454 229533.A0A1C3YLD3 pp 229533.I1RTI7
61455 229533.A0A1C3YLD3 pp 229533.Q4I948
61456 229533.A0A1C3YLD3 pp 229533.Q4ICA8
61457 229533.A0A1C3YLD3 pp 229533.I1SA27
61458 229533.A0A1C3YLD3 pp 229533.I1S439
61459 229533.A0A1C3YLD3 pp 229533.A0A1C3YLW0
61460 229533.A0A1C3YLD3 pp 229533.I1RYV3
61461 229533.A0A1C3YLD3 pp 229533.I1RQ93
61462 229533.A0A1C3YLD3 pp 229533.I1RAQ4
61463 229533.A0A1C3YLD3 pp 229533.V6REE9
61464 229533.A0A1C3YLD3 pp 229533.I1RDB1
61465 229533.A0A1C3YLD3 pp 229533.I1RY98
61466 229533.A0A1C3YLD3 pp 229533.I1S2C1

61467 229533.A0A1C3YLD3 pp 229533.I1RTU1
61468 229533.A0A1C3YLD3 pp 229533.I1RCP3
61469 229533.A0A1C3YLD3 pp 229533.I1S9M0
61470 229533.A0A1C3YLD3 pp 229533.I1RIX0
61471 229533.A0A1C3YLD3 pp 229533.I1RJX2
61472 229533.A0A1C3YLD3 pp 229533.I1RSJ2
61473 229533.A0A0E0SGQ4 pp 229533.A0A1C3YN56
61474 229533.A0A0E0SGQ4 pp 229533.I1RLM0
61475 229533.A0A0E0SGQ4 pp 229533.I1RUP5
61476 229533.A0A0E0SGQ4 pp 229533.A0A1C3YHP2
61477 229533.A0A0E0SGQ4 pp 229533.I1RNG4
61478 229533.A0A0E0SGQ4 pp 229533.I1RSX9
61479 229533.A0A0E0SGQ4 pp 229533.I1RND4
61480 229533.A0A0E0SGQ4 pp 229533.V6RRR4
61481 229533.A0A0E0SGQ4 pp 229533.I1RD35
61482 229533.A0A0E0SGQ4 pp 229533.A0A1C3YJU0
61483 229533.A0A0E0SGQ4 pp 229533.A0A1C3YKE3
61484 229533.A0A0E0SGQ4 pp 229533.I1RGY2
61485 229533.A0A0E0SGQ4 pp 229533.I1RHQ0
61486 229533.A0A0E0SGQ4 pp 229533.I1RRG1
61487 229533.A0A0E0SGQ4 pp 229533.I1RJS0
61488 229533.A0A0E0SGQ4 pp 229533.A0A1C3YM97
61489 229533.A0A0E0SGQ4 pp 229533.I1S183
61490 229533.I1RZ12 pp 229533.I1S224
61491 229533.I1RZ12 pp 229533.I1S0R1
61492 229533.I1RZ12 pp 229533.I1S004
61493 229533.I1RZ12 pp 229533.Q4I7N9
61494 229533.I1RZ12 pp 229533.V6REE9
61495 229533.I1RZ12 pp 229533.Q5I2J3
61496 229533.I1RZ12 pp 229533.V6QV09
61497 229533.I1RZ12 pp 229533.I1S2E9
61498 229533.I1RZ12 pp 229533.I1RZ63
61499 229533.I1RZ12 pp 229533.I1S549
61500 229533.I1RZ12 pp 229533.I1S267
61501 229533.I1RZ12 pp 229533.I1S0J7
61502 229533.I1RZ12 pp 229533.I1S098
61503 229533.I1RZ12 pp 229533.I1RZC3
61504 229533.I1RZ12 pp 229533.V6RGI1
61505 229533.I1RRK8 pp 229533.Q4IAS1
61506 229533.I1RRK8 pp 229533.I1RT98
61507 229533.I1RRK8 pp 229533.Q4I2W2
61508 229533.I1RRK8 pp 229533.I1RZP8
61509 229533.I1RRK8 pp 229533.I1RZR6
61510 229533.I1RRK8 pp 229533.I1S9U2
61511 229533.I1RRK8 pp 229533.I1RZJ6
61512 229533.I1RRK8 pp 229533.I1S0M9
61513 229533.I1RRK8 pp 229533.I1RS41
61514 229533.I1RRK8 pp 229533.I1RSJ4
61515 229533.I1RRK8 pp 229533.Q4HZ47
61516 229533.I1RRK8 pp 229533.I1S0J1
61517 229533.I1RRK8 pp 229533.I1RUG1
61518 229533.I1RRK8 pp 229533.Q4HYB8
61519 229533.I1RRK8 pp 229533.Q4IJU0
61520 229533.I1RRK8 pp 229533.I1S4H6
61521 229533.I1RRK8 pp 229533.I1RWE1
61522 229533.I1RRK8 pp 229533.I1S8F0
61523 229533.I1RRK8 pp 229533.Q4IBU4
61524 229533.I1RRK8 pp 229533.I1RZH5
61525 229533.I1RZN1 pp 229533.V6R382
61526 229533.I1RZN1 pp 229533.I1S2B0
61527 229533.I1RZN1 pp 229533.V6RLM7
61528 229533.I1RZN1 pp 229533.I1S3H8
61529 229533.I1RZN1 pp 229533.I1S5C5
61530 229533.I1RZN1 pp 229533.I1S8H4
61531 229533.I1RZN1 pp 229533.I1RZT6
61532 229533.I1RZN1 pp 229533.V6RK96
61533 229533.I1RZN1 pp 229533.I1SAV3
61534 229533.I1RZN1 pp 229533.I1S334
61535 229533.I1RIE9 pp 229533.I1RPI4
61536 229533.I1RIE9 pp 229533.I1RJ49
61537 229533.I1RIE9 pp 229533.I1RNE9
61538 229533.I1RIE9 pp 229533.I1RYH1
61539 229533.I1RTG0 pp 229533.I1RWT6

61540 229533.I1RTG0 pp 229533.I1RYL5
61541 229533.I1RTG0 pp 229533.I1S267
61542 229533.I1RTG0 pp 229533.Q4IEA7
61543 229533.I1RTG0 pp 229533.Q4I7N9
61544 229533.I1RTG0 pp 229533.Q4I785
61545 229533.I1RTG0 pp 229533.I1RX77
61546 229533.I1RTG0 pp 229533.I1S224
61547 229533.I1RTG0 pp 229533.I1RX27
61548 229533.I1RTG0 pp 229533.I1RZ12
61549 229533.I1RTG0 pp 229533.I1RZC3
61550 229533.I1RTG0 pp 229533.I1RVN2
61551 229533.I1RTG0 pp 229533.I1RY58
61552 229533.I1RTG0 pp 229533.I1RY44
61553 229533.I1RTG0 pp 229533.I1S098
61554 229533.I1RTG0 pp 229533.I1RV91
61555 229533.I1RTG0 pp 229533.I1S286
61556 229533.I1RTG0 pp 229533.I1S2E9
61557 229533.I1RTG0 pp 229533.I1RZ63
61558 229533.I1RTG0 pp 229533.I1RX38
61559 229533.I1RTG0 pp 229533.I1S004
61560 229533.I1RTG0 pp 229533.I1S0J7
61561 229533.I1RTG0 pp 229533.I1S549
61562 229533.I1RFV5 pp 229533.I1RRK8
61563 229533.I1RFV5 pp 229533.I1S8F0
61564 229533.I1RFV5 pp 229533.Q4IB65
61565 229533.I1RFV5 pp 229533.Q4IPZ1
61566 229533.I1RFV5 pp 229533.Q4I2W2
61567 229533.I1RFV5 pp 229533.I1S4H6
61568 229533.A0A1C3YLD1 pp 229533.I1RZA8
61569 229533.A0A1C3YLD1 pp 229533.I1RY16
61570 229533.A0A1C3YLD1 pp 229533.I1RI11
61571 229533.A0A1C3YLD1 pp 229533.I1RRG1
61572 229533.A0A1C3YLD1 pp 229533.I1S694
61573 229533.I1RXR1 pp 229533.I1RZZ9
61574 229533.I1RXR1 pp 229533.I1RZ39
61575 229533.I1RXR1 pp 229533.Q4I624
61576 229533.I1RXR1 pp 229533.I1S1C7
61577 229533.I1RXR1 pp 229533.O74638
61578 229533.I1RXR1 pp 229533.V6R042
61579 229533.I1RXR1 pp 229533.I1S9Y1
61580 229533.I1RXR1 pp 229533.I1S5R1
61581 229533.I1RXR1 pp 229533.I1S7W5
61582 229533.I1RXR1 pp 229533.I1S2H4
61583 229533.I1RXR1 pp 229533.V6QVH8
61584 229533.I1RXR1 pp 229533.I1S2B9
61585 229533.I1RXR1 pp 229533.I1RYW3
61586 229533.I1RXR1 pp 229533.I1S0J1
61587 229533.I1RXR1 pp 229533.I1RYX5
61588 229533.I1RXR1 pp 229533.I1S6K9
61589 229533.I1RXR1 pp 229533.I1S6Y8
61590 229533.I1RXR1 pp 229533.I1S0L4
61591 229533.I1RXR1 pp 229533.I1RZM8
61592 229533.I1RXR1 pp 229533.V6RCZ8
61593 229533.I1RXR1 pp 229533.I1RXW3
61594 229533.I1RXR1 pp 229533.V6R3J1
61595 229533.I1RXR1 pp 229533.I1RZK5
61596 229533.I1RXR1 pp 229533.Q4IJK2
61597 229533.I1RXR1 pp 229533.I1RXV9
61598 229533.I1RXR1 pp 229533.I1S5B0
61599 229533.I1RXR1 pp 229533.I1S0T2
61600 229533.I1RXR1 pp 229533.V6QUE6
61601 229533.I1RXR1 pp 229533.I1S0K2
61602 229533.I1RXR1 pp 229533.I1S338
61603 229533.I1RXR1 pp 229533.I1S4S7
61604 229533.I1RXR1 pp 229533.I1S419
61605 229533.I1RXR1 pp 229533.V6RTJ8
61606 229533.A0A1C3YI31 pp 229533.I1RRX6
61607 229533.A0A1C3YI31 pp 229533.I1RW44
61608 229533.A0A1C3YI31 pp 229533.I1S224
61609 229533.A0A1C3YI31 pp 229533.I1S549
61610 229533.A0A1C3YI31 pp 229533.I1RRN5
61611 229533.A0A1C3YI31 pp 229533.A0A1C3YJH8
61612 229533.A0A1C3YI31 pp 229533.I1RD18

61613	229533.A0A1C3YI31	pp	229533.I1RBG2
61614	229533.A0A1C3YI31	pp	229533.I1RQA3
61615	229533.A0A1C3YI31	pp	229533.I1RX70
61616	229533.A0A1C3YI31	pp	229533.I1RZ12
61617	229533.A0A1C3YI31	pp	229533.I1RY58
61618	229533.A0A1C3YI31	pp	229533.I1RWT6
61619	229533.A0A1C3YI31	pp	229533.I1RZT1
61620	229533.A0A1C3YI31	pp	229533.I1RW83
61621	229533.A0A1C3YI31	pp	229533.I1RFZ3
61622	229533.A0A1C3YI31	pp	229533.I1RY44
61623	229533.A0A1C3YI31	pp	229533.I1RTG0
61624	229533.A0A1C3YI31	pp	229533.I1S2E9
61625	229533.A0A1C3YI31	pp	229533.I1S3E6
61626	229533.A0A1C3YI31	pp	229533.I1RV91
61627	229533.A0A1C3YI31	pp	229533.I1RC17
61628	229533.A0A1C3YI31	pp	229533.I1RME5
61629	229533.A0A1C3YI31	pp	229533.I1RMA4
61630	229533.A0A1C3YI31	pp	229533.A0A1C3YM91
61631	229533.A0A1C3YI31	pp	229533.I1RJK7
61632	229533.A0A1C3YI31	pp	229533.I1RDV9
61633	229533.A0A1C3YI31	pp	229533.I1REE1
61634	229533.A0A1C3YI31	pp	229533.I1RRW0
61635	229533.A0A1C3YI31	pp	229533.I1RB41
61636	229533.A0A1C3YI31	pp	229533.A0A1C3YM29
61637	229533.A0A1C3YI31	pp	229533.I1RQS4
61638	229533.I1RSY2	pp	229533.I1RVU7
61639	229533.I1RSY2	pp	229533.I1RVW0
61640	229533.I1RSY2	pp	229533.I1S9Y1
61641	229533.I1RSY2	pp	229533.I1RXC2
61642	229533.I1RSY2	pp	229533.I1S2H4
61643	229533.I1RSY2	pp	229533.I1RYQ1
61644	229533.I1RSY2	pp	229533.I1S4P6
61645	229533.I1RSY2	pp	229533.I1RTA1
61646	229533.I1RSY2	pp	229533.V6RAC9
61647	229533.I1RSY2	pp	229533.I1RXV3
61648	229533.I1RSY2	pp	229533.I1RZ39
61649	229533.I1RSY2	pp	229533.I1RSZ3
61650	229533.I1RSY2	pp	229533.Q4I665
61651	229533.I1RSY2	pp	229533.Q4I624
61652	229533.I1RSY2	pp	229533.I1RZ29
61653	229533.I1RSY2	pp	229533.I1S0J1
61654	229533.I1RSY2	pp	229533.I1RVX0
61655	229533.I1RSY2	pp	229533.V6RGN5
61656	229533.I1RSY2	pp	229533.I1RWE8
61657	229533.I1RSY2	pp	229533.I1S5R6
61658	229533.I1RSY2	pp	229533.I1RWI5
61659	229533.I1RSY2	pp	229533.V6R042
61660	229533.I1RSY2	pp	229533.I1S0D5
61661	229533.I1RSY2	pp	229533.I1S5B0
61662	229533.I1RSY2	pp	229533.Q4HZK7
61663	229533.I1RSY2	pp	229533.I1RTN6
61664	229533.I1RSY2	pp	229533.V6RCZ8
61665	229533.I1RSY2	pp	229533.I1RZ95
61666	229533.I1RSY2	pp	229533.V6QUE6
61667	229533.I1RSY2	pp	229533.Q4IJW4
61668	229533.I1RSY2	pp	229533.I1S4S7
61669	229533.I1RSY2	pp	229533.I1RYW3
61670	229533.I1RSY2	pp	229533.V6RTJ8
61671	229533.I1RSY2	pp	229533.I1RZM8
61672	229533.I1RSY2	pp	229533.I1RZ94
61673	229533.I1RSY2	pp	229533.I1RX46
61674	229533.I1RSY2	pp	229533.I1RWH8
61675	229533.I1RSY2	pp	229533.I1RZZ1
61676	229533.I1RSY2	pp	229533.I1RVX9
61677	229533.I1RSY2	pp	229533.I1RZK6
61678	229533.I1RSY2	pp	229533.I1S267
61679	229533.I1RSY2	pp	229533.I1RXD7
61680	229533.I1RSY2	pp	229533.I1RVZ9
61681	229533.I1RSY2	pp	229533.I1S0R7
61682	229533.I1RSY2	pp	229533.Q4IJK2
61683	229533.I1RSY2	pp	229533.I1S0G0
61684	229533.I1RSY2	pp	229533.I1RZB5
61685	229533.I1RSY2	pp	229533.I1RXC4

61686	229533.A0A0E0RR40	pp	229533.I1S2K7
61687	229533.A0A0E0RR40	pp	229533.I1RQ72
61688	229533.A0A0E0RR40	pp	229533.I1RB41
61689	229533.A0A0E0RR40	pp	229533.I1RX20
61690	229533.A0A0E0RR40	pp	229533.A0A1C3YM29
61691	229533.A0A0E0RR40	pp	229533.A0A1C3YJH8
61692	229533.A0A0E0RR40	pp	229533.I1RQS4
61693	229533.A0A0E0RR40	pp	229533.I1RV66
61694	229533.A0A0E0RR40	pp	229533.Q4IR87
61695	229533.A0A0E0RR40	pp	229533.A0A1C3YJV7
61696	229533.A0A0E0RR40	pp	229533.I1RGF1
61697	229533.A0A0E0RR40	pp	229533.I1RRN5
61698	229533.A0A0E0RR40	pp	229533.I1RX70
61699	229533.A0A0E0RR40	pp	229533.I1RHQ8
61700	229533.A0A0E0RR40	pp	229533.I1RQA3
61701	229533.A0A0E0RR40	pp	229533.I1RZ63
61702	229533.A0A0E0RR40	pp	229533.I1S8E0
61703	229533.A0A0E0RR40	pp	229533.A0A0E0SA11
61704	229533.A0A0E0RR40	pp	229533.A0A1C3YHP8
61705	229533.A0A0E0RR40	pp	229533.A0A1C3YN39
61706	229533.A0A0E0RR40	pp	229533.I1RC17
61707	229533.A0A0E0RR40	pp	229533.I1RAU6
61708	229533.A0A0E0RR40	pp	229533.I1S275
61709	229533.A0A0E0RR40	pp	229533.I1RPA6
61710	229533.A0A0E0RR40	pp	229533.I1RME5
61711	229533.A0A0E0RR40	pp	229533.I1S0J7
61712	229533.A0A0E0RR40	pp	229533.A0A1C3YM91
61713	229533.A0A0E0RR40	pp	229533.I1RCB3
61714	229533.A0A0E0RR40	pp	229533.I1RXH6
61715	229533.A0A0E0RR40	pp	229533.I1RHU7
61716	229533.A0A0E0RR40	pp	229533.I1S224
61717	229533.A0A0E0RR40	pp	229533.I1RQB4
61718	229533.A0A0E0RR40	pp	229533.I1RC46
61719	229533.A0A0E0RR40	pp	229533.I1S9L0
61720	229533.A0A0E0RR40	pp	229533.I1REE1
61721	229533.A0A0E0RR40	pp	229533.I1RRX6
61722	229533.A0A0E0RR40	pp	229533.A0A1C3YIG3
61723	229533.A0A0E0RR40	pp	229533.I1RMA4
61724	229533.A0A0E0RR40	pp	229533.I1RRW0
61725	229533.A0A0E0RR40	pp	229533.I1S549
61726	229533.A0A0E0RR40	pp	229533.I1RC22
61727	229533.A0A0E0RR40	pp	229533.I1RC91
61728	229533.A0A0E0RR40	pp	229533.I1RWT6
61729	229533.A0A0E0RR40	pp	229533.A0A1C3YKT6
61730	229533.A0A0E0RR40	pp	229533.I1RJK7
61731	229533.A0A0E0RR40	pp	229533.I1RCD1
61732	229533.A0A0E0RR40	pp	229533.I1RY44
61733	229533.A0A0E0RR40	pp	229533.A0A0E0SH88
61734	229533.A0A0E0RR40	pp	229533.I1RNF4
61735	229533.A0A0E0RR40	pp	229533.I1RJV4
61736	229533.A0A0E0RR40	pp	229533.A0A1C3YJG7
61737	229533.A0A0E0RR40	pp	229533.I1RZ12
61738	229533.A0A0E0RR40	pp	229533.I1RAY4
61739	229533.A0A0E0RR40	pp	229533.I1RX77
61740	229533.A0A0E0RR40	pp	229533.A0A0E0SGM9
61741	229533.A0A0E0RR40	pp	229533.I1RLU2
61742	229533.A0A0E0RR40	pp	229533.A0A0E0SQG9
61743	229533.A0A0E0RR40	pp	229533.I1RTG0
61744	229533.A0A0E0RR40	pp	229533.I1S2E9
61745	229533.A0A0E0RR40	pp	229533.I1RLX7
61746	229533.A0A0E0RR40	pp	229533.I1RX04
61747	229533.A0A0E0RR40	pp	229533.A0A1C3YL20
61748	229533.A0A1C3YLN5	pp	229533.I1RY49
61749	229533.A0A1C3YLN5	pp	229533.I1RQE1
61750	229533.A0A1C3YLN5	pp	229533.I1RXB6
61751	229533.A0A1C3YLN5	pp	229533.V6R382
61752	229533.Q4IK03	pp	229533.Q4IPZ1
61753	229533.Q4IK03	pp	229533.V6R1X4
61754	229533.I1RXH2	pp	229533.I1S094
61755	229533.I1RXH2	pp	229533.I1S6H3
61756	229533.I1RXH2	pp	229533.I1S3H8
61757	229533.I1RXH2	pp	229533.I1S0A5
61758	229533.I1RXH2	pp	229533.I1RZ25

61759 229533.I1RXH2 pp 229533.I1RZ56
61760 229533.I1RXH2 pp 229533.I1S8F8
61761 229533.I1RXH2 pp 229533.I1S334
61762 229533.A0A098DCF9 pp 229533.A0A1C3YJ86
61763 229533.A0A098DCF9 pp 229533.A0A1C3YHQ1
61764 229533.A0A098DCF9 pp 229533.I1RZ83
61765 229533.A0A098DCF9 pp 229533.A0A1C3YJI6
61766 229533.A0A098DCF9 pp 229533.V6RAC9
61767 229533.A0A098DCF9 pp 229533.I1RRK8
61768 229533.A0A098DCF9 pp 229533.I1RFV5
61769 229533.A0A098DCF9 pp 229533.I1RSY2
61770 229533.A0A098DCF9 pp 229533.A0A0E0RMM5
61771 229533.A0A098DCF9 pp 229533.Q4IK03
61772 229533.A0A098DCF9 pp 229533.I1RBE7
61773 229533.I1RVK5 pp 229533.I1RZS8
61774 229533.I1RVK5 pp 229533.I1S7X9
61775 229533.I1RVK5 pp 229533.I1S4M5
61776 229533.I1RVK5 pp 229533.V6QXG1
61777 229533.I1RVK5 pp 229533.I1RX81
61778 229533.I1RVK5 pp 229533.I1RXF2
61779 229533.I1RVK5 pp 229533.I1RX55
61780 229533.I1RVK5 pp 229533.I1S7M2
61781 229533.I1RVK5 pp 229533.I1S7M3
61782 229533.I1RVK5 pp 229533.I1RZG7
61783 229533.I1RZ19 pp 229533.Q4IR08
61784 229533.I1RZ19 pp 229533.Q4IP51
61785 229533.I1RLD3 pp 229533.Q4IPN9
61786 229533.I1RRX6 pp 229533.I1RZC3
61787 229533.I1RRX6 pp 229533.I1RVN2
61788 229533.I1RRX6 pp 229533.I1S224
61789 229533.I1RRX6 pp 229533.I1RZ63
61790 229533.I1RRX6 pp 229533.I1RY44
61791 229533.I1RRX6 pp 229533.I1RY58
61792 229533.I1RRX6 pp 229533.I1S0J7
61793 229533.I1RRX6 pp 229533.I1S2E9
61794 229533.I1RRX6 pp 229533.I1RV91
61795 229533.I1RRX6 pp 229533.I1S549
61796 229533.I1RRX6 pp 229533.I1S098
61797 229533.I1RRX6 pp 229533.I1RWT6
61798 229533.I1RRX6 pp 229533.I1RX38
61799 229533.I1RRX6 pp 229533.I1S004
61800 229533.I1RRX6 pp 229533.Q4I7N9
61801 229533.I1RRX6 pp 229533.I1S267
61802 229533.I1RRX6 pp 229533.I1RTG0
61803 229533.I1RRX6 pp 229533.I1RZ12
61804 229533.A0A098DCD9 pp 229533.I1RLD3
61805 229533.I1RWD2 pp 229533.I1RZ56
61806 229533.I1RWD2 pp 229533.I1RY81
61807 229533.I1RWD2 pp 229533.I1S094
61808 229533.I1RWD2 pp 229533.I1S0A5
61809 229533.I1RWD2 pp 229533.I1S3T7
61810 229533.I1RWD2 pp 229533.I1S6H3
61811 229533.I1S224 pp 229533.I1S2E9
61812 229533.I1S224 pp 229533.I1S7X9
61813 229533.I1S224 pp 229533.V6RGI1
61814 229533.I1S224 pp 229533.V6RVG3
61815 229533.I1S224 pp 229533.V6RKY7
61816 229533.I1S224 pp 229533.I1S3T6
61817 229533.I1S224 pp 229533.V6REE9
61818 229533.I1S224 pp 229533.I1S3H8
61819 229533.I1S224 pp 229533.I1S267
61820 229533.I1S224 pp 229533.I1S549
61821 229533.I1S224 pp 229533.I1S439
61822 229533.I1S224 pp 229533.Q4IER8
61823 229533.I1S224 pp 229533.V6QV09
61824 229533.I1S224 pp 229533.Q4I7N9
61825 229533.I1S224 pp 229533.Q4I963
61826 229533.I1S224 pp 229533.I1S334
61827 229533.I1RKJ7 pp 229533.I1RUY6
61828 229533.I1RKJ7 pp 229533.I1RXX0
61829 229533.I1RKJ7 pp 229533.Q4ICA8
61830 229533.I1RKJ7 pp 229533.I1RSJ2
61831 229533.I1RKJ7 pp 229533.I1RYU0

61832 229533.I1RKJ7 pp 229533.I1RNJ7
61833 229533.I1RKJ7 pp 229533.I1RY98
61834 229533.I1RKJ7 pp 229533.V6REE9
61835 229533.I1RKJ7 pp 229533.I1RYV3
61836 229533.I1RKJ7 pp 229533.I1RSE0
61837 229533.I1RKJ7 pp 229533.I1RQ93
61838 229533.I1RKJ7 pp 229533.I1RQ88
61839 229533.I1RKJ7 pp 229533.I1RN65
61840 229533.I1RKJ7 pp 229533.I1S7X9
61841 229533.I1RKJ7 pp 229533.I1RZA9
61842 229533.I1RKJ7 pp 229533.I1SAA0
61843 229533.I1RKJ7 pp 229533.I1S0F3
61844 229533.I1RKJ7 pp 229533.I1SA27
61845 229533.I1RKJ7 pp 229533.I1S0J8
61846 229533.I1RKJ7 pp 229533.I1S2A8
61847 229533.I1RKJ7 pp 229533.V6QVM5
61848 229533.I1RKJ7 pp 229533.I1RTI7
61849 229533.I1RKJ7 pp 229533.V6RGI1
61850 229533.I1RKJ7 pp 229533.I1RTE6
61851 229533.I1RKJ7 pp 229533.I1RPK0
61852 229533.I1RKJ7 pp 229533.V6QT89
61853 229533.I1RKJ7 pp 229533.Q4HTS9
61854 229533.I1RKJ7 pp 229533.I1RNW8
61855 229533.I1RND2 pp 229533.I1S8S9
61856 229533.I1RND2 pp 229533.I1RQ28
61857 229533.I1RND2 pp 229533.I1RQL4
61858 229533.I1RND2 pp 229533.I1RRT1
61859 229533.I1RND2 pp 229533.V6QVM5
61860 229533.I1RND2 pp 229533.I1RNJ7
61861 229533.A0A098D4B3 pp 229533.I1RPW0
61862 229533.A0A098D4B3 pp 229533.A0A1C3YM91
61863 229533.A0A098D4B3 pp 229533.I1RY58
61864 229533.A0A098D4B3 pp 229533.I1RRW0
61865 229533.A0A098D4B3 pp 229533.A0A1C3YHP8
61866 229533.A0A098D4B3 pp 229533.I1RZC3
61867 229533.A0A098D4B3 pp 229533.I1RMA4
61868 229533.A0A098D4B3 pp 229533.A0A0E0SQG9
61869 229533.A0A098D4B3 pp 229533.I1RVN2
61870 229533.A0A098D4B3 pp 229533.A0A098D6T7
61871 229533.A0A098D4B3 pp 229533.A0A1C3YM29
61872 229533.A0A098D4B3 pp 229533.I1RBV5
61873 229533.A0A098D4B3 pp 229533.A0A1C3YKJ6
61874 229533.A0A098D4B3 pp 229533.I1RBM3
61875 229533.A0A098D4B3 pp 229533.I1RY32
61876 229533.A0A098D4B3 pp 229533.A0A0E0S9G3
61877 229533.A0A098D4B3 pp 229533.I1S098
61878 229533.A0A098D4B3 pp 229533.A0A1C3YN69
61879 229533.A0A098D4B3 pp 229533.I1S2E9
61880 229533.A0A098D4B3 pp 229533.I1RV91
61881 229533.A0A098D4B3 pp 229533.I1RY44
61882 229533.A0A098D4B3 pp 229533.A0A098E2M3
61883 229533.A0A098D4B3 pp 229533.I1RFL7
61884 229533.A0A098D4B3 pp 229533.I1RME5
61885 229533.A0A098D4B3 pp 229533.I1RZ63
61886 229533.A0A098D4B3 pp 229533.I1REE1
61887 229533.A0A098D4B3 pp 229533.A0A1C3YK37
61888 229533.A0A098D4B3 pp 229533.I1RPK4
61889 229533.A0A098D4B3 pp 229533.I1RPU4
61890 229533.A0A098D4B3 pp 229533.I1RX38
61891 229533.A0A098D4B3 pp 229533.I1S549
61892 229533.A0A098D4B3 pp 229533.Q4I7N9
61893 229533.A0A098D4B3 pp 229533.I1RQA3
61894 229533.A0A098D4B3 pp 229533.I1RKK0
61895 229533.A0A098D4B3 pp 229533.I1RDV9
61896 229533.A0A098D4B3 pp 229533.I1RJK7
61897 229533.A0A098D4B3 pp 229533.I1S004
61898 229533.A0A098D4B3 pp 229533.I1S267
61899 229533.A0A098D4B3 pp 229533.A0A1C3YI39
61900 229533.A0A098D4B3 pp 229533.I1S0J7
61901 229533.A0A098D4B3 pp 229533.I1RWT6
61902 229533.A0A098D4B3 pp 229533.I1RZ12
61903 229533.A0A098D4B3 pp 229533.I1RTG0
61904 229533.A0A098D4B3 pp 229533.A0A1C3YI31

61905 229533.A0A098D4B3 pp 229533.A0A0E0RR40
61906 229533.A0A098D4B3 pp 229533.I1RAN0
61907 229533.A0A098D4B3 pp 229533.A0A0E0SA11
61908 229533.A0A098D4B3 pp 229533.A0A098D5E5
61909 229533.A0A098D4B3 pp 229533.I1RD18
61910 229533.A0A098D4B3 pp 229533.I1RRX6
61911 229533.A0A098D4B3 pp 229533.I1S224
61912 229533.A0A098DCB9 pp 229533.A0A1C3YJG7
61913 229533.A0A098DCB9 pp 229533.I1RQL4
61914 229533.I1RQ50 pp 229533.I1S0S4
61915 229533.I1RQ50 pp 229533.I1RTC2
61916 229533.I1RI40 pp 229533.I1S6H3
61917 229533.I1RI40 pp 229533.I1S094
61918 229533.I1RI40 pp 229533.I1RRL5
61919 229533.I1RI40 pp 229533.I1S334
61920 229533.I1RI40 pp 229533.I1RLA2
61921 229533.I1RI40 pp 229533.I1RUY5
61922 229533.I1RI40 pp 229533.I1RNM7
61923 229533.I1RI40 pp 229533.I1RST9
61924 229533.I1RI40 pp 229533.I1S8F8
61925 229533.I1RI40 pp 229533.I1RZ25
61926 229533.I1RI40 pp 229533.I1S3H8
61927 229533.I1RI40 pp 229533.I1RNG4
61928 229533.I1RI40 pp 229533.I1RKB2
61929 229533.I1RI40 pp 229533.I1RZ56
61930 229533.I1RI40 pp 229533.I1S0A5
61931 229533.I1RI40 pp 229533.I1RPV8
61932 229533.I1RI40 pp 229533.I1RJS1
61933 229533.I1RRX5 pp 229533.I1S256
61934 229533.I1RRX5 pp 229533.I1RS90
61935 229533.I1RRX5 pp 229533.V6RN69
61936 229533.I1RRX5 pp 229533.I1S147
61937 229533.I1RRX5 pp 229533.I1S5R6
61938 229533.I1RRX5 pp 229533.V6RRM0
61939 229533.I1RRX5 pp 229533.I1RUG3
61940 229533.I1RRX5 pp 229533.I1S0M2
61941 229533.I1RRX5 pp 229533.I1RXU9
61942 229533.I1RRX5 pp 229533.I1RSQ3
61943 229533.I1RRX5 pp 229533.V6R042
61944 229533.I1RRX5 pp 229533.I1S1C7
61945 229533.I1RRX5 pp 229533.I1S1W3
61946 229533.I1RRX5 pp 229533.I1RYW3
61947 229533.I1RRX5 pp 229533.I1RYC8
61948 229533.I1RRX5 pp 229533.I1S0Q7
61949 229533.I1RRX5 pp 229533.I1RYH7
61950 229533.I1RRX5 pp 229533.I1S0J1
61951 229533.I1RRX5 pp 229533.V6RRH2
61952 229533.I1RRX5 pp 229533.V6R1X4
61953 229533.I1RRX5 pp 229533.I1RXR1
61954 229533.I1RRX5 pp 229533.Q4I624
61955 229533.I1RRX5 pp 229533.V6RM02
61956 229533.I1RRX5 pp 229533.I1RSY2
61957 229533.I1RRX5 pp 229533.I1S7W5
61958 229533.I1RRX5 pp 229533.I1S8R2
61959 229533.I1RRX5 pp 229533.Q4I7K4
61960 229533.I1RRX5 pp 229533.I1S0R8
61961 229533.I1RRX5 pp 229533.I1RYA0
61962 229533.I1RRX5 pp 229533.I1RWD3
61963 229533.I1RRX5 pp 229533.I1RT03
61964 229533.I1RRX5 pp 229533.I1RVX0
61965 229533.I1RRX5 pp 229533.I1S5X9
61966 229533.I1RRX5 pp 229533.Q4HXF6
61967 229533.I1RRX5 pp 229533.V6R3J1
61968 229533.I1RRX5 pp 229533.V6RLM7
61969 229533.I1RRX5 pp 229533.V6RIV7
61970 229533.I1RRX5 pp 229533.I1S855
61971 229533.I1RRX5 pp 229533.I1S1F3
61972 229533.I1RRX5 pp 229533.I1RX39
61973 229533.I1RRX5 pp 229533.I1RSX0
61974 229533.I1RRX5 pp 229533.I1RYW9
61975 229533.I1RRX5 pp 229533.I1S0L4
61976 229533.I1RRX5 pp 229533.I1RTD5
61977 229533.I1RRX5 pp 229533.I1RWF5

61978	229533.I1RRX5	pp	229533.I1S2H4
61979	229533.I1RRX5	pp	229533.I1RX00
61980	229533.I1RRX5	pp	229533.I1RT40
61981	229533.I1RRX5	pp	229533.I1RYI5
61982	229533.I1RRX5	pp	229533.I1RXD9
61983	229533.I1RRX5	pp	229533.I1RZ29
61984	229533.I1RRX5	pp	229533.I1RYK0
61985	229533.I1RRX5	pp	229533.Q4HXF0
61986	229533.I1RRX5	pp	229533.I1S299
61987	229533.I1RRX5	pp	229533.I1RZZ9
61988	229533.I1RRX5	pp	229533.I1S464
61989	229533.I1RRX5	pp	229533.I1RSQ4
61990	229533.I1RRX5	pp	229533.I1RSZ7
61991	229533.I1RRX5	pp	229533.I1S5R1
61992	229533.I1RRX5	pp	229533.I1RY62
61993	229533.I1RRX5	pp	229533.I1RWI5
61994	229533.I1RRX5	pp	229533.I1RVR2
61995	229533.I1RRX5	pp	229533.I1RY92
61996	229533.I1RRX5	pp	229533.I1S9Y1
61997	229533.I1RRX5	pp	229533.I1S253
61998	229533.I1RRX5	pp	229533.V6QUE6
61999	229533.I1RRX5	pp	229533.I1S3T7
62000	229533.I1RRX5	pp	229533.I1RWA3
62001	229533.I1RRX5	pp	229533.V6RCZ8
62002	229533.I1RRX5	pp	229533.I1RZ39
62003	229533.I1RRX5	pp	229533.I1S5B0
62004	229533.I1RRX5	pp	229533.I1RYX5
62005	229533.I1RRX5	pp	229533.I1RUR8
62006	229533.I1RRX5	pp	229533.I1RXF0
62007	229533.I1RRX5	pp	229533.I1RXE6
62008	229533.I1RRX5	pp	229533.I1S6K9
62009	229533.I1RRX5	pp	229533.I1S8F8
62010	229533.I1RRX5	pp	229533.I1S8S9
62011	229533.I1RRX5	pp	229533.I1RUY6
62012	229533.I1RRX5	pp	229533.I1RZM8
62013	229533.I1RRX5	pp	229533.I1S887
62014	229533.I1RRX5	pp	229533.V6RJP8
62015	229533.I1RRX5	pp	229533.I1RZK5
62016	229533.I1RRX5	pp	229533.I1RXT0
62017	229533.I1RRX5	pp	229533.I1RX46
62018	229533.I1RRX5	pp	229533.I1S0C1
62019	229533.I1RRX5	pp	229533.Q4I5G1
62020	229533.I1RRX5	pp	229533.I1S854
62021	229533.I1RRX5	pp	229533.I1RS18
62022	229533.I1RRX5	pp	229533.V6RTJ8
62023	229533.I1RRX5	pp	229533.I1S0K2
62024	229533.I1RRX5	pp	229533.I1RXB6
62025	229533.I1RRX5	pp	229533.I1RXD7
62026	229533.I1RRX5	pp	229533.I1S212
62027	229533.I1RRX5	pp	229533.Q4IJK2
62028	229533.I1RRX5	pp	229533.I1RWB0
62029	229533.I1RRX5	pp	229533.V6RBN2
62030	229533.I1RRX5	pp	229533.I1RRY9
62031	229533.I1RRX5	pp	229533.I1RYA2
62032	229533.I1RRX5	pp	229533.V6RUT5
62033	229533.I1RRX5	pp	229533.I1RS05
62034	229533.I1RRX5	pp	229533.I1S053
62035	229533.I1RRX5	pp	229533.I1RS29
62036	229533.I1RRX5	pp	229533.I1RWC9
62037	229533.I1RRX5	pp	229533.V6QXN0
62038	229533.I1RRX5	pp	229533.I1S8C1
62039	229533.I1RRX5	pp	229533.I1RSQ7
62040	229533.I1RRX5	pp	229533.I1S0P0
62041	229533.I1RRX5	pp	229533.I1S4S7
62042	229533.I1RRX5	pp	229533.I1RYT8
62043	229533.I1RRX5	pp	229533.I1S0B7
62044	229533.I1RRX5	pp	229533.I1S4S4
62045	229533.I1RRX5	pp	229533.Q4I4A4
62046	229533.I1RRX5	pp	229533.I1RY21
62047	229533.I1RL41	pp	229533.I1RLA7
62048	229533.I1RL41	pp	229533.I1RR33
62049	229533.I1RL41	pp	229533.I1RQL4
62050	229533.I1RL41	pp	229533.I1S5R5

62051 229533.I1RL41 pp 229533.V6RPN0
62052 229533.I1RL41 pp 229533.I1S0F0
62053 229533.I1S4B7 pp 229533.V6RK96
62054 229533.I1S4B7 pp 229533.V6RM02
62055 229533.I1RA49 pp 229533.I1RMR9
62056 229533.I1RA49 pp 229533.I1RYT8
62057 229533.I1RA49 pp 229533.I1RG62
62058 229533.I1RA49 pp 229533.I1RWA3
62059 229533.I1RA49 pp 229533.I1RMK7
62060 229533.I1RA49 pp 229533.I1RCC5
62061 229533.I1RA49 pp 229533.I1RNM7
62062 229533.I1RA49 pp 229533.I1RX39
62063 229533.I1RA49 pp 229533.I1RBE6
62064 229533.I1RA49 pp 229533.I1RBA6
62065 229533.I1RA49 pp 229533.I1RY21
62066 229533.I1RA49 pp 229533.I1RXT0
62067 229533.I1RA49 pp 229533.I1RKB7
62068 229533.I1RA49 pp 229533.I1RMQ6
62069 229533.I1RA49 pp 229533.I1RY92
62070 229533.I1RA49 pp 229533.I1RAV5
62071 229533.I1RA49 pp 229533.V6R3J1
62072 229533.I1RA49 pp 229533.I1RYH7
62073 229533.I1RA49 pp 229533.I1RLP1
62074 229533.I1RA49 pp 229533.I1RAW3
62075 229533.I1RA49 pp 229533.I1RAE5
62076 229533.I1RA49 pp 229533.I1RGC3
62077 229533.I1RA49 pp 229533.I1RS05
62078 229533.I1RA49 pp 229533.I1S0K2
62079 229533.I1RA49 pp 229533.I1RRS8
62080 229533.I1RA49 pp 229533.I1RCR6
62081 229533.I1RA49 pp 229533.I1S0R8
62082 229533.I1RA49 pp 229533.I1RUR8
62083 229533.I1RA49 pp 229533.I1RA52
62084 229533.I1RA49 pp 229533.I1RXD9
62085 229533.I1RA49 pp 229533.I1RRX5
62086 229533.I1RA49 pp 229533.I1RYW9
62087 229533.I1RA49 pp 229533.I1RS29
62088 229533.I1RA49 pp 229533.I1RAE3
62089 229533.I1RA49 pp 229533.I1S5X9
62090 229533.I1RA49 pp 229533.I1S0P0
62091 229533.I1RA49 pp 229533.I1S464
62092 229533.I1RA49 pp 229533.I1RKB2
62093 229533.I1RA49 pp 229533.I1RPP5
62094 229533.I1RA49 pp 229533.I1RXE6
62095 229533.I1RA49 pp 229533.I1RE66
62096 229533.I1RA49 pp 229533.I1RYA2
62097 229533.I1RA49 pp 229533.I1RMV0
62098 229533.I1RA49 pp 229533.I1RE65
62099 229533.I1RA49 pp 229533.I1RPR9
62100 229533.I1RA49 pp 229533.I1RP02
62101 229533.I1RA49 pp 229533.I1RPK0
62102 229533.I1RA49 pp 229533.I1RFS5
62103 229533.I1RA49 pp 229533.I1RAE4
62104 229533.I1RA49 pp 229533.I1RFJ6
62105 229533.I1RA49 pp 229533.I1RWB0
62106 229533.I1RA49 pp 229533.I1RZ43
62107 229533.I1RA49 pp 229533.I1S7W5
62108 229533.I1RA49 pp 229533.I1RB38
62109 229533.I1RA49 pp 229533.I1S8S9
62110 229533.I1RA49 pp 229533.I1RHC8
62111 229533.I1RA49 pp 229533.I1S0Q7
62112 229533.I1RA49 pp 229533.I1RP88
62113 229533.I1RA49 pp 229533.I1RAA2
62114 229533.I1RA49 pp 229533.I1RNY6
62115 229533.I1RA49 pp 229533.I1RAM9
62116 229533.I1RA49 pp 229533.I1RAE2
62117 229533.I1RA49 pp 229533.V6RJP8
62118 229533.I1RA49 pp 229533.I1RFJ5
62119 229533.I1RA49 pp 229533.I1RBT6
62120 229533.I1RR33 pp 229533.V6RQW4
62121 229533.I1RWW7 pp 229533.I1S236
62122 229533.I1RWW7 pp 229533.I1S5I8
62123 229533.I1RWW7 pp 229533.I1RZH8

62124 229533.I1RWW7 pp 229533.I1S0T4
62125 229533.I1RWW7 pp 229533.I1S3G4
62126 229533.I1RWW7 pp 229533.V6QV31
62127 229533.I1RWW7 pp 229533.I1RZA7
62128 229533.I1RWW7 pp 229533.I1S6P5
62129 229533.I1RWW7 pp 229533.I1S1W8
62130 229533.I1RWW7 pp 229533.I1S237
62131 229533.I1RWW7 pp 229533.I1SAE8
62132 229533.I1RWW7 pp 229533.V6RUB1
62133 229533.I1S5J9 pp 229533.I1S8F8
62134 229533.I1S5J9 pp 229533.I1S6H3
62135 229533.I1RAI4 pp 229533.I1RUR8
62136 229533.I1RAI4 pp 229533.I1RMR9
62137 229533.I1RAI4 pp 229533.I1RYT8
62138 229533.I1RAI4 pp 229533.I1RG62
62139 229533.I1RAI4 pp 229533.I1RS05
62140 229533.I1RAI4 pp 229533.I1RKB7
62141 229533.I1RAI4 pp 229533.I1RCR6
62142 229533.I1RAI4 pp 229533.I1RMQ6
62143 229533.I1RAI4 pp 229533.I1RLP1
62144 229533.I1RAI4 pp 229533.I1RXD9
62145 229533.I1RAI4 pp 229533.I1RY92
62146 229533.I1RAI4 pp 229533.I1RY21
62147 229533.I1RAI4 pp 229533.I1RX39
62148 229533.I1RAI4 pp 229533.I1RMK7
62149 229533.I1RAI4 pp 229533.I1RWA3
62150 229533.I1RAI4 pp 229533.I1RBE6
62151 229533.I1RAI4 pp 229533.I1RNM7
62152 229533.I1RAI4 pp 229533.V6R3J1
62153 229533.I1RAI4 pp 229533.I1RCC5
62154 229533.I1RAI4 pp 229533.I1RYW9
62155 229533.I1RAI4 pp 229533.I1RAW3
62156 229533.I1RAI4 pp 229533.I1RXT0
62157 229533.I1RAI4 pp 229533.I1RGC3
62158 229533.I1RAI4 pp 229533.I1S0K2
62159 229533.I1RAI4 pp 229533.I1S0P0
62160 229533.I1RAI4 pp 229533.I1RE66
62161 229533.I1RAI4 pp 229533.I1S464
62162 229533.I1RAI4 pp 229533.I1RBT6
62163 229533.I1RAI4 pp 229533.I1RYH7
62164 229533.I1RAI4 pp 229533.I1RRX5
62165 229533.I1RAI4 pp 229533.I1S8S9
62166 229533.I1RAI4 pp 229533.I1RAM9
62167 229533.I1RAI4 pp 229533.I1RAV5
62168 229533.I1RAI4 pp 229533.I1S0Q7
62169 229533.I1RAI4 pp 229533.I1RKB2
62170 229533.I1RAI4 pp 229533.I1RPR9
62171 229533.I1RAI4 pp 229533.I1RYA2
62172 229533.I1RAI4 pp 229533.I1RNY6
62173 229533.I1RAI4 pp 229533.I1RWB0
62174 229533.I1RAI4 pp 229533.I1RHC8
62175 229533.I1RAI4 pp 229533.I1RE65
62176 229533.I1RAI4 pp 229533.I1RFJ6
62177 229533.I1RAI4 pp 229533.I1RP02
62178 229533.I1RAI4 pp 229533.I1RS29
62179 229533.I1RAI4 pp 229533.I1S5X9
62180 229533.I1RAI4 pp 229533.I1RFS5
62181 229533.I1RAI4 pp 229533.I1RXE6
62182 229533.I1RAI4 pp 229533.I1RPK0
62183 229533.I1RAI4 pp 229533.I1RPP5
62184 229533.I1RAI4 pp 229533.I1RMV0
62185 229533.I1RAI4 pp 229533.I1S0R8
62186 229533.I1RAI4 pp 229533.I1RB38
62187 229533.I1RAI4 pp 229533.I1S7W5
62188 229533.I1RAI4 pp 229533.V6RJP8
62189 229533.A0A098DCB5 pp 229533.I1RLA7
62190 229533.A0A098DCB5 pp 229533.I1RL41
62191 229533.A0A098DCB5 pp 229533.I1RR33
62192 229533.A0A1C3YMC7 pp 229533.A0A1C3YN89
62193 229533.I1R9Y4 pp 229533.I1RCR8
62194 229533.I1R9Y4 pp 229533.I1RUG3
62195 229533.I1R9Y4 pp 229533.I1RNC1
62196 229533.I1R9Y4 pp 229533.I1S0S5

62197	229533.I1R9Y4	pp	229533.I1RN84
62198	229533.I1R9Y4	pp	229533.V6R042
62199	229533.I1R9Y4	pp	229533.I1RSQ3
62200	229533.I1R9Y4	pp	229533.I1RPU8
62201	229533.I1R9Y4	pp	229533.I1S0Q7
62202	229533.I1R9Y4	pp	229533.I1RD22
62203	229533.I1R9Y4	pp	229533.I1RFS5
62204	229533.I1R9Y4	pp	229533.I1RB38
62205	229533.I1R9Y4	pp	229533.I1RE01
62206	229533.I1R9Y4	pp	229533.I1RAE4
62207	229533.I1R9Y4	pp	229533.I1S0J1
62208	229533.I1R9Y4	pp	229533.I1S147
62209	229533.I1R9Y4	pp	229533.I1S256
62210	229533.I1R9Y4	pp	229533.I1S7W5
62211	229533.I1R9Y4	pp	229533.I1RRN2
62212	229533.I1R9Y4	pp	229533.I1RPK0
62213	229533.I1R9Y4	pp	229533.V6RLM7
62214	229533.I1R9Y4	pp	229533.I1RS90
62215	229533.I1R9Y4	pp	229533.V6RRM0
62216	229533.I1R9Y4	pp	229533.I1S5R6
62217	229533.I1R9Y4	pp	229533.I1RE52
62218	229533.I1R9Y4	pp	229533.I1RE65
62219	229533.I1R9Y4	pp	229533.I1RWD3
62220	229533.I1R9Y4	pp	229533.I1RG11
62221	229533.I1R9Y4	pp	229533.Q4I7K4
62222	229533.I1R9Y4	pp	229533.I1RFL4
62223	229533.I1R9Y4	pp	229533.I1RMV0
62224	229533.I1R9Y4	pp	229533.I1S5X9
62225	229533.I1R9Y4	pp	229533.I1S1W9
62226	229533.I1R9Y4	pp	229533.I1S8R2
62227	229533.I1R9Y4	pp	229533.I1RQE7
62228	229533.I1R9Y4	pp	229533.I1S1W3
62229	229533.I1R9Y4	pp	229533.I1RVZ2
62230	229533.I1R9Y4	pp	229533.I1RB92
62231	229533.I1R9Y4	pp	229533.I1RKB2
62232	229533.I1R9Y4	pp	229533.V6RIV7
62233	229533.I1R9Y4	pp	229533.I1S0R8
62234	229533.I1R9Y4	pp	229533.I1RCH5
62235	229533.I1R9Y4	pp	229533.I1RTA1
62236	229533.I1R9Y4	pp	229533.I1RMJ3
62237	229533.I1R9Y4	pp	229533.I1RXR1
62238	229533.I1R9Y4	pp	229533.I1RFT1
62239	229533.I1R9Y4	pp	229533.V6RRH2
62240	229533.I1R9Y4	pp	229533.I1RYH7
62241	229533.I1R9Y4	pp	229533.I1RT03
62242	229533.I1R9Y4	pp	229533.I1RVX0
62243	229533.I1R9Y4	pp	229533.I1RYP2
62244	229533.I1R9Y4	pp	229533.V6RM02
62245	229533.I1R9Y4	pp	229533.I1RMD6
62246	229533.I1R9Y4	pp	229533.I1RBT6
62247	229533.I1R9Y4	pp	229533.I1S464
62248	229533.I1R9Y4	pp	229533.Q4I624
62249	229533.I1R9Y4	pp	229533.I1S299
62250	229533.I1R9Y4	pp	229533.I1S1C7
62251	229533.I1R9Y4	pp	229533.I1RSY2
62252	229533.I1R9Y4	pp	229533.I1RYI5
62253	229533.I1R9Y4	pp	229533.I1RYW3
62254	229533.I1R9Y4	pp	229533.I1RPP3
62255	229533.I1R9Y4	pp	229533.I1RSX0
62256	229533.I1R9Y4	pp	229533.V6R3J1
62257	229533.I1R9Y4	pp	229533.I1RN67
62258	229533.I1R9Y4	pp	229533.I1RBY9
62259	229533.I1R9Y4	pp	229533.I1RRI2
62260	229533.I1R9Y4	pp	229533.I1RA70
62261	229533.I1R9Y4	pp	229533.I1RY62
62262	229533.I1R9Y4	pp	229533.I1RBC7
62263	229533.I1R9Y4	pp	229533.V6RSF3
62264	229533.I1R9Y4	pp	229533.I1RAB4
62265	229533.I1R9Y4	pp	229533.I1RGA4
62266	229533.I1R9Y4	pp	229533.I1RBA6
62267	229533.I1R9Y4	pp	229533.I1RAE3
62268	229533.I1R9Y4	pp	229533.I1RX39
62269	229533.I1R9Y4	pp	229533.I1RJV3

62270 229533.I1R9Y4 pp 229533.I1S855
62271 229533.I1R9Y4 pp 229533.I1S1F3
62272 229533.I1R9Y4 pp 229533.I1RBE6
62273 229533.I1R9Y4 pp 229533.I1RSZ7
62274 229533.I1R9Y4 pp 229533.I1RCC5
62275 229533.I1R9Y4 pp 229533.I1RYW9
62276 229533.I1R9Y4 pp 229533.I1RFJ6
62277 229533.I1R9Y4 pp 229533.I1RF85
62278 229533.I1R9Y4 pp 229533.I1RXU9
62279 229533.I1R9Y4 pp 229533.I1RT40
62280 229533.I1R9Y4 pp 229533.I1RPV3
62281 229533.I1R9Y4 pp 229533.I1RFS3
62282 229533.I1R9Y4 pp 229533.I1RN88
62283 229533.I1R9Y4 pp 229533.I1RXD9
62284 229533.I1R9Y4 pp 229533.I1RTD5
62285 229533.I1R9Y4 pp 229533.I1RAW0
62286 229533.I1R9Y4 pp 229533.I1RWA3
62287 229533.I1R9Y4 pp 229533.I1RC29
62288 229533.I1R9Y4 pp 229533.I1RWF5
62289 229533.I1R9Y4 pp 229533.I1RBF3
62290 229533.I1R9Y4 pp 229533.I1RSQ4
62291 229533.I1R9Y4 pp 229533.I1RR72
62292 229533.I1R9Y4 pp 229533.I1RR68
62293 229533.I1R9Y4 pp 229533.I1RVN1
62294 229533.I1R9Y4 pp 229533.I1RMR9
62295 229533.I1R9Y4 pp 229533.I1R9Z5
62296 229533.I1R9Y4 pp 229533.I1RUR8
62297 229533.I1R9Y4 pp 229533.I1RC66
62298 229533.I1R9Y4 pp 229533.I1S8F8
62299 229533.I1R9Y4 pp 229533.I1RY92
62300 229533.I1R9Y4 pp 229533.I1RZ39
62301 229533.I1R9Y4 pp 229533.I1RBY4
62302 229533.I1R9Y4 pp 229533.I1RZM1
62303 229533.I1R9Y4 pp 229533.I1RE76
62304 229533.I1R9Y4 pp 229533.I1RFI4
62305 229533.I1R9Y4 pp 229533.I1S277
62306 229533.I1R9Y4 pp 229533.I1RUY6
62307 229533.I1R9Y4 pp 229533.I1RH91
62308 229533.I1R9Y4 pp 229533.I1S253
62309 229533.I1R9Y4 pp 229533.I1RA90
62310 229533.I1R9Y4 pp 229533.I1RA11
62311 229533.I1R9Y4 pp 229533.I1S9Y1
62312 229533.I1R9Y4 pp 229533.I1RRT8
62313 229533.I1R9Y4 pp 229533.I1RA52
62314 229533.I1R9Y4 pp 229533.I1RYX5
62315 229533.I1R9Y4 pp 229533.I1RAW3
62316 229533.I1R9Y4 pp 229533.I1RGC3
62317 229533.I1R9Y4 pp 229533.V6RJP8
62318 229533.I1R9Y4 pp 229533.I1RZZ9
62319 229533.I1R9Y4 pp 229533.I1RWI5
62320 229533.I1R9Y4 pp 229533.I1S5R1
62321 229533.I1R9Y4 pp 229533.I1RXF0
62322 229533.I1R9Y4 pp 229533.I1RZM8
62323 229533.I1R9Y4 pp 229533.O74638
62324 229533.I1R9Y4 pp 229533.I1RXC2
62325 229533.I1R9Y4 pp 229533.I1RS29
62326 229533.I1R9Y4 pp 229533.V6QXN0
62327 229533.I1R9Y4 pp 229533.I1RNY6
62328 229533.I1R9Y4 pp 229533.I1RH67
62329 229533.I1R9Y4 pp 229533.I1S2H4
62330 229533.I1R9Y4 pp 229533.I1S6K9
62331 229533.I1R9Y4 pp 229533.V6RBN2
62332 229533.I1R9Y4 pp 229533.I1S0K2
62333 229533.I1R9Y4 pp 229533.I1RKP1
62334 229533.I1R9Y4 pp 229533.V6RCZ8
62335 229533.I1R9Y4 pp 229533.I1S3T7
62336 229533.I1R9Y4 pp 229533.I1RBE4
62337 229533.I1R9Y4 pp 229533.I1S0L4
62338 229533.I1R9Y4 pp 229533.I1RPR1
62339 229533.I1R9Y4 pp 229533.Q4IJM4
62340 229533.I1R9Y4 pp 229533.I1RK95
62341 229533.I1R9Y4 pp 229533.I1RA29
62342 229533.I1R9Y4 pp 229533.I1REE2

62343	229533.I1R9Y4	pp	229533.I1RXC4
62344	229533.I1R9Y4	pp	229533.I1S0A0
62345	229533.I1R9Y4	pp	229533.I1RJY7
62346	229533.I1R9Y4	pp	229533.V6QUE6
62347	229533.I1R9Y4	pp	229533.Q4I5G1
62348	229533.I1R9Y4	pp	229533.I1RAV5
62349	229533.I1R9Y4	pp	229533.V6RK96
62350	229533.I1R9Y4	pp	229533.I1RS18
62351	229533.I1R9Y4	pp	229533.I1RP02
62352	229533.I1R9Y4	pp	229533.I1RFJ5
62353	229533.I1R9Y4	pp	229533.I1RMR7
62354	229533.I1R9Y4	pp	229533.I1REK8
62355	229533.I1R9Y4	pp	229533.I1RCR5
62356	229533.I1R9Y4	pp	229533.I1RXE6
62357	229533.I1R9Y4	pp	229533.I1RAM9
62358	229533.I1R9Y4	pp	229533.I1S1J7
62359	229533.I1R9Y4	pp	229533.I1S8S9
62360	229533.I1R9Y4	pp	229533.I1S212
62361	229533.I1R9Y4	pp	229533.I1S5B0
62362	229533.I1R9Y4	pp	229533.I1RMG4
62363	229533.I1R9Y4	pp	229533.I1RPR9
62364	229533.I1R9Y4	pp	229533.I1RMW4
62365	229533.I1R9Y4	pp	229533.Q4IJK2
62366	229533.I1R9Y4	pp	229533.I1RBL7
62367	229533.I1R9Y4	pp	229533.I1S887
62368	229533.I1R9Y4	pp	229533.I1RWC9
62369	229533.I1R9Y4	pp	229533.I1RPP5
62370	229533.I1R9Y4	pp	229533.I1S0C1
62371	229533.I1R9Y4	pp	229533.I1RRY9
62372	229533.I1R9Y4	pp	229533.I1RS05
62373	229533.I1R9Y4	pp	229533.I1RCM5
62374	229533.I1R9Y4	pp	229533.I1RCU2
62375	229533.I1R9Y4	pp	229533.I1RZK5
62376	229533.I1R9Y4	pp	229533.I1RBD5
62377	229533.I1R9Y4	pp	229533.I1S053
62378	229533.I1R9Y4	pp	229533.I1RP86
62379	229533.I1R9Y4	pp	229533.I1RRG3
62380	229533.I1R9Y4	pp	229533.I1S4S7
62381	229533.I1R9Y4	pp	229533.I1RWA4
62382	229533.I1R9Y4	pp	229533.I1RZ43
62383	229533.I1R9Y4	pp	229533.I1RLP1
62384	229533.I1R9Y4	pp	229533.I1RSQ7
62385	229533.I1R9Y4	pp	229533.I1RXD7
62386	229533.I1R9Y4	pp	229533.I1RZM4
62387	229533.I1R9Y4	pp	229533.I1RRX5
62388	229533.I1R9Y4	pp	229533.I1RWB0
62389	229533.I1R9Y4	pp	229533.I1RY12
62390	229533.I1R9Y4	pp	229533.I1RQE8
62391	229533.I1R9Y4	pp	229533.V6RUT5
62392	229533.I1R9Y4	pp	229533.I1S0P0
62393	229533.I1R9Y4	pp	229533.I1S854
62394	229533.I1R9Y4	pp	229533.I1RJU3
62395	229533.I1R9Y4	pp	229533.I1S8C1
62396	229533.I1R9Y4	pp	229533.I1RXT0
62397	229533.I1R9Y4	pp	229533.I1RKB7
62398	229533.I1R9Y4	pp	229533.I1RE66
62399	229533.I1R9Y4	pp	229533.I1RMQ6
62400	229533.I1R9Y4	pp	229533.I1RX46
62401	229533.I1R9Y4	pp	229533.I1RA85
62402	229533.I1R9Y4	pp	229533.I1RY21
62403	229533.I1R9Y4	pp	229533.I1RAX6
62404	229533.I1R9Y4	pp	229533.I1RD52
62405	229533.I1R9Y4	pp	229533.I1RAU9
62406	229533.I1R9Y4	pp	229533.I1S4S4
62407	229533.I1R9Y4	pp	229533.I1RA49
62408	229533.I1R9Y4	pp	229533.I1RYJ7
62409	229533.I1R9Y4	pp	229533.V6RTJ8
62410	229533.I1R9Y4	pp	229533.I1RR24
62411	229533.I1R9Y4	pp	229533.I1RNM7
62412	229533.I1R9Y4	pp	229533.I1RPA4
62413	229533.I1R9Y4	pp	229533.I1RAI4
62414	229533.I1R9Y4	pp	229533.I1RYT8
62415	229533.I1R9Y4	pp	229533.I1RMK7

62416	229533.I1R9Y4	pp	229533.I1RG62
62417	229533.I1R9Y4	pp	229533.I1RA35
62418	229533.I1R9Y4	pp	229533.I1RBT1
62419	229533.I1R9Y4	pp	229533.I1RHK2
62420	229533.I1RWU2	pp	229533.I1S1X9
62421	229533.I1RWU2	pp	229533.Q4HXF6
62422	229533.I1RWU2	pp	229533.Q4I1Y1
62423	229533.I1RWU2	pp	229533.I1S0B1
62424	229533.I1RWU2	pp	229533.I1RZQ5
62425	229533.I1RWU2	pp	229533.Q4IPH4
62426	229533.I1RWU2	pp	229533.I1S0Z9
62427	229533.I1RWU2	pp	229533.I1RX47
62428	229533.I1RWU2	pp	229533.I1S7M3
62429	229533.I1RWU2	pp	229533.V6QVD5
62430	229533.I1RWU2	pp	229533.Q4IE79
62431	229533.I1RWU2	pp	229533.I1RYB1
62432	229533.I1RWU2	pp	229533.Q4IB70
62433	229533.I1RWU2	pp	229533.I1S4M5
62434	229533.I1RWU2	pp	229533.Q4IAA0
62435	229533.I1RWU2	pp	229533.I1S044
62436	229533.I1RWU2	pp	229533.I1S056
62437	229533.I1RWU2	pp	229533.Q4IRI9
62438	229533.I1RWU2	pp	229533.Q4IBK5
62439	229533.I1RWU2	pp	229533.Q4IPB8
62440	229533.I1RWU2	pp	229533.I1S9L5
62441	229533.I1RWU2	pp	229533.I1RX24
62442	229533.I1RWU2	pp	229533.I1S0R2
62443	229533.I1RWU2	pp	229533.Q4IPB3
62444	229533.I1RWU2	pp	229533.I1RWX8
62445	229533.I1RWU2	pp	229533.I1S2Y5
62446	229533.I1RWU2	pp	229533.I1RX81
62447	229533.I1RWU2	pp	229533.I1S246
62448	229533.I1RWU2	pp	229533.I1RZG7
62449	229533.I1RWU2	pp	229533.I1S9L6
62450	229533.I1RWU2	pp	229533.Q4IF76
62451	229533.I1RWU2	pp	229533.I1RXF2
62452	229533.I1RWU2	pp	229533.V6QXG1
62453	229533.I1RWU2	pp	229533.Q4IJH1
62454	229533.I1RWU2	pp	229533.I1S7M2
62455	229533.I1RWU2	pp	229533.Q4I5R9
62456	229533.I1RRT3	pp	229533.I1RZ34
62457	229533.I1RRT3	pp	229533.I1RZ78
62458	229533.I1RRT3	pp	229533.I1S252
62459	229533.I1RRT3	pp	229533.V6RV57
62460	229533.I1RRT3	pp	229533.I1RST0
62461	229533.I1RRT3	pp	229533.I1S8J1
62462	229533.I1RRT3	pp	229533.I1RUP6
62463	229533.I1RRT3	pp	229533.I1RYK0
62464	229533.I1RRT3	pp	229533.V6R729
62465	229533.I1RRT3	pp	229533.Q4I1Y5
62466	229533.I1RRT3	pp	229533.I1S4S9
62467	229533.I1RRT3	pp	229533.V6RPQ8
62468	229533.I1RRT3	pp	229533.I1RY58
62469	229533.I1RRT3	pp	229533.I1RT36
62470	229533.I1RRT3	pp	229533.I1S0P6
62471	229533.I1RRT3	pp	229533.I1RZT6
62472	229533.I1RRT3	pp	229533.I1S984
62473	229533.I1RRT3	pp	229533.I1RSJ2
62474	229533.I1RRT3	pp	229533.I1S574
62475	229533.I1RRT3	pp	229533.I1RXV9
62476	229533.I1RRT3	pp	229533.I1RUM7
62477	229533.I1RX80	pp	229533.Q4IBU4
62478	229533.I1RX80	pp	229533.I1S9D4
62479	229533.I1RX80	pp	229533.I1RZC3
62480	229533.I1RX80	pp	229533.I1S2E2
62481	229533.I1RPP5	pp	229533.I1RZS5
62482	229533.I1RPP5	pp	229533.I1S0Q7
62483	229533.I1RPP5	pp	229533.I1S0L4
62484	229533.I1RPP5	pp	229533.I1RRX5
62485	229533.I1RPP5	pp	229533.I1RVQ0
62486	229533.I1RPP5	pp	229533.I1RPR9
62487	229533.I1RPP5	pp	229533.Q4IB65
62488	229533.I1RPP5	pp	229533.I1RXT0

62489	229533.I1RPP5	pp	229533.I1RQR6
62490	229533.I1RPP5	pp	229533.V6R3J1
62491	229533.I1RPP5	pp	229533.I1RZP8
62492	229533.I1RPP5	pp	229533.I1RWN6
62493	229533.I1RPP5	pp	229533.Q4IPZ1
62494	229533.I1RPP5	pp	229533.I1RPY8
62495	229533.I1RPP5	pp	229533.I1S0R9
62496	229533.I1RYX3	pp	229533.I1S1X2
62497	229533.I1RYX3	pp	229533.Q4IEV4
62498	229533.I1RYX3	pp	229533.Q4IMD1
62499	229533.I1RYX3	pp	229533.I1S109
62500	229533.I1RYX3	pp	229533.Q4I5R3
62501	229533.I1RYX3	pp	229533.Q4IER8
62502	229533.I1RYX3	pp	229533.Q4IB50
62503	229533.I1RYX3	pp	229533.Q4IAK7
62504	229533.I1RSC0	pp	229533.I1S0K2
62505	229533.I1RSC0	pp	229533.I1RWA3
62506	229533.I1RSC0	pp	229533.I1S464
62507	229533.I1RSC0	pp	229533.I1RYT8
62508	229533.I1RSC0	pp	229533.I1S5X9
62509	229533.I1RSC0	pp	229533.I1S0F1
62510	229533.I1RSC0	pp	229533.I1RY92
62511	229533.I1RSC0	pp	229533.I1S8S9
62512	229533.I1RSC0	pp	229533.I1S0P0
62513	229533.I1RSC0	pp	229533.V6RJP8
62514	229533.I1RSC0	pp	229533.I1RW26
62515	229533.I1RSC0	pp	229533.I1S458
62516	229533.I1RSC0	pp	229533.I1S1N7
62517	229533.I1RSC0	pp	229533.I1RY21
62518	229533.I1RSC0	pp	229533.I1S5R5
62519	229533.I1RSC0	pp	229533.I1RXD9
62520	229533.I1RDJ1	pp	229533.I1RR39
62521	229533.I1RDJ1	pp	229533.I1RVW0
62522	229533.I1RDJ1	pp	229533.I1RVV5
62523	229533.I1RDJ1	pp	229533.I1RQE0
62524	229533.I1RDJ1	pp	229533.I1RZ13
62525	229533.I1RDJ1	pp	229533.I1RZT6
62526	229533.I1RDJ1	pp	229533.I1RKB5
62527	229533.I1RDJ1	pp	229533.I1S252
62528	229533.I1RDJ1	pp	229533.I1RJW1
62529	229533.I1RDJ1	pp	229533.I1RZ78
62530	229533.I1RDJ1	pp	229533.I1S984
62531	229533.I1RDJ1	pp	229533.I1RIX0
62532	229533.I1RDJ1	pp	229533.I1RST0
62533	229533.I1RDJ1	pp	229533.I1RZ34
62534	229533.I1RDJ1	pp	229533.I1RSJ2
62535	229533.I1RDJ1	pp	229533.I1S574
62536	229533.I1RDJ1	pp	229533.I1RP86
62537	229533.I1RDJ1	pp	229533.I1RK44
62538	229533.I1RDJ1	pp	229533.I1RJL9
62539	229533.I1RDJ1	pp	229533.I1RIE8
62540	229533.I1RDJ1	pp	229533.I1RP81
62541	229533.I1RDJ1	pp	229533.I1S272
62542	229533.I1RDJ1	pp	229533.I1RPT0
62543	229533.I1RDJ1	pp	229533.I1RZV2
62544	229533.I1RDJ1	pp	229533.Q4INS6
62545	229533.I1RDJ1	pp	229533.V6RPQ8
62546	229533.I1RDJ1	pp	229533.I1RQ75
62547	229533.I1RDJ1	pp	229533.I1RNU7
62548	229533.I1RDJ1	pp	229533.I1S4S9
62549	229533.I1RDJ1	pp	229533.I1RFZ0
62550	229533.I1RDJ1	pp	229533.Q4I1Y5
62551	229533.I1RDJ1	pp	229533.I1RNA1
62552	229533.I1RDJ1	pp	229533.I1RWI8
62553	229533.I1RDJ1	pp	229533.I1RP99
62554	229533.I1RMQ6	pp	229533.I1RRX5
62555	229533.I1RMQ6	pp	229533.I1S0T7
62556	229533.I1RMQ6	pp	229533.I1RXT0
62557	229533.I1RMQ6	pp	229533.I1S0P0
62558	229533.I1RMQ6	pp	229533.I1RS05
62559	229533.I1RMQ6	pp	229533.V6RTJ8
62560	229533.I1RMQ6	pp	229533.I1S5C6
62561	229533.I1RMQ6	pp	229533.I1RWG0

229533.I1RMQ6 pp 229533.I1S4S1
229533.I1RMQ6 pp 229533.I1RY21
229533.I1RMQ6 pp 229533.I1RX17
229533.I1RMQ6 pp 229533.I1RYC7
229533.I1RMQ6 pp 229533.I1RVU1
229533.I1RMQ6 pp 229533.I1S0U0
229533.I1RMQ6 pp 229533.I1RPN1
229533.I1RMQ6 pp 229533.I1RNM7
229533.I1RMQ6 pp 229533.V6QUG0
229533.I1RMQ6 pp 229533.V6R7V8
229533.I1RMQ6 pp 229533.I1S8F8
229533.I1RMQ6 pp 229533.I1RZ68
229533.I1RMQ6 pp 229533.I1RYN2
229533.I1RMQ6 pp 229533.I1RRK9
229533.I1RMQ6 pp 229533.I1RYT8
229533.I1RMQ6 pp 229533.I1RW13
229533.I1RMQ6 pp 229533.I1RRS8
229533.I1RMQ6 pp 229533.V6RJP8
229533.I1RMQ6 pp 229533.I1RP02
229533.I1RMQ6 pp 229533.I1S8S9
229533.I1RMQ6 pp 229533.I1RXE6
229533.I1RMQ6 pp 229533.I1RQA5
229533.I1RMQ6 pp 229533.I1RPR9
229533.I1RMQ6 pp 229533.I1RMQ7
229533.I1RMQ6 pp 229533.I1RWF5
229533.I1RMQ6 pp 229533.I1S0K2
229533.I1RMQ6 pp 229533.I1RPP5
229533.I1RMQ6 pp 229533.I1RXD9
229533.I1RMQ6 pp 229533.I1RZ43
229533.I1RMQ6 pp 229533.I1RP38
229533.I1RMQ6 pp 229533.I1RS29
229533.I1RMQ6 pp 229533.I1RUL3
229533.I1RMQ6 pp 229533.I1S2A1
229533.I1RMQ6 pp 229533.V6R3J1
229533.I1RMQ6 pp 229533.Q4I5G1
229533.I1RMQ6 pp 229533.I1RUQ3
229533.I1RMQ6 pp 229533.I1S212
229533.I1RMQ6 pp 229533.Q4HVQ9
229533.I1RMQ6 pp 229533.I1RYM5
229533.I1RMQ6 pp 229533.I1RWB0
229533.I1RMQ6 pp 229533.I1RXG6
229533.I1RMQ6 pp 229533.V6RM66
229533.I1RMQ6 pp 229533.I1S464
229533.I1RMQ6 pp 229533.I1S128
229533.I1RMQ6 pp 229533.I1RX39
229533.I1RMQ6 pp 229533.I1S1F3
229533.I1RMQ6 pp 229533.I1RTA1
229533.I1RMQ6 pp 229533.I1S0Q7
229533.I1RMQ6 pp 229533.I1RNN1
229533.I1RMQ6 pp 229533.I1S8P5
229533.I1RMQ6 pp 229533.I1RX21
229533.I1RMQ6 pp 229533.I1RPY8
229533.I1RMQ6 pp 229533.I1RN84
229533.I1RMQ6 pp 229533.I1RSQ4
229533.I1RMQ6 pp 229533.I1S0V7
229533.I1RMQ6 pp 229533.I1S3T7
229533.I1RMQ6 pp 229533.I1RPY0
229533.I1RMQ6 pp 229533.I1S147
229533.I1RMQ6 pp 229533.I1S1A0
229533.I1RMQ6 pp 229533.I1RWA3
229533.I1RMQ6 pp 229533.I1RY92
229533.I1RMQ6 pp 229533.I1RUR8
229533.I1RMQ6 pp 229533.V6RRM0
229533.I1RMQ6 pp 229533.I1RZ39
229533.I1RMQ6 pp 229533.I1RMV0
229533.I1RMQ6 pp 229533.I1RRT8
229533.I1RMQ6 pp 229533.I1RVX8
229533.I1RMQ6 pp 229533.I1RY11
229533.I1RMQ6 pp 229533.I1S7W5
229533.I1RMQ6 pp 229533.I1RTA0
229533.I1RMQ6 pp 229533.I1RYC8
229533.I1RMQ6 pp 229533.V6RLM7
229533.I1RMQ6 pp 229533.I1RU95

62635 229533.I1RMQ6 pp 229533.I1RZR5
62636 229533.I1RMQ6 pp 229533.I1RRI2
62637 229533.I1RMQ6 pp 229533.I1S0R8
62638 229533.I1RMQ6 pp 229533.I1RQ21
62639 229533.I1RMQ6 pp 229533.I1S5X9
62640 229533.I1RMQ6 pp 229533.I1S5U0
62641 229533.I1RMQ6 pp 229533.I1RYA0
62642 229533.I1RMQ6 pp 229533.I1S266
62643 229533.I1RMQ6 pp 229533.I1RWT0
62644 229533.I1RMQ6 pp 229533.I1RNJ5
62645 229533.A0A1C3YKX4 pp 229533.I1RNG4
62646 229533.A0A1C3YKX4 pp 229533.A0A1C3YN26
62647 229533.A0A1C3YKX4 pp 229533.I1RUY5
62648 229533.A0A1C3YKX4 pp 229533.I1RDU0
62649 229533.I1RUZ8 pp 229533.I1RY77
62650 229533.I1RUZ8 pp 229533.I1S0F0
62651 229533.I1RUZ8 pp 229533.I1S1Y7
62652 229533.I1RUZ8 pp 229533.I1RZE1
62653 229533.I1RUZ8 pp 229533.Q4I7L0
62654 229533.I1RUZ8 pp 229533.I1RY99
62655 229533.A0A1C3YLE3 pp 229533.I1RAI4
62656 229533.A0A1C3YLE3 pp 229533.I1RMQ6
62657 229533.A0A1C3YLE3 pp 229533.I1RKB7
62658 229533.A0A1C3YLE3 pp 229533.I1R9Y4
62659 229533.A0A1C3YLE3 pp 229533.I1RZS5
62660 229533.A0A1C3YLE3 pp 229533.I1RRX5
62661 229533.A0A1C3YLE3 pp 229533.I1RB86
62662 229533.A0A1C3YLE3 pp 229533.I1RCU2
62663 229533.A0A1C3YLE3 pp 229533.I1RA49
62664 229533.A0A1C3YLE3 pp 229533.Q4IB65
62665 229533.A0A1C3YLE3 pp 229533.I1RXT0
62666 229533.A0A1C3YLE3 pp 229533.I1RPP5
62667 229533.A0A1C3YLE3 pp 229533.I1RAV5
62668 229533.A0A1C3YLE3 pp 229533.I1RCC5
62669 229533.A0A1C3YLE3 pp 229533.I1RAM9
62670 229533.A0A1C3YLE3 pp 229533.I1RBE6
62671 229533.A0A1C3YLE3 pp 229533.V6R3J1
62672 229533.A0A1C3YLE3 pp 229533.Q4IPZ1
62673 229533.A0A1C3YLE3 pp 229533.I1RNJ5
62674 229533.A0A1C3YLE3 pp 229533.I1RZP8
62675 229533.A0A1C3YLE3 pp 229533.I1S5B1
62676 229533.A0A1C3YLE3 pp 229533.I1RPY8
62677 229533.A0A1C3YLE3 pp 229533.I1RF85
62678 229533.I1RHF1 pp 229533.I1RND4
62679 229533.I1RHF1 pp 229533.I1S9T8
62680 229533.I1RHF1 pp 229533.V6RV57
62681 229533.I1RHF1 pp 229533.V6RLM7
62682 229533.I1RHF1 pp 229533.I1RW06
62683 229533.I1RHF1 pp 229533.I1RYK0
62684 229533.I1RKI7 pp 229533.I1RRG3
62685 229533.I1RKI7 pp 229533.V6RUT5
62686 229533.I1RKI7 pp 229533.I1RM30
62687 229533.I1RKI7 pp 229533.I1S1B3
62688 229533.I1RKI7 pp 229533.I1RPI5
62689 229533.I1RKI7 pp 229533.I1RZS5
62690 229533.I1RKI7 pp 229533.I1RM67
62691 229533.I1RKI7 pp 229533.I1RVR0
62692 229533.I1RKI7 pp 229533.I1RYI7
62693 229533.I1RKI7 pp 229533.I1RN82
62694 229533.I1RKI7 pp 229533.V6QY25
62695 229533.I1RKI7 pp 229533.I1S3K6
62696 229533.I1RKI7 pp 229533.I1S2C1
62697 229533.I1RKI7 pp 229533.I1S0X0
62698 229533.I1RKI7 pp 229533.I1S887
62699 229533.I1RKI7 pp 229533.I1RZB2
62700 229533.I1RKI7 pp 229533.I1RN46
62701 229533.I1RKI7 pp 229533.I1RSH3
62702 229533.I1RKI7 pp 229533.I1RMM1
62703 229533.I1RKI7 pp 229533.Q4HZE0
62704 229533.I1RKI7 pp 229533.I1RW76
62705 229533.I1RKI7 pp 229533.I1RTA1
62706 229533.I1RKI7 pp 229533.Q4HXF0
62707 229533.I1RKI7 pp 229533.I1RNG4

62708 229533.I1RKI7 pp 229533.I1S1W3
62709 229533.I1RKI7 pp 229533.O74638
62710 229533.I1RKI7 pp 229533.V6R382
62711 229533.I1RKI7 pp 229533.I1RPK0
62712 229533.I1RKI7 pp 229533.I1RZ60
62713 229533.I1RKI7 pp 229533.I1S1U6
62714 229533.I1RKI7 pp 229533.I1RP69
62715 229533.I1RKI7 pp 229533.V6QVH8
62716 229533.I1RKI7 pp 229533.I1RND7
62717 229533.I1RKI7 pp 229533.I1RXR1
62718 229533.I1RKI7 pp 229533.I1RUE2
62719 229533.I1RKI7 pp 229533.I1RXU9
62720 229533.A0A098DSZ8 pp 229533.A0A1C3YLB0
62721 229533.A0A098DSZ8 pp 229533.I1RAM9
62722 229533.A0A098DSZ8 pp 229533.I1RAV5
62723 229533.A0A098DSZ8 pp 229533.I1RA49
62724 229533.A0A098DSZ8 pp 229533.I1RXT0
62725 229533.A0A098DSZ8 pp 229533.I1RRX5
62726 229533.A0A098DSZ8 pp 229533.A0A0E0SJS1
62727 229533.A0A098DSZ8 pp 229533.I1RMQ6
62728 229533.A0A098DSZ8 pp 229533.I1RKB7
62729 229533.A0A098DSZ8 pp 229533.I1R9Y4
62730 229533.A0A098DSZ8 pp 229533.I1RB86
62731 229533.A0A098DSZ8 pp 229533.I1RZS5
62732 229533.A0A098DSZ8 pp 229533.I1RAI4
62733 229533.A0A098DSZ8 pp 229533.I1RCU2
62734 229533.A0A098DSZ8 pp 229533.I1RZP8
62735 229533.A0A098DSZ8 pp 229533.I1RF85
62736 229533.A0A098DSZ8 pp 229533.I1RNJ5
62737 229533.A0A098DSZ8 pp 229533.A0A1C3YI20
62738 229533.A0A098DSZ8 pp 229533.Q4IPZ1
62739 229533.A0A098DSZ8 pp 229533.I1RCC5
62740 229533.A0A098DSZ8 pp 229533.I1RPY8
62741 229533.A0A098DSZ8 pp 229533.A0A1C3YHG7
62742 229533.A0A098DSZ8 pp 229533.A0A0E0RP23
62743 229533.A0A098DSZ8 pp 229533.A0A1C3YIE7
62744 229533.A0A098DSZ8 pp 229533.I1RBE6
62745 229533.A0A098DSZ8 pp 229533.V6R3J1
62746 229533.A0A098DSZ8 pp 229533.Q4IB65
62747 229533.A0A098DSZ8 pp 229533.A0A1C3YIU6
62748 229533.I1RK45 pp 229533.Q4ILH3
62749 229533.I1RK45 pp 229533.I1RNA1
62750 229533.I1RK45 pp 229533.Q4I073
62751 229533.I1RK45 pp 229533.Q4I0K3
62752 229533.I1RK45 pp 229533.V6RPQ8
62753 229533.I1RK45 pp 229533.I1RQ75
62754 229533.I1RK45 pp 229533.I1RZ78
62755 229533.I1RK45 pp 229533.Q4I1Y5
62756 229533.I1RK45 pp 229533.I1RNU7
62757 229533.I1RK45 pp 229533.I1S252
62758 229533.I1RK45 pp 229533.I1RST0
62759 229533.I1RK45 pp 229533.I1RQE0
62760 229533.I1RK45 pp 229533.I1RZ34
62761 229533.I1RK45 pp 229533.I1S574
62762 229533.I1RK45 pp 229533.I1S4S9
62763 229533.I1RK45 pp 229533.I1RPT0
62764 229533.I1RK45 pp 229533.I1S984
62765 229533.I1RK45 pp 229533.I1RNK8
62766 229533.I1RK45 pp 229533.I1RP81
62767 229533.I1RK45 pp 229533.Q4HXT5
62768 229533.I1RK45 pp 229533.I1RZT6
62769 229533.I1RK45 pp 229533.I1RSJ2
62770 229533.I1RK45 pp 229533.I1RP86
62771 229533.I1RK45 pp 229533.I1RKB5
62772 229533.I1RK45 pp 229533.I1RR39
62773 229533.I1RK45 pp 229533.Q4IB63
62774 229533.I1S8H9 pp 229533.V6RLM7
62775 229533.I1S8H9 pp 229533.V6R9Z3
62776 229533.I1S8H9 pp 229533.I1S8R2
62777 229533.I1S201 pp 229533.Q4IMD1
62778 229533.I1S201 pp 229533.Q4I5W5
62779 229533.I1S201 pp 229533.Q4HYQ4
62780 229533.I1S201 pp 229533.Q4HW67

62781	229533.I1S201	pp	229533.Q4ICA8
62782	229533.I1S201	pp	229533.Q4I5I4
62783	229533.I1S201	pp	229533.Q4HYB8
62784	229533.I1S201	pp	229533.Q4IJU0
62785	229533.I1S2P8	pp	229533.V6R382
62786	229533.I1RAV5	pp	229533.I1S1F3
62787	229533.I1RAV5	pp	229533.I1RL81
62788	229533.I1RAV5	pp	229533.I1RYE5
62789	229533.I1RAV5	pp	229533.I1RSQ4
62790	229533.I1RAV5	pp	229533.I1RYW9
62791	229533.I1RAV5	pp	229533.I1RC18
62792	229533.I1RAV5	pp	229533.I1RFI4
62793	229533.I1RAV5	pp	229533.I1RAW3
62794	229533.I1RAV5	pp	229533.I1S299
62795	229533.I1RAV5	pp	229533.I1RGC3
62796	229533.I1RAV5	pp	229533.I1RX39
62797	229533.I1RAV5	pp	229533.I1RUR8
62798	229533.I1RAV5	pp	229533.I1S464
62799	229533.I1RAV5	pp	229533.I1RC34
62800	229533.I1RAV5	pp	229533.I1RBE6
62801	229533.I1RAV5	pp	229533.I1S3T7
62802	229533.I1RAV5	pp	229533.I1RKB2
62803	229533.I1RAV5	pp	229533.I1RWA3
62804	229533.I1RAV5	pp	229533.I1RCC5
62805	229533.I1RAV5	pp	229533.V6R3J1
62806	229533.I1RAV5	pp	229533.Q4HVQ9
62807	229533.I1RAV5	pp	229533.V6RRM0
62808	229533.I1RAV5	pp	229533.I1RGB0
62809	229533.I1RAV5	pp	229533.I1RY57
62810	229533.I1RAV5	pp	229533.I1RAW9
62811	229533.I1RAV5	pp	229533.I1RE65
62812	229533.I1RAV5	pp	229533.I1RFS5
62813	229533.I1RAV5	pp	229533.I1RY92
62814	229533.I1RAV5	pp	229533.I1RXD9
62815	229533.I1RAV5	pp	229533.I1RWT0
62816	229533.I1RAV5	pp	229533.I1RFJ6
62817	229533.I1RAV5	pp	229533.I1S0Q7
62818	229533.I1RAV5	pp	229533.V6R1X4
62819	229533.I1RAV5	pp	229533.I1S0R9
62820	229533.I1RAV5	pp	229533.I1RYC8
62821	229533.I1RAV5	pp	229533.I1RNJ5
62822	229533.I1RAV5	pp	229533.I1RN84
62823	229533.I1RAV5	pp	229533.I1RY21
62824	229533.I1RAV5	pp	229533.I1RSU1
62825	229533.I1RAV5	pp	229533.I1RQ72
62826	229533.I1RAV5	pp	229533.I1RE52
62827	229533.I1RAV5	pp	229533.I1RBT6
62828	229533.I1RAV5	pp	229533.I1RMI1
62829	229533.I1RAV5	pp	229533.I1RPK0
62830	229533.I1RAV5	pp	229533.I1RX17
62831	229533.I1RAV5	pp	229533.I1RB38
62832	229533.I1RAV5	pp	229533.I1RMQ6
62833	229533.I1RAV5	pp	229533.I1S5X9
62834	229533.I1RAV5	pp	229533.I1RYA0
62835	229533.I1RAV5	pp	229533.I1RKB7
62836	229533.I1RAV5	pp	229533.I1RPZ8
62837	229533.I1RAV5	pp	229533.I1RI27
62838	229533.I1RAV5	pp	229533.I1S2C1
62839	229533.I1RAV5	pp	229533.I1S0R8
62840	229533.I1RAV5	pp	229533.I1RXT0
62841	229533.I1RAV5	pp	229533.I1RS05
62842	229533.I1RAV5	pp	229533.I1RYN2
62843	229533.I1RAV5	pp	229533.I1S8R2
62844	229533.I1RAV5	pp	229533.I1RRS8
62845	229533.I1RAV5	pp	229533.I1RZ43
62846	229533.I1RAV5	pp	229533.I1S0P0
62847	229533.I1RAV5	pp	229533.I1RE66
62848	229533.I1RAV5	pp	229533.I1RG62
62849	229533.I1RAV5	pp	229533.I1RYT8
62850	229533.I1RAV5	pp	229533.I1RRX5
62851	229533.I1RAV5	pp	229533.I1S4R0
62852	229533.I1RAV5	pp	229533.I1RGU0
62853	229533.I1RAV5	pp	229533.I1RMK7

62854	229533.I1RAV5	pp	229533.I1S8S9
62855	229533.I1RAV5	pp	229533.I1RNM7
62856	229533.I1RAV5	pp	229533.I1S0R1
62857	229533.I1RAV5	pp	229533.V6RJP8
62858	229533.I1RAV5	pp	229533.I1RUQ3
62859	229533.I1RAV5	pp	229533.I1RP02
62860	229533.I1RAV5	pp	229533.I1RXE6
62861	229533.I1RAV5	pp	229533.I1S8F8
62862	229533.I1RAV5	pp	229533.I1RPP5
62863	229533.I1RAV5	pp	229533.I1RQR6
62864	229533.I1RAV5	pp	229533.I1RS29
62865	229533.I1RAV5	pp	229533.I1RQS2
62866	229533.I1RAV5	pp	229533.I1RG33
62867	229533.I1RAV5	pp	229533.I1S0K2
62868	229533.I1RAV5	pp	229533.I1RGB1
62869	229533.I1RAV5	pp	229533.I1S212
62870	229533.I1RAV5	pp	229533.I1REE2
62871	229533.A0A0E0S0Q5	pp	229533.I1RK03
62872	229533.A0A0E0S0Q5	pp	229533.A0A1C3YIL8
62873	229533.A0A0E0S0Q5	pp	229533.A0A1C3YLZ8
62874	229533.A0A0E0S0Q5	pp	229533.A0A0E0SPV0
62875	229533.A0A0E0S0Q5	pp	229533.I1RWW7
62876	229533.I1RAK6	pp	229533.I1RY99
62877	229533.I1RAK6	pp	229533.I1S1Y7
62878	229533.I1RAK6	pp	229533.I1RZE1
62879	229533.I1RAK6	pp	229533.I1S0F0
62880	229533.I1RAK6	pp	229533.I1RY77
62881	229533.I1RAK6	pp	229533.I1RCN2
62882	229533.I1RAK6	pp	229533.I1RB73
62883	229533.I1RAK6	pp	229533.Q4I7L0
62884	229533.I1RAK6	pp	229533.I1RC82
62885	229533.I1RAK6	pp	229533.I1RFQ0
62886	229533.A0A1C3YLB0	pp	229533.I1RRK9
62887	229533.A0A1C3YLB0	pp	229533.I1RG62
62888	229533.A0A1C3YLB0	pp	229533.I1RYT8
62889	229533.A0A1C3YLB0	pp	229533.I1RYX5
62890	229533.A0A1C3YLB0	pp	229533.I1RGC3
62891	229533.A0A1C3YLB0	pp	229533.I1RAW3
62892	229533.A0A1C3YLB0	pp	229533.I1RF87
62893	229533.A0A1C3YLB0	pp	229533.I1RA52
62894	229533.A0A1C3YLB0	pp	229533.I1RYN2
62895	229533.A0A1C3YLB0	pp	229533.I1S277
62896	229533.A0A1C3YLB0	pp	229533.I1RI27
62897	229533.A0A1C3YLB0	pp	229533.I1RX21
62898	229533.A0A1C3YLB0	pp	229533.I1RFI4
62899	229533.A0A1C3YLB0	pp	229533.I1REF0
62900	229533.A0A1C3YLB0	pp	229533.I1R9Y4
62901	229533.A0A1C3YLB0	pp	229533.I1RMQ6
62902	229533.A0A1C3YLB0	pp	229533.I1S3U5
62903	229533.A0A1C3YLB0	pp	229533.I1RKB7
62904	229533.A0A1C3YLB0	pp	229533.I1RPM4
62905	229533.A0A1C3YLB0	pp	229533.I1S0U0
62906	229533.A0A1C3YLB0	pp	229533.I1RCC5
62907	229533.A0A1C3YLB0	pp	229533.I1RMY6
62908	229533.A0A1C3YLB0	pp	229533.I1RB64
62909	229533.A0A1C3YLB0	pp	229533.I1RBS0
62910	229533.A0A1C3YLB0	pp	229533.I1RX17
62911	229533.A0A1C3YLB0	pp	229533.I1RNM7
62912	229533.A0A1C3YLB0	pp	229533.I1RY92
62913	229533.A0A1C3YLB0	pp	229533.I1RY21
62914	229533.A0A1C3YLB0	pp	229533.I1S0P0
62915	229533.A0A1C3YLB0	pp	229533.I1RMK7
62916	229533.A0A1C3YLB0	pp	229533.I1RDC3
62917	229533.A0A1C3YLB0	pp	229533.I1RE66
62918	229533.A0A1C3YLB0	pp	229533.I1RTQ5
62919	229533.A0A1C3YLB0	pp	229533.I1RAW9
62920	229533.A0A1C3YLB0	pp	229533.I1S3T7
62921	229533.A0A1C3YLB0	pp	229533.I1RYE5
62922	229533.A0A1C3YLB0	pp	229533.I1RWA3
62923	229533.A0A1C3YLB0	pp	229533.I1RAI4
62924	229533.A0A1C3YLB0	pp	229533.I1RJB3
62925	229533.A0A1C3YLB0	pp	229533.I1RKP1
62926	229533.A0A1C3YLB0	pp	229533.I1S0R8

229533.A0A1C3YLB0	pp	229533.I1RGJ4
229533.A0A1C3YLB0	pp	229533.I1S1V6
229533.A0A1C3YLB0	pp	229533.I1RG33
229533.A0A1C3YLB0	pp	229533.I1RWK3
229533.A0A1C3YLB0	pp	229533.I1S0K2
229533.A0A1C3YLB0	pp	229533.I1RYW9
229533.A0A1C3YLB0	pp	229533.I1S0T7
229533.A0A1C3YLB0	pp	229533.I1S5X9
229533.A0A1C3YLB0	pp	229533.I1RWG0
229533.A0A1C3YLB0	pp	229533.I1RCU2
229533.A0A1C3YLB0	pp	229533.I1S1F3
229533.A0A1C3YLB0	pp	229533.I1RKQ6
229533.A0A1C3YLB0	pp	229533.A0A1C3YMN6
229533.A0A1C3YLB0	pp	229533.I1RS29
229533.A0A1C3YLB0	pp	229533.I1RL81
229533.A0A1C3YLB0	pp	229533.I1RRX5
229533.A0A1C3YLB0	pp	229533.I1S464
229533.A0A1C3YLB0	pp	229533.I1RPP5
229533.A0A1C3YLB0	pp	229533.I1RYA0
229533.A0A1C3YLB0	pp	229533.I1RBM4
229533.A0A1C3YLB0	pp	229533.I1RL35
229533.A0A1C3YLB0	pp	229533.I1RWC9
229533.A0A1C3YLB0	pp	229533.I1RTD5
229533.A0A1C3YLB0	pp	229533.I1REZ6
229533.A0A1C3YLB0	pp	229533.A0A1C3YLM6
229533.A0A1C3YLB0	pp	229533.I1RUQ3
229533.A0A1C3YLB0	pp	229533.I1RXD9
229533.A0A1C3YLB0	pp	229533.I1RMI1
229533.A0A1C3YLB0	pp	229533.I1S266
229533.A0A1C3YLB0	pp	229533.I1RHG0
229533.A0A1C3YLB0	pp	229533.I1RF85
229533.A0A1C3YLB0	pp	229533.I1RLP1
229533.A0A1C3YLB0	pp	229533.I1RS05
229533.A0A1C3YLB0	pp	229533.I1RAV5
229533.A0A1C3YLB0	pp	229533.A0A1C3YM60
229533.A0A1C3YLB0	pp	229533.I1RPR9
229533.A0A1C3YLB0	pp	229533.I1RQS2
229533.A0A1C3YLB0	pp	229533.Q4I5G1
229533.A0A1C3YLB0	pp	229533.A0A1C3YMC1
229533.A0A1C3YLB0	pp	229533.I1RK39
229533.A0A1C3YLB0	pp	229533.I1RLN1
229533.A0A1C3YLB0	pp	229533.I1RXP4
229533.A0A1C3YLB0	pp	229533.I1RGI8
229533.A0A1C3YLB0	pp	229533.I1S1G0
229533.A0A1C3YLB0	pp	229533.I1RU95
229533.A0A1C3YLB0	pp	229533.I1RHX8
229533.A0A1C3YLB0	pp	229533.V6R3J1
229533.A0A1C3YLB0	pp	229533.I1S7W5
229533.A0A1C3YLB0	pp	229533.I1RB38
229533.A0A1C3YLB0	pp	229533.A0A1C3YLB6
229533.A0A1C3YLB0	pp	229533.I1RXT0
229533.A0A1C3YLB0	pp	229533.I1RS01
229533.A0A1C3YLB0	pp	229533.I1RBT6
229533.A0A1C3YLB0	pp	229533.I1RMV0
229533.A0A1C3YLB0	pp	229533.A0A1C3YLB3
229533.A0A1C3YLB0	pp	229533.I1RE52
229533.A0A1C3YLB0	pp	229533.I1S8S9
229533.A0A1C3YLB0	pp	229533.I1RAM9
229533.A0A1C3YLB0	pp	229533.I1RTN6
229533.A0A1C3YLB0	pp	229533.I1RJW0
229533.A0A1C3YLB0	pp	229533.I1RIF9
229533.A0A1C3YLB0	pp	229533.I1S8F8
229533.A0A1C3YLB0	pp	229533.I1RZ43
229533.A0A1C3YLB0	pp	229533.I1RA49
229533.A0A1C3YLB0	pp	229533.I1RYC8
229533.A0A1C3YLB0	pp	229533.I1RSF1
229533.A0A1C3YLB0	pp	229533.I1RBU8
229533.A0A1C3YLB0	pp	229533.I1RP38
229533.A0A1C3YLB0	pp	229533.I1RJV4
229533.A0A1C3YLB0	pp	229533.I1S5U0
229533.A0A1C3YLB0	pp	229533.I1RBE6
229533.A0A1C3YLB0	pp	229533.I1RWT0
229533.A0A1C3YLB0	pp	229533.I1S212

63000 229533.A0A1C3YLB0 pp 229533.I1RX39
63001 229533.A0A1C3YLB0 pp 229533.I1RFJ6
63002 229533.A0A1C3YLB0 pp 229533.V6RJP8
63003 229533.A0A1C3YLB0 pp 229533.I1RTL0
63004 229533.A0A1C3YLB0 pp 229533.I1RJF5
63005 229533.A0A1C3YLB0 pp 229533.I1RRY9
63006 229533.A0A1C3YLB0 pp 229533.I1RWB0
63007 229533.A0A1C3YLB0 pp 229533.I1RL71
63008 229533.A0A1C3YLB0 pp 229533.I1RPP1
63009 229533.A0A1C3YLB0 pp 229533.I1RZ68
63010 229533.A0A1C3YLB0 pp 229533.I1RYC7
63011 229533.A0A1C3YLB0 pp 229533.I1RFS5
63012 229533.A0A1C3YLB0 pp 229533.A0A1C3YLE3
63013 229533.A0A1C3YLB0 pp 229533.I1REE2
63014 229533.A0A1C3YLB0 pp 229533.I1S2E7
63015 229533.A0A1C3YLB0 pp 229533.I1S0Q7
63016 229533.A0A1C3YLB0 pp 229533.I1RXE6
63017 229533.A0A1C3YLB0 pp 229533.I1RE65
63018 229533.A0A1C3YLB0 pp 229533.I1RW13
63019 229533.A0A1C3YLB0 pp 229533.I1RP02
63020 229533.A0A1C3YLB0 pp 229533.I1RI95
63021 229533.A0A1C3YLD0 pp 229533.I1RZP4
63022 229533.A0A1C3YLD0 pp 229533.A0A1C3YM29
63023 229533.A0A1C3YLD0 pp 229533.I1RB01
63024 229533.A0A1C3YLD0 pp 229533.I1S109
63025 229533.A0A1C3YLD0 pp 229533.I1S087
63026 229533.A0A1C3YLD0 pp 229533.Q4IQC1
63027 229533.A0A1C3YLD0 pp 229533.I1S7A3
63028 229533.A0A1C3YLD0 pp 229533.I1S472
63029 229533.A0A1C3YLD0 pp 229533.Q4ICM0
63030 229533.A0A1C3YLD0 pp 229533.I1RCC7
63031 229533.A0A1C3YLD0 pp 229533.I1RB14
63032 229533.A0A1C3YLD0 pp 229533.I1RDY3
63033 229533.A0A1C3YLD0 pp 229533.Q4I7L0
63034 229533.A0A1C3YLD0 pp 229533.I1RCN2
63035 229533.A0A1C3YLD0 pp 229533.I1RB73
63036 229533.I1RXA9 pp 229533.I1S1W3
63037 229533.I1RXA9 pp 229533.I1S0J8
63038 229533.I1RXA9 pp 229533.I1RXU9
63039 229533.I1RXA9 pp 229533.I1S7W5
63040 229533.I1RXA9 pp 229533.V6R382
63041 229533.I1RXA9 pp 229533.I1S237
63042 229533.I1RXA9 pp 229533.I1S209
63043 229533.I1RXA9 pp 229533.I1RXB1
63044 229533.I1RXA9 pp 229533.I1RXR1
63045 229533.I1RXA9 pp 229533.I1S887
63046 229533.I1RXA9 pp 229533.I1RXB6
63047 229533.I1RXA9 pp 229533.V6RUT5
63048 229533.I1RXA9 pp 229533.I1S236
63049 229533.A0A1C3YI20 pp 229533.I1RRI2
63050 229533.A0A1C3YI20 pp 229533.I1RCG5
63051 229533.A0A1C3YI20 pp 229533.A0A1C3YJI9
63052 229533.A0A1C3YI20 pp 229533.I1RB92
63053 229533.A0A1C3YI20 pp 229533.V6RIV7
63054 229533.A0A1C3YI20 pp 229533.I1S1C7
63055 229533.A0A1C3YI20 pp 229533.I1RSY2
63056 229533.A0A1C3YI20 pp 229533.I1RYW3
63057 229533.A0A1C3YI20 pp 229533.I1RVX0
63058 229533.A0A1C3YI20 pp 229533.I1RCW6
63059 229533.A0A1C3YI20 pp 229533.A0A1C3YK33
63060 229533.A0A1C3YI20 pp 229533.I1RT03
63061 229533.A0A1C3YI20 pp 229533.I1RVX8
63062 229533.A0A1C3YI20 pp 229533.I1RXR1
63063 229533.A0A1C3YI20 pp 229533.I1RCH5
63064 229533.A0A1C3YI20 pp 229533.I1RAW0
63065 229533.A0A1C3YI20 pp 229533.I1S064
63066 229533.A0A1C3YI20 pp 229533.I1S0R8
63067 229533.A0A1C3YI20 pp 229533.I1S7W5
63068 229533.A0A1C3YI20 pp 229533.I1RF85
63069 229533.A0A1C3YI20 pp 229533.I1RXU9
63070 229533.A0A1C3YI20 pp 229533.I1RQE7
63071 229533.A0A1C3YI20 pp 229533.A0A1C3YKY0
63072 229533.A0A1C3YI20 pp 229533.I1RFJ6

63073	229533.A0A1C3YI20	pp	229533.I1S0J1
63074	229533.A0A1C3YI20	pp	229533.I1S8R2
63075	229533.A0A1C3YI20	pp	229533.I1RNL5
63076	229533.A0A1C3YI20	pp	229533.I1S855
63077	229533.A0A1C3YI20	pp	229533.I1RFL4
63078	229533.A0A1C3YI20	pp	229533.I1RJV3
63079	229533.A0A1C3YI20	pp	229533.A0A1C3YJU7
63080	229533.A0A1C3YI20	pp	229533.I1RE01
63081	229533.A0A1C3YI20	pp	229533.I1RYA0
63082	229533.A0A1C3YI20	pp	229533.I1RNK6
63083	229533.A0A1C3YI20	pp	229533.I1REC7
63084	229533.A0A1C3YI20	pp	229533.I1RKB6
63085	229533.A0A1C3YI20	pp	229533.I1RC84
63086	229533.A0A1C3YI20	pp	229533.A0A1C3YLC1
63087	229533.A0A1C3YI20	pp	229533.I1RTA0
63088	229533.A0A1C3YI20	pp	229533.I1RAU7
63089	229533.A0A1C3YI20	pp	229533.I1RB38
63090	229533.A0A1C3YI20	pp	229533.I1RCR2
63091	229533.A0A1C3YI20	pp	229533.A0A1C3YLB6
63092	229533.A0A1C3YI20	pp	229533.I1RQ21
63093	229533.A0A1C3YI20	pp	229533.I1S9Z4
63094	229533.A0A1C3YI20	pp	229533.I1RMV0
63095	229533.A0A1C3YI20	pp	229533.Q4I624
63096	229533.A0A1C3YI20	pp	229533.I1RBT6
63097	229533.A0A1C3YI20	pp	229533.Q4I7K4
63098	229533.A0A1C3YI20	pp	229533.I1RLV0
63099	229533.A0A1C3YI20	pp	229533.I1RMD6
63100	229533.A0A1C3YI20	pp	229533.I1S1W3
63101	229533.A0A1C3YI20	pp	229533.I1RWD3
63102	229533.A0A1C3YI20	pp	229533.I1RYH7
63103	229533.A0A1C3YI20	pp	229533.A0A1C3YJ04
63104	229533.A0A1C3YI20	pp	229533.I1RE52
63105	229533.A0A1C3YI20	pp	229533.V6RLM7
63106	229533.A0A1C3YI20	pp	229533.I1S1W9
63107	229533.A0A1C3YI20	pp	229533.V6RRH2
63108	229533.A0A1C3YI20	pp	229533.I1RYC8
63109	229533.A0A1C3YI20	pp	229533.I1S2C4
63110	229533.A0A1C3YI20	pp	229533.I1RPK0
63111	229533.A0A1C3YI20	pp	229533.I1RA70
63112	229533.A0A1C3YI20	pp	229533.I1S0Q7
63113	229533.A0A1C3YI20	pp	229533.I1RPV3
63114	229533.A0A1C3YI20	pp	229533.I1RHC8
63115	229533.A0A1C3YI20	pp	229533.I1RL04
63116	229533.A0A1C3YI20	pp	229533.I1RCC5
63117	229533.A0A1C3YI20	pp	229533.I1RSZ7
63118	229533.A0A1C3YI20	pp	229533.I1RMQ5
63119	229533.A0A1C3YI20	pp	229533.V6RMW6
63120	229533.A0A1C3YI20	pp	229533.I1RUG3
63121	229533.A0A1C3YI20	pp	229533.I1RPU8
63122	229533.A0A1C3YI20	pp	229533.I1RGC3
63123	229533.A0A1C3YI20	pp	229533.I1RNC1
63124	229533.A0A1C3YI20	pp	229533.I1RAW3
63125	229533.A0A1C3YI20	pp	229533.I1RYX5
63126	229533.A0A1C3YI20	pp	229533.I1RBS5
63127	229533.A0A1C3YI20	pp	229533.I1RCR8
63128	229533.A0A1C3YI20	pp	229533.V6R042
63129	229533.A0A1C3YI20	pp	229533.I1RG11
63130	229533.A0A1C3YI20	pp	229533.I1R9Y8
63131	229533.A0A1C3YI20	pp	229533.I1RE65
63132	229533.A0A1C3YI20	pp	229533.I1RSQ3
63133	229533.A0A1C3YI20	pp	229533.V6R1X4
63134	229533.A0A1C3YI20	pp	229533.I1S5R6
63135	229533.A0A1C3YI20	pp	229533.I1RA52
63136	229533.A0A1C3YI20	pp	229533.I1RFI4
63137	229533.A0A1C3YI20	pp	229533.I1RS90
63138	229533.A0A1C3YI20	pp	229533.I1RBY4
63139	229533.A0A1C3YI20	pp	229533.I1R9M0
63140	229533.A0A1C3YI20	pp	229533.I1RUR8
63141	229533.A0A1C3YI20	pp	229533.I1RN84
63142	229533.A0A1C3YI20	pp	229533.A0A1C3YIR8
63143	229533.A0A1C3YI20	pp	229533.V6RRM0
63144	229533.A0A1C3YI20	pp	229533.I1RA29
63145	229533.A0A1C3YI20	pp	229533.I1S0L4

63146 229533.A0A1C3YI20 pp 229533.I1S2B8
63147 229533.A0A1C3YI20 pp 229533.I1RRN2
63148 229533.A0A1C3YI20 pp 229533.I1RMR9
63149 229533.A0A1C3YI20 pp 229533.I1S3T7
63150 229533.A0A1C3YI20 pp 229533.I1S256
63151 229533.A0A1C3YI20 pp 229533.A0A1C3YJK3
63152 229533.A0A1C3YI20 pp 229533.I1S2H4
63153 229533.A0A1C3YI20 pp 229533.A0A1C3YJI5
63154 229533.A0A1C3YI20 pp 229533.I1RW43
63155 229533.A0A1C3YI20 pp 229533.A0A1C3YKM9
63156 229533.A0A1C3YI20 pp 229533.I1S147
63157 229533.A0A1C3YI20 pp 229533.I1RZ39
63158 229533.A0A1C3YI20 pp 229533.I1RAE4
63159 229533.A0A1C3YI20 pp 229533.I1S3G4
63160 229533.A0A1C3YI20 pp 229533.I1RD22
63161 229533.A0A1C3YI20 pp 229533.I1RDE7
63162 229533.A0A1C3YI20 pp 229533.I1RY92
63163 229533.A0A1C3YI20 pp 229533.O74638
63164 229533.A0A1C3YI20 pp 229533.I1S0V7
63165 229533.A0A1C3YI20 pp 229533.I1RFS5
63166 229533.A0A1C3YI20 pp 229533.I1RRT8
63167 229533.A0A1C3YI20 pp 229533.I1RC66
63168 229533.A0A1C3YI20 pp 229533.A0A1C3YJD4
63169 229533.A0A1C3YI20 pp 229533.I1RVN1
63170 229533.A0A1C3YI20 pp 229533.I1RC29
63171 229533.A0A1C3YI20 pp 229533.I1S5R1
63172 229533.A0A1C3YI20 pp 229533.I1REC6
63173 229533.A0A1C3YI20 pp 229533.I1RX00
63174 229533.A0A1C3YI20 pp 229533.I1RN88
63175 229533.A0A1C3YI20 pp 229533.I1RSQ4
63176 229533.A0A1C3YI20 pp 229533.A0A1C3YI53
63177 229533.A0A1C3YI20 pp 229533.I1RA11
63178 229533.A0A1C3YI20 pp 229533.I1RUN0
63179 229533.A0A1C3YI20 pp 229533.I1RFS3
63180 229533.A0A1C3YI20 pp 229533.I1S9Y1
63181 229533.A0A1C3YI20 pp 229533.I1RZZ9
63182 229533.A0A1C3YI20 pp 229533.I1RWF5
63183 229533.A0A1C3YI20 pp 229533.I1RWI5
63184 229533.A0A1C3YI20 pp 229533.I1RTD5
63185 229533.A0A1C3YI20 pp 229533.I1S8P5
63186 229533.A0A1C3YI20 pp 229533.A0A1C3YJI6
63187 229533.A0A1C3YI20 pp 229533.I1RA62
63188 229533.A0A1C3YI20 pp 229533.I1RYW9
63189 229533.A0A1C3YI20 pp 229533.A0A1C3YJP4
63190 229533.A0A1C3YI20 pp 229533.I1RXD9
63191 229533.A0A1C3YI20 pp 229533.I1RPP3
63192 229533.A0A1C3YI20 pp 229533.I1RFL3
63193 229533.A0A1C3YI20 pp 229533.I1S1F3
63194 229533.A0A1C3YI20 pp 229533.I1RYI5
63195 229533.A0A1C3YI20 pp 229533.I1RT40
63196 229533.A0A1C3YI20 pp 229533.I1RAE3
63197 229533.A0A1C3YI20 pp 229533.A0A1C3YLA6
63198 229533.A0A1C3YI20 pp 229533.I1RYK0
63199 229533.A0A1C3YI20 pp 229533.I1RAB4
63200 229533.A0A1C3YI20 pp 229533.I1RBE6
63201 229533.A0A1C3YI20 pp 229533.I1RL81
63202 229533.A0A1C3YI20 pp 229533.V6R3U5
63203 229533.A0A1C3YI20 pp 229533.A0A1C3YN78
63204 229533.A0A1C3YI20 pp 229533.I1S299
63205 229533.A0A1C3YI20 pp 229533.I1RX39
63206 229533.A0A1C3YI20 pp 229533.I1RBA6
63207 229533.A0A1C3YI20 pp 229533.I1S464
63208 229533.A0A1C3YI20 pp 229533.I1RBS0
63209 229533.A0A1C3YI20 pp 229533.I1RGA4
63210 229533.A0A1C3YI20 pp 229533.I1RC31
63211 229533.A0A1C3YI20 pp 229533.I1RBC7
63212 229533.A0A1C3YI20 pp 229533.I1RFT1
63213 229533.A0A1C3YI20 pp 229533.I1RMJ3
63214 229533.A0A1C3YI20 pp 229533.I1RY62
63215 229533.A0A1C3YI20 pp 229533.I1RC05
63216 229533.A0A1C3YI20 pp 229533.V6R3J1
63217 229533.A0A1C3YI20 pp 229533.I1RN67
63218 229533.A0A1C3YI20 pp 229533.I1RNG4

63219 229533.A0A1C3YI20 pp 229533.I1RKB2
63220 229533.A0A1C3YI20 pp 229533.I1RSX0
63221 229533.A0A1C3YI20 pp 229533.V6QXN0
63222 229533.A0A1C3YI20 pp 229533.I1RC34
63223 229533.A0A1C3YI20 pp 229533.I1RS29
63224 229533.A0A1C3YI20 pp 229533.A0A1C3YIU6
63225 229533.A0A1C3YI20 pp 229533.A0A1C3YLE2
63226 229533.A0A1C3YI20 pp 229533.I1S0Q5
63227 229533.A0A1C3YI20 pp 229533.Q4HVQ9
63228 229533.A0A1C3YI20 pp 229533.A0A1C3YLK8
63229 229533.A0A1C3YI20 pp 229533.V6RUY9
63230 229533.A0A1C3YI20 pp 229533.I1RPP5
63231 229533.A0A1C3YI20 pp 229533.I1RUQ3
63232 229533.A0A1C3YI20 pp 229533.Q4IJK2
63233 229533.A0A1C3YI20 pp 229533.I1RCR5
63234 229533.A0A1C3YI20 pp 229533.I1RFJ5
63235 229533.A0A1C3YI20 pp 229533.A0A1C3YM60
63236 229533.A0A1C3YI20 pp 229533.A0A1C3YL55
63237 229533.A0A1C3YI20 pp 229533.Q4HXF0
63238 229533.A0A1C3YI20 pp 229533.I1RK95
63239 229533.A0A1C3YI20 pp 229533.I1RAM9
63240 229533.A0A1C3YI20 pp 229533.I1RDU0
63241 229533.A0A1C3YI20 pp 229533.I1S0V6
63242 229533.A0A1C3YI20 pp 229533.I1RG33
63243 229533.A0A1C3YI20 pp 229533.I1RAA2
63244 229533.A0A1C3YI20 pp 229533.I1RRY9
63245 229533.A0A1C3YI20 pp 229533.I1RAV5
63246 229533.A0A1C3YI20 pp 229533.I1S4Q4
63247 229533.A0A1C3YI20 pp 229533.I1S0K2
63248 229533.A0A1C3YI20 pp 229533.Q4I5G1
63249 229533.A0A1C3YI20 pp 229533.I1RG14
63250 229533.A0A1C3YI20 pp 229533.I1RQW8
63251 229533.A0A1C3YI20 pp 229533.I1RWC9
63252 229533.A0A1C3YI20 pp 229533.I1RYA2
63253 229533.A0A1C3YI20 pp 229533.V6RJP8
63254 229533.A0A1C3YI20 pp 229533.I1RBL7
63255 229533.A0A1C3YI20 pp 229533.I1RJY7
63256 229533.A0A1C3YI20 pp 229533.I1RUY6
63257 229533.A0A1C3YI20 pp 229533.I1REL7
63258 229533.A0A1C3YI20 pp 229533.I1RPR9
63259 229533.A0A1C3YI20 pp 229533.A0A1C3YJR3
63260 229533.A0A1C3YI20 pp 229533.A0A1C3YKY6
63261 229533.A0A1C3YI20 pp 229533.A0A1C3YJ93
63262 229533.A0A1C3YI20 pp 229533.I1RY12
63263 229533.A0A1C3YI20 pp 229533.A0A1C3YK68
63264 229533.A0A1C3YI20 pp 229533.I1RZK5
63265 229533.A0A1C3YI20 pp 229533.I1RZM1
63266 229533.A0A1C3YI20 pp 229533.I1RCM5
63267 229533.A0A1C3YI20 pp 229533.I1S0C1
63268 229533.A0A1C3YI20 pp 229533.I1RWB0
63269 229533.A0A1C3YI20 pp 229533.I1S8F8
63270 229533.A0A1C3YI20 pp 229533.I1R9Z5
63271 229533.A0A1C3YI20 pp 229533.A0A1C3YIK4
63272 229533.A0A1C3YI20 pp 229533.I1RS18
63273 229533.A0A1C3YI20 pp 229533.I1RQA5
63274 229533.A0A1C3YI20 pp 229533.I1RK57
63275 229533.A0A1C3YI20 pp 229533.I1S887
63276 229533.A0A1C3YI20 pp 229533.A0A1C3YKV3
63277 229533.A0A1C3YI20 pp 229533.I1RXB6
63278 229533.A0A1C3YI20 pp 229533.I1RPR1
63279 229533.A0A1C3YI20 pp 229533.A0A1C3YIV7
63280 229533.A0A1C3YI20 pp 229533.A0A1C3YKR5
63281 229533.A0A1C3YI20 pp 229533.I1RJ17
63282 229533.A0A1C3YI20 pp 229533.A0A1C3YIX6
63283 229533.A0A1C3YI20 pp 229533.V6RK96
63284 229533.A0A1C3YI20 pp 229533.I1RAZ3
63285 229533.A0A1C3YI20 pp 229533.I1RXC1
63286 229533.A0A1C3YI20 pp 229533.V6RCZ8
63287 229533.A0A1C3YI20 pp 229533.A0A1C3YLB0
63288 229533.A0A1C3YI20 pp 229533.I1RU76
63289 229533.A0A1C3YI20 pp 229533.I1RBE4
63290 229533.A0A1C3YI20 pp 229533.I1RM91
63291 229533.A0A1C3YI20 pp 229533.A0A1C3YLB3

63292	229533.A0A1C3YI20	pp	229533.I1RAQ4
63293	229533.A0A1C3YI20	pp	229533.I1S6K9
63294	229533.A0A1C3YI20	pp	229533.A0A1C3YJ89
63295	229533.A0A1C3YI20	pp	229533.I1S0Q4
63296	229533.A0A1C3YI20	pp	229533.A0A1C3YJW9
63297	229533.A0A1C3YI20	pp	229533.I1S0A0
63298	229533.A0A1C3YI20	pp	229533.I1S8S9
63299	229533.A0A1C3YI20	pp	229533.I1S0X0
63300	229533.A0A1C3YI20	pp	229533.I1RP02
63301	229533.A0A1C3YI20	pp	229533.A0A1C3YK34
63302	229533.A0A1C3YI20	pp	229533.I1RNY6
63303	229533.A0A1C3YI20	pp	229533.A0A1C3YKB0
63304	229533.A0A1C3YI20	pp	229533.A0A1C3YLE3
63305	229533.A0A1C3YI20	pp	229533.I1S2D3
63306	229533.A0A1C3YI20	pp	229533.A0A1C3YIJ9
63307	229533.A0A1C3YI20	pp	229533.V6QUE6
63308	229533.A0A1C3YI20	pp	229533.I1RSQ7
63309	229533.A0A1C3YI20	pp	229533.I1RZM4
63310	229533.A0A1C3YI20	pp	229533.I1RXF0
63311	229533.A0A1C3YI20	pp	229533.I1RFC0
63312	229533.A0A1C3YI20	pp	229533.I1RZM8
63313	229533.A0A1C3YI20	pp	229533.I1RRV4
63314	229533.A0A1C3YI20	pp	229533.I1REE2
63315	229533.A0A1C3YI20	pp	229533.I1RRX5
63316	229533.A0A1C3YI20	pp	229533.A0A1C3YK13
63317	229533.A0A1C3YI20	pp	229533.I1S5B0
63318	229533.A0A1C3YI20	pp	229533.I1S4S7
63319	229533.A0A1C3YI20	pp	229533.I1RYI7
63320	229533.A0A1C3YI20	pp	229533.I1RXE6
63321	229533.A0A1C3YI20	pp	229533.I1RMK7
63322	229533.A0A1C3YI20	pp	229533.I1RW54
63323	229533.A0A1C3YI20	pp	229533.I1RPN4
63324	229533.A0A1C3YI20	pp	229533.I1RUY5
63325	229533.A0A1C3YI20	pp	229533.I1RCK9
63326	229533.A0A1C3YI20	pp	229533.I1RA94
63327	229533.A0A1C3YI20	pp	229533.I1RAI4
63328	229533.A0A1C3YI20	pp	229533.I1S1W8
63329	229533.A0A1C3YI20	pp	229533.Q4IJT5
63330	229533.A0A1C3YI20	pp	229533.I1RMR7
63331	229533.A0A1C3YI20	pp	229533.I1RBD5
63332	229533.A0A1C3YI20	pp	229533.I1RAH8
63333	229533.A0A1C3YI20	pp	229533.A0A1C3YM63
63334	229533.A0A1C3YI20	pp	229533.I1RG62
63335	229533.A0A1C3YI20	pp	229533.I1RCU2
63336	229533.A0A1C3YI20	pp	229533.I1RYX6
63337	229533.A0A1C3YI20	pp	229533.Q4HVW2
63338	229533.A0A1C3YI20	pp	229533.I1RD52
63339	229533.A0A1C3YI20	pp	229533.I1RAR3
63340	229533.A0A1C3YI20	pp	229533.I1RA85
63341	229533.A0A1C3YI20	pp	229533.I1S0P0
63342	229533.A0A1C3YI20	pp	229533.I1RPA4
63343	229533.A0A1C3YI20	pp	229533.I1RMQ6
63344	229533.A0A1C3YI20	pp	229533.I1RNM7
63345	229533.A0A1C3YI20	pp	229533.I1RKB7
63346	229533.A0A1C3YI20	pp	229533.I1RE66
63347	229533.A0A1C3YI20	pp	229533.I1RC49
63348	229533.A0A1C3YI20	pp	229533.I1S8C1
63349	229533.A0A1C3YI20	pp	229533.I1RHK2
63350	229533.A0A1C3YI20	pp	229533.A0A1C3YLR6
63351	229533.A0A1C3YI20	pp	229533.I1RQE8
63352	229533.A0A1C3YI20	pp	229533.A0A1C3YK51
63353	229533.A0A1C3YI20	pp	229533.A0A1C3YN26
63354	229533.A0A1C3YI20	pp	229533.I1RC11
63355	229533.A0A1C3YI20	pp	229533.I1RRS8
63356	229533.A0A1C3YI20	pp	229533.A0A1C3YIP5
63357	229533.A0A1C3YI20	pp	229533.V6RUT5
63358	229533.A0A1C3YI20	pp	229533.I1RBT1
63359	229533.A0A1C3YI20	pp	229533.I1R9Y4
63360	229533.A0A1C3YI20	pp	229533.I1RXD7
63361	229533.A0A1C3YI20	pp	229533.I1RA35
63362	229533.A0A1C3YI20	pp	229533.I1RYU6
63363	229533.A0A1C3YI20	pp	229533.Q4IR09
63364	229533.A0A1C3YI20	pp	229533.A0A1C3YL54

63365 229533.A0A1C3YI20 pp 229533.I1RLP1
63366 229533.A0A1C3YI20 pp 229533.I1RR24
63367 229533.A0A1C3YI20 pp 229533.I1RCR6
63368 229533.A0A1C3YI20 pp 229533.I1RP86
63369 229533.A0A1C3YI20 pp 229533.I1RM67
63370 229533.A0A1C3YI20 pp 229533.V6RTJ8
63371 229533.A0A1C3YI20 pp 229533.I1S053
63372 229533.A0A1C3YI20 pp 229533.I1S4S4
63373 229533.A0A1C3YI20 pp 229533.A0A1C3YK11
63374 229533.A0A1C3YI20 pp 229533.I1RT16
63375 229533.A0A1C3YI20 pp 229533.I1RY21
63376 229533.A0A1C3YI20 pp 229533.I1RA49
63377 229533.A0A1C3YI20 pp 229533.I1RAU9
63378 229533.A0A1C3YI20 pp 229533.I1RAX6
63379 229533.A0A1C3YI20 pp 229533.I1RX46
63380 229533.A0A1C3YI20 pp 229533.A0A1C3YIG9
63381 229533.A0A1C3YI20 pp 229533.I1RXT0
63382 229533.A0A1C3YI20 pp 229533.I1S854
63383 229533.A0A1C3YI20 pp 229533.A0A1C3YIW9
63384 229533.A0A098D437 pp 229533.I1RRX5
63385 229533.A0A098D437 pp 229533.I1RA49
63386 229533.A0A098D437 pp 229533.I1RAI4
63387 229533.A0A098D437 pp 229533.I1R9Y4
63388 229533.A0A098D437 pp 229533.I1RPP5
63389 229533.A0A098D437 pp 229533.I1RMQ6
63390 229533.A0A098D437 pp 229533.A0A1C3YLE3
63391 229533.A0A098D437 pp 229533.A0A098DSZ8
63392 229533.A0A098D437 pp 229533.I1RAV5
63393 229533.A0A098D437 pp 229533.A0A1C3YLB0
63394 229533.A0A098D437 pp 229533.V6R3J1
63395 229533.A0A098D437 pp 229533.A0A1C3YI20
63396 229533.I1RPJ1 pp 229533.Q4IPI4
63397 229533.I1RPJ1 pp 229533.I1S1V2
63398 229533.I1RPJ1 pp 229533.I1RW71
63399 229533.I1RPJ1 pp 229533.Q4I7L0
63400 229533.I1RU33 pp 229533.I1S0R9
63401 229533.I1S268 pp 229533.V6RTJ8
63402 229533.I1S268 pp 229533.V6QUE6
63403 229533.I1S268 pp 229533.I1S7I5
63404 229533.I1S268 pp 229533.I1S5B0
63405 229533.I1S268 pp 229533.I1S4S7
63406 229533.I1S268 pp 229533.V6R042
63407 229533.I1S268 pp 229533.Q4IK03
63408 229533.I1S268 pp 229533.I1S2B8
63409 229533.I1S268 pp 229533.V6RCZ8
63410 229533.I1S268 pp 229533.I1S5R1
63411 229533.I1RMF1 pp 229533.I1RWL3
63412 229533.I1RMF1 pp 229533.I1RW54
63413 229533.I1RMF1 pp 229533.V6R5T7
63414 229533.I1RMF1 pp 229533.I1S3T6
63415 229533.I1RMF1 pp 229533.I1RQV7
63416 229533.I1RMF1 pp 229533.I1S4I7
63417 229533.I1RMF1 pp 229533.I1RYP5
63418 229533.I1RMF1 pp 229533.V6RWB3
63419 229533.I1RMF1 pp 229533.I1RW64
63420 229533.I1RMF1 pp 229533.I1S968
63421 229533.I1RMF1 pp 229533.I1S2F7
63422 229533.I1RMF1 pp 229533.I1RZ25
63423 229533.I1RMF1 pp 229533.I1RW55
63424 229533.I1RMF1 pp 229533.I1RY81
63425 229533.I1RMF1 pp 229533.I1S8D3
63426 229533.I1RMF1 pp 229533.I1RZ38
63427 229533.I1RMF1 pp 229533.I1RST9
63428 229533.I1RMF1 pp 229533.I1S267
63429 229533.I1RMF1 pp 229533.I1S223
63430 229533.A0A098D3V5 pp 229533.I1S268
63431 229533.I1RKP3 pp 229533.I1RP81
63432 229533.I1RKP3 pp 229533.I1RUM7
63433 229533.I1RKP3 pp 229533.Q4INS6
63434 229533.I1RKP3 pp 229533.I1S4S9
63435 229533.I1RKP3 pp 229533.I1RPT0
63436 229533.I1RKP3 pp 229533.I1RQE0
63437 229533.I1RKP3 pp 229533.I1RR39

63438	229533.I1RKP3	pp	229533.I1RP86
63439	229533.I1RKP3	pp	229533.I1RZT6
63440	229533.I1RKP3	pp	229533.I1S574
63441	229533.I1RKP3	pp	229533.I1S984
63442	229533.I1RKP3	pp	229533.I1RQ75
63443	229533.I1RKP3	pp	229533.I1RSJ2
63444	229533.I1RKP3	pp	229533.I1RNA1
63445	229533.I1RKP3	pp	229533.I1RZ34
63446	229533.I1RKP3	pp	229533.V6RPQ8
63447	229533.I1RKP3	pp	229533.I1S252
63448	229533.I1RKP3	pp	229533.I1RZ13
63449	229533.I1RKP3	pp	229533.I1S272
63450	229533.I1RKP3	pp	229533.I1RZ78
63451	229533.I1RKP3	pp	229533.I1RNU7
63452	229533.I1RKP3	pp	229533.Q4I1Y5
63453	229533.I1RKP3	pp	229533.I1RST0
63454	229533.I1RQ16	pp	229533.Q4I0J6
63455	229533.I1RQ16	pp	229533.I1S1D3
63456	229533.I1RQ16	pp	229533.V6R227
63457	229533.I1RQ16	pp	229533.I1RZG7
63458	229533.I1RQ16	pp	229533.I1RXT9
63459	229533.I1RQ16	pp	229533.I1S1C8
63460	229533.I1RQ16	pp	229533.I1RRB0
63461	229533.I1RQ16	pp	229533.Q4IBX0
63462	229533.I1RQ16	pp	229533.I1S0G3
63463	229533.I1RQ16	pp	229533.I1RQJ0
63464	229533.I1RQ16	pp	229533.I1S1V5
63465	229533.I1RQ16	pp	229533.I1RQ80
63466	229533.I1RQ16	pp	229533.Q4IMM0
63467	229533.I1RAU1	pp	229533.I1S1V1
63468	229533.I1RAU1	pp	229533.I1RBW7
63469	229533.I1RAU1	pp	229533.I1RS28
63470	229533.I1RAU1	pp	229533.I1RBH8
63471	229533.I1RAU1	pp	229533.I1RSD4
63472	229533.I1RAU1	pp	229533.V6QXG1
63473	229533.I1RAU1	pp	229533.I1RXF2
63474	229533.I1RAU1	pp	229533.I1RK92
63475	229533.I1RAU1	pp	229533.I1RG57
63476	229533.I1RAU1	pp	229533.I1RBV3
63477	229533.I1RAU1	pp	229533.I1RK90
63478	229533.I1RAU1	pp	229533.I1RSA4
63479	229533.I1RAU1	pp	229533.I1RND5
63480	229533.I1RAU1	pp	229533.I1S4M5
63481	229533.I1RAU1	pp	229533.I1RG90
63482	229533.I1RAU1	pp	229533.I1RCS2
63483	229533.I1RAU1	pp	229533.I1RBG5
63484	229533.I1RAU1	pp	229533.I1S7M3
63485	229533.I1RAU1	pp	229533.I1S7M2
63486	229533.I1RAU1	pp	229533.I1RQG9
63487	229533.I1RAU1	pp	229533.I1S7X9
63488	229533.I1RAU1	pp	229533.I1RNY7
63489	229533.I1RAU1	pp	229533.I1RB50
63490	229533.I1RAU1	pp	229533.I1RX81
63491	229533.I1RAU1	pp	229533.I1RCI4
63492	229533.I1RAU1	pp	229533.I1RZG7
63493	229533.I1S9C6	pp	229533.V6RM02
63494	229533.I1S9C6	pp	229533.V6RK96
63495	229533.A0A1C3YN20	pp	229533.I1S077
63496	229533.A0A1C3YN20	pp	229533.I1RT41
63497	229533.A0A1C3YN20	pp	229533.I1RJT5
63498	229533.A0A1C3YN20	pp	229533.I1RKG4
63499	229533.A0A1C3YN20	pp	229533.I1RBD6
63500	229533.A0A1C3YN20	pp	229533.I1RWM7
63501	229533.A0A1C3YIA6	pp	229533.Q4IBK5
63502	229533.A0A1C3YIA6	pp	229533.I1RK72
63503	229533.A0A1C3YIA6	pp	229533.I1RRL0
63504	229533.A0A1C3YIA6	pp	229533.I1RCS2
63505	229533.A0A1C3YIA6	pp	229533.I1RSD4
63506	229533.A0A1C3YIA6	pp	229533.I1RFQ1
63507	229533.A0A1C3YIA6	pp	229533.I1S2Y5
63508	229533.A0A1C3YIA6	pp	229533.I1RPY5
63509	229533.A0A1C3YIA6	pp	229533.I1RS75
63510	229533.A0A1C3YIA6	pp	229533.I1RFT1

63511 229533.A0A1C3YIA6 pp 229533.I1RS28
63512 229533.A0A1C3YIA6 pp 229533.I1RM25
63513 229533.A0A1C3YIA6 pp 229533.I1S4M5
63514 229533.A0A1C3YIA6 pp 229533.I1RT17
63515 229533.A0A1C3YIA6 pp 229533.I1RWU2
63516 229533.A0A1C3YIA6 pp 229533.A0A1C3YK13
63517 229533.A0A1C3YIA6 pp 229533.I1RS83
63518 229533.A0A1C3YIA6 pp 229533.I1S9L5
63519 229533.A0A1C3YIA6 pp 229533.I1RK19
63520 229533.A0A1C3YIA6 pp 229533.I1RTS2
63521 229533.A0A1C3YIA6 pp 229533.I1RCA7
63522 229533.A0A1C3YIA6 pp 229533.Q4IRI9
63523 229533.A0A1C3YIA6 pp 229533.I1RZG7
63524 229533.A0A1C3YIA6 pp 229533.I1R9N6
63525 229533.A0A1C3YIA6 pp 229533.I1RYU4
63526 229533.A0A1C3YIA6 pp 229533.I1RCQ6
63527 229533.A0A1C3YIA6 pp 229533.Q4IAA0
63528 229533.A0A1C3YIA6 pp 229533.I1RX51
63529 229533.A0A1C3YIA6 pp 229533.I1RPY4
63530 229533.A0A1C3YIA6 pp 229533.I1RAF3
63531 229533.A0A1C3YIA6 pp 229533.I1RD56
63532 229533.A0A1C3YIA6 pp 229533.Q4IPB8
63533 229533.A0A1C3YIA6 pp 229533.I1RWX8
63534 229533.A0A1C3YIA6 pp 229533.V6QXG1
63535 229533.A0A1C3YIA6 pp 229533.Q4IPB3
63536 229533.A0A1C3YIA6 pp 229533.I1RBH8
63537 229533.A0A1C3YIA6 pp 229533.I1RQR3
63538 229533.A0A1C3YIA6 pp 229533.I1RQZ2
63539 229533.A0A1C3YIA6 pp 229533.I1S9L6
63540 229533.A0A1C3YIA6 pp 229533.I1RND5
63541 229533.A0A1C3YIA6 pp 229533.I1RSA4
63542 229533.A0A1C3YIA6 pp 229533.I1RX81
63543 229533.A0A1C3YIA6 pp 229533.A0A1C3YNC3
63544 229533.A0A1C3YIA6 pp 229533.V6RY33
63545 229533.A0A1C3YIA6 pp 229533.I1RDG0
63546 229533.A0A1C3YIA6 pp 229533.I1RYJ9
63547 229533.A0A1C3YIA6 pp 229533.I1RSA8
63548 229533.A0A1C3YIA6 pp 229533.Q4IE79
63549 229533.A0A1C3YIA6 pp 229533.I1RG57
63550 229533.A0A1C3YIA6 pp 229533.I1RB50
63551 229533.A0A1C3YIA6 pp 229533.I1S0Z9
63552 229533.A0A1C3YIA6 pp 229533.I1RAC1
63553 229533.A0A1C3YIA6 pp 229533.I1RMV5
63554 229533.A0A1C3YIA6 pp 229533.I1RBV3
63555 229533.A0A1C3YIA6 pp 229533.Q4I1Y1
63556 229533.A0A1C3YIA6 pp 229533.I1RX47
63557 229533.A0A1C3YIA6 pp 229533.I1RG90
63558 229533.A0A1C3YIA6 pp 229533.I1S7M3
63559 229533.A0A1C3YIA6 pp 229533.I1RCI4
63560 229533.A0A1C3YIA6 pp 229533.I1RMH5
63561 229533.A0A1C3YIA6 pp 229533.A0A1C3YL11
63562 229533.A0A1C3YIA6 pp 229533.I1RC84
63563 229533.A0A1C3YIA6 pp 229533.I1S0B1
63564 229533.A0A1C3YIA6 pp 229533.A0A1C3YK19
63565 229533.A0A1C3YIA6 pp 229533.I1RCN5
63566 229533.A0A1C3YIA6 pp 229533.I1RPA0
63567 229533.A0A1C3YIA6 pp 229533.I1RYB1
63568 229533.A0A1C3YIA6 pp 229533.I1RK92
63569 229533.A0A1C3YIA6 pp 229533.A0A1C3YJW0
63570 229533.A0A1C3YIA6 pp 229533.V6QVD5
63571 229533.A0A1C3YIA6 pp 229533.I1RXF2
63572 229533.A0A1C3YIA6 pp 229533.I1S7M2
63573 229533.A0A1C3YIA6 pp 229533.I1RBZ8
63574 229533.A0A1C3YIA6 pp 229533.I1RZL5
63575 229533.A0A1C3YIA6 pp 229533.I1RFP9
63576 229533.A0A1C3YIA6 pp 229533.I1RCR5
63577 229533.A0A1C3YIA6 pp 229533.I1RT47
63578 229533.A0A1C3YIA6 pp 229533.I1RST1
63579 229533.A0A1C3YIA6 pp 229533.I1RA36
63580 229533.A0A1C3YIA6 pp 229533.I1RYW3
63581 229533.A0A1C3YIA6 pp 229533.A0A1C3YKM6
63582 229533.A0A1C3YIA6 pp 229533.I1S056
63583 229533.A0A1C3YIA6 pp 229533.I1RZ11

63584 229533.A0A1C3YIA6 pp 229533.I1RK90
63585 229533.A0A1C3YIA6 pp 229533.I1RU69
63586 229533.A0A1C3YIA6 pp 229533.I1RQP8
63587 229533.I1RW60 pp 229533.Q4I7L0
63588 229533.I1RW60 pp 229533.I1RY99
63589 229533.I1RW60 pp 229533.I1SB04
63590 229533.I1RW60 pp 229533.Q4IEV4
63591 229533.I1RW60 pp 229533.I1S109
63592 229533.I1RW60 pp 229533.I1S4S7
63593 229533.I1RVU7 pp 229533.V6R382
63594 229533.I1RVU7 pp 229533.I1S4R1
63595 229533.I1RVU7 pp 229533.I1S8W6
63596 229533.I1S0S8 pp 229533.Q4I2D7
63597 229533.I1S0S8 pp 229533.Q4ICM0
63598 229533.I1S0S8 pp 229533.I1S0Z3
63599 229533.I1S0S8 pp 229533.Q4IPI4
63600 229533.I1S0S8 pp 229533.Q4INI2
63601 229533.I1S0S8 pp 229533.Q4HZB8
63602 229533.I1S0S8 pp 229533.I1S270
63603 229533.I1RB00 pp 229533.Q4IBX0
63604 229533.I1RB00 pp 229533.I1RQJ0
63605 229533.I1RB00 pp 229533.I1RMG2
63606 229533.I1RB00 pp 229533.I1RJH5
63607 229533.I1RB00 pp 229533.I1S1C8
63608 229533.I1RB00 pp 229533.I1RFJ2
63609 229533.I1RB00 pp 229533.I1RJI8
63610 229533.I1RB00 pp 229533.I1RE46
63611 229533.I1RB00 pp 229533.I1REG5
63612 229533.I1RB00 pp 229533.I1RRB0
63613 229533.I1RB00 pp 229533.I1RLX6
63614 229533.I1RB00 pp 229533.I1RP94
63615 229533.I1RB00 pp 229533.Q4I0J6
63616 229533.I1RB00 pp 229533.I1RM36
63617 229533.I1RB00 pp 229533.I1RQ80
63618 229533.I1RB00 pp 229533.I1RI75
63619 229533.I1RB00 pp 229533.Q4HZS8
63620 229533.I1RB00 pp 229533.Q4IER8
63621 229533.I1RB00 pp 229533.I1RNC0
63622 229533.I1RB00 pp 229533.I1S9J6
63623 229533.I1RB00 pp 229533.I1RSI4
63624 229533.I1RB00 pp 229533.I1RN31
63625 229533.I1RB00 pp 229533.I1RYP9
63626 229533.I1RB00 pp 229533.Q4IMM0
63627 229533.I1RB00 pp 229533.Q5I2J3
63628 229533.I1RB00 pp 229533.I1RFX8
63629 229533.I1RB00 pp 229533.I1RJE5
63630 229533.I1RB00 pp 229533.I1RDX8
63631 229533.I1RB00 pp 229533.I1RGE7
63632 229533.I1RB00 pp 229533.I1RLE7
63633 229533.I1RB00 pp 229533.I1RBW0
63634 229533.I1RB00 pp 229533.I1RMB7
63635 229533.I1RB00 pp 229533.I1RG77
63636 229533.I1RB00 pp 229533.I1RD18
63637 229533.I1RB00 pp 229533.I1RCL7
63638 229533.I1RB00 pp 229533.I1RN55
63639 229533.I1RB00 pp 229533.I1RLX4
63640 229533.I1RB00 pp 229533.I1RSC7
63641 229533.I1RB00 pp 229533.I1RR21
63642 229533.I1RB00 pp 229533.I1S283
63643 229533.I1RB00 pp 229533.I1RC54
63644 229533.I1RB00 pp 229533.I1RCM6
63645 229533.I1RB00 pp 229533.I1S0G3
63646 229533.I1RB00 pp 229533.I1RN46
63647 229533.I1RB00 pp 229533.I1RFN0
63648 229533.I1RB00 pp 229533.I1S461
63649 229533.I1RB00 pp 229533.V6R382
63650 229533.I1RB00 pp 229533.I1RQ13
63651 229533.I1RB00 pp 229533.I1RBX2
63652 229533.I1RB00 pp 229533.I1RD70
63653 229533.I1RB00 pp 229533.I1RYR2
63654 229533.I1RB00 pp 229533.I1RFW1
63655 229533.I1RB00 pp 229533.I1RW16
63656 229533.I1RB00 pp 229533.I1RPW0

63657 229533.I1RB00 pp 229533.I1S1D3
63658 229533.I1RB00 pp 229533.I1RDX0
63659 229533.I1RB00 pp 229533.I1RQ16
63660 229533.I1RB00 pp 229533.I1RLR8
63661 229533.I1RB00 pp 229533.I1RMP0
63662 229533.I1RB00 pp 229533.I1RJI7
63663 229533.I1RB00 pp 229533.I1RFZ2
63664 229533.I1RB00 pp 229533.I1RBL4
63665 229533.I1RB00 pp 229533.I1RN57
63666 229533.I1RB00 pp 229533.I1RNG6
63667 229533.I1RB00 pp 229533.I1S2F2
63668 229533.I1RB00 pp 229533.I1RKI1
63669 229533.I1RB00 pp 229533.I1RR95
63670 229533.I1RB00 pp 229533.I1S0N5
63671 229533.I1RB00 pp 229533.I1RX64
63672 229533.I1RB00 pp 229533.V6R3J1
63673 229533.I1RB00 pp 229533.I1RBW1
63674 229533.I1RB00 pp 229533.I1RD19
63675 229533.I1RB00 pp 229533.I1RQZ5
63676 229533.I1RB00 pp 229533.I1RCF0
63677 229533.I1RB00 pp 229533.I1S1V5
63678 229533.I1RB00 pp 229533.I1RYX9
63679 229533.I1RB00 pp 229533.I1RSJ4
63680 229533.I1RB00 pp 229533.I1RFR6
63681 229533.I1RB00 pp 229533.I1RLU1
63682 229533.I1RB00 pp 229533.I1RPX8
63683 229533.I1RB00 pp 229533.I1RC35
63684 229533.I1RB00 pp 229533.I1RQM0
63685 229533.I1RB00 pp 229533.I1S4P2
63686 229533.I1RB00 pp 229533.V6RLW4
63687 229533.I1RB00 pp 229533.I1RUG1
63688 229533.I1S4S5 pp 229533.V6RRM0
63689 229533.I1S4S5 pp 229533.I1S6P6
63690 229533.I1S4S5 pp 229533.V6RLM7
63691 229533.I1S4S5 pp 229533.V6R9Z3
63692 229533.I1S4S5 pp 229533.I1S8F8
63693 229533.I1S4S5 pp 229533.I1S6H3
63694 229533.I1S4S5 pp 229533.I1S8R2
63695 229533.I1RY90 pp 229533.Q4IPI4
63696 229533.I1RY90 pp 229533.I1S1V2
63697 229533.I1RY90 pp 229533.Q4I7L0
63698 229533.V6QVH2 pp 229533.V6R9Z3
63699 229533.V6QVH2 pp 229533.V6RLM7
63700 229533.I1RD70 pp 229533.I1RN31
63701 229533.I1RD70 pp 229533.I1S4P4
63702 229533.I1RD70 pp 229533.I1RFU3
63703 229533.I1RD70 pp 229533.I1RQM0
63704 229533.I1RD70 pp 229533.I1S382
63705 229533.I1RD70 pp 229533.I1RZ02
63706 229533.I1RD70 pp 229533.I1RNC0
63707 229533.I1RD70 pp 229533.I1RL04
63708 229533.I1RD70 pp 229533.I1RFR6
63709 229533.I1RD70 pp 229533.I1RI75
63710 229533.I1RD70 pp 229533.I1RTQ9
63711 229533.I1RD70 pp 229533.I1S1V5
63712 229533.I1RD70 pp 229533.Q4HZS8
63713 229533.I1RD70 pp 229533.I1REC6
63714 229533.I1RD70 pp 229533.I1RJS4
63715 229533.I1RD70 pp 229533.I1RZG7
63716 229533.I1RD70 pp 229533.I1RX38
63717 229533.I1RD70 pp 229533.I1RN54
63718 229533.I1RD70 pp 229533.I1RMB7
63719 229533.I1RD70 pp 229533.I1RL24
63720 229533.I1RD70 pp 229533.I1S951
63721 229533.I1RD70 pp 229533.I1RLV3
63722 229533.I1RD70 pp 229533.I1RDG8
63723 229533.I1RD70 pp 229533.I1S2F2
63724 229533.I1RD70 pp 229533.I1RK32
63725 229533.I1RD70 pp 229533.I1RQ70
63726 229533.I1RD70 pp 229533.I1RMP2
63727 229533.I1RD70 pp 229533.I1RSK0
63728 229533.I1RD70 pp 229533.I1RMT4
63729 229533.I1RD70 pp 229533.I1REF9

63730	229533.I1RD70	pp	229533.I1RTT3
63731	229533.I1RD70	pp	229533.I1RYF8
63732	229533.I1RD70	pp	229533.I1S635
63733	229533.I1RD70	pp	229533.I1RWG6
63734	229533.I1RD70	pp	229533.I1RTC2
63735	229533.I1RD70	pp	229533.I1RRT4
63736	229533.I1RD70	pp	229533.Q5I2J3
63737	229533.I1RD70	pp	229533.Q4IMM0
63738	229533.I1RD70	pp	229533.I1RQN9
63739	229533.I1RD70	pp	229533.I1RDE9
63740	229533.I1RD70	pp	229533.V6R3J1
63741	229533.I1RD70	pp	229533.I1S2A8
63742	229533.I1RD70	pp	229533.I1RFZ2
63743	229533.I1RD70	pp	229533.I1S0Z2
63744	229533.I1RD70	pp	229533.I1RQ80
63745	229533.I1RD70	pp	229533.I1RY00
63746	229533.I1RD70	pp	229533.I1RML6
63747	229533.I1RD70	pp	229533.Q4IMD1
63748	229533.I1RD70	pp	229533.I1RPV5
63749	229533.I1RD70	pp	229533.I1RWB3
63750	229533.I1RD70	pp	229533.I1RQ67
63751	229533.I1RD70	pp	229533.I1RFW1
63752	229533.I1RD70	pp	229533.I1RDP1
63753	229533.I1RD70	pp	229533.I1S2F3
63754	229533.I1RD70	pp	229533.V6QV09
63755	229533.I1RD70	pp	229533.I1RI34
63756	229533.I1RD70	pp	229533.Q4ICM0
63757	229533.I1RD70	pp	229533.I1RQA3
63758	229533.I1RD70	pp	229533.I1RR95
63759	229533.I1RD70	pp	229533.I1S077
63760	229533.I1RD70	pp	229533.I1RQ16
63761	229533.I1RD70	pp	229533.I1RE19
63762	229533.I1RD70	pp	229533.I1RUG2
63763	229533.I1RD70	pp	229533.I1S201
63764	229533.I1RD70	pp	229533.I1RYZ9
63765	229533.I1RD70	pp	229533.I1RSH3
63766	229533.I1RD70	pp	229533.I1RPW0
63767	229533.I1RD70	pp	229533.I1S2E9
63768	229533.I1RD70	pp	229533.I1RZV9
63769	229533.I1RD70	pp	229533.I1RWM6
63770	229533.I1RD70	pp	229533.I1RN65
63771	229533.I1RD70	pp	229533.I1S3S4
63772	229533.I1RD70	pp	229533.I1RNG8
63773	229533.I1RD70	pp	229533.I1RWI8
63774	229533.I1RD70	pp	229533.I1RRB0
63775	229533.I1RD70	pp	229533.I1RQL9
63776	229533.I1RD70	pp	229533.I1S0S6
63777	229533.I1RD70	pp	229533.I1REC7
63778	229533.I1RD70	pp	229533.I1RLF0
63779	229533.I1RD70	pp	229533.I1RS38
63780	229533.I1RD70	pp	229533.I1RE37
63781	229533.I1RD70	pp	229533.I1RJS5
63782	229533.I1RD70	pp	229533.I1S287
63783	229533.I1RD70	pp	229533.I1RFJ2
63784	229533.I1RD70	pp	229533.I1RF02
63785	229533.I1RD70	pp	229533.Q4IBX0
63786	229533.I1RD70	pp	229533.I1RMJ8
63787	229533.I1RD70	pp	229533.I1RQJ0
63788	229533.I1RD70	pp	229533.I1RLX6
63789	229533.I1RD70	pp	229533.I1RHI1
63790	229533.I1RD70	pp	229533.I1S050
63791	229533.I1RD70	pp	229533.I1RTE6
63792	229533.I1RD70	pp	229533.V6REE9
63793	229533.I1RD70	pp	229533.I1RRP7
63794	229533.I1RD70	pp	229533.I1RSW5
63795	229533.I1RD70	pp	229533.I1RME7
63796	229533.I1RD70	pp	229533.I1RRF9
63797	229533.I1RD70	pp	229533.I1RDE8
63798	229533.I1RD70	pp	229533.I1RZQ6
63799	229533.I1RD70	pp	229533.I1RWT6
63800	229533.I1RD70	pp	229533.I1RN92
63801	229533.I1RD70	pp	229533.I1RKR8
63802	229533.I1RD70	pp	229533.V6R227

63803 229533.I1RD70 pp 229533.I1RTD7
63804 229533.I1RD70 pp 229533.I1RSL6
63805 229533.I1RD70 pp 229533.I1S096
63806 229533.I1RD70 pp 229533.I1S3D2
63807 229533.I1RD70 pp 229533.I1RRH3
63808 229533.I1RD70 pp 229533.V6RGI1
63809 229533.I1RD70 pp 229533.I1RJK8
63810 229533.I1RD70 pp 229533.I1RQ49
63811 229533.I1RD70 pp 229533.I1RPU4
63812 229533.I1RD70 pp 229533.I1RY73
63813 229533.I1RD70 pp 229533.I1S5B5
63814 229533.I1RD70 pp 229533.I1RU02
63815 229533.I1RD70 pp 229533.I1S1D3
63816 229533.I1RD70 pp 229533.I1RSC7
63817 229533.I1RD70 pp 229533.I1RZH4
63818 229533.I1RD70 pp 229533.I1RN43
63819 229533.I1RD70 pp 229533.I1RJH5
63820 229533.I1RD70 pp 229533.I1RNI2
63821 229533.I1RD70 pp 229533.Q4IER8
63822 229533.I1RD70 pp 229533.I1RXB5
63823 229533.I1RD70 pp 229533.I1RHM4
63824 229533.I1RD70 pp 229533.Q4I7X1
63825 229533.I1RD70 pp 229533.I1RW16
63826 229533.I1RD70 pp 229533.Q4I877
63827 229533.I1RD70 pp 229533.I1RZ91
63828 229533.I1RD70 pp 229533.I1RWQ0
63829 229533.I1RD70 pp 229533.V6QX58
63830 229533.I1RD70 pp 229533.I1RE46
63831 229533.I1RD70 pp 229533.Q4I0J6
63832 229533.I1RD70 pp 229533.I1RY44
63833 229533.I1RD70 pp 229533.I1RZP6
63834 229533.I1RD70 pp 229533.Q4HTT2
63835 229533.I1RD70 pp 229533.I1RMG2
63836 229533.I1RD70 pp 229533.I1S1C8
63837 229533.I1RD70 pp 229533.I1RLX4
63838 229533.I1RD70 pp 229533.I1RXT9
63839 229533.I1RD70 pp 229533.Q4I5U9
63840 229533.I1RD70 pp 229533.I1RGD4
63841 229533.I1RD70 pp 229533.Q4INS6
63842 229533.I1RD70 pp 229533.I1RSI1
63843 229533.A0A1C3YKT1 pp 229533.I1RUJ0
63844 229533.A0A1C3YKT1 pp 229533.Q4INI2
63845 229533.A0A1C3YKT1 pp 229533.I1RYM4
63846 229533.A0A1C3YKT1 pp 229533.Q4I2D7
63847 229533.A0A1C3YKT1 pp 229533.I1RAZ9
63848 229533.A0A1C3YMK3 pp 229533.I1S9T8
63849 229533.A0A1C3YMK3 pp 229533.I1RFV9
63850 229533.A0A1C3YMK3 pp 229533.V6RLM7
63851 229533.A0A1C3YMK3 pp 229533.I1RND4
63852 229533.A0A1C3YMK3 pp 229533.I1RW06
63853 229533.A0A1C3YMK3 pp 229533.V6RV57
63854 229533.A0A1C3YMK3 pp 229533.I1RYK0
63855 229533.A0A1C3YI93 pp 229533.I1RFN0
63856 229533.A0A1C3YI93 pp 229533.Q4I0J6
63857 229533.A0A1C3YI93 pp 229533.I1REG5
63858 229533.A0A1C3YI93 pp 229533.Q4IMM0
63859 229533.A0A1C3YI93 pp 229533.I1RJH5
63860 229533.A0A1C3YI93 pp 229533.I1RSC7
63861 229533.A0A1C3YI93 pp 229533.I1S1D3
63862 229533.A0A1C3YI93 pp 229533.I1RE46
63863 229533.A0A1C3YI93 pp 229533.I1RMG2
63864 229533.A0A1C3YI93 pp 229533.I1RNG6
63865 229533.A0A1C3YI93 pp 229533.I1RMP0
63866 229533.A0A1C3YI93 pp 229533.I1RQJ0
63867 229533.A0A1C3YI93 pp 229533.Q4IBX0
63868 229533.A0A1C3YI93 pp 229533.I1RN46
63869 229533.A0A1C3YI93 pp 229533.I1RFZ2
63870 229533.A0A1C3YI93 pp 229533.I1RQ80
63871 229533.A0A1C3YI93 pp 229533.A0A1C3YKW5
63872 229533.A0A1C3YI93 pp 229533.I1RYX9
63873 229533.A0A1C3YI93 pp 229533.I1RCM6
63874 229533.A0A1C3YI93 pp 229533.V6R3J1
63875 229533.A0A1C3YI93 pp 229533.I1RCL7

63876 229533.A0A1C3YI93 pp 229533.I1REU7
63877 229533.A0A1C3YI93 pp 229533.I1RQ16
63878 229533.A0A1C3YI93 pp 229533.I1RB00
63879 229533.A0A1C3YI93 pp 229533.A0A1C3YIH7
63880 229533.A0A1C3YI93 pp 229533.I1RMB7
63881 229533.A0A1C3YI93 pp 229533.A0A1C3YLR6
63882 229533.A0A1C3YI93 pp 229533.A0A1C3YIP6
63883 229533.A0A1C3YI93 pp 229533.I1RGE7
63884 229533.A0A1C3YI93 pp 229533.I1RQ13
63885 229533.A0A1C3YI93 pp 229533.I1RC35
63886 229533.A0A1C3YI93 pp 229533.I1RSJ4
63887 229533.A0A1C3YI93 pp 229533.I1RFR6
63888 229533.A0A1C3YI93 pp 229533.I1S0G3
63889 229533.A0A1C3YI93 pp 229533.I1RR21
63890 229533.A0A1C3YI93 pp 229533.I1S2F2
63891 229533.A0A1C3YI93 pp 229533.I1RD18
63892 229533.I1RSW2 pp 229533.Q4IJH1
63893 229533.I1RSW2 pp 229533.Q4I1Y1
63894 229533.I1RSW2 pp 229533.I1S1W7
63895 229533.I1RSW2 pp 229533.I1S9L0
63896 229533.I1RSW2 pp 229533.I1RU69
63897 229533.I1RSW2 pp 229533.Q4IF76
63898 229533.I1RSW2 pp 229533.I1RZG7
63899 229533.I1RSW2 pp 229533.Q4HXF6
63900 229533.I1RSW2 pp 229533.Q4IB70
63901 229533.I1RSW2 pp 229533.I1RYJ9
63902 229533.I1RSW2 pp 229533.Q4IPB3
63903 229533.I1RSW2 pp 229533.Q4IBK5
63904 229533.I1RSW2 pp 229533.Q4IE79
63905 229533.I1RSW2 pp 229533.Q4IRI9
63906 229533.I1RSW2 pp 229533.Q4IPB8
63907 229533.I1RSW2 pp 229533.I1RX47
63908 229533.I1RR95 pp 229533.I1RRC2
63909 229533.I1RR95 pp 229533.I1RTG5
63910 229533.I1RR95 pp 229533.I1RWX5
63911 229533.I1RR95 pp 229533.V6QX21
63912 229533.I1RR95 pp 229533.I1RTA0
63913 229533.I1RR95 pp 229533.V6QUV7
63914 229533.I1RR95 pp 229533.I1RYW3
63915 229533.I1RR95 pp 229533.I1S7X9
63916 229533.I1RR95 pp 229533.Q4IPI4
63917 229533.I1RR95 pp 229533.I1S8P5
63918 229533.I1RR95 pp 229533.I1S4Z7
63919 229533.I1RR95 pp 229533.Q4I7L0
63920 229533.I1RR95 pp 229533.I1SAA0
63921 229533.I1RR95 pp 229533.I1RRM9
63922 229533.I1RR95 pp 229533.I1S074
63923 229533.I1RR95 pp 229533.I1RRB0
63924 229533.I1RR95 pp 229533.V6RPN0
63925 229533.I1RR95 pp 229533.Q4ICA8
63926 229533.I1RR95 pp 229533.Q4IBX0
63927 229533.I1RR95 pp 229533.Q4I0J6
63928 229533.I1RR95 pp 229533.I1RZB7
63929 229533.I1RR95 pp 229533.I1RWD5
63930 229533.I1RR95 pp 229533.I1S0Z3
63931 229533.I1RR95 pp 229533.Q4I963
63932 229533.I1RR95 pp 229533.Q4HTT2
63933 229533.I1RR95 pp 229533.Q4IER8
63934 229533.I1RR95 pp 229533.I1RUK7
63935 229533.I1RR95 pp 229533.I1RW71
63936 229533.I1RR95 pp 229533.I1RRF9
63937 229533.I1RR95 pp 229533.V6REE9
63938 229533.I1RR95 pp 229533.Q4I9B7
63939 229533.I1RR95 pp 229533.I1S1C8
63940 229533.I1RR95 pp 229533.I1RSI4
63941 229533.I1RR95 pp 229533.I1RYL5
63942 229533.I1RR95 pp 229533.Q4HZS8
63943 229533.I1RR95 pp 229533.I1RZU6
63944 229533.I1RR95 pp 229533.Q5I2J3
63945 229533.I1RR95 pp 229533.I1RT41
63946 229533.I1RR95 pp 229533.I1RTQ9
63947 229533.I1RMB7 pp 229533.I1S2F2
63948 229533.I1RMB7 pp 229533.I1RV78

63949 229533.I1RMB7 pp 229533.I1RSJ4
63950 229533.I1RMB7 pp 229533.I1RSA7
63951 229533.I1RMB7 pp 229533.I1RZ29
63952 229533.I1RMB7 pp 229533.I1RMP0
63953 229533.I1RMB7 pp 229533.V6RLW4
63954 229533.I1RMB7 pp 229533.I1RYQ1
63955 229533.I1RMB7 pp 229533.I1RSL6
63956 229533.I1RMB7 pp 229533.I1RRS7
63957 229533.I1RMB7 pp 229533.I1RYR2
63958 229533.I1RMB7 pp 229533.I1RSC7
63959 229533.I1RMB7 pp 229533.I1RZ52
63960 229533.I1RMB7 pp 229533.I1RYX9
63961 229533.I1RMB7 pp 229533.I1RUG1
63962 229533.I1RMB7 pp 229533.I1RWX7
63963 229533.I1RMB7 pp 229533.Q4IMD1
63964 229533.I1RMB7 pp 229533.I1RN57
63965 229533.I1RMB7 pp 229533.I1RSL8
63966 229533.I1RMB7 pp 229533.I1RQJ4
63967 229533.I1RMB7 pp 229533.I1S1D3
63968 229533.I1RMB7 pp 229533.I1RW16
63969 229533.I1RMB7 pp 229533.I1RW03
63970 229533.I1RMB7 pp 229533.I1RN31
63971 229533.I1RMB7 pp 229533.I1RX64
63972 229533.I1RMB7 pp 229533.I1S283
63973 229533.I1RMB7 pp 229533.I1RRT1
63974 229533.I1RMB7 pp 229533.I1RW71
63975 229533.I1RMB7 pp 229533.I1RPQ1
63976 229533.I1RMB7 pp 229533.I1RWM6
63977 229533.I1RMB7 pp 229533.Q4IBX0
63978 229533.I1RMB7 pp 229533.I1RYV1
63979 229533.I1RMB7 pp 229533.Q4IMM0
63980 229533.I1RMB7 pp 229533.I1RWB3
63981 229533.I1RMB7 pp 229533.I1RN46
63982 229533.I1RMB7 pp 229533.I1RSI4
63983 229533.I1RMB7 pp 229533.I1RN55
63984 229533.I1RMB7 pp 229533.Q4IE79
63985 229533.I1RMB7 pp 229533.I1RRK8
63986 229533.I1RMB7 pp 229533.I1RMX3
63987 229533.I1RMB7 pp 229533.Q4I0J6
63988 229533.I1RMB7 pp 229533.Q4HTT2
63989 229533.A0A0E0RN56 pp 229533.I1RSW2
63990 229533.A0A1C3YJ83 pp 229533.I1RYX5
63991 229533.A0A1C3YJ83 pp 229533.I1RN04
63992 229533.A0A1C3YJ83 pp 229533.I1RFV2
63993 229533.A0A1C3YJ83 pp 229533.I1RMV0
63994 229533.A0A1C3YJ83 pp 229533.I1RHJ2
63995 229533.A0A1C3YJ83 pp 229533.I1RQM9
63996 229533.A0A1C3YJ83 pp 229533.I1RNJ7
63997 229533.A0A1C3YJ83 pp 229533.I1S5Z5
63998 229533.A0A1C3YJ83 pp 229533.I1RRF7
63999 229533.A0A1C3YJ83 pp 229533.I1RPW8
64000 229533.A0A1C3YJ83 pp 229533.I1RBA6
64001 229533.A0A1C3YJ83 pp 229533.I1RCM8
64002 229533.A0A1C3YJ83 pp 229533.I1S2B9
64003 229533.A0A1C3YJ83 pp 229533.I1RZN1
64004 229533.A0A1C3YJ83 pp 229533.I1RAP4
64005 229533.A0A1C3YJ83 pp 229533.A0A1C3YLC3
64006 229533.A0A1C3YJ83 pp 229533.A0A1C3YMU9
64007 229533.A0A1C3YJ83 pp 229533.I1RW80
64008 229533.A0A1C3YJ83 pp 229533.I1RKF3
64009 229533.A0A1C3YJ83 pp 229533.I1RLP1
64010 229533.A0A1C3YJ83 pp 229533.I1RQ42
64011 229533.A0A1C3YJ83 pp 229533.I1RFH9
64012 229533.A0A1C3YJ83 pp 229533.I1RR61
64013 229533.A0A1C3YJ83 pp 229533.I1RRC4
64014 229533.A0A1C3YJ83 pp 229533.I1RWB0
64015 229533.A0A1C3YJ83 pp 229533.I1SAK7
64016 229533.A0A1C3YJ83 pp 229533.I1RCE6
64017 229533.A0A1C3YJ83 pp 229533.I1RMG4
64018 229533.A0A1C3YJ83 pp 229533.I1R9Y2
64019 229533.A0A1C3YJ83 pp 229533.I1RCG6
64020 229533.A0A1C3YJ83 pp 229533.I1RW91
64021 229533.A0A1C3YJ83 pp 229533.I1RRN4

64022 229533.I1RID2 pp 229533.I1RWI6
64023 229533.I1RID2 pp 229533.I1RPE5
64024 229533.A0A0E0RTV8 pp 229533.I1RZV9
64025 229533.A0A0E0RTV8 pp 229533.I1RRB0
64026 229533.A0A0E0RTV8 pp 229533.I1RMM9
64027 229533.A0A0E0RTV8 pp 229533.I1R994
64028 229533.A0A0E0RTV8 pp 229533.I1RUQ5
64029 229533.A0A0E0RTV8 pp 229533.I1RN08
64030 229533.A0A0E0RTV8 pp 229533.Q4IBX0
64031 229533.A0A0E0RTV8 pp 229533.I1RFJ2
64032 229533.A0A0E0RTV8 pp 229533.A0A1C3YJY9
64033 229533.A0A0E0RTV8 pp 229533.Q4IER8
64034 229533.A0A0E0RTV8 pp 229533.I1RN46
64035 229533.A0A0E0RTV8 pp 229533.I1RPU4
64036 229533.A0A0E0RTV8 pp 229533.I1RKK0
64037 229533.A0A0E0RTV8 pp 229533.Q4I0J6
64038 229533.A0A0E0RTV8 pp 229533.I1S439
64039 229533.A0A0E0RTV8 pp 229533.I1RA12
64040 229533.A0A0E0RTV8 pp 229533.A0A1C3YK37
64041 229533.A0A0E0RTV8 pp 229533.A0A1C3YJW9
64042 229533.A0A0E0RTV8 pp 229533.I1RQJ0
64043 229533.A0A0E0RTV8 pp 229533.I1RAH4
64044 229533.A0A0E0RTV8 pp 229533.I1RYH9
64045 229533.A0A0E0RTV8 pp 229533.I1RAM7
64046 229533.A0A0E0RTV8 pp 229533.I1RCM6
64047 229533.A0A0E0RTV8 pp 229533.I1RE46
64048 229533.A0A0E0RTV8 pp 229533.I1RCL7
64049 229533.A0A0E0RTV8 pp 229533.I1RB00
64050 229533.A0A0E0RTV8 pp 229533.I1RCK4
64051 229533.A0A0E0RTV8 pp 229533.I1RAL2
64052 229533.A0A0E0RTV8 pp 229533.I1RFC3
64053 229533.A0A0E0RTV8 pp 229533.I1S1C8
64054 229533.A0A0E0RTV8 pp 229533.I1RC95
64055 229533.A0A0E0RTV8 pp 229533.Q4IR08
64056 229533.A0A0E0RTV8 pp 229533.I1RMB7
64057 229533.A0A0E0RTV8 pp 229533.I1S224
64058 229533.A0A0E0RTV8 pp 229533.A0A1C3YMF2
64059 229533.A0A0E0RTV8 pp 229533.A0A0E0SAC4
64060 229533.A0A0E0RTV8 pp 229533.I1RFX8
64061 229533.A0A0E0RTV8 pp 229533.I1RUG2
64062 229533.A0A0E0RTV8 pp 229533.I1RN31
64063 229533.A0A0E0RTV8 pp 229533.A0A1C3YIR5
64064 229533.A0A0E0RTV8 pp 229533.A0A0E0SA11
64065 229533.A0A0E0RTV8 pp 229533.I1RDB1
64066 229533.A0A0E0RTV8 pp 229533.A0A1C3YLR6
64067 229533.A0A0E0RTV8 pp 229533.A0A1C3YIP6
64068 229533.A0A0E0RTV8 pp 229533.I1RNC0
64069 229533.A0A0E0RTV8 pp 229533.I1RAW7
64070 229533.A0A0E0RTV8 pp 229533.I1RL24
64071 229533.A0A0E0RTV8 pp 229533.I1R9X7
64072 229533.A0A0E0RTV8 pp 229533.V6R382
64073 229533.A0A0E0RTV8 pp 229533.I1RQ80
64074 229533.A0A0E0RTV8 pp 229533.I1RW16
64075 229533.A0A0E0RTV8 pp 229533.I1RUQ1
64076 229533.A0A0E0RTV8 pp 229533.I1RCU7
64077 229533.A0A0E0RTV8 pp 229533.V6QV09
64078 229533.A0A0E0RTV8 pp 229533.A0A1C3YKF2
64079 229533.A0A0E0RTV8 pp 229533.I1RD18
64080 229533.A0A0E0RTV8 pp 229533.I1RLE7
64081 229533.A0A0E0RTV8 pp 229533.I1RBD6
64082 229533.A0A0E0RTV8 pp 229533.I1RSI4
64083 229533.A0A0E0RTV8 pp 229533.I1S2M1
64084 229533.A0A0E0RTV8 pp 229533.A0A0E0SR03
64085 229533.A0A0E0RTV8 pp 229533.I1RZE4
64086 229533.A0A0E0RTV8 pp 229533.I1RQ26
64087 229533.A0A0E0RTV8 pp 229533.I1RC54
64088 229533.A0A0E0RTV8 pp 229533.V6RGI1
64089 229533.A0A0E0RTV8 pp 229533.I1RFN0
64090 229533.A0A0E0RTV8 pp 229533.I1RG45
64091 229533.A0A0E0RTV8 pp 229533.I1RQ16
64092 229533.A0A0E0RTV8 pp 229533.I1RPW0
64093 229533.A0A0E0RTV8 pp 229533.I1RYZ9
64094 229533.A0A0E0RTV8 pp 229533.I1RRY3

64095 229533.A0A0E0RTV8 pp 229533.I1RRW0
64096 229533.A0A0E0RTV8 pp 229533.I1RLX6
64097 229533.A0A0E0RTV8 pp 229533.I1RSC7
64098 229533.A0A0E0RTV8 pp 229533.A0A1C3YI93
64099 229533.A0A0E0RTV8 pp 229533.I1RRP7
64100 229533.A0A0E0RTV8 pp 229533.I1RL35
64101 229533.A0A0E0RTV8 pp 229533.I1RD70
64102 229533.A0A0E0RTV8 pp 229533.I1S0M2
64103 229533.A0A0E0RTV8 pp 229533.I1RM99
64104 229533.A0A0E0RTV8 pp 229533.A0A1C3YI28
64105 229533.A0A0E0RTV8 pp 229533.I1S478
64106 229533.A0A0E0RTV8 pp 229533.I1S2F2
64107 229533.A0A0E0RTV8 pp 229533.I1RZT1
64108 229533.A0A0E0RTV8 pp 229533.V6RLW4
64109 229533.A0A0E0RTV8 pp 229533.I1S4R1
64110 229533.A0A0E0RTV8 pp 229533.I1RBL4
64111 229533.A0A0E0RTV8 pp 229533.A0A1C3YHL0
64112 229533.A0A0E0RTV8 pp 229533.A0A0E0SNY0
64113 229533.A0A0E0RTV8 pp 229533.I1RFC2
64114 229533.A0A0E0RTV8 pp 229533.A0A1C3YI10
64115 229533.A0A0E0RTV8 pp 229533.I1RUG1
64116 229533.A0A0E0RTV8 pp 229533.I1RAN2
64117 229533.A0A0E0RTV8 pp 229533.I1RSP7
64118 229533.A0A0E0RTV8 pp 229533.A0A0E0S0W9
64119 229533.A0A0E0RTV8 pp 229533.V6RVG3
64120 229533.A0A0E0RTV8 pp 229533.I1RQM0
64121 229533.A0A0E0RTV8 pp 229533.I1RMM1
64122 229533.A0A0E0RTV8 pp 229533.I1RCA5
64123 229533.A0A0E0RTV8 pp 229533.I1RMP0
64124 229533.A0A0E0RTV8 pp 229533.V6QX58
64125 229533.A0A0E0RTV8 pp 229533.I1RY44
64126 229533.A0A0E0RTV8 pp 229533.I1RUN8
64127 229533.A0A0E0RTV8 pp 229533.A0A1C3YI39
64128 229533.A0A0E0RTV8 pp 229533.I1RFZ2
64129 229533.A0A0E0RTV8 pp 229533.I1RFW1
64130 229533.A0A0E0RTV8 pp 229533.A0A1C3YJD4
64131 229533.A0A0E0RTV8 pp 229533.A0A0E0SLZ3
64132 229533.A0A0E0RTV8 pp 229533.V6RKY7
64133 229533.A0A0E0RTV8 pp 229533.I1RC35
64134 229533.A0A0E0RTV8 pp 229533.I1RSJ4
64135 229533.A0A0E0RTV8 pp 229533.I1RFL7
64136 229533.A0A0E0RTV8 pp 229533.I1S1W4
64137 229533.A0A0E0RTV8 pp 229533.I1RLU1
64138 229533.A0A0E0RTV8 pp 229533.I1RYX9
64139 229533.A0A0E0RTV8 pp 229533.I1RFR6
64140 229533.A0A0E0RTV8 pp 229533.I1S1V5
64141 229533.A0A0E0RTV8 pp 229533.I1R9Y9
64142 229533.A0A0E0RTV8 pp 229533.I1RA43
64143 229533.A0A0E0RTV8 pp 229533.V6R3J1
64144 229533.A0A0E0RTV8 pp 229533.A0A1C3YL99
64145 229533.A0A0E0RTV8 pp 229533.A0A1C3YKJ6
64146 229533.A0A0E0RTV8 pp 229533.A0A1C3YIL4
64147 229533.A0A0E0RTV8 pp 229533.I1S2E9
64148 229533.I1RJK3 pp 229533.I1S0K9
64149 229533.I1RJK3 pp 229533.I1RNM8
64150 229533.I1RJK3 pp 229533.I1RN84
64151 229533.I1RJK3 pp 229533.I1RMH2
64152 229533.I1RJK3 pp 229533.I1RJS9
64153 229533.I1RJK3 pp 229533.I1RRP3
64154 229533.I1RJK3 pp 229533.I1S549
64155 229533.I1RJK3 pp 229533.I1S8R4
64156 229533.I1RJK3 pp 229533.I1RN88
64157 229533.I1RJK3 pp 229533.I1RQA2
64158 229533.I1RJK3 pp 229533.I1S8C1
64159 229533.I1RJK3 pp 229533.I1RJY7
64160 229533.I1RJK3 pp 229533.I1S3L2
64161 229533.I1RIC8 pp 229533.I1S0N8
64162 229533.I1RIJ5 pp 229533.I1RPM6
64163 229533.I1RIJ5 pp 229533.I1RR12
64164 229533.I1RIJ5 pp 229533.I1RJU9
64165 229533.I1RIJ5 pp 229533.I1RY49
64166 229533.I1RIJ5 pp 229533.I1RLM3
64167 229533.I1RIJ5 pp 229533.I1RVX9

64168 229533.I1RIJ5 pp 229533.I1RM26
64169 229533.I1RIJ5 pp 229533.I1RPW2
64170 229533.I1RIJ5 pp 229533.I1RPD6
64171 229533.I1RIJ5 pp 229533.I1RNG4
64172 229533.I1RBN0 pp 229533.I1RZ18
64173 229533.I1RBN0 pp 229533.I1RC31
64174 229533.I1RBN0 pp 229533.Q4I0K3
64175 229533.I1RBN0 pp 229533.I1RST0
64176 229533.I1RBN0 pp 229533.I1RZ34
64177 229533.I1RBN0 pp 229533.I1RZ78
64178 229533.I1RBN0 pp 229533.I1RZ17
64179 229533.I1RBN0 pp 229533.I1S0B4
64180 229533.I1RBN0 pp 229533.V6RPQ8
64181 229533.I1RBN0 pp 229533.I1RLL8
64182 229533.I1RBN0 pp 229533.I1RK44
64183 229533.I1RBN0 pp 229533.I1RFD5
64184 229533.I1RBN0 pp 229533.I1S252
64185 229533.I1RBN0 pp 229533.Q4I073
64186 229533.I1RBN0 pp 229533.I1RJL9
64187 229533.I1RBN0 pp 229533.I1RMA0
64188 229533.I1RBN0 pp 229533.I1RNA1
64189 229533.I1RBN0 pp 229533.Q4ILH3
64190 229533.I1RBN0 pp 229533.I1RD96
64191 229533.I1RBN0 pp 229533.I1RPT0
64192 229533.I1RBN0 pp 229533.I1RRF6
64193 229533.I1RBN0 pp 229533.I1RQ75
64194 229533.I1RBN0 pp 229533.I1RMG4
64195 229533.I1RBN0 pp 229533.I1S4S9
64196 229533.I1RBN0 pp 229533.I1REU0
64197 229533.I1RBN0 pp 229533.I1RNU7
64198 229533.I1RBN0 pp 229533.I1RX03
64199 229533.I1RBN0 pp 229533.Q4I1Y5
64200 229533.I1RBN0 pp 229533.Q4IB63
64201 229533.I1RBN0 pp 229533.I1RJW1
64202 229533.I1RBN0 pp 229533.I1S574
64203 229533.I1RBN0 pp 229533.I1RZT6
64204 229533.I1RBN0 pp 229533.I1RP81
64205 229533.I1RBN0 pp 229533.I1RKB5
64206 229533.I1RBN0 pp 229533.I1RIX0
64207 229533.I1RBN0 pp 229533.I1RP86
64208 229533.I1RBN0 pp 229533.Q4HXT5
64209 229533.I1RBN0 pp 229533.I1RRC4
64210 229533.I1RBN0 pp 229533.I1S984
64211 229533.I1RBN0 pp 229533.I1RNK8
64212 229533.I1RBN0 pp 229533.I1RR39
64213 229533.I1RBN0 pp 229533.I1RQE0
64214 229533.I1RBN0 pp 229533.I1RSJ2
64215 229533.I1RBN0 pp 229533.I1RIE8
64216 229533.I1RIC6 pp 229533.I1RW64
64217 229533.A0A098DYK1 pp 229533.I1RQ42
64218 229533.A0A098DYK1 pp 229533.I1SAK7
64219 229533.A0A098DYK1 pp 229533.I1RFH9
64220 229533.A0A098DYK1 pp 229533.I1RCE6
64221 229533.A0A098DYK1 pp 229533.I1R9Y2
64222 229533.A0A098DYK1 pp 229533.I1RW91
64223 229533.A0A098DYK1 pp 229533.I1RNJ7
64224 229533.A0A098DYK1 pp 229533.I1RFV2
64225 229533.A0A098DYK1 pp 229533.I1RCM8
64226 229533.A0A098DYK1 pp 229533.I1RPW8
64227 229533.A0A098DYK1 pp 229533.I1RMG4
64228 229533.A0A098DYK1 pp 229533.A0A1C3YHX6
64229 229533.A0A098DYK1 pp 229533.A0A1C3YLC3
64230 229533.A0A098DYK1 pp 229533.I1RCG6
64231 229533.A0A098DYK1 pp 229533.I1RZN1
64232 229533.A0A098DYK1 pp 229533.A0A1C3YMU9
64233 229533.A0A098DYK1 pp 229533.I1RKF3
64234 229533.A0A098DYK1 pp 229533.I1RRN4
64235 229533.A0A098DYK1 pp 229533.I1RRC4
64236 229533.A0A098DYK1 pp 229533.A0A098E1Y1
64237 229533.A0A098DYK1 pp 229533.I1RBA6
64238 229533.A0A098DYK1 pp 229533.I1RYX5
64239 229533.A0A098DYK1 pp 229533.I1RW80
64240 229533.A0A098DYK1 pp 229533.I1RN04

64241 229533.A0A098DYK1 pp 229533.A0A0E0SFV6
64242 229533.A0A098DYK1 pp 229533.I1RR61
64243 229533.A0A098DYK1 pp 229533.I1RQM9
64244 229533.A0A098DYK1 pp 229533.I1RMV0
64245 229533.A0A098DYK1 pp 229533.I1RHJ2
64246 229533.A0A098DYK1 pp 229533.I1S2B9
64247 229533.A0A098DYK1 pp 229533.I1S5Z5
64248 229533.A0A098DYK1 pp 229533.I1RRF7
64249 229533.A0A1C3YJJ1 pp 229533.I1RTE0
64250 229533.A0A1C3YJJ1 pp 229533.I1RCT2
64251 229533.A0A1C3YJJ1 pp 229533.I1RD10
64252 229533.A0A1C3YJJ1 pp 229533.I1RTG5
64253 229533.A0A1C3YJJ1 pp 229533.I1RNW8
64254 229533.A0A1C3YJJ1 pp 229533.I1RWM7
64255 229533.A0A1C3YJJ1 pp 229533.A0A1C3YLH6
64256 229533.A0A1C3YJJ1 pp 229533.V6R476
64257 229533.A0A1C3YJJ1 pp 229533.I1RZL3
64258 229533.A0A1C3YJJ1 pp 229533.I1RQN9
64259 229533.A0A1C3YJJ1 pp 229533.A0A1C3YLK5
64260 229533.A0A1C3YJJ1 pp 229533.I1S045
64261 229533.A0A1C3YJJ1 pp 229533.I1RKG4
64262 229533.A0A1C3YJJ1 pp 229533.V6RB81
64263 229533.A0A1C3YJJ1 pp 229533.I1RKR9
64264 229533.A0A1C3YJJ1 pp 229533.I1S0Z3
64265 229533.A0A1C3YJJ1 pp 229533.I1RMP2
64266 229533.A0A1C3YJJ1 pp 229533.I1RQP5
64267 229533.A0A1C3YJJ1 pp 229533.I1S2C1
64268 229533.A0A1C3YJJ1 pp 229533.V6QUU6
64269 229533.A0A1C3YJJ1 pp 229533.I1S4X2
64270 229533.A0A1C3YJJ1 pp 229533.Q4I963
64271 229533.A0A1C3YJJ1 pp 229533.I1RN81
64272 229533.A0A1C3YJJ1 pp 229533.A0A1C3YN20
64273 229533.A0A1C3YJJ1 pp 229533.I1RJK8
64274 229533.A0A1C3YJJ1 pp 229533.I1RMR5
64275 229533.A0A1C3YJJ1 pp 229533.I1S270
64276 229533.A0A1C3YJJ1 pp 229533.Q4ILI9
64277 229533.A0A1C3YJJ1 pp 229533.I1RZX9
64278 229533.A0A1C3YJJ1 pp 229533.I1RT41
64279 229533.A0A1C3YJJ1 pp 229533.I1RRF9
64280 229533.A0A1C3YJJ1 pp 229533.I1S254
64281 229533.A0A1C3YJJ1 pp 229533.I1RWI6
64282 229533.A0A1C3YJJ1 pp 229533.V6RR76
64283 229533.A0A1C3YJJ1 pp 229533.I1RBK6
64284 229533.A0A1C3YJJ1 pp 229533.I1S1B1
64285 229533.A0A098DVU3 pp 229533.I1RBA6
64286 229533.A0A098DVU3 pp 229533.I1S2B9
64287 229533.A0A098DVU3 pp 229533.A0A1C3YJ83
64288 229533.A0A098DVU3 pp 229533.I1RMV0
64289 229533.A0A098DVU3 pp 229533.A0A098DYK1
64290 229533.A0A098DVU3 pp 229533.I1RYX5
64291 229533.A0A1C3YLR6 pp 229533.I1RSZ7
64292 229533.A0A1C3YLR6 pp 229533.I1RRJ6
64293 229533.A0A1C3YLR6 pp 229533.I1RWI5
64294 229533.A0A1C3YLR6 pp 229533.I1RYW6
64295 229533.A0A1C3YLR6 pp 229533.I1RFS3
64296 229533.A0A1C3YLR6 pp 229533.I1RSQ4
64297 229533.A0A1C3YLR6 pp 229533.I1RC35
64298 229533.A0A1C3YLR6 pp 229533.I1RL04
64299 229533.A0A1C3YLR6 pp 229533.I1RZZ9
64300 229533.A0A1C3YLR6 pp 229533.I1S004
64301 229533.A0A1C3YLR6 pp 229533.I1RGA6
64302 229533.A0A1C3YLR6 pp 229533.I1RBL4
64303 229533.A0A1C3YLR6 pp 229533.I1RBF3
64304 229533.A0A1C3YLR6 pp 229533.I1RR72
64305 229533.A0A1C3YLR6 pp 229533.I1RZ86
64306 229533.A0A1C3YLR6 pp 229533.I1RDE7
64307 229533.A0A1C3YLR6 pp 229533.I1RZ39
64308 229533.A0A1C3YLR6 pp 229533.I1RX72
64309 229533.A0A1C3YLR6 pp 229533.I1S253
64310 229533.A0A1C3YLR6 pp 229533.I1RJZ7
64311 229533.A0A1C3YLR6 pp 229533.V6RLW4
64312 229533.A0A1C3YLR6 pp 229533.I1RWS4
64313 229533.A0A1C3YLR6 pp 229533.I1RTA1

64314	229533.A0A1C3YLR6	pp	229533.I1S1V5
64315	229533.A0A1C3YLR6	pp	229533.I1RKB2
64316	229533.A0A1C3YLR6	pp	229533.I1S2B8
64317	229533.A0A1C3YLR6	pp	229533.I1RBY4
64318	229533.A0A1C3YLR6	pp	229533.I1S9Y1
64319	229533.A0A1C3YLR6	pp	229533.I1RUG1
64320	229533.A0A1C3YLR6	pp	229533.I1RYP2
64321	229533.A0A1C3YLR6	pp	229533.I1RBX1
64322	229533.A0A1C3YLR6	pp	229533.I1RSJ4
64323	229533.A0A1C3YLR6	pp	229533.I1S277
64324	229533.A0A1C3YLR6	pp	229533.I1RFR6
64325	229533.A0A1C3YLR6	pp	229533.I1RVW0
64326	229533.A0A1C3YLR6	pp	229533.I1RA52
64327	229533.A0A1C3YLR6	pp	229533.I1RFT1
64328	229533.A0A1C3YLR6	pp	229533.I1RWF9
64329	229533.A0A1C3YLR6	pp	229533.I1RYX9
64330	229533.A0A1C3YLR6	pp	229533.I1S5R1
64331	229533.A0A1C3YLR6	pp	229533.I1RAH9
64332	229533.A0A1C3YLR6	pp	229533.I1R9Y8
64333	229533.A0A1C3YLR6	pp	229533.O74638
64334	229533.A0A1C3YLR6	pp	229533.I1S998
64335	229533.A0A1C3YLR6	pp	229533.I1RMP0
64336	229533.A0A1C3YLR6	pp	229533.I1RYK0
64337	229533.A0A1C3YLR6	pp	229533.I1RPP3
64338	229533.A0A1C3YLR6	pp	229533.I1S3G4
64339	229533.A0A1C3YLR6	pp	229533.I1RZ83
64340	229533.A0A1C3YLR6	pp	229533.I1S1F3
64341	229533.A0A1C3YLR6	pp	229533.I1RFZ2
64342	229533.A0A1C3YLR6	pp	229533.I1S2H4
64343	229533.A0A1C3YLR6	pp	229533.I1RT40
64344	229533.A0A1C3YLR6	pp	229533.I1RW26
64345	229533.A0A1C3YLR6	pp	229533.I1RE53
64346	229533.A0A1C3YLR6	pp	229533.I1S2F2
64347	229533.A0A1C3YLR6	pp	229533.I1RBW1
64348	229533.A0A1C3YLR6	pp	229533.I1RN67
64349	229533.A0A1C3YLR6	pp	229533.I1RF02
64350	229533.A0A1C3YLR6	pp	229533.I1RNG4
64351	229533.A0A1C3YLR6	pp	229533.I1RTD5
64352	229533.A0A1C3YLR6	pp	229533.I1RZB2
64353	229533.A0A1C3YLR6	pp	229533.I1RC31
64354	229533.A0A1C3YLR6	pp	229533.Q4HXF0
64355	229533.A0A1C3YLR6	pp	229533.I1RPD6
64356	229533.A0A1C3YLR6	pp	229533.I1RYQ1
64357	229533.A0A1C3YLR6	pp	229533.I1S1L2
64358	229533.A0A1C3YLR6	pp	229533.I1RYR2
64359	229533.A0A1C3YLR6	pp	229533.I1RQE7
64360	229533.A0A1C3YLR6	pp	229533.I1RDJ8
64361	229533.A0A1C3YLR6	pp	229533.I1RCH5
64362	229533.A0A1C3YLR6	pp	229533.I1S0Q5
64363	229533.A0A1C3YLR6	pp	229533.I1S0N5
64364	229533.A0A1C3YLR6	pp	229533.I1RAN1
64365	229533.A0A1C3YLR6	pp	229533.A0A1C3YM67
64366	229533.A0A1C3YLR6	pp	229533.I1RSP7
64367	229533.A0A1C3YLR6	pp	229533.V6R3J1
64368	229533.A0A1C3YLR6	pp	229533.I1RSS4
64369	229533.A0A1C3YLR6	pp	229533.I1RSS7
64370	229533.A0A1C3YLR6	pp	229533.I1RBA4
64371	229533.A0A1C3YLR6	pp	229533.I1RYW3
64372	229533.A0A1C3YLR6	pp	229533.I1RVX0
64373	229533.A0A1C3YLR6	pp	229533.I1RX64
64374	229533.A0A1C3YLR6	pp	229533.I1RD19
64375	229533.A0A1C3YLR6	pp	229533.Q4IMD1
64376	229533.A0A1C3YLR6	pp	229533.I1RRR6
64377	229533.A0A1C3YLR6	pp	229533.I1RA70
64378	229533.A0A1C3YLR6	pp	229533.I1RBC7
64379	229533.A0A1C3YLR6	pp	229533.I1RSY2
64380	229533.A0A1C3YLR6	pp	229533.I1S2C4
64381	229533.A0A1C3YLR6	pp	229533.I1RB92
64382	229533.A0A1C3YLR6	pp	229533.I1RGA4
64383	229533.A0A1C3YLR6	pp	229533.I1RXG6
64384	229533.A0A1C3YLR6	pp	229533.I1RNC1
64385	229533.A0A1C3YLR6	pp	229533.I1RKB6
64386	229533.A0A1C3YLR6	pp	229533.I1RTN6

64387 229533.A0A1C3YLR6 pp 229533.V6RRH2
64388 229533.A0A1C3YLR6 pp 229533.I1RG11
64389 229533.A0A1C3YLR6 pp 229533.I1S0D5
64390 229533.A0A1C3YLR6 pp 229533.Q4I5V2
64391 229533.A0A1C3YLR6 pp 229533.I1RUG3
64392 229533.A0A1C3YLR6 pp 229533.I1RZ98
64393 229533.A0A1C3YLR6 pp 229533.I1RSC7
64394 229533.A0A1C3YLR6 pp 229533.V6RN69
64395 229533.A0A1C3YLR6 pp 229533.I1RD70
64396 229533.A0A1C3YLR6 pp 229533.I1RUN9
64397 229533.A0A1C3YLR6 pp 229533.I1RNM7
64398 229533.A0A1C3YLR6 pp 229533.I1RZT1
64399 229533.A0A1C3YLR6 pp 229533.I1RPA4
64400 229533.A0A1C3YLR6 pp 229533.I1RAW0
64401 229533.A0A1C3YLR6 pp 229533.I1RBS5
64402 229533.A0A1C3YLR6 pp 229533.I1S1D3
64403 229533.A0A1C3YLR6 pp 229533.V6R042
64404 229533.A0A1C3YLR6 pp 229533.I1RST8
64405 229533.A0A1C3YLR6 pp 229533.V6RMW6
64406 229533.A0A1C3YLR6 pp 229533.I1RCQ4
64407 229533.A0A1C3YLR6 pp 229533.I1RE01
64408 229533.A0A1C3YLR6 pp 229533.I1RN57
64409 229533.A0A1C3YLR6 pp 229533.V6RLM7
64410 229533.A0A1C3YLR6 pp 229533.I1S0B7
64411 229533.A0A1C3YLR6 pp 229533.I1RE84
64412 229533.A0A1C3YLR6 pp 229533.I1RRT1
64413 229533.A0A1C3YLR6 pp 229533.I1RSU2
64414 229533.A0A1C3YLR6 pp 229533.I1RQ75
64415 229533.A0A1C3YLR6 pp 229533.I1RE52
64416 229533.A0A1C3YLR6 pp 229533.V6R382
64417 229533.A0A1C3YLR6 pp 229533.I1S9Z4
64418 229533.A0A1C3YLR6 pp 229533.I1S1W8
64419 229533.A0A1C3YLR6 pp 229533.I1RD22
64420 229533.A0A1C3YLR6 pp 229533.I1RNA2
64421 229533.A0A1C3YLR6 pp 229533.I1RFX8
64422 229533.A0A1C3YLR6 pp 229533.I1S987
64423 229533.A0A1C3YLR6 pp 229533.I1RRS7
64424 229533.A0A1C3YLR6 pp 229533.V6RRM0
64425 229533.A0A1C3YLR6 pp 229533.I1S7L6
64426 229533.A0A1C3YLR6 pp 229533.I1RW16
64427 229533.A0A1C3YLR6 pp 229533.I1RB00
64428 229533.A0A1C3YLR6 pp 229533.A0A1C3YN26
64429 229533.A0A1C3YLR6 pp 229533.I1RC54
64430 229533.A0A1C3YLR6 pp 229533.I1RBW0
64431 229533.A0A1C3YLR6 pp 229533.I1RFN0
64432 229533.A0A1C3YLR6 pp 229533.I1RLE7
64433 229533.A0A1C3YLR6 pp 229533.I1RJU3
64434 229533.A0A1C3YLR6 pp 229533.I1RAH4
64435 229533.A0A1C3YLR6 pp 229533.I1RMB7
64436 229533.A0A1C3YLR6 pp 229533.I1RP86
64437 229533.A0A1C3YLR6 pp 229533.I1RY49
64438 229533.A0A1C3YLR6 pp 229533.I1RCM7
64439 229533.A0A1C3YLR6 pp 229533.I1RKI2
64440 229533.A0A1C3YLR6 pp 229533.I1RN31
64441 229533.A0A1C3YLR6 pp 229533.I1S8E0
64442 229533.A0A1C3YLR6 pp 229533.I1RVX9
64443 229533.A0A1C3YLR6 pp 229533.I1RRG3
64444 229533.A0A1C3YLR6 pp 229533.I1RAW7
64445 229533.A0A1C3YLR6 pp 229533.I1S283
64446 229533.A0A1C3YLR6 pp 229533.I1RAJ6
64447 229533.A0A1C3YLR6 pp 229533.A0A1C3YML3
64448 229533.A0A1C3YLR6 pp 229533.I1RRY1
64449 229533.A0A1C3YLR6 pp 229533.I1RSI4
64450 229533.A0A1C3YLR6 pp 229533.I1RM36
64451 229533.A0A1C3YLR6 pp 229533.I1RAL9
64452 229533.A0A1C3YLR6 pp 229533.I1RWM6
64453 229533.A0A1C3YLR6 pp 229533.I1RPN4
64454 229533.A0A1C3YLR6 pp 229533.I1S4S4
64455 229533.A0A1C3YLR6 pp 229533.I1RUR0
64456 229533.A0A1C3YLR6 pp 229533.V6RTJ8
64457 229533.A0A1C3YLR6 pp 229533.I1RPQ1
64458 229533.A0A1C3YLR6 pp 229533.I1RW71
64459 229533.A0A1C3YLR6 pp 229533.I1RM67

64460 229533.A0A1C3YLR6 pp 229533.I1RRX5
64461 229533.A0A1C3YLR6 pp 229533.Q4IJK2
64462 229533.A0A1C3YLR6 pp 229533.I1RD18
64463 229533.A0A1C3YLR6 pp 229533.I1RR24
64464 229533.A0A1C3YLR6 pp 229533.I1RT33
64465 229533.A0A1C3YLR6 pp 229533.I1RD52
64466 229533.A0A1C3YLR6 pp 229533.A0A1C3YM63
64467 229533.A0A1C3YLR6 pp 229533.I1RVZ9
64468 229533.A0A1C3YLR6 pp 229533.Q4I0J6
64469 229533.A0A1C3YLR6 pp 229533.I1RG14
64470 229533.A0A1C3YLR6 pp 229533.I1RYJ6
64471 229533.A0A1C3YLR6 pp 229533.V6RBN2
64472 229533.A0A1C3YLR6 pp 229533.I1RLX6
64473 229533.A0A1C3YLR6 pp 229533.I1RXX0
64474 229533.A0A1C3YLR6 pp 229533.I1RWH8
64475 229533.A0A1C3YLR6 pp 229533.I1RJL9
64476 229533.A0A1C3YLR6 pp 229533.I1S3S3
64477 229533.A0A1C3YLR6 pp 229533.I1RCM6
64478 229533.A0A1C3YLR6 pp 229533.I1RXF0
64479 229533.A0A1C3YLR6 pp 229533.I1RN55
64480 229533.A0A1C3YLR6 pp 229533.I1RM26
64481 229533.A0A1C3YLR6 pp 229533.I1RK95
64482 229533.A0A1C3YLR6 pp 229533.I1RFJ2
64483 229533.A0A1C3YLR6 pp 229533.I1RZM8
64484 229533.A0A1C3YLR6 pp 229533.I1S097
64485 229533.A0A1C3YLR6 pp 229533.A0A1C3YN86
64486 229533.A0A1C3YLR6 pp 229533.I1RBC1
64487 229533.A0A1C3YLR6 pp 229533.Q4IBX0
64488 229533.A0A1C3YLR6 pp 229533.I1RPI0
64489 229533.A0A1C3YLR6 pp 229533.I1RC61
64490 229533.A0A1C3YLR6 pp 229533.I1RXX1
64491 229533.A0A1C3YLR6 pp 229533.I1S4Q4
64492 229533.A0A1C3YLR6 pp 229533.V6RCZ8
64493 229533.A0A1C3YLR6 pp 229533.I1RBL7
64494 229533.A0A1C3YLR6 pp 229533.I1RE00
64495 229533.A0A1C3YLR6 pp 229533.I1RE37
64496 229533.A0A1C3YLR6 pp 229533.I1RRY9
64497 229533.A0A1C3YLR6 pp 229533.I1REK8
64498 229533.A0A1C3YLR6 pp 229533.I1RBE4
64499 229533.A0A1C3YLR6 pp 229533.Q4INS6
64500 229533.A0A1C3YLR6 pp 229533.A0A1C3YMT2
64501 229533.A0A1C3YLR6 pp 229533.I1RBE7
64502 229533.A0A1C3YLR6 pp 229533.I1S0Q4
64503 229533.A0A1C3YLR6 pp 229533.V6RCG5
64504 229533.A0A1C3YLR6 pp 229533.I1RJT5
64505 229533.A0A1C3YLR6 pp 229533.I1RE46
64506 229533.A0A1C3YLR6 pp 229533.I1RN46
64507 229533.A0A1C3YLR6 pp 229533.I1RE72
64508 229533.A0A1C3YLR6 pp 229533.I1RXB6
64509 229533.A0A1C3YLR6 pp 229533.I1S9J6
64510 229533.A0A1C3YLR6 pp 229533.I1RQH8
64511 229533.A0A1C3YLR6 pp 229533.I1RRV4
64512 229533.A0A1C3YLR6 pp 229533.I1RS18
64513 229533.A0A1C3YLR6 pp 229533.I1RB27
64514 229533.A0A1C3YLR6 pp 229533.I1RCV1
64515 229533.A0A1C3YLR6 pp 229533.I1RYI7
64516 229533.A0A1C3YLR6 pp 229533.I1RAZ3
64517 229533.A0A1C3YLR6 pp 229533.I1RSX2
64518 229533.A0A1C3YLR6 pp 229533.V6QUE6
64519 229533.A0A1C3YLR6 pp 229533.I1RCN8
64520 229533.A0A1C3YLR6 pp 229533.I1RA12
64521 229533.A0A1C3YLR6 pp 229533.I1S835
64522 229533.A0A1C3YLR6 pp 229533.Q4I5I4
64523 229533.A0A1C3YLR6 pp 229533.Q4IR08
64524 229533.A0A1C3YLR6 pp 229533.I1RA94
64525 229533.A0A1C3YLR6 pp 229533.I1S5B0
64526 229533.A0A1C3YLR6 pp 229533.I1S209
64527 229533.I1RDE4 pp 229533.I1RP86
64528 229533.I1RDE4 pp 229533.I1RKB5
64529 229533.I1RDE4 pp 229533.I1RR39
64530 229533.I1RDE4 pp 229533.I1RJW1
64531 229533.I1RDE4 pp 229533.I1RIX0
64532 229533.I1RDE4 pp 229533.I1S984

64533 229533.I1RDE4 pp 229533.I1S574
64534 229533.I1RDE4 pp 229533.I1RQE0
64535 229533.I1RDE4 pp 229533.Q4INS6
64536 229533.I1RDE4 pp 229533.I1RZV2
64537 229533.I1RDE4 pp 229533.I1RJL9
64538 229533.I1RDE4 pp 229533.I1RST0
64539 229533.I1RDE4 pp 229533.I1RIE8
64540 229533.I1RDE4 pp 229533.I1RZT6
64541 229533.I1RDE4 pp 229533.I1RSJ2
64542 229533.I1RDE4 pp 229533.I1S4S9
64543 229533.I1RDE4 pp 229533.I1RPT0
64544 229533.I1RDE4 pp 229533.I1RZ13
64545 229533.I1RDE4 pp 229533.I1RZ78
64546 229533.I1RDE4 pp 229533.I1RP81
64547 229533.I1RDE4 pp 229533.I1RK44
64548 229533.I1RDE4 pp 229533.I1RNU7
64549 229533.I1RDE4 pp 229533.I1RFZ0
64550 229533.I1RDE4 pp 229533.I1S252
64551 229533.I1RDE4 pp 229533.I1RZ34
64552 229533.I1RDE4 pp 229533.I1RNA1
64553 229533.I1RDE4 pp 229533.Q4I1Y5
64554 229533.I1RDE4 pp 229533.V6RPQ8
64555 229533.I1RDE4 pp 229533.I1S272
64556 229533.I1RDE4 pp 229533.I1RDF6
64557 229533.I1RDE4 pp 229533.I1RQ75
64558 229533.A0A1C3YIT6 pp 229533.A0A1C3YLC2
64559 229533.A0A1C3YIT6 pp 229533.I1RBE7
64560 229533.A0A1C3YIT6 pp 229533.I1RM26
64561 229533.A0A1C3YIT6 pp 229533.I1RMW3
64562 229533.A0A1C3YIT6 pp 229533.I1RFV9
64563 229533.A0A1C3YIT6 pp 229533.I1RLM2
64564 229533.A0A1C3YIT6 pp 229533.I1RG09
64565 229533.A0A1C3YIT6 pp 229533.I1RCR9
64566 229533.A0A1C3YIT6 pp 229533.A0A1C3YK58
64567 229533.A0A1C3YIT6 pp 229533.I1RPD6
64568 229533.A0A1C3YIT6 pp 229533.I1RJU9
64569 229533.A0A1C3YIT6 pp 229533.A0A1C3YJW4
64570 229533.A0A1C3YIT6 pp 229533.I1RNG4
64571 229533.A0A1C3YIT6 pp 229533.I1RLM3
64572 229533.A0A1C3YIT6 pp 229533.I1RPM6
64573 229533.A0A1C3YIT6 pp 229533.I1RA94
64574 229533.A0A1C3YIT6 pp 229533.I1RSL2
64575 229533.A0A1C3YIT6 pp 229533.I1RY49
64576 229533.A0A1C3YIT6 pp 229533.I1RPW2
64577 229533.A0A1C3YIT6 pp 229533.I1RVX9
64578 229533.A0A1C3YIT6 pp 229533.I1RR12
64579 229533.I1RJS9 pp 229533.I1RTB7
64580 229533.I1RJS9 pp 229533.I1RW91
64581 229533.I1RJS9 pp 229533.I1RPS7
64582 229533.I1RJS9 pp 229533.I1RQ42
64583 229533.I1RJS9 pp 229533.I1S0S6
64584 229533.I1RJS9 pp 229533.I1RWL6
64585 229533.I1RJS9 pp 229533.I1RQN9
64586 229533.I1RJS9 pp 229533.I1RWF5
64587 229533.I1RJS9 pp 229533.I1RMR5
64588 229533.I1RJS9 pp 229533.I1RUK7
64589 229533.I1RJS9 pp 229533.I1RRP3
64590 229533.I1RJS9 pp 229533.I1RN04
64591 229533.I1RJS9 pp 229533.I1S0K9
64592 229533.I1RJS9 pp 229533.I1S7X9
64593 229533.I1RJS9 pp 229533.I1S0Y1
64594 229533.I1RJS9 pp 229533.P0C431
64595 229533.I1RJS9 pp 229533.I1RQA2
64596 229533.I1RJS9 pp 229533.I1RT36
64597 229533.I1RJS9 pp 229533.I1S549
64598 229533.I1RJS9 pp 229533.I1RJT0
64599 229533.I1RJS9 pp 229533.I1RS64
64600 229533.I1RJS9 pp 229533.I1RNM8
64601 229533.I1RJS9 pp 229533.I1RMH2
64602 229533.I1RZT3 pp 229533.I1S268
64603 229533.A0A098DQ30 pp 229533.I1RD70
64604 229533.A0A098DQ30 pp 229533.I1RDE8
64605 229533.A0A098DQ30 pp 229533.I1RMA7

64606	229533.A0A098DQ30	pp	229533.V6QUV7
64607	229533.A0A098DQ30	pp	229533.I1RYW3
64608	229533.A0A098DQ30	pp	229533.I1RRC2
64609	229533.A0A098DQ30	pp	229533.A0A098DRX7
64610	229533.A0A098DQ30	pp	229533.I1RTG5
64611	229533.A0A098DQ30	pp	229533.I1RK44
64612	229533.A0A098DQ30	pp	229533.V6QX21
64613	229533.A0A098DQ30	pp	229533.I1RTA0
64614	229533.A0A098DQ30	pp	229533.I1RAI0
64615	229533.A0A098DQ30	pp	229533.I1RQ32
64616	229533.A0A098DQ30	pp	229533.I1S4Z7
64617	229533.A0A098DQ30	pp	229533.A0A1C3YMB7
64618	229533.A0A098DQ30	pp	229533.I1RFR6
64619	229533.A0A098DQ30	pp	229533.A0A1C3YI53
64620	229533.A0A098DQ30	pp	229533.I1RMQ5
64621	229533.A0A098DQ30	pp	229533.I1RRM9
64622	229533.A0A098DQ30	pp	229533.I1RN84
64623	229533.A0A098DQ30	pp	229533.I1SAA0
64624	229533.A0A098DQ30	pp	229533.I1RJZ7
64625	229533.A0A098DQ30	pp	229533.Q4I7L0
64626	229533.A0A098DQ30	pp	229533.I1RP28
64627	229533.A0A098DQ30	pp	229533.I1RMP2
64628	229533.A0A098DQ30	pp	229533.Q4IPI4
64629	229533.A0A098DQ30	pp	229533.A0A1C3YM61
64630	229533.A0A098DQ30	pp	229533.I1RFZ2
64631	229533.A0A098DQ30	pp	229533.I1RN88
64632	229533.A0A098DQ30	pp	229533.A0A098E425
64633	229533.A0A098DQ30	pp	229533.I1RFW1
64634	229533.A0A098DQ30	pp	229533.A0A1C3YLK5
64635	229533.A0A098DQ30	pp	229533.I1S074
64636	229533.A0A098DQ30	pp	229533.I1S7X9
64637	229533.A0A098DQ30	pp	229533.I1RQ93
64638	229533.A0A098DQ30	pp	229533.A0A1C3YIH7
64639	229533.A0A098DQ30	pp	229533.Q4IER8
64640	229533.A0A098DQ30	pp	229533.I1RRB0
64641	229533.A0A098DQ30	pp	229533.I1RAS5
64642	229533.A0A098DQ30	pp	229533.Q4ICA8
64643	229533.A0A098DQ30	pp	229533.A0A0E0SLZ3
64644	229533.A0A098DQ30	pp	229533.I1S8P5
64645	229533.A0A098DQ30	pp	229533.Q4IBX0
64646	229533.A0A098DQ30	pp	229533.I1RQG4
64647	229533.A0A098DQ30	pp	229533.I1RCM6
64648	229533.A0A098DQ30	pp	229533.I1RAH9
64649	229533.A0A098DQ30	pp	229533.I1RE19
64650	229533.A0A098DQ30	pp	229533.A0A098DX26
64651	229533.A0A098DQ30	pp	229533.A0A1C3YLE2
64652	229533.A0A098DQ30	pp	229533.A0A0E0SJN8
64653	229533.A0A098DQ30	pp	229533.I1RWD5
64654	229533.A0A098DQ30	pp	229533.A0A1C3YN54
64655	229533.A0A098DQ30	pp	229533.Q4I0J6
64656	229533.A0A098DQ30	pp	229533.I1S0Z3
64657	229533.A0A098DQ30	pp	229533.I1REG5
64658	229533.A0A098DQ30	pp	229533.A0A1C3YI44
64659	229533.A0A098DQ30	pp	229533.V6RPN0
64660	229533.A0A098DQ30	pp	229533.I1RYL5
64661	229533.A0A098DQ30	pp	229533.I1RRF9
64662	229533.A0A098DQ30	pp	229533.I1RA72
64663	229533.A0A098DQ30	pp	229533.I1RPL2
64664	229533.A0A098DQ30	pp	229533.I1S1C8
64665	229533.A0A098DQ30	pp	229533.Q4HTT2
64666	229533.A0A098DQ30	pp	229533.I1RUK7
64667	229533.A0A098DQ30	pp	229533.I1RB00
64668	229533.A0A098DQ30	pp	229533.I1RT41
64669	229533.A0A098DQ30	pp	229533.I1RZU6
64670	229533.A0A098DQ30	pp	229533.A0A1C3YIP6
64671	229533.A0A098DQ30	pp	229533.I1RTQ9
64672	229533.A0A098DQ30	pp	229533.Q5I2J3
64673	229533.A0A098DQ30	pp	229533.I1RN81
64674	229533.A0A098DQ30	pp	229533.A0A1C3YN26
64675	229533.A0A098DQ30	pp	229533.I1RR39
64676	229533.A0A098DQ30	pp	229533.A0A0E0SNZ9
64677	229533.A0A098DQ30	pp	229533.Q4I9B7
64678	229533.A0A098DQ30	pp	229533.I1RQ80

229533.A0A098DQ30 pp 229533.A0A098DQ41
229533.A0A098DQ30 pp 229533.I1RW71
229533.A0A098DQ30 pp 229533.I1RJW1
229533.A0A098DQ30 pp 229533.I1RAU9
229533.Q4IB70 pp 229533.Q4IJH1
229533.Q4IB70 pp 229533.Q4IBK5
229533.Q4IB70 pp 229533.Q4IPB3
229533.Q4IB70 pp 229533.Q4IF76
229533.Q4IB70 pp 229533.Q4IPB8
229533.Q4IB70 pp 229533.V6QXG1
229533.Q4IB70 pp 229533.V6QVD5
229533.Q4IB70 pp 229533.V6RY33
229533.Q4IB70 pp 229533.Q4IRI9
229533.Q4IB70 pp 229533.Q4IE79
229533.I1RAM1 pp 229533.I1S1C8
229533.I1RAM1 pp 229533.Q4ICM0
229533.I1RAM1 pp 229533.I1RCD9
229533.I1RAM1 pp 229533.I1RRB0
229533.I1RAM1 pp 229533.I1RT41
229533.I1RAM1 pp 229533.I1RTG3
229533.I1RAM1 pp 229533.I1RQL1
229533.I1RAM1 pp 229533.I1S0Z3
229533.I1RAM1 pp 229533.I1RRT4
229533.I1RAM1 pp 229533.I1RKG4
229533.I1RAM1 pp 229533.I1RCF0
229533.I1RAM1 pp 229533.I1RX54
229533.I1RAM1 pp 229533.I1RRH3
229533.I1RAM1 pp 229533.I1RTG5
229533.I1RAM1 pp 229533.I1RBX2
229533.I1S574 pp 229533.I1SAS7
229533.I1S574 pp 229533.I1SAS8
229533.I1S574 pp 229533.Q4I1Y5
229533.I1S574 pp 229533.V6RAN2
229533.I1S574 pp 229533.I1S575
229533.I1REI3 pp 229533.I1RP86
229533.I1REI3 pp 229533.I1RMJ3
229533.I1REI3 pp 229533.I1RVX9
229533.I1REI3 pp 229533.I1RVU7
229533.I1REI3 pp 229533.I1RM26
229533.I1REI3 pp 229533.I1RNG4
229533.I1REI3 pp 229533.I1RK47
229533.I1REI3 pp 229533.V6RPN0
229533.I1REI3 pp 229533.I1S0V6
229533.I1REI3 pp 229533.I1RHR3
229533.I1REI3 pp 229533.I1S5R5
229533.I1REI3 pp 229533.I1RN84
229533.I1REI3 pp 229533.I1S0V7
229533.A0A098DWY7 pp 229533.I1REG5
229533.A0A098DWY7 pp 229533.Q4I0J6
229533.A0A098DWY7 pp 229533.A0A1C3YID3
229533.A0A098DWY7 pp 229533.Q4HY20
229533.A0A098DWY7 pp 229533.I1S0G1
229533.A0A098DWY7 pp 229533.I1RAH5
229533.A0A098DWY7 pp 229533.I1RQJ0
229533.A0A098DWY7 pp 229533.I1RUN9
229533.A0A098DWY7 pp 229533.I1RMN3
229533.A0A098DWY7 pp 229533.A0A1C3YKT4
229533.A0A098DWY7 pp 229533.I1RCK4
229533.A0A098DWY7 pp 229533.I1RMJ1
229533.A0A098DWY7 pp 229533.I1RXT9
229533.A0A098DWY7 pp 229533.I1RCM6
229533.A0A098DWY7 pp 229533.I1RD70
229533.A0A098DWY7 pp 229533.I1RLX4
229533.A0A098DWY7 pp 229533.A0A1C3YI93
229533.A0A098DWY7 pp 229533.A0A1C3YIU7
229533.A0A098DWY7 pp 229533.I1RMG2
229533.A0A098DWY7 pp 229533.I1RBF2
229533.A0A098DWY7 pp 229533.I1RNG2
229533.A0A098DWY7 pp 229533.I1S1V5
229533.A0A098DWY7 pp 229533.I1S2B6
229533.A0A098DWY7 pp 229533.Q4ILI9
229533.A0A098DWY7 pp 229533.I1S0G3
229533.A0A098DWY7 pp 229533.I1RE18

64752 229533.A0A098DWY7 pp 229533.A0A0E0RR64
64753 229533.A0A098DWY7 pp 229533.A0A0E0SDU6
64754 229533.A0A098DWY7 pp 229533.I1RQD6
64755 229533.A0A098DWY7 pp 229533.I1RQF7
64756 229533.A0A098DWY7 pp 229533.I1RGC1
64757 229533.A0A098DWY7 pp 229533.V6RXW9
64758 229533.A0A098DWY7 pp 229533.I1RAH7
64759 229533.A0A098DWY7 pp 229533.I1RB00
64760 229533.A0A098DWY7 pp 229533.I1RX87
64761 229533.A0A098DWY7 pp 229533.I1RFZ2
64762 229533.A0A098DWY7 pp 229533.I1RY40
64763 229533.A0A098DWY7 pp 229533.I1RWZ9
64764 229533.A0A098DWY7 pp 229533.A0A0E0RS85
64765 229533.A0A098DWY7 pp 229533.I1RZK3
64766 229533.A0A098DWY7 pp 229533.A0A0E0SGH2
64767 229533.A0A098DWY7 pp 229533.A0A1C3YIP6
64768 229533.A0A098DWY7 pp 229533.I1RCF0
64769 229533.A0A098DWY7 pp 229533.A0A0E0SLZ3
64770 229533.A0A098DWY7 pp 229533.I1RAW7
64771 229533.A0A098DWY7 pp 229533.I1RQ80
64772 229533.A0A098DWY7 pp 229533.I1S0V5
64773 229533.A0A098DWY7 pp 229533.I1RCQ0
64774 229533.A0A098DWY7 pp 229533.I1RQF9
64775 229533.A0A098DWY7 pp 229533.A0A1C3YIH7
64776 229533.A0A098DWY7 pp 229533.I1RP87
64777 229533.A0A098DWY7 pp 229533.I1S287
64778 229533.A0A098DWY7 pp 229533.I1RN70
64779 229533.I1RNA1 pp 229533.I1RVW7
64780 229533.I1RNA1 pp 229533.I1RV02
64781 229533.I1RNA1 pp 229533.I1S328
64782 229533.I1RNA1 pp 229533.V6RAN2
64783 229533.I1RNA1 pp 229533.I1RYA5
64784 229533.I1RNA1 pp 229533.V6R729
64785 229533.I1RNA1 pp 229533.I1S272
64786 229533.I1RNA1 pp 229533.I1SAS7
64787 229533.I1RNA1 pp 229533.I1RNU7
64788 229533.I1RNA1 pp 229533.I1RPT0
64789 229533.I1RNA1 pp 229533.I1RTP4
64790 229533.I1RNA1 pp 229533.Q4I1Y5
64791 229533.I1RNA1 pp 229533.I1S984
64792 229533.I1RNA1 pp 229533.I1RV01
64793 229533.I1RNA1 pp 229533.I1RR39
64794 229533.I1RNA1 pp 229533.I1S2Y3
64795 229533.I1RNA1 pp 229533.I1SAS8
64796 229533.I1RNA1 pp 229533.I1RRT3
64797 229533.I1RNA1 pp 229533.I1S2Y4
64798 229533.I1RNA1 pp 229533.I1RZ13
64799 229533.I1RN31 pp 229533.V6QV09
64800 229533.I1RN31 pp 229533.I1S224
64801 229533.I1RN31 pp 229533.I1RNC0
64802 229533.I1RN31 pp 229533.I1S0S6
64803 229533.I1RN31 pp 229533.I1RS37
64804 229533.I1RN31 pp 229533.I1RQ80
64805 229533.I1RN31 pp 229533.I1RYH9
64806 229533.I1RN31 pp 229533.I1RYZ9
64807 229533.I1RN31 pp 229533.I1RNT2
64808 229533.I1RN31 pp 229533.I1RZ94
64809 229533.I1RN31 pp 229533.I1RQ92
64810 229533.I1RN31 pp 229533.I1S219
64811 229533.I1RN31 pp 229533.Q4ICM0
64812 229533.I1RN31 pp 229533.I1RTC2
64813 229533.I1RN31 pp 229533.I1S439
64814 229533.I1RN31 pp 229533.I1RNG8
64815 229533.I1RN31 pp 229533.I1RR49
64816 229533.I1RN31 pp 229533.I1RN46
64817 229533.I1RN31 pp 229533.I1RQ26
64818 229533.I1RN31 pp 229533.V6REE9
64819 229533.I1RN31 pp 229533.I1RSI4
64820 229533.I1RN31 pp 229533.I1RWL6
64821 229533.I1RN31 pp 229533.I1RRB0
64822 229533.I1RN31 pp 229533.I1RPU4
64823 229533.I1RN31 pp 229533.Q4IBX0
64824 229533.I1RN31 pp 229533.I1S1C8

64825	229533.I1RN31	pp	229533.I1S0P4
64826	229533.I1RN31	pp	229533.Q4I7X1
64827	229533.I1RN31	pp	229533.I1RN65
64828	229533.I1RN31	pp	229533.Q4I0J6
64829	229533.I1RN31	pp	229533.Q4I877
64830	229533.I1RN31	pp	229533.I1RUG2
64831	229533.I1RN31	pp	229533.I1RN55
64832	229533.I1RN31	pp	229533.I1RN43
64833	229533.I1RN31	pp	229533.P0C431
64834	229533.I1RN31	pp	229533.I1S604
64835	229533.I1RN31	pp	229533.I1RWB3
64836	229533.I1RN31	pp	229533.I1RZH4
64837	229533.I1RN31	pp	229533.I1RY73
64838	229533.I1RN31	pp	229533.I1RPZ0
64839	229533.I1RN31	pp	229533.I1RZV9
64840	229533.I1RN31	pp	229533.V6RLW4
64841	229533.I1RN31	pp	229533.I1S1V5
64842	229533.I1RN31	pp	229533.I1RRN0
64843	229533.I1RN31	pp	229533.I1RSA7
64844	229533.I1RN31	pp	229533.I1S382
64845	229533.I1RN31	pp	229533.I1S2A8
64846	229533.I1RN31	pp	229533.I1S7X9
64847	229533.I1RN31	pp	229533.I1RWJ1
64848	229533.I1RN31	pp	229533.I1RQ67
64849	229533.I1RN31	pp	229533.I1S951
64850	229533.I1RN31	pp	229533.I1RVA7
64851	229533.I1RN31	pp	229533.I1RYR2
64852	229533.I1RN31	pp	229533.I1RX38
64853	229533.I1RN31	pp	229533.I1S2F2
64854	229533.I1RN31	pp	229533.I1RYX9
64855	229533.I1RN31	pp	229533.I1RW15
64856	229533.I1RN31	pp	229533.I1RRP7
64857	229533.I1RN31	pp	229533.Q4IMD1
64858	229533.I1RN31	pp	229533.I1RZP8
64859	229533.I1RN31	pp	229533.I1RX64
64860	229533.I1RN31	pp	229533.I1RQN9
64861	229533.I1RN31	pp	229533.V6R3J1
64862	229533.I1RN31	pp	229533.I1RTE6
64863	229533.I1RN31	pp	229533.I1S2E9
64864	229533.I1RN31	pp	229533.I1RZE4
64865	229533.I1RN31	pp	229533.I1RPW0
64866	229533.I1RN31	pp	229533.I1RQJ4
64867	229533.I1RN31	pp	229533.V6RKY7
64868	229533.I1RN31	pp	229533.I1RSP7
64869	229533.I1RN31	pp	229533.I1RXU0
64870	229533.I1RN31	pp	229533.Q4HZB8
64871	229533.I1RN31	pp	229533.I1S1W4
64872	229533.I1RN31	pp	229533.I1RSC7
64873	229533.I1RN31	pp	229533.I1S4P6
64874	229533.I1RN31	pp	229533.V6RVG3
64875	229533.I1RN31	pp	229533.I1RRW0
64876	229533.I1RN31	pp	229533.I1RY44
64877	229533.I1RN31	pp	229533.I1S1D3
64878	229533.I1RN31	pp	229533.Q4IB89
64879	229533.I1RN31	pp	229533.I1RWQ0
64880	229533.I1RN31	pp	229533.V6RGI1
64881	229533.I1RN31	pp	229533.I1RN57
64882	229533.I1RN31	pp	229533.I1RSL6
64883	229533.I1S0K9	pp	229533.I1S7X9
64884	229533.I1S0K9	pp	229533.I1S549
64885	229533.I1S0K9	pp	229533.P0C431
64886	229533.I1S0K9	pp	229533.I1S0Y1
64887	229533.I1S0K9	pp	229533.I1S0S6
64888	229533.I1R9B1	pp	229533.I1RZ56
64889	229533.I1R9B1	pp	229533.I1S3H8
64890	229533.I1R9B1	pp	229533.I1S0A5
64891	229533.I1R9B1	pp	229533.I1RPV8
64892	229533.I1R9B1	pp	229533.I1RJS1
64893	229533.I1R9B1	pp	229533.I1RZ25
64894	229533.I1R9B1	pp	229533.I1RGZ3
64895	229533.I1R9B1	pp	229533.I1RNG4
64896	229533.I1R9B1	pp	229533.I1RKB2
64897	229533.I1R9B1	pp	229533.I1S6H3

64898	229533.I1R9B1	pp	229533.I1RUY5
64899	229533.I1R9B1	pp	229533.I1S8F8
64900	229533.I1R9B1	pp	229533.I1RST9
64901	229533.I1R9B1	pp	229533.I1RLA2
64902	229533.I1R9B1	pp	229533.I1RDU0
64903	229533.I1R9B1	pp	229533.I1S334
64904	229533.I1R9B1	pp	229533.I1S094
64905	229533.I1R9B1	pp	229533.I1RDN3
64906	229533.I1R9B1	pp	229533.I1RA13
64907	229533.I1R9B1	pp	229533.I1RRL5
64908	229533.I1R9B1	pp	229533.I1RNM7
64909	229533.I1RC35	pp	229533.I1RQ16
64910	229533.I1RC35	pp	229533.I1RG77
64911	229533.I1RC35	pp	229533.I1RCL7
64912	229533.I1RC35	pp	229533.I1RVZ9
64913	229533.I1RC35	pp	229533.I1RJI7
64914	229533.I1RC35	pp	229533.I1RQJ0
64915	229533.I1RC35	pp	229533.I1RCM6
64916	229533.I1RC35	pp	229533.Q4I0J6
64917	229533.I1RC35	pp	229533.I1RN46
64918	229533.I1RC35	pp	229533.Q4IBX0
64919	229533.I1RC35	pp	229533.I1RDX8
64920	229533.I1RC35	pp	229533.I1RLX4
64921	229533.I1RC35	pp	229533.I1RX64
64922	229533.I1RC35	pp	229533.I1RJI8
64923	229533.I1RC35	pp	229533.I1RLE7
64924	229533.I1RC35	pp	229533.I1RD19
64925	229533.I1RC35	pp	229533.I1RD18
64926	229533.I1RC35	pp	229533.I1S2F2
64927	229533.I1RC35	pp	229533.I1RRH3
64928	229533.I1RC35	pp	229533.I1RFN0
64929	229533.I1RC35	pp	229533.I1RSC7
64930	229533.I1RC35	pp	229533.I1RC54
64931	229533.I1RC35	pp	229533.I1RR21
64932	229533.I1RC35	pp	229533.I1RJE5
64933	229533.I1RC35	pp	229533.I1S283
64934	229533.I1RC35	pp	229533.I1RGE7
64935	229533.I1RC35	pp	229533.I1RD70
64936	229533.I1RC35	pp	229533.I1RQ13
64937	229533.I1RC35	pp	229533.I1RPX8
64938	229533.I1RC35	pp	229533.I1S1V5
64939	229533.I1RC35	pp	229533.I1RYP9
64940	229533.I1RC35	pp	229533.I1RSJ4
64941	229533.I1RC35	pp	229533.I1RUQ1
64942	229533.I1RC35	pp	229533.I1RFR6
64943	229533.I1RC35	pp	229533.I1RP94
64944	229533.I1RC35	pp	229533.I1RMS4
64945	229533.I1RC35	pp	229533.I1RW16
64946	229533.I1RC35	pp	229533.I1RYX9
64947	229533.I1RC35	pp	229533.I1RNC0
64948	229533.I1RC35	pp	229533.I1S1D3
64949	229533.I1RC35	pp	229533.V6R382
64950	229533.I1RC35	pp	229533.I1RQM0
64951	229533.I1RC35	pp	229533.I1RUG1
64952	229533.I1RC35	pp	229533.I1RQ80
64953	229533.I1RC35	pp	229533.I1S095
64954	229533.I1RC35	pp	229533.I1RSV0
64955	229533.I1RC35	pp	229533.I1RYR2
64956	229533.I1RC35	pp	229533.I1RNG6
64957	229533.I1RC35	pp	229533.I1RN31
64958	229533.I1RC35	pp	229533.I1RFX8
64959	229533.I1RC35	pp	229533.I1RMB7
64960	229533.I1RC35	pp	229533.Q4IMM0
64961	229533.I1RC35	pp	229533.I1RFZ2
64962	229533.I1RC35	pp	229533.I1RM36
64963	229533.I1RC35	pp	229533.I1RLX6
64964	229533.I1RC35	pp	229533.V6R3J1
64965	229533.I1RFP9	pp	229533.I1RST1
64966	229533.I1RFP9	pp	229533.I1S0B6
64967	229533.I1RFP9	pp	229533.I1RPW9
64968	229533.I1RFP9	pp	229533.Q4I1Y1
64969	229533.I1RFP9	pp	229533.Q4IB70
64970	229533.I1RFP9	pp	229533.I1RK90

64971 229533.I1RFP9 pp 229533.I1RG57
64972 229533.I1RFP9 pp 229533.I1RK23
64973 229533.I1RFP9 pp 229533.Q4IE79
64974 229533.I1RFP9 pp 229533.I1RX47
64975 229533.I1RFP9 pp 229533.I1RQP8
64976 229533.I1RFP9 pp 229533.Q4IPB8
64977 229533.I1RFP9 pp 229533.I1RU69
64978 229533.I1RFP9 pp 229533.I1RMV5
64979 229533.I1RFP9 pp 229533.Q4IPH4
64980 229533.I1RFP9 pp 229533.I1S9L6
64981 229533.I1RFP9 pp 229533.I1S0Z9
64982 229533.I1RFP9 pp 229533.V6QXG1
64983 229533.I1RFP9 pp 229533.I1RG90
64984 229533.I1RFP9 pp 229533.I1RX51
64985 229533.I1RFP9 pp 229533.I1S0B1
64986 229533.I1RFP9 pp 229533.I1RND5
64987 229533.I1RFP9 pp 229533.I1RME9
64988 229533.I1RFP9 pp 229533.I1RYB1
64989 229533.I1RFP9 pp 229533.I1RSA4
64990 229533.I1RFP9 pp 229533.I1RX24
64991 229533.I1RFP9 pp 229533.I1RV70
64992 229533.I1RFP9 pp 229533.I1S1X9
64993 229533.I1RFP9 pp 229533.I1RM25
64994 229533.I1RFP9 pp 229533.I1RJF9
64995 229533.I1RFP9 pp 229533.I1S4M5
64996 229533.I1RFP9 pp 229533.I1RX81
64997 229533.I1RFP9 pp 229533.I1S9L5
64998 229533.I1RFP9 pp 229533.I1RPL3
64999 229533.I1RFP9 pp 229533.I1RZG7
65000 229533.I1RFP9 pp 229533.Q4IRI9
65001 229533.I1RFP9 pp 229533.Q4IPB3
65002 229533.I1RFP9 pp 229533.Q4I7F9
65003 229533.I1RFP9 pp 229533.I1RRL0
65004 229533.I1RFP9 pp 229533.I1RPY4
65005 229533.I1RFP9 pp 229533.I1RT17
65006 229533.I1RFP9 pp 229533.I1RS75
65007 229533.I1RFP9 pp 229533.Q4IBK5
65008 229533.I1RFP9 pp 229533.I1RPY5
65009 229533.I1RFP9 pp 229533.I1RSL9
65010 229533.I1RFP9 pp 229533.I1RFQ1
65011 229533.I1RFP9 pp 229533.I1S056
65012 229533.I1RFP9 pp 229533.I1RS28
65013 229533.I1RFP9 pp 229533.I1RSD4
65014 229533.I1RFP9 pp 229533.I1S1C6
65015 229533.I1RFP9 pp 229533.I1RPZ3
65016 229533.I1RFP9 pp 229533.I1S2Y5
65017 229533.I1S984 pp 229533.I1SAS7
65018 229533.I1S984 pp 229533.I1SAS8
65019 229533.I1S984 pp 229533.Q4I1Y5
65020 229533.I1S984 pp 229533.V6RAN2
65021 229533.I1S984 pp 229533.V6R729
65022 229533.A0A0E0RS85 pp 229533.Q4ICM0
65023 229533.A0A0E0RS85 pp 229533.I1RAW7
65024 229533.A0A0E0RS85 pp 229533.I1RQG9
65025 229533.A0A0E0RS85 pp 229533.I1RQ80
65026 229533.A0A0E0RS85 pp 229533.I1RML9
65027 229533.A0A0E0RS85 pp 229533.I1RP96
65028 229533.A0A0E0RS85 pp 229533.I1RLX6
65029 229533.A0A0E0RS85 pp 229533.I1RQ26
65030 229533.A0A0E0RS85 pp 229533.I1RD18
65031 229533.A0A0E0RS85 pp 229533.I1RUQ6
65032 229533.A0A0E0RS85 pp 229533.A0A1C3YII4
65033 229533.A0A0E0RS85 pp 229533.A0A0E0SA11
65034 229533.A0A0E0RS85 pp 229533.A0A1C3YIA8
65035 229533.A0A0E0RS85 pp 229533.A0A1C3YIP6
65036 229533.A0A0E0RS85 pp 229533.I1RWD4
65037 229533.A0A0E0RS85 pp 229533.I1RDB1
65038 229533.A0A0E0RS85 pp 229533.I1RFX8
65039 229533.A0A0E0RS85 pp 229533.I1RK32
65040 229533.A0A0E0RS85 pp 229533.A0A0E0SAC4
65041 229533.A0A0E0RS85 pp 229533.I1RPU4
65042 229533.A0A0E0RS85 pp 229533.I1RY40
65043 229533.A0A0E0RS85 pp 229533.I1RAM6

65044 229533.A0A0E0RS85 pp 229533.A0A1C3YLR6
65045 229533.A0A0E0RS85 pp 229533.Q4IMM0
65046 229533.A0A0E0RS85 pp 229533.I1S1C8
65047 229533.A0A0E0RS85 pp 229533.I1RN31
65048 229533.A0A0E0RS85 pp 229533.I1RC95
65049 229533.A0A0E0RS85 pp 229533.I1RNC0
65050 229533.A0A0E0RS85 pp 229533.I1RRT4
65051 229533.A0A0E0RS85 pp 229533.I1RCK4
65052 229533.A0A0E0RS85 pp 229533.I1S096
65053 229533.A0A0E0RS85 pp 229533.A0A1C3YKW5
65054 229533.A0A0E0RS85 pp 229533.I1RYH9
65055 229533.A0A0E0RS85 pp 229533.Q4IER8
65056 229533.A0A0E0RS85 pp 229533.I1RB00
65057 229533.A0A0E0RS85 pp 229533.I1RJH5
65058 229533.A0A0E0RS85 pp 229533.I1RAH4
65059 229533.A0A0E0RS85 pp 229533.A0A1C3YK37
65060 229533.A0A0E0RS85 pp 229533.I1RN08
65061 229533.A0A0E0RS85 pp 229533.A0A0E0RTV8
65062 229533.A0A0E0RS85 pp 229533.I1RUQ5
65063 229533.A0A0E0RS85 pp 229533.V6R227
65064 229533.A0A0E0RS85 pp 229533.I1S224
65065 229533.A0A0E0RS85 pp 229533.I1RWT6
65066 229533.A0A0E0RS85 pp 229533.I1RC22
65067 229533.A0A0E0RS85 pp 229533.I1RKE9
65068 229533.A0A0E0RS85 pp 229533.I1RQJ0
65069 229533.A0A0E0RS85 pp 229533.I1RXE5
65070 229533.A0A0E0RS85 pp 229533.V6R3J1
65071 229533.A0A0E0RS85 pp 229533.I1RN46
65072 229533.A0A0E0RS85 pp 229533.I1S439
65073 229533.A0A0E0RS85 pp 229533.Q4I0J6
65074 229533.A0A0E0RS85 pp 229533.A0A1C3YIJ4
65075 229533.A0A0E0RS85 pp 229533.I1RRB0
65076 229533.A0A0E0RS85 pp 229533.I1RE46
65077 229533.A0A0E0RS85 pp 229533.I1S2E9
65078 229533.A0A0E0RS85 pp 229533.I1RAM7
65079 229533.A0A0E0RS85 pp 229533.A0A1C3YK01
65080 229533.A0A0E0RS85 pp 229533.Q4IBX0
65081 229533.A0A0E0RS85 pp 229533.I1RBV5
65082 229533.A0A0E0RS85 pp 229533.I1RZH4
65083 229533.A0A0E0RS85 pp 229533.A0A1C3YIE7
65084 229533.A0A0E0RS85 pp 229533.A0A0E0SNM6
65085 229533.A0A0E0RS85 pp 229533.I1RDC9
65086 229533.A0A0E0RS85 pp 229533.A0A1C3YKJ6
65087 229533.A0A0E0RS85 pp 229533.Q4IR18
65088 229533.A0A0E0RS85 pp 229533.I1RHI1
65089 229533.A0A0E0RS85 pp 229533.I1RV91
65090 229533.A0A0E0RS85 pp 229533.I1RCM6
65091 229533.A0A0E0RS85 pp 229533.I1RAL2
65092 229533.A0A0E0RS85 pp 229533.V6QV38
65093 229533.A0A0E0RS85 pp 229533.A0A1C3YJV9
65094 229533.A0A0E0RS85 pp 229533.I1RQA3
65095 229533.A0A0E0RS85 pp 229533.I1RMP0
65096 229533.A0A0E0RS85 pp 229533.I1RMM9
65097 229533.A0A0E0RS85 pp 229533.A0A1C3YI44
65098 229533.A0A0E0RS85 pp 229533.I1RFZ2
65099 229533.A0A0E0RS85 pp 229533.A0A1C3YL99
65100 229533.A0A0E0RS85 pp 229533.I1RAN0
65101 229533.A0A0E0RS85 pp 229533.I1RLX4
65102 229533.A0A0E0RS85 pp 229533.I1RPX5
65103 229533.A0A0E0RS85 pp 229533.I1RRW0
65104 229533.A0A0E0RS85 pp 229533.I1RQM0
65105 229533.A0A0E0RS85 pp 229533.A0A1C3YI28
65106 229533.A0A0E0RS85 pp 229533.I1S0M2
65107 229533.A0A0E0RS85 pp 229533.I1RBR5
65108 229533.A0A0E0RS85 pp 229533.I1RP28
65109 229533.A0A0E0RS85 pp 229533.I1S1W4
65110 229533.A0A0E0RS85 pp 229533.I1RVY0
65111 229533.A0A0E0RS85 pp 229533.I1RY44
65112 229533.A0A0E0RS85 pp 229533.A0A1C3YLP8
65113 229533.A0A0E0RS85 pp 229533.I1RQG4
65114 229533.A0A0E0RS85 pp 229533.V6RLW4
65115 229533.A0A0E0RS85 pp 229533.I1RYX9
65116 229533.A0A0E0RS85 pp 229533.A0A1C3YJZ2

65117 229533.A0A0E0RS85 pp 229533.A0A0E0SHR3
65118 229533.A0A0E0RS85 pp 229533.V6RVG3
65119 229533.A0A0E0RS85 pp 229533.I1RAN3
65120 229533.A0A0E0RS85 pp 229533.I1RC35
65121 229533.A0A0E0RS85 pp 229533.I1RZM2
65122 229533.A0A0E0RS85 pp 229533.V6RKY7
65123 229533.A0A0E0RS85 pp 229533.I1RUN8
65124 229533.A0A0E0RS85 pp 229533.I1RW16
65125 229533.A0A0E0RS85 pp 229533.I1RBD6
65126 229533.A0A0E0RS85 pp 229533.I1RFW1
65127 229533.A0A0E0RS85 pp 229533.A0A0E0SLZ3
65128 229533.A0A0E0RS85 pp 229533.I1RX38
65129 229533.A0A0E0RS85 pp 229533.I1RFR6
65130 229533.A0A0E0RS85 pp 229533.I1RL04
65131 229533.A0A0E0RS85 pp 229533.I1RLU1
65132 229533.A0A0E0RS85 pp 229533.I1RA56
65133 229533.A0A0E0RS85 pp 229533.I1RFN0
65134 229533.A0A0E0RS85 pp 229533.I1R9Y9
65135 229533.A0A0E0RS85 pp 229533.I1RA43
65136 229533.A0A0E0RS85 pp 229533.I1S1D3
65137 229533.A0A0E0RS85 pp 229533.I1RAN2
65138 229533.A0A0E0RS85 pp 229533.I1S2A8
65139 229533.A0A0E0RS85 pp 229533.V6RGI1
65140 229533.A0A0E0RS85 pp 229533.A0A1C3YI10
65141 229533.A0A0E0RS85 pp 229533.I1RBL4
65142 229533.A0A0E0RS85 pp 229533.A0A0E0SNY0
65143 229533.A0A0E0RS85 pp 229533.A0A1C3YHL0
65144 229533.A0A0E0RS85 pp 229533.A0A1C3YI81
65145 229533.A0A0E0RS85 pp 229533.I1RRP7
65146 229533.A0A0E0RS85 pp 229533.I1RXT9
65147 229533.A0A0E0RS85 pp 229533.I1RD70
65148 229533.A0A0E0RS85 pp 229533.I1RBM3
65149 229533.A0A0E0RS85 pp 229533.I1RRY3
65150 229533.A0A0E0RS85 pp 229533.A0A1C3YKG7
65151 229533.A0A0E0RS85 pp 229533.A0A0E0S9G3
65152 229533.A0A0E0RS85 pp 229533.I1RQ16
65153 229533.A0A0E0RS85 pp 229533.I1RMQ5
65154 229533.A0A0E0RS85 pp 229533.I1RPW0
65155 229533.A0A098DQK4 pp 229533.A0A1C3YLZ8
65156 229533.A0A098DQK4 pp 229533.I1RMG4
65157 229533.A0A098DQK4 pp 229533.I1RWW7
65158 229533.A0A098DQK4 pp 229533.I1S267
65159 229533.A0A098DQK4 pp 229533.A0A0E0SPV0
65160 229533.A0A098DCA3 pp 229533.A0A1C3YLZ7
65161 229533.A0A098DCA3 pp 229533.I1RRT3
65162 229533.A0A098DCA3 pp 229533.I1RDJ1
65163 229533.A0A098DCA3 pp 229533.Q4HZS8
65164 229533.A0A098DCA3 pp 229533.I1RW07
65165 229533.A0A098DCA3 pp 229533.I1RZ13
65166 229533.A0A098DCA3 pp 229533.I1RK45
65167 229533.A0A098DCA3 pp 229533.I1RI02
65168 229533.A0A098DCA3 pp 229533.I1RLC7
65169 229533.A0A098DCA3 pp 229533.A0A1C3YK73
65170 229533.A0A098DCA3 pp 229533.I1RIL3
65171 229533.A0A098DCA3 pp 229533.I1RRQ1
65172 229533.A0A098DCA3 pp 229533.I1RGL4
65173 229533.A0A098DCA3 pp 229533.I1RLM7
65174 229533.A0A098DCA3 pp 229533.I1S352
65175 229533.A0A098DCA3 pp 229533.I1RU29
65176 229533.A0A098DCA3 pp 229533.I1RKP3
65177 229533.A0A098DCA3 pp 229533.I1RIL8
65178 229533.A0A098DCA3 pp 229533.V6R4J1
65179 229533.A0A098DCA3 pp 229533.I1RCA0
65180 229533.A0A098DCA3 pp 229533.I1RBF4
65181 229533.A0A098DCA3 pp 229533.I1RDU4
65182 229533.A0A098DCA3 pp 229533.A0A0E0SPV2
65183 229533.A0A098DCA3 pp 229533.I1RI03
65184 229533.A0A098DCA3 pp 229533.A0A0E0RZM5
65185 229533.A0A098DCA3 pp 229533.I1S272
65186 229533.A0A098DCA3 pp 229533.I1RNA5
65187 229533.A0A098DCA3 pp 229533.I1RGF5
65188 229533.A0A098DCA3 pp 229533.I1RR95
65189 229533.A0A098DCA3 pp 229533.I1RLN6

65190 229533.A0A098DCA3 pp 229533.I1RCC2
65191 229533.A0A098DCA3 pp 229533.I1RBN0
65192 229533.A0A098DCA3 pp 229533.I1RBJ5
65193 229533.A0A098DCA3 pp 229533.I1RDE4
65194 229533.A0A098DCA3 pp 229533.A0A098DQ30
65195 229533.A0A098DCA3 pp 229533.I1S574
65196 229533.A0A098DCA3 pp 229533.A0A0E0RQ52
65197 229533.A0A098DCA3 pp 229533.I1S569
65198 229533.A0A098DCA3 pp 229533.I1RNA1
65199 229533.A0A098DCA3 pp 229533.I1R9D1
65200 229533.A0A098DCA3 pp 229533.I1RE72
65201 229533.A0A098DCA3 pp 229533.I1S984
65202 229533.A0A098DCA3 pp 229533.I1R9J6
65203 229533.A0A1C3YI68 pp 229533.I1RB17
65204 229533.I1RFD6 pp 229533.I1S268
65205 229533.I1S277 pp 229533.Q4I5G1
65206 229533.I1S277 pp 229533.Q4HZ95
65207 229533.I1S277 pp 229533.Q4IPZ1
65208 229533.I1S277 pp 229533.Q4IK03
65209 229533.I1S277 pp 229533.I1S6K9
65210 229533.I1S277 pp 229533.I1S7W5
65211 229533.I1S277 pp 229533.Q4IJK2
65212 229533.I1S277 pp 229533.V6RDZ4
65213 229533.I1S277 pp 229533.V6RCZ8
65214 229533.I1S277 pp 229533.Q4I665
65215 229533.I1S277 pp 229533.I1S2B8
65216 229533.I1S277 pp 229533.O74638
65217 229533.I1S277 pp 229533.I1S2C1
65218 229533.I1S277 pp 229533.Q4I099
65219 229533.I1S277 pp 229533.I1S3G4
65220 229533.I1S277 pp 229533.I1S3S3
65221 229533.I1S277 pp 229533.V6RM66
65222 229533.I1S277 pp 229533.I1S4S4
65223 229533.I1S277 pp 229533.Q4IB65
65224 229533.I1S277 pp 229533.V6R3J1
65225 229533.I1S277 pp 229533.V6RTJ8
65226 229533.A0A1C3YM53 pp 229533.I1RA36
65227 229533.A0A1C3YM53 pp 229533.I1S7M3
65228 229533.A0A1C3YM53 pp 229533.I1RB50
65229 229533.A0A1C3YM53 pp 229533.I1S7M2
65230 229533.A0A1C3YM53 pp 229533.I1RX81
65231 229533.A0A1C3YM53 pp 229533.I1RZG7
65232 229533.A0A1C3YM53 pp 229533.I1RSD4
65233 229533.A0A1C3YM53 pp 229533.I1RCI4
65234 229533.A0A1C3YM53 pp 229533.I1RDK3
65235 229533.A0A1C3YM53 pp 229533.I1RS28
65236 229533.A0A1C3YM53 pp 229533.I1RBH8
65237 229533.A0A1C3YM53 pp 229533.I1RXF2
65238 229533.A0A1C3YM53 pp 229533.I1RG90
65239 229533.A0A1C3YM53 pp 229533.I1RSA4
65240 229533.A0A1C3YM53 pp 229533.I1RBV3
65241 229533.A0A1C3YM53 pp 229533.I1S4M5
65242 229533.A0A1C3YM53 pp 229533.I1RND5
65243 229533.A0A1C3YM53 pp 229533.V6QXG1
65244 229533.A0A1C3YM53 pp 229533.I1S7X9
65245 229533.A0A1C3YM53 pp 229533.I1RCS2
65246 229533.A0A1C3YM53 pp 229533.I1RG57
65247 229533.A0A1C3YM53 pp 229533.I1RK90
65248 229533.A0A1C3YM53 pp 229533.A0A1C3YNC3
65249 229533.A0A1C3YM53 pp 229533.I1RK92
65250 229533.A0A1C3YIG4 pp 229533.I1RRQ7
65251 229533.I1RSK5 pp 229533.I1RVZ2
65252 229533.I1RSK5 pp 229533.I1S8R4
65253 229533.I1RSK5 pp 229533.I1S673
65254 229533.I1RSK5 pp 229533.V6RLM7
65255 229533.I1RSK5 pp 229533.I1RW43
65256 229533.I1RSK5 pp 229533.V6R382
65257 229533.I1RSK5 pp 229533.I1RSS7
65258 229533.I1RSK5 pp 229533.I1RZ29
65259 229533.I1RSK5 pp 229533.I1RYK0
65260 229533.I1RSK5 pp 229533.I1RXY7
65261 229533.I1RSK5 pp 229533.I1RVR2
65262 229533.I1RSK5 pp 229533.I1S274

65263	229533.I1RSK5	pp	229533.I1RZ92
65264	229533.I1RSK5	pp	229533.I1RUR8
65265	229533.I1RSK5	pp	229533.V6RM02
65266	229533.I1RSK5	pp	229533.I1S0K0
65267	229533.I1RSK5	pp	229533.I1RYU6
65268	229533.I1RSK5	pp	229533.I1RY49
65269	229533.I1RSK5	pp	229533.I1RVX9
65270	229533.I1RSK5	pp	229533.I1S3L2
65271	229533.I1RSK5	pp	229533.I1S241
65272	229533.I1RSK5	pp	229533.I1RY21
65273	229533.I1RSK5	pp	229533.I1RZM4
65274	229533.I1RSK5	pp	229533.I1RUI6
65275	229533.I1RSK5	pp	229533.I1RYX6
65276	229533.I1RSK5	pp	229533.I1RW54
65277	229533.I1RSK5	pp	229533.I1RZK8
65278	229533.I1RSK5	pp	229533.I1S8C1
65279	229533.I1RSK5	pp	229533.V6RDH3
65280	229533.I1RSK5	pp	229533.I1S0N3
65281	229533.I1RSK5	pp	229533.V6RK96
65282	229533.I1RSK5	pp	229533.I1RXS0
65283	229533.I1RSK5	pp	229533.I1RX59
65284	229533.I1RSK5	pp	229533.I1RYL5
65285	229533.A0A098DSW7	pp	229533.I1RMG2
65286	229533.A0A098DSW7	pp	229533.A0A0E0SNZ9
65287	229533.A0A098DSW7	pp	229533.I1RNP1
65288	229533.A0A098DSW7	pp	229533.I1RB00
65289	229533.A0A098DSW7	pp	229533.I1RQF9
65290	229533.A0A098DSW7	pp	229533.A0A098DWY7
65291	229533.A0A098DSW7	pp	229533.A0A1C3YI93
65292	229533.A0A098DSW7	pp	229533.I1RQ16
65293	229533.A0A098DSW7	pp	229533.I1SB04
65294	229533.A0A098DSW7	pp	229533.I1RFR6
65295	229533.A0A098DSW7	pp	229533.A0A1C3YM02
65296	229533.I1RXB2	pp	229533.I1S1W3
65297	229533.I1RXB2	pp	229533.I1S0J8
65298	229533.I1RXB2	pp	229533.I1RXB6
65299	229533.I1RXB2	pp	229533.V6RUT5
65300	229533.I1RXB2	pp	229533.I1S887
65301	229533.I1RXB2	pp	229533.I1RXR1
65302	229533.I1RXB2	pp	229533.I1RXU9
65303	229533.I1RXB2	pp	229533.V6R382
65304	229533.I1REC2	pp	229533.I1RN04
65305	229533.I1REC2	pp	229533.I1RW91
65306	229533.I1REC2	pp	229533.I1S0C6
65307	229533.I1REC2	pp	229533.I1SAF7
65308	229533.I1REC2	pp	229533.I1RQ64
65309	229533.I1REC2	pp	229533.I1S182
65310	229533.I1REC2	pp	229533.I1RW80
65311	229533.I1REC2	pp	229533.I1RPS7
65312	229533.I1REC2	pp	229533.I1RQ42
65313	229533.I1REC2	pp	229533.I1RMD2
65314	229533.I1REC2	pp	229533.I1RPW8
65315	229533.I1REC2	pp	229533.I1RR61
65316	229533.I1RSP1	pp	229533.I1RZE1
65317	229533.I1RSP1	pp	229533.I1RVA7
65318	229533.I1RSP1	pp	229533.I1RYJ8
65319	229533.I1RSP1	pp	229533.I1S2A8
65320	229533.I1RSP1	pp	229533.I1S8Z2
65321	229533.I1RSP1	pp	229533.I1RW71
65322	229533.I1S0G3	pp	229533.I1SB04
65323	229533.I1S4B6	pp	229533.V6RM02
65324	229533.I1S4B6	pp	229533.V6RK96
65325	229533.I1RRB0	pp	229533.I1S1D3
65326	229533.I1RRB0	pp	229533.I1RW16
65327	229533.I1RRB0	pp	229533.I1RWM7
65328	229533.I1RRB0	pp	229533.I1RTG5
65329	229533.I1RRB0	pp	229533.I1S1V5
65330	229533.I1RRB0	pp	229533.Q4IMD1
65331	229533.I1RRB0	pp	229533.Q4ICM0
65332	229533.I1RRB0	pp	229533.I1RX54
65333	229533.I1RRB0	pp	229533.I1S1C8
65334	229533.I1RRB0	pp	229533.Q4I9B7
65335	229533.I1RRB0	pp	229533.Q4IPI4

65336	229533.I1RRB0	pp	229533.Q5I2J3
65337	229533.I1RRB0	pp	229533.I1RRI3
65338	229533.I1RRB0	pp	229533.I1RRT4
65339	229533.I1RRB0	pp	229533.I1S096
65340	229533.I1RRB0	pp	229533.I1S0Z3
65341	229533.I1RRB0	pp	229533.Q4IBX0
65342	229533.I1RRB0	pp	229533.I1S270
65343	229533.I1RRB0	pp	229533.I1RZF7
65344	229533.I1RRB0	pp	229533.I1RX84
65345	229533.I1RRB0	pp	229533.Q4HZS8
65346	229533.I1RRB0	pp	229533.I1RT41
65347	229533.I1RRB0	pp	229533.I1RTQ9
65348	229533.I1RA72	pp	229533.I1RZ60
65349	229533.I1RA72	pp	229533.I1RE19
65350	229533.I1RA72	pp	229533.I1RQG4
65351	229533.I1RA72	pp	229533.I1RR95
65352	229533.I1RA72	pp	229533.I1RSH0
65353	229533.I1RA72	pp	229533.I1S074
65354	229533.I1RA72	pp	229533.I1S8P5
65355	229533.I1RA72	pp	229533.I1RFW1
65356	229533.I1RA72	pp	229533.I1RKR9
65357	229533.I1RA72	pp	229533.I1RJZ7
65358	229533.I1RA72	pp	229533.Q4IPI4
65359	229533.I1RA72	pp	229533.I1RY50
65360	229533.I1RA72	pp	229533.I1RMP2
65361	229533.I1RA72	pp	229533.I1RRT8
65362	229533.I1RA72	pp	229533.I1RFR6
65363	229533.I1RA72	pp	229533.I1S7X9
65364	229533.I1RA72	pp	229533.I1RKR7
65365	229533.I1RA72	pp	229533.I1RCD4
65366	229533.I1RA72	pp	229533.I1RKI1
65367	229533.I1RA72	pp	229533.I1S4Z7
65368	229533.I1RA72	pp	229533.I1S2G9
65369	229533.I1RA72	pp	229533.I1RRE5
65370	229533.I1RA72	pp	229533.I1RMF2
65371	229533.I1RA72	pp	229533.I1RBD6
65372	229533.I1RA72	pp	229533.V6QUV7
65373	229533.I1RA72	pp	229533.I1RG11
65374	229533.I1RA72	pp	229533.I1RZV1
65375	229533.I1RA72	pp	229533.I1RRF0
65376	229533.I1RA72	pp	229533.I1RWH4
65377	229533.I1RA72	pp	229533.I1RPK0
65378	229533.I1RA72	pp	229533.I1RAI0
65379	229533.I1RA72	pp	229533.I1RD70
65380	229533.I1RA72	pp	229533.I1RK44
65381	229533.I1RA72	pp	229533.I1S238
65382	229533.I1RA72	pp	229533.I1RST4
65383	229533.I1RA72	pp	229533.I1RMQ5
65384	229533.I1RA72	pp	229533.I1RN84
65385	229533.I1RA72	pp	229533.I1RTA3
65386	229533.I1RA72	pp	229533.I1RSL8
65387	229533.I1RA72	pp	229533.I1RTA0
65388	229533.I1RA72	pp	229533.I1RZU6
65389	229533.I1RA72	pp	229533.V6QX21
65390	229533.I1RA72	pp	229533.Q4I2W2
65391	229533.I1RA72	pp	229533.I1RYW3
65392	229533.I1RA72	pp	229533.I1RRT4
65393	229533.I1RA72	pp	229533.Q5I2J3
65394	229533.I1RA72	pp	229533.I1RWX5
65395	229533.I1RA72	pp	229533.I1RZ12
65396	229533.I1RA72	pp	229533.I1RTG5
65397	229533.I1RA72	pp	229533.I1RW71
65398	229533.I1RA72	pp	229533.V6RTJ8
65399	229533.I1RA72	pp	229533.V6QUU6
65400	229533.I1RA72	pp	229533.I1RZB7
65401	229533.I1RA72	pp	229533.I1S270
65402	229533.I1RA72	pp	229533.I1RAU9
65403	229533.I1RA72	pp	229533.I1RBS4
65404	229533.I1RA72	pp	229533.I1S096
65405	229533.I1RA72	pp	229533.I1RMT4
65406	229533.I1RA72	pp	229533.I1RPH1
65407	229533.I1RA72	pp	229533.I1RZ02
65408	229533.I1RA72	pp	229533.I1RI75

65409 229533.I1RA72 pp 229533.Q4HZS8
65410 229533.I1RA72 pp 229533.I1RTQ9
65411 229533.I1RA72 pp 229533.I1S5R5
65412 229533.I1RA72 pp 229533.I1RN81
65413 229533.I1RA72 pp 229533.I1RB00
65414 229533.I1RA72 pp 229533.I1S1C8
65415 229533.I1RA72 pp 229533.I1RT41
65416 229533.I1RA72 pp 229533.Q4HTT2
65417 229533.I1RA72 pp 229533.I1RA98
65418 229533.I1RA72 pp 229533.I1S0Z3
65419 229533.I1RA72 pp 229533.I1RZK4
65420 229533.I1RA72 pp 229533.I1RRF9
65421 229533.I1RA72 pp 229533.I1REG5
65422 229533.I1RA72 pp 229533.I1RUK7
65423 229533.I1RA72 pp 229533.V6RR76
65424 229533.I1RA72 pp 229533.V6RPN0
65425 229533.I1RA72 pp 229533.I1RZH4
65426 229533.I1RA72 pp 229533.I1RPL2
65427 229533.I1RA72 pp 229533.I1RWD5
65428 229533.I1RA72 pp 229533.Q4IER8
65429 229533.I1RA72 pp 229533.I1RYL5
65430 229533.I1RA72 pp 229533.V6REE9
65431 229533.I1RA72 pp 229533.I1RT65
65432 229533.I1RA72 pp 229533.I1RJK7
65433 229533.I1RA72 pp 229533.I1RRB0
65434 229533.I1RA72 pp 229533.I1RBE7
65435 229533.I1RA72 pp 229533.I1RBC4
65436 229533.I1RUR8 pp 229533.I1RVR2
65437 229533.I1RUR8 pp 229533.V6R3J1
65438 229533.I1RUR8 pp 229533.I1RY35
65439 229533.I1RUR8 pp 229533.V6RUY9
65440 229533.I1RUR8 pp 229533.I1RZV5
65441 229533.I1RUR8 pp 229533.Q4HVQ9
65442 229533.I1RUR8 pp 229533.I1S3T7
65443 229533.I1RUR8 pp 229533.I1S968
65444 229533.I1RUR8 pp 229533.I1RWF5
65445 229533.I1RUR8 pp 229533.I1RYW9
65446 229533.I1RUR8 pp 229533.I1RYK0
65447 229533.I1RUR8 pp 229533.I1S2C4
65448 229533.I1RUR8 pp 229533.I1RZ38
65449 229533.I1RUR8 pp 229533.I1S299
65450 229533.I1RUR8 pp 229533.I1RY62
65451 229533.I1RUR8 pp 229533.I1RYA0
65452 229533.I1RUR8 pp 229533.I1S8R2
65453 229533.I1RUR8 pp 229533.I1S256
65454 229533.I1RUR8 pp 229533.V6RRM0
65455 229533.I1RUR8 pp 229533.V6RM02
65456 229533.I1RUR8 pp 229533.I1S0V7
65457 229533.I1RUR8 pp 229533.I1S210
65458 229533.I1RUR8 pp 229533.I1RWL3
65459 229533.I1RUR8 pp 229533.I1S3L2
65460 229533.I1RUR8 pp 229533.I1RW54
65461 229533.I1RUR8 pp 229533.I1S8E0
65462 229533.I1RUR8 pp 229533.I1RY21
65463 229533.I1RUR8 pp 229533.I1RYC8
65464 229533.I1RUR8 pp 229533.I1RXY7
65465 229533.I1RUR8 pp 229533.I1S8R4
65466 229533.I1RUR8 pp 229533.I1S3T6
65467 229533.I1RUR8 pp 229533.I1S0K0
65468 229533.I1RUR8 pp 229533.V6R382
65469 229533.I1RUR8 pp 229533.I1S8C1
65470 229533.I1RUR8 pp 229533.I1RYQ4
65471 229533.I1RUR8 pp 229533.I1RYA2
65472 229533.I1RUR8 pp 229533.I1S0K2
65473 229533.I1RUR8 pp 229533.I1RYP5
65474 229533.I1RUR8 pp 229533.I1RY12
65475 229533.I1RUR8 pp 229533.I1S223
65476 229533.I1RUR8 pp 229533.I1RUY4
65477 229533.I1RUR8 pp 229533.I1RXS0
65478 229533.I1RUR8 pp 229533.I1RXZ4
65479 229533.I1RUR8 pp 229533.I1S8F8
65480 229533.I1RUR8 pp 229533.I1RZM4
65481 229533.I1RUR8 pp 229533.I1RXE6

65482	229533.I1RUR8	pp	229533.I1S2F7
65483	229533.I1RUR8	pp	229533.I1RY81
65484	229533.I1RUR8	pp	229533.I1S0V6
65485	229533.I1RUR8	pp	229533.V6RK96
65486	229533.I1RUR8	pp	229533.I1S267
65487	229533.I1RW16	pp	229533.V6RKY7
65488	229533.I1RW16	pp	229533.I1S1W4
65489	229533.I1RW16	pp	229533.V6RVG3
65490	229533.I1RW16	pp	229533.I1RY44
65491	229533.I1RW16	pp	229533.I1S4P6
65492	229533.I1RW16	pp	229533.Q4IB89
65493	229533.I1RW16	pp	229533.I1S1D3
65494	229533.I1RW16	pp	229533.I1RWQ0
65495	229533.I1RW16	pp	229533.V6RGI1
65496	229533.I1RW16	pp	229533.I1S7X9
65497	229533.I1RW16	pp	229533.I1RZP8
65498	229533.I1RW16	pp	229533.Q4IMD1
65499	229533.I1RW16	pp	229533.I1RZ25
65500	229533.I1RW16	pp	229533.I1RYX9
65501	229533.I1RW16	pp	229533.I1RXU0
65502	229533.I1RW16	pp	229533.I1RYR2
65503	229533.I1RW16	pp	229533.V6R3J1
65504	229533.I1RW16	pp	229533.I1RZE4
65505	229533.I1RW16	pp	229533.V6RLW4
65506	229533.I1RW16	pp	229533.I1S382
65507	229533.I1RW16	pp	229533.I1RX64
65508	229533.I1RW16	pp	229533.Q4I7X1
65509	229533.I1RW16	pp	229533.I1S951
65510	229533.I1RW16	pp	229533.I1S2E9
65511	229533.I1RW16	pp	229533.I1S0P4
65512	229533.I1RW16	pp	229533.I1RX38
65513	229533.I1RW16	pp	229533.I1S604
65514	229533.I1RW16	pp	229533.I1S2F2
65515	229533.I1RW16	pp	229533.P0C431
65516	229533.I1RW16	pp	229533.Q4I877
65517	229533.I1RW16	pp	229533.I1S1V5
65518	229533.I1RW16	pp	229533.Q4I0J6
65519	229533.I1RW16	pp	229533.I1RWJ1
65520	229533.I1RW16	pp	229533.I1RY73
65521	229533.I1RW16	pp	229533.I1S2A8
65522	229533.I1RW16	pp	229533.I1RZH4
65523	229533.I1RW16	pp	229533.I1S050
65524	229533.I1RW16	pp	229533.I1RWG6
65525	229533.I1RW16	pp	229533.I1S439
65526	229533.I1RW16	pp	229533.I1S1C8
65527	229533.I1RW16	pp	229533.I1RWB3
65528	229533.I1RW16	pp	229533.Q4IBX0
65529	229533.I1RW16	pp	229533.V6REE9
65530	229533.I1RW16	pp	229533.I1RZV9
65531	229533.I1RW16	pp	229533.V6QV09
65532	229533.I1RW16	pp	229533.I1S224
65533	229533.I1RW16	pp	229533.I1S0S6
65534	229533.I1RW16	pp	229533.I1RWL6
65535	229533.I1RW16	pp	229533.I1RYH9
65536	229533.I1RW16	pp	229533.I1RYZ9
65537	229533.I1RW16	pp	229533.I1S219
65538	229533.I1RW16	pp	229533.Q4ICM0
65539	229533.I1RW16	pp	229533.I1RZ94
65540	229533.I1S1V5	pp	229533.V6RGI1
65541	229533.I1S1V5	pp	229533.I1S1W4
65542	229533.I1S1V5	pp	229533.I1S2A8
65543	229533.I1S1V5	pp	229533.I1S2E9
65544	229533.I1S1V5	pp	229533.V6RKY7
65545	229533.I1S1V5	pp	229533.V6RVG3
65546	229533.I1S1V5	pp	229533.Q4IMM0
65547	229533.I1S1V5	pp	229533.V6R3J1
65548	229533.I1S1V5	pp	229533.V6QV38
65549	229533.I1S1V5	pp	229533.I1S439
65550	229533.I1S1V5	pp	229533.V6RLW4
65551	229533.I1S1V5	pp	229533.Q4IR18
65552	229533.I1S1V5	pp	229533.Q4IER8
65553	229533.I1S1V5	pp	229533.Q4I0J6
65554	229533.I1S1V5	pp	229533.I1S224

65555 229533.I1S1V5 pp 229533.Q4ICM0
65556 229533.I1S1V5 pp 229533.V6R227
65557 229533.I1S1V5 pp 229533.Q4IBX0
65558 229533.I1RWK0 pp 229533.Q4IEA7
65559 229533.I1RWK0 pp 229533.I1S286
65560 229533.I1RWK0 pp 229533.I1RX77
65561 229533.I1S1C8 pp 229533.Q5I2J3
65562 229533.I1S1C8 pp 229533.Q4I9B7
65563 229533.I1S1C8 pp 229533.Q4HZS8
65564 229533.I1S1C8 pp 229533.Q4IBX0
65565 229533.I1S1C8 pp 229533.I1S2F2
65566 229533.I1S1C8 pp 229533.Q4ICM0
65567 229533.I1S1C8 pp 229533.I1S1V5
65568 229533.I1S1C8 pp 229533.Q4I061
65569 229533.I1S1C8 pp 229533.Q4I0J6
65570 229533.I1S1C8 pp 229533.I1S1D3
65571 229533.I1RQJ0 pp 229533.I1S1C8
65572 229533.I1RQJ0 pp 229533.I1RVV7
65573 229533.I1RQJ0 pp 229533.I1RTD3
65574 229533.I1RQJ0 pp 229533.I1RXT9
65575 229533.I1RQJ0 pp 229533.I1S7R5
65576 229533.I1RQJ0 pp 229533.I1RSU1
65577 229533.I1RQJ0 pp 229533.I1RWE8
65578 229533.I1RQJ0 pp 229533.I1RYX9
65579 229533.I1RQJ0 pp 229533.Q4I0J6
65580 229533.I1RQJ0 pp 229533.I1RUE2
65581 229533.I1RQJ0 pp 229533.I1S1V5
65582 229533.I1RQJ0 pp 229533.I1S0S8
65583 229533.I1RQJ0 pp 229533.I1S111
65584 229533.I1RQJ0 pp 229533.V6RJ21
65585 229533.I1RQJ0 pp 229533.I1RSJ4
65586 229533.I1RQJ0 pp 229533.I1RUG1
65587 229533.I1RQJ0 pp 229533.V6R223
65588 229533.I1RQJ0 pp 229533.I1RRB0
65589 229533.I1RQJ0 pp 229533.I1RZ02
65590 229533.I1RQJ0 pp 229533.Q4IBX0
65591 229533.I1RQJ0 pp 229533.Q4INI2
65592 229533.I1RQJ0 pp 229533.V6R3J1
65593 229533.I1RQJ0 pp 229533.V6RLW4
65594 229533.A0A1C3YL22 pp 229533.I1RX81
65595 229533.A0A1C3YL22 pp 229533.I1RCI4
65596 229533.A0A1C3YL22 pp 229533.I1RZG7
65597 229533.A0A1C3YL22 pp 229533.I1RG57
65598 229533.A0A1C3YL22 pp 229533.I1RK90
65599 229533.A0A1C3YL22 pp 229533.I1S4M5
65600 229533.A0A1C3YL22 pp 229533.I1RCS2
65601 229533.A0A1C3YL22 pp 229533.I1RB50
65602 229533.A0A1C3YL22 pp 229533.I1RBH8
65603 229533.A0A1C3YL22 pp 229533.I1S7M2
65604 229533.A0A1C3YL22 pp 229533.I1RSA4
65605 229533.A0A1C3YL22 pp 229533.I1S7X9
65606 229533.A0A1C3YL22 pp 229533.I1RA36
65607 229533.A0A1C3YL22 pp 229533.I1RND5
65608 229533.A0A1C3YL22 pp 229533.I1S7M3
65609 229533.A0A1C3YL22 pp 229533.V6QXG1
65610 229533.A0A1C3YL22 pp 229533.I1RBV3
65611 229533.A0A1C3YL22 pp 229533.I1RXF2
65612 229533.A0A1C3YL22 pp 229533.I1RS28
65613 229533.A0A1C3YL22 pp 229533.A0A1C3YNC3
65614 229533.A0A1C3YL22 pp 229533.I1RSD4
65615 229533.A0A1C3YL22 pp 229533.I1RG90
65616 229533.A0A1C3YL22 pp 229533.I1RK92
65617 229533.I1RFR6 pp 229533.V6R3J1
65618 229533.I1RFR6 pp 229533.I1RR95
65619 229533.I1RFR6 pp 229533.Q5I2J3
65620 229533.I1RFR6 pp 229533.I1RMP0
65621 229533.I1RFR6 pp 229533.I1RSC7
65622 229533.I1RFR6 pp 229533.I1RFZ2
65623 229533.I1RFR6 pp 229533.I1RYX9
65624 229533.I1RFR6 pp 229533.I1S1D3
65625 229533.I1RFR6 pp 229533.I1RSJ4
65626 229533.I1RFR6 pp 229533.Q4I9B7
65627 229533.I1RFR6 pp 229533.I1RLU1

65628 229533.I1RFR6 pp 229533.I1RSI4
65629 229533.I1RFR6 pp 229533.I1S0G3
65630 229533.I1RFR6 pp 229533.I1S1V5
65631 229533.I1RFR6 pp 229533.V6R382
65632 229533.I1RFR6 pp 229533.I1RW16
65633 229533.I1RFR6 pp 229533.I1S2F2
65634 229533.I1RFR6 pp 229533.I1RMB7
65635 229533.I1RFR6 pp 229533.V6RLW4
65636 229533.I1RFR6 pp 229533.I1RFX8
65637 229533.I1RFR6 pp 229533.I1RX64
65638 229533.I1RFR6 pp 229533.I1RRP7
65639 229533.I1RFR6 pp 229533.I1RN31
65640 229533.I1RFR6 pp 229533.I1RUG1
65641 229533.I1RFR6 pp 229533.I1RI75
65642 229533.I1RFR6 pp 229533.I1RNC0
65643 229533.I1RFR6 pp 229533.I1RQ16
65644 229533.I1RFR6 pp 229533.I1RRB0
65645 229533.I1RFR6 pp 229533.Q4IMM0
65646 229533.I1RFR6 pp 229533.I1RPW0
65647 229533.I1RFR6 pp 229533.Q4HZS8
65648 229533.I1RFR6 pp 229533.I1S1C8
65649 229533.I1RFR6 pp 229533.I1RN46
65650 229533.I1RFR6 pp 229533.Q4IBX0
65651 229533.I1RFR6 pp 229533.I1RJH5
65652 229533.I1RFR6 pp 229533.I1RRT4
65653 229533.I1RFR6 pp 229533.Q4I0J6
65654 229533.I1RFR6 pp 229533.I1RQJ0
65655 229533.I1RFR6 pp 229533.I1RG77
65656 229533.A0A1C3YLP4 pp 229533.I1RQ75
65657 229533.A0A1C3YLP4 pp 229533.I1RYF2
65658 229533.A0A1C3YLP4 pp 229533.I1RXF0
65659 229533.A0A1C3YLP4 pp 229533.Q4HZE0
65660 229533.A0A1C3YLP4 pp 229533.I1RJL9
65661 229533.A0A1C3YLP4 pp 229533.I1S272
65662 229533.A0A1C3YLP4 pp 229533.Q4HY20
65663 229533.A0A1C3YLP4 pp 229533.I1RQ38
65664 229533.A0A1C3YLP4 pp 229533.I1RZM0
65665 229533.A0A1C3YLP4 pp 229533.I1RD39
65666 229533.A0A1C3YLP4 pp 229533.V6QUV7
65667 229533.A0A1C3YLP4 pp 229533.I1RX25
65668 229533.A0A1C3YLP4 pp 229533.I1RSQ2
65669 229533.A0A1C3YLP4 pp 229533.I1RYL5
65670 229533.A0A1C3YLP4 pp 229533.I1RZV2
65671 229533.A0A1C3YLP4 pp 229533.I1RYB8
65672 229533.A0A1C3YLP4 pp 229533.I1S4Z7
65673 229533.A0A1C3YLP4 pp 229533.I1SAM9
65674 229533.A0A1C3YLP4 pp 229533.Q4IJT0
65675 229533.A0A1C3YLP4 pp 229533.V6RPQ8
65676 229533.A0A1C3YLP4 pp 229533.I1RDF6
65677 229533.A0A1C3YLP4 pp 229533.I1RMM6
65678 229533.A0A1C3YLP4 pp 229533.I1RSM8
65679 229533.A0A1C3YLP4 pp 229533.I1RRR5
65680 229533.A0A1C3YLP4 pp 229533.I1RAH0
65681 229533.A0A1C3YLP4 pp 229533.I1R9N7
65682 229533.A0A1C3YLP4 pp 229533.I1RNM4
65683 229533.A0A1C3YLP4 pp 229533.A0A1C3YM63
65684 229533.I1RCM6 pp 229533.I1RMP0
65685 229533.I1RCM6 pp 229533.I1RRT4
65686 229533.I1RCM6 pp 229533.I1RFZ2
65687 229533.I1RCM6 pp 229533.I1RY40
65688 229533.I1RCM6 pp 229533.I1RYX9
65689 229533.I1RCM6 pp 229533.I1RCY0
65690 229533.I1RCM6 pp 229533.Q4IMM0
65691 229533.I1RCM6 pp 229533.I1RSI4
65692 229533.I1RCM6 pp 229533.I1S382
65693 229533.I1RCM6 pp 229533.I1S2F3
65694 229533.I1RCM6 pp 229533.I1RNC0
65695 229533.I1RCM6 pp 229533.I1RM36
65696 229533.I1RCM6 pp 229533.I1RFU3
65697 229533.I1RCM6 pp 229533.I1RUG1
65698 229533.I1RCM6 pp 229533.I1RR95
65699 229533.I1RCM6 pp 229533.I1RQM0
65700 229533.I1RCM6 pp 229533.I1RFW1

65701	229533.I1RCM6	pp	229533.I1RVA7
65702	229533.I1RCM6	pp	229533.Q4ICM0
65703	229533.I1RCM6	pp	229533.I1RQ80
65704	229533.I1RCM6	pp	229533.I1RDG8
65705	229533.I1RCM6	pp	229533.Q4HZS8
65706	229533.I1RCM6	pp	229533.I1RN31
65707	229533.I1RCM6	pp	229533.V6R3J1
65708	229533.I1RCM6	pp	229533.V6RLW4
65709	229533.I1RCM6	pp	229533.I1RI75
65710	229533.I1RCM6	pp	229533.I1RP94
65711	229533.I1RCM6	pp	229533.I1RTA9
65712	229533.I1RCM6	pp	229533.I1RD18
65713	229533.I1RCM6	pp	229533.I1S2E9
65714	229533.I1RCM6	pp	229533.I1RMB7
65715	229533.I1RCM6	pp	229533.I1S2F2
65716	229533.I1RCM6	pp	229533.I1S224
65717	229533.I1RCM6	pp	229533.I1RLX6
65718	229533.I1RCM6	pp	229533.I1RDX8
65719	229533.I1RCM6	pp	229533.I1RPX8
65720	229533.I1RCM6	pp	229533.I1S0Z2
65721	229533.I1RCM6	pp	229533.I1S2A8
65722	229533.I1RCM6	pp	229533.I1RX38
65723	229533.I1RCM6	pp	229533.I1S951
65724	229533.I1RCM6	pp	229533.I1RZ86
65725	229533.I1RCM6	pp	229533.I1S283
65726	229533.I1RCM6	pp	229533.V6R382
65727	229533.I1RCM6	pp	229533.I1RLE7
65728	229533.I1RCM6	pp	229533.I1REG5
65729	229533.I1RCM6	pp	229533.I1RZH4
65730	229533.I1RCM6	pp	229533.I1RCW8
65731	229533.I1RCM6	pp	229533.I1S1D3
65732	229533.I1RCM6	pp	229533.I1S1V5
65733	229533.I1RCM6	pp	229533.I1S0P4
65734	229533.I1RCM6	pp	229533.I1RWT6
65735	229533.I1RCM6	pp	229533.I1RFR6
65736	229533.I1RCM6	pp	229533.I1RQ49
65737	229533.I1RCM6	pp	229533.I1RY44
65738	229533.I1RCM6	pp	229533.I1RSJ4
65739	229533.I1RCM6	pp	229533.I1RK32
65740	229533.I1RCM6	pp	229533.I1RFN0
65741	229533.I1RCM6	pp	229533.Q4I0J6
65742	229533.I1RCM6	pp	229533.I1RPU4
65743	229533.I1RCM6	pp	229533.I1RJI8
65744	229533.I1RCM6	pp	229533.I1RJH5
65745	229533.I1RCM6	pp	229533.V6QX58
65746	229533.I1RCM6	pp	229533.I1S096
65747	229533.I1RCM6	pp	229533.I1RSC7
65748	229533.I1RCM6	pp	229533.I1RXT9
65749	229533.I1RCM6	pp	229533.I1RE46
65750	229533.I1RCM6	pp	229533.I1RRW0
65751	229533.I1RCM6	pp	229533.I1S1C8
65752	229533.I1RCM6	pp	229533.I1RJK7
65753	229533.I1RCM6	pp	229533.I1RMX3
65754	229533.I1RCM6	pp	229533.Q4I877
65755	229533.I1RCM6	pp	229533.I1RW16
65756	229533.I1RCM6	pp	229533.I1RQJ0
65757	229533.I1RCM6	pp	229533.Q4I7X1
65758	229533.I1RCM6	pp	229533.I1RLX4
65759	229533.I1RCM6	pp	229533.I1RN46
65760	229533.I1RCM6	pp	229533.I1RQS4
65761	229533.I1RCM6	pp	229533.I1RQ16
65762	229533.I1RCM6	pp	229533.I1RG77
65763	229533.I1RCM6	pp	229533.I1RUG2
65764	229533.I1RCM6	pp	229533.I1RFJ2
65765	229533.I1RCM6	pp	229533.I1RJI7
65766	229533.I1RCM6	pp	229533.Q4IBX0
65767	229533.I1RCM6	pp	229533.I1RI34
65768	229533.I1RCM6	pp	229533.I1RQA3
65769	229533.I1RCM6	pp	229533.I1RN65
65770	229533.I1RCM6	pp	229533.I1RRB0
65771	229533.I1RCM6	pp	229533.I1RPW0
65772	229533.I1RCM6	pp	229533.I1RWB3
65773	229533.I1RCM6	pp	229533.Q4IER8

65774 229533.I1RCM6 pp 229533.I1RYP9
65775 229533.I1RCM6 pp 229533.I1RD70
65776 229533.I1RCM6 pp 229533.I1RRP7
65777 229533.I1RCM6 pp 229533.I1RHI1
65778 229533.I1RCM6 pp 229533.I1RJE5
65779 229533.I1RCM6 pp 229533.I1RX64
65780 229533.I1RCM6 pp 229533.I1RZV9
65781 229533.I1RCM6 pp 229533.I1RU02
65782 229533.I1RCM6 pp 229533.I1RD19
65783 229533.A0A098DNR8 pp 229533.I1RNG4
65784 229533.A0A098DNR8 pp 229533.I1RCR9
65785 229533.A0A098DNR8 pp 229533.A0A1C3YLB6
65786 229533.A0A098DNR8 pp 229533.A0A1C3YLP4
65787 229533.I1RN03 pp 229533.Q4IBK5
65788 229533.I1RN03 pp 229533.Q4IPH4
65789 229533.I1RN03 pp 229533.I1RPY4
65790 229533.I1RN03 pp 229533.I1S2Y5
65791 229533.I1RN03 pp 229533.I1RS28
65792 229533.I1RN03 pp 229533.I1RPY5
65793 229533.I1RN03 pp 229533.Q4IPB3
65794 229533.I1RN03 pp 229533.Q4I5R9
65795 229533.I1RN03 pp 229533.Q4HXF6
65796 229533.I1RN03 pp 229533.I1RX81
65797 229533.I1RN03 pp 229533.Q4IE79
65798 229533.I1RN03 pp 229533.I1RS66
65799 229533.I1RN03 pp 229533.I1RQC5
65800 229533.I1RN03 pp 229533.Q4I1Y1
65801 229533.I1RN03 pp 229533.I1RN59
65802 229533.I1RN03 pp 229533.Q4IPB8
65803 229533.I1RN03 pp 229533.I1RRW3
65804 229533.I1RSJ4 pp 229533.I1RYR2
65805 229533.I1RSJ4 pp 229533.Q4IBX0
65806 229533.I1RSJ4 pp 229533.I1RUG1
65807 229533.I1RSJ4 pp 229533.Q4I0J6
65808 229533.I1RSJ4 pp 229533.Q4IE79
65809 229533.I1RSJ4 pp 229533.I1RWM6
65810 229533.I1RSJ4 pp 229533.I1S1D3
65811 229533.I1RSJ4 pp 229533.I1RYX9
65812 229533.I1RSJ4 pp 229533.Q4IQM6
65813 229533.I1RSJ4 pp 229533.V6RLW4
65814 229533.I1RSJ4 pp 229533.I1S283
65815 229533.I1RSJ4 pp 229533.I1S2F2
65816 229533.I1RSJ4 pp 229533.I1S236
65817 229533.I1RSJ4 pp 229533.V6R3J1
65818 229533.A0A1C3YJQ6 pp 229533.I1RYQ1
65819 229533.A0A1C3YJQ6 pp 229533.I1S8W6
65820 229533.A0A1C3YJQ6 pp 229533.I1RKL2
65821 229533.A0A1C3YJQ6 pp 229533.I1RSZ3
65822 229533.A0A1C3YJQ6 pp 229533.I1REF2
65823 229533.A0A1C3YJQ6 pp 229533.I1RJQ3
65824 229533.A0A1C3YJQ6 pp 229533.I1RY29
65825 229533.A0A1C3YJQ6 pp 229533.Q4HVU7
65826 229533.A0A1C3YJQ6 pp 229533.Q4HZQ1
65827 229533.A0A1C3YJQ6 pp 229533.I1RY80
65828 229533.A0A1C3YJQ6 pp 229533.A0A1C3YK80
65829 229533.A0A1C3YJQ6 pp 229533.A0A1C3YMH3
65830 229533.A0A1C3YJQ6 pp 229533.I1RWI8
65831 229533.A0A1C3YJQ6 pp 229533.A0A1C3YN25
65832 229533.A0A1C3YJQ6 pp 229533.I1S4Q7
65833 229533.A0A1C3YJQ6 pp 229533.I1RKA7
65834 229533.A0A1C3YJQ6 pp 229533.I1RQB7
65835 229533.A0A1C3YJQ6 pp 229533.A0A1C3YNA1
65836 229533.A0A1C3YJQ6 pp 229533.I1RRH5
65837 229533.A0A1C3YJQ6 pp 229533.I1S4T3
65838 229533.A0A1C3YJQ6 pp 229533.I1SA55
65839 229533.A0A1C3YJQ6 pp 229533.I1RBZ1
65840 229533.A0A1C3YJQ6 pp 229533.I1RPN1
65841 229533.A0A1C3YJQ6 pp 229533.I1RZ94
65842 229533.A0A1C3YJQ6 pp 229533.A0A1C3YMJ3
65843 229533.A0A1C3YJQ6 pp 229533.A0A1C3YN55
65844 229533.A0A1C3YJQ6 pp 229533.I1RB17
65845 229533.A0A1C3YJQ6 pp 229533.V6RG79
65846 229533.A0A1C3YJQ6 pp 229533.I1RN57

```
229533.A0A1C3YJQ6   pp  229533.I1RMQ7
229533.A0A1C3YJQ6   pp  229533.I1R9X4
229533.A0A1C3YJQ6   pp  229533.I1RFY0
229533.A0A1C3YJQ6   pp  229533.A0A1C3YJW8
229533.A0A1C3YJQ6   pp  229533.I1RW83
229533.A0A1C3YJQ6   pp  229533.V6RN69
229533.A0A1C3YJQ6   pp  229533.A0A1C3YK00
229533.A0A1C3YJQ6   pp  229533.I1S5Z2
229533.A0A1C3YJQ6   pp  229533.I1RDX0
229533.A0A1C3YJQ6   pp  229533.I1S0C2
229533.A0A1C3YJQ6   pp  229533.I1RQP5
229533.A0A1C3YJQ6   pp  229533.I1RAE8
229533.A0A1C3YJQ6   pp  229533.I1RSP7
229533.A0A1C3YJQ6   pp  229533.A0A1C3YN82
229533.A0A1C3YJQ6   pp  229533.I1RSI0
229533.A0A1C3YJQ6   pp  229533.I1RUL3
229533.A0A1C3YJQ6   pp  229533.I1RUG4
229533.A0A1C3YJQ6   pp  229533.I1RR61
229533.A0A1C3YJQ6   pp  229533.I1RDH0
229533.A0A1C3YJQ6   pp  229533.I1RT09
229533.A0A1C3YJQ6   pp  229533.I1RPP5
229533.A0A1C3YJQ6   pp  229533.I1RDA8
229533.A0A1C3YJQ6   pp  229533.I1RN55
229533.I1RXT9   pp  229533.I1RY40
229533.I1RXT9   pp  229533.Q4I877
229533.I1RXT9   pp  229533.Q4IMM0
229533.I1RXT9   pp  229533.Q4IER8
229533.I1RXT9   pp  229533.I1S5S3
229533.I1RXT9   pp  229533.Q4I0J6
229533.I1RXT9   pp  229533.I1RYE4
229533.I1RXT9   pp  229533.I1S6S3
229533.I1RXT9   pp  229533.I1RZH4
229533.I1RXT9   pp  229533.Q4ICM0
229533.I1RXT9   pp  229533.Q4IJK2
229533.I1RXT9   pp  229533.I1S5B5
229533.I1RXT9   pp  229533.I1S096
229533.I1RXT9   pp  229533.I1RZV9
229533.I1RXT9   pp  229533.I1S1V5
229533.I1RXT9   pp  229533.V6R3J1
229533.I1RXT9   pp  229533.I1S0N4
229533.I1RXT9   pp  229533.I1S2A8
229533.I1RXT9   pp  229533.Q4IBX0
229533.I1RXT9   pp  229533.I1S247
229533.I1RXT9   pp  229533.Q4HXF6
229533.I1RP20   pp  229533.I1S7J6
229533.I1RP20   pp  229533.I1S4S8
229533.I1RP20   pp  229533.I1RSI0
229533.I1RP20   pp  229533.I1RT05
229533.I1RP20   pp  229533.I1RTE0
229533.I1RP20   pp  229533.I1RRH5
229533.I1RP20   pp  229533.I1RTE9
229533.I1RP20   pp  229533.I1RQ40
229533.I1RP20   pp  229533.I1S0R2
229533.I1RP20   pp  229533.I1RQN0
229533.I1RP20   pp  229533.I1RZP1
229533.I1RKD2   pp  229533.I1RW71
229533.I1RKD2   pp  229533.I1RY03
229533.I1RKD2   pp  229533.I1RZX0
229533.I1RKD2   pp  229533.I1S0H1
229533.I1RQ80   pp  229533.I1RY44
229533.I1RQ80   pp  229533.I1RRH3
229533.I1RQ80   pp  229533.I1S1D3
229533.I1RQ80   pp  229533.I1RW16
229533.I1RQ80   pp  229533.I1RRW0
229533.I1RQ80   pp  229533.I1RQA3
229533.I1RQ80   pp  229533.I1RXT9
229533.I1RQ80   pp  229533.I1S382
229533.I1RQ80   pp  229533.I1RRP7
229533.I1RQ80   pp  229533.I1RZV9
229533.I1RQ80   pp  229533.Q4IER8
229533.I1RQ80   pp  229533.I1RR95
229533.I1RQ80   pp  229533.Q4IMD1
229533.I1RQ80   pp  229533.I1S5B5
```

65920	229533.I1RQ80	pp	229533.I1RQM0
65921	229533.I1RQ80	pp	229533.I1RTE5
65922	229533.I1RQ80	pp	229533.V6R3J1
65923	229533.I1RQ80	pp	229533.I1RX38
65924	229533.I1RQ80	pp	229533.I1RWT6
65925	229533.I1RQ80	pp	229533.I1S951
65926	229533.I1RQ80	pp	229533.Q4I7L0
65927	229533.I1RQ80	pp	229533.I1S2E9
65928	229533.I1RQ80	pp	229533.I1S0P4
65929	229533.I1RQ80	pp	229533.Q4I0J6
65930	229533.I1RQ80	pp	229533.Q4IBX0
65931	229533.I1RQ80	pp	229533.I1RQJ0
65932	229533.I1RQ80	pp	229533.Q4I877
65933	229533.I1RQ80	pp	229533.I1S1V5
65934	229533.I1RQ80	pp	229533.I1RRB0
65935	229533.I1RQ80	pp	229533.I1S2A8
65936	229533.I1RQ80	pp	229533.I1RQS4
65937	229533.I1RQ80	pp	229533.I1S096
65938	229533.I1RQ80	pp	229533.I1RYX9
65939	229533.I1RQ80	pp	229533.I1RZH4
65940	229533.I1RQ80	pp	229533.I1RY40
65941	229533.I1RQ80	pp	229533.Q4I7X1
65942	229533.I1RQ80	pp	229533.I1S1C8
65943	229533.I1RQ80	pp	229533.Q4HZS8
65944	229533.I1RQ80	pp	229533.I1S224
65945	229533.I1RQ80	pp	229533.I1S0Z2
65946	229533.I1RQ80	pp	229533.I1RRT4
65947	229533.I1RQ80	pp	229533.Q4IMM0
65948	229533.I1RQ80	pp	229533.I1S2F3
65949	229533.I1RQ80	pp	229533.I1RZU6
65950	229533.I1RQ80	pp	229533.Q4ICM0
65951	229533.I1RD18	pp	229533.V6RKY7
65952	229533.I1RD18	pp	229533.I1RY44
65953	229533.I1RD18	pp	229533.I1S1W4
65954	229533.I1RD18	pp	229533.I1S0D1
65955	229533.I1RD18	pp	229533.V6RVG3
65956	229533.I1RD18	pp	229533.V6R382
65957	229533.I1RD18	pp	229533.I1RZC3
65958	229533.I1RD18	pp	229533.I1RW16
65959	229533.I1RD18	pp	229533.I1RSC7
65960	229533.I1RD18	pp	229533.I1RMA4
65961	229533.I1RD18	pp	229533.I1S0M2
65962	229533.I1RD18	pp	229533.I1RVN2
65963	229533.I1RD18	pp	229533.I1S1D3
65964	229533.I1RD18	pp	229533.I1RFN0
65965	229533.I1RD18	pp	229533.I1RRW0
65966	229533.I1RD18	pp	229533.I1RPW0
65967	229533.I1RD18	pp	229533.I1RQ16
65968	229533.I1RD18	pp	229533.I1S0J7
65969	229533.I1RD18	pp	229533.V6RGI1
65970	229533.I1RD18	pp	229533.I1S004
65971	229533.I1RD18	pp	229533.I1RZE4
65972	229533.I1RD18	pp	229533.I1RY58
65973	229533.I1RD18	pp	229533.I1RRP7
65974	229533.I1RD18	pp	229533.I1RRY3
65975	229533.I1RD18	pp	229533.I1RD70
65976	229533.I1RD18	pp	229533.I1RFL7
65977	229533.I1RD18	pp	229533.I1S549
65978	229533.I1RD18	pp	229533.I1RUN8
65979	229533.I1RD18	pp	229533.I1RX64
65980	229533.I1RD18	pp	229533.I1RFW1
65981	229533.I1RD18	pp	229533.I1RFZ2
65982	229533.I1RD18	pp	229533.I1RMP0
65983	229533.I1RD18	pp	229533.I1S2F2
65984	229533.I1RD18	pp	229533.I1S2E9
65985	229533.I1RD18	pp	229533.V6R3J1
65986	229533.I1RD18	pp	229533.I1RX38
65987	229533.I1RD18	pp	229533.I1RYX9
65988	229533.I1RD18	pp	229533.I1RPK4
65989	229533.I1RD18	pp	229533.I1RZ63
65990	229533.I1RD18	pp	229533.I1S098
65991	229533.I1RD18	pp	229533.I1RWT6
65992	229533.I1RD18	pp	229533.I1S1V5

65993 229533.I1RD18 pp 229533.I1RV91
65994 229533.I1RD18 pp 229533.I1RQM0
65995 229533.I1RD18 pp 229533.Q4I0J6
65996 229533.I1RD18 pp 229533.I1RSJ4
65997 229533.I1RD18 pp 229533.I1RFR6
65998 229533.I1RD18 pp 229533.I1RUG1
65999 229533.I1RD18 pp 229533.I1RLU1
66000 229533.I1RD18 pp 229533.I1RME5
66001 229533.I1RD18 pp 229533.I1S267
66002 229533.I1RD18 pp 229533.I1RKK0
66003 229533.I1RD18 pp 229533.I1REE1
66004 229533.I1RD18 pp 229533.I1RJK7
66005 229533.I1RD18 pp 229533.I1RNP7
66006 229533.I1RD18 pp 229533.I1RKK1
66007 229533.I1RD18 pp 229533.I1RPU4
66008 229533.I1RD18 pp 229533.Q4IER8
66009 229533.I1RD18 pp 229533.I1RQJ0
66010 229533.I1RD18 pp 229533.I1RDV9
66011 229533.I1RD18 pp 229533.I1S209
66012 229533.I1RD18 pp 229533.Q4IR08
66013 229533.I1RD18 pp 229533.I1RN46
66014 229533.I1RD18 pp 229533.I1RN08
66015 229533.I1RD18 pp 229533.I1RUQ5
66016 229533.I1RD18 pp 229533.Q4IBX0
66017 229533.I1RD18 pp 229533.I1S439
66018 229533.I1RD18 pp 229533.I1RQS4
66019 229533.I1RD18 pp 229533.Q4IMM0
66020 229533.I1RD18 pp 229533.Q4I7N9
66021 229533.I1RD18 pp 229533.I1S9Z3
66022 229533.I1RD18 pp 229533.I1RQA3
66023 229533.I1RD18 pp 229533.I1RQ26
66024 229533.I1RD18 pp 229533.I1RMM9
66025 229533.I1RD18 pp 229533.I1RZ12
66026 229533.I1RD18 pp 229533.I1RLX6
66027 229533.I1RD18 pp 229533.I1RTG0
66028 229533.I1RD18 pp 229533.I1RN31
66029 229533.I1RD18 pp 229533.I1RNC0
66030 229533.I1RD18 pp 229533.I1RQ80
66031 229533.I1RD18 pp 229533.I1RMB7
66032 229533.I1RD18 pp 229533.I1RYH9
66033 229533.I1RD18 pp 229533.I1RFX8
66034 229533.I1RD18 pp 229533.I1RQ13
66035 229533.I1RD18 pp 229533.I1S224
66036 229533.I1RD18 pp 229533.I1RKJ7
66037 229533.I1RD18 pp 229533.I1RDB1
66038 229533.I1RJQ3 pp 229533.I1RKL2
66039 229533.I1RJQ3 pp 229533.I1RRH5
66040 229533.I1RJQ3 pp 229533.I1S8W6
66041 229533.I1RJQ3 pp 229533.I1RLT2
66042 229533.I1RJQ3 pp 229533.I1RW83
66043 229533.I1RJQ3 pp 229533.I1RP37
66044 229533.I1RJQ3 pp 229533.I1RMH6
66045 229533.I1RJQ3 pp 229533.I1S4T3
66046 229533.I1RJQ3 pp 229533.I1RW08
66047 229533.I1RJQ3 pp 229533.I1RQB7
66048 229533.A0A1C3YLN4 pp 229533.I1RQN9
66049 229533.A0A1C3YLN4 pp 229533.I1S286
66050 229533.A0A1C3YLN4 pp 229533.I1RFW0
66051 229533.A0A1C3YLN4 pp 229533.I1RD66
66052 229533.A0A1C3YLN4 pp 229533.I1RX77
66053 229533.A0A1C3YLN4 pp 229533.I1RSS1
66054 229533.A0A1C3YLN4 pp 229533.I1RMB2
66055 229533.A0A1C3YLN4 pp 229533.Q4IEA7
66056 229533.I1RAW7 pp 229533.I1RML9
66057 229533.I1RAW7 pp 229533.I1RLX6
66058 229533.I1RAW7 pp 229533.I1RP96
66059 229533.I1RAW7 pp 229533.I1RQG9
66060 229533.I1RAW7 pp 229533.I1RY40
66061 229533.I1RAW7 pp 229533.Q4IMM0
66062 229533.I1RAW7 pp 229533.I1RWD4
66063 229533.I1RAW7 pp 229533.Q4ICM0
66064 229533.I1RAW7 pp 229533.I1RUQ6
66065 229533.I1RAW7 pp 229533.I1RD18

66066	229533.I1RAW7	pp	229533.I1RQ80
66067	229533.I1RAW7	pp	229533.I1RRT4
66068	229533.I1RAW7	pp	229533.I1RPU4
66069	229533.I1RAW7	pp	229533.I1RK32
66070	229533.I1RAW7	pp	229533.I1RQ26
66071	229533.I1RAW7	pp	229533.I1RDB1
66072	229533.I1RAW7	pp	229533.I1S193
66073	229533.I1RAW7	pp	229533.I1S0P4
66074	229533.I1RAW7	pp	229533.I1RWT6
66075	229533.I1RAW7	pp	229533.I1RNC0
66076	229533.I1RAW7	pp	229533.I1RN31
66077	229533.I1RAW7	pp	229533.I1S1C8
66078	229533.I1RAW7	pp	229533.I1S096
66079	229533.I1RAW7	pp	229533.I1RFX8
66080	229533.I1RAW7	pp	229533.I1RKE9
66081	229533.I1RAW7	pp	229533.I1RCK4
66082	229533.I1RAW7	pp	229533.Q4I0J6
66083	229533.I1RAW7	pp	229533.I1RC95
66084	229533.I1RAW7	pp	229533.I1RJH5
66085	229533.I1RAW7	pp	229533.I1RB00
66086	229533.I1RAW7	pp	229533.I1RYH9
66087	229533.I1RAW7	pp	229533.I1RE46
66088	229533.I1RAW7	pp	229533.I1RXE5
66089	229533.I1RAW7	pp	229533.I1RC22
66090	229533.I1RAW7	pp	229533.V6R227
66091	229533.I1RAW7	pp	229533.I1S224
66092	229533.I1RAW7	pp	229533.I1RZH4
66093	229533.I1RAW7	pp	229533.I1RDC9
66094	229533.I1RAW7	pp	229533.I1RUQ5
66095	229533.I1RAW7	pp	229533.V6QV38
66096	229533.I1RAW7	pp	229533.V6R3J1
66097	229533.I1RAW7	pp	229533.I1RQJ0
66098	229533.I1RAW7	pp	229533.I1RBV5
66099	229533.I1RAW7	pp	229533.I1RHI1
66100	229533.I1RAW7	pp	229533.I1S2E9
66101	229533.I1RAW7	pp	229533.I1S439
66102	229533.I1RAW7	pp	229533.I1RN46
66103	229533.I1RAW7	pp	229533.I1RV91
66104	229533.I1RAW7	pp	229533.I1RMM9
66105	229533.I1RAW7	pp	229533.Q4IR18
66106	229533.I1RAW7	pp	229533.Q4IER8
66107	229533.I1RAW7	pp	229533.Q4IBX0
66108	229533.I1RAW7	pp	229533.I1RN08
66109	229533.I1RAW7	pp	229533.I1RFZ2
66110	229533.I1RAW7	pp	229533.I1RRB0
66111	229533.I1RAW7	pp	229533.I1RQA3
66112	229533.I1RAW7	pp	229533.I1RYX9
66113	229533.I1RAW7	pp	229533.I1RP28
66114	229533.I1RAW7	pp	229533.I1RMP0
66115	229533.I1RAW7	pp	229533.I1RCM6
66116	229533.I1RAW7	pp	229533.I1RBL4
66117	229533.I1RAW7	pp	229533.I1RPX5
66118	229533.I1RAW7	pp	229533.I1RQG4
66119	229533.I1RAW7	pp	229533.I1RRQ3
66120	229533.I1RAW7	pp	229533.I1RZM2
66121	229533.I1RAW7	pp	229533.I1RLX4
66122	229533.I1RAW7	pp	229533.I1RFW1
66123	229533.I1RAW7	pp	229533.I1RQM0
66124	229533.I1RAW7	pp	229533.I1RX38
66125	229533.I1RAW7	pp	229533.I1RMQ5
66126	229533.I1RAW7	pp	229533.I1RBR5
66127	229533.I1RAW7	pp	229533.I1RVY0
66128	229533.I1RAW7	pp	229533.Q4I7L0
66129	229533.I1RAW7	pp	229533.I1RFR6
66130	229533.I1RAW7	pp	229533.I1S0M2
66131	229533.I1RAW7	pp	229533.V6RLW4
66132	229533.I1RAW7	pp	229533.I1RLU1
66133	229533.I1RAW7	pp	229533.I1REX8
66134	229533.I1RAW7	pp	229533.I1RRW0
66135	229533.I1RAW7	pp	229533.I1RC35
66136	229533.I1RAW7	pp	229533.I1S2A8
66137	229533.I1RAW7	pp	229533.I1RL04
66138	229533.I1RAW7	pp	229533.I1RUN8

66139 229533.I1RAW7 pp 229533.I1S1W4
66140 229533.I1RAW7 pp 229533.V6RKY7
66141 229533.I1RAW7 pp 229533.V6RGI1
66142 229533.I1RAW7 pp 229533.I1RFN0
66143 229533.I1RAW7 pp 229533.I1RXT9
66144 229533.I1RAW7 pp 229533.I1RY44
66145 229533.I1RAW7 pp 229533.I1RRP7
66146 229533.I1RAW7 pp 229533.V6RVG3
66147 229533.I1RAW7 pp 229533.I1RBM3
66148 229533.I1RAW7 pp 229533.I1RBD6
66149 229533.I1RAW7 pp 229533.I1RRY3
66150 229533.I1RAW7 pp 229533.I1RW16
66151 229533.I1RAW7 pp 229533.I1S1D3
66152 229533.I1RAW7 pp 229533.I1RPW0
66153 229533.I1RAW7 pp 229533.I1RQ16
66154 229533.I1RAW7 pp 229533.I1RD70
66155 229533.A0A098DC94 pp 229533.A0A1C3YJG7
66156 229533.A0A098DC94 pp 229533.I1RQL4
66157 229533.I1RMF9 pp 229533.I1RXF4
66158 229533.I1RMF9 pp 229533.I1RWD1
66159 229533.I1RMF9 pp 229533.Q4HY20
66160 229533.I1RMF9 pp 229533.I1RSK3
66161 229533.I1RMF9 pp 229533.I1RRL4
66162 229533.I1RYX9 pp 229533.I1S1D3
66163 229533.I1RYX9 pp 229533.Q4HYB8
66164 229533.I1RYX9 pp 229533.V6R382
66165 229533.I1RYX9 pp 229533.Q4I0J6
66166 229533.I1RYX9 pp 229533.Q4IB89
66167 229533.I1RYX9 pp 229533.V6RGI1
66168 229533.I1RYX9 pp 229533.V6RLW4
66169 229533.I1RYX9 pp 229533.I1S604
66170 229533.I1RYX9 pp 229533.I1S209
66171 229533.I1RYX9 pp 229533.Q4IR08
66172 229533.I1RYX9 pp 229533.I1S998
66173 229533.I1RYX9 pp 229533.I1S461
66174 229533.I1RYX9 pp 229533.Q4IBX0
66175 229533.I1RYX9 pp 229533.I1S987
66176 229533.I1RYX9 pp 229533.Q4INS6
66177 229533.I1RYX9 pp 229533.Q4IEA5
66178 229533.I1RYX9 pp 229533.I1RZ86
66179 229533.I1RYX9 pp 229533.I1RZ94
66180 229533.I1RYX9 pp 229533.Q4IMM0
66181 229533.I1RYX9 pp 229533.I1S0D5
66182 229533.I1RYX9 pp 229533.Q4IC11
66183 229533.I1RYX9 pp 229533.I1S283
66184 229533.I1RYX9 pp 229533.V6R3J1
66185 229533.I1RYX9 pp 229533.I1S1V5
66186 229533.I1RYX9 pp 229533.I1RZP8
66187 229533.I1RYX9 pp 229533.Q4IP51
66188 229533.I1RYX9 pp 229533.Q4I5R9
66189 229533.I1RYX9 pp 229533.I1S2F2
66190 229533.I1RYX9 pp 229533.Q4IQM6
66191 229533.I1S1A3 pp 229533.I1S8F8
66192 229533.I1S1A3 pp 229533.V6RRM0
66193 229533.I1S1A3 pp 229533.I1S248
66194 229533.I1S1A3 pp 229533.Q4I1Y5
66195 229533.I1S1A3 pp 229533.I1S2E9
66196 229533.I1S1A3 pp 229533.Q4IBS9
66197 229533.I1RFN0 pp 229533.I1RHR0
66198 229533.I1RFN0 pp 229533.I1RQ67
66199 229533.I1RFN0 pp 229533.I1RW71
66200 229533.I1RFN0 pp 229533.I1RWM6
66201 229533.I1RFN0 pp 229533.I1RYR2
66202 229533.I1RFN0 pp 229533.I1RYX9
66203 229533.I1RFN0 pp 229533.I1RNT2
66204 229533.I1RFN0 pp 229533.I1RFZ2
66205 229533.I1RFN0 pp 229533.Q4IMM0
66206 229533.I1RFN0 pp 229533.I1RLE7
66207 229533.I1RFN0 pp 229533.Q4IC11
66208 229533.I1RFN0 pp 229533.I1RQ13
66209 229533.I1RFN0 pp 229533.I1RUG1
66210 229533.I1RFN0 pp 229533.I1S2F2
66211 229533.I1RFN0 pp 229533.I1S0M2

66212 229533.I1RFN0 pp 229533.I1S283
66213 229533.I1RFN0 pp 229533.I1RMB7
66214 229533.I1RFN0 pp 229533.I1RFR6
66215 229533.I1RFN0 pp 229533.I1RSC7
66216 229533.I1RFN0 pp 229533.I1S1V5
66217 229533.I1RFN0 pp 229533.I1RSJ4
66218 229533.I1RFN0 pp 229533.I1RSL6
66219 229533.I1RFN0 pp 229533.Q4I0J6
66220 229533.I1RFN0 pp 229533.I1RRK8
66221 229533.I1RFN0 pp 229533.I1RMX3
66222 229533.I1RFN0 pp 229533.I1RN65
66223 229533.I1RFN0 pp 229533.I1S1D3
66224 229533.I1RFN0 pp 229533.I1RHM4
66225 229533.I1RFN0 pp 229533.I1RUG2
66226 229533.I1RFN0 pp 229533.I1RQ16
66227 229533.I1RFN0 pp 229533.Q4IBX0
66228 229533.I1RFN0 pp 229533.I1RQJ0
66229 229533.I1RFN0 pp 229533.I1RN46
66230 229533.I1RCR3 pp 229533.I1RM26
66231 229533.I1RCR3 pp 229533.I1RPM6
66232 229533.I1RCR3 pp 229533.I1RJU9
66233 229533.I1RCR3 pp 229533.I1RPW2
66234 229533.I1RCR3 pp 229533.I1RLM3
66235 229533.I1RCR3 pp 229533.I1RFV9
66236 229533.I1RCR3 pp 229533.I1RVX9
66237 229533.I1RCR3 pp 229533.I1RR12
66238 229533.I1RCR3 pp 229533.I1RY49
66239 229533.I1RCR3 pp 229533.I1RTM8
66240 229533.I1RCR3 pp 229533.I1RCR9
66241 229533.I1RCR3 pp 229533.I1RNG4
66242 229533.I1RCR3 pp 229533.I1RPD6
66243 229533.I1RDA8 pp 229533.I1RQF7
66244 229533.I1RDA8 pp 229533.I1RJM5
66245 229533.I1RDA8 pp 229533.I1RFH2
66246 229533.I1RDA8 pp 229533.I1RSB3
66247 229533.I1RDA8 pp 229533.I1S4T3
66248 229533.I1RDA8 pp 229533.I1S8W6
66249 229533.I1RDA8 pp 229533.I1RRH8
66250 229533.I1RDA8 pp 229533.I1RRH5
66251 229533.I1RDA8 pp 229533.I1S4Q7
66252 229533.I1RDA8 pp 229533.I1RZ18
66253 229533.I1RDA8 pp 229533.I1RMN2
66254 229533.A0A098D635 pp 229533.I1RST4
66255 229533.A0A098D635 pp 229533.A0A1C3YJ12
66256 229533.A0A098D635 pp 229533.I1RXU0
66257 229533.A0A098D635 pp 229533.I1RLX4
66258 229533.A0A098D635 pp 229533.I1RRW0
66259 229533.A0A098D635 pp 229533.A0A1C3YKA6
66260 229533.A0A098D635 pp 229533.A0A0E0RS85
66261 229533.A0A098D635 pp 229533.I1RY44
66262 229533.A0A098D635 pp 229533.I1RW16
66263 229533.A0A098D635 pp 229533.I1RSL8
66264 229533.A0A098D635 pp 229533.I1RRF0
66265 229533.A0A098D635 pp 229533.I1S2A8
66266 229533.A0A098D635 pp 229533.A0A1C3YI10
66267 229533.A0A098D635 pp 229533.I1R9Q0
66268 229533.A0A098D635 pp 229533.I1RRP7
66269 229533.A0A098D635 pp 229533.I1RKI1
66270 229533.A0A098D635 pp 229533.A0A1C3YMS7
66271 229533.A0A098D635 pp 229533.I1S1D3
66272 229533.A0A098D635 pp 229533.I1RD70
66273 229533.A0A098D635 pp 229533.A0A1C3YKG7
66274 229533.A0A098D635 pp 229533.I1RWG6
66275 229533.A0A098D635 pp 229533.A0A1C3YHN3
66276 229533.A0A098D635 pp 229533.I1RBW1
66277 229533.A0A098D635 pp 229533.I1S382
66278 229533.A0A098D635 pp 229533.A0A098DDT3
66279 229533.A0A098D635 pp 229533.V6REZ4
66280 229533.A0A098D635 pp 229533.I1S2F2
66281 229533.A0A098D635 pp 229533.I1RKR9
66282 229533.A0A098D635 pp 229533.I1RX38
66283 229533.A0A098D635 pp 229533.I1S951
66284 229533.A0A098D635 pp 229533.A0A098D6T7

66285	229533.A0A098D635	pp	229533.I1RI34
66286	229533.A0A098D635	pp	229533.Q4IPI4
66287	229533.A0A098D635	pp	229533.I1RQ16
66288	229533.A0A098D635	pp	229533.I1RVY0
66289	229533.A0A098D635	pp	229533.A0A1C3YMF9
66290	229533.A0A098D635	pp	229533.I1RSJ4
66291	229533.A0A098D635	pp	229533.I1RLU1
66292	229533.A0A098D635	pp	229533.I1RPW0
66293	229533.A0A098D635	pp	229533.I1RC54
66294	229533.A0A098D635	pp	229533.V6RLW4
66295	229533.A0A098D635	pp	229533.I1RFN0
66296	229533.A0A098D635	pp	229533.I1RCD4
66297	229533.A0A098D635	pp	229533.A0A1C3YHH5
66298	229533.A0A098D635	pp	229533.I1RBM3
66299	229533.A0A098D635	pp	229533.I1RAN3
66300	229533.A0A098D635	pp	229533.I1RSL6
66301	229533.A0A098D635	pp	229533.I1RC35
66302	229533.A0A098D635	pp	229533.I1RFR6
66303	229533.A0A098D635	pp	229533.I1S2E9
66304	229533.A0A098D635	pp	229533.I1RBV5
66305	229533.A0A098D635	pp	229533.A0A0E0SNM6
66306	229533.A0A098D635	pp	229533.I1RRR9
66307	229533.A0A098D635	pp	229533.A0A1C3YKJ6
66308	229533.A0A098D635	pp	229533.I1S1V5
66309	229533.A0A098D635	pp	229533.I1RXB5
66310	229533.A0A098D635	pp	229533.V6REE9
66311	229533.A0A098D635	pp	229533.I1RFS3
66312	229533.A0A098D635	pp	229533.I1RT65
66313	229533.A0A098D635	pp	229533.A0A1C3YJP1
66314	229533.A0A098D635	pp	229533.I1RFZ2
66315	229533.A0A098D635	pp	229533.I1RMP0
66316	229533.A0A098D635	pp	229533.V6R3J1
66317	229533.A0A098D635	pp	229533.A0A098DF94
66318	229533.A0A098D635	pp	229533.A0A1C3YIF4
66319	229533.A0A098D635	pp	229533.A0A0E0RTV8
66320	229533.A0A098D635	pp	229533.I1RN46
66321	229533.A0A098D635	pp	229533.I1REN3
66322	229533.A0A098D635	pp	229533.I1RE37
66323	229533.A0A098D635	pp	229533.Q4IMD1
66324	229533.A0A098D635	pp	229533.A0A0E0SDE1
66325	229533.A0A098D635	pp	229533.A0A098DQF9
66326	229533.A0A098D635	pp	229533.I1RFJ2
66327	229533.A0A098D635	pp	229533.Q4IBX0
66328	229533.A0A098D635	pp	229533.Q4I7X1
66329	229533.A0A098D635	pp	229533.I1RYX9
66330	229533.A0A098D635	pp	229533.A0A1C3YIB8
66331	229533.A0A098D635	pp	229533.I1S604
66332	229533.A0A098D635	pp	229533.A0A1C3YK37
66333	229533.A0A098D635	pp	229533.I1RN65
66334	229533.A0A098D635	pp	229533.I1RAH9
66335	229533.A0A098D635	pp	229533.A0A0E0SMG8
66336	229533.A0A098D635	pp	229533.A0A0E0SLZ3
66337	229533.A0A098D635	pp	229533.I1RB49
66338	229533.A0A098D635	pp	229533.I1RWT6
66339	229533.A0A098D635	pp	229533.I1RBL7
66340	229533.A0A098D635	pp	229533.I1RZV9
66341	229533.A0A098D635	pp	229533.I1RWB3
66342	229533.A0A098D635	pp	229533.Q4I0J6
66343	229533.A0A098D635	pp	229533.I1RCM6
66344	229533.A0A098D635	pp	229533.I1RQA3
66345	229533.A0A098D635	pp	229533.A0A0E0SA11
66346	229533.A0A098D635	pp	229533.I1RK32
66347	229533.A0A098D635	pp	229533.I1S0Z3
66348	229533.A0A098D635	pp	229533.A0A098DZN5
66349	229533.A0A098D635	pp	229533.I1RNT2
66350	229533.A0A098D635	pp	229533.I1RN31
66351	229533.A0A098D635	pp	229533.A0A1C3YLR6
66352	229533.A0A098D635	pp	229533.I1RFU3
66353	229533.A0A098D635	pp	229533.I1RCL7
66354	229533.A0A098D635	pp	229533.I1RAL2
66355	229533.A0A098D635	pp	229533.I1RCT9
66356	229533.A0A098D635	pp	229533.I1S0P4
66357	229533.A0A098D635	pp	229533.I1RPU4

66358 229533.A0A098D635 pp 229533.I1S1C8
66359 229533.A0A098D635 pp 229533.V6RPN0
66360 229533.A0A098D635 pp 229533.I1RS37
66361 229533.A0A098D635 pp 229533.I1RE46
66362 229533.A0A098D635 pp 229533.A0A1C3YJL7
66363 229533.A0A098D635 pp 229533.I1RNC0
66364 229533.A0A098D635 pp 229533.I1RPZ0
66365 229533.A0A098D635 pp 229533.I1RMB7
66366 229533.A0A098D635 pp 229533.A0A1C3YHY5
66367 229533.A0A098D635 pp 229533.I1RLX6
66368 229533.A0A098D635 pp 229533.I1RB00
66369 229533.A0A098D635 pp 229533.I1RPK5
66370 229533.A0A098D635 pp 229533.I1RBW0
66371 229533.A0A098D635 pp 229533.I1RBI1
66372 229533.A0A098D635 pp 229533.I1RAH4
66373 229533.A0A098D635 pp 229533.I1S224
66374 229533.A0A098D635 pp 229533.A0A1C3YIA8
66375 229533.A0A098D635 pp 229533.A0A098DSW9
66376 229533.A0A098D635 pp 229533.I1RQ92
66377 229533.A0A098D635 pp 229533.I1RZH4
66378 229533.A0A098D635 pp 229533.A0A1C3YIP6
66379 229533.A0A098D635 pp 229533.I1RTC2
66380 229533.A0A098D635 pp 229533.I1RDG8
66381 229533.A0A098D635 pp 229533.I1RD18
66382 229533.A0A098D635 pp 229533.I1RT41
66383 229533.A0A098D635 pp 229533.I1RCU7
66384 229533.A0A098D635 pp 229533.I1RFX8
66385 229533.A0A098D635 pp 229533.I1S270
66386 229533.A0A098D635 pp 229533.I1RAW7
66387 229533.A0A098D635 pp 229533.I1RN27
66388 229533.A0A098D635 pp 229533.I1RQ80
66389 229533.A0A098D635 pp 229533.I1S0Z2
66390 229533.A0A098D635 pp 229533.I1RSI4
66391 229533.A0A098D635 pp 229533.I1R976
66392 229533.A0A098D635 pp 229533.A0A098DTS6
66393 229533.A0A098D635 pp 229533.A0A098DVZ4
66394 229533.A0A098D635 pp 229533.A0A1C3YKW5
66395 229533.I1RWM7 pp 229533.I1S2B6
66396 229533.I1RWM7 pp 229533.Q4HZ95
66397 229533.I1RWM7 pp 229533.I1S1C8
66398 229533.I1RWM7 pp 229533.I1SAM5
66399 229533.I1RWM7 pp 229533.I1S1V4
66400 229533.I1RWM7 pp 229533.I1S2B7
66401 229533.I1RZW1 pp 229533.I1S248
66402 229533.A0A1C3YJI7 pp 229533.I1RJQ4
66403 229533.A0A1C3YJI7 pp 229533.A0A1C3YK00
66404 229533.A0A1C3YJI7 pp 229533.I1RRH5
66405 229533.A0A1C3YJI7 pp 229533.I1RNV7
66406 229533.A0A1C3YJI7 pp 229533.I1RJQ3
66407 229533.A0A1C3YJI7 pp 229533.A0A1C3YJQ6
66408 229533.A0A1C3YJI7 pp 229533.I1RB17
66409 229533.A0A1C3YJI7 pp 229533.I1R9X8
66410 229533.A0A1C3YJI7 pp 229533.I1RDY1
66411 229533.A0A1C3YJI7 pp 229533.I1RKL2
66412 229533.A0A1C3YJI7 pp 229533.A0A1C3YMH3
66413 229533.A0A1C3YJI7 pp 229533.I1RKQ0
66414 229533.A0A1C3YJI7 pp 229533.I1RMH6
66415 229533.A0A1C3YJI7 pp 229533.I1RBA8
66416 229533.A0A1C3YJI7 pp 229533.A0A1C3YK80
66417 229533.A0A1C3YJI7 pp 229533.I1RBZ1
66418 229533.A0A1C3YJI7 pp 229533.I1RQB7
66419 229533.A0A098DNR3 pp 229533.A0A1C3YJQ6
66420 229533.A0A098DNR3 pp 229533.I1RP20
66421 229533.A0A098DNR3 pp 229533.I1RB17
66422 229533.A0A098DNR3 pp 229533.I1RRH5
66423 229533.A0A098DNR3 pp 229533.I1RJQ3
66424 229533.A0A098DNR3 pp 229533.I1RMF9
66425 229533.A0A098DNR3 pp 229533.A0A1C3YMH3
66426 229533.A0A098DNR3 pp 229533.A0A1C3YJ51
66427 229533.A0A098DNR3 pp 229533.I1S4T3
66428 229533.A0A098DNR3 pp 229533.I1RDA8
66429 229533.A0A098DNR3 pp 229533.I1RQB7
66430 229533.A0A098DNR3 pp 229533.A0A1C3YJI7

66431	229533.I1S2F2	pp	229533.Q4IBX0
66432	229533.I1S2F2	pp	229533.Q4I0J6
66433	229533.I1S2F2	pp	229533.Q4IMD1
66434	229533.I1S2F2	pp	229533.V6RLW4
66435	229533.I1S2F2	pp	229533.Q4HTT2
66436	229533.I1RF55	pp	229533.I1RFV2
66437	229533.I1RF55	pp	229533.I1RF60
66438	229533.I1RF55	pp	229533.I1RF58
66439	229533.I1RF55	pp	229533.I1RMG4
66440	229533.I1RF55	pp	229533.I1RF57
66441	229533.I1RF55	pp	229533.I1RF62
66442	229533.I1RF55	pp	229533.I1RF61
66443	229533.I1RF55	pp	229533.I1RF63
66444	229533.I1RF55	pp	229533.I1RF56
66445	229533.I1RF55	pp	229533.I1RF59
66446	229533.I1RF55	pp	229533.I1RZN1
66447	229533.I1RVA8	pp	229533.I1S287
66448	229533.I1RVA8	pp	229533.I1RW52
66449	229533.I1RVA8	pp	229533.I1RVN1
66450	229533.I1RVA8	pp	229533.I1SB04
66451	229533.I1RVA8	pp	229533.I1RWU9
66452	229533.I1RKI1	pp	229533.I1RX87
66453	229533.I1RKI1	pp	229533.Q4I2D7
66454	229533.I1RKI1	pp	229533.I1S1V2
66455	229533.I1RKI1	pp	229533.I1RR74
66456	229533.I1RKI1	pp	229533.Q4ICM0
66457	229533.I1RKI1	pp	229533.Q4INI2
66458	229533.I1RKI1	pp	229533.I1RW71
66459	229533.I1RKI1	pp	229533.I1RMG2
66460	229533.I1RKI1	pp	229533.I1S270
66461	229533.I1RKI1	pp	229533.Q4I948
66462	229533.I1RKI1	pp	229533.I1RT41
66463	229533.I1RKI1	pp	229533.Q5I2J3
66464	229533.I1RKI1	pp	229533.I1RWI6
66465	229533.I1RKI1	pp	229533.I1RRF9
66466	229533.I1RKI1	pp	229533.V6RPN0
66467	229533.I1RKI1	pp	229533.I1RQP3
66468	229533.I1RKI1	pp	229533.I1RQF7
66469	229533.I1RKI1	pp	229533.I1RQM8
66470	229533.I1RKI1	pp	229533.Q4ICA8
66471	229533.I1RKI1	pp	229533.I1S9S4
66472	229533.I1RKI1	pp	229533.I1S0Z3
66473	229533.I1RKI1	pp	229533.I1RX68
66474	229533.I1RKI1	pp	229533.I1RQP5
66475	229533.I1RKI1	pp	229533.I1RSD7
66476	229533.I1RKI1	pp	229533.I1RXS3
66477	229533.I1RKI1	pp	229533.I1S7X9
66478	229533.I1RKI1	pp	229533.I1S559
66479	229533.I1RKI1	pp	229533.I1RQJ0
66480	229533.I1RKI1	pp	229533.I1RQP1
66481	229533.I1RKI1	pp	229533.I1RSH3
66482	229533.I1RKI1	pp	229533.I1RQG4
66483	229533.I1RKI1	pp	229533.I1RZ60
66484	229533.I1RKI1	pp	229533.Q4I963
66485	229533.I1RKI1	pp	229533.I1RZK3
66486	229533.I1RKI1	pp	229533.I1RNG4
66487	229533.I1RKI1	pp	229533.I1RVY0
66488	229533.I1RKI1	pp	229533.I1RRF0
66489	229533.I1RKI1	pp	229533.I1S0S8
66490	229533.I1RKI1	pp	229533.I1RYM4
66491	229533.I1RKI1	pp	229533.I1RZ84
66492	229533.I1RKI1	pp	229533.I1S7W2
66493	229533.I1RKI1	pp	229533.I1RTE0
66494	229533.I1RKI1	pp	229533.I1RZL3
66495	229533.I1RKI1	pp	229533.I1RKR9
66496	229533.I1RKI1	pp	229533.Q4IPI4
66497	229533.I1RKI1	pp	229533.I1RUJ0
66498	229533.I1RKI1	pp	229533.I1RSZ6
66499	229533.I1RKI1	pp	229533.I1S248
66500	229533.I1RKI1	pp	229533.V6R2A1
66501	229533.I1RKI1	pp	229533.I1RMQ5
66502	229533.I1RKI1	pp	229533.I1S247
66503	229533.I1RKI1	pp	229533.I1RZV1

66504	229533.I1RKI1	pp	229533.I1RX69
66505	229533.I1RKI1	pp	229533.Q4HY90
66506	229533.I1RKI1	pp	229533.I1RTG5
66507	229533.I1RKI1	pp	229533.I1RYW3
66508	229533.I1RZK4	pp	229533.I1S0X7
66509	229533.I1RZK4	pp	229533.I1S248
66510	229533.I1RZK4	pp	229533.Q5I2J3
66511	229533.I1RZK4	pp	229533.I1S074
66512	229533.I1RZK4	pp	229533.I1S278
66513	229533.I1RB19	pp	229533.I1RB20
66514	229533.I1RRW1	pp	229533.I1RVA8
66515	229533.I1RRW1	pp	229533.V6RXW9
66516	229533.I1RRW1	pp	229533.I1RWU9
66517	229533.I1RSC7	pp	229533.I1S9J6
66518	229533.I1RSC7	pp	229533.Q4IBX0
66519	229533.I1RSC7	pp	229533.I1S183
66520	229533.I1RSC7	pp	229533.I1RVZ9
66521	229533.I1RSC7	pp	229533.I1RUG2
66522	229533.I1RSC7	pp	229533.I1S788
66523	229533.I1RSC7	pp	229533.Q4I0J6
66524	229533.I1RSC7	pp	229533.I1S461
66525	229533.I1RSC7	pp	229533.Q4INS6
66526	229533.I1RSC7	pp	229533.I1S1W8
66527	229533.I1RSC7	pp	229533.I1RU61
66528	229533.I1RSC7	pp	229533.I1RU52
66529	229533.I1RSC7	pp	229533.I1RSK0
66530	229533.I1RSC7	pp	229533.I1S209
66531	229533.I1RSC7	pp	229533.I1RZ94
66532	229533.I1RSC7	pp	229533.I1S283
66533	229533.I1RSC7	pp	229533.I1S5Z3
66534	229533.I1RSC7	pp	229533.I1S987
66535	229533.I1RSC7	pp	229533.I1RSI4
66536	229533.I1RSC7	pp	229533.Q4IR08
66537	229533.I1RSC7	pp	229533.I1RUR0
66538	229533.I1RSC7	pp	229533.I1RUP7
66539	229533.I1RSC7	pp	229533.I1RX64
66540	229533.I1RSC7	pp	229533.I1RYJ6
66541	229533.I1RSC7	pp	229533.Q4IMM0
66542	229533.I1RSC7	pp	229533.I1RWM6
66543	229533.I1RSC7	pp	229533.Q4I5R9
66544	229533.I1RSC7	pp	229533.I1RSJ1
66545	229533.I1RSC7	pp	229533.Q4IJ84
66546	229533.I1RSC7	pp	229533.I1S0R7
66547	229533.I1RSC7	pp	229533.I1RSY2
66548	229533.I1RSC7	pp	229533.I1RTN6
66549	229533.I1RSC7	pp	229533.Q4IB89
66550	229533.I1RSC7	pp	229533.I1RZP8
66551	229533.I1RSC7	pp	229533.I1S289
66552	229533.I1RSC7	pp	229533.I1RY58
66553	229533.I1RSC7	pp	229533.I1RSP7
66554	229533.I1RSC7	pp	229533.I1RSR9
66555	229533.I1RSC7	pp	229533.I1RSL8
66556	229533.I1RSC7	pp	229533.I1S276
66557	229533.I1RSC7	pp	229533.I1RUG4
66558	229533.I1RSC7	pp	229533.I1S0N5
66559	229533.I1RSC7	pp	229533.I1RW16
66560	229533.I1RSC7	pp	229533.I1S0D5
66561	229533.I1RSC7	pp	229533.I1S4P6
66562	229533.I1RSC7	pp	229533.I1RZ86
66563	229533.I1RSC7	pp	229533.I1SAD2
66564	229533.I1RSC7	pp	229533.I1RZ98
66565	229533.I1RSC7	pp	229533.I1RVW0
66566	229533.I1RSC7	pp	229533.V6R382
66567	229533.I1RSC7	pp	229533.I1RSN7
66568	229533.I1RSC7	pp	229533.I1RSJ4
66569	229533.I1RSC7	pp	229533.I1RT36
66570	229533.I1RSC7	pp	229533.I1S998
66571	229533.I1RSC7	pp	229533.I1RYX9
66572	229533.I1RSC7	pp	229533.I1RUG1
66573	229533.I1RSC7	pp	229533.I1RWS4
66574	229533.I1RSC7	pp	229533.I1RWI8
66575	229533.I1RSC7	pp	229533.I1RYR2
66576	229533.I1RSC7	pp	229533.I1S523

66577 229533.I1RSC7 pp 229533.I1RY29
66578 229533.I1RSC7 pp 229533.I1RZI4
66579 229533.I1RSC7 pp 229533.I1RSZ3
66580 229533.I1RSC7 pp 229533.V6RLW4
66581 229533.I1RSC7 pp 229533.I1RYQ1
66582 229533.I1RSC7 pp 229533.I1RW15
66583 229533.I1RSC7 pp 229533.I1S2F2
66584 229533.I1RSC7 pp 229533.I1S1F3
66585 229533.I1RSC7 pp 229533.I1S8G4
66586 229533.I1RSC7 pp 229533.I1S3G4
66587 229533.I1RSC7 pp 229533.I1RZ19
66588 229533.I1RSC7 pp 229533.I1RUF8
66589 229533.I1RSC7 pp 229533.I1RXI6
66590 229533.I1RSC7 pp 229533.Q4IB96
66591 229533.I1RSC7 pp 229533.I1RX22
66592 229533.I1RB20 pp 229533.I1RSG0
66593 229533.I1RB20 pp 229533.I1RZC3
66594 229533.I1RPE7 pp 229533.I1RY49
66595 229533.I1RPE7 pp 229533.I1RR12
66596 229533.I1RPE7 pp 229533.I1RPM6
66597 229533.I1RPE7 pp 229533.I1RVX9
66598 229533.I1RPE7 pp 229533.I1RPW2
66599 229533.A0A1C3YJ50 pp 229533.I1S7S9
66600 229533.A0A1C3YJ50 pp 229533.I1RQ88
66601 229533.A0A1C3YJ50 pp 229533.I1RGC2
66602 229533.A0A1C3YJ50 pp 229533.I1RAI9
66603 229533.A0A1C3YJ50 pp 229533.I1RS41
66604 229533.A0A0E0SNZ9 pp 229533.I1RP61
66605 229533.A0A0E0SNZ9 pp 229533.Q4I2W2
66606 229533.A0A0E0SNZ9 pp 229533.I1S339
66607 229533.A0A0E0SNZ9 pp 229533.I1RVA5
66608 229533.A0A0E0SNZ9 pp 229533.A0A1C3YKV0
66609 229533.A0A0E0SNZ9 pp 229533.I1RF69
66610 229533.A0A0E0SNZ9 pp 229533.I1RA72
66611 229533.A0A0E0SNZ9 pp 229533.Q4I877
66612 229533.A0A0E0SNZ9 pp 229533.I1RLS5
66613 229533.A0A0E0SNZ9 pp 229533.I1RZK4
66614 229533.A0A0E0SNZ9 pp 229533.I1S0S8
66615 229533.A0A0E0SNZ9 pp 229533.I1RTE0
66616 229533.A0A0E0SNZ9 pp 229533.I1S0Z3
66617 229533.A0A0E0SNZ9 pp 229533.I1RZ84
66618 229533.A0A0E0SNZ9 pp 229533.I1REG5
66619 229533.A0A0E0SNZ9 pp 229533.A0A1C3YI93
66620 229533.A0A0E0SNZ9 pp 229533.I1RQP5
66621 229533.A0A0E0SNZ9 pp 229533.I1RQ16
66622 229533.A0A0E0SNZ9 pp 229533.V6R2L8
66623 229533.A0A0E0SNZ9 pp 229533.I1RTG5
66624 229533.A0A0E0SNZ9 pp 229533.A0A1C3YIH7
66625 229533.A0A0E0SNZ9 pp 229533.A0A1C3YI44
66626 229533.A0A0E0SNZ9 pp 229533.A0A1C3YLY5
66627 229533.A0A0E0SNZ9 pp 229533.I1S9S4
66628 229533.A0A0E0SNZ9 pp 229533.I1S9H9
66629 229533.A0A0E0SNZ9 pp 229533.I1RZ60
66630 229533.A0A0E0SNZ9 pp 229533.A0A0E0SQG9
66631 229533.A0A0E0SNZ9 pp 229533.I1RR95
66632 229533.A0A0E0SNZ9 pp 229533.I1RXA1
66633 229533.A0A0E0SNZ9 pp 229533.A0A1C3YLK5
66634 229533.A0A0E0SNZ9 pp 229533.I1RJS2
66635 229533.A0A0E0SNZ9 pp 229533.I1RNP1
66636 229533.A0A0E0SNZ9 pp 229533.I1RCF0
66637 229533.A0A0E0SNZ9 pp 229533.I1RRB0
66638 229533.A0A0E0SNZ9 pp 229533.I1RKI1
66639 229533.A0A0E0SNZ9 pp 229533.I1RR74
66640 229533.A0A0E0SNZ9 pp 229533.I1RT08
66641 229533.A0A0E0SNZ9 pp 229533.I1SA96
66642 229533.A0A0E0SNZ9 pp 229533.I1RQJ0
66643 229533.A0A0E0SNZ9 pp 229533.Q4HZS8
66644 229533.A0A0E0SNZ9 pp 229533.I1RQW2
66645 229533.A0A0E0SNZ9 pp 229533.I1RI75
66646 229533.A0A0E0SNZ9 pp 229533.I1RTN1
66647 229533.A0A0E0SNZ9 pp 229533.I1RQ70
66648 229533.A0A0E0SNZ9 pp 229533.I1S2R1
66649 229533.A0A0E0SNZ9 pp 229533.I1RJH5

66650 229533.A0A0E0SNZ9 pp 229533.I1RPD2
66651 229533.A0A0E0SNZ9 pp 229533.I1RMG2
66652 229533.A0A0E0SNZ9 pp 229533.A0A1C3YI22
66653 229533.A0A0E0SNZ9 pp 229533.I1RT41
66654 229533.A0A0E0SNZ9 pp 229533.I1S927
66655 229533.A0A0E0SNZ9 pp 229533.I1RB00
66656 229533.A0A0E0SNZ9 pp 229533.I1S330
66657 229533.A0A0E0SNZ9 pp 229533.Q5I2J3
66658 229533.A0A0E0SNZ9 pp 229533.A0A1C3YLL9
66659 229533.A0A0E0SNZ9 pp 229533.I1S0G3
66660 229533.I1RK67 pp 229533.I1S248
66661 229533.I1RK67 pp 229533.I1RYB7
66662 229533.I1RK67 pp 229533.I1RMA7
66663 229533.I1RK67 pp 229533.I1RRH1
66664 229533.I1RK67 pp 229533.I1RPH1
66665 229533.I1RK67 pp 229533.I1RMR7
66666 229533.I1RK67 pp 229533.I1S1A3
66667 229533.I1RK67 pp 229533.I1RZK4
66668 229533.I1RK67 pp 229533.I1RM12
66669 229533.I1RK67 pp 229533.I1S074
66670 229533.I1RWU9 pp 229533.I1SAM5
66671 229533.I1RWU9 pp 229533.I1S287
66672 229533.I1RWU9 pp 229533.I1RY40
66673 229533.I1RWU9 pp 229533.I1RX87
66674 229533.I1RWU9 pp 229533.Q4HX89
66675 229533.A0A098DNP5 pp 229533.I1RVA8
66676 229533.A0A098DNP5 pp 229533.I1RRW1
66677 229533.A0A098DNP5 pp 229533.V6RXW9
66678 229533.A0A098DNP5 pp 229533.I1RWU9
66679 229533.I1RCF0 pp 229533.Q4I2D7
66680 229533.I1RCF0 pp 229533.I1RQJ0
66681 229533.I1RCF0 pp 229533.I1RRB0
66682 229533.I1RCF0 pp 229533.Q4INI2
66683 229533.I1RCF0 pp 229533.I1RQ70
66684 229533.I1RCF0 pp 229533.I1RQ16
66685 229533.I1RCF0 pp 229533.I1RN55
66686 229533.I1RCF0 pp 229533.I1RT41
66687 229533.I1RCF0 pp 229533.I1S247
66688 229533.I1RCF0 pp 229533.I1RUJ0
66689 229533.I1RCF0 pp 229533.I1S270
66690 229533.I1RCF0 pp 229533.I1REC7
66691 229533.I1RCF0 pp 229533.I1RYM4
66692 229533.I1RCF0 pp 229533.I1RQF7
66693 229533.I1RCF0 pp 229533.I1RKI1
66694 229533.I1RCF0 pp 229533.I1RRF0
66695 229533.I1RCF0 pp 229533.I1RKR9
66696 229533.I1RCF0 pp 229533.I1RD70
66697 229533.I1RCF0 pp 229533.I1RRT4
66698 229533.I1RCF0 pp 229533.I1RWD5
66699 229533.I1RCF0 pp 229533.Q4IPI4
66700 229533.I1RCF0 pp 229533.Q4ICM0
66701 229533.I1RCF0 pp 229533.I1RD02
66702 229533.I1RCF0 pp 229533.I1S0S8
66703 229533.I1RCF0 pp 229533.I1RTQ9
66704 229533.I1RCF0 pp 229533.I1REC6
66705 229533.I1RCF0 pp 229533.Q4INZ9
66706 229533.I1RCF0 pp 229533.I1RFK0
66707 229533.I1RCF0 pp 229533.I1RR74
66708 229533.I1RCF0 pp 229533.Q4IN00
66709 229533.I1RCF0 pp 229533.I1RX54
66710 229533.I1RCF0 pp 229533.I1RT65
66711 229533.I1RCF0 pp 229533.Q4I877
66712 229533.I1RCF0 pp 229533.I1S0Z3
66713 229533.I1RCF0 pp 229533.I1RST4
66714 229533.I1RCF0 pp 229533.I1RJH5
66715 229533.I1RCF0 pp 229533.I1S096
66716 229533.I1RCF0 pp 229533.Q4HZB8
66717 229533.I1RCF0 pp 229533.I1RRI4
66718 229533.I1RCF0 pp 229533.I1RN57
66719 229533.I1RCF0 pp 229533.I1RMG2
66720 229533.I1RPH1 pp 229533.I1RX77
66721 229533.I1RPH1 pp 229533.I1RX87
66722 229533.I1RPH1 pp 229533.I1S278

66723	229533.I1RPH1	pp	229533.I1RV66
66724	229533.I1RPH1	pp	229533.I1RQL4
66725	229533.I1RPH1	pp	229533.Q5I2J3
66726	229533.I1RPH1	pp	229533.I1RSS1
66727	229533.I1RPH1	pp	229533.I1S074
66728	229533.I1RPH1	pp	229533.I1S248
66729	229533.I1RPH1	pp	229533.I1RQD6
66730	229533.I1RPH1	pp	229533.I1RZC3
66731	229533.I1RPH1	pp	229533.I1S0X7
66732	229533.I1RPH1	pp	229533.I1RY32
66733	229533.I1RPH1	pp	229533.I1RQB4
66734	229533.I1RHQ4	pp	229533.I1S303
66735	229533.I1RHQ4	pp	229533.I1RPV5
66736	229533.I1RHQ4	pp	229533.I1RLN1
66737	229533.I1RHQ4	pp	229533.I1SAS4
66738	229533.I1RHQ4	pp	229533.I1S2I7
66739	229533.A0A098DXS1	pp	229533.I1RWG6
66740	229533.A0A098DXS1	pp	229533.I1S2A8
66741	229533.A0A098DXS1	pp	229533.V6REZ4
66742	229533.A0A098DXS1	pp	229533.A0A1C3YI10
66743	229533.A0A098DXS1	pp	229533.I1RRW0
66744	229533.A0A098DXS1	pp	229533.I1R9Q0
66745	229533.A0A098DXS1	pp	229533.A0A1C3YHN3
66746	229533.A0A098DXS1	pp	229533.A0A1C3YJ12
66747	229533.A0A098DXS1	pp	229533.A0A1C3YKG7
66748	229533.A0A098DXS1	pp	229533.I1RFR6
66749	229533.A0A098DXS1	pp	229533.I1RLU1
66750	229533.A0A098DXS1	pp	229533.I1RRF0
66751	229533.A0A098DXS1	pp	229533.I1RXU0
66752	229533.A0A098DXS1	pp	229533.I1RRP7
66753	229533.A0A098DXS1	pp	229533.I1RSJ4
66754	229533.A0A098DXS1	pp	229533.I1RLX4
66755	229533.A0A098DXS1	pp	229533.I1RD70
66756	229533.A0A098DXS1	pp	229533.A0A1C3YHH5
66757	229533.A0A098DXS1	pp	229533.I1RQ16
66758	229533.A0A098DXS1	pp	229533.I1S1V5
66759	229533.A0A098DXS1	pp	229533.I1RBM3
66760	229533.A0A098DXS1	pp	229533.I1RBW1
66761	229533.A0A098DXS1	pp	229533.I1RI34
66762	229533.A0A098DXS1	pp	229533.I1RSL6
66763	229533.A0A098DXS1	pp	229533.I1RPW0
66764	229533.A0A098DXS1	pp	229533.I1S2F2
66765	229533.A0A098DXS1	pp	229533.I1RST4
66766	229533.A0A098DXS1	pp	229533.I1S951
66767	229533.A0A098DXS1	pp	229533.A0A1C3YMF9
66768	229533.A0A098DXS1	pp	229533.I1RFN0
66769	229533.A0A098DXS1	pp	229533.I1RKR9
66770	229533.A0A098DXS1	pp	229533.I1RC54
66771	229533.A0A098DXS1	pp	229533.Q4IPI4
66772	229533.A0A098DXS1	pp	229533.I1RX38
66773	229533.A0A098DXS1	pp	229533.I1RSL8
66774	229533.A0A098DXS1	pp	229533.I1RFS3
66775	229533.A0A098DXS1	pp	229533.A0A0E0SNM6
66776	229533.A0A098DXS1	pp	229533.I1RRR9
66777	229533.A0A098DXS1	pp	229533.A0A1C3YKJ6
66778	229533.A0A098DXS1	pp	229533.A0A1C3YMS7
66779	229533.A0A098DXS1	pp	229533.A0A1C3YJP1
66780	229533.A0A098DXS1	pp	229533.I1S2E9
66781	229533.A0A098DXS1	pp	229533.I1RAN3
66782	229533.A0A098DXS1	pp	229533.I1RC35
66783	229533.A0A098DXS1	pp	229533.I1S1D3
66784	229533.A0A098DXS1	pp	229533.I1RCD4
66785	229533.A0A098DXS1	pp	229533.I1RVY0
66786	229533.A0A098DXS1	pp	229533.I1RBV5
66787	229533.A0A098DXS1	pp	229533.Q4IMD1
66788	229533.A0A098DXS1	pp	229533.I1RW16
66789	229533.A0A098DXS1	pp	229533.V6R3J1
66790	229533.A0A098DXS1	pp	229533.V6RLW4
66791	229533.A0A098DXS1	pp	229533.I1RY44
66792	229533.A0A098DXS1	pp	229533.A0A0E0RS85
66793	229533.A0A098DXS1	pp	229533.A0A1C3YKA6
66794	229533.A0A098DXS1	pp	229533.I1S382
66795	229533.A0A098DXS1	pp	229533.I1RKI1

66796	229533.A0A098DXS1	pp	229533.I1REN3
66797	229533.A0A098DXS1	pp	229533.A0A1C3YK37
66798	229533.A0A098DXS1	pp	229533.A0A0E0SMG8
66799	229533.A0A098DXS1	pp	229533.I1RB49
66800	229533.A0A098DXS1	pp	229533.A0A0E0SLZ3
66801	229533.A0A098DXS1	pp	229533.I1RAH9
66802	229533.A0A098DXS1	pp	229533.I1S604
66803	229533.A0A098DXS1	pp	229533.I1RT65
66804	229533.A0A098DXS1	pp	229533.I1RYX9
66805	229533.A0A098DXS1	pp	229533.I1RFZ2
66806	229533.A0A098DXS1	pp	229533.I1RMP0
66807	229533.A0A098DXS1	pp	229533.Q4I7X1
66808	229533.A0A098DXS1	pp	229533.A0A098DZN5
66809	229533.A0A098DXS1	pp	229533.V6REE9
66810	229533.A0A098DXS1	pp	229533.I1S1C8
66811	229533.A0A098DXS1	pp	229533.A0A1C3YIF4
66812	229533.A0A098DXS1	pp	229533.I1S0Z2
66813	229533.A0A098DXS1	pp	229533.I1RCT9
66814	229533.A0A098DXS1	pp	229533.A0A0E0SA11
66815	229533.A0A098DXS1	pp	229533.I1RK32
66816	229533.A0A098DXS1	pp	229533.A0A0E0RTV8
66817	229533.A0A098DXS1	pp	229533.I1RXB5
66818	229533.A0A098DXS1	pp	229533.I1RPU4
66819	229533.A0A098DXS1	pp	229533.I1RZV9
66820	229533.A0A098DXS1	pp	229533.I1RCU7
66821	229533.A0A098DXS1	pp	229533.I1RPK5
66822	229533.A0A098DXS1	pp	229533.I1RD18
66823	229533.A0A098DXS1	pp	229533.I1RBI1
66824	229533.A0A098DXS1	pp	229533.I1R976
66825	229533.A0A098DXS1	pp	229533.I1S270
66826	229533.A0A098DXS1	pp	229533.I1RBL7
66827	229533.A0A098DXS1	pp	229533.I1RCM6
66828	229533.A0A098DXS1	pp	229533.I1RPZ0
66829	229533.A0A098DXS1	pp	229533.I1RWB3
66830	229533.A0A098DXS1	pp	229533.A0A1C3YHY5
66831	229533.A0A098DXS1	pp	229533.I1RMB7
66832	229533.A0A098DXS1	pp	229533.I1RAH4
66833	229533.A0A098DXS1	pp	229533.I1RBW0
66834	229533.A0A098DXS1	pp	229533.I1RB00
66835	229533.A0A098DXS1	pp	229533.I1S224
66836	229533.A0A098DXS1	pp	229533.I1RCL7
66837	229533.A0A098DXS1	pp	229533.I1RAL2
66838	229533.A0A098DXS1	pp	229533.I1RQA3
66839	229533.A0A098DXS1	pp	229533.A0A1C3YIB8
66840	229533.A0A098DXS1	pp	229533.I1RZH4
66841	229533.A0A098DXS1	pp	229533.I1RN65
66842	229533.A0A098DXS1	pp	229533.I1RFX8
66843	229533.A0A098DXS1	pp	229533.V6RPN0
66844	229533.A0A098DXS1	pp	229533.I1RDG8
66845	229533.A0A098DXS1	pp	229533.I1RT41
66846	229533.A0A098DXS1	pp	229533.I1RE46
66847	229533.A0A098DXS1	pp	229533.A0A1C3YLR6
66848	229533.A0A098DXS1	pp	229533.I1RN31
66849	229533.A0A098DXS1	pp	229533.I1RE37
66850	229533.A0A098DXS1	pp	229533.A0A0E0SDE1
66851	229533.A0A098DXS1	pp	229533.Q4IBX0
66852	229533.A0A098DXS1	pp	229533.Q4I0J6
66853	229533.A0A098DXS1	pp	229533.I1RFJ2
66854	229533.A0A098DXS1	pp	229533.I1RNC0
66855	229533.A0A098DXS1	pp	229533.I1S0Z3
66856	229533.A0A098DXS1	pp	229533.I1RS37
66857	229533.A0A098DXS1	pp	229533.I1RN46
66858	229533.A0A098DXS1	pp	229533.A0A1C3YIA8
66859	229533.A0A098DXS1	pp	229533.I1S0P4
66860	229533.A0A098DXS1	pp	229533.A0A1C3YJL7
66861	229533.A0A098DXS1	pp	229533.I1RSI4
66862	229533.A0A098DXS1	pp	229533.I1RFU3
66863	229533.A0A098DXS1	pp	229533.I1RWT6
66864	229533.A0A098DXS1	pp	229533.I1RLX6
66865	229533.A0A098DXS1	pp	229533.A0A1C3YKW5
66866	229533.A0A098DXS1	pp	229533.I1RQ80
66867	229533.A0A098DXS1	pp	229533.I1RNT2
66868	229533.A0A098DXS1	pp	229533.I1RN27

66869 229533.A0A098DXS1 pp 229533.I1RAW7
66870 229533.A0A098DXS1 pp 229533.I1RTC2
66871 229533.A0A098DXS1 pp 229533.A0A1C3YIP6
66872 229533.A0A098DXS1 pp 229533.I1RQ92
66873 229533.A0A0E0RZZ3 pp 229533.I1RMU9
66874 229533.A0A0E0RZZ3 pp 229533.I1RDT5
66875 229533.A0A0E0RZZ3 pp 229533.I1RF85
66876 229533.A0A0E0RZZ3 pp 229533.I1R9R5
66877 229533.A0A0E0RZZ3 pp 229533.A0A1C3YMD8
66878 229533.A0A0E0RZZ3 pp 229533.A0A1C3YIB0
66879 229533.A0A0E0RZZ3 pp 229533.I1RXY7
66880 229533.A0A0E0RZZ3 pp 229533.I1RFK3
66881 229533.A0A0E0RZZ3 pp 229533.A0A1C3YJW4
66882 229533.A0A0E0RZZ3 pp 229533.I1RMU3
66883 229533.A0A0E0RZZ3 pp 229533.I1RE53
66884 229533.A0A0E0RZZ3 pp 229533.I1RNG4
66885 229533.A0A0E0RZZ3 pp 229533.I1RYP2
66886 229533.A0A0E0RZZ3 pp 229533.A0A1C3YHG7
66887 229533.A0A0E0RZZ3 pp 229533.A0A1C3YN78
66888 229533.A0A0E0RZZ3 pp 229533.I1RKB2
66889 229533.A0A0E0RZZ3 pp 229533.I1RJS2
66890 229533.A0A0E0RZZ3 pp 229533.A0A1C3YLC2
66891 229533.A0A0E0RZZ3 pp 229533.A0A1C3YKY6
66892 229533.A0A0E0RZZ3 pp 229533.I1RCV1
66893 229533.A0A0E0RZZ3 pp 229533.I1RMD3
66894 229533.A0A0E0RZZ3 pp 229533.V6RR76
66895 229533.A0A0E0RZZ3 pp 229533.I1RJT5
66896 229533.A0A0E0RZZ3 pp 229533.I1RXE6
66897 229533.A0A0E0RZZ3 pp 229533.A0A1C3YIJ9
66898 229533.A0A0E0RZZ3 pp 229533.I1RH67
66899 229533.A0A0E0RZZ3 pp 229533.I1RNM7
66900 229533.A0A0E0RZZ3 pp 229533.I1RN81
66901 229533.A0A0E0RZZ3 pp 229533.A0A0E0SNZ9
66902 229533.A0A0E0RZZ3 pp 229533.I1RMM8
66903 229533.A0A0E0RZZ3 pp 229533.I1RY49
66904 229533.A0A0E0RZZ3 pp 229533.I1S2C1
66905 229533.A0A0E0RZZ3 pp 229533.A0A1C3YKZ8
66906 229533.A0A0E0RZZ3 pp 229533.I1RCU2
66907 229533.A0A0E0RZZ3 pp 229533.A0A1C3YJL7
66908 229533.A0A0E0RZZ3 pp 229533.I1RP86
66909 229533.I1RN41 pp 229533.I1RPU4
66910 229533.I1RN41 pp 229533.I1RU37
66911 229533.I1S1L2 pp 229533.V6RRF6
66912 229533.I1RZB7 pp 229533.Q4HZS8
66913 229533.I1RZB7 pp 229533.I1S238
66914 229533.I1RZB7 pp 229533.Q5I2J3
66915 229533.I1RLM5 pp 229533.I1RSJ3
66916 229533.I1RLM5 pp 229533.V6RRM0
66917 229533.I1RLM5 pp 229533.I1RN80
66918 229533.I1RLM5 pp 229533.I1RW43
66919 229533.I1RLM5 pp 229533.I1RVX9
66920 229533.I1RLM5 pp 229533.I1RM26
66921 229533.I1RLM5 pp 229533.I1RVR2
66922 229533.I1RLM5 pp 229533.I1RY49
66923 229533.I1RLM5 pp 229533.I1RVR7
66924 229533.Q4ICM0 pp 229533.V6REE9
66925 229533.Q4ICM0 pp 229533.V6QV09
66926 229533.Q4ICM0 pp 229533.Q4INI2
66927 229533.A0A1C3YM48 pp 229533.I1RDK2
66928 229533.A0A1C3YM48 pp 229533.I1RK67
66929 229533.A0A1C3YM48 pp 229533.I1RJT8
66930 229533.A0A1C3YM48 pp 229533.I1RMA7
66931 229533.A0A1C3YM48 pp 229533.I1S248
66932 229533.A0A1C3YM48 pp 229533.I1RYB7
66933 229533.A0A1C3YM48 pp 229533.I1RW84
66934 229533.A0A1C3YM48 pp 229533.I1RJT0
66935 229533.A0A1C3YM48 pp 229533.I1S1A3
66936 229533.A0A1C3YM48 pp 229533.I1RNA3
66937 229533.A0A1C3YM48 pp 229533.I1RW38
66938 229533.A0A1C3YM48 pp 229533.I1RLT6
66939 229533.A0A1C3YM48 pp 229533.Q4IBS9
66940 229533.A0A1C3YM48 pp 229533.I1S074
66941 229533.A0A1C3YM48 pp 229533.I1RYI3

66942 229533.A0A1C3YM48 pp 229533.I1R9X6
66943 229533.A0A1C3YM48 pp 229533.I1RMR7
66944 229533.I1RPV5 pp 229533.I1RUV5
66945 229533.I1RPV5 pp 229533.Q4I5W5
66946 229533.I1RPV5 pp 229533.Q4HW67
66947 229533.I1RPV5 pp 229533.Q4HYQ4
66948 229533.I1RPV5 pp 229533.I1RRG3
66949 229533.I1RPV5 pp 229533.I1S1J0
66950 229533.I1RPV5 pp 229533.Q4HYB8
66951 229533.I1RPV5 pp 229533.Q4IJU0
66952 229533.I1RPV5 pp 229533.Q4IMD1
66953 229533.I1RPV5 pp 229533.Q4ICA8
66954 229533.I1RPV5 pp 229533.I1RQP1
66955 229533.I1RPV5 pp 229533.I1RRT0
66956 229533.I1RPV5 pp 229533.I1RZR6
66957 229533.I1RPV5 pp 229533.I1RZB5
66958 229533.I1RPV5 pp 229533.Q4I5I4
66959 229533.A0A1C3YJ82 pp 229533.I1RVR7
66960 229533.A0A1C3YJ82 pp 229533.I1RF80
66961 229533.A0A1C3YJ82 pp 229533.I1RYX6
66962 229533.I1RMG2 pp 229533.I1RNH7
66963 229533.I1RMG2 pp 229533.I1RRT4
66964 229533.I1RMG2 pp 229533.I1RYM4
66965 229533.I1RMG2 pp 229533.Q4IPI4
66966 229533.I1RMG2 pp 229533.I1RWD5
66967 229533.I1RMG2 pp 229533.I1RX84
66968 229533.I1RMG2 pp 229533.Q4ICM0
66969 229533.I1RMG2 pp 229533.I1S0S8
66970 229533.I1RMG2 pp 229533.Q4IB70
66971 229533.I1RMG2 pp 229533.I1RRF0
66972 229533.I1RMG2 pp 229533.I1RT41
66973 229533.I1RMG2 pp 229533.I1RSH3
66974 229533.I1RMG2 pp 229533.I1S270
66975 229533.I1RMG2 pp 229533.Q4I2D7
66976 229533.I1RMG2 pp 229533.Q4INI2
66977 229533.I1RMG2 pp 229533.I1RT65
66978 229533.I1RMG2 pp 229533.I1RQ70
66979 229533.I1RMG2 pp 229533.I1RQJ0
66980 229533.I1RMG2 pp 229533.I1RRI4
66981 229533.I1RMG2 pp 229533.I1RR74
66982 229533.I1RMG2 pp 229533.I1S0Z3
66983 229533.I1RMG2 pp 229533.I1RRB0
66984 229533.I1RMG2 pp 229533.I1RUJ0
66985 229533.I1RMG2 pp 229533.Q4I877
66986 229533.I1RMG2 pp 229533.I1RST4
66987 229533.I1RMG2 pp 229533.I1RQ16
66988 229533.I1RMG2 pp 229533.I1RTQ9
66989 229533.I1RMG2 pp 229533.I1RX87
66990 229533.I1REL7 pp 229533.I1RJU3
66991 229533.I1REL7 pp 229533.V6RTJ8
66992 229533.I1REL7 pp 229533.I1S4S7
66993 229533.I1REL7 pp 229533.Q4IJK2
66994 229533.I1REL7 pp 229533.Q4I948
66995 229533.I1REL7 pp 229533.I1RRX5
66996 229533.I1REL7 pp 229533.I1RFC0
66997 229533.I1REL7 pp 229533.I1RPN4
66998 229533.I1REL7 pp 229533.I1RPV5
66999 229533.I1REL7 pp 229533.I1RSQ7
67000 229533.I1REL7 pp 229533.I1S4Q4
67001 229533.I1REL7 pp 229533.V6RBN2
67002 229533.I1REL7 pp 229533.I1REZ6
67003 229533.I1REL7 pp 229533.I1S0B7
67004 229533.I1REL7 pp 229533.I1S3S3
67005 229533.I1REL7 pp 229533.V6R1K4
67006 229533.I1REL7 pp 229533.I1RQS9
67007 229533.I1REL7 pp 229533.I1RM35
67008 229533.I1REL7 pp 229533.I1RXD7
67009 229533.I1REL7 pp 229533.I1S1J7
67010 229533.I1REL7 pp 229533.I1RME6
67011 229533.I1REL7 pp 229533.I1RX46
67012 229533.I1REL7 pp 229533.I1RRY9
67013 229533.I1REL7 pp 229533.I1S4Z8
67014 229533.I1REL7 pp 229533.I1RMQ4

67015 229533.I1REL7 pp 229533.I1RZZ9
67016 229533.I1REL7 pp 229533.I1RSQ4
67017 229533.I1REL7 pp 229533.I1RS18
67018 229533.I1REL7 pp 229533.I1RJ17
67019 229533.I1REL7 pp 229533.I1RF82
67020 229533.I1REL7 pp 229533.I1S8G4
67021 229533.I1REL7 pp 229533.Q4IJ56
67022 229533.I1REL7 pp 229533.I1RZM8
67023 229533.I1REL7 pp 229533.I1RGA6
67024 229533.I1REL7 pp 229533.I1S5R1
67025 229533.I1REL7 pp 229533.I1RUV5
67026 229533.I1REL7 pp 229533.I1RS47
67027 229533.I1REL7 pp 229533.I1RXF0
67028 229533.I1REL7 pp 229533.O74638
67029 229533.I1REL7 pp 229533.Q4HZ42
67030 229533.I1REL7 pp 229533.I1RFL3
67031 229533.I1REL7 pp 229533.V6RCZ8
67032 229533.I1REL7 pp 229533.I1RTD3
67033 229533.I1REL7 pp 229533.I1RT40
67034 229533.I1REL7 pp 229533.I1S2H4
67035 229533.I1REL7 pp 229533.I1RTD5
67036 229533.I1REL7 pp 229533.I1S0L4
67037 229533.I1REL7 pp 229533.I1RRV4
67038 229533.I1REL7 pp 229533.V6QUE6
67039 229533.I1REL7 pp 229533.Q4HXF0
67040 229533.I1REL7 pp 229533.I1RGA4
67041 229533.I1REL7 pp 229533.I1S1J0
67042 229533.I1REL7 pp 229533.I1S5B0
67043 229533.I1REL7 pp 229533.I1RQM7
67044 229533.I1REL7 pp 229533.I1RFT1
67045 229533.I1REL7 pp 229533.I1RTA1
67046 229533.I1REL7 pp 229533.I1RQE7
67047 229533.I1REL7 pp 229533.I1RFL4
67048 229533.I1REL7 pp 229533.I1RL04
67049 229533.I1REL7 pp 229533.I1RSZ7
67050 229533.I1REL7 pp 229533.I1RYI5
67051 229533.I1REL7 pp 229533.I1RPP3
67052 229533.I1REL7 pp 229533.V6RRH2
67053 229533.I1REL7 pp 229533.I1RVX0
67054 229533.I1REL7 pp 229533.I1RQG4
67055 229533.I1REL7 pp 229533.I1RKY2
67056 229533.I1REL7 pp 229533.I1S0J1
67057 229533.I1REL7 pp 229533.I1RJV3
67058 229533.I1REL7 pp 229533.I1RNG4
67059 229533.I1REL7 pp 229533.I1RN67
67060 229533.I1REL7 pp 229533.I1S3D5
67061 229533.I1REL7 pp 229533.I1RMI8
67062 229533.I1REL7 pp 229533.V6R057
67063 229533.I1REL7 pp 229533.I1RG11
67064 229533.I1REL7 pp 229533.I1RWE8
67065 229533.I1REL7 pp 229533.I1RX22
67066 229533.I1REL7 pp 229533.Q4I624
67067 229533.I1REL7 pp 229533.I1RYW3
67068 229533.I1REL7 pp 229533.I1RNC1
67069 229533.I1REL7 pp 229533.I1RHQ4
67070 229533.I1REL7 pp 229533.I1RUG3
67071 229533.I1REL7 pp 229533.V6R042
67072 229533.I1REL7 pp 229533.I1SAN9
67073 229533.I1REL7 pp 229533.I1RRV1
67074 229533.I1REL7 pp 229533.I1S5R6
67075 229533.I1REL7 pp 229533.I1RGW8
67076 229533.A0A0E0S1S6 pp 229533.I1RJT0
67077 229533.A0A0E0S1S6 pp 229533.I1S1A3
67078 229533.A0A0E0S1S6 pp 229533.I1RMA7
67079 229533.A0A0E0S1S6 pp 229533.I1RLT6
67080 229533.A0A0E0S1S6 pp 229533.I1RW84
67081 229533.A0A0E0S1S6 pp 229533.I1RZW1
67082 229533.A0A0E0S1S6 pp 229533.I1RYB7
67083 229533.A0A0E0S1S6 pp 229533.I1RZK4
67084 229533.A0A0E0S1S6 pp 229533.I1RDK2
67085 229533.A0A0E0S1S6 pp 229533.I1RJT8
67086 229533.A0A0E0S1S6 pp 229533.I1S248
67087 229533.A0A0E0S1S6 pp 229533.I1RK67

67088 229533.A0A0E0S1S6 pp 229533.I1RPH1
67089 229533.A0A0E0S1S6 pp 229533.I1RMR7
67090 229533.A0A0E0S1S6 pp 229533.I1S074
67091 229533.A0A0E0S1S6 pp 229533.I1R9X6
67092 229533.A0A0E0S1S6 pp 229533.Q4IBS9
67093 229533.A0A0E0S1S6 pp 229533.A0A1C3YM48
67094 229533.A0A0E0S1S6 pp 229533.I1RW83
67095 229533.A0A0E0S1S6 pp 229533.I1S2G7
67096 229533.Q5I2J3 pp 229533.V6RPN0
67097 229533.Q5I2J3 pp 229533.V6QUU6
67098 229533.Q5I2J3 pp 229533.V6QUV7
67099 229533.Q5I2J3 pp 229533.V6RTJ8
67100 229533.Q5I2J3 pp 229533.V6R3J1
67101 229533.Q5I2J3 pp 229533.V6RR76
67102 229533.Q5I2J3 pp 229533.V6REE9
67103 229533.Q5I2J3 pp 229533.V6QX21
67104 229533.I1RNW1 pp 229533.V6QWD8
67105 229533.I1RNW1 pp 229533.I1RVR7
67106 229533.I1RNW1 pp 229533.I1RZ29
67107 229533.I1RNW1 pp 229533.I1RVX9
67108 229533.I1RNW1 pp 229533.V6RUB1
67109 229533.I1RNW1 pp 229533.I1RPM6
67110 229533.I1RNW1 pp 229533.I1RZA7
67111 229533.I1RNW1 pp 229533.I1S6P5
67112 229533.I1RNW1 pp 229533.I1S3G4
67113 229533.I1RNW1 pp 229533.I1S1W8
67114 229533.I1RTU0 pp 229533.I1S746
67115 229533.I1RTU0 pp 229533.I1RV21
67116 229533.I1RTU0 pp 229533.I1RVX9
67117 229533.I1RTU0 pp 229533.I1S3X2
67118 229533.I1RTU0 pp 229533.I1RY49
67119 229533.I1RRT4 pp 229533.I1RXT9
67120 229533.I1RRT4 pp 229533.I1S1C8
67121 229533.I1RRT4 pp 229533.I1RT41
67122 229533.I1RRT4 pp 229533.I1S0Z3
67123 229533.I1RRT4 pp 229533.I1RWM7
67124 229533.I1RRT4 pp 229533.I1S1V5
67125 229533.I1RRT4 pp 229533.I1RSE9
67126 229533.I1RRT4 pp 229533.Q4ICM0
67127 229533.I1RRT4 pp 229533.Q5I2J3
67128 229533.I1RRT4 pp 229533.I1RX54
67129 229533.I1RRT4 pp 229533.I1RZB7
67130 229533.I1RRT4 pp 229533.Q4I9B7
67131 229533.A0A0E0SA26 pp 229533.A0A1C3YK58
67132 229533.A0A0E0SA26 pp 229533.I1RPM6
67133 229533.A0A0E0SA26 pp 229533.I1RA94
67134 229533.A0A0E0SA26 pp 229533.I1RAG9
67135 229533.A0A0E0SA26 pp 229533.I1RRV4
67136 229533.A0A0E0SA26 pp 229533.I1RYI7
67137 229533.A0A0E0SA26 pp 229533.A0A0E0SBC4
67138 229533.A0A0E0SA26 pp 229533.I1RGL3
67139 229533.A0A0E0SA26 pp 229533.I1RBL7
67140 229533.A0A0E0SA26 pp 229533.I1RMM8
67141 229533.A0A0E0SA26 pp 229533.I1RP97
67142 229533.A0A0E0SA26 pp 229533.I1RZQ1
67143 229533.A0A0E0SA26 pp 229533.I1RM32
67144 229533.A0A0E0SA26 pp 229533.I1RVR7
67145 229533.A0A0E0SA26 pp 229533.I1RF80
67146 229533.A0A0E0SA26 pp 229533.V6RUB1
67147 229533.A0A0E0SA26 pp 229533.I1S6P5
67148 229533.A0A0E0SA26 pp 229533.I1RRZ6
67149 229533.A0A0E0SA26 pp 229533.I1RZA7
67150 229533.A0A0E0SA26 pp 229533.I1RFS3
67151 229533.A0A0E0SA26 pp 229533.I1RXY9
67152 229533.A0A0E0SA26 pp 229533.I1RNB4
67153 229533.A0A0E0SA26 pp 229533.I1RNG4
67154 229533.V6R3J1 pp 229533.V6RY56
67155 229533.V6R3J1 pp 229533.V6RLM7
67156 229533.V6R3J1 pp 229533.V6RUY9
67157 229533.V6R3J1 pp 229533.V6RUT5
67158 229533.V6R3J1 pp 229533.V6RTJ8
67159 229533.V6R3J1 pp 229533.V6RCZ8
67160 229533.I1RJF2 pp 229533.I1RPD6

67161 229533.I1RJF2 pp 229533.I1RPM6
67162 229533.I1RJF2 pp 229533.I1RNG4
67163 229533.I1RJF2 pp 229533.I1RLM3
67164 229533.I1RJF2 pp 229533.I1RM26
67165 229533.I1RJF2 pp 229533.I1RR12
67166 229533.I1RJF2 pp 229533.I1RJU9
67167 229533.I1RJF2 pp 229533.I1RVX9
67168 229533.I1RJF2 pp 229533.I1RKL9
67169 229533.I1RJF2 pp 229533.I1RY49
67170 229533.I1RJF2 pp 229533.I1S2D2
67171 229533.I1RJF2 pp 229533.V6RH92
67172 229533.I1RJF2 pp 229533.I1RPW2
67173 229533.I1RQF9 pp 229533.I1S4P6
67174 229533.I1RQF9 pp 229533.I1RUN9
67175 229533.I1RQF9 pp 229533.Q4HY20
67176 229533.I1RQF9 pp 229533.V6RXW9
67177 229533.I1RQF9 pp 229533.I1S1U6
67178 229533.I1RQF9 pp 229533.I1RQJ0
67179 229533.I1RQF9 pp 229533.Q4ILI9
67180 229533.I1RQF9 pp 229533.I1RQJ4
67181 229533.I1RQF9 pp 229533.I1RX95
67182 229533.I1RQF9 pp 229533.I1S287
67183 229533.I1RQF9 pp 229533.I1S2B6
67184 229533.I1RQF9 pp 229533.I1S0V5
67185 229533.I1RQF9 pp 229533.I1RWZ9
67186 229533.I1RQF9 pp 229533.I1S0G3
67187 229533.I1RQF9 pp 229533.I1RZK3
67188 229533.A0A1C3YIF5 pp 229533.I1RX00
67189 229533.A0A1C3YIF5 pp 229533.I1RS50
67190 229533.A0A1C3YIF5 pp 229533.I1RNL5
67191 229533.A0A1C3YIF5 pp 229533.I1RPR9
67192 229533.A0A1C3YL17 pp 229533.I1RVR7
67193 229533.A0A1C3YL17 pp 229533.I1RYX6
67194 229533.A0A1C3YL17 pp 229533.I1RF80
67195 229533.A0A1C3YIP6 pp 229533.I1S287
67196 229533.A0A1C3YIP6 pp 229533.I1S4Y4
67197 229533.A0A1C3YIP6 pp 229533.A0A1C3YLL9
67198 229533.A0A1C3YIP6 pp 229533.Q5I2J3
67199 229533.A0A1C3YIP6 pp 229533.I1RY40
67200 229533.A0A1C3YIP6 pp 229533.I1S3S4
67201 229533.A0A1C3YIP6 pp 229533.I1RRT4
67202 229533.A0A1C3YIP6 pp 229533.Q4IMM0
67203 229533.A0A1C3YIP6 pp 229533.I1RWM6
67204 229533.A0A1C3YIP6 pp 229533.I1RY98
67205 229533.A0A1C3YIP6 pp 229533.I1S0Z2
67206 229533.A0A1C3YIP6 pp 229533.I1REU2
67207 229533.A0A1C3YIP6 pp 229533.I1RCY0
67208 229533.A0A1C3YIP6 pp 229533.I1RYZ9
67209 229533.A0A1C3YIP6 pp 229533.I1S2B7
67210 229533.A0A1C3YIP6 pp 229533.I1S635
67211 229533.A0A1C3YIP6 pp 229533.Q4ICM0
67212 229533.A0A1C3YIP6 pp 229533.I1RMT4
67213 229533.A0A1C3YIP6 pp 229533.I1RNC0
67214 229533.A0A1C3YIP6 pp 229533.I1RFS6
67215 229533.A0A1C3YIP6 pp 229533.I1RY00
67216 229533.A0A1C3YIP6 pp 229533.V6QV09
67217 229533.A0A1C3YIP6 pp 229533.A0A1C3YJL7
67218 229533.A0A1C3YIP6 pp 229533.I1RSI4
67219 229533.A0A1C3YIP6 pp 229533.I1RZ02
67220 229533.A0A1C3YIP6 pp 229533.I1RS37
67221 229533.A0A1C3YIP6 pp 229533.I1S201
67222 229533.A0A1C3YIP6 pp 229533.I1RFU3
67223 229533.A0A1C3YIP6 pp 229533.I1RLX6
67224 229533.A0A1C3YIP6 pp 229533.I1S2F3
67225 229533.A0A1C3YIP6 pp 229533.A0A1C3YKW5
67226 229533.A0A1C3YIP6 pp 229533.I1RQ80
67227 229533.A0A1C3YIP6 pp 229533.I1RN31
67228 229533.A0A1C3YIP6 pp 229533.A0A1C3YLR6
67229 229533.A0A1C3YIP6 pp 229533.I1RTQ9
67230 229533.A0A1C3YIP6 pp 229533.I1RFX8
67231 229533.A0A1C3YIP6 pp 229533.I1RAW7
67232 229533.A0A1C3YIP6 pp 229533.I1RI75
67233 229533.A0A1C3YIP6 pp 229533.I1RN27

67234	229533.A0A1C3YIP6	pp	229533.I1RT41
67235	229533.A0A1C3YIP6	pp	229533.I1RDG8
67236	229533.A0A1C3YIP6	pp	229533.I1RPU4
67237	229533.A0A1C3YIP6	pp	229533.I1RD18
67238	229533.A0A1C3YIP6	pp	229533.A0A1C3YKT4
67239	229533.A0A1C3YIP6	pp	229533.I1RAH4
67240	229533.A0A1C3YIP6	pp	229533.I1RUR0
67241	229533.A0A1C3YIP6	pp	229533.I1RB00
67242	229533.A0A1C3YIP6	pp	229533.I1S096
67243	229533.A0A1C3YIP6	pp	229533.I1S224
67244	229533.A0A1C3YIP6	pp	229533.I1RMB7
67245	229533.A0A1C3YIP6	pp	229533.I1RN92
67246	229533.A0A1C3YIP6	pp	229533.I1RNM1
67247	229533.A0A1C3YIP6	pp	229533.I1RRF9
67248	229533.A0A1C3YIP6	pp	229533.I1RQ70
67249	229533.A0A1C3YIP6	pp	229533.I1RK32
67250	229533.A0A1C3YIP6	pp	229533.I1S1C8
67251	229533.A0A1C3YIP6	pp	229533.I1RSI1
67252	229533.A0A1C3YIP6	pp	229533.I1RCK4
67253	229533.A0A1C3YIP6	pp	229533.Q4I0J6
67254	229533.A0A1C3YIP6	pp	229533.I1RSK0
67255	229533.A0A1C3YIP6	pp	229533.I1RDJ1
67256	229533.A0A1C3YIP6	pp	229533.Q4INS6
67257	229533.A0A1C3YIP6	pp	229533.I1R976
67258	229533.A0A1C3YIP6	pp	229533.I1S0Z3
67259	229533.A0A1C3YIP6	pp	229533.I1RL24
67260	229533.A0A1C3YIP6	pp	229533.I1RMG2
67261	229533.A0A1C3YIP6	pp	229533.I1RE46
67262	229533.A0A1C3YIP6	pp	229533.I1RZG7
67263	229533.A0A1C3YIP6	pp	229533.Q4I877
67264	229533.A0A1C3YIP6	pp	229533.Q4HZS8
67265	229533.A0A1C3YIP6	pp	229533.Q4HTT2
67266	229533.A0A1C3YIP6	pp	229533.I1RCQ2
67267	229533.A0A1C3YIP6	pp	229533.V6REE9
67268	229533.A0A1C3YIP6	pp	229533.I1RA72
67269	229533.A0A1C3YIP6	pp	229533.Q4I7X1
67270	229533.A0A1C3YIP6	pp	229533.I1RZH4
67271	229533.A0A1C3YIP6	pp	229533.I1RN43
67272	229533.A0A1C3YIP6	pp	229533.I1RNI2
67273	229533.A0A1C3YIP6	pp	229533.A0A1C3YN54
67274	229533.A0A1C3YIP6	pp	229533.A0A1C3YK37
67275	229533.A0A1C3YIP6	pp	229533.I1RBI1
67276	229533.A0A1C3YIP6	pp	229533.I1S3D2
67277	229533.A0A1C3YIP6	pp	229533.I1RQJ0
67278	229533.A0A1C3YIP6	pp	229533.I1RN46
67279	229533.A0A1C3YIP6	pp	229533.Q4IBX0
67280	229533.A0A1C3YIP6	pp	229533.I1S604
67281	229533.A0A1C3YIP6	pp	229533.I1RPI0
67282	229533.A0A1C3YIP6	pp	229533.I1RJH5
67283	229533.A0A1C3YIP6	pp	229533.I1RS38
67284	229533.A0A1C3YIP6	pp	229533.I1RHI1
67285	229533.A0A1C3YIP6	pp	229533.I1RFJ2
67286	229533.A0A1C3YIP6	pp	229533.I1RY73
67287	229533.A0A1C3YIP6	pp	229533.I1RM09
67288	229533.A0A1C3YIP6	pp	229533.I1RE37
67289	229533.A0A1C3YIP6	pp	229533.I1RLF0
67290	229533.A0A1C3YIP6	pp	229533.I1RRB0
67291	229533.A0A1C3YIP6	pp	229533.A0A1C3YM86
67292	229533.A0A1C3YIP6	pp	229533.I1RCW8
67293	229533.A0A1C3YIP6	pp	229533.I1RN65
67294	229533.A0A1C3YIP6	pp	229533.I1RWP4
67295	229533.A0A1C3YIP6	pp	229533.I1RZV9
67296	229533.A0A1C3YIP6	pp	229533.Q4IER8
67297	229533.A0A1C3YIP6	pp	229533.I1S077
67298	229533.A0A1C3YIP6	pp	229533.I1RUG2
67299	229533.A0A1C3YIP6	pp	229533.V6R227
67300	229533.A0A1C3YIP6	pp	229533.I1RXB5
67301	229533.A0A1C3YIP6	pp	229533.I1RU02
67302	229533.A0A1C3YIP6	pp	229533.I1RAN0
67303	229533.A0A1C3YIP6	pp	229533.I1RWT6
67304	229533.A0A1C3YIP6	pp	229533.I1RJK8
67305	229533.A0A1C3YIP6	pp	229533.I1RAL2
67306	229533.A0A1C3YIP6	pp	229533.A0A1C3YKY3

67307 229533.A0A1C3YIP6 pp 229533.I1RVA7
67308 229533.A0A1C3YIP6 pp 229533.I1RQA3
67309 229533.A0A1C3YIP6 pp 229533.I1RDP1
67310 229533.A0A1C3YIP6 pp 229533.I1S5B5
67311 229533.A0A1C3YIP6 pp 229533.Q4IMD1
67312 229533.A0A1C3YIP6 pp 229533.I1RKQ0
67313 229533.A0A1C3YIP6 pp 229533.A0A1C3YLK5
67314 229533.A0A1C3YIP6 pp 229533.I1RCF0
67315 229533.A0A1C3YIP6 pp 229533.I1RPV5
67316 229533.A0A1C3YIP6 pp 229533.I1RCM6
67317 229533.A0A1C3YIP6 pp 229533.I1RWB3
67318 229533.A0A1C3YIP6 pp 229533.V6R3J1
67319 229533.A0A1C3YIP6 pp 229533.I1RB49
67320 229533.A0A1C3YIP6 pp 229533.I1RBV5
67321 229533.A0A1C3YIP6 pp 229533.I1RQL9
67322 229533.A0A1C3YIP6 pp 229533.I1RFW1
67323 229533.A0A1C3YIP6 pp 229533.I1S2F2
67324 229533.A0A1C3YIP6 pp 229533.I1S951
67325 229533.A0A1C3YIP6 pp 229533.I1RWI8
67326 229533.A0A1C3YIP6 pp 229533.I1RP92
67327 229533.A0A1C3YIP6 pp 229533.A0A1C3YL99
67328 229533.A0A1C3YIP6 pp 229533.I1S2E9
67329 229533.A0A1C3YIP6 pp 229533.I1RR95
67330 229533.A0A1C3YIP6 pp 229533.A0A1C3YJP1
67331 229533.A0A1C3YIP6 pp 229533.I1RE19
67332 229533.A0A1C3YIP6 pp 229533.I1RT81
67333 229533.A0A1C3YIP6 pp 229533.I1RX38
67334 229533.A0A1C3YIP6 pp 229533.I1RN54
67335 229533.A0A1C3YIP6 pp 229533.I1RSH3
67336 229533.A0A1C3YIP6 pp 229533.I1S382
67337 229533.A0A1C3YIP6 pp 229533.A0A1C3YKJ6
67338 229533.A0A1C3YIP6 pp 229533.I1RF02
67339 229533.A0A1C3YIP6 pp 229533.I1RJS4
67340 229533.A0A1C3YIP6 pp 229533.Q4IPI4
67341 229533.A0A1C3YIP6 pp 229533.I1S1V5
67342 229533.A0A1C3YIP6 pp 229533.I1RML6
67343 229533.A0A1C3YIP6 pp 229533.I1RFR6
67344 229533.A0A1C3YIP6 pp 229533.I1RUG1
67345 229533.A0A1C3YIP6 pp 229533.I1RFZ2
67346 229533.A0A1C3YIP6 pp 229533.I1RLU1
67347 229533.A0A1C3YIP6 pp 229533.A0A1C3YM61
67348 229533.A0A1C3YIP6 pp 229533.I1RQM0
67349 229533.A0A1C3YIP6 pp 229533.V6RLW4
67350 229533.A0A1C3YIP6 pp 229533.I1R9Q0
67351 229533.A0A1C3YIP6 pp 229533.A0A1C3YKC8
67352 229533.A0A1C3YIP6 pp 229533.I1RTT3
67353 229533.A0A1C3YIP6 pp 229533.I1RDE9
67354 229533.A0A1C3YIP6 pp 229533.I1RAN3
67355 229533.A0A1C3YIP6 pp 229533.I1REF9
67356 229533.A0A1C3YIP6 pp 229533.I1S2A8
67357 229533.A0A1C3YIP6 pp 229533.I1RMP2
67358 229533.A0A1C3YIP6 pp 229533.A0A1C3YLQ7
67359 229533.A0A1C3YIP6 pp 229533.I1RWG6
67360 229533.A0A1C3YIP6 pp 229533.I1RBL4
67361 229533.A0A1C3YIP6 pp 229533.Q4HTT1
67362 229533.A0A1C3YIP6 pp 229533.I1RCD4
67363 229533.A0A1C3YIP6 pp 229533.I1RC35
67364 229533.A0A1C3YIP6 pp 229533.I1RAH9
67365 229533.A0A1C3YIP6 pp 229533.I1RLV3
67366 229533.A0A1C3YIP6 pp 229533.A0A1C3YJ12
67367 229533.A0A1C3YIP6 pp 229533.I1RLX4
67368 229533.A0A1C3YIP6 pp 229533.I1RZ91
67369 229533.A0A1C3YIP6 pp 229533.I1RSC7
67370 229533.A0A1C3YIP6 pp 229533.A0A1C3YKA6
67371 229533.A0A1C3YIP6 pp 229533.I1RRW0
67372 229533.A0A1C3YIP6 pp 229533.I1REC6
67373 229533.A0A1C3YIP6 pp 229533.I1RL04
67374 229533.A0A1C3YIP6 pp 229533.I1RGD4
67375 229533.A0A1C3YIP6 pp 229533.I1RFN0
67376 229533.A0A1C3YIP6 pp 229533.I1RC54
67377 229533.A0A1C3YIP6 pp 229533.V6RGI1
67378 229533.A0A1C3YIP6 pp 229533.I1RD23
67379 229533.A0A1C3YIP6 pp 229533.I1RCT5

67380 229533.A0A1C3YIP6 pp 229533.V6QX58
67381 229533.A0A1C3YIP6 pp 229533.I1RY44
67382 229533.A0A1C3YIP6 pp 229533.I1RSL6
67383 229533.A0A1C3YIP6 pp 229533.I1RXT9
67384 229533.A0A1C3YIP6 pp 229533.I1RBD6
67385 229533.A0A1C3YIP6 pp 229533.I1RW16
67386 229533.A0A1C3YIP6 pp 229533.I1RBM3
67387 229533.A0A1C3YIP6 pp 229533.A0A1C3YM29
67388 229533.A0A1C3YIP6 pp 229533.I1S1D3
67389 229533.A0A1C3YIP6 pp 229533.I1RD10
67390 229533.A0A1C3YIP6 pp 229533.Q4IB89
67391 229533.A0A1C3YIP6 pp 229533.I1RPW0
67392 229533.A0A1C3YIP6 pp 229533.I1RCT2
67393 229533.A0A1C3YIP6 pp 229533.I1RRR6
67394 229533.A0A1C3YIP6 pp 229533.A0A1C3YMF9
67395 229533.A0A1C3YIP6 pp 229533.I1RBW7
67396 229533.A0A1C3YIP6 pp 229533.I1RI34
67397 229533.A0A1C3YIP6 pp 229533.I1RQ16
67398 229533.A0A1C3YIP6 pp 229533.I1RQ49
67399 229533.A0A1C3YIP6 pp 229533.I1RBX2
67400 229533.A0A1C3YIP6 pp 229533.I1RRP7
67401 229533.A0A1C3YIP6 pp 229533.I1RDE8
67402 229533.A0A1C3YIP6 pp 229533.I1RSW5
67403 229533.A0A1C3YIP6 pp 229533.I1RMJ8
67404 229533.A0A1C3YIP6 pp 229533.I1RJS5
67405 229533.A0A1C3YIP6 pp 229533.A0A1C3YKG7
67406 229533.A0A1C3YIP6 pp 229533.I1REC7
67407 229533.A0A1C3YIP6 pp 229533.I1RRH3
67408 229533.A0A1C3YIP6 pp 229533.I1RZQ6
67409 229533.A0A1C3YIP6 pp 229533.I1RTD7
67410 229533.A0A1C3YIP6 pp 229533.I1RME7
67411 229533.A0A1C3YIP6 pp 229533.I1RTE6
67412 229533.I1RLP2 pp 229533.I1RRJ8
67413 229533.I1RLP2 pp 229533.I1RQ31
67414 229533.I1RLP2 pp 229533.I1RS47
67415 229533.I1RLP2 pp 229533.I1RUQ2
67416 229533.I1RLP2 pp 229533.I1RRT2
67417 229533.I1RLP2 pp 229533.I1RWD4
67418 229533.I1RLP2 pp 229533.I1RT80
67419 229533.I1RLP2 pp 229533.V6QYF0
67420 229533.I1RLP2 pp 229533.I1RRI2
67421 229533.I1RLP2 pp 229533.I1RNV6
67422 229533.I1RLH7 pp 229533.I1RYX6
67423 229533.I1RLH7 pp 229533.I1RVR7
67424 229533.I1RFZ2 pp 229533.Q4HZS8
67425 229533.I1RFZ2 pp 229533.I1RI75
67426 229533.I1RFZ2 pp 229533.I1RM36
67427 229533.I1RFZ2 pp 229533.I1RKI2
67428 229533.I1RFZ2 pp 229533.I1RLX6
67429 229533.I1RFZ2 pp 229533.I1RNC0
67430 229533.I1RFZ2 pp 229533.I1S236
67431 229533.I1RFZ2 pp 229533.I1RS37
67432 229533.I1RFZ2 pp 229533.I1RN31
67433 229533.I1RFZ2 pp 229533.Q4I5R9
67434 229533.I1RFZ2 pp 229533.Q4IP51
67435 229533.I1RFZ2 pp 229533.I1S224
67436 229533.I1RFZ2 pp 229533.I1RMB7
67437 229533.I1RFZ2 pp 229533.Q4I7X1
67438 229533.I1RFZ2 pp 229533.Q4IE79
67439 229533.I1RFZ2 pp 229533.I1RZB7
67440 229533.I1RFZ2 pp 229533.I1RQ80
67441 229533.I1RFZ2 pp 229533.Q4I877
67442 229533.I1RFZ2 pp 229533.I1RPU4
67443 229533.I1RFZ2 pp 229533.I1RZH4
67444 229533.I1RFZ2 pp 229533.I1RJH5
67445 229533.I1RFZ2 pp 229533.Q4IR08
67446 229533.I1RFZ2 pp 229533.I1RWT6
67447 229533.I1RFZ2 pp 229533.I1RSI4
67448 229533.I1RFZ2 pp 229533.Q4I0J6
67449 229533.I1RFZ2 pp 229533.Q4IMM0
67450 229533.I1RFZ2 pp 229533.I1S096
67451 229533.I1RFZ2 pp 229533.I1S209
67452 229533.I1RFZ2 pp 229533.I1RRT4

67453	229533.I1RFZ2	pp	229533.I1S283
67454	229533.I1RFZ2	pp	229533.I1S5B5
67455	229533.I1RFZ2	pp	229533.Q4IER8
67456	229533.I1RFZ2	pp	229533.I1RN65
67457	229533.I1RFZ2	pp	229533.I1RMX3
67458	229533.I1RFZ2	pp	229533.I1RUG2
67459	229533.I1RFZ2	pp	229533.I1S1C8
67460	229533.I1RFZ2	pp	229533.I1RQ93
67461	229533.I1RFZ2	pp	229533.I1S2A8
67462	229533.I1RFZ2	pp	229533.I1RQA3
67463	229533.I1RFZ2	pp	229533.I1RHI1
67464	229533.I1RFZ2	pp	229533.I1RZV9
67465	229533.I1RFZ2	pp	229533.I1RX38
67466	229533.I1RFZ2	pp	229533.I1RJK7
67467	229533.I1RFZ2	pp	229533.I1RVZ9
67468	229533.I1RFZ2	pp	229533.I1RMN1
67469	229533.I1RFZ2	pp	229533.I1S951
67470	229533.I1RFZ2	pp	229533.I1RN46
67471	229533.I1RFZ2	pp	229533.I1RR95
67472	229533.I1RFZ2	pp	229533.I1RQ67
67473	229533.I1RFZ2	pp	229533.I1S2F2
67474	229533.I1RFZ2	pp	229533.I1S1V5
67475	229533.I1RFZ2	pp	229533.I1RQJ0
67476	229533.I1RFZ2	pp	229533.I1S148
67477	229533.I1RFZ2	pp	229533.I1RJS4
67478	229533.I1RFZ2	pp	229533.I1SAA0
67479	229533.I1RFZ2	pp	229533.I1RQS4
67480	229533.I1RFZ2	pp	229533.Q4IBX0
67481	229533.I1RFZ2	pp	229533.V6R3J1
67482	229533.I1RFZ2	pp	229533.I1RSJ4
67483	229533.I1RFZ2	pp	229533.Q4IMD1
67484	229533.I1RFZ2	pp	229533.I1RRB0
67485	229533.I1RFZ2	pp	229533.I1RVA7
67486	229533.I1RFZ2	pp	229533.I1S237
67487	229533.I1RFZ2	pp	229533.I1RLU1
67488	229533.I1RFZ2	pp	229533.I1RYR2
67489	229533.I1RFZ2	pp	229533.I1RYX9
67490	229533.I1RFZ2	pp	229533.I1RUG1
67491	229533.I1RFZ2	pp	229533.I1RZ86
67492	229533.I1RFZ2	pp	229533.I1S2E9
67493	229533.I1RFZ2	pp	229533.I1RQM0
67494	229533.I1RFZ2	pp	229533.I1RMP0
67495	229533.I1RFZ2	pp	229533.I1RLX4
67496	229533.I1RFZ2	pp	229533.I1RY44
67497	229533.I1RFZ2	pp	229533.I1RRW0
67498	229533.I1RFZ2	pp	229533.I1RS03
67499	229533.I1RFZ2	pp	229533.I1S382
67500	229533.I1RFZ2	pp	229533.I1RSC7
67501	229533.I1RFZ2	pp	229533.I1RXT9
67502	229533.I1RFZ2	pp	229533.I1RRH3
67503	229533.I1RFZ2	pp	229533.I1RX64
67504	229533.I1RFZ2	pp	229533.V6RLW4
67505	229533.I1RFZ2	pp	229533.I1RQJ4
67506	229533.I1RFZ2	pp	229533.I1RMS4
67507	229533.I1RFZ2	pp	229533.V6R382
67508	229533.I1RFZ2	pp	229533.I1RRP7
67509	229533.I1RFZ2	pp	229533.I1RW16
67510	229533.I1RFZ2	pp	229533.I1RPW0
67511	229533.I1RFZ2	pp	229533.I1RJS5
67512	229533.I1RFZ2	pp	229533.I1S1D3
67513	229533.I1RFZ2	pp	229533.I1RI34
67514	229533.I1RFZ2	pp	229533.I1RUG4
67515	229533.I1RFZ2	pp	229533.I1RQ16
67516	229533.I1RFZ2	pp	229533.I1RQ49
67517	229533.I1RFZ2	pp	229533.I1S020
67518	229533.I1RFZ2	pp	229533.I1RYQ0
67519	229533.I1RQP3	pp	229533.I1S270
67520	229533.I1RQP3	pp	229533.Q4HTT1
67521	229533.I1RQP3	pp	229533.I1RT41
67522	229533.I1RQP3	pp	229533.Q4IPI4
67523	229533.I1RQP3	pp	229533.I1RS41
67524	229533.I1RQP3	pp	229533.I1RU52
67525	229533.I1RQP3	pp	229533.I1RZV9

67526	229533.I1RQP3	pp	229533.Q4I948
67527	229533.I1RQP3	pp	229533.I1RZI4
67528	229533.I1RQP3	pp	229533.I1RRF0
67529	229533.I1RQP3	pp	229533.I1S086
67530	229533.I1RQP3	pp	229533.Q4IAK7
67531	229533.I1RQP3	pp	229533.Q4IB96
67532	229533.I1RQP3	pp	229533.Q4IER8
67533	229533.I1RQP3	pp	229533.I1RST4
67534	229533.I1RQP3	pp	229533.Q4IJ84
67535	229533.I1RQP3	pp	229533.I1RZR6
67536	229533.I1RQP3	pp	229533.Q4IL82
67537	229533.I1RQP3	pp	229533.I1RSI1
67538	229533.I1RQP3	pp	229533.I1RT65
67539	229533.I1RQP3	pp	229533.Q4IQX3
67540	229533.I1RQP3	pp	229533.Q4ICA8
67541	229533.I1RQP3	pp	229533.I1S0Z3
67542	229533.I1RQP3	pp	229533.I1RRM8
67543	229533.I1RQP3	pp	229533.I1RR13
67544	229533.I1RQP3	pp	229533.Q4INS6
67545	229533.I1RQP3	pp	229533.I1RUF8
67546	229533.I1RQP3	pp	229533.Q4I7S1
67547	229533.I1RQP3	pp	229533.Q4HTT2
67548	229533.I1RIH2	pp	229533.I1RVR7
67549	229533.I1RIH2	pp	229533.I1RYX6
67550	229533.I1RPM5	pp	229533.I1RQU9
67551	229533.I1RPM5	pp	229533.I1RVR7
67552	229533.I1RPM5	pp	229533.I1RPM6
67553	229533.I1RX54	pp	229533.I1S0Y1
67554	229533.I1RX54	pp	229533.Q4ICM0
67555	229533.A0A1C3YKW8	pp	229533.I1RYX6
67556	229533.A0A1C3YKW8	pp	229533.I1RQU9
67557	229533.A0A1C3YKW8	pp	229533.I1RNM7
67558	229533.A0A1C3YKW8	pp	229533.I1RF80
67559	229533.A0A1C3YKW8	pp	229533.I1RVR7
67560	229533.A0A1C3YKW8	pp	229533.I1RB38
67561	229533.A0A1C3YKW8	pp	229533.I1S8F8
67562	229533.A0A1C3YKW8	pp	229533.I1RXE6
67563	229533.A0A1C3YKW8	pp	229533.V6RRM0
67564	229533.A0A1C3YKW8	pp	229533.I1RLZ6
67565	229533.A0A1C3YKW8	pp	229533.I1RGI4
67566	229533.A0A0E0SLZ3	pp	229533.Q4IMM0
67567	229533.A0A0E0SLZ3	pp	229533.A0A1C3YM61
67568	229533.A0A0E0SLZ3	pp	229533.A0A1C3YIP6
67569	229533.A0A0E0SLZ3	pp	229533.Q4ICM0
67570	229533.A0A0E0SLZ3	pp	229533.I1RFZ2
67571	229533.A0A0E0SLZ3	pp	229533.I1RMP0
67572	229533.A0A0E0SLZ3	pp	229533.V6R3J1
67573	229533.A0A0E0SLZ3	pp	229533.I1RNG6
67574	229533.A0A0E0SLZ3	pp	229533.A0A1C3YM63
67575	229533.A0A0E0SLZ3	pp	229533.A0A1C3YLK5
67576	229533.A0A0E0SLZ3	pp	229533.I1RQ80
67577	229533.A0A0E0SLZ3	pp	229533.I1RAW7
67578	229533.A0A0E0SLZ3	pp	229533.A0A1C3YJ33
67579	229533.A0A0E0SLZ3	pp	229533.I1RR95
67580	229533.A0A0E0SLZ3	pp	229533.A0A1C3YKW5
67581	229533.A0A0E0SLZ3	pp	229533.I1RFW1
67582	229533.A0A0E0SLZ3	pp	229533.I1S2F3
67583	229533.A0A0E0SLZ3	pp	229533.I1RWM6
67584	229533.A0A0E0SLZ3	pp	229533.A0A1C3YL99
67585	229533.A0A0E0SLZ3	pp	229533.A0A1C3YIA8
67586	229533.A0A0E0SLZ3	pp	229533.A0A1C3YJP1
67587	229533.A0A0E0SLZ3	pp	229533.V6RM66
67588	229533.A0A0E0SLZ3	pp	229533.I1RAH9
67589	229533.A0A0E0SLZ3	pp	229533.I1RCY0
67590	229533.A0A0E0SLZ3	pp	229533.I1RF02
67591	229533.A0A0E0SLZ3	pp	229533.A0A1C3YKJ6
67592	229533.A0A0E0SLZ3	pp	229533.I1RLX6
67593	229533.A0A0E0SLZ3	pp	229533.A0A0E0SNM6
67594	229533.A0A0E0SLZ3	pp	229533.A0A1C3YIH7
67595	229533.A0A0E0SLZ3	pp	229533.A0A1C3YIE7
67596	229533.A0A0E0SLZ3	pp	229533.I1RBV5
67597	229533.A0A0E0SLZ3	pp	229533.I1S2E9
67598	229533.A0A0E0SLZ3	pp	229533.I1RNC0

67599	229533.A0A0E0SLZ3	pp	229533.I1RS37
67600	229533.A0A0E0SLZ3	pp	229533.I1RDX8
67601	229533.A0A0E0SLZ3	pp	229533.I1RZH4
67602	229533.A0A0E0SLZ3	pp	229533.I1RMB7
67603	229533.A0A0E0SLZ3	pp	229533.I1RBI1
67604	229533.A0A0E0SLZ3	pp	229533.I1RAH4
67605	229533.A0A0E0SLZ3	pp	229533.I1RB00
67606	229533.A0A0E0SLZ3	pp	229533.A0A1C3YI55
67607	229533.A0A0E0SLZ3	pp	229533.I1RN31
67608	229533.A0A0E0SLZ3	pp	229533.I1RQ49
67609	229533.A0A0E0SLZ3	pp	229533.A0A1C3YLR6
67610	229533.A0A0E0SLZ3	pp	229533.I1RQM0
67611	229533.A0A0E0SLZ3	pp	229533.I1RE46
67612	229533.A0A0E0SLZ3	pp	229533.I1RUG1
67613	229533.A0A0E0SLZ3	pp	229533.I1RZR5
67614	229533.A0A0E0SLZ3	pp	229533.I1RZG7
67615	229533.A0A0E0SLZ3	pp	229533.I1RBD6
67616	229533.A0A0E0SLZ3	pp	229533.I1RX38
67617	229533.A0A0E0SLZ3	pp	229533.A0A1C3YI39
67618	229533.A0A0E0SLZ3	pp	229533.I1RI75
67619	229533.A0A0E0SLZ3	pp	229533.I1RW16
67620	229533.A0A0E0SLZ3	pp	229533.I1RP94
67621	229533.A0A0E0SLZ3	pp	229533.I1S2F2
67622	229533.A0A0E0SLZ3	pp	229533.I1RSL6
67623	229533.A0A0E0SLZ3	pp	229533.I1RD18
67624	229533.A0A0E0SLZ3	pp	229533.I1RCQ4
67625	229533.A0A0E0SLZ3	pp	229533.I1RCK4
67626	229533.A0A0E0SLZ3	pp	229533.I1RC91
67627	229533.A0A0E0SLZ3	pp	229533.Q4HZS8
67628	229533.A0A0E0SLZ3	pp	229533.A0A0E0SNY0
67629	229533.A0A0E0SLZ3	pp	229533.A0A1C3YLS3
67630	229533.A0A0E0SLZ3	pp	229533.I1RBL4
67631	229533.A0A0E0SLZ3	pp	229533.I1RNA2
67632	229533.A0A0E0SLZ3	pp	229533.A0A1C3YLQ7
67633	229533.A0A0E0SLZ3	pp	229533.I1RLE7
67634	229533.A0A0E0SLZ3	pp	229533.I1RRS7
67635	229533.A0A0E0SLZ3	pp	229533.A0A1C3YJD4
67636	229533.A0A0E0SLZ3	pp	229533.A0A1C3YI10
67637	229533.A0A0E0SLZ3	pp	229533.I1S2A8
67638	229533.A0A0E0SLZ3	pp	229533.I1RC35
67639	229533.A0A0E0SLZ3	pp	229533.I1RPX8
67640	229533.A0A0E0SLZ3	pp	229533.I1RMX3
67641	229533.A0A0E0SLZ3	pp	229533.I1S1V5
67642	229533.A0A0E0SLZ3	pp	229533.I1RY09
67643	229533.A0A0E0SLZ3	pp	229533.I1S1C8
67644	229533.A0A0E0SLZ3	pp	229533.Q4I0J6
67645	229533.A0A0E0SLZ3	pp	229533.I1RBM3
67646	229533.A0A0E0SLZ3	pp	229533.I1RJS4
67647	229533.A0A0E0SLZ3	pp	229533.I1RR21
67648	229533.A0A0E0SLZ3	pp	229533.I1RXT9
67649	229533.A0A0E0SLZ3	pp	229533.I1SAA0
67650	229533.A0A0E0SLZ3	pp	229533.I1RY44
67651	229533.A0A0E0SLZ3	pp	229533.I1RC54
67652	229533.A0A0E0SLZ3	pp	229533.A0A0E0SQV5
67653	229533.A0A0E0SLZ3	pp	229533.I1RTE5
67654	229533.A0A0E0SLZ3	pp	229533.I1RPU4
67655	229533.A0A0E0SLZ3	pp	229533.I1RLU1
67656	229533.A0A0E0SLZ3	pp	229533.I1RFN0
67657	229533.A0A0E0SLZ3	pp	229533.I1RSJ4
67658	229533.A0A0E0SLZ3	pp	229533.I1RCW8
67659	229533.A0A0E0SLZ3	pp	229533.I1REG5
67660	229533.A0A0E0SLZ3	pp	229533.I1RS50
67661	229533.A0A0E0SLZ3	pp	229533.A0A1C3YIA9
67662	229533.A0A0E0SLZ3	pp	229533.Q4I0M9
67663	229533.A0A0E0SLZ3	pp	229533.I1RCU7
67664	229533.A0A0E0SLZ3	pp	229533.I1RJI8
67665	229533.A0A0E0SLZ3	pp	229533.I1RUR0
67666	229533.A0A0E0SLZ3	pp	229533.A0A1C3YK37
67667	229533.A0A0E0SLZ3	pp	229533.I1S1D3
67668	229533.A0A0E0SLZ3	pp	229533.I1RFR6
67669	229533.A0A0E0SLZ3	pp	229533.I1RY73
67670	229533.A0A0E0SLZ3	pp	229533.I1RI34
67671	229533.A0A0E0SLZ3	pp	229533.I1RSC7

67672 229533.A0A0E0SLZ3 pp 229533.Q4IBX0
67673 229533.A0A0E0SLZ3 pp 229533.I1RJH5
67674 229533.A0A0E0SLZ3 pp 229533.I1RUG2
67675 229533.A0A0E0SLZ3 pp 229533.Q4INW6
67676 229533.A0A0E0SLZ3 pp 229533.Q4HTT2
67677 229533.A0A0E0SLZ3 pp 229533.I1RLX4
67678 229533.A0A0E0SLZ3 pp 229533.A0A1C3YM29
67679 229533.A0A0E0SLZ3 pp 229533.A0A1C3YJW9
67680 229533.A0A0E0SLZ3 pp 229533.I1RRB0
67681 229533.A0A0E0SLZ3 pp 229533.I1RE37
67682 229533.A0A0E0SLZ3 pp 229533.I1S050
67683 229533.A0A0E0SLZ3 pp 229533.I1RQ16
67684 229533.A0A0E0SLZ3 pp 229533.V6RXB3
67685 229533.A0A0E0SLZ3 pp 229533.I1RJS5
67686 229533.A0A0E0SLZ3 pp 229533.I1RBE4
67687 229533.A0A0E0SLZ3 pp 229533.I1RZV9
67688 229533.A0A0E0SLZ3 pp 229533.I1RGE7
67689 229533.A0A0E0SLZ3 pp 229533.I1RJE5
67690 229533.A0A0E0SLZ3 pp 229533.I1RPI0
67691 229533.A0A0E0SLZ3 pp 229533.I1RN46
67692 229533.A0A0E0SLZ3 pp 229533.I1RQJ0
67693 229533.A0A0E0SLZ3 pp 229533.A0A1C3YI44
67694 229533.A0A0E0SLZ3 pp 229533.I1RJI7
67695 229533.A0A0E0SLZ3 pp 229533.I1RAL2
67696 229533.A0A0E0SLZ3 pp 229533.Q4INS6
67697 229533.A0A0E0SLZ3 pp 229533.I1RCL7
67698 229533.A0A0E0SLZ3 pp 229533.I1RD70
67699 229533.A0A0E0SLZ3 pp 229533.I1RYP9
67700 229533.A0A0E0SLZ3 pp 229533.I1RDH9
67701 229533.A0A0E0SLZ3 pp 229533.I1RWB3
67702 229533.A0A0E0SLZ3 pp 229533.A0A1C3YI93
67703 229533.A0A0E0SLZ3 pp 229533.I1RHI1
67704 229533.A0A0E0SLZ3 pp 229533.I1RRP7
67705 229533.A0A0E0SLZ3 pp 229533.I1RCM6
67706 229533.A0A0E0SLZ3 pp 229533.I1RPW0
67707 229533.A0A0E0SLZ3 pp 229533.I1RDY1
67708 229533.A0A0E0SLZ3 pp 229533.I1RD19
67709 229533.A0A0E0SLZ3 pp 229533.I1RN65
67710 229533.A0A0E0SLZ3 pp 229533.I1RX64
67711 229533.A0A0E0SLZ3 pp 229533.Q4IER8
67712 229533.A0A0E0SLZ3 pp 229533.I1RQ93
67713 229533.A0A0E0SLZ3 pp 229533.I1S5B5
67714 229533.A0A0E0SLZ3 pp 229533.I1RAN0
67715 229533.A0A0E0SLZ3 pp 229533.I1RU02
67716 229533.A0A0E0SLZ3 pp 229533.I1RTN6
67717 229533.I1RUV4 pp 229533.I1RVX9
67718 229533.I1RUV4 pp 229533.I1RY49
67719 229533.A0A1C3YKW5 pp 229533.I1RW16
67720 229533.A0A1C3YKW5 pp 229533.I1RBD6
67721 229533.A0A1C3YKW5 pp 229533.V6R382
67722 229533.A0A1C3YKW5 pp 229533.I1S2M1
67723 229533.A0A1C3YKW5 pp 229533.I1R9X7
67724 229533.A0A1C3YKW5 pp 229533.I1RA43
67725 229533.A0A1C3YKW5 pp 229533.I1S1W4
67726 229533.A0A1C3YKW5 pp 229533.I1R9Y9
67727 229533.A0A1C3YKW5 pp 229533.V6RKY7
67728 229533.A0A1C3YKW5 pp 229533.I1RY44
67729 229533.A0A1C3YKW5 pp 229533.V6QX58
67730 229533.A0A1C3YKW5 pp 229533.V6RVG3
67731 229533.A0A1C3YKW5 pp 229533.I1S0M2
67732 229533.A0A1C3YKW5 pp 229533.I1RSC7
67733 229533.A0A1C3YKW5 pp 229533.I1RM99
67734 229533.A0A1C3YKW5 pp 229533.I1RRW0
67735 229533.A0A1C3YKW5 pp 229533.I1RZQ6
67736 229533.A0A1C3YKW5 pp 229533.I1RSP7
67737 229533.A0A1C3YKW5 pp 229533.I1RFN0
67738 229533.A0A1C3YKW5 pp 229533.I1RZT1
67739 229533.A0A1C3YKW5 pp 229533.I1RC54
67740 229533.A0A1C3YKW5 pp 229533.V6RGI1
67741 229533.A0A1C3YKW5 pp 229533.I1RD70
67742 229533.A0A1C3YKW5 pp 229533.I1RMM1
67743 229533.A0A1C3YKW5 pp 229533.I1RRP7
67744 229533.A0A1C3YKW5 pp 229533.I1RPW0

67745	229533.A0A1C3YKW5	pp	229533.I1RQ16
67746	229533.A0A1C3YKW5	pp	229533.I1S2E9
67747	229533.A0A1C3YKW5	pp	229533.I1RRY3
67748	229533.A0A1C3YKW5	pp	229533.A0A1C3YL99
67749	229533.A0A1C3YKW5	pp	229533.I1RG45
67750	229533.A0A1C3YKW5	pp	229533.I1RYX9
67751	229533.A0A1C3YKW5	pp	229533.I1RZE4
67752	229533.A0A1C3YKW5	pp	229533.I1RR95
67753	229533.A0A1C3YKW5	pp	229533.I1RFL7
67754	229533.A0A1C3YKW5	pp	229533.I1RKK0
67755	229533.A0A1C3YKW5	pp	229533.I1RFW1
67756	229533.A0A1C3YKW5	pp	229533.V6R3J1
67757	229533.A0A1C3YKW5	pp	229533.I1RMP0
67758	229533.A0A1C3YKW5	pp	229533.I1RFZ2
67759	229533.A0A1C3YKW5	pp	229533.I1RAN2
67760	229533.A0A1C3YKW5	pp	229533.I1RPU4
67761	229533.A0A1C3YKW5	pp	229533.I1RC35
67762	229533.A0A1C3YKW5	pp	229533.Q4IR08
67763	229533.A0A1C3YKW5	pp	229533.I1S1C8
67764	229533.A0A1C3YKW5	pp	229533.I1RBL4
67765	229533.A0A1C3YKW5	pp	229533.I1RUN8
67766	229533.A0A1C3YKW5	pp	229533.I1S4R1
67767	229533.A0A1C3YKW5	pp	229533.I1RCA5
67768	229533.A0A1C3YKW5	pp	229533.I1S2F2
67769	229533.A0A1C3YKW5	pp	229533.I1RCK4
67770	229533.A0A1C3YKW5	pp	229533.I1RC95
67771	229533.A0A1C3YKW5	pp	229533.I1RQM0
67772	229533.A0A1C3YKW5	pp	229533.I1RUG2
67773	229533.A0A1C3YKW5	pp	229533.I1RUG1
67774	229533.A0A1C3YKW5	pp	229533.I1S1V5
67775	229533.A0A1C3YKW5	pp	229533.I1RFR6
67776	229533.A0A1C3YKW5	pp	229533.I1RSJ4
67777	229533.A0A1C3YKW5	pp	229533.I1RAL2
67778	229533.A0A1C3YKW5	pp	229533.I1RCL7
67779	229533.A0A1C3YKW5	pp	229533.I1RCM6
67780	229533.A0A1C3YKW5	pp	229533.I1RQJ0
67781	229533.A0A1C3YKW5	pp	229533.I1RN46
67782	229533.A0A1C3YKW5	pp	229533.V6RLW4
67783	229533.A0A1C3YKW5	pp	229533.I1RLU1
67784	229533.A0A1C3YKW5	pp	229533.I1RMM9
67785	229533.A0A1C3YKW5	pp	229533.I1RAM7
67786	229533.A0A1C3YKW5	pp	229533.I1RE46
67787	229533.A0A1C3YKW5	pp	229533.Q4I0J6
67788	229533.A0A1C3YKW5	pp	229533.I1S439
67789	229533.A0A1C3YKW5	pp	229533.Q4IBX0
67790	229533.A0A1C3YKW5	pp	229533.I1RZV9
67791	229533.A0A1C3YKW5	pp	229533.I1RQ26
67792	229533.A0A1C3YKW5	pp	229533.A0A1C3YM63
67793	229533.A0A1C3YKW5	pp	229533.I1RA12
67794	229533.A0A1C3YKW5	pp	229533.I1RFJ2
67795	229533.A0A1C3YKW5	pp	229533.I1RUQ1
67796	229533.A0A1C3YKW5	pp	229533.I1RSI4
67797	229533.A0A1C3YKW5	pp	229533.I1R994
67798	229533.A0A1C3YKW5	pp	229533.Q4HZS8
67799	229533.A0A1C3YKW5	pp	229533.I1RRB0
67800	229533.A0A1C3YKW5	pp	229533.V6QV09
67801	229533.A0A1C3YKW5	pp	229533.I1RAW7
67802	229533.A0A1C3YKW5	pp	229533.Q4IER8
67803	229533.A0A1C3YKW5	pp	229533.I1RNC0
67804	229533.A0A1C3YKW5	pp	229533.A0A1C3YLR6
67805	229533.A0A1C3YKW5	pp	229533.I1RN31
67806	229533.A0A1C3YKW5	pp	229533.I1RQ80
67807	229533.A0A1C3YKW5	pp	229533.I1RN08
67808	229533.A0A1C3YKW5	pp	229533.I1RLX6
67809	229533.A0A1C3YKW5	pp	229533.I1RFX8
67810	229533.A0A1C3YKW5	pp	229533.I1RUQ5
67811	229533.A0A1C3YKW5	pp	229533.A0A1C3YMF2
67812	229533.A0A1C3YKW5	pp	229533.I1RYZ9
67813	229533.A0A1C3YKW5	pp	229533.I1RAH4
67814	229533.A0A1C3YKW5	pp	229533.I1RMB7
67815	229533.A0A1C3YKW5	pp	229533.I1RL24
67816	229533.A0A1C3YKW5	pp	229533.I1RYH9
67817	229533.A0A1C3YKW5	pp	229533.I1S224

229533.A0A1C3YKW5 pp 229533.I1RB00
229533.A0A1C3YKW5 pp 229533.I1RDB1
229533.A0A1C3YKW5 pp 229533.I1RLE7
229533.A0A1C3YKW5 pp 229533.I1RCU7
229533.A0A1C3YKW5 pp 229533.I1RD18
229533.A0A098D3N9 pp 229533.I1RQ16
229533.A0A098D3N9 pp 229533.A0A1C3YN20
229533.A0A098D3N9 pp 229533.I1S0S8
229533.A0A098D3N9 pp 229533.I1RB00
229533.A0A098D3N9 pp 229533.I1RD70
229533.A0A098D3N9 pp 229533.A0A1C3YI93
229533.A0A098D3N9 pp 229533.I1RMB7
229533.A0A098D3N9 pp 229533.A0A0E0RTV8
229533.A0A098D3N9 pp 229533.A0A1C3YJJ1
229533.A0A098D3N9 pp 229533.A0A1C3YLR6
229533.A0A098D3N9 pp 229533.I1RZT3
229533.A0A098D3N9 pp 229533.I1RAM1
229533.A0A098D3N9 pp 229533.A0A098DWY7
229533.A0A098D3N9 pp 229533.Q4I0J6
229533.A0A098D3N9 pp 229533.I1RN31
229533.A0A098D3N9 pp 229533.I1RC35
229533.A0A098D3N9 pp 229533.A0A0E0RS85
229533.A0A098D3N9 pp 229533.I1RFD6
229533.A0A098D3N9 pp 229533.Q4INI2
229533.A0A098D3N9 pp 229533.I1REG5
229533.A0A098D3N9 pp 229533.A0A098DSW7
229533.A0A098D3N9 pp 229533.I1RDY5
229533.A0A098D3N9 pp 229533.I1RB08
229533.A0A098D3N9 pp 229533.Q4IBX0
229533.A0A098D3N9 pp 229533.I1S0G3
229533.A0A098D3N9 pp 229533.I1RRB0
229533.A0A098D3N9 pp 229533.I1RA72
229533.A0A098D3N9 pp 229533.I1RW16
229533.A0A098D3N9 pp 229533.I1S1V5
229533.A0A098D3N9 pp 229533.I1S1C8
229533.A0A098D3N9 pp 229533.I1RQJ0
229533.A0A098D3N9 pp 229533.I1RFR6
229533.A0A098D3N9 pp 229533.A0A1C3YI44
229533.A0A098D3N9 pp 229533.I1RCM6
229533.A0A098D3N9 pp 229533.I1RSJ4
229533.A0A098D3N9 pp 229533.I1RXT9
229533.A0A098D3N9 pp 229533.I1RQ80
229533.A0A098D3N9 pp 229533.I1RD18
229533.A0A098D3N9 pp 229533.I1RAW7
229533.A0A098D3N9 pp 229533.I1RYX9
229533.A0A098D3N9 pp 229533.I1RFN0
229533.A0A098D3N9 pp 229533.A0A098D635
229533.A0A098D3N9 pp 229533.I1RWM7
229533.A0A098D3N9 pp 229533.I1S2F2
229533.A0A098D3N9 pp 229533.I1RKI1
229533.A0A098D3N9 pp 229533.I1RSC7
229533.A0A098D3N9 pp 229533.A0A0E0SNZ9
229533.A0A098D3N9 pp 229533.I1RCF0
229533.A0A098D3N9 pp 229533.A0A098DXS1
229533.A0A098D3N9 pp 229533.I1RZB7
229533.A0A098D3N9 pp 229533.Q4ICM0
229533.A0A098D3N9 pp 229533.I1RMG2
229533.A0A098D3N9 pp 229533.Q5I2J3
229533.A0A098D3N9 pp 229533.I1RRT4
229533.A0A098D3N9 pp 229533.V6R3J1
229533.A0A098D3N9 pp 229533.I1RQF9
229533.A0A098D3N9 pp 229533.A0A1C3YIP6
229533.A0A098D3N9 pp 229533.I1RFZ2
229533.A0A098D3N9 pp 229533.A0A1C3YIH7
229533.A0A098D3N9 pp 229533.A0A1C3YLK5
229533.A0A098D3N9 pp 229533.I1RX54
229533.A0A098D3N9 pp 229533.A0A0E0SLZ3
229533.A0A098D3N9 pp 229533.A0A1C3YKW5
229533.I1RSL2 pp 229533.V6RRM0
229533.I1RSL2 pp 229533.I1RWM4
229533.I1RSL2 pp 229533.I1RXH1
229533.I1RSL2 pp 229533.I1RXH3
229533.A0A1C3YMM2 pp 229533.I1RQU9

67891 229533.A0A1C3YMM2 pp 229533.I1RW71
67892 229533.A0A1C3YMM2 pp 229533.I1RLZ6
67893 229533.A0A1C3YMM2 pp 229533.I1RGI4
67894 229533.A0A1C3YMM2 pp 229533.I1RCH9
67895 229533.A0A1C3YMM2 pp 229533.I1RK24
67896 229533.A0A1C3YMM2 pp 229533.I1S8P5
67897 229533.A0A1C3YMM2 pp 229533.V6QUE6
67898 229533.A0A1C3YMM2 pp 229533.I1RF80
67899 229533.A0A1C3YMM2 pp 229533.I1RBS0
67900 229533.A0A1C3YMM2 pp 229533.I1RVR7
67901 229533.A0A1C3YMM2 pp 229533.I1RTA0
67902 229533.A0A098DC44 pp 229533.Q4ICA8
67903 229533.A0A098DC44 pp 229533.I1RRG3
67904 229533.A0A098DC44 pp 229533.I1RQP3
67905 229533.A0A098DC44 pp 229533.I1RBW0
67906 229533.A0A098DC44 pp 229533.I1RQP1
67907 229533.A0A098DC44 pp 229533.Q4IPI4
67908 229533.A0A098DC44 pp 229533.I1RBW1
67909 229533.A0A098DC44 pp 229533.A0A1C3YM91
67910 229533.A0A098DC44 pp 229533.I1RSC7
67911 229533.A0A098DC44 pp 229533.A0A1C3YN69
67912 229533.A0A098DC44 pp 229533.I1RY58
67913 229533.I1R9V5 pp 229533.I1S7X9
67914 229533.I1RJ72 pp 229533.I1RM26
67915 229533.I1RJ72 pp 229533.I1RPM6
67916 229533.I1RJ72 pp 229533.I1S3G4
67917 229533.I1RJ72 pp 229533.I1S6P5
67918 229533.I1RJ72 pp 229533.I1RZA7
67919 229533.I1RJ72 pp 229533.I1S1W8
67920 229533.I1RJ72 pp 229533.I1RVR7
67921 229533.I1RJ72 pp 229533.I1RNW1
67922 229533.I1RJ72 pp 229533.V6QWD8
67923 229533.I1RJ72 pp 229533.I1RZ29
67924 229533.I1RJ72 pp 229533.I1RNG4
67925 229533.I1RJ72 pp 229533.I1RVX9
67926 229533.I1RJ72 pp 229533.V6RUB1
67927 229533.A0A098DVJ5 pp 229533.A0A1C3YKJ7
67928 229533.A0A098DVJ5 pp 229533.I1RJU7
67929 229533.A0A098DVJ5 pp 229533.A0A1C3YID5
67930 229533.A0A098DVJ5 pp 229533.A0A098E488
67931 229533.A0A098DVJ5 pp 229533.A0A098E1K1
67932 229533.A0A098DVJ5 pp 229533.I1RDY1
67933 229533.A0A098DVJ5 pp 229533.A0A0E0SCW3
67934 229533.A0A098DVJ5 pp 229533.I1RYV6
67935 229533.A0A098DVJ5 pp 229533.A0A1C3YMH5
67936 229533.A0A098DVJ5 pp 229533.A0A0E0SMG8
67937 229533.A0A098DVJ5 pp 229533.I1R9Z0
67938 229533.A0A1C3YIF6 pp 229533.A0A1C3YJ16
67939 229533.A0A1C3YIF6 pp 229533.I1RMH0
67940 229533.A0A1C3YIF6 pp 229533.V6R6F6
67941 229533.A0A1C3YIF6 pp 229533.A0A1C3YK94
67942 229533.A0A098D3N0 pp 229533.I1R9P0
67943 229533.A0A098D3N0 pp 229533.A0A098DVJ5
67944 229533.I1RSB5 pp 229533.I1RWQ3
67945 229533.A0A1C3YLN6 pp 229533.I1RY49
67946 229533.A0A1C3YLN6 pp 229533.I1RPW2
67947 229533.A0A1C3YLN6 pp 229533.I1S3X2
67948 229533.A0A1C3YLN6 pp 229533.I1RHG1
67949 229533.A0A1C3YLN6 pp 229533.I1RPD6
67950 229533.A0A1C3YLN6 pp 229533.I1RR12
67951 229533.A0A1C3YLN6 pp 229533.I1RF35
67952 229533.A0A1C3YLN6 pp 229533.I1S746
67953 229533.A0A1C3YLN6 pp 229533.I1RNG4
67954 229533.A0A1C3YLN6 pp 229533.I1R9B5
67955 229533.A0A1C3YLN6 pp 229533.I1RVX9
67956 229533.A0A1C3YLN6 pp 229533.I1RV21
67957 229533.A0A1C3YLN6 pp 229533.I1RG29
67958 229533.A0A1C3YLN6 pp 229533.I1RLM3
67959 229533.A0A1C3YLN6 pp 229533.I1RBH0
67960 229533.A0A1C3YLN6 pp 229533.I1RBE7
67961 229533.A0A1C3YLN6 pp 229533.I1RPM6
67962 229533.A0A1C3YLN6 pp 229533.I1RA94
67963 229533.A0A1C3YLN6 pp 229533.I1RM26

67964 229533.A0A1C3YLN6 pp 229533.I1RFV9
67965 229533.A0A1C3YLN6 pp 229533.I1RJU9
67966 229533.Q4I2W2 pp 229533.Q4IBU4
67967 229533.Q4I2W2 pp 229533.V6RB81
67968 229533.Q4I2W2 pp 229533.Q4IJW4
67969 229533.Q4I2W2 pp 229533.Q4I6B0
67970 229533.Q4I2W2 pp 229533.Q5I2J3
67971 229533.I1RVR7 pp 229533.I1S3G4
67972 229533.I1RVR7 pp 229533.V6R5P0
67973 229533.I1RVR7 pp 229533.I1S1W8
67974 229533.I1RVR7 pp 229533.I1S318
67975 229533.I1RVR7 pp 229533.I1RYM5
67976 229533.I1RZS4 pp 229533.Q4HZ95
67977 229533.I1RZS4 pp 229533.Q4I6B0
67978 229533.I1RZS4 pp 229533.Q4IJW4
67979 229533.I1RZS4 pp 229533.Q4IB65
67980 229533.I1RZS4 pp 229533.Q4IPZ1
67981 229533.I1RZS4 pp 229533.Q4I099
67982 229533.I1RZS4 pp 229533.Q4IK03
67983 229533.I1RZS4 pp 229533.Q4I7T5
67984 229533.I1RZS4 pp 229533.V6R3J1
67985 229533.I1RZS4 pp 229533.I1S088
67986 229533.I1RZS4 pp 229533.Q4HYR2
67987 229533.I1RZS4 pp 229533.Q4I2W2
67988 229533.I1RZS4 pp 229533.V6R1X4
67989 229533.I1RZS4 pp 229533.Q4IBU4
67990 229533.I1RZS4 pp 229533.I1S8F0
67991 229533.I1RZS4 pp 229533.Q4I375
67992 229533.A0A1C3YN33 pp 229533.I1RQU9
67993 229533.A0A1C3YN33 pp 229533.I1RW71
67994 229533.A0A1C3YN33 pp 229533.I1RTA0
67995 229533.A0A1C3YN33 pp 229533.V6QUE6
67996 229533.A0A1C3YN33 pp 229533.I1RLZ6
67997 229533.A0A1C3YN33 pp 229533.I1RCH9
67998 229533.A0A1C3YN33 pp 229533.I1RK24
67999 229533.A0A1C3YN33 pp 229533.I1RVR7
68000 229533.A0A1C3YN33 pp 229533.I1RF80
68001 229533.A0A1C3YN33 pp 229533.I1RBS0
68002 229533.A0A1C3YN33 pp 229533.I1S8P5
68003 229533.A0A1C3YN33 pp 229533.I1RGI4
68004 229533.I1RG10 pp 229533.I1S9H4
68005 229533.I1RG10 pp 229533.I1RH14
68006 229533.I1RJR8 pp 229533.I1RLA2
68007 229533.I1RJR8 pp 229533.I1RKK0
68008 229533.I1RJR8 pp 229533.I1RX70
68009 229533.I1RJR8 pp 229533.I1S048
68010 229533.I1RJR8 pp 229533.I1RST9
68011 229533.I1RJR8 pp 229533.I1RYH9
68012 229533.I1RJR8 pp 229533.I1RSK9
68013 229533.I1RJR8 pp 229533.I1RRL5
68014 229533.I1RJR8 pp 229533.I1RM09
68015 229533.I1RJR8 pp 229533.I1S1W8
68016 229533.I1RJR8 pp 229533.V6RJP8
68017 229533.I1RJR8 pp 229533.I1S334
68018 229533.I1RJR8 pp 229533.I1RYT8
68019 229533.I1RJR8 pp 229533.I1RQV7
68020 229533.I1RJR8 pp 229533.I1RQV8
68021 229533.I1RJR8 pp 229533.I1RSE2
68022 229533.I1RJR8 pp 229533.I1RUQ5
68023 229533.I1RJR8 pp 229533.Q4IJW4
68024 229533.I1RJR8 pp 229533.I1RSG0
68025 229533.I1RJR8 pp 229533.I1RWT6
68026 229533.I1RJR8 pp 229533.I1S439
68027 229533.I1RJR8 pp 229533.V6RHX2
68028 229533.I1RJR8 pp 229533.I1RNP7
68029 229533.I1RJR8 pp 229533.I1SAB4
68030 229533.I1RJR8 pp 229533.I1S267
68031 229533.I1RJR8 pp 229533.I1RS07
68032 229533.I1RJR8 pp 229533.Q4I6B0
68033 229533.I1RJR8 pp 229533.I1RQA3
68034 229533.I1RJR8 pp 229533.I1RPK5
68035 229533.I1RJR8 pp 229533.I1RLU1
68036 229533.I1RJR8 pp 229533.I1S2E9

68037 229533.I1RJR8 pp 229533.I1RTG2
68038 229533.I1RJR8 pp 229533.I1RUN8
68039 229533.I1RJR8 pp 229533.I1RY32
68040 229533.I1RJR8 pp 229533.I1RZ25
68041 229533.I1RJR8 pp 229533.I1S3G4
68042 229533.I1RJR8 pp 229533.I1S9T6
68043 229533.I1RJR8 pp 229533.I1RNJ5
68044 229533.I1RJR8 pp 229533.I1RMP0
68045 229533.I1RJR8 pp 229533.I1RTM8
68046 229533.I1RJR8 pp 229533.I1RM41
68047 229533.I1RJR8 pp 229533.I1S3H8
68048 229533.I1RJR8 pp 229533.I1S5X9
68049 229533.I1RJR8 pp 229533.I1S1D7
68050 229533.I1RJR8 pp 229533.I1RZ95
68051 229533.I1RJR8 pp 229533.I1RZE4
68052 229533.I1RJR8 pp 229533.I1RY44
68053 229533.I1RJR8 pp 229533.I1RZC3
68054 229533.I1RJR8 pp 229533.V6RKY7
68055 229533.I1RJR8 pp 229533.V6RVG3
68056 229533.I1RF80 pp 229533.I1RPM5
68057 229533.I1RF80 pp 229533.I1RGI5
68058 229533.I1RF80 pp 229533.I1RJ72
68059 229533.I1RF80 pp 229533.I1RNW1
68060 229533.I1RF80 pp 229533.I1S318
68061 229533.I1RF80 pp 229533.I1RLH7
68062 229533.I1RF80 pp 229533.I1RGI4
68063 229533.I1RF80 pp 229533.V6R5P0
68064 229533.I1RF80 pp 229533.I1RVR7
68065 229533.I1RF80 pp 229533.I1RLM5
68066 229533.I1RF80 pp 229533.I1S3G4
68067 229533.I1RF80 pp 229533.I1RNG4
68068 229533.I1RF80 pp 229533.I1RQU9
68069 229533.I1RF80 pp 229533.I1S1W8
68070 229533.I1RF80 pp 229533.I1RJU9
68071 229533.I1RF80 pp 229533.I1RIH2
68072 229533.I1RF80 pp 229533.I1RLM3
68073 229533.I1RF80 pp 229533.I1RYM5
68074 229533.I1RF80 pp 229533.I1RPM6
68075 229533.I1RF80 pp 229533.I1RLZ6
68076 229533.A0A098DNM6 pp 229533.A0A0E0SH57
68077 229533.A0A098DNM6 pp 229533.I1RPM6
68078 229533.A0A098DNM6 pp 229533.I1RGI4
68079 229533.A0A098DNM6 pp 229533.I1RJU9
68080 229533.A0A098DNM6 pp 229533.I1RLM5
68081 229533.A0A098DNM6 pp 229533.A0A1C3YJ82
68082 229533.A0A098DNM6 pp 229533.I1RNG4
68083 229533.A0A098DNM6 pp 229533.I1RNW1
68084 229533.A0A098DNM6 pp 229533.A0A0E0SA26
68085 229533.A0A098DNM6 pp 229533.I1S318
68086 229533.A0A098DNM6 pp 229533.I1RYM5
68087 229533.A0A098DNM6 pp 229533.A0A1C3YL17
68088 229533.A0A098DNM6 pp 229533.I1RLH7
68089 229533.A0A098DNM6 pp 229533.I1RIH2
68090 229533.A0A098DNM6 pp 229533.I1RPM5
68091 229533.A0A098DNM6 pp 229533.A0A1C3YKW8
68092 229533.A0A098DNM6 pp 229533.I1RLZ6
68093 229533.A0A098DNM6 pp 229533.I1RLM3
68094 229533.A0A098DNM6 pp 229533.A0A1C3YMM2
68095 229533.A0A098DNM6 pp 229533.I1RQU9
68096 229533.A0A098DNM6 pp 229533.I1RJ72
68097 229533.A0A098DNM6 pp 229533.V6R5P0
68098 229533.A0A098DNM6 pp 229533.I1RVR7
68099 229533.A0A098DNM6 pp 229533.I1RCR9
68100 229533.A0A098DNM6 pp 229533.I1RGI5
68101 229533.A0A098DNM6 pp 229533.A0A1C3YN33
68102 229533.A0A098DNM6 pp 229533.I1S1W8
68103 229533.A0A098DNM6 pp 229533.I1S3G4
68104 229533.A0A098DNM6 pp 229533.I1RF80
68105 229533.Q4IA56 pp 229533.V6R3J1
68106 229533.Q4IA56 pp 229533.V6RM66
68107 229533.I1RMH9 pp 229533.I1RNG4
68108 229533.I1RMH9 pp 229533.I1RPW2
68109 229533.I1RMH9 pp 229533.I1RR12

68110	229533.I1RMH9	pp	229533.I1RPD6
68111	229533.I1RMH9	pp	229533.I1RY49
68112	229533.I1RMH9	pp	229533.I1RPM6
68113	229533.I1RMH9	pp	229533.I1S2H3
68114	229533.I1RMH9	pp	229533.I1RVX9
68115	229533.I1RC71	pp	229533.I1RWC1
68116	229533.I1RC71	pp	229533.I1RRW0
68117	229533.I1RC71	pp	229533.I1RZC3
68118	229533.I1RC71	pp	229533.I1RST9
68119	229533.I1RC71	pp	229533.I1RKB3
68120	229533.I1RC71	pp	229533.I1RZ95
68121	229533.I1RC71	pp	229533.I1RRN5
68122	229533.I1RC71	pp	229533.I1RGI2
68123	229533.I1RC71	pp	229533.I1RWM4
68124	229533.I1RC71	pp	229533.I1RSI3
68125	229533.I1RC71	pp	229533.V6RHX2
68126	229533.I1RC71	pp	229533.I1RI71
68127	229533.I1RC71	pp	229533.I1RSE2
68128	229533.I1RC71	pp	229533.I1S0M4
68129	229533.I1RC71	pp	229533.Q4IB89
68130	229533.I1RC71	pp	229533.I1RS07
68131	229533.I1RC71	pp	229533.I1RQS4
68132	229533.I1RC71	pp	229533.I1RY81
68133	229533.I1RC71	pp	229533.I1RWT6
68134	229533.I1RC71	pp	229533.I1RZ89
68135	229533.I1RC71	pp	229533.I1RD37
68136	229533.I1RC71	pp	229533.I1RM41
68137	229533.I1RC71	pp	229533.I1RZX3
68138	229533.I1RC71	pp	229533.I1S0D1
68139	229533.I1RC71	pp	229533.I1RSG0
68140	229533.I1RC71	pp	229533.I1S267
68141	229533.I1RC71	pp	229533.I1REW3
68142	229533.I1RC71	pp	229533.I1RRY3
68143	229533.I1RC71	pp	229533.I1RMA8
68144	229533.I1RC71	pp	229533.I1RRK5
68145	229533.I1RC71	pp	229533.I1RY44
68146	229533.I1RC71	pp	229533.I1S1D7
68147	229533.I1RC71	pp	229533.I1RR45
68148	229533.I1RC71	pp	229533.Q4IBU4
68149	229533.I1RC71	pp	229533.I1RE59
68150	229533.I1RC71	pp	229533.I1RJK7
68151	229533.I1RC71	pp	229533.I1RV91
68152	229533.I1RC71	pp	229533.I1RQA3
68153	229533.I1RC71	pp	229533.I1RCH5
68154	229533.I1RC71	pp	229533.Q4HYR2
68155	229533.I1RC71	pp	229533.I1RY32
68156	229533.I1RC71	pp	229533.I1RQV8
68157	229533.I1RC71	pp	229533.I1S9T6
68158	229533.I1RC71	pp	229533.Q4IPX8
68159	229533.I1RC71	pp	229533.I1RJU4
68160	229533.I1RC71	pp	229533.I1RTM8
68161	229533.I1RC71	pp	229533.I1RUQ6
68162	229533.I1RC71	pp	229533.I1RSP5
68163	229533.I1RC71	pp	229533.I1RH91
68164	229533.I1RC71	pp	229533.I1RK02
68165	229533.I1RC71	pp	229533.V6RCJ5
68166	229533.I1RC71	pp	229533.I1S968
68167	229533.I1RC71	pp	229533.I1RTA6
68168	229533.I1RC71	pp	229533.I1RPN6
68169	229533.I1RC71	pp	229533.I1S2E9
68170	229533.I1RC71	pp	229533.I1RMK9
68171	229533.I1RC71	pp	229533.I1S969
68172	229533.I1RC71	pp	229533.I1RF24
68173	229533.I1RC71	pp	229533.I1S3H8
68174	229533.I1RC71	pp	229533.I1RG61
68175	229533.I1RC71	pp	229533.I1SAB4
68176	229533.I1RC71	pp	229533.I1RLA2
68177	229533.I1RC71	pp	229533.I1RN52
68178	229533.I1RC71	pp	229533.I1RZ25
68179	229533.I1RC71	pp	229533.I1S048
68180	229533.I1RC71	pp	229533.I1S334
68181	229533.I1RC71	pp	229533.I1RJR8
68182	229533.I1RC71	pp	229533.I1S1W5

68183	229533.I1RC71	pp	229533.I1RHQ0
68184	229533.I1RC71	pp	229533.I1S0J7
68185	229533.I1RC71	pp	229533.I1RMA2
68186	229533.I1RC71	pp	229533.I1RRL5
68187	229533.I1RC71	pp	229533.I1S224
68188	229533.I1RC71	pp	229533.I1RK10
68189	229533.I1RC71	pp	229533.I1RPX5
68190	229533.I1RC71	pp	229533.I1RS35
68191	229533.I1RC71	pp	229533.I1RFI4
68192	229533.I1RC71	pp	229533.I1RQV7
68193	229533.I1RC71	pp	229533.I1RYP5
68194	229533.I1RC71	pp	229533.I1RK55
68195	229533.I1RC71	pp	229533.I1RPE5
68196	229533.I1RH91	pp	229533.I1RN84
68197	229533.I1RH91	pp	229533.I1RY44
68198	229533.I1RH91	pp	229533.I1RZC3
68199	229533.I1RH91	pp	229533.I1RKZ7
68200	229533.I1RH91	pp	229533.I1RHC8
68201	229533.I1RH91	pp	229533.I1S599
68202	229533.I1RH91	pp	229533.Q4I1Y5
68203	229533.I1RH91	pp	229533.V6RLM7
68204	229533.I1RH91	pp	229533.I1RL76
68205	229533.I1RH91	pp	229533.I1S332
68206	229533.I1RH91	pp	229533.I1RM41
68207	229533.I1RH91	pp	229533.I1RMJ3
68208	229533.I1RH91	pp	229533.I1S2Z7
68209	229533.I1RH91	pp	229533.I1S0I3
68210	229533.I1RH91	pp	229533.I1S6M5
68211	229533.I1RH91	pp	229533.I1RNK9
68212	229533.I1RH91	pp	229533.I1S0V7
68213	229533.I1RH91	pp	229533.I1RR45
68214	229533.I1RH91	pp	229533.I1RKZ2
68215	229533.I1RH91	pp	229533.I1S1F3
68216	229533.I1RH91	pp	229533.I1RNV4
68217	229533.I1RH91	pp	229533.I1RUS5
68218	229533.I1RH91	pp	229533.I1S2E9
68219	229533.I1RH91	pp	229533.V6RUY9
68220	229533.I1RH91	pp	229533.I1RZ25
68221	229533.I1RH91	pp	229533.I1RUR8
68222	229533.I1RH91	pp	229533.V6RXR0
68223	229533.I1RH91	pp	229533.I1S3G4
68224	229533.I1RH91	pp	229533.I1RYW9
68225	229533.I1RH91	pp	229533.I1RK10
68226	229533.I1RH91	pp	229533.V6RHD0
68227	229533.I1RH91	pp	229533.I1RWF5
68228	229533.I1RH91	pp	229533.I1RZA8
68229	229533.I1RH91	pp	229533.I1RNG4
68230	229533.I1RH91	pp	229533.I1RJB0
68231	229533.I1RH91	pp	229533.I1RY32
68232	229533.I1RH91	pp	229533.I1RI11
68233	229533.I1RH91	pp	229533.I1SAB4
68234	229533.I1RH91	pp	229533.I1RS07
68235	229533.I1RH91	pp	229533.I1RYA8
68236	229533.I1RH91	pp	229533.I1RRF5
68237	229533.I1RH91	pp	229533.I1RWM4
68238	229533.I1RH91	pp	229533.Q4HYR2
68239	229533.I1RH91	pp	229533.I1RQA3
68240	229533.I1RH91	pp	229533.I1S694
68241	229533.I1RH91	pp	229533.I1S423
68242	229533.I1RH91	pp	229533.I1RYA2
68243	229533.I1RH91	pp	229533.I1RST9
68244	229533.I1RH91	pp	229533.I1RW43
68245	229533.I1RH91	pp	229533.I1RNY6
68246	229533.I1RH91	pp	229533.I1RZ29
68247	229533.I1RH91	pp	229533.I1RY12
68248	229533.I1RH91	pp	229533.I1S3M4
68249	229533.I1RH91	pp	229533.I1RSP5
68250	229533.I1RH91	pp	229533.I1RJY7
68251	229533.I1RH91	pp	229533.I1RWT6
68252	229533.I1RH91	pp	229533.I1RIE8
68253	229533.I1RH91	pp	229533.I1RIM6
68254	229533.I1RH91	pp	229533.I1RM26
68255	229533.I1RH91	pp	229533.V6QU65

68256 229533.I1RH91 pp 229533.I1RPW1
68257 229533.I1RH91 pp 229533.I1RRK5
68258 229533.I1RH91 pp 229533.I1S267
68259 229533.I1RH91 pp 229533.I1S0V6
68260 229533.I1RH91 pp 229533.I1S0M4
68261 229533.I1RH91 pp 229533.I1RNM7
68262 229533.I1RH91 pp 229533.I1RSG0
68263 229533.I1RH91 pp 229533.I1RVB2
68264 229533.I1RH91 pp 229533.I1S2F6
68265 229533.I1RH91 pp 229533.I1RYX6
68266 229533.I1RH91 pp 229533.I1RJR8
68267 229533.I1RH91 pp 229533.I1RQE1
68268 229533.I1RH91 pp 229533.I1RR12
68269 229533.I1RH91 pp 229533.I1S8C1
68270 229533.I1RH91 pp 229533.I1RP86
68271 229533.I1RH91 pp 229533.I1SAF0
68272 229533.I1RH91 pp 229533.I1S048
68273 229533.I1RH91 pp 229533.I1RWZ9
68274 229533.I1RH91 pp 229533.I1RLA2
68275 229533.I1RH91 pp 229533.I1RKZ8
68276 229533.I1RH91 pp 229533.I1RYK1
68277 229533.I1RH91 pp 229533.I1RY49
68278 229533.I1RH91 pp 229533.I1RPI4
68279 229533.I1RH91 pp 229533.I1RYQ4
68280 229533.I1RH91 pp 229533.I1RY16
68281 229533.I1RH91 pp 229533.I1RN52
68282 229533.I1RH91 pp 229533.I1RVX9
68283 229533.I1RH91 pp 229533.I1RMM8
68284 229533.I1RH91 pp 229533.I1RJS7
68285 229533.I1RH91 pp 229533.I1RYX4
68286 229533.I1RH91 pp 229533.I1S1W8
68287 229533.I1RH91 pp 229533.V6RXE7
68288 229533.I1RH91 pp 229533.I1RQ60
68289 229533.I1RSX1 pp 229533.I1RW25
68290 229533.I1RSX1 pp 229533.I1RT80
68291 229533.I1RSX1 pp 229533.V6RCG5
68292 229533.I1RSX1 pp 229533.V6QYF0
68293 229533.I1RSX1 pp 229533.I1RZF6
68294 229533.I1RSX1 pp 229533.Q4I5Z5
68295 229533.I1RSX1 pp 229533.V6R227
68296 229533.I1RSX1 pp 229533.I1S0L3
68297 229533.I1RSX1 pp 229533.I1RUQ2
68298 229533.I1RSX1 pp 229533.Q4IEK8
68299 229533.I1RSX1 pp 229533.I1RWD4
68300 229533.I1RSX1 pp 229533.Q4IR18
68301 229533.I1RSX1 pp 229533.I1RZZ9
68302 229533.I1RSX1 pp 229533.Q4HZ42
68303 229533.I1RSX1 pp 229533.I1RSX2
68304 229533.I1RSX1 pp 229533.I1S296
68305 229533.I1RSX1 pp 229533.I1S4P6
68306 229533.I1RSX1 pp 229533.I1RUG3
68307 229533.A0A0E0S409 pp 229533.A0A1C3YLC2
68308 229533.A0A0E0S409 pp 229533.I1RMX6
68309 229533.A0A1C3YJF8 pp 229533.I1RII1
68310 229533.A0A1C3YJF8 pp 229533.I1RYE2
68311 229533.A0A1C3YJF8 pp 229533.I1RJW5
68312 229533.A0A1C3YJF8 pp 229533.I1RCV9
68313 229533.A0A1C3YJF8 pp 229533.I1RL03
68314 229533.I1RIA2 pp 229533.I1RIA9
68315 229533.I1RIA2 pp 229533.V6RG22
68316 229533.I1RIA2 pp 229533.Q00909
68317 229533.I1RIA2 pp 229533.I1RZD0
68318 229533.I1RIA2 pp 229533.I1RY35
68319 229533.I1RIA2 pp 229533.I1RIA5
68320 229533.I1RIA2 pp 229533.I1RY81
68321 229533.I1S7L6 pp 229533.V6R949
68322 229533.I1S7L6 pp 229533.V6RN44
68323 229533.A0A1C3YJ38 pp 229533.I1RRE0
68324 229533.A0A1C3YJ38 pp 229533.I1RYQ5
68325 229533.A0A1C3YJ38 pp 229533.I1RN70
68326 229533.I1RAX2 pp 229533.I1RKB3
68327 229533.I1RAX2 pp 229533.I1S7A5
68328 229533.I1RAX2 pp 229533.I1S3H8

68329 229533.I1RAX2 pp 229533.I1RF24
68330 229533.I1RAX2 pp 229533.I1RST9
68331 229533.I1RAX2 pp 229533.I1RZK4
68332 229533.I1RAX2 pp 229533.I1RBY9
68333 229533.I1RAX2 pp 229533.I1S9T6
68334 229533.I1RAX2 pp 229533.Q4IBU4
68335 229533.I1RAX2 pp 229533.I1S7M8
68336 229533.I1RAX2 pp 229533.I1RAY4
68337 229533.I1RAX2 pp 229533.I1S0V7
68338 229533.I1RAX2 pp 229533.I1RRK5
68339 229533.I1RAX2 pp 229533.I1RSI3
68340 229533.I1RAX2 pp 229533.V6RHX2
68341 229533.I1RAX2 pp 229533.I1RGI2
68342 229533.I1RAX2 pp 229533.I1RK02
68343 229533.I1RAX2 pp 229533.I1RS07
68344 229533.I1RAX2 pp 229533.I1RX80
68345 229533.I1RAX2 pp 229533.I1RJH3
68346 229533.I1RAX2 pp 229533.I1RHQ0
68347 229533.I1RAX2 pp 229533.I1RSE2
68348 229533.I1RAX2 pp 229533.I1RMK9
68349 229533.I1RAX2 pp 229533.I1S2K7
68350 229533.I1RAX2 pp 229533.I1RRL5
68351 229533.I1RAX2 pp 229533.I1S267
68352 229533.I1RAX2 pp 229533.I1RQV7
68353 229533.I1RAX2 pp 229533.I1RNF4
68354 229533.I1RAX2 pp 229533.I1RHQ8
68355 229533.I1RAX2 pp 229533.I1RTM8
68356 229533.I1RAX2 pp 229533.I1S969
68357 229533.I1RAX2 pp 229533.I1RD37
68358 229533.I1RAX2 pp 229533.I1RK10
68359 229533.I1RAX2 pp 229533.I1S0M4
68360 229533.I1RAX2 pp 229533.I1RZ89
68361 229533.I1RAX2 pp 229533.I1RMA2
68362 229533.I1RAX2 pp 229533.I1RQ72
68363 229533.I1RAX2 pp 229533.I1RQV8
68364 229533.I1RAX2 pp 229533.I1RQS4
68365 229533.I1RAX2 pp 229533.I1RYP5
68366 229533.I1RAX2 pp 229533.I1S224
68367 229533.I1RAX2 pp 229533.I1RY32
68368 229533.I1RAX2 pp 229533.I1S334
68369 229533.I1RAX2 pp 229533.I1RN19
68370 229533.I1RAX2 pp 229533.I1RC71
68371 229533.I1RAX2 pp 229533.I1RK55
68372 229533.I1RAX2 pp 229533.I1S0V6
68373 229533.I1RAX2 pp 229533.I1S968
68374 229533.I1RAX2 pp 229533.I1RXH6
68375 229533.I1RAX2 pp 229533.I1RRW0
68376 229533.I1RAX2 pp 229533.I1RM41
68377 229533.I1RAX2 pp 229533.I1RWC1
68378 229533.I1RAX2 pp 229533.I1RSP5
68379 229533.I1RAX2 pp 229533.I1RSG0
68380 229533.I1RAX2 pp 229533.I1RZ95
68381 229533.I1RAX2 pp 229533.I1RY81
68382 229533.I1RAX2 pp 229533.I1S2B6
68383 229533.I1RAX2 pp 229533.I1S1D7
68384 229533.I1RAX2 pp 229533.I1RH91
68385 229533.I1RAX2 pp 229533.I1RPH1
68386 229533.I1RAX2 pp 229533.I1REW3
68387 229533.I1RAX2 pp 229533.I1RZC3
68388 229533.I1RAX2 pp 229533.I1RS35
68389 229533.I1RAX2 pp 229533.I1RPN6
68390 229533.I1RAX2 pp 229533.I1SAB4
68391 229533.I1RAX2 pp 229533.I1RG61
68392 229533.I1RAX2 pp 229533.I1RZX3
68393 229533.I1RAX2 pp 229533.Q4IPX8
68394 229533.I1RAX2 pp 229533.I1RLA2
68395 229533.I1RAX2 pp 229533.I1S296
68396 229533.I1RAX2 pp 229533.I1RC46
68397 229533.I1RAX2 pp 229533.I1RJR8
68398 229533.I1RAX2 pp 229533.I1S048
68399 229533.I1RAX2 pp 229533.I1RZ25
68400 229533.I1RAX2 pp 229533.I1RJK7
68401 229533.I1RAX2 pp 229533.I1RR45

68402 229533.I1RAX2 pp 229533.I1RE59
68403 229533.A0A1C3YLX5 pp 229533.I1S0D5
68404 229533.A0A1C3YLX5 pp 229533.I1RU02
68405 229533.A0A1C3YLX5 pp 229533.Q4IER8
68406 229533.A0A1C3YLX5 pp 229533.I1RAD3
68407 229533.A0A1C3YLX5 pp 229533.I1RHP9
68408 229533.A0A1C3YLX5 pp 229533.I1R9W6
68409 229533.A0A1C3YLX5 pp 229533.I1RYX9
68410 229533.A0A1C3YLX5 pp 229533.Q4IR08
68411 229533.A0A1C3YLX5 pp 229533.I1RFW1
68412 229533.A0A1C3YLX5 pp 229533.I1RPR8
68413 229533.A0A1C3YLX5 pp 229533.Q4I6A4
68414 229533.A0A1C3YLX5 pp 229533.I1RI51
68415 229533.A0A1C3YLX5 pp 229533.I1RB73
68416 229533.A0A1C3YLX5 pp 229533.I1RCW8
68417 229533.A0A1C3YLX5 pp 229533.I1RTG3
68418 229533.A0A1C3YLX5 pp 229533.I1RAX3
68419 229533.Q4IBR4 pp 229533.Q4IFH8
68420 229533.Q4IBR4 pp 229533.Q4IR08
68421 229533.Q4IBR4 pp 229533.Q4IER8
68422 229533.Q4IBR4 pp 229533.Q4IF76
68423 229533.I1S7S4 pp 229533.I1S7S5
68424 229533.A0A098D3K8 pp 229533.Q4I2W2
68425 229533.A0A098D3K8 pp 229533.I1RZS4
68426 229533.A0A098D3K8 pp 229533.I1RY44
68427 229533.A0A098D3K8 pp 229533.I1RWT6
68428 229533.A0A098D3K8 pp 229533.I1S267
68429 229533.A0A098D3K8 pp 229533.A0A098D5E5
68430 229533.A0A098D3K8 pp 229533.I1RJR8
68431 229533.A0A098D3K8 pp 229533.Q4IA56
68432 229533.A0A098D3K8 pp 229533.I1RC71
68433 229533.A0A098D3K8 pp 229533.I1RH91
68434 229533.A0A098D3K8 pp 229533.A0A0E0SA11
68435 229533.A0A098D3K8 pp 229533.A0A098D6T7
68436 229533.A0A098D3K8 pp 229533.I1RQA3
68437 229533.A0A098D3K8 pp 229533.I1S7L6
68438 229533.A0A098D3K8 pp 229533.I1RNG4
68439 229533.A0A098D3K8 pp 229533.I1RAX2
68440 229533.A0A098D3K8 pp 229533.I1RV91
68441 229533.A0A098D3K8 pp 229533.I1S2E9
68442 229533.A0A1C3YL18 pp 229533.I1RUR0
68443 229533.A0A1C3YL18 pp 229533.I1RRT1
68444 229533.A0A1C3YL18 pp 229533.I1RWM6
68445 229533.A0A1C3YL18 pp 229533.Q4HTT2
68446 229533.A0A1C3YL18 pp 229533.Q4INS6
68447 229533.A0A1C3YL18 pp 229533.I1RAU6
68448 229533.A0A1C3YL18 pp 229533.I1RRG3
68449 229533.A0A1C3YL18 pp 229533.I1RK19
68450 229533.A0A1C3YL18 pp 229533.Q4IR08
68451 229533.A0A1C3YL18 pp 229533.I1RCW8
68452 229533.A0A1C3YL18 pp 229533.I1RSC7
68453 229533.A0A1C3YL18 pp 229533.I1RN91
68454 229533.A0A1C3YL18 pp 229533.Q4ICA8
68455 229533.A0A1C3YL18 pp 229533.V6R9X9
68456 229533.A0A1C3YL18 pp 229533.A0A1C3YLC6
68457 229533.A0A1C3YL18 pp 229533.Q4IER8
68458 229533.A0A1C3YL18 pp 229533.I1RC54
68459 229533.A0A1C3YL18 pp 229533.I1S3L3
68460 229533.A0A1C3YL18 pp 229533.I1RQP1
68461 229533.A0A1C3YL18 pp 229533.I1RBC8
68462 229533.A0A1C3YL18 pp 229533.I1RMS4
68463 229533.A0A1C3YL18 pp 229533.I1RDY2
68464 229533.A0A1C3YL18 pp 229533.I1RU02
68465 229533.A0A1C3YL18 pp 229533.I1S3W0
68466 229533.A0A1C3YL18 pp 229533.Q4HYB8
68467 229533.A0A1C3YL18 pp 229533.A0A1C3YLS3
68468 229533.A0A1C3YL18 pp 229533.Q4IMD1
68469 229533.A0A1C3YL18 pp 229533.Q4IB89
68470 229533.A0A1C3YL18 pp 229533.I1RUM5
68471 229533.A0A1C3YL18 pp 229533.I1RZ86
68472 229533.A0A1C3YL18 pp 229533.I1RFW1
68473 229533.A0A1C3YL18 pp 229533.I1RTS2
68474 229533.A0A1C3YL18 pp 229533.I1RND9

229533.A0A1C3YL18	pp	229533.I1RSD4
229533.A0A1C3YL18	pp	229533.I1RA99
229533.A0A1C3YL18	pp	229533.I1RRN6
229533.A0A1C3YL18	pp	229533.I1S0D5
229533.A0A1C3YL18	pp	229533.I1RMU1
229533.A0A1C3YL18	pp	229533.Q4IBR4
229533.Q4IR18	pp	229533.V6R227
229533.Q4IR18	pp	229533.V6QYF0
229533.I1RMJ4	pp	229533.I1RPM6
229533.I1RMJ4	pp	229533.I1RY49
229533.I1RMJ4	pp	229533.I1RV21
229533.I1RMJ4	pp	229533.I1RNG4
229533.I1RMJ4	pp	229533.I1RR12
229533.I1RMJ4	pp	229533.I1RVX9
229533.I1RMJ4	pp	229533.I1RPW2
229533.I1RMJ4	pp	229533.I1RPD6
229533.I1RMJ4	pp	229533.I1S3X2
229533.I1RMJ4	pp	229533.I1S746
229533.A0A098DZL8	pp	229533.I1RMQ0
229533.A0A098DZL8	pp	229533.Q4I948
229533.A0A098DZL8	pp	229533.I1RQ28
229533.A0A098DZL8	pp	229533.I1S9U2
229533.A0A098DZL8	pp	229533.I1RVZ0
229533.A0A098DZL8	pp	229533.I1RC06
229533.A0A098DZL8	pp	229533.A0A0E0S7B3
229533.A0A098DZL8	pp	229533.Q4ICA8
229533.A0A098DZL8	pp	229533.I1RTF4
229533.A0A098DZL8	pp	229533.I1RQ65
229533.A0A098DZL8	pp	229533.I1RT98
229533.A0A098DZL8	pp	229533.Q4IAS1
229533.A0A098DZL8	pp	229533.Q4HZK7
229533.A0A098DZL8	pp	229533.I1RMN6
229533.A0A098DZL8	pp	229533.I1RMK3
229533.A0A098DZL8	pp	229533.I1RYF2
229533.A0A098DZL8	pp	229533.I1S2C0
229533.A0A098DZL8	pp	229533.I1RNJ2
229533.A0A098DZL8	pp	229533.I1RKJ6
229533.A0A098DZL8	pp	229533.I1RMR1
229533.A0A098DZL8	pp	229533.I1RK01
229533.I1RQJ4	pp	229533.I1S8E2
229533.I1RQJ4	pp	229533.I1S283
229533.I1RQJ4	pp	229533.O74638
229533.I1RQJ4	pp	229533.I1RRT1
229533.I1RQJ4	pp	229533.I1RUG1
229533.I1RQJ4	pp	229533.Q4IC11
229533.I1RQJ4	pp	229533.I1RYQ1
229533.I1RQJ4	pp	229533.I1RSA7
229533.I1RQJ4	pp	229533.I1RZN1
229533.I1RQJ4	pp	229533.V6RLW4
229533.I1RQJ4	pp	229533.Q4IEA5
229533.I1RQJ4	pp	229533.Q4IQM6
229533.I1RQJ4	pp	229533.I1RSR9
229533.I1RQJ4	pp	229533.I1RZ94
229533.I1RQJ4	pp	229533.I1RZ86
229533.I1RQJ4	pp	229533.I1RZP8
229533.I1RQJ4	pp	229533.I1RTN6
229533.I1RQJ4	pp	229533.I1RRS7
229533.I1RQJ4	pp	229533.I1RSS7
229533.I1RQJ4	pp	229533.I1RSC7
229533.I1RQJ4	pp	229533.I1RZB5
229533.I1RQJ4	pp	229533.I1RTK5
229533.I1RQJ4	pp	229533.I1RSP7
229533.I1RQJ4	pp	229533.I1RY73
229533.I1RQJ4	pp	229533.I1RSN7
229533.I1RQJ4	pp	229533.I1S461
229533.I1RQJ4	pp	229533.I1RYR2
229533.I1RQJ4	pp	229533.I1RZ19
229533.I1RQJ4	pp	229533.Q4IBX0
229533.I1RQJ4	pp	229533.I1S604
229533.I1RQJ4	pp	229533.I1RYX9
229533.I1RQJ4	pp	229533.V6R382
229533.I1RQJ4	pp	229533.I1RW16
229533.I1RQJ4	pp	229533.I1S209

229533.I1RQJ4 pp 229533.I1RUG2
229533.I1RQJ4 pp 229533.I1RSI4
229533.I1RQJ4 pp 229533.I1RYR1
229533.I1RQJ4 pp 229533.Q4IP51
229533.I1RPD9 pp 229533.I1RR40
229533.I1RPD9 pp 229533.I1S5J8
229533.I1RPD9 pp 229533.I1RUP6
229533.I1RXH3 pp 229533.I1S4D0
229533.I1RXH3 pp 229533.I1S2C4
229533.I1RXH3 pp 229533.V6RY56
229533.I1RXH3 pp 229533.V6RRM0
229533.I1RXH3 pp 229533.I1RYY2
229533.I1RQ05 pp 229533.Q4IR08
229533.I1RQ05 pp 229533.I1RTE7
229533.I1RQ05 pp 229533.Q4IBR4
229533.I1RQ05 pp 229533.I1RXX1
229533.I1RQ05 pp 229533.Q4I7L0
229533.I1RQ05 pp 229533.I1S604
229533.I1RQ05 pp 229533.Q4IER8
229533.I1RQ05 pp 229533.I1RY25
229533.A0A1C3YHT2 pp 229533.I1RCN6
229533.A0A1C3YHT2 pp 229533.I1RW76
229533.A0A1C3YHT2 pp 229533.I1RZ60
229533.I1RDZ3 pp 229533.I1RPT0
229533.I1RDZ3 pp 229533.I1RIN3
229533.I1RDZ3 pp 229533.I1RMP5
229533.I1RDZ3 pp 229533.I1S1W7
229533.I1RDZ3 pp 229533.I1SA27
229533.I1RDZ3 pp 229533.I1REG4
229533.I1RDZ3 pp 229533.I1RJA7
229533.I1RFW9 pp 229533.I1S1Y3
229533.I1RFW9 pp 229533.I1RZC9
229533.I1S1W7 pp 229533.Q4I7N9
229533.I1S1W7 pp 229533.V6R0U9
229533.I1RVU8 pp 229533.I1S268
229533.A0A0E0S1I7 pp 229533.A0A1C3YHV5
229533.A0A0E0S1I7 pp 229533.I1RJL5
229533.A0A0E0S1I7 pp 229533.I1RY59
229533.A0A0E0S1I7 pp 229533.I1RWC6
229533.A0A0E0S1I7 pp 229533.I1S575
229533.A0A0E0S1I7 pp 229533.I1RFW9
229533.A0A0E0S1I7 pp 229533.I1R9Z0
229533.A0A0E0S1I7 pp 229533.I1RB89
229533.I1S0C6 pp 229533.Q4HYB8
229533.I1S0C6 pp 229533.I1S276
229533.I1S0C6 pp 229533.Q4IPC8
229533.I1S0C6 pp 229533.Q4I5W5
229533.I1S0C6 pp 229533.V6RGU1
229533.I1S0C6 pp 229533.I1S182
229533.I1S0C6 pp 229533.V6QUE6
229533.I1S0C6 pp 229533.Q4I5I4
229533.I1S0C6 pp 229533.V6QWX8
229533.I1RJA7 pp 229533.I1SA27
229533.I1RJA7 pp 229533.I1RMP5
229533.I1RJA7 pp 229533.I1S1W7
229533.I1RJA7 pp 229533.I1RPT0
229533.A0A098DBX7 pp 229533.I1RAX3
229533.A0A098DBX7 pp 229533.Q4HYB8
229533.A0A098DBX7 pp 229533.Q4IR08
229533.A0A098DBX7 pp 229533.Q4IJU0
229533.A0A098DBX7 pp 229533.A0A1C3YLX5
229533.A0A098DBX7 pp 229533.Q4IBR4
229533.A0A098DBX7 pp 229533.Q4IER8
229533.A0A098DBX7 pp 229533.A0A098DZL8
229533.A0A098DBX7 pp 229533.I1RQ05
229533.A0A098DBX7 pp 229533.I1RFW1
229533.A0A098DBX7 pp 229533.I1RTG3
229533.A0A098DBX7 pp 229533.Q4I7L0
229533.A0A098DBX7 pp 229533.A0A098DU58
229533.A0A098DBX7 pp 229533.I1RST8
229533.A0A0E0SN72 pp 229533.I1RTD7
229533.A0A0E0SN72 pp 229533.I1RPJ3
229533.A0A0E0SN72 pp 229533.I1RQA2

68621 229533.A0A0E0SN72 pp 229533.A0A1C3YII4
68622 229533.A0A0E0SN72 pp 229533.I1RRP3
68623 229533.A0A0E0SN72 pp 229533.I1RQ86
68624 229533.A0A0E0SN72 pp 229533.I1RA35
68625 229533.A0A0E0SN72 pp 229533.I1S8C5
68626 229533.I1RLX5 pp 229533.I1RZ91
68627 229533.I1RLX5 pp 229533.I1RW15
68628 229533.I1RLX5 pp 229533.I1RPD9
68629 229533.I1RLX5 pp 229533.I1RS38
68630 229533.I1RLX5 pp 229533.I1RQX9
68631 229533.I1RZV1 pp 229533.Q4INI2
68632 229533.I1RZV1 pp 229533.I1S270
68633 229533.I1RZV1 pp 229533.Q5I2J3
68634 229533.I1RZV1 pp 229533.Q4I963
68635 229533.I1RZV1 pp 229533.I1S8E0
68636 229533.I1RZV1 pp 229533.I1S7W2
68637 229533.I1RZV1 pp 229533.Q4IPI4
68638 229533.I1RZV1 pp 229533.Q4I2D7
68639 229533.I1RZV1 pp 229533.I1S0Z3
68640 229533.I1RZV1 pp 229533.I1S193
68641 229533.I1RE54 pp 229533.I1REN3
68642 229533.I1RE54 pp 229533.I1RPM7
68643 229533.I1RE54 pp 229533.I1RMV1
68644 229533.I1RE54 pp 229533.I1RK71
68645 229533.I1RE54 pp 229533.I1S276
68646 229533.I1RE54 pp 229533.V6QV38
68647 229533.I1RE54 pp 229533.I1S5Z2
68648 229533.I1RE54 pp 229533.I1RJL2
68649 229533.I1RE54 pp 229533.I1RVL8
68650 229533.I1RE54 pp 229533.Q4I5U9
68651 229533.I1RE54 pp 229533.I1RZB5
68652 229533.I1RE54 pp 229533.V6QWX8
68653 229533.I1RE54 pp 229533.I1RVY0
68654 229533.I1RE54 pp 229533.I1RXE5
68655 229533.I1RE54 pp 229533.I1RUG0
68656 229533.I1RE54 pp 229533.I1RWF3
68657 229533.I1RE54 pp 229533.I1S9H8
68658 229533.I1RE54 pp 229533.I1S5Z5
68659 229533.I1RE54 pp 229533.I1RRG3
68660 229533.I1RE54 pp 229533.I1RT35
68661 229533.I1RE54 pp 229533.I1RRT0
68662 229533.I1RE54 pp 229533.I1RTC2
68663 229533.I1RE54 pp 229533.I1RKR8
68664 229533.I1RE54 pp 229533.Q4I5W5
68665 229533.I1RE54 pp 229533.Q4HYB8
68666 229533.I1RE54 pp 229533.Q4I5I4
68667 229533.I1RE54 pp 229533.I1RMQ7
68668 229533.I1RE54 pp 229533.I1RSW9
68669 229533.I1RE54 pp 229533.I1RP96
68670 229533.I1RE54 pp 229533.V6QUE6
68671 229533.I1RE54 pp 229533.I1RML9
68672 229533.I1RE54 pp 229533.Q4IPC8
68673 229533.I1RE54 pp 229533.Q4IJU0
68674 229533.I1RE54 pp 229533.I1RR25
68675 229533.I1RE54 pp 229533.I1RKF3
68676 229533.I1RE54 pp 229533.I1RG11
68677 229533.I1RE54 pp 229533.I1RXU0
68678 229533.A0A0E0S7W0 pp 229533.I1RC82
68679 229533.A0A0E0S7W0 pp 229533.I1RRT2
68680 229533.A0A0E0S7W0 pp 229533.Q4IR18
68681 229533.A0A0E0S7W0 pp 229533.I1RQ65
68682 229533.A0A0E0S7W0 pp 229533.I1RWD4
68683 229533.A0A0E0S7W0 pp 229533.I1RQJ5
68684 229533.A0A0E0S7W0 pp 229533.I1RRT8
68685 229533.A0A0E0S7W0 pp 229533.I1RTF4
68686 229533.A0A0E0S7W0 pp 229533.I1RD31
68687 229533.A0A0E0S7W0 pp 229533.I1RZS6
68688 229533.A0A0E0S7W0 pp 229533.V6QYF0
68689 229533.A0A0E0S7W0 pp 229533.I1S021
68690 229533.A0A0E0S7W0 pp 229533.V6R227
68691 229533.A0A0E0S7W0 pp 229533.I1RWT7
68692 229533.A0A0E0S7W0 pp 229533.I1RCL3
68693 229533.A0A0E0S7W0 pp 229533.I1RRH8

68694 229533.A0A0E0S7W0 pp 229533.I1RAZ8
68695 229533.A0A0E0S7W0 pp 229533.I1RDG0
68696 229533.A0A0E0S7W0 pp 229533.I1RRR6
68697 229533.A0A0E0S7W0 pp 229533.I1RSD8
68698 229533.I1S3D5 pp 229533.I1SAF3
68699 229533.A0A0E0S173 pp 229533.I1RNB8
68700 229533.A0A0E0S173 pp 229533.I1RG90
68701 229533.A0A0E0S173 pp 229533.I1RK32
68702 229533.A0A0E0S173 pp 229533.I1S0Z2
68703 229533.A0A0E0S173 pp 229533.I1RT35
68704 229533.A0A0E0S173 pp 229533.A0A1C3YIA8
68705 229533.A0A098DQH7 pp 229533.V6R0U9
68706 229533.A0A098DQH7 pp 229533.A0A098DTH7
68707 229533.A0A098DQH7 pp 229533.I1REG4
68708 229533.A0A098DQH7 pp 229533.I1RVD9
68709 229533.A0A098DQH7 pp 229533.A0A1C3YKA2
68710 229533.A0A098DQH7 pp 229533.I1RVL7
68711 229533.A0A098DQH7 pp 229533.I1RIN3
68712 229533.A0A098DQH7 pp 229533.I1RIV2
68713 229533.A0A098DQH7 pp 229533.I1RVM4
68714 229533.A0A098DQH7 pp 229533.I1S9Z4
68715 229533.A0A098DQH7 pp 229533.I1RHK0
68716 229533.A0A098DQH7 pp 229533.A0A1C3YLG9
68717 229533.A0A098DQH7 pp 229533.I1RDZ3
68718 229533.A0A098DQH7 pp 229533.A0A1C3YHQ6
68719 229533.A0A098DQH7 pp 229533.A0A098E2M9
68720 229533.A0A098DQH7 pp 229533.A0A098E3B9
68721 229533.A0A098DQH7 pp 229533.A0A098E4D0
68722 229533.A0A098DQH7 pp 229533.I1RPR1
68723 229533.A0A098DQH7 pp 229533.A0A1C3YIP3
68724 229533.A0A098DQH7 pp 229533.I1S1W7
68725 229533.A0A098DQH7 pp 229533.A0A1C3YJ35
68726 229533.A0A098DQH7 pp 229533.I1RF49
68727 229533.A0A098DQH7 pp 229533.I1S1L0
68728 229533.A0A098DQH7 pp 229533.I1RQ48
68729 229533.A0A098DQH7 pp 229533.I1RJA7
68730 229533.A0A098DQH7 pp 229533.A0A098E0Z2
68731 229533.A0A098DQH7 pp 229533.I1RK69
68732 229533.A0A098DQH7 pp 229533.I1S5U5
68733 229533.I1RQZ3 pp 229533.I1RXU0
68734 229533.I1RQZ3 pp 229533.Q4HYB8
68735 229533.I1RQZ3 pp 229533.Q4IPC8
68736 229533.I1RQZ3 pp 229533.I1RTC2
68737 229533.I1RQZ3 pp 229533.I1RRG3
68738 229533.I1RQZ3 pp 229533.I1RWF3
68739 229533.I1RQZ3 pp 229533.V6QUE6
68740 229533.I1RQZ3 pp 229533.Q4I5I4
68741 229533.I1RQZ3 pp 229533.I1RUG0
68742 229533.I1RQZ3 pp 229533.I1RRT0
68743 229533.I1RQZ3 pp 229533.I1S276
68744 229533.I1RQZ3 pp 229533.V6QWX8
68745 229533.I1RQZ3 pp 229533.I1RSZ3
68746 229533.I1RQZ3 pp 229533.I1RZB5
68747 229533.I1RQZ3 pp 229533.I1RR25
68748 229533.I1RQZ3 pp 229533.Q4I5W5
68749 229533.I1RQZ3 pp 229533.I1RVY0
68750 229533.I1RQZ3 pp 229533.I1RVL8
68751 229533.I1RWX8 pp 229533.I1S7M3
68752 229533.I1RWX8 pp 229533.Q4IPH4
68753 229533.I1RWX8 pp 229533.I1S0B1
68754 229533.I1RWX8 pp 229533.I1RX51
68755 229533.I1RWX8 pp 229533.I1RYJ9
68756 229533.I1RWX8 pp 229533.I1S0Z9
68757 229533.I1RWX8 pp 229533.V6QXG1
68758 229533.I1RWX8 pp 229533.I1RYB1
68759 229533.I1RWX8 pp 229533.V6RIS2
68760 229533.I1RWX8 pp 229533.I1S1X9
68761 229533.I1RWX8 pp 229533.I1RXE5
68762 229533.I1RWX8 pp 229533.V6QVD5
68763 229533.I1RWX8 pp 229533.Q4IAA0
68764 229533.I1RWX8 pp 229533.V6RY33
68765 229533.I1RWX8 pp 229533.I1RZQ5
68766 229533.I1RWX8 pp 229533.I1RYI9

68767	229533.I1RWX8	pp	229533.Q4HXF6
68768	229533.I1RWX8	pp	229533.Q4IP34
68769	229533.I1RWX8	pp	229533.I1S8F0
68770	229533.I1RWX8	pp	229533.Q4I7F9
68771	229533.I1RWX8	pp	229533.I1RZ11
68772	229533.I1RWX8	pp	229533.I1RZZ1
68773	229533.I1RWX8	pp	229533.Q4IE79
68774	229533.I1RWX8	pp	229533.I1S9L6
68775	229533.I1RWX8	pp	229533.I1RXF2
68776	229533.I1RWX8	pp	229533.I1S7M2
68777	229533.I1RWX8	pp	229533.I1S0R7
68778	229533.I1RWX8	pp	229533.I1RYW3
68779	229533.I1RWX8	pp	229533.V6QV38
68780	229533.I1RWX8	pp	229533.I1S5S2
68781	229533.I1RWX8	pp	229533.I1RX25
68782	229533.I1RWX8	pp	229533.Q4IB65
68783	229533.I1RWX8	pp	229533.Q4IB70
68784	229533.I1RWX8	pp	229533.I1RZB5
68785	229533.I1RWX8	pp	229533.I1RX24
68786	229533.I1RWX8	pp	229533.I1RX81
68787	229533.I1RWX8	pp	229533.I1S4M5
68788	229533.I1RWX8	pp	229533.Q4I1Y1
68789	229533.I1RWX8	pp	229533.I1S9L5
68790	229533.I1RWX8	pp	229533.I1S0B6
68791	229533.I1RWX8	pp	229533.Q4IPB3
68792	229533.I1RWX8	pp	229533.I1S524
68793	229533.I1RWX8	pp	229533.I1RZG7
68794	229533.I1RWX8	pp	229533.Q4IBK5
68795	229533.I1RWX8	pp	229533.I1S2Y5
68796	229533.I1RMR1	pp	229533.I1RZZ1
68797	229533.I1RMR1	pp	229533.Q4I948
68798	229533.I1RMR1	pp	229533.I1RUG4
68799	229533.I1RMR1	pp	229533.I1RW25
68800	229533.I1RMR1	pp	229533.I1RSS4
68801	229533.I1RMR1	pp	229533.Q4ICA8
68802	229533.I1RMR1	pp	229533.I1RX64
68803	229533.I1RMR1	pp	229533.I1RQM6
68804	229533.I1RMR1	pp	229533.I1RT04
68805	229533.I1RMR1	pp	229533.I1S0R7
68806	229533.I1RMR1	pp	229533.I1RRZ0
68807	229533.I1RMR1	pp	229533.I1RT33
68808	229533.A0A1C3YMB4	pp	229533.I1S984
68809	229533.A0A1C3YMB4	pp	229533.I1RC10
68810	229533.A0A1C3YMB4	pp	229533.I1RG48
68811	229533.A0A1C3YMB4	pp	229533.I1RCK4
68812	229533.I1S3N1	pp	229533.Q4I5I4
68813	229533.I1S3N1	pp	229533.V6QWX8
68814	229533.I1S3N1	pp	229533.Q4I5W5
68815	229533.I1S3N1	pp	229533.Q4IPC8
68816	229533.I1S3N1	pp	229533.Q4HYB8
68817	229533.I1S3N1	pp	229533.V6QUE6
68818	229533.I1RRT2	pp	229533.I1RT33
68819	229533.I1RRT2	pp	229533.I1S2M6
68820	229533.I1RRT2	pp	229533.I1RS47
68821	229533.I1RRT2	pp	229533.Q4HZ42
68822	229533.I1RRT2	pp	229533.I1S6F0
68823	229533.I1RRT2	pp	229533.I1RVQ0
68824	229533.I1RRT2	pp	229533.I1RSI4
68825	229533.I1RRT2	pp	229533.V6R283
68826	229533.I1RRT2	pp	229533.Q4IAS1
68827	229533.I1RRT2	pp	229533.I1RT62
68828	229533.I1RRT2	pp	229533.Q4IQW0
68829	229533.I1RRT2	pp	229533.I1RRZ0
68830	229533.I1RRT2	pp	229533.I1RSQ7
68831	229533.I1RRT2	pp	229533.I1S0F6
68832	229533.I1RRT2	pp	229533.V6QW22
68833	229533.I1RRT2	pp	229533.I1S1B6
68834	229533.I1RRT2	pp	229533.I1RWD4
68835	229533.I1RRT2	pp	229533.I1S0L3
68836	229533.I1RRT2	pp	229533.Q4IR18
68837	229533.I1RRT2	pp	229533.I1RZ99
68838	229533.I1RRT2	pp	229533.Q4IEV5
68839	229533.I1RRT2	pp	229533.I1S0D4

68840 229533.I1RRT2 pp 229533.I1RWF7
68841 229533.I1RRT2 pp 229533.Q4I830
68842 229533.I1RRT2 pp 229533.I1RT40
68843 229533.I1RRT2 pp 229533.Q4IFI0
68844 229533.I1RRT2 pp 229533.I1RSX1
68845 229533.I1RRT2 pp 229533.I1RV91
68846 229533.I1RRT2 pp 229533.I1RUQ2
68847 229533.I1RRT2 pp 229533.I1RW71
68848 229533.I1RRT2 pp 229533.Q4INZ9
68849 229533.I1RRT2 pp 229533.I1S6Y8
68850 229533.I1RRT2 pp 229533.I1RXD7
68851 229533.I1RRT2 pp 229533.Q4HXF0
68852 229533.I1RRT2 pp 229533.Q4I662
68853 229533.I1RRT2 pp 229533.Q4IPI1
68854 229533.I1RRT2 pp 229533.I1RWJ4
68855 229533.I1RRT2 pp 229533.I1RTI6
68856 229533.I1RRT2 pp 229533.I1S0T2
68857 229533.I1RRT2 pp 229533.I1RW61
68858 229533.I1RRT2 pp 229533.I1RX46
68859 229533.I1RRT2 pp 229533.I1S5R1
68860 229533.I1RRT2 pp 229533.I1S0R7
68861 229533.I1RRT2 pp 229533.I1RYT5
68862 229533.I1RRT2 pp 229533.I1RW25
68863 229533.I1RRT2 pp 229533.I1RRT8
68864 229533.I1RRT2 pp 229533.I1RT04
68865 229533.I1RRT2 pp 229533.I1RWJ8
68866 229533.I1RRT2 pp 229533.I1RZS6
68867 229533.I1RRT2 pp 229533.I1S9U2
68868 229533.I1RRT2 pp 229533.I1S0J7
68869 229533.I1RRT2 pp 229533.Q4IQK7
68870 229533.I1RRT2 pp 229533.I1S0L4
68871 229533.I1RRT2 pp 229533.V6QYF0
68872 229533.I1RRT2 pp 229533.I1S6U8
68873 229533.I1RRT2 pp 229533.Q4HVW2
68874 229533.I1RRT2 pp 229533.I1RX24
68875 229533.I1RRT2 pp 229533.I1SA32
68876 229533.I1RRT2 pp 229533.I1S8E2
68877 229533.I1RRT2 pp 229533.I1S2H4
68878 229533.I1RRT2 pp 229533.Q4IJH1
68879 229533.I1RRT2 pp 229533.I1RW46
68880 229533.I1RRT2 pp 229533.I1RT05
68881 229533.I1RRT2 pp 229533.I1RTF4
68882 229533.I1RRT2 pp 229533.Q4IF76
68883 229533.I1RRT2 pp 229533.I1RS18
68884 229533.I1RRT2 pp 229533.Q4HZI0
68885 229533.I1RRT2 pp 229533.I1S060
68886 229533.I1RRT2 pp 229533.I1RT80
68887 229533.I1RRT2 pp 229533.Q4IAA0
68888 229533.I1RRT2 pp 229533.Q4I8P5
68889 229533.I1RRT2 pp 229533.I1RZZ9
68890 229533.I1RRT2 pp 229533.Q4IEK8
68891 229533.I1RRT2 pp 229533.I1S253
68892 229533.I1RRT2 pp 229533.I1RS17
68893 229533.I1RRT2 pp 229533.I1S419
68894 229533.I1RRT2 pp 229533.Q4I327
68895 229533.I1RRT2 pp 229533.V6RIV9
68896 229533.I1RRT2 pp 229533.Q4IJ56
68897 229533.I1RRT2 pp 229533.Q4IBS2
68898 229533.I1RRT2 pp 229533.I1S4T3
68899 229533.I1RRT2 pp 229533.V6QUE6
68900 229533.I1RRT2 pp 229533.I1SA33
68901 229533.I1RRT2 pp 229533.I1RZM8
68902 229533.I1RRT2 pp 229533.Q4IN63
68903 229533.I1RRT2 pp 229533.I1RZF6
68904 229533.I1RRT2 pp 229533.V6R227
68905 229533.I1RRT2 pp 229533.I1RW99
68906 229533.I1RRT2 pp 229533.I1S0J1
68907 229533.I1RRT2 pp 229533.I1RWT7
68908 229533.I1RRT2 pp 229533.I1S9T1
68909 229533.I1RRT2 pp 229533.I1RUG3
68910 229533.I1RRT2 pp 229533.V6RGN5
68911 229533.I1RRT2 pp 229533.I1S1U7
68912 229533.I1RRT2 pp 229533.V6RCG5

68913 229533.I1RRT2 pp 229533.I1RTG6
68914 229533.I1RRT2 pp 229533.I1RSI6
68915 229533.I1RRT2 pp 229533.I1RSR3
68916 229533.I1RRT2 pp 229533.I1RSI0
68917 229533.I1RRT2 pp 229533.V6RGY3
68918 229533.I1RRT2 pp 229533.I1S2E8
68919 229533.I1RRT2 pp 229533.I1RZE5
68920 229533.I1RRT2 pp 229533.I1S021
68921 229533.I1RRT2 pp 229533.I1S1J6
68922 229533.I1RRT2 pp 229533.I1RSM3
68923 229533.I1RRT2 pp 229533.I1RVZ0
68924 229533.I1RRT2 pp 229533.I1RZK6
68925 229533.I1RRT2 pp 229533.I1RZW8
68926 229533.I1RRT2 pp 229533.I1RTD9
68927 229533.I1RRT2 pp 229533.I1S296
68928 229533.I1RRT2 pp 229533.I1RZZ1
68929 229533.I1RRT2 pp 229533.I1S2U2
68930 229533.I1RRT2 pp 229533.I1RTE9
68931 229533.I1RRT2 pp 229533.I1RSS4
68932 229533.I1RRT2 pp 229533.I1RSD8
68933 229533.I1RRT2 pp 229533.Q4HZ68
68934 229533.I1RRT2 pp 229533.Q4HWE2
68935 229533.I1RRT2 pp 229533.I1RZB5
68936 229533.I1RRT2 pp 229533.I1S2C0
68937 229533.I1RRT2 pp 229533.Q4HZK7
68938 229533.I1RRT2 pp 229533.I1S1X9
68939 229533.I1RRT2 pp 229533.I1S558
68940 229533.I1RRT2 pp 229533.I1RY63
68941 229533.I1RRT2 pp 229533.I1S8R3
68942 229533.A0A098E4D0 pp 229533.I1RR40
68943 229533.A0A098E4D0 pp 229533.I1RP90
68944 229533.A0A098E4D0 pp 229533.I1RJA7
68945 229533.A0A098E4D0 pp 229533.I1RPD9
68946 229533.A0A098E4D0 pp 229533.A0A1C3YLG9
68947 229533.A0A098E4D0 pp 229533.I1S1W7
68948 229533.A0A098E4D0 pp 229533.I1RDP9
68949 229533.A0A098E4D0 pp 229533.I1RDZ3
68950 229533.I1RTF0 pp 229533.I1S039
68951 229533.I1RTF0 pp 229533.Q4ICG5
68952 229533.I1RTF0 pp 229533.I1S1W5
68953 229533.I1RTF0 pp 229533.I1RWI6
68954 229533.I1RTF0 pp 229533.V6R9X9
68955 229533.I1RTF0 pp 229533.I1RXU1
68956 229533.I1RTF0 pp 229533.I1S9T1
68957 229533.I1RTF0 pp 229533.Q4IBU4
68958 229533.I1RTF0 pp 229533.I1RWP9
68959 229533.I1RTF0 pp 229533.I1RZA4
68960 229533.I1RTF0 pp 229533.I1RXF5
68961 229533.I1RTF0 pp 229533.V6RQ48
68962 229533.I1RTF0 pp 229533.V6RKE4
68963 229533.I1RDZ6 pp 229533.I1RVL8
68964 229533.I1RDZ6 pp 229533.I1RPM7
68965 229533.I1RDZ6 pp 229533.I1RVY0
68966 229533.I1RDZ6 pp 229533.I1RXU0
68967 229533.I1RDZ6 pp 229533.I1RK71
68968 229533.I1RDZ6 pp 229533.Q4IPC8
68969 229533.I1RDZ6 pp 229533.I1RG11
68970 229533.I1RDZ6 pp 229533.Q4HYB8
68971 229533.I1RDZ6 pp 229533.I1S276
68972 229533.I1RDZ6 pp 229533.I1RR25
68973 229533.I1RDZ6 pp 229533.I1RSZ3
68974 229533.I1RDZ6 pp 229533.Q4I5W5
68975 229533.I1RDZ6 pp 229533.I1RKB1
68976 229533.I1RDZ6 pp 229533.I1RE30
68977 229533.I1RDZ6 pp 229533.I1RWF3
68978 229533.I1RDZ6 pp 229533.I1RK79
68979 229533.I1RDZ6 pp 229533.I1RE14
68980 229533.I1RDZ6 pp 229533.I1RTC2
68981 229533.I1RDZ6 pp 229533.I1RRG3
68982 229533.I1RDZ6 pp 229533.Q4I5I4
68983 229533.I1RDZ6 pp 229533.V6QWX8
68984 229533.I1RDZ6 pp 229533.I1RRT0
68985 229533.I1RDZ6 pp 229533.V6QUE6

68986 229533.I1RDZ6 pp 229533.I1RJL2
68987 229533.I1RDZ6 pp 229533.I1RUG0
68988 229533.I1RDZ6 pp 229533.I1RZB5
68989 229533.I1RWD4 pp 229533.Q4HVW2
68990 229533.I1RWD4 pp 229533.I1RZZ9
68991 229533.I1RWD4 pp 229533.I1RZW8
68992 229533.I1RWD4 pp 229533.I1S8R3
68993 229533.I1RWD4 pp 229533.Q4I5Z5
68994 229533.I1RWD4 pp 229533.I1S5R1
68995 229533.I1RWD4 pp 229533.I1S2H4
68996 229533.I1RWD4 pp 229533.I1S1V5
68997 229533.I1RWD4 pp 229533.I1RWJ8
68998 229533.I1RWD4 pp 229533.V6QYF0
68999 229533.I1RWD4 pp 229533.I1S951
69000 229533.I1RWD4 pp 229533.I1S1J6
69001 229533.I1RWD4 pp 229533.Q4IFI0
69002 229533.I1RWD4 pp 229533.I1S0L4
69003 229533.I1RWD4 pp 229533.V6R227
69004 229533.I1RWD4 pp 229533.V6R057
69005 229533.I1RWD4 pp 229533.I1RWT7
69006 229533.I1RWD4 pp 229533.I1S382
69007 229533.I1RWD4 pp 229533.Q4I7X1
69008 229533.I1RWD4 pp 229533.I1RZM8
69009 229533.I1RWD4 pp 229533.Q4IEV5
69010 229533.I1RWD4 pp 229533.V6RCG5
69011 229533.I1RWD4 pp 229533.V6RCZ8
69012 229533.I1RWD4 pp 229533.V6R1K4
69013 229533.I1RWD4 pp 229533.I1S296
69014 229533.I1RWD4 pp 229533.I1S0D4
69015 229533.I1RWD4 pp 229533.Q4IAS1
69016 229533.I1RWD4 pp 229533.Q4IR18
69017 229533.I1RWD4 pp 229533.Q4I327
69018 229533.I1RWD4 pp 229533.I1S2G7
69019 229533.I1RWD4 pp 229533.Q4IAA0
69020 229533.I1RWD4 pp 229533.I1S5B0
69021 229533.I1RWD4 pp 229533.I1SAE3
69022 229533.I1RWD4 pp 229533.I1S0S0
69023 229533.I1RWD4 pp 229533.V6QW22
69024 229533.I1RWD4 pp 229533.I1S0L3
69025 229533.I1RWD4 pp 229533.V6RIV9
69026 229533.I1RWD4 pp 229533.Q4IQ72
69027 229533.I1R9U5 pp 229533.I1RC10
69028 229533.I1R9U5 pp 229533.I1RCP6
69029 229533.A0A098DBW9 pp 229533.I1RDC9
69030 229533.A0A098DBW9 pp 229533.I1RZV1
69031 229533.A0A098DBW9 pp 229533.A0A0E0SQJ6
69032 229533.A0A098DBW9 pp 229533.I1RB36
69033 229533.A0A098DBW9 pp 229533.I1RWU3
69034 229533.A0A098DBW9 pp 229533.I1RWX8
69035 229533.A0A098DBW9 pp 229533.I1RXE5
69036 229533.A0A098DBW9 pp 229533.I1RMA5
69037 229533.A0A098DBW9 pp 229533.A0A1C3YJZ2
69038 229533.A0A098DBW9 pp 229533.I1RKR6
69039 229533.A0A098DBW9 pp 229533.A0A1C3YK01
69040 229533.A0A098DBW9 pp 229533.I1RGW0
69041 229533.A0A098DBW9 pp 229533.V6QV38
69042 229533.A0A098DNM0 pp 229533.I1RUG4
69043 229533.A0A098DNM0 pp 229533.I1RDJ8
69044 229533.A0A098DNM0 pp 229533.I1RN55
69045 229533.A0A098DNM0 pp 229533.I1RSS4
69046 229533.A0A098DNM0 pp 229533.I1RSR9
69047 229533.A0A098DNM0 pp 229533.I1RDX0
69048 229533.A0A098DNM0 pp 229533.I1RRR2
69049 229533.A0A098DNM0 pp 229533.I1RR60
69050 229533.A0A098DNM0 pp 229533.I1S209
69051 229533.A0A098DNM0 pp 229533.I1RQM6
69052 229533.A0A098DNM0 pp 229533.I1RRT1
69053 229533.A0A098DNM0 pp 229533.I1RRV1
69054 229533.A0A098DNM0 pp 229533.I1RCA5
69055 229533.A0A098DNM0 pp 229533.I1RZ94
69056 229533.A0A098DNM0 pp 229533.A0A1C3YL18
69057 229533.A0A098DNM0 pp 229533.I1RQJ4
69058 229533.A0A098DNM0 pp 229533.I1RP26

69059 229533.A0A098DNM0 pp 229533.Q4I2M7
69060 229533.A0A098DNM0 pp 229533.I1RSP7
69061 229533.A0A098DNM0 pp 229533.I1RPQ1
69062 229533.A0A098DNM0 pp 229533.Q4HTT2
69063 229533.A0A098DNM0 pp 229533.I1RYQ1
69064 229533.A0A098DNM0 pp 229533.A0A1C3YLB0
69065 229533.A0A098DNM0 pp 229533.A0A0E0RW49
69066 229533.A0A098DNM0 pp 229533.I1RA12
69067 229533.A0A098DNM0 pp 229533.I1S3S6
69068 229533.A0A098DNM0 pp 229533.A0A1C3YN55
69069 229533.A0A098DNM0 pp 229533.A0A1C3YM43
69070 229533.A0A098DNM0 pp 229533.I1RMR1
69071 229533.A0A098DNM0 pp 229533.I1S4P6
69072 229533.A0A098DNM0 pp 229533.I1S0R7
69073 229533.A0A098DNM0 pp 229533.I1RN57
69074 229533.A0A098DNM0 pp 229533.I1RT33
69075 229533.A0A098DNM0 pp 229533.Q4I1J3
69076 229533.A0A098DNM0 pp 229533.A0A0E0RLR6
69077 229533.A0A1C3YLG9 pp 229533.I1S4A5
69078 229533.A0A1C3YLG9 pp 229533.I1RAE2
69079 229533.A0A1C3YLG9 pp 229533.I1RA99
69080 229533.A0A1C3YLG9 pp 229533.I1RJA7
69081 229533.A0A1C3YLG9 pp 229533.I1RZ35
69082 229533.A0A1C3YLG9 pp 229533.I1RM78
69083 229533.A0A1C3YLG9 pp 229533.I1SA27
69084 229533.A0A1C3YLG9 pp 229533.I1RLI4
69085 229533.A0A1C3YLG9 pp 229533.I1RDP6
69086 229533.A0A1C3YLG9 pp 229533.I1RIN3
69087 229533.A0A1C3YLG9 pp 229533.I1RXN7
69088 229533.A0A1C3YLG9 pp 229533.I1RSJ0
69089 229533.A0A1C3YLG9 pp 229533.I1S325
69090 229533.A0A1C3YLG9 pp 229533.I1RPT0
69091 229533.A0A1C3YLG9 pp 229533.I1S3H6
69092 229533.A0A1C3YLG9 pp 229533.I1REG4
69093 229533.A0A1C3YLG9 pp 229533.I1RKK6
69094 229533.A0A1C3YLG9 pp 229533.I1RBT2
69095 229533.A0A1C3YLG9 pp 229533.I1RDZ3
69096 229533.A0A1C3YLG9 pp 229533.I1S1W7
69097 229533.A0A1C3YLG9 pp 229533.I1RN54
69098 229533.I1RTF4 pp 229533.I1S021
69099 229533.I1RTF4 pp 229533.I1RW99
69100 229533.I1RTF4 pp 229533.Q4I327
69101 229533.I1RTF4 pp 229533.V6QUE6
69102 229533.I1RTF4 pp 229533.Q4IP34
69103 229533.I1RTF4 pp 229533.V6RGN5
69104 229533.I1RTF4 pp 229533.I1S0M9
69105 229533.I1RTF4 pp 229533.Q4IJ56
69106 229533.I1RTF4 pp 229533.Q4IBS2
69107 229533.I1RTF4 pp 229533.I1RX08
69108 229533.I1RTF4 pp 229533.I1RWD3
69109 229533.I1RTF4 pp 229533.V6RCG5
69110 229533.I1RTF4 pp 229533.Q4I7K4
69111 229533.I1RTF4 pp 229533.V6RIV9
69112 229533.I1RTF4 pp 229533.I1RZM8
69113 229533.I1RTF4 pp 229533.I1S4T3
69114 229533.I1RTF4 pp 229533.Q4IAA0
69115 229533.I1RTF4 pp 229533.I1RZB5
69116 229533.I1RTF4 pp 229533.Q4IEK8
69117 229533.I1RTF4 pp 229533.I1RX14
69118 229533.I1RTF4 pp 229533.I1RW46
69119 229533.I1RTF4 pp 229533.I1S060
69120 229533.I1RTF4 pp 229533.I1S8R3
69121 229533.I1RTF4 pp 229533.I1S0J1
69122 229533.I1RTF4 pp 229533.Q4IN63
69123 229533.I1RTF4 pp 229533.Q4HZK7
69124 229533.I1RTF4 pp 229533.I1RUG3
69125 229533.I1RTF4 pp 229533.V6R227
69126 229533.I1RTF4 pp 229533.I1RY63
69127 229533.I1RTF4 pp 229533.I1RZF6
69128 229533.I1RTF4 pp 229533.I1S0C1
69129 229533.I1RTF4 pp 229533.I1S1X9
69130 229533.I1RTF4 pp 229533.I1S2C0
69131 229533.I1RTF4 pp 229533.I1RUQ2

69132 229533.I1RTF4 pp 229533.I1RZW8
69133 229533.I1RTF4 pp 229533.Q4I7F9
69134 229533.I1RTF4 pp 229533.I1RXD7
69135 229533.I1RTF4 pp 229533.Q4I948
69136 229533.I1RTF4 pp 229533.I1S2E8
69137 229533.I1RTF4 pp 229533.Q4INZ9
69138 229533.I1RTF4 pp 229533.I1S1U7
69139 229533.I1RTF4 pp 229533.I1RWT7
69140 229533.I1RTF4 pp 229533.Q4IR18
69141 229533.I1RTF4 pp 229533.Q4IQW0
69142 229533.I1RTF4 pp 229533.I1S855
69143 229533.I1RTF4 pp 229533.I1RZK6
69144 229533.I1RTF4 pp 229533.I1S2M6
69145 229533.I1RTF4 pp 229533.Q4IAS1
69146 229533.I1RTF4 pp 229533.I1RZZ1
69147 229533.I1RTF4 pp 229533.Q4HZ42
69148 229533.I1RTF4 pp 229533.Q4HZ68
69149 229533.I1RTF4 pp 229533.Q4IPI1
69150 229533.I1RTF4 pp 229533.I1S296
69151 229533.I1RTF4 pp 229533.I1S854
69152 229533.I1RTF4 pp 229533.Q4IEV5
69153 229533.I1RTF4 pp 229533.I1S1J6
69154 229533.I1RTF4 pp 229533.I1S0L3
69155 229533.I1RTF4 pp 229533.Q4I5Z5
69156 229533.I1RTF4 pp 229533.I1RX46
69157 229533.I1RTF4 pp 229533.I1RVZ0
69158 229533.I1RTF4 pp 229533.Q4HZ47
69159 229533.I1RTF4 pp 229533.Q4I830
69160 229533.I1RTF4 pp 229533.I1S0M3
69161 229533.I1RTF4 pp 229533.I1RW71
69162 229533.I1RTF4 pp 229533.I1RWD4
69163 229533.I1RTF4 pp 229533.I1RVQ0
69164 229533.I1RTF4 pp 229533.V6QW22
69165 229533.I1RTF4 pp 229533.I1RZ99
69166 229533.I1RTF4 pp 229533.I1S6F0
69167 229533.I1RTF4 pp 229533.I1RZE5
69168 229533.I1RTF4 pp 229533.V6R283
69169 229533.I1RTF4 pp 229533.I1RWF7
69170 229533.I1RTF4 pp 229533.I1S0R7
69171 229533.I1RTF4 pp 229533.I1S9U2
69172 229533.I1RTF4 pp 229533.I1S8E2
69173 229533.I1RTF4 pp 229533.Q4HW67
69174 229533.I1RTF4 pp 229533.I1RWJ8
69175 229533.I1RTF4 pp 229533.Q4IJH1
69176 229533.I1RTF4 pp 229533.Q4HVW2
69177 229533.I1RTF4 pp 229533.I1S5R1
69178 229533.I1RTF4 pp 229533.I1S6U8
69179 229533.I1RTF4 pp 229533.Q4IF76
69180 229533.I1RTF4 pp 229533.V6QYF0
69181 229533.I1RTF4 pp 229533.I1RZS6
69182 229533.I1RTF4 pp 229533.I1RWI5
69183 229533.I1RTF4 pp 229533.I1RX24
69184 229533.I1RTF4 pp 229533.I1RZG3
69185 229533.I1RTF4 pp 229533.Q4I662
69186 229533.I1RTF4 pp 229533.I1RW61
69187 229533.I1RTF4 pp 229533.I1RW25
69188 229533.I1RTF4 pp 229533.Q4IFI0
69189 229533.I1RMN2 pp 229533.I1S4Q7
69190 229533.I1RMN2 pp 229533.I1RZ18
69191 229533.I1RMN2 pp 229533.I1RRH8
69192 229533.I1RMN2 pp 229533.I1RQF7
69193 229533.I1RMN2 pp 229533.I1RZ87
69194 229533.V6R9X9 pp 229533.V6RKE4
69195 229533.V6R9X9 pp 229533.V6RQ48
69196 229533.I1RSM3 pp 229533.V6R227
69197 229533.I1RSM3 pp 229533.I1S4T3
69198 229533.I1RSM3 pp 229533.I1RWT7
69199 229533.I1RSM3 pp 229533.I1RT80
69200 229533.I1RSM3 pp 229533.I1RTF4
69201 229533.I1RSM3 pp 229533.I1S5B0
69202 229533.I1RSM3 pp 229533.I1RZS6
69203 229533.I1RSM3 pp 229533.I1RW25
69204 229533.I1RSM3 pp 229533.I1RWD4

69205	229533.I1RSM3	pp	229533.V6QYF0
69206	229533.I1RSM3	pp	229533.I1S021
69207	229533.I1RSM3	pp	229533.I1RZZ9
69208	229533.I1RSM3	pp	229533.I1S0L4
69209	229533.I1RSM3	pp	229533.I1S0F5
69210	229533.I1RSM3	pp	229533.Q4IR18
69211	229533.I1RSM3	pp	229533.I1S0L3
69212	229533.I1RFI6	pp	229533.Q4ICA8
69213	229533.I1RFI6	pp	229533.I1RQP3
69214	229533.I1RFI6	pp	229533.I1RRG3
69215	229533.I1RFI6	pp	229533.I1RVP2
69216	229533.I1RFI6	pp	229533.I1RMJ8
69217	229533.I1RFI6	pp	229533.I1RVU0
69218	229533.I1RFI6	pp	229533.I1RY58
69219	229533.I1RFI6	pp	229533.I1RSC7
69220	229533.I1RFI6	pp	229533.I1RQP1
69221	229533.I1RFI6	pp	229533.I1RPR8
69222	229533.I1RFI6	pp	229533.Q4IPI4
69223	229533.A0A1C3YJK1	pp	229533.I1RHK0
69224	229533.A0A1C3YJK1	pp	229533.I1S9Z4
69225	229533.A0A1C3YJK1	pp	229533.I1RVL7
69226	229533.A0A1C3YJK1	pp	229533.A0A1C3YKA2
69227	229533.A0A1C3YJK1	pp	229533.I1RLX5
69228	229533.A0A1C3YJK1	pp	229533.I1RK69
69229	229533.A0A1C3YJK1	pp	229533.I1RVM4
69230	229533.A0A1C3YJK1	pp	229533.I1RPD9
69231	229533.A0A1C3YJK1	pp	229533.I1S5U5
69232	229533.A0A1C3YJK1	pp	229533.I1RQ48
69233	229533.I1R9S9	pp	229533.I1RHZ5
69234	229533.I1R9S9	pp	229533.I1RXS0
69235	229533.I1R9S9	pp	229533.I1RC48
69236	229533.I1R9S9	pp	229533.I1S0K0
69237	229533.I1R9S9	pp	229533.I1S8R4
69238	229533.I1R9S9	pp	229533.I1RQP2
69239	229533.I1R9S9	pp	229533.I1S3L2
69240	229533.A0A098DBU0	pp	229533.I1S249
69241	229533.A0A098DBU0	pp	229533.I1RMN2
69242	229533.A0A098DBU0	pp	229533.I1RFI6
69243	229533.A0A098DBU0	pp	229533.I1RPR8
69244	229533.A0A098DNI7	pp	229533.Q4HTT2
69245	229533.A0A098DNI7	pp	229533.I1RYV3
69246	229533.A0A098DNI7	pp	229533.Q4I7L0
69247	229533.A0A098DNI7	pp	229533.I1S4Z5
69248	229533.A0A098DNI7	pp	229533.I1RBJ0
69249	229533.I1RAZ8	pp	229533.I1RD01
69250	229533.I1RAZ8	pp	229533.I1RZS6
69251	229533.I1RAZ8	pp	229533.I1RX24
69252	229533.I1RAZ8	pp	229533.I1RM06
69253	229533.I1RAZ8	pp	229533.I1RBG9
69254	229533.I1RAZ8	pp	229533.I1RNA0
69255	229533.I1RAZ8	pp	229533.I1RG51
69256	229533.I1RAZ8	pp	229533.V6QYF0
69257	229533.I1RAZ8	pp	229533.I1RRQ6
69258	229533.I1RAZ8	pp	229533.I1RC32
69259	229533.I1RAZ8	pp	229533.I1REX4
69260	229533.I1RAZ8	pp	229533.I1RWJ4
69261	229533.I1RAZ8	pp	229533.I1RFS3
69262	229533.I1RAZ8	pp	229533.Q4IF76
69263	229533.I1RAZ8	pp	229533.I1RW25
69264	229533.I1RAZ8	pp	229533.I1RPW9
69265	229533.I1RAZ8	pp	229533.I1RBY4
69266	229533.I1RAZ8	pp	229533.Q4IFI0
69267	229533.I1RAZ8	pp	229533.I1S0L4
69268	229533.I1RAZ8	pp	229533.Q4I662
69269	229533.I1RAZ8	pp	229533.I1S9U2
69270	229533.I1RAZ8	pp	229533.I1S6Y8
69271	229533.I1RAZ8	pp	229533.I1RJZ7
69272	229533.I1RAZ8	pp	229533.I1RN51
69273	229533.I1RAZ8	pp	229533.I1RPM4
69274	229533.I1RAZ8	pp	229533.I1RR24
69275	229533.I1RAZ8	pp	229533.I1RW61
69276	229533.I1RAZ8	pp	229533.I1RRM5
69277	229533.I1RAZ8	pp	229533.I1RQS8

69278	229533.I1RAZ8	pp	229533.I1RD64
69279	229533.I1RAZ8	pp	229533.I1RBN1
69280	229533.I1RAZ8	pp	229533.I1RDA0
69281	229533.I1RAZ8	pp	229533.I1RCS1
69282	229533.I1RAZ8	pp	229533.I1S2H4
69283	229533.I1RAZ8	pp	229533.I1S2M6
69284	229533.I1RAZ8	pp	229533.Q4HVW2
69285	229533.I1RAZ8	pp	229533.I1RFW5
69286	229533.I1RAZ8	pp	229533.I1S5R1
69287	229533.I1RAZ8	pp	229533.I1RFQ7
69288	229533.I1RAZ8	pp	229533.I1RMK3
69289	229533.I1RAZ8	pp	229533.I1RN63
69290	229533.I1RAZ8	pp	229533.I1RXB1
69291	229533.I1RAZ8	pp	229533.Q4IJH1
69292	229533.I1RAZ8	pp	229533.I1RRT8
69293	229533.I1RAZ8	pp	229533.V6R283
69294	229533.I1RAZ8	pp	229533.I1RM67
69295	229533.I1RAZ8	pp	229533.I1RT80
69296	229533.I1RAZ8	pp	229533.I1RZV6
69297	229533.I1RAZ8	pp	229533.I1RTF4
69298	229533.I1RAZ8	pp	229533.Q4HW67
69299	229533.I1RAZ8	pp	229533.I1S9Y1
69300	229533.I1RAZ8	pp	229533.I1RWJ8
69301	229533.I1RAZ8	pp	229533.V6R1K4
69302	229533.I1RAZ8	pp	229533.I1RNV6
69303	229533.I1RAZ8	pp	229533.I1RM82
69304	229533.I1RAZ8	pp	229533.I1RJU3
69305	229533.I1RAZ8	pp	229533.I1RQM6
69306	229533.I1RAZ8	pp	229533.I1RPU0
69307	229533.I1RAZ8	pp	229533.I1RKJ6
69308	229533.I1RAZ8	pp	229533.I1RQ65
69309	229533.I1RAZ8	pp	229533.I1RBT1
69310	229533.I1RAZ8	pp	229533.I1RD31
69311	229533.I1RAZ8	pp	229533.I1RD52
69312	229533.I1RAZ8	pp	229533.I1S0L3
69313	229533.I1RAZ8	pp	229533.I1RZZ9
69314	229533.I1RAZ8	pp	229533.I1RZ99
69315	229533.I1RAZ8	pp	229533.I1RS47
69316	229533.I1RAZ8	pp	229533.I1RQJ5
69317	229533.I1RAZ8	pp	229533.Q4IR18
69318	229533.I1RAZ8	pp	229533.I1RRJ8
69319	229533.I1RAZ8	pp	229533.V6QW22
69320	229533.I1RAZ8	pp	229533.Q4HZ42
69321	229533.I1RAZ8	pp	229533.I1RT98
69322	229533.I1RAZ8	pp	229533.I1RSQ7
69323	229533.I1RAZ8	pp	229533.Q4INZ9
69324	229533.I1RAZ8	pp	229533.I1RT33
69325	229533.I1RAZ8	pp	229533.I1RT40
69326	229533.I1RAZ8	pp	229533.I1S6F0
69327	229533.I1RAZ8	pp	229533.Q4I830
69328	229533.I1RAZ8	pp	229533.I1RSX1
69329	229533.I1RAZ8	pp	229533.I1RRQ3
69330	229533.I1RAZ8	pp	229533.I1RFG0
69331	229533.I1RAZ8	pp	229533.Q4IQW0
69332	229533.I1RAZ8	pp	229533.I1RP27
69333	229533.I1RAZ8	pp	229533.I1RJ74
69334	229533.I1RAZ8	pp	229533.I1RNH1
69335	229533.I1RAZ8	pp	229533.I1RVQ0
69336	229533.I1RAZ8	pp	229533.I1RKL3
69337	229533.I1RAZ8	pp	229533.I1RW71
69338	229533.I1RAZ8	pp	229533.I1RJV4
69339	229533.I1RAZ8	pp	229533.I1RRP3
69340	229533.I1RAZ8	pp	229533.I1RBC9
69341	229533.I1RAZ8	pp	229533.I1RSV2
69342	229533.I1RAZ8	pp	229533.I1S1B6
69343	229533.I1RAZ8	pp	229533.I1RBS6
69344	229533.I1RAZ8	pp	229533.I1RS30
69345	229533.I1RAZ8	pp	229533.I1RZK6
69346	229533.I1RAZ8	pp	229533.I1RWD4
69347	229533.I1RAZ8	pp	229533.I1RFN1
69348	229533.I1RAZ8	pp	229533.I1RBC7
69349	229533.I1RAZ8	pp	229533.I1S0T2
69350	229533.I1RAZ8	pp	229533.I1RC82

69351 229533.I1RAZ8 pp 229533.I1RZZ1
69352 229533.I1RAZ8 pp 229533.I1RCH5
69353 229533.I1RAZ8 pp 229533.I1RXD7
69354 229533.I1RAZ8 pp 229533.I1RQS5
69355 229533.I1RAZ8 pp 229533.I1RE87
69356 229533.I1RAZ8 pp 229533.I1RUQ2
69357 229533.I1RAZ8 pp 229533.I1S2E8
69358 229533.I1RAZ8 pp 229533.I1RRT2
69359 229533.I1RAZ8 pp 229533.Q4I7F9
69360 229533.I1RAZ8 pp 229533.I1RQE7
69361 229533.I1RAZ8 pp 229533.Q4HXF0
69362 229533.I1RAZ8 pp 229533.I1RT04
69363 229533.I1RAZ8 pp 229533.Q4IPI1
69364 229533.I1RAZ8 pp 229533.I1RJL4
69365 229533.I1RAZ8 pp 229533.I1RTI6
69366 229533.I1RAZ8 pp 229533.I1RJZ6
69367 229533.I1RAZ8 pp 229533.I1RLV9
69368 229533.I1RAZ8 pp 229533.I1RFL4
69369 229533.I1RAZ8 pp 229533.I1RSD8
69370 229533.I1RAZ8 pp 229533.Q4IAS1
69371 229533.I1RAZ8 pp 229533.I1RN18
69372 229533.I1RAZ8 pp 229533.I1S0R7
69373 229533.I1RAZ8 pp 229533.Q4HZ68
69374 229533.I1RAZ8 pp 229533.I1S0C1
69375 229533.I1RAZ8 pp 229533.I1RJW6
69376 229533.I1RAZ8 pp 229533.I1S854
69377 229533.I1RAZ8 pp 229533.I1RTE9
69378 229533.I1RAZ8 pp 229533.I1RPJ3
69379 229533.I1RAZ8 pp 229533.I1RC06
69380 229533.I1RAZ8 pp 229533.I1S296
69381 229533.I1RAZ8 pp 229533.I1RRE2
69382 229533.I1RAZ8 pp 229533.I1RF08
69383 229533.I1RAZ8 pp 229533.I1RN99
69384 229533.I1RAZ8 pp 229533.I1RKQ5
69385 229533.I1RAZ8 pp 229533.I1S2C0
69386 229533.I1RAZ8 pp 229533.I1RZB5
69387 229533.I1RAZ8 pp 229533.I1RVY2
69388 229533.I1RAZ8 pp 229533.V6R057
69389 229533.I1RAZ8 pp 229533.I1RB92
69390 229533.I1RAZ8 pp 229533.I1RUG2
69391 229533.I1RAZ8 pp 229533.I1RQ28
69392 229533.I1RAZ8 pp 229533.Q4HZK7
69393 229533.I1RAZ8 pp 229533.I1RK95
69394 229533.I1RAZ8 pp 229533.I1RD17
69395 229533.I1RAZ8 pp 229533.I1RZE5
69396 229533.I1RAZ8 pp 229533.I1REK8
69397 229533.I1RAZ8 pp 229533.I1RE00
69398 229533.I1RAZ8 pp 229533.I1S8R3
69399 229533.I1RAZ8 pp 229533.I1RCG4
69400 229533.I1RAZ8 pp 229533.I1RDG0
69401 229533.I1RAZ8 pp 229533.I1RRR6
69402 229533.I1RAZ8 pp 229533.I1RY63
69403 229533.I1RAZ8 pp 229533.I1RFJ9
69404 229533.I1RAZ8 pp 229533.I1S1X9
69405 229533.I1RAZ8 pp 229533.I1RNK2
69406 229533.I1RAZ8 pp 229533.I1RF19
69407 229533.I1RAZ8 pp 229533.I1S1J6
69408 229533.I1RAZ8 pp 229533.I1RVZ0
69409 229533.I1RAZ8 pp 229533.I1RRI2
69410 229533.I1RAZ8 pp 229533.I1RZW8
69411 229533.I1RAZ8 pp 229533.I1RS18
69412 229533.I1RAZ8 pp 229533.I1RSS4
69413 229533.I1RAZ8 pp 229533.I1RRH8
69414 229533.I1RAZ8 pp 229533.I1RKC6
69415 229533.I1RAZ8 pp 229533.I1RMD5
69416 229533.I1RAZ8 pp 229533.Q4I5Z5
69417 229533.I1RAZ8 pp 229533.I1RWD3
69418 229533.I1RAZ8 pp 229533.Q4I7K4
69419 229533.I1RAZ8 pp 229533.I1RSR8
69420 229533.I1RAZ8 pp 229533.I1RB27
69421 229533.I1RAZ8 pp 229533.I1RTD9
69422 229533.I1RAZ8 pp 229533.V6RCG5
69423 229533.I1RAZ8 pp 229533.I1RSM3

229533.I1RAZ8 pp 229533.Q4IJ56
229533.I1RAZ8 pp 229533.I1RSI6
229533.I1RAZ8 pp 229533.I1S0M9
229533.I1RAZ8 pp 229533.I1S1U7
229533.I1RAZ8 pp 229533.Q4I327
229533.I1RAZ8 pp 229533.I1RNY2
229533.I1RAZ8 pp 229533.I1RNU6
229533.I1RAZ8 pp 229533.I1RS17
229533.I1RAZ8 pp 229533.I1RWT7
229533.I1RAZ8 pp 229533.I1RI82
229533.I1RAZ8 pp 229533.I1RSL8
229533.I1RAZ8 pp 229533.I1S0J1
229533.I1RAZ8 pp 229533.I1RSR3
229533.I1RAZ8 pp 229533.I1S021
229533.I1RAZ8 pp 229533.I1RNH6
229533.I1RAZ8 pp 229533.I1RMN7
229533.I1RAZ8 pp 229533.V6RGY3
229533.I1RAZ8 pp 229533.I1RBL7
229533.I1RAZ8 pp 229533.I1RE01
229533.I1RAZ8 pp 229533.I1S274
229533.I1RAZ8 pp 229533.I1RC72
229533.I1RAZ8 pp 229533.I1RCL3
229533.I1RAZ8 pp 229533.I1S060
229533.I1RAZ8 pp 229533.I1RJV3
229533.I1RAZ8 pp 229533.I1RW46
229533.I1RAZ8 pp 229533.I1RMC0
229533.I1RAZ8 pp 229533.I1RX14
229533.I1RAZ8 pp 229533.I1S855
229533.I1RAZ8 pp 229533.I1RUG3
229533.I1RAZ8 pp 229533.I1RQ31
229533.I1RAZ8 pp 229533.V6R227
229533.I1RAZ8 pp 229533.Q4IN63
229533.I1RAZ8 pp 229533.I1RQA2
229533.I1RAZ8 pp 229533.I1RZM8
229533.I1RAZ8 pp 229533.I1S558
229533.I1RAZ8 pp 229533.I1RZF6
229533.I1RAZ8 pp 229533.I1RRR7
229533.I1RAZ8 pp 229533.V6RGN5
229533.I1RAZ8 pp 229533.I1RM96
229533.I1RAZ8 pp 229533.Q4IEK8
229533.I1RAZ8 pp 229533.Q4IAA0
229533.I1RAZ8 pp 229533.I1S044
229533.I1RAZ8 pp 229533.Q4IP34
229533.I1RAZ8 pp 229533.I1RG11
229533.I1RAZ8 pp 229533.I1RC33
229533.I1RAZ8 pp 229533.V6RIV9
229533.I1RAZ8 pp 229533.I1RW99
229533.I1RAZ8 pp 229533.I1RC27
229533.I1RAZ8 pp 229533.I1RBI8
229533.I1RAZ8 pp 229533.I1S4T3
229533.I1RAZ8 pp 229533.Q4IBS2
229533.I1RAZ8 pp 229533.I1S419
229533.I1RAZ8 pp 229533.I1RRU9
229533.I1RAZ8 pp 229533.I1RD22
229533.I1RAZ8 pp 229533.I1RG56
229533.I1RAZ8 pp 229533.I1RPY1
229533.I1RAZ8 pp 229533.I1RMN6
229533.I1RAZ8 pp 229533.V6QUE6
229533.I1RAZ8 pp 229533.I1RGC2
229533.I1RAZ8 pp 229533.I1RR51
229533.A0A0E0SIV1 pp 229533.A0A1C3YN55
229533.A0A0E0SIV1 pp 229533.I1RWF3
229533.A0A0E0SIV1 pp 229533.I1RK79
229533.A0A0E0SIV1 pp 229533.I1RCB7
229533.A0A0E0SIV1 pp 229533.I1RXU0
229533.A0A0E0SIV1 pp 229533.I1RK71
229533.A0A0E0SIV1 pp 229533.I1RG11
229533.A0A0E0SIV1 pp 229533.I1RE30
229533.A0A0E0SIV1 pp 229533.I1RPM7
229533.A0A0E0SIV1 pp 229533.A0A1C3YIR8
229533.A0A0E0SIV1 pp 229533.I1RC24
229533.A0A0E0SIV1 pp 229533.I1RVL8
229533.A0A0E0SIV1 pp 229533.Q4IPC8

69497 229533.A0A0E0SIV1 pp 229533.I1S276
69498 229533.A0A0E0SIV1 pp 229533.Q4HYB8
69499 229533.A0A0E0SIV1 pp 229533.I1RVY0
69500 229533.A0A0E0SIV1 pp 229533.I1RR25
69501 229533.A0A0E0SIV1 pp 229533.I1RE14
69502 229533.A0A0E0SIV1 pp 229533.I1RSZ3
69503 229533.A0A0E0SIV1 pp 229533.I1RTC2
69504 229533.A0A0E0SIV1 pp 229533.V6QWX8
69505 229533.A0A0E0SIV1 pp 229533.A0A1C3YI23
69506 229533.A0A0E0SIV1 pp 229533.Q4I5I4
69507 229533.A0A0E0SIV1 pp 229533.Q4I5W5
69508 229533.A0A0E0SIV1 pp 229533.I1RAR2
69509 229533.A0A0E0SIV1 pp 229533.V6QUE6
69510 229533.A0A0E0SIV1 pp 229533.I1RJL2
69511 229533.A0A0E0SIV1 pp 229533.I1RUG0
69512 229533.A0A0E0SIV1 pp 229533.I1RZB5
69513 229533.A0A0E0SIV1 pp 229533.I1RRT0
69514 229533.A0A0E0SIV1 pp 229533.I1RKB1
69515 229533.A0A0E0SIV1 pp 229533.I1RC15
69516 229533.A0A0E0SIV1 pp 229533.I1RRG3
69517 229533.V6R227 pp 229533.V6R283
69518 229533.V6R227 pp 229533.V6RCG5
69519 229533.V6R227 pp 229533.V6RIV9
69520 229533.V6R227 pp 229533.V6RGN5
69521 229533.V6R227 pp 229533.V6RGY3
69522 229533.Q4I7S1 pp 229533.Q4IAK7
69523 229533.Q4I7S1 pp 229533.Q4IL82
69524 229533.Q4I7S1 pp 229533.V6QWX8
69525 229533.Q4I7S1 pp 229533.Q4IMN9
69526 229533.Q4I7S1 pp 229533.Q4IJ11
69527 229533.Q4I7S1 pp 229533.V6QUE6
69528 229533.Q4I7S1 pp 229533.Q4IMD1
69529 229533.Q4I7S1 pp 229533.Q4IEV4
69530 229533.Q4I7S1 pp 229533.Q4IPI4
69531 229533.Q4I7S1 pp 229533.Q4IB96
69532 229533.Q4I7S1 pp 229533.Q4ICA8
69533 229533.Q4I7S1 pp 229533.Q4I948
69534 229533.I1R9R8 pp 229533.I1RMM8
69535 229533.I1R9R8 pp 229533.I1RBE4
69536 229533.I1R9R8 pp 229533.I1RW06
69537 229533.A0A098D3D9 pp 229533.Q4IR18
69538 229533.A0A098D3D9 pp 229533.I1RPD9
69539 229533.A0A098D3D9 pp 229533.I1RDZ3
69540 229533.A0A098D3D9 pp 229533.I1S1W7
69541 229533.A0A098D3D9 pp 229533.I1RJA7
69542 229533.A0A098D3D9 pp 229533.V6R0U9
69543 229533.A0A098D3D9 pp 229533.I1RLX5
69544 229533.A0A098D3D9 pp 229533.A0A0E0S7W0
69545 229533.A0A098D3D9 pp 229533.A0A1C3YI39
69546 229533.A0A098D3D9 pp 229533.A0A098DQH7
69547 229533.A0A098D3D9 pp 229533.I1RRT2
69548 229533.A0A098D3D9 pp 229533.A0A098E4D0
69549 229533.A0A098D3D9 pp 229533.I1RWD4
69550 229533.A0A098D3D9 pp 229533.I1RSL8
69551 229533.A0A098D3D9 pp 229533.A0A1C3YLG9
69552 229533.A0A098D3D9 pp 229533.I1RTF4
69553 229533.A0A098D3D9 pp 229533.I1RSM3
69554 229533.A0A098D3D9 pp 229533.A0A1C3YJK1
69555 229533.A0A098D3D9 pp 229533.I1RAZ8
69556 229533.A0A098D3D9 pp 229533.V6R227
69557 229533.I1RRZ9 pp 229533.I1RTD0
69558 229533.I1RRZ9 pp 229533.I1RTC1
69559 229533.I1RAF4 pp 229533.V6RSM7
69560 229533.A0A1C3YKB5 pp 229533.I1RJZ7
69561 229533.A0A1C3YKB5 pp 229533.A0A1C3YMQ5
69562 229533.A0A1C3YKB5 pp 229533.I1RZD9
69563 229533.A0A1C3YKB5 pp 229533.A0A1C3YL20
69564 229533.A0A1C3YKB5 pp 229533.I1RCD4
69565 229533.A0A1C3YKB5 pp 229533.I1RSB0
69566 229533.A0A1C3YKB5 pp 229533.I1RZV4
69567 229533.A0A1C3YKB5 pp 229533.I1S095
69568 229533.A0A1C3YKB5 pp 229533.I1RTG9
69569 229533.A0A1C3YKB5 pp 229533.I1S0K7

69570 229533.A0A1C3YKB5 pp 229533.A0A1C3YLH7
69571 229533.A0A1C3YKB5 pp 229533.I1RD16
69572 229533.A0A1C3YKB5 pp 229533.A0A1C3YKT9
69573 229533.A0A1C3YKB5 pp 229533.I1RW71
69574 229533.A0A1C3YKB5 pp 229533.I1RT41
69575 229533.A0A1C3YKB5 pp 229533.I1RN81
69576 229533.A0A1C3YKB5 pp 229533.I1RTE6
69577 229533.A0A1C3YKB5 pp 229533.I1S5K9
69578 229533.A0A1C3YKB5 pp 229533.I1RMR5
69579 229533.A0A1C3YKB5 pp 229533.I1S1V3
69580 229533.A0A1C3YKB5 pp 229533.I1RPP0
69581 229533.A0A1C3YKB5 pp 229533.I1RB15
69582 229533.A0A1C3YKB5 pp 229533.A0A1C3YL89
69583 229533.A0A1C3YKB5 pp 229533.V6QUU6
69584 229533.A0A1C3YKB5 pp 229533.I1R991
69585 229533.A0A1C3YKB5 pp 229533.I1S0Z3
69586 229533.A0A1C3YKB5 pp 229533.I1S1B1
69587 229533.A0A1C3YKB5 pp 229533.I1RRQ0
69588 229533.A0A1C3YKB5 pp 229533.I1RX09
69589 229533.A0A1C3YKB5 pp 229533.V6RR76
69590 229533.A0A1C3YKB5 pp 229533.A0A1C3YMD9
69591 229533.A0A1C3YKB5 pp 229533.I1RXB5
69592 229533.A0A1C3YKB5 pp 229533.V6REE9
69593 229533.I1RTI6 pp 229533.I1S558
69594 229533.I1RTI6 pp 229533.I1S2C0
69595 229533.I1RTI6 pp 229533.I1RWJ4
69596 229533.I1RTI6 pp 229533.I1S9U2
69597 229533.I1RTI6 pp 229533.I1RZ35
69598 229533.I1RTI6 pp 229533.I1RZS6
69599 229533.I1RTI6 pp 229533.Q4I662
69600 229533.I1RTI6 pp 229533.Q4IQW0
69601 229533.I1RTI6 pp 229533.Q4IJH1
69602 229533.I1RTI6 pp 229533.I1RZ11
69603 229533.I1RTI6 pp 229533.Q4IEK8
69604 229533.I1RTI6 pp 229533.V6R283
69605 229533.I1RTI6 pp 229533.V6R227
69606 229533.A0A1C3YKV4 pp 229533.Q4I948
69607 229533.A0A1C3YKV4 pp 229533.I1RFN9
69608 229533.A0A1C3YKV4 pp 229533.Q4I650
69609 229533.A0A1C3YKV4 pp 229533.I1RZU9
69610 229533.A0A1C3YKV4 pp 229533.Q4ICA8
69611 229533.A0A1C3YKV4 pp 229533.I1RRG3
69612 229533.A0A1C3YKV4 pp 229533.Q4HTT2
69613 229533.A0A1C3YKV4 pp 229533.Q4I5V3
69614 229533.A0A1C3YKV4 pp 229533.I1SB04
69615 229533.A0A1C3YKV4 pp 229533.Q4IPI4
69616 229533.A0A1C3YKV4 pp 229533.Q4IMD1
69617 229533.A0A1C3YKV4 pp 229533.Q4IB50
69618 229533.A0A1C3YKV4 pp 229533.I1RFL7
69619 229533.A0A1C3YKV4 pp 229533.I1RQP1
69620 229533.A0A1C3YKV4 pp 229533.Q4HY90
69621 229533.A0A1C3YKV4 pp 229533.I1RZF3
69622 229533.A0A1C3YKV4 pp 229533.Q4I7S1
69623 229533.A0A1C3YKV4 pp 229533.A0A1C3YM29
69624 229533.I1RAI9 pp 229533.I1RTN1
69625 229533.I1RAI9 pp 229533.I1S604
69626 229533.I1RAI9 pp 229533.I1RCB3
69627 229533.I1RAI9 pp 229533.I1RV79
69628 229533.I1RAI9 pp 229533.I1S9T1
69629 229533.I1RAI9 pp 229533.I1RG11
69630 229533.I1RAI9 pp 229533.Q4IER8
69631 229533.I1RAI9 pp 229533.I1RQP3
69632 229533.I1RAI9 pp 229533.I1S339
69633 229533.I1RAI9 pp 229533.Q4I5I4
69634 229533.I1RAI9 pp 229533.I1RK79
69635 229533.I1RAI9 pp 229533.I1RVA5
69636 229533.I1RAI9 pp 229533.I1RF69
69637 229533.I1RAI9 pp 229533.V6QUE6
69638 229533.I1RAI9 pp 229533.I1RLS5
69639 229533.I1RAI9 pp 229533.I1RS41
69640 229533.I1RAI9 pp 229533.I1RJL2
69641 229533.I1RAI9 pp 229533.I1RU02
69642 229533.I1RAI9 pp 229533.I1RQX6

69643 229533.I1RAI9 pp 229533.I1RZB5
69644 229533.I1RAI9 pp 229533.V6R2L8
69645 229533.I1RAI9 pp 229533.Q4I7N9
69646 229533.I1RAI9 pp 229533.I1S0C6
69647 229533.I1RAI9 pp 229533.I1RJX2
69648 229533.I1RAI9 pp 229533.I1RPD2
69649 229533.I1RAI9 pp 229533.Q4HTT1
69650 229533.I1RAI9 pp 229533.I1RSH9
69651 229533.I1RAI9 pp 229533.I1RVX1
69652 229533.I1RAI9 pp 229533.I1RW23
69653 229533.I1RAI9 pp 229533.I1RPK5
69654 229533.I1RAI9 pp 229533.I1RT08
69655 229533.I1RAI9 pp 229533.I1RMQ7
69656 229533.I1RAI9 pp 229533.I1RR25
69657 229533.I1RAI9 pp 229533.I1RB59
69658 229533.I1RAI9 pp 229533.I1RRT0
69659 229533.I1RAI9 pp 229533.Q4IJU0
69660 229533.I1RAI9 pp 229533.I1RNC0
69661 229533.I1RAI9 pp 229533.I1RZG3
69662 229533.I1RAI9 pp 229533.Q4HYB8
69663 229533.I1RAI9 pp 229533.I1SA96
69664 229533.I1RAI9 pp 229533.I1RDD9
69665 229533.I1RAI9 pp 229533.I1RCB7
69666 229533.I1RAI9 pp 229533.I1S002
69667 229533.I1RAI9 pp 229533.I1RP61
69668 229533.I1RAI9 pp 229533.I1RCW8
69669 229533.I1RAI9 pp 229533.Q4HTT2
69670 229533.I1RAI9 pp 229533.I1RCN2
69671 229533.I1RAI9 pp 229533.I1RZA9
69672 229533.I1RAI9 pp 229533.Q4I5U9
69673 229533.I1RAI9 pp 229533.Q4IJ11
69674 229533.I1RAI9 pp 229533.I1RT01
69675 229533.I1RAI9 pp 229533.I1RBC7
69676 229533.I1RAI9 pp 229533.Q4I5W5
69677 229533.I1RAI9 pp 229533.I1RC16
69678 229533.I1RAI9 pp 229533.I1RWT1
69679 229533.I1RAI9 pp 229533.I1RQW2
69680 229533.I1RAI9 pp 229533.I1RW90
69681 229533.I1RAI9 pp 229533.I1RXA1
69682 229533.I1RAI9 pp 229533.I1RQ88
69683 229533.I1RAI9 pp 229533.I1RB73
69684 229533.I1RAI9 pp 229533.I1RV82
69685 229533.I1RAI9 pp 229533.Q4IB50
69686 229533.I1RAI9 pp 229533.I1RTH2
69687 229533.I1RAI9 pp 229533.I1RSW9
69688 229533.I1RAI9 pp 229533.I1RC24
69689 229533.I1RAI9 pp 229533.I1S0V1
69690 229533.I1RAI9 pp 229533.I1RRG3
69691 229533.I1RAI9 pp 229533.I1S1K9
69692 229533.I1RAI9 pp 229533.Q4IF44
69693 229533.I1RAI9 pp 229533.Q4HYQ4
69694 229533.I1RAI9 pp 229533.V6QWX8
69695 229533.I1RAI9 pp 229533.I1S2R6
69696 229533.I1RAI9 pp 229533.I1RFW1
69697 229533.I1RAI9 pp 229533.I1S2R1
69698 229533.I1RAI9 pp 229533.I1RIC5
69699 229533.I1RAI9 pp 229533.I1S9H9
69700 229533.I1RAI9 pp 229533.I1RKJ7
69701 229533.I1RAI9 pp 229533.I1RZR6
69702 229533.I1RAI9 pp 229533.I1S330
69703 229533.Q4HY90 pp 229533.Q4I650
69704 229533.Q4HY90 pp 229533.Q4IB96
69705 229533.Q4HY90 pp 229533.Q4IPI4
69706 229533.Q4HY90 pp 229533.Q4I5V3
69707 229533.Q4HY90 pp 229533.Q4IAK7
69708 229533.Q4HY90 pp 229533.Q4HYQ4
69709 229533.Q4HY90 pp 229533.Q4IMD1
69710 229533.Q4HY90 pp 229533.Q4I948
69711 229533.Q4HY90 pp 229533.Q4ICA8
69712 229533.Q4HY90 pp 229533.Q4IEV4
69713 229533.Q4HY90 pp 229533.Q4IJU0
69714 229533.Q4HY90 pp 229533.Q4IER8
69715 229533.Q4HY90 pp 229533.Q4I7S1

69716	229533.I1S0K7	pp	229533.I1S4T3
69717	229533.I1RQB3	pp	229533.I1RT28
69718	229533.I1RQB3	pp	229533.I1RRN5
69719	229533.I1S0N5	pp	229533.Q4IF76
69720	229533.I1S0N5	pp	229533.Q4HTT2
69721	229533.I1S0N5	pp	229533.V6QUE6
69722	229533.I1S0N5	pp	229533.I1SB04
69723	229533.I1S0N5	pp	229533.Q4I5R3
69724	229533.I1S0N5	pp	229533.V6RLW4
69725	229533.I1S0N5	pp	229533.Q4IPI4
69726	229533.I1S0N5	pp	229533.I1S3W0
69727	229533.I1S0N5	pp	229533.I1S283
69728	229533.I1S0N5	pp	229533.Q4HTT1
69729	229533.I1S0N5	pp	229533.Q4IER8
69730	229533.I1S0N5	pp	229533.Q4IMD1
69731	229533.I1S0N5	pp	229533.I1S0Z3
69732	229533.I1S0N5	pp	229533.I1S1V2
69733	229533.I1S0N5	pp	229533.Q4HYB8
69734	229533.I1S0N5	pp	229533.Q4ICA8
69735	229533.I1S0N5	pp	229533.Q4IJH1
69736	229533.I1S0N5	pp	229533.I1S209
69737	229533.I1S0N5	pp	229533.Q4I7L0
69738	229533.I1S0N5	pp	229533.I1S3L3
69739	229533.I1RAV2	pp	229533.I1RFL7
69740	229533.I1RAV2	pp	229533.Q4IB50
69741	229533.I1RAV2	pp	229533.Q4IMD1
69742	229533.I1RAV2	pp	229533.Q4IPI4
69743	229533.I1RAV2	pp	229533.I1RQP1
69744	229533.I1RAV2	pp	229533.Q4I5V3
69745	229533.I1RAV2	pp	229533.I1SB04
69746	229533.I1RAV2	pp	229533.I1RFN9
69747	229533.I1RAV2	pp	229533.Q4I650
69748	229533.I1RAV2	pp	229533.I1RZF3
69749	229533.I1RAV2	pp	229533.Q4ICA8
69750	229533.I1RAV2	pp	229533.Q4I7S1
69751	229533.I1RAV2	pp	229533.Q4I948
69752	229533.I1RAV2	pp	229533.Q4HTT2
69753	229533.I1RAV2	pp	229533.Q4HY90
69754	229533.I1RAV2	pp	229533.I1RZU9
69755	229533.I1RAV2	pp	229533.I1RRG3
69756	229533.I1RLV1	pp	229533.I1RVY0
69757	229533.I1RLV1	pp	229533.I1RSZ3
69758	229533.I1RLV1	pp	229533.Q4I5W5
69759	229533.I1RLV1	pp	229533.I1RXU0
69760	229533.I1RLV1	pp	229533.I1RRG3
69761	229533.I1RLV1	pp	229533.Q4IPC8
69762	229533.I1RLV1	pp	229533.I1RTC2
69763	229533.I1RLV1	pp	229533.I1RVL8
69764	229533.I1RLV1	pp	229533.V6QWX8
69765	229533.I1RLV1	pp	229533.Q4HYB8
69766	229533.I1RLV1	pp	229533.I1RWF3
69767	229533.I1RLV1	pp	229533.I1RUG0
69768	229533.I1RLV1	pp	229533.I1RPM7
69769	229533.I1RLV1	pp	229533.I1RR25
69770	229533.I1RLV1	pp	229533.V6QUE6
69771	229533.I1RLV1	pp	229533.I1S276
69772	229533.I1RLV1	pp	229533.I1RRT0
69773	229533.I1RLV1	pp	229533.I1RZB5
69774	229533.I1RLV1	pp	229533.Q4I5I4
69775	229533.I1RQB2	pp	229533.I1RT28
69776	229533.I1RQB2	pp	229533.I1RRN5
69777	229533.I1RFN9	pp	229533.I1RZU9
69778	229533.I1RFN9	pp	229533.I1RWD0
69779	229533.I1RFN9	pp	229533.Q4HY90
69780	229533.I1RFN9	pp	229533.Q4I948
69781	229533.I1RFN9	pp	229533.Q4I5V3
69782	229533.I1RFN9	pp	229533.I1RPF4
69783	229533.I1RFN9	pp	229533.Q4I7N9
69784	229533.I1RFN9	pp	229533.I1RZF3
69785	229533.I1RFN9	pp	229533.I1SB04
69786	229533.I1RFN9	pp	229533.Q4HVW2
69787	229533.I1RFN9	pp	229533.I1RW91
69788	229533.I1RFN9	pp	229533.Q4ICA8

69789	229533.I1RFN9	pp	229533.Q4I7S1
69790	229533.I1RFN9	pp	229533.Q4IPI4
69791	229533.I1RFN9	pp	229533.Q4I650
69792	229533.I1RFN9	pp	229533.I1RT41
69793	229533.I1RFN9	pp	229533.I1S0Z3
69794	229533.I1RFN9	pp	229533.I1RPR8
69795	229533.I1RFN9	pp	229533.I1RKL2
69796	229533.I1RFN9	pp	229533.Q4IAK7
69797	229533.I1RFN9	pp	229533.I1RW28
69798	229533.I1RFN9	pp	229533.Q4IEV4
69799	229533.I1RFN9	pp	229533.Q4IMD1
69800	229533.I1RR25	pp	229533.Q4IJU0
69801	229533.I1RR25	pp	229533.I1RT35
69802	229533.I1RR25	pp	229533.I1RXE5
69803	229533.I1RR25	pp	229533.V6RCZ8
69804	229533.I1RR25	pp	229533.I1RT01
69805	229533.I1RR25	pp	229533.Q4HYI0
69806	229533.I1RR25	pp	229533.I1RYS4
69807	229533.I1RR25	pp	229533.I1RU02
69808	229533.I1RR25	pp	229533.Q4IER8
69809	229533.I1RR25	pp	229533.Q4HTT2
69810	229533.I1RR25	pp	229533.I1RRT0
69811	229533.I1RR25	pp	229533.I1RZB5
69812	229533.I1RR25	pp	229533.I1S255
69813	229533.I1RR25	pp	229533.Q4I5I4
69814	229533.I1RR25	pp	229533.I1RWT6
69815	229533.I1RR25	pp	229533.I1RVZ4
69816	229533.I1RR25	pp	229533.I1RZL5
69817	229533.I1RR25	pp	229533.I1S9U1
69818	229533.I1RR25	pp	229533.I1RYY5
69819	229533.I1RR25	pp	229533.I1RSI1
69820	229533.I1RR25	pp	229533.V6QUE6
69821	229533.I1RR25	pp	229533.Q4HYQ4
69822	229533.I1RR25	pp	229533.V6QV38
69823	229533.I1RR25	pp	229533.Q4IJM4
69824	229533.I1RR25	pp	229533.I1RZ42
69825	229533.I1RR25	pp	229533.I1RRG3
69826	229533.I1RR25	pp	229533.I1S5Z2
69827	229533.I1RR25	pp	229533.I1RRW3
69828	229533.I1RR25	pp	229533.I1RZU9
69829	229533.I1RR25	pp	229533.I1RZR6
69830	229533.I1RR25	pp	229533.Q4HYB8
69831	229533.I1RR25	pp	229533.I1RTC2
69832	229533.I1RR25	pp	229533.I1S182
69833	229533.I1RR25	pp	229533.I1RVJ1
69834	229533.I1RR25	pp	229533.Q4IQT8
69835	229533.I1RR25	pp	229533.I1RUG0
69836	229533.I1RR25	pp	229533.I1RW90
69837	229533.I1RR25	pp	229533.Q4IQX3
69838	229533.I1RR25	pp	229533.I1RY44
69839	229533.I1RR25	pp	229533.I1RRN6
69840	229533.I1RR25	pp	229533.I1RR61
69841	229533.I1RR25	pp	229533.V6QWX8
69842	229533.I1RR25	pp	229533.Q4I5U9
69843	229533.I1RR25	pp	229533.I1RW91
69844	229533.I1RR25	pp	229533.I1RW23
69845	229533.I1RR25	pp	229533.I1RWF3
69846	229533.I1RR25	pp	229533.I1RUG4
69847	229533.I1RR25	pp	229533.I1RSA4
69848	229533.I1RR25	pp	229533.I1RS66
69849	229533.I1RR25	pp	229533.I1RWL3
69850	229533.I1RR25	pp	229533.Q4I7S1
69851	229533.I1RR25	pp	229533.I1S4M5
69852	229533.I1RR25	pp	229533.Q4IPZ1
69853	229533.I1RR25	pp	229533.I1S3N1
69854	229533.I1RR25	pp	229533.V6R0U9
69855	229533.I1RR25	pp	229533.I1RXU0
69856	229533.I1RR25	pp	229533.Q4IPA4
69857	229533.I1RR25	pp	229533.I1S2C4
69858	229533.I1RR25	pp	229533.I1RZM2
69859	229533.I1RR25	pp	229533.I1RSG8
69860	229533.I1RR25	pp	229533.Q4IPC8
69861	229533.I1RR25	pp	229533.I1S276

69862 229533.I1RR25 pp 229533.I1RX63
69863 229533.I1RR25 pp 229533.I1RRF7
69864 229533.I1RR25 pp 229533.I1RZU4
69865 229533.I1RR25 pp 229533.I1S0C6
69866 229533.I1RR25 pp 229533.I1RS43
69867 229533.I1RR25 pp 229533.I1RS30
69868 229533.I1RR25 pp 229533.I1RV91
69869 229533.I1RR25 pp 229533.Q4IJ11
69870 229533.I1RR25 pp 229533.I1S3N0
69871 229533.I1RR25 pp 229533.I1RS41
69872 229533.I1RR25 pp 229533.I1S2E9
69873 229533.I1RR25 pp 229533.Q4I5W5
69874 229533.I1RR25 pp 229533.Q4IMD1
69875 229533.I1RR25 pp 229533.I1RRM8
69876 229533.I1RR25 pp 229533.I1RSZ3
69877 229533.I1RR25 pp 229533.I1RVY0
69878 229533.I1RR25 pp 229533.I1RTH2
69879 229533.I1RR25 pp 229533.I1RW80
69880 229533.I1RR25 pp 229533.I1RVL8
69881 229533.I1RR25 pp 229533.Q4HTT1
69882 229533.I1RR25 pp 229533.I1S0J3
69883 229533.I1RR25 pp 229533.I1S9Y1
69884 229533.I1RD16 pp 229533.I1S0G1
69885 229533.I1RD16 pp 229533.I1S0K7
69886 229533.A0A1C3YMA2 pp 229533.I1RGD5
69887 229533.A0A1C3YMA2 pp 229533.I1RNR0
69888 229533.A0A1C3YMA2 pp 229533.I1RP29
69889 229533.A0A1C3YMA2 pp 229533.I1RZF7
69890 229533.A0A1C3YMA2 pp 229533.I1S927
69891 229533.A0A1C3YMA2 pp 229533.I1RKT9
69892 229533.A0A1C3YMA2 pp 229533.I1RK86
69893 229533.A0A1C3YMA2 pp 229533.I1RZK4
69894 229533.I1RAP2 pp 229533.I1RSZ3
69895 229533.I1RAP2 pp 229533.V6RAC9
69896 229533.I1RAP2 pp 229533.I1RQP1
69897 229533.I1RAP2 pp 229533.I1RCN2
69898 229533.I1RAP2 pp 229533.Q4IPI4
69899 229533.I1RAP2 pp 229533.I1RVY0
69900 229533.I1RAP2 pp 229533.I1RRX1
69901 229533.I1RAP2 pp 229533.I1RVL8
69902 229533.I1RAP2 pp 229533.I1RC73
69903 229533.I1RAP2 pp 229533.Q4I7L0
69904 229533.I1RAP2 pp 229533.I1RWF3
69905 229533.I1RAP2 pp 229533.Q4IMD1
69906 229533.I1RAP2 pp 229533.Q4HYB8
69907 229533.I1RAP2 pp 229533.I1RDH0
69908 229533.I1RAP2 pp 229533.I1RB73
69909 229533.I1RAP2 pp 229533.Q4IAS1
69910 229533.I1RAP2 pp 229533.I1RC24
69911 229533.I1RAP2 pp 229533.Q4IL82
69912 229533.I1RAP2 pp 229533.I1S276
69913 229533.I1RAP2 pp 229533.I1RRF0
69914 229533.I1RAP2 pp 229533.I1RS41
69915 229533.I1RAP2 pp 229533.I1RPM7
69916 229533.I1RAP2 pp 229533.I1RYV4
69917 229533.I1RAP2 pp 229533.I1RSG8
69918 229533.I1RAP2 pp 229533.I1REN3
69919 229533.I1RAP2 pp 229533.I1RM67
69920 229533.I1RAP2 pp 229533.I1RK95
69921 229533.I1RAP2 pp 229533.Q4I948
69922 229533.I1RAP2 pp 229533.I1RK79
69923 229533.I1RAP2 pp 229533.Q4IJH1
69924 229533.I1RAP2 pp 229533.I1RNH1
69925 229533.I1RAP2 pp 229533.I1RZB5
69926 229533.I1RAP2 pp 229533.Q4ICA8
69927 229533.I1RAP2 pp 229533.I1RKB1
69928 229533.I1RAP2 pp 229533.I1RP96
69929 229533.I1RAP2 pp 229533.I1RXE5
69930 229533.I1RAP2 pp 229533.I1RT65
69931 229533.I1RAP2 pp 229533.I1RFQ0
69932 229533.I1RAP2 pp 229533.I1RC15
69933 229533.I1RAP2 pp 229533.I1RYT8
69934 229533.I1RAP2 pp 229533.I1S0F0

69935 229533.I1RAP2 pp 229533.I1RQP3
69936 229533.I1RAP2 pp 229533.I1RAR2
69937 229533.A0A098DX13 pp 229533.A0A1C3YM67
69938 229533.A0A098DX13 pp 229533.I1RVV5
69939 229533.A0A098DX13 pp 229533.I1S2E9
69940 229533.A0A098DX13 pp 229533.I1RB74
69941 229533.A0A098DX13 pp 229533.I1RFW1
69942 229533.A0A098DX13 pp 229533.Q4IEV4
69943 229533.A0A098DX13 pp 229533.A0A1C3YLC6
69944 229533.A0A098DX13 pp 229533.I1RPR8
69945 229533.A0A098DX13 pp 229533.I1RQ88
69946 229533.A0A098DX13 pp 229533.I1RB73
69947 229533.A0A098DX13 pp 229533.Q4IB50
69948 229533.A0A098DX13 pp 229533.Q4I5V3
69949 229533.A0A098DX13 pp 229533.I1RV91
69950 229533.A0A098DX13 pp 229533.I1RDV4
69951 229533.A0A098DX13 pp 229533.I1RC24
69952 229533.A0A098DX13 pp 229533.I1RQP1
69953 229533.A0A098DX13 pp 229533.A0A1C3YIR8
69954 229533.A0A098DX13 pp 229533.I1S7I5
69955 229533.A0A098DX13 pp 229533.A0A1C3YKX5
69956 229533.A0A098DX13 pp 229533.I1RTG3
69957 229533.A0A098DX13 pp 229533.I1RW28
69958 229533.A0A098DX13 pp 229533.Q4HTT1
69959 229533.A0A098DX13 pp 229533.I1RBC7
69960 229533.A0A098DX13 pp 229533.Q4IL82
69961 229533.A0A098DX13 pp 229533.A0A1C3YJD4
69962 229533.A0A098DX13 pp 229533.Q4I5W5
69963 229533.A0A098DX13 pp 229533.I1RRJ6
69964 229533.A0A098DX13 pp 229533.Q4IPI4
69965 229533.A0A098DX13 pp 229533.Q4I7L0
69966 229533.A0A098DX13 pp 229533.Q4IMD1
69967 229533.A0A098DX13 pp 229533.I1RG11
69968 229533.A0A098DX13 pp 229533.A0A1C3YJW9
69969 229533.A0A098DX13 pp 229533.I1RCN2
69970 229533.A0A098DX13 pp 229533.I1RVY0
69971 229533.A0A098DX13 pp 229533.Q4HTT2
69972 229533.A0A098DX13 pp 229533.Q4HYB8
69973 229533.A0A098DX13 pp 229533.A0A1C3YLA2
69974 229533.A0A098DX13 pp 229533.I1RCB7
69975 229533.A0A098DX13 pp 229533.I1RQP3
69976 229533.A0A098DX13 pp 229533.A0A1C3YHP3
69977 229533.A0A098DX13 pp 229533.I1RE51
69978 229533.A0A098DX13 pp 229533.I1RK79
69979 229533.A0A098DX13 pp 229533.Q4IB89
69980 229533.A0A098DX13 pp 229533.I1RC17
69981 229533.A0A098DX13 pp 229533.A0A098E3M9
69982 229533.A0A098DX13 pp 229533.I1RSD1
69983 229533.A0A098DX13 pp 229533.I1RBJ0
69984 229533.A0A098DX13 pp 229533.I1RMJ8
69985 229533.A0A098DX13 pp 229533.I1S1U7
69986 229533.A0A098DX13 pp 229533.Q4I5I4
69987 229533.A0A098DX13 pp 229533.I1RS41
69988 229533.A0A098DX13 pp 229533.I1RY44
69989 229533.A0A098DX13 pp 229533.A0A1C3YJZ4
69990 229533.A0A098DX13 pp 229533.I1S4P6
69991 229533.A0A098DX13 pp 229533.V6QUE6
69992 229533.A0A098DX13 pp 229533.I1S020
69993 229533.A0A098DX13 pp 229533.I1RRN5
69994 229533.A0A098DX13 pp 229533.I1RDJ8
69995 229533.A0A098DX13 pp 229533.Q4I5R3
69996 229533.A0A098DX13 pp 229533.I1RR25
69997 229533.A0A098DX13 pp 229533.I1RAI9
69998 229533.A0A098DX13 pp 229533.I1RRT0
69999 229533.A0A098DX13 pp 229533.I1RM36
70000 229533.A0A098DX13 pp 229533.Q4HYQ4
70001 229533.A0A098DX13 pp 229533.I1S0N5
70002 229533.A0A098DX13 pp 229533.I1RQ19
70003 229533.A0A098DX13 pp 229533.I1S219
70004 229533.A0A098DX13 pp 229533.I1RWT6
70005 229533.A0A098DX13 pp 229533.I1RG34
70006 229533.A0A098DX13 pp 229533.I1RCW8
70007 229533.A0A098DX13 pp 229533.I1RRG3

70008	229533.A0A098DX13	pp	229533.A0A1C3YJR3
70009	229533.A0A098DX13	pp	229533.I1S788
70010	229533.A0A098DX13	pp	229533.A0A0E0SQV5
70011	229533.A0A098DX13	pp	229533.Q4IJP1
70012	229533.A0A098DX13	pp	229533.I1RPF4
70013	229533.A0A098DX13	pp	229533.Q4ICA8
70014	229533.A0A098DX13	pp	229533.I1RD01
70015	229533.A0A098DX13	pp	229533.I1RAN8
70016	229533.A0A098DX13	pp	229533.I1RZR6
70017	229533.A0A098DX13	pp	229533.I1RU02
70018	229533.A0A098DX13	pp	229533.A0A0E0SA11
70019	229533.A0A098DX13	pp	229533.I1RRT1
70020	229533.A0A098DX13	pp	229533.I1RJL2
70021	229533.A0A098DX13	pp	229533.I1RC32
70022	229533.A0A098DX13	pp	229533.I1S1V2
70023	229533.A0A098DX13	pp	229533.Q4IER8
70024	229533.A0A098DX13	pp	229533.I1RD69
70025	229533.A0A098DX13	pp	229533.A0A1C3YI23
70026	229533.A0A098DX13	pp	229533.I1RLJ8
70027	229533.A0A098DX13	pp	229533.I1RCL7
70028	229533.A0A098DX13	pp	229533.I1RZB5
70029	229533.A0A098DX13	pp	229533.Q4I948
70030	229533.A0A098DX13	pp	229533.I1RQA3
70031	229533.A0A098DX13	pp	229533.I1S9J6
70032	229533.A0A098DX13	pp	229533.I1RYV3
70033	229533.A0A098DX13	pp	229533.A0A1C3YK01
70034	229533.A0A098DX13	pp	229533.Q4IJU0
70035	229533.I1S9Z3	pp	229533.V6R9X9
70036	229533.I1S9Z3	pp	229533.Q4ICG5
70037	229533.I1S9Z3	pp	229533.I1S9Z4
70038	229533.I1R9C7	pp	229533.I1RE30
70039	229533.I1R9C7	pp	229533.I1RPM7
70040	229533.I1R9C7	pp	229533.I1RK71
70041	229533.I1R9C7	pp	229533.I1RC24
70042	229533.I1R9C7	pp	229533.I1S276
70043	229533.I1R9C7	pp	229533.I1RK79
70044	229533.I1R9C7	pp	229533.I1RVY0
70045	229533.I1R9C7	pp	229533.I1RVL8
70046	229533.I1R9C7	pp	229533.I1RR25
70047	229533.I1R9C7	pp	229533.I1RWF3
70048	229533.I1R9C7	pp	229533.I1RG11
70049	229533.I1R9C7	pp	229533.Q4HYB8
70050	229533.I1R9C7	pp	229533.Q4IPC8
70051	229533.I1R9C7	pp	229533.Q4I5W5
70052	229533.I1R9C7	pp	229533.V6QWX8
70053	229533.I1R9C7	pp	229533.I1RXU0
70054	229533.I1R9C7	pp	229533.I1RCB7
70055	229533.I1R9C7	pp	229533.I1RSZ3
70056	229533.I1R9C7	pp	229533.I1RUG0
70057	229533.I1R9C7	pp	229533.I1RKB1
70058	229533.I1R9C7	pp	229533.I1RZB5
70059	229533.I1R9C7	pp	229533.I1RJL2
70060	229533.I1R9C7	pp	229533.I1RRG3
70061	229533.I1R9C7	pp	229533.V6QUE6
70062	229533.I1R9C7	pp	229533.I1RTC2
70063	229533.I1R9C7	pp	229533.I1RAR2
70064	229533.I1R9C7	pp	229533.I1RC15
70065	229533.I1R9C7	pp	229533.I1RE14
70066	229533.I1R9C7	pp	229533.Q4I5I4
70067	229533.I1R9C7	pp	229533.I1RRT0
70068	229533.Q4I650	pp	229533.Q4ICA8
70069	229533.Q4I650	pp	229533.Q4I948
70070	229533.Q4I650	pp	229533.Q4IMD1
70071	229533.Q4I650	pp	229533.Q4I7S1
70072	229533.Q4I650	pp	229533.Q4IAK7
70073	229533.Q4I650	pp	229533.Q4IPI4
70074	229533.I1R9R3	pp	229533.I1RZ29
70075	229533.I1RSD1	pp	229533.I1S1W3
70076	229533.I1RSD1	pp	229533.I1RY44
70077	229533.I1RSD1	pp	229533.I1RWT6
70078	229533.I1RSD1	pp	229533.I1S4Z5
70079	229533.I1RSD1	pp	229533.Q4I5R3
70080	229533.I1RSD1	pp	229533.I1S8V4

70081 229533.I1RSD1 pp 229533.I1RSL8
70082 229533.I1RSD1 pp 229533.I1S8J7
70083 229533.I1RSD1 pp 229533.I1SAG3
70084 229533.I1RSD1 pp 229533.Q4IMD1
70085 229533.I1RSD1 pp 229533.I1RU02
70086 229533.I1RSD1 pp 229533.I1S193
70087 229533.I1RSD1 pp 229533.I1RVV5
70088 229533.I1RSD1 pp 229533.I1RYV3
70089 229533.I1RSD1 pp 229533.Q4IAK7
70090 229533.I1RSD1 pp 229533.Q4IB50
70091 229533.I1RSD1 pp 229533.I1RTG5
70092 229533.I1RSD1 pp 229533.Q4IER8
70093 229533.I1RSD1 pp 229533.I1S2E9
70094 229533.I1RSD1 pp 229533.V6RUT5
70095 229533.I1RSD1 pp 229533.Q4HTT1
70096 229533.I1RSD1 pp 229533.I1RV91
70097 229533.I1RSD1 pp 229533.Q4I2W2
70098 229533.I1RSD1 pp 229533.I1S8V3
70099 229533.I1RJU5 pp 229533.I1S2D7
70100 229533.I1RJU5 pp 229533.Q4HZB8
70101 229533.I1RJU5 pp 229533.I1RM09
70102 229533.I1RJU5 pp 229533.I1RQS5
70103 229533.I1RJU5 pp 229533.I1RTE6
70104 229533.I1RJU5 pp 229533.V6REE9
70105 229533.I1RJU5 pp 229533.I1RPS0
70106 229533.I1RJU5 pp 229533.I1S0L8
70107 229533.I1RJU5 pp 229533.I1RQM7
70108 229533.I1RJU5 pp 229533.I1RJU6
70109 229533.I1RJU5 pp 229533.I1RP88
70110 229533.I1RJU5 pp 229533.I1RNJ2
70111 229533.I1RJU5 pp 229533.I1S4Z8
70112 229533.I1RJU5 pp 229533.I1RKD7
70113 229533.I1RJU5 pp 229533.I1RPA7
70114 229533.I1RJU5 pp 229533.I1RN72
70115 229533.A0A1C3YMA4 pp 229533.I1RBA4
70116 229533.A0A1C3YMA4 pp 229533.I1RDJ8
70117 229533.A0A1C3YMA4 pp 229533.I1S209
70118 229533.A0A1C3YMA4 pp 229533.I1RVZ9
70119 229533.A0A1C3YMA4 pp 229533.I1RSC7
70120 229533.A0A0E0SGL9 pp 229533.A0A1C3YKQ9
70121 229533.Q4HYB8 pp 229533.Q4I5U9
70122 229533.Q4HYB8 pp 229533.Q4IEV4
70123 229533.Q4HYB8 pp 229533.Q4IR08
70124 229533.Q4HYB8 pp 229533.Q4IJ11
70125 229533.Q4HYB8 pp 229533.Q4IQM6
70126 229533.Q4HYB8 pp 229533.Q4I5W5
70127 229533.Q4HYB8 pp 229533.Q4IPC8
70128 229533.Q4HYB8 pp 229533.Q4I5I4
70129 229533.Q4HYB8 pp 229533.Q4IN63
70130 229533.Q4HYB8 pp 229533.V6QUE6
70131 229533.Q4HYB8 pp 229533.Q4I7N9
70132 229533.Q4HYB8 pp 229533.Q4I948
70133 229533.Q4HYB8 pp 229533.Q4IB50
70134 229533.Q4HYB8 pp 229533.Q4IMD1
70135 229533.Q4HYB8 pp 229533.Q4I099
70136 229533.Q4HYB8 pp 229533.Q4ICA8
70137 229533.Q4HYB8 pp 229533.V6QWX8
70138 229533.Q4HYB8 pp 229533.Q4IJU0
70139 229533.Q4HYB8 pp 229533.Q4I5R3
70140 229533.Q4HYB8 pp 229533.Q4IER8
70141 229533.Q4HYB8 pp 229533.Q4HYQ4
70142 229533.Q4HYB8 pp 229533.Q4IQX3
70143 229533.Q4HYB8 pp 229533.V6QYF0
70144 229533.I1RQQ7 pp 229533.I1S4M5
70145 229533.I1RQQ7 pp 229533.I1RSA4
70146 229533.I1RQQ7 pp 229533.I1S7X9
70147 229533.I1RQQ7 pp 229533.I1S7M2
70148 229533.I1RQQ7 pp 229533.V6QXG1
70149 229533.I1RQQ7 pp 229533.I1RXF2
70150 229533.I1RQQ7 pp 229533.I1S8C5
70151 229533.I1RQQ7 pp 229533.I1RX81
70152 229533.I1RQQ7 pp 229533.I1RS28
70153 229533.I1RQQ7 pp 229533.I1RSD4

229533.I1RQQ7 pp 229533.I1RZG7
229533.I1RQQ7 pp 229533.I1S7M3
229533.A0A098DBN7 pp 229533.I1RJU5
229533.A0A098DBN7 pp 229533.I1RQQ7
229533.I1RK79 pp 229533.I1RZL5
229533.I1RK79 pp 229533.I1RZU9
229533.I1RK79 pp 229533.I1S5Z2
229533.I1RK79 pp 229533.V6QV38
229533.I1RK79 pp 229533.Q4IER8
229533.I1RK79 pp 229533.I1RYS4
229533.I1RK79 pp 229533.I1RU02
229533.I1RK79 pp 229533.I1RTK6
229533.I1RK79 pp 229533.I1RN17
229533.I1RK79 pp 229533.I1S9J6
229533.I1RK79 pp 229533.I1RZB5
229533.I1RK79 pp 229533.I1RYY5
229533.I1RK79 pp 229533.I1RQ28
229533.I1RK79 pp 229533.Q4IJU0
229533.I1RK79 pp 229533.Q4IJM4
229533.I1RK79 pp 229533.I1RK95
229533.I1RK79 pp 229533.I1RXE5
229533.I1RK79 pp 229533.I1RM65
229533.I1RK79 pp 229533.I1RN07
229533.I1RK79 pp 229533.I1RT99
229533.I1RK79 pp 229533.I1RQZ2
229533.I1RK79 pp 229533.I1S255
229533.I1RK79 pp 229533.I1RWT6
229533.I1RK79 pp 229533.I1RPV5
229533.I1RK79 pp 229533.I1RZZ1
229533.I1RK79 pp 229533.I1S9U1
229533.I1RK79 pp 229533.I1RT01
229533.I1RK79 pp 229533.I1RPK5
229533.I1RK79 pp 229533.I1RQA3
229533.I1RK79 pp 229533.I1S0M9
229533.I1RK79 pp 229533.Q4HTT2
229533.I1RK79 pp 229533.I1RSI1
229533.I1RK79 pp 229533.I1RS66
229533.I1RK79 pp 229533.I1S7M3
229533.I1RK79 pp 229533.I1RX14
229533.I1RK79 pp 229533.I1RMQ7
229533.I1RK79 pp 229533.I1RW90
229533.I1RK79 pp 229533.I1RRT0
229533.I1RK79 pp 229533.Q4IJH1
229533.I1RK79 pp 229533.I1RYB9
229533.I1RK79 pp 229533.I1RNP4
229533.I1RK79 pp 229533.I1RUG0
229533.I1RK79 pp 229533.I1RVZ4
229533.I1RK79 pp 229533.I1RVJ1
229533.I1RK79 pp 229533.Q4I5I4
229533.I1RK79 pp 229533.I1RQ42
229533.I1RK79 pp 229533.V6QWX8
229533.I1RK79 pp 229533.Q4IPN9
229533.I1RK79 pp 229533.I1RPW8
229533.I1RK79 pp 229533.I1RW91
229533.I1RK79 pp 229533.V6QUE6
229533.I1RK79 pp 229533.Q4HYQ4
229533.I1RK79 pp 229533.Q4HYI0
229533.I1RK79 pp 229533.I1RWL3
229533.I1RK79 pp 229533.I1RZR6
229533.I1RK79 pp 229533.I1RRW3
229533.I1RK79 pp 229533.I1RRG3
229533.I1RK79 pp 229533.I1RNH1
229533.I1RK79 pp 229533.I1S201
229533.I1RK79 pp 229533.I1S182
229533.I1RK79 pp 229533.I1RKB1
229533.I1RK79 pp 229533.I1RP21
229533.I1RK79 pp 229533.I1RTC2
229533.I1RK79 pp 229533.I1S8V0
229533.I1RK79 pp 229533.I1RM36
229533.I1RK79 pp 229533.I1RR25
229533.I1RK79 pp 229533.I1RUP7
229533.I1RK79 pp 229533.I1S0N5
229533.I1RK79 pp 229533.I1RWF3

229533.I1RK79 pp 229533.Q4IQX3
229533.I1RK79 pp 229533.I1RUG4
229533.I1RK79 pp 229533.I1RQZ3
229533.I1RK79 pp 229533.I1RZ42
229533.I1RK79 pp 229533.I1RLV1
229533.I1RK79 pp 229533.I1RKQ5
229533.I1RK79 pp 229533.I1S7M2
229533.I1RK79 pp 229533.I1RNB8
229533.I1RK79 pp 229533.I1S0B1
229533.I1RK79 pp 229533.I1RUQ2
229533.I1RK79 pp 229533.I1RKC6
229533.I1RK79 pp 229533.I1S0J1
229533.I1RK79 pp 229533.I1RR61
229533.I1RK79 pp 229533.I1RUP9
229533.I1RK79 pp 229533.I1RP96
229533.I1RK79 pp 229533.I1RW23
229533.I1RK79 pp 229533.I1RML9
229533.I1RK79 pp 229533.I1S0E8
229533.I1RK79 pp 229533.Q4HYB8
229533.I1RK79 pp 229533.I1RMV1
229533.I1RK79 pp 229533.I1S0R7
229533.I1RK79 pp 229533.V6RGN5
229533.I1RK79 pp 229533.I1S276
229533.I1RK79 pp 229533.I1RNQ6
229533.I1RK79 pp 229533.I1RK92
229533.I1RK79 pp 229533.I1RS41
229533.I1RK79 pp 229533.I1RTG6
229533.I1RK79 pp 229533.I1S2C4
229533.I1RK79 pp 229533.I1RZU4
229533.I1RK79 pp 229533.I1RPM7
229533.I1RK79 pp 229533.I1RRF7
229533.I1RK79 pp 229533.I1RLV3
229533.I1RK79 pp 229533.I1RT36
229533.I1RK79 pp 229533.Q4I7S1
229533.I1RK79 pp 229533.I1S0C6
229533.I1RK79 pp 229533.I1RVL8
229533.I1RK79 pp 229533.I1RRI2
229533.I1RK79 pp 229533.I1RSG8
229533.I1RK79 pp 229533.I1RXU0
229533.I1RK79 pp 229533.I1RRT8
229533.I1RK79 pp 229533.I1RTD7
229533.I1RK79 pp 229533.I1RY44
229533.I1RK79 pp 229533.I1RRN6
229533.I1RK79 pp 229533.I1S3N1
229533.I1RK79 pp 229533.I1S9Y1
229533.I1RK79 pp 229533.I1S8U9
229533.I1RK79 pp 229533.I1RSA4
229533.I1RK79 pp 229533.I1S0J3
229533.I1RK79 pp 229533.Q4I5U9
229533.I1RK79 pp 229533.I1RSI6
229533.I1RK79 pp 229533.I1RM42
229533.I1RK79 pp 229533.I1S4M5
229533.I1RK79 pp 229533.I1RRJ8
229533.I1RK79 pp 229533.I1S8E2
229533.I1RK79 pp 229533.I1RVY0
229533.I1RK79 pp 229533.Q4IF76
229533.I1RK79 pp 229533.I1S0R5
229533.I1RK79 pp 229533.I1RQ65
229533.I1RK79 pp 229533.I1RZM2
229533.I1RK79 pp 229533.I1RX63
229533.I1RK79 pp 229533.I1RLR0
229533.I1RK79 pp 229533.Q4IMD1
229533.I1RK79 pp 229533.I1S214
229533.I1RK79 pp 229533.Q4HTT1
229533.I1RK79 pp 229533.I1RN04
229533.I1RK79 pp 229533.I1RS43
229533.I1RK79 pp 229533.I1RRM8
229533.I1RK79 pp 229533.Q4I5W5
229533.I1RK79 pp 229533.I1RRT2
229533.I1RK79 pp 229533.Q4I099
229533.I1RK79 pp 229533.I1RW80
229533.I1RK79 pp 229533.I1S2E9
229533.I1RK79 pp 229533.I1RS30

70300	229533.I1RK79	pp	229533.I1RTH2
70301	229533.I1RK79	pp	229533.I1S3N0
70302	229533.I1RK79	pp	229533.I1RQ88
70303	229533.I1RK79	pp	229533.I1RSZ3
70304	229533.I1RK79	pp	229533.I1RWT1
70305	229533.I1RK79	pp	229533.I1RV91
70306	229533.I1RK79	pp	229533.I1RXF2
70307	229533.I1RK79	pp	229533.Q4IJ11
70308	229533.A0A098DND1	pp	229533.I1RHP6
70309	229533.I1RCF3	pp	229533.I1RPS2
70310	229533.I1RCF3	pp	229533.I1RMI7
70311	229533.I1RCF3	pp	229533.I1RXX0
70312	229533.I1RCF3	pp	229533.I1RCF6
70313	229533.I1RCF3	pp	229533.I1RN63
70314	229533.I1RCF3	pp	229533.I1RRZ0
70315	229533.I1RCF3	pp	229533.I1S444
70316	229533.I1RCF3	pp	229533.I1RSX2
70317	229533.I1RCF3	pp	229533.I1RD80
70318	229533.A0A1C3YI59	pp	229533.A0A1C3YI60
70319	229533.A0A1C3YI59	pp	229533.I1RJH1
70320	229533.A0A1C3YI59	pp	229533.I1RMA1
70321	229533.A0A1C3YI59	pp	229533.Q4IMN9
70322	229533.I1RHQ5	pp	229533.I1RR25
70323	229533.I1RHQ5	pp	229533.Q4IPC8
70324	229533.I1RHQ5	pp	229533.Q4HYB8
70325	229533.I1RHQ5	pp	229533.I1RXU0
70326	229533.I1RHQ5	pp	229533.I1RPM7
70327	229533.I1RHQ5	pp	229533.I1RK79
70328	229533.I1RHQ5	pp	229533.I1RK71
70329	229533.I1RHQ5	pp	229533.I1RWF3
70330	229533.I1RHQ5	pp	229533.I1RVL8
70331	229533.I1RHQ5	pp	229533.I1RSZ3
70332	229533.I1RHQ5	pp	229533.V6QUE6
70333	229533.I1RHQ5	pp	229533.I1S276
70334	229533.I1RHQ5	pp	229533.Q4I5W5
70335	229533.I1RHQ5	pp	229533.I1RVY0
70336	229533.I1RHQ5	pp	229533.I1RZB5
70337	229533.I1RHQ5	pp	229533.I1RKB1
70338	229533.I1RHQ5	pp	229533.I1RRG3
70339	229533.I1RHQ5	pp	229533.I1RTC2
70340	229533.I1RHQ5	pp	229533.I1RJL2
70341	229533.I1RHQ5	pp	229533.Q4I5I4
70342	229533.I1RHQ5	pp	229533.V6QWX8
70343	229533.I1RHQ5	pp	229533.I1RRT0
70344	229533.I1RHQ5	pp	229533.I1RUG0
70345	229533.I1R9Z1	pp	229533.I1RS35
70346	229533.I1R9Z1	pp	229533.I1S7A5
70347	229533.I1R9Z1	pp	229533.I1RMK9
70348	229533.I1R9Z1	pp	229533.I1RLZ4
70349	229533.I1R9Z1	pp	229533.I1S9D4
70350	229533.I1R9Z1	pp	229533.I1RZV5
70351	229533.I1R9Z1	pp	229533.I1RPN6
70352	229533.I1R9Z1	pp	229533.I1RAX2
70353	229533.I1R9Z1	pp	229533.I1RY32
70354	229533.I1R9Z1	pp	229533.I1RE59
70355	229533.I1R9Z1	pp	229533.I1RD37
70356	229533.I1R9Z1	pp	229533.I1RJH3
70357	229533.I1R9Z1	pp	229533.I1RAT6
70358	229533.I1R9Z1	pp	229533.Q4IBU4
70359	229533.I1R9Z1	pp	229533.I1S2E2
70360	229533.I1R9Z1	pp	229533.I1RZC3
70361	229533.I1RK55	pp	229533.I1RLA2
70362	229533.I1RK55	pp	229533.I1RNM7
70363	229533.I1RK55	pp	229533.I1S6H3
70364	229533.I1RK55	pp	229533.I1S048
70365	229533.I1RK55	pp	229533.I1RMA2
70366	229533.I1RK55	pp	229533.V6R5T7
70367	229533.I1RK55	pp	229533.I1RMF1
70368	229533.I1RK55	pp	229533.I1S210
70369	229533.I1RK55	pp	229533.I1RQV7
70370	229533.I1RK55	pp	229533.I1S094
70371	229533.I1RK55	pp	229533.I1S5D0
70372	229533.I1RK55	pp	229533.I1RW54

70373 229533.I1RK55 pp 229533.V6QVV9
70374 229533.I1RK55 pp 229533.I1RQV8
70375 229533.I1RK55 pp 229533.I1RSG0
70376 229533.I1RK55 pp 229533.I1S223
70377 229533.I1RK55 pp 229533.V6RCC0
70378 229533.I1RK55 pp 229533.I1RPE5
70379 229533.I1RK55 pp 229533.I1RQJ7
70380 229533.I1RK55 pp 229533.I1RYP5
70381 229533.I1RK55 pp 229533.I1S8C1
70382 229533.I1RK55 pp 229533.I1RRS6
70383 229533.I1RK55 pp 229533.V6RWB3
70384 229533.I1RK55 pp 229533.I1RYU6
70385 229533.I1RK55 pp 229533.I1S3T6
70386 229533.I1RK55 pp 229533.V6RHX2
70387 229533.I1RK55 pp 229533.I1RY81
70388 229533.I1RK55 pp 229533.I1S130
70389 229533.I1RK55 pp 229533.I1RW55
70390 229533.I1RK55 pp 229533.I1S2F7
70391 229533.I1RK55 pp 229533.I1S8F8
70392 229533.I1RK55 pp 229533.I1RPR1
70393 229533.I1RK55 pp 229533.I1S659
70394 229533.I1RK55 pp 229533.I1S968
70395 229533.I1RK55 pp 229533.I1S8D3
70396 229533.I1RK55 pp 229533.I1RUR8
70397 229533.I1RK55 pp 229533.I1RST9
70398 229533.I1RK55 pp 229533.I1RSP5
70399 229533.I1RK55 pp 229533.I1RY32
70400 229533.I1RK55 pp 229533.I1RWM4
70401 229533.I1RK55 pp 229533.V6RCJ5
70402 229533.I1RK55 pp 229533.I1S267
70403 229533.I1RK55 pp 229533.I1SAB4
70404 229533.I1RK55 pp 229533.I1RR68
70405 229533.I1RK55 pp 229533.I1RZ25
70406 229533.I1RK55 pp 229533.I1S3T7
70407 229533.I1RK55 pp 229533.I1RY35
70408 229533.I1RK55 pp 229533.I1RZ38
70409 229533.I1RK55 pp 229533.I1S299
70410 229533.I1RK55 pp 229533.I1RMK9
70411 229533.I1RK55 pp 229533.I1S8R2
70412 229533.I1RK55 pp 229533.I1S0A5
70413 229533.I1RK55 pp 229533.I1RPV8
70414 229533.I1RK55 pp 229533.I1RSA5
70415 229533.I1RK55 pp 229533.I1RM41
70416 229533.I1RK55 pp 229533.I1RL81
70417 229533.I1RK55 pp 229533.I1RYA0
70418 229533.I1RK55 pp 229533.I1RZ89
70419 229533.I1RK55 pp 229533.I1S6V0
70420 229533.I1RK55 pp 229533.I1RYC8
70421 229533.I1RK55 pp 229533.I1RZC3
70422 229533.I1RK55 pp 229533.I1RZ56
70423 229533.I1RK55 pp 229533.I1RVX8
70424 229533.I1RK55 pp 229533.I1SAT2
70425 229533.I1RK55 pp 229533.I1S2C4
70426 229533.Q4IB89 pp 229533.Q4INS6
70427 229533.Q4IB89 pp 229533.Q4IEV4
70428 229533.A0A1C3YI60 pp 229533.A0A1C3YIR8
70429 229533.A0A1C3YI60 pp 229533.I1RX41
70430 229533.A0A1C3YI60 pp 229533.I1RA00
70431 229533.A0A1C3YI60 pp 229533.I1RBC7
70432 229533.A0A1C3YI60 pp 229533.A0A1C3YM89
70433 229533.A0A1C3YI60 pp 229533.A0A1C3YK88
70434 229533.A0A1C3YI60 pp 229533.Q4I5I4
70435 229533.A0A1C3YI60 pp 229533.I1S9Y1
70436 229533.A0A1C3YI60 pp 229533.A0A1C3YLC9
70437 229533.A0A1C3YI60 pp 229533.A0A1C3YM33
70438 229533.A0A1C3YI60 pp 229533.V6QUE6
70439 229533.A0A1C3YI60 pp 229533.I1RB71
70440 229533.A0A1C3YI60 pp 229533.I1RAR2
70441 229533.A0A1C3YI60 pp 229533.I1RYE2
70442 229533.A0A1C3YI60 pp 229533.I1RK95
70443 229533.A0A1C3YI60 pp 229533.I1RRG3
70444 229533.A0A1C3YI60 pp 229533.I1S8Y5
70445 229533.A0A1C3YI60 pp 229533.I1RMV1

70446 229533.A0A1C3YI60 pp 229533.I1S255
70447 229533.A0A1C3YI60 pp 229533.A0A1C3YN92
70448 229533.A0A1C3YI60 pp 229533.I1RRN6
70449 229533.A0A1C3YI60 pp 229533.I1S9U1
70450 229533.A0A1C3YI60 pp 229533.I1RY71
70451 229533.A0A1C3YI60 pp 229533.I1S0Z2
70452 229533.A0A1C3YI60 pp 229533.I1RXE5
70453 229533.A0A1C3YI60 pp 229533.I1RQL1
70454 229533.A0A1C3YI60 pp 229533.I1RCP3
70455 229533.A0A1C3YI60 pp 229533.I1RWF3
70456 229533.A0A1C3YI60 pp 229533.I1RJI3
70457 229533.A0A1C3YI60 pp 229533.I1RK32
70458 229533.A0A1C3YI60 pp 229533.I1RVY0
70459 229533.I1RMJ8 pp 229533.I1S0D5
70460 229533.I1RMJ8 pp 229533.I1S461
70461 229533.I1RMJ8 pp 229533.I1RPS0
70462 229533.I1RMJ8 pp 229533.I1RZT1
70463 229533.I1RMJ8 pp 229533.I1RY58
70464 229533.I1RMJ8 pp 229533.I1RSP7
70465 229533.I1RMJ8 pp 229533.Q4IER8
70466 229533.I1RMJ8 pp 229533.I1RQA3
70467 229533.I1RMJ8 pp 229533.I1RZK9
70468 229533.I1RMJ8 pp 229533.Q4INS6
70469 229533.I1RMJ8 pp 229533.I1RXZ4
70470 229533.I1RMJ8 pp 229533.I1RRS7
70471 229533.I1RMJ8 pp 229533.I1S209
70472 229533.I1RMJ8 pp 229533.I1RMX3
70473 229533.I1RMJ8 pp 229533.Q4HTT2
70474 229533.I1RMJ8 pp 229533.I1RSC7
70475 229533.I1RMJ8 pp 229533.Q4IJ84
70476 229533.I1RMJ8 pp 229533.I1RU61
70477 229533.I1RMJ8 pp 229533.I1RY44
70478 229533.I1RMJ8 pp 229533.I1RQS4
70479 229533.I1RMJ8 pp 229533.I1RRW0
70480 229533.I1RMJ8 pp 229533.I1RZ86
70481 229533.I1RMJ8 pp 229533.I1RWT6
70482 229533.I1RMJ8 pp 229533.I1RSK0
70483 229533.I1RMJ8 pp 229533.I1S4P6
70484 229533.I1RMJ8 pp 229533.I1S788
70485 229533.I1RMJ8 pp 229533.I1RSL8
70486 229533.I1RMJ8 pp 229533.I1S004
70487 229533.I1RMJ8 pp 229533.I1S224
70488 229533.I1RMJ8 pp 229533.Q4HTT1
70489 229533.I1RMJ8 pp 229533.I1RN57
70490 229533.I1RMJ8 pp 229533.I1RQ67
70491 229533.I1RMJ8 pp 229533.Q4I5R3
70492 229533.I1RMJ8 pp 229533.I1RYQ1
70493 229533.I1RMJ8 pp 229533.I1S987
70494 229533.I1RMJ8 pp 229533.Q4IB89
70495 229533.I1RMJ8 pp 229533.I1RV91
70496 229533.I1RMJ8 pp 229533.I1RRJ6
70497 229533.I1RMJ8 pp 229533.I1RY29
70498 229533.I1RMJ8 pp 229533.I1S998
70499 229533.I1RMJ8 pp 229533.I1RW16
70500 229533.I1RMJ8 pp 229533.I1RVV5
70501 229533.I1RMJ8 pp 229533.Q4I8C9
70502 229533.I1RMJ8 pp 229533.I1RN31
70503 229533.I1RMJ8 pp 229533.I1RVW0
70504 229533.I1RMJ8 pp 229533.I1RUR0
70505 229533.I1RMJ8 pp 229533.I1RXI6
70506 229533.I1RMJ8 pp 229533.I1RRT1
70507 229533.I1RMJ8 pp 229533.I1S2E9
70508 229533.I1RMJ8 pp 229533.I1RWI8
70509 229533.A0A1C3YLC9 pp 229533.I1RK32
70510 229533.A0A1C3YLC9 pp 229533.I1S0Z2
70511 229533.A0A1C3YLC9 pp 229533.A0A1C3YN92
70512 229533.A0A1C3YLC9 pp 229533.A0A1C3YM89
70513 229533.A0A1C3YLC9 pp 229533.I1RY71
70514 229533.A0A1C3YLC9 pp 229533.I1RX41
70515 229533.A0A1C3YLC9 pp 229533.A0A1C3YM33
70516 229533.A0A1C3YLC9 pp 229533.I1RQL1
70517 229533.I1RJH3 pp 229533.I1RS35
70518 229533.I1RJH3 pp 229533.I1RSH8

70519 229533.I1RJH3 pp 229533.I1RPN6
70520 229533.I1RJH3 pp 229533.I1RZV5
70521 229533.I1RJH3 pp 229533.I1RY32
70522 229533.I1RJH3 pp 229533.I1RLZ4
70523 229533.I1RJH3 pp 229533.I1RMK9
70524 229533.I1RJH3 pp 229533.I1S0F1
70525 229533.I1RJH3 pp 229533.I1RZC3
70526 229533.I1RJH3 pp 229533.Q4IBU4
70527 229533.I1RPC5 pp 229533.I1RPE5
70528 229533.I1RDZ4 pp 229533.I1RMM9
70529 229533.I1RDZ4 pp 229533.I1RRF0
70530 229533.I1RDZ4 pp 229533.I1RRY3
70531 229533.I1RDZ4 pp 229533.I1S0D8
70532 229533.I1RDZ4 pp 229533.I1RMJ8
70533 229533.I1RDZ4 pp 229533.I1RUQ5
70534 229533.I1RDZ4 pp 229533.I1RYY5
70535 229533.I1RDZ4 pp 229533.I1S439
70536 229533.I1RDZ4 pp 229533.I1RZE4
70537 229533.I1RDZ4 pp 229533.I1RN08
70538 229533.I1RDZ4 pp 229533.I1RY58
70539 229533.I1RDZ4 pp 229533.I1RQP3
70540 229533.I1RDZ4 pp 229533.I1S0M2
70541 229533.I1RDZ4 pp 229533.I1RKK0
70542 229533.I1RDZ4 pp 229533.I1RQA3
70543 229533.I1RDZ4 pp 229533.I1RQP1
70544 229533.I1RDZ4 pp 229533.I1S2E9
70545 229533.I1RDZ4 pp 229533.Q4I948
70546 229533.I1RDZ4 pp 229533.Q4ICA8
70547 229533.I1RDZ4 pp 229533.I1RY44
70548 229533.I1RDZ4 pp 229533.I1S2G7
70549 229533.I1RDZ4 pp 229533.Q4IJM4
70550 229533.I1RDZ4 pp 229533.V6RVG3
70551 229533.I1RDZ4 pp 229533.I1RN27
70552 229533.I1RDZ4 pp 229533.I1RX70
70553 229533.I1RDZ4 pp 229533.I1RRN5
70554 229533.I1RDZ4 pp 229533.V6RKY7
70555 229533.I1RDZ4 pp 229533.I1RQ26
70556 229533.I1RDZ4 pp 229533.I1S1W4
70557 229533.I1RDZ4 pp 229533.I1RYH9
70558 229533.I1RDZ4 pp 229533.I1RWT6
70559 229533.I1RDZ4 pp 229533.I1RUN8
70560 229533.I1RDZ4 pp 229533.Q4IB89
70561 229533.I1RDZ4 pp 229533.Q4IPI4
70562 229533.I1RDZ4 pp 229533.I1RNP7
70563 229533.I1RDZ4 pp 229533.I1RJ88
70564 229533.I1RDZ4 pp 229533.I1RV91
70565 229533.I1RDZ4 pp 229533.I1S0X7
70566 229533.I1RDZ4 pp 229533.Q4HTT1
70567 229533.A0A1C3YIE8 pp 229533.I1RAB7
70568 229533.I1RM65 pp 229533.Q4HYB8
70569 229533.I1RM65 pp 229533.I1RWF3
70570 229533.I1RM65 pp 229533.I1RVL8
70571 229533.I1RM65 pp 229533.Q4IPC8
70572 229533.I1RM65 pp 229533.I1RSZ3
70573 229533.I1RM65 pp 229533.Q4I5W5
70574 229533.I1RM65 pp 229533.I1RXU0
70575 229533.I1RM65 pp 229533.I1RNB3
70576 229533.I1RM65 pp 229533.I1RUG0
70577 229533.I1RM65 pp 229533.I1RVY0
70578 229533.I1RM65 pp 229533.I1RR25
70579 229533.I1RM65 pp 229533.V6QWX8
70580 229533.I1RM65 pp 229533.I1S276
70581 229533.I1RM65 pp 229533.I1RZB5
70582 229533.I1RM65 pp 229533.I1RRT0
70583 229533.I1RM65 pp 229533.I1RPM7
70584 229533.I1RM65 pp 229533.V6QUE6
70585 229533.I1RM65 pp 229533.I1RRG3
70586 229533.I1RM65 pp 229533.I1RTC2
70587 229533.I1RM65 pp 229533.Q4I5I4
70588 229533.I1RN19 pp 229533.I1S2E2
70589 229533.I1RN19 pp 229533.Q4IBU4
70590 229533.I1RN19 pp 229533.I1S9D4
70591 229533.I1RN19 pp 229533.I1RZC3

70592 229533.I1RN19 pp 229533.I1RS35
70593 229533.I1RN19 pp 229533.I1RY32
70594 229533.I1RN19 pp 229533.I1RPN6
70595 229533.I1S1U7 pp 229533.I1SAA0
70596 229533.I1S1U7 pp 229533.I1S274
70597 229533.I1S1U7 pp 229533.Q4I662
70598 229533.I1S1U7 pp 229533.Q4HTT1
70599 229533.I1S1U7 pp 229533.I1S490
70600 229533.I1S1U7 pp 229533.V6R283
70601 229533.I1S1U7 pp 229533.Q4IF76
70602 229533.I1S1U7 pp 229533.I1SA47
70603 229533.I1S1U7 pp 229533.Q4IEV4
70604 229533.I1S1U7 pp 229533.I1S2E8
70605 229533.I1S1U7 pp 229533.I1S2C0
70606 229533.I1S1U7 pp 229533.Q4IMD1
70607 229533.I1S1U7 pp 229533.V6QUE6
70608 229533.I1S1U7 pp 229533.V6RGN5
70609 229533.I1S1U7 pp 229533.I1S9U2
70610 229533.I1S1U7 pp 229533.Q4HZ42
70611 229533.I1S1U7 pp 229533.I1S1X9
70612 229533.I1S1U7 pp 229533.Q4IEK8
70613 229533.I1S1U7 pp 229533.V6QYF0
70614 229533.I1S1U7 pp 229533.Q4I5R3
70615 229533.I1S1U7 pp 229533.Q4HTT2
70616 229533.I1S1U7 pp 229533.Q4IJH1
70617 229533.I1S1U7 pp 229533.Q4IQK7
70618 229533.I1S1U7 pp 229533.Q4IER8
70619 229533.I1RCM1 pp 229533.I1S4T3
70620 229533.I1RCM1 pp 229533.I1RTA3
70621 229533.I1RCM1 pp 229533.I1RCP1
70622 229533.I1RCM1 pp 229533.I1RE19
70623 229533.I1RCM1 pp 229533.I1RUP6
70624 229533.I1RCM1 pp 229533.I1RRF9
70625 229533.I1RCM1 pp 229533.I1RTB7
70626 229533.I1RW82 pp 229533.I1S3T6
70627 229533.I1RW82 pp 229533.I1S210
70628 229533.I1RW82 pp 229533.I1S267
70629 229533.I1RW82 pp 229533.I1S2F7
70630 229533.I1RW82 pp 229533.I1S489
70631 229533.I1RW82 pp 229533.I1S048
70632 229533.I1RW82 pp 229533.Q4HWE2
70633 229533.I1RW82 pp 229533.I1SAB4
70634 229533.I1RW82 pp 229533.I1S223
70635 229533.I1RW82 pp 229533.Q4IB89
70636 229533.I1RW82 pp 229533.I1RZ25
70637 229533.I1RW82 pp 229533.I1RZ38
70638 229533.A0A0E0RUJ7 pp 229533.I1RCB7
70639 229533.A0A0E0RUJ7 pp 229533.Q4IPC8
70640 229533.A0A0E0RUJ7 pp 229533.I1RG11
70641 229533.A0A0E0RUJ7 pp 229533.I1RE54
70642 229533.A0A0E0RUJ7 pp 229533.Q4HYB8
70643 229533.A0A0E0RUJ7 pp 229533.A0A1C3YKR1
70644 229533.A0A0E0RUJ7 pp 229533.I1RWF3
70645 229533.A0A0E0RUJ7 pp 229533.A0A1C3YN55
70646 229533.A0A0E0RUJ7 pp 229533.I1RK79
70647 229533.A0A0E0RUJ7 pp 229533.I1S276
70648 229533.A0A0E0RUJ7 pp 229533.I1RPM7
70649 229533.A0A0E0RUJ7 pp 229533.I1RSZ3
70650 229533.A0A0E0RUJ7 pp 229533.A0A1C3YII3
70651 229533.A0A0E0RUJ7 pp 229533.I1RXU0
70652 229533.A0A0E0RUJ7 pp 229533.Q4I5W5
70653 229533.A0A0E0RUJ7 pp 229533.I1S9Y0
70654 229533.A0A0E0RUJ7 pp 229533.I1RK71
70655 229533.A0A0E0RUJ7 pp 229533.I1RR25
70656 229533.A0A0E0RUJ7 pp 229533.I1RE30
70657 229533.A0A0E0RUJ7 pp 229533.I1RC24
70658 229533.A0A0E0RUJ7 pp 229533.A0A1C3YIR8
70659 229533.A0A0E0RUJ7 pp 229533.I1RVL8
70660 229533.A0A0E0RUJ7 pp 229533.I1RVY0
70661 229533.A0A0E0RUJ7 pp 229533.A0A1C3YI23
70662 229533.A0A0E0RUJ7 pp 229533.I1RE14
70663 229533.A0A0E0RUJ7 pp 229533.Q4I5I4
70664 229533.A0A0E0RUJ7 pp 229533.V6QUE6

70665 229533.A0A0E0RUJ7 pp 229533.I1RAR2
70666 229533.A0A0E0RUJ7 pp 229533.I1RC15
70667 229533.A0A0E0RUJ7 pp 229533.I1RRT0
70668 229533.A0A0E0RUJ7 pp 229533.I1RJL2
70669 229533.A0A0E0RUJ7 pp 229533.I1RZB5
70670 229533.A0A0E0RUJ7 pp 229533.I1RKB1
70671 229533.A0A0E0RUJ7 pp 229533.I1RTC2
70672 229533.A0A0E0RUJ7 pp 229533.I1RRG3
70673 229533.A0A0E0RUJ7 pp 229533.I1RUG0
70674 229533.A0A0E0RUJ7 pp 229533.V6QWX8
70675 229533.I1RC54 pp 229533.I1RW24
70676 229533.I1RC54 pp 229533.I1RYR2
70677 229533.I1RC54 pp 229533.I1RKQ0
70678 229533.I1RC54 pp 229533.I1RNG4
70679 229533.I1RC54 pp 229533.I1RFZ2
70680 229533.I1RC54 pp 229533.I1RSN7
70681 229533.I1RC54 pp 229533.I1RMG6
70682 229533.I1RC54 pp 229533.I1RX22
70683 229533.I1RC54 pp 229533.Q4IEV4
70684 229533.I1RC54 pp 229533.I1S8G4
70685 229533.I1RC54 pp 229533.I1RYX9
70686 229533.I1RC54 pp 229533.I1RY29
70687 229533.I1RC54 pp 229533.I1RWI8
70688 229533.I1RC54 pp 229533.I1S237
70689 229533.I1RC54 pp 229533.I1RVV5
70690 229533.I1RC54 pp 229533.I1S2F2
70691 229533.I1RC54 pp 229533.I1RPR8
70692 229533.I1RC54 pp 229533.I1RCA5
70693 229533.I1RC54 pp 229533.I1S004
70694 229533.I1RC54 pp 229533.I1RF02
70695 229533.I1RC54 pp 229533.I1RVW0
70696 229533.I1RC54 pp 229533.I1RUG1
70697 229533.I1RC54 pp 229533.I1S3G4
70698 229533.I1RC54 pp 229533.I1RW28
70699 229533.I1RC54 pp 229533.I1S998
70700 229533.I1RC54 pp 229533.I1RYQ1
70701 229533.I1RC54 pp 229533.I1RDC0
70702 229533.I1RC54 pp 229533.I1RQ67
70703 229533.I1RC54 pp 229533.Q4I7L0
70704 229533.I1RC54 pp 229533.I1RRJ6
70705 229533.I1RC54 pp 229533.I1RHD2
70706 229533.I1RC54 pp 229533.V6R3J1
70707 229533.I1RC54 pp 229533.O74638
70708 229533.I1RC54 pp 229533.I1S523
70709 229533.I1RC54 pp 229533.I1RF70
70710 229533.I1RC54 pp 229533.I1RFR6
70711 229533.I1RC54 pp 229533.I1RJV8
70712 229533.I1RC54 pp 229533.I1RSJ4
70713 229533.I1RC54 pp 229533.Q4HTT1
70714 229533.I1RC54 pp 229533.I1RJZ7
70715 229533.I1RC54 pp 229533.I1RDV4
70716 229533.I1RC54 pp 229533.I1RW16
70717 229533.I1RC54 pp 229533.V6RLW4
70718 229533.I1RC54 pp 229533.I1RE76
70719 229533.I1RC54 pp 229533.I1RZ86
70720 229533.I1RC54 pp 229533.I1RN57
70721 229533.I1RC54 pp 229533.I1RSA7
70722 229533.I1RC54 pp 229533.V6R382
70723 229533.I1RC54 pp 229533.V6RSF3
70724 229533.I1RC54 pp 229533.I1RX64
70725 229533.I1RC54 pp 229533.I1RMI8
70726 229533.I1RC54 pp 229533.Q4HZK7
70727 229533.I1RC54 pp 229533.Q4IB89
70728 229533.I1RC54 pp 229533.I1RG11
70729 229533.I1RC54 pp 229533.I1S4P6
70730 229533.I1RC54 pp 229533.I1RFN0
70731 229533.I1RC54 pp 229533.I1RCB4
70732 229533.I1RC54 pp 229533.Q4IJ84
70733 229533.I1RC54 pp 229533.I1RSC7
70734 229533.I1RC54 pp 229533.I1RRS7
70735 229533.I1RC54 pp 229533.I1RQI6
70736 229533.I1RC54 pp 229533.I1RNA2
70737 229533.I1RC54 pp 229533.I1RZ98

70738	229533.I1RC54	pp	229533.I1RZP8
70739	229533.I1RC54	pp	229533.I1RP99
70740	229533.I1RC54	pp	229533.I1RKI2
70741	229533.I1RC54	pp	229533.I1RTN6
70742	229533.I1RC54	pp	229533.I1S236
70743	229533.I1RC54	pp	229533.I1RHR0
70744	229533.I1RC54	pp	229533.Q4HWE2
70745	229533.I1RC54	pp	229533.I1RD19
70746	229533.I1RC54	pp	229533.I1RSP7
70747	229533.I1RC54	pp	229533.I1RRN5
70748	229533.I1RC54	pp	229533.I1RM36
70749	229533.I1RC54	pp	229533.I1RQJ4
70750	229533.I1RC54	pp	229533.I1RW03
70751	229533.I1RC54	pp	229533.I1RDJ8
70752	229533.I1RC54	pp	229533.I1RZT1
70753	229533.I1RC54	pp	229533.I1RPQ1
70754	229533.I1RC54	pp	229533.I1RDH0
70755	229533.I1RC54	pp	229533.I1RY58
70756	229533.I1RC54	pp	229533.I1RWM6
70757	229533.I1RC54	pp	229533.I1RQL4
70758	229533.I1RC54	pp	229533.I1RMJ8
70759	229533.I1RC54	pp	229533.I1RDX0
70760	229533.I1RC54	pp	229533.I1RE55
70761	229533.I1RC54	pp	229533.I1RQE7
70762	229533.I1RC54	pp	229533.I1RSR9
70763	229533.I1RC54	pp	229533.I1S0A6
70764	229533.I1RC54	pp	229533.I1RRY1
70765	229533.I1RC54	pp	229533.I1RVX9
70766	229533.I1RC54	pp	229533.I1RJM9
70767	229533.I1RC54	pp	229533.I1S1W8
70768	229533.I1RC54	pp	229533.I1S0D5
70769	229533.I1RC54	pp	229533.I1RKB6
70770	229533.I1RC54	pp	229533.I1RUG4
70771	229533.I1RC54	pp	229533.I1RQ13
70772	229533.I1RC54	pp	229533.I1RFX8
70773	229533.I1RC54	pp	229533.I1RN31
70774	229533.I1RC54	pp	229533.Q4IP51
70775	229533.I1RC54	pp	229533.I1RTC2
70776	229533.I1RC54	pp	229533.I1S987
70777	229533.I1RC54	pp	229533.I1RD01
70778	229533.I1RC54	pp	229533.I1RDJ1
70779	229533.I1RC54	pp	229533.I1RMB7
70780	229533.I1RC54	pp	229533.I1RS81
70781	229533.I1RC54	pp	229533.I1RSK0
70782	229533.I1RC54	pp	229533.I1RQS9
70783	229533.I1RC54	pp	229533.I1RZ94
70784	229533.I1RC54	pp	229533.I1RD18
70785	229533.I1RC54	pp	229533.I1RCQ4
70786	229533.I1RC54	pp	229533.I1RSI4
70787	229533.I1RC54	pp	229533.I1RNC2
70788	229533.I1RC54	pp	229533.I1RRT1
70789	229533.I1RC54	pp	229533.I1RG34
70790	229533.I1RC54	pp	229533.I1S788
70791	229533.I1RC54	pp	229533.I1RZ02
70792	229533.I1RC54	pp	229533.I1S283
70793	229533.I1RC54	pp	229533.Q4IC11
70794	229533.I1RC54	pp	229533.I1RT16
70795	229533.I1RC54	pp	229533.I1RQJ0
70796	229533.I1RC54	pp	229533.I1S5Z3
70797	229533.I1RC54	pp	229533.I1RSJ1
70798	229533.I1RC54	pp	229533.I1RE46
70799	229533.I1RC54	pp	229533.I1RUR0
70800	229533.I1RC54	pp	229533.I1RN46
70801	229533.I1RC54	pp	229533.I1S461
70802	229533.I1RC54	pp	229533.I1S209
70803	229533.I1RC54	pp	229533.Q4I0J6
70804	229533.I1RC54	pp	229533.Q4IR08
70805	229533.I1RC54	pp	229533.Q4IBX0
70806	229533.I1RC54	pp	229533.I1RN55
70807	229533.I1RC54	pp	229533.Q4INS6
70808	229533.I1RC54	pp	229533.I1RM26
70809	229533.I1RC54	pp	229533.I1RFJ2
70810	229533.I1RC54	pp	229533.I1RG77

70811 229533.I1RC54 pp 229533.I1RMG7
70812 229533.I1RC54 pp 229533.I1RSI0
70813 229533.I1RC54 pp 229533.I1RQH8
70814 229533.I1RC54 pp 229533.I1RLJ8
70815 229533.I1RC54 pp 229533.I1RK95
70816 229533.I1RC54 pp 229533.I1RVZ9
70817 229533.I1RC54 pp 229533.I1RCL7
70818 229533.I1RC54 pp 229533.I1RPI0
70819 229533.I1RC54 pp 229533.I1RCM6
70820 229533.I1RBJ1 pp 229533.Q4IJU0
70821 229533.I1RBJ1 pp 229533.Q4IJM4
70822 229533.I1RBJ1 pp 229533.V6RGB5
70823 229533.I1RBJ1 pp 229533.I1REN3
70824 229533.I1RBJ1 pp 229533.I1RFQ5
70825 229533.I1RBJ1 pp 229533.I1RKF3
70826 229533.I1RBJ1 pp 229533.Q4I948
70827 229533.I1RBJ1 pp 229533.I1RZB5
70828 229533.I1RBJ1 pp 229533.I1RYY5
70829 229533.I1RBJ1 pp 229533.I1RN07
70830 229533.I1RBJ1 pp 229533.I1RE31
70831 229533.I1RBJ1 pp 229533.I1RJL2
70832 229533.I1RBJ1 pp 229533.Q4IER8
70833 229533.I1RBJ1 pp 229533.I1RW69
70834 229533.I1RBJ1 pp 229533.I1S0N8
70835 229533.I1RBJ1 pp 229533.I1RT01
70836 229533.I1RBJ1 pp 229533.I1RC15
70837 229533.I1RBJ1 pp 229533.I1RW90
70838 229533.I1RBJ1 pp 229533.I1S5Z2
70839 229533.I1RBJ1 pp 229533.I1RS66
70840 229533.I1RBJ1 pp 229533.I1RT35
70841 229533.I1RBJ1 pp 229533.V6QV38
70842 229533.I1RBJ1 pp 229533.I1RCU3
70843 229533.I1RBJ1 pp 229533.I1RXE5
70844 229533.I1RBJ1 pp 229533.I1RC16
70845 229533.I1RBJ1 pp 229533.I1RCG6
70846 229533.I1RBJ1 pp 229533.I1RSI1
70847 229533.I1RBJ1 pp 229533.I1RMQ7
70848 229533.I1RBJ1 pp 229533.Q4IR09
70849 229533.I1RBJ1 pp 229533.Q4I1B1
70850 229533.I1RBJ1 pp 229533.I1RH36
70851 229533.I1RBJ1 pp 229533.I1RRG3
70852 229533.I1RBJ1 pp 229533.I1RE14
70853 229533.I1RBJ1 pp 229533.Q4HTT2
70854 229533.I1RBJ1 pp 229533.I1RDC9
70855 229533.I1RBJ1 pp 229533.I1RRT0
70856 229533.I1RBJ1 pp 229533.Q4HYI0
70857 229533.I1RBJ1 pp 229533.Q4I5R3
70858 229533.I1RBJ1 pp 229533.Q4HYQ4
70859 229533.I1RBJ1 pp 229533.V6QUE6
70860 229533.I1RBJ1 pp 229533.P0C431
70861 229533.I1RBJ1 pp 229533.I1RSI4
70862 229533.I1RBJ1 pp 229533.I1RTC2
70863 229533.I1RBJ1 pp 229533.I1RBW4
70864 229533.I1RBJ1 pp 229533.V6R0U9
70865 229533.I1RBJ1 pp 229533.I1RR25
70866 229533.I1RBJ1 pp 229533.I1RG90
70867 229533.I1RBJ1 pp 229533.I1S182
70868 229533.I1RBJ1 pp 229533.I1RVZ4
70869 229533.I1RBJ1 pp 229533.Q4I5I4
70870 229533.I1RBJ1 pp 229533.I1RQ72
70871 229533.I1RBJ1 pp 229533.I1REF5
70872 229533.I1RBJ1 pp 229533.I1RFI5
70873 229533.I1RBJ1 pp 229533.I1RK79
70874 229533.I1RBJ1 pp 229533.I1RZR6
70875 229533.I1RBJ1 pp 229533.Q4HZS8
70876 229533.I1RBJ1 pp 229533.I1RK90
70877 229533.I1RBJ1 pp 229533.I1RCQ6
70878 229533.I1RBJ1 pp 229533.I1RT41
70879 229533.I1RBJ1 pp 229533.V6RDZ4
70880 229533.I1RBJ1 pp 229533.I1RPW8
70881 229533.I1RBJ1 pp 229533.I1RK71
70882 229533.I1RBJ1 pp 229533.I1RML9
70883 229533.I1RBJ1 pp 229533.I1RQ42

70884	229533.I1RBJ1	pp	229533.I1RVJ1
70885	229533.I1RBJ1	pp	229533.I1RP96
70886	229533.I1RBJ1	pp	229533.I1RT16
70887	229533.I1RBJ1	pp	229533.I1RS41
70888	229533.I1RBJ1	pp	229533.I1RPM7
70889	229533.I1RBJ1	pp	229533.I1RQA9
70890	229533.I1RBJ1	pp	229533.I1RZU4
70891	229533.I1RBJ1	pp	229533.I1RNI4
70892	229533.I1RBJ1	pp	229533.I1RW91
70893	229533.I1RBJ1	pp	229533.I1RUQ6
70894	229533.I1RBJ1	pp	229533.I1RKB1
70895	229533.I1RBJ1	pp	229533.Q4IQT8
70896	229533.I1RBJ1	pp	229533.I1S276
70897	229533.I1RBJ1	pp	229533.I1S0E8
70898	229533.I1RBJ1	pp	229533.V6QWX8
70899	229533.I1RBJ1	pp	229533.Q4HYB8
70900	229533.I1RBJ1	pp	229533.I1RUG0
70901	229533.I1RBJ1	pp	229533.I1RKR8
70902	229533.I1RBJ1	pp	229533.I1RZ42
70903	229533.I1RBJ1	pp	229533.I1RCB7
70904	229533.I1RBJ1	pp	229533.I1RMV1
70905	229533.I1RBJ1	pp	229533.I1RRF7
70906	229533.I1RBJ1	pp	229533.I1RXU0
70907	229533.I1RBJ1	pp	229533.I1RG11
70908	229533.I1RBJ1	pp	229533.I1RNB8
70909	229533.I1RBJ1	pp	229533.I1RWF3
70910	229533.I1RBJ1	pp	229533.I1RJW8
70911	229533.I1RBJ1	pp	229533.I1RSA4
70912	229533.I1RBJ1	pp	229533.I1RNQ6
70913	229533.I1RBJ1	pp	229533.Q4IPC8
70914	229533.I1RBJ1	pp	229533.Q4I5U9
70915	229533.I1RBJ1	pp	229533.I1RFL7
70916	229533.I1RBJ1	pp	229533.I1RVL8
70917	229533.I1RBJ1	pp	229533.Q4HTT1
70918	229533.I1RBJ1	pp	229533.I1RN04
70919	229533.I1RBJ1	pp	229533.I1RVY0
70920	229533.I1RBJ1	pp	229533.I1RJL3
70921	229533.I1RBJ1	pp	229533.I1RE30
70922	229533.I1RBJ1	pp	229533.I1RS43
70923	229533.I1RBJ1	pp	229533.I1RWI0
70924	229533.I1RBJ1	pp	229533.I1RNF4
70925	229533.I1RBJ1	pp	229533.I1RPU7
70926	229533.I1RBJ1	pp	229533.I1RC24
70927	229533.I1RBJ1	pp	229533.I1S0J3
70928	229533.I1RBJ1	pp	229533.I1RBR5
70929	229533.I1RBJ1	pp	229533.I1RSZ3
70930	229533.I1RBJ1	pp	229533.I1RQ88
70931	229533.I1RBJ1	pp	229533.Q4HW67
70932	229533.I1RBJ1	pp	229533.Q4IJ11
70933	229533.I1RBJ1	pp	229533.Q4IB50
70934	229533.I1RBJ1	pp	229533.I1RSW9
70935	229533.I1RBJ1	pp	229533.I1RW80
70936	229533.I1RBJ1	pp	229533.I1RTH2
70937	229533.I1RBJ1	pp	229533.Q4I5W5
70938	229533.I1RBJ1	pp	229533.Q4IMD1
70939	229533.I1RBJ1	pp	229533.I1RE19
70940	229533.I1RBJ1	pp	229533.I1RFW1
70941	229533.I1RBJ1	pp	229533.I1RZM2
70942	229533.A0A098DBH9	pp	229533.A0A1C3YI59
70943	229533.A0A098DBH9	pp	229533.A0A1C3YI60
70944	229533.A0A098DBH9	pp	229533.A0A1C3YM33
70945	229533.A0A098DBH9	pp	229533.A0A1C3YLC9
70946	229533.A0A098DBH9	pp	229533.I1RK32
70947	229533.A0A098DBH9	pp	229533.I1RCM1
70948	229533.A0A098DBH9	pp	229533.I1S0Z2
70949	229533.A0A098DBH9	pp	229533.A0A0E0S4D3
70950	229533.A0A098DBH9	pp	229533.I1S8Y5
70951	229533.I1S7A5	pp	229533.I1S9D4
70952	229533.I1S7A5	pp	229533.Q4IBU4
70953	229533.I1S4P6	pp	229533.I1S998
70954	229533.I1S4P6	pp	229533.V6RVG3
70955	229533.I1S4P6	pp	229533.Q4HXF6
70956	229533.I1S4P6	pp	229533.Q4IB89

70957 229533.I1S4P6 pp 229533.Q4I073
70958 229533.I1S4P6 pp 229533.Q4HTT1
70959 229533.I1S4P6 pp 229533.Q4IMD1
70960 229533.I1S4P6 pp 229533.Q4INS6
70961 229533.I1S4P6 pp 229533.Q4I5R9
70962 229533.I1S4P6 pp 229533.I1S987
70963 229533.I1S4P6 pp 229533.Q4IEV4
70964 229533.I1S4P6 pp 229533.I1S788
70965 229533.I1S4P6 pp 229533.Q4I2M7
70966 229533.I1S4P6 pp 229533.Q4HTT2
70967 229533.I1S4P6 pp 229533.Q4I1J3
70968 229533.I1S968 pp 229533.V6RCJ5
70969 229533.I1S968 pp 229533.I1SAT2
70970 229533.I1S968 pp 229533.V6QVV9
70971 229533.I1S968 pp 229533.V6RWB3
70972 229533.I1S968 pp 229533.I1SAB4
70973 229533.I1S968 pp 229533.V6RCC0
70974 229533.I1S968 pp 229533.V6RHX2
70975 229533.I1S968 pp 229533.V6R5T7
70976 229533.I1RIN5 pp 229533.I1S272
70977 229533.I1RIN5 pp 229533.V6RPQ8
70978 229533.I1RIN5 pp 229533.I1RZ13
70979 229533.I1RTC7 pp 229533.I1S522
70980 229533.I1RTC7 pp 229533.V6QWP4
70981 229533.I1RTC7 pp 229533.I1RZA5
70982 229533.I1S020 pp 229533.Q4HTT2
70983 229533.I1S020 pp 229533.Q4I6A4
70984 229533.I1S020 pp 229533.Q4I0J6
70985 229533.I1S020 pp 229533.Q4IER8
70986 229533.I1S020 pp 229533.Q4I7L0
70987 229533.I1S020 pp 229533.Q4IR08
70988 229533.I1S020 pp 229533.Q4HYB8
70989 229533.I1S020 pp 229533.V6R382
70990 229533.I1S020 pp 229533.V6RLW4
70991 229533.I1S020 pp 229533.Q4HTT1
70992 229533.I1S020 pp 229533.Q4IBX0
70993 229533.I1RMK9 pp 229533.I1S2F7
70994 229533.I1RMK9 pp 229533.I1RZS4
70995 229533.I1RMK9 pp 229533.I1S3T6
70996 229533.I1RMK9 pp 229533.I1RQ48
70997 229533.I1RMK9 pp 229533.I1S0Y1
70998 229533.I1RMK9 pp 229533.I1S223
70999 229533.I1RMK9 pp 229533.I1RZ38
71000 229533.I1RMK9 pp 229533.I1S968
71001 229533.I1RMK9 pp 229533.I1S969
71002 229533.I1RMK9 pp 229533.I1RZV5
71003 229533.I1RMK9 pp 229533.I1RPN6
71004 229533.I1RMK9 pp 229533.I1RXT2
71005 229533.I1RMK9 pp 229533.I1SAB4
71006 229533.I1RMK9 pp 229533.I1RYP5
71007 229533.I1RMK9 pp 229533.I1RY32
71008 229533.I1RMK9 pp 229533.I1RZC3
71009 229533.I1RMK9 pp 229533.I1RX25
71010 229533.I1RMK9 pp 229533.Q4IBU4
71011 229533.I1RMK9 pp 229533.I1RS35
71012 229533.I1RMK9 pp 229533.I1S8Q3
71013 229533.I1RMK9 pp 229533.I1RW55
71014 229533.I1RMK9 pp 229533.I1S210
71015 229533.I1RMK9 pp 229533.I1S267
71016 229533.I1RMK9 pp 229533.I1S560
71017 229533.I1RMK9 pp 229533.I1RY81
71018 229533.I1RMK9 pp 229533.I1RQV7
71019 229533.I1RMK9 pp 229533.I1S2C1
71020 229533.I1RHL8 pp 229533.I1S276
71021 229533.I1RHL8 pp 229533.I1RPM7
71022 229533.I1RHL8 pp 229533.I1RK79
71023 229533.I1RHL8 pp 229533.I1RR25
71024 229533.I1RHL8 pp 229533.I1RK71
71025 229533.I1RHL8 pp 229533.I1RVY0
71026 229533.I1RHL8 pp 229533.I1RXU0
71027 229533.I1RHL8 pp 229533.I1RVL8
71028 229533.I1RHL8 pp 229533.I1RSZ3
71029 229533.I1RHL8 pp 229533.Q4HYB8

71030	229533.I1RHL8	pp	229533.Q4IPC8
71031	229533.I1RHL8	pp	229533.I1RWF3
71032	229533.I1RHL8	pp	229533.Q4I5W5
71033	229533.I1RHL8	pp	229533.I1RUG0
71034	229533.I1RHL8	pp	229533.I1RKB1
71035	229533.I1RHL8	pp	229533.Q4I5I4
71036	229533.I1RHL8	pp	229533.I1RTC2
71037	229533.I1RHL8	pp	229533.I1RRG3
71038	229533.I1RHL8	pp	229533.V6QUE6
71039	229533.I1RHL8	pp	229533.I1RJL2
71040	229533.I1RHL8	pp	229533.V6QWX8
71041	229533.I1RHL8	pp	229533.I1RZB5
71042	229533.I1RHL8	pp	229533.I1RRT0
71043	229533.I1RC24	pp	229533.I1RQA3
71044	229533.I1RC24	pp	229533.I1RMV1
71045	229533.I1RC24	pp	229533.I1RW23
71046	229533.I1RC24	pp	229533.I1RE31
71047	229533.I1RC24	pp	229533.I1RQZ2
71048	229533.I1RC24	pp	229533.Q4IJM4
71049	229533.I1RC24	pp	229533.I1RR61
71050	229533.I1RC24	pp	229533.I1RM65
71051	229533.I1RC24	pp	229533.I1RYY5
71052	229533.I1RC24	pp	229533.Q4IJU0
71053	229533.I1RC24	pp	229533.V6R0U9
71054	229533.I1RC24	pp	229533.I1RPV5
71055	229533.I1RC24	pp	229533.I1RN07
71056	229533.I1RC24	pp	229533.I1S276
71057	229533.I1RC24	pp	229533.I1RDC9
71058	229533.I1RC24	pp	229533.I1RZB5
71059	229533.I1RC24	pp	229533.I1RFQ5
71060	229533.I1RC24	pp	229533.Q4IER8
71061	229533.I1RC24	pp	229533.I1REN3
71062	229533.I1RC24	pp	229533.I1RMQ7
71063	229533.I1RC24	pp	229533.I1RG90
71064	229533.I1RC24	pp	229533.I1RJL2
71065	229533.I1RC24	pp	229533.I1RYS4
71066	229533.I1RC24	pp	229533.I1RUG4
71067	229533.I1RC24	pp	229533.I1RJY3
71068	229533.I1RC24	pp	229533.I1RZH4
71069	229533.I1RC24	pp	229533.Q4HYB8
71070	229533.I1RC24	pp	229533.I1RU02
71071	229533.I1RC24	pp	229533.I1RR25
71072	229533.I1RC24	pp	229533.I1S2C4
71073	229533.I1RC24	pp	229533.Q4IQT8
71074	229533.I1RC24	pp	229533.I1S8V0
71075	229533.I1RC24	pp	229533.I1RD38
71076	229533.I1RC24	pp	229533.I1RZU9
71077	229533.I1RC24	pp	229533.I1S9U1
71078	229533.I1RC24	pp	229533.I1RK79
71079	229533.I1RC24	pp	229533.I1RRT0
71080	229533.I1RC24	pp	229533.I1RNB8
71081	229533.I1RC24	pp	229533.I1RK71
71082	229533.I1RC24	pp	229533.I1RPM7
71083	229533.I1RC24	pp	229533.I1RWT6
71084	229533.I1RC24	pp	229533.I1RQZ3
71085	229533.I1RC24	pp	229533.V6QV38
71086	229533.I1RC24	pp	229533.I1RS41
71087	229533.I1RC24	pp	229533.I1RZ42
71088	229533.I1RC24	pp	229533.I1RT01
71089	229533.I1RC24	pp	229533.I1RE14
71090	229533.I1RC24	pp	229533.I1S255
71091	229533.I1RC24	pp	229533.I1RK92
71092	229533.I1RC24	pp	229533.Q4HTT2
71093	229533.I1RC24	pp	229533.I1RWF3
71094	229533.I1RC24	pp	229533.I1RT99
71095	229533.I1RC24	pp	229533.I1RZL5
71096	229533.I1RC24	pp	229533.I1RZU4
71097	229533.I1RC24	pp	229533.Q4HYI0
71098	229533.I1RC24	pp	229533.I1RDZ6
71099	229533.I1RC24	pp	229533.I1S3N1
71100	229533.I1RC24	pp	229533.I1RXE5
71101	229533.I1RC24	pp	229533.I1RJW8
71102	229533.I1RC24	pp	229533.I1RCG6

71103	229533.I1RC24	pp	229533.I1RJY2
71104	229533.I1RC24	pp	229533.I1S8U9
71105	229533.I1RC24	pp	229533.I1RSA4
71106	229533.I1RC24	pp	229533.I1RRF7
71107	229533.I1RC24	pp	229533.I1RNQ6
71108	229533.I1RC24	pp	229533.I1RCB7
71109	229533.I1RC24	pp	229533.Q4IPC8
71110	229533.I1RC24	pp	229533.I1RSI1
71111	229533.I1RC24	pp	229533.I1RG11
71112	229533.I1RC24	pp	229533.Q4I7S1
71113	229533.I1RC24	pp	229533.Q4I5U9
71114	229533.I1RC24	pp	229533.I1RXU0
71115	229533.I1RC24	pp	229533.I1RVL8
71116	229533.I1RC24	pp	229533.V6QUE6
71117	229533.I1RC24	pp	229533.I1RCW8
71118	229533.I1RC24	pp	229533.I1RY44
71119	229533.I1RC24	pp	229533.I1RLV3
71120	229533.I1RC24	pp	229533.I1S0C6
71121	229533.I1RC24	pp	229533.I1RVJ1
71122	229533.I1RC24	pp	229533.Q4I099
71123	229533.I1RC24	pp	229533.I1RM42
71124	229533.I1RC24	pp	229533.Q4I5I4
71125	229533.I1RC24	pp	229533.I1RHL7
71126	229533.I1RC24	pp	229533.I1RCQ6
71127	229533.I1RC24	pp	229533.Q4HTT1
71128	229533.I1RC24	pp	229533.I1RNP4
71129	229533.I1RC24	pp	229533.I1RN04
71130	229533.I1RC24	pp	229533.I1S9Y1
71131	229533.I1RC24	pp	229533.I1RUR0
71132	229533.I1RC24	pp	229533.I1RQ42
71133	229533.I1RC24	pp	229533.I1RLR0
71134	229533.I1RC24	pp	229533.Q4IPN9
71135	229533.I1RC24	pp	229533.I1RW91
71136	229533.I1RC24	pp	229533.I1RD36
71137	229533.I1RC24	pp	229533.I1RC98
71138	229533.I1RC24	pp	229533.I1RS66
71139	229533.I1RC24	pp	229533.V6QWX8
71140	229533.I1RC24	pp	229533.I1RVY0
71141	229533.I1RC24	pp	229533.I1RWL3
71142	229533.I1RC24	pp	229533.I1S0R5
71143	229533.I1RC24	pp	229533.I1RUG0
71144	229533.I1RC24	pp	229533.I1S182
71145	229533.I1RC24	pp	229533.I1S0J3
71146	229533.I1RC24	pp	229533.I1RRG3
71147	229533.I1RC24	pp	229533.Q4HYQ4
71148	229533.I1RC24	pp	229533.I1S214
71149	229533.I1RC24	pp	229533.I1RZR6
71150	229533.I1RC24	pp	229533.I1RW90
71151	229533.I1RC24	pp	229533.I1RZM2
71152	229533.I1RC24	pp	229533.Q4IJ11
71153	229533.I1RC24	pp	229533.I1RRW3
71154	229533.I1RC24	pp	229533.I1RE30
71155	229533.I1RC24	pp	229533.I1RFW1
71156	229533.I1RC24	pp	229533.I1RHL8
71157	229533.I1RC24	pp	229533.I1RX63
71158	229533.I1RC24	pp	229533.I1RLV1
71159	229533.I1RC24	pp	229533.I1S4M5
71160	229533.I1RC24	pp	229533.Q4IPA4
71161	229533.I1RC24	pp	229533.I1RDD3
71162	229533.I1RC24	pp	229533.I1RKQ5
71163	229533.I1RC24	pp	229533.I1RTC2
71164	229533.I1RC24	pp	229533.I1RTH2
71165	229533.I1RC24	pp	229533.I1RFL7
71166	229533.I1RC24	pp	229533.I1RW80
71167	229533.I1RC24	pp	229533.I1RPW8
71168	229533.I1RC24	pp	229533.I1RP21
71169	229533.I1RC24	pp	229533.Q4IQX3
71170	229533.I1RC24	pp	229533.I1S3N0
71171	229533.I1RC24	pp	229533.I1RS30
71172	229533.I1RC24	pp	229533.I1S201
71173	229533.I1RC24	pp	229533.I1RQ88
71174	229533.I1RC24	pp	229533.I1RRM8
71175	229533.I1RC24	pp	229533.I1RS43

71176	229533.I1RC24	pp	229533.Q4I5W5
71177	229533.I1RC24	pp	229533.I1RP96
71178	229533.I1RC24	pp	229533.I1RSZ3
71179	229533.I1RC24	pp	229533.Q4IMD1
71180	229533.I1RC24	pp	229533.I1RML9
71181	229533.I1RC24	pp	229533.I1S2E9
71182	229533.I1RC24	pp	229533.I1RHQ5
71183	229533.I1RC24	pp	229533.I1RKB1
71184	229533.I1RC24	pp	229533.I1RGR8
71185	229533.I1RGR8	pp	229533.I1RK71
71186	229533.I1RGR8	pp	229533.I1S276
71187	229533.I1RGR8	pp	229533.I1RPM7
71188	229533.I1RGR8	pp	229533.I1RSZ3
71189	229533.I1RGR8	pp	229533.Q4HYB8
71190	229533.I1RGR8	pp	229533.I1RXU0
71191	229533.I1RGR8	pp	229533.Q4I5W5
71192	229533.I1RGR8	pp	229533.I1RWF3
71193	229533.I1RGR8	pp	229533.I1RR25
71194	229533.I1RGR8	pp	229533.I1RK79
71195	229533.I1RGR8	pp	229533.I1RVL8
71196	229533.I1RGR8	pp	229533.Q4IPC8
71197	229533.I1RGR8	pp	229533.I1RU37
71198	229533.I1RGR8	pp	229533.I1RN41
71199	229533.I1RGR8	pp	229533.I1RVY0
71200	229533.I1RGR8	pp	229533.V6QWX8
71201	229533.I1RGR8	pp	229533.I1RRT0
71202	229533.I1RGR8	pp	229533.I1RTC2
71203	229533.I1RGR8	pp	229533.I1RRG3
71204	229533.I1RGR8	pp	229533.V6QUE6
71205	229533.I1RGR8	pp	229533.I1RUG0
71206	229533.I1RGR8	pp	229533.I1RKB1
71207	229533.I1RGR8	pp	229533.I1RGZ0
71208	229533.I1RGR8	pp	229533.Q4I5I4
71209	229533.I1RGR8	pp	229533.I1RJL2
71210	229533.I1RGR8	pp	229533.I1RZB5
71211	229533.I1RYP5	pp	229533.I1S8D3
71212	229533.I1RYP5	pp	229533.I1S267
71213	229533.I1RYP5	pp	229533.I1S8F8
71214	229533.I1RYP5	pp	229533.I1RYU6
71215	229533.I1RYP5	pp	229533.I1S6H3
71216	229533.I1RYP5	pp	229533.I1S8C1
71217	229533.I1RYP5	pp	229533.I1S094
71218	229533.I1RYP5	pp	229533.V6RHX2
71219	229533.I1RYP5	pp	229533.I1S2F7
71220	229533.I1RYP5	pp	229533.I1S223
71221	229533.I1RYP5	pp	229533.I1S5D0
71222	229533.I1RYP5	pp	229533.I1S3T6
71223	229533.I1RYP5	pp	229533.I1S048
71224	229533.I1RYP5	pp	229533.V6QVV9
71225	229533.I1RYP5	pp	229533.V6R5T7
71226	229533.I1RYP5	pp	229533.I1S130
71227	229533.I1RYP5	pp	229533.I1S659
71228	229533.I1RYP5	pp	229533.V6RWB3
71229	229533.I1RYP5	pp	229533.I1S210
71230	229533.I1RYP5	pp	229533.V6RCC0
71231	229533.I1RYP5	pp	229533.I1S2C4
71232	229533.I1RYP5	pp	229533.I1RZC3
71233	229533.I1RYP5	pp	229533.I1RZ89
71234	229533.I1RYP5	pp	229533.I1SAT2
71235	229533.I1RYP5	pp	229533.I1S0A5
71236	229533.I1RYP5	pp	229533.V6RCJ5
71237	229533.I1RYP5	pp	229533.I1RZ25
71238	229533.I1RYP5	pp	229533.I1S968
71239	229533.I1RYP5	pp	229533.I1RZ56
71240	229533.I1RYP5	pp	229533.I1S6V0
71241	229533.I1RYP5	pp	229533.I1RZ38
71242	229533.I1RYP5	pp	229533.I1S3T7
71243	229533.I1RYP5	pp	229533.I1SAB4
71244	229533.I1RYP5	pp	229533.I1S299
71245	229533.I1RYP5	pp	229533.I1S8R2
71246	229533.I1RB73	pp	229533.I1RIW5
71247	229533.I1RB73	pp	229533.Q4IR08
71248	229533.I1RB73	pp	229533.I1RSH4

229533.I1RB73	pp	229533.I1RYX3
229533.I1RB73	pp	229533.Q4I6A4
229533.I1RB73	pp	229533.I1RWT2
229533.I1RB73	pp	229533.I1S0F0
229533.I1RB73	pp	229533.Q4HTT2
229533.I1RB73	pp	229533.I1RSI1
229533.I1RB73	pp	229533.Q4ICA8
229533.I1RB73	pp	229533.Q4I5R3
229533.I1RB73	pp	229533.I1S364
229533.I1RB73	pp	229533.I1S5T5
229533.I1RB73	pp	229533.I1RCW8
229533.I1RB73	pp	229533.Q4IER8
229533.I1RB73	pp	229533.I1S2B6
229533.I1RB73	pp	229533.I1RFQ5
229533.I1RB73	pp	229533.I1RWB3
229533.I1RB73	pp	229533.I1RQ93
229533.I1RB73	pp	229533.I1RCK7
229533.I1RB73	pp	229533.I1RU02
229533.I1RB73	pp	229533.I1S2M5
229533.I1RB73	pp	229533.I1RPU1
229533.I1RB73	pp	229533.I1RUZ8
229533.I1RB73	pp	229533.I1RPJ1
229533.I1RB73	pp	229533.I1RE37
229533.I1RB73	pp	229533.I1RMS5
229533.I1RB73	pp	229533.I1RYN7
229533.I1RB73	pp	229533.I1RT16
229533.I1RB73	pp	229533.Q4I061
229533.I1RB73	pp	229533.I1RQL4
229533.I1RB73	pp	229533.I1RW60
229533.I1RB73	pp	229533.I1RPN4
229533.I1RB73	pp	229533.Q4IQX3
229533.I1RB73	pp	229533.I1RPF4
229533.I1RB73	pp	229533.I1RJX2
229533.I1RB73	pp	229533.I1RJR2
229533.I1RB73	pp	229533.I1RY98
229533.I1RB73	pp	229533.I1RC32
229533.I1RB73	pp	229533.I1RFQ0
229533.I1RB73	pp	229533.I1RNT2
229533.I1RB73	pp	229533.I1S1V2
229533.I1RB73	pp	229533.I1RDP5
229533.I1RB73	pp	229533.I1RY99
229533.I1RB73	pp	229533.I1RC98
229533.I1RB73	pp	229533.I1RSL6
229533.I1RB73	pp	229533.I1RYV4
229533.I1RB73	pp	229533.I1RXI8
229533.I1RB73	pp	229533.I1S0N5
229533.I1RB73	pp	229533.I1S724
229533.I1RB73	pp	229533.I1RGR2
229533.I1RB73	pp	229533.I1RWD0
229533.I1RB73	pp	229533.I1RL41
229533.I1RB73	pp	229533.I1RYW3
229533.I1RB73	pp	229533.I1RX64
229533.I1RB73	pp	229533.I1S109
229533.I1RB73	pp	229533.I1S1Y7
229533.I1RB73	pp	229533.I1RZT1
229533.I1RB73	pp	229533.I1S1U7
229533.I1RB73	pp	229533.I1RCD8
229533.I1RB73	pp	229533.I1RHP9
229533.I1RB73	pp	229533.Q4IEV4
229533.I1RB73	pp	229533.Q4IMD1
229533.I1RB73	pp	229533.I1RBR4
229533.I1RB73	pp	229533.I1REC7
229533.I1RB73	pp	229533.I1RQ88
229533.I1RB73	pp	229533.I1S247
229533.I1RB73	pp	229533.I1RRM8
229533.I1RB73	pp	229533.I1S0J3
229533.I1RB73	pp	229533.I1RFW1
229533.I1RB73	pp	229533.I1RW28
229533.I1RB73	pp	229533.I1SB04
229533.I1RB73	pp	229533.Q4HTT1
229533.I1RB73	pp	229533.I1S3D7
229533.I1RB73	pp	229533.I1RPY4
229533.I1RB73	pp	229533.Q4IB50

71322	229533.I1RB73	pp	229533.I1SAN0
71323	229533.I1RB73	pp	229533.I1REC6
71324	229533.I1RB73	pp	229533.I1RPR8
71325	229533.I1RB73	pp	229533.Q4I7L0
71326	229533.I1RB73	pp	229533.I1RZA9
71327	229533.I1RB73	pp	229533.I1RVY0
71328	229533.I1RB73	pp	229533.I1RY90
71329	229533.I1RB73	pp	229533.I1SAA0
71330	229533.I1RB73	pp	229533.I1RY77
71331	229533.I1RHU8	pp	229533.I1RRN5
71332	229533.I1RHU8	pp	229533.I1RIM7
71333	229533.I1RHU8	pp	229533.I1RTP2
71334	229533.I1RHU8	pp	229533.I1SAB4
71335	229533.I1RHU8	pp	229533.I1RM07
71336	229533.I1RHU8	pp	229533.I1S267
71337	229533.I1RHU8	pp	229533.I1RQ48
71338	229533.I1RHU8	pp	229533.I1RTM8
71339	229533.I1RHU8	pp	229533.Q4IB89
71340	229533.I1RHU8	pp	229533.I1RST9
71341	229533.I1RHU8	pp	229533.I1RW82
71342	229533.I1RHU8	pp	229533.I1RRN4
71343	229533.I1RHU8	pp	229533.I1RZ38
71344	229533.I1RHU8	pp	229533.I1RZ25
71345	229533.I1RHU8	pp	229533.I1RMW8
71346	229533.I1RHU8	pp	229533.I1S489
71347	229533.I1RHU8	pp	229533.I1RR68
71348	229533.I1RHU8	pp	229533.I1RRC4
71349	229533.I1RHU8	pp	229533.I1S210
71350	229533.I1RHU8	pp	229533.I1RW55
71351	229533.I1RHU8	pp	229533.I1RLA2
71352	229533.I1RHU8	pp	229533.I1S2F7
71353	229533.I1RHU8	pp	229533.I1RMW9
71354	229533.I1RHU8	pp	229533.I1S048
71355	229533.I1RHU8	pp	229533.I1S3T6
71356	229533.I1RHU8	pp	229533.I1RIF7
71357	229533.I1RHU8	pp	229533.I1S223
71358	229533.Q4IB50	pp	229533.Q4IPC8
71359	229533.Q4IB50	pp	229533.V6QX21
71360	229533.Q4IB50	pp	229533.Q4IJ11
71361	229533.Q4IB50	pp	229533.Q4IMD1
71362	229533.Q4IB50	pp	229533.Q4IR08
71363	229533.Q4IB50	pp	229533.Q4IJP1
71364	229533.Q4IB50	pp	229533.Q4IER8
71365	229533.I1RX63	pp	229533.Q4I5I4
71366	229533.I1RX63	pp	229533.V6QWX8
71367	229533.I1RX63	pp	229533.Q4IPC8
71368	229533.I1RX63	pp	229533.I1RZB5
71369	229533.I1RX63	pp	229533.V6QUE6
71370	229533.I1RX63	pp	229533.Q4HYB8
71371	229533.I1RX63	pp	229533.I1RXU0
71372	229533.I1RX63	pp	229533.Q4I5W5
71373	229533.I1RX63	pp	229533.I1S276
71374	229533.A0A1C3YKX5	pp	229533.I1RE54
71375	229533.A0A1C3YKX5	pp	229533.Q4I5U9
71376	229533.A0A1C3YKX5	pp	229533.I1RWE1
71377	229533.A0A1C3YKX5	pp	229533.I1RXU0
71378	229533.A0A1C3YKX5	pp	229533.I1RCB7
71379	229533.A0A1C3YKX5	pp	229533.I1RG11
71380	229533.A0A1C3YKX5	pp	229533.I1RW23
71381	229533.A0A1C3YKX5	pp	229533.I1RAI9
71382	229533.A0A1C3YKX5	pp	229533.I1RR25
71383	229533.A0A1C3YKX5	pp	229533.Q4HY90
71384	229533.A0A1C3YKX5	pp	229533.I1S0R9
71385	229533.A0A1C3YKX5	pp	229533.V6RNM4
71386	229533.A0A1C3YKX5	pp	229533.I1RK79
71387	229533.A0A1C3YKX5	pp	229533.I1RN57
71388	229533.A0A1C3YKX5	pp	229533.I1RK71
71389	229533.A0A1C3YKX5	pp	229533.I1RS41
71390	229533.A0A1C3YKX5	pp	229533.Q4HYB8
71391	229533.A0A1C3YKX5	pp	229533.I1RHM4
71392	229533.A0A1C3YKX5	pp	229533.I1RRT0
71393	229533.A0A1C3YKX5	pp	229533.I1RC18
71394	229533.A0A1C3YKX5	pp	229533.I1RFW1

71395 229533.A0A1C3YKX5 pp 229533.I1RAY2
71396 229533.A0A1C3YKX5 pp 229533.I1RC15
71397 229533.A0A1C3YKX5 pp 229533.I1RSI1
71398 229533.A0A1C3YKX5 pp 229533.I1RQ88
71399 229533.A0A1C3YKX5 pp 229533.I1RJL2
71400 229533.A0A1C3YKX5 pp 229533.I1RSZ3
71401 229533.A0A1C3YKX5 pp 229533.I1RZB5
71402 229533.A0A1C3YKX5 pp 229533.I1RU02
71403 229533.A0A1C3YKX5 pp 229533.I1RT01
71404 229533.A0A1C3YKX5 pp 229533.I1RC16
71405 229533.A0A1C3YKX5 pp 229533.I1RCW8
71406 229533.A0A1C3YKX5 pp 229533.Q4IJ11
71407 229533.A0A1C3YKX5 pp 229533.Q4HTT2
71408 229533.A0A1C3YKX5 pp 229533.Q4IER8
71409 229533.A0A1C3YKX5 pp 229533.I1RBC7
71410 229533.A0A1C3YKX5 pp 229533.Q4I5W5
71411 229533.A0A1C3YKX5 pp 229533.I1RRG3
71412 229533.A0A1C3YKX5 pp 229533.Q4I5I4
71413 229533.A0A1C3YKX5 pp 229533.Q4IQM6
71414 229533.A0A1C3YKX5 pp 229533.I1RSI4
71415 229533.A0A1C3YKX5 pp 229533.V6QUE6
71416 229533.A0A1C3YKX5 pp 229533.Q4HYQ4
71417 229533.A0A1C3YKX5 pp 229533.I1RTC2
71418 229533.A0A1C3YKX5 pp 229533.I1RLV3
71419 229533.A0A1C3YKX5 pp 229533.I1RRK8
71420 229533.A0A1C3YKX5 pp 229533.Q4HTT1
71421 229533.A0A1C3YKX5 pp 229533.A0A1C3YMB9
71422 229533.A0A1C3YKX5 pp 229533.Q4IR08
71423 229533.A0A1C3YKX5 pp 229533.I1RZR6
71424 229533.A0A1C3YKX5 pp 229533.V6RLW4
71425 229533.A0A1C3YKX5 pp 229533.I1RPV5
71426 229533.A0A1C3YKX5 pp 229533.I1S201
71427 229533.A0A1C3YKX5 pp 229533.I1RC24
71428 229533.A0A1C3YKX5 pp 229533.I1RKB1
71429 229533.A0A1C3YKX5 pp 229533.I1RAE0
71430 229533.A0A1C3YKX5 pp 229533.I1RN55
71431 229533.A0A1C3YKX5 pp 229533.I1RAH4
71432 229533.A0A1C3YKX5 pp 229533.I1RW90
71433 229533.A0A1C3YKX5 pp 229533.I1RFX8
71434 229533.A0A1C3YKX5 pp 229533.I1RUG0
71435 229533.A0A1C3YKX5 pp 229533.V6QWX8
71436 229533.A0A1C3YKX5 pp 229533.I1RNI4
71437 229533.I1RTG3 pp 229533.I1S8P5
71438 229533.I1RTG3 pp 229533.Q4HTT1
71439 229533.I1RTG3 pp 229533.I1RTG5
71440 229533.I1RTG3 pp 229533.Q4IMD1
71441 229533.I1RTG3 pp 229533.V6R1K4
71442 229533.I1RTG3 pp 229533.Q4IR08
71443 229533.I1RTG3 pp 229533.V6R057
71444 229533.I1RTG3 pp 229533.I1S246
71445 229533.I1RTG3 pp 229533.I1S037
71446 229533.I1RTG3 pp 229533.Q4HTT2
71447 229533.I1RTG3 pp 229533.V6RBN2
71448 229533.I1RTG3 pp 229533.I1S1Z2
71449 229533.A0A1C3YJZ4 pp 229533.I1RZE1
71450 229533.A0A1C3YJZ4 pp 229533.I1RSL8
71451 229533.A0A1C3YJZ4 pp 229533.I1RDY5
71452 229533.A0A1C3YJZ4 pp 229533.A0A1C3YKG7
71453 229533.A0A1C3YJZ4 pp 229533.I1RRE0
71454 229533.A0A1C3YJZ4 pp 229533.I1RB43
71455 229533.A0A1C3YJZ4 pp 229533.Q4I963
71456 229533.A0A1C3YJZ4 pp 229533.I1RSP1
71457 229533.A0A1C3YJZ4 pp 229533.I1RQP5
71458 229533.A0A1C3YJZ4 pp 229533.Q4IMD1
71459 229533.A0A1C3YJZ4 pp 229533.I1RC00
71460 229533.A0A1C3YJZ4 pp 229533.A0A1C3YMK1
71461 229533.A0A1C3YJZ4 pp 229533.I1RFW3
71462 229533.A0A1C3YJZ4 pp 229533.I1RYJ8
71463 229533.A0A1C3YJZ4 pp 229533.Q4HTT2
71464 229533.A0A1C3YJZ4 pp 229533.I1RZ63
71465 229533.A0A1C3YJZ4 pp 229533.I1S2A8
71466 229533.A0A1C3YJZ4 pp 229533.I1RVY0
71467 229533.A0A1C3YJZ4 pp 229533.I1REX4

71468 229533.A0A1C3YJZ4 pp 229533.I1RTG3
71469 229533.A0A1C3YJZ4 pp 229533.Q4HTT1
71470 229533.Q4IL82 pp 229533.Q4IQX3
71471 229533.Q4IL82 pp 229533.Q4IPI4
71472 229533.I1RZF7 pp 229533.V6RB81
71473 229533.I1RZF7 pp 229533.I1S444
71474 229533.I1RZF7 pp 229533.I1S279
71475 229533.I1RZF7 pp 229533.I1S927
71476 229533.I1RZF7 pp 229533.I1S0Y1
71477 229533.I1RDV4 pp 229533.I1RF02
71478 229533.I1RDV4 pp 229533.I1RWM6
71479 229533.I1RDV4 pp 229533.I1RRT1
71480 229533.I1RDV4 pp 229533.I1RYV3
71481 229533.I1RDV4 pp 229533.I1RVW0
71482 229533.I1RDV4 pp 229533.Q4HTT1
71483 229533.I1RDV4 pp 229533.I1RXZ4
71484 229533.I1RDV4 pp 229533.I1RRJ6
71485 229533.I1RDV4 pp 229533.Q4IB89
71486 229533.I1RDV4 pp 229533.I1RZT1
71487 229533.I1RDV4 pp 229533.I1RFS6
71488 229533.I1RDV4 pp 229533.I1RMJ8
71489 229533.I1RDV4 pp 229533.I1S4P6
71490 229533.I1RDV4 pp 229533.Q4INS6
71491 229533.I1RZ89 pp 229533.Q4IBU4
71492 229533.I1RZ89 pp 229533.I1S223
71493 229533.I1RZ89 pp 229533.I1S2F7
71494 229533.I1RZ89 pp 229533.I1S3T6
71495 229533.I1RZ89 pp 229533.I1S969
71496 229533.I1RZ89 pp 229533.I1S968
71497 229533.I1RZ89 pp 229533.I1SAB4
71498 229533.I1RZ89 pp 229533.I1S210
71499 229533.I1RZ89 pp 229533.I1RZC3
71500 229533.I1RZ89 pp 229533.I1S267
71501 229533.A0A1C3YJY8 pp 229533.I1RRN5
71502 229533.A0A1C3YJY8 pp 229533.A0A1C3YN27
71503 229533.I1RPR8 pp 229533.I1RYM3
71504 229533.I1RPR8 pp 229533.Q4HTT1
71505 229533.I1RPR8 pp 229533.Q4IJP1
71506 229533.I1RPR8 pp 229533.I1RYX3
71507 229533.I1RPR8 pp 229533.Q4HTT2
71508 229533.I1RPR8 pp 229533.I1RRT1
71509 229533.I1RPR8 pp 229533.Q4ICA8
71510 229533.I1RPR8 pp 229533.Q4IPI4
71511 229533.I1RPR8 pp 229533.I1RW60
71512 229533.I1RPR8 pp 229533.Q4IJH1
71513 229533.I1RPR8 pp 229533.Q4IR08
71514 229533.I1RPR8 pp 229533.I1RVY0
71515 229533.I1RPR8 pp 229533.Q4I948
71516 229533.I1RPR8 pp 229533.I1SB04
71517 229533.I1RPR8 pp 229533.Q4IB96
71518 229533.I1RPR8 pp 229533.Q4IMD1
71519 229533.I1RPR8 pp 229533.Q4IF76
71520 229533.I1RPR8 pp 229533.I1RZ87
71521 229533.I1RPR8 pp 229533.Q4IAK7
71522 229533.I1RPR8 pp 229533.Q4IER8
71523 229533.I1RPR8 pp 229533.Q4IB89
71524 229533.I1RPR8 pp 229533.Q4I7L0
71525 229533.I1RPR8 pp 229533.Q4HY90
71526 229533.I1RPR8 pp 229533.I1RWD0
71527 229533.I1RPR8 pp 229533.Q4I5V3
71528 229533.I1RPR8 pp 229533.I1RSU7
71529 229533.I1RPR8 pp 229533.I1S1U7
71530 229533.I1RPR8 pp 229533.I1S4P6
71531 229533.I1RPR8 pp 229533.I1RZF3
71532 229533.I1RPR8 pp 229533.I1S0D5
71533 229533.I1RPR8 pp 229533.Q4I7S1
71534 229533.I1RHL7 pp 229533.I1RSZ3
71535 229533.I1RHL7 pp 229533.I1RTC2
71536 229533.I1RHL7 pp 229533.Q4I5W5
71537 229533.I1RHL7 pp 229533.I1RVY0
71538 229533.I1RHL7 pp 229533.I1RVL8
71539 229533.I1RHL7 pp 229533.Q4HYB8
71540 229533.I1RHL7 pp 229533.I1RWF3

229533.I1RHL7 pp 229533.I1RUG0
229533.I1RHL7 pp 229533.I1RRG3
229533.I1RHL7 pp 229533.I1S276
229533.I1RHL7 pp 229533.Q4IPC8
229533.I1RHL7 pp 229533.V6QWX8
229533.I1RHL7 pp 229533.I1RPM7
229533.I1RHL7 pp 229533.V6QUE6
229533.I1RHL7 pp 229533.I1RXU0
229533.I1RHL7 pp 229533.I1RK71
229533.I1RHL7 pp 229533.Q4I5I4
229533.I1RHL7 pp 229533.I1RRT0
229533.I1RHL7 pp 229533.I1RK79
229533.I1RHL7 pp 229533.I1RR25
229533.I1RHL7 pp 229533.I1RKB1
229533.I1RHL7 pp 229533.I1RJL2
229533.I1RHL7 pp 229533.I1RZB5
229533.A0A1C3YK91 pp 229533.I1RBJ0
229533.A0A1C3YK91 pp 229533.I1RQE0
229533.A0A1C3YK91 pp 229533.I1REJ0
229533.A0A1C3YK91 pp 229533.I1RZT0
229533.A0A1C3YK91 pp 229533.I1S3K8
229533.A0A1C3YM91 pp 229533.I1RWT6
229533.A0A1C3YM91 pp 229533.I1S267
229533.A0A1C3YM91 pp 229533.I1RB75
229533.A0A1C3YM91 pp 229533.Q4IJM4
229533.A0A1C3YM91 pp 229533.I1RDV9
229533.A0A1C3YM91 pp 229533.I1RZH4
229533.A0A1C3YM91 pp 229533.I1RMQ7
229533.A0A1C3YM91 pp 229533.I1RG77
229533.A0A1C3YM91 pp 229533.I1RKK0
229533.A0A1C3YM91 pp 229533.I1RQA3
229533.A0A1C3YM91 pp 229533.I1RPU4
229533.A0A1C3YM91 pp 229533.I1RC17
229533.A0A1C3YM91 pp 229533.I1RNC0
229533.A0A1C3YM91 pp 229533.I1RST9
229533.A0A1C3YM91 pp 229533.I1RCM6
229533.A0A1C3YM91 pp 229533.I1S1Z9
229533.A0A1C3YM91 pp 229533.Q4I7N9
229533.A0A1C3YM91 pp 229533.I1RLJ8
229533.A0A1C3YM91 pp 229533.I1RC95
229533.A0A1C3YM91 pp 229533.I1S334
229533.A0A1C3YM91 pp 229533.Q4INS6
229533.A0A1C3YM91 pp 229533.I1RBW0
229533.A0A1C3YM91 pp 229533.I1RV65
229533.A0A1C3YM91 pp 229533.I1RMS2
229533.A0A1C3YM91 pp 229533.I1RMM9
229533.A0A1C3YM91 pp 229533.I1RYH9
229533.A0A1C3YM91 pp 229533.I1RXZ4
229533.A0A1C3YM91 pp 229533.I1S224
229533.A0A1C3YM91 pp 229533.I1RME5
229533.A0A1C3YM91 pp 229533.Q4HTT2
229533.A0A1C3YM91 pp 229533.I1RN08
229533.A0A1C3YM91 pp 229533.I1RAN0
229533.A0A1C3YM91 pp 229533.I1RRX6
229533.A0A1C3YM91 pp 229533.I1RC22
229533.A0A1C3YM91 pp 229533.P0C431
229533.A0A1C3YM91 pp 229533.I1RCB3
229533.A0A1C3YM91 pp 229533.I1RRL5
229533.A0A1C3YM91 pp 229533.I1RSG0
229533.A0A1C3YM91 pp 229533.I1S1V3
229533.A0A1C3YM91 pp 229533.I1REE1
229533.A0A1C3YM91 pp 229533.I1RDB1
229533.A0A1C3YM91 pp 229533.I1S0D4
229533.A0A1C3YM91 pp 229533.I1RDJ8
229533.A0A1C3YM91 pp 229533.I1RDH0
229533.A0A1C3YM91 pp 229533.I1RSK0
229533.A0A1C3YM91 pp 229533.I1RY58
229533.A0A1C3YM91 pp 229533.I1RZ12
229533.A0A1C3YM91 pp 229533.I1RFS6
229533.A0A1C3YM91 pp 229533.I1RZ94
229533.A0A1C3YM91 pp 229533.I1RL24
229533.A0A1C3YM91 pp 229533.I1RZT1
229533.A0A1C3YM91 pp 229533.I1RD69

71614 229533.A0A1C3YM91 pp 229533.I1RQ26
71615 229533.A0A1C3YM91 pp 229533.I1RTG0
71616 229533.A0A1C3YM91 pp 229533.I1RRP7
71617 229533.A0A1C3YM91 pp 229533.I1RMJ8
71618 229533.A0A1C3YM91 pp 229533.I1RPA7
71619 229533.A0A1C3YM91 pp 229533.I1RD18
71620 229533.A0A1C3YM91 pp 229533.V6QV09
71621 229533.A0A1C3YM91 pp 229533.I1RDQ9
71622 229533.A0A1C3YM91 pp 229533.I1RG34
71623 229533.A0A1C3YM91 pp 229533.I1S0D5
71624 229533.A0A1C3YM91 pp 229533.I1S788
71625 229533.A0A1C3YM91 pp 229533.I1RQ80
71626 229533.A0A1C3YM91 pp 229533.I1RPM7
71627 229533.A0A1C3YM91 pp 229533.I1RU52
71628 229533.A0A1C3YM91 pp 229533.Q4IJ84
71629 229533.A0A1C3YM91 pp 229533.I1RYZ9
71630 229533.A0A1C3YM91 pp 229533.I1RUR0
71631 229533.A0A1C3YM91 pp 229533.I1RZK9
71632 229533.A0A1C3YM91 pp 229533.I1RM41
71633 229533.A0A1C3YM91 pp 229533.A0A1C3YMC9
71634 229533.A0A1C3YM91 pp 229533.Q4IB89
71635 229533.A0A1C3YM91 pp 229533.I1R9Y7
71636 229533.A0A1C3YM91 pp 229533.I1RPW0
71637 229533.A0A1C3YM91 pp 229533.I1RW03
71638 229533.A0A1C3YM91 pp 229533.I1R9Y9
71639 229533.A0A1C3YM91 pp 229533.I1S1W4
71640 229533.A0A1C3YM91 pp 229533.I1RVN2
71641 229533.A0A1C3YM91 pp 229533.A0A1C3YN69
71642 229533.A0A1C3YM91 pp 229533.I1RRN5
71643 229533.A0A1C3YM91 pp 229533.I1RC54
71644 229533.A0A1C3YM91 pp 229533.A0A1C3YN55
71645 229533.A0A1C3YM91 pp 229533.V6RGI1
71646 229533.A0A1C3YM91 pp 229533.I1S4P6
71647 229533.A0A1C3YM91 pp 229533.I1RDZ4
71648 229533.A0A1C3YM91 pp 229533.I1RSI3
71649 229533.A0A1C3YM91 pp 229533.I1RY44
71650 229533.A0A1C3YM91 pp 229533.I1RQD1
71651 229533.A0A1C3YM91 pp 229533.I1RZ63
71652 229533.A0A1C3YM91 pp 229533.I1RBM3
71653 229533.A0A1C3YM91 pp 229533.I1RRJ6
71654 229533.A0A1C3YM91 pp 229533.I1RQ24
71655 229533.A0A1C3YM91 pp 229533.I1RFI6
71656 229533.A0A1C3YM91 pp 229533.I1RZC3
71657 229533.A0A1C3YM91 pp 229533.I1S0J7
71658 229533.A0A1C3YM91 pp 229533.I1RAN2
71659 229533.A0A1C3YM91 pp 229533.I1S0M2
71660 229533.A0A1C3YM91 pp 229533.Q4HTT1
71661 229533.A0A1C3YM91 pp 229533.A0A1C3YMD7
71662 229533.A0A1C3YM91 pp 229533.I1RC71
71663 229533.A0A1C3YM91 pp 229533.I1RMA4
71664 229533.A0A1C3YM91 pp 229533.I1RRW0
71665 229533.A0A1C3YM91 pp 229533.I1RDV4
71666 229533.A0A1C3YM91 pp 229533.I1RZI4
71667 229533.A0A1C3YM91 pp 229533.I1S0M6
71668 229533.A0A1C3YM91 pp 229533.I1S549
71669 229533.A0A1C3YM91 pp 229533.I1RMP0
71670 229533.A0A1C3YM91 pp 229533.I1RVW0
71671 229533.A0A1C3YM91 pp 229533.I1RFZ2
71672 229533.A0A1C3YM91 pp 229533.I1RBV5
71673 229533.A0A1C3YM91 pp 229533.I1S004
71674 229533.A0A1C3YM91 pp 229533.I1RVV5
71675 229533.A0A1C3YM91 pp 229533.I1S3H8
71676 229533.A0A1C3YM91 pp 229533.I1RB59
71677 229533.A0A1C3YM91 pp 229533.I1RX38
71678 229533.A0A1C3YM91 pp 229533.I1RC00
71679 229533.A0A1C3YM91 pp 229533.I1S2E9
71680 229533.A0A1C3YM91 pp 229533.I1RTB2
71681 229533.A0A1C3YM91 pp 229533.I1RAH9
71682 229533.A0A1C3YM91 pp 229533.I1S098
71683 229533.A0A1C3YM91 pp 229533.I1RB74
71684 229533.A0A1C3YM91 pp 229533.I1RV91
71685 229533.A0A1C3YM91 pp 229533.I1RFL7
71686 229533.A0A1C3YM91 pp 229533.I1RKQ0

229533.A0A1C3YM91 pp 229533.I1RBW1
229533.A0A1C3YM91 pp 229533.I1RPF5
229533.A0A1C3YM91 pp 229533.I1RPK4
229533.A0A1C3YM91 pp 229533.I1RY32
229533.A0A1C3YM91 pp 229533.I1S7X9
229533.A0A1C3YM91 pp 229533.A0A1C3YMJ3
229533.A0A1C3YM91 pp 229533.I1RE19
229533.A0A1C3YM91 pp 229533.I1RAX2
229533.I1S048 pp 229533.I1S694
229533.I1S048 pp 229533.I1S6V0
229533.I1S048 pp 229533.I1SAB4
229533.I1S048 pp 229533.I1S0A5
229533.I1S048 pp 229533.V6RHX2
229533.I1S048 pp 229533.I1S8D3
229533.I1S048 pp 229533.I1S0D1
229533.I1S048 pp 229533.V6RCJ5
229533.I1S048 pp 229533.I1S968
229533.I1S048 pp 229533.I1S969
229533.I1S048 pp 229533.I1S2F7
229533.I1S048 pp 229533.I1S223
229533.I1S048 pp 229533.I1S267
229533.I1S048 pp 229533.V6RWB3
229533.I1S048 pp 229533.I1S210
229533.I1S048 pp 229533.V6RCC0
229533.I1S048 pp 229533.I1S3T6
229533.I1S048 pp 229533.V6QVV9
229533.I1S048 pp 229533.I1S5D0
229533.I1S048 pp 229533.I1S094
229533.I1S048 pp 229533.V6RH92
229533.I1S048 pp 229533.V6R5T7
229533.I1S048 pp 229533.I1S6H3
229533.A0A098DBJ5 pp 229533.I1RAS5
229533.A0A098DBJ5 pp 229533.I1S0D5
229533.A0A098DBJ5 pp 229533.I1RE46
229533.A0A098DBJ5 pp 229533.I1RBN9
229533.A0A098DBJ5 pp 229533.A0A1C3YHN3
229533.A0A098DBJ5 pp 229533.Q4IB89
229533.A0A098DBJ5 pp 229533.A0A098E4E9
229533.A0A098DBJ5 pp 229533.I1RYR2
229533.A0A098DBJ5 pp 229533.I1RRS7
229533.A0A098DBJ5 pp 229533.I1RXI6
229533.A0A098DBJ5 pp 229533.I1RMX3
229533.A0A098DBJ5 pp 229533.I1RU61
229533.A0A098DBJ5 pp 229533.I1RYX9
229533.A0A098DBJ5 pp 229533.I1RSC7
229533.A0A098DBJ5 pp 229533.I1RKQ0
229533.A0A098DBJ5 pp 229533.I1S461
229533.A0A098DBJ5 pp 229533.Q4IQM6
229533.A0A098DBJ5 pp 229533.I1R9W7
229533.A0A098DBJ5 pp 229533.I1RFJ2
229533.A0A098DBJ5 pp 229533.I1RUG2
229533.A0A098DBJ5 pp 229533.I1RJV8
229533.A0A098DBJ5 pp 229533.A0A098DSW9
229533.A0A098DBJ5 pp 229533.I1RMJ8
229533.A0A098DBJ5 pp 229533.V6RLW4
229533.A0A098DBJ5 pp 229533.I1RAH4
229533.A0A098DBJ5 pp 229533.I1RFX8
229533.A0A098DBJ5 pp 229533.I1RSK0
229533.A0A098DBJ5 pp 229533.A0A098DDT3
229533.A0A098DBJ5 pp 229533.I1S283
229533.A0A098DBJ5 pp 229533.I1RSA7
229533.A0A098DBJ5 pp 229533.A0A1C3YI10
229533.A0A1C3YL79 pp 229533.I1S267
229533.A0A1C3YL79 pp 229533.I1RST9
229533.A0A1C3YL79 pp 229533.I1RNM7
229533.A0A1C3YL79 pp 229533.I1RWF9
229533.A0A1C3YL79 pp 229533.I1RH91
229533.A0A1C3YL79 pp 229533.I1RBY9
229533.I1RD36 pp 229533.Q4I5W5
229533.I1RD36 pp 229533.Q4HYB8
229533.I1RD36 pp 229533.I1RSZ3
229533.I1RD36 pp 229533.Q4IPC8
229533.I1RD36 pp 229533.I1RG11

71760	229533.I1RD36	pp	229533.I1RR25
71761	229533.I1RD36	pp	229533.I1S276
71762	229533.I1RD36	pp	229533.I1RPM7
71763	229533.I1RD36	pp	229533.I1RK71
71764	229533.I1RD36	pp	229533.I1RXU0
71765	229533.I1RD36	pp	229533.I1RVY0
71766	229533.I1RD36	pp	229533.I1RUG0
71767	229533.I1RD36	pp	229533.I1RWF3
71768	229533.I1RD36	pp	229533.V6QWX8
71769	229533.I1RD36	pp	229533.I1RK79
71770	229533.I1RD36	pp	229533.I1RTC2
71771	229533.I1RD36	pp	229533.I1RE30
71772	229533.I1RD36	pp	229533.I1RVL8
71773	229533.I1RD36	pp	229533.I1RJL2
71774	229533.I1RD36	pp	229533.I1RZB5
71775	229533.I1RD36	pp	229533.V6QUE6
71776	229533.I1RD36	pp	229533.I1RRG3
71777	229533.I1RD36	pp	229533.I1RKB1
71778	229533.I1RD36	pp	229533.Q4I5I4
71779	229533.I1RD36	pp	229533.I1RE14
71780	229533.I1RD36	pp	229533.I1RRT0
71781	229533.I1RAQ3	pp	229533.I1RTD0
71782	229533.I1RAQ3	pp	229533.I1RE15
71783	229533.I1RAQ3	pp	229533.I1RE20
71784	229533.I1RAQ3	pp	229533.I1REI8
71785	229533.I1RAQ3	pp	229533.I1S9Z3
71786	229533.I1RCB7	pp	229533.I1RVL8
71787	229533.I1RCB7	pp	229533.I1RNP4
71788	229533.I1RCB7	pp	229533.I1RCQ6
71789	229533.I1RCB7	pp	229533.I1RQ42
71790	229533.I1RCB7	pp	229533.I1RVY0
71791	229533.I1RCB7	pp	229533.I1RLR0
71792	229533.I1RCB7	pp	229533.I1RHL7
71793	229533.I1RCB7	pp	229533.I1S0R5
71794	229533.I1RCB7	pp	229533.I1RGC1
71795	229533.I1RCB7	pp	229533.I1RVJ1
71796	229533.I1RCB7	pp	229533.Q4HTT1
71797	229533.I1RCB7	pp	229533.I1RPW8
71798	229533.I1RCB7	pp	229533.I1S0J3
71799	229533.I1RCB7	pp	229533.I1RN04
71800	229533.I1RCB7	pp	229533.I1RS43
71801	229533.I1RCB7	pp	229533.Q4I099
71802	229533.I1RCB7	pp	229533.I1RS30
71803	229533.I1RCB7	pp	229533.V6QWX8
71804	229533.I1RCB7	pp	229533.I1RUG0
71805	229533.I1RCB7	pp	229533.I1S182
71806	229533.I1RCB7	pp	229533.I1RJL3
71807	229533.I1RCB7	pp	229533.I1RW90
71808	229533.I1RCB7	pp	229533.Q4IB50
71809	229533.I1RCB7	pp	229533.I1RD36
71810	229533.I1RCB7	pp	229533.I1S3N0
71811	229533.I1RCB7	pp	229533.I1RWL3
71812	229533.I1RCB7	pp	229533.I1RE30
71813	229533.I1RCB7	pp	229533.I1RW91
71814	229533.I1RCB7	pp	229533.I1RZR6
71815	229533.I1RCB7	pp	229533.I1S4M5
71816	229533.I1RCB7	pp	229533.I1RTC2
71817	229533.I1RCB7	pp	229533.I1RNH1
71818	229533.I1RCB7	pp	229533.I1RTH2
71819	229533.I1RCB7	pp	229533.I1RLV1
71820	229533.I1RCB7	pp	229533.I1RKQ5
71821	229533.I1RCB7	pp	229533.I1RHQ5
71822	229533.I1RCB7	pp	229533.I1RML9
71823	229533.I1RCB7	pp	229533.I1RZM2
71824	229533.I1RCB7	pp	229533.I1RFL7
71825	229533.I1RCB7	pp	229533.I1RP96
71826	229533.I1RCB7	pp	229533.Q4IMD1
71827	229533.I1RCB7	pp	229533.Q4IJ11
71828	229533.I1RCB7	pp	229533.Q4I5W5
71829	229533.I1RCB7	pp	229533.Q4HYQ4
71830	229533.I1RCB7	pp	229533.I1RGR8
71831	229533.I1RCB7	pp	229533.I1RRM8
71832	229533.I1RCB7	pp	229533.I1RHL8

229533.I1RCB7	pp	229533.I1RFW1
229533.I1RCB7	pp	229533.Q4IQX3
229533.I1RCB7	pp	229533.I1RX63
229533.I1RCB7	pp	229533.I1RRG3
229533.I1RCB7	pp	229533.I1S2E9
229533.I1RCB7	pp	229533.I1RW80
229533.I1RCB7	pp	229533.I1RSZ3
229533.I1RCB7	pp	229533.I1RV91
229533.I1RCB7	pp	229533.I1RG90
229533.I1RCB7	pp	229533.Q4IJM4
229533.I1RCB7	pp	229533.Q4IJU0
229533.I1RCB7	pp	229533.I1RFQ5
229533.I1RCB7	pp	229533.I1RQ88
229533.I1RCB7	pp	229533.I1RKB1
229533.I1RCB7	pp	229533.I1RE31
229533.I1RCB7	pp	229533.I1RQZ2
229533.I1RCB7	pp	229533.Q4IER8
229533.I1RCB7	pp	229533.I1RW23
229533.I1RCB7	pp	229533.I1RMV1
229533.I1RCB7	pp	229533.I1RS41
229533.I1RCB7	pp	229533.I1RZB5
229533.I1RCB7	pp	229533.I1S0E8
229533.I1RCB7	pp	229533.I1RPM7
229533.I1RCB7	pp	229533.I1RK71
229533.I1RCB7	pp	229533.I1RQA3
229533.I1RCB7	pp	229533.I1RU02
229533.I1RCB7	pp	229533.I1S2C4
229533.I1RCB7	pp	229533.I1RNB8
229533.I1RCB7	pp	229533.I1RR61
229533.I1RCB7	pp	229533.I1RJL2
229533.I1RCB7	pp	229533.I1RK79
229533.I1RCB7	pp	229533.I1RZ42
229533.I1RCB7	pp	229533.I1RQZ3
229533.I1RCB7	pp	229533.I1REN3
229533.I1RCB7	pp	229533.I1RRT0
229533.I1RCB7	pp	229533.I1RUG4
229533.I1RCB7	pp	229533.I1RD38
229533.I1RCB7	pp	229533.I1RYY5
229533.I1RCB7	pp	229533.I1RR25
229533.I1RCB7	pp	229533.I1RN07
229533.I1RCB7	pp	229533.Q4HYI0
229533.I1RCB7	pp	229533.Q4HYB8
229533.I1RCB7	pp	229533.I1RK92
229533.I1RCB7	pp	229533.I1S3N1
229533.I1RCB7	pp	229533.I1RM65
229533.I1RCB7	pp	229533.V6QV38
229533.I1RCB7	pp	229533.I1RDC9
229533.I1RCB7	pp	229533.Q4IQT8
229533.I1RCB7	pp	229533.I1RMQ7
229533.I1RCB7	pp	229533.Q4HTT2
229533.I1RCB7	pp	229533.I1RDZ6
229533.I1RCB7	pp	229533.I1S276
229533.I1RCB7	pp	229533.I1RCW8
229533.I1RCB7	pp	229533.I1S255
229533.I1RCB7	pp	229533.I1RE14
229533.I1RCB7	pp	229533.I1RXU0
229533.I1RCB7	pp	229533.I1RZL5
229533.I1RCB7	pp	229533.I1RRF7
229533.I1RCB7	pp	229533.Q4IPC8
229533.I1RCB7	pp	229533.I1RWT6
229533.I1RCB7	pp	229533.I1RZU4
229533.I1RCB7	pp	229533.I1RNQ6
229533.I1RCB7	pp	229533.I1RT99
229533.I1RCB7	pp	229533.I1RWF3
229533.I1RCB7	pp	229533.I1RT01
229533.I1RCB7	pp	229533.I1RG11
229533.I1RCB7	pp	229533.V6QUE6
229533.I1RCB7	pp	229533.I1S0C6
229533.I1RCB7	pp	229533.I1RY44
229533.I1RCB7	pp	229533.I1RVZ4
229533.I1RCB7	pp	229533.I1RSI1
229533.I1RCB7	pp	229533.Q4I5U9
229533.I1RCB7	pp	229533.I1RXE5

71906 229533.I1RCB7 pp 229533.Q4I5I4
71907 229533.I1RCB7 pp 229533.I1RCG6
71908 229533.I1RCB7 pp 229533.I1RSA4
71909 229533.I1RCB7 pp 229533.I1RJW8
71910 229533.I1RLR0 pp 229533.I1RRG3
71911 229533.I1RLR0 pp 229533.I1RVY0
71912 229533.I1RLR0 pp 229533.I1RTC2
71913 229533.I1RLR0 pp 229533.I1RUG0
71914 229533.I1RLR0 pp 229533.Q4I5I4
71915 229533.I1RLR0 pp 229533.I1RXU0
71916 229533.I1RLR0 pp 229533.Q4IPC8
71917 229533.I1RLR0 pp 229533.V6QWX8
71918 229533.I1RLR0 pp 229533.Q4HYB8
71919 229533.I1RLR0 pp 229533.V6QUE6
71920 229533.I1RLR0 pp 229533.I1RSZ3
71921 229533.I1RLR0 pp 229533.Q4I5W5
71922 229533.I1RLR0 pp 229533.I1RZB5
71923 229533.I1RLR0 pp 229533.I1RRT0
71924 229533.I1RLR0 pp 229533.I1RVL8
71925 229533.I1RLR0 pp 229533.I1RWF3
71926 229533.I1RLR0 pp 229533.I1RR25
71927 229533.I1RLR0 pp 229533.I1RPM7
71928 229533.I1RLR0 pp 229533.I1S276
71929 229533.A0A098DBF8 pp 229533.A0A098DVZ4
71930 229533.A0A098DBF8 pp 229533.I1RW71
71931 229533.A0A098DBF8 pp 229533.I1RJZ7
71932 229533.I1RRJ6 pp 229533.Q4IB89
71933 229533.I1RRJ6 pp 229533.I1S183
71934 229533.I1RRJ6 pp 229533.Q4HTT2
71935 229533.I1RRJ6 pp 229533.I1S4P6
71936 229533.I1RRJ6 pp 229533.Q4INS6
71937 229533.I1RRJ6 pp 229533.I1RWM6
71938 229533.I1RRJ6 pp 229533.I1RY58
71939 229533.I1RRJ6 pp 229533.I1RYQ1
71940 229533.I1RRJ6 pp 229533.I1RSC7
71941 229533.I1RRJ6 pp 229533.I1RYB1
71942 229533.I1RRJ6 pp 229533.I1RUF8
71943 229533.I1RRJ6 pp 229533.I1RZK9
71944 229533.I1RRJ6 pp 229533.I1RXZ4
71945 229533.I1RRJ6 pp 229533.I1S209
71946 229533.I1RRJ6 pp 229533.Q4IPN9
71947 229533.I1RRJ6 pp 229533.I1S004
71948 229533.I1RRJ6 pp 229533.I1RX64
71949 229533.I1RRJ6 pp 229533.I1S0D5
71950 229533.I1RRJ6 pp 229533.I1RW15
71951 229533.I1RRJ6 pp 229533.I1RZ86
71952 229533.I1RRJ6 pp 229533.I1SAD2
71953 229533.I1RRJ6 pp 229533.I1S214
71954 229533.I1RRJ6 pp 229533.I1S9G7
71955 229533.I1RRJ6 pp 229533.I1RRT1
71956 229533.I1RRJ6 pp 229533.Q4HTT1
71957 229533.I1RRJ6 pp 229533.I1S987
71958 229533.I1RRJ6 pp 229533.I1RVV5
71959 229533.I1RRJ6 pp 229533.I1RY29
71960 229533.I1RRJ6 pp 229533.I1RSJ1
71961 229533.I1RRJ6 pp 229533.I1RWJ1
71962 229533.I1RRJ6 pp 229533.I1RWI8
71963 229533.I1RRJ6 pp 229533.I1RUR0
71964 229533.I1RRJ6 pp 229533.I1S788
71965 229533.I1RRJ6 pp 229533.I1S998
71966 229533.I1RWX1 pp 229533.I1SA55
71967 229533.I1RWX1 pp 229533.I1S0C2
71968 229533.I1RWX1 pp 229533.Q4HZQ1
71969 229533.A0A098DQJ3 pp 229533.A0A098DXB1
71970 229533.A0A098DQJ3 pp 229533.Q4IA56
71971 229533.A0A098DQJ3 pp 229533.I1RIS2
71972 229533.A0A098DQJ3 pp 229533.I1RCJ6
71973 229533.A0A098DQJ3 pp 229533.I1RRF5
71974 229533.A0A098DQJ3 pp 229533.Q4I7L0
71975 229533.A0A098DQJ3 pp 229533.A0A1C3YIP7
71976 229533.A0A098DQJ3 pp 229533.A0A1C3YHH5
71977 229533.I1RRC6 pp 229533.I1RXF0
71978 229533.I1RRC6 pp 229533.V6QUV7

71979 229533.I1RRC6 pp 229533.I1S235
71980 229533.I1RRC6 pp 229533.I1RYL5
71981 229533.I1RRC6 pp 229533.I1RT00
71982 229533.I1RRC6 pp 229533.I1RZF7
71983 229533.I1RRC6 pp 229533.I1RV63
71984 229533.I1RRC6 pp 229533.I1RY40
71985 229533.I1RRC6 pp 229533.I1RZK1
71986 229533.I1RRC6 pp 229533.Q4I7T5
71987 229533.I1RRC6 pp 229533.I1RT62
71988 229533.I1RRC6 pp 229533.I1RZM0
71989 229533.I1RRC6 pp 229533.I1RX25
71990 229533.Q4I5W5 pp 229533.Q4IER8
71991 229533.Q4I5W5 pp 229533.V6QUE6
71992 229533.Q4I5W5 pp 229533.Q4IJ11
71993 229533.Q4I5W5 pp 229533.Q4I7S1
71994 229533.Q4I5W5 pp 229533.Q4IPC8
71995 229533.Q4I5W5 pp 229533.Q4IJU0
71996 229533.Q4I5W5 pp 229533.V6QWX8
71997 229533.Q4I5W5 pp 229533.Q4IQX3
71998 229533.Q4I5W5 pp 229533.Q4IPN9
71999 229533.Q4I5W5 pp 229533.Q4IMD1
72000 229533.Q4I5W5 pp 229533.V6QV38
72001 229533.Q4I5W5 pp 229533.Q4IB50
72002 229533.A0A1C3YIJ7 pp 229533.I1RQV8
72003 229533.A0A1C3YIJ7 pp 229533.I1RL81
72004 229533.A0A1C3YIJ7 pp 229533.I1RSA5
72005 229533.A0A1C3YIJ7 pp 229533.I1R9F2
72006 229533.A0A1C3YIJ7 pp 229533.I1S968
72007 229533.A0A1C3YIJ7 pp 229533.I1RYU6
72008 229533.A0A1C3YIJ7 pp 229533.I1RCU2
72009 229533.A0A1C3YIJ7 pp 229533.I1RHT5
72010 229533.A0A1C3YIJ7 pp 229533.I1RFB0
72011 229533.A0A1C3YIJ7 pp 229533.V6RCJ5
72012 229533.A0A1C3YIJ7 pp 229533.I1RKB2
72013 229533.A0A1C3YIJ7 pp 229533.I1RRS6
72014 229533.A0A1C3YIJ7 pp 229533.I1RIJ6
72015 229533.A0A1C3YIJ7 pp 229533.I1S969
72016 229533.A0A1C3YIJ7 pp 229533.V6R5T7
72017 229533.A0A1C3YIJ7 pp 229533.I1RY35
72018 229533.A0A1C3YIJ7 pp 229533.V6RCC0
72019 229533.A0A1C3YIJ7 pp 229533.I1RHH5
72020 229533.A0A1C3YIJ7 pp 229533.I1RW54
72021 229533.A0A1C3YIJ7 pp 229533.I1RYP5
72022 229533.A0A1C3YIJ7 pp 229533.I1RGU3
72023 229533.A0A1C3YIJ7 pp 229533.I1RWL3
72024 229533.A0A1C3YIJ7 pp 229533.I1RQV7
72025 229533.A0A1C3YIJ7 pp 229533.I1RK55
72026 229533.A0A1C3YIJ7 pp 229533.A0A1C3YK51
72027 229533.A0A1C3YIJ7 pp 229533.I1RMA2
72028 229533.A0A1C3YIJ7 pp 229533.I1RST9
72029 229533.A0A1C3YIJ7 pp 229533.I1RNM7
72030 229533.A0A1C3YIJ7 pp 229533.I1RW55
72031 229533.A0A1C3YIJ7 pp 229533.A0A1C3YIJ9
72032 229533.A0A1C3YIJ7 pp 229533.I1RYC8
72033 229533.A0A1C3YIJ7 pp 229533.I1RJS1
72034 229533.A0A1C3YIJ7 pp 229533.I1S8D3
72035 229533.A0A1C3YIJ7 pp 229533.I1RF85
72036 229533.A0A1C3YIJ7 pp 229533.I1RPM6
72037 229533.A0A1C3YIJ7 pp 229533.I1S8F8
72038 229533.A0A1C3YIJ7 pp 229533.I1RPV8
72039 229533.A0A1C3YIJ7 pp 229533.I1RQJ7
72040 229533.A0A1C3YIJ7 pp 229533.V6RHX2
72041 229533.A0A1C3YIJ7 pp 229533.I1RAV0
72042 229533.A0A1C3YIJ7 pp 229533.I1RXE6
72043 229533.A0A1C3YIJ7 pp 229533.I1S6V0
72044 229533.A0A1C3YIJ7 pp 229533.I1RYA0
72045 229533.A0A1C3YIJ7 pp 229533.I1S2C4
72046 229533.A0A1C3YIJ7 pp 229533.I1RE52
72047 229533.A0A1C3YIJ7 pp 229533.I1RIM7
72048 229533.A0A1C3YIJ7 pp 229533.I1RB05
72049 229533.A0A1C3YIJ7 pp 229533.I1RVB1
72050 229533.A0A1C3YIJ7 pp 229533.I1RG33
72051 229533.A0A1C3YIJ7 pp 229533.V6RWB3

72052 229533.A0A1C3YIJ7 pp 229533.I1RY81
72053 229533.A0A1C3YIP7 pp 229533.Q4IA56
72054 229533.A0A1C3YIP7 pp 229533.I1RS37
72055 229533.A0A1C3YIP7 pp 229533.I1RIS2
72056 229533.A0A1C3YIP7 pp 229533.I1RA31
72057 229533.A0A1C3YIP7 pp 229533.I1RAW4
72058 229533.A0A1C3YIP7 pp 229533.I1S537
72059 229533.A0A1C3YIP7 pp 229533.Q4I7L0
72060 229533.A0A1C3YIP7 pp 229533.I1RRF5
72061 229533.I1RW93 pp 229533.I1S9Z3
72062 229533.I1RQV8 pp 229533.I1S968
72063 229533.I1RQV8 pp 229533.I1RZ38
72064 229533.I1RQV8 pp 229533.I1RY32
72065 229533.I1RQV8 pp 229533.I1RZ25
72066 229533.I1RQV8 pp 229533.I1RR68
72067 229533.I1RQV8 pp 229533.I1S0A5
72068 229533.I1RQV8 pp 229533.I1RYI5
72069 229533.I1RQV8 pp 229533.I1RTA1
72070 229533.I1RQV8 pp 229533.I1RSA5
72071 229533.I1RQV8 pp 229533.I1RZC3
72072 229533.I1RQV8 pp 229533.I1RVZ2
72073 229533.I1RQV8 pp 229533.V6RCJ5
72074 229533.I1RQV8 pp 229533.I1SAB4
72075 229533.I1RQV8 pp 229533.I1S9Z4
72076 229533.I1RQV8 pp 229533.I1RRI1
72077 229533.I1RQV8 pp 229533.I1SAT2
72078 229533.I1RQV8 pp 229533.I1S2C4
72079 229533.I1RQV8 pp 229533.I1RY35
72080 229533.I1RQV8 pp 229533.I1RZ56
72081 229533.I1RQV8 pp 229533.I1RRS6
72082 229533.I1RQV8 pp 229533.I1RZX3
72083 229533.I1RQV8 pp 229533.I1S969
72084 229533.I1RQV8 pp 229533.I1S094
72085 229533.I1RQV8 pp 229533.I1RYU6
72086 229533.I1RQV8 pp 229533.I1S4S7
72087 229533.I1RQV8 pp 229533.I1RW54
72088 229533.I1RQV8 pp 229533.I1S048
72089 229533.I1RQV8 pp 229533.I1S6H3
72090 229533.I1RQV8 pp 229533.I1S210
72091 229533.I1RQV8 pp 229533.I1RYP5
72092 229533.I1RQV8 pp 229533.I1RWL3
72093 229533.I1RQV8 pp 229533.I1S223
72094 229533.I1RQV8 pp 229533.I1S5D0
72095 229533.I1RQV8 pp 229533.I1RW55
72096 229533.I1RQV8 pp 229533.I1RWM4
72097 229533.I1RQV8 pp 229533.I1S4I7
72098 229533.I1RQV8 pp 229533.I1RUY5
72099 229533.I1RQV8 pp 229533.I1S267
72100 229533.I1RQV8 pp 229533.I1RST9
72101 229533.I1RQV8 pp 229533.I1RWC1
72102 229533.I1RQV8 pp 229533.I1S3T6
72103 229533.I1RQV8 pp 229533.I1RSG0
72104 229533.I1RQV8 pp 229533.I1S2F7
72105 229533.I1RQV8 pp 229533.V6RWB3
72106 229533.I1RQV8 pp 229533.V6RHX2
72107 229533.I1RQV8 pp 229533.I1RY81
72108 229533.I1RQV8 pp 229533.I1S659
72109 229533.I1RA84 pp 229533.I1RCU3
72110 229533.I1RA84 pp 229533.I1RS66
72111 229533.I1RA84 pp 229533.I1RUG0
72112 229533.I1RA84 pp 229533.Q4IJM4
72113 229533.I1RA84 pp 229533.I1RW90
72114 229533.I1RA84 pp 229533.I1RNI4
72115 229533.I1RA84 pp 229533.I1RQ42
72116 229533.I1RA84 pp 229533.I1RVJ1
72117 229533.I1RA84 pp 229533.I1RQA9
72118 229533.I1RA84 pp 229533.I1RW91
72119 229533.I1RA84 pp 229533.I1RE31
72120 229533.I1RA84 pp 229533.Q4I948
72121 229533.I1RA84 pp 229533.I1RCQ6
72122 229533.I1RA84 pp 229533.V6QV38
72123 229533.I1RA84 pp 229533.V6QWX8
72124 229533.I1RA84 pp 229533.I1RPW8

72125	229533.I1RA84	pp	229533.Q4IR09
72126	229533.I1RA84	pp	229533.I1RC15
72127	229533.I1RA84	pp	229533.I1S182
72128	229533.I1RA84	pp	229533.I1RW69
72129	229533.I1RA84	pp	229533.I1S0N8
72130	229533.I1RA84	pp	229533.I1S5Z2
72131	229533.I1RA84	pp	229533.I1RZR6
72132	229533.I1RA84	pp	229533.I1RTC2
72133	229533.I1RA84	pp	229533.I1RRT0
72134	229533.I1RA84	pp	229533.I1RMQ7
72135	229533.I1RA84	pp	229533.I1RDC9
72136	229533.I1RA84	pp	229533.I1RBW4
72137	229533.I1RA84	pp	229533.Q4IER8
72138	229533.I1RA84	pp	229533.Q4HYQ4
72139	229533.I1RA84	pp	229533.Q4I5R3
72140	229533.I1RA84	pp	229533.I1RRG3
72141	229533.I1RA84	pp	229533.I1RFI5
72142	229533.I1RA84	pp	229533.I1RAR2
72143	229533.I1RA84	pp	229533.I1RZB5
72144	229533.I1RA84	pp	229533.I1RT16
72145	229533.I1RA84	pp	229533.I1RKB1
72146	229533.I1RA84	pp	229533.I1RJL2
72147	229533.I1RA84	pp	229533.V6QUE6
72148	229533.I1RA84	pp	229533.I1RN07
72149	229533.I1RA84	pp	229533.I1RYY5
72150	229533.I1RA84	pp	229533.I1RP96
72151	229533.I1RA84	pp	229533.I1RML9
72152	229533.I1RA84	pp	229533.I1RAT7
72153	229533.I1RA84	pp	229533.Q4I5I4
72154	229533.I1RA84	pp	229533.I1RAE0
72155	229533.I1RA84	pp	229533.I1RT35
72156	229533.I1RA84	pp	229533.I1RC16
72157	229533.I1RA84	pp	229533.I1RVZ4
72158	229533.I1RA84	pp	229533.I1RT01
72159	229533.I1RA84	pp	229533.I1REN3
72160	229533.I1RA84	pp	229533.Q4HYI0
72161	229533.I1RA84	pp	229533.I1RFQ5
72162	229533.I1RA84	pp	229533.Q4IJU0
72163	229533.I1RA84	pp	229533.I1RSI1
72164	229533.I1RA84	pp	229533.Q4HTT2
72165	229533.I1RA84	pp	229533.I1RCG6
72166	229533.I1RA84	pp	229533.I1RXE5
72167	229533.I1RA84	pp	229533.I1RR25
72168	229533.I1RA84	pp	229533.I1RG90
72169	229533.I1RA84	pp	229533.I1RE14
72170	229533.I1RA84	pp	229533.I1RWI0
72171	229533.I1RA84	pp	229533.I1RNF4
72172	229533.I1RA84	pp	229533.V6R0U9
72173	229533.I1RA84	pp	229533.I1RS43
72174	229533.I1RA84	pp	229533.I1RBR5
72175	229533.I1RA84	pp	229533.I1S0E8
72176	229533.I1RA84	pp	229533.I1RVY0
72177	229533.I1RA84	pp	229533.I1RJL3
72178	229533.I1RA84	pp	229533.I1RE30
72179	229533.I1RA84	pp	229533.I1RFL7
72180	229533.I1RA84	pp	229533.I1RMV1
72181	229533.I1RA84	pp	229533.Q4HW67
72182	229533.I1RA84	pp	229533.I1S276
72183	229533.I1RA84	pp	229533.Q4HTT1
72184	229533.I1RA84	pp	229533.I1RZU4
72185	229533.I1RA84	pp	229533.I1RN04
72186	229533.I1RA84	pp	229533.V6RDZ4
72187	229533.I1RA84	pp	229533.I1RFW1
72188	229533.I1RA84	pp	229533.I1RBC7
72189	229533.I1RA84	pp	229533.I1RZM2
72190	229533.I1RA84	pp	229533.I1RVL8
72191	229533.I1RA84	pp	229533.I1RSW9
72192	229533.I1RA84	pp	229533.I1RS41
72193	229533.I1RA84	pp	229533.I1RTH2
72194	229533.I1RA84	pp	229533.I1RPM7
72195	229533.I1RA84	pp	229533.I1S0J3
72196	229533.I1RA84	pp	229533.I1RK71
72197	229533.I1RA84	pp	229533.I1RC24

72198 229533.I1RA84 pp 229533.I1RK79
72199 229533.I1RA84 pp 229533.I1RK90
72200 229533.I1RA84 pp 229533.Q4IMD1
72201 229533.I1RA84 pp 229533.Q4I5W5
72202 229533.I1RA84 pp 229533.Q4IB50
72203 229533.I1RA84 pp 229533.I1RW80
72204 229533.I1RA84 pp 229533.I1RKR8
72205 229533.I1RA84 pp 229533.I1REF5
72206 229533.I1RA84 pp 229533.Q4HYB8
72207 229533.I1RA84 pp 229533.I1RWF3
72208 229533.I1RA84 pp 229533.Q4IJ11
72209 229533.I1RA84 pp 229533.Q4I5U9
72210 229533.I1RA84 pp 229533.Q4IQT8
72211 229533.I1RA84 pp 229533.I1RPU7
72212 229533.I1RA84 pp 229533.I1RSA4
72213 229533.I1RA84 pp 229533.I1RRF7
72214 229533.I1RA84 pp 229533.I1RJW8
72215 229533.I1RA84 pp 229533.I1RSZ3
72216 229533.I1RA84 pp 229533.I1RQ88
72217 229533.I1RA84 pp 229533.Q4IPC8
72218 229533.I1RA84 pp 229533.I1RZ42
72219 229533.I1RA84 pp 229533.I1RG11
72220 229533.I1RA84 pp 229533.I1RQ72
72221 229533.I1RA84 pp 229533.I1RNB8
72222 229533.I1RA84 pp 229533.I1RNQ6
72223 229533.I1RA84 pp 229533.I1RXU0
72224 229533.I1RA84 pp 229533.I1RCB7
72225 229533.I1RIS2 pp 229533.I1RRF5
72226 229533.I1RIS2 pp 229533.Q4IA56
72227 229533.I1RIS2 pp 229533.Q4I7L0
72228 229533.Q4I5V3 pp 229533.Q4IPI4
72229 229533.Q4I5V3 pp 229533.Q4IAK7
72230 229533.Q4I5V3 pp 229533.Q4I7L0
72231 229533.Q4I5V3 pp 229533.Q4IEV4
72232 229533.Q4I5V3 pp 229533.Q4ICA8
72233 229533.Q4I5V3 pp 229533.Q4IB96
72234 229533.Q4I5V3 pp 229533.Q4I7S1
72235 229533.Q4I5V3 pp 229533.Q4IMD1
72236 229533.Q4I5V3 pp 229533.Q4I948
72237 229533.A0A098DXB1 pp 229533.I1RK49
72238 229533.A0A098DXB1 pp 229533.I1RRC4
72239 229533.A0A098DXB1 pp 229533.I1RED2
72240 229533.A0A098DXB1 pp 229533.A0A0E0SHL3
72241 229533.A0A098DXB1 pp 229533.I1REI2
72242 229533.A0A098DXB1 pp 229533.I1RIS2
72243 229533.A0A098DXB1 pp 229533.A0A0E0RQT8
72244 229533.A0A098DXB1 pp 229533.A0A1C3YJ07
72245 229533.A0A098DXB1 pp 229533.I1S368
72246 229533.A0A098DXB1 pp 229533.A0A0E0SIE3
72247 229533.A0A098DXB1 pp 229533.I1RFY0
72248 229533.A0A098DXB1 pp 229533.Q4IA56
72249 229533.A0A098DXB1 pp 229533.I1RW69
72250 229533.A0A098DXB1 pp 229533.I1RDZ5
72251 229533.A0A098DXB1 pp 229533.I1RQM4
72252 229533.A0A098DXB1 pp 229533.I1RTP2
72253 229533.A0A098DXB1 pp 229533.A0A1C3YHY5
72254 229533.A0A098DXB1 pp 229533.I1S2B0
72255 229533.A0A098DXB1 pp 229533.A0A1C3YLQ4
72256 229533.A0A098DXB1 pp 229533.I1S2L1
72257 229533.A0A098DXB1 pp 229533.A0A1C3YIP7
72258 229533.A0A098DXB1 pp 229533.A0A1C3YHH5
72259 229533.A0A098DXB1 pp 229533.A0A0E0RSR0
72260 229533.A0A098DXB1 pp 229533.A0A1C3YIX3
72261 229533.A0A098DXB1 pp 229533.A0A1C3YMF6
72262 229533.A0A098DXB1 pp 229533.I1RWN0
72263 229533.A0A098DXB1 pp 229533.A0A1C3YJJ7
72264 229533.A0A098DXB1 pp 229533.Q4I7L0
72265 229533.A0A098DXB1 pp 229533.I1REB5
72266 229533.A0A098DXB1 pp 229533.I1RE88
72267 229533.A0A098DXB1 pp 229533.I1S360
72268 229533.A0A098DXB1 pp 229533.I1RRF5
72269 229533.A0A098DXB1 pp 229533.I1RCN6
72270 229533.I1RCN2 pp 229533.I1RSL6

72271	229533.I1RCN2	pp	229533.I1RY99
72272	229533.I1RCN2	pp	229533.I1RWD0
72273	229533.I1RCN2	pp	229533.I1RX64
72274	229533.I1RCN2	pp	229533.I1RXI8
72275	229533.I1RCN2	pp	229533.I1RGR2
72276	229533.I1RCN2	pp	229533.I1RZT1
72277	229533.I1RCN2	pp	229533.Q4IEV4
72278	229533.I1RCN2	pp	229533.I1S1Y7
72279	229533.I1RCN2	pp	229533.I1RHP9
72280	229533.I1RCN2	pp	229533.I1RYW3
72281	229533.I1RCN2	pp	229533.I1S247
72282	229533.I1RCN2	pp	229533.I1REC7
72283	229533.I1RCN2	pp	229533.I1RQ88
72284	229533.I1RCN2	pp	229533.I1RL41
72285	229533.I1RCN2	pp	229533.I1S1U7
72286	229533.I1RCN2	pp	229533.I1RW28
72287	229533.I1RCN2	pp	229533.I1S0N5
72288	229533.I1RCN2	pp	229533.I1RRM8
72289	229533.I1RCN2	pp	229533.I1RFW1
72290	229533.I1RCN2	pp	229533.I1S109
72291	229533.I1RCN2	pp	229533.I1S3D7
72292	229533.I1RCN2	pp	229533.Q4IMD1
72293	229533.I1RCN2	pp	229533.Q4IB50
72294	229533.I1RCN2	pp	229533.I1SAN0
72295	229533.I1RCN2	pp	229533.Q4I7L0
72296	229533.I1RCN2	pp	229533.I1SAA0
72297	229533.I1RCN2	pp	229533.I1RZA9
72298	229533.I1RCN2	pp	229533.I1RPR8
72299	229533.I1RCN2	pp	229533.I1RY77
72300	229533.I1RCN2	pp	229533.I1SB04
72301	229533.I1RCN2	pp	229533.I1S0J3
72302	229533.I1RCN2	pp	229533.I1RY90
72303	229533.I1RCN2	pp	229533.Q4HTT1
72304	229533.I1RCN2	pp	229533.I1REC6
72305	229533.I1RCN2	pp	229533.I1RPY4
72306	229533.I1RCN2	pp	229533.I1RIW5
72307	229533.I1RCN2	pp	229533.Q4HTT2
72308	229533.I1RCN2	pp	229533.I1RVY0
72309	229533.I1RCN2	pp	229533.I1RQ93
72310	229533.I1RCN2	pp	229533.I1S0F0
72311	229533.I1RCN2	pp	229533.Q4I6A4
72312	229533.I1RCN2	pp	229533.I1RU02
72313	229533.I1RCN2	pp	229533.I1S2B6
72314	229533.I1RCN2	pp	229533.Q4I5R3
72315	229533.I1RCN2	pp	229533.I1RPU1
72316	229533.I1RCN2	pp	229533.I1RYX3
72317	229533.I1RCN2	pp	229533.I1RUZ8
72318	229533.I1RCN2	pp	229533.I1RSH4
72319	229533.I1RCN2	pp	229533.Q4IER8
72320	229533.I1RCN2	pp	229533.I1S5T5
72321	229533.I1RCN2	pp	229533.Q4I061
72322	229533.I1RCN2	pp	229533.I1RE37
72323	229533.I1RCN2	pp	229533.I1RCW8
72324	229533.I1RCN2	pp	229533.I1RFQ5
72325	229533.I1RCN2	pp	229533.I1S364
72326	229533.I1RCN2	pp	229533.I1RPJ1
72327	229533.I1RCN2	pp	229533.I1RT16
72328	229533.I1RCN2	pp	229533.I1RWB3
72329	229533.I1RCN2	pp	229533.I1RYN7
72330	229533.I1RCN2	pp	229533.Q4ICA8
72331	229533.I1RCN2	pp	229533.I1S2M5
72332	229533.I1RCN2	pp	229533.I1RSI1
72333	229533.I1RCN2	pp	229533.I1RW60
72334	229533.I1RCN2	pp	229533.I1RY98
72335	229533.I1RCN2	pp	229533.I1RQL4
72336	229533.I1RCN2	pp	229533.I1RYV4
72337	229533.I1RCN2	pp	229533.I1S1V2
72338	229533.I1RCN2	pp	229533.I1RMS5
72339	229533.I1RCN2	pp	229533.Q4IPI1
72340	229533.I1RCN2	pp	229533.I1RNT2
72341	229533.I1RCN2	pp	229533.I1RJX2
72342	229533.I1RCN2	pp	229533.I1RJR2
72343	229533.I1RCN2	pp	229533.I1RDP5

72344 229533.I1RCN2 pp 229533.Q4IQX3
72345 229533.I1RCN2 pp 229533.I1RFQ0
72346 229533.I1RCN2 pp 229533.I1S724
72347 229533.I1RCN2 pp 229533.I1RPN4
72348 229533.I1RCN2 pp 229533.I1RPF4
72349 229533.A0A098DBF3 pp 229533.A0A098DQJ3
72350 229533.A0A098DBF3 pp 229533.Q4I7L0
72351 229533.A0A098DBF3 pp 229533.Q4IA56
72352 229533.A0A098DBF3 pp 229533.A0A1C3YIP7
72353 229533.A0A098DBF3 pp 229533.I1RIS2
72354 229533.A0A098DBF3 pp 229533.A0A1C3YHH5
72355 229533.A0A098DBF3 pp 229533.A0A098DXB1
72356 229533.I1RQ41 pp 229533.I1S235
72357 229533.I1RQ41 pp 229533.I1RYL5
72358 229533.I1RQ41 pp 229533.I1RYI7
72359 229533.I1RQ41 pp 229533.I1RXF0
72360 229533.I1RQ41 pp 229533.I1RZF7
72361 229533.I1RQ41 pp 229533.V6QUV7
72362 229533.I1RQ41 pp 229533.I1RQC4
72363 229533.I1RQ41 pp 229533.I1RQN8
72364 229533.I1RQ41 pp 229533.I1RTF5
72365 229533.I1RQ41 pp 229533.I1RZM0
72366 229533.I1RQ41 pp 229533.I1S1W5
72367 229533.I1RQ41 pp 229533.I1RRC6
72368 229533.I1RQ41 pp 229533.I1RX25
72369 229533.A0A098DBB2 pp 229533.A0A098DRI1
72370 229533.A0A098DBB2 pp 229533.A0A1C3YL16
72371 229533.A0A0E0RMZ6 pp 229533.I1S095
72372 229533.A0A0E0RMZ6 pp 229533.I1RZV4
72373 229533.A0A0E0RMZ6 pp 229533.I1RB15
72374 229533.A0A0E0RMZ6 pp 229533.A0A1C3YLH7
72375 229533.A0A0E0RMZ6 pp 229533.I1S1C8
72376 229533.A0A0E0RMZ6 pp 229533.I1RZD9
72377 229533.A0A0E0RMZ6 pp 229533.I1RSB0
72378 229533.A0A0E0RMZ6 pp 229533.I1R991
72379 229533.A0A0E0RMZ6 pp 229533.A0A1C3YMD9
72380 229533.A0A0E0RMZ6 pp 229533.I1RTG9
72381 229533.I1RVA7 pp 229533.V6R3J1
72382 229533.I1RVA7 pp 229533.I1RW82
72383 229533.I1RVA7 pp 229533.I1RWZ9
72384 229533.I1RVA7 pp 229533.I1S287
72385 229533.I1RVA7 pp 229533.I1RX96
72386 229533.I1RVA7 pp 229533.Q4HWB3
72387 229533.I1RVA7 pp 229533.I1RZW7
72388 229533.I1RVA7 pp 229533.I1RYJ8
72389 229533.I1RVA7 pp 229533.V6QYF0
72390 229533.I1RVA7 pp 229533.I1RYM2
72391 229533.I1RVA7 pp 229533.Q4IER8
72392 229533.I1RVA7 pp 229533.Q4HTT2
72393 229533.I1RVA7 pp 229533.I1RW24
72394 229533.I1RVA7 pp 229533.V6RLW4
72395 229533.I1RVA7 pp 229533.I1S2A8
72396 229533.I1RVA7 pp 229533.Q4HTT1
72397 229533.I1RVA7 pp 229533.I1S549
72398 229533.I1RVA7 pp 229533.I1RW16
72399 229533.I1RVA7 pp 229533.Q4I9U7
72400 229533.I1RVA7 pp 229533.Q4HYB8
72401 229533.I1RVA7 pp 229533.I1RXT9
72402 229533.I1RVA7 pp 229533.I1RYF8
72403 229533.I1RVA7 pp 229533.I1RZT1
72404 229533.I1RVA7 pp 229533.I1S2F2
72405 229533.A0A1C3YJ00 pp 229533.A0A1C3YJS4
72406 229533.A0A1C3YJ00 pp 229533.I1S130
72407 229533.A0A1C3YJ00 pp 229533.A0A1C3YKK4
72408 229533.A0A1C3YJ00 pp 229533.A0A1C3YM33
72409 229533.A0A1C3YJ00 pp 229533.I1RVN0
72410 229533.A0A1C3YJ00 pp 229533.I1RCJ0
72411 229533.A0A1C3YJ00 pp 229533.I1RRN8
72412 229533.A0A1C3YJ00 pp 229533.I1S409
72413 229533.Q4I948 pp 229533.Q4IR09
72414 229533.Q4I948 pp 229533.Q4IQX3
72415 229533.Q4I948 pp 229533.Q4IEV4
72416 229533.Q4I948 pp 229533.Q4ICA8

229533.Q4I948 pp 229533.Q4IER8
229533.Q4I948 pp 229533.Q4IB96
229533.Q4I948 pp 229533.Q4IPI4
229533.Q4I948 pp 229533.Q4IMD1
229533.Q4I948 pp 229533.Q4IL82
229533.Q4I948 pp 229533.V6RLW4
229533.Q4I948 pp 229533.Q4IAK7
229533.I1RRS3 pp 229533.I1S409
229533.Q4IEV4 pp 229533.Q4IMD1
229533.Q4IEV4 pp 229533.Q4IJH1
229533.Q4IEV4 pp 229533.Q4IF76
229533.Q4IEV4 pp 229533.Q4IPI4
229533.I1RQJ7 pp 229533.I1RRS6
229533.I1RQJ7 pp 229533.I1S969
229533.I1RQJ7 pp 229533.V6RCC0
229533.I1RQJ7 pp 229533.I1RSA5
229533.I1RQJ7 pp 229533.I1RQV8
229533.I1RQJ7 pp 229533.I1RYP5
229533.I1RQJ7 pp 229533.V6R5T7
229533.I1RQJ7 pp 229533.I1RYU6
229533.I1RQJ7 pp 229533.I1RY35
229533.I1RQJ7 pp 229533.I1RWL3
229533.I1RQJ7 pp 229533.I1RQV7
229533.I1RQJ7 pp 229533.I1RW54
229533.I1RQJ7 pp 229533.V6RCJ5
229533.I1RQJ7 pp 229533.I1S968
229533.I1RQJ7 pp 229533.I1S8D3
229533.I1RQJ7 pp 229533.I1S2C4
229533.I1RQJ7 pp 229533.I1S8F8
229533.I1RQJ7 pp 229533.I1RXE6
229533.I1RQJ7 pp 229533.I1RYC8
229533.I1RQJ7 pp 229533.I1RW55
229533.I1RQJ7 pp 229533.V6RHX2
229533.I1RQJ7 pp 229533.I1S6V0
229533.I1RQJ7 pp 229533.I1RVB1
229533.I1RQJ7 pp 229533.I1RY81
229533.I1RQJ7 pp 229533.I1RST9
229533.I1RQJ7 pp 229533.V6RWB3
229533.I1RQJ7 pp 229533.I1RYA0
229533.I1S2I7 pp 229533.I1S303
229533.I1S2I7 pp 229533.Q4IMJ1
229533.A0A1C3YIJ1 pp 229533.I1RPW0
229533.A0A1C3YIJ1 pp 229533.I1RAE8
229533.A0A1C3YIJ1 pp 229533.I1RD45
229533.A0A1C3YIJ1 pp 229533.I1RWP9
229533.A0A1C3YIJ1 pp 229533.I1RS69
229533.A0A1C3YIJ1 pp 229533.I1RFK0
229533.A0A1C3YIJ1 pp 229533.I1RE78
229533.A0A1C3YIJ1 pp 229533.I1S287
229533.A0A1C3YIJ1 pp 229533.A0A1C3YJH6
229533.A0A1C3YIJ1 pp 229533.I1RMK1
229533.A0A1C3YIJ1 pp 229533.A0A1C3YKT4
229533.I1RCH8 pp 229533.I1S409
229533.I1RCH8 pp 229533.I1RCJ0
229533.I1RCH8 pp 229533.I1RRN8
229533.I1RCH8 pp 229533.I1S130
229533.I1RCH8 pp 229533.I1RVN0
229533.I1RCL7 pp 229533.Q4IBX0
229533.I1RCL7 pp 229533.I1RRP7
229533.I1RCL7 pp 229533.I1RDX0
229533.I1RCL7 pp 229533.I1RU02
229533.I1RCL7 pp 229533.I1RFJ2
229533.I1RCL7 pp 229533.I1RUG2
229533.I1RCL7 pp 229533.I1RG77
229533.I1RCL7 pp 229533.I1RN08
229533.I1RCL7 pp 229533.I1RZP8
229533.I1RCL7 pp 229533.I1RN55
229533.I1RCL7 pp 229533.Q4IER8
229533.I1RCL7 pp 229533.I1S209
229533.I1RCL7 pp 229533.I1RCM6
229533.I1RCL7 pp 229533.I1RQA3
229533.I1RCL7 pp 229533.I1RQJ4
229533.I1RCL7 pp 229533.V6RGI1

229533.I1RCL7	pp	229533.I1RX64
229533.I1RCL7	pp	229533.I1RYJ8
229533.I1RCL7	pp	229533.I1S1W4
229533.I1RCL7	pp	229533.V6RVG3
229533.I1RCL7	pp	229533.I1RSP7
229533.I1RCL7	pp	229533.I1RD19
229533.I1RCL7	pp	229533.I1S439
229533.I1RCL7	pp	229533.I1RRS7
229533.I1RCL7	pp	229533.I1RN46
229533.I1RCL7	pp	229533.Q4I0J6
229533.I1RCL7	pp	229533.I1RMX3
229533.I1RCL7	pp	229533.I1RY44
229533.I1RCL7	pp	229533.Q4I6A4
229533.I1RCL7	pp	229533.I1S461
229533.I1RCL7	pp	229533.Q4HTT2
229533.I1RCL7	pp	229533.I1S020
229533.I1RCL7	pp	229533.I1RDB1
229533.I1RCL7	pp	229533.I1RN31
229533.I1RCL7	pp	229533.I1RSC7
229533.I1RCL7	pp	229533.I1RRJ6
229533.I1RCL7	pp	229533.I1RF70
229533.I1RCL7	pp	229533.V6R382
229533.I1RCL7	pp	229533.I1RSA7
229533.I1RCL7	pp	229533.I1RW16
229533.I1RCL7	pp	229533.I1RY73
229533.I1RCL7	pp	229533.I1S604
229533.I1RCL7	pp	229533.I1RMB7
229533.I1RCL7	pp	229533.I1RYH9
229533.I1RCL7	pp	229533.I1RMS4
229533.I1RCL7	pp	229533.I1RWT6
229533.I1RCL7	pp	229533.I1RE46
229533.I1RCL7	pp	229533.I1RCY2
229533.I1RCL7	pp	229533.I1RQ81
229533.I1RCL7	pp	229533.I1RCW8
229533.I1RCL7	pp	229533.V6RLW4
229533.I1RCL7	pp	229533.Q4IMD1
229533.I1RCL7	pp	229533.I1RPZ0
229533.I1RCL7	pp	229533.I1RM36
229533.I1RCL7	pp	229533.I1RFX8
229533.I1RCL7	pp	229533.I1RZ94
229533.I1RCL7	pp	229533.I1RN57
229533.I1RCL7	pp	229533.I1RQ67
229533.I1RCL7	pp	229533.I1RZ86
229533.I1RCL7	pp	229533.I1RKI2
229533.I1RCL7	pp	229533.I1S2E9
229533.I1RCL7	pp	229533.I1RSL8
229533.I1RCL7	pp	229533.I1RKK7
229533.I1RCL7	pp	229533.Q4I7L0
229533.I1RCL7	pp	229533.I1RSK0
229533.I1RCL7	pp	229533.I1RLX6
229533.I1RCL7	pp	229533.I1RQ13
229533.I1RCL7	pp	229533.I1RQ92
229533.I1RCL7	pp	229533.I1RFR6
229533.I1RCL7	pp	229533.I1RFZ2
229533.I1RCL7	pp	229533.Q4HTT1
229533.I1RCL7	pp	229533.I1S283
229533.I1RCL7	pp	229533.I1RYR2
229533.I1RCL7	pp	229533.I1RRT1
229533.I1RCL7	pp	229533.I1RST8
229533.I1RCL7	pp	229533.I1RUN8
229533.I1RCL7	pp	229533.Q4IJP1
229533.I1RCL7	pp	229533.I1RSN7
229533.I1RCL7	pp	229533.Q4IMM0
229533.I1RCL7	pp	229533.I1RKQ0
229533.I1RCL7	pp	229533.I1RZG7
229533.I1RCL7	pp	229533.I1RYX9
229533.I1RCL7	pp	229533.I1RYQ1
229533.I1RCL7	pp	229533.I1RFW1
229533.I1RCL7	pp	229533.Q4IP51
229533.I1RCL7	pp	229533.I1RSI4
229533.A0A098DBD1	pp	229533.A0A1C3YJ00
229533.A0A098DBD1	pp	229533.I1RRS3
229533.A0A098DBD1	pp	229533.A0A1C3YM33

72563	229533.A0A098DBD1	pp	229533.I1RCH8
72564	229533.A0A1C3YMC4	pp	229533.I1RZ81
72565	229533.A0A1C3YMC4	pp	229533.I1S8S9
72566	229533.A0A1C3YMC4	pp	229533.I1S458
72567	229533.A0A1C3YMC4	pp	229533.I1S1N7
72568	229533.A0A1C3YMC4	pp	229533.I1RN32
72569	229533.A0A1C3YMC4	pp	229533.I1RY92
72570	229533.A0A1C3YMC4	pp	229533.I1SAB4
72571	229533.A0A1C3YMC4	pp	229533.I1RFJ6
72572	229533.A0A1C3YMC4	pp	229533.I1RY21
72573	229533.A0A1C3YMC4	pp	229533.I1S3T6
72574	229533.A0A1C3YMC4	pp	229533.I1S5R5
72575	229533.A0A1C3YMC4	pp	229533.I1RX38
72576	229533.A0A1C3YMC4	pp	229533.I1RS05
72577	229533.A0A1C3YMC4	pp	229533.I1S0K2
72578	229533.A0A1C3YMC4	pp	229533.I1RU33
72579	229533.A0A1C3YMC4	pp	229533.I1S5X9
72580	229533.A0A1C3YMC4	pp	229533.I1RSC0
72581	229533.A0A1C3YMC4	pp	229533.I1RS29
72582	229533.A0A1C3YMC4	pp	229533.I1R9N8
72583	229533.A0A1C3YMC4	pp	229533.I1RE39
72584	229533.A0A1C3YMC4	pp	229533.I1RE65
72585	229533.A0A1C3YMC4	pp	229533.I1RMK7
72586	229533.A0A1C3YMC4	pp	229533.I1RP02
72587	229533.A0A1C3YMC4	pp	229533.I1S210
72588	229533.A0A1C3YMC4	pp	229533.I1RKF5
72589	229533.A0A1C3YMC4	pp	229533.I1RHT5
72590	229533.A0A1C3YMC4	pp	229533.I1RBB1
72591	229533.A0A1C3YMC4	pp	229533.I1RZ38
72592	229533.A0A1C3YMC4	pp	229533.I1RFB0
72593	229533.A0A1C3YMC4	pp	229533.I1RVT8
72594	229533.A0A1C3YMC4	pp	229533.A0A1C3YMS7
72595	229533.A0A1C3YMC4	pp	229533.I1RHJ0
72596	229533.A0A1C3YMC4	pp	229533.I1RWA3
72597	229533.A0A1C3YMC4	pp	229533.I1RIM7
72598	229533.A0A1C3YMC4	pp	229533.I1S2F7
72599	229533.A0A1C3YMC4	pp	229533.I1S223
72600	229533.A0A1C3YMC4	pp	229533.V6RPN0
72601	229533.A0A1C3YMC4	pp	229533.I1RYT8
72602	229533.A0A1C3YMC4	pp	229533.I1S0I9
72603	229533.A0A1C3YMC4	pp	229533.I1RA52
72604	229533.A0A1C3YMC4	pp	229533.V6QUG0
72605	229533.A0A1C3YMC4	pp	229533.I1S464
72606	229533.A0A1C3YMC4	pp	229533.I1RGU3
72607	229533.A0A1C3YMC4	pp	229533.I1S0P0
72608	229533.A0A1C3YMC4	pp	229533.I1RXD9
72609	229533.A0A1C3YMC4	pp	229533.I1RN42
72610	229533.A0A1C3YMC4	pp	229533.I1RE66
72611	229533.A0A1C3YMC4	pp	229533.I1RF24
72612	229533.A0A1C3YMC4	pp	229533.I1RW55
72613	229533.A0A1C3YMC4	pp	229533.V6RJP8
72614	229533.A0A1C3YMC4	pp	229533.I1RKF4
72615	229533.A0A1C3YJX7	pp	229533.I1RKG1
72616	229533.A0A098DKA5	pp	229533.I1S4P6
72617	229533.A0A098DKA5	pp	229533.I1RWM6
72618	229533.A0A098DKA5	pp	229533.Q4IB89
72619	229533.A0A098DKA5	pp	229533.I1RF02
72620	229533.A0A098DKA5	pp	229533.I1RFS6
72621	229533.A0A098DKA5	pp	229533.I1RRJ6
72622	229533.A0A098DKA5	pp	229533.I1RDJ8
72623	229533.A0A098DKA5	pp	229533.A0A098DX13
72624	229533.A0A098DKA5	pp	229533.I1RC54
72625	229533.A0A098DKA5	pp	229533.I1RD23
72626	229533.A0A098DKA5	pp	229533.I1RVW0
72627	229533.A0A098DKA5	pp	229533.A0A1C3YM91
72628	229533.A0A098DKA5	pp	229533.I1RBJ0
72629	229533.A0A098DKA5	pp	229533.I1RMJ8
72630	229533.A0A098DKA5	pp	229533.I1RRT1
72631	229533.A0A098DKA5	pp	229533.A0A1C3YI39
72632	229533.A0A098DKA5	pp	229533.I1RZT1
72633	229533.A0A098DKA5	pp	229533.I1RD01
72634	229533.A0A098DKA5	pp	229533.I1RBN9
72635	229533.A0A098DKA5	pp	229533.I1RXZ4

72636 229533.A0A098DKA5 pp 229533.Q4INS6
72637 229533.A0A098DKA5 pp 229533.Q4HTT1
72638 229533.A0A098DKA5 pp 229533.A0A1C3YJ79
72639 229533.A0A098DKA5 pp 229533.I1RYV3
72640 229533.A0A098DKA5 pp 229533.A0A1C3YLQ7
72641 229533.I1RXU3 pp 229533.I1S489
72642 229533.I1RXU3 pp 229533.I1RZ25
72643 229533.I1RNN7 pp 229533.I1RS07
72644 229533.I1RNN7 pp 229533.Q4IMJ1
72645 229533.A0A098DBA3 pp 229533.I1RXU3
72646 229533.I1RRT0 pp 229533.V6RIS2
72647 229533.I1RRT0 pp 229533.V6QUE6
72648 229533.I1RRT0 pp 229533.Q4IPC8
72649 229533.I1RRT0 pp 229533.Q4HTT2
72650 229533.I1RRT0 pp 229533.Q4I5I4
72651 229533.I1RRT0 pp 229533.I1S0C6
72652 229533.I1RRT0 pp 229533.Q4HYI0
72653 229533.I1RRT0 pp 229533.I1S8U9
72654 229533.I1RRT0 pp 229533.I1S3N1
72655 229533.I1RRT0 pp 229533.I1RXU0
72656 229533.I1RRT0 pp 229533.Q4I7S1
72657 229533.I1RRT0 pp 229533.I1RWX8
72658 229533.I1RRT0 pp 229533.I1S7M3
72659 229533.I1RRT0 pp 229533.I1RT01
72660 229533.I1RRT0 pp 229533.I1RSA4
72661 229533.I1RRT0 pp 229533.I1RWF3
72662 229533.I1RRT0 pp 229533.I1S1W4
72663 229533.I1RRT0 pp 229533.I1RXE5
72664 229533.I1RRT0 pp 229533.I1RWT6
72665 229533.I1RRT0 pp 229533.I1RZB5
72666 229533.I1RRT0 pp 229533.I1S255
72667 229533.I1RRT0 pp 229533.I1RS41
72668 229533.I1RRT0 pp 229533.I1RT99
72669 229533.I1RRT0 pp 229533.I1RY44
72670 229533.I1RRT0 pp 229533.Q4IER8
72671 229533.I1RRT0 pp 229533.I1RZ42
72672 229533.I1RRT0 pp 229533.I1RSI1
72673 229533.I1RRT0 pp 229533.I1RYS4
72674 229533.I1RRT0 pp 229533.Q4HYB8
72675 229533.I1RRT0 pp 229533.I1RW80
72676 229533.I1RRT0 pp 229533.I1RU02
72677 229533.I1RRT0 pp 229533.I1S8F0
72678 229533.I1RRT0 pp 229533.I1S2C4
72679 229533.I1RRT0 pp 229533.Q4IMD1
72680 229533.I1RRT0 pp 229533.Q4IQT8
72681 229533.I1RRT0 pp 229533.Q4I5W5
72682 229533.I1RRT0 pp 229533.I1RTD7
72683 229533.I1RRT0 pp 229533.I1RS28
72684 229533.I1RRT0 pp 229533.I1RUG4
72685 229533.I1RRT0 pp 229533.V6QV38
72686 229533.I1RRT0 pp 229533.I1RYY5
72687 229533.I1RRT0 pp 229533.I1S8V0
72688 229533.I1RRT0 pp 229533.Q4IQX3
72689 229533.I1RRT0 pp 229533.I1RZL5
72690 229533.I1RRT0 pp 229533.I1RWT1
72691 229533.I1RRT0 pp 229533.I1RZU4
72692 229533.I1RRT0 pp 229533.Q4IJU0
72693 229533.I1RRT0 pp 229533.I1S5S2
72694 229533.I1RRT0 pp 229533.V6R0U9
72695 229533.I1RRT0 pp 229533.I1RZU9
72696 229533.I1RRT0 pp 229533.I1S276
72697 229533.I1RRT0 pp 229533.I1S3N0
72698 229533.I1RRT0 pp 229533.Q4IJ11
72699 229533.I1RRT0 pp 229533.I1S2E9
72700 229533.I1RRT0 pp 229533.Q4IJM4
72701 229533.I1RRT0 pp 229533.I1RXF2
72702 229533.I1RRT0 pp 229533.I1S0B1
72703 229533.I1RRT0 pp 229533.I1S7M2
72704 229533.I1RRT0 pp 229533.I1S182
72705 229533.I1RRT0 pp 229533.I1RW23
72706 229533.I1RRT0 pp 229533.I1RS30
72707 229533.I1RRT0 pp 229533.I1RS43
72708 229533.I1RRT0 pp 229533.I1RTH2

72709 229533.I1RRT0 pp 229533.I1RYI9
72710 229533.I1RRT0 pp 229533.I1S201
72711 229533.I1RRT0 pp 229533.I1RSZ3
72712 229533.I1RRT0 pp 229533.I1RZR6
72713 229533.I1RRT0 pp 229533.I1RUG0
72714 229533.I1RRT0 pp 229533.I1RRW3
72715 229533.I1RRT0 pp 229533.I1S214
72716 229533.I1RRT0 pp 229533.I1RX63
72717 229533.I1RRT0 pp 229533.I1S4M5
72718 229533.I1RRT0 pp 229533.I1S0J3
72719 229533.I1RRT0 pp 229533.I1RZM2
72720 229533.I1RRT0 pp 229533.I1RTC2
72721 229533.I1RRT0 pp 229533.I1RW91
72722 229533.I1RRT0 pp 229533.Q4HTT1
72723 229533.I1RRT0 pp 229533.I1RW90
72724 229533.I1RRT0 pp 229533.Q4IPN9
72725 229533.I1RRT0 pp 229533.Q4HYQ4
72726 229533.I1RRT0 pp 229533.V6QWX8
72727 229533.I1RRT0 pp 229533.I1RVL8
72728 229533.I1RRT0 pp 229533.I1RWL3
72729 229533.I1RRT0 pp 229533.Q4I099
72730 229533.I1RRT0 pp 229533.I1RVY0
72731 229533.I1RRT0 pp 229533.I1S0R5
72732 229533.I1RRT0 pp 229533.I1S524
72733 229533.I1RRT0 pp 229533.I1RVJ1
72734 229533.A0A1C3YKD1 pp 229533.V6RGI1
72735 229533.A0A1C3YKD1 pp 229533.I1RZB7
72736 229533.A0A1C3YKD1 pp 229533.I1RB26
72737 229533.A0A1C3YKD1 pp 229533.I1RA98
72738 229533.A0A1C3YKD1 pp 229533.I1RA00
72739 229533.I1RT61 pp 229533.I1RT63
72740 229533.I1RT61 pp 229533.I1RT62
72741 229533.I1RE03 pp 229533.I1RG99
72742 229533.I1RE03 pp 229533.Q4I6S5
72743 229533.I1RE03 pp 229533.I1RMF2
72744 229533.I1RE03 pp 229533.I1RW76
72745 229533.I1RE03 pp 229533.I1RYU1
72746 229533.I1RE03 pp 229533.I1RQF7
72747 229533.I1RE03 pp 229533.I1RFS7
72748 229533.I1RE03 pp 229533.I1RFV4
72749 229533.I1RE03 pp 229533.I1RT38
72750 229533.I1RE03 pp 229533.I1RZJ9
72751 229533.I1RE03 pp 229533.I1RFK0
72752 229533.I1RE03 pp 229533.I1RK33
72753 229533.I1RE03 pp 229533.I1RJT0
72754 229533.I1RE03 pp 229533.I1S278
72755 229533.I1RE03 pp 229533.I1S1V4
72756 229533.I1RE03 pp 229533.I1SAM5
72757 229533.I1RE03 pp 229533.I1S6S4
72758 229533.I1RE03 pp 229533.I1RNA3
72759 229533.I1RE03 pp 229533.I1RPU5
72760 229533.I1RE03 pp 229533.I1RQP7
72761 229533.I1RE03 pp 229533.I1S830
72762 229533.I1RE03 pp 229533.I1S0K5
72763 229533.I1R9P5 pp 229533.I1RT61
72764 229533.A0A1C3YJ31 pp 229533.Q4HYQ4
72765 229533.A0A1C3YM67 pp 229533.I1RK95
72766 229533.A0A1C3YM67 pp 229533.I1RJ33
72767 229533.A0A1C3YM67 pp 229533.I1RJL2
72768 229533.A0A1C3YM67 pp 229533.I1S3L3
72769 229533.A0A1C3YM67 pp 229533.I1RDH0
72770 229533.A0A1C3YM67 pp 229533.Q4ICA8
72771 229533.A0A1C3YM67 pp 229533.I1RMQ7
72772 229533.A0A1C3YM67 pp 229533.I1RVY0
72773 229533.A0A1C3YM67 pp 229533.I1RSC7
72774 229533.A0A1C3YM67 pp 229533.I1RE46
72775 229533.A0A1C3YM67 pp 229533.Q4IJH1
72776 229533.A0A1C3YM67 pp 229533.Q4HTT1
72777 229533.A0A1C3YM67 pp 229533.I1RU02
72778 229533.A0A1C3YM67 pp 229533.I1RRG3
72779 229533.A0A1C3YM67 pp 229533.Q4IER8
72780 229533.A0A1C3YM67 pp 229533.Q4IMD1
72781 229533.A0A1C3YM67 pp 229533.I1RFW1

229533.A0A1C3YM67	pp	229533.Q4I7L0
229533.A0A1C3YM67	pp	229533.I1RZB5
229533.A0A1C3YM67	pp	229533.I1S3W0
229533.A0A1C3YM67	pp	229533.I1RCW8
229533.A0A1C3YM67	pp	229533.I1RWI8
229533.A0A1C3YM67	pp	229533.V6QUE6
229533.A0A1C3YM67	pp	229533.I1RQR3
229533.A0A1C3YM67	pp	229533.I1RY58
229533.A0A1C3YM67	pp	229533.I1RUF8
229533.A0A1C3YM67	pp	229533.Q4HTT2
229533.A0A1C3YM67	pp	229533.I1RB00
229533.A0A1C3YM67	pp	229533.I1RSZ3
229533.A0A1C3YM67	pp	229533.I1RK79
229533.A0A1C3YM67	pp	229533.I1RFP4
229533.A0A1C3YM67	pp	229533.I1RQP1
229533.A0A1C3YM67	pp	229533.I1RSD4
229533.A0A1C3YM67	pp	229533.I1S283
229533.A0A1C3YM67	pp	229533.Q4HYB8
229533.A0A1C3YM67	pp	229533.Q4IF76
229533.A0A1C3YM67	pp	229533.V6RLW4
229533.A0A1C3YM67	pp	229533.I1RFG0
229533.A0A098DNA4	pp	229533.I1RK55
229533.A0A098DNA4	pp	229533.V6RCJ5
229533.A0A098DNA4	pp	229533.I1RIJ6
229533.A0A098DNA4	pp	229533.I1RC46
229533.A0A098DNA4	pp	229533.I1RSA5
229533.A0A098DNA4	pp	229533.I1RRR9
229533.A0A098DNA4	pp	229533.I1RW82
229533.A0A098DNA4	pp	229533.I1SAT2
229533.A0A098DNA4	pp	229533.I1RWF5
229533.A0A098DNA4	pp	229533.I1RN84
229533.A0A098DNA4	pp	229533.I1S0A5
229533.A0A098DNA4	pp	229533.I1S968
229533.A0A098DNA4	pp	229533.I1RMK9
229533.A0A098DNA4	pp	229533.I1RB05
229533.A0A098DNA4	pp	229533.I1RZ25
229533.A0A098DNA4	pp	229533.I1RYP5
229533.A0A098DNA4	pp	229533.I1RHU8
229533.A0A098DNA4	pp	229533.I1RDN3
229533.A0A098DNA4	pp	229533.A0A1C3YLE2
229533.A0A098DNA4	pp	229533.I1RVX8
229533.A0A098DNA4	pp	229533.I1S2C4
229533.A0A098DNA4	pp	229533.A0A1C3YHU3
229533.A0A098DNA4	pp	229533.V6RCC0
229533.A0A098DNA4	pp	229533.I1RH83
229533.A0A098DNA4	pp	229533.A0A0E0RSI1
229533.A0A098DNA4	pp	229533.I1S6V0
229533.A0A098DNA4	pp	229533.I1RW54
229533.A0A098DNA4	pp	229533.I1RJS1
229533.A0A098DNA4	pp	229533.I1S5D0
229533.A0A098DNA4	pp	229533.I1RZ56
229533.A0A098DNA4	pp	229533.I1RWL3
229533.A0A098DNA4	pp	229533.I1RZ89
229533.A0A098DNA4	pp	229533.I1RLA2
229533.A0A098DNA4	pp	229533.I1RMA2
229533.A0A098DNA4	pp	229533.I1RQV7
229533.A0A098DNA4	pp	229533.I1RPV8
229533.A0A098DNA4	pp	229533.I1S048
229533.A0A098DNA4	pp	229533.A0A1C3YL79
229533.A0A098DNA4	pp	229533.I1RE52
229533.A0A098DNA4	pp	229533.A0A1C3YLB6
229533.A0A098DNA4	pp	229533.V6R5T7
229533.A0A098DNA4	pp	229533.I1S6H3
229533.A0A098DNA4	pp	229533.I1S094
229533.A0A098DNA4	pp	229533.A0A1C3YIJ7
229533.A0A098DNA4	pp	229533.I1R9F2
229533.A0A098DNA4	pp	229533.I1RQV8
229533.A0A098DNA4	pp	229533.I1RRS6
229533.A0A098DNA4	pp	229533.I1RYU6
229533.A0A098DNA4	pp	229533.I1S659
229533.A0A098DNA4	pp	229533.I1S969
229533.A0A098DNA4	pp	229533.I1RY81
229533.A0A098DNA4	pp	229533.V6RWB3

72855 229533.A0A098DNA4 pp 229533.I1RA13
72856 229533.A0A098DNA4 pp 229533.A0A1C3YIB8
72857 229533.A0A098DNA4 pp 229533.I1RY12
72858 229533.A0A098DNA4 pp 229533.V6RHX2
72859 229533.A0A098DNA4 pp 229533.I1RQJ7
72860 229533.A0A098DNA4 pp 229533.I1S267
72861 229533.A0A098DNA4 pp 229533.A0A1C3YMC4
72862 229533.A0A098DNA4 pp 229533.A0A0E0S530
72863 229533.A0A098DNA4 pp 229533.I1RWM4
72864 229533.A0A098DNA4 pp 229533.I1RPM6
72865 229533.A0A098DNA4 pp 229533.I1RCT9
72866 229533.A0A098DNA4 pp 229533.I1RIB5
72867 229533.A0A098DNA4 pp 229533.I1RST9
72868 229533.A0A098DNA4 pp 229533.I1S8D3
72869 229533.A0A1C3YJH6 pp 229533.I1S2B6
72870 229533.A0A1C3YJH6 pp 229533.I1RJK8
72871 229533.A0A1C3YJH6 pp 229533.I1RWR7
72872 229533.A0A1C3YJH6 pp 229533.A0A1C3YLP9
72873 229533.A0A1C3YJH6 pp 229533.I1RAW0
72874 229533.A0A1C3YJH6 pp 229533.I1RQ33
72875 229533.A0A1C3YJH6 pp 229533.I1RRM3
72876 229533.A0A1C3YJH6 pp 229533.I1RMK1
72877 229533.A0A1C3YJH6 pp 229533.A0A1C3YKT4
72878 229533.A0A1C3YJH6 pp 229533.I1S830
72879 229533.A0A1C3YJH6 pp 229533.I1S5R6
72880 229533.A0A1C3YJH6 pp 229533.I1RRI1
72881 229533.A0A1C3YJH6 pp 229533.I1RMF2
72882 229533.A0A1C3YJH6 pp 229533.I1RFK0
72883 229533.A0A1C3YJH6 pp 229533.I1RZV3
72884 229533.A0A1C3YJH6 pp 229533.I1RQ32
72885 229533.A0A1C3YJH6 pp 229533.I1RWP9
72886 229533.A0A1C3YJH6 pp 229533.I1RQ85
72887 229533.A0A1C3YJH6 pp 229533.I1RQD6
72888 229533.A0A1C3YJH6 pp 229533.I1RS69
72889 229533.A0A1C3YJH6 pp 229533.I1S287
72890 229533.A0A1C3YJH6 pp 229533.I1RTA1
72891 229533.A0A1C3YJH6 pp 229533.I1S4S7
72892 229533.A0A1C3YJH6 pp 229533.I1RVZ2
72893 229533.A0A1C3YJX6 pp 229533.I1RRV1
72894 229533.A0A1C3YJX6 pp 229533.I1S8V3
72895 229533.A0A1C3YIE3 pp 229533.I1RNA3
72896 229533.A0A1C3YIE3 pp 229533.I1S1V4
72897 229533.A0A1C3YIE3 pp 229533.I1RBF2
72898 229533.A0A1C3YIE3 pp 229533.I1S830
72899 229533.A0A1C3YIE3 pp 229533.I1RYU1
72900 229533.A0A1C3YIE3 pp 229533.I1S6S4
72901 229533.A0A1C3YIE3 pp 229533.I1R9X6
72902 229533.A0A1C3YIE3 pp 229533.I1RNP1
72903 229533.A0A1C3YIE3 pp 229533.I1S278
72904 229533.A0A1C3YIE3 pp 229533.I1RPU5
72905 229533.A0A1C3YIE3 pp 229533.A0A1C3YME0
72906 229533.A0A1C3YIE3 pp 229533.A0A1C3YN54
72907 229533.A0A1C3YIE3 pp 229533.I1RQP7
72908 229533.A0A1C3YIE3 pp 229533.I1S0K5
72909 229533.A0A1C3YIE3 pp 229533.I1RXF5
72910 229533.A0A1C3YIE3 pp 229533.A0A1C3YKU7
72911 229533.A0A1C3YIE3 pp 229533.I1RK33
72912 229533.A0A1C3YIE3 pp 229533.I1S074
72913 229533.A0A1C3YIE3 pp 229533.I1RMV6
72914 229533.A0A1C3YIE3 pp 229533.I1RE03
72915 229533.A0A1C3YIE3 pp 229533.I1RZ60
72916 229533.A0A1C3YIE3 pp 229533.I1RXV4
72917 229533.A0A1C3YIE3 pp 229533.I1RFV4
72918 229533.A0A1C3YIE3 pp 229533.I1RZK3
72919 229533.A0A1C3YIE3 pp 229533.I1RAH7
72920 229533.A0A1C3YIE3 pp 229533.Q4I6S5
72921 229533.A0A1C3YIE3 pp 229533.I1RFS7
72922 229533.A0A1C3YIE3 pp 229533.A0A1C3YN81
72923 229533.A0A1C3YIE3 pp 229533.I1SAM5
72924 229533.A0A1C3YIE3 pp 229533.I1RMF2
72925 229533.A0A1C3YIE3 pp 229533.I1RTQ9
72926 229533.I1RXN8 pp 229533.I1RY33
72927 229533.I1RFV4 pp 229533.I1S0V5

229533.I1RFV4	pp	229533.V6R9X9
229533.I1RFV4	pp	229533.I1RYU1
229533.I1RFV4	pp	229533.I1S830
229533.I1RFV4	pp	229533.I1S278
229533.I1RFV4	pp	229533.I1RZ60
229533.I1RFV4	pp	229533.I1RW76
229533.I1RFV4	pp	229533.I1S1V4
229533.I1RFV4	pp	229533.I1RQP7
229533.I1RFV4	pp	229533.I1RG99
229533.I1RFV4	pp	229533.I1RZK3
229533.I1RFV4	pp	229533.I1RK33
229533.I1RFV4	pp	229533.I1RQD6
229533.I1RFV4	pp	229533.I1RPU5
229533.I1RFV4	pp	229533.I1SAM5
229533.I1RFV4	pp	229533.I1RNA3
229533.I1RFV4	pp	229533.I1RNH7
229533.I1RFV4	pp	229533.I1RPG2
229533.I1RFV4	pp	229533.I1RQH7
229533.I1RFV4	pp	229533.I1S0K5
229533.I1RFV4	pp	229533.I1RQF7
229533.I1RFV4	pp	229533.I1RFV8
229533.I1RFV4	pp	229533.I1S6S4
229533.I1RFV4	pp	229533.Q4INI2
229533.I1RFV4	pp	229533.I1RJT8
229533.I1RFV4	pp	229533.I1RMF2
229533.I1RFV4	pp	229533.I1RX87
229533.I1RVV5	pp	229533.I1S0D5
229533.I1RVV5	pp	229533.I1RZT1
229533.I1RVV5	pp	229533.I1RYV3
229533.I1RVV5	pp	229533.Q4INS6
229533.I1RVV5	pp	229533.I1S209
229533.I1RVV5	pp	229533.I1S4P6
229533.I1RVV5	pp	229533.I1RW03
229533.I1RVV5	pp	229533.I1RXZ4
229533.I1RVV5	pp	229533.Q4HTT1
229533.I1RVV5	pp	229533.I1RVW0
229533.I1RVV5	pp	229533.Q4IB89
229533.I1RVV5	pp	229533.I1RWM6
229533.I1RVV5	pp	229533.I1S461
229533.I1S9H8	pp	229533.V6QWX8
229533.I1RPU5	pp	229533.I1S1V4
229533.I1RPU5	pp	229533.I1SAM5
229533.I1RPU5	pp	229533.I1RQP7
229533.I1RPU5	pp	229533.I1RZ60
229533.I1RPU5	pp	229533.Q4I6S5
229533.I1RPU5	pp	229533.I1RYU1
229533.I1RPU5	pp	229533.I1S278
229533.I1RPU5	pp	229533.I1RW76
229533.I1RPU5	pp	229533.I1S050
229533.I1RPU5	pp	229533.I1S830
229533.I1RPU5	pp	229533.I1S0K5
229533.I1RPU5	pp	229533.I1S2B6
229533.I1RPU5	pp	229533.I1RTD0
229533.I1RPU5	pp	229533.I1S6S4
229533.I1RPU5	pp	229533.I1RQD6
229533.Q4IJU0	pp	229533.Q4IQX3
229533.Q4IJU0	pp	229533.V6QUE6
229533.Q4IJU0	pp	229533.V6RNM4
229533.Q4IJU0	pp	229533.Q4IR08
229533.Q4IJU0	pp	229533.Q4IMD1
229533.Q4IJU0	pp	229533.V6RLW4
229533.Q4IJU0	pp	229533.Q4IQM6
229533.Q4IJU0	pp	229533.V6QWX8
229533.Q4IJU0	pp	229533.Q4IN63
229533.I1R9W6	pp	229533.I1RBJ0
229533.I1R9W6	pp	229533.I1S1U7
229533.I1R9W6	pp	229533.I1RA56
229533.I1R9W6	pp	229533.I1RPY4
229533.I1R9W6	pp	229533.Q4I7S1
229533.I1R9W6	pp	229533.I1RVY0
229533.I1R9W6	pp	229533.Q4IER8
229533.I1R9W6	pp	229533.I1S0J7
229533.I1R9W6	pp	229533.I1RU02

73001 229533.I1R9W6 pp 229533.I1RCN2
73002 229533.I1R9W6 pp 229533.V6R6F6
73003 229533.I1R9W6 pp 229533.I1RPX5
73004 229533.I1R9W6 pp 229533.I1RZU9
73005 229533.I1R9W6 pp 229533.I1RGW0
73006 229533.I1R9W6 pp 229533.I1RB73
73007 229533.I1R9W6 pp 229533.Q4IB50
73008 229533.I1R9W6 pp 229533.I1RCL8
73009 229533.I1R9W6 pp 229533.I1RQE9
73010 229533.I1R9W6 pp 229533.V6QV38
73011 229533.I1R9W6 pp 229533.I1RDC9
73012 229533.I1R9W6 pp 229533.I1S0N5
73013 229533.I1R9W6 pp 229533.Q4I5R3
73014 229533.I1R9W6 pp 229533.I1RPR8
73015 229533.I1R9W6 pp 229533.I1RB27
73016 229533.I1R9W6 pp 229533.I1RFW1
73017 229533.I1R9W6 pp 229533.I1RSI1
73018 229533.I1R9W6 pp 229533.I1RXE5
73019 229533.I1R9W6 pp 229533.I1RCW8
73020 229533.I1R9W6 pp 229533.Q4IEV4
73021 229533.I1R9W6 pp 229533.I1RQ19
73022 229533.I1R9W6 pp 229533.Q4IMD1
73023 229533.I1R9W6 pp 229533.Q4HTT2
73024 229533.I1R9W6 pp 229533.I1RK15
73025 229533.I1R9W6 pp 229533.I1RW28
73026 229533.I1R9W6 pp 229533.I1RKR6
73027 229533.I1R9W6 pp 229533.Q4IR08
73028 229533.I1R9W6 pp 229533.I1RE51
73029 229533.I1R9W6 pp 229533.I1RYV3
73030 229533.I1R9W6 pp 229533.I1RC32
73031 229533.I1R9W6 pp 229533.I1RYV4
73032 229533.I1R9W6 pp 229533.I1S1W5
73033 229533.I1R9W6 pp 229533.I1RMA5
73034 229533.I1R9W6 pp 229533.I1RWU3
73035 229533.I1R9W6 pp 229533.I1REF2
73036 229533.I1R9W6 pp 229533.I1RGE1
73037 229533.I1R9W6 pp 229533.I1RT16
73038 229533.I1R9W6 pp 229533.I1RNG8
73039 229533.I1R9W6 pp 229533.I1RUQ6
73040 229533.I1R9W6 pp 229533.I1RB36
73041 229533.A0A0E0SGB3 pp 229533.I1RNM7
73042 229533.A0A0E0SGB3 pp 229533.I1RRL5
73043 229533.A0A0E0SGB3 pp 229533.I1RLA2
73044 229533.A0A0E0SGB3 pp 229533.I1RDN3
73045 229533.A0A0E0SGB3 pp 229533.I1S6H3
73046 229533.A0A0E0SGB3 pp 229533.I1S334
73047 229533.A0A0E0SGB3 pp 229533.I1S0A5
73048 229533.A0A0E0SGB3 pp 229533.I1RA13
73049 229533.A0A0E0SGB3 pp 229533.I1RDU0
73050 229533.A0A0E0SGB3 pp 229533.I1S094
73051 229533.A0A0E0SGB3 pp 229533.I1S8F8
73052 229533.A0A0E0SGB3 pp 229533.I1RNG4
73053 229533.A0A0E0SGB3 pp 229533.I1RST9
73054 229533.A0A0E0SGB3 pp 229533.I1S3H8
73055 229533.A0A0E0SGB3 pp 229533.A0A1C3YKN4
73056 229533.A0A0E0SGB3 pp 229533.I1RGZ3
73057 229533.A0A0E0SGB3 pp 229533.I1RUY5
73058 229533.A0A0E0SGB3 pp 229533.I1RPV8
73059 229533.A0A0E0SGB3 pp 229533.I1RZ56
73060 229533.A0A0E0SGB3 pp 229533.I1RKB2
73061 229533.A0A0E0SGB3 pp 229533.I1RZ25
73062 229533.A0A0E0SGB3 pp 229533.I1RJS1
73063 229533.I1RSZ1 pp 229533.I1RWB4
73064 229533.I1RSZ1 pp 229533.Q4I6S5
73065 229533.I1RSZ1 pp 229533.I1RXF5
73066 229533.I1RSZ1 pp 229533.I1RYU1
73067 229533.I1RSZ1 pp 229533.I1S0R4
73068 229533.I1RSZ1 pp 229533.I1RWT6
73069 229533.I1RSZ1 pp 229533.I1S0R3
73070 229533.I1RQP1 pp 229533.Q4IJM4
73071 229533.I1RQP1 pp 229533.I1RRN5
73072 229533.I1RQP1 pp 229533.I1S086
73073 229533.I1RQP1 pp 229533.I1S270

73074	229533.I1RQP1	pp	229533.Q4HTT1
73075	229533.I1RQP1	pp	229533.Q4I948
73076	229533.I1RQP1	pp	229533.I1S438
73077	229533.I1RQP1	pp	229533.Q4IL82
73078	229533.I1RQP1	pp	229533.I1RT65
73079	229533.I1RQP1	pp	229533.I1RT41
73080	229533.I1RQP1	pp	229533.I1S437
73081	229533.I1RQP1	pp	229533.I1S0N5
73082	229533.I1RQP1	pp	229533.I1RUQ1
73083	229533.I1RQP1	pp	229533.Q4IJ84
73084	229533.I1RQP1	pp	229533.I1S9J6
73085	229533.I1RQP1	pp	229533.I1RZF5
73086	229533.I1RQP1	pp	229533.I1RRF0
73087	229533.I1RQP1	pp	229533.Q4IER8
73088	229533.I1RQP1	pp	229533.Q4IPI4
73089	229533.I1RQP1	pp	229533.I1S0Z3
73090	229533.I1RQP1	pp	229533.I1RU52
73091	229533.I1RQP1	pp	229533.I1S0J3
73092	229533.I1RQP1	pp	229533.I1RTB2
73093	229533.I1RQP1	pp	229533.I1RYR0
73094	229533.I1RQP1	pp	229533.I1RSI1
73095	229533.I1RQP1	pp	229533.I1RR13
73096	229533.I1RQP1	pp	229533.I1RTN6
73097	229533.I1RQP1	pp	229533.I1S0F0
73098	229533.I1RQP1	pp	229533.Q4IB96
73099	229533.I1RQP1	pp	229533.I1RU02
73100	229533.I1RQP1	pp	229533.I1RZI4
73101	229533.I1RQP1	pp	229533.Q4IAK7
73102	229533.I1RQP1	pp	229533.I1RUR0
73103	229533.I1RQP1	pp	229533.I1RYI1
73104	229533.I1RQP1	pp	229533.I1RST4
73105	229533.I1RQP1	pp	229533.I1S201
73106	229533.I1RQP1	pp	229533.Q4IQX3
73107	229533.I1RQP1	pp	229533.I1RSZ3
73108	229533.I1RQP1	pp	229533.Q4ICA8
73109	229533.I1RQP1	pp	229533.I1RQP3
73110	229533.I1RQP1	pp	229533.I1RRM8
73111	229533.I1RQP1	pp	229533.Q4HTT2
73112	229533.I1S830	pp	229533.I1SAM5
73113	229533.I1S830	pp	229533.Q4I6S5
73114	229533.I1RI51	pp	229533.I1RKR6
73115	229533.I1RI51	pp	229533.V6QV38
73116	229533.I1RI51	pp	229533.I1RQE9
73117	229533.I1RI51	pp	229533.Q4HTT2
73118	229533.I1RI51	pp	229533.I1RXE5
73119	229533.I1RI51	pp	229533.Q4IER8
73120	229533.I1RI51	pp	229533.I1RYV4
73121	229533.I1RI51	pp	229533.I1RK15
73122	229533.I1RI51	pp	229533.I1RQ19
73123	229533.I1RI51	pp	229533.I1RUQ6
73124	229533.I1RI51	pp	229533.I1RWU3
73125	229533.I1RI51	pp	229533.I1RU02
73126	229533.I1RI51	pp	229533.Q4I5R3
73127	229533.I1RI51	pp	229533.I1RYV3
73128	229533.I1RI51	pp	229533.I1RT16
73129	229533.I1RI51	pp	229533.I1RMA5
73130	229533.I1RI51	pp	229533.I1RPR8
73131	229533.I1RI51	pp	229533.Q4IMD1
73132	229533.I1RI51	pp	229533.I1RPY4
73133	229533.I1RI51	pp	229533.I1S0N5
73134	229533.I1RI51	pp	229533.I1RW28
73135	229533.I1RI51	pp	229533.I1RPX5
73136	229533.I1RI51	pp	229533.I1RVY0
73137	229533.I1RI51	pp	229533.Q4IEV4
73138	229533.I1RD45	pp	229533.I1S7X9
73139	229533.I1RD45	pp	229533.I1RJS2
73140	229533.I1RD45	pp	229533.V6RB81
73141	229533.I1RD45	pp	229533.I1RSJ0
73142	229533.I1RD45	pp	229533.I1SAM5
73143	229533.I1RD45	pp	229533.I1RND7
73144	229533.I1RD45	pp	229533.I1S0K5
73145	229533.I1RD45	pp	229533.I1RWB4
73146	229533.I1RD45	pp	229533.I1RSR8

73147	229533.I1RD45	pp	229533.I1RQ32
73148	229533.I1RD45	pp	229533.I1RMF2
73149	229533.I1RD45	pp	229533.I1S1V7
73150	229533.I1RD45	pp	229533.I1RSH3
73151	229533.I1RD45	pp	229533.I1RQP5
73152	229533.I1RD45	pp	229533.I1RYU1
73153	229533.I1RD45	pp	229533.I1S287
73154	229533.I1RD45	pp	229533.I1RFK0
73155	229533.I1RD45	pp	229533.I1RE20
73156	229533.I1RD45	pp	229533.I1RPK5
73157	229533.I1RD45	pp	229533.I1RTQ9
73158	229533.I1RD45	pp	229533.I1RY40
73159	229533.I1RT01	pp	229533.V6QUE6
73160	229533.I1RT01	pp	229533.I1RZR6
73161	229533.I1RT01	pp	229533.I1S0C6
73162	229533.I1RT01	pp	229533.Q4I7N9
73163	229533.I1RT01	pp	229533.Q4IJ11
73164	229533.I1RT01	pp	229533.Q4I5I4
73165	229533.I1RT01	pp	229533.I1RTH2
73166	229533.I1RT01	pp	229533.I1S0S6
73167	229533.I1RT01	pp	229533.Q4HYB8
73168	229533.I1RT01	pp	229533.Q4I5W5
73169	229533.I1RT01	pp	229533.Q4IJU0
73170	229533.I1RT01	pp	229533.I1RT35
73171	229533.I1RT01	pp	229533.Q4HTT1
73172	229533.I1RT01	pp	229533.I1RZB5
73173	229533.I1RT01	pp	229533.I1RZG3
73174	229533.I1RT01	pp	229533.Q4IER8
73175	229533.I1RT01	pp	229533.Q4HTT2
73176	229533.I1RT01	pp	229533.I1RW90
73177	229533.I1RT01	pp	229533.Q4HYQ4
73178	229533.I1RT01	pp	229533.V6QWX8
73179	229533.I1RNA3	pp	229533.I1RXF5
73180	229533.I1RNA3	pp	229533.I1RZ60
73181	229533.I1RNA3	pp	229533.I1RYU1
73182	229533.I1RNA3	pp	229533.I1S278
73183	229533.I1RNA3	pp	229533.Q4I6S5
73184	229533.I1RNA3	pp	229533.I1SAM5
73185	229533.I1RNA3	pp	229533.I1S830
73186	229533.I1RNA3	pp	229533.I1S6S4
73187	229533.I1RNA3	pp	229533.I1RPU5
73188	229533.I1RNA3	pp	229533.I1S0K5
73189	229533.I1RNA3	pp	229533.I1S1V4
73190	229533.I1RNA3	pp	229533.I1RNP1
73191	229533.A0A1C3YLM3	pp	229533.I1RW37
73192	229533.A0A1C3YLM3	pp	229533.I1RSI3
73193	229533.A0A1C3YLM3	pp	229533.I1S267
73194	229533.A0A1C3YLM3	pp	229533.I1RMV6
73195	229533.A0A1C3YLM3	pp	229533.I1RD44
73196	229533.A0A1C3YLM3	pp	229533.I1RKA1
73197	229533.A0A1C3YLM3	pp	229533.I1S0F5
73198	229533.A0A1C3YLM3	pp	229533.A0A1C3YME0
73199	229533.A0A1C3YLM3	pp	229533.I1S0P7
73200	229533.A0A1C3YLM3	pp	229533.I1RNG8
73201	229533.A0A1C3YLM3	pp	229533.I1S0J7
73202	229533.A0A1C3YLM3	pp	229533.I1RX50
73203	229533.A0A1C3YLM3	pp	229533.I1S1W5
73204	229533.A0A1C3YLM3	pp	229533.I1S039
73205	229533.A0A1C3YLM3	pp	229533.I1S9X9
73206	229533.I1RZB5	pp	229533.I1S0R7
73207	229533.I1RZB5	pp	229533.I1S201
73208	229533.I1RZB5	pp	229533.I1S6F0
73209	229533.I1RZB5	pp	229533.Q4HYQ4
73210	229533.I1RZB5	pp	229533.Q4IJH1
73211	229533.I1RZB5	pp	229533.I1RZR6
73212	229533.I1RZB5	pp	229533.Q4IQX3
73213	229533.I1RZB5	pp	229533.Q4INZ9
73214	229533.I1RZB5	pp	229533.I1RZG3
73215	229533.I1RZB5	pp	229533.I1S9U1
73216	229533.I1RZB5	pp	229533.V6QYF0
73217	229533.I1RZB5	pp	229533.Q4IPN9
73218	229533.I1RZB5	pp	229533.V6QWX8
73219	229533.I1RZB5	pp	229533.I1S182

73220	229533.I1RZB5	pp	229533.I1S255
73221	229533.I1RZB5	pp	229533.Q4HTT2
73222	229533.I1RZB5	pp	229533.V6R227
73223	229533.I1RZB5	pp	229533.Q4IFI0
73224	229533.I1RZB5	pp	229533.V6QUE6
73225	229533.I1RZB5	pp	229533.I1S0M9
73226	229533.I1RZB5	pp	229533.Q4IEK8
73227	229533.I1RZB5	pp	229533.Q4ICA8
73228	229533.I1RZB5	pp	229533.Q4I5I4
73229	229533.I1RZB5	pp	229533.Q4HYI0
73230	229533.I1RZB5	pp	229533.I1RZL5
73231	229533.I1RZB5	pp	229533.Q4IAA0
73232	229533.I1RZB5	pp	229533.I1RZU9
73233	229533.I1RZB5	pp	229533.V6QV38
73234	229533.I1RZB5	pp	229533.Q4IER8
73235	229533.I1RZB5	pp	229533.Q4IJ56
73236	229533.I1RZB5	pp	229533.I1S5Z2
73237	229533.I1RZB5	pp	229533.I1RZZ1
73238	229533.I1RZB5	pp	229533.I1S9J6
73239	229533.I1RZB5	pp	229533.Q4I1Y1
73240	229533.I1RZB5	pp	229533.I1RZH4
73241	229533.I1RZB5	pp	229533.I1RZK6
73242	229533.I1RZB5	pp	229533.Q4IJU0
73243	229533.I1RZB5	pp	229533.Q4IJM4
73244	229533.I1RZB5	pp	229533.I1RZM2
73245	229533.I1RZB5	pp	229533.Q4IPA4
73246	229533.I1RZB5	pp	229533.Q4IQW0
73247	229533.I1RZB5	pp	229533.I1S3N0
73248	229533.I1RZB5	pp	229533.Q4IJ11
73249	229533.I1RZB5	pp	229533.Q4I948
73250	229533.I1RZB5	pp	229533.I1S2E9
73251	229533.I1RZB5	pp	229533.I1S2G7
73252	229533.I1RZB5	pp	229533.I1S214
73253	229533.I1RZB5	pp	229533.Q4I5W5
73254	229533.I1RZB5	pp	229533.I1S8E2
73255	229533.I1RZB5	pp	229533.Q4IMD1
73256	229533.I1RZB5	pp	229533.Q4IF76
73257	229533.I1RZB5	pp	229533.Q4IB50
73258	229533.I1RZB5	pp	229533.Q4I830
73259	229533.I1RZB5	pp	229533.I1S4M5
73260	229533.I1RZB5	pp	229533.I1S2G9
73261	229533.I1RZB5	pp	229533.I1S0J3
73262	229533.I1RZB5	pp	229533.Q4HTT1
73263	229533.I1RZB5	pp	229533.I1S056
73264	229533.I1RZB5	pp	229533.I1RZS6
73265	229533.I1RZB5	pp	229533.Q4I099
73266	229533.I1RZB5	pp	229533.I1S9Y1
73267	229533.I1RZB5	pp	229533.Q4IN63
73268	229533.I1RZB5	pp	229533.I1S0R5
73269	229533.I1RZB5	pp	229533.I1S8U9
73270	229533.I1RZB5	pp	229533.I1S0J1
73271	229533.I1RZB5	pp	229533.I1SAN0
73272	229533.I1RZB5	pp	229533.I1S471
73273	229533.I1RZB5	pp	229533.I1S3N1
73274	229533.I1RZB5	pp	229533.V6RGN5
73275	229533.I1RZB5	pp	229533.Q4IPC8
73276	229533.I1RZB5	pp	229533.I1S1W4
73277	229533.I1RZB5	pp	229533.Q4I5U9
73278	229533.I1RZB5	pp	229533.I1S2C0
73279	229533.I1RZB5	pp	229533.Q4IQT8
73280	229533.I1RZB5	pp	229533.I1RZU4
73281	229533.I1RZB5	pp	229533.Q4I7S1
73282	229533.I1RZB5	pp	229533.Q4HZK7
73283	229533.I1RZB5	pp	229533.I1S0C6
73284	229533.I1RZB5	pp	229533.I1RZP8
73285	229533.I1RZB5	pp	229533.I1S276
73286	229533.I1RZB5	pp	229533.I1S2E8
73287	229533.I1RZB5	pp	229533.I1S238
73288	229533.I1RZB5	pp	229533.Q4HYB8
73289	229533.I1RZB5	pp	229533.I1S0E8
73290	229533.I1RZB5	pp	229533.I1S8V0
73291	229533.I1RZB5	pp	229533.I1S1X9
73292	229533.I1RZB5	pp	229533.I1S2C4

73293	229533.I1RZB5	pp	229533.I1S0N5
73294	229533.A0A0E0SQN6	pp	229533.I1RYU1
73295	229533.A0A0E0SQN6	pp	229533.A0A1C3YKT4
73296	229533.A0A0E0SQN6	pp	229533.I1S287
73297	229533.A0A0E0SQN6	pp	229533.I1RQK5
73298	229533.A0A0E0SQN6	pp	229533.I1RW83
73299	229533.A0A0E0SQN6	pp	229533.I1RFK0
73300	229533.A0A0E0SQN6	pp	229533.A0A1C3YHW1
73301	229533.I1RQ88	pp	229533.Q4I7N9
73302	229533.I1RQ88	pp	229533.Q4IJU0
73303	229533.I1RQ88	pp	229533.I1RWT1
73304	229533.I1RQ88	pp	229533.I1RS41
73305	229533.I1RQ88	pp	229533.I1RQA3
73306	229533.I1RQ88	pp	229533.I1RR25
73307	229533.I1RQ88	pp	229533.I1RW23
73308	229533.I1RQ88	pp	229533.I1RVX1
73309	229533.I1RQ88	pp	229533.I1S339
73310	229533.I1RQ88	pp	229533.I1RT08
73311	229533.I1RQ88	pp	229533.I1RVA5
73312	229533.I1RQ88	pp	229533.Q4I5U9
73313	229533.I1RQ88	pp	229533.V6R2L8
73314	229533.I1RQ88	pp	229533.Q4HTT1
73315	229533.I1RQ88	pp	229533.I1RY44
73316	229533.I1RQ88	pp	229533.I1RU02
73317	229533.I1RQ88	pp	229533.Q4IER8
73318	229533.I1RQ88	pp	229533.I1S2R6
73319	229533.I1RQ88	pp	229533.Q4IB50
73320	229533.I1RQ88	pp	229533.I1RSI1
73321	229533.I1RQ88	pp	229533.Q4HYB8
73322	229533.I1RQ88	pp	229533.I1RZB5
73323	229533.I1RQ88	pp	229533.I1RT35
73324	229533.I1RQ88	pp	229533.I1RSH9
73325	229533.I1RQ88	pp	229533.I1RZA9
73326	229533.I1RQ88	pp	229533.Q4I5W5
73327	229533.I1RQ88	pp	229533.I1RT01
73328	229533.I1RQ88	pp	229533.I1SA96
73329	229533.I1RQ88	pp	229533.I1RWT6
73330	229533.I1RQ88	pp	229533.Q4IMD1
73331	229533.I1RQ88	pp	229533.I1RSW9
73332	229533.I1RQ88	pp	229533.Q4I5R3
73333	229533.I1RQ88	pp	229533.I1RXA1
73334	229533.I1RQ88	pp	229533.I1RTH2
73335	229533.I1RQ88	pp	229533.I1RTN1
73336	229533.I1RQ88	pp	229533.I1S002
73337	229533.I1RQ88	pp	229533.I1RV79
73338	229533.I1RQ88	pp	229533.I1RRT0
73339	229533.I1RQ88	pp	229533.I1S9H9
73340	229533.I1RQ88	pp	229533.Q4I5I4
73341	229533.I1RQ88	pp	229533.I1RZG3
73342	229533.I1RQ88	pp	229533.I1RV82
73343	229533.I1RQ88	pp	229533.Q4HTT2
73344	229533.I1RQ88	pp	229533.V6QUE6
73345	229533.I1RQ88	pp	229533.I1RQX6
73346	229533.I1RQ88	pp	229533.I1S0V1
73347	229533.I1RQ88	pp	229533.I1S9T1
73348	229533.I1RQ88	pp	229533.V6QWX8
73349	229533.I1RQ88	pp	229533.I1S2E9
73350	229533.I1RQ88	pp	229533.I1RW90
73351	229533.I1RQ88	pp	229533.Q4IJ11
73352	229533.I1RQ88	pp	229533.I1S2R1
73353	229533.I1RQ88	pp	229533.I1S1K9
73354	229533.I1RQ88	pp	229533.I1RQP3
73355	229533.I1RQ88	pp	229533.I1S330
73356	229533.I1RQ88	pp	229533.I1RZR6
73357	229533.I1RQ88	pp	229533.I1RRG3
73358	229533.I1RQ88	pp	229533.Q4HYQ4
73359	229533.I1RQ88	pp	229533.I1RQW2
73360	229533.I1RXV4	pp	229533.I1S4N7
73361	229533.I1RXV4	pp	229533.I1RYZ0
73362	229533.I1RXV4	pp	229533.I1S278
73363	229533.I1RXV4	pp	229533.I1S039
73364	229533.I1RXV4	pp	229533.I1S4N8
73365	229533.I1RXV4	pp	229533.I1S6S4

73366 229533.I1RXV4 pp 229533.I1S0J7
73367 229533.I1RXV4 pp 229533.I1S0P7
73368 229533.I1RXV4 pp 229533.I1S1W5
73369 229533.I1RXV4 pp 229533.I1S9X9
73370 229533.A0A0E0RMX5 pp 229533.A0A1C3YHW3
73371 229533.A0A0E0RMX5 pp 229533.I1RMK1
73372 229533.A0A0E0RMX5 pp 229533.I1RMA4
73373 229533.A0A0E0RMX5 pp 229533.I1S287
73374 229533.A0A0E0RMX5 pp 229533.A0A1C3YIJ1
73375 229533.A0A0E0RMX5 pp 229533.A0A1C3YKT4
73376 229533.A0A0E0RMX5 pp 229533.I1RYU1
73377 229533.A0A0E0RMX5 pp 229533.A0A1C3YHW1
73378 229533.A0A0E0RMX5 pp 229533.A0A1C3YKD1
73379 229533.A0A0E0RMX5 pp 229533.I1S034
73380 229533.A0A0E0RMX5 pp 229533.I1REK3
73381 229533.A0A0E0RMX5 pp 229533.A0A1C3YJH6
73382 229533.A0A0E0RMX5 pp 229533.I1RFK0
73383 229533.A0A0E0S0X2 pp 229533.A0A1C3YLB6
73384 229533.A0A0E0S0X2 pp 229533.A0A1C3YIB4
73385 229533.A0A0E0S0X2 pp 229533.I1RTM8
73386 229533.A0A0E0S0X2 pp 229533.I1RNG4
73387 229533.Q4ICA8 pp 229533.Q4IPI4
73388 229533.Q4ICA8 pp 229533.Q4IL82
73389 229533.Q4ICA8 pp 229533.Q4IJM4
73390 229533.Q4ICA8 pp 229533.Q4IEV4
73391 229533.Q4ICA8 pp 229533.Q4IER8
73392 229533.Q4ICA8 pp 229533.Q4IMD1
73393 229533.Q4ICA8 pp 229533.Q4IJ84
73394 229533.Q4ICA8 pp 229533.V6QUE6
73395 229533.Q4ICA8 pp 229533.Q4IQX3
73396 229533.Q4ICA8 pp 229533.V6RDP1
73397 229533.Q4ICA8 pp 229533.Q4IJ56
73398 229533.I1RW28 pp 229533.Q4IAK7
73399 229533.I1RW28 pp 229533.Q4I7L0
73400 229533.I1RW28 pp 229533.Q4IB96
73401 229533.I1RW28 pp 229533.Q4IMD1
73402 229533.I1RW28 pp 229533.Q4HYB8
73403 229533.I1RW28 pp 229533.I1S4P6
73404 229533.I1RW28 pp 229533.Q4I5V3
73405 229533.I1RW28 pp 229533.Q4IB89
73406 229533.I1RW28 pp 229533.I1S1U7
73407 229533.I1RW28 pp 229533.I1SB04
73408 229533.I1RW28 pp 229533.Q4IPI4
73409 229533.I1RW28 pp 229533.Q4IF76
73410 229533.I1RW28 pp 229533.I1S0D5
73411 229533.I1RW28 pp 229533.Q4HTT1
73412 229533.I1RW28 pp 229533.Q4HY90
73413 229533.I1RW28 pp 229533.I1RZF3
73414 229533.I1RW28 pp 229533.I1S605
73415 229533.I1RW28 pp 229533.Q4I7S1
73416 229533.I1RW28 pp 229533.I1RWD0
73417 229533.I1RW28 pp 229533.Q4IJH1
73418 229533.I1RW28 pp 229533.Q4HTT2
73419 229533.I1RW28 pp 229533.I1RW60
73420 229533.I1RW28 pp 229533.I1RYX3
73421 229533.I1RW28 pp 229533.Q4I948
73422 229533.I1RW28 pp 229533.Q4IER8
73423 229533.I1RW28 pp 229533.Q4ICA8
73424 229533.I1RSI1 pp 229533.Q4IR08
73425 229533.I1RSI1 pp 229533.I1RT16
73426 229533.I1RSI1 pp 229533.Q4IER8
73427 229533.I1RSI1 pp 229533.I1RZR6
73428 229533.I1RSI1 pp 229533.Q4I5R3
73429 229533.I1RSI1 pp 229533.Q4HTT2
73430 229533.I1RSI1 pp 229533.Q4HYQ4
73431 229533.I1RSI1 pp 229533.Q4I5I4
73432 229533.I1RSI1 pp 229533.I1RZB5
73433 229533.I1RSI1 pp 229533.I1RSI4
73434 229533.I1RSI1 pp 229533.V6QWX8
73435 229533.I1RSI1 pp 229533.Q4ICA8
73436 229533.I1RSI1 pp 229533.Q4IJU0
73437 229533.I1RSI1 pp 229533.Q4I7N9
73438 229533.I1RSI1 pp 229533.Q4I5W5

73439	229533.I1RSI1	pp	229533.I1S0F0
73440	229533.I1RSI1	pp	229533.I1RVA7
73441	229533.I1RSI1	pp	229533.Q4I7L0
73442	229533.I1RSI1	pp	229533.Q4HW67
73443	229533.I1RSI1	pp	229533.Q4IB50
73444	229533.I1RSI1	pp	229533.Q4IMD1
73445	229533.I1RSI1	pp	229533.I1RY99
73446	229533.I1RSI1	pp	229533.I1RVY0
73447	229533.I1RSI1	pp	229533.I1SB04
73448	229533.I1RSI1	pp	229533.Q4HTT1
73449	229533.I1RSI1	pp	229533.I1RWE1
73450	229533.I1RSI1	pp	229533.Q4HY90
73451	229533.I1RSI1	pp	229533.I1S0N5
73452	229533.I1RSI1	pp	229533.Q4HYB8
73453	229533.I1S1X7	pp	229533.I1S4N7
73454	229533.I1S1X7	pp	229533.I1S4N8
73455	229533.I1S1X7	pp	229533.I1S287
73456	229533.I1S1X7	pp	229533.I1S9X9
73457	229533.I1RJL2	pp	229533.I1RVJ1
73458	229533.I1RJL2	pp	229533.I1RT41
73459	229533.I1RJL2	pp	229533.I1S0R5
73460	229533.I1RJL2	pp	229533.I1RN04
73461	229533.I1RJL2	pp	229533.I1RLR0
73462	229533.I1RJL2	pp	229533.I1RVY0
73463	229533.I1RJL2	pp	229533.I1RS66
73464	229533.I1RJL2	pp	229533.I1S9Y1
73465	229533.I1RJL2	pp	229533.Q4IPN9
73466	229533.I1RJL2	pp	229533.I1RNA0
73467	229533.I1RJL2	pp	229533.Q4HTT1
73468	229533.I1RJL2	pp	229533.Q4I099
73469	229533.I1RJL2	pp	229533.I1RW90
73470	229533.I1RJL2	pp	229533.Q4IJH1
73471	229533.I1RJL2	pp	229533.I1RNP4
73472	229533.I1RJL2	pp	229533.I1S0J3
73473	229533.I1RJL2	pp	229533.I1RLV3
73474	229533.I1RJL2	pp	229533.I1RQ42
73475	229533.I1RJL2	pp	229533.I1RVL8
73476	229533.I1RJL2	pp	229533.I1RM42
73477	229533.I1RJL2	pp	229533.I1RWL3
73478	229533.I1RJL2	pp	229533.I1RJU3
73479	229533.I1RJL2	pp	229533.I1RX63
73480	229533.I1RJL2	pp	229533.I1RKB1
73481	229533.I1RJL2	pp	229533.I1RUG0
73482	229533.I1RJL2	pp	229533.I1S8V3
73483	229533.I1RJL2	pp	229533.I1RRW3
73484	229533.I1RJL2	pp	229533.I1RZR6
73485	229533.I1RJL2	pp	229533.I1RM36
73486	229533.I1RJL2	pp	229533.V6QWX8
73487	229533.I1RJL2	pp	229533.I1S214
73488	229533.I1RJL2	pp	229533.Q4IF76
73489	229533.I1RJL2	pp	229533.I1S8F0
73490	229533.I1RJL2	pp	229533.I1RW91
73491	229533.I1RJL2	pp	229533.Q4IQX3
73492	229533.I1RJL2	pp	229533.I1RRM8
73493	229533.I1RJL2	pp	229533.I1RNH1
73494	229533.I1RJL2	pp	229533.I1S182
73495	229533.I1RJL2	pp	229533.I1RPW8
73496	229533.I1RJL2	pp	229533.Q4I5W5
73497	229533.I1RJL2	pp	229533.I1RS30
73498	229533.I1RJL2	pp	229533.I1RML9
73499	229533.I1RJL2	pp	229533.I1S2E9
73500	229533.I1RJL2	pp	229533.I1RTC2
73501	229533.I1RJL2	pp	229533.I1S3N0
73502	229533.I1RJL2	pp	229533.Q4IMD1
73503	229533.I1RJL2	pp	229533.I1RWT1
73504	229533.I1RJL2	pp	229533.I1S4M5
73505	229533.I1RJL2	pp	229533.I1RP96
73506	229533.I1RJL2	pp	229533.Q4HYQ4
73507	229533.I1RJL2	pp	229533.I1S9Y0
73508	229533.I1RJL2	pp	229533.I1RQ88
73509	229533.I1RJL2	pp	229533.I1RSZ3
73510	229533.I1RJL2	pp	229533.I1RW80
73511	229533.I1RJL2	pp	229533.I1RZM2

73512	229533.I1RJL2	pp	229533.I1S201
73513	229533.I1RJL2	pp	229533.I1RMU8
73514	229533.I1RJL2	pp	229533.I1RV91
73515	229533.I1RJL2	pp	229533.I1RRZ0
73516	229533.I1RJL2	pp	229533.Q4IJU0
73517	229533.I1RJL2	pp	229533.I1RRG3
73518	229533.I1RJL2	pp	229533.I1RTH2
73519	229533.I1RJL2	pp	229533.I1RLV1
73520	229533.I1RJL2	pp	229533.I1S8V0
73521	229533.I1RJL2	pp	229533.I1RP21
73522	229533.I1RJL2	pp	229533.I1RK95
73523	229533.I1RJL2	pp	229533.I1RKQ5
73524	229533.I1RJL2	pp	229533.I1RN17
73525	229533.I1RJL2	pp	229533.Q4IJM4
73526	229533.I1RJL2	pp	229533.I1RXF2
73527	229533.I1RJL2	pp	229533.I1RR25
73528	229533.I1RJL2	pp	229533.Q4IJ11
73529	229533.I1RJL2	pp	229533.I1RN65
73530	229533.I1RJL2	pp	229533.I1RUG4
73531	229533.I1RJL2	pp	229533.I1S0N5
73532	229533.I1RJL2	pp	229533.I1S7M2
73533	229533.I1RJL2	pp	229533.I1RQZ2
73534	229533.I1RJL2	pp	229533.I1RPV5
73535	229533.I1RJL2	pp	229533.I1RW23
73536	229533.I1RJL2	pp	229533.I1RZ59
73537	229533.I1RJL2	pp	229533.I1RR61
73538	229533.I1RJL2	pp	229533.V6QV38
73539	229533.I1RJL2	pp	229533.I1RZH4
73540	229533.I1RJL2	pp	229533.I1S0E8
73541	229533.I1RJL2	pp	229533.I1RQA3
73542	229533.I1RJL2	pp	229533.Q4IQT8
73543	229533.I1RJL2	pp	229533.I1RS41
73544	229533.I1RJL2	pp	229533.I1RPM7
73545	229533.I1RJL2	pp	229533.I1RMV1
73546	229533.I1RJL2	pp	229533.I1RK71
73547	229533.I1RJL2	pp	229533.Q4HYB8
73548	229533.I1RJL2	pp	229533.I1RZU4
73549	229533.I1RJL2	pp	229533.I1S276
73550	229533.I1RJL2	pp	229533.I1RRT0
73551	229533.I1RJL2	pp	229533.I1RX14
73552	229533.I1RJL2	pp	229533.I1S2C4
73553	229533.I1RJL2	pp	229533.I1RZU9
73554	229533.I1RJL2	pp	229533.I1RJY2
73555	229533.I1RJL2	pp	229533.I1RMQ7
73556	229533.I1RJL2	pp	229533.V6RGN5
73557	229533.I1RJL2	pp	229533.I1RZL5
73558	229533.I1RJL2	pp	229533.I1RJY3
73559	229533.I1RJL2	pp	229533.I1RK92
73560	229533.I1RJL2	pp	229533.I1S255
73561	229533.I1RJL2	pp	229533.I1RU02
73562	229533.I1RJL2	pp	229533.V6RIS2
73563	229533.I1RJL2	pp	229533.I1RYS4
73564	229533.I1RJL2	pp	229533.Q4IPC8
73565	229533.I1RJL2	pp	229533.I1S238
73566	229533.I1RJL2	pp	229533.I1RWT6
73567	229533.I1RJL2	pp	229533.I1RSG8
73568	229533.I1RJL2	pp	229533.I1RNQ6
73569	229533.I1RJL2	pp	229533.I1S8V4
73570	229533.I1RJL2	pp	229533.I1RRF7
73571	229533.I1RJL2	pp	229533.I1RX64
73572	229533.I1RJL2	pp	229533.I1RRN6
73573	229533.I1RJL2	pp	229533.I1S9U1
73574	229533.I1RJL2	pp	229533.Q4IER8
73575	229533.I1RJL2	pp	229533.I1RSI1
73576	229533.I1RJL2	pp	229533.I1RZB5
73577	229533.I1RJL2	pp	229533.Q4I5I4
73578	229533.I1RJL2	pp	229533.I1RM65
73579	229533.I1RJL2	pp	229533.I1S0C6
73580	229533.I1RJL2	pp	229533.V6QUE6
73581	229533.I1RJL2	pp	229533.I1RYY5
73582	229533.I1RJL2	pp	229533.I1RY44
73583	229533.I1RJL2	pp	229533.I1RN07
73584	229533.I1RJL2	pp	229533.I1S0Z3

73585 229533.I1RJL2 pp 229533.I1RSA4
73586 229533.I1RJL2 pp 229533.I1RXE5
73587 229533.I1RJL2 pp 229533.I1RXU0
73588 229533.I1RJL2 pp 229533.I1RK79
73589 229533.I1RJL2 pp 229533.I1S9J6
73590 229533.I1RJL2 pp 229533.Q4I7S1
73591 229533.I1RJL2 pp 229533.I1RWF3
73592 229533.I1RJL2 pp 229533.I1S3N1
73593 229533.I1RJL2 pp 229533.I1RMD5
73594 229533.I1RJL2 pp 229533.I1RT99
73595 229533.I1RJL2 pp 229533.I1RT01
73596 229533.I1RJL2 pp 229533.Q4HYI0
73597 229533.I1RJL2 pp 229533.I1S8U9
73598 229533.I1RJL2 pp 229533.I1RZ42
73599 229533.I1RJL2 pp 229533.I1RNB8
73600 229533.I1RJL2 pp 229533.Q4HTT2
73601 229533.I1RJL2 pp 229533.I1RQZ3
73602 229533.I1RJL2 pp 229533.I1S7M3
73603 229533.I1RJL2 pp 229533.I1RPK5
73604 229533.A0A098DB79 pp 229533.I1RSC7
73605 229533.A0A098DB79 pp 229533.A0A098DNT9
73606 229533.A0A098DB79 pp 229533.A0A098E4K9
73607 229533.A0A098DB79 pp 229533.I1S209
73608 229533.A0A098DB79 pp 229533.I1RDH0
73609 229533.A0A098DB79 pp 229533.I1REN3
73610 229533.A0A098DB79 pp 229533.A0A1C3YJA2
73611 229533.A0A098DB79 pp 229533.A0A1C3YMC9
73612 229533.A0A098DB79 pp 229533.I1RMQ7
73613 229533.A0A098DB79 pp 229533.I1RKI2
73614 229533.A0A098DB79 pp 229533.A0A1C3YN55
73615 229533.A0A098DB79 pp 229533.I1RZT1
73616 229533.A0A098DB79 pp 229533.I1RSZ3
73617 229533.A0A098DB79 pp 229533.I1RMS2
73618 229533.A0A098DB79 pp 229533.I1RUF8
73619 229533.A0A098DB79 pp 229533.I1RRN5
73620 229533.A0A098DB79 pp 229533.I1RTC2
73621 229533.A0A098DB79 pp 229533.A0A1C3YJT5
73622 229533.A0A098DB79 pp 229533.I1RZ94
73623 229533.A0A098DB79 pp 229533.A0A1C3YM61
73624 229533.A0A098DB79 pp 229533.A0A1C3YI39
73625 229533.A0A098DB79 pp 229533.I1RRJ6
73626 229533.A0A1C3YHM4 pp 229533.I1S0R4
73627 229533.A0A1C3YHM4 pp 229533.I1RXF5
73628 229533.A0A1C3YHM4 pp 229533.I1RWB4
73629 229533.A0A1C3YHM4 pp 229533.I1S0R3
73630 229533.A0A1C3YHM4 pp 229533.I1RYU1
73631 229533.A0A1C3YHM4 pp 229533.I1RQ32
73632 229533.A0A1C3YHM4 pp 229533.A0A1C3YII7
73633 229533.A0A1C3YHM4 pp 229533.A0A1C3YHW1
73634 229533.A0A1C3YHM4 pp 229533.I1RSZ1
73635 229533.A0A1C3YHM4 pp 229533.I1RE15
73636 229533.A0A1C3YHM4 pp 229533.Q4I6S5
73637 229533.A0A1C3YHM4 pp 229533.I1RMF2
73638 229533.A0A1C3YHM4 pp 229533.I1RIX1
73639 229533.A0A1C3YHM4 pp 229533.I1RQA3
73640 229533.A0A1C3YHM4 pp 229533.I1RG99
73641 229533.A0A1C3YHM4 pp 229533.I1RFK0
73642 229533.A0A1C3YHM4 pp 229533.I1RD63
73643 229533.A0A1C3YHM4 pp 229533.I1RWT6
73644 229533.A0A1C3YHM4 pp 229533.A0A1C3YK51
73645 229533.A0A1C3YJ56 pp 229533.I1RP86
73646 229533.A0A1C3YJ56 pp 229533.I1RGY2
73647 229533.A0A1C3YJ56 pp 229533.A0A1C3YL54
73648 229533.A0A1C3YJ56 pp 229533.I1RZM4
73649 229533.A0A1C3YJ56 pp 229533.I1RGB2
73650 229533.A0A1C3YJ56 pp 229533.A0A1C3YKR5
73651 229533.A0A1C3YJ56 pp 229533.I1S8C1
73652 229533.A0A1C3YJ56 pp 229533.I1S183
73653 229533.A0A1C3YJ56 pp 229533.I1RQE8
73654 229533.A0A1C3YJ56 pp 229533.V6RK96
73655 229533.A0A1C3YJ56 pp 229533.I1RR44
73656 229533.A0A1C3YJ56 pp 229533.I1RJY7
73657 229533.A0A1C3YJ56 pp 229533.A0A1C3YLE2

73658 229533.A0A1C3YJ56 pp 229533.A0A1C3YN26
73659 229533.A0A1C3YJ56 pp 229533.I1RCZ8
73660 229533.A0A1C3YJ56 pp 229533.V6RV57
73661 229533.A0A1C3YJ56 pp 229533.I1RC11
73662 229533.A0A1C3YJ56 pp 229533.I1RBE4
73663 229533.A0A1C3YJ56 pp 229533.I1RUY6
73664 229533.A0A1C3YJ56 pp 229533.I1S148
73665 229533.A0A1C3YJ56 pp 229533.I1RCM5
73666 229533.A0A1C3YJ56 pp 229533.V6RPS5
73667 229533.A0A1C3YJ56 pp 229533.A0A1C3YM97
73668 229533.A0A1C3YJ56 pp 229533.I1RAQ4
73669 229533.A0A1C3YJ56 pp 229533.I1RY12
73670 229533.A0A1C3YJ56 pp 229533.I1RUY5
73671 229533.A0A1C3YJ56 pp 229533.I1RYK0
73672 229533.A0A1C3YJ56 pp 229533.I1RA62
73673 229533.A0A1C3YJ56 pp 229533.I1RMJ3
73674 229533.A0A1C3YJ56 pp 229533.I1RH82
73675 229533.A0A1C3YJ56 pp 229533.I1RDU0
73676 229533.A0A1C3YJ56 pp 229533.I1RGC3
73677 229533.A0A1C3YJ56 pp 229533.I1RNG4
73678 229533.A0A1C3YJ56 pp 229533.I1RUR8
73679 229533.A0A1C3YJ56 pp 229533.I1RY62
73680 229533.A0A1C3YJ56 pp 229533.I1RSQ4
73681 229533.A0A1C3YJ56 pp 229533.I1RM54
73682 229533.A0A1C3YJ56 pp 229533.I1RMC5
73683 229533.A0A1C3YJ56 pp 229533.I1RN84
73684 229533.A0A1C3YJ56 pp 229533.I1RC29
73685 229533.A0A1C3YJ56 pp 229533.I1S3F7
73686 229533.A0A1C3YJ56 pp 229533.I1RQI4
73687 229533.A0A1C3YJ56 pp 229533.I1S0S5
73688 229533.A0A1C3YJ56 pp 229533.I1RN88
73689 229533.A0A1C3YJ56 pp 229533.I1RND4
73690 229533.A0A1C3YJ56 pp 229533.I1RCG5
73691 229533.A0A1C3YJ56 pp 229533.I1R9Y8
73692 229533.A0A1C3YJ56 pp 229533.I1S256
73693 229533.A0A1C3YJ56 pp 229533.I1RYW3
73694 229533.A0A1C3YJ56 pp 229533.I1S147
73695 229533.A0A1C3YJ56 pp 229533.I1RM55
73696 229533.A0A1C3YJ56 pp 229533.I1S149
73697 229533.A0A1C3YJ56 pp 229533.V6RLM7
73698 229533.I1S4Y4 pp 229533.V6RIS2
73699 229533.I1S4Y4 pp 229533.V6RY33
73700 229533.I1S4Y4 pp 229533.I1S7M3
73701 229533.I1S4Y4 pp 229533.I1S7M2
73702 229533.I1S4Y4 pp 229533.Q4I7F9
73703 229533.Q4IAK7 pp 229533.Q4IB70
73704 229533.Q4IAK7 pp 229533.Q4IMD1
73705 229533.Q4IAK7 pp 229533.Q4IB50
73706 229533.Q4IAK7 pp 229533.Q4ICA8
73707 229533.Q4IAK7 pp 229533.Q4IEV4
73708 229533.Q4IAK7 pp 229533.Q4IPI4
73709 229533.I1RQ70 pp 229533.I1RT41
73710 229533.I1RQ70 pp 229533.I1RX84
73711 229533.I1RQ70 pp 229533.I1RTQ9
73712 229533.I1RQ70 pp 229533.I1S0K5
73713 229533.I1RQ70 pp 229533.I1RTC1
73714 229533.I1RQ70 pp 229533.I1S287
73715 229533.I1RQ70 pp 229533.V6RHZ8
73716 229533.I1RQ70 pp 229533.I1RRB0
73717 229533.I1RQ70 pp 229533.Q4HYZ2
73718 229533.I1RQ70 pp 229533.I1RW76
73719 229533.I1RQ70 pp 229533.V6RR76
73720 229533.I1RQ70 pp 229533.I1RYW8
73721 229533.I1RQ70 pp 229533.Q4I6S5
73722 229533.I1RQ70 pp 229533.I1RZ60
73723 229533.I1RQ70 pp 229533.Q4ILI9
73724 229533.I1RQ70 pp 229533.I1S0Z3
73725 229533.I1RQ70 pp 229533.I1S6S4
73726 229533.I1RQ70 pp 229533.I1RYU1
73727 229533.I1RQ70 pp 229533.I1RWB4
73728 229533.I1RQ70 pp 229533.I1S1V7
73729 229533.I1RQ70 pp 229533.I1RSZ6
73730 229533.I1RQ70 pp 229533.I1RTG5

73731	229533.I1RQ70	pp	229533.I1RSJ0
73732	229533.A0A1C3YLB8	pp	229533.I1S0H2
73733	229533.A0A1C3YLB8	pp	229533.I1RXB6
73734	229533.A0A1C3YLB8	pp	229533.I1RKP5
73735	229533.A0A1C3YLB8	pp	229533.I1RB11
73736	229533.A0A1C3YLB8	pp	229533.I1RB58
73737	229533.I1S9J6	pp	229533.Q4I7L0
73738	229533.I1S9J6	pp	229533.Q4ICA8
73739	229533.I1S9J6	pp	229533.V6QUE6
73740	229533.I1S9J6	pp	229533.Q4IMD1
73741	229533.I1S9J6	pp	229533.Q4HTT1
73742	229533.I1S9J6	pp	229533.Q4HTT2
73743	229533.I1S9J6	pp	229533.V6RLW4
73744	229533.I1S9J6	pp	229533.Q4IJH1
73745	229533.I1S9J6	pp	229533.Q4IF76
73746	229533.I1S9J6	pp	229533.Q4HYB8
73747	229533.I1S9J6	pp	229533.Q4IER8
73748	229533.I1RDE9	pp	229533.Q4IN00
73749	229533.I1RDE9	pp	229533.I1RSD4
73750	229533.I1RDE9	pp	229533.I1RG57
73751	229533.I1RDE9	pp	229533.I1RS28
73752	229533.I1RDE9	pp	229533.I1RK90
73753	229533.I1RDE9	pp	229533.V6QXG1
73754	229533.I1RDE9	pp	229533.I1RK92
73755	229533.I1RDE9	pp	229533.I1RSA4
73756	229533.I1RDE9	pp	229533.I1RG90
73757	229533.I1RDE9	pp	229533.I1RND5
73758	229533.I1RDE9	pp	229533.I1RS64
73759	229533.I1RDE9	pp	229533.I1RXB5
73760	229533.I1RDE9	pp	229533.I1RXF2
73761	229533.I1RDE9	pp	229533.I1RWG6
73762	229533.I1RDE9	pp	229533.I1RWD5
73763	229533.I1RDE9	pp	229533.I1RHI1
73764	229533.I1RDE9	pp	229533.I1S082
73765	229533.I1RDE9	pp	229533.I1S092
73766	229533.I1RDE9	pp	229533.I1S4M5
73767	229533.I1RDE9	pp	229533.I1RPA0
73768	229533.I1RDE9	pp	229533.I1RQD4
73769	229533.I1RDE9	pp	229533.I1S7M3
73770	229533.I1RDE9	pp	229533.V6QXP4
73771	229533.I1RDE9	pp	229533.I1S7X9
73772	229533.I1RDE9	pp	229533.I1RZG7
73773	229533.I1RDE9	pp	229533.I1RX81
73774	229533.I1RDE9	pp	229533.I1RW57
73775	229533.I1RDE9	pp	229533.I1RSR2
73776	229533.I1RDE9	pp	229533.I1RFS6
73777	229533.I1RDE9	pp	229533.I1S7M2
73778	229533.V6QX58	pp	229533.V6QXG1
73779	229533.V6QX58	pp	229533.V6QXP4
73780	229533.I1RWQ8	pp	229533.I1RX04
73781	229533.I1RWQ8	pp	229533.I1RXD7
73782	229533.I1RWQ8	pp	229533.Q4I624
73783	229533.I1RWQ8	pp	229533.I1S0L3
73784	229533.I1RWQ8	pp	229533.I1S5B0
73785	229533.I1RWQ8	pp	229533.I1RZM8
73786	229533.I1RWQ8	pp	229533.I1S4S7
73787	229533.I1RWQ8	pp	229533.V6RCG5
73788	229533.I1RWQ8	pp	229533.V6QW22
73789	229533.I1RWQ8	pp	229533.V6RCZ8
73790	229533.I1RWQ8	pp	229533.V6RIV9
73791	229533.I1RWQ8	pp	229533.I1S0L4
73792	229533.I1RWQ8	pp	229533.I1S265
73793	229533.I1RWQ8	pp	229533.I1S1W9
73794	229533.I1RWQ8	pp	229533.I1S5R1
73795	229533.I1RWQ8	pp	229533.I1RZZ9
73796	229533.I1RHP9	pp	229533.Q4HTT2
73797	229533.I1RHP9	pp	229533.Q4I5R3
73798	229533.I1RHP9	pp	229533.Q4IR08
73799	229533.I1RHP9	pp	229533.I1RQ19
73800	229533.I1RHP9	pp	229533.I1RSI1
73801	229533.I1RHP9	pp	229533.I1RXE5
73802	229533.I1RHP9	pp	229533.I1RU02
73803	229533.I1RHP9	pp	229533.Q4IER8

73804 229533.I1RHP9 pp 229533.I1RK15
73805 229533.I1RHP9 pp 229533.I1RZU9
73806 229533.I1RHP9 pp 229533.I1RQE9
73807 229533.I1RHP9 pp 229533.I1RKR6
73808 229533.I1RHP9 pp 229533.V6QV38
73809 229533.I1RHP9 pp 229533.I1RT16
73810 229533.I1RHP9 pp 229533.I1RUQ6
73811 229533.I1RHP9 pp 229533.I1RMA5
73812 229533.I1RHP9 pp 229533.I1RYV3
73813 229533.I1RHP9 pp 229533.Q4IEV4
73814 229533.I1RHP9 pp 229533.I1RYV4
73815 229533.I1RHP9 pp 229533.Q4I7S1
73816 229533.I1RHP9 pp 229533.Q4IMD1
73817 229533.I1RHP9 pp 229533.I1RWU3
73818 229533.I1RHP9 pp 229533.Q4IB50
73819 229533.I1RHP9 pp 229533.I1S1U7
73820 229533.I1RHP9 pp 229533.I1RW28
73821 229533.I1RHP9 pp 229533.I1RPX5
73822 229533.I1RHP9 pp 229533.I1S0N5
73823 229533.I1RHP9 pp 229533.I1RVY0
73824 229533.I1RHP9 pp 229533.I1RPR8
73825 229533.I1RHP9 pp 229533.I1RPY4
73826 229533.I1RKA0 pp 229533.I1RRI2
73827 229533.I1RKA0 pp 229533.I1RQN9
73828 229533.I1RKA0 pp 229533.I1RRJ8
73829 229533.I1RKA0 pp 229533.I1RS47
73830 229533.I1RKA0 pp 229533.I1RSR3
73831 229533.I1RKA0 pp 229533.I1S7X9
73832 229533.I1RKA0 pp 229533.I1S0S6
73833 229533.I1RKA0 pp 229533.P0C431
73834 229533.I1RKA0 pp 229533.I1RTB7
73835 229533.I1RAR5 pp 229533.I1RBY9
73836 229533.I1RAR5 pp 229533.I1RWF9
73837 229533.I1RAR5 pp 229533.I1RH91
73838 229533.I1RAR5 pp 229533.I1RNM7
73839 229533.I1RAR5 pp 229533.I1S3T6
73840 229533.I1RAR5 pp 229533.I1RSD7
73841 229533.I1RAR5 pp 229533.I1S223
73842 229533.I1RAR5 pp 229533.I1RST9
73843 229533.I1RAR5 pp 229533.I1S267
73844 229533.I1RLJ8 pp 229533.I1RRJ6
73845 229533.I1RLJ8 pp 229533.I1RWM6
73846 229533.I1RLJ8 pp 229533.I1RVW0
73847 229533.I1RLJ8 pp 229533.Q4HTT1
73848 229533.I1RLJ8 pp 229533.Q4IB89
73849 229533.I1RLJ8 pp 229533.I1RMJ8
73850 229533.I1RLJ8 pp 229533.I1RZT1
73851 229533.I1RLJ8 pp 229533.I1RRT1
73852 229533.I1RLJ8 pp 229533.I1S4P6
73853 229533.I1RLJ8 pp 229533.I1RXZ4
73854 229533.I1RLJ8 pp 229533.Q4INS6
73855 229533.I1RLJ8 pp 229533.I1RYV3
73856 229533.A0A0E0RMT9 pp 229533.I1RPA6
73857 229533.A0A0E0RMT9 pp 229533.A0A1C3YL99
73858 229533.A0A0E0RMT9 pp 229533.A0A0E0RS12
73859 229533.A0A0E0RMT9 pp 229533.I1RHI1
73860 229533.A0A0E0RMT9 pp 229533.I1RDE9
73861 229533.A0A0E0RMT9 pp 229533.V6QX58
73862 229533.A0A0E0RMT9 pp 229533.I1RBD6
73863 229533.A0A0E0RMT9 pp 229533.I1RAR5
73864 229533.A0A1C3YIC5 pp 229533.I1S6S4
73865 229533.A0A1C3YIC5 pp 229533.I1S1V4
73866 229533.A0A1C3YIC5 pp 229533.I1S4S9
73867 229533.A0A1C3YIC5 pp 229533.I1RK33
73868 229533.A0A1C3YIC5 pp 229533.I1RE03
73869 229533.A0A1C3YIC5 pp 229533.A0A1C3YKU7
73870 229533.A0A1C3YIC5 pp 229533.I1RE18
73871 229533.A0A1C3YIC5 pp 229533.I1RPU5
73872 229533.A0A1C3YIC5 pp 229533.I1S0K5
73873 229533.A0A1C3YIC5 pp 229533.I1RFS7
73874 229533.A0A1C3YIC5 pp 229533.A0A1C3YN81
73875 229533.A0A1C3YIC5 pp 229533.A0A1C3YIE3
73876 229533.A0A1C3YIC5 pp 229533.I1RFV4

73877	229533.A0A1C3YIC5	pp	229533.I1RMF2
73878	229533.A0A1C3YIC5	pp	229533.A0A1C3YM48
73879	229533.A0A1C3YIC5	pp	229533.I1RYU1
73880	229533.A0A1C3YIC5	pp	229533.I1RMR7
73881	229533.A0A1C3YIC5	pp	229533.I1S830
73882	229533.A0A1C3YIC5	pp	229533.I1RXF5
73883	229533.A0A1C3YIC5	pp	229533.I1RC52
73884	229533.A0A1C3YIC5	pp	229533.I1RK86
73885	229533.A0A1C3YIC5	pp	229533.I1S278
73886	229533.A0A1C3YIC5	pp	229533.I1RNA3
73887	229533.A0A1C3YIC5	pp	229533.Q4I6S5
73888	229533.A0A1C3YIC5	pp	229533.I1RZJ9
73889	229533.A0A1C3YIC5	pp	229533.I1RJT0
73890	229533.I1RHC7	pp	229533.I1RNG4
73891	229533.I1RC67	pp	229533.I1S1W3
73892	229533.I1RC67	pp	229533.I1RPK0
73893	229533.I1RC67	pp	229533.I1RTG7
73894	229533.I1RC67	pp	229533.I1RN84
73895	229533.I1RC67	pp	229533.I1RHJ2
73896	229533.I1RC67	pp	229533.I1RMJ3
73897	229533.I1RC67	pp	229533.I1RYP2
73898	229533.I1RC67	pp	229533.I1S0S5
73899	229533.I1RC67	pp	229533.I1RXR1
73900	229533.I1RC67	pp	229533.I1RR68
73901	229533.I1RC67	pp	229533.I1RE79
73902	229533.I1RC67	pp	229533.I1RXU9
73903	229533.I1RC67	pp	229533.I1RCN8
73904	229533.I1RC67	pp	229533.V6QWB1
73905	229533.I1RC67	pp	229533.I1RKZ4
73906	229533.I1RC67	pp	229533.I1RN46
73907	229533.I1RC67	pp	229533.V6RUT5
73908	229533.I1RC67	pp	229533.I1RQH5
73909	229533.I1RC67	pp	229533.I1S2A1
73910	229533.I1RC67	pp	229533.I1S887
73911	229533.I1RC67	pp	229533.V6R7V8
73912	229533.I1RC67	pp	229533.I1S8Z2
73913	229533.I1RCG7	pp	229533.V6R1K4
73914	229533.I1RCG7	pp	229533.V6R729
73915	229533.I1RCG7	pp	229533.I1RS47
73916	229533.I1RCG7	pp	229533.I1RPT0
73917	229533.I1RCG7	pp	229533.I1RMQ6
73918	229533.I1RCG7	pp	229533.I1RCQ1
73919	229533.I1RCG7	pp	229533.I1RSX9
73920	229533.I1RCG7	pp	229533.I1RMW4
73921	229533.I1RCG7	pp	229533.I1RZE7
73922	229533.I1RCG7	pp	229533.I1RTT0
73923	229533.I1RCG7	pp	229533.I1RNU7
73924	229533.I1RCG7	pp	229533.I1RVW6
73925	229533.I1RCG7	pp	229533.I1RVX8
73926	229533.I1RCG7	pp	229533.I1RWT0
73927	229533.I1RCG7	pp	229533.I1RSU2
73928	229533.I1RCG7	pp	229533.V6RVJ3
73929	229533.I1RCG7	pp	229533.I1RPM6
73930	229533.I1RCG7	pp	229533.V6RQW4
73931	229533.I1RCG7	pp	229533.I1RJU4
73932	229533.I1RCG7	pp	229533.I1S064
73933	229533.I1RCG7	pp	229533.I1RQ12
73934	229533.I1RCG7	pp	229533.Q4I5G1
73935	229533.I1RCG7	pp	229533.I1RXB6
73936	229533.I1RCG7	pp	229533.I1RE90
73937	229533.I1RCG7	pp	229533.I1RCN8
73938	229533.I1S788	pp	229533.I1S987
73939	229533.I1S788	pp	229533.Q4IB89
73940	229533.I1S788	pp	229533.Q4INS6
73941	229533.I1S788	pp	229533.Q4IPN9
73942	229533.I1S788	pp	229533.Q4HTT2
73943	229533.I1S788	pp	229533.I1S998
73944	229533.I1S788	pp	229533.Q4HTT1
73945	229533.I1S788	pp	229533.I1S9G7
73946	229533.I1RRJ0	pp	229533.I1RSG8
73947	229533.I1RRJ0	pp	229533.I1RS38
73948	229533.I1RRJ0	pp	229533.I1RWB4
73949	229533.I1RB90	pp	229533.I1RZQ6

73950 229533.I1RB90 pp 229533.I1RRB5
73951 229533.I1RB90 pp 229533.I1RZM1
73952 229533.I1RB90 pp 229533.I1RWT2
73953 229533.I1RB90 pp 229533.I1RSV0
73954 229533.I1RB90 pp 229533.I1RE49
73955 229533.I1RM59 pp 229533.I1RNG4
73956 229533.I1RN14 pp 229533.I1S1W8
73957 229533.I1RN14 pp 229533.I1S3G4
73958 229533.I1RN14 pp 229533.I1RY87
73959 229533.I1RN14 pp 229533.I1S132
73960 229533.I1RN14 pp 229533.I1RNG4
73961 229533.I1RN14 pp 229533.I1RN16
73962 229533.I1RN14 pp 229533.I1S2M8
73963 229533.A0A1C3YI23 pp 229533.I1RG90
73964 229533.A0A1C3YI23 pp 229533.I1RFQ5
73965 229533.A0A1C3YI23 pp 229533.Q4IJU0
73966 229533.A0A1C3YI23 pp 229533.I1S0B1
73967 229533.A0A1C3YI23 pp 229533.I1R9Y7
73968 229533.A0A1C3YI23 pp 229533.Q4IPZ1
73969 229533.A0A1C3YI23 pp 229533.I1RAI9
73970 229533.A0A1C3YI23 pp 229533.I1S0N5
73971 229533.A0A1C3YI23 pp 229533.I1REK8
73972 229533.A0A1C3YI23 pp 229533.I1S5Z2
73973 229533.A0A1C3YI23 pp 229533.I1RCR5
73974 229533.A0A1C3YI23 pp 229533.I1REN3
73975 229533.A0A1C3YI23 pp 229533.I1R9C7
73976 229533.A0A1C3YI23 pp 229533.I1RPA0
73977 229533.A0A1C3YI23 pp 229533.I1S5X9
73978 229533.A0A1C3YI23 pp 229533.I1RUG4
73979 229533.A0A1C3YI23 pp 229533.I1S8V0
73980 229533.A0A1C3YI23 pp 229533.V6QV38
73981 229533.A0A1C3YI23 pp 229533.I1RR25
73982 229533.A0A1C3YI23 pp 229533.I1RE31
73983 229533.A0A1C3YI23 pp 229533.I1RFL4
73984 229533.A0A1C3YI23 pp 229533.I1RW23
73985 229533.A0A1C3YI23 pp 229533.I1S276
73986 229533.A0A1C3YI23 pp 229533.A0A1C3YL10
73987 229533.A0A1C3YI23 pp 229533.I1RWR7
73988 229533.A0A1C3YI23 pp 229533.I1RMV1
73989 229533.A0A1C3YI23 pp 229533.I1S0E8
73990 229533.A0A1C3YI23 pp 229533.I1S2C4
73991 229533.A0A1C3YI23 pp 229533.I1RWN6
73992 229533.A0A1C3YI23 pp 229533.I1RJL2
73993 229533.A0A1C3YI23 pp 229533.I1RYS4
73994 229533.A0A1C3YI23 pp 229533.I1RZZ1
73995 229533.A0A1C3YI23 pp 229533.I1RRY9
73996 229533.A0A1C3YI23 pp 229533.I1RA36
73997 229533.A0A1C3YI23 pp 229533.I1RCH5
73998 229533.A0A1C3YI23 pp 229533.I1RPV5
73999 229533.A0A1C3YI23 pp 229533.I1RZL5
74000 229533.A0A1C3YI23 pp 229533.I1RAW0
74001 229533.A0A1C3YI23 pp 229533.V6QXG1
74002 229533.A0A1C3YI23 pp 229533.I1RR61
74003 229533.A0A1C3YI23 pp 229533.I1RDZ6
74004 229533.A0A1C3YI23 pp 229533.Q4I6B0
74005 229533.A0A1C3YI23 pp 229533.I1RWF3
74006 229533.A0A1C3YI23 pp 229533.I1RZU4
74007 229533.A0A1C3YI23 pp 229533.I1RJY2
74008 229533.A0A1C3YI23 pp 229533.I1RWX8
74009 229533.A0A1C3YI23 pp 229533.Q4IJM4
74010 229533.A0A1C3YI23 pp 229533.I1S1C7
74011 229533.A0A1C3YI23 pp 229533.I1RA38
74012 229533.A0A1C3YI23 pp 229533.I1RT03
74013 229533.A0A1C3YI23 pp 229533.A0A1C3YN55
74014 229533.A0A1C3YI23 pp 229533.I1RWN5
74015 229533.A0A1C3YI23 pp 229533.I1RYW3
74016 229533.A0A1C3YI23 pp 229533.I1RT99
74017 229533.A0A1C3YI23 pp 229533.I1RQZ2
74018 229533.A0A1C3YI23 pp 229533.I1RD38
74019 229533.A0A1C3YI23 pp 229533.I1RB92
74020 229533.A0A1C3YI23 pp 229533.I1RT01
74021 229533.A0A1C3YI23 pp 229533.I1RK79
74022 229533.A0A1C3YI23 pp 229533.I1RSI1

74023 229533.A0A1C3YI23 pp 229533.I1RK95
74024 229533.A0A1C3YI23 pp 229533.I1RZU9
74025 229533.A0A1C3YI23 pp 229533.I1RY03
74026 229533.A0A1C3YI23 pp 229533.A0A1C3YKF1
74027 229533.A0A1C3YI23 pp 229533.A0A1C3YIN6
74028 229533.A0A1C3YI23 pp 229533.I1RY44
74029 229533.A0A1C3YI23 pp 229533.I1RRI2
74030 229533.A0A1C3YI23 pp 229533.I1RXE5
74031 229533.A0A1C3YI23 pp 229533.I1RS41
74032 229533.A0A1C3YI23 pp 229533.I1RM65
74033 229533.A0A1C3YI23 pp 229533.I1RPM7
74034 229533.A0A1C3YI23 pp 229533.I1RSA4
74035 229533.A0A1C3YI23 pp 229533.I1RN07
74036 229533.A0A1C3YI23 pp 229533.I1RCG6
74037 229533.A0A1C3YI23 pp 229533.I1RJW8
74038 229533.A0A1C3YI23 pp 229533.I1RRN6
74039 229533.A0A1C3YI23 pp 229533.I1RCW8
74040 229533.A0A1C3YI23 pp 229533.I1RK71
74041 229533.A0A1C3YI23 pp 229533.I1RJV3
74042 229533.A0A1C3YI23 pp 229533.Q4IER8
74043 229533.A0A1C3YI23 pp 229533.I1S0J1
74044 229533.A0A1C3YI23 pp 229533.I1RBJ1
74045 229533.A0A1C3YI23 pp 229533.I1S9U1
74046 229533.A0A1C3YI23 pp 229533.I1RSG8
74047 229533.A0A1C3YI23 pp 229533.I1RA70
74048 229533.A0A1C3YI23 pp 229533.I1S504
74049 229533.A0A1C3YI23 pp 229533.A0A1C3YKV3
74050 229533.A0A1C3YI23 pp 229533.I1RA55
74051 229533.A0A1C3YI23 pp 229533.I1S8Y0
74052 229533.A0A1C3YI23 pp 229533.I1S8R3
74053 229533.A0A1C3YI23 pp 229533.I1RNB8
74054 229533.A0A1C3YI23 pp 229533.A0A1C3YJR3
74055 229533.A0A1C3YI23 pp 229533.A0A1C3YKB0
74056 229533.A0A1C3YI23 pp 229533.I1S255
74057 229533.A0A1C3YI23 pp 229533.I1RTH6
74058 229533.A0A1C3YI23 pp 229533.Q4HYB8
74059 229533.A0A1C3YI23 pp 229533.I1RQ81
74060 229533.A0A1C3YI23 pp 229533.I1RZB5
74061 229533.A0A1C3YI23 pp 229533.I1RQZ3
74062 229533.A0A1C3YI23 pp 229533.I1RJY3
74063 229533.A0A1C3YI23 pp 229533.Q4IQT8
74064 229533.A0A1C3YI23 pp 229533.I1RZ42
74065 229533.A0A1C3YI23 pp 229533.I1RU02
74066 229533.A0A1C3YI23 pp 229533.I1RCM5
74067 229533.A0A1C3YI23 pp 229533.I1RC15
74068 229533.A0A1C3YI23 pp 229533.I1RRT0
74069 229533.A0A1C3YI23 pp 229533.I1RE01
74070 229533.A0A1C3YI23 pp 229533.I1RDC9
74071 229533.A0A1C3YI23 pp 229533.I1RS18
74072 229533.A0A1C3YI23 pp 229533.I1S0C6
74073 229533.A0A1C3YI23 pp 229533.I1RZK5
74074 229533.A0A1C3YI23 pp 229533.V6QUE6
74075 229533.A0A1C3YI23 pp 229533.I1RMQ7
74076 229533.A0A1C3YI23 pp 229533.I1RCR8
74077 229533.A0A1C3YI23 pp 229533.A0A1C3YJZ9
74078 229533.A0A1C3YI23 pp 229533.A0A1C3YK34
74079 229533.A0A1C3YI23 pp 229533.I1RUP9
74080 229533.A0A1C3YI23 pp 229533.I1RI82
74081 229533.A0A1C3YI23 pp 229533.I1RAR2
74082 229533.A0A1C3YI23 pp 229533.I1RE14
74083 229533.A0A1C3YI23 pp 229533.Q4I5I4
74084 229533.A0A1C3YI23 pp 229533.I1RNP4
74085 229533.A0A1C3YI23 pp 229533.I1RJI3
74086 229533.A0A1C3YI23 pp 229533.I1RFQ0
74087 229533.A0A1C3YI23 pp 229533.I1RM42
74088 229533.A0A1C3YI23 pp 229533.I1RBM3
74089 229533.A0A1C3YI23 pp 229533.I1RD22
74090 229533.A0A1C3YI23 pp 229533.I1S7M3
74091 229533.A0A1C3YI23 pp 229533.I1RE65
74092 229533.A0A1C3YI23 pp 229533.A0A1C3YMI5
74093 229533.A0A1C3YI23 pp 229533.I1RC17
74094 229533.A0A1C3YI23 pp 229533.I1RRF7
74095 229533.A0A1C3YI23 pp 229533.Q4HYI0

229533.A0A1C3YI23	pp	229533.I1RHL7
229533.A0A1C3YI23	pp	229533.I1RLV3
229533.A0A1C3YI23	pp	229533.V6RIS2
229533.A0A1C3YI23	pp	229533.I1S3N1
229533.A0A1C3YI23	pp	229533.I1RNQ6
229533.A0A1C3YI23	pp	229533.I1RG11
229533.A0A1C3YI23	pp	229533.Q4I099
229533.A0A1C3YI23	pp	229533.A0A1C3YLW6
229533.A0A1C3YI23	pp	229533.I1S8U9
229533.A0A1C3YI23	pp	229533.I1RVL8
229533.A0A1C3YI23	pp	229533.Q4HTT2
229533.A0A1C3YI23	pp	229533.I1RUG3
229533.A0A1C3YI23	pp	229533.A0A1C3YI60
229533.A0A1C3YI23	pp	229533.I1RCQ6
229533.A0A1C3YI23	pp	229533.I1S8Y1
229533.A0A1C3YI23	pp	229533.I1RXU0
229533.A0A1C3YI23	pp	229533.A0A1C3YJ12
229533.A0A1C3YI23	pp	229533.I1RZM8
229533.A0A1C3YI23	pp	229533.A0A1C3YKX5
229533.A0A1C3YI23	pp	229533.Q4I7S1
229533.A0A1C3YI23	pp	229533.Q4HTT1
229533.A0A1C3YI23	pp	229533.I1S2H4
229533.A0A1C3YI23	pp	229533.V6RMW6
229533.A0A1C3YI23	pp	229533.I1RRI1
229533.A0A1C3YI23	pp	229533.I1RC29
229533.A0A1C3YI23	pp	229533.I1RB71
229533.A0A1C3YI23	pp	229533.A0A1C3YMY3
229533.A0A1C3YI23	pp	229533.A0A1C3YJZ2
229533.A0A1C3YI23	pp	229533.V6R042
229533.A0A1C3YI23	pp	229533.Q4IPN9
229533.A0A1C3YI23	pp	229533.I1RSQ3
229533.A0A1C3YI23	pp	229533.I1RN04
229533.A0A1C3YI23	pp	229533.I1RCB7
229533.A0A1C3YI23	pp	229533.I1RBR5
229533.A0A1C3YI23	pp	229533.I1S9Y1
229533.A0A1C3YI23	pp	229533.I1R9M0
229533.A0A1C3YI23	pp	229533.I1RWL3
229533.A0A1C3YI23	pp	229533.I1RLR0
229533.A0A1C3YI23	pp	229533.I1RD36
229533.A0A1C3YI23	pp	229533.I1RZZ9
229533.A0A1C3YI23	pp	229533.I1RS66
229533.A0A1C3YI23	pp	229533.I1RVY0
229533.A0A1C3YI23	pp	229533.I1RRT8
229533.A0A1C3YI23	pp	229533.I1S5R1
229533.A0A1C3YI23	pp	229533.I1RXZ3
229533.A0A1C3YI23	pp	229533.I1S2B8
229533.A0A1C3YI23	pp	229533.I1RA84
229533.A0A1C3YI23	pp	229533.A0A1C3YIR8
229533.A0A1C3YI23	pp	229533.I1RW91
229533.A0A1C3YI23	pp	229533.I1S0R5
229533.A0A1C3YI23	pp	229533.I1RR24
229533.A0A1C3YI23	pp	229533.I1S0X6
229533.A0A1C3YI23	pp	229533.I1RVJ1
229533.A0A1C3YI23	pp	229533.V6QWX8
229533.A0A1C3YI23	pp	229533.I1RC00
229533.A0A1C3YI23	pp	229533.I1RQ42
229533.A0A1C3YI23	pp	229533.I1S214
229533.A0A1C3YI23	pp	229533.I1RTH2
229533.A0A1C3YI23	pp	229533.I1RBS0
229533.A0A1C3YI23	pp	229533.I1RZR6
229533.A0A1C3YI23	pp	229533.I1R9Q0
229533.A0A1C3YI23	pp	229533.I1RYI9
229533.A0A1C3YI23	pp	229533.A0A1C3YKM9
229533.A0A1C3YI23	pp	229533.I1S0J3
229533.A0A1C3YI23	pp	229533.I1RLV1
229533.A0A1C3YI23	pp	229533.I1RE30
229533.A0A1C3YI23	pp	229533.I1RE66
229533.A0A1C3YI23	pp	229533.I1RZ39
229533.A0A1C3YI23	pp	229533.I1RKQ5
229533.A0A1C3YI23	pp	229533.I1RUG0
229533.A0A1C3YI23	pp	229533.I1RNH1
229533.A0A1C3YI23	pp	229533.I1RX63
229533.A0A1C3YI23	pp	229533.I1S503

74169 229533.A0A1C3YI23 pp 229533.I1RAB5
74170 229533.A0A1C3YI23 pp 229533.I1RRW3
74171 229533.A0A1C3YI23 pp 229533.I1RC24
74172 229533.A0A1C3YI23 pp 229533.A0A1C3YLK8
74173 229533.A0A1C3YI23 pp 229533.I1RJU3
74174 229533.A0A1C3YI23 pp 229533.I1RW90
74175 229533.A0A1C3YI23 pp 229533.Q4I5W5
74176 229533.A0A1C3YI23 pp 229533.I1RB50
74177 229533.A0A1C3YI23 pp 229533.V6R3X5
74178 229533.A0A1C3YI23 pp 229533.I1RPW8
74179 229533.A0A1C3YI23 pp 229533.Q4IMD1
74180 229533.A0A1C3YI23 pp 229533.I1RWK3
74181 229533.A0A1C3YI23 pp 229533.I1RAD1
74182 229533.A0A1C3YI23 pp 229533.I1RN67
74183 229533.A0A1C3YI23 pp 229533.I1RJN9
74184 229533.A0A1C3YI23 pp 229533.I1RX46
74185 229533.A0A1C3YI23 pp 229533.I1RZM2
74186 229533.A0A1C3YI23 pp 229533.I1RS30
74187 229533.A0A1C3YI23 pp 229533.A0A1C3YK88
74188 229533.A0A1C3YI23 pp 229533.I1RS43
74189 229533.A0A1C3YI23 pp 229533.I1RW80
74190 229533.A0A1C3YI23 pp 229533.Q4HYQ4
74191 229533.A0A1C3YI23 pp 229533.I1S182
74192 229533.A0A1C3YI23 pp 229533.I1RMU8
74193 229533.A0A1C3YI23 pp 229533.A0A1C3YKM6
74194 229533.A0A1C3YI23 pp 229533.I1RHL8
74195 229533.A0A1C3YI23 pp 229533.A0A1C3YM43
74196 229533.A0A1C3YI23 pp 229533.A0A1C3YIP5
74197 229533.A0A1C3YI23 pp 229533.I1S3N0
74198 229533.A0A1C3YI23 pp 229533.I1RFW1
74199 229533.A0A1C3YI23 pp 229533.I1RGA4
74200 229533.A0A1C3YI23 pp 229533.A0A1C3YJT5
74201 229533.A0A1C3YI23 pp 229533.I1RAX6
74202 229533.A0A1C3YI23 pp 229533.I1RRG3
74203 229533.A0A1C3YI23 pp 229533.I1RBC7
74204 229533.A0A1C3YI23 pp 229533.I1RBV3
74205 229533.A0A1C3YI23 pp 229533.I1RTC2
74206 229533.A0A1C3YI23 pp 229533.I1RKB1
74207 229533.A0A1C3YI23 pp 229533.A0A1C3YK13
74208 229533.A0A1C3YI23 pp 229533.I1RA46
74209 229533.A0A1C3YI23 pp 229533.I1RDD3
74210 229533.A0A1C3YI23 pp 229533.A0A1C3YMK1
74211 229533.A0A1C3YI23 pp 229533.I1RHQ5
74212 229533.A0A1C3YI23 pp 229533.I1S0R7
74213 229533.A0A1C3YI23 pp 229533.V6RTJ8
74214 229533.A0A1C3YI23 pp 229533.I1RP96
74215 229533.A0A1C3YI23 pp 229533.I1RFT1
74216 229533.A0A1C3YI23 pp 229533.I1RJV4
74217 229533.A0A1C3YI23 pp 229533.A0A1C3YLP8
74218 229533.A0A1C3YI23 pp 229533.I1RSQ7
74219 229533.A0A1C3YI23 pp 229533.I1RML9
74220 229533.A0A1C3YI23 pp 229533.I1RV91
74221 229533.A0A1C3YI23 pp 229533.I1S7M2
74222 229533.A0A1C3YI23 pp 229533.I1RFL7
74223 229533.A0A1C3YI23 pp 229533.I1RBW4
74224 229533.A0A1C3YI23 pp 229533.I1S201
74225 229533.A0A1C3YI23 pp 229533.I1RC20
74226 229533.A0A1C3YI23 pp 229533.I1RAV6
74227 229533.A0A1C3YI23 pp 229533.I1RXF2
74228 229533.A0A1C3YI23 pp 229533.I1RP21
74229 229533.A0A1C3YI23 pp 229533.Q4IJ11
74230 229533.A0A1C3YI23 pp 229533.I1RSZ3
74231 229533.A0A1C3YI23 pp 229533.I1S2E9
74232 229533.A0A1C3YI23 pp 229533.I1RTD5
74233 229533.A0A1C3YI23 pp 229533.I1RGR8
74234 229533.A0A1C3YI23 pp 229533.I1RQ88
74235 229533.A0A1C3YI23 pp 229533.I1RXD7
74236 229533.I1RGX1 pp 229533.I1RUR8
74237 229533.I1RGX1 pp 229533.V6RUY9
74238 229533.I1RGX1 pp 229533.I1RN84
74239 229533.I1RGX1 pp 229533.V6RV57
74240 229533.I1RGX1 pp 229533.I1S0S5
74241 229533.I1RGX1 pp 229533.I1RMJ3

74242 229533.I1RGX1 pp 229533.I1RSQ4
74243 229533.I1RGX1 pp 229533.I1RND4
74244 229533.I1RGX1 pp 229533.I1RYW3
74245 229533.I1RGX1 pp 229533.I1RYK0
74246 229533.I1RGX1 pp 229533.I1RZM4
74247 229533.I1RGX1 pp 229533.I1RNG4
74248 229533.I1RGX1 pp 229533.I1RQE8
74249 229533.I1RGX1 pp 229533.V6RLM7
74250 229533.I1RGX1 pp 229533.I1RH82
74251 229533.I1RGX1 pp 229533.I1RUY5
74252 229533.I1RGX1 pp 229533.I1RY12
74253 229533.I1RGX1 pp 229533.I1S8C1
74254 229533.I1RGX1 pp 229533.V6RK96
74255 229533.I1RGX1 pp 229533.I1RP86
74256 229533.I1RGX1 pp 229533.I1RUY6
74257 229533.I1RGX1 pp 229533.I1RJY7
74258 229533.I1RGX1 pp 229533.I1S183
74259 229533.Q4I5I4 pp 229533.Q4I5R3
74260 229533.Q4I5I4 pp 229533.V6QUE6
74261 229533.Q4I5I4 pp 229533.Q4ICA8
74262 229533.Q4I5I4 pp 229533.Q4I7X1
74263 229533.Q4I5I4 pp 229533.Q4IPC8
74264 229533.Q4I5I4 pp 229533.Q4IJU0
74265 229533.Q4I5I4 pp 229533.V6RDZ4
74266 229533.Q4I5I4 pp 229533.V6QV38
74267 229533.Q4I5I4 pp 229533.V6QVD5
74268 229533.Q4I5I4 pp 229533.Q4I5U9
74269 229533.Q4I5I4 pp 229533.Q4I5W5
74270 229533.Q4I5I4 pp 229533.Q4IPA4
74271 229533.Q4I5I4 pp 229533.Q4IB50
74272 229533.Q4I5I4 pp 229533.Q4IER8
74273 229533.Q4I5I4 pp 229533.V6QWX8
74274 229533.Q4I5I4 pp 229533.Q4IJ11
74275 229533.Q4I5I4 pp 229533.Q4IPN9
74276 229533.A0A1C3YMV8 pp 229533.I1S6C7
74277 229533.A0A1C3YMV8 pp 229533.I1RZN1
74278 229533.A0A1C3YMV8 pp 229533.I1RIM5
74279 229533.A0A1C3YMV8 pp 229533.I1RNG4
74280 229533.A0A1C3YMV8 pp 229533.I1RFV2
74281 229533.I1RYN7 pp 229533.Q4IPI4
74282 229533.I1RYN7 pp 229533.Q4HTT1
74283 229533.I1RYN7 pp 229533.Q4I7L0
74284 229533.I1RYN7 pp 229533.I1S1V2
74285 229533.I1RYN7 pp 229533.Q4IER8
74286 229533.A0A1C3YIV0 pp 229533.I1RBA4
74287 229533.A0A1C3YIV0 pp 229533.I1RXR1
74288 229533.A0A1C3YIV0 pp 229533.I1RBF3
74289 229533.A0A1C3YIV0 pp 229533.A0A1C3YKF3
74290 229533.A0A1C3YIV0 pp 229533.I1RXU9
74291 229533.A0A1C3YIV0 pp 229533.I1RTG7
74292 229533.A0A1C3YIV0 pp 229533.I1S1W3
74293 229533.A0A1C3YIV0 pp 229533.A0A1C3YKY6
74294 229533.A0A1C3YIV0 pp 229533.I1RYP2
74295 229533.A0A1C3YIV0 pp 229533.I1S887
74296 229533.A0A1C3YIV0 pp 229533.I1RPK0
74297 229533.A0A1C3YIV0 pp 229533.A0A1C3YLC1
74298 229533.A0A1C3YIV0 pp 229533.I1RMJ3
74299 229533.A0A1C3YIV0 pp 229533.A0A1C3YLE2
74300 229533.A0A1C3YIV0 pp 229533.A0A1C3YJZ0
74301 229533.A0A1C3YIV0 pp 229533.I1RCN8
74302 229533.A0A1C3YIV0 pp 229533.I1RHJ2
74303 229533.A0A1C3YIV0 pp 229533.I1RKZ4
74304 229533.A0A1C3YIV0 pp 229533.I1RR68
74305 229533.A0A1C3YIV0 pp 229533.I1RN46
74306 229533.A0A1C3YIV0 pp 229533.I1RN84
74307 229533.A0A1C3YIV0 pp 229533.I1S2A1
74308 229533.A0A1C3YIV0 pp 229533.I1RBE4
74309 229533.A0A1C3YIV0 pp 229533.I1S0S5
74310 229533.A0A1C3YIV0 pp 229533.V6R7V8
74311 229533.A0A1C3YIV0 pp 229533.I1RE79
74312 229533.A0A1C3YIV0 pp 229533.I1S8Z2
74313 229533.A0A1C3YIV0 pp 229533.I1RBD5
74314 229533.A0A1C3YIV0 pp 229533.I1RA85

229533.A0A1C3YIV0 pp 229533.V6RUT5
229533.A0A1C3YIV0 pp 229533.A0A1C3YJL7
229533.A0A1C3YHS2 pp 229533.I1RDQ9
229533.A0A1C3YHS2 pp 229533.I1RWB4
229533.A0A1C3YHS2 pp 229533.I1RNM1
229533.A0A1C3YHS2 pp 229533.I1RQ32
229533.A0A1C3YHS2 pp 229533.Q4I5V3
229533.A0A1C3YHS2 pp 229533.I1RN27
229533.A0A1C3YHS2 pp 229533.I1RRJ0
229533.A0A1C3YHS2 pp 229533.I1RMT6
229533.A0A1C3YHS2 pp 229533.A0A1C3YK01
229533.A0A1C3YHS2 pp 229533.A0A1C3YI81
229533.A0A1C3YHS2 pp 229533.A0A1C3YHX4
229533.A0A1C3YHS2 pp 229533.Q4I7S1
229533.A0A1C3YHS2 pp 229533.Q4IPC8
229533.A0A1C3YHS2 pp 229533.I1RN49
229533.I1RK33 pp 229533.I1S6S4
229533.I1RK33 pp 229533.I1RW84
229533.I1RK33 pp 229533.I1S1V4
229533.I1RK33 pp 229533.I1RMV6
229533.I1RK33 pp 229533.I1RM18
229533.I1RK33 pp 229533.I1RYU1
229533.I1RK33 pp 229533.I1S830
229533.I1RK33 pp 229533.I1RZ93
229533.I1RK33 pp 229533.I1RPU5
229533.I1RK33 pp 229533.I1RMF2
229533.I1RK33 pp 229533.I1RZ60
229533.I1RK33 pp 229533.Q4I6S5
229533.I1RK33 pp 229533.I1RR93
229533.I1RK33 pp 229533.I1S278
229533.I1RK33 pp 229533.I1RNA3
229533.A0A098E375 pp 229533.I1S8C1
229533.A0A098E375 pp 229533.I1RQE8
229533.A0A098E375 pp 229533.I1RC11
229533.A0A098E375 pp 229533.I1RCM5
229533.A0A098E375 pp 229533.A0A1C3YN26
229533.A0A098E375 pp 229533.I1RAQ4
229533.A0A098E375 pp 229533.I1RY12
229533.A0A098E375 pp 229533.I1RP86
229533.A0A098E375 pp 229533.I1RUY5
229533.A0A098E375 pp 229533.I1RZM4
229533.A0A098E375 pp 229533.I1RH82
229533.A0A098E375 pp 229533.I1RDU0
229533.A0A098E375 pp 229533.I1RGB2
229533.A0A098E375 pp 229533.I1RJY7
229533.A0A098E375 pp 229533.I1RC29
229533.A0A098E375 pp 229533.I1S183
229533.A0A098E375 pp 229533.I1R9Y8
229533.A0A098E375 pp 229533.A0A1C3YKR5
229533.A0A098E375 pp 229533.I1RUR8
229533.A0A098E375 pp 229533.A0A1C3YLE2
229533.A0A098E375 pp 229533.V6RV57
229533.A0A098E375 pp 229533.I1RBE4
229533.A0A098E375 pp 229533.I1RMJ3
229533.A0A098E375 pp 229533.V6RK96
229533.A0A098E375 pp 229533.I1RNG4
229533.A0A098E375 pp 229533.I1RYW3
229533.A0A098E375 pp 229533.I1RUY6
229533.A0A098E375 pp 229533.I1RCG5
229533.A0A098E375 pp 229533.I1RYK0
229533.A0A098E375 pp 229533.I1RSQ4
229533.A0A098E375 pp 229533.A0A1C3YHU3
229533.A0A098E375 pp 229533.A0A0E0RU55
229533.A0A098E375 pp 229533.I1RN84
229533.A0A098E375 pp 229533.I1S0S5
229533.A0A098E375 pp 229533.I1RND4
229533.A0A098E375 pp 229533.V6RLM7
229533.A0A0E0RTW3 pp 229533.I1RWU3
229533.A0A0E0RTW3 pp 229533.A0A0E0SPD4
229533.A0A0E0RTW3 pp 229533.I1RPY4
229533.A0A0E0RTW3 pp 229533.A0A1C3YJZ2
229533.A0A0E0RTW3 pp 229533.I1RYV4
229533.A0A0E0RTW3 pp 229533.I1RPR8

74388 229533.A0A0E0RTW3 pp 229533.Q4IMD1
74389 229533.A0A0E0RTW3 pp 229533.I1RB36
74390 229533.A0A0E0RTW3 pp 229533.I1RUQ6
74391 229533.A0A0E0RTW3 pp 229533.I1RW28
74392 229533.A0A0E0RTW3 pp 229533.I1REF2
74393 229533.A0A0E0RTW3 pp 229533.I1RGW0
74394 229533.A0A0E0RTW3 pp 229533.I1RVY0
74395 229533.A0A0E0RTW3 pp 229533.Q4IEV4
74396 229533.A0A0E0RTW3 pp 229533.I1RT16
74397 229533.A0A0E0RTW3 pp 229533.I1RMA5
74398 229533.A0A0E0RTW3 pp 229533.A0A0E0SQJ6
74399 229533.A0A0E0RTW3 pp 229533.I1RFW1
74400 229533.A0A0E0RTW3 pp 229533.I1RPX5
74401 229533.A0A0E0RTW3 pp 229533.I1RGE1
74402 229533.A0A0E0RTW3 pp 229533.I1RKR6
74403 229533.A0A0E0RTW3 pp 229533.I1RBJ0
74404 229533.A0A0E0RTW3 pp 229533.A0A1C3YLX5
74405 229533.A0A0E0RTW3 pp 229533.I1RQE9
74406 229533.A0A0E0RTW3 pp 229533.I1S0N5
74407 229533.A0A0E0RTW3 pp 229533.A0A1C3YHV5
74408 229533.A0A0E0RTW3 pp 229533.I1RE51
74409 229533.A0A0E0RTW3 pp 229533.Q4HTT2
74410 229533.A0A0E0RTW3 pp 229533.V6QV38
74411 229533.A0A0E0RTW3 pp 229533.A0A1C3YJE9
74412 229533.A0A0E0RTW3 pp 229533.I1RXE5
74413 229533.A0A0E0RTW3 pp 229533.A0A0E0SQV5
74414 229533.A0A0E0RTW3 pp 229533.A0A1C3YJ12
74415 229533.A0A0E0RTW3 pp 229533.I1RA56
74416 229533.A0A0E0RTW3 pp 229533.Q4IER8
74417 229533.A0A0E0RTW3 pp 229533.A0A1C3YL16
74418 229533.A0A0E0RTW3 pp 229533.I1RCW8
74419 229533.A0A0E0RTW3 pp 229533.I1RK15
74420 229533.A0A0E0RTW3 pp 229533.I1RU02
74421 229533.A0A0E0RTW3 pp 229533.I1RQ19
74422 229533.A0A0E0RTW3 pp 229533.A0A1C3YK01
74423 229533.A0A0E0RTW3 pp 229533.A0A1C3YIJ2
74424 229533.A0A0E0RTW3 pp 229533.I1RDC9
74425 229533.A0A0E0RTW3 pp 229533.Q4I5R3
74426 229533.A0A0E0RTW3 pp 229533.I1RYV3
74427 229533.I1S219 pp 229533.Q4IMD1
74428 229533.I1S219 pp 229533.Q4INZ9
74429 229533.I1S219 pp 229533.Q4HTT1
74430 229533.I1S219 pp 229533.Q4IER8
74431 229533.I1S219 pp 229533.Q4HVW2
74432 229533.I1S219 pp 229533.Q4HTT2
74433 229533.I1RD01 pp 229533.I1RV91
74434 229533.I1RD01 pp 229533.I1S2E9
74435 229533.I1RD01 pp 229533.I1RPF5
74436 229533.I1RD01 pp 229533.I1RWI8
74437 229533.I1RD01 pp 229533.I1RY29
74438 229533.I1RD01 pp 229533.I1RVV5
74439 229533.I1RD01 pp 229533.I1RJV8
74440 229533.I1RD01 pp 229533.I1RQ67
74441 229533.I1RD01 pp 229533.I1RR68
74442 229533.I1RD01 pp 229533.I1RY44
74443 229533.I1RD01 pp 229533.I1RYQ1
74444 229533.I1RD01 pp 229533.I1S7X9
74445 229533.I1RD01 pp 229533.I1S004
74446 229533.I1RD01 pp 229533.I1S4P6
74447 229533.I1RD01 pp 229533.I1RKQ0
74448 229533.I1RD01 pp 229533.I1RSP7
74449 229533.I1RD01 pp 229533.I1RDV4
74450 229533.I1RD01 pp 229533.I1RD19
74451 229533.I1RD01 pp 229533.I1S0X7
74452 229533.I1RD01 pp 229533.I1RZ86
74453 229533.I1RD01 pp 229533.I1RTN6
74454 229533.I1RD01 pp 229533.I1RXI6
74455 229533.I1RD01 pp 229533.I1RZP8
74456 229533.I1RD01 pp 229533.Q4HTT1
74457 229533.I1RD01 pp 229533.I1RHD2
74458 229533.I1RD01 pp 229533.I1RF70
74459 229533.I1RD01 pp 229533.I1RX64
74460 229533.I1RD01 pp 229533.I1S998

74461	229533.I1RD01	pp	229533.I1S788
74462	229533.I1RD01	pp	229533.I1S0D5
74463	229533.I1RD01	pp	229533.I1RRJ6
74464	229533.I1RD01	pp	229533.I1RYV4
74465	229533.I1RD01	pp	229533.I1RSR9
74466	229533.I1RD01	pp	229533.I1RRT1
74467	229533.I1RD01	pp	229533.I1RMJ8
74468	229533.I1RD01	pp	229533.I1RWT5
74469	229533.I1RD01	pp	229533.I1RZ98
74470	229533.I1RD01	pp	229533.I1RZ94
74471	229533.I1RD01	pp	229533.I1RZT1
74472	229533.I1RD01	pp	229533.I1RY58
74473	229533.I1RD01	pp	229533.I1RSC7
74474	229533.I1RD01	pp	229533.I1RMB7
74475	229533.I1RD01	pp	229533.I1RSK0
74476	229533.I1RD01	pp	229533.I1RDJ8
74477	229533.I1RD01	pp	229533.Q4IB89
74478	229533.I1RD01	pp	229533.I1S987
74479	229533.I1RD01	pp	229533.I1RUG4
74480	229533.I1RD01	pp	229533.I1RP99
74481	229533.I1RD01	pp	229533.I1RDZ4
74482	229533.I1RD01	pp	229533.I1RU61
74483	229533.I1RD01	pp	229533.I1RKI2
74484	229533.I1RD01	pp	229533.I1RQH8
74485	229533.I1RD01	pp	229533.I1RG77
74486	229533.I1RD01	pp	229533.I1RWM6
74487	229533.I1RD01	pp	229533.Q4HTT2
74488	229533.I1RD01	pp	229533.I1RG34
74489	229533.I1RD01	pp	229533.I1RPQ1
74490	229533.I1RD01	pp	229533.I1RFS6
74491	229533.I1RD01	pp	229533.Q4INS6
74492	229533.I1RD01	pp	229533.I1RLJ8
74493	229533.I1RD01	pp	229533.I1S209
74494	229533.I1RD01	pp	229533.I1RXZ4
74495	229533.A0A1C3YHS1	pp	229533.Q4I0C5
74496	229533.A0A1C3YHS1	pp	229533.A0A1C3YKN4
74497	229533.A0A1C3YHS1	pp	229533.I1S2C5
74498	229533.A0A1C3YHS1	pp	229533.A0A1C3YL34
74499	229533.I1RD46	pp	229533.I1RX83
74500	229533.I1RM36	pp	229533.Q4ICA8
74501	229533.I1RM36	pp	229533.I1RMQ7
74502	229533.I1RM36	pp	229533.I1RRG3
74503	229533.I1RM36	pp	229533.Q4IJH1
74504	229533.I1RM36	pp	229533.Q4I0J6
74505	229533.I1RM36	pp	229533.I1RST8
74506	229533.I1RM36	pp	229533.Q4HTT1
74507	229533.I1RM36	pp	229533.I1RN46
74508	229533.I1RM36	pp	229533.I1RQ80
74509	229533.I1RM36	pp	229533.I1RVY0
74510	229533.I1RM36	pp	229533.V6R382
74511	229533.I1RM36	pp	229533.I1S2F2
74512	229533.I1RM36	pp	229533.I1RZ94
74513	229533.I1RM36	pp	229533.I1RYX9
74514	229533.I1RM36	pp	229533.Q4I7L0
74515	229533.I1RM36	pp	229533.I1S3L3
74516	229533.I1RM36	pp	229533.I1RWI8
74517	229533.I1RM36	pp	229533.I1RSC7
74518	229533.I1RM36	pp	229533.Q4IMD1
74519	229533.I1RM36	pp	229533.Q4IBX0
74520	229533.I1RM36	pp	229533.I1RN55
74521	229533.I1RM36	pp	229533.I1RQJ4
74522	229533.I1RM36	pp	229533.I1RQ13
74523	229533.I1RM36	pp	229533.I1RU02
74524	229533.I1RM36	pp	229533.I1RN57
74525	229533.I1RM36	pp	229533.I1RMB7
74526	229533.I1RM36	pp	229533.Q4IER8
74527	229533.I1RM36	pp	229533.I1RZB5
74528	229533.I1RM36	pp	229533.I1RUR0
74529	229533.I1RM36	pp	229533.I1S283
74530	229533.I1RM36	pp	229533.I1RSZ3
74531	229533.I1RM36	pp	229533.Q4HTT2
74532	229533.I1RM36	pp	229533.I1RUF8
74533	229533.I1RM36	pp	229533.I1RQP1

74534 229533.I1RM36 pp 229533.V6QUE6
74535 229533.I1RM36 pp 229533.I1S3W0
74536 229533.I1RM36 pp 229533.I1S020
74537 229533.I1RM36 pp 229533.I1RQR3
74538 229533.I1RM36 pp 229533.I1RY58
74539 229533.I1RM36 pp 229533.Q4IF76
74540 229533.I1RM36 pp 229533.I1RRN5
74541 229533.I1RM36 pp 229533.V6RLW4
74542 229533.I1RM36 pp 229533.Q4HYB8
74543 229533.I1RM36 pp 229533.I1RSD4
74544 229533.I1RM36 pp 229533.I1RZP8
74545 229533.I1RM36 pp 229533.I1S0J3
74546 229533.I1RA32 pp 229533.I1S6D7
74547 229533.I1RA32 pp 229533.I1RMJ3
74548 229533.I1RA32 pp 229533.I1RSQ2
74549 229533.I1RA32 pp 229533.I1RJD3
74550 229533.I1RA32 pp 229533.I1RSF1
74551 229533.I1RA32 pp 229533.I1RKP1
74552 229533.I1RA32 pp 229533.I1S3G4
74553 229533.I1RA32 pp 229533.I1RQ38
74554 229533.I1RA32 pp 229533.I1RBU8
74555 229533.I1RA32 pp 229533.V6QX21
74556 229533.I1RA32 pp 229533.I1S8Z2
74557 229533.I1RA32 pp 229533.I1S123
74558 229533.I1RA32 pp 229533.I1RBF3
74559 229533.I1RA32 pp 229533.I1RYB8
74560 229533.I1RA32 pp 229533.I1RLW5
74561 229533.I1RA32 pp 229533.I1S9T5
74562 229533.I1RA32 pp 229533.I1S1W8
74563 229533.I1RA32 pp 229533.I1RN84
74564 229533.I1RA32 pp 229533.I1RGG6
74565 229533.I1RA32 pp 229533.I1S4Z7
74566 229533.I1RA32 pp 229533.I1RE48
74567 229533.I1RA32 pp 229533.I1S5H2
74568 229533.I1RA32 pp 229533.I1RT75
74569 229533.I1RA32 pp 229533.I1RG71
74570 229533.I1RA32 pp 229533.I1RM49
74571 229533.I1RSF0 pp 229533.I1RWT7
74572 229533.I1RDX3 pp 229533.I1RP25
74573 229533.I1RDX3 pp 229533.I1RQN9
74574 229533.I1RDX3 pp 229533.I1S7X9
74575 229533.I1RDX3 pp 229533.V6REE9
74576 229533.I1RDX3 pp 229533.I1S0S6
74577 229533.I1RDX3 pp 229533.I1RTE6
74578 229533.I1RDX3 pp 229533.I1RN65
74579 229533.A0A0E0SES1 pp 229533.Q4HTT1
74580 229533.A0A0E0SES1 pp 229533.I1RCN2
74581 229533.A0A0E0SES1 pp 229533.I1RC98
74582 229533.A0A0E0SES1 pp 229533.I1S1Y7
74583 229533.A0A0E0SES1 pp 229533.I1RB73
74584 229533.A0A0E0SES1 pp 229533.A0A1C3YMS7
74585 229533.A0A0E0SES1 pp 229533.I1RSL8
74586 229533.A0A0E0SES1 pp 229533.I1RQL4
74587 229533.A0A0E0SES1 pp 229533.Q4IQX3
74588 229533.A0A0E0SES1 pp 229533.Q4I7L0
74589 229533.A0A0E0SES1 pp 229533.I1RFW1
74590 229533.A0A0E0SES1 pp 229533.A0A0E0SF53
74591 229533.A0A0E0SES1 pp 229533.Q4IER8
74592 229533.A0A0E0SES1 pp 229533.Q4IMD1
74593 229533.A0A0E0SES1 pp 229533.I1S830
74594 229533.A0A0E0SES1 pp 229533.I1S0F0
74595 229533.A0A0E0SES1 pp 229533.I1RRM8
74596 229533.A0A0E0SES1 pp 229533.V6RPN0
74597 229533.A0A0E0SES1 pp 229533.I1RCX7
74598 229533.A0A0E0SES1 pp 229533.Q4HTT2
74599 229533.A0A0E0SES1 pp 229533.I1RD69
74600 229533.A0A0E0SES1 pp 229533.A0A1C3YN58
74601 229533.A0A0E0SES1 pp 229533.I1RFQ0
74602 229533.A0A0E0SES1 pp 229533.Q4I5R3
74603 229533.A0A0E0SES1 pp 229533.I1S5R5
74604 229533.A0A0E0SES1 pp 229533.A0A1C3YK75
74605 229533.A0A0E0SES1 pp 229533.I1RQ86
74606 229533.I1RJN7 pp 229533.I1S0S5

74607 229533.I1RJN7 pp 229533.I1RND4
74608 229533.I1RJN7 pp 229533.V6RLM7
74609 229533.I1RJN7 pp 229533.I1RSA3
74610 229533.I1RJN7 pp 229533.I1RN84
74611 229533.I1RJN7 pp 229533.I1RSQ4
74612 229533.I1RJN7 pp 229533.I1RUR8
74613 229533.I1RJN7 pp 229533.I1RYW3
74614 229533.I1RJN7 pp 229533.I1RMJ3
74615 229533.I1RJN7 pp 229533.I1RNG4
74616 229533.I1RJN7 pp 229533.I1S183
74617 229533.I1RJN7 pp 229533.V6RV57
74618 229533.I1RJN7 pp 229533.I1RYK0
74619 229533.I1RJN7 pp 229533.I1RY12
74620 229533.I1RJN7 pp 229533.V6RK96
74621 229533.I1RJN7 pp 229533.I1RUY6
74622 229533.I1RJN7 pp 229533.I1S8C1
74623 229533.I1RJN7 pp 229533.I1RUY5
74624 229533.I1RJN7 pp 229533.I1RZM4
74625 229533.I1RJN7 pp 229533.I1RP86
74626 229533.I1RJN7 pp 229533.I1RJY7
74627 229533.I1RJN7 pp 229533.I1RQE8
74628 229533.I1S2V2 pp 229533.I1S3S5
74629 229533.I1S2V2 pp 229533.I1S821
74630 229533.I1S2V2 pp 229533.Q4I7G0
74631 229533.A0A1C3YHR0 pp 229533.I1RSF0
74632 229533.A0A1C3YHR0 pp 229533.I1S0B1
74633 229533.A0A1C3YHR0 pp 229533.I1S7W5
74634 229533.I1RAS3 pp 229533.I1RN50
74635 229533.I1RAS3 pp 229533.I1RX83
74636 229533.I1RAS3 pp 229533.I1RD46
74637 229533.I1RAS3 pp 229533.I1RCP6
74638 229533.I1RAS3 pp 229533.I1RZM0
74639 229533.I1RU88 pp 229533.I1RWT6
74640 229533.I1RPF4 pp 229533.I1RVY0
74641 229533.I1RPF4 pp 229533.Q4HTT1
74642 229533.I1RPF4 pp 229533.I1RRT1
74643 229533.I1RPF4 pp 229533.Q4ICA8
74644 229533.I1RPF4 pp 229533.Q4IPI4
74645 229533.I1RPF4 pp 229533.I1SB04
74646 229533.I1RPF4 pp 229533.I1RYX3
74647 229533.I1RPF4 pp 229533.Q4HTT2
74648 229533.I1RPF4 pp 229533.Q4I5V3
74649 229533.I1RPF4 pp 229533.I1RW60
74650 229533.I1RPF4 pp 229533.I1S4P6
74651 229533.I1RPF4 pp 229533.I1RSU7
74652 229533.I1RPF4 pp 229533.Q4I948
74653 229533.I1RPF4 pp 229533.I1RZF3
74654 229533.I1RPF4 pp 229533.Q4HY90
74655 229533.I1RPF4 pp 229533.Q4IB89
74656 229533.I1RPF4 pp 229533.I1S0D5
74657 229533.I1RPF4 pp 229533.Q4IMD1
74658 229533.A0A098DIL3 pp 229533.I1RD80
74659 229533.A0A098DIL3 pp 229533.I1RE01
74660 229533.A0A098DIL3 pp 229533.I1RPK0
74661 229533.A0A098DIL3 pp 229533.I1RZV3
74662 229533.A0A098DIL3 pp 229533.I1S1W9
74663 229533.A0A098DIL3 pp 229533.I1RPU2
74664 229533.A0A098DIL3 pp 229533.A0A1C3YIB6
74665 229533.A0A098DIL3 pp 229533.A0A098DTE3
74666 229533.A0A098DIL3 pp 229533.I1RUG3
74667 229533.A0A098DIL3 pp 229533.A0A0E0SKJ9
74668 229533.A0A098DIL3 pp 229533.I1RNC1
74669 229533.A0A098DIL3 pp 229533.I1RRM3
74670 229533.A0A098DIL3 pp 229533.I1S5R6
74671 229533.A0A098DIL3 pp 229533.V6RMW6
74672 229533.A0A098DIL3 pp 229533.I1RQE7
74673 229533.A0A098DIL3 pp 229533.I1RPP3
74674 229533.A0A098DIL3 pp 229533.I1RCH5
74675 229533.A0A098DIL3 pp 229533.I1RSQ3
74676 229533.A0A098DIL3 pp 229533.V6R042
74677 229533.A0A098DIL3 pp 229533.I1RYI5
74678 229533.A0A098DIL3 pp 229533.I1RVX0
74679 229533.A0A098DIL3 pp 229533.I1RDF7

74680 229533.A0A098DIL3 pp 229533.I1RBR1
74681 229533.A0A098DIL3 pp 229533.I1S444
74682 229533.A0A098DIL3 pp 229533.I1S0W4
74683 229533.A0A098DIL3 pp 229533.I1RQ33
74684 229533.A0A098DIL3 pp 229533.I1RSA5
74685 229533.A0A098DIL3 pp 229533.I1RD22
74686 229533.A0A098DIL3 pp 229533.I1S437
74687 229533.A0A098DIL3 pp 229533.I1RZ39
74688 229533.A0A098DIL3 pp 229533.A0A1C3YJL2
74689 229533.A0A098DIL3 pp 229533.I1RT40
74690 229533.A0A098DIL3 pp 229533.I1RTE9
74691 229533.A0A098DIL3 pp 229533.A0A1C3YJI6
74692 229533.A0A098DIL3 pp 229533.I1RE89
74693 229533.A0A098DIL3 pp 229533.A0A1C3YKM9
74694 229533.A0A098DIL3 pp 229533.I1RA11
74695 229533.A0A098DIL3 pp 229533.A0A098E3Y1
74696 229533.A0A098DIL3 pp 229533.A0A1C3YLK8
74697 229533.A0A098DIL3 pp 229533.I1RAW0
74698 229533.A0A098DIL3 pp 229533.I1RGA4
74699 229533.A0A098DIL3 pp 229533.I1RZ78
74700 229533.A0A098DIL3 pp 229533.I1RXC2
74701 229533.A0A098DIL3 pp 229533.I1RVZ2
74702 229533.A0A098DIL3 pp 229533.I1S5R1
74703 229533.A0A098DIL3 pp 229533.I1RSZ7
74704 229533.A0A098DIL3 pp 229533.I1RTA1
74705 229533.A0A098DIL3 pp 229533.I1RCD4
74706 229533.A0A098DIL3 pp 229533.I1RRI1
74707 229533.A0A098DIL3 pp 229533.I1RJN9
74708 229533.A0A098DIL3 pp 229533.I1RPE0
74709 229533.A0A098DIL3 pp 229533.A0A1C3YK34
74710 229533.A0A098DIL3 pp 229533.I1S2B8
74711 229533.A0A098DIL3 pp 229533.I1R9M0
74712 229533.A0A098DIL3 pp 229533.I1RYR0
74713 229533.A0A098DIL3 pp 229533.I1RKM2
74714 229533.A0A098DIL3 pp 229533.I1RL20
74715 229533.A0A098DIL3 pp 229533.I1RXC4
74716 229533.A0A098DIL3 pp 229533.A0A1C3YKV3
74717 229533.A0A098DIL3 pp 229533.Q4IJK2
74718 229533.A0A098DIL3 pp 229533.A0A1C3YJN3
74719 229533.A0A098DIL3 pp 229533.I1RQ85
74720 229533.A0A098DIL3 pp 229533.I1REK8
74721 229533.A0A098DIL3 pp 229533.I1RXD7
74722 229533.A0A098DIL3 pp 229533.I1RBR2
74723 229533.A0A098DIL3 pp 229533.A0A1C3YKB0
74724 229533.A0A098DIL3 pp 229533.I1RVS4
74725 229533.A0A098DIL3 pp 229533.V6REE9
74726 229533.A0A098DIL3 pp 229533.I1RZM8
74727 229533.A0A098DIL3 pp 229533.V6RCZ8
74728 229533.A0A098DIL3 pp 229533.A0A1C3YI19
74729 229533.A0A098DIL3 pp 229533.V6RTJ8
74730 229533.A0A098DIL3 pp 229533.I1RZK5
74731 229533.A0A098DIL3 pp 229533.I1RCU8
74732 229533.A0A098DIL3 pp 229533.I1S438
74733 229533.A0A098DIL3 pp 229533.I1S5B0
74734 229533.A0A098DIL3 pp 229533.I1RWR7
74735 229533.A0A098DIL3 pp 229533.A0A098DSB7
74736 229533.A0A098DIL3 pp 229533.A0A1C3YJV6
74737 229533.A0A098DIL3 pp 229533.A0A1C3YI30
74738 229533.A0A098DIL3 pp 229533.I1RQD2
74739 229533.A0A098DIL3 pp 229533.A0A098DR13
74740 229533.A0A098DIL3 pp 229533.I1RJU3
74741 229533.A0A098DIL3 pp 229533.I1S4S7
74742 229533.A0A098DIL3 pp 229533.A0A098DR40
74743 229533.A0A098DIL3 pp 229533.I1S4S4
74744 229533.A0A098DIL3 pp 229533.I1RD52
74745 229533.A0A1C3YIM5 pp 229533.I1RZX4
74746 229533.I1RUY4 pp 229533.I1S0S5
74747 229533.I1RUY4 pp 229533.V6RK96
74748 229533.I1RUY4 pp 229533.V6RLM7
74749 229533.I1RUY4 pp 229533.I1RUY6
74750 229533.I1RUY4 pp 229533.I1RYK0
74751 229533.I1RUY4 pp 229533.V6RV57
74752 229533.I1RUY4 pp 229533.I1S183

74753 229533.I1RUY4 pp 229533.I1S8C1
74754 229533.I1RUY4 pp 229533.I1RYW3
74755 229533.I1RUY4 pp 229533.I1RZM4
74756 229533.I1RUY4 pp 229533.I1RUY5
74757 229533.I1RUY4 pp 229533.I1RY12
74758 229533.I1RG34 pp 229533.Q4HTT1
74759 229533.I1RG34 pp 229533.I1RRJ6
74760 229533.I1RG34 pp 229533.I1RRT1
74761 229533.I1RG34 pp 229533.I1RWM6
74762 229533.I1RG34 pp 229533.I1RVW0
74763 229533.I1RG34 pp 229533.I1RXZ4
74764 229533.I1RG34 pp 229533.I1RZT1
74765 229533.I1RG34 pp 229533.Q4IB89
74766 229533.I1RG34 pp 229533.I1RMJ8
74767 229533.I1RG34 pp 229533.I1S4P6
74768 229533.I1RG34 pp 229533.I1RYV3
74769 229533.I1RG34 pp 229533.Q4INS6
74770 229533.I1RY12 pp 229533.I1S3T7
74771 229533.I1RY12 pp 229533.I1RYQ4
74772 229533.I1RY12 pp 229533.Q4IR09
74773 229533.I1RY12 pp 229533.I1RZ25
74774 229533.I1RY12 pp 229533.I1SAB4
74775 229533.I1RY12 pp 229533.I1S048
74776 229533.I1RY12 pp 229533.I1S210
74777 229533.I1RY12 pp 229533.I1RY62
74778 229533.I1RY12 pp 229533.V6RXR0
74779 229533.I1RY12 pp 229533.V6RHD0
74780 229533.I1RY12 pp 229533.I1RYK0
74781 229533.I1RY12 pp 229533.I1S223
74782 229533.I1RY12 pp 229533.I1S8C1
74783 229533.I1RY12 pp 229533.I1RZ38
74784 229533.I1RY12 pp 229533.I1S746
74785 229533.I1RY12 pp 229533.I1S2C4
74786 229533.I1RY12 pp 229533.I1S299
74787 229533.I1RY12 pp 229533.V6RUY9
74788 229533.I1RY12 pp 229533.I1S2F7
74789 229533.I1RY12 pp 229533.I1S3T6
74790 229533.I1RY12 pp 229533.V6QU65
74791 229533.I1RY12 pp 229533.V6RV57
74792 229533.I1RY12 pp 229533.I1S0V6
74793 229533.I1RY12 pp 229533.V6RRM0
74794 229533.I1RY12 pp 229533.I1RY74
74795 229533.I1RY12 pp 229533.I1S3V2
74796 229533.I1RY12 pp 229533.V6RM02
74797 229533.I1RY12 pp 229533.V6RN69
74798 229533.I1RY12 pp 229533.I1S8R2
74799 229533.I1RY12 pp 229533.I1S256
74800 229533.I1RY12 pp 229533.I1RZK8
74801 229533.I1RY12 pp 229533.I1S267
74802 229533.I1RY12 pp 229533.I1S0V7
74803 229533.I1RY12 pp 229533.I1S147
74804 229533.I1RY12 pp 229533.V6RLM7
74805 229533.I1RC32 pp 229533.I1RZE1
74806 229533.I1RC32 pp 229533.I1RC72
74807 229533.I1RC32 pp 229533.I1RNU6
74808 229533.I1RC32 pp 229533.I1RCL3
74809 229533.I1RC32 pp 229533.I1S274
74810 229533.I1RC32 pp 229533.V6RGN5
74811 229533.I1RC32 pp 229533.I1S2C0
74812 229533.I1RC32 pp 229533.I1RPJ3
74813 229533.I1RC32 pp 229533.I1RTD7
74814 229533.I1RC32 pp 229533.I1RY99
74815 229533.I1RC32 pp 229533.I1RMN6
74816 229533.I1RC32 pp 229533.I1RD22
74817 229533.I1RC32 pp 229533.I1RRI2
74818 229533.I1RC32 pp 229533.I1RD64
74819 229533.I1RC32 pp 229533.I1RSI6
74820 229533.I1RC32 pp 229533.I1SA47
74821 229533.I1RC32 pp 229533.I1RP08
74822 229533.I1RC32 pp 229533.I1RKC6
74823 229533.I1RC32 pp 229533.I1RNL7
74824 229533.I1RC32 pp 229533.I1RM96
74825 229533.I1RC32 pp 229533.I1SAA0

74826	229533.I1RC32	pp	229533.I1RSD8
74827	229533.I1RC32	pp	229533.I1S1X9
74828	229533.I1RC32	pp	229533.I1RC82
74829	229533.I1RC32	pp	229533.I1RW28
74830	229533.I1RC32	pp	229533.I1RKJ6
74831	229533.I1RC32	pp	229533.I1RFN1
74832	229533.I1RC32	pp	229533.I1S1U6
74833	229533.I1RC32	pp	229533.O74638
74834	229533.I1RC32	pp	229533.Q4IMD1
74835	229533.I1RC32	pp	229533.I1RGY5
74836	229533.I1RC32	pp	229533.Q4IF76
74837	229533.I1RC32	pp	229533.I1RRT2
74838	229533.I1RC32	pp	229533.I1RVJ6
74839	229533.I1RC32	pp	229533.I1S2E8
74840	229533.I1RC32	pp	229533.I1RJL4
74841	229533.I1RC32	pp	229533.I1S490
74842	229533.I1RC32	pp	229533.I1RHP9
74843	229533.I1RC32	pp	229533.Q4IEV4
74844	229533.I1RC32	pp	229533.Q4HTT2
74845	229533.I1RC32	pp	229533.I1S9F8
74846	229533.I1RC32	pp	229533.I1RS47
74847	229533.I1RC32	pp	229533.I1RQH8
74848	229533.I1RC32	pp	229533.Q4HZ42
74849	229533.I1RC32	pp	229533.I1RQ31
74850	229533.I1RC32	pp	229533.I1RVY0
74851	229533.I1RC32	pp	229533.I1RNH6
74852	229533.I1RC32	pp	229533.I1RS30
74853	229533.I1RC32	pp	229533.I1RCN2
74854	229533.I1RC32	pp	229533.I1RX24
74855	229533.I1RC32	pp	229533.I1RZF6
74856	229533.I1RC32	pp	229533.I1RWG5
74857	229533.I1RC32	pp	229533.I1RFG0
74858	229533.I1RC32	pp	229533.Q4HTT1
74859	229533.I1RC32	pp	229533.I1RKL3
74860	229533.I1RC32	pp	229533.I1RRJ8
74861	229533.I1RC32	pp	229533.I1RG56
74862	229533.I1RC32	pp	229533.I1RZS6
74863	229533.I1RC32	pp	229533.I1RQ19
74864	229533.I1RC32	pp	229533.I1RFW1
74865	229533.I1RC32	pp	229533.V6R283
74866	229533.I1RC32	pp	229533.Q4I662
74867	229533.I1RC32	pp	229533.I1RFS3
74868	229533.I1RC32	pp	229533.Q4IEK8
74869	229533.I1RC32	pp	229533.I1RQ81
74870	229533.I1RC32	pp	229533.Q4IJP1
74871	229533.I1RC32	pp	229533.I1RPR8
74872	229533.I1RC32	pp	229533.Q4I5R3
74873	229533.I1RC32	pp	229533.V6QUE6
74874	229533.I1RC32	pp	229533.I1RSR3
74875	229533.I1RC32	pp	229533.I1RQ93
74876	229533.I1RC32	pp	229533.I1RNH1
74877	229533.I1RC32	pp	229533.I1RCY5
74878	229533.I1RC32	pp	229533.I1RKQ9
74879	229533.I1RC32	pp	229533.I1RW71
74880	229533.I1RC32	pp	229533.I1RYV3
74881	229533.I1RC32	pp	229533.I1RZL5
74882	229533.I1RC32	pp	229533.I1S9U2
74883	229533.I1RC32	pp	229533.I1RZ99
74884	229533.I1RC32	pp	229533.I1RQA2
74885	229533.I1RC32	pp	229533.I1RZE5
74886	229533.I1RC32	pp	229533.Q4IER8
74887	229533.I1RC32	pp	229533.I1RM67
74888	229533.I1RC32	pp	229533.I1RSC6
74889	229533.I1RC32	pp	229533.I1RE00
74890	229533.I1RC32	pp	229533.I1RT80
74891	229533.I1RC32	pp	229533.I1RNV6
74892	229533.I1RC32	pp	229533.I1RM06
74893	229533.I1RC32	pp	229533.Q4IJH1
74894	229533.I1RC32	pp	229533.I1RQZ2
74895	229533.I1RC32	pp	229533.I1RG51
74896	229533.I1RC32	pp	229533.I1RYV4
74897	229533.I1RC32	pp	229533.I1RDG0
74898	229533.I1RC32	pp	229533.I1RCG8

74899 229533.I1RC32 pp 229533.I1RK95
74900 229533.I1RC32 pp 229533.I1RTF4
74901 229533.I1RC32 pp 229533.I1RMK3
74902 229533.I1RC32 pp 229533.I1RKQ5
74903 229533.I1RC32 pp 229533.I1RZZ1
74904 229533.I1RC32 pp 229533.I1RQM6
74905 229533.I1RC32 pp 229533.I1RDA0
74906 229533.I1RC32 pp 229533.Q4IQK7
74907 229533.I1RC32 pp 229533.V6QYF0
74908 229533.I1RC32 pp 229533.I1S0R7
74909 229533.I1RA86 pp 229533.I1RAB3
74910 229533.I1RA86 pp 229533.I1RG60
74911 229533.I1RA86 pp 229533.I1RYL5
74912 229533.I1RA86 pp 229533.I1RPK5
74913 229533.I1RA86 pp 229533.I1RDH7
74914 229533.I1RA86 pp 229533.I1S3S5
74915 229533.I1RA86 pp 229533.I1RPL2
74916 229533.I1RA86 pp 229533.I1RQA5
74917 229533.I1RA86 pp 229533.I1S821
74918 229533.I1RA86 pp 229533.I1RMQ5
74919 229533.I1RA86 pp 229533.I1RB53
74920 229533.I1RA86 pp 229533.I1RFY0
74921 229533.I1RA86 pp 229533.I1S0Z8
74922 229533.I1RA86 pp 229533.I1RBY6
74923 229533.I1RA86 pp 229533.I1RBY7
74924 229533.I1RA86 pp 229533.Q4I7G0
74925 229533.I1RA86 pp 229533.I1RRT6
74926 229533.I1RA86 pp 229533.I1RJT9
74927 229533.I1RA86 pp 229533.I1RW71
74928 229533.I1RA86 pp 229533.I1RQL4
74929 229533.I1RA86 pp 229533.I1REB8
74930 229533.I1RA86 pp 229533.I1RL79
74931 229533.I1RA86 pp 229533.I1RT62
74932 229533.I1RA86 pp 229533.I1RZD3
74933 229533.I1RA86 pp 229533.I1RF94
74934 229533.I1RA86 pp 229533.I1RQH1
74935 229533.I1RA86 pp 229533.I1RRF8
74936 229533.I1RA86 pp 229533.I1RAM6
74937 229533.I1RA86 pp 229533.I1RME4
74938 229533.I1RA86 pp 229533.I1RTU8
74939 229533.I1RA86 pp 229533.I1RMQ3
74940 229533.I1RA86 pp 229533.I1RMB1
74941 229533.I1RA86 pp 229533.I1RPV0
74942 229533.I1RA86 pp 229533.I1RZK1
74943 229533.I1RA86 pp 229533.I1RU27
74944 229533.I1RA86 pp 229533.I1RBT8
74945 229533.I1RA86 pp 229533.I1REC7
74946 229533.I1RA86 pp 229533.I1RGC6
74947 229533.I1RA86 pp 229533.I1RZS8
74948 229533.I1RA86 pp 229533.I1REG1
74949 229533.I1RA86 pp 229533.I1RQG4
74950 229533.I1RA86 pp 229533.I1RBS5
74951 229533.I1RA86 pp 229533.I1RP28
74952 229533.I1RA86 pp 229533.I1RNG4
74953 229533.I1RA86 pp 229533.I1RB59
74954 229533.I1RA86 pp 229533.I1REC6
74955 229533.I1RA86 pp 229533.I1RJZ7
74956 229533.I1RA86 pp 229533.I1S0F9
74957 229533.A0A1C3YHR3 pp 229533.I1S071
74958 229533.A0A1C3YHR3 pp 229533.A0A1C3YIM5
74959 229533.A0A1C3YHR3 pp 229533.I1RCN0
74960 229533.A0A1C3YHR3 pp 229533.I1S0Z8
74961 229533.A0A1C3YHR3 pp 229533.I1RED5
74962 229533.I1RMB9 pp 229533.I1RNG4
74963 229533.A0A098DBR4 pp 229533.Q4ICA8
74964 229533.A0A098DBR4 pp 229533.Q4I7S1
74965 229533.A0A098DBR4 pp 229533.A0A1C3YM29
74966 229533.A0A098DBR4 pp 229533.A0A1C3YKV4
74967 229533.A0A098DBR4 pp 229533.Q4IMD1
74968 229533.A0A098DBR4 pp 229533.Q4IAK7
74969 229533.A0A098DBR4 pp 229533.Q4HTT2
74970 229533.A0A098DBR4 pp 229533.I1RAV2
74971 229533.A0A098DBR4 pp 229533.I1RFN9

74972 229533.A0A098DBR4 pp 229533.I1S270
74973 229533.A0A098DBR4 pp 229533.Q4I948
74974 229533.A0A098DBR4 pp 229533.Q4HTT1
74975 229533.A0A098DBR4 pp 229533.Q4HY90
74976 229533.A0A098DBR4 pp 229533.I1RKR9
74977 229533.A0A098DBR4 pp 229533.Q4I650
74978 229533.I1RA20 pp 229533.I1RMP2
74979 229533.I1RA20 pp 229533.I1S0S6
74980 229533.I1RA20 pp 229533.I1RQL4
74981 229533.I1RA20 pp 229533.I1RE19
74982 229533.I1RA20 pp 229533.I1RLX6
74983 229533.I1RPN8 pp 229533.I1RQG4
74984 229533.I1RPN8 pp 229533.I1RU27
74985 229533.I1RPN8 pp 229533.I1RZD3
74986 229533.I1RPN8 pp 229533.I1RQW1
74987 229533.I1RPN8 pp 229533.I1RZK1
74988 229533.I1RPN8 pp 229533.I1RYL5
74989 229533.I1RPN8 pp 229533.I1RZS8
74990 229533.I1RPN8 pp 229533.I1S0F9
74991 229533.I1RPN8 pp 229533.Q4I7G0
74992 229533.I1RPN8 pp 229533.I1RPV0
74993 229533.I1RPN8 pp 229533.I1S0Z8
74994 229533.I1RPN8 pp 229533.I1RQA5
74995 229533.I1RPN8 pp 229533.I1S3S5
74996 229533.I1RPN8 pp 229533.I1RQL4
74997 229533.I1RPN8 pp 229533.I1S821
74998 229533.I1RPN8 pp 229533.I1RRF8
74999 229533.I1RPN8 pp 229533.I1RQH1
75000 229533.I1RPN8 pp 229533.I1S3I4
75001 229533.I1RPN8 pp 229533.I1RTU8
75002 229533.I1RPN8 pp 229533.I1RT62
75003 229533.I1RPN8 pp 229533.I1RRT6
75004 229533.I1RPN8 pp 229533.I1RW71
75005 229533.I1RVC7 pp 229533.I1S3M2
75006 229533.I1RVC7 pp 229533.I1S303
75007 229533.I1RVC7 pp 229533.I1S069
75008 229533.I1RVC7 pp 229533.I1S2I7
75009 229533.I1RVS4 pp 229533.I1RWC2
75010 229533.I1RVS4 pp 229533.I1S037
75011 229533.A0A1C3YM35 pp 229533.I1S3G4
75012 229533.A0A1C3YM35 pp 229533.I1RE58
75013 229533.A0A1C3YM35 pp 229533.I1RC74
75014 229533.A0A1C3YM35 pp 229533.I1RVR2
75015 229533.A0A1C3YM35 pp 229533.I1RY49
75016 229533.A0A1C3YM35 pp 229533.I1S1W8
75017 229533.A0A1C3YM35 pp 229533.I1RJZ7
75018 229533.A0A1C3YM35 pp 229533.I1RMJ3
75019 229533.A0A1C3YM35 pp 229533.I1RZM4
75020 229533.A0A1C3YM35 pp 229533.I1RQE8
75021 229533.A0A1C3YM35 pp 229533.I1RVZ3
75022 229533.A0A1C3YM35 pp 229533.I1RSK5
75023 229533.A0A1C3YM35 pp 229533.I1RW71
75024 229533.A0A1C3YM35 pp 229533.I1S0N3
75025 229533.A0A1C3YM35 pp 229533.I1RVX9
75026 229533.A0A1C3YM35 pp 229533.I1RNG4
75027 229533.A0A1C3YM35 pp 229533.V6RM02
75028 229533.A0A1C3YM35 pp 229533.I1RX59
75029 229533.A0A1C3YM35 pp 229533.V6RDH3
75030 229533.A0A1C3YM35 pp 229533.V6RTJ8
75031 229533.A0A1C3YM35 pp 229533.I1RA94
75032 229533.A0A1C3YM35 pp 229533.I1RCT7
75033 229533.A0A1C3YM35 pp 229533.I1RM26
75034 229533.A0A1C3YM35 pp 229533.I1RAH3
75035 229533.A0A1C3YM35 pp 229533.I1RFK3
75036 229533.A0A1C3YM35 pp 229533.I1RBE7
75037 229533.A0A1C3YM35 pp 229533.V6RK96
75038 229533.A0A1C3YM35 pp 229533.I1RZK8
75039 229533.A0A1C3YM35 pp 229533.I1RN84
75040 229533.A0A1C3YM35 pp 229533.I1RC48
75041 229533.A0A1C3YM35 pp 229533.I1RXY7
75042 229533.A0A1C3YM35 pp 229533.I1RA90
75043 229533.A0A098E4F8 pp 229533.I1RBT8
75044 229533.A0A098E4F8 pp 229533.I1RZK1

75045 229533.A0A098E4F8 pp 229533.I1S0F9
75046 229533.A0A098E4F8 pp 229533.I1RU27
75047 229533.A0A098E4F8 pp 229533.I1RB59
75048 229533.A0A098E4F8 pp 229533.I1REC6
75049 229533.A0A098E4F8 pp 229533.I1REC7
75050 229533.A0A098E4F8 pp 229533.I1RGC6
75051 229533.A0A098E4F8 pp 229533.I1RJZ7
75052 229533.A0A098E4F8 pp 229533.I1RQG4
75053 229533.A0A098E4F8 pp 229533.I1REG1
75054 229533.A0A098E4F8 pp 229533.I1R9Y8
75055 229533.A0A098E4F8 pp 229533.I1RL79
75056 229533.A0A098E4F8 pp 229533.I1RBS5
75057 229533.A0A098E4F8 pp 229533.Q4I7G0
75058 229533.A0A098E4F8 pp 229533.I1RNG4
75059 229533.A0A098E4F8 pp 229533.I1RMQ5
75060 229533.A0A098E4F8 pp 229533.I1RP28
75061 229533.A0A098E4F8 pp 229533.I1RBY6
75062 229533.A0A098E4F8 pp 229533.I1REB8
75063 229533.A0A098E4F8 pp 229533.I1RZD3
75064 229533.A0A098E4F8 pp 229533.I1RMB1
75065 229533.A0A098E4F8 pp 229533.I1S0Z8
75066 229533.A0A098E4F8 pp 229533.I1RQH1
75067 229533.A0A098E4F8 pp 229533.A0A0E0SK74
75068 229533.A0A098E4F8 pp 229533.I1RQL4
75069 229533.A0A098E4F8 pp 229533.I1RME4
75070 229533.A0A098E4F8 pp 229533.I1RMQ3
75071 229533.A0A098E4F8 pp 229533.I1RAM6
75072 229533.A0A098E4F8 pp 229533.I1RW71
75073 229533.A0A098E4F8 pp 229533.I1RPV0
75074 229533.A0A098E4F8 pp 229533.A0A1C3YLT3
75075 229533.A0A098E4F8 pp 229533.I1RT62
75076 229533.A0A098E4F8 pp 229533.A0A1C3YK11
75077 229533.A0A098E4F8 pp 229533.I1RRF8
75078 229533.A0A098E4F8 pp 229533.I1RZS8
75079 229533.A0A098E4F8 pp 229533.I1RTU8
75080 229533.A0A098E4F8 pp 229533.A0A1C3YI83
75081 229533.A0A098E4F8 pp 229533.I1S3S5
75082 229533.A0A098E4F8 pp 229533.A0A1C3YIK4
75083 229533.A0A098E4F8 pp 229533.I1RF94
75084 229533.A0A098E4F8 pp 229533.I1RRT6
75085 229533.A0A098E4F8 pp 229533.I1RJT9
75086 229533.A0A098E4F8 pp 229533.I1RYL5
75087 229533.A0A098E4F8 pp 229533.I1RQA5
75088 229533.A0A098E4F8 pp 229533.A0A0E0RVF4
75089 229533.A0A098E4F8 pp 229533.I1RG60
75090 229533.A0A098E4F8 pp 229533.I1RAB3
75091 229533.A0A098E4F8 pp 229533.A0A1C3YHZ0
75092 229533.A0A098E4F8 pp 229533.I1S821
75093 229533.A0A098E4F8 pp 229533.I1RFY0
75094 229533.A0A098E4F8 pp 229533.I1RBY7
75095 229533.A0A098E4F8 pp 229533.I1RB53
75096 229533.A0A098E4F8 pp 229533.I1RPK5
75097 229533.A0A098E4F8 pp 229533.I1RDH7
75098 229533.A0A098E4F8 pp 229533.I1RPL2
75099 229533.I1RD69 pp 229533.I1RE67
75100 229533.I1RD69 pp 229533.I1RFY2
75101 229533.I1RD69 pp 229533.I1S364
75102 229533.I1RD69 pp 229533.I1S224
75103 229533.I1RD69 pp 229533.I1S724
75104 229533.I1RD69 pp 229533.I1RZV4
75105 229533.I1RD69 pp 229533.I1S095
75106 229533.I1RD69 pp 229533.I1RJK7
75107 229533.I1RD69 pp 229533.I1RQA3
75108 229533.I1RD69 pp 229533.I1RQS4
75109 229533.I1RD69 pp 229533.I1RFS6
75110 229533.I1RD69 pp 229533.I1RMS5
75111 229533.I1RD69 pp 229533.I1RDP5
75112 229533.I1RD69 pp 229533.I1S259
75113 229533.I1RD69 pp 229533.I1RU02
75114 229533.I1RD69 pp 229533.Q4IER8
75115 229533.I1RD69 pp 229533.Q4I5R3
75116 229533.I1RD69 pp 229533.I1RIW5
75117 229533.I1RD69 pp 229533.Q4HTT2

75118 229533.I1RD69 pp 229533.I1RRX1
75119 229533.I1RD69 pp 229533.I1RXZ4
75120 229533.I1RD69 pp 229533.I1RQ19
75121 229533.I1RD69 pp 229533.I1S5T5
75122 229533.I1RD69 pp 229533.I1RZD9
75123 229533.I1RD69 pp 229533.I1RWT6
75124 229533.I1RD69 pp 229533.Q4IB50
75125 229533.I1RD69 pp 229533.I1S004
75126 229533.I1RD69 pp 229533.Q4IMD1
75127 229533.I1RD69 pp 229533.I1RV91
75128 229533.I1RD69 pp 229533.Q4HTT1
75129 229533.I1RD69 pp 229533.I1RFX9
75130 229533.I1RD69 pp 229533.I1RTG9
75131 229533.I1RD69 pp 229533.I1RSB0
75132 229533.I1RD69 pp 229533.I1RFW1
75133 229533.I1RD69 pp 229533.V6R3J1
75134 229533.I1RD69 pp 229533.I1RG59
75135 229533.I1RD69 pp 229533.I1RGR2
75136 229533.I1RD69 pp 229533.I1RP88
75137 229533.I1RD69 pp 229533.I1RRN5
75138 229533.I1RD69 pp 229533.I1RDJ8
75139 229533.I1RD69 pp 229533.I1S2E9
75140 229533.I1RD69 pp 229533.I1RMJ8
75141 229533.I1RD69 pp 229533.I1RL41
75142 229533.I1RD69 pp 229533.I1RY44
75143 229533.I1RD69 pp 229533.Q4IB89
75144 229533.I1RD69 pp 229533.I1RZT1
75145 229533.I1RD69 pp 229533.I1S4P6
75146 229533.I1RD69 pp 229533.I1RRW0
75147 229533.I1RD69 pp 229533.I1RXI8
75148 229533.A0A0E0SL81 pp 229533.A0A1C3YLT3
75149 229533.A0A0E0SL81 pp 229533.I1RW71
75150 229533.A0A0E0SL81 pp 229533.I1S821
75151 229533.A0A0E0SL81 pp 229533.I1RT62
75152 229533.A0A0E0SL81 pp 229533.I1RQL4
75153 229533.A0A0E0SL81 pp 229533.I1RAB3
75154 229533.A0A0E0SL81 pp 229533.I1RQH1
75155 229533.A0A0E0SL81 pp 229533.I1RP83
75156 229533.A0A0E0SL81 pp 229533.A0A1C3YK11
75157 229533.A0A0E0SL81 pp 229533.I1S3I4
75158 229533.A0A0E0SL81 pp 229533.I1RG60
75159 229533.A0A0E0SL81 pp 229533.I1RJT9
75160 229533.A0A0E0SL81 pp 229533.I1RB53
75161 229533.A0A0E0SL81 pp 229533.I1RAM6
75162 229533.A0A0E0SL81 pp 229533.I1RPK5
75163 229533.A0A0E0SL81 pp 229533.A0A1C3YIK4
75164 229533.A0A0E0SL81 pp 229533.I1RRT6
75165 229533.A0A0E0SL81 pp 229533.I1RF94
75166 229533.A0A0E0SL81 pp 229533.I1RRF8
75167 229533.A0A0E0SL81 pp 229533.I1RBY7
75168 229533.A0A0E0SL81 pp 229533.I1RTU8
75169 229533.A0A0E0SL81 pp 229533.I1RPL2
75170 229533.A0A0E0SL81 pp 229533.I1RDH7
75171 229533.A0A0E0SL81 pp 229533.I1RYL5
75172 229533.A0A0E0SL81 pp 229533.I1RFY0
75173 229533.A0A0E0SL81 pp 229533.A0A1C3YHZ0
75174 229533.A0A0E0SL81 pp 229533.I1RP28
75175 229533.A0A0E0SL81 pp 229533.A0A1C3YI83
75176 229533.A0A0E0SL81 pp 229533.I1RPV0
75177 229533.A0A0E0SL81 pp 229533.I1S3S5
75178 229533.A0A0E0SL81 pp 229533.I1RZS8
75179 229533.A0A0E0SL81 pp 229533.I1RQG1
75180 229533.A0A0E0SL81 pp 229533.I1RQA5
75181 229533.A0A0E0SL81 pp 229533.I1RNG4
75182 229533.A0A0E0SL81 pp 229533.I1RMQ3
75183 229533.A0A0E0SL81 pp 229533.I1RJZ7
75184 229533.A0A0E0SL81 pp 229533.I1RBY6
75185 229533.A0A0E0SL81 pp 229533.I1R9Y8
75186 229533.A0A0E0SL81 pp 229533.I1RQG4
75187 229533.A0A0E0SL81 pp 229533.I1S0Z8
75188 229533.A0A0E0SL81 pp 229533.I1REC6
75189 229533.A0A0E0SL81 pp 229533.I1RMQ5
75190 229533.A0A0E0SL81 pp 229533.Q4I7G0

75191 229533.A0A0E0SL81 pp 229533.I1RLV3
75192 229533.A0A0E0SL81 pp 229533.I1RB59
75193 229533.A0A0E0SL81 pp 229533.I1RL79
75194 229533.A0A0E0SL81 pp 229533.I1REB8
75195 229533.A0A0E0SL81 pp 229533.I1RGC6
75196 229533.A0A0E0SL81 pp 229533.I1REC7
75197 229533.A0A0E0SL81 pp 229533.I1RQW1
75198 229533.A0A0E0SL81 pp 229533.I1REG1
75199 229533.A0A0E0SL81 pp 229533.I1RMB1
75200 229533.A0A0E0SL81 pp 229533.I1RBS5
75201 229533.A0A0E0SL81 pp 229533.I1RME4
75202 229533.A0A0E0SL81 pp 229533.I1RZD3
75203 229533.A0A0E0SL81 pp 229533.I1S0F9
75204 229533.A0A0E0SL81 pp 229533.I1RZK1
75205 229533.A0A0E0SL81 pp 229533.I1RU27
75206 229533.A0A0E0SL81 pp 229533.I1RBT8
75207 229533.Q4HYQ4 pp 229533.Q4I5W5
75208 229533.Q4HYQ4 pp 229533.Q4I5R3
75209 229533.Q4HYQ4 pp 229533.V6R3J1
75210 229533.Q4HYQ4 pp 229533.Q4I099
75211 229533.Q4HYQ4 pp 229533.Q4IMD1
75212 229533.Q4HYQ4 pp 229533.Q4IPC8
75213 229533.Q4HYQ4 pp 229533.Q4I5U9
75214 229533.Q4HYQ4 pp 229533.Q4I5I4
75215 229533.Q4HYQ4 pp 229533.Q4IB50
75216 229533.Q4HYQ4 pp 229533.Q4IJ11
75217 229533.Q4HYQ4 pp 229533.Q4IR08
75218 229533.Q4HYQ4 pp 229533.V6QUE6
75219 229533.Q4HYQ4 pp 229533.Q4IJU0
75220 229533.Q4HYQ4 pp 229533.Q4I7N9
75221 229533.Q4HYQ4 pp 229533.V6QWX8
75222 229533.Q4HYQ4 pp 229533.Q4I7S1
75223 229533.Q4HYQ4 pp 229533.Q4IER8
75224 229533.I1RKH9 pp 229533.I1S050
75225 229533.I1RUI6 pp 229533.I1RXY7
75226 229533.I1RUI6 pp 229533.I1S0N3
75227 229533.I1RUI6 pp 229533.V6RM02
75228 229533.I1RUI6 pp 229533.I1RX59
75229 229533.I1RUI6 pp 229533.I1S1W8
75230 229533.I1RUI6 pp 229533.I1RVR2
75231 229533.I1RUI6 pp 229533.I1RZK8
75232 229533.I1RUI6 pp 229533.I1RZM4
75233 229533.I1RUI6 pp 229533.I1S3G4
75234 229533.I1RUI6 pp 229533.V6RK96
75235 229533.I1RUI6 pp 229533.I1RVX9
75236 229533.I1RUI6 pp 229533.I1RW71
75237 229533.I1RUI6 pp 229533.I1RY49
75238 229533.A0A098DB16 pp 229533.A0A0E0SMM8
75239 229533.A0A098DB16 pp 229533.A0A0E0SJS1
75240 229533.A0A098DB16 pp 229533.I1RW71
75241 229533.A0A098DB16 pp 229533.A0A098DPS3
75242 229533.A0A098DB16 pp 229533.I1RJZ7
75243 229533.I1RM03 pp 229533.I1RMQ3
75244 229533.I1RM03 pp 229533.I1RZS8
75245 229533.I1RM03 pp 229533.I1RQG4
75246 229533.I1RM03 pp 229533.I1S0F9
75247 229533.I1RM03 pp 229533.I1RPV0
75248 229533.I1RM03 pp 229533.I1RP28
75249 229533.I1RM03 pp 229533.I1RW71
75250 229533.I1RM03 pp 229533.I1RMQ5
75251 229533.I1RM03 pp 229533.I1RNG4
75252 229533.I1RM03 pp 229533.I1S0Z8
75253 229533.I1RM03 pp 229533.I1RME4
75254 229533.I1RM03 pp 229533.I1RT62
75255 229533.I1RM03 pp 229533.I1RMB1
75256 229533.I1RM03 pp 229533.I1RZD3
75257 229533.I1RM03 pp 229533.I1RQL4
75258 229533.I1RM03 pp 229533.Q4I7G0
75259 229533.I1RM03 pp 229533.I1RRT6
75260 229533.I1RM03 pp 229533.I1RU27
75261 229533.I1RM03 pp 229533.I1RQH1
75262 229533.I1RM03 pp 229533.I1RRF8
75263 229533.I1RM03 pp 229533.I1RZK1

75264	229533.I1RM03	pp	229533.I1S821
75265	229533.I1RM03	pp	229533.I1RPL2
75266	229533.I1RM03	pp	229533.I1RPK5
75267	229533.I1RM03	pp	229533.I1RYL5
75268	229533.I1RM03	pp	229533.I1S3S5
75269	229533.I1RM03	pp	229533.I1RTU8
75270	229533.I1RM03	pp	229533.I1RQA5
75271	229533.I1RRT1	pp	229533.I1S072
75272	229533.I1RRT1	pp	229533.I1RWT6
75273	229533.I1RRT1	pp	229533.Q4INS6
75274	229533.I1RRT1	pp	229533.I1S209
75275	229533.I1RRT1	pp	229533.Q4I1J3
75276	229533.I1RRT1	pp	229533.I1S461
75277	229533.I1RRT1	pp	229533.Q4IR08
75278	229533.I1RRT1	pp	229533.I1S183
75279	229533.I1RRT1	pp	229533.I1RVZ9
75280	229533.I1RRT1	pp	229533.I1RYB1
75281	229533.I1RRT1	pp	229533.I1S0F0
75282	229533.I1RRT1	pp	229533.I1S0Q4
75283	229533.I1RRT1	pp	229533.I1RWM6
75284	229533.I1RRT1	pp	229533.I1RS81
75285	229533.I1RRT1	pp	229533.Q4I2M7
75286	229533.I1RRT1	pp	229533.I1RRY1
75287	229533.I1RRT1	pp	229533.I1RSI4
75288	229533.I1RRT1	pp	229533.I1RSJ1
75289	229533.I1RRT1	pp	229533.I1RSC7
75290	229533.I1RRT1	pp	229533.I1RZ98
75291	229533.I1RRT1	pp	229533.I1S0B2
75292	229533.I1RRT1	pp	229533.I1RSR9
75293	229533.I1RRT1	pp	229533.I1S4P6
75294	229533.I1RRT1	pp	229533.I1S788
75295	229533.I1RRT1	pp	229533.I1RY44
75296	229533.I1RRT1	pp	229533.I1S0D5
75297	229533.I1RRT1	pp	229533.I1S283
75298	229533.I1RRT1	pp	229533.Q4IB89
75299	229533.I1RRT1	pp	229533.I1S5Z3
75300	229533.I1RRT1	pp	229533.I1RUR0
75301	229533.I1RRT1	pp	229533.I1RTN6
75302	229533.I1RRT1	pp	229533.I1RX64
75303	229533.I1RRT1	pp	229533.I1RZ94
75304	229533.I1RRT1	pp	229533.I1RZT1
75305	229533.I1RRT1	pp	229533.I1RZP8
75306	229533.I1RRT1	pp	229533.I1RSP7
75307	229533.I1RRT1	pp	229533.I1RW28
75308	229533.I1RRT1	pp	229533.Q4IEV4
75309	229533.I1RRT1	pp	229533.I1RYQ1
75310	229533.I1RRT1	pp	229533.I1RW03
75311	229533.I1RRT1	pp	229533.I1S216
75312	229533.I1RRT1	pp	229533.I1RUG4
75313	229533.I1RRT1	pp	229533.I1S0A6
75314	229533.I1RRT1	pp	229533.I1RY29
75315	229533.I1RRT1	pp	229533.I1RWI8
75316	229533.I1RRT1	pp	229533.I1RSY2
75317	229533.I1RRT1	pp	229533.I1S3S6
75318	229533.I1RRT1	pp	229533.I1RVV5
75319	229533.I1RRT1	pp	229533.I1RYR2
75320	229533.I1RRT1	pp	229533.I1S2E9
75321	229533.I1RRT1	pp	229533.I1S0Q5
75322	229533.I1RRT1	pp	229533.I1RV91
75323	229533.I1RRT1	pp	229533.I1RX22
75324	229533.I1RRT1	pp	229533.I1RTB2
75325	229533.I1RRT1	pp	229533.I1RSN7
75326	229533.I1RRT1	pp	229533.I1RSZ3
75327	229533.I1RRT1	pp	229533.I1RYX9
75328	229533.I1RRT1	pp	229533.I1RVW0
75329	229533.I1RRT1	pp	229533.I1S8G4
75330	229533.I1RRT1	pp	229533.I1S523
75331	229533.I1RRT1	pp	229533.I1S6Y6
75332	229533.I1RRT1	pp	229533.Q4HTT1
75333	229533.I1RRT1	pp	229533.I1S2F2
75334	229533.I1RRT1	pp	229533.V6RLW4
75335	229533.I1RRT1	pp	229533.I1RZ86
75336	229533.I1RRT1	pp	229533.I1RSD0

75337 229533.I1RC73 pp 229533.I1RD01
75338 229533.I1RC73 pp 229533.I1RLR8
75339 229533.I1RC73 pp 229533.Q4IAS1
75340 229533.I1RC73 pp 229533.I1RYV4
75341 229533.I1RC73 pp 229533.Q4IJH1
75342 229533.I1RC73 pp 229533.I1RS41
75343 229533.Q4IJP1 pp 229533.Q4IMD1
75344 229533.I1RN43 pp 229533.I1S5Z5
75345 229533.I1RN43 pp 229533.Q4I877
75346 229533.I1RN43 pp 229533.I1RW16
75347 229533.I1RN43 pp 229533.V6REE9
75348 229533.I1RN43 pp 229533.I1RQ50
75349 229533.I1RN43 pp 229533.I1RRF7
75350 229533.I1S0N3 pp 229533.V6RLM7
75351 229533.I1S0N3 pp 229533.V6RSF3
75352 229533.I1S0N3 pp 229533.I1S3G4
75353 229533.I1S0N3 pp 229533.Q4HXF0
75354 229533.I1S0N3 pp 229533.I1S0Y7
75355 229533.I1S0N3 pp 229533.V6RM02
75356 229533.I1S0N3 pp 229533.V6R382
75357 229533.I1S0N3 pp 229533.V6R5D2
75358 229533.I1S0N3 pp 229533.I1S241
75359 229533.I1S0N3 pp 229533.I1S1W8
75360 229533.I1S0N3 pp 229533.V6RK96
75361 229533.I1S0N3 pp 229533.I1S8E9
75362 229533.A0A1C3YN12 pp 229533.I1RBY6
75363 229533.A0A1C3YN12 pp 229533.I1S0Z8
75364 229533.A0A1C3YN12 pp 229533.I1RNG4
75365 229533.A0A1C3YN12 pp 229533.I1RBS5
75366 229533.A0A1C3YN12 pp 229533.I1RMQ5
75367 229533.A0A1C3YN12 pp 229533.I1RZD3
75368 229533.A0A1C3YN12 pp 229533.I1RP28
75369 229533.A0A1C3YN12 pp 229533.I1RQG4
75370 229533.A0A1C3YN12 pp 229533.I1RJZ7
75371 229533.A0A1C3YN12 pp 229533.I1R9Y8
75372 229533.A0A1C3YN12 pp 229533.I1RME4
75373 229533.A0A1C3YN12 pp 229533.I1RB59
75374 229533.A0A1C3YN12 pp 229533.I1S0F9
75375 229533.A0A1C3YN12 pp 229533.I1RL79
75376 229533.A0A1C3YN12 pp 229533.I1RPV0
75377 229533.A0A1C3YN12 pp 229533.I1REC6
75378 229533.A0A1C3YN12 pp 229533.I1RMB1
75379 229533.A0A1C3YN12 pp 229533.I1RBT8
75380 229533.A0A1C3YN12 pp 229533.I1RZS8
75381 229533.A0A1C3YN12 pp 229533.Q4I7G0
75382 229533.A0A1C3YN12 pp 229533.I1REB8
75383 229533.A0A1C3YN12 pp 229533.I1REC7
75384 229533.A0A1C3YN12 pp 229533.I1RMQ3
75385 229533.A0A1C3YN12 pp 229533.I1RZK1
75386 229533.A0A1C3YN12 pp 229533.I1RU27
75387 229533.A0A1C3YN12 pp 229533.I1RGC6
75388 229533.A0A1C3YN12 pp 229533.I1REG1
75389 229533.A0A1C3YN12 pp 229533.I1RQL4
75390 229533.A0A1C3YN12 pp 229533.I1RPK5
75391 229533.A0A1C3YN12 pp 229533.I1RT62
75392 229533.A0A1C3YN12 pp 229533.I1RW71
75393 229533.A0A1C3YN12 pp 229533.I1RRT6
75394 229533.A0A1C3YN12 pp 229533.I1RJT9
75395 229533.A0A1C3YN12 pp 229533.I1RDH7
75396 229533.A0A1C3YN12 pp 229533.I1RPL2
75397 229533.A0A1C3YN12 pp 229533.I1RQH1
75398 229533.A0A1C3YN12 pp 229533.I1RF94
75399 229533.A0A1C3YN12 pp 229533.I1RFY0
75400 229533.A0A1C3YN12 pp 229533.I1RG60
75401 229533.A0A1C3YN12 pp 229533.I1RRF8
75402 229533.A0A1C3YN12 pp 229533.I1S821
75403 229533.A0A1C3YN12 pp 229533.I1RBY7
75404 229533.A0A1C3YN12 pp 229533.I1RB53
75405 229533.A0A1C3YN12 pp 229533.I1RTU8
75406 229533.A0A1C3YN12 pp 229533.I1RAB3
75407 229533.A0A1C3YN12 pp 229533.I1RAM6
75408 229533.A0A1C3YN12 pp 229533.I1RYL5
75409 229533.A0A1C3YN12 pp 229533.I1S3S5

75410	229533.A0A1C3YN12	pp	229533.I1RQA5
75411	229533.I1RSD0	pp	229533.I1RY29
75412	229533.I1RSD0	pp	229533.I1RZ98
75413	229533.I1RSD0	pp	229533.I1RZP1
75414	229533.I1RSD0	pp	229533.I1RSJ1
75415	229533.I1RSD0	pp	229533.I1RX22
75416	229533.I1RSD0	pp	229533.Q4INS6
75417	229533.I1RSD0	pp	229533.I1RW24
75418	229533.I1RSD0	pp	229533.I1RTN6
75419	229533.I1RSD0	pp	229533.I1S8G4
75420	229533.I1RSD0	pp	229533.I1RWI8
75421	229533.I1SA27	pp	229533.V6R0U9
75422	229533.I1RT16	pp	229533.I1S0F0
75423	229533.I1RT16	pp	229533.Q4IR08
75424	229533.I1RT16	pp	229533.Q4IER8
75425	229533.I1RT16	pp	229533.Q4HTT2
75426	229533.I1RT16	pp	229533.I1S0D5
75427	229533.I1RT16	pp	229533.Q4ICA8
75428	229533.I1RT16	pp	229533.I1S0Z3
75429	229533.I1RT16	pp	229533.I1RT41
75430	229533.I1RT16	pp	229533.I1S0N5
75431	229533.I1RT16	pp	229533.Q4I948
75432	229533.I1RT16	pp	229533.V6R3J1
75433	229533.I1RT16	pp	229533.I1S1V2
75434	229533.I1RT16	pp	229533.I1RTB2
75435	229533.I1RT16	pp	229533.I1RYN7
75436	229533.I1RT16	pp	229533.Q4IMD1
75437	229533.I1RT16	pp	229533.I1S951
75438	229533.I1RT16	pp	229533.I1S7I5
75439	229533.I1RT16	pp	229533.I1RY99
75440	229533.I1RT16	pp	229533.I1S382
75441	229533.I1RT16	pp	229533.Q4IPI4
75442	229533.I1RT16	pp	229533.Q4HTT1
75443	229533.I1RT16	pp	229533.I1RVY0
75444	229533.A0A0E0SFP4	pp	229533.I1RZ29
75445	229533.I1RDI8	pp	229533.I1REC6
75446	229533.I1RDI8	pp	229533.I1S0F9
75447	229533.I1RDI8	pp	229533.I1RJZ7
75448	229533.I1RDI8	pp	229533.I1RII8
75449	229533.I1RDI8	pp	229533.I1RNG4
75450	229533.I1RDI8	pp	229533.I1RP28
75451	229533.I1RDI8	pp	229533.I1REG1
75452	229533.I1RDI8	pp	229533.I1REC7
75453	229533.I1RDI8	pp	229533.I1RZD3
75454	229533.I1RDI8	pp	229533.I1RME4
75455	229533.I1RDI8	pp	229533.I1RQG4
75456	229533.I1RDI8	pp	229533.I1RZK1
75457	229533.I1RDI8	pp	229533.I1RLN6
75458	229533.I1RDI8	pp	229533.I1RMB1
75459	229533.I1RDI8	pp	229533.Q4I7G0
75460	229533.I1RDI8	pp	229533.I1RU27
75461	229533.I1RDI8	pp	229533.I1REB8
75462	229533.I1RDI8	pp	229533.I1S1Q6
75463	229533.I1RDI8	pp	229533.I1RL79
75464	229533.I1RDI8	pp	229533.I1S0Z8
75465	229533.I1RDI8	pp	229533.I1RZS8
75466	229533.I1RDI8	pp	229533.I1RPV0
75467	229533.I1RDI8	pp	229533.I1RMQ5
75468	229533.I1RDI8	pp	229533.I1RGC6
75469	229533.I1RDI8	pp	229533.I1RF94
75470	229533.I1RDI8	pp	229533.I1RG60
75471	229533.I1RDI8	pp	229533.I1RMQ3
75472	229533.I1RDI8	pp	229533.I1RT62
75473	229533.I1RDI8	pp	229533.I1RYL5
75474	229533.I1RDI8	pp	229533.I1RJA2
75475	229533.I1RDI8	pp	229533.I1RRF8
75476	229533.I1RDI8	pp	229533.I1RW71
75477	229533.I1RDI8	pp	229533.I1RTU8
75478	229533.I1RDI8	pp	229533.I1RQL4
75479	229533.I1RDI8	pp	229533.I1S3S5
75480	229533.I1RDI8	pp	229533.I1RQH1
75481	229533.I1RDI8	pp	229533.I1RPL2
75482	229533.I1RDI8	pp	229533.I1RQA5

75483 229533.I1RDI8 pp 229533.I1RFY0
75484 229533.I1RDI8 pp 229533.I1RJT9
75485 229533.I1RDI8 pp 229533.I1RI16
75486 229533.I1RDI8 pp 229533.I1RPK5
75487 229533.I1RDI8 pp 229533.I1RRT6
75488 229533.I1RDI8 pp 229533.I1S821
75489 229533.I1RDI8 pp 229533.I1RFA9
75490 229533.I1RDC0 pp 229533.I1S3G4
75491 229533.I1RDC0 pp 229533.I1RNA2
75492 229533.I1RDC0 pp 229533.Q4IB89
75493 229533.I1RDC0 pp 229533.I1RSP7
75494 229533.I1RDC0 pp 229533.I1RZ86
75495 229533.I1RDC0 pp 229533.I1RTN6
75496 229533.I1RDC0 pp 229533.I1S998
75497 229533.I1RDC0 pp 229533.I1RP99
75498 229533.I1RDC0 pp 229533.I1RQI6
75499 229533.I1RDC0 pp 229533.I1RSC7
75500 229533.I1RDC0 pp 229533.I1RVW0
75501 229533.I1RDC0 pp 229533.I1S523
75502 229533.I1RDC0 pp 229533.I1S1W8
75503 229533.I1RDC0 pp 229533.I1RZ98
75504 229533.I1RDC0 pp 229533.I1RRT1
75505 229533.I1RDC0 pp 229533.I1RY29
75506 229533.I1RDC0 pp 229533.I1RPI0
75507 229533.I1RDC0 pp 229533.I1RYB7
75508 229533.I1RDC0 pp 229533.I1RFX8
75509 229533.I1RDC0 pp 229533.Q4INS6
75510 229533.I1RDC0 pp 229533.I1RKI2
75511 229533.I1RDC0 pp 229533.I1S987
75512 229533.I1RDC0 pp 229533.I1RM09
75513 229533.I1RDC0 pp 229533.I1RSJ1
75514 229533.I1RDC0 pp 229533.I1RE55
75515 229533.I1RDC0 pp 229533.I1RRY1
75516 229533.I1RDC0 pp 229533.I1S209
75517 229533.A0A098D386 pp 229533.I1RDJ8
75518 229533.A0A098D386 pp 229533.I1RAI9
75519 229533.A0A098D386 pp 229533.Q4HY90
75520 229533.A0A098D386 pp 229533.I1S0N5
75521 229533.A0A098D386 pp 229533.I1RS41
75522 229533.A0A098D386 pp 229533.I1RR25
75523 229533.A0A098D386 pp 229533.A0A098D6T7
75524 229533.A0A098D386 pp 229533.I1S1Y7
75525 229533.A0A098D386 pp 229533.A0A098DX13
75526 229533.A0A098D386 pp 229533.A0A098E3M9
75527 229533.A0A098D386 pp 229533.I1RSD1
75528 229533.A0A098D386 pp 229533.Q4HYB8
75529 229533.A0A098D386 pp 229533.A0A1C3YHP3
75530 229533.A0A098D386 pp 229533.I1RK79
75531 229533.A0A098D386 pp 229533.I1RRN5
75532 229533.A0A098D386 pp 229533.Q4IB89
75533 229533.A0A098D386 pp 229533.I1RMJ8
75534 229533.A0A098D386 pp 229533.I1RDZ4
75535 229533.A0A098D386 pp 229533.I1S1U7
75536 229533.A0A098D386 pp 229533.I1RC54
75537 229533.A0A098D386 pp 229533.I1RBJ1
75538 229533.A0A098D386 pp 229533.I1S4P6
75539 229533.A0A098D386 pp 229533.I1S020
75540 229533.A0A098D386 pp 229533.I1RBJ0
75541 229533.A0A098D386 pp 229533.I1RC24
75542 229533.A0A098D386 pp 229533.I1RB73
75543 229533.A0A098D386 pp 229533.I1RG11
75544 229533.A0A098D386 pp 229533.Q4IB50
75545 229533.A0A098D386 pp 229533.A0A1C3YJD4
75546 229533.A0A098D386 pp 229533.A0A1C3YKX5
75547 229533.A0A098D386 pp 229533.A0A1C3YIR8
75548 229533.A0A098D386 pp 229533.I1RTG3
75549 229533.A0A098D386 pp 229533.A0A1C3YJZ4
75550 229533.A0A098D386 pp 229533.I1RY44
75551 229533.A0A098D386 pp 229533.I1S7I5
75552 229533.A0A098D386 pp 229533.Q4IL82
75553 229533.A0A098D386 pp 229533.I1RDV4
75554 229533.A0A098D386 pp 229533.A0A1C3YI39
75555 229533.A0A098D386 pp 229533.I1RPR8

75556	229533.A0A098D386	pp	229533.I1RC00
75557	229533.A0A098D386	pp	229533.A0A1C3YM91
75558	229533.A0A098D386	pp	229533.A0A1C3YLA2
75559	229533.A0A098D386	pp	229533.Q4IPI4
75560	229533.A0A098D386	pp	229533.I1RCB7
75561	229533.A0A098D386	pp	229533.I1RRJ6
75562	229533.A0A098D386	pp	229533.Q4I5W5
75563	229533.A0A098D386	pp	229533.Q4I7L0
75564	229533.A0A098D386	pp	229533.I1RVY0
75565	229533.A0A098D386	pp	229533.Q4IMD1
75566	229533.A0A098D386	pp	229533.I1RA84
75567	229533.A0A098D386	pp	229533.Q4I5V3
75568	229533.A0A098D386	pp	229533.I1RCN2
75569	229533.A0A098D386	pp	229533.A0A1C3YLC6
75570	229533.A0A098D386	pp	229533.I1RFW1
75571	229533.A0A098D386	pp	229533.I1RVA7
75572	229533.A0A098D386	pp	229533.Q4I948
75573	229533.A0A098D386	pp	229533.Q4IEV4
75574	229533.A0A098D386	pp	229533.I1RQA3
75575	229533.A0A098D386	pp	229533.I1RCL7
75576	229533.A0A098D386	pp	229533.A0A098DKA5
75577	229533.A0A098D386	pp	229533.I1RRT0
75578	229533.A0A098D386	pp	229533.I1S2E9
75579	229533.A0A098D386	pp	229533.Q4I5R3
75580	229533.A0A098D386	pp	229533.A0A1C3YM67
75581	229533.A0A098D386	pp	229533.I1RCW8
75582	229533.A0A098D386	pp	229533.I1RYV3
75583	229533.A0A098D386	pp	229533.I1RVV5
75584	229533.A0A098D386	pp	229533.Q4IJU0
75585	229533.A0A098D386	pp	229533.A0A1C3YJR3
75586	229533.A0A098D386	pp	229533.I1RWT6
75587	229533.A0A098D386	pp	229533.I1RQP1
75588	229533.A0A098D386	pp	229533.I1RQ19
75589	229533.A0A098D386	pp	229533.I1RV91
75590	229533.A0A098D386	pp	229533.I1RT01
75591	229533.A0A098D386	pp	229533.A0A1C3YK01
75592	229533.A0A098D386	pp	229533.I1RZB5
75593	229533.A0A098D386	pp	229533.I1RQ88
75594	229533.A0A098D386	pp	229533.I1RBC7
75595	229533.A0A098D386	pp	229533.Q4IER8
75596	229533.A0A098D386	pp	229533.Q4ICA8
75597	229533.A0A098D386	pp	229533.I1RW28
75598	229533.A0A098D386	pp	229533.I1S0Z3
75599	229533.A0A098D386	pp	229533.I1RSI1
75600	229533.A0A098D386	pp	229533.I1RJL2
75601	229533.A0A098D386	pp	229533.I1RU02
75602	229533.A0A098D386	pp	229533.Q4IAK7
75603	229533.A0A098D386	pp	229533.I1S9J6
75604	229533.A0A098D386	pp	229533.I1RE51
75605	229533.A0A098D386	pp	229533.A0A0E0SQV5
75606	229533.A0A098D386	pp	229533.A0A1C3YJW9
75607	229533.A0A098D386	pp	229533.Q4HTT2
75608	229533.A0A098D386	pp	229533.I1RLJ8
75609	229533.A0A098D386	pp	229533.V6QUE6
75610	229533.A0A098D386	pp	229533.A0A0E0SA11
75611	229533.A0A098D386	pp	229533.I1S788
75612	229533.A0A098D386	pp	229533.A0A1C3YI23
75613	229533.A0A098D386	pp	229533.I1RC17
75614	229533.A0A098D386	pp	229533.Q4I5I4
75615	229533.A0A098D386	pp	229533.I1RYN7
75616	229533.A0A098D386	pp	229533.I1RAN8
75617	229533.A0A098D386	pp	229533.A0A098D5E5
75618	229533.A0A098D386	pp	229533.I1S219
75619	229533.A0A098D386	pp	229533.I1RD01
75620	229533.A0A098D386	pp	229533.I1RM36
75621	229533.A0A098D386	pp	229533.A0A0E0SES1
75622	229533.A0A098D386	pp	229533.I1RUQ6
75623	229533.A0A098D386	pp	229533.I1RPF4
75624	229533.A0A098D386	pp	229533.I1S1V2
75625	229533.A0A098D386	pp	229533.I1RG34
75626	229533.A0A098D386	pp	229533.I1RC32
75627	229533.A0A098D386	pp	229533.A0A098DBR4
75628	229533.A0A098D386	pp	229533.I1RRG3

229533.A0A098D386	pp	229533.I1RD69
229533.A0A098D386	pp	229533.A0A0E0SPD4
229533.A0A098D386	pp	229533.Q4HYQ4
229533.A0A098D386	pp	229533.I1RRT1
229533.A0A098D386	pp	229533.Q4IJP1
229533.A0A098D386	pp	229533.I1RZR6
229533.A0A098D386	pp	229533.I1RT16
229533.A0A098D386	pp	229533.I1RT41
229533.I1RP66	pp	229533.I1RW71
229533.I1RP66	pp	229533.I1RT62
229533.I1RP66	pp	229533.I1RTU8
229533.I1RP66	pp	229533.I1RRF8
229533.I1RP66	pp	229533.I1S6L5
229533.I1RP66	pp	229533.I1RQL4
229533.I1RP66	pp	229533.I1RQH1
229533.I1RP66	pp	229533.I1S9I6
229533.I1RP66	pp	229533.I1RRT6
229533.I1RP66	pp	229533.I1S3S5
229533.I1RP66	pp	229533.I1RQA5
229533.I1RP66	pp	229533.I1S821
229533.I1RP66	pp	229533.I1RYL5
229533.I1RP66	pp	229533.I1RPK5
229533.I1RP66	pp	229533.I1RZK1
229533.I1RP66	pp	229533.I1RPL2
229533.I1RP66	pp	229533.I1RX98
229533.I1RP66	pp	229533.I1RU27
229533.I1RP66	pp	229533.I1S1J6
229533.I1RP66	pp	229533.I1RV58
229533.I1RP66	pp	229533.I1S2R6
229533.I1RP66	pp	229533.I1S0F9
229533.I1RP66	pp	229533.I1RZD3
229533.I1RP66	pp	229533.I1S0Z8
229533.I1RP66	pp	229533.I1RQG4
229533.I1RP66	pp	229533.Q4I7G0
229533.I1RP66	pp	229533.I1RZS8
229533.I1RP66	pp	229533.I1RPV0
229533.A0A0E0SBC4	pp	229533.I1RC33
229533.A0A0E0SBC4	pp	229533.I1S8V4
229533.A0A0E0SBC4	pp	229533.I1RXJ2
229533.A0A0E0SBC4	pp	229533.I1RKZ7
229533.A0A0E0SBC4	pp	229533.V6RKN5
229533.A0A0E0SBC4	pp	229533.A0A1C3YMI5
229533.A0A0E0SBC4	pp	229533.I1RE01
229533.A0A0E0SBC4	pp	229533.A0A1C3YK33
229533.A0A0E0SBC4	pp	229533.V6RLM7
229533.A0A0E0SBC4	pp	229533.I1S8R4
229533.A0A0E0SBC4	pp	229533.I1RCR3
229533.A0A0E0SBC4	pp	229533.I1RMJ3
229533.A0A0E0SBC4	pp	229533.I1RN84
229533.A0A0E0SBC4	pp	229533.I1RFT1
229533.A0A0E0SBC4	pp	229533.I1RBS6
229533.A0A0E0SBC4	pp	229533.V6RMW6
229533.A0A0E0SBC4	pp	229533.I1RZA7
229533.A0A0E0SBC4	pp	229533.I1S685
229533.A0A0E0SBC4	pp	229533.I1RSZ7
229533.A0A0E0SBC4	pp	229533.I1RPP3
229533.A0A0E0SBC4	pp	229533.I1RN67
229533.A0A0E0SBC4	pp	229533.I1RFS3
229533.A0A0E0SBC4	pp	229533.I1RIJ5
229533.A0A0E0SBC4	pp	229533.A0A1C3YIT6
229533.A0A0E0SBC4	pp	229533.I1RY74
229533.A0A0E0SBC4	pp	229533.I1RPM4
229533.A0A0E0SBC4	pp	229533.I1RNG4
229533.A0A0E0SBC4	pp	229533.I1S6P5
229533.A0A0E0SBC4	pp	229533.I1RQE7
229533.A0A0E0SBC4	pp	229533.I1RNW6
229533.A0A0E0SBC4	pp	229533.I1RVX0
229533.A0A0E0SBC4	pp	229533.I1RVR2
229533.A0A0E0SBC4	pp	229533.I1RNB4
229533.A0A0E0SBC4	pp	229533.I1RBS0
229533.A0A0E0SBC4	pp	229533.I1RJZ7
229533.A0A0E0SBC4	pp	229533.I1S2B8
229533.A0A0E0SBC4	pp	229533.V6RM02

75702	229533.A0A0E0SBC4	pp	229533.I1RB92
75703	229533.A0A0E0SBC4	pp	229533.I1RG90
75704	229533.A0A0E0SBC4	pp	229533.I1RPD6
75705	229533.A0A0E0SBC4	pp	229533.I1RNW2
75706	229533.A0A0E0SBC4	pp	229533.I1RSQ9
75707	229533.A0A0E0SBC4	pp	229533.I1RE76
75708	229533.A0A0E0SBC4	pp	229533.A0A0E0SMT9
75709	229533.A0A0E0SBC4	pp	229533.I1S684
75710	229533.A0A0E0SBC4	pp	229533.I1RAU7
75711	229533.A0A0E0SBC4	pp	229533.I1RCG5
75712	229533.A0A0E0SBC4	pp	229533.I1RYW3
75713	229533.A0A0E0SBC4	pp	229533.I1S3X7
75714	229533.A0A0E0SBC4	pp	229533.I1RVZ3
75715	229533.A0A0E0SBC4	pp	229533.I1RV91
75716	229533.A0A0E0SBC4	pp	229533.A0A1C3YMA1
75717	229533.A0A0E0SBC4	pp	229533.I1RSQ4
75718	229533.A0A0E0SBC4	pp	229533.I1S8V3
75719	229533.A0A0E0SBC4	pp	229533.V6RN31
75720	229533.A0A0E0SBC4	pp	229533.V6RUY9
75721	229533.A0A0E0SBC4	pp	229533.A0A1C3YLE2
75722	229533.A0A0E0SBC4	pp	229533.I1RNV4
75723	229533.A0A0E0SBC4	pp	229533.I1R9W4
75724	229533.A0A0E0SBC4	pp	229533.I1RZ29
75725	229533.A0A0E0SBC4	pp	229533.I1SAJ3
75726	229533.A0A0E0SBC4	pp	229533.I1RJW6
75727	229533.A0A0E0SBC4	pp	229533.I1RBY9
75728	229533.A0A0E0SBC4	pp	229533.A0A1C3YIG2
75729	229533.A0A0E0SBC4	pp	229533.I1RIL9
75730	229533.A0A0E0SBC4	pp	229533.I1RDE7
75731	229533.A0A0E0SBC4	pp	229533.V6RUB1
75732	229533.A0A0E0SBC4	pp	229533.V6R3X5
75733	229533.A0A0E0SBC4	pp	229533.A0A1C3YIK4
75734	229533.A0A0E0SBC4	pp	229533.I1RNQ5
75735	229533.A0A0E0SBC4	pp	229533.I1RSS7
75736	229533.A0A0E0SBC4	pp	229533.I1RJF2
75737	229533.A0A0E0SBC4	pp	229533.I1RCR5
75738	229533.A0A0E0SBC4	pp	229533.I1S3G4
75739	229533.A0A0E0SBC4	pp	229533.I1RJ73
75740	229533.A0A0E0SBC4	pp	229533.A0A1C3YLN6
75741	229533.A0A0E0SBC4	pp	229533.A0A1C3YK58
75742	229533.A0A0E0SBC4	pp	229533.I1RZM8
75743	229533.A0A0E0SBC4	pp	229533.A0A0E0SC46
75744	229533.A0A0E0SBC4	pp	229533.A0A1C3YJW4
75745	229533.A0A0E0SBC4	pp	229533.I1S0A0
75746	229533.A0A0E0SBC4	pp	229533.I1RY03
75747	229533.A0A0E0SBC4	pp	229533.I1RFU8
75748	229533.A0A0E0SBC4	pp	229533.I1RMH9
75749	229533.A0A0E0SBC4	pp	229533.I1RBE4
75750	229533.A0A0E0SBC4	pp	229533.V6RCZ8
75751	229533.A0A0E0SBC4	pp	229533.I1RZK5
75752	229533.A0A0E0SBC4	pp	229533.A0A1C3YKY4
75753	229533.A0A0E0SBC4	pp	229533.I1RXY4
75754	229533.A0A0E0SBC4	pp	229533.I1RPW1
75755	229533.A0A0E0SBC4	pp	229533.I1RVX9
75756	229533.A0A0E0SBC4	pp	229533.I1S0K0
75757	229533.A0A0E0SBC4	pp	229533.A0A1C3YKX1
75758	229533.A0A0E0SBC4	pp	229533.I1RC48
75759	229533.A0A0E0SBC4	pp	229533.I1RHZ5
75760	229533.A0A0E0SBC4	pp	229533.I1S1J7
75761	229533.A0A0E0SBC4	pp	229533.A0A1C3YLP6
75762	229533.A0A0E0SBC4	pp	229533.I1S019
75763	229533.A0A0E0SBC4	pp	229533.I1RBL7
75764	229533.A0A0E0SBC4	pp	229533.I1R9F4
75765	229533.A0A0E0SBC4	pp	229533.I1S5B0
75766	229533.A0A0E0SBC4	pp	229533.I1RPE7
75767	229533.A0A0E0SBC4	pp	229533.I1RAX6
75768	229533.A0A0E0SBC4	pp	229533.I1RDQ2
75769	229533.A0A0E0SBC4	pp	229533.I1RPM6
75770	229533.A0A0E0SBC4	pp	229533.I1RT77
75771	229533.A0A0E0SBC4	pp	229533.I1RW99
75772	229533.A0A0E0SBC4	pp	229533.I1S1W8
75773	229533.A0A0E0SBC4	pp	229533.I1RRG3
75774	229533.A0A0E0SBC4	pp	229533.I1R9B6

229533.A0A0E0SBC4 pp 229533.I1RQP2
229533.A0A0E0SBC4 pp 229533.I1RXS0
229533.A0A0E0SBC4 pp 229533.A0A1C3YK13
229533.A0A0E0SBC4 pp 229533.I1RTU0
229533.A0A0E0SBC4 pp 229533.I1S686
229533.A0A0E0SBC4 pp 229533.I1RW71
229533.A0A0E0SBC4 pp 229533.I1RBC9
229533.A0A0E0SBC4 pp 229533.I1RMJ4
229533.A0A0E0SBC4 pp 229533.I1RM26
229533.A0A0E0SBC4 pp 229533.I1RNW1
229533.A0A0E0SBC4 pp 229533.I1RK03
229533.A0A0E0SBC4 pp 229533.I1RBT1
229533.A0A0E0SBC4 pp 229533.I1RC20
229533.A0A0E0SBC4 pp 229533.A0A1C3YHX8
229533.A0A0E0SBC4 pp 229533.I1RQ60
229533.A0A0E0SBC4 pp 229533.I1RMM8
229533.A0A0E0SBC4 pp 229533.I1RYQ4
229533.A0A0E0SBC4 pp 229533.I1RF19
229533.A0A0E0SBC4 pp 229533.V6RQW4
229533.A0A0E0SBC4 pp 229533.A0A1C3YN47
229533.A0A0E0SBC4 pp 229533.I1RK93
229533.A0A0E0SBC4 pp 229533.I1RBE7
229533.A0A0E0SBC4 pp 229533.I1RAG9
229533.A0A0E0SBC4 pp 229533.V6QWD8
229533.A0A0E0SBC4 pp 229533.I1RUV4
229533.A0A0E0SBC4 pp 229533.I1RD52
229533.A0A0E0SBC4 pp 229533.I1RY49
229533.A0A0E0SBC4 pp 229533.I1RAR3
229533.A0A0E0SBC4 pp 229533.I1RYX6
229533.A0A0E0SBC4 pp 229533.I1S0L3
229533.A0A0E0SBC4 pp 229533.A0A1C3YL54
229533.A0A0E0SBC4 pp 229533.I1S1K5
229533.A0A0E0SBC4 pp 229533.I1RMK4
229533.A0A0E0SBC4 pp 229533.I1RM67
229533.A0A0E0SBC4 pp 229533.I1RM80
229533.A0A0E0SBC4 pp 229533.I1RR24
229533.A0A0E0SBC4 pp 229533.I1RP86
229533.A0A0E0SBC4 pp 229533.I1S3L2
229533.A0A0E0SBC4 pp 229533.I1RQE1
229533.A0A0E0SBC4 pp 229533.I1RRG1
229533.A0A0E0SBC4 pp 229533.I1RJ72
229533.I1RW87 pp 229533.I1RZS8
229533.I1RW87 pp 229533.Q4I7G0
229533.I1RW87 pp 229533.I1S0F9
229533.I1RW87 pp 229533.I1RZK1
229533.I1RW87 pp 229533.I1S821
229533.I1RW87 pp 229533.I1S0Z8
229533.I1RW87 pp 229533.I1S3S5
229533.I1RW87 pp 229533.I1RYL5
229533.I1RW87 pp 229533.I1RZD3
229533.I1RFZ0 pp 229533.I1S0S5
229533.I1RFZ0 pp 229533.I1RZV2
229533.I1RFZ0 pp 229533.I1S272
229533.I1RFZ0 pp 229533.I1RZ13
229533.I1RWL4 pp 229533.V6RM02
229533.I1RWL4 pp 229533.V6RN69
229533.I1RBC3 pp 229533.P0C431
229533.I1RBC3 pp 229533.I1S0S6
229533.I1RBC3 pp 229533.I1RTB7
229533.I1RBC3 pp 229533.I1S7X9
229533.I1RBC3 pp 229533.I1RH68
229533.I1RBC3 pp 229533.I1RQN9
229533.I1S685 pp 229533.V6RKN5
229533.I1RJ73 pp 229533.I1RNW2
229533.I1RJ73 pp 229533.V6RKN5
229533.I1RJ73 pp 229533.I1RM26
229533.I1RJ73 pp 229533.I1S1W8
229533.I1RJ73 pp 229533.I1S019
229533.I1RJ73 pp 229533.I1RNW0
229533.I1RJ73 pp 229533.I1S686
229533.I1RJ73 pp 229533.I1S3G4
229533.I1RJ73 pp 229533.I1RZ20
229533.I1RJ73 pp 229533.I1RNB4

75848	229533.I1RJ73	pp	229533.I1RK93
75849	229533.I1RJ73	pp	229533.I1RVX9
75850	229533.I1RJ73	pp	229533.I1RNG4
75851	229533.I1RKK8	pp	229533.I1RMQ5
75852	229533.I1RKK8	pp	229533.I1RPV0
75853	229533.I1RKK8	pp	229533.I1RMQ3
75854	229533.I1RKK8	pp	229533.I1S0Z8
75855	229533.I1RKK8	pp	229533.I1RP28
75856	229533.I1RKK8	pp	229533.I1RNG4
75857	229533.I1RKK8	pp	229533.I1RQG4
75858	229533.I1RKK8	pp	229533.I1S0F9
75859	229533.I1RKK8	pp	229533.I1RZS8
75860	229533.I1RKK8	pp	229533.I1RL79
75861	229533.I1RKK8	pp	229533.I1RZK1
75862	229533.I1RKK8	pp	229533.I1RMB1
75863	229533.I1RKK8	pp	229533.I1RU27
75864	229533.I1RKK8	pp	229533.I1RZD3
75865	229533.I1RKK8	pp	229533.Q4I7G0
75866	229533.I1RKK8	pp	229533.I1RPL2
75867	229533.I1RKK8	pp	229533.I1RRT6
75868	229533.I1RKK8	pp	229533.I1RPK5
75869	229533.I1RKK8	pp	229533.I1RQH1
75870	229533.I1RKK8	pp	229533.I1RME4
75871	229533.I1RKK8	pp	229533.I1RQA5
75872	229533.I1RKK8	pp	229533.I1RTU8
75873	229533.I1RKK8	pp	229533.I1RQL4
75874	229533.I1RKK8	pp	229533.I1S821
75875	229533.I1RKK8	pp	229533.I1RRF8
75876	229533.I1RKK8	pp	229533.I1RT62
75877	229533.I1RKK8	pp	229533.I1S3S5
75878	229533.I1RKK8	pp	229533.I1RW71
75879	229533.I1RKK8	pp	229533.I1RYL5
75880	229533.I1RAR3	pp	229533.I1S0B7
75881	229533.I1RAR3	pp	229533.I1S1W8
75882	229533.I1RAR3	pp	229533.V6QWD8
75883	229533.I1RAR3	pp	229533.I1RYX6
75884	229533.I1RAR3	pp	229533.I1RYQ4
75885	229533.I1RAR3	pp	229533.I1RY49
75886	229533.I1RAR3	pp	229533.I1RFV2
75887	229533.I1RAR3	pp	229533.I1RDQ2
75888	229533.I1RAR3	pp	229533.I1RQE1
75889	229533.I1RAR3	pp	229533.I1RVX9
75890	229533.I1RAR3	pp	229533.I1RQ60
75891	229533.I1RAR3	pp	229533.I1RPW1
75892	229533.I1RAR3	pp	229533.I1RKZ7
75893	229533.I1RAR3	pp	229533.I1RG66
75894	229533.I1RAR3	pp	229533.I1RMK4
75895	229533.I1RAR3	pp	229533.I1RLM3
75896	229533.I1RAR3	pp	229533.I1RK03
75897	229533.I1RAR3	pp	229533.I1RBE7
75898	229533.I1RAR3	pp	229533.V6RQW4
75899	229533.I1RAR3	pp	229533.I1S0A0
75900	229533.I1RAR3	pp	229533.I1RPM6
75901	229533.I1RAR3	pp	229533.I1RM26
75902	229533.I1RAR3	pp	229533.I1RCL0
75903	229533.I1RAR3	pp	229533.I1RVP5
75904	229533.I1RAR3	pp	229533.I1RJU9
75905	229533.I1RAR3	pp	229533.I1S3G4
75906	229533.I1RAR3	pp	229533.I1S253
75907	229533.I1RAR3	pp	229533.I1RR72
75908	229533.I1RAR3	pp	229533.I1RW43
75909	229533.I1RAR3	pp	229533.I1RHG5
75910	229533.I1RAR3	pp	229533.I1RIH0
75911	229533.I1RAR3	pp	229533.I1RDE7
75912	229533.I1RAR3	pp	229533.I1S0I3
75913	229533.I1RAR3	pp	229533.I1RZ29
75914	229533.I1RAR3	pp	229533.I1RDJ8
75915	229533.I1RAR3	pp	229533.I1RSQ9
75916	229533.I1RAR3	pp	229533.V6QVH8
75917	229533.I1RAR3	pp	229533.I1S1F3
75918	229533.I1RAR3	pp	229533.I1RNB4
75919	229533.I1RAR3	pp	229533.I1RY74
75920	229533.I1RAR3	pp	229533.I1RMU9

75921 229533.I1RAR3 pp 229533.I1RPD6
75922 229533.I1RAR3 pp 229533.I1RS43
75923 229533.I1RAR3 pp 229533.I1RNG4
75924 229533.I1RAR3 pp 229533.I1RZN1
75925 229533.I1RAR3 pp 229533.V6RKN5
75926 229533.I1RAR3 pp 229533.I1RNV4
75927 229533.I1RAR3 pp 229533.Q4HXF0
75928 229533.I1RAR3 pp 229533.I1RCG5
75929 229533.I1RAR3 pp 229533.I1SAJ3
75930 229533.I1RAR3 pp 229533.V6RLM7
75931 229533.I1RAR3 pp 229533.I1RHJ2
75932 229533.I1RAR3 pp 229533.I1RFK3
75933 229533.A0A1C3YMA0 pp 229533.I1RWA3
75934 229533.A0A098DMZ8 pp 229533.I1RDE6
75935 229533.A0A098DMZ8 pp 229533.V6QXG1
75936 229533.I1S2M7 pp 229533.I1S2M8
75937 229533.I1S2M7 pp 229533.I1S3G4
75938 229533.I1S2M7 pp 229533.I1S2N0
75939 229533.I1RDE7 pp 229533.I1RYX6
75940 229533.I1RDE7 pp 229533.I1S686
75941 229533.I1RDE7 pp 229533.I1S1W8
75942 229533.I1RDE7 pp 229533.I1RK93
75943 229533.I1RDE7 pp 229533.I1RME6
75944 229533.I1RDE7 pp 229533.I1RY49
75945 229533.I1RDE7 pp 229533.I1RJ72
75946 229533.I1RDE7 pp 229533.I1RP86
75947 229533.I1RDE7 pp 229533.I1RQE1
75948 229533.I1RDE7 pp 229533.I1RYQ4
75949 229533.I1RDE7 pp 229533.V6QWD8
75950 229533.I1RDE7 pp 229533.I1RMK4
75951 229533.I1RDE7 pp 229533.I1RMM8
75952 229533.I1RDE7 pp 229533.I1RXY4
75953 229533.I1RDE7 pp 229533.I1RVX9
75954 229533.I1RDE7 pp 229533.I1RDU0
75955 229533.I1RDE7 pp 229533.I1RQS9
75956 229533.I1RDE7 pp 229533.I1RH91
75957 229533.I1RDE7 pp 229533.V6RBN2
75958 229533.I1RDE7 pp 229533.I1RW43
75959 229533.I1RDE7 pp 229533.I1RSK5
75960 229533.I1RDE7 pp 229533.I1RNW1
75961 229533.I1RDE7 pp 229533.I1S4S4
75962 229533.I1RDE7 pp 229533.I1RG66
75963 229533.I1RDE7 pp 229533.I1RZ29
75964 229533.I1RDE7 pp 229533.I1S5B0
75965 229533.I1RDE7 pp 229533.I1RPM6
75966 229533.I1RDE7 pp 229533.I1RJ73
75967 229533.I1RDE7 pp 229533.V6RQW4
75968 229533.I1RDE7 pp 229533.O74638
75969 229533.I1RDE7 pp 229533.I1S3G4
75970 229533.I1RDE7 pp 229533.I1RK03
75971 229533.I1RDE7 pp 229533.I1RUY5
75972 229533.I1RDE7 pp 229533.I1RWF9
75973 229533.I1RDE7 pp 229533.I1S019
75974 229533.I1RDE7 pp 229533.I1RM26
75975 229533.I1RDE7 pp 229533.I1RKB2
75976 229533.I1RDE7 pp 229533.I1RIY0
75977 229533.I1RDE7 pp 229533.I1RKD3
75978 229533.I1RDE7 pp 229533.I1RL04
75979 229533.I1RDE7 pp 229533.I1RTA1
75980 229533.I1RDE7 pp 229533.I1RZ52
75981 229533.I1RDE7 pp 229533.I1RPW1
75982 229533.I1RDE7 pp 229533.I1RSZ7
75983 229533.I1RDE7 pp 229533.I1RX22
75984 229533.I1RDE7 pp 229533.I1RW69
75985 229533.I1RDE7 pp 229533.I1RPD6
75986 229533.I1RDE7 pp 229533.Q4HXF0
75987 229533.I1RDE7 pp 229533.I1RNB4
75988 229533.I1RDE7 pp 229533.I1RZV5
75989 229533.I1RDE7 pp 229533.I1RNW6
75990 229533.I1RDE7 pp 229533.I1RZ83
75991 229533.I1RDE7 pp 229533.I1RP97
75992 229533.I1RDE7 pp 229533.I1RKB6
75993 229533.I1RDE7 pp 229533.I1RZ84

229533.I1RDE7	pp	229533.I1RN67
229533.I1RDE7	pp	229533.V6RKN5
229533.I1RDE7	pp	229533.I1S685
229533.I1RDE7	pp	229533.I1RNG4
229533.I1RDE7	pp	229533.I1S0I3
229533.I1RDE7	pp	229533.I1S684
229533.I1RDE7	pp	229533.I1SAN9
229533.I1RDE7	pp	229533.I1S064
229533.I1RDE7	pp	229533.V6RRM0
229533.I1RDE7	pp	229533.I1RNW2
229533.I1RDE7	pp	229533.V6RN31
229533.I1RCZ9	pp	229533.I1RK93
229533.I1RCZ9	pp	229533.I1RNG4
229533.I1RCZ9	pp	229533.V6RKN5
229533.I1RXF7	pp	229533.I1S2B4
229533.I1RXF7	pp	229533.P0C431
229533.I1RXF7	pp	229533.I1S0S6
229533.I1RXF7	pp	229533.I1S7X9
229533.I1RMX1	pp	229533.I1S074
229533.I1RK93	pp	229533.V6RKN5
229533.I1RK93	pp	229533.I1S3G4
229533.I1RK93	pp	229533.I1RNW2
229533.I1RK93	pp	229533.I1RM26
229533.I1RK93	pp	229533.I1RZ20
229533.I1RK93	pp	229533.I1S019
229533.I1RK93	pp	229533.I1RNW0
229533.I1RK93	pp	229533.I1RNG4
229533.I1RK93	pp	229533.I1S686
229533.I1RK93	pp	229533.I1RVX9
229533.I1RK93	pp	229533.I1RNB4
229533.I1RK93	pp	229533.I1S1W8
229533.I1RK93	pp	229533.I1RZV5
229533.I1RM81	pp	229533.I1S074
229533.A0A098D381	pp	229533.A0A0E0SBC4
229533.A0A098D381	pp	229533.V6RKN5
229533.A0A098D381	pp	229533.I1S685
229533.A0A098D381	pp	229533.I1RJ73
229533.A0A098D381	pp	229533.I1RAR3
229533.A0A098D381	pp	229533.I1RDE7
229533.A0A098D381	pp	229533.I1RCZ9
229533.A0A098D381	pp	229533.I1RK93
229533.I1RFB6	pp	229533.I1RWW4
229533.I1RFB6	pp	229533.I1RT14
229533.I1RFB6	pp	229533.I1RNG4
229533.I1RFB6	pp	229533.I1RSX2
229533.I1RFB6	pp	229533.I1RXX0
229533.I1RFB6	pp	229533.V6RM02
229533.I1RFB6	pp	229533.I1RV48
229533.I1RFB6	pp	229533.I1RST9
229533.I1RQA5	pp	229533.I1RUL3
229533.I1RQA5	pp	229533.Q4I963
229533.I1RQA5	pp	229533.I1RQE0
229533.I1RQA5	pp	229533.V6RDH3
229533.I1RQA5	pp	229533.I1RYL5
229533.I1RQA5	pp	229533.V6RY56
229533.I1RQA5	pp	229533.I1S235
229533.I1RQA5	pp	229533.I1RW87
229533.I1RQA5	pp	229533.I1RYS6
229533.I1RQA5	pp	229533.I1RRV4
229533.I1RQA5	pp	229533.I1RUQ3
229533.I1RQA5	pp	229533.V6RCZ8
229533.I1RQA5	pp	229533.Q4IA56
229533.I1RQA5	pp	229533.I1RT41
229533.I1RQA5	pp	229533.I1RW54
229533.I1RQA5	pp	229533.I1RQR6
229533.I1RQA5	pp	229533.I1RRT6
229533.I1RQA5	pp	229533.I1RQL4
229533.I1RQA5	pp	229533.I1S0Z3
229533.I1RQA5	pp	229533.I1RYI9
229533.I1RQA5	pp	229533.I1RTU8
229533.I1RQA5	pp	229533.I1RQH1
229533.I1RQA5	pp	229533.I1RZS4
229533.I1RQA5	pp	229533.I1S0B7

76067 229533.I1RQA5 pp 229533.I1S8F8
76068 229533.I1RQA5 pp 229533.I1RRS8
76069 229533.I1RQA5 pp 229533.I1RW71
76070 229533.I1RQA5 pp 229533.I1RWX5
76071 229533.I1RQA5 pp 229533.I1RT38
76072 229533.I1RQA5 pp 229533.I1RZS8
76073 229533.I1RQA5 pp 229533.I1S0R9
76074 229533.I1RQA5 pp 229533.Q4HXF6
76075 229533.I1RQA5 pp 229533.I1RYW3
76076 229533.I1RQA5 pp 229533.I1RSY2
76077 229533.I1RQA5 pp 229533.I1RSQ4
76078 229533.I1RQA5 pp 229533.I1S247
76079 229533.I1RQA5 pp 229533.I1S0Z8
76080 229533.I1RQA5 pp 229533.I1S0F9
76081 229533.I1RQA5 pp 229533.I1RZD3
76082 229533.I1RQA5 pp 229533.I1RRM9
76083 229533.I1RQA5 pp 229533.I1RWH4
76084 229533.I1RQA5 pp 229533.I1S2V2
76085 229533.I1RQA5 pp 229533.I1RWK9
76086 229533.I1RQA5 pp 229533.I1RVR2
76087 229533.I1RQA5 pp 229533.I1RZ95
76088 229533.I1RQA5 pp 229533.V6RLM7
76089 229533.I1RQA5 pp 229533.V6R3J1
76090 229533.I1RQA5 pp 229533.Q4I099
76091 229533.I1RQA5 pp 229533.I1RZH5
76092 229533.I1RQA5 pp 229533.I1S1F3
76093 229533.I1RQA5 pp 229533.I1RQG4
76094 229533.I1RCS6 pp 229533.I1RRK5
76095 229533.I1RCS6 pp 229533.I1RKK2
76096 229533.I1RCS6 pp 229533.I1RN52
76097 229533.I1RCS6 pp 229533.I1RMD3
76098 229533.I1RCS6 pp 229533.I1RMQ6
76099 229533.I1RCS6 pp 229533.I1RDE0
76100 229533.I1RCS6 pp 229533.I1REW3
76101 229533.I1RCS6 pp 229533.I1RR45
76102 229533.I1RCS6 pp 229533.I1RWF9
76103 229533.I1RCS6 pp 229533.I1RSK5
76104 229533.I1RCS6 pp 229533.I1RJV8
76105 229533.I1RCS6 pp 229533.I1S6D1
76106 229533.I1RCS6 pp 229533.I1RRM8
76107 229533.I1RI31 pp 229533.I1RNG4
76108 229533.A0A098DRZ4 pp 229533.A0A098E4F8
76109 229533.A0A098DRZ4 pp 229533.I1RA86
76110 229533.A0A098DRZ4 pp 229533.I1RWK9
76111 229533.A0A098DRZ4 pp 229533.I1REC6
76112 229533.A0A098DRZ4 pp 229533.I1R9Y8
76113 229533.A0A098DRZ4 pp 229533.I1REC7
76114 229533.A0A098DRZ4 pp 229533.I1RWH4
76115 229533.A0A098DRZ4 pp 229533.A0A0E0SK74
76116 229533.A0A098DRZ4 pp 229533.I1S2V2
76117 229533.A0A098DRZ4 pp 229533.I1RQ21
76118 229533.A0A098DRZ4 pp 229533.I1RDI8
76119 229533.A0A098DRZ4 pp 229533.A0A1C3YN12
76120 229533.A0A098DRZ4 pp 229533.A0A1C3YK11
76121 229533.A0A098DRZ4 pp 229533.I1RBS5
76122 229533.A0A098DRZ4 pp 229533.I1RM03
76123 229533.A0A098DRZ4 pp 229533.I1RQL4
76124 229533.A0A098DRZ4 pp 229533.I1RPN8
76125 229533.A0A098DRZ4 pp 229533.A0A0E0SL81
76126 229533.A0A098DRZ4 pp 229533.I1RBC4
76127 229533.A0A098DRZ4 pp 229533.I1RKK8
76128 229533.A0A098DRZ4 pp 229533.I1RYL5
76129 229533.A0A098DRZ4 pp 229533.I1RW87
76130 229533.A0A098DRZ4 pp 229533.I1RP66
76131 229533.A0A098DRZ4 pp 229533.I1S235
76132 229533.A0A1C3YK11 pp 229533.A0A1C3YK94
76133 229533.A0A1C3YK11 pp 229533.I1RA94
76134 229533.A0A1C3YK11 pp 229533.I1RZM8
76135 229533.A0A1C3YK11 pp 229533.I1RXF0
76136 229533.A0A1C3YK11 pp 229533.I1RVP5
76137 229533.A0A1C3YK11 pp 229533.I1S6K9
76138 229533.A0A1C3YK11 pp 229533.A0A1C3YKB0
76139 229533.A0A1C3YK11 pp 229533.I1S821

76140	229533.A0A1C3YK11	pp	229533.I1S0A0
76141	229533.A0A1C3YK11	pp	229533.I1S235
76142	229533.A0A1C3YK11	pp	229533.A0A1C3YK34
76143	229533.A0A1C3YK11	pp	229533.I1RYS6
76144	229533.A0A1C3YK11	pp	229533.I1RDH7
76145	229533.A0A1C3YK11	pp	229533.I1RPL2
76146	229533.A0A1C3YK11	pp	229533.I1RP66
76147	229533.A0A1C3YK11	pp	229533.I1RM26
76148	229533.A0A1C3YK11	pp	229533.I1RFY0
76149	229533.A0A1C3YK11	pp	229533.V6QUE6
76150	229533.A0A1C3YK11	pp	229533.I1RQE0
76151	229533.A0A1C3YK11	pp	229533.A0A1C3YLI1
76152	229533.A0A1C3YK11	pp	229533.I1RAB3
76153	229533.A0A1C3YK11	pp	229533.I1RS29
76154	229533.A0A1C3YK11	pp	229533.I1RGB2
76155	229533.A0A1C3YK11	pp	229533.I1RKK8
76156	229533.A0A1C3YK11	pp	229533.I1RZZ1
76157	229533.A0A1C3YK11	pp	229533.I1RUY5
76158	229533.A0A1C3YK11	pp	229533.I1RK95
76159	229533.A0A1C3YK11	pp	229533.Q4IJW4
76160	229533.A0A1C3YK11	pp	229533.I1RBC4
76161	229533.A0A1C3YK11	pp	229533.I1RQ14
76162	229533.A0A1C3YK11	pp	229533.I1RW87
76163	229533.A0A1C3YK11	pp	229533.I1RG60
76164	229533.A0A1C3YK11	pp	229533.A0A1C3YM63
76165	229533.A0A1C3YK11	pp	229533.I1RBY7
76166	229533.A0A1C3YK11	pp	229533.I1RDU0
76167	229533.A0A1C3YK11	pp	229533.A0A1C3YKY6
76168	229533.A0A1C3YK11	pp	229533.A0A1C3YN12
76169	229533.A0A1C3YK11	pp	229533.I1S7L6
76170	229533.A0A1C3YK11	pp	229533.I1RW25
76171	229533.A0A1C3YK11	pp	229533.A0A1C3YLT3
76172	229533.A0A1C3YK11	pp	229533.A0A1C3YLP6
76173	229533.A0A1C3YK11	pp	229533.I1RRX5
76174	229533.A0A1C3YK11	pp	229533.A0A1C3YKF3
76175	229533.A0A1C3YK11	pp	229533.I1RBL7
76176	229533.A0A1C3YK11	pp	229533.I1RF94
76177	229533.A0A1C3YK11	pp	229533.I1RVX9
76178	229533.A0A1C3YK11	pp	229533.A0A1C3YLR6
76179	229533.A0A1C3YK11	pp	229533.I1RDI8
76180	229533.A0A1C3YK11	pp	229533.I1RPA4
76181	229533.A0A1C3YK11	pp	229533.I1RNM7
76182	229533.A0A1C3YK11	pp	229533.I1RQH1
76183	229533.A0A1C3YK11	pp	229533.I1S3S5
76184	229533.A0A1C3YK11	pp	229533.I1RYL5
76185	229533.A0A1C3YK11	pp	229533.I1RMW4
76186	229533.A0A1C3YK11	pp	229533.A0A1C3YLB0
76187	229533.A0A1C3YK11	pp	229533.I1RBB3
76188	229533.A0A1C3YK11	pp	229533.I1RBU8
76189	229533.A0A1C3YK11	pp	229533.I1RRF8
76190	229533.A0A1C3YK11	pp	229533.V6RTJ8
76191	229533.A0A1C3YK11	pp	229533.I1RW54
76192	229533.A0A1C3YK11	pp	229533.I1S1W8
76193	229533.A0A1C3YK11	pp	229533.I1RQA5
76194	229533.A0A1C3YK11	pp	229533.I1RE66
76195	229533.A0A1C3YK11	pp	229533.I1RM03
76196	229533.A0A1C3YK11	pp	229533.I1RRT6
76197	229533.A0A1C3YK11	pp	229533.I1RTU8
76198	229533.A0A1C3YK11	pp	229533.I1S147
76199	229533.A0A1C3YK11	pp	229533.I1RMK7
76200	229533.A0A1C3YK11	pp	229533.I1RYU6
76201	229533.A0A1C3YK11	pp	229533.I1RRN2
76202	229533.A0A1C3YK11	pp	229533.I1RJT9
76203	229533.A0A1C3YK11	pp	229533.I1S4S4
76204	229533.A0A1C3YK11	pp	229533.I1RM67
76205	229533.A0A1C3YK11	pp	229533.I1RD22
76206	229533.A0A1C3YK11	pp	229533.I1RP86
76207	229533.A0A1C3YK11	pp	229533.I1RWH4
76208	229533.A0A1C3YK11	pp	229533.I1S0Z8
76209	229533.A0A1C3YK11	pp	229533.I1RJD3
76210	229533.A0A1C3YK11	pp	229533.I1RRG1
76211	229533.A0A1C3YK11	pp	229533.I1RL79
76212	229533.A0A1C3YK11	pp	229533.I1RFL4

76213	229533.A0A1C3YK11	pp	229533.I1RBT1
76214	229533.A0A1C3YK11	pp	229533.I1RE65
76215	229533.A0A1C3YK11	pp	229533.I1R9Y4
76216	229533.A0A1C3YK11	pp	229533.I1S5R6
76217	229533.A0A1C3YK11	pp	229533.Q4I7G0
76218	229533.A0A1C3YK11	pp	229533.A0A1C3YN26
76219	229533.A0A1C3YK11	pp	229533.V6RRM0
76220	229533.A0A1C3YK11	pp	229533.I1RAM6
76221	229533.A0A1C3YK11	pp	229533.I1RKB7
76222	229533.A0A1C3YK11	pp	229533.I1RZS8
76223	229533.A0A1C3YK11	pp	229533.I1RVX0
76224	229533.A0A1C3YK11	pp	229533.V6RPQ8
76225	229533.A0A1C3YK11	pp	229533.I1S9T5
76226	229533.A0A1C3YK11	pp	229533.I1RPV0
76227	229533.A0A1C3YK11	pp	229533.I1RPN8
76228	229533.A0A1C3YK11	pp	229533.I1RSY2
76229	229533.A0A1C3YK11	pp	229533.I1RQ21
76230	229533.A0A1C3YK11	pp	229533.I1RS06
76231	229533.A0A1C3YK11	pp	229533.I1RFK3
76232	229533.A0A1C3YK11	pp	229533.V6RN69
76233	229533.A0A1C3YK11	pp	229533.I1RSF1
76234	229533.A0A1C3YK11	pp	229533.I1RE01
76235	229533.A0A1C3YK11	pp	229533.I1RBA4
76236	229533.A0A1C3YK11	pp	229533.I1REB8
76237	229533.A0A1C3YK11	pp	229533.I1RUG3
76238	229533.A0A1C3YK11	pp	229533.A0A1C3YLB6
76239	229533.A0A1C3YK11	pp	229533.I1RMQ3
76240	229533.A0A1C3YK11	pp	229533.I1S123
76241	229533.A0A1C3YK11	pp	229533.I1RSD1
76242	229533.A0A1C3YK11	pp	229533.I1RE79
76243	229533.A0A1C3YK11	pp	229533.V6RLM7
76244	229533.A0A1C3YK11	pp	229533.I1RZ95
76245	229533.A0A1C3YK11	pp	229533.Q4HXF6
76246	229533.A0A1C3YK11	pp	229533.I1RKB6
76247	229533.A0A1C3YK11	pp	229533.I1S0S5
76248	229533.A0A1C3YK11	pp	229533.I1RMQ5
76249	229533.A0A1C3YK11	pp	229533.I1RBS5
76250	229533.A0A1C3YK11	pp	229533.I1RPK0
76251	229533.A0A1C3YK11	pp	229533.A0A1C3YM29
76252	229533.A0A1C3YK11	pp	229533.I1RE52
76253	229533.A0A1C3YK11	pp	229533.I1RBY6
76254	229533.A0A1C3YK11	pp	229533.I1RSU2
76255	229533.A0A1C3YK11	pp	229533.I1REC7
76256	229533.A0A1C3YK11	pp	229533.I1RME4
76257	229533.A0A1C3YK11	pp	229533.I1REG1
76258	229533.A0A1C3YK11	pp	229533.I1RAW0
76259	229533.A0A1C3YK11	pp	229533.Q4IPZ1
76260	229533.A0A1C3YK11	pp	229533.I1REC6
76261	229533.A0A1C3YK11	pp	229533.I1RA86
76262	229533.A0A1C3YK11	pp	229533.I1RFS3
76263	229533.A0A1C3YK11	pp	229533.I1RZD3
76264	229533.A0A1C3YK11	pp	229533.I1RPV3
76265	229533.A0A1C3YK11	pp	229533.I1S0R9
76266	229533.A0A1C3YK11	pp	229533.I1RHG5
76267	229533.A0A1C3YK11	pp	229533.I1RBF3
76268	229533.A0A1C3YK11	pp	229533.I1RMB1
76269	229533.A0A1C3YK11	pp	229533.A0A1C3YLK8
76270	229533.A0A1C3YK11	pp	229533.I1RWK9
76271	229533.A0A1C3YK11	pp	229533.I1RQ67
76272	229533.A0A1C3YK11	pp	229533.I1S9Y1
76273	229533.A0A1C3YK11	pp	229533.I1RRI2
76274	229533.A0A1C3YK11	pp	229533.I1S2V2
76275	229533.A0A1C3YK11	pp	229533.I1RBT8
76276	229533.A0A1C3YK11	pp	229533.A0A1C3YMC1
76277	229533.A0A1C3YK11	pp	229533.I1RSX0
76278	229533.A0A1C3YK11	pp	229533.I1RSS7
76279	229533.A0A1C3YK11	pp	229533.I1RU27
76280	229533.A0A1C3YK11	pp	229533.I1S5R1
76281	229533.A0A1C3YK11	pp	229533.I1RZK1
76282	229533.A0A1C3YK11	pp	229533.I1RZH5
76283	229533.A0A1C3YK11	pp	229533.I1RRT8
76284	229533.A0A1C3YK11	pp	229533.I1RN67
76285	229533.A0A1C3YK11	pp	229533.I1RXD9

76286 229533.A0A1C3YK11 pp 229533.I1RKI7
76287 229533.A0A1C3YK11 pp 229533.I1RNG4
76288 229533.A0A1C3YK11 pp 229533.I1RRM9
76289 229533.A0A1C3YK11 pp 229533.I1RP28
76290 229533.A0A1C3YK11 pp 229533.A0A1C3YL55
76291 229533.A0A1C3YK11 pp 229533.I1S3G4
76292 229533.A0A1C3YK11 pp 229533.I1RC18
76293 229533.A0A1C3YK11 pp 229533.I1RJT3
76294 229533.A0A1C3YK11 pp 229533.I1RKP1
76295 229533.A0A1C3YK11 pp 229533.I1RAB4
76296 229533.A0A1C3YK11 pp 229533.I1RWI5
76297 229533.A0A1C3YK11 pp 229533.Q4HXF0
76298 229533.A0A1C3YK11 pp 229533.I1R9W4
76299 229533.A0A1C3YK11 pp 229533.I1RZH6
76300 229533.A0A1C3YK11 pp 229533.I1RSQ4
76301 229533.A0A1C3YK11 pp 229533.I1RYP2
76302 229533.A0A1C3YK11 pp 229533.I1RFI4
76303 229533.A0A1C3YK11 pp 229533.I1S0F9
76304 229533.A0A1C3YK11 pp 229533.I1RYI5
76305 229533.A0A1C3YK11 pp 229533.I1S277
76306 229533.A0A1C3YK11 pp 229533.I1RPP3
76307 229533.A0A1C3YK11 pp 229533.I1RT38
76308 229533.A0A1C3YK11 pp 229533.I1RZ39
76309 229533.A0A1C3YK11 pp 229533.I1R9Y8
76310 229533.A0A1C3YK11 pp 229533.I1RQG4
76311 229533.A0A1C3YK11 pp 229533.I1RB59
76312 229533.A0A1C3YK11 pp 229533.A0A1C3YKM9
76313 229533.A0A1C3YK11 pp 229533.I1RSK5
76314 229533.I1RB88 pp 229533.I1RCI3
76315 229533.I1RB88 pp 229533.Q4IQX3
76316 229533.I1RB88 pp 229533.I1S4M5
76317 229533.I1RB88 pp 229533.I1RFQ1
76318 229533.I1RB88 pp 229533.I1RBU1
76319 229533.I1RB88 pp 229533.I1RCA7
76320 229533.I1RB88 pp 229533.V6RY33
76321 229533.I1RB88 pp 229533.I1RRS7
76322 229533.I1RB88 pp 229533.Q4IB96
76323 229533.I1RTU8 pp 229533.I1S2V2
76324 229533.I1RTU8 pp 229533.I1S235
76325 229533.I1RTU8 pp 229533.I1RWK9
76326 229533.I1RTU8 pp 229533.I1RWH4
76327 229533.I1RTU8 pp 229533.I1RW87
76328 229533.I1RTU8 pp 229533.I1RYL5
76329 229533.I1RI52 pp 229533.Q4I7L0
76330 229533.I1REV6 pp 229533.I1RT14
76331 229533.I1REV6 pp 229533.I1RXX0
76332 229533.I1REV6 pp 229533.I1RSX2
76333 229533.I1REV6 pp 229533.I1RWW4
76334 229533.I1REV6 pp 229533.I1RNG4
76335 229533.I1REV6 pp 229533.V6RM02
76336 229533.I1REV6 pp 229533.I1RST9
76337 229533.I1REV6 pp 229533.I1RV48
76338 229533.I1RF94 pp 229533.I1RW87
76339 229533.I1RF94 pp 229533.I1RYL5
76340 229533.I1RF94 pp 229533.I1RM03
76341 229533.I1RF94 pp 229533.I1RP66
76342 229533.I1RF94 pp 229533.I1S235
76343 229533.I1RF94 pp 229533.I1RQA5
76344 229533.I1RF94 pp 229533.I1S2V2
76345 229533.I1RF94 pp 229533.I1RWH4
76346 229533.I1RF94 pp 229533.I1RKK8
76347 229533.I1RF94 pp 229533.I1RPN8
76348 229533.I1RF94 pp 229533.I1RWK9
76349 229533.I1RF94 pp 229533.I1RP28
76350 229533.I1RF94 pp 229533.I1RQ21
76351 229533.I1RRT6 pp 229533.I1RZD3
76352 229533.I1RRT6 pp 229533.I1S2V2
76353 229533.I1RRT6 pp 229533.Q4I624
76354 229533.I1RRT6 pp 229533.Q4HXF6
76355 229533.I1RRT6 pp 229533.I1RWK9
76356 229533.I1RRT6 pp 229533.I1RYL5
76357 229533.I1RRT6 pp 229533.I1RWH4
76358 229533.I1RRT6 pp 229533.I1RW87

76359	229533.I1RRT6	pp	229533.I1S235
76360	229533.I1RRT6	pp	229533.I1RYS6
76361	229533.I1RRT6	pp	229533.I1RWD7
76362	229533.I1RW41	pp	229533.P0C431
76363	229533.I1RW41	pp	229533.I1S0S6
76364	229533.I1RW41	pp	229533.I1S7X9
76365	229533.I1RJT9	pp	229533.I1S235
76366	229533.I1RJT9	pp	229533.I1RQH1
76367	229533.I1RJT9	pp	229533.I1RPK5
76368	229533.I1RJT9	pp	229533.I1RM03
76369	229533.I1RJT9	pp	229533.I1RP66
76370	229533.I1RJT9	pp	229533.I1RRT6
76371	229533.I1RJT9	pp	229533.Q4HXF6
76372	229533.I1RJT9	pp	229533.I1RPL2
76373	229533.I1RJT9	pp	229533.I1RT62
76374	229533.I1RJT9	pp	229533.I1RP28
76375	229533.I1RJT9	pp	229533.I1RKK8
76376	229533.I1RJT9	pp	229533.I1RW87
76377	229533.I1RJT9	pp	229533.I1RQA5
76378	229533.I1RJT9	pp	229533.I1RWK9
76379	229533.I1RJT9	pp	229533.I1RYL5
76380	229533.I1RJT9	pp	229533.I1RYS6
76381	229533.I1RJT9	pp	229533.I1S2V2
76382	229533.I1RJT9	pp	229533.I1RPV0
76383	229533.I1RJT9	pp	229533.Q4ICI6
76384	229533.I1RJT9	pp	229533.I1RZS8
76385	229533.I1RJT9	pp	229533.I1RZD3
76386	229533.I1RJT9	pp	229533.I1RWH4
76387	229533.I1RJT9	pp	229533.I1RQ21
76388	229533.I1RJT9	pp	229533.I1RME4
76389	229533.I1RJT9	pp	229533.I1RPN8
76390	229533.I1RJT9	pp	229533.Q4I624
76391	229533.I1RJT9	pp	229533.I1RMQ5
76392	229533.I1S0S4	pp	229533.Q4I877
76393	229533.I1S0S4	pp	229533.I1S5Z5
76394	229533.I1RQH1	pp	229533.I1RRV1
76395	229533.I1RQH1	pp	229533.I1S9U6
76396	229533.I1RQH1	pp	229533.Q4I1Y5
76397	229533.I1RQH1	pp	229533.I1RS90
76398	229533.I1RQH1	pp	229533.I1RZ95
76399	229533.I1RQH1	pp	229533.I1S2C0
76400	229533.I1RQH1	pp	229533.I1RWH4
76401	229533.I1RQH1	pp	229533.I1S238
76402	229533.I1RQH1	pp	229533.I1S2G9
76403	229533.I1RQH1	pp	229533.V6RRH2
76404	229533.I1RQH1	pp	229533.I1RYW3
76405	229533.I1RQH1	pp	229533.I1S5S8
76406	229533.I1RQH1	pp	229533.I1RRM9
76407	229533.I1RQH1	pp	229533.I1RS47
76408	229533.I1RQH1	pp	229533.I1RSH0
76409	229533.I1RQH1	pp	229533.I1S2V2
76410	229533.I1RQH1	pp	229533.I1S235
76411	229533.I1RQH1	pp	229533.I1RZG0
76412	229533.I1RQH1	pp	229533.I1S0Z3
76413	229533.I1RQH1	pp	229533.I1RWK9
76414	229533.I1RQH1	pp	229533.I1RQQ6
76415	229533.I1RQH1	pp	229533.I1RYL5
76416	229533.I1RQH1	pp	229533.I1RRU9
76417	229533.I1RQH1	pp	229533.I1RZH4
76418	229533.I1RQH1	pp	229533.V6RBN2
76419	229533.I1RQH1	pp	229533.I1RW87
76420	229533.I1RQH1	pp	229533.I1RRT6
76421	229533.I1RQH1	pp	229533.I1RW71
76422	229533.I1RQH1	pp	229533.I1RQQ8
76423	229533.I1RQH1	pp	229533.I1RQH2
76424	229533.I1RQH1	pp	229533.I1RT41
76425	229533.I1RQH1	pp	229533.I1RSW4
76426	229533.I1RQL4	pp	229533.I1S594
76427	229533.I1RQL4	pp	229533.I1RSF2
76428	229533.I1RQL4	pp	229533.I1RVY0
76429	229533.I1RQL4	pp	229533.I1RSU6
76430	229533.I1RQL4	pp	229533.I1RRX1
76431	229533.I1RQL4	pp	229533.I1S2V2

76432	229533.I1RQL4	pp	229533.I1RZ86
76433	229533.I1RQL4	pp	229533.I1RRN0
76434	229533.I1RQL4	pp	229533.I1RWK9
76435	229533.I1RQL4	pp	229533.I1S296
76436	229533.I1RQL4	pp	229533.I1RTE6
76437	229533.I1RQL4	pp	229533.I1RUC4
76438	229533.I1RQL4	pp	229533.I1S7X9
76439	229533.I1RQL4	pp	229533.I1RQS1
76440	229533.I1RQL4	pp	229533.I1RQN9
76441	229533.I1RQL4	pp	229533.Q4HZB8
76442	229533.I1RQL4	pp	229533.I1RUP1
76443	229533.I1RQL4	pp	229533.I1S2K7
76444	229533.I1RQL4	pp	229533.Q4I7G0
76445	229533.I1RQL4	pp	229533.I1S0L8
76446	229533.I1RQL4	pp	229533.I1RT75
76447	229533.I1RQL4	pp	229533.I1RSQ1
76448	229533.I1RQL4	pp	229533.I1S247
76449	229533.I1RQL4	pp	229533.I1RS60
76450	229533.I1RQL4	pp	229533.I1RXI8
76451	229533.I1RQL4	pp	229533.Q4IN00
76452	229533.I1RQL4	pp	229533.I1RWQ0
76453	229533.I1RQL4	pp	229533.I1RW88
76454	229533.I1RQL4	pp	229533.I1S4Q2
76455	229533.I1RQL4	pp	229533.I1S7K6
76456	229533.I1RQL4	pp	229533.I1RTL1
76457	229533.I1RQL4	pp	229533.I1RWH4
76458	229533.I1RQL4	pp	229533.I1RWX5
76459	229533.I1RQL4	pp	229533.I1RSI3
76460	229533.I1RQL4	pp	229533.I1S0Z2
76461	229533.I1RQL4	pp	229533.I1RU27
76462	229533.I1RQL4	pp	229533.I1RT36
76463	229533.I1RQL4	pp	229533.I1S0W5
76464	229533.I1RQL4	pp	229533.I1RY98
76465	229533.I1RQL4	pp	229533.I1SA77
76466	229533.I1RQL4	pp	229533.I1RW87
76467	229533.I1RQL4	pp	229533.I1RWW4
76468	229533.I1RQL4	pp	229533.I1S0C0
76469	229533.I1RQL4	pp	229533.I1RXH6
76470	229533.I1RQL4	pp	229533.I1RYL5
76471	229533.I1RQL4	pp	229533.I1S749
76472	229533.I1RQL4	pp	229533.I1RTB7
76473	229533.I1RQL4	pp	229533.V6REE9
76474	229533.I1RQL4	pp	229533.I1S724
76475	229533.I1RQL4	pp	229533.I1S4H6
76476	229533.I1RQL4	pp	229533.P0C431
76477	229533.I1RQL4	pp	229533.I1S0S6
76478	229533.I1RQL4	pp	229533.I1S2B6
76479	229533.I1RQL4	pp	229533.I1S3S5
76480	229533.I1RQL4	pp	229533.I1S235
76481	229533.I1RQL4	pp	229533.Q4INZ9
76482	229533.I1RQL4	pp	229533.I1RZK4
76483	229533.I1RQL4	pp	229533.I1RT14
76484	229533.I1RQL4	pp	229533.I1S821
76485	229533.I1RQL4	pp	229533.I1S5T5
76486	229533.I1RQL4	pp	229533.I1S364
76487	229533.I1RQL4	pp	229533.I1RS57
76488	229533.I1R982	pp	229533.V6RM02
76489	229533.I1R982	pp	229533.I1RA90
76490	229533.I1R982	pp	229533.I1RV48
76491	229533.I1R982	pp	229533.I1RST9
76492	229533.I1R982	pp	229533.I1RAU3
76493	229533.I1R982	pp	229533.I1RSX2
76494	229533.I1R982	pp	229533.I1RNG4
76495	229533.I1R982	pp	229533.I1RT14
76496	229533.I1R982	pp	229533.I1RWW4
76497	229533.I1R982	pp	229533.I1RXX0
76498	229533.I1RRF8	pp	229533.I1S2V2
76499	229533.I1RRF8	pp	229533.V6RTJ8
76500	229533.I1RRF8	pp	229533.I1RWK9
76501	229533.I1RRF8	pp	229533.I1RRZ0
76502	229533.I1RRF8	pp	229533.I1RW87
76503	229533.I1RRF8	pp	229533.I1S235
76504	229533.I1RRF8	pp	229533.I1RWH4

76505	229533.I1RRF8	pp	229533.I1RYL5
76506	229533.A0A1C3YJ24	pp	229533.I1REK8
76507	229533.A0A1C3YJ24	pp	229533.I1RJU3
76508	229533.A0A1C3YJ24	pp	229533.I1RCH5
76509	229533.A0A1C3YJ24	pp	229533.V6RIV9
76510	229533.A0A1C3YJ24	pp	229533.A0A1C3YKV3
76511	229533.A0A1C3YJ24	pp	229533.I1RS17
76512	229533.I1RM07	pp	229533.V6RN69
76513	229533.I1RM07	pp	229533.I1RPU2
76514	229533.I1RM07	pp	229533.I1RW64
76515	229533.I1RM07	pp	229533.I1RW82
76516	229533.I1RM07	pp	229533.I1RVQ0
76517	229533.I1RM07	pp	229533.Q4I8B6
76518	229533.I1RM07	pp	229533.Q4HX89
76519	229533.I1RM07	pp	229533.Q4I8E9
76520	229533.I1RM07	pp	229533.I1RM09
76521	229533.A0A1C3YHZ0	pp	229533.I1RWH4
76522	229533.A0A1C3YHZ0	pp	229533.I1S235
76523	229533.A0A1C3YHZ0	pp	229533.I1RRH6
76524	229533.A0A1C3YHZ0	pp	229533.I1RW87
76525	229533.A0A1C3YHZ0	pp	229533.I1RP66
76526	229533.A0A1C3YHZ0	pp	229533.I1RPN8
76527	229533.A0A1C3YHZ0	pp	229533.A0A1C3YK11
76528	229533.A0A1C3YHZ0	pp	229533.A0A1C3YN12
76529	229533.A0A1C3YHZ0	pp	229533.I1RDI8
76530	229533.A0A1C3YHZ0	pp	229533.I1RYL5
76531	229533.A0A1C3YHZ0	pp	229533.I1RKK8
76532	229533.A0A1C3YHZ0	pp	229533.V6R1X4
76533	229533.A0A1C3YHZ0	pp	229533.I1RBC4
76534	229533.A0A1C3YHZ0	pp	229533.I1RBS5
76535	229533.A0A1C3YHZ0	pp	229533.I1RQ21
76536	229533.A0A1C3YHZ0	pp	229533.I1RM03
76537	229533.A0A1C3YHZ0	pp	229533.I1REC7
76538	229533.A0A1C3YHZ0	pp	229533.I1RWK9
76539	229533.A0A1C3YHZ0	pp	229533.I1RA86
76540	229533.A0A1C3YHZ0	pp	229533.I1RT38
76541	229533.A0A1C3YHZ0	pp	229533.I1S2V2
76542	229533.A0A1C3YHZ0	pp	229533.I1RVN1
76543	229533.A0A1C3YHZ0	pp	229533.I1R9Y8
76544	229533.A0A1C3YHZ0	pp	229533.I1REC6
76545	229533.A0A1C3YHZ0	pp	229533.I1RT28
76546	229533.I1RRY4	pp	229533.I1RWT6
76547	229533.I1RVB7	pp	229533.I1RY87
76548	229533.I1RVB7	pp	229533.I1RVB8
76549	229533.I1RVB7	pp	229533.I1S3G4
76550	229533.I1RVB7	pp	229533.I1S1W8
76551	229533.A0A098DN31	pp	229533.I1RWK9
76552	229533.A0A098DN31	pp	229533.I1S2V2
76553	229533.A0A098DN31	pp	229533.I1RQ21
76554	229533.A0A098DN31	pp	229533.I1R9Y8
76555	229533.A0A098DN31	pp	229533.A0A0E0SK74
76556	229533.A0A098DN31	pp	229533.I1REC6
76557	229533.A0A098DN31	pp	229533.I1RWH4
76558	229533.A0A098DN31	pp	229533.I1REC7
76559	229533.A0A098DN31	pp	229533.I1RA86
76560	229533.A0A098DN31	pp	229533.I1RPN8
76561	229533.A0A098DN31	pp	229533.A0A098E4F8
76562	229533.A0A098DN31	pp	229533.A0A0E0SL81
76563	229533.A0A098DN31	pp	229533.I1RQL4
76564	229533.A0A098DN31	pp	229533.I1RM03
76565	229533.A0A098DN31	pp	229533.A0A1C3YK11
76566	229533.A0A098DN31	pp	229533.A0A1C3YN12
76567	229533.A0A098DN31	pp	229533.I1RBS5
76568	229533.A0A098DN31	pp	229533.I1RDI8
76569	229533.A0A098DN31	pp	229533.I1S235
76570	229533.A0A098DN31	pp	229533.I1RP66
76571	229533.A0A098DN31	pp	229533.I1RW87
76572	229533.A0A098DN31	pp	229533.I1RYL5
76573	229533.A0A098DN31	pp	229533.I1RBC4
76574	229533.A0A098DN31	pp	229533.A0A098DRS5
76575	229533.A0A098DN31	pp	229533.I1RKK8
76576	229533.A0A1C3YHY9	pp	229533.I1RRS7
76577	229533.A0A1C3YHY9	pp	229533.I1RBU1

76578 229533.I1RWL8 pp 229533.I1S0Q7
76579 229533.I1RWL8 pp 229533.V6RM02
76580 229533.I1RWL8 pp 229533.V6RN69
76581 229533.I1RWL8 pp 229533.I1S489
76582 229533.I1RDH7 pp 229533.I1RQL4
76583 229533.I1RDH7 pp 229533.I1RP66
76584 229533.I1RDH7 pp 229533.I1S235
76585 229533.I1RDH7 pp 229533.I1RW87
76586 229533.I1RDH7 pp 229533.I1RM03
76587 229533.I1RDH7 pp 229533.I1RWK9
76588 229533.I1RDH7 pp 229533.I1RDI8
76589 229533.I1RDH7 pp 229533.I1RYL5
76590 229533.I1RDH7 pp 229533.I1REC6
76591 229533.I1RDH7 pp 229533.I1S2V2
76592 229533.I1RDH7 pp 229533.I1RKK8
76593 229533.I1RDH7 pp 229533.I1RPN8
76594 229533.I1RDH7 pp 229533.I1RWH4
76595 229533.I1RDH7 pp 229533.I1RQ21
76596 229533.I1RDH7 pp 229533.I1REC7
76597 229533.I1RPL2 pp 229533.I1RYW3
76598 229533.I1RPL2 pp 229533.I1RZD3
76599 229533.I1RPL2 pp 229533.I1RZS8
76600 229533.I1RPL2 pp 229533.I1S238
76601 229533.I1RPL2 pp 229533.I1RZE4
76602 229533.I1RPL2 pp 229533.I1RWH4
76603 229533.I1RPL2 pp 229533.I1RQ21
76604 229533.I1RPL2 pp 229533.I1RQF2
76605 229533.I1RPL2 pp 229533.I1RPN8
76606 229533.I1RPL2 pp 229533.I1RQG4
76607 229533.I1RPL2 pp 229533.I1S0M2
76608 229533.I1RPL2 pp 229533.I1RTA0
76609 229533.I1RPL2 pp 229533.I1S9U6
76610 229533.I1RPL2 pp 229533.I1RRM9
76611 229533.I1RPL2 pp 229533.I1RRV1
76612 229533.I1RPL2 pp 229533.I1S8P5
76613 229533.I1RPL2 pp 229533.I1S2V2
76614 229533.I1RPL2 pp 229533.I1RZ95
76615 229533.I1RPL2 pp 229533.I1RWK9
76616 229533.I1RPL2 pp 229533.I1RUF8
76617 229533.I1RPL2 pp 229533.Q4IL82
76618 229533.I1RPL2 pp 229533.I1S2G9
76619 229533.I1RPL2 pp 229533.I1RZ25
76620 229533.I1RPL2 pp 229533.I1RX50
76621 229533.I1RPL2 pp 229533.V6R3J1
76622 229533.I1RPL2 pp 229533.I1RW87
76623 229533.I1RPL2 pp 229533.I1RR95
76624 229533.I1RPL2 pp 229533.I1S5S8
76625 229533.I1RPL2 pp 229533.I1RYL5
76626 229533.I1RPL2 pp 229533.I1RT75
76627 229533.I1RPL2 pp 229533.I1RQQ6
76628 229533.I1RPL2 pp 229533.I1RSH0
76629 229533.I1RPL2 pp 229533.I1RQ81
76630 229533.I1RPL2 pp 229533.I1S0Z3
76631 229533.I1RPL2 pp 229533.I1RQA5
76632 229533.I1RPL2 pp 229533.I1S235
76633 229533.I1RPL2 pp 229533.I1S099
76634 229533.I1RPL2 pp 229533.V6RBN2
76635 229533.I1RPL2 pp 229533.I1RT41
76636 229533.I1RPL2 pp 229533.I1RQ26
76637 229533.I1RPL2 pp 229533.Q4HTT2
76638 229533.I1RPL2 pp 229533.I1RW91
76639 229533.I1RPL2 pp 229533.I1RW71
76640 229533.I1RPL2 pp 229533.I1RZH4
76641 229533.I1RPL2 pp 229533.V6RTJ8
76642 229533.I1RPL2 pp 229533.Q4HZS8
76643 229533.I1RPL2 pp 229533.I1RQH1
76644 229533.I1RPL2 pp 229533.Q5I2J3
76645 229533.I1RPL2 pp 229533.I1RX80
76646 229533.I1RPL2 pp 229533.I1RRT6
76647 229533.I1RPL2 pp 229533.I1RQQ8
76648 229533.V6RIE5 pp 229533.V6RQW4
76649 229533.V6RIE5 pp 229533.V6RY56
76650 229533.V6RIE5 pp 229533.V6RM02

76651 229533.I1RBY7 pp 229533.I1RKK8
76652 229533.I1RBY7 pp 229533.I1RDI8
76653 229533.I1RBY7 pp 229533.I1S235
76654 229533.I1RBY7 pp 229533.I1RM03
76655 229533.I1RBY7 pp 229533.I1RQL4
76656 229533.I1RBY7 pp 229533.I1RW87
76657 229533.I1RBY7 pp 229533.I1RP66
76658 229533.I1RBY7 pp 229533.I1RM27
76659 229533.I1RBY7 pp 229533.I1RYL5
76660 229533.I1RBY7 pp 229533.I1S2V2
76661 229533.I1RBY7 pp 229533.I1REC6
76662 229533.I1RBY7 pp 229533.I1REC7
76663 229533.I1RBY7 pp 229533.I1RWK9
76664 229533.I1RBY7 pp 229533.I1RSJ0
76665 229533.I1RBY7 pp 229533.I1RQ21
76666 229533.I1RBY7 pp 229533.I1RWH4
76667 229533.I1RBY7 pp 229533.I1RPN8
76668 229533.A0A1C3YIK4 pp 229533.I1RZM4
76669 229533.A0A1C3YIK4 pp 229533.I1RRG3
76670 229533.A0A1C3YIK4 pp 229533.I1RRX5
76671 229533.A0A1C3YIK4 pp 229533.I1S4S7
76672 229533.A0A1C3YIK4 pp 229533.I1RYU3
76673 229533.A0A1C3YIK4 pp 229533.I1S7L6
76674 229533.A0A1C3YIK4 pp 229533.I1RPN4
76675 229533.A0A1C3YIK4 pp 229533.I1RT33
76676 229533.A0A1C3YIK4 pp 229533.I1RYT8
76677 229533.A0A1C3YIK4 pp 229533.I1S0P0
76678 229533.A0A1C3YIK4 pp 229533.I1RYU6
76679 229533.A0A1C3YIK4 pp 229533.A0A1C3YN12
76680 229533.A0A1C3YIK4 pp 229533.I1RS18
76681 229533.A0A1C3YIK4 pp 229533.A0A1C3YK34
76682 229533.A0A1C3YIK4 pp 229533.A0A1C3YLR6
76683 229533.A0A1C3YIK4 pp 229533.I1RRS8
76684 229533.A0A1C3YIK4 pp 229533.I1RDI8
76685 229533.A0A1C3YIK4 pp 229533.I1RME6
76686 229533.A0A1C3YIK4 pp 229533.I1RPL2
76687 229533.A0A1C3YIK4 pp 229533.I1RP86
76688 229533.A0A1C3YIK4 pp 229533.I1RAM6
76689 229533.A0A1C3YIK4 pp 229533.A0A1C3YKV3
76690 229533.A0A1C3YIK4 pp 229533.A0A1C3YN26
76691 229533.A0A1C3YIK4 pp 229533.I1RS05
76692 229533.A0A1C3YIK4 pp 229533.I1RP66
76693 229533.A0A1C3YIK4 pp 229533.I1RMC8
76694 229533.A0A1C3YIK4 pp 229533.A0A1C3YJL7
76695 229533.A0A1C3YIK4 pp 229533.A0A1C3YJN3
76696 229533.A0A1C3YIK4 pp 229533.A0A1C3YKB0
76697 229533.A0A1C3YIK4 pp 229533.I1S8F8
76698 229533.A0A1C3YIK4 pp 229533.I1RG14
76699 229533.A0A1C3YIK4 pp 229533.I1RBT1
76700 229533.A0A1C3YIK4 pp 229533.I1RMQ6
76701 229533.A0A1C3YIK4 pp 229533.I1RMM8
76702 229533.A0A1C3YIK4 pp 229533.I1RJU3
76703 229533.A0A1C3YIK4 pp 229533.I1RYI9
76704 229533.A0A1C3YIK4 pp 229533.V6RJP8
76705 229533.A0A1C3YIK4 pp 229533.I1RQE8
76706 229533.A0A1C3YIK4 pp 229533.I1RM03
76707 229533.A0A1C3YIK4 pp 229533.I1RA90
76708 229533.A0A1C3YIK4 pp 229533.I1RXF0
76709 229533.A0A1C3YIK4 pp 229533.I1RVP5
76710 229533.A0A1C3YIK4 pp 229533.I1S235
76711 229533.A0A1C3YIK4 pp 229533.I1RM67
76712 229533.A0A1C3YIK4 pp 229533.A0A1C3YK11
76713 229533.A0A1C3YIK4 pp 229533.I1S4S4
76714 229533.A0A1C3YIK4 pp 229533.I1RY21
76715 229533.A0A1C3YIK4 pp 229533.I1S8Z2
76716 229533.A0A1C3YIK4 pp 229533.I1R9Y2
76717 229533.A0A1C3YIK4 pp 229533.I1RQH1
76718 229533.A0A1C3YIK4 pp 229533.I1RJR8
76719 229533.A0A1C3YIK4 pp 229533.I1RBE4
76720 229533.A0A1C3YIK4 pp 229533.I1RNM7
76721 229533.A0A1C3YIK4 pp 229533.V6RCZ8
76722 229533.A0A1C3YIK4 pp 229533.I1RCE6
76723 229533.A0A1C3YIK4 pp 229533.I1S6K9

76724	229533.A0A1C3YIK4	pp	229533.I1RMK7
76725	229533.A0A1C3YIK4	pp	229533.I1RRG1
76726	229533.A0A1C3YIK4	pp	229533.I1RR24
76727	229533.A0A1C3YIK4	pp	229533.V6RK96
76728	229533.A0A1C3YIK4	pp	229533.I1RKK8
76729	229533.A0A1C3YIK4	pp	229533.I1RW54
76730	229533.A0A1C3YIK4	pp	229533.I1RXC4
76731	229533.A0A1C3YIK4	pp	229533.I1RFY0
76732	229533.A0A1C3YIK4	pp	229533.V6QUE6
76733	229533.A0A1C3YIK4	pp	229533.I1RK95
76734	229533.A0A1C3YIK4	pp	229533.I1RAV5
76735	229533.A0A1C3YIK4	pp	229533.I1RT40
76736	229533.A0A1C3YIK4	pp	229533.I1S5B0
76737	229533.A0A1C3YIK4	pp	229533.I1RQE0
76738	229533.A0A1C3YIK4	pp	229533.I1R9W4
76739	229533.A0A1C3YIK4	pp	229533.I1REK8
76740	229533.A0A1C3YIK4	pp	229533.A0A1C3YJ21
76741	229533.A0A1C3YIK4	pp	229533.A0A1C3YLU6
76742	229533.A0A1C3YIK4	pp	229533.I1RPT0
76743	229533.A0A1C3YIK4	pp	229533.Q4HXF0
76744	229533.A0A1C3YIK4	pp	229533.V6R3J1
76745	229533.A0A1C3YIK4	pp	229533.I1RYL5
76746	229533.A0A1C3YIK4	pp	229533.I1RA94
76747	229533.A0A1C3YIK4	pp	229533.I1RBC4
76748	229533.A0A1C3YIK4	pp	229533.Q4I6B0
76749	229533.A0A1C3YIK4	pp	229533.I1RQA5
76750	229533.A0A1C3YIK4	pp	229533.I1RBU8
76751	229533.A0A1C3YIK4	pp	229533.I1RSX0
76752	229533.A0A1C3YIK4	pp	229533.A0A1C3YKY6
76753	229533.A0A1C3YIK4	pp	229533.A0A1C3YJ14
76754	229533.A0A1C3YIK4	pp	229533.I1RBL7
76755	229533.A0A1C3YIK4	pp	229533.I1RJN9
76756	229533.A0A1C3YIK4	pp	229533.I1RW87
76757	229533.A0A1C3YIK4	pp	229533.I1RTK5
76758	229533.A0A1C3YIK4	pp	229533.I1RAD4
76759	229533.A0A1C3YIK4	pp	229533.I1R9M0
76760	229533.A0A1C3YIK4	pp	229533.I1RTA1
76761	229533.A0A1C3YIK4	pp	229533.A0A1C3YLK8
76762	229533.A0A1C3YIK4	pp	229533.I1RKB2
76763	229533.A0A1C3YIK4	pp	229533.I1RZB2
76764	229533.A0A1C3YIK4	pp	229533.I1S2B8
76765	229533.A0A1C3YIK4	pp	229533.I1RBE6
76766	229533.A0A1C3YIK4	pp	229533.I1RGA4
76767	229533.A0A1C3YIK4	pp	229533.I1RPP3
76768	229533.A0A1C3YIK4	pp	229533.I1RYI5
76769	229533.A0A1C3YIK4	pp	229533.I1R9Y8
76770	229533.A0A1C3YIK4	pp	229533.I1RA52
76771	229533.A0A1C3YIK4	pp	229533.I1REC6
76772	229533.A0A1C3YIK4	pp	229533.I1RFI4
76773	229533.A0A1C3YIK4	pp	229533.I1RFS3
76774	229533.A0A1C3YIK4	pp	229533.I1RQG4
76775	229533.A0A1C3YIK4	pp	229533.I1RL04
76776	229533.A0A1C3YIK4	pp	229533.I1RPV3
76777	229533.A0A1C3YIK4	pp	229533.I1RSQ4
76778	229533.A0A1C3YIK4	pp	229533.I1RBF3
76779	229533.A0A1C3YIK4	pp	229533.I1RP28
76780	229533.A0A1C3YIK4	pp	229533.I1RZZ9
76781	229533.A0A1C3YIK4	pp	229533.I1RC18
76782	229533.A0A1C3YIK4	pp	229533.I1S1F3
76783	229533.A0A1C3YIK4	pp	229533.I1RWI5
76784	229533.A0A1C3YIK4	pp	229533.I1RBY4
76785	229533.A0A1C3YIK4	pp	229533.A0A1C3YIR8
76786	229533.A0A1C3YIK4	pp	229533.I1RRN2
76787	229533.A0A1C3YIK4	pp	229533.I1RIU0
76788	229533.A0A1C3YIK4	pp	229533.I1RZ39
76789	229533.A0A1C3YIK4	pp	229533.A0A1C3YLB6
76790	229533.A0A1C3YIK4	pp	229533.I1RE52
76791	229533.A0A1C3YIK4	pp	229533.A0A1C3YKM9
76792	229533.A0A1C3YIK4	pp	229533.V6RLM7
76793	229533.A0A1C3YIK4	pp	229533.I1RBM3
76794	229533.A0A1C3YIK4	pp	229533.I1RDE7
76795	229533.A0A1C3YIK4	pp	229533.Q4I665
76796	229533.A0A1C3YIK4	pp	229533.I1RY92

76797 229533.A0A1C3YIK4 pp 229533.I1RWH4
76798 229533.A0A1C3YIK4 pp 229533.I1RSU2
76799 229533.A0A1C3YIK4 pp 229533.I1RFS5
76800 229533.A0A1C3YIK4 pp 229533.I1RSF1
76801 229533.A0A1C3YIK4 pp 229533.I1RXC2
76802 229533.A0A1C3YIK4 pp 229533.I1RQ21
76803 229533.A0A1C3YIK4 pp 229533.I1RG11
76804 229533.A0A1C3YIK4 pp 229533.I1RY57
76805 229533.A0A1C3YIK4 pp 229533.I1RPN8
76806 229533.A0A1C3YIK4 pp 229533.I1RA11
76807 229533.A0A1C3YIK4 pp 229533.V6RRM0
76808 229533.A0A1C3YIK4 pp 229533.I1RCR8
76809 229533.A0A1C3YIK4 pp 229533.I1RWK9
76810 229533.A0A1C3YIK4 pp 229533.I1RMQ5
76811 229533.A0A1C3YIK4 pp 229533.I1RCH5
76812 229533.A0A1C3YIK4 pp 229533.I1S9Y1
76813 229533.A0A1C3YIK4 pp 229533.I1S5X9
76814 229533.A0A1C3YIK4 pp 229533.I1S8A6
76815 229533.A0A1C3YIK4 pp 229533.V6R042
76816 229533.A0A1C3YIK4 pp 229533.A0A1C3YK08
76817 229533.A0A1C3YIK4 pp 229533.I1S2V2
76818 229533.A0A1C3YIK4 pp 229533.I1RBS5
76819 229533.A0A1C3YIK4 pp 229533.A0A1C3YJK3
76820 229533.A0A1C3YIK4 pp 229533.I1RSY2
76821 229533.A0A1C3YIK4 pp 229533.I1RB92
76822 229533.A0A1C3YIK4 pp 229533.I1RSQ3
76823 229533.A0A1C3YIK4 pp 229533.I1RKP1
76824 229533.A0A1C3YIK4 pp 229533.I1RVX0
76825 229533.A0A1C3YIK4 pp 229533.I1RZ95
76826 229533.A0A1C3YIK4 pp 229533.I1S2H4
76827 229533.A0A1C3YIK4 pp 229533.I1RT03
76828 229533.A0A1C3YIK4 pp 229533.I1RA86
76829 229533.A0A1C3YIK4 pp 229533.I1S1C7
76830 229533.A0A1C3YIK4 pp 229533.I1RBA4
76831 229533.A0A1C3YIK4 pp 229533.I1RSS4
76832 229533.A0A1C3YIK4 pp 229533.I1S0R9
76833 229533.A0A1C3YIK4 pp 229533.V6RRH2
76834 229533.A0A1C3YIK4 pp 229533.I1RA70
76835 229533.A0A1C3YIK4 pp 229533.I1RJV3
76836 229533.A0A1C3YIK4 pp 229533.I1RNU7
76837 229533.A0A1C3YIK4 pp 229533.I1RHG5
76838 229533.A0A1C3YIK4 pp 229533.V6RDZ4
76839 229533.A0A1C3YIK4 pp 229533.I1REC7
76840 229533.A0A1C3YIK4 pp 229533.I1RQC0
76841 229533.A0A1C3YIK4 pp 229533.I1RKB6
76842 229533.I1RUF9 pp 229533.I1S2Q0
76843 229533.I1RUF9 pp 229533.I1S3S7
76844 229533.I1RUF9 pp 229533.I1S1K2
76845 229533.I1RZL6 pp 229533.V6RMW6
76846 229533.I1RZL6 pp 229533.V6RIV9
76847 229533.I1RIN2 pp 229533.I1RNG4
76848 229533.I1RG60 pp 229533.I1S235
76849 229533.I1RG60 pp 229533.I1RP66
76850 229533.I1RG60 pp 229533.I1RW87
76851 229533.I1RG60 pp 229533.I1RQA5
76852 229533.I1RG60 pp 229533.I1RWK9
76853 229533.I1RG60 pp 229533.I1RYL5
76854 229533.I1RG60 pp 229533.I1RM03
76855 229533.I1RG60 pp 229533.I1RKK8
76856 229533.I1RG60 pp 229533.I1RPN8
76857 229533.I1RG60 pp 229533.I1RP28
76858 229533.I1RG60 pp 229533.I1S2V2
76859 229533.I1RG60 pp 229533.I1RWH4
76860 229533.I1RG60 pp 229533.I1RQ21
76861 229533.I1RP13 pp 229533.I1RPB1
76862 229533.I1RP13 pp 229533.I1S3S7
76863 229533.I1RP13 pp 229533.I1RT87
76864 229533.I1RP13 pp 229533.I1S1K2
76865 229533.I1RP13 pp 229533.I1S2Q0
76866 229533.I1RP13 pp 229533.I1S8M4
76867 229533.A0A1C3YLT3 pp 229533.I1RA86
76868 229533.A0A1C3YLT3 pp 229533.I1RQ21
76869 229533.A0A1C3YLT3 pp 229533.I1RWH4

229533.A0A1C3YLT3 pp 229533.I1RE65
229533.A0A1C3YLT3 pp 229533.I1RPN8
229533.A0A1C3YLT3 pp 229533.I1R9Y8
229533.A0A1C3YLT3 pp 229533.I1RSZ7
229533.A0A1C3YLT3 pp 229533.I1REC7
229533.A0A1C3YLT3 pp 229533.V6R1X4
229533.A0A1C3YLT3 pp 229533.I1RKK8
229533.A0A1C3YLT3 pp 229533.I1RBC4
229533.A0A1C3YLT3 pp 229533.I1RYL5
229533.A0A1C3YLT3 pp 229533.I1RW87
229533.A0A1C3YLT3 pp 229533.I1REC6
229533.A0A1C3YLT3 pp 229533.I1S2V2
229533.A0A1C3YLT3 pp 229533.I1RDF7
229533.A0A1C3YLT3 pp 229533.I1RWK9
229533.A0A1C3YLT3 pp 229533.I1RJN9
229533.A0A1C3YLT3 pp 229533.I1RBS5
229533.A0A1C3YLT3 pp 229533.I1RP66
229533.A0A1C3YLT3 pp 229533.I1RM03
229533.A0A1C3YLT3 pp 229533.I1S235
229533.A0A1C3YLT3 pp 229533.I1RQL4
229533.A0A1C3YLT3 pp 229533.I1RDI8
229533.A0A1C3YLT3 pp 229533.A0A1C3YN12
229533.I1RJ14 pp 229533.V6RN69
229533.I1RJ14 pp 229533.I1RTN1
229533.I1RJ14 pp 229533.V6RM02
229533.I1RJ14 pp 229533.I1RTN8
229533.I1RJ14 pp 229533.I1RND4
229533.I1RJ14 pp 229533.V6REA4
229533.I1RJ14 pp 229533.V6RM09
229533.I1RJ14 pp 229533.I1S2L3
229533.I1RJ14 pp 229533.V6QTX7
229533.I1RJ14 pp 229533.V6R556
229533.I1RJ14 pp 229533.I1RQW2
229533.I1RJ14 pp 229533.I1RME1
229533.A0A098DRS5 pp 229533.I1S3S5
229533.A0A098DRS5 pp 229533.Q4I1Y1
229533.A0A098DRS5 pp 229533.I1RQA5
229533.A0A098DRS5 pp 229533.I1S2B6
229533.A0A098DRS5 pp 229533.I1RW87
229533.A0A098DRS5 pp 229533.I1RBY7
229533.A0A098DRS5 pp 229533.I1RYZ0
229533.A0A098DRS5 pp 229533.I1RYS6
229533.A0A098DRS5 pp 229533.I1RHB0
229533.A0A098DRS5 pp 229533.A0A1C3YN54
229533.A0A098DRS5 pp 229533.I1RAB3
229533.A0A098DRS5 pp 229533.A0A1C3YME9
229533.A0A098DRS5 pp 229533.Q4IE79
229533.A0A098DRS5 pp 229533.I1RYL5
229533.A0A098DRS5 pp 229533.I1RBC4
229533.A0A098DRS5 pp 229533.A0A0E0RVF4
229533.A0A098DRS5 pp 229533.V6REE9
229533.A0A098DRS5 pp 229533.I1RKK8
229533.A0A098DRS5 pp 229533.I1RPK5
229533.A0A098DRS5 pp 229533.A0A1C3YIK4
229533.A0A098DRS5 pp 229533.V6RR76
229533.A0A098DRS5 pp 229533.A0A1C3YHZ0
229533.A0A098DRS5 pp 229533.A0A1C3YLU7
229533.A0A098DRS5 pp 229533.I1S235
229533.A0A098DRS5 pp 229533.A0A1C3YK75
229533.A0A098DRS5 pp 229533.I1RBL8
229533.A0A098DRS5 pp 229533.I1RQA3
229533.A0A098DRS5 pp 229533.I1S821
229533.A0A098DRS5 pp 229533.I1RG60
229533.A0A098DRS5 pp 229533.A0A098DRZ4
229533.A0A098DRS5 pp 229533.I1RDH7
229533.A0A098DRS5 pp 229533.I1RPL2
229533.A0A098DRS5 pp 229533.I1RMG2
229533.A0A098DRS5 pp 229533.I1RTA6
229533.A0A098DRS5 pp 229533.A0A1C3YN89
229533.A0A098DRS5 pp 229533.A0A1C3YJV7
229533.A0A098DRS5 pp 229533.I1RP66
229533.A0A098DRS5 pp 229533.I1RN72
229533.A0A098DRS5 pp 229533.A0A1C3YM54

76943 229533.A0A098DRS5 pp 229533.A0A1C3YK11
76944 229533.A0A098DRS5 pp 229533.I1S0Z3
76945 229533.A0A098DRS5 pp 229533.I1RPI2
76946 229533.A0A098DRS5 pp 229533.I1S275
76947 229533.A0A098DRS5 pp 229533.A0A1C3YN12
76948 229533.A0A098DRS5 pp 229533.I1RWT6
76949 229533.A0A098DRS5 pp 229533.A0A1C3YN26
76950 229533.A0A098DRS5 pp 229533.I1RAM6
76951 229533.A0A098DRS5 pp 229533.A0A0E0SA11
76952 229533.A0A098DRS5 pp 229533.I1RPA7
76953 229533.A0A098DRS5 pp 229533.I1RQH1
76954 229533.A0A098DRS5 pp 229533.A0A1C3YLT3
76955 229533.A0A098DRS5 pp 229533.A0A1C3YLH7
76956 229533.A0A098DRS5 pp 229533.I1RT41
76957 229533.A0A098DRS5 pp 229533.I1RZG3
76958 229533.A0A098DRS5 pp 229533.Q4IPB3
76959 229533.A0A098DRS5 pp 229533.A0A1C3YN58
76960 229533.A0A098DRS5 pp 229533.I1RF94
76961 229533.A0A098DRS5 pp 229533.A0A1C3YIP6
76962 229533.A0A098DRS5 pp 229533.I1RQC5
76963 229533.A0A098DRS5 pp 229533.I1S0F5
76964 229533.A0A098DRS5 pp 229533.I1RTQ9
76965 229533.A0A098DRS5 pp 229533.I1RWX5
76966 229533.A0A098DRS5 pp 229533.I1RDI8
76967 229533.A0A098DRS5 pp 229533.I1RJT9
76968 229533.A0A098DRS5 pp 229533.I1RN81
76969 229533.A0A098DRS5 pp 229533.Q4INZ9
76970 229533.A0A098DRS5 pp 229533.I1RRF8
76971 229533.A0A098DRS5 pp 229533.I1RZS4
76972 229533.A0A098DRS5 pp 229533.Q4IN00
76973 229533.A0A098DRS5 pp 229533.I1RRT6
76974 229533.A0A098DRS5 pp 229533.Q4INI2
76975 229533.A0A098DRS5 pp 229533.A0A0E0SL81
76976 229533.A0A098DRS5 pp 229533.A0A098DVZ4
76977 229533.A0A098DRS5 pp 229533.Q4I5R9
76978 229533.A0A098DRS5 pp 229533.I1RYW3
76979 229533.A0A098DRS5 pp 229533.I1RTU8
76980 229533.A0A098DRS5 pp 229533.I1RPS0
76981 229533.A0A098DRS5 pp 229533.I1RQL4
76982 229533.A0A098DRS5 pp 229533.I1S247
76983 229533.A0A098DRS5 pp 229533.I1RM03
76984 229533.A0A098DRS5 pp 229533.I1RA86
76985 229533.A0A098DRS5 pp 229533.I1RMB1
76986 229533.A0A098DRS5 pp 229533.I1RME4
76987 229533.A0A098DRS5 pp 229533.I1S0Z8
76988 229533.A0A098DRS5 pp 229533.I1RZK1
76989 229533.A0A098DRS5 pp 229533.I1RZD3
76990 229533.A0A098DRS5 pp 229533.I1RU27
76991 229533.A0A098DRS5 pp 229533.I1RQ72
76992 229533.A0A098DRS5 pp 229533.I1RT36
76993 229533.A0A098DRS5 pp 229533.I1RC55
76994 229533.A0A098DRS5 pp 229533.I1RPP7
76995 229533.A0A098DRS5 pp 229533.I1RPN8
76996 229533.A0A098DRS5 pp 229533.Q4IPH4
76997 229533.A0A098DRS5 pp 229533.A0A098E4F8
76998 229533.A0A098DRS5 pp 229533.I1RCB9
76999 229533.A0A098DRS5 pp 229533.I1RMQ3
77000 229533.A0A098DRS5 pp 229533.I1RTE6
77001 229533.A0A098DRS5 pp 229533.A0A0E0SK74
77002 229533.A0A098DRS5 pp 229533.I1RUP2
77003 229533.A0A098DRS5 pp 229533.V6QUV7
77004 229533.A0A098DRS5 pp 229533.Q4I624
77005 229533.A0A098DRS5 pp 229533.A0A1C3YK30
77006 229533.A0A098DRS5 pp 229533.I1REG1
77007 229533.A0A098DRS5 pp 229533.I1RMQ5
77008 229533.A0A098DRS5 pp 229533.I1RD70
77009 229533.A0A098DRS5 pp 229533.I1RBS5
77010 229533.A0A098DRS5 pp 229533.I1RY44
77011 229533.A0A098DRS5 pp 229533.I1RHW9
77012 229533.A0A098DRS5 pp 229533.Q4I7G0
77013 229533.A0A098DRS5 pp 229533.I1RL79
77014 229533.A0A098DRS5 pp 229533.I1S282
77015 229533.A0A098DRS5 pp 229533.I1RPV0

77016	229533.A0A098DRS5	pp	229533.I1RZ95
77017	229533.A0A098DRS5	pp	229533.I1RZS8
77018	229533.A0A098DRS5	pp	229533.I1REB8
77019	229533.A0A098DRS5	pp	229533.Q4HXF6
77020	229533.A0A098DRS5	pp	229533.I1RT94
77021	229533.A0A098DRS5	pp	229533.I1RWH4
77022	229533.A0A098DRS5	pp	229533.I1RBY6
77023	229533.A0A098DRS5	pp	229533.Q4HZB8
77024	229533.A0A098DRS5	pp	229533.A0A1C3YI20
77025	229533.A0A098DRS5	pp	229533.A0A1C3YN27
77026	229533.A0A098DRS5	pp	229533.A0A1C3YI83
77027	229533.A0A098DRS5	pp	229533.I1RQ21
77028	229533.A0A098DRS5	pp	229533.I1S0F9
77029	229533.A0A098DRS5	pp	229533.I1RB73
77030	229533.A0A098DRS5	pp	229533.Q4IPB8
77031	229533.A0A098DRS5	pp	229533.A0A1C3YI53
77032	229533.A0A098DRS5	pp	229533.I1RRM9
77033	229533.A0A098DRS5	pp	229533.I1RKI1
77034	229533.A0A098DRS5	pp	229533.A0A098DUK3
77035	229533.A0A098DRS5	pp	229533.I1RCD4
77036	229533.A0A098DRS5	pp	229533.I1S2V2
77037	229533.A0A098DRS5	pp	229533.I1RCN2
77038	229533.A0A098DRS5	pp	229533.A0A1C3YKU8
77039	229533.A0A098DRS5	pp	229533.I1RWK9
77040	229533.A0A098DRS5	pp	229533.I1RXY9
77041	229533.A0A098DRS5	pp	229533.A0A1C3YK08
77042	229533.A0A098DRS5	pp	229533.A0A1C3YMQ5
77043	229533.A0A098DRS5	pp	229533.I1RSF2
77044	229533.A0A098DRS5	pp	229533.I1RAM2
77045	229533.A0A098DRS5	pp	229533.I1RTG9
77046	229533.A0A098DRS5	pp	229533.I1R9Y8
77047	229533.A0A098DRS5	pp	229533.I1RSQ1
77048	229533.A0A098DRS5	pp	229533.I1RB59
77049	229533.A0A098DRS5	pp	229533.A0A1C3YI39
77050	229533.A0A098DRS5	pp	229533.I1S2E9
77051	229533.A0A098DRS5	pp	229533.A0A1C3YIE7
77052	229533.A0A098DRS5	pp	229533.V6R3J1
77053	229533.A0A098DRS5	pp	229533.I1S0L8
77054	229533.A0A098DRS5	pp	229533.I1RE19
77055	229533.A0A098DRS5	pp	229533.A0A0E0SMG8
77056	229533.A0A098DRS5	pp	229533.A0A1C3YLK5
77057	229533.A0A098DRS5	pp	229533.I1S7X9
77058	229533.A0A098DRS5	pp	229533.Q4IBK5
77059	229533.A0A098DRS5	pp	229533.A0A1C3YM82
77060	229533.A0A098DRS5	pp	229533.I1RQG4
77061	229533.A0A098DRS5	pp	229533.I1RCF0
77062	229533.A0A098DRS5	pp	229533.A0A1C3YIH5
77063	229533.A0A098DRS5	pp	229533.I1RP28
77064	229533.A0A098DRS5	pp	229533.I1RMP2
77065	229533.A0A098DRS5	pp	229533.I1RN67
77066	229533.A0A098DRS5	pp	229533.I1RNF4
77067	229533.A0A0E0RVF4	pp	229533.V6RLW4
77068	229533.A0A0E0RVF4	pp	229533.A0A1C3YHL0
77069	229533.A0A0E0RVF4	pp	229533.A0A1C3YMG8
77070	229533.A0A0E0RVF4	pp	229533.I1RAI9
77071	229533.A0A0E0RVF4	pp	229533.I1RPW0
77072	229533.A0A0E0RVF4	pp	229533.I1REC6
77073	229533.A0A0E0RVF4	pp	229533.I1S247
77074	229533.A0A0E0RVF4	pp	229533.I1RYX9
77075	229533.A0A0E0RVF4	pp	229533.I1RWK9
77076	229533.A0A0E0RVF4	pp	229533.Q4IQM6
77077	229533.A0A0E0RVF4	pp	229533.Q4HXF6
77078	229533.A0A0E0RVF4	pp	229533.I1S2V2
77079	229533.A0A0E0RVF4	pp	229533.I1REC7
77080	229533.A0A0E0RVF4	pp	229533.I1REG1
77081	229533.A0A0E0RVF4	pp	229533.A0A1C3YKJ6
77082	229533.A0A0E0RVF4	pp	229533.I1RX38
77083	229533.A0A0E0RVF4	pp	229533.I1RQ88
77084	229533.A0A0E0RVF4	pp	229533.I1RBV5
77085	229533.A0A0E0RVF4	pp	229533.I1RMJ8
77086	229533.A0A0E0RVF4	pp	229533.I1RA86
77087	229533.A0A0E0RVF4	pp	229533.I1S0F9
77088	229533.A0A0E0RVF4	pp	229533.I1RZP8

77089 229533.A0A0E0RVF4 pp 229533.I1RMQ3
77090 229533.A0A0E0RVF4 pp 229533.I1RBS5
77091 229533.A0A0E0RVF4 pp 229533.A0A0E0SK74
77092 229533.A0A0E0RVF4 pp 229533.I1RFX8
77093 229533.A0A0E0RVF4 pp 229533.Q4IEA5
77094 229533.A0A0E0RVF4 pp 229533.A0A1C3YN12
77095 229533.A0A0E0RVF4 pp 229533.I1RAH4
77096 229533.A0A0E0RVF4 pp 229533.I1RF94
77097 229533.A0A0E0RVF4 pp 229533.I1RWH4
77098 229533.A0A0E0RVF4 pp 229533.I1RSI4
77099 229533.A0A0E0RVF4 pp 229533.I1RDI8
77100 229533.A0A0E0RVF4 pp 229533.Q4IP51
77101 229533.A0A0E0RVF4 pp 229533.I1RRS7
77102 229533.A0A0E0RVF4 pp 229533.Q4IC11
77103 229533.A0A0E0RVF4 pp 229533.I1S0Z8
77104 229533.A0A0E0RVF4 pp 229533.A0A0E0SL81
77105 229533.A0A0E0RVF4 pp 229533.I1RHR0
77106 229533.A0A0E0RVF4 pp 229533.I1RL24
77107 229533.A0A0E0RVF4 pp 229533.I1RPN8
77108 229533.A0A0E0RVF4 pp 229533.I1RYZ9
77109 229533.A0A0E0RVF4 pp 229533.I1RTU8
77110 229533.A0A0E0RVF4 pp 229533.I1RM03
77111 229533.A0A0E0RVF4 pp 229533.V6RGI1
77112 229533.A0A0E0RVF4 pp 229533.I1RJQ3
77113 229533.A0A0E0RVF4 pp 229533.I1RRT6
77114 229533.A0A0E0RVF4 pp 229533.I1RJT9
77115 229533.A0A0E0RVF4 pp 229533.I1S283
77116 229533.A0A0E0RVF4 pp 229533.I1RFJ2
77117 229533.A0A0E0RVF4 pp 229533.I1RE46
77118 229533.A0A0E0RVF4 pp 229533.I1RYS6
77119 229533.A0A0E0RVF4 pp 229533.I1S461
77120 229533.A0A0E0RVF4 pp 229533.I1RKK8
77121 229533.A0A0E0RVF4 pp 229533.I1RBC4
77122 229533.A0A0E0RVF4 pp 229533.I1RP66
77123 229533.A0A0E0RVF4 pp 229533.I1RG60
77124 229533.A0A0E0RVF4 pp 229533.I1RW87
77125 229533.A0A0E0RVF4 pp 229533.I1RZS3
77126 229533.A0A0E0RVF4 pp 229533.I1RPU4
77127 229533.A0A0E0RVF4 pp 229533.A0A1C3YK37
77128 229533.A0A0E0RVF4 pp 229533.I1RYL5
77129 229533.A0A0E0RVF4 pp 229533.I1R9X8
77130 229533.A0A0E0RVF4 pp 229533.I1S2B6
77131 229533.A0A0E0RVF4 pp 229533.I1RQA5
77132 229533.A0A0E0SCK7 pp 229533.I1RRS7
77133 229533.A0A0E0SCK7 pp 229533.I1RBU1
77134 229533.I1RT62 pp 229533.I1RUF8
77135 229533.I1RT62 pp 229533.I1S2E9
77136 229533.I1RT62 pp 229533.V6RGI1
77137 229533.I1RT62 pp 229533.I1RWH4
77138 229533.I1RT62 pp 229533.Q4I7T5
77139 229533.I1RT62 pp 229533.I1S2V2
77140 229533.I1RT62 pp 229533.I1RT63
77141 229533.I1RT62 pp 229533.I1S0Z8
77142 229533.I1RT62 pp 229533.I1RZD3
77143 229533.I1RT62 pp 229533.I1RWK9
77144 229533.I1RT62 pp 229533.Q4I7L0
77145 229533.I1RT62 pp 229533.I1RY44
77146 229533.I1RT62 pp 229533.I1S0M6
77147 229533.I1RT62 pp 229533.I1RYZ9
77148 229533.I1RT62 pp 229533.I1RWT0
77149 229533.I1RT62 pp 229533.I1RW87
77150 229533.I1RT62 pp 229533.I1S235
77151 229533.I1RT62 pp 229533.V6QV09
77152 229533.I1RT62 pp 229533.Q4IJM4
77153 229533.I1RT62 pp 229533.I1RWT6
77154 229533.I1RT62 pp 229533.I1RYY5
77155 229533.I1RT62 pp 229533.I1RYZ0
77156 229533.I1RT62 pp 229533.I1RYL5
77157 229533.V6RN69 pp 229533.V6RVJ3
77158 229533.A0A0E0RM13 pp 229533.A0A1C3YJK3
77159 229533.A0A0E0RM13 pp 229533.I1S277
77160 229533.A0A0E0RM13 pp 229533.I1RT75
77161 229533.A0A0E0RM13 pp 229533.I1RPA4

77162 229533.I1RAB3 pp 229533.I1RYL5
77163 229533.I1RAB3 pp 229533.I1RW87
77164 229533.I1RAB3 pp 229533.I1RKK8
77165 229533.I1RAB3 pp 229533.I1RBC4
77166 229533.I1RAB3 pp 229533.I1RM03
77167 229533.I1RAB3 pp 229533.I1RWH4
77168 229533.I1RAB3 pp 229533.I1S235
77169 229533.I1RAB3 pp 229533.I1RQL4
77170 229533.I1RAB3 pp 229533.I1RP66
77171 229533.I1RAB3 pp 229533.I1RPN8
77172 229533.I1RAB3 pp 229533.I1RBS5
77173 229533.I1RAB3 pp 229533.V6RUT5
77174 229533.I1RAB3 pp 229533.I1REC7
77175 229533.I1RAB3 pp 229533.I1RDI8
77176 229533.I1RAB3 pp 229533.I1RQ21
77177 229533.I1RAB3 pp 229533.I1S2V2
77178 229533.I1RAB3 pp 229533.I1REC6
77179 229533.I1RAB3 pp 229533.I1RWK9
77180 229533.I1RPD3 pp 229533.I1RWV4
77181 229533.I1RPD3 pp 229533.V6QW25
77182 229533.I1RPD3 pp 229533.I1RQ61
77183 229533.I1RDD1 pp 229533.O74638
77184 229533.I1S0F9 pp 229533.I1S235
77185 229533.I1S0F9 pp 229533.I1S2V2
77186 229533.A0A098DN22 pp 229533.I1REB8
77187 229533.A0A098DN22 pp 229533.I1RL79
77188 229533.A0A098DN22 pp 229533.Q4I7G0
77189 229533.A0A098DN22 pp 229533.I1RZD3
77190 229533.A0A098DN22 pp 229533.I1RME4
77191 229533.A0A098DN22 pp 229533.I1RGC6
77192 229533.A0A098DN22 pp 229533.I1RBT8
77193 229533.A0A098DN22 pp 229533.A0A0E0SK74
77194 229533.A0A098DN22 pp 229533.I1RMQ3
77195 229533.A0A098DN22 pp 229533.I1RZK1
77196 229533.A0A098DN22 pp 229533.I1RPV0
77197 229533.A0A098DN22 pp 229533.I1RZS8
77198 229533.A0A098DN22 pp 229533.A0A098DRS5
77199 229533.A0A098DN22 pp 229533.A0A0E0RVF4
77200 229533.A0A098DN22 pp 229533.I1RU27
77201 229533.A0A098DN22 pp 229533.I1RAB3
77202 229533.A0A098DN22 pp 229533.I1RQA5
77203 229533.A0A098DN22 pp 229533.I1RMB1
77204 229533.A0A098DN22 pp 229533.I1S3S5
77205 229533.A0A098DN22 pp 229533.I1REG1
77206 229533.A0A098DN22 pp 229533.I1RYL5
77207 229533.A0A098DN22 pp 229533.A0A1C3YI83
77208 229533.A0A098DN22 pp 229533.I1RBS5
77209 229533.A0A098DN22 pp 229533.I1REC7
77210 229533.A0A098DN22 pp 229533.I1RMQ5
77211 229533.A0A098DN22 pp 229533.I1RB59
77212 229533.A0A098DN22 pp 229533.I1REC6
77213 229533.A0A098DN22 pp 229533.I1S0Z8
77214 229533.A0A098DN22 pp 229533.I1R9Y8
77215 229533.A0A098DN22 pp 229533.I1RJZ7
77216 229533.A0A098DN22 pp 229533.I1RBY6
77217 229533.A0A098DN22 pp 229533.I1RBY7
77218 229533.A0A098DN22 pp 229533.I1RB53
77219 229533.A0A098DN22 pp 229533.I1RDH7
77220 229533.A0A098DN22 pp 229533.I1RNG4
77221 229533.A0A098DN22 pp 229533.I1S0F9
77222 229533.A0A098DN22 pp 229533.I1RP28
77223 229533.A0A098DN22 pp 229533.I1RG60
77224 229533.A0A098DN22 pp 229533.I1RPL2
77225 229533.A0A098DN22 pp 229533.A0A1C3YIK4
77226 229533.A0A098DN22 pp 229533.I1RPK5
77227 229533.A0A098DN22 pp 229533.I1RQG4
77228 229533.A0A098DN22 pp 229533.A0A1C3YK11
77229 229533.A0A098DN22 pp 229533.I1S821
77230 229533.A0A098DN22 pp 229533.I1RW71
77231 229533.A0A098DN22 pp 229533.A0A1C3YLT3
77232 229533.A0A098DN22 pp 229533.A0A1C3YHZ0
77233 229533.A0A098DN22 pp 229533.A0A098DQ41
77234 229533.A0A098DN22 pp 229533.A0A098DN31

229533.A0A098DN22 pp 229533.I1RFY0
229533.A0A098DN22 pp 229533.I1RTU8
229533.A0A098DN22 pp 229533.I1RT62
229533.A0A098DN22 pp 229533.I1RRF8
229533.A0A098DN22 pp 229533.I1RRT6
229533.A0A098DN22 pp 229533.I1RF94
229533.A0A098DN22 pp 229533.I1RJT9
229533.A0A098DN22 pp 229533.A0A098DRZ4
229533.A0A098DN22 pp 229533.I1RAM6
229533.A0A098DN22 pp 229533.I1RQH1
229533.A0A098DN22 pp 229533.I1RQL4
229533.I1S769 pp 229533.V6QX21
229533.I1RB59 pp 229533.Q4HXF6
229533.I1RB59 pp 229533.I1RMQ5
229533.I1RB59 pp 229533.I1RQ21
229533.I1RB59 pp 229533.I1RBS5
229533.I1RB59 pp 229533.Q4HWE2
229533.I1RB59 pp 229533.I1RTA0
229533.I1RB59 pp 229533.I1RWH4
229533.I1RB59 pp 229533.I1RRS7
229533.I1RB59 pp 229533.I1S247
229533.I1RB59 pp 229533.V6RGI1
229533.I1RB59 pp 229533.I1RPN8
229533.I1RB59 pp 229533.Q4HYB8
229533.I1RB59 pp 229533.I1RMQ3
229533.I1RB59 pp 229533.I1RZD3
229533.I1RB59 pp 229533.I1RPW0
229533.I1RB59 pp 229533.I1REG1
229533.I1RB59 pp 229533.I1S0Z8
229533.I1RB59 pp 229533.Q4I624
229533.I1RB59 pp 229533.I1RME4
229533.I1RB59 pp 229533.I1RQG4
229533.I1RB59 pp 229533.I1RYX9
229533.I1RB59 pp 229533.I1RBC4
229533.I1RB59 pp 229533.I1RPU4
229533.I1RB59 pp 229533.I1REC7
229533.I1RB59 pp 229533.Q4I948
229533.I1RB59 pp 229533.I1RQ88
229533.I1RB59 pp 229533.I1RP28
229533.I1RB59 pp 229533.I1RWK9
229533.I1RB59 pp 229533.I1RKK8
229533.I1RB59 pp 229533.I1RBV5
229533.I1RB59 pp 229533.I1S2V2
229533.I1RB59 pp 229533.I1RYS6
229533.I1RB59 pp 229533.I1RG60
229533.I1RB59 pp 229533.I1RP66
229533.I1RB59 pp 229533.I1RX38
229533.I1RB59 pp 229533.Q4IQM6
229533.I1RB59 pp 229533.I1REC6
229533.I1RB59 pp 229533.I1S461
229533.I1RB59 pp 229533.I1S439
229533.I1RB59 pp 229533.I1RPL2
229533.I1RB59 pp 229533.I1RE46
229533.I1RB59 pp 229533.Q4ICA8
229533.I1RB59 pp 229533.V6RLW4
229533.I1RB59 pp 229533.I1RC66
229533.I1RB59 pp 229533.I1RYL5
229533.I1RB59 pp 229533.I1S0F9
229533.I1RB59 pp 229533.I1RWD7
229533.I1RB59 pp 229533.I1RWT6
229533.I1RB59 pp 229533.I1RC22
229533.I1RB59 pp 229533.I1RW87
229533.I1RB59 pp 229533.I1RRM9
229533.I1RB59 pp 229533.I1RBL8
229533.I1RB59 pp 229533.I1RTU8
229533.I1RB59 pp 229533.I1S2B6
229533.I1RB59 pp 229533.I1RQA5
229533.I1RB59 pp 229533.I1RSI4
229533.I1RB59 pp 229533.I1RFX8
229533.I1RB59 pp 229533.I1RDI8
229533.I1RB59 pp 229533.I1S0F5
229533.I1RB59 pp 229533.I1RZG3
229533.I1RB59 pp 229533.I1RF94

77308 229533.I1RB59 pp 229533.I1RRT6
77309 229533.I1RB59 pp 229533.I1RJT9
77310 229533.I1RB59 pp 229533.I1S283
77311 229533.I1RB59 pp 229533.I1RM03
77312 229533.I1RB59 pp 229533.I1RPI2
77313 229533.A0A098DMY9 pp 229533.I1RNG4
77314 229533.I1R9A3 pp 229533.I1RNG4
77315 229533.I1REC6 pp 229533.P0C431
77316 229533.I1REC6 pp 229533.I1RVW6
77317 229533.I1REC6 pp 229533.I1S821
77318 229533.I1REC6 pp 229533.I1RMG2
77319 229533.I1REC6 pp 229533.I1RWT6
77320 229533.I1REC6 pp 229533.Q4ICA8
77321 229533.I1REC6 pp 229533.Q4IE79
77322 229533.I1REC6 pp 229533.I1S275
77323 229533.I1REC6 pp 229533.I1S0Z3
77324 229533.I1REC6 pp 229533.I1RYZ0
77325 229533.I1REC6 pp 229533.I1S235
77326 229533.I1REC6 pp 229533.V6RR76
77327 229533.I1REC6 pp 229533.I1RHB0
77328 229533.I1REC6 pp 229533.I1RYS6
77329 229533.I1REC6 pp 229533.I1RPL2
77330 229533.I1REC6 pp 229533.I1RP66
77331 229533.I1REC6 pp 229533.I1RW87
77332 229533.I1REC6 pp 229533.Q4I1Y1
77333 229533.I1REC6 pp 229533.I1RG60
77334 229533.I1REC6 pp 229533.I1RPK5
77335 229533.I1REC6 pp 229533.I1RQA5
77336 229533.I1REC6 pp 229533.Q4I948
77337 229533.I1REC6 pp 229533.I1RKK8
77338 229533.I1REC6 pp 229533.I1S2B6
77339 229533.I1REC6 pp 229533.I1RT41
77340 229533.I1REC6 pp 229533.Q4INI2
77341 229533.I1REC6 pp 229533.I1RQA3
77342 229533.I1REC6 pp 229533.V6REE9
77343 229533.I1REC6 pp 229533.I1RM03
77344 229533.I1REC6 pp 229533.I1RTU8
77345 229533.I1REC6 pp 229533.I1RQL4
77346 229533.I1REC6 pp 229533.I1RQH1
77347 229533.I1REC6 pp 229533.I1RYL5
77348 229533.I1REC6 pp 229533.I1S0S6
77349 229533.I1REC6 pp 229533.I1S3S5
77350 229533.I1REC6 pp 229533.Q4IPB3
77351 229533.I1REC6 pp 229533.I1RNJ2
77352 229533.I1REC6 pp 229533.I1RN81
77353 229533.I1REC6 pp 229533.I1RTQ9
77354 229533.I1REC6 pp 229533.I1RJT9
77355 229533.I1REC6 pp 229533.I1RZE7
77356 229533.I1REC6 pp 229533.I1RRT6
77357 229533.I1REC6 pp 229533.Q4INZ9
77358 229533.I1REC6 pp 229533.Q4I5R9
77359 229533.I1REC6 pp 229533.I1RRF8
77360 229533.I1REC6 pp 229533.I1RPA7
77361 229533.I1REC6 pp 229533.I1S0F5
77362 229533.I1REC6 pp 229533.I1RTA6
77363 229533.I1REC6 pp 229533.I1RT36
77364 229533.I1REC6 pp 229533.I1RPI2
77365 229533.I1REC6 pp 229533.I1RQC5
77366 229533.I1REC6 pp 229533.I1S0Z8
77367 229533.I1REC6 pp 229533.V6QUV7
77368 229533.I1REC6 pp 229533.I1RPN8
77369 229533.I1REC6 pp 229533.I1RN72
77370 229533.I1REC6 pp 229533.I1RT94
77371 229533.I1REC6 pp 229533.I1RZG3
77372 229533.I1REC6 pp 229533.I1REG1
77373 229533.I1REC6 pp 229533.I1RWH4
77374 229533.I1REC6 pp 229533.I1RF94
77375 229533.I1REC6 pp 229533.I1RQ21
77376 229533.I1REC6 pp 229533.I1RMQ5
77377 229533.I1REC6 pp 229533.I1RU27
77378 229533.I1REC6 pp 229533.I1RZK1
77379 229533.I1REC6 pp 229533.I1RZ95
77380 229533.I1REC6 pp 229533.I1RTE6

77381 229533.I1REC6 pp 229533.Q4IPB8
77382 229533.I1REC6 pp 229533.I1RPP7
77383 229533.I1REC6 pp 229533.Q4I624
77384 229533.I1REC6 pp 229533.I1RZS4
77385 229533.I1REC6 pp 229533.I1S282
77386 229533.I1REC6 pp 229533.I1RWX5
77387 229533.I1REC6 pp 229533.I1RY44
77388 229533.I1REC6 pp 229533.I1RZD3
77389 229533.I1REC6 pp 229533.Q4IN00
77390 229533.I1REC6 pp 229533.I1RME4
77391 229533.I1REC6 pp 229533.Q4HXF6
77392 229533.I1REC6 pp 229533.I1RYW3
77393 229533.I1REC6 pp 229533.Q4HZB8
77394 229533.I1REC6 pp 229533.I1RMB1
77395 229533.I1REC6 pp 229533.I1RZS8
77396 229533.I1REC6 pp 229533.I1RPS0
77397 229533.I1REC6 pp 229533.I1RPV0
77398 229533.I1REC6 pp 229533.I1S247
77399 229533.I1REC6 pp 229533.Q4IPH4
77400 229533.I1REC6 pp 229533.Q4I7G0
77401 229533.I1REC6 pp 229533.I1RUP2
77402 229533.I1REC6 pp 229533.I1RHW9
77403 229533.I1REC6 pp 229533.I1RMQ3
77404 229533.I1REC6 pp 229533.I1S2E9
77405 229533.I1REC6 pp 229533.I1RL79
77406 229533.I1REC6 pp 229533.I1RQ72
77407 229533.I1REC6 pp 229533.V6R3J1
77408 229533.I1REC6 pp 229533.I1RSQ1
77409 229533.I1REC6 pp 229533.I1S0L8
77410 229533.I1REC6 pp 229533.I1RQG4
77411 229533.I1REC6 pp 229533.I1RNF4
77412 229533.I1REC6 pp 229533.Q4IBK5
77413 229533.I1REC6 pp 229533.I1RP28
77414 229533.I1REC6 pp 229533.I1RMP2
77415 229533.I1REC6 pp 229533.I1RN67
77416 229533.I1REC6 pp 229533.I1RTG9
77417 229533.I1REC6 pp 229533.I1S7X9
77418 229533.I1REC6 pp 229533.I1RWK9
77419 229533.I1REC6 pp 229533.I1RRM9
77420 229533.I1REC6 pp 229533.I1S0F9
77421 229533.I1REC6 pp 229533.I1S2V2
77422 229533.I1REC6 pp 229533.I1RKI1
77423 229533.I1REC6 pp 229533.I1RXY9
77424 229533.I1REC6 pp 229533.I1RSF2
77425 229533.I1RGY4 pp 229533.I1RNG4
77426 229533.A0A1C3YN77 pp 229533.I1RP37
77427 229533.A0A1C3YN77 pp 229533.I1RD39
77428 229533.A0A1C3YN77 pp 229533.I1RRN5
77429 229533.A0A1C3YN77 pp 229533.I1RS73
77430 229533.A0A1C3YN77 pp 229533.I1RS35
77431 229533.A0A1C3YN77 pp 229533.I1RXF0
77432 229533.A0A1C3YN77 pp 229533.I1RI81
77433 229533.A0A1C3YN77 pp 229533.I1RRK8
77434 229533.A0A1C3YN77 pp 229533.I1RT28
77435 229533.A0A1C3YN77 pp 229533.I1RUQ8
77436 229533.A0A1C3YN77 pp 229533.I1RZP1
77437 229533.A0A1C3YN77 pp 229533.I1REC1
77438 229533.A0A1C3YN77 pp 229533.I1RXZ6
77439 229533.A0A1C3YN77 pp 229533.I1S8D1
77440 229533.A0A1C3YN77 pp 229533.I1RPA4
77441 229533.A0A1C3YN77 pp 229533.I1RNC2
77442 229533.A0A1C3YN77 pp 229533.V6QWP4
77443 229533.A0A098DMY4 pp 229533.A0A1C3YN77
77444 229533.I1RZJ9 pp 229533.I1S0K5
77445 229533.I1RZJ9 pp 229533.I1S0L1
77446 229533.I1RZJ9 pp 229533.I1S9U1
77447 229533.I1RZJ9 pp 229533.Q4ICM9
77448 229533.I1S821 pp 229533.Q4I7G0
77449 229533.I1S821 pp 229533.I1S9I5
77450 229533.I1S821 pp 229533.V6RCZ8
77451 229533.I1S821 pp 229533.V6R9Z0
77452 229533.I1S821 pp 229533.Q4IJK2
77453 229533.I1RC58 pp 229533.I1S0Y1

77454 229533.I1RC58 pp 229533.I1RFV2
77455 229533.I1RC58 pp 229533.I1RSX2
77456 229533.I1RC58 pp 229533.I1RN66
77457 229533.I1RC58 pp 229533.I1RCN6
77458 229533.I1RC58 pp 229533.I1RWC2
77459 229533.I1RC58 pp 229533.I1RNR9
77460 229533.I1RC58 pp 229533.I1REK1
77461 229533.I1RC58 pp 229533.I1RM77
77462 229533.I1RC58 pp 229533.I1RRE1
77463 229533.I1RC58 pp 229533.I1RZN1
77464 229533.I1RC58 pp 229533.Q4I1T9
77465 229533.I1RC58 pp 229533.I1S368
77466 229533.I1RC58 pp 229533.I1S0H5
77467 229533.I1RC58 pp 229533.I1RLT3
77468 229533.I1RC58 pp 229533.V6QW25
77469 229533.I1RC58 pp 229533.I1RMG4
77470 229533.I1RC58 pp 229533.I1RT41
77471 229533.I1RC58 pp 229533.I1RWV4
77472 229533.I1RC58 pp 229533.I1RJ78
77473 229533.I1RC58 pp 229533.I1RPD3
77474 229533.I1RC58 pp 229533.I1RQ61
77475 229533.A0A1C3YM93 pp 229533.I1RQQ7
77476 229533.A0A1C3YM93 pp 229533.I1RJU5
77477 229533.I1RPZ5 pp 229533.V6RRM0
77478 229533.I1RPZ5 pp 229533.V6RM02
77479 229533.I1RPZ5 pp 229533.I1RVR2
77480 229533.I1RPZ5 pp 229533.V6RUY9
77481 229533.I1RPZ5 pp 229533.I1RXH3
77482 229533.I1RPZ5 pp 229533.I1RRG1
77483 229533.I1RPZ5 pp 229533.I1RSL2
77484 229533.I1RPZ5 pp 229533.I1RWM4
77485 229533.I1RPZ5 pp 229533.I1RXB1
77486 229533.A0A1C3YM92 pp 229533.I1RWL2
77487 229533.I1RGI5 pp 229533.I1S360
77488 229533.I1RGI5 pp 229533.I1RVR7
77489 229533.I1RGI5 pp 229533.I1S8P5
77490 229533.I1RGI5 pp 229533.I1RIT5
77491 229533.I1RGI5 pp 229533.I1S0H5
77492 229533.I1RGI5 pp 229533.I1S3A6
77493 229533.I1RGI5 pp 229533.I1RPM6
77494 229533.I1RGI5 pp 229533.I1S2B4
77495 229533.I1RGI5 pp 229533.I1S2L1
77496 229533.I1RGI5 pp 229533.I1RWM4
77497 229533.I1RGI5 pp 229533.V6QUE6
77498 229533.I1RGI5 pp 229533.I1RXJ2
77499 229533.I1RGI5 pp 229533.I1RIL9
77500 229533.I1RGI5 pp 229533.I1RK24
77501 229533.I1RGI5 pp 229533.I1RTW0
77502 229533.I1RGI5 pp 229533.I1RM80
77503 229533.I1RGI5 pp 229533.I1RQU9
77504 229533.I1RGI5 pp 229533.I1S7J8
77505 229533.I1RGI5 pp 229533.I1RW71
77506 229533.I1RGI5 pp 229533.I1RJX3
77507 229533.I1RGI5 pp 229533.I1S2R2
77508 229533.I1RGI5 pp 229533.I1S1K5
77509 229533.I1RGI5 pp 229533.I1RTA0
77510 229533.I1RGI5 pp 229533.I1RJ04
77511 229533.I1RGI5 pp 229533.I1RLZ6
77512 229533.I1RGI5 pp 229533.I1RLC9
77513 229533.I1R975 pp 229533.I1RFQ0
77514 229533.I1R975 pp 229533.I1RD23
77515 229533.I1R975 pp 229533.I1RMH5
77516 229533.I1RFC0 pp 229533.I1S4S4
77517 229533.I1RFC0 pp 229533.I1RM67
77518 229533.I1RFC0 pp 229533.I1S9Y1
77519 229533.I1RFC0 pp 229533.I1S5R1
77520 229533.I1RFC0 pp 229533.I1RRT8
77521 229533.I1RFC0 pp 229533.I1RZ39
77522 229533.I1RFC0 pp 229533.I1RSQ4
77523 229533.I1RFC0 pp 229533.I1RZZ9
77524 229533.I1RFC0 pp 229533.I1S2H4
77525 229533.I1RFC0 pp 229533.I1RT33
77526 229533.I1RFC0 pp 229533.O74638

77527 229533.I1RFC0 pp 229533.Q4IEV5
77528 229533.I1RFC0 pp 229533.I1RXF0
77529 229533.I1RFC0 pp 229533.I1RFS3
77530 229533.I1RFC0 pp 229533.I1RNC1
77531 229533.I1RFC0 pp 229533.I1RSZ7
77532 229533.I1RFC0 pp 229533.I1RYW3
77533 229533.I1RFC0 pp 229533.I1RL04
77534 229533.I1RFC0 pp 229533.I1RUG3
77535 229533.I1RFC0 pp 229533.I1RT40
77536 229533.I1RFC0 pp 229533.I1RYB9
77537 229533.I1RFC0 pp 229533.I1RK83
77538 229533.I1RFC0 pp 229533.Q4HXF0
77539 229533.I1RFC0 pp 229533.I1RRI2
77540 229533.I1RFC0 pp 229533.I1RJV3
77541 229533.I1RFC0 pp 229533.Q4IJK2
77542 229533.I1RFC0 pp 229533.V6RCZ8
77543 229533.I1RFC0 pp 229533.V6R042
77544 229533.I1RFC0 pp 229533.I1RXD7
77545 229533.I1RFC0 pp 229533.I1RSQ3
77546 229533.I1RFC0 pp 229533.I1RX46
77547 229533.I1RFC0 pp 229533.I1RFL4
77548 229533.I1RFC0 pp 229533.I1RSS4
77549 229533.I1RFC0 pp 229533.I1RGA4
77550 229533.I1RFC0 pp 229533.I1RQE7
77551 229533.I1RFC0 pp 229533.V6QUE6
77552 229533.I1RFC0 pp 229533.I1RFT1
77553 229533.I1RFC0 pp 229533.I1RVX0
77554 229533.I1RFC0 pp 229533.I1RSQ7
77555 229533.I1RFC0 pp 229533.I1RG11
77556 229533.I1RFC0 pp 229533.I1RPP3
77557 229533.I1RFC0 pp 229533.I1S5B0
77558 229533.I1RFC0 pp 229533.I1RK95
77559 229533.I1RFC0 pp 229533.I1S6E5
77560 229533.I1RFC0 pp 229533.I1RN67
77561 229533.I1RFC0 pp 229533.I1S0J1
77562 229533.I1RFC0 pp 229533.I1RS18
77563 229533.A0A1C3YIX0 pp 229533.I1RQA5
77564 229533.A0A1C3YIX0 pp 229533.Q4ICM9
77565 229533.A0A1C3YIX0 pp 229533.I1RZS4
77566 229533.A0A1C3YIX0 pp 229533.I1RAM9
77567 229533.A0A1C3YIX0 pp 229533.I1RD38
77568 229533.A0A1C3YIX0 pp 229533.Q4I7T5
77569 229533.A0A1C3YIX0 pp 229533.I1RAW0
77570 229533.A0A1C3YIX0 pp 229533.I1RTA1
77571 229533.A0A1C3YIX0 pp 229533.Q4IB65
77572 229533.A0A1C3YIX0 pp 229533.A0A1C3YLP6
77573 229533.A0A1C3YIX0 pp 229533.Q4IK03
77574 229533.A0A1C3YIX0 pp 229533.Q4IJW4
77575 229533.A0A1C3YIX0 pp 229533.I1RM62
77576 229533.A0A1C3YIX0 pp 229533.Q4I6B0
77577 229533.A0A1C3YIX0 pp 229533.I1RC18
77578 229533.A0A1C3YIX0 pp 229533.I1RZD3
77579 229533.A0A1C3YIX0 pp 229533.I1RNJ5
77580 229533.A0A1C3YIX0 pp 229533.I1RCH5
77581 229533.A0A1C3YIX0 pp 229533.I1RBC4
77582 229533.A0A1C3YIX0 pp 229533.Q4IPZ1
77583 229533.A0A1C3YIX0 pp 229533.I1RT38
77584 229533.A0A1C3YIX0 pp 229533.I1RVN1
77585 229533.A0A1C3YIX0 pp 229533.Q4HZ95
77586 229533.A0A1C3YIX0 pp 229533.I1RXZ3
77587 229533.A0A1C3YIX0 pp 229533.I1RSQ4
77588 229533.A0A1C3YIX0 pp 229533.I1RPY8
77589 229533.A0A1C3YIX0 pp 229533.Q4I375
77590 229533.A0A1C3YIX0 pp 229533.I1RXT3
77591 229533.A0A1C3YIX0 pp 229533.I1RB86
77592 229533.A0A1C3YIX0 pp 229533.I1RBF3
77593 229533.A0A1C3YIX0 pp 229533.I1RWH4
77594 229533.A0A1C3YIX0 pp 229533.I1RPA4
77595 229533.A0A1C3YIX0 pp 229533.Q4I099
77596 229533.A0A1C3YIX0 pp 229533.I1RQR6
77597 229533.A0A1C3YIX0 pp 229533.I1RFS5
77598 229533.A0A1C3YIX0 pp 229533.I1RAN6
77599 229533.A0A1C3YIX0 pp 229533.I1RQ21

77600 229533.A0A1C3YIX0 pp 229533.I1RT99
77601 229533.A0A1C3YIX0 pp 229533.I1RC86
77602 229533.A0A1C3YIX0 pp 229533.I1RUG0
77603 229533.A0A1C3YIX0 pp 229533.I1R9Y8
77604 229533.A0A1C3YIX0 pp 229533.I1S0R5
77605 229533.A0A1C3YIX0 pp 229533.I1RJT3
77606 229533.A0A1C3YIX0 pp 229533.I1RUQ2
77607 229533.A0A1C3YIX0 pp 229533.I1RZH5
77608 229533.A0A1C3YIX0 pp 229533.V6R3J1
77609 229533.A0A1C3YIX0 pp 229533.I1S8F0
77610 229533.A0A1C3YIX0 pp 229533.I1RSS7
77611 229533.I1RBY6 pp 229533.I1RQ21
77612 229533.I1RBY6 pp 229533.I1RWH4
77613 229533.I1RBY6 pp 229533.I1REC7
77614 229533.I1RBY6 pp 229533.I1RPN8
77615 229533.I1RBY6 pp 229533.I1S2V2
77616 229533.I1RBY6 pp 229533.I1REC6
77617 229533.I1RBY6 pp 229533.I1S235
77618 229533.I1RBY6 pp 229533.I1RWK9
77619 229533.I1RBY6 pp 229533.I1RP66
77620 229533.I1RBY6 pp 229533.I1RYL5
77621 229533.I1RBY6 pp 229533.I1RW87
77622 229533.I1RBY6 pp 229533.I1RKK8
77623 229533.I1RBY6 pp 229533.I1RQL4
77624 229533.I1RBY6 pp 229533.I1RM03
77625 229533.I1RBY6 pp 229533.I1RDI8
77626 229533.A0A1C3YMN2 pp 229533.I1R9F4
77627 229533.A0A1C3YMN2 pp 229533.I1S1K5
77628 229533.A0A1C3YMN2 pp 229533.I1RXJ2
77629 229533.A0A1C3YMN2 pp 229533.I1RIL9
77630 229533.A0A1C3YMN2 pp 229533.I1RY11
77631 229533.A0A1C3YMN2 pp 229533.I1RTW0
77632 229533.A0A1C3YMN2 pp 229533.I1RLC9
77633 229533.A0A1C3YMN2 pp 229533.I1RM80
77634 229533.A0A1C3YMN2 pp 229533.I1RIT5
77635 229533.I1REC7 pp 229533.I1RN67
77636 229533.I1REC7 pp 229533.I1RNF4
77637 229533.I1REC7 pp 229533.I1RP28
77638 229533.I1REC7 pp 229533.I1RQG4
77639 229533.I1REC7 pp 229533.I1RMP2
77640 229533.I1REC7 pp 229533.Q4IBK5
77641 229533.I1REC7 pp 229533.I1S7X9
77642 229533.I1REC7 pp 229533.I1RHF8
77643 229533.I1REC7 pp 229533.I1S0L8
77644 229533.I1REC7 pp 229533.I1REC9
77645 229533.I1REC7 pp 229533.I1S2E9
77646 229533.I1REC7 pp 229533.I1S0F9
77647 229533.I1REC7 pp 229533.V6R3J1
77648 229533.I1REC7 pp 229533.I1RKI1
77649 229533.I1REC7 pp 229533.I1RXY9
77650 229533.I1REC7 pp 229533.I1RTG9
77651 229533.I1REC7 pp 229533.Q4IPB8
77652 229533.I1REC7 pp 229533.I1RSQ1
77653 229533.I1REC7 pp 229533.I1RQ21
77654 229533.I1REC7 pp 229533.I1S2V2
77655 229533.I1REC7 pp 229533.I1RSF2
77656 229533.I1REC7 pp 229533.I1RMQ3
77657 229533.I1REC7 pp 229533.Q4HZB8
77658 229533.I1REC7 pp 229533.I1S2B9
77659 229533.I1REC7 pp 229533.I1RPN8
77660 229533.I1REC7 pp 229533.I1RWK9
77661 229533.I1REC7 pp 229533.I1RRM9
77662 229533.I1REC7 pp 229533.I1RZS8
77663 229533.I1REC7 pp 229533.I1RPV0
77664 229533.I1REC7 pp 229533.I1RQ72
77665 229533.I1REC7 pp 229533.I1RT36
77666 229533.I1REC7 pp 229533.Q4IPH4
77667 229533.I1REC7 pp 229533.I1S0Z8
77668 229533.I1REC7 pp 229533.I1RY44
77669 229533.I1REC7 pp 229533.I1RWH4
77670 229533.I1REC7 pp 229533.I1RT94
77671 229533.I1REC7 pp 229533.I1S282
77672 229533.I1REC7 pp 229533.I1RUP2

77673	229533.I1REC7	pp	229533.I1RZD3
77674	229533.I1REC7	pp	229533.I1RMQ5
77675	229533.I1REC7	pp	229533.I1RME4
77676	229533.I1REC7	pp	229533.I1RZ95
77677	229533.I1REC7	pp	229533.I1S247
77678	229533.I1REC7	pp	229533.I1RZK1
77679	229533.I1REC7	pp	229533.I1RPP7
77680	229533.I1REC7	pp	229533.I1RU27
77681	229533.I1REC7	pp	229533.Q4I624
77682	229533.I1REC7	pp	229533.I1RTE6
77683	229533.I1REC7	pp	229533.Q4HXF6
77684	229533.I1REC7	pp	229533.I1RMB1
77685	229533.I1REC7	pp	229533.I1S0S6
77686	229533.I1REC7	pp	229533.I1RQH1
77687	229533.I1REC7	pp	229533.I1RL79
77688	229533.I1REC7	pp	229533.I1RYW3
77689	229533.I1REC7	pp	229533.I1RPS0
77690	229533.I1REC7	pp	229533.I1RHW9
77691	229533.I1REC7	pp	229533.Q4I7G0
77692	229533.I1REC7	pp	229533.I1S0C2
77693	229533.I1REC7	pp	229533.I1REG1
77694	229533.I1REC7	pp	229533.Q4IN00
77695	229533.I1REC7	pp	229533.I1RNJ2
77696	229533.I1REC7	pp	229533.I1RM03
77697	229533.I1REC7	pp	229533.I1RWX5
77698	229533.I1REC7	pp	229533.Q4I5R9
77699	229533.I1REC7	pp	229533.I1RQL4
77700	229533.I1REC7	pp	229533.V6QUV7
77701	229533.I1REC7	pp	229533.I1RRT6
77702	229533.I1REC7	pp	229533.I1RZS4
77703	229533.I1REC7	pp	229533.I1RF94
77704	229533.I1REC7	pp	229533.I1S0F5
77705	229533.I1REC7	pp	229533.I1RZG3
77706	229533.I1REC7	pp	229533.I1RJT9
77707	229533.I1REC7	pp	229533.Q4INZ9
77708	229533.I1REC7	pp	229533.I1RT41
77709	229533.I1REC7	pp	229533.I1RTU8
77710	229533.I1REC7	pp	229533.I1RPK5
77711	229533.I1REC7	pp	229533.I1RQC5
77712	229533.I1REC7	pp	229533.I1RN72
77713	229533.I1REC7	pp	229533.I1S275
77714	229533.I1REC7	pp	229533.I1RRF8
77715	229533.I1REC7	pp	229533.Q4INI2
77716	229533.I1REC7	pp	229533.I1RTA6
77717	229533.I1REC7	pp	229533.I1RMG2
77718	229533.I1REC7	pp	229533.I1RI44
77719	229533.I1REC7	pp	229533.I1RFY0
77720	229533.I1REC7	pp	229533.I1RZE7
77721	229533.I1REC7	pp	229533.I1RWT6
77722	229533.I1REC7	pp	229533.I1RN81
77723	229533.I1REC7	pp	229533.Q4ICA8
77724	229533.I1REC7	pp	229533.I1S821
77725	229533.I1REC7	pp	229533.I1RTQ9
77726	229533.I1REC7	pp	229533.P0C431
77727	229533.I1REC7	pp	229533.I1RVW6
77728	229533.I1REC7	pp	229533.I1RP66
77729	229533.I1REC7	pp	229533.I1S0Z3
77730	229533.I1REC7	pp	229533.I1S235
77731	229533.I1REC7	pp	229533.Q4IPB3
77732	229533.I1REC7	pp	229533.I1RPL2
77733	229533.I1REC7	pp	229533.Q4IE79
77734	229533.I1REC7	pp	229533.V6RR76
77735	229533.I1REC7	pp	229533.I1RPA7
77736	229533.I1REC7	pp	229533.I1RW87
77737	229533.I1REC7	pp	229533.I1RPI2
77738	229533.I1REC7	pp	229533.I1RYZ0
77739	229533.I1REC7	pp	229533.I1RYS6
77740	229533.I1REC7	pp	229533.I1S2B6
77741	229533.I1REC7	pp	229533.I1S0I8
77742	229533.I1REC7	pp	229533.I1RQA5
77743	229533.I1REC7	pp	229533.I1RG60
77744	229533.I1REC7	pp	229533.Q4I1Y1
77745	229533.I1REC7	pp	229533.I1RQA3

77746 229533.I1REC7 pp 229533.I1S3S5
77747 229533.I1REC7 pp 229533.Q4I948
77748 229533.I1REC7 pp 229533.V6REE9
77749 229533.I1REC7 pp 229533.I1RKK8
77750 229533.I1REC7 pp 229533.I1RYL5
77751 229533.I1REC7 pp 229533.I1RHB0
77752 229533.Q4I7T5 pp 229533.Q4ICM9
77753 229533.Q4I7T5 pp 229533.Q4IBU4
77754 229533.Q4I7T5 pp 229533.Q4IB65
77755 229533.Q4I7T5 pp 229533.Q4IJW4
77756 229533.Q4I7T5 pp 229533.Q4IPZ1
77757 229533.Q4I7T5 pp 229533.Q4IK03
77758 229533.Q4I7T5 pp 229533.Q4IN00
77759 229533.Q4I7T5 pp 229533.V6RM66
77760 229533.I1RWV4 pp 229533.V6QW25
77761 229533.I1RMQ3 pp 229533.I1S2V2
77762 229533.I1RMQ3 pp 229533.I1RP28
77763 229533.I1RMQ3 pp 229533.I1RPN8
77764 229533.I1RMQ3 pp 229533.I1RWH4
77765 229533.I1RMQ3 pp 229533.I1S235
77766 229533.I1RMQ3 pp 229533.I1RQ21
77767 229533.I1RMQ3 pp 229533.I1RWK9
77768 229533.I1RMQ3 pp 229533.I1RQA5
77769 229533.I1RMQ3 pp 229533.I1RYL5
77770 229533.I1RMQ3 pp 229533.I1RP66
77771 229533.I1RMQ3 pp 229533.I1RW87
77772 229533.I1S6A2 pp 229533.V6RK96
77773 229533.I1S6A2 pp 229533.I1S8R4
77774 229533.I1S6A2 pp 229533.I1S9T6
77775 229533.I1RXN6 pp 229533.I1RY11
77776 229533.I1RXN6 pp 229533.I1S1K5
77777 229533.I1RGC6 pp 229533.I1RPN8
77778 229533.I1RGC6 pp 229533.I1RWH4
77779 229533.I1RGC6 pp 229533.I1S2E8
77780 229533.I1RGC6 pp 229533.V6RGI1
77781 229533.I1RGC6 pp 229533.I1S0Z8
77782 229533.I1RGC6 pp 229533.I1RZD3
77783 229533.I1RGC6 pp 229533.I1RY44
77784 229533.I1RGC6 pp 229533.I1RNY8
77785 229533.I1RGC6 pp 229533.I1S2V2
77786 229533.I1RGC6 pp 229533.I1RME4
77787 229533.I1RGC6 pp 229533.I1RK75
77788 229533.I1RGC6 pp 229533.I1RPV0
77789 229533.I1RGC6 pp 229533.I1RWK9
77790 229533.I1RGC6 pp 229533.I1RQ21
77791 229533.I1RGC6 pp 229533.I1RQA3
77792 229533.I1RGC6 pp 229533.I1S2C0
77793 229533.I1RGC6 pp 229533.Q4I7T5
77794 229533.I1RGC6 pp 229533.Q4I7L0
77795 229533.I1RGC6 pp 229533.I1RQ65
77796 229533.I1RGC6 pp 229533.I1RYN4
77797 229533.I1RGC6 pp 229533.I1RKK8
77798 229533.I1RGC6 pp 229533.I1RK95
77799 229533.I1RGC6 pp 229533.I1RPK5
77800 229533.I1RGC6 pp 229533.I1RKR6
77801 229533.I1RGC6 pp 229533.I1RQ81
77802 229533.I1RGC6 pp 229533.I1S2E9
77803 229533.I1RGC6 pp 229533.I1RSI0
77804 229533.I1RGC6 pp 229533.Q4IJM4
77805 229533.I1RGC6 pp 229533.I1RUF8
77806 229533.I1RGC6 pp 229533.I1RZM1
77807 229533.I1RGC6 pp 229533.I1RRT2
77808 229533.I1RGC6 pp 229533.I1RP66
77809 229533.I1RGC6 pp 229533.Q4I2J8
77810 229533.I1RGC6 pp 229533.I1RWT6
77811 229533.I1RGC6 pp 229533.Q4I283
77812 229533.I1RGC6 pp 229533.I1RPC2
77813 229533.I1RGC6 pp 229533.I1RYZ0
77814 229533.I1RGC6 pp 229533.I1RQ31
77815 229533.I1RGC6 pp 229533.I1RWD7
77816 229533.I1RGC6 pp 229533.I1S0M6
77817 229533.I1RGC6 pp 229533.I1RYL5
77818 229533.I1RGC6 pp 229533.I1RRM9

77819 229533.I1RGC6 pp 229533.I1RW87
77820 229533.I1RGC6 pp 229533.I1S235
77821 229533.I1RGC6 pp 229533.I1RTF4
77822 229533.I1RGC6 pp 229533.I1RHB0
77823 229533.I1RGC6 pp 229533.V6QV09
77824 229533.I1RGC6 pp 229533.I1RYY5
77825 229533.I1RGC6 pp 229533.I1RJT9
77826 229533.I1RGC6 pp 229533.I1RT62
77827 229533.I1RGC6 pp 229533.I1RYZ9
77828 229533.I1RGC6 pp 229533.I1RRC6
77829 229533.I1RGC6 pp 229533.I1RRT6
77830 229533.I1RGC6 pp 229533.I1RM49
77831 229533.I1RGC6 pp 229533.I1RL24
77832 229533.I1RGC6 pp 229533.I1RM03
77833 229533.I1RGC6 pp 229533.I1RZG7
77834 229533.I1RFF3 pp 229533.I1RVR2
77835 229533.I1RFF3 pp 229533.V6RM02
77836 229533.I1RFF3 pp 229533.V6RUY9
77837 229533.I1RFF3 pp 229533.I1RMM8
77838 229533.I1RIT9 pp 229533.I1RNG4
77839 229533.A0A098DAX6 pp 229533.A0A0E0SP71
77840 229533.A0A098DAX6 pp 229533.I1RGI5
77841 229533.A0A098DAX6 pp 229533.A0A1C3YMN2
77842 229533.A0A098DAX6 pp 229533.A0A0E0SBC4
77843 229533.A0A098DAX6 pp 229533.I1RXN6
77844 229533.A0A098DAX6 pp 229533.V6RDP1
77845 229533.A0A098DAX6 pp 229533.I1RJU9
77846 229533.A0A098DAX6 pp 229533.I1RLM3
77847 229533.Q4IMJ1 pp 229533.V6QVH8
77848 229533.A0A0E0SK74 pp 229533.I1RRG3
77849 229533.A0A0E0SK74 pp 229533.A0A0E0SL81
77850 229533.A0A0E0SK74 pp 229533.A0A1C3YLT3
77851 229533.A0A0E0SK74 pp 229533.I1RQL4
77852 229533.A0A0E0SK74 pp 229533.I1RRX5
77853 229533.A0A0E0SK74 pp 229533.I1RM03
77854 229533.A0A0E0SK74 pp 229533.I1RQH1
77855 229533.A0A0E0SK74 pp 229533.I1RW71
77856 229533.A0A0E0SK74 pp 229533.I1S0F5
77857 229533.A0A0E0SK74 pp 229533.I1RRF8
77858 229533.A0A0E0SK74 pp 229533.I1RYU6
77859 229533.A0A0E0SK74 pp 229533.I1RBL8
77860 229533.A0A0E0SK74 pp 229533.I1S235
77861 229533.A0A0E0SK74 pp 229533.I1RTU8
77862 229533.A0A0E0SK74 pp 229533.I1RJT9
77863 229533.A0A0E0SK74 pp 229533.I1S7L6
77864 229533.A0A0E0SK74 pp 229533.I1RRT6
77865 229533.A0A0E0SK74 pp 229533.I1RBE4
77866 229533.A0A0E0SK74 pp 229533.I1S821
77867 229533.A0A0E0SK74 pp 229533.I1RP86
77868 229533.A0A0E0SK74 pp 229533.A0A1C3YN12
77869 229533.A0A0E0SK74 pp 229533.I1RT41
77870 229533.A0A0E0SK74 pp 229533.A0A1C3YLR6
77871 229533.A0A0E0SK74 pp 229533.I1RF94
77872 229533.A0A0E0SK74 pp 229533.A0A1C3YJL7
77873 229533.A0A0E0SK74 pp 229533.I1RDI8
77874 229533.A0A0E0SK74 pp 229533.I1RDE6
77875 229533.A0A0E0SK74 pp 229533.I1RAM6
77876 229533.A0A0E0SK74 pp 229533.I1RM67
77877 229533.A0A0E0SK74 pp 229533.I1RYL5
77878 229533.A0A0E0SK74 pp 229533.A0A1C3YI30
77879 229533.A0A0E0SK74 pp 229533.I1RBC4
77880 229533.A0A0E0SK74 pp 229533.A0A1C3YK11
77881 229533.A0A0E0SK74 pp 229533.I1RIB9
77882 229533.A0A0E0SK74 pp 229533.A0A1C3YIK4
77883 229533.A0A0E0SK74 pp 229533.I1RKK8
77884 229533.A0A0E0SK74 pp 229533.I1RG60
77885 229533.A0A0E0SK74 pp 229533.I1RPL2
77886 229533.A0A0E0SK74 pp 229533.I1RDH7
77887 229533.A0A0E0SK74 pp 229533.I1S3S5
77888 229533.A0A0E0SK74 pp 229533.I1RP66
77889 229533.A0A0E0SK74 pp 229533.I1RY63
77890 229533.A0A0E0SK74 pp 229533.I1RQA5
77891 229533.A0A0E0SK74 pp 229533.I1RYS6

77892	229533.A0A0E0SK74	pp	229533.I1RUM7
77893	229533.A0A0E0SK74	pp	229533.A0A1C3YKY6
77894	229533.A0A0E0SK74	pp	229533.A0A1C3YJ14
77895	229533.A0A0E0SK74	pp	229533.I1RXX0
77896	229533.A0A0E0SK74	pp	229533.I1RWD7
77897	229533.A0A0E0SK74	pp	229533.I1RW87
77898	229533.A0A0E0SK74	pp	229533.I1RAB3
77899	229533.A0A0E0SK74	pp	229533.I1RQG4
77900	229533.A0A0E0SK74	pp	229533.I1RYP2
77901	229533.A0A0E0SK74	pp	229533.A0A1C3YHZ0
77902	229533.A0A0E0SK74	pp	229533.I1RGA4
77903	229533.A0A0E0SK74	pp	229533.I1REC6
77904	229533.A0A0E0SK74	pp	229533.I1RB59
77905	229533.A0A0E0SK74	pp	229533.I1RBY7
77906	229533.A0A0E0SK74	pp	229533.I1RBF3
77907	229533.A0A0E0SK74	pp	229533.I1RJZ7
77908	229533.A0A0E0SK74	pp	229533.I1RA62
77909	229533.A0A0E0SK74	pp	229533.I1R9Y8
77910	229533.A0A0E0SK74	pp	229533.I1RP28
77911	229533.A0A0E0SK74	pp	229533.I1RL79
77912	229533.A0A0E0SK74	pp	229533.I1RC31
77913	229533.A0A0E0SK74	pp	229533.A0A0E0SKE0
77914	229533.A0A0E0SK74	pp	229533.I1RAM2
77915	229533.A0A0E0SK74	pp	229533.I1RA52
77916	229533.A0A0E0SK74	pp	229533.I1REB8
77917	229533.A0A0E0SK74	pp	229533.I1RC66
77918	229533.A0A0E0SK74	pp	229533.A0A1C3YI20
77919	229533.A0A0E0SK74	pp	229533.I1RSQ4
77920	229533.A0A0E0SK74	pp	229533.A0A0E0SMR8
77921	229533.A0A0E0SK74	pp	229533.A0A1C3YL55
77922	229533.A0A0E0SK74	pp	229533.A0A1C3YI83
77923	229533.A0A0E0SK74	pp	229533.I1RPV0
77924	229533.A0A0E0SK74	pp	229533.Q4HXF6
77925	229533.A0A0E0SK74	pp	229533.I1RZS8
77926	229533.A0A0E0SK74	pp	229533.I1S0F9
77927	229533.A0A0E0SK74	pp	229533.I1RSY2
77928	229533.A0A0E0SK74	pp	229533.A0A1C3YN27
77929	229533.A0A0E0SK74	pp	229533.I1RK77
77930	229533.A0A0E0SK74	pp	229533.I1RQ21
77931	229533.A0A0E0SK74	pp	229533.I1S247
77932	229533.A0A0E0SK74	pp	229533.I1RMQ5
77933	229533.A0A0E0SK74	pp	229533.I1RBA4
77934	229533.A0A0E0SK74	pp	229533.O74638
77935	229533.A0A0E0SK74	pp	229533.I1RPU8
77936	229533.A0A0E0SK74	pp	229533.I1RME4
77937	229533.A0A0E0SK74	pp	229533.I1RBS5
77938	229533.A0A0E0SK74	pp	229533.I1RZ95
77939	229533.A0A0E0SK74	pp	229533.I1S2V2
77940	229533.A0A0E0SK74	pp	229533.I1RZD3
77941	229533.A0A0E0SK74	pp	229533.I1RWK9
77942	229533.A0A0E0SK74	pp	229533.I1RWH4
77943	229533.A0A0E0SK74	pp	229533.I1RPS2
77944	229533.A0A0E0SK74	pp	229533.I1RBY6
77945	229533.A0A0E0SK74	pp	229533.I1RSU2
77946	229533.A0A0E0SK74	pp	229533.A0A1C3YK08
77947	229533.A0A0E0SK74	pp	229533.A0A1C3YJK3
77948	229533.A0A0E0SK74	pp	229533.I1RD80
77949	229533.A0A0E0SK74	pp	229533.Q4I624
77950	229533.A0A0E0SK74	pp	229533.I1RMQ3
77951	229533.A0A0E0SK74	pp	229533.I1RCG5
77952	229533.A0A0E0SK74	pp	229533.A0A1C3YLB6
77953	229533.A0A0E0SK74	pp	229533.V6RLM7
77954	229533.A0A0E0SK74	pp	229533.I1RG11
77955	229533.A0A0E0SK74	pp	229533.I1S0Z8
77956	229533.A0A0E0SK74	pp	229533.I1RMB1
77957	229533.A0A0E0SK74	pp	229533.I1RA86
77958	229533.A0A0E0SK74	pp	229533.I1RPN8
77959	229533.A0A0E0SK74	pp	229533.I1RZK1
77960	229533.A0A0E0SK74	pp	229533.Q4I7G0
77961	229533.A0A0E0SK74	pp	229533.I1RU27
77962	229533.A0A0E0SK74	pp	229533.I1RHW9
77963	229533.A0A0E0SK74	pp	229533.I1RKB6
77964	229533.A0A0E0SK74	pp	229533.I1RTE9

229533.A0A0E0SK74 pp 229533.I1REC7
229533.A0A0E0SK74 pp 229533.I1RC55
229533.A0A0E0SK74 pp 229533.A0A1C3YK30
229533.A0A0E0SK74 pp 229533.I1REG1
229533.A0A0E0SK74 pp 229533.I1RGC6
229533.I1RZI8 pp 229533.Q4IK03
229533.I1RZI8 pp 229533.Q4IPZ1
229533.I1RZI8 pp 229533.Q4IB65
229533.I1RZI8 pp 229533.I1S8Y1
229533.I1RZI8 pp 229533.Q4I6B0
229533.I1RZI8 pp 229533.Q4I099
229533.I1RZI8 pp 229533.Q4IJW4
229533.I1RZI8 pp 229533.Q4HVU7
229533.I1RZS8 pp 229533.I1S2V2
229533.I1RZS8 pp 229533.Q4I7T5
229533.I1RZS8 pp 229533.V6R7V8
229533.I1RZS8 pp 229533.I1S2A1
229533.I1RZS8 pp 229533.I1S235
229533.I1RZS8 pp 229533.Q4I624
229533.I1RZS8 pp 229533.I1S0D7
229533.I1RZS8 pp 229533.V6RQ28
229533.I1RAF9 pp 229533.I1RJX2
229533.I1RAF9 pp 229533.I1RMC5
229533.I1RAF9 pp 229533.I1RMC2
229533.I1RAF9 pp 229533.I1S274
229533.I1RAF9 pp 229533.I1RE47
229533.I1RAF9 pp 229533.I1S3F7
229533.I1RAF9 pp 229533.V6QVM5
229533.I1REB8 pp 229533.I1S235
229533.I1REB8 pp 229533.I1S2V2
229533.I1REB8 pp 229533.I1RM03
229533.I1REB8 pp 229533.I1RWK9
229533.I1REB8 pp 229533.I1RP66
229533.I1REB8 pp 229533.I1REC7
229533.I1REB8 pp 229533.I1REC6
229533.I1REB8 pp 229533.I1RQL4
229533.I1REB8 pp 229533.I1RWH4
229533.I1REB8 pp 229533.I1RPN8
229533.I1REB8 pp 229533.I1RYL5
229533.I1REB8 pp 229533.I1RW87
229533.I1REB8 pp 229533.I1RKK8
229533.I1REB8 pp 229533.I1RQ21
229533.I1RW10 pp 229533.I1S9T6
229533.I1RW10 pp 229533.I1S3L2
229533.I1RW10 pp 229533.I1RZM4
229533.I1RW10 pp 229533.I1S0K0
229533.I1RW10 pp 229533.I1RXS0
229533.I1RW10 pp 229533.I1S8R4
229533.I1RW10 pp 229533.I1S673
229533.I1RW10 pp 229533.V6RK96
229533.I1RVY1 pp 229533.I1S0K0
229533.I1RVY1 pp 229533.I1S9T6
229533.I1RVY1 pp 229533.I1RXS0
229533.I1RVY1 pp 229533.V6RK96
229533.I1RVY1 pp 229533.I1RZM4
229533.I1RVY1 pp 229533.I1S3L2
229533.I1RVY1 pp 229533.I1S673
229533.I1RVY1 pp 229533.I1S8R4
229533.I1RN58 pp 229533.Q4IJH1
229533.I1RN58 pp 229533.Q4HVW2
229533.I1RN58 pp 229533.Q4IJ56
229533.I1RN58 pp 229533.I1RRU9
229533.I1RN58 pp 229533.I1RQ81
229533.I1RN58 pp 229533.I1RN65
229533.I1RN58 pp 229533.I1RNE7
229533.I1RN58 pp 229533.Q4IAA0
229533.I1RN58 pp 229533.I1RSM2
229533.I1RN58 pp 229533.I1S6F1
229533.I1RN58 pp 229533.V6RTJ8
229533.I1RN58 pp 229533.I1RWD7
229533.I1RN58 pp 229533.I1RT35
229533.I1RN58 pp 229533.I1RW71
229533.I1RN58 pp 229533.I1RZH4

78038	229533.I1RN58	pp	229533.I1RN61
78039	229533.I1RN58	pp	229533.I1RQ31
78040	229533.I1RN58	pp	229533.I1RWH4
78041	229533.I1RN58	pp	229533.Q4IEK8
78042	229533.I1RN58	pp	229533.I1S0X5
78043	229533.I1RN58	pp	229533.I1RNB8
78044	229533.I1RN58	pp	229533.I1RPU3
78045	229533.I1RN58	pp	229533.I1RZM1
78046	229533.I1RN58	pp	229533.I1RPP4
78047	229533.I1RN58	pp	229533.I1RT04
78048	229533.I1RN58	pp	229533.I1S2E9
78049	229533.I1RN58	pp	229533.Q4IF76
78050	229533.I1RN58	pp	229533.I1RWI5
78051	229533.I1RN58	pp	229533.Q4IAS1
78052	229533.I1RN58	pp	229533.I1RP27
78053	229533.I1RN58	pp	229533.Q4I7K4
78054	229533.I1RN58	pp	229533.I1RWD3
78055	229533.I1RN58	pp	229533.I1RNN1
78056	229533.I1RN58	pp	229533.I1RSV2
78057	229533.I1RN58	pp	229533.I1RZS7
78058	229533.I1RN58	pp	229533.I1RWN6
78059	229533.I1RN58	pp	229533.I1RY44
78060	229533.I1RN58	pp	229533.I1RSK1
78061	229533.I1RN58	pp	229533.Q4HW67
78062	229533.I1RN58	pp	229533.Q4HZ42
78063	229533.I1RN58	pp	229533.Q4HZ68
78064	229533.I1RN58	pp	229533.I1RN67
78065	229533.I1RN58	pp	229533.V6RMW6
78066	229533.I1RN58	pp	229533.I1RRV1
78067	229533.I1RN58	pp	229533.I1S0N4
78068	229533.I1RN58	pp	229533.I1RSQ3
78069	229533.I1RN58	pp	229533.I1RTH6
78070	229533.I1REA6	pp	229533.I1RP88
78071	229533.Q4I099	pp	229533.Q4I5I4
78072	229533.Q4I099	pp	229533.V6QUE6
78073	229533.Q4I099	pp	229533.Q4IK03
78074	229533.Q4I099	pp	229533.Q4IB65
78075	229533.Q4I099	pp	229533.Q4ICM9
78076	229533.Q4I099	pp	229533.Q4I7T5
78077	229533.Q4I099	pp	229533.Q4IJW4
78078	229533.Q4I099	pp	229533.Q4I6B0
78079	229533.Q4I099	pp	229533.V6R1X4
78080	229533.Q4I099	pp	229533.V6R3J1
78081	229533.Q4I099	pp	229533.Q4I375
78082	229533.Q4I099	pp	229533.Q4IJ11
78083	229533.Q4I099	pp	229533.Q4I5U9
78084	229533.Q4I099	pp	229533.Q4I5W5
78085	229533.Q4I099	pp	229533.Q4IPZ1
78086	229533.I1S4H6	pp	229533.Q4I2W2
78087	229533.I1S4H6	pp	229533.I1S8F0
78088	229533.I1RL79	pp	229533.I1RM03
78089	229533.I1RL79	pp	229533.I1RPN8
78090	229533.I1RL79	pp	229533.I1RQ21
78091	229533.I1RL79	pp	229533.I1RQL4
78092	229533.I1RL79	pp	229533.I1RWK9
78093	229533.I1RL79	pp	229533.I1RWH4
78094	229533.I1RL79	pp	229533.I1RYL5
78095	229533.I1RL79	pp	229533.I1S2V2
78096	229533.I1RL79	pp	229533.I1RW87
78097	229533.I1RL79	pp	229533.I1RP66
78098	229533.I1RL79	pp	229533.I1S235
78099	229533.I1RQR6	pp	229533.I1RUG0
78100	229533.I1RQR6	pp	229533.I1RY92
78101	229533.I1RQR6	pp	229533.I1RT38
78102	229533.I1RQR6	pp	229533.I1RVN1
78103	229533.I1RQR6	pp	229533.I1RRS8
78104	229533.I1RQR6	pp	229533.I1S458
78105	229533.I1RQR6	pp	229533.I1RXZ3
78106	229533.I1RQR6	pp	229533.Q4IB65
78107	229533.I1RQR6	pp	229533.I1S0R5
78108	229533.I1RQR6	pp	229533.I1S277
78109	229533.I1RQR6	pp	229533.I1S0R1
78110	229533.I1RQR6	pp	229533.I1RYT8

229533.I1RQR6	pp	229533.Q4IJW4
229533.I1RQR6	pp	229533.Q4I099
229533.I1RQR6	pp	229533.I1RWA3
229533.I1RQR6	pp	229533.I1S8F0
229533.I1RQR6	pp	229533.I1S0P0
229533.I1RQR6	pp	229533.I1RY21
229533.I1RQR6	pp	229533.V6R3J1
229533.I1RQR6	pp	229533.Q4IK03
229533.I1RQR6	pp	229533.I1S464
229533.I1RQR6	pp	229533.I1RZI8
229533.I1RQR6	pp	229533.Q4I7T5
229533.I1RQR6	pp	229533.I1RS05
229533.I1RQR6	pp	229533.Q4I6B0
229533.I1RQR6	pp	229533.I1RSC0
229533.I1RQR6	pp	229533.I1S0R8
229533.I1RQR6	pp	229533.Q4ICM9
229533.I1RQR6	pp	229533.I1RXD9
229533.I1RQR6	pp	229533.I1RZH5
229533.I1RQR6	pp	229533.I1RZS4
229533.I1RQR6	pp	229533.I1RS29
229533.I1RQR6	pp	229533.I1S8S9
229533.I1RQR6	pp	229533.I1S0K2
229533.I1RQR6	pp	229533.I1S5X9
229533.I1RQR6	pp	229533.Q4I2W2
229533.I1RQR6	pp	229533.I1RZD3
229533.I1RQR6	pp	229533.I1RXE6
229533.I1RQR6	pp	229533.Q4IPZ1
229533.I1RQR6	pp	229533.I1S0Q7
229533.I1RQR6	pp	229533.Q4HZ95
229533.I1RQR6	pp	229533.Q4I375
229533.I1RQR6	pp	229533.I1RT99
229533.I1RQR6	pp	229533.V6RJP8
229533.I1RPV0	pp	229533.I1S235
229533.I1RPV0	pp	229533.I1RYL5
229533.I1RPV0	pp	229533.I1S0Z8
229533.I1RPV0	pp	229533.I1RW87
229533.I1RPV0	pp	229533.I1RRT6
229533.I1RPV0	pp	229533.I1RWH4
229533.I1RPV0	pp	229533.I1RT62
229533.I1RPV0	pp	229533.Q4I7T5
229533.I1RPV0	pp	229533.I1RQL4
229533.I1RPV0	pp	229533.I1RZD3
229533.I1RPV0	pp	229533.V6RCJ5
229533.I1RPV0	pp	229533.I1RQ21
229533.I1RPV0	pp	229533.I1S2V2
229533.I1RPV0	pp	229533.I1RWK9
229533.A0A1C3YI83	pp	229533.I1RWK9
229533.A0A1C3YI83	pp	229533.I1REC6
229533.A0A1C3YI83	pp	229533.I1S2V2
229533.A0A1C3YI83	pp	229533.I1R9Y8
229533.A0A1C3YI83	pp	229533.I1RBS5
229533.A0A1C3YI83	pp	229533.I1RC66
229533.A0A1C3YI83	pp	229533.Q4I7T5
229533.A0A1C3YI83	pp	229533.I1RWH4
229533.A0A1C3YI83	pp	229533.I1RPN8
229533.A0A1C3YI83	pp	229533.I1RPV0
229533.A0A1C3YI83	pp	229533.I1RJT9
229533.A0A1C3YI83	pp	229533.I1RRT6
229533.A0A1C3YI83	pp	229533.I1RQ21
229533.A0A1C3YI83	pp	229533.I1RQL4
229533.A0A1C3YI83	pp	229533.I1RA86
229533.A0A1C3YI83	pp	229533.I1RM03
229533.A0A1C3YI83	pp	229533.I1RT62
229533.A0A1C3YI83	pp	229533.I1REC7
229533.A0A1C3YI83	pp	229533.I1RGC6
229533.A0A1C3YI83	pp	229533.Q4I624
229533.A0A1C3YI83	pp	229533.A0A1C3YK11
229533.A0A1C3YI83	pp	229533.I1RBC4
229533.A0A1C3YI83	pp	229533.A0A1C3YN12
229533.A0A1C3YI83	pp	229533.I1RKK8
229533.A0A1C3YI83	pp	229533.I1RDI8
229533.A0A1C3YI83	pp	229533.I1RPK5
229533.A0A1C3YI83	pp	229533.I1S235

78184 229533.A0A1C3YI83 pp 229533.I1RP66
78185 229533.A0A1C3YI83 pp 229533.I1RYL5
78186 229533.A0A1C3YI83 pp 229533.I1RW87
78187 229533.I1RHA8 pp 229533.I1RVR2
78188 229533.I1RHA8 pp 229533.I1RQE8
78189 229533.I1RHA8 pp 229533.I1RQP2
78190 229533.I1RHA8 pp 229533.I1S9T6
78191 229533.I1RHA8 pp 229533.I1S3L2
78192 229533.I1RHA8 pp 229533.I1S0K0
78193 229533.I1RHA8 pp 229533.I1RSX9
78194 229533.I1RHA8 pp 229533.I1S673
78195 229533.I1RHA8 pp 229533.I1RHZ5
78196 229533.I1RHA8 pp 229533.I1RXS0
78197 229533.I1RHA8 pp 229533.I1RZM4
78198 229533.I1RHA8 pp 229533.I1RMY1
78199 229533.I1RHA8 pp 229533.V6RK96
78200 229533.I1RHA8 pp 229533.I1S8R4
78201 229533.I1RCQ7 pp 229533.V6QX21
78202 229533.I1RCQ7 pp 229533.I1S769
78203 229533.I1RBT8 pp 229533.I1S2V2
78204 229533.I1RBT8 pp 229533.I1RUL3
78205 229533.I1RBT8 pp 229533.I1RW87
78206 229533.I1RBT8 pp 229533.I1RWK9
78207 229533.I1RBT8 pp 229533.V6QUG0
78208 229533.I1RBT8 pp 229533.I1RYL5
78209 229533.I1RBT8 pp 229533.I1S4S1
78210 229533.I1RBT8 pp 229533.I1S2A1
78211 229533.I1RBT8 pp 229533.I1RZD3
78212 229533.I1RBT8 pp 229533.V6R7V8
78213 229533.I1RBT8 pp 229533.I1RDI8
78214 229533.I1RBT8 pp 229533.I1RFI9
78215 229533.I1RBT8 pp 229533.I1RPN8
78216 229533.I1RBT8 pp 229533.I1RPY0
78217 229533.I1RBT8 pp 229533.I1RKK8
78218 229533.I1RBT8 pp 229533.I1RFY0
78219 229533.I1RBT8 pp 229533.I1RWH4
78220 229533.I1RBT8 pp 229533.I1RGY6
78221 229533.I1RBT8 pp 229533.I1S235
78222 229533.I1RBT8 pp 229533.I1RM03
78223 229533.I1RBT8 pp 229533.I1RPN1
78224 229533.I1RBT8 pp 229533.V6QWB1
78225 229533.I1RBT8 pp 229533.Q4I7T5
78226 229533.I1RBT8 pp 229533.I1RQ21
78227 229533.I1RBT8 pp 229533.I1RKZ4
78228 229533.I1RBT8 pp 229533.I1RF33
78229 229533.I1RBT8 pp 229533.I1RZS8
78230 229533.I1RBT8 pp 229533.I1RP66
78231 229533.I1RBT8 pp 229533.I1RPK5
78232 229533.I1RBT8 pp 229533.I1RKB6
78233 229533.I1RAD7 pp 229533.I1RBP3
78234 229533.I1S0H7 pp 229533.V6QVH8
78235 229533.I1S0H7 pp 229533.Q4IMJ1
78236 229533.I1S0H7 pp 229533.I1S334
78237 229533.I1S0H7 pp 229533.I1S3H8
78238 229533.I1S0H7 pp 229533.Q4HTS9
78239 229533.I1S0H7 pp 229533.Q4HXF0
78240 229533.I1S0J8 pp 229533.V6RLM7
78241 229533.I1S0J8 pp 229533.I1S237
78242 229533.I1S0J8 pp 229533.V6R382
78243 229533.I1S0J8 pp 229533.I1S236
78244 229533.I1S0J8 pp 229533.V6QVM5
78245 229533.I1S0J8 pp 229533.V6RQW4
78246 229533.I1S0J8 pp 229533.O74638
78247 229533.I1RJ78 pp 229533.I1RQ61
78248 229533.I1RJ78 pp 229533.I1RPD3
78249 229533.I1RJ78 pp 229533.I1RM23
78250 229533.I1RJ78 pp 229533.I1RWV4
78251 229533.I1RJ78 pp 229533.I1RN66
78252 229533.I1RJ78 pp 229533.I1RM77
78253 229533.I1RJ78 pp 229533.V6QW25
78254 229533.I1RJ78 pp 229533.I1RMG4
78255 229533.A0A1C3YMU9 pp 229533.I1RKR8
78256 229533.A0A1C3YMU9 pp 229533.I1RA65

78257 229533.A0A1C3YMU9 pp 229533.I1RPW8
78258 229533.A0A1C3YMU9 pp 229533.I1RH75
78259 229533.A0A1C3YMU9 pp 229533.I1RCH4
78260 229533.A0A1C3YMU9 pp 229533.I1RPQ2
78261 229533.A0A1C3YMU9 pp 229533.I1RAP1
78262 229533.A0A1C3YMU9 pp 229533.I1RW80
78263 229533.A0A1C3YMU9 pp 229533.I1RFI5
78264 229533.A0A1C3YMU9 pp 229533.I1RRF7
78265 229533.A0A1C3YMU9 pp 229533.I1RSM8
78266 229533.A0A1C3YMU9 pp 229533.I1RR61
78267 229533.A0A1C3YMU9 pp 229533.I1RED3
78268 229533.A0A1C3YMU9 pp 229533.I1RN04
78269 229533.A0A1C3YMU9 pp 229533.I1RZ94
78270 229533.A0A1C3YMU9 pp 229533.I1RED2
78271 229533.A0A1C3YMU9 pp 229533.I1S5Z5
78272 229533.A0A1C3YMU9 pp 229533.I1RQ42
78273 229533.A0A1C3YMU9 pp 229533.I1S9H8
78274 229533.A0A1C3YMU9 pp 229533.I1RKF3
78275 229533.A0A1C3YMU9 pp 229533.I1RR25
78276 229533.A0A1C3YMU9 pp 229533.I1RCG6
78277 229533.A0A1C3YMU9 pp 229533.I1RLI4
78278 229533.A0A1C3YMU9 pp 229533.I1RW90
78279 229533.A0A1C3YMU9 pp 229533.I1RW91
78280 229533.A0A1C3YMU9 pp 229533.V6QWX8
78281 229533.I1RU27 pp 229533.I1RWK9
78282 229533.I1RU27 pp 229533.I1S2V2
78283 229533.I1RU27 pp 229533.I1RW87
78284 229533.I1RU27 pp 229533.I1S235
78285 229533.I1RU27 pp 229533.I1RWH4
78286 229533.I1RU27 pp 229533.I1RYL5
78287 229533.I1RAD5 pp 229533.I1RY16
78288 229533.I1RAD5 pp 229533.I1RRG1
78289 229533.I1RAD5 pp 229533.I1RZA8
78290 229533.I1RAD5 pp 229533.I1S694
78291 229533.I1RAD5 pp 229533.I1RI11
78292 229533.I1RQ61 pp 229533.I1RWI6
78293 229533.I1RQ61 pp 229533.I1S368
78294 229533.I1RQ61 pp 229533.V6QW25
78295 229533.I1RQ61 pp 229533.I1RWC2
78296 229533.I1RQ61 pp 229533.I1RRE1
78297 229533.I1RQ61 pp 229533.I1RWV4
78298 229533.A0A1C3YK88 pp 229533.V6QUE6
78299 229533.A0A1C3YK88 pp 229533.I1RJY2
78300 229533.A0A1C3YK88 pp 229533.I1RG11
78301 229533.A0A1C3YK88 pp 229533.I1RK79
78302 229533.A0A1C3YK88 pp 229533.I1RJI3
78303 229533.A0A1C3YK88 pp 229533.I1RY03
78304 229533.A0A1C3YK88 pp 229533.I1RRI2
78305 229533.A0A1C3YK88 pp 229533.I1S9U1
78306 229533.A0A1C3YK88 pp 229533.I1RRN6
78307 229533.A0A1C3YK88 pp 229533.I1RZB5
78308 229533.A0A1C3YK88 pp 229533.V6QWX8
78309 229533.A0A1C3YK88 pp 229533.I1S255
78310 229533.A0A1C3YK88 pp 229533.I1RJY3
78311 229533.A0A1C3YK88 pp 229533.I1RJM5
78312 229533.A0A1C3YK88 pp 229533.I1RW90
78313 229533.A0A1C3YK88 pp 229533.I1RC24
78314 229533.A0A1C3YK88 pp 229533.I1RRT0
78315 229533.A0A1C3YK88 pp 229533.I1RPJ3
78316 229533.A0A1C3YK88 pp 229533.I1RJL2
78317 229533.A0A1C3YK88 pp 229533.I1S0J1
78318 229533.A0A1C3YK88 pp 229533.Q4I8X0
78319 229533.A0A1C3YK88 pp 229533.V6R3X5
78320 229533.A0A1C3YK88 pp 229533.I1RVY0
78321 229533.A0A1C3YK88 pp 229533.I1RN17
78322 229533.A0A1C3YK88 pp 229533.I1RR25
78323 229533.A0A1C3YK88 pp 229533.I1S9Y1
78324 229533.A0A1C3YK88 pp 229533.I1RK95
78325 229533.A0A1C3YK88 pp 229533.I1RC74
78326 229533.A0A1C3YK88 pp 229533.I1RG51
78327 229533.A0A1C3YK88 pp 229533.I1S9U2
78328 229533.A0A1C3YK88 pp 229533.I1RB71
78329 229533.A0A1C3YK88 pp 229533.I1RS30

78330	229533.A0A1C3YK88	pp	229533.I1RAV6
78331	229533.A0A1C3YK88	pp	229533.I1RKQ5
78332	229533.A0A1C3YK88	pp	229533.I1RNH1
78333	229533.A0A1C3YK88	pp	229533.I1RE30
78334	229533.A0A1C3YK88	pp	229533.I1RRG3
78335	229533.A0A1C3YK88	pp	229533.I1RJL4
78336	229533.A0A1C3YK88	pp	229533.I1RBC7
78337	229533.I1RME4	pp	229533.I1RPL2
78338	229533.I1RME4	pp	229533.I1RZJ9
78339	229533.I1RME4	pp	229533.I1S9U1
78340	229533.I1RME4	pp	229533.I1RP66
78341	229533.I1RME4	pp	229533.I1S235
78342	229533.I1RME4	pp	229533.I1RT62
78343	229533.I1RME4	pp	229533.I1S1Y9
78344	229533.I1RME4	pp	229533.I1RRT6
78345	229533.I1RME4	pp	229533.I1RQL4
78346	229533.I1RME4	pp	229533.I1RYS6
78347	229533.I1RME4	pp	229533.I1RMR1
78348	229533.I1RME4	pp	229533.I1RQA5
78349	229533.I1RME4	pp	229533.I1RW87
78350	229533.I1RME4	pp	229533.I1RYJ7
78351	229533.I1RME4	pp	229533.I1RPA7
78352	229533.I1RME4	pp	229533.Q4I7T5
78353	229533.I1RME4	pp	229533.I1RWD7
78354	229533.I1RME4	pp	229533.I1S2V2
78355	229533.I1RME4	pp	229533.I1RYV3
78356	229533.I1RME4	pp	229533.I1RPK5
78357	229533.I1RME4	pp	229533.Q4IBD0
78358	229533.I1RME4	pp	229533.I1RWK9
78359	229533.I1RME4	pp	229533.Q4HXF6
78360	229533.I1RME4	pp	229533.I1RYL5
78361	229533.I1RME4	pp	229533.I1S247
78362	229533.I1RME4	pp	229533.I1RQ21
78363	229533.I1RME4	pp	229533.I1RSL8
78364	229533.I1RME4	pp	229533.I1RZD3
78365	229533.I1RME4	pp	229533.I1RXT9
78366	229533.I1RME4	pp	229533.I1S0Z8
78367	229533.I1RME4	pp	229533.Q4I624
78368	229533.I1RME4	pp	229533.I1RWH4
78369	229533.I1RME4	pp	229533.I1RPN8
78370	229533.Q4I375	pp	229533.V6RM66
78371	229533.Q4I375	pp	229533.Q4IPZ1
78372	229533.Q4I375	pp	229533.Q4IJW4
78373	229533.Q4I375	pp	229533.Q4ICM9
78374	229533.Q4I375	pp	229533.Q4IB65
78375	229533.Q4I375	pp	229533.Q4I6B0
78376	229533.Q4I375	pp	229533.Q4I7T5
78377	229533.A0A1C3YJT8	pp	229533.I1S9T6
78378	229533.A0A1C3YJT8	pp	229533.I1S0K0
78379	229533.A0A1C3YJT8	pp	229533.I1S673
78380	229533.A0A1C3YJT8	pp	229533.A0A1C3YKN4
78381	229533.A0A1C3YJT8	pp	229533.I1RQE8
78382	229533.A0A1C3YJT8	pp	229533.I1S3L2
78383	229533.A0A1C3YJT8	pp	229533.I1RQP2
78384	229533.A0A1C3YJT8	pp	229533.I1RZM4
78385	229533.A0A1C3YJT8	pp	229533.A0A1C3YJU5
78386	229533.A0A1C3YJT8	pp	229533.I1RSX9
78387	229533.A0A1C3YJT8	pp	229533.I1RXS0
78388	229533.A0A1C3YJT8	pp	229533.I1RVR2
78389	229533.A0A1C3YJT8	pp	229533.I1RMY1
78390	229533.A0A1C3YJT8	pp	229533.I1RHZ5
78391	229533.A0A1C3YJT8	pp	229533.I1S8R4
78392	229533.A0A1C3YJT8	pp	229533.I1RC48
78393	229533.A0A1C3YJT8	pp	229533.V6RK96
78394	229533.Q4HZ47	pp	229533.Q4HZ68
78395	229533.Q4HZ47	pp	229533.Q4I830
78396	229533.Q4HZ47	pp	229533.Q4I5Z5
78397	229533.Q4HZ47	pp	229533.Q4IPI1
78398	229533.Q4HZ47	pp	229533.Q4I662
78399	229533.Q4HZ47	pp	229533.Q4IJH1
78400	229533.Q4HZ47	pp	229533.Q4IFI0
78401	229533.Q4HZ47	pp	229533.Q4IJ56
78402	229533.Q4HZ47	pp	229533.Q4I327

78403 229533.Q4HZ47 pp 229533.Q4IEK8
78404 229533.Q4HZ47 pp 229533.Q4IBS2
78405 229533.Q4HZ47 pp 229533.Q4IEV5
78406 229533.Q4HZ47 pp 229533.Q4IAS1
78407 229533.I1RZK1 pp 229533.I1S2V2
78408 229533.I1RZK1 pp 229533.I1S235
78409 229533.I1RZK1 pp 229533.I1S0Z8
78410 229533.I1RU32 pp 229533.Q4IBX0
78411 229533.I1RU32 pp 229533.Q4IMM0
78412 229533.I1RSS7 pp 229533.V6RLM7
78413 229533.I1RSS7 pp 229533.V6R382
78414 229533.I1RSS7 pp 229533.I1S9Z4
78415 229533.I1RSS7 pp 229533.I1S1W3
78416 229533.I1RSS7 pp 229533.I1RXA9
78417 229533.I1RSS7 pp 229533.I1S237
78418 229533.I1RSS7 pp 229533.V6QT89
78419 229533.I1RSS7 pp 229533.I1RXY7
78420 229533.I1RSS7 pp 229533.V6RM02
78421 229533.I1RSS7 pp 229533.I1S8R4
78422 229533.I1RSS7 pp 229533.Q4HXF0
78423 229533.I1RSS7 pp 229533.I1RVR2
78424 229533.I1RSS7 pp 229533.Q4I099
78425 229533.I1RSS7 pp 229533.I1RTG7
78426 229533.I1RSS7 pp 229533.Q4HZ95
78427 229533.I1RSS7 pp 229533.I1RUR8
78428 229533.I1RSS7 pp 229533.I1RZ52
78429 229533.I1RSS7 pp 229533.I1RX64
78430 229533.I1RSS7 pp 229533.I1S0Y7
78431 229533.I1RSS7 pp 229533.I1RWS4
78432 229533.I1RSS7 pp 229533.I1RXB2
78433 229533.I1RSS7 pp 229533.I1RZ83
78434 229533.I1RSS7 pp 229533.I1S0J8
78435 229533.I1RSS7 pp 229533.I1S3G4
78436 229533.I1RSS7 pp 229533.V6RK96
78437 229533.I1RSS7 pp 229533.V6RG22
78438 229533.I1RSS7 pp 229533.I1RVJ6
78439 229533.I1RSS7 pp 229533.I1RXS0
78440 229533.I1RSS7 pp 229533.I1RT09
78441 229533.I1RSS7 pp 229533.I1RVP5
78442 229533.I1RSS7 pp 229533.I1RXB6
78443 229533.I1RSS7 pp 229533.I1RVY2
78444 229533.I1RSS7 pp 229533.I1S0K0
78445 229533.I1RSS7 pp 229533.V6RUT5
78446 229533.I1RSS7 pp 229533.I1S0N3
78447 229533.I1RSS7 pp 229533.I1RXB1
78448 229533.I1RSS7 pp 229533.I1RYU0
78449 229533.I1RSS7 pp 229533.I1S1W8
78450 229533.I1RSS7 pp 229533.I1S236
78451 229533.I1RSS7 pp 229533.I1RZM4
78452 229533.I1RSS7 pp 229533.I1S8E0
78453 229533.I1RSS7 pp 229533.I1S8C1
78454 229533.I1RSS7 pp 229533.I1RW71
78455 229533.I1RSS7 pp 229533.I1RYJ6
78456 229533.I1RSS7 pp 229533.I1RY49
78457 229533.I1RSS7 pp 229533.I1S3L2
78458 229533.I1RSS7 pp 229533.V6RH92
78459 229533.I1RM77 pp 229533.I1RWV4
78460 229533.I1RM77 pp 229533.I1RPD3
78461 229533.I1RM77 pp 229533.I1RQ61
78462 229533.I1RM77 pp 229533.V6QW25
78463 229533.I1RM77 pp 229533.I1RMG4
78464 229533.I1RM77 pp 229533.I1RN66
78465 229533.I1RZD3 pp 229533.I1S2A1
78466 229533.I1RZD3 pp 229533.Q4HXF6
78467 229533.I1RZD3 pp 229533.V6R7V8
78468 229533.I1RZD3 pp 229533.I1S235
78469 229533.I1RZD3 pp 229533.Q4I7T5
78470 229533.I1RZD3 pp 229533.Q4I624
78471 229533.I1RZD3 pp 229533.I1S2V2
78472 229533.A0A0E0S0E1 pp 229533.I1RPT4
78473 229533.A0A0E0S0E1 pp 229533.I1RU32
78474 229533.I1S1W3 pp 229533.I1S2B9
78475 229533.I1S1W3 pp 229533.V6QVH8

78476	229533.I1S1W3	pp	229533.I1S7W5
78477	229533.I1S1W3	pp	229533.Q4I624
78478	229533.I1S1W3	pp	229533.V6R042
78479	229533.I1S1W3	pp	229533.O74638
78480	229533.I1S1W3	pp	229533.I1S9Y1
78481	229533.I1S1W3	pp	229533.I1S237
78482	229533.I1S1W3	pp	229533.I1S6Y8
78483	229533.I1S1W3	pp	229533.Q4IMJ1
78484	229533.I1S1W3	pp	229533.I1S5R1
78485	229533.I1S1W3	pp	229533.V6R3J1
78486	229533.I1S1W3	pp	229533.V6RCZ8
78487	229533.I1S1W3	pp	229533.V6QUE6
78488	229533.I1S1W3	pp	229533.Q4IJK2
78489	229533.I1S1W3	pp	229533.I1S2H4
78490	229533.I1S1W3	pp	229533.Q4I2J8
78491	229533.I1S1W3	pp	229533.I1S419
78492	229533.I1S1W3	pp	229533.I1S384
78493	229533.I1S1W3	pp	229533.I1S5B0
78494	229533.I1S1W3	pp	229533.I1S6K9
78495	229533.I1S1W3	pp	229533.I1S338
78496	229533.I1S1W3	pp	229533.I1S236
78497	229533.I1S1W3	pp	229533.I1S4S7
78498	229533.I1S1W3	pp	229533.V6RTJ8
78499	229533.I1S1W3	pp	229533.V6RUT5
78500	229533.I1RS30	pp	229533.I1RT05
78501	229533.I1RS30	pp	229533.Q4HZI0
78502	229533.I1RS30	pp	229533.I1RT80
78503	229533.I1RS30	pp	229533.Q4IB65
78504	229533.I1RS30	pp	229533.V6QWX8
78505	229533.I1RS30	pp	229533.I1RTD7
78506	229533.I1RS30	pp	229533.I1RTF4
78507	229533.I1RS30	pp	229533.I1S8E2
78508	229533.I1RS30	pp	229533.Q4IJH1
78509	229533.I1RS30	pp	229533.I1RTD9
78510	229533.I1RS30	pp	229533.I1S9U2
78511	229533.I1RS30	pp	229533.I1RSG8
78512	229533.I1RS30	pp	229533.I1RSR8
78513	229533.I1RS30	pp	229533.I1S6U8
78514	229533.I1RS30	pp	229533.Q4HZK7
78515	229533.I1RS30	pp	229533.I1S9Y1
78516	229533.I1RS30	pp	229533.V6QYF0
78517	229533.I1RS30	pp	229533.I1RW90
78518	229533.I1RS30	pp	229533.I1S2C0
78519	229533.I1RS30	pp	229533.I1RZS6
78520	229533.I1RS30	pp	229533.I1RZB5
78521	229533.I1RS30	pp	229533.I1RX46
78522	229533.I1RS30	pp	229533.I1RVY0
78523	229533.I1RS30	pp	229533.Q4IFI0
78524	229533.I1RS30	pp	229533.I1RTE9
78525	229533.I1RS30	pp	229533.I1S268
78526	229533.I1RS30	pp	229533.I1S0R7
78527	229533.I1RS30	pp	229533.I1RWH5
78528	229533.I1RS30	pp	229533.I1S274
78529	229533.I1RS30	pp	229533.V6RGY3
78530	229533.I1RS30	pp	229533.V6R283
78531	229533.I1RS30	pp	229533.I1S1U7
78532	229533.I1RS30	pp	229533.I1RSR3
78533	229533.I1RS30	pp	229533.I1RUQ2
78534	229533.I1RS30	pp	229533.I1RWF7
78535	229533.I1RS30	pp	229533.V6RGN5
78536	229533.I1RS30	pp	229533.I1RW71
78537	229533.I1RS30	pp	229533.I1RX14
78538	229533.I1RS30	pp	229533.I1RSD8
78539	229533.I1RS30	pp	229533.Q4IQT8
78540	229533.I1RS30	pp	229533.I1RZ99
78541	229533.I1RS30	pp	229533.I1RZZ1
78542	229533.I1RS30	pp	229533.I1RSC6
78543	229533.I1RS30	pp	229533.I1S0J1
78544	229533.I1RS30	pp	229533.I1RZK6
78545	229533.I1RS30	pp	229533.Q4IPZ1
78546	229533.I1RS30	pp	229533.I1S0M9
78547	229533.I1RS30	pp	229533.I1RYB9
78548	229533.I1RS30	pp	229533.Q4IN63

78549 229533.I1RS30 pp 229533.Q4IJ56
78550 229533.I1RS30 pp 229533.I1RZF6
78551 229533.I1RS30 pp 229533.I1RSI6
78552 229533.I1RS30 pp 229533.Q4IEK8
78553 229533.I1RS30 pp 229533.I1S9U1
78554 229533.I1RS30 pp 229533.V6QUE6
78555 229533.I1RS30 pp 229533.I1S255
78556 229533.I1RS30 pp 229533.I1RTG6
78557 229533.I1RMB1 pp 229533.I1RWK9
78558 229533.I1RMB1 pp 229533.I1S2V2
78559 229533.I1RMB1 pp 229533.I1RPN8
78560 229533.I1RMB1 pp 229533.I1RQL4
78561 229533.I1RMB1 pp 229533.I1RWH4
78562 229533.I1RMB1 pp 229533.I1RQ21
78563 229533.I1RMB1 pp 229533.I1RYL5
78564 229533.I1RMB1 pp 229533.I1RW87
78565 229533.I1RMB1 pp 229533.I1RP66
78566 229533.I1RMB1 pp 229533.I1S235
78567 229533.I1RMR9 pp 229533.I1RYH7
78568 229533.I1RMR9 pp 229533.I1RRX5
78569 229533.I1RMR9 pp 229533.I1RS50
78570 229533.I1RMR9 pp 229533.V6R3J1
78571 229533.I1RMR9 pp 229533.I1RPR9
78572 229533.I1RMR9 pp 229533.I1RZ43
78573 229533.A0A0E0RLF6 pp 229533.Q4HTT2
78574 229533.A0A0E0RLF6 pp 229533.I1RBJ0
78575 229533.A0A0E0RLF6 pp 229533.I1RYV3
78576 229533.I1REG1 pp 229533.I1RW87
78577 229533.I1REG1 pp 229533.I1RM03
78578 229533.I1REG1 pp 229533.I1RYL5
78579 229533.I1REG1 pp 229533.I1RQA5
78580 229533.I1REG1 pp 229533.I1RP66
78581 229533.I1REG1 pp 229533.I1RQL4
78582 229533.I1REG1 pp 229533.I1S235
78583 229533.I1REG1 pp 229533.I1S2V2
78584 229533.I1REG1 pp 229533.I1RKK8
78585 229533.I1REG1 pp 229533.I1RQ21
78586 229533.I1REG1 pp 229533.I1RP28
78587 229533.I1REG1 pp 229533.I1RWK9
78588 229533.I1REG1 pp 229533.I1RWH4
78589 229533.I1REG1 pp 229533.I1RPN8
78590 229533.A0A0E0S067 pp 229533.I1RN32
78591 229533.A0A0E0S067 pp 229533.I1RMR9
78592 229533.I1RCP7 pp 229533.I1S268
78593 229533.I1RCP7 pp 229533.Q4HXF0
78594 229533.I1RCP7 pp 229533.I1S0H7
78595 229533.I1RCP7 pp 229533.I1S3V2
78596 229533.I1RCP7 pp 229533.I1RTG6
78597 229533.I1RCP7 pp 229533.I1S1W3
78598 229533.I1RCP7 pp 229533.I1S3H8
78599 229533.I1RCP7 pp 229533.I1RMC5
78600 229533.I1RCP7 pp 229533.I1RNG4
78601 229533.I1RCP7 pp 229533.I1RW78
78602 229533.I1RCP7 pp 229533.O74638
78603 229533.I1RCP7 pp 229533.Q4HTS9
78604 229533.I1RCP7 pp 229533.Q4IMJ1
78605 229533.I1RCP7 pp 229533.V6RSF3
78606 229533.I1RCP7 pp 229533.I1RTG7
78607 229533.I1RCP7 pp 229533.I1RNY6
78608 229533.I1RCP7 pp 229533.I1RMC2
78609 229533.I1RCP7 pp 229533.I1S3F7
78610 229533.I1RCP7 pp 229533.I1RIH0
78611 229533.I1RCP7 pp 229533.V6QVH8
78612 229533.I1RCP7 pp 229533.I1RZ78
78613 229533.I1RCP7 pp 229533.I1RMK1
78614 229533.I1RCP7 pp 229533.I1RVR2
78615 229533.I1RCP7 pp 229533.I1RXV9
78616 229533.I1RCP7 pp 229533.I1RYI7
78617 229533.I1RCP7 pp 229533.I1RW06
78618 229533.I1RCP7 pp 229533.I1RK47
78619 229533.I1RCP7 pp 229533.I1REI0
78620 229533.I1RCP7 pp 229533.I1S334
78621 229533.I1RCP7 pp 229533.I1RTU1

78622 229533.I1RCP7 pp 229533.I1RZK6
78623 229533.I1RCP7 pp 229533.I1RRL5
78624 229533.I1RCP7 pp 229533.I1RJT2
78625 229533.I1RCP7 pp 229533.V6RUT5
78626 229533.I1RPX9 pp 229533.I1RZH5
78627 229533.I1RPX9 pp 229533.Q4IBU4
78628 229533.I1RPX9 pp 229533.I1RT38
78629 229533.I1RPX9 pp 229533.I1RTF0
78630 229533.I1RBS5 pp 229533.I1RPL2
78631 229533.I1RBS5 pp 229533.I1RDH7
78632 229533.I1RBS5 pp 229533.I1S821
78633 229533.I1RBS5 pp 229533.I1RP66
78634 229533.I1RBS5 pp 229533.I1RYS6
78635 229533.I1RBS5 pp 229533.I1RKK8
78636 229533.I1RBS5 pp 229533.I1RWD7
78637 229533.I1RBS5 pp 229533.I1S235
78638 229533.I1RBS5 pp 229533.I1RBY7
78639 229533.I1RBS5 pp 229533.I1RW87
78640 229533.I1RBS5 pp 229533.I1RG60
78641 229533.I1RBS5 pp 229533.I1RQA5
78642 229533.I1RBS5 pp 229533.I1S3S5
78643 229533.I1RBS5 pp 229533.I1RW71
78644 229533.I1RBS5 pp 229533.I1RY63
78645 229533.I1RBS5 pp 229533.I1RYL5
78646 229533.I1RBS5 pp 229533.I1RQH1
78647 229533.I1RBS5 pp 229533.I1RRX5
78648 229533.I1RBS5 pp 229533.I1RQL4
78649 229533.I1RBS5 pp 229533.I1RRG3
78650 229533.I1RBS5 pp 229533.I1RPU8
78651 229533.I1RBS5 pp 229533.I1RM03
78652 229533.I1RBS5 pp 229533.I1RMQ5
78653 229533.I1RBS5 pp 229533.I1S0Z8
78654 229533.I1RBS5 pp 229533.I1RDE6
78655 229533.I1RBS5 pp 229533.I1S0F5
78656 229533.I1RBS5 pp 229533.I1RP86
78657 229533.I1RBS5 pp 229533.I1RTU8
78658 229533.I1RBS5 pp 229533.I1RPN8
78659 229533.I1RBS5 pp 229533.I1RRF8
78660 229533.I1RBS5 pp 229533.I1RRT6
78661 229533.I1RBS5 pp 229533.I1RF94
78662 229533.I1RBS5 pp 229533.I1RYU6
78663 229533.I1RBS5 pp 229533.I1RG11
78664 229533.I1RBS5 pp 229533.I1S7L6
78665 229533.I1RBS5 pp 229533.I1RDI8
78666 229533.I1RBS5 pp 229533.I1RBY6
78667 229533.I1RBS5 pp 229533.I1RSU2
78668 229533.I1RBS5 pp 229533.I1RJT9
78669 229533.I1RBS5 pp 229533.I1RM67
78670 229533.I1RBS5 pp 229533.I1RSY2
78671 229533.I1RBS5 pp 229533.I1RWH4
78672 229533.I1RBS5 pp 229533.I1RME4
78673 229533.I1RBS5 pp 229533.I1RPS2
78674 229533.I1RBS5 pp 229533.I1S247
78675 229533.I1RBS5 pp 229533.V6RLM7
78676 229533.I1RBS5 pp 229533.I1RZD3
78677 229533.I1RBS5 pp 229533.I1REB8
78678 229533.I1RBS5 pp 229533.I1RZK1
78679 229533.I1RBS5 pp 229533.I1RMQ3
78680 229533.I1RBS5 pp 229533.I1RU27
78681 229533.I1RBS5 pp 229533.I1RQ21
78682 229533.I1RBS5 pp 229533.I1RL79
78683 229533.I1RBS5 pp 229533.Q4HXF6
78684 229533.I1RBS5 pp 229533.I1RPV0
78685 229533.I1RBS5 pp 229533.I1RZS8
78686 229533.I1RBS5 pp 229533.I1RC55
78687 229533.I1RBS5 pp 229533.I1RTE9
78688 229533.I1RBS5 pp 229533.Q4I7G0
78689 229533.I1RBS5 pp 229533.I1RCG5
78690 229533.I1RBS5 pp 229533.Q4I624
78691 229533.I1RBS5 pp 229533.I1RZ95
78692 229533.I1RBS5 pp 229533.I1RHW9
78693 229533.I1RBS5 pp 229533.I1REG1
78694 229533.I1RBS5 pp 229533.I1RYP2

78695	229533.I1RBS5	pp	229533.I1RMB1
78696	229533.I1RBS5	pp	229533.I1RKB6
78697	229533.I1RBS5	pp	229533.I1REC7
78698	229533.I1RBS5	pp	229533.I1REC6
78699	229533.I1RBS5	pp	229533.I1RQG4
78700	229533.I1RBS5	pp	229533.I1RGA4
78701	229533.I1RBS5	pp	229533.I1RJZ7
78702	229533.I1RBS5	pp	229533.I1RC31
78703	229533.I1RBS5	pp	229533.I1RP28
78704	229533.I1RBS5	pp	229533.I1RSQ4
78705	229533.I1RBS5	pp	229533.I1RK77
78706	229533.I1RBS5	pp	229533.I1S2V2
78707	229533.I1RBS5	pp	229533.I1S0F9
78708	229533.I1RBS5	pp	229533.I1RC66
78709	229533.I1RBS5	pp	229533.O74638
78710	229533.I1RBS5	pp	229533.I1RWK9
78711	229533.A0A0E0RLL5	pp	229533.A0A1C3YJC0
78712	229533.A0A0E0RLL5	pp	229533.A0A1C3YM61
78713	229533.A0A0E0RLL5	pp	229533.I1RTA6
78714	229533.I1S0M3	pp	229533.Q4IJ56
78715	229533.I1S0M3	pp	229533.Q4IPI1
78716	229533.I1S0M3	pp	229533.Q4I327
78717	229533.I1S0M3	pp	229533.Q4IQW0
78718	229533.I1RX21	pp	229533.I1RZI8
78719	229533.I1RX21	pp	229533.Q4IK03
78720	229533.I1RX21	pp	229533.I1S8Y1
78721	229533.I1RX21	pp	229533.I1S0R9
78722	229533.I1RX21	pp	229533.Q4I6B0
78723	229533.I1S0Z8	pp	229533.I1S2V2
78724	229533.I1S0Z8	pp	229533.Q4I624
78725	229533.I1S0Z8	pp	229533.I1S235
78726	229533.I1S0Z8	pp	229533.V6QUV7
78727	229533.I1REK6	pp	229533.I1RWM6
78728	229533.I1REK6	pp	229533.I1RMB7
78729	229533.I1REK6	pp	229533.I1RYV1
78730	229533.I1REK6	pp	229533.I1RW71
78731	229533.I1REK6	pp	229533.I1RZT1
78732	229533.I1REK6	pp	229533.I1RF02
78733	229533.I1REK6	pp	229533.I1RW03
78734	229533.I1REK6	pp	229533.I1RJZ7
78735	229533.I1REK6	pp	229533.I1S2F2
78736	229533.A0A098D350	pp	229533.I1RQA5
78737	229533.A0A098D350	pp	229533.A0A098DRZ4
78738	229533.A0A098D350	pp	229533.I1RAM6
78739	229533.A0A098D350	pp	229533.A0A1C3YK11
78740	229533.A0A098D350	pp	229533.I1RTU8
78741	229533.A0A098D350	pp	229533.I1S3S5
78742	229533.A0A098D350	pp	229533.I1RF94
78743	229533.A0A098D350	pp	229533.I1RRT6
78744	229533.A0A098D350	pp	229533.I1RJT9
78745	229533.A0A098D350	pp	229533.I1RQH1
78746	229533.A0A098D350	pp	229533.I1RQL4
78747	229533.A0A098D350	pp	229533.I1RRF8
78748	229533.A0A098D350	pp	229533.A0A1C3YHZ0
78749	229533.A0A098D350	pp	229533.A0A098DN31
78750	229533.A0A098D350	pp	229533.I1RFY0
78751	229533.A0A098D350	pp	229533.I1RDH7
78752	229533.A0A098D350	pp	229533.I1RYL5
78753	229533.A0A098D350	pp	229533.I1RPL2
78754	229533.A0A098D350	pp	229533.I1RBY7
78755	229533.A0A098D350	pp	229533.A0A1C3YIK4
78756	229533.A0A098D350	pp	229533.I1RW71
78757	229533.A0A098D350	pp	229533.I1RB53
78758	229533.A0A098D350	pp	229533.I1RG60
78759	229533.A0A098D350	pp	229533.A0A1C3YLT3
78760	229533.A0A098D350	pp	229533.A0A098DRS5
78761	229533.A0A098D350	pp	229533.A0A0E0RVF4
78762	229533.A0A098D350	pp	229533.A0A098DQ41
78763	229533.A0A098D350	pp	229533.I1RT62
78764	229533.A0A098D350	pp	229533.I1RAB3
78765	229533.A0A098D350	pp	229533.I1RPK5
78766	229533.A0A098D350	pp	229533.I1S0F9
78767	229533.A0A098D350	pp	229533.I1RB59

78768 229533.A0A098D350 pp 229533.I1REC6
78769 229533.A0A098D350 pp 229533.I1R9Y8
78770 229533.A0A098D350 pp 229533.I1S821
78771 229533.A0A098D350 pp 229533.I1RJZ7
78772 229533.A0A098D350 pp 229533.I1RBY6
78773 229533.A0A098D350 pp 229533.I1REC7
78774 229533.A0A098D350 pp 229533.I1RMQ3
78775 229533.A0A098D350 pp 229533.I1RGC6
78776 229533.A0A098D350 pp 229533.I1RQG4
78777 229533.A0A098D350 pp 229533.A0A0E0SK74
78778 229533.A0A098D350 pp 229533.I1RZS8
78779 229533.A0A098D350 pp 229533.I1REB8
78780 229533.A0A098D350 pp 229533.I1RNG4
78781 229533.A0A098D350 pp 229533.I1RL79
78782 229533.A0A098D350 pp 229533.I1RPV0
78783 229533.A0A098D350 pp 229533.A0A1C3YI83
78784 229533.A0A098D350 pp 229533.I1RP28
78785 229533.A0A098D350 pp 229533.I1RBT8
78786 229533.A0A098D350 pp 229533.Q4I7G0
78787 229533.A0A098D350 pp 229533.I1RU27
78788 229533.A0A098D350 pp 229533.I1RME4
78789 229533.A0A098D350 pp 229533.I1RZK1
78790 229533.A0A098D350 pp 229533.I1RZD3
78791 229533.A0A098D350 pp 229533.I1RMB1
78792 229533.A0A098D350 pp 229533.I1REG1
78793 229533.A0A098D350 pp 229533.I1RMQ5
78794 229533.A0A098D350 pp 229533.I1RBS5
78795 229533.A0A098D350 pp 229533.I1S0Z8
78796 229533.A0A098E5I5 pp 229533.I1RWI6
78797 229533.A0A098E5I5 pp 229533.I1RPE5
78798 229533.I1RK25 pp 229533.I1RNH4
78799 229533.I1RK25 pp 229533.I1S224
78800 229533.I1RK25 pp 229533.I1RRM1
78801 229533.I1RK25 pp 229533.Q4I963
78802 229533.A0A1C3YHP3 pp 229533.I1S0N5
78803 229533.A0A1C3YHP3 pp 229533.I1RC80
78804 229533.A0A1C3YHP3 pp 229533.A0A1C3YM29
78805 229533.A0A1C3YHP3 pp 229533.I1RPU1
78806 229533.A0A1C3YHP3 pp 229533.I1S3R6
78807 229533.A0A1C3YHP3 pp 229533.V6RHA0
78808 229533.A0A1C3YHP3 pp 229533.I1RE51
78809 229533.A0A1C3YHP3 pp 229533.A0A1C3YI77
78810 229533.A0A1C3YHP3 pp 229533.I1RRD9
78811 229533.A0A1C3YHP3 pp 229533.V6R5G9
78812 229533.A0A1C3YHP3 pp 229533.Q4HTT2
78813 229533.A0A1C3YHP3 pp 229533.I1RC90
78814 229533.A0A1C3YHP3 pp 229533.Q4IB50
78815 229533.A0A1C3YHP3 pp 229533.A0A1C3YLA2
78816 229533.A0A1C3YHP3 pp 229533.I1RCZ0
78817 229533.A0A1C3YHP3 pp 229533.Q4I7X1
78818 229533.A0A1C3YHP3 pp 229533.A0A1C3YL18
78819 229533.A0A1C3YHP3 pp 229533.I1S7I5
78820 229533.A0A1C3YHP3 pp 229533.I1RCW8
78821 229533.A0A1C3YHP3 pp 229533.I1S0T8
78822 229533.A0A1C3YHP3 pp 229533.I1RAV3
78823 229533.A0A1C3YHP3 pp 229533.I1S0Z3
78824 229533.A0A1C3YHP3 pp 229533.I1RRF0
78825 229533.A0A1C3YHP3 pp 229533.I1RRP6
78826 229533.A0A1C3YHP3 pp 229533.I1RCS8
78827 229533.A0A1C3YHP3 pp 229533.Q4IER8
78828 229533.A0A1C3YHP3 pp 229533.A0A1C3YLS3
78829 229533.A0A1C3YHP3 pp 229533.I1RSN4
78830 229533.A0A1C3YHP3 pp 229533.I1RXX9
78831 229533.A0A1C3YHP3 pp 229533.I1RU02
78832 229533.A0A1C3YHP3 pp 229533.I1RED9
78833 229533.A0A1C3YHP3 pp 229533.Q4IPI4
78834 229533.A0A1C3YHP3 pp 229533.I1RQY1
78835 229533.A0A1C3YHP3 pp 229533.I1RSQ5
78836 229533.A0A1C3YHP3 pp 229533.I1RF01
78837 229533.A0A1C3YHP3 pp 229533.I1RFW1
78838 229533.A0A1C3YHP3 pp 229533.I1RMY4
78839 229533.A0A1C3YHP3 pp 229533.I1RT41
78840 229533.A0A1C3YHP3 pp 229533.I1RY90

78841 229533.A0A1C3YHP3 pp 229533.I1RAN8
78842 229533.A0A1C3YHP3 pp 229533.Q4HTT1
78843 229533.A0A1C3YHP3 pp 229533.I1S1V2
78844 229533.A0A1C3YHP3 pp 229533.I1RMU2
78845 229533.A0A1C3YHP3 pp 229533.I1RP56
78846 229533.A0A1C3YHP3 pp 229533.I1RB29
78847 229533.A0A1C3YHP3 pp 229533.A0A1C3YJR8
78848 229533.A0A1C3YHP3 pp 229533.I1RTI0
78849 229533.A0A1C3YHP3 pp 229533.I1RT16
78850 229533.A0A1C3YHP3 pp 229533.I1RPJ1
78851 229533.A0A1C3YHP3 pp 229533.I1RYN7
78852 229533.A0A1C3YHP3 pp 229533.A0A1C3YJU1
78853 229533.A0A1C3YHP3 pp 229533.I1RYC1
78854 229533.I1RR29 pp 229533.I1RSX9
78855 229533.I1RR29 pp 229533.I1RXS0
78856 229533.I1RR29 pp 229533.I1S8R4
78857 229533.I1RR29 pp 229533.I1S0K0
78858 229533.I1RR29 pp 229533.I1S9T6
78859 229533.I1RR29 pp 229533.I1S673
78860 229533.I1RR29 pp 229533.I1RZM4
78861 229533.I1RR29 pp 229533.I1RVR2
78862 229533.I1RR29 pp 229533.I1S3L2
78863 229533.I1RR29 pp 229533.V6RK96
78864 229533.I1S0R4 pp 229533.I1SAN9
78865 229533.I1S0R4 pp 229533.Q4I1Y5
78866 229533.I1RLV7 pp 229533.I1S5X9
78867 229533.I1RLV7 pp 229533.I1RS50
78868 229533.I1RLV7 pp 229533.I1S056
78869 229533.I1RLV7 pp 229533.I1S8Y0
78870 229533.I1RLV7 pp 229533.I1S8Y1
78871 229533.I1RLV7 pp 229533.Q4IPZ1
78872 229533.A0A1C3YLA2 pp 229533.I1RT16
78873 229533.A0A1C3YLA2 pp 229533.I1RRP6
78874 229533.A0A1C3YLA2 pp 229533.I1RPJ1
78875 229533.A0A1C3YLA2 pp 229533.A0A1C3YLC6
78876 229533.A0A1C3YLA2 pp 229533.I1RCS8
78877 229533.A0A1C3YLA2 pp 229533.I1RTI0
78878 229533.A0A1C3YLA2 pp 229533.I1RYN7
78879 229533.A0A1C3YLA2 pp 229533.I1RMU2
78880 229533.A0A1C3YLA2 pp 229533.I1S7I5
78881 229533.A0A1C3YLA2 pp 229533.I1RP56
78882 229533.A0A1C3YLA2 pp 229533.Q4IMD1
78883 229533.A0A1C3YLA2 pp 229533.I1RFW1
78884 229533.A0A1C3YLA2 pp 229533.Q4I7L0
78885 229533.A0A1C3YLA2 pp 229533.I1RB29
78886 229533.A0A1C3YLA2 pp 229533.I1RSN4
78887 229533.A0A1C3YLA2 pp 229533.A0A1C3YMC9
78888 229533.A0A1C3YLA2 pp 229533.I1S270
78889 229533.A0A1C3YLA2 pp 229533.I1RMY4
78890 229533.A0A1C3YLA2 pp 229533.Q4HTT1
78891 229533.A0A1C3YLA2 pp 229533.I1RTH9
78892 229533.A0A1C3YLA2 pp 229533.I1RF01
78893 229533.A0A1C3YLA2 pp 229533.I1RSQ5
78894 229533.A0A1C3YLA2 pp 229533.I1RCW8
78895 229533.A0A1C3YLA2 pp 229533.I1S0Z3
78896 229533.A0A1C3YLA2 pp 229533.I1S1V2
78897 229533.A0A1C3YLA2 pp 229533.I1RAV3
78898 229533.A0A1C3YLA2 pp 229533.I1RYC1
78899 229533.A0A1C3YLA2 pp 229533.I1RQY1
78900 229533.A0A1C3YLA2 pp 229533.Q4I7X1
78901 229533.A0A1C3YLA2 pp 229533.I1RKR9
78902 229533.A0A1C3YLA2 pp 229533.A0A1C3YM21
78903 229533.A0A1C3YLA2 pp 229533.Q4I7S1
78904 229533.A0A1C3YLA2 pp 229533.I1RRN5
78905 229533.A0A1C3YLA2 pp 229533.I1RC80
78906 229533.A0A1C3YLA2 pp 229533.I1S0N5
78907 229533.A0A1C3YLA2 pp 229533.Q4HTT2
78908 229533.A0A1C3YLA2 pp 229533.Q4IPI4
78909 229533.A0A1C3YLA2 pp 229533.I1RY90
78910 229533.A0A1C3YLA2 pp 229533.I1RPU1
78911 229533.A0A1C3YLA2 pp 229533.I1RU02
78912 229533.A0A1C3YLA2 pp 229533.I1RRD9
78913 229533.A0A1C3YLA2 pp 229533.I1RCN2

78914 229533.A0A1C3YLA2 pp 229533.I1RE51
78915 229533.A0A1C3YLA2 pp 229533.I1RB73
78916 229533.A0A1C3YLA2 pp 229533.I1RT41
78917 229533.A0A1C3YLA2 pp 229533.Q4IER8
78918 229533.A0A1C3YLA2 pp 229533.V6RHA0
78919 229533.A0A1C3YLA2 pp 229533.I1RRF0
78920 229533.A0A1C3YLA2 pp 229533.I1RT65
78921 229533.A0A1C3YLA2 pp 229533.I1RC90
78922 229533.A0A1C3YLA2 pp 229533.I1RED9
78923 229533.A0A1C3YLA2 pp 229533.I1S3R6
78924 229533.A0A1C3YLA2 pp 229533.I1RKI1
78925 229533.A0A1C3YLA2 pp 229533.I1RXX9
78926 229533.A0A1C3YLA2 pp 229533.I1S0T8
78927 229533.A0A1C3YLA2 pp 229533.I1RCZ0
78928 229533.A0A1C3YLA2 pp 229533.V6R5G9
78929 229533.A0A1C3YLA2 pp 229533.I1RMS2
78930 229533.A0A1C3YLA2 pp 229533.I1RST4
78931 229533.I1RZU9 pp 229533.V6QUE6
78932 229533.I1RZU9 pp 229533.Q4HYQ4
78933 229533.I1RZU9 pp 229533.Q4HY90
78934 229533.I1RZU9 pp 229533.Q4IAK7
78935 229533.I1RZU9 pp 229533.Q4IJ11
78936 229533.I1RZU9 pp 229533.Q4IMD1
78937 229533.I1RZU9 pp 229533.Q4ICA8
78938 229533.I1RZU9 pp 229533.Q4I948
78939 229533.I1RZU9 pp 229533.Q4I5W5
78940 229533.I1RZU9 pp 229533.Q4I5V3
78941 229533.I1RZU9 pp 229533.Q4IL82
78942 229533.I1RZU9 pp 229533.Q4IPI4
78943 229533.I1RZU9 pp 229533.V6QWX8
78944 229533.I1RZU9 pp 229533.I1SB04
78945 229533.Q4IPI4 pp 229533.V6R5G9
78946 229533.Q4IPI4 pp 229533.Q5I2J3
78947 229533.Q4IPI4 pp 229533.Q4IQX3
78948 229533.Q4IPI4 pp 229533.V6RHA0
78949 229533.Q4IPI4 pp 229533.V6RB81
78950 229533.I1RIP9 pp 229533.I1RMB7
78951 229533.I1RIP9 pp 229533.I1RZT1
78952 229533.I1RIP9 pp 229533.I1RW03
78953 229533.I1RIP9 pp 229533.I1S2F2
78954 229533.I1RIP9 pp 229533.I1RJZ7
78955 229533.I1RIP9 pp 229533.I1RWM6
78956 229533.I1RIP9 pp 229533.I1RW71
78957 229533.I1RIP9 pp 229533.I1RYV1
78958 229533.I1S749 pp 229533.I1S9T6
78959 229533.I1S7I5 pp 229533.V6R5G9
78960 229533.I1S7I5 pp 229533.Q4HTT2
78961 229533.I1S7I5 pp 229533.Q4HTT1
78962 229533.I1S7I5 pp 229533.V6RHA0
78963 229533.I1RX14 pp 229533.I1S4M5
78964 229533.I1RX14 pp 229533.Q4IEV5
78965 229533.I1RX14 pp 229533.Q4HZ42
78966 229533.I1RX14 pp 229533.I1RZS6
78967 229533.I1RX14 pp 229533.V6RGN5
78968 229533.I1RX14 pp 229533.Q4IAS1
78969 229533.I1RX14 pp 229533.I1S0J1
78970 229533.I1RX14 pp 229533.Q4IQW0
78971 229533.I1RX14 pp 229533.Q4I327
78972 229533.I1RX14 pp 229533.Q4IAA0
78973 229533.I1RX14 pp 229533.I1S0M9
78974 229533.I1RX14 pp 229533.I1RX24
78975 229533.I1RX14 pp 229533.Q4IF76
78976 229533.I1RX14 pp 229533.I1S1B6
78977 229533.I1RX14 pp 229533.I1S1X9
78978 229533.I1RX14 pp 229533.Q4IJ56
78979 229533.I1RX14 pp 229533.I1S0M3
78980 229533.I1RX14 pp 229533.I1S4T3
78981 229533.I1RX14 pp 229533.V6RGY0
78982 229533.I1RX14 pp 229533.I1RZ99
78983 229533.I1RX14 pp 229533.V6QUE6
78984 229533.I1RX14 pp 229533.Q4IBS2
78985 229533.I1RX14 pp 229533.Q4HZ47
78986 229533.I1RX14 pp 229533.V6QYF0

229533.I1RX14	pp	229533.Q4IPI1
229533.I1RX14	pp	229533.Q4IJH1
229533.I1RX14	pp	229533.I1S0R7
229533.I1RX14	pp	229533.I1S9U2
229533.I1RX14	pp	229533.I1S021
229533.I1RX14	pp	229533.I1RZE5
229533.I1RX14	pp	229533.I1S2U2
229533.I1RX14	pp	229533.I1RZB5
229533.I1RX14	pp	229533.I1RZF6
229533.I1RX14	pp	229533.Q4I5Z5
229533.I1RX14	pp	229533.I1S9U1
229533.I1S0R3	pp	229533.Q4I1Y5
229533.I1S0R3	pp	229533.I1SAN9
229533.I1RZM9	pp	229533.Q4I5G1
229533.A0A1C3YLC6	pp	229533.V6R5G9
229533.A0A1C3YLC6	pp	229533.I1RE51
229533.A0A1C3YLC6	pp	229533.I1S3R6
229533.A0A1C3YLC6	pp	229533.V6RHA0
229533.A0A1C3YLC6	pp	229533.I1RAV3
229533.A0A1C3YLC6	pp	229533.I1RMY4
229533.A0A1C3YLC6	pp	229533.I1S0Z3
229533.A0A1C3YLC6	pp	229533.I1RRD9
229533.A0A1C3YLC6	pp	229533.Q4HTT2
229533.A0A1C3YLC6	pp	229533.Q4I7X1
229533.A0A1C3YLC6	pp	229533.I1RB29
229533.A0A1C3YLC6	pp	229533.I1RUM5
229533.A0A1C3YLC6	pp	229533.I1RMU2
229533.A0A1C3YLC6	pp	229533.I1RCW8
229533.A0A1C3YLC6	pp	229533.I1RCS8
229533.A0A1C3YLC6	pp	229533.I1RP56
229533.A0A1C3YLC6	pp	229533.A0A1C3YM29
229533.A0A1C3YLC6	pp	229533.I1RPU1
229533.A0A1C3YLC6	pp	229533.I1RRP6
229533.A0A1C3YLC6	pp	229533.I1RYN7
229533.A0A1C3YLC6	pp	229533.A0A1C3YLS3
229533.A0A1C3YLC6	pp	229533.I1RPJ1
229533.A0A1C3YLC6	pp	229533.I1S0N5
229533.A0A1C3YLC6	pp	229533.I1RT16
229533.A0A1C3YLC6	pp	229533.I1S7I5
229533.A0A1C3YLC6	pp	229533.I1RC80
229533.A0A1C3YLC6	pp	229533.I1RCZ0
229533.A0A1C3YLC6	pp	229533.I1RED9
229533.A0A1C3YLC6	pp	229533.I1RTI0
229533.A0A1C3YLC6	pp	229533.I1RT41
229533.A0A1C3YLC6	pp	229533.I1RXX9
229533.A0A1C3YLC6	pp	229533.I1RRF0
229533.A0A1C3YLC6	pp	229533.I1S0T8
229533.A0A1C3YLC6	pp	229533.I1RC90
229533.A0A1C3YLC6	pp	229533.Q4IPI4
229533.A0A1C3YLC6	pp	229533.I1RY90
229533.A0A1C3YLC6	pp	229533.Q4IER8
229533.A0A1C3YLC6	pp	229533.Q4IB50
229533.A0A1C3YLC6	pp	229533.I1RYC1
229533.A0A1C3YLC6	pp	229533.I1RU02
229533.A0A1C3YLC6	pp	229533.I1RQY1
229533.A0A1C3YLC6	pp	229533.I1S1V2
229533.A0A1C3YLC6	pp	229533.Q4HTT1
229533.A0A1C3YLC6	pp	229533.I1RF01
229533.A0A1C3YLC6	pp	229533.I1RSQ5
229533.A0A1C3YLC6	pp	229533.I1RFW1
229533.A0A1C3YLC6	pp	229533.I1RSN4
229533.A0A1C3YLC6	pp	229533.I1RAN8
229533.A0A1C3YKV6	pp	229533.I1S182
229533.I1RBE2	pp	229533.Q4IN52
229533.I1RBE2	pp	229533.I1RTI6
229533.I1RBE2	pp	229533.I1RSU2
229533.I1RBE2	pp	229533.Q4I1M9
229533.I1RB66	pp	229533.I1RB68
229533.A0A098E4D4	pp	229533.I1S3H8
229533.A0A098E4D4	pp	229533.Q4IMJ1
229533.A0A098E4D4	pp	229533.I1RNG4
229533.A0A098E4D4	pp	229533.I1S0H7
229533.A0A098E4D4	pp	229533.V6QVH8

79060	229533.A0A098E4D4	pp	229533.I1R9U7
79061	229533.A0A098E4D4	pp	229533.I1RCP7
79062	229533.A0A098E4D4	pp	229533.I1RIH0
79063	229533.A0A098E4D4	pp	229533.Q4HXF0
79064	229533.A0A098E4D4	pp	229533.I1S334
79065	229533.A0A098E4D4	pp	229533.Q4HTS9
79066	229533.A0A098E4D4	pp	229533.I1RW06
79067	229533.A0A098E4D4	pp	229533.I1REI0
79068	229533.A0A098E4D4	pp	229533.I1RJT2
79069	229533.A0A098E4D4	pp	229533.I1RRL5
79070	229533.A0A098E4D4	pp	229533.I1RTU1
79071	229533.A0A098E4D4	pp	229533.I1RFB4
79072	229533.A0A098E4D4	pp	229533.I1RK47
79073	229533.A0A098E4D4	pp	229533.I1RBE4
79074	229533.I1RYV1	pp	229533.I1S2F2
79075	229533.I1RAN8	pp	229533.I1RCS8
79076	229533.I1RAN8	pp	229533.I1RN31
79077	229533.I1RAN8	pp	229533.I1RB29
79078	229533.I1RAN8	pp	229533.I1RQY1
79079	229533.I1RAN8	pp	229533.I1RT41
79080	229533.I1RAN8	pp	229533.I1S270
79081	229533.I1RAN8	pp	229533.I1RPJ1
79082	229533.I1RAN8	pp	229533.I1RT16
79083	229533.I1RAN8	pp	229533.I1S1V2
79084	229533.I1RAN8	pp	229533.I1RED9
79085	229533.I1RAN8	pp	229533.I1RLX6
79086	229533.I1RAN8	pp	229533.I1RMU2
79087	229533.I1RAN8	pp	229533.I1RYN7
79088	229533.I1RAN8	pp	229533.I1RMY4
79089	229533.I1RAN8	pp	229533.I1RT65
79090	229533.I1RAN8	pp	229533.I1RYC1
79091	229533.I1RAN8	pp	229533.I1S0T8
79092	229533.I1RAN8	pp	229533.I1RPU1
79093	229533.I1RAN8	pp	229533.Q4I7L0
79094	229533.I1RAN8	pp	229533.I1RZU9
79095	229533.I1RAN8	pp	229533.I1RRD9
79096	229533.I1RAN8	pp	229533.I1RU02
79097	229533.I1RAN8	pp	229533.V6RHA0
79098	229533.I1RAN8	pp	229533.I1RE51
79099	229533.I1RAN8	pp	229533.I1RQ93
79100	229533.I1RAN8	pp	229533.I1RJQ5
79101	229533.I1RAN8	pp	229533.I1S3R6
79102	229533.I1RAN8	pp	229533.I1SAA0
79103	229533.I1RAN8	pp	229533.Q4IER8
79104	229533.I1RAN8	pp	229533.V6R5G9
79105	229533.I1RAN8	pp	229533.Q4I7X1
79106	229533.I1RAN8	pp	229533.I1RKR9
79107	229533.I1RAN8	pp	229533.I1RSN4
79108	229533.I1RAN8	pp	229533.I1RB73
79109	229533.I1RAN8	pp	229533.I1RC90
79110	229533.I1RAN8	pp	229533.Q4IPI4
79111	229533.I1RAN8	pp	229533.I1RKI1
79112	229533.I1RAN8	pp	229533.I1RY77
79113	229533.I1RAN8	pp	229533.I1RXX9
79114	229533.I1RAN8	pp	229533.Q4HTT2
79115	229533.I1RAN8	pp	229533.I1RY90
79116	229533.I1RAN8	pp	229533.I1RRP6
79117	229533.I1RAN8	pp	229533.I1RCN2
79118	229533.I1RAN8	pp	229533.I1RCW8
79119	229533.I1RAN8	pp	229533.I1S7I5
79120	229533.I1RAN8	pp	229533.I1S0Z3
79121	229533.I1RAN8	pp	229533.I1RP56
79122	229533.I1RAN8	pp	229533.I1RVY0
79123	229533.I1RAN8	pp	229533.Q4IMD1
79124	229533.I1RAN8	pp	229533.I1RFW1
79125	229533.I1RAN8	pp	229533.Q4HTT1
79126	229533.I1RAN8	pp	229533.I1RF01
79127	229533.I1RAN8	pp	229533.I1RSQ5
79128	229533.I1RAN8	pp	229533.I1RST4
79129	229533.I1RAN8	pp	229533.I1RTI0
79130	229533.I1RAN8	pp	229533.I1RRF0
79131	229533.I1RAN8	pp	229533.I1RY99
79132	229533.I1RAN8	pp	229533.I1RAV3

79133 229533.I1RAN8 pp 229533.I1RCZ0
79134 229533.I1RAN8 pp 229533.I1RW16
79135 229533.I1RAN8 pp 229533.Q4I7S1
79136 229533.I1RAN8 pp 229533.I1S4Z5
79137 229533.I1RAN8 pp 229533.I1RTH9
79138 229533.I1RAN8 pp 229533.I1RSL8
79139 229533.I1RNP8 pp 229533.Q4IJW4
79140 229533.I1RNP8 pp 229533.I1RT38
79141 229533.I1RNP8 pp 229533.Q4IPZ1
79142 229533.I1RNP8 pp 229533.Q4IB65
79143 229533.I1RNP8 pp 229533.Q4I6B0
79144 229533.I1RNP8 pp 229533.Q4I099
79145 229533.I1RNP8 pp 229533.Q4IK03
79146 229533.I1REK9 pp 229533.I1RX25
79147 229533.I1REK9 pp 229533.I1RQ41
79148 229533.I1REK9 pp 229533.I1RRC6
79149 229533.I1REK9 pp 229533.I1RXF0
79150 229533.I1REK9 pp 229533.I1S1V1
79151 229533.A0A1C3YID6 pp 229533.I1RYU6
79152 229533.A0A1C3YID6 pp 229533.A0A1C3YLB6
79153 229533.A0A1C3YID6 pp 229533.I1RE52
79154 229533.A0A1C3YID6 pp 229533.I1RBE2
79155 229533.A0A1C3YID6 pp 229533.A0A1C3YN84
79156 229533.A0A1C3YID6 pp 229533.Q4IN52
79157 229533.I1RV78 pp 229533.I1S2F2
79158 229533.I1RV78 pp 229533.I1RWM6
79159 229533.I1RV78 pp 229533.I1RW03
79160 229533.I1RV78 pp 229533.I1RZT1
79161 229533.I1RV78 pp 229533.I1RW71
79162 229533.I1RV78 pp 229533.I1RYV1
79163 229533.Q4IN52 pp 229533.V6RM66
79164 229533.I1S1V2 pp 229533.Q4HTT1
79165 229533.I1S1V2 pp 229533.Q4IPI4
79166 229533.I1S1V2 pp 229533.Q4I7X1
79167 229533.I1S1V2 pp 229533.I1S270
79168 229533.I1S1V2 pp 229533.V6R5G9
79169 229533.I1S1V2 pp 229533.Q4HTT2
79170 229533.I1S1V2 pp 229533.I1S4Z5
79171 229533.I1S1V2 pp 229533.Q4IER8
79172 229533.I1S1V2 pp 229533.I1S1V3
79173 229533.I1S1V2 pp 229533.I1S3R6
79174 229533.I1S1V2 pp 229533.V6RHA0
79175 229533.I1S255 pp 229533.V6QWX8
79176 229533.I1S255 pp 229533.I1S9Y1
79177 229533.I1S255 pp 229533.V6QUE6
79178 229533.I1S255 pp 229533.I1S9U1
79179 229533.I1RWX7 pp 229533.I1S2F2
79180 229533.I1RWX7 pp 229533.I1RZT1
79181 229533.I1RWX7 pp 229533.I1RYV1
79182 229533.I1RL40 pp 229533.I1RNG4
79183 229533.A0A098D332 pp 229533.A0A1C3YHP3
79184 229533.A0A098D332 pp 229533.A0A1C3YLA2
79185 229533.A0A098D332 pp 229533.Q4IPI4
79186 229533.A0A098D332 pp 229533.I1S7I5
79187 229533.A0A098D332 pp 229533.A0A1C3YLC6
79188 229533.A0A098D332 pp 229533.I1RE51
79189 229533.A0A098D332 pp 229533.I1RAN8
79190 229533.A0A098D332 pp 229533.I1S1V2
79191 229533.A0A1C3YJC8 pp 229533.I1S182
79192 229533.I1RE92 pp 229533.I1RFI3
79193 229533.I1RE92 pp 229533.I1RPI4
79194 229533.I1RE92 pp 229533.I1RIG4
79195 229533.I1RE92 pp 229533.I1RFI5
79196 229533.I1RE92 pp 229533.I1RKR7
79197 229533.I1RE92 pp 229533.I1RXU0
79198 229533.I1RE92 pp 229533.V6RXW9
79199 229533.I1RVZ4 pp 229533.I1RZB5
79200 229533.I1RVZ4 pp 229533.V6QWX8
79201 229533.I1RVZ4 pp 229533.I1RX64
79202 229533.I1RVZ4 pp 229533.I1S276
79203 229533.I1RVZ4 pp 229533.I1RW90
79204 229533.I1RVZ4 pp 229533.V6QUE6
79205 229533.I1RVZ4 pp 229533.Q4I613

79206	229533.I1RVZ4	pp	229533.I1S0Z9
79207	229533.I1RVZ4	pp	229533.Q4I5U9
79208	229533.I1RVZ4	pp	229533.Q4I5I4
79209	229533.I1RC06	pp	229533.I1RU69
79210	229533.I1RC06	pp	229533.I1RMK3
79211	229533.I1RC06	pp	229533.Q4HVW2
79212	229533.I1RC06	pp	229533.I1RQM6
79213	229533.I1RC06	pp	229533.I1RZJ6
79214	229533.I1RC06	pp	229533.I1RDA0
79215	229533.I1RC06	pp	229533.I1RT80
79216	229533.I1RC06	pp	229533.I1S8E2
79217	229533.I1RC06	pp	229533.I1RT98
79218	229533.I1RC06	pp	229533.I1S0R7
79219	229533.I1RC06	pp	229533.Q4IJH1
79220	229533.I1RC06	pp	229533.I1RUQ2
79221	229533.I1RC06	pp	229533.I1RTF4
79222	229533.I1RC06	pp	229533.Q4IPI1
79223	229533.I1RC06	pp	229533.I1RKJ6
79224	229533.I1RC06	pp	229533.I1RM67
79225	229533.I1RC06	pp	229533.I1RJL4
79226	229533.I1RC06	pp	229533.I1S9U2
79227	229533.I1RC06	pp	229533.I1RQS5
79228	229533.I1RC06	pp	229533.I1S9Y0
79229	229533.I1RC06	pp	229533.I1RRT2
79230	229533.I1RC06	pp	229533.I1RFS3
79231	229533.I1RC06	pp	229533.Q4I662
79232	229533.I1RC06	pp	229533.I1RKQ5
79233	229533.I1RC06	pp	229533.I1RGC9
79234	229533.I1RC06	pp	229533.Q4IAS1
79235	229533.I1RC06	pp	229533.I1RZS6
79236	229533.I1RC06	pp	229533.I1RMN6
79237	229533.I1RC06	pp	229533.I1RC27
79238	229533.I1RC06	pp	229533.I1RLV9
79239	229533.I1RC06	pp	229533.I1RX14
79240	229533.I1RC06	pp	229533.I1RM06
79241	229533.I1RC06	pp	229533.I1RC72
79242	229533.I1RC06	pp	229533.I1RRQ6
79243	229533.I1RC06	pp	229533.I1RC82
79244	229533.I1RC06	pp	229533.Q4IQW0
79245	229533.I1RC06	pp	229533.I1S060
79246	229533.I1RC06	pp	229533.I1RFN1
79247	229533.I1RC06	pp	229533.Q4IAA0
79248	229533.I1RC06	pp	229533.I1RHM4
79249	229533.I1RC06	pp	229533.V6QYF0
79250	229533.I1RC06	pp	229533.V6R283
79251	229533.I1RC06	pp	229533.I1RNA0
79252	229533.I1RC06	pp	229533.I1RFW5
79253	229533.I1RC06	pp	229533.I1RRJ8
79254	229533.I1RC06	pp	229533.I1RX24
79255	229533.I1RC06	pp	229533.I1RNU6
79256	229533.I1RC06	pp	229533.I1RM25
79257	229533.I1RC06	pp	229533.I1RN51
79258	229533.I1RC06	pp	229533.I1RC33
79259	229533.I1RC06	pp	229533.Q4IEK8
79260	229533.I1RC06	pp	229533.Q4IJ56
79261	229533.I1RC06	pp	229533.I1RR00
79262	229533.I1RC06	pp	229533.I1RQJ5
79263	229533.I1RC06	pp	229533.Q4IFI0
79264	229533.I1RC06	pp	229533.I1RKL3
79265	229533.I1RC06	pp	229533.Q4I830
79266	229533.I1RC06	pp	229533.I1RPY1
79267	229533.I1RC06	pp	229533.I1S021
79268	229533.I1RC06	pp	229533.I1S6F0
79269	229533.I1RC06	pp	229533.I1RNH6
79270	229533.I1RC06	pp	229533.I1S4T3
79271	229533.I1RC06	pp	229533.I1RRR7
79272	229533.I1RC06	pp	229533.I1RW26
79273	229533.I1RC06	pp	229533.Q4HZ47
79274	229533.I1RC06	pp	229533.V6R227
79275	229533.I1RC06	pp	229533.I1RNY2
79276	229533.I1RC06	pp	229533.Q4I327
79277	229533.I1RC06	pp	229533.Q4IEV5
79278	229533.I1RC06	pp	229533.I1RQ65

79279	229533.I1RC06	pp	229533.Q4HZ42
79280	229533.I1RC06	pp	229533.Q4IN63
79281	229533.I1RC06	pp	229533.I1RQ31
79282	229533.I1RC06	pp	229533.I1RRI2
79283	229533.I1RC06	pp	229533.I1RTD9
79284	229533.I1RC06	pp	229533.I1RD56
79285	229533.I1RC06	pp	229533.I1RMC0
79286	229533.I1RC06	pp	229533.I1RMD5
79287	229533.I1RC06	pp	229533.I1RSI6
79288	229533.I1RC06	pp	229533.I1S2C0
79289	229533.I1RC06	pp	229533.I1RZZ1
79290	229533.I1RC06	pp	229533.I1RN99
79291	229533.I1RC06	pp	229533.I1RTE9
79292	229533.I1RC06	pp	229533.I1RQ28
79293	229533.I1RC06	pp	229533.Q4IBS2
79294	229533.I1RC06	pp	229533.I1RPJ3
79295	229533.I1RC06	pp	229533.I1S1X9
79296	229533.I1RC06	pp	229533.I1RCL3
79297	229533.I1RC06	pp	229533.I1RQA2
79298	229533.I1RC06	pp	229533.I1RZ59
79299	229533.I1RC06	pp	229533.I1RFJ9
79300	229533.I1RC06	pp	229533.I1RF19
79301	229533.I1RC06	pp	229533.V6RGY3
79302	229533.I1RC06	pp	229533.I1RCN5
79303	229533.I1RC06	pp	229533.Q4I5Z5
79304	229533.I1RC06	pp	229533.I1RSM3
79305	229533.I1RC06	pp	229533.I1RE00
79306	229533.I1RC06	pp	229533.I1RKC6
79307	229533.I1RC06	pp	229533.I1RVZ0
79308	229533.I1RC06	pp	229533.I1RJZ6
79309	229533.I1RC06	pp	229533.Q4HZ68
79310	229533.I1RC06	pp	229533.I1RF08
79311	229533.I1RJT2	pp	229533.Q4IMJ1
79312	229533.I1RJT2	pp	229533.I1S334
79313	229533.I1RJT2	pp	229533.Q4HTS9
79314	229533.I1RJT2	pp	229533.I1RTU1
79315	229533.I1RJT2	pp	229533.I1RW06
79316	229533.I1RJT2	pp	229533.I1RK47
79317	229533.I1RJT2	pp	229533.Q4HXF0
79318	229533.I1RJT2	pp	229533.V6QVH8
79319	229533.I1RJT2	pp	229533.I1RRL5
79320	229533.I1RJT2	pp	229533.I1RUP9
79321	229533.I1RJT2	pp	229533.I1S0H7
79322	229533.I1RJT2	pp	229533.I1S3H8
79323	229533.I1RJT2	pp	229533.I1RNG4
79324	229533.I1RQP7	pp	229533.V6QXP4
79325	229533.I1RQP7	pp	229533.I1RZK3
79326	229533.I1RQP7	pp	229533.I1RWZ9
79327	229533.I1RQP7	pp	229533.I1RY40
79328	229533.I1RQP7	pp	229533.I1RXT7
79329	229533.I1RQP7	pp	229533.I1S4N5
79330	229533.I1RQP7	pp	229533.Q4I6S5
79331	229533.I1RQP7	pp	229533.I1RYP0
79332	229533.I1RQP7	pp	229533.I1SAM5
79333	229533.I1RQP7	pp	229533.I1S287
79334	229533.I1RQP7	pp	229533.V6RR76
79335	229533.I1RQP7	pp	229533.I1S0Y1
79336	229533.I1RQP7	pp	229533.Q4HY20
79337	229533.I1RQP7	pp	229533.I1RTQ9
79338	229533.I1RQP7	pp	229533.I1S0V5
79339	229533.I1RQP7	pp	229533.Q4I1B1
79340	229533.I1RQP7	pp	229533.I1RSH3
79341	229533.I1RQP7	pp	229533.I1RX87
79342	229533.I1RQP7	pp	229533.I1RW76
79343	229533.I1RQP7	pp	229533.I1S6S4
79344	229533.I1RQP7	pp	229533.I1RTF2
79345	229533.I1RQP7	pp	229533.I1S2B6
79346	229533.I1RQP7	pp	229533.I1RUN9
79347	229533.I1RQP7	pp	229533.I1S0K5
79348	229533.I1RQP7	pp	229533.I1RZS5
79349	229533.I1RQP7	pp	229533.I1RS34
79350	229533.I1RQP7	pp	229533.I1S830
79351	229533.I1RQP7	pp	229533.I1S1V4

229533.I1RQP7 pp 229533.I1RSJ0
229533.I1RQP7 pp 229533.I1S9X9
229533.I1RQP7 pp 229533.I1RXF5
229533.I1RQP7 pp 229533.I1S278
229533.I1RQP7 pp 229533.I1S051
229533.I1RQP7 pp 229533.V6RXW9
229533.I1RQP7 pp 229533.I1S1V7
229533.I1RQP7 pp 229533.Q4I327
229533.I1RQP7 pp 229533.I1RRC9
229533.I1RQP7 pp 229533.I1S1U6
229533.I1RQP7 pp 229533.I1RRC8
229533.I1RQP7 pp 229533.I1RZ84
229533.I1RQP7 pp 229533.Q4I1H6
229533.I1RFJ1 pp 229533.I1RM55
229533.I1RFJ1 pp 229533.I1RMR8
229533.I1RFJ1 pp 229533.I1RMC5
229533.I1RFJ1 pp 229533.I1RMC2
229533.I1RFJ1 pp 229533.I1RSQ4
229533.I1RFJ1 pp 229533.I1S3F7
229533.I1RFJ1 pp 229533.I1RSX8
229533.I1RFJ1 pp 229533.I1RUJ0
229533.I1RFJ1 pp 229533.I1RJU6
229533.I1RFJ1 pp 229533.I1RSX7
229533.I1S2U2 pp 229533.I1S9U2
229533.I1S2U2 pp 229533.V6R283
229533.I1S2U2 pp 229533.Q4IJ56
229533.I1S2U2 pp 229533.Q4IQW0
229533.I1S2U2 pp 229533.V6QYF0
229533.A0A0E0S065 pp 229533.Q4INS6
229533.A0A0E0S065 pp 229533.I1RW03
229533.A0A0E0S065 pp 229533.I1RZT1
229533.A0A0E0S065 pp 229533.I1RWB3
229533.A0A0E0S065 pp 229533.I1REK6
229533.A0A0E0S065 pp 229533.A0A0E0SDE1
229533.A0A0E0S065 pp 229533.A0A1C3YJ79
229533.A0A0E0S065 pp 229533.I1RE37
229533.A0A0E0S065 pp 229533.I1RF02
229533.A0A0E0S065 pp 229533.I1RIP9
229533.A0A0E0S065 pp 229533.I1RVV5
229533.A0A0E0S065 pp 229533.A0A1C3YLQ7
229533.A0A0E0S065 pp 229533.I1RWM6
229533.A0A0E0S065 pp 229533.I1S2F2
229533.A0A0E0S065 pp 229533.I1RYV1
229533.A0A0E0S065 pp 229533.I1RV78
229533.A0A0E0S065 pp 229533.I1RWX7
229533.A0A0E0S065 pp 229533.A0A1C3YI39
229533.A0A0E0S065 pp 229533.I1RJZ7
229533.A0A0E0S065 pp 229533.I1RAH9
229533.A0A0E0S065 pp 229533.I1RW71
229533.A0A0E0S065 pp 229533.I1RMB7
229533.I1REG5 pp 229533.I1RQ16
229533.I1REG5 pp 229533.I1RRB0
229533.I1REG5 pp 229533.I1RWT1
229533.I1REG5 pp 229533.I1RPK5
229533.I1REG5 pp 229533.I1RFJ2
229533.I1REG5 pp 229533.Q4IBX0
229533.I1REG5 pp 229533.Q4I0J6
229533.I1REG5 pp 229533.I1S2N3
229533.I1REG5 pp 229533.I1RJH5
229533.I1REG5 pp 229533.I1RQJ0
229533.I1REG5 pp 229533.I1RIG4
229533.I1REG5 pp 229533.I1RNZ8
229533.I1REG5 pp 229533.I1S1C8
229533.I1REG5 pp 229533.I1RR95
229533.I1REG5 pp 229533.I1RZ84
229533.I1REG5 pp 229533.I1RX84
229533.I1REG5 pp 229533.I1RLS9
229533.I1REG5 pp 229533.I1RJI1
229533.I1REG5 pp 229533.I1RXM0
229533.I1REG5 pp 229533.I1RFR6
229533.I1REG5 pp 229533.I1RU80
229533.I1REG5 pp 229533.Q4HZS8
229533.I1REG5 pp 229533.Q5I2J3

79425 229533.I1REG5 pp 229533.I1RFZ2
79426 229533.I1REG5 pp 229533.I1RTM8
79427 229533.I1RV75 pp 229533.I1S182
79428 229533.A0A1C3YN54 pp 229533.I1RNG8
79429 229533.A0A1C3YN54 pp 229533.I1RY40
79430 229533.A0A1C3YN54 pp 229533.I1S287
79431 229533.A0A1C3YN54 pp 229533.Q5I2J3
79432 229533.A0A1C3YN54 pp 229533.I1REU2
79433 229533.A0A1C3YN54 pp 229533.I1RX84
79434 229533.A0A1C3YN54 pp 229533.I1RP29
79435 229533.A0A1C3YN54 pp 229533.Q4HYZ2
79436 229533.A0A1C3YN54 pp 229533.I1RG01
79437 229533.A0A1C3YN54 pp 229533.I1RT41
79438 229533.A0A1C3YN54 pp 229533.I1RNB9
79439 229533.A0A1C3YN54 pp 229533.I1RQ70
79440 229533.A0A1C3YN54 pp 229533.Q4ICM0
79441 229533.A0A1C3YN54 pp 229533.I1RIG4
79442 229533.A0A1C3YN54 pp 229533.I1RNM1
79443 229533.A0A1C3YN54 pp 229533.I1RE20
79444 229533.A0A1C3YN54 pp 229533.I1RQP7
79445 229533.A0A1C3YN54 pp 229533.Q4ILI9
79446 229533.A0A1C3YN54 pp 229533.I1RBS4
79447 229533.A0A1C3YN54 pp 229533.I1RI75
79448 229533.A0A1C3YN54 pp 229533.I1RTQ9
79449 229533.A0A1C3YN54 pp 229533.Q4HZS8
79450 229533.A0A1C3YN54 pp 229533.I1RMT3
79451 229533.A0A1C3YN54 pp 229533.I1RM71
79452 229533.A0A1C3YN54 pp 229533.I1S0Z3
79453 229533.A0A1C3YN54 pp 229533.I1RA72
79454 229533.A0A1C3YN54 pp 229533.I1RS34
79455 229533.A0A1C3YN54 pp 229533.Q4I877
79456 229533.A0A1C3YN54 pp 229533.I1S1C8
79457 229533.A0A1C3YN54 pp 229533.I1RMG2
79458 229533.A0A1C3YN54 pp 229533.I1S842
79459 229533.A0A1C3YN54 pp 229533.I1RG99
79460 229533.A0A1C3YN54 pp 229533.I1RFK0
79461 229533.A0A1C3YN54 pp 229533.V6REE9
79462 229533.A0A1C3YN54 pp 229533.I1RPK5
79463 229533.A0A1C3YN54 pp 229533.I1RYW8
79464 229533.A0A1C3YN54 pp 229533.I1RW76
79465 229533.A0A1C3YN54 pp 229533.I1RMN3
79466 229533.A0A1C3YN54 pp 229533.I1RN70
79467 229533.A0A1C3YN54 pp 229533.V6RB81
79468 229533.A0A1C3YN54 pp 229533.I1RR95
79469 229533.A0A1C3YN54 pp 229533.I1RCE7
79470 229533.A0A1C3YN54 pp 229533.I1RRB0
79471 229533.A0A1C3YN54 pp 229533.I1RP92
79472 229533.A0A1C3YN54 pp 229533.I1RCF0
79473 229533.A0A1C3YN54 pp 229533.I1SAM5
79474 229533.A0A1C3YN54 pp 229533.I1RTC1
79475 229533.A0A1C3YN54 pp 229533.I1RYU1
79476 229533.A0A1C3YN54 pp 229533.I1RWP9
79477 229533.A0A1C3YN54 pp 229533.I1RKR7
79478 229533.A0A1C3YN54 pp 229533.I1RCD4
79479 229533.A0A1C3YN54 pp 229533.I1RVA8
79480 229533.A0A1C3YN54 pp 229533.I1RD45
79481 229533.A0A1C3YN54 pp 229533.I1S1V7
79482 229533.A0A1C3YN54 pp 229533.I1S7X9
79483 229533.A0A1C3YN54 pp 229533.I1RQ32
79484 229533.A0A1C3YN54 pp 229533.I1RQM0
79485 229533.A0A1C3YN54 pp 229533.I1RCN6
79486 229533.A0A1C3YN54 pp 229533.I1RMF2
79487 229533.A0A1C3YN54 pp 229533.I1RC46
79488 229533.A0A1C3YN54 pp 229533.I1REC6
79489 229533.A0A1C3YN54 pp 229533.I1RPC6
79490 229533.A0A1C3YN54 pp 229533.I1RZQ6
79491 229533.A0A1C3YN54 pp 229533.I1RD70
79492 229533.A0A1C3YN54 pp 229533.I1RXF5
79493 229533.A0A1C3YN54 pp 229533.I1RSJ0
79494 229533.A0A1C3YN54 pp 229533.I1REC7
79495 229533.A0A1C3YN54 pp 229533.A0A1C3YN84
79496 229533.A0A1C3YN54 pp 229533.I1RWB4
79497 229533.A0A1C3YN54 pp 229533.I1RTE6

79498 229533.A0A1C3YN54 pp 229533.I1RTG5
79499 229533.A0A1C3YN54 pp 229533.A0A1C3YN81
79500 229533.A0A1C3YN54 pp 229533.I1RMJ1
79501 229533.I1RGS6 pp 229533.I1S1V1
79502 229533.I1RGS6 pp 229533.I1RX25
79503 229533.I1RGS6 pp 229533.I1RXF0
79504 229533.I1RGS6 pp 229533.I1RQ41
79505 229533.I1RGS6 pp 229533.I1RRC6
79506 229533.I1RU52 pp 229533.I1RY58
79507 229533.I1RU52 pp 229533.Q4IPI4
79508 229533.I1RU52 pp 229533.Q4ICA8
79509 229533.A0A1C3YLP6 pp 229533.I1RVJ6
79510 229533.A0A1C3YLP6 pp 229533.I1RJY7
79511 229533.A0A1C3YLP6 pp 229533.I1RT09
79512 229533.A0A1C3YLP6 pp 229533.I1RBE4
79513 229533.A0A1C3YLP6 pp 229533.V6RK96
79514 229533.A0A1C3YLP6 pp 229533.I1RA90
79515 229533.A0A1C3YLP6 pp 229533.I1RXS0
79516 229533.A0A1C3YLP6 pp 229533.I1S0K0
79517 229533.A0A1C3YLP6 pp 229533.I1S236
79518 229533.A0A1C3YLP6 pp 229533.V6RG22
79519 229533.A0A1C3YLP6 pp 229533.I1RXB6
79520 229533.A0A1C3YLP6 pp 229533.I1RMK4
79521 229533.A0A1C3YLP6 pp 229533.I1RYJ6
79522 229533.A0A1C3YLP6 pp 229533.I1RYU0
79523 229533.A0A1C3YLP6 pp 229533.I1RHZ5
79524 229533.A0A1C3YLP6 pp 229533.I1RBH9
79525 229533.A0A1C3YLP6 pp 229533.I1RIY0
79526 229533.A0A1C3YLP6 pp 229533.I1RDU5
79527 229533.A0A1C3YLP6 pp 229533.I1RA94
79528 229533.A0A1C3YLP6 pp 229533.I1RVP5
79529 229533.A0A1C3YLP6 pp 229533.V6RUT5
79530 229533.A0A1C3YLP6 pp 229533.I1RC61
79531 229533.A0A1C3YLP6 pp 229533.I1RC48
79532 229533.A0A1C3YLP6 pp 229533.I1RD42
79533 229533.A0A1C3YLP6 pp 229533.I1RZM4
79534 229533.A0A1C3YLP6 pp 229533.I1RBT7
79535 229533.A0A1C3YLP6 pp 229533.I1RW71
79536 229533.A0A1C3YLP6 pp 229533.I1S0N3
79537 229533.A0A1C3YLP6 pp 229533.I1RP86
79538 229533.A0A1C3YLP6 pp 229533.I1RK03
79539 229533.A0A1C3YLP6 pp 229533.A0A1C3YLZ8
79540 229533.A0A1C3YLP6 pp 229533.I1RM67
79541 229533.A0A1C3YLP6 pp 229533.I1S1W8
79542 229533.A0A1C3YLP6 pp 229533.V6RH92
79543 229533.A0A1C3YLP6 pp 229533.I1RQP2
79544 229533.A0A1C3YLP6 pp 229533.I1S8C1
79545 229533.A0A1C3YLP6 pp 229533.I1RAJ6
79546 229533.A0A1C3YLP6 pp 229533.I1RN84
79547 229533.A0A1C3YLP6 pp 229533.I1S9Z4
79548 229533.A0A1C3YLP6 pp 229533.I1S3L2
79549 229533.A0A1C3YLP6 pp 229533.I1S8R4
79550 229533.A0A1C3YLP6 pp 229533.I1RY49
79551 229533.A0A1C3YLP6 pp 229533.I1S1W3
79552 229533.A0A1C3YLP6 pp 229533.V6RLM7
79553 229533.A0A1C3YLP6 pp 229533.I1RAY9
79554 229533.A0A1C3YLP6 pp 229533.I1RQJ4
79555 229533.A0A1C3YLP6 pp 229533.I1RXY7
79556 229533.A0A1C3YLP6 pp 229533.I1S8E0
79557 229533.A0A1C3YLP6 pp 229533.I1RQE8
79558 229533.A0A1C3YLP6 pp 229533.I1RX64
79559 229533.A0A1C3YLP6 pp 229533.I1RL49
79560 229533.A0A1C3YLP6 pp 229533.V6QT89
79561 229533.A0A1C3YLP6 pp 229533.V6R382
79562 229533.A0A1C3YLP6 pp 229533.Q4HZ95
79563 229533.A0A1C3YLP6 pp 229533.I1RC11
79564 229533.A0A1C3YLP6 pp 229533.I1RHG5
79565 229533.A0A1C3YLP6 pp 229533.I1RBA4
79566 229533.A0A1C3YLP6 pp 229533.A0A1C3YLR6
79567 229533.A0A1C3YLP6 pp 229533.I1S237
79568 229533.A0A1C3YLP6 pp 229533.I1RXA9
79569 229533.A0A1C3YLP6 pp 229533.I1R9W4
79570 229533.A0A1C3YLP6 pp 229533.V6RM02

79571 229533.A0A1C3YLP6 pp 229533.Q4HXF0
79572 229533.A0A1C3YLP6 pp 229533.I1RXB2
79573 229533.A0A1C3YLP6 pp 229533.I1S3G4
79574 229533.A0A1C3YLP6 pp 229533.I1RP97
79575 229533.A0A1C3YLP6 pp 229533.I1RZ83
79576 229533.A0A1C3YLP6 pp 229533.I1S0J8
79577 229533.A0A1C3YLP6 pp 229533.I1RNG4
79578 229533.A0A1C3YLP6 pp 229533.I1RE53
79579 229533.A0A1C3YLP6 pp 229533.I1RTG7
79580 229533.A0A1C3YLP6 pp 229533.I1RMJ3
79581 229533.A0A1C3YLP6 pp 229533.I1RBD7
79582 229533.A0A1C3YLP6 pp 229533.I1RSK5
79583 229533.A0A1C3YLP6 pp 229533.I1RJZ7
79584 229533.A0A1C3YLP6 pp 229533.I1RWS4
79585 229533.A0A1C3YLP6 pp 229533.I1RUR8
79586 229533.A0A1C3YLP6 pp 229533.I1S0Y7
79587 229533.A0A1C3YLP6 pp 229533.I1RZ52
79588 229533.A0A1C3YLP6 pp 229533.Q4I099
79589 229533.A0A1C3YLP6 pp 229533.I1RVR2
79590 229533.A0A098E562 pp 229533.I1RX04
79591 229533.Q4HYI0 pp 229533.Q4IJM4
79592 229533.Q4HYI0 pp 229533.V6QUE6
79593 229533.Q4HYI0 pp 229533.V6QWX8
79594 229533.A0A098DE06 pp 229533.I1RC29
79595 229533.A0A098DE06 pp 229533.I1RNA0
79596 229533.A0A098DE06 pp 229533.V6QYF0
79597 229533.A0A098DE06 pp 229533.I1RX24
79598 229533.A0A098DE06 pp 229533.A0A098DVE3
79599 229533.A0A098DE06 pp 229533.I1RPM4
79600 229533.A0A098DE06 pp 229533.I1RG51
79601 229533.A0A098DE06 pp 229533.I1RC32
79602 229533.A0A098DE06 pp 229533.I1R9G0
79603 229533.A0A098DE06 pp 229533.I1RQK4
79604 229533.A0A098DE06 pp 229533.I1RAC2
79605 229533.A0A098DE06 pp 229533.I1RBY4
79606 229533.A0A098DE06 pp 229533.Q4IFI0
79607 229533.A0A098DE06 pp 229533.I1RL23
79608 229533.A0A098DE06 pp 229533.A0A1C3YIN1
79609 229533.A0A098DE06 pp 229533.I1RDA0
79610 229533.A0A098DE06 pp 229533.I1S253
79611 229533.A0A098DE06 pp 229533.I1S6U8
79612 229533.A0A098DE06 pp 229533.I1RMK3
79613 229533.A0A098DE06 pp 229533.I1RPH9
79614 229533.A0A098DE06 pp 229533.I1RTF4
79615 229533.A0A098DE06 pp 229533.Q4IJH1
79616 229533.A0A098DE06 pp 229533.I1RNV6
79617 229533.A0A098DE06 pp 229533.I1RT80
79618 229533.A0A098DE06 pp 229533.A0A1C3YKC0
79619 229533.A0A098DE06 pp 229533.I1RZZ9
79620 229533.A0A098DE06 pp 229533.I1S9U2
79621 229533.A0A098DE06 pp 229533.I1RD64
79622 229533.A0A098DE06 pp 229533.I1RGF6
79623 229533.A0A098DE06 pp 229533.I1RM67
79624 229533.A0A098DE06 pp 229533.I1RRT8
79625 229533.A0A098DE06 pp 229533.I1RRJ8
79626 229533.A0A098DE06 pp 229533.I1RKJ6
79627 229533.A0A098DE06 pp 229533.I1RPU0
79628 229533.A0A098DE06 pp 229533.I1RZS6
79629 229533.A0A098DE06 pp 229533.I1RM06
79630 229533.A0A098DE06 pp 229533.I1RZ99
79631 229533.A0A098DE06 pp 229533.I1RKQ5
79632 229533.A0A098DE06 pp 229533.I1RWF7
79633 229533.A0A098DE06 pp 229533.I1RLV9
79634 229533.A0A098DE06 pp 229533.I1S1U7
79635 229533.A0A098DE06 pp 229533.I1RR00
79636 229533.A0A098DE06 pp 229533.A0A098DRR8
79637 229533.A0A098DE06 pp 229533.I1RCL3
79638 229533.A0A098DE06 pp 229533.A0A0E0RP78
79639 229533.A0A098DE06 pp 229533.I1RQA2
79640 229533.A0A098DE06 pp 229533.I1RQ28
79641 229533.A0A098DE06 pp 229533.I1RW71
79642 229533.A0A098DE06 pp 229533.I1RSC6
79643 229533.A0A098DE06 pp 229533.I1RKL3

79644	229533.A0A098DE06	pp	229533.I1RB27
79645	229533.A0A098DE06	pp	229533.I1R9X7
79646	229533.A0A098DE06	pp	229533.I1RA48
79647	229533.A0A098DE06	pp	229533.I1RFW5
79648	229533.A0A098DE06	pp	229533.I1R9V7
79649	229533.A0A098DE06	pp	229533.I1S1X9
79650	229533.A0A098DE06	pp	229533.I1RNU6
79651	229533.A0A098DE06	pp	229533.I1RQH2
79652	229533.A0A098DE06	pp	229533.Q4IJ56
79653	229533.A0A098DE06	pp	229533.A0A0E0S7B3
79654	229533.A0A098DE06	pp	229533.I1S2E8
79655	229533.A0A098DE06	pp	229533.I1S2U2
79656	229533.A0A098DE06	pp	229533.V6R283
79657	229533.A0A098DE06	pp	229533.I1S0J1
79658	229533.A0A098DE06	pp	229533.I1RC72
79659	229533.A0A098DE06	pp	229533.I1RSD8
79660	229533.A0A098DE06	pp	229533.I1RD17
79661	229533.A0A098DE06	pp	229533.I1RC03
79662	229533.A0A098DE06	pp	229533.I1RFJ9
79663	229533.A0A098DE06	pp	229533.A0A0E0S621
79664	229533.A0A098DE06	pp	229533.I1RX14
79665	229533.A0A098DE06	pp	229533.I1RQ65
79666	229533.A0A098DE06	pp	229533.A0A1C3YKW9
79667	229533.A0A098DE06	pp	229533.I1RNH1
79668	229533.A0A098DE06	pp	229533.I1RRR7
79669	229533.A0A098DE06	pp	229533.A0A1C3YN63
79670	229533.A0A098DE06	pp	229533.I1RF19
79671	229533.A0A098DE06	pp	229533.A0A1C3YIP5
79672	229533.A0A098DE06	pp	229533.A0A1C3YK72
79673	229533.A0A098DE06	pp	229533.I1S9U1
79674	229533.A0A098DE06	pp	229533.I1RZE5
79675	229533.A0A098DE06	pp	229533.I1RS30
79676	229533.A0A098DE06	pp	229533.I1RAZ8
79677	229533.A0A098DE06	pp	229533.I1RKC6
79678	229533.A0A098DE06	pp	229533.I1RZF6
79679	229533.A0A098DE06	pp	229533.I1RUQ2
79680	229533.A0A098DE06	pp	229533.A0A0E0SMM8
79681	229533.A0A098DE06	pp	229533.I1RM96
79682	229533.A0A098DE06	pp	229533.I1RC33
79683	229533.A0A098DE06	pp	229533.I1RG56
79684	229533.A0A098DE06	pp	229533.I1RPY1
79685	229533.A0A098DE06	pp	229533.I1RNK2
79686	229533.A0A098DE06	pp	229533.I1RVZ0
79687	229533.A0A098DE06	pp	229533.I1RTE9
79688	229533.A0A098DE06	pp	229533.I1S0R7
79689	229533.A0A098DE06	pp	229533.Q4HZK7
79690	229533.A0A098DE06	pp	229533.I1RAF8
79691	229533.A0A098DE06	pp	229533.A0A098DE18
79692	229533.A0A098DE06	pp	229533.A0A0E0SJN2
79693	229533.A0A098DE06	pp	229533.I1RSI6
79694	229533.A0A098DE06	pp	229533.I1RX08
79695	229533.A0A098DE06	pp	229533.Q4IQW0
79696	229533.A0A098DE06	pp	229533.I1RFN1
79697	229533.A0A098DE06	pp	229533.I1RMN7
79698	229533.A0A098DE06	pp	229533.I1RC82
79699	229533.A0A098DE06	pp	229533.I1RNH6
79700	229533.A0A098DE06	pp	229533.I1S021
79701	229533.A0A098DE06	pp	229533.I1RRI2
79702	229533.A0A098DE06	pp	229533.I1RTD7
79703	229533.A0A098DE06	pp	229533.I1S1B6
79704	229533.A0A098DE06	pp	229533.I1RJL4
79705	229533.A0A098DE06	pp	229533.I1RMD5
79706	229533.A0A098DE06	pp	229533.I1RNY2
79707	229533.A0A098DE06	pp	229533.A0A1C3YMW0
79708	229533.A0A098DE06	pp	229533.Q4IN63
79709	229533.A0A098DE06	pp	229533.I1RSR3
79710	229533.A0A098DE06	pp	229533.I1RMN6
79711	229533.A0A098DE06	pp	229533.V6QUE6
79712	229533.A0A098DE06	pp	229533.I1RQ31
79713	229533.A0A098DE06	pp	229533.A0A1C3YJG0
79714	229533.A0A098DE06	pp	229533.V6RGN5
79715	229533.A0A098DE06	pp	229533.I1RTG6
79716	229533.A0A098DE06	pp	229533.I1RC27

79717 229533.I1RMN3 pp 229533.I1RXF5
79718 229533.I1RMN3 pp 229533.I1S830
79719 229533.I1RMN3 pp 229533.I1RUN9
79720 229533.I1RMN3 pp 229533.I1RTQ9
79721 229533.I1RMN3 pp 229533.I1SAM5
79722 229533.I1RMN3 pp 229533.I1RNM3
79723 229533.I1RMN3 pp 229533.I1RSW4
79724 229533.I1RMN3 pp 229533.I1RQF7
79725 229533.I1RMN3 pp 229533.I1RQ75
79726 229533.I1RMN3 pp 229533.I1S1U6
79727 229533.I1RMN3 pp 229533.I1S4N5
79728 229533.I1RMN3 pp 229533.I1RQN8
79729 229533.I1RMN3 pp 229533.I1RX87
79730 229533.I1RMN3 pp 229533.I1RQC3
79731 229533.I1RMN3 pp 229533.I1RRC8
79732 229533.I1RMN3 pp 229533.I1RZP1
79733 229533.I1RMN3 pp 229533.I1RZS5
79734 229533.I1RMN3 pp 229533.I1RNG2
79735 229533.I1RMN3 pp 229533.I1RZK3
79736 229533.I1RMN3 pp 229533.I1RZ84
79737 229533.I1RMN3 pp 229533.I1S1V4
79738 229533.I1RMN3 pp 229533.I1RZ60
79739 229533.I1RMN3 pp 229533.I1RP87
79740 229533.I1RMN3 pp 229533.Q4HY20
79741 229533.I1RMN3 pp 229533.I1RPL2
79742 229533.I1RMN3 pp 229533.I1RW76
79743 229533.I1RMN3 pp 229533.I1RXT7
79744 229533.I1RMN3 pp 229533.V6RXW9
79745 229533.I1RMN3 pp 229533.I1RN70
79746 229533.I1RMN3 pp 229533.I1RNB9
79747 229533.I1RMN3 pp 229533.I1RSH3
79748 229533.I1RMN3 pp 229533.I1S278
79749 229533.I1RMN3 pp 229533.I1RYP0
79750 229533.I1RMN3 pp 229533.I1RX50
79751 229533.I1RMN3 pp 229533.I1RX95
79752 229533.I1RMN3 pp 229533.V6QXP4
79753 229533.I1RMN3 pp 229533.I1RXF0
79754 229533.I1RMN3 pp 229533.I1RQD6
79755 229533.I1RMN3 pp 229533.I1S051
79756 229533.I1RMN3 pp 229533.I1RTF2
79757 229533.I1RMN3 pp 229533.I1RQP7
79758 229533.I1RMN3 pp 229533.I1RPC6
79759 229533.I1RMN3 pp 229533.I1RY40
79760 229533.I1RMN3 pp 229533.I1RRC9
79761 229533.I1RMN3 pp 229533.I1RWZ9
79762 229533.I1RMN3 pp 229533.I1RRC4
79763 229533.I1RMN3 pp 229533.I1RS34
79764 229533.I1RMN3 pp 229533.I1S287
79765 229533.I1RMN3 pp 229533.I1RQP5
79766 229533.I1RMN3 pp 229533.I1RPY3
79767 229533.I1RMN3 pp 229533.I1S9S4
79768 229533.I1RMN3 pp 229533.I1S2B6
79769 229533.I1RMN3 pp 229533.I1S0V5
79770 229533.I1RMN3 pp 229533.I1RQC4
79771 229533.I1RMN3 pp 229533.I1RQF9
79772 229533.A0A098E5B3 pp 229533.A0A0E0SP71
79773 229533.A0A098E5B3 pp 229533.I1RY11
79774 229533.I1RFK0 pp 229533.I1S183
79775 229533.I1RFK0 pp 229533.I1S2B6
79776 229533.I1RFK0 pp 229533.I1S0Z3
79777 229533.I1RFK0 pp 229533.I1RX87
79778 229533.I1RFK0 pp 229533.I1S5B5
79779 229533.I1RFK0 pp 229533.I1RMG2
79780 229533.I1RFK0 pp 229533.I1S213
79781 229533.I1RFK0 pp 229533.I1RNP1
79782 229533.I1RFK0 pp 229533.I1RQC4
79783 229533.I1RFK0 pp 229533.I1RIG4
79784 229533.I1RFK0 pp 229533.I1RQH7
79785 229533.I1RFK0 pp 229533.I1S1V4
79786 229533.I1RFK0 pp 229533.V6RKK5
79787 229533.I1RFK0 pp 229533.I1RMN3
79788 229533.I1RFK0 pp 229533.I1RQP7
79789 229533.I1RFK0 pp 229533.I1RS69

79790	229533.I1RFK0	pp	229533.I1S0V5
79791	229533.I1RFK0	pp	229533.I1RTD0
79792	229533.I1RFK0	pp	229533.I1RJZ6
79793	229533.I1RFK0	pp	229533.Q4ICM0
79794	229533.I1RFK0	pp	229533.I1RQF7
79795	229533.I1RFK0	pp	229533.Q4I2D7
79796	229533.I1RFK0	pp	229533.I1RT41
79797	229533.I1RFK0	pp	229533.I1RQ70
79798	229533.I1RFK0	pp	229533.I1RR74
79799	229533.I1RFK0	pp	229533.I1RTQ9
79800	229533.I1RFK0	pp	229533.I1S287
79801	229533.I1RFK0	pp	229533.I1RNH7
79802	229533.I1RFK0	pp	229533.I1S830
79803	229533.I1RFK0	pp	229533.I1RJS4
79804	229533.I1RFK0	pp	229533.I1RXF5
79805	229533.I1RFK0	pp	229533.Q4IN63
79806	229533.I1RFK0	pp	229533.I1RKI1
79807	229533.I1RFK0	pp	229533.I1RYM4
79808	229533.I1RFK0	pp	229533.I1RKR7
79809	229533.I1RFK0	pp	229533.I1RJS5
79810	229533.I1RFK0	pp	229533.I1RWB4
79811	229533.I1RFK0	pp	229533.I1RPG2
79812	229533.I1RFK0	pp	229533.I1RQD6
79813	229533.I1RFK0	pp	229533.I1S1V7
79814	229533.I1RFK0	pp	229533.I1RGC1
79815	229533.I1RFK0	pp	229533.I1RFV4
79816	229533.I1RFK0	pp	229533.I1RSJ0
79817	229533.I1RFK0	pp	229533.I1RSZ1
79818	229533.I1RFK0	pp	229533.I1RQ32
79819	229533.I1RFK0	pp	229533.I1RYU1
79820	229533.I1RFK0	pp	229533.I1RUJ0
79821	229533.I1RFK0	pp	229533.I1RND7
79822	229533.I1RFK0	pp	229533.I1RW76
79823	229533.I1RFK0	pp	229533.I1RG99
79824	229533.I1RFK0	pp	229533.I1RMF2
79825	229533.I1RFK0	pp	229533.I1SAM5
79826	229533.I1RFK0	pp	229533.I1RZK3
79827	229533.I1RTU1	pp	229533.V6QVM5
79828	229533.I1RTU1	pp	229533.V6RUT5
79829	229533.I1RTU1	pp	229533.I1S0J8
79830	229533.I1RTU1	pp	229533.Q4IMJ1
79831	229533.I1RTU1	pp	229533.Q4HXF0
79832	229533.I1RTU1	pp	229533.V6QT89
79833	229533.I1RTU1	pp	229533.I1RXM6
79834	229533.I1RTU1	pp	229533.I1S338
79835	229533.I1RTU1	pp	229533.V6QVH8
79836	229533.I1RTU1	pp	229533.V6R382
79837	229533.I1RTU1	pp	229533.I1RYU0
79838	229533.I1RTU1	pp	229533.I1RXV9
79839	229533.I1RTU1	pp	229533.I1S1W3
79840	229533.I1RTU1	pp	229533.I1S0H7
79841	229533.I1RTU1	pp	229533.Q4HTS9
79842	229533.Q4HY20	pp	229533.V6QXP4
79843	229533.Q4HY20	pp	229533.Q4IB63
79844	229533.Q4HY20	pp	229533.V6RXW9
79845	229533.I1RZ99	pp	229533.Q4I327
79846	229533.I1RZ99	pp	229533.Q4HZK7
79847	229533.I1RZ99	pp	229533.Q4IN63
79848	229533.I1RZ99	pp	229533.I1S4T3
79849	229533.I1RZ99	pp	229533.I1S1U7
79850	229533.I1RZ99	pp	229533.V6RGY3
79851	229533.I1RZ99	pp	229533.I1S274
79852	229533.I1RZ99	pp	229533.I1S060
79853	229533.I1RZ99	pp	229533.I1S296
79854	229533.I1RZ99	pp	229533.Q4IBS2
79855	229533.I1RZ99	pp	229533.Q4HZ68
79856	229533.I1RZ99	pp	229533.I1S0J1
79857	229533.I1RZ99	pp	229533.I1RZS6
79858	229533.I1RZ99	pp	229533.Q4IQW0
79859	229533.I1RZ99	pp	229533.I1S1X9
79860	229533.I1RZ99	pp	229533.I1RZZ9
79861	229533.I1RZ99	pp	229533.I1S021
79862	229533.I1RZ99	pp	229533.I1RZF6

229533.I1RZ99 pp 229533.I1S1B6
229533.I1RZ99 pp 229533.Q4IAS1
229533.I1RZ99 pp 229533.V6RGN5
229533.I1RZ99 pp 229533.V6R227
229533.I1RZ99 pp 229533.V6QYF0
229533.I1RZ99 pp 229533.I1RZJ6
229533.I1RZ99 pp 229533.V6R283
229533.I1RZ99 pp 229533.I1S9U2
229533.I1RZ99 pp 229533.Q4IQK7
229533.I1RZ99 pp 229533.Q4IJ56
229533.I1RZ99 pp 229533.I1RZE5
229533.I1RZ99 pp 229533.Q4IJH1
229533.I1RZ99 pp 229533.Q4IFI0
229533.I1RFB4 pp 229533.I1RJT2
229533.I1RFB4 pp 229533.I1RRL5
229533.I1RFB4 pp 229533.I1RK47
229533.I1RFB4 pp 229533.I1RW06
229533.I1RFB4 pp 229533.I1S334
229533.I1RFB4 pp 229533.I1S0H7
229533.I1RFB4 pp 229533.I1RTU1
229533.I1RFB4 pp 229533.Q4HXF0
229533.I1RFB4 pp 229533.I1S3H8
229533.I1RFB4 pp 229533.I1RNG4
229533.I1RFB4 pp 229533.I1RIH0
229533.I1RFB4 pp 229533.Q4IMJ1
229533.I1RFB4 pp 229533.Q4HTS9
229533.I1RFB4 pp 229533.V6QVH8
229533.I1RHL9 pp 229533.I1RK44
229533.I1RHL9 pp 229533.I1RZM4
229533.I1RHL9 pp 229533.V6R2Z2
229533.I1RHL9 pp 229533.V6RAM6
229533.I1RHL9 pp 229533.V6RK96
229533.I1RHL9 pp 229533.I1RNH4
229533.I1RHL9 pp 229533.I1RIW5
229533.I1RHL9 pp 229533.V6QU99
229533.I1RHL9 pp 229533.I1RYM5
229533.I1RHL9 pp 229533.I1S9T6
229533.I1RHL9 pp 229533.I1RRM1
229533.I1RHL9 pp 229533.I1SAI4
229533.I1RHL9 pp 229533.V6R756
229533.I1RHL9 pp 229533.I1RHU0
229533.I1RHL9 pp 229533.I1RMU5
229533.I1RHL9 pp 229533.I1RQE8
229533.I1RHL9 pp 229533.V6R5D2
229533.I1RHL9 pp 229533.V6R0W1
229533.A0A0E0S6D3 pp 229533.I1RT16
229533.A0A0E0S6D3 pp 229533.I1S182
229533.A0A0E0S6D3 pp 229533.I1RSZ3
229533.A0A0E0S6D3 pp 229533.A0A1C3YHX4
229533.A0A0E0S6D3 pp 229533.I1RW28
229533.A0A0E0S6D3 pp 229533.I1RB36
229533.A0A0E0S6D3 pp 229533.I1RW80
229533.A0A0E0S6D3 pp 229533.Q4IMD1
229533.A0A0E0S6D3 pp 229533.I1RW90
229533.A0A0E0S6D3 pp 229533.Q4IJ11
229533.A0A0E0S6D3 pp 229533.I1SB04
229533.A0A0E0S6D3 pp 229533.I1RRM8
229533.A0A0E0S6D3 pp 229533.A0A0E0SQJ6
229533.A0A0E0S6D3 pp 229533.Q4I5W5
229533.A0A0E0S6D3 pp 229533.I1RML9
229533.A0A0E0S6D3 pp 229533.Q4IAK7
229533.A0A0E0S6D3 pp 229533.I1RC24
229533.A0A0E0S6D3 pp 229533.I1RUG0
229533.A0A0E0S6D3 pp 229533.I1S1V2
229533.A0A0E0S6D3 pp 229533.V6QWX8
229533.A0A0E0S6D3 pp 229533.I1RQ42
229533.A0A0E0S6D3 pp 229533.A0A1C3YLP8
229533.A0A0E0S6D3 pp 229533.I1RRG3
229533.A0A0E0S6D3 pp 229533.A0A1C3YKV4
229533.A0A0E0S6D3 pp 229533.Q4IL82
229533.A0A0E0S6D3 pp 229533.Q4IPI4
229533.A0A0E0S6D3 pp 229533.A0A1C3YIR8
229533.A0A0E0S6D3 pp 229533.I1RZM2

79936 229533.A0A0E0S6D3 pp 229533.I1RT41
79937 229533.A0A0E0S6D3 pp 229533.Q4HYQ4
79938 229533.A0A0E0S6D3 pp 229533.I1RA84
79939 229533.A0A0E0S6D3 pp 229533.I1RPR8
79940 229533.A0A0E0S6D3 pp 229533.I1RVY0
79941 229533.A0A0E0S6D3 pp 229533.I1RPW8
79942 229533.A0A0E0S6D3 pp 229533.I1RBR5
79943 229533.A0A0E0S6D3 pp 229533.Q4I5V3
79944 229533.A0A0E0S6D3 pp 229533.I1RCB7
79945 229533.A0A0E0S6D3 pp 229533.I1RC15
79946 229533.A0A0E0S6D3 pp 229533.A0A1C3YLA2
79947 229533.A0A0E0S6D3 pp 229533.I1RZ42
79948 229533.A0A0E0S6D3 pp 229533.I1RSU7
79949 229533.A0A0E0S6D3 pp 229533.I1RN04
79950 229533.A0A0E0S6D3 pp 229533.I1RFN9
79951 229533.A0A0E0S6D3 pp 229533.Q4I7S1
79952 229533.A0A0E0S6D3 pp 229533.A0A1C3YJZ2
79953 229533.A0A0E0S6D3 pp 229533.A0A1C3YI23
79954 229533.A0A0E0S6D3 pp 229533.I1RAN8
79955 229533.A0A0E0S6D3 pp 229533.I1RBZ7
79956 229533.A0A0E0S6D3 pp 229533.I1RDC9
79957 229533.A0A0E0S6D3 pp 229533.I1RG11
79958 229533.A0A0E0S6D3 pp 229533.I1RRT0
79959 229533.A0A0E0S6D3 pp 229533.I1RQP3
79960 229533.A0A0E0S6D3 pp 229533.I1RBJ1
79961 229533.A0A0E0S6D3 pp 229533.A0A0E0S9G7
79962 229533.A0A0E0S6D3 pp 229533.I1RE51
79963 229533.A0A0E0S6D3 pp 229533.I1RK71
79964 229533.A0A0E0S6D3 pp 229533.I1RJS5
79965 229533.A0A0E0S6D3 pp 229533.Q4ICA8
79966 229533.A0A0E0S6D3 pp 229533.I1RZF3
79967 229533.A0A0E0S6D3 pp 229533.I1RXE5
79968 229533.A0A0E0S6D3 pp 229533.I1RAR2
79969 229533.A0A0E0S6D3 pp 229533.I1RJL2
79970 229533.A0A0E0S6D3 pp 229533.I1RZB5
79971 229533.A0A0E0S6D3 pp 229533.I1RAV2
79972 229533.A0A0E0S6D3 pp 229533.I1RKR6
79973 229533.A0A0E0S6D3 pp 229533.Q4I948
79974 229533.A0A0E0S6D3 pp 229533.V6QUE6
79975 229533.A0A0E0S6D3 pp 229533.I1RR25
79976 229533.A0A0E0S6D3 pp 229533.I1RK79
79977 229533.A0A0E0S6D3 pp 229533.I1RRF0
79978 229533.A0A0E0S6D3 pp 229533.I1RC80
79979 229533.A0A0E0S6D3 pp 229533.Q4HY90
79980 229533.A0A0E0S6D3 pp 229533.I1RPU1
79981 229533.A0A0E0S6D3 pp 229533.A0A1C3YK01
79982 229533.A0A098DP84 pp 229533.I1RWX4
79983 229533.A0A098DP84 pp 229533.A0A098DVZ4
79984 229533.A0A098DP84 pp 229533.I1RBE4
79985 229533.A0A098DP84 pp 229533.I1S1D1
79986 229533.I1RVJ4 pp 229533.I1S0K2
79987 229533.I1RVJ4 pp 229533.I1RY21
79988 229533.A0A0E0RP78 pp 229533.Q4IQK7
79989 229533.A0A0E0RP78 pp 229533.I1RNA0
79990 229533.A0A0E0RP78 pp 229533.I1RBG9
79991 229533.A0A0E0RP78 pp 229533.V6QYF0
79992 229533.A0A0E0RP78 pp 229533.I1RX24
79993 229533.A0A0E0RP78 pp 229533.I1RZS6
79994 229533.A0A0E0RP78 pp 229533.I1RM06
79995 229533.A0A0E0RP78 pp 229533.I1RC32
79996 229533.A0A0E0RP78 pp 229533.I1RYT5
79997 229533.A0A0E0RP78 pp 229533.I1REX4
79998 229533.A0A0E0RP78 pp 229533.I1RFS3
79999 229533.A0A0E0RP78 pp 229533.Q4I662
80000 229533.A0A0E0RP78 pp 229533.Q4IF76
80001 229533.A0A0E0RP78 pp 229533.I1RG51
80002 229533.A0A0E0RP78 pp 229533.I1RN31
80003 229533.A0A0E0RP78 pp 229533.I1RQM6
80004 229533.A0A0E0RP78 pp 229533.I1RJZ7
80005 229533.A0A0E0RP78 pp 229533.I1RD64
80006 229533.A0A0E0RP78 pp 229533.I1S9U2
80007 229533.A0A0E0RP78 pp 229533.Q4IMJ1
80008 229533.A0A0E0RP78 pp 229533.A0A1C3YHT9

80009 229533.A0A0E0RP78 pp 229533.I1RPU0
80010 229533.A0A0E0RP78 pp 229533.I1RAC2
80011 229533.A0A0E0RP78 pp 229533.I1RKJ6
80012 229533.A0A0E0RP78 pp 229533.I1S9Y1
80013 229533.A0A0E0RP78 pp 229533.Q4IJH1
80014 229533.A0A0E0RP78 pp 229533.I1RTF4
80015 229533.A0A0E0RP78 pp 229533.I1RFQ7
80016 229533.A0A0E0RP78 pp 229533.O74638
80017 229533.A0A0E0RP78 pp 229533.I1RT80
80018 229533.A0A0E0RP78 pp 229533.I1RRI2
80019 229533.A0A0E0RP78 pp 229533.I1RTC2
80020 229533.A0A0E0RP78 pp 229533.A0A1C3YLE9
80021 229533.A0A0E0RP78 pp 229533.I1RKL3
80022 229533.A0A0E0RP78 pp 229533.I1RNV6
80023 229533.A0A0E0RP78 pp 229533.A0A1C3YIN1
80024 229533.A0A0E0RP78 pp 229533.I1RW71
80025 229533.A0A0E0RP78 pp 229533.I1RDA0
80026 229533.A0A0E0RP78 pp 229533.I1RRJ8
80027 229533.A0A0E0RP78 pp 229533.I1RMK3
80028 229533.A0A0E0RP78 pp 229533.I1S274
80029 229533.A0A0E0RP78 pp 229533.I1S1U7
80030 229533.A0A0E0RP78 pp 229533.I1RZ99
80031 229533.A0A0E0RP78 pp 229533.Q4INZ9
80032 229533.A0A0E0RP78 pp 229533.I1RQJ5
80033 229533.A0A0E0RP78 pp 229533.I1REF2
80034 229533.A0A0E0RP78 pp 229533.V6R283
80035 229533.A0A0E0RP78 pp 229533.I1RSR3
80036 229533.A0A0E0RP78 pp 229533.I1RBC7
80037 229533.A0A0E0RP78 pp 229533.I1RFN1
80038 229533.A0A0E0RP78 pp 229533.V6RGY3
80039 229533.A0A0E0RP78 pp 229533.I1RC82
80040 229533.A0A0E0RP78 pp 229533.I1RC03
80041 229533.A0A0E0RP78 pp 229533.A0A1C3YL39
80042 229533.A0A0E0RP78 pp 229533.A0A0E0SGM4
80043 229533.A0A0E0RP78 pp 229533.I1RFW5
80044 229533.A0A0E0RP78 pp 229533.A0A0E0S7B3
80045 229533.A0A0E0RP78 pp 229533.I1RS47
80046 229533.A0A0E0RP78 pp 229533.I1RCN5
80047 229533.A0A0E0RP78 pp 229533.I1RQA2
80048 229533.A0A0E0RP78 pp 229533.Q4IQW0
80049 229533.A0A0E0RP78 pp 229533.I1RCL3
80050 229533.A0A0E0RP78 pp 229533.Q4HZ47
80051 229533.A0A0E0RP78 pp 229533.I1RKQ5
80052 229533.A0A0E0RP78 pp 229533.A0A1C3YK72
80053 229533.A0A0E0RP78 pp 229533.I1RS30
80054 229533.A0A0E0RP78 pp 229533.I1RNU6
80055 229533.A0A0E0RP78 pp 229533.I1RWQ0
80056 229533.A0A0E0RP78 pp 229533.Q4IAS1
80057 229533.A0A0E0RP78 pp 229533.Q4IJ56
80058 229533.A0A0E0RP78 pp 229533.I1RNH1
80059 229533.A0A0E0RP78 pp 229533.I1RQS5
80060 229533.A0A0E0RP78 pp 229533.I1RRQ3
80061 229533.A0A0E0RP78 pp 229533.I1RRT2
80062 229533.A0A0E0RP78 pp 229533.I1R9X7
80063 229533.A0A0E0RP78 pp 229533.I1RWD3
80064 229533.A0A0E0RP78 pp 229533.A0A1C3YN63
80065 229533.A0A0E0RP78 pp 229533.I1RSS4
80066 229533.A0A0E0RP78 pp 229533.Q4HXF0
80067 229533.A0A0E0RP78 pp 229533.I1RB27
80068 229533.A0A0E0RP78 pp 229533.I1RA48
80069 229533.A0A0E0RP78 pp 229533.Q4I7K4
80070 229533.A0A0E0RP78 pp 229533.I1R9V7
80071 229533.A0A0E0RP78 pp 229533.I1S0R7
80072 229533.A0A0E0RP78 pp 229533.I1S1X9
80073 229533.A0A0E0RP78 pp 229533.I1RW16
80074 229533.A0A0E0RP78 pp 229533.A0A1C3YLF5
80075 229533.A0A0E0RP78 pp 229533.I1RAF8
80076 229533.A0A0E0RP78 pp 229533.I1RK95
80077 229533.A0A0E0RP78 pp 229533.I1RDG0
80078 229533.A0A0E0RP78 pp 229533.I1RN58
80079 229533.A0A0E0RP78 pp 229533.I1RAS2
80080 229533.A0A0E0RP78 pp 229533.I1S060
80081 229533.A0A0E0RP78 pp 229533.Q4IN63

80082	229533.A0A0E0RP78	pp	229533.I1RN99
80083	229533.A0A0E0RP78	pp	229533.A0A1C3YJG0
80084	229533.A0A0E0RP78	pp	229533.A0A1C3YKS2
80085	229533.A0A0E0RP78	pp	229533.I1RSD8
80086	229533.A0A0E0RP78	pp	229533.I1RC72
80087	229533.A0A0E0RP78	pp	229533.A0A0E0S621
80088	229533.A0A0E0RP78	pp	229533.I1RQ31
80089	229533.A0A0E0RP78	pp	229533.I1RMQ0
80090	229533.A0A0E0RP78	pp	229533.I1S021
80091	229533.A0A0E0RP78	pp	229533.I1RE31
80092	229533.A0A0E0RP78	pp	229533.I1RNH6
80093	229533.A0A0E0RP78	pp	229533.A0A1C3YJR3
80094	229533.A0A0E0RP78	pp	229533.A0A1C3YIN3
80095	229533.A0A0E0RP78	pp	229533.I1S2E8
80096	229533.A0A0E0RP78	pp	229533.I1RZF6
80097	229533.A0A0E0RP78	pp	229533.I1RKR8
80098	229533.A0A0E0RP78	pp	229533.Q4I327
80099	229533.A0A0E0RP78	pp	229533.Q4HZ68
80100	229533.A0A0E0RP78	pp	229533.I1RNY2
80101	229533.A0A0E0RP78	pp	229533.V6R227
80102	229533.A0A0E0RP78	pp	229533.I1RC06
80103	229533.A0A0E0RP78	pp	229533.I1RKC6
80104	229533.A0A0E0RP78	pp	229533.I1RAZ8
80105	229533.A0A0E0RP78	pp	229533.I1RG56
80106	229533.A0A0E0RP78	pp	229533.Q4HZB8
80107	229533.A0A0E0RP78	pp	229533.I1RC27
80108	229533.A0A0E0RP78	pp	229533.I1RVZ0
80109	229533.A0A0E0RP78	pp	229533.V6RGN5
80110	229533.A0A0E0RP78	pp	229533.I1RE00
80111	229533.A0A0E0RP78	pp	229533.I1RM96
80112	229533.A0A0E0RP78	pp	229533.I1RF19
80113	229533.A0A0E0RP78	pp	229533.I1RMN6
80114	229533.A0A0E0RP78	pp	229533.Q4IEK8
80115	229533.A0A0E0RP78	pp	229533.I1RTE9
80116	229533.A0A0E0RP78	pp	229533.I1RC33
80117	229533.A0A0E0RP78	pp	229533.I1RSI6
80118	229533.A0A0E0RP78	pp	229533.V6QUE6
80119	229533.A0A0E0RP78	pp	229533.A0A1C3YIK9
80120	229533.A0A0E0RP78	pp	229533.I1S2C0
80121	229533.A0A0E0RP78	pp	229533.Q4IBS2
80122	229533.A0A098DLB3	pp	229533.I1S051
80123	229533.A0A098DLB3	pp	229533.I1RMN3
80124	229533.A0A098DLB3	pp	229533.I1RKI1
80125	229533.A0A098DLB3	pp	229533.A0A1C3YIG8
80126	229533.A0A098DLB3	pp	229533.I1RE44
80127	229533.A0A098DLB3	pp	229533.I1RGC1
80128	229533.A0A098DLB3	pp	229533.I1RYM4
80129	229533.A0A098DLB3	pp	229533.A0A1C3YN54
80130	229533.A0A098DLB3	pp	229533.I1RT41
80131	229533.A0A098DLB3	pp	229533.V6RXW9
80132	229533.A0A098DLB3	pp	229533.I1RJY4
80133	229533.A0A098DLB3	pp	229533.I1RB00
80134	229533.A0A098DLB3	pp	229533.I1RUJ0
80135	229533.A0A098DLB3	pp	229533.I1RCV3
80136	229533.A0A098DLB3	pp	229533.I1RMM1
80137	229533.A0A098DLB3	pp	229533.I1S1U6
80138	229533.A0A098DLB3	pp	229533.I1RWZ9
80139	229533.A0A098DLB3	pp	229533.I1S9S4
80140	229533.A0A098DLB3	pp	229533.A0A0E0RV94
80141	229533.A0A098DLB3	pp	229533.A0A1C3YIE7
80142	229533.A0A098DLB3	pp	229533.I1RP87
80143	229533.A0A098DLB3	pp	229533.I1S0Z3
80144	229533.A0A098DLB3	pp	229533.I1RBS3
80145	229533.A0A098DLB3	pp	229533.I1RMG2
80146	229533.A0A098DLB3	pp	229533.I1RTQ9
80147	229533.A0A098DLB3	pp	229533.I1RXT7
80148	229533.A0A098DLB3	pp	229533.I1RZ84
80149	229533.A0A098DLB3	pp	229533.I1RCF0
80150	229533.A0A098DLB3	pp	229533.Q4I2D7
80151	229533.A0A098DLB3	pp	229533.I1RYP1
80152	229533.A0A098DLB3	pp	229533.Q4HY20
80153	229533.A0A098DLB3	pp	229533.A0A1C3YI93
80154	229533.A0A098DLB3	pp	229533.I1RKA7

80155	229533.A0A098DLB3	pp	229533.A0A1C3YLK5
80156	229533.A0A098DLB3	pp	229533.I1RIG4
80157	229533.A0A098DLB3	pp	229533.I1RS79
80158	229533.A0A098DLB3	pp	229533.I1S0G3
80159	229533.A0A098DLB3	pp	229533.I1RYP0
80160	229533.A0A098DLB3	pp	229533.I1RDD0
80161	229533.A0A098DLB3	pp	229533.A0A0E0SMG8
80162	229533.A0A098DLB3	pp	229533.A0A098DSW7
80163	229533.A0A098DLB3	pp	229533.I1RQP7
80164	229533.A0A098DLB3	pp	229533.I1RBS4
80165	229533.A0A098DLB3	pp	229533.A0A0E0SNZ9
80166	229533.A0A098DLB3	pp	229533.A0A1C3YL89
80167	229533.A0A098DLB3	pp	229533.I1RUN9
80168	229533.A0A098DLB3	pp	229533.I1RKR7
80169	229533.A0A098DLB3	pp	229533.I1RN70
80170	229533.A0A098DLB3	pp	229533.I1RAH5
80171	229533.A0A098DLB3	pp	229533.I1S287
80172	229533.A0A098DLB3	pp	229533.I1RAZ9
80173	229533.A0A098DLB3	pp	229533.A0A1C3YKT4
80174	229533.I1RJE7	pp	229533.I1RQX9
80175	229533.I1RJE7	pp	229533.I1SAI4
80176	229533.I1RJE7	pp	229533.V6R0W1
80177	229533.I1RJE7	pp	229533.V6RAM6
80178	229533.I1RJE7	pp	229533.V6QU99
80179	229533.I1RJE7	pp	229533.V6R2Z2
80180	229533.I1RJE7	pp	229533.V6R5D2
80181	229533.I1RJE7	pp	229533.I1RNG4
80182	229533.I1RJE7	pp	229533.V6R756
80183	229533.I1RBS3	pp	229533.I1RQC3
80184	229533.I1RBS3	pp	229533.I1RDD0
80185	229533.I1RBS3	pp	229533.I1RXT7
80186	229533.I1RBS3	pp	229533.I1RT00
80187	229533.I1RBS3	pp	229533.I1RBS4
80188	229533.I1RBS3	pp	229533.I1RX87
80189	229533.I1RBS3	pp	229533.I1RZ84
80190	229533.I1RBS3	pp	229533.I1S2B6
80191	229533.I1RBS3	pp	229533.I1S051
80192	229533.I1RBS3	pp	229533.I1RMN3
80193	229533.I1RBS3	pp	229533.I1RFP5
80194	229533.I1RBS3	pp	229533.I1RQD6
80195	229533.I1RBS3	pp	229533.I1RE44
80196	229533.I1RBS3	pp	229533.I1RJP3
80197	229533.I1RBS3	pp	229533.I1RSW4
80198	229533.I1RBS3	pp	229533.I1RGC1
80199	229533.I1RBS3	pp	229533.I1RNL7
80200	229533.I1RBS3	pp	229533.I1S0V5
80201	229533.I1RBS3	pp	229533.I1RRC8
80202	229533.I1RBS3	pp	229533.I1RG01
80203	229533.I1RBS3	pp	229533.I1RSH3
80204	229533.I1RBS3	pp	229533.I1RQC4
80205	229533.I1RBS3	pp	229533.V6QU20
80206	229533.I1RBS3	pp	229533.I1RQN8
80207	229533.I1RBS3	pp	229533.I1RN70
80208	229533.I1RBS3	pp	229533.I1S1U6
80209	229533.I1RBS3	pp	229533.I1RPY3
80210	229533.I1RBS3	pp	229533.I1S287
80211	229533.I1RBS3	pp	229533.I1RP87
80212	229533.I1RBS3	pp	229533.I1RWZ9
80213	229533.I1RBS3	pp	229533.I1RRC9
80214	229533.I1RBS3	pp	229533.I1RY40
80215	229533.I1RBS3	pp	229533.I1RTF5
80216	229533.I1RBS3	pp	229533.I1RMM1
80217	229533.I1RBS3	pp	229533.I1RTF2
80218	229533.I1RBS3	pp	229533.I1RNM3
80219	229533.I1RBS3	pp	229533.I1RUN9
80220	229533.I1RBS3	pp	229533.I1RQP7
80221	229533.I1RBS3	pp	229533.V6QXP4
80222	229533.I1RBS3	pp	229533.I1RIG4
80223	229533.I1RBS3	pp	229533.I1RLR7
80224	229533.I1RBS3	pp	229533.I1RYP0
80225	229533.I1RBS3	pp	229533.I1RX95
80226	229533.I1RBS3	pp	229533.I1RRI4
80227	229533.I1RBS3	pp	229533.I1RCV3

80228 229533.I1RBS3 pp 229533.Q4HY20
80229 229533.I1RBS3 pp 229533.V6RXW9
80230 229533.I1RSC6 pp 229533.I1RSI6
80231 229533.I1RSC6 pp 229533.V6RCG5
80232 229533.I1RSC6 pp 229533.V6RGY0
80233 229533.I1RSC6 pp 229533.I1RX08
80234 229533.I1RSC6 pp 229533.I1RUG3
80235 229533.I1RSC6 pp 229533.V6RGN5
80236 229533.I1RSC6 pp 229533.I1RW99
80237 229533.I1RSC6 pp 229533.I1S1U7
80238 229533.I1RSC6 pp 229533.I1S021
80239 229533.I1RSC6 pp 229533.I1S0J1
80240 229533.I1RSC6 pp 229533.I1RX14
80241 229533.I1RSC6 pp 229533.I1RSR3
80242 229533.I1RSC6 pp 229533.V6QW22
80243 229533.I1RSC6 pp 229533.I1RZ99
80244 229533.I1RSC6 pp 229533.I1RZF6
80245 229533.I1RSC6 pp 229533.I1RW71
80246 229533.I1RSC6 pp 229533.I1RSD8
80247 229533.I1RSC6 pp 229533.I1S274
80248 229533.I1RSC6 pp 229533.I1S0M3
80249 229533.I1RSC6 pp 229533.I1S0L3
80250 229533.I1RSC6 pp 229533.V6RIV9
80251 229533.I1RSC6 pp 229533.I1RTE9
80252 229533.I1RSC6 pp 229533.I1RVQ0
80253 229533.I1RSC6 pp 229533.I1S253
80254 229533.I1RSC6 pp 229533.I1RTD9
80255 229533.I1RSC6 pp 229533.I1RVZ0
80256 229533.I1RSC6 pp 229533.I1S0R7
80257 229533.I1RSC6 pp 229533.I1RTF4
80258 229533.I1RSC6 pp 229533.I1RUQ2
80259 229533.I1RSC6 pp 229533.I1RWJ8
80260 229533.I1RSC6 pp 229533.I1RT80
80261 229533.I1RSC6 pp 229533.I1S9U2
80262 229533.I1RSC6 pp 229533.I1RZS6
80263 229533.I1RSC6 pp 229533.I1RZZ9
80264 229533.I1RSC6 pp 229533.I1RW25
80265 229533.I1RSC6 pp 229533.V6QYF0
80266 229533.Q4INI2 pp 229533.Q4INZ9
80267 229533.Q4INI2 pp 229533.V6RR76
80268 229533.Q4INI2 pp 229533.Q4IPI4
80269 229533.I1RJG4 pp 229533.I1RVI8
80270 229533.I1RJG4 pp 229533.V6R0W1
80271 229533.I1RJG4 pp 229533.V6R5D2
80272 229533.I1RJG4 pp 229533.V6RH92
80273 229533.I1RJG4 pp 229533.I1SAI4
80274 229533.I1RJG4 pp 229533.V6QU99
80275 229533.I1RJG4 pp 229533.V6R2Z2
80276 229533.I1RJG4 pp 229533.V6RAM6
80277 229533.I1RJG4 pp 229533.I1S147
80278 229533.I1RJG4 pp 229533.V6R756
80279 229533.A0A098DMQ3 pp 229533.I1RDD0
80280 229533.I1S9S4 pp 229533.V6RXW9
80281 229533.I1RJ13 pp 229533.I1RNG4
80282 229533.I1RJ13 pp 229533.I1RS87
80283 229533.A0A1C3YI44 pp 229533.I1RPK0
80284 229533.A0A1C3YI44 pp 229533.I1RW16
80285 229533.A0A1C3YI44 pp 229533.Q4I877
80286 229533.A0A1C3YI44 pp 229533.I1RA72
80287 229533.A0A1C3YI44 pp 229533.I1RFK0
80288 229533.A0A1C3YI44 pp 229533.A0A1C3YII1
80289 229533.A0A1C3YI44 pp 229533.I1RWT6
80290 229533.A0A1C3YI44 pp 229533.I1S0Z3
80291 229533.A0A1C3YI44 pp 229533.V6QX58
80292 229533.A0A1C3YI44 pp 229533.I1RST4
80293 229533.A0A1C3YI44 pp 229533.I1RY44
80294 229533.A0A1C3YI44 pp 229533.I1RRF0
80295 229533.A0A1C3YI44 pp 229533.I1S096
80296 229533.A0A1C3YI44 pp 229533.I1RCF0
80297 229533.A0A1C3YI44 pp 229533.I1RMG2
80298 229533.A0A1C3YI44 pp 229533.A0A1C3YK75
80299 229533.A0A1C3YI44 pp 229533.A0A1C3YLK5
80300 229533.A0A1C3YI44 pp 229533.I1RAW7

80301 229533.A0A1C3YI44 pp 229533.I1RIG4
80302 229533.A0A1C3YI44 pp 229533.I1RRF9
80303 229533.A0A1C3YI44 pp 229533.I1RXT9
80304 229533.A0A1C3YI44 pp 229533.I1RE19
80305 229533.A0A1C3YI44 pp 229533.I1RUJ0
80306 229533.A0A1C3YI44 pp 229533.I1RR95
80307 229533.A0A1C3YI44 pp 229533.I1RAZ9
80308 229533.A0A1C3YI44 pp 229533.I1RP92
80309 229533.A0A1C3YI44 pp 229533.I1RQ80
80310 229533.A0A1C3YI44 pp 229533.A0A1C3YLL9
80311 229533.A0A1C3YI44 pp 229533.Q4ICM0
80312 229533.A0A1C3YI44 pp 229533.I1RRT4
80313 229533.A0A1C3YI44 pp 229533.I1RND7
80314 229533.A0A1C3YI44 pp 229533.I1RQ16
80315 229533.A0A1C3YI44 pp 229533.I1S270
80316 229533.A0A1C3YI44 pp 229533.A0A1C3YM61
80317 229533.A0A1C3YI44 pp 229533.Q5I2J3
80318 229533.A0A1C3YI44 pp 229533.A0A1C3YIP6
80319 229533.A0A1C3YI44 pp 229533.Q4INI2
80320 229533.A0A1C3YI44 pp 229533.I1S9S4
80321 229533.A0A1C3YI44 pp 229533.I1RQA3
80322 229533.A0A1C3YI44 pp 229533.I1RZK3
80323 229533.A0A1C3YI44 pp 229533.I1RMP2
80324 229533.A0A1C3YI44 pp 229533.I1RTA3
80325 229533.A0A1C3YI44 pp 229533.I1RRB0
80326 229533.A0A1C3YI44 pp 229533.I1RNM1
80327 229533.A0A1C3YI44 pp 229533.I1RZU6
80328 229533.A0A1C3YI44 pp 229533.I1REU2
80329 229533.A0A1C3YI44 pp 229533.I1RR74
80330 229533.A0A1C3YI44 pp 229533.A0A1C3YIF4
80331 229533.A0A1C3YI44 pp 229533.I1RTQ9
80332 229533.A0A1C3YI44 pp 229533.A0A1C3YIG8
80333 229533.A0A1C3YI44 pp 229533.I1RI75
80334 229533.A0A1C3YI44 pp 229533.A0A1C3YN54
80335 229533.A0A1C3YI44 pp 229533.I1RT65
80336 229533.A0A1C3YI44 pp 229533.I1RD70
80337 229533.A0A1C3YI44 pp 229533.Q4I2D7
80338 229533.A0A1C3YI44 pp 229533.I1S0S8
80339 229533.A0A1C3YI44 pp 229533.I1RMF2
80340 229533.A0A1C3YI44 pp 229533.Q4HZS8
80341 229533.A0A1C3YI44 pp 229533.I1S2E9
80342 229533.A0A1C3YI44 pp 229533.A0A1C3YN58
80343 229533.A0A1C3YI44 pp 229533.Q4IPI4
80344 229533.A0A1C3YI44 pp 229533.I1RAT7
80345 229533.A0A1C3YI44 pp 229533.I1RWD5
80346 229533.A0A1C3YI44 pp 229533.I1RDE9
80347 229533.A0A1C3YI44 pp 229533.I1RYM4
80348 229533.A0A1C3YI44 pp 229533.I1RKR9
80349 229533.A0A1C3YI44 pp 229533.I1RX54
80350 229533.A0A1C3YI44 pp 229533.A0A1C3YL06
80351 229533.A0A1C3YI44 pp 229533.I1RKI1
80352 229533.A0A1C3YI44 pp 229533.I1RB35
80353 229533.A0A1C3YI44 pp 229533.I1RSH3
80354 229533.A0A1C3YI44 pp 229533.I1S1V5
80355 229533.A0A1C3YI44 pp 229533.I1RN31
80356 229533.A0A1C3YI44 pp 229533.I1RQF7
80357 229533.A0A1C3YI44 pp 229533.I1RLX6
80358 229533.A0A1C3YI44 pp 229533.I1RD02
80359 229533.A0A1C3YI44 pp 229533.I1RBS4
80360 229533.A0A1C3YI44 pp 229533.I1RT41
80361 229533.A0A1C3YJY5 pp 229533.I1RCN2
80362 229533.A0A1C3YJY5 pp 229533.I1RC91
80363 229533.A0A1C3YJY5 pp 229533.I1RB73
80364 229533.I1RF16 pp 229533.I1RHU0
80365 229533.I1RF16 pp 229533.I1RZM4
80366 229533.I1RF16 pp 229533.I1S9T6
80367 229533.I1RF16 pp 229533.V6R2Z2
80368 229533.I1RF16 pp 229533.V6RAM6
80369 229533.I1RF16 pp 229533.V6QU99
80370 229533.I1RF16 pp 229533.V6R5D2
80371 229533.I1RF16 pp 229533.V6RK96
80372 229533.I1RF16 pp 229533.I1RK44
80373 229533.I1RF16 pp 229533.I1RIW5

80374	229533.I1RF16	pp	229533.I1RYM5
80375	229533.I1RF16	pp	229533.I1RMU5
80376	229533.I1RF16	pp	229533.I1RRM1
80377	229533.I1RF16	pp	229533.I1RNH4
80378	229533.I1RF16	pp	229533.I1RQE8
80379	229533.I1RF16	pp	229533.V6R756
80380	229533.I1RF16	pp	229533.I1SAI4
80381	229533.I1RF16	pp	229533.V6R0W1
80382	229533.I1RTQ9	pp	229533.I1S1V7
80383	229533.I1RTQ9	pp	229533.I1RZQ6
80384	229533.I1RTQ9	pp	229533.I1RWB4
80385	229533.I1RTQ9	pp	229533.I1RXF5
80386	229533.I1RTQ9	pp	229533.I1RW76
80387	229533.I1RTQ9	pp	229533.I1SAM5
80388	229533.I1RTQ9	pp	229533.I1RWP9
80389	229533.I1RTQ9	pp	229533.V6RB81
80390	229533.I1RTQ9	pp	229533.I1RVA8
80391	229533.I1RTQ9	pp	229533.I1S7X9
80392	229533.I1RTQ9	pp	229533.I1RYU1
80393	229533.I1RTQ9	pp	229533.I1RYW8
80394	229533.I1RTQ9	pp	229533.I1S842
80395	229533.I1RTQ9	pp	229533.V6REE9
80396	229533.I1RTQ9	pp	229533.Q4ILI9
80397	229533.I1RTQ9	pp	229533.I1S1C8
80398	229533.I1RTQ9	pp	229533.I1RX84
80399	229533.I1RTQ9	pp	229533.I1S224
80400	229533.I1RTQ9	pp	229533.I1S0Z3
80401	229533.I1RTQ9	pp	229533.I1S287
80402	229533.I1RTQ9	pp	229533.Q4HZS8
80403	229533.I1RTQ9	pp	229533.Q5I2J3
80404	229533.I1RTQ9	pp	229533.Q4ICM0
80405	229533.I1RTQ9	pp	229533.Q4I877
80406	229533.I1RTQ9	pp	229533.I1RY40
80407	229533.I1RTQ9	pp	229533.Q4HYZ2
80408	229533.I1RDC4	pp	229533.I1RSV4
80409	229533.I1RYP0	pp	229533.I1S2B6
80410	229533.I1RYP0	pp	229533.I1RZS5
80411	229533.I1RYP0	pp	229533.I1RZK3
80412	229533.I1RYP0	pp	229533.I1S9S4
80413	229533.I1RYP0	pp	229533.V6RXW9
80414	229533.I1RYP0	pp	229533.I1RZ84
80415	229533.I1RYP0	pp	229533.Q4HY20
80416	229533.I1RYP0	pp	229533.I1SAM5
80417	229533.I1RYP0	pp	229533.I1S051
80418	229533.I1RYP0	pp	229533.I1S287
80419	229533.I1RYP0	pp	229533.I1S278
80420	229533.I1RYP0	pp	229533.I1S1U6
80421	229533.I1RYP0	pp	229533.V6QXP4
80422	229533.I1RYP0	pp	229533.I1S0V5
80423	229533.I1RY96	pp	229533.V6R756
80424	229533.I1RY96	pp	229533.I1SAI4
80425	229533.I1RY96	pp	229533.V6QU99
80426	229533.I1RY96	pp	229533.V6R0W1
80427	229533.I1RY96	pp	229533.V6R2Z2
80428	229533.I1RY96	pp	229533.V6RAM6
80429	229533.I1RY96	pp	229533.V6R5D2
80430	229533.I1RAH5	pp	229533.I1RRR5
80431	229533.I1RAH5	pp	229533.I1RXT7
80432	229533.I1RAH5	pp	229533.I1S051
80433	229533.I1RAH5	pp	229533.I1RX87
80434	229533.I1RAH5	pp	229533.I1RYP0
80435	229533.I1RAH5	pp	229533.I1RX95
80436	229533.I1RAH5	pp	229533.I1RBS4
80437	229533.I1RAH5	pp	229533.I1RDD0
80438	229533.I1RAH5	pp	229533.I1RZ21
80439	229533.I1RAH5	pp	229533.I1RGC1
80440	229533.I1RAH5	pp	229533.I1RY40
80441	229533.I1RAH5	pp	229533.I1RYF2
80442	229533.I1RAH5	pp	229533.I1RWZ9
80443	229533.I1RAH5	pp	229533.I1RP87
80444	229533.I1RAH5	pp	229533.I1RNM3
80445	229533.I1RAH5	pp	229533.I1RBS3
80446	229533.I1RAH5	pp	229533.I1S2B6

80447 229533.I1RAH5 pp 229533.I1RAK7
80448 229533.I1RAH5 pp 229533.I1RPY3
80449 229533.I1RAH5 pp 229533.Q4HY20
80450 229533.I1RAH5 pp 229533.I1RN70
80451 229533.I1RAH5 pp 229533.I1S287
80452 229533.I1RAH5 pp 229533.Q4IN00
80453 229533.I1RAH5 pp 229533.I1RCV3
80454 229533.I1RAH5 pp 229533.I1RZ84
80455 229533.I1RAH5 pp 229533.I1S0V5
80456 229533.I1RAH5 pp 229533.V6RXW9
80457 229533.I1RAH5 pp 229533.I1RT00
80458 229533.I1RAH5 pp 229533.I1RRE1
80459 229533.I1RAH5 pp 229533.I1RPW0
80460 229533.I1RAH5 pp 229533.I1RBF2
80461 229533.I1RAH5 pp 229533.I1S1U6
80462 229533.I1RAH5 pp 229533.I1RQC4
80463 229533.I1RAH5 pp 229533.I1RMN3
80464 229533.I1RAH5 pp 229533.I1RE44
80465 229533.I1RAH5 pp 229533.I1RXF0
80466 229533.I1RAH5 pp 229533.I1RIG4
80467 229533.I1RAH5 pp 229533.I1RUN9
80468 229533.I1RAH5 pp 229533.I1RQP7
80469 229533.I1RAH5 pp 229533.I1RWQ8
80470 229533.I1RGF6 pp 229533.I1RSD8
80471 229533.I1RGF6 pp 229533.I1RRT2
80472 229533.I1RGF6 pp 229533.Q4IQW0
80473 229533.I1RGF6 pp 229533.I1RJL4
80474 229533.I1RGF6 pp 229533.I1RZS6
80475 229533.I1RGF6 pp 229533.I1RRQ3
80476 229533.I1RGF6 pp 229533.I1RS47
80477 229533.I1RGF6 pp 229533.I1RNH6
80478 229533.I1RGF6 pp 229533.I1RQ65
80479 229533.I1RGF6 pp 229533.I1RQ31
80480 229533.I1RGF6 pp 229533.I1RMN6
80481 229533.I1RGF6 pp 229533.V6R283
80482 229533.I1RGF6 pp 229533.I1RSI6
80483 229533.I1RGF6 pp 229533.I1RKL3
80484 229533.I1RGF6 pp 229533.I1S021
80485 229533.I1RGF6 pp 229533.I1RRJ8
80486 229533.I1RGF6 pp 229533.I1RM96
80487 229533.I1RGF6 pp 229533.I1RZF6
80488 229533.I1RGF6 pp 229533.I1RNU6
80489 229533.I1RGF6 pp 229533.I1RZZ9
80490 229533.I1RGF6 pp 229533.I1RQA2
80491 229533.I1RGF6 pp 229533.I1RX14
80492 229533.I1RGF6 pp 229533.Q4IJ56
80493 229533.I1RGF6 pp 229533.I1RRI2
80494 229533.I1RGF6 pp 229533.I1RPU0
80495 229533.I1RGF6 pp 229533.I1RNH1
80496 229533.I1RGF6 pp 229533.I1RSR3
80497 229533.I1RGF6 pp 229533.I1S9U2
80498 229533.I1RGF6 pp 229533.I1RNV6
80499 229533.I1RGF6 pp 229533.I1RVZ0
80500 229533.I1RGF6 pp 229533.I1RPH9
80501 229533.I1RGF6 pp 229533.I1RKQ5
80502 229533.I1RGF6 pp 229533.I1RT80
80503 229533.I1RGF6 pp 229533.I1RK95
80504 229533.I1RGF6 pp 229533.I1RM06
80505 229533.I1RGF6 pp 229533.V6QYF0
80506 229533.I1RK17 pp 229533.Q4HYR2
80507 229533.A0A1C3YMB6 pp 229533.I1RMQ7
80508 229533.A0A1C3YMB6 pp 229533.I1RC17
80509 229533.A0A1C3YMB6 pp 229533.I1S193
80510 229533.A0A1C3YMB6 pp 229533.Q4IJM4
80511 229533.A0A1C3YMB6 pp 229533.I1RYY5
80512 229533.A0A1C3YMB6 pp 229533.I1RAT7
80513 229533.A0A1C3YMB6 pp 229533.I1R9N1
80514 229533.A0A1C3YMB6 pp 229533.I1RFL7
80515 229533.A0A1C3YMB6 pp 229533.I1RK75
80516 229533.A0A1C3YMB6 pp 229533.I1S098
80517 229533.A0A1C3YMB6 pp 229533.I1RD15
80518 229533.A0A1C3YMB6 pp 229533.I1S0E8
80519 229533.A0A1C3YMB6 pp 229533.I1RPC2

80520 229533.A0A1C3YMB6 pp 229533.Q4I7L0
80521 229533.A0A1C3YMB6 pp 229533.I1RBJ0
80522 229533.I1RMS5 pp 229533.I1SAI4
80523 229533.I1RMS5 pp 229533.V6QU99
80524 229533.I1RMS5 pp 229533.I1RQL4
80525 229533.I1RMS5 pp 229533.V6QWD8
80526 229533.I1RMS5 pp 229533.I1RMU5
80527 229533.I1RMS5 pp 229533.V6R0W1
80528 229533.I1RMS5 pp 229533.I1S0F0
80529 229533.I1RMS5 pp 229533.V6RPN0
80530 229533.I1RMS5 pp 229533.V6R756
80531 229533.I1RMS5 pp 229533.I1S0H2
80532 229533.I1RMS5 pp 229533.I1RV06
80533 229533.I1RMS5 pp 229533.V6R2Z2
80534 229533.I1RMS5 pp 229533.I1S5R5
80535 229533.I1RMS5 pp 229533.V6R0H4
80536 229533.I1RMS5 pp 229533.I1RYM5
80537 229533.I1RMS5 pp 229533.I1S405
80538 229533.I1RMS5 pp 229533.I1S269
80539 229533.I1RMS5 pp 229533.I1RXH1
80540 229533.I1RMS5 pp 229533.Q4IJP1
80541 229533.I1RMS5 pp 229533.V6RAM6
80542 229533.I1RMS5 pp 229533.V6R5D2
80543 229533.I1RNL4 pp 229533.I1RTM8
80544 229533.I1RUN9 pp 229533.I1RZ84
80545 229533.I1RUN9 pp 229533.I1S2B6
80546 229533.I1RUN9 pp 229533.I1S9S4
80547 229533.I1RUN9 pp 229533.V6RXW9
80548 229533.I1RUN9 pp 229533.I1S051
80549 229533.I1RUN9 pp 229533.Q4HY20
80550 229533.I1RUN9 pp 229533.Q4I5I4
80551 229533.I1RUN9 pp 229533.I1RX87
80552 229533.I1RUN9 pp 229533.I1RXT7
80553 229533.I1RUN9 pp 229533.I1S287
80554 229533.I1RUN9 pp 229533.O74638
80555 229533.I1RUN9 pp 229533.I1RY40
80556 229533.I1RUN9 pp 229533.I1RWZ9
80557 229533.I1RUN9 pp 229533.I1RYP0
80558 229533.I1RQ24 pp 229533.I1RQP3
80559 229533.I1RQ24 pp 229533.Q4ICA8
80560 229533.I1RQ24 pp 229533.I1RY58
80561 229533.I1RQ24 pp 229533.Q4IPI4
80562 229533.I1RQ24 pp 229533.I1RQP1
80563 229533.I1RQ24 pp 229533.I1RRG3
80564 229533.I1RQ24 pp 229533.I1RSC7
80565 229533.A0A098DMP5 pp 229533.A0A1C3YN30
80566 229533.A0A098DMP5 pp 229533.I1RYU5
80567 229533.A0A098DMP5 pp 229533.Q4HXF0
80568 229533.A0A098DMP5 pp 229533.V6R1K4
80569 229533.A0A098DMP5 pp 229533.I1RZ20
80570 229533.A0A098DMP5 pp 229533.V6R057
80571 229533.A0A098DMP5 pp 229533.I1RCB4
80572 229533.A0A098DMP5 pp 229533.A0A098E0A5
80573 229533.A0A098DMP5 pp 229533.I1RPU8
80574 229533.A0A098DMP5 pp 229533.I1RS17
80575 229533.A0A0E0SFV6 pp 229533.I1S182
80576 229533.A0A0E0SFV6 pp 229533.I1RED3
80577 229533.A0A0E0SFV6 pp 229533.I1S9H8
80578 229533.A0A0E0SFV6 pp 229533.I1RN04
80579 229533.A0A0E0SFV6 pp 229533.I1RE30
80580 229533.A0A0E0SFV6 pp 229533.A0A1C3YLP8
80581 229533.A0A0E0SFV6 pp 229533.I1S5Z5
80582 229533.A0A0E0SFV6 pp 229533.I1RVL8
80583 229533.A0A0E0SFV6 pp 229533.I1RC24
80584 229533.A0A0E0SFV6 pp 229533.I1RRG3
80585 229533.A0A0E0SFV6 pp 229533.I1RW90
80586 229533.A0A0E0SFV6 pp 229533.I1RZM2
80587 229533.A0A0E0SFV6 pp 229533.A0A1C3YIR8
80588 229533.A0A0E0SFV6 pp 229533.I1RW91
80589 229533.A0A0E0SFV6 pp 229533.I1RSM8
80590 229533.A0A0E0SFV6 pp 229533.I1RBC7
80591 229533.A0A0E0SFV6 pp 229533.I1RKB1
80592 229533.A0A0E0SFV6 pp 229533.I1RQ42

80593 229533.A0A0E0SFV6 pp 229533.A0A0E0SHL3
80594 229533.A0A0E0SFV6 pp 229533.I1RVY0
80595 229533.A0A0E0SFV6 pp 229533.V6QWX8
80596 229533.A0A0E0SFV6 pp 229533.I1RPM7
80597 229533.A0A0E0SFV6 pp 229533.I1RPQ2
80598 229533.A0A0E0SFV6 pp 229533.I1RPW8
80599 229533.A0A0E0SFV6 pp 229533.A0A1C3YLK8
80600 229533.A0A0E0SFV6 pp 229533.I1RW80
80601 229533.A0A0E0SFV6 pp 229533.I1RK71
80602 229533.A0A0E0SFV6 pp 229533.I1RSZ3
80603 229533.A0A0E0SFV6 pp 229533.I1RZB5
80604 229533.A0A0E0SFV6 pp 229533.A0A1C3YMU9
80605 229533.A0A0E0SFV6 pp 229533.I1REN3
80606 229533.A0A0E0SFV6 pp 229533.I1RH75
80607 229533.A0A0E0SFV6 pp 229533.I1RAP1
80608 229533.A0A0E0SFV6 pp 229533.A0A0E0SMY1
80609 229533.A0A0E0SFV6 pp 229533.I1RJL2
80610 229533.A0A0E0SFV6 pp 229533.I1RUG4
80611 229533.A0A0E0SFV6 pp 229533.I1RLI4
80612 229533.A0A0E0SFV6 pp 229533.I1RFI5
80613 229533.A0A0E0SFV6 pp 229533.I1RR25
80614 229533.A0A0E0SFV6 pp 229533.I1RG11
80615 229533.A0A0E0SFV6 pp 229533.V6QUE6
80616 229533.A0A0E0SFV6 pp 229533.I1RAR2
80617 229533.A0A0E0SFV6 pp 229533.A0A1C3YHX6
80618 229533.A0A0E0SFV6 pp 229533.I1RK79
80619 229533.A0A0E0SFV6 pp 229533.I1RKF3
80620 229533.A0A0E0SFV6 pp 229533.A0A1C3YJR3
80621 229533.A0A0E0SFV6 pp 229533.I1RCB7
80622 229533.A0A0E0SFV6 pp 229533.I1RED2
80623 229533.A0A0E0SFV6 pp 229533.A0A1C3YI23
80624 229533.A0A0E0SFV6 pp 229533.I1RXU0
80625 229533.A0A0E0SFV6 pp 229533.I1RKR8
80626 229533.A0A0E0SFV6 pp 229533.I1RA65
80627 229533.A0A0E0SFV6 pp 229533.I1RMV1
80628 229533.A0A0E0SFV6 pp 229533.I1RWF3
80629 229533.A0A0E0SFV6 pp 229533.I1RCH4
80630 229533.A0A0E0SFV6 pp 229533.I1RR61
80631 229533.A0A0E0SFV6 pp 229533.A0A1C3YN55
80632 229533.A0A0E0SFV6 pp 229533.I1RCG6
80633 229533.A0A0E0SFV6 pp 229533.A0A1C3YJ83
80634 229533.A0A0E0SFV6 pp 229533.I1RZ42
80635 229533.A0A0E0SFV6 pp 229533.I1S276
80636 229533.A0A0E0SFV6 pp 229533.I1RC15
80637 229533.A0A0E0SFV6 pp 229533.I1RDC9
80638 229533.A0A0E0SFV6 pp 229533.I1RRT0
80639 229533.I1RPY3 pp 229533.I1RZ39
80640 229533.I1RPY3 pp 229533.I1RXC2
80641 229533.I1RPY3 pp 229533.I1S5R1
80642 229533.I1RPY3 pp 229533.I1RSZ7
80643 229533.I1RPY3 pp 229533.I1RRI1
80644 229533.I1RPY3 pp 229533.I1RR24
80645 229533.I1RPY3 pp 229533.I1S549
80646 229533.I1RPY3 pp 229533.I1RTA1
80647 229533.I1RPY3 pp 229533.I1RPY4
80648 229533.I1RPY3 pp 229533.I1RXT7
80649 229533.I1RPY3 pp 229533.I1S2B8
80650 229533.I1RPY3 pp 229533.I1RQD6
80651 229533.I1RPY3 pp 229533.I1S4S7
80652 229533.I1RPY3 pp 229533.I1S051
80653 229533.I1RPY3 pp 229533.I1RYI5
80654 229533.I1RPY3 pp 229533.I1S287
80655 229533.I1RPY3 pp 229533.I1RT40
80656 229533.I1RPY3 pp 229533.I1S0W4
80657 229533.I1RPY3 pp 229533.I1RWR7
80658 229533.I1RPY3 pp 229533.I1RXD7
80659 229533.I1RPY3 pp 229533.I1S1U6
80660 229533.I1RPY3 pp 229533.I1RTD5
80661 229533.I1RPY3 pp 229533.I1RSA5
80662 229533.I1RPY3 pp 229533.Q4IJK2
80663 229533.I1RPY3 pp 229533.I1RXD6
80664 229533.I1RPY3 pp 229533.I1RQE7
80665 229533.I1RPY3 pp 229533.I1RQF9

80666	229533.I1RPY3	pp	229533.I1S1C7
80667	229533.I1RPY3	pp	229533.V6RTJ8
80668	229533.I1RPY3	pp	229533.I1RX46
80669	229533.I1RPY3	pp	229533.I1RT03
80670	229533.I1RPY3	pp	229533.I1S2B6
80671	229533.I1RPY3	pp	229533.I1S9S4
80672	229533.I1RPY3	pp	229533.I1RVZ2
80673	229533.I1RPY3	pp	229533.I1RZK5
80674	229533.I1RPY3	pp	229533.I1RZ84
80675	229533.I1RPY3	pp	229533.I1S1B2
80676	229533.I1RPY3	pp	229533.I1RVX0
80677	229533.I1RPY3	pp	229533.I1S5R6
80678	229533.I1RPY3	pp	229533.I1RRY9
80679	229533.I1RPY3	pp	229533.I1RUF7
80680	229533.I1RPY3	pp	229533.I1RT36
80681	229533.I1RPY3	pp	229533.I1RQ33
80682	229533.I1RPY3	pp	229533.I1RUG3
80683	229533.I1RPY3	pp	229533.I1RQD2
80684	229533.I1RPY3	pp	229533.I1RZM8
80685	229533.I1RPY3	pp	229533.Q4HY20
80686	229533.I1RPY3	pp	229533.I1S821
80687	229533.I1RPY3	pp	229533.I1RXC4
80688	229533.I1RPY3	pp	229533.I1S0J1
80689	229533.I1RPY3	pp	229533.I1RSQ3
80690	229533.I1RPY3	pp	229533.I1RRM3
80691	229533.I1RPY3	pp	229533.I1S1W9
80692	229533.I1RPY3	pp	229533.V6R042
80693	229533.I1RPY3	pp	229533.V6RMW6
80694	229533.I1RPY3	pp	229533.V6RXW9
80695	229533.I1RPY3	pp	229533.I1RZV3
80696	229533.I1RPY3	pp	229533.I1RSF1
80697	229533.I1RPY3	pp	229533.I1RQ85
80698	229533.I1RQ28	pp	229533.I1S2M6
80699	229533.I1RQ28	pp	229533.I1RWF7
80700	229533.I1RQ28	pp	229533.V6R283
80701	229533.I1RQ28	pp	229533.I1RW71
80702	229533.I1RQ28	pp	229533.Q4I830
80703	229533.I1RQ28	pp	229533.I1RRJ8
80704	229533.I1RQ28	pp	229533.I1RRP3
80705	229533.I1RQ28	pp	229533.Q4HZ47
80706	229533.I1RQ28	pp	229533.I1RRQ3
80707	229533.I1RQ28	pp	229533.Q4IEV5
80708	229533.I1RQ28	pp	229533.I1RS30
80709	229533.I1RQ28	pp	229533.I1RS47
80710	229533.I1RQ28	pp	229533.I1S0D4
80711	229533.I1RQ28	pp	229533.I1RQ65
80712	229533.I1RQ28	pp	229533.I1RZJ6
80713	229533.I1RQ28	pp	229533.Q4HZ42
80714	229533.I1RQ28	pp	229533.I1RT33
80715	229533.I1RQ28	pp	229533.I1S0R7
80716	229533.I1RQ28	pp	229533.I1RT98
80717	229533.I1RQ28	pp	229533.Q4IEK8
80718	229533.I1RQ28	pp	229533.Q4IPI1
80719	229533.I1RQ28	pp	229533.I1RVH7
80720	229533.I1RQ28	pp	229533.Q4IAS1
80721	229533.I1RQ28	pp	229533.I1S4T3
80722	229533.I1RQ28	pp	229533.I1RT05
80723	229533.I1RQ28	pp	229533.Q4IQW0
80724	229533.I1RQ28	pp	229533.Q4IF76
80725	229533.I1RQ28	pp	229533.I1RRR7
80726	229533.I1RQ28	pp	229533.I1S253
80727	229533.I1RQ28	pp	229533.V6R227
80728	229533.I1RQ28	pp	229533.Q4I662
80729	229533.I1RQ28	pp	229533.I1S6U8
80730	229533.I1RQ28	pp	229533.I1RWJ4
80731	229533.I1RQ28	pp	229533.Q4HVW2
80732	229533.I1RQ28	pp	229533.I1RZF6
80733	229533.I1RQ28	pp	229533.Q4IJH1
80734	229533.I1RQ28	pp	229533.I1RZS6
80735	229533.I1RQ28	pp	229533.Q4I327
80736	229533.I1RQ28	pp	229533.I1RTF4
80737	229533.I1RQ28	pp	229533.I1S021
80738	229533.I1RQ28	pp	229533.Q4IQK7

80739 229533.I1RQ28 pp 229533.V6QYF0
80740 229533.I1RQ28 pp 229533.I1RYB9
80741 229533.I1RQ28 pp 229533.I1S8E2
80742 229533.I1RQ28 pp 229533.I1S9F8
80743 229533.I1RQ28 pp 229533.I1RT80
80744 229533.I1RQ28 pp 229533.I1RRT8
80745 229533.I1RQ28 pp 229533.I1RQ31
80746 229533.I1RQ28 pp 229533.Q4IFI0
80747 229533.I1RQ28 pp 229533.I1S9U2
80748 229533.I1RQ28 pp 229533.I1RX08
80749 229533.I1RQ28 pp 229533.I1RQS8
80750 229533.I1RQ28 pp 229533.I1RSI6
80751 229533.I1RQ28 pp 229533.I1RZE5
80752 229533.I1RQ28 pp 229533.I1RSL8
80753 229533.I1RQ28 pp 229533.I1S0M3
80754 229533.I1RQ28 pp 229533.Q4IBS2
80755 229533.I1RQ28 pp 229533.V6QUE6
80756 229533.I1RQ28 pp 229533.I1RVZ0
80757 229533.I1RQ28 pp 229533.Q4I5Z5
80758 229533.I1RQ28 pp 229533.V6RGN5
80759 229533.I1RQ28 pp 229533.I1S0J1
80760 229533.I1RQ28 pp 229533.I1RYJ8
80761 229533.I1RQ28 pp 229533.Q4HZ68
80762 229533.I1RQ28 pp 229533.I1S060
80763 229533.I1RQ28 pp 229533.I1RZK6
80764 229533.I1RQ28 pp 229533.I1RX14
80765 229533.I1RQ28 pp 229533.I1RRI2
80766 229533.I1RQ28 pp 229533.Q4IAA0
80767 229533.I1RQ28 pp 229533.I1S296
80768 229533.I1RQ28 pp 229533.Q4IJ56
80769 229533.I1RQ28 pp 229533.I1S2E8
80770 229533.I1RQ28 pp 229533.I1RTD7
80771 229533.I1RQ28 pp 229533.I1RY63
80772 229533.I1RQ28 pp 229533.I1RSD8
80773 229533.I1RQ28 pp 229533.I1RTD9
80774 229533.I1RQ28 pp 229533.I1S0M9
80775 229533.I1RQ28 pp 229533.I1RSR8
80776 229533.I1RQ28 pp 229533.I1RSC6
80777 229533.I1RQ28 pp 229533.I1RSS4
80778 229533.I1RQ28 pp 229533.I1RTE9
80779 229533.I1RQ28 pp 229533.I1S558
80780 229533.I1RQ28 pp 229533.I1RQA2
80781 229533.I1RQ28 pp 229533.V6RGY3
80782 229533.I1RQ28 pp 229533.I1RSR3
80783 229533.I1S007 pp 229533.I1S0M4
80784 229533.I1RMV1 pp 229533.Q4HYI0
80785 229533.I1RMV1 pp 229533.I1RZ42
80786 229533.I1RMV1 pp 229533.I1RWF3
80787 229533.I1RMV1 pp 229533.I1RXE5
80788 229533.I1RMV1 pp 229533.Q4IPC8
80789 229533.I1RMV1 pp 229533.I1RRT0
80790 229533.I1RMV1 pp 229533.I1RSD1
80791 229533.I1RMV1 pp 229533.I1RRF7
80792 229533.I1RMV1 pp 229533.I1S276
80793 229533.I1RMV1 pp 229533.V6QV38
80794 229533.I1RMV1 pp 229533.I1RVL8
80795 229533.I1RMV1 pp 229533.V6QUE6
80796 229533.I1RMV1 pp 229533.I1RZB5
80797 229533.I1RMV1 pp 229533.I1RXU0
80798 229533.I1RMV1 pp 229533.I1RPM7
80799 229533.I1RMV1 pp 229533.I1RTC2
80800 229533.I1RMV1 pp 229533.I1S182
80801 229533.I1RMV1 pp 229533.I1RR25
80802 229533.I1RMV1 pp 229533.I1RRG3
80803 229533.I1RMV1 pp 229533.Q4IER8
80804 229533.I1RMV1 pp 229533.I1RR61
80805 229533.I1RMV1 pp 229533.I1RUG4
80806 229533.I1RMV1 pp 229533.I1RZM2
80807 229533.I1RMV1 pp 229533.I1RW90
80808 229533.I1RMV1 pp 229533.I1RW80
80809 229533.I1RMV1 pp 229533.I1RP96
80810 229533.I1RMV1 pp 229533.Q4I5W5
80811 229533.I1RMV1 pp 229533.Q4IJH1

80812 229533.I1RMV1 pp 229533.Q4IF76
80813 229533.I1RMV1 pp 229533.I1RSZ3
80814 229533.I1RMV1 pp 229533.V6QWX8
80815 229533.I1RMV1 pp 229533.I1RW91
80816 229533.I1RMV1 pp 229533.I1RPW8
80817 229533.I1RMV1 pp 229533.I1RN04
80818 229533.I1RMV1 pp 229533.I1RVY0
80819 229533.I1RMV1 pp 229533.I1RQ42
80820 229533.A0A098DWE6 pp 229533.I1RKR7
80821 229533.A0A098DWE6 pp 229533.I1RTD0
80822 229533.A0A098DWE6 pp 229533.A0A1C3YK51
80823 229533.A0A098DWE6 pp 229533.I1RWP9
80824 229533.A0A098DWE6 pp 229533.I1RE15
80825 229533.A0A098DWE6 pp 229533.I1RMF2
80826 229533.A0A098DWE6 pp 229533.A0A1C3YIR6
80827 229533.A0A098DWE6 pp 229533.I1RYU1
80828 229533.A0A098DWE6 pp 229533.I1S2W9
80829 229533.A0A098DWE6 pp 229533.I1RW76
80830 229533.A0A098DWE6 pp 229533.I1RXF5
80831 229533.A0A098DWE6 pp 229533.I1SAT1
80832 229533.A0A098DWE6 pp 229533.I1RIG4
80833 229533.A0A098DWE6 pp 229533.I1RV63
80834 229533.A0A098DWE6 pp 229533.A0A0E0SGM3
80835 229533.A0A098DWE6 pp 229533.A0A1C3YHW1
80836 229533.A0A098DWE6 pp 229533.I1SAM5
80837 229533.A0A098DWE6 pp 229533.I1RPK5
80838 229533.A0A098DWE6 pp 229533.I1RBF2
80839 229533.A0A098DWE6 pp 229533.I1S2B6
80840 229533.A0A098DWE6 pp 229533.I1RAS9
80841 229533.A0A098DWE6 pp 229533.I1RQH7
80842 229533.A0A098DWE6 pp 229533.I1RN82
80843 229533.A0A098DWE6 pp 229533.I1RE18
80844 229533.A0A098DWE6 pp 229533.I1RE20
80845 229533.A0A098DWE6 pp 229533.I1RBS4
80846 229533.A0A098DWE6 pp 229533.A0A0E0SDU6
80847 229533.A0A098DWE6 pp 229533.A0A1C3YID3
80848 229533.I1S567 pp 229533.Q4HTT2
80849 229533.I1S567 pp 229533.Q4I7L0
80850 229533.I1RX95 pp 229533.I1RXD6
80851 229533.I1RX95 pp 229533.I1S0W4
80852 229533.I1RX95 pp 229533.I1RYI5
80853 229533.I1RX95 pp 229533.I1S287
80854 229533.I1RX95 pp 229533.I1RXT7
80855 229533.I1RX95 pp 229533.I1RXC2
80856 229533.I1RX95 pp 229533.I1RXD7
80857 229533.I1RX95 pp 229533.I1S4S7
80858 229533.I1RX95 pp 229533.I1S438
80859 229533.I1RX95 pp 229533.I1S549
80860 229533.I1RX95 pp 229533.I1S5R1
80861 229533.I1RX95 pp 229533.I1S2B8
80862 229533.I1RX95 pp 229533.V6RTJ8
80863 229533.I1RX95 pp 229533.I1RZV3
80864 229533.I1RX95 pp 229533.Q4IJK2
80865 229533.I1RX95 pp 229533.V6RMW6
80866 229533.I1RX95 pp 229533.I1RZ39
80867 229533.I1RX95 pp 229533.V6RXW9
80868 229533.I1RX95 pp 229533.I1S051
80869 229533.I1RX95 pp 229533.I1S1U6
80870 229533.I1RX95 pp 229533.I1S0J1
80871 229533.I1RX95 pp 229533.I1RZ84
80872 229533.I1RX95 pp 229533.I1S1W9
80873 229533.I1RX95 pp 229533.V6R042
80874 229533.I1RX95 pp 229533.I1RXC4
80875 229533.I1RX95 pp 229533.I1S821
80876 229533.I1RX95 pp 229533.V6R9Z0
80877 229533.I1RX95 pp 229533.V6RCZ8
80878 229533.I1RX95 pp 229533.Q4HY20
80879 229533.I1RX95 pp 229533.I1RZM8
80880 229533.I1RX95 pp 229533.I1S1C7
80881 229533.I1RX95 pp 229533.I1S9S4
80882 229533.I1RX95 pp 229533.I1RZK5
80883 229533.I1RX95 pp 229533.I1S5R6
80884 229533.I1RX95 pp 229533.I1S2B6

229533.I1RX95 pp 229533.I1S1B2
229533.I1RX95 pp 229533.I1S437
229533.I1S5Y0 pp 229533.I1SAK2
229533.I1S768 pp 229533.I1S7H3
229533.A0A098DMN4 pp 229533.I1RJU3
229533.A0A098DMN4 pp 229533.A0A1C3YKV3
229533.A0A098DMN4 pp 229533.I1RS17
229533.A0A098DMN4 pp 229533.V6RIV9
229533.A0A098DMN4 pp 229533.I1RCH5
229533.A0A098DMN4 pp 229533.I1REK8
229533.I1RWZ9 pp 229533.I1RYW8
229533.I1RWZ9 pp 229533.I1S9S4
229533.I1RWZ9 pp 229533.I1S2B6
229533.I1RWZ9 pp 229533.V6R042
229533.I1RWZ9 pp 229533.I1RXT7
229533.I1RWZ9 pp 229533.I1S1U6
229533.I1RWZ9 pp 229533.V6RXW9
229533.I1RWZ9 pp 229533.V6RKE4
229533.I1RWZ9 pp 229533.I1RXF0
229533.I1RWZ9 pp 229533.I1RYW3
229533.I1RWZ9 pp 229533.I1RYP0
229533.I1RWZ9 pp 229533.I1RZ84
229533.I1RWZ9 pp 229533.I1RZ60
229533.I1RWZ9 pp 229533.I1S051
229533.I1RWZ9 pp 229533.V6RR76
229533.I1RWZ9 pp 229533.I1RZA4
229533.I1RWZ9 pp 229533.V6RQ48
229533.I1RWZ9 pp 229533.I1RY40
229533.I1RWZ9 pp 229533.Q4HY20
229533.I1RWZ9 pp 229533.I1S0V5
229533.I1RWZ9 pp 229533.I1RX87
229533.I1RWZ9 pp 229533.I1S287
229533.A0A0E0RTH0 pp 229533.I1SAI4
229533.A0A0E0RTH0 pp 229533.I1RDV8
229533.A0A0E0RTH0 pp 229533.I1RAG3
229533.A0A0E0RTH0 pp 229533.I1RIW5
229533.A0A0E0RTH0 pp 229533.A0A1C3YIE0
229533.A0A0E0RTH0 pp 229533.I1RMU5
229533.A0A0E0RTH0 pp 229533.V6R5D2
229533.A0A0E0RTH0 pp 229533.V6R0W1
229533.A0A0E0RTH0 pp 229533.I1RQE8
229533.A0A0E0RTH0 pp 229533.A0A1C3YKB7
229533.A0A0E0RTH0 pp 229533.I1RZM4
229533.A0A0E0RTH0 pp 229533.V6R2Z2
229533.A0A0E0RTH0 pp 229533.I1RHU0
229533.A0A0E0RTH0 pp 229533.A0A1C3YKN4
229533.A0A0E0RTH0 pp 229533.V6RAM6
229533.A0A0E0RTH0 pp 229533.V6QU99
229533.A0A0E0RTH0 pp 229533.I1S9T6
229533.A0A0E0RTH0 pp 229533.A0A1C3YHF4
229533.A0A0E0RTH0 pp 229533.I1RNH4
229533.A0A0E0RTH0 pp 229533.A0A1C3YIR5
229533.A0A0E0RTH0 pp 229533.I1RYM5
229533.A0A0E0RTH0 pp 229533.A0A1C3YIP2
229533.A0A0E0RTH0 pp 229533.V6R756
229533.A0A0E0RTH0 pp 229533.I1RRM1
229533.A0A0E0RTH0 pp 229533.I1RK44
229533.A0A0E0RTH0 pp 229533.V6RK96
229533.A0A0E0RTH0 pp 229533.A0A0E0SH57
229533.A0A1C3YMD7 pp 229533.Q4ICA8
229533.A0A1C3YMD7 pp 229533.I1RBW0
229533.A0A1C3YMD7 pp 229533.I1RY58
229533.A0A1C3YMD7 pp 229533.I1RQP3
229533.A0A1C3YMD7 pp 229533.I1RRG3
229533.A0A1C3YMD7 pp 229533.I1RSC7
229533.A0A1C3YMD7 pp 229533.A0A1C3YN69
229533.A0A1C3YMD7 pp 229533.Q4IPI4
229533.A0A1C3YMD7 pp 229533.I1RBW1
229533.A0A1C3YMD7 pp 229533.I1RQP1
229533.I1S9W0 pp 229533.V6R3U5
229533.I1RNM3 pp 229533.I1RQF9
229533.I1RNM3 pp 229533.I1RXW3
229533.I1RNM3 pp 229533.I1S287

80958	229533.I1RNM3	pp	229533.I1RPY3
80959	229533.I1RNM3	pp	229533.I1RXF0
80960	229533.I1RNM3	pp	229533.I1RP87
80961	229533.I1RNM3	pp	229533.I1RUN9
80962	229533.I1RNM3	pp	229533.I1S9S4
80963	229533.I1RNM3	pp	229533.I1RWZ9
80964	229533.I1RNM3	pp	229533.I1S051
80965	229533.I1RNM3	pp	229533.I1RXV9
80966	229533.I1RNM3	pp	229533.I1RQD6
80967	229533.I1RNM3	pp	229533.I1RXT7
80968	229533.I1RNM3	pp	229533.V6RXW9
80969	229533.I1RNM3	pp	229533.I1RX95
80970	229533.I1RNM3	pp	229533.I1RZ84
80971	229533.I1RNM3	pp	229533.I1RSL5
80972	229533.I1RNM3	pp	229533.I1RT00
80973	229533.I1RNM3	pp	229533.Q4HY20
80974	229533.I1RNM3	pp	229533.I1RYL5
80975	229533.I1RNM3	pp	229533.I1S2B6
80976	229533.I1RW23	pp	229533.Q4I7N9
80977	229533.I1RW23	pp	229533.I1S755
80978	229533.I1RW23	pp	229533.Q4IJU0
80979	229533.I1RW23	pp	229533.I1RZB5
80980	229533.I1RW23	pp	229533.Q4HYB8
80981	229533.I1RW23	pp	229533.V6QWX8
80982	229533.I1RW23	pp	229533.I1RW90
80983	229533.I1RW23	pp	229533.Q4HTT2
80984	229533.I1RW23	pp	229533.I1RZG3
80985	229533.I1RW23	pp	229533.V6QUE6
80986	229533.I1RW23	pp	229533.Q4I5I4
80987	229533.I1RW23	pp	229533.Q4IJ11
80988	229533.I1RW23	pp	229533.Q4I5W5
80989	229533.I1RW23	pp	229533.I1RZR6
80990	229533.I1RW23	pp	229533.Q4HYQ4
80991	229533.I1RNH9	pp	229533.I1RRJ8
80992	229533.I1RNH9	pp	229533.I1S255
80993	229533.I1RNH9	pp	229533.I1S0J1
80994	229533.I1RNH9	pp	229533.I1S060
80995	229533.I1RNH9	pp	229533.Q4IJ56
80996	229533.I1RNH9	pp	229533.Q4IEV5
80997	229533.I1RNH9	pp	229533.I1RTD7
80998	229533.I1RNH9	pp	229533.I1RZ99
80999	229533.I1RNH9	pp	229533.I1S4T6
81000	229533.I1RNH9	pp	229533.I1RT98
81001	229533.I1RNH9	pp	229533.I1RRI2
81002	229533.I1RNH9	pp	229533.V6RGY0
81003	229533.I1RNH9	pp	229533.I1RNY2
81004	229533.I1RNH9	pp	229533.V6RGY3
81005	229533.I1RNH9	pp	229533.I1RQ28
81006	229533.I1RNH9	pp	229533.V6RGN5
81007	229533.I1RNH9	pp	229533.I1RQA2
81008	229533.I1RNH9	pp	229533.I1S0M3
81009	229533.I1RNH9	pp	229533.I1RVZ0
81010	229533.I1RNH9	pp	229533.I1RNK2
81011	229533.I1RG67	pp	229533.I1RK46
81012	229533.I1RJ34	pp	229533.Q4I7L0
81013	229533.I1RJ34	pp	229533.I1RYV3
81014	229533.I1RJ34	pp	229533.Q4HTT2
81015	229533.I1RJ34	pp	229533.I1S4Z5
81016	229533.A0A1C3YJ19	pp	229533.I1RG67
81017	229533.I1RXT7	pp	229533.I1RYP0
81018	229533.I1RXT7	pp	229533.I1RZ84
81019	229533.I1RXT7	pp	229533.V6RXW9
81020	229533.I1RXT7	pp	229533.I1S9S4
81021	229533.I1RXT7	pp	229533.I1S1U6
81022	229533.I1RXT7	pp	229533.I1S287
81023	229533.I1RXT7	pp	229533.I1S051
81024	229533.I1RXT7	pp	229533.Q4HY20
81025	229533.I1RXT7	pp	229533.I1S2B6
81026	229533.I1RR61	pp	229533.I1RVY0
81027	229533.I1RR61	pp	229533.I1RW25
81028	229533.I1RR61	pp	229533.I1RRH5
81029	229533.I1RR61	pp	229533.I1RR62
81030	229533.I1RR61	pp	229533.V6QWX8

81031	229533.I1RR61	pp	229533.I1RZM2
81032	229533.I1RR61	pp	229533.I1S196
81033	229533.I1RR61	pp	229533.I1RW91
81034	229533.I1RR61	pp	229533.I1RVL8
81035	229533.I1RR61	pp	229533.I1RRG3
81036	229533.I1RR61	pp	229533.I1S2G9
81037	229533.I1RR61	pp	229533.I1S5Z5
81038	229533.I1RR61	pp	229533.I1S276
81039	229533.I1RR61	pp	229533.I1RW90
81040	229533.I1RR61	pp	229533.I1RTC2
81041	229533.I1RR61	pp	229533.I1RRT0
81042	229533.I1RR61	pp	229533.I1RZB5
81043	229533.I1RR61	pp	229533.I1S9H8
81044	229533.I1RR61	pp	229533.I1RZ42
81045	229533.I1RR61	pp	229533.V6QUE6
81046	229533.I1RR61	pp	229533.I1S182
81047	229533.I1RR61	pp	229533.I1RRF7
81048	229533.I1RR61	pp	229533.I1S0C6
81049	229533.I1RR61	pp	229533.I1RSZ3
81050	229533.I1RR61	pp	229533.I1RW80
81051	229533.I1RR61	pp	229533.I1RXU0
81052	229533.I1RR61	pp	229533.I1RWF3
81053	229533.I1RAS2	pp	229533.Q4HZ42
81054	229533.I1RAS2	pp	229533.I1RN67
81055	229533.I1RAS2	pp	229533.Q4HW67
81056	229533.I1RAS2	pp	229533.I1RSV2
81057	229533.I1RAS2	pp	229533.I1RNB8
81058	229533.I1RAS2	pp	229533.Q4IAS1
81059	229533.I1RAS2	pp	229533.I1RPP4
81060	229533.I1RAS2	pp	229533.I1RC82
81061	229533.I1RAS2	pp	229533.I1RCJ7
81062	229533.I1RAS2	pp	229533.I1RP27
81063	229533.I1RAS2	pp	229533.I1RWD3
81064	229533.I1RAS2	pp	229533.Q4I7K4
81065	229533.I1RAS2	pp	229533.I1S2E9
81066	229533.I1RAS2	pp	229533.I1RT04
81067	229533.I1RAS2	pp	229533.I1RD22
81068	229533.I1RAS2	pp	229533.I1RNE7
81069	229533.I1RAS2	pp	229533.I1RJZ7
81070	229533.I1RAS2	pp	229533.I1RWH4
81071	229533.I1RAS2	pp	229533.I1RFQ7
81072	229533.I1RAS2	pp	229533.I1RPU3
81073	229533.I1RAS2	pp	229533.I1RCI8
81074	229533.I1RAS2	pp	229533.I1S0X5
81075	229533.I1RAS2	pp	229533.I1S0N4
81076	229533.I1RAS2	pp	229533.Q4IJH1
81077	229533.I1RAS2	pp	229533.I1RM82
81078	229533.I1RAS2	pp	229533.I1RRV1
81079	229533.I1RAS2	pp	229533.Q4HVW2
81080	229533.I1RAS2	pp	229533.I1RWI5
81081	229533.I1RAS2	pp	229533.I1RSQ3
81082	229533.I1RAS2	pp	229533.Q4IF76
81083	229533.I1RAS2	pp	229533.V6RMW6
81084	229533.I1RAS2	pp	229533.I1RT35
81085	229533.I1RAS2	pp	229533.I1RFT6
81086	229533.I1RAS2	pp	229533.Q4HZ68
81087	229533.I1RAS2	pp	229533.I1RNN1
81088	229533.I1RAS2	pp	229533.Q4IEK8
81089	229533.I1RAS2	pp	229533.I1RZS7
81090	229533.I1RAS2	pp	229533.I1RY44
81091	229533.I1RAS2	pp	229533.I1RAU7
81092	229533.I1RAS2	pp	229533.I1RCM3
81093	229533.I1RAS2	pp	229533.I1RZM1
81094	229533.I1RAS2	pp	229533.I1RSK1
81095	229533.I1RAS2	pp	229533.I1RJV3
81096	229533.I1RAS2	pp	229533.I1RZH4
81097	229533.I1RAS2	pp	229533.I1RAV4
81098	229533.I1RAS2	pp	229533.I1RTH6
81099	229533.I1RAS2	pp	229533.I1RW71
81100	229533.I1RAS2	pp	229533.I1RWN6
81101	229533.I1RAS2	pp	229533.Q4IJ56
81102	229533.I1RAS2	pp	229533.I1RQ31
81103	229533.I1RAS2	pp	229533.I1S6F1

81104	229533.I1RAS2	pp	229533.I1RQ81
81105	229533.I1RAS2	pp	229533.V6RTJ8
81106	229533.I1RAS2	pp	229533.I1RN61
81107	229533.I1RAS2	pp	229533.I1RE00
81108	229533.I1RAS2	pp	229533.Q4IAA0
81109	229533.I1RAS2	pp	229533.I1RB27
81110	229533.I1RAS2	pp	229533.I1RWD7
81111	229533.I1RAS2	pp	229533.I1RSM2
81112	229533.I1RAS2	pp	229533.I1RFT2
81113	229533.I1RAS2	pp	229533.I1RN65
81114	229533.I1RAS2	pp	229533.I1RBC4
81115	229533.I1RAS2	pp	229533.I1RRU9
81116	229533.I1RAS2	pp	229533.I1RB53
81117	229533.I1RP89	pp	229533.I1RWH8
81118	229533.I1RP89	pp	229533.I1S768
81119	229533.I1RP89	pp	229533.I1S7H3
81120	229533.I1RDD0	pp	229533.I1RWZ9
81121	229533.I1RDD0	pp	229533.I1RRI4
81122	229533.I1RDD0	pp	229533.I1RIG4
81123	229533.I1RDD0	pp	229533.V6RXW9
81124	229533.I1RDD0	pp	229533.I1RXF0
81125	229533.I1RDD0	pp	229533.I1RSE4
81126	229533.I1RDD0	pp	229533.I1S287
81127	229533.I1RDD0	pp	229533.I1RQP7
81128	229533.I1RDD0	pp	229533.I1RN70
81129	229533.I1RDD0	pp	229533.I1RPY3
81130	229533.I1RDD0	pp	229533.I1RQD6
81131	229533.I1RDD0	pp	229533.I1RYL5
81132	229533.I1RDD0	pp	229533.I1RP87
81133	229533.I1RDD0	pp	229533.I1RNG2
81134	229533.I1RDD0	pp	229533.I1RNM3
81135	229533.I1RDD0	pp	229533.I1RT28
81136	229533.I1RDD0	pp	229533.I1RUN9
81137	229533.I1RDD0	pp	229533.I1S2B6
81138	229533.I1RDD0	pp	229533.I1RX95
81139	229533.I1RDD0	pp	229533.I1RZK1
81140	229533.I1RDD0	pp	229533.I1S1U6
81141	229533.I1RDD0	pp	229533.I1RX87
81142	229533.I1RDD0	pp	229533.I1S9S4
81143	229533.I1RDD0	pp	229533.Q4HY20
81144	229533.I1RDD0	pp	229533.I1RGC1
81145	229533.I1RDD0	pp	229533.I1RT00
81146	229533.I1RDD0	pp	229533.I1RYP0
81147	229533.I1RDD0	pp	229533.I1RZM0
81148	229533.I1RDD0	pp	229533.I1RE44
81149	229533.I1RDD0	pp	229533.I1RRR5
81150	229533.I1RDD0	pp	229533.I1RZ84
81151	229533.I1RDD0	pp	229533.I1RMN3
81152	229533.I1RDD0	pp	229533.I1S051
81153	229533.I1RZI4	pp	229533.Q4IPI4
81154	229533.I1RZI4	pp	229533.Q4ICA8
81155	229533.I1R9C0	pp	229533.V6R2Z2
81156	229533.I1R9C0	pp	229533.I1RZV5
81157	229533.I1R9C0	pp	229533.V6RAM6
81158	229533.I1R9C0	pp	229533.V6QU99
81159	229533.I1R9C0	pp	229533.V6R0W1
81160	229533.I1R9C0	pp	229533.V6R5D2
81161	229533.I1RXF5	pp	229533.I1S0K5
81162	229533.I1RXF5	pp	229533.V6RQ48
81163	229533.I1RXF5	pp	229533.Q4IPH4
81164	229533.I1RXF5	pp	229533.Q4ICG5
81165	229533.I1RXF5	pp	229533.I1RZS5
81166	229533.I1RXF5	pp	229533.I1S278
81167	229533.I1RXF5	pp	229533.Q4ILI9
81168	229533.I1RXF5	pp	229533.I1S1V4
81169	229533.I1RXF5	pp	229533.V6RTJ8
81170	229533.I1RXF5	pp	229533.I1SAM5
81171	229533.I1RXF5	pp	229533.I1S6S4
81172	229533.I1RXF5	pp	229533.I1RYU1
81173	229533.I1RXF5	pp	229533.I1RYP0
81174	229533.I1RXF5	pp	229533.I1S2E9
81175	229533.I1RXF5	pp	229533.I1RZA4
81176	229533.I1RXF5	pp	229533.I1RYB7

81177	229533.I1RXF5	pp	229533.V6RKE4
81178	229533.I1RXF5	pp	229533.I1S830
81179	229533.I1RXF5	pp	229533.I1S9Z3
81180	229533.I1RXF5	pp	229533.V6R9X9
81181	229533.I1RXF5	pp	229533.Q4I6S5
81182	229533.I1RXF5	pp	229533.I1RXU1
81183	229533.I1RXF5	pp	229533.I1RY44
81184	229533.I1RSH4	pp	229533.Q4IN63
81185	229533.I1RSH4	pp	229533.I1S7M3
81186	229533.I1RSH4	pp	229533.I1RST1
81187	229533.I1RSH4	pp	229533.I1S0B6
81188	229533.I1RSH4	pp	229533.I1S1V2
81189	229533.I1RSH4	pp	229533.I1S7M2
81190	229533.I1RSH4	pp	229533.I1S0B1
81191	229533.I1RSH4	pp	229533.I1RXF2
81192	229533.I1RSH4	pp	229533.I1RY99
81193	229533.I1RSH4	pp	229533.I1S4S7
81194	229533.I1RSX4	pp	229533.V6RQW4
81195	229533.I1RSX4	pp	229533.Q4HXF0
81196	229533.I1RPP0	pp	229533.V6R476
81197	229533.I1RPP0	pp	229533.I1RSK0
81198	229533.I1RPP0	pp	229533.I1S254
81199	229533.I1RPP0	pp	229533.I1RZT7
81200	229533.I1RPP0	pp	229533.V6QUU6
81201	229533.I1RPP0	pp	229533.I1RRU8
81202	229533.I1RPP0	pp	229533.V6RR76
81203	229533.I1RPP0	pp	229533.I1RY27
81204	229533.I1RPP0	pp	229533.I1RPP7
81205	229533.I1RPP0	pp	229533.I1RRW2
81206	229533.I1RPP0	pp	229533.I1RWI6
81207	229533.A0A1C3YM79	pp	229533.I1RYB1
81208	229533.A0A1C3YM79	pp	229533.I1RNH6
81209	229533.A0A1C3YM79	pp	229533.I1RG11
81210	229533.A0A1C3YM79	pp	229533.I1RT47
81211	229533.A0A1C3YM79	pp	229533.I1RMN6
81212	229533.A0A1C3YM79	pp	229533.I1RN09
81213	229533.A0A1C3YM79	pp	229533.I1RNB8
81214	229533.A0A1C3YM79	pp	229533.I1RC15
81215	229533.A0A1C3YM79	pp	229533.I1RAC1
81216	229533.A0A1C3YM79	pp	229533.I1S060
81217	229533.A0A1C3YM79	pp	229533.I1S0J1
81218	229533.A0A1C3YM79	pp	229533.I1RBZ8
81219	229533.A0A1C3YM79	pp	229533.I1RJL2
81220	229533.A0A1C3YM79	pp	229533.I1RBI8
81221	229533.A0A1C3YM79	pp	229533.I1RT35
81222	229533.A0A1C3YM79	pp	229533.Q4IJH1
81223	229533.A0A1C3YM79	pp	229533.I1RMH5
81224	229533.A0A1C3YM79	pp	229533.Q4IB70
81225	229533.A0A1C3YM79	pp	229533.Q4IF76
81226	229533.A0A1C3YM79	pp	229533.I1RDG0
81227	229533.A0A1C3YM79	pp	229533.I1S1X9
81228	229533.A0A1C3YM79	pp	229533.I1RPU0
81229	229533.A0A1C3YM79	pp	229533.I1RE31
81230	229533.A0A1C3YM79	pp	229533.I1RX24
81231	229533.A0A1C3YM79	pp	229533.I1RS66
81232	229533.A0A1C3YM79	pp	229533.I1RC06
81233	229533.A0A1C3YM79	pp	229533.I1RM06
81234	229533.A0A1C3YM79	pp	229533.I1RZJ6
81235	229533.A0A1C3YM79	pp	229533.A0A1C3YMY3
81236	229533.A0A1C3YM79	pp	229533.I1RT98
81237	229533.A0A1C3YM79	pp	229533.I1RBU6
81238	229533.A0A1C3YM79	pp	229533.I1S1C6
81239	229533.A0A1C3YM79	pp	229533.Q4IAS1
81240	229533.A0A1C3YM79	pp	229533.I1RAB5
81241	229533.A0A1C3YM79	pp	229533.I1RKK4
81242	229533.A0A1C3YM79	pp	229533.I1RBW4
81243	229533.A0A1C3YM79	pp	229533.I1RQJ5
81244	229533.A0A1C3YM79	pp	229533.I1RCS2
81245	229533.A0A1C3YM79	pp	229533.I1S246
81246	229533.A0A1C3YM79	pp	229533.I1RQ65
81247	229533.I1RMD5	pp	229533.I1RW61
81248	229533.I1RMD5	pp	229533.Q4HZ68
81249	229533.I1RMD5	pp	229533.I1RWJ4

81250 229533.I1RMD5 pp 229533.I1RZZ1
81251 229533.I1RMD5 pp 229533.I1RNK2
81252 229533.I1RMD5 pp 229533.I1RPM4
81253 229533.I1RMD5 pp 229533.Q4I662
81254 229533.I1RMD5 pp 229533.Q4I5Z5
81255 229533.I1RMD5 pp 229533.I1RY63
81256 229533.I1RMD5 pp 229533.I1RQE7
81257 229533.I1RMD5 pp 229533.I1RN51
81258 229533.I1RMD5 pp 229533.I1RRQ6
81259 229533.I1RMD5 pp 229533.Q4IFI0
81260 229533.I1RMD5 pp 229533.V6QYF0
81261 229533.I1RMD5 pp 229533.I1RSR3
81262 229533.I1RMD5 pp 229533.I1RZE5
81263 229533.I1RMD5 pp 229533.V6RGY3
81264 229533.I1RMD5 pp 229533.I1RNA0
81265 229533.I1RMD5 pp 229533.I1RN99
81266 229533.I1RMD5 pp 229533.I1S9U2
81267 229533.I1RMD5 pp 229533.I1RQ28
81268 229533.I1RMD5 pp 229533.I1S0M3
81269 229533.I1RMD5 pp 229533.I1RZS6
81270 229533.I1RMD5 pp 229533.Q4IF76
81271 229533.I1RMD5 pp 229533.I1RW25
81272 229533.I1RMD5 pp 229533.I1RTD9
81273 229533.I1RMD5 pp 229533.I1RS47
81274 229533.I1RMD5 pp 229533.I1RWJ8
81275 229533.I1RMD5 pp 229533.I1RSR8
81276 229533.I1RMD5 pp 229533.I1S558
81277 229533.I1RMD5 pp 229533.I1RQA2
81278 229533.I1RMD5 pp 229533.I1RQ65
81279 229533.I1RMD5 pp 229533.I1RRI2
81280 229533.I1RMD5 pp 229533.Q4HZ42
81281 229533.I1RMD5 pp 229533.I1S5R1
81282 229533.I1RMD5 pp 229533.I1RPU0
81283 229533.I1RMD5 pp 229533.Q4IJH1
81284 229533.I1RMD5 pp 229533.Q4IEV5
81285 229533.I1RMD5 pp 229533.I1RZW8
81286 229533.I1RMD5 pp 229533.I1RR24
81287 229533.I1RMD5 pp 229533.I1RUQ9
81288 229533.I1RMD5 pp 229533.I1RRT8
81289 229533.I1RMD5 pp 229533.V6R227
81290 229533.I1RMD5 pp 229533.I1RTF4
81291 229533.I1RMD5 pp 229533.I1S0D4
81292 229533.I1RMD5 pp 229533.I1RRT2
81293 229533.I1RMD5 pp 229533.I1S8R3
81294 229533.I1RMD5 pp 229533.I1S0L3
81295 229533.I1RMD5 pp 229533.I1RZB5
81296 229533.I1RMD5 pp 229533.Q4IPI1
81297 229533.I1RMD5 pp 229533.Q4IBS2
81298 229533.I1RMD5 pp 229533.Q4HVW2
81299 229533.I1RMD5 pp 229533.I1RMK3
81300 229533.I1RMD5 pp 229533.Q4IQW0
81301 229533.I1RMD5 pp 229533.I1RN63
81302 229533.I1RMD5 pp 229533.I1S0J1
81303 229533.I1RMD5 pp 229533.I1S2C0
81304 229533.I1RMD5 pp 229533.I1RW46
81305 229533.I1RMD5 pp 229533.I1RPJ3
81306 229533.I1RMD5 pp 229533.V6RGN5
81307 229533.I1RMD5 pp 229533.I1RW99
81308 229533.I1RMD5 pp 229533.I1S0M9
81309 229533.I1RMD5 pp 229533.I1RTE9
81310 229533.I1RMD5 pp 229533.Q4IJ56
81311 229533.I1RMD5 pp 229533.I1RRP3
81312 229533.I1RMD5 pp 229533.I1RT98
81313 229533.I1RMD5 pp 229533.I1S4T3
81314 229533.I1RMD5 pp 229533.I1RS30
81315 229533.I1RMD5 pp 229533.I1RUG3
81316 229533.I1RMD5 pp 229533.Q4I830
81317 229533.I1RMD5 pp 229533.I1RQ31
81318 229533.I1RMD5 pp 229533.V6RIV9
81319 229533.I1RMD5 pp 229533.Q4IN63
81320 229533.I1RMD5 pp 229533.I1RNU6
81321 229533.I1RMD5 pp 229533.I1RPY1
81322 229533.I1RMD5 pp 229533.I1RS17

81323 229533.I1RMD5 pp 229533.I1RW26
81324 229533.I1RMD5 pp 229533.Q4I327
81325 229533.I1RMD5 pp 229533.I1RZM8
81326 229533.I1RMD5 pp 229533.I1RSQ7
81327 229533.I1RMD5 pp 229533.I1S6F0
81328 229533.I1RMD5 pp 229533.Q4IAA0
81329 229533.I1RMD5 pp 229533.Q4IEK8
81330 229533.I1RMD5 pp 229533.I1RNH6
81331 229533.I1RMD5 pp 229533.I1RTI6
81332 229533.I1RMD5 pp 229533.I1RS18
81333 229533.I1RMD5 pp 229533.I1RT04
81334 229533.I1RMD5 pp 229533.I1S0R7
81335 229533.I1RMD5 pp 229533.I1RVQ0
81336 229533.I1RMD5 pp 229533.I1RRJ8
81337 229533.I1RMD5 pp 229533.I1RSI6
81338 229533.I1RMD5 pp 229533.V6QW22
81339 229533.I1RMD5 pp 229533.I1RMN6
81340 229533.I1RMD5 pp 229533.Q4IAS1
81341 229533.I1RMD5 pp 229533.V6RCG5
81342 229533.I1RMD5 pp 229533.I1RUQ2
81343 229533.I1RMD5 pp 229533.I1RXD7
81344 229533.A0A1C3YI55 pp 229533.I1RD45
81345 229533.A0A1C3YI55 pp 229533.A0A1C3YLH6
81346 229533.A0A1C3YI55 pp 229533.I1RTD1
81347 229533.A0A1C3YI55 pp 229533.I1RMP2
81348 229533.A0A1C3YI55 pp 229533.A0A1C3YLL6
81349 229533.A0A1C3YI55 pp 229533.I1REZ0
81350 229533.A0A1C3YI55 pp 229533.I1RC35
81351 229533.A0A1C3YI55 pp 229533.I1RST4
81352 229533.A0A1C3YI55 pp 229533.V6R476
81353 229533.A0A1C3YI55 pp 229533.V6RB81
81354 229533.A0A1C3YI55 pp 229533.I1RFR6
81355 229533.A0A1C3YI55 pp 229533.I1RNG6
81356 229533.A0A1C3YI55 pp 229533.I1RB26
81357 229533.A0A1C3YI55 pp 229533.I1RSB0
81358 229533.A0A1C3YI55 pp 229533.I1S7X9
81359 229533.A0A1C3YI55 pp 229533.I1RRW2
81360 229533.A0A1C3YI55 pp 229533.A0A1C3YI93
81361 229533.A0A1C3YI55 pp 229533.I1RE19
81362 229533.A0A1C3YI55 pp 229533.I1RTG9
81363 229533.A0A1C3YI55 pp 229533.I1RTE0
81364 229533.A0A1C3YI55 pp 229533.A0A1C3YM95
81365 229533.A0A1C3YI55 pp 229533.Q4IPI4
81366 229533.A0A1C3YI55 pp 229533.I1RKI1
81367 229533.A0A1C3YI55 pp 229533.I1RKR7
81368 229533.A0A1C3YI55 pp 229533.I1RRF0
81369 229533.A0A1C3YI55 pp 229533.I1REF5
81370 229533.A0A1C3YI55 pp 229533.I1S0Z3
81371 229533.A0A1C3YI55 pp 229533.I1RKR9
81372 229533.A0A1C3YI55 pp 229533.I1RZD9
81373 229533.A0A1C3YI55 pp 229533.Q4I2W2
81374 229533.A0A1C3YI55 pp 229533.Q4I0J6
81375 229533.A0A1C3YI55 pp 229533.I1RX09
81376 229533.A0A1C3YI55 pp 229533.I1RIG4
81377 229533.A0A1C3YI55 pp 229533.I1RWA5
81378 229533.A0A1C3YI55 pp 229533.I1RMU8
81379 229533.A0A1C3YI55 pp 229533.I1RB00
81380 229533.A0A1C3YI55 pp 229533.V6RR76
81381 229533.A0A1C3YI55 pp 229533.I1RN65
81382 229533.A0A1C3YI55 pp 229533.I1RT41
81383 229533.A0A1C3YI55 pp 229533.A0A1C3YMD9
81384 229533.A0A1C3YI55 pp 229533.I1RWA9
81385 229533.A0A1C3YI55 pp 229533.I1RRF9
81386 229533.A0A1C3YI55 pp 229533.I1RTQ9
81387 229533.A0A1C3YI55 pp 229533.I1S4X2
81388 229533.A0A1C3YI55 pp 229533.I1RN81
81389 229533.A0A1C3YI55 pp 229533.I1RT65
81390 229533.A0A1C3YI55 pp 229533.A0A1C3YN54
81391 229533.A0A1C3YI55 pp 229533.I1S270
81392 229533.A0A1C3YI55 pp 229533.I1RBS4
81393 229533.A0A1C3YI55 pp 229533.I1RXB5
81394 229533.A0A1C3YI55 pp 229533.A0A1C3YIF4
81395 229533.A0A1C3YI55 pp 229533.I1R991

81396 229533.A0A1C3YI55 pp 229533.I1RB15
81397 229533.A0A1C3YI55 pp 229533.V6QUU6
81398 229533.A0A1C3YI55 pp 229533.I1S095
81399 229533.A0A1C3YI55 pp 229533.I1RZV4
81400 229533.A0A1C3YI55 pp 229533.I1RNP1
81401 229533.A0A1C3YI55 pp 229533.I1S254
81402 229533.A0A1C3YI55 pp 229533.A0A1C3YID2
81403 229533.A0A1C3YI55 pp 229533.I1RSI7
81404 229533.A0A1C3YI55 pp 229533.A0A1C3YLH7
81405 229533.A0A1C3YI55 pp 229533.I1RB98
81406 229533.A0A1C3YI55 pp 229533.I1RPP0
81407 229533.A0A1C3YI55 pp 229533.A0A1C3YL89
81408 229533.I1RQ91 pp 229533.I1RYV3
81409 229533.I1RQ91 pp 229533.Q4HTT2
81410 229533.I1RNQ6 pp 229533.I1S2G9
81411 229533.I1RNQ6 pp 229533.Q4I5I4
81412 229533.I1RNQ6 pp 229533.I1RW90
81413 229533.I1RNQ6 pp 229533.V6QWX8
81414 229533.I1RNQ6 pp 229533.Q4I5W5
81415 229533.I1RNQ6 pp 229533.I1RUG0
81416 229533.I1RNQ6 pp 229533.I1RZB5
81417 229533.I1RNQ6 pp 229533.V6QUE6
81418 229533.I1RNQ6 pp 229533.I1RR25
81419 229533.I1RNQ6 pp 229533.I1RRG3
81420 229533.I1RNQ6 pp 229533.I1RZR6
81421 229533.I1RNQ6 pp 229533.Q4IPC8
81422 229533.I1RNQ6 pp 229533.I1RTC2
81423 229533.I1RNQ6 pp 229533.I1RRT0
81424 229533.I1RNQ6 pp 229533.Q4IQT8
81425 229533.I1RNQ6 pp 229533.I1S5Z2
81426 229533.A0A1C3YL67 pp 229533.I1RUF8
81427 229533.A0A1C3YL67 pp 229533.I1RFL7
81428 229533.A0A1C3YL67 pp 229533.I1RCH7
81429 229533.A0A1C3YL67 pp 229533.I1RQ13
81430 229533.A0A1C3YL67 pp 229533.A0A1C3YMB6
81431 229533.A0A1C3YL67 pp 229533.Q4I7L0
81432 229533.A0A1C3YL67 pp 229533.I1RK75
81433 229533.A0A1C3YL67 pp 229533.I1RYY5
81434 229533.A0A1C3YL67 pp 229533.I1RN54
81435 229533.A0A1C3YL67 pp 229533.Q4IJM4
81436 229533.A0A1C3YL67 pp 229533.I1RTT3
81437 229533.A0A1C3YL67 pp 229533.I1RZM1
81438 229533.A0A1C3YL67 pp 229533.I1S077
81439 229533.A0A1C3YL67 pp 229533.I1RT62
81440 229533.A0A1C3YL67 pp 229533.I1RMQ7
81441 229533.A0A1C3YL67 pp 229533.I1RGC6
81442 229533.A0A1C3YL67 pp 229533.Q4I7X1
81443 229533.I1RT05 pp 229533.Q4I830
81444 229533.I1RT05 pp 229533.I1RWS4
81445 229533.I1RT05 pp 229533.I1RW71
81446 229533.I1RT05 pp 229533.I1S2L2
81447 229533.I1RT05 pp 229533.I1RZH6
81448 229533.I1RT05 pp 229533.Q4IJH1
81449 229533.I1RT05 pp 229533.V6R283
81450 229533.I1RT05 pp 229533.I1RTF4
81451 229533.I1RT05 pp 229533.Q4HZ42
81452 229533.I1RT05 pp 229533.Q4HVW2
81453 229533.I1RT05 pp 229533.I1S253
81454 229533.I1RT05 pp 229533.I1RT80
81455 229533.I1RT05 pp 229533.I1RYJ6
81456 229533.I1RT05 pp 229533.I1RW26
81457 229533.I1RT05 pp 229533.I1RT33
81458 229533.I1RT05 pp 229533.I1RW68
81459 229533.I1RT05 pp 229533.I1S0R7
81460 229533.I1RT05 pp 229533.Q4I5R9
81461 229533.I1RT05 pp 229533.Q4IEK8
81462 229533.I1RT05 pp 229533.Q4IF76
81463 229533.I1RT05 pp 229533.I1S5R1
81464 229533.I1RT05 pp 229533.I1S0F1
81465 229533.I1RT05 pp 229533.V6RCZ8
81466 229533.I1RT05 pp 229533.O74638
81467 229533.I1RT05 pp 229533.Q4IPI1
81468 229533.I1RT05 pp 229533.V6RGN5

81469	229533.I1RT05	pp	229533.Q4IQK7
81470	229533.I1RT05	pp	229533.I1RUG3
81471	229533.I1RT05	pp	229533.Q4I8X0
81472	229533.I1RT05	pp	229533.V6QYF0
81473	229533.I1RT05	pp	229533.Q4IJ56
81474	229533.I1RT05	pp	229533.I1RZS6
81475	229533.I1RT05	pp	229533.I1S5B0
81476	229533.I1RT05	pp	229533.I1RZF6
81477	229533.I1RT05	pp	229533.I1RW61
81478	229533.I1RT05	pp	229533.Q4IBS2
81479	229533.I1RT05	pp	229533.Q4HXF6
81480	229533.I1RT05	pp	229533.I1RWJ4
81481	229533.I1RT05	pp	229533.I1RY63
81482	229533.I1RT05	pp	229533.I1S558
81483	229533.I1RT05	pp	229533.I1S2E8
81484	229533.I1RT05	pp	229533.Q4IPH4
81485	229533.I1RT05	pp	229533.Q4HZ68
81486	229533.I1RT05	pp	229533.I1RWH8
81487	229533.I1RT05	pp	229533.I1RTE9
81488	229533.I1RT05	pp	229533.V6QWZ7
81489	229533.I1RT05	pp	229533.I1RVZ0
81490	229533.I1RT05	pp	229533.I1S274
81491	229533.I1RT05	pp	229533.I1S2C0
81492	229533.I1RF62	pp	229533.I1RF63
81493	229533.I1RF62	pp	229533.I1RMH0
81494	229533.I1RF62	pp	229533.I1RLH5
81495	229533.V6QXG1	pp	229533.V6RIS2
81496	229533.I1RCV3	pp	229533.I1RTF2
81497	229533.I1RCV3	pp	229533.I1RPC6
81498	229533.I1RCV3	pp	229533.I1RXT7
81499	229533.I1RCV3	pp	229533.I1RX95
81500	229533.I1RCV3	pp	229533.I1SAM5
81501	229533.I1RCV3	pp	229533.Q4HY20
81502	229533.I1RCV3	pp	229533.I1RNB9
81503	229533.I1RCV3	pp	229533.I1RYP0
81504	229533.I1RCV3	pp	229533.I1RNH7
81505	229533.I1RCV3	pp	229533.V6QXP4
81506	229533.I1RCV3	pp	229533.I1RP87
81507	229533.I1RCV3	pp	229533.I1RSH3
81508	229533.I1RCV3	pp	229533.I1RXF5
81509	229533.I1RCV3	pp	229533.V6RXW9
81510	229533.I1RCV3	pp	229533.I1RWZ9
81511	229533.I1RCV3	pp	229533.I1RIG4
81512	229533.I1RCV3	pp	229533.I1RQP7
81513	229533.I1RCV3	pp	229533.I1S287
81514	229533.I1RCV3	pp	229533.I1RY40
81515	229533.I1RCV3	pp	229533.I1S1V4
81516	229533.I1RCV3	pp	229533.I1RZK3
81517	229533.I1RCV3	pp	229533.I1RW76
81518	229533.I1RCV3	pp	229533.I1RZS5
81519	229533.I1RCV3	pp	229533.I1RN70
81520	229533.I1RCV3	pp	229533.I1RNG2
81521	229533.I1RCV3	pp	229533.I1RPY3
81522	229533.I1RCV3	pp	229533.I1S2B6
81523	229533.I1RCV3	pp	229533.I1RQN8
81524	229533.I1RCV3	pp	229533.I1RDD0
81525	229533.I1RCV3	pp	229533.I1RFP5
81526	229533.I1RCV3	pp	229533.I1RSW4
81527	229533.I1RCV3	pp	229533.I1RG01
81528	229533.I1RCV3	pp	229533.I1S278
81529	229533.I1RCV3	pp	229533.I1RE18
81530	229533.I1RCV3	pp	229533.I1S1U6
81531	229533.I1RCV3	pp	229533.I1S051
81532	229533.I1RCV3	pp	229533.I1RR74
81533	229533.I1RCV3	pp	229533.I1S0V5
81534	229533.I1RCV3	pp	229533.I1RRC9
81535	229533.I1RCV3	pp	229533.I1RS34
81536	229533.I1RCV3	pp	229533.I1RUN9
81537	229533.I1RCV3	pp	229533.I1RPG2
81538	229533.I1RCV3	pp	229533.I1S9S4
81539	229533.I1RCV3	pp	229533.I1RCV4
81540	229533.I1RCV3	pp	229533.I1RQD6
81541	229533.I1RCV3	pp	229533.I1RQC4

81542 229533.I1RCV3 pp 229533.I1RX87
81543 229533.I1RCV3 pp 229533.I1RQF7
81544 229533.I1RCV3 pp 229533.I1RZ84
81545 229533.I1RCV3 pp 229533.I1RGC1
81546 229533.I1RCV3 pp 229533.I1RRC8
81547 229533.I1RCV3 pp 229533.I1RMN3
81548 229533.I1RCV3 pp 229533.I1RE44
81549 229533.I1RJY3 pp 229533.I1RQ65
81550 229533.I1RJY3 pp 229533.I1RRG3
81551 229533.I1RJY3 pp 229533.V6QUE6
81552 229533.I1RJY3 pp 229533.I1RRN6
81553 229533.I1RJY3 pp 229533.I1S255
81554 229533.I1RJY3 pp 229533.I1RKQ5
81555 229533.I1RJY3 pp 229533.I1RS30
81556 229533.I1RJY3 pp 229533.I1S9U1
81557 229533.I1RJY3 pp 229533.I1RR25
81558 229533.I1RJY3 pp 229533.I1RK95
81559 229533.I1RJY3 pp 229533.I1RN17
81560 229533.I1RJY3 pp 229533.I1RZB5
81561 229533.I1RJY3 pp 229533.I1RSI6
81562 229533.I1RJY3 pp 229533.I1RW90
81563 229533.I1RJY3 pp 229533.V6QWX8
81564 229533.I1RJY3 pp 229533.I1RVY0
81565 229533.I1RJY3 pp 229533.I1S9Y1
81566 229533.I1RJY3 pp 229533.I1RK79
81567 229533.A0A0E0RQ82 pp 229533.V6RAM6
81568 229533.A0A0E0RQ82 pp 229533.I1S0A5
81569 229533.A0A0E0RQ82 pp 229533.I1RF64
81570 229533.A0A0E0RQ82 pp 229533.V6R2Z2
81571 229533.A0A0E0RQ82 pp 229533.I1RAC0
81572 229533.A0A0E0RQ82 pp 229533.A0A1C3YKB7
81573 229533.A0A0E0RQ82 pp 229533.I1RVI8
81574 229533.A0A0E0RQ82 pp 229533.I1RI19
81575 229533.A0A0E0RQ82 pp 229533.V6R0W1
81576 229533.A0A0E0RQ82 pp 229533.I1RDN3
81577 229533.A0A0E0RQ82 pp 229533.V6R5D2
81578 229533.A0A0E0RQ82 pp 229533.I1RJG4
81579 229533.A0A0E0RQ82 pp 229533.I1SAI4
81580 229533.A0A0E0RQ82 pp 229533.I1RQI4
81581 229533.A0A0E0RQ82 pp 229533.I1RR44
81582 229533.A0A0E0RQ82 pp 229533.I1RB03
81583 229533.A0A0E0RQ82 pp 229533.V6R756
81584 229533.A0A0E0RQ82 pp 229533.A0A1C3YIP2
81585 229533.A0A0E0RQ82 pp 229533.I1RCH2
81586 229533.A0A0E0RQ82 pp 229533.A0A1C3YIR5
81587 229533.A0A0E0RQ82 pp 229533.I1RA13
81588 229533.A0A0E0RQ82 pp 229533.V6QU99
81589 229533.A0A0E0RQ82 pp 229533.I1RIF9
81590 229533.A0A0E0RQ82 pp 229533.I1S2L0
81591 229533.I1SA28 pp 229533.Q4HTT2
81592 229533.A0A1C3YJ42 pp 229533.I1S7S4
81593 229533.A0A1C3YJ42 pp 229533.I1RWX8
81594 229533.A0A1C3YJ42 pp 229533.I1RT35
81595 229533.A0A1C3YJ42 pp 229533.I1RBH8
81596 229533.A0A1C3YJ42 pp 229533.I1RC84
81597 229533.A0A1C3YJ42 pp 229533.I1RA36
81598 229533.A0A1C3YJ42 pp 229533.I1RMH5
81599 229533.A0A1C3YJ42 pp 229533.I1RZL5
81600 229533.A0A1C3YJ42 pp 229533.I1RT70
81601 229533.A0A1C3YJ42 pp 229533.I1RK92
81602 229533.A0A1C3YJ42 pp 229533.I1S7M3
81603 229533.A0A1C3YJ42 pp 229533.A0A1C3YJW0
81604 229533.A0A1C3YJ42 pp 229533.I1RPA0
81605 229533.A0A1C3YJ42 pp 229533.I1RQD8
81606 229533.A0A1C3YJ42 pp 229533.Q4I7F9
81607 229533.A0A1C3YJ42 pp 229533.I1RG17
81608 229533.A0A1C3YJ42 pp 229533.I1RQZ2
81609 229533.A0A1C3YJ42 pp 229533.I1RPY7
81610 229533.A0A1C3YJ42 pp 229533.I1RT47
81611 229533.A0A1C3YJ42 pp 229533.I1RN09
81612 229533.A0A1C3YJ42 pp 229533.I1RYB1
81613 229533.A0A1C3YJ42 pp 229533.Q4IP34
81614 229533.A0A1C3YJ42 pp 229533.I1S4M5

81615	229533.A0A1C3YJ42	pp	229533.V6QVD5
81616	229533.A0A1C3YJ42	pp	229533.I1RSD4
81617	229533.A0A1C3YJ42	pp	229533.I1RK19
81618	229533.A0A1C3YJ42	pp	229533.I1RA46
81619	229533.A0A1C3YJ42	pp	229533.A0A1C3YNC3
81620	229533.A0A1C3YJ42	pp	229533.I1RNB8
81621	229533.A0A1C3YJ42	pp	229533.I1RSH9
81622	229533.A0A1C3YJ42	pp	229533.I1S0B1
81623	229533.A0A1C3YJ42	pp	229533.Q4I650
81624	229533.A0A1C3YJ42	pp	229533.I1RG90
81625	229533.A0A1C3YJ42	pp	229533.I1RB50
81626	229533.A0A1C3YJ42	pp	229533.I1RBV3
81627	229533.A0A1C3YJ42	pp	229533.I1RW90
81628	229533.A0A1C3YJ42	pp	229533.I1RSA4
81629	229533.A0A1C3YJ42	pp	229533.I1S0B6
81630	229533.A0A1C3YJ42	pp	229533.I1S7M2
81631	229533.A0A1C3YJ42	pp	229533.A0A1C3YL11
81632	229533.A0A1C3YJ42	pp	229533.I1RCQ6
81633	229533.A0A1C3YJ42	pp	229533.I1RXF2
81634	229533.A0A1C3YJ42	pp	229533.I1RTS2
81635	229533.A0A1C3YJ42	pp	229533.A0A1C3YKM6
81636	229533.A0A1C3YJ42	pp	229533.I1RX81
81637	229533.A0A1C3YJ42	pp	229533.I1RCD6
81638	229533.A0A1C3YJ42	pp	229533.I1RMV5
81639	229533.I1RAK7	pp	229533.I1RBT0
81640	229533.I1RAK7	pp	229533.V6RXW9
81641	229533.I1RAK7	pp	229533.Q4HY20
81642	229533.I1RAK7	pp	229533.I1RAL1
81643	229533.I1RAK7	pp	229533.I1RZ84
81644	229533.I1RAK7	pp	229533.I1RT00
81645	229533.I1RAK7	pp	229533.I1S2B6
81646	229533.I1RAK7	pp	229533.I1RPY3
81647	229533.I1RAK7	pp	229533.I1RN70
81648	229533.I1RAK7	pp	229533.I1RYL5
81649	229533.I1RAK7	pp	229533.I1RMN3
81650	229533.I1RAK7	pp	229533.V6QXP4
81651	229533.I1RAK7	pp	229533.I1RD46
81652	229533.I1RAK7	pp	229533.I1RWZ9
81653	229533.I1RAK7	pp	229533.I1RXV9
81654	229533.I1RAK7	pp	229533.I1RXT7
81655	229533.I1RAK7	pp	229533.I1S9S4
81656	229533.I1RAK7	pp	229533.I1RUN9
81657	229533.I1RAK7	pp	229533.I1RX95
81658	229533.I1RAK7	pp	229533.I1RQF9
81659	229533.I1RAK7	pp	229533.I1RNM4
81660	229533.I1RAK7	pp	229533.V6RPQ8
81661	229533.I1RAK7	pp	229533.I1RXW3
81662	229533.I1RAK7	pp	229533.I1RDD0
81663	229533.I1RAK7	pp	229533.I1RBS4
81664	229533.I1RAK7	pp	229533.I1RXF0
81665	229533.I1RAK7	pp	229533.I1RX17
81666	229533.I1RAK7	pp	229533.I1RIG4
81667	229533.I1RAK7	pp	229533.I1RBS3
81668	229533.I1RAK7	pp	229533.I1RP87
81669	229533.I1RAK7	pp	229533.I1S287
81670	229533.I1RAK7	pp	229533.I1RSL5
81671	229533.I1RAK7	pp	229533.I1RQD6
81672	229533.I1RAK7	pp	229533.I1S051
81673	229533.I1RAK7	pp	229533.I1RGC1
81674	229533.A0A1C3YJW0	pp	229533.A0A1C3YL11
81675	229533.A0A1C3YJW0	pp	229533.I1RZD2
81676	229533.A0A1C3YJW0	pp	229533.I1RB50
81677	229533.A0A1C3YJW0	pp	229533.Q4IF76
81678	229533.A0A1C3YJW0	pp	229533.Q4IJH1
81679	229533.A0A1C3YJW0	pp	229533.I1RMV5
81680	229533.A0A1C3YJW0	pp	229533.I1RCQ6
81681	229533.A0A1C3YJW0	pp	229533.I1RCD6
81682	229533.A0A1C3YJW0	pp	229533.I1RX81
81683	229533.A0A1C3YJW0	pp	229533.A0A1C3YKM6
81684	229533.A0A1C3YJW0	pp	229533.I1RBV3
81685	229533.A0A1C3YJW0	pp	229533.I1RQD8
81686	229533.A0A1C3YJW0	pp	229533.I1RBU6
81687	229533.A0A1C3YJW0	pp	229533.I1RG17

81688	229533.A0A1C3YJW0	pp	229533.I1RBH8
81689	229533.A0A1C3YJW0	pp	229533.I1RT47
81690	229533.A0A1C3YJW0	pp	229533.A0A1C3YM79
81691	229533.A0A1C3YJW0	pp	229533.I1RXF2
81692	229533.A0A1C3YJW0	pp	229533.I1RSH9
81693	229533.A0A1C3YJW0	pp	229533.I1RSD4
81694	229533.A0A1C3YJW0	pp	229533.I1RN09
81695	229533.A0A1C3YJW0	pp	229533.I1S7M2
81696	229533.A0A1C3YJW0	pp	229533.A0A1C3YNC3
81697	229533.A0A1C3YJW0	pp	229533.Q4IP34
81698	229533.A0A1C3YJW0	pp	229533.I1RNB8
81699	229533.A0A1C3YJW0	pp	229533.I1RCS2
81700	229533.A0A1C3YJW0	pp	229533.Q4IB70
81701	229533.A0A1C3YJW0	pp	229533.I1S7M3
81702	229533.A0A1C3YJW0	pp	229533.I1RKK4
81703	229533.A0A1C3YJW0	pp	229533.I1RMH5
81704	229533.A0A1C3YJW0	pp	229533.I1RSA4
81705	229533.A0A1C3YJW0	pp	229533.I1S4M5
81706	229533.A0A1C3YJW0	pp	229533.I1RC84
81707	229533.A0A1C3YJW0	pp	229533.I1RBZ8
81708	229533.A0A1C3YJW0	pp	229533.I1RA36
81709	229533.A0A1C3YJW0	pp	229533.I1S0B6
81710	229533.A0A1C3YJW0	pp	229533.I1RWX8
81711	229533.A0A1C3YJW0	pp	229533.I1RT35
81712	229533.A0A1C3YJW0	pp	229533.I1RPA0
81713	229533.A0A1C3YJW0	pp	229533.I1RZL5
81714	229533.A0A1C3YJW0	pp	229533.I1RK92
81715	229533.A0A1C3YJW0	pp	229533.A0A1C3YL18
81716	229533.A0A1C3YJW0	pp	229533.V6QVD5
81717	229533.A0A1C3YJW0	pp	229533.I1RYB1
81718	229533.A0A1C3YJW0	pp	229533.I1RPY7
81719	229533.A0A1C3YJW0	pp	229533.I1RG90
81720	229533.A0A1C3YJW0	pp	229533.Q4I7F9
81721	229533.A0A1C3YJW0	pp	229533.I1RYJ9
81722	229533.A0A1C3YJW0	pp	229533.I1RDG0
81723	229533.A0A1C3YJW0	pp	229533.I1RQZ2
81724	229533.I1RZ84	pp	229533.Q4HY20
81725	229533.I1RZ84	pp	229533.V6RR76
81726	229533.I1RZ84	pp	229533.I1S1U6
81727	229533.I1RZ84	pp	229533.I1S9S4
81728	229533.I1RZ84	pp	229533.I1S051
81729	229533.I1RZ84	pp	229533.I1S0Z3
81730	229533.I1RZ84	pp	229533.V6RXW9
81731	229533.I1RZ84	pp	229533.Q4INI2
81732	229533.I1RZ84	pp	229533.I1S0V5
81733	229533.I1RZ84	pp	229533.I1S287
81734	229533.I1RL23	pp	229533.I1RRT2
81735	229533.I1RL23	pp	229533.Q4IQW0
81736	229533.I1RL23	pp	229533.I1RMN6
81737	229533.I1RL23	pp	229533.I1RSI6
81738	229533.I1RL23	pp	229533.I1RRJ8
81739	229533.I1RL23	pp	229533.I1RQ65
81740	229533.I1RL23	pp	229533.V6R283
81741	229533.I1RL23	pp	229533.I1RRI2
81742	229533.I1RL23	pp	229533.I1RM96
81743	229533.I1RL23	pp	229533.I1RNU6
81744	229533.I1RL23	pp	229533.I1RSD8
81745	229533.I1RL23	pp	229533.I1RRQ3
81746	229533.I1RL23	pp	229533.I1RS47
81747	229533.I1RL23	pp	229533.Q4IJ56
81748	229533.I1RL23	pp	229533.I1RNH1
81749	229533.I1RL23	pp	229533.I1RZS6
81750	229533.I1RL23	pp	229533.I1RPH9
81751	229533.I1RL23	pp	229533.I1RZZ9
81752	229533.I1RL23	pp	229533.I1RT80
81753	229533.I1RL23	pp	229533.I1RX14
81754	229533.I1RL23	pp	229533.I1RNV6
81755	229533.I1RL23	pp	229533.I1RZF6
81756	229533.I1RL23	pp	229533.I1RNH6
81757	229533.I1RL23	pp	229533.I1S021
81758	229533.I1RL23	pp	229533.I1RSR3
81759	229533.I1RL23	pp	229533.I1S9U2
81760	229533.I1RL23	pp	229533.I1RQA2

81761	229533.I1RL23	pp	229533.I1RQ31
81762	229533.I1RL23	pp	229533.I1RPU0
81763	229533.I1RL23	pp	229533.I1RVZ0
81764	229533.I1RL23	pp	229533.I1RM06
81765	229533.I1RL23	pp	229533.V6QYF0
81766	229533.I1S0T1	pp	229533.V6R0W1
81767	229533.I1S0T1	pp	229533.I1SAI4
81768	229533.I1S0T1	pp	229533.V6R2Z2
81769	229533.I1S0T1	pp	229533.V6QU99
81770	229533.I1S0T1	pp	229533.V6RAM6
81771	229533.I1S0T1	pp	229533.V6R5D2
81772	229533.I1S0T1	pp	229533.V6R756
81773	229533.I1R9N1	pp	229533.I1S0Z3
81774	229533.I1R9N1	pp	229533.Q4ICA8
81775	229533.I1R9N1	pp	229533.I1RY44
81776	229533.I1R9N1	pp	229533.I1S1W4
81777	229533.I1R9N1	pp	229533.I1RBJ1
81778	229533.I1R9N1	pp	229533.I1RWT6
81779	229533.I1R9N1	pp	229533.I1RMQ7
81780	229533.I1R9N1	pp	229533.V6QV09
81781	229533.I1R9N1	pp	229533.I1RRT0
81782	229533.I1R9N1	pp	229533.V6QUE6
81783	229533.I1R9N1	pp	229533.I1RC95
81784	229533.I1R9N1	pp	229533.I1RFL7
81785	229533.I1R9N1	pp	229533.V6RGI1
81786	229533.I1R9N1	pp	229533.I1S0E8
81787	229533.I1R9N1	pp	229533.I1RB74
81788	229533.I1R9N1	pp	229533.I1RC17
81789	229533.I1R9N1	pp	229533.Q4ICM0
81790	229533.I1R9N1	pp	229533.I1RQA3
81791	229533.I1R9N1	pp	229533.I1RG11
81792	229533.I1R9N1	pp	229533.I1RXU0
81793	229533.I1R9N1	pp	229533.I1RRN5
81794	229533.I1R9N1	pp	229533.I1RD15
81795	229533.I1R9N1	pp	229533.I1RUF8
81796	229533.I1R9N1	pp	229533.Q4IJM4
81797	229533.I1R9N1	pp	229533.I1RBC7
81798	229533.I1R9N1	pp	229533.I1RYZ9
81799	229533.I1R9N1	pp	229533.I1RB41
81800	229533.I1R9N1	pp	229533.I1RPM7
81801	229533.I1R9N1	pp	229533.I1R9Y7
81802	229533.I1R9N1	pp	229533.I1RB75
81803	229533.I1R9N1	pp	229533.I1RKB1
81804	229533.I1R9N1	pp	229533.I1RGC6
81805	229533.I1R9N1	pp	229533.I1REN3
81806	229533.I1R9N1	pp	229533.I1S0N5
81807	229533.I1R9N1	pp	229533.I1RSZ3
81808	229533.I1R9N1	pp	229533.I1RA31
81809	229533.I1R9N1	pp	229533.I1RHB0
81810	229533.I1R9N1	pp	229533.I1S2E9
81811	229533.I1R9N1	pp	229533.I1RAT7
81812	229533.I1R9N1	pp	229533.I1RYY5
81813	229533.I1R9N1	pp	229533.I1RR25
81814	229533.I1R9N1	pp	229533.I1RT41
81815	229533.I1R9N1	pp	229533.I1RZB5
81816	229533.I1R9N1	pp	229533.I1RYH9
81817	229533.I1R9N1	pp	229533.I1RA84
81818	229533.I1R9N1	pp	229533.I1RJL2
81819	229533.I1R9N1	pp	229533.Q4IPI4
81820	229533.I1R9N1	pp	229533.I1S2G7
81821	229533.I1R9N1	pp	229533.I1RT62
81822	229533.I1R9N1	pp	229533.I1RL24
81823	229533.I1R9N1	pp	229533.I1RQ13
81824	229533.I1R9N1	pp	229533.Q4I7L0
81825	229533.I1R9N1	pp	229533.I1RPA7
81826	229533.I1R9N1	pp	229533.V6QWX8
81827	229533.I1R9N1	pp	229533.I1RW90
81828	229533.I1S4E0	pp	229533.I1S694
81829	229533.I1RIV0	pp	229533.I1RN13
81830	229533.I1RIV0	pp	229533.I1RM26
81831	229533.I1RIV0	pp	229533.I1RXF8
81832	229533.I1RIV0	pp	229533.I1RKJ8
81833	229533.I1RIV0	pp	229533.I1RPU8

81834 229533.I1RIV0 pp 229533.I1RVX9
81835 229533.I1RIV0 pp 229533.I1RIV2
81836 229533.I1RIV0 pp 229533.I1RML0
81837 229533.I1RIV0 pp 229533.I1S064
81838 229533.I1R9G0 pp 229533.I1RSI6
81839 229533.I1R9G0 pp 229533.I1RCL3
81840 229533.I1R9G0 pp 229533.I1RSD8
81841 229533.I1R9G0 pp 229533.I1RRI2
81842 229533.I1R9G0 pp 229533.I1RRQ3
81843 229533.I1R9G0 pp 229533.I1RS47
81844 229533.I1R9G0 pp 229533.I1RBY4
81845 229533.I1R9G0 pp 229533.I1RC29
81846 229533.I1R9G0 pp 229533.I1RBG9
81847 229533.I1R9G0 pp 229533.V6R283
81848 229533.I1R9G0 pp 229533.I1RQ65
81849 229533.I1R9G0 pp 229533.I1RZS6
81850 229533.I1R9G0 pp 229533.I1RD64
81851 229533.I1R9G0 pp 229533.I1RKL3
81852 229533.I1R9G0 pp 229533.I1RC03
81853 229533.I1R9G0 pp 229533.I1RM96
81854 229533.I1R9G0 pp 229533.I1RC72
81855 229533.I1R9G0 pp 229533.I1RNU6
81856 229533.I1R9G0 pp 229533.I1RZZ9
81857 229533.I1R9G0 pp 229533.I1RG56
81858 229533.I1R9G0 pp 229533.I1RRJ8
81859 229533.I1R9G0 pp 229533.I1RMN6
81860 229533.I1R9G0 pp 229533.I1R9X7
81861 229533.I1R9G0 pp 229533.I1RRT2
81862 229533.I1R9G0 pp 229533.I1RZF6
81863 229533.I1R9G0 pp 229533.I1RC27
81864 229533.I1R9G0 pp 229533.I1RJL4
81865 229533.I1R9G0 pp 229533.I1RX14
81866 229533.I1R9G0 pp 229533.I1RA48
81867 229533.I1R9G0 pp 229533.I1RQA2
81868 229533.I1R9G0 pp 229533.I1RK95
81869 229533.I1R9G0 pp 229533.I1RFN1
81870 229533.I1R9G0 pp 229533.I1RBC7
81871 229533.I1R9G0 pp 229533.Q4IJ56
81872 229533.I1R9G0 pp 229533.I1RC82
81873 229533.I1R9G0 pp 229533.Q4IQW0
81874 229533.I1R9G0 pp 229533.I1RB27
81875 229533.I1R9G0 pp 229533.I1RQ31
81876 229533.I1R9G0 pp 229533.I1RSR3
81877 229533.I1R9G0 pp 229533.I1RAF8
81878 229533.I1R9G0 pp 229533.I1S9U2
81879 229533.I1R9G0 pp 229533.I1RGU3
81880 229533.I1R9G0 pp 229533.I1RNH1
81881 229533.I1R9G0 pp 229533.I1S021
81882 229533.I1R9G0 pp 229533.I1RKQ5
81883 229533.I1R9G0 pp 229533.I1RPH9
81884 229533.I1R9G0 pp 229533.I1RNH6
81885 229533.I1R9G0 pp 229533.I1RAC2
81886 229533.I1R9G0 pp 229533.I1RG51
81887 229533.I1R9G0 pp 229533.V6QYF0
81888 229533.I1R9G0 pp 229533.I1RKY6
81889 229533.I1R9G0 pp 229533.I1RNV6
81890 229533.I1R9G0 pp 229533.I1RVZ0
81891 229533.I1R9G0 pp 229533.I1R9V7
81892 229533.I1R9G0 pp 229533.I1RT80
81893 229533.I1R9G0 pp 229533.I1RM06
81894 229533.I1R9G0 pp 229533.I1RPU0
81895 229533.I1R9G0 pp 229533.I1RTF5
81896 229533.I1R9G0 pp 229533.I1RDA0
81897 229533.I1RMG9 pp 229533.Q4HYR2
81898 229533.I1RMG9 pp 229533.I1RMH0
81899 229533.I1RMG9 pp 229533.I1RN14
81900 229533.I1RMG9 pp 229533.I1RN13
81901 229533.A0A1C3YJY1 pp 229533.I1RW25
81902 229533.A0A1C3YJY1 pp 229533.I1RW76
81903 229533.A0A1C3YJY1 pp 229533.I1S7W2
81904 229533.A0A1C3YJY1 pp 229533.I1RAQ3
81905 229533.A0A1C3YJY1 pp 229533.I1S9Z3
81906 229533.A0A1C3YJY1 pp 229533.I1S0F5

81907 229533.A0A1C3YJY1 pp 229533.V6RKE4
81908 229533.A0A1C3YJY1 pp 229533.V6RQ48
81909 229533.A0A1C3YJY1 pp 229533.I1RTF0
81910 229533.A0A1C3YJY1 pp 229533.V6R9X9
81911 229533.A0A1C3YJY1 pp 229533.I1RZA4
81912 229533.A0A1C3YJY1 pp 229533.I1RZV1
81913 229533.A0A1C3YJY1 pp 229533.I1RW93
81914 229533.A0A1C3YJY1 pp 229533.I1RAS9
81915 229533.A0A1C3YJY1 pp 229533.I1REC4
81916 229533.A0A1C3YJY1 pp 229533.I1RSP4
81917 229533.A0A1C3YJY1 pp 229533.I1RPK5
81918 229533.A0A1C3YJY1 pp 229533.I1RCK2
81919 229533.A0A1C3YJY1 pp 229533.I1REI8
81920 229533.A0A1C3YJY1 pp 229533.I1RBS4
81921 229533.A0A1C3YJY1 pp 229533.I1RE20
81922 229533.A0A1C3YJY1 pp 229533.I1RE15
81923 229533.A0A1C3YJY1 pp 229533.A0A1C3YK51
81924 229533.A0A1C3YJY1 pp 229533.I1RXF5
81925 229533.A0A1C3YJY1 pp 229533.I1RTD0
81926 229533.A0A1C3YJY1 pp 229533.I1RIG4
81927 229533.A0A1C3YJY1 pp 229533.I1RUN9
81928 229533.I1RW76 pp 229533.I1S0X0
81929 229533.I1RW76 pp 229533.I1S278
81930 229533.I1RW76 pp 229533.I1RWM7
81931 229533.I1RW76 pp 229533.I1RXF5
81932 229533.I1RW76 pp 229533.Q4ILI9
81933 229533.I1RW76 pp 229533.I1S830
81934 229533.I1RW76 pp 229533.I1RZ60
81935 229533.I1RW76 pp 229533.I1RWT6
81936 229533.I1RW76 pp 229533.I1RY44
81937 229533.I1RW76 pp 229533.I1RX09
81938 229533.I1RW76 pp 229533.I1S2B6
81939 229533.I1RW76 pp 229533.I1RZA4
81940 229533.I1RW76 pp 229533.I1S0K3
81941 229533.I1RW76 pp 229533.I1RXU1
81942 229533.I1RW76 pp 229533.I1RW84
81943 229533.I1RW76 pp 229533.I1S2E9
81944 229533.I1RW76 pp 229533.I1RZ84
81945 229533.I1RW76 pp 229533.I1S2W9
81946 229533.I1RW76 pp 229533.I1RYW8
81947 229533.I1RW76 pp 229533.I1S0V5
81948 229533.I1RW76 pp 229533.I1RZB4
81949 229533.I1RW76 pp 229533.I1RYP0
81950 229533.I1RW76 pp 229533.I1SAM5
81951 229533.I1RW76 pp 229533.I1S7X9
81952 229533.I1RW76 pp 229533.I1S287
81953 229533.I1RW76 pp 229533.V6RKE4
81954 229533.I1RW76 pp 229533.I1S150
81955 229533.I1RW76 pp 229533.I1RZK3
81956 229533.I1RW76 pp 229533.I1S0Y1
81957 229533.I1RW76 pp 229533.I1RX87
81958 229533.I1RW76 pp 229533.V6RQ48
81959 229533.I1RW76 pp 229533.I1S1V4
81960 229533.I1RW76 pp 229533.I1RZS5
81961 229533.I1RW76 pp 229533.I1S6S4
81962 229533.I1RW76 pp 229533.I1S2C1
81963 229533.I1RW76 pp 229533.I1RWP9
81964 229533.I1RW76 pp 229533.Q4INI2
81965 229533.I1RW76 pp 229533.I1RYH6
81966 229533.I1RW76 pp 229533.Q4ICG5
81967 229533.I1RSH9 pp 229533.I1RTS2
81968 229533.I1RSH9 pp 229533.I1S2E9
81969 229533.I1RSH9 pp 229533.I1RZG7
81970 229533.I1RSH9 pp 229533.I1RY44
81971 229533.I1RSH9 pp 229533.I1RZL5
81972 229533.I1RSH9 pp 229533.I1RX81
81973 229533.I1RSH9 pp 229533.I1RT01
81974 229533.I1RSH9 pp 229533.Q4I7N9
81975 229533.I1RSH9 pp 229533.I1RT35
81976 229533.I1RSH9 pp 229533.I1S0B6
81977 229533.I1RSH9 pp 229533.I1RW23
81978 229533.I1RSH9 pp 229533.I1S7S4
81979 229533.I1RSH9 pp 229533.I1S0B1

81980	229533.I1RSH9	pp	229533.I1RT47
81981	229533.I1RSH9	pp	229533.I1S4M5
81982	229533.I1RSH9	pp	229533.I1RWT6
81983	229533.I1RSH9	pp	229533.Q4IP34
81984	229533.I1RSH9	pp	229533.V6QVD5
81985	229533.I1RN70	pp	229533.I1RQN8
81986	229533.I1RN70	pp	229533.I1S1U6
81987	229533.I1RN70	pp	229533.I1RQC4
81988	229533.I1RN70	pp	229533.I1S9S4
81989	229533.I1RN70	pp	229533.I1RQE7
81990	229533.I1RN70	pp	229533.I1RYW8
81991	229533.I1RN70	pp	229533.I1S2B6
81992	229533.I1RN70	pp	229533.I1RZ84
81993	229533.I1RN70	pp	229533.I1RQC3
81994	229533.I1RN70	pp	229533.I1RTD0
81995	229533.I1RN70	pp	229533.I1RX95
81996	229533.I1RN70	pp	229533.Q4HY20
81997	229533.I1RN70	pp	229533.I1RTQ9
81998	229533.I1RN70	pp	229533.V6RXW9
81999	229533.I1RN70	pp	229533.I1S5R1
82000	229533.I1RN70	pp	229533.I1RYP0
82001	229533.I1RN70	pp	229533.I1RTF2
82002	229533.I1RN70	pp	229533.I1RX87
82003	229533.I1RN70	pp	229533.I1RXT7
82004	229533.I1RN70	pp	229533.I1RNM3
82005	229533.I1RN70	pp	229533.I1S0V5
82006	229533.I1RN70	pp	229533.I1RQF9
82007	229533.I1RN70	pp	229533.V6RRM0
82008	229533.I1RN70	pp	229533.I1S051
82009	229533.I1RN70	pp	229533.I1RUN9
82010	229533.I1RN70	pp	229533.I1RSH3
82011	229533.I1RN70	pp	229533.I1RQD6
82012	229533.I1RN70	pp	229533.I1RPY3
82013	229533.I1RN70	pp	229533.I1RWZ9
82014	229533.I1RN70	pp	229533.I1S287
82015	229533.I1RN70	pp	229533.I1RXJ0
82016	229533.I1RN70	pp	229533.I1RQP7
82017	229533.I1RN70	pp	229533.I1RP87
82018	229533.I1RN70	pp	229533.I1RY40
82019	229533.I1RN70	pp	229533.I1RZ60
82020	229533.I1RT26	pp	229533.I1RY16
82021	229533.I1RT26	pp	229533.I1RZA8
82022	229533.I1RT26	pp	229533.I1S694
82023	229533.I1RS62	pp	229533.Q4HTT2
82024	229533.I1RS62	pp	229533.I1RYV3
82025	229533.I1RTA3	pp	229533.Q5I2J3
82026	229533.I1RTA3	pp	229533.I1RTB7
82027	229533.I1RTA3	pp	229533.I1S8X0
82028	229533.I1RTA3	pp	229533.I1RZU6
82029	229533.I1RTA3	pp	229533.I1S0S6
82030	229533.I1RTA3	pp	229533.I1RUK7
82031	229533.I1RTA3	pp	229533.I1S7X9
82032	229533.I1RTA3	pp	229533.I1RWJ8
82033	229533.I1RTA3	pp	229533.I1RXB8
82034	229533.I1RTA3	pp	229533.I1RW57
82035	229533.I1RTA3	pp	229533.I1S092
82036	229533.I1RTA3	pp	229533.I1RTE0
82037	229533.I1RTA3	pp	229533.I1RW46
82038	229533.I1RTA3	pp	229533.I1RWF4
82039	229533.I1RTA3	pp	229533.I1RY58
82040	229533.I1RTA3	pp	229533.I1RZL9
82041	229533.I1RTA3	pp	229533.Q4IN00
82042	229533.I1RTA3	pp	229533.I1RTG5
82043	229533.I1RTA3	pp	229533.Q4HZB8
82044	229533.I1S2W0	pp	229533.I1S694
82045	229533.I1S3A5	pp	229533.V6RQW4
82046	229533.I1S3A5	pp	229533.Q4HXF0
82047	229533.I1S926	pp	229533.Q4I7L0
82048	229533.I1S926	pp	229533.Q4HTT2
82049	229533.I1RJK6	pp	229533.I1RWI3
82050	229533.I1RJK6	pp	229533.I1RNP9
82051	229533.I1RJK6	pp	229533.I1RMM1
82052	229533.I1RIK8	pp	229533.V6R5D2

82053 229533.I1RIK8 pp 229533.I1SAI4
82054 229533.I1RIK8 pp 229533.V6R2Z2
82055 229533.I1RIK8 pp 229533.V6R0W1
82056 229533.I1RIK8 pp 229533.V6R756
82057 229533.I1RIK8 pp 229533.V6QU99
82058 229533.I1RIK8 pp 229533.V6RAM6
82059 229533.I1RNA0 pp 229533.I1RQK4
82060 229533.I1RNA0 pp 229533.I1RW25
82061 229533.I1RNA0 pp 229533.I1RX24
82062 229533.I1RNA0 pp 229533.V6QYF0
82063 229533.I1RNA0 pp 229533.I1RRQ6
82064 229533.I1RNA0 pp 229533.I1RYT5
82065 229533.I1RNA0 pp 229533.I1RR72
82066 229533.I1RNA0 pp 229533.I1RZS6
82067 229533.I1RNA0 pp 229533.I1RW61
82068 229533.I1RNA0 pp 229533.Q4HZ42
82069 229533.I1RNA0 pp 229533.I1S0L3
82070 229533.I1RNA0 pp 229533.I1RS47
82071 229533.I1RNA0 pp 229533.I1RPM4
82072 229533.I1RNA0 pp 229533.I1RQS8
82073 229533.I1RNA0 pp 229533.I1RQ65
82074 229533.I1RNA0 pp 229533.Q4I662
82075 229533.I1RNA0 pp 229533.I1RR24
82076 229533.I1RNA0 pp 229533.Q4IEV5
82077 229533.I1RNA0 pp 229533.I1RS30
82078 229533.I1RNA0 pp 229533.I1S9U2
82079 229533.I1RNA0 pp 229533.I1RRP3
82080 229533.I1RNA0 pp 229533.I1RPU0
82081 229533.I1RNA0 pp 229533.Q4HZ47
82082 229533.I1RNA0 pp 229533.I1RPW9
82083 229533.I1RNA0 pp 229533.I1RWJ8
82084 229533.I1RNA0 pp 229533.I1RVQ0
82085 229533.I1RNA0 pp 229533.I1RRQ3
82086 229533.I1RNA0 pp 229533.I1RRT8
82087 229533.I1RNA0 pp 229533.I1RNH1
82088 229533.I1RNA0 pp 229533.I1RT80
82089 229533.I1RNA0 pp 229533.I1S6F0
82090 229533.I1RNA0 pp 229533.I1S8E2
82091 229533.I1RNA0 pp 229533.I1RW71
82092 229533.I1RNA0 pp 229533.I1RR00
82093 229533.I1RNA0 pp 229533.I1RTF4
82094 229533.I1RNA0 pp 229533.Q4I830
82095 229533.I1RNA0 pp 229533.Q4IJH1
82096 229533.I1RNA0 pp 229533.Q4HVW2
82097 229533.I1RNA0 pp 229533.I1S464
82098 229533.I1RNA0 pp 229533.Q4IFI0
82099 229533.I1RNA0 pp 229533.Q4IQW0
82100 229533.I1RNA0 pp 229533.I1RSQ7
82101 229533.I1RNA0 pp 229533.I1RRJ8
82102 229533.I1RNA0 pp 229533.Q4IAS1
82103 229533.I1RNA0 pp 229533.I1S253
82104 229533.I1RNA0 pp 229533.I1RRZ0
82105 229533.I1RNA0 pp 229533.V6QW22
82106 229533.I1RNA0 pp 229533.I1S6U8
82107 229533.I1RNA0 pp 229533.Q4IPI1
82108 229533.I1RNA0 pp 229533.I1RT05
82109 229533.I1RNA0 pp 229533.Q4IF76
82110 229533.I1RNA0 pp 229533.I1RZ99
82111 229533.I1RNA0 pp 229533.I1RWF7
82112 229533.I1RNA0 pp 229533.I1RQS5
82113 229533.I1RNA0 pp 229533.I1RX46
82114 229533.I1RNA0 pp 229533.I1RQJ5
82115 229533.I1RNA0 pp 229533.I1RRT2
82116 229533.I1RNA0 pp 229533.I1S5X9
82117 229533.I1RNA0 pp 229533.I1RWH5
82118 229533.I1RNA0 pp 229533.I1RSD8
82119 229533.I1RNA0 pp 229533.V6R283
82120 229533.I1RNA0 pp 229533.I1RT04
82121 229533.I1RNA0 pp 229533.I1RS29
82122 229533.I1RNA0 pp 229533.I1S0R7
82123 229533.I1RNA0 pp 229533.I1RQE7
82124 229533.I1RNA0 pp 229533.I1S2M6
82125 229533.I1RNA0 pp 229533.I1S2E8

82126	229533.I1RNA0	pp	229533.I1RUQ2
82127	229533.I1RNA0	pp	229533.I1S1B6
82128	229533.I1RNA0	pp	229533.I1RZF6
82129	229533.I1RNA0	pp	229533.I1RZZ1
82130	229533.I1RNA0	pp	229533.I1RXD7
82131	229533.I1RNA0	pp	229533.V6RJP8
82132	229533.I1RNA0	pp	229533.I1RZJ6
82133	229533.I1RNA0	pp	229533.Q4INZ9
82134	229533.I1RNA0	pp	229533.V6R227
82135	229533.I1RNA0	pp	229533.I1RZK6
82136	229533.I1RNA0	pp	229533.I1RSR3
82137	229533.I1RNA0	pp	229533.I1RT98
82138	229533.I1RNA0	pp	229533.V6RGY3
82139	229533.I1RNA0	pp	229533.I1RRR7
82140	229533.I1RNA0	pp	229533.Q4HZ68
82141	229533.I1RNA0	pp	229533.I1S296
82142	229533.I1RNA0	pp	229533.I1RPY1
82143	229533.I1RNA0	pp	229533.I1RSM3
82144	229533.I1RNA0	pp	229533.I1S4T3
82145	229533.I1RNA0	pp	229533.I1RVZ0
82146	229533.I1RNA0	pp	229533.I1RQA2
82147	229533.I1RNA0	pp	229533.Q4HZK7
82148	229533.I1RNA0	pp	229533.V6RIV9
82149	229533.I1RNA0	pp	229533.I1RZE5
82150	229533.I1RNA0	pp	229533.I1RNK2
82151	229533.I1RNA0	pp	229533.Q4IEK8
82152	229533.I1RNA0	pp	229533.I1RZB5
82153	229533.I1RNA0	pp	229533.I1RSC6
82154	229533.I1RNA0	pp	229533.I1S8R3
82155	229533.I1RNA0	pp	229533.I1RNU6
82156	229533.I1RNA0	pp	229533.I1RS17
82157	229533.I1RNA0	pp	229533.I1RY63
82158	229533.I1RNA0	pp	229533.I1S2C0
82159	229533.I1RNA0	pp	229533.I1S1X9
82160	229533.I1RNA0	pp	229533.Q4IJ56
82161	229533.I1RNA0	pp	229533.I1RTE9
82162	229533.I1RNA0	pp	229533.I1S0M9
82163	229533.I1RNA0	pp	229533.I1RNF5
82164	229533.I1RNA0	pp	229533.I1RQ28
82165	229533.I1RNA0	pp	229533.Q4I8X0
82166	229533.I1RNA0	pp	229533.I1RTD9
82167	229533.I1RNA0	pp	229533.V6RCG5
82168	229533.I1RNA0	pp	229533.I1RX14
82169	229533.I1RNA0	pp	229533.Q4IAA0
82170	229533.I1RNA0	pp	229533.I1RSR8
82171	229533.I1RNA0	pp	229533.I1RZM8
82172	229533.I1RNA0	pp	229533.Q4IN63
82173	229533.I1RNA0	pp	229533.I1RQ31
82174	229533.I1RNA0	pp	229533.I1RUG3
82175	229533.I1RNA0	pp	229533.I1RZW8
82176	229533.I1RNA0	pp	229533.I1S0J1
82177	229533.I1RNA0	pp	229533.I1RRI2
82178	229533.I1RNA0	pp	229533.I1RW46
82179	229533.I1RNA0	pp	229533.V6RGN5
82180	229533.I1RNA0	pp	229533.I1RW99
82181	229533.I1RNA0	pp	229533.V6QUE6
82182	229533.I1RNA0	pp	229533.I1S021
82183	229533.I1RNA0	pp	229533.I1RYB9
82184	229533.I1RNA0	pp	229533.Q4IBS2
82185	229533.I1RNA0	pp	229533.Q4I327
82186	229533.I1RNA0	pp	229533.I1RNH6
82187	229533.I1RNA0	pp	229533.I1RNY2
82188	229533.I1RNA0	pp	229533.I1RSI6
82189	229533.I1RNA0	pp	229533.I1RX08
82190	229533.I1RK72	pp	229533.I1RT91
82191	229533.I1RK72	pp	229533.I1RK96
82192	229533.I1RK72	pp	229533.I1RQR3
82193	229533.I1RK72	pp	229533.I1RYW3
82194	229533.I1RK72	pp	229533.I1RK90
82195	229533.I1RK72	pp	229533.I1S1X9
82196	229533.I1RK72	pp	229533.I1RX81
82197	229533.I1RK72	pp	229533.I1RX24
82198	229533.I1RK72	pp	229533.I1RU69

229533.I1RK72	pp	229533.I1RN59
229533.I1RK72	pp	229533.I1RZQ5
229533.I1RK72	pp	229533.Q4HXF6
229533.I1RK72	pp	229533.I1RPA0
229533.I1RK72	pp	229533.I1RK92
229533.I1RK72	pp	229533.I1RQP8
229533.I1RK72	pp	229533.I1RST1
229533.I1RK72	pp	229533.I1RPY4
229533.I1RK72	pp	229533.I1RZG7
229533.I1RK72	pp	229533.I1RZZ1
229533.I1RK72	pp	229533.I1RPW9
229533.I1RK72	pp	229533.I1S4M5
229533.I1RK72	pp	229533.Q4IB70
229533.I1RK72	pp	229533.Q4IPB3
229533.I1RK72	pp	229533.I1RM25
229533.I1RK72	pp	229533.I1S0Z9
229533.I1RK72	pp	229533.I1S524
229533.I1RK72	pp	229533.I1RUM5
229533.I1RK72	pp	229533.I1RWU2
229533.I1RK72	pp	229533.I1S0B1
229533.I1RK72	pp	229533.I1RPY7
229533.I1RK72	pp	229533.I1RMV5
229533.I1RK72	pp	229533.I1S9L5
229533.I1RK72	pp	229533.I1RND5
229533.I1RK72	pp	229533.Q4IRI9
229533.I1RK72	pp	229533.I1RSA4
229533.I1RK72	pp	229533.I1RN91
229533.I1RK72	pp	229533.Q4I1Y1
229533.I1RK72	pp	229533.I1RME9
229533.I1RK72	pp	229533.I1RMU1
229533.I1RK72	pp	229533.I1RRL0
229533.I1RK72	pp	229533.I1S5S2
229533.I1RK72	pp	229533.Q4IBK5
229533.I1RK72	pp	229533.I1RMW7
229533.I1RK72	pp	229533.I1S2Y5
229533.I1RK72	pp	229533.I1RXF2
229533.I1RK72	pp	229533.I1RSD4
229533.I1RK72	pp	229533.I1S7M2
229533.I1RK72	pp	229533.I1RS75
229533.I1RK72	pp	229533.I1RSA8
229533.I1RK72	pp	229533.I1RS28
229533.I1RK72	pp	229533.I1RPY5
229533.I1RK72	pp	229533.Q4IE79
229533.I1RK72	pp	229533.V6RY33
229533.I1RK72	pp	229533.I1RX47
229533.I1RK72	pp	229533.I1S9L6
229533.I1RK72	pp	229533.I1RQC5
229533.I1RK72	pp	229533.I1RT17
229533.I1RK72	pp	229533.I1S7M3
229533.I1RK72	pp	229533.I1RX51
229533.I1RK72	pp	229533.I1RS83
229533.I1RK72	pp	229533.I1S8F8
229533.I1RK72	pp	229533.V6RIS2
229533.I1RK72	pp	229533.I1RND9
229533.I1RK72	pp	229533.I1RT47
229533.I1RK72	pp	229533.V6QXG1
229533.I1RK72	pp	229533.I1RYI9
229533.I1RK72	pp	229533.I1RWX8
229533.I1RK72	pp	229533.I1RN09
229533.I1RK72	pp	229533.I1RZ11
229533.I1RK72	pp	229533.I1RQD8
229533.I1S051	pp	229533.I1S2B6
229533.I1S051	pp	229533.I1S9S4
229533.I1S051	pp	229533.I1S287
229533.I1S051	pp	229533.V6QXP4
229533.I1S051	pp	229533.Q4HY20
229533.I1S051	pp	229533.V6RXW9
229533.I1RRL0	pp	229533.I1RT47
229533.I1RRL0	pp	229533.I1RS75
229533.I1RRL0	pp	229533.Q4I5R9
229533.I1RRL0	pp	229533.I1RSL9
229533.I1RRL0	pp	229533.I1RS28
229533.I1RRL0	pp	229533.I1S1C6

82272	229533.I1RRL0	pp	229533.I1S9L5
82273	229533.I1RRL0	pp	229533.I1S056
82274	229533.I1RRL0	pp	229533.Q4IBK5
82275	229533.I1RRL0	pp	229533.Q4IRI9
82276	229533.I1RRL0	pp	229533.I1RT17
82277	229533.I1RRL0	pp	229533.Q4IPB3
82278	229533.I1RRL0	pp	229533.Q4IAA0
82279	229533.I1RRL0	pp	229533.Q4I7F9
82280	229533.I1RRL0	pp	229533.I1RWU2
82281	229533.I1RRL0	pp	229533.I1RZG7
82282	229533.I1RRL0	pp	229533.I1S4M5
82283	229533.I1RRL0	pp	229533.I1RSA4
82284	229533.I1RRL0	pp	229533.I1RZQ5
82285	229533.I1RRL0	pp	229533.Q4HZB8
82286	229533.I1RRL0	pp	229533.Q4HXF6
82287	229533.I1RRL0	pp	229533.I1RX51
82288	229533.I1RRL0	pp	229533.I1S1X9
82289	229533.I1RRL0	pp	229533.I1RWX8
82290	229533.I1RRL0	pp	229533.I1RX24
82291	229533.I1RRL0	pp	229533.I1S0B1
82292	229533.I1RRL0	pp	229533.V6QXG1
82293	229533.I1RRL0	pp	229533.I1RYJ9
82294	229533.I1RRL0	pp	229533.Q4IPB8
82295	229533.I1RRL0	pp	229533.I1RYB1
82296	229533.I1RRL0	pp	229533.I1RX47
82297	229533.I1RRL0	pp	229533.I1S0Z9
82298	229533.I1RRL0	pp	229533.I1S9L6
82299	229533.I1RRL0	pp	229533.I1RSW2
82300	229533.I1RRL0	pp	229533.I1RSD4
82301	229533.I1RRL0	pp	229533.Q4IPH4
82302	229533.I1RRL0	pp	229533.I1S7M3
82303	229533.I1RRL0	pp	229533.I1S2Y5
82304	229533.I1RRL0	pp	229533.I1RT30
82305	229533.I1RRL0	pp	229533.Q4I1Y1
82306	229533.I1RRL0	pp	229533.I1RSA8
82307	229533.I1RRL0	pp	229533.Q4IJH1
82308	229533.I1RRL0	pp	229533.I1RU69
82309	229533.I1RRL0	pp	229533.I1RXF2
82310	229533.I1RRL0	pp	229533.Q4IE79
82311	229533.I1RRL0	pp	229533.Q4IF76
82312	229533.I1RRL0	pp	229533.Q4IB70
82313	229533.I1RRL0	pp	229533.I1S7M2
82314	229533.I1RRL0	pp	229533.I1S9L0
82315	229533.I1RRL0	pp	229533.I1S0B6
82316	229533.I1RRL0	pp	229533.I1RST1
82317	229533.I1RRL0	pp	229533.I1RYW3
82318	229533.V6RB81	pp	229533.V6RR76
82319	229533.Q4IQK7	pp	229533.V6RGN5
82320	229533.Q4IQK7	pp	229533.V6QYF0
82321	229533.Q4IQK7	pp	229533.V6R227
82322	229533.Q4IQK7	pp	229533.V6RCZ8
82323	229533.Q4IQK7	pp	229533.V6R283
82324	229533.I1S0L6	pp	229533.Q4HTT2
82325	229533.I1S132	pp	229533.I1S2M7
82326	229533.I1RZ38	pp	229533.I1S2F7
82327	229533.I1RZ38	pp	229533.V6RHX2
82328	229533.I1RZ38	pp	229533.I1S267
82329	229533.I1RZ38	pp	229533.I1S3T6
82330	229533.I1RZ38	pp	229533.I1S5J9
82331	229533.I1RZ38	pp	229533.I1S659
82332	229533.I1RZ38	pp	229533.V6RWB3
82333	229533.I1RZ38	pp	229533.V6RCC0
82334	229533.I1RZ38	pp	229533.I1SAI4
82335	229533.I1RZ38	pp	229533.V6R5T7
82336	229533.I1RZ38	pp	229533.I1S6H3
82337	229533.I1RZ38	pp	229533.I1S8D3
82338	229533.I1RZ38	pp	229533.I1S5D0
82339	229533.I1RZ38	pp	229533.I1S048
82340	229533.I1RZ38	pp	229533.V6QU99
82341	229533.I1RZ38	pp	229533.I1S6V0
82342	229533.I1RZ38	pp	229533.I1S969
82343	229533.I1RZ38	pp	229533.I1S223
82344	229533.I1RZ38	pp	229533.V6R9L8

82345 229533.I1RZ38 pp 229533.V6QVV9
82346 229533.I1RZ38 pp 229533.V6R0W1
82347 229533.I1RZ38 pp 229533.I1S2C4
82348 229533.I1RZ38 pp 229533.I1RZ56
82349 229533.I1RZ38 pp 229533.V6R1D2
82350 229533.I1RZ38 pp 229533.I1S094
82351 229533.I1RZ38 pp 229533.I1SAT2
82352 229533.I1RZ38 pp 229533.V6QZ48
82353 229533.I1RZ38 pp 229533.V6RAM6
82354 229533.I1RZ38 pp 229533.I1RZ89
82355 229533.I1RZ38 pp 229533.V6R2Z2
82356 229533.I1RZ38 pp 229533.I1S968
82357 229533.I1RZ38 pp 229533.V6R756
82358 229533.I1RZ38 pp 229533.V6RCJ5
82359 229533.I1RZ38 pp 229533.I1S0A5
82360 229533.I1S1U6 pp 229533.Q4HY20
82361 229533.I1S1U6 pp 229533.I1S1U7
82362 229533.I1S1U6 pp 229533.V6QXP4
82363 229533.I1S1U6 pp 229533.V6RXW9
82364 229533.I1S1U6 pp 229533.I1S287
82365 229533.I1S1U6 pp 229533.I1S2B6
82366 229533.I1S1U6 pp 229533.V6QY25
82367 229533.I1S1U6 pp 229533.I1S278
82368 229533.I1RAC3 pp 229533.I1RAL5
82369 229533.A0A0E0RTW2 pp 229533.I1RZM4
82370 229533.A0A0E0RTW2 pp 229533.V6R2Z2
82371 229533.A0A0E0RTW2 pp 229533.A0A1C3YKN4
82372 229533.A0A0E0RTW2 pp 229533.I1RDV8
82373 229533.A0A0E0RTW2 pp 229533.V6R0W1
82374 229533.A0A0E0RTW2 pp 229533.A0A1C3YIP2
82375 229533.A0A0E0RTW2 pp 229533.I1RQE8
82376 229533.A0A0E0RTW2 pp 229533.V6RAM6
82377 229533.A0A0E0RTW2 pp 229533.V6R756
82378 229533.A0A0E0RTW2 pp 229533.I1S9T6
82379 229533.A0A0E0RTW2 pp 229533.A0A1C3YKB7
82380 229533.A0A0E0RTW2 pp 229533.I1SAI4
82381 229533.A0A0E0RTW2 pp 229533.I1RRM1
82382 229533.A0A0E0RTW2 pp 229533.A0A1C3YHF4
82383 229533.A0A0E0RTW2 pp 229533.V6QU99
82384 229533.A0A0E0RTW2 pp 229533.I1RNH4
82385 229533.A0A0E0RTW2 pp 229533.I1RMU5
82386 229533.A0A0E0RTW2 pp 229533.A0A1C3YIR5
82387 229533.A0A0E0RTW2 pp 229533.V6R5D2
82388 229533.A0A0E0RTW2 pp 229533.I1RHU0
82389 229533.A0A0E0RTW2 pp 229533.I1RYM5
82390 229533.A0A0E0RTW2 pp 229533.A0A1C3YIE0
82391 229533.A0A0E0RTW2 pp 229533.I1RAG3
82392 229533.A0A0E0RTW2 pp 229533.I1RIW5
82393 229533.A0A0E0RTW2 pp 229533.A0A0E0SH57
82394 229533.A0A0E0RTW2 pp 229533.I1RK44
82395 229533.A0A0E0RTW2 pp 229533.V6RK96
82396 229533.I1S625 pp 229533.Q4I7L0
82397 229533.I1S625 pp 229533.Q4HTT2
82398 229533.I1S253 pp 229533.V6QUE6
82399 229533.I1S253 pp 229533.V6R227
82400 229533.I1S253 pp 229533.I1S2L2
82401 229533.I1S253 pp 229533.V6QYF0
82402 229533.I1S253 pp 229533.I1S9U2
82403 229533.I1S253 pp 229533.Q4I5M4
82404 229533.I1S253 pp 229533.V6QWZ7
82405 229533.I1S253 pp 229533.Q4I8X0
82406 229533.I1S253 pp 229533.I1S3G4
82407 229533.I1S253 pp 229533.I1S4S7
82408 229533.I1S253 pp 229533.I1S2E8
82409 229533.I1S253 pp 229533.Q4IQ72
82410 229533.I1S253 pp 229533.V6RGY0
82411 229533.I1S253 pp 229533.I1S9Y1
82412 229533.I1S253 pp 229533.Q4IQW0
82413 229533.I1S253 pp 229533.Q4I1M9
82414 229533.I1S253 pp 229533.I1S274
82415 229533.I1S253 pp 229533.I1S558
82416 229533.I1RN09 pp 229533.I1RPY4
82417 229533.I1RN09 pp 229533.I1RX81

229533.I1RN09	pp	229533.I1RZG7
229533.I1RN09	pp	229533.I1RTS2
229533.I1RN09	pp	229533.I1RZQ5
229533.I1RN09	pp	229533.I1RWW5
229533.I1RN09	pp	229533.V6QXG1
229533.I1RN09	pp	229533.I1RQR3
229533.I1RN09	pp	229533.Q4IF76
229533.I1RN09	pp	229533.V6QVD5
229533.I1RN09	pp	229533.Q4IJH1
229533.I1RN09	pp	229533.I1S8F8
229533.I1RN09	pp	229533.I1RYB1
229533.I1RN09	pp	229533.I1S2Y5
229533.I1RN09	pp	229533.I1RSA8
229533.I1RN09	pp	229533.I1RT35
229533.I1RN09	pp	229533.I1S246
229533.I1RN09	pp	229533.I1RZD0
229533.I1RN09	pp	229533.I1RZ11
229533.I1RN09	pp	229533.I1RYJ9
229533.I1RN09	pp	229533.I1RYI9
229533.I1RN09	pp	229533.Q4IB70
229533.I1RN09	pp	229533.Q4IP34
229533.I1RN09	pp	229533.I1RZT1
229533.I1RN09	pp	229533.I1RT47
229533.I1RN09	pp	229533.I1RSD4
229533.I1RN09	pp	229533.I1RPA0
229533.I1RN09	pp	229533.I1S056
229533.I1RN09	pp	229533.I1RS28
229533.I1RN09	pp	229533.I1RPY5
229533.I1RN09	pp	229533.I1RSA4
229533.I1RN09	pp	229533.I1RND5
229533.I1RN50	pp	229533.I1RYX6
229533.A0A0E0SGM3	pp	229533.I1RPK0
229533.A0A0E0SGM3	pp	229533.I1RBS4
229533.A0A0E0SGM3	pp	229533.A0A1C3YJY1
229533.A0A0E0SGM3	pp	229533.I1RE20
229533.A0A0E0SGM3	pp	229533.I1REC4
229533.A0A0E0SGM3	pp	229533.I1RQ38
229533.A0A0E0SGM3	pp	229533.I1RSP4
229533.A0A0E0SGM3	pp	229533.I1RAS9
229533.A0A0E0SGM3	pp	229533.A0A1C3YIN0
229533.A0A0E0SGM3	pp	229533.I1RTF0
229533.A0A0E0SGM3	pp	229533.I1S2A8
229533.A0A0E0SGM3	pp	229533.I1RCK2
229533.A0A0E0SGM3	pp	229533.I1RNR3
229533.A0A0E0SGM3	pp	229533.I1S2W9
229533.A0A0E0SGM3	pp	229533.V6RQ48
229533.A0A0E0SGM3	pp	229533.V6R9X9
229533.A0A0E0SGM3	pp	229533.I1RZA4
229533.A0A0E0SGM3	pp	229533.I1RSQ2
229533.A0A0E0SGM3	pp	229533.I1RUK7
229533.A0A0E0SGM3	pp	229533.I1RWP9
229533.A0A0E0SGM3	pp	229533.A0A1C3YKG7
229533.A0A0E0SGM3	pp	229533.I1RIG4
229533.A0A0E0SGM3	pp	229533.I1RRZ9
229533.A0A0E0SGM3	pp	229533.A0A1C3YN54
229533.A0A0E0SGM3	pp	229533.I1RIX1
229533.A0A0E0SGM3	pp	229533.I1RXF5
229533.A0A0E0SGM3	pp	229533.I1RTD0
229533.A0A0E0SGM3	pp	229533.I1RE15
229533.A0A0E0SGM3	pp	229533.I1RPK5
229533.A0A0E0SGM3	pp	229533.I1RYB8
229533.A0A0E0SGM3	pp	229533.A0A1C3YHZ2
229533.A0A0E0SGM3	pp	229533.I1RXU1
229533.A0A0E0SGM3	pp	229533.A0A1C3YK51
229533.A0A0E0SGM3	pp	229533.Q4ICG5
229533.A0A0E0SGM3	pp	229533.I1RTQ9
229533.A0A0E0SGM3	pp	229533.I1RW76
229533.A0A0E0SGM3	pp	229533.I1S9Z3
229533.A0A0E0SGM3	pp	229533.V6RKE4
229533.A0A0E0SGM3	pp	229533.I1REK1
229533.A0A1C3YID0	pp	229533.I1RS52
229533.I1RS52	pp	229533.Q4ICG8
229533.I1RS52	pp	229533.I1RWG5

82491	229533.I1RS52	pp	229533.Q4I0M9
82492	229533.I1RS52	pp	229533.I1RZH4
82493	229533.I1RS52	pp	229533.I1RZ02
82494	229533.I1RS52	pp	229533.I1RZG0
82495	229533.I1RS52	pp	229533.Q4I6S5
82496	229533.I1RS52	pp	229533.Q4INW6
82497	229533.I1RWY5	pp	229533.I1S694
82498	229533.I1RWY5	pp	229533.I1RZA8
82499	229533.I1RWY5	pp	229533.I1RY16
82500	229533.I1RAC0	pp	229533.I1RVI8
82501	229533.I1RAC0	pp	229533.V6R756
82502	229533.I1RAC0	pp	229533.I1RI19
82503	229533.I1RAC0	pp	229533.V6R0W1
82504	229533.I1RAC0	pp	229533.V6R5D2
82505	229533.I1RAC0	pp	229533.I1RDN3
82506	229533.I1RAC0	pp	229533.I1RJG4
82507	229533.I1RAC0	pp	229533.I1SAI4
82508	229533.I1RAC0	pp	229533.V6RAM6
82509	229533.I1RAC0	pp	229533.V6QU99
82510	229533.I1RAC0	pp	229533.V6R2Z2
82511	229533.I1RAD0	pp	229533.I1S8D2
82512	229533.A0A1C3YIR6	pp	229533.I1RXF5
82513	229533.A0A1C3YIR6	pp	229533.I1RUK7
82514	229533.A0A1C3YIR6	pp	229533.I1RIG4
82515	229533.A0A1C3YIR6	pp	229533.I1RTQ9
82516	229533.A0A1C3YIR6	pp	229533.I1RE15
82517	229533.A0A1C3YIR6	pp	229533.A0A1C3YN54
82518	229533.A0A1C3YIR6	pp	229533.A0A1C3YK51
82519	229533.A0A1C3YIR6	pp	229533.I1RZA4
82520	229533.A0A1C3YIR6	pp	229533.A0A1C3YKG7
82521	229533.A0A1C3YIR6	pp	229533.I1RTD0
82522	229533.A0A1C3YIR6	pp	229533.I1RPK0
82523	229533.A0A1C3YIR6	pp	229533.I1RIX1
82524	229533.A0A1C3YIR6	pp	229533.I1RWP9
82525	229533.A0A1C3YIR6	pp	229533.I1RPK5
82526	229533.A0A1C3YIR6	pp	229533.I1S2A8
82527	229533.A0A1C3YIR6	pp	229533.I1S2W9
82528	229533.A0A1C3YIR6	pp	229533.I1RXU1
82529	229533.A0A1C3YIR6	pp	229533.V6RQ48
82530	229533.A0A1C3YIR6	pp	229533.I1RAS9
82531	229533.A0A1C3YIR6	pp	229533.I1RBS4
82532	229533.A0A1C3YIR6	pp	229533.I1RE20
82533	229533.A0A1C3YIR6	pp	229533.I1RNR3
82534	229533.A0A1C3YIR6	pp	229533.A0A1C3YJY1
82535	229533.A0A1C3YIR6	pp	229533.I1REK1
82536	229533.A0A1C3YIR6	pp	229533.I1S025
82537	229533.A0A1C3YIR6	pp	229533.V6RKE4
82538	229533.A0A1C3YIR6	pp	229533.I1RW76
82539	229533.I1RDX6	pp	229533.I1S471
82540	229533.I1RDX6	pp	229533.I1RND9
82541	229533.I1RDX6	pp	229533.I1RX47
82542	229533.I1RDX6	pp	229533.I1RT35
82543	229533.I1RDX6	pp	229533.I1RRW3
82544	229533.I1RDX6	pp	229533.I1RDY2
82545	229533.I1RDX6	pp	229533.I1S5Z2
82546	229533.I1RDX6	pp	229533.I1RQP5
82547	229533.I1RDX6	pp	229533.I1RLU5
82548	229533.I1RDX6	pp	229533.I1RN91
82549	229533.I1RDX6	pp	229533.I1RMU1
82550	229533.I1RDX6	pp	229533.I1S246
82551	229533.I1RDX6	pp	229533.I1RN60
82552	229533.I1RDX6	pp	229533.I1RPY7
82553	229533.I1RDX6	pp	229533.Q4IAA0
82554	229533.I1RDX6	pp	229533.I1RS03
82555	229533.I1RDX6	pp	229533.I1RUM5
82556	229533.I1RDX6	pp	229533.I1RNB8
82557	229533.I1RAU2	pp	229533.I1RXF5
82558	229533.I1RAU2	pp	229533.V6RJP8
82559	229533.I1RAU2	pp	229533.I1RW76
82560	229533.I1RAU2	pp	229533.I1RE15
82561	229533.I1RAU2	pp	229533.I1RTD0
82562	229533.I1RAU2	pp	229533.I1RNP1
82563	229533.I1RAU2	pp	229533.I1RIG4

82564	229533.I1RAU2	pp	229533.I1RBS4
82565	229533.I1RAU2	pp	229533.I1RE20
82566	229533.I1RCS3	pp	229533.Q4I7L0
82567	229533.I1RCS3	pp	229533.I1RYV3
82568	229533.I1RCS3	pp	229533.I1S4Z5
82569	229533.I1RCS3	pp	229533.Q4HTT2
82570	229533.I1RCS3	pp	229533.Q4IQM6
82571	229533.I1RCS3	pp	229533.Q4I5R3
82572	229533.A0A0E0S8Y8	pp	229533.I1RPA0
82573	229533.A0A0E0S8Y8	pp	229533.I1RU69
82574	229533.A0A0E0S8Y8	pp	229533.I1RN59
82575	229533.A0A0E0S8Y8	pp	229533.I1RQP8
82576	229533.A0A0E0S8Y8	pp	229533.I1RMW7
82577	229533.A0A0E0S8Y8	pp	229533.A0A1C3YKF1
82578	229533.A0A0E0S8Y8	pp	229533.I1RYW3
82579	229533.A0A0E0S8Y8	pp	229533.I1RK90
82580	229533.A0A0E0S8Y8	pp	229533.I1RB50
82581	229533.A0A0E0S8Y8	pp	229533.I1RT47
82582	229533.A0A0E0S8Y8	pp	229533.I1RG57
82583	229533.A0A0E0S8Y8	pp	229533.I1RG17
82584	229533.A0A0E0S8Y8	pp	229533.I1RS83
82585	229533.A0A0E0S8Y8	pp	229533.Q4I1Y1
82586	229533.A0A0E0S8Y8	pp	229533.V6RIS2
82587	229533.A0A0E0S8Y8	pp	229533.I1RAC1
82588	229533.A0A0E0S8Y8	pp	229533.I1S0B1
82589	229533.A0A0E0S8Y8	pp	229533.I1RCI4
82590	229533.A0A0E0S8Y8	pp	229533.I1S0Z9
82591	229533.A0A0E0S8Y8	pp	229533.I1S4Y4
82592	229533.A0A0E0S8Y8	pp	229533.I1RG90
82593	229533.A0A0E0S8Y8	pp	229533.I1RX47
82594	229533.A0A0E0S8Y8	pp	229533.I1RMV5
82595	229533.A0A0E0S8Y8	pp	229533.I1RBV3
82596	229533.A0A0E0S8Y8	pp	229533.I1RCI3
82597	229533.A0A0E0S8Y8	pp	229533.I1RCR5
82598	229533.A0A0E0S8Y8	pp	229533.I1RCQ6
82599	229533.A0A0E0S8Y8	pp	229533.I1S7M3
82600	229533.A0A0E0S8Y8	pp	229533.I1R9N6
82601	229533.A0A0E0S8Y8	pp	229533.I1S524
82602	229533.A0A0E0S8Y8	pp	229533.I1RSA8
82603	229533.A0A0E0S8Y8	pp	229533.Q4IPB3
82604	229533.A0A0E0S8Y8	pp	229533.Q4IE79
82605	229533.A0A0E0S8Y8	pp	229533.I1RZZ1
82606	229533.A0A0E0S8Y8	pp	229533.I1S5S2
82607	229533.A0A0E0S8Y8	pp	229533.I1RPY4
82608	229533.A0A0E0S8Y8	pp	229533.I1S7M2
82609	229533.A0A0E0S8Y8	pp	229533.I1RZG7
82610	229533.A0A0E0S8Y8	pp	229533.I1RXF2
82611	229533.A0A0E0S8Y8	pp	229533.I1RZQ5
82612	229533.A0A0E0S8Y8	pp	229533.I1RE31
82613	229533.A0A0E0S8Y8	pp	229533.Q4INS6
82614	229533.A0A0E0S8Y8	pp	229533.Q4HXF6
82615	229533.A0A0E0S8Y8	pp	229533.I1RSA4
82616	229533.A0A0E0S8Y8	pp	229533.I1RND5
82617	229533.A0A0E0S8Y8	pp	229533.A0A1C3YMY3
82618	229533.A0A0E0S8Y8	pp	229533.I1RG13
82619	229533.A0A0E0S8Y8	pp	229533.I1RT91
82620	229533.A0A0E0S8Y8	pp	229533.I1RME9
82621	229533.A0A0E0S8Y8	pp	229533.Q4IB70
82622	229533.A0A0E0S8Y8	pp	229533.I1RK96
82623	229533.A0A0E0S8Y8	pp	229533.I1RX51
82624	229533.A0A0E0S8Y8	pp	229533.I1RQR3
82625	229533.A0A0E0S8Y8	pp	229533.I1RQC5
82626	229533.A0A0E0S8Y8	pp	229533.I1RAE1
82627	229533.A0A0E0S8Y8	pp	229533.I1RD03
82628	229533.A0A0E0S8Y8	pp	229533.I1RC84
82629	229533.A0A0E0S8Y8	pp	229533.A0A1C3YL11
82630	229533.A0A0E0S8Y8	pp	229533.I1RCN5
82631	229533.A0A0E0S8Y8	pp	229533.I1RX81
82632	229533.A0A0E0S8Y8	pp	229533.V6QXG1
82633	229533.A0A0E0S8Y8	pp	229533.I1RVX0
82634	229533.A0A0E0S8Y8	pp	229533.I1RA36
82635	229533.A0A0E0S8Y8	pp	229533.I1RBZ8
82636	229533.A0A0E0S8Y8	pp	229533.I1RND9

82637 229533.A0A0E0S8Y8 pp 229533.A0A0E0SC46
82638 229533.A0A0E0S8Y8 pp 229533.I1RST1
82639 229533.A0A0E0S8Y8 pp 229533.I1RWX8
82640 229533.A0A0E0S8Y8 pp 229533.I1RK92
82641 229533.A0A0E0S8Y8 pp 229533.V6RY33
82642 229533.A0A0E0S8Y8 pp 229533.I1RZ11
82643 229533.A0A0E0S8Y8 pp 229533.A0A1C3YNC3
82644 229533.A0A0E0S8Y8 pp 229533.I1RLU5
82645 229533.A0A0E0S8Y8 pp 229533.I1RQD8
82646 229533.A0A0E0S8Y8 pp 229533.I1RD56
82647 229533.A0A0E0S8Y8 pp 229533.I1RBH8
82648 229533.A0A0E0S8Y8 pp 229533.I1RAD1
82649 229533.A0A0E0S8Y8 pp 229533.I1RYI9
82650 229533.A0A0E0S8Y8 pp 229533.I1S9L6
82651 229533.A0A0E0S8Y8 pp 229533.I1RAB5
82652 229533.A0A0E0S8Y8 pp 229533.I1S2Y5
82653 229533.A0A0E0S8Y8 pp 229533.I1RN09
82654 229533.A0A0E0S8Y8 pp 229533.I1RSD4
82655 229533.A0A0E0S8Y8 pp 229533.A0A1C3YI62
82656 229533.A0A0E0S8Y8 pp 229533.I1RS75
82657 229533.A0A0E0S8Y8 pp 229533.I1RCA7
82658 229533.A0A0E0S8Y8 pp 229533.I1S9L5
82659 229533.A0A0E0S8Y8 pp 229533.I1RPY5
82660 229533.A0A0E0S8Y8 pp 229533.A0A1C3YHK3
82661 229533.A0A0E0S8Y8 pp 229533.I1RS28
82662 229533.A0A0E0S8Y8 pp 229533.I1RM25
82663 229533.A0A0E0S8Y8 pp 229533.I1RFQ1
82664 229533.A0A0E0S8Y8 pp 229533.I1S4M5
82665 229533.A0A0E0S8Y8 pp 229533.Q4IBK5
82666 229533.A0A0E0S8Y8 pp 229533.A0A1C3YIA6
82667 229533.A0A0E0S8Y8 pp 229533.I1RFT1
82668 229533.A0A0E0S8Y8 pp 229533.I1RK72
82669 229533.A0A0E0S8Y8 pp 229533.I1RSH9
82670 229533.A0A0E0S8Y8 pp 229533.I1RCS2
82671 229533.A0A0E0S8Y8 pp 229533.A0A1C3YK13
82672 229533.A0A0E0S8Y8 pp 229533.I1RRL0
82673 229533.A0A0E0S8Y8 pp 229533.I1RWU2
82674 229533.A0A0E0S8Y8 pp 229533.Q4IRI9
82675 229533.I1RAC9 pp 229533.V6REE9
82676 229533.I1RAC9 pp 229533.I1RTE6
82677 229533.I1RAC9 pp 229533.I1RAD0
82678 229533.I1RAC9 pp 229533.I1RN65
82679 229533.I1RAC9 pp 229533.I1S7X9
82680 229533.I1RAC9 pp 229533.I1RCD4
82681 229533.A0A1C3YIH7 pp 229533.I1RZ84
82682 229533.A0A1C3YIH7 pp 229533.I1RQ16
82683 229533.A0A1C3YIH7 pp 229533.I1RCM6
82684 229533.A0A1C3YIH7 pp 229533.Q4IBX0
82685 229533.A0A1C3YIH7 pp 229533.I1RB00
82686 229533.A0A1C3YIH7 pp 229533.I1RRB0
82687 229533.A0A1C3YIH7 pp 229533.I1RFJ2
82688 229533.A0A1C3YIH7 pp 229533.I1RB26
82689 229533.A0A1C3YIH7 pp 229533.I1R9R7
82690 229533.A0A1C3YIH7 pp 229533.I1RA72
82691 229533.A0A1C3YIH7 pp 229533.I1RPK5
82692 229533.A0A1C3YIH7 pp 229533.E6PBP1
82693 229533.A0A1C3YIH7 pp 229533.Q4I0J6
82694 229533.A0A1C3YIH7 pp 229533.I1RIG4
82695 229533.A0A1C3YIH7 pp 229533.I1RQJ0
82696 229533.A0A1C3YIH7 pp 229533.I1RJH5
82697 229533.A0A1C3YIH7 pp 229533.I1RWT1
82698 229533.A0A1C3YIH7 pp 229533.I1S1C8
82699 229533.A0A1C3YIH7 pp 229533.A0A1C3YIU5
82700 229533.A0A1C3YIH7 pp 229533.A0A1C3YL28
82701 229533.A0A1C3YIH7 pp 229533.I1RD33
82702 229533.A0A1C3YIH7 pp 229533.I1RJI1
82703 229533.A0A1C3YIH7 pp 229533.I1S2N3
82704 229533.A0A1C3YIH7 pp 229533.I1RLS9
82705 229533.A0A1C3YIH7 pp 229533.I1RNZ8
82706 229533.A0A1C3YIH7 pp 229533.I1RBS4
82707 229533.A0A1C3YIH7 pp 229533.I1RFR6
82708 229533.A0A1C3YIH7 pp 229533.I1RFZ2
82709 229533.A0A1C3YIH7 pp 229533.Q4HZS8

229533.A0A1C3YIH7 pp 229533.I1RX84
229533.A0A1C3YIH7 pp 229533.I1RTM8
229533.A0A1C3YIH7 pp 229533.I1RU80
229533.A0A1C3YIH7 pp 229533.I1RR95
229533.A0A1C3YIH7 pp 229533.A0A1C3YLK5
229533.A0A1C3YIH7 pp 229533.A0A1C3YM61
229533.A0A1C3YIH7 pp 229533.I1RXM0
229533.A0A1C3YIH7 pp 229533.Q5I2J3
229533.I1RI19 pp 229533.I1RVI8
229533.I1RI19 pp 229533.V6RH92
229533.I1RI19 pp 229533.V6R0W1
229533.I1RI19 pp 229533.V6R5D2
229533.I1RI19 pp 229533.V6QU99
229533.I1RI19 pp 229533.I1SAI4
229533.I1RI19 pp 229533.V6RAM6
229533.I1RI19 pp 229533.I1RJG4
229533.I1RI19 pp 229533.V6R2Z2
229533.I1RI19 pp 229533.V6R756
229533.I1RI19 pp 229533.I1S147
229533.I1RK90 pp 229533.I1S6S4
229533.I1RK90 pp 229533.I1RST1
229533.I1RK90 pp 229533.I1RPW9
229533.I1RK90 pp 229533.I1RN03
229533.I1RK90 pp 229533.I1RN59
229533.I1RK90 pp 229533.I1RWW5
229533.I1RK90 pp 229533.I1RU69
229533.I1RK90 pp 229533.I1RMV5
229533.I1RK90 pp 229533.V6QWX8
229533.I1RK90 pp 229533.I1RZG7
229533.I1RK90 pp 229533.I1S0J3
229533.I1RK90 pp 229533.I1RPY4
229533.I1RK90 pp 229533.Q4HVW2
229533.I1RK90 pp 229533.Q4IPB3
229533.I1RK90 pp 229533.I1RND9
229533.I1RK90 pp 229533.I1RX81
229533.I1RK90 pp 229533.I1RX24
229533.I1RK90 pp 229533.I1RT95
229533.I1RK90 pp 229533.Q4I7F9
229533.I1RK90 pp 229533.I1S1X9
229533.I1RK90 pp 229533.I1RK96
229533.I1RK90 pp 229533.I1RQC5
229533.I1RK90 pp 229533.I1RQR3
229533.I1RK90 pp 229533.I1RME9
229533.I1RK90 pp 229533.I1S1C6
229533.I1RK90 pp 229533.I1RYI9
229533.I1RK90 pp 229533.I1S9L6
229533.I1RK90 pp 229533.I1RLU5
229533.I1RK90 pp 229533.Q4I5R9
229533.I1RK90 pp 229533.I1RT91
229533.I1RK90 pp 229533.I1RMS2
229533.I1RK90 pp 229533.Q4IBK5
229533.I1RK90 pp 229533.Q4HXF6
229533.I1RK90 pp 229533.I1RS75
229533.I1RK90 pp 229533.I1RLU0
229533.I1RK90 pp 229533.I1RSL9
229533.I1RK90 pp 229533.Q4IB70
229533.I1RK90 pp 229533.I1RT17
229533.I1RK90 pp 229533.I1RWU2
229533.I1RK90 pp 229533.I1RPA0
229533.I1RK90 pp 229533.I1S2Y5
229533.I1RK90 pp 229533.I1RK92
229533.I1RK90 pp 229533.I1RPZ3
229533.I1RK90 pp 229533.I1S4M5
229533.I1RK90 pp 229533.I1RXF2
229533.I1RK90 pp 229533.I1RSZ3
229533.I1RK90 pp 229533.I1RQP8
229533.I1RK90 pp 229533.I1S9L5
229533.I1RK90 pp 229533.I1RYW3
229533.I1RK90 pp 229533.I1RS28
229533.I1RK90 pp 229533.I1S7M2
229533.I1RK90 pp 229533.I1RVK5
229533.I1RK90 pp 229533.I1S5S2
229533.I1RK90 pp 229533.I1RPY5

82783	229533.I1RK90	pp	229533.I1RM25
82784	229533.I1RK90	pp	229533.I1RSD4
82785	229533.I1RK90	pp	229533.I1RYJ9
82786	229533.I1RK90	pp	229533.Q4I1Y1
82787	229533.I1RK90	pp	229533.I1S0Z9
82788	229533.I1RK90	pp	229533.I1RWX8
82789	229533.I1RK90	pp	229533.Q4IAA0
82790	229533.I1RK90	pp	229533.I1S0B1
82791	229533.I1RK90	pp	229533.I1RPY7
82792	229533.I1RK90	pp	229533.I1RYB1
82793	229533.I1RK90	pp	229533.Q4IRI9
82794	229533.I1RK90	pp	229533.Q4IPH4
82795	229533.I1RK90	pp	229533.V6QXG1
82796	229533.I1RK90	pp	229533.I1RZ11
82797	229533.I1RK90	pp	229533.I1RX51
82798	229533.I1RK90	pp	229533.I1RT47
82799	229533.I1RK90	pp	229533.I1RQD8
82800	229533.I1RK90	pp	229533.I1RSA4
82801	229533.I1RK90	pp	229533.I1RZQ5
82802	229533.I1RK90	pp	229533.I1RPL3
82803	229533.I1RK90	pp	229533.I1RND5
82804	229533.I1RK90	pp	229533.V6QX58
82805	229533.I1RK90	pp	229533.I1RS83
82806	229533.I1RK90	pp	229533.I1S471
82807	229533.I1RK90	pp	229533.Q4IE79
82808	229533.I1RK90	pp	229533.Q4IPB8
82809	229533.I1RK90	pp	229533.Q4IP34
82810	229533.I1RK90	pp	229533.I1RSA8
82811	229533.I1RK90	pp	229533.I1RQQ7
82812	229533.I1RK90	pp	229533.I1RRL0
82813	229533.I1RK90	pp	229533.I1S7M3
82814	229533.I1RK90	pp	229533.V6RY33
82815	229533.I1RK90	pp	229533.I1RXU0
82816	229533.I1RK90	pp	229533.I1RX47
82817	229533.I1S3A2	pp	229533.I1S4Z5
82818	229533.I1S3A2	pp	229533.Q4I7L0
82819	229533.I1S3A2	pp	229533.Q4HTT2
82820	229533.I1RGI2	pp	229533.V6RHX2
82821	229533.I1RGI2	pp	229533.I1S267
82822	229533.I1RGI2	pp	229533.I1RZC3
82823	229533.I1RGI2	pp	229533.I1RQ48
82824	229533.I1RGI2	pp	229533.I1RPH3
82825	229533.I1RGI2	pp	229533.V6RHZ8
82826	229533.I1RGI2	pp	229533.I1RSG0
82827	229533.I1RGI2	pp	229533.I1RM41
82828	229533.I1RGI2	pp	229533.I1RQV8
82829	229533.I1RGI2	pp	229533.I1RNV4
82830	229533.I1RGI2	pp	229533.I1RQ70
82831	229533.I1RP87	pp	229533.I1RZD2
82832	229533.I1RP87	pp	229533.I1RQC3
82833	229533.I1RP87	pp	229533.I1RYP0
82834	229533.I1RP87	pp	229533.I1RXT7
82835	229533.I1RP87	pp	229533.I1S8P5
82836	229533.I1RP87	pp	229533.I1RX95
82837	229533.I1RP87	pp	229533.I1S9S4
82838	229533.I1RP87	pp	229533.I1RY40
82839	229533.I1RP87	pp	229533.I1RWZ9
82840	229533.I1RP87	pp	229533.I1RPY3
82841	229533.I1RP87	pp	229533.V6R1K4
82842	229533.I1RP87	pp	229533.I1S287
82843	229533.I1RP87	pp	229533.I1RYW8
82844	229533.I1RP87	pp	229533.I1RQD6
82845	229533.I1RP87	pp	229533.I1S051
82846	229533.I1RP87	pp	229533.I1S1U6
82847	229533.I1RP87	pp	229533.I1RQN8
82848	229533.I1RP87	pp	229533.V6RXW9
82849	229533.I1RP87	pp	229533.I1RSH3
82850	229533.I1RP87	pp	229533.I1S2B6
82851	229533.I1RP87	pp	229533.I1RQC4
82852	229533.I1RP87	pp	229533.I1RQF9
82853	229533.I1RP87	pp	229533.I1RTF2
82854	229533.I1RP87	pp	229533.I1S0V5
82855	229533.I1RP87	pp	229533.I1RQP7

82856	229533.I1RP87	pp	229533.I1RZ84
82857	229533.I1RP87	pp	229533.I1RUN9
82858	229533.I1RP87	pp	229533.I1RTA0
82859	229533.I1RP87	pp	229533.Q4HY20
82860	229533.I1RP87	pp	229533.I1RT00
82861	229533.I1RIV7	pp	229533.Q4I7L0
82862	229533.I1RIV7	pp	229533.I1S4Z5
82863	229533.I1RIV7	pp	229533.I1RYV3
82864	229533.I1RIV7	pp	229533.Q4HTT2
82865	229533.A0A1C3YHW3	pp	229533.A0A1C3YIA6
82866	229533.A0A1C3YHW3	pp	229533.I1RKK4
82867	229533.A0A1C3YHW3	pp	229533.I1RK19
82868	229533.A0A1C3YHW3	pp	229533.I1RWU2
82869	229533.A0A1C3YHW3	pp	229533.A0A1C3YHW5
82870	229533.A0A1C3YHW3	pp	229533.A0A1C3YMC1
82871	229533.A0A1C3YHW3	pp	229533.A0A1C3YJI0
82872	229533.A0A1C3YHW3	pp	229533.A0A1C3YMN3
82873	229533.A0A1C3YHW3	pp	229533.I1RYV4
82874	229533.A0A1C3YHW3	pp	229533.I1RT17
82875	229533.A0A1C3YHW3	pp	229533.A0A1C3YMK1
82876	229533.A0A1C3YHW3	pp	229533.I1RCQ6
82877	229533.A0A1C3YHW3	pp	229533.I1RTS2
82878	229533.A0A1C3YHW3	pp	229533.I1RXF8
82879	229533.A0A1C3YHW3	pp	229533.I1S9L0
82880	229533.A0A1C3YHW3	pp	229533.I1RBU6
82881	229533.A0A1C3YHW3	pp	229533.I1RC73
82882	229533.A0A1C3YHW3	pp	229533.I1RAP1
82883	229533.A0A1C3YHW3	pp	229533.I1RX24
82884	229533.A0A1C3YHW3	pp	229533.I1RYM9
82885	229533.A0A1C3YHW3	pp	229533.I1RBZ8
82886	229533.A0A1C3YHW3	pp	229533.V6QVD5
82887	229533.A0A1C3YHW3	pp	229533.I1RYB1
82888	229533.A0A1C3YHW3	pp	229533.I1RKJ8
82889	229533.A0A1C3YHW3	pp	229533.Q4I1Y1
82890	229533.A0A1C3YHW3	pp	229533.I1RML0
82891	229533.A0A1C3YHW3	pp	229533.I1RST1
82892	229533.A0A1C3YHW3	pp	229533.I1S0B1
82893	229533.A0A1C3YHW3	pp	229533.I1RAC1
82894	229533.A0A1C3YHW3	pp	229533.I1RAP2
82895	229533.A0A1C3YHW3	pp	229533.I1RZD2
82896	229533.A0A1C3YHW3	pp	229533.I1RPY7
82897	229533.A0A1C3YHW3	pp	229533.I1RU69
82898	229533.A0A1C3YHW3	pp	229533.I1RSA4
82899	229533.A0A1C3YHW3	pp	229533.I1RT35
82900	229533.A0A1C3YHW3	pp	229533.I1S0Z9
82901	229533.A0A1C3YHW3	pp	229533.A0A1C3YMB5
82902	229533.A0A1C3YHW3	pp	229533.A0A1C3YJ42
82903	229533.A0A1C3YHW3	pp	229533.I1RYJ9
82904	229533.A0A1C3YHW3	pp	229533.V6RY33
82905	229533.A0A1C3YHW3	pp	229533.Q4IF76
82906	229533.A0A1C3YHW3	pp	229533.A0A1C3YL25
82907	229533.A0A1C3YHW3	pp	229533.I1S2A8
82908	229533.A0A1C3YHW3	pp	229533.A0A1C3YM79
82909	229533.A0A1C3YHW3	pp	229533.Q4I7F9
82910	229533.A0A1C3YHW3	pp	229533.A0A1C3YN69
82911	229533.A0A1C3YHW3	pp	229533.A0A1C3YL40
82912	229533.A0A1C3YHW3	pp	229533.Q4IJH1
82913	229533.A0A1C3YHW3	pp	229533.Q4IAA0
82914	229533.A0A1C3YHW3	pp	229533.I1RDG0
82915	229533.A0A1C3YHW3	pp	229533.I1RNB8
82916	229533.A0A1C3YHW3	pp	229533.I1RQ45
82917	229533.A0A1C3YHW3	pp	229533.Q4HXF6
82918	229533.A0A1C3YHW3	pp	229533.I1RG13
82919	229533.A0A1C3YHW3	pp	229533.I1RM25
82920	229533.A0A1C3YHW3	pp	229533.I1RZ11
82921	229533.A0A1C3YHW3	pp	229533.I1S1X9
82922	229533.A0A1C3YHW3	pp	229533.I1RGC2
82923	229533.A0A1C3YHW3	pp	229533.I1RFG0
82924	229533.A0A1C3YHW3	pp	229533.I1RYC3
82925	229533.A0A1C3YHW3	pp	229533.I1RQR3
82926	229533.A0A1C3YHW3	pp	229533.I1RAF3
82927	229533.A0A1C3YHW3	pp	229533.I1RN09
82928	229533.A0A1C3YHW3	pp	229533.I1RS41

82929 229533.A0A1C3YHW3 pp 229533.I1RG17
82930 229533.A0A1C3YHW3 pp 229533.I1S044
82931 229533.A0A1C3YHW3 pp 229533.I1RD56
82932 229533.A0A1C3YHW3 pp 229533.A0A1C3YJT5
82933 229533.A0A1C3YHW3 pp 229533.I1S246
82934 229533.A0A1C3YHW3 pp 229533.Q4IB70
82935 229533.A0A1C3YHW3 pp 229533.Q4IBK5
82936 229533.A0A1C3YHW3 pp 229533.Q4IPC8
82937 229533.A0A1C3YHW3 pp 229533.I1S5Z2
82938 229533.A0A1C3YHW3 pp 229533.I1RMA4
82939 229533.A0A1C3YHW3 pp 229533.I1RCN5
82940 229533.A0A1C3YHW3 pp 229533.A0A1C3YK19
82941 229533.A0A1C3YHW3 pp 229533.I1RSA8
82942 229533.A0A1C3YHW3 pp 229533.I1RMH5
82943 229533.A0A1C3YHW3 pp 229533.A0A1C3YJW0
82944 229533.I1RMM1 pp 229533.I1S1U6
82945 229533.I1RMM1 pp 229533.I1RW23
82946 229533.I1RMM1 pp 229533.I1RPC6
82947 229533.I1RMM1 pp 229533.I1S1D3
82948 229533.I1RMM1 pp 229533.I1RQ49
82949 229533.I1RMM1 pp 229533.I1RS41
82950 229533.I1RMM1 pp 229533.I1RRC8
82951 229533.I1RMM1 pp 229533.I1RQN8
82952 229533.I1RMM1 pp 229533.V6QUV7
82953 229533.I1RMM1 pp 229533.I1RXT9
82954 229533.I1RMM1 pp 229533.Q4HY20
82955 229533.I1RMM1 pp 229533.I1RQC3
82956 229533.I1RMM1 pp 229533.I1S4N5
82957 229533.I1RMM1 pp 229533.Q4I1H6
82958 229533.I1RMM1 pp 229533.I1RT01
82959 229533.I1RMM1 pp 229533.I1RZK3
82960 229533.I1RMM1 pp 229533.Q4I6S5
82961 229533.I1RMM1 pp 229533.I1SAM5
82962 229533.I1RMM1 pp 229533.V6QW25
82963 229533.I1RMM1 pp 229533.I1RMP0
82964 229533.I1RMM1 pp 229533.I1RW24
82965 229533.I1RMM1 pp 229533.I1RYP7
82966 229533.I1RMM1 pp 229533.I1RQ88
82967 229533.I1RMM1 pp 229533.I1RWQ1
82968 229533.I1RMM1 pp 229533.I1RTF2
82969 229533.I1RMM1 pp 229533.Q4ILI9
82970 229533.I1RMM1 pp 229533.I1RNB9
82971 229533.I1RMM1 pp 229533.I1RRC9
82972 229533.I1RMM1 pp 229533.Q4HYQ4
82973 229533.I1RMM1 pp 229533.I1RZR6
82974 229533.I1RMM1 pp 229533.I1RQP7
82975 229533.I1RMM1 pp 229533.I1RMN3
82976 229533.I1RMM1 pp 229533.I1RS34
82977 229533.I1RMM1 pp 229533.I1RQC4
82978 229533.I1RMM1 pp 229533.V6QXP4
82979 229533.I1RMM1 pp 229533.I1RWZ9
82980 229533.I1RMM1 pp 229533.I1RY40
82981 229533.I1RMM1 pp 229533.I1RSW4
82982 229533.I1RMM1 pp 229533.I1RS35
82983 229533.I1RMM1 pp 229533.I1S287
82984 229533.I1RMM1 pp 229533.I1RX87
82985 229533.I1RMM1 pp 229533.I1S2C1
82986 229533.I1RGC1 pp 229533.I1S287
82987 229533.I1RGC1 pp 229533.I1RN70
82988 229533.I1RGC1 pp 229533.I1RP87
82989 229533.I1RGC1 pp 229533.I1RPY3
82990 229533.I1RGC1 pp 229533.I1RQF9
82991 229533.I1RGC1 pp 229533.I1S5R1
82992 229533.I1RGC1 pp 229533.I1RX87
82993 229533.I1RGC1 pp 229533.I1S0V5
82994 229533.I1RGC1 pp 229533.I1S0Z6
82995 229533.I1RGC1 pp 229533.I1RTD0
82996 229533.I1RGC1 pp 229533.I1RQF7
82997 229533.I1RGC1 pp 229533.I1RX95
82998 229533.I1RGC1 pp 229533.I1RQC3
82999 229533.I1RGC1 pp 229533.I1RIG4
83000 229533.I1RGC1 pp 229533.I1RXF0
83001 229533.I1RGC1 pp 229533.I1RYP0

83002	229533.I1RGC1	pp	229533.I1RXT7
83003	229533.I1RGC1	pp	229533.I1RQP7
83004	229533.I1RGC1	pp	229533.I1RY40
83005	229533.I1RGC1	pp	229533.I1RPG2
83006	229533.I1RGC1	pp	229533.I1RWZ9
83007	229533.I1RGC1	pp	229533.I1S2B6
83008	229533.I1RGC1	pp	229533.I1RT00
83009	229533.I1RGC1	pp	229533.I1RTF2
83010	229533.I1RGC1	pp	229533.I1RNM3
83011	229533.I1RGC1	pp	229533.I1RJY4
83012	229533.I1RGC1	pp	229533.I1RZ84
83013	229533.I1RGC1	pp	229533.I1RUN9
83014	229533.I1RGC1	pp	229533.I1RQD6
83015	229533.I1RGC1	pp	229533.I1S051
83016	229533.I1RGC1	pp	229533.I1RRI4
83017	229533.I1RGC1	pp	229533.Q4HY20
83018	229533.I1RGC1	pp	229533.V6QUV7
83019	229533.I1RGC1	pp	229533.I1RKR7
83020	229533.I1RGC1	pp	229533.I1S1U6
83021	229533.I1RGC1	pp	229533.V6RXW9
83022	229533.I1RGC1	pp	229533.I1RYW8
83023	229533.I1RGC1	pp	229533.I1RQN8
83024	229533.I1RGC1	pp	229533.I1RQC4
83025	229533.I1RGC1	pp	229533.I1S9S4
83026	229533.I1RGC1	pp	229533.I1RMN3
83027	229533.A0A098DAR0	pp	229533.I1RMN6
83028	229533.A0A098DAR0	pp	229533.Q4IBS2
83029	229533.A0A098DAR0	pp	229533.I1RC27
83030	229533.A0A098DAR0	pp	229533.V6RGN5
83031	229533.A0A098DAR0	pp	229533.I1RN58
83032	229533.A0A098DAR0	pp	229533.I1RQ31
83033	229533.A0A098DAR0	pp	229533.I1RTE9
83034	229533.A0A098DAR0	pp	229533.I1RSI6
83035	229533.A0A098DAR0	pp	229533.A0A1C3YJG0
83036	229533.A0A098DAR0	pp	229533.I1RG56
83037	229533.A0A098DAR0	pp	229533.I1RNH6
83038	229533.A0A098DAR0	pp	229533.I1S2C0
83039	229533.A0A098DAR0	pp	229533.I1RAZ8
83040	229533.A0A098DAR0	pp	229533.I1RPY1
83041	229533.A0A098DAR0	pp	229533.I1S4T3
83042	229533.A0A098DAR0	pp	229533.I1S021
83043	229533.A0A098DAR0	pp	229533.I1RM96
83044	229533.A0A098DAR0	pp	229533.I1RKC6
83045	229533.A0A098DAR0	pp	229533.I1RNK2
83046	229533.A0A098DAR0	pp	229533.Q4HZ47
83047	229533.A0A098DAR0	pp	229533.I1RVZ0
83048	229533.A0A098DAR0	pp	229533.Q4IEK8
83049	229533.A0A098DAR0	pp	229533.I1RS30
83050	229533.A0A098DAR0	pp	229533.I1RC33
83051	229533.A0A098DAR0	pp	229533.I1RE00
83052	229533.A0A098DAR0	pp	229533.I1RFJ9
83053	229533.A0A098DAR0	pp	229533.I1RNH1
83054	229533.A0A098DAR0	pp	229533.I1S0M3
83055	229533.A0A098DAR0	pp	229533.A0A1C3YJR3
83056	229533.A0A098DAR0	pp	229533.I1RZF6
83057	229533.A0A098DAR0	pp	229533.I1RRQ3
83058	229533.A0A098DAR0	pp	229533.A0A1C3YN63
83059	229533.A0A098DAR0	pp	229533.V6R227
83060	229533.A0A098DAR0	pp	229533.I1RS47
83061	229533.A0A098DAR0	pp	229533.I1RF19
83062	229533.A0A098DAR0	pp	229533.I1S060
83063	229533.A0A098DAR0	pp	229533.I1RQ65
83064	229533.A0A098DAR0	pp	229533.I1RX14
83065	229533.A0A098DAR0	pp	229533.I1S296
83066	229533.A0A098DAR0	pp	229533.V6R283
83067	229533.A0A098DAR0	pp	229533.Q4HZ68
83068	229533.A0A098DAR0	pp	229533.I1S0J1
83069	229533.A0A098DAR0	pp	229533.I1RC03
83070	229533.A0A098DAR0	pp	229533.A0A0E0SGM4
83071	229533.A0A098DAR0	pp	229533.A0A0E0S7B3
83072	229533.A0A098DAR0	pp	229533.I1RC72
83073	229533.A0A098DAR0	pp	229533.I1RC06
83074	229533.A0A098DAR0	pp	229533.I1RKL3

83075	229533.A0A098DAR0	pp	229533.I1S2U2
83076	229533.A0A098DAR0	pp	229533.A0A0E0S621
83077	229533.A0A098DAR0	pp	229533.I1RNU6
83078	229533.A0A098DAR0	pp	229533.I1RW71
83079	229533.A0A098DAR0	pp	229533.Q4IJ56
83080	229533.A0A098DAR0	pp	229533.I1RSD8
83081	229533.A0A098DAR0	pp	229533.I1RRJ8
83082	229533.A0A098DAR0	pp	229533.A0A098DE06
83083	229533.A0A098DAR0	pp	229533.I1S2E8
83084	229533.A0A098DAR0	pp	229533.I1R9X7
83085	229533.A0A098DAR0	pp	229533.I1RB27
83086	229533.A0A098DAR0	pp	229533.I1RZ99
83087	229533.A0A098DAR0	pp	229533.I1RAN5
83088	229533.A0A098DAR0	pp	229533.A0A0E0RP78
83089	229533.A0A098DAR0	pp	229533.I1RCN5
83090	229533.A0A098DAR0	pp	229533.I1RSC6
83091	229533.A0A098DAR0	pp	229533.I1RPU0
83092	229533.A0A098DAR0	pp	229533.I1RSS4
83093	229533.A0A098DAR0	pp	229533.I1RQA2
83094	229533.A0A098DAR0	pp	229533.I1RKJ6
83095	229533.A0A098DAR0	pp	229533.I1RCL3
83096	229533.A0A098DAR0	pp	229533.I1RA48
83097	229533.A0A098DAR0	pp	229533.I1R9V7
83098	229533.A0A098DAR0	pp	229533.I1RM67
83099	229533.A0A098DAR0	pp	229533.I1S1X9
83100	229533.A0A098DAR0	pp	229533.I1S274
83101	229533.A0A098DAR0	pp	229533.I1RD64
83102	229533.A0A098DAR0	pp	229533.I1RGF6
83103	229533.A0A098DAR0	pp	229533.A0A098DRR8
83104	229533.A0A098DAR0	pp	229533.I1RSR3
83105	229533.A0A098DAR0	pp	229533.V6RGY3
83106	229533.A0A098DAR0	pp	229533.I1RQ28
83107	229533.A0A098DAR0	pp	229533.I1RK95
83108	229533.A0A098DAR0	pp	229533.I1S9U2
83109	229533.A0A098DAR0	pp	229533.I1RDG0
83110	229533.A0A098DAR0	pp	229533.I1RN99
83111	229533.A0A098DAR0	pp	229533.I1S1U7
83112	229533.A0A098DAR0	pp	229533.I1RPW9
83113	229533.A0A098DAR0	pp	229533.I1RNH9
83114	229533.A0A098DAR0	pp	229533.I1RAS2
83115	229533.A0A098DAR0	pp	229533.I1RKQ5
83116	229533.A0A098DAR0	pp	229533.Q4IF76
83117	229533.A0A098DAR0	pp	229533.A0A0E0RNT7
83118	229533.A0A098DAR0	pp	229533.I1RLV9
83119	229533.A0A098DAR0	pp	229533.Q4IAS1
83120	229533.A0A098DAR0	pp	229533.I1RMD5
83121	229533.A0A098DAR0	pp	229533.I1RT05
83122	229533.A0A098DAR0	pp	229533.I1RRI2
83123	229533.A0A098DAR0	pp	229533.I1RJL4
83124	229533.A0A098DAR0	pp	229533.Q4IPI1
83125	229533.A0A098DAR0	pp	229533.I1RQM6
83126	229533.A0A098DAR0	pp	229533.A0A1C3YMW0
83127	229533.A0A098DAR0	pp	229533.I1RL23
83128	229533.A0A098DAR0	pp	229533.I1RAC2
83129	229533.A0A098DAR0	pp	229533.I1RTF4
83130	229533.A0A098DAR0	pp	229533.I1R9G0
83131	229533.A0A098DAR0	pp	229533.I1RC32
83132	229533.A0A098DAR0	pp	229533.I1RFS3
83133	229533.A0A098DAR0	pp	229533.I1RRT2
83134	229533.A0A098DAR0	pp	229533.I1RFN1
83135	229533.A0A098DAR0	pp	229533.Q4IJH1
83136	229533.A0A098DAR0	pp	229533.I1RC82
83137	229533.A0A098DAR0	pp	229533.Q4IQW0
83138	229533.A0A098DAR0	pp	229533.I1RG51
83139	229533.A0A098DAR0	pp	229533.I1RC29
83140	229533.A0A098DAR0	pp	229533.I1RT80
83141	229533.A0A098DAR0	pp	229533.I1RNA0
83142	229533.A0A098DAR0	pp	229533.I1RNV6
83143	229533.A0A098DAR0	pp	229533.Q4IQK7
83144	229533.A0A098DAR0	pp	229533.A0A098DE18
83145	229533.A0A098DAR0	pp	229533.A0A1C3YIN1
83146	229533.A0A098DAR0	pp	229533.I1S253
83147	229533.A0A098DAR0	pp	229533.I1RAF8

83148	229533.A0A098DAR0	pp	229533.I1RBG9
83149	229533.A0A098DAR0	pp	229533.I1RDA0
83150	229533.A0A098DAR0	pp	229533.V6QYF0
83151	229533.A0A098DAR0	pp	229533.I1REF2
83152	229533.A0A098DAR0	pp	229533.A0A1C3YLF5
83153	229533.A0A098DAR0	pp	229533.I1RMK3
83154	229533.A0A098DAR0	pp	229533.I1RUQ2
83155	229533.A0A098DAR0	pp	229533.I1RX24
83156	229533.A0A098DAR0	pp	229533.I1RJZ7
83157	229533.A0A098DAR0	pp	229533.I1RZS6
83158	229533.A0A098DAR0	pp	229533.A0A1C3YK72
83159	229533.A0A098DAR0	pp	229533.Q4IFI0
83160	229533.A0A098DAR0	pp	229533.I1RM06
83161	229533.I1RF24	pp	229533.I1SAT2
83162	229533.I1RF24	pp	229533.I1RPV8
83163	229533.I1RF24	pp	229533.I1RZC3
83164	229533.I1RF24	pp	229533.I1S0V7
83165	229533.I1RF24	pp	229533.I1RZ56
83166	229533.I1RF24	pp	229533.I1RZ89
83167	229533.I1RF24	pp	229533.V6R2Z2
83168	229533.I1RF24	pp	229533.I1S6V0
83169	229533.I1RF24	pp	229533.I1RMK9
83170	229533.I1RF24	pp	229533.I1RRR9
83171	229533.I1RF24	pp	229533.I1RY32
83172	229533.I1RF24	pp	229533.I1RW82
83173	229533.I1RF24	pp	229533.I1S2C4
83174	229533.I1RF24	pp	229533.I1RH83
83175	229533.I1RF24	pp	229533.I1S968
83176	229533.I1RF24	pp	229533.I1RLZ4
83177	229533.I1RF24	pp	229533.I1S0A5
83178	229533.I1RF24	pp	229533.I1RMT6
83179	229533.I1RF24	pp	229533.I1RJS1
83180	229533.I1RF24	pp	229533.I1RR68
83181	229533.I1RF24	pp	229533.V6R756
83182	229533.I1RF24	pp	229533.I1RSA5
83183	229533.I1RF24	pp	229533.I1RHU8
83184	229533.I1RF24	pp	229533.I1RZV5
83185	229533.I1RF24	pp	229533.I1RTM8
83186	229533.I1RF24	pp	229533.I1RZX3
83187	229533.I1RF24	pp	229533.I1RM41
83188	229533.I1RF24	pp	229533.I1RH91
83189	229533.I1RF24	pp	229533.I1RSP5
83190	229533.I1RF24	pp	229533.I1RZ25
83191	229533.I1RF24	pp	229533.V6RCJ5
83192	229533.I1RF24	pp	229533.I1RZ38
83193	229533.I1RF24	pp	229533.I1RK55
83194	229533.I1RF24	pp	229533.I1RWC1
83195	229533.I1RF24	pp	229533.I1RY12
83196	229533.I1RF24	pp	229533.I1RST9
83197	229533.I1RF24	pp	229533.I1RIJ6
83198	229533.I1RF24	pp	229533.I1RIB5
83199	229533.I1RF24	pp	229533.V6R5D2
83200	229533.I1RF24	pp	229533.I1S3T6
83201	229533.I1RF24	pp	229533.I1RRM1
83202	229533.I1RF24	pp	229533.I1RW64
83203	229533.I1RF24	pp	229533.I1S8D3
83204	229533.I1RF24	pp	229533.Q4HYR2
83205	229533.I1RF24	pp	229533.I1RWM4
83206	229533.I1RF24	pp	229533.V6RWB3
83207	229533.I1RF24	pp	229533.I1S267
83208	229533.I1RF24	pp	229533.I1S2F7
83209	229533.I1RF24	pp	229533.I1RPM6
83210	229533.I1RF24	pp	229533.V6RHX2
83211	229533.I1RF24	pp	229533.I1RNH4
83212	229533.I1RF24	pp	229533.I1S659
83213	229533.I1RF24	pp	229533.I1RSG0
83214	229533.I1RF24	pp	229533.I1RQV8
83215	229533.I1RF24	pp	229533.I1RLA2
83216	229533.I1RF24	pp	229533.I1RYU6
83217	229533.I1RF24	pp	229533.I1RY81
83218	229533.I1RF24	pp	229533.I1S048
83219	229533.I1RF24	pp	229533.V6RAM6
83220	229533.I1RF24	pp	229533.V6QU99

83221 229533.I1RF24 pp 229533.I1S6H3
83222 229533.I1RF24 pp 229533.V6QVV9
83223 229533.I1RF24 pp 229533.I1RWL3
83224 229533.I1RF24 pp 229533.V6R5T7
83225 229533.I1RF24 pp 229533.I1RJR8
83226 229533.I1RF24 pp 229533.I1RW54
83227 229533.I1RF24 pp 229533.I1SAI4
83228 229533.I1RF24 pp 229533.I1S223
83229 229533.I1RF24 pp 229533.I1RRS6
83230 229533.I1RF24 pp 229533.I1S969
83231 229533.I1RF24 pp 229533.V6R0W1
83232 229533.I1RF24 pp 229533.I1RMF1
83233 229533.I1RF24 pp 229533.I1S094
83234 229533.I1RF24 pp 229533.I1RQV7
83235 229533.I1RF24 pp 229533.I1S5D0
83236 229533.I1RF24 pp 229533.I1RYP5
83237 229533.I1RF24 pp 229533.I1RMA2
83238 229533.I1RF24 pp 229533.V6RCC0
83239 229533.A0A1C3YHW5 pp 229533.I1RPY7
83240 229533.A0A1C3YHW5 pp 229533.I1S0B1
83241 229533.A0A1C3YHW5 pp 229533.V6QVD5
83242 229533.A0A1C3YHW5 pp 229533.I1RYB1
83243 229533.A0A1C3YHW5 pp 229533.I1RG90
83244 229533.A0A1C3YHW5 pp 229533.I1RA36
83245 229533.A0A1C3YHW5 pp 229533.I1RZL5
83246 229533.A0A1C3YHW5 pp 229533.I1RK92
83247 229533.A0A1C3YHW5 pp 229533.I1RMH5
83248 229533.A0A1C3YHW5 pp 229533.I1RSH9
83249 229533.A0A1C3YHW5 pp 229533.I1RNB8
83250 229533.A0A1C3YHW5 pp 229533.I1RSD4
83251 229533.A0A1C3YHW5 pp 229533.I1RBH8
83252 229533.A0A1C3YHW5 pp 229533.I1RSA4
83253 229533.A0A1C3YHW5 pp 229533.I1RCQ6
83254 229533.A0A1C3YHW5 pp 229533.I1RT35
83255 229533.A0A1C3YHW5 pp 229533.I1RTS2
83256 229533.A0A1C3YHW5 pp 229533.I1RCD6
83257 229533.A0A1C3YHW5 pp 229533.I1RMV5
83258 229533.A0A1C3YHW5 pp 229533.I1RWX8
83259 229533.A0A1C3YHW5 pp 229533.A0A1C3YJW0
83260 229533.A0A1C3YHW5 pp 229533.I1RB50
83261 229533.A0A1C3YHW5 pp 229533.I1RQZ2
83262 229533.A0A1C3YHW5 pp 229533.I1S0B6
83263 229533.A0A1C3YHW5 pp 229533.I1S7M3
83264 229533.A0A1C3YHW5 pp 229533.Q4I7F9
83265 229533.A0A1C3YHW5 pp 229533.Q4IP34
83266 229533.A0A1C3YHW5 pp 229533.A0A1C3YL11
83267 229533.A0A1C3YHW5 pp 229533.I1S4M5
83268 229533.A0A1C3YHW5 pp 229533.I1RK19
83269 229533.A0A1C3YHW5 pp 229533.I1RQD8
83270 229533.A0A1C3YHW5 pp 229533.I1RT47
83271 229533.A0A1C3YHW5 pp 229533.A0A1C3YNC3
83272 229533.A0A1C3YHW5 pp 229533.I1RN09
83273 229533.A0A1C3YHW5 pp 229533.I1RG17
83274 229533.A0A1C3YHW5 pp 229533.I1RXF2
83275 229533.A0A1C3YHW5 pp 229533.I1S7M2
83276 229533.A0A1C3YHW5 pp 229533.I1RBV3
83277 229533.A0A1C3YHW5 pp 229533.A0A1C3YKM6
83278 229533.A0A1C3YHW5 pp 229533.A0A1C3YI62
83279 229533.I1RKD4 pp 229533.I1S064
83280 229533.I1RKD4 pp 229533.I1RKJ8
83281 229533.I1RKD4 pp 229533.I1RM26
83282 229533.I1RKD4 pp 229533.I1RVX9
83283 229533.I1RKD4 pp 229533.I1RPU8
83284 229533.I1RKD4 pp 229533.I1RML0
83285 229533.I1RKD4 pp 229533.I1RXF8
83286 229533.A0A1C3YM08 pp 229533.I1RCL8
83287 229533.A0A1C3YM08 pp 229533.I1RVC8
83288 229533.A0A1C3YM08 pp 229533.I1SAG3
83289 229533.A0A1C3YM08 pp 229533.I1RJ33
83290 229533.A0A1C3YM08 pp 229533.I1SA77
83291 229533.A0A1C3YJL9 pp 229533.V6RAM6
83292 229533.A0A1C3YJL9 pp 229533.V6QU99
83293 229533.A0A1C3YJL9 pp 229533.I1S9T6

83294 229533.A0A1C3YJL9 pp 229533.I1RAG3
83295 229533.A0A1C3YJL9 pp 229533.I1RIW5
83296 229533.A0A1C3YJL9 pp 229533.I1RZM4
83297 229533.A0A1C3YJL9 pp 229533.I1RK44
83298 229533.A0A1C3YJL9 pp 229533.V6R2Z2
83299 229533.A0A1C3YJL9 pp 229533.V6RK96
83300 229533.A0A1C3YJL9 pp 229533.I1RDV8
83301 229533.A0A1C3YJL9 pp 229533.I1RHU0
83302 229533.A0A1C3YJL9 pp 229533.V6R756
83303 229533.A0A1C3YJL9 pp 229533.A0A1C3YKN4
83304 229533.A0A1C3YJL9 pp 229533.V6R0W1
83305 229533.A0A1C3YJL9 pp 229533.I1RRM1
83306 229533.A0A1C3YJL9 pp 229533.I1RQE8
83307 229533.A0A1C3YJL9 pp 229533.I1RNH4
83308 229533.A0A1C3YJL9 pp 229533.A0A1C3YKB7
83309 229533.A0A1C3YJL9 pp 229533.I1RYM5
83310 229533.A0A1C3YJL9 pp 229533.I1RMU5
83311 229533.A0A1C3YJL9 pp 229533.I1SAI4
83312 229533.A0A1C3YJL9 pp 229533.V6R5D2
83313 229533.I1REZ0 pp 229533.V6R476
83314 229533.I1REZ0 pp 229533.I1RN81
83315 229533.I1REZ0 pp 229533.V6QUU6
83316 229533.I1REZ0 pp 229533.I1RZT7
83317 229533.I1REZ0 pp 229533.I1RKR7
83318 229533.I1REZ0 pp 229533.I1RY27
83319 229533.I1REZ0 pp 229533.I1S254
83320 229533.I1REZ0 pp 229533.I1RIG4
83321 229533.I1REZ0 pp 229533.V6RR76
83322 229533.I1REZ0 pp 229533.I1RRW2
83323 229533.I1RQ54 pp 229533.Q4HTT2
83324 229533.I1RQ54 pp 229533.I1RYV3
83325 229533.I1SA77 pp 229533.I1SAG3
83326 229533.A0A1C3YJM7 pp 229533.I1RQE8
83327 229533.A0A1C3YJM7 pp 229533.V6R0W1
83328 229533.A0A1C3YJM7 pp 229533.V6R5D2
83329 229533.A0A1C3YJM7 pp 229533.I1SAI4
83330 229533.A0A1C3YJM7 pp 229533.I1RMU5
83331 229533.A0A1C3YJM7 pp 229533.V6QU99
83332 229533.A0A1C3YJM7 pp 229533.I1S9T6
83333 229533.A0A1C3YJM7 pp 229533.I1RAG3
83334 229533.A0A1C3YJM7 pp 229533.I1RIW5
83335 229533.A0A1C3YJM7 pp 229533.I1RDV8
83336 229533.A0A1C3YJM7 pp 229533.I1RK44
83337 229533.A0A1C3YJM7 pp 229533.A0A1C3YKN4
83338 229533.A0A1C3YJM7 pp 229533.V6RAM6
83339 229533.A0A1C3YJM7 pp 229533.V6R2Z2
83340 229533.A0A1C3YJM7 pp 229533.V6RK96
83341 229533.A0A1C3YJM7 pp 229533.V6R756
83342 229533.A0A1C3YJM7 pp 229533.I1RRM1
83343 229533.A0A1C3YJM7 pp 229533.I1RZM4
83344 229533.A0A1C3YJM7 pp 229533.I1RHU0
83345 229533.A0A1C3YJM7 pp 229533.I1RYM5
83346 229533.A0A1C3YJM7 pp 229533.A0A1C3YKB7
83347 229533.A0A1C3YJM7 pp 229533.I1RNH4
83348 229533.I1S7X9 pp 229533.V6RLH0
83349 229533.I1S7X9 pp 229533.Q5I2J3
83350 229533.I1S7X9 pp 229533.V6RR76
83351 229533.I1S7X9 pp 229533.V6QX58
83352 229533.I1S7X9 pp 229533.P0C431
83353 229533.I1S7X9 pp 229533.V6RGI1
83354 229533.I1S7X9 pp 229533.Q4HZS8
83355 229533.I1S7X9 pp 229533.V6QV09
83356 229533.I1S7X9 pp 229533.V6REE9
83357 229533.I1S7X9 pp 229533.Q4ICG5
83358 229533.I1S7X9 pp 229533.Q4IER8
83359 229533.I1S7X9 pp 229533.Q4IB89
83360 229533.I1S7X9 pp 229533.V6R476
83361 229533.I1S7X9 pp 229533.Q4IPI4
83362 229533.I1S7X9 pp 229533.V6QUU6
83363 229533.I1S7X9 pp 229533.Q4ICM0
83364 229533.I1S7X9 pp 229533.Q4I2W2
83365 229533.I1RJ33 pp 229533.I1SA77
83366 229533.I1RJ33 pp 229533.I1RM36

83367 229533.I1RJ33 pp 229533.I1RVC8
83368 229533.I1RJ33 pp 229533.I1SAG3
83369 229533.I1RJ33 pp 229533.I1S9J6
83370 229533.I1RJ33 pp 229533.I1S0N5
83371 229533.I1RSX9 pp 229533.V6RJ29
83372 229533.I1RSX9 pp 229533.I1RY16
83373 229533.I1RSX9 pp 229533.I1RZM4
83374 229533.I1RSX9 pp 229533.I1RVX9
83375 229533.I1RSX9 pp 229533.Q4HXT5
83376 229533.I1RSX9 pp 229533.V6RK96
83377 229533.I1RSX9 pp 229533.V6QU63
83378 229533.I1RSX9 pp 229533.I1RYZ2
83379 229533.I1RSX9 pp 229533.Q4HVU7
83380 229533.I1RSX9 pp 229533.I1RYV4
83381 229533.I1RSX9 pp 229533.V6RW66
83382 229533.I1RSX9 pp 229533.I1RXB6
83383 229533.I1RSX9 pp 229533.I1S6A2
83384 229533.I1RSX9 pp 229533.Q4HVQ9
83385 229533.I1RSX9 pp 229533.V6RPN0
83386 229533.I1RSX9 pp 229533.I1S3D8
83387 229533.I1RSX9 pp 229533.I1S9T6
83388 229533.I1RSX9 pp 229533.V6RVJ3
83389 229533.I1RSX9 pp 229533.I1RZA8
83390 229533.I1RSX9 pp 229533.I1S694
83391 229533.I1RSX9 pp 229533.I1RVR2
83392 229533.I1RSX9 pp 229533.I1S673
83393 229533.I1RSX9 pp 229533.V6RAG6
83394 229533.I1RSX9 pp 229533.V6RK40
83395 229533.I1RSX9 pp 229533.I1RVY1
83396 229533.I1RSX9 pp 229533.I1RW10
83397 229533.I1RSX9 pp 229533.I1S064
83398 229533.I1RSX9 pp 229533.Q4HZB8
83399 229533.I1RSX9 pp 229533.V6RM02
83400 229533.I1RSX9 pp 229533.I1S0C9
83401 229533.A0A1C3YNB6 pp 229533.I1RNG4
83402 229533.A0A1C3YNB6 pp 229533.I1RE53
83403 229533.A0A1C3YNB6 pp 229533.V6RQW4
83404 229533.A0A1C3YNB6 pp 229533.Q4HXF0
83405 229533.A0A1C3YNB6 pp 229533.I1RM55
83406 229533.A0A1C3YNB6 pp 229533.I1RCV1
83407 229533.A0A1C3YNB6 pp 229533.I1RJT5
83408 229533.A0A098D302 pp 229533.I1RE92
83409 229533.A0A098D302 pp 229533.I1RQP7
83410 229533.A0A098D302 pp 229533.I1REG5
83411 229533.A0A098D302 pp 229533.V6RXW9
83412 229533.A0A098D302 pp 229533.A0A1C3YN54
83413 229533.A0A098D302 pp 229533.A0A1C3YKT4
83414 229533.A0A098D302 pp 229533.I1RPK5
83415 229533.A0A098D302 pp 229533.I1RMN3
83416 229533.A0A098D302 pp 229533.I1RFK0
83417 229533.A0A098D302 pp 229533.Q4HY20
83418 229533.A0A098D302 pp 229533.I1S2W9
83419 229533.A0A098D302 pp 229533.A0A098DLB3
83420 229533.A0A098D302 pp 229533.I1RBS3
83421 229533.A0A098D302 pp 229533.I1S287
83422 229533.A0A098D302 pp 229533.Q4INI2
83423 229533.A0A098D302 pp 229533.I1S9S4
83424 229533.A0A098D302 pp 229533.A0A1C3YI44
83425 229533.A0A098D302 pp 229533.I1RTQ9
83426 229533.A0A098D302 pp 229533.I1RYP0
83427 229533.A0A098D302 pp 229533.I1RAH5
83428 229533.A0A098D302 pp 229533.I1RUN9
83429 229533.A0A098D302 pp 229533.I1RPY3
83430 229533.A0A098D302 pp 229533.A0A098DWE6
83431 229533.A0A098D302 pp 229533.I1RX95
83432 229533.A0A098D302 pp 229533.I1RWZ9
83433 229533.A0A098D302 pp 229533.I1RNM3
83434 229533.A0A098D302 pp 229533.I1RE20
83435 229533.A0A098D302 pp 229533.I1RXT7
83436 229533.A0A098D302 pp 229533.I1RDD0
83437 229533.A0A098D302 pp 229533.I1RXF5
83438 229533.A0A098D302 pp 229533.I1RPP0
83439 229533.A0A098D302 pp 229533.I1RW71

229533.A0A098D302 pp 229533.A0A1C3YI55
229533.A0A098D302 pp 229533.A0A1C3YK51
229533.A0A098D302 pp 229533.I1RCV3
229533.A0A098D302 pp 229533.I1RAK7
229533.A0A098D302 pp 229533.I1RZ84
229533.A0A098D302 pp 229533.I1RTD0
229533.A0A098D302 pp 229533.I1RZA4
229533.A0A098D302 pp 229533.A0A1C3YLH6
229533.A0A098D302 pp 229533.A0A1C3YJY1
229533.A0A098D302 pp 229533.I1RW76
229533.A0A098D302 pp 229533.I1REN4
229533.A0A098D302 pp 229533.V6RQ48
229533.A0A098D302 pp 229533.I1RN70
229533.A0A098D302 pp 229533.I1RTA3
229533.A0A098D302 pp 229533.I1RJK6
229533.A0A098D302 pp 229533.I1S051
229533.A0A098D302 pp 229533.V6RB81
229533.A0A098D302 pp 229533.I1S1U6
229533.A0A098D302 pp 229533.I1RC46
229533.A0A098D302 pp 229533.V6RKE4
229533.A0A098D302 pp 229533.A0A0E0SGM3
229533.A0A098D302 pp 229533.I1RWP9
229533.A0A098D302 pp 229533.A0A1C3YIR6
229533.A0A098D302 pp 229533.I1RAU2
229533.A0A098D302 pp 229533.A0A1C3YIH7
229533.A0A098D302 pp 229533.I1RP87
229533.A0A098D302 pp 229533.I1RMM1
229533.A0A098D302 pp 229533.I1RGC1
229533.A0A098D302 pp 229533.I1RJZ7
229533.A0A098D302 pp 229533.I1REZ0
229533.A0A098D302 pp 229533.I1S7X9
229533.A0A098D302 pp 229533.I1RE15
229533.A0A098D302 pp 229533.A0A1C3YLK5
229533.I1SAG3 pp 229533.Q4IER8
229533.I1SAG3 pp 229533.Q4I5R3
229533.I1RE31 pp 229533.I1RN61
229533.I1RE31 pp 229533.I1RRG3
229533.I1RE31 pp 229533.I1RQC9
229533.I1RE31 pp 229533.I1RLU5
229533.I1RE31 pp 229533.I1S0B6
229533.I1RE31 pp 229533.I1S4Y4
229533.I1RE31 pp 229533.I1RRT0
229533.I1RE31 pp 229533.I1RS66
229533.I1RE31 pp 229533.I1RZB5
229533.I1RE31 pp 229533.I1RT35
229533.I1RE31 pp 229533.I1RJL2
229533.I1RE31 pp 229533.I1S4M5
229533.I1RE31 pp 229533.I1S0N7
229533.I1RE31 pp 229533.I1RSD4
229533.I1RE31 pp 229533.I1SA47
229533.I1RE31 pp 229533.I1RFN1
229533.I1RE31 pp 229533.I1RK79
229533.I1RE31 pp 229533.I1RNB8
229533.I1RE31 pp 229533.I1RRV1
229533.I1RE31 pp 229533.I1RSA4
229533.I1RE31 pp 229533.I1RK92
229533.I1RE31 pp 229533.I1S471
229533.I1RE31 pp 229533.I1RG11
229533.I1RE31 pp 229533.I1RT47
229533.I1RE31 pp 229533.I1RR25
229533.I1RE31 pp 229533.I1RG90
229533.A0A098E017 pp 229533.I1R9Z0
229533.A0A098E017 pp 229533.I1RCY6
229533.A0A098E017 pp 229533.A0A1C3YM02
229533.A0A098E017 pp 229533.I1RJL5
229533.A0A098E017 pp 229533.I1RJS4
229533.A0A098E017 pp 229533.A0A1C3YMF9
229533.A0A098E017 pp 229533.I1RA22
229533.A0A098E017 pp 229533.I1RBD7
229533.A0A098E017 pp 229533.I1RKK6
229533.A0A098E017 pp 229533.A0A1C3YMH5
229533.A0A098E017 pp 229533.A0A0E0S658
229533.A0A098E017 pp 229533.I1RDY1

229533.A0A098E017 pp 229533.I1RMH6
229533.A0A098E017 pp 229533.I1RB17
229533.A0A098E017 pp 229533.A0A1C3YIU4
229533.A0A098E017 pp 229533.I1RWC6
229533.A0A098E017 pp 229533.A0A0E0SFM9
229533.A0A098E017 pp 229533.A0A1C3YID5
229533.A0A0E0S460 pp 229533.I1SAG3
229533.A0A0E0S460 pp 229533.I1RCL8
229533.A0A0E0S460 pp 229533.A0A1C3YKV0
229533.A0A0E0S460 pp 229533.I1SA77
229533.A0A0E0S460 pp 229533.A0A1C3YM08
229533.A0A098DAQ4 pp 229533.A0A1C3YM08
229533.A0A098DAQ4 pp 229533.I1SA77
229533.A0A098DAQ4 pp 229533.I1RJ33
229533.A0A098DAQ4 pp 229533.I1SAG3
229533.A0A098DAQ4 pp 229533.A0A0E0S460
229533.I1S9M9 pp 229533.Q4IAA0
229533.A0A1C3YKJ7 pp 229533.I1RQH1
229533.A0A1C3YKJ7 pp 229533.I1R9Z0
229533.A0A1C3YKJ7 pp 229533.I1RDD8
229533.A0A1C3YKJ7 pp 229533.I1RSH0
229533.A0A1C3YKJ7 pp 229533.I1RB74
229533.A0A1C3YKJ7 pp 229533.I1S0C7
229533.A0A1C3YKJ7 pp 229533.I1RP28
229533.A0A1C3YKJ7 pp 229533.I1RQG4
229533.A0A1C3YKJ7 pp 229533.I1RRM9
229533.A0A1C3YKJ7 pp 229533.I1RBD7
229533.A0A1C3YKJ7 pp 229533.I1RKK0
229533.A0A1C3YKJ7 pp 229533.I1RJS4
229533.A0A1C3YKJ7 pp 229533.I1S0M2
229533.A0A1C3YKJ7 pp 229533.I1RCQ3
229533.A0A1C3YKJ7 pp 229533.I1RMQ5
229533.A0A1C3YKJ7 pp 229533.I1S2G9
229533.A0A1C3YKJ7 pp 229533.I1RCY6
229533.A0A1C3YKJ7 pp 229533.I1RAM6
229533.A0A1C3YKJ7 pp 229533.A0A1C3YMH5
229533.A0A1C3YKJ7 pp 229533.I1RB17
229533.A0A1C3YKJ7 pp 229533.I1RLU7
229533.A0A1C3YKJ7 pp 229533.I1RE17
229533.A0A1C3YKJ7 pp 229533.I1RPL2
229533.A0A1C3YKJ7 pp 229533.I1RWI1
229533.A0A1C3YKJ7 pp 229533.I1RMM9
229533.A0A1C3YKJ7 pp 229533.I1RQ81
229533.A0A1C3YKJ7 pp 229533.I1RZ27
229533.A0A1C3YKJ7 pp 229533.I1RWC6
229533.A0A1C3YKJ7 pp 229533.I1RDY1
229533.A0A1C3YKJ7 pp 229533.I1RSJ0
229533.A0A1C3YKJ7 pp 229533.I1RCL5
229533.A0A1C3YKJ7 pp 229533.I1S238
229533.A0A1C3YKJ7 pp 229533.I1R9Y7
229533.A0A1C3YKJ7 pp 229533.I1RGB5
229533.A0A1C3YKJ7 pp 229533.I1RB75
229533.A0A098DV37 pp 229533.I1RMH0
229533.A0A098DV37 pp 229533.A0A1C3YJ16
229533.I1RI07 pp 229533.I1RZ25
229533.A0A098E1Y1 pp 229533.I1RPQ2
229533.A0A098E1Y1 pp 229533.A0A1C3YLK8
229533.A0A098E1Y1 pp 229533.I1RW80
229533.A0A098E1Y1 pp 229533.I1S0P5
229533.A0A098E1Y1 pp 229533.A0A1C3YMU9
229533.A0A098E1Y1 pp 229533.I1RSM8
229533.A0A098E1Y1 pp 229533.I1RRF7
229533.A0A098E1Y1 pp 229533.I1RFI5
229533.A0A098E1Y1 pp 229533.I1RG11
229533.A0A098E1Y1 pp 229533.I1RKL2
229533.A0A098E1Y1 pp 229533.I1RKB1
229533.A0A098E1Y1 pp 229533.V6QUE6
229533.A0A098E1Y1 pp 229533.I1RSZ3
229533.A0A098E1Y1 pp 229533.I1S0K1
229533.A0A098E1Y1 pp 229533.I1RH75
229533.A0A098E1Y1 pp 229533.I1RP96
229533.A0A098E1Y1 pp 229533.I1RML9
229533.A0A098E1Y1 pp 229533.I1RAR2

83586	229533.A0A098E1Y1	pp	229533.A0A0E0RZF3
83587	229533.A0A098E1Y1	pp	229533.I1RAP1
83588	229533.A0A098E1Y1	pp	229533.I1RCB7
83589	229533.A0A098E1Y1	pp	229533.A0A1C3YI23
83590	229533.A0A098E1Y1	pp	229533.A0A1C3YK80
83591	229533.A0A098E1Y1	pp	229533.I1RIF7
83592	229533.A0A098E1Y1	pp	229533.I1RDC9
83593	229533.A0A098E1Y1	pp	229533.I1RQW1
83594	229533.A0A098E1Y1	pp	229533.I1RXU0
83595	229533.A0A098E1Y1	pp	229533.I1S276
83596	229533.A0A098E1Y1	pp	229533.I1RRT0
83597	229533.A0A098E1Y1	pp	229533.I1RCH4
83598	229533.A0A098E1Y1	pp	229533.I1R9X3
83599	229533.A0A098E1Y1	pp	229533.I1S5Z5
83600	229533.A0A098E1Y1	pp	229533.I1SAH9
83601	229533.A0A098E1Y1	pp	229533.I1RWF3
83602	229533.A0A098E1Y1	pp	229533.A0A1C3YIR8
83603	229533.A0A098E1Y1	pp	229533.I1RW91
83604	229533.A0A098E1Y1	pp	229533.I1RRG3
83605	229533.A0A098E1Y1	pp	229533.I1RND9
83606	229533.A0A098E1Y1	pp	229533.A0A1C3YN55
83607	229533.A0A098E1Y1	pp	229533.I1RZM2
83608	229533.A0A098E1Y1	pp	229533.I1RLI4
83609	229533.A0A098E1Y1	pp	229533.I1RCG6
83610	229533.A0A098E1Y1	pp	229533.A0A1C3YHX6
83611	229533.A0A098E1Y1	pp	229533.I1RK79
83612	229533.A0A098E1Y1	pp	229533.V6QWX8
83613	229533.A0A098E1Y1	pp	229533.I1RA99
83614	229533.A0A098E1Y1	pp	229533.I1S9H8
83615	229533.A0A098E1Y1	pp	229533.A0A1C3YJ83
83616	229533.A0A098E1Y1	pp	229533.I1RTC2
83617	229533.A0A098E1Y1	pp	229533.I1RE30
83618	229533.A0A098E1Y1	pp	229533.A0A1C3YIB4
83619	229533.A0A098E1Y1	pp	229533.I1RUM5
83620	229533.A0A098E1Y1	pp	229533.I1RKR8
83621	229533.A0A098E1Y1	pp	229533.I1RA65
83622	229533.A0A098E1Y1	pp	229533.A0A1C3YLP8
83623	229533.A0A098E1Y1	pp	229533.I1RC15
83624	229533.A0A098E1Y1	pp	229533.A0A0E0RN05
83625	229533.A0A098E1Y1	pp	229533.I1RJS4
83626	229533.A0A098E1Y1	pp	229533.I1RPM7
83627	229533.A0A098E1Y1	pp	229533.A0A0E0RRM0
83628	229533.A0A098E1Y1	pp	229533.I1RDY2
83629	229533.A0A098E1Y1	pp	229533.A0A1C3YMH5
83630	229533.A0A098E1Y1	pp	229533.I1RC24
83631	229533.A0A098E1Y1	pp	229533.I1RPW8
83632	229533.A0A098E1Y1	pp	229533.I1RN91
83633	229533.A0A098E1Y1	pp	229533.I1RVY0
83634	229533.A0A098E1Y1	pp	229533.I1RZB5
83635	229533.A0A098E1Y1	pp	229533.A0A0E0SA25
83636	229533.A0A098E1Y1	pp	229533.I1RUE3
83637	229533.A0A098E1Y1	pp	229533.I1RED2
83638	229533.A0A098E1Y1	pp	229533.I1RK71
83639	229533.A0A098E1Y1	pp	229533.I1S0X1
83640	229533.A0A098E1Y1	pp	229533.I1REN3
83641	229533.A0A098E1Y1	pp	229533.I1RR25
83642	229533.A0A098E1Y1	pp	229533.A0A0E0SMY1
83643	229533.A0A098E1Y1	pp	229533.I1RMU1
83644	229533.A0A098E1Y1	pp	229533.I1RP12
83645	229533.A0A098E1Y1	pp	229533.I1RMH6
83646	229533.A0A098E1Y1	pp	229533.I1RR61
83647	229533.A0A098E1Y1	pp	229533.I1RKF3
83648	229533.A0A098E1Y1	pp	229533.I1RNG5
83649	229533.A0A098E1Y1	pp	229533.I1RW90
83650	229533.A0A098E1Y1	pp	229533.I1RQ42
83651	229533.A0A098E1Y1	pp	229533.A0A0E0SHL3
83652	229533.A0A098E1Y1	pp	229533.A0A1C3YN82
83653	229533.A0A098E1Y1	pp	229533.I1RED3
83654	229533.A0A098E1Y1	pp	229533.I1RN04
83655	229533.A0A098E1Y1	pp	229533.I1RJL2
83656	229533.A0A098E1Y1	pp	229533.I1RTY9
83657	229533.A0A098E1Y1	pp	229533.I1RZ94
83658	229533.A0A098E1Y1	pp	229533.I1RMV1

```
229533.A0A098E1Y1	pp	229533.A0A1C3YJZ2
229533.A0A098E1Y1	pp	229533.I1RGK3
229533.A0A098E1Y1	pp	229533.I1RVL8
229533.A0A098E1Y1	pp	229533.A0A0E0S658
229533.A0A098E1Y1	pp	229533.A0A0E0SFV6
229533.I1RCM0	pp	229533.I1RYV3
229533.I1RCM0	pp	229533.Q4HTT2
229533.A0A1C3YLP9	pp	229533.I1RD63
229533.A0A1C3YLP9	pp	229533.I1RMF9
229533.A0A1C3YLP9	pp	229533.Q4I1H6
229533.A0A1C3YLP9	pp	229533.I1RRZ9
229533.A0A1C3YLP9	pp	229533.I1RYK6
229533.A0A1C3YLP9	pp	229533.I1RB26
229533.A0A1C3YLP9	pp	229533.I1RC52
229533.A0A1C3YLP9	pp	229533.I1RY63
229533.A0A1C3YLP9	pp	229533.I1RND7
229533.A0A1C3YLP9	pp	229533.I1RYW4
229533.A0A1C3YLP9	pp	229533.I1RCH2
229533.A0A1C3YLP9	pp	229533.I1RCS2
229533.A0A1C3YLP9	pp	229533.I1RJY4
229533.A0A1C3YLP9	pp	229533.I1RWG0
229533.A0A1C3YLP9	pp	229533.I1RMR5
229533.A0A1C3YLP9	pp	229533.I1RN81
229533.A0A1C3YLP9	pp	229533.I1RTD0
229533.A0A1C3YLP9	pp	229533.I1RN04
229533.A0A1C3YHP7	pp	229533.A0A1C3YMH5
229533.A0A1C3YHP7	pp	229533.I1RKK4
229533.I1RBK8	pp	229533.I1RCW8
229533.I1RBK8	pp	229533.I1RU02
229533.I1RBK8	pp	229533.Q4IER8
229533.I1RBK8	pp	229533.I1RMJ8
229533.I1RBK8	pp	229533.Q4IB89
229533.I1RBK8	pp	229533.I1RJQ3
229533.I1RBK8	pp	229533.I1RFW1
229533.I1RBK8	pp	229533.Q4IMD1
229533.A0A098DAG6	pp	229533.I1RAI9
229533.A0A098DAG6	pp	229533.I1RS41
229533.A0A098DAG6	pp	229533.I1RGC2
229533.A0A098DAG6	pp	229533.I1S7S9
229533.A0A098DAG6	pp	229533.I1RQ88
229533.A0A098DV25	pp	229533.I1RMF9
229533.A0A098DV25	pp	229533.I1RC97
229533.A0A098DV25	pp	229533.A0A1C3YLP9
229533.I1RVC4	pp	229533.I1RY33
229533.I1RTU5	pp	229533.I1SAI4
229533.I1RTU5	pp	229533.V6R756
229533.I1RTU5	pp	229533.V6RAM6
229533.I1RTU5	pp	229533.V6R2Z2
229533.I1RTU5	pp	229533.V6R5D2
229533.I1RTU5	pp	229533.V6R0W1
229533.I1RTU5	pp	229533.V6QU99
229533.A0A1C3YHY1	pp	229533.I1RMH6
229533.A0A1C3YHY1	pp	229533.A0A1C3YK80
229533.A0A1C3YHY1	pp	229533.A0A1C3YK00
229533.A0A1C3YHY1	pp	229533.I1RJQ3
229533.A0A1C3YHY1	pp	229533.I1RKK6
229533.A0A1C3YHY1	pp	229533.I1RSJ0
229533.A0A1C3YHY1	pp	229533.A0A1C3YJI7
229533.I1REJ3	pp	229533.V6RHZ8
229533.I1REJ3	pp	229533.I1RPH3
229533.I1REJ3	pp	229533.I1RNV4
229533.I1REJ3	pp	229533.I1RF38
229533.I1REJ3	pp	229533.I1RGI2
229533.I1REJ3	pp	229533.I1RQ70
229533.I1S566	pp	229533.Q4HTT2
229533.I1S566	pp	229533.Q4I7L0
229533.Q4HXT5	pp	229533.Q4I0K3
229533.Q4HXT5	pp	229533.Q4ILH3
229533.Q4HXT5	pp	229533.Q4I073
229533.Q4HXT5	pp	229533.Q4I785
229533.Q4HXT5	pp	229533.Q4IB63
229533.I1RES8	pp	229533.Q4HTT2
229533.I1RES8	pp	229533.I1S4Z5
```

83732 229533.I1RES8 pp 229533.Q4I7L0
83733 229533.I1RES8 pp 229533.I1RYV3
83734 229533.I1RC15 pp 229533.Q4I7S1
83735 229533.I1RC15 pp 229533.Q4HYI0
83736 229533.I1RC15 pp 229533.I1RCB7
83737 229533.I1RC15 pp 229533.Q4I5I4
83738 229533.I1RC15 pp 229533.I1S3N1
83739 229533.I1RC15 pp 229533.I1RV65
83740 229533.I1RC15 pp 229533.I1RVZ4
83741 229533.I1RC15 pp 229533.I1RD38
83742 229533.I1RC15 pp 229533.I1RM65
83743 229533.I1RC15 pp 229533.I1S0C6
83744 229533.I1RC15 pp 229533.I1RK79
83745 229533.I1RC15 pp 229533.I1S2C4
83746 229533.I1RC15 pp 229533.I1RDZ6
83747 229533.I1RC15 pp 229533.I1S0G0
83748 229533.I1RC15 pp 229533.V6QUE6
83749 229533.I1RC15 pp 229533.Q4IER8
83750 229533.I1RC15 pp 229533.I1RJL2
83751 229533.I1RC15 pp 229533.Q4IPC8
83752 229533.I1RC15 pp 229533.V6QV38
83753 229533.I1RC15 pp 229533.I1RW99
83754 229533.I1RC15 pp 229533.I1RR61
83755 229533.I1RC15 pp 229533.I1RZB5
83756 229533.I1RC15 pp 229533.I1RG11
83757 229533.I1RC15 pp 229533.I1RMV1
83758 229533.I1RC15 pp 229533.I1RPM7
83759 229533.I1RC15 pp 229533.I1RK71
83760 229533.I1RC15 pp 229533.I1RQZ2
83761 229533.I1RC15 pp 229533.I1RZ59
83762 229533.I1RC15 pp 229533.I1RRF7
83763 229533.I1RC15 pp 229533.I1RZU9
83764 229533.I1RC15 pp 229533.Q4IJU0
83765 229533.I1RC15 pp 229533.I1S276
83766 229533.I1RC15 pp 229533.Q4HYB8
83767 229533.I1RC15 pp 229533.I1RRP7
83768 229533.I1RC15 pp 229533.I1RRT0
83769 229533.I1RC15 pp 229533.I1RE54
83770 229533.I1RC15 pp 229533.I1RDC9
83771 229533.I1RC15 pp 229533.I1RR25
83772 229533.I1RC15 pp 229533.I1REN3
83773 229533.I1RC15 pp 229533.I1RNB8
83774 229533.I1RC15 pp 229533.I1RZL5
83775 229533.I1RC15 pp 229533.I1RML9
83776 229533.I1RC15 pp 229533.I1RUG4
83777 229533.I1RC15 pp 229533.I1RXU0
83778 229533.I1RC15 pp 229533.I1RQZ3
83779 229533.I1RC15 pp 229533.Q4IQX3
83780 229533.I1RC15 pp 229533.Q4I099
83781 229533.I1RC15 pp 229533.I1RRM8
83782 229533.I1RC15 pp 229533.I1RZ42
83783 229533.I1RC15 pp 229533.I1RCG6
83784 229533.I1RC15 pp 229533.I1RXE5
83785 229533.I1RC15 pp 229533.I1RC16
83786 229533.I1RC15 pp 229533.Q4I5W5
83787 229533.I1RC15 pp 229533.I1RVL8
83788 229533.I1RC15 pp 229533.I1S9Y0
83789 229533.I1RC15 pp 229533.I1RWF3
83790 229533.I1RC15 pp 229533.I1RN04
83791 229533.I1RC15 pp 229533.I1RVJ1
83792 229533.I1RC15 pp 229533.I1RW80
83793 229533.I1RC15 pp 229533.I1RVY0
83794 229533.I1RC15 pp 229533.I1RT35
83795 229533.I1RC15 pp 229533.I1RLR0
83796 229533.I1RC15 pp 229533.I1RT99
83797 229533.I1RC15 pp 229533.I1RKB1
83798 229533.I1RC15 pp 229533.I1RLV1
83799 229533.I1RC15 pp 229533.I1S0R5
83800 229533.I1RC15 pp 229533.I1RZM2
83801 229533.I1RC15 pp 229533.I1RNP4
83802 229533.I1RC15 pp 229533.V6QWX8
83803 229533.I1RC15 pp 229533.I1RD36
83804 229533.I1RC15 pp 229533.I1RHL8

229533.I1RC15 pp 229533.I1RQ42
229533.I1RC15 pp 229533.I1RFW1
229533.I1RC15 pp 229533.I1RHL7
229533.I1RC15 pp 229533.I1RW91
229533.I1RC15 pp 229533.I1RTC2
229533.I1RC15 pp 229533.I1RRG3
229533.I1RC15 pp 229533.I1RW90
229533.I1RC15 pp 229533.I1RSZ3
229533.I1RC15 pp 229533.I1RUG0
229533.I1RC15 pp 229533.I1RGC9
229533.I1RC15 pp 229533.I1RHQ5
229533.I1RC15 pp 229533.I1RGR8
229533.I1RC15 pp 229533.I1S182
229533.I1RC15 pp 229533.I1RP96
229533.I1RC15 pp 229533.I1RE30
229533.I1RC15 pp 229533.I1RC24
229533.I1RC15 pp 229533.I1RC98
229533.I1RC15 pp 229533.I1S3N0
229533.I1RC15 pp 229533.I1RX63
229533.I1RC15 pp 229533.I1RPW8
229533.I1RC15 pp 229533.I1RRW3
229533.I1RDB2 pp 229533.V6R949
229533.I1RDB2 pp 229533.I1RT32
229533.I1RDB2 pp 229533.I1RTB7
229533.I1RDB2 pp 229533.I1RND8
229533.A0A0E0S1T0 pp 229533.I1RG90
229533.A0A0E0S1T0 pp 229533.I1S0B1
229533.A0A0E0S1T0 pp 229533.A0A1C3YIM4
229533.A0A0E0S1T0 pp 229533.I1S1X9
229533.A0A0E0S1T0 pp 229533.I1RQR3
229533.A0A0E0S1T0 pp 229533.I1RT91
229533.A0A0E0S1T0 pp 229533.Q4HXF6
229533.A0A0E0S1T0 pp 229533.I1RZQ5
229533.A0A0E0S1T0 pp 229533.I1RPL3
229533.A0A0E0S1T0 pp 229533.I1S471
229533.A0A0E0S1T0 pp 229533.I1RK92
229533.A0A0E0S1T0 pp 229533.I1RYW2
229533.A0A0E0S1T0 pp 229533.V6RIS2
229533.A0A0E0S1T0 pp 229533.I1RTM0
229533.A0A0E0S1T0 pp 229533.I1RBZ8
229533.A0A0E0S1T0 pp 229533.I1RCN5
229533.A0A0E0S1T0 pp 229533.I1RDE9
229533.A0A0E0S1T0 pp 229533.I1RWW5
229533.A0A0E0S1T0 pp 229533.I1RT47
229533.A0A0E0S1T0 pp 229533.A0A1C3YK19
229533.A0A0E0S1T0 pp 229533.I1RC84
229533.A0A0E0S1T0 pp 229533.I1RN09
229533.A0A0E0S1T0 pp 229533.I1RU69
229533.A0A0E0S1T0 pp 229533.A0A1C3YM09
229533.A0A0E0S1T0 pp 229533.I1RKR9
229533.A0A0E0S1T0 pp 229533.I1RJG6
229533.A0A0E0S1T0 pp 229533.I1RMV5
229533.A0A0E0S1T0 pp 229533.I1RX24
229533.A0A0E0S1T0 pp 229533.I1R9A0
229533.A0A0E0S1T0 pp 229533.I1RX47
229533.A0A0E0S1T0 pp 229533.I1RH64
229533.A0A0E0S1T0 pp 229533.I1RPI3
229533.A0A0E0S1T0 pp 229533.Q4IPB8
229533.A0A0E0S1T0 pp 229533.I1RDC1
229533.A0A0E0S1T0 pp 229533.I1RG57
229533.A0A0E0S1T0 pp 229533.I1RPY4
229533.A0A0E0S1T0 pp 229533.A0A1C3YIT2
229533.A0A0E0S1T0 pp 229533.I1RXI7
229533.A0A0E0S1T0 pp 229533.I1RBH8
229533.A0A0E0S1T0 pp 229533.I1RK90
229533.A0A0E0S1T0 pp 229533.I1S0S0
229533.A0A0E0S1T0 pp 229533.I1RT17
229533.A0A0E0S1T0 pp 229533.I1RYW3
229533.A0A0E0S1T0 pp 229533.Q4IQW0
229533.A0A0E0S1T0 pp 229533.A0A1C3YI62
229533.A0A0E0S1T0 pp 229533.I1R9Z0
229533.A0A0E0S1T0 pp 229533.V6QXG1
229533.A0A0E0S1T0 pp 229533.I1RX51

83878	229533.A0A0E0S1T0	pp	229533.I1RQP8
83879	229533.A0A0E0S1T0	pp	229533.I1RSA4
83880	229533.A0A0E0S1T0	pp	229533.I1RND5
83881	229533.A0A0E0S1T0	pp	229533.A0A1C3YIE7
83882	229533.A0A0E0S1T0	pp	229533.A0A0E0S8Y8
83883	229533.A0A0E0S1T0	pp	229533.I1RXF2
83884	229533.A0A0E0S1T0	pp	229533.I1RVK5
83885	229533.A0A0E0S1T0	pp	229533.I1S5S2
83886	229533.A0A0E0S1T0	pp	229533.V6QX58
83887	229533.A0A0E0S1T0	pp	229533.I1RND2
83888	229533.A0A0E0S1T0	pp	229533.I1RM25
83889	229533.A0A0E0S1T0	pp	229533.I1RBV3
83890	229533.A0A0E0S1T0	pp	229533.I1S9L6
83891	229533.A0A0E0S1T0	pp	229533.Q4IPH4
83892	229533.A0A0E0S1T0	pp	229533.Q4IQ72
83893	229533.A0A0E0S1T0	pp	229533.I1RNB8
83894	229533.A0A0E0S1T0	pp	229533.I1RRL0
83895	229533.A0A0E0S1T0	pp	229533.I1RWU2
83896	229533.A0A0E0S1T0	pp	229533.I1RJF9
83897	229533.A0A0E0S1T0	pp	229533.I1RS28
83898	229533.A0A0E0S1T0	pp	229533.I1RAF3
83899	229533.A0A0E0S1T0	pp	229533.A0A1C3YNC3
83900	229533.A0A0E0S1T0	pp	229533.I1RND9
83901	229533.A0A0E0S1T0	pp	229533.A0A1C3YHK3
83902	229533.A0A0E0S1T0	pp	229533.I1RFQ1
83903	229533.A0A0E0S1T0	pp	229533.I1S4M5
83904	229533.A0A0E0S1T0	pp	229533.I1RSD4
83905	229533.A0A0E0S1T0	pp	229533.I1RFT1
83906	229533.A0A0E0S1T0	pp	229533.I1RME9
83907	229533.A0A0E0S1T0	pp	229533.I1RCA7
83908	229533.A0A0E0S1T0	pp	229533.A0A0E0SC46
83909	229533.A0A0E0S1T0	pp	229533.I1RST1
83910	229533.A0A0E0S1T0	pp	229533.I1RG13
83911	229533.A0A0E0S1T0	pp	229533.I1RCS2
83912	229533.A0A0E0S1T0	pp	229533.I1R9X1
83913	229533.A0A0E0S1T0	pp	229533.I1RAU1
83914	229533.A0A0E0S1T0	pp	229533.A0A1C3YIA6
83915	229533.A0A0E0S1T0	pp	229533.I1RSA8
83916	229533.A0A0E0S1T0	pp	229533.I1S7M3
83917	229533.A0A0E0S1T0	pp	229533.I1RPZ3
83918	229533.A0A0E0S1T0	pp	229533.Q4IBK5
83919	229533.A0A0E0S1T0	pp	229533.I1S1C6
83920	229533.A0A0E0S1T0	pp	229533.I1RPA0
83921	229533.A0A0E0S1T0	pp	229533.I1RQQ7
83922	229533.A0A0E0S1T0	pp	229533.I1RN49
83923	229533.A0A0E0S1T0	pp	229533.I1RVG8
83924	229533.A0A0E0S1T0	pp	229533.I1RYI9
83925	229533.A0A0E0S1T0	pp	229533.I1RE24
83926	229533.A0A0E0S1T0	pp	229533.I1RA36
83927	229533.A0A0E0S1T0	pp	229533.I1RT35
83928	229533.A0A0E0S1T0	pp	229533.I1RWX8
83929	229533.A0A0E0S1T0	pp	229533.Q4I5R9
83930	229533.A0A0E0S1T0	pp	229533.Q4IB70
83931	229533.A0A0E0S1T0	pp	229533.I1RAD1
83932	229533.A0A0E0S1T0	pp	229533.A0A0E0SMM8
83933	229533.A0A0E0S1T0	pp	229533.Q4IAA0
83934	229533.A0A0E0S1T0	pp	229533.Q4I1Y1
83935	229533.A0A0E0S1T0	pp	229533.I1RAC1
83936	229533.A0A0E0S1T0	pp	229533.Q4IRI9
83937	229533.A0A0E0S1T0	pp	229533.I1S7M2
83938	229533.A0A0E0S1T0	pp	229533.I1RCR5
83939	229533.A0A0E0S1T0	pp	229533.I1RFP9
83940	229533.A0A0E0S1T0	pp	229533.A0A1C3YI68
83941	229533.A0A0E0S1T0	pp	229533.A0A1C3YM53
83942	229533.A0A0E0S1T0	pp	229533.I1RD56
83943	229533.A0A0E0S1T0	pp	229533.I1RK72
83944	229533.A0A0E0S1T0	pp	229533.I1S0Z9
83945	229533.A0A0E0S1T0	pp	229533.I1RPY5
83946	229533.A0A0E0S1T0	pp	229533.A0A1C3YL11
83947	229533.A0A0E0S1T0	pp	229533.I1RM67
83948	229533.A0A0E0S1T0	pp	229533.I1RRW3
83949	229533.A0A0E0S1T0	pp	229533.I1RLU5
83950	229533.A0A0E0S1T0	pp	229533.I1RYB1

83951 229533.A0A0E0S1T0 pp 229533.I1RG17
83952 229533.A0A0E0S1T0 pp 229533.I1S3T0
83953 229533.A0A0E0S1T0 pp 229533.I1RZ11
83954 229533.A0A0E0S1T0 pp 229533.I1RWD4
83955 229533.A0A0E0S1T0 pp 229533.I1RB50
83956 229533.A0A0E0S1T0 pp 229533.A0A1C3YKS6
83957 229533.A0A0E0S1T0 pp 229533.I1RQD8
83958 229533.A0A0E0S1T0 pp 229533.I1RMW7
83959 229533.A0A0E0S1T0 pp 229533.I1RQC5
83960 229533.A0A0E0S1T0 pp 229533.A0A1C3YL22
83961 229533.A0A0E0S1T0 pp 229533.A0A1C3YK13
83962 229533.A0A0E0S1T0 pp 229533.I1S9L5
83963 229533.A0A0E0S1T0 pp 229533.Q4IE79
83964 229533.A0A0E0S1T0 pp 229533.I1RS75
83965 229533.A0A0E0S1T0 pp 229533.I1RN03
83966 229533.A0A0E0S1T0 pp 229533.I1RN59
83967 229533.A0A0E0S1T0 pp 229533.Q4IFI0
83968 229533.A0A0E0S1T0 pp 229533.I1S2Y5
83969 229533.A0A0E0S1T0 pp 229533.I1RCI4
83970 229533.A0A0E0S1T0 pp 229533.I1RX81
83971 229533.A0A0E0S1T0 pp 229533.I1RZG7
83972 229533.A0A0E0S1T0 pp 229533.Q4IPB3
83973 229533.A0A0E0S1T0 pp 229533.I1R9N6
83974 229533.A0A0E0S1T0 pp 229533.I1RCQ6
83975 229533.A0A0E0S658 pp 229533.I1S1Y5
83976 229533.A0A0E0S658 pp 229533.A0A0E0SMG8
83977 229533.A0A0E0S658 pp 229533.I1RJS4
83978 229533.A0A0E0S658 pp 229533.A0A1C3YK80
83979 229533.A0A0E0S658 pp 229533.A0A1C3YMH5
83980 229533.A0A0E0S658 pp 229533.A0A1C3YI56
83981 229533.A0A0E0S658 pp 229533.I1S0P5
83982 229533.A0A0E0S658 pp 229533.I1RMH6
83983 229533.A0A0E0S658 pp 229533.I1RZ27
83984 229533.A0A0E0S658 pp 229533.I1RDY1
83985 229533.A0A0E0S658 pp 229533.I1RDX9
83986 229533.A0A0E0S658 pp 229533.A0A0E0SFM9
83987 229533.A0A098E4X9 pp 229533.I1RCM7
83988 229533.I1RUG0 pp 229533.Q4HYQ4
83989 229533.I1RUG0 pp 229533.I1RVJ1
83990 229533.I1RUG0 pp 229533.V6QWX8
83991 229533.I1RUG0 pp 229533.Q4HZ95
83992 229533.I1RUG0 pp 229533.I1RZU9
83993 229533.I1RUG0 pp 229533.I1RVY0
83994 229533.I1RUG0 pp 229533.I1S5Z2
83995 229533.I1RUG0 pp 229533.I1S0C6
83996 229533.I1RUG0 pp 229533.Q4IJU0
83997 229533.I1RUG0 pp 229533.I1RVL8
83998 229533.I1RUG0 pp 229533.I1RZB5
83999 229533.I1RUG0 pp 229533.Q4IPC8
84000 229533.I1RUG0 pp 229533.Q4I375
84001 229533.I1RUG0 pp 229533.V6QUE6
84002 229533.I1RUG0 pp 229533.I1S3N1
84003 229533.I1RUG0 pp 229533.Q4I5W5
84004 229533.I1RUG0 pp 229533.Q4I7S1
84005 229533.I1RUG0 pp 229533.Q4I5I4
84006 229533.I1RUG0 pp 229533.Q4IJ11
84007 229533.I1RUG0 pp 229533.I1RXU0
84008 229533.I1RUG0 pp 229533.I1S3N0
84009 229533.I1RUG0 pp 229533.I1RW69
84010 229533.I1RUG0 pp 229533.I1S9H8
84011 229533.I1RUG0 pp 229533.I1RYR1
84012 229533.I1RUG0 pp 229533.Q4HYB8
84013 229533.I1RUG0 pp 229533.I1RX63
84014 229533.I1RUG0 pp 229533.Q4I5U9
84015 229533.I1RUG0 pp 229533.V6RDZ4
84016 229533.I1RPH3 pp 229533.V6RHZ8
84017 229533.I1RPH3 pp 229533.I1RQ70
84018 229533.I1RK19 pp 229533.I1RPY7
84019 229533.I1RK19 pp 229533.I1RYJ9
84020 229533.I1RK19 pp 229533.I1RSD4
84021 229533.I1RK19 pp 229533.I1RYB1
84022 229533.I1RK19 pp 229533.V6QVD5
84023 229533.I1RK19 pp 229533.I1RQZ2

84024 229533.I1RK19 pp 229533.I1RSH9
84025 229533.I1RK19 pp 229533.Q4I7F9
84026 229533.I1RK19 pp 229533.I1RMH5
84027 229533.I1RK19 pp 229533.I1RNB8
84028 229533.I1RK19 pp 229533.I1RQD8
84029 229533.I1RK19 pp 229533.Q4IB70
84030 229533.I1RK19 pp 229533.I1RN09
84031 229533.I1RK19 pp 229533.I1RT35
84032 229533.I1RK19 pp 229533.I1RZL5
84033 229533.I1RK19 pp 229533.Q4IP34
84034 229533.I1RK19 pp 229533.I1RX81
84035 229533.I1RK19 pp 229533.I1RMV5
84036 229533.I1RK19 pp 229533.I1RKK4
84037 229533.I1RK19 pp 229533.I1S0B6
84038 229533.I1RK19 pp 229533.I1RSA4
84039 229533.I1RDQ1 pp 229533.I1RNG4
84040 229533.A0A098DWB1 pp 229533.I1S0P5
84041 229533.A0A098DWB1 pp 229533.A0A1C3YIU4
84042 229533.A0A098DWB1 pp 229533.A0A098E1H5
84043 229533.A0A098DWB1 pp 229533.A0A0E0RV05
84044 229533.A0A098DWB1 pp 229533.I1S0F0
84045 229533.A0A098DWB1 pp 229533.Q4IMD1
84046 229533.A0A098DWB1 pp 229533.A0A0E0SQV5
84047 229533.A0A098DWB1 pp 229533.I1RCY6
84048 229533.A0A098DWB1 pp 229533.I1RQB7
84049 229533.A0A098DWB1 pp 229533.I1R9Z0
84050 229533.A0A098DWB1 pp 229533.I1S8W6
84051 229533.A0A098DWB1 pp 229533.I1RB17
84052 229533.A0A098DWB1 pp 229533.I1RMK8
84053 229533.A0A098DWB1 pp 229533.I1RCW8
84054 229533.A0A098DWB1 pp 229533.Q4IER8
84055 229533.A0A098DWB1 pp 229533.A0A098E1Y1
84056 229533.A0A098DWB1 pp 229533.I1RWC6
84057 229533.A0A098DWB1 pp 229533.I1RDY1
84058 229533.A0A098DWB1 pp 229533.A0A0E0SMG8
84059 229533.A0A098DWB1 pp 229533.I1RBD7
84060 229533.A0A098DWB1 pp 229533.I1RFW1
84061 229533.A0A098DWB1 pp 229533.I1RZ27
84062 229533.A0A098DWB1 pp 229533.A0A0E0SFM9
84063 229533.A0A098DWB1 pp 229533.I1R9V4
84064 229533.A0A098DWB1 pp 229533.I1RU02
84065 229533.A0A098DWB1 pp 229533.I1S1Y5
84066 229533.A0A098DWB1 pp 229533.A0A098E488
84067 229533.A0A098DWB1 pp 229533.A0A098E1K1
84068 229533.A0A098DWB1 pp 229533.A0A0E0S658
84069 229533.A0A098DWB1 pp 229533.I1RDX9
84070 229533.A0A098DWB1 pp 229533.A0A1C3YMH5
84071 229533.A0A098DWB1 pp 229533.A0A098E017
84072 229533.A0A098DWB1 pp 229533.A0A1C3YI56
84073 229533.A0A098DWB1 pp 229533.I1RJS4
84074 229533.A0A098DWB1 pp 229533.I1RFN2
84075 229533.A0A098DWB1 pp 229533.A0A1C3YID5
84076 229533.A0A098DWB1 pp 229533.I1RKK6
84077 229533.A0A098DWB1 pp 229533.A0A0E0SED1
84078 229533.A0A098DWB1 pp 229533.I1RJL5
84079 229533.A0A098DWB1 pp 229533.A0A1C3YKJ7
84080 229533.A0A098DWB1 pp 229533.A0A0E0RUB0
84081 229533.I1RC16 pp 229533.I1RT99
84082 229533.I1RC16 pp 229533.V6RIV9
84083 229533.I1RC16 pp 229533.I1RT01
84084 229533.I1RC16 pp 229533.I1RQC5
84085 229533.I1RC16 pp 229533.V6QUE6
84086 229533.I1RC16 pp 229533.I1RSQ7
84087 229533.I1RC16 pp 229533.I1RN67
84088 229533.I1RC16 pp 229533.I1RXD7
84089 229533.I1RC16 pp 229533.I1RFL7
84090 229533.I1RC16 pp 229533.V6R042
84091 229533.I1RC16 pp 229533.I1RPP4
84092 229533.I1RC16 pp 229533.I1RPP3
84093 229533.I1RC16 pp 229533.V6RCG5
84094 229533.I1RC16 pp 229533.I1RE54
84095 229533.I1RC16 pp 229533.I1RTC2
84096 229533.I1RC16 pp 229533.V6QV09

84097 229533.I1RC16 pp 229533.I1RRG3
84098 229533.I1RC16 pp 229533.Q4IJ11
84099 229533.I1RC16 pp 229533.V6RMW6
84100 229533.I1RC16 pp 229533.Q4HYQ4
84101 229533.I1RC16 pp 229533.I1RE01
84102 229533.I1RC16 pp 229533.I1RQ88
84103 229533.I1RC16 pp 229533.I1RMG3
84104 229533.I1RC16 pp 229533.I1RSZ3
84105 229533.I1RC16 pp 229533.I1RWK3
84106 229533.I1RC16 pp 229533.I1RI82
84107 229533.I1RC16 pp 229533.Q4HYB8
84108 229533.I1RC16 pp 229533.I1S0L3
84109 229533.I1RC16 pp 229533.I1RVQ0
84110 229533.I1RC16 pp 229533.I1RG11
84111 229533.I1RC16 pp 229533.I1RD52
84112 229533.I1RC16 pp 229533.I1RJV4
84113 229533.I1RC16 pp 229533.I1RZM8
84114 229533.I1RC16 pp 229533.V6RTJ8
84115 229533.I1RC16 pp 229533.I1RW99
84116 229533.I1RC16 pp 229533.I1RNC1
84117 229533.I1RC16 pp 229533.I1RUG3
84118 229533.I1RC16 pp 229533.I1RE27
84119 229533.I1RC16 pp 229533.Q4I5U9
84120 229533.I1RC16 pp 229533.I1S7A8
84121 229533.I1RC16 pp 229533.Q4IJU0
84122 229533.I1RC16 pp 229533.I1RQE7
84123 229533.I1RC16 pp 229533.I1RR25
84124 229533.I1RC16 pp 229533.I1RD22
84125 229533.I1RC16 pp 229533.I1RPM4
84126 229533.I1RC16 pp 229533.Q4I5I4
84127 229533.I1RC16 pp 229533.Q4I624
84128 229533.I1RC16 pp 229533.I1RQ23
84129 229533.I1RC16 pp 229533.I1RD17
84130 229533.I1RC16 pp 229533.I1RW23
84131 229533.I1RC16 pp 229533.I1REK8
84132 229533.I1RC16 pp 229533.I1RC95
84133 229533.I1RC16 pp 229533.Q4I099
84134 229533.I1RC16 pp 229533.Q4I5W5
84135 229533.I1RC16 pp 229533.I1REN3
84136 229533.I1RC16 pp 229533.I1RX46
84137 229533.I1RC16 pp 229533.I1RW25
84138 229533.I1RC16 pp 229533.I1S0L4
84139 229533.I1RC16 pp 229533.I1RVJ1
84140 229533.I1RC16 pp 229533.I1RHB0
84141 229533.I1RC16 pp 229533.I1S0R5
84142 229533.I1RC16 pp 229533.I1RJU3
84143 229533.I1RC16 pp 229533.I1RJW6
84144 229533.I1RC16 pp 229533.I1RRY9
84145 229533.I1RC16 pp 229533.I1RTH6
84146 229533.I1RC16 pp 229533.I1RJV3
84147 229533.I1RC16 pp 229533.I1RRM5
84148 229533.I1RC16 pp 229533.I1RR24
84149 229533.I1RC16 pp 229533.I1RUG0
84150 229533.I1RC16 pp 229533.I1RPM7
84151 229533.I1RC16 pp 229533.I1S8R3
84152 229533.I1RC16 pp 229533.I1RCG4
84153 229533.I1RC16 pp 229533.I1RZJ5
84154 229533.I1RC16 pp 229533.I1RK71
84155 229533.I1RC16 pp 229533.I1RZW8
84156 229533.I1RC16 pp 229533.V6QWX8
84157 229533.I1RC16 pp 229533.I1S5R1
84158 229533.I1RC16 pp 229533.I1RC66
84159 229533.I1RC16 pp 229533.I1RRT8
84160 229533.I1RC16 pp 229533.I1RK79
84161 229533.I1RC16 pp 229533.I1RD38
84162 229533.I1RC16 pp 229533.I1RWJ8
84163 229533.I1RC16 pp 229533.I1RZZ9
84164 229533.I1RC16 pp 229533.I1RS41
84165 229533.I1RC16 pp 229533.I1RW90
84166 229533.I1RC16 pp 229533.I1S9Y1
84167 229533.I1RKK6 pp 229533.I1RSJ0
84168 229533.I1RKK6 pp 229533.I1RNR6
84169 229533.I1RKK6 pp 229533.I1RWL9

84170	229533.I1RDT5	pp	229533.I1S4D0
84171	229533.I1RDT5	pp	229533.V6RY56
84172	229533.I1RDT5	pp	229533.I1RJT5
84173	229533.I1RDT5	pp	229533.I1RE53
84174	229533.I1RDT5	pp	229533.I1S029
84175	229533.I1RDT5	pp	229533.Q4HXF0
84176	229533.I1RK36	pp	229533.Q4HTT2
84177	229533.I1RK36	pp	229533.Q4I7L0
84178	229533.I1RK36	pp	229533.I1RYV3
84179	229533.I1RK36	pp	229533.I1S4Z5
84180	229533.I1R9W2	pp	229533.I1RRU9
84181	229533.I1R9W2	pp	229533.I1RD09
84182	229533.I1R9W2	pp	229533.I1RSJ2
84183	229533.I1R9W2	pp	229533.I1RNK8
84184	229533.I1R9W2	pp	229533.I1RJL9
84185	229533.I1R9W2	pp	229533.I1RVJ8
84186	229533.I1R9W2	pp	229533.I1RBN0
84187	229533.I1R9W2	pp	229533.I1RBQ4
84188	229533.I1R9W2	pp	229533.Q4I0K3
84189	229533.I1R9W2	pp	229533.I1RST0
84190	229533.I1R9W2	pp	229533.V6QVH8
84191	229533.I1R9W2	pp	229533.I1RSM8
84192	229533.I1R9W2	pp	229533.I1RNM4
84193	229533.V6QVD5	pp	229533.V6RY33
84194	229533.I1RMH6	pp	229533.I1S0P5
84195	229533.I1RMH6	pp	229533.I1S4T3
84196	229533.I1S7H9	pp	229533.Q4HTT2
84197	229533.I1S7H9	pp	229533.Q4I5R3
84198	229533.I1S7H9	pp	229533.Q4I7L0
84199	229533.I1RA55	pp	229533.I1RRT0
84200	229533.I1RA55	pp	229533.I1RWL3
84201	229533.I1RA55	pp	229533.I1RRG3
84202	229533.I1RA55	pp	229533.I1RNB8
84203	229533.I1RA55	pp	229533.I1RA84
84204	229533.I1RA55	pp	229533.I1RT35
84205	229533.I1RA55	pp	229533.I1RBJ1
84206	229533.I1RA55	pp	229533.I1RCB7
84207	229533.I1RA55	pp	229533.Q4IJU0
84208	229533.I1RA55	pp	229533.Q4I5I4
84209	229533.I1RA55	pp	229533.I1RVJ1
84210	229533.I1RA55	pp	229533.Q4I5W5
84211	229533.I1RA55	pp	229533.I1RR25
84212	229533.I1RA55	pp	229533.I1RAE0
84213	229533.I1RA55	pp	229533.I1RG11
84214	229533.I1RA55	pp	229533.I1RK79
84215	229533.I1RA55	pp	229533.I1RC24
84216	229533.I1RA55	pp	229533.I1RZB5
84217	229533.I1RA55	pp	229533.I1S2C4
84218	229533.I1RA55	pp	229533.I1RJL2
84219	229533.I1RUA7	pp	229533.I1SAF3
84220	229533.A0A0E0SFM9	pp	229533.I1RPY1
84221	229533.A0A0E0SFM9	pp	229533.I1S4T3
84222	229533.A0A0E0SFM9	pp	229533.I1RM96
84223	229533.A0A0E0SFM9	pp	229533.I1RC33
84224	229533.A0A0E0SFM9	pp	229533.I1RSI6
84225	229533.A0A0E0SFM9	pp	229533.I1RSD8
84226	229533.A0A0E0SFM9	pp	229533.I1S2E8
84227	229533.A0A0E0SFM9	pp	229533.I1RM25
84228	229533.A0A0E0SFM9	pp	229533.Q4I327
84229	229533.A0A0E0SFM9	pp	229533.I1RNU6
84230	229533.A0A0E0SFM9	pp	229533.I1RQE7
84231	229533.A0A0E0SFM9	pp	229533.I1R9X7
84232	229533.A0A0E0SFM9	pp	229533.I1S471
84233	229533.A0A0E0SFM9	pp	229533.Q4HZ68
84234	229533.A0A0E0SFM9	pp	229533.I1S060
84235	229533.A0A0E0SFM9	pp	229533.I1S296
84236	229533.A0A0E0SFM9	pp	229533.I1RC27
84237	229533.A0A0E0SFM9	pp	229533.I1RSD4
84238	229533.A0A0E0SFM9	pp	229533.V6RIS2
84239	229533.A0A0E0SFM9	pp	229533.I1RC72
84240	229533.A0A0E0SFM9	pp	229533.V6RGN5
84241	229533.A0A0E0SFM9	pp	229533.I1RAU7
84242	229533.A0A0E0SFM9	pp	229533.I1RKC6

84243 229533.A0A0E0SFM9 pp 229533.I1S0J1
84244 229533.A0A0E0SFM9 pp 229533.I1RPJ3
84245 229533.A0A0E0SFM9 pp 229533.I1RRR7
84246 229533.A0A0E0SFM9 pp 229533.I1RTE9
84247 229533.A0A0E0SFM9 pp 229533.I1S2C0
84248 229533.A0A0E0SFM9 pp 229533.I1RMN6
84249 229533.A0A0E0SFM9 pp 229533.I1RSS4
84250 229533.A0A0E0SFM9 pp 229533.V6RY33
84251 229533.A0A0E0SFM9 pp 229533.Q4HWE2
84252 229533.A0A0E0SFM9 pp 229533.A0A0E0SJN2
84253 229533.A0A0E0SFM9 pp 229533.I1RRI2
84254 229533.A0A0E0SFM9 pp 229533.I1RQ65
84255 229533.A0A0E0SFM9 pp 229533.I1S1X9
84256 229533.A0A0E0SFM9 pp 229533.I1RRQ3
84257 229533.A0A0E0SFM9 pp 229533.I1RFG0
84258 229533.A0A0E0SFM9 pp 229533.I1RA22
84259 229533.A0A0E0SFM9 pp 229533.A0A1C3YN63
84260 229533.A0A0E0SFM9 pp 229533.I1R9Z0
84261 229533.A0A0E0SFM9 pp 229533.I1RQS5
84262 229533.A0A0E0SFM9 pp 229533.I1RRT2
84263 229533.A0A0E0SFM9 pp 229533.V6RGY3
84264 229533.A0A0E0SFM9 pp 229533.A0A1C3YLF5
84265 229533.A0A0E0SFM9 pp 229533.I1RCN5
84266 229533.A0A0E0SFM9 pp 229533.I1RAF8
84267 229533.A0A0E0SFM9 pp 229533.I1RCL3
84268 229533.A0A0E0SFM9 pp 229533.I1RBG9
84269 229533.A0A0E0SFM9 pp 229533.I1RK96
84270 229533.A0A0E0SFM9 pp 229533.I1RKL3
84271 229533.A0A0E0SFM9 pp 229533.I1RZ40
84272 229533.A0A0E0SFM9 pp 229533.I1RRJ8
84273 229533.A0A0E0SFM9 pp 229533.I1RX24
84274 229533.A0A0E0SFM9 pp 229533.I1RBC7
84275 229533.A0A0E0SFM9 pp 229533.V6R283
84276 229533.A0A0E0SFM9 pp 229533.I1RFN1
84277 229533.A0A0E0SFM9 pp 229533.I1RZS6
84278 229533.A0A0E0SFM9 pp 229533.I1RC82
84279 229533.A0A0E0SFM9 pp 229533.I1RFS3
84280 229533.A0A0E0SFM9 pp 229533.I1S524
84281 229533.A0A0E0SFM9 pp 229533.I1RC03
84282 229533.A0A0E0SFM9 pp 229533.A0A0E0SGM4
84283 229533.A0A0E0SFM9 pp 229533.I1RBD7
84284 229533.A0A0E0SFM9 pp 229533.Q4IQW0
84285 229533.A0A0E0SFM9 pp 229533.Q4HZ42
84286 229533.A0A0E0SFM9 pp 229533.I1RLV9
84287 229533.A0A0E0SFM9 pp 229533.I1RS47
84288 229533.A0A0E0SFM9 pp 229533.Q4IAS1
84289 229533.A0A0E0SFM9 pp 229533.I1SAN0
84290 229533.A0A0E0SFM9 pp 229533.I1RJZ7
84291 229533.A0A0E0SFM9 pp 229533.A0A1C3YNC3
84292 229533.A0A0E0SFM9 pp 229533.V6QUE6
84293 229533.A0A0E0SFM9 pp 229533.Q4IBS2
84294 229533.A0A0E0SFM9 pp 229533.I1RJL4
84295 229533.A0A0E0SFM9 pp 229533.A0A1C3YIN1
84296 229533.A0A0E0SFM9 pp 229533.A0A1C3YI56
84297 229533.A0A0E0SFM9 pp 229533.I1RA48
84298 229533.A0A0E0SFM9 pp 229533.I1R9V7
84299 229533.A0A0E0SFM9 pp 229533.I1RK95
84300 229533.A0A0E0SFM9 pp 229533.A0A1C3YHT9
84301 229533.A0A0E0SFM9 pp 229533.A0A1C3YJR3
84302 229533.A0A0E0SFM9 pp 229533.I1RZF6
84303 229533.A0A0E0SFM9 pp 229533.Q4IF76
84304 229533.A0A0E0SFM9 pp 229533.A0A1C3YMH5
84305 229533.A0A0E0SFM9 pp 229533.V6R227
84306 229533.A0A0E0SFM9 pp 229533.I1RQ28
84307 229533.A0A0E0SFM9 pp 229533.I1RD64
84308 229533.A0A0E0SFM9 pp 229533.I1RDG0
84309 229533.A0A0E0SFM9 pp 229533.I1RKK6
84310 229533.A0A0E0SFM9 pp 229533.I1RN99
84311 229533.A0A0E0SFM9 pp 229533.I1RZZ1
84312 229533.A0A0E0SFM9 pp 229533.I1RG56
84313 229533.A0A0E0SFM9 pp 229533.I1RBI8
84314 229533.A0A0E0SFM9 pp 229533.I1RKJ6
84315 229533.A0A0E0SFM9 pp 229533.Q4IEK8

229533.A0A0E0SFM9 pp 229533.I1RJS4
229533.A0A0E0SFM9 pp 229533.I1RC06
229533.A0A0E0SFM9 pp 229533.I1RAZ8
229533.A0A0E0SFM9 pp 229533.I1S0Z9
229533.A0A0E0SFM9 pp 229533.I1RMC0
229533.A0A0E0SFM9 pp 229533.Q4IJ56
229533.A0A0E0SFM9 pp 229533.I1RQ31
229533.A0A0E0SFM9 pp 229533.I1S021
229533.A0A0E0SFM9 pp 229533.I1RNH6
229533.A0A0E0SFM9 pp 229533.I1RVZ0
229533.A0A0E0SFM9 pp 229533.I1RE00
229533.A0A0E0SFM9 pp 229533.I1RB27
229533.A0A0E0SFM9 pp 229533.I1RF19
229533.A0A0E0SFM9 pp 229533.I1RDY1
229533.A0A0E0SFM9 pp 229533.I1RZB5
229533.A0A0E0SFM9 pp 229533.I1RD56
229533.A0A0E0SFM9 pp 229533.I1RMD5
229533.A0A0E0SFM9 pp 229533.I1RLU5
229533.A0A0E0SFM9 pp 229533.I1RT33
229533.A0A0E0SFM9 pp 229533.I1RKQ5
229533.A0A0E0SFM9 pp 229533.I1RWC6
229533.A0A0E0SFM9 pp 229533.I1RAV6
229533.A0A0E0SFM9 pp 229533.I1RBL7
229533.A0A0E0SFM9 pp 229533.Q4IPI1
229533.A0A0E0SFM9 pp 229533.I1RRH5
229533.A0A0E0SFM9 pp 229533.I1RD52
229533.A0A0E0SFM9 pp 229533.I1RZ27
229533.A0A0E0SFM9 pp 229533.A0A1C3YIP5
229533.A0A0E0SFM9 pp 229533.Q4HZ47
229533.A0A0E0SFM9 pp 229533.I1RNA0
229533.A0A0E0SFM9 pp 229533.I1RSR3
229533.A0A0E0SFM9 pp 229533.V6QYF0
229533.A0A0E0SFM9 pp 229533.I1RAN5
229533.A0A0E0SFM9 pp 229533.A0A1C3YK72
229533.A0A0E0SFM9 pp 229533.I1RNH1
229533.A0A0E0SFM9 pp 229533.I1RQA2
229533.A0A0E0SFM9 pp 229533.I1S6F0
229533.A0A0E0SFM9 pp 229533.I1RKK4
229533.A0A0E0SFM9 pp 229533.I1RM06
229533.A0A0E0SFM9 pp 229533.I1RW71
229533.A0A0E0SFM9 pp 229533.I1RUQ2
229533.A0A0E0SFM9 pp 229533.I1RT98
229533.A0A0E0SFM9 pp 229533.I1RG51
229533.A0A0E0SFM9 pp 229533.Q4IFI0
229533.A0A0E0SFM9 pp 229533.I1RQJ5
229533.A0A0E0SFM9 pp 229533.A0A1C3YKJ7
229533.A0A0E0SFM9 pp 229533.I1RAC2
229533.A0A0E0SFM9 pp 229533.I1RZJ6
229533.A0A0E0SFM9 pp 229533.Q4INZ9
229533.A0A0E0SFM9 pp 229533.Q4IJH1
229533.A0A0E0SFM9 pp 229533.I1RTF4
229533.A0A0E0SFM9 pp 229533.I1RT80
229533.A0A0E0SFM9 pp 229533.I1RDA0
229533.A0A0E0SFM9 pp 229533.Q4HVW2
229533.A0A0E0SFM9 pp 229533.I1RMK3
229533.A0A0E0SFM9 pp 229533.I1RT05
229533.A0A0E0SFM9 pp 229533.I1RQM6
229533.A0A0E0SFM9 pp 229533.A0A1C3YID5
229533.A0A0E0SFM9 pp 229533.I1S9U2
229533.A0A0E0SFM9 pp 229533.I1RPW9
229533.A0A0E0SFM9 pp 229533.I1RM67
229533.I1R9R5 pp 229533.Q4I8E9
229533.A0A1C3YK80 pp 229533.A0A1C3YKZ0
229533.A0A1C3YK80 pp 229533.I1S0P5
229533.A0A1C3YK80 pp 229533.I1S4T3
229533.A0A1C3YK80 pp 229533.I1RJQ3
229533.A0A1C3YK80 pp 229533.I1RDY1
229533.A0A1C3YK80 pp 229533.I1RKK4
229533.A0A1C3YK80 pp 229533.I1RBK8
229533.A0A1C3YK80 pp 229533.I1RKL2
229533.A0A1C3YK80 pp 229533.I1RQ48
229533.A0A1C3YK80 pp 229533.I1S8W6
229533.A0A1C3YK80 pp 229533.I1RMH6

84389	229533.A0A1C3YMD8	pp	229533.V6QVH8
84390	229533.A0A1C3YMD8	pp	229533.I1RPV8
84391	229533.A0A1C3YMD8	pp	229533.I1RY74
84392	229533.A0A1C3YMD8	pp	229533.I1RIH0
84393	229533.A0A1C3YMD8	pp	229533.I1RF40
84394	229533.A0A1C3YMD8	pp	229533.I1RJU6
84395	229533.A0A1C3YMD8	pp	229533.I1RZ56
84396	229533.A0A1C3YMD8	pp	229533.I1RJS1
84397	229533.A0A1C3YMD8	pp	229533.I1RF85
84398	229533.A0A1C3YMD8	pp	229533.A0A1C3YN78
84399	229533.A0A1C3YMD8	pp	229533.I1S0A5
84400	229533.A0A1C3YMD8	pp	229533.I1S0A0
84401	229533.A0A1C3YMD8	pp	229533.V6R9Z3
84402	229533.A0A1C3YMD8	pp	229533.I1S3G4
84403	229533.A0A1C3YMD8	pp	229533.I1S2C4
84404	229533.A0A1C3YMD8	pp	229533.I1RCU2
84405	229533.A0A1C3YMD8	pp	229533.I1S6H3
84406	229533.A0A1C3YMD8	pp	229533.Q4I1M9
84407	229533.A0A1C3YMD8	pp	229533.I1RM26
84408	229533.A0A1C3YMD8	pp	229533.I1RM12
84409	229533.A0A1C3YMD8	pp	229533.I1RA13
84410	229533.A0A1C3YMD8	pp	229533.I1S094
84411	229533.A0A1C3YMD8	pp	229533.I1R9Y4
84412	229533.A0A1C3YMD8	pp	229533.I1S6P6
84413	229533.A0A1C3YMD8	pp	229533.I1RDN3
84414	229533.A0A1C3YMD8	pp	229533.I1RE36
84415	229533.A0A1C3YMD8	pp	229533.I1S1W8
84416	229533.A0A1C3YMD8	pp	229533.I1RVX9
84417	229533.A0A1C3YMH5	pp	229533.I1R9P0
84418	229533.A0A1C3YMH5	pp	229533.I1RWC6
84419	229533.A0A1C3YMH5	pp	229533.I1RJU7
84420	229533.A0A1C3YMH5	pp	229533.I1RKK4
84421	229533.A0A1C3YMH5	pp	229533.I1RDY1
84422	229533.A0A1C3YMH5	pp	229533.I1RZ27
84423	229533.A0A1C3YMH5	pp	229533.I1RWI1
84424	229533.A0A1C3YMH5	pp	229533.I1RBD7
84425	229533.A0A1C3YMH5	pp	229533.I1SAN0
84426	229533.A0A1C3YMH5	pp	229533.I1RY59
84427	229533.A0A1C3YMH5	pp	229533.I1RKK6
84428	229533.A0A1C3YMH5	pp	229533.I1R9Z0
84429	229533.A0A1C3YMH5	pp	229533.I1RJS4
84430	229533.A0A1C3YMH5	pp	229533.I1RLU7
84431	229533.A0A1C3YMH5	pp	229533.I1RFN2
84432	229533.A0A1C3YMH5	pp	229533.I1RCY6
84433	229533.I1RAE0	pp	229533.I1RG11
84434	229533.I1RAE0	pp	229533.Q4IPC8
84435	229533.I1RAE0	pp	229533.I1RCB7
84436	229533.I1RAE0	pp	229533.I1RT35
84437	229533.I1RAE0	pp	229533.I1RXU0
84438	229533.I1RAE0	pp	229533.I1RC16
84439	229533.I1RAE0	pp	229533.I1RWF3
84440	229533.I1RAE0	pp	229533.Q4HTS9
84441	229533.I1RAE0	pp	229533.I1RXE5
84442	229533.I1RAE0	pp	229533.Q4I5U9
84443	229533.I1RAE0	pp	229533.I1RBJ1
84444	229533.I1RAE0	pp	229533.I1RMQ7
84445	229533.I1RAE0	pp	229533.I1RKR8
84446	229533.I1RAE0	pp	229533.Q4HYB8
84447	229533.I1RAE0	pp	229533.I1RAR2
84448	229533.I1RAE0	pp	229533.Q4I5I4
84449	229533.I1RAE0	pp	229533.V6QUE6
84450	229533.I1RAE0	pp	229533.I1RN43
84451	229533.I1RAE0	pp	229533.I1RRT0
84452	229533.I1RAE0	pp	229533.I1RVZ4
84453	229533.I1RAE0	pp	229533.Q4I5R3
84454	229533.I1RAE0	pp	229533.I1RZR6
84455	229533.I1RAE0	pp	229533.I1RNB8
84456	229533.I1RAE0	pp	229533.I1RC15
84457	229533.I1RAE0	pp	229533.I1RK79
84458	229533.I1RAE0	pp	229533.I1RE30
84459	229533.I1RAE0	pp	229533.I1RZB5
84460	229533.I1RAE0	pp	229533.I1S5Z2
84461	229533.I1RAE0	pp	229533.I1S9H8

84462 229533.I1RAE0 pp 229533.I1RJL2
84463 229533.I1RAE0 pp 229533.I1RPM7
84464 229533.I1RAE0 pp 229533.I1RRG3
84465 229533.I1RAE0 pp 229533.I1RK71
84466 229533.I1RAE0 pp 229533.Q4IJU0
84467 229533.I1RAE0 pp 229533.I1S5Z5
84468 229533.I1RAE0 pp 229533.I1REN3
84469 229533.I1RAE0 pp 229533.I1RS41
84470 229533.I1RAE0 pp 229533.V6QV38
84471 229533.I1RAE0 pp 229533.I1RTC2
84472 229533.I1RAE0 pp 229533.I1RKF3
84473 229533.I1RAE0 pp 229533.I1RR25
84474 229533.I1RAE0 pp 229533.V6QWX8
84475 229533.I1RAE0 pp 229533.I1RZU4
84476 229533.I1RAE0 pp 229533.I1S276
84477 229533.I1RAE0 pp 229533.I1RMV1
84478 229533.I1RAE0 pp 229533.I1RUG0
84479 229533.I1RAE0 pp 229533.I1RSW9
84480 229533.I1RAE0 pp 229533.I1RVY0
84481 229533.I1RAE0 pp 229533.I1RC24
84482 229533.I1RAE0 pp 229533.Q4I5W5
84483 229533.I1RAE0 pp 229533.I1RVJ1
84484 229533.I1RAE0 pp 229533.I1RVL8
84485 229533.I1RAE0 pp 229533.I1RQ88
84486 229533.I1RAE0 pp 229533.I1RP96
84487 229533.I1RAE0 pp 229533.I1RML9
84488 229533.I1S3R6 pp 229533.I1S7I5
84489 229533.I1S3R6 pp 229533.Q4IPI4
84490 229533.A0A0E0SED1 pp 229533.I1S0P5
84491 229533.A0A0E0SED1 pp 229533.I1RWI1
84492 229533.A0A0E0SED1 pp 229533.I1RBY7
84493 229533.I1RZU4 pp 229533.Q4I5W5
84494 229533.I1RZU4 pp 229533.I1S2C4
84495 229533.I1RZU4 pp 229533.Q4I5I4
84496 229533.I1RZU4 pp 229533.Q4IJU0
84497 229533.A0A098E1H5 pp 229533.I1RC54
84498 229533.A0A098E1H5 pp 229533.I1S279
84499 229533.A0A098E1H5 pp 229533.I1RFN2
84500 229533.A0A098E1H5 pp 229533.I1RML0
84501 229533.A0A098E1H5 pp 229533.I1S0P5
84502 229533.A0A098E1H5 pp 229533.Q4IMD1
84503 229533.A0A098E1H5 pp 229533.A0A098E454
84504 229533.A0A098E1H5 pp 229533.I1RMT5
84505 229533.A0A098E1H5 pp 229533.I1RHM5
84506 229533.A0A098E1H5 pp 229533.A0A1C3YM61
84507 229533.A0A098E1H5 pp 229533.A0A0E0SF67
84508 229533.A0A098E1H5 pp 229533.I1RD70
84509 229533.A0A098E1H5 pp 229533.I1RRH1
84510 229533.A0A098E1H5 pp 229533.A0A1C3YIL2
84511 229533.A0A098E1H5 pp 229533.I1RMY3
84512 229533.A0A098E1H5 pp 229533.A0A0E0SMG8
84513 229533.A0A098E1H5 pp 229533.I1RJS4
84514 229533.A0A098E1H5 pp 229533.A0A1C3YI56
84515 229533.A0A098E1H5 pp 229533.A0A1C3YMH5
84516 229533.A0A098E1H5 pp 229533.A0A1C3YK06
84517 229533.A0A098E1H5 pp 229533.I1RRJ6
84518 229533.A0A098E1H5 pp 229533.A0A1C3YIZ6
84519 229533.A0A098E1H5 pp 229533.A0A0E0S658
84520 229533.A0A098E1H5 pp 229533.I1RPP5
84521 229533.A0A098E1H5 pp 229533.I1RWM6
84522 229533.A0A098E1H5 pp 229533.I1RK61
84523 229533.A0A098E1H5 pp 229533.I1RRF9
84524 229533.A0A098E1H5 pp 229533.I1RA50
84525 229533.A0A098E1H5 pp 229533.I1RZV9
84526 229533.A0A098E1H5 pp 229533.A0A1C3YIP6
84527 229533.A0A098E1H5 pp 229533.I1S788
84528 229533.A0A098E1H5 pp 229533.A0A1C3YID5
84529 229533.A0A098E1H5 pp 229533.I1RDY1
84530 229533.A0A098E1H5 pp 229533.I1RWC6
84531 229533.A0A098E1H5 pp 229533.I1R9C1
84532 229533.A0A098E1H5 pp 229533.A0A0E0SNZ9
84533 229533.I1RZU5 pp 229533.I1S1Y5
84534 229533.A0A098DDW3 pp 229533.I1RK71

229533.A0A098DDW3 pp 229533.Q4HYB8
229533.A0A098DDW3 pp 229533.A0A1C3YN55
229533.A0A098DDW3 pp 229533.I1RWF3
229533.A0A098DDW3 pp 229533.I1RCB7
229533.A0A098DDW3 pp 229533.I1RXU0
229533.A0A098DDW3 pp 229533.I1RR25
229533.A0A098DDW3 pp 229533.I1RG11
229533.A0A098DDW3 pp 229533.A0A1C3YI56
229533.A0A098DDW3 pp 229533.A0A1C3YIR8
229533.A0A098DDW3 pp 229533.A0A1C3YMH5
229533.A0A098DDW3 pp 229533.I1RK79
229533.A0A098DDW3 pp 229533.A0A098E1K1
229533.A0A098DDW3 pp 229533.I1S276
229533.A0A098DDW3 pp 229533.Q4IPC8
229533.A0A098DDW3 pp 229533.I1RPM7
229533.A0A098DDW3 pp 229533.I1RJS4
229533.A0A098DDW3 pp 229533.A0A0E0SMG8
229533.A0A098DDW3 pp 229533.I1RVL8
229533.A0A098DDW3 pp 229533.I1RE30
229533.A0A098DDW3 pp 229533.I1RS60
229533.A0A098DDW3 pp 229533.A0A1C3YI23
229533.A0A098DDW3 pp 229533.I1RE14
229533.A0A098DDW3 pp 229533.I1RVY0
229533.A0A098DDW3 pp 229533.I1RAR2
229533.A0A098DDW3 pp 229533.V6QUE6
229533.A0A098DDW3 pp 229533.I1RC24
229533.A0A098DDW3 pp 229533.A0A0E0SFM9
229533.A0A098DDW3 pp 229533.I1RSZ3
229533.A0A098DDW3 pp 229533.I1RRG3
229533.A0A098DDW3 pp 229533.Q4I5W5
229533.A0A098DDW3 pp 229533.A0A0E0SCW3
229533.A0A098DDW3 pp 229533.Q4I5I4
229533.A0A098DDW3 pp 229533.I1RKB1
229533.A0A098DDW3 pp 229533.A0A098DUU3
229533.A0A098DDW3 pp 229533.A0A1C3YKJ7
229533.A0A098DDW3 pp 229533.I1RUG0
229533.A0A098DDW3 pp 229533.A0A098DWB1
229533.A0A098DDW3 pp 229533.A0A0E0S658
229533.A0A098DDW3 pp 229533.I1RZB5
229533.A0A098DDW3 pp 229533.I1RJL2
229533.A0A098DDW3 pp 229533.I1RWC6
229533.A0A098DDW3 pp 229533.V6QWX8
229533.A0A098DDW3 pp 229533.A0A1C3YID5
229533.A0A098DDW3 pp 229533.A0A0E0RN05
229533.A0A098DDW3 pp 229533.I1RC15
229533.A0A098DDW3 pp 229533.I1RRT0
229533.A0A098DDW3 pp 229533.I1RTC2
229533.I1RSR1 pp 229533.I1S7X9
229533.A0A0E0RZU7 pp 229533.I1RG90
229533.A0A0E0RZU7 pp 229533.I1S0B1
229533.A0A0E0RZU7 pp 229533.I1RYB1
229533.A0A0E0RZU7 pp 229533.I1RIR9
229533.A0A0E0RZU7 pp 229533.I1S7M3
229533.A0A0E0RZU7 pp 229533.I1RBV3
229533.A0A0E0RZU7 pp 229533.I1RXF2
229533.A0A0E0RZU7 pp 229533.I1S7M2
229533.A0A0E0RZU7 pp 229533.A0A1C3YJW0
229533.A0A0E0RZU7 pp 229533.A0A1C3YJ29
229533.A0A0E0RZU7 pp 229533.I1RT47
229533.A0A0E0RZU7 pp 229533.I1RN09
229533.A0A0E0RZU7 pp 229533.I1RG17
229533.A0A0E0RZU7 pp 229533.Q4IP34
229533.A0A0E0RZU7 pp 229533.A0A1C3YI62
229533.A0A0E0RZU7 pp 229533.I1RSL9
229533.A0A0E0RZU7 pp 229533.I1RSD4
229533.A0A0E0RZU7 pp 229533.A0A1C3YHK3
229533.A0A0E0RZU7 pp 229533.I1RWM6
229533.A0A0E0RZU7 pp 229533.I1RNI1
229533.A0A0E0RZU7 pp 229533.I1RVT9
229533.A0A0E0RZU7 pp 229533.I1S4M5
229533.A0A0E0RZU7 pp 229533.I1RSA4
229533.A0A0E0RZU7 pp 229533.I1RK19
229533.A0A0E0RZU7 pp 229533.I1RKK4

84608 229533.A0A0E0RZU7 pp 229533.I1RMY3
84609 229533.A0A0E0RZU7 pp 229533.A0A1C3YNC3
84610 229533.A0A0E0RZU7 pp 229533.I1RBH8
84611 229533.A0A0E0RZU7 pp 229533.A0A1C3YHQ4
84612 229533.A0A0E0RZU7 pp 229533.I1RC84
84613 229533.A0A0E0RZU7 pp 229533.I1RB50
84614 229533.A0A0E0RZU7 pp 229533.A0A1C3YL18
84615 229533.A0A0E0RZU7 pp 229533.I1RPA0
84616 229533.A0A0E0RZU7 pp 229533.I1RK92
84617 229533.A0A0E0RZU7 pp 229533.A0A1C3YL11
84618 229533.A0A0E0RZU7 pp 229533.I1RA36
84619 229533.A0A0E0RZU7 pp 229533.I1S1X9
84620 229533.A0A0E0RZU7 pp 229533.I1RX24
84621 229533.A0A0E0RZU7 pp 229533.I1RZG7
84622 229533.A0A0E0RZU7 pp 229533.I1RX81
84623 229533.A0A0E0RZU7 pp 229533.I1RTS2
84624 229533.A0A0E0RZU7 pp 229533.I1RMV5
84625 229533.A0A0E0RZU7 pp 229533.I1RCQ6
84626 229533.A0A1C3YI77 pp 229533.I1S7I5
84627 229533.A0A1C3YI77 pp 229533.Q4IPI4
84628 229533.A0A1C3YI77 pp 229533.A0A1C3YLC6
84629 229533.A0A1C3YI77 pp 229533.A0A1C3YLA2
84630 229533.A0A1C3YI77 pp 229533.I1RAN8
84631 229533.A0A1C3YI77 pp 229533.I1S1V2
84632 229533.A0A1C3YI77 pp 229533.I1RE51
84633 229533.I1RKL6 pp 229533.I1RZU5
84634 229533.I1RKL6 pp 229533.I1RQ53
84635 229533.A0A1C3YI56 pp 229533.I1RJS4
84636 229533.A0A1C3YI56 pp 229533.I1RFN2
84637 229533.A0A1C3YI56 pp 229533.I1S1Y5
84638 229533.A0A1C3YI56 pp 229533.A0A1C3YKJ7
84639 229533.A0A1C3YI56 pp 229533.I1RCY6
84640 229533.A0A1C3YI56 pp 229533.A0A1C3YIU4
84641 229533.A0A1C3YI56 pp 229533.A0A1C3YMH5
84642 229533.A0A1C3YI56 pp 229533.A0A1C3YID5
84643 229533.A0A1C3YI56 pp 229533.A0A1C3YIU2
84644 229533.A0A1C3YI56 pp 229533.I1RY39
84645 229533.A0A1C3YI56 pp 229533.I1RSJ0
84646 229533.A0A1C3YI56 pp 229533.I1RWI1
84647 229533.A0A1C3YI56 pp 229533.I1RKK6
84648 229533.A0A1C3YI56 pp 229533.I1RMH6
84649 229533.A0A1C3YI56 pp 229533.A0A1C3YK00
84650 229533.A0A1C3YI56 pp 229533.I1S227
84651 229533.A0A1C3YI56 pp 229533.I1RZ27
84652 229533.A0A1C3YI56 pp 229533.I1RB17
84653 229533.A0A1C3YI56 pp 229533.I1S8W6
84654 229533.A0A1C3YI56 pp 229533.I1RDY1
84655 229533.A0A1C3YI56 pp 229533.A0A1C3YK80
84656 229533.A0A1C3YIC1 pp 229533.I1RZU5
84657 229533.A0A1C3YIC1 pp 229533.I1RKL6
84658 229533.I1RJD4 pp 229533.I1RNG4
84659 229533.I1RRD9 pp 229533.I1S7I5
84660 229533.I1RRD9 pp 229533.I1S1V2
84661 229533.I1RRD9 pp 229533.Q4IPI4
84662 229533.A0A098E1K1 pp 229533.I1R9V4
84663 229533.A0A098E1K1 pp 229533.A0A098E488
84664 229533.A0A098E1K1 pp 229533.I1RWC6
84665 229533.A0A098E1K1 pp 229533.I1RQB7
84666 229533.A0A098E1K1 pp 229533.A0A1C3YID5
84667 229533.A0A098E1K1 pp 229533.A0A1C3YKJ7
84668 229533.A0A098E1K1 pp 229533.A0A0E0SFM9
84669 229533.A0A098E1K1 pp 229533.I1RKK6
84670 229533.A0A098E1K1 pp 229533.I1RJS4
84671 229533.A0A098E1K1 pp 229533.A0A1C3YI56
84672 229533.A0A098E1K1 pp 229533.A0A1C3YMH5
84673 229533.A0A098E1K1 pp 229533.A0A0E0SJ79
84674 229533.A0A098E1K1 pp 229533.I1RWI1
84675 229533.A0A098E1K1 pp 229533.I1R9Z0
84676 229533.A0A098E1K1 pp 229533.I1RY59
84677 229533.Q4IJ11 pp 229533.V6QWX8
84678 229533.Q4IJ11 pp 229533.V6QUE6
84679 229533.Q4IJ11 pp 229533.Q4IJU0
84680 229533.I1S996 pp 229533.Q4I7L0

84681 229533.I1RTS2 pp 229533.Q4IB70
84682 229533.I1RTS2 pp 229533.Q4IP34
84683 229533.I1RTS2 pp 229533.V6QVD5
84684 229533.I1RTS2 pp 229533.I1S0B6
84685 229533.I1RTS2 pp 229533.I1RZL5
84686 229533.I1RTS2 pp 229533.I1RX81
84687 229533.I1RTS2 pp 229533.Q4I7F9
84688 229533.I1RTS2 pp 229533.I1RYJ9
84689 229533.I1RTS2 pp 229533.I1RYB1
84690 229533.A0A1C3YID5 pp 229533.I1RB75
84691 229533.A0A1C3YID5 pp 229533.I1RWI1
84692 229533.A0A1C3YID5 pp 229533.I1RZ27
84693 229533.A0A1C3YID5 pp 229533.I1RJS4
84694 229533.A0A1C3YID5 pp 229533.I1RWC6
84695 229533.A0A1C3YID5 pp 229533.I1RKK6
84696 229533.A0A1C3YID5 pp 229533.A0A1C3YMH5
84697 229533.A0A1C3YID5 pp 229533.I1RRS2
84698 229533.A0A1C3YID5 pp 229533.I1RY59
84699 229533.A0A1C3YID5 pp 229533.I1R9Z0
84700 229533.A0A1C3YID5 pp 229533.I1RDY1
84701 229533.A0A1C3YID5 pp 229533.I1RTE5
84702 229533.A0A1C3YID5 pp 229533.A0A1C3YKJ7
84703 229533.A0A1C3YID5 pp 229533.A0A1C3YIU4
84704 229533.A0A1C3YID5 pp 229533.I1RS60
84705 229533.A0A1C3YID5 pp 229533.I1RJU7
84706 229533.A0A098D2Y7 pp 229533.A0A098E017
84707 229533.A0A098D2Y7 pp 229533.A0A1C3YKJ7
84708 229533.A0A098D2Y7 pp 229533.I1RDY1
84709 229533.A0A098D2Y7 pp 229533.A0A098E1Y1
84710 229533.A0A098D2Y7 pp 229533.I1RBK8
84711 229533.A0A098D2Y7 pp 229533.A0A1C3YHY1
84712 229533.A0A098D2Y7 pp 229533.I1RJS4
84713 229533.A0A098D2Y7 pp 229533.A0A0E0S658
84714 229533.A0A098D2Y7 pp 229533.I1S0P5
84715 229533.A0A098D2Y7 pp 229533.A0A098DWB1
84716 229533.A0A098D2Y7 pp 229533.I1RKK6
84717 229533.A0A098D2Y7 pp 229533.I1RMH6
84718 229533.A0A098D2Y7 pp 229533.A0A0E0SFM9
84719 229533.A0A098D2Y7 pp 229533.A0A1C3YK80
84720 229533.A0A098D2Y7 pp 229533.A0A1C3YMH5
84721 229533.A0A098D2Y7 pp 229533.A0A0E0SED1
84722 229533.A0A098D2Y7 pp 229533.A0A098E1H5
84723 229533.A0A098D2Y7 pp 229533.A0A098DDW3
84724 229533.A0A098D2Y7 pp 229533.A0A1C3YI56
84725 229533.A0A098D2Y7 pp 229533.A0A098E1K1
84726 229533.A0A098D2Y7 pp 229533.A0A1C3YID5
84727 229533.Q4I8C9 pp 229533.Q4IB50
84728 229533.Q4I8C9 pp 229533.Q4IJP1
84729 229533.I1REH5 pp 229533.I1RNW2
84730 229533.I1RCD6 pp 229533.I1RT47
84731 229533.I1RCD6 pp 229533.I1RQD8
84732 229533.I1RCD6 pp 229533.I1S7M3
84733 229533.I1RCD6 pp 229533.I1S7M2
84734 229533.I1RCD6 pp 229533.I1RXF2
84735 229533.I1RCD6 pp 229533.I1RSA4
84736 229533.I1RCD6 pp 229533.I1RT35
84737 229533.I1RCD6 pp 229533.I1S0B6
84738 229533.I1RCD6 pp 229533.I1RWX8
84739 229533.I1RCD6 pp 229533.Q4I963
84740 229533.I1RCD6 pp 229533.I1RFP9
84741 229533.I1RCD6 pp 229533.I1RK19
84742 229533.I1RCD6 pp 229533.I1RWU2
84743 229533.I1RCD6 pp 229533.I1RNB8
84744 229533.I1RCD6 pp 229533.I1S4M5
84745 229533.I1RCD6 pp 229533.I1S0B1
84746 229533.I1RCD6 pp 229533.I1RMV5
84747 229533.I1RCD6 pp 229533.I1RG90
84748 229533.I1RCD6 pp 229533.I1RSD4
84749 229533.I1RCD6 pp 229533.I1RX81
84750 229533.I1RCD6 pp 229533.I1RSH9
84751 229533.I1RCD6 pp 229533.Q4I7F9
84752 229533.I1RCD6 pp 229533.Q4HW67
84753 229533.I1RCD6 pp 229533.I1RCQ6

84754 229533.I1RCD6 pp 229533.I1RTS2
84755 229533.I1RCD6 pp 229533.Q4IR09
84756 229533.I1RCD6 pp 229533.I1RPA0
84757 229533.I1RCD6 pp 229533.I1RZG7
84758 229533.I1RCD6 pp 229533.I1RK92
84759 229533.I1RCD6 pp 229533.Q4IP34
84760 229533.A0A098DAM1 pp 229533.Q4HYQ4
84761 229533.A0A098DAM1 pp 229533.I1RRG3
84762 229533.A0A098DAM1 pp 229533.I1RC15
84763 229533.A0A098DAM1 pp 229533.I1RUG0
84764 229533.A0A098DAM1 pp 229533.I1RRT0
84765 229533.A0A098DAM1 pp 229533.I1RC16
84766 229533.A0A098DAM1 pp 229533.V6QWX8
84767 229533.A0A098DAM1 pp 229533.I1RT35
84768 229533.A0A098DAM1 pp 229533.A0A1C3YI23
84769 229533.A0A098DAM1 pp 229533.I1RA55
84770 229533.A0A098DAM1 pp 229533.I1RNB8
84771 229533.A0A098DAM1 pp 229533.Q4I5I4
84772 229533.A0A098DAM1 pp 229533.Q4HYB8
84773 229533.A0A098DAM1 pp 229533.I1RAE0
84774 229533.A0A098DAM1 pp 229533.I1RK79
84775 229533.A0A098DAM1 pp 229533.I1RZU4
84776 229533.A0A098DAM1 pp 229533.I1RBJ1
84777 229533.A0A098DAM1 pp 229533.Q4IJU0
84778 229533.A0A098DAM1 pp 229533.I1RCB7
84779 229533.A0A098DAM1 pp 229533.I1RG11
84780 229533.A0A098DAM1 pp 229533.I1RR25
84781 229533.A0A098DAM1 pp 229533.Q4IJ11
84782 229533.A0A098DAM1 pp 229533.I1RZB5
84783 229533.A0A098DAM1 pp 229533.A0A098DC45
84784 229533.A0A098DAM1 pp 229533.Q4I5W5
84785 229533.A0A098DAM1 pp 229533.I1R9Y7
84786 229533.A0A098DAM1 pp 229533.I1RJL2
84787 229533.A0A098DAM1 pp 229533.I1RA84
84788 229533.A0A098DAM1 pp 229533.I1RC24
84789 229533.A0A098DAM1 pp 229533.A0A1C3YIR8
84790 229533.I1RGL3 pp 229533.I1RMU9
84791 229533.I1RGL3 pp 229533.I1S8E0
84792 229533.I1RGL3 pp 229533.V6R382
84793 229533.I1RGL3 pp 229533.I1S0J7
84794 229533.I1RGL3 pp 229533.I1RPK0
84795 229533.I1RGL3 pp 229533.I1RP97
84796 229533.I1RGL3 pp 229533.V6RLM7
84797 229533.I1RGL3 pp 229533.I1RZ63
84798 229533.I1RGL3 pp 229533.I1RRZ6
84799 229533.I1RGL3 pp 229533.I1RTJ2
84800 229533.I1RGL3 pp 229533.I1RM49
84801 229533.I1RGL3 pp 229533.I1RTG7
84802 229533.I1RGL3 pp 229533.I1RZQ1
84803 229533.I1RGL3 pp 229533.I1RSU2
84804 229533.I1RGL3 pp 229533.I1RK75
84805 229533.I1RGL3 pp 229533.V6RKK5
84806 229533.I1RH67 pp 229533.I1S6H3
84807 229533.I1RH67 pp 229533.I1S1W8
84808 229533.I1RH67 pp 229533.I1S2E2
84809 229533.I1RH67 pp 229533.I1S0A0
84810 229533.I1RH67 pp 229533.I1S094
84811 229533.I1RH67 pp 229533.I1RVX9
84812 229533.I1RH67 pp 229533.V6R9Z3
84813 229533.I1RH67 pp 229533.I1RM12
84814 229533.I1RH67 pp 229533.I1RM26
84815 229533.I1RH67 pp 229533.I1S0A5
84816 229533.I1RH67 pp 229533.I1S6P6
84817 229533.I1RH67 pp 229533.I1RY74
84818 229533.I1RH67 pp 229533.I1RZ56
84819 229533.I1RH67 pp 229533.V6QVH8
84820 229533.I1RH67 pp 229533.I1S3G4
84821 229533.I1RH67 pp 229533.I1RJS1
84822 229533.I1RH67 pp 229533.I1RPV8
84823 229533.I1RH67 pp 229533.I1RIH0
84824 229533.I1RH67 pp 229533.I1S2C4
84825 229533.I1RD89 pp 229533.I1RZN1
84826 229533.I1RD89 pp 229533.I1RNG4

84827	229533.I1RD89	pp	229533.I1RFV2
84828	229533.I1S0T8	pp	229533.I1S7I5
84829	229533.I1S0T8	pp	229533.Q4IPI4
84830	229533.I1S0T8	pp	229533.I1S1V2
84831	229533.A0A098DAL6	pp	229533.I1RNG4
84832	229533.I1RN36	pp	229533.I1RZD9
84833	229533.I1RN36	pp	229533.I1RZQ6
84834	229533.I1RN36	pp	229533.I1RTG9
84835	229533.I1RN36	pp	229533.I1RSW5
84836	229533.I1RN36	pp	229533.I1S1V3
84837	229533.I1RN36	pp	229533.I1RSB0
84838	229533.I1RN36	pp	229533.I1RT41
84839	229533.I1RN36	pp	229533.I1RW71
84840	229533.I1RN36	pp	229533.I1RN81
84841	229533.I1RN36	pp	229533.I1S095
84842	229533.I1RN36	pp	229533.I1S5K9
84843	229533.I1RN36	pp	229533.I1RRQ0
84844	229533.I1RN36	pp	229533.I1RZV4
84845	229533.I1RN36	pp	229533.V6RR76
84846	229533.I1RN36	pp	229533.I1RXB5
84847	229533.I1RN36	pp	229533.I1S0Z3
84848	229533.I1RN36	pp	229533.I1RX09
84849	229533.I1RN36	pp	229533.V6REE9
84850	229533.I1RAV3	pp	229533.I1RE51
84851	229533.I1RAV3	pp	229533.I1S7I5
84852	229533.I1RAV3	pp	229533.Q4IPI4
84853	229533.I1RAV3	pp	229533.I1S1V2
84854	229533.I1RJZ8	pp	229533.I1RNG4
84855	229533.I1RRZ6	pp	229533.V6RKK5
84856	229533.I1RRZ6	pp	229533.I1RTJ2
84857	229533.I1RRZ6	pp	229533.I1RZ63
84858	229533.I1RRZ6	pp	229533.I1RTG7
84859	229533.I1RRZ6	pp	229533.I1RZQ1
84860	229533.I1RRZ6	pp	229533.I1S8E0
84861	229533.I1RRZ6	pp	229533.V6RLM7
84862	229533.I1RRZ6	pp	229533.I1S0J7
84863	229533.I1RRZ6	pp	229533.I1RSU2
84864	229533.I1RRZ6	pp	229533.V6R382
84865	229533.A0A098DLY3	pp	229533.A0A0E0S6X1
84866	229533.A0A098DLY3	pp	229533.I1RTG7
84867	229533.A0A098DLY3	pp	229533.I1RMC2
84868	229533.A0A098DLY3	pp	229533.I1RGL3
84869	229533.A0A098DLY3	pp	229533.I1RK03
84870	229533.A0A098DLY3	pp	229533.A0A1C3YI72
84871	229533.A0A098DLY3	pp	229533.I1RBE4
84872	229533.A0A098DLY3	pp	229533.I1RC61
84873	229533.A0A098DLY3	pp	229533.I1RRZ6
84874	229533.A0A098DLY3	pp	229533.I1S8E0
84875	229533.A0A098DLY3	pp	229533.I1S0Y7
84876	229533.A0A098DLY3	pp	229533.A0A1C3YL54
84877	229533.A0A098DLY3	pp	229533.I1RM54
84878	229533.A0A098DLY3	pp	229533.I1RAY9
84879	229533.A0A098DLY3	pp	229533.A0A1C3YL20
84880	229533.A0A098DLY3	pp	229533.I1RPK0
84881	229533.A0A098DLY3	pp	229533.A0A1C3YI26
84882	229533.A0A098DLY3	pp	229533.I1RP97
84883	229533.A0A098DLY3	pp	229533.I1RMG6
84884	229533.A0A098DLY3	pp	229533.I1RW00
84885	229533.A0A098DLY3	pp	229533.I1RNG4
84886	229533.A0A098DLY3	pp	229533.I1RYJ6
84887	229533.A0A098DLY3	pp	229533.I1RWF9
84888	229533.A0A098DLY3	pp	229533.I1RWS4
84889	229533.A0A098DPD6	pp	229533.I1RUG0
84890	229533.A0A098DPD6	pp	229533.I1RB73
84891	229533.A0A098DPD6	pp	229533.I1SB04
84892	229533.A0A098DPD6	pp	229533.Q4IB50
84893	229533.A0A098DPD6	pp	229533.A0A1C3YN58
84894	229533.A0A098DPD6	pp	229533.I1RCN2
84895	229533.A0A098DPD6	pp	229533.I1RY77
84896	229533.A0A098DPD6	pp	229533.A0A1C3YJD4
84897	229533.A0A098DPD6	pp	229533.A0A1C3YKP8
84898	229533.A0A098DPD6	pp	229533.A0A1C3YIA5
84899	229533.A0A098DPD6	pp	229533.A0A1C3YLP1

84900 229533.A0A098DPD6 pp 229533.I1RY99
84901 229533.A0A098DPD6 pp 229533.V6R3J1
84902 229533.A0A098DPD6 pp 229533.A0A1C3YM68
84903 229533.A0A098DPD6 pp 229533.I1RQ48
84904 229533.A0A098DPD6 pp 229533.A0A0E0RNL0
84905 229533.A0A098DPD6 pp 229533.A0A098DTF7
84906 229533.A0A098DPD6 pp 229533.Q4I7L0
84907 229533.A0A098DPD6 pp 229533.I1RZF3
84908 229533.A0A098DPD6 pp 229533.A0A1C3YJW9
84909 229533.A0A098DPD6 pp 229533.A0A1C3YK75
84910 229533.A0A098DPD6 pp 229533.A0A098E3M9
84911 229533.A0A098DPD6 pp 229533.Q4I5R3
84912 229533.A0A098DPD6 pp 229533.I1RVJ7
84913 229533.A0A098DPD6 pp 229533.I1RKQ9
84914 229533.I1RRP6 pp 229533.I1S1V2
84915 229533.I1RRP6 pp 229533.I1S7I5
84916 229533.I1RRP6 pp 229533.Q4IPI4
84917 229533.I1RJE9 pp 229533.I1RSE0
84918 229533.A0A098E0D1 pp 229533.I1RMQ0
84919 229533.A0A098E0D1 pp 229533.Q4I7F9
84920 229533.A0A098E0D1 pp 229533.I1RK15
84921 229533.A0A098E0D1 pp 229533.I1RT35
84922 229533.A0A098E0D1 pp 229533.A0A1C3YL25
84923 229533.A0A098E0D1 pp 229533.I1RE00
84924 229533.A0A098E0D1 pp 229533.I1RBE4
84925 229533.A0A098E0D1 pp 229533.A0A1C3YJW0
84926 229533.A0A098E0D1 pp 229533.I1RN58
84927 229533.A0A098E0D1 pp 229533.I1S7M3
84928 229533.A0A098E0D1 pp 229533.I1RWX8
84929 229533.A0A098E0D1 pp 229533.A0A1C3YJ42
84930 229533.A0A098E0D1 pp 229533.I1RB50
84931 229533.A0A098E0D1 pp 229533.I1R9X2
84932 229533.A0A098E0D1 pp 229533.A0A1C3YL11
84933 229533.A0A098E0D1 pp 229533.I1S0B6
84934 229533.A0A098E0D1 pp 229533.I1RE51
84935 229533.A0A098E0D1 pp 229533.I1RPA0
84936 229533.A0A098E0D1 pp 229533.I1RYJ8
84937 229533.A0A098E0D1 pp 229533.I1RA36
84938 229533.A0A098E0D1 pp 229533.I1RX81
84939 229533.A0A098E0D1 pp 229533.I1RCD6
84940 229533.A0A098E0D1 pp 229533.I1RQD8
84941 229533.A0A098E0D1 pp 229533.I1S7M2
84942 229533.A0A098E0D1 pp 229533.I1RCQ6
84943 229533.A0A098E0D1 pp 229533.I1RT47
84944 229533.A0A098E0D1 pp 229533.I1RNB8
84945 229533.A0A098E0D1 pp 229533.I1S246
84946 229533.A0A098E0D1 pp 229533.I1RSH9
84947 229533.A0A098E0D1 pp 229533.I1RK92
84948 229533.A0A098E0D1 pp 229533.I1RAV4
84949 229533.A0A098E0D1 pp 229533.I1RC84
84950 229533.A0A098E0D1 pp 229533.I1RAS2
84951 229533.A0A098E0D1 pp 229533.I1RG90
84952 229533.A0A098E0D1 pp 229533.Q4I7K4
84953 229533.A0A098E0D1 pp 229533.I1RWD3
84954 229533.A0A098E0D1 pp 229533.I1RZS7
84955 229533.A0A098E0D1 pp 229533.I1RFQ1
84956 229533.A0A098E0D1 pp 229533.I1RMV5
84957 229533.A0A098E0D1 pp 229533.I1RXF2
84958 229533.A0A098E0D1 pp 229533.I1RSL8
84959 229533.A0A098E0D1 pp 229533.I1RSD4
84960 229533.A0A098E0D1 pp 229533.I1RZ40
84961 229533.A0A098E0D1 pp 229533.A0A1C3YKM6
84962 229533.A0A098E0D1 pp 229533.I1S4M5
84963 229533.A0A098E0D1 pp 229533.I1RSA4
84964 229533.A0A098E0D1 pp 229533.A0A1C3YJ90
84965 229533.A0A098E0D1 pp 229533.Q4IB50
84966 229533.A0A098E0D1 pp 229533.A0A1C3YL59
84967 229533.A0A098E0D1 pp 229533.I1RCA7
84968 229533.A0A098E0D1 pp 229533.I1RRI3
84969 229533.A0A098E0D1 pp 229533.A0A0E0SC46
84970 229533.A0A098E0D1 pp 229533.A0A1C3YL15
84971 229533.A0A098E0D1 pp 229533.A0A1C3YNC3
84972 229533.A0A098DUZ4 pp 229533.Q4IPH4

84973 229533.A0A098DUZ4 pp 229533.I1RSA8
84974 229533.A0A098DUZ4 pp 229533.I1S7M3
84975 229533.A0A098DUZ4 pp 229533.I1RT47
84976 229533.A0A098DUZ4 pp 229533.I1RZ11
84977 229533.A0A098DUZ4 pp 229533.I1RQD8
84978 229533.A0A098DUZ4 pp 229533.I1RS83
84979 229533.A0A098DUZ4 pp 229533.V6RIS2
84980 229533.A0A098DUZ4 pp 229533.I1RFQ1
84981 229533.A0A098DUZ4 pp 229533.I1RSD4
84982 229533.A0A098DUZ4 pp 229533.I1RWX8
84983 229533.A0A098DUZ4 pp 229533.I1RSA4
84984 229533.A0A098DUZ4 pp 229533.I1RBH8
84985 229533.A0A098DUZ4 pp 229533.V6RY33
84986 229533.A0A098DUZ4 pp 229533.I1RC84
84987 229533.A0A098DUZ4 pp 229533.I1RK92
84988 229533.A0A098DUZ4 pp 229533.I1RPA0
84989 229533.A0A098DUZ4 pp 229533.I1RMW7
84990 229533.A0A098DUZ4 pp 229533.I1RA36
84991 229533.A0A098DUZ4 pp 229533.I1RB50
84992 229533.A0A098DUZ4 pp 229533.I1RZZ1
84993 229533.A0A098DUZ4 pp 229533.A0A1C3YL11
84994 229533.A0A098DUZ4 pp 229533.A0A0E0SC46
84995 229533.A0A098DUZ4 pp 229533.Q4I7F9
84996 229533.A0A098DUZ4 pp 229533.I1S524
84997 229533.A0A098DUZ4 pp 229533.I1RCQ6
84998 229533.A0A098DUZ4 pp 229533.I1RBV3
84999 229533.A0A098DUZ4 pp 229533.I1RG90
85000 229533.A0A098DUZ4 pp 229533.A0A1C3YM29
85001 229533.A0A098DUZ4 pp 229533.I1S0B1
85002 229533.A0A098DUZ4 pp 229533.I1S7M2
85003 229533.A0A098DUZ4 pp 229533.I1RX81
85004 229533.A0A098DUZ4 pp 229533.I1RXF2
85005 229533.A0A098DUZ4 pp 229533.I1RK96
85006 229533.A0A098DUZ4 pp 229533.I1RMV5
85007 229533.A0A098DUZ4 pp 229533.I1S5S2
85008 229533.A0A098DUZ4 pp 229533.I1RCI3
85009 229533.A0A098DUZ4 pp 229533.A0A1C3YI62
85010 229533.A0A098DUZ4 pp 229533.I1RAD1
85011 229533.A0A098DUZ4 pp 229533.A0A0E0RUQ7
85012 229533.A0A098DUZ4 pp 229533.I1RS75
85013 229533.A0A098DUZ4 pp 229533.I1S4M5
85014 229533.A0A098DUZ4 pp 229533.I1RCA7
85015 229533.A0A098DUZ4 pp 229533.A0A1C3YNC3
85016 229533.I1RWQ0 pp 229533.I1RZQ6
85017 229533.I1RWQ0 pp 229533.I1S050
85018 229533.I1RWQ0 pp 229533.I1RZD9
85019 229533.I1RWQ0 pp 229533.I1S0S6
85020 229533.I1RWQ0 pp 229533.I1S095
85021 229533.I1RWQ0 pp 229533.Q4HZB8
85022 229533.I1RCS8 pp 229533.I1S1V2
85023 229533.I1RCS8 pp 229533.I1RE51
85024 229533.I1RCS8 pp 229533.Q4IPI4
85025 229533.I1RCS8 pp 229533.I1S230
85026 229533.I1RCS8 pp 229533.I1S7I5
85027 229533.I1RCI4 pp 229533.Q4I1Y1
85028 229533.I1RCI4 pp 229533.I1RCN5
85029 229533.I1RCI4 pp 229533.I1RVK5
85030 229533.I1RCI4 pp 229533.I1RG57
85031 229533.I1RCI4 pp 229533.I1RQP8
85032 229533.I1RCI4 pp 229533.I1RYW3
85033 229533.I1RCI4 pp 229533.I1RK90
85034 229533.I1RCI4 pp 229533.I1RRH3
85035 229533.I1RCI4 pp 229533.I1RFP9
85036 229533.I1RCI4 pp 229533.Q4IPH4
85037 229533.I1RCI4 pp 229533.V6QVD5
85038 229533.I1RCI4 pp 229533.I1RCR5
85039 229533.I1RCI4 pp 229533.I1S0B1
85040 229533.I1RCI4 pp 229533.I1RPY7
85041 229533.I1RCI4 pp 229533.I1RG90
85042 229533.I1RCI4 pp 229533.I1RYB1
85043 229533.I1RCI4 pp 229533.Q4HXF6
85044 229533.I1RCI4 pp 229533.I1S0Z9
85045 229533.I1RCI4 pp 229533.I1RT91

85046	229533.I1RCI4	pp	229533.I1S1X9
85047	229533.I1RCI4	pp	229533.I1RQR3
85048	229533.I1RCI4	pp	229533.I1RPL3
85049	229533.I1RCI4	pp	229533.Q4I7F9
85050	229533.I1RCI4	pp	229533.Q4HWE2
85051	229533.I1RCI4	pp	229533.V6RIS2
85052	229533.I1RCI4	pp	229533.I1RZQ5
85053	229533.I1RCI4	pp	229533.Q4IP34
85054	229533.I1RCI4	pp	229533.I1RSA8
85055	229533.I1RCI4	pp	229533.V6RY33
85056	229533.I1RCI4	pp	229533.I1RE24
85057	229533.I1RCI4	pp	229533.Q4IPB8
85058	229533.I1RCI4	pp	229533.I1RE33
85059	229533.I1RCI4	pp	229533.I1RQQ7
85060	229533.I1RCI4	pp	229533.I1S471
85061	229533.I1RCI4	pp	229533.I1RND5
85062	229533.I1RCI4	pp	229533.I1S056
85063	229533.I1RCI4	pp	229533.I1RSA4
85064	229533.I1RCI4	pp	229533.V6QX58
85065	229533.I1RCI4	pp	229533.I1RQD8
85066	229533.I1RCI4	pp	229533.I1RZ11
85067	229533.I1RCI4	pp	229533.I1RWX8
85068	229533.I1RCI4	pp	229533.I1RN09
85069	229533.I1RCI4	pp	229533.I1RG17
85070	229533.I1RCI4	pp	229533.I1RT47
85071	229533.I1RCI4	pp	229533.V6QXG1
85072	229533.I1RCI4	pp	229533.I1RX51
85073	229533.I1RCI4	pp	229533.I1RPA0
85074	229533.I1RCI4	pp	229533.I1RPY8
85075	229533.I1RCI4	pp	229533.I1S7M3
85076	229533.I1RCI4	pp	229533.Q4IAA0
85077	229533.I1RCI4	pp	229533.I1RX47
85078	229533.I1RCI4	pp	229533.Q4IRI9
85079	229533.I1RCI4	pp	229533.I1RK92
85080	229533.I1RCI4	pp	229533.I1RDC1
85081	229533.I1RCI4	pp	229533.I1RM25
85082	229533.I1RCI4	pp	229533.Q4IB70
85083	229533.I1RCI4	pp	229533.I1RNB8
85084	229533.I1RCI4	pp	229533.Q4IE79
85085	229533.I1RCI4	pp	229533.I1RQC5
85086	229533.I1RCI4	pp	229533.I1RX24
85087	229533.I1RCI4	pp	229533.I1RPY5
85088	229533.I1RCI4	pp	229533.I1RX81
85089	229533.I1RCI4	pp	229533.I1S9L5
85090	229533.I1RCI4	pp	229533.I1RS28
85091	229533.I1RCI4	pp	229533.I1RFQ1
85092	229533.I1RCI4	pp	229533.I1S524
85093	229533.I1RCI4	pp	229533.I1RK72
85094	229533.I1RCI4	pp	229533.I1RSD4
85095	229533.I1RCI4	pp	229533.I1RWU2
85096	229533.I1RCI4	pp	229533.Q4IPB3
85097	229533.I1RCI4	pp	229533.I1S4M5
85098	229533.I1RCI4	pp	229533.I1RRL0
85099	229533.I1RCI4	pp	229533.I1RJF9
85100	229533.I1RCI4	pp	229533.I1RPY4
85101	229533.I1RCI4	pp	229533.I1RFT1
85102	229533.I1RCI4	pp	229533.I1RCQ6
85103	229533.I1RCI4	pp	229533.I1RST1
85104	229533.I1RCI4	pp	229533.Q4HVW2
85105	229533.I1RCI4	pp	229533.I1RZG7
85106	229533.I1RCI4	pp	229533.I1RDE9
85107	229533.I1RCI4	pp	229533.I1RPZ3
85108	229533.I1RCI4	pp	229533.I1RS75
85109	229533.I1RCI4	pp	229533.I1RCS2
85110	229533.I1RCI4	pp	229533.I1RT17
85111	229533.I1RCI4	pp	229533.I1S2Y5
85112	229533.I1RCI4	pp	229533.I1RYI9
85113	229533.I1RCI4	pp	229533.I1RU69
85114	229533.I1RCI4	pp	229533.I1S1C6
85115	229533.I1RCI4	pp	229533.I1RN03
85116	229533.I1RCI4	pp	229533.Q4IBK5
85117	229533.I1RCI4	pp	229533.I1RJG6
85118	229533.I1RCI4	pp	229533.Q4I5R9

229533.I1RCI4	pp	229533.I1RN59
229533.I1RCI4	pp	229533.Q4IFI0
229533.I1RCI4	pp	229533.I1RXF2
229533.I1RCI4	pp	229533.I1RMV5
229533.I1RCI4	pp	229533.I1S5S2
229533.I1RCI4	pp	229533.I1S7M2
229533.I1RCI4	pp	229533.I1RLU5
229533.I1RCI4	pp	229533.I1S9L6
229533.I1RCI4	pp	229533.I1RD56
229533.I1RCI4	pp	229533.I1RG13
229533.I1RCI4	pp	229533.I1RND9
229533.I1RCI4	pp	229533.I1RME9
229533.I1RCI4	pp	229533.I1RT95
229533.I1R9B2	pp	229533.I1RNG4
229533.I1RQM0	pp	229533.V6RVG3
229533.I1RQM0	pp	229533.I1RUQ5
229533.I1RQM0	pp	229533.I1RZQ6
229533.I1RQM0	pp	229533.Q4I0J6
229533.I1RQM0	pp	229533.I1RTG9
229533.I1RQM0	pp	229533.I1S1D3
229533.I1RQM0	pp	229533.Q4IB89
229533.I1RQM0	pp	229533.I1RRP7
229533.I1RQM0	pp	229533.I1S439
229533.I1RQM0	pp	229533.I1S0Z7
229533.I1RQM0	pp	229533.I1RX38
229533.I1RQM0	pp	229533.Q4IBX0
229533.I1RQM0	pp	229533.I1S382
229533.I1RQM0	pp	229533.Q4I7X1
229533.I1RQM0	pp	229533.I1S951
229533.I1RQM0	pp	229533.I1RUN8
229533.I1RQM0	pp	229533.I1RSB0
229533.I1RQM0	pp	229533.I1RS10
229533.I1RQM0	pp	229533.I1RTQ9
229533.I1RQM0	pp	229533.I1S1V5
229533.I1RQM0	pp	229533.I1S095
229533.I1RQM0	pp	229533.I1RZD9
229533.I1RQM0	pp	229533.I1RZ65
229533.I1RQM0	pp	229533.I1RZV4
229533.I1RW54	pp	229533.I1S1Y7
229533.I1RW54	pp	229533.I1S6V0
229533.I1RW54	pp	229533.I1S2C4
229533.I1RW54	pp	229533.I1S9Z4
229533.I1RW54	pp	229533.I1SAB4
229533.I1RW54	pp	229533.I1S4P6
229533.I1RW54	pp	229533.I1S0V7
229533.I1RW54	pp	229533.I1RZX3
229533.I1RW54	pp	229533.V6RLM7
229533.I1RW54	pp	229533.I1RWZ3
229533.I1RW54	pp	229533.I1S3T7
229533.I1RW54	pp	229533.I1S147
229533.I1RW54	pp	229533.I1S968
229533.I1RW54	pp	229533.I1S2Q4
229533.I1RW54	pp	229533.V6RCJ5
229533.I1RW54	pp	229533.I1RWI5
229533.I1RW54	pp	229533.I1RWF5
229533.I1RW54	pp	229533.I1RZ39
229533.I1RW54	pp	229533.V6R042
229533.I1RW54	pp	229533.I1RYM5
229533.I1RW54	pp	229533.I1RZ38
229533.I1RW54	pp	229533.I1RY81
229533.I1RW54	pp	229533.I1RYI5
229533.I1RW54	pp	229533.I1S267
229533.I1RW54	pp	229533.V6RY56
229533.I1RW54	pp	229533.V6RWB3
229533.I1RW54	pp	229533.I1S8D3
229533.I1RW54	pp	229533.I1RWM4
229533.I1RW54	pp	229533.I1S4G3
229533.I1RW54	pp	229533.V6R3J1
229533.I1RW54	pp	229533.I1S0I9
229533.I1RW54	pp	229533.I1S4D0
229533.I1RW54	pp	229533.I1RZ25
229533.I1RW54	pp	229533.I1S2F7
229533.I1RW54	pp	229533.I1RY12

85192 229533.I1RW54 pp 229533.V6RCZ8
85193 229533.I1RW54 pp 229533.I1S3T6
85194 229533.I1RW54 pp 229533.I1RW55
85195 229533.I1RW54 pp 229533.I1S3B2
85196 229533.I1RW54 pp 229533.I1S0V6
85197 229533.I1RW54 pp 229533.V6R5T7
85198 229533.I1RW54 pp 229533.I1S210
85199 229533.I1RW54 pp 229533.I1S0N3
85200 229533.I1RW54 pp 229533.I1S029
85201 229533.I1RW54 pp 229533.V6RCC0
85202 229533.I1RW54 pp 229533.I1S048
85203 229533.I1RW54 pp 229533.I1RWL3
85204 229533.I1RW54 pp 229533.I1RYP5
85205 229533.I1RW54 pp 229533.V6RHX2
85206 229533.I1RW54 pp 229533.Q4IR09
85207 229533.I1RW54 pp 229533.I1S223
85208 229533.I1RW54 pp 229533.I1S4S7
85209 229533.I1RW54 pp 229533.I1RYQ4
85210 229533.I1RW54 pp 229533.I1S969
85211 229533.I1RW54 pp 229533.I1RY67
85212 229533.I1RW54 pp 229533.I1RYU6
85213 229533.I1RW54 pp 229533.I1S8C1
85214 229533.I1RW54 pp 229533.I1S1B8
85215 229533.I1RSN4 pp 229533.Q4IPI4
85216 229533.I1RSN4 pp 229533.I1S1V2
85217 229533.I1RSN4 pp 229533.I1S7I5
85218 229533.A0A1C3YML2 pp 229533.I1RMH5
85219 229533.A0A1C3YML2 pp 229533.I1RZG7
85220 229533.I1RCI5 pp 229533.I1S092
85221 229533.I1RCI5 pp 229533.I1RCP1
85222 229533.I1RCI5 pp 229533.I1S2I4
85223 229533.I1RCI5 pp 229533.V6RDH3
85224 229533.I1RCI5 pp 229533.I1RV70
85225 229533.I1RCI5 pp 229533.I1RIY8
85226 229533.I1RCI5 pp 229533.I1RW57
85227 229533.I1RCI5 pp 229533.I1RZQ6
85228 229533.I1RXX9 pp 229533.I1S7I5
85229 229533.I1RXX9 pp 229533.Q4IPI4
85230 229533.I1RXX9 pp 229533.I1S1V2
85231 229533.I1RFV8 pp 229533.I1RFV9
85232 229533.I1S1J6 pp 229533.I1S9I6
85233 229533.I1S1J6 pp 229533.V6QYF0
85234 229533.I1S1J6 pp 229533.Q4HVW2
85235 229533.I1S1J6 pp 229533.Q4I662
85236 229533.I1S1J6 pp 229533.V6R283
85237 229533.I1S1J6 pp 229533.I1S6L5
85238 229533.I1S1J6 pp 229533.V6R227
85239 229533.I1S1J6 pp 229533.V6RGN5
85240 229533.I1S1J6 pp 229533.V6QUE6
85241 229533.I1S1J6 pp 229533.I1S296
85242 229533.I1S1J6 pp 229533.Q4IJ56
85243 229533.I1S1J6 pp 229533.Q4I5Z5
85244 229533.I1RG13 pp 229533.I1RZG7
85245 229533.I1RG13 pp 229533.I1RPY4
85246 229533.I1RG13 pp 229533.I1RMV5
85247 229533.I1RG13 pp 229533.I1RSW2
85248 229533.I1RG13 pp 229533.I1RX81
85249 229533.I1RG13 pp 229533.I1S0R3
85250 229533.I1RG13 pp 229533.I1S7M3
85251 229533.I1RG13 pp 229533.I1RWH4
85252 229533.I1RG13 pp 229533.I1RSA8
85253 229533.I1RG13 pp 229533.I1RZ11
85254 229533.I1RG13 pp 229533.I1RXF2
85255 229533.I1RG13 pp 229533.I1RYI9
85256 229533.I1RG13 pp 229533.I1S7M2
85257 229533.I1RG13 pp 229533.I1RS28
85258 229533.I1RG13 pp 229533.I1RT47
85259 229533.I1RG13 pp 229533.I1RPA0
85260 229533.I1RG13 pp 229533.I1RPY5
85261 229533.I1RG13 pp 229533.I1RS75
85262 229533.I1RG13 pp 229533.I1RSD4
85263 229533.I1RG13 pp 229533.I1RZQ5
85264 229533.I1RG13 pp 229533.I1S2Y5

85265 229533.I1RG13 pp 229533.I1RSH9
85266 229533.I1RG13 pp 229533.I1RK72
85267 229533.I1RG13 pp 229533.I1RQC5
85268 229533.I1RG13 pp 229533.Q4IBK5
85269 229533.I1RG13 pp 229533.I1RT91
85270 229533.I1RG13 pp 229533.I1RQR3
85271 229533.I1RG13 pp 229533.I1RND5
85272 229533.I1RG13 pp 229533.I1RSA4
85273 229533.I1RG13 pp 229533.I1RN59
85274 229533.I1RG13 pp 229533.I1S7S4
85275 229533.I1RG13 pp 229533.V6QXG1
85276 229533.I1RG13 pp 229533.I1S0B1
85277 229533.I1RG13 pp 229533.I1RPY7
85278 229533.I1RG13 pp 229533.I1RWX8
85279 229533.I1RG13 pp 229533.I1RK90
85280 229533.I1RG13 pp 229533.I1RG90
85281 229533.I1RG13 pp 229533.I1RG57
85282 229533.I1RG13 pp 229533.I1S0R4
85283 229533.I1RZD9 pp 229533.I1RZX9
85284 229533.I1RZD9 pp 229533.I1S2B7
85285 229533.I1RZD9 pp 229533.I1S1V3
85286 229533.I1RZD9 pp 229533.I1S5K9
85287 229533.I1RZD9 pp 229533.V6RR76
85288 229533.I1RZD9 pp 229533.I1S0Z7
85289 229533.I1RZD9 pp 229533.P0C431
85290 229533.I1RZD9 pp 229533.I1S095
85291 229533.I1RZD9 pp 229533.V6REE9
85292 229533.I1RZD9 pp 229533.I1S1B1
85293 229533.I1RZD9 pp 229533.I1RZQ6
85294 229533.I1RZD9 pp 229533.I1RZM1
85295 229533.I1RZD9 pp 229533.I1S0L8
85296 229533.I1RZD9 pp 229533.V6RB81
85297 229533.I1RZD9 pp 229533.I1S7X9
85298 229533.I1RZD9 pp 229533.Q4I2M7
85299 229533.I1S939 pp 229533.V6RE46
85300 229533.I1S939 pp 229533.Q4I2X5
85301 229533.A0A1C3YIC0 pp 229533.I1S0L1
85302 229533.A0A1C3YIC0 pp 229533.I1RW71
85303 229533.A0A1C3YIC0 pp 229533.I1RJZ7
85304 229533.I1RED9 pp 229533.I1S1V2
85305 229533.I1RED9 pp 229533.Q4IPI4
85306 229533.I1RED9 pp 229533.I1S7I5
85307 229533.I1RPM8 pp 229533.Q4I061
85308 229533.I1RPM8 pp 229533.I1RSV0
85309 229533.I1RPM8 pp 229533.I1RS10
85310 229533.I1RAY6 pp 229533.I1RTF2
85311 229533.I1RAY6 pp 229533.I1RRC9
85312 229533.I1S0Z9 pp 229533.Q4ICG5
85313 229533.I1S0Z9 pp 229533.Q4IFI0
85314 229533.I1S0Z9 pp 229533.Q4IPB3
85315 229533.I1S0Z9 pp 229533.Q4IJH1
85316 229533.I1S0Z9 pp 229533.Q4IPI1
85317 229533.I1S0Z9 pp 229533.I1S7M3
85318 229533.I1S0Z9 pp 229533.I1S1C6
85319 229533.I1S0Z9 pp 229533.Q4IB70
85320 229533.I1S0Z9 pp 229533.Q4I1Y1
85321 229533.I1S0Z9 pp 229533.Q4IRI9
85322 229533.I1S0Z9 pp 229533.I1S7M2
85323 229533.I1S0Z9 pp 229533.Q4I7F9
85324 229533.I1S0Z9 pp 229533.Q4IE79
85325 229533.I1S0Z9 pp 229533.I1S2Y5
85326 229533.I1S0Z9 pp 229533.V6QVD5
85327 229533.I1S0Z9 pp 229533.Q4HZ42
85328 229533.I1S0Z9 pp 229533.I1S2E8
85329 229533.I1S0Z9 pp 229533.I1S246
85330 229533.I1S0Z9 pp 229533.Q4IAA0
85331 229533.I1S0Z9 pp 229533.V6RY33
85332 229533.I1S0Z9 pp 229533.I1S8E2
85333 229533.I1S0Z9 pp 229533.Q4I830
85334 229533.I1S0Z9 pp 229533.Q4IF76
85335 229533.I1S0Z9 pp 229533.Q4IPB8
85336 229533.I1S0Z9 pp 229533.I1S4M5
85337 229533.I1S0Z9 pp 229533.I1S9Y0

85338 229533.I1S0Z9 pp 229533.V6QXG1
85339 229533.I1S0Z9 pp 229533.Q4IBK5
85340 229533.I1S0Z9 pp 229533.Q4I662
85341 229533.I1RQY1 pp 229533.Q4IPI4
85342 229533.I1RQY1 pp 229533.I1S1V2
85343 229533.I1RQY1 pp 229533.I1S7I5
85344 229533.I1RJS2 pp 229533.I1S0X6
85345 229533.I1RJS2 pp 229533.I1RY40
85346 229533.I1RJS2 pp 229533.I1RJU3
85347 229533.I1RJS2 pp 229533.I1RVQ0
85348 229533.I1RJS2 pp 229533.I1RW25
85349 229533.I1RJS2 pp 229533.I1RS10
85350 229533.I1RJS2 pp 229533.I1S0L3
85351 229533.I1RJS2 pp 229533.I1S254
85352 229533.I1RJS2 pp 229533.I1RTB7
85353 229533.I1RJS2 pp 229533.I1RT19
85354 229533.I1RJS2 pp 229533.V6QW22
85355 229533.I1RJS2 pp 229533.I1RT41
85356 229533.I1RJS2 pp 229533.I1RZX9
85357 229533.I1RJS2 pp 229533.Q4ICM0
85358 229533.I1RJS2 pp 229533.I1RN81
85359 229533.I1RJS2 pp 229533.I1RWJ8
85360 229533.I1RJS2 pp 229533.V6RTJ8
85361 229533.I1RJS2 pp 229533.I1S0S6
85362 229533.I1RJS2 pp 229533.I1RPI2
85363 229533.I1RJS2 pp 229533.V6QUU6
85364 229533.I1RJS2 pp 229533.I1RSV0
85365 229533.I1RJS2 pp 229533.I1RRN0
85366 229533.I1RJS2 pp 229533.I1RQH1
85367 229533.I1RJS2 pp 229533.I1S2I7
85368 229533.I1RJS2 pp 229533.I1RSD7
85369 229533.I1RJS2 pp 229533.I1RU89
85370 229533.I1RJS2 pp 229533.Q4IJK2
85371 229533.I1RJS2 pp 229533.I1RRM9
85372 229533.I1RJS2 pp 229533.V6R476
85373 229533.I1RJS2 pp 229533.I1RPL2
85374 229533.I1RJS2 pp 229533.V6RR76
85375 229533.I1RJS2 pp 229533.I1RRT8
85376 229533.I1RJS2 pp 229533.I1RNF9
85377 229533.I1RJS2 pp 229533.I1S0L4
85378 229533.I1RJS2 pp 229533.I1RQA5
85379 229533.I1RJS2 pp 229533.I1S5B0
85380 229533.I1RJS2 pp 229533.I1S0Z3
85381 229533.I1RJS2 pp 229533.I1RPM8
85382 229533.I1RJS2 pp 229533.P0C431
85383 229533.I1RJS2 pp 229533.I1RYZ7
85384 229533.I1RJS2 pp 229533.I1RZM1
85385 229533.I1RJS2 pp 229533.I1RKI0
85386 229533.I1RJS2 pp 229533.V6RCG5
85387 229533.I1RJS2 pp 229533.I1RNQ5
85388 229533.I1RJS2 pp 229533.I1RPM4
85389 229533.I1RJS2 pp 229533.I1RR73
85390 229533.I1RJS2 pp 229533.I1RLU6
85391 229533.I1RJS2 pp 229533.I1S1B1
85392 229533.I1RJS2 pp 229533.I1RZD9
85393 229533.I1RJS2 pp 229533.I1RSW5
85394 229533.I1RJS2 pp 229533.Q4IJ79
85395 229533.I1RJS2 pp 229533.I1RQN9
85396 229533.I1RJS2 pp 229533.I1RRI2
85397 229533.I1RJS2 pp 229533.I1RUF8
85398 229533.I1RJS2 pp 229533.I1RP28
85399 229533.I1RJS2 pp 229533.I1RJW6
85400 229533.I1RJS2 pp 229533.I1RN67
85401 229533.I1RJS2 pp 229533.I1RQG4
85402 229533.I1RJS2 pp 229533.I1S0L8
85403 229533.I1RJS2 pp 229533.I1RZQ6
85404 229533.I1RJS2 pp 229533.I1RXS3
85405 229533.I1RJS2 pp 229533.I1RNC1
85406 229533.I1RJS2 pp 229533.Q4IBU4
85407 229533.I1RJS2 pp 229533.I1RUG3
85408 229533.I1RJS2 pp 229533.I1RTG5
85409 229533.I1RJS2 pp 229533.I1RSQ3
85410 229533.I1RJS2 pp 229533.I1RNN7

85411 229533.I1RJS2 pp 229533.I1RQE7
85412 229533.I1RJS2 pp 229533.I1RJV3
85413 229533.I1RJS2 pp 229533.I1RW46
85414 229533.I1RJS2 pp 229533.I1RN18
85415 229533.I1RJS2 pp 229533.I1RJS3
85416 229533.I1RJS2 pp 229533.V6RMW6
85417 229533.I1RJS2 pp 229533.I1RMP5
85418 229533.I1RJS2 pp 229533.I1RMQ5
85419 229533.I1RJS2 pp 229533.I1RS17
85420 229533.I1RJS2 pp 229533.I1RU37
85421 229533.I1RSQ5 pp 229533.I1S1V2
85422 229533.I1RSQ5 pp 229533.Q4IPI4
85423 229533.I1RSQ5 pp 229533.I1S7I5
85424 229533.I1RSQ5 pp 229533.I1RUN0
85425 229533.I1RNK2 pp 229533.V6RGN5
85426 229533.I1RNK2 pp 229533.I1RX08
85427 229533.I1RNK2 pp 229533.Q4IBS2
85428 229533.I1RNK2 pp 229533.V6QUE6
85429 229533.I1RNK2 pp 229533.I1RQ31
85430 229533.I1RNK2 pp 229533.Q4I7X1
85431 229533.I1RNK2 pp 229533.I1RSI6
85432 229533.I1RNK2 pp 229533.I1S060
85433 229533.I1RNK2 pp 229533.I1RX14
85434 229533.I1RNK2 pp 229533.I1RNY2
85435 229533.I1RNK2 pp 229533.I1S274
85436 229533.I1RNK2 pp 229533.I1S0J1
85437 229533.I1RNK2 pp 229533.I1RZF6
85438 229533.I1RNK2 pp 229533.I1RSR3
85439 229533.I1RNK2 pp 229533.I1S021
85440 229533.I1RNK2 pp 229533.Q4HZK7
85441 229533.I1RNK2 pp 229533.I1RQA2
85442 229533.I1RNK2 pp 229533.I1S2C0
85443 229533.I1RNK2 pp 229533.I1RRR7
85444 229533.I1RNK2 pp 229533.V6R227
85445 229533.I1RNK2 pp 229533.I1RPY1
85446 229533.I1RNK2 pp 229533.I1RTE9
85447 229533.I1RNK2 pp 229533.I1RPJ3
85448 229533.I1RNK2 pp 229533.Q4IJ56
85449 229533.I1RNK2 pp 229533.Q4IEK8
85450 229533.I1RNK2 pp 229533.I1RNU6
85451 229533.I1RNK2 pp 229533.I1RSR8
85452 229533.I1RNK2 pp 229533.I1RTD9
85453 229533.I1RNK2 pp 229533.I1RW71
85454 229533.I1RNK2 pp 229533.I1RSC6
85455 229533.I1RNK2 pp 229533.I1S6F0
85456 229533.I1RNK2 pp 229533.I1RQ28
85457 229533.I1RNK2 pp 229533.I1RRI2
85458 229533.I1RNK2 pp 229533.Q4HZ68
85459 229533.I1RNK2 pp 229533.I1RSD8
85460 229533.I1RNK2 pp 229533.I1RR00
85461 229533.I1RNK2 pp 229533.I1S2E8
85462 229533.I1RNK2 pp 229533.I1RVZ0
85463 229533.I1RNK2 pp 229533.I1RQJ5
85464 229533.I1RNK2 pp 229533.Q4IQW0
85465 229533.I1RNK2 pp 229533.I1RRJ8
85466 229533.I1RNK2 pp 229533.I1RZE5
85467 229533.I1RNK2 pp 229533.V6R283
85468 229533.I1RNK2 pp 229533.V6QVD5
85469 229533.I1RNK2 pp 229533.I1RRT2
85470 229533.I1RNK2 pp 229533.Q4I7L0
85471 229533.I1RNK2 pp 229533.I1RRZ0
85472 229533.I1RNK2 pp 229533.Q4IPI1
85473 229533.I1RNK2 pp 229533.I1S1X9
85474 229533.I1RNK2 pp 229533.I1RTD0
85475 229533.I1RNK2 pp 229533.I1RUQ2
85476 229533.I1RNK2 pp 229533.I1S9U2
85477 229533.I1RNK2 pp 229533.I1RS47
85478 229533.I1RNK2 pp 229533.Q4IFI0
85479 229533.I1RNK2 pp 229533.I1RPU0
85480 229533.I1RNK2 pp 229533.Q4HZ42
85481 229533.I1RNK2 pp 229533.I1RRT8
85482 229533.I1RNK2 pp 229533.I1RX24
85483 229533.I1RNK2 pp 229533.I1RNV6

229533.I1RNK2	pp	229533.I1RYH9
229533.I1RNK2	pp	229533.V6QYF0
229533.I1RNK2	pp	229533.I1RQ65
229533.I1RNK2	pp	229533.I1RT80
229533.I1RNK2	pp	229533.I1RQ13
229533.I1RNK2	pp	229533.I1RTF4
229533.I1RNK2	pp	229533.Q4IJH1
229533.I1RNK2	pp	229533.I1RRP3
229533.I1RNK2	pp	229533.I1RS30
229533.I1RNK2	pp	229533.I1S8E2
229533.I1RNK2	pp	229533.I1RZS6
229533.I1RNK2	pp	229533.I1RRQ3
229533.I1RNK2	pp	229533.Q4HVW2
229533.I1RNK2	pp	229533.Q4I662
229533.I1RNK2	pp	229533.I1RT05
229533.A0A0E0SQI9	pp	229533.I1RJZ7
229533.A0A0E0SQI9	pp	229533.I1S4S4
229533.A0A0E0SQI9	pp	229533.I1RZP2
229533.A0A0E0SQI9	pp	229533.I1S7I5
229533.A0A0E0SQI9	pp	229533.I1RZM1
229533.A0A0E0SQI9	pp	229533.I1RFI5
229533.A0A0E0SQI9	pp	229533.I1RWQ8
229533.A0A0E0SQI9	pp	229533.I1S265
229533.A0A0E0SQI9	pp	229533.I1RAU6
229533.A0A0E0SQI9	pp	229533.I1RD22
229533.A0A0E0SQI9	pp	229533.A0A1C3YL15
229533.A0A0E0SQI9	pp	229533.I1S0F5
229533.A0A0E0SQI9	pp	229533.I1RNC1
229533.A0A0E0SQI9	pp	229533.I1RN67
229533.A0A0E0SQI9	pp	229533.I1SAP6
229533.A0A0E0SQI9	pp	229533.I1RW71
229533.A0A0E0SQI9	pp	229533.I1RPY7
229533.A0A0E0SQI9	pp	229533.I1RG53
229533.A0A0E0SQI9	pp	229533.I1RSI0
229533.A0A0E0SQI9	pp	229533.I1RLX7
229533.A0A0E0SQI9	pp	229533.I1RQC9
229533.A0A0E0SQI9	pp	229533.I1RK00
229533.A0A0E0SQI9	pp	229533.Q4IJK2
229533.A0A0E0SQI9	pp	229533.A0A1C3YK03
229533.A0A0E0SQI9	pp	229533.I1RQP8
229533.A0A0E0SQI9	pp	229533.A0A1C3YLK2
229533.I1RF01	pp	229533.I1S1V2
229533.I1RF01	pp	229533.I1S7I5
229533.I1RF01	pp	229533.Q4IPI4
229533.P0C431	pp	229533.Q4ICG5
229533.P0C431	pp	229533.V6RR76
229533.P0C431	pp	229533.V6REE9
229533.P0C431	pp	229533.Q4IER8
229533.A0A098DUY3	pp	229533.A0A0E0SGX3
229533.A0A098DUY3	pp	229533.A0A1C3YHY5
229533.A0A098DUY3	pp	229533.I1RBC0
229533.A0A098DUY3	pp	229533.I1RCM8
229533.A0A098DUY3	pp	229533.I1S927
229533.I1RMY4	pp	229533.Q4IPI4
229533.I1RMY4	pp	229533.I1S1V2
229533.I1RMY4	pp	229533.I1S7I5
229533.I1RXG6	pp	229533.I1S2H3
229533.I1RXG6	pp	229533.I1S4F4
229533.I1RXG6	pp	229533.Q4HVQ9
229533.I1RXG6	pp	229533.I1S1Y7
229533.I1RXG6	pp	229533.I1RYR7
229533.I1RXG6	pp	229533.I1RXY7
229533.A0A098DMJ4	pp	229533.I1RE19
229533.A0A098DMJ4	pp	229533.I1RMP2
229533.A0A098DMJ4	pp	229533.I1RQL4
229533.A0A0E0S6X1	pp	229533.V6QVH8
229533.A0A0E0S6X1	pp	229533.A0A1C3YJ26
229533.A0A0E0S6X1	pp	229533.I1RMF4
229533.A0A0E0S6X1	pp	229533.I1RK83
229533.A0A0E0S6X1	pp	229533.I1RMG6
229533.A0A0E0S6X1	pp	229533.I1RPI5
229533.A0A0E0S6X1	pp	229533.I1S338
229533.A0A0E0S6X1	pp	229533.V6RUT5

85557 229533.A0A0E0S6X1 pp 229533.I1RRZ6
85558 229533.A0A0E0S6X1 pp 229533.I1S1W3
85559 229533.A0A0E0S6X1 pp 229533.I1RCP7
85560 229533.A0A0E0S6X1 pp 229533.I1RTU1
85561 229533.A0A0E0S6X1 pp 229533.Q4HTS9
85562 229533.A0A0E0S6X1 pp 229533.A0A1C3YJL7
85563 229533.A0A0E0S6X1 pp 229533.Q4HXF0
85564 229533.A0A0E0S6X1 pp 229533.V6R382
85565 229533.A0A0E0S6X1 pp 229533.I1RP97
85566 229533.A0A0E0S6X1 pp 229533.I1RPK0
85567 229533.A0A0E0S6X1 pp 229533.A0A1C3YL54
85568 229533.A0A0E0S6X1 pp 229533.A0A1C3YLC1
85569 229533.A0A0E0S6X1 pp 229533.I1RTG6
85570 229533.A0A0E0S6X1 pp 229533.A0A0E0SH52
85571 229533.A0A0E0S6X1 pp 229533.A0A1C3YLD3
85572 229533.A0A0E0S6X1 pp 229533.O74638
85573 229533.A0A0E0S6X1 pp 229533.I1RBE4
85574 229533.A0A0E0S6X1 pp 229533.A0A1C3YI05
85575 229533.A0A0E0S6X1 pp 229533.I1RJX2
85576 229533.A0A0E0S6X1 pp 229533.I1RN46
85577 229533.A0A0E0S6X1 pp 229533.I1RTG7
85578 229533.A0A0E0S6X1 pp 229533.I1RGL3
85579 229533.A0A0E0S6X1 pp 229533.I1RK47
85580 229533.A0A0E0S6X1 pp 229533.I1RXV9
85581 229533.I1RNI1 pp 229533.I1RT47
85582 229533.I1RNI1 pp 229533.I1RUG0
85583 229533.I1RNI1 pp 229533.I1RYU6
85584 229533.I1RNI1 pp 229533.I1RPA0
85585 229533.I1RNI1 pp 229533.I1RWX8
85586 229533.I1RNI1 pp 229533.I1RPZ1
85587 229533.I1RNI1 pp 229533.I1RX81
85588 229533.I1RNI1 pp 229533.I1RSD4
85589 229533.I1RXD0 pp 229533.I1RZV3
85590 229533.I1RZ59 pp 229533.I1RZJ6
85591 229533.I1RZ59 pp 229533.Q4IAS1
85592 229533.I1RZ59 pp 229533.I1S0J1
85593 229533.I1RZ59 pp 229533.I1S060
85594 229533.I1RZ59 pp 229533.I1S1X9
85595 229533.I1RYZ7 pp 229533.I1S0L8
85596 229533.I1RYZ7 pp 229533.V6R476
85597 229533.I1RYZ7 pp 229533.Q4ICM0
85598 229533.I1RYZ7 pp 229533.I1RZX9
85599 229533.I1RYZ7 pp 229533.P0C431
85600 229533.I1RYZ7 pp 229533.V6RR76
85601 229533.I1RYZ7 pp 229533.I1S0S6
85602 229533.I1RYZ7 pp 229533.I1RZM1
85603 229533.I1RYZ7 pp 229533.Q4I061
85604 229533.A0A098DUW8 pp 229533.I1RDF6
85605 229533.A0A098DUW8 pp 229533.V6RPQ8
85606 229533.A0A098DUW8 pp 229533.I1RZ13
85607 229533.A0A098DUW8 pp 229533.I1S272
85608 229533.A0A098DMH7 pp 229533.A0A1C3YJG7
85609 229533.A0A098DMH7 pp 229533.I1S549
85610 229533.A0A098DMH7 pp 229533.I1RXB5
85611 229533.A0A098DMH7 pp 229533.I1RWM0
85612 229533.A0A0E0SNY0 pp 229533.I1RTQ9
85613 229533.A0A0E0SNY0 pp 229533.I1RDB1
85614 229533.A0A0E0SNY0 pp 229533.I1RUN8
85615 229533.A0A0E0SNY0 pp 229533.I1R9Q0
85616 229533.A0A0E0SNY0 pp 229533.I1RB15
85617 229533.A0A0E0SNY0 pp 229533.I1RNM7
85618 229533.A0A0E0SNY0 pp 229533.I1R976
85619 229533.A0A0E0SNY0 pp 229533.I1S1V5
85620 229533.A0A0E0SNY0 pp 229533.I1RZD9
85621 229533.A0A0E0SNY0 pp 229533.I1RSB0
85622 229533.A0A0E0SNY0 pp 229533.I1R991
85623 229533.A0A0E0SNY0 pp 229533.I1RC35
85624 229533.A0A0E0SNY0 pp 229533.A0A1C3YKB5
85625 229533.A0A0E0SNY0 pp 229533.I1RB00
85626 229533.A0A0E0SNY0 pp 229533.I1RZV4
85627 229533.A0A0E0SNY0 pp 229533.I1RX38
85628 229533.A0A0E0SNY0 pp 229533.I1RAW7
85629 229533.A0A0E0SNY0 pp 229533.I1RN27

85630 229533.A0A0E0SNY0 pp 229533.I1RDG8
85631 229533.A0A0E0SNY0 pp 229533.I1RC86
85632 229533.A0A0E0SNY0 pp 229533.I1RFU3
85633 229533.A0A0E0SNY0 pp 229533.I1S951
85634 229533.A0A0E0SNY0 pp 229533.I1S382
85635 229533.A0A0E0SNY0 pp 229533.I1RD18
85636 229533.A0A0E0SNY0 pp 229533.I1RQ80
85637 229533.A0A0E0SNY0 pp 229533.I1S095
85638 229533.A0A0E0SNY0 pp 229533.I1RNC0
85639 229533.A0A0E0SNY0 pp 229533.I1RTG9
85640 229533.A0A0E0SNY0 pp 229533.A0A1C3YKJ6
85641 229533.A0A0E0SNY0 pp 229533.I1RBV5
85642 229533.A0A0E0SNY0 pp 229533.I1RM09
85643 229533.A0A0E0SNY0 pp 229533.A0A1C3YKW5
85644 229533.A0A0E0SNY0 pp 229533.A0A1C3YLH7
85645 229533.A0A0E0SNY0 pp 229533.A0A1C3YK37
85646 229533.A0A0E0SNY0 pp 229533.I1S0Z7
85647 229533.A0A0E0SNY0 pp 229533.I1RZ99
85648 229533.A0A0E0SNY0 pp 229533.A0A1C3YJ12
85649 229533.A0A0E0SNY0 pp 229533.A0A1C3YIP6
85650 229533.A0A0E0SNY0 pp 229533.I1RZ65
85651 229533.A0A0E0SNY0 pp 229533.A0A1C3YKN5
85652 229533.A0A0E0SNY0 pp 229533.I1RRP7
85653 229533.A0A0E0SNY0 pp 229533.Q4I7X1
85654 229533.A0A0E0SNY0 pp 229533.A0A1C3YHH5
85655 229533.A0A0E0SNY0 pp 229533.I1RD70
85656 229533.A0A0E0SNY0 pp 229533.I1RC95
85657 229533.A0A0E0SNY0 pp 229533.I1RFZ2
85658 229533.A0A0E0SNY0 pp 229533.I1RN36
85659 229533.A0A0E0SNY0 pp 229533.I1RAX0
85660 229533.A0A0E0SNY0 pp 229533.A0A1C3YMD9
85661 229533.A0A0E0SNY0 pp 229533.I1RPU4
85662 229533.A0A0E0SNY0 pp 229533.I1RCM6
85663 229533.A0A0E0SNY0 pp 229533.I1RAM7
85664 229533.A0A0E0SNY0 pp 229533.A0A1C3YL40
85665 229533.A0A0E0SNY0 pp 229533.I1RBI1
85666 229533.A0A0E0SNY0 pp 229533.I1RI34
85667 229533.A0A0E0SNY0 pp 229533.I1RB38
85668 229533.A0A0E0SNY0 pp 229533.A0A1C3YMF9
85669 229533.A0A0E0SNY0 pp 229533.I1RS10
85670 229533.A0A0E0SNY0 pp 229533.A0A1C3YN54
85671 229533.A0A0E0SNY0 pp 229533.I1RUQ5
85672 229533.A0A0E0SNY0 pp 229533.I1RZQ6
85673 229533.A0A0E0SNY0 pp 229533.I1RPW0
85674 229533.A0A0E0SNY0 pp 229533.Q4IBX0
85675 229533.A0A0E0SNY0 pp 229533.I1S439
85676 229533.A0A0E0SNY0 pp 229533.I1S1D3
85677 229533.A0A0E0SNY0 pp 229533.I1RZ98
85678 229533.A0A0E0SNY0 pp 229533.V6RVG3
85679 229533.A0A0E0SNY0 pp 229533.Q4I0J6
85680 229533.Q4HWE2 pp 229533.Q4I5I4
85681 229533.Q4HWE2 pp 229533.Q4I7X1
85682 229533.Q4HWE2 pp 229533.V6R042
85683 229533.Q4HWE2 pp 229533.Q4IBS2
85684 229533.Q4HWE2 pp 229533.V6RIS2
85685 229533.Q4HWE2 pp 229533.Q4IPC8
85686 229533.Q4HWE2 pp 229533.Q4IJ56
85687 229533.Q4HWE2 pp 229533.Q4IQX3
85688 229533.Q4HWE2 pp 229533.Q4IQW0
85689 229533.Q4HWE2 pp 229533.Q4IEK8
85690 229533.Q4HWE2 pp 229533.V6R227
85691 229533.Q4HWE2 pp 229533.Q4IPI1
85692 229533.Q4HWE2 pp 229533.Q4IR18
85693 229533.Q4HWE2 pp 229533.Q4IR09
85694 229533.Q4HWE2 pp 229533.Q4IF76
85695 229533.Q4HWE2 pp 229533.Q4I5W5
85696 229533.Q4HWE2 pp 229533.Q4I830
85697 229533.Q4HWE2 pp 229533.Q4IPN9
85698 229533.Q4HWE2 pp 229533.Q4IFI0
85699 229533.Q4HWE2 pp 229533.Q4I662
85700 229533.Q4HWE2 pp 229533.V6QYF0
85701 229533.Q4HWE2 pp 229533.Q4I613
85702 229533.I1RP56 pp 229533.I1S1V2

85703 229533.I1RP56 pp 229533.I1S7I5
85704 229533.I1RP56 pp 229533.Q4IPI4
85705 229533.I1RIT3 pp 229533.I1RQ48
85706 229533.I1RKP6 pp 229533.I1RZ94
85707 229533.I1RB29 pp 229533.I1S1V2
85708 229533.I1RB29 pp 229533.Q4IPI4
85709 229533.I1RB29 pp 229533.I1S7I5
85710 229533.I1RB29 pp 229533.I1RE51
85711 229533.I1REH7 pp 229533.I1RZP6
85712 229533.A0A098DMI7 pp 229533.I1RE66
85713 229533.A0A098DMI7 pp 229533.I1RKP6
85714 229533.I1S8W5 pp 229533.I1S8W6
85715 229533.I1RGC9 pp 229533.I1RQ65
85716 229533.I1RGC9 pp 229533.I1RT35
85717 229533.I1RGC9 pp 229533.I1RNB8
85718 229533.I1RGC9 pp 229533.I1RQJ5
85719 229533.I1RGC9 pp 229533.I1S0J1
85720 229533.I1RGC9 pp 229533.I1S060
85721 229533.I1RGC9 pp 229533.Q4IAS1
85722 229533.I1RGC9 pp 229533.I1RMN6
85723 229533.I1RGC9 pp 229533.I1RZJ6
85724 229533.I1RGC9 pp 229533.I1RT98
85725 229533.I1RGC9 pp 229533.I1RX24
85726 229533.I1RGC9 pp 229533.I1RNH6
85727 229533.I1RGC9 pp 229533.I1S1X9
85728 229533.I1RGC9 pp 229533.I1RYB1
85729 229533.I1RGC9 pp 229533.I1RM06
85730 229533.I1RGC9 pp 229533.I1RPU0
85731 229533.I1RGC9 pp 229533.I1RJL2
85732 229533.I1RGC9 pp 229533.I1RMH5
85733 229533.I1RP97 pp 229533.I1RPK0
85734 229533.I1RP97 pp 229533.V6R382
85735 229533.I1RP97 pp 229533.V6RN69
85736 229533.I1RP97 pp 229533.I1RRZ6
85737 229533.I1RP97 pp 229533.I1S064
85738 229533.I1RP97 pp 229533.I1RSS4
85739 229533.I1RP97 pp 229533.I1RTN3
85740 229533.I1RP97 pp 229533.I1RZH3
85741 229533.I1RP97 pp 229533.I1RZ52
85742 229533.I1RP97 pp 229533.I1RY74
85743 229533.I1RP97 pp 229533.I1RTI7
85744 229533.I1RP97 pp 229533.I1RWI5
85745 229533.I1RP97 pp 229533.I1RWQ6
85746 229533.I1RP97 pp 229533.I1S217
85747 229533.I1RP97 pp 229533.I1RXY9
85748 229533.I1RP97 pp 229533.I1RWF9
85749 229533.I1RP97 pp 229533.I1S0N3
85750 229533.I1RP97 pp 229533.I1RX72
85751 229533.I1RP97 pp 229533.I1RW26
85752 229533.I1RP97 pp 229533.I1RYB7
85753 229533.I1RP97 pp 229533.I1S0Y7
85754 229533.I1RP97 pp 229533.I1RSK5
85755 229533.I1RP97 pp 229533.I1RZ83
85756 229533.I1RP97 pp 229533.I1RXV3
85757 229533.I1RP97 pp 229533.I1RZF7
85758 229533.I1RP97 pp 229533.I1RT75
85759 229533.I1RP97 pp 229533.V6RK96
85760 229533.I1RP97 pp 229533.I1RXB6
85761 229533.I1RP97 pp 229533.I1RW43
85762 229533.I1RP97 pp 229533.I1RTG7
85763 229533.I1RP97 pp 229533.I1RPY2
85764 229533.I1RP97 pp 229533.I1RZB2
85765 229533.I1RP97 pp 229533.I1RRS8
85766 229533.I1RP97 pp 229533.I1RSS7
85767 229533.I1RP97 pp 229533.I1S7L6
85768 229533.I1RP97 pp 229533.I1RWH1
85769 229533.I1RP97 pp 229533.I1RT09
85770 229533.I1RP97 pp 229533.I1RVY2
85771 229533.I1RP97 pp 229533.I1RS83
85772 229533.I1RP97 pp 229533.I1RVP5
85773 229533.I1RP97 pp 229533.I1RQE8
85774 229533.I1RP97 pp 229533.I1S8E0
85775 229533.I1RP97 pp 229533.I1RXB1

85776 229533.I1RP97 pp 229533.I1RZM4
85777 229533.I1RP97 pp 229533.I1RR94
85778 229533.I1RP97 pp 229533.I1RZ92
85779 229533.I1RP97 pp 229533.I1RT33
85780 229533.I1RTF1 pp 229533.I1RZC5
85781 229533.I1RTF1 pp 229533.I1RZ14
85782 229533.A0A098DMH0 pp 229533.I1RTF1
85783 229533.I1RTI0 pp 229533.Q4IPI4
85784 229533.I1RTI0 pp 229533.I1S1V2
85785 229533.I1RTI0 pp 229533.I1S7I5
85786 229533.I1S1C3 pp 229533.I1S3G4
85787 229533.I1S1C3 pp 229533.I1S1W8
85788 229533.I1RBE4 pp 229533.I1RFK7
85789 229533.I1RBE4 pp 229533.I1RCS1
85790 229533.I1RBE4 pp 229533.I1RYU5
85791 229533.I1RBE4 pp 229533.I1RIV0
85792 229533.I1RBE4 pp 229533.I1S1W8
85793 229533.I1RBE4 pp 229533.I1RDH5
85794 229533.I1RBE4 pp 229533.I1RRL5
85795 229533.I1RBE4 pp 229533.I1RW54
85796 229533.I1RBE4 pp 229533.I1RJT2
85797 229533.I1RBE4 pp 229533.I1REI0
85798 229533.I1RBE4 pp 229533.I1RBW0
85799 229533.I1RBE4 pp 229533.I1RW06
85800 229533.I1RBE4 pp 229533.I1S334
85801 229533.I1RBE4 pp 229533.I1RMB7
85802 229533.I1RBE4 pp 229533.I1RQ44
85803 229533.I1RBE4 pp 229533.I1RBT1
85804 229533.I1RBE4 pp 229533.I1RBJ8
85805 229533.I1RBE4 pp 229533.I1RJU3
85806 229533.I1RBE4 pp 229533.I1RVM5
85807 229533.I1RBE4 pp 229533.I1RZW7
85808 229533.I1RBE4 pp 229533.I1RD52
85809 229533.I1RBE4 pp 229533.I1RQE8
85810 229533.I1RBE4 pp 229533.I1RLX6
85811 229533.I1RBE4 pp 229533.Q4INZ9
85812 229533.I1RBE4 pp 229533.Q4HYQ4
85813 229533.I1RBE4 pp 229533.I1RYJ7
85814 229533.I1RBE4 pp 229533.I1RJN7
85815 229533.I1RBE4 pp 229533.I1RCU2
85816 229533.I1RBE4 pp 229533.I1RYJ6
85817 229533.I1RBE4 pp 229533.I1RXB1
85818 229533.I1RBE4 pp 229533.I1S3S3
85819 229533.I1RBE4 pp 229533.I1RSB7
85820 229533.I1RBE4 pp 229533.I1RTU1
85821 229533.I1RBE4 pp 229533.I1RZM4
85822 229533.I1RBE4 pp 229533.Q4IJK2
85823 229533.I1RBE4 pp 229533.I1S2M8
85824 229533.I1RBE4 pp 229533.I1RP86
85825 229533.I1RBE4 pp 229533.I1S4S7
85826 229533.I1RBE4 pp 229533.I1RW71
85827 229533.I1RBE4 pp 229533.I1RK95
85828 229533.I1RBE4 pp 229533.I1RX46
85829 229533.I1RBE4 pp 229533.I1RYX6
85830 229533.I1RBE4 pp 229533.I1RXV9
85831 229533.I1RBE4 pp 229533.I1RK47
85832 229533.I1RBE4 pp 229533.I1S0T2
85833 229533.I1RBE4 pp 229533.I1RE00
85834 229533.I1RBE4 pp 229533.I1RQ14
85835 229533.I1RBE4 pp 229533.I1RDU0
85836 229533.I1RBE4 pp 229533.I1RWM6
85837 229533.I1RBE4 pp 229533.V6RTJ8
85838 229533.I1RBE4 pp 229533.I1RFV2
85839 229533.I1RBE4 pp 229533.I1RK03
85840 229533.I1RBE4 pp 229533.I1RE37
85841 229533.I1RBE4 pp 229533.I1REK8
85842 229533.I1RBE4 pp 229533.I1RT33
85843 229533.I1RBE4 pp 229533.I1RCL0
85844 229533.I1RBE4 pp 229533.I1RQH1
85845 229533.I1RBE4 pp 229533.V6RDH3
85846 229533.I1RBE4 pp 229533.I1RVY2
85847 229533.I1RBE4 pp 229533.I1RQE0
85848 229533.I1RBE4 pp 229533.V6RBN2

85849	229533.I1RBE4	pp	229533.I1RFB4
85850	229533.I1RBE4	pp	229533.I1RZK5
85851	229533.I1RBE4	pp	229533.I1RBE7
85852	229533.I1RBE4	pp	229533.I1RVR0
85853	229533.I1RBE4	pp	229533.I1RCV1
85854	229533.I1RBE4	pp	229533.I1RTD3
85855	229533.I1RBE4	pp	229533.I1RS18
85856	229533.I1RBE4	pp	229533.I1RFM6
85857	229533.I1RBE4	pp	229533.V6RCZ8
85858	229533.I1RBE4	pp	229533.I1S2M0
85859	229533.I1RBE4	pp	229533.I1S3K6
85860	229533.I1RBE4	pp	229533.I1RRY9
85861	229533.I1RBE4	pp	229533.I1RUY4
85862	229533.I1RBE4	pp	229533.Q4IJ56
85863	229533.I1RBE4	pp	229533.I1RPL2
85864	229533.I1RBE4	pp	229533.I1RIY0
85865	229533.I1RBE4	pp	229533.V6QUE6
85866	229533.I1RBE4	pp	229533.I1RXX1
85867	229533.I1RBE4	pp	229533.I1RYI7
85868	229533.I1RBE4	pp	229533.I1RZM1
85869	229533.I1RBE4	pp	229533.I1RRV4
85870	229533.I1RBE4	pp	229533.Q4IEK8
85871	229533.I1RBE4	pp	229533.V6RK96
85872	229533.I1RBE4	pp	229533.I1S5B0
85873	229533.I1RBE4	pp	229533.I1RM30
85874	229533.I1RBE4	pp	229533.I1RS83
85875	229533.I1RBE4	pp	229533.I1S419
85876	229533.I1RBE4	pp	229533.I1S0A0
85877	229533.I1RBE4	pp	229533.I1RF82
85878	229533.I1RBE4	pp	229533.I1RWP4
85879	229533.I1RBE4	pp	229533.I1RCN8
85880	229533.I1RBE4	pp	229533.I1RUY5
85881	229533.I1RBE4	pp	229533.I1RC61
85882	229533.I1RBE4	pp	229533.I1RR51
85883	229533.I1RBE4	pp	229533.I1RL04
85884	229533.I1RBE4	pp	229533.Q4IMJ1
85885	229533.I1RBE4	pp	229533.I1RWM4
85886	229533.I1RBE4	pp	229533.I1RBY4
85887	229533.I1RBE4	pp	229533.I1S6Y8
85888	229533.I1RBE4	pp	229533.I1RVJ6
85889	229533.I1RBE4	pp	229533.I1RPR1
85890	229533.I1RBE4	pp	229533.I1RJZ7
85891	229533.I1RBE4	pp	229533.I1RZ52
85892	229533.I1RBE4	pp	229533.I1RBF3
85893	229533.I1RBE4	pp	229533.I1RN88
85894	229533.I1RBE4	pp	229533.I1RDE7
85895	229533.I1RBE4	pp	229533.I1S8P5
85896	229533.I1RBE4	pp	229533.I1RGA6
85897	229533.I1RBE4	pp	229533.I1RTG7
85898	229533.I1RBE4	pp	229533.I1RTI7
85899	229533.I1RBE4	pp	229533.I1RZ39
85900	229533.I1RBE4	pp	229533.I1RYK0
85901	229533.I1RBE4	pp	229533.I1RW43
85902	229533.I1RBE4	pp	229533.Q4IF76
85903	229533.I1RBE4	pp	229533.I1RC67
85904	229533.I1RBE4	pp	229533.I1RSQ4
85905	229533.I1RBE4	pp	229533.I1S299
85906	229533.I1RBE4	pp	229533.I1RKD4
85907	229533.I1RBE4	pp	229533.I1RWF9
85908	229533.I1RBE4	pp	229533.I1RZZ9
85909	229533.I1RBE4	pp	229533.I1RRM9
85910	229533.I1RBE4	pp	229533.Q4I5M4
85911	229533.I1RBE4	pp	229533.V6R949
85912	229533.I1RBE4	pp	229533.I1RC31
85913	229533.I1RBE4	pp	229533.I1RWI5
85914	229533.I1RBE4	pp	229533.O74638
85915	229533.I1RBE4	pp	229533.I1RW00
85916	229533.I1RBE4	pp	229533.I1RPD6
85917	229533.I1RBE4	pp	229533.I1RY62
85918	229533.I1RBE4	pp	229533.I1RM82
85919	229533.I1RBE4	pp	229533.I1RRT8
85920	229533.I1RBE4	pp	229533.I1S2H4
85921	229533.I1RBE4	pp	229533.V6RUY9

85922	229533.I1RBE4	pp	229533.I1RBW1
85923	229533.I1RBE4	pp	229533.I1S3H8
85924	229533.I1RBE4	pp	229533.Q4HXF0
85925	229533.I1RBE4	pp	229533.I1RP28
85926	229533.I1RBE4	pp	229533.I1S2F2
85927	229533.I1RBE4	pp	229533.I1RZ83
85928	229533.I1RBE4	pp	229533.I1RN67
85929	229533.I1RBE4	pp	229533.I1RP97
85930	229533.I1RBE4	pp	229533.I1S3G4
85931	229533.I1RBE4	pp	229533.I1RNG4
85932	229533.I1RBE4	pp	229533.I1RZ20
85933	229533.I1RBE4	pp	229533.I1RXW3
85934	229533.I1RBE4	pp	229533.I1RSS7
85935	229533.I1RBE4	pp	229533.I1RKB2
85936	229533.I1RBE4	pp	229533.I1RS47
85937	229533.I1RBE4	pp	229533.I1RX96
85938	229533.I1RBE4	pp	229533.V6RG79
85939	229533.I1RBE4	pp	229533.I1RTD5
85940	229533.I1RBE4	pp	229533.I1RWS4
85941	229533.I1RBE4	pp	229533.V6R3J1
85942	229533.I1RBE4	pp	229533.I1RQG4
85943	229533.I1RBE4	pp	229533.I1S0H7
85944	229533.I1RBE4	pp	229533.I1RZB2
85945	229533.I1RBE4	pp	229533.I1RF02
85946	229533.I1RBE4	pp	229533.I1RJU4
85947	229533.I1RBE4	pp	229533.I1RFN1
85948	229533.I1RBE4	pp	229533.I1RCP7
85949	229533.I1RBE4	pp	229533.I1RT40
85950	229533.I1RBE4	pp	229533.I1RKB6
85951	229533.I1RBE4	pp	229533.I1RP69
85952	229533.I1RBE4	pp	229533.I1RM55
85953	229533.I1RBE4	pp	229533.I1RF85
85954	229533.I1RBE4	pp	229533.I1RZN1
85955	229533.I1RBE4	pp	229533.I1S2E8
85956	229533.I1RBE4	pp	229533.V6RRH2
85957	229533.I1RBE4	pp	229533.I1S0J1
85958	229533.I1RBE4	pp	229533.V6QVH8
85959	229533.I1RBE4	pp	229533.I1S064
85960	229533.I1RBE4	pp	229533.I1RTA0
85961	229533.I1RBE4	pp	229533.I1RI98
85962	229533.I1RBE4	pp	229533.Q4IN00
85963	229533.I1RBE4	pp	229533.I1RWK4
85964	229533.I1RBE4	pp	229533.I1RQE7
85965	229533.I1RBE4	pp	229533.I1RHJ2
85966	229533.I1RBE4	pp	229533.I1S8R2
85967	229533.I1RBE4	pp	229533.I1S0I3
85968	229533.I1RBE4	pp	229533.V6R382
85969	229533.I1RBE4	pp	229533.I1RRI2
85970	229533.I1RBE4	pp	229533.I1S132
85971	229533.I1RBE4	pp	229533.I1RIH0
85972	229533.I1RBE4	pp	229533.I1RCG5
85973	229533.I1RBE4	pp	229533.I1RJJ4
85974	229533.I1RBE4	pp	229533.I1RSS4
85975	229533.I1RBE4	pp	229533.I1RW16
85976	229533.I1RBE4	pp	229533.V6RLM7
85977	229533.I1RBE4	pp	229533.V6RSF3
85978	229533.I1RBE4	pp	229533.V6RRM0
85979	229533.I1RBE4	pp	229533.I1RPK0
85980	229533.I1RBE4	pp	229533.I1S5R6
85981	229533.I1RBE4	pp	229533.I1RSL5
85982	229533.I1RBE4	pp	229533.I1RNC1
85983	229533.I1RBE4	pp	229533.Q4HTS9
85984	229533.I1RBE4	pp	229533.I1RGX1
85985	229533.I1RBE4	pp	229533.Q4HZB8
85986	229533.I1RBE4	pp	229533.I1RMQ5
85987	229533.I1RBE4	pp	229533.I1RBS5
85988	229533.I1RBE4	pp	229533.I1S0S5
85989	229533.I1RBE4	pp	229533.I1RZQ1
85990	229533.I1RBE4	pp	229533.I1RCB4
85991	229533.I1RBE4	pp	229533.I1RD22
85992	229533.I1RBE4	pp	229533.I1S256
85993	229533.I1RBE4	pp	229533.I1RG11
85994	229533.I1RBE4	pp	229533.I1RSU2

85995 229533.I1RYB1 pp 229533.Q4IF76
85996 229533.I1RYB1 pp 229533.I1S7X9
85997 229533.I1RYB1 pp 229533.I1S8E2
85998 229533.I1RYB1 pp 229533.V6RY33
85999 229533.I1RYB1 pp 229533.I1S9Y0
86000 229533.I1RYB1 pp 229533.I1S4M5
86001 229533.I1RYB1 pp 229533.I1SB04
86002 229533.I1RYB1 pp 229533.I1S0N7
86003 229533.I1RYB1 pp 229533.I1S060
86004 229533.I1RYB1 pp 229533.Q4IAS1
86005 229533.I1RYB1 pp 229533.I1S0M9
86006 229533.I1RYB1 pp 229533.I1S2E8
86007 229533.I1RYB1 pp 229533.I1RZV6
86008 229533.I1RYB1 pp 229533.I1RZF6
86009 229533.I1RYB1 pp 229533.I1S1C6
86010 229533.I1RYB1 pp 229533.I1S0B1
86011 229533.I1RYB1 pp 229533.I1RYI9
86012 229533.I1RYB1 pp 229533.V6QVD5
86013 229533.I1RYB1 pp 229533.I1S246
86014 229533.I1RYB1 pp 229533.Q4IBS2
86015 229533.I1RYB1 pp 229533.Q4ICG5
86016 229533.I1RYB1 pp 229533.I1S7M3
86017 229533.I1RYB1 pp 229533.I1RYJ9
86018 229533.I1RYB1 pp 229533.I1RZG7
86019 229533.I1RYB1 pp 229533.Q4IJH1
86020 229533.I1RYB1 pp 229533.Q4IPI1
86021 229533.I1RYB1 pp 229533.I1S044
86022 229533.I1RYB1 pp 229533.I1RZ11
86023 229533.I1RYB1 pp 229533.I1S7M2
86024 229533.I1RYB1 pp 229533.Q4IRI9
86025 229533.I1RYB1 pp 229533.Q4IB70
86026 229533.I1RYB1 pp 229533.I1RZD2
86027 229533.I1RYB1 pp 229533.I1RZK6
86028 229533.I1RYB1 pp 229533.Q4IER8
86029 229533.I1RYB1 pp 229533.I1RZ59
86030 229533.I1RL60 pp 229533.I1RQ48
86031 229533.I1RE59 pp 229533.I1RPN6
86032 229533.I1RE59 pp 229533.I1RX04
86033 229533.I1RE59 pp 229533.I1RX77
86034 229533.I1RE59 pp 229533.I1RQL4
86035 229533.I1RE59 pp 229533.I1RPQ1
86036 229533.I1RE59 pp 229533.I1RV66
86037 229533.I1RE59 pp 229533.I1RX87
86038 229533.I1RE59 pp 229533.I1REJ0
86039 229533.I1RE59 pp 229533.I1RPA6
86040 229533.I1RE59 pp 229533.I1RS35
86041 229533.I1RE59 pp 229533.I1RSS1
86042 229533.I1RE59 pp 229533.I1RJT1
86043 229533.I1RE59 pp 229533.V6RKK5
86044 229533.I1RE59 pp 229533.I1S278
86045 229533.I1RE59 pp 229533.I1RZV5
86046 229533.I1RE59 pp 229533.I1RJH3
86047 229533.I1RE59 pp 229533.I1RMX3
86048 229533.I1RE59 pp 229533.I1RY32
86049 229533.I1RE59 pp 229533.I1SAM5
86050 229533.I1RE59 pp 229533.I1S0X7
86051 229533.I1RE59 pp 229533.I1RMK9
86052 229533.I1RE59 pp 229533.I1RZ89
86053 229533.I1RE59 pp 229533.I1RNY8
86054 229533.I1RE59 pp 229533.I1RZP1
86055 229533.I1RE59 pp 229533.I1RGF1
86056 229533.I1RE59 pp 229533.I1RLZ4
86057 229533.I1RE59 pp 229533.I1RFW0
86058 229533.I1RE59 pp 229533.I1S248
86059 229533.I1RE59 pp 229533.I1RQD6
86060 229533.I1RE59 pp 229533.I1RZS4
86061 229533.I1RE59 pp 229533.Q4IBU4
86062 229533.I1RE59 pp 229533.I1RZC3
86063 229533.I1RE59 pp 229533.I1RQB4
86064 229533.I1RE59 pp 229533.I1RVN2
86065 229533.I1RE59 pp 229533.I1RMA7
86066 229533.A0A0E0S3A4 pp 229533.I1RMD9
86067 229533.A0A0E0S3A4 pp 229533.I1RKP5

86068 229533.A0A0E0S3A4 pp 229533.I1RB58
86069 229533.A0A0E0S3A4 pp 229533.I1S0H2
86070 229533.A0A0E0S3A4 pp 229533.I1RT23
86071 229533.A0A0E0S3A4 pp 229533.I1RK35
86072 229533.A0A0E0S3A4 pp 229533.I1RRS6
86073 229533.I1RC46 pp 229533.I1S2K7
86074 229533.I1RC46 pp 229533.I1RZV5
86075 229533.I1RC46 pp 229533.I1RD37
86076 229533.I1RC46 pp 229533.I1RKR7
86077 229533.I1RC46 pp 229533.Q4IBU4
86078 229533.I1RC46 pp 229533.I1S968
86079 229533.I1RC46 pp 229533.I1RY32
86080 229533.I1RC46 pp 229533.I1RW76
86081 229533.I1RC46 pp 229533.I1RTG9
86082 229533.I1RC46 pp 229533.Q4I2W2
86083 229533.I1RC46 pp 229533.I1RC71
86084 229533.I1RC46 pp 229533.I1RHQ8
86085 229533.I1RC46 pp 229533.I1S0L4
86086 229533.I1RC46 pp 229533.I1RHJ0
86087 229533.I1RC46 pp 229533.I1RSB0
86088 229533.I1RC46 pp 229533.I1S7X9
86089 229533.I1RC46 pp 229533.I1SAB4
86090 229533.I1RC46 pp 229533.I1S296
86091 229533.I1RC46 pp 229533.I1RZ38
86092 229533.I1RC46 pp 229533.I1RFB0
86093 229533.I1RC46 pp 229533.V6R476
86094 229533.I1RC46 pp 229533.I1RZC3
86095 229533.I1RC46 pp 229533.I1RQ72
86096 229533.I1RC46 pp 229533.I1RLZ4
86097 229533.I1RC46 pp 229533.I1RQ48
86098 229533.I1RC46 pp 229533.I1RD45
86099 229533.I1RC46 pp 229533.I1RNF4
86100 229533.I1RC46 pp 229533.I1RSI3
86101 229533.I1RC46 pp 229533.I1RZD9
86102 229533.I1RC46 pp 229533.I1RXB5
86103 229533.I1RC46 pp 229533.I1RK55
86104 229533.I1RC46 pp 229533.I1RF24
86105 229533.I1RC46 pp 229533.I1S2B6
86106 229533.I1RC46 pp 229533.I1RIM7
86107 229533.I1RC46 pp 229533.I1RX25
86108 229533.I1RC46 pp 229533.I1RS35
86109 229533.I1RC46 pp 229533.V6RR76
86110 229533.I1RC46 pp 229533.I1RX77
86111 229533.I1RC46 pp 229533.I1RZK4
86112 229533.I1RC46 pp 229533.I1S3T6
86113 229533.I1RC46 pp 229533.I1RE59
86114 229533.I1RC46 pp 229533.I1S2F7
86115 229533.I1RC46 pp 229533.I1RPN6
86116 229533.I1RC46 pp 229533.V6QUU6
86117 229533.I1RC46 pp 229533.I1RNP1
86118 229533.I1RC46 pp 229533.I1RPH1
86119 229533.I1RC46 pp 229533.I1RQL4
86120 229533.I1RC46 pp 229533.I1RMU8
86121 229533.I1RC46 pp 229533.I1RY81
86122 229533.I1RC46 pp 229533.I1RW55
86123 229533.I1RC46 pp 229533.I1RMA2
86124 229533.I1RC46 pp 229533.I1RIG4
86125 229533.I1RC46 pp 229533.I1S969
86126 229533.I1RC46 pp 229533.I1S210
86127 229533.I1RC46 pp 229533.I1RQV7
86128 229533.I1RC46 pp 229533.I1S223
86129 229533.I1RC46 pp 229533.I1RGU3
86130 229533.I1RC46 pp 229533.I1S267
86131 229533.I1RC46 pp 229533.I1RKB3
86132 229533.I1RC46 pp 229533.I1RTQ9
86133 229533.I1RC46 pp 229533.I1RN81
86134 229533.I1RC46 pp 229533.I1RYP5
86135 229533.I1RC46 pp 229533.I1S254
86136 229533.I1RC46 pp 229533.I1RHT5
86137 229533.I1RC46 pp 229533.I1S095
86138 229533.I1RC46 pp 229533.I1RZV4
86139 229533.I1RC46 pp 229533.I1RXH6
86140 229533.I1RKB3 pp 229533.I1RPN6

86141 229533.I1RKB3 pp 229533.I1RX87
86142 229533.I1RKB3 pp 229533.I1RV66
86143 229533.I1RKB3 pp 229533.I1RX77
86144 229533.I1RKB3 pp 229533.I1S278
86145 229533.I1RKB3 pp 229533.I1RS35
86146 229533.I1RKB3 pp 229533.I1RPA6
86147 229533.I1RKB3 pp 229533.I1RSS1
86148 229533.I1RKB3 pp 229533.I1RQL4
86149 229533.I1RKB3 pp 229533.I1RZV5
86150 229533.I1RKB3 pp 229533.I1S248
86151 229533.I1RKB3 pp 229533.I1RY32
86152 229533.I1RKB3 pp 229533.I1RQD6
86153 229533.I1RKB3 pp 229533.I1RLZ4
86154 229533.I1RKB3 pp 229533.I1RMK9
86155 229533.I1RKB3 pp 229533.I1RZP1
86156 229533.I1RKB3 pp 229533.I1S0X7
86157 229533.I1RKB3 pp 229533.I1SAM5
86158 229533.I1RKB3 pp 229533.I1RZC3
86159 229533.I1RKB3 pp 229533.I1RQB4
86160 229533.I1RKB3 pp 229533.I1RZ89
86161 229533.I1RKB3 pp 229533.Q4IBU4
86162 229533.I1RKB3 pp 229533.I1RMA7
86163 229533.I1RKB3 pp 229533.I1RNY8
86164 229533.I1RRB5 pp 229533.I1RTA0
86165 229533.I1RRB5 pp 229533.I1RZQ6
86166 229533.I1RRB5 pp 229533.I1RZM1
86167 229533.I1RRB5 pp 229533.I1RSV0
86168 229533.I1RRB5 pp 229533.I1RSW5
86169 229533.I1RRB5 pp 229533.I1RWT6
86170 229533.I1RRB5 pp 229533.I1RWT2
86171 229533.I1RRB5 pp 229533.I1RS10
86172 229533.A0A1C3YJU1 pp 229533.I1RAN8
86173 229533.A0A1C3YJU1 pp 229533.I1S1V2
86174 229533.A0A1C3YJU1 pp 229533.A0A1C3YLC6
86175 229533.A0A1C3YJU1 pp 229533.I1RE51
86176 229533.A0A1C3YJU1 pp 229533.A0A1C3YLA2
86177 229533.A0A1C3YJU1 pp 229533.Q4IPI4
86178 229533.A0A1C3YJU1 pp 229533.I1S7I5
86179 229533.I1RP79 pp 229533.I1RYU6
86180 229533.I1RK35 pp 229533.I1RMD9
86181 229533.I1RK35 pp 229533.I1RRS6
86182 229533.I1RK35 pp 229533.I1RT23
86183 229533.I1RK35 pp 229533.I1RKP5
86184 229533.I1RTG7 pp 229533.Q4I5R9
86185 229533.I1RTG7 pp 229533.I1S236
86186 229533.I1RTG7 pp 229533.Q4HZQ1
86187 229533.I1RTG7 pp 229533.I1S7L6
86188 229533.I1RTG7 pp 229533.I1S338
86189 229533.I1RTG7 pp 229533.I1RW69
86190 229533.I1RTG7 pp 229533.I1RTU1
86191 229533.I1RTG7 pp 229533.I1RYB9
86192 229533.I1RTG7 pp 229533.I1RXB1
86193 229533.I1RTG7 pp 229533.I1SAN9
86194 229533.I1RTG7 pp 229533.Q4HVW2
86195 229533.I1RTG7 pp 229533.I1RVY2
86196 229533.I1RTG7 pp 229533.Q4IPB8
86197 229533.I1RTG7 pp 229533.Q4HTS9
86198 229533.I1RTG7 pp 229533.V6R382
86199 229533.I1RTG7 pp 229533.I1S6E5
86200 229533.I1RTG7 pp 229533.I1RXV3
86201 229533.I1RTG7 pp 229533.I1RW90
86202 229533.I1RTG7 pp 229533.Q4IAS1
86203 229533.I1RTG7 pp 229533.Q4HZ68
86204 229533.I1RTG7 pp 229533.Q4IMJ1
86205 229533.I1RTG7 pp 229533.V6QVH8
86206 229533.I1RTG7 pp 229533.Q4HXF0
86207 229533.I1RTG7 pp 229533.Q4IPZ1
86208 229533.I1RTG7 pp 229533.I1RY74
86209 229533.I1RTG7 pp 229533.I1S237
86210 229533.I1RTG7 pp 229533.O74638
86211 229533.I1RTG7 pp 229533.I1RWF9
86212 229533.I1RTG7 pp 229533.I1RZ83
86213 229533.I1RFQ0 pp 229533.I1RZB5

86214	229533.I1RFQ0	pp	229533.I1RG90
86215	229533.I1RFQ0	pp	229533.I1S1Y7
86216	229533.I1RFQ0	pp	229533.I1RMH5
86217	229533.I1RFQ0	pp	229533.I1RYJ9
86218	229533.I1RFQ0	pp	229533.I1RK92
86219	229533.I1RFQ0	pp	229533.I1RSA4
86220	229533.I1RFQ0	pp	229533.I1S0B1
86221	229533.I1RFQ0	pp	229533.I1RUZ8
86222	229533.I1RFQ0	pp	229533.I1RPU1
86223	229533.I1RFQ0	pp	229533.I1S0F0
86224	229533.I1RFQ0	pp	229533.I1RRT0
86225	229533.I1RFQ0	pp	229533.Q4IAA0
86226	229533.I1RFQ0	pp	229533.I1S471
86227	229533.I1RFQ0	pp	229533.I1RSD1
86228	229533.I1RFQ0	pp	229533.I1RS28
86229	229533.I1RFQ0	pp	229533.V6RIS2
86230	229533.I1RFQ0	pp	229533.Q4I7L0
86231	229533.I1RFQ0	pp	229533.I1RY99
86232	229533.I1RFQ0	pp	229533.I1RY90
86233	229533.I1RFQ0	pp	229533.I1S0B6
86234	229533.I1RFQ0	pp	229533.I1SB04
86235	229533.I1RFQ0	pp	229533.I1RYU4
86236	229533.I1RFQ0	pp	229533.Q4IQX3
86237	229533.I1RFQ0	pp	229533.I1RY77
86238	229533.I1RFQ0	pp	229533.Q4IMD1
86239	229533.I1RFQ0	pp	229533.I1RRM8
86240	229533.I1RFQ0	pp	229533.I1RYN7
86241	229533.I1RFQ0	pp	229533.I1S0N7
86242	229533.I1RFQ0	pp	229533.I1RPJ1
86243	229533.I1RFQ0	pp	229533.I1RLU5
86244	229533.I1RFQ0	pp	229533.I1RP28
86245	229533.I1RFQ0	pp	229533.I1RQK2
86246	229533.I1RFQ0	pp	229533.I1S246
86247	229533.I1RFQ0	pp	229533.I1S4M5
86248	229533.I1RM25	pp	229533.I1RST1
86249	229533.I1RM25	pp	229533.I1RZV6
86250	229533.I1RM25	pp	229533.I1RX24
86251	229533.I1RM25	pp	229533.I1RR73
86252	229533.I1RM25	pp	229533.I1RND6
86253	229533.I1RM25	pp	229533.Q4IF76
86254	229533.I1RM25	pp	229533.I1RRQ3
86255	229533.I1RM25	pp	229533.I1S8E2
86256	229533.I1RM25	pp	229533.Q4IEV5
86257	229533.I1RM25	pp	229533.I1RQ65
86258	229533.I1RM25	pp	229533.I1RME9
86259	229533.I1RM25	pp	229533.I1RMV5
86260	229533.I1RM25	pp	229533.Q4IBK5
86261	229533.I1RM25	pp	229533.Q4HZ42
86262	229533.I1RM25	pp	229533.I1RSW2
86263	229533.I1RM25	pp	229533.I1RRJ8
86264	229533.I1RM25	pp	229533.Q4I830
86265	229533.I1RM25	pp	229533.Q4I662
86266	229533.I1RM25	pp	229533.I1S4M5
86267	229533.I1RM25	pp	229533.I1S9Y0
86268	229533.I1RM25	pp	229533.I1RWU2
86269	229533.I1RM25	pp	229533.I1RPY4
86270	229533.I1RM25	pp	229533.Q4IAS1
86271	229533.I1RM25	pp	229533.I1RZS6
86272	229533.I1RM25	pp	229533.I1S2C0
86273	229533.I1RM25	pp	229533.I1RT17
86274	229533.I1RM25	pp	229533.I1S056
86275	229533.I1RM25	pp	229533.I1RXF2
86276	229533.I1RM25	pp	229533.I1RSA4
86277	229533.I1RM25	pp	229533.I1S471
86278	229533.I1RM25	pp	229533.I1S0B1
86279	229533.I1RM25	pp	229533.I1RX47
86280	229533.I1RM25	pp	229533.I1RND5
86281	229533.I1RM25	pp	229533.Q4HZ68
86282	229533.I1RM25	pp	229533.I1RYJ9
86283	229533.I1RM25	pp	229533.V6QXG1
86284	229533.I1RM25	pp	229533.I1RX51
86285	229533.I1RM25	pp	229533.I1RM96
86286	229533.I1RM25	pp	229533.I1RRR7

86287	229533.I1RM25	pp	229533.Q4IN63
86288	229533.I1RM25	pp	229533.I1RPL3
86289	229533.I1RM25	pp	229533.V6R042
86290	229533.I1RM25	pp	229533.I1S2E8
86291	229533.I1RM25	pp	229533.Q4IFI0
86292	229533.I1RM25	pp	229533.I1RWE1
86293	229533.I1RM25	pp	229533.I1RSD8
86294	229533.I1RM25	pp	229533.I1S0J1
86295	229533.I1RM25	pp	229533.Q4IPB8
86296	229533.I1RM25	pp	229533.I1RT80
86297	229533.I1RM25	pp	229533.Q4ICG5
86298	229533.I1RM25	pp	229533.I1S1X9
86299	229533.I1RM25	pp	229533.I1RS28
86300	229533.I1RM25	pp	229533.I1RZG7
86301	229533.I1RM25	pp	229533.I1RSD4
86302	229533.I1RM25	pp	229533.V6QYF0
86303	229533.I1RM25	pp	229533.Q4IJH1
86304	229533.I1RM25	pp	229533.I1S060
86305	229533.I1RM25	pp	229533.I1S2Y5
86306	229533.I1RM25	pp	229533.I1RQC5
86307	229533.I1RM25	pp	229533.I1RM67
86308	229533.I1RM25	pp	229533.I1RRL0
86309	229533.I1RM25	pp	229533.I1RX81
86310	229533.I1RM25	pp	229533.I1RWB5
86311	229533.I1RM25	pp	229533.I1RNB8
86312	229533.I1RM25	pp	229533.I1RPU0
86313	229533.I1RM25	pp	229533.I1S246
86314	229533.I1RM25	pp	229533.I1RS75
86315	229533.I1RM25	pp	229533.I1RMN6
86316	229533.I1RM25	pp	229533.I1RNU6
86317	229533.I1RM25	pp	229533.I1RWH5
86318	229533.I1RM25	pp	229533.I1RT47
86319	229533.I1RM25	pp	229533.I1S9U2
86320	229533.I1RM25	pp	229533.I1RW71
86321	229533.I1RM25	pp	229533.I1RPW9
86322	229533.I1RM25	pp	229533.I1RQJ5
86323	229533.I1RM25	pp	229533.Q4IEK8
86324	229533.I1RM25	pp	229533.I1RN59
86325	229533.I1RM25	pp	229533.V6RGN5
86326	229533.I1RM25	pp	229533.Q4HVW2
86327	229533.I1RM25	pp	229533.Q4IPI1
86328	229533.I1RM25	pp	229533.I1RZF6
86329	229533.I1RM25	pp	229533.I1RVZ0
86330	229533.I1RM25	pp	229533.I1RX46
86331	229533.I1RM25	pp	229533.I1RPZ3
86332	229533.I1RM25	pp	229533.I1S1C6
86333	229533.I1RM25	pp	229533.I1RPY5
86334	229533.I1RM25	pp	229533.I1RZJ6
86335	229533.I1RM25	pp	229533.Q4I7F9
86336	229533.I1RM25	pp	229533.Q4IAA0
86337	229533.I1RM25	pp	229533.I1RZK6
86338	229533.I1RM25	pp	229533.Q4IRI9
86339	229533.I1RM25	pp	229533.I1S7M2
86340	229533.I1RM25	pp	229533.I1RN99
86341	229533.I1RM25	pp	229533.I1RT98
86342	229533.I1RM25	pp	229533.Q4IB70
86343	229533.I1RM25	pp	229533.I1S0M9
86344	229533.I1RM25	pp	229533.I1RPA0
86345	229533.I1RM25	pp	229533.I1S9L0
86346	229533.I1RM25	pp	229533.Q4IJ56
86347	229533.I1RM25	pp	229533.I1RNH6
86348	229533.I1RM25	pp	229533.I1RSR8
86349	229533.I1RM25	pp	229533.Q4IBS2
86350	229533.I1RM25	pp	229533.Q4I1Y1
86351	229533.I1RM25	pp	229533.I1S021
86352	229533.I1RM25	pp	229533.Q4IE79
86353	229533.I1RM25	pp	229533.I1S7M3
86354	229533.V6RQW4	pp	229533.V6RVJ3
86355	229533.V6RQW4	pp	229533.V6RY56
86356	229533.V6RQW4	pp	229533.V6RRM0
86357	229533.V6RQW4	pp	229533.V6RSF3
86358	229533.I1RKK4	pp	229533.I1RYB1
86359	229533.I1RKK4	pp	229533.V6RY33

86360	229533.I1RKK4	pp	229533.I1RWX8
86361	229533.I1RKK4	pp	229533.I1RSA4
86362	229533.I1RKK4	pp	229533.I1RRQ4
86363	229533.I1RKK4	pp	229533.I1RYJ9
86364	229533.I1RKK4	pp	229533.I1RSD4
86365	229533.I1RKK4	pp	229533.I1RQD8
86366	229533.I1RKK4	pp	229533.Q4I7F9
86367	229533.I1RKK4	pp	229533.I1RN09
86368	229533.I1RKK4	pp	229533.I1RZY7
86369	229533.I1RKK4	pp	229533.I1RPA0
86370	229533.I1RKK4	pp	229533.I1RTS2
86371	229533.I1RKK4	pp	229533.I1RMH5
86372	229533.I1RKK4	pp	229533.Q4INI2
86373	229533.I1RKK4	pp	229533.V6RIS2
86374	229533.I1RKK4	pp	229533.I1RVN3
86375	229533.I1RKK4	pp	229533.I1RMW7
86376	229533.I1RKK4	pp	229533.Q4IB50
86377	229533.I1RKK4	pp	229533.I1RKL2
86378	229533.I1RNU6	pp	229533.I1RTD7
86379	229533.I1RNU6	pp	229533.I1RRI2
86380	229533.I1RNU6	pp	229533.I1RSR8
86381	229533.I1RNU6	pp	229533.I1RTD9
86382	229533.I1RNU6	pp	229533.I1RPJ3
86383	229533.I1RNU6	pp	229533.Q4IAA0
86384	229533.I1RNU6	pp	229533.Q4HZK7
86385	229533.I1RNU6	pp	229533.I1S060
86386	229533.I1RNU6	pp	229533.I1RQA2
86387	229533.I1RNU6	pp	229533.I1S2C0
86388	229533.I1RNU6	pp	229533.I1S558
86389	229533.I1RNU6	pp	229533.I1S0M9
86390	229533.I1RNU6	pp	229533.I1RTE9
86391	229533.I1RNU6	pp	229533.I1S296
86392	229533.I1RNU6	pp	229533.I1RSR3
86393	229533.I1RNU6	pp	229533.Q4IJ56
86394	229533.I1RNU6	pp	229533.Q4IEK8
86395	229533.I1RNU6	pp	229533.Q4HZ68
86396	229533.I1RNU6	pp	229533.I1RNY2
86397	229533.I1RNU6	pp	229533.I1S4T3
86398	229533.I1RNU6	pp	229533.V6RGY3
86399	229533.I1RNU6	pp	229533.I1RPY1
86400	229533.I1RNU6	pp	229533.Q4I327
86401	229533.I1RNU6	pp	229533.I1S1U7
86402	229533.I1RNU6	pp	229533.I1RRR7
86403	229533.I1RNU6	pp	229533.I1S274
86404	229533.I1RNU6	pp	229533.I1RQ31
86405	229533.I1RNU6	pp	229533.I1RZK6
86406	229533.I1RNU6	pp	229533.I1S021
86407	229533.I1RNU6	pp	229533.I1RZF6
86408	229533.I1RNU6	pp	229533.I1S2E8
86409	229533.I1RNU6	pp	229533.V6R227
86410	229533.I1RNU6	pp	229533.V6RGY0
86411	229533.I1RNU6	pp	229533.I1RSD8
86412	229533.I1RNU6	pp	229533.I1S9U1
86413	229533.I1RNU6	pp	229533.I1S2U2
86414	229533.I1RNU6	pp	229533.I1RWE8
86415	229533.I1RNU6	pp	229533.I1RX08
86416	229533.I1RNU6	pp	229533.I1RU69
86417	229533.I1RNU6	pp	229533.I1RQ28
86418	229533.I1RNU6	pp	229533.I1S1X9
86419	229533.I1RNU6	pp	229533.I1RSI6
86420	229533.I1RNU6	pp	229533.I1RSS4
86421	229533.I1RNU6	pp	229533.I1RQM6
86422	229533.I1RNU6	pp	229533.Q4IBS2
86423	229533.I1RNU6	pp	229533.I1RRK8
86424	229533.I1RNU6	pp	229533.I1RSC6
86425	229533.I1RNU6	pp	229533.Q4IF76
86426	229533.I1RNU6	pp	229533.I1RQR3
86427	229533.I1RNU6	pp	229533.Q4HVW2
86428	229533.I1RNU6	pp	229533.I1RZE5
86429	229533.I1RNU6	pp	229533.V6QUE6
86430	229533.I1RNU6	pp	229533.I1S253
86431	229533.I1RNU6	pp	229533.V6QWZ7
86432	229533.I1RNU6	pp	229533.I1S6U8

86433 229533.I1RNU6 pp 229533.V6RGN5
86434 229533.I1RNU6 pp 229533.I1S0J1
86435 229533.I1RNU6 pp 229533.I1RNV6
86436 229533.I1RNU6 pp 229533.I1RT80
86437 229533.I1RNU6 pp 229533.I1RX14
86438 229533.I1RNU6 pp 229533.I1S0M3
86439 229533.I1RNU6 pp 229533.I1RTF4
86440 229533.I1RNU6 pp 229533.I1RPH9
86441 229533.I1RNU6 pp 229533.I1RVZ0
86442 229533.I1RNU6 pp 229533.I1S9Y1
86443 229533.I1RNU6 pp 229533.I1RRT8
86444 229533.I1RNU6 pp 229533.Q4IJH1
86445 229533.I1RNU6 pp 229533.I1RPU0
86446 229533.I1RNU6 pp 229533.Q4INZ9
86447 229533.I1RNU6 pp 229533.I1RPW9
86448 229533.I1RNU6 pp 229533.I1S8E2
86449 229533.I1RNU6 pp 229533.I1RZJ6
86450 229533.I1RNU6 pp 229533.I1S9U2
86451 229533.I1RNU6 pp 229533.I1RT98
86452 229533.I1RNU6 pp 229533.I1RRJ8
86453 229533.I1RNU6 pp 229533.I1RWJ4
86454 229533.I1RNU6 pp 229533.I1RUQ2
86455 229533.I1RNU6 pp 229533.I1S2L2
86456 229533.I1RNU6 pp 229533.I1S0R7
86457 229533.I1RNU6 pp 229533.I1RW71
86458 229533.I1RNU6 pp 229533.I1RRT2
86459 229533.I1RNU6 pp 229533.Q4I662
86460 229533.I1RNU6 pp 229533.Q4I830
86461 229533.I1RNU6 pp 229533.I1RQS5
86462 229533.I1RNU6 pp 229533.I1S0M6
86463 229533.I1RNU6 pp 229533.I1RX24
86464 229533.I1RNU6 pp 229533.Q4IPI1
86465 229533.I1RNU6 pp 229533.I1RZS6
86466 229533.I1RNU6 pp 229533.I1RRQ3
86467 229533.I1RNU6 pp 229533.I1RR00
86468 229533.I1RNU6 pp 229533.V6QYF0
86469 229533.I1RNU6 pp 229533.I1S6F0
86470 229533.I1RNU6 pp 229533.Q4IAS1
86471 229533.I1RNU6 pp 229533.I1RS30
86472 229533.I1RNU6 pp 229533.Q4IQK7
86473 229533.I1RNU6 pp 229533.Q4IQW0
86474 229533.I1RNU6 pp 229533.V6R283
86475 229533.I1RNU6 pp 229533.Q4HZ47
86476 229533.I1RNU6 pp 229533.I1RWF7
86477 229533.I1RNU6 pp 229533.Q4IFI0
86478 229533.I1RNU6 pp 229533.I1RQ65
86479 229533.I1RNU6 pp 229533.I1RZ99
86480 229533.I1RNU6 pp 229533.I1RT33
86481 229533.I1RNU6 pp 229533.I1RZG3
86482 229533.I1RNU6 pp 229533.I1RQJ5
86483 229533.I1RNU6 pp 229533.Q4HZ42
86484 229533.I1S0Z3 pp 229533.Q4I2W2
86485 229533.I1S0Z3 pp 229533.Q4HY90
86486 229533.I1S0Z3 pp 229533.Q4I7S1
86487 229533.I1S0Z3 pp 229533.Q4IAK7
86488 229533.I1S0Z3 pp 229533.V6QUV7
86489 229533.I1S0Z3 pp 229533.Q4IEV4
86490 229533.I1S0Z3 pp 229533.I1S248
86491 229533.I1S0Z3 pp 229533.Q4IB96
86492 229533.I1S0Z3 pp 229533.V6RB81
86493 229533.I1S0Z3 pp 229533.I1S559
86494 229533.I1S0Z3 pp 229533.I1S247
86495 229533.I1S0Z3 pp 229533.Q4IL82
86496 229533.I1S0Z3 pp 229533.I1S7W2
86497 229533.I1S0Z3 pp 229533.Q4ILI9
86498 229533.I1S0Z3 pp 229533.I1S7X9
86499 229533.I1S0Z3 pp 229533.V6R476
86500 229533.I1S0Z3 pp 229533.Q4IPI4
86501 229533.I1S0Z3 pp 229533.Q4HTT1
86502 229533.I1S0Z3 pp 229533.Q4HTT2
86503 229533.I1S0Z3 pp 229533.Q4I5V3
86504 229533.I1S0Z3 pp 229533.Q4ICA8
86505 229533.I1S0Z3 pp 229533.I1S9S4

86506 229533.I1S0Z3 pp 229533.V6REE9
86507 229533.I1S0Z3 pp 229533.V6RR76
86508 229533.I1S0Z3 pp 229533.V6RPN0
86509 229533.I1S0Z3 pp 229533.I1S1B1
86510 229533.I1S0Z3 pp 229533.V6QUE6
86511 229533.I1S0Z3 pp 229533.Q4IJM4
86512 229533.I1S0Z3 pp 229533.Q4HZS8
86513 229533.I1S0Z3 pp 229533.Q4I963
86514 229533.I1S0Z3 pp 229533.Q4I948
86515 229533.I1S0Z3 pp 229533.Q4IER8
86516 229533.I1S0Z3 pp 229533.V6RTJ8
86517 229533.I1S0Z3 pp 229533.I1S270
86518 229533.I1S0Z3 pp 229533.I1S1V2
86519 229533.I1S0Z3 pp 229533.Q4ICM0
86520 229533.I1S0Z3 pp 229533.I1S287
86521 229533.I1S0Z3 pp 229533.Q5I2J3
86522 229533.I1S0Z3 pp 229533.I1S8E0
86523 229533.I1S0Z3 pp 229533.I1S2B7
86524 229533.I1S0Z3 pp 229533.Q4I2D7
86525 229533.I1S0Z3 pp 229533.Q4INI2
86526 229533.I1S0Z3 pp 229533.I1S1W5
86527 229533.I1S0Z3 pp 229533.I1S224
86528 229533.I1S0Z3 pp 229533.V6QUU6
86529 229533.I1RCM8 pp 229533.I1S927
86530 229533.I1RCM8 pp 229533.I1RK49
86531 229533.I1RCM8 pp 229533.V6RXE7
86532 229533.I1RCM8 pp 229533.I1RXX1
86533 229533.I1RCM8 pp 229533.I1RRC4
86534 229533.I1RCM8 pp 229533.I1RM11
86535 229533.I1RCM8 pp 229533.I1RQV7
86536 229533.I1RCM8 pp 229533.I1RED2
86537 229533.I1RCM8 pp 229533.I1RRN4
86538 229533.I1RCM8 pp 229533.I1REI2
86539 229533.I1RCM8 pp 229533.I1RSM8
86540 229533.I1RCM8 pp 229533.I1S1Z9
86541 229533.I1RCM8 pp 229533.I1RRG7
86542 229533.I1RCM8 pp 229533.I1RX67
86543 229533.I1RCM8 pp 229533.I1REC9
86544 229533.I1RCM8 pp 229533.I1RH75
86545 229533.I1RCM8 pp 229533.I1S8X0
86546 229533.I1RCM8 pp 229533.V6RPN0
86547 229533.I1RCM8 pp 229533.I1RMH4
86548 229533.I1RCM8 pp 229533.I1RPQ2
86549 229533.I1RCM8 pp 229533.I1RQ48
86550 229533.I1RCM8 pp 229533.I1RTM8
86551 229533.I1RCM8 pp 229533.I1RQM9
86552 229533.I1RCM8 pp 229533.I1RW82
86553 229533.I1RCM8 pp 229533.I1RR68
86554 229533.I1RCM8 pp 229533.I1RED3
86555 229533.I1RCM8 pp 229533.I1RTP2
86556 229533.I1RCM8 pp 229533.I1RZV5
86557 229533.I1RCM8 pp 229533.I1RUP5
86558 229533.I1RCM8 pp 229533.I1RHU8
86559 229533.I1RCM8 pp 229533.I1S2B0
86560 229533.I1RKE9 pp 229533.I1RW83
86561 229533.I1RKE9 pp 229533.I1RMQ7
86562 229533.I1RKE9 pp 229533.Q4INS6
86563 229533.I1RKE9 pp 229533.I1RSN7
86564 229533.I1RKE9 pp 229533.I1RWT6
86565 229533.I1RKE9 pp 229533.I1RX64
86566 229533.I1RKE9 pp 229533.I1RY44
86567 229533.I1RKE9 pp 229533.I1RSP7
86568 229533.I1RKE9 pp 229533.I1RZP8
86569 229533.I1RKE9 pp 229533.I1S2E9
86570 229533.I1RKE9 pp 229533.I1S951
86571 229533.I1RKE9 pp 229533.I1RY29
86572 229533.I1RKE9 pp 229533.Q4I7X1
86573 229533.I1RKE9 pp 229533.I1RV91
86574 229533.I1RKE9 pp 229533.I1S1V5
86575 229533.I1RKE9 pp 229533.I1RSR9
86576 229533.I1RKE9 pp 229533.I1RW24
86577 229533.I1RKE9 pp 229533.I1RPQ1
86578 229533.I1RKE9 pp 229533.I1RQA3

86579	229533.I1RKE9	pp	229533.Q4IJM4
86580	229533.I1RKE9	pp	229533.I1RQ13
86581	229533.I1RKE9	pp	229533.I1S382
86582	229533.I1S296	pp	229533.V6RGY3
86583	229533.I1S296	pp	229533.Q4HXF0
86584	229533.I1S296	pp	229533.I1S2C0
86585	229533.I1S296	pp	229533.O74638
86586	229533.I1S296	pp	229533.Q4IR18
86587	229533.I1S296	pp	229533.Q4HZ68
86588	229533.I1S296	pp	229533.Q4IAS1
86589	229533.I1S296	pp	229533.I1S4T3
86590	229533.I1S296	pp	229533.Q4IBS2
86591	229533.I1S296	pp	229533.Q4IQW0
86592	229533.I1S296	pp	229533.Q4I327
86593	229533.I1S296	pp	229533.V6R283
86594	229533.I1S296	pp	229533.Q4HZ42
86595	229533.I1S296	pp	229533.Q4IJ56
86596	229533.I1S296	pp	229533.I1S6U8
86597	229533.I1S296	pp	229533.Q4I830
86598	229533.I1S296	pp	229533.Q4HVW2
86599	229533.I1S296	pp	229533.V6QYF0
86600	229533.I1S296	pp	229533.Q4IJH1
86601	229533.I1S296	pp	229533.Q4IEK8
86602	229533.I1S296	pp	229533.I1S9U2
86603	229533.I1S296	pp	229533.V6R227
86604	229533.I1S296	pp	229533.Q4IQK7
86605	229533.I1S296	pp	229533.Q4IPI1
86606	229533.I1S296	pp	229533.Q4IFI0
86607	229533.I1RQ83	pp	229533.I1RW18
86608	229533.I1RQM9	pp	229533.I1RRG7
86609	229533.I1RQM9	pp	229533.I1RRN4
86610	229533.I1RQM9	pp	229533.I1RSU2
86611	229533.I1RQM9	pp	229533.V6RXE7
86612	229533.I1RQM9	pp	229533.I1RSM8
86613	229533.I1RQM9	pp	229533.I1RUP5
86614	229533.I1RQM9	pp	229533.I1RSX9
86615	229533.I1RQM9	pp	229533.I1RTP2
86616	229533.I1RQM9	pp	229533.I1S267
86617	229533.I1RQM9	pp	229533.I1RRC4
86618	229533.I1RY63	pp	229533.Q4IRI9
86619	229533.I1RY63	pp	229533.Q4IEK8
86620	229533.I1RY63	pp	229533.Q4IJ56
86621	229533.I1RY63	pp	229533.I1RZK6
86622	229533.I1RY63	pp	229533.I1S0F1
86623	229533.I1RY63	pp	229533.I1RZM8
86624	229533.I1RY63	pp	229533.I1RZF6
86625	229533.I1RY63	pp	229533.I1RZZ1
86626	229533.I1RY63	pp	229533.V6R227
86627	229533.I1RY63	pp	229533.V6QW22
86628	229533.I1RY63	pp	229533.I1S217
86629	229533.I1RY63	pp	229533.Q4HZ47
86630	229533.I1RY63	pp	229533.V6RBN2
86631	229533.I1RY63	pp	229533.I1S021
86632	229533.I1RY63	pp	229533.I1S0L3
86633	229533.I1RY63	pp	229533.I1RZB5
86634	229533.I1RY63	pp	229533.V6RCG5
86635	229533.I1RY63	pp	229533.V6QWZ7
86636	229533.I1RY63	pp	229533.V6QYF0
86637	229533.I1RY63	pp	229533.I1S060
86638	229533.I1RY63	pp	229533.V6RGN5
86639	229533.I1RY63	pp	229533.Q4I8X0
86640	229533.I1RY63	pp	229533.I1RYJ6
86641	229533.I1RY63	pp	229533.I1S0R7
86642	229533.I1RY63	pp	229533.I1S274
86643	229533.I1RY63	pp	229533.I1S2E8
86644	229533.I1RY63	pp	229533.I1S4T3
86645	229533.I1RY63	pp	229533.Q4I1M9
86646	229533.I1RY63	pp	229533.Q4IPI1
86647	229533.I1RY63	pp	229533.I1RZS6
86648	229533.I1RY63	pp	229533.I1S2C0
86649	229533.I1RY63	pp	229533.I1S5R1
86650	229533.I1RY63	pp	229533.V6RIV9
86651	229533.I1RY63	pp	229533.I1RZW8

86652 229533.I1RY63 pp 229533.I1S253
86653 229533.I1RY63 pp 229533.I1RZH6
86654 229533.I1RY63 pp 229533.I1RZB2
86655 229533.I1RY63 pp 229533.I1S558
86656 229533.I1RY63 pp 229533.Q4I327
86657 229533.I1RY63 pp 229533.V6R283
86658 229533.I1RW18 pp 229533.Q4HVW2
86659 229533.I1RE49 pp 229533.I1RST4
86660 229533.I1RE49 pp 229533.I1RWT2
86661 229533.I1RE49 pp 229533.I1RQP5
86662 229533.I1RE49 pp 229533.I1RT65
86663 229533.I1RE49 pp 229533.V6RQW4
86664 229533.I1RE49 pp 229533.Q4I963
86665 229533.I1RE49 pp 229533.I1RZQ6
86666 229533.I1RE49 pp 229533.I1RPH3
86667 229533.I1RE49 pp 229533.I1RQX3
86668 229533.I1RE49 pp 229533.I1RRF0
86669 229533.I1RE49 pp 229533.I1RQ70
86670 229533.I1RE49 pp 229533.I1S270
86671 229533.I1RE49 pp 229533.I1RYX4
86672 229533.I1RE49 pp 229533.I1RRB5
86673 229533.I1RE49 pp 229533.Q4INI2
86674 229533.I1RE49 pp 229533.Q4IPI4
86675 229533.I1RE49 pp 229533.I1S7W2
86676 229533.I1RE49 pp 229533.I1RT41
86677 229533.I1RE49 pp 229533.I1RZL3
86678 229533.I1RE49 pp 229533.I1RZM1
86679 229533.I1RE49 pp 229533.I1RZV1
86680 229533.I1RE49 pp 229533.I1RKI1
86681 229533.I1RE49 pp 229533.I1RKR9
86682 229533.I1RE49 pp 229533.I1S0Z3
86683 229533.A0A098DM81 pp 229533.I1RCM8
86684 229533.A0A098DM81 pp 229533.I1RQM9
86685 229533.A0A098DM81 pp 229533.A0A1C3YHY5
86686 229533.I1RYU4 pp 229533.Q4IB70
86687 229533.I1RYU4 pp 229533.Q4IAA0
86688 229533.I1RYU4 pp 229533.I1S471
86689 229533.I1RYU4 pp 229533.I1S246
86690 229533.I1RYU4 pp 229533.I1S0N7
86691 229533.A0A1C3YL10 pp 229533.I1S0F1
86692 229533.A0A1C3YL10 pp 229533.I1RT33
86693 229533.A0A1C3YL10 pp 229533.I1RT80
86694 229533.A0A1C3YL10 pp 229533.I1S4S4
86695 229533.A0A1C3YL10 pp 229533.I1RZF6
86696 229533.A0A1C3YL10 pp 229533.I1RB27
86697 229533.A0A1C3YL10 pp 229533.I1RK83
86698 229533.A0A1C3YL10 pp 229533.I1RQA2
86699 229533.A0A1C3YL10 pp 229533.Q4IPN9
86700 229533.A0A1C3YL10 pp 229533.I1RK81
86701 229533.A0A1C3YL10 pp 229533.I1RPE5
86702 229533.A0A1C3YL10 pp 229533.I1RD03
86703 229533.A0A1C3YL10 pp 229533.I1RA38
86704 229533.A0A1C3YL10 pp 229533.I1RRE0
86705 229533.A0A1C3YL10 pp 229533.I1RK95
86706 229533.A0A1C3YL10 pp 229533.I1REF2
86707 229533.A0A1C3YL10 pp 229533.A0A1C3YN36
86708 229533.A0A1C3YL10 pp 229533.I1RYB9
86709 229533.A0A1C3YL10 pp 229533.A0A1C3YN57
86710 229533.A0A1C3YL10 pp 229533.I1RAZ8
86711 229533.A0A1C3YL10 pp 229533.I1RRG3
86712 229533.A0A1C3YL10 pp 229533.I1RBC1
86713 229533.A0A1C3YL10 pp 229533.I1S6E5
86714 229533.A0A1C3YL10 pp 229533.Q4IJ56
86715 229533.A0A1C3YL10 pp 229533.I1S299
86716 229533.A0A1C3YL10 pp 229533.I1RA63
86717 229533.A0A1C3YL10 pp 229533.V6RM66
86718 229533.A0A1C3YL10 pp 229533.I1RCZ8
86719 229533.A0A1C3YL10 pp 229533.I1RBZ3
86720 229533.A0A1C3YL10 pp 229533.I1RPJ3
86721 229533.A0A1C3YL10 pp 229533.I1RR72
86722 229533.A0A1C3YL10 pp 229533.I1RC81
86723 229533.A0A1C3YL10 pp 229533.I1RSD8
86724 229533.A0A1C3YL10 pp 229533.I1RXF6

229533.A0A1C3YL10 pp 229533.I1S8R2
229533.A0A1C3YL10 pp 229533.I1RRQ3
229533.A0A1C3YL10 pp 229533.I1RW64
229533.A0A1C3YL10 pp 229533.I1S9Y1
229533.A0A1C3YL10 pp 229533.I1RFV3
229533.A0A1C3YL10 pp 229533.Q4ICI6
229533.A0A1C3YL10 pp 229533.I1RSS4
229533.A0A1C3YL10 pp 229533.I1RRV1
229533.A0A1C3YL10 pp 229533.Q4HWE2
229533.A0A1C3YL10 pp 229533.I1S5R6
229533.A0A1C3YL10 pp 229533.I1RD64
229533.A0A1C3YL10 pp 229533.Q4I1N3
229533.A0A1C3YL10 pp 229533.V6RLM7
229533.A0A1C3YL10 pp 229533.Q4I8X0
229533.A0A1C3YL10 pp 229533.I1RML0
229533.A0A1C3YL10 pp 229533.V6QT89
229533.I1RRM7 pp 229533.I1RZB2
229533.I1RFH9 pp 229533.I1RWF9
229533.I1RFH9 pp 229533.I1S5S2
229533.I1RFH9 pp 229533.I1RJU6
229533.I1RFH9 pp 229533.I1RPD6
229533.I1RFH9 pp 229533.I1RHY5
229533.I1RFH9 pp 229533.I1S0S5
229533.I1RFH9 pp 229533.I1RZ40
229533.I1RFH9 pp 229533.I1RXV3
229533.I1RFH9 pp 229533.I1RP97
229533.I1RFH9 pp 229533.I1RH91
229533.I1RFH9 pp 229533.V6RN69
229533.I1RFH9 pp 229533.I1RN77
229533.I1RFH9 pp 229533.O74638
229533.I1RFH9 pp 229533.I1RSM8
229533.I1RFH9 pp 229533.I1RZQ6
229533.I1RFH9 pp 229533.V6RUT5
229533.I1RFH9 pp 229533.I1S3V2
229533.I1RFH9 pp 229533.I1RM55
229533.I1RFH9 pp 229533.I1S1W3
229533.I1RFH9 pp 229533.I1RH75
229533.I1RFH9 pp 229533.I1RHV1
229533.I1RFH9 pp 229533.I1RMM8
229533.I1RFH9 pp 229533.I1RMF4
229533.I1RFH9 pp 229533.I1RMW7
229533.I1RFH9 pp 229533.I1RPQ2
229533.I1RFH9 pp 229533.I1RNE7
229533.I1RFH9 pp 229533.I1RRG1
229533.I1RFH9 pp 229533.I1RYI6
229533.I1RFH9 pp 229533.I1RMT0
229533.I1RFH9 pp 229533.I1RFJ1
229533.A0A098DM63 pp 229533.I1RT35
229533.A0A098DM63 pp 229533.I1RNB8
229533.A0A098DM63 pp 229533.I1RG90
229533.A0A1C3YJF1 pp 229533.I1RMP5
229533.A0A1C3YJF1 pp 229533.I1S439
229533.A0A1C3YJF1 pp 229533.I1RC95
229533.A0A1C3YJF1 pp 229533.I1RCI2
229533.A0A1C3YJF1 pp 229533.I1RB75
229533.A0A1C3YJF1 pp 229533.I1RZE4
229533.A0A1C3YJF1 pp 229533.I1RU37
229533.A0A1C3YJF1 pp 229533.I1RC17
229533.A0A1C3YJF1 pp 229533.I1RKK0
229533.A0A1C3YJF1 pp 229533.I1S2B4
229533.A0A1C3YJF1 pp 229533.I1RQA3
229533.A0A1C3YJF1 pp 229533.Q4IJM4
229533.A0A1C3YJF1 pp 229533.I1RGZ0
229533.A0A1C3YJF1 pp 229533.I1S0M2
229533.A0A1C3YJF1 pp 229533.V6RVG3
229533.A0A1C3YJF1 pp 229533.I1RG07
229533.A0A1C3YJF1 pp 229533.I1RIT1
229533.A0A1C3YJF1 pp 229533.I1RY44
229533.A0A1C3YJF1 pp 229533.V6RKY7
229533.A0A1C3YJF1 pp 229533.I1RGR8
229533.A0A1C3YJF1 pp 229533.I1RV91
229533.A0A1C3YJF1 pp 229533.I1S2E9
229533.A0A1C3YJF1 pp 229533.I1RWT6

86798	229533.A0A1C3YJF1	pp	229533.I1RBZ9
86799	229533.A0A1C3YJF1	pp	229533.I1S1W4
86800	229533.A0A1C3YJF1	pp	229533.I1RC97
86801	229533.A0A1C3YJF1	pp	229533.I1RQ26
86802	229533.A0A1C3YJF1	pp	229533.I1R9Y9
86803	229533.A0A1C3YJF1	pp	229533.I1RAM7
86804	229533.A0A1C3YJF1	pp	229533.I1RTQ0
86805	229533.A0A1C3YJF1	pp	229533.I1RB74
86806	229533.A0A1C3YJF1	pp	229533.I1RA43
86807	229533.A0A1C3YJF1	pp	229533.I1RNP7
86808	229533.A0A1C3YJF1	pp	229533.I1RRY3
86809	229533.A0A1C3YJF1	pp	229533.I1RN41
86810	229533.A0A1C3YJF1	pp	229533.I1RMM9
86811	229533.A0A1C3YJF1	pp	229533.I1RWY3
86812	229533.A0A1C3YJF1	pp	229533.I1RUQ5
86813	229533.A0A1C3YJF1	pp	229533.I1RYZ7
86814	229533.A0A1C3YJF1	pp	229533.I1RC22
86815	229533.A0A1C3YJF1	pp	229533.I1RNF9
86816	229533.A0A1C3YJF1	pp	229533.I1RAN2
86817	229533.A0A1C3YJF1	pp	229533.I1RZ63
86818	229533.A0A1C3YJF1	pp	229533.I1RDB1
86819	229533.A0A1C3YJF1	pp	229533.I1RDZ2
86820	229533.A0A1C3YJF1	pp	229533.I1RN08
86821	229533.A0A1C3YJF1	pp	229533.I1RX70
86822	229533.A0A1C3YJF1	pp	229533.A0A1C3YJW1
86823	229533.A0A1C3YJF1	pp	229533.I1RYH9
86824	229533.A0A1C3YJF1	pp	229533.I1RK75
86825	229533.A0A1C3YJF1	pp	229533.I1RUN8
86826	229533.A0A1C3YJF1	pp	229533.I1RZX9
86827	229533.I1RT41	pp	229533.I1RWX5
86828	229533.I1RT41	pp	229533.I1RW84
86829	229533.I1RT41	pp	229533.I1RUN2
86830	229533.I1RT41	pp	229533.I1S086
86831	229533.I1RT41	pp	229533.I1RW32
86832	229533.I1RT41	pp	229533.I1S247
86833	229533.I1RT41	pp	229533.I1S0N5
86834	229533.I1RT41	pp	229533.I1RYW3
86835	229533.I1RT41	pp	229533.V6R476
86836	229533.I1RT41	pp	229533.V6RN31
86837	229533.I1RT41	pp	229533.I1S248
86838	229533.I1RT41	pp	229533.I1RTG5
86839	229533.I1RT41	pp	229533.Q4I2W2
86840	229533.I1RT41	pp	229533.Q4HY90
86841	229533.I1RT41	pp	229533.I1RZL3
86842	229533.I1RT41	pp	229533.I1RTE0
86843	229533.I1RT41	pp	229533.V6RGY0
86844	229533.I1RT41	pp	229533.I1RZ91
86845	229533.I1RT41	pp	229533.Q4I5V3
86846	229533.I1RT41	pp	229533.Q4IPI4
86847	229533.I1RT41	pp	229533.I1RWM7
86848	229533.I1RT41	pp	229533.I1RZ84
86849	229533.I1RT41	pp	229533.I1S7W2
86850	229533.I1RT41	pp	229533.Q4IBU4
86851	229533.I1RT41	pp	229533.Q4IL82
86852	229533.I1RT41	pp	229533.I1RXS3
86853	229533.I1RT41	pp	229533.Q4IEV4
86854	229533.I1RT41	pp	229533.V6QW25
86855	229533.I1RT41	pp	229533.Q4IER8
86856	229533.I1RT41	pp	229533.I1S7X9
86857	229533.I1RT41	pp	229533.I1S1B1
86858	229533.I1RT41	pp	229533.I1RYL5
86859	229533.I1RT41	pp	229533.V6REE9
86860	229533.I1RT41	pp	229533.Q4I650
86861	229533.I1RT41	pp	229533.Q4HTT2
86862	229533.I1RT41	pp	229533.V6QUU6
86863	229533.I1RT41	pp	229533.Q4IJM4
86864	229533.I1RT41	pp	229533.Q4ILI9
86865	229533.I1RT41	pp	229533.I1S1W5
86866	229533.I1RT41	pp	229533.I1RWI6
86867	229533.I1RT41	pp	229533.I1S0V5
86868	229533.I1RT41	pp	229533.V6QUE6
86869	229533.I1RT41	pp	229533.Q4I2D7
86870	229533.I1RT41	pp	229533.V6RPN0

86871 229533.I1RT41 pp 229533.I1S039
86872 229533.I1RT41 pp 229533.I1S788
86873 229533.I1RT41 pp 229533.Q4ICM0
86874 229533.I1RT41 pp 229533.I1S270
86875 229533.I1RT41 pp 229533.Q4HZS8
86876 229533.I1RT41 pp 229533.Q4HYZ2
86877 229533.I1RT41 pp 229533.I1RX09
86878 229533.I1RT41 pp 229533.I1S2B7
86879 229533.I1RT41 pp 229533.I1S1V2
86880 229533.I1RT41 pp 229533.I1RZH4
86881 229533.I1RT41 pp 229533.I1S034
86882 229533.I1RT41 pp 229533.I1RTH6
86883 229533.I1RT41 pp 229533.I1RZV5
86884 229533.I1RT41 pp 229533.V6QUV7
86885 229533.I1RT41 pp 229533.I1RVY0
86886 229533.I1RT41 pp 229533.Q4I7S1
86887 229533.I1RT41 pp 229533.I1S0S8
86888 229533.I1RT41 pp 229533.V6QV09
86889 229533.I1RT41 pp 229533.I1RUJ0
86890 229533.I1RT41 pp 229533.I1RZ60
86891 229533.I1RT41 pp 229533.I1RX69
86892 229533.I1RT41 pp 229533.I1RZV1
86893 229533.I1RT41 pp 229533.I1RT81
86894 229533.I1RT41 pp 229533.I1RYM4
86895 229533.I1RT41 pp 229533.I1S559
86896 229533.I1RT41 pp 229533.I1RZD6
86897 229533.I1RT41 pp 229533.I1RUP5
86898 229533.I1RT41 pp 229533.Q4IMD1
86899 229533.I1RT41 pp 229533.Q4IAK7
86900 229533.I1RT41 pp 229533.I1S077
86901 229533.I1RT41 pp 229533.I1RWG6
86902 229533.I1RT41 pp 229533.Q00909
86903 229533.I1RT41 pp 229533.I1RX68
86904 229533.I1RT41 pp 229533.Q4HTT1
86905 229533.I1RT41 pp 229533.I1S9S4
86906 229533.I1RT41 pp 229533.I1S2D3
86907 229533.I1RT41 pp 229533.Q4IB96
86908 229533.I1RT41 pp 229533.I1RWA9
86909 229533.I1RT41 pp 229533.V6RB81
86910 229533.I1RT41 pp 229533.P0C431
86911 229533.I1RT41 pp 229533.V6RR76
86912 229533.I1RT41 pp 229533.Q4I948
86913 229533.I1RT41 pp 229533.Q4ICA8
86914 229533.I1RT41 pp 229533.Q4I963
86915 229533.I1RT41 pp 229533.I1S287
86916 229533.I1RT41 pp 229533.I1S0X0
86917 229533.I1RT41 pp 229533.Q4INI2
86918 229533.I1RT41 pp 229533.I1RTQ9
86919 229533.I1RT41 pp 229533.V6RTJ8
86920 229533.I1RT41 pp 229533.I1S8E0
86921 229533.I1RT41 pp 229533.I1S224
86922 229533.I1RT41 pp 229533.I1RZ02
86923 229533.I1RT41 pp 229533.I1RT72
86924 229533.I1RT41 pp 229533.I1RW71
86925 229533.I1RT41 pp 229533.I1S0S6
86926 229533.I1RT41 pp 229533.Q5I2J3
86927 229533.I1RYZ4 pp 229533.I1S444
86928 229533.A0A1C3YLK9 pp 229533.I1RQM6
86929 229533.A0A1C3YLK9 pp 229533.I1RNH9
86930 229533.A0A1C3YLK9 pp 229533.I1RMK3
86931 229533.A0A1C3YLK9 pp 229533.I1RDA0
86932 229533.A0A1C3YLK9 pp 229533.I1RTF4
86933 229533.A0A1C3YLK9 pp 229533.I1RT80
86934 229533.A0A1C3YLK9 pp 229533.I1RCL3
86935 229533.A0A1C3YLK9 pp 229533.I1RM67
86936 229533.A0A1C3YLK9 pp 229533.I1RKJ6
86937 229533.A0A1C3YLK9 pp 229533.I1RPU0
86938 229533.A0A1C3YLK9 pp 229533.I1RD64
86939 229533.A0A1C3YLK9 pp 229533.I1S9U2
86940 229533.A0A1C3YLK9 pp 229533.I1RG51
86941 229533.A0A1C3YLK9 pp 229533.I1RSD8
86942 229533.A0A1C3YLK9 pp 229533.I1RQ28
86943 229533.A0A1C3YLK9 pp 229533.I1RMQ0

86944 229533.A0A1C3YLK9 pp 229533.I1RZS6
86945 229533.A0A1C3YLK9 pp 229533.I1RFJ9
86946 229533.A0A1C3YLK9 pp 229533.I1RKC6
86947 229533.A0A1C3YLK9 pp 229533.I1RN99
86948 229533.A0A1C3YLK9 pp 229533.I1RB71
86949 229533.A0A1C3YLK9 pp 229533.I1RSS4
86950 229533.A0A1C3YLK9 pp 229533.I1RC03
86951 229533.A0A1C3YLK9 pp 229533.I1RSC6
86952 229533.A0A1C3YLK9 pp 229533.I1RB27
86953 229533.A0A1C3YLK9 pp 229533.I1R9X7
86954 229533.A0A1C3YLK9 pp 229533.I1RRJ8
86955 229533.A0A1C3YLK9 pp 229533.I1RVZ0
86956 229533.A0A1C3YLK9 pp 229533.I1RNA0
86957 229533.A0A1C3YLK9 pp 229533.I1RNH6
86958 229533.A0A1C3YLK9 pp 229533.I1RBG9
86959 229533.A0A1C3YLK9 pp 229533.I1RA48
86960 229533.A0A1C3YLK9 pp 229533.I1R9V7
86961 229533.A0A1C3YLK9 pp 229533.I1RNU6
86962 229533.A0A1C3YLK9 pp 229533.I1RAF8
86963 229533.A0A1C3YLK9 pp 229533.I1S0M9
86964 229533.A0A1C3YLK9 pp 229533.I1RW71
86965 229533.A0A1C3YLK9 pp 229533.I1RQ31
86966 229533.A0A1C3YLK9 pp 229533.I1RUQ2
86967 229533.A0A1C3YLK9 pp 229533.I1RM96
86968 229533.A0A1C3YLK9 pp 229533.A0A1C3YN63
86969 229533.A0A1C3YLK9 pp 229533.I1RG56
86970 229533.A0A1C3YLK9 pp 229533.I1RPY1
86971 229533.A0A1C3YLK9 pp 229533.I1RSI6
86972 229533.A0A1C3YLK9 pp 229533.A0A1C3YMW0
86973 229533.A0A1C3YLK9 pp 229533.I1RS30
86974 229533.A0A1C3YLK9 pp 229533.I1RRR7
86975 229533.A0A1C3YLK9 pp 229533.I1RMN6
86976 229533.A0A1C3YLK9 pp 229533.I1RKQ5
86977 229533.A0A1C3YLK9 pp 229533.I1RQ65
86978 229533.A0A1C3YLK9 pp 229533.I1RFN1
86979 229533.A0A1C3YLK9 pp 229533.I1RLV9
86980 229533.A0A1C3YLK9 pp 229533.A0A1C3YLL3
86981 229533.A0A1C3YLK9 pp 229533.I1RT33
86982 229533.A0A1C3YLK9 pp 229533.I1RC72
86983 229533.A0A1C3YLK9 pp 229533.I1RQ80
86984 229533.A0A098DUW2 pp 229533.I1RQK2
86985 229533.A0A098DUW2 pp 229533.I1RLU5
86986 229533.A0A098DUW2 pp 229533.I1RIT3
86987 229533.A0A098DUW2 pp 229533.I1S246
86988 229533.A0A098DUW2 pp 229533.I1RGC9
86989 229533.A0A098DUW2 pp 229533.A0A1C3YJW0
86990 229533.A0A098DUW2 pp 229533.I1RL60
86991 229533.A0A098DUW2 pp 229533.I1RYJ9
86992 229533.A0A098DUW2 pp 229533.I1RS75
86993 229533.A0A098DUW2 pp 229533.I1RE00
86994 229533.A0A098DUW2 pp 229533.I1RFQ0
86995 229533.A0A098DUW2 pp 229533.I1RKK4
86996 229533.A0A098DUW2 pp 229533.A0A1C3YL25
86997 229533.A0A098DUW2 pp 229533.I1RK19
86998 229533.A0A098DUW2 pp 229533.I1RE31
86999 229533.A0A098DUW2 pp 229533.A0A0E0RZU7
87000 229533.A0A098DUW2 pp 229533.I1RTS2
87001 229533.A0A098DUW2 pp 229533.Q4IAA0
87002 229533.A0A098DUW2 pp 229533.I1RYU4
87003 229533.A0A098DUW2 pp 229533.I1S0N7
87004 229533.A0A098DUW2 pp 229533.I1RQ28
87005 229533.A0A098DUW2 pp 229533.I1RMH5
87006 229533.A0A098DUW2 pp 229533.I1S471
87007 229533.A0A098DUW2 pp 229533.I1RM49
87008 229533.A0A098DUW2 pp 229533.I1RBZ8
87009 229533.A0A098DUW2 pp 229533.A0A1C3YK80
87010 229533.A0A098DUW2 pp 229533.A0A1C3YM64
87011 229533.I1RN81 pp 229533.I1RZD9
87012 229533.I1RN81 pp 229533.I1RQ32
87013 229533.I1RN81 pp 229533.I1RRN0
87014 229533.I1RN81 pp 229533.I1RVN1
87015 229533.I1RN81 pp 229533.V6R476
87016 229533.I1RN81 pp 229533.I1RX68

229533.I1RN81 pp 229533.I1RYZ7
229533.I1RN81 pp 229533.I1RR95
229533.I1RN81 pp 229533.I1RQN9
229533.I1RN81 pp 229533.I1S7W2
229533.I1RN81 pp 229533.I1RZV5
229533.I1RN81 pp 229533.V6RB81
229533.I1RN81 pp 229533.I1RPU7
229533.I1RN81 pp 229533.I1S559
229533.I1RN81 pp 229533.I1RNF4
229533.I1RN81 pp 229533.I1RTG5
229533.I1RN81 pp 229533.I1RW76
229533.I1RN81 pp 229533.I1RRW2
229533.I1RN81 pp 229533.I1RQ72
229533.I1RN81 pp 229533.I1RZ84
229533.I1RN81 pp 229533.I1S7X9
229533.I1RN81 pp 229533.I1RW84
229533.I1RN81 pp 229533.Q4HZS8
229533.I1RN81 pp 229533.I1RWM7
229533.I1RN81 pp 229533.I1RX69
229533.I1RN81 pp 229533.I1RTE0
229533.I1RN81 pp 229533.Q4INI2
229533.I1RN81 pp 229533.Q4HZE0
229533.I1RN81 pp 229533.I1RQA9
229533.I1RN81 pp 229533.V6QUU6
229533.I1RN81 pp 229533.I1RPP0
229533.I1RN81 pp 229533.I1RWZ9
229533.I1RN81 pp 229533.I1RY98
229533.I1RN81 pp 229533.I1S270
229533.I1RN81 pp 229533.I1S2C1
229533.I1RN81 pp 229533.I1S1V3
229533.I1RN81 pp 229533.Q5I2J3
229533.I1RN81 pp 229533.I1RQ70
229533.I1RN81 pp 229533.I1RYH6
229533.I1RN81 pp 229533.I1RT41
229533.I1RN81 pp 229533.I1RTB7
229533.I1RN81 pp 229533.I1S254
229533.I1RN81 pp 229533.I1RZX9
229533.I1RN81 pp 229533.I1RPA6
229533.I1RN81 pp 229533.I1RWL6
229533.I1RN81 pp 229533.I1S0S6
229533.I1RN81 pp 229533.I1S034
229533.I1RN81 pp 229533.I1RYW8
229533.I1RN81 pp 229533.I1S095
229533.I1RN81 pp 229533.I1S096
229533.I1RN81 pp 229533.I1RQM8
229533.I1RN81 pp 229533.I1RW38
229533.I1RN81 pp 229533.I1RNF9
229533.I1RN81 pp 229533.I1RS38
229533.I1RN81 pp 229533.Q4HYZ2
229533.I1RN81 pp 229533.I1RRF9
229533.I1RN81 pp 229533.I1S4X2
229533.I1RN81 pp 229533.I1RQP7
229533.I1RN81 pp 229533.I1RWI6
229533.I1RN81 pp 229533.I1S2B7
229533.I1RN81 pp 229533.V6REE9
229533.I1RN81 pp 229533.I1RQP5
229533.I1RN81 pp 229533.I1S0N8
229533.I1RN81 pp 229533.Q4ILI9
229533.I1RN81 pp 229533.Q4I963
229533.I1RN81 pp 229533.I1RSD7
229533.I1RN81 pp 229533.I1S0X0
229533.I1RN81 pp 229533.I1S1B1
229533.I1RN81 pp 229533.P0C431
229533.I1RN81 pp 229533.I1RWA5
229533.I1RN81 pp 229533.I1RWA9
229533.I1RN81 pp 229533.I1S0Z3
229533.I1RN81 pp 229533.I1RRI4
229533.I1RZX9 pp 229533.P0C431
229533.I1RZX9 pp 229533.V6QUU6
229533.I1RZX9 pp 229533.I1S0S6
229533.I1RZX9 pp 229533.Q4I061
229533.I1RZX9 pp 229533.V6RR76
229533.I1RZX9 pp 229533.V6R476

87090 229533.I1RL92 pp 229533.I1S2R5
87091 229533.I1RFH8 pp 229533.I1RW99
87092 229533.I1RFH8 pp 229533.I1RFL4
87093 229533.I1RFH8 pp 229533.I1RRT2
87094 229533.I1RFH8 pp 229533.I1RN18
87095 229533.I1RFH8 pp 229533.I1RMN6
87096 229533.I1RFH8 pp 229533.I1RJW6
87097 229533.I1RFH8 pp 229533.I1RSI6
87098 229533.I1RFH8 pp 229533.V6RCG5
87099 229533.I1RFH8 pp 229533.I1RNH6
87100 229533.I1RFH8 pp 229533.I1RUQ2
87101 229533.I1RFH8 pp 229533.I1RZM8
87102 229533.I1RFH8 pp 229533.I1RT40
87103 229533.I1RFH8 pp 229533.I1RR24
87104 229533.I1RFH8 pp 229533.I1RQ31
87105 229533.I1RFH8 pp 229533.I1S5R1
87106 229533.I1RFH8 pp 229533.I1S0L3
87107 229533.I1RFH8 pp 229533.I1RRT8
87108 229533.I1RFH8 pp 229533.I1RS47
87109 229533.I1RFH8 pp 229533.V6RIV9
87110 229533.I1RFH8 pp 229533.I1RUG3
87111 229533.I1RFH8 pp 229533.I1RVQ0
87112 229533.I1RFH8 pp 229533.I1RRJ8
87113 229533.I1RFH8 pp 229533.I1RMK3
87114 229533.I1RFH8 pp 229533.I1RWJ8
87115 229533.I1RFH8 pp 229533.V6QW22
87116 229533.I1RFH8 pp 229533.I1RS17
87117 229533.I1RFH8 pp 229533.I1RSQ7
87118 229533.I1RFH8 pp 229533.I1RW46
87119 229533.I1RFH8 pp 229533.I1RTF4
87120 229533.I1RFH8 pp 229533.I1RSR3
87121 229533.I1RFH8 pp 229533.I1RW71
87122 229533.I1RFH8 pp 229533.I1RZZ9
87123 229533.I1RFH8 pp 229533.I1RW25
87124 229533.I1RFH8 pp 229533.I1RZW8
87125 229533.I1RFH8 pp 229533.I1RRI2
87126 229533.I1RFH8 pp 229533.I1RWK3
87127 229533.I1RFH8 pp 229533.I1RRM5
87128 229533.I1RFH8 pp 229533.I1RZS6
87129 229533.I1RFH8 pp 229533.I1RQE7
87130 229533.I1RFH8 pp 229533.I1RN63
87131 229533.I1RFH8 pp 229533.I1S0L4
87132 229533.I1RFH8 pp 229533.I1RNA0
87133 229533.I1RFH8 pp 229533.I1RW61
87134 229533.I1RFH8 pp 229533.V6QYF0
87135 229533.I1RFH8 pp 229533.I1RPM4
87136 229533.A0A1C3YIJ4 pp 229533.I1RCA5
87137 229533.A0A1C3YIJ4 pp 229533.I1R976
87138 229533.A0A1C3YIJ4 pp 229533.I1RHF4
87139 229533.A0A1C3YIJ4 pp 229533.I1RZ94
87140 229533.A0A1C3YIJ4 pp 229533.I1RQ13
87141 229533.A0A1C3YIJ4 pp 229533.I1S2G7
87142 229533.A0A1C3YIJ4 pp 229533.I1RDG8
87143 229533.A0A1C3YIJ4 pp 229533.I1S382
87144 229533.A0A1C3YIJ4 pp 229533.I1RBW0
87145 229533.A0A1C3YIJ4 pp 229533.I1RV91
87146 229533.A0A1C3YIJ4 pp 229533.A0A1C3YKJ6
87147 229533.A0A1C3YIJ4 pp 229533.I1S2E9
87148 229533.A0A1C3YIJ4 pp 229533.I1RY29
87149 229533.A0A1C3YIJ4 pp 229533.I1RVZ6
87150 229533.A0A1C3YIJ4 pp 229533.I1RPX5
87151 229533.A0A1C3YIJ4 pp 229533.I1RFU3
87152 229533.A0A1C3YIJ4 pp 229533.I1RX70
87153 229533.A0A1C3YIJ4 pp 229533.I1R9Q0
87154 229533.A0A1C3YIJ4 pp 229533.I1S1V5
87155 229533.A0A1C3YIJ4 pp 229533.A0A1C3YJH8
87156 229533.A0A1C3YIJ4 pp 229533.I1RPQ1
87157 229533.A0A1C3YIJ4 pp 229533.I1RQA3
87158 229533.A0A1C3YIJ4 pp 229533.I1RA10
87159 229533.A0A1C3YIJ4 pp 229533.I1RMQ7
87160 229533.A0A1C3YIJ4 pp 229533.A0A1C3YLS8
87161 229533.A0A1C3YIJ4 pp 229533.I1S951
87162 229533.A0A1C3YIJ4 pp 229533.A0A1C3YN69

229533.A0A1C3YIJ4 pp 229533.I1RN55
229533.A0A1C3YIJ4 pp 229533.I1RN57
229533.A0A1C3YIJ4 pp 229533.I1RG77
229533.A0A1C3YIJ4 pp 229533.A0A1C3YMF9
229533.A0A1C3YIJ4 pp 229533.I1RA56
229533.A0A1C3YIJ4 pp 229533.I1RRN5
229533.A0A1C3YIJ4 pp 229533.I1RB41
229533.A0A1C3YIJ4 pp 229533.A0A1C3YM29
229533.A0A1C3YIJ4 pp 229533.I1RBW1
229533.A0A1C3YIJ4 pp 229533.I1RW83
229533.A0A1C3YIJ4 pp 229533.A0A1C3YL52
229533.A0A1C3YIJ4 pp 229533.I1RFZ3
229533.A0A1C3YIJ4 pp 229533.I1RAW7
229533.A0A1C3YIJ4 pp 229533.A0A1C3YJT5
229533.A0A1C3YIJ4 pp 229533.I1RKE9
229533.A0A1C3YIJ4 pp 229533.I1S091
229533.A0A1C3YIJ4 pp 229533.I1RRQ4
229533.A0A1C3YIJ4 pp 229533.A0A1C3YK00
229533.A0A1C3YIJ4 pp 229533.I1RY44
229533.A0A1C3YIJ4 pp 229533.I1RFL7
229533.A0A1C3YIJ4 pp 229533.I1RSP7
229533.A0A1C3YIJ4 pp 229533.I1RA12
229533.A0A1C3YIJ4 pp 229533.I1RWT6
229533.A0A1C3YIJ4 pp 229533.I1RWX5
229533.A0A1C3YIJ4 pp 229533.I1RCB3
229533.A0A1C3YIJ4 pp 229533.I1RBN9
229533.A0A1C3YIJ4 pp 229533.Q4I7X1
229533.A0A1C3YIJ4 pp 229533.I1RZP8
229533.A0A1C3YIJ4 pp 229533.I1S0N8
229533.A0A1C3YIJ4 pp 229533.A0A1C3YJ12
229533.A0A1C3YIJ4 pp 229533.I1RSR9
229533.A0A1C3YIJ4 pp 229533.I1RC17
229533.I1S558 pp 229533.Q4IQW0
229533.I1S558 pp 229533.Q4IPI1
229533.I1S558 pp 229533.Q4IAS1
229533.I1S558 pp 229533.I1S6F0
229533.I1S558 pp 229533.V6QYF0
229533.I1S558 pp 229533.Q4IEV5
229533.I1S558 pp 229533.Q4IFI0
229533.I1S558 pp 229533.V6R283
229533.I1S558 pp 229533.Q4I5M4
229533.I1S558 pp 229533.Q4IAA0
229533.I1S558 pp 229533.Q4IBS2
229533.I1S558 pp 229533.Q4I8X0
229533.I1S558 pp 229533.I1S9U2
229533.I1S558 pp 229533.Q4I662
229533.I1S558 pp 229533.V6QUE6
229533.I1S558 pp 229533.Q4IJH1
229533.I1S558 pp 229533.V6RGY3
229533.I1S558 pp 229533.Q4IJ56
229533.I1S558 pp 229533.Q4HVW2
229533.I1S558 pp 229533.V6R227
229533.I1S558 pp 229533.Q4IF76
229533.I1S558 pp 229533.Q4I327
229533.I1S558 pp 229533.Q4HZK7
229533.I1S558 pp 229533.Q4I1M9
229533.I1S558 pp 229533.V6QWZ7
229533.I1S558 pp 229533.Q4HZ68
229533.I1S558 pp 229533.Q4IEK8
229533.I1SAJ0 pp 229533.V6QU99
229533.I1SAJ0 pp 229533.V6R0W1
229533.I1SAJ0 pp 229533.V6R2Z2
229533.I1SAJ0 pp 229533.V6R057
229533.I1SAJ0 pp 229533.V6RAM6
229533.I1SAJ0 pp 229533.V6R1K4
229533.I1SAJ0 pp 229533.Q4HXF0
229533.I1SAJ0 pp 229533.V6R756
229533.I1SAJ0 pp 229533.V6R5D2
229533.I1RNF9 pp 229533.P0C431
229533.I1RNF9 pp 229533.Q4I061
229533.I1RNF9 pp 229533.I1RSV0
229533.I1RNF9 pp 229533.I1RZM1
229533.I1RNF9 pp 229533.V6RR76

87236	229533.I1RNF9	pp	229533.I1RYZ7
87237	229533.I1RNF9	pp	229533.I1RWJ6
87238	229533.I1RNF9	pp	229533.V6R476
87239	229533.I1RNF9	pp	229533.I1S0S6
87240	229533.I1RNF9	pp	229533.I1RNV6
87241	229533.I1RNF9	pp	229533.I1RTB7
87242	229533.I1RNF9	pp	229533.I1RZD9
87243	229533.I1RNF9	pp	229533.I1RY40
87244	229533.I1RNF9	pp	229533.I1RS10
87245	229533.I1RNF9	pp	229533.I1RQN9
87246	229533.I1RNF9	pp	229533.I1S0L8
87247	229533.A0A1C3YIB7	pp	229533.I1S1W5
87248	229533.A0A1C3YIB7	pp	229533.I1RYN9
87249	229533.A0A1C3YIB7	pp	229533.A0A1C3YII1
87250	229533.A0A1C3YIB7	pp	229533.I1RUN8
87251	229533.V6QU63	pp	229533.V6RRR4
87252	229533.I1RD73	pp	229533.I1RDC0
87253	229533.I1RD73	pp	229533.Q4INS6
87254	229533.I1RD73	pp	229533.I1S523
87255	229533.I1RD73	pp	229533.I1RZ98
87256	229533.I1RD73	pp	229533.I1RFJ2
87257	229533.I1RS10	pp	229533.I1RZD9
87258	229533.I1RS10	pp	229533.Q4IJ79
87259	229533.I1RS10	pp	229533.I1RVU9
87260	229533.I1RS10	pp	229533.I1S0E4
87261	229533.I1RS10	pp	229533.I1RZV4
87262	229533.I1RS10	pp	229533.I1S095
87263	229533.I1RS10	pp	229533.I1RZQ6
87264	229533.I1RS10	pp	229533.I1RW78
87265	229533.I1RS10	pp	229533.I1RYZ7
87266	229533.I1RS10	pp	229533.I1RSW5
87267	229533.I1RS10	pp	229533.Q4IQC1
87268	229533.I1RS10	pp	229533.I1RZX9
87269	229533.I1RSL0	pp	229533.Q4HWE2
87270	229533.I1RSL0	pp	229533.I1RW82
87271	229533.I1RSL0	pp	229533.Q4IQX3
87272	229533.I1RR31	pp	229533.I1RW71
87273	229533.I1RR31	pp	229533.I1RR32
87274	229533.A0A1C3YMD9	pp	229533.V6RR76
87275	229533.A0A1C3YMD9	pp	229533.I1RRQ0
87276	229533.A0A1C3YMD9	pp	229533.I1RPK5
87277	229533.A0A1C3YMD9	pp	229533.P0C431
87278	229533.A0A1C3YMD9	pp	229533.I1RX09
87279	229533.A0A1C3YMD9	pp	229533.I1RFV6
87280	229533.A0A1C3YMD9	pp	229533.I1RCJ5
87281	229533.A0A1C3YMD9	pp	229533.I1S1B1
87282	229533.A0A1C3YMD9	pp	229533.V6REE9
87283	229533.A0A1C3YMD9	pp	229533.I1RWL6
87284	229533.A0A1C3YMD9	pp	229533.I1RWC9
87285	229533.A0A1C3YMD9	pp	229533.I1S2B7
87286	229533.A0A1C3YMD9	pp	229533.I1S5K9
87287	229533.A0A1C3YMD9	pp	229533.I1RN72
87288	229533.A0A1C3YMD9	pp	229533.I1S0Z7
87289	229533.A0A1C3YMD9	pp	229533.I1RYW8
87290	229533.A0A1C3YMD9	pp	229533.I1RCK9
87291	229533.A0A1C3YMD9	pp	229533.I1RS10
87292	229533.A0A1C3YMD9	pp	229533.I1S1V3
87293	229533.A0A1C3YMD9	pp	229533.I1RNG8
87294	229533.A0A1C3YMD9	pp	229533.I1RA34
87295	229533.A0A1C3YMD9	pp	229533.I1RZX9
87296	229533.A0A1C3YMD9	pp	229533.I1RZQ6
87297	229533.A0A1C3YMD9	pp	229533.I1RN81
87298	229533.A0A1C3YMD9	pp	229533.I1RE19
87299	229533.A0A1C3YMD9	pp	229533.I1RMR5
87300	229533.A0A1C3YMD9	pp	229533.I1RPS0
87301	229533.A0A1C3YMD9	pp	229533.I1RRC2
87302	229533.A0A1C3YMD9	pp	229533.I1S0L8
87303	229533.A0A1C3YMD9	pp	229533.I1RQN9
87304	229533.A0A1C3YMD9	pp	229533.I1RD69
87305	229533.A0A1C3YMD9	pp	229533.I1S7X9
87306	229533.A0A1C3YMD9	pp	229533.I1RWT5
87307	229533.A0A1C3YMD9	pp	229533.I1RRG8
87308	229533.A0A1C3YMD9	pp	229533.Q4I2M7

87309 229533.A0A1C3YMD9 pp 229533.I1RD10
87310 229533.A0A1C3YMD9 pp 229533.I1RN36
87311 229533.A0A1C3YMD9 pp 229533.I1RQM0
87312 229533.A0A1C3YMD9 pp 229533.V6RB81
87313 229533.A0A1C3YMD9 pp 229533.I1RC46
87314 229533.A0A1C3YMD9 pp 229533.I1RMP2
87315 229533.A0A1C3YMD9 pp 229533.I1RCD4
87316 229533.A0A1C3YMD9 pp 229533.I1RRX1
87317 229533.A0A1C3YMD9 pp 229533.A0A1C3YMQ5
87318 229533.I1RCN5 pp 229533.I1S1C6
87319 229533.I1RCN5 pp 229533.Q4IPI1
87320 229533.I1RCN5 pp 229533.I1RPU0
87321 229533.I1RCN5 pp 229533.I1RX46
87322 229533.I1RCN5 pp 229533.I1RM67
87323 229533.I1RCN5 pp 229533.I1S9U2
87324 229533.I1RCN5 pp 229533.I1RPW9
87325 229533.I1RCN5 pp 229533.I1RX14
87326 229533.I1RCN5 pp 229533.I1RPZ3
87327 229533.I1RCN5 pp 229533.I1S246
87328 229533.I1RCN5 pp 229533.I1RZG7
87329 229533.I1RCN5 pp 229533.I1RG51
87330 229533.I1RCN5 pp 229533.I1RCQ6
87331 229533.I1RCN5 pp 229533.I1RK72
87332 229533.I1RCN5 pp 229533.I1S0M9
87333 229533.I1RCN5 pp 229533.I1RWH5
87334 229533.I1RCN5 pp 229533.I1RPY5
87335 229533.I1RCN5 pp 229533.V6QYF0
87336 229533.I1RCN5 pp 229533.I1RFW5
87337 229533.I1RCN5 pp 229533.Q4IJ56
87338 229533.I1RCN5 pp 229533.I1S2Y5
87339 229533.I1RCN5 pp 229533.I1RNA0
87340 229533.I1RCN5 pp 229533.I1RX81
87341 229533.I1RCN5 pp 229533.Q4IRI9
87342 229533.I1RCN5 pp 229533.I1RS75
87343 229533.I1RCN5 pp 229533.I1RM06
87344 229533.I1RCN5 pp 229533.I1RW71
87345 229533.I1RCN5 pp 229533.Q4IFI0
87346 229533.I1RCN5 pp 229533.I1RZ99
87347 229533.I1RCN5 pp 229533.Q4IAA0
87348 229533.I1RCN5 pp 229533.I1RQJ5
87349 229533.I1RCN5 pp 229533.I1RQC5
87350 229533.I1RCN5 pp 229533.Q4IEK8
87351 229533.I1RCN5 pp 229533.I1RRH5
87352 229533.I1RCN5 pp 229533.Q4ICG5
87353 229533.I1RCN5 pp 229533.V6R227
87354 229533.I1RCN5 pp 229533.I1RZF6
87355 229533.I1RCN5 pp 229533.I1RN59
87356 229533.I1RCN5 pp 229533.I1RNV6
87357 229533.I1RCN5 pp 229533.I1RT98
87358 229533.I1RCN5 pp 229533.I1RZJ6
87359 229533.I1RCN5 pp 229533.I1RQ31
87360 229533.I1RCN5 pp 229533.I1RT80
87361 229533.I1RCN5 pp 229533.Q4IJH1
87362 229533.I1RCN5 pp 229533.Q4IE79
87363 229533.I1RCN5 pp 229533.I1S7M2
87364 229533.I1RCN5 pp 229533.I1RTF4
87365 229533.I1RCN5 pp 229533.I1RJZ6
87366 229533.I1RCN5 pp 229533.I1RKQ5
87367 229533.I1RCN5 pp 229533.I1S021
87368 229533.I1RCN5 pp 229533.Q4I7F9
87369 229533.I1RCN5 pp 229533.I1S7M3
87370 229533.I1RCN5 pp 229533.I1RNH6
87371 229533.I1RCN5 pp 229533.Q4HVW2
87372 229533.I1RCN5 pp 229533.I1RDA0
87373 229533.I1RCN5 pp 229533.I1RSC6
87374 229533.I1RCN5 pp 229533.I1RZK6
87375 229533.I1RCN5 pp 229533.I1S9L0
87376 229533.I1RCN5 pp 229533.I1RK23
87377 229533.I1RCN5 pp 229533.I1RK95
87378 229533.I1RCN5 pp 229533.I1RGC2
87379 229533.I1RCN5 pp 229533.I1RDG0
87380 229533.I1RCN5 pp 229533.Q4IBK5
87381 229533.I1RCN5 pp 229533.I1RRL0

87382	229533.I1RCN5	pp	229533.Q4IBS2
87383	229533.I1RCN5	pp	229533.I1RN99
87384	229533.I1RCN5	pp	229533.I1RQ28
87385	229533.I1RCN5	pp	229533.I1S060
87386	229533.I1RCN5	pp	229533.V6R283
87387	229533.I1RCN5	pp	229533.I1RVZ0
87388	229533.I1RCN5	pp	229533.I1S0J1
87389	229533.I1RCN5	pp	229533.I1RNK2
87390	229533.I1RCN5	pp	229533.I1RCS2
87391	229533.I1RCN5	pp	229533.I1RFJ9
87392	229533.I1RCN5	pp	229533.I1S4M5
87393	229533.I1RCN5	pp	229533.I1RKL3
87394	229533.I1RCN5	pp	229533.I1RFP9
87395	229533.I1RCN5	pp	229533.I1RSD4
87396	229533.I1RCN5	pp	229533.I1RS28
87397	229533.I1RCN5	pp	229533.I1RF08
87398	229533.I1RCN5	pp	229533.I1RFQ1
87399	229533.I1RCN5	pp	229533.I1RWU2
87400	229533.I1RCN5	pp	229533.I1RSR8
87401	229533.I1RCN5	pp	229533.I1RNB8
87402	229533.I1RCN5	pp	229533.I1RK83
87403	229533.I1RCN5	pp	229533.Q4I1Y1
87404	229533.I1RCN5	pp	229533.Q4I830
87405	229533.I1RCN5	pp	229533.I1RNU6
87406	229533.I1RCN5	pp	229533.I1RWB5
87407	229533.I1RCN5	pp	229533.I1RRJ8
87408	229533.I1RCN5	pp	229533.I1RQA2
87409	229533.I1RCN5	pp	229533.I1RRQ3
87410	229533.I1RCN5	pp	229533.Q4IB70
87411	229533.I1RCN5	pp	229533.I1RHM4
87412	229533.I1RCN5	pp	229533.I1RFG0
87413	229533.I1RCN5	pp	229533.I1RPA0
87414	229533.I1RCN5	pp	229533.I1RX51
87415	229533.I1RCN5	pp	229533.I1RME9
87416	229533.I1RCN5	pp	229533.I1RPY1
87417	229533.I1RCN5	pp	229533.Q4HZ42
87418	229533.I1RCN5	pp	229533.V6QXG1
87419	229533.I1RCN5	pp	229533.I1RSR3
87420	229533.I1RCN5	pp	229533.I1RJL4
87421	229533.I1RCN5	pp	229533.I1S4T3
87422	229533.I1RCN5	pp	229533.I1RLV9
87423	229533.I1RCN5	pp	229533.I1RSA4
87424	229533.I1RCN5	pp	229533.I1RS47
87425	229533.I1RCN5	pp	229533.I1RFN1
87426	229533.I1RCN5	pp	229533.I1S9Y0
87427	229533.I1RCN5	pp	229533.I1RQ65
87428	229533.I1RCN5	pp	229533.Q4IEV5
87429	229533.I1RCN5	pp	229533.I1RM96
87430	229533.I1RCN5	pp	229533.I1RND5
87431	229533.I1RCN5	pp	229533.I1S1B6
87432	229533.I1RCN5	pp	229533.Q4IPB8
87433	229533.I1RCN5	pp	229533.I1RFS3
87434	229533.I1RCN5	pp	229533.I1RXF2
87435	229533.I1RCN5	pp	229533.I1RT17
87436	229533.I1RCN5	pp	229533.I1RWE1
87437	229533.I1RCN5	pp	229533.Q4IAS1
87438	229533.I1RCN5	pp	229533.Q4I662
87439	229533.I1RCN5	pp	229533.Q4IN63
87440	229533.I1RCN5	pp	229533.I1RRR7
87441	229533.I1RCN5	pp	229533.I1RK96
87442	229533.I1RCN5	pp	229533.I1RPY4
87443	229533.I1RCN5	pp	229533.I1S1X9
87444	229533.I1RCN5	pp	229533.I1RX24
87445	229533.I1RCN5	pp	229533.I1RG90
87446	229533.I1RCN5	pp	229533.I1RYJ9
87447	229533.I1RCN5	pp	229533.I1RX47
87448	229533.I1RCN5	pp	229533.I1RZS6
87449	229533.I1RCN5	pp	229533.I1RJZ7
87450	229533.I1RCN5	pp	229533.I1S0B1
87451	229533.I1RCN5	pp	229533.Q4I327
87452	229533.I1RCN5	pp	229533.I1RMV5
87453	229533.I1RCN5	pp	229533.I1RSI6
87454	229533.I1RCN5	pp	229533.I1RML0

87455	229533.I1RCN5	pp	229533.V6R042
87456	229533.I1RCN5	pp	229533.Q4HZ68
87457	229533.I1RCN5	pp	229533.Q4IF76
87458	229533.I1RCN5	pp	229533.I1RSW2
87459	229533.I1RCN5	pp	229533.I1S296
87460	229533.I1RCN5	pp	229533.I1RMN6
87461	229533.I1RCN5	pp	229533.I1RT47
87462	229533.I1RCN5	pp	229533.I1S8E2
87463	229533.I1RCN5	pp	229533.I1RD22
87464	229533.I1RCN5	pp	229533.I1RK90
87465	229533.I1RCN5	pp	229533.I1RND6
87466	229533.I1RCN5	pp	229533.I1S056
87467	229533.I1RCN5	pp	229533.I1RRI2
87468	229533.I1RCN5	pp	229533.I1S471
87469	229533.I1RCN5	pp	229533.I1S2C0
87470	229533.I1RCN5	pp	229533.I1RR73
87471	229533.I1RCN5	pp	229533.I1RKJ6
87472	229533.I1RCN5	pp	229533.V6RGN5
87473	229533.I1RCN5	pp	229533.I1RG57
87474	229533.I1RCN5	pp	229533.I1RTE9
87475	229533.I1RCN5	pp	229533.I1RZV6
87476	229533.I1RCN5	pp	229533.I1RSD8
87477	229533.I1RCN5	pp	229533.I1S274
87478	229533.I1RCN5	pp	229533.I1RD64
87479	229533.I1RCN5	pp	229533.I1RST1
87480	229533.I1RCN5	pp	229533.I1S2E8
87481	229533.I1RCN5	pp	229533.V6RGY3
87482	229533.I1RCN5	pp	229533.I1RK92
87483	229533.I1RCN5	pp	229533.I1RPL3
87484	229533.I1S095	pp	229533.I1S0L8
87485	229533.I1S095	pp	229533.I1S7X9
87486	229533.I1S095	pp	229533.V6RB81
87487	229533.I1S095	pp	229533.Q4I2M7
87488	229533.I1S095	pp	229533.I1S2B7
87489	229533.I1S095	pp	229533.I1S1V3
87490	229533.I1S095	pp	229533.I1S1B1
87491	229533.I1S095	pp	229533.I1S5K9
87492	229533.I1S095	pp	229533.I1S0Z7
87493	229533.I1S095	pp	229533.P0C431
87494	229533.I1S095	pp	229533.V6REE9
87495	229533.I1S095	pp	229533.V6RR76
87496	229533.A0A098DUV8	pp	229533.I1RYU5
87497	229533.A0A098DUV8	pp	229533.V6R1K4
87498	229533.A0A098DUV8	pp	229533.A0A1C3YN30
87499	229533.A0A098DUV8	pp	229533.Q4HXF0
87500	229533.A0A098DUV8	pp	229533.I1SAJ0
87501	229533.A0A098DUV8	pp	229533.V6R057
87502	229533.A0A098DUV8	pp	229533.I1RZ20
87503	229533.A0A098DUV8	pp	229533.A0A098E0A5
87504	229533.A0A098DUV8	pp	229533.I1RCB4
87505	229533.A0A098DUV8	pp	229533.I1RPU8
87506	229533.A0A098DUV8	pp	229533.I1RS17
87507	229533.I1RPI2	pp	229533.I1RYS6
87508	229533.I1RPI2	pp	229533.I1S247
87509	229533.I1RPI2	pp	229533.V6RGI1
87510	229533.I1RPI2	pp	229533.I1RT62
87511	229533.I1RPI2	pp	229533.I1RZJ1
87512	229533.I1RF48	pp	229533.I1RNU7
87513	229533.I1RF48	pp	229533.I1RPU8
87514	229533.I1RF48	pp	229533.I1RGY2
87515	229533.I1RF48	pp	229533.I1RPT0
87516	229533.I1RZV4	pp	229533.V6REE9
87517	229533.I1RZV4	pp	229533.I1S1V3
87518	229533.I1RZV4	pp	229533.I1S5K9
87519	229533.I1RZV4	pp	229533.I1S1B1
87520	229533.I1RZV4	pp	229533.I1S7X9
87521	229533.I1RZV4	pp	229533.V6RB81
87522	229533.I1RZV4	pp	229533.I1S0L8
87523	229533.I1RZV4	pp	229533.I1S2B7
87524	229533.I1RZV4	pp	229533.Q4I2M7
87525	229533.I1RZV4	pp	229533.I1S0Z7
87526	229533.I1RFU9	pp	229533.I1RJD9
87527	229533.V6RK40	pp	229533.V6RRR4

87528	229533.I1RWJ6	pp	229533.I1RZE4
87529	229533.I1RWJ6	pp	229533.Q4IJM4
87530	229533.I1RWJ6	pp	229533.I1S0M2
87531	229533.I1RWJ6	pp	229533.I1RZ63
87532	229533.I1RWJ6	pp	229533.I1RX70
87533	229533.I1RWJ6	pp	229533.I1RYH9
87534	229533.I1RWJ6	pp	229533.I1S1W4
87535	229533.I1RWJ6	pp	229533.I1RYZ7
87536	229533.I1RWJ6	pp	229533.I1RZX9
87537	229533.I1RWJ6	pp	229533.I1RWT6
87538	229533.I1RWJ6	pp	229533.I1RY44
87539	229533.I1RWJ6	pp	229533.I1S2E9
87540	229533.I1RWJ6	pp	229533.V6RKY7
87541	229533.I1RWJ6	pp	229533.I1S439
87542	229533.I1RWJ6	pp	229533.V6RVG3
87543	229533.A0A098E454	pp	229533.A0A0E0SJV3
87544	229533.A0A098E454	pp	229533.I1RB17
87545	229533.A0A098E454	pp	229533.A0A1C3YIU2
87546	229533.A0A098E454	pp	229533.I1RWH6
87547	229533.A0A098E454	pp	229533.I1RFN2
87548	229533.A0A098E454	pp	229533.I1R9V4
87549	229533.A0A098E454	pp	229533.A0A098E488
87550	229533.A0A098E454	pp	229533.A0A1C3YJK8
87551	229533.A0A098E454	pp	229533.A0A1C3YI56
87552	229533.A0A098E454	pp	229533.A0A1C3YMH5
87553	229533.I1RYI7	pp	229533.I1RYW6
87554	229533.I1RYI7	pp	229533.I1SAJ3
87555	229533.I1RYI7	pp	229533.I1RZ52
87556	229533.I1RYI7	pp	229533.I1RZQ1
87557	229533.I1RYI7	pp	229533.I1RZ78
87558	229533.I1RYI7	pp	229533.I1S2E8
87559	229533.I1RYI7	pp	229533.V6RG79
87560	229533.I1RYI7	pp	229533.I1RZ29
87561	229533.I1RYI7	pp	229533.V6QVH8
87562	229533.I1RYI7	pp	229533.V6RSF3
87563	229533.I1RYI7	pp	229533.I1S3K6
87564	229533.I1RYI7	pp	229533.I1RZ20
87565	229533.I1RYI7	pp	229533.O74638
87566	229533.I1RYI7	pp	229533.Q4I5V2
87567	229533.I1RYI7	pp	229533.I1S4Q4
87568	229533.I1RYI7	pp	229533.I1S0N3
87569	229533.I1RYI7	pp	229533.I1RYU0
87570	229533.I1RYI7	pp	229533.I1RZZ1
87571	229533.I1RYI7	pp	229533.Q4HXF0
87572	229533.I1RYI7	pp	229533.V6RQW4
87573	229533.I1RYI7	pp	229533.V6QUE6
87574	229533.I1RYI7	pp	229533.I1RZM4
87575	229533.I1RYI7	pp	229533.V6RK96
87576	229533.I1RYI7	pp	229533.I1RYU5
87577	229533.I1RYI7	pp	229533.V6RBN2
87578	229533.I1RYI7	pp	229533.I1S835
87579	229533.I1RYI7	pp	229533.I1RYX6
87580	229533.I1RYI7	pp	229533.I1RYJ7
87581	229533.I1RYI7	pp	229533.I1S338
87582	229533.I1RFX2	pp	229533.I1RNG4
87583	229533.A0A1C3YL54	pp	229533.I1RK95
87584	229533.A0A1C3YL54	pp	229533.Q4IQC1
87585	229533.A0A1C3YL54	pp	229533.I1RDU0
87586	229533.A0A1C3YL54	pp	229533.I1RCM5
87587	229533.A0A1C3YL54	pp	229533.Q4HYR2
87588	229533.A0A1C3YL54	pp	229533.I1RZK5
87589	229533.A0A1C3YL54	pp	229533.I1RZZ1
87590	229533.A0A1C3YL54	pp	229533.A0A1C3YM97
87591	229533.A0A1C3YL54	pp	229533.I1RCG4
87592	229533.A0A1C3YL54	pp	229533.I1RFU8
87593	229533.A0A1C3YL54	pp	229533.Q4IJK2
87594	229533.A0A1C3YL54	pp	229533.I1RGB2
87595	229533.A0A1C3YL54	pp	229533.I1RPT0
87596	229533.A0A1C3YL54	pp	229533.I1RNQ5
87597	229533.A0A1C3YL54	pp	229533.I1RY03
87598	229533.A0A1C3YL54	pp	229533.I1RBE4
87599	229533.A0A1C3YL54	pp	229533.I1RPP5
87600	229533.A0A1C3YL54	pp	229533.I1RN46

87601 229533.A0A1C3YL54 pp 229533.I1S8S9
87602 229533.A0A1C3YL54 pp 229533.V6RCZ8
87603 229533.A0A1C3YL54 pp 229533.I1RI82
87604 229533.A0A1C3YL54 pp 229533.I1RZM8
87605 229533.A0A1C3YL54 pp 229533.I1RRY9
87606 229533.A0A1C3YL54 pp 229533.I1S439
87607 229533.A0A1C3YL54 pp 229533.I1REK8
87608 229533.A0A1C3YL54 pp 229533.I1RBL7
87609 229533.A0A1C3YL54 pp 229533.I1RBE7
87610 229533.A0A1C3YL54 pp 229533.I1RCR5
87611 229533.A0A1C3YL54 pp 229533.V6RPS5
87612 229533.A0A1C3YL54 pp 229533.I1RY12
87613 229533.A0A1C3YL54 pp 229533.I1RFD4
87614 229533.A0A1C3YL54 pp 229533.I1RW99
87615 229533.A0A1C3YL54 pp 229533.A0A1C3YM86
87616 229533.A0A1C3YL54 pp 229533.I1RUY4
87617 229533.A0A1C3YL54 pp 229533.A0A1C3YLC2
87618 229533.A0A1C3YL54 pp 229533.I1RF19
87619 229533.A0A1C3YL54 pp 229533.I1RJY7
87620 229533.A0A1C3YL54 pp 229533.I1RBC9
87621 229533.A0A1C3YL54 pp 229533.I1RUY5
87622 229533.A0A1C3YL54 pp 229533.I1S5B0
87623 229533.A0A1C3YL54 pp 229533.Q4I8P5
87624 229533.A0A1C3YL54 pp 229533.I1RH82
87625 229533.A0A1C3YL54 pp 229533.I1RS18
87626 229533.A0A1C3YL54 pp 229533.I1RRG3
87627 229533.A0A1C3YL54 pp 229533.I1S0L3
87628 229533.A0A1C3YL54 pp 229533.A0A1C3YMC0
87629 229533.A0A1C3YL54 pp 229533.V6QUE6
87630 229533.A0A1C3YL54 pp 229533.I1RC95
87631 229533.A0A1C3YL54 pp 229533.I1RYX6
87632 229533.A0A1C3YL54 pp 229533.I1RR44
87633 229533.A0A1C3YL54 pp 229533.I1REE2
87634 229533.A0A1C3YL54 pp 229533.I1RR12
87635 229533.A0A1C3YL54 pp 229533.I1SA32
87636 229533.A0A1C3YL54 pp 229533.I1RTG4
87637 229533.A0A1C3YL54 pp 229533.I1S1W8
87638 229533.A0A1C3YL54 pp 229533.I1RD52
87639 229533.A0A1C3YL54 pp 229533.I1S8C1
87640 229533.A0A1C3YL54 pp 229533.I1S504
87641 229533.A0A1C3YL54 pp 229533.I1RB92
87642 229533.A0A1C3YL54 pp 229533.I1RXD7
87643 229533.A0A1C3YL54 pp 229533.I1RDU5
87644 229533.A0A1C3YL54 pp 229533.I1RVX0
87645 229533.A0A1C3YL54 pp 229533.I1RT03
87646 229533.A0A1C3YL54 pp 229533.V6RUT5
87647 229533.A0A1C3YL54 pp 229533.I1RQE1
87648 229533.A0A1C3YL54 pp 229533.I1RC11
87649 229533.A0A1C3YL54 pp 229533.I1RW25
87650 229533.A0A1C3YL54 pp 229533.I1RQE7
87651 229533.A0A1C3YL54 pp 229533.I1RCH5
87652 229533.A0A1C3YL54 pp 229533.I1RB07
87653 229533.A0A1C3YL54 pp 229533.I1RZ70
87654 229533.A0A1C3YL54 pp 229533.I1S0R7
87655 229533.A0A1C3YL54 pp 229533.I1RP86
87656 229533.A0A1C3YL54 pp 229533.I1RJU3
87657 229533.A0A1C3YL54 pp 229533.I1RJN7
87658 229533.A0A1C3YL54 pp 229533.I1RFL4
87659 229533.A0A1C3YL54 pp 229533.I1RAU7
87660 229533.A0A1C3YL54 pp 229533.A0A1C3YN26
87661 229533.A0A1C3YL54 pp 229533.Q4IR09
87662 229533.A0A1C3YL54 pp 229533.I1RBT1
87663 229533.A0A1C3YL54 pp 229533.I1RKB5
87664 229533.A0A1C3YL54 pp 229533.I1S149
87665 229533.A0A1C3YL54 pp 229533.I1RD17
87666 229533.A0A1C3YL54 pp 229533.I1RPI5
87667 229533.A0A1C3YL54 pp 229533.V6RTJ8
87668 229533.A0A1C3YL54 pp 229533.I1RR24
87669 229533.A0A1C3YL54 pp 229533.Q4I624
87670 229533.A0A1C3YL54 pp 229533.I1RCU4
87671 229533.A0A1C3YL54 pp 229533.V6RH92
87672 229533.A0A1C3YL54 pp 229533.I1RWJ8
87673 229533.A0A1C3YL54 pp 229533.I1RAX6

87674	229533.A0A1C3YL54	pp	229533.I1RGY2
87675	229533.A0A1C3YL54	pp	229533.I1RX46
87676	229533.A0A1C3YL54	pp	229533.I1RNU7
87677	229533.A0A1C3YL54	pp	229533.I1RSQ7
87678	229533.A0A1C3YL54	pp	229533.I1S338
87679	229533.A0A1C3YL54	pp	229533.I1RGL3
87680	229533.A0A1C3YL54	pp	229533.I1RPS2
87681	229533.A0A1C3YL54	pp	229533.I1RCR8
87682	229533.A0A1C3YL54	pp	229533.I1RH92
87683	229533.A0A1C3YL54	pp	229533.I1RQI4
87684	229533.A0A1C3YL54	pp	229533.I1S8R3
87685	229533.A0A1C3YL54	pp	229533.V6RLM7
87686	229533.A0A1C3YL54	pp	229533.I1RUG3
87687	229533.A0A1C3YL54	pp	229533.I1RTH6
87688	229533.A0A1C3YL54	pp	229533.I1RA70
87689	229533.A0A1C3YL54	pp	229533.I1RRI2
87690	229533.A0A1C3YL54	pp	229533.I1RPK0
87691	229533.A0A1C3YL54	pp	229533.I1RNC1
87692	229533.A0A1C3YL54	pp	229533.I1RCG5
87693	229533.A0A1C3YL54	pp	229533.A0A1C3YLB6
87694	229533.A0A1C3YL54	pp	229533.I1S0J1
87695	229533.A0A1C3YL54	pp	229533.I1RFF3
87696	229533.A0A1C3YL54	pp	229533.I1RYW3
87697	229533.A0A1C3YL54	pp	229533.I1RE01
87698	229533.A0A1C3YL54	pp	229533.I1RTG6
87699	229533.A0A1C3YL54	pp	229533.I1RW46
87700	229533.A0A1C3YL54	pp	229533.I1RD22
87701	229533.A0A1C3YL54	pp	229533.I1S256
87702	229533.A0A1C3YL54	pp	229533.I1S1C7
87703	229533.A0A1C3YL54	pp	229533.I1RAW0
87704	229533.A0A1C3YL54	pp	229533.I1RRZ6
87705	229533.A0A1C3YL54	pp	229533.I1S147
87706	229533.A0A1C3YL54	pp	229533.I1RGX1
87707	229533.A0A1C3YL54	pp	229533.I1RWN6
87708	229533.A0A1C3YL54	pp	229533.I1RPZ5
87709	229533.A0A1C3YL54	pp	229533.I1RJV3
87710	229533.A0A1C3YL54	pp	229533.V6RVG3
87711	229533.A0A1C3YL54	pp	229533.I1R9M0
87712	229533.A0A1C3YL54	pp	229533.I1S1W3
87713	229533.A0A1C3YL54	pp	229533.I1RUR8
87714	229533.A0A1C3YL54	pp	229533.I1SA33
87715	229533.A0A1C3YL54	pp	229533.I1RSU2
87716	229533.A0A1C3YL54	pp	229533.I1RC33
87717	229533.A0A1C3YL54	pp	229533.V6R3X5
87718	229533.A0A1C3YL54	pp	229533.V6RIV9
87719	229533.A0A1C3YL54	pp	229533.I1RWN5
87720	229533.A0A1C3YL54	pp	229533.I1RC29
87721	229533.A0A1C3YL54	pp	229533.I1RRT3
87722	229533.A0A1C3YL54	pp	229533.I1RE22
87723	229533.A0A1C3YL54	pp	229533.I1RTG7
87724	229533.A0A1C3YL54	pp	229533.I1RH91
87725	229533.A0A1C3YL54	pp	229533.I1RFS3
87726	229533.A0A1C3YL54	pp	229533.I1RGZ3
87727	229533.A0A1C3YL54	pp	229533.V6RMW6
87728	229533.A0A1C3YL54	pp	229533.I1RZ39
87729	229533.A0A1C3YL54	pp	229533.I1RPM4
87730	229533.A0A1C3YL54	pp	229533.V6R042
87731	229533.A0A1C3YL54	pp	229533.I1RC66
87732	229533.A0A1C3YL54	pp	229533.I1RN88
87733	229533.A0A1C3YL54	pp	229533.I1RSQ3
87734	229533.A0A1C3YL54	pp	229533.I1RSQ4
87735	229533.A0A1C3YL54	pp	229533.I1RN84
87736	229533.A0A1C3YL54	pp	229533.I1RWN7
87737	229533.A0A1C3YL54	pp	229533.I1RGC3
87738	229533.A0A1C3YL54	pp	229533.I1RZZ9
87739	229533.A0A1C3YL54	pp	229533.I1RVZ3
87740	229533.A0A1C3YL54	pp	229533.I1RRV1
87741	229533.A0A1C3YL54	pp	229533.I1RBS6
87742	229533.A0A1C3YL54	pp	229533.I1S0L4
87743	229533.A0A1C3YL54	pp	229533.V6R3J1
87744	229533.A0A1C3YL54	pp	229533.I1RFI4
87745	229533.A0A1C3YL54	pp	229533.I1S2H4
87746	229533.A0A1C3YL54	pp	229533.I1RBY4

87747 229533.A0A1C3YL54 pp 229533.I1RRM5
87748 229533.A0A1C3YL54 pp 229533.I1S2B8
87749 229533.A0A1C3YL54 pp 229533.I1S3G4
87750 229533.A0A1C3YL54 pp 229533.I1S148
87751 229533.A0A1C3YL54 pp 229533.I1RBY9
87752 229533.A0A1C3YL54 pp 229533.I1RKB2
87753 229533.A0A1C3YL54 pp 229533.I1RA62
87754 229533.A0A1C3YL54 pp 229533.I1RA11
87755 229533.A0A1C3YL54 pp 229533.I1RX72
87756 229533.A0A1C3YL54 pp 229533.I1S503
87757 229533.A0A1C3YL54 pp 229533.I1S5R1
87758 229533.A0A1C3YL54 pp 229533.I1RJW6
87759 229533.A0A1C3YL54 pp 229533.A0A1C3YLK8
87760 229533.A0A1C3YL54 pp 229533.I1RRZ0
87761 229533.A0A1C3YL54 pp 229533.I1RRT8
87762 229533.A0A1C3YL54 pp 229533.I1RWK3
87763 229533.A0A1C3YL54 pp 229533.I1RC31
87764 229533.A0A1C3YL54 pp 229533.I1RY62
87765 229533.A0A1C3YL54 pp 229533.I1RBS0
87766 229533.A0A1C3YL54 pp 229533.I1S9Y1
87767 229533.A0A1C3YL54 pp 229533.I1S0F6
87768 229533.A0A1C3YL54 pp 229533.I1RPP3
87769 229533.A0A1C3YL54 pp 229533.I1RYK0
87770 229533.A0A1C3YL54 pp 229533.I1RN67
87771 229533.A0A1C3YL54 pp 229533.I1RNG4
87772 229533.A0A1C3YL54 pp 229533.I1RFT1
87773 229533.A0A1C3YL54 pp 229533.I1RT04
87774 229533.A0A1C3YL54 pp 229533.A0A1C3YLE2
87775 229533.A0A1C3YL54 pp 229533.I1RMJ3
87776 229533.A0A1C3YL54 pp 229533.I1RP97
87777 229533.A0A1C3YL54 pp 229533.I1RTD5
87778 229533.A0A1C3YL54 pp 229533.I1RR68
87779 229533.A0A1C3YL54 pp 229533.I1RWF5
87780 229533.A0A1C3YL54 pp 229533.I1RT40
87781 229533.A0A1C3YL54 pp 229533.I1RJV4
87782 229533.A0A1C3YL54 pp 229533.I1RV91
87783 229533.A0A1C3YL54 pp 229533.V6RV57
87784 229533.A0A1C3YL54 pp 229533.I1RBA6
87785 229533.A0A1C3YL54 pp 229533.I1RGA4
87786 229533.I1RSB3 pp 229533.I1S0P5
87787 229533.I1RSB3 pp 229533.I1S0Z1
87788 229533.I1RSB3 pp 229533.I1S969
87789 229533.A0A098D2X2 pp 229533.I1RW71
87790 229533.A0A098D2X2 pp 229533.I1RJZ7
87791 229533.I1RLU7 pp 229533.I1RRS2
87792 229533.I1RPP7 pp 229533.I1RQL4
87793 229533.I1RPP7 pp 229533.Q4IR08
87794 229533.I1RPP7 pp 229533.I1RWL9
87795 229533.I1RPP7 pp 229533.I1RRF9
87796 229533.I1RPP7 pp 229533.V6REE9
87797 229533.I1RPP7 pp 229533.Q4I5R9
87798 229533.I1RPP7 pp 229533.I1RYN2
87799 229533.I1RPP7 pp 229533.I1RX09
87800 229533.I1RPP7 pp 229533.I1S7X9
87801 229533.I1RPP7 pp 229533.I1RYH6
87802 229533.I1RPP7 pp 229533.Q4I7K4
87803 229533.I1RPP7 pp 229533.I1RWD3
87804 229533.I1RPP7 pp 229533.I1RTE6
87805 229533.I1RPP7 pp 229533.I1S1B3
87806 229533.I1S208 pp 229533.I1S969
87807 229533.I1S208 pp 229533.I1S209
87808 229533.I1RCZ3 pp 229533.I1RPI3
87809 229533.I1RCZ3 pp 229533.I1RK81
87810 229533.I1RCZ3 pp 229533.I1S7A2
87811 229533.I1RCZ3 pp 229533.I1S274
87812 229533.I1RCZ3 pp 229533.I1REK8
87813 229533.I1RCZ3 pp 229533.V6R223
87814 229533.I1RCZ3 pp 229533.I1S4Q4
87815 229533.I1RFG0 pp 229533.Q4HXF6
87816 229533.I1RFG0 pp 229533.I1S0N5
87817 229533.I1RFG0 pp 229533.Q4HZ68
87818 229533.I1RFG0 pp 229533.I1RSD8
87819 229533.I1RFG0 pp 229533.I1RRR7

87820	229533.I1RFG0	pp	229533.I1RKC6
87821	229533.I1RFG0	pp	229533.V6RY33
87822	229533.I1RFG0	pp	229533.I1RYJ9
87823	229533.I1RFG0	pp	229533.I1RPL3
87824	229533.I1RFG0	pp	229533.I1RMV1
87825	229533.I1RFG0	pp	229533.V6QVD5
87826	229533.I1RFG0	pp	229533.I1RQE7
87827	229533.I1RFG0	pp	229533.I1S1X9
87828	229533.I1RFG0	pp	229533.I1S4T3
87829	229533.I1RFG0	pp	229533.I1RFP4
87830	229533.I1RFG0	pp	229533.I1S2E8
87831	229533.I1RFG0	pp	229533.I1RM96
87832	229533.I1RFG0	pp	229533.I1RSS4
87833	229533.I1RFG0	pp	229533.I1RX51
87834	229533.I1RFG0	pp	229533.I1RNU6
87835	229533.I1RFG0	pp	229533.I1RZT1
87836	229533.I1RFG0	pp	229533.I1RM25
87837	229533.I1RFG0	pp	229533.I1RQR3
87838	229533.I1RFG0	pp	229533.I1S1U7
87839	229533.I1RFG0	pp	229533.I1RNB8
87840	229533.I1RFG0	pp	229533.V6RGY3
87841	229533.I1RFG0	pp	229533.I1RTE9
87842	229533.I1RFG0	pp	229533.Q4IN63
87843	229533.I1RFG0	pp	229533.I1S060
87844	229533.I1RFG0	pp	229533.I1RRL0
87845	229533.I1RFG0	pp	229533.Q4I327
87846	229533.I1RFG0	pp	229533.I1S2C0
87847	229533.I1RFG0	pp	229533.Q4IPB8
87848	229533.I1RFG0	pp	229533.I1RK79
87849	229533.I1RFG0	pp	229533.I1RRI2
87850	229533.I1RFG0	pp	229533.I1S0J1
87851	229533.I1RFG0	pp	229533.I1S471
87852	229533.I1RFG0	pp	229533.I1RSD4
87853	229533.I1RFG0	pp	229533.I1RX47
87854	229533.I1RFG0	pp	229533.V6RGN5
87855	229533.I1RFG0	pp	229533.I1RSW2
87856	229533.I1RFG0	pp	229533.V6RIS2
87857	229533.I1RFG0	pp	229533.I1RWU2
87858	229533.I1RFG0	pp	229533.I1RJZ7
87859	229533.I1RFG0	pp	229533.I1S0N4
87860	229533.I1RFG0	pp	229533.Q4I830
87861	229533.I1RFG0	pp	229533.I1RKL3
87862	229533.I1RFG0	pp	229533.I1RVY0
87863	229533.I1RFG0	pp	229533.I1RSI6
87864	229533.I1RFG0	pp	229533.I1RRJ8
87865	229533.I1RFG0	pp	229533.I1RMN6
87866	229533.I1RFG0	pp	229533.I1RST1
87867	229533.I1RFG0	pp	229533.V6R283
87868	229533.I1RFG0	pp	229533.I1RX24
87869	229533.I1RFG0	pp	229533.Q4IBK5
87870	229533.I1RFG0	pp	229533.I1RZS6
87871	229533.I1RFG0	pp	229533.I1S0J3
87872	229533.I1RFG0	pp	229533.I1RKJ6
87873	229533.I1RFG0	pp	229533.I1RFS3
87874	229533.I1RFG0	pp	229533.I1RU69
87875	229533.I1RFG0	pp	229533.I1RT17
87876	229533.I1RFG0	pp	229533.I1RG56
87877	229533.I1RFG0	pp	229533.I1RFN1
87878	229533.I1RFG0	pp	229533.Q4IEK8
87879	229533.I1RFG0	pp	229533.Q4HZ42
87880	229533.I1RFG0	pp	229533.Q4IAS1
87881	229533.I1RFG0	pp	229533.I1RS47
87882	229533.I1RFG0	pp	229533.I1RQ65
87883	229533.I1RFG0	pp	229533.I1RRT2
87884	229533.I1RFG0	pp	229533.Q4IJ56
87885	229533.I1RFG0	pp	229533.I1RJL4
87886	229533.I1RFG0	pp	229533.I1S267
87887	229533.I1RFG0	pp	229533.Q4IAA0
87888	229533.I1RFG0	pp	229533.Q4HXF0
87889	229533.I1RFG0	pp	229533.I1RS94
87890	229533.I1RFG0	pp	229533.I1RSR3
87891	229533.I1RFG0	pp	229533.Q4IRI9
87892	229533.I1RFG0	pp	229533.I1RZ40

87893 229533.I1RFG0 pp 229533.I1RVZ0
87894 229533.I1RFG0 pp 229533.I1RQA2
87895 229533.I1RFG0 pp 229533.I1S0Z9
87896 229533.I1RFG0 pp 229533.I1RS83
87897 229533.I1RFG0 pp 229533.I1RFQ5
87898 229533.I1RFG0 pp 229533.I1RZB5
87899 229533.I1RFG0 pp 229533.I1S044
87900 229533.I1RFG0 pp 229533.I1RMH5
87901 229533.I1RFG0 pp 229533.I1RYB1
87902 229533.I1RFG0 pp 229533.Q4I1Y1
87903 229533.I1RFG0 pp 229533.I1RJL2
87904 229533.I1RFG0 pp 229533.V6QUE6
87905 229533.I1RFG0 pp 229533.I1RZE5
87906 229533.I1RFG0 pp 229533.I1RMD5
87907 229533.I1RFG0 pp 229533.I1S9J6
87908 229533.I1RFG0 pp 229533.I1RZ11
87909 229533.I1RFG0 pp 229533.I1RK95
87910 229533.I1RFG0 pp 229533.I1RN99
87911 229533.I1RFG0 pp 229533.I1RMC0
87912 229533.I1RFG0 pp 229533.Q4IBS2
87913 229533.I1RFG0 pp 229533.I1RQ28
87914 229533.I1RFG0 pp 229533.V6R227
87915 229533.I1RFG0 pp 229533.I1RZK6
87916 229533.I1RFG0 pp 229533.I1RZF6
87917 229533.I1RFG0 pp 229533.Q4IE79
87918 229533.I1RFG0 pp 229533.I1RJZ6
87919 229533.I1RFG0 pp 229533.V6QYF0
87920 229533.I1RFG0 pp 229533.I1RNA0
87921 229533.I1RFG0 pp 229533.I1RQ31
87922 229533.I1RFG0 pp 229533.I1RSA8
87923 229533.I1RFG0 pp 229533.I1RQM6
87924 229533.I1RFG0 pp 229533.I1RMK3
87925 229533.I1RFG0 pp 229533.I1RM06
87926 229533.I1RFG0 pp 229533.I1RNH6
87927 229533.I1RFG0 pp 229533.I1RT33
87928 229533.I1RFG0 pp 229533.I1RG51
87929 229533.I1RFG0 pp 229533.Q4IPB3
87930 229533.I1RFG0 pp 229533.I1S021
87931 229533.I1RFG0 pp 229533.I1S6F0
87932 229533.I1RFG0 pp 229533.I1RLU5
87933 229533.I1RFG0 pp 229533.I1RPW9
87934 229533.I1RFG0 pp 229533.I1RPZ3
87935 229533.I1RFG0 pp 229533.I1RNH1
87936 229533.I1RFG0 pp 229533.I1RKQ5
87937 229533.I1RFG0 pp 229533.I1S9U2
87938 229533.I1RFG0 pp 229533.I1RYI9
87939 229533.I1RFG0 pp 229533.I1RQJ5
87940 229533.I1RFG0 pp 229533.I1RW71
87941 229533.I1RFG0 pp 229533.I1RM67
87942 229533.I1RFG0 pp 229533.I1RFQ7
87943 229533.I1RFG0 pp 229533.I1S246
87944 229533.I1RFG0 pp 229533.Q4IPI1
87945 229533.I1RFG0 pp 229533.I1RTF4
87946 229533.I1RFG0 pp 229533.I1RRG3
87947 229533.I1RFG0 pp 229533.I1RM36
87948 229533.I1RFG0 pp 229533.I1S0R7
87949 229533.I1RFG0 pp 229533.I1RUQ2
87950 229533.I1RFG0 pp 229533.I1RT80
87951 229533.I1RFG0 pp 229533.I1RT98
87952 229533.A0A098DM23 pp 229533.I1RCB7
87953 229533.A0A098DM23 pp 229533.I1RXU0
87954 229533.A0A098DM23 pp 229533.I1RG11
87955 229533.A0A098DM23 pp 229533.Q4IPC8
87956 229533.A0A098DM23 pp 229533.Q4HYB8
87957 229533.A0A098DM23 pp 229533.A0A1C3YN55
87958 229533.A0A098DM23 pp 229533.I1RVY0
87959 229533.A0A098DM23 pp 229533.I1RWF3
87960 229533.A0A098DM23 pp 229533.I1RE14
87961 229533.A0A098DM23 pp 229533.I1RR25
87962 229533.A0A098DM23 pp 229533.I1RK79
87963 229533.A0A098DM23 pp 229533.I1RSZ3
87964 229533.A0A098DM23 pp 229533.A0A1C3YI23
87965 229533.A0A098DM23 pp 229533.I1RAR2

87966 229533.A0A098DM23 pp 229533.I1RK71
87967 229533.A0A098DM23 pp 229533.V6QUE6
87968 229533.A0A098DM23 pp 229533.I1RPM7
87969 229533.A0A098DM23 pp 229533.Q4I5W5
87970 229533.A0A098DM23 pp 229533.Q4I5I4
87971 229533.A0A098DM23 pp 229533.I1S276
87972 229533.A0A098DM23 pp 229533.I1RRT0
87973 229533.A0A098DM23 pp 229533.I1RKB1
87974 229533.A0A098DM23 pp 229533.I1RE30
87975 229533.A0A098DM23 pp 229533.A0A1C3YIR8
87976 229533.A0A098DM23 pp 229533.I1RC15
87977 229533.A0A098DM23 pp 229533.I1RRG3
87978 229533.A0A098DM23 pp 229533.I1RZB5
87979 229533.A0A098DM23 pp 229533.I1RC24
87980 229533.A0A098DM23 pp 229533.I1RVL8
87981 229533.A0A098DM23 pp 229533.I1RTC2
87982 229533.A0A098DM23 pp 229533.I1RJL2
87983 229533.A0A098DM23 pp 229533.A0A0E0RN05
87984 229533.A0A098DM23 pp 229533.V6QWX8
87985 229533.A0A098DM23 pp 229533.I1RUG0
87986 229533.I1S2F3 pp 229533.V6REZ4
87987 229533.I1S2F3 pp 229533.I1S5B5
87988 229533.I1S193 pp 229533.Q4I7X1
87989 229533.I1S193 pp 229533.Q4I7L0
87990 229533.I1S193 pp 229533.Q4I5R3
87991 229533.I1S193 pp 229533.Q4IER8
87992 229533.I1S193 pp 229533.I1S284
87993 229533.I1S193 pp 229533.V6QXG1
87994 229533.A0A1C3YI39 pp 229533.I1S077
87995 229533.A0A1C3YI39 pp 229533.A0A1C3YJW1
87996 229533.A0A1C3YI39 pp 229533.I1RQA3
87997 229533.A0A1C3YI39 pp 229533.I1RWB3
87998 229533.A0A1C3YI39 pp 229533.I1RQ93
87999 229533.A0A1C3YI39 pp 229533.I1RLJ8
88000 229533.A0A1C3YI39 pp 229533.A0A1C3YL18
88001 229533.A0A1C3YI39 pp 229533.I1S5B5
88002 229533.A0A1C3YI39 pp 229533.I1S0G0
88003 229533.A0A1C3YI39 pp 229533.I1RAN0
88004 229533.A0A1C3YI39 pp 229533.I1RB75
88005 229533.A0A1C3YI39 pp 229533.I1RJK7
88006 229533.A0A1C3YI39 pp 229533.I1RDY1
88007 229533.A0A1C3YI39 pp 229533.I1RQS4
88008 229533.A0A1C3YI39 pp 229533.I1RDV9
88009 229533.A0A1C3YI39 pp 229533.A0A1C3YLP6
88010 229533.A0A1C3YI39 pp 229533.I1RWT7
88011 229533.A0A1C3YI39 pp 229533.I1RMS2
88012 229533.A0A1C3YI39 pp 229533.I1RQP5
88013 229533.A0A1C3YI39 pp 229533.I1RZV9
88014 229533.A0A1C3YI39 pp 229533.I1RE37
88015 229533.A0A1C3YI39 pp 229533.I1RCK4
88016 229533.A0A1C3YI39 pp 229533.I1S9J6
88017 229533.A0A1C3YI39 pp 229533.I1RRF9
88018 229533.A0A1C3YI39 pp 229533.I1RYY5
88019 229533.A0A1C3YI39 pp 229533.I1S0P4
88020 229533.A0A1C3YI39 pp 229533.Q4HTT2
88021 229533.A0A1C3YI39 pp 229533.I1RTE5
88022 229533.A0A1C3YI39 pp 229533.I1RE46
88023 229533.A0A1C3YI39 pp 229533.I1RME5
88024 229533.A0A1C3YI39 pp 229533.I1S0F0
88025 229533.A0A1C3YI39 pp 229533.I1RWT6
88026 229533.A0A1C3YI39 pp 229533.Q4INS6
88027 229533.A0A1C3YI39 pp 229533.I1RMQ7
88028 229533.A0A1C3YI39 pp 229533.A0A1C3YI64
88029 229533.A0A1C3YI39 pp 229533.A0A1C3YN58
88030 229533.A0A1C3YI39 pp 229533.A0A1C3YLR6
88031 229533.A0A1C3YI39 pp 229533.I1RKK0
88032 229533.A0A1C3YI39 pp 229533.I1RC17
88033 229533.A0A1C3YI39 pp 229533.I1S209
88034 229533.A0A1C3YI39 pp 229533.A0A1C3YLU7
88035 229533.A0A1C3YI39 pp 229533.I1S7M3
88036 229533.A0A1C3YI39 pp 229533.A0A1C3YK75
88037 229533.A0A1C3YI39 pp 229533.I1S987
88038 229533.A0A1C3YI39 pp 229533.I1REE1

88039 229533.A0A1C3YI39 pp 229533.I1RZ12
88040 229533.A0A1C3YI39 pp 229533.I1RRX6
88041 229533.A0A1C3YI39 pp 229533.I1RRT1
88042 229533.A0A1C3YI39 pp 229533.I1RYU3
88043 229533.A0A1C3YI39 pp 229533.I1RBX4
88044 229533.A0A1C3YI39 pp 229533.I1RD18
88045 229533.A0A1C3YI39 pp 229533.I1RV66
88046 229533.A0A1C3YI39 pp 229533.I1RQ26
88047 229533.A0A1C3YI39 pp 229533.A0A1C3YK82
88048 229533.A0A1C3YI39 pp 229533.I1S224
88049 229533.A0A1C3YI39 pp 229533.I1RTG0
88050 229533.A0A1C3YI39 pp 229533.I1RMB7
88051 229533.A0A1C3YI39 pp 229533.A0A1C3YJH8
88052 229533.A0A1C3YI39 pp 229533.I1RCU7
88053 229533.A0A1C3YI39 pp 229533.I1RBW0
88054 229533.A0A1C3YI39 pp 229533.Q4IJH1
88055 229533.A0A1C3YI39 pp 229533.I1RX70
88056 229533.A0A1C3YI39 pp 229533.A0A1C3YJT5
88057 229533.A0A1C3YI39 pp 229533.I1RG34
88058 229533.A0A1C3YI39 pp 229533.I1RZ94
88059 229533.A0A1C3YI39 pp 229533.A0A1C3YKW5
88060 229533.A0A1C3YI39 pp 229533.I1RAL9
88061 229533.A0A1C3YI39 pp 229533.I1RM49
88062 229533.A0A1C3YI39 pp 229533.I1RD69
88063 229533.A0A1C3YI39 pp 229533.I1RD01
88064 229533.A0A1C3YI39 pp 229533.I1RB50
88065 229533.A0A1C3YI39 pp 229533.I1RM36
88066 229533.A0A1C3YI39 pp 229533.I1RWM6
88067 229533.A0A1C3YI39 pp 229533.I1RCQ6
88068 229533.A0A1C3YI39 pp 229533.I1RRY1
88069 229533.A0A1C3YI39 pp 229533.I1RMW7
88070 229533.A0A1C3YI39 pp 229533.I1RQL4
88071 229533.A0A1C3YI39 pp 229533.I1RKI2
88072 229533.A0A1C3YI39 pp 229533.I1RD31
88073 229533.A0A1C3YI39 pp 229533.V6RTJ8
88074 229533.A0A1C3YI39 pp 229533.I1RNT2
88075 229533.A0A1C3YI39 pp 229533.I1S7M2
88076 229533.A0A1C3YI39 pp 229533.I1RIR9
88077 229533.A0A1C3YI39 pp 229533.I1RY40
88078 229533.A0A1C3YI39 pp 229533.I1RYV1
88079 229533.A0A1C3YI39 pp 229533.I1RUG4
88080 229533.A0A1C3YI39 pp 229533.A0A1C3YL53
88081 229533.A0A1C3YI39 pp 229533.I1RK75
88082 229533.A0A1C3YI39 pp 229533.I1RKB1
88083 229533.A0A1C3YI39 pp 229533.I1S0N5
88084 229533.A0A1C3YI39 pp 229533.A0A1C3YJ51
88085 229533.A0A1C3YI39 pp 229533.V6REZ4
88086 229533.A0A1C3YI39 pp 229533.I1RG90
88087 229533.A0A1C3YI39 pp 229533.I1RB41
88088 229533.A0A1C3YI39 pp 229533.I1RYI9
88089 229533.A0A1C3YI39 pp 229533.I1RAU7
88090 229533.A0A1C3YI39 pp 229533.I1RRN5
88091 229533.A0A1C3YI39 pp 229533.I1RP99
88092 229533.A0A1C3YI39 pp 229533.I1S1Y7
88093 229533.A0A1C3YI39 pp 229533.I1REK6
88094 229533.A0A1C3YI39 pp 229533.I1RZT1
88095 229533.A0A1C3YI39 pp 229533.I1RMJ8
88096 229533.A0A1C3YI39 pp 229533.I1RRH8
88097 229533.A0A1C3YI39 pp 229533.I1RYW3
88098 229533.A0A1C3YI39 pp 229533.I1RRR6
88099 229533.A0A1C3YI39 pp 229533.I1S276
88100 229533.A0A1C3YI39 pp 229533.I1RY58
88101 229533.A0A1C3YI39 pp 229533.I1RNE4
88102 229533.A0A1C3YI39 pp 229533.I1RQC0
88103 229533.A0A1C3YI39 pp 229533.I1RDH0
88104 229533.A0A1C3YI39 pp 229533.I1RW03
88105 229533.A0A1C3YI39 pp 229533.I1RDJ8
88106 229533.A0A1C3YI39 pp 229533.I1REC7
88107 229533.A0A1C3YI39 pp 229533.A0A1C3YMC9
88108 229533.A0A1C3YI39 pp 229533.I1RDQ9
88109 229533.A0A1C3YI39 pp 229533.I1RJS5
88110 229533.A0A1C3YI39 pp 229533.I1RWX5
88111 229533.A0A1C3YI39 pp 229533.I1RTA0

229533.A0A1C3YI39	pp	229533.I1RPM7
229533.A0A1C3YI39	pp	229533.Q4HZB8
229533.A0A1C3YI39	pp	229533.I1RMK8
229533.A0A1C3YI39	pp	229533.I1S0D5
229533.A0A1C3YI39	pp	229533.Q4IB89
229533.A0A1C3YI39	pp	229533.I1S2C0
229533.A0A1C3YI39	pp	229533.I1RTA3
229533.A0A1C3YI39	pp	229533.I1S4P6
229533.A0A1C3YI39	pp	229533.I1S1D3
229533.A0A1C3YI39	pp	229533.I1RSL8
229533.A0A1C3YI39	pp	229533.A0A1C3YN55
229533.A0A1C3YI39	pp	229533.A0A1C3YL06
229533.A0A1C3YI39	pp	229533.I1RY44
229533.A0A1C3YI39	pp	229533.I1RBC8
229533.A0A1C3YI39	pp	229533.I1RQ49
229533.A0A1C3YI39	pp	229533.A0A1C3YLT9
229533.A0A1C3YI39	pp	229533.I1S998
229533.A0A1C3YI39	pp	229533.V6RGI1
229533.A0A1C3YI39	pp	229533.I1S0J7
229533.A0A1C3YI39	pp	229533.I1RC54
229533.A0A1C3YI39	pp	229533.I1R9M0
229533.A0A1C3YI39	pp	229533.A0A1C3YM91
229533.A0A1C3YI39	pp	229533.I1RCN2
229533.A0A1C3YI39	pp	229533.A0A1C3YL34
229533.A0A1C3YI39	pp	229533.I1S573
229533.A0A1C3YI39	pp	229533.I1S0M2
229533.A0A1C3YI39	pp	229533.I1SAD2
229533.A0A1C3YI39	pp	229533.A0A1C3YK57
229533.A0A1C3YI39	pp	229533.I1RQB4
229533.A0A1C3YI39	pp	229533.Q4HTT1
229533.A0A1C3YI39	pp	229533.I1RRW0
229533.A0A1C3YI39	pp	229533.I1S471
229533.A0A1C3YI39	pp	229533.I1RSC7
229533.A0A1C3YI39	pp	229533.I1RMA4
229533.A0A1C3YI39	pp	229533.I1S004
229533.A0A1C3YI39	pp	229533.I1RSL6
229533.A0A1C3YI39	pp	229533.I1RB73
229533.A0A1C3YI39	pp	229533.I1RZ86
229533.A0A1C3YI39	pp	229533.I1RVW0
229533.A0A1C3YI39	pp	229533.I1RDV4
229533.A0A1C3YI39	pp	229533.I1REC6
229533.A0A1C3YI39	pp	229533.I1RZ63
229533.A0A1C3YI39	pp	229533.I1S2F2
229533.A0A1C3YI39	pp	229533.Q4IF76
229533.A0A1C3YI39	pp	229533.A0A1C3YLQ7
229533.A0A1C3YI39	pp	229533.I1RUF8
229533.A0A1C3YI39	pp	229533.I1RV78
229533.A0A1C3YI39	pp	229533.I1RC00
229533.A0A1C3YI39	pp	229533.V6R3J1
229533.A0A1C3YI39	pp	229533.I1RBW1
229533.A0A1C3YI39	pp	229533.I1RIU0
229533.A0A1C3YI39	pp	229533.I1RSS7
229533.A0A1C3YI39	pp	229533.I1RTT3
229533.A0A1C3YI39	pp	229533.I1RN54
229533.A0A1C3YI39	pp	229533.I1RYT7
229533.A0A1C3YI39	pp	229533.Q4IMD1
229533.A0A1C3YI39	pp	229533.A0A1C3YM61
229533.A0A1C3YI39	pp	229533.I1RJS4
229533.A0A1C3YI39	pp	229533.I1RMY3
229533.A0A1C3YI39	pp	229533.I1RMP2
229533.A0A1C3YI39	pp	229533.I1SAA0
229533.A0A1C3YI39	pp	229533.A0A1C3YJG7
229533.A0A1C3YI39	pp	229533.I1RWI8
229533.A0A1C3YI39	pp	229533.I1RAH9
229533.A0A1C3YI39	pp	229533.A0A1C3YIE7
229533.A0A1C3YI39	pp	229533.I1RFG0
229533.A0A1C3YI39	pp	229533.I1RFL7
229533.A0A1C3YI39	pp	229533.I1S8P5
229533.A0A1C3YI39	pp	229533.I1RB74
229533.A0A1C3YI39	pp	229533.I1S098
229533.A0A1C3YI39	pp	229533.I1S2E9
229533.A0A1C3YI39	pp	229533.I1RWX7
229533.A0A1C3YI39	pp	229533.I1RIP9

88185 229533.A0A1C3YI39 pp 229533.I1RE19
88186 229533.A0A1C3YI39 pp 229533.I1RXF2
88187 229533.A0A1C3YI39 pp 229533.A0A1C3YM67
88188 229533.A0A1C3YI39 pp 229533.I1RVV5
88189 229533.A0A1C3YI39 pp 229533.I1RPF5
88190 229533.A0A1C3YI39 pp 229533.I1RV91
88191 229533.A0A1C3YI39 pp 229533.I1RSZ3
88192 229533.A0A1C3YI39 pp 229533.I1RF02
88193 229533.I1RDA7 pp 229533.I1RTF1
88194 229533.A0A1C3YI72 pp 229533.A0A1C3YL56
88195 229533.A0A1C3YI72 pp 229533.I1RWF9
88196 229533.A0A1C3YI72 pp 229533.I1RWS4
88197 229533.A0A1C3YI72 pp 229533.I1RXB6
88198 229533.A0A1C3YI72 pp 229533.I1S0S5
88199 229533.A0A1C3YI72 pp 229533.A0A1C3YL20
88200 229533.A0A1C3YI72 pp 229533.I1RHY5
88201 229533.A0A1C3YI72 pp 229533.I1RC61
88202 229533.A0A1C3YI72 pp 229533.I1RY74
88203 229533.A0A1C3YI72 pp 229533.I1RA94
88204 229533.A0A1C3YI72 pp 229533.I1RNG4
88205 229533.A0A1C3YI72 pp 229533.I1RMC2
88206 229533.A0A1C3YI72 pp 229533.I1RBE7
88207 229533.A0A1C3YI72 pp 229533.I1S8E0
88208 229533.A0A1C3YI72 pp 229533.V6RQW4
88209 229533.A0A1C3YI72 pp 229533.I1RW00
88210 229533.A0A1C3YI72 pp 229533.I1RBJ8
88211 229533.A0A1C3YI72 pp 229533.I1RZ29
88212 229533.A0A1C3YI72 pp 229533.I1RYJ6
88213 229533.A0A1C3YI72 pp 229533.I1RN77
88214 229533.A0A1C3YI72 pp 229533.I1RMK7
88215 229533.A0A1C3YI72 pp 229533.I1RHV1
88216 229533.I1RDA6 pp 229533.I1RW71
88217 229533.I1RDA6 pp 229533.I1RJZ7
88218 229533.I1RK83 pp 229533.I1RQ65
88219 229533.I1RK83 pp 229533.Q4IEV5
88220 229533.I1RK83 pp 229533.I1S8E2
88221 229533.I1RK83 pp 229533.I1RTG7
88222 229533.I1RK83 pp 229533.I1RND6
88223 229533.I1RK83 pp 229533.I1RR73
88224 229533.I1RK83 pp 229533.I1RZ39
88225 229533.I1RK83 pp 229533.I1RN48
88226 229533.I1RK83 pp 229533.I1RZV6
88227 229533.I1RK83 pp 229533.I1RKJ6
88228 229533.I1RK83 pp 229533.Q4I662
88229 229533.I1RK83 pp 229533.I1S3G4
88230 229533.I1RK83 pp 229533.I1RX24
88231 229533.I1RK83 pp 229533.I1S0J1
88232 229533.I1RK83 pp 229533.I1S060
88233 229533.I1RK83 pp 229533.I1RSL8
88234 229533.I1RK83 pp 229533.I1S7S9
88235 229533.I1RK83 pp 229533.I1RZS6
88236 229533.I1RK83 pp 229533.I1RPK0
88237 229533.I1RK83 pp 229533.I1RWB5
88238 229533.I1RK83 pp 229533.I1RM96
88239 229533.I1RK83 pp 229533.Q4IN63
88240 229533.I1RK83 pp 229533.V6R382
88241 229533.I1RK83 pp 229533.I1RLV9
88242 229533.I1RK83 pp 229533.I1RRR7
88243 229533.I1RK83 pp 229533.I1RRZ0
88244 229533.I1RK83 pp 229533.I1RKL3
88245 229533.I1RK83 pp 229533.Q4I327
88246 229533.I1RK83 pp 229533.I1RRQ3
88247 229533.I1RK83 pp 229533.V6R042
88248 229533.I1RK83 pp 229533.I1RPP3
88249 229533.I1RK83 pp 229533.I1RT80
88250 229533.I1RK83 pp 229533.V6RMW6
88251 229533.I1RK83 pp 229533.I1S2C0
88252 229533.I1RK83 pp 229533.I1RSD8
88253 229533.I1RK83 pp 229533.I1RMN6
88254 229533.I1RK83 pp 229533.I1RM67
88255 229533.I1RK83 pp 229533.I1S2E8
88256 229533.I1RK83 pp 229533.I1RPU0
88257 229533.I1RK83 pp 229533.I1S1X9

88258	229533.I1RK83	pp	229533.I1RPW9
88259	229533.I1RK83	pp	229533.I1RT98
88260	229533.I1RK83	pp	229533.I1RNA0
88261	229533.I1RK83	pp	229533.I1S0R7
88262	229533.I1RK83	pp	229533.I1S1W8
88263	229533.I1RK83	pp	229533.Q4IPI1
88264	229533.I1RK83	pp	229533.Q4IJ56
88265	229533.I1RK83	pp	229533.Q4IFI0
88266	229533.I1RK83	pp	229533.I1S0M9
88267	229533.I1RK83	pp	229533.I1RX46
88268	229533.I1RK83	pp	229533.I1RWH5
88269	229533.I1RK83	pp	229533.I1RZF6
88270	229533.I1RK83	pp	229533.I1RSR8
88271	229533.I1RK83	pp	229533.I1RQJ5
88272	229533.I1RK83	pp	229533.I1S021
88273	229533.I1RK83	pp	229533.I1RYB9
88274	229533.I1RK83	pp	229533.I1RZB5
88275	229533.I1RK83	pp	229533.I1RNH6
88276	229533.I1RK83	pp	229533.I1RZZ1
88277	229533.I1RK83	pp	229533.I1RZK6
88278	229533.I1RK83	pp	229533.I1S6E5
88279	229533.I1RK83	pp	229533.I1RVZ0
88280	229533.I1RK83	pp	229533.I1S044
88281	229533.A0A098DU58	pp	229533.Q4I7L0
88282	229533.A0A098DU58	pp	229533.I1S2A8
88283	229533.A0A098DU58	pp	229533.I1RTG3
88284	229533.A0A098DU58	pp	229533.A0A1C3YI39
88285	229533.A0A098DU58	pp	229533.Q4IMD1
88286	229533.A0A098DU58	pp	229533.I1RMB7
88287	229533.A0A098DU58	pp	229533.I1RD01
88288	229533.A0A098DU58	pp	229533.I1RQ80
88289	229533.A0A098DU58	pp	229533.I1RU02
88290	229533.A0A098DU58	pp	229533.Q4IER8
88291	229533.A0A098DU58	pp	229533.I1RFW1
88292	229533.A0A098DU58	pp	229533.A0A1C3YKG7
88293	229533.A0A098DU58	pp	229533.A0A1C3YLX5
88294	229533.A0A098DU58	pp	229533.A0A1C3YJ51
88295	229533.A0A098DU58	pp	229533.Q4IR08
88296	229533.A0A098DU58	pp	229533.Q4HTT2
88297	229533.A0A098DU58	pp	229533.I1RCW8
88298	229533.A0A098DU58	pp	229533.A0A0E0SQV5
88299	229533.A0A098DU58	pp	229533.I1RAX3
88300	229533.I1RVN1	pp	229533.I1RZS4
88301	229533.I1RVN1	pp	229533.I1RYW3
88302	229533.I1RVN1	pp	229533.Q4IPZ1
88303	229533.I1RVN1	pp	229533.Q4HZ95
88304	229533.I1RVN1	pp	229533.V6RDZ4
88305	229533.I1RVN1	pp	229533.V6R1X4
88306	229533.I1RVN1	pp	229533.I1RZH5
88307	229533.I1RVN1	pp	229533.I1S8F0
88308	229533.I1RVN1	pp	229533.I1RZB2
88309	229533.I1RVN1	pp	229533.Q4I099
88310	229533.I1RVN1	pp	229533.I1S0J1
88311	229533.I1RVN1	pp	229533.Q4IB65
88312	229533.I1RVN1	pp	229533.V6RPQ8
88313	229533.I1RVN1	pp	229533.Q4IJW4
88314	229533.I1RVN1	pp	229533.I1S277
88315	229533.I1RVN1	pp	229533.Q4IK03
88316	229533.I1RVN1	pp	229533.I1S088
88317	229533.I1RVN1	pp	229533.Q4I665
88318	229533.I1RVN1	pp	229533.I1S4H6
88319	229533.I1RVN1	pp	229533.I1S0B7
88320	229533.I1RVN1	pp	229533.I1S4S4
88321	229533.I1RVN1	pp	229533.Q4I6B0
88322	229533.I1RVN1	pp	229533.V6RTJ8
88323	229533.I1RVN1	pp	229533.Q4I5G1
88324	229533.I1RVN1	pp	229533.I1RXF0
88325	229533.I1RVN1	pp	229533.I1S8X1
88326	229533.I1RVN1	pp	229533.V6RR76
88327	229533.I1RVN1	pp	229533.I1RX46
88328	229533.A0A1C3YN89	pp	229533.I1RYH6
88329	229533.A0A1C3YN89	pp	229533.I1S1B3
88330	229533.A0A1C3YN89	pp	229533.I1RX09

88331 229533.A0A1C3YN89 pp 229533.I1RYN2
88332 229533.A0A1C3YN89 pp 229533.I1RA52
88333 229533.A0A1C3YN89 pp 229533.V6REE9
88334 229533.A0A1C3YN89 pp 229533.I1REC6
88335 229533.A0A1C3YN89 pp 229533.I1S7X9
88336 229533.A0A1C3YN89 pp 229533.I1RDG9
88337 229533.A0A1C3YN89 pp 229533.I1RCD4
88338 229533.A0A1C3YN89 pp 229533.I1RTE6
88339 229533.A0A1C3YN89 pp 229533.I1RBD2
88340 229533.A0A1C3YN89 pp 229533.Q4I5R9
88341 229533.A0A1C3YN89 pp 229533.I1RPP0
88342 229533.A0A1C3YN89 pp 229533.I1RE37
88343 229533.A0A1C3YN89 pp 229533.I1RN65
88344 229533.A0A1C3YN89 pp 229533.I1RRF9
88345 229533.A0A1C3YN89 pp 229533.I1RWD3
88346 229533.A0A1C3YN89 pp 229533.Q4IR08
88347 229533.A0A1C3YN89 pp 229533.I1RQL4
88348 229533.A0A1C3YN89 pp 229533.I1RM09
88349 229533.A0A1C3YN89 pp 229533.I1RWL9
88350 229533.A0A1C3YN89 pp 229533.I1RFL7
88351 229533.A0A1C3YN89 pp 229533.I1REC7
88352 229533.A0A1C3YN89 pp 229533.Q4I7K4
88353 229533.I1S0Y7 pp 229533.I1S3G4
88354 229533.I1S0Y7 pp 229533.V6R382
88355 229533.I1S0Y7 pp 229533.I1S1W8
88356 229533.I1S0Y7 pp 229533.I1S8E0
88357 229533.I1S0Y7 pp 229533.V6RK96
88358 229533.I1S0Y7 pp 229533.I1S673
88359 229533.I1R9I3 pp 229533.I1RNG4
88360 229533.A0A0E0RQ72 pp 229533.I1RRK8
88361 229533.A0A0E0RQ72 pp 229533.Q4HYB8
88362 229533.A0A0E0RQ72 pp 229533.I1RA48
88363 229533.A0A0E0RQ72 pp 229533.I1S0J1
88364 229533.A0A0E0RQ72 pp 229533.Q4IJU0
88365 229533.A0A0E0RQ72 pp 229533.I1RCH2
88366 229533.A0A0E0RQ72 pp 229533.I1S0M9
88367 229533.A0A0E0RQ72 pp 229533.I1RNW8
88368 229533.A0A0E0RQ72 pp 229533.I1RHM4
88369 229533.A0A0E0RQ72 pp 229533.I1RM96
88370 229533.A0A0E0RQ72 pp 229533.I1S149
88371 229533.A0A0E0RQ72 pp 229533.I1RSI1
88372 229533.A0A0E0RQ72 pp 229533.I1RSR8
88373 229533.A0A0E0RQ72 pp 229533.I1RWE1
88374 229533.A0A0E0RQ72 pp 229533.A0A0E0S2K9
88375 229533.A0A0E0RQ72 pp 229533.I1RS41
88376 229533.A0A0E0RQ72 pp 229533.V6RPS5
88377 229533.A0A0E0RQ72 pp 229533.I1RQJ5
88378 229533.A0A0E0RQ72 pp 229533.A0A0E0S0W9
88379 229533.A0A0E0RQ72 pp 229533.I1RM09
88380 229533.A0A0E0RQ72 pp 229533.I1RAY2
88381 229533.A0A0E0RQ72 pp 229533.A0A1C3YKX5
88382 229533.A0A0E0RQ72 pp 229533.I1RQ65
88383 229533.A0A0E0RQ72 pp 229533.I1RZS6
88384 229533.A0A0E0RQ72 pp 229533.I1RT98
88385 229533.A0A0E0RQ72 pp 229533.I1RBY4
88386 229533.A0A0E0RQ72 pp 229533.I1RLV9
88387 229533.A0A0E0RQ72 pp 229533.I1RPU0
88388 229533.A0A0E0RQ72 pp 229533.I1RKJ6
88389 229533.A0A0E0RQ72 pp 229533.I1RFQ7
88390 229533.A0A0E0RQ72 pp 229533.Q4IAS1
88391 229533.A0A1C3YLB3 pp 229533.V6RZD4
88392 229533.A0A1C3YLB3 pp 229533.I1RL81
88393 229533.A0A1C3YLB3 pp 229533.I1RLZ4
88394 229533.A0A1C3YLB3 pp 229533.I1RWF5
88395 229533.A0A1C3YLB3 pp 229533.I1S968
88396 229533.A0A1C3YLB3 pp 229533.I1RR68
88397 229533.A0A1C3YLB3 pp 229533.A0A1C3YLE2
88398 229533.A0A1C3YLB3 pp 229533.I1RMJ3
88399 229533.A0A1C3YLB3 pp 229533.I1RZV5
88400 229533.A0A1C3YLB3 pp 229533.I1RKB2
88401 229533.A0A1C3YLB3 pp 229533.A0A1C3YM60
88402 229533.A0A1C3YLB3 pp 229533.I1S299
88403 229533.A0A1C3YLB3 pp 229533.I1RFI4

88404 229533.A0A1C3YLB3 pp 229533.I1RSQ4
88405 229533.A0A1C3YLB3 pp 229533.V6R3J1
88406 229533.A0A1C3YLB3 pp 229533.I1RAV0
88407 229533.A0A1C3YLB3 pp 229533.I1RKP1
88408 229533.A0A1C3YLB3 pp 229533.I1S3T7
88409 229533.A0A1C3YLB3 pp 229533.Q4HVQ9
88410 229533.A0A1C3YLB3 pp 229533.I1RC34
88411 229533.A0A1C3YLB3 pp 229533.I1RPU8
88412 229533.A0A1C3YLB3 pp 229533.I1S2Q4
88413 229533.A0A1C3YLB3 pp 229533.I1RN88
88414 229533.A0A1C3YLB3 pp 229533.I1RN84
88415 229533.A0A1C3YLB3 pp 229533.I1RY35
88416 229533.A0A1C3YLB3 pp 229533.I1RB38
88417 229533.A0A1C3YLB3 pp 229533.I1S147
88418 229533.A0A1C3YLB3 pp 229533.I1RPK0
88419 229533.A0A1C3YLB3 pp 229533.I1RC29
88420 229533.A0A1C3YLB3 pp 229533.I1RD22
88421 229533.A0A1C3YLB3 pp 229533.V6RLM7
88422 229533.A0A1C3YLB3 pp 229533.I1RUR8
88423 229533.A0A1C3YLB3 pp 229533.I1S0V7
88424 229533.A0A1C3YLB3 pp 229533.I1RE52
88425 229533.A0A1C3YLB3 pp 229533.I1RGC3
88426 229533.A0A1C3YLB3 pp 229533.I1RK55
88427 229533.A0A1C3YLB3 pp 229533.V6RRM0
88428 229533.A0A1C3YLB3 pp 229533.I1RA52
88429 229533.A0A1C3YLB3 pp 229533.I1RYC8
88430 229533.A0A1C3YLB3 pp 229533.I1RAW0
88431 229533.A0A1C3YLB3 pp 229533.I1RSF1
88432 229533.A0A1C3YLB3 pp 229533.I1RF85
88433 229533.A0A1C3YLB3 pp 229533.I1S8R2
88434 229533.A0A1C3YLB3 pp 229533.I1RYA0
88435 229533.A0A1C3YLB3 pp 229533.I1S4S5
88436 229533.A0A1C3YLB3 pp 229533.I1RNM7
88437 229533.A0A1C3YLB3 pp 229533.I1RCU2
88438 229533.A0A1C3YLB3 pp 229533.I1RMK7
88439 229533.A0A1C3YLB3 pp 229533.V6RF62
88440 229533.A0A1C3YLB3 pp 229533.I1S8H9
88441 229533.A0A1C3YLB3 pp 229533.V6QVH2
88442 229533.A0A1C3YLB3 pp 229533.I1RMA2
88443 229533.A0A1C3YLB3 pp 229533.I1RRX5
88444 229533.A0A1C3YLB3 pp 229533.A0A1C3YN04
88445 229533.A0A1C3YLB3 pp 229533.I1RW54
88446 229533.A0A1C3YLB3 pp 229533.I1S8F8
88447 229533.A0A1C3YLB3 pp 229533.I1RYT8
88448 229533.A0A1C3YLB3 pp 229533.I1RDN3
88449 229533.A0A1C3YLB3 pp 229533.I1S8C1
88450 229533.A0A1C3YLB3 pp 229533.I1RYP5
88451 229533.A0A1C3YLB3 pp 229533.I1RNY6
88452 229533.A0A1C3YLB3 pp 229533.I1RYU6
88453 229533.A0A1C3YLB3 pp 229533.I1RPR1
88454 229533.A0A1C3YLB3 pp 229533.I1REE2
88455 229533.A0A1C3YLB3 pp 229533.I1RJC5
88456 229533.A0A1C3YLB3 pp 229533.I1R9Y4
88457 229533.A0A1C3YLB3 pp 229533.I1RJY7
88458 229533.A0A1C3YLB3 pp 229533.I1RVI8
88459 229533.A0A1C3YLB3 pp 229533.I1RXE6
88460 229533.A0A1C3YLB3 pp 229533.I1RWM4
88461 229533.A0A1C3YLB3 pp 229533.I1S8S9
88462 229533.A0A1C3YLB3 pp 229533.I1RMQ6
88463 229533.A0A1C3YLB3 pp 229533.I1RY21
88464 229533.A0A1C3YLB3 pp 229533.I1S0V6
88465 229533.A0A1C3YLB3 pp 229533.I1RG33
88466 229533.A0A1C3YLB3 pp 229533.I1S0K2
88467 229533.A0A1C3YLB3 pp 229533.I1RAM9
88468 229533.A0A1C3YLB3 pp 229533.I1RUQ3
88469 229533.A0A1C3YLB3 pp 229533.I1RCM5
88470 229533.A0A1C3YLB3 pp 229533.I1RA13
88471 229533.A0A1C3YLB3 pp 229533.V6R3V4
88472 229533.A0A1C3YLB3 pp 229533.I1RBU8
88473 229533.A0A1C3YLB3 pp 229533.I1RY81
88474 229533.A0A1C3YLB3 pp 229533.A0A1C3YLC2
88475 229533.A0A1C3YLB3 pp 229533.I1RAV5
88476 229533.A0A1C3YLB3 pp 229533.I1RWD2

88477	229533.A0A1C3YLB3	pp	229533.I1RY12
88478	229533.I1RAY9	pp	229533.V6R1X4
88479	229533.I1RAY9	pp	229533.I1RSS7
88480	229533.I1RAY9	pp	229533.I1RZM4
88481	229533.I1RAY9	pp	229533.I1RRV4
88482	229533.I1RAY9	pp	229533.I1RNG4
88483	229533.I1RAY9	pp	229533.I1S673
88484	229533.I1RAY9	pp	229533.I1RMY1
88485	229533.I1RAY9	pp	229533.V6R382
88486	229533.I1RAY9	pp	229533.V6RK96
88487	229533.I1RAY9	pp	229533.I1RZ83
88488	229533.I1RAY9	pp	229533.I1RQE8
88489	229533.I1RAY9	pp	229533.I1RP97
88490	229533.I1RAY9	pp	229533.I1RUY6
88491	229533.I1RAY9	pp	229533.I1RBJ8
88492	229533.I1RAY9	pp	229533.I1RXV3
88493	229533.I1RAY9	pp	229533.I1RW00
88494	229533.I1RAY9	pp	229533.I1RMK4
88495	229533.I1RAY9	pp	229533.I1RXY7
88496	229533.I1RAY9	pp	229533.I1S8E0
88497	229533.I1RAY9	pp	229533.I1S3G4
88498	229533.I1RAY9	pp	229533.I1S1W8
88499	229533.I1RAY9	pp	229533.I1S0N3
88500	229533.I1RAY9	pp	229533.I1S0Y7
88501	229533.A0A1C3YMK1	pp	229533.I1RY40
88502	229533.A0A1C3YMK1	pp	229533.I1RW71
88503	229533.A0A1C3YMK1	pp	229533.I1RWH5
88504	229533.A0A1C3YMK1	pp	229533.I1RQJ5
88505	229533.A0A1C3YMK1	pp	229533.I1S246
88506	229533.A0A1C3YMK1	pp	229533.I1RRG3
88507	229533.A0A1C3YMK1	pp	229533.I1RZG7
88508	229533.A0A1C3YMK1	pp	229533.I1RM06
88509	229533.A0A1C3YMK1	pp	229533.Q4IPI1
88510	229533.A0A1C3YMK1	pp	229533.I1REX4
88511	229533.A0A1C3YMK1	pp	229533.A0A1C3YMW0
88512	229533.A0A1C3YMK1	pp	229533.I1RG51
88513	229533.A0A1C3YMK1	pp	229533.I1RX46
88514	229533.A0A1C3YMK1	pp	229533.I1S219
88515	229533.A0A1C3YMK1	pp	229533.I1RPU0
88516	229533.A0A1C3YMK1	pp	229533.I1S0R7
88517	229533.A0A1C3YMK1	pp	229533.I1RCU7
88518	229533.A0A1C3YMK1	pp	229533.I1RQC9
88519	229533.A0A1C3YMK1	pp	229533.I1RT98
88520	229533.A0A1C3YMK1	pp	229533.I1RFQ7
88521	229533.A0A1C3YMK1	pp	229533.I1RT80
88522	229533.A0A1C3YMK1	pp	229533.I1RW25
88523	229533.A0A1C3YMK1	pp	229533.I1RNT2
88524	229533.A0A1C3YMK1	pp	229533.I1RDG0
88525	229533.A0A1C3YMK1	pp	229533.Q4IJK2
88526	229533.A0A1C3YMK1	pp	229533.I1RTE5
88527	229533.A0A1C3YMK1	pp	229533.Q4IRI9
88528	229533.A0A1C3YMK1	pp	229533.I1RB27
88529	229533.A0A1C3YMK1	pp	229533.I1RZK6
88530	229533.A0A1C3YMK1	pp	229533.I1RZF6
88531	229533.A0A1C3YMK1	pp	229533.I1RG56
88532	229533.A0A1C3YMK1	pp	229533.I1RZZ1
88533	229533.A0A1C3YMK1	pp	229533.I1RRT0
88534	229533.A0A1C3YMK1	pp	229533.I1RYJ8
88535	229533.A0A1C3YMK1	pp	229533.I1S0P4
88536	229533.A0A1C3YMK1	pp	229533.I1RRE0
88537	229533.A0A1C3YMK1	pp	229533.I1RBL7
88538	229533.A0A1C3YMK1	pp	229533.I1S9L0
88539	229533.A0A1C3YMK1	pp	229533.I1RZM1
88540	229533.A0A1C3YMK1	pp	229533.V6QUE6
88541	229533.A0A1C3YMK1	pp	229533.I1RLU6
88542	229533.A0A1C3YMK1	pp	229533.I1RCK4
88543	229533.A0A1C3YMK1	pp	229533.I1RAN5
88544	229533.A0A1C3YMK1	pp	229533.I1RQ31
88545	229533.A0A1C3YMK1	pp	229533.I1RZV9
88546	229533.A0A1C3YMK1	pp	229533.I1RNQ5
88547	229533.A0A1C3YMK1	pp	229533.I1S021
88548	229533.A0A1C3YMK1	pp	229533.I1RZB5
88549	229533.A0A1C3YMK1	pp	229533.I1S0M9

88550	229533.A0A1C3YMK1	pp	229533.I1RNH6
88551	229533.A0A1C3YMK1	pp	229533.I1RKL3
88552	229533.A0A1C3YMK1	pp	229533.I1RB43
88553	229533.A0A1C3YMK1	pp	229533.I1S5B5
88554	229533.A0A1C3YMK1	pp	229533.I1RYB9
88555	229533.A0A1C3YMK1	pp	229533.I1RAZ8
88556	229533.A0A1C3YMK1	pp	229533.I1RRJ8
88557	229533.A0A1C3YMK1	pp	229533.I1RK83
88558	229533.A0A1C3YMK1	pp	229533.I1RC03
88559	229533.A0A1C3YMK1	pp	229533.I1RE00
88560	229533.A0A1C3YMK1	pp	229533.I1RJL2
88561	229533.A0A1C3YMK1	pp	229533.I1RCS2
88562	229533.A0A1C3YMK1	pp	229533.I1RFJ9
88563	229533.A0A1C3YMK1	pp	229533.I1RSR8
88564	229533.A0A1C3YMK1	pp	229533.I1RBC7
88565	229533.A0A1C3YMK1	pp	229533.I1RSI0
88566	229533.A0A1C3YMK1	pp	229533.I1RGA4
88567	229533.A0A1C3YMK1	pp	229533.I1RDY1
88568	229533.A0A1C3YMK1	pp	229533.I1S6E5
88569	229533.A0A1C3YMK1	pp	229533.I1RAF3
88570	229533.A0A1C3YMK1	pp	229533.I1RT17
88571	229533.A0A1C3YMK1	pp	229533.I1RA48
88572	229533.A0A1C3YMK1	pp	229533.I1RQ65
88573	229533.A0A1C3YMK1	pp	229533.I1RP27
88574	229533.A0A1C3YMK1	pp	229533.I1R9V7
88575	229533.A0A1C3YMK1	pp	229533.I1RN67
88576	229533.A0A1C3YMK1	pp	229533.I1RK95
88577	229533.A0A1C3YMK1	pp	229533.I1RLV9
88578	229533.A0A1C3YMK1	pp	229533.I1RMY3
88579	229533.A0A1C3YMK1	pp	229533.I1RPP4
88580	229533.A0A1C3YMK1	pp	229533.I1RRQ3
88581	229533.A0A1C3YMK1	pp	229533.I1RYT7
88582	229533.A0A1C3YMK1	pp	229533.I1RE87
88583	229533.A0A1C3YMK1	pp	229533.I1RSV2
88584	229533.A0A1C3YMK1	pp	229533.I1RZE1
88585	229533.A0A1C3YMK1	pp	229533.I1RT04
88586	229533.A0A1C3YMK1	pp	229533.I1RWU2
88587	229533.A0A1C3YMK1	pp	229533.I1RC72
88588	229533.A0A1C3YMK1	pp	229533.I1S060
88589	229533.A0A1C3YMK1	pp	229533.I1S0J1
88590	229533.A0A1C3YMK1	pp	229533.I1RFS3
88591	229533.A0A1C3YMK1	pp	229533.I1RAF8
88592	229533.A0A1C3YMK1	pp	229533.I1RFW3
88593	229533.A0A1C3YMK1	pp	229533.I1RC00
88594	229533.A0A1C3YMK1	pp	229533.I1RJZ7
88595	229533.A0A1C3YMK1	pp	229533.I1RA84
88596	229533.A0A1C3YMK1	pp	229533.I1RSL8
88597	229533.A0A1C3YMK1	pp	229533.I1RST1
88598	229533.A0A1C3YMK1	pp	229533.I1RQ49
88599	229533.A0A1C3YMK1	pp	229533.I1RDY5
88600	229533.A0A1C3YMK1	pp	229533.I1S7S9
88601	229533.A0A1C3YMK1	pp	229533.I1RBG9
88602	229533.A0A1C3YMK1	pp	229533.I1RG11
88603	229533.A0A1C3YMK1	pp	229533.I1RZV6
88604	229533.A0A1C3YMK1	pp	229533.I1RKJ6
88605	229533.A0A1C3YMK1	pp	229533.I1RMN6
88606	229533.A0A1C3YMK1	pp	229533.I1RD22
88607	229533.A0A1C3YMK1	pp	229533.V6R042
88608	229533.A0A1C3YMK1	pp	229533.I1RX24
88609	229533.A0A1C3YMK1	pp	229533.I1RJS4
88610	229533.A0A1C3YMK1	pp	229533.I1RRT8
88611	229533.A0A1C3YMK1	pp	229533.I1RAW0
88612	229533.A0A1C3YMK1	pp	229533.I1RZS6
88613	229533.A0A1C3YMK1	pp	229533.I1S8E2
88614	229533.A0A1C3YMK1	pp	229533.I1RJS5
88615	229533.A0A1C3YMK1	pp	229533.I1RC81
88616	229533.A0A1C3YMK1	pp	229533.I1RNC1
88617	229533.A0A1C3YMK1	pp	229533.I1RR73
88618	229533.A0A1C3YMK1	pp	229533.I1RCL3
88619	229533.A0A1C3YMK1	pp	229533.I1RIR9
88620	229533.A0A1C3YMK1	pp	229533.I1S2A8
88621	229533.A0A1C3YMK1	pp	229533.I1RBZ3
88622	229533.A0A1C3YMK1	pp	229533.V6R057

229533.A0A1C3YMK1 pp 229533.I1RND6
229533.A0A1C3YMK1 pp 229533.I1RTH6
229533.A0A1C3YMK1 pp 229533.I1RA70
229533.A0A1C3YMK1 pp 229533.I1RBJ1
229533.A0A1C3YMK1 pp 229533.V6REZ4
229533.A0A1C3YMK1 pp 229533.I1RRR7
229533.A0A1C3YMK1 pp 229533.I1S2C0
229533.A0A1C3YMK1 pp 229533.I1RR25
229533.A0A1C3YMK1 pp 229533.V6RY33
229533.A0A1C3YMK1 pp 229533.I1RAU7
229533.A0A1C3YMK1 pp 229533.V6RNM4
229533.A0A1C3YMK1 pp 229533.I1RM96
229533.A0A1C3YMK1 pp 229533.I1RK79
229533.A0A1C3YMK1 pp 229533.V6R0U9
229533.A0A1C3YMK1 pp 229533.I1S1X9
229533.A0A1C3YMK1 pp 229533.I1RWB5
229533.A0A1C3YMK1 pp 229533.I1R9X7
229533.A0A1C3YMK1 pp 229533.I1RNE4
229533.A0A1C3YMK1 pp 229533.I1RRH3
229533.A0A1C3YMK1 pp 229533.I1RFL4
229533.A0A1C3YMK1 pp 229533.I1S2E8
229533.A0A1C3YMK1 pp 229533.I1RSD8
229533.A0A1C3YMK1 pp 229533.I1RRI2
229533.I1RVI8 pp 229533.V6R0W1
229533.I1RVI8 pp 229533.I1S3T7
229533.I1RVI8 pp 229533.I1S694
229533.I1RVI8 pp 229533.I1RZA8
229533.I1RVI8 pp 229533.V6RAM6
229533.I1RVI8 pp 229533.V6R5D2
229533.I1RVI8 pp 229533.I1RW54
229533.I1RVI8 pp 229533.I1SAI4
229533.I1RVI8 pp 229533.I1S2Q4
229533.I1RVI8 pp 229533.V6QU99
229533.I1RVI8 pp 229533.I1RY16
229533.I1RVI8 pp 229533.V6RWB3
229533.I1RVI8 pp 229533.V6R756
229533.I1RVI8 pp 229533.I1S299
229533.I1RVI8 pp 229533.I1S8C1
229533.I1RVI8 pp 229533.V6R2Z2
229533.I1RVI8 pp 229533.I1S8R2
229533.I1RVI8 pp 229533.I1RYA0
229533.I1RVI8 pp 229533.I1S0A5
229533.I1RVI8 pp 229533.I1S8F8
229533.I1RVI8 pp 229533.I1RYM5
229533.I1RVI8 pp 229533.I1RY81
229533.I1RVI8 pp 229533.I1RYC8
229533.I1RVI8 pp 229533.I1RWM4
229533.I1RVI8 pp 229533.I1S8D3
229533.A0A1C3YI26 pp 229533.V6RK96
229533.A0A1C3YI26 pp 229533.I1RZM4
229533.A0A1C3YI26 pp 229533.V6R382
229533.A0A1C3YI26 pp 229533.A0A1C3YKY4
229533.A0A1C3YI26 pp 229533.I1RAY9
229533.A0A1C3YI26 pp 229533.I1RZ83
229533.A0A1C3YI26 pp 229533.I1RMY1
229533.A0A1C3YI26 pp 229533.I1RRV4
229533.A0A1C3YI26 pp 229533.I1RXY7
229533.A0A1C3YI26 pp 229533.I1RA90
229533.A0A1C3YI26 pp 229533.I1RNG4
229533.A0A1C3YI26 pp 229533.I1S673
229533.A0A1C3YI26 pp 229533.I1RUY6
229533.A0A1C3YI26 pp 229533.A0A1C3YL20
229533.A0A1C3YI26 pp 229533.I1S0N3
229533.A0A1C3YI26 pp 229533.I1S8E0
229533.A0A1C3YI26 pp 229533.A0A1C3YLP6
229533.A0A1C3YI26 pp 229533.A0A1C3YJU5
229533.A0A1C3YI26 pp 229533.I1RXV3
229533.A0A1C3YI26 pp 229533.I1RMK4
229533.A0A1C3YI26 pp 229533.I1RBJ8
229533.A0A1C3YI26 pp 229533.I1RW00
229533.A0A1C3YI26 pp 229533.I1RQE8
229533.A0A1C3YI26 pp 229533.I1RP97
229533.A0A1C3YI26 pp 229533.I1RAJ6

88696	229533.A0A1C3YI26	pp	229533.I1S3G4
88697	229533.A0A1C3YI26	pp	229533.I1RSS7
88698	229533.A0A1C3YI26	pp	229533.A0A1C3YL56
88699	229533.A0A1C3YI26	pp	229533.I1S1W8
88700	229533.A0A1C3YI26	pp	229533.I1S0Y7
88701	229533.Q4IR08	pp	229533.V6RLW4
88702	229533.Q4IR08	pp	229533.V6RIS2
88703	229533.Q4IF76	pp	229533.V6QVD5
88704	229533.Q4IF76	pp	229533.Q4IPB3
88705	229533.Q4IF76	pp	229533.Q4INZ9
88706	229533.Q4IF76	pp	229533.Q4IQW0
88707	229533.Q4IF76	pp	229533.V6QYF0
88708	229533.Q4IF76	pp	229533.V6QUE6
88709	229533.Q4IF76	pp	229533.Q4IMJ1
88710	229533.Q4IF76	pp	229533.V6RGN5
88711	229533.Q4IF76	pp	229533.Q4IPI1
88712	229533.Q4IF76	pp	229533.V6R227
88713	229533.Q4IF76	pp	229533.V6RY33
88714	229533.Q4IF76	pp	229533.V6RGY3
88715	229533.Q4IF76	pp	229533.Q4IN63
88716	229533.Q4IF76	pp	229533.V6RIS2
88717	229533.Q4IF76	pp	229533.Q4IJ56
88718	229533.Q4IF76	pp	229533.Q4IPB8
88719	229533.Q4IF76	pp	229533.Q4IJH1
88720	229533.Q4IF76	pp	229533.Q4IR08
88721	229533.Q4IF76	pp	229533.Q4IRI9
88722	229533.Q4IF76	pp	229533.V6R283
88723	229533.Q4IF76	pp	229533.Q4IFI0
88724	229533.I1RBD2	pp	229533.I1RNV6
88725	229533.I1RBD2	pp	229533.I1RT80
88726	229533.I1RBD2	pp	229533.I1S253
88727	229533.I1RBD2	pp	229533.I1RBG1
88728	229533.I1RBD2	pp	229533.V6RIS2
88729	229533.I1RBD2	pp	229533.Q4IR08
88730	229533.I1RBD2	pp	229533.Q4I1M9
88731	229533.I1RBD2	pp	229533.I1RYH6
88732	229533.I1RBD2	pp	229533.I1RS19
88733	229533.I1RBD2	pp	229533.I1RD64
88734	229533.I1RBD2	pp	229533.I1RM06
88735	229533.I1RBD2	pp	229533.V6RGY0
88736	229533.I1RBD2	pp	229533.I1RKF9
88737	229533.I1RBD2	pp	229533.I1S021
88738	229533.I1RBD2	pp	229533.I1RN81
88739	229533.I1RBD2	pp	229533.I1REF2
88740	229533.I1RBD2	pp	229533.I1RS47
88741	229533.I1RBD2	pp	229533.I1RC82
88742	229533.I1RBD2	pp	229533.V6RGY3
88743	229533.I1RBD2	pp	229533.Q4I327
88744	229533.I1RBD2	pp	229533.I1S558
88745	229533.I1RBD2	pp	229533.Q4ILI9
88746	229533.I1RBD2	pp	229533.I1RRQ3
88747	229533.I1RBD2	pp	229533.I1RQA2
88748	229533.I1RBD2	pp	229533.I1RZ99
88749	229533.I1RBD2	pp	229533.I1RQJ5
88750	229533.I1RBD2	pp	229533.I1RQ31
88751	229533.I1RBD2	pp	229533.I1RTE9
88752	229533.I1RBD2	pp	229533.I1RW71
88753	229533.I1RBD2	pp	229533.I1RS81
88754	229533.I1RBD2	pp	229533.I1RPJ3
88755	229533.I1RBD2	pp	229533.I1RPP7
88756	229533.I1RBD2	pp	229533.Q4I8X0
88757	229533.I1RBD2	pp	229533.I1RM96
88758	229533.I1RBD2	pp	229533.I1RE37
88759	229533.I1RBD2	pp	229533.I1RKL3
88760	229533.I1RBD2	pp	229533.V6RR76
88761	229533.I1RBD2	pp	229533.I1RZH6
88762	229533.I1RBD2	pp	229533.I1RD10
88763	229533.I1RBD2	pp	229533.I1RYD3
88764	229533.I1RBD2	pp	229533.I1RY63
88765	229533.I1RBD2	pp	229533.I1RK95
88766	229533.I1RBD2	pp	229533.I1RX09
88767	229533.I1RBD2	pp	229533.I1RNU6
88768	229533.I1RBD2	pp	229533.I1RKL0

88769 229533.I1RBD2 pp 229533.I1RLX7
88770 229533.I1RBD2 pp 229533.I1S2E8
88771 229533.I1RBD2 pp 229533.I1RZZ1
88772 229533.I1RBD2 pp 229533.I1RSD8
88773 229533.I1RBD2 pp 229533.V6RBN2
88774 229533.I1RW00 pp 229533.I1RYJ6
88775 229533.I1RW00 pp 229533.I1RWF9
88776 229533.I1RW00 pp 229533.I1RWS4
88777 229533.I1RW00 pp 229533.I1S0Y7
88778 229533.I1RW00 pp 229533.I1S8E0
88779 229533.I1RQ87 pp 229533.I1RVQ0
88780 229533.I1RQ87 pp 229533.V6RIV9
88781 229533.I1RQ87 pp 229533.I1RRM5
88782 229533.I1RQ87 pp 229533.I1RW99
88783 229533.I1RQ87 pp 229533.V6RMW6
88784 229533.Q4IJH1 pp 229533.Q4IN63
88785 229533.Q4IJH1 pp 229533.Q4IR08
88786 229533.Q4IJH1 pp 229533.V6RIS2
88787 229533.Q4IJH1 pp 229533.Q4IRI9
88788 229533.Q4IJH1 pp 229533.Q4IPB3
88789 229533.Q4IJH1 pp 229533.V6RGY3
88790 229533.Q4IJH1 pp 229533.Q4IPB8
88791 229533.Q4IJH1 pp 229533.V6RY33
88792 229533.Q4IJH1 pp 229533.V6R283
88793 229533.Q4IJH1 pp 229533.V6QUE6
88794 229533.Q4IJH1 pp 229533.Q4IQK7
88795 229533.Q4IJH1 pp 229533.V6R227
88796 229533.Q4IJH1 pp 229533.Q4INZ9
88797 229533.Q4IJH1 pp 229533.Q4IPI1
88798 229533.Q4IJH1 pp 229533.V6RGN5
88799 229533.Q4IJH1 pp 229533.Q4IQW0
88800 229533.Q4IJH1 pp 229533.V6QYF0
88801 229533.Q4IJH1 pp 229533.V6QVD5
88802 229533.I1RYJ6 pp 229533.I1RYM5
88803 229533.I1RYJ6 pp 229533.V6RCZ8
88804 229533.I1RYJ6 pp 229533.I1RZB2
88805 229533.I1RYJ6 pp 229533.I1S253
88806 229533.I1RYJ6 pp 229533.I1S9Y1
88807 229533.I1RYJ6 pp 229533.I1S0F1
88808 229533.I1RYJ6 pp 229533.I1S5B0
88809 229533.I1RYJ6 pp 229533.I1S5R1
88810 229533.I1RYJ6 pp 229533.V6R382
88811 229533.I1RYJ6 pp 229533.I1RZQ1
88812 229533.I1RYJ6 pp 229533.I1S274
88813 229533.I1RYJ6 pp 229533.I1S3F7
88814 229533.I1RYJ6 pp 229533.I1S0J8
88815 229533.I1RYJ6 pp 229533.I1S0T4
88816 229533.A0A1C3YMB9 pp 229533.I1RQ65
88817 229533.A0A1C3YMB9 pp 229533.I1RAY2
88818 229533.A0A1C3YMB9 pp 229533.I1RPU0
88819 229533.A0A1C3YMB9 pp 229533.I1RZS6
88820 229533.A0A1C3YMB9 pp 229533.I1RKJ6
88821 229533.A0A1C3YMB9 pp 229533.I1RM09
88822 229533.A0A1C3YMB9 pp 229533.I1RRK8
88823 229533.A0A1C3YMB9 pp 229533.I1RHM4
88824 229533.A0A1C3YMB9 pp 229533.I1S0M9
88825 229533.A0A1C3YMB9 pp 229533.Q4HYB8
88826 229533.A0A1C3YMB9 pp 229533.I1RT98
88827 229533.A0A1C3YMB9 pp 229533.Q4IAS1
88828 229533.A0A1C3YMB9 pp 229533.I1RLV9
88829 229533.A0A1C3YMB9 pp 229533.I1S0J1
88830 229533.A0A1C3YMB9 pp 229533.I1RWE1
88831 229533.A0A1C3YMB9 pp 229533.I1RA48
88832 229533.A0A1C3YMB9 pp 229533.I1RQJ5
88833 229533.A0A1C3YMB9 pp 229533.Q4IJU0
88834 229533.A0A1C3YMB9 pp 229533.I1RM96
88835 229533.A0A1C3YMB9 pp 229533.I1RSR8
88836 229533.A0A1C3YMB9 pp 229533.I1RNW8
88837 229533.A0A1C3YJ01 pp 229533.I1RI11
88838 229533.A0A1C3YJ01 pp 229533.I1S694
88839 229533.A0A1C3YJ01 pp 229533.I1RZA8
88840 229533.A0A1C3YJ01 pp 229533.I1RY16
88841 229533.I1RWS4 pp 229533.I1S253

88842 229533.I1RWS4 pp 229533.I1S3F7
88843 229533.I1RWS4 pp 229533.I1S0J8
88844 229533.I1RWS4 pp 229533.I1RZB2
88845 229533.I1RWS4 pp 229533.I1RZQ1
88846 229533.I1RWS4 pp 229533.I1S9Y1
88847 229533.I1RWS4 pp 229533.I1RY63
88848 229533.I1RWS4 pp 229533.I1S5R1
88849 229533.I1RWS4 pp 229533.V6R382
88850 229533.I1RWS4 pp 229533.I1S274
88851 229533.I1RWS4 pp 229533.I1S0T4
88852 229533.I1RWS4 pp 229533.I1S5B0
88853 229533.I1RWS4 pp 229533.V6RCZ8
88854 229533.I1RWS4 pp 229533.I1RYM5
88855 229533.I1RWS4 pp 229533.I1S0F1
88856 229533.I1RP54 pp 229533.I1RVB6
88857 229533.I1RPY1 pp 229533.I1S6F0
88858 229533.I1RPY1 pp 229533.Q4I830
88859 229533.I1RPY1 pp 229533.I1RRP3
88860 229533.I1RPY1 pp 229533.I1RS30
88861 229533.I1RPY1 pp 229533.I1RRQ3
88862 229533.I1RPY1 pp 229533.Q4IEV5
88863 229533.I1RPY1 pp 229533.I1RQ65
88864 229533.I1RPY1 pp 229533.I1RRG3
88865 229533.I1RPY1 pp 229533.I1RS47
88866 229533.I1RPY1 pp 229533.I1S0D4
88867 229533.I1RPY1 pp 229533.I1RT98
88868 229533.I1RPY1 pp 229533.V6R283
88869 229533.I1RPY1 pp 229533.Q4HZ42
88870 229533.I1RPY1 pp 229533.I1RUQ2
88871 229533.I1RPY1 pp 229533.I1RZ99
88872 229533.I1RPY1 pp 229533.Q4I662
88873 229533.I1RPY1 pp 229533.I1RQJ5
88874 229533.I1RPY1 pp 229533.I1RWJ4
88875 229533.I1RPY1 pp 229533.I1S0R7
88876 229533.I1RPY1 pp 229533.I1RSQ7
88877 229533.I1RPY1 pp 229533.I1RT04
88878 229533.I1RPY1 pp 229533.I1RW61
88879 229533.I1RPY1 pp 229533.I1RRT2
88880 229533.I1RPY1 pp 229533.I1RQS5
88881 229533.I1RPY1 pp 229533.I1RZS6
88882 229533.I1RPY1 pp 229533.V6QW22
88883 229533.I1RPY1 pp 229533.I1RX24
88884 229533.I1RPY1 pp 229533.I1RX46
88885 229533.I1RPY1 pp 229533.I1RRJ8
88886 229533.I1RPY1 pp 229533.I1RRQ6
88887 229533.I1RPY1 pp 229533.I1RRT8
88888 229533.I1RPY1 pp 229533.Q4IAS1
88889 229533.I1RPY1 pp 229533.I1S9U2
88890 229533.I1RPY1 pp 229533.Q4IPI1
88891 229533.I1RPY1 pp 229533.I1RW25
88892 229533.I1RPY1 pp 229533.I1S0J1
88893 229533.I1RPY1 pp 229533.V6QYF0
88894 229533.I1RPY1 pp 229533.I1RX14
88895 229533.I1RPY1 pp 229533.Q4IQW0
88896 229533.I1RPY1 pp 229533.Q4IAA0
88897 229533.I1RPY1 pp 229533.Q4IFI0
88898 229533.I1RPY1 pp 229533.I1RX08
88899 229533.I1RPY1 pp 229533.I1RSI6
88900 229533.I1RPY1 pp 229533.I1RT05
88901 229533.I1RPY1 pp 229533.I1RQM6
88902 229533.I1RPY1 pp 229533.Q4IBS2
88903 229533.I1RPY1 pp 229533.I1S0M9
88904 229533.I1RPY1 pp 229533.I1S253
88905 229533.I1RPY1 pp 229533.Q4IJ56
88906 229533.I1RPY1 pp 229533.Q4HVW2
88907 229533.I1RPY1 pp 229533.I1RW99
88908 229533.I1RPY1 pp 229533.I1RQ81
88909 229533.I1RPY1 pp 229533.V6RGN5
88910 229533.I1RPY1 pp 229533.Q4IEK8
88911 229533.I1RPY1 pp 229533.I1RTF4
88912 229533.I1RPY1 pp 229533.I1S4T3
88913 229533.I1RPY1 pp 229533.I1RZZ1
88914 229533.I1RPY1 pp 229533.Q4IJH1

88915 229533.I1RPY1 pp 229533.I1RRI2
88916 229533.I1RPY1 pp 229533.I1RRR7
88917 229533.I1RPY1 pp 229533.I1RZK6
88918 229533.I1RPY1 pp 229533.V6R227
88919 229533.I1RPY1 pp 229533.I1S2E8
88920 229533.I1RPY1 pp 229533.I1RSD8
88921 229533.I1RPY1 pp 229533.I1RQE7
88922 229533.I1RPY1 pp 229533.I1RTD7
88923 229533.I1RPY1 pp 229533.I1RTD9
88924 229533.I1RPY1 pp 229533.I1RQ28
88925 229533.I1RPY1 pp 229533.I1RZF6
88926 229533.I1RPY1 pp 229533.I1RSR8
88927 229533.I1RPY1 pp 229533.Q4I327
88928 229533.I1RPY1 pp 229533.I1S021
88929 229533.I1RPY1 pp 229533.I1S1X9
88930 229533.I1RPY1 pp 229533.I1S2C0
88931 229533.I1RPY1 pp 229533.I1RTE9
88932 229533.I1RPY1 pp 229533.I1RQ31
88933 229533.I1RPY1 pp 229533.I1RSC6
88934 229533.I1RPY1 pp 229533.I1S0M3
88935 229533.I1RPY1 pp 229533.I1RZE5
88936 229533.I1RPY1 pp 229533.I1RZB5
88937 229533.I1RPY1 pp 229533.Q4IN63
88938 229533.I1RPY1 pp 229533.Q4HZK7
88939 229533.I1RPY1 pp 229533.Q4HZ68
88940 229533.I1RPY1 pp 229533.Q4I5Z5
88941 229533.I1RPY1 pp 229533.I1RVZ0
88942 229533.I1RPY1 pp 229533.I1S558
88943 229533.I1RPY1 pp 229533.I1RQA2
88944 229533.I1RPY1 pp 229533.I1RSR3
88945 229533.I1RNG5 pp 229533.I1RS94
88946 229533.I1RNG5 pp 229533.I1RYT7
88947 229533.I1RNG5 pp 229533.I1RW80
88948 229533.I1RNG5 pp 229533.I1RPV8
88949 229533.I1RNG5 pp 229533.I1SAT2
88950 229533.I1RNG5 pp 229533.I1S0A5
88951 229533.I1RNG5 pp 229533.V6REZ4
88952 229533.I1RNG5 pp 229533.I1RZ56
88953 229533.I1RNG5 pp 229533.I1S279
88954 229533.I1RNG5 pp 229533.I1RW91
88955 229533.I1RNG5 pp 229533.I1RQ49
88956 229533.I1RNG5 pp 229533.I1S5D0
88957 229533.I1RNG5 pp 229533.I1RNT2
88958 229533.I1RNG5 pp 229533.I1S6H3
88959 229533.I1RNG5 pp 229533.I1S094
88960 229533.I1RNG5 pp 229533.I1RY40
88961 229533.I1RNG5 pp 229533.I1RQV7
88962 229533.I1RNG5 pp 229533.I1S969
88963 229533.I1RNG5 pp 229533.I1RPW8
88964 229533.I1RNG5 pp 229533.I1RRS6
88965 229533.I1RNG5 pp 229533.I1S659
88966 229533.I1RNG5 pp 229533.I1S267
88967 229533.I1RNG5 pp 229533.I1RQQ5
88968 229533.I1RNG5 pp 229533.I1RZV9
88969 229533.I1RNG5 pp 229533.I1S0P4
88970 229533.I1RNG5 pp 229533.I1RST9
88971 229533.A0A1C3YJ29 pp 229533.I1RZF6
88972 229533.A0A1C3YJ29 pp 229533.I1RNH6
88973 229533.A0A1C3YJ29 pp 229533.I1S0M9
88974 229533.A0A1C3YJ29 pp 229533.I1RRU9
88975 229533.A0A1C3YJ29 pp 229533.I1RGC2
88976 229533.A0A1C3YJ29 pp 229533.Q4IJ56
88977 229533.A0A1C3YJ29 pp 229533.I1RB50
88978 229533.A0A1C3YJ29 pp 229533.I1RZK6
88979 229533.A0A1C3YJ29 pp 229533.I1RPU0
88980 229533.A0A1C3YJ29 pp 229533.Q4IRI9
88981 229533.A0A1C3YJ29 pp 229533.I1R9V7
88982 229533.A0A1C3YJ29 pp 229533.I1RDG0
88983 229533.A0A1C3YJ29 pp 229533.I1RA48
88984 229533.A0A1C3YJ29 pp 229533.I1S060
88985 229533.A0A1C3YJ29 pp 229533.I1S1C6
88986 229533.A0A1C3YJ29 pp 229533.I1RS28
88987 229533.A0A1C3YJ29 pp 229533.I1RRL0

88988 229533.A0A1C3YJ29 pp 229533.I1RYB1
88989 229533.A0A1C3YJ29 pp 229533.I1RFP9
88990 229533.A0A1C3YJ29 pp 229533.I1RSR8
88991 229533.A0A1C3YJ29 pp 229533.Q4IPI1
88992 229533.A0A1C3YJ29 pp 229533.I1RSD4
88993 229533.A0A1C3YJ29 pp 229533.I1RZG7
88994 229533.A0A1C3YJ29 pp 229533.I1RAN5
88995 229533.A0A1C3YJ29 pp 229533.I1RWB5
88996 229533.A0A1C3YJ29 pp 229533.I1RBL7
88997 229533.A0A1C3YJ29 pp 229533.I1RX81
88998 229533.A0A1C3YJ29 pp 229533.I1RA36
88999 229533.A0A1C3YJ29 pp 229533.I1RPA0
89000 229533.A0A1C3YJ29 pp 229533.I1RQJ5
89001 229533.A0A1C3YJ29 pp 229533.I1RMN6
89002 229533.A0A1C3YJ29 pp 229533.I1RM96
89003 229533.A0A1C3YJ29 pp 229533.I1RX51
89004 229533.A0A1C3YJ29 pp 229533.I1RSA4
89005 229533.A0A1C3YJ29 pp 229533.Q4ICG5
89006 229533.A0A1C3YJ29 pp 229533.I1RX47
89007 229533.A0A1C3YJ29 pp 229533.I1RCS2
89008 229533.A0A1C3YJ29 pp 229533.I1RT47
89009 229533.A0A1C3YJ29 pp 229533.I1RC03
89010 229533.A0A1C3YJ29 pp 229533.I1RKL3
89011 229533.A0A1C3YJ29 pp 229533.I1RSD8
89012 229533.A0A1C3YJ29 pp 229533.I1S2E8
89013 229533.A0A1C3YJ29 pp 229533.I1RWU2
89014 229533.A0A1C3YJ29 pp 229533.I1RXU1
89015 229533.A0A1C3YJ29 pp 229533.I1RG90
89016 229533.A0A1C3YJ29 pp 229533.I1S4M5
89017 229533.A0A1C3YJ29 pp 229533.I1RFS3
89018 229533.A0A1C3YJ29 pp 229533.V6RQ48
89019 229533.A0A1C3YJ29 pp 229533.I1RBG9
89020 229533.A0A1C3YJ29 pp 229533.I1RAU7
89021 229533.A0A1C3YJ29 pp 229533.I1RK90
89022 229533.A0A1C3YJ29 pp 229533.V6RKE4
89023 229533.A0A1C3YJ29 pp 229533.I1RG57
89024 229533.A0A1C3YJ29 pp 229533.I1RZA4
89025 229533.A0A1C3YJ29 pp 229533.I1RRQ3
89026 229533.A0A1C3YJ29 pp 229533.I1RNR3
89027 229533.A0A1C3YJ29 pp 229533.I1RK92
89028 229533.A0A1C3YJ29 pp 229533.I1RQ65
89029 229533.A0A1C3YJ29 pp 229533.I1S8E2
89030 229533.A0A1C3YJ29 pp 229533.I1RAF8
89031 229533.A0A1C3YJ29 pp 229533.A0A1C3YNC3
89032 229533.A0A1C3YJ29 pp 229533.I1RR73
89033 229533.A0A1C3YJ29 pp 229533.I1RBV3
89034 229533.A0A1C3YJ29 pp 229533.I1RLV9
89035 229533.A0A1C3YJ29 pp 229533.I1S9Y1
89036 229533.A0A1C3YJ29 pp 229533.I1RKJ6
89037 229533.A0A1C3YJ29 pp 229533.I1RD64
89038 229533.A0A1C3YJ29 pp 229533.I1RST1
89039 229533.I1S8R2 pp 229533.V6R3J1
89040 229533.I1S8R2 pp 229533.I1S968
89041 229533.I1S8R2 pp 229533.V6RF62
89042 229533.I1S8R2 pp 229533.V6RRM0
89043 229533.I1S8R2 pp 229533.Q4HVQ9
89044 229533.I1S8R2 pp 229533.V6QVH2
89045 229533.I1S8R2 pp 229533.V6R3V4
89046 229533.I1S8R2 pp 229533.I1S8R4
89047 229533.I1S8R2 pp 229533.V6RZD4
89048 229533.I1S8R2 pp 229533.V6RM66
89049 229533.I1RRN8 pp 229533.I1S130
89050 229533.I1RNB7 pp 229533.I1RQ49
89051 229533.I1RNB7 pp 229533.I1S0H1
89052 229533.I1RNB7 pp 229533.I1RTE5
89053 229533.I1RNB7 pp 229533.I1RNG5
89054 229533.A0A098DLV6 pp 229533.Q4IPI4
89055 229533.A0A098DLV6 pp 229533.I1S7I5
89056 229533.A0A098DLV6 pp 229533.A0A1C3YLC6
89057 229533.A0A098DLV6 pp 229533.I1RE51
89058 229533.A0A098DLV6 pp 229533.A0A1C3YHP3
89059 229533.A0A098DLV6 pp 229533.I1S1V2
89060 229533.A0A098DLV6 pp 229533.A0A1C3YLA2

89061 229533.A0A098DLV6 pp 229533.I1RAN8
89062 229533.A0A1C3YIZ7 pp 229533.I1RZ25
89063 229533.I1RJA4 pp 229533.I1RL80
89064 229533.V6RIS2 pp 229533.V6RY33
89065 229533.A0A098DLU8 pp 229533.I1RG07
89066 229533.I1S044 pp 229533.I1S2C0
89067 229533.I1S044 pp 229533.V6RNM4
89068 229533.I1S044 pp 229533.I1S2E8
89069 229533.I1S044 pp 229533.V6R042
89070 229533.I1S044 pp 229533.V6RMW6
89071 229533.I1S044 pp 229533.V6R0U9
89072 229533.I1S044 pp 229533.V6REZ4
89073 229533.I1S044 pp 229533.I1S6E5
89074 229533.I1S044 pp 229533.I1S060
89075 229533.I1S044 pp 229533.I1S0J1
89076 229533.I1S044 pp 229533.V6RY33
89077 229533.I1S044 pp 229533.Q4IF76
89078 229533.I1S044 pp 229533.I1S7S9
89079 229533.I1S044 pp 229533.Q4IJK2
89080 229533.I1S044 pp 229533.I1S0M9
89081 229533.I1S044 pp 229533.I1S8E2
89082 229533.I1S044 pp 229533.I1S9L0
89083 229533.I1S044 pp 229533.Q4I948
89084 229533.I1S044 pp 229533.Q4IRI9
89085 229533.I1S044 pp 229533.I1S0R7
89086 229533.I1S044 pp 229533.I1S246
89087 229533.I1S044 pp 229533.Q4IBS2
89088 229533.I1S044 pp 229533.Q4IPI1
89089 229533.I1S044 pp 229533.Q4IJH1
89090 229533.I1S044 pp 229533.Q4IFI0
89091 229533.I1RZT1 pp 229533.I1S1X4
89092 229533.I1RZT1 pp 229533.V6QU65
89093 229533.I1RZT1 pp 229533.I1RZZ1
89094 229533.I1RZT1 pp 229533.I1RZV9
89095 229533.I1RZT1 pp 229533.I1S077
89096 229533.I1RZT1 pp 229533.Q4IJH1
89097 229533.I1RZT1 pp 229533.Q4HTT2
89098 229533.I1RZT1 pp 229533.I1S5B5
89099 229533.I1RZT1 pp 229533.Q4I7F9
89100 229533.I1RZT1 pp 229533.I1S0P4
89101 229533.I1RZT1 pp 229533.I1S123
89102 229533.I1RZT1 pp 229533.V6QV09
89103 229533.I1RZT1 pp 229533.I1RZY9
89104 229533.I1RZT1 pp 229533.Q4I7N9
89105 229533.I1RZT1 pp 229533.I1S987
89106 229533.I1RZT1 pp 229533.I1S267
89107 229533.I1RZT1 pp 229533.Q4IN00
89108 229533.I1RZT1 pp 229533.V6REZ4
89109 229533.I1RZT1 pp 229533.I1S4P6
89110 229533.I1RZT1 pp 229533.I1S2C0
89111 229533.I1RZT1 pp 229533.I1S9T5
89112 229533.I1RZT1 pp 229533.V6RGI1
89113 229533.I1RZT1 pp 229533.Q4IB89
89114 229533.I1RZT1 pp 229533.I1S0D5
89115 229533.I1RZT1 pp 229533.Q4IF76
89116 229533.I1RZT1 pp 229533.I1S4Z5
89117 229533.I1RZT1 pp 229533.I1S2E9
89118 229533.I1RZT1 pp 229533.I1S0N7
89119 229533.I1RZT1 pp 229533.I1SAD2
89120 229533.I1RZT1 pp 229533.I1S004
89121 229533.I1RZT1 pp 229533.I1S998
89122 229533.I1RZT1 pp 229533.I1S549
89123 229533.I1RZT1 pp 229533.V6R3J1
89124 229533.I1RZT1 pp 229533.V6RHD0
89125 229533.A0A0E0S631 pp 229533.I1RRY9
89126 229533.A0A0E0S631 pp 229533.I1RK95
89127 229533.A0A0E0S631 pp 229533.I1RUP7
89128 229533.A0A0E0S631 pp 229533.I1RTC2
89129 229533.A0A0E0S631 pp 229533.I1RSG8
89130 229533.A0A0E0S631 pp 229533.I1RK79
89131 229533.A0A0E0S631 pp 229533.I1RXU0
89132 229533.A0A0E0S631 pp 229533.I1RVY0
89133 229533.A0A0E0S631 pp 229533.I1RTD5

89134	229533.A0A1C3YK58	pp	229533.I1SAE8
89135	229533.A0A1C3YK58	pp	229533.I1S0P4
89136	229533.A0A1C3YK58	pp	229533.I1REA5
89137	229533.A0A1C3YK58	pp	229533.I1S8F8
89138	229533.A0A1C3YK58	pp	229533.I1RKU0
89139	229533.A0A1C3YK58	pp	229533.I1RJT5
89140	229533.A0A1C3YK58	pp	229533.A0A1C3YM86
89141	229533.A0A1C3YK58	pp	229533.I1RUQ3
89142	229533.A0A1C3YK58	pp	229533.A0A1C3YKF3
89143	229533.A0A1C3YK58	pp	229533.I1RXX1
89144	229533.A0A1C3YK58	pp	229533.I1RJ03
89145	229533.A0A1C3YK58	pp	229533.A0A1C3YLC2
89146	229533.A0A1C3YK58	pp	229533.I1S0K2
89147	229533.A0A1C3YK58	pp	229533.I1RZV9
89148	229533.A0A1C3YK58	pp	229533.I1RG33
89149	229533.A0A1C3YK58	pp	229533.I1RCV1
89150	229533.A0A1C3YK58	pp	229533.I1RGU6
89151	229533.A0A1C3YK58	pp	229533.I1S084
89152	229533.A0A1C3YK58	pp	229533.I1RPM6
89153	229533.A0A1C3YK58	pp	229533.V6R3V4
89154	229533.A0A1C3YK58	pp	229533.I1RCT7
89155	229533.A0A1C3YK58	pp	229533.I1RA94
89156	229533.A0A1C3YK58	pp	229533.I1RZH8
89157	229533.A0A1C3YK58	pp	229533.I1RCM5
89158	229533.A0A1C3YK58	pp	229533.I1RXE6
89159	229533.A0A1C3YK58	pp	229533.I1RZY1
89160	229533.A0A1C3YK58	pp	229533.I1RCK4
89161	229533.A0A1C3YK58	pp	229533.I1S0V6
89162	229533.A0A1C3YK58	pp	229533.I1RMH9
89163	229533.A0A1C3YK58	pp	229533.I1RH30
89164	229533.A0A1C3YK58	pp	229533.I1RC48
89165	229533.A0A1C3YK58	pp	229533.I1S8S9
89166	229533.A0A1C3YK58	pp	229533.I1RPR1
89167	229533.A0A1C3YK58	pp	229533.I1RP86
89168	229533.A0A1C3YK58	pp	229533.I1RAQ4
89169	229533.A0A1C3YK58	pp	229533.I1RNY6
89170	229533.A0A1C3YK58	pp	229533.V6RY56
89171	229533.A0A1C3YK58	pp	229533.I1RD21
89172	229533.A0A1C3YK58	pp	229533.I1RNW1
89173	229533.A0A1C3YK58	pp	229533.I1RIL0
89174	229533.A0A1C3YK58	pp	229533.I1S2V3
89175	229533.A0A1C3YK58	pp	229533.I1RJ72
89176	229533.A0A1C3YK58	pp	229533.I1RYM5
89177	229533.A0A1C3YK58	pp	229533.I1RR12
89178	229533.A0A1C3YK58	pp	229533.I1RCU2
89179	229533.A0A1C3YK58	pp	229533.I1S4S5
89180	229533.A0A1C3YK58	pp	229533.I1RNM7
89181	229533.A0A1C3YK58	pp	229533.I1RLQ5
89182	229533.A0A1C3YK58	pp	229533.I1S1P8
89183	229533.A0A1C3YK58	pp	229533.I1RQX9
89184	229533.A0A1C3YK58	pp	229533.I1RCU7
89185	229533.A0A1C3YK58	pp	229533.I1RMM8
89186	229533.A0A1C3YK58	pp	229533.I1RY49
89187	229533.A0A1C3YK58	pp	229533.I1RC39
89188	229533.A0A1C3YK58	pp	229533.V6QWD8
89189	229533.A0A1C3YK58	pp	229533.I1RHT4
89190	229533.A0A1C3YK58	pp	229533.A0A1C3YKZ8
89191	229533.A0A1C3YK58	pp	229533.I1RC74
89192	229533.A0A1C3YK58	pp	229533.I1RY40
89193	229533.A0A1C3YK58	pp	229533.I1RAG9
89194	229533.A0A1C3YK58	pp	229533.I1RM66
89195	229533.A0A1C3YK58	pp	229533.I1RV23
89196	229533.A0A1C3YK58	pp	229533.I1RPW2
89197	229533.A0A1C3YK58	pp	229533.A0A1C3YN26
89198	229533.A0A1C3YK58	pp	229533.I1RNT2
89199	229533.A0A1C3YK58	pp	229533.I1RWW7
89200	229533.A0A1C3YK58	pp	229533.I1RIA1
89201	229533.A0A1C3YK58	pp	229533.V6RRM0
89202	229533.A0A1C3YK58	pp	229533.I1RZV5
89203	229533.A0A1C3YK58	pp	229533.I1S0V7
89204	229533.A0A1C3YK58	pp	229533.I1S3K8
89205	229533.A0A1C3YK58	pp	229533.I1RC11
89206	229533.A0A1C3YK58	pp	229533.I1RNP4

89207 229533.A0A1C3YK58 pp 229533.I1S147
89208 229533.A0A1C3YK58 pp 229533.I1S024
89209 229533.A0A1C3YK58 pp 229533.I1RGP5
89210 229533.A0A1C3YK58 pp 229533.I1RZ20
89211 229533.A0A1C3YK58 pp 229533.I1RL81
89212 229533.A0A1C3YK58 pp 229533.I1RLZ4
89213 229533.A0A1C3YK58 pp 229533.I1S273
89214 229533.A0A1C3YK58 pp 229533.I1RYC8
89215 229533.A0A1C3YK58 pp 229533.I1RMU5
89216 229533.A0A1C3YK58 pp 229533.V6RN31
89217 229533.A0A1C3YK58 pp 229533.I1RE63
89218 229533.A0A1C3YK58 pp 229533.V6RLM7
89219 229533.A0A1C3YK58 pp 229533.A0A1C3YKN4
89220 229533.A0A1C3YK58 pp 229533.I1RYR3
89221 229533.A0A1C3YK58 pp 229533.I1RCG5
89222 229533.A0A1C3YK58 pp 229533.I1RNE4
89223 229533.A0A1C3YK58 pp 229533.I1RB38
89224 229533.A0A1C3YK58 pp 229533.I1RD80
89225 229533.A0A1C3YK58 pp 229533.I1RYW9
89226 229533.A0A1C3YK58 pp 229533.I1RQ49
89227 229533.A0A1C3YK58 pp 229533.V6REZ4
89228 229533.A0A1C3YK58 pp 229533.I1RS94
89229 229533.A0A1C3YK58 pp 229533.I1RF85
89230 229533.A0A1C3YK58 pp 229533.I1RAR1
89231 229533.A0A1C3YK58 pp 229533.I1R9B8
89232 229533.A0A1C3YK58 pp 229533.I1S3N0
89233 229533.A0A1C3YK58 pp 229533.A0A1C3YKW4
89234 229533.A0A1C3YK58 pp 229533.V6RF62
89235 229533.A0A1C3YK58 pp 229533.I1RNG4
89236 229533.A0A1C3YK58 pp 229533.I1RW43
89237 229533.A0A1C3YK58 pp 229533.A0A1C3YNB1
89238 229533.A0A1C3YK58 pp 229533.I1RPD6
89239 229533.A0A1C3YK58 pp 229533.I1RYA0
89240 229533.A0A1C3YK58 pp 229533.I1RZT0
89241 229533.A0A1C3YK58 pp 229533.A0A1C3YKV2
89242 229533.A0A1C3YK58 pp 229533.I1S0I3
89243 229533.A0A1C3YK58 pp 229533.I1RNB4
89244 229533.A0A1C3YK58 pp 229533.I1RSQ4
89245 229533.A0A1C3YK58 pp 229533.I1RAN1
89246 229533.A0A1C3YK58 pp 229533.I1RZ29
89247 229533.A0A1C3YK58 pp 229533.I1RZA7
89248 229533.A0A1C3YK58 pp 229533.I1RA62
89249 229533.A0A1C3YK58 pp 229533.I1RKB2
89250 229533.A0A1C3YK58 pp 229533.I1RVZ3
89251 229533.A0A1C3YK58 pp 229533.V6RV57
89252 229533.A0A1C3YK58 pp 229533.I1S202
89253 229533.A0A1C3YK58 pp 229533.I1RFI4
89254 229533.A0A1C3YK58 pp 229533.I1RMJ3
89255 229533.A0A1C3YK58 pp 229533.I1RCR3
89256 229533.A0A1C3YK58 pp 229533.I1RA52
89257 229533.A0A1C3YK58 pp 229533.I1RYT7
89258 229533.A0A1C3YK58 pp 229533.I1RE58
89259 229533.A0A1C3YK58 pp 229533.I1RGC3
89260 229533.A0A1C3YK58 pp 229533.V6RUB1
89261 229533.A0A1C3YK58 pp 229533.V6QV31
89262 229533.A0A1C3YK58 pp 229533.I1RDU6
89263 229533.A0A1C3YK58 pp 229533.I1S6P5
89264 229533.A0A1C3YK58 pp 229533.I1RC29
89265 229533.A0A1C3YK58 pp 229533.I1S3X8
89266 229533.A0A1C3YK58 pp 229533.I1RDJ7
89267 229533.A0A1C3YK58 pp 229533.A0A1C3YLW6
89268 229533.I1RHZ3 pp 229533.I1RNG4
89269 229533.I1S3T7 pp 229533.I1S8S9
89270 229533.I1S3T7 pp 229533.I1S8C1
89271 229533.I1S3T7 pp 229533.V6RZD4
89272 229533.I1S3T7 pp 229533.I1S8F8
89273 229533.I1S3T7 pp 229533.Q4HVQ9
89274 229533.I1S3T7 pp 229533.V6QVH2
89275 229533.I1S3T7 pp 229533.I1S4S5
89276 229533.I1S3T7 pp 229533.V6R3J1
89277 229533.I1S3T7 pp 229533.V6RF62
89278 229533.I1S3T7 pp 229533.V6R3V4
89279 229533.I1S3T7 pp 229533.I1S8H9

89280 229533.I1S3T7 pp 229533.V6RLM7
89281 229533.I1S3T7 pp 229533.I1S968
89282 229533.I1S3T7 pp 229533.V6RRM0
89283 229533.I1S3T7 pp 229533.I1S8R2
89284 229533.I1RUP7 pp 229533.Q4IBX0
89285 229533.I1RUP7 pp 229533.V6QUE6
89286 229533.I1RUP7 pp 229533.I1RVL8
89287 229533.I1RUP7 pp 229533.I1S9Y1
89288 229533.I1RUP7 pp 229533.I1RVY0
89289 229533.I1RUP7 pp 229533.I1SAN0
89290 229533.I1RUP7 pp 229533.Q4I0J6
89291 229533.I1RUP7 pp 229533.I1RX64
89292 229533.I1RUP7 pp 229533.I1RUP9
89293 229533.A0A1C3YKL6 pp 229533.I1RR52
89294 229533.A0A1C3YKL6 pp 229533.I1RDY1
89295 229533.I1RBI0 pp 229533.I1RW71
89296 229533.I1RBI0 pp 229533.I1RJZ7
89297 229533.I1RBI0 pp 229533.I1RCG5
89298 229533.A0A1C3YKJ3 pp 229533.I1RST9
89299 229533.A0A1C3YKJ3 pp 229533.I1RZV9
89300 229533.A0A1C3YKJ3 pp 229533.I1RCK4
89301 229533.A0A1C3YKJ3 pp 229533.I1RGD8
89302 229533.A0A1C3YKJ3 pp 229533.I1S8F8
89303 229533.A0A1C3YKJ3 pp 229533.I1RUY5
89304 229533.A0A1C3YKJ3 pp 229533.I1S0P4
89305 229533.A0A1C3YKJ3 pp 229533.I1RNG5
89306 229533.A0A1C3YKJ3 pp 229533.I1RNT2
89307 229533.A0A1C3YKJ3 pp 229533.I1RDU0
89308 229533.A0A1C3YKJ3 pp 229533.I1S094
89309 229533.A0A1C3YKJ3 pp 229533.I1RA50
89310 229533.A0A1C3YKJ3 pp 229533.I1RA13
89311 229533.A0A1C3YKJ3 pp 229533.I1RCL0
89312 229533.A0A1C3YKJ3 pp 229533.I1S241
89313 229533.A0A1C3YKJ3 pp 229533.I1RCT7
89314 229533.A0A1C3YKJ3 pp 229533.I1RY40
89315 229533.A0A1C3YKJ3 pp 229533.I1RCU7
89316 229533.A0A1C3YKJ3 pp 229533.V6RS91
89317 229533.A0A1C3YKJ3 pp 229533.I1RLA2
89318 229533.A0A1C3YKJ3 pp 229533.I1S6H3
89319 229533.A0A1C3YKJ3 pp 229533.I1RC74
89320 229533.A0A1C3YKJ3 pp 229533.A0A1C3YLB7
89321 229533.A0A1C3YKJ3 pp 229533.I1RNM7
89322 229533.A0A1C3YKJ3 pp 229533.I1RM10
89323 229533.A0A1C3YKJ3 pp 229533.I1RRL5
89324 229533.A0A1C3YKJ3 pp 229533.I1S279
89325 229533.A0A1C3YKJ3 pp 229533.I1RPV8
89326 229533.A0A1C3YKJ3 pp 229533.I1RDN3
89327 229533.A0A1C3YKJ3 pp 229533.I1S334
89328 229533.A0A1C3YKJ3 pp 229533.I1RZ56
89329 229533.A0A1C3YKJ3 pp 229533.I1RNE4
89330 229533.A0A1C3YKJ3 pp 229533.I1RJS1
89331 229533.A0A1C3YKJ3 pp 229533.A0A1C3YL19
89332 229533.A0A1C3YKJ3 pp 229533.I1RZ25
89333 229533.A0A1C3YKJ3 pp 229533.I1RYT7
89334 229533.A0A1C3YKJ3 pp 229533.I1RBY9
89335 229533.A0A1C3YKJ3 pp 229533.I1RS94
89336 229533.A0A1C3YKJ3 pp 229533.I1S3H8
89337 229533.A0A1C3YKJ3 pp 229533.I1RKB2
89338 229533.A0A1C3YKJ3 pp 229533.I1RNG4
89339 229533.A0A1C3YKJ3 pp 229533.A0A1C3YM61
89340 229533.A0A1C3YKJ3 pp 229533.I1RZ38
89341 229533.A0A1C3YKJ3 pp 229533.I1S0A5
89342 229533.A0A1C3YKJ3 pp 229533.I1RE58
89343 229533.A0A1C3YKJ3 pp 229533.A0A1C3YKN4
89344 229533.A0A1C3YKJ3 pp 229533.I1RGZ3
89345 229533.I1RLX3 pp 229533.I1RNG4
89346 229533.I1RLX3 pp 229533.I1RWG5
89347 229533.I1RYH6 pp 229533.V6RLH0
89348 229533.I1RYH6 pp 229533.I1S2C1
89349 229533.I1RYH6 pp 229533.V6RR76
89350 229533.I1RYH6 pp 229533.I1S2E8
89351 229533.I1RYH6 pp 229533.I1S7X9
89352 229533.I1RYH6 pp 229533.I1RZZ1

89353 229533.I1RYH6 pp 229533.Q4IR08
89354 229533.I1RYH6 pp 229533.V6RIS2
89355 229533.I1RYH6 pp 229533.I1RYI7
89356 229533.A0A098DLU6 pp 229533.I1RSG8
89357 229533.A0A098DLU6 pp 229533.I1RK79
89358 229533.A0A098DLU6 pp 229533.I1RXU0
89359 229533.A0A098DLU6 pp 229533.A0A0E0S631
89360 229533.A0A098DLU6 pp 229533.I1RTC2
89361 229533.A0A098DLU6 pp 229533.I1RVY0
89362 229533.A0A098DLU6 pp 229533.I1RUP7
89363 229533.A0A098DLU6 pp 229533.I1RTD5
89364 229533.A0A098DLU6 pp 229533.I1RK95
89365 229533.A0A098DLU6 pp 229533.I1RRY9
89366 229533.I1REE2 pp 229533.I1RUR8
89367 229533.I1REE2 pp 229533.I1RY35
89368 229533.I1REE2 pp 229533.Q4HVQ9
89369 229533.I1REE2 pp 229533.I1RN88
89370 229533.I1REE2 pp 229533.I1RZV5
89371 229533.I1REE2 pp 229533.V6R3J1
89372 229533.I1REE2 pp 229533.I1S2Q4
89373 229533.I1REE2 pp 229533.I1RR68
89374 229533.I1REE2 pp 229533.I1RKP1
89375 229533.I1REE2 pp 229533.I1S3T7
89376 229533.I1REE2 pp 229533.I1S968
89377 229533.I1REE2 pp 229533.I1RXD9
89378 229533.I1REE2 pp 229533.I1RWF5
89379 229533.I1REE2 pp 229533.I1RY92
89380 229533.I1REE2 pp 229533.I1RSQ4
89381 229533.I1REE2 pp 229533.I1RL81
89382 229533.I1REE2 pp 229533.I1S8R2
89383 229533.I1REE2 pp 229533.I1RFI4
89384 229533.I1REE2 pp 229533.V6RRM0
89385 229533.I1REE2 pp 229533.I1RK55
89386 229533.I1REE2 pp 229533.V6RZD4
89387 229533.I1REE2 pp 229533.I1RLZ4
89388 229533.I1REE2 pp 229533.I1RGC3
89389 229533.I1REE2 pp 229533.I1S299
89390 229533.I1REE2 pp 229533.I1S147
89391 229533.I1REE2 pp 229533.I1S0V7
89392 229533.I1REE2 pp 229533.I1S464
89393 229533.I1REE2 pp 229533.I1RYW9
89394 229533.I1REE2 pp 229533.I1RMJ3
89395 229533.I1REE2 pp 229533.I1RY21
89396 229533.I1REE2 pp 229533.I1RN84
89397 229533.I1REE2 pp 229533.I1RMQ6
89398 229533.I1REE2 pp 229533.I1RKB2
89399 229533.I1REE2 pp 229533.I1RPU8
89400 229533.I1REE2 pp 229533.I1RSF1
89401 229533.I1REE2 pp 229533.I1RVI8
89402 229533.I1REE2 pp 229533.I1RYP5
89403 229533.I1REE2 pp 229533.I1RZ38
89404 229533.I1REE2 pp 229533.I1RYC8
89405 229533.I1REE2 pp 229533.I1RJC5
89406 229533.I1REE2 pp 229533.I1RG62
89407 229533.I1REE2 pp 229533.I1RF85
89408 229533.I1REE2 pp 229533.V6RF62
89409 229533.I1REE2 pp 229533.V6RLM7
89410 229533.I1REE2 pp 229533.I1RYA0
89411 229533.I1REE2 pp 229533.I1RPK0
89412 229533.I1REE2 pp 229533.I1RYT8
89413 229533.I1REE2 pp 229533.Q4IPX8
89414 229533.I1REE2 pp 229533.I1RMA2
89415 229533.I1REE2 pp 229533.I1RW54
89416 229533.I1REE2 pp 229533.I1RMK7
89417 229533.I1REE2 pp 229533.V6R3V4
89418 229533.I1REE2 pp 229533.I1S0V6
89419 229533.I1REE2 pp 229533.I1RNM7
89420 229533.I1REE2 pp 229533.I1RYU6
89421 229533.I1REE2 pp 229533.I1S0P0
89422 229533.I1REE2 pp 229533.I1S8S9
89423 229533.I1REE2 pp 229533.I1S8C1
89424 229533.I1REE2 pp 229533.I1RPR1
89425 229533.I1REE2 pp 229533.I1RJY7

229533.I1REE2 pp 229533.I1RNY6
229533.I1REE2 pp 229533.I1RRX5
229533.I1REE2 pp 229533.V6QVH2
229533.I1REE2 pp 229533.I1S8F8
229533.I1REE2 pp 229533.I1RUQ3
229533.I1REE2 pp 229533.I1RY81
229533.I1REE2 pp 229533.I1RG33
229533.I1REE2 pp 229533.I1S4S5
229533.I1REE2 pp 229533.I1S0K2
229533.I1REE2 pp 229533.I1RWD2
229533.I1REE2 pp 229533.I1S8H9
229533.I1REE2 pp 229533.I1RWM4
229533.I1REE2 pp 229533.I1RXE6
229533.I1REE2 pp 229533.I1RY12
229533.A0A1C3YJG0 pp 229533.I1RM67
229533.A0A1C3YJG0 pp 229533.I1RKJ6
229533.A0A1C3YJG0 pp 229533.I1S9U2
229533.A0A1C3YJG0 pp 229533.I1RPU0
229533.A0A1C3YJG0 pp 229533.I1RGF6
229533.A0A1C3YJG0 pp 229533.I1RD64
229533.A0A1C3YJG0 pp 229533.I1RT05
229533.A0A1C3YJG0 pp 229533.Q4IF76
229533.A0A1C3YJG0 pp 229533.I1RMK3
229533.A0A1C3YJG0 pp 229533.I1RDA0
229533.A0A1C3YJG0 pp 229533.I1RNV6
229533.A0A1C3YJG0 pp 229533.I1S6U8
229533.A0A1C3YJG0 pp 229533.I1RT80
229533.A0A1C3YJG0 pp 229533.Q4IJH1
229533.A0A1C3YJG0 pp 229533.I1RAC2
229533.A0A1C3YJG0 pp 229533.I1R9G0
229533.A0A1C3YJG0 pp 229533.I1RA35
229533.A0A1C3YJG0 pp 229533.Q4IMJ1
229533.A0A1C3YJG0 pp 229533.I1RL23
229533.A0A1C3YJG0 pp 229533.I1RS19
229533.A0A1C3YJG0 pp 229533.I1RWJ4
229533.A0A1C3YJG0 pp 229533.I1RN51
229533.A0A1C3YJG0 pp 229533.Q4IFI0
229533.A0A1C3YJG0 pp 229533.I1RG51
229533.A0A1C3YJG0 pp 229533.I1RC32
229533.A0A1C3YJG0 pp 229533.I1RM06
229533.A0A1C3YJG0 pp 229533.I1RNH1
229533.A0A1C3YJG0 pp 229533.I1RPJ3
229533.A0A1C3YJG0 pp 229533.I1RS30
229533.A0A1C3YJG0 pp 229533.I1RZS6
229533.A0A1C3YJG0 pp 229533.I1RTE9
229533.A0A1C3YJG0 pp 229533.I1RRP3
229533.A0A1C3YJG0 pp 229533.I1RX24
229533.A0A1C3YJG0 pp 229533.I1RZE5
229533.A0A1C3YJG0 pp 229533.A0A1C3YK72
229533.A0A1C3YJG0 pp 229533.V6QWZ7
229533.A0A1C3YJG0 pp 229533.I1RW26
229533.A0A1C3YJG0 pp 229533.V6QYF0
229533.A0A1C3YJG0 pp 229533.I1RBG9
229533.A0A1C3YJG0 pp 229533.I1RQ65
229533.A0A1C3YJG0 pp 229533.Q4IEV5
229533.A0A1C3YJG0 pp 229533.I1RE00
229533.A0A1C3YJG0 pp 229533.I1RVZ0
229533.A0A1C3YJG0 pp 229533.I1RNA0
229533.A0A1C3YJG0 pp 229533.Q4INW6
229533.A0A1C3YJG0 pp 229533.I1RS47
229533.A0A1C3YJG0 pp 229533.I1RRT2
229533.A0A1C3YJG0 pp 229533.Q4IQK7
229533.A0A1C3YJG0 pp 229533.I1RC03
229533.A0A1C3YJG0 pp 229533.I1RNK2
229533.A0A1C3YJG0 pp 229533.Q4IPI1
229533.A0A1C3YJG0 pp 229533.I1RAZ8
229533.A0A1C3YJG0 pp 229533.I1RKC6
229533.A0A1C3YJG0 pp 229533.I1RJL4
229533.A0A1C3YJG0 pp 229533.A0A1C3YMG7
229533.A0A1C3YJG0 pp 229533.I1S296
229533.A0A1C3YJG0 pp 229533.V6R283
229533.A0A1C3YJG0 pp 229533.Q4IAS1
229533.A0A1C3YJG0 pp 229533.I1RSD8

89499 229533.A0A1C3YJG0 pp 229533.I1RWF7
89500 229533.A0A1C3YJG0 pp 229533.I1RLV9
89501 229533.A0A1C3YJG0 pp 229533.I1S2U2
89502 229533.A0A1C3YJG0 pp 229533.I1RQJ5
89503 229533.A0A1C3YJG0 pp 229533.I1RKQ5
89504 229533.A0A1C3YJG0 pp 229533.I1RZ99
89505 229533.A0A1C3YJG0 pp 229533.A0A1C3YKR0
89506 229533.A0A1C3YJG0 pp 229533.I1S1B6
89507 229533.A0A1C3YJG0 pp 229533.I1RDG0
89508 229533.A0A1C3YJG0 pp 229533.I1RK95
89509 229533.A0A1C3YJG0 pp 229533.I1RC82
89510 229533.A0A1C3YJG0 pp 229533.I1RRJ8
89511 229533.A0A1C3YJG0 pp 229533.I1RY63
89512 229533.A0A1C3YJG0 pp 229533.I1RFN1
89513 229533.A0A1C3YJG0 pp 229533.I1REF2
89514 229533.A0A1C3YJG0 pp 229533.I1RAF8
89515 229533.A0A1C3YJG0 pp 229533.I1RW71
89516 229533.A0A1C3YJG0 pp 229533.I1S1X9
89517 229533.A0A1C3YJG0 pp 229533.A0A1C3YLF5
89518 229533.A0A1C3YJG0 pp 229533.A0A1C3YN63
89519 229533.A0A1C3YJG0 pp 229533.I1RKL3
89520 229533.A0A1C3YJG0 pp 229533.I1R9V7
89521 229533.A0A1C3YJG0 pp 229533.I1RA48
89522 229533.A0A1C3YJG0 pp 229533.I1RCL3
89523 229533.A0A1C3YJG0 pp 229533.I1RMN6
89524 229533.A0A1C3YJG0 pp 229533.I1RCW1
89525 229533.A0A1C3YJG0 pp 229533.I1RRQ3
89526 229533.A0A1C3YJG0 pp 229533.I1RG56
89527 229533.A0A1C3YJG0 pp 229533.I1RQA2
89528 229533.A0A1C3YJG0 pp 229533.V6RGN5
89529 229533.A0A1C3YJG0 pp 229533.I1RCN5
89530 229533.A0A1C3YJG0 pp 229533.I1RRR7
89531 229533.A0A1C3YJG0 pp 229533.I1S558
89532 229533.A0A1C3YJG0 pp 229533.I1RAN5
89533 229533.A0A1C3YJG0 pp 229533.I1RSR3
89534 229533.A0A1C3YJG0 pp 229533.A0A1C3YL07
89535 229533.A0A1C3YJG0 pp 229533.I1RC27
89536 229533.A0A1C3YJG0 pp 229533.I1RNY2
89537 229533.A0A1C3YJG0 pp 229533.V6R227
89538 229533.A0A1C3YJG0 pp 229533.A0A1C3YJR3
89539 229533.A0A1C3YJG0 pp 229533.Q4I327
89540 229533.A0A1C3YJG0 pp 229533.V6RGY3
89541 229533.A0A1C3YJG0 pp 229533.I1S0J1
89542 229533.A0A1C3YJG0 pp 229533.I1S1U7
89543 229533.A0A1C3YJG0 pp 229533.I1RMN7
89544 229533.A0A1C3YJG0 pp 229533.I1RC72
89545 229533.A0A1C3YJG0 pp 229533.I1S274
89546 229533.A0A1C3YJG0 pp 229533.I1S021
89547 229533.A0A1C3YJG0 pp 229533.I1RNH6
89548 229533.A0A1C3YJG0 pp 229533.I1S060
89549 229533.A0A1C3YJG0 pp 229533.I1RQ31
89550 229533.A0A1C3YJG0 pp 229533.I1RB27
89551 229533.A0A1C3YJG0 pp 229533.Q4I0M9
89552 229533.A0A1C3YJG0 pp 229533.I1RSI6
89553 229533.A0A1C3YJG0 pp 229533.I1RRI2
89554 229533.A0A1C3YJG0 pp 229533.Q4I8X0
89555 229533.A0A1C3YJG0 pp 229533.I1RTD7
89556 229533.A0A1C3YJG0 pp 229533.I1R9X7
89557 229533.A0A1C3YJG0 pp 229533.I1RBD2
89558 229533.A0A1C3YJG0 pp 229533.Q4IEK8
89559 229533.A0A1C3YJG0 pp 229533.I1RNF5
89560 229533.A0A1C3YJG0 pp 229533.Q4IJ56
89561 229533.A0A1C3YJG0 pp 229533.I1RM96
89562 229533.A0A1C3YJG0 pp 229533.I1RNU6
89563 229533.I1RYL5 pp 229533.I1S235
89564 229533.I1RYL5 pp 229533.I1S0Z3
89565 229533.I1RYL5 pp 229533.I1S821
89566 229533.I1RYL5 pp 229533.V6REZ4
89567 229533.I1RYL5 pp 229533.Q4HZS8
89568 229533.I1RYL5 pp 229533.I1RYW3
89569 229533.I1RYL5 pp 229533.I1RZD3
89570 229533.I1RYL5 pp 229533.I1S3S5
89571 229533.I1RYL5 pp 229533.I1RZM1

229533.I1RYL5 pp 229533.Q4IEA7
229533.I1RYL5 pp 229533.I1S247
229533.I1RYL5 pp 229533.I1RZV9
229533.I1RYL5 pp 229533.Q4I7G0
229533.I1RYL5 pp 229533.V6QX21
229533.I1RYL5 pp 229533.I1S7W4
229533.I1RYL5 pp 229533.I1RZK1
229533.I1RYL5 pp 229533.I1RYT7
229533.I1RYL5 pp 229533.I1S4Z7
229533.I1RYL5 pp 229533.I1RZ94
229533.I1RYL5 pp 229533.I1S2K7
229533.I1RYL5 pp 229533.Q4I5R9
229533.I1RYL5 pp 229533.I1S0P4
229533.I1RYL5 pp 229533.I1RYS6
229533.I1RYL5 pp 229533.I1S0F9
229533.I1RYL5 pp 229533.I1S0Z8
229533.I1RYL5 pp 229533.I1RZS8
229533.I1RYL5 pp 229533.Q5I2J3
229533.I1RYL5 pp 229533.I1S286
229533.I1RYL5 pp 229533.V6QUV7
229533.I1RYL5 pp 229533.Q4I624
229533.I1RYL5 pp 229533.I1S8P5
229533.I1RYL5 pp 229533.I1S282
229533.I1RYL5 pp 229533.Q4IN00
229533.I1RYL5 pp 229533.I1RZT6
229533.I1RYL5 pp 229533.I1RZM0
229533.I1RYL5 pp 229533.Q4HXF6
229533.I1RYL5 pp 229533.V6R223
229533.I1RYL5 pp 229533.I1S2V2
229533.I1RYL5 pp 229533.Q4I1Y5
229533.I1RXM0 pp 229533.I1S2K2
229533.I1RXM0 pp 229533.I1SAE8
229533.I1RXM0 pp 229533.I1S308
229533.I1RXM0 pp 229533.I1RZH8
229533.I1RND8 pp 229533.I1RT72
229533.I1RND8 pp 229533.I1RXX1
229533.I1RND8 pp 229533.I1S4P6
229533.I1RND8 pp 229533.I1RRY6
229533.I1RND8 pp 229533.I1RSJ3
229533.I1RND8 pp 229533.I1RXT3
229533.I1RND8 pp 229533.V6R949
229533.I1RND8 pp 229533.I1RR67
229533.I1RND8 pp 229533.I1S7S9
229533.I1RYT5 pp 229533.V6R283
229533.I1RYT5 pp 229533.I1RZ99
229533.I1RYT5 pp 229533.I1RZS6
229533.I1RYT5 pp 229533.I1S0L4
229533.I1RYT5 pp 229533.Q4IFI0
229533.I1RYT5 pp 229533.Q4I624
229533.I1RYT5 pp 229533.Q4IEK8
229533.I1RYT5 pp 229533.V6R227
229533.I1RYT5 pp 229533.I1S0R7
229533.I1RYT5 pp 229533.I1RZE5
229533.I1S2N3 pp 229533.I1S308
229533.I1S2N3 pp 229533.I1S3I4
229533.I1S2N3 pp 229533.I1SAE8
229533.A0A098D2S7 pp 229533.I1RND8
229533.I1RH92 pp 229533.I1RN88
229533.I1RH92 pp 229533.I1RWN7
229533.I1RH92 pp 229533.I1S148
229533.I1RH92 pp 229533.I1S147
229533.I1RH92 pp 229533.I1RZ70
229533.I1RH92 pp 229533.I1S8D2
229533.I1RH92 pp 229533.I1S8D1
229533.I1RH92 pp 229533.I1RYX6
229533.I1RH92 pp 229533.I1RHU0
229533.I1RH92 pp 229533.I1RQE1
229533.I1RH92 pp 229533.I1RNM7
229533.I1RH92 pp 229533.V6RH92
229533.I1RH92 pp 229533.I1RWM4
229533.A0A1C3YM70 pp 229533.I1RBW0
229533.A0A1C3YM70 pp 229533.Q4ICA8
229533.A0A1C3YM70 pp 229533.I1RQP3

89645 229533.A0A1C3YM70 pp 229533.I1RRG3
89646 229533.A0A1C3YM70 pp 229533.I1RQP1
89647 229533.A0A1C3YM70 pp 229533.Q4IPI4
89648 229533.A0A1C3YM70 pp 229533.A0A1C3YN69
89649 229533.A0A1C3YM70 pp 229533.I1RBW1
89650 229533.A0A1C3YM70 pp 229533.I1RSC7
89651 229533.A0A1C3YM70 pp 229533.A0A1C3YM91
89652 229533.A0A1C3YM70 pp 229533.I1RY58
89653 229533.E6PBP1 pp 229533.I1RPT0
89654 229533.E6PBP1 pp 229533.I0LNZ1
89655 229533.E6PBP1 pp 229533.I1RIK1
89656 229533.E6PBP1 pp 229533.I1RLQ8
89657 229533.E6PBP1 pp 229533.V6RUY9
89658 229533.E6PBP1 pp 229533.I1RGP4
89659 229533.E6PBP1 pp 229533.I1RZH8
89660 229533.E6PBP1 pp 229533.I1S308
89661 229533.E6PBP1 pp 229533.I1RFL7
89662 229533.E6PBP1 pp 229533.I1RHL0
89663 229533.E6PBP1 pp 229533.I1RIU7
89664 229533.E6PBP1 pp 229533.I1S2K2
89665 229533.E6PBP1 pp 229533.I1RDW1
89666 229533.E6PBP1 pp 229533.I1RPL0
89667 229533.E6PBP1 pp 229533.I1R9F8
89668 229533.E6PBP1 pp 229533.I1RDT7
89669 229533.E6PBP1 pp 229533.I1R9D3
89670 229533.E6PBP1 pp 229533.I1REG5
89671 229533.E6PBP1 pp 229533.I1RHQ0
89672 229533.E6PBP1 pp 229533.I1SAE8
89673 229533.E6PBP1 pp 229533.I1REH0
89674 229533.A0A098D2Q3 pp 229533.I1RPA6
89675 229533.I1RWE1 pp 229533.V6RPS5
89676 229533.I1RWE1 pp 229533.Q4IJU0
89677 229533.I1RWE1 pp 229533.I1S149
89678 229533.I1RWE1 pp 229533.Q4IAS1
89679 229533.I1RWE1 pp 229533.I1RZS6
89680 229533.I1RWE1 pp 229533.I1S0M9
89681 229533.I1RWE1 pp 229533.I1S0J1
89682 229533.I1RWE1 pp 229533.Q4HYB8
89683 229533.I1RN37 pp 229533.I1RSE9
89684 229533.I1RN37 pp 229533.I1S0Z2
89685 229533.I1RN37 pp 229533.I1RS13
89686 229533.I1RN37 pp 229533.I1S0D4
89687 229533.I1RB45 pp 229533.I1RR68
89688 229533.I1RB45 pp 229533.I1S0Z2
89689 229533.I1RB45 pp 229533.I1RZT6
89690 229533.I1RB45 pp 229533.I1S0D4
89691 229533.I1RB45 pp 229533.I1RK32
89692 229533.I1RB45 pp 229533.I1RN37
89693 229533.I1RWN7 pp 229533.I1S147
89694 229533.I1RWN7 pp 229533.I1S8D2
89695 229533.I1RWN7 pp 229533.I1S148
89696 229533.I1RWN7 pp 229533.I1RZ70
89697 229533.I1RWN7 pp 229533.I1S8D1
89698 229533.I1RWN7 pp 229533.V6RH92
89699 229533.I1RWN7 pp 229533.I1RYX6
89700 229533.A0A1C3YJW9 pp 229533.I1R9Z5
89701 229533.A0A1C3YJW9 pp 229533.A0A1C3YKB0
89702 229533.A0A1C3YJW9 pp 229533.I1RCW8
89703 229533.A0A1C3YJW9 pp 229533.I1RZM8
89704 229533.A0A1C3YJW9 pp 229533.I1RXF0
89705 229533.A0A1C3YJW9 pp 229533.I1S6K9
89706 229533.A0A1C3YJW9 pp 229533.Q4HTT2
89707 229533.A0A1C3YJW9 pp 229533.V6RCZ8
89708 229533.A0A1C3YJW9 pp 229533.I1RPR1
89709 229533.A0A1C3YJW9 pp 229533.I1S836
89710 229533.A0A1C3YJW9 pp 229533.I1REE2
89711 229533.A0A1C3YJW9 pp 229533.A0A1C3YK34
89712 229533.A0A1C3YJW9 pp 229533.A0A1C3YKV3
89713 229533.A0A1C3YJW9 pp 229533.I1S887
89714 229533.A0A1C3YJW9 pp 229533.I1RS18
89715 229533.A0A1C3YJW9 pp 229533.Q4IJK2
89716 229533.A0A1C3YJW9 pp 229533.V6QUE6
89717 229533.A0A1C3YJW9 pp 229533.I1RCM5

89718	229533.A0A1C3YJW9	pp	229533.I1RZK5
89719	229533.A0A1C3YJW9	pp	229533.I1S5B0
89720	229533.A0A1C3YJW9	pp	229533.I1S0K2
89721	229533.A0A1C3YJW9	pp	229533.I1RCM6
89722	229533.A0A1C3YJW9	pp	229533.I1RU02
89723	229533.A0A1C3YJW9	pp	229533.I1RWB0
89724	229533.A0A1C3YJW9	pp	229533.I1RA85
89725	229533.A0A1C3YJW9	pp	229533.A0A1C3YKY6
89726	229533.A0A1C3YJW9	pp	229533.I1RK95
89727	229533.A0A1C3YJW9	pp	229533.I1RM26
89728	229533.A0A1C3YJW9	pp	229533.I1RD52
89729	229533.A0A1C3YJW9	pp	229533.I1RPR9
89730	229533.A0A1C3YJW9	pp	229533.A0A1C3YL54
89731	229533.A0A1C3YJW9	pp	229533.I1RLP1
89732	229533.A0A1C3YJW9	pp	229533.I1RAV5
89733	229533.A0A1C3YJW9	pp	229533.I1RCR5
89734	229533.A0A1C3YJW9	pp	229533.I1RBL7
89735	229533.A0A1C3YJW9	pp	229533.I1RYV3
89736	229533.A0A1C3YJW9	pp	229533.I1RXD7
89737	229533.A0A1C3YJW9	pp	229533.V6RUT5
89738	229533.A0A1C3YJW9	pp	229533.I1RWC9
89739	229533.A0A1C3YJW9	pp	229533.I1RAA2
89740	229533.A0A1C3YJW9	pp	229533.I1RRY9
89741	229533.A0A1C3YJW9	pp	229533.A0A1C3YLB3
89742	229533.A0A1C3YJW9	pp	229533.I1RAM9
89743	229533.A0A1C3YJW9	pp	229533.I1RBD5
89744	229533.A0A1C3YJW9	pp	229533.A0A1C3YLB0
89745	229533.A0A1C3YJW9	pp	229533.A0A1C3YKW5
89746	229533.A0A1C3YJW9	pp	229533.I1RVX9
89747	229533.A0A1C3YJW9	pp	229533.I1RX46
89748	229533.A0A1C3YJW9	pp	229533.Q4IER8
89749	229533.A0A1C3YJW9	pp	229533.A0A1C3YK13
89750	229533.A0A1C3YJW9	pp	229533.I1S4S7
89751	229533.A0A1C3YJW9	pp	229533.I1RR24
89752	229533.A0A1C3YJW9	pp	229533.I1RAU9
89753	229533.A0A1C3YJW9	pp	229533.I1RSQ7
89754	229533.A0A1C3YJW9	pp	229533.I1RAX6
89755	229533.A0A1C3YJW9	pp	229533.I1RA49
89756	229533.A0A1C3YJW9	pp	229533.V6RTJ8
89757	229533.A0A1C3YJW9	pp	229533.I1S1D3
89758	229533.A0A1C3YJW9	pp	229533.A0A1C3YLC1
89759	229533.A0A1C3YJW9	pp	229533.I1RRX5
89760	229533.A0A1C3YJW9	pp	229533.I1RE01
89761	229533.A0A1C3YJW9	pp	229533.I1RNM7
89762	229533.A0A1C3YJW9	pp	229533.I1S0J1
89763	229533.A0A1C3YJW9	pp	229533.I1RSQ3
89764	229533.A0A1C3YJW9	pp	229533.I1RD69
89765	229533.A0A1C3YJW9	pp	229533.V6R042
89766	229533.A0A1C3YJW9	pp	229533.I1S7W5
89767	229533.A0A1C3YJW9	pp	229533.I1RFS5
89768	229533.A0A1C3YJW9	pp	229533.V6RLM7
89769	229533.A0A1C3YJW9	pp	229533.I1RD22
89770	229533.A0A1C3YJW9	pp	229533.I1S1W8
89771	229533.A0A1C3YJW9	pp	229533.I1RAI4
89772	229533.A0A1C3YJW9	pp	229533.I1RRN2
89773	229533.A0A1C3YJW9	pp	229533.I1RMV0
89774	229533.A0A1C3YJW9	pp	229533.I1RMK7
89775	229533.A0A1C3YJW9	pp	229533.I1S256
89776	229533.A0A1C3YJW9	pp	229533.I1S1W3
89777	229533.A0A1C3YJW9	pp	229533.I1R9Y4
89778	229533.A0A1C3YJW9	pp	229533.A0A1C3YLR6
89779	229533.A0A1C3YJW9	pp	229533.I1RBT1
89780	229533.A0A1C3YJW9	pp	229533.I1RS90
89781	229533.A0A1C3YJW9	pp	229533.I1RY21
89782	229533.A0A1C3YJW9	pp	229533.I1RMQ6
89783	229533.A0A1C3YJW9	pp	229533.I1RCR8
89784	229533.A0A1C3YJW9	pp	229533.I1S5R6
89785	229533.A0A1C3YJW9	pp	229533.I1RG11
89786	229533.A0A1C3YJW9	pp	229533.I1RNC1
89787	229533.A0A1C3YJW9	pp	229533.I1S4S4
89788	229533.A0A1C3YJW9	pp	229533.I1RFL4
89789	229533.A0A1C3YJW9	pp	229533.I1RFW1
89790	229533.A0A1C3YJW9	pp	229533.I1RUG3

89791 229533.A0A1C3YJW9 pp 229533.I1S8R2
89792 229533.A0A1C3YJW9 pp 229533.I1RKB2
89793 229533.A0A1C3YJW9 pp 229533.I1RQE7
89794 229533.A0A1C3YJW9 pp 229533.I1RFT1
89795 229533.A0A1C3YJW9 pp 229533.I1RYH7
89796 229533.A0A1C3YJW9 pp 229533.I1RCH5
89797 229533.A0A1C3YJW9 pp 229533.Q4I624
89798 229533.A0A1C3YJW9 pp 229533.I1S299
89799 229533.A0A1C3YJW9 pp 229533.I1RXR1
89800 229533.A0A1C3YJW9 pp 229533.I1S1C7
89801 229533.A0A1C3YJW9 pp 229533.I1RNG4
89802 229533.A0A1C3YJW9 pp 229533.I1RPP3
89803 229533.A0A1C3YJW9 pp 229533.I1RT03
89804 229533.A0A1C3YJW9 pp 229533.I1RN67
89805 229533.A0A1C3YJW9 pp 229533.I1RY62
89806 229533.A0A1C3YJW9 pp 229533.I1RVX0
89807 229533.A0A1C3YJW9 pp 229533.I1RYW3
89808 229533.A0A1C3YJW9 pp 229533.I1RB92
89809 229533.A0A1C3YJW9 pp 229533.I1RQC1
89810 229533.A0A1C3YJW9 pp 229533.I1RRI2
89811 229533.A0A1C3YJW9 pp 229533.I1S1F3
89812 229533.A0A1C3YJW9 pp 229533.V6RUY9
89813 229533.A0A1C3YJW9 pp 229533.I1RA70
89814 229533.A0A1C3YJW9 pp 229533.I1RFZ2
89815 229533.A0A1C3YJW9 pp 229533.A0A1C3YLK8
89816 229533.A0A1C3YJW9 pp 229533.I1RJV3
89817 229533.A0A1C3YJW9 pp 229533.I1RMP0
89818 229533.A0A1C3YJW9 pp 229533.I1RXU9
89819 229533.A0A1C3YJW9 pp 229533.I1RT40
89820 229533.A0A1C3YJW9 pp 229533.I1RSX0
89821 229533.A0A1C3YJW9 pp 229533.I1RAW0
89822 229533.A0A1C3YJW9 pp 229533.I1RTD5
89823 229533.A0A1C3YJW9 pp 229533.I1RBJ0
89824 229533.A0A1C3YJW9 pp 229533.Q4IMD1
89825 229533.A0A1C3YJW9 pp 229533.A0A1C3YM60
89826 229533.A0A1C3YJW9 pp 229533.A0A1C3YK33
89827 229533.A0A1C3YJW9 pp 229533.I1RBC7
89828 229533.A0A1C3YJW9 pp 229533.I1R9M0
89829 229533.A0A1C3YJW9 pp 229533.I1RBY4
89830 229533.A0A1C3YJW9 pp 229533.I1RGA4
89831 229533.A0A1C3YJW9 pp 229533.I1RBA6
89832 229533.A0A1C3YJW9 pp 229533.I1RA52
89833 229533.A0A1C3YJW9 pp 229533.I1RBE6
89834 229533.A0A1C3YJW9 pp 229533.I1RYX5
89835 229533.A0A1C3YJW9 pp 229533.I1RPV3
89836 229533.A0A1C3YJW9 pp 229533.I1RAW3
89837 229533.A0A1C3YJW9 pp 229533.I1RFS3
89838 229533.A0A1C3YJW9 pp 229533.I1RWI5
89839 229533.A0A1C3YJW9 pp 229533.I1RN88
89840 229533.A0A1C3YJW9 pp 229533.I1RZZ9
89841 229533.A0A1C3YJW9 pp 229533.I1RSQ4
89842 229533.A0A1C3YJW9 pp 229533.I1RC29
89843 229533.A0A1C3YJW9 pp 229533.I1RC66
89844 229533.A0A1C3YJW9 pp 229533.Q4HTT1
89845 229533.A0A1C3YJW9 pp 229533.I1RZ39
89846 229533.A0A1C3YJW9 pp 229533.A0A1C3YKM9
89847 229533.A0A1C3YJW9 pp 229533.I1S2H4
89848 229533.A0A1C3YJW9 pp 229533.Q4IB50
89849 229533.A0A1C3YJW9 pp 229533.I1S3T7
89850 229533.A0A1C3YJW9 pp 229533.I1SB04
89851 229533.A0A1C3YJW9 pp 229533.I1S9Y1
89852 229533.A0A1C3YJW9 pp 229533.I1S0L4
89853 229533.A0A1C3YJW9 pp 229533.I1RRT8
89854 229533.A0A1C3YJW9 pp 229533.I1RA11
89855 229533.A0A1C3YJW9 pp 229533.Q4I7L0
89856 229533.A0A1C3YJW9 pp 229533.I1S5R1
89857 229533.A0A1C3YJW9 pp 229533.I1S3G4
89858 229533.A0A1C3YJW9 pp 229533.O74638
89859 229533.Q4I4A4 pp 229533.V6RRF6
89860 229533.A0A0E0RXI2 pp 229533.I1RSR2
89861 229533.A0A0E0RXI2 pp 229533.I1RK32
89862 229533.A0A0E0RXI2 pp 229533.I1S0Z2
89863 229533.A0A0E0RXI2 pp 229533.I1RA83

89864 229533.A0A0E0RXI2 pp 229533.I1RB45
89865 229533.A0A0E0RXI2 pp 229533.I1RRD2
89866 229533.A0A0E0RXI2 pp 229533.A0A1C3YJC9
89867 229533.A0A0E0RXI2 pp 229533.I1RN37
89868 229533.A0A0E0RXI2 pp 229533.I1S0D4
89869 229533.A0A0E0RXI2 pp 229533.A0A1C3YLG0
89870 229533.A0A0E0RXI2 pp 229533.I1RAL5
89871 229533.A0A098DLP1 pp 229533.I1RXM0
89872 229533.A0A098DLP1 pp 229533.A0A098DVZ4
89873 229533.A0A098DLP1 pp 229533.I1S2N3
89874 229533.A0A098DLP1 pp 229533.E6PBP1
89875 229533.A0A098DLP1 pp 229533.A0A098DVI8
89876 229533.A0A098DLP1 pp 229533.A0A1C3YL54
89877 229533.A0A098DLP1 pp 229533.I1RVR2
89878 229533.A0A098DLP1 pp 229533.A0A1C3YN26
89879 229533.A0A098DLP1 pp 229533.V6RM02
89880 229533.A0A098DLP1 pp 229533.I1RMM8
89881 229533.A0A098DLP1 pp 229533.A0A1C3YKN4
89882 229533.A0A098DLP1 pp 229533.A0A1C3YK33
89883 229533.A0A098DLP1 pp 229533.V6RUY9
89884 229533.I1S0Z2 pp 229533.I1S604
89885 229533.I1RKL0 pp 229533.I1RQE7
89886 229533.I1RKL0 pp 229533.I1RYV3
89887 229533.I1RKL0 pp 229533.Q4HZK7
89888 229533.I1RKL0 pp 229533.I1S2E8
89889 229533.I1RKL0 pp 229533.Q4IB50
89890 229533.I1RKL0 pp 229533.I1RX64
89891 229533.I1RKL0 pp 229533.I1RYM5
89892 229533.I1RKL0 pp 229533.Q4I5R3
89893 229533.I1RHV9 pp 229533.I1RUP5
89894 229533.I1RHV9 pp 229533.I1RJS0
89895 229533.I1S8D1 pp 229533.I1S8D2
89896 229533.I1S8D1 pp 229533.V6RH92
89897 229533.I1RII1 pp 229533.I1SAT1
89898 229533.I1RII1 pp 229533.I1RJW5
89899 229533.I1RII1 pp 229533.I1S836
89900 229533.I1RII1 pp 229533.I1RQC1
89901 229533.A0A1C3YJ44 pp 229533.A0A1C3YJF8
89902 229533.I1RWJ4 pp 229533.V6QYF0
89903 229533.I1RWJ4 pp 229533.Q4I5M4
89904 229533.I1RWJ4 pp 229533.I1RZS6
89905 229533.I1RWJ4 pp 229533.Q4I1M9
89906 229533.I1RWJ4 pp 229533.Q4IF76
89907 229533.I1RWJ4 pp 229533.I1S9U2
89908 229533.I1RWJ4 pp 229533.Q4IEV5
89909 229533.I1RWJ4 pp 229533.V6R283
89910 229533.I1RWJ4 pp 229533.I1S6F0
89911 229533.I1RWJ4 pp 229533.Q4IAS1
89912 229533.I1RWJ4 pp 229533.Q4IJH1
89913 229533.I1RWJ4 pp 229533.Q4IPI1
89914 229533.I1RWJ4 pp 229533.V6R227
89915 229533.I1RWJ4 pp 229533.I1S253
89916 229533.I1RWJ4 pp 229533.Q4IQW0
89917 229533.I1RWJ4 pp 229533.Q4HVW2
89918 229533.I1RWJ4 pp 229533.Q4IJ56
89919 229533.I1RWJ4 pp 229533.Q4IEK8
89920 229533.I1RWJ4 pp 229533.I1S4T3
89921 229533.I1RWJ4 pp 229533.Q4I8X0
89922 229533.I1RWJ4 pp 229533.Q4IFI0
89923 229533.I1RWJ4 pp 229533.Q4IAA0
89924 229533.I1RWJ4 pp 229533.I1S274
89925 229533.I1RWJ4 pp 229533.V6QUE6
89926 229533.I1RWJ4 pp 229533.I1RY63
89927 229533.I1RWJ4 pp 229533.V6RGY3
89928 229533.I1RWJ4 pp 229533.Q4IBS2
89929 229533.I1RWJ4 pp 229533.I1S021
89930 229533.I1RWJ4 pp 229533.Q4HZK7
89931 229533.I1RWJ4 pp 229533.Q4I327
89932 229533.I1RWJ4 pp 229533.Q4HZ68
89933 229533.I1RWJ4 pp 229533.V6QWZ7
89934 229533.I1RK32 pp 229533.I1RY71
89935 229533.I1RK32 pp 229533.I1S0D4
89936 229533.I1RK32 pp 229533.I1RQ80

89937	229533.I1RK32	pp	229533.I1RN31
89938	229533.I1RK32	pp	229533.I1S1V5
89939	229533.I1RK32	pp	229533.I1S0Z2
89940	229533.I1RK32	pp	229533.I1RN27
89941	229533.I1RK32	pp	229533.I1RQG9
89942	229533.I1RK32	pp	229533.I1RY98
89943	229533.I1RK32	pp	229533.I1S0S6
89944	229533.I1RK32	pp	229533.I1RQL1
89945	229533.I1RK32	pp	229533.I1RN37
89946	229533.I1RK32	pp	229533.I1RWM0
89947	229533.I1RK32	pp	229533.I1RW16
89948	229533.I1RK32	pp	229533.I1S604
89949	229533.I1S060	pp	229533.Q4IJ56
89950	229533.I1S060	pp	229533.I1S0M9
89951	229533.I1S060	pp	229533.V6QUE6
89952	229533.I1S060	pp	229533.I1S296
89953	229533.I1S060	pp	229533.V6R042
89954	229533.I1S060	pp	229533.I1S0J1
89955	229533.I1S060	pp	229533.V6RGN5
89956	229533.I1S060	pp	229533.Q4IBS2
89957	229533.I1S060	pp	229533.Q4I327
89958	229533.I1S060	pp	229533.I1S0Z9
89959	229533.I1S060	pp	229533.Q4IN63
89960	229533.I1S060	pp	229533.Q4HZ68
89961	229533.I1S060	pp	229533.V6RGY3
89962	229533.I1S060	pp	229533.Q4IEK8
89963	229533.I1S060	pp	229533.Q4IFI0
89964	229533.I1S060	pp	229533.Q4HWE2
89965	229533.I1S060	pp	229533.V6R227
89966	229533.I1S060	pp	229533.I1S1X9
89967	229533.I1S060	pp	229533.I1S8E2
89968	229533.I1S060	pp	229533.Q4IAA0
89969	229533.I1S060	pp	229533.Q4HTT1
89970	229533.I1S060	pp	229533.Q4HVW2
89971	229533.I1S060	pp	229533.V6QYF0
89972	229533.I1S060	pp	229533.I1S4T3
89973	229533.I1S060	pp	229533.Q4IF76
89974	229533.I1S060	pp	229533.I1S9U2
89975	229533.I1S060	pp	229533.Q4HZ42
89976	229533.I1S060	pp	229533.Q4IJH1
89977	229533.I1S060	pp	229533.Q4I662
89978	229533.I1S060	pp	229533.Q4HZ47
89979	229533.I1S060	pp	229533.I1S9Y0
89980	229533.I1S060	pp	229533.Q4IQW0
89981	229533.I1S060	pp	229533.I1S6F0
89982	229533.I1S060	pp	229533.Q4IPI1
89983	229533.I1S060	pp	229533.Q4I830
89984	229533.I1S060	pp	229533.Q4IAS1
89985	229533.I1S060	pp	229533.V6R283
89986	229533.I1S0D4	pp	229533.Q4I662
89987	229533.I1S0D4	pp	229533.Q4IAS1
89988	229533.I1S0D4	pp	229533.Q4IR18
89989	229533.I1S0D4	pp	229533.I1S0J1
89990	229533.I1S0D4	pp	229533.I1S2C0
89991	229533.I1S0D4	pp	229533.Q4HXF0
89992	229533.I1S0D4	pp	229533.V6RGN5
89993	229533.I1S0D4	pp	229533.V6RVG3
89994	229533.I1S0D4	pp	229533.Q4HVW2
89995	229533.I1S0D4	pp	229533.Q4IPI1
89996	229533.I1S0D4	pp	229533.Q4IFI0
89997	229533.I1S0D4	pp	229533.I1S0Z2
89998	229533.I1S0D4	pp	229533.V6R227
89999	229533.I1S0D4	pp	229533.Q4HZ68
90000	229533.I1S0D4	pp	229533.I1S0L3
90001	229533.I1S0D4	pp	229533.Q4HZ47
90002	229533.I1S0D4	pp	229533.Q4IBS2
90003	229533.I1RSY1	pp	229533.I1S024
90004	229533.I1RSY1	pp	229533.I1RZY1
90005	229533.I1RSY1	pp	229533.I1S036
90006	229533.I1RSY1	pp	229533.I1S2P0
90007	229533.I1RSY1	pp	229533.I1S7J8
90008	229533.I1RSY1	pp	229533.I1S5N3
90009	229533.I1RSY1	pp	229533.I1RZM4

90010 229533.I1RSY1 pp 229533.V6RK96
90011 229533.I1RSY1 pp 229533.I1S273
90012 229533.I1RB07 pp 229533.I1RZ70
90013 229533.I1RB07 pp 229533.I1S147
90014 229533.I1RB07 pp 229533.I1RNM7
90015 229533.I1RB07 pp 229533.I1RH92
90016 229533.I1RB07 pp 229533.I1RBY9
90017 229533.I1RB07 pp 229533.I1S148
90018 229533.I1RB07 pp 229533.I1S8D1
90019 229533.I1RB07 pp 229533.I1RDU5
90020 229533.I1RB07 pp 229533.I1RQE1
90021 229533.I1RB07 pp 229533.I1S8D2
90022 229533.I1RB07 pp 229533.I1RHU0
90023 229533.I1RB07 pp 229533.I1RN88
90024 229533.I1RB07 pp 229533.I1RWM4
90025 229533.I1RB07 pp 229533.I1RWN7
90026 229533.I1RB07 pp 229533.I1RYX6
90027 229533.I1RB07 pp 229533.V6RH92
90028 229533.I1RB07 pp 229533.I1RGC3
90029 229533.A0A098E3M9 pp 229533.I1RU02
90030 229533.A0A098E3M9 pp 229533.I1S0K2
90031 229533.A0A098E3M9 pp 229533.I1S5B0
90032 229533.A0A098E3M9 pp 229533.I1RCM5
90033 229533.A0A098E3M9 pp 229533.I1RZK5
90034 229533.A0A098E3M9 pp 229533.I1S887
90035 229533.A0A098E3M9 pp 229533.V6QUE6
90036 229533.A0A098E3M9 pp 229533.I1RBL7
90037 229533.A0A098E3M9 pp 229533.I1S836
90038 229533.A0A098E3M9 pp 229533.A0A0E0RTV8
90039 229533.A0A098E3M9 pp 229533.A0A1C3YHX6
90040 229533.A0A098E3M9 pp 229533.I1RPR9
90041 229533.A0A098E3M9 pp 229533.I1RAM9
90042 229533.A0A098E3M9 pp 229533.I1RAA2
90043 229533.A0A098E3M9 pp 229533.I1RRY9
90044 229533.A0A098E3M9 pp 229533.A0A1C3YLB3
90045 229533.A0A098E3M9 pp 229533.I1REE2
90046 229533.A0A098E3M9 pp 229533.I1RCR5
90047 229533.A0A098E3M9 pp 229533.I1RYV3
90048 229533.A0A098E3M9 pp 229533.V6RCZ8
90049 229533.A0A098E3M9 pp 229533.I1RWC9
90050 229533.A0A098E3M9 pp 229533.I1S6K9
90051 229533.A0A098E3M9 pp 229533.I1RM26
90052 229533.A0A098E3M9 pp 229533.I1RWB0
90053 229533.A0A098E3M9 pp 229533.I1RRU8
90054 229533.A0A098E3M9 pp 229533.A0A1C3YKY6
90055 229533.A0A098E3M9 pp 229533.I1RAV5
90056 229533.A0A098E3M9 pp 229533.Q4HTT2
90057 229533.A0A098E3M9 pp 229533.Q4IER8
90058 229533.A0A098E3M9 pp 229533.A0A098E4W0
90059 229533.A0A098E3M9 pp 229533.I1RPR1
90060 229533.A0A098E3M9 pp 229533.A0A1C3YLB0
90061 229533.A0A098E3M9 pp 229533.I1RK95
90062 229533.A0A098E3M9 pp 229533.I1R9Z5
90063 229533.A0A098E3M9 pp 229533.I1RZM8
90064 229533.A0A098E3M9 pp 229533.A0A0E0SQV5
90065 229533.A0A098E3M9 pp 229533.Q4IJK2
90066 229533.A0A098E3M9 pp 229533.A0A1C3YKV3
90067 229533.A0A098E3M9 pp 229533.I1RCM6
90068 229533.A0A098E3M9 pp 229533.I1RXF0
90069 229533.A0A098E3M9 pp 229533.I1RS18
90070 229533.A0A098E3M9 pp 229533.I1RCW8
90071 229533.A0A098E3M9 pp 229533.A0A1C3YK34
90072 229533.A0A098E3M9 pp 229533.A0A1C3YJR3
90073 229533.A0A098E3M9 pp 229533.A0A1C3YKB0
90074 229533.A0A098E3M9 pp 229533.I1RBT1
90075 229533.A0A098E3M9 pp 229533.V6RTJ8
90076 229533.A0A098E3M9 pp 229533.I1S9T2
90077 229533.A0A098E3M9 pp 229533.I1RA49
90078 229533.A0A098E3M9 pp 229533.I1RVX9
90079 229533.A0A098E3M9 pp 229533.I1R9Y4
90080 229533.A0A098E3M9 pp 229533.A0A1C3YHX8
90081 229533.A0A098E3M9 pp 229533.A0A1C3YLR6
90082 229533.A0A098E3M9 pp 229533.I1RAI4

90083 229533.A0A098E3M9 pp 229533.Q4I5R3
90084 229533.A0A098E3M9 pp 229533.I1S1W8
90085 229533.A0A098E3M9 pp 229533.I1RX46
90086 229533.A0A098E3M9 pp 229533.I1RR24
90087 229533.A0A098E3M9 pp 229533.A0A0E0RSD7
90088 229533.A0A098E3M9 pp 229533.A0A0E0SFN0
90089 229533.A0A098E3M9 pp 229533.A0A0E0S6V8
90090 229533.A0A098E3M9 pp 229533.V6RUT5
90091 229533.A0A098E3M9 pp 229533.I1RMK7
90092 229533.A0A098E3M9 pp 229533.I1RNM7
90093 229533.A0A098E3M9 pp 229533.I1S4S4
90094 229533.A0A098E3M9 pp 229533.I1RD69
90095 229533.A0A098E3M9 pp 229533.I1RXD7
90096 229533.A0A098E3M9 pp 229533.I1RY21
90097 229533.A0A098E3M9 pp 229533.I1RLP1
90098 229533.A0A098E3M9 pp 229533.I1RMQ6
90099 229533.A0A098E3M9 pp 229533.A0A1C3YL54
90100 229533.A0A098E3M9 pp 229533.A0A0E0SES1
90101 229533.A0A098E3M9 pp 229533.A0A0E0SAR4
90102 229533.A0A098E3M9 pp 229533.I1RAX6
90103 229533.A0A098E3M9 pp 229533.I1RAU9
90104 229533.A0A098E3M9 pp 229533.I1RBD5
90105 229533.A0A098E3M9 pp 229533.I1RD52
90106 229533.A0A098E3M9 pp 229533.I1RA85
90107 229533.A0A098E3M9 pp 229533.A0A1C3YIP5
90108 229533.A0A098E3M9 pp 229533.I1RYH7
90109 229533.A0A098E3M9 pp 229533.A0A1C3YK13
90110 229533.A0A098E3M9 pp 229533.I1S4S7
90111 229533.A0A098E3M9 pp 229533.A0A1C3YKW5
90112 229533.A0A098E3M9 pp 229533.I1RXU9
90113 229533.A0A098E3M9 pp 229533.I1RVX0
90114 229533.A0A098E3M9 pp 229533.A0A1C3YK33
90115 229533.A0A098E3M9 pp 229533.I1RBJ0
90116 229533.A0A098E3M9 pp 229533.I1RT03
90117 229533.A0A098E3M9 pp 229533.I1RSQ7
90118 229533.A0A098E3M9 pp 229533.I1RAW0
90119 229533.A0A098E3M9 pp 229533.I1RJV3
90120 229533.A0A098E3M9 pp 229533.I1RXR1
90121 229533.A0A098E3M9 pp 229533.I1RA70
90122 229533.A0A098E3M9 pp 229533.I1RB92
90123 229533.A0A098E3M9 pp 229533.I1RRX5
90124 229533.A0A098E3M9 pp 229533.I1RQC1
90125 229533.A0A098E3M9 pp 229533.I1RFL4
90126 229533.A0A098E3M9 pp 229533.A0A1C3YKS8
90127 229533.A0A098E3M9 pp 229533.I1S1C7
90128 229533.A0A098E3M9 pp 229533.I1RE01
90129 229533.A0A098E3M9 pp 229533.I1RCH5
90130 229533.A0A098E3M9 pp 229533.I1S1D3
90131 229533.A0A098E3M9 pp 229533.I1RYW3
90132 229533.A0A098E3M9 pp 229533.I1RQE7
90133 229533.A0A098E3M9 pp 229533.I1RRI2
90134 229533.A0A098E3M9 pp 229533.A0A1C3YLC1
90135 229533.A0A098E3M9 pp 229533.I1S7W5
90136 229533.A0A098E3M9 pp 229533.I1S8R2
90137 229533.A0A098E3M9 pp 229533.I1S0J1
90138 229533.A0A098E3M9 pp 229533.Q4I624
90139 229533.A0A098E3M9 pp 229533.I1S5R6
90140 229533.A0A098E3M9 pp 229533.I1RRV1
90141 229533.A0A098E3M9 pp 229533.I1S2H4
90142 229533.A0A098E3M9 pp 229533.I1RS90
90143 229533.A0A098E3M9 pp 229533.A0A1C3YLP1
90144 229533.A0A098E3M9 pp 229533.A0A0E0SPS2
90145 229533.A0A098E3M9 pp 229533.I1S3G4
90146 229533.A0A098E3M9 pp 229533.A0A0E0SC46
90147 229533.A0A098E3M9 pp 229533.Q4I7L0
90148 229533.A0A098E3M9 pp 229533.V6R042
90149 229533.A0A098E3M9 pp 229533.I1RSQ3
90150 229533.A0A098E3M9 pp 229533.I1RG11
90151 229533.A0A098E3M9 pp 229533.I1RUG3
90152 229533.A0A098E3M9 pp 229533.I1RFS5
90153 229533.A0A098E3M9 pp 229533.I1S0L4
90154 229533.A0A098E3M9 pp 229533.I1RY99
90155 229533.A0A098E3M9 pp 229533.I1S3T7

90156	229533.A0A098E3M9	pp	229533.I1RNC1
90157	229533.A0A098E3M9	pp	229533.I1S256
90158	229533.A0A098E3M9	pp	229533.I1RCR8
90159	229533.A0A098E3M9	pp	229533.I1RRN2
90160	229533.A0A098E3M9	pp	229533.I1RD22
90161	229533.A0A098E3M9	pp	229533.I1S1W3
90162	229533.A0A098E3M9	pp	229533.I1S9Y1
90163	229533.A0A098E3M9	pp	229533.I1RPV3
90164	229533.A0A098E3M9	pp	229533.I1RBE6
90165	229533.A0A098E3M9	pp	229533.O74638
90166	229533.A0A098E3M9	pp	229533.I1RBA6
90167	229533.A0A098E3M9	pp	229533.V6RLM7
90168	229533.A0A098E3M9	pp	229533.I1RGA4
90169	229533.A0A098E3M9	pp	229533.I1RRT8
90170	229533.A0A098E3M9	pp	229533.I1RBC7
90171	229533.A0A098E3M9	pp	229533.I1RMV0
90172	229533.A0A098E3M9	pp	229533.A0A1C3YLK8
90173	229533.A0A098E3M9	pp	229533.I1S5R1
90174	229533.A0A098E3M9	pp	229533.A0A1C3YIR8
90175	229533.A0A098E3M9	pp	229533.I1RC66
90176	229533.A0A098E3M9	pp	229533.Q4IMD1
90177	229533.A0A098E3M9	pp	229533.A0A1C3YI20
90178	229533.A0A098E3M9	pp	229533.I1RA52
90179	229533.A0A098E3M9	pp	229533.I1RYX5
90180	229533.A0A098E3M9	pp	229533.I1RA11
90181	229533.A0A098E3M9	pp	229533.A0A1C3YKM9
90182	229533.A0A098E3M9	pp	229533.I1RZ39
90183	229533.A0A098E3M9	pp	229533.I1RSX0
90184	229533.A0A098E3M9	pp	229533.I1RB73
90185	229533.A0A098E3M9	pp	229533.I1SB04
90186	229533.A0A098E3M9	pp	229533.I1RAW3
90187	229533.A0A098E3M9	pp	229533.I1R9M0
90188	229533.A0A098E3M9	pp	229533.I1RCN2
90189	229533.A0A098E3M9	pp	229533.V6RUY9
90190	229533.A0A098E3M9	pp	229533.I1RBY4
90191	229533.A0A098E3M9	pp	229533.A0A1C3YM60
90192	229533.A0A098E3M9	pp	229533.A0A1C3YJI6
90193	229533.A0A098E3M9	pp	229533.Q4IB50
90194	229533.A0A098E3M9	pp	229533.Q4HTT1
90195	229533.A0A098E3M9	pp	229533.I1RT40
90196	229533.A0A098E3M9	pp	229533.I1RC29
90197	229533.A0A098E3M9	pp	229533.I1RSQ4
90198	229533.A0A098E3M9	pp	229533.I1RTD5
90199	229533.A0A098E3M9	pp	229533.I1RZZ9
90200	229533.A0A098E3M9	pp	229533.I1RMP0
90201	229533.A0A098E3M9	pp	229533.I1RN88
90202	229533.A0A098E3M9	pp	229533.I1RFZ2
90203	229533.A0A098E3M9	pp	229533.I1RFS3
90204	229533.A0A098E3M9	pp	229533.I1RWI5
90205	229533.A0A098E3M9	pp	229533.I1S1F3
90206	229533.A0A098E3M9	pp	229533.I1RN67
90207	229533.A0A098E3M9	pp	229533.I1RNG4
90208	229533.A0A098E3M9	pp	229533.I1RY62
90209	229533.A0A098E3M9	pp	229533.I1S299
90210	229533.A0A098E3M9	pp	229533.I1RFT1
90211	229533.A0A098E3M9	pp	229533.I1RPP3
90212	229533.A0A098E3M9	pp	229533.A0A0E0SLZ3
90213	229533.A0A098E3M9	pp	229533.I1RKB2
90214	229533.A0A098E3M9	pp	229533.I1RFW1
90215	229533.I1RMS1	pp	229533.I1RPA6
90216	229533.I1RMS1	pp	229533.I1RNF4
90217	229533.I1RMS1	pp	229533.I1RQ72
90218	229533.A0A1C3YJD4	pp	229533.Q4IJK2
90219	229533.A0A1C3YJD4	pp	229533.I1RCM6
90220	229533.A0A1C3YJD4	pp	229533.I1RBL7
90221	229533.A0A1C3YJD4	pp	229533.I1S0K2
90222	229533.A0A1C3YJD4	pp	229533.V6QUE6
90223	229533.A0A1C3YJD4	pp	229533.I1RPR9
90224	229533.A0A1C3YJD4	pp	229533.I1RRY9
90225	229533.A0A1C3YJD4	pp	229533.I1S836
90226	229533.A0A1C3YJD4	pp	229533.I1REE2
90227	229533.A0A1C3YJD4	pp	229533.I1RWC9
90228	229533.A0A1C3YJD4	pp	229533.I1RWB0

90229	229533.A0A1C3YJD4	pp	229533.V6RCZ8
90230	229533.A0A1C3YJD4	pp	229533.A0A1C3YKY6
90231	229533.A0A1C3YJD4	pp	229533.I1RCR5
90232	229533.A0A1C3YJD4	pp	229533.I1S6K9
90233	229533.A0A1C3YJD4	pp	229533.I1RYV3
90234	229533.A0A1C3YJD4	pp	229533.Q4IER8
90235	229533.A0A1C3YJD4	pp	229533.Q4HTT2
90236	229533.A0A1C3YJD4	pp	229533.I1RPR1
90237	229533.A0A1C3YJD4	pp	229533.A0A1C3YLB0
90238	229533.A0A1C3YJD4	pp	229533.I1RM26
90239	229533.A0A1C3YJD4	pp	229533.I1RAV5
90240	229533.A0A1C3YJD4	pp	229533.I1RK95
90241	229533.A0A1C3YJD4	pp	229533.I1RU02
90242	229533.A0A1C3YJD4	pp	229533.I1RCM5
90243	229533.A0A1C3YJD4	pp	229533.I1RZM8
90244	229533.A0A1C3YJD4	pp	229533.I1RZK5
90245	229533.A0A1C3YJD4	pp	229533.A0A1C3YLB3
90246	229533.A0A1C3YJD4	pp	229533.I1RCW8
90247	229533.A0A1C3YJD4	pp	229533.A0A1C3YJR3
90248	229533.A0A1C3YJD4	pp	229533.I1R9Z5
90249	229533.A0A1C3YJD4	pp	229533.A0A1C3YKB0
90250	229533.A0A1C3YJD4	pp	229533.I1RXF0
90251	229533.A0A1C3YJD4	pp	229533.A0A1C3YLR6
90252	229533.A0A1C3YJD4	pp	229533.I1RAI4
90253	229533.A0A1C3YJD4	pp	229533.I1S887
90254	229533.A0A1C3YJD4	pp	229533.I1S1W8
90255	229533.A0A1C3YJD4	pp	229533.A0A1C3YKV3
90256	229533.A0A1C3YJD4	pp	229533.I1RAM9
90257	229533.A0A1C3YJD4	pp	229533.I1RS18
90258	229533.A0A1C3YJD4	pp	229533.A0A1C3YK34
90259	229533.A0A1C3YJD4	pp	229533.I1RAA2
90260	229533.A0A1C3YJD4	pp	229533.I1RMK7
90261	229533.A0A1C3YJD4	pp	229533.I1S5B0
90262	229533.A0A1C3YJD4	pp	229533.I1RNM7
90263	229533.A0A1C3YJD4	pp	229533.I1RSQ7
90264	229533.A0A1C3YJD4	pp	229533.Q4I624
90265	229533.A0A1C3YJD4	pp	229533.I1RD69
90266	229533.A0A1C3YJD4	pp	229533.I1RRX5
90267	229533.A0A1C3YJD4	pp	229533.I1RBD5
90268	229533.A0A1C3YJD4	pp	229533.I1RYH7
90269	229533.A0A1C3YJD4	pp	229533.A0A1C3YKW5
90270	229533.A0A1C3YJD4	pp	229533.I1RAX6
90271	229533.A0A1C3YJD4	pp	229533.I1RR24
90272	229533.A0A1C3YJD4	pp	229533.I1S4S4
90273	229533.A0A1C3YJD4	pp	229533.I1RAU9
90274	229533.A0A1C3YJD4	pp	229533.I1RVX0
90275	229533.A0A1C3YJD4	pp	229533.I1RT03
90276	229533.A0A1C3YJD4	pp	229533.I1RA49
90277	229533.A0A1C3YJD4	pp	229533.V6RTJ8
90278	229533.A0A1C3YJD4	pp	229533.I1RXR1
90279	229533.A0A1C3YJD4	pp	229533.I1RY21
90280	229533.A0A1C3YJD4	pp	229533.I1RMQ6
90281	229533.A0A1C3YJD4	pp	229533.I1RVX9
90282	229533.A0A1C3YJD4	pp	229533.I1RB92
90283	229533.A0A1C3YJD4	pp	229533.I1RFL4
90284	229533.A0A1C3YJD4	pp	229533.I1RBT1
90285	229533.A0A1C3YJD4	pp	229533.I1RCH5
90286	229533.A0A1C3YJD4	pp	229533.I1RX46
90287	229533.A0A1C3YJD4	pp	229533.I1R9Y4
90288	229533.A0A1C3YJD4	pp	229533.I1RA85
90289	229533.A0A1C3YJD4	pp	229533.I1RD52
90290	229533.A0A1C3YJD4	pp	229533.A0A1C3YK13
90291	229533.A0A1C3YJD4	pp	229533.I1S8R2
90292	229533.A0A1C3YJD4	pp	229533.I1RQE7
90293	229533.A0A1C3YJD4	pp	229533.V6RUT5
90294	229533.A0A1C3YJD4	pp	229533.I1RXU9
90295	229533.A0A1C3YJD4	pp	229533.I1S4S7
90296	229533.A0A1C3YJD4	pp	229533.I1S1C7
90297	229533.A0A1C3YJD4	pp	229533.I1RXD7
90298	229533.A0A1C3YJD4	pp	229533.I1RLP1
90299	229533.A0A1C3YJD4	pp	229533.A0A1C3YK33
90300	229533.A0A1C3YJD4	pp	229533.A0A1C3YL54
90301	229533.A0A1C3YJD4	pp	229533.I1RBJ0

229533.A0A1C3YJD4 pp 229533.I1RAW0
229533.A0A1C3YJD4 pp 229533.I1RJV3
229533.A0A1C3YJD4 pp 229533.I1S0J1
229533.A0A1C3YJD4 pp 229533.I1RYW3
229533.A0A1C3YJD4 pp 229533.I1RRI2
229533.A0A1C3YJD4 pp 229533.I1RA70
229533.A0A1C3YJD4 pp 229533.I1S5R6
229533.A0A1C3YJD4 pp 229533.I1RQC1
229533.A0A1C3YJD4 pp 229533.I1RBY4
229533.A0A1C3YJD4 pp 229533.Q4HTT1
229533.A0A1C3YJD4 pp 229533.I1RS90
229533.A0A1C3YJD4 pp 229533.I1S1W3
229533.A0A1C3YJD4 pp 229533.V6RLM7
229533.A0A1C3YJD4 pp 229533.I1RC29
229533.A0A1C3YJD4 pp 229533.I1RG11
229533.A0A1C3YJD4 pp 229533.I1RN88
229533.A0A1C3YJD4 pp 229533.I1RFS3
229533.A0A1C3YJD4 pp 229533.I1RMV0
229533.A0A1C3YJD4 pp 229533.I1S1D3
229533.A0A1C3YJD4 pp 229533.I1RFS5
229533.A0A1C3YJD4 pp 229533.I1RPV3
229533.A0A1C3YJD4 pp 229533.I1S2H4
229533.A0A1C3YJD4 pp 229533.I1S3G4
229533.A0A1C3YJD4 pp 229533.I1S256
229533.A0A1C3YJD4 pp 229533.I1RE01
229533.A0A1C3YJD4 pp 229533.A0A1C3YLC1
229533.A0A1C3YJD4 pp 229533.I1S7W5
229533.A0A1C3YJD4 pp 229533.I1RRN2
229533.A0A1C3YJD4 pp 229533.I1RD22
229533.A0A1C3YJD4 pp 229533.A0A1C3YKM9
229533.A0A1C3YJD4 pp 229533.V6R042
229533.A0A1C3YJD4 pp 229533.Q4I7L0
229533.A0A1C3YJD4 pp 229533.I1S0L4
229533.A0A1C3YJD4 pp 229533.I1RZ39
229533.A0A1C3YJD4 pp 229533.I1SB04
229533.A0A1C3YJD4 pp 229533.I1RSQ3
229533.A0A1C3YJD4 pp 229533.Q4IB50
229533.A0A1C3YJD4 pp 229533.I1S3T7
229533.A0A1C3YJD4 pp 229533.I1S9Y1
229533.A0A1C3YJD4 pp 229533.I1RUG3
229533.A0A1C3YJD4 pp 229533.I1RSQ4
229533.A0A1C3YJD4 pp 229533.I1RNC1
229533.A0A1C3YJD4 pp 229533.I1RZZ9
229533.A0A1C3YJD4 pp 229533.I1RCR8
229533.A0A1C3YJD4 pp 229533.I1RA52
229533.A0A1C3YJD4 pp 229533.I1RWI5
229533.A0A1C3YJD4 pp 229533.I1RYX5
229533.A0A1C3YJD4 pp 229533.O74638
229533.A0A1C3YJD4 pp 229533.I1RAW3
229533.A0A1C3YJD4 pp 229533.I1RRT8
229533.A0A1C3YJD4 pp 229533.I1RMP0
229533.A0A1C3YJD4 pp 229533.I1RC66
229533.A0A1C3YJD4 pp 229533.I1R9M0
229533.A0A1C3YJD4 pp 229533.I1S5R1
229533.A0A1C3YJD4 pp 229533.I1RT40
229533.A0A1C3YJD4 pp 229533.I1RFZ2
229533.A0A1C3YJD4 pp 229533.I1S1F3
229533.A0A1C3YJD4 pp 229533.I1RA11
229533.A0A1C3YJD4 pp 229533.I1RPP3
229533.A0A1C3YJD4 pp 229533.I1RTD5
229533.A0A1C3YJD4 pp 229533.I1RN67
229533.A0A1C3YJD4 pp 229533.I1RNG4
229533.A0A1C3YJD4 pp 229533.I1RFW1
229533.A0A1C3YJD4 pp 229533.I1RKB2
229533.A0A1C3YJD4 pp 229533.I1RY62
229533.A0A1C3YJD4 pp 229533.I1RBE6
229533.A0A1C3YJD4 pp 229533.I1RGA4
229533.A0A1C3YJD4 pp 229533.I1RBA6
229533.A0A1C3YJD4 pp 229533.I1RBC7
229533.A0A1C3YJD4 pp 229533.A0A1C3YLK8
229533.A0A1C3YJD4 pp 229533.I1S299
229533.A0A1C3YJD4 pp 229533.Q4IMD1
229533.A0A1C3YJD4 pp 229533.I1RFT1

90375 229533.A0A1C3YJD4 pp 229533.I1RSX0
90376 229533.A0A1C3YJD4 pp 229533.A0A1C3YM60
90377 229533.A0A1C3YJD4 pp 229533.V6RUY9
90378 229533.A0A1C3YJD4 pp 229533.A0A1C3YJI6
90379 229533.I1RLW8 pp 229533.I1S2Q0
90380 229533.I1RLW8 pp 229533.I1S1K2
90381 229533.I1RLW8 pp 229533.I1S3S7
90382 229533.I1RF57 pp 229533.I1RF61
90383 229533.I1RF57 pp 229533.I1RF63
90384 229533.I1RF57 pp 229533.I1RF58
90385 229533.I1RF57 pp 229533.I1RF62
90386 229533.I1RF57 pp 229533.I1RF60
90387 229533.A0A098DKA9 pp 229533.I1RQ38
90388 229533.A0A098DKA9 pp 229533.I1RYB8
90389 229533.A0A098DKA9 pp 229533.I1RN37
90390 229533.A0A098DKA9 pp 229533.I1RB45
90391 229533.A0A098DKA9 pp 229533.I1S0D4
90392 229533.A0A098D2S9 pp 229533.I1RN37
90393 229533.A0A098D2S9 pp 229533.I1RB45
90394 229533.A0A098D2S9 pp 229533.A0A1C3YJW9
90395 229533.A0A098D2S9 pp 229533.A0A0E0RXI2
90396 229533.A0A098D2S9 pp 229533.I1S0Z2
90397 229533.A0A098D2S9 pp 229533.I1RII1
90398 229533.A0A098D2S9 pp 229533.I1RMK8
90399 229533.A0A098D2S9 pp 229533.I1RK32
90400 229533.A0A098D2S9 pp 229533.I1S0D4
90401 229533.A0A098D2S9 pp 229533.A0A098E3M9
90402 229533.A0A098D2S9 pp 229533.A0A1C3YJD4
90403 229533.A0A098D2S9 pp 229533.A0A098DKA9
90404 229533.I1S8D2 pp 229533.V6RH92
90405 229533.I1RAB6 pp 229533.I1RMJ3
90406 229533.I1RAB6 pp 229533.I1RFL7
90407 229533.I1RAB6 pp 229533.I1RK24
90408 229533.I1RAB6 pp 229533.I1RLT3
90409 229533.I1RAB6 pp 229533.I1RLP9
90410 229533.I1RAB6 pp 229533.I1RYI9
90411 229533.I1RAB6 pp 229533.I1S7J8
90412 229533.I1RAB6 pp 229533.I1RJX3
90413 229533.I1RAB6 pp 229533.I1RKX6
90414 229533.I1RAB6 pp 229533.I1S0H5
90415 229533.I1RAB6 pp 229533.I1RSH0
90416 229533.I1RAB6 pp 229533.I1RSY1
90417 229533.I1RAB6 pp 229533.I1REI2
90418 229533.I1RAB6 pp 229533.I1S273
90419 229533.I1RAB6 pp 229533.I1RCH9
90420 229533.I1RAB6 pp 229533.I1RE24
90421 229533.I1RAB6 pp 229533.I1S2P0
90422 229533.I1RAB6 pp 229533.I1RDU9
90423 229533.I1RAB6 pp 229533.I1RJ04
90424 229533.I1RAB6 pp 229533.I1S5N3
90425 229533.I1RVY0 pp 229533.I1S0J3
90426 229533.I1RVY0 pp 229533.I1S3G4
90427 229533.I1RVY0 pp 229533.I1S9Y1
90428 229533.I1RVY0 pp 229533.I1S7I5
90429 229533.I1RVY0 pp 229533.I1S951
90430 229533.I1RVY0 pp 229533.I1SAA0
90431 229533.I1RVY0 pp 229533.I1S490
90432 229533.I1RVY0 pp 229533.I1SAN0
90433 229533.I1RVY0 pp 229533.I1S1V5
90434 229533.I1RVY0 pp 229533.Q4IF76
90435 229533.I1RVY0 pp 229533.Q4IMD1
90436 229533.I1RVY0 pp 229533.Q4HTT1
90437 229533.I1RVY0 pp 229533.Q4I5W5
90438 229533.I1RVY0 pp 229533.I1S549
90439 229533.I1RVY0 pp 229533.I1S382
90440 229533.I1RVY0 pp 229533.I1RYE2
90441 229533.I1RVY0 pp 229533.I1RW80
90442 229533.I1RVY0 pp 229533.Q4IEV4
90443 229533.I1RVY0 pp 229533.I1S268
90444 229533.I1RVY0 pp 229533.I1RX63
90445 229533.I1RVY0 pp 229533.I1RZM2
90446 229533.I1RVY0 pp 229533.Q4IPI4
90447 229533.I1RVY0 pp 229533.I1RW28

90448 229533.I1RVY0 pp 229533.I1RWX5
90449 229533.I1RVY0 pp 229533.I1S3D7
90450 229533.I1RVY0 pp 229533.I1S0N5
90451 229533.I1RVY0 pp 229533.I1S276
90452 229533.I1RVY0 pp 229533.I1S3N1
90453 229533.I1RVY0 pp 229533.I1S3N0
90454 229533.I1RVY0 pp 229533.Q4IPC8
90455 229533.I1RVY0 pp 229533.I1RXU0
90456 229533.I1RVY0 pp 229533.Q4I7S1
90457 229533.I1RVY0 pp 229533.I1S0C6
90458 229533.I1RVY0 pp 229533.I1S1U7
90459 229533.I1RVY0 pp 229533.I1RZ42
90460 229533.I1RVY0 pp 229533.I1S270
90461 229533.I1RVY0 pp 229533.I1RWF3
90462 229533.I1RVY0 pp 229533.I1S6S6
90463 229533.I1RVY0 pp 229533.Q4IJH1
90464 229533.I1RVY0 pp 229533.I1RYV4
90465 229533.I1RVY0 pp 229533.I1RW90
90466 229533.I1RVY0 pp 229533.I1S1W8
90467 229533.I1RVY0 pp 229533.Q4HYQ4
90468 229533.I1RVY0 pp 229533.I1S7Q8
90469 229533.I1RVY0 pp 229533.I1RW91
90470 229533.I1RVY0 pp 229533.V6QWX8
90471 229533.I1RVY0 pp 229533.I1S0F5
90472 229533.I1RVY0 pp 229533.I1S540
90473 229533.I1RVY0 pp 229533.I1RXB6
90474 229533.I1RVY0 pp 229533.I1S182
90475 229533.I1RVY0 pp 229533.Q4IQX3
90476 229533.I1RVY0 pp 229533.Q4IER8
90477 229533.I1RVY0 pp 229533.I1S0F0
90478 229533.I1RVY0 pp 229533.Q4IAA0
90479 229533.I1RVY0 pp 229533.I1RZB5
90480 229533.I1RVY0 pp 229533.I1S9J6
90481 229533.I1RVY0 pp 229533.V6RPN0
90482 229533.I1RVY0 pp 229533.I1RW71
90483 229533.I1RVY0 pp 229533.I1S9U1
90484 229533.I1RVY0 pp 229533.Q4HYI0
90485 229533.I1RVY0 pp 229533.I1S0G0
90486 229533.I1RVY0 pp 229533.I1RWG5
90487 229533.I1RVY0 pp 229533.I1RXX1
90488 229533.I1RVY0 pp 229533.Q4I7X1
90489 229533.I1RVY0 pp 229533.V6QV38
90490 229533.I1RVY0 pp 229533.Q4HTT2
90491 229533.I1RVY0 pp 229533.I1S8F8
90492 229533.I1RVY0 pp 229533.I1RZU9
90493 229533.I1RVY0 pp 229533.I1S255
90494 229533.I1RVY0 pp 229533.I1S5Z2
90495 229533.I1RVY0 pp 229533.I1S5R5
90496 229533.I1RVY0 pp 229533.I1RWJ3
90497 229533.I1RVY0 pp 229533.I1S0Z3
90498 229533.I1RVY0 pp 229533.Q4IE79
90499 229533.I1RVY0 pp 229533.V6QUE6
90500 229533.I1RVY0 pp 229533.Q4ICA8
90501 229533.I1RVY0 pp 229533.I1RXE5
90502 229533.I1RVY0 pp 229533.Q4I5I4
90503 229533.I1RVY0 pp 229533.Q4I948
90504 229533.A0A1C3YIZ5 pp 229533.I1RN84
90505 229533.A0A1C3YIZ5 pp 229533.A0A1C3YJR2
90506 229533.A0A1C3YIZ5 pp 229533.A0A1C3YLE2
90507 229533.A0A1C3YIZ5 pp 229533.I1RN88
90508 229533.A0A1C3YIZ5 pp 229533.I1RWF5
90509 229533.A0A1C3YIZ5 pp 229533.I1RGC3
90510 229533.A0A1C3YIZ5 pp 229533.I1S8C1
90511 229533.A0A1C3YIZ5 pp 229533.I1RF57
90512 229533.A0A1C3YIZ5 pp 229533.I1RJY7
90513 229533.A0A1C3YIZ5 pp 229533.I1RY12
90514 229533.I1RPU7 pp 229533.I1S6N6
90515 229533.I1RPU7 pp 229533.I1RRW0
90516 229533.I1RPU7 pp 229533.I1RY44
90517 229533.I1RPU7 pp 229533.I1RTE6
90518 229533.I1RPU7 pp 229533.I1RV91
90519 229533.I1RPU7 pp 229533.I1RX09
90520 229533.I1RPU7 pp 229533.V6RR76

90521 229533.I1RPU7 pp 229533.I1S2E9
90522 229533.I1RPU7 pp 229533.I1RW84
90523 229533.I1RPU7 pp 229533.I1RRI4
90524 229533.I1RPU7 pp 229533.Q4HXT6
90525 229533.I1RPU7 pp 229533.I1S224
90526 229533.I1RPU7 pp 229533.I1RWT6
90527 229533.I1RPU7 pp 229533.V6REE9
90528 229533.I1RPU7 pp 229533.I1RUI0
90529 229533.I1RPU7 pp 229533.I1RQA3
90530 229533.I1RPU7 pp 229533.I1RW38
90531 229533.I1RXC3 pp 229533.I1RYE5
90532 229533.I1RXC3 pp 229533.I1RZS8
90533 229533.I1RXC3 pp 229533.I1RZD3
90534 229533.A0A098DLM6 pp 229533.I1RWX5
90535 229533.A0A098DLM6 pp 229533.A0A098DTE3
90536 229533.A0A098DLM6 pp 229533.A0A1C3YIR8
90537 229533.A0A098DLM6 pp 229533.I1RRT8
90538 229533.A0A098DLM6 pp 229533.I1RYW3
90539 229533.A0A098DLM6 pp 229533.A0A1C3YNB1
90540 229533.A0A098DLM6 pp 229533.I1RSY1
90541 229533.A0A098DLM6 pp 229533.I1RMJ3
90542 229533.A0A098DLM6 pp 229533.A0A1C3YLL1
90543 229533.A0A098DLM6 pp 229533.A0A098DS79
90544 229533.A0A098DLM6 pp 229533.I1S273
90545 229533.A0A098DLM6 pp 229533.I1RBS0
90546 229533.A0A098DLM6 pp 229533.I1RKM2
90547 229533.A0A098DLM6 pp 229533.I1RAB6
90548 229533.A0A098DLM6 pp 229533.A0A1C3YLU7
90549 229533.A0A098DLM6 pp 229533.V6QUE6
90550 229533.A0A098DLM6 pp 229533.I1RW71
90551 229533.A0A0E0SMG8 pp 229533.I1S0S8
90552 229533.A0A0E0SMG8 pp 229533.I1RCZ0
90553 229533.A0A0E0SMG8 pp 229533.I1RRF0
90554 229533.A0A0E0SMG8 pp 229533.I1RUJ0
90555 229533.A0A0E0SMG8 pp 229533.I1RY14
90556 229533.A0A0E0SMG8 pp 229533.I1S247
90557 229533.A0A0E0SMG8 pp 229533.I1RZ84
90558 229533.A0A0E0SMG8 pp 229533.I1RSZ6
90559 229533.A0A0E0SMG8 pp 229533.I1RTE0
90560 229533.A0A0E0SMG8 pp 229533.I1RYW3
90561 229533.A0A0E0SMG8 pp 229533.I1RMQ5
90562 229533.A0A0E0SMG8 pp 229533.A0A1C3YLA2
90563 229533.A0A0E0SMG8 pp 229533.I1REC7
90564 229533.A0A0E0SMG8 pp 229533.Q4HY90
90565 229533.A0A0E0SMG8 pp 229533.A0A1C3YMH5
90566 229533.A0A0E0SMG8 pp 229533.I1RYM4
90567 229533.A0A0E0SMG8 pp 229533.I1RX69
90568 229533.A0A0E0SMG8 pp 229533.I1RTG5
90569 229533.A0A0E0SMG8 pp 229533.A0A1C3YMS7
90570 229533.A0A0E0SMG8 pp 229533.I1RB89
90571 229533.A0A0E0SMG8 pp 229533.I1RBL4
90572 229533.A0A0E0SMG8 pp 229533.I1REC6
90573 229533.A0A0E0SMG8 pp 229533.I1RAH7
90574 229533.A0A0E0SMG8 pp 229533.I1RZV1
90575 229533.A0A0E0SMG8 pp 229533.I1RFS3
90576 229533.A0A0E0SMG8 pp 229533.I1RZL3
90577 229533.A0A0E0SMG8 pp 229533.I1RVY0
90578 229533.A0A0E0SMG8 pp 229533.I1RX68
90579 229533.A0A0E0SMG8 pp 229533.I1RJZ7
90580 229533.A0A0E0SMG8 pp 229533.I1R9Z0
90581 229533.A0A0E0SMG8 pp 229533.I1RJS4
90582 229533.A0A0E0SMG8 pp 229533.I1S248
90583 229533.A0A0E0SMG8 pp 229533.I1S7W2
90584 229533.A0A0E0SMG8 pp 229533.I1RXS3
90585 229533.A0A0E0SMG8 pp 229533.I1RKR9
90586 229533.A0A0E0SMG8 pp 229533.Q4IPI4
90587 229533.A0A0E0SMG8 pp 229533.I1S559
90588 229533.A0A0E0SMG8 pp 229533.I1RZ60
90589 229533.A0A0E0SMG8 pp 229533.I1RAZ9
90590 229533.A0A0E0SMG8 pp 229533.A0A1C3YM61
90591 229533.A0A0E0SMG8 pp 229533.I1RE49
90592 229533.A0A0E0SMG8 pp 229533.I1RKL2
90593 229533.A0A0E0SMG8 pp 229533.I1RBB0

90594 229533.A0A0E0SMG8 pp 229533.I1RNG4
90595 229533.A0A0E0SMG8 pp 229533.I1RSH3
90596 229533.A0A0E0SMG8 pp 229533.I1RZK3
90597 229533.A0A0E0SMG8 pp 229533.I1RQJ0
90598 229533.A0A0E0SMG8 pp 229533.Q4I948
90599 229533.A0A0E0SMG8 pp 229533.I1RB25
90600 229533.A0A0E0SMG8 pp 229533.I1RQP1
90601 229533.A0A0E0SMG8 pp 229533.A0A1C3YLK5
90602 229533.A0A0E0SMG8 pp 229533.I1S7X9
90603 229533.A0A0E0SMG8 pp 229533.I1RQG4
90604 229533.A0A0E0SMG8 pp 229533.I1RCF0
90605 229533.A0A0E0SMG8 pp 229533.A0A1C3YI44
90606 229533.A0A0E0SMG8 pp 229533.I1S9S4
90607 229533.A0A0E0SMG8 pp 229533.I1S0Z7
90608 229533.A0A0E0SMG8 pp 229533.I1RQM8
90609 229533.A0A0E0SMG8 pp 229533.I1RMG2
90610 229533.A0A0E0SMG8 pp 229533.I1RBL7
90611 229533.A0A0E0SMG8 pp 229533.I1RAS5
90612 229533.A0A0E0SMG8 pp 229533.Q4ICA8
90613 229533.A0A0E0SMG8 pp 229533.I1RZ27
90614 229533.A0A0E0SMG8 pp 229533.I1RDY1
90615 229533.A0A0E0SMG8 pp 229533.I1RRF9
90616 229533.A0A0E0SMG8 pp 229533.I1S0Z3
90617 229533.A0A0E0SMG8 pp 229533.I1RQP5
90618 229533.A0A0E0SMG8 pp 229533.A0A1C3YIG8
90619 229533.A0A0E0SMG8 pp 229533.I1RD63
90620 229533.A0A0E0SMG8 pp 229533.I1RFK0
90621 229533.A0A0E0SMG8 pp 229533.I1RE51
90622 229533.A0A0E0SMG8 pp 229533.I1RWI6
90623 229533.A0A0E0SMG8 pp 229533.Q4I963
90624 229533.A0A0E0SMG8 pp 229533.I1RQP3
90625 229533.A0A0E0SMG8 pp 229533.V6RPN0
90626 229533.A0A0E0SMG8 pp 229533.I1RBR0
90627 229533.A0A0E0SMG8 pp 229533.I1RA72
90628 229533.A0A0E0SMG8 pp 229533.I1RCZ1
90629 229533.A0A0E0SMG8 pp 229533.I1RIG4
90630 229533.A0A0E0SMG8 pp 229533.I1RSD7
90631 229533.A0A0E0SMG8 pp 229533.I1RBS4
90632 229533.A0A0E0SMG8 pp 229533.I1RX87
90633 229533.A0A0E0SMG8 pp 229533.I1RBT0
90634 229533.A0A0E0SMG8 pp 229533.I1RQF7
90635 229533.A0A0E0SMG8 pp 229533.A0A1C3YI55
90636 229533.A0A0E0SMG8 pp 229533.I1RR74
90637 229533.A0A0E0SMG8 pp 229533.I1S1V2
90638 229533.A0A0E0SMG8 pp 229533.A0A1C3YID5
90639 229533.A0A0E0SMG8 pp 229533.A0A1C3YIQ1
90640 229533.A0A0E0SMG8 pp 229533.A0A0E0SNZ9
90641 229533.A0A0E0SMG8 pp 229533.A0A1C3YKJ7
90642 229533.A0A0E0SMG8 pp 229533.I1RT41
90643 229533.A0A0E0SMG8 pp 229533.I1RB00
90644 229533.A0A0E0SMG8 pp 229533.I1S270
90645 229533.A0A0E0SMG8 pp 229533.I1RDD8
90646 229533.A0A0E0SMG8 pp 229533.Q4I2D7
90647 229533.A0A0E0SMG8 pp 229533.Q5I2J3
90648 229533.A0A0E0SMG8 pp 229533.Q4INI2
90649 229533.A0A0E0SMG8 pp 229533.I1RAN8
90650 229533.A0A0E0SMG8 pp 229533.Q4ICM0
90651 229533.A0A0E0SMG8 pp 229533.I1RRH5
90652 229533.A0A0E0SMG8 pp 229533.I1RJU7
90653 229533.A0A0E0SMG8 pp 229533.I1RY00
90654 229533.A0A0E0SMG8 pp 229533.A0A1C3YLL9
90655 229533.A0A0E0SMG8 pp 229533.I1RKK4
90656 229533.A0A0E0SMG8 pp 229533.I1RW71
90657 229533.I1S299 pp 229533.V6R3J1
90658 229533.I1S299 pp 229533.Q4HVQ9
90659 229533.I1S299 pp 229533.V6RM66
90660 229533.I1S299 pp 229533.I1S3T7
90661 229533.I1S299 pp 229533.V6RZD4
90662 229533.I1S299 pp 229533.I1S8R2
90663 229533.I1S299 pp 229533.V6RF62
90664 229533.I1S299 pp 229533.I1S968
90665 229533.I1S299 pp 229533.V6QVH2
90666 229533.I1S299 pp 229533.I1S8R4

90667 229533.I1S299 pp 229533.I1S2Q4
90668 229533.I1S299 pp 229533.V6RRM0
90669 229533.I1S299 pp 229533.I1S8C1
90670 229533.I1S299 pp 229533.I1S3L2
90671 229533.I1S299 pp 229533.I1S8H9
90672 229533.I1S299 pp 229533.V6R3V4
90673 229533.I1S299 pp 229533.I1S4S5
90674 229533.I1S299 pp 229533.I1S2A1
90675 229533.I1S299 pp 229533.I1S8F8
90676 229533.A0A1C3YJZ2 pp 229533.I1RMV1
90677 229533.A0A1C3YJZ2 pp 229533.A0A1C3YM21
90678 229533.A0A1C3YJZ2 pp 229533.I1RKR6
90679 229533.A0A1C3YJZ2 pp 229533.A0A1C3YMF9
90680 229533.A0A1C3YJZ2 pp 229533.A0A1C3YK01
90681 229533.A0A1C3YJZ2 pp 229533.I1REN3
90682 229533.A0A1C3YJZ2 pp 229533.I1RR25
90683 229533.A0A1C3YJZ2 pp 229533.I1RUG4
90684 229533.A0A1C3YJZ2 pp 229533.I1RK79
90685 229533.A0A1C3YJZ2 pp 229533.I1RZB5
90686 229533.A0A1C3YJZ2 pp 229533.I1RJL2
90687 229533.A0A1C3YJZ2 pp 229533.I1RPM7
90688 229533.A0A1C3YJZ2 pp 229533.I1RDC9
90689 229533.A0A1C3YJZ2 pp 229533.I1RR61
90690 229533.A0A1C3YJZ2 pp 229533.I1RRT0
90691 229533.A0A1C3YJZ2 pp 229533.I1RC15
90692 229533.A0A1C3YJZ2 pp 229533.I1RA56
90693 229533.A0A1C3YJZ2 pp 229533.I1RK71
90694 229533.A0A1C3YJZ2 pp 229533.I1RXE5
90695 229533.A0A1C3YJZ2 pp 229533.I1RN04
90696 229533.A0A1C3YJZ2 pp 229533.I1RQE9
90697 229533.A0A1C3YJZ2 pp 229533.I1RCG6
90698 229533.A0A1C3YJZ2 pp 229533.I1RFW6
90699 229533.A0A1C3YJZ2 pp 229533.I1RZU9
90700 229533.A0A1C3YJZ2 pp 229533.I1RHR6
90701 229533.A0A1C3YJZ2 pp 229533.I1RWF3
90702 229533.A0A1C3YJZ2 pp 229533.I1S276
90703 229533.A0A1C3YJZ2 pp 229533.I1RVY0
90704 229533.A0A1C3YJZ2 pp 229533.I1RWX8
90705 229533.A0A1C3YJZ2 pp 229533.V6QV38
90706 229533.A0A1C3YJZ2 pp 229533.I1RA84
90707 229533.A0A1C3YJZ2 pp 229533.I1RDG8
90708 229533.A0A1C3YJZ2 pp 229533.A0A1C3YN55
90709 229533.A0A1C3YJZ2 pp 229533.I1RBR5
90710 229533.A0A1C3YJZ2 pp 229533.I1RK15
90711 229533.A0A1C3YJZ2 pp 229533.I1RBJ1
90712 229533.A0A1C3YJZ2 pp 229533.I1RQ42
90713 229533.A0A1C3YJZ2 pp 229533.I1RCK7
90714 229533.A0A1C3YJZ2 pp 229533.I1RTC2
90715 229533.A0A1C3YJZ2 pp 229533.I1RCB7
90716 229533.A0A1C3YJZ2 pp 229533.I1RPX5
90717 229533.A0A1C3YJZ2 pp 229533.I1RE30
90718 229533.A0A1C3YJZ2 pp 229533.I1RXU0
90719 229533.A0A1C3YJZ2 pp 229533.I1R9W6
90720 229533.A0A1C3YJZ2 pp 229533.I1RFU3
90721 229533.A0A1C3YJZ2 pp 229533.I1RNV7
90722 229533.A0A1C3YJZ2 pp 229533.I1RPW8
90723 229533.A0A1C3YJZ2 pp 229533.Q4I7S1
90724 229533.A0A1C3YJZ2 pp 229533.I1S382
90725 229533.A0A1C3YJZ2 pp 229533.I1RGE1
90726 229533.A0A1C3YJZ2 pp 229533.Q4I7X1
90727 229533.A0A1C3YJZ2 pp 229533.I1RMA5
90728 229533.A0A1C3YJZ2 pp 229533.V6QWX8
90729 229533.A0A1C3YJZ2 pp 229533.Q4HTT2
90730 229533.A0A1C3YJZ2 pp 229533.I1S3D7
90731 229533.A0A1C3YJZ2 pp 229533.I1RW91
90732 229533.A0A1C3YJZ2 pp 229533.V6QUE6
90733 229533.A0A1C3YJZ2 pp 229533.I1RW90
90734 229533.A0A1C3YJZ2 pp 229533.I1RSZ3
90735 229533.A0A1C3YJZ2 pp 229533.I1RHP9
90736 229533.A0A1C3YJZ2 pp 229533.I1RI51
90737 229533.A0A1C3YJZ2 pp 229533.I1RAR2
90738 229533.A0A1C3YJZ2 pp 229533.I1RP96
90739 229533.A0A1C3YJZ2 pp 229533.I1RGW0

90740 229533.A0A1C3YJZ2 pp 229533.I1RG11
90741 229533.A0A1C3YJZ2 pp 229533.I1RRF7
90742 229533.A0A1C3YJZ2 pp 229533.I1RC24
90743 229533.A0A1C3YJZ2 pp 229533.I1RML9
90744 229533.A0A1C3YJZ2 pp 229533.I1R9Q0
90745 229533.A0A1C3YJZ2 pp 229533.I1RJ40
90746 229533.A0A1C3YJZ2 pp 229533.I1RW80
90747 229533.A0A1C3YJZ2 pp 229533.I1S1V5
90748 229533.A0A1C3YJZ2 pp 229533.I1RBZ7
90749 229533.A0A1C3YJZ2 pp 229533.A0A1C3YL11
90750 229533.A0A1C3YJZ2 pp 229533.I1RB36
90751 229533.A0A1C3YJZ2 pp 229533.I1RRM8
90752 229533.A0A1C3YJZ2 pp 229533.Q4IQX3
90753 229533.A0A1C3YJZ2 pp 229533.I1RWU3
90754 229533.A0A1C3YJZ2 pp 229533.I1S951
90755 229533.A0A1C3YJZ2 pp 229533.I1RC98
90756 229533.A0A1C3YJZ2 pp 229533.I1RVL8
90757 229533.A0A1C3YJZ2 pp 229533.I1RRG3
90758 229533.A0A1C3YJZ2 pp 229533.Q4IMD1
90759 229533.A0A1C3YJZ2 pp 229533.I1RAW7
90760 229533.A0A1C3YJZ2 pp 229533.I1RUQ6
90761 229533.A0A1C3YJZ2 pp 229533.I1RZM2
90762 229533.A0A1C3YJZ2 pp 229533.I1R976
90763 229533.A0A1C3YJZ2 pp 229533.I1RKB1
90764 229533.A0A1C3YJZ2 pp 229533.A0A1C3YLP8
90765 229533.A0A1C3YJZ2 pp 229533.I1RBC7
90766 229533.I1RFT8 pp 229533.Q4IJM4
90767 229533.I1RFT8 pp 229533.I1RZV3
90768 229533.I1RCL4 pp 229533.I1S0A0
90769 229533.I1RCL4 pp 229533.I1RRV4
90770 229533.I1RCL4 pp 229533.I1RK15
90771 229533.I1RCL4 pp 229533.I1RMQ4
90772 229533.I1RCL4 pp 229533.I1RPN4
90773 229533.I1RCL4 pp 229533.I1RF82
90774 229533.I1RCL4 pp 229533.I1RYD3
90775 229533.I1RCL4 pp 229533.I1RK95
90776 229533.I1RCL4 pp 229533.I1RDD3
90777 229533.I1RCL4 pp 229533.V6RBN2
90778 229533.I1RCL4 pp 229533.I1S8U9
90779 229533.I1RCL4 pp 229533.I1RTD3
90780 229533.I1RCL4 pp 229533.I1RWH8
90781 229533.I1RCL4 pp 229533.V6R1K4
90782 229533.I1RCL4 pp 229533.I1RM35
90783 229533.I1RCL4 pp 229533.I1RME6
90784 229533.I1RCL4 pp 229533.I1RPU8
90785 229533.I1RCL4 pp 229533.I1REL7
90786 229533.I1RCL4 pp 229533.Q4IPI1
90787 229533.I1RCL4 pp 229533.V6RPQ8
90788 229533.I1RCL4 pp 229533.I1S4Q4
90789 229533.I1RCL4 pp 229533.I1RG11
90790 229533.I1RCL4 pp 229533.I1RE00
90791 229533.I1RCL4 pp 229533.V6R2A1
90792 229533.I1RCL4 pp 229533.I1S0B7
90793 229533.I1RCL4 pp 229533.I1RMM8
90794 229533.I1RCL4 pp 229533.I1RI34
90795 229533.I1RCL4 pp 229533.I1RL04
90796 229533.I1RCL4 pp 229533.I1RKY2
90797 229533.I1RCL4 pp 229533.I1RFL4
90798 229533.I1RCL4 pp 229533.V6R057
90799 229533.I1RCL4 pp 229533.I1RQS9
90800 229533.I1RCL4 pp 229533.I1S4Z8
90801 229533.I1RCL4 pp 229533.I1S216
90802 229533.I1RCL4 pp 229533.I1S8G4
90803 229533.I1RCL4 pp 229533.I1S4S4
90804 229533.I1RCL4 pp 229533.I1RSQ4
90805 229533.I1RCL4 pp 229533.I1RS17
90806 229533.I1RCL4 pp 229533.V6RRH2
90807 229533.I1RCL4 pp 229533.I1S8V0
90808 229533.I1RCL4 pp 229533.I1RTA0
90809 229533.I1RCL4 pp 229533.I1RWE8
90810 229533.I1RCL4 pp 229533.O74638
90811 229533.I1RCL4 pp 229533.I1S9Y1
90812 229533.I1RCL4 pp 229533.I1RCL5

90813	229533.I1RCL4	pp	229533.I1RMI8
90814	229533.I1RCL4	pp	229533.I1RRP7
90815	229533.I1RCL4	pp	229533.I1RCW6
90816	229533.I1RCL4	pp	229533.I1RVZ7
90817	229533.I1RCL4	pp	229533.I1RVZ2
90818	229533.I1RCL4	pp	229533.I1RX22
90819	229533.I1RCL4	pp	229533.I1S8P5
90820	229533.I1RCL4	pp	229533.I1RP87
90821	229533.I1RCL4	pp	229533.Q4HXF0
90822	229533.I1RCL4	pp	229533.I1RQM7
90823	229533.A0A0E0SQJ6	pp	229533.I1RCK7
90824	229533.A0A0E0SQJ6	pp	229533.I1RY44
90825	229533.A0A0E0SQJ6	pp	229533.I1RXE5
90826	229533.A0A0E0SQJ6	pp	229533.I1RWX8
90827	229533.A0A0E0SQJ6	pp	229533.V6QV38
90828	229533.A0A0E0SQJ6	pp	229533.I1RA56
90829	229533.A0A0E0SQJ6	pp	229533.A0A1C3YJX1
90830	229533.A0A0E0SQJ6	pp	229533.I1RDC9
90831	229533.A0A0E0SQJ6	pp	229533.I1RJ40
90832	229533.A0A0E0SQJ6	pp	229533.A0A1C3YJ12
90833	229533.A0A0E0SQJ6	pp	229533.Q4I7S1
90834	229533.A0A0E0SQJ6	pp	229533.I1RGC6
90835	229533.A0A0E0SQJ6	pp	229533.I1RQE9
90836	229533.A0A0E0SQJ6	pp	229533.I1RZU9
90837	229533.A0A0E0SQJ6	pp	229533.Q4HTT2
90838	229533.A0A0E0SQJ6	pp	229533.A0A1C3YLI5
90839	229533.A0A0E0SQJ6	pp	229533.A0A1C3YJE9
90840	229533.A0A0E0SQJ6	pp	229533.A0A1C3YJC9
90841	229533.A0A0E0SQJ6	pp	229533.I1RFV3
90842	229533.A0A0E0SQJ6	pp	229533.A0A1C3YIJ2
90843	229533.A0A0E0SQJ6	pp	229533.A0A1C3YK01
90844	229533.A0A0E0SQJ6	pp	229533.I1RKR6
90845	229533.A0A0E0SQJ6	pp	229533.I1REF5
90846	229533.A0A0E0SQJ6	pp	229533.I1RGE1
90847	229533.A0A0E0SQJ6	pp	229533.I1RNV7
90848	229533.A0A0E0SQJ6	pp	229533.I1S3D7
90849	229533.A0A0E0SQJ6	pp	229533.A0A1C3YM21
90850	229533.A0A0E0SQJ6	pp	229533.I1RDJ8
90851	229533.A0A0E0SQJ6	pp	229533.A0A1C3YHV5
90852	229533.A0A0E0SQJ6	pp	229533.I1S2E9
90853	229533.A0A0E0SQJ6	pp	229533.I1RMA5
90854	229533.A0A0E0SQJ6	pp	229533.I1RPX5
90855	229533.A0A0E0SQJ6	pp	229533.I1RHR6
90856	229533.A0A0E0SQJ6	pp	229533.A0A1C3YLT1
90857	229533.A0A0E0SQJ6	pp	229533.I1R9W6
90858	229533.A0A0E0SQJ6	pp	229533.I1RT62
90859	229533.A0A0E0SQJ6	pp	229533.I1RUQ6
90860	229533.A0A0E0SQJ6	pp	229533.I1RVY0
90861	229533.A0A0E0SQJ6	pp	229533.A0A1C3YJZ2
90862	229533.A0A0E0SQJ6	pp	229533.I1RE42
90863	229533.A0A0E0SQJ6	pp	229533.I1RB36
90864	229533.A0A0E0SQJ6	pp	229533.I1RWU3
90865	229533.A0A0E0SQJ6	pp	229533.I1RHP9
90866	229533.A0A0E0SQJ6	pp	229533.A0A1C3YJH8
90867	229533.A0A0E0SQJ6	pp	229533.I1RI51
90868	229533.A0A0E0SQJ6	pp	229533.I1R9Q0
90869	229533.A0A0E0SQJ6	pp	229533.I1RGW0
90870	229533.A0A0E0SQJ6	pp	229533.I1S0F1
90871	229533.I1RHV8	pp	229533.I1RPL0
90872	229533.I1RGW0	pp	229533.I1S3D7
90873	229533.I1RGW0	pp	229533.I1RNV7
90874	229533.I1RGW0	pp	229533.I1RHP9
90875	229533.I1RGW0	pp	229533.I1RJ40
90876	229533.I1RGW0	pp	229533.I1RI51
90877	229533.I1RGW0	pp	229533.I1RXE5
90878	229533.I1RGW0	pp	229533.I1RWU3
90879	229533.I1RGW0	pp	229533.I1RK15
90880	229533.I1RGW0	pp	229533.I1RUQ6
90881	229533.I1RGW0	pp	229533.I1RWX8
90882	229533.I1RGW0	pp	229533.V6QV38
90883	229533.I1RGW0	pp	229533.I1RPX5
90884	229533.I1RGW0	pp	229533.I1RHR6
90885	229533.I1RGW0	pp	229533.I1RQE9

90886 229533.I1RGW0 pp 229533.I1RMV5
90887 229533.I1RGW0 pp 229533.I1RVY0
90888 229533.I1RGW0 pp 229533.I1RKR6
90889 229533.I1RZV6 pp 229533.V6RGN5
90890 229533.I1RZV6 pp 229533.I1S044
90891 229533.I1RZV6 pp 229533.I1S9Z2
90892 229533.I1RZV6 pp 229533.I1S0M9
90893 229533.I1RZV6 pp 229533.I1S021
90894 229533.I1RZV6 pp 229533.Q4IAA0
90895 229533.I1RZV6 pp 229533.Q4IN63
90896 229533.I1RZV6 pp 229533.I1S0J1
90897 229533.I1RZV6 pp 229533.Q4I327
90898 229533.I1RZV6 pp 229533.V6RMW6
90899 229533.I1RZV6 pp 229533.Q4IEK8
90900 229533.I1RZV6 pp 229533.I1S2C0
90901 229533.I1RZV6 pp 229533.V6R042
90902 229533.I1RZV6 pp 229533.Q4I8Q4
90903 229533.I1RZV6 pp 229533.Q4IPI1
90904 229533.I1RZV6 pp 229533.I1S6E5
90905 229533.I1RZV6 pp 229533.I1S1X9
90906 229533.I1RZV6 pp 229533.I1S060
90907 229533.I1RZV6 pp 229533.Q4IAS1
90908 229533.I1RZV6 pp 229533.I1S0R7
90909 229533.I1RZV6 pp 229533.Q4IFI0
90910 229533.I1RZV6 pp 229533.I1S7S9
90911 229533.I1RZV6 pp 229533.I1RZZ1
90912 229533.I1RZV6 pp 229533.I1S8E2
90913 229533.I1RZV6 pp 229533.I1S2E8
90914 229533.I1RZV6 pp 229533.Q4I662
90915 229533.Q4I5R3 pp 229533.Q4IB50
90916 229533.Q4I5R3 pp 229533.Q4I7L0
90917 229533.Q4I5R3 pp 229533.Q4I8C9
90918 229533.Q4I5R3 pp 229533.Q4I7N9
90919 229533.Q4I5R3 pp 229533.Q4IMD1
90920 229533.Q4I5R3 pp 229533.Q4IER8
90921 229533.Q4I5R3 pp 229533.Q4IAK7
90922 229533.Q4I5R3 pp 229533.Q4IJP1
90923 229533.I1RPX5 pp 229533.Q4IBU4
90924 229533.I1RPX5 pp 229533.I1RY44
90925 229533.I1RPX5 pp 229533.Q4HTT2
90926 229533.I1RPX5 pp 229533.I1RQE9
90927 229533.I1RPX5 pp 229533.I1RXE5
90928 229533.I1RPX5 pp 229533.I1S1V5
90929 229533.I1RPX5 pp 229533.V6QV38
90930 229533.I1RPX5 pp 229533.I1RWT6
90931 229533.I1RPX5 pp 229533.I1RVY0
90932 229533.I1RPX5 pp 229533.Q4I2W2
90933 229533.I1RPX5 pp 229533.I1RX70
90934 229533.I1RPX5 pp 229533.I1RWU3
90935 229533.I1RPX5 pp 229533.I1RZS4
90936 229533.I1RPX5 pp 229533.I1RZH5
90937 229533.I1RPX5 pp 229533.I1RV91
90938 229533.I1RPX5 pp 229533.I1RQA3
90939 229533.I1RPX5 pp 229533.I1S2E9
90940 229533.I1RS82 pp 229533.I1RYE9
90941 229533.I1RS82 pp 229533.I1RYV2
90942 229533.A0A1C3YKR1 pp 229533.I1S1X2
90943 229533.A0A1C3YKR1 pp 229533.I1RQ19
90944 229533.A0A1C3YKR1 pp 229533.I1RA91
90945 229533.A0A1C3YKR1 pp 229533.I1RVV9
90946 229533.A0A1C3YKR1 pp 229533.I1RY28
90947 229533.I1RA56 pp 229533.I1RDC9
90948 229533.I1RA56 pp 229533.I1RY44
90949 229533.I1RA56 pp 229533.I1RWT6
90950 229533.I1RA56 pp 229533.I1RXE5
90951 229533.I1RA56 pp 229533.Q4I2W2
90952 229533.I1RA56 pp 229533.V6QV38
90953 229533.I1RA56 pp 229533.I1RZS4
90954 229533.I1RA56 pp 229533.I1RQE9
90955 229533.I1RA56 pp 229533.I1RKR6
90956 229533.I1RA56 pp 229533.I1RHP9
90957 229533.I1RA56 pp 229533.I1S2E9
90958 229533.I1RA56 pp 229533.Q4HTT2

90959 229533.I1RA56 pp 229533.I1RV91
90960 229533.I1RA56 pp 229533.I1RI51
90961 229533.I1RA56 pp 229533.I1RQA3
90962 229533.I1RA56 pp 229533.I1RTK1
90963 229533.I1RA56 pp 229533.I1S2D5
90964 229533.I1RA56 pp 229533.I1RZH5
90965 229533.I1RA56 pp 229533.I1RB36
90966 229533.I1RA56 pp 229533.I1RC71
90967 229533.I1RA56 pp 229533.I1RMA5
90968 229533.I1RA56 pp 229533.I1RAW7
90969 229533.I1RA56 pp 229533.I1S1V5
90970 229533.I1RA56 pp 229533.I1RGE1
90971 229533.I1RA56 pp 229533.I1RWU3
90972 229533.I1RA56 pp 229533.I1RNV7
90973 229533.I1RA56 pp 229533.I1RGW0
90974 229533.I1RA56 pp 229533.I1RVY0
90975 229533.A0A1C3YLB1 pp 229533.I1RS82
90976 229533.A0A1C3YLB1 pp 229533.I1RNG4
90977 229533.I1RDC9 pp 229533.I1RQE9
90978 229533.I1RDC9 pp 229533.I1RG11
90979 229533.I1RDC9 pp 229533.I1REN3
90980 229533.I1RDC9 pp 229533.I1RUG4
90981 229533.I1RDC9 pp 229533.I1RMV1
90982 229533.I1RDC9 pp 229533.I1RK71
90983 229533.I1RDC9 pp 229533.I1RR25
90984 229533.I1RDC9 pp 229533.Q4HTT2
90985 229533.I1RDC9 pp 229533.I1RPM7
90986 229533.I1RDC9 pp 229533.Q4I7S1
90987 229533.I1RDC9 pp 229533.V6QV38
90988 229533.I1RDC9 pp 229533.I1RQA3
90989 229533.I1RDC9 pp 229533.I1RKR6
90990 229533.I1RDC9 pp 229533.I1S276
90991 229533.I1RDC9 pp 229533.I1RR61
90992 229533.I1RDC9 pp 229533.I1RK79
90993 229533.I1RDC9 pp 229533.I1RXU0
90994 229533.I1RDC9 pp 229533.Q4I7X1
90995 229533.I1RDC9 pp 229533.I1RZB5
90996 229533.I1RDC9 pp 229533.I1RZU9
90997 229533.I1RDC9 pp 229533.I1RJL2
90998 229533.I1RDC9 pp 229533.I1RVY0
90999 229533.I1RDC9 pp 229533.V6QWX8
91000 229533.I1RDC9 pp 229533.I1RDG8
91001 229533.I1RDC9 pp 229533.I1RWF3
91002 229533.I1RDC9 pp 229533.I1RHR6
91003 229533.I1RDC9 pp 229533.I1RWX8
91004 229533.I1RDC9 pp 229533.I1RGW0
91005 229533.I1RDC9 pp 229533.I1RW90
91006 229533.I1RDC9 pp 229533.I1RY44
91007 229533.I1RDC9 pp 229533.I1RI51
91008 229533.I1RDC9 pp 229533.I1RXE5
91009 229533.I1RDC9 pp 229533.I1S382
91010 229533.I1RDC9 pp 229533.I1S3D7
91011 229533.I1RDC9 pp 229533.I1RE30
91012 229533.I1RDC9 pp 229533.I1RPX5
91013 229533.I1RDC9 pp 229533.I1RK15
91014 229533.I1RDC9 pp 229533.I1RWT6
91015 229533.I1RDC9 pp 229533.I1RFU3
91016 229533.I1RDC9 pp 229533.I1RQ42
91017 229533.I1RDC9 pp 229533.I1RMA5
91018 229533.I1RDC9 pp 229533.I1RVL8
91019 229533.I1RDC9 pp 229533.I1RGE1
91020 229533.I1RDC9 pp 229533.I1RRT0
91021 229533.I1RDC9 pp 229533.I1RNV7
91022 229533.I1RDC9 pp 229533.I1RPW8
91023 229533.I1RDC9 pp 229533.V6QUE6
91024 229533.I1RDC9 pp 229533.I1RZM2
91025 229533.I1RDC9 pp 229533.I1RWU3
91026 229533.I1RDC9 pp 229533.I1RN04
91027 229533.I1RDC9 pp 229533.I1RMP9
91028 229533.I1RDC9 pp 229533.I1RVN2
91029 229533.I1RDC9 pp 229533.I1RRF7
91030 229533.I1RDC9 pp 229533.I1RRG3
91031 229533.I1RDC9 pp 229533.I1RJ40

91032	229533.I1RDC9	pp	229533.I1S1V5
91033	229533.I1RDC9	pp	229533.I1RTC2
91034	229533.I1RDC9	pp	229533.I1S951
91035	229533.I1RDC9	pp	229533.Q4IMD1
91036	229533.I1RDC9	pp	229533.I1RML9
91037	229533.I1RDC9	pp	229533.I1S2E9
91038	229533.I1RDC9	pp	229533.I1RW80
91039	229533.I1RDC9	pp	229533.I1RP96
91040	229533.I1RDC9	pp	229533.I1RW91
91041	229533.I1RDC9	pp	229533.I1RKB1
91042	229533.I1RDC9	pp	229533.I1RV91
91043	229533.I1RDC9	pp	229533.I1RUQ6
91044	229533.I1RDC9	pp	229533.I1RHP9
91045	229533.I1RDC9	pp	229533.I1RSZ3
91046	229533.I1RUQ6	pp	229533.I1S1V5
91047	229533.I1RUQ6	pp	229533.I1RWU3
91048	229533.I1RUQ6	pp	229533.Q4HTT1
91049	229533.I1RUQ6	pp	229533.I1RX70
91050	229533.I1RUQ6	pp	229533.I1RV91
91051	229533.I1RUQ6	pp	229533.I1S2E9
91052	229533.I1RUQ6	pp	229533.I1RVY0
91053	229533.I1RUQ6	pp	229533.I1RZH5
91054	229533.I1RUQ6	pp	229533.Q4I2W2
91055	229533.I1RUQ6	pp	229533.I1S091
91056	229533.I1RUQ6	pp	229533.V6QV38
91057	229533.I1RUQ6	pp	229533.I1RZS4
91058	229533.I1RUQ6	pp	229533.I1RY44
91059	229533.I1RUQ6	pp	229533.Q4I7N9
91060	229533.I1RUQ6	pp	229533.I1RWT6
91061	229533.I1RUQ6	pp	229533.I1RXE5
91062	229533.I1RUQ6	pp	229533.Q4HTT2
91063	229533.I1RUQ6	pp	229533.I1S0M2
91064	229533.V6QV38	pp	229533.V6R2A1
91065	229533.V6QV38	pp	229533.V6QWX8
91066	229533.I1RS81	pp	229533.I1RYQ1
91067	229533.I1RS81	pp	229533.I1S604
91068	229533.I1RS81	pp	229533.I1RSA7
91069	229533.I1RS81	pp	229533.Q4IR08
91070	229533.I1RS81	pp	229533.Q4INS6
91071	229533.I1RS81	pp	229533.I1S4P6
91072	229533.I1RS81	pp	229533.I1RVW0
91073	229533.I1RS81	pp	229533.I1RZZ1
91074	229533.I1RS81	pp	229533.I1S2E8
91075	229533.I1RS81	pp	229533.I1S0D5
91076	229533.I1RS81	pp	229533.I1RSS4
91077	229533.I1RWU3	pp	229533.Q4I7N9
91078	229533.I1RWU3	pp	229533.V6R2A1
91079	229533.I1RWU3	pp	229533.I1RXE5
91080	229533.I1RWU3	pp	229533.V6QV38
91081	229533.I1RWU3	pp	229533.Q4IFH8
91082	229533.I1RWU3	pp	229533.Q4HTT2
91083	229533.I1RWU3	pp	229533.I1RZX0
91084	229533.I1RMK3	pp	229533.I1RRJ8
91085	229533.I1RMK3	pp	229533.I1RSQ7
91086	229533.I1RMK3	pp	229533.I1RWF7
91087	229533.I1RMK3	pp	229533.I1RW71
91088	229533.I1RMK3	pp	229533.I1S2L2
91089	229533.I1RMK3	pp	229533.V6QW22
91090	229533.I1RMK3	pp	229533.I1RR00
91091	229533.I1RMK3	pp	229533.Q4I830
91092	229533.I1RMK3	pp	229533.V6R283
91093	229533.I1RMK3	pp	229533.I1RN63
91094	229533.I1RMK3	pp	229533.I1S2M6
91095	229533.I1RMK3	pp	229533.Q4IEV5
91096	229533.I1RMK3	pp	229533.I1RQ65
91097	229533.I1RMK3	pp	229533.I1RWH5
91098	229533.I1RMK3	pp	229533.I1S0L3
91099	229533.I1RMK3	pp	229533.Q4IQW0
91100	229533.I1RMK3	pp	229533.I1RQS5
91101	229533.I1RMK3	pp	229533.I1RS47
91102	229533.I1RMK3	pp	229533.I1S1B6
91103	229533.I1RMK3	pp	229533.I1S6F0
91104	229533.I1RMK3	pp	229533.I1RRT2

91105	229533.I1RMK3	pp	229533.Q4HZ42
91106	229533.I1RMK3	pp	229533.I1RRQ3
91107	229533.I1RMK3	pp	229533.Q4IPI1
91108	229533.I1RMK3	pp	229533.I1RXD7
91109	229533.I1RMK3	pp	229533.I1RVQ0
91110	229533.I1RMK3	pp	229533.Q4IAS1
91111	229533.I1RMK3	pp	229533.I1RUQ2
91112	229533.I1RMK3	pp	229533.I1RW25
91113	229533.I1RMK3	pp	229533.I1RNH1
91114	229533.I1RMK3	pp	229533.I1RRP3
91115	229533.I1RMK3	pp	229533.Q4IFI0
91116	229533.I1RMK3	pp	229533.I1RZS6
91117	229533.I1RMK3	pp	229533.I1RS30
91118	229533.I1RMK3	pp	229533.I1RT04
91119	229533.I1RMK3	pp	229533.Q4HZ47
91120	229533.I1RMK3	pp	229533.I1S0R7
91121	229533.I1RMK3	pp	229533.I1RWD4
91122	229533.I1RMK3	pp	229533.I1RT98
91123	229533.I1RMK3	pp	229533.I1RX24
91124	229533.I1RMK3	pp	229533.I1RZJ6
91125	229533.I1RMK3	pp	229533.I1RNV6
91126	229533.I1RMK3	pp	229533.V6QYF0
91127	229533.I1RMK3	pp	229533.I1RWJ4
91128	229533.I1RMK3	pp	229533.I1RNA0
91129	229533.I1RMK3	pp	229533.Q4IJH1
91130	229533.I1RMK3	pp	229533.Q4I662
91131	229533.I1RMK3	pp	229533.Q4IBS2
91132	229533.I1RMK3	pp	229533.V6QUE6
91133	229533.I1RMK3	pp	229533.I1RQM6
91134	229533.I1RMK3	pp	229533.I1RTF4
91135	229533.I1RMK3	pp	229533.I1RMN6
91136	229533.I1RMK3	pp	229533.I1RPM4
91137	229533.I1RMK3	pp	229533.I1RZZ9
91138	229533.I1RMK3	pp	229533.I1S9U2
91139	229533.I1RMK3	pp	229533.I1RW61
91140	229533.I1RMK3	pp	229533.Q4IF76
91141	229533.I1RMK3	pp	229533.V6RCG5
91142	229533.I1RMK3	pp	229533.I1RT05
91143	229533.I1RMK3	pp	229533.I1RR24
91144	229533.I1RMK3	pp	229533.V6R227
91145	229533.I1RMK3	pp	229533.I1RX08
91146	229533.I1RMK3	pp	229533.I1RSI6
91147	229533.I1RMK3	pp	229533.I1RS20
91148	229533.I1RMK3	pp	229533.Q4I327
91149	229533.I1RMK3	pp	229533.I1RRR7
91150	229533.I1RMK3	pp	229533.I1RQS8
91151	229533.I1RMK3	pp	229533.I1RNY2
91152	229533.I1RMK3	pp	229533.I1RRT8
91153	229533.I1RMK3	pp	229533.I1RZF6
91154	229533.I1RMK3	pp	229533.I1S9U1
91155	229533.I1RMK3	pp	229533.I1RWJ8
91156	229533.I1RMK3	pp	229533.I1RPU0
91157	229533.I1RMK3	pp	229533.Q4IEK8
91158	229533.I1RMK3	pp	229533.I1RNH6
91159	229533.I1RMK3	pp	229533.I1RMN7
91160	229533.I1RMK3	pp	229533.Q4HVW2
91161	229533.I1RMK3	pp	229533.I1S021
91162	229533.I1RMK3	pp	229533.I1S4T3
91163	229533.I1RMK3	pp	229533.I1S6U8
91164	229533.I1RMK3	pp	229533.I1RPY1
91165	229533.I1RMK3	pp	229533.I1RQ31
91166	229533.I1RMK3	pp	229533.V6RIV9
91167	229533.I1RMK3	pp	229533.I1S2E8
91168	229533.I1RMK3	pp	229533.Q4IN63
91169	229533.I1RMK3	pp	229533.I1RT80
91170	229533.I1RMK3	pp	229533.I1RZM8
91171	229533.I1RMK3	pp	229533.Q4IAA0
91172	229533.I1RMK3	pp	229533.Q4HZ68
91173	229533.I1RMK3	pp	229533.I1RQE7
91174	229533.I1RMK3	pp	229533.Q4I8X0
91175	229533.I1RMK3	pp	229533.Q4IJ56
91176	229533.I1RMK3	pp	229533.I1RSD8
91177	229533.I1RMK3	pp	229533.I1RN18

```
229533.I1RMK3	pp	229533.I1RQ81
229533.I1RMK3	pp	229533.I1S296
229533.I1RMK3	pp	229533.I1RZE5
229533.I1RMK3	pp	229533.I1S1U7
229533.I1RMK3	pp	229533.I1RNU6
229533.I1RMK3	pp	229533.I1S0M3
229533.I1RMK3	pp	229533.I1RS17
229533.I1RMK3	pp	229533.I1RSR3
229533.I1RMK3	pp	229533.I1S274
229533.I1RMK3	pp	229533.I1S0J1
229533.I1RMK3	pp	229533.I1RX14
229533.I1RMK3	pp	229533.I1RQA2
229533.I1RMK3	pp	229533.I1RSI0
229533.I1RMK3	pp	229533.I1S558
229533.I1RMK3	pp	229533.I1S060
229533.I1RMK3	pp	229533.I1RVZ0
229533.I1RMK3	pp	229533.I1S2C0
229533.I1RMK3	pp	229533.I1RNK2
229533.I1RMK3	pp	229533.V6RGN5
229533.I1RMK3	pp	229533.Q4I5Z5
229533.I1RMK3	pp	229533.I1RW46
229533.I1RMK3	pp	229533.I1RTE9
229533.I1RMK3	pp	229533.I1RN99
229533.I1RMK3	pp	229533.I1RPJ3
229533.I1RMK3	pp	229533.I1RW99
229533.I1RMK3	pp	229533.I1RQ28
229533.I1RMK3	pp	229533.I1S8R3
229533.I1RMK3	pp	229533.I1S1X9
229533.I1RMK3	pp	229533.Q4HZK7
229533.I1RMK3	pp	229533.I1RSC6
229533.I1RMK3	pp	229533.I1RRI2
229533.I1RMK3	pp	229533.I1RY63
229533.I1RMK3	pp	229533.I1RZW8
229533.I1RMK3	pp	229533.I1RZZ1
229533.I1RMK3	pp	229533.I1RSR8
229533.I1RMK3	pp	229533.I1RTD9
229533.I1RJU7	pp	229533.I1RRS2
229533.I1RJU7	pp	229533.I1RRN4
229533.I1RJU7	pp	229533.I1RY28
229533.I1RJU7	pp	229533.I1RQL4
229533.I1RJU7	pp	229533.I1RY59
229533.I1REE9	pp	229533.I1RPA6
229533.A0A0E0SPD4	pp	229533.I1R9Q0
229533.A0A0E0SPD4	pp	229533.I1RGW0
229533.A0A0E0SPD4	pp	229533.I1RI51
229533.A0A0E0SPD4	pp	229533.I1RJZ7
229533.A0A0E0SPD4	pp	229533.I1R9W6
229533.A0A0E0SPD4	pp	229533.I1S3D7
229533.A0A0E0SPD4	pp	229533.I1RVY0
229533.A0A0E0SPD4	pp	229533.I1RPX5
229533.A0A0E0SPD4	pp	229533.I1RMA5
229533.A0A0E0SPD4	pp	229533.A0A1C3YI55
229533.A0A0E0SPD4	pp	229533.A0A1C3YHV5
229533.A0A0E0SPD4	pp	229533.I1RQA3
229533.A0A0E0SPD4	pp	229533.I1RWU3
229533.A0A0E0SPD4	pp	229533.I1RKR6
229533.A0A0E0SPD4	pp	229533.I1RZU9
229533.A0A0E0SPD4	pp	229533.I1RGE1
229533.A0A0E0SPD4	pp	229533.Q4HTT1
229533.A0A0E0SPD4	pp	229533.I1RNV7
229533.A0A0E0SPD4	pp	229533.A0A1C3YJZ2
229533.A0A0E0SPD4	pp	229533.I1RFW1
229533.A0A0E0SPD4	pp	229533.I1RQE9
229533.A0A0E0SPD4	pp	229533.A0A1C3YL11
229533.A0A0E0SPD4	pp	229533.V6QV38
229533.A0A0E0SPD4	pp	229533.I1RW71
229533.A0A0E0SPD4	pp	229533.I1RB36
229533.A0A0E0SPD4	pp	229533.I1RUQ6
229533.A0A0E0SPD4	pp	229533.A0A1C3YJE9
229533.A0A0E0SPD4	pp	229533.Q4IER8
229533.A0A0E0SPD4	pp	229533.I1RWX8
229533.A0A0E0SPD4	pp	229533.I1S0P4
229533.A0A0E0SPD4	pp	229533.I1RV91
```

91251 229533.A0A0E0SPD4 pp 229533.A0A0E0SQJ6
91252 229533.A0A0E0SPD4 pp 229533.I1RHP9
91253 229533.A0A0E0SPD4 pp 229533.I1S2E9
91254 229533.A0A0E0SPD4 pp 229533.V6RKY7
91255 229533.A0A0E0SPD4 pp 229533.I1RY44
91256 229533.A0A0E0SPD4 pp 229533.A0A1C3YK01
91257 229533.A0A0E0SPD4 pp 229533.A0A1C3YIJ2
91258 229533.A0A0E0SPD4 pp 229533.I1RXE5
91259 229533.A0A0E0SPD4 pp 229533.I1RCK7
91260 229533.A0A0E0SPD4 pp 229533.I1RWT6
91261 229533.A0A0E0SPD4 pp 229533.I1RDC9
91262 229533.A0A0E0SPD4 pp 229533.I1RA56
91263 229533.A0A0E0SPD4 pp 229533.A0A1C3YL52
91264 229533.A0A0E0SPD4 pp 229533.Q4HTT2
91265 229533.A0A0E0SPD4 pp 229533.A0A1C3YJ12
91266 229533.A0A098DUK6 pp 229533.I1RE51
91267 229533.A0A098DUK6 pp 229533.I1S1V2
91268 229533.A0A098DUK6 pp 229533.I1RAN8
91269 229533.A0A098DUK6 pp 229533.A0A1C3YLA2
91270 229533.A0A098DUK6 pp 229533.A0A1C3YHP3
91271 229533.A0A098DUK6 pp 229533.A0A1C3YLC6
91272 229533.A0A098DUK6 pp 229533.Q4IPI4
91273 229533.A0A098DUK6 pp 229533.I1S7I5
91274 229533.I1RJL5 pp 229533.I1S0P5
91275 229533.I1RJL5 pp 229533.I1RY59
91276 229533.I1RJL5 pp 229533.I1RWC6
91277 229533.I1RJL5 pp 229533.I1S1Y5
91278 229533.I1RJL5 pp 229533.I1RZU5
91279 229533.I1RB36 pp 229533.I1RXE5
91280 229533.I1RB36 pp 229533.I1RWX8
91281 229533.I1RB36 pp 229533.I1RQA3
91282 229533.I1RB36 pp 229533.I1RY44
91283 229533.I1RB36 pp 229533.I1RCK7
91284 229533.I1RB36 pp 229533.V6R2A1
91285 229533.I1RB36 pp 229533.I1RK15
91286 229533.I1RB36 pp 229533.I1RKR6
91287 229533.I1RB36 pp 229533.I1RGC6
91288 229533.I1RB36 pp 229533.I1RZU9
91289 229533.I1RB36 pp 229533.I1RWT6
91290 229533.I1RB36 pp 229533.I1RQE9
91291 229533.I1RB36 pp 229533.I1RNV0
91292 229533.I1RB36 pp 229533.I1RDC9
91293 229533.I1RB36 pp 229533.V6QV38
91294 229533.I1RB36 pp 229533.I1RUQ6
91295 229533.I1RB36 pp 229533.I1RUC9
91296 229533.I1RB36 pp 229533.I1RWU3
91297 229533.I1RB36 pp 229533.I1RZF3
91298 229533.I1RB36 pp 229533.I1RV91
91299 229533.I1RB36 pp 229533.I1RJ40
91300 229533.I1RB36 pp 229533.I1RGW0
91301 229533.I1RB36 pp 229533.I1RJ18
91302 229533.I1RB36 pp 229533.I1RI51
91303 229533.I1RB36 pp 229533.I1RHP9
91304 229533.I1RB36 pp 229533.I1S2E9
91305 229533.I1RB36 pp 229533.I1RT62
91306 229533.I1RB36 pp 229533.Q4HTT2
91307 229533.I1RB36 pp 229533.I1RMA5
91308 229533.I1RB36 pp 229533.I1RNV7
91309 229533.I1RB36 pp 229533.I1S3D7
91310 229533.I1RB36 pp 229533.Q4I7S1
91311 229533.I1RB36 pp 229533.I1RGE1
91312 229533.I1RB36 pp 229533.I1RHR6
91313 229533.I1RB36 pp 229533.I1RVY0
91314 229533.I1RB36 pp 229533.I1RPX5
91315 229533.I1RB36 pp 229533.I1RWU2
91316 229533.I1RE51 pp 229533.V6R5G9
91317 229533.I1RE51 pp 229533.V6RHA0
91318 229533.I1RE51 pp 229533.Q4I7X1
91319 229533.I1RE51 pp 229533.I1RRD9
91320 229533.I1RE51 pp 229533.I1S3R6
91321 229533.I1RE51 pp 229533.I1RT65
91322 229533.I1RE51 pp 229533.I1S0F0
91323 229533.I1RE51 pp 229533.I1S0Z3

91324	229533.I1RE51	pp	229533.I1RU02
91325	229533.I1RE51	pp	229533.I1RQ93
91326	229533.I1RE51	pp	229533.Q4IER8
91327	229533.I1RE51	pp	229533.I1S0T8
91328	229533.I1RE51	pp	229533.I1RQE9
91329	229533.I1RE51	pp	229533.I1RMU2
91330	229533.I1RE51	pp	229533.I1RPU1
91331	229533.I1RE51	pp	229533.I1RYN7
91332	229533.I1RE51	pp	229533.I1RYC1
91333	229533.I1RE51	pp	229533.I1RT16
91334	229533.I1RE51	pp	229533.I1RMY4
91335	229533.I1RE51	pp	229533.Q4HTT2
91336	229533.I1RE51	pp	229533.I1RPJ1
91337	229533.I1RE51	pp	229533.I1RF01
91338	229533.I1RE51	pp	229533.I1RNV7
91339	229533.I1RE51	pp	229533.I1RP56
91340	229533.I1RE51	pp	229533.I1RFV7
91341	229533.I1RE51	pp	229533.I1RSQ5
91342	229533.I1RE51	pp	229533.I1RED9
91343	229533.I1RE51	pp	229533.I1RFW1
91344	229533.I1RE51	pp	229533.I1RTA6
91345	229533.I1RE51	pp	229533.I1S270
91346	229533.I1RE51	pp	229533.I1S1V2
91347	229533.I1RE51	pp	229533.I1RRP6
91348	229533.I1RE51	pp	229533.I1RT41
91349	229533.I1RE51	pp	229533.I1RKI1
91350	229533.I1RE51	pp	229533.I1S7I5
91351	229533.I1RE51	pp	229533.I1RXX9
91352	229533.I1RE51	pp	229533.I1RQY1
91353	229533.I1RE51	pp	229533.I1RRS7
91354	229533.I1RE51	pp	229533.I1RST4
91355	229533.I1RE51	pp	229533.I1RI51
91356	229533.I1RE51	pp	229533.I1SAA0
91357	229533.I1RE51	pp	229533.I1S4Z5
91358	229533.I1RE51	pp	229533.I1RTH9
91359	229533.I1RE51	pp	229533.I1RSN4
91360	229533.I1RE51	pp	229533.I1RG50
91361	229533.I1RE51	pp	229533.Q4HTT1
91362	229533.I1RE51	pp	229533.I1RRF0
91363	229533.I1RE51	pp	229533.I1S0N5
91364	229533.I1RE51	pp	229533.I1RY90
91365	229533.I1RE51	pp	229533.I1RHP9
91366	229533.I1RE51	pp	229533.I1RTI0
91367	229533.I1RE51	pp	229533.Q4IMD1
91368	229533.I1RE51	pp	229533.I1RKR9
91369	229533.I1RE51	pp	229533.Q4IPI4
91370	229533.Q4I5R9	pp	229533.V6RMW6
91371	229533.Q4I5R9	pp	229533.V6QXG1
91372	229533.Q4I5R9	pp	229533.Q4IJ56
91373	229533.Q4I5R9	pp	229533.V6R283
91374	229533.Q4I5R9	pp	229533.V6QX21
91375	229533.Q4I5R9	pp	229533.Q4IN00
91376	229533.Q4I5R9	pp	229533.Q4INZ9
91377	229533.Q4I5R9	pp	229533.V6QYF0
91378	229533.Q4I5R9	pp	229533.Q4IRI9
91379	229533.A0A098DLE3	pp	229533.I1RWM4
91380	229533.A0A098DLE3	pp	229533.I1RNM7
91381	229533.I1RRG4	pp	229533.I1RVY0
91382	229533.I1RGE1	pp	229533.V6QV38
91383	229533.I1RGE1	pp	229533.I1RGE7
91384	229533.I1RGE1	pp	229533.I1RGF1
91385	229533.I1RGE1	pp	229533.I1RGF3
91386	229533.I1RGE1	pp	229533.I1RQE9
91387	229533.I1RGE1	pp	229533.I1RI51
91388	229533.I1RGE1	pp	229533.I1RGW0
91389	229533.I1RGE1	pp	229533.I1RGE8
91390	229533.I1RGE1	pp	229533.I1RGF0
91391	229533.I1RGE1	pp	229533.I1RPX5
91392	229533.I1RGE1	pp	229533.I1RVY0
91393	229533.I1RGE1	pp	229533.I1S6V3
91394	229533.I1RGE1	pp	229533.I1RNV7
91395	229533.I1RGE1	pp	229533.I1RKR6
91396	229533.I1RGE1	pp	229533.I1RGF2

91397 229533.I1RGE1 pp 229533.I1RGF4
91398 229533.I1RGE1 pp 229533.I1RXE5
91399 229533.I1RGE1 pp 229533.I1RWU3
91400 229533.I1RGE1 pp 229533.I1RGE9
91401 229533.I1RGE1 pp 229533.I1RHP9
91402 229533.I1RGE1 pp 229533.I1RUQ6
91403 229533.I1RGE1 pp 229533.I1RMA5
91404 229533.A0A1C3YKL2 pp 229533.I1RTB7
91405 229533.A0A1C3YKL2 pp 229533.I1RH68
91406 229533.A0A1C3YKL2 pp 229533.I1S0S6
91407 229533.A0A1C3YKL2 pp 229533.I1RQN9
91408 229533.A0A1C3YKL2 pp 229533.P0C431
91409 229533.A0A1C3YKL2 pp 229533.I1RCD4
91410 229533.A0A1C3YKL2 pp 229533.I1RTE6
91411 229533.A0A1C3YKL2 pp 229533.V6REE9
91412 229533.I1RQ19 pp 229533.Q4I7N9
91413 229533.I1RQ19 pp 229533.Q4I5I4
91414 229533.I1RQ19 pp 229533.I1RYV3
91415 229533.I1RQ19 pp 229533.I1RQE9
91416 229533.I1RQ19 pp 229533.I1RQA3
91417 229533.I1RQ19 pp 229533.I1RQ93
91418 229533.I1RQ19 pp 229533.Q4HTT1
91419 229533.I1RQ19 pp 229533.Q4HTT2
91420 229533.I1RQ19 pp 229533.Q4IER8
91421 229533.I1RQ19 pp 229533.Q4IJP1
91422 229533.I1RQ19 pp 229533.I1RU02
91423 229533.I1RQ19 pp 229533.Q4I7L0
91424 229533.I1RQ19 pp 229533.I1RZR6
91425 229533.I1RQ19 pp 229533.Q4HYQ4
91426 229533.I1RQ19 pp 229533.I1RWT6
91427 229533.I1RQ19 pp 229533.I1SAA0
91428 229533.I1RQ19 pp 229533.I1SB04
91429 229533.I1RQ19 pp 229533.I1S1U7
91430 229533.I1RQ19 pp 229533.Q4IMD1
91431 229533.I1RQ19 pp 229533.I1RY44
91432 229533.I1RQ19 pp 229533.I1S2E9
91433 229533.I1RQ19 pp 229533.I1S4Z5
91434 229533.I1RQ19 pp 229533.Q4IB50
91435 229533.I1RQ19 pp 229533.I1RV91
91436 229533.I1RQ19 pp 229533.Q4I8C9
91437 229533.I1RQ19 pp 229533.I1RS62
91438 229533.I1RQ19 pp 229533.I1S1X2
91439 229533.I1RQ19 pp 229533.Q4HYB8
91440 229533.I1RQ19 pp 229533.I1RSD1
91441 229533.A0A1C3YHM5 pp 229533.A0A1C3YM29
91442 229533.A0A1C3YHM5 pp 229533.A0A1C3YLT9
91443 229533.I1RMA5 pp 229533.V6QV38
91444 229533.I1RMA5 pp 229533.I1RWU3
91445 229533.I1RMA5 pp 229533.I1RVY0
91446 229533.I1RMA5 pp 229533.I1RPX5
91447 229533.I1RMA5 pp 229533.I1RXE5
91448 229533.I1RMA5 pp 229533.I1RQE9
91449 229533.I1RMA5 pp 229533.I1RMV5
91450 229533.I1RMA5 pp 229533.I1RNV7
91451 229533.I1RMA5 pp 229533.I1RUQ6
91452 229533.I1RMA5 pp 229533.I1RWX8
91453 229533.I1RMA5 pp 229533.I1S3D7
91454 229533.I1REE5 pp 229533.I1RNM7
91455 229533.I1REE5 pp 229533.I1RWM4
91456 229533.A0A1C3YIG8 pp 229533.I1S0S8
91457 229533.A0A1C3YIG8 pp 229533.Q4ICM0
91458 229533.A0A1C3YIG8 pp 229533.I1S247
91459 229533.A0A1C3YIG8 pp 229533.I1RCF0
91460 229533.A0A1C3YIG8 pp 229533.A0A1C3YLK5
91461 229533.A0A1C3YIG8 pp 229533.A0A1C3YKT1
91462 229533.A0A1C3YIG8 pp 229533.A0A1C3YLL9
91463 229533.A0A1C3YIG8 pp 229533.I1RKI1
91464 229533.A0A1C3YIG8 pp 229533.I1S270
91465 229533.A0A1C3YIG8 pp 229533.I1RMG2
91466 229533.A0A1C3YIG8 pp 229533.I1RZV1
91467 229533.A0A1C3YIG8 pp 229533.Q4INI2
91468 229533.A0A1C3YIG8 pp 229533.I1RR74
91469 229533.A0A1C3YIG8 pp 229533.I1RFK0

91470 229533.A0A1C3YIG8 pp 229533.I1S0Z3
91471 229533.A0A1C3YIG8 pp 229533.I1RT41
91472 229533.A0A1C3YIG8 pp 229533.I1S7W2
91473 229533.A0A1C3YIY6 pp 229533.I1RLV9
91474 229533.A0A1C3YIY6 pp 229533.I1RVZ0
91475 229533.A0A1C3YIY6 pp 229533.I1S4T3
91476 229533.A0A1C3YIY6 pp 229533.I1RAN5
91477 229533.A0A1C3YIY6 pp 229533.I1RKJ6
91478 229533.A0A1C3YIY6 pp 229533.I1RMN6
91479 229533.A0A1C3YIY6 pp 229533.I1RPU0
91480 229533.A0A1C3YIY6 pp 229533.I1RAU7
91481 229533.A0A1C3YIY6 pp 229533.I1RQJ5
91482 229533.A0A1C3YIY6 pp 229533.I1RRR7
91483 229533.A0A1C3YIY6 pp 229533.I1RM06
91484 229533.A0A1C3YIY6 pp 229533.I1RPP3
91485 229533.A0A1C3YIY6 pp 229533.I1RBG9
91486 229533.A0A1C3YIY6 pp 229533.I1RRY9
91487 229533.A0A1C3YIY6 pp 229533.Q4I327
91488 229533.A0A1C3YIY6 pp 229533.I1RB27
91489 229533.A0A1C3YIY6 pp 229533.I1RWN6
91490 229533.A0A1C3YIY6 pp 229533.I1RR73
91491 229533.A0A1C3YIY6 pp 229533.I1RZK6
91492 229533.A0A1C3YIY6 pp 229533.I1RQ65
91493 229533.A0A1C3YIY6 pp 229533.V6R042
91494 229533.A0A1C3YIY6 pp 229533.I1S9U2
91495 229533.A0A1C3YIY6 pp 229533.I1RZZ1
91496 229533.A0A1C3YIY6 pp 229533.I1RZS6
91497 229533.A0A1C3YIY6 pp 229533.I1RZF6
91498 229533.A0A1C3YIY6 pp 229533.I1S2E8
91499 229533.A0A1C3YIY6 pp 229533.I1S2C0
91500 229533.A0A1C3YIY6 pp 229533.I1RRH5
91501 229533.A0A1C3YIY6 pp 229533.I1RNH6
91502 229533.A0A1C3YIY6 pp 229533.V6R3X5
91503 229533.A0A1C3YIY6 pp 229533.I1S021
91504 229533.A0A1C3YIY6 pp 229533.V6RMW6
91505 229533.A0A1C3YIY6 pp 229533.I1RFW5
91506 229533.A0A1C3YIY6 pp 229533.I1S504
91507 229533.A0A1C3YIY6 pp 229533.I1RJZ6
91508 229533.A0A1C3YIY6 pp 229533.A0A1C3YKM9
91509 229533.A0A1C3YIY6 pp 229533.I1RRQ3
91510 229533.A0A1C3YIY6 pp 229533.I1R9V7
91511 229533.A0A1C3YIY6 pp 229533.I1S0J1
91512 229533.A0A1C3YIY6 pp 229533.I1RFS3
91513 229533.A0A1C3YIY6 pp 229533.I1S0M9
91514 229533.A0A1C3YIY6 pp 229533.I1RM96
91515 229533.A0A1C3YIY6 pp 229533.I1RX46
91516 229533.A0A1C3YIY6 pp 229533.I1RSD8
91517 229533.A0A1C3YIY6 pp 229533.I1RKL3
91518 229533.A0A1C3YIY6 pp 229533.I1RT80
91519 229533.A0A1C3YIY6 pp 229533.I1RE00
91520 229533.A0A1C3YIY6 pp 229533.I1RAF8
91521 229533.A0A1C3YIY6 pp 229533.I1RSR8
91522 229533.A0A1C3YIY6 pp 229533.I1RJV3
91523 229533.A0A1C3YIY6 pp 229533.I1RTH6
91524 229533.A0A1C3YIY6 pp 229533.I1RA48
91525 229533.A0A1C3YIY6 pp 229533.I1RC03
91526 229533.A0A1C3YIY6 pp 229533.Q4IN63
91527 229533.I1RYM4 pp 229533.I1RZV1
91528 229533.I1RYM4 pp 229533.I1S247
91529 229533.I1RYM4 pp 229533.I1S270
91530 229533.I1RYM4 pp 229533.Q4ICM0
91531 229533.I1RYM4 pp 229533.I1S0S8
91532 229533.I1RYM4 pp 229533.I1S0G1
91533 229533.I1RYM4 pp 229533.I1S7W2
91534 229533.I1RYM4 pp 229533.I1S1X2
91535 229533.I1RYM4 pp 229533.I1S0Z3
91536 229533.I1RYM4 pp 229533.Q4INI2
91537 229533.I1RXE5 pp 229533.Q4I7S1
91538 229533.I1RXE5 pp 229533.I1RXU0
91539 229533.I1RXE5 pp 229533.I1RZB5
91540 229533.I1RXE5 pp 229533.V6QV38
91541 229533.I1RXE5 pp 229533.Q4I5I4
91542 229533.I1RXE5 pp 229533.Q4I7X1

229533.I1RXE5 pp 229533.I1S276
229533.I1RXE5 pp 229533.Q4HTT2
229533.I1RXE5 pp 229533.Q4HYB8
229533.I1RXE5 pp 229533.I1S1V5
229533.I1RXE5 pp 229533.I1S382
229533.I1RXE5 pp 229533.I1RZU9
229533.I1RXE5 pp 229533.V6QUE6
229533.I1RXE5 pp 229533.I1S951
229533.I1RXE5 pp 229533.V6QWX8
229533.I1RXE5 pp 229533.I1S3D7
229533.I1RXE5 pp 229533.Q4IMD1
229533.I1RXE5 pp 229533.I1RZM2
229533.I1RXE5 pp 229533.Q4HYQ4
229533.I1RXE5 pp 229533.Q4IQX3
229533.A0A0E0RZ26 pp 229533.I1RKB2
229533.A0A0E0RZ26 pp 229533.A0A1C3YKN4
229533.A0A0E0RZ26 pp 229533.I1RNG4
229533.A0A0E0RZ26 pp 229533.I1RZ56
229533.A0A0E0RZ26 pp 229533.I1S3H8
229533.A0A0E0RZ26 pp 229533.I1RJS1
229533.A0A0E0RZ26 pp 229533.I1S0A5
229533.A0A0E0RZ26 pp 229533.I1RZ25
229533.A0A0E0RZ26 pp 229533.I1RPV8
229533.A0A0E0RZ26 pp 229533.I1RGZ3
229533.A0A0E0RZ26 pp 229533.I1RA13
229533.A0A0E0RZ26 pp 229533.I1RDU0
229533.A0A0E0RZ26 pp 229533.I1RUY5
229533.A0A0E0RZ26 pp 229533.I1S094
229533.A0A0E0RZ26 pp 229533.I1RST9
229533.A0A0E0RZ26 pp 229533.I1S8F8
229533.A0A0E0RZ26 pp 229533.I1S6H3
229533.A0A0E0RZ26 pp 229533.I1RLA2
229533.A0A0E0RZ26 pp 229533.I1RRL5
229533.A0A0E0RZ26 pp 229533.I1RNM7
229533.A0A0E0RZ26 pp 229533.I1RDN3
229533.A0A0E0RZ26 pp 229533.I1S334
229533.I1RT30 pp 229533.I1S5R6
229533.I1RT30 pp 229533.V6R9P0
229533.I1RD56 pp 229533.Q4IPI1
229533.I1RD56 pp 229533.I1RQJ5
229533.I1RD56 pp 229533.I1RW71
229533.I1RD56 pp 229533.I1S246
229533.I1RD56 pp 229533.Q4ICG5
229533.I1RD56 pp 229533.I1RWH5
229533.I1RD56 pp 229533.I1RK23
229533.I1RD56 pp 229533.I1RS75
229533.I1RD56 pp 229533.Q4IFI0
229533.I1RD56 pp 229533.Q4IE79
229533.I1RD56 pp 229533.I1RFW5
229533.I1RD56 pp 229533.I1S7M3
229533.I1RD56 pp 229533.I1S2Y5
229533.I1RD56 pp 229533.I1RQC5
229533.I1RD56 pp 229533.I1RNH6
229533.I1RD56 pp 229533.I1S7M2
229533.I1RD56 pp 229533.Q4IJH1
229533.I1RD56 pp 229533.I1RT80
229533.I1RD56 pp 229533.I1S021
229533.I1RD56 pp 229533.I1RT98
229533.I1RD56 pp 229533.I1RGC2
229533.I1RD56 pp 229533.I1RZJ6
229533.I1RD56 pp 229533.Q4IBS2
229533.I1RD56 pp 229533.I1RN59
229533.I1RD56 pp 229533.Q4HVW2
229533.I1RD56 pp 229533.I1RPZ3
229533.I1RD56 pp 229533.I1RPW9
229533.I1RD56 pp 229533.I1RX46
229533.I1RD56 pp 229533.I1S1C6
229533.I1RD56 pp 229533.Q4IJ56
229533.I1RD56 pp 229533.I1S0M9
229533.I1RD56 pp 229533.I1RPU0
229533.I1RD56 pp 229533.I1S9U2
229533.I1RD56 pp 229533.Q4IEK8
229533.I1RD56 pp 229533.I1RM67

91616	229533.I1RD56	pp	229533.I1RX81
91617	229533.I1RD56	pp	229533.Q4IAA0
91618	229533.I1RD56	pp	229533.Q4I1Y1
91619	229533.I1RD56	pp	229533.V6QYF0
91620	229533.I1RD56	pp	229533.I1RK72
91621	229533.I1RD56	pp	229533.Q4IRI9
91622	229533.I1RD56	pp	229533.I1RZG7
91623	229533.I1RD56	pp	229533.I1RSR8
91624	229533.I1RD56	pp	229533.I1RPA0
91625	229533.I1RD56	pp	229533.I1RPY5
91626	229533.I1RD56	pp	229533.I1RX47
91627	229533.I1RD56	pp	229533.I1RZF6
91628	229533.I1RD56	pp	229533.Q4IB70
91629	229533.I1RD56	pp	229533.I1S9L0
91630	229533.I1RD56	pp	229533.I1RQ65
91631	229533.I1RD56	pp	229533.I1S471
91632	229533.I1RD56	pp	229533.I1RN99
91633	229533.I1RD56	pp	229533.I1RFG0
91634	229533.I1RD56	pp	229533.I1S056
91635	229533.I1RD56	pp	229533.I1RK95
91636	229533.I1RD56	pp	229533.V6RGN5
91637	229533.I1RD56	pp	229533.I1RRQ3
91638	229533.I1RD56	pp	229533.I1RMN6
91639	229533.I1RD56	pp	229533.I1RME9
91640	229533.I1RD56	pp	229533.I1S8E2
91641	229533.I1RD56	pp	229533.I1RDG0
91642	229533.I1RD56	pp	229533.I1RZK6
91643	229533.I1RD56	pp	229533.I1RT47
91644	229533.I1RD56	pp	229533.I1RR73
91645	229533.I1RD56	pp	229533.Q4I7F9
91646	229533.I1RD56	pp	229533.I1RND6
91647	229533.I1RD56	pp	229533.I1RWB5
91648	229533.I1RD56	pp	229533.I1RFP9
91649	229533.I1RD56	pp	229533.I1RJZ6
91650	229533.I1RD56	pp	229533.I1RF08
91651	229533.I1RD56	pp	229533.I1RNU6
91652	229533.I1RD56	pp	229533.I1RNB8
91653	229533.I1RD56	pp	229533.I1RVZ0
91654	229533.I1RD56	pp	229533.I1RRL0
91655	229533.I1RD56	pp	229533.Q4IF76
91656	229533.I1RD56	pp	229533.I1RXF2
91657	229533.I1RD56	pp	229533.I1RD64
91658	229533.I1RD56	pp	229533.I1S060
91659	229533.I1RD56	pp	229533.I1RT17
91660	229533.I1RD56	pp	229533.I1RFQ1
91661	229533.I1RD56	pp	229533.I1RST1
91662	229533.I1RD56	pp	229533.I1RS28
91663	229533.I1RD56	pp	229533.I1RZV6
91664	229533.I1RD56	pp	229533.I1RSD4
91665	229533.I1RD56	pp	229533.Q4IAS1
91666	229533.I1RD56	pp	229533.I1RKJ6
91667	229533.I1RD56	pp	229533.I1RLV9
91668	229533.I1RD56	pp	229533.I1RX24
91669	229533.I1RD56	pp	229533.I1S0J1
91670	229533.I1RD56	pp	229533.I1RK96
91671	229533.I1RD56	pp	229533.I1S9Y0
91672	229533.I1RD56	pp	229533.I1RZS6
91673	229533.I1RD56	pp	229533.Q4I830
91674	229533.I1RD56	pp	229533.Q4IPB8
91675	229533.I1RD56	pp	229533.I1RPY4
91676	229533.I1RD56	pp	229533.I1RWU2
91677	229533.I1RD56	pp	229533.I1RRR7
91678	229533.I1RD56	pp	229533.I1RWE1
91679	229533.I1RD56	pp	229533.I1RKL3
91680	229533.I1RD56	pp	229533.I1RX51
91681	229533.I1RD56	pp	229533.V6QXG1
91682	229533.I1RD56	pp	229533.Q4I662
91683	229533.I1RD56	pp	229533.I1RRJ8
91684	229533.I1RD56	pp	229533.I1RFS3
91685	229533.I1RD56	pp	229533.I1S4M5
91686	229533.I1RD56	pp	229533.I1RM96
91687	229533.I1RD56	pp	229533.Q4IBK5
91688	229533.I1RD56	pp	229533.I1RMV5

91689 229533.I1RD56 pp 229533.I1RSW2
91690 229533.I1RD56 pp 229533.I1RSA4
91691 229533.I1RD56 pp 229533.I1RND5
91692 229533.I1RD56 pp 229533.I1RJZ7
91693 229533.I1RD56 pp 229533.Q4HZ42
91694 229533.I1RD56 pp 229533.I1S2C0
91695 229533.I1RD56 pp 229533.I1RG57
91696 229533.I1RD56 pp 229533.V6R042
91697 229533.I1RD56 pp 229533.I1RK90
91698 229533.I1RD56 pp 229533.Q4IEV5
91699 229533.I1RD56 pp 229533.Q4IN63
91700 229533.I1RD56 pp 229533.I1S1X9
91701 229533.I1RD56 pp 229533.I1RK92
91702 229533.I1RD56 pp 229533.I1RPL3
91703 229533.I1RD56 pp 229533.I1RSD8
91704 229533.I1RD56 pp 229533.I1RG90
91705 229533.I1RD56 pp 229533.I1S2E8
91706 229533.I1RD56 pp 229533.Q4HZ68
91707 229533.I1RD56 pp 229533.I1S0B1
91708 229533.I1RD56 pp 229533.I1RYJ9
91709 229533.A0A1C3YL16 pp 229533.I1RD43
91710 229533.A0A1C3YL16 pp 229533.I1RPY4
91711 229533.A0A1C3YL16 pp 229533.I1S2Y5
91712 229533.A0A1C3YL16 pp 229533.A0A1C3YN58
91713 229533.A0A1C3YL16 pp 229533.I1RCN2
91714 229533.A0A1C3YL16 pp 229533.I1RB73
91715 229533.A0A1C3YL16 pp 229533.I1RI51
91716 229533.A0A1C3YL16 pp 229533.I1RNV7
91717 229533.A0A1C3YL16 pp 229533.I1S3D7
91718 229533.A0A1C3YL16 pp 229533.I1R9W6
91719 229533.A0A1C3YL16 pp 229533.I1S6S3
91720 229533.A0A1C3YL16 pp 229533.I1RX54
91721 229533.A0A1C3YL16 pp 229533.I1RHP9
91722 229533.A0A1C3YL16 pp 229533.I1RYE4
91723 229533.A0A1C3YL16 pp 229533.I1RAX8
91724 229533.A0A1C3YL16 pp 229533.I1RJV7
91725 229533.A0A1C3YL16 pp 229533.I1RVA7
91726 229533.A0A1C3YL16 pp 229533.I1RQ93
91727 229533.A0A1C3YL16 pp 229533.A0A1C3YNC1
91728 229533.A0A1C3YL16 pp 229533.I1RW36
91729 229533.A0A1C3YL16 pp 229533.I1RUU3
91730 229533.A0A1C3YL16 pp 229533.I1RQE9
91731 229533.A0A1C3YL16 pp 229533.I1RII1
91732 229533.A0A1C3YL16 pp 229533.I1SAA0
91733 229533.A0A1C3YL16 pp 229533.I1RPY5
91734 229533.A0A1C3YL16 pp 229533.I1RQF6
91735 229533.A0A1C3YL16 pp 229533.I1RCK7
91736 229533.A0A1C3YL16 pp 229533.I1RLP4
91737 229533.A0A1C3YL16 pp 229533.A0A1C3YMF9
91738 229533.I1RJY4 pp 229533.I1RZ84
91739 229533.I1RJY4 pp 229533.I1RRN4
91740 229533.I1RJY4 pp 229533.I1RKR7
91741 229533.I1RJY4 pp 229533.I1S287
91742 229533.I1RJY4 pp 229533.I1RRC4
91743 229533.A0A1C3YK01 pp 229533.I1RAU6
91744 229533.A0A1C3YK01 pp 229533.Q4I7X1
91745 229533.A0A1C3YK01 pp 229533.Q4HTT2
91746 229533.A0A1C3YK01 pp 229533.A0A1C3YK75
91747 229533.A0A1C3YK01 pp 229533.Q4I948
91748 229533.A0A1C3YK01 pp 229533.A0A1C3YKH8
91749 229533.A0A1C3YK01 pp 229533.I1RQA3
91750 229533.A0A1C3YK01 pp 229533.I1RKR6
91751 229533.A0A1C3YK01 pp 229533.I1RAW7
91752 229533.A0A1C3YK01 pp 229533.I1RZU9
91753 229533.A0A1C3YK01 pp 229533.I1RWX8
91754 229533.A0A1C3YK01 pp 229533.Q4ICA8
91755 229533.A0A1C3YK01 pp 229533.I1RW71
91756 229533.A0A1C3YK01 pp 229533.I1RQE9
91757 229533.A0A1C3YK01 pp 229533.I1RB36
91758 229533.A0A1C3YK01 pp 229533.I1RXE5
91759 229533.A0A1C3YK01 pp 229533.I1S0Z3
91760 229533.A0A1C3YK01 pp 229533.I1RUQ6
91761 229533.A0A1C3YK01 pp 229533.I1RNJ2

91762 229533.A0A1C3YK01 pp 229533.V6QV38
91763 229533.A0A1C3YK01 pp 229533.I1RQA8
91764 229533.A0A1C3YK01 pp 229533.I1RCK7
91765 229533.A0A1C3YK01 pp 229533.I1RRZ9
91766 229533.A0A1C3YK01 pp 229533.I1RK15
91767 229533.A0A1C3YK01 pp 229533.I1RWT6
91768 229533.A0A1C3YK01 pp 229533.I1RDG8
91769 229533.A0A1C3YK01 pp 229533.I1RT41
91770 229533.A0A1C3YK01 pp 229533.Q4IER8
91771 229533.A0A1C3YK01 pp 229533.A0A1C3YN58
91772 229533.A0A1C3YK01 pp 229533.I1RRH5
91773 229533.A0A1C3YK01 pp 229533.I1RFU3
91774 229533.A0A1C3YK01 pp 229533.I1RBH2
91775 229533.A0A1C3YK01 pp 229533.I1RMA5
91776 229533.A0A1C3YK01 pp 229533.I1RWU3
91777 229533.A0A1C3YK01 pp 229533.I1R976
91778 229533.A0A1C3YK01 pp 229533.I1RDC9
91779 229533.A0A1C3YK01 pp 229533.I1RGE1
91780 229533.A0A1C3YK01 pp 229533.A0A1C3YLW0
91781 229533.A0A1C3YK01 pp 229533.I1RVY0
91782 229533.A0A1C3YK01 pp 229533.A0A1C3YKP8
91783 229533.A0A1C3YK01 pp 229533.I1RNV7
91784 229533.A0A1C3YK01 pp 229533.I1RPF4
91785 229533.A0A1C3YK01 pp 229533.I1RFN9
91786 229533.A0A1C3YK01 pp 229533.I1RJZ7
91787 229533.A0A1C3YK01 pp 229533.I1RPS0
91788 229533.A0A1C3YK01 pp 229533.A0A1C3YLG0
91789 229533.A0A1C3YK01 pp 229533.I1RCN2
91790 229533.A0A1C3YK01 pp 229533.I1RZA9
91791 229533.A0A1C3YK01 pp 229533.I1RJX2
91792 229533.A0A1C3YK01 pp 229533.I1RWD0
91793 229533.A0A1C3YK01 pp 229533.A0A1C3YL11
91794 229533.A0A1C3YK01 pp 229533.A0A1C3YLD3
91795 229533.A0A1C3YK01 pp 229533.I1RKJ7
91796 229533.A0A1C3YK01 pp 229533.I1S382
91797 229533.A0A1C3YK01 pp 229533.I1RHR6
91798 229533.A0A1C3YK01 pp 229533.I1RMM1
91799 229533.A0A1C3YK01 pp 229533.Q4IPI4
91800 229533.A0A1C3YK01 pp 229533.I1RY44
91801 229533.A0A1C3YK01 pp 229533.I1R9Y1
91802 229533.A0A1C3YK01 pp 229533.Q4IB96
91803 229533.A0A1C3YK01 pp 229533.I1R9W6
91804 229533.A0A1C3YK01 pp 229533.I1RFQ1
91805 229533.A0A1C3YK01 pp 229533.Q4IB89
91806 229533.A0A1C3YK01 pp 229533.I1RA56
91807 229533.A0A1C3YK01 pp 229533.I1RPX5
91808 229533.A0A1C3YK01 pp 229533.I1S3D7
91809 229533.A0A1C3YK01 pp 229533.Q4HTT1
91810 229533.A0A1C3YK01 pp 229533.I1S1V5
91811 229533.A0A1C3YK01 pp 229533.A0A1C3YKV4
91812 229533.A0A1C3YK01 pp 229533.Q4I5V3
91813 229533.A0A1C3YK01 pp 229533.I1RFW1
91814 229533.A0A1C3YK01 pp 229533.I1RZF3
91815 229533.A0A1C3YK01 pp 229533.A0A1C3YM82
91816 229533.A0A1C3YK01 pp 229533.I1RPR8
91817 229533.A0A1C3YK01 pp 229533.I1RJ40
91818 229533.A0A1C3YK01 pp 229533.Q4IAK7
91819 229533.A0A1C3YK01 pp 229533.I1S951
91820 229533.A0A1C3YK01 pp 229533.Q4IMD1
91821 229533.A0A1C3YK01 pp 229533.Q4I7S1
91822 229533.A0A1C3YK01 pp 229533.A0A1C3YM29
91823 229533.A0A1C3YK01 pp 229533.A0A1C3YKT6
91824 229533.A0A1C3YK01 pp 229533.Q4HY90
91825 229533.A0A1C3YK01 pp 229533.I1RA40
91826 229533.A0A1C3YK01 pp 229533.I1RW28
91827 229533.A0A1C3YK01 pp 229533.V6R2A1
91828 229533.A0A1C3YK01 pp 229533.A0A1C3YMF9
91829 229533.A0A1C3YK01 pp 229533.I1RV91
91830 229533.A0A1C3YK01 pp 229533.I1RAV2
91831 229533.A0A1C3YK01 pp 229533.I1R9Q0
91832 229533.A0A1C3YK01 pp 229533.I1RGW0
91833 229533.A0A1C3YK01 pp 229533.Q4IEV4
91834 229533.A0A1C3YK01 pp 229533.I1RB73

91835 229533.A0A1C3YK01 pp 229533.I1S2E9
91836 229533.A0A1C3YK01 pp 229533.I1RHP9
91837 229533.A0A1C3YK01 pp 229533.I1SB04
91838 229533.A0A1C3YK01 pp 229533.I1RI51
91839 229533.A0A1C3YK01 pp 229533.I1RBR5
91840 229533.Q4IPI1 pp 229533.V6QV38
91841 229533.Q4IPI1 pp 229533.V6RMW6
91842 229533.Q4IPI1 pp 229533.V6R283
91843 229533.Q4IPI1 pp 229533.V6R227
91844 229533.Q4IPI1 pp 229533.V6RGN5
91845 229533.Q4IPI1 pp 229533.V6RGY3
91846 229533.Q4IPI1 pp 229533.V6QYF0
91847 229533.Q4IPI1 pp 229533.V6R042
91848 229533.Q4IPI1 pp 229533.Q4IQW0
91849 229533.I1RKR6 pp 229533.V6RKK5
91850 229533.I1RKR6 pp 229533.V6R2A1
91851 229533.I1RKR6 pp 229533.I1RU02
91852 229533.I1RKR6 pp 229533.Q4IER8
91853 229533.I1RKR6 pp 229533.I1RQE9
91854 229533.I1RKR6 pp 229533.I1RWX8
91855 229533.I1RKR6 pp 229533.I1RZU9
91856 229533.I1RKR6 pp 229533.I1RXE5
91857 229533.I1RKR6 pp 229533.I1RSQ4
91858 229533.I1RKR6 pp 229533.I1RPX5
91859 229533.I1RKR6 pp 229533.V6QV38
91860 229533.I1RKR6 pp 229533.I1RWU2
91861 229533.I1RKR6 pp 229533.I1RT62
91862 229533.I1RKR6 pp 229533.I1RNV0
91863 229533.I1RKR6 pp 229533.I1RSI1
91864 229533.I1RKR6 pp 229533.I1RN49
91865 229533.I1RKR6 pp 229533.I1RMA5
91866 229533.I1RKR6 pp 229533.Q4I7S1
91867 229533.I1RKR6 pp 229533.I1RUQ6
91868 229533.I1RKR6 pp 229533.I1RUC9
91869 229533.I1RKR6 pp 229533.I1RWU3
91870 229533.I1RKR6 pp 229533.I1S3D7
91871 229533.I1RKR6 pp 229533.I1RRH5
91872 229533.I1RKR6 pp 229533.I1RVY0
91873 229533.I1RKR6 pp 229533.I1RNV7
91874 229533.I1RUJ0 pp 229533.I1S7W2
91875 229533.I1RUJ0 pp 229533.Q4ICM0
91876 229533.I1RUJ0 pp 229533.I1S270
91877 229533.I1RUJ0 pp 229533.I1S247
91878 229533.I1RUJ0 pp 229533.Q4INI2
91879 229533.I1RUJ0 pp 229533.I1S0S8
91880 229533.I1RUJ0 pp 229533.I1S0Z3
91881 229533.A0A098D2M3 pp 229533.I1RVY0
91882 229533.A0A098D2M3 pp 229533.I1RPU7
91883 229533.A0A098D2M3 pp 229533.A0A0E0SMG8
91884 229533.A0A098D2M3 pp 229533.A0A1C3YJZ2
91885 229533.A0A098D2M3 pp 229533.Q4IMD1
91886 229533.A0A098D2M3 pp 229533.I1RFW1
91887 229533.A0A098D2M3 pp 229533.A0A0E0SQJ6
91888 229533.A0A098D2M3 pp 229533.I1RGW0
91889 229533.A0A098D2M3 pp 229533.Q4I5R3
91890 229533.A0A098D2M3 pp 229533.I1RPX5
91891 229533.A0A098D2M3 pp 229533.A0A1C3YKR1
91892 229533.A0A098D2M3 pp 229533.I1RA56
91893 229533.A0A098D2M3 pp 229533.I1RDC9
91894 229533.A0A098D2M3 pp 229533.I1RUQ6
91895 229533.A0A098D2M3 pp 229533.V6QV38
91896 229533.A0A098D2M3 pp 229533.I1RWU3
91897 229533.A0A098D2M3 pp 229533.I1RJU7
91898 229533.A0A098D2M3 pp 229533.A0A0E0SPD4
91899 229533.A0A098D2M3 pp 229533.I1RB36
91900 229533.A0A098D2M3 pp 229533.I1RU02
91901 229533.A0A098D2M3 pp 229533.I1RE51
91902 229533.A0A098D2M3 pp 229533.I1RDD8
91903 229533.A0A098D2M3 pp 229533.Q4HTT2
91904 229533.A0A098D2M3 pp 229533.I1RGE1
91905 229533.A0A098D2M3 pp 229533.Q4IER8
91906 229533.A0A098D2M3 pp 229533.I1RQ19
91907 229533.A0A098D2M3 pp 229533.I1RMA5

91908 229533.A0A098D2M3 pp 229533.I1RYV3
91909 229533.A0A098D2M3 pp 229533.I1RCW8
91910 229533.A0A098D2M3 pp 229533.I1RY28
91911 229533.A0A098D2M3 pp 229533.A0A0E0SQV5
91912 229533.A0A098D2M3 pp 229533.I1RXE5
91913 229533.A0A098D2M3 pp 229533.I1RB75
91914 229533.A0A098D2M3 pp 229533.A0A1C3YL16
91915 229533.A0A098D2M3 pp 229533.A0A1C3YK01
91916 229533.A0A098D2M3 pp 229533.I1RKR6
91917 229533.I1R9L4 pp 229533.I1RNG4
91918 229533.A0A1C3YJ15 pp 229533.I1RKF7
91919 229533.A0A1C3YJ15 pp 229533.I1RYG4
91920 229533.I1RV20 pp 229533.I1RZ94
91921 229533.I1RT33 pp 229533.V6RGN5
91922 229533.I1RT33 pp 229533.I1RUG3
91923 229533.I1RT33 pp 229533.Q4I327
91924 229533.I1RT33 pp 229533.I1S5R6
91925 229533.I1RT33 pp 229533.V6R042
91926 229533.I1RT33 pp 229533.V6RMW6
91927 229533.I1RT33 pp 229533.Q4I8X0
91928 229533.I1RT33 pp 229533.Q4HZ42
91929 229533.I1RT33 pp 229533.V6RLM7
91930 229533.I1RT33 pp 229533.I1RWS4
91931 229533.I1RT33 pp 229533.I1S4T3
91932 229533.I1RT33 pp 229533.I1S558
91933 229533.I1RT33 pp 229533.I1RYI5
91934 229533.I1RT33 pp 229533.V6RGY3
91935 229533.I1RT33 pp 229533.V6QT89
91936 229533.I1RT33 pp 229533.I1S0J1
91937 229533.I1RT33 pp 229533.I1S274
91938 229533.I1RT33 pp 229533.I1RUG4
91939 229533.I1RT33 pp 229533.I1RWF9
91940 229533.I1RT33 pp 229533.Q4I830
91941 229533.I1RT33 pp 229533.I1S5R1
91942 229533.I1RT33 pp 229533.Q4HZ68
91943 229533.I1RT33 pp 229533.O74638
91944 229533.I1RT33 pp 229533.I1S1X9
91945 229533.I1RT33 pp 229533.I1S2E8
91946 229533.I1RT33 pp 229533.I1S9Y1
91947 229533.I1RT33 pp 229533.I1RTE9
91948 229533.I1RT33 pp 229533.I1S2C0
91949 229533.I1RT33 pp 229533.I1S2H4
91950 229533.I1RT33 pp 229533.Q4IAS1
91951 229533.I1RT33 pp 229533.I1S2B8
91952 229533.I1RT33 pp 229533.Q4I662
91953 229533.I1RT33 pp 229533.I1RZB2
91954 229533.I1RT33 pp 229533.I1RTD5
91955 229533.I1RT33 pp 229533.I1RWJ4
91956 229533.I1RT33 pp 229533.Q4I1M9
91957 229533.I1RT33 pp 229533.Q4HXF0
91958 229533.I1RT33 pp 229533.I1RT75
91959 229533.I1RT33 pp 229533.Q4IF76
91960 229533.I1RT33 pp 229533.I1RZZ9
91961 229533.I1RT33 pp 229533.I1RW61
91962 229533.I1RT33 pp 229533.I1RW26
91963 229533.I1RT33 pp 229533.I1RWI5
91964 229533.I1RT33 pp 229533.I1RX24
91965 229533.I1RT33 pp 229533.I1S253
91966 229533.I1RT33 pp 229533.Q4IBS2
91967 229533.I1RT33 pp 229533.Q4IEK8
91968 229533.I1RT33 pp 229533.I1RZB5
91969 229533.I1RT33 pp 229533.V6QUE6
91970 229533.I1RT33 pp 229533.I1RVZ0
91971 229533.I1RT33 pp 229533.I1RYJ8
91972 229533.I1RT33 pp 229533.V6R227
91973 229533.I1RT33 pp 229533.I1RZF6
91974 229533.I1RT33 pp 229533.Q4ICA8
91975 229533.I1RT33 pp 229533.I1RXF0
91976 229533.I1RT33 pp 229533.I1RZZ1
91977 229533.I1RT33 pp 229533.Q4IJ56
91978 229533.I1RT33 pp 229533.I1RZK6
91979 229533.I1RT33 pp 229533.V6RTJ8
91980 229533.I1RT33 pp 229533.I1RXB6

91981 229533.I1RT33 pp 229533.Q4I948
91982 229533.I1RT33 pp 229533.I1S0F1
91983 229533.I1RT33 pp 229533.Q4INZ9
91984 229533.I1RT33 pp 229533.I1RY63
91985 229533.I1RT33 pp 229533.Q4HVW2
91986 229533.I1RT33 pp 229533.I1RXD7
91987 229533.I1RT33 pp 229533.I1RYJ6
91988 229533.I1RT33 pp 229533.I1RT80
91989 229533.I1RT33 pp 229533.I1S6F0
91990 229533.I1RT33 pp 229533.I1S0R7
91991 229533.I1RT33 pp 229533.Q4IPI1
91992 229533.I1RT33 pp 229533.I1RTF4
91993 229533.I1RT33 pp 229533.Q4IJH1
91994 229533.I1RT33 pp 229533.Q4IFI0
91995 229533.I1RT33 pp 229533.I1RW25
91996 229533.I1RT33 pp 229533.I1RX46
91997 229533.I1RT33 pp 229533.V6QYF0
91998 229533.I1RT33 pp 229533.I1S4S4
91999 229533.I1RYG4 pp 229533.I1S0H1
92000 229533.I1RDD8 pp 229533.I1RZX0
92001 229533.A0A1C3YHW7 pp 229533.I1RZK6
92002 229533.A0A1C3YHW7 pp 229533.I1S0M9
92003 229533.A0A1C3YHW7 pp 229533.I1R9V7
92004 229533.A0A1C3YHW7 pp 229533.I1RA48
92005 229533.A0A1C3YHW7 pp 229533.Q4IRI9
92006 229533.A0A1C3YHW7 pp 229533.I1RDG0
92007 229533.A0A1C3YHW7 pp 229533.I1RAN5
92008 229533.A0A1C3YHW7 pp 229533.I1RGC2
92009 229533.A0A1C3YHW7 pp 229533.I1RYB1
92010 229533.A0A1C3YHW7 pp 229533.I1RPA0
92011 229533.A0A1C3YHW7 pp 229533.I1RFP9
92012 229533.A0A1C3YHW7 pp 229533.I1RNH6
92013 229533.A0A1C3YHW7 pp 229533.I1RBL7
92014 229533.A0A1C3YHW7 pp 229533.I1RA36
92015 229533.A0A1C3YHW7 pp 229533.Q4ICG5
92016 229533.A0A1C3YHW7 pp 229533.I1RK90
92017 229533.A0A1C3YHW7 pp 229533.I1RW32
92018 229533.A0A1C3YHW7 pp 229533.I1RS38
92019 229533.A0A1C3YHW7 pp 229533.I1RG57
92020 229533.A0A1C3YHW7 pp 229533.I1RTD0
92021 229533.A0A1C3YHW7 pp 229533.I1RSR8
92022 229533.A0A1C3YHW7 pp 229533.A0A1C3YK51
92023 229533.A0A1C3YHW7 pp 229533.I1RZG7
92024 229533.A0A1C3YHW7 pp 229533.I1RX51
92025 229533.A0A1C3YHW7 pp 229533.I1RZF6
92026 229533.A0A1C3YHW7 pp 229533.I1RM96
92027 229533.A0A1C3YHW7 pp 229533.I1RSA4
92028 229533.A0A1C3YHW7 pp 229533.I1RX81
92029 229533.A0A1C3YHW7 pp 229533.Q4IJ56
92030 229533.A0A1C3YHW7 pp 229533.I1RE20
92031 229533.A0A1C3YHW7 pp 229533.I1RPU0
92032 229533.A0A1C3YHW7 pp 229533.V6RY33
92033 229533.A0A1C3YHW7 pp 229533.I1RG90
92034 229533.A0A1C3YHW7 pp 229533.I1REK3
92035 229533.A0A1C3YHW7 pp 229533.I1S060
92036 229533.A0A1C3YHW7 pp 229533.I1RB50
92037 229533.A0A1C3YHW7 pp 229533.I1RAU7
92038 229533.A0A1C3YHW7 pp 229533.I1RQJ5
92039 229533.A0A1C3YHW7 pp 229533.I1RRL0
92040 229533.A0A1C3YHW7 pp 229533.I1RSD8
92041 229533.A0A1C3YHW7 pp 229533.I1RS28
92042 229533.A0A1C3YHW7 pp 229533.I1S1C6
92043 229533.A0A1C3YHW7 pp 229533.I1S2E8
92044 229533.A0A1C3YHW7 pp 229533.Q4IPI1
92045 229533.A0A1C3YHW7 pp 229533.I1RSD4
92046 229533.A0A1C3YHW7 pp 229533.I1RZA4
92047 229533.A0A1C3YHW7 pp 229533.I1RWB5
92048 229533.A0A1C3YHW7 pp 229533.I1RW84
92049 229533.A0A1C3YHW7 pp 229533.I1RT47
92050 229533.A0A1C3YHW7 pp 229533.I1RK92
92051 229533.A0A1C3YHW7 pp 229533.I1RMN6
92052 229533.A0A1C3YHW7 pp 229533.I1RR73
92053 229533.A0A1C3YHW7 pp 229533.A0A1C3YIN1

92054 229533.A0A1C3YHW7 pp 229533.I1RPH7
92055 229533.A0A1C3YHW7 pp 229533.I1RX47
92056 229533.A0A1C3YHW7 pp 229533.V6RKE4
92057 229533.A0A1C3YHW7 pp 229533.V6R042
92058 229533.A0A1C3YHW7 pp 229533.I1RRQ3
92059 229533.A0A1C3YHW7 pp 229533.I1RE15
92060 229533.A0A1C3YHW7 pp 229533.I1RFS3
92061 229533.A0A1C3YHW7 pp 229533.A0A1C3YNC3
92062 229533.A0A1C3YHW7 pp 229533.I1RBG9
92063 229533.A0A1C3YHW7 pp 229533.I1RQ65
92064 229533.A0A1C3YHW7 pp 229533.I1RKJ6
92065 229533.A0A1C3YHW7 pp 229533.I1RCS2
92066 229533.A0A1C3YHW7 pp 229533.I1RWU2
92067 229533.A0A1C3YHW7 pp 229533.I1RST1
92068 229533.A0A1C3YHW7 pp 229533.A0A1C3YIN0
92069 229533.A0A1C3YHW7 pp 229533.I1RKL3
92070 229533.A0A1C3YHW7 pp 229533.I1RNR3
92071 229533.A0A1C3YHW7 pp 229533.I1S4M5
92072 229533.A0A1C3YHW7 pp 229533.I1RLV9
92073 229533.A0A1C3YHW7 pp 229533.V6RQ48
92074 229533.A0A1C3YHW7 pp 229533.I1RXU1
92075 229533.A0A1C3YHW7 pp 229533.I1S8E2
92076 229533.A0A1C3YHW7 pp 229533.I1RAF8
92077 229533.A0A1C3YHW7 pp 229533.I1RW76
92078 229533.A0A1C3YHW7 pp 229533.I1RBV3
92079 229533.I1RKF7 pp 229533.I1S0B1
92080 229533.I1RKF7 pp 229533.I1RQK2
92081 229533.I1RKF7 pp 229533.I1RW23
92082 229533.I1RKF7 pp 229533.I1RYG4
92083 229533.I1RKF7 pp 229533.I1RWT7
92084 229533.I1RKF7 pp 229533.I1RPR4
92085 229533.I1S0H1 pp 229533.Q4IFH8
92086 229533.A0A0E0RXU0 pp 229533.I1RFM2
92087 229533.A0A0E0RXU0 pp 229533.A0A1C3YMS7
92088 229533.A0A0E0RXU0 pp 229533.I1SAS3
92089 229533.A0A0E0RXU0 pp 229533.I1SAS4
92090 229533.A0A0E0RXU0 pp 229533.I1RNT1
92091 229533.A0A0E0RXU0 pp 229533.I1RQ48
92092 229533.A0A0E0RXU0 pp 229533.I1RCA0
92093 229533.A0A0E0RXU0 pp 229533.I1RJG1
92094 229533.A0A0E0RXU0 pp 229533.I1RF79
92095 229533.A0A0E0RXU0 pp 229533.I1RR90
92096 229533.A0A0E0RXU0 pp 229533.A0A1C3YLL7
92097 229533.A0A0E0RXU0 pp 229533.I1RY35
92098 229533.A0A0E0RXU0 pp 229533.V6RR38
92099 229533.A0A0E0RXU0 pp 229533.I1RZZ3
92100 229533.A0A0E0RXU0 pp 229533.I1RRQ1
92101 229533.A0A0E0RXU0 pp 229533.V6RPN0
92102 229533.A0A0E0RXU0 pp 229533.I1RQ76
92103 229533.A0A0E0RXU0 pp 229533.I1RT23
92104 229533.A0A0E0RXU0 pp 229533.I1S1Q3
92105 229533.A0A0E0RXU0 pp 229533.I1RI22
92106 229533.A0A0E0RXU0 pp 229533.A0A0E0SP71
92107 229533.A0A0E0RXU0 pp 229533.I1RCC2
92108 229533.I1RZX0 pp 229533.I1S0H1
92109 229533.I1RZX0 pp 229533.I1S7W5
92110 229533.A0A098D2D2 pp 229533.A0A1C3YJ15
92111 229533.A0A098D2D2 pp 229533.I1RYG4
92112 229533.A0A098D2D2 pp 229533.I1RDD8
92113 229533.A0A098D2D2 pp 229533.I1RDY2
92114 229533.A0A098D2D2 pp 229533.I1RKF7
92115 229533.A0A098D2D2 pp 229533.I1RKL4
92116 229533.A0A098D2D2 pp 229533.I1S0H1
92117 229533.A0A098D2D2 pp 229533.I1RZX0
92118 229533.Q4I2D7 pp 229533.Q4INI2
92119 229533.Q4I2D7 pp 229533.Q4ICM0
92120 229533.I1REP6 pp 229533.I1SA62
92121 229533.I1REP6 pp 229533.I1S2L9
92122 229533.I1REP6 pp 229533.I1S3C0
92123 229533.I1REP6 pp 229533.I1RU13
92124 229533.I1REP6 pp 229533.I1S6J8
92125 229533.I1RSL9 pp 229533.I1RZF6
92126 229533.I1RSL9 pp 229533.I1S1C6

229533.I1RSL9 pp 229533.Q4IPI1
229533.I1RSL9 pp 229533.Q4IRI9
229533.I1RSL9 pp 229533.I1RWU2
229533.I1RSL9 pp 229533.Q4IJ56
229533.I1RSL9 pp 229533.I1S4M5
229533.I1RSL9 pp 229533.Q4ICG5
229533.I1RSL9 pp 229533.I1S0M9
229533.I1RSL9 pp 229533.I1RSR8
229533.I1RSL9 pp 229533.I1RX81
229533.I1RSL9 pp 229533.I1RZG7
229533.I1RSL9 pp 229533.I1S9Y1
229533.I1RSL9 pp 229533.I1RYB1
229533.I1RSL9 pp 229533.I1RST1
229533.I1RSL9 pp 229533.I1RT47
229533.I1RSL9 pp 229533.I1RZK6
229533.I1RSL9 pp 229533.I1RXU1
229533.I1RSL9 pp 229533.I1RX51
229533.I1RSL9 pp 229533.I1RX47
229533.I1RSL9 pp 229533.V6RKE4
229533.I1RSL9 pp 229533.V6RQ48
229533.I1RSL9 pp 229533.I1RZA4
229533.I1RSL9 pp 229533.I1RWB5
229533.I1RSL9 pp 229533.I1S060
229533.I1RSL9 pp 229533.I1S2E8
229533.I1RSL9 pp 229533.I1S8E2
229533.I1S1K2 pp 229533.I1S1L0
229533.I1S1K2 pp 229533.I1S1K7
229533.I1S1K2 pp 229533.I1S6L6
229533.A0A098DD65 pp 229533.I1RU13
229533.A0A098DD65 pp 229533.I1R9P9
229533.A0A098DD65 pp 229533.A0A0E0RSU2
229533.A0A098DD65 pp 229533.I1RVX2
229533.A0A098DD65 pp 229533.I1S3C0
229533.A0A098DD65 pp 229533.I1S6J8
229533.A0A098DD65 pp 229533.I1S2L9
229533.A0A098DD65 pp 229533.I1SA62
229533.A0A098DD65 pp 229533.A0A0E0S9X0
229533.A0A098DD65 pp 229533.A0A098E1U3
229533.I1S3S7 pp 229533.I1S6L6
229533.I1SAJ3 pp 229533.Q4HXF0
229533.I1SAJ3 pp 229533.V6RLM7
229533.I1SAJ3 pp 229533.V6RQW4
229533.I1SAJ3 pp 229533.V6RN31
229533.I1RKA7 pp 229533.I1RX84
229533.I1RKA7 pp 229533.Q4HZQ1
229533.I1RKA7 pp 229533.I1RSP7
229533.I1RKA7 pp 229533.I1RSQ1
229533.I1RKA7 pp 229533.I1S0C2
229533.I1RKA7 pp 229533.Q4I174
229533.I1RKA7 pp 229533.Q4IN00
229533.I1RKA7 pp 229533.I1S193
229533.I1RKA7 pp 229533.I1RUG4
229533.I1RKA7 pp 229533.V6RN69
229533.I1RKA7 pp 229533.I1RY80
229533.I1RKA7 pp 229533.I1RPP5
229533.A0A1C3YIH6 pp 229533.I1R9P9
229533.A0A1C3YIH6 pp 229533.I1S3C0
229533.A0A1C3YIH6 pp 229533.I1SA62
229533.A0A1C3YIH6 pp 229533.I1S6J8
229533.A0A1C3YIH6 pp 229533.I1RU13
229533.A0A1C3YIH6 pp 229533.I1RVX2
229533.A0A1C3YIH6 pp 229533.I1S2L9
229533.A0A1C3YIH6 pp 229533.A0A1C3YMJ1
229533.I1RLS1 pp 229533.I1RZ11
229533.I1RLS1 pp 229533.I1RSD4
229533.I1RLS1 pp 229533.I1RQ80
229533.I1RLS1 pp 229533.Q4I7X1
229533.I1RLS1 pp 229533.Q4I7L0
229533.I1RK41 pp 229533.I1S8M4
229533.I1RK41 pp 229533.I1RUF9
229533.I1RK41 pp 229533.I1S6L6
229533.I1RK41 pp 229533.I1RPB1
229533.I1RK41 pp 229533.I1RP13

92200 229533.I1RIG4 pp 229533.V6RKE4
92201 229533.I1RIG4 pp 229533.I1S7X9
92202 229533.I1RIG4 pp 229533.V6RB81
92203 229533.I1RIG4 pp 229533.I1RTM8
92204 229533.I1RIG4 pp 229533.I1RJZ7
92205 229533.I1RIG4 pp 229533.I1RMM1
92206 229533.I1RIG4 pp 229533.I1RKI1
92207 229533.I1RIG4 pp 229533.I1RJK6
92208 229533.I1RIG4 pp 229533.I1RW76
92209 229533.I1RIG4 pp 229533.I1RP87
92210 229533.I1RIG4 pp 229533.I1RXU1
92211 229533.I1RIG4 pp 229533.I1RN70
92212 229533.I1RIG4 pp 229533.I1RWP9
92213 229533.I1RIG4 pp 229533.I1S051
92214 229533.I1RIG4 pp 229533.I1S1U6
92215 229533.I1RIG4 pp 229533.I1RNR3
92216 229533.I1RIG4 pp 229533.V6RQ48
92217 229533.I1RIG4 pp 229533.I1RQK5
92218 229533.I1RIG4 pp 229533.I1RS79
92219 229533.I1RIG4 pp 229533.I1RTA3
92220 229533.I1RIG4 pp 229533.I1RYP0
92221 229533.I1RIG4 pp 229533.I1RXF5
92222 229533.I1RIG4 pp 229533.I1RZ84
92223 229533.I1RIG4 pp 229533.I1RZA4
92224 229533.I1RIG4 pp 229533.I1RX95
92225 229533.I1RIG4 pp 229533.I1RNM3
92226 229533.I1RIG4 pp 229533.I1RUN9
92227 229533.I1RIG4 pp 229533.I1RX84
92228 229533.I1RIG4 pp 229533.I1RTQ9
92229 229533.I1RIG4 pp 229533.I1RXT7
92230 229533.I1RIG4 pp 229533.I1RQ70
92231 229533.I1RIG4 pp 229533.I1RW71
92232 229533.I1RIG4 pp 229533.I1RJY4
92233 229533.I1RIG4 pp 229533.I1RWZ9
92234 229533.I1RIG4 pp 229533.Q4INI2
92235 229533.I1RIG4 pp 229533.I1RT41
92236 229533.I1RIG4 pp 229533.I1RTD0
92237 229533.I1RIG4 pp 229533.I1S287
92238 229533.I1RIG4 pp 229533.I1RPP0
92239 229533.I1RIG4 pp 229533.I1RPY3
92240 229533.I1RIG4 pp 229533.Q4HY20
92241 229533.I1RIG4 pp 229533.V6RXW9
92242 229533.I1RIG4 pp 229533.I1RPK5
92243 229533.I1RIG4 pp 229533.I1S0Z3
92244 229533.I1RIG4 pp 229533.V6R9X9
92245 229533.I1RIG4 pp 229533.I1RQP7
92246 229533.I1RIG4 pp 229533.I1RWI6
92247 229533.I1RIG4 pp 229533.I1S2W9
92248 229533.I1RIG4 pp 229533.I1RMN3
92249 229533.I1RIG4 pp 229533.I1S9S4
92250 229533.I1RV76 pp 229533.I1RVX2
92251 229533.I1RV76 pp 229533.I1S6J8
92252 229533.I1RV76 pp 229533.I1SA62
92253 229533.I1RV76 pp 229533.I1S3C0
92254 229533.I1RV76 pp 229533.I1S2L9
92255 229533.I1RF95 pp 229533.I1RK41
92256 229533.I1RF95 pp 229533.I1S2J3
92257 229533.I1RT87 pp 229533.I1RUF9
92258 229533.A0A098DHZ1 pp 229533.I1S3C0
92259 229533.A0A098DHZ1 pp 229533.I1S2L9
92260 229533.A0A098DHZ1 pp 229533.I1S6J8
92261 229533.A0A098DHZ1 pp 229533.A0A0E0RSU2
92262 229533.A0A098DHZ1 pp 229533.I1RVX2
92263 229533.A0A098DHZ1 pp 229533.A0A0E0S9X0
92264 229533.A0A098DHZ1 pp 229533.I1SA62
92265 229533.A0A098DHZ1 pp 229533.I1RU13
92266 229533.I1RFU5 pp 229533.I1RFU6
92267 229533.A0A1C3YKK5 pp 229533.I1RA70
92268 229533.A0A1C3YKK5 pp 229533.I1RZK5
92269 229533.A0A1C3YKK5 pp 229533.I1RTH6
92270 229533.A0A1C3YKK5 pp 229533.I1RK83
92271 229533.A0A1C3YKK5 pp 229533.I1S2C0
92272 229533.A0A1C3YKK5 pp 229533.I1RAN5

92273 229533.A0A1C3YKK5 pp 229533.I1RCN5
92274 229533.A0A1C3YKK5 pp 229533.I1RVZ0
92275 229533.A0A1C3YKK5 pp 229533.I1RD03
92276 229533.A0A1C3YKK5 pp 229533.I1RYB1
92277 229533.A0A1C3YKK5 pp 229533.I1RBZ3
92278 229533.A0A1C3YKK5 pp 229533.I1RC81
92279 229533.A0A1C3YKK5 pp 229533.I1RJV3
92280 229533.A0A1C3YKK5 pp 229533.I1RBL7
92281 229533.A0A1C3YKK5 pp 229533.I1S6E5
92282 229533.A0A1C3YKK5 pp 229533.I1RDX0
92283 229533.A0A1C3YKK5 pp 229533.I1RE00
92284 229533.A0A1C3YKK5 pp 229533.I1RAU7
92285 229533.A0A1C3YKK5 pp 229533.I1RAW0
92286 229533.A0A1C3YKK5 pp 229533.I1RSD8
92287 229533.A0A1C3YKK5 pp 229533.I1RSR8
92288 229533.A0A1C3YKK5 pp 229533.I1RZZ1
92289 229533.A0A1C3YKK5 pp 229533.Q4IN63
92290 229533.A0A1C3YKK5 pp 229533.I1RZK6
92291 229533.A0A1C3YKK5 pp 229533.A0A1C3YLK2
92292 229533.A0A1C3YKK5 pp 229533.I1RQJ4
92293 229533.A0A1C3YKK5 pp 229533.I1RJZ6
92294 229533.A0A1C3YKK5 pp 229533.I1S2E8
92295 229533.A0A1C3YKK5 pp 229533.Q4I327
92296 229533.A0A1C3YKK5 pp 229533.I1RYB9
92297 229533.A0A1C3YKK5 pp 229533.I1RU69
92298 229533.A0A1C3YKK5 pp 229533.I1S1X9
92299 229533.A0A1C3YKK5 pp 229533.I1RT80
92300 229533.A0A1C3YKK5 pp 229533.I1R9V7
92301 229533.A0A1C3YKK5 pp 229533.A0A1C3YKM9
92302 229533.A0A1C3YKK5 pp 229533.I1RA48
92303 229533.A0A1C3YKK5 pp 229533.I1RDG0
92304 229533.A0A1C3YKK5 pp 229533.I1S021
92305 229533.A0A1C3YKK5 pp 229533.I1RNH6
92306 229533.A0A1C3YKK5 pp 229533.I1S8E2
92307 229533.A0A1C3YKK5 pp 229533.V6RMW6
92308 229533.A0A1C3YKK5 pp 229533.I1RND6
92309 229533.A0A1C3YKK5 pp 229533.I1RGC2
92310 229533.A0A1C3YKK5 pp 229533.I1RMN6
92311 229533.A0A1C3YKK5 pp 229533.I1RR73
92312 229533.A0A1C3YKK5 pp 229533.I1RD22
92313 229533.A0A1C3YKK5 pp 229533.V6R042
92314 229533.A0A1C3YKK5 pp 229533.I1S044
92315 229533.A0A1C3YKK5 pp 229533.Q4IFI0
92316 229533.A0A1C3YKK5 pp 229533.I1RM96
92317 229533.A0A1C3YKK5 pp 229533.V6RGN5
92318 229533.A0A1C3YKK5 pp 229533.I1RZF6
92319 229533.A0A1C3YKK5 pp 229533.I1RW25
92320 229533.A0A1C3YKK5 pp 229533.I1RRR7
92321 229533.A0A1C3YKK5 pp 229533.I1S504
92322 229533.A0A1C3YKK5 pp 229533.A0A1C3YMK1
92323 229533.A0A1C3YKK5 pp 229533.I1RT04
92324 229533.A0A1C3YKK5 pp 229533.I1S0R7
92325 229533.A0A1C3YKK5 pp 229533.I1S7S9
92326 229533.A0A1C3YKK5 pp 229533.A0A1C3YKW9
92327 229533.A0A1C3YKK5 pp 229533.I1S060
92328 229533.A0A1C3YKK5 pp 229533.I1RDY5
92329 229533.A0A1C3YKK5 pp 229533.Q4I662
92330 229533.A0A1C3YKK5 pp 229533.Q4I613
92331 229533.A0A1C3YKK5 pp 229533.I1RRQ3
92332 229533.A0A1C3YKK5 pp 229533.I1RFS3
92333 229533.A0A1C3YKK5 pp 229533.I1S0J1
92334 229533.A0A1C3YKK5 pp 229533.I1RX24
92335 229533.A0A1C3YKK5 pp 229533.I1RBG9
92336 229533.A0A1C3YKK5 pp 229533.I1RC72
92337 229533.A0A1C3YKK5 pp 229533.I1RD56
92338 229533.A0A1C3YKK5 pp 229533.I1S0M9
92339 229533.A0A1C3YKK5 pp 229533.I1RM25
92340 229533.A0A1C3YKK5 pp 229533.I1RNA0
92341 229533.A0A1C3YKK5 pp 229533.I1RM06
92342 229533.A0A1C3YKK5 pp 229533.A0A1C3YL15
92343 229533.A0A1C3YKK5 pp 229533.I1RWB5
92344 229533.A0A1C3YKK5 pp 229533.I1RQ65
92345 229533.A0A1C3YKK5 pp 229533.I1RZS6

92346 229533.A0A1C3YKK5 pp 229533.I1RB27
92347 229533.A0A1C3YKK5 pp 229533.I1RLV9
92348 229533.A0A1C3YKK5 pp 229533.I1RN48
92349 229533.A0A1C3YKK5 pp 229533.I1RZV6
92350 229533.A0A1C3YKK5 pp 229533.I1RRZ0
92351 229533.A0A1C3YKK5 pp 229533.I1RC03
92352 229533.A0A1C3YKK5 pp 229533.I1RPU0
92353 229533.A0A1C3YKK5 pp 229533.I1RWH5
92354 229533.A0A1C3YKK5 pp 229533.I1RRT2
92355 229533.A0A1C3YKK5 pp 229533.I1RKJ6
92356 229533.A0A1C3YKK5 pp 229533.I1RFW5
92357 229533.A0A1C3YKK5 pp 229533.I1RX46
92358 229533.A0A1C3YKK5 pp 229533.I1RM67
92359 229533.A0A1C3YKK5 pp 229533.I1RKL3
92360 229533.A0A1C3YKK5 pp 229533.I1RIS3
92361 229533.A0A1C3YKK5 pp 229533.I1RPP3
92362 229533.A0A1C3YKK5 pp 229533.Q4IPI1
92363 229533.A0A1C3YKK5 pp 229533.I1RPW9
92364 229533.A0A1C3YKK5 pp 229533.I1RQJ5
92365 229533.A0A1C3YKK5 pp 229533.I1RGA4
92366 229533.A0A1C3YKK5 pp 229533.I1RKE7
92367 229533.A0A1C3YKK5 pp 229533.I1RAF8
92368 229533.A0A1C3YKK5 pp 229533.I1RT98
92369 229533.A0A098DY45 pp 229533.A0A0E0S9X0
92370 229533.A0A098DY45 pp 229533.I1S6J8
92371 229533.A0A098DY45 pp 229533.I1SA62
92372 229533.A0A098DY45 pp 229533.I1S2L9
92373 229533.A0A098DY45 pp 229533.A0A098E1U3
92374 229533.A0A098DY45 pp 229533.I1RVX2
92375 229533.A0A098DY45 pp 229533.I1S3C0
92376 229533.A0A098DY45 pp 229533.A0A0E0RSU2
92377 229533.A0A098DY45 pp 229533.I1R9P9
92378 229533.A0A098DY45 pp 229533.I1RU13
92379 229533.I0LNZ1 pp 229533.I1RDT7
92380 229533.I0LNZ1 pp 229533.I1R9F8
92381 229533.I0LNZ1 pp 229533.I1RDW1
92382 229533.I0LNZ1 pp 229533.I1REH0
92383 229533.I0LNZ1 pp 229533.I1RPI4
92384 229533.I0LNZ1 pp 229533.I1R9D3
92385 229533.I0LNZ1 pp 229533.I1S308
92386 229533.I0LNZ1 pp 229533.I1S2N3
92387 229533.I0LNZ1 pp 229533.I1RGY2
92388 229533.I0LNZ1 pp 229533.I1RHL0
92389 229533.I0LNZ1 pp 229533.I1RXM0
92390 229533.I0LNZ1 pp 229533.I1RPT0
92391 229533.I1RV34 pp 229533.I1S2L9
92392 229533.I1RV34 pp 229533.I1S3C0
92393 229533.I1RV34 pp 229533.I1RVX2
92394 229533.I1RV34 pp 229533.I1SA62
92395 229533.I1RV34 pp 229533.I1S6J8
92396 229533.I1RIM9 pp 229533.I1S6J8
92397 229533.I1RIM9 pp 229533.I1SA62
92398 229533.I1RIM9 pp 229533.I1RU13
92399 229533.I1RIM9 pp 229533.I1RVX2
92400 229533.I1RIM9 pp 229533.I1S2L9
92401 229533.I1RIM9 pp 229533.I1S3C0
92402 229533.I1RBS4 pp 229533.I1S7X9
92403 229533.I1RBS4 pp 229533.I1RJK6
92404 229533.I1RBS4 pp 229533.I1RR95
92405 229533.I1RBS4 pp 229533.I1REZ0
92406 229533.I1RBS4 pp 229533.I1RTM8
92407 229533.I1RBS4 pp 229533.I1RXU1
92408 229533.I1RBS4 pp 229533.V6RKE4
92409 229533.I1RBS4 pp 229533.I1RCD4
92410 229533.I1RBS4 pp 229533.I1RMM1
92411 229533.I1RBS4 pp 229533.I1RKR9
92412 229533.I1RBS4 pp 229533.I1RE15
92413 229533.I1RBS4 pp 229533.I1RP87
92414 229533.I1RBS4 pp 229533.I1RJZ7
92415 229533.I1RBS4 pp 229533.V6RB81
92416 229533.I1RBS4 pp 229533.I1RWP9
92417 229533.I1RBS4 pp 229533.I1RC46
92418 229533.I1RBS4 pp 229533.I1RGC1

92419 229533.I1RBS4 pp 229533.I1RW76
92420 229533.I1RBS4 pp 229533.I1RNR3
92421 229533.I1RBS4 pp 229533.I1REN4
92422 229533.I1RBS4 pp 229533.I1RN70
92423 229533.I1RBS4 pp 229533.I1RKR7
92424 229533.I1RBS4 pp 229533.V6RQ48
92425 229533.I1RBS4 pp 229533.I1S051
92426 229533.I1RBS4 pp 229533.I1RXF5
92427 229533.I1RBS4 pp 229533.I1RKI1
92428 229533.I1RBS4 pp 229533.I1RS79
92429 229533.I1RBS4 pp 229533.I1RZ84
92430 229533.I1RBS4 pp 229533.I1RTA3
92431 229533.I1RBS4 pp 229533.I1RUN9
92432 229533.I1RBS4 pp 229533.I1RNM3
92433 229533.I1RBS4 pp 229533.I1RCV3
92434 229533.I1RBS4 pp 229533.I1RC00
92435 229533.I1RBS4 pp 229533.I1RIG3
92436 229533.I1RBS4 pp 229533.I1RQK5
92437 229533.I1RBS4 pp 229533.I1RX95
92438 229533.I1RBS4 pp 229533.I1RZA4
92439 229533.I1RBS4 pp 229533.I1RW71
92440 229533.I1RBS4 pp 229533.I1RYP0
92441 229533.I1RBS4 pp 229533.I1RJY4
92442 229533.I1RBS4 pp 229533.I1S1U6
92443 229533.I1RBS4 pp 229533.Q4INI2
92444 229533.I1RBS4 pp 229533.I1RXT7
92445 229533.I1RBS4 pp 229533.I1RQ70
92446 229533.I1RBS4 pp 229533.I1REK1
92447 229533.I1RBS4 pp 229533.Q5I2J3
92448 229533.I1RBS4 pp 229533.I1RDD0
92449 229533.I1RBS4 pp 229533.I1RE20
92450 229533.I1RBS4 pp 229533.I1RWZ9
92451 229533.I1RBS4 pp 229533.Q4HZS8
92452 229533.I1RBS4 pp 229533.I1REC4
92453 229533.I1RBS4 pp 229533.I1RPY3
92454 229533.I1RBS4 pp 229533.I1RFK0
92455 229533.I1RBS4 pp 229533.Q4HY20
92456 229533.I1RBS4 pp 229533.I1S287
92457 229533.I1RBS4 pp 229533.I1RX84
92458 229533.I1RBS4 pp 229533.I1RTQ9
92459 229533.I1RBS4 pp 229533.I1RTD0
92460 229533.I1RBS4 pp 229533.I1RPK5
92461 229533.I1RBS4 pp 229533.I1REG5
92462 229533.I1RBS4 pp 229533.V6RXW9
92463 229533.I1RBS4 pp 229533.I1RT41
92464 229533.I1RBS4 pp 229533.I1RE92
92465 229533.I1RBS4 pp 229533.I1RQP7
92466 229533.I1RBS4 pp 229533.I1S0Z3
92467 229533.I1RBS4 pp 229533.I1RWI6
92468 229533.I1RBS4 pp 229533.I1S224
92469 229533.I1RBS4 pp 229533.V6REE9
92470 229533.I1RBS4 pp 229533.I1RPP0
92471 229533.I1RBS4 pp 229533.I1RMN3
92472 229533.I1RBS4 pp 229533.I1S2W9
92473 229533.I1RBS4 pp 229533.I1S9S4
92474 229533.I1RBS4 pp 229533.V6R9X9
92475 229533.I1RBS4 pp 229533.Q4I061
92476 229533.I1RDW1 pp 229533.I1RPT0
92477 229533.I1RDW1 pp 229533.I1S4D0
92478 229533.I1RDW1 pp 229533.V6RAN2
92479 229533.I1RDW1 pp 229533.I1RXM0
92480 229533.I1RDW1 pp 229533.V6RY56
92481 229533.I1RDW1 pp 229533.I1S2P8
92482 229533.I1RDW1 pp 229533.I1S2N3
92483 229533.I1RDW1 pp 229533.I1RGY2
92484 229533.I1RDW1 pp 229533.I1RJQ5
92485 229533.I1RDW1 pp 229533.I1REH0
92486 229533.I1RDW1 pp 229533.I1RHL0
92487 229533.I1RDW1 pp 229533.I1RHQ0
92488 229533.I1RDW1 pp 229533.I1S308
92489 229533.I1RDW1 pp 229533.I1RZ78
92490 229533.A0A098DHF1 pp 229533.I1S6J8
92491 229533.A0A098DHF1 pp 229533.I1SA62

229533.A0A098DHF1	pp	229533.I1S2L9
229533.A0A098DHF1	pp	229533.A0A098E1U3
229533.A0A098DHF1	pp	229533.A0A0E0S9X0
229533.A0A098DHF1	pp	229533.I1RVX2
229533.A0A098DHF1	pp	229533.I1S3C0
229533.A0A098DHF1	pp	229533.A0A0E0RSU2
229533.A0A098DHF1	pp	229533.I1RU13
229533.A0A098DHF1	pp	229533.I1R9P9
229533.I1S1V3	pp	229533.V6RR76
229533.I1S1V3	pp	229533.P0C431
229533.I1RT98	pp	229533.I1RZS6
229533.I1RT98	pp	229533.V6QYF0
229533.I1RT98	pp	229533.Q4IFI0
229533.I1RT98	pp	229533.Q4I662
229533.I1RT98	pp	229533.I1RWJ4
229533.I1RT98	pp	229533.I1S8E2
229533.I1RT98	pp	229533.Q4IJH1
229533.I1RT98	pp	229533.I1RTF4
229533.I1RT98	pp	229533.Q4IF76
229533.I1RT98	pp	229533.Q4HVW2
229533.I1RT98	pp	229533.I1RXR7
229533.I1RT98	pp	229533.I1RU69
229533.I1RT98	pp	229533.I1RZZ1
229533.I1RT98	pp	229533.I1S9U2
229533.I1RT98	pp	229533.I1RZK6
229533.I1RT98	pp	229533.I1RWH5
229533.I1RT98	pp	229533.I1RZV6
229533.I1RT98	pp	229533.Q4HZ68
229533.I1RT98	pp	229533.I1S2E8
229533.I1RT98	pp	229533.Q4HZ42
229533.I1RT98	pp	229533.I1RVZ0
229533.I1RT98	pp	229533.I1S0J1
229533.I1RT98	pp	229533.Q4I830
229533.I1RT98	pp	229533.Q4HZ47
229533.I1RT98	pp	229533.Q4I5Z5
229533.I1RT98	pp	229533.I1RUQ9
229533.I1RT98	pp	229533.I1RW26
229533.I1RT98	pp	229533.I1RZF6
229533.I1RT98	pp	229533.I1RWE1
229533.I1RT98	pp	229533.I1S6F0
229533.I1RT98	pp	229533.I1RZ59
229533.I1RT98	pp	229533.Q4HZK7
229533.I1RT98	pp	229533.I1S0R7
229533.I1RT98	pp	229533.I1S2C0
229533.I1RT98	pp	229533.I1RTE9
229533.I1RT98	pp	229533.I1S4T3
229533.I1RT98	pp	229533.Q4IEK8
229533.I1RT98	pp	229533.I1RZJ6
229533.I1RT98	pp	229533.I1RTI6
229533.I1RT98	pp	229533.I1S9Y0
229533.I1RT98	pp	229533.Q4IAS1
229533.I1RT98	pp	229533.V6RMW6
229533.I1RT98	pp	229533.V6RGY3
229533.I1RT98	pp	229533.I1RTD9
229533.I1RT98	pp	229533.Q4I327
229533.I1RT98	pp	229533.I1RX46
229533.I1RT98	pp	229533.I1S558
229533.I1RT98	pp	229533.Q4IJ56
229533.I1RT98	pp	229533.Q4IPI1
229533.I1RT98	pp	229533.I1RY63
229533.I1RT98	pp	229533.I1S0M9
229533.I1RT98	pp	229533.I1S021
229533.I1RT98	pp	229533.I1RWB5
229533.I1RT98	pp	229533.Q4HWE2
229533.I1RT98	pp	229533.I1RYB9
229533.I1RT98	pp	229533.V6RGN5
229533.I1RT98	pp	229533.Q4IAA0
229533.I1RT98	pp	229533.I1S060
229533.I1RT98	pp	229533.I1S044
229533.I1RT98	pp	229533.Q4I8X0
229533.I1RT98	pp	229533.I1RX14
229533.I1RT98	pp	229533.Q4IBS2
229533.I1RJI9	pp	229533.I1S2L9

92565	229533.I1RJI9	pp	229533.I1S6J8
92566	229533.I1RJI9	pp	229533.I1SA62
92567	229533.I1RJI9	pp	229533.I1S3C0
92568	229533.I1RJI9	pp	229533.I1RU13
92569	229533.I1RJI9	pp	229533.I1RVX2
92570	229533.I1R9F8	pp	229533.I1RPT0
92571	229533.I1R9F8	pp	229533.I1RDT7
92572	229533.I1R9F8	pp	229533.I1S2Q5
92573	229533.I1R9F8	pp	229533.I1RGY2
92574	229533.I1R9F8	pp	229533.I1S308
92575	229533.I1R9F8	pp	229533.I1RPI4
92576	229533.I1R9F8	pp	229533.I1REH0
92577	229533.I1R9F8	pp	229533.I1RNU7
92578	229533.I1R9F8	pp	229533.I1S2N3
92579	229533.I1R9F8	pp	229533.I1RPU8
92580	229533.I1R9F8	pp	229533.I1RXM0
92581	229533.I1R9F8	pp	229533.I1RHL0
92582	229533.I1R9F8	pp	229533.I1RDW1
92583	229533.I1S0E3	pp	229533.I1S0Z5
92584	229533.I1S841	pp	229533.I1S842
92585	229533.A0A098DDZ6	pp	229533.I1R9P9
92586	229533.A0A098DDZ6	pp	229533.I1RU13
92587	229533.A0A098DDZ6	pp	229533.I1S2L9
92588	229533.A0A098DDZ6	pp	229533.I1S6J8
92589	229533.A0A1C3YIY5	pp	229533.I1RNG4
92590	229533.A0A1C3YJ49	pp	229533.I1RVX2
92591	229533.A0A1C3YJ49	pp	229533.I1S3C0
92592	229533.A0A1C3YJ49	pp	229533.I1S6J8
92593	229533.A0A1C3YJ49	pp	229533.I1SA62
92594	229533.A0A1C3YJ49	pp	229533.I1S2L9
92595	229533.A0A1C3YJ49	pp	229533.I1RU13
92596	229533.I1RAZ9	pp	229533.I1RR74
92597	229533.I1RAZ9	pp	229533.I1RCF0
92598	229533.I1RAZ9	pp	229533.Q4ICM0
92599	229533.I1RAZ9	pp	229533.I1S0Z3
92600	229533.I1RAZ9	pp	229533.I1S247
92601	229533.I1RAZ9	pp	229533.I1RMG2
92602	229533.I1RAZ9	pp	229533.I1RKI1
92603	229533.I1RAZ9	pp	229533.I1S7W2
92604	229533.I1RAZ9	pp	229533.I1RFK0
92605	229533.I1RAZ9	pp	229533.Q4INI2
92606	229533.I1RAZ9	pp	229533.I1RT41
92607	229533.I1RAZ9	pp	229533.I1S0S8
92608	229533.I1RAZ9	pp	229533.I1S270
92609	229533.A0A1C3YIF2	pp	229533.I1RU13
92610	229533.A0A1C3YIF2	pp	229533.I1S2L9
92611	229533.A0A1C3YIF2	pp	229533.I1SA62
92612	229533.A0A1C3YIF2	pp	229533.I1RVX2
92613	229533.A0A1C3YIF2	pp	229533.I1S6J8
92614	229533.A0A1C3YIF2	pp	229533.I1S3C0
92615	229533.I1S1F7	pp	229533.I1S3C0
92616	229533.I1S1F7	pp	229533.I1S2L9
92617	229533.I1S1F7	pp	229533.I1S6J8
92618	229533.I1S1F7	pp	229533.I1SA62
92619	229533.A0A098D250	pp	229533.I1REP6
92620	229533.A0A098D250	pp	229533.A0A098DD65
92621	229533.A0A098D250	pp	229533.I1SAJ3
92622	229533.A0A098D250	pp	229533.A0A1C3YIH6
92623	229533.A0A098D250	pp	229533.I1RV76
92624	229533.A0A098D250	pp	229533.A0A098DHZ1
92625	229533.A0A098D250	pp	229533.A0A098DY45
92626	229533.A0A098D250	pp	229533.I1RV34
92627	229533.A0A098D250	pp	229533.I1RIM9
92628	229533.A0A098D250	pp	229533.A0A098DHF1
92629	229533.A0A098D250	pp	229533.I1RJI9
92630	229533.A0A098D250	pp	229533.I1S6Z3
92631	229533.A0A098D250	pp	229533.A0A098DDZ6
92632	229533.A0A098D250	pp	229533.A0A1C3YJ49
92633	229533.A0A098D250	pp	229533.A0A1C3YIF2
92634	229533.A0A098D250	pp	229533.I1S1F7
92635	229533.I1RTP5	pp	229533.I1RYQ4
92636	229533.I1RIS0	pp	229533.I1S0E3
92637	229533.I1RIS0	pp	229533.I1RW37

92638	229533.I1RIS0	pp	229533.I1RR07
92639	229533.I1RHL0	pp	229533.I1RPT0
92640	229533.I1RHL0	pp	229533.I1S2N3
92641	229533.I1RHL0	pp	229533.I1S308
92642	229533.I1RHL0	pp	229533.I1RXM0
92643	229533.I1RHL0	pp	229533.I1RPI4
92644	229533.I1RL09	pp	229533.I1S0E3
92645	229533.I1RL09	pp	229533.I1RW37
92646	229533.I1RL09	pp	229533.I1RR07
92647	229533.I1REU9	pp	229533.V6RJP8
92648	229533.I1REU9	pp	229533.I1RTP5
92649	229533.I1REU9	pp	229533.I1RTJ2
92650	229533.I1REU9	pp	229533.I1RS05
92651	229533.I1REU9	pp	229533.I1RS29
92652	229533.I1REU9	pp	229533.I1RH75
92653	229533.I1REU9	pp	229533.I1RGQ2
92654	229533.I1REU9	pp	229533.I1S5X9
92655	229533.I1REU9	pp	229533.I1RYT8
92656	229533.I1REU9	pp	229533.I1RXD9
92657	229533.I1REU9	pp	229533.I1RYQ4
92658	229533.A0A1C3YM23	pp	229533.I1RWH5
92659	229533.A0A1C3YM23	pp	229533.A0A1C3YMB5
92660	229533.A0A1C3YM23	pp	229533.I1RWB5
92661	229533.A0A1C3YM23	pp	229533.I1RN28
92662	229533.A0A1C3YM23	pp	229533.I1RFZ8
92663	229533.A0A1C3YM23	pp	229533.I1S044
92664	229533.A0A1C3YM23	pp	229533.I1S0N7
92665	229533.A0A1C3YM23	pp	229533.I1RGC2
92666	229533.A0A1C3YM23	pp	229533.I1RBS3
92667	229533.A0A1C3YM23	pp	229533.I1RE45
92668	229533.A0A1C3YM23	pp	229533.A0A1C3YMK1
92669	229533.A0A1C3YM23	pp	229533.I1S7S9
92670	229533.A0A1C3YM23	pp	229533.Q4HWE2
92671	229533.A0A1C3YM23	pp	229533.I1RCS0
92672	229533.A0A1C3YM23	pp	229533.I1RDG0
92673	229533.A0A1C3YM23	pp	229533.I1RK70
92674	229533.A0A1C3YM23	pp	229533.I1RA18
92675	229533.A0A1C3YM23	pp	229533.I1RCU3
92676	229533.A0A1C3YM23	pp	229533.I1RX64
92677	229533.A0A1C3YM23	pp	229533.V6RNM4
92678	229533.A0A098DL83	pp	229533.A0A1C3YJG7
92679	229533.A0A098DL83	pp	229533.A0A0E0RR40
92680	229533.A0A098DL83	pp	229533.I1REC6
92681	229533.A0A098DL83	pp	229533.I1RTG5
92682	229533.A0A098DL83	pp	229533.A0A098DRS5
92683	229533.A0A098DL83	pp	229533.I1REC7
92684	229533.A0A098D234	pp	229533.I1RDG0
92685	229533.A0A098D234	pp	229533.A0A1C3YM23
92686	229533.I1S160	pp	229533.I1S163
92687	229533.I1RZW7	pp	229533.I1S287
92688	229533.I1RZW7	pp	229533.Q4I5R9
92689	229533.A0A098DC95	pp	229533.A0A1C3YMT3
92690	229533.A0A098DC95	pp	229533.I1RSY7
92691	229533.A0A098DC95	pp	229533.I1RVH7
92692	229533.A0A098DC95	pp	229533.I1RLY2
92693	229533.A0A098DC95	pp	229533.I1RT54
92694	229533.A0A098DC95	pp	229533.I1RF73
92695	229533.A0A098DC95	pp	229533.I1RI39
92696	229533.A0A098DC95	pp	229533.I1S4A4
92697	229533.A0A098DC95	pp	229533.I1RR89
92698	229533.A0A098DC95	pp	229533.I1RIJ1
92699	229533.A0A098DC95	pp	229533.I1RH94
92700	229533.A0A098DC95	pp	229533.I1R9L9
92701	229533.A0A098DC95	pp	229533.I1S320
92702	229533.A0A098DC95	pp	229533.I1RYJ1
92703	229533.A0A098DC95	pp	229533.I1RJ55
92704	229533.A0A098DC95	pp	229533.A0A1C3YMT0
92705	229533.A0A098DC95	pp	229533.I1S407
92706	229533.A0A098DC95	pp	229533.V6R5G9
92707	229533.A0A098DC95	pp	229533.I1RLU4
92708	229533.A0A098DC95	pp	229533.I1S6W3
92709	229533.A0A098DC95	pp	229533.I1RWE3
92710	229533.A0A098DC95	pp	229533.I1RDC7

92711 229533.A0A098DC95 pp 229533.I1RIZ7
92712 229533.A0A098DC95 pp 229533.I1RVW8
92713 229533.A0A098DC95 pp 229533.I1RHM8
92714 229533.A0A098DC95 pp 229533.I1RXT4
92715 229533.A0A098DC95 pp 229533.A0A098DJ33
92716 229533.A0A098DC95 pp 229533.I1RB60
92717 229533.A0A098DC95 pp 229533.I1RAN9
92718 229533.A0A098DC95 pp 229533.I1RFY7
92719 229533.A0A098DC95 pp 229533.I1RWF6
92720 229533.A0A098DC95 pp 229533.I1RYC1
92721 229533.A0A098DC95 pp 229533.A0A098DIZ0
92722 229533.A0A098DC95 pp 229533.A0A1C3YKM3
92723 229533.A0A098DC95 pp 229533.I1RMU2
92724 229533.A0A0E0RXA9 pp 229533.I1RPT0
92725 229533.A0A0E0RXA9 pp 229533.A0A1C3YM97
92726 229533.A0A0E0RXA9 pp 229533.I0LNZ1
92727 229533.A0A0E0RXA9 pp 229533.V6RY56
92728 229533.A0A0E0RXA9 pp 229533.I1S4D0
92729 229533.A0A0E0RXA9 pp 229533.I1S163
92730 229533.A0A0E0RXA9 pp 229533.E6PBP1
92731 229533.A0A0E0RXA9 pp 229533.I1RDW1
92732 229533.A0A0E0RXA9 pp 229533.I1RJQ5
92733 229533.A0A0E0RXA9 pp 229533.I1R9F8
92734 229533.A0A0E0RXA9 pp 229533.V6RAN2
92735 229533.A0A0E0RXA9 pp 229533.I1RDT7
92736 229533.A0A0E0RXA9 pp 229533.I1R9D3
92737 229533.A0A0E0RXA9 pp 229533.A0A1C3YHP2
92738 229533.A0A0E0RXA9 pp 229533.I1RHQ0
92739 229533.A0A0E0RXA9 pp 229533.I1RXM0
92740 229533.A0A0E0RXA9 pp 229533.I1RZ78
92741 229533.A0A0E0RXA9 pp 229533.I1S2P8
92742 229533.A0A0E0RXA9 pp 229533.I1REH0
92743 229533.A0A0E0RXA9 pp 229533.I1S2N3
92744 229533.A0A0E0RXA9 pp 229533.I1RHL0
92745 229533.A0A0E0RXA9 pp 229533.I1S308
92746 229533.A0A0E0RXA9 pp 229533.I1S160
92747 229533.A0A0E0RXA9 pp 229533.I1RGY2
92748 229533.I1RYU0 pp 229533.I1S5R6
92749 229533.I1RYU0 pp 229533.I1S3V2
92750 229533.I1RYU0 pp 229533.I1S1W8
92751 229533.I1RYU0 pp 229533.V6QT89
92752 229533.I1RYU0 pp 229533.I1S3G4
92753 229533.I1RYU0 pp 229533.V6RLM7
92754 229533.I1RYU0 pp 229533.V6R7V8
92755 229533.I1RYU0 pp 229533.I1S2A1
92756 229533.I1RYU0 pp 229533.I1RZ21
92757 229533.I1RYU0 pp 229533.Q4HTS9
92758 229533.I1RYU0 pp 229533.I1S8F8
92759 229533.A0A1C3YMT0 pp 229533.I1RFY7
92760 229533.A0A1C3YMT0 pp 229533.I1RB60
92761 229533.A0A1C3YMT0 pp 229533.I1RWF6
92762 229533.A0A1C3YMT0 pp 229533.I1RHM8
92763 229533.A0A1C3YMT0 pp 229533.I1RVW8
92764 229533.A0A1C3YMT0 pp 229533.V6RUY9
92765 229533.A0A1C3YMT0 pp 229533.I1RIJ1
92766 229533.A0A1C3YMT0 pp 229533.I1RYJ1
92767 229533.A0A1C3YMT0 pp 229533.I1R9L9
92768 229533.A0A1C3YMT0 pp 229533.I1S320
92769 229533.A0A1C3YMT0 pp 229533.I1RR89
92770 229533.A0A1C3YMT0 pp 229533.I1S4A4
92771 229533.A0A1C3YMT0 pp 229533.I1RH94
92772 229533.A0A1C3YMT0 pp 229533.I1RMU2
92773 229533.A0A1C3YMT0 pp 229533.I1RF73
92774 229533.A0A1C3YMT0 pp 229533.I1RI39
92775 229533.A0A1C3YMT0 pp 229533.I1RT54
92776 229533.A0A1C3YMT0 pp 229533.I1RN16
92777 229533.A0A1C3YMT0 pp 229533.I1RVH7
92778 229533.A0A1C3YMT0 pp 229533.I1RYC1
92779 229533.A0A1C3YMT0 pp 229533.I1RAN9
92780 229533.A0A1C3YMT0 pp 229533.I1RLY2
92781 229533.A0A1C3YMT0 pp 229533.A0A1C3YMT3
92782 229533.A0A1C3YMT0 pp 229533.I1RSY7
92783 229533.A0A1C3YMT0 pp 229533.V6R5G9

92784	229533.A0A1C3YMT0	pp	229533.I1RXT4
92785	229533.A0A1C3YMT0	pp	229533.I1RIZ7
92786	229533.A0A1C3YMT0	pp	229533.I1RDC7
92787	229533.A0A1C3YMT0	pp	229533.I1RLU4
92788	229533.A0A1C3YMT0	pp	229533.I1S407
92789	229533.A0A1C3YMT0	pp	229533.I1RJ55
92790	229533.A0A1C3YMT0	pp	229533.I1S6W3
92791	229533.A0A1C3YMT0	pp	229533.I1RWE3
92792	229533.I1RX55	pp	229533.I1S0Y1
92793	229533.I1S407	pp	229533.I1S4A4
92794	229533.I1S407	pp	229533.V6R5G9
92795	229533.I1S407	pp	229533.I1S6W3
92796	229533.I1RD82	pp	229533.I1S9X9
92797	229533.I1RD82	pp	229533.I1S0P7
92798	229533.I1RD82	pp	229533.I1RGD4
92799	229533.I1RD82	pp	229533.I1RNG8
92800	229533.I1RD82	pp	229533.I1RKA1
92801	229533.I1RD82	pp	229533.I1RNG4
92802	229533.I1RA90	pp	229533.I1RY12
92803	229533.I1RA90	pp	229533.I1RXV9
92804	229533.I1RA90	pp	229533.I1RFM6
92805	229533.I1RA90	pp	229533.I1RS18
92806	229533.I1RA90	pp	229533.I1RT09
92807	229533.I1RA90	pp	229533.I1RW69
92808	229533.I1RA90	pp	229533.I1RXS0
92809	229533.I1RA90	pp	229533.I1RUI6
92810	229533.I1RA90	pp	229533.V6RK96
92811	229533.I1RA90	pp	229533.I1RVY2
92812	229533.I1RA90	pp	229533.I1RHZ5
92813	229533.I1RA90	pp	229533.I1RRV4
92814	229533.I1RA90	pp	229533.I1RQE0
92815	229533.I1RA90	pp	229533.I1RJT5
92816	229533.I1RA90	pp	229533.I1RBE7
92817	229533.I1RA90	pp	229533.I1RYI7
92818	229533.I1RA90	pp	229533.I1S0A0
92819	229533.I1RA90	pp	229533.I1RCL0
92820	229533.I1RA90	pp	229533.I1RJY7
92821	229533.I1RA90	pp	229533.I1RK15
92822	229533.I1RA90	pp	229533.I1S1W8
92823	229533.I1RA90	pp	229533.I1RBC1
92824	229533.I1RA90	pp	229533.I1RUY6
92825	229533.I1RA90	pp	229533.I1RQP2
92826	229533.I1RA90	pp	229533.I1RMG4
92827	229533.I1RA90	pp	229533.I1RC48
92828	229533.I1RA90	pp	229533.I1RMY1
92829	229533.I1RA90	pp	229533.I1S0N3
92830	229533.I1RA90	pp	229533.I1RNM7
92831	229533.I1RA90	pp	229533.I1RHK2
92832	229533.I1RA90	pp	229533.I1RBE4
92833	229533.I1RA90	pp	229533.I1RVP5
92834	229533.I1RA90	pp	229533.I1RAQ4
92835	229533.I1RA90	pp	229533.I1REV6
92836	229533.I1RA90	pp	229533.I1RY49
92837	229533.I1RA90	pp	229533.I1S8E0
92838	229533.I1RA90	pp	229533.I1RRG1
92839	229533.I1RA90	pp	229533.I1RC61
92840	229533.I1RA90	pp	229533.I1S835
92841	229533.I1RA90	pp	229533.I1RA94
92842	229533.I1RA90	pp	229533.I1S3L2
92843	229533.I1RA90	pp	229533.I1RP86
92844	229533.I1RA90	pp	229533.I1RPW2
92845	229533.I1RA90	pp	229533.I1RQE8
92846	229533.I1RA90	pp	229533.Q4IR09
92847	229533.I1RA90	pp	229533.I1RMM8
92848	229533.I1RA90	pp	229533.I1RMK4
92849	229533.I1RA90	pp	229533.V6RTJ8
92850	229533.I1RA90	pp	229533.I1RYJ7
92851	229533.I1RA90	pp	229533.I1RAJ6
92852	229533.I1RA90	pp	229533.V6RSF3
92853	229533.I1RA90	pp	229533.I1S4S4
92854	229533.I1RA90	pp	229533.I1RDE6
92855	229533.I1RA90	pp	229533.I1S0K0
92856	229533.I1RA90	pp	229533.I1RFB6

92857	229533.I1RA90	pp	229533.I1S0C2
92858	229533.I1RA90	pp	229533.I1RIH0
92859	229533.I1RA90	pp	229533.I1RY21
92860	229533.I1RA90	pp	229533.I1RCG5
92861	229533.I1RA90	pp	229533.V6RM02
92862	229533.I1RA90	pp	229533.Q4I5V2
92863	229533.I1RA90	pp	229533.I1RRX5
92864	229533.I1RA90	pp	229533.V6R2A1
92865	229533.I1RA90	pp	229533.I1RZM4
92866	229533.I1RA90	pp	229533.V6R382
92867	229533.I1RA90	pp	229533.V6RLM7
92868	229533.I1RA90	pp	229533.V6QVH8
92869	229533.I1RA90	pp	229533.I1RXB1
92870	229533.I1RA90	pp	229533.I1S241
92871	229533.I1RA90	pp	229533.I1RY74
92872	229533.I1RA90	pp	229533.I1RPK0
92873	229533.I1RA90	pp	229533.I1RVT8
92874	229533.I1RA90	pp	229533.I1RFV2
92875	229533.I1RA90	pp	229533.I1RSL5
92876	229533.I1RA90	pp	229533.I1RAN1
92877	229533.I1RA90	pp	229533.I1S8C1
92878	229533.I1RA90	pp	229533.I1RHG5
92879	229533.I1RA90	pp	229533.I1S0Y7
92880	229533.I1RA90	pp	229533.V6R5D2
92881	229533.I1RA90	pp	229533.V6RG79
92882	229533.I1RA90	pp	229533.I1RXY7
92883	229533.I1RA90	pp	229533.I1RAY9
92884	229533.I1RA90	pp	229533.I1RPU8
92885	229533.I1RA90	pp	229533.I1S673
92886	229533.I1RA90	pp	229533.I1RUR8
92887	229533.I1RA90	pp	229533.I1RW43
92888	229533.I1RA90	pp	229533.I1RN84
92889	229533.I1RA90	pp	229533.I1S8R4
92890	229533.I1RA90	pp	229533.I1RZ83
92891	229533.I1RA90	pp	229533.I1RSK5
92892	229533.I1RA90	pp	229533.I1RNG4
92893	229533.I1RA90	pp	229533.I1RE53
92894	229533.I1RA90	pp	229533.V6RN69
92895	229533.I1RA90	pp	229533.Q4I174
92896	229533.I1RA90	pp	229533.I1RVN1
92897	229533.I1RA90	pp	229533.Q4HXF0
92898	229533.I1RA90	pp	229533.I1RSQ4
92899	229533.I1RA90	pp	229533.I1S2H4
92900	229533.I1RA90	pp	229533.I1RP97
92901	229533.I1RA90	pp	229533.I1S3G4
92902	229533.I1RA90	pp	229533.Q4HZ34
92903	229533.I1RA90	pp	229533.I1RR68
92904	229533.I1RA90	pp	229533.I1RYK0
92905	229533.I1RA90	pp	229533.I1RVR2
92906	229533.I1RA90	pp	229533.I1RBF3
92907	229533.I1RA90	pp	229533.V6RIE5
92908	229533.I1RA90	pp	229533.I1RTI7
92909	229533.I1RA90	pp	229533.I1RZN1
92910	229533.I1RA90	pp	229533.I1RWF9
92911	229533.I1RA90	pp	229533.I1RVZ2
92912	229533.I1RA90	pp	229533.I1RMJ3
92913	229533.I1RA90	pp	229533.I1RSS7
92914	229533.I1RA90	pp	229533.I1RXW3
92915	229533.I1RJ55	pp	229533.I1RR89
92916	229533.I1RJ55	pp	229533.I1S4A4
92917	229533.I1RJ55	pp	229533.I1RT54
92918	229533.I1RJ55	pp	229533.I1RLY2
92919	229533.I1RJ55	pp	229533.I1RSY7
92920	229533.I1RJ55	pp	229533.I1RWF6
92921	229533.I1RJ55	pp	229533.I1RVH7
92922	229533.I1RJ55	pp	229533.I1RMU2
92923	229533.I1RJ55	pp	229533.I1RVW8
92924	229533.I1RJ55	pp	229533.I1RYC1
92925	229533.I1RJ55	pp	229533.I1S6W3
92926	229533.I1RJ55	pp	229533.I1S320
92927	229533.I1RJ55	pp	229533.V6R5G9
92928	229533.I1RJ55	pp	229533.I1RLU4
92929	229533.I1RJ55	pp	229533.I1RWE3

92930 229533.I1RJ55 pp 229533.I1S407
92931 229533.I1RJ55 pp 229533.I1RYJ1
92932 229533.I1RJ55 pp 229533.I1RXT4
92933 229533.A0A0E0RX98 pp 229533.I1RN04
92934 229533.A0A0E0RX98 pp 229533.I1RRF7
92935 229533.A0A0E0RX98 pp 229533.I1RCG6
92936 229533.A0A0E0RX98 pp 229533.I1RW91
92937 229533.A0A0E0RX98 pp 229533.I1RPW8
92938 229533.I1RFY7 pp 229533.I1RHM8
92939 229533.I1RFY7 pp 229533.I1RWF6
92940 229533.I1RFY7 pp 229533.I1RVW8
92941 229533.I1RFY7 pp 229533.I1S6W3
92942 229533.I1RFY7 pp 229533.I1RWE3
92943 229533.I1RFY7 pp 229533.I1RXT4
92944 229533.I1RFY7 pp 229533.I1RLU4
92945 229533.I1RFY7 pp 229533.I1RIZ7
92946 229533.I1RFY7 pp 229533.I1RYC1
92947 229533.I1RFY7 pp 229533.I1RVH7
92948 229533.I1RFY7 pp 229533.I1RSY7
92949 229533.I1RFY7 pp 229533.I1RLY2
92950 229533.I1RFY7 pp 229533.I1RMU2
92951 229533.I1RFY7 pp 229533.I1RT54
92952 229533.I1RFY7 pp 229533.I1RH94
92953 229533.I1RFY7 pp 229533.I1S320
92954 229533.I1RFY7 pp 229533.I1RYJ1
92955 229533.I1RFY7 pp 229533.I1RJ55
92956 229533.I1RFY7 pp 229533.I1RI39
92957 229533.I1RFY7 pp 229533.I1S407
92958 229533.I1RFY7 pp 229533.I1S4A4
92959 229533.I1RFY7 pp 229533.I1RIJ1
92960 229533.I1RFY7 pp 229533.I1RR89
92961 229533.I1RFY7 pp 229533.V6R5G9
92962 229533.I1RVY2 pp 229533.I1RYI7
92963 229533.I1RVY2 pp 229533.I1RZM4
92964 229533.I1RVY2 pp 229533.V6RQW4
92965 229533.I1RVY2 pp 229533.V6RK96
92966 229533.I1RVY2 pp 229533.I1RXA9
92967 229533.I1RVY2 pp 229533.I1RW69
92968 229533.I1RVY2 pp 229533.I1RYX6
92969 229533.I1RVY2 pp 229533.Q4HXF0
92970 229533.I1RVY2 pp 229533.I1RXB1
92971 229533.I1RVY2 pp 229533.I1RZN1
92972 229533.I1RVY2 pp 229533.I1S0N3
92973 229533.I1RVY2 pp 229533.I1RXW3
92974 229533.I1RVY2 pp 229533.I1RYJ7
92975 229533.I1RVY2 pp 229533.I1RXV9
92976 229533.I1RVY2 pp 229533.I1RZ29
92977 229533.I1RVY2 pp 229533.O74638
92978 229533.I1RVY2 pp 229533.V6RM02
92979 229533.I1RVY2 pp 229533.V6QVH8
92980 229533.I1RVY2 pp 229533.V6RSF3
92981 229533.I1RVY2 pp 229533.I1RXB2
92982 229533.I1RXT3 pp 229533.I1RZM8
92983 229533.I1RXT3 pp 229533.I1S0L4
92984 229533.I1RXT3 pp 229533.I1RZZ9
92985 229533.I1RXT3 pp 229533.V6RIV9
92986 229533.I1RXT3 pp 229533.V6RTJ8
92987 229533.I1RXT3 pp 229533.I1RZM1
92988 229533.I1RXT3 pp 229533.V6R042
92989 229533.I1RXT3 pp 229533.I1S2B8
92990 229533.I1RXT3 pp 229533.V6RMW6
92991 229533.I1RXT3 pp 229533.I1RZW8
92992 229533.I1RXT3 pp 229533.Q4IJK2
92993 229533.I1RXT3 pp 229533.V6QW22
92994 229533.I1RXT3 pp 229533.I1S0L3
92995 229533.I1RB60 pp 229533.I1RIJ1
92996 229533.I1RB60 pp 229533.I1RHM8
92997 229533.I1RB60 pp 229533.I1RVW8
92998 229533.I1RB60 pp 229533.I1S4A4
92999 229533.I1RB60 pp 229533.I1RI39
93000 229533.I1RB60 pp 229533.I1RR89
93001 229533.I1RB60 pp 229533.I1RT54
93002 229533.I1RB60 pp 229533.I1RF73

93003 229533.I1RB60 pp 229533.I1RSY7
93004 229533.I1RB60 pp 229533.I1RLY2
93005 229533.I1RB60 pp 229533.I1RVH7
93006 229533.I1RB60 pp 229533.I1S407
93007 229533.I1RB60 pp 229533.I1RJ55
93008 229533.I1RB60 pp 229533.V6R5G9
93009 229533.I1RB60 pp 229533.I1S320
93010 229533.I1RB60 pp 229533.I1RYJ1
93011 229533.I1RB60 pp 229533.I1RYC1
93012 229533.I1RB60 pp 229533.I1RH94
93013 229533.I1RB60 pp 229533.I1RMU2
93014 229533.I1RB60 pp 229533.I1RIZ7
93015 229533.I1RB60 pp 229533.I1RDC7
93016 229533.I1RB60 pp 229533.I1RXT4
93017 229533.I1RB60 pp 229533.I1RWE3
93018 229533.I1RB60 pp 229533.I1RWF6
93019 229533.I1RB60 pp 229533.I1RLU4
93020 229533.I1RB60 pp 229533.I1RFY7
93021 229533.I1RB60 pp 229533.I1S6W3
93022 229533.I1RAD4 pp 229533.I1RRS8
93023 229533.I1RAD4 pp 229533.I1S2C1
93024 229533.I1RAD4 pp 229533.Q4I4A4
93025 229533.I1RAD4 pp 229533.I1RTF0
93026 229533.I1RAD4 pp 229533.I1RJR8
93027 229533.I1RAD4 pp 229533.V6RM66
93028 229533.I1RAD4 pp 229533.Q4IBU4
93029 229533.I1RAD4 pp 229533.I1RFS5
93030 229533.I1RAD4 pp 229533.I1S8A6
93031 229533.I1RAD4 pp 229533.I1RPX9
93032 229533.I1RAD4 pp 229533.I1RC18
93033 229533.I1RAD4 pp 229533.I1RZH5
93034 229533.I1RAD4 pp 229533.Q4I2W2
93035 229533.I1RAD4 pp 229533.I1RNJ5
93036 229533.I1RAD4 pp 229533.I1S8F0
93037 229533.I1RAD4 pp 229533.Q4I6B0
93038 229533.I1RAD4 pp 229533.I1S0R9
93039 229533.I1RAD4 pp 229533.Q4I099
93040 229533.I1RAD4 pp 229533.I1RQ82
93041 229533.I1RAD4 pp 229533.Q4HZK7
93042 229533.I1RAD4 pp 229533.I1RPX5
93043 229533.I1RAD4 pp 229533.I1RJT1
93044 229533.I1RAD4 pp 229533.I1RY57
93045 229533.I1RB86 pp 229533.I1RPP5
93046 229533.I1RB86 pp 229533.I1RZG7
93047 229533.I1RB86 pp 229533.I1S5X9
93048 229533.I1RB86 pp 229533.I1S0K2
93049 229533.I1RB86 pp 229533.Q4I099
93050 229533.I1RB86 pp 229533.I1RPL3
93051 229533.I1RB86 pp 229533.Q4I6B0
93052 229533.I1RB86 pp 229533.I1RKF5
93053 229533.I1RB86 pp 229533.I1RPY0
93054 229533.I1RB86 pp 229533.I1RWA3
93055 229533.I1RB86 pp 229533.Q4HZ95
93056 229533.I1RB86 pp 229533.I1RMQ6
93057 229533.I1RB86 pp 229533.I1RYT8
93058 229533.I1RB86 pp 229533.I1S9E7
93059 229533.I1RB86 pp 229533.I1RQY8
93060 229533.I1RB86 pp 229533.I1RY21
93061 229533.I1RB86 pp 229533.Q4I1Y1
93062 229533.I1RB86 pp 229533.Q4IPZ1
93063 229533.I1RB86 pp 229533.I1RST1
93064 229533.I1RB86 pp 229533.I1RCI4
93065 229533.I1RB86 pp 229533.I1RFJ6
93066 229533.I1RB86 pp 229533.V6RJP8
93067 229533.I1RB86 pp 229533.I1RBT6
93068 229533.I1RB86 pp 229533.I1RCS2
93069 229533.I1RB86 pp 229533.I1RNJ5
93070 229533.I1RB86 pp 229533.I1RRL0
93071 229533.I1RB86 pp 229533.I1RN32
93072 229533.I1RB86 pp 229533.Q4IJW4
93073 229533.I1RB86 pp 229533.I1RC86
93074 229533.I1RB86 pp 229533.I1S464
93075 229533.I1RB86 pp 229533.I1RT38

93076 229533.I1RB86 pp 229533.I1RXD9
93077 229533.I1RB86 pp 229533.Q4IB65
93078 229533.I1RB86 pp 229533.Q4I7T5
93079 229533.I1RB86 pp 229533.I1RX47
93080 229533.I1RB86 pp 229533.I1RX98
93081 229533.I1RB86 pp 229533.I1RKF4
93082 229533.I1RB86 pp 229533.I1RQR6
93083 229533.I1RB86 pp 229533.I1S0Q7
93084 229533.I1RB86 pp 229533.I1RBE6
93085 229533.I1RB86 pp 229533.I1RPZ3
93086 229533.A0A098DUG3 pp 229533.I1RYU0
93087 229533.A0A098DUG3 pp 229533.A0A1C3YKN4
93088 229533.A0A098DUG3 pp 229533.I1RNG4
93089 229533.A0A098DUG3 pp 229533.I1RXV3
93090 229533.A0A098DUG3 pp 229533.A0A1C3YKT2
93091 229533.A0A098DUG3 pp 229533.A0A1C3YJ36
93092 229533.A0A098DUG3 pp 229533.I1RVZ3
93093 229533.A0A098DUG3 pp 229533.I1S2C4
93094 229533.A0A098DUG3 pp 229533.A0A0E0SH88
93095 229533.I1R9L9 pp 229533.I1RFY7
93096 229533.I1R9L9 pp 229533.I1RHM8
93097 229533.I1R9L9 pp 229533.I1RWF6
93098 229533.I1R9L9 pp 229533.I1RVW8
93099 229533.I1R9L9 pp 229533.I1RR89
93100 229533.I1R9L9 pp 229533.I1RF73
93101 229533.I1R9L9 pp 229533.I1RIJ1
93102 229533.I1R9L9 pp 229533.I1RI39
93103 229533.I1R9L9 pp 229533.I1S4A4
93104 229533.I1R9L9 pp 229533.I1RVH7
93105 229533.I1R9L9 pp 229533.I1RLY2
93106 229533.I1R9L9 pp 229533.I1RT54
93107 229533.I1R9L9 pp 229533.I1RSY7
93108 229533.I1R9L9 pp 229533.I1RJ55
93109 229533.I1R9L9 pp 229533.V6R5G9
93110 229533.I1R9L9 pp 229533.I1S407
93111 229533.I1R9L9 pp 229533.I1RYJ1
93112 229533.I1R9L9 pp 229533.I1S320
93113 229533.I1R9L9 pp 229533.I1RH94
93114 229533.I1R9L9 pp 229533.I1RMU2
93115 229533.I1R9L9 pp 229533.I1RAN9
93116 229533.I1R9L9 pp 229533.I1RIZ7
93117 229533.I1R9L9 pp 229533.I1RYC1
93118 229533.I1R9L9 pp 229533.I1RXT4
93119 229533.I1R9L9 pp 229533.I1RLU4
93120 229533.I1R9L9 pp 229533.I1RWE3
93121 229533.I1R9L9 pp 229533.I1S6W3
93122 229533.I1R9L9 pp 229533.I1RDC7
93123 229533.I1R9L9 pp 229533.I1RB60
93124 229533.I1S320 pp 229533.V6R5G9
93125 229533.I1S320 pp 229533.I1S407
93126 229533.I1S320 pp 229533.I1S6W3
93127 229533.I1S320 pp 229533.I1S4A4
93128 229533.Q4I6B0 pp 229533.Q4IP51
93129 229533.Q4I6B0 pp 229533.Q4IJW4
93130 229533.Q4I6B0 pp 229533.Q4IB65
93131 229533.Q4I6B0 pp 229533.Q4IPZ1
93132 229533.Q4I6B0 pp 229533.Q4I7T5
93133 229533.Q4I6B0 pp 229533.Q4IK03
93134 229533.Q4I6B0 pp 229533.V6RJP8
93135 229533.I1RH94 pp 229533.I1RHM8
93136 229533.I1RH94 pp 229533.I1RXT4
93137 229533.I1RH94 pp 229533.I1RIZ7
93138 229533.I1RH94 pp 229533.I1RLU4
93139 229533.I1RH94 pp 229533.I1S6W3
93140 229533.I1RH94 pp 229533.I1RWE3
93141 229533.I1RH94 pp 229533.I1RVH7
93142 229533.I1RH94 pp 229533.I1RYC1
93143 229533.I1RH94 pp 229533.I1RMU2
93144 229533.I1RH94 pp 229533.I1RYJ1
93145 229533.I1RH94 pp 229533.I1RLY2
93146 229533.I1RH94 pp 229533.I1RSY7
93147 229533.I1RH94 pp 229533.I1RT54
93148 229533.I1RH94 pp 229533.I1S320

229533.I1RH94	pp	229533.V6R5G9
229533.I1RH94	pp	229533.I1RIJ1
229533.I1RH94	pp	229533.I1RR89
229533.I1RH94	pp	229533.I1RJ55
229533.I1RH94	pp	229533.I1RI39
229533.I1RH94	pp	229533.I1RVW8
229533.I1RH94	pp	229533.I1S407
229533.I1RH94	pp	229533.I1S4A4
229533.I1RH94	pp	229533.I1RWF6
229533.A0A098DUA4	pp	229533.I1RMP0
229533.A0A098DUA4	pp	229533.I1S549
229533.A0A098DUA4	pp	229533.I1RQL4
229533.A0A098DUA4	pp	229533.A0A1C3YJG7
229533.I1S4A4	pp	229533.I1S6W3
229533.I1S4A4	pp	229533.V6R5G9
229533.A0A1C3YI98	pp	229533.I1RYI5
229533.A0A1C3YI98	pp	229533.I1RWV9
229533.I1RDH5	pp	229533.I1RYE4
229533.I1RDH5	pp	229533.I1RW06
229533.I1RDH5	pp	229533.Q4I5R9
229533.I1RDH5	pp	229533.I1RPY0
229533.I1RDH5	pp	229533.I1RJV7
229533.I1RDH5	pp	229533.I1RVA7
229533.I1RDH5	pp	229533.I1S287
229533.I1RDH5	pp	229533.I1RQF6
229533.I1RXB1	pp	229533.V6RM02
229533.I1RXB1	pp	229533.Q4HXF0
229533.I1RXB1	pp	229533.I1RXW3
229533.I1RXB1	pp	229533.V6QVH8
229533.I1RXB1	pp	229533.I1RZM4
229533.I1RXB1	pp	229533.I1RYJ7
229533.I1RXB1	pp	229533.O74638
229533.I1RXB1	pp	229533.I1RYX6
229533.I1RXB1	pp	229533.I1RZN1
229533.I1RXB1	pp	229533.I1RXH3
229533.I1RXB1	pp	229533.V6RSF3
229533.I1RXB1	pp	229533.I1RYI7
229533.I1RXB1	pp	229533.I1RXB2
229533.I1RXB1	pp	229533.V6RRM0
229533.I1RXB1	pp	229533.I1RZ29
229533.I1RXB1	pp	229533.V6RQW4
229533.I1RXB1	pp	229533.I1RXV9
229533.I1RXB1	pp	229533.V6RK96
229533.I1RXB1	pp	229533.I1S0N3
229533.I1RUK0	pp	229533.I1RZD0
229533.I1RUK0	pp	229533.I1S0K0
229533.I1RUK0	pp	229533.I1S3L2
229533.I1RUK0	pp	229533.I1RY81
229533.I1RUK0	pp	229533.I1S8R4
229533.I1RUK0	pp	229533.I1RXS0
229533.I1RUK0	pp	229533.I1RWV9
229533.I1RSK9	pp	229533.Q4I6B0
229533.I1RSK9	pp	229533.I1S5X9
229533.I1RSK9	pp	229533.V6RJP8
229533.I1RAN9	pp	229533.I1RWE3
229533.I1RAN9	pp	229533.I1RYV3
229533.I1RAN9	pp	229533.I1S320
229533.I1RAN9	pp	229533.I1RLU4
229533.I1RAN9	pp	229533.I1S6W3
229533.I1RAN9	pp	229533.I1RH94
229533.I1RAN9	pp	229533.I1S407
229533.I1RAN9	pp	229533.I1RXT4
229533.I1RAN9	pp	229533.I1RJ55
229533.I1RAN9	pp	229533.I1RT54
229533.I1RAN9	pp	229533.I1RIZ7
229533.I1RAN9	pp	229533.I1S4Z5
229533.I1RAN9	pp	229533.I1RBJ0
229533.I1RAN9	pp	229533.Q4HTT2
229533.I1RAN9	pp	229533.Q4I7L0
229533.I1RAN9	pp	229533.I1RVH7
229533.I1RAN9	pp	229533.I1RDC7
229533.I1RAN9	pp	229533.I1RFY7
229533.I1RAN9	pp	229533.I1RDI6

93222 229533.I1RAN9 pp 229533.I1RB60
93223 229533.I1RAN9 pp 229533.I1RLY2
93224 229533.I1RAN9 pp 229533.I1RSY7
93225 229533.I1RAN9 pp 229533.I1S4A4
93226 229533.I1RAN9 pp 229533.I1RR89
93227 229533.I1RAN9 pp 229533.I1RIJ1
93228 229533.A0A1C3YJJ3 pp 229533.I1REF6
93229 229533.A0A1C3YJJ3 pp 229533.I1S1B1
93230 229533.A0A1C3YJJ3 pp 229533.I1RCA3
93231 229533.A0A1C3YJJ3 pp 229533.A0A1C3YL20
93232 229533.A0A1C3YJJ3 pp 229533.I1S8E0
93233 229533.A0A1C3YJJ3 pp 229533.I1RCF2
93234 229533.A0A1C3YJJ3 pp 229533.I1RWA9
93235 229533.A0A1C3YJJ3 pp 229533.I1RWA5
93236 229533.A0A1C3YJJ3 pp 229533.I1RT52
93237 229533.I1RYJ7 pp 229533.I1S0D5
93238 229533.I1RYJ7 pp 229533.I1S3G4
93239 229533.I1RYJ7 pp 229533.I1RZ29
93240 229533.I1RYJ7 pp 229533.O74638
93241 229533.I1RYJ7 pp 229533.V6RSF3
93242 229533.I1RYJ7 pp 229533.V6QVH8
93243 229533.I1RYJ7 pp 229533.I1RYW6
93244 229533.I1RYJ7 pp 229533.V6RQW4
93245 229533.I1RYJ7 pp 229533.I1RZM4
93246 229533.I1RYJ7 pp 229533.V6RM02
93247 229533.I1RYJ7 pp 229533.I1S0N3
93248 229533.I1RYJ7 pp 229533.Q4HXF0
93249 229533.I1RYJ7 pp 229533.V6RK96
93250 229533.I1RYJ7 pp 229533.V6RLW2
93251 229533.I1RYJ7 pp 229533.I1S1W8
93252 229533.I1RIJ1 pp 229533.I1S6W3
93253 229533.I1RIJ1 pp 229533.I1RWE3
93254 229533.I1RIJ1 pp 229533.I1RXT4
93255 229533.I1RIJ1 pp 229533.I1S320
93256 229533.I1RIJ1 pp 229533.I1RQQ0
93257 229533.I1RIJ1 pp 229533.I1RVH7
93258 229533.I1RIJ1 pp 229533.I1RT54
93259 229533.I1RIJ1 pp 229533.I1RJ55
93260 229533.I1RIJ1 pp 229533.I1S407
93261 229533.I1RIJ1 pp 229533.I1RIZ7
93262 229533.I1RIJ1 pp 229533.I1RIU7
93263 229533.I1RIJ1 pp 229533.I1S4A4
93264 229533.I1RIJ1 pp 229533.I1RLY2
93265 229533.I1RIJ1 pp 229533.I1RLQ8
93266 229533.I1RIJ1 pp 229533.I1RSY7
93267 229533.I1RIJ1 pp 229533.I1RLU4
93268 229533.I1RIJ1 pp 229533.I1RR89
93269 229533.A0A098DU81 pp 229533.I1RUK0
93270 229533.A0A098DU81 pp 229533.I1RFJ3
93271 229533.A0A098DU81 pp 229533.I1RAW4
93272 229533.A0A098DU81 pp 229533.A0A1C3YIP7
93273 229533.I1RXV9 pp 229533.I1RYJ7
93274 229533.I1RXV9 pp 229533.V6R382
93275 229533.I1RXV9 pp 229533.V6RQW4
93276 229533.I1RXV9 pp 229533.V6RK96
93277 229533.I1RXV9 pp 229533.I1RYI7
93278 229533.I1RXV9 pp 229533.I1S1W3
93279 229533.I1RXV9 pp 229533.I1S8F8
93280 229533.I1RXV9 pp 229533.I1S0N3
93281 229533.I1RXV9 pp 229533.I1S9Z4
93282 229533.I1RXV9 pp 229533.I1S0V7
93283 229533.I1RXV9 pp 229533.Q4IER0
93284 229533.I1RXV9 pp 229533.I1S147
93285 229533.I1RXV9 pp 229533.I1S887
93286 229533.I1RXV9 pp 229533.V6RM02
93287 229533.I1RXV9 pp 229533.I1RZM4
93288 229533.I1RXV9 pp 229533.V6QVH8
93289 229533.I1RXV9 pp 229533.I1S1W8
93290 229533.I1RXV9 pp 229533.V6R057
93291 229533.I1RXV9 pp 229533.I1S256
93292 229533.I1RXV9 pp 229533.V6RUT5
93293 229533.I1RXV9 pp 229533.Q4IMJ1
93294 229533.I1RXV9 pp 229533.V6RSF3

229533.I1RXV9 pp 229533.I1RYW9
229533.I1RXV9 pp 229533.I1RY62
229533.I1RXV9 pp 229533.I1RYW6
229533.I1RXV9 pp 229533.I1S3G4
229533.I1RXV9 pp 229533.Q4HXF0
229533.I1RXV9 pp 229533.O74638
229533.A0A098DIZ0 pp 229533.I1RJ55
229533.A0A098DIZ0 pp 229533.I1S407
229533.A0A098DIZ0 pp 229533.A0A1C3YMT0
229533.A0A098DIZ0 pp 229533.V6R5G9
229533.A0A098DIZ0 pp 229533.I1RH94
229533.A0A098DIZ0 pp 229533.I1S320
229533.A0A098DIZ0 pp 229533.I1R9L9
229533.A0A098DIZ0 pp 229533.I1RYJ1
229533.A0A098DIZ0 pp 229533.I1RAN9
229533.A0A098DIZ0 pp 229533.I1RYC1
229533.A0A098DIZ0 pp 229533.A0A1C3YKM3
229533.A0A098DIZ0 pp 229533.I1RMU2
229533.A0A098DIZ0 pp 229533.I1S6W3
229533.A0A098DIZ0 pp 229533.I1RWE3
229533.A0A098DIZ0 pp 229533.I1RLU4
229533.A0A098DIZ0 pp 229533.I1RDC7
229533.A0A098DIZ0 pp 229533.I1RIZ7
229533.A0A098DIZ0 pp 229533.A0A098DJ33
229533.A0A098DIZ0 pp 229533.I1RXT4
229533.A0A098DIZ0 pp 229533.I1RHM8
229533.A0A098DIZ0 pp 229533.I1RB60
229533.A0A098DIZ0 pp 229533.I1RWF6
229533.A0A098DIZ0 pp 229533.I1RFY7
229533.A0A098DIZ0 pp 229533.I1RVW8
229533.A0A098DIZ0 pp 229533.I1RIJ1
229533.A0A098DIZ0 pp 229533.I1RI39
229533.A0A098DIZ0 pp 229533.I1RSY7
229533.A0A098DIZ0 pp 229533.I1RF73
229533.A0A098DIZ0 pp 229533.A0A1C3YMT3
229533.A0A098DIZ0 pp 229533.I1S4A4
229533.A0A098DIZ0 pp 229533.I1RLY2
229533.A0A098DIZ0 pp 229533.I1RR89
229533.A0A098DIZ0 pp 229533.I1RVH7
229533.A0A098DIZ0 pp 229533.I1RT54
229533.A0A1C3YHV3 pp 229533.I1RBA6
229533.A0A1C3YHV3 pp 229533.I1RAU9
229533.A0A1C3YHV3 pp 229533.I1RDH6
229533.A0A1C3YHV3 pp 229533.I1RPY0
229533.A0A1C3YHV3 pp 229533.I1RBW1
229533.A0A1C3YHV3 pp 229533.I1S3F7
229533.A0A1C3YHV3 pp 229533.A0A1C3YK00
229533.A0A1C3YHV3 pp 229533.I1RJU6
229533.A0A1C3YHV3 pp 229533.I1RMC5
229533.A0A1C3YHV3 pp 229533.I1RC14
229533.A0A1C3YHV3 pp 229533.I1RNE7
229533.A0A1C3YHV3 pp 229533.I1RMC2
229533.A0A1C3YHV3 pp 229533.I1RBB3
229533.A0A1C3YHV3 pp 229533.I1RRW9
229533.A0A1C3YHV3 pp 229533.I1RRJ3
229533.A0A1C3YHV3 pp 229533.I1RZ21
229533.A0A1C3YHV3 pp 229533.I1RMW4
229533.A0A1C3YHV3 pp 229533.I1RYI6
229533.A0A1C3YHV3 pp 229533.I1RMV8
229533.A0A1C3YHV3 pp 229533.I1RM55
229533.A0A1C3YHV3 pp 229533.I1RE47
229533.A0A1C3YHV3 pp 229533.I1RYX5
229533.A0A1C3YHV3 pp 229533.I1S3V2
229533.A0A1C3YHV3 pp 229533.I1RAA0
229533.A0A1C3YHV3 pp 229533.I1RM54
229533.A0A1C3YHV3 pp 229533.V6RGI1
229533.A0A1C3YHV3 pp 229533.I1RMM8
229533.A0A1C3YHV3 pp 229533.I1S0N5
229533.I1RRK1 pp 229533.I1RS94
229533.A0A1C3YKP5 pp 229533.I1RPA4
229533.A0A1C3YKP5 pp 229533.I1S5X9
229533.A0A1C3YKP5 pp 229533.Q4I099
229533.A0A1C3YKP5 pp 229533.I1S277

93368 229533.A0A1C3YKP5 pp 229533.I1RNJ5
93369 229533.A0A1C3YKP5 pp 229533.I1RSK9
93370 229533.A0A1C3YKP5 pp 229533.I1RS01
93371 229533.A0A1C3YKP5 pp 229533.I1RYT8
93372 229533.A0A1C3YKP5 pp 229533.Q4I5G1
93373 229533.A0A1C3YKP5 pp 229533.I1RAV5
93374 229533.A0A1C3YKP5 pp 229533.Q4IPZ1
93375 229533.A0A1C3YKP5 pp 229533.I1RGC5
93376 229533.A0A1C3YKP5 pp 229533.I1RMQ6
93377 229533.A0A1C3YKP5 pp 229533.I1RKB7
93378 229533.A0A1C3YKP5 pp 229533.I1RPY8
93379 229533.A0A1C3YKP5 pp 229533.I1RWT0
93380 229533.A0A1C3YKP5 pp 229533.I1RX17
93381 229533.A0A1C3YKP5 pp 229533.I1RK80
93382 229533.A0A1C3YKP5 pp 229533.A0A1C3YLB0
93383 229533.A0A1C3YKP5 pp 229533.I1RZN1
93384 229533.A0A1C3YKP5 pp 229533.I1S268
93385 229533.A0A1C3YKP5 pp 229533.I1RAM9
93386 229533.A0A1C3YKP5 pp 229533.I1RB86
93387 229533.A0A1C3YKP5 pp 229533.I1RZM9
93388 229533.A0A1C3YKP5 pp 229533.I1RX39
93389 229533.A0A1C3YKP5 pp 229533.I1RBE6
93390 229533.A0A1C3YKP5 pp 229533.I1RFS5
93391 229533.A0A1C3YKP5 pp 229533.I1S212
93392 229533.A0A1C3YKP5 pp 229533.I1RAD4
93393 229533.A0A1C3YKP5 pp 229533.A0A1C3YMC1
93394 229533.A0A1C3YKP5 pp 229533.V6R3J1
93395 229533.A0A1C3YKP5 pp 229533.A0A1C3YLC3
93396 229533.A0A1C3YKP5 pp 229533.Q4IK03
93397 229533.A0A1C3YKP5 pp 229533.Q4IJW4
93398 229533.A0A1C3YKP5 pp 229533.V6RJP8
93399 229533.A0A1C3YKP5 pp 229533.A0A1C3YLL3
93400 229533.A0A1C3YKP5 pp 229533.I1RFV2
93401 229533.A0A1C3YKP5 pp 229533.I1S1F3
93402 229533.A0A1C3YKP5 pp 229533.I1RC18
93403 229533.A0A1C3YKP5 pp 229533.I1RA12
93404 229533.A0A1C3YKP5 pp 229533.I1RM37
93405 229533.I1RR89 pp 229533.I1RVH7
93406 229533.I1RR89 pp 229533.I1S4A4
93407 229533.I1RR89 pp 229533.I1RWF6
93408 229533.I1RR89 pp 229533.I1RSY7
93409 229533.I1RR89 pp 229533.I1RT54
93410 229533.I1RR89 pp 229533.I1RXT4
93411 229533.I1RR89 pp 229533.I1RYC1
93412 229533.I1RR89 pp 229533.I1RVW8
93413 229533.I1RR89 pp 229533.I1S6W3
93414 229533.I1RR89 pp 229533.I1RWE3
93415 229533.I1RR89 pp 229533.I1RYJ1
93416 229533.I1RR89 pp 229533.I1S407
93417 229533.I1RR89 pp 229533.V6R5G9
93418 229533.I1RR89 pp 229533.I1S320
93419 229533.Q4IJW4 pp 229533.V6RJP8
93420 229533.Q4IJW4 pp 229533.V6QWP4
93421 229533.Q4IJW4 pp 229533.Q4IPZ1
93422 229533.Q4IJW4 pp 229533.Q4IK03
93423 229533.I1RHD4 pp 229533.V6RHD3
93424 229533.I1RHD4 pp 229533.I1RNH4
93425 229533.A0A1C3YMT3 pp 229533.I1RR89
93426 229533.A0A1C3YMT3 pp 229533.I1RIJ1
93427 229533.A0A1C3YMT3 pp 229533.I1S4A4
93428 229533.A0A1C3YMT3 pp 229533.I1RF73
93429 229533.A0A1C3YMT3 pp 229533.I1RT54
93430 229533.A0A1C3YMT3 pp 229533.I1RI39
93431 229533.A0A1C3YMT3 pp 229533.I1RLY2
93432 229533.A0A1C3YMT3 pp 229533.I1RVH7
93433 229533.A0A1C3YMT3 pp 229533.I1RFY7
93434 229533.A0A1C3YMT3 pp 229533.I1RSY7
93435 229533.A0A1C3YMT3 pp 229533.I1RWF6
93436 229533.A0A1C3YMT3 pp 229533.I1RB60
93437 229533.A0A1C3YMT3 pp 229533.I1RVW8
93438 229533.A0A1C3YMT3 pp 229533.I1RHM8
93439 229533.A0A1C3YMT3 pp 229533.I1RMU2
93440 229533.A0A1C3YMT3 pp 229533.I1RYC1

93441 229533.A0A1C3YMT3 pp 229533.I1RXT4
93442 229533.A0A1C3YMT3 pp 229533.I1RAN9
93443 229533.A0A1C3YMT3 pp 229533.I1RIZ7
93444 229533.A0A1C3YMT3 pp 229533.I1RDC7
93445 229533.A0A1C3YMT3 pp 229533.I1RWE3
93446 229533.A0A1C3YMT3 pp 229533.I1RLU4
93447 229533.A0A1C3YMT3 pp 229533.V6R5G9
93448 229533.A0A1C3YMT3 pp 229533.I1S407
93449 229533.A0A1C3YMT3 pp 229533.I1RJ55
93450 229533.A0A1C3YMT3 pp 229533.I1S6W3
93451 229533.A0A1C3YMT3 pp 229533.I1RYJ1
93452 229533.A0A1C3YMT3 pp 229533.I1R9L9
93453 229533.A0A1C3YMT3 pp 229533.I1RH94
93454 229533.A0A1C3YMT3 pp 229533.I1S320
93455 229533.I1RY57 pp 229533.I1S8A6
93456 229533.I1RY57 pp 229533.I1S0R9
93457 229533.I1RY57 pp 229533.Q4I099
93458 229533.I1RY57 pp 229533.I1S5X9
93459 229533.I1RY57 pp 229533.I1S217
93460 229533.I1RY57 pp 229533.V6R3J1
93461 229533.I1RY57 pp 229533.V6RVG3
93462 229533.I1RY57 pp 229533.Q4IBR4
93463 229533.I1RY57 pp 229533.I1S277
93464 229533.I1RY57 pp 229533.I1S439
93465 229533.I1RY57 pp 229533.Q4IJM4
93466 229533.I1RY57 pp 229533.Q4I6B0
93467 229533.A0A1C3YHL7 pp 229533.I1RDY1
93468 229533.I1RDC7 pp 229533.I1RT54
93469 229533.I1RDC7 pp 229533.I1S407
93470 229533.I1RDC7 pp 229533.I1RLY2
93471 229533.I1RDC7 pp 229533.I1RV63
93472 229533.I1RDC7 pp 229533.I1RVH7
93473 229533.I1RDC7 pp 229533.I1S320
93474 229533.I1RDC7 pp 229533.I1RWE3
93475 229533.I1RDC7 pp 229533.I1RJ55
93476 229533.I1RDC7 pp 229533.I1RH94
93477 229533.I1RDC7 pp 229533.I1RLU4
93478 229533.I1RDC7 pp 229533.I1RIZ7
93479 229533.I1RDC7 pp 229533.I1S6W3
93480 229533.I1RDC7 pp 229533.I1RXT4
93481 229533.I1RDC7 pp 229533.I1S4A4
93482 229533.I1RDC7 pp 229533.I1RIJ1
93483 229533.I1RDC7 pp 229533.I1RFY7
93484 229533.I1RDC7 pp 229533.I1RSY7
93485 229533.I1RDC7 pp 229533.I1RR89
93486 229533.I1RZJ8 pp 229533.I1SAS4
93487 229533.I1RZJ8 pp 229533.I1SAS3
93488 229533.I1RZJ8 pp 229533.I1S1Q3
93489 229533.I1RZJ8 pp 229533.V6RR38
93490 229533.I1RZJ8 pp 229533.I1S0Y2
93491 229533.I1RSY7 pp 229533.I1RVW8
93492 229533.I1RSY7 pp 229533.V6R5G9
93493 229533.I1RSY7 pp 229533.I1RUY6
93494 229533.I1RSY7 pp 229533.I1RYJ1
93495 229533.I1RSY7 pp 229533.I1S4A4
93496 229533.I1RSY7 pp 229533.I1S320
93497 229533.I1RSY7 pp 229533.I1RYC1
93498 229533.I1RSY7 pp 229533.I1RT54
93499 229533.I1RSY7 pp 229533.I1RWE3
93500 229533.I1RSY7 pp 229533.I1RWF6
93501 229533.I1RSY7 pp 229533.I1RVH7
93502 229533.I1RSY7 pp 229533.I1RXT4
93503 229533.I1RSY7 pp 229533.I1S407
93504 229533.I1RSY7 pp 229533.I1S6W3
93505 229533.I1RRZ3 pp 229533.I1S0R9
93506 229533.A0A1C3YNA1 pp 229533.I1RGY6
93507 229533.A0A1C3YNA1 pp 229533.I1RPN1
93508 229533.A0A1C3YNA1 pp 229533.I1S4Q7
93509 229533.A0A1C3YNA1 pp 229533.I1RF33
93510 229533.A0A1C3YNA1 pp 229533.I1S287
93511 229533.A0A1C3YNA1 pp 229533.I1RQF7
93512 229533.A0A1C3YNA1 pp 229533.I1RPY0
93513 229533.A0A1C3YNA1 pp 229533.I1RFY0

93514 229533.A0A1C3YNA1 pp 229533.I1RFH2
93515 229533.A0A1C3YNA1 pp 229533.I1RDJ8
93516 229533.A0A1C3YNA1 pp 229533.I1RMN2
93517 229533.A0A1C3YNA1 pp 229533.I1RFI9
93518 229533.A0A1C3YNA1 pp 229533.I1RDA8
93519 229533.A0A1C3YNA1 pp 229533.I1RBN6
93520 229533.A0A1C3YNA1 pp 229533.I1RUL3
93521 229533.A0A1C3YNA1 pp 229533.I1RZ87
93522 229533.I1RLU4 pp 229533.I1RR89
93523 229533.I1RLU4 pp 229533.I1S4A4
93524 229533.I1RLU4 pp 229533.I1RVH7
93525 229533.I1RLU4 pp 229533.I1RT54
93526 229533.I1RLU4 pp 229533.I1RSY7
93527 229533.I1RLU4 pp 229533.I1RVW8
93528 229533.I1RLU4 pp 229533.I1RWF6
93529 229533.I1RLU4 pp 229533.I1RLY2
93530 229533.I1RLU4 pp 229533.I1RMU2
93531 229533.I1RLU4 pp 229533.I1RYC1
93532 229533.I1RLU4 pp 229533.I1RXT4
93533 229533.I1RLU4 pp 229533.I1S6W3
93534 229533.I1RLU4 pp 229533.I1RWE3
93535 229533.I1RLU4 pp 229533.I1S320
93536 229533.I1RLU4 pp 229533.V6R5G9
93537 229533.I1RLU4 pp 229533.I1S407
93538 229533.I1RLU4 pp 229533.I1RYJ1
93539 229533.A0A0E0RX65 pp 229533.I1RXT3
93540 229533.A0A0E0RX65 pp 229533.I1RAD4
93541 229533.A0A0E0RX65 pp 229533.I1S834
93542 229533.A0A0E0RX65 pp 229533.Q4I6B0
93543 229533.A0A0E0RX65 pp 229533.I1S5X9
93544 229533.A0A0E0RX65 pp 229533.I1RRS8
93545 229533.A0A0E0RX65 pp 229533.I1RNJ5
93546 229533.A0A0E0RX65 pp 229533.I1RSK9
93547 229533.A0A0E0RX65 pp 229533.I1S0R9
93548 229533.A0A0E0RX65 pp 229533.I1RNN3
93549 229533.A0A0E0RX65 pp 229533.I1S2C1
93550 229533.A0A0E0RX65 pp 229533.I1RC18
93551 229533.A0A0E0RX65 pp 229533.A0A1C3YKP5
93552 229533.A0A0E0RX65 pp 229533.Q4IJW4
93553 229533.A0A0E0RX65 pp 229533.I1RY57
93554 229533.A0A0E0RX65 pp 229533.I1RSJ3
93555 229533.A0A0E0RX65 pp 229533.I1RRZ3
93556 229533.I1RCA3 pp 229533.I1RWA9
93557 229533.I1RCA3 pp 229533.I1RWA5
93558 229533.I1RCA3 pp 229533.I1RCF2
93559 229533.I1RCA3 pp 229533.I1S1B1
93560 229533.I1RCF2 pp 229533.I1RMR5
93561 229533.I1RCF2 pp 229533.I1RWA5
93562 229533.I1RCF2 pp 229533.I1RJS2
93563 229533.I1RCF2 pp 229533.I1RWA9
93564 229533.I1RCF2 pp 229533.I1S1B1
93565 229533.I1RWE3 pp 229533.I1RWF6
93566 229533.I1RWE3 pp 229533.I1S407
93567 229533.I1RWE3 pp 229533.V6R5G9
93568 229533.I1RWE3 pp 229533.I1S4A4
93569 229533.I1RWE3 pp 229533.I1RYC1
93570 229533.I1RWE3 pp 229533.I1S320
93571 229533.I1RWE3 pp 229533.I1RYJ1
93572 229533.I1RWE3 pp 229533.I1S6W3
93573 229533.I1RWE3 pp 229533.I1RXT4
93574 229533.I1S6W3 pp 229533.V6R5G9
93575 229533.A0A1C3YN47 pp 229533.I1RXB6
93576 229533.A0A1C3YN47 pp 229533.I1S064
93577 229533.A0A1C3YN47 pp 229533.I1RCN8
93578 229533.A0A1C3YN47 pp 229533.I1RXY7
93579 229533.A0A1C3YN47 pp 229533.I1RCV1
93580 229533.A0A1C3YN47 pp 229533.I1RMW4
93581 229533.A0A1C3YN47 pp 229533.I1RFK3
93582 229533.A0A1C3YN47 pp 229533.I1RE79
93583 229533.A0A1C3YN47 pp 229533.I1RCT7
93584 229533.A0A1C3YN47 pp 229533.I1RW11
93585 229533.A0A1C3YN47 pp 229533.I1RCL0
93586 229533.A0A1C3YN47 pp 229533.V6RS91

93587	229533.A0A1C3YN47	pp	229533.Q4I1N3
93588	229533.A0A1C3YN47	pp	229533.I1RCG7
93589	229533.A0A1C3YN47	pp	229533.I1RZ29
93590	229533.A0A1C3YN47	pp	229533.I1RBF3
93591	229533.A0A1C3YI94	pp	229533.A0A1C3YJJ3
93592	229533.A0A1C3YI94	pp	229533.I1RWA5
93593	229533.A0A1C3YI94	pp	229533.I1REK3
93594	229533.A0A1C3YI94	pp	229533.A0A1C3YLP9
93595	229533.A0A1C3YI94	pp	229533.A0A1C3YJJ1
93596	229533.A0A1C3YI94	pp	229533.V6RR76
93597	229533.A0A1C3YI94	pp	229533.V6QUU6
93598	229533.A0A1C3YI94	pp	229533.I1S2B7
93599	229533.A0A1C3YI94	pp	229533.I1RWM7
93600	229533.A0A1C3YI94	pp	229533.I1RWA9
93601	229533.A0A1C3YI94	pp	229533.I1RMR5
93602	229533.A0A1C3YI94	pp	229533.I1S1B1
93603	229533.A0A1C3YI94	pp	229533.I1RN81
93604	229533.A0A1C3YI94	pp	229533.I1RJS2
93605	229533.A0A1C3YI94	pp	229533.I1RCA3
93606	229533.A0A1C3YI94	pp	229533.I1RCF2
93607	229533.A0A1C3YI94	pp	229533.I1S1W7
93608	229533.I1RT54	pp	229533.I1S4A4
93609	229533.I1RT54	pp	229533.V6R5G9
93610	229533.I1RT54	pp	229533.I1S320
93611	229533.I1RT54	pp	229533.I1RYJ1
93612	229533.I1RT54	pp	229533.I1RVW8
93613	229533.I1RT54	pp	229533.I1RUY6
93614	229533.I1RT54	pp	229533.I1RXT4
93615	229533.I1RT54	pp	229533.I1RWE3
93616	229533.I1RT54	pp	229533.I1S407
93617	229533.I1RT54	pp	229533.I1RVH7
93618	229533.I1RT54	pp	229533.I1S6W3
93619	229533.I1RT54	pp	229533.I1RWF6
93620	229533.I1RT54	pp	229533.I1RYC1
93621	229533.I1RGQ2	pp	229533.I1RQQ0
93622	229533.I1RGQ2	pp	229533.I1RTJ2
93623	229533.I1RGQ2	pp	229533.I1RYQ4
93624	229533.I1RGQ2	pp	229533.I1RH75
93625	229533.I1RGQ2	pp	229533.I1RTP5
93626	229533.I1RZQ1	pp	229533.I1S0B7
93627	229533.I1RZQ1	pp	229533.Q4IR09
93628	229533.I1RZQ1	pp	229533.V6RN16
93629	229533.I1RZQ1	pp	229533.I1S694
93630	229533.I1RZQ1	pp	229533.I1S065
93631	229533.I1RZQ1	pp	229533.V6QVH8
93632	229533.I1RZQ1	pp	229533.V6RLM7
93633	229533.I1RZQ1	pp	229533.V6RQW4
93634	229533.I1RXT4	pp	229533.I1S407
93635	229533.I1RXT4	pp	229533.V6R5G9
93636	229533.I1RXT4	pp	229533.I1S4A4
93637	229533.I1RXT4	pp	229533.I1S320
93638	229533.I1RXT4	pp	229533.I1RYJ1
93639	229533.I1RXT4	pp	229533.I1S6W3
93640	229533.I1RXT4	pp	229533.I1RYC1
93641	229533.I1RPY8	pp	229533.I1RYT8
93642	229533.I1RPY8	pp	229533.I1S9E7
93643	229533.I1RPY8	pp	229533.Q4IPZ1
93644	229533.I1RPY8	pp	229533.I1RT38
93645	229533.I1RPY8	pp	229533.Q4HZ95
93646	229533.I1RPY8	pp	229533.Q4I6B0
93647	229533.I1RPY8	pp	229533.I1RQY8
93648	229533.I1RPY8	pp	229533.I1S0K2
93649	229533.I1RPY8	pp	229533.Q4I1Y1
93650	229533.I1RPY8	pp	229533.Q4I7T5
93651	229533.I1RPY8	pp	229533.I1RZG7
93652	229533.I1RPY8	pp	229533.I1S5X9
93653	229533.I1RPY8	pp	229533.I1S464
93654	229533.I1RPY8	pp	229533.I1RQR6
93655	229533.I1RPY8	pp	229533.Q4I099
93656	229533.I1RPY8	pp	229533.I1RX98
93657	229533.I1RPY8	pp	229533.I1RXE6
93658	229533.I1RPY8	pp	229533.Q4IB65
93659	229533.I1RPY8	pp	229533.I1RPZ3

93660 229533.I1RPY8 pp 229533.I1RY21
93661 229533.I1RPY8 pp 229533.I1RWA3
93662 229533.I1RPY8 pp 229533.Q4I375
93663 229533.I1RPY8 pp 229533.I1RST1
93664 229533.I1RPY8 pp 229533.Q4IJW4
93665 229533.I1RPY8 pp 229533.I1RXD9
93666 229533.I1RPY8 pp 229533.V6RJP8
93667 229533.I1RPY8 pp 229533.I1RX47
93668 229533.I1RPY8 pp 229533.I1S0Q7
93669 229533.I1RPY8 pp 229533.I1RRL0
93670 229533.A0A1C3YLA7 pp 229533.I1RZV5
93671 229533.A0A1C3YLA7 pp 229533.I1RSD7
93672 229533.A0A1C3YLA7 pp 229533.I1RLZ4
93673 229533.A0A1C3YI05 pp 229533.V6RLM7
93674 229533.A0A1C3YI05 pp 229533.I1RPK0
93675 229533.A0A1C3YI05 pp 229533.I1RMQ5
93676 229533.A0A1C3YI05 pp 229533.I1RPU8
93677 229533.A0A1C3YI05 pp 229533.Q4HTS9
93678 229533.A0A1C3YI05 pp 229533.I1RN84
93679 229533.A0A1C3YI05 pp 229533.I1S1W3
93680 229533.A0A1C3YI05 pp 229533.I1RNM3
93681 229533.A0A1C3YI05 pp 229533.I1RUP2
93682 229533.A0A1C3YI05 pp 229533.I1S2C4
93683 229533.A0A1C3YI05 pp 229533.I1RGB5
93684 229533.A0A1C3YI05 pp 229533.V6R382
93685 229533.A0A1C3YI05 pp 229533.I1RAK7
93686 229533.A0A1C3YI05 pp 229533.I1RA10
93687 229533.A0A1C3YI05 pp 229533.V6RSF3
93688 229533.A0A1C3YI05 pp 229533.V6QVH8
93689 229533.A0A1C3YI05 pp 229533.I1RVR2
93690 229533.A0A1C3YI05 pp 229533.I1SAJ3
93691 229533.A0A1C3YI05 pp 229533.I1S4Z7
93692 229533.A0A1C3YI05 pp 229533.I1RYW3
93693 229533.A0A1C3YI05 pp 229533.A0A1C3YLE2
93694 229533.A0A1C3YI05 pp 229533.I1RCP7
93695 229533.A0A1C3YI05 pp 229533.I1RYW6
93696 229533.A0A1C3YI05 pp 229533.I1RQ30
93697 229533.A0A1C3YI05 pp 229533.I1RQG7
93698 229533.A0A1C3YI05 pp 229533.A0A1C3YLU6
93699 229533.A0A1C3YI05 pp 229533.Q4HXF0
93700 229533.A0A1C3YI05 pp 229533.I1RIH0
93701 229533.A0A1C3YI05 pp 229533.A0A1C3YLA6
93702 229533.A0A1C3YI05 pp 229533.I1RZ52
93703 229533.A0A1C3YI05 pp 229533.V6RM02
93704 229533.A0A1C3YI05 pp 229533.I1RTI7
93705 229533.A0A1C3YI05 pp 229533.I1RRT2
93706 229533.A0A1C3YI05 pp 229533.I1RSS7
93707 229533.A0A1C3YI05 pp 229533.I1RVX0
93708 229533.A0A1C3YI05 pp 229533.I1RXW3
93709 229533.A0A1C3YI05 pp 229533.I1RQG4
93710 229533.A0A1C3YI05 pp 229533.V6R5D2
93711 229533.A0A1C3YI05 pp 229533.I1RN67
93712 229533.A0A1C3YI05 pp 229533.V6RK96
93713 229533.A0A1C3YI05 pp 229533.I1RC48
93714 229533.A0A1C3YI05 pp 229533.I1RMG6
93715 229533.A0A1C3YI05 pp 229533.I1RBE4
93716 229533.A0A1C3YI05 pp 229533.I1S4D0
93717 229533.A0A1C3YI05 pp 229533.I1RA90
93718 229533.A0A1C3YI05 pp 229533.A0A1C3YM54
93719 229533.A0A1C3YI05 pp 229533.I1RTG7
93720 229533.A0A1C3YI05 pp 229533.I1RCV1
93721 229533.A0A1C3YI05 pp 229533.I1S0N3
93722 229533.A0A1C3YI05 pp 229533.I1RSQ4
93723 229533.A0A1C3YI05 pp 229533.I1RYI7
93724 229533.A0A1C3YI05 pp 229533.I1RYL5
93725 229533.A0A1C3YI05 pp 229533.I1RW69
93726 229533.A0A1C3YI05 pp 229533.O74638
93727 229533.A0A1C3YI05 pp 229533.I1RIY0
93728 229533.A0A1C3YI05 pp 229533.I1RN17
93729 229533.A0A1C3YI05 pp 229533.I1RB53
93730 229533.A0A1C3YI05 pp 229533.I1RBE7
93731 229533.A0A1C3YI05 pp 229533.I1RFM6
93732 229533.A0A1C3YI05 pp 229533.I1RMG7

229533.A0A1C3YI05	pp	229533.I1RVY2
229533.A0A1C3YI05	pp	229533.I1RXV9
229533.A0A1C3YI05	pp	229533.I1RK47
229533.A0A1C3YI05	pp	229533.V6RY56
229533.A0A1C3YI05	pp	229533.V6RQW4
229533.A0A1C3YI05	pp	229533.I1RM26
229533.A0A1C3YI05	pp	229533.I1RMM8
229533.A0A1C3YI05	pp	229533.I1RQE8
229533.A0A1C3YI05	pp	229533.V6RUT5
229533.A0A1C3YI05	pp	229533.I1RYJ7
229533.A0A1C3YI05	pp	229533.A0A1C3YJL7
229533.A0A1C3YI05	pp	229533.I1RFQ7
229533.A0A1C3YI05	pp	229533.I1RTU1
229533.A0A1C3YI05	pp	229533.A0A1C3YJJ2
229533.A0A1C3YI05	pp	229533.I1RTG4
229533.A0A1C3YI05	pp	229533.I1RVX9
229533.A0A1C3YI05	pp	229533.I1RW54
229533.A0A1C3YI05	pp	229533.I1S029
229533.A0A1C3YI05	pp	229533.I1RZM4
229533.A0A1C3YI05	pp	229533.A0A1C3YLE9
229533.A0A1C3YI05	pp	229533.I1RXB1
229533.A0A098DJ33	pp	229533.I1RT54
229533.A0A098DJ33	pp	229533.A0A1C3YMT3
229533.A0A098DJ33	pp	229533.I1RSY7
229533.A0A098DJ33	pp	229533.V6R5G9
229533.A0A098DJ33	pp	229533.I1RJ55
229533.A0A098DJ33	pp	229533.A0A1C3YMT0
229533.A0A098DJ33	pp	229533.I1S407
229533.A0A098DJ33	pp	229533.I1RYJ1
229533.A0A098DJ33	pp	229533.I1RB60
229533.A0A098DJ33	pp	229533.I1RH94
229533.A0A098DJ33	pp	229533.I1RFY7
229533.A0A098DJ33	pp	229533.I1R9L9
229533.A0A098DJ33	pp	229533.I1S320
229533.A0A098DJ33	pp	229533.I1RWF6
229533.A0A098DJ33	pp	229533.I1RHM8
229533.A0A098DJ33	pp	229533.A0A1C3YKM3
229533.A0A098DJ33	pp	229533.I1RMU2
229533.A0A098DJ33	pp	229533.I1RVW8
229533.A0A098DJ33	pp	229533.I1RAN9
229533.A0A098DJ33	pp	229533.I1RYC1
229533.A0A098DJ33	pp	229533.I1RR89
229533.A0A098DJ33	pp	229533.I1RIJ1
229533.A0A098DJ33	pp	229533.I1RIZ7
229533.A0A098DJ33	pp	229533.I1RF73
229533.A0A098DJ33	pp	229533.I1RXT4
229533.A0A098DJ33	pp	229533.I1RI39
229533.A0A098DJ33	pp	229533.I1RLU4
229533.A0A098DJ33	pp	229533.I1RWE3
229533.A0A098DJ33	pp	229533.I1S4A4
229533.A0A098DJ33	pp	229533.I1S6W3
229533.A0A098DJ33	pp	229533.I1RVH7
229533.A0A098DJ33	pp	229533.I1RDC7
229533.A0A098DJ33	pp	229533.I1RLY2
229533.I1RQ69	pp	229533.I1RQ70
229533.A0A1C3YIY0	pp	229533.I1RBH7
229533.I1RJU8	pp	229533.I1S1F3
229533.I1RJU8	pp	229533.I1RY21
229533.I1RJU8	pp	229533.I1RMQ6
229533.I1RSL5	pp	229533.I1S2C4
229533.I1RSL5	pp	229533.V6RSF3
229533.I1RSL5	pp	229533.V6QVH8
229533.I1RSL5	pp	229533.O74638
229533.I1RSL5	pp	229533.I1SAJ3
229533.I1RSL5	pp	229533.V6RM02
229533.I1RSL5	pp	229533.I1RZ52
229533.I1RSL5	pp	229533.I1RTI7
229533.I1RSL5	pp	229533.I1RVY2
229533.I1RSL5	pp	229533.V6RLM7
229533.I1RSL5	pp	229533.I1RUP2
229533.I1RSL5	pp	229533.I1RYW6
229533.I1RSL5	pp	229533.I1RVR2
229533.I1RSL5	pp	229533.I1RW69

93806 229533.I1RSL5 pp 229533.V6RY56
93807 229533.I1RSL5 pp 229533.I1RXW3
93808 229533.I1RSL5 pp 229533.Q4HXF0
93809 229533.I1RSL5 pp 229533.V6RQW4
93810 229533.I1RSL5 pp 229533.I1RVX9
93811 229533.I1RSL5 pp 229533.I1S0N3
93812 229533.I1RSL5 pp 229533.V6RK96
93813 229533.I1RSL5 pp 229533.I1RYI7
93814 229533.I1RSL5 pp 229533.I1RXV9
93815 229533.I1RSL5 pp 229533.I1RZM4
93816 229533.I1RSL5 pp 229533.I1RYJ7
93817 229533.I1RSL5 pp 229533.I1S4D0
93818 229533.I1RSL5 pp 229533.I1S029
93819 229533.I1RSL5 pp 229533.I1RXB1
93820 229533.I1S0K5 pp 229533.I1S278
93821 229533.I1S0K5 pp 229533.I1S1V4
93822 229533.I1S0K5 pp 229533.I1S6S4
93823 229533.I1S0K5 pp 229533.I1SAM5
93824 229533.I1S0K5 pp 229533.I1S2B6
93825 229533.I1S0K5 pp 229533.Q4I6S5
93826 229533.I1S0K5 pp 229533.I1S830
93827 229533.I1RIZ7 pp 229533.I1S320
93828 229533.I1RIZ7 pp 229533.I1RVH7
93829 229533.I1RIZ7 pp 229533.I1RJ55
93830 229533.I1RIZ7 pp 229533.I1S407
93831 229533.I1RIZ7 pp 229533.I1RLY2
93832 229533.I1RIZ7 pp 229533.I1RV63
93833 229533.I1RIZ7 pp 229533.I1S4A4
93834 229533.I1RIZ7 pp 229533.I1RT54
93835 229533.I1RIZ7 pp 229533.I1RXT4
93836 229533.I1RIZ7 pp 229533.I1RWE3
93837 229533.I1RIZ7 pp 229533.I1RLU4
93838 229533.I1RIZ7 pp 229533.I1RSY7
93839 229533.I1RIZ7 pp 229533.I1S6W3
93840 229533.I1RIZ7 pp 229533.I1RR89
93841 229533.A0A0E0SFL4 pp 229533.I1RWB3
93842 229533.A0A0E0SFL4 pp 229533.I1RPY1
93843 229533.I1RDJ4 pp 229533.I1S444
93844 229533.I1RDJ4 pp 229533.I1RZF7
93845 229533.I1RLY2 pp 229533.I1S6W3
93846 229533.I1RLY2 pp 229533.I1RYC1
93847 229533.I1RLY2 pp 229533.I1RXT4
93848 229533.I1RLY2 pp 229533.I1RWE3
93849 229533.I1RLY2 pp 229533.I1RYJ1
93850 229533.I1RLY2 pp 229533.I1S320
93851 229533.I1RLY2 pp 229533.I1RMU2
93852 229533.I1RLY2 pp 229533.V6R5G9
93853 229533.I1RLY2 pp 229533.I1S407
93854 229533.I1RLY2 pp 229533.I1RVH7
93855 229533.I1RLY2 pp 229533.I1RT54
93856 229533.I1RLY2 pp 229533.I1RSY7
93857 229533.I1RLY2 pp 229533.I1RVW8
93858 229533.I1RLY2 pp 229533.I1RR89
93859 229533.I1RLY2 pp 229533.I1S4A4
93860 229533.I1RLY2 pp 229533.I1RWF6
93861 229533.I1S7R4 pp 229533.I1S7R5
93862 229533.A0A098D203 pp 229533.A0A098DC95
93863 229533.A0A098D203 pp 229533.A0A1C3YMT0
93864 229533.A0A098D203 pp 229533.I1S407
93865 229533.A0A098D203 pp 229533.I1RJ55
93866 229533.A0A098D203 pp 229533.I1RFY7
93867 229533.A0A098D203 pp 229533.I1RB60
93868 229533.A0A098D203 pp 229533.I1R9L9
93869 229533.A0A098D203 pp 229533.I1S320
93870 229533.A0A098D203 pp 229533.I1RH94
93871 229533.A0A098D203 pp 229533.I1S4A4
93872 229533.A0A098D203 pp 229533.I1RAN9
93873 229533.A0A098D203 pp 229533.I1RIJ1
93874 229533.A0A098D203 pp 229533.A0A098DIZ0
93875 229533.A0A098D203 pp 229533.I1RR89
93876 229533.A0A098D203 pp 229533.A0A1C3YMT3
93877 229533.A0A098D203 pp 229533.I1RDC7
93878 229533.A0A098D203 pp 229533.I1RSY7

93879	229533.A0A098D203	pp	229533.I1RLU4
93880	229533.A0A098D203	pp	229533.I1RWE3
93881	229533.A0A098D203	pp	229533.I1S6W3
93882	229533.A0A098D203	pp	229533.I1RT54
93883	229533.A0A098D203	pp	229533.I1RXT4
93884	229533.A0A098D203	pp	229533.A0A098DJ33
93885	229533.A0A098D203	pp	229533.I1RIZ7
93886	229533.A0A098D203	pp	229533.I1RLY2
93887	229533.A0A098D203	pp	229533.I1RVH7
93888	229533.I1RXW3	pp	229533.I1S3G4
93889	229533.I1RXW3	pp	229533.I1RYW6
93890	229533.I1RXW3	pp	229533.V6QVH8
93891	229533.I1RXW3	pp	229533.O74638
93892	229533.I1RXW3	pp	229533.Q4HXF0
93893	229533.I1RXW3	pp	229533.V6RM02
93894	229533.I1RXW3	pp	229533.I1S256
93895	229533.I1RXW3	pp	229533.I1RY62
93896	229533.I1RXW3	pp	229533.V6RSF3
93897	229533.I1RXW3	pp	229533.I1S9Z4
93898	229533.I1RXW3	pp	229533.I1RZM4
93899	229533.I1RXW3	pp	229533.I1S1W3
93900	229533.I1RXW3	pp	229533.I1S147
93901	229533.I1RXW3	pp	229533.I1RYJ7
93902	229533.I1RXW3	pp	229533.I1RYI7
93903	229533.I1RXW3	pp	229533.V6RUT5
93904	229533.I1RXW3	pp	229533.V6RK96
93905	229533.I1RXW3	pp	229533.I1S1W8
93906	229533.I1RXW3	pp	229533.I1S0N3
93907	229533.I1RXW3	pp	229533.I1S887
93908	229533.I1RXW3	pp	229533.I1S8F8
93909	229533.I1RLM3	pp	229533.I1S108
93910	229533.I1RLM3	pp	229533.I1S3X7
93911	229533.I1RLM3	pp	229533.I1S1W8
93912	229533.I1RLM3	pp	229533.I1RVZ3
93913	229533.I1RLM3	pp	229533.I1RMJ4
93914	229533.I1RLM3	pp	229533.I1S3G4
93915	229533.I1RLM3	pp	229533.I1S1K5
93916	229533.I1RLM3	pp	229533.I1RXH1
93917	229533.I1RLM3	pp	229533.I1RVR7
93918	229533.I1RLM3	pp	229533.I1RSY4
93919	229533.I1RLM3	pp	229533.I1RU20
93920	229533.I1RLM3	pp	229533.I1S2S8
93921	229533.I1RLM3	pp	229533.I1S350
93922	229533.I1RLM3	pp	229533.I1RLM5
93923	229533.I1RLM3	pp	229533.I1RSL2
93924	229533.I1RLM3	pp	229533.I1RRJ7
93925	229533.I1RLM3	pp	229533.I1RXV8
93926	229533.I1RLM3	pp	229533.I1RTU0
93927	229533.I1RLM3	pp	229533.I1RZW1
93928	229533.I1RLM3	pp	229533.I1RXJ2
93929	229533.I1RLM3	pp	229533.I1RUJ7
93930	229533.I1RLM3	pp	229533.I1RPE7
93931	229533.I1RLM3	pp	229533.I1RUV4
93932	229533.I1RLM3	pp	229533.I1RNG4
93933	229533.I1RLM3	pp	229533.I1RTR2
93934	229533.I1RLM3	pp	229533.I1RYU0
93935	229533.I1RLM3	pp	229533.I1RQM4
93936	229533.I1RLM3	pp	229533.I1RLZ7
93937	229533.I1RLM3	pp	229533.I1RSE0
93938	229533.I1RLM3	pp	229533.I1RMH9
93939	229533.I1RLM3	pp	229533.I1RV54
93940	229533.I1RLM3	pp	229533.I1S167
93941	229533.I1RLM3	pp	229533.I1RPY2
93942	229533.I1RLM3	pp	229533.I1RTW0
93943	229533.I1RLM3	pp	229533.V6RN31
93944	229533.I1RLM3	pp	229533.I1RR45
93945	229533.I1RLM3	pp	229533.I1RM80
93946	229533.I1RLM3	pp	229533.I1RRK5
93947	229533.I1RZP1	pp	229533.I1S0S5
93948	229533.I1RZP1	pp	229533.Q4IBU4
93949	229533.I1RZP1	pp	229533.I1S2E9
93950	229533.I1RZP1	pp	229533.V6QWP4
93951	229533.I1RZP1	pp	229533.I1S8D1

93952 229533.I1RZP1 pp 229533.V6RS91
93953 229533.I1RTC1 pp 229533.Q4ILI9
93954 229533.I1RTC1 pp 229533.I1RYW8
93955 229533.I1RTC1 pp 229533.V6RGI1
93956 229533.I1RTC1 pp 229533.I1RTQ9
93957 229533.I1RTC1 pp 229533.Q4HYZ2
93958 229533.I1RTC1 pp 229533.I1RTG5
93959 229533.I1RTC1 pp 229533.I1RX84
93960 229533.I1RTC1 pp 229533.I1RZ60
93961 229533.I1RJU9 pp 229533.I1RLC8
93962 229533.I1RJU9 pp 229533.I1S2S8
93963 229533.I1RJU9 pp 229533.I1RLM5
93964 229533.I1RJU9 pp 229533.I1RYU0
93965 229533.I1RJU9 pp 229533.I1RSL2
93966 229533.I1RJU9 pp 229533.I1RLM2
93967 229533.I1RJU9 pp 229533.I1RXJ2
93968 229533.I1RJU9 pp 229533.I1RZW1
93969 229533.I1RJU9 pp 229533.I1RR45
93970 229533.I1RJU9 pp 229533.I1RRK5
93971 229533.I1RJU9 pp 229533.I1RV54
93972 229533.I1RJU9 pp 229533.I1RVR7
93973 229533.I1RJU9 pp 229533.I1RLM3
93974 229533.I1RJU9 pp 229533.I1RTR2
93975 229533.I1RJU9 pp 229533.I1RMH9
93976 229533.I1RJU9 pp 229533.I1S350
93977 229533.I1RJU9 pp 229533.I1RKG6
93978 229533.I1RJU9 pp 229533.I1RM80
93979 229533.I1RJU9 pp 229533.I1S3G4
93980 229533.I1RJU9 pp 229533.I1S167
93981 229533.I1RJU9 pp 229533.I1RSY4
93982 229533.I1RJU9 pp 229533.I1RUV4
93983 229533.I1RJU9 pp 229533.I1RXV8
93984 229533.I1RJU9 pp 229533.I1RKG4
93985 229533.I1RJU9 pp 229533.I1RLZ7
93986 229533.I1RJU9 pp 229533.I1RNG4
93987 229533.I1RJU9 pp 229533.I1RPE7
93988 229533.I1RJU9 pp 229533.V6RN31
93989 229533.I1RJU9 pp 229533.I1RSE0
93990 229533.I1RJU9 pp 229533.I1S3X7
93991 229533.I1RJU9 pp 229533.I1RRJ7
93992 229533.I1RJU9 pp 229533.I1S108
93993 229533.I1RJU9 pp 229533.I1RUJ7
93994 229533.I1RJU9 pp 229533.I1RLD2
93995 229533.I1RJU9 pp 229533.I1S1K5
93996 229533.I1RJU9 pp 229533.I1RTU0
93997 229533.I1RJU9 pp 229533.I1RQM4
93998 229533.I1RJU9 pp 229533.I1RTW0
93999 229533.I1RJU9 pp 229533.I1RVZ3
94000 229533.I1RJU9 pp 229533.I1RMJ4
94001 229533.I1RJU9 pp 229533.I1RPY2
94002 229533.I1RJU9 pp 229533.I1RXH1
94003 229533.I1RJU9 pp 229533.I1RLC9
94004 229533.I1RJU9 pp 229533.I1S1W8
94005 229533.I1RJU9 pp 229533.I1RU20
94006 229533.A0A098D1R3 pp 229533.I1RLM3
94007 229533.A0A098D1R3 pp 229533.I1RJU9
94008 229533.A0A098D1R3 pp 229533.A0A0E0SP71
94009 229533.I1RN77 pp 229533.I1RSX7
94010 229533.I1RN77 pp 229533.I1RWF9
94011 229533.I1RN77 pp 229533.I1S0S5
94012 229533.I1RE68 pp 229533.I1RVA8
94013 229533.I1RE68 pp 229533.I1RE78
94014 229533.I1RFW3 pp 229533.I1RYJ8
94015 229533.I1RFW3 pp 229533.I1S471
94016 229533.I1RFW3 pp 229533.I1RLU5
94017 229533.I1RFW3 pp 229533.I1RVA7
94018 229533.I1RFW3 pp 229533.I1RZE1
94019 229533.I1RFW3 pp 229533.I1RYD3
94020 229533.I1RFW3 pp 229533.I1RSP1
94021 229533.I1RFW3 pp 229533.I1RXB1
94022 229533.I1RFW3 pp 229533.I1RWI5
94023 229533.I1RFW3 pp 229533.I1S2A8
94024 229533.I1RFW3 pp 229533.I1S1J7

94025 229533.I1RFW3 pp 229533.I1RRE0
94026 229533.I1RFW3 pp 229533.I1RZT1
94027 229533.I1RFW3 pp 229533.I1RVY2
94028 229533.I1S166 pp 229533.I1S9Z4
94029 229533.I1S166 pp 229533.I1S3K7
94030 229533.I1S166 pp 229533.I1S2J4
94031 229533.I1S166 pp 229533.I1S1L0
94032 229533.I1S166 pp 229533.I1S5U5
94033 229533.A0A0E0SFJ9 pp 229533.I1RB98
94034 229533.A0A0E0SFJ9 pp 229533.I1RWB4
94035 229533.A0A0E0SFJ9 pp 229533.I1RFK0
94036 229533.A0A0E0SFJ9 pp 229533.I1RS56
94037 229533.A0A0E0SFJ9 pp 229533.A0A1C3YHW1
94038 229533.A0A0E0SFJ9 pp 229533.I1RQP7
94039 229533.A0A0E0SFJ9 pp 229533.I1S287
94040 229533.A0A0E0SFJ9 pp 229533.I1RSI6
94041 229533.A0A0E0SFJ9 pp 229533.I1RYU1
94042 229533.A0A0E0SFJ9 pp 229533.A0A1C3YKT4
94043 229533.A0A0E0SFJ9 pp 229533.I1RMF2
94044 229533.A0A0E0SFJ9 pp 229533.I1RTQ9
94045 229533.A0A0E0SFJ9 pp 229533.I1RQ32
94046 229533.A0A0E0SFJ9 pp 229533.I1RD45
94047 229533.A0A0E0SFJ9 pp 229533.I1RQ70
94048 229533.A0A0E0SFJ9 pp 229533.A0A1C3YN54
94049 229533.A0A0E0SFJ9 pp 229533.I1S1V7
94050 229533.A0A0E0SFJ9 pp 229533.Q4IJ79
94051 229533.I1RDL0 pp 229533.I1SAS4
94052 229533.I1RDL0 pp 229533.I1SAS3
94053 229533.I1RDL0 pp 229533.I1RSU2
94054 229533.I1RDL0 pp 229533.I1RPY0
94055 229533.I1RDL0 pp 229533.I1RFM2
94056 229533.I1RDL0 pp 229533.I1RJG1
94057 229533.I1RDL0 pp 229533.I1S0Y2
94058 229533.I1RDL0 pp 229533.I1RFP1
94059 229533.I1RDL0 pp 229533.I1RMH4
94060 229533.I1RDL0 pp 229533.I1S1Q3
94061 229533.I1RDL0 pp 229533.I1RPI4
94062 229533.I1RDL0 pp 229533.I1RE53
94063 229533.I1RDL0 pp 229533.V6RR38
94064 229533.I1RDL0 pp 229533.I1RQ48
94065 229533.I1RF58 pp 229533.Q00909
94066 229533.I1RF58 pp 229533.I1S1T5
94067 229533.I1RF58 pp 229533.I1RF61
94068 229533.I1RF58 pp 229533.I1S9Z4
94069 229533.I1RF58 pp 229533.I1RF62
94070 229533.I1RF58 pp 229533.I1RVL7
94071 229533.I1RF58 pp 229533.I1RIV2
94072 229533.I1RF58 pp 229533.I1RFC3
94073 229533.I1RF58 pp 229533.I1RHK0
94074 229533.I1RF58 pp 229533.I1S8H5
94075 229533.I1RF58 pp 229533.I1RF59
94076 229533.I1RF58 pp 229533.I1S1L0
94077 229533.I1RF58 pp 229533.I1RPR1
94078 229533.I1RF58 pp 229533.I1RQ48
94079 229533.I1RF58 pp 229533.I1S2J4
94080 229533.I1RF58 pp 229533.I1RK69
94081 229533.I1RF58 pp 229533.I1RF63
94082 229533.I1RF58 pp 229533.I1RVD9
94083 229533.I1RF58 pp 229533.I1S3K7
94084 229533.I1RF58 pp 229533.I1RVM4
94085 229533.I1RF58 pp 229533.I1S5U5
94086 229533.I1RF58 pp 229533.I1RF60
94087 229533.I1RYW8 pp 229533.I1RZK3
94088 229533.I1RYW8 pp 229533.I1RZV4
94089 229533.I1RYW8 pp 229533.Q4HYZ2
94090 229533.I1RYW8 pp 229533.I1RZ60
94091 229533.I1RYW8 pp 229533.I1S095
94092 229533.I1RYW8 pp 229533.V6RR76
94093 229533.I1RYW8 pp 229533.Q4ILI9
94094 229533.I1RYW8 pp 229533.I1RZD9
94095 229533.A0A1C3YJV4 pp 229533.I1RAI1
94096 229533.A0A1C3YJV4 pp 229533.I1RSZ3
94097 229533.A0A1C3YJV4 pp 229533.I1RCH7

94098 229533.A0A1C3YJV4 pp 229533.I1S246
94099 229533.A0A1C3YJV4 pp 229533.I1RN60
94100 229533.A0A1C3YJV4 pp 229533.A0A1C3YL25
94101 229533.A0A1C3YJV4 pp 229533.I1RCK4
94102 229533.A0A1C3YMF1 pp 229533.I1S3G4
94103 229533.A0A1C3YMF1 pp 229533.I1RQ60
94104 229533.A0A1C3YMF1 pp 229533.I1RYQ4
94105 229533.A0A1C3YMF1 pp 229533.I1RBE7
94106 229533.A0A1C3YMF1 pp 229533.I1SAF0
94107 229533.A0A1C3YMF1 pp 229533.I1RKZ7
94108 229533.A0A1C3YMF1 pp 229533.I1RPE5
94109 229533.A0A1C3YMF1 pp 229533.I1RUS5
94110 229533.A0A1C3YMF1 pp 229533.I1RHC8
94111 229533.A0A1C3YMF1 pp 229533.I1RNY6
94112 229533.A0A1C3YMF1 pp 229533.I1RY35
94113 229533.A0A1C3YMF1 pp 229533.I1RYA2
94114 229533.A0A1C3YMF1 pp 229533.I1RKZ2
94115 229533.A0A1C3YMF1 pp 229533.I1RW54
94116 229533.A0A1C3YMF1 pp 229533.I1S599
94117 229533.A0A1C3YMF1 pp 229533.I1RG66
94118 229533.A0A1C3YMF1 pp 229533.I1S1W8
94119 229533.A0A1C3YMF1 pp 229533.Q4HVQ9
94120 229533.A0A1C3YMF1 pp 229533.I1RNV4
94121 229533.A0A1C3YMF1 pp 229533.I1S267
94122 229533.A0A1C3YMF1 pp 229533.I1S2C4
94123 229533.A0A1C3YMF1 pp 229533.I1RYK1
94124 229533.A0A1C3YMF1 pp 229533.I1RCR6
94125 229533.A0A1C3YMF1 pp 229533.I1RJB0
94126 229533.A0A1C3YMF1 pp 229533.I1RQE1
94127 229533.A0A1C3YMF1 pp 229533.I1RNG4
94128 229533.A0A1C3YMF1 pp 229533.I1RYA8
94129 229533.A0A1C3YMF1 pp 229533.I1S6M5
94130 229533.A0A1C3YMF1 pp 229533.I1RYW9
94131 229533.A0A1C3YMF1 pp 229533.I1RWL3
94132 229533.A0A1C3YMF1 pp 229533.I1RDQ2
94133 229533.A0A1C3YMF1 pp 229533.I1RVB2
94134 229533.A0A1C3YMF1 pp 229533.I1RYX6
94135 229533.A0A1C3YMF1 pp 229533.I1RD67
94136 229533.A0A1C3YMF1 pp 229533.I1R9B6
94137 229533.A0A1C3YMF1 pp 229533.I1RRF5
94138 229533.I1RFT5 pp 229533.I1S290
94139 229533.I1RFT5 pp 229533.I1RXV3
94140 229533.I1RFL8 pp 229533.I1RTF5
94141 229533.I1RFL8 pp 229533.I1RJH8
94142 229533.I1RFL8 pp 229533.I1RQ82
94143 229533.I1RFL8 pp 229533.I1RJL5
94144 229533.I1RFL8 pp 229533.I1S9G7
94145 229533.I1RFL8 pp 229533.I1RNL7
94146 229533.I1RFL8 pp 229533.I1RX25
94147 229533.I1RFL8 pp 229533.I1RZJ5
94148 229533.I1RFL8 pp 229533.I1RRR5
94149 229533.I1RFL8 pp 229533.I1RJH7
94150 229533.I1RFL8 pp 229533.Q4HY20
94151 229533.I1RAB2 pp 229533.I1S344
94152 229533.I1RAB2 pp 229533.I1RE68
94153 229533.I1RAB2 pp 229533.I1RN41
94154 229533.I1RAB2 pp 229533.I1RYP7
94155 229533.I1RAB2 pp 229533.I1RNG8
94156 229533.I1RAB2 pp 229533.I1RE78
94157 229533.I1RAB2 pp 229533.I1RR07
94158 229533.A0A1C3YLM7 pp 229533.I1RFI9
94159 229533.A0A1C3YLM7 pp 229533.I1S287
94160 229533.A0A1C3YLM7 pp 229533.I1RGY6
94161 229533.A0A1C3YLM7 pp 229533.I1RPY0
94162 229533.A0A1C3YLM7 pp 229533.I1RF33
94163 229533.A0A1C3YLM7 pp 229533.I1RPN1
94164 229533.A0A1C3YLM7 pp 229533.I1RUL3
94165 229533.A0A1C3YLM7 pp 229533.I1RDJ8
94166 229533.A0A1C3YLM7 pp 229533.I1RFY0
94167 229533.I1RUB2 pp 229533.I1RVD9
94168 229533.I1RUB2 pp 229533.I1RVD4
94169 229533.I1RUB2 pp 229533.Q4HWQ2
94170 229533.I1RUB2 pp 229533.I1S2J4

94171	229533.I1RUB2	pp	229533.I1S3K7
94172	229533.I1RUB2	pp	229533.I1S1L0
94173	229533.I1RUB2	pp	229533.I1S1K2
94174	229533.I1RUB2	pp	229533.I1S5U5
94175	229533.I1RUB2	pp	229533.I1RVM4
94176	229533.I1RUB2	pp	229533.I1SAJ7
94177	229533.I1RUB2	pp	229533.I1S8P4
94178	229533.I1RUB2	pp	229533.I1S8P5
94179	229533.I1RUB2	pp	229533.I1RVL7
94180	229533.I1RUB2	pp	229533.I1S9Z4
94181	229533.I1RUB2	pp	229533.I1RUB5
94182	229533.I1RUB2	pp	229533.I1RV24
94183	229533.I1RX96	pp	229533.I1RYE4
94184	229533.I1RX96	pp	229533.I1S287
94185	229533.I1RX96	pp	229533.Q4I5R9
94186	229533.I1RB98	pp	229533.I1RC46
94187	229533.I1RB98	pp	229533.I1RMF2
94188	229533.I1RB98	pp	229533.I1RQ32
94189	229533.I1RB98	pp	229533.I1RKR7
94190	229533.I1RB98	pp	229533.V6RB81
94191	229533.I1RB98	pp	229533.I1RRZ3
94192	229533.I1RB98	pp	229533.I1S7X9
94193	229533.I1RB98	pp	229533.I1RG99
94194	229533.I1RB98	pp	229533.I1S1V7
94195	229533.I1RB98	pp	229533.I1S834
94196	229533.I1RB98	pp	229533.I1RYU1
94197	229533.I1RB98	pp	229533.I1RQK5
94198	229533.I1RB98	pp	229533.I1RSJ0
94199	229533.I1RB98	pp	229533.I1RQ70
94200	229533.I1RB98	pp	229533.I1RK33
94201	229533.I1RB98	pp	229533.I1RX09
94202	229533.I1RB98	pp	229533.I1RFK0
94203	229533.I1RB98	pp	229533.I1RMK1
94204	229533.I1RBE7	pp	229533.I1RME3
94205	229533.I1RBE7	pp	229533.I1RVB2
94206	229533.I1RBE7	pp	229533.I1S788
94207	229533.I1RBE7	pp	229533.I1RMJ4
94208	229533.I1RBE7	pp	229533.I1S2D5
94209	229533.I1RBE7	pp	229533.I1RQ60
94210	229533.I1RBE7	pp	229533.I1RPW2
94211	229533.I1RBE7	pp	229533.I1RCS1
94212	229533.I1RBE7	pp	229533.I1RYJ7
94213	229533.I1RBE7	pp	229533.I1RVD9
94214	229533.I1RBE7	pp	229533.I1S8E0
94215	229533.I1RBE7	pp	229533.I1RYK1
94216	229533.I1RBE7	pp	229533.I1RY49
94217	229533.I1RBE7	pp	229533.I1RTG4
94218	229533.I1RBE7	pp	229533.I1RMM8
94219	229533.I1RBE7	pp	229533.I1RYU5
94220	229533.I1RBE7	pp	229533.I1S1W8
94221	229533.I1RBE7	pp	229533.I1RQE8
94222	229533.I1RBE7	pp	229533.I1RMK4
94223	229533.I1RBE7	pp	229533.I1RTY0
94224	229533.I1RBE7	pp	229533.I1RYQ4
94225	229533.I1RBE7	pp	229533.I1RME6
94226	229533.I1RBE7	pp	229533.I1S686
94227	229533.I1RBE7	pp	229533.I1S0T2
94228	229533.I1RBE7	pp	229533.I1RCR6
94229	229533.I1RBE7	pp	229533.I1RJ72
94230	229533.I1RBE7	pp	229533.I1RRY1
94231	229533.I1RBE7	pp	229533.I1RBW0
94232	229533.I1RBE7	pp	229533.I1RXY4
94233	229533.I1RBE7	pp	229533.I1RP86
94234	229533.I1RBE7	pp	229533.I1RNQ1
94235	229533.I1RBE7	pp	229533.I1S029
94236	229533.I1RBE7	pp	229533.I1RBF1
94237	229533.I1RBE7	pp	229533.I1RMH9
94238	229533.I1RBE7	pp	229533.I1RQE1
94239	229533.I1RBE7	pp	229533.I1S1B8
94240	229533.I1RBE7	pp	229533.I1RFH9
94241	229533.I1RBE7	pp	229533.I1SAF0
94242	229533.I1RBE7	pp	229533.I1RI02
94243	229533.I1RBE7	pp	229533.I1RK93

94244 229533.I1RBE7 pp 229533.I1RVX9
94245 229533.I1RBE7 pp 229533.I1RPI4
94246 229533.I1RBE7 pp 229533.I1RUV4
94247 229533.I1RBE7 pp 229533.I1S3K8
94248 229533.I1RBE7 pp 229533.I1RDQ2
94249 229533.I1RBE7 pp 229533.V6RQW4
94250 229533.I1RBE7 pp 229533.I1RE86
94251 229533.I1RBE7 pp 229533.I1RYX6
94252 229533.I1RBE7 pp 229533.Q5I2J3
94253 229533.I1RBE7 pp 229533.I1RYJ6
94254 229533.I1RBE7 pp 229533.I1RYM6
94255 229533.I1RBE7 pp 229533.I1RC83
94256 229533.I1RBE7 pp 229533.I1S1D1
94257 229533.I1RBE7 pp 229533.Q4I5G1
94258 229533.I1RBE7 pp 229533.I1RPW1
94259 229533.I1RBE7 pp 229533.Q4HYQ4
94260 229533.I1RBE7 pp 229533.I1RD67
94261 229533.I1RBE7 pp 229533.I1RFM6
94262 229533.I1RBE7 pp 229533.I1RVB7
94263 229533.I1RBE7 pp 229533.I1RXB1
94264 229533.I1RBE7 pp 229533.I1RM26
94265 229533.I1RBE7 pp 229533.I1RJ73
94266 229533.I1RBE7 pp 229533.I1RCL0
94267 229533.I1RBE7 pp 229533.I1RPE7
94268 229533.I1RBE7 pp 229533.I1RYA2
94269 229533.I1RBE7 pp 229533.I1SAJ7
94270 229533.I1RBE7 pp 229533.I1RZT6
94271 229533.I1RBE7 pp 229533.I1RPI0
94272 229533.I1RBE7 pp 229533.I1RIY0
94273 229533.I1RBE7 pp 229533.V6RN16
94274 229533.I1RBE7 pp 229533.I1RCU2
94275 229533.I1RBE7 pp 229533.I1RQE0
94276 229533.I1RBE7 pp 229533.V6RY56
94277 229533.I1RBE7 pp 229533.I1RYL5
94278 229533.I1RBE7 pp 229533.I1RVY2
94279 229533.I1RBE7 pp 229533.I1RQC4
94280 229533.I1RBE7 pp 229533.I1RU76
94281 229533.I1RBE7 pp 229533.I1S241
94282 229533.I1RBE7 pp 229533.I1RNW1
94283 229533.I1RBE7 pp 229533.I1S4G3
94284 229533.I1RBE7 pp 229533.V6RDH3
94285 229533.I1RBE7 pp 229533.I1RN61
94286 229533.I1RBE7 pp 229533.I1RUQ3
94287 229533.I1RBE7 pp 229533.I1RZM4
94288 229533.I1RBE7 pp 229533.V6QU65
94289 229533.I1RBE7 pp 229533.I1RYI7
94290 229533.I1RBE7 pp 229533.I1S0N3
94291 229533.I1RBE7 pp 229533.I1RMG7
94292 229533.I1RBE7 pp 229533.I1RTU0
94293 229533.I1RBE7 pp 229533.I1RCV1
94294 229533.I1RBE7 pp 229533.I1RUY5
94295 229533.I1RBE7 pp 229533.I1RPM6
94296 229533.I1RBE7 pp 229533.I1RBW1
94297 229533.I1RBE7 pp 229533.I1RXV9
94298 229533.I1RBE7 pp 229533.I1RDU0
94299 229533.I1RBE7 pp 229533.I1RIE8
94300 229533.I1RBE7 pp 229533.V6QV31
94301 229533.I1RBE7 pp 229533.I1S2M7
94302 229533.I1RBE7 pp 229533.I1S2Q4
94303 229533.I1RBE7 pp 229533.I1RR51
94304 229533.I1RBE7 pp 229533.I1S4D0
94305 229533.I1RBE7 pp 229533.I1RUI6
94306 229533.I1RBE7 pp 229533.I1RFC3
94307 229533.I1RBE7 pp 229533.I1RN14
94308 229533.I1RBE7 pp 229533.I1S3G4
94309 229533.I1RBE7 pp 229533.I1S019
94310 229533.I1RBE7 pp 229533.I1RF49
94311 229533.I1RBE7 pp 229533.I1S419
94312 229533.I1RBE7 pp 229533.I1RVN1
94313 229533.I1RBE7 pp 229533.I1RZ29
94314 229533.I1RBE7 pp 229533.I1RG66
94315 229533.I1RBE7 pp 229533.I1RF38
94316 229533.I1RBE7 pp 229533.I1RJF2

94317 229533.I1RBE7 pp 229533.V6RK96
94318 229533.I1RBE7 pp 229533.I1RJL9
94319 229533.I1RBE7 pp 229533.I1S0A0
94320 229533.I1RBE7 pp 229533.I1S1L0
94321 229533.I1RBE7 pp 229533.I1RW69
94322 229533.I1RBE7 pp 229533.I1RXV3
94323 229533.I1RBE7 pp 229533.I1RVW0
94324 229533.I1RBE7 pp 229533.I1RD81
94325 229533.I1RBE7 pp 229533.I1S183
94326 229533.I1RBE7 pp 229533.I1RUS5
94327 229533.I1RBE7 pp 229533.I1RZT0
94328 229533.I1RBE7 pp 229533.I1RVZ3
94329 229533.I1RBE7 pp 229533.I1RQ81
94330 229533.I1RBE7 pp 229533.I1RN88
94331 229533.I1RBE7 pp 229533.I1S2J4
94332 229533.I1RBE7 pp 229533.I1RSK5
94333 229533.I1RBE7 pp 229533.I1S5R1
94334 229533.I1RBE7 pp 229533.I1RH91
94335 229533.I1RBE7 pp 229533.I1S1C3
94336 229533.I1RBE7 pp 229533.I1RW43
94337 229533.I1RBE7 pp 229533.I1S4B6
94338 229533.I1RBE7 pp 229533.O74638
94339 229533.I1RBE7 pp 229533.I1RYA8
94340 229533.I1RBE7 pp 229533.I1RJB0
94341 229533.I1RBE7 pp 229533.I1S6Y8
94342 229533.I1RBE7 pp 229533.I1RJQ5
94343 229533.I1RBE7 pp 229533.I1S065
94344 229533.I1RBE7 pp 229533.I1RDE7
94345 229533.I1RBE7 pp 229533.V6RXR0
94346 229533.I1RBE7 pp 229533.I1RFI8
94347 229533.I1RBE7 pp 229533.I1S9C6
94348 229533.I1RBE7 pp 229533.I1RZ21
94349 229533.I1RBE7 pp 229533.I1RZ86
94350 229533.I1RBE7 pp 229533.I1RVY0
94351 229533.I1RBE7 pp 229533.I1RP97
94352 229533.I1RBE7 pp 229533.I1S3X7
94353 229533.I1RBE7 pp 229533.I1RJJ7
94354 229533.I1RBE7 pp 229533.I1RT75
94355 229533.I1RBE7 pp 229533.I1RZ52
94356 229533.I1RBE7 pp 229533.I1RBY9
94357 229533.I1RBE7 pp 229533.I1RXW3
94358 229533.I1RBE7 pp 229533.V6RUY9
94359 229533.I1RBE7 pp 229533.I1S5L5
94360 229533.I1RBE7 pp 229533.Q4HVQ9
94361 229533.I1RBE7 pp 229533.I1RS47
94362 229533.I1RBE7 pp 229533.I1RNW6
94363 229533.I1RBE7 pp 229533.I1S3K7
94364 229533.I1RBE7 pp 229533.I1RC34
94365 229533.I1RBE7 pp 229533.I1RNB4
94366 229533.I1RBE7 pp 229533.I1RPD6
94367 229533.I1RBE7 pp 229533.I1RY62
94368 229533.I1RBE7 pp 229533.I1S6M5
94369 229533.I1RBE7 pp 229533.I1RCR3
94370 229533.I1RBE7 pp 229533.I1RZ20
94371 229533.I1RBE7 pp 229533.I1RF60
94372 229533.I1RBE7 pp 229533.I1RE53
94373 229533.I1RBE7 pp 229533.I1RNG7
94374 229533.I1RBE7 pp 229533.I1RNG4
94375 229533.I1RBE7 pp 229533.I1RKB2
94376 229533.I1RBE7 pp 229533.I1SAJ3
94377 229533.I1RBE7 pp 229533.I1RQC3
94378 229533.I1RBE7 pp 229533.I1RMG6
94379 229533.I1RBE7 pp 229533.I1RX72
94380 229533.I1RBE7 pp 229533.V6RHD0
94381 229533.I1RBE7 pp 229533.I1S5C5
94382 229533.I1RBE7 pp 229533.I1RYW9
94383 229533.I1RBE7 pp 229533.I1RWS4
94384 229533.I1RBE7 pp 229533.I1RYW3
94385 229533.I1RBE7 pp 229533.I1RIH0
94386 229533.I1RBE7 pp 229533.I1RE63
94387 229533.I1RBE7 pp 229533.Q4HW67
94388 229533.I1RBE7 pp 229533.I1RCG5
94389 229533.I1RBE7 pp 229533.V6RN31

94390 229533.I1RBE7 pp 229533.I1RCB4
94391 229533.I1RBE7 pp 229533.I1S256
94392 229533.I1RBE7 pp 229533.I1S1T5
94393 229533.I1RBE7 pp 229533.I1RNA2
94394 229533.I1RBE7 pp 229533.I1RYK0
94395 229533.I1RBE7 pp 229533.V6RSF3
94396 229533.I1RBE7 pp 229533.I1S2C4
94397 229533.I1RBE7 pp 229533.I1S4B7
94398 229533.I1RBE7 pp 229533.I1RRF5
94399 229533.I1RBE7 pp 229533.I1RQ75
94400 229533.I1RBE7 pp 229533.I1S685
94401 229533.I1RBE7 pp 229533.I1S0D5
94402 229533.I1RBE7 pp 229533.I1RNW2
94403 229533.I1RBE7 pp 229533.I1RG11
94404 229533.I1RBE7 pp 229533.I1S684
94405 229533.I1RBE7 pp 229533.I1RFK3
94406 229533.I1RBE7 pp 229533.V6RRM0
94407 229533.I1RBE7 pp 229533.I1RF53
94408 229533.I1RBE7 pp 229533.I1RWZ3
94409 229533.I1RBE7 pp 229533.I1RQ30
94410 229533.I1RBE7 pp 229533.I1S8H5
94411 229533.I1RBE7 pp 229533.Q4I1Y5
94412 229533.I1RBE7 pp 229533.I1RIJ5
94413 229533.I1RBE7 pp 229533.I1RF85
94414 229533.I1RBE7 pp 229533.I1S3V2
94415 229533.I1RBE7 pp 229533.I1RNV4
94416 229533.I1RBE7 pp 229533.I1RC54
94417 229533.I1RBE7 pp 229533.I1S569
94418 229533.I1RBE7 pp 229533.I1RKZ7
94419 229533.I1RBE7 pp 229533.I1RUG3
94420 229533.I1RBE7 pp 229533.I1S0I3
94421 229533.I1RBE7 pp 229533.I1RHC8
94422 229533.I1RBE7 pp 229533.I1RKZ2
94423 229533.I1RBE7 pp 229533.I1RPU8
94424 229533.I1RBE7 pp 229533.I1RE79
94425 229533.I1RBE7 pp 229533.I1RZ70
94426 229533.I1RBE7 pp 229533.V6QVH8
94427 229533.I1RBE7 pp 229533.I1RZQ1
94428 229533.I1RBE7 pp 229533.I1S0S5
94429 229533.I1RBE7 pp 229533.I1SAN9
94430 229533.I1RBE7 pp 229533.I1RXY7
94431 229533.I1RBE7 pp 229533.I1RSL5
94432 229533.I1RBE7 pp 229533.I1S4P6
94433 229533.I1RBE7 pp 229533.I1S599
94434 229533.I1RBE7 pp 229533.I1RRV1
94435 229533.I1RBE7 pp 229533.I1RDY5
94436 229533.I1RBE7 pp 229533.I1RGG7
94437 229533.I1RBE7 pp 229533.I1S064
94438 229533.I1RBE7 pp 229533.V6RLM7
94439 229533.I1RBE7 pp 229533.I1RR05
94440 229533.I1RBE7 pp 229533.I1RIV2
94441 229533.I1RA83 pp 229533.I1RW53
94442 229533.I1RA83 pp 229533.I1S9S6
94443 229533.I1RA83 pp 229533.I1RM74
94444 229533.I1RA83 pp 229533.I1RS64
94445 229533.I1RA83 pp 229533.I1RAL5
94446 229533.I1RA83 pp 229533.I1RWN2
94447 229533.I1RA83 pp 229533.I1RNB8
94448 229533.I1RA83 pp 229533.I1RBP9
94449 229533.I1RA83 pp 229533.I1RG90
94450 229533.I1RA83 pp 229533.I1RZY6
94451 229533.I1RA83 pp 229533.I1S1X2
94452 229533.I1RA83 pp 229533.I1RT35
94453 229533.I1RA83 pp 229533.I1RSR2
94454 229533.I1RA83 pp 229533.I1RT65
94455 229533.A0A098DME4 pp 229533.A0A0E0S3A4
94456 229533.A0A098DME4 pp 229533.I1RB58
94457 229533.A0A098DME4 pp 229533.I1RKP5
94458 229533.A0A098DME4 pp 229533.I1RK35
94459 229533.A0A098DME4 pp 229533.I1RIA5
94460 229533.A0A098DME4 pp 229533.A0A0E0SP71
94461 229533.A0A098DME4 pp 229533.I1RJU9
94462 229533.A0A098DME4 pp 229533.I1RLM3

94463	229533.I1RL11	pp	229533.I1RQ48
94464	229533.I1RL11	pp	229533.I1S1L0
94465	229533.I1RL11	pp	229533.I1S3K7
94466	229533.I1RL11	pp	229533.I1RVD9
94467	229533.I1RL11	pp	229533.I1S5U5
94468	229533.I1RL11	pp	229533.I1SAJ7
94469	229533.I1RL11	pp	229533.I1RVM4
94470	229533.I1RL11	pp	229533.I1S9Z4
94471	229533.I1RL11	pp	229533.I1RVL7
94472	229533.I1RL11	pp	229533.I1RPR1
94473	229533.A0A098DL10	pp	229533.A0A098DME4
94474	229533.I1S6D1	pp	229533.V6RS91
94475	229533.I1RAL5	pp	229533.I1RW53
94476	229533.I1RAL5	pp	229533.I1RV65
94477	229533.I1RAL5	pp	229533.I1S282
94478	229533.I1RAL5	pp	229533.I1RBP9
94479	229533.I1RAL5	pp	229533.I1RSR2
94480	229533.I1RA28	pp	229533.I1S3G4
94481	229533.I1RA28	pp	229533.I1RW43
94482	229533.I1RA28	pp	229533.I1S2D5
94483	229533.I1RA28	pp	229533.V6R5D2
94484	229533.I1RA28	pp	229533.Q4HVQ9
94485	229533.I1RA28	pp	229533.I1RCR6
94486	229533.I1RA28	pp	229533.I1S1W8
94487	229533.I1RA28	pp	229533.I1RTG4
94488	229533.I1RA28	pp	229533.I1RPD6
94489	229533.I1RA28	pp	229533.I1RR05
94490	229533.I1RA28	pp	229533.I1RC48
94491	229533.I1RA28	pp	229533.I1RBE7
94492	229533.A0A098DAD6	pp	229533.I1RA83
94493	229533.A0A098DAD6	pp	229533.I1RAL5
94494	229533.A0A098DL75	pp	229533.V6RN31
94495	229533.A0A098DL75	pp	229533.A0A098E1U3
94496	229533.I1RY87	pp	229533.I1S2J4
94497	229533.I1RY87	pp	229533.I1S1L0
94498	229533.I1RY87	pp	229533.I1S1T5
94499	229533.I1RY87	pp	229533.I1S3K7
94500	229533.I1RY87	pp	229533.I1S1C3
94501	229533.I1RY87	pp	229533.I1S2M7
94502	229533.I1RY87	pp	229533.V6RPQ8
94503	229533.I1RY87	pp	229533.I1S8H5
94504	229533.I1RY87	pp	229533.I1SAJ7
94505	229533.I1S1W8	pp	229533.V6QWD8
94506	229533.I1S1W8	pp	229533.I1S2D5
94507	229533.I1S1W8	pp	229533.I1S8Z2
94508	229533.I1S1W8	pp	229533.V6RS91
94509	229533.I1S1W8	pp	229533.I1S686
94510	229533.I1S1W8	pp	229533.V6R1K4
94511	229533.I1S1W8	pp	229533.I1SAF0
94512	229533.I1S1W8	pp	229533.I1S3K8
94513	229533.I1S1W8	pp	229533.I1S9G7
94514	229533.I1S1W8	pp	229533.I1S241
94515	229533.I1S1W8	pp	229533.I1S4S7
94516	229533.I1S1W8	pp	229533.I1SAJ7
94517	229533.I1S1W8	pp	229533.V6RTJ8
94518	229533.I1S1W8	pp	229533.V6QU65
94519	229533.I1S1W8	pp	229533.V6RDH3
94520	229533.I1S1W8	pp	229533.V6RQW4
94521	229533.I1S1W8	pp	229533.I1S209
94522	229533.I1S1W8	pp	229533.V6RY56
94523	229533.I1S1W8	pp	229533.I1S3K6
94524	229533.I1S1W8	pp	229533.I1S5R5
94525	229533.I1S1W8	pp	229533.I1S2M7
94526	229533.I1S1W8	pp	229533.I1S4D0
94527	229533.I1S1W8	pp	229533.V6RXR0
94528	229533.I1S1W8	pp	229533.V6QUE6
94529	229533.I1S1W8	pp	229533.I1S4G3
94530	229533.I1S1W8	pp	229533.I1S4Z7
94531	229533.I1S1W8	pp	229533.V6RBN2
94532	229533.I1S1W8	pp	229533.I1S6P5
94533	229533.I1S1W8	pp	229533.I1S3G4
94534	229533.I1S1W8	pp	229533.V6RKY8
94535	229533.I1S1W8	pp	229533.V6RPN0

94536	229533.I1S1W8	pp	229533.O74638
94537	229533.I1S1W8	pp	229533.I1S2Q4
94538	229533.I1S1W8	pp	229533.I1S8F8
94539	229533.I1S1W8	pp	229533.I1S5R1
94540	229533.I1S1W8	pp	229533.I1S6M5
94541	229533.I1S1W8	pp	229533.I1S4B6
94542	229533.I1S1W8	pp	229533.V6RG22
94543	229533.I1S1W8	pp	229533.I1S253
94544	229533.I1S1W8	pp	229533.I1S2J4
94545	229533.I1S1W8	pp	229533.V6RK96
94546	229533.I1S1W8	pp	229533.I1S9C6
94547	229533.I1S1W8	pp	229533.V6R3J1
94548	229533.I1S1W8	pp	229533.I1S277
94549	229533.I1S1W8	pp	229533.I1S4Z8
94550	229533.I1S1W8	pp	229533.V6RUB1
94551	229533.I1S1W8	pp	229533.V6RSF3
94552	229533.I1S1W8	pp	229533.I1S9T5
94553	229533.I1S1W8	pp	229533.V6QV31
94554	229533.I1S1W8	pp	229533.I1S2C4
94555	229533.I1S1W8	pp	229533.Q4HXF0
94556	229533.I1S1W8	pp	229533.I1S3K7
94557	229533.I1S1W8	pp	229533.I1SAJ3
94558	229533.I1S1W8	pp	229533.I1S684
94559	229533.I1S1W8	pp	229533.V6RUY9
94560	229533.I1S1W8	pp	229533.V6QVH8
94561	229533.I1S1W8	pp	229533.V6QX21
94562	229533.I1S1W8	pp	229533.I1S4B7
94563	229533.I1S1W8	pp	229533.Q4I624
94564	229533.I1S1W8	pp	229533.V6RRM0
94565	229533.I1S1W8	pp	229533.V6RHD0
94566	229533.I1S1W8	pp	229533.I1S8H5
94567	229533.I1S1W8	pp	229533.V6RLM7
94568	229533.I1S1W8	pp	229533.I1S256
94569	229533.I1S1W8	pp	229533.I1S685
94570	229533.I1S1W8	pp	229533.V6R057
94571	229533.I1S1W8	pp	229533.I1S599
94572	229533.I1RK44	pp	229533.V6RAN2
94573	229533.I1RK44	pp	229533.I1RNA1
94574	229533.I1RK44	pp	229533.Q5I2J3
94575	229533.I1RK44	pp	229533.V6R4J1
94576	229533.I1RK44	pp	229533.I1RP86
94577	229533.I1RK44	pp	229533.I1RNA5
94578	229533.I1RK44	pp	229533.I1S097
94579	229533.I1RK44	pp	229533.I1RLM7
94580	229533.I1RK44	pp	229533.I1S574
94581	229533.I1RK44	pp	229533.I1RR95
94582	229533.I1RK44	pp	229533.I1RLN6
94583	229533.I1RK44	pp	229533.I1S1L2
94584	229533.I1RK44	pp	229533.Q4HZS8
94585	229533.I1RK44	pp	229533.I1S569
94586	229533.I1RK44	pp	229533.I1S984
94587	229533.I1RK44	pp	229533.I1RNU7
94588	229533.I1RK44	pp	229533.I1RRT3
94589	229533.I1RK44	pp	229533.I1RW07
94590	229533.I1RK44	pp	229533.I1RLC7
94591	229533.I1RK44	pp	229533.V6R729
94592	229533.I1RK44	pp	229533.I1RKP3
94593	229533.I1RK44	pp	229533.I1RU29
94594	229533.I1RK44	pp	229533.I1RZ13
94595	229533.I1RK44	pp	229533.I1RRQ1
94596	229533.I1RK44	pp	229533.I1RK45
94597	229533.I1RK44	pp	229533.I1S272
94598	229533.I1RK44	pp	229533.I1S352
94599	229533.A0A098DKW7	pp	229533.I1RBY9
94600	229533.A0A098DKW7	pp	229533.I1RK44
94601	229533.I1S3G4	pp	229533.I1S5R5
94602	229533.I1S3G4	pp	229533.V6RG22
94603	229533.I1S3G4	pp	229533.V6RDH3
94604	229533.I1S3G4	pp	229533.I1S4D0
94605	229533.I1S3G4	pp	229533.I1S3K6
94606	229533.I1S3G4	pp	229533.V6QUE6
94607	229533.I1S3G4	pp	229533.V6RBN2
94608	229533.I1S3G4	pp	229533.V6RK96

94609 229533.I1S3G4 pp 229533.I1SAJ7
94610 229533.I1S3G4 pp 229533.I1S9G7
94611 229533.I1S3G4 pp 229533.V6RPN0
94612 229533.I1S3G4 pp 229533.V6QU65
94613 229533.I1S3G4 pp 229533.V6RQW4
94614 229533.I1S3G4 pp 229533.I1S8F8
94615 229533.I1S3G4 pp 229533.I1S4G3
94616 229533.I1S3G4 pp 229533.V6RY56
94617 229533.I1S3G4 pp 229533.V6RS91
94618 229533.I1S3G4 pp 229533.I1S4S7
94619 229533.I1S3G4 pp 229533.V6QWD8
94620 229533.I1S3G4 pp 229533.I1S8Z2
94621 229533.I1S3G4 pp 229533.V6RTJ8
94622 229533.I1S3G4 pp 229533.I1SAF0
94623 229533.I1S3G4 pp 229533.V6R1K4
94624 229533.I1S3G4 pp 229533.I1S599
94625 229533.I1S3G4 pp 229533.V6RRM0
94626 229533.I1S3G4 pp 229533.I1S3K8
94627 229533.I1S3G4 pp 229533.I1S686
94628 229533.I1S3G4 pp 229533.V6QX21
94629 229533.I1S3G4 pp 229533.V6RSF3
94630 229533.I1S3G4 pp 229533.I1S684
94631 229533.I1S3G4 pp 229533.I1S685
94632 229533.I1S3G4 pp 229533.V6RLM7
94633 229533.I1S3G4 pp 229533.V6R057
94634 229533.I1S3G4 pp 229533.V6QVH8
94635 229533.I1S3G4 pp 229533.I1S6M5
94636 229533.I1S3G4 pp 229533.I1S8H5
94637 229533.I1S3G4 pp 229533.I1SAJ3
94638 229533.I1S3G4 pp 229533.I1S9T5
94639 229533.I1S3G4 pp 229533.V6QV31
94640 229533.I1S3G4 pp 229533.Q4HXF0
94641 229533.I1S3G4 pp 229533.I1S5R1
94642 229533.I1S3G4 pp 229533.Q4I624
94643 229533.I1S3G4 pp 229533.V6R3J1
94644 229533.I1S3G4 pp 229533.O74638
94645 229533.I1S3G4 pp 229533.I1S3K7
94646 229533.I1S3G4 pp 229533.V6RUB1
94647 229533.I1S3G4 pp 229533.V6RUY9
94648 229533.I1S3G4 pp 229533.I1S4B7
94649 229533.I1S3G4 pp 229533.V6RKY8
94650 229533.I1S3G4 pp 229533.I1S6P5
94651 229533.I1S3G4 pp 229533.I1S4B6
94652 229533.I1S3G4 pp 229533.I1S4Z7
94653 229533.I1S3G4 pp 229533.V6RXR0
94654 229533.I1S3G4 pp 229533.V6RHD0
94655 229533.I1S3G4 pp 229533.I1S4Z8
94656 229533.I1S3G4 pp 229533.I1S9C6
94657 229533.I1REK1 pp 229533.V6QW25
94658 229533.I1REK1 pp 229533.I1RXF5
94659 229533.I1REK1 pp 229533.I1RK86
94660 229533.I1REK1 pp 229533.I1RM09
94661 229533.I1REK1 pp 229533.I1RIG4
94662 229533.I1REK1 pp 229533.I1RM48
94663 229533.I1REK1 pp 229533.V6RR76
94664 229533.I1REK1 pp 229533.I1RWQ1
94665 229533.I1REK1 pp 229533.I1RM77
94666 229533.I1REK1 pp 229533.I1RFK0
94667 229533.I1REK1 pp 229533.I1RX09
94668 229533.I1REK1 pp 229533.V6RQ48
94669 229533.I1REK1 pp 229533.I1RNR3
94670 229533.I1REK1 pp 229533.I1RND7
94671 229533.I1REK1 pp 229533.I1RYU1
94672 229533.I1REK1 pp 229533.I1RTF0
94673 229533.I1REK1 pp 229533.I1RZA4
94674 229533.I1REK1 pp 229533.I1RKR7
94675 229533.I1REK1 pp 229533.I1RXU1
94676 229533.I1REK1 pp 229533.I1RG57
94677 229533.I1REK1 pp 229533.I1RPD3
94678 229533.I1REK1 pp 229533.I1RQ61
94679 229533.I1REK1 pp 229533.I1RWV4
94680 229533.I1REK1 pp 229533.V6RKE4
94681 229533.I1REK1 pp 229533.I1RTD0

94682 229533.I1REK1 pp 229533.I1RK96
94683 229533.I1REK1 pp 229533.I1RSP4
94684 229533.I1REK1 pp 229533.I1RJ78
94685 229533.I1REK1 pp 229533.I1RN81
94686 229533.I1REK1 pp 229533.I1RQ70
94687 229533.A0A098DAA6 pp 229533.I1RAI9
94688 229533.A0A098DAA6 pp 229533.I1RS41
94689 229533.A0A098DAA6 pp 229533.I1RGC2
94690 229533.A0A098DAA6 pp 229533.I1S7S9
94691 229533.A0A098DAA6 pp 229533.I1RQ88
94692 229533.A0A098DL05 pp 229533.I1RE53
94693 229533.I1RFS7 pp 229533.I1SAM5
94694 229533.I1RFS7 pp 229533.I1S1V4
94695 229533.I1RFS7 pp 229533.I1RNA3
94696 229533.I1RFS7 pp 229533.I1RMF2
94697 229533.I1RFS7 pp 229533.I1RQ70
94698 229533.I1RFS7 pp 229533.Q4I6S5
94699 229533.I1RFS7 pp 229533.I1RW76
94700 229533.I1RFS7 pp 229533.I1RQP7
94701 229533.I1RFS7 pp 229533.I1RXV4
94702 229533.I1RFS7 pp 229533.I1S278
94703 229533.I1RFS7 pp 229533.I1RFV4
94704 229533.I1RFS7 pp 229533.I1S0K5
94705 229533.I1RFS7 pp 229533.Q4INS6
94706 229533.I1RFS7 pp 229533.I1RK90
94707 229533.I1RFS7 pp 229533.I1RPU5
94708 229533.I1RFS7 pp 229533.I1RXF5
94709 229533.I1RFS7 pp 229533.I1S830
94710 229533.I1RFS7 pp 229533.I1RK33
94711 229533.I1RFS7 pp 229533.I1RYU1
94712 229533.A0A098DC24 pp 229533.I1S9Z4
94713 229533.A0A098DC24 pp 229533.I1RPR1
94714 229533.A0A098DC24 pp 229533.A0A098E3B9
94715 229533.A0A098DC24 pp 229533.A0A098E2M9
94716 229533.A0A098DC24 pp 229533.A0A1C3YIP3
94717 229533.A0A098DC24 pp 229533.I1RFC3
94718 229533.A0A098DC24 pp 229533.I1RVL7
94719 229533.A0A098DC24 pp 229533.I1RIV2
94720 229533.A0A098DC24 pp 229533.I1S8H5
94721 229533.A0A098DC24 pp 229533.I1RHK0
94722 229533.A0A098DC24 pp 229533.I1S3K7
94723 229533.A0A098DC24 pp 229533.A0A098E0Z2
94724 229533.A0A098DC24 pp 229533.I1RK69
94725 229533.A0A098DC24 pp 229533.I1S5U5
94726 229533.A0A098DC24 pp 229533.I1RQ48
94727 229533.A0A098DC24 pp 229533.A0A1C3YJ35
94728 229533.A0A098DC24 pp 229533.I1S1L0
94729 229533.A0A098DC24 pp 229533.I1RVD9
94730 229533.A0A098DC24 pp 229533.I1S2J4
94731 229533.A0A098DC24 pp 229533.I1RF49
94732 229533.A0A098DC24 pp 229533.A0A1C3YKA2
94733 229533.A0A098DC24 pp 229533.I1RVM4
94734 229533.I1S2D5 pp 229533.I1S3G4
94735 229533.I1S2D5 pp 229533.V6RQW4
94736 229533.I1S2D5 pp 229533.Q4HVQ9
94737 229533.I1RQS6 pp 229533.I1RRT1
94738 229533.I1RQS6 pp 229533.I1RZ99
94739 229533.I1RQS6 pp 229533.V6RGI1
94740 229533.A0A098D1P1 pp 229533.I1S166
94741 229533.A0A098D1P1 pp 229533.I1RF58
94742 229533.A0A098D1P1 pp 229533.A0A1C3YLB6
94743 229533.A0A098D1P1 pp 229533.A0A1C3YMF1
94744 229533.A0A098D1P1 pp 229533.I1RUB2
94745 229533.A0A098D1P1 pp 229533.I1RBE7
94746 229533.A0A098D1P1 pp 229533.I1RNG4
94747 229533.A0A098D1P1 pp 229533.I1RL11
94748 229533.A0A098D1P1 pp 229533.I1RA28
94749 229533.A0A098D1P1 pp 229533.I1RY87
94750 229533.A0A098D1P1 pp 229533.I1S1W8
94751 229533.A0A098D1P1 pp 229533.I1S3G4
94752 229533.A0A098D1P1 pp 229533.A0A098DC24
94753 229533.A0A098D1P1 pp 229533.I1S2D5
94754 229533.I1RGV6 pp 229533.I1S444

94755 229533.I1RGV6 pp 229533.I1RZF7
94756 229533.I1RPW8 pp 229533.Q4HYI0
94757 229533.I1RPW8 pp 229533.I1RU42
94758 229533.I1RPW8 pp 229533.I1RXU0
94759 229533.I1RPW8 pp 229533.I1S8C5
94760 229533.I1RPW8 pp 229533.I1RRF7
94761 229533.I1RPW8 pp 229533.I1RXE5
94762 229533.I1RPW8 pp 229533.I1RRT0
94763 229533.I1RPW8 pp 229533.I1S5Y0
94764 229533.I1RPW8 pp 229533.I1RQW1
94765 229533.I1RPW8 pp 229533.I1S6R1
94766 229533.I1RPW8 pp 229533.V6QUE6
94767 229533.I1RPW8 pp 229533.I1S276
94768 229533.I1RPW8 pp 229533.I1S0C6
94769 229533.I1RPW8 pp 229533.I1RZ42
94770 229533.I1RPW8 pp 229533.I1RWF3
94771 229533.I1RPW8 pp 229533.V6QV38
94772 229533.I1RPW8 pp 229533.I1S0X1
94773 229533.I1RPW8 pp 229533.I1S0K1
94774 229533.I1RPW8 pp 229533.I1RZB5
94775 229533.I1RPW8 pp 229533.I1RUE3
94776 229533.I1RPW8 pp 229533.I1RSZ3
94777 229533.I1RPW8 pp 229533.I1RZM2
94778 229533.I1RPW8 pp 229533.I1RSM8
94779 229533.I1RPW8 pp 229533.I1S2W7
94780 229533.I1RPW8 pp 229533.I1RR61
94781 229533.I1RPW8 pp 229533.I1RW80
94782 229533.I1RPW8 pp 229533.V6RGU1
94783 229533.I1RPW8 pp 229533.I1RWG8
94784 229533.I1RPW8 pp 229533.I1RUY1
94785 229533.I1RPW8 pp 229533.I1RV19
94786 229533.I1RPW8 pp 229533.I1S9H8
94787 229533.I1RPW8 pp 229533.I1RU81
94788 229533.I1RPW8 pp 229533.I1S9B5
94789 229533.I1RPW8 pp 229533.I1RR25
94790 229533.I1RPW8 pp 229533.I1S182
94791 229533.I1RPW8 pp 229533.I1RUG4
94792 229533.I1RPW8 pp 229533.I1RTY9
94793 229533.I1RPW8 pp 229533.I1S2X3
94794 229533.I1RPW8 pp 229533.I1RUM5
94795 229533.I1RPW8 pp 229533.I1RW90
94796 229533.I1RPW8 pp 229533.V6QWX8
94797 229533.I1RPW8 pp 229533.I1SAH9
94798 229533.I1RPW8 pp 229533.I1RTC2
94799 229533.I1RPW8 pp 229533.I1RW91
94800 229533.I1RPW8 pp 229533.I1S5Z5
94801 229533.I1RPW8 pp 229533.I1RVL8
94802 229533.I1RPW8 pp 229533.I1RRG3
94803 229533.I1RPW8 pp 229533.I1RZ94
94804 229533.I1RPW8 pp 229533.I1RUW5
94805 229533.I1RPW8 pp 229533.I1RVY0
94806 229533.I1RPW8 pp 229533.I1RQ42
94807 229533.I1RUX3 pp 229533.I1S7X9
94808 229533.I1RMJ1 pp 229533.I1RTQ9
94809 229533.I1RMJ1 pp 229533.I1RN81
94810 229533.I1RMJ1 pp 229533.I1RQ70
94811 229533.I1RMJ1 pp 229533.I1RQF7
94812 229533.I1RMJ1 pp 229533.I1S1Z9
94813 229533.I1RMJ1 pp 229533.I1RMM1
94814 229533.I1RMJ1 pp 229533.Q4HYZ2
94815 229533.I1RMJ1 pp 229533.I1RT41
94816 229533.I1RMJ1 pp 229533.I1RW76
94817 229533.I1RMJ1 pp 229533.I1RZ60
94818 229533.I1RMJ1 pp 229533.I1RZK3
94819 229533.I1RMJ1 pp 229533.I1RTC1
94820 229533.I1RMJ1 pp 229533.I1RX84
94821 229533.I1RMJ1 pp 229533.I1RYW8
94822 229533.I1RMJ1 pp 229533.I1RZF7
94823 229533.I1RMJ1 pp 229533.I1S0V5
94824 229533.I1RMJ1 pp 229533.Q4ILI9
94825 229533.I1RMJ1 pp 229533.I1RTG5
94826 229533.I1RMJ1 pp 229533.V6RR76
94827 229533.I1RMJ1 pp 229533.I1S830

94828 229533.A0A0E0SKE0 pp 229533.I1RNV4
94829 229533.A0A0E0SKE0 pp 229533.I1S444
94830 229533.A0A0E0SKE0 pp 229533.I1RRT3
94831 229533.A0A0E0SKE0 pp 229533.V6R729
94832 229533.A0A0E0SKE0 pp 229533.I1RZ21
94833 229533.A0A0E0SKE0 pp 229533.I1RCE5
94834 229533.A0A0E0SKE0 pp 229533.I1SAC4
94835 229533.A0A0E0SKE0 pp 229533.I1RSA3
94836 229533.A0A0E0SKE0 pp 229533.I1RD80
94837 229533.A0A0E0SKE0 pp 229533.I1RZM3
94838 229533.A0A0E0SKE0 pp 229533.I1RPS2
94839 229533.A0A0E0SKE0 pp 229533.Q4HTS9
94840 229533.A0A0E0SKE0 pp 229533.I1RUP6
94841 229533.A0A0E0SKE0 pp 229533.I1RWP9
94842 229533.A0A0E0SKE0 pp 229533.I1RA62
94843 229533.A0A0E0SKE0 pp 229533.A0A1C3YHZ7
94844 229533.A0A0E0SKE0 pp 229533.I1RJ42
94845 229533.A0A0E0SKE0 pp 229533.A0A1C3YJZ0
94846 229533.A0A0E0SKE0 pp 229533.I1RUM7
94847 229533.A0A0E0SKE0 pp 229533.V6R3R0
94848 229533.A0A0E0SKE0 pp 229533.V6RUG6
94849 229533.A0A0E0SKE0 pp 229533.I1RQE0
94850 229533.A0A0E0SKE0 pp 229533.A0A1C3YM97
94851 229533.A0A0E0SKE0 pp 229533.I1RP86
94852 229533.A0A0E0SKE0 pp 229533.I1RIX0
94853 229533.A0A0E0SKE0 pp 229533.I1RKB5
94854 229533.A0A0E0SKE0 pp 229533.I1R9B6
94855 229533.A0A0E0SKE0 pp 229533.I1RJ82
94856 229533.A0A0E0SKE0 pp 229533.I1RSJ2
94857 229533.A0A0E0SKE0 pp 229533.A0A1C3YK51
94858 229533.V6QWB1 pp 229533.V6RRM0
94859 229533.V6QWB1 pp 229533.V6R7V8
94860 229533.I1S182 pp 229533.V6QUE6
94861 229533.I1S182 pp 229533.I1S5Y0
94862 229533.I1S182 pp 229533.Q4HYI0
94863 229533.I1S182 pp 229533.V6RGU1
94864 229533.I1S182 pp 229533.I1S2W7
94865 229533.I1S182 pp 229533.I1S2X3
94866 229533.I1S182 pp 229533.I1S276
94867 229533.A0A1C3YL35 pp 229533.I1RWT6
94868 229533.A0A1C3YL35 pp 229533.I1RX70
94869 229533.A0A1C3YL35 pp 229533.Q4I1H6
94870 229533.A0A1C3YL35 pp 229533.I1RBW1
94871 229533.A0A1C3YL35 pp 229533.I1RBW0
94872 229533.A0A1C3YL35 pp 229533.I1RRI3
94873 229533.A0A1C3YL35 pp 229533.I1RFZ3
94874 229533.A0A1C3YL35 pp 229533.I1RQA3
94875 229533.A0A0E0SB93 pp 229533.I1RK90
94876 229533.A0A0E0SB93 pp 229533.I1S0K5
94877 229533.A0A0E0SB93 pp 229533.I1RPU5
94878 229533.A0A0E0SB93 pp 229533.A0A1C3YKU7
94879 229533.A0A0E0SB93 pp 229533.I1RE03
94880 229533.A0A0E0SB93 pp 229533.I1RMF2
94881 229533.A0A0E0SB93 pp 229533.I1RQ70
94882 229533.A0A0E0SB93 pp 229533.I1S1V4
94883 229533.A0A0E0SB93 pp 229533.I1RFV4
94884 229533.A0A0E0SB93 pp 229533.I1RNA3
94885 229533.A0A0E0SB93 pp 229533.A0A1C3YN81
94886 229533.A0A0E0SB93 pp 229533.A0A1C3YIE3
94887 229533.A0A0E0SB93 pp 229533.I1SAM5
94888 229533.A0A0E0SB93 pp 229533.A0A1C3YIC5
94889 229533.A0A0E0SB93 pp 229533.I1RQP7
94890 229533.A0A0E0SB93 pp 229533.I1S278
94891 229533.A0A0E0SB93 pp 229533.I1RW76
94892 229533.A0A0E0SB93 pp 229533.Q4INS6
94893 229533.A0A0E0SB93 pp 229533.I1RXF5
94894 229533.A0A0E0SB93 pp 229533.I1S830
94895 229533.A0A1C3YLA0 pp 229533.A0A1C3YM76
94896 229533.A0A098DAB5 pp 229533.A0A1C3YKJ6
94897 229533.A0A098DAB5 pp 229533.I1RQ88
94898 229533.A0A098DAB5 pp 229533.I1RBV5
94899 229533.A0A098DAB5 pp 229533.I1RAI9
94900 229533.A0A098DAB5 pp 229533.I1RPW0

94901 229533.A0A098DAB5 pp 229533.I1RX38
94902 229533.A0A098DAB5 pp 229533.A0A0E0SNZ9
94903 229533.A0A098DAB5 pp 229533.I1RPU4
94904 229533.A0A098DAB5 pp 229533.A0A1C3YK37
94905 229533.I1REL1 pp 229533.I1RSJ0
94906 229533.I1REL1 pp 229533.I1RJI0
94907 229533.I1REL1 pp 229533.I1RNR6
94908 229533.I1REL1 pp 229533.I1RKK6
94909 229533.I1REL1 pp 229533.I1RWL9
94910 229533.I1REL1 pp 229533.I1RGZ5
94911 229533.I1S0S5 pp 229533.I1S6D1
94912 229533.I1S0S5 pp 229533.Q4I5V2
94913 229533.I1S0S5 pp 229533.Q4IPC8
94914 229533.I1S0S5 pp 229533.V6RS91
94915 229533.I1S0S5 pp 229533.I1S835
94916 229533.A0A098DKU2 pp 229533.I1RZ78
94917 229533.A0A098DKU2 pp 229533.I1RPS2
94918 229533.A0A098DKU2 pp 229533.I1RQE0
94919 229533.A0A098DKU2 pp 229533.I1RA62
94920 229533.A0A098DKU2 pp 229533.I1RC31
94921 229533.A0A098DKU2 pp 229533.A0A0E0SKE0
94922 229533.A0A098DKU2 pp 229533.I1RKB5
94923 229533.A0A098DKU2 pp 229533.I1RUM7
94924 229533.A0A098DKU2 pp 229533.A0A098DR13
94925 229533.I1RYU1 pp 229533.I1S1V7
94926 229533.I1RYU1 pp 229533.I1S287
94927 229533.I1RYU1 pp 229533.I1S6S4
94928 229533.I1RYU1 pp 229533.I1S830
94929 229533.I1RGZ5 pp 229533.I1RSJ0
94930 229533.I1RGZ5 pp 229533.I1RWL9
94931 229533.I1RGZ5 pp 229533.I1RJI0
94932 229533.I1RGZ5 pp 229533.I1RKK6
94933 229533.I1RGZ5 pp 229533.I1RNR6
94934 229533.I1S746 pp 229533.V6RN31
94935 229533.I1RCG6 pp 229533.I1S9B5
94936 229533.I1RCG6 pp 229533.I1RGM9
94937 229533.I1RCG6 pp 229533.I1RPW8
94938 229533.I1RCG6 pp 229533.I1RU81
94939 229533.I1RCG6 pp 229533.I1RIF7
94940 229533.I1RCG6 pp 229533.I1S182
94941 229533.I1RCG6 pp 229533.I1RND9
94942 229533.I1RCG6 pp 229533.I1RZM2
94943 229533.I1RCG6 pp 229533.I1RTC2
94944 229533.I1RCG6 pp 229533.I1S9H8
94945 229533.I1RCG6 pp 229533.I1RE30
94946 229533.I1RCG6 pp 229533.I1S5Z5
94947 229533.I1RCG6 pp 229533.I1RRG3
94948 229533.I1RCG6 pp 229533.I1RUM5
94949 229533.I1RCG6 pp 229533.I1S0K1
94950 229533.I1RCG6 pp 229533.I1RSZ3
94951 229533.I1RCG6 pp 229533.I1RPS7
94952 229533.I1RCG6 pp 229533.I1RSM8
94953 229533.I1RCG6 pp 229533.I1RML9
94954 229533.I1RCG6 pp 229533.I1RED3
94955 229533.I1RCG6 pp 229533.I1RH75
94956 229533.I1RCG6 pp 229533.I1RP96
94957 229533.I1RCG6 pp 229533.I1RKB1
94958 229533.I1RCG6 pp 229533.I1RHQ9
94959 229533.I1RCG6 pp 229533.I1RZ94
94960 229533.I1RCG6 pp 229533.I1RGK3
94961 229533.I1RCG6 pp 229533.I1RPQ2
94962 229533.I1RCG6 pp 229533.I1RUW5
94963 229533.I1RCG6 pp 229533.I1RV19
94964 229533.I1RCG6 pp 229533.I1RN04
94965 229533.I1RCG6 pp 229533.I1RVL8
94966 229533.I1RCG6 pp 229533.I1RVY0
94967 229533.I1RCG6 pp 229533.I1RRS4
94968 229533.I1RCG6 pp 229533.I1S2X3
94969 229533.I1RCG6 pp 229533.I1RUY1
94970 229533.I1RCG6 pp 229533.I1RQ42
94971 229533.I1RCG6 pp 229533.I1RW80
94972 229533.I1RCG6 pp 229533.V6QWX8
94973 229533.I1RCG6 pp 229533.I1RDN5

94974 229533.I1RCG6 pp 229533.I1S6R1
94975 229533.I1RCG6 pp 229533.I1RN91
94976 229533.I1RCG6 pp 229533.I1RDN0
94977 229533.I1RCG6 pp 229533.I1RW90
94978 229533.I1RCG6 pp 229533.I1SAH9
94979 229533.I1RCG6 pp 229533.I1S0K9
94980 229533.I1RCG6 pp 229533.I1RH44
94981 229533.I1RCG6 pp 229533.I1RW91
94982 229533.I1RCG6 pp 229533.I1RJS9
94983 229533.I1RCG6 pp 229533.I1RXE5
94984 229533.I1RCG6 pp 229533.I1REC2
94985 229533.I1RCG6 pp 229533.I1RD09
94986 229533.I1RCG6 pp 229533.I1RRT0
94987 229533.I1RCG6 pp 229533.O74638
94988 229533.I1RCG6 pp 229533.Q4HYI0
94989 229533.I1RCG6 pp 229533.I1RU42
94990 229533.I1RCG6 pp 229533.I1RFQ9
94991 229533.I1RCG6 pp 229533.I1RDC9
94992 229533.I1RCG6 pp 229533.I1RKR8
94993 229533.I1RCG6 pp 229533.I1RXU0
94994 229533.I1RCG6 pp 229533.I1RZ42
94995 229533.I1RCG6 pp 229533.I1RWF3
94996 229533.I1RCG6 pp 229533.I1RNG5
94997 229533.I1RCG6 pp 229533.I1RMV1
94998 229533.I1RCG6 pp 229533.I1RCH4
94999 229533.I1RCG6 pp 229533.I1RRF7
95000 229533.I1RCG6 pp 229533.I1S5Y0
95001 229533.I1RCG6 pp 229533.I1RP12
95002 229533.I1RCG6 pp 229533.Q4I948
95003 229533.I1RCG6 pp 229533.I1RMH2
95004 229533.I1RCG6 pp 229533.I1RWG8
95005 229533.I1RCG6 pp 229533.I1RED2
95006 229533.I1RCG6 pp 229533.I1S0C6
95007 229533.I1RCG6 pp 229533.I1RG11
95008 229533.I1RCG6 pp 229533.I1RR61
95009 229533.I1RCG6 pp 229533.V6QUE6
95010 229533.I1RCG6 pp 229533.I1REN3
95011 229533.I1RCG6 pp 229533.I1S2W7
95012 229533.I1RCG6 pp 229533.I1RMU1
95013 229533.I1RCG6 pp 229533.I1RKF3
95014 229533.I1RCG6 pp 229533.I1RUG4
95015 229533.I1RCG6 pp 229533.I1S0X1
95016 229533.I1RCG6 pp 229533.I1RIA9
95017 229533.I1RCG6 pp 229533.I1RR25
95018 229533.I1RCG6 pp 229533.V6QV38
95019 229533.I1RCG6 pp 229533.I1RTY9
95020 229533.I1RCG6 pp 229533.I1S276
95021 229533.I1RCG6 pp 229533.I1RJL2
95022 229533.I1RCG6 pp 229533.I1RZB5
95023 229533.I1RCG6 pp 229533.I1RK79
95024 229533.I1RCG6 pp 229533.I1RQW1
95025 229533.I1RCG6 pp 229533.I1RUE3
95026 229533.I1RCG6 pp 229533.V6RGU1
95027 229533.I1RCG6 pp 229533.I1RLI4
95028 229533.I1RCG6 pp 229533.I1RK71
95029 229533.I1RCG6 pp 229533.I1RPM7
95030 229533.I1RCG6 pp 229533.I1RDY2
95031 229533.I1RCG6 pp 229533.I1RGV3
95032 229533.I1RGR7 pp 229533.I1RW06
95033 229533.I1RGR7 pp 229533.V6QVM5
95034 229533.I1RGR7 pp 229533.V6RN31
95035 229533.I1RGR7 pp 229533.V6QVH8
95036 229533.I1RGR7 pp 229533.I1RMX0
95037 229533.I1RMU3 pp 229533.V6QV38
95038 229533.I1RMU3 pp 229533.I1RRY9
95039 229533.I1RMU3 pp 229533.I1RXF0
95040 229533.I1RMU3 pp 229533.I1RZ14
95041 229533.I1RMU3 pp 229533.I1RPW1
95042 229533.I1RMU3 pp 229533.I1RSF1
95043 229533.I1RMU3 pp 229533.Q4IPI1
95044 229533.I1RMU3 pp 229533.I1RZF6
95045 229533.I1RMU3 pp 229533.I1S2G9
95046 229533.I1RZD1 pp 229533.I1S1Y9

95047 229533.I1RQE0 pp 229533.I1RYI8
95048 229533.I1RQE0 pp 229533.V6RUG6
95049 229533.I1RQE0 pp 229533.I1RZN1
95050 229533.I1RQE0 pp 229533.I1S3G4
95051 229533.I1RQE0 pp 229533.I1S4F4
95052 229533.I1RQE0 pp 229533.I1RYK0
95053 229533.I1RQE0 pp 229533.I1RW43
95054 229533.I1RQE0 pp 229533.I1S5L5
95055 229533.I1RQE0 pp 229533.V6R3J1
95056 229533.I1RQE0 pp 229533.I1RZ20
95057 229533.I1RQE0 pp 229533.O74638
95058 229533.I1RQE0 pp 229533.V6RLM7
95059 229533.I1RQE0 pp 229533.I1RZ95
95060 229533.I1RQE0 pp 229533.I1RZ13
95061 229533.I1RQE0 pp 229533.I1RZM3
95062 229533.I1RQE0 pp 229533.I1S2H3
95063 229533.I1RQE0 pp 229533.I1SAC4
95064 229533.I1RQE0 pp 229533.I1S9Z4
95065 229533.I1RQE0 pp 229533.I1RRT3
95066 229533.I1RQE0 pp 229533.Q4I1Y5
95067 229533.I1RQE0 pp 229533.I1RZ78
95068 229533.I1RQE0 pp 229533.I1S2C4
95069 229533.I1RQE0 pp 229533.I1RWL3
95070 229533.I1RQE0 pp 229533.I1RU18
95071 229533.I1RQE0 pp 229533.I1S272
95072 229533.I1RQE0 pp 229533.I1S4S4
95073 229533.I1RQE0 pp 229533.I1RVV3
95074 229533.I1RQE0 pp 229533.I1RUY1
95075 229533.I1RQE0 pp 229533.I1RYU5
95076 229533.I1RQE0 pp 229533.I1S332
95077 229533.I1RQE0 pp 229533.I1S1W8
95078 229533.I1RQE0 pp 229533.I1RSB7
95079 229533.I1RQE0 pp 229533.I1RVX9
95080 229533.I1RQE0 pp 229533.I1RUY5
95081 229533.I1RQE0 pp 229533.I1S2X3
95082 229533.I1RQE0 pp 229533.I1RU42
95083 229533.I1RQE0 pp 229533.I1RUM7
95084 229533.I1RQE0 pp 229533.I1S5Y0
95085 229533.I1RQE0 pp 229533.I1RU76
95086 229533.I1RQE0 pp 229533.I1S2W7
95087 229533.I1RQE0 pp 229533.V6R3R0
95088 229533.I1RCE7 pp 229533.Q4HYZ2
95089 229533.I1RCE7 pp 229533.I1RCN6
95090 229533.I1RCE7 pp 229533.I1RTG5
95091 229533.I1RCE7 pp 229533.I1RX84
95092 229533.I1RCE7 pp 229533.I1RZ60
95093 229533.I1RCE7 pp 229533.I1RMJ1
95094 229533.I1RCE7 pp 229533.I1RYW8
95095 229533.I1RCE7 pp 229533.I1RE03
95096 229533.I1RCE7 pp 229533.V6RGI1
95097 229533.I1RCE7 pp 229533.V6RR76
95098 229533.I1RCE7 pp 229533.I1RN81
95099 229533.I1RCE7 pp 229533.Q4ILI9
95100 229533.I1RCE7 pp 229533.I1RQF7
95101 229533.I1RCE7 pp 229533.I1RQ70
95102 229533.I1RCE7 pp 229533.I1RKR7
95103 229533.I1RCE7 pp 229533.I1RTQ9
95104 229533.I1RUE2 pp 229533.I1S111
95105 229533.I1RUE2 pp 229533.I1S338
95106 229533.I1RUE2 pp 229533.I1S7R5
95107 229533.I1RUE2 pp 229533.V6RJ21
95108 229533.I1RUE2 pp 229533.I1RYV4
95109 229533.I1RUE2 pp 229533.I1RXB6
95110 229533.I1RUE2 pp 229533.I1RUP2
95111 229533.I1RUE2 pp 229533.I1RWE8
95112 229533.I1RUE2 pp 229533.V6R223
95113 229533.I1RUE2 pp 229533.I1RVV7
95114 229533.I1RUE2 pp 229533.I1S2A1
95115 229533.I1RUE2 pp 229533.I1RZH6
95116 229533.A0A098DKT8 pp 229533.A0A0E0SP71
95117 229533.A0A098DKT8 pp 229533.I1RJU9
95118 229533.A0A098DKT8 pp 229533.I1RLM3
95119 229533.I1S693 pp 229533.V6QVM5

95120 229533.I1S693 pp 229533.V6RN31
95121 229533.I1RGA0 pp 229533.I1RUI0
95122 229533.I1RW91 pp 229533.V6QWX8
95123 229533.I1RW91 pp 229533.I1S9H8
95124 229533.I1RW91 pp 229533.I1S182
95125 229533.I1RW91 pp 229533.V6RGU1
95126 229533.I1RW91 pp 229533.I1S276
95127 229533.I1RW91 pp 229533.I1RWG8
95128 229533.I1RW91 pp 229533.I1RZM2
95129 229533.I1RW91 pp 229533.I1SAH9
95130 229533.I1RW91 pp 229533.I1S9B5
95131 229533.I1RW91 pp 229533.I1S2W7
95132 229533.I1RW91 pp 229533.I1S0K9
95133 229533.I1RW91 pp 229533.I1RWF3
95134 229533.I1RW91 pp 229533.I1S2X3
95135 229533.I1RW91 pp 229533.I1S5Z5
95136 229533.I1RW91 pp 229533.Q4IMD1
95137 229533.I1RW91 pp 229533.I1S0X1
95138 229533.I1RW91 pp 229533.I1S6R1
95139 229533.I1RW91 pp 229533.I1RXE5
95140 229533.I1RW91 pp 229533.I1RZB5
95141 229533.I1RW91 pp 229533.I1S0C6
95142 229533.I1RW91 pp 229533.I1RZ42
95143 229533.I1RW91 pp 229533.I1S0K1
95144 229533.I1RW91 pp 229533.Q4HYI0
95145 229533.I1RW91 pp 229533.I1S5Y0
95146 229533.I1RW91 pp 229533.V6QUE6
95147 229533.I1RW91 pp 229533.I1RXU0
95148 229533.I1RNR6 pp 229533.I1RWL9
95149 229533.I1RNR6 pp 229533.I1RSJ0
95150 229533.I1RNR6 pp 229533.I1RSN9
95151 229533.A0A0E0SF70 pp 229533.I1RCP1
95152 229533.I1RTG2 pp 229533.I1S1D0
95153 229533.I1RTG2 pp 229533.V6QW25
95154 229533.Q4HZE0 pp 229533.V6QUU6
95155 229533.Q4HZE0 pp 229533.V6RR76
95156 229533.Q4HZE0 pp 229533.V6R476
95157 229533.Q4HZE0 pp 229533.Q4INI2
95158 229533.Q4HZE0 pp 229533.Q4IJT0
95159 229533.I1RN04 pp 229533.I1SAH9
95160 229533.I1RN04 pp 229533.I1RW91
95161 229533.I1RN04 pp 229533.I1S0K9
95162 229533.I1RN04 pp 229533.I1RSZ3
95163 229533.I1RN04 pp 229533.I1S2X3
95164 229533.I1RN04 pp 229533.V6QWX8
95165 229533.I1RN04 pp 229533.I1RVY0
95166 229533.I1RN04 pp 229533.I1RW90
95167 229533.I1RN04 pp 229533.I1S0K1
95168 229533.I1RN04 pp 229533.I1S182
95169 229533.I1RN04 pp 229533.I1RP96
95170 229533.I1RN04 pp 229533.I1RQ42
95171 229533.I1RN04 pp 229533.I1RUW5
95172 229533.I1RN04 pp 229533.I1S9H8
95173 229533.I1RN04 pp 229533.I1RPM7
95174 229533.I1RN04 pp 229533.I1RVL8
95175 229533.I1RN04 pp 229533.I1RUE3
95176 229533.I1RN04 pp 229533.I1RPS7
95177 229533.I1RN04 pp 229533.I1RPW8
95178 229533.I1RN04 pp 229533.V6QV38
95179 229533.I1RN04 pp 229533.I1RV19
95180 229533.I1RN04 pp 229533.I1RPQ2
95181 229533.I1RN04 pp 229533.I1RQW1
95182 229533.I1RN04 pp 229533.I1RU81
95183 229533.I1RN04 pp 229533.I1RUY1
95184 229533.I1RN04 pp 229533.I1S5Z5
95185 229533.I1RN04 pp 229533.I1RRG3
95186 229533.I1RN04 pp 229533.Q4IMD1
95187 229533.I1RN04 pp 229533.I1S276
95188 229533.I1RN04 pp 229533.I1RW80
95189 229533.I1RN04 pp 229533.I1RZB5
95190 229533.I1RN04 pp 229533.I1RZM2
95191 229533.I1RN04 pp 229533.I1RPG2
95192 229533.I1RN04 pp 229533.I1S9B5

95193 229533.I1RN04 pp 229533.I1RSM8
95194 229533.I1RN04 pp 229533.I1S0X1
95195 229533.I1RN04 pp 229533.I1RTC2
95196 229533.I1RN04 pp 229533.I1S2W7
95197 229533.I1RN04 pp 229533.Q4HYI0
95198 229533.I1RN04 pp 229533.I1RUG4
95199 229533.I1RN04 pp 229533.V6RGU1
95200 229533.I1RN04 pp 229533.I1RP12
95201 229533.I1RN04 pp 229533.I1RR25
95202 229533.I1RN04 pp 229533.I1RTY9
95203 229533.I1RN04 pp 229533.I1RXU0
95204 229533.I1RN04 pp 229533.I1RU42
95205 229533.I1RN04 pp 229533.I1RWF3
95206 229533.I1RN04 pp 229533.I1RWG8
95207 229533.I1RN04 pp 229533.I1S6R1
95208 229533.I1RN04 pp 229533.I1RXE5
95209 229533.I1RN04 pp 229533.I1RR61
95210 229533.I1RN04 pp 229533.V6QUE6
95211 229533.I1RN04 pp 229533.I1RRT0
95212 229533.I1RN04 pp 229533.I1RNG5
95213 229533.I1RN04 pp 229533.I1RZ42
95214 229533.I1RN04 pp 229533.I1S0C6
95215 229533.I1RN04 pp 229533.I1S8C5
95216 229533.I1RN04 pp 229533.I1S5Y0
95217 229533.I1RN04 pp 229533.I1RRF7
95218 229533.I1RVT1 pp 229533.V6QVM5
95219 229533.I1RVT1 pp 229533.I1RW06
95220 229533.I1RVT1 pp 229533.V6RN31
95221 229533.A0A098DVM4 pp 229533.I1RWB4
95222 229533.A0A098DVM4 pp 229533.I1RE03
95223 229533.A0A098DVM4 pp 229533.I1RMV6
95224 229533.A0A098DVM4 pp 229533.I1RSJ0
95225 229533.A0A098DVM4 pp 229533.A0A1C3YIE3
95226 229533.A0A098DVM4 pp 229533.I1RFV4
95227 229533.A0A098DVM4 pp 229533.I1RPU5
95228 229533.A0A098DVM4 pp 229533.I1RSZ1
95229 229533.A0A098DVM4 pp 229533.I1RZQ6
95230 229533.A0A098DVM4 pp 229533.I1S830
95231 229533.A0A098DVM4 pp 229533.I1RD45
95232 229533.A0A098DVM4 pp 229533.I1RQ32
95233 229533.A0A098DVM4 pp 229533.I1RNA3
95234 229533.A0A098DVM4 pp 229533.A0A1C3YHW1
95235 229533.A0A098DVM4 pp 229533.A0A0E0SQN6
95236 229533.A0A098DVM4 pp 229533.I1RMF2
95237 229533.A0A098DVM4 pp 229533.A0A0E0RMX5
95238 229533.A0A098DVM4 pp 229533.I1RXF5
95239 229533.A0A098DVM4 pp 229533.I1RBA6
95240 229533.A0A098DVM4 pp 229533.I1RE15
95241 229533.A0A098DVM4 pp 229533.A0A1C3YME0
95242 229533.A0A098DVM4 pp 229533.I1S6S4
95243 229533.A0A098DVM4 pp 229533.I1RE49
95244 229533.A0A098DVM4 pp 229533.A0A1C3YN81
95245 229533.A0A098DVM4 pp 229533.A0A1C3YHM4
95246 229533.A0A098DVM4 pp 229533.I1RFS7
95247 229533.A0A098DVM4 pp 229533.I1RQ70
95248 229533.A0A098DVM4 pp 229533.I1RTQ9
95249 229533.A0A098DVM4 pp 229533.I1RAS9
95250 229533.A0A098DVM4 pp 229533.I1S1V7
95251 229533.A0A098DVM4 pp 229533.I1RRB5
95252 229533.A0A098DVM4 pp 229533.A0A1C3YN54
95253 229533.A0A098DVM4 pp 229533.A0A098DWE6
95254 229533.A0A098DVM4 pp 229533.A0A1C3YIC5
95255 229533.A0A098DVM4 pp 229533.I1RZM1
95256 229533.A0A098DVM4 pp 229533.I1RB90
95257 229533.A0A098DVM4 pp 229533.I1REK1
95258 229533.A0A098DVM4 pp 229533.I1REC4
95259 229533.A0A098DVM4 pp 229533.A0A0E0SFJ9
95260 229533.A0A098DVM4 pp 229533.I1RFK0
95261 229533.A0A098DVM4 pp 229533.I1RE20
95262 229533.A0A098DVM4 pp 229533.I1RB98
95263 229533.A0A098DVM4 pp 229533.I1S287
95264 229533.A0A098DVM4 pp 229533.I1RK33
95265 229533.A0A098DVM4 pp 229533.I1RWT2

95266 229533.A0A098DVM4 pp 229533.A0A1C3YK51
95267 229533.A0A098DVM4 pp 229533.I1RTD0
95268 229533.I1RJI0 pp 229533.I1RSJ0
95269 229533.I1RJI0 pp 229533.I1RKK6
95270 229533.I1RJI0 pp 229533.I1RNR6
95271 229533.I1RJI0 pp 229533.I1RWL9
95272 229533.I1RJI0 pp 229533.I1RZ09
95273 229533.I1RQ42 pp 229533.I1S9Z3
95274 229533.I1RQ42 pp 229533.I1S182
95275 229533.I1RQ42 pp 229533.I1S9H8
95276 229533.I1RQ42 pp 229533.I1RUM5
95277 229533.I1RQ42 pp 229533.I1RVY0
95278 229533.I1RQ42 pp 229533.I1S0K9
95279 229533.I1RQ42 pp 229533.I1RTC2
95280 229533.I1RQ42 pp 229533.I1RVL8
95281 229533.I1RQ42 pp 229533.I1RRG3
95282 229533.I1RQ42 pp 229533.I1RW90
95283 229533.I1RQ42 pp 229533.I1RZM2
95284 229533.I1RQ42 pp 229533.I1S2X3
95285 229533.I1RQ42 pp 229533.I1S5Z5
95286 229533.I1RQ42 pp 229533.I1RWF3
95287 229533.I1RQ42 pp 229533.V6QWX8
95288 229533.I1RQ42 pp 229533.I1RUY1
95289 229533.I1RQ42 pp 229533.I1RYT7
95290 229533.I1RQ42 pp 229533.I1RW80
95291 229533.I1RQ42 pp 229533.I1RZ42
95292 229533.I1RQ42 pp 229533.I1RSZ3
95293 229533.I1RQ42 pp 229533.I1RW91
95294 229533.I1RQ42 pp 229533.I1S5Y0
95295 229533.I1RQ42 pp 229533.I1RR25
95296 229533.I1RQ42 pp 229533.I1RRT0
95297 229533.I1RQ42 pp 229533.I1RR61
95298 229533.I1RQ42 pp 229533.I1RRF7
95299 229533.I1RQ42 pp 229533.I1RUG4
95300 229533.I1RQ42 pp 229533.I1RWG8
95301 229533.I1RQ42 pp 229533.I1RZ11
95302 229533.I1RQ42 pp 229533.V6QUE6
95303 229533.I1RQ42 pp 229533.I1S276
95304 229533.I1RQ42 pp 229533.I1S0C6
95305 229533.I1RQ42 pp 229533.I1RXU0
95306 229533.I1RQ42 pp 229533.I1RU42
95307 229533.I1RQ42 pp 229533.V6QV38
95308 229533.I1RQ42 pp 229533.Q4HYI0
95309 229533.I1RQ42 pp 229533.V6RGU1
95310 229533.I1RQ42 pp 229533.I1RZB5
95311 229533.I1RQ42 pp 229533.I1S2W7
95312 229533.I1RC19 pp 229533.I1S251
95313 229533.I1RC19 pp 229533.I1RRF7
95314 229533.I1RC19 pp 229533.I1RN04
95315 229533.I1RC19 pp 229533.I1RZX0
95316 229533.I1RC19 pp 229533.I1S0H1
95317 229533.I1RC19 pp 229533.I1RDY2
95318 229533.I1RC19 pp 229533.I1RCG6
95319 229533.I1RC19 pp 229533.I1RPW8
95320 229533.I1RC19 pp 229533.I1RNG5
95321 229533.I1RC19 pp 229533.I1RW91
95322 229533.I1RRW2 pp 229533.I1RW25
95323 229533.I1RRW2 pp 229533.V6QUU6
95324 229533.I1RRW2 pp 229533.I1S254
95325 229533.I1RRW2 pp 229533.V6RR76
95326 229533.I1RRW2 pp 229533.V6R476
95327 229533.I1RRW2 pp 229533.Q4HZE0
95328 229533.I1RZ42 pp 229533.Q4HYI0
95329 229533.I1RZ42 pp 229533.I1S182
95330 229533.I1RZ42 pp 229533.I1S2W7
95331 229533.I1RZ42 pp 229533.V6RGU1
95332 229533.I1RZ42 pp 229533.I1RZB5
95333 229533.I1RZ42 pp 229533.I1S0C6
95334 229533.I1RZ42 pp 229533.I1S2X3
95335 229533.I1RZ42 pp 229533.I1S276
95336 229533.I1R9G7 pp 229533.I1RFF2
95337 229533.I1RW78 pp 229533.I1RWJ5
95338 229533.I1RW78 pp 229533.Q4HXT5

95339	229533.I1RW78	pp	229533.I1S0S1
95340	229533.I1RW78	pp	229533.V6R382
95341	229533.I1RW78	pp	229533.I1S755
95342	229533.I1RJ85	pp	229533.V6RN31
95343	229533.I1RJ85	pp	229533.V6QVM5
95344	229533.I1RJ85	pp	229533.I1RW06
95345	229533.I1R9H5	pp	229533.I1RNG4
95346	229533.I1RZ65	pp	229533.I1S0Z7
95347	229533.I1S8Q2	pp	229533.V6RN31
95348	229533.I1S8Q2	pp	229533.V6QVM5
95349	229533.I1RW80	pp	229533.Q4HTT2
95350	229533.I1RW80	pp	229533.Q4HYI0
95351	229533.I1RW80	pp	229533.I1RXU0
95352	229533.I1RW80	pp	229533.Q4I7S1
95353	229533.I1RW80	pp	229533.V6QUE6
95354	229533.I1RW80	pp	229533.I1S0C6
95355	229533.I1RW80	pp	229533.I1S5Y0
95356	229533.I1RW80	pp	229533.I1RWF3
95357	229533.I1RW80	pp	229533.I1RZ42
95358	229533.I1RW80	pp	229533.V6QV38
95359	229533.I1RW80	pp	229533.I1RXE5
95360	229533.I1RW80	pp	229533.I1RZM2
95361	229533.I1RW80	pp	229533.I1S2W7
95362	229533.I1RW80	pp	229533.Q4HYQ4
95363	229533.I1RW80	pp	229533.I1S276
95364	229533.I1RW80	pp	229533.I1S0X1
95365	229533.I1RW80	pp	229533.I1RZU9
95366	229533.I1RW80	pp	229533.I1S5Z5
95367	229533.I1RW80	pp	229533.I1S182
95368	229533.I1RW80	pp	229533.V6RGU1
95369	229533.I1RW80	pp	229533.I1S0K1
95370	229533.I1RW80	pp	229533.I1S9H8
95371	229533.I1RW80	pp	229533.I1RZB5
95372	229533.I1RW80	pp	229533.Q4I5W5
95373	229533.I1RW80	pp	229533.I1S2X3
95374	229533.I1RW80	pp	229533.I1RWG8
95375	229533.I1RW80	pp	229533.I1RW91
95376	229533.I1RW80	pp	229533.I1RW90
95377	229533.I1RW80	pp	229533.I1RZ94
95378	229533.I1RW80	pp	229533.I1SAH9
95379	229533.I1RW80	pp	229533.V6QWX8
95380	229533.I1RBD6	pp	229533.I1RQD4
95381	229533.I1RBD6	pp	229533.I1RN65
95382	229533.I1RBD6	pp	229533.I1RCM6
95383	229533.I1RBD6	pp	229533.V6REE9
95384	229533.I1RBD6	pp	229533.Q4IER8
95385	229533.I1RBD6	pp	229533.V6RR76
95386	229533.I1RBD6	pp	229533.I1RW99
95387	229533.I1RBD6	pp	229533.I1RQ73
95388	229533.I1RBD6	pp	229533.I1RPU4
95389	229533.I1RBD6	pp	229533.I1RN28
95390	229533.I1RBD6	pp	229533.Q4I7X1
95391	229533.I1RBD6	pp	229533.I1RQ81
95392	229533.I1RBD6	pp	229533.I1RRF9
95393	229533.I1RBD6	pp	229533.V6QUU6
95394	229533.I1RBD6	pp	229533.I1RN31
95395	229533.I1RBD6	pp	229533.I1RN81
95396	229533.I1RBD6	pp	229533.I1S0Z3
95397	229533.I1RBD6	pp	229533.I1RW25
95398	229533.I1RBD6	pp	229533.I1RT41
95399	229533.I1RBD6	pp	229533.I1RH68
95400	229533.I1RBD6	pp	229533.I1RM67
95401	229533.I1RBD6	pp	229533.I1S0S6
95402	229533.I1RBD6	pp	229533.Q5I2J3
95403	229533.I1RBD6	pp	229533.I1RQE7
95404	229533.I1RBD6	pp	229533.I1RN49
95405	229533.I1RBD6	pp	229533.Q4IPN9
95406	229533.I1RBD6	pp	229533.I1RTA3
95407	229533.I1RBD6	pp	229533.I1RD70
95408	229533.I1RBD6	pp	229533.I1RQ80
95409	229533.I1RBD6	pp	229533.Q4IPC8
95410	229533.I1RBD6	pp	229533.I1RZG0
95411	229533.I1RBD6	pp	229533.I1RP04

229533.I1RBD6 pp 229533.I1RTG5
229533.I1RBD6 pp 229533.I1RXT9
229533.I1RBD6 pp 229533.I1RRT8
229533.I1RBD6 pp 229533.I1RRT3
229533.I1RBD6 pp 229533.I1RST4
229533.I1RBD6 pp 229533.I1RCD4
229533.I1RBD6 pp 229533.I1RZP2
229533.I1RBD6 pp 229533.I1RGQ9
229533.I1RBD6 pp 229533.I1S1V5
229533.I1RBD6 pp 229533.I1RCW7
229533.I1RBD6 pp 229533.I1RR95
229533.I1RBD6 pp 229533.I1RDE9
229533.I1RBD6 pp 229533.V6QX58
229533.I1RBD6 pp 229533.I1RE45
229533.I1RBD6 pp 229533.I1RE19
229533.I1RBD6 pp 229533.I1RJL4
229533.I1RBD6 pp 229533.I1RKR9
229533.I1RBD6 pp 229533.I1RW16
229533.I1RBD6 pp 229533.I1S7X9
229533.I1RBD6 pp 229533.I1RTK5
229533.I1RBD6 pp 229533.Q4IF44
229533.I1RBD6 pp 229533.I1RFZ2
229533.I1RBD6 pp 229533.Q4IPA4
229533.I1RBD6 pp 229533.I1S214
229533.I1RBD6 pp 229533.I1RMP2
229533.I1RSJ0 pp 229533.I1RTQ9
229533.I1RSJ0 pp 229533.I1RWL9
229533.I1RSJ0 pp 229533.I1RWB4
229533.I1RSJ0 pp 229533.I1S1V7
229533.I1RSJ0 pp 229533.I1RYU1
229533.I1RSJ0 pp 229533.I1S287
229533.A0A098D1N3 pp 229533.I1RPW8
229533.A0A098D1N3 pp 229533.I1S182
229533.A0A098D1N3 pp 229533.I1R9B6
229533.A0A098D1N3 pp 229533.I1S5P3
229533.A0A098D1N3 pp 229533.I1RCG6
229533.A0A098D1N3 pp 229533.I1RQE0
229533.A0A098D1N3 pp 229533.I1RW91
229533.A0A098D1N3 pp 229533.I1RN04
229533.A0A098D1N3 pp 229533.I1RQ42
229533.A0A098D1N3 pp 229533.I1RZ42
229533.A0A098D1N3 pp 229533.I1RW80
229533.I1RFI3 pp 229533.I1RJZ4
229533.I1RFI3 pp 229533.V6R3U5
229533.I1S7C3 pp 229533.I1SAT1
229533.Q4I613 pp 229533.Q4IR18
229533.I1S6S4 pp 229533.I1SAM5
229533.I1S6S4 pp 229533.I1S830
229533.I1S6S4 pp 229533.Q4I6S5
229533.I1S6S4 pp 229533.Q4INS6
229533.I1RCW8 pp 229533.I1RJZ7
229533.I1RCW8 pp 229533.Q4IPI4
229533.I1RCW8 pp 229533.Q4HTT1
229533.I1RCW8 pp 229533.I1S549
229533.I1RCW8 pp 229533.I1S7X9
229533.I1RCW8 pp 229533.I1RVY0
229533.I1RCW8 pp 229533.Q4I7L0
229533.I1RCW8 pp 229533.I1RI51
229533.I1RCW8 pp 229533.I1SB04
229533.I1RCW8 pp 229533.I1RGY5
229533.I1RCW8 pp 229533.Q4IB50
229533.I1RCW8 pp 229533.V6R3J1
229533.I1RCW8 pp 229533.Q4IL82
229533.I1RCW8 pp 229533.Q4IMD1
229533.I1RCW8 pp 229533.I1RSZ3
229533.I1RCW8 pp 229533.I1RQ88
229533.I1RCW8 pp 229533.Q4I5W5
229533.I1RCW8 pp 229533.I1RK79
229533.I1RCW8 pp 229533.I1RP08
229533.I1RCW8 pp 229533.I1RTH2
229533.I1RCW8 pp 229533.I1RV91
229533.I1RCW8 pp 229533.I1RQP1
229533.I1RCW8 pp 229533.I1RY44

229533.I1RCW8	pp	229533.I1S2E9
229533.I1RCW8	pp	229533.I1RR25
229533.I1RCW8	pp	229533.I1S020
229533.I1RCW8	pp	229533.I1S0N5
229533.I1RCW8	pp	229533.I1RHP9
229533.I1RCW8	pp	229533.Q4IBR4
229533.I1RCW8	pp	229533.Q4IJ11
229533.I1RCW8	pp	229533.I1RS41
229533.I1RCW8	pp	229533.I1S4Z5
229533.I1RCW8	pp	229533.I1RD70
229533.I1RCW8	pp	229533.I1REX4
229533.I1RCW8	pp	229533.I1S1V2
229533.I1RCW8	pp	229533.I1RSD1
229533.I1RCW8	pp	229533.I1RX64
229533.I1RCW8	pp	229533.Q4HYB8
229533.I1RCW8	pp	229533.I1S219
229533.I1RCW8	pp	229533.V6QWX8
229533.I1RCW8	pp	229533.I1RD69
229533.I1RCW8	pp	229533.I1RW71
229533.I1RCW8	pp	229533.I1RNV7
229533.I1RCW8	pp	229533.I1RG11
229533.I1RCW8	pp	229533.Q4IJP1
229533.I1RCW8	pp	229533.I1RYV3
229533.I1RCW8	pp	229533.Q4HYQ4
229533.I1RCW8	pp	229533.I1RRG3
229533.I1RCW8	pp	229533.I1RYB1
229533.I1RCW8	pp	229533.Q4IJU0
229533.I1RCW8	pp	229533.I1S259
229533.I1RCW8	pp	229533.I1RZR6
229533.I1RCW8	pp	229533.I1RRU8
229533.I1RCW8	pp	229533.I1RM36
229533.I1RCW8	pp	229533.I1RJL2
229533.I1RCW8	pp	229533.I1RQE9
229533.I1RCW8	pp	229533.I1RKR6
229533.I1RCW8	pp	229533.Q4I5R3
229533.I1RCW8	pp	229533.Q4I948
229533.I1RCW8	pp	229533.I1RZB5
229533.I1RCW8	pp	229533.Q4IR08
229533.I1RCW8	pp	229533.I1RRT0
229533.I1RCW8	pp	229533.I1RWT6
229533.I1RCW8	pp	229533.I1S9J6
229533.I1RCW8	pp	229533.I1RQA3
229533.I1RCW8	pp	229533.Q4HTT2
229533.I1RCW8	pp	229533.Q4I5I4
229533.I1RCW8	pp	229533.I1RE51
229533.I1RCW8	pp	229533.I1SAY2
229533.I1RCW8	pp	229533.I1RQ19
229533.I1RCW8	pp	229533.Q4ICA8
229533.I1RCW8	pp	229533.I1RWT2
229533.I1RCW8	pp	229533.V6QUE6
229533.I1RCW8	pp	229533.Q4I6A4
229533.I1RED1	pp	229533.I1RPE5
229533.I1RED1	pp	229533.I1RWI6
229533.I1RMK8	pp	229533.I1RQC1
229533.I1RMK8	pp	229533.Q4IMD1
229533.I1RMK8	pp	229533.Q4I7L0
229533.I1RMK8	pp	229533.I1RPK5
229533.I1RMK8	pp	229533.V6RGI1
229533.I1RMK8	pp	229533.I1RZT1
229533.I1RMK8	pp	229533.Q4HTT2
229533.I1RMK8	pp	229533.I1SAY2
229533.I1RMK8	pp	229533.I1RWT2
229533.I1RMK8	pp	229533.I1S836
229533.I1RU02	pp	229533.I1S7X9
229533.I1RU02	pp	229533.Q4I7L0
229533.I1RU02	pp	229533.Q4I5W5
229533.I1RU02	pp	229533.I1RVY0
229533.I1RU02	pp	229533.V6R3J1
229533.I1RU02	pp	229533.Q4IMD1
229533.I1RU02	pp	229533.Q4IPI4
229533.I1RU02	pp	229533.Q4IL82
229533.I1RU02	pp	229533.I1RV91
229533.I1RU02	pp	229533.I1S549

95558 229533.I1RU02 pp 229533.Q4IJ11
95559 229533.I1RU02 pp 229533.Q4IB50
95560 229533.I1RU02 pp 229533.I1S2E9
95561 229533.I1RU02 pp 229533.I1SB04
95562 229533.I1RU02 pp 229533.I1RX64
95563 229533.I1RU02 pp 229533.I1S020
95564 229533.I1RU02 pp 229533.Q4HTT1
95565 229533.I1RU02 pp 229533.Q4HYB8
95566 229533.I1RU02 pp 229533.I1RY44
95567 229533.I1RU02 pp 229533.Q4IJP1
95568 229533.I1RU02 pp 229533.I1RZR6
95569 229533.I1RU02 pp 229533.Q4I5R3
95570 229533.I1RU02 pp 229533.I1S259
95571 229533.I1RU02 pp 229533.Q4HYQ4
95572 229533.I1RU02 pp 229533.I1S4Z5
95573 229533.I1RU02 pp 229533.Q4IBR4
95574 229533.I1RU02 pp 229533.I1RWT2
95575 229533.I1RU02 pp 229533.I1S0N5
95576 229533.I1RU02 pp 229533.I1RW71
95577 229533.I1RU02 pp 229533.Q4I5I4
95578 229533.I1RU02 pp 229533.I1S1V2
95579 229533.I1RU02 pp 229533.V6QUE6
95580 229533.I1RU02 pp 229533.V6QWX8
95581 229533.I1RU02 pp 229533.Q4ICA8
95582 229533.I1RU02 pp 229533.Q4IR08
95583 229533.I1RU02 pp 229533.Q4I6A4
95584 229533.I1RU02 pp 229533.I1RWT6
95585 229533.I1RU02 pp 229533.I1S219
95586 229533.I1RU02 pp 229533.I1SAY2
95587 229533.I1RU02 pp 229533.Q4HTT2
95588 229533.I1RU02 pp 229533.I1RYB1
95589 229533.I1RU02 pp 229533.I1RYV3
95590 229533.I1RU02 pp 229533.Q4IJU0
95591 229533.I1RU02 pp 229533.Q4I948
95592 229533.I1RU02 pp 229533.I1RZB5
95593 229533.I1RU02 pp 229533.I1S9J6
95594 229533.I1S2Z8 pp 229533.V6RN31
95595 229533.I1S2Z8 pp 229533.V6QVM5
95596 229533.I1RY09 pp 229533.I1RZ94
95597 229533.I1RY09 pp 229533.I1RZH4
95598 229533.I1RY09 pp 229533.Q4I7L0
95599 229533.I1S1D1 pp 229533.V6R382
95600 229533.A0A1C3YL99 pp 229533.I1RP04
95601 229533.A0A1C3YL99 pp 229533.I1RWI0
95602 229533.A0A1C3YL99 pp 229533.I1RD45
95603 229533.A0A1C3YL99 pp 229533.I1RKR9
95604 229533.A0A1C3YL99 pp 229533.I1RE45
95605 229533.A0A1C3YL99 pp 229533.I1S214
95606 229533.A0A1C3YL99 pp 229533.I1RZP2
95607 229533.A0A1C3YL99 pp 229533.I1RGQ9
95608 229533.A0A1C3YL99 pp 229533.I1RFZ2
95609 229533.A0A1C3YL99 pp 229533.Q4IPA4
95610 229533.A0A1C3YL99 pp 229533.I1RZG0
95611 229533.A0A1C3YL99 pp 229533.I1RRT8
95612 229533.A0A1C3YL99 pp 229533.I1RFW1
95613 229533.A0A1C3YL99 pp 229533.I1RRT3
95614 229533.A0A1C3YL99 pp 229533.I1RZP7
95615 229533.A0A1C3YL99 pp 229533.I1S7X9
95616 229533.A0A1C3YL99 pp 229533.I1S1V5
95617 229533.A0A1C3YL99 pp 229533.I1RCD4
95618 229533.A0A1C3YL99 pp 229533.I1RKR7
95619 229533.A0A1C3YL99 pp 229533.I1RDE9
95620 229533.A0A1C3YL99 pp 229533.I1RTK5
95621 229533.A0A1C3YL99 pp 229533.I1RB74
95622 229533.A0A1C3YL99 pp 229533.I1RJL4
95623 229533.A0A1C3YL99 pp 229533.I1RMP2
95624 229533.A0A1C3YL99 pp 229533.I1RR95
95625 229533.A0A1C3YL99 pp 229533.I1RE19
95626 229533.A0A1C3YL99 pp 229533.Q4IF44
95627 229533.A0A1C3YL99 pp 229533.I1RW16
95628 229533.A0A1C3YL99 pp 229533.I1RBD6
95629 229533.A0A1C3YL99 pp 229533.I1RTG5
95630 229533.A0A1C3YL99 pp 229533.I1RXT9

95631 229533.A0A1C3YL99 pp 229533.I1RAX8
95632 229533.A0A1C3YL99 pp 229533.I1RQE7
95633 229533.A0A1C3YL99 pp 229533.I1RT41
95634 229533.A0A1C3YL99 pp 229533.I1RD70
95635 229533.A0A1C3YL99 pp 229533.Q4IPC8
95636 229533.A0A1C3YL99 pp 229533.I1RST4
95637 229533.A0A1C3YL99 pp 229533.I1RN31
95638 229533.A0A1C3YL99 pp 229533.I1RZQ6
95639 229533.A0A1C3YL99 pp 229533.I1RTA3
95640 229533.A0A1C3YL99 pp 229533.I1RPA6
95641 229533.A0A1C3YL99 pp 229533.I1RRV1
95642 229533.A0A1C3YL99 pp 229533.I1RW25
95643 229533.A0A1C3YL99 pp 229533.I1RT19
95644 229533.A0A1C3YL99 pp 229533.A0A1C3YLZ8
95645 229533.A0A1C3YL99 pp 229533.V6QX58
95646 229533.A0A1C3YL99 pp 229533.Q4HZS8
95647 229533.A0A1C3YL99 pp 229533.I1S224
95648 229533.A0A1C3YL99 pp 229533.I1RCW7
95649 229533.A0A1C3YL99 pp 229533.A0A1C3YLH6
95650 229533.A0A1C3YL99 pp 229533.V6QUU6
95651 229533.A0A1C3YL99 pp 229533.I1RQ80
95652 229533.A0A1C3YL99 pp 229533.I1RN81
95653 229533.A0A1C3YL99 pp 229533.Q4IPN9
95654 229533.A0A1C3YL99 pp 229533.I1RAW7
95655 229533.A0A1C3YL99 pp 229533.Q5I2J3
95656 229533.A0A1C3YL99 pp 229533.I1RM67
95657 229533.A0A1C3YL99 pp 229533.I1S5P3
95658 229533.A0A1C3YL99 pp 229533.I1RA46
95659 229533.A0A1C3YL99 pp 229533.I1RQD4
95660 229533.A0A1C3YL99 pp 229533.I1RN49
95661 229533.A0A1C3YL99 pp 229533.I1RN65
95662 229533.A0A1C3YL99 pp 229533.I1RCM6
95663 229533.A0A1C3YL99 pp 229533.I1RJK7
95664 229533.A0A1C3YL99 pp 229533.I1RQ73
95665 229533.A0A1C3YL99 pp 229533.I1RB53
95666 229533.A0A1C3YL99 pp 229533.V6RR76
95667 229533.A0A1C3YL99 pp 229533.A0A1C3YN20
95668 229533.A0A1C3YL99 pp 229533.V6REE9
95669 229533.A0A1C3YL99 pp 229533.I1RA72
95670 229533.A0A1C3YL99 pp 229533.I1RQ81
95671 229533.A0A1C3YL99 pp 229533.I1RW99
95672 229533.A0A1C3YL99 pp 229533.Q4IER8
95673 229533.A0A1C3YL99 pp 229533.I1RN28
95674 229533.A0A1C3YL99 pp 229533.I1S0Z3
95675 229533.A0A1C3YL99 pp 229533.I1RPU4
95676 229533.A0A1C3YL99 pp 229533.I1RZP6
95677 229533.A0A1C3YL99 pp 229533.Q4I7X1
95678 229533.A0A1C3YL99 pp 229533.I1RRF9
95679 229533.A0A0E0SQV5 pp 229533.I1RAX3
95680 229533.A0A0E0SQV5 pp 229533.Q4ICA8
95681 229533.A0A0E0SQV5 pp 229533.A0A1C3YJR3
95682 229533.A0A0E0SQV5 pp 229533.I1SAY2
95683 229533.A0A0E0SQV5 pp 229533.I1RAR2
95684 229533.A0A0E0SQV5 pp 229533.I1RWT6
95685 229533.A0A0E0SQV5 pp 229533.I1RQ19
95686 229533.A0A0E0SQV5 pp 229533.V6QUE6
95687 229533.A0A0E0SQV5 pp 229533.I1RRT0
95688 229533.A0A0E0SQV5 pp 229533.Q4I5I4
95689 229533.A0A0E0SQV5 pp 229533.Q4I5R3
95690 229533.A0A0E0SQV5 pp 229533.I1RWT2
95691 229533.A0A0E0SQV5 pp 229533.I1S259
95692 229533.A0A0E0SQV5 pp 229533.I1RE51
95693 229533.A0A0E0SQV5 pp 229533.I1RYB1
95694 229533.A0A0E0SQV5 pp 229533.I1S9J6
95695 229533.A0A0E0SQV5 pp 229533.Q4IR08
95696 229533.A0A0E0SQV5 pp 229533.I1RZB5
95697 229533.A0A0E0SQV5 pp 229533.I1RYV3
95698 229533.A0A0E0SQV5 pp 229533.Q4HTT2
95699 229533.A0A0E0SQV5 pp 229533.A0A1C3YJW9
95700 229533.A0A0E0SQV5 pp 229533.I1RQA3
95701 229533.A0A0E0SQV5 pp 229533.Q4I948
95702 229533.A0A0E0SQV5 pp 229533.I1RJL2
95703 229533.A0A0E0SQV5 pp 229533.A0A1C3YIP6

95704 229533.A0A0E0SQV5 pp 229533.I1RZR6
95705 229533.A0A0E0SQV5 pp 229533.Q4I6A4
95706 229533.A0A0E0SQV5 pp 229533.A0A1C3YK75
95707 229533.A0A0E0SQV5 pp 229533.A0A1C3YI23
95708 229533.A0A0E0SQV5 pp 229533.Q4IJP1
95709 229533.A0A0E0SQV5 pp 229533.I1RCL7
95710 229533.A0A0E0SQV5 pp 229533.I1RCM6
95711 229533.A0A0E0SQV5 pp 229533.I1RRU8
95712 229533.A0A0E0SQV5 pp 229533.I1RKR6
95713 229533.A0A0E0SQV5 pp 229533.I1RQE9
95714 229533.A0A0E0SQV5 pp 229533.I1RNV7
95715 229533.A0A0E0SQV5 pp 229533.I1RRG3
95716 229533.A0A0E0SQV5 pp 229533.Q4IJU0
95717 229533.A0A0E0SQV5 pp 229533.Q4HYQ4
95718 229533.A0A0E0SQV5 pp 229533.A0A1C3YL18
95719 229533.A0A0E0SQV5 pp 229533.I1RY44
95720 229533.A0A0E0SQV5 pp 229533.I1RW71
95721 229533.A0A0E0SQV5 pp 229533.I1RP08
95722 229533.A0A0E0SQV5 pp 229533.A0A1C3YL77
95723 229533.A0A0E0SQV5 pp 229533.I1RM36
95724 229533.A0A0E0SQV5 pp 229533.I1S219
95725 229533.A0A0E0SQV5 pp 229533.A0A1C3YN58
95726 229533.A0A0E0SQV5 pp 229533.A0A1C3YJ34
95727 229533.A0A0E0SQV5 pp 229533.I1RSD1
95728 229533.A0A0E0SQV5 pp 229533.I1RD69
95729 229533.A0A0E0SQV5 pp 229533.Q4HYB8
95730 229533.A0A0E0SQV5 pp 229533.I1S1V2
95731 229533.A0A0E0SQV5 pp 229533.V6QWX8
95732 229533.A0A0E0SQV5 pp 229533.I1REX4
95733 229533.A0A0E0SQV5 pp 229533.I1S4Z5
95734 229533.A0A0E0SQV5 pp 229533.I1RAN8
95735 229533.A0A0E0SQV5 pp 229533.A0A1C3YLA2
95736 229533.A0A0E0SQV5 pp 229533.I1RG11
95737 229533.A0A0E0SQV5 pp 229533.I1S020
95738 229533.A0A0E0SQV5 pp 229533.I1RCB7
95739 229533.A0A0E0SQV5 pp 229533.I1RTH2
95740 229533.A0A0E0SQV5 pp 229533.Q4IBR4
95741 229533.A0A0E0SQV5 pp 229533.V6R3J1
95742 229533.A0A0E0SQV5 pp 229533.A0A1C3YLW7
95743 229533.A0A0E0SQV5 pp 229533.I1RMC2
95744 229533.A0A0E0SQV5 pp 229533.I1RS41
95745 229533.A0A0E0SQV5 pp 229533.A0A1C3YLX5
95746 229533.A0A0E0SQV5 pp 229533.I1RD70
95747 229533.A0A0E0SQV5 pp 229533.I1RBJ0
95748 229533.A0A0E0SQV5 pp 229533.Q4I5W5
95749 229533.A0A0E0SQV5 pp 229533.I1S0N5
95750 229533.A0A0E0SQV5 pp 229533.I1RAI9
95751 229533.A0A0E0SQV5 pp 229533.Q4IMD1
95752 229533.A0A0E0SQV5 pp 229533.I1RR25
95753 229533.A0A0E0SQV5 pp 229533.I1RBC7
95754 229533.A0A0E0SQV5 pp 229533.A0A1C3YHP3
95755 229533.A0A0E0SQV5 pp 229533.I1RK79
95756 229533.A0A0E0SQV5 pp 229533.I1R9W6
95757 229533.A0A0E0SQV5 pp 229533.A0A1C3YKA7
95758 229533.A0A0E0SQV5 pp 229533.I1RX64
95759 229533.A0A0E0SQV5 pp 229533.I1RQP1
95760 229533.A0A0E0SQV5 pp 229533.I1RV91
95761 229533.A0A0E0SQV5 pp 229533.I1RBK8
95762 229533.A0A0E0SQV5 pp 229533.I1RQ88
95763 229533.A0A0E0SQV5 pp 229533.I1RSZ3
95764 229533.A0A0E0SQV5 pp 229533.I1RGY5
95765 229533.A0A0E0SQV5 pp 229533.I1S7X9
95766 229533.A0A0E0SQV5 pp 229533.Q4I7L0
95767 229533.A0A0E0SQV5 pp 229533.A0A1C3YLC6
95768 229533.A0A0E0SQV5 pp 229533.I1RHP9
95769 229533.A0A0E0SQV5 pp 229533.I1RJZ7
95770 229533.A0A0E0SQV5 pp 229533.I1S2E9
95771 229533.A0A0E0SQV5 pp 229533.I1RVY0
95772 229533.A0A0E0SQV5 pp 229533.Q4IL82
95773 229533.A0A0E0SQV5 pp 229533.Q4IJ11
95774 229533.A0A0E0SQV5 pp 229533.I1RCN2
95775 229533.A0A0E0SQV5 pp 229533.A0A1C3YM67
95776 229533.A0A0E0SQV5 pp 229533.A0A1C3YIR8

95777 229533.A0A0E0SQV5 pp 229533.I1RC24
95778 229533.A0A0E0SQV5 pp 229533.Q4IPI4
95779 229533.A0A0E0SQV5 pp 229533.I1RI51
95780 229533.A0A0E0SQV5 pp 229533.A0A1C3YJD4
95781 229533.A0A0E0SQV5 pp 229533.Q4IB50
95782 229533.A0A0E0SQV5 pp 229533.Q4HTT1
95783 229533.A0A0E0SQV5 pp 229533.I1SB04
95784 229533.A0A0E0SQV5 pp 229533.A0A1C3YKX5
95785 229533.A0A0E0SQV5 pp 229533.I1RB73
95786 229533.A0A0E0SQV5 pp 229533.I1S549
95787 229533.I1S1V7 pp 229533.I1S287
95788 229533.I1S1V7 pp 229533.V6RGI1
95789 229533.I1S1V7 pp 229533.I1S9Z3
95790 229533.A0A1C3YM95 pp 229533.I1RSI7
95791 229533.A0A1C3YM95 pp 229533.I1RR21
95792 229533.A0A1C3YM95 pp 229533.V6QUU6
95793 229533.A0A1C3YM95 pp 229533.V6RR76
95794 229533.A0A1C3YM95 pp 229533.I1RNG6
95795 229533.A0A1C3YM95 pp 229533.I1RWA9
95796 229533.A0A1C3YM95 pp 229533.I1RTD1
95797 229533.A0A1C3YM95 pp 229533.I1RD10
95798 229533.A0A1C3YM95 pp 229533.I1RTE0
95799 229533.A0A1C3YM95 pp 229533.V6R476
95800 229533.A0A1C3YM95 pp 229533.I1S045
95801 229533.A0A1C3YM95 pp 229533.I1S1B1
95802 229533.A0A1C3YM95 pp 229533.I1S254
95803 229533.A0A1C3YM95 pp 229533.I1RA34
95804 229533.A0A1C3YM95 pp 229533.I1RWI6
95805 229533.A0A1C3YM95 pp 229533.I1RN81
95806 229533.A0A1C3YM95 pp 229533.I1S4X2
95807 229533.A0A1C3YM95 pp 229533.I1RGE7
95808 229533.A0A098DUM6 pp 229533.I1RPU8
95809 229533.A0A098DUM6 pp 229533.I1RUP2
95810 229533.A0A098DUM6 pp 229533.A0A1C3YM54
95811 229533.A0A098DUM6 pp 229533.I1RCV1
95812 229533.A0A098DUM6 pp 229533.I1S2A1
95813 229533.A0A098DUM6 pp 229533.I1RKI7
95814 229533.A0A098DUM6 pp 229533.I1S338
95815 229533.A0A098DUM6 pp 229533.I1RPI5
95816 229533.A0A098DUM6 pp 229533.I1RKI3
95817 229533.A0A098DUM6 pp 229533.I1RQG7
95818 229533.I1RAX3 pp 229533.I1RZP8
95819 229533.I1RAX3 pp 229533.I1RCL7
95820 229533.I1RAX3 pp 229533.I1RU02
95821 229533.I1RAX3 pp 229533.I1RQJ4
95822 229533.I1RAX3 pp 229533.I1RSP7
95823 229533.I1RAX3 pp 229533.Q4IER8
95824 229533.I1RAX3 pp 229533.I1S020
95825 229533.I1RAX3 pp 229533.I1RCW8
95826 229533.I1RAX3 pp 229533.I1RN57
95827 229533.I1RAX3 pp 229533.V6R382
95828 229533.I1RAX3 pp 229533.I1RMS4
95829 229533.I1RAX3 pp 229533.I1S209
95830 229533.I1RAX3 pp 229533.I1RRK4
95831 229533.I1RAX3 pp 229533.Q4IR08
95832 229533.I1RAX3 pp 229533.I1RMX3
95833 229533.I1RAX3 pp 229533.I1RSC7
95834 229533.I1RAX3 pp 229533.I1RWT2
95835 229533.I1RAX3 pp 229533.I1RN46
95836 229533.I1RAX3 pp 229533.Q4IBR4
95837 229533.I1RAX3 pp 229533.I1RBW0
95838 229533.I1RAX3 pp 229533.I1RCH7
95839 229533.I1RAX3 pp 229533.V6RLW4
95840 229533.I1RAX3 pp 229533.I1RFJ2
95841 229533.I1RAX3 pp 229533.I1RN55
95842 229533.I1RAX3 pp 229533.I1RG77
95843 229533.I1RAX3 pp 229533.I1RFX8
95844 229533.I1RAX3 pp 229533.Q4I7L0
95845 229533.I1RAX3 pp 229533.I1RQ13
95846 229533.I1RAX3 pp 229533.I1RBW1
95847 229533.I1RAX3 pp 229533.I1S283
95848 229533.I1RAX3 pp 229533.I1RNK1
95849 229533.I1RAX3 pp 229533.I1RYX9

95850 229533.I1RAX3 pp 229533.I1RYR2
95851 229533.I1RAX3 pp 229533.I1RQ05
95852 229533.I1RAX3 pp 229533.Q4IQM6
95853 229533.I1RAX3 pp 229533.I1RFW1
95854 229533.I1RNG6 pp 229533.I1RTD1
95855 229533.I1RNG6 pp 229533.Q4I0J6
95856 229533.I1RNG6 pp 229533.V6R476
95857 229533.I1RNG6 pp 229533.I1RWA9
95858 229533.I1S0A0 pp 229533.V6RGB5
95859 229533.I1S0A0 pp 229533.V6RQW4
95860 229533.I1S0A0 pp 229533.V6RY56
95861 229533.I1S0A0 pp 229533.I1S2C8
95862 229533.I1S0A0 pp 229533.O74638
95863 229533.I1S0A0 pp 229533.I1S5B0
95864 229533.I1S0A0 pp 229533.I1S1W8
95865 229533.I1S0A0 pp 229533.I1S4D0
95866 229533.I1S0A0 pp 229533.Q4HXF0
95867 229533.I1S0A0 pp 229533.V6QUE6
95868 229533.I1S0A0 pp 229533.I1S0I3
95869 229533.I1S0A0 pp 229533.V6RK96
95870 229533.I1S0A0 pp 229533.I1S3G4
95871 229533.I1S0A0 pp 229533.V6R382
95872 229533.I1S0A0 pp 229533.I1SAJ3
95873 229533.I1S0A0 pp 229533.I1S5L5
95874 229533.I1S0A0 pp 229533.V6RLM7
95875 229533.I1S0F0 pp 229533.I1S0J3
95876 229533.I1S0F0 pp 229533.Q4I7L0
95877 229533.I1S0F0 pp 229533.Q4ICA8
95878 229533.I1S0F0 pp 229533.I1SB04
95879 229533.I1S0F0 pp 229533.I1S5T5
95880 229533.I1S0F0 pp 229533.I1SAN0
95881 229533.I1S0F0 pp 229533.Q4IQX3
95882 229533.I1S0F0 pp 229533.Q4IER8
95883 229533.I1S0F0 pp 229533.Q4IMD1
95884 229533.I1S0F0 pp 229533.I1S364
95885 229533.I1S0F0 pp 229533.I1S724
95886 229533.I1S0F0 pp 229533.Q4HYQ4
95887 229533.I1RIU1 pp 229533.I1RSI0
95888 229533.I1RIU1 pp 229533.Q4HVU7
95889 229533.I1RIU1 pp 229533.V6QWB1
95890 229533.A0A1C3YM11 pp 229533.I1S9Q3
95891 229533.A0A1C3YM11 pp 229533.I1RSX9
95892 229533.A0A1C3YM11 pp 229533.I1RBY8
95893 229533.A0A1C3YM11 pp 229533.I1S2A1
95894 229533.A0A1C3YM11 pp 229533.I1RFD1
95895 229533.A0A1C3YM11 pp 229533.Q4HVU7
95896 229533.I1RPL5 pp 229533.I1RYV3
95897 229533.I1RPL5 pp 229533.Q4I7L0
95898 229533.I1RPL5 pp 229533.I1S4Z5
95899 229533.I1RPL5 pp 229533.I1RWT2
95900 229533.I1RPL5 pp 229533.Q4HTT2
95901 229533.I1R9G1 pp 229533.I1RNM7
95902 229533.I1R9G1 pp 229533.I1RHP6
95903 229533.I1R9G1 pp 229533.I1RUC0
95904 229533.I1R9G1 pp 229533.I1RWM4
95905 229533.I1S0X0 pp 229533.V6QUU6
95906 229533.I1S0X0 pp 229533.I1S8E0
95907 229533.I1S0X0 pp 229533.I1S0Z3
95908 229533.I1S0X0 pp 229533.I1S254
95909 229533.I1S0X0 pp 229533.V6R476
95910 229533.I1S0X0 pp 229533.V6RR76
95911 229533.A0A1C3YHW1 pp 229533.I1S287
95912 229533.A0A1C3YHW1 pp 229533.A0A1C3YK51
95913 229533.A0A1C3YHW1 pp 229533.A0A1C3YIC5
95914 229533.A0A1C3YHW1 pp 229533.I1RTD0
95915 229533.A0A1C3YHW1 pp 229533.I1RQ70
95916 229533.A0A1C3YHW1 pp 229533.A0A1C3YME0
95917 229533.A0A1C3YHW1 pp 229533.I1RFS7
95918 229533.A0A1C3YHW1 pp 229533.I1RFK0
95919 229533.A0A1C3YHW1 pp 229533.I1RFV4
95920 229533.A0A1C3YHW1 pp 229533.A0A1C3YN54
95921 229533.A0A1C3YHW1 pp 229533.I1RTQ9
95922 229533.A0A1C3YHW1 pp 229533.A0A1C3YIE3

95923	229533.A0A1C3YHW1	pp	229533.I1RD45
95924	229533.A0A1C3YHW1	pp	229533.I1RQ32
95925	229533.A0A1C3YHW1	pp	229533.I1REC4
95926	229533.A0A1C3YHW1	pp	229533.I1REK1
95927	229533.A0A1C3YHW1	pp	229533.I1RE20
95928	229533.A0A1C3YHW1	pp	229533.I1RSZ1
95929	229533.A0A1C3YHW1	pp	229533.I1RWB4
95930	229533.A0A1C3YHW1	pp	229533.I1RE15
95931	229533.A0A1C3YHW1	pp	229533.I1RB98
95932	229533.A0A1C3YHW1	pp	229533.I1RE03
95933	229533.A0A1C3YHW1	pp	229533.I1RK33
95934	229533.A0A1C3YHW1	pp	229533.I1S830
95935	229533.A0A1C3YHW1	pp	229533.I1RMF2
95936	229533.A0A1C3YHW1	pp	229533.I1RMV6
95937	229533.A0A1C3YHW1	pp	229533.I1RAS9
95938	229533.A0A1C3YHW1	pp	229533.I1S6S4
95939	229533.A0A1C3YHW1	pp	229533.I1RSJ0
95940	229533.A0A1C3YHW1	pp	229533.I1RXF5
95941	229533.A0A1C3YHW1	pp	229533.I1RPU5
95942	229533.A0A1C3YHW1	pp	229533.I1RKR7
95943	229533.A0A1C3YHW1	pp	229533.I1S1V7
95944	229533.A0A1C3YHW1	pp	229533.A0A1C3YN81
95945	229533.A0A1C3YHW1	pp	229533.I1RNA3
95946	229533.A0A1C3YHW1	pp	229533.I1RBA6
95947	229533.A0A098DUZ1	pp	229533.I1RQ28
95948	229533.A0A098DUZ1	pp	229533.A0A0E0SF53
95949	229533.A0A098DUZ1	pp	229533.I1RYV3
95950	229533.A0A098DUZ1	pp	229533.Q4IER8
95951	229533.A0A098DUZ1	pp	229533.I1RMQ7
95952	229533.A0A098DUZ1	pp	229533.Q4HTT2
95953	229533.A0A098DUZ1	pp	229533.I1RWT2
95954	229533.A0A098DUZ1	pp	229533.I1RBJ0
95955	229533.A0A098DUZ1	pp	229533.I1S4Z5
95956	229533.A0A098DUZ1	pp	229533.A0A1C3YJ34
95957	229533.A0A098DUZ1	pp	229533.Q4I7L0
95958	229533.A0A098DUZ1	pp	229533.I1RFW1
95959	229533.A0A098DUZ1	pp	229533.Q4IMD1
95960	229533.I1RUI0	pp	229533.I1S0N8
95961	229533.I1RUI0	pp	229533.I1S757
95962	229533.A0A098DU14	pp	229533.I1RQ69
95963	229533.A0A098DU14	pp	229533.I1S0K5
95964	229533.A0A098DU14	pp	229533.A0A1C3YN54
95965	229533.A0A098DU14	pp	229533.A0A0E0RUL1
95966	229533.A0A098DU14	pp	229533.I1RMG2
95967	229533.A0A098DU14	pp	229533.I1RJK8
95968	229533.A0A098DU14	pp	229533.I1RFK0
95969	229533.A0A098DU14	pp	229533.I1RPK5
95970	229533.A0A098DU14	pp	229533.I1RTC1
95971	229533.A0A098DU14	pp	229533.V6RR76
95972	229533.A0A098DU14	pp	229533.I1RRB0
95973	229533.A0A098DU14	pp	229533.I1RBS4
95974	229533.A0A098DU14	pp	229533.A0A0E0SFJ9
95975	229533.A0A098DU14	pp	229533.I1RYW8
95976	229533.A0A098DU14	pp	229533.I1RX84
95977	229533.A0A098DU14	pp	229533.A0A1C3YIQ1
95978	229533.A0A098DU14	pp	229533.A0A1C3YKT4
95979	229533.A0A098DU14	pp	229533.A0A0E0SNZ9
95980	229533.A0A098DU14	pp	229533.I1RB98
95981	229533.A0A098DU14	pp	229533.I1RT41
95982	229533.A0A098DU14	pp	229533.I1RIG4
95983	229533.A0A098DU14	pp	229533.I1S287
95984	229533.A0A098DU14	pp	229533.I1RN81
95985	229533.A0A098DU14	pp	229533.Q4ILI9
95986	229533.A0A098DU14	pp	229533.I1RTQ9
95987	229533.A0A098DU14	pp	229533.I1RMK1
95988	229533.A0A098DU14	pp	229533.A0A1C3YIP6
95989	229533.A0A098DU14	pp	229533.A0A1C3YLL9
95990	229533.A0A098DU14	pp	229533.I1REK1
95991	229533.A0A098DU14	pp	229533.I1RFS7
95992	229533.A0A098DU14	pp	229533.I1RMF2
95993	229533.A0A098DU14	pp	229533.I1RTG5
95994	229533.A0A098DU14	pp	229533.I1RQ32
95995	229533.A0A098DU14	pp	229533.Q4HYZ2

95996	229533.A0A098DU14	pp	229533.I1RMJ1
95997	229533.A0A098DU14	pp	229533.A0A0E0SB93
95998	229533.A0A098DU14	pp	229533.I1RWB4
95999	229533.A0A098DU14	pp	229533.I1RYU1
96000	229533.A0A098DU14	pp	229533.I1RB26
96001	229533.A0A098DU14	pp	229533.I1RCE7
96002	229533.A0A098DU14	pp	229533.I1RBX2
96003	229533.A0A098DU14	pp	229533.I1RSZ6
96004	229533.A0A098DU14	pp	229533.A0A1C3YL28
96005	229533.A0A098DU14	pp	229533.I1RD70
96006	229533.A0A098DU14	pp	229533.A0A098DVM4
96007	229533.A0A098DU14	pp	229533.I1RW76
96008	229533.A0A098DU14	pp	229533.I1RZ60
96009	229533.A0A098DU14	pp	229533.I1RDE8
96010	229533.A0A098DU14	pp	229533.I1RSJ0
96011	229533.A0A098DU14	pp	229533.I1S6S4
96012	229533.A0A098DU14	pp	229533.A0A0E0SGH2
96013	229533.A0A098DU14	pp	229533.I1RKT9
96014	229533.A0A098DU14	pp	229533.A0A1C3YLS7
96015	229533.A0A098DU14	pp	229533.A0A1C3YM61
96016	229533.A0A098DU14	pp	229533.A0A0E0S0W9
96017	229533.A0A098DU14	pp	229533.I1S1V7
96018	229533.A0A098DU14	pp	229533.A0A1C3YJG7
96019	229533.A0A098DU14	pp	229533.Q4I6S5
96020	229533.A0A098DU14	pp	229533.I1RCN6
96021	229533.A0A098DU14	pp	229533.A0A1C3YLK5
96022	229533.A0A098DU14	pp	229533.A0A1C3YHW1
96023	229533.A0A098DU14	pp	229533.I1RCF0
96024	229533.Q4ILI9	pp	229533.V6RR76
96025	229533.Q4ILI9	pp	229533.V6QW25
96026	229533.Q4ILI9	pp	229533.V6R476
96027	229533.Q4ILI9	pp	229533.V6QUU6
96028	229533.I1S1Y7	pp	229533.Q4HTT2
96029	229533.I1S1Y7	pp	229533.Q4HTT1
96030	229533.I1S1Y7	pp	229533.Q4HY90
96031	229533.I1S1Y7	pp	229533.Q4I7L0
96032	229533.I1S1Y7	pp	229533.Q4IER8
96033	229533.I1S1Y7	pp	229533.Q4IMD1
96034	229533.I1S1Y7	pp	229533.I1S2E9
96035	229533.I1RMW4	pp	229533.I1S0K2
96036	229533.I1RMW4	pp	229533.I1S2A1
96037	229533.I1RMW4	pp	229533.I1RUQ3
96038	229533.I1RMW4	pp	229533.I1RSI0
96039	229533.I1RMW4	pp	229533.I1RSI1
96040	229533.I1RMW4	pp	229533.I1RUL3
96041	229533.I1RMW4	pp	229533.I1RQ12
96042	229533.I1RMW4	pp	229533.Q4I5G1
96043	229533.I1RMW4	pp	229533.V6RQW4
96044	229533.I1RMW4	pp	229533.I1RXB6
96045	229533.I1RMW4	pp	229533.I1S0A0
96046	229533.I1RMW4	pp	229533.I1RYI7
96047	229533.I1RMW4	pp	229533.I1S128
96048	229533.I1RMW4	pp	229533.I1RNM7
96049	229533.I1RMW4	pp	229533.I1S064
96050	229533.I1RMW4	pp	229533.Q4HVU7
96051	229533.I1RMW4	pp	229533.V6R7V8
96052	229533.I1RMW4	pp	229533.V6RLM7
96053	229533.I1RMW4	pp	229533.I1RYJ7
96054	229533.I1RMW4	pp	229533.I1RSX9
96055	229533.I1RMW4	pp	229533.I1RVU1
96056	229533.I1RMW4	pp	229533.I1RVX9
96057	229533.I1RMW4	pp	229533.V6RPQ8
96058	229533.I1RMW4	pp	229533.I1S0L3
96059	229533.I1RMW4	pp	229533.V6RN69
96060	229533.I1RMW4	pp	229533.V6R1K4
96061	229533.I1RMW4	pp	229533.I1S0C2
96062	229533.I1RMW4	pp	229533.I1RPN1
96063	229533.I1RMW4	pp	229533.I1RTG2
96064	229533.I1RMW4	pp	229533.I1RWT0
96065	229533.I1RMW4	pp	229533.V6RSF3
96066	229533.I1RMW4	pp	229533.V6RVJ3
96067	229533.I1RMW4	pp	229533.I1RWA3
96068	229533.I1RMW4	pp	229533.I1RZZ9

96069 229533.I1RMW4 pp 229533.I1RRX5
96070 229533.I1RMW4 pp 229533.I1RZD0
96071 229533.I1RV48 pp 229533.V6RIE5
96072 229533.I1RV48 pp 229533.V6RGI1
96073 229533.I1S6Y1 pp 229533.Q4I7L0
96074 229533.I1S6Y1 pp 229533.Q4HTT2
96075 229533.I1RMX0 pp 229533.V6QVH8
96076 229533.I1RMX0 pp 229533.V6RN31
96077 229533.I1RMX0 pp 229533.V6QVM5
96078 229533.I1RMX0 pp 229533.I1RVR2
96079 229533.I1RMX0 pp 229533.I1RW06
96080 229533.A0A1C3YME3 pp 229533.I1RUM7
96081 229533.A0A1C3YME3 pp 229533.I1RYN9
96082 229533.A0A1C3YME3 pp 229533.I1RKR7
96083 229533.A0A1C3YME3 pp 229533.V6RG06
96084 229533.Q4IMD1 pp 229533.V6R3J1
96085 229533.Q4IMD1 pp 229533.V6QV38
96086 229533.Q4IMD1 pp 229533.Q4IQX3
96087 229533.Q4IMD1 pp 229533.Q4IPI4
96088 229533.Q4IMD1 pp 229533.V6R0U9
96089 229533.Q4IMD1 pp 229533.Q4IN63
96090 229533.Q4IMD1 pp 229533.Q4IR08
96091 229533.Q4IMD1 pp 229533.V6QWX8
96092 229533.Q4IMD1 pp 229533.V6QUE6
96093 229533.A0A098E4M0 pp 229533.V6RCC0
96094 229533.A0A098E4M0 pp 229533.A0A0E0S530
96095 229533.A0A098E4M0 pp 229533.I1S8D3
96096 229533.A0A098E4M0 pp 229533.V6R5T7
96097 229533.I1RCQ6 pp 229533.Q4I1Y1
96098 229533.I1RCQ6 pp 229533.I1RZB5
96099 229533.I1RCQ6 pp 229533.I1RK79
96100 229533.I1RCQ6 pp 229533.I1RG57
96101 229533.I1RCQ6 pp 229533.I1RJL2
96102 229533.I1RCQ6 pp 229533.I1RYW3
96103 229533.I1RCQ6 pp 229533.I1RQP8
96104 229533.I1RCQ6 pp 229533.I1RK90
96105 229533.I1RCQ6 pp 229533.I1RPA0
96106 229533.I1RCQ6 pp 229533.I1RRQ4
96107 229533.I1RCQ6 pp 229533.I1RFP9
96108 229533.I1RCQ6 pp 229533.I1RCR5
96109 229533.I1RCQ6 pp 229533.I1RK92
96110 229533.I1RCQ6 pp 229533.I1RF91
96111 229533.I1RCQ6 pp 229533.I1RZL5
96112 229533.I1RCQ6 pp 229533.Q4IPH4
96113 229533.I1RCQ6 pp 229533.I1S8V0
96114 229533.I1RCQ6 pp 229533.V6RIS2
96115 229533.I1RCQ6 pp 229533.I1RG90
96116 229533.I1RCQ6 pp 229533.I1RZV9
96117 229533.I1RCQ6 pp 229533.I1RR25
96118 229533.I1RCQ6 pp 229533.Q4IP34
96119 229533.I1RCQ6 pp 229533.I1RS83
96120 229533.I1RCQ6 pp 229533.I1S0Z9
96121 229533.I1RCQ6 pp 229533.Q4IB70
96122 229533.I1RCQ6 pp 229533.I1RG11
96123 229533.I1RCQ6 pp 229533.I1RND5
96124 229533.I1RCQ6 pp 229533.I1RT47
96125 229533.I1RCQ6 pp 229533.I1RYB1
96126 229533.I1RCQ6 pp 229533.I1RN09
96127 229533.I1RCQ6 pp 229533.I1RSA4
96128 229533.I1RCQ6 pp 229533.V6QVD5
96129 229533.I1RCQ6 pp 229533.I1RPY7
96130 229533.I1RCQ6 pp 229533.I1RXT9
96131 229533.I1RCQ6 pp 229533.I1RQD8
96132 229533.I1RCQ6 pp 229533.I1RSH9
96133 229533.I1RCQ6 pp 229533.I1S1X9
96134 229533.I1RCQ6 pp 229533.I1RZ11
96135 229533.I1RCQ6 pp 229533.I1RNB8
96136 229533.I1RCQ6 pp 229533.I1RM25
96137 229533.I1RCQ6 pp 229533.I1RQR3
96138 229533.I1RCQ6 pp 229533.I1RS06
96139 229533.I1RCQ6 pp 229533.I1RSA8
96140 229533.I1RCQ6 pp 229533.I1S8U9
96141 229533.I1RCQ6 pp 229533.Q4IAA0

96142	229533.I1RCQ6	pp	229533.I1RMM5
96143	229533.I1RCQ6	pp	229533.I1RT91
96144	229533.I1RCQ6	pp	229533.I1RQZ2
96145	229533.I1RCQ6	pp	229533.I1S7M3
96146	229533.I1RCQ6	pp	229533.Q4HXF6
96147	229533.I1RCQ6	pp	229533.I1RRL0
96148	229533.I1RCQ6	pp	229533.Q4IRI9
96149	229533.I1RCQ6	pp	229533.I1RX47
96150	229533.I1RCQ6	pp	229533.I1RZQ5
96151	229533.I1RCQ6	pp	229533.V6RY33
96152	229533.I1RCQ6	pp	229533.I1RE31
96153	229533.I1RCQ6	pp	229533.I1RSH4
96154	229533.I1RCQ6	pp	229533.I1S289
96155	229533.I1RCQ6	pp	229533.I1RZZ1
96156	229533.I1RCQ6	pp	229533.I1RPL3
96157	229533.I1RCQ6	pp	229533.I1RK72
96158	229533.I1RCQ6	pp	229533.I1RRT0
96159	229533.I1RCQ6	pp	229533.I1RT35
96160	229533.I1RCQ6	pp	229533.I1RVT9
96161	229533.I1RCQ6	pp	229533.I1RWX8
96162	229533.I1RCQ6	pp	229533.I1RFQ1
96163	229533.I1RCQ6	pp	229533.I1S471
96164	229533.I1RCQ6	pp	229533.Q4I7F9
96165	229533.I1RCQ6	pp	229533.I1RPY5
96166	229533.I1RCQ6	pp	229533.V6QXG1
96167	229533.I1RCQ6	pp	229533.I1RSD4
96168	229533.I1RCQ6	pp	229533.I1RS28
96169	229533.I1RCQ6	pp	229533.I1RX51
96170	229533.I1RCQ6	pp	229533.I1RMW7
96171	229533.I1RCQ6	pp	229533.I1RN59
96172	229533.I1RCQ6	pp	229533.I1RST1
96173	229533.I1RCQ6	pp	229533.I1RME9
96174	229533.I1RCQ6	pp	229533.I1S0B6
96175	229533.I1RCQ6	pp	229533.I1RFQ0
96176	229533.I1RCQ6	pp	229533.I1RU69
96177	229533.I1RCQ6	pp	229533.I1RCU7
96178	229533.I1RCQ6	pp	229533.I1S214
96179	229533.I1RCQ6	pp	229533.I1RJG6
96180	229533.I1RCQ6	pp	229533.I1RG13
96181	229533.I1RCQ6	pp	229533.I1RMY3
96182	229533.I1RCQ6	pp	229533.I1S4Y4
96183	229533.I1RCQ6	pp	229533.I1RS03
96184	229533.I1RCQ6	pp	229533.I1RMV5
96185	229533.I1RCQ6	pp	229533.I1RPZ3
96186	229533.I1RCQ6	pp	229533.I1RWU2
96187	229533.I1RCQ6	pp	229533.I1RX25
96188	229533.I1RCQ6	pp	229533.I1RFT1
96189	229533.I1RCQ6	pp	229533.I1REX2
96190	229533.I1RCQ6	pp	229533.I1RX24
96191	229533.I1RCQ6	pp	229533.I1RDD3
96192	229533.I1RCQ6	pp	229533.I1S1C6
96193	229533.I1RCQ6	pp	229533.I1RYI9
96194	229533.I1RCQ6	pp	229533.I1S4M5
96195	229533.I1RCQ6	pp	229533.I1RKF8
96196	229533.I1RCQ6	pp	229533.I1RJF9
96197	229533.I1RCQ6	pp	229533.I1RX81
96198	229533.I1RCQ6	pp	229533.I1S0R7
96199	229533.I1RCQ6	pp	229533.I1RSF6
96200	229533.I1RCQ6	pp	229533.I1RK96
96201	229533.I1RCQ6	pp	229533.I1S9L5
96202	229533.I1RCQ6	pp	229533.Q4IPN9
96203	229533.I1RCQ6	pp	229533.I1RXF2
96204	229533.I1RCQ6	pp	229533.I1RCS2
96205	229533.I1RCQ6	pp	229533.I1RZG7
96206	229533.I1RCQ6	pp	229533.I1RPY4
96207	229533.I1RCQ6	pp	229533.I1S7M2
96208	229533.I1RCQ6	pp	229533.I1RQB5
96209	229533.I1RCQ6	pp	229533.I1RJM7
96210	229533.I1RCQ6	pp	229533.I1S524
96211	229533.I1RCQ6	pp	229533.I1S5S2
96212	229533.I1RCQ6	pp	229533.Q4IBK5
96213	229533.I1RCQ6	pp	229533.I1S246
96214	229533.I1RCQ6	pp	229533.I1S9L6

96215 229533.I1RCQ6 pp 229533.Q4IPB3
96216 229533.I1RCQ6 pp 229533.I1RS75
96217 229533.I1RCQ6 pp 229533.I1RD56
96218 229533.I1RCQ6 pp 229533.I1RRG3
96219 229533.I1RCQ6 pp 229533.I1RLU5
96220 229533.I1RCQ6 pp 229533.I1RT17
96221 229533.I1RCQ6 pp 229533.I1S2Y5
96222 229533.I1RCQ6 pp 229533.I1RND9
96223 229533.I1RCQ6 pp 229533.I1RY40
96224 229533.I1RCQ6 pp 229533.I1RQC5
96225 229533.I1RDA5 pp 229533.I1RWT2
96226 229533.I1RDA5 pp 229533.Q4HTT2
96227 229533.I1RDA5 pp 229533.I1S4Z5
96228 229533.I1RDA5 pp 229533.I1RNC0
96229 229533.I1RDA5 pp 229533.I1RYV3
96230 229533.I1RDA5 pp 229533.Q4I7L0
96231 229533.I1S1B1 pp 229533.V6RB81
96232 229533.I1S1B1 pp 229533.Q4ILI9
96233 229533.I1S1B1 pp 229533.V6RR76
96234 229533.I1S1B1 pp 229533.V6QUU6
96235 229533.I1S1B1 pp 229533.V6R476
96236 229533.I1S1B1 pp 229533.I1S4X2
96237 229533.A0A0E0RSR0 pp 229533.I1RCM8
96238 229533.A0A0E0RSR0 pp 229533.I1RFV2
96239 229533.A0A0E0RSR0 pp 229533.I1RPZ1
96240 229533.A0A0E0RSR0 pp 229533.I1RBN1
96241 229533.A0A0E0RSR0 pp 229533.I1REY8
96242 229533.A0A0E0RSR0 pp 229533.I1RQE8
96243 229533.A0A0E0RSR0 pp 229533.I1RMM8
96244 229533.A0A0E0RSR0 pp 229533.I1RRC4
96245 229533.A0A0E0RSR0 pp 229533.A0A1C3YIB8
96246 229533.A0A0E0RSR0 pp 229533.I1RZM4
96247 229533.A0A0E0RSR0 pp 229533.I1RK49
96248 229533.A0A0E0RSR0 pp 229533.A0A1C3YLC3
96249 229533.A0A0E0RSR0 pp 229533.A0A1C3YJZ0
96250 229533.A0A0E0RSR0 pp 229533.I1RQ48
96251 229533.A0A0E0RSR0 pp 229533.I1RW82
96252 229533.A0A0E0RSR0 pp 229533.V6RK96
96253 229533.A0A0E0RSR0 pp 229533.A0A1C3YHY5
96254 229533.A0A0E0RSR0 pp 229533.I1RCT9
96255 229533.A0A0E0RSR0 pp 229533.A0A0E0SHL3
96256 229533.A0A0E0RSR0 pp 229533.I1RRN4
96257 229533.A0A0E0RSR0 pp 229533.I1RRQ7
96258 229533.A0A0E0RSR0 pp 229533.A0A1C3YJ07
96259 229533.A0A0E0RSR0 pp 229533.I1RED2
96260 229533.A0A0E0RSR0 pp 229533.I1RF24
96261 229533.A0A0E0RSR0 pp 229533.I1S8X0
96262 229533.A0A0E0RSR0 pp 229533.I1S0Y1
96263 229533.A0A0E0RSR0 pp 229533.I1RR68
96264 229533.A0A0E0RSR0 pp 229533.I1RK02
96265 229533.A0A0E0RSR0 pp 229533.I1REI2
96266 229533.A0A0E0RSR0 pp 229533.I1RRR9
96267 229533.A0A0E0RSR0 pp 229533.I1RE52
96268 229533.A0A0E0RSR0 pp 229533.A0A1C3YIX3
96269 229533.A0A0E0RSR0 pp 229533.I1RZN1
96270 229533.A0A0E0RSR0 pp 229533.I1RPK4
96271 229533.A0A0E0RSR0 pp 229533.I1R9N7
96272 229533.A0A0E0RSR0 pp 229533.I1RPX7
96273 229533.A0A0E0RSR0 pp 229533.A0A1C3YLB6
96274 229533.A0A0E0RSR0 pp 229533.I1RHJ2
96275 229533.A0A0E0RSR0 pp 229533.I1S2B0
96276 229533.A0A0E0RSR0 pp 229533.I1SAB4
96277 229533.A0A0E0RSR0 pp 229533.I1RHU8
96278 229533.A0A0E0RSR0 pp 229533.I1RQM9
96279 229533.A0A0E0RSR0 pp 229533.I1S5X9
96280 229533.A0A0E0RSR0 pp 229533.I1RTP2
96281 229533.A0A0E0RSR0 pp 229533.I1RMA7
96282 229533.I1RBP0 pp 229533.V6QVM5
96283 229533.I1RBP0 pp 229533.V6RN31
96284 229533.I1RBP0 pp 229533.I1RW06
96285 229533.A0A0E0RWV5 pp 229533.I1REL7
96286 229533.A0A0E0RWV5 pp 229533.I1SAF3
96287 229533.A0A0E0RWV5 pp 229533.I1RGW8

96288	229533.I1R9Z2	pp	229533.I1RP20
96289	229533.I1R9Z2	pp	229533.I1RBH8
96290	229533.I1R9Z2	pp	229533.I1RGB6
96291	229533.I1R9Z2	pp	229533.I1RQN0
96292	229533.I1R9Z2	pp	229533.I1S0R2
96293	229533.I1R9Z2	pp	229533.I1RS35
96294	229533.I1R9Z2	pp	229533.I1RXJ6
96295	229533.I1R9Z2	pp	229533.I1RD69
96296	229533.I1R9Z2	pp	229533.I1RDN5
96297	229533.I1R9Z2	pp	229533.I1RBF8
96298	229533.I1R9Z2	pp	229533.I1RKA9
96299	229533.I1R9Z2	pp	229533.I1RHI5
96300	229533.I1R9Z2	pp	229533.I1RID3
96301	229533.I1R9Z2	pp	229533.I1RMD2
96302	229533.I1R9Z2	pp	229533.I1RRJ9
96303	229533.I1R9Z2	pp	229533.I1RQ64
96304	229533.I1R9Z2	pp	229533.I1REI2
96305	229533.I1R9Z2	pp	229533.I1RG90
96306	229533.I1R9Z2	pp	229533.I1RUG9
96307	229533.I1R9Z2	pp	229533.I1RG59
96308	229533.I1R9Z2	pp	229533.I1S4S8
96309	229533.I1R9Z2	pp	229533.I1RTS6
96310	229533.I1R9Z2	pp	229533.I1SAF7
96311	229533.I1R9Z2	pp	229533.I1RE33
96312	229533.I1RVH7	pp	229533.I1S407
96313	229533.I1RVH7	pp	229533.Q4I7L0
96314	229533.I1RVH7	pp	229533.I1RYV3
96315	229533.I1RVH7	pp	229533.I1RWT2
96316	229533.I1RVH7	pp	229533.Q4HTT2
96317	229533.I1RVH7	pp	229533.I1S5C4
96318	229533.I1RVH7	pp	229533.Q4IER8
96319	229533.I1RVH7	pp	229533.I1S320
96320	229533.I1RVH7	pp	229533.I1S4Z5
96321	229533.I1RVH7	pp	229533.I1S746
96322	229533.I1RVH7	pp	229533.I1S4A4
96323	229533.I1RVH7	pp	229533.I1RXT4
96324	229533.I1RVH7	pp	229533.I1S0F5
96325	229533.I1RVH7	pp	229533.I1S6W3
96326	229533.I1RVH7	pp	229533.Q4IMD1
96327	229533.I1RVH7	pp	229533.I1RWE3
96328	229533.A0A098DAA4	pp	229533.I1S0S5
96329	229533.A0A098DAA4	pp	229533.I1RMU3
96330	229533.A0A098DAA4	pp	229533.I1RUE2
96331	229533.A0A098DAA4	pp	229533.I1RTG2
96332	229533.A0A098DAA4	pp	229533.I1RRZ6
96333	229533.A0A098DAA4	pp	229533.I1RCG5
96334	229533.A0A098DAA4	pp	229533.A0A0E0SH57
96335	229533.A0A098DAA4	pp	229533.I1RGL3
96336	229533.A0A098DAA4	pp	229533.I1RW78
96337	229533.A0A098DAA4	pp	229533.I1RJZ7
96338	229533.A0A098DAA4	pp	229533.I1RFI3
96339	229533.A0A098DAA4	pp	229533.I1RBF3
96340	229533.A0A098DAA4	pp	229533.I1RFY0
96341	229533.A0A098DAA4	pp	229533.I1RT77
96342	229533.A0A098DAA4	pp	229533.I1RW71
96343	229533.A0A098DAA4	pp	229533.I1RBA4
96344	229533.A0A098DAA4	pp	229533.I1S1D1
96345	229533.A0A098DAA4	pp	229533.I1RWM4
96346	229533.A0A098DAA4	pp	229533.A0A098DUM6
96347	229533.A0A098DAA4	pp	229533.I1S0A0
96348	229533.A0A098DAA4	pp	229533.A0A1C3YM11
96349	229533.A0A098DAA4	pp	229533.I1RM67
96350	229533.A0A098DAA4	pp	229533.I1RUI0
96351	229533.A0A098DAA4	pp	229533.I1RMW4
96352	229533.A0A098DAA4	pp	229533.A0A1C3YME3
96353	229533.A0A098DAA4	pp	229533.I1RYM5
96354	229533.A0A098DAA4	pp	229533.I1RCQ6
96355	229533.A0A098DAA4	pp	229533.I1R9Z2
96356	229533.I1RMF2	pp	229533.I1S5B5
96357	229533.I1RMF2	pp	229533.I1RMN3
96358	229533.I1RMF2	pp	229533.I1S278
96359	229533.I1RMF2	pp	229533.I1S2B6
96360	229533.I1RMF2	pp	229533.I1RMK1

96361	229533.I1RMF2	pp	229533.I1RQP7
96362	229533.I1RMF2	pp	229533.I1RSJ0
96363	229533.I1RMF2	pp	229533.I1RQQ6
96364	229533.I1RMF2	pp	229533.V6QUU6
96365	229533.I1RMF2	pp	229533.I1RPU5
96366	229533.I1RMF2	pp	229533.I1S830
96367	229533.I1RMF2	pp	229533.I1S0K5
96368	229533.I1RMF2	pp	229533.I1S2B9
96369	229533.I1RMF2	pp	229533.I1RWB4
96370	229533.I1RMF2	pp	229533.I1S1W5
96371	229533.I1RMF2	pp	229533.I1S1Y9
96372	229533.I1RMF2	pp	229533.I1RNA3
96373	229533.I1RMF2	pp	229533.I1S6S4
96374	229533.I1RMF2	pp	229533.I1S1V4
96375	229533.I1RMF2	pp	229533.I1RNB9
96376	229533.I1RMF2	pp	229533.V6R476
96377	229533.I1RMF2	pp	229533.I1S254
96378	229533.I1RMF2	pp	229533.V6RR76
96379	229533.I1RMF2	pp	229533.Q4I6S5
96380	229533.I1RMF2	pp	229533.I1RQ70
96381	229533.I1RMF2	pp	229533.Q4I877
96382	229533.I1RMF2	pp	229533.Q4I1B1
96383	229533.I1RMF2	pp	229533.I1RQ32
96384	229533.I1RMF2	pp	229533.I1RSZ1
96385	229533.I1RMF2	pp	229533.I1S9X9
96386	229533.I1RMF2	pp	229533.Q5I2J3
96387	229533.I1RMF2	pp	229533.I1S1V7
96388	229533.I1RMF2	pp	229533.Q4HZS8
96389	229533.I1RMF2	pp	229533.I1RTQ9
96390	229533.I1RMF2	pp	229533.I1RT41
96391	229533.I1RMF2	pp	229533.I1RN81
96392	229533.I1RMF2	pp	229533.I1RQD6
96393	229533.I1RMF2	pp	229533.I1RNG8
96394	229533.I1RMF2	pp	229533.I1RZ60
96395	229533.I1RMF2	pp	229533.I1RR95
96396	229533.I1RMF2	pp	229533.I1RW76
96397	229533.I1RMF2	pp	229533.I1S287
96398	229533.I1RMF2	pp	229533.I1SAM5
96399	229533.I1RMF2	pp	229533.I1RYU1
96400	229533.I1RR65	pp	229533.Q4IPZ1
96401	229533.I1RR65	pp	229533.V6R7V8
96402	229533.I1RR65	pp	229533.I1S2A1
96403	229533.I1RR65	pp	229533.I1RYT8
96404	229533.I1RR65	pp	229533.I1RWA3
96405	229533.I1RR65	pp	229533.I1S0C2
96406	229533.I1RR65	pp	229533.V6QUG0
96407	229533.I1RR65	pp	229533.V6RN69
96408	229533.I1RR65	pp	229533.I1RXD9
96409	229533.I1RR65	pp	229533.I1S0R8
96410	229533.I1RR65	pp	229533.V6QWB1
96411	229533.I1RR65	pp	229533.I1RS29
96412	229533.I1RR65	pp	229533.I1S5C6
96413	229533.I1RR65	pp	229533.I1RT09
96414	229533.I1RR65	pp	229533.I1S8F0
96415	229533.I1RR65	pp	229533.I1RS05
96416	229533.I1RR65	pp	229533.I1S5X9
96417	229533.I1RY25	pp	229533.I1S5S3
96418	229533.I1RY25	pp	229533.I1S491
96419	229533.I1RY25	pp	229533.I1RYX4
96420	229533.I1RY25	pp	229533.I1RY71
96421	229533.I1RG29	pp	229533.I1S147
96422	229533.I1RG29	pp	229533.I1RY12
96423	229533.I1RG29	pp	229533.I1RTU0
96424	229533.I1RG29	pp	229533.I1RWF5
96425	229533.I1RG29	pp	229533.I1S746
96426	229533.I1RG29	pp	229533.V6RN31
96427	229533.I1RG29	pp	229533.I1S3X2
96428	229533.I1RG29	pp	229533.I1RHG1
96429	229533.I1RG29	pp	229533.I1RW06
96430	229533.I1RG29	pp	229533.I1RVH7
96431	229533.I1RG29	pp	229533.I1RMJ4
96432	229533.I1RG29	pp	229533.I1RV21
96433	229533.A0A0E0RWX7	pp	229533.I1S5S8

229533.A0A0E0RWX7 pp 229533.I1RZV4
229533.A0A0E0RWX7 pp 229533.I1S095
229533.A0A0E0RWX7 pp 229533.A0A1C3YID2
229533.A0A0E0RWX7 pp 229533.I1S7X9
229533.A0A0E0RWX7 pp 229533.I1RB26
229533.A0A0E0RWX7 pp 229533.I1S254
229533.A0A0E0RWX7 pp 229533.I1RX09
229533.A0A0E0RWX7 pp 229533.A0A1C3YHZ2
229533.A0A0E0RWX7 pp 229533.A0A1C3YLH7
229533.A0A0E0RWX7 pp 229533.A0A1C3YLL6
229533.A0A0E0RWX7 pp 229533.I1RZ65
229533.A0A0E0RWX7 pp 229533.V6RR76
229533.A0A0E0RWX7 pp 229533.I1RN36
229533.A0A0E0RWX7 pp 229533.I1S1B1
229533.A0A0E0RWX7 pp 229533.A0A1C3YL20
229533.A0A0E0RWX7 pp 229533.A0A1C3YLH6
229533.A0A0E0RWX7 pp 229533.A0A0E0SNY0
229533.A0A0E0RWX7 pp 229533.A0A1C3YLS3
229533.A0A0E0RWX7 pp 229533.I1RKR7
229533.A0A0E0RWX7 pp 229533.I1S0Z7
229533.A0A0E0RWX7 pp 229533.I1RB15
229533.A0A0E0RWX7 pp 229533.A0A1C3YMD9
229533.A0A0E0RWX7 pp 229533.V6R476
229533.A0A0E0RWX7 pp 229533.I1RD10
229533.A0A0E0RWX7 pp 229533.I1RSB0
229533.A0A0E0RWX7 pp 229533.I1R991
229533.A0A0E0RWX7 pp 229533.V6QUU6
229533.A0A0E0RWX7 pp 229533.I1RTG9
229533.A0A0E0RWX7 pp 229533.I1RMR5
229533.A0A0E0RWX7 pp 229533.I1RN81
229533.A0A0E0RWX7 pp 229533.I1RZD9
229533.A0A0E0RWX7 pp 229533.A0A1C3YKB5
229533.A0A0E0RWX7 pp 229533.A0A1C3YMQ5
229533.A0A0E0RWX7 pp 229533.I1RQM0
229533.A0A0E0RWX7 pp 229533.I1S9U6
229533.A0A1C3YIS1 pp 229533.Q4IJP1
229533.A0A1C3YIS1 pp 229533.A0A1C3YN58
229533.A0A1C3YIS1 pp 229533.Q4I5R3
229533.A0A1C3YIS1 pp 229533.I1RMQ7
229533.A0A1C3YIS1 pp 229533.Q4HTT2
229533.A0A1C3YIS1 pp 229533.I1RWT2
229533.A0A1C3YIS1 pp 229533.Q4IMD1
229533.A0A1C3YIS1 pp 229533.I1RQ28
229533.A0A1C3YIS1 pp 229533.A0A1C3YJT5
229533.A0A1C3YIS1 pp 229533.I1RYV3
229533.A0A1C3YIS1 pp 229533.Q4IER8
229533.A0A1C3YIS1 pp 229533.Q4IB50
229533.A0A1C3YIS1 pp 229533.Q4I7L0
229533.A0A1C3YIS1 pp 229533.I1RFW1
229533.A0A1C3YIS1 pp 229533.I1S4Z5
229533.A0A1C3YIS1 pp 229533.Q4I8C9
229533.A0A1C3YIS1 pp 229533.A0A1C3YJ34
229533.A0A1C3YIS1 pp 229533.I1RBJ0
229533.Q4I0J6 pp 229533.Q4IBX0
229533.Q4I0J6 pp 229533.V6RLW4
229533.Q4I0J6 pp 229533.Q4IER8
229533.Q4I0J6 pp 229533.V6R382
229533.Q4I0J6 pp 229533.Q4IMM0
229533.Q4I0J6 pp 229533.V6R3J1
229533.I1RTE7 pp 229533.I1S491
229533.I1RTE7 pp 229533.Q4I4A4
229533.I1RTE7 pp 229533.I1RX77
229533.I1RTE7 pp 229533.I1RY71
229533.I1RTE7 pp 229533.V6RR38
229533.I1RTE7 pp 229533.I1RXX1
229533.A0A098DA98 pp 229533.I1RY25
229533.A0A098DA98 pp 229533.I1RYJ8
229533.A0A098DA98 pp 229533.I1RTE7
229533.Q4IBX0 pp 229533.V6R3J1
229533.Q4IBX0 pp 229533.Q4IMM0
229533.Q4IBX0 pp 229533.V6R382
229533.Q4IBX0 pp 229533.V6R6F6
229533.Q4IBX0 pp 229533.V6RLW4

96507 229533.I1RWA5 pp 229533.V6RR76
96508 229533.I1RWA5 pp 229533.I1S1B1
96509 229533.I1RWA5 pp 229533.I1RWI6
96510 229533.I1RWA5 pp 229533.V6R476
96511 229533.I1RWA5 pp 229533.I1RWA9
96512 229533.I1RWA5 pp 229533.I1RZA4
96513 229533.I1RWA5 pp 229533.Q4ICG5
96514 229533.I1RWA5 pp 229533.V6RQ48
96515 229533.I1RWA5 pp 229533.V6QUU6
96516 229533.I1RWA5 pp 229533.I1S635
96517 229533.I1RWA5 pp 229533.I1S254
96518 229533.I1RWA5 pp 229533.V6RKE4
96519 229533.A0A098E425 pp 229533.I1RB73
96520 229533.A0A098E425 pp 229533.I1S0J3
96521 229533.A0A098E425 pp 229533.I1RP08
96522 229533.A0A098E425 pp 229533.A0A1C3YIR8
96523 229533.A0A098E425 pp 229533.I1RDH0
96524 229533.A0A098E425 pp 229533.A0A1C3YM29
96525 229533.A0A098E425 pp 229533.I1RCZ5
96526 229533.A0A098E425 pp 229533.Q4I7L0
96527 229533.A0A098E425 pp 229533.I1S237
96528 229533.A0A098E425 pp 229533.I1RGY5
96529 229533.A0A098E425 pp 229533.I1S1U7
96530 229533.A0A098E425 pp 229533.I1RR95
96531 229533.A0A098E425 pp 229533.O74638
96532 229533.A0A098E425 pp 229533.Q4IMD1
96533 229533.A0A098E425 pp 229533.A0A1C3YLK8
96534 229533.A0A098E425 pp 229533.I1RBJ0
96535 229533.A0A098E425 pp 229533.I1RFX9
96536 229533.A0A098E425 pp 229533.I1RBD7
96537 229533.A0A098E425 pp 229533.Q4I5R3
96538 229533.A0A098E425 pp 229533.A0A0E0SLZ3
96539 229533.A0A098E425 pp 229533.I1RFZ2
96540 229533.A0A098E425 pp 229533.I1RYE2
96541 229533.A0A098E425 pp 229533.I1RB17
96542 229533.A0A098E425 pp 229533.I1RA91
96543 229533.A0A098E425 pp 229533.I1RE51
96544 229533.A0A098E425 pp 229533.I1RQ19
96545 229533.A0A098E425 pp 229533.V6QUE6
96546 229533.A0A098E425 pp 229533.A0A1C3YI39
96547 229533.A0A098E425 pp 229533.I1RZA9
96548 229533.A0A098E425 pp 229533.Q4ICA8
96549 229533.A0A098E425 pp 229533.I1RCN2
96550 229533.A0A098E425 pp 229533.I1RE67
96551 229533.A0A098E425 pp 229533.A0A1C3YK75
96552 229533.A0A098E425 pp 229533.A0A1C3YM21
96553 229533.A0A098E425 pp 229533.A0A1C3YJ26
96554 229533.A0A098E425 pp 229533.I1RYV3
96555 229533.A0A098E425 pp 229533.I1RVY0
96556 229533.A0A098E425 pp 229533.Q4HTT2
96557 229533.A0A098E425 pp 229533.I1RKI2
96558 229533.A0A098E425 pp 229533.I1S236
96559 229533.A0A098E425 pp 229533.I1RFQ5
96560 229533.A0A098E425 pp 229533.I1RYZ0
96561 229533.A0A098E425 pp 229533.I1S209
96562 229533.A0A098E425 pp 229533.A0A1C3YHX6
96563 229533.A0A098E425 pp 229533.I1RBH9
96564 229533.A0A098E425 pp 229533.I1RAN8
96565 229533.A0A098E425 pp 229533.I1RI75
96566 229533.A0A098E425 pp 229533.I1S5B0
96567 229533.A0A098E425 pp 229533.A0A1C3YLW0
96568 229533.A0A098E425 pp 229533.I1RY98
96569 229533.A0A098E425 pp 229533.V6QWX8
96570 229533.A0A098E425 pp 229533.A0A1C3YL16
96571 229533.A0A098E425 pp 229533.Q4HZS8
96572 229533.A0A098E425 pp 229533.I1RC32
96573 229533.A0A098E425 pp 229533.I1RFY2
96574 229533.A0A098E425 pp 229533.A0A1C3YI64
96575 229533.A0A098E425 pp 229533.A0A1C3YN58
96576 229533.A0A098E425 pp 229533.I1RKJ7
96577 229533.A0A098E425 pp 229533.A0A1C3YLD3
96578 229533.A0A098E425 pp 229533.I1RJX2
96579 229533.V6QWD8 pp 229533.V6RN31

96580	229533.V6QWD8	pp	229533.V6RUB1
96581	229533.I1RBH9	pp	229533.I1RBW0
96582	229533.I1RBH9	pp	229533.I1RN46
96583	229533.I1RBH9	pp	229533.I1RK47
96584	229533.I1RBH9	pp	229533.Q4IBX0
96585	229533.I1RBH9	pp	229533.I1RFX8
96586	229533.I1RBH9	pp	229533.I1RFJ2
96587	229533.I1RBH9	pp	229533.I1S8E0
96588	229533.I1RBH9	pp	229533.I1RVV7
96589	229533.I1RBH9	pp	229533.I1RQ93
96590	229533.I1RBH9	pp	229533.I1RE46
96591	229533.I1RBH9	pp	229533.I1RSI4
96592	229533.I1RBH9	pp	229533.I1RTU1
96593	229533.I1RBH9	pp	229533.V6RLW4
96594	229533.I1RBH9	pp	229533.I1RXA9
96595	229533.I1RBH9	pp	229533.I1RTG7
96596	229533.I1RBH9	pp	229533.I1RXB6
96597	229533.I1RBH9	pp	229533.I1S0J8
96598	229533.I1RBH9	pp	229533.I1RWW7
96599	229533.I1RBH9	pp	229533.Q4I0J6
96600	229533.I1RBH9	pp	229533.I1RBW1
96601	229533.I1RBH9	pp	229533.I1S1W3
96602	229533.I1RBH9	pp	229533.I1SAA0
96603	229533.I1RBH9	pp	229533.Q4I7L0
96604	229533.I1RBH9	pp	229533.I1RSS7
96605	229533.I1RBH9	pp	229533.Q4IMD1
96606	229533.I1RBH9	pp	229533.I1RFZ2
96607	229533.I1RBH9	pp	229533.I1RC54
96608	229533.I1RBH9	pp	229533.I1RPK0
96609	229533.I1RBH9	pp	229533.V6R382
96610	229533.Q4HYZ2	pp	229533.V6QW25
96611	229533.Q4HYZ2	pp	229533.V6RR76
96612	229533.Q4HYZ2	pp	229533.Q4ILI9
96613	229533.Q4HYZ2	pp	229533.V6R476
96614	229533.Q4HYZ2	pp	229533.Q4IBX0
96615	229533.I1RLZ4	pp	229533.I1S094
96616	229533.I1RLZ4	pp	229533.I1S1W8
96617	229533.I1RLZ4	pp	229533.I1RMA2
96618	229533.I1RLZ4	pp	229533.I1S6H3
96619	229533.I1RLZ4	pp	229533.I1RNM7
96620	229533.I1RLZ4	pp	229533.I1RLZ6
96621	229533.I1RLZ4	pp	229533.I1RY16
96622	229533.I1RLZ4	pp	229533.I1RYM5
96623	229533.I1RLZ4	pp	229533.I1S8C1
96624	229533.I1RLZ4	pp	229533.I1RY81
96625	229533.I1RLZ4	pp	229533.I1RPN6
96626	229533.I1RLZ4	pp	229533.I1RNH4
96627	229533.I1RLZ4	pp	229533.I1RQU9
96628	229533.I1RLZ4	pp	229533.I1RST9
96629	229533.I1RLZ4	pp	229533.I1S3K0
96630	229533.I1RLZ4	pp	229533.I1S694
96631	229533.I1RLZ4	pp	229533.I1S8F8
96632	229533.I1RLZ4	pp	229533.I1RRM1
96633	229533.I1RLZ4	pp	229533.I1RUR8
96634	229533.I1RLZ4	pp	229533.I1S0H2
96635	229533.I1RLZ4	pp	229533.I1RUY5
96636	229533.I1RLZ4	pp	229533.I1S3T7
96637	229533.I1RLZ4	pp	229533.I1SAB4
96638	229533.I1RLZ4	pp	229533.I1S0A5
96639	229533.I1RLZ4	pp	229533.I1RYK0
96640	229533.I1RLZ4	pp	229533.I1RMH4
96641	229533.I1RLZ4	pp	229533.I1RNG4
96642	229533.I1RLZ4	pp	229533.I1RMK9
96643	229533.I1RLZ4	pp	229533.I1S3G4
96644	229533.I1RLZ4	pp	229533.I1RLZ8
96645	229533.I1RLZ4	pp	229533.I1S0R9
96646	229533.I1RLZ4	pp	229533.V6RLM7
96647	229533.I1RLZ4	pp	229533.I1RZA8
96648	229533.I1RLZ4	pp	229533.I1RZ56
96649	229533.I1RLZ4	pp	229533.I1RPV8
96650	229533.I1RLZ4	pp	229533.Q4IBU4
96651	229533.I1RLZ4	pp	229533.I1RZC3
96652	229533.I1RBD7	pp	229533.V6RLW4

96653 229533.I1RBD7 pp 229533.I1RJL5
96654 229533.I1RBD7 pp 229533.I1RZU5
96655 229533.I1RBD7 pp 229533.I1RMH6
96656 229533.I1RBD7 pp 229533.I1S0J8
96657 229533.I1RBD7 pp 229533.I1RTG7
96658 229533.I1RBD7 pp 229533.Q4I7L0
96659 229533.I1RBD7 pp 229533.I1RBW1
96660 229533.I1RBD7 pp 229533.I1S1Y5
96661 229533.I1RBD7 pp 229533.I1RFZ2
96662 229533.I1RBD7 pp 229533.I1RJS4
96663 229533.I1RBD7 pp 229533.I1RXA9
96664 229533.I1RBD7 pp 229533.I1RN46
96665 229533.I1RBD7 pp 229533.I1SAA0
96666 229533.I1RBD7 pp 229533.I1RSS7
96667 229533.I1RBD7 pp 229533.V6R382
96668 229533.I1RBD7 pp 229533.I1RWW7
96669 229533.I1RBD7 pp 229533.I1RPK0
96670 229533.I1RBD7 pp 229533.Q4IBX0
96671 229533.I1RBD7 pp 229533.I1RQ93
96672 229533.I1RBD7 pp 229533.I1RWC6
96673 229533.I1RBD7 pp 229533.I1RFJ2
96674 229533.I1RBD7 pp 229533.I1S1W3
96675 229533.I1RBD7 pp 229533.I1RDY1
96676 229533.I1RBD7 pp 229533.I1RBW0
96677 229533.I1RBD7 pp 229533.I1RCY6
96678 229533.I1RBD7 pp 229533.I1RC54
96679 229533.I1RBD7 pp 229533.Q4IMD1
96680 229533.I1RBD7 pp 229533.I1RSI4
96681 229533.I1RBD7 pp 229533.I1RFX8
96682 229533.I1RBD7 pp 229533.I1RXB6
96683 229533.I1RBD7 pp 229533.I1RE46
96684 229533.I1RBD7 pp 229533.Q4I0J6
96685 229533.I1RYP2 pp 229533.I1S4S7
96686 229533.I1RYP2 pp 229533.V6QX21
96687 229533.I1RYP2 pp 229533.I1S5B0
96688 229533.I1RYP2 pp 229533.I1RYZ0
96689 229533.I1RYP2 pp 229533.V6RCZ8
96690 229533.I1RYP2 pp 229533.I1S5R1
96691 229533.I1RYP2 pp 229533.I1S123
96692 229533.I1RYP2 pp 229533.I1S9T5
96693 229533.I1RYP2 pp 229533.I1S8Z2
96694 229533.I1RYP2 pp 229533.V6RN44
96695 229533.I1RYP2 pp 229533.I1S1W9
96696 229533.I1RYP2 pp 229533.V6RPQ8
96697 229533.I1RYP2 pp 229533.V6R949
96698 229533.I1RYP2 pp 229533.I1S6D1
96699 229533.I1RYP2 pp 229533.I1S2B8
96700 229533.I1RYP2 pp 229533.I1S4Z7
96701 229533.I1RM62 pp 229533.Q4HZ95
96702 229533.I1RM62 pp 229533.I1RNJ5
96703 229533.I1RM62 pp 229533.I1RQR6
96704 229533.I1RM62 pp 229533.Q4I375
96705 229533.I1RM62 pp 229533.I1RZJ9
96706 229533.I1RM62 pp 229533.Q4I7T5
96707 229533.I1RM62 pp 229533.Q4I099
96708 229533.A0A0E0RUL1 pp 229533.I1S842
96709 229533.A0A0E0RUL1 pp 229533.I1RBF2
96710 229533.A0A0E0RUL1 pp 229533.Q4HYZ2
96711 229533.A0A0E0RUL1 pp 229533.A0A1C3YID2
96712 229533.A0A0E0RUL1 pp 229533.I1S254
96713 229533.A0A0E0RUL1 pp 229533.I1RCN6
96714 229533.A0A0E0RUL1 pp 229533.I1RZK3
96715 229533.A0A0E0RUL1 pp 229533.A0A1C3YLS7
96716 229533.A0A0E0RUL1 pp 229533.A0A1C3YLB5
96717 229533.A0A0E0RUL1 pp 229533.I1RZ60
96718 229533.A0A0E0RUL1 pp 229533.I1S287
96719 229533.A0A0E0RUL1 pp 229533.I1RQ88
96720 229533.A0A0E0RUL1 pp 229533.I1RCE7
96721 229533.A0A0E0RUL1 pp 229533.I1RCQ0
96722 229533.A0A0E0RUL1 pp 229533.A0A1C3YKX2
96723 229533.A0A0E0RUL1 pp 229533.I1S0V5
96724 229533.A0A0E0RUL1 pp 229533.V6RR76
96725 229533.A0A0E0RUL1 pp 229533.I1RX84

229533.A0A0E0RUL1	pp	229533.A0A1C3YHZ2
229533.A0A0E0RUL1	pp	229533.A0A1C3YKJ3
229533.A0A0E0RUL1	pp	229533.A0A1C3YL28
229533.A0A0E0RUL1	pp	229533.I1RQ70
229533.A0A0E0RUL1	pp	229533.I1RA25
229533.A0A0E0RUL1	pp	229533.Q4ILI9
229533.A0A0E0RUL1	pp	229533.I1RTQ9
229533.A0A0E0RUL1	pp	229533.I1RN81
229533.A0A0E0RUL1	pp	229533.I1RMR5
229533.A0A0E0RUL1	pp	229533.A0A0E0SNH8
229533.A0A0E0RUL1	pp	229533.I1RSS4
229533.A0A0E0RUL1	pp	229533.I1RQF7
229533.A0A0E0RUL1	pp	229533.I1RYW8
229533.A0A0E0RUL1	pp	229533.I1RT41
229533.A0A0E0RUL1	pp	229533.I1RAH7
229533.A0A0E0RUL1	pp	229533.I1RMJ1
229533.A0A0E0RUL1	pp	229533.I1RKR7
229533.A0A0E0RUL1	pp	229533.I1RTG5
229533.A0A0E0RUL1	pp	229533.I1RB98
229533.A0A0E0RUL1	pp	229533.I1RMT3
229533.A0A0E0RUL1	pp	229533.I1RTC1
229533.A0A0E0RUL1	pp	229533.A0A0E0SGH2
229533.A0A0E0RUL1	pp	229533.I1RMN3
229533.A0A0E0RUL1	pp	229533.V6R476
229533.A0A0E0RUL1	pp	229533.A0A1C3YN54
229533.A0A0E0RUL1	pp	229533.A0A0E0SDU6
229533.I1RFW1	pp	229533.I1S2E9
229533.I1RFW1	pp	229533.Q4IL82
229533.I1RFW1	pp	229533.I1RVH7
229533.I1RFW1	pp	229533.I1RHP9
229533.I1RFW1	pp	229533.I1RQP1
229533.I1RFW1	pp	229533.Q4IJ11
229533.I1RFW1	pp	229533.I1RPR8
229533.I1RFW1	pp	229533.Q4I7L0
229533.I1RFW1	pp	229533.I1RPF5
229533.I1RFW1	pp	229533.I1RV91
229533.I1RFW1	pp	229533.I1RQ88
229533.I1RFW1	pp	229533.I1S1V5
229533.I1RFW1	pp	229533.I1RGY5
229533.I1RFW1	pp	229533.I1RI51
229533.I1RFW1	pp	229533.I1SB04
229533.I1RFW1	pp	229533.Q4HTT1
229533.I1RFW1	pp	229533.I1RW28
229533.I1RFW1	pp	229533.I1RSZ3
229533.I1RFW1	pp	229533.I1S549
229533.I1RFW1	pp	229533.Q4IB50
229533.I1RFW1	pp	229533.I1RWD0
229533.I1RFW1	pp	229533.I1RR95
229533.I1RFW1	pp	229533.I1RKL2
229533.I1RFW1	pp	229533.I1RTH2
229533.I1RFW1	pp	229533.Q4IPI4
229533.I1RFW1	pp	229533.I1RVL8
229533.I1RFW1	pp	229533.I1S1Y7
229533.I1RFW1	pp	229533.I1RX64
229533.I1RFW1	pp	229533.I1RVY0
229533.I1RFW1	pp	229533.V6R3J1
229533.I1RFW1	pp	229533.I1RK79
229533.I1RFW1	pp	229533.I1RJZ7
229533.I1RFW1	pp	229533.I1S1U7
229533.I1RFW1	pp	229533.I1SAG3
229533.I1RFW1	pp	229533.Q4IEV4
229533.I1RFW1	pp	229533.I1RMJ8
229533.I1RFW1	pp	229533.I1S276
229533.I1RFW1	pp	229533.Q4IMD1
229533.I1RFW1	pp	229533.I1S7X9
229533.I1RFW1	pp	229533.I1RPM7
229533.I1RFW1	pp	229533.I1RS41
229533.I1RFW1	pp	229533.I1RVA7
229533.I1RFW1	pp	229533.Q4I5W5
229533.I1RFW1	pp	229533.I1RFZ2
229533.I1RFW1	pp	229533.I1RSD1
229533.I1RFW1	pp	229533.I1S4Z5
229533.I1RFW1	pp	229533.I1RMV1

96799 229533.I1RFW1 pp 229533.Q4HY90
96800 229533.I1RFW1 pp 229533.I1RG11
96801 229533.I1RFW1 pp 229533.I1RXU0
96802 229533.I1RFW1 pp 229533.I1S0N5
96803 229533.I1RFW1 pp 229533.Q4HYB8
96804 229533.I1RFW1 pp 229533.I1RR25
96805 229533.I1RFW1 pp 229533.I1RSU7
96806 229533.I1RFW1 pp 229533.I1RY99
96807 229533.I1RFW1 pp 229533.I1RSD4
96808 229533.I1RFW1 pp 229533.I1RP08
96809 229533.I1RFW1 pp 229533.I1RXT9
96810 229533.I1RFW1 pp 229533.I1S020
96811 229533.I1RFW1 pp 229533.Q4IBR4
96812 229533.I1RFW1 pp 229533.Q4HZS8
96813 229533.I1RFW1 pp 229533.I1RY44
96814 229533.I1RFW1 pp 229533.I1S1V2
96815 229533.I1RFW1 pp 229533.I1RYV4
96816 229533.I1RFW1 pp 229533.V6QWX8
96817 229533.I1RFW1 pp 229533.I1RMY7
96818 229533.I1RFW1 pp 229533.I1RFX8
96819 229533.I1RFW1 pp 229533.I1RT41
96820 229533.I1RFW1 pp 229533.I1RQ80
96821 229533.I1RFW1 pp 229533.I1S224
96822 229533.I1RFW1 pp 229533.I1RRG3
96823 229533.I1RFW1 pp 229533.Q4HYQ4
96824 229533.I1RFW1 pp 229533.I1RT16
96825 229533.I1RFW1 pp 229533.I1S219
96826 229533.I1RFW1 pp 229533.I1RYN7
96827 229533.I1RFW1 pp 229533.V6REE9
96828 229533.I1RFW1 pp 229533.I1RNV7
96829 229533.I1RFW1 pp 229533.Q4IJP1
96830 229533.I1RFW1 pp 229533.I1S9J6
96831 229533.I1RFW1 pp 229533.I1RM36
96832 229533.I1RFW1 pp 229533.I1RZ87
96833 229533.I1RFW1 pp 229533.I1RQ05
96834 229533.I1RFW1 pp 229533.I1RW71
96835 229533.I1RFW1 pp 229533.I1RKB1
96836 229533.I1RFW1 pp 229533.I1RZR6
96837 229533.I1RFW1 pp 229533.Q5I2J3
96838 229533.I1RFW1 pp 229533.I1S8J7
96839 229533.I1RFW1 pp 229533.I1RQE9
96840 229533.I1RFW1 pp 229533.I1RKR6
96841 229533.I1RFW1 pp 229533.I1RYV3
96842 229533.I1RFW1 pp 229533.I1RZB5
96843 229533.I1RFW1 pp 229533.Q4IJU0
96844 229533.I1RFW1 pp 229533.Q4I948
96845 229533.I1RFW1 pp 229533.I1RJL2
96846 229533.I1RFW1 pp 229533.I1RRU8
96847 229533.I1RFW1 pp 229533.Q4IR08
96848 229533.I1RFW1 pp 229533.I1RQA3
96849 229533.I1RFW1 pp 229533.I1RPU1
96850 229533.I1RFW1 pp 229533.I1RYB1
96851 229533.I1RFW1 pp 229533.I1RQP3
96852 229533.I1RFW1 pp 229533.V6QUE6
96853 229533.I1RFW1 pp 229533.I1RYX3
96854 229533.I1RFW1 pp 229533.Q4I6A4
96855 229533.I1RFW1 pp 229533.I1S193
96856 229533.I1RFW1 pp 229533.Q4HTT2
96857 229533.I1RFW1 pp 229533.I1RWT6
96858 229533.I1RFW1 pp 229533.I1RT01
96859 229533.I1RFW1 pp 229533.I1RQ19
96860 229533.I1RFW1 pp 229533.Q4I5I4
96861 229533.I1RFW1 pp 229533.P0C431
96862 229533.I1RFW1 pp 229533.I1RWT2
96863 229533.I1RFW1 pp 229533.I1S259
96864 229533.I1RFW1 pp 229533.Q4ICA8
96865 229533.I1RFW1 pp 229533.Q4I0J6
96866 229533.I1RFW1 pp 229533.I1S0Z3
96867 229533.I1RFW1 pp 229533.I1S0F0
96868 229533.I1RFW1 pp 229533.I1RSI1
96869 229533.I1RFW1 pp 229533.I1RRT0
96870 229533.I1RFW1 pp 229533.Q4I5R3
96871 229533.I1RFW1 pp 229533.I1SAY2

229533.I1S241	pp	229533.V6QWD8
229533.I1S241	pp	229533.V6RHD0
229533.I1S241	pp	229533.V6RTJ8
229533.I1S241	pp	229533.V6QV31
229533.I1S241	pp	229533.V6RM02
229533.I1S241	pp	229533.V6RDH3
229533.I1S241	pp	229533.I1S3G4
229533.I1S241	pp	229533.V6RXR0
229533.I1S241	pp	229533.V6RK96
229533.I1S241	pp	229533.Q4I1N3
229533.I1S241	pp	229533.V6QU65
229533.I1S4X2	pp	229533.V6RR76
229533.I1S236	pp	229533.V6R382
229533.I1S236	pp	229533.Q4I7L0
229533.I1S236	pp	229533.I1S237
229533.I1S236	pp	229533.I1SAA0
229533.I1S236	pp	229533.Q4I0J6
229533.I1S236	pp	229533.Q4IBX0
229533.I1S236	pp	229533.Q4IMD1
229533.I1S236	pp	229533.V6RLW4
229533.A0A098DA90	pp	229533.I1RBA4
229533.A0A098DA90	pp	229533.V6QWD8
229533.A0A098DA90	pp	229533.I1RXY7
229533.A0A098DA90	pp	229533.I1S0J1
229533.A0A098DA90	pp	229533.I1RYP2
229533.A0A098DA90	pp	229533.I1S241
229533.A0A098DA90	pp	229533.I1RA94
229533.I1SAA0	pp	229533.Q4I7L0
229533.I1SAA0	pp	229533.Q4HTT2
229533.I1SAA0	pp	229533.Q4I5R3
229533.I1SAA0	pp	229533.Q4ICA8
229533.I1SAA0	pp	229533.V6QUE6
229533.I1SAA0	pp	229533.O74638
229533.I1SAA0	pp	229533.Q4IMD1
229533.I1SAA0	pp	229533.V6QWX8
229533.I1SAA0	pp	229533.Q4HZS8
229533.I1RQB7	pp	229533.I1S8W6
229533.I1RQB7	pp	229533.I1S1Y5
229533.I1RQB7	pp	229533.I1RRH5
229533.I1S237	pp	229533.V6R382
229533.I1S237	pp	229533.V6RLW4
229533.I1S237	pp	229533.Q4IMD1
229533.I1S237	pp	229533.Q4IBX0
229533.I1S237	pp	229533.I1SAA0
229533.I1S237	pp	229533.Q4I7L0
229533.I1S237	pp	229533.Q4I0J6
229533.I1RTM8	pp	229533.I1RWT1
229533.I1RTM8	pp	229533.I1RY32
229533.I1RTM8	pp	229533.I1S224
229533.I1RTM8	pp	229533.I1RX84
229533.I1RTM8	pp	229533.V6QVV9
229533.I1RTM8	pp	229533.I1RZ25
229533.I1RTM8	pp	229533.I1RZC3
229533.I1RTM8	pp	229533.I1RW82
229533.I1RTM8	pp	229533.I1S267
229533.I1RTM8	pp	229533.I1SAB4
229533.I1RQ93	pp	229533.V6QUE6
229533.I1RQ93	pp	229533.Q4HTT2
229533.I1RQ93	pp	229533.I1S209
229533.I1RQ93	pp	229533.I1S5B0
229533.I1RQ93	pp	229533.Q4I5R3
229533.I1RQ93	pp	229533.Q4ICA8
229533.I1RQ93	pp	229533.I1RY98
229533.I1RQ93	pp	229533.I1RYZ0
229533.I1RQ93	pp	229533.V6QWX8
229533.I1RQ93	pp	229533.Q4HZS8
229533.I1RQ93	pp	229533.I1S1U7
229533.I1RQ93	pp	229533.I1RYV3
229533.I1RQ93	pp	229533.Q4IMD1
229533.I1RQ93	pp	229533.I1S236
229533.I1RQ93	pp	229533.I1S237
229533.I1RQ93	pp	229533.I1RR95
229533.I1RQ93	pp	229533.I1RYE2

229533.I1RQ93 pp 229533.I1S0J3
229533.I1RQ93 pp 229533.Q4I7L0
229533.I1RQ93 pp 229533.O74638
229533.I1RQ93 pp 229533.I1RVY0
229533.I1RQ93 pp 229533.I1RZA9
229533.I1RJH6 pp 229533.I1RNG5
229533.Q4HZ95 pp 229533.Q4IPZ1
229533.Q4HZ95 pp 229533.Q4I099
229533.Q4HZ95 pp 229533.Q4I6B0
229533.Q4HZ95 pp 229533.Q4IB65
229533.Q4HZ95 pp 229533.Q4IJW4
229533.Q4HZ95 pp 229533.V6RJP8
229533.Q4HZ95 pp 229533.Q4IK03
229533.Q4HZ95 pp 229533.Q4ICM9
229533.Q4HZ95 pp 229533.Q4I7T5
229533.Q4HZ95 pp 229533.Q4I375
229533.A0A1C3YMB7 pp 229533.I1RPU7
229533.A0A1C3YMB7 pp 229533.I1RSN5
229533.A0A1C3YMB7 pp 229533.I1S237
229533.A0A1C3YMB7 pp 229533.I1RQA9
229533.A0A1C3YMB7 pp 229533.I1RR94
229533.A0A1C3YMB7 pp 229533.I1RQL4
229533.A0A1C3YMB7 pp 229533.I1S236
229533.A0A1C3YMB7 pp 229533.I1RBD7
229533.A0A1C3YMB7 pp 229533.I1S757
229533.A0A1C3YMB7 pp 229533.I1RWM0
229533.A0A1C3YMB7 pp 229533.I1S0N8
229533.A0A1C3YMB7 pp 229533.I1RA31
229533.A0A1C3YMB7 pp 229533.I1RBH9
229533.I1RUR4 pp 229533.I1RXH3
229533.I1RSN5 pp 229533.I1S237
229533.Q4IER8 pp 229533.Q4IMD1
229533.Q4IER8 pp 229533.Q4IJ11
229533.Q4IER8 pp 229533.Q4IEV4
229533.Q4IER8 pp 229533.V6R3J1
229533.Q4IER8 pp 229533.V6REE9
229533.Q4IER8 pp 229533.Q4IL82
229533.Q4IER8 pp 229533.Q4IJU0
229533.Q4IER8 pp 229533.Q4IPI4
229533.Q4IER8 pp 229533.Q4IR08
229533.Q4IER8 pp 229533.V6QWX8
229533.Q4IER8 pp 229533.Q5I2J3
229533.Q4IER8 pp 229533.Q4IJP1
229533.Q4IER8 pp 229533.V6QUE6
229533.A0A098DA88 pp 229533.I1RQB7
229533.A0A098DA88 pp 229533.I1S8W6
229533.A0A098DA88 pp 229533.I1RJH6
229533.A0A098DA88 pp 229533.A0A1C3YK00
229533.A0A098DA88 pp 229533.I1RC02
229533.A0A098DA88 pp 229533.I1RB17
229533.A0A098DA88 pp 229533.I1RRN1
229533.A0A098D1L0 pp 229533.Q4I0J6
229533.A0A098D1L0 pp 229533.Q4IBX0
229533.A0A098D1L0 pp 229533.A0A098E425
229533.A0A098D1L0 pp 229533.I1RBH9
229533.A0A098D1L0 pp 229533.I1RBD7
229533.A0A098D1L0 pp 229533.I1RFW1
229533.A0A098D1L0 pp 229533.I1S236
229533.A0A098D1L0 pp 229533.I1SAA0
229533.A0A098D1L0 pp 229533.I1S237
229533.A0A098D1L0 pp 229533.I1RQ93
229533.A0A098D1L0 pp 229533.A0A1C3YMB7
229533.A0A098D1L0 pp 229533.I1RSN5
229533.A0A098D1L0 pp 229533.Q4IER8
229533.I1RSP5 pp 229533.I1S267
229533.I1RSP5 pp 229533.I1RST9
229533.I1RSP5 pp 229533.I1RZC3
229533.I1RSP5 pp 229533.I1S969
229533.I1RSP5 pp 229533.I1S968
229533.I1RSP5 pp 229533.I1RY32
229533.I1RSP5 pp 229533.I1RZ25
229533.I1RSP5 pp 229533.I1RWF9
229533.I1RSP5 pp 229533.I1SAB4

97018 229533.I1RSP5 pp 229533.I1RYP5
97019 229533.A0A098DKH5 pp 229533.I1RW71
97020 229533.A0A098DKH5 pp 229533.I1RJZ7
97021 229533.A0A098E4K9 pp 229533.I1SAD2
97022 229533.A0A098E4K9 pp 229533.I1RJQ3
97023 229533.A0A098E4K9 pp 229533.A0A0E0SIK8
97024 229533.A0A098E4K9 pp 229533.I1RPF5
97025 229533.A0A098D1J8 pp 229533.I1RM27
97026 229533.I1S267 pp 229533.V6QUV7
97027 229533.I1S267 pp 229533.Q4IF76
97028 229533.I1S267 pp 229533.I1S2E9
97029 229533.I1S267 pp 229533.I1SAT2
97030 229533.I1S267 pp 229533.I1S6V0
97031 229533.I1S267 pp 229533.I1SAH9
97032 229533.I1S267 pp 229533.V6RCJ5
97033 229533.I1S267 pp 229533.I1S2Z7
97034 229533.I1S267 pp 229533.I1S4I8
97035 229533.I1S267 pp 229533.I1S659
97036 229533.I1S267 pp 229533.I1S694
97037 229533.I1S267 pp 229533.Q4HYR2
97038 229533.I1S267 pp 229533.I1S2Q4
97039 229533.I1S267 pp 229533.I1S2F7
97040 229533.I1S267 pp 229533.I1S3H8
97041 229533.I1S267 pp 229533.V6RWB3
97042 229533.I1S267 pp 229533.I1SAB4
97043 229533.I1S267 pp 229533.I1S4I7
97044 229533.I1S267 pp 229533.I1S4G3
97045 229533.I1S267 pp 229533.I1S968
97046 229533.I1S267 pp 229533.I1S9T6
97047 229533.I1S267 pp 229533.I1S3T6
97048 229533.I1S267 pp 229533.V6RH92
97049 229533.I1S267 pp 229533.I1S8D3
97050 229533.I1S267 pp 229533.I1S3M4
97051 229533.I1S267 pp 229533.V6RHX2
97052 229533.I1S267 pp 229533.I1S334
97053 229533.I1S267 pp 229533.V6R5T7
97054 229533.I1S267 pp 229533.Q4IJH1
97055 229533.I1S267 pp 229533.I1S969
97056 229533.I1S267 pp 229533.V6RCC0
97057 229533.I1S267 pp 229533.I1S423
97058 229533.I1S267 pp 229533.I1S6H3
97059 229533.I1S267 pp 229533.I1S2F6
97060 229533.I1S267 pp 229533.I1S5D0
97061 229533.I1S267 pp 229533.V6QVV9
97062 229533.I1RE52 pp 229533.V6RPQ8
97063 229533.I1RE52 pp 229533.V6RAN2
97064 229533.I1RE52 pp 229533.I1RST9
97065 229533.I1RE52 pp 229533.V6RRM0
97066 229533.I1RE52 pp 229533.I1RXE6
97067 229533.I1RE52 pp 229533.I1RIB5
97068 229533.I1RE52 pp 229533.I1RRN4
97069 229533.I1RE52 pp 229533.I1REE2
97070 229533.I1RE52 pp 229533.I1RSU2
97071 229533.I1RE52 pp 229533.I1S267
97072 229533.I1RE52 pp 229533.I1SAT2
97073 229533.I1RE52 pp 229533.I1RIM7
97074 229533.I1RE52 pp 229533.I1RHJ2
97075 229533.I1RE52 pp 229533.V6RHX2
97076 229533.I1RE52 pp 229533.I1S3T6
97077 229533.I1RE52 pp 229533.I1S8F8
97078 229533.I1RE52 pp 229533.I1RQW1
97079 229533.I1RE52 pp 229533.I1S8D3
97080 229533.I1RE52 pp 229533.I1RN84
97081 229533.I1RE52 pp 229533.I1RNG5
97082 229533.I1RE52 pp 229533.I1RVX8
97083 229533.I1RE52 pp 229533.I1RPR1
97084 229533.I1RE52 pp 229533.I1RH83
97085 229533.I1RE52 pp 229533.I1S2F7
97086 229533.I1RE52 pp 229533.I1RW55
97087 229533.I1RE52 pp 229533.I1RUE3
97088 229533.I1RE52 pp 229533.I1RHU8
97089 229533.I1RE52 pp 229533.I1RRC4
97090 229533.I1RE52 pp 229533.I1RHJ0

97091	229533.I1RE52	pp	229533.I1S0K2
97092	229533.I1RE52	pp	229533.I1REK8
97093	229533.I1RE52	pp	229533.V6RWB3
97094	229533.I1RE52	pp	229533.I1S969
97095	229533.I1RE52	pp	229533.I1RP12
97096	229533.I1RE52	pp	229533.I1RR00
97097	229533.I1RE52	pp	229533.I1RRS6
97098	229533.I1RE52	pp	229533.I1S8R2
97099	229533.I1RE52	pp	229533.I1RTY9
97100	229533.I1RE52	pp	229533.I1RSA5
97101	229533.I1RE52	pp	229533.I1S299
97102	229533.I1RE52	pp	229533.I1RF24
97103	229533.I1RE52	pp	229533.I1REI2
97104	229533.I1RE52	pp	229533.I1RE53
97105	229533.I1RE52	pp	229533.I1RJY7
97106	229533.I1RE52	pp	229533.I1RRX5
97107	229533.I1RE52	pp	229533.I1RY81
97108	229533.I1RE52	pp	229533.I1RW82
97109	229533.I1RE52	pp	229533.I1RGU3
97110	229533.I1RE52	pp	229533.I1S223
97111	229533.I1RE52	pp	229533.I1S659
97112	229533.I1RE52	pp	229533.I1S0X1
97113	229533.I1RE52	pp	229533.I1S8C1
97114	229533.I1RE52	pp	229533.I1RIF7
97115	229533.I1RE52	pp	229533.I1RFB0
97116	229533.I1RE52	pp	229533.I1RRG3
97117	229533.I1RE52	pp	229533.I1S6V0
97118	229533.I1RE52	pp	229533.I1RHT5
97119	229533.I1RE52	pp	229533.I1S0K1
97120	229533.I1RE52	pp	229533.I1RTA1
97121	229533.I1RE52	pp	229533.I1RQV8
97122	229533.I1RE52	pp	229533.I1S8S9
97123	229533.I1RE52	pp	229533.I1RMJ3
97124	229533.I1RE52	pp	229533.I1RQJ7
97125	229533.I1RE52	pp	229533.I1S968
97126	229533.I1RE52	pp	229533.I1RYU6
97127	229533.I1RE52	pp	229533.I1RZ38
97128	229533.I1RE52	pp	229533.I1S5D0
97129	229533.I1RE52	pp	229533.I1S8D2
97130	229533.I1RE52	pp	229533.I1RRR9
97131	229533.I1RE52	pp	229533.I1RW54
97132	229533.I1RE52	pp	229533.I1RZ25
97133	229533.I1RE52	pp	229533.I1RWF5
97134	229533.I1RE52	pp	229533.I1RMU8
97135	229533.I1RE52	pp	229533.I1RKD3
97136	229533.I1RE52	pp	229533.I1RMA2
97137	229533.I1RE52	pp	229533.I1S210
97138	229533.I1RE52	pp	229533.I1RJU3
97139	229533.I1RE52	pp	229533.I1RQV7
97140	229533.I1RE52	pp	229533.I1RSK5
97141	229533.I1RE52	pp	229533.I1RMQ6
97142	229533.I1RE52	pp	229533.I1RMK7
97143	229533.I1RE52	pp	229533.I1RGK3
97144	229533.I1RE52	pp	229533.I1RSQ4
97145	229533.I1RE52	pp	229533.V6RCJ5
97146	229533.I1RE52	pp	229533.V6R5T7
97147	229533.I1RE52	pp	229533.I1RFI4
97148	229533.I1RE52	pp	229533.I1S3T7
97149	229533.I1RE52	pp	229533.I1RNM7
97150	229533.I1RE52	pp	229533.I1SAH9
97151	229533.I1RE52	pp	229533.I1S0B8
97152	229533.I1RE52	pp	229533.I1RY21
97153	229533.I1RE52	pp	229533.I1RK55
97154	229533.I1RE52	pp	229533.I1RYP5
97155	229533.I1RE52	pp	229533.I1S048
97156	229533.I1RE52	pp	229533.V6RCC0
97157	229533.I1RE52	pp	229533.I1RGC3
97158	229533.I1RE52	pp	229533.I1RIJ6
97159	229533.I1RE52	pp	229533.O74638
97160	229533.I1RE52	pp	229533.I1SAB4
97161	229533.I1RE52	pp	229533.I1RUR8
97162	229533.I1RE52	pp	229533.I1RLA2
97163	229533.I1RF59	pp	229533.I1S3H8

97164 229533.I1RF59 pp 229533.I1S0A5
97165 229533.I1RF59 pp 229533.I1RNG4
97166 229533.I1RF59 pp 229533.I1RF60
97167 229533.I1RF59 pp 229533.I1RGZ3
97168 229533.I1RF59 pp 229533.I1RKB2
97169 229533.I1RF59 pp 229533.I1RJS1
97170 229533.I1RF59 pp 229533.I1S673
97171 229533.I1RF59 pp 229533.I1RNM7
97172 229533.I1RF59 pp 229533.I1RF61
97173 229533.I1RF59 pp 229533.I1RPV8
97174 229533.I1RF59 pp 229533.I1RZ25
97175 229533.I1RF59 pp 229533.I1S334
97176 229533.I1RF59 pp 229533.I1RRL5
97177 229533.I1RF59 pp 229533.I1RF63
97178 229533.I1RF59 pp 229533.I1RF62
97179 229533.I1RF59 pp 229533.I1S6H3
97180 229533.I1RF59 pp 229533.I1S094
97181 229533.I1RF59 pp 229533.I1RLA2
97182 229533.I1RF59 pp 229533.I1RF64
97183 229533.I1RF59 pp 229533.I1RMY1
97184 229533.I1RF59 pp 229533.I1RZ56
97185 229533.I1RF59 pp 229533.I1RST9
97186 229533.I1RF59 pp 229533.I1S8F8
97187 229533.I1RF59 pp 229533.I1RUY5
97188 229533.Q4I8X0 pp 229533.V6QWZ7
97189 229533.Q4I8X0 pp 229533.Q4IFI0
97190 229533.I1RWC1 pp 229533.I1RZX3
97191 229533.I1RWC1 pp 229533.I1RZC3
97192 229533.I1RWC1 pp 229533.I1RWT0
97193 229533.I1RWC1 pp 229533.I1SAB4
97194 229533.I1RWC1 pp 229533.V6RHX2
97195 229533.I1RWC1 pp 229533.I1S267
97196 229533.I1RWC1 pp 229533.I1RZ25
97197 229533.I1RWC1 pp 229533.I1RY32
97198 229533.I1RXX0 pp 229533.I1S2H3
97199 229533.I1RXX0 pp 229533.V6RIE5
97200 229533.I1RXX0 pp 229533.I1RYU0
97201 229533.I1RXX0 pp 229533.I1RXX1
97202 229533.I1RXX0 pp 229533.I1S4F4
97203 229533.I1RXX0 pp 229533.V6QT89
97204 229533.I1RXX0 pp 229533.I1S0H5
97205 229533.I1RXX0 pp 229533.I1S3H8
97206 229533.I1RXX0 pp 229533.I1S334
97207 229533.A0A0E0S530 pp 229533.I1RA13
97208 229533.A0A0E0S530 pp 229533.I1S2F7
97209 229533.A0A0E0S530 pp 229533.I1RDU0
97210 229533.A0A0E0S530 pp 229533.I1RY81
97211 229533.A0A0E0S530 pp 229533.I1S659
97212 229533.A0A0E0S530 pp 229533.I1S094
97213 229533.A0A0E0S530 pp 229533.I1RQJ7
97214 229533.A0A0E0S530 pp 229533.I1RWM4
97215 229533.A0A0E0S530 pp 229533.I1RLA2
97216 229533.A0A0E0S530 pp 229533.I1RUY5
97217 229533.A0A0E0S530 pp 229533.I1S048
97218 229533.A0A0E0S530 pp 229533.I1S3T6
97219 229533.A0A0E0S530 pp 229533.I1RPM6
97220 229533.A0A0E0S530 pp 229533.I1S6H3
97221 229533.A0A0E0S530 pp 229533.I1RW54
97222 229533.A0A0E0S530 pp 229533.I1S5D0
97223 229533.A0A0E0S530 pp 229533.I1S267
97224 229533.A0A0E0S530 pp 229533.I1RWL3
97225 229533.A0A0E0S530 pp 229533.I1RIM7
97226 229533.A0A0E0S530 pp 229533.I1RHT5
97227 229533.A0A0E0S530 pp 229533.I1RQV7
97228 229533.A0A0E0S530 pp 229533.I1RMA2
97229 229533.A0A0E0S530 pp 229533.A0A1C3YMQ9
97230 229533.A0A0E0S530 pp 229533.I1RW55
97231 229533.A0A0E0S530 pp 229533.I1S4S7
97232 229533.A0A0E0S530 pp 229533.A0A1C3YIP3
97233 229533.A0A0E0S530 pp 229533.I1RST9
97234 229533.A0A0E0S530 pp 229533.I1S210
97235 229533.A0A0E0S530 pp 229533.I1S969
97236 229533.A0A0E0S530 pp 229533.I1RDN3

97237 229533.A0A0E0S530 pp 229533.I1RYP5
97238 229533.A0A0E0S530 pp 229533.I1RIB5
97239 229533.A0A0E0S530 pp 229533.I1RRS6
97240 229533.A0A0E0S530 pp 229533.A0A1C3YLB6
97241 229533.A0A0E0S530 pp 229533.I1RE52
97242 229533.A0A0E0S530 pp 229533.I1S9Z4
97243 229533.A0A0E0S530 pp 229533.A0A1C3YHN8
97244 229533.A0A0E0S530 pp 229533.I1S223
97245 229533.A0A0E0S530 pp 229533.I1RHJ0
97246 229533.A0A0E0S530 pp 229533.I1RGU3
97247 229533.A0A0E0S530 pp 229533.I1RZX3
97248 229533.A0A0E0S530 pp 229533.I1RPV8
97249 229533.A0A0E0S530 pp 229533.A0A1C3YIJ7
97250 229533.A0A0E0S530 pp 229533.I1RZ56
97251 229533.A0A0E0S530 pp 229533.I1RQ33
97252 229533.A0A0E0S530 pp 229533.I1RYU6
97253 229533.A0A0E0S530 pp 229533.I1R9F2
97254 229533.A0A0E0S530 pp 229533.I1S2C4
97255 229533.A0A0E0S530 pp 229533.I1RPU2
97256 229533.A0A0E0S530 pp 229533.A0A1C3YLZ6
97257 229533.A0A0E0S530 pp 229533.I1RAV0
97258 229533.A0A0E0S530 pp 229533.I1RH83
97259 229533.A0A0E0S530 pp 229533.I1RJS1
97260 229533.A0A0E0S530 pp 229533.I1RK55
97261 229533.A0A0E0S530 pp 229533.I1SAB4
97262 229533.A0A0E0S530 pp 229533.I1SAT2
97263 229533.A0A0E0S530 pp 229533.I1RY35
97264 229533.A0A0E0S530 pp 229533.I1RKD3
97265 229533.A0A0E0S530 pp 229533.I1RRI1
97266 229533.A0A0E0S530 pp 229533.I1RTA1
97267 229533.A0A0E0S530 pp 229533.I1RYI5
97268 229533.A0A0E0S530 pp 229533.I1RZ38
97269 229533.A0A0E0S530 pp 229533.I1RFB0
97270 229533.A0A0E0S530 pp 229533.I1RVZ2
97271 229533.A0A0E0S530 pp 229533.I1RF24
97272 229533.A0A0E0S530 pp 229533.I1S0A5
97273 229533.A0A0E0S530 pp 229533.I1RSA5
97274 229533.A0A0E0S530 pp 229533.A0A1C3YJI6
97275 229533.A0A0E0S530 pp 229533.I1S968
97276 229533.A0A0E0S530 pp 229533.I1RR68
97277 229533.A0A0E0S530 pp 229533.I1RZ25
97278 229533.I1REI2 pp 229533.I1RRC4
97279 229533.I1REI2 pp 229533.I1RK02
97280 229533.I1REI2 pp 229533.I1RPX7
97281 229533.I1REI2 pp 229533.I1RZN1
97282 229533.I1REI2 pp 229533.I1RRR9
97283 229533.I1REI2 pp 229533.I1RFV2
97284 229533.I1REI2 pp 229533.I1RZM4
97285 229533.I1REI2 pp 229533.I1RW82
97286 229533.I1REI2 pp 229533.I1REY8
97287 229533.I1REI2 pp 229533.I1RPQ2
97288 229533.I1REI2 pp 229533.I1RQE8
97289 229533.I1REI2 pp 229533.I1RMM8
97290 229533.I1REI2 pp 229533.I1RPZ1
97291 229533.I1REI2 pp 229533.I1RF24
97292 229533.I1REI2 pp 229533.I1RQ48
97293 229533.I1REI2 pp 229533.I1RRQ7
97294 229533.I1REI2 pp 229533.I1S368
97295 229533.I1REI2 pp 229533.I1S0Y1
97296 229533.I1REI2 pp 229533.I1RQM9
97297 229533.I1REI2 pp 229533.I1RRN4
97298 229533.I1REI2 pp 229533.I1SAB4
97299 229533.I1REI2 pp 229533.I1S8X0
97300 229533.I1REI2 pp 229533.V6RK96
97301 229533.I1REI2 pp 229533.I1RK49
97302 229533.I1REI2 pp 229533.I1SAF7
97303 229533.I1REI2 pp 229533.I1RN32
97304 229533.I1REI2 pp 229533.I1RS41
97305 229533.I1REI2 pp 229533.I1RHJ2
97306 229533.I1REI2 pp 229533.I1S2B0
97307 229533.I1REI2 pp 229533.I1RZ81
97308 229533.I1REI2 pp 229533.I1RTP2
97309 229533.I1REI2 pp 229533.I1RHU8

97310 229533.A0A1C3YMK4 pp 229533.I1RBL7
97311 229533.A0A1C3YMK4 pp 229533.I1S5R5
97312 229533.A0A1C3YMK4 pp 229533.I1R9G2
97313 229533.A0A1C3YMK4 pp 229533.A0A1C3YMS7
97314 229533.A0A1C3YMK4 pp 229533.I1RVY0
97315 229533.A0A1C3YMK4 pp 229533.Q4I8X0
97316 229533.A0A1C3YMK4 pp 229533.I1RFS3
97317 229533.A0A1C3YMK4 pp 229533.I1RHD9
97318 229533.A0A1C3YMK4 pp 229533.I1RGP4
97319 229533.A0A1C3YMK4 pp 229533.I1RNG4
97320 229533.A0A1C3YMK4 pp 229533.I1RF02
97321 229533.A0A1C3YMK4 pp 229533.V6RPN0
97322 229533.I1RUF8 pp 229533.I1RWI8
97323 229533.I1RUF8 pp 229533.I1RY29
97324 229533.I1RUF8 pp 229533.I1S0J3
97325 229533.I1RUF8 pp 229533.I1SAD2
97326 229533.I1RUF8 pp 229533.I1RZ94
97327 229533.I1RUF8 pp 229533.I1S098
97328 229533.I1RUF8 pp 229533.I1S788
97329 229533.I1RUF8 pp 229533.Q4I7L0
97330 229533.I1RUF8 pp 229533.I1S2E9
97331 229533.I1RUF8 pp 229533.I1S0E8
97332 229533.I1RUF8 pp 229533.I1RY44
97333 229533.I1RUF8 pp 229533.I1RWT6
97334 229533.I1RUF8 pp 229533.I1RZM1
97335 229533.I1RUF8 pp 229533.I1RY58
97336 229533.I1RUF8 pp 229533.Q4INS6
97337 229533.I1RUF8 pp 229533.I1RXU0
97338 229533.I1RUF8 pp 229533.I1S0N5
97339 229533.I1RUF8 pp 229533.Q4HTT2
97340 229533.I1RUF8 pp 229533.I1RUG4
97341 229533.I1RUF8 pp 229533.I1S9J6
97342 229533.I1RUF8 pp 229533.I1RYY5
97343 229533.I1RUF8 pp 229533.I1RZT1
97344 229533.I1RUF8 pp 229533.Q4IJM4
97345 229533.I1RUF8 pp 229533.I1S276
97346 229533.I1RUF8 pp 229533.I1S209
97347 229533.A0A1C3YN25 pp 229533.I1RF53
97348 229533.A0A1C3YN25 pp 229533.I1S2H3
97349 229533.A0A1C3YN25 pp 229533.I1S4F4
97350 229533.A0A1C3YN25 pp 229533.I1RF85
97351 229533.A0A1C3YN25 pp 229533.I1RB38
97352 229533.A0A1C3YN25 pp 229533.I1RKB2
97353 229533.A0A1C3YN25 pp 229533.I1S0V7
97354 229533.A0A1C3YN25 pp 229533.V6RG79
97355 229533.A0A1C3YN25 pp 229533.I1RCB4
97356 229533.A0A1C3YN25 pp 229533.I1RPD6
97357 229533.A0A1C3YN25 pp 229533.I1RYY2
97358 229533.A0A1C3YN25 pp 229533.I1RW43
97359 229533.A0A1C3YN25 pp 229533.I1RDJ7
97360 229533.A0A1C3YN25 pp 229533.I1S3G4
97361 229533.A0A1C3YN25 pp 229533.I1RMG6
97362 229533.A0A1C3YN25 pp 229533.V6RRM0
97363 229533.A0A1C3YN25 pp 229533.I1R9B8
97364 229533.A0A1C3YN25 pp 229533.I1RNK1
97365 229533.A0A1C3YN25 pp 229533.I1S8F8
97366 229533.A0A1C3YN25 pp 229533.I1RD81
97367 229533.A0A1C3YN25 pp 229533.I1RW26
97368 229533.A0A1C3YN25 pp 229533.I1RNG4
97369 229533.A0A1C3YN25 pp 229533.I1RE63
97370 229533.A0A1C3YN25 pp 229533.V6RY56
97371 229533.A0A1C3YN25 pp 229533.I1RYW9
97372 229533.A0A1C3YN25 pp 229533.I1RQE0
97373 229533.A0A1C3YN25 pp 229533.I1RBE7
97374 229533.A0A1C3YN25 pp 229533.I1RF38
97375 229533.A0A1C3YN25 pp 229533.I1RNY6
97376 229533.A0A1C3YN25 pp 229533.I1RZ20
97377 229533.A0A1C3YN25 pp 229533.I1RPR1
97378 229533.A0A1C3YN25 pp 229533.I1S5L5
97379 229533.A0A1C3YN25 pp 229533.I1RU76
97380 229533.A0A1C3YN25 pp 229533.I1RDE4
97381 229533.A0A1C3YN25 pp 229533.I1RN80
97382 229533.A0A1C3YN25 pp 229533.I1RRV4

97383 229533.A0A1C3YN25 pp 229533.I1RR68
97384 229533.A0A1C3YN25 pp 229533.I1RC83
97385 229533.A0A1C3YN25 pp 229533.A0A1C3YN86
97386 229533.A0A1C3YN25 pp 229533.I1RXE6
97387 229533.A0A1C3YN25 pp 229533.Q4HVQ9
97388 229533.A0A1C3YN25 pp 229533.I1RCU2
97389 229533.A0A1C3YN25 pp 229533.I1RA06
97390 229533.A0A1C3YN25 pp 229533.I1RMG7
97391 229533.A0A1C3YN25 pp 229533.I1RA28
97392 229533.A0A1C3YN25 pp 229533.I1RB46
97393 229533.A0A1C3YN25 pp 229533.I1RXV9
97394 229533.A0A1C3YN25 pp 229533.I1RXG6
97395 229533.A0A1C3YN25 pp 229533.I1RVX9
97396 229533.A0A1C3YN25 pp 229533.V6RQW4
97397 229533.A0A1C3YN25 pp 229533.I1RQX9
97398 229533.A0A1C3YN25 pp 229533.I1RNG7
97399 229533.A0A1C3YN25 pp 229533.I1REV2
97400 229533.A0A1C3YN25 pp 229533.I1RNM7
97401 229533.A0A1C3YN25 pp 229533.I1RZ29
97402 229533.A0A1C3YN25 pp 229533.I1RFI4
97403 229533.A0A1C3YN25 pp 229533.I1RM26
97404 229533.A0A1C3YN25 pp 229533.I1RYU5
97405 229533.A0A1C3YN25 pp 229533.I1RPW2
97406 229533.A0A1C3YN25 pp 229533.I1S1W8
97407 229533.A0A1C3YN25 pp 229533.I1RXH3
97408 229533.A0A1C3YN25 pp 229533.I1RCL4
97409 229533.I1S6S6 pp 229533.V6RPN0
97410 229533.I1S6S6 pp 229533.Q4I8X0
97411 229533.I1RNZ1 pp 229533.I1RPU3
97412 229533.I1RH48 pp 229533.I1RVY0
97413 229533.I1RH48 pp 229533.I1RNG4
97414 229533.I1RH48 pp 229533.Q4I8X0
97415 229533.I1RH48 pp 229533.V6RPN0
97416 229533.I1RH48 pp 229533.I1S5R5
97417 229533.I1RSX2 pp 229533.I1S334
97418 229533.I1RSX2 pp 229533.I1RYU0
97419 229533.I1RSX2 pp 229533.I1S4F4
97420 229533.I1RSX2 pp 229533.I1S2H3
97421 229533.I1RSX2 pp 229533.I1S0H5
97422 229533.I1RSX2 pp 229533.I1S3H8
97423 229533.I1RSX2 pp 229533.V6QT89
97424 229533.I1RSX2 pp 229533.V6RIE5
97425 229533.I1RY21 pp 229533.I1RY57
97426 229533.I1RY21 pp 229533.I1S299
97427 229533.I1RY21 pp 229533.I1S3T7
97428 229533.I1RY21 pp 229533.I1S458
97429 229533.I1RY21 pp 229533.I1RZP1
97430 229533.I1RY21 pp 229533.I1RYA0
97431 229533.I1RY21 pp 229533.I1S464
97432 229533.I1RY21 pp 229533.I1RY92
97433 229533.I1RY21 pp 229533.I1S266
97434 229533.I1RY21 pp 229533.I1S1F3
97435 229533.I1RY21 pp 229533.I1S5X9
97436 229533.I1RY21 pp 229533.I1RYW9
97437 229533.I1RY21 pp 229533.I1S2P5
97438 229533.I1RY21 pp 229533.I1RZJ3
97439 229533.I1RY21 pp 229533.I1S0R9
97440 229533.I1RY21 pp 229533.I1RYQ1
97441 229533.I1RY21 pp 229533.I1S0R8
97442 229533.I1RY21 pp 229533.I1S5U0
97443 229533.I1RY21 pp 229533.V6RRM0
97444 229533.I1RY21 pp 229533.I1S8R2
97445 229533.I1RY21 pp 229533.Q4I174
97446 229533.I1RY21 pp 229533.I1RYC8
97447 229533.I1RY21 pp 229533.Q4HVQ9
97448 229533.I1RY21 pp 229533.V6R3J1
97449 229533.I1RY21 pp 229533.I1RYQ3
97450 229533.I1RY21 pp 229533.Q4HZ34
97451 229533.I1RY21 pp 229533.I1S029
97452 229533.I1RY21 pp 229533.V6RLM7
97453 229533.I1RY21 pp 229533.I1RYT8
97454 229533.I1RY21 pp 229533.I1S0P0
97455 229533.I1RY21 pp 229533.I1S0Q7

97456 229533.I1RY21 pp 229533.I1S0V7
97457 229533.I1RY21 pp 229533.I1S8C1
97458 229533.I1RY21 pp 229533.I1S0U0
97459 229533.I1RY21 pp 229533.I1S8Z2
97460 229533.I1RY21 pp 229533.I1RYC7
97461 229533.I1RY21 pp 229533.I1RYU6
97462 229533.I1RY21 pp 229533.I1S0R1
97463 229533.I1RY21 pp 229533.I1RYN2
97464 229533.I1RY21 pp 229533.I1S0T7
97465 229533.I1RY21 pp 229533.V6RKK5
97466 229533.I1RY21 pp 229533.I1S0K2
97467 229533.I1RY21 pp 229533.I1S212
97468 229533.I1RY21 pp 229533.V6RY56
97469 229533.I1RY21 pp 229533.I1S5C6
97470 229533.I1RY21 pp 229533.V6RXW9
97471 229533.I1RY21 pp 229533.I1S5R5
97472 229533.I1RY21 pp 229533.I1S8S9
97473 229533.I1RY21 pp 229533.I1RZ43
97474 229533.I1RY21 pp 229533.Q4I5G1
97475 229533.I1RY21 pp 229533.I1S8F8
97476 229533.I1RY21 pp 229533.I1RZ68
97477 229533.I1RY21 pp 229533.I1RYI6
97478 229533.I1RY21 pp 229533.I1S0V6
97479 229533.I1RY21 pp 229533.V6RJP8
97480 229533.I1RY21 pp 229533.I1S4D0
97481 229533.A0A1C3YJA2 pp 229533.I1RPF5
97482 229533.A0A1C3YJA2 pp 229533.I1SAD2
97483 229533.A0A0E0SEV4 pp 229533.A0A1C3YMB7
97484 229533.I1RIQ7 pp 229533.V6RPN0
97485 229533.I1RIQ7 pp 229533.I1S5R5
97486 229533.I1RIQ7 pp 229533.Q4I8X0
97487 229533.I1RIQ7 pp 229533.I1RNG4
97488 229533.I1RIQ7 pp 229533.I1RVY0
97489 229533.A0A0E0SEY5 pp 229533.I1RMR5
97490 229533.A0A0E0SEY5 pp 229533.I1RJS2
97491 229533.V6R5T7 pp 229533.V6RWB3
97492 229533.A0A1C3YJT5 pp 229533.V6QWX8
97493 229533.A0A1C3YJT5 pp 229533.A0A1C3YL06
97494 229533.A0A1C3YJT5 pp 229533.I1RVH7
97495 229533.A0A1C3YJT5 pp 229533.Q4IPI4
97496 229533.A0A1C3YJT5 pp 229533.I1RBW0
97497 229533.A0A1C3YJT5 pp 229533.I1RA84
97498 229533.A0A1C3YJT5 pp 229533.Q4I7L0
97499 229533.A0A1C3YJT5 pp 229533.I1RBW1
97500 229533.A0A1C3YJT5 pp 229533.I1RZ63
97501 229533.A0A1C3YJT5 pp 229533.I1RPQ1
97502 229533.A0A1C3YJT5 pp 229533.I1S0J7
97503 229533.A0A1C3YJT5 pp 229533.I1S549
97504 229533.A0A1C3YJT5 pp 229533.I1RW90
97505 229533.A0A1C3YJT5 pp 229533.A0A1C3YL67
97506 229533.A0A1C3YJT5 pp 229533.I1RE19
97507 229533.A0A1C3YJT5 pp 229533.I1RMY7
97508 229533.A0A1C3YJT5 pp 229533.I1RM49
97509 229533.A0A1C3YJT5 pp 229533.I1RBC7
97510 229533.A0A1C3YJT5 pp 229533.I1S004
97511 229533.A0A1C3YJT5 pp 229533.A0A1C3YK57
97512 229533.A0A1C3YJT5 pp 229533.I1SAG3
97513 229533.A0A1C3YJT5 pp 229533.I1RUF8
97514 229533.A0A1C3YJT5 pp 229533.I1RX70
97515 229533.A0A1C3YJT5 pp 229533.I1SAD2
97516 229533.A0A1C3YJT5 pp 229533.I1RQ13
97517 229533.A0A1C3YJT5 pp 229533.I1S0J3
97518 229533.A0A1C3YJT5 pp 229533.I1RC24
97519 229533.A0A1C3YJT5 pp 229533.I1RA87
97520 229533.A0A1C3YJT5 pp 229533.I1RY29
97521 229533.A0A1C3YJT5 pp 229533.I1RAT7
97522 229533.A0A1C3YJT5 pp 229533.I1RKI2
97523 229533.A0A1C3YJT5 pp 229533.I1RPC2
97524 229533.A0A1C3YJT5 pp 229533.I1RYC3
97525 229533.A0A1C3YJT5 pp 229533.I1S2E9
97526 229533.A0A1C3YJT5 pp 229533.I1RG77
97527 229533.A0A1C3YJT5 pp 229533.I1S098
97528 229533.A0A1C3YJT5 pp 229533.I1RRN5

97529	229533.A0A1C3YJT5	pp	229533.I1RSN7
97530	229533.A0A1C3YJT5	pp	229533.I1RA10
97531	229533.A0A1C3YJT5	pp	229533.I1RMP2
97532	229533.A0A1C3YJT5	pp	229533.I1RDH0
97533	229533.A0A1C3YJT5	pp	229533.I1RWI8
97534	229533.A0A1C3YJT5	pp	229533.I1RSZ3
97535	229533.A0A1C3YJT5	pp	229533.I1RPF5
97536	229533.A0A1C3YJT5	pp	229533.A0A1C3YM61
97537	229533.A0A1C3YJT5	pp	229533.I1S2G7
97538	229533.A0A1C3YJT5	pp	229533.I1R9N1
97539	229533.A0A1C3YJT5	pp	229533.I1RB74
97540	229533.A0A1C3YJT5	pp	229533.A0A1C3YLE9
97541	229533.A0A1C3YJT5	pp	229533.I1RFL7
97542	229533.A0A1C3YJT5	pp	229533.I1RTC2
97543	229533.A0A1C3YJT5	pp	229533.I1RY58
97544	229533.A0A1C3YJT5	pp	229533.I1RQA3
97545	229533.A0A1C3YJT5	pp	229533.A0A1C3YMJ3
97546	229533.A0A1C3YJT5	pp	229533.I1RRG3
97547	229533.A0A1C3YJT5	pp	229533.A0A1C3YN36
97548	229533.A0A1C3YJT5	pp	229533.I1S0E8
97549	229533.A0A1C3YJT5	pp	229533.I1RZP8
97550	229533.A0A1C3YJT5	pp	229533.I1RJL2
97551	229533.A0A1C3YJT5	pp	229533.I1RTA3
97552	229533.A0A1C3YJT5	pp	229533.I1RBJ0
97553	229533.A0A1C3YJT5	pp	229533.I1RRF0
97554	229533.A0A1C3YJT5	pp	229533.I1S209
97555	229533.A0A1C3YJT5	pp	229533.I1S8J7
97556	229533.A0A1C3YJT5	pp	229533.I1RCT9
97557	229533.A0A1C3YJT5	pp	229533.I1RBN9
97558	229533.A0A1C3YJT5	pp	229533.I1RZB5
97559	229533.A0A1C3YJT5	pp	229533.I1RC17
97560	229533.A0A1C3YJT5	pp	229533.I1RG11
97561	229533.A0A1C3YJT5	pp	229533.I1RHB0
97562	229533.A0A1C3YJT5	pp	229533.I1RZT1
97563	229533.A0A1C3YJT5	pp	229533.V6RGI1
97564	229533.A0A1C3YJT5	pp	229533.I1RYY5
97565	229533.A0A1C3YJT5	pp	229533.I1RNE4
97566	229533.A0A1C3YJT5	pp	229533.I1RD19
97567	229533.A0A1C3YJT5	pp	229533.I1RRF9
97568	229533.A0A1C3YJT5	pp	229533.I1RS41
97569	229533.A0A1C3YJT5	pp	229533.I1RSR9
97570	229533.A0A1C3YJT5	pp	229533.I1RDQ9
97571	229533.A0A1C3YJT5	pp	229533.I1RSP7
97572	229533.A0A1C3YJT5	pp	229533.I1RK79
97573	229533.A0A1C3YJT5	pp	229533.I1RWT2
97574	229533.A0A1C3YJT5	pp	229533.I1RR25
97575	229533.A0A1C3YJT5	pp	229533.I1S193
97576	229533.A0A1C3YJT5	pp	229533.I1RMS2
97577	229533.A0A1C3YJT5	pp	229533.A0A1C3YJZ9
97578	229533.A0A1C3YJT5	pp	229533.I1RWT6
97579	229533.A0A1C3YJT5	pp	229533.I1REN3
97580	229533.A0A1C3YJT5	pp	229533.I1S1W4
97581	229533.A0A1C3YJT5	pp	229533.I1RUG4
97582	229533.A0A1C3YJT5	pp	229533.V6QUE6
97583	229533.A0A1C3YJT5	pp	229533.I1R9Y7
97584	229533.A0A1C3YJT5	pp	229533.I1RB75
97585	229533.A0A1C3YJT5	pp	229533.I1RFQ5
97586	229533.A0A1C3YJT5	pp	229533.I1RB41
97587	229533.A0A1C3YJT5	pp	229533.I1RD15
97588	229533.A0A1C3YJT5	pp	229533.A0A1C3YMB6
97589	229533.A0A1C3YJT5	pp	229533.I1RBJ1
97590	229533.A0A1C3YJT5	pp	229533.I1RXU0
97591	229533.A0A1C3YJT5	pp	229533.A0A1C3YM91
97592	229533.A0A1C3YJT5	pp	229533.I1RY44
97593	229533.A0A1C3YJT5	pp	229533.A0A1C3YM29
97594	229533.A0A1C3YJT5	pp	229533.I1RK75
97595	229533.A0A1C3YJT5	pp	229533.I1RN55
97596	229533.A0A1C3YJT5	pp	229533.I1RCB7
97597	229533.A0A1C3YJT5	pp	229533.Q4IJM4
97598	229533.A0A1C3YJT5	pp	229533.I1RKE9
97599	229533.A0A1C3YJT5	pp	229533.Q4INS6
97600	229533.A0A1C3YJT5	pp	229533.I1RA12
97601	229533.A0A1C3YJT5	pp	229533.A0A1C3YN55

97602 229533.A0A1C3YJT5 pp 229533.I1REZ5
97603 229533.A0A1C3YJT5 pp 229533.A0A1C3YMC9
97604 229533.A0A1C3YJT5 pp 229533.A0A1C3YN69
97605 229533.A0A1C3YJT5 pp 229533.Q4HTT2
97606 229533.A0A1C3YJT5 pp 229533.I1RRT0
97607 229533.A0A1C3YJT5 pp 229533.I1RN57
97608 229533.A0A1C3YJT5 pp 229533.I1RSC7
97609 229533.A0A1C3YJT5 pp 229533.Q4IB89
97610 229533.A0A1C3YJT5 pp 229533.I1RMQ7
97611 229533.I1REV3 pp 229533.I1S5R5
97612 229533.I1REV3 pp 229533.I1RVY0
97613 229533.I1REV3 pp 229533.Q4I8X0
97614 229533.I1REV3 pp 229533.V6RPN0
97615 229533.I1REV3 pp 229533.I1RNG4
97616 229533.I1REV3 pp 229533.I1RFS3
97617 229533.A0A098DA87 pp 229533.I1RRN5
97618 229533.A0A098DA87 pp 229533.A0A098E4K9
97619 229533.A0A098DA87 pp 229533.A0A098DNT9
97620 229533.A0A098DA87 pp 229533.I1S209
97621 229533.A0A098DA87 pp 229533.I1RMS2
97622 229533.A0A098DA87 pp 229533.I1RSC7
97623 229533.A0A098DA87 pp 229533.I1RZT1
97624 229533.A0A098DA87 pp 229533.I1REN3
97625 229533.A0A098DA87 pp 229533.A0A1C3YN55
97626 229533.A0A098DA87 pp 229533.I1RDH0
97627 229533.A0A098DA87 pp 229533.I1RMQ7
97628 229533.A0A098DA87 pp 229533.A0A1C3YMC9
97629 229533.A0A098DA87 pp 229533.I1RUF8
97630 229533.A0A098DA87 pp 229533.I1RRJ6
97631 229533.A0A098DA87 pp 229533.I1RSZ3
97632 229533.A0A098DA87 pp 229533.A0A1C3YI39
97633 229533.A0A098DA87 pp 229533.A0A1C3YJA2
97634 229533.A0A098DA87 pp 229533.I1RZ94
97635 229533.A0A098DA87 pp 229533.I1RKI2
97636 229533.A0A098DA87 pp 229533.A0A1C3YM61
97637 229533.A0A098DA87 pp 229533.A0A1C3YJT5
97638 229533.A0A098DA87 pp 229533.I1RTC2
97639 229533.I1S6V0 pp 229533.I1S9Z4
97640 229533.I1S6V0 pp 229533.I1SAT2
97641 229533.I1S6V0 pp 229533.I1SAB4
97642 229533.I1S6V0 pp 229533.I1S968
97643 229533.I1S6V0 pp 229533.I1S969
97644 229533.I1RB05 pp 229533.I1S0A5
97645 229533.I1RB05 pp 229533.I1RYI5
97646 229533.I1RB05 pp 229533.I1RFB0
97647 229533.I1RB05 pp 229533.I1RF24
97648 229533.I1RB05 pp 229533.I1RSA5
97649 229533.I1RB05 pp 229533.I1RVZ2
97650 229533.I1RB05 pp 229533.I1RZ38
97651 229533.I1RB05 pp 229533.I1RTA1
97652 229533.I1RB05 pp 229533.I1RR68
97653 229533.I1RB05 pp 229533.I1S968
97654 229533.I1RB05 pp 229533.I1SAB4
97655 229533.I1RB05 pp 229533.I1RRI1
97656 229533.I1RB05 pp 229533.I1RK55
97657 229533.I1RB05 pp 229533.I1RZ25
97658 229533.I1RB05 pp 229533.I1S9Z4
97659 229533.I1RB05 pp 229533.I1RKD3
97660 229533.I1RB05 pp 229533.I1RPV8
97661 229533.I1RB05 pp 229533.I1RZ56
97662 229533.I1RB05 pp 229533.I1RY35
97663 229533.I1RB05 pp 229533.I1RPU2
97664 229533.I1RB05 pp 229533.I1RE52
97665 229533.I1RB05 pp 229533.I1RYU6
97666 229533.I1RB05 pp 229533.I1RGU3
97667 229533.I1RB05 pp 229533.I1S2C4
97668 229533.I1RB05 pp 229533.I1SAT2
97669 229533.I1RB05 pp 229533.I1RYP5
97670 229533.I1RB05 pp 229533.I1RQ33
97671 229533.I1RB05 pp 229533.I1RQV8
97672 229533.I1RB05 pp 229533.I1RZX3
97673 229533.I1RB05 pp 229533.I1S223
97674 229533.I1RB05 pp 229533.I1S210

97675 229533.I1RB05 pp 229533.I1RDN3
97676 229533.I1RB05 pp 229533.I1S969
97677 229533.I1RB05 pp 229533.I1RRS6
97678 229533.I1RB05 pp 229533.I1RJS1
97679 229533.I1RB05 pp 229533.I1RHJ0
97680 229533.I1RB05 pp 229533.I1RMA2
97681 229533.I1RB05 pp 229533.I1RH83
97682 229533.I1RB05 pp 229533.I1S5D0
97683 229533.I1RB05 pp 229533.I1RQV7
97684 229533.I1RB05 pp 229533.I1S4S7
97685 229533.I1RB05 pp 229533.I1RHT5
97686 229533.I1RB05 pp 229533.I1RWL3
97687 229533.I1RB05 pp 229533.I1RW54
97688 229533.I1RB05 pp 229533.I1RW55
97689 229533.I1RB05 pp 229533.I1RIM7
97690 229533.I1RB05 pp 229533.I1S048
97691 229533.I1RB05 pp 229533.I1S6H3
97692 229533.I1RB05 pp 229533.I1S267
97693 229533.I1RB05 pp 229533.I1RPM6
97694 229533.I1RB05 pp 229533.I1S659
97695 229533.I1RB05 pp 229533.I1RY81
97696 229533.I1RB05 pp 229533.I1RUY5
97697 229533.I1RB05 pp 229533.I1RLA2
97698 229533.I1RB05 pp 229533.I1S2F7
97699 229533.I1RB05 pp 229533.I1S094
97700 229533.I1RB05 pp 229533.I1RDU0
97701 229533.I1RB05 pp 229533.I1S3T6
97702 229533.I1RB05 pp 229533.I1RST9
97703 229533.I1RB05 pp 229533.I1RWM4
97704 229533.I1RB05 pp 229533.I1RQJ7
97705 229533.I1RB05 pp 229533.I1RIB5
97706 229533.I1RTB7 pp 229533.I1RZM1
97707 229533.I1RTB7 pp 229533.I1S096
97708 229533.I1RTB7 pp 229533.I1RZH4
97709 229533.I1RTB7 pp 229533.I1S0W5
97710 229533.I1RTB7 pp 229533.I1RUA4
97711 229533.I1RTB7 pp 229533.I1S0C0
97712 229533.I1RTB7 pp 229533.V6RR76
97713 229533.I1RTB7 pp 229533.I1S336
97714 229533.I1RTB7 pp 229533.Q4ICG5
97715 229533.I1RTB7 pp 229533.I1RWL6
97716 229533.I1RTB7 pp 229533.I1RZX9
97717 229533.I1RTB7 pp 229533.I1S0S6
97718 229533.I1RTB7 pp 229533.I1RY98
97719 229533.I1RTB7 pp 229533.I1S282
97720 229533.I1RTB7 pp 229533.I1RXD5
97721 229533.I1RTB7 pp 229533.I1RUE4
97722 229533.I1RTB7 pp 229533.I1RTH6
97723 229533.I1RTB7 pp 229533.I1S2A8
97724 229533.I1RTB7 pp 229533.I1S1B6
97725 229533.I1RTB7 pp 229533.I1S7X9
97726 229533.I1RTB7 pp 229533.I1RYZ7
97727 229533.I1RTB7 pp 229533.I1RW41
97728 229533.I1RTB7 pp 229533.I1RXF7
97729 229533.I1RTB7 pp 229533.I1S6S9
97730 229533.I1RTB7 pp 229533.I1S0K9
97731 229533.A0A1C3YMW5 pp 229533.I1RFS3
97732 229533.A0A1C3YMW5 pp 229533.I1RVY0
97733 229533.A0A1C3YMW5 pp 229533.I1RNG4
97734 229533.A0A1C3YMW5 pp 229533.Q4I8X0
97735 229533.A0A1C3YMW5 pp 229533.I1RBL7
97736 229533.A0A1C3YMW5 pp 229533.V6RPN0
97737 229533.A0A1C3YMW5 pp 229533.I1S5R5
97738 229533.I1RY81 pp 229533.I1S8R2
97739 229533.I1RY81 pp 229533.I1RYA0
97740 229533.I1RY81 pp 229533.V6RCJ5
97741 229533.I1RY81 pp 229533.I1RZ56
97742 229533.I1RY81 pp 229533.I1S2C4
97743 229533.I1RY81 pp 229533.I1RYC8
97744 229533.I1RY81 pp 229533.I1RZ89
97745 229533.I1RY81 pp 229533.I1S6V0
97746 229533.I1RY81 pp 229533.I1RZC3
97747 229533.I1RY81 pp 229533.I1S3T7

97748 229533.I1RY81 pp 229533.I1S2Q4
97749 229533.I1RY81 pp 229533.I1S0A5
97750 229533.I1RY81 pp 229533.I1SAB4
97751 229533.I1RY81 pp 229533.I1RZV5
97752 229533.I1RY81 pp 229533.I1RZ38
97753 229533.I1RY81 pp 229533.I1RZD0
97754 229533.I1RY81 pp 229533.I1S968
97755 229533.I1RY81 pp 229533.I1SAT2
97756 229533.I1RY81 pp 229533.V6RG22
97757 229533.I1RY81 pp 229533.V6RWB3
97758 229533.I1RY81 pp 229533.I1S8D3
97759 229533.I1RY81 pp 229533.I1S3T6
97760 229533.I1RY81 pp 229533.I1S299
97761 229533.I1RY81 pp 229533.I1RZ25
97762 229533.I1RY81 pp 229533.I1S2F7
97763 229533.I1RY81 pp 229533.I1S267
97764 229533.I1RY81 pp 229533.I1S659
97765 229533.I1RY81 pp 229533.V6RPN0
97766 229533.I1RY81 pp 229533.V6R5T7
97767 229533.I1RY81 pp 229533.I1S8F8
97768 229533.I1RY81 pp 229533.I1S048
97769 229533.I1RY81 pp 229533.I1S210
97770 229533.I1RY81 pp 229533.V6RHX2
97771 229533.I1RY81 pp 229533.I1S5D0
97772 229533.I1RY81 pp 229533.I1S094
97773 229533.I1RY81 pp 229533.V6RR38
97774 229533.I1RY81 pp 229533.I1S6H3
97775 229533.I1RY81 pp 229533.V6RCC0
97776 229533.I1RY81 pp 229533.I1RZZ3
97777 229533.I1RY81 pp 229533.I1RYP5
97778 229533.I1RY81 pp 229533.I1RZ92
97779 229533.I1RY81 pp 229533.I1S2M5
97780 229533.I1RY81 pp 229533.I1S130
97781 229533.I1RY81 pp 229533.I1S223
97782 229533.I1RY81 pp 229533.I1RYU6
97783 229533.I1RY81 pp 229533.I1S8C1
97784 229533.I1RY81 pp 229533.V6QVV9
97785 229533.A0A1C3YNB3 pp 229533.I1RMY1
97786 229533.A0A1C3YNB3 pp 229533.V6R4J6
97787 229533.A0A1C3YNB3 pp 229533.I1RVR2
97788 229533.A0A1C3YNB3 pp 229533.I1RZ25
97789 229533.A0A1C3YNB3 pp 229533.I1S673
97790 229533.Q4IMM0 pp 229533.V6R6F6
97791 229533.I1RQN9 pp 229533.I1RRX1
97792 229533.I1RQN9 pp 229533.I1RRN0
97793 229533.I1RQN9 pp 229533.I1S0K9
97794 229533.I1RQN9 pp 229533.I1RW41
97795 229533.I1RQN9 pp 229533.I1RS64
97796 229533.I1RQN9 pp 229533.I1RYZ7
97797 229533.I1RQN9 pp 229533.I1S6S9
97798 229533.I1RQN9 pp 229533.I1S282
97799 229533.I1RQN9 pp 229533.I1S2A8
97800 229533.I1RQN9 pp 229533.I1RW16
97801 229533.I1RQN9 pp 229533.I1S1B6
97802 229533.I1RQN9 pp 229533.I1RUE4
97803 229533.I1RQN9 pp 229533.I1RZD9
97804 229533.I1RQN9 pp 229533.I1S0W5
97805 229533.I1RQN9 pp 229533.I1S7X9
97806 229533.I1RQN9 pp 229533.I1S336
97807 229533.I1RQN9 pp 229533.I1RXF7
97808 229533.I1RQN9 pp 229533.I1S050
97809 229533.I1RQN9 pp 229533.I1S1B1
97810 229533.I1RQN9 pp 229533.I1RUA4
97811 229533.I1RQN9 pp 229533.P0C431
97812 229533.I1RQN9 pp 229533.I1RXD5
97813 229533.I1RQN9 pp 229533.I1RZM1
97814 229533.I1RQN9 pp 229533.Q4ICG5
97815 229533.I1RQN9 pp 229533.I1RT19
97816 229533.I1RQN9 pp 229533.I1RZH4
97817 229533.I1RQN9 pp 229533.I1RWL6
97818 229533.I1RQN9 pp 229533.V6RR76
97819 229533.I1RQN9 pp 229533.I1RZX9
97820 229533.I1RQN9 pp 229533.I1S0C0

97821 229533.I1RQN9 pp 229533.I1RY98
97822 229533.I1RQN9 pp 229533.I1S1V3
97823 229533.I1RQN9 pp 229533.I1S095
97824 229533.I1RQN9 pp 229533.I1RTB7
97825 229533.I1RQN9 pp 229533.I1S0S6
97826 229533.A0A1C3YJP9 pp 229533.I1RAP1
97827 229533.A0A1C3YJP9 pp 229533.I1RYV4
97828 229533.A0A1C3YJP9 pp 229533.Q4IAS1
97829 229533.A0A1C3YJP9 pp 229533.I1RAP2
97830 229533.A0A1C3YJP9 pp 229533.I1RA31
97831 229533.A0A1C3YJP9 pp 229533.Q4IJH1
97832 229533.A0A1C3YJP9 pp 229533.I1RZP8
97833 229533.A0A1C3YJP9 pp 229533.I1RSG9
97834 229533.I1RMA2 pp 229533.I1RNM7
97835 229533.I1RMA2 pp 229533.I1S223
97836 229533.I1RMA2 pp 229533.I1S210
97837 229533.I1RMA2 pp 229533.V6R5T7
97838 229533.I1RMA2 pp 229533.I1RRS6
97839 229533.I1RMA2 pp 229533.I1RQV7
97840 229533.I1RMA2 pp 229533.I1RQ76
97841 229533.I1RMA2 pp 229533.I1S8C1
97842 229533.I1RMA2 pp 229533.I1S5D0
97843 229533.I1RMA2 pp 229533.I1RW54
97844 229533.I1RMA2 pp 229533.I1RYU6
97845 229533.I1RMA2 pp 229533.I1S130
97846 229533.I1RMA2 pp 229533.I1RMF1
97847 229533.I1RMA2 pp 229533.I1S2M5
97848 229533.I1RMA2 pp 229533.I1RQV8
97849 229533.I1RMA2 pp 229533.I1RVI8
97850 229533.I1RMA2 pp 229533.I1S094
97851 229533.I1RMA2 pp 229533.I1S048
97852 229533.I1RMA2 pp 229533.I1RZ92
97853 229533.I1RMA2 pp 229533.V6RCC0
97854 229533.I1RMA2 pp 229533.I1RWL3
97855 229533.I1RMA2 pp 229533.I1RYP5
97856 229533.I1RMA2 pp 229533.I1RST9
97857 229533.I1RMA2 pp 229533.I1RPE5
97858 229533.I1RMA2 pp 229533.V6QVV9
97859 229533.I1RMA2 pp 229533.I1S3T6
97860 229533.I1RMA2 pp 229533.I1RZZ3
97861 229533.I1RMA2 pp 229533.I1S6H3
97862 229533.I1RMA2 pp 229533.I1RW55
97863 229533.I1RMA2 pp 229533.I1RWM4
97864 229533.I1RMA2 pp 229533.V6RPN0
97865 229533.I1RMA2 pp 229533.I1RPR1
97866 229533.I1RMA2 pp 229533.V6RG22
97867 229533.I1RMA2 pp 229533.I1S8D3
97868 229533.I1RMA2 pp 229533.I1RXX1
97869 229533.I1RMA2 pp 229533.I1RWD2
97870 229533.I1RMA2 pp 229533.I1RR68
97871 229533.I1RMA2 pp 229533.V6RWB3
97872 229533.I1RMA2 pp 229533.I1RZV5
97873 229533.I1RMA2 pp 229533.I1S267
97874 229533.I1RMA2 pp 229533.I1S2F7
97875 229533.I1RMA2 pp 229533.I1RZ25
97876 229533.I1RMA2 pp 229533.I1S659
97877 229533.I1RMA2 pp 229533.V6RHX2
97878 229533.I1RMA2 pp 229533.I1RMK9
97879 229533.I1RMA2 pp 229533.I1S8F8
97880 229533.I1RMA2 pp 229533.I1RZ38
97881 229533.I1RMA2 pp 229533.I1RQJ7
97882 229533.I1RMA2 pp 229533.I1RY81
97883 229533.I1RMA2 pp 229533.I1RY32
97884 229533.I1RMA2 pp 229533.I1S299
97885 229533.I1RMA2 pp 229533.I1S968
97886 229533.I1RMA2 pp 229533.I1RQ48
97887 229533.I1RMA2 pp 229533.I1S0A5
97888 229533.I1RMA2 pp 229533.I1RSA5
97889 229533.I1RMA2 pp 229533.V6RCJ5
97890 229533.I1RMA2 pp 229533.I1RZD0
97891 229533.I1RMA2 pp 229533.I1S3T7
97892 229533.I1RMA2 pp 229533.I1RYC8
97893 229533.I1RMA2 pp 229533.I1SAB4

97894 229533.I1RMA2 pp 229533.I1S2Q4
97895 229533.I1RMA2 pp 229533.I1RPV8
97896 229533.I1RMA2 pp 229533.I1RZ56
97897 229533.I1RMA2 pp 229533.I1RY35
97898 229533.I1RMA2 pp 229533.I1RZ89
97899 229533.I1RMA2 pp 229533.I1S6V0
97900 229533.I1RMA2 pp 229533.I1RZC3
97901 229533.I1RMA2 pp 229533.I1RUR8
97902 229533.I1RMA2 pp 229533.I1S2C4
97903 229533.I1RMA2 pp 229533.I1SAT2
97904 229533.I1RMA2 pp 229533.I1RYA0
97905 229533.I1RMA2 pp 229533.I1RVX8
97906 229533.I1RMA2 pp 229533.I1S8R2
97907 229533.A0A098DA75 pp 229533.Q4IBX0
97908 229533.A0A098DA75 pp 229533.A0A1C3YM21
97909 229533.A0A098DA75 pp 229533.P0C431
97910 229533.A0A098DA75 pp 229533.I1S0S6
97911 229533.A0A098DA75 pp 229533.I1RTB7
97912 229533.A0A098DA75 pp 229533.I1RH68
97913 229533.A0A098DA75 pp 229533.Q4IMM0
97914 229533.A0A098DA75 pp 229533.I1RQN9
97915 229533.I1RQV7 pp 229533.I1S223
97916 229533.I1RQV7 pp 229533.I1S5D0
97917 229533.I1RQV7 pp 229533.V6RCC0
97918 229533.I1RQV7 pp 229533.I1RRS6
97919 229533.I1RQV7 pp 229533.I1RW54
97920 229533.I1RQV7 pp 229533.I1S969
97921 229533.I1RQV7 pp 229533.V6R5T7
97922 229533.I1RQV7 pp 229533.I1S4S7
97923 229533.I1RQV7 pp 229533.I1RYP5
97924 229533.I1RQV7 pp 229533.V6QVV9
97925 229533.I1RQV7 pp 229533.I1RWL3
97926 229533.I1RQV7 pp 229533.I1S048
97927 229533.I1RQV7 pp 229533.I1S210
97928 229533.I1RQV7 pp 229533.I1S6H3
97929 229533.I1RQV7 pp 229533.I1S659
97930 229533.I1RQV7 pp 229533.I1RVF2
97931 229533.I1RQV7 pp 229533.I1S0K1
97932 229533.I1RQV7 pp 229533.I1S3T6
97933 229533.I1RQV7 pp 229533.I1RYU6
97934 229533.I1RQV7 pp 229533.I1S094
97935 229533.I1RQV7 pp 229533.I1RQV8
97936 229533.I1RQV7 pp 229533.I1RY81
97937 229533.I1RQV7 pp 229533.I1S267
97938 229533.I1RQV7 pp 229533.I1S000
97939 229533.I1RQV7 pp 229533.I1S4I8
97940 229533.I1RQV7 pp 229533.I1S8D3
97941 229533.I1RQV7 pp 229533.V6RWB3
97942 229533.I1RQV7 pp 229533.I1S4I7
97943 229533.I1RQV7 pp 229533.I1RSG0
97944 229533.I1RQV7 pp 229533.I1S2F7
97945 229533.I1RQV7 pp 229533.I1RUY5
97946 229533.I1RQV7 pp 229533.V6RHX2
97947 229533.I1RQV7 pp 229533.I1RST9
97948 229533.I1RQV7 pp 229533.I1RWM4
97949 229533.I1RQV7 pp 229533.I1RW55
97950 229533.I1RQV7 pp 229533.I1SAB4
97951 229533.I1RQV7 pp 229533.I1RRI1
97952 229533.I1RQV7 pp 229533.I1RY35
97953 229533.I1RQV7 pp 229533.I1RUE3
97954 229533.I1RQV7 pp 229533.I1SAH9
97955 229533.I1RQV7 pp 229533.I1RVZ2
97956 229533.I1RQV7 pp 229533.I1RQW1
97957 229533.I1RQV7 pp 229533.I1RTA1
97958 229533.I1RQV7 pp 229533.I1RZ38
97959 229533.I1RQV7 pp 229533.I1RYI5
97960 229533.I1RQV7 pp 229533.V6RCJ5
97961 229533.I1RQV7 pp 229533.I1RZ25
97962 229533.I1RQV7 pp 229533.I1RVX8
97963 229533.I1RQV7 pp 229533.I1S0A5
97964 229533.I1RQV7 pp 229533.I1RSA5
97965 229533.I1RQV7 pp 229533.I1S2C4
97966 229533.I1RQV7 pp 229533.I1S6V0

97967 229533.I1RQV7 pp 229533.I1S968
97968 229533.I1RQV7 pp 229533.I1RY32
97969 229533.I1RQV7 pp 229533.I1RZ56
97970 229533.I1RQV7 pp 229533.I1S0X1
97971 229533.I1RQV7 pp 229533.I1RR68
97972 229533.I1RQV7 pp 229533.I1S9Z4
97973 229533.I1RQV7 pp 229533.I1RZX3
97974 229533.I1RQV7 pp 229533.I1RZV3
97975 229533.I1RQV7 pp 229533.I1RZC3
97976 229533.I1RQV7 pp 229533.I1RTY9
97977 229533.I1RQV7 pp 229533.I1RSU2
97978 229533.I1RQV7 pp 229533.I1SAT2
97979 229533.I1RQV7 pp 229533.I1RZ89
97980 229533.I1RNM1 pp 229533.I1RTG5
97981 229533.I1RNM1 pp 229533.Q4HYQ4
97982 229533.I1RNM1 pp 229533.I1RX87
97983 229533.I1RNM1 pp 229533.I1S1C8
97984 229533.I1RNM1 pp 229533.Q4IPC8
97985 229533.I1RNM1 pp 229533.I1S1U6
97986 229533.I1RNM1 pp 229533.I1RTQ9
97987 229533.I1RK10 pp 229533.I1RW43
97988 229533.I1RK10 pp 229533.I1RY32
97989 229533.I1RK10 pp 229533.I1RSG0
97990 229533.I1RK10 pp 229533.I1RZC3
97991 229533.I1RK10 pp 229533.I1S267
97992 229533.I1RK10 pp 229533.I1RM41
97993 229533.I1RZX3 pp 229533.I1S224
97994 229533.I1RZX3 pp 229533.V6R5T7
97995 229533.I1RZX3 pp 229533.I1S048
97996 229533.I1RZX3 pp 229533.V6RCC0
97997 229533.I1RZX3 pp 229533.I1S6V0
97998 229533.I1RZX3 pp 229533.V6QVV9
97999 229533.I1RZX3 pp 229533.V6RHX2
98000 229533.I1RZX3 pp 229533.I1SAB4
98001 229533.I1RZX3 pp 229533.V6RCJ5
98002 229533.I1RZX3 pp 229533.I1S8D3
98003 229533.I1RZX3 pp 229533.I1S2E9
98004 229533.I1RZX3 pp 229533.I1S267
98005 229533.I1RZX3 pp 229533.V6RWB3
98006 229533.A0A098DTX5 pp 229533.I1RZ56
98007 229533.A0A098DTX5 pp 229533.I1RB05
98008 229533.A0A098DTX5 pp 229533.I1RZ89
98009 229533.A0A098DTX5 pp 229533.I1RHU8
98010 229533.A0A098DTX5 pp 229533.I1RH83
98011 229533.A0A098DTX5 pp 229533.I1RJS1
98012 229533.A0A098DTX5 pp 229533.A0A0E0RSR0
98013 229533.A0A098DTX5 pp 229533.I1SAT2
98014 229533.A0A098DTX5 pp 229533.I1RPV8
98015 229533.A0A098DTX5 pp 229533.I1RE52
98016 229533.A0A098DTX5 pp 229533.A0A0E0RSI1
98017 229533.A0A098DTX5 pp 229533.A0A1C3YLB6
98018 229533.A0A098DTX5 pp 229533.I1S0A5
98019 229533.A0A098DTX5 pp 229533.I1RZC3
98020 229533.A0A098DTX5 pp 229533.I1S6V0
98021 229533.A0A098DTX5 pp 229533.I1RAV0
98022 229533.A0A098DTX5 pp 229533.I1RW82
98023 229533.A0A098DTX5 pp 229533.I1S2C4
98024 229533.A0A098DTX5 pp 229533.I1RMK9
98025 229533.A0A098DTX5 pp 229533.I1RLZ4
98026 229533.A0A098DTX5 pp 229533.I1RZX3
98027 229533.A0A098DTX5 pp 229533.I1RAX2
98028 229533.A0A098DTX5 pp 229533.I1RZ25
98029 229533.A0A098DTX5 pp 229533.I1RSA5
98030 229533.A0A098DTX5 pp 229533.I1RZV5
98031 229533.A0A098DTX5 pp 229533.I1RM41
98032 229533.A0A098DTX5 pp 229533.I1RY32
98033 229533.A0A098DTX5 pp 229533.I1RTM8
98034 229533.A0A098DTX5 pp 229533.I1RH91
98035 229533.A0A098DTX5 pp 229533.I1S968
98036 229533.A0A098DTX5 pp 229533.I1RR68
98037 229533.A0A098DTX5 pp 229533.V6RCJ5
98038 229533.A0A098DTX5 pp 229533.I1RRR9
98039 229533.A0A098DTX5 pp 229533.I1RC71

98040 229533.A0A098DTX5 pp 229533.I1RK55
98041 229533.A0A098DTX5 pp 229533.I1RSP5
98042 229533.A0A098DTX5 pp 229533.I1RIJ6
98043 229533.A0A098DTX5 pp 229533.I1RC46
98044 229533.A0A098DTX5 pp 229533.I1S267
98045 229533.A0A098DTX5 pp 229533.V6RHX2
98046 229533.A0A098DTX5 pp 229533.A0A0E0S530
98047 229533.A0A098DTX5 pp 229533.I1RWM4
98048 229533.A0A098DTX5 pp 229533.I1RWC1
98049 229533.A0A098DTX5 pp 229533.A0A1C3YMC4
98050 229533.A0A098DTX5 pp 229533.I1RCT9
98051 229533.A0A098DTX5 pp 229533.I1REI2
98052 229533.A0A098DTX5 pp 229533.I1RPM6
98053 229533.A0A098DTX5 pp 229533.I1RST9
98054 229533.A0A098DTX5 pp 229533.I1RY81
98055 229533.A0A098DTX5 pp 229533.I1RIB5
98056 229533.A0A098DTX5 pp 229533.I1S8D3
98057 229533.A0A098DTX5 pp 229533.I1S659
98058 229533.A0A098DTX5 pp 229533.I1RA13
98059 229533.A0A098DTX5 pp 229533.A0A1C3YIB8
98060 229533.A0A098DTX5 pp 229533.I1RYP5
98061 229533.A0A098DTX5 pp 229533.V6RCC0
98062 229533.A0A098DTX5 pp 229533.V6RWB3
98063 229533.A0A098DTX5 pp 229533.I1RY12
98064 229533.A0A098DTX5 pp 229533.I1RDN3
98065 229533.A0A098DTX5 pp 229533.I1S5D0
98066 229533.A0A098DTX5 pp 229533.I1RSG0
98067 229533.A0A098DTX5 pp 229533.I1RW54
98068 229533.A0A098DTX5 pp 229533.I1S969
98069 229533.A0A098DTX5 pp 229533.I1RQV7
98070 229533.A0A098DTX5 pp 229533.I1RRS6
98071 229533.A0A098DTX5 pp 229533.I1RMA2
98072 229533.A0A098DTX5 pp 229533.I1R9F2
98073 229533.A0A098DTX5 pp 229533.V6R5T7
98074 229533.A0A098DTX5 pp 229533.I1RQV8
98075 229533.A0A098DTX5 pp 229533.I1S6H3
98076 229533.A0A098DTX5 pp 229533.I1RYU6
98077 229533.A0A098DTX5 pp 229533.I1RWL3
98078 229533.A0A098DTX5 pp 229533.I1S048
98079 229533.A0A098DTX5 pp 229533.I1RJR8
98080 229533.A0A098DTX5 pp 229533.I1RLA2
98081 229533.A0A098DTX5 pp 229533.I1S094
98082 229533.I1R9F3 pp 229533.I1RBY9
98083 229533.I1R9F3 pp 229533.I1RNM7
98084 229533.I1R9F3 pp 229533.I1RWF9
98085 229533.I1R9F3 pp 229533.I1S267
98086 229533.I1R9F3 pp 229533.I1RH91
98087 229533.I1R9F3 pp 229533.I1RST9
98088 229533.I1RIJ6 pp 229533.I1S0A5
98089 229533.I1RIJ6 pp 229533.I1RY35
98090 229533.I1RIJ6 pp 229533.I1RKD3
98091 229533.I1RIJ6 pp 229533.I1RTA1
98092 229533.I1RIJ6 pp 229533.I1RYI5
98093 229533.I1RIJ6 pp 229533.I1RVZ2
98094 229533.I1RIJ6 pp 229533.I1SAB4
98095 229533.I1RIJ6 pp 229533.I1RSA5
98096 229533.I1RIJ6 pp 229533.I1RRI1
98097 229533.I1RIJ6 pp 229533.I1RK55
98098 229533.I1RIJ6 pp 229533.I1RZ38
98099 229533.I1RIJ6 pp 229533.I1RR68
98100 229533.I1RIJ6 pp 229533.I1RZ25
98101 229533.I1RIJ6 pp 229533.I1S210
98102 229533.I1RIJ6 pp 229533.I1RQV7
98103 229533.I1RIJ6 pp 229533.I1RJS1
98104 229533.I1RIJ6 pp 229533.I1RMA2
98105 229533.I1RIJ6 pp 229533.I1S2C4
98106 229533.I1RIJ6 pp 229533.I1S968
98107 229533.I1RIJ6 pp 229533.I1RW54
98108 229533.I1RIJ6 pp 229533.I1S5D0
98109 229533.I1RIJ6 pp 229533.I1RQ33
98110 229533.I1RIJ6 pp 229533.I1RZ56
98111 229533.I1RIJ6 pp 229533.I1RZX3
98112 229533.I1RIJ6 pp 229533.I1RPU2

98113 229533.I1RIJ6 pp 229533.I1RWL3
98114 229533.I1RIJ6 pp 229533.I1RPV8
98115 229533.I1RIJ6 pp 229533.I1S094
98116 229533.I1RIJ6 pp 229533.I1S9Z4
98117 229533.I1RIJ6 pp 229533.I1S6H3
98118 229533.I1RIJ6 pp 229533.I1S223
98119 229533.I1RIJ6 pp 229533.I1S969
98120 229533.I1RIJ6 pp 229533.I1SAT2
98121 229533.I1RIJ6 pp 229533.I1RLA2
98122 229533.I1RIJ6 pp 229533.I1S048
98123 229533.I1RIJ6 pp 229533.I1RYU6
98124 229533.I1RIJ6 pp 229533.I1RYP5
98125 229533.I1RIJ6 pp 229533.I1RIM7
98126 229533.I1RIJ6 pp 229533.I1S267
98127 229533.I1RIJ6 pp 229533.I1S4S7
98128 229533.I1RIJ6 pp 229533.I1RRS6
98129 229533.I1RIJ6 pp 229533.I1S3T6
98130 229533.I1RIJ6 pp 229533.I1RY81
98131 229533.I1RIJ6 pp 229533.I1S659
98132 229533.I1RIJ6 pp 229533.I1RPM6
98133 229533.I1RIJ6 pp 229533.I1RUY5
98134 229533.I1RIJ6 pp 229533.I1RQJ7
98135 229533.I1RIJ6 pp 229533.I1S2F7
98136 229533.I1RIJ6 pp 229533.I1RWM4
98137 229533.I1RIJ6 pp 229533.I1RST9
98138 229533.I1RIJ6 pp 229533.I1RW55
98139 229533.A0A0E0RUB0 pp 229533.I1RA22
98140 229533.A0A0E0RUB0 pp 229533.A0A0E0SH87
98141 229533.A0A0E0RUB0 pp 229533.A0A1C3YJS7
98142 229533.A0A0E0RUB0 pp 229533.A0A1C3YK06
98143 229533.A0A0E0RUB0 pp 229533.I1S1Y5
98144 229533.A0A0E0RUB0 pp 229533.I1RZF5
98145 229533.A0A0E0RUB0 pp 229533.I1RT72
98146 229533.A0A0E0RUB0 pp 229533.A0A0E0RVD7
98147 229533.A0A0E0RUB0 pp 229533.I1RW54
98148 229533.I1S0M4 pp 229533.I1S267
98149 229533.I1RND5 pp 229533.I1S0B1
98150 229533.I1RND5 pp 229533.I1RS28
98151 229533.I1RND5 pp 229533.I1RQQ7
98152 229533.I1RND5 pp 229533.I1RPY5
98153 229533.I1RND5 pp 229533.I1RST1
98154 229533.I1RND5 pp 229533.I1RS75
98155 229533.I1RND5 pp 229533.I1RYJ9
98156 229533.I1RND5 pp 229533.I1RZD2
98157 229533.I1RND5 pp 229533.I1RSD4
98158 229533.I1RND5 pp 229533.I1RPA0
98159 229533.I1RND5 pp 229533.I1S0Z9
98160 229533.I1RND5 pp 229533.I1RVK5
98161 229533.I1RND5 pp 229533.I1RQP8
98162 229533.I1RND5 pp 229533.I1RRL0
98163 229533.I1RND5 pp 229533.I1S2Y5
98164 229533.I1RND5 pp 229533.Q4IPH4
98165 229533.I1RND5 pp 229533.I1RU69
98166 229533.I1RND5 pp 229533.Q4IPB3
98167 229533.I1RND5 pp 229533.I1RPL3
98168 229533.I1RND5 pp 229533.I1RYW3
98169 229533.I1RND5 pp 229533.Q4IBK5
98170 229533.I1RND5 pp 229533.I1RZG7
98171 229533.I1RND5 pp 229533.Q4IAA0
98172 229533.I1RND5 pp 229533.I1RPY4
98173 229533.I1RND5 pp 229533.I1S9L5
98174 229533.I1RND5 pp 229533.Q4I1Y1
98175 229533.I1RND5 pp 229533.I1RZQ5
98176 229533.I1RND5 pp 229533.I1RSA4
98177 229533.I1RND5 pp 229533.I1RNQ5
98178 229533.I1RND5 pp 229533.I1RX24
98179 229533.I1RND5 pp 229533.Q4IRI9
98180 229533.I1RND5 pp 229533.Q4HXF6
98181 229533.I1RND5 pp 229533.I1RWX8
98182 229533.I1RND5 pp 229533.I1RWU2
98183 229533.I1RND5 pp 229533.V6QX58
98184 229533.I1RND5 pp 229533.I1S4M5
98185 229533.I1RND5 pp 229533.I1RX81

98186 229533.I1RND5 pp 229533.I1RZ11
98187 229533.I1RND5 pp 229533.I1RT35
98188 229533.I1RND5 pp 229533.I1S1X9
98189 229533.I1RND5 pp 229533.I1RWH4
98190 229533.I1RND5 pp 229533.I1RQR3
98191 229533.I1RND5 pp 229533.I1S7S4
98192 229533.I1RND5 pp 229533.V6QXG1
98193 229533.I1RND5 pp 229533.I1RT91
98194 229533.I1RND5 pp 229533.Q4IB70
98195 229533.I1RND5 pp 229533.I1RND9
98196 229533.I1RND5 pp 229533.I1S1C6
98197 229533.I1RND5 pp 229533.I1RX51
98198 229533.I1RND5 pp 229533.I1RX47
98199 229533.I1RND5 pp 229533.I1RT47
98200 229533.I1RND5 pp 229533.I1RQC5
98201 229533.I1RND5 pp 229533.I1RYI9
98202 229533.I1RND5 pp 229533.Q4I5R9
98203 229533.I1RND5 pp 229533.Q4IE79
98204 229533.I1RND5 pp 229533.I1RWP4
98205 229533.I1RND5 pp 229533.I1S9L6
98206 229533.I1RND5 pp 229533.I1RXF2
98207 229533.I1RND5 pp 229533.I1RSA8
98208 229533.I1RND5 pp 229533.Q4IPB8
98209 229533.I1RND5 pp 229533.V6RIS2
98210 229533.I1RND5 pp 229533.I1RT17
98211 229533.I1RND5 pp 229533.I1S7M2
98212 229533.I1RND5 pp 229533.I1RPZ3
98213 229533.I1RND5 pp 229533.I1S471
98214 229533.I1RND5 pp 229533.I1S5S2
98215 229533.I1RND5 pp 229533.I1RRW3
98216 229533.I1RND5 pp 229533.I1S7M3
98217 229533.I1RY67 pp 229533.I1RZF5
98218 229533.I1S8D3 pp 229533.I1S968
98219 229533.I1S8D3 pp 229533.I1S969
98220 229533.I1S8D3 pp 229533.I1SAB4
98221 229533.I1S8D3 pp 229533.V6R5T7
98222 229533.I1S8D3 pp 229533.I1SAT2
98223 229533.I1S8D3 pp 229533.V6RTJ8
98224 229533.I1S8D3 pp 229533.I1S9Z4
98225 229533.I1S8D3 pp 229533.V6RWB3
98226 229533.A0A1C3YMK2 pp 229533.I1RBL7
98227 229533.A0A1C3YMK2 pp 229533.A0A1C3YMS7
98228 229533.A0A1C3YMK2 pp 229533.Q4I8X0
98229 229533.A0A1C3YMK2 pp 229533.V6RPN0
98230 229533.A0A1C3YMK2 pp 229533.I1S5R5
98231 229533.A0A1C3YMK2 pp 229533.I1RFS3
98232 229533.A0A1C3YMK2 pp 229533.I1RNG4
98233 229533.A0A1C3YMK2 pp 229533.I1RVY0
98234 229533.I1RD23 pp 229533.I1RDJ8
98235 229533.I1RD23 pp 229533.I1RDV4
98236 229533.I1RD23 pp 229533.I1RMJ8
98237 229533.I1RD23 pp 229533.I1RD70
98238 229533.I1RD23 pp 229533.I1RVV5
98239 229533.I1RD23 pp 229533.Q4IB89
98240 229533.I1RD23 pp 229533.I1RRJ6
98241 229533.I1RD23 pp 229533.I1RN65
98242 229533.I1RD23 pp 229533.I1RLJ8
98243 229533.I1RD23 pp 229533.V6RR76
98244 229533.I1RD23 pp 229533.I1RG34
98245 229533.I1RD23 pp 229533.I1RZH4
98246 229533.I1RD23 pp 229533.I1RN81
98247 229533.I1RDF8 pp 229533.I1RSV9
98248 229533.I1RDF8 pp 229533.I1RW54
98249 229533.I1RDF8 pp 229533.I1RZF5
98250 229533.I1RDF8 pp 229533.I1RT72
98251 229533.I1RAF3 pp 229533.I1RT17
98252 229533.I1RAF3 pp 229533.Q4I5R9
98253 229533.I1RAF3 pp 229533.I1RYU4
98254 229533.I1RAF3 pp 229533.I1RCQ6
98255 229533.I1RAF3 pp 229533.Q4IPB3
98256 229533.I1RAF3 pp 229533.I1RPY4
98257 229533.I1RAF3 pp 229533.I1RZG7
98258 229533.I1RAF3 pp 229533.I1RX24

98259 229533.I1RAF3 pp 229533.I1RMV5
98260 229533.I1RAF3 pp 229533.I1RCI4
98261 229533.I1RAF3 pp 229533.I1RN59
98262 229533.I1RAF3 pp 229533.I1RU69
98263 229533.I1RAF3 pp 229533.Q4IF76
98264 229533.I1RAF3 pp 229533.I1RGC2
98265 229533.I1RAF3 pp 229533.I1S044
98266 229533.I1RAF3 pp 229533.I1S056
98267 229533.I1RAF3 pp 229533.I1RN60
98268 229533.I1RAF3 pp 229533.I1RB50
98269 229533.I1RAF3 pp 229533.Q4IJH1
98270 229533.I1RAF3 pp 229533.I1RZQ5
98271 229533.I1RAF3 pp 229533.Q4HXF6
98272 229533.I1RAF3 pp 229533.I1RDG0
98273 229533.I1RAF3 pp 229533.I1RST1
98274 229533.I1RAF3 pp 229533.I1RFQ1
98275 229533.I1RAF3 pp 229533.I1RS28
98276 229533.I1RAF3 pp 229533.I1S1X9
98277 229533.I1RAF3 pp 229533.I1RRL0
98278 229533.I1RAF3 pp 229533.Q4IRI9
98279 229533.I1RAF3 pp 229533.I1RG90
98280 229533.I1RAF3 pp 229533.I1S0Z9
98281 229533.I1RAF3 pp 229533.Q4IPH4
98282 229533.I1RAF3 pp 229533.Q4IAA0
98283 229533.I1RAF3 pp 229533.I1S246
98284 229533.I1RAF3 pp 229533.I1RM25
98285 229533.I1RAF3 pp 229533.I1RND5
98286 229533.I1RAF3 pp 229533.I1RSA4
98287 229533.I1RAF3 pp 229533.Q4IBK5
98288 229533.I1RAF3 pp 229533.I1RK90
98289 229533.I1RAF3 pp 229533.I1RG57
98290 229533.I1RAF3 pp 229533.I1RCS2
98291 229533.I1RAF3 pp 229533.Q4I1Y1
98292 229533.I1RAF3 pp 229533.V6QXG1
98293 229533.I1RAF3 pp 229533.I1RCA7
98294 229533.I1RAF3 pp 229533.I1RFG0
98295 229533.I1RAF3 pp 229533.Q4IPB8
98296 229533.I1RAF3 pp 229533.Q4IB70
98297 229533.I1RAF3 pp 229533.I1RQC5
98298 229533.I1RAF3 pp 229533.I1RD56
98299 229533.I1RAF3 pp 229533.I1RCN5
98300 229533.I1RAF3 pp 229533.Q4IE79
98301 229533.I1RAF3 pp 229533.I1S9L0
98302 229533.I1RAF3 pp 229533.I1RLU5
98303 229533.I1S7Q8 pp 229533.Q4I8X0
98304 229533.I1S7Q8 pp 229533.V6RPN0
98305 229533.I1RC02 pp 229533.I1S0H1
98306 229533.I1RC02 pp 229533.I1S8W6
98307 229533.I1RC02 pp 229533.I1RT72
98308 229533.I1RC02 pp 229533.I1RZF5
98309 229533.I1RC02 pp 229533.I1RW54
98310 229533.A0A0E0RVH6 pp 229533.I1RVY0
98311 229533.A0A0E0RVH6 pp 229533.I1RFS3
98312 229533.A0A0E0RVH6 pp 229533.I1S5R5
98313 229533.A0A0E0RVH6 pp 229533.I1RNG4
98314 229533.A0A0E0RVH6 pp 229533.I1RBL7
98315 229533.A0A0E0RVH6 pp 229533.A0A1C3YMS7
98316 229533.A0A0E0RVH6 pp 229533.Q4I8X0
98317 229533.A0A0E0RVH6 pp 229533.V6RPN0
98318 229533.I1RZQ5 pp 229533.I1S0B1
98319 229533.I1RZQ5 pp 229533.I1S0Z9
98320 229533.I1RZQ5 pp 229533.I1S9L5
98321 229533.I1RZQ5 pp 229533.I1S9L0
98322 229533.I1RZQ5 pp 229533.Q4I1Y1
98323 229533.I1RZQ5 pp 229533.V6QXG1
98324 229533.I1RZQ5 pp 229533.I1S4M5
98325 229533.I1RZQ5 pp 229533.Q4IRI9
98326 229533.I1RZQ5 pp 229533.Q4IAA0
98327 229533.I1RZQ5 pp 229533.I1S471
98328 229533.I1RZQ5 pp 229533.I1S7M2
98329 229533.I1RZQ5 pp 229533.Q4IPB3
98330 229533.I1RZQ5 pp 229533.I1S2Y5
98331 229533.I1RZQ5 pp 229533.I1S7M3

98332 229533.I1RZQ5 pp 229533.I1S9L6
98333 229533.I1RAU6 pp 229533.I1RB41
98334 229533.I1RAU6 pp 229533.I1RY44
98335 229533.I1RAU6 pp 229533.I1S2E9
98336 229533.I1RAU6 pp 229533.I1RV91
98337 229533.I1RAU6 pp 229533.I1RCA7
98338 229533.I1RAU6 pp 229533.I1RFQ1
98339 229533.I1RAU6 pp 229533.I1S0J7
98340 229533.I1RAU6 pp 229533.I1RZ63
98341 229533.I1RAU6 pp 229533.I1RJZ7
98342 229533.I1RAU6 pp 229533.I1S091
98343 229533.I1RAU6 pp 229533.I1RJK7
98344 229533.I1RAU6 pp 229533.I1RWT6
98345 229533.I1RAU6 pp 229533.I1S246
98346 229533.I1RAU6 pp 229533.I1RQA3
98347 229533.I1RAU6 pp 229533.I1RW71
98348 229533.I1RAU6 pp 229533.I1RC91
98349 229533.I1RMW6 pp 229533.I1RW54
98350 229533.I1RMW6 pp 229533.I1RT72
98351 229533.I1RMW6 pp 229533.I1RRJ7
98352 229533.I1RMW6 pp 229533.I1RZF5
98353 229533.I1RMW6 pp 229533.I1RWN8
98354 229533.A0A0E0RWU6 pp 229533.I1RLM3
98355 229533.A0A0E0RWU6 pp 229533.I1RJU9
98356 229533.A0A0E0RWU6 pp 229533.V6RDP1
98357 229533.A0A0E0RWU6 pp 229533.A0A0E0SP71
98358 229533.A0A0E0RWU6 pp 229533.A0A1C3YMN2
98359 229533.A0A0E0RWU6 pp 229533.I1RGI5
98360 229533.A0A0E0RWU6 pp 229533.I1RXN6
98361 229533.A0A1C3YHC6 pp 229533.I1RBL7
98362 229533.A0A1C3YHC6 pp 229533.V6RPN0
98363 229533.A0A1C3YHC6 pp 229533.Q4I8X0
98364 229533.A0A1C3YHC6 pp 229533.A0A1C3YMS7
98365 229533.A0A1C3YHC6 pp 229533.I1S5R5
98366 229533.A0A1C3YHC6 pp 229533.I1RVY0
98367 229533.A0A1C3YHC6 pp 229533.I1RFS3
98368 229533.A0A1C3YHC6 pp 229533.I1RNG4
98369 229533.I1RDE6 pp 229533.I1RVP5
98370 229533.I1RDE6 pp 229533.I1RN84
98371 229533.I1RDE6 pp 229533.I1RND2
98372 229533.I1RDE6 pp 229533.I1RSU2
98373 229533.I1RDE6 pp 229533.V6RN69
98374 229533.I1RDE6 pp 229533.I1RHG5
98375 229533.I1RDE6 pp 229533.I1S256
98376 229533.I1RDE6 pp 229533.I1RJ14
98377 229533.I1RDE6 pp 229533.I1RJY7
98378 229533.I1RDE6 pp 229533.I1RWF5
98379 229533.I1RDE6 pp 229533.I1RWL8
98380 229533.I1RDE6 pp 229533.I1RKB2
98381 229533.I1RDE6 pp 229533.I1RY12
98382 229533.I1RDE6 pp 229533.I1RYX6
98383 229533.I1RDE6 pp 229533.I1RMJ3
98384 229533.I1RDE6 pp 229533.I1RER6
98385 229533.I1RDE6 pp 229533.I1RWL4
98386 229533.I1RDE6 pp 229533.V6RM02
98387 229533.I1RDE6 pp 229533.I1S8C1
98388 229533.I1RDE6 pp 229533.I1RVZ9
98389 229533.I1RDE6 pp 229533.I1RY62
98390 229533.I1RDE6 pp 229533.I1RN88
98391 229533.I1RDE6 pp 229533.I1RGZ3
98392 229533.I1RDE6 pp 229533.I1RUR8
98393 229533.I1RDE6 pp 229533.I1RGC3
98394 229533.I1RDE6 pp 229533.I1RFH9
98395 229533.I1RYU6 pp 229533.I1S223
98396 229533.I1RYU6 pp 229533.I1S969
98397 229533.I1RYU6 pp 229533.V6RCC0
98398 229533.I1RYU6 pp 229533.I1RZ38
98399 229533.I1RYU6 pp 229533.I1S4T6
98400 229533.I1RYU6 pp 229533.I1S8D2
98401 229533.I1RYU6 pp 229533.I1S5X9
98402 229533.I1RYU6 pp 229533.I1S968
98403 229533.I1RYU6 pp 229533.I1S9S4
98404 229533.I1RYU6 pp 229533.V6RQR4

98405 229533.I1RYU6 pp 229533.I1S5D0
98406 229533.I1RYU6 pp 229533.I1S2F7
98407 229533.I1RYU6 pp 229533.V6R5T7
98408 229533.I1RYU6 pp 229533.I1S3T7
98409 229533.I1RYU6 pp 229533.V6RWB3
98410 229533.I1RYU6 pp 229533.I1S210
98411 229533.I1RYU6 pp 229533.I1SAB4
98412 229533.I1RYU6 pp 229533.I1S8D3
98413 229533.I1RYU6 pp 229533.V6RHX2
98414 229533.I1RYU6 pp 229533.V6RCJ5
98415 229533.I1RYU6 pp 229533.I1S0K2
98416 229533.I1RYU6 pp 229533.I1RZ15
98417 229533.I1RYU6 pp 229533.I1S6V0
98418 229533.I1RYU6 pp 229533.I1S659
98419 229533.I1RYU6 pp 229533.I1SAT2
98420 229533.I1RYU6 pp 229533.I1S3T6
98421 229533.I1RYU6 pp 229533.I1S267
98422 229533.A0A1C3YJG5 pp 229533.A0A1C3YK06
98423 229533.A0A1C3YJG5 pp 229533.A0A1C3YJS7
98424 229533.A0A1C3YJG5 pp 229533.I1RW54
98425 229533.A0A1C3YJG5 pp 229533.A0A1C3YJK8
98426 229533.A0A1C3YJG5 pp 229533.I1RT72
98427 229533.A0A1C3YJG5 pp 229533.I1RZF5
98428 229533.A0A0E0RWU5 pp 229533.I1RVB6
98429 229533.A0A0E0RWU5 pp 229533.I1REM2
98430 229533.A0A0E0RWU5 pp 229533.A0A0E0S6U6
98431 229533.I1RS28 pp 229533.I1S524
98432 229533.I1RS28 pp 229533.Q4IPB3
98433 229533.I1RS28 pp 229533.I1RZG7
98434 229533.I1RS28 pp 229533.I1RZQ5
98435 229533.I1RS28 pp 229533.I1RX81
98436 229533.I1RS28 pp 229533.I1S4M5
98437 229533.I1RS28 pp 229533.Q4HXF6
98438 229533.I1RS28 pp 229533.I1S1X9
98439 229533.I1RS28 pp 229533.Q4I1Y1
98440 229533.I1RS28 pp 229533.V6QX58
98441 229533.I1RS28 pp 229533.I1RX24
98442 229533.I1RS28 pp 229533.I1RSA4
98443 229533.I1RS28 pp 229533.I1RST1
98444 229533.I1RS28 pp 229533.V6QXG1
98445 229533.I1RS28 pp 229533.Q4IB70
98446 229533.I1RS28 pp 229533.I1RYB1
98447 229533.I1RS28 pp 229533.I1RWX8
98448 229533.I1RS28 pp 229533.I1RT91
98449 229533.I1RS28 pp 229533.I1RT35
98450 229533.I1RS28 pp 229533.I1S9L0
98451 229533.I1RS28 pp 229533.I1RX51
98452 229533.I1RS28 pp 229533.I1S0B1
98453 229533.I1RS28 pp 229533.I1RS75
98454 229533.I1RS28 pp 229533.I1RT95
98455 229533.I1RS28 pp 229533.I1S2Y5
98456 229533.I1RS28 pp 229533.I1S0Z9
98457 229533.I1RS28 pp 229533.Q4IPH4
98458 229533.I1RS28 pp 229533.I1RSD4
98459 229533.I1RS28 pp 229533.Q4IBK5
98460 229533.I1RS28 pp 229533.I1RVK5
98461 229533.I1RS28 pp 229533.I1S9L6
98462 229533.I1RS28 pp 229533.I1RU69
98463 229533.I1RS28 pp 229533.Q4IAA0
98464 229533.I1RS28 pp 229533.Q4IPB8
98465 229533.I1RS28 pp 229533.I1RYW3
98466 229533.I1RS28 pp 229533.I1S7M3
98467 229533.I1RS28 pp 229533.I1S9L5
98468 229533.I1RS28 pp 229533.I1RX47
98469 229533.I1RS28 pp 229533.Q4IRI9
98470 229533.I1RS28 pp 229533.I1RT47
98471 229533.I1RS28 pp 229533.Q4IE79
98472 229533.I1RS28 pp 229533.I1RYI9
98473 229533.I1RS28 pp 229533.I1RSA8
98474 229533.I1RS28 pp 229533.I1RWU2
98475 229533.I1RS28 pp 229533.V6RIS2
98476 229533.I1RS28 pp 229533.I1S7M2
98477 229533.I1RS28 pp 229533.I1RS83

98478 229533.I1RS28 pp 229533.I1S5S2
98479 229533.I1RS28 pp 229533.I1S471
98480 229533.I1RS28 pp 229533.I1RXF2
98481 229533.I1RS28 pp 229533.I1RZ11
98482 229533.I1RS28 pp 229533.I1RT17
98483 229533.I1RS28 pp 229533.I1S1C6
98484 229533.I1RS28 pp 229533.I1RSL9
98485 229533.I1RS28 pp 229533.I1S056
98486 229533.I1RS28 pp 229533.Q4I5R9
98487 229533.A0A1C3YLK5 pp 229533.I1RRQ0
98488 229533.A0A1C3YLK5 pp 229533.I1REG5
98489 229533.A0A1C3YLK5 pp 229533.I1RFK0
98490 229533.A0A1C3YLK5 pp 229533.I1RWT6
98491 229533.A0A1C3YLK5 pp 229533.I1S0Z3
98492 229533.A0A1C3YLK5 pp 229533.I1RCM6
98493 229533.A0A1C3YLK5 pp 229533.Q4HTT2
98494 229533.A0A1C3YLK5 pp 229533.I1RQA3
98495 229533.A0A1C3YLK5 pp 229533.I1RQJ0
98496 229533.A0A1C3YLK5 pp 229533.I1RRB0
98497 229533.A0A1C3YLK5 pp 229533.I1RMG2
98498 229533.A0A1C3YLK5 pp 229533.I1RXB5
98499 229533.A0A1C3YLK5 pp 229533.I1RJH5
98500 229533.A0A1C3YLK5 pp 229533.I1RT65
98501 229533.A0A1C3YLK5 pp 229533.I1RUK7
98502 229533.A0A1C3YLK5 pp 229533.I1RIG4
98503 229533.A0A1C3YLK5 pp 229533.I1S1C8
98504 229533.A0A1C3YLK5 pp 229533.Q4I877
98505 229533.A0A1C3YLK5 pp 229533.I1RWD5
98506 229533.A0A1C3YLK5 pp 229533.I1RRF9
98507 229533.A0A1C3YLK5 pp 229533.I1S096
98508 229533.A0A1C3YLK5 pp 229533.A0A1C3YLL9
98509 229533.A0A1C3YLK5 pp 229533.I1RA72
98510 229533.A0A1C3YLK5 pp 229533.I1RR74
98511 229533.A0A1C3YLK5 pp 229533.I1REU2
98512 229533.A0A1C3YLK5 pp 229533.I1RN31
98513 229533.A0A1C3YLK5 pp 229533.I1RQ70
98514 229533.A0A1C3YLK5 pp 229533.A0A1C3YN58
98515 229533.A0A1C3YLK5 pp 229533.I1RNM1
98516 229533.A0A1C3YLK5 pp 229533.I1S270
98517 229533.A0A1C3YLK5 pp 229533.I1RZU6
98518 229533.A0A1C3YLK5 pp 229533.I1RB00
98519 229533.A0A1C3YLK5 pp 229533.I1RYU3
98520 229533.A0A1C3YLK5 pp 229533.I1RT41
98521 229533.A0A1C3YLK5 pp 229533.I1RY40
98522 229533.A0A1C3YLK5 pp 229533.Q4INI2
98523 229533.A0A1C3YLK5 pp 229533.Q4HZS8
98524 229533.A0A1C3YLK5 pp 229533.Q5I2J3
98525 229533.A0A1C3YLK5 pp 229533.I1S5K9
98526 229533.A0A1C3YLK5 pp 229533.I1RX87
98527 229533.A0A1C3YLK5 pp 229533.I1RRT4
98528 229533.A0A1C3YLK5 pp 229533.Q4IN00
98529 229533.A0A1C3YLK5 pp 229533.I1RI75
98530 229533.A0A1C3YLK5 pp 229533.I1RBS4
98531 229533.A0A1C3YLK5 pp 229533.I1RQ80
98532 229533.A0A1C3YLK5 pp 229533.I1RLX6
98533 229533.A0A1C3YLK5 pp 229533.Q4I2D7
98534 229533.A0A1C3YLK5 pp 229533.I1RD22
98535 229533.A0A1C3YLK5 pp 229533.I1RST4
98536 229533.A0A1C3YLK5 pp 229533.I1RPG2
98537 229533.A0A1C3YLK5 pp 229533.I1RRH3
98538 229533.A0A1C3YLK5 pp 229533.Q4INZ9
98539 229533.A0A1C3YLK5 pp 229533.I1RZ84
98540 229533.A0A1C3YLK5 pp 229533.I1RRF0
98541 229533.A0A1C3YLK5 pp 229533.Q4ICM0
98542 229533.A0A1C3YLK5 pp 229533.I1REC6
98543 229533.A0A1C3YLK5 pp 229533.I1RMF2
98544 229533.A0A1C3YLK5 pp 229533.I1RBX2
98545 229533.A0A1C3YLK5 pp 229533.I1RND7
98546 229533.A0A1C3YLK5 pp 229533.I1S0S8
98547 229533.A0A1C3YLK5 pp 229533.Q4HXT6
98548 229533.A0A1C3YLK5 pp 229533.I1RAM1
98549 229533.A0A1C3YLK5 pp 229533.I1RUJ0
98550 229533.A0A1C3YLK5 pp 229533.I1RD70

98551 229533.A0A1C3YLK5 pp 229533.I1RTG5
98552 229533.A0A1C3YLK5 pp 229533.I1REC7
98553 229533.A0A1C3YLK5 pp 229533.I1RQC0
98554 229533.A0A1C3YLK5 pp 229533.I1RTI7
98555 229533.A0A1C3YLK5 pp 229533.Q4IPI4
98556 229533.A0A1C3YLK5 pp 229533.I1RKR9
98557 229533.A0A1C3YLK5 pp 229533.I1RTA3
98558 229533.A0A1C3YLK5 pp 229533.I1RDE9
98559 229533.A0A1C3YLK5 pp 229533.I1RW16
98560 229533.A0A1C3YLK5 pp 229533.I1RY44
98561 229533.A0A1C3YLK5 pp 229533.I1RGC4
98562 229533.A0A1C3YLK5 pp 229533.I1RFZ2
98563 229533.A0A1C3YLK5 pp 229533.V6QX58
98564 229533.A0A1C3YLK5 pp 229533.I1RIU0
98565 229533.A0A1C3YLK5 pp 229533.I1RZ63
98566 229533.A0A1C3YLK5 pp 229533.I1RKI1
98567 229533.A0A1C3YLK5 pp 229533.I1RMP2
98568 229533.A0A1C3YLK5 pp 229533.I1RYM4
98569 229533.A0A1C3YLK5 pp 229533.A0A1C3YM61
98570 229533.A0A1C3YLK5 pp 229533.Q4HZB8
98571 229533.A0A1C3YLK5 pp 229533.I1RX54
98572 229533.A0A1C3YLK5 pp 229533.I1RB35
98573 229533.A0A1C3YLK5 pp 229533.I1RXT9
98574 229533.A0A1C3YLK5 pp 229533.I1RJQ5
98575 229533.A0A1C3YLK5 pp 229533.I1S2E9
98576 229533.A0A1C3YLK5 pp 229533.I1RP92
98577 229533.A0A1C3YLK5 pp 229533.I1RCF0
98578 229533.A0A1C3YLK5 pp 229533.I1RAZ9
98579 229533.A0A1C3YLK5 pp 229533.I1RR95
98580 229533.A0A1C3YLK5 pp 229533.I1RD02
98581 229533.A0A1C3YLK5 pp 229533.I1RE19
98582 229533.A0A1C3YLK5 pp 229533.I1RSM8
98583 229533.I1RBG6 pp 229533.I1RTG3
98584 229533.I1RBG6 pp 229533.I1RCD9
98585 229533.A0A098D1J5 pp 229533.I1S267
98586 229533.A0A098D1J5 pp 229533.I1RE52
98587 229533.A0A098D1J5 pp 229533.Q4I8X0
98588 229533.A0A098D1J5 pp 229533.A0A0E0S530
98589 229533.A0A098D1J5 pp 229533.A0A1C3YMK4
98590 229533.A0A098D1J5 pp 229533.I1S6S6
98591 229533.A0A098D1J5 pp 229533.I1RH48
98592 229533.A0A098D1J5 pp 229533.I1RY21
98593 229533.A0A098D1J5 pp 229533.I1RIQ7
98594 229533.A0A098D1J5 pp 229533.V6R5T7
98595 229533.A0A098D1J5 pp 229533.I1REV3
98596 229533.A0A098D1J5 pp 229533.I1S6V0
98597 229533.A0A098D1J5 pp 229533.I1RB05
98598 229533.A0A098D1J5 pp 229533.V6RWB3
98599 229533.A0A098D1J5 pp 229533.A0A1C3YMW5
98600 229533.A0A098D1J5 pp 229533.I1RY81
98601 229533.A0A098D1J5 pp 229533.I1RPY0
98602 229533.A0A098D1J5 pp 229533.I1RMA2
98603 229533.A0A098D1J5 pp 229533.I1RQV7
98604 229533.A0A098D1J5 pp 229533.I1RZX3
98605 229533.A0A098D1J5 pp 229533.I1RST9
98606 229533.A0A098D1J5 pp 229533.I1RIJ6
98607 229533.A0A098D1J5 pp 229533.V6RCC0
98608 229533.A0A098D1J5 pp 229533.I1S8D3
98609 229533.A0A098D1J5 pp 229533.A0A1C3YMK2
98610 229533.A0A098D1J5 pp 229533.I1S7Q8
98611 229533.A0A098D1J5 pp 229533.A0A0E0RVH6
98612 229533.A0A098D1J5 pp 229533.I1RAU6
98613 229533.A0A098D1J5 pp 229533.A0A1C3YHC6
98614 229533.A0A098D1J5 pp 229533.I1RYU6
98615 229533.A0A098D1J5 pp 229533.A0A1C3YLK5
98616 229533.I1RZF5 pp 229533.I1S0I9
98617 229533.I1RZF5 pp 229533.I1S1Y7
98618 229533.I1RZF5 pp 229533.I1S437
98619 229533.I1RZF5 pp 229533.I1S438
98620 229533.I1RPY4 pp 229533.I1RX24
98621 229533.I1RPY4 pp 229533.Q4IPB3
98622 229533.I1RPY4 pp 229533.I1RQC5
98623 229533.I1RPY4 pp 229533.I1RX81

98624	229533.I1RPY4	pp	229533.I1RST1
98625	229533.I1RPY4	pp	229533.I1RU69
98626	229533.I1RPY4	pp	229533.I1RXF2
98627	229533.I1RPY4	pp	229533.I1S9L5
98628	229533.I1RPY4	pp	229533.I1RZG7
98629	229533.I1RPY4	pp	229533.I1S5S2
98630	229533.I1RPY4	pp	229533.I1RRL0
98631	229533.I1RPY4	pp	229533.I1S7M2
98632	229533.I1RPY4	pp	229533.I1S4M5
98633	229533.I1RPY4	pp	229533.V6RY33
98634	229533.I1RPY4	pp	229533.I1RWU2
98635	229533.I1RPY4	pp	229533.I1RT17
98636	229533.I1RPY4	pp	229533.I1RPZ3
98637	229533.I1RPY4	pp	229533.I1RS75
98638	229533.I1RPY4	pp	229533.I1S1C6
98639	229533.I1RPY4	pp	229533.Q4IPB8
98640	229533.I1RPY4	pp	229533.I1RYI9
98641	229533.I1RPY4	pp	229533.Q4IBK5
98642	229533.I1RPY4	pp	229533.I1RSA4
98643	229533.I1RPY4	pp	229533.Q4ICG8
98644	229533.I1RPY4	pp	229533.I1RT35
98645	229533.I1RPY4	pp	229533.I1RRW3
98646	229533.I1RPY4	pp	229533.I1RWX8
98647	229533.I1RPY4	pp	229533.I1S9L6
98648	229533.I1RPY4	pp	229533.Q4HXF6
98649	229533.I1RPY4	pp	229533.V6QXG1
98650	229533.I1RPY4	pp	229533.Q4I5R9
98651	229533.I1RPY4	pp	229533.I1S8F8
98652	229533.I1RPY4	pp	229533.I1S0B1
98653	229533.I1RPY4	pp	229533.I1RX51
98654	229533.I1RPY4	pp	229533.I1S1X9
98655	229533.I1RPY4	pp	229533.I1S0Z9
98656	229533.I1RPY4	pp	229533.I1S7M3
98657	229533.I1RPY4	pp	229533.Q4I1Y1
98658	229533.I1RPY4	pp	229533.I1RQR3
98659	229533.I1RPY4	pp	229533.I1RT91
98660	229533.I1RPY4	pp	229533.I1RQP8
98661	229533.I1RPY4	pp	229533.Q4IE79
98662	229533.I1RPY4	pp	229533.Q4I7F9
98663	229533.I1RPY4	pp	229533.I1RX47
98664	229533.I1RPY4	pp	229533.I1RSA8
98665	229533.I1RPY4	pp	229533.I1RZQ5
98666	229533.I1RPY4	pp	229533.I1RYW3
98667	229533.I1RPY4	pp	229533.Q4IB70
98668	229533.I1RPY4	pp	229533.Q4IPH4
98669	229533.I1RPY4	pp	229533.Q4IP34
98670	229533.I1RPY4	pp	229533.Q4IAA0
98671	229533.I1RPY4	pp	229533.V6RIS2
98672	229533.I1RPY4	pp	229533.I1RS28
98673	229533.I1RPY4	pp	229533.I1S471
98674	229533.I1RPY4	pp	229533.Q4IRI9
98675	229533.I1RPY4	pp	229533.I1S064
98676	229533.I1RPY4	pp	229533.I1S1C8
98677	229533.I1RPY4	pp	229533.Q4I0M9
98678	229533.I1RPY4	pp	229533.I1RSD4
98679	229533.I1RPY4	pp	229533.I1RZ11
98680	229533.I1RPY4	pp	229533.I1RT47
98681	229533.I1RPY4	pp	229533.I1RQD8
98682	229533.I1RCP3	pp	229533.I1RYL5
98683	229533.I1RCP3	pp	229533.I1RZ35
98684	229533.I1RCP3	pp	229533.I1RLI4
98685	229533.I1RCP3	pp	229533.I1RJM9
98686	229533.I1RCP3	pp	229533.I1RXN7
98687	229533.I1RCP3	pp	229533.I1S4A5
98688	229533.I1RCP3	pp	229533.I1S3H6
98689	229533.I1RCP3	pp	229533.I1RMG1
98690	229533.I1RCP3	pp	229533.I1RZA9
98691	229533.I1RCP3	pp	229533.I1RQL1
98692	229533.I1RCP3	pp	229533.I1RDP6
98693	229533.I1RCP3	pp	229533.I1RQN9
98694	229533.I1RCP3	pp	229533.I1S325
98695	229533.I1RCP3	pp	229533.I1RX41
98696	229533.I1RCP3	pp	229533.I1RM78

98697 229533.I1RCP3 pp 229533.I1RY71
98698 229533.I1RCP3 pp 229533.I1RJX2
98699 229533.I1RCP3 pp 229533.I1RN54
98700 229533.I1RCP3 pp 229533.I1RKJ7
98701 229533.A0A098DBA1 pp 229533.I1S1U6
98702 229533.A0A098DBA1 pp 229533.I1S1C8
98703 229533.A0A098DBA1 pp 229533.A0A1C3YN54
98704 229533.A0A098DBA1 pp 229533.I1RX87
98705 229533.A0A098DBA1 pp 229533.I1RTG5
98706 229533.A0A098DBA1 pp 229533.A0A1C3YLK5
98707 229533.A0A098DBA1 pp 229533.I1RTQ9
98708 229533.A0A098DBA1 pp 229533.A0A1C3YK06
98709 229533.A0A098DBA1 pp 229533.I1RBT0
98710 229533.A0A098DBA1 pp 229533.A0A1C3YI44
98711 229533.A0A098DBA1 pp 229533.A0A0E0RVD7
98712 229533.A0A098DBA1 pp 229533.A0A1C3YIP6
98713 229533.V6R0U9 pp 229533.V6REZ4
98714 229533.Q4IRI9 pp 229533.V6QXG1
98715 229533.A0A098E488 pp 229533.A0A0E0RVD7
98716 229533.A0A098E488 pp 229533.A0A0E0SH87
98717 229533.A0A098E488 pp 229533.A0A1C3YK06
98718 229533.A0A098E488 pp 229533.I1R9V4
98719 229533.A0A098E488 pp 229533.A0A0E0SJV3
98720 229533.A0A098E488 pp 229533.A0A1C3YJK8
98721 229533.A0A098E488 pp 229533.I1RWH6
98722 229533.A0A098E488 pp 229533.I1RZF5
98723 229533.A0A098E488 pp 229533.A0A1C3YIU2
98724 229533.A0A098E488 pp 229533.I1RJH7
98725 229533.A0A098E488 pp 229533.A0A1C3YMH5
98726 229533.A0A098E488 pp 229533.A0A1C3YJS7
98727 229533.A0A098E488 pp 229533.A0A1C3YKJ7
98728 229533.A0A098E488 pp 229533.I1RW54
98729 229533.A0A098E488 pp 229533.I1RT72
98730 229533.A0A098E488 pp 229533.A0A1C3YID5
98731 229533.A0A098E488 pp 229533.I1RJU7
98732 229533.I1RSE9 pp 229533.I1RZ60
98733 229533.I1RSE9 pp 229533.I1S251
98734 229533.A0A0E0S621 pp 229533.V6RGN5
98735 229533.A0A0E0S621 pp 229533.I1RW99
98736 229533.A0A0E0S621 pp 229533.V6QUE6
98737 229533.A0A0E0S621 pp 229533.I1RMN6
98738 229533.A0A0E0S621 pp 229533.Q4IBS2
98739 229533.A0A0E0S621 pp 229533.I1RX08
98740 229533.A0A0E0S621 pp 229533.I1RSI6
98741 229533.A0A0E0S621 pp 229533.I1RQ31
98742 229533.A0A0E0S621 pp 229533.A0A1C3YJG0
98743 229533.A0A0E0S621 pp 229533.I1RMC0
98744 229533.A0A0E0S621 pp 229533.Q4IN63
98745 229533.A0A0E0S621 pp 229533.I1RNY2
98746 229533.A0A0E0S621 pp 229533.Q4I327
98747 229533.A0A0E0S621 pp 229533.I1RMN7
98748 229533.A0A0E0S621 pp 229533.I1S021
98749 229533.A0A0E0S621 pp 229533.I1RNH6
98750 229533.A0A0E0S621 pp 229533.I1RZF6
98751 229533.A0A0E0S621 pp 229533.A0A1C3YJR3
98752 229533.A0A0E0S621 pp 229533.I1S9U1
98753 229533.A0A0E0S621 pp 229533.I1R9X7
98754 229533.A0A0E0S621 pp 229533.I1RX14
98755 229533.A0A0E0S621 pp 229533.V6R227
98756 229533.A0A0E0S621 pp 229533.I1S060
98757 229533.A0A0E0S621 pp 229533.I1RRR7
98758 229533.A0A0E0S621 pp 229533.A0A1C3YKB0
98759 229533.A0A0E0S621 pp 229533.I1RCN4
98760 229533.A0A0E0S621 pp 229533.A0A1C3YKW9
98761 229533.A0A0E0S621 pp 229533.I1RE01
98762 229533.A0A0E0S621 pp 229533.I1RC72
98763 229533.A0A0E0S621 pp 229533.I1S0J1
98764 229533.A0A0E0S621 pp 229533.I1S4T3
98765 229533.A0A0E0S621 pp 229533.I1RBI8
98766 229533.A0A0E0S621 pp 229533.I1RBL7
98767 229533.A0A0E0S621 pp 229533.I1RPY1
98768 229533.A0A0E0S621 pp 229533.I1S274
98769 229533.A0A0E0S621 pp 229533.I1RG56

98770	229533.A0A0E0S621	pp	229533.A0A1C3YK36
98771	229533.A0A0E0S621	pp	229533.I1RSM3
98772	229533.A0A0E0S621	pp	229533.I1RC33
98773	229533.A0A0E0S621	pp	229533.I1RSR3
98774	229533.A0A0E0S621	pp	229533.Q4IEK8
98775	229533.A0A0E0S621	pp	229533.V6RGY3
98776	229533.A0A0E0S621	pp	229533.I1RM96
98777	229533.A0A0E0S621	pp	229533.I1S1U7
98778	229533.A0A0E0S621	pp	229533.I1RC27
98779	229533.A0A0E0S621	pp	229533.A0A1C3YL07
98780	229533.A0A0E0S621	pp	229533.I1S0M9
98781	229533.A0A0E0S621	pp	229533.I1RVZ0
98782	229533.A0A0E0S621	pp	229533.I1RNK2
98783	229533.A0A0E0S621	pp	229533.I1RQA2
98784	229533.A0A0E0S621	pp	229533.I1RCN5
98785	229533.A0A0E0S621	pp	229533.Q4IJ56
98786	229533.A0A0E0S621	pp	229533.I1RCL3
98787	229533.A0A0E0S621	pp	229533.I1S2C0
98788	229533.A0A0E0S621	pp	229533.I1RPJ3
98789	229533.A0A0E0S621	pp	229533.I1RAZ8
98790	229533.A0A0E0S621	pp	229533.I1RKC6
98791	229533.A0A0E0S621	pp	229533.I1RNU6
98792	229533.A0A0E0S621	pp	229533.I1RZE5
98793	229533.A0A0E0S621	pp	229533.I1RTE9
98794	229533.A0A0E0S621	pp	229533.I1RMD5
98795	229533.A0A0E0S621	pp	229533.Q4IAA0
98796	229533.A0A0E0S621	pp	229533.I1RNH9
98797	229533.A0A0E0S621	pp	229533.I1RTD9
98798	229533.A0A0E0S621	pp	229533.I1RF19
98799	229533.A0A0E0S621	pp	229533.I1RB27
98800	229533.A0A0E0S621	pp	229533.I1S0M3
98801	229533.A0A0E0S621	pp	229533.I1RSR8
98802	229533.A0A0E0S621	pp	229533.I1RUQ2
98803	229533.A0A0E0S621	pp	229533.I1RRI2
98804	229533.A0A0E0S621	pp	229533.I1RFJ9
98805	229533.A0A0E0S621	pp	229533.A0A1C3YIJ3
98806	229533.A0A0E0S621	pp	229533.I1RE00
98807	229533.A0A0E0S621	pp	229533.Q4INZ9
98808	229533.A0A0E0S621	pp	229533.I1RY63
98809	229533.A0A0E0S621	pp	229533.A0A0E0SJN2
98810	229533.A0A0E0S621	pp	229533.I1RT98
98811	229533.A0A0E0S621	pp	229533.I1RC06
98812	229533.A0A0E0S621	pp	229533.I1RF08
98813	229533.A0A0E0S621	pp	229533.I1S1X9
98814	229533.A0A0E0S621	pp	229533.I1RAF8
98815	229533.A0A0E0S621	pp	229533.I1R9V7
98816	229533.A0A0E0S621	pp	229533.I1RA48
98817	229533.A0A0E0S621	pp	229533.Q4HZ68
98818	229533.A0A0E0S621	pp	229533.Q4IJP1
98819	229533.A0A0E0S621	pp	229533.I1S296
98820	229533.A0A0E0S621	pp	229533.I1RSS4
98821	229533.A0A0E0S621	pp	229533.I1RS47
98822	229533.A0A0E0S621	pp	229533.I1RSC6
98823	229533.A0A0E0S621	pp	229533.I1RD52
98824	229533.A0A0E0S621	pp	229533.A0A1C3YLK9
98825	229533.A0A0E0S621	pp	229533.A0A0E0S7B3
98826	229533.A0A0E0S621	pp	229533.I1RFW5
98827	229533.A0A0E0S621	pp	229533.I1RN99
98828	229533.A0A0E0S621	pp	229533.I1RRH5
98829	229533.A0A0E0S621	pp	229533.I1RDG0
98830	229533.A0A0E0S621	pp	229533.I1RQ28
98831	229533.A0A0E0S621	pp	229533.A0A0E0SGM4
98832	229533.A0A0E0S621	pp	229533.I1RGF6
98833	229533.A0A0E0S621	pp	229533.I1RT33
98834	229533.A0A0E0S621	pp	229533.I1RD64
98835	229533.A0A0E0S621	pp	229533.I1RPW9
98836	229533.A0A0E0S621	pp	229533.I1S9U2
98837	229533.A0A0E0S621	pp	229533.I1RK95
98838	229533.A0A0E0S621	pp	229533.I1RMQ0
98839	229533.A0A0E0S621	pp	229533.I1RSD8
98840	229533.A0A0E0S621	pp	229533.Q4HZ42
98841	229533.A0A0E0S621	pp	229533.I1S2U2
98842	229533.A0A0E0S621	pp	229533.I1RM82

98843	229533.A0A0E0S621	pp	229533.I1S9Y1
98844	229533.A0A0E0S621	pp	229533.I1RQ65
98845	229533.A0A0E0S621	pp	229533.I1RKJ6
98846	229533.A0A0E0S621	pp	229533.A0A1C3YKR0
98847	229533.A0A0E0S621	pp	229533.I1RZZ1
98848	229533.A0A0E0S621	pp	229533.Q4IEV5
98849	229533.A0A0E0S621	pp	229533.I1RJZ6
98850	229533.A0A0E0S621	pp	229533.I1RQE7
98851	229533.A0A0E0S621	pp	229533.I1RPU0
98852	229533.A0A0E0S621	pp	229533.I1S0D4
98853	229533.A0A0E0S621	pp	229533.I1RRT8
98854	229533.A0A0E0S621	pp	229533.I1S2E8
98855	229533.A0A0E0S621	pp	229533.I1RS30
98856	229533.A0A0E0S621	pp	229533.I1S1B6
98857	229533.A0A0E0S621	pp	229533.I1RM67
98858	229533.A0A0E0S621	pp	229533.I1RNV6
98859	229533.A0A0E0S621	pp	229533.I1RRP3
98860	229533.A0A0E0S621	pp	229533.A0A1C3YK72
98861	229533.A0A0E0S621	pp	229533.A0A1C3YIP5
98862	229533.A0A0E0S621	pp	229533.I1RT80
98863	229533.A0A0E0S621	pp	229533.A0A1C3YN63
98864	229533.A0A0E0S621	pp	229533.I1RC82
98865	229533.A0A0E0S621	pp	229533.I1RFN1
98866	229533.A0A0E0S621	pp	229533.I1RTF4
98867	229533.A0A0E0S621	pp	229533.I1RFG0
98868	229533.A0A0E0S621	pp	229533.Q4IJH1
98869	229533.A0A0E0S621	pp	229533.I1RBC7
98870	229533.A0A0E0S621	pp	229533.Q4IQW0
98871	229533.A0A0E0S621	pp	229533.I1RRQ3
98872	229533.A0A0E0S621	pp	229533.I1S6F0
98873	229533.A0A0E0S621	pp	229533.I1RJV4
98874	229533.A0A0E0S621	pp	229533.Q4IAS1
98875	229533.A0A0E0S621	pp	229533.I1RNH1
98876	229533.A0A0E0S621	pp	229533.I1RW71
98877	229533.A0A0E0S621	pp	229533.I1RLV9
98878	229533.A0A0E0S621	pp	229533.I1RBC9
98879	229533.A0A0E0S621	pp	229533.I1RKQ5
98880	229533.A0A0E0S621	pp	229533.I1S8E2
98881	229533.A0A0E0S621	pp	229533.I1RKL3
98882	229533.A0A0E0S621	pp	229533.A0A0E0S7W0
98883	229533.A0A0E0S621	pp	229533.I1RMK3
98884	229533.A0A0E0S621	pp	229533.I1RX46
98885	229533.A0A0E0S621	pp	229533.Q4I830
98886	229533.A0A0E0S621	pp	229533.I1RE87
98887	229533.A0A0E0S621	pp	229533.I1RRT2
98888	229533.A0A0E0S621	pp	229533.Q4HVW2
98889	229533.A0A0E0S621	pp	229533.I1RR00
98890	229533.A0A0E0S621	pp	229533.I1RBN1
98891	229533.A0A0E0S621	pp	229533.I1RWF7
98892	229533.A0A0E0S621	pp	229533.I1RDA0
98893	229533.A0A0E0S621	pp	229533.I1S6U8
98894	229533.A0A0E0S621	pp	229533.A0A0E0SPV0
98895	229533.A0A0E0S621	pp	229533.I1RQS5
98896	229533.A0A0E0S621	pp	229533.I1RZ99
98897	229533.A0A0E0S621	pp	229533.A0A1C3YMW0
98898	229533.A0A0E0S621	pp	229533.I1RQJ5
98899	229533.A0A0E0S621	pp	229533.A0A1C3YIN1
98900	229533.A0A0E0S621	pp	229533.Q4IPI1
98901	229533.A0A0E0S621	pp	229533.I1RBT1
98902	229533.A0A0E0S621	pp	229533.A0A0E0SFM9
98903	229533.A0A0E0S621	pp	229533.Q4IF76
98904	229533.A0A0E0S621	pp	229533.I1RRJ8
98905	229533.A0A0E0S621	pp	229533.I1RJL4
98906	229533.A0A0E0S621	pp	229533.I1RN63
98907	229533.A0A0E0S621	pp	229533.I1RT05
98908	229533.A0A0E0S621	pp	229533.A0A1C3YLF5
98909	229533.A0A0E0S621	pp	229533.I1S0R7
98910	229533.A0A0E0S621	pp	229533.I1RT04
98911	229533.A0A0E0S621	pp	229533.I1RQM6
98912	229533.A0A0E0S621	pp	229533.I1R9G0
98913	229533.A0A0E0S621	pp	229533.I1RC03
98914	229533.A0A0E0S621	pp	229533.A0A1C3YIS7
98915	229533.A0A0E0S621	pp	229533.I1RX24

98916 229533.A0A0E0S621 pp 229533.V6R283
98917 229533.A0A0E0S621 pp 229533.V6QYF0
98918 229533.A0A0E0S621 pp 229533.I1RBG9
98919 229533.A0A0E0S621 pp 229533.I1RL23
98920 229533.A0A0E0S621 pp 229533.Q4IFI0
98921 229533.A0A0E0S621 pp 229533.I1RNA0
98922 229533.A0A0E0S621 pp 229533.I1RC29
98923 229533.A0A0E0S621 pp 229533.I1RM06
98924 229533.A0A0E0S621 pp 229533.I1RZS6
98925 229533.A0A0E0S621 pp 229533.I1RN51
98926 229533.A0A0E0S621 pp 229533.I1RJZ7
98927 229533.A0A0E0S621 pp 229533.I1RAC2
98928 229533.A0A0E0S621 pp 229533.I1RW25
98929 229533.A0A0E0S621 pp 229533.I1RW61
98930 229533.A0A0E0S621 pp 229533.I1RC32
98931 229533.A0A0E0S621 pp 229533.I1RG51
98932 229533.A0A0E0S621 pp 229533.Q4I662
98933 229533.A0A0E0S621 pp 229533.I1RFS3
98934 229533.A0A098DK85 pp 229533.A0A1C3YLW9
98935 229533.A0A098DK85 pp 229533.A0A1C3YLT9
98936 229533.A0A098DK85 pp 229533.I1RHQ4
98937 229533.A0A0E0SNH8 pp 229533.A0A1C3YM63
98938 229533.A0A0E0SNH8 pp 229533.I1RX84
98939 229533.A0A0E0SNH8 pp 229533.A0A1C3YK51
98940 229533.A0A0E0SNH8 pp 229533.I1S4Y4
98941 229533.A0A0E0SNH8 pp 229533.I1RZX9
98942 229533.A0A0E0SNH8 pp 229533.I1RPD3
98943 229533.A0A0E0SNH8 pp 229533.I1RD01
98944 229533.A0A0E0SNH8 pp 229533.I1RE19
98945 229533.A0A0E0SNH8 pp 229533.I1RJS2
98946 229533.A0A0E0SNH8 pp 229533.I1RNF5
98947 229533.A0A0E0SNH8 pp 229533.I1RNF9
98948 229533.A0A0E0SNH8 pp 229533.I1RJ78
98949 229533.A0A0E0SNH8 pp 229533.I1RU89
98950 229533.A0A0E0SNH8 pp 229533.I1RXX1
98951 229533.A0A0E0SNH8 pp 229533.I1RCE7
98952 229533.A0A0E0SNH8 pp 229533.I1RCI5
98953 229533.A0A0E0SNH8 pp 229533.A0A1C3YN68
98954 229533.A0A0E0SNH8 pp 229533.I1RM77
98955 229533.A0A0E0SNH8 pp 229533.I1S7X9
98956 229533.A0A0E0SNH8 pp 229533.I1RYZ7
98957 229533.A0A0E0SNH8 pp 229533.A0A1C3YIN0
98958 229533.A0A0E0SNH8 pp 229533.I1RYW8
98959 229533.A0A0E0SNH8 pp 229533.I1RCN6
98960 229533.A0A0E0SNH8 pp 229533.I1RTM8
98961 229533.A0A0E0SNH8 pp 229533.I1RPM8
98962 229533.A0A0E0SNH8 pp 229533.I1RWV4
98963 229533.A0A0E0SNH8 pp 229533.A0A1C3YI05
98964 229533.A0A0E0SNH8 pp 229533.I1S549
98965 229533.A0A0E0SNH8 pp 229533.I1RE57
98966 229533.A0A0E0SNH8 pp 229533.I1RJS3
98967 229533.A0A0E0SNH8 pp 229533.A0A1C3YLS7
98968 229533.A0A0E0SNH8 pp 229533.I1RMP2
98969 229533.A0A0E0SNH8 pp 229533.I1RYT5
98970 229533.A0A0E0SNH8 pp 229533.Q4IJ79
98971 229533.A0A0E0SNH8 pp 229533.I1RE15
98972 229533.A0A0E0SNH8 pp 229533.A0A1C3YII1
98973 229533.A0A0E0SNH8 pp 229533.I1RZQ6
98974 229533.A0A0E0SNH8 pp 229533.Q4IN00
98975 229533.A0A0E0SNH8 pp 229533.I1RNR3
98976 229533.A0A0E0SNH8 pp 229533.A0A1C3YL06
98977 229533.A0A0E0SNH8 pp 229533.I1RII1
98978 229533.A0A0E0SNH8 pp 229533.I1RRH2
98979 229533.I1RBW6 pp 229533.I1RPI4
98980 229533.I1RBW6 pp 229533.I1S0Y2
98981 229533.I1RBW6 pp 229533.I1RLE2
98982 229533.I1RBW6 pp 229533.I1RP58
98983 229533.I1RBW6 pp 229533.I1RC95
98984 229533.I1RBW6 pp 229533.I1RJS0
98985 229533.I1RBW6 pp 229533.I1S5S8
98986 229533.I1RBW6 pp 229533.I1RUP5
98987 229533.I1RBW6 pp 229533.I1RCY0
98988 229533.I1RBW6 pp 229533.I1RZM1

98989 229533.I1S6J7 pp 229533.I1S7X9
98990 229533.I1RT17 pp 229533.I1RU69
98991 229533.I1RT17 pp 229533.Q4IJH1
98992 229533.I1RT17 pp 229533.Q4IF76
98993 229533.I1RT17 pp 229533.I1RX47
98994 229533.I1RT17 pp 229533.Q4IE79
98995 229533.I1RT17 pp 229533.I1RZG7
98996 229533.I1RT17 pp 229533.Q4IPB3
98997 229533.I1RT17 pp 229533.I1RZQ5
98998 229533.I1RT17 pp 229533.I1S056
98999 229533.I1RT17 pp 229533.I1S044
99000 229533.I1RT17 pp 229533.Q4IPH4
99001 229533.I1RT17 pp 229533.Q4IBK5
99002 229533.I1RT17 pp 229533.Q4IPB8
99003 229533.I1RT17 pp 229533.Q4IB70
99004 229533.I1RT17 pp 229533.I1S471
99005 229533.I1RT17 pp 229533.Q4IRI9
99006 229533.I1RT17 pp 229533.Q4I5R9
99007 229533.I1RT17 pp 229533.I1RT95
99008 229533.I1RT17 pp 229533.V6QXG1
99009 229533.I1RT17 pp 229533.I1S0Z9
99010 229533.I1RT17 pp 229533.Q4IAA0
99011 229533.I1RT17 pp 229533.Q4I1Y1
99012 229533.I1RT17 pp 229533.Q4HXF6
99013 229533.I1RT17 pp 229533.I1S2Y5
99014 229533.A0A098DTH7 pp 229533.I1RDZ3
99015 229533.A0A098DTH7 pp 229533.A0A0E0SCW3
99016 229533.A0A098DTH7 pp 229533.I1RIN3
99017 229533.A0A098DTH7 pp 229533.A0A1C3YHQ6
99018 229533.A0A098DTH7 pp 229533.V6R0U9
99019 229533.A0A098DTH7 pp 229533.I1REG4
99020 229533.A0A098DTH7 pp 229533.A0A0E0RS12
99021 229533.A0A098DTH7 pp 229533.I1RCP3
99022 229533.A0A098DTH7 pp 229533.A0A0E0SKT0
99023 229533.I1REU2 pp 229533.I1RTQ9
99024 229533.I1REU2 pp 229533.I1RTG5
99025 229533.I1REU2 pp 229533.I1RX87
99026 229533.I1REU2 pp 229533.I1S1U6
99027 229533.I1REU2 pp 229533.I1S1C8
99028 229533.A0A098D1G8 pp 229533.I1RCP3
99029 229533.A0A098D1G8 pp 229533.V6R0U9
99030 229533.A0A098D1G8 pp 229533.A0A0E0S621
99031 229533.A0A098D1G8 pp 229533.A0A0E0SNH8
99032 229533.A0A098D1G8 pp 229533.A0A098DTH7
99033 229533.A0A098D1G8 pp 229533.I1RM67
99034 229533.I1RD33 pp 229533.I1REG5
99035 229533.I1RD33 pp 229533.I1RFL7
99036 229533.I1RD33 pp 229533.I1RKF7
99037 229533.I1RG61 pp 229533.I1RSG0
99038 229533.I1RG61 pp 229533.I1RQV8
99039 229533.I1RG61 pp 229533.I1RQ48
99040 229533.I1RG61 pp 229533.I1RZC3
99041 229533.I1RG61 pp 229533.I1S267
99042 229533.I1RG61 pp 229533.I1RM41
99043 229533.I1RG61 pp 229533.V6RHX2
99044 229533.I1S0E9 pp 229533.I1S1V1
99045 229533.I1RMR5 pp 229533.V6RR76
99046 229533.I1RMR5 pp 229533.I1RT41
99047 229533.I1RMR5 pp 229533.V6QUU6
99048 229533.I1RMR5 pp 229533.I1RN81
99049 229533.I1RMR5 pp 229533.I1S0G1
99050 229533.I1RMR5 pp 229533.I1S4X2
99051 229533.I1RMR5 pp 229533.I1RWI6
99052 229533.I1RMR5 pp 229533.I1S0S6
99053 229533.I1RMR5 pp 229533.I1RXT2
99054 229533.I1RMR5 pp 229533.I1RTB7
99055 229533.I1RMR5 pp 229533.Q4ILI9
99056 229533.I1RMR5 pp 229533.I1S1B1
99057 229533.I1RMR5 pp 229533.P0C431
99058 229533.I1RMR5 pp 229533.I1RYY6
99059 229533.I1RMR5 pp 229533.I1S254
99060 229533.I1RMR5 pp 229533.I1S095
99061 229533.I1RMR5 pp 229533.Q4I1H6

99062 229533.I1RMR5 pp 229533.I1RZD9
99063 229533.I1RMR5 pp 229533.I1S034
99064 229533.I1RMR5 pp 229533.I1RQN9
99065 229533.I1RMR5 pp 229533.I1RTG5
99066 229533.I1RMR5 pp 229533.I1RRN0
99067 229533.I1RMR5 pp 229533.V6R476
99068 229533.I1RMR5 pp 229533.I1RZH3
99069 229533.I1RMR5 pp 229533.I1S5R2
99070 229533.I1RMR5 pp 229533.V6RB81
99071 229533.I1RMR5 pp 229533.I1S0K9
99072 229533.I1RMR5 pp 229533.I1S8F9
99073 229533.I1RST1 pp 229533.I1RX24
99074 229533.I1RST1 pp 229533.I1RX81
99075 229533.I1RST1 pp 229533.I1S1X9
99076 229533.I1RST1 pp 229533.I1S0Y2
99077 229533.I1RST1 pp 229533.Q4HXF6
99078 229533.I1RST1 pp 229533.Q4I7F9
99079 229533.I1RST1 pp 229533.I1RZG7
99080 229533.I1RST1 pp 229533.I1RZQ5
99081 229533.I1RST1 pp 229533.Q4IPH4
99082 229533.I1RST1 pp 229533.I1RSW2
99083 229533.I1RST1 pp 229533.Q4IPB3
99084 229533.I1RST1 pp 229533.I1S0Z9
99085 229533.I1RST1 pp 229533.Q4I1Y1
99086 229533.I1RST1 pp 229533.I1RYW3
99087 229533.I1RST1 pp 229533.Q4IF76
99088 229533.I1RST1 pp 229533.I1RYJ9
99089 229533.I1RST1 pp 229533.I1S0B1
99090 229533.I1RST1 pp 229533.I1RYB1
99091 229533.I1RST1 pp 229533.I1RU69
99092 229533.I1RST1 pp 229533.I1S2Y5
99093 229533.I1RST1 pp 229533.Q4IJH1
99094 229533.I1RST1 pp 229533.Q4IPB8
99095 229533.I1RST1 pp 229533.I1S9L0
99096 229533.I1RST1 pp 229533.I1RT17
99097 229533.I1RST1 pp 229533.I1S9L6
99098 229533.I1RST1 pp 229533.Q4IP34
99099 229533.I1RST1 pp 229533.Q4IB70
99100 229533.I1RST1 pp 229533.I1S4M5
99101 229533.I1RST1 pp 229533.I1S044
99102 229533.I1RST1 pp 229533.V6RY33
99103 229533.I1RST1 pp 229533.Q4I5R9
99104 229533.I1RST1 pp 229533.I1RWU2
99105 229533.I1RST1 pp 229533.I1RX51
99106 229533.I1RST1 pp 229533.Q4IRI9
99107 229533.I1RST1 pp 229533.I1RT47
99108 229533.I1RST1 pp 229533.I1S1C6
99109 229533.I1RST1 pp 229533.I1S9L5
99110 229533.I1RST1 pp 229533.Q4IAA0
99111 229533.I1RST1 pp 229533.I1RZ11
99112 229533.I1RST1 pp 229533.V6QXG1
99113 229533.I1RST1 pp 229533.I1S7M2
99114 229533.I1RST1 pp 229533.Q4IBK5
99115 229533.I1RST1 pp 229533.I1RWX8
99116 229533.I1RST1 pp 229533.I1S246
99117 229533.I1RST1 pp 229533.I1RXF2
99118 229533.I1RST1 pp 229533.I1RX47
99119 229533.I1RST1 pp 229533.Q4IE79
99120 229533.I1RST1 pp 229533.Q4HW67
99121 229533.I1RST1 pp 229533.I1S471
99122 229533.I1RST1 pp 229533.I1S7M3
99123 229533.I1S0I9 pp 229533.V6RJP8
99124 229533.I1S0I9 pp 229533.I1S458
99125 229533.A0A098D1B8 pp 229533.I1RMR5
99126 229533.I1RFW6 pp 229533.I1RPG1
99127 229533.I1RFW6 pp 229533.I1RYV4
99128 229533.I1RFW6 pp 229533.I1RSG9
99129 229533.A0A0E0RVD7 pp 229533.A0A1C3YJK8
99130 229533.A0A0E0RVD7 pp 229533.I1RC02
99131 229533.A0A0E0RVD7 pp 229533.I1RPK0
99132 229533.A0A0E0RVD7 pp 229533.I1S0Z3
99133 229533.A0A0E0RVD7 pp 229533.I1RYI1
99134 229533.A0A0E0RVD7 pp 229533.A0A1C3YJG5

99135 229533.A0A0E0RVD7 pp 229533.I1RDF8
99136 229533.A0A0E0RVD7 pp 229533.I1S1C8
99137 229533.A0A0E0RVD7 pp 229533.I1RAV9
99138 229533.A0A0E0RVD7 pp 229533.I1RUQ1
99139 229533.A0A0E0RVD7 pp 229533.I1RNM1
99140 229533.A0A0E0RVD7 pp 229533.I1RT41
99141 229533.A0A0E0RVD7 pp 229533.A0A1C3YK06
99142 229533.A0A0E0RVD7 pp 229533.I1RD61
99143 229533.A0A0E0RVD7 pp 229533.I1S0I9
99144 229533.A0A0E0RVD7 pp 229533.A0A1C3YJN2
99145 229533.A0A0E0RVD7 pp 229533.A0A1C3YIZ6
99146 229533.A0A0E0RVD7 pp 229533.I1REU2
99147 229533.A0A0E0RVD7 pp 229533.A0A1C3YJS7
99148 229533.A0A0E0RVD7 pp 229533.A0A1C3YM61
99149 229533.A0A0E0RVD7 pp 229533.I1RRB0
99150 229533.A0A0E0RVD7 pp 229533.I1RT72
99151 229533.A0A0E0RVD7 pp 229533.A0A0E0SJV3
99152 229533.A0A0E0RVD7 pp 229533.I1RD48
99153 229533.A0A0E0RVD7 pp 229533.I1S1Y7
99154 229533.A0A0E0RVD7 pp 229533.I1RQP1
99155 229533.A0A0E0RVD7 pp 229533.I1RY67
99156 229533.A0A0E0RVD7 pp 229533.I1RMW6
99157 229533.A0A0E0RVD7 pp 229533.A0A0E0SH87
99158 229533.A0A0E0RVD7 pp 229533.I1RP92
99159 229533.I1REI8 pp 229533.I1S368
99160 229533.I1REI8 pp 229533.I1RR94
99161 229533.I1REI8 pp 229533.I1RJ45
99162 229533.I1REI8 pp 229533.I1RMU5
99163 229533.I1REI8 pp 229533.I1RHV1
99164 229533.I1REI8 pp 229533.I1RU98
99165 229533.I1REI8 pp 229533.I1RTD0
99166 229533.I1REI8 pp 229533.I1RW83
99167 229533.I1REI8 pp 229533.I1RU06
99168 229533.I1REI8 pp 229533.I1S9Z3
99169 229533.I1REI8 pp 229533.I1RHY5
99170 229533.I1RN59 pp 229533.I1S9L5
99171 229533.I1RN59 pp 229533.I1RT17
99172 229533.I1RN59 pp 229533.I1S471
99173 229533.I1RN59 pp 229533.Q4IAA0
99174 229533.I1RN59 pp 229533.I1RRL0
99175 229533.I1RN59 pp 229533.I1RPZ3
99176 229533.I1RN59 pp 229533.Q4IBK5
99177 229533.I1RN59 pp 229533.I1S2Y5
99178 229533.I1RN59 pp 229533.Q4I5R9
99179 229533.I1RN59 pp 229533.I1RT47
99180 229533.I1RN59 pp 229533.I1RSD4
99181 229533.I1RN59 pp 229533.I1RYI9
99182 229533.I1RN59 pp 229533.I1RZ11
99183 229533.I1RN59 pp 229533.I1RPY5
99184 229533.I1RN59 pp 229533.I1RS75
99185 229533.I1RN59 pp 229533.I1RT91
99186 229533.I1RN59 pp 229533.I1RS28
99187 229533.I1RN59 pp 229533.I1S9L6
99188 229533.I1RN59 pp 229533.I1RQR3
99189 229533.I1RN59 pp 229533.Q4IPB8
99190 229533.I1RN59 pp 229533.I1RQC5
99191 229533.I1RN59 pp 229533.I1RX81
99192 229533.I1RN59 pp 229533.I1S1X9
99193 229533.I1RN59 pp 229533.I1RX24
99194 229533.I1RN59 pp 229533.I1RX51
99195 229533.I1RN59 pp 229533.V6QXG1
99196 229533.I1RN59 pp 229533.Q4IRI9
99197 229533.I1RN59 pp 229533.I1S4M5
99198 229533.I1RN59 pp 229533.I1RWX8
99199 229533.I1RN59 pp 229533.I1RT35
99200 229533.I1RN59 pp 229533.Q4HXF6
99201 229533.I1RN59 pp 229533.I1RZG7
99202 229533.I1RN59 pp 229533.I1RYW3
99203 229533.I1RN59 pp 229533.I1RZQ5
99204 229533.I1RN59 pp 229533.I1RND5
99205 229533.I1RN59 pp 229533.I1RWW5
99206 229533.I1RN59 pp 229533.I1S7M2
99207 229533.I1RN59 pp 229533.I1RPY4

99208 229533.I1RN59 pp 229533.I1RSA4
99209 229533.I1RN59 pp 229533.Q4IPB3
99210 229533.I1RN59 pp 229533.I1RQP8
99211 229533.I1RN59 pp 229533.I1RPL3
99212 229533.I1RN59 pp 229533.I1RXF2
99213 229533.I1RN59 pp 229533.I1RPA0
99214 229533.I1RN59 pp 229533.I1RT70
99215 229533.I1RN59 pp 229533.Q4IE79
99216 229533.I1RN59 pp 229533.I1RSA8
99217 229533.I1RN59 pp 229533.Q4IPH4
99218 229533.I1RN59 pp 229533.I1RST1
99219 229533.I1RN59 pp 229533.I1RX47
99220 229533.I1RN59 pp 229533.I1S0Z9
99221 229533.I1RN59 pp 229533.Q4IB70
99222 229533.I1RN59 pp 229533.I1S0B1
99223 229533.I1RN59 pp 229533.I1RYJ9
99224 229533.I1RN59 pp 229533.I1S7M3
99225 229533.I1RN59 pp 229533.Q4I1Y1
99226 229533.A0A098DK43 pp 229533.V6RV57
99227 229533.A0A098DK43 pp 229533.I1RW06
99228 229533.A0A098DK43 pp 229533.I1RYK0
99229 229533.A0A098DK43 pp 229533.A0A098DVZ4
99230 229533.A0A098DK43 pp 229533.I1S9T8
99231 229533.A0A098DK43 pp 229533.V6RLM7
99232 229533.A0A098DK43 pp 229533.A0A1C3YJI4
99233 229533.A0A098DK43 pp 229533.I1RFV9
99234 229533.A0A098DK43 pp 229533.I1RND4
99235 229533.I1RT72 pp 229533.I1S0Z3
99236 229533.I1RT72 pp 229533.I1RZ48
99237 229533.I1RT72 pp 229533.I1RY67
99238 229533.I1RT72 pp 229533.I1RZF5
99239 229533.I1RT72 pp 229533.I1S0I9
99240 229533.I1RT72 pp 229533.I1S1Y7
99241 229533.I1RD68 pp 229533.I1RKT7
99242 229533.I1RCA9 pp 229533.I1RY73
99243 229533.I1RCA9 pp 229533.I1S604
99244 229533.I1RCA9 pp 229533.I1RLX6
99245 229533.I1RCA9 pp 229533.I1S2E9
99246 229533.I1RCA9 pp 229533.I1RPQ1
99247 229533.I1RCA9 pp 229533.Q4I6A4
99248 229533.I1RCA9 pp 229533.I1RNK1
99249 229533.I1RCA9 pp 229533.I1RZP8
99250 229533.I1RCA9 pp 229533.I1RSI4
99251 229533.I1RCA9 pp 229533.I1RN57
99252 229533.I1RCA9 pp 229533.I1RPZ0
99253 229533.I1RCA9 pp 229533.I1RDX0
99254 229533.I1RCA9 pp 229533.I1RSL8
99255 229533.I1RCA9 pp 229533.Q4I7L0
99256 229533.I1RCA9 pp 229533.I1RYX9
99257 229533.I1RCA9 pp 229533.I1RE46
99258 229533.I1RCA9 pp 229533.I1RW16
99259 229533.I1RCA9 pp 229533.I1RRP7
99260 229533.I1RCA9 pp 229533.I1RQA3
99261 229533.I1RCA9 pp 229533.I1RKK7
99262 229533.I1RCA9 pp 229533.I1RQ05
99263 229533.I1RCA9 pp 229533.I1RCL7
99264 229533.I1RCA9 pp 229533.I1RN55
99265 229533.I1RCA9 pp 229533.I1RY44
99266 229533.I1RCA9 pp 229533.I1RYR2
99267 229533.I1RCA9 pp 229533.I1RZ94
99268 229533.I1RCA9 pp 229533.I1RUG2
99269 229533.I1RCA9 pp 229533.I1RQ92
99270 229533.I1RCA9 pp 229533.I1RN99
99271 229533.I1RCA9 pp 229533.I1RWT6
99272 229533.I1RCA9 pp 229533.V6RLW4
99273 229533.I1RCA9 pp 229533.I1RCY2
99274 229533.I1RCA9 pp 229533.I1RQJ4
99275 229533.I1RCA9 pp 229533.I1RSP7
99276 229533.I1RCA9 pp 229533.I1RD19
99277 229533.I1RCA9 pp 229533.I1RFJ2
99278 229533.I1RCA9 pp 229533.I1RSA7
99279 229533.I1RCA9 pp 229533.I1RFX8
99280 229533.I1RCA9 pp 229533.I1RN31

229533.I1RED2 pp 229533.I1RPW8
229533.I1RED2 pp 229533.I1RRC4
229533.I1RED2 pp 229533.I1REI2
229533.I1RED2 pp 229533.I1RNJ7
229533.I1RED2 pp 229533.I1RKF3
229533.I1RED2 pp 229533.I1RFV2
229533.I1RED2 pp 229533.I1RW91
229533.I1RED2 pp 229533.I1RW80
229533.I1RED2 pp 229533.I1RQ42
229533.I1RED2 pp 229533.I1RFH9
229533.I1RED2 pp 229533.I1RRG7
229533.I1RED2 pp 229533.I1RZN1
229533.I1RED2 pp 229533.I1S368
229533.I1RED2 pp 229533.I1RRN4
229533.I1RED2 pp 229533.I1S5Z5
229533.I1RED2 pp 229533.I1RFN8
229533.I1RED2 pp 229533.I1RK49
229533.I1RED2 pp 229533.I1S8X0
229533.I1RED2 pp 229533.I1RMG4
229533.I1RED2 pp 229533.I1RQM9
229533.I1RED2 pp 229533.I1RRF7
229533.I1RED2 pp 229533.I1S2B9
229533.I1RED2 pp 229533.I1RN04
229533.I1RED2 pp 229533.I1RHJ2
229533.I1RED2 pp 229533.I1RWN0
229533.I1RED2 pp 229533.I1RYX5
229533.I1RED2 pp 229533.I1RR61
229533.I1RED2 pp 229533.I1RTP2
229533.I1RED2 pp 229533.I1S2B0
229533.I1RJI1 pp 229533.I1RKF7
229533.I1RJI1 pp 229533.I1S0B1
229533.A0A0E0SEI0 pp 229533.I1RSE9
229533.A0A0E0SEI0 pp 229533.A0A1C3YLA2
229533.A0A0E0SEI0 pp 229533.I1S0B1
229533.A0A0E0SEI0 pp 229533.A0A1C3YHP3
229533.A0A0E0SEI0 pp 229533.I1S251
229533.A0A0E0SEI0 pp 229533.A0A1C3YLC6
229533.A0A0E0SEI0 pp 229533.Q4IPI4
229533.A0A0E0SEI0 pp 229533.I1RD33
229533.A0A0E0SEI0 pp 229533.I1S7I5
229533.A0A0E0SEI0 pp 229533.I1RE51
229533.A0A0E0SEI0 pp 229533.I1RKF7
229533.A0A0E0SEI0 pp 229533.I1S1V2
229533.A0A0E0SEI0 pp 229533.I1RAN8
229533.A0A0E0SEI0 pp 229533.I1RJI1
229533.I1RHY5 pp 229533.I1S0S5
229533.I1RHY5 pp 229533.I1RJ45
229533.I1RHY5 pp 229533.I1RJU6
229533.I1RHY5 pp 229533.I1RN77
229533.I1RHY5 pp 229533.I1RWF9
229533.I1RHY5 pp 229533.I1S368
229533.I1RHY5 pp 229533.I1RU06
229533.I1RHY5 pp 229533.I1RSX7
229533.I1RHY5 pp 229533.I1RR94
229533.A0A098DUK7 pp 229533.I1RDF8
229533.A0A098DUK7 pp 229533.A0A0E0RVD7
229533.A0A098DUK7 pp 229533.I1RZF5
229533.A0A098DUK7 pp 229533.I1RT72
229533.A0A098DUK7 pp 229533.I1RW54
229533.A0A098DUK7 pp 229533.A0A1C3YJN2
229533.A0A098DUK7 pp 229533.I1RZD0
229533.A0A098DUK7 pp 229533.A0A1C3YJS7
229533.A0A098DUK7 pp 229533.A0A0E0SH87
229533.A0A098DUK7 pp 229533.A0A1C3YK06
229533.A0A098DTU9 pp 229533.I1RG90
229533.A0A098DTU9 pp 229533.V6QXG1
229533.A0A098DTU9 pp 229533.I1RND5
229533.A0A098DTU9 pp 229533.I1RSA4
229533.A0A098DTU9 pp 229533.I1RCI4
229533.A0A098DTU9 pp 229533.I1RLU5
229533.A0A098DTU9 pp 229533.I1RAF3
229533.A0A098DTU9 pp 229533.I1RZQ5
229533.A0A098DTU9 pp 229533.I1RZG7

99354 229533.A0A098DTU9 pp 229533.I1RCS2
99355 229533.A0A098DTU9 pp 229533.I1RRL0
99356 229533.A0A098DTU9 pp 229533.I1R9N6
99357 229533.A0A098DTU9 pp 229533.I1RCQ6
99358 229533.A0A098DTU9 pp 229533.I1RS28
99359 229533.A0A098DTU9 pp 229533.I1RFQ1
99360 229533.A0A098DTU9 pp 229533.I1RPY4
99361 229533.A0A098DTU9 pp 229533.I1RCA7
99362 229533.A0A098DTU9 pp 229533.Q4IRI9
99363 229533.A0A098DTU9 pp 229533.A0A0E0S1T0
99364 229533.A0A098DTU9 pp 229533.Q4IAA0
99365 229533.A0A098DTU9 pp 229533.A0A1C3YI62
99366 229533.A0A098DTU9 pp 229533.I1RT17
99367 229533.A0A098DTU9 pp 229533.I1RA36
99368 229533.A0A098DTU9 pp 229533.A0A0E0RUQ7
99369 229533.A0A098DTU9 pp 229533.A0A0E0SC46
99370 229533.A0A098DTU9 pp 229533.I1RST1
99371 229533.A0A098DTU9 pp 229533.I1RK90
99372 229533.A0A098DTU9 pp 229533.I1RN59
99373 229533.A0A098DTU9 pp 229533.I1RG57
99374 229533.A0A098DTU9 pp 229533.I1RB50
99375 229533.A0A098DTU9 pp 229533.Q4I1Y1
99376 229533.A0A098DTU9 pp 229533.I1RQC5
99377 229533.A0A098DTU9 pp 229533.I1RAC1
99378 229533.A0A098DTU9 pp 229533.I1RMV5
99379 229533.I1RU06 pp 229533.I1RU98
99380 229533.I1RU06 pp 229533.I1S368
99381 229533.A0A1C3YKM4 pp 229533.Q4I099
99382 229533.A0A1C3YKM4 pp 229533.I1S5X9
99383 229533.A0A1C3YKM4 pp 229533.I1RJD6
99384 229533.A0A1C3YKM4 pp 229533.A0A1C3YMC1
99385 229533.A0A1C3YKM4 pp 229533.Q4IK03
99386 229533.A0A1C3YKM4 pp 229533.I1RFS5
99387 229533.A0A1C3YKM4 pp 229533.I1RBE6
99388 229533.A0A1C3YKM4 pp 229533.I1S268
99389 229533.A0A1C3YKM4 pp 229533.I1RZN1
99390 229533.A0A1C3YKM4 pp 229533.A0A1C3YLC3
99391 229533.A0A1C3YKM4 pp 229533.A0A1C3YKP5
99392 229533.A0A1C3YKM4 pp 229533.I1RNL0
99393 229533.A0A1C3YKM4 pp 229533.I1S1F3
99394 229533.A0A1C3YKM4 pp 229533.A0A1C3YLL3
99395 229533.A0A1C3YKM4 pp 229533.I1RAM9
99396 229533.A0A1C3YKM4 pp 229533.Q4IJW4
99397 229533.A0A1C3YKM4 pp 229533.I1RFV2
99398 229533.A0A1C3YKM4 pp 229533.I1RWT0
99399 229533.A0A1C3YKM4 pp 229533.I1RX17
99400 229533.A0A1C3YKM4 pp 229533.I1RAV5
99401 229533.A0A1C3YKM4 pp 229533.I1RMQ6
99402 229533.A0A1C3YKM4 pp 229533.Q4I5G1
99403 229533.I1RWN0 pp 229533.V6RXE7
99404 229533.I1RWN0 pp 229533.I1S108
99405 229533.I1RWN0 pp 229533.I1RZN1
99406 229533.I1RWN0 pp 229533.I1S2B0
99407 229533.I1RWN0 pp 229533.I1RYH6
99408 229533.I1RWN0 pp 229533.I1S368
99409 229533.I1RWN0 pp 229533.I1S2L1
99410 229533.I1RWN0 pp 229533.I1S360
99411 229533.A0A1C3YMN5 pp 229533.I1RE19
99412 229533.A0A1C3YMN5 pp 229533.I1REN4
99413 229533.A0A1C3YMN5 pp 229533.I1RCI5
99414 229533.A0A1C3YMN5 pp 229533.Q4INZ9
99415 229533.A0A1C3YMN5 pp 229533.I1RTA3
99416 229533.A0A1C3YMN5 pp 229533.I1RMP2
99417 229533.A0A1C3YMN5 pp 229533.Q4IN00
99418 229533.A0A1C3YMN5 pp 229533.I1RRF9
99419 229533.A0A1C3YMN5 pp 229533.Q4HZB8
99420 229533.A0A1C3YLL3 pp 229533.I1RM37
99421 229533.A0A1C3YLL3 pp 229533.I1S9Z4
99422 229533.A0A1C3YLL3 pp 229533.I1RNU6
99423 229533.A0A1C3YLL3 pp 229533.I1RQD2
99424 229533.A0A1C3YLL3 pp 229533.I1RD22
99425 229533.A0A1C3YLL3 pp 229533.I1RVZ0
99426 229533.A0A1C3YLL3 pp 229533.I1S5R6

99427	229533.A0A1C3YLL3	pp	229533.Q4IJK2
99428	229533.A0A1C3YLL3	pp	229533.I1RE79
99429	229533.A0A1C3YLL3	pp	229533.I1RNC1
99430	229533.A0A1C3YLL3	pp	229533.I1RVX0
99431	229533.A0A1C3YLL3	pp	229533.I1RAW0
99432	229533.A0A1C3YLL3	pp	229533.V6RMW6
99433	229533.A0A1C3YLL3	pp	229533.V6R042
99434	229533.A0A1C3YLL3	pp	229533.I1RR72
99435	229533.A0A1C3YLL3	pp	229533.I1RZK5
99436	229533.A0A1C3YLL3	pp	229533.V6QYF0
99437	229533.A0A1C3YLL3	pp	229533.I1RM67
99438	229533.A0A1C3YLL3	pp	229533.I1RSQ3
99439	229533.A0A1C3YLL3	pp	229533.I1S0S5
99440	229533.A0A1C3YLL3	pp	229533.I1RZV3
99441	229533.A0A1C3YLL3	pp	229533.I1S253
99442	229533.A0A1C3YLL3	pp	229533.I1RX46
99443	229533.A0A1C3YLL3	pp	229533.I1RSC5
99444	229533.A0A1C3YLL3	pp	229533.I1RZ39
99445	229533.A0A1C3YLL3	pp	229533.V6RTJ8
99446	229533.A0A1C3YLL3	pp	229533.I1S2B8
99447	229533.A0A1C3YLL3	pp	229533.I1RGA4
99448	229533.A0A1C3YLL3	pp	229533.I1RDF7
99449	229533.A0A1C3YLL3	pp	229533.I1RSA5
99450	229533.A0A1C3YLL3	pp	229533.I1RAX6
99451	229533.A0A1C3YLL3	pp	229533.I1S9Q3
99452	229533.A0A1C3YLL3	pp	229533.I1R9M0
99453	229533.A0A1C3YLL3	pp	229533.I1RFV2
99454	229533.A0A1C3YLL3	pp	229533.I1RTA1
99455	229533.A0A1C3YLL3	pp	229533.I1RPP3
99456	229533.A0A1C3YLL3	pp	229533.I1RZN1
99457	229533.A0A1C3YLL3	pp	229533.I1RYI5
99458	229533.A0A1C3YJK8	pp	229533.A0A1C3YJS7
99459	229533.A0A1C3YJK8	pp	229533.A0A1C3YK06
99460	229533.A0A1C3YJK8	pp	229533.I1RWH6
99461	229533.A0A1C3YJK8	pp	229533.I1RT72
99462	229533.A0A1C3YJK8	pp	229533.I1RZF5
99463	229533.A0A1C3YJK8	pp	229533.I1RW54
99464	229533.A0A0E0SHL3	pp	229533.A0A1C3YLQ4
99465	229533.A0A0E0SHL3	pp	229533.I1S2B9
99466	229533.A0A0E0SHL3	pp	229533.I1RN04
99467	229533.A0A0E0SHL3	pp	229533.I1RRF7
99468	229533.A0A0E0SHL3	pp	229533.I1RYX5
99469	229533.A0A0E0SHL3	pp	229533.I1RR61
99470	229533.A0A0E0SHL3	pp	229533.I1RQM9
99471	229533.A0A0E0SHL3	pp	229533.A0A1C3YHY5
99472	229533.A0A0E0SHL3	pp	229533.I1S2B0
99473	229533.A0A0E0SHL3	pp	229533.I1RFN8
99474	229533.A0A0E0SHL3	pp	229533.A0A1C3YJC4
99475	229533.A0A0E0SHL3	pp	229533.A0A1C3YJZ0
99476	229533.A0A0E0SHL3	pp	229533.A0A1C3YIX3
99477	229533.A0A0E0SHL3	pp	229533.I1RHJ2
99478	229533.A0A0E0SHL3	pp	229533.I1RTP2
99479	229533.A0A0E0SHL3	pp	229533.I1RCG6
99480	229533.A0A0E0SHL3	pp	229533.A0A1C3YJ07
99481	229533.A0A0E0SHL3	pp	229533.I1S5Z5
99482	229533.A0A0E0SHL3	pp	229533.I1RWN0
99483	229533.A0A0E0SHL3	pp	229533.I1RRG7
99484	229533.A0A0E0SHL3	pp	229533.I1RMG4
99485	229533.A0A0E0SHL3	pp	229533.I1S8X0
99486	229533.A0A0E0SHL3	pp	229533.I1RRN4
99487	229533.A0A0E0SHL3	pp	229533.I1RW80
99488	229533.A0A0E0SHL3	pp	229533.A0A1C3YLC3
99489	229533.A0A0E0SHL3	pp	229533.I1RCM8
99490	229533.A0A0E0SHL3	pp	229533.I1RPW8
99491	229533.A0A0E0SHL3	pp	229533.I1S368
99492	229533.A0A0E0SHL3	pp	229533.I1RNJ7
99493	229533.A0A0E0SHL3	pp	229533.I1RZN1
99494	229533.A0A0E0SHL3	pp	229533.I1RRC4
99495	229533.A0A0E0SHL3	pp	229533.I1RED2
99496	229533.A0A0E0SHL3	pp	229533.I1RAJ0
99497	229533.A0A0E0SHL3	pp	229533.I1RBA6
99498	229533.A0A0E0SHL3	pp	229533.I1RFV2
99499	229533.A0A0E0SHL3	pp	229533.I1REI2

99500 229533.A0A0E0SHL3 pp 229533.A0A1C3YMU9
99501 229533.A0A0E0SHL3 pp 229533.I1R9Y2
99502 229533.A0A0E0SHL3 pp 229533.I1RCE6
99503 229533.A0A0E0SHL3 pp 229533.I1RKF3
99504 229533.A0A0E0SHL3 pp 229533.I1RFH9
99505 229533.A0A0E0SHL3 pp 229533.I1RK49
99506 229533.A0A0E0SHL3 pp 229533.I1RQ42
99507 229533.A0A0E0SHL3 pp 229533.A0A1C3YHX6
99508 229533.A0A0E0SHL3 pp 229533.I1RW91
99509 229533.I1RN88 pp 229533.V6R1K4
99510 229533.I1RN88 pp 229533.I1RYX6
99511 229533.I1RN88 pp 229533.I1S4S7
99512 229533.I1RN88 pp 229533.I1S8C1
99513 229533.I1RN88 pp 229533.I1RNM7
99514 229533.I1RN88 pp 229533.I1RQE1
99515 229533.I1RN88 pp 229533.I1RP86
99516 229533.I1RN88 pp 229533.Q4HZS8
99517 229533.I1RN88 pp 229533.I1RSQ7
99518 229533.I1RN88 pp 229533.I1RXD7
99519 229533.I1RN88 pp 229533.V6QVV9
99520 229533.I1RN88 pp 229533.I1RRX5
99521 229533.I1RN88 pp 229533.V6RH92
99522 229533.I1RN88 pp 229533.I1RX46
99523 229533.I1RN88 pp 229533.Q4IA56
99524 229533.I1RN88 pp 229533.I1S1W8
99525 229533.I1RN88 pp 229533.I1RR44
99526 229533.I1RN88 pp 229533.I1S3L2
99527 229533.I1RN88 pp 229533.I1S4Q4
99528 229533.I1RN88 pp 229533.I1RY21
99529 229533.I1RN88 pp 229533.I1S8F8
99530 229533.I1RN88 pp 229533.I1S8S9
99531 229533.I1RN88 pp 229533.I1RT82
99532 229533.I1RN88 pp 229533.V6RPS5
99533 229533.I1RN88 pp 229533.I1RPR1
99534 229533.I1RN88 pp 229533.I1S8D2
99535 229533.I1RN88 pp 229533.I1RYW9
99536 229533.I1RN88 pp 229533.I1RYA2
99537 229533.I1RN88 pp 229533.I1S1J7
99538 229533.I1RN88 pp 229533.I1RNG4
99539 229533.I1RN88 pp 229533.I1RY12
99540 229533.I1RN88 pp 229533.I1RUY5
99541 229533.I1RN88 pp 229533.I1RNY6
99542 229533.I1RN88 pp 229533.I1S299
99543 229533.I1RN88 pp 229533.I1RXV9
99544 229533.I1RN88 pp 229533.V6RUY9
99545 229533.I1RN88 pp 229533.I1S8P5
99546 229533.I1RN88 pp 229533.Q4HXF0
99547 229533.I1RN88 pp 229533.I1RPP3
99548 229533.I1RN88 pp 229533.Q4IJK2
99549 229533.I1RN88 pp 229533.I1RWF5
99550 229533.I1RN88 pp 229533.I1RUQ3
99551 229533.I1RN88 pp 229533.I1RR95
99552 229533.I1RN88 pp 229533.I1RYK0
99553 229533.I1RN88 pp 229533.V6R3J1
99554 229533.I1RN88 pp 229533.I1RNC1
99555 229533.I1RN88 pp 229533.I1RTA1
99556 229533.I1RN88 pp 229533.I1RXW3
99557 229533.I1RN88 pp 229533.I1S3T7
99558 229533.I1RN88 pp 229533.I1RQI4
99559 229533.I1RN88 pp 229533.I1S5R6
99560 229533.I1RN88 pp 229533.O74638
99561 229533.I1RN88 pp 229533.I1RRT8
99562 229533.I1RN88 pp 229533.V6RRM0
99563 229533.I1RN88 pp 229533.I1S148
99564 229533.I1RN88 pp 229533.I1S147
99565 229533.I1RN88 pp 229533.V6RLM7
99566 229533.I1RN88 pp 229533.I1RVR2
99567 229533.I1RN88 pp 229533.V6RM02
99568 229533.I1RN88 pp 229533.I1RSZ7
99569 229533.I1RN88 pp 229533.I1RS17
99570 229533.I1RN88 pp 229533.I1RWN7
99571 229533.I1RN88 pp 229533.I1RPK0
99572 229533.I1RN88 pp 229533.I1RZ70

99573 229533.I1RN88 pp 229533.I1RZ52
99574 229533.I1RN88 pp 229533.I1RVX0
99575 229533.I1RN88 pp 229533.I1RUR8
99576 229533.I1RN88 pp 229533.I1RTA0
99577 229533.I1RN88 pp 229533.I1RQE7
99578 229533.I1RN88 pp 229533.I1S8D1
99579 229533.I1RN88 pp 229533.I1S0J1
99580 229533.I1RN88 pp 229533.I1RSQ4
99581 229533.I1RN88 pp 229533.I1S8R2
99582 229533.I1RN88 pp 229533.I1RZ39
99583 229533.I1RN88 pp 229533.I1S149
99584 229533.I1RN88 pp 229533.V6R057
99585 229533.I1RN88 pp 229533.I1S3G4
99586 229533.I1RN88 pp 229533.V6R1X4
99587 229533.I1RN88 pp 229533.I1S8R4
99588 229533.I1RN88 pp 229533.I1RRI2
99589 229533.I1RN88 pp 229533.V6RRH2
99590 229533.I1RN88 pp 229533.I1RYW3
99591 229533.I1RN88 pp 229533.I1RQ30
99592 229533.I1S8Y1 pp 229533.Q4I5G1
99593 229533.I1S8Y1 pp 229533.Q4IB65
99594 229533.I1S8Y1 pp 229533.Q4IK03
99595 229533.I1RAI8 pp 229533.I1RWG6
99596 229533.I1RAI8 pp 229533.I1RW41
99597 229533.I1RAI8 pp 229533.I1RAL2
99598 229533.I1RAI8 pp 229533.I1RBW7
99599 229533.I1RAI8 pp 229533.I1RXB5
99600 229533.I1RAI8 pp 229533.I1RZH4
99601 229533.I1RAI8 pp 229533.I1RXF7
99602 229533.I1RAI8 pp 229533.I1RCJ5
99603 229533.I1RAI8 pp 229533.I1RKA0
99604 229533.I1RAI8 pp 229533.I1S1V1
99605 229533.I1RAI8 pp 229533.I1RS64
99606 229533.I1RAI8 pp 229533.I1S1B6
99607 229533.I1RU98 pp 229533.I1S368
99608 229533.I1RJ45 pp 229533.I1S368
99609 229533.I1RJ45 pp 229533.I1RU06
99610 229533.I1RJ45 pp 229533.I1RWN0
99611 229533.I1RJ45 pp 229533.I1RR94
99612 229533.I1RJ45 pp 229533.I1RU98
99613 229533.I1S8X0 pp 229533.I1S8X1
99614 229533.I1S8X0 pp 229533.V6RN16
99615 229533.I1S8X0 pp 229533.V6RDP1
99616 229533.A0A1C3YK06 pp 229533.I1RNM1
99617 229533.A0A1C3YK06 pp 229533.I1RB00
99618 229533.A0A1C3YK06 pp 229533.I1REU2
99619 229533.A0A1C3YK06 pp 229533.I1RT41
99620 229533.A0A1C3YK06 pp 229533.A0A1C3YM61
99621 229533.A0A1C3YK06 pp 229533.I1RT72
99622 229533.A0A1C3YK06 pp 229533.I1RD48
99623 229533.A0A1C3YK06 pp 229533.I1RY67
99624 229533.A0A1C3YK06 pp 229533.I1S1Y7
99625 229533.A0A1C3YK06 pp 229533.I1RC02
99626 229533.A0A1C3YK06 pp 229533.I1RZF5
99627 229533.A0A1C3YK06 pp 229533.I1S0I9
99628 229533.A0A1C3YK06 pp 229533.I1RMW6
99629 229533.A0A1C3YK06 pp 229533.I1RP92
99630 229533.A0A1C3YK06 pp 229533.I1RRN5
99631 229533.A0A1C3YK06 pp 229533.I1RDF8
99632 229533.A0A1C3YK06 pp 229533.I1RRB0
99633 229533.A0A1C3YK06 pp 229533.I1S1C8
99634 229533.A0A1C3YK06 pp 229533.I1RPK0
99635 229533.A0A1C3YK06 pp 229533.I1RCT5
99636 229533.A0A1C3YK06 pp 229533.I1S0Z3
99637 229533.I1RR94 pp 229533.I1RU98
99638 229533.I1RR94 pp 229533.I1S2E9
99639 229533.I1RR94 pp 229533.I1RV91
99640 229533.I1RR94 pp 229533.I1RY44
99641 229533.I1RR94 pp 229533.I1RU06
99642 229533.I1RR94 pp 229533.I1RWT6
99643 229533.I1RR94 pp 229533.I1RUI0
99644 229533.I1RR94 pp 229533.I1RTE6
99645 229533.I1RR94 pp 229533.V6REE9

99646 229533.I1RR94 pp 229533.I1S368
99647 229533.I1RR94 pp 229533.I1RX09
99648 229533.A0A0E0SJV3 pp 229533.I1R9V4
99649 229533.A0A0E0SJV3 pp 229533.I1RZF5
99650 229533.A0A0E0SJV3 pp 229533.I1RT72
99651 229533.A0A0E0SJV3 pp 229533.I1RAQ6
99652 229533.A0A0E0SJV3 pp 229533.I1RW54
99653 229533.A0A0E0SJV3 pp 229533.A0A1C3YJK8
99654 229533.A0A0E0SJV3 pp 229533.A0A1C3YJS7
99655 229533.A0A0E0SJV3 pp 229533.A0A1C3YK06
99656 229533.A0A098DK39 pp 229533.A0A1C3YIR8
99657 229533.A0A098DK39 pp 229533.I1S0V4
99658 229533.A0A098DK39 pp 229533.I1RXF8
99659 229533.A0A098DK39 pp 229533.A0A1C3YMK1
99660 229533.A0A098DK39 pp 229533.Q4IMD1
99661 229533.A0A098DK39 pp 229533.V6REZ4
99662 229533.A0A098DK39 pp 229533.I1RML0
99663 229533.A0A098DK39 pp 229533.I1RDG0
99664 229533.A0A098DK39 pp 229533.I1RKJ8
99665 229533.A0A098DK39 pp 229533.I1S0P4
99666 229533.A0A098DK39 pp 229533.I1RYD9
99667 229533.A0A098DK39 pp 229533.Q4HTT2
99668 229533.A0A098DK39 pp 229533.I1RE89
99669 229533.A0A098DK39 pp 229533.I1RCU7
99670 229533.A0A098DK39 pp 229533.I1S044
99671 229533.A0A098DK39 pp 229533.A0A0E0S035
99672 229533.A0A098DK39 pp 229533.I1RGC2
99673 229533.A0A1C3YJJ7 pp 229533.I1RQM4
99674 229533.A0A1C3YJJ7 pp 229533.I1RDZ5
99675 229533.A0A1C3YJJ7 pp 229533.A0A1C3YLC3
99676 229533.A0A1C3YJJ7 pp 229533.I1RV85
99677 229533.A0A1C3YJJ7 pp 229533.I1RFV2
99678 229533.A0A1C3YJJ7 pp 229533.I1RZN1
99679 229533.A0A1C3YJJ7 pp 229533.A0A1C3YMF6
99680 229533.A0A1C3YJJ7 pp 229533.I1S360
99681 229533.A0A1C3YJJ7 pp 229533.I1RWN0
99682 229533.A0A1C3YJJ7 pp 229533.I1RE88
99683 229533.A0A1C3YJJ7 pp 229533.I1REB5
99684 229533.A0A1C3YJJ7 pp 229533.I1RTP2
99685 229533.A0A1C3YJJ7 pp 229533.I1REI4
99686 229533.A0A1C3YJJ7 pp 229533.A0A1C3YLQ4
99687 229533.A0A1C3YJJ7 pp 229533.I1S2L1
99688 229533.A0A1C3YJJ7 pp 229533.I1S368
99689 229533.A0A1C3YJJ7 pp 229533.I1S108
99690 229533.A0A1C3YJJ7 pp 229533.I1RMG4
99691 229533.I1S8Y0 pp 229533.Q4IB65
99692 229533.A0A1C3YIZ2 pp 229533.I1RBW7
99693 229533.A0A1C3YIZ2 pp 229533.I1RWG6
99694 229533.A0A1C3YIZ2 pp 229533.I1RCJ5
99695 229533.I1RD48 pp 229533.I1RT72
99696 229533.I1RD48 pp 229533.I1RW54
99697 229533.I1RD48 pp 229533.I1RZF5
99698 229533.V6RRM0 pp 229533.V6RZD4
99699 229533.V6RRM0 pp 229533.V6RUB1
99700 229533.I1S2B0 pp 229533.I1S368
99701 229533.I1S2B0 pp 229533.V6RXE7
99702 229533.I1RNE2 pp 229533.I1S209
99703 229533.I1RNE2 pp 229533.I1RZ19
99704 229533.I1RNE2 pp 229533.I1RSY2
99705 229533.I1RNE2 pp 229533.I1RZP8
99706 229533.I1RNE2 pp 229533.I1RVZ9
99707 229533.I1RNE2 pp 229533.V6RAC9
99708 229533.I1RNE2 pp 229533.Q4IPX8
99709 229533.I1RNE2 pp 229533.I1RYQ1
99710 229533.I1RNE2 pp 229533.I1RQJ4
99711 229533.I1RNE2 pp 229533.V6RLM7
99712 229533.A0A098E2F0 pp 229533.I1RPR1
99713 229533.A0A098E2F0 pp 229533.I1RBE4
99714 229533.A0A098E2F0 pp 229533.A0A1C3YIJ9
99715 229533.A0A098E2F0 pp 229533.I1RNY6
99716 229533.A0A098E2F0 pp 229533.I1RAQ4
99717 229533.A0A098E2F0 pp 229533.A0A1C3YLB3
99718 229533.A0A098E2F0 pp 229533.I1S0V6

99719	229533.A0A098E2F0	pp	229533.A0A1C3YK58
99720	229533.A0A098E2F0	pp	229533.I1RMD3
99721	229533.A0A098E2F0	pp	229533.I1RER8
99722	229533.A0A098E2F0	pp	229533.A0A1C3YM54
99723	229533.A0A098E2F0	pp	229533.I1S8F8
99724	229533.A0A098E2F0	pp	229533.A0A1C3YIK4
99725	229533.A0A098E2F0	pp	229533.A0A1C3YKR5
99726	229533.A0A098E2F0	pp	229533.I1RYA2
99727	229533.A0A098E2F0	pp	229533.A0A098E320
99728	229533.A0A098E2F0	pp	229533.I1RBE7
99729	229533.A0A098E2F0	pp	229533.I1RXE6
99730	229533.A0A098E2F0	pp	229533.V6RQW4
99731	229533.A0A098E2F0	pp	229533.I1RRM1
99732	229533.A0A098E2F0	pp	229533.I1RWM4
99733	229533.A0A098E2F0	pp	229533.I1RAV5
99734	229533.A0A098E2F0	pp	229533.I1RJY7
99735	229533.A0A098E2F0	pp	229533.I1RN80
99736	229533.A0A098E2F0	pp	229533.I1REE2
99737	229533.A0A098E2F0	pp	229533.A0A1C3YI01
99738	229533.A0A098E2F0	pp	229533.I1RY12
99739	229533.A0A098E2F0	pp	229533.I1RNH4
99740	229533.A0A098E2F0	pp	229533.I1RQE1
99741	229533.A0A098E2F0	pp	229533.I1RUQ3
99742	229533.A0A098E2F0	pp	229533.A0A0E0RU85
99743	229533.A0A098E2F0	pp	229533.I1RP86
99744	229533.A0A098E2F0	pp	229533.I1S0K2
99745	229533.A0A098E2F0	pp	229533.I1RCR6
99746	229533.A0A098E2F0	pp	229533.A0A1C3YLC2
99747	229533.A0A098E2F0	pp	229533.I1RCM5
99748	229533.A0A098E2F0	pp	229533.I1RG33
99749	229533.A0A098E2F0	pp	229533.I1RIJ2
99750	229533.A0A098E2F0	pp	229533.A0A0E0RT42
99751	229533.A0A098E2F0	pp	229533.A0A0E0SAR4
99752	229533.A0A098E2F0	pp	229533.I1RMQ6
99753	229533.A0A098E2F0	pp	229533.I1RR12
99754	229533.A0A098E2F0	pp	229533.I1S1D4
99755	229533.A0A098E2F0	pp	229533.I1S8C1
99756	229533.A0A098E2F0	pp	229533.A0A1C3YN26
99757	229533.A0A098E2F0	pp	229533.I1R9Y4
99758	229533.A0A098E2F0	pp	229533.I1RRX5
99759	229533.A0A098E2F0	pp	229533.I1RN84
99760	229533.A0A098E2F0	pp	229533.I1RBT1
99761	229533.A0A098E2F0	pp	229533.I1RC11
99762	229533.A0A098E2F0	pp	229533.I1RRG3
99763	229533.A0A098E2F0	pp	229533.A0A1C3YLR6
99764	229533.A0A098E2F0	pp	229533.I1RCU2
99765	229533.A0A098E2F0	pp	229533.I1S3K8
99766	229533.A0A098E2F0	pp	229533.I1RHC8
99767	229533.A0A098E2F0	pp	229533.A0A1C3YKC2
99768	229533.A0A098E2F0	pp	229533.A0A0E0RNI4
99769	229533.A0A098E2F0	pp	229533.I1RLM5
99770	229533.A0A098E2F0	pp	229533.A0A1C3YIB0
99771	229533.A0A098E2F0	pp	229533.I1RYC8
99772	229533.A0A098E2F0	pp	229533.I1RMK7
99773	229533.A0A098E2F0	pp	229533.I1RY21
99774	229533.A0A098E2F0	pp	229533.A0A1C3YK11
99775	229533.A0A098E2F0	pp	229533.I1S1W8
99776	229533.A0A098E2F0	pp	229533.A0A1C3YJ04
99777	229533.A0A098E2F0	pp	229533.I1RAR9
99778	229533.A0A098E2F0	pp	229533.I1RUP2
99779	229533.A0A098E2F0	pp	229533.I1RL81
99780	229533.A0A098E2F0	pp	229533.A0A1C3YJW3
99781	229533.A0A098E2F0	pp	229533.I1RE52
99782	229533.A0A098E2F0	pp	229533.I1RPD6
99783	229533.A0A098E2F0	pp	229533.V6RLM7
99784	229533.A0A098E2F0	pp	229533.I1RNM7
99785	229533.A0A098E2F0	pp	229533.I1RC31
99786	229533.A0A098E2F0	pp	229533.I1S0V7
99787	229533.A0A098E2F0	pp	229533.I1RNG4
99788	229533.A0A098E2F0	pp	229533.A0A1C3YLB6
99789	229533.A0A098E2F0	pp	229533.A0A1C3YJP4
99790	229533.A0A098E2F0	pp	229533.I1S147
99791	229533.A0A098E2F0	pp	229533.I1RB38

99792 229533.A0A098E2F0 pp 229533.A0A1C3YHU3
99793 229533.A0A098E2F0 pp 229533.I1S2H3
99794 229533.A0A098E2F0 pp 229533.I1RYK0
99795 229533.A0A098E2F0 pp 229533.I1RSU2
99796 229533.A0A098E2F0 pp 229533.I1RF85
99797 229533.A0A098E2F0 pp 229533.I1RZA7
99798 229533.A0A098E2F0 pp 229533.A0A1C3YKW4
99799 229533.A0A098E2F0 pp 229533.A0A1C3YI20
99800 229533.A0A098E2F0 pp 229533.I1RCG5
99801 229533.A0A098E2F0 pp 229533.A0A0E0RU55
99802 229533.A0A098E2F0 pp 229533.I1RNB5
99803 229533.A0A098E2F0 pp 229533.I1RZ29
99804 229533.A0A098E2F0 pp 229533.I1S299
99805 229533.A0A098E2F0 pp 229533.I1RSQ4
99806 229533.A0A098E2F0 pp 229533.I1RQG7
99807 229533.A0A098E2F0 pp 229533.I1S8R2
99808 229533.A0A098E2F0 pp 229533.I1S4F4
99809 229533.A0A098E2F0 pp 229533.I1RYA0
99810 229533.A0A098E2F0 pp 229533.I1R9Y8
99811 229533.A0A098E2F0 pp 229533.I1RKB2
99812 229533.A0A098E2F0 pp 229533.I1RGC3
99813 229533.A0A098E2F0 pp 229533.I1RFI4
99814 229533.A0A098E2F0 pp 229533.I1RMJ3
99815 229533.A0A098E2F0 pp 229533.I1RBE6
99816 229533.A0A098E2F0 pp 229533.V6RUB1
99817 229533.A0A098E2F0 pp 229533.A0A1C3YHG7
99818 229533.A0A098E2F0 pp 229533.I1RA52
99819 229533.A0A098E2F0 pp 229533.I1RUR8
99820 229533.A0A098E2F0 pp 229533.I1S3T7
99821 229533.A0A098E2F0 pp 229533.I1RBF3
99822 229533.A0A098E2F0 pp 229533.I1REV2
99823 229533.A0A098E2F0 pp 229533.I1RC34
99824 229533.A0A098E2F0 pp 229533.Q4HVQ9
99825 229533.A0A098E2F0 pp 229533.I1S3G4
99826 229533.A0A098E2F0 pp 229533.A0A1C3YLE2
99827 229533.A0A098E2F0 pp 229533.A0A0E0SMT9
99828 229533.A0A098E2F0 pp 229533.I1S6P5
99829 229533.A0A098E2F0 pp 229533.O74638
99830 229533.A0A098E2F0 pp 229533.I1RC29
99831 229533.A0A098E2F0 pp 229533.A0A1C3YKN4
99832 229533.A0A098E2F0 pp 229533.A0A1C3YJI5
99833 229533.A0A098E2F0 pp 229533.I1RYW9
99834 229533.A0A098E2F0 pp 229533.I1RN88
99835 229533.A0A098E2F0 pp 229533.I1RZT0
99836 229533.A0A098E2F0 pp 229533.I1RDE7
99837 229533.A0A098E2F0 pp 229533.I1RLC1
99838 229533.I1RPX7 pp 229533.I1S559
99839 229533.I1RPX7 pp 229533.I1S7W2
99840 229533.I1RPX7 pp 229533.I1RTA3
99841 229533.I1RPX7 pp 229533.I1RSD7
99842 229533.I1RPX7 pp 229533.I1RRC4
99843 229533.I1RPX7 pp 229533.I1S270
99844 229533.I1RPX7 pp 229533.I1RRF9
99845 229533.I1RPX7 pp 229533.I1RRN4
99846 229533.A0A0E0SH87 pp 229533.I1RMW6
99847 229533.A0A0E0SH87 pp 229533.I1RY67
99848 229533.A0A0E0SH87 pp 229533.I1RQP1
99849 229533.A0A0E0SH87 pp 229533.I1RD48
99850 229533.A0A0E0SH87 pp 229533.A0A1C3YK06
99851 229533.A0A0E0SH87 pp 229533.I1RT41
99852 229533.A0A0E0SH87 pp 229533.I1RB00
99853 229533.A0A0E0SH87 pp 229533.I1RQP2
99854 229533.A0A0E0SH87 pp 229533.I1RUQ1
99855 229533.A0A0E0SH87 pp 229533.I1RAV9
99856 229533.A0A0E0SH87 pp 229533.I1RD61
99857 229533.A0A0E0SH87 pp 229533.I1RYI1
99858 229533.A0A0E0SH87 pp 229533.Q4I0J6
99859 229533.A0A0E0SH87 pp 229533.I1S0I9
99860 229533.A0A0E0SH87 pp 229533.I1RZF5
99861 229533.A0A0E0SH87 pp 229533.I1S0Z3
99862 229533.A0A0E0SH87 pp 229533.I1RT72
99863 229533.A0A0E0SH87 pp 229533.A0A1C3YJS7
99864 229533.A0A0E0SH87 pp 229533.I1RPK0

99865 229533.A0A0E0SH87 pp 229533.I1RDF8
99866 229533.A0A0E0SH87 pp 229533.A0A1C3YJG5
99867 229533.A0A0E0SH87 pp 229533.A0A0E0SJV3
99868 229533.A0A0E0SH87 pp 229533.Q4IBX0
99869 229533.A0A0E0SH87 pp 229533.I1RC02
99870 229533.A0A0E0SH87 pp 229533.A0A1C3YJK8
99871 229533.A0A0E0SH87 pp 229533.A0A1C3YJV9
99872 229533.A0A0E0SH87 pp 229533.A0A1C3YJN2
99873 229533.A0A0E0SH87 pp 229533.I1S1Y7
99874 229533.A0A1C3YHY5 pp 229533.I1S2B0
99875 229533.A0A1C3YHY5 pp 229533.I1RD38
99876 229533.A0A1C3YHY5 pp 229533.I1RTP2
99877 229533.A0A1C3YHY5 pp 229533.I1RHJ2
99878 229533.A0A1C3YHY5 pp 229533.A0A1C3YIX3
99879 229533.A0A1C3YHY5 pp 229533.I1RW16
99880 229533.A0A1C3YHY5 pp 229533.I1RQ48
99881 229533.A0A1C3YHY5 pp 229533.I1RAU3
99882 229533.A0A1C3YHY5 pp 229533.I1RR68
99883 229533.A0A1C3YHY5 pp 229533.I1RWN0
99884 229533.A0A1C3YHY5 pp 229533.I1RAJ0
99885 229533.A0A1C3YHY5 pp 229533.A0A1C3YMF6
99886 229533.A0A1C3YHY5 pp 229533.I1RK02
99887 229533.A0A1C3YHY5 pp 229533.A0A1C3YLQ4
99888 229533.A0A1C3YHY5 pp 229533.I1REC9
99889 229533.A0A1C3YHY5 pp 229533.I1RM32
99890 229533.A0A1C3YHY5 pp 229533.Q4I099
99891 229533.A0A1C3YHY5 pp 229533.I1RRC4
99892 229533.A0A1C3YHY5 pp 229533.I1REI2
99893 229533.A0A1C3YHY5 pp 229533.I1RED2
99894 229533.A0A1C3YHY5 pp 229533.I1RR72
99895 229533.A0A1C3YHY5 pp 229533.I1RK49
99896 229533.A0A1C3YHY5 pp 229533.I1RJZ7
99897 229533.A0A1C3YHY5 pp 229533.I1RMG4
99898 229533.A0A1C3YHY5 pp 229533.I1S0R5
99899 229533.A0A1C3YHY5 pp 229533.I1RFN8
99900 229533.A0A1C3YHY5 pp 229533.A0A1C3YJF7
99901 229533.A0A1C3YHY5 pp 229533.I1RQM9
99902 229533.A0A1C3YHY5 pp 229533.I1RN31
99903 229533.A0A1C3YHY5 pp 229533.I1RT99
99904 229533.A0A1C3YHY5 pp 229533.V6RXE7
99905 229533.A0A1C3YHY5 pp 229533.A0A1C3YJC4
99906 229533.A0A1C3YHY5 pp 229533.A0A1C3YJZ0
99907 229533.A0A1C3YHY5 pp 229533.I1RBL4
99908 229533.A0A1C3YHY5 pp 229533.A0A1C3YJ07
99909 229533.A0A1C3YHY5 pp 229533.I1S0Y1
99910 229533.A0A1C3YHY5 pp 229533.I1RCH4
99911 229533.A0A1C3YHY5 pp 229533.I1RUG0
99912 229533.A0A1C3YHY5 pp 229533.I1S8X0
99913 229533.A0A1C3YHY5 pp 229533.I1RRG7
99914 229533.A0A1C3YHY5 pp 229533.A0A1C3YJE2
99915 229533.A0A1C3YHY5 pp 229533.I1RPZ1
99916 229533.A0A1C3YHY5 pp 229533.I1RRN4
99917 229533.A0A1C3YHY5 pp 229533.I1RCM8
99918 229533.A0A1C3YHY5 pp 229533.I1RLX6
99919 229533.A0A1C3YHY5 pp 229533.I1S368
99920 229533.I1RQD4 pp 229533.V6QX58
99921 229533.I1RNY1 pp 229533.V6QXP4
99922 229533.I1RP92 pp 229533.I1RTG5
99923 229533.I1RP92 pp 229533.I1S1U6
99924 229533.I1RP92 pp 229533.I1RX87
99925 229533.I1RP92 pp 229533.I1S1C8
99926 229533.I1RP92 pp 229533.I1RTQ9
99927 229533.I1RA92 pp 229533.I1S0P5
99928 229533.I1RA92 pp 229533.I1RDX9
99929 229533.I1RA92 pp 229533.I1RZU5
99930 229533.A0A1C3YMP2 pp 229533.I1RHV1
99931 229533.A0A1C3YMP2 pp 229533.I1RHY5
99932 229533.A0A1C3YMP2 pp 229533.I1RU98
99933 229533.A0A1C3YMP2 pp 229533.I1RU06
99934 229533.A0A1C3YMP2 pp 229533.I1RWN0
99935 229533.A0A1C3YMP2 pp 229533.I1RR94
99936 229533.A0A1C3YMP2 pp 229533.I1RJ45
99937 229533.A0A1C3YMP2 pp 229533.I1S368

99938 229533.A0A1C3YMP2 pp 229533.I1REI8
99939 229533.A0A098DK04 pp 229533.V6RRM0
99940 229533.A0A098DK04 pp 229533.A0A098E2F0
99941 229533.A0A098DK04 pp 229533.I1RVX9
99942 229533.A0A098DK04 pp 229533.I1RW43
99943 229533.A0A098DK04 pp 229533.I1RY49
99944 229533.A0A098DK04 pp 229533.I1RM26
99945 229533.I1REB5 pp 229533.I1RWN0
99946 229533.I1REB5 pp 229533.I1RQM4
99947 229533.I1REB5 pp 229533.I1S2L1
99948 229533.I1REB5 pp 229533.I1REI4
99949 229533.I1REB5 pp 229533.I1S368
99950 229533.I1REB5 pp 229533.I1RFV2
99951 229533.I1REB5 pp 229533.I1RMG4
99952 229533.I1REB5 pp 229533.I1S360
99953 229533.I1REB5 pp 229533.I1RV85
99954 229533.I1REB5 pp 229533.I1RTP2
99955 229533.I1REB5 pp 229533.I1RZN1
99956 229533.I1RZ41 pp 229533.Q4HY20
99957 229533.A0A1C3YJN2 pp 229533.A0A1C3YK06
99958 229533.A0A1C3YJN2 pp 229533.V6RAC9
99959 229533.A0A1C3YJN2 pp 229533.A0A1C3YJS7
99960 229533.A0A1C3YJN2 pp 229533.I1RZF5
99961 229533.A0A1C3YJN2 pp 229533.I1RW54
99962 229533.A0A1C3YJN2 pp 229533.I1RT72
99963 229533.A0A1C3YLQ4 pp 229533.A0A1C3YMP2
99964 229533.A0A1C3YLQ4 pp 229533.I1RVA8
99965 229533.A0A1C3YLQ4 pp 229533.I1RFV2
99966 229533.A0A1C3YLQ4 pp 229533.I1RZN1
99967 229533.A0A1C3YLQ4 pp 229533.I1RCJ2
99968 229533.A0A1C3YLQ4 pp 229533.I1RMK6
99969 229533.A0A1C3YLQ4 pp 229533.I1RK02
99970 229533.A0A1C3YLQ4 pp 229533.I1REB5
99971 229533.A0A1C3YLQ4 pp 229533.I1S360
99972 229533.A0A1C3YLQ4 pp 229533.I1RE88
99973 229533.A0A1C3YLQ4 pp 229533.I1RKZ8
99974 229533.A0A1C3YLQ4 pp 229533.V6RXE7
99975 229533.A0A1C3YLQ4 pp 229533.I1RDZ5
99976 229533.A0A1C3YLQ4 pp 229533.I1RQM4
99977 229533.A0A1C3YLQ4 pp 229533.I1RJ45
99978 229533.A0A1C3YLQ4 pp 229533.I1RWN0
99979 229533.A0A1C3YLQ4 pp 229533.A0A1C3YMF6
99980 229533.A0A1C3YLQ4 pp 229533.I1S2L1
99981 229533.A0A1C3YLQ4 pp 229533.I1S368
99982 229533.A0A1C3YLQ4 pp 229533.I1RED2
99983 229533.A0A1C3YLQ4 pp 229533.I1S2B0
99984 229533.A0A1C3YLQ4 pp 229533.I1RMG4
99985 229533.A0A1C3YLQ4 pp 229533.I1RTP2
99986 229533.A0A1C3YJS7 pp 229533.I1RT72
99987 229533.A0A1C3YJS7 pp 229533.I1RPK0
99988 229533.A0A1C3YJS7 pp 229533.I1RMW6
99989 229533.A0A1C3YJS7 pp 229533.I1RNR0
99990 229533.A0A1C3YJS7 pp 229533.I1RC02
99991 229533.A0A1C3YJS7 pp 229533.I1RZF5
99992 229533.A0A1C3YJS7 pp 229533.I1RDF8
99993 229533.A0A1C3YJS7 pp 229533.I1RSM3
99994 229533.A0A1C3YJS7 pp 229533.I1RD48
99995 229533.A0A1C3YJS7 pp 229533.I1RY67
99996 229533.A0A1C3YJS7 pp 229533.I1S1Y7
99997 229533.A0A1C3YJS7 pp 229533.I1S0Z3
99998 229533.A0A1C3YJS7 pp 229533.I1RT41
99999 229533.A0A1C3YJS7 pp 229533.A0A1C3YK06
100000 229533.A0A1C3YJS7 pp 229533.I1S0I9
100001 229533.A0A098DJX8 pp 229533.A0A1C3YIH7
100002 229533.A0A098DJX8 pp 229533.A0A1C3YJG7
100003 229533.A0A098DJX8 pp 229533.I1RFL7
100004 229533.A0A098DJX8 pp 229533.I1REG5
100005 229533.I1RZL9 pp 229533.I1S270
100006 229533.I1RB06 pp 229533.I1RQN9
100007 229533.I1RB06 pp 229533.I1S0S6
100008 229533.I1RB06 pp 229533.I1RFX9
100009 229533.I1RB06 pp 229533.P0C431
100010 229533.I1RB06 pp 229533.I1RH68

100011	229533.I1RB06	pp	229533.I1RTB7
100012	229533.I1RB06	pp	229533.I1RB17
100013	229533.I1RB06	pp	229533.I1RFY2
100014	229533.I1RB06	pp	229533.I1RY98
100015	229533.I1RB06	pp	229533.I1RE67
100016	229533.I1RB06	pp	229533.I1RCZ5
100017	229533.I1RB06	pp	229533.I1RWM0
100018	229533.I1RB06	pp	229533.I1RYE2
100019	229533.I1RDZ5	pp	229533.I1REI4
100020	229533.I1RDZ5	pp	229533.I1REI2
100021	229533.I1RDZ5	pp	229533.I1RMG4
100022	229533.I1RDZ5	pp	229533.I1RK49
100023	229533.I1RDZ5	pp	229533.V6R398
100024	229533.I1RDZ5	pp	229533.I1RPA4
100025	229533.I1RDZ5	pp	229533.I1S368
100026	229533.I1RDZ5	pp	229533.I1RKZ8
100027	229533.I1RDZ5	pp	229533.I1RVS1
100028	229533.I1RDZ5	pp	229533.I1RJS0
100029	229533.I1RDZ5	pp	229533.I1RFV2
100030	229533.I1RDZ5	pp	229533.I1S130
100031	229533.I1RDZ5	pp	229533.V6RXE7
100032	229533.I1RDZ5	pp	229533.I1S2L1
100033	229533.I1RDZ5	pp	229533.I1RWN0
100034	229533.I1RDZ5	pp	229533.V6RG04
100035	229533.I1RDZ5	pp	229533.I1S108
100036	229533.I1RDZ5	pp	229533.I1RQM4
100037	229533.I1RDZ5	pp	229533.I1REB5
100038	229533.I1RDZ5	pp	229533.I1RF55
100039	229533.I1RDZ5	pp	229533.I1RE88
100040	229533.I1RDZ5	pp	229533.I1RTP2
100041	229533.I1RDZ5	pp	229533.I1RQM9
100042	229533.I1RDZ5	pp	229533.I1S360
100043	229533.I1RDZ5	pp	229533.I1RUP5
100044	229533.I1RDZ5	pp	229533.I1RZN1
100045	229533.I1RDZ5	pp	229533.I1RV85
100046	229533.I1RZH8	pp	229533.I1SAE8
100047	229533.I1RZH8	pp	229533.I1S6P5
100048	229533.I1RZH8	pp	229533.I1RZY1
100049	229533.I1RZH8	pp	229533.V6RUB1
100050	229533.I1RZH8	pp	229533.I1S024
100051	229533.I1RZH8	pp	229533.V6QV31
100052	229533.I1RZH8	pp	229533.I1S2N3
100053	229533.A0A098DAF7	pp	229533.A0A1C3YK06
100054	229533.A0A098DAF7	pp	229533.A0A0E0SH87
100055	229533.A0A098DAF7	pp	229533.A0A1C3YJS7
100056	229533.A0A098DAF7	pp	229533.A0A098DPD6
100057	229533.A0A098DAF7	pp	229533.I1RZF5
100058	229533.A0A098DAF7	pp	229533.I1RVJ7
100059	229533.A0A098DAF7	pp	229533.A0A0E0RVD7
100060	229533.A0A098DAF7	pp	229533.I1RW54
100061	229533.A0A098DAF7	pp	229533.I1RT72
100062	229533.I1RHV1	pp	229533.I1S0S5
100063	229533.I1RHV1	pp	229533.I1RWF9
100064	229533.I1RHV1	pp	229533.I1S368
100065	229533.I1RHV1	pp	229533.I1RJ45
100066	229533.I1RHV1	pp	229533.I1RJU6
100067	229533.I1RHV1	pp	229533.I1RHY5
100068	229533.I1RHV1	pp	229533.I1RSX7
100069	229533.I1RHV1	pp	229533.I1RN77
100070	229533.I1RHV1	pp	229533.I1RR94
100071	229533.I1RHV1	pp	229533.I1RU06
100072	229533.I1RMU5	pp	229533.I1RZA7
100073	229533.I1RMU5	pp	229533.V6RUB1
100074	229533.I1RMU5	pp	229533.I1RNB4
100075	229533.I1RMU5	pp	229533.I1RW83
100076	229533.I1RMU5	pp	229533.V6QWD8
100077	229533.I1RMU5	pp	229533.I1S2V3
100078	229533.I1RMU5	pp	229533.I1S3N0
100079	229533.I1RMU5	pp	229533.I1RZ29
100080	229533.I1RMU5	pp	229533.I1S0G7
100081	229533.I1RMU5	pp	229533.I1S3G4
100082	229533.I1RMU5	pp	229533.I1S1W8
100083	229533.I1RMU5	pp	229533.I1RXH1

100084 229533.I1RMU5 pp 229533.V6RN31
100085 229533.I1RMU5 pp 229533.I1RNP4
100086 229533.I1RMU5 pp 229533.I1S6P5
100087 229533.I1RMU5 pp 229533.I1S3X8
100088 229533.I1RMU5 pp 229533.I1RSL2
100089 229533.A0A098D1C1 pp 229533.I1REI8
100090 229533.A0A098D1C1 pp 229533.A0A1C3YII1
100091 229533.A0A098D1C1 pp 229533.I1RED2
100092 229533.A0A098D1C1 pp 229533.I1RHY5
100093 229533.A0A098D1C1 pp 229533.I1RU06
100094 229533.A0A098D1C1 pp 229533.I1RWN0
100095 229533.A0A098D1C1 pp 229533.A0A0E0SHL3
100096 229533.A0A098D1C1 pp 229533.I1RU98
100097 229533.A0A098D1C1 pp 229533.I1RJ45
100098 229533.A0A098D1C1 pp 229533.I1RR94
100099 229533.A0A098D1C1 pp 229533.A0A1C3YJJ7
100100 229533.A0A098D1C1 pp 229533.I1S2B0
100101 229533.A0A098D1C1 pp 229533.I1S368
100102 229533.A0A098D1C1 pp 229533.A0A1C3YHY5
100103 229533.A0A098D1C1 pp 229533.A0A1C3YMP2
100104 229533.A0A098D1C1 pp 229533.I1REB5
100105 229533.A0A098D1C1 pp 229533.A0A0E0RQT8
100106 229533.A0A098D1C1 pp 229533.A0A1C3YLQ4
100107 229533.A0A098D1C1 pp 229533.I1RDZ5
100108 229533.A0A098D1C1 pp 229533.I1RHV1
100109 229533.V6QT89 pp 229533.V6RKK5
100110 229533.V6QT89 pp 229533.V6R382
100111 229533.V6QT89 pp 229533.V6RQW4
100112 229533.V6QT89 pp 229533.V6QVM5
100113 229533.Q4IP51 pp 229533.V6RGI1
100114 229533.Q4IP51 pp 229533.V6RLW4
100115 229533.Q4IP51 pp 229533.Q4IQM6
100116 229533.A0A098DA13 pp 229533.I1RNM1
100117 229533.A0A098DA13 pp 229533.I1RT41
100118 229533.A0A098DA13 pp 229533.I1RB00
100119 229533.A0A098DA13 pp 229533.A0A0E0RTV8
100120 229533.A0A098DA13 pp 229533.A0A0E0RUB0
100121 229533.A0A098DA13 pp 229533.A0A1C3YK51
100122 229533.A0A098DA13 pp 229533.I1RY67
100123 229533.A0A098DA13 pp 229533.I1RBE7
100124 229533.A0A098DA13 pp 229533.A0A1C3YN54
100125 229533.A0A098DA13 pp 229533.I1RDF8
100126 229533.A0A098DA13 pp 229533.I1RC02
100127 229533.A0A098DA13 pp 229533.A0A098DPS3
100128 229533.A0A098DA13 pp 229533.I1RMW6
100129 229533.A0A098DA13 pp 229533.I1RDE6
100130 229533.A0A098DA13 pp 229533.A0A1C3YJG5
100131 229533.A0A098DA13 pp 229533.I1S1C8
100132 229533.A0A098DA13 pp 229533.I1RZF5
100133 229533.A0A098DA13 pp 229533.A0A098DBA1
100134 229533.A0A098DA13 pp 229533.A0A098E488
100135 229533.A0A098DA13 pp 229533.I1S0Z3
100136 229533.A0A098DA13 pp 229533.A0A1C3YKW5
100137 229533.A0A098DA13 pp 229533.I1RBW6
100138 229533.A0A098DA13 pp 229533.A0A1C3YIP6
100139 229533.A0A098DA13 pp 229533.I1REU2
100140 229533.A0A098DA13 pp 229533.A0A1C3YJR3
100141 229533.A0A098DA13 pp 229533.I1S0I9
100142 229533.A0A098DA13 pp 229533.A0A0E0RVD7
100143 229533.A0A098DA13 pp 229533.I1REG5
100144 229533.A0A098DA13 pp 229533.I1RT72
100145 229533.A0A098DA13 pp 229533.I1S1Y7
100146 229533.A0A098DA13 pp 229533.A0A098DUK7
100147 229533.A0A098DA13 pp 229533.I1RTQ9
100148 229533.A0A098DA13 pp 229533.I1RRB0
100149 229533.A0A098DA13 pp 229533.A0A1C3YJK8
100150 229533.A0A098DA13 pp 229533.I1RN88
100151 229533.A0A098DA13 pp 229533.I1RD70
100152 229533.A0A098DA13 pp 229533.I1RYW3
100153 229533.A0A098DA13 pp 229533.A0A1C3YHP8
100154 229533.A0A098DA13 pp 229533.A0A1C3YK06
100155 229533.A0A098DA13 pp 229533.A0A0E0SJV3
100156 229533.A0A098DA13 pp 229533.I1RD48

100157 229533.A0A098DA13 pp 229533.A0A1C3YIH7
100158 229533.A0A098DA13 pp 229533.I1RNB8
100159 229533.A0A098DA13 pp 229533.A0A0E0SH87
100160 229533.A0A098DA13 pp 229533.I1RP92
100161 229533.A0A098DA13 pp 229533.I1RPK0
100162 229533.A0A098DA13 pp 229533.A0A1C3YLK5
100163 229533.A0A098DA13 pp 229533.A0A1C3YJN2
100164 229533.A0A098DA13 pp 229533.A0A1C3YJS7
100165 229533.A0A098DA13 pp 229533.A0A1C3YM61
100166 229533.A0A098DA13 pp 229533.A0A098DAF7
100167 229533.A0A098DA13 pp 229533.I1RFZ2
100168 229533.A0A098DA13 pp 229533.V6QT89
100169 229533.A0A098DAD8 pp 229533.I1RYM5
100170 229533.A0A098DAD8 pp 229533.A0A1C3YKE0
100171 229533.A0A098DAD8 pp 229533.I1RUL3
100172 229533.A0A098DAD8 pp 229533.I1S2A1
100173 229533.A0A098DAD8 pp 229533.I1S0K2
100174 229533.A0A098DAD8 pp 229533.I1RCV1
100175 229533.A0A098DAD8 pp 229533.I1RAE8
100176 229533.A0A098DAD8 pp 229533.I1S8S9
100177 229533.A0A098DAD8 pp 229533.I1RAM9
100178 229533.A0A098DAD8 pp 229533.V6RJP8
100179 229533.A0A098DAD8 pp 229533.A0A098DGA1
100180 229533.A0A098DAD8 pp 229533.I1RS29
100181 229533.A0A098DAD8 pp 229533.I1RZW7
100182 229533.A0A098DAD8 pp 229533.I1S267
100183 229533.A0A098DAD8 pp 229533.I1RVV7
100184 229533.A0A098DAD8 pp 229533.I1RY21
100185 229533.A0A098DAD8 pp 229533.I1RJT5
100186 229533.A0A098DAD8 pp 229533.I1S5C6
100187 229533.A0A098DAD8 pp 229533.I1RHK2
100188 229533.A0A098DAD8 pp 229533.I1RFY0
100189 229533.A0A098DAD8 pp 229533.I1RYT8
100190 229533.A0A098DAD8 pp 229533.A0A1C3YKT4
100191 229533.A0A098DAD8 pp 229533.V6R7V8
100192 229533.A0A098DAD8 pp 229533.I1RB86
100193 229533.A0A098DAD8 pp 229533.I1RMQ6
100194 229533.A0A098DAD8 pp 229533.I1RST9
100195 229533.A0A098DAD8 pp 229533.A0A0E0SAR4
100196 229533.A0A098DAD8 pp 229533.I1S0P0
100197 229533.A0A098DAD8 pp 229533.I1RPN1
100198 229533.A0A098DAD8 pp 229533.I1RDH5
100199 229533.A0A098DAD8 pp 229533.I1RMK7
100200 229533.A0A098DAD8 pp 229533.I1RE66
100201 229533.A0A098DAD8 pp 229533.I1RYD3
100202 229533.A0A098DAD8 pp 229533.A0A1C3YKZ8
100203 229533.A0A098DAD8 pp 229533.A0A1C3YHV3
100204 229533.A0A098DAD8 pp 229533.A0A0E0SH57
100205 229533.A0A098DAD8 pp 229533.A0A1C3YLB6
100206 229533.A0A098DAD8 pp 229533.I1S287
100207 229533.A0A098DAD8 pp 229533.I1RS05
100208 229533.A0A098DAD8 pp 229533.I1S5X9
100209 229533.A0A098DAD8 pp 229533.I1RZJ8
100210 229533.A0A098DAD8 pp 229533.A0A1C3YNA1
100211 229533.A0A098DAD8 pp 229533.I1RFK3
100212 229533.A0A098DAD8 pp 229533.I1RFJ6
100213 229533.A0A098DAD8 pp 229533.A0A1C3YJK5
100214 229533.A0A098DAD8 pp 229533.A0A1C3YJ17
100215 229533.A0A098DAD8 pp 229533.A0A0E0S8Z0
100216 229533.A0A098DAD8 pp 229533.I1RY92
100217 229533.A0A098DAD8 pp 229533.I1RE65
100218 229533.A0A098DAD8 pp 229533.I1RBT8
100219 229533.A0A098DAD8 pp 229533.I1RPY8
100220 229533.A0A098DAD8 pp 229533.I1RT88
100221 229533.A0A098DAD8 pp 229533.I1RG46
100222 229533.A0A098DAD8 pp 229533.I1RA52
100223 229533.A0A098DAD8 pp 229533.I1RGY6
100224 229533.A0A098DAD8 pp 229533.I1RFI9
100225 229533.A0A098DAD8 pp 229533.I1S3L8
100226 229533.A0A098DAD8 pp 229533.I1RE53
100227 229533.A0A098DAD8 pp 229533.I1RBF3
100228 229533.A0A098DAD8 pp 229533.I1S464
100229 229533.A0A098DAD8 pp 229533.I1RWA3

100230 229533.A0A098DAD8 pp 229533.I1RBE6
100231 229533.A0A098DAD8 pp 229533.A0A1C3YL46
100232 229533.A0A098DAD8 pp 229533.I1RWF9
100233 229533.A0A098DAD8 pp 229533.I1RZ90
100234 229533.A0A098DAD8 pp 229533.I1RDL0
100235 229533.A0A098DAD8 pp 229533.A0A1C3YN70
100236 229533.A0A098DAD8 pp 229533.I1RF33
100237 229533.A0A098DAD8 pp 229533.A0A1C3YLM7
100238 229533.A0A098DAD8 pp 229533.I1RX96
100239 229533.A0A098DAD8 pp 229533.I1RGJ3
100240 229533.A0A098DAD8 pp 229533.I1RXD9
100241 229533.A0A1C3YJJ2 pp 229533.I1S4Q4
100242 229533.A0A1C3YJJ2 pp 229533.V6RM02
100243 229533.A0A1C3YJJ2 pp 229533.I1RSS4
100244 229533.A0A1C3YJJ2 pp 229533.I1RAQ4
100245 229533.A0A1C3YJJ2 pp 229533.I1RNM8
100246 229533.A0A1C3YJJ2 pp 229533.I1RB92
100247 229533.A0A1C3YJJ2 pp 229533.I1RA90
100248 229533.A0A1C3YJJ2 pp 229533.Q4IJK2
100249 229533.A0A1C3YJJ2 pp 229533.I1RM35
100250 229533.A0A1C3YJJ2 pp 229533.I1RUY6
100251 229533.A0A1C3YJJ2 pp 229533.I1RCH5
100252 229533.A0A1C3YJJ2 pp 229533.I1RVY2
100253 229533.A0A1C3YJJ2 pp 229533.V6RBN2
100254 229533.A0A1C3YJJ2 pp 229533.I1RWE8
100255 229533.A0A1C3YJJ2 pp 229533.I1RMH2
100256 229533.A0A1C3YJJ2 pp 229533.A0A1C3YLP6
100257 229533.A0A1C3YJJ2 pp 229533.I1RJL9
100258 229533.A0A1C3YJJ2 pp 229533.I1RIY0
100259 229533.A0A1C3YJJ2 pp 229533.V6RRH2
100260 229533.A0A1C3YJJ2 pp 229533.A0A1C3YKB0
100261 229533.A0A1C3YJJ2 pp 229533.I1RSL5
100262 229533.A0A1C3YJJ2 pp 229533.I1REL7
100263 229533.A0A1C3YJJ2 pp 229533.A0A1C3YLI1
100264 229533.A0A1C3YJJ2 pp 229533.I1RQA2
100265 229533.A0A1C3YJJ2 pp 229533.A0A1C3YKY0
100266 229533.A0A1C3YJJ2 pp 229533.I1RXY7
100267 229533.A0A1C3YJJ2 pp 229533.I1RFM6
100268 229533.A0A1C3YJJ2 pp 229533.A0A1C3YKV3
100269 229533.A0A1C3YJJ2 pp 229533.I1RAH6
100270 229533.A0A1C3YJJ2 pp 229533.I1RQ75
100271 229533.A0A1C3YJJ2 pp 229533.V6QUE6
100272 229533.A0A1C3YJJ2 pp 229533.I1RAZ3
100273 229533.A0A1C3YJJ2 pp 229533.A0A1C3YN86
100274 229533.A0A1C3YJJ2 pp 229533.I1RA70
100275 229533.A0A1C3YJJ2 pp 229533.V6RLM7
100276 229533.A0A1C3YJJ2 pp 229533.I1RRV4
100277 229533.A0A1C3YJJ2 pp 229533.I1S0N3
100278 229533.A0A1C3YJJ2 pp 229533.I1RW69
100279 229533.A0A1C3YJJ2 pp 229533.I1RYI7
100280 229533.A0A1C3YJJ2 pp 229533.I1RCW6
100281 229533.A0A1C3YJJ2 pp 229533.A0A1C3YK68
100282 229533.A0A1C3YJJ2 pp 229533.I1RS18
100283 229533.A0A1C3YJJ2 pp 229533.I1S2B8
100284 229533.A0A1C3YJJ2 pp 229533.I1RBE4
100285 229533.A0A1C3YJJ2 pp 229533.I1RR72
100286 229533.A0A1C3YJJ2 pp 229533.I1RML4
100287 229533.A0A1C3YJJ2 pp 229533.V6R382
100288 229533.A0A1C3YJJ2 pp 229533.I1RPU8
100289 229533.A0A1C3YJJ2 pp 229533.A0A1C3YLB6
100290 229533.A0A1C3YJJ2 pp 229533.I1RBF3
100291 229533.A0A1C3YJJ2 pp 229533.A0A1C3YK34
100292 229533.A0A1C3YJJ2 pp 229533.I1S0B7
100293 229533.A0A1C3YJJ2 pp 229533.I1RVR2
100294 229533.A0A1C3YJJ2 pp 229533.V6RMW6
100295 229533.A0A1C3YJJ2 pp 229533.V6RK96
100296 229533.A0A1C3YJJ2 pp 229533.I1RM67
100297 229533.A0A1C3YJJ2 pp 229533.I1RC48
100298 229533.A0A1C3YJJ2 pp 229533.I1RNC1
100299 229533.A0A1C3YJJ2 pp 229533.I1RMM8
100300 229533.A0A1C3YJJ2 pp 229533.I1RAA8
100301 229533.A0A1C3YJJ2 pp 229533.I1RFV3
100302 229533.A0A1C3YJJ2 pp 229533.I1RZ52

100303	229533.A0A1C3YJJ2	pp	229533.I1RL04
100304	229533.A0A1C3YJJ2	pp	229533.I1RKI3
100305	229533.A0A1C3YJJ2	pp	229533.I1RW43
100306	229533.A0A1C3YJJ2	pp	229533.I1RR24
100307	229533.A0A1C3YJJ2	pp	229533.I1RZ39
100308	229533.A0A1C3YJJ2	pp	229533.A0A1C3YKM9
100309	229533.A0A1C3YJJ2	pp	229533.I1RPN4
100310	229533.A0A1C3YJJ2	pp	229533.I1RYI5
100311	229533.A0A1C3YJJ2	pp	229533.I1S2H4
100312	229533.A0A1C3YJJ2	pp	229533.Q4IR09
100313	229533.A0A1C3YJJ2	pp	229533.I1RSK5
100314	229533.A0A1C3YJJ2	pp	229533.I1RRX5
100315	229533.A0A1C3YJJ2	pp	229533.I1RDE7
100316	229533.A0A1C3YJJ2	pp	229533.I1RQE8
100317	229533.A0A1C3YJJ2	pp	229533.I1RZM4
100318	229533.A0A1C3YJJ2	pp	229533.I1RWS4
100319	229533.A0A1C3YJJ2	pp	229533.I1S0K9
100320	229533.A0A1C3YJJ2	pp	229533.I1S4S4
100321	229533.A0A1C3YJJ2	pp	229533.I1RSQ4
100322	229533.A0A1C3YJJ2	pp	229533.I1RJS9
100323	229533.A0A1C3YJJ2	pp	229533.I1RJK3
100324	229533.A0A1C3YJJ2	pp	229533.I1RAJ6
100325	229533.A0A1C3YJJ2	pp	229533.I1RXB1
100326	229533.A0A1C3YJJ2	pp	229533.I1RZZ9
100327	229533.A0A1C3YJJ2	pp	229533.I1REK4
100328	229533.A0A1C3YJJ2	pp	229533.I1RYJ6
100329	229533.A0A1C3YJJ2	pp	229533.I1R9Y4
100330	229533.A0A1C3YJJ2	pp	229533.I1RVN1
100331	229533.A0A1C3YJJ2	pp	229533.V6RTJ8
100332	229533.A0A1C3YJJ2	pp	229533.I1RGA6
100333	229533.A0A1C3YJJ2	pp	229533.I1RKI7
100334	229533.A0A1C3YJJ2	pp	229533.I1RT33
100335	229533.A0A1C3YJJ2	pp	229533.I1RZB2
100336	229533.A0A1C3YJJ2	pp	229533.I1RPI5
100337	229533.A0A1C3YJJ2	pp	229533.I1RRP3
100338	229533.A0A1C3YJJ2	pp	229533.I1RN67
100339	229533.A0A1C3YJJ2	pp	229533.I1S338
100340	229533.A0A1C3YJJ2	pp	229533.I1RSS7
100341	229533.A0A1C3YJJ2	pp	229533.I1RNG4
100342	229533.A0A1C3YJJ2	pp	229533.I1RZ83
100343	229533.A0A1C3YJJ2	pp	229533.I1RP97
100344	229533.A0A1C3YJJ2	pp	229533.I1RSJ1
100345	229533.A0A1C3YJJ2	pp	229533.I1RW26
100346	229533.A0A1C3YJJ2	pp	229533.I1RC05
100347	229533.A0A1C3YJJ2	pp	229533.I1S3S3
100348	229533.A0A1C3YJJ2	pp	229533.Q4HXF0
100349	229533.A0A098D9X8	pp	229533.A0A1C3YJJ2
100350	229533.I1S287	pp	229533.I1S2W1
100351	229533.I1S287	pp	229533.I1S6S3
100352	229533.I1S287	pp	229533.I1S9X9
100353	229533.I1S287	pp	229533.I1S2B6
100354	229533.I1S287	pp	229533.I1S7S5
100355	229533.I1S287	pp	229533.I1S830
100356	229533.I1S287	pp	229533.I1SAM5
100357	229533.I1S287	pp	229533.V6RXW9
100358	229533.I1S287	pp	229533.Q4I5R9
100359	229533.I1S287	pp	229533.I1S9S4
100360	229533.I1S287	pp	229533.I1S3B7
100361	229533.I1S287	pp	229533.Q4HY20
100362	229533.I1S287	pp	229533.I1S4N5
100363	229533.I1S287	pp	229533.I1S7X9
100364	229533.A0A098DNS0	pp	229533.I1S0A0
100365	229533.A0A098DNS0	pp	229533.I1RZH4
100366	229533.A0A098DNS0	pp	229533.I1RHI1
100367	229533.A0A098DNS0	pp	229533.I1S209
100368	229533.A0A098DNS0	pp	229533.I1RXX1
100369	229533.A0A098DNS0	pp	229533.A0A1C3YK94
100370	229533.A0A098DNS0	pp	229533.I1RA94
100371	229533.A0A098DNS0	pp	229533.A0A1C3YLP6
100372	229533.A0A098DNS0	pp	229533.I1RIY0
100373	229533.A0A098DNS0	pp	229533.A0A1C3YHQ3
100374	229533.A0A098DNS0	pp	229533.A0A0E0SH52
100375	229533.A0A098DNS0	pp	229533.A0A1C3YIL8

100376 229533.A0A098DNS0 pp 229533.I1RR51
100377 229533.A0A098DNS0 pp 229533.I1RHZ5
100378 229533.A0A098DNS0 pp 229533.A0A1C3YKX1
100379 229533.A0A098DNS0 pp 229533.I1RMD3
100380 229533.A0A098DNS0 pp 229533.I1RC48
100381 229533.A0A098DNS0 pp 229533.I1RA90
100382 229533.A0A098DNS0 pp 229533.I1RBE4
100383 229533.A0A098DNS0 pp 229533.A0A1C3YN86
100384 229533.A0A098DNS0 pp 229533.I1RA12
100385 229533.A0A098DNS0 pp 229533.I1RQA2
100386 229533.A0A098DNS0 pp 229533.A0A1C3YKY4
100387 229533.A0A098DNS0 pp 229533.A0A1C3YJZ0
100388 229533.A0A098DNS0 pp 229533.I1RVP5
100389 229533.A0A098DNS0 pp 229533.I1RYB9
100390 229533.A0A098DNS0 pp 229533.I1RFM6
100391 229533.A0A098DNS0 pp 229533.I1RBH9
100392 229533.A0A098DNS0 pp 229533.Q4I0J6
100393 229533.A0A098DNS0 pp 229533.A0A1C3YK68
100394 229533.A0A098DNS0 pp 229533.A0A1C3YMG4
100395 229533.A0A098DNS0 pp 229533.I1RCN8
100396 229533.A0A098DNS0 pp 229533.A0A1C3YIK4
100397 229533.A0A098DNS0 pp 229533.I1S887
100398 229533.A0A098DNS0 pp 229533.I1RPL2
100399 229533.A0A098DNS0 pp 229533.I1S604
100400 229533.A0A098DNS0 pp 229533.A0A1C3YKR5
100401 229533.A0A098DNS0 pp 229533.I1RQA5
100402 229533.A0A098DNS0 pp 229533.I1RK83
100403 229533.A0A098DNS0 pp 229533.I1S0N3
100404 229533.A0A098DNS0 pp 229533.I1S4D0
100405 229533.A0A098DNS0 pp 229533.I1RHR3
100406 229533.A0A098DNS0 pp 229533.I1RWH1
100407 229533.A0A098DNS0 pp 229533.I1RE46
100408 229533.A0A098DNS0 pp 229533.I1RAQ4
100409 229533.A0A098DNS0 pp 229533.I1RMW4
100410 229533.A0A098DNS0 pp 229533.I1RAZ3
100411 229533.A0A098DNS0 pp 229533.I1S250
100412 229533.A0A098DNS0 pp 229533.I1RMG4
100413 229533.A0A098DNS0 pp 229533.A0A0E0RTV8
100414 229533.A0A098DNS0 pp 229533.I1RN46
100415 229533.A0A098DNS0 pp 229533.I1RVY2
100416 229533.A0A098DNS0 pp 229533.I1RW17
100417 229533.A0A098DNS0 pp 229533.V6RK96
100418 229533.A0A098DNS0 pp 229533.Q4IK03
100419 229533.A0A098DNS0 pp 229533.V6RY56
100420 229533.A0A098DNS0 pp 229533.I1S0K0
100421 229533.A0A098DNS0 pp 229533.I1RK03
100422 229533.A0A098DNS0 pp 229533.I1RT09
100423 229533.A0A098DNS0 pp 229533.I1RKI2
100424 229533.A0A098DNS0 pp 229533.I1RXS0
100425 229533.A0A098DNS0 pp 229533.A0A1C3YLI1
100426 229533.A0A098DNS0 pp 229533.V6RQW4
100427 229533.A0A098DNS0 pp 229533.I1RME6
100428 229533.A0A098DNS0 pp 229533.Q4IBX0
100429 229533.A0A098DNS0 pp 229533.I1RCL7
100430 229533.A0A098DNS0 pp 229533.I1RPI5
100431 229533.A0A098DNS0 pp 229533.V6RUT5
100432 229533.A0A098DNS0 pp 229533.I1RQH1
100433 229533.A0A098DNS0 pp 229533.I1RP86
100434 229533.A0A098DNS0 pp 229533.I1RFJ2
100435 229533.A0A098DNS0 pp 229533.A0A1C3YJL7
100436 229533.A0A098DNS0 pp 229533.I1RDQ2
100437 229533.A0A098DNS0 pp 229533.I1S236
100438 229533.A0A098DNS0 pp 229533.A0A1C3YJN7
100439 229533.A0A098DNS0 pp 229533.I1RBC1
100440 229533.A0A098DNS0 pp 229533.I1RQE1
100441 229533.A0A098DNS0 pp 229533.A0A098DVI8
100442 229533.A0A098DNS0 pp 229533.I1RJY7
100443 229533.A0A098DNS0 pp 229533.I1RMG7
100444 229533.A0A098DNS0 pp 229533.I1RYJ6
100445 229533.A0A098DNS0 pp 229533.I1RN55
100446 229533.A0A098DNS0 pp 229533.I1RMK4
100447 229533.A0A098DNS0 pp 229533.I1S338
100448 229533.A0A098DNS0 pp 229533.I1S0T2

229533.A0A098DNS0 pp 229533.V6R1K4
229533.A0A098DNS0 pp 229533.I1RBI6
229533.A0A098DNS0 pp 229533.I1RCY0
229533.A0A098DNS0 pp 229533.A0A1C3YJJ2
229533.A0A098DNS0 pp 229533.I1RYM6
229533.A0A098DNS0 pp 229533.A0A098DVZ4
229533.A0A098DNS0 pp 229533.I1S5I8
229533.A0A098DNS0 pp 229533.I1RZM4
229533.A0A098DNS0 pp 229533.I1RYX7
229533.A0A098DNS0 pp 229533.A0A1C3YLC3
229533.A0A098DNS0 pp 229533.A0A1C3YL20
229533.A0A098DNS0 pp 229533.I1S1D1
229533.A0A098DNS0 pp 229533.I1RD18
229533.A0A098DNS0 pp 229533.I1RA85
229533.A0A098DNS0 pp 229533.I1RM67
229533.A0A098DNS0 pp 229533.I1RFV2
229533.A0A098DNS0 pp 229533.A0A1C3YKW5
229533.A0A098DNS0 pp 229533.I1RBE3
229533.A0A098DNS0 pp 229533.A0A1C3YK11
229533.A0A098DNS0 pp 229533.I1RAJ6
229533.A0A098DNS0 pp 229533.A0A1C3YHX9
229533.A0A098DNS0 pp 229533.I1S283
229533.A0A098DNS0 pp 229533.I1RSI4
229533.A0A098DNS0 pp 229533.A0A098DQ41
229533.A0A098DNS0 pp 229533.I1RRG3
229533.A0A098DNS0 pp 229533.I1S8C1
229533.A0A098DNS0 pp 229533.I1R9B6
229533.A0A098DNS0 pp 229533.A0A1C3YKS6
229533.A0A098DNS0 pp 229533.I1RXB1
229533.A0A098DNS0 pp 229533.I1S8E0
229533.A0A098DNS0 pp 229533.I1RMT4
229533.A0A098DNS0 pp 229533.I1RAR3
229533.A0A098DNS0 pp 229533.I1RBD5
229533.A0A098DNS0 pp 229533.I1RNK8
229533.A0A098DNS0 pp 229533.I1S863
229533.A0A098DNS0 pp 229533.I1RMM8
229533.A0A098DNS0 pp 229533.A0A1C3YLR6
229533.A0A098DNS0 pp 229533.I1RAA8
229533.A0A098DNS0 pp 229533.I1RQE8
229533.A0A098DNS0 pp 229533.A0A1C3YI64
229533.A0A098DNS0 pp 229533.I1RX17
229533.A0A098DNS0 pp 229533.I1S3L2
229533.A0A098DNS0 pp 229533.I1RZ02
229533.A0A098DNS0 pp 229533.I1RAH4
229533.A0A098DNS0 pp 229533.I1RKI3
229533.A0A098DNS0 pp 229533.I1RYQ4
229533.A0A098DNS0 pp 229533.I1RBW0
229533.A0A098DNS0 pp 229533.A0A1C3YN30
229533.A0A098DNS0 pp 229533.A0A1C3YN26
229533.A0A098DNS0 pp 229533.I1S029
229533.A0A098DNS0 pp 229533.I1RAM6
229533.A0A098DNS0 pp 229533.I1RPA4
229533.A0A098DNS0 pp 229533.I1RFX8
229533.A0A098DNS0 pp 229533.I1RFH9
229533.A0A098DNS0 pp 229533.I1S1E4
229533.A0A098DNS0 pp 229533.I1RQ60
229533.A0A098DNS0 pp 229533.I1RTG4
229533.A0A098DNS0 pp 229533.A0A1C3YKM5
229533.A0A098DNS0 pp 229533.V6RRM0
229533.A0A098DNS0 pp 229533.I1S1W8
229533.A0A098DNS0 pp 229533.I1RC54
229533.A0A098DNS0 pp 229533.I1RBC8
229533.A0A098DNS0 pp 229533.I1RMQ5
229533.A0A098DNS0 pp 229533.I1RBS5
229533.A0A098DNS0 pp 229533.I1RQP2
229533.A0A098DNS0 pp 229533.I1RPU8
229533.A0A098DNS0 pp 229533.I1S8R4
229533.A0A098DNS0 pp 229533.I1RML4
229533.A0A098DNS0 pp 229533.I1RSU2
229533.A0A098DNS0 pp 229533.I1S1W3
229533.A0A098DNS0 pp 229533.I1RQ13
229533.A0A098DNS0 pp 229533.I1RAY9
229533.A0A098DNS0 pp 229533.I1S1W9

100522	229533.A0A098DNS0	pp	229533.I1SAN9
100523	229533.A0A098DNS0	pp	229533.I1S274
100524	229533.A0A098DNS0	pp	229533.I1RN84
100525	229533.A0A098DNS0	pp	229533.I1RBD6
100526	229533.A0A098DNS0	pp	229533.I1S9Z4
100527	229533.A0A098DNS0	pp	229533.I1RXU9
100528	229533.A0A098DNS0	pp	229533.I1RMS4
100529	229533.A0A098DNS0	pp	229533.I1RKZ7
100530	229533.A0A098DNS0	pp	229533.I1RSC7
100531	229533.A0A098DNS0	pp	229533.Q4ILH3
100532	229533.A0A098DNS0	pp	229533.I1RZ98
100533	229533.A0A098DNS0	pp	229533.V6R382
100534	229533.A0A098DNS0	pp	229533.I1RBZ3
100535	229533.A0A098DNS0	pp	229533.A0A1C3YMK0
100536	229533.A0A098DNS0	pp	229533.I1RCW1
100537	229533.A0A098DNS0	pp	229533.V6RLM7
100538	229533.A0A098DNS0	pp	229533.A0A1C3YI05
100539	229533.A0A098DNS0	pp	229533.A0A1C3YIB0
100540	229533.A0A098DNS0	pp	229533.A0A1C3YLB2
100541	229533.A0A098DNS0	pp	229533.I1RXY7
100542	229533.A0A098DNS0	pp	229533.I1RZP8
100543	229533.A0A098DNS0	pp	229533.I1RSL5
100544	229533.A0A098DNS0	pp	229533.I1RPK0
100545	229533.A0A098DNS0	pp	229533.I1RS17
100546	229533.A0A098DNS0	pp	229533.I1RRZ6
100547	229533.A0A098DNS0	pp	229533.I1RX64
100548	229533.A0A098DNS0	pp	229533.V6QT89
100549	229533.A0A098DNS0	pp	229533.I1RN57
100550	229533.A0A098DNS0	pp	229533.I1RWT0
100551	229533.A0A098DNS0	pp	229533.I1RW78
100552	229533.A0A098DNS0	pp	229533.I1RQJ4
100553	229533.A0A098DNS0	pp	229533.I1S1D3
100554	229533.A0A098DNS0	pp	229533.I1RPL1
100555	229533.A0A098DNS0	pp	229533.I1RNV4
100556	229533.A0A098DNS0	pp	229533.I1RHG5
100557	229533.A0A098DNS0	pp	229533.I1RNM8
100558	229533.A0A098DNS0	pp	229533.A0A0E0SIE1
100559	229533.A0A098DNS0	pp	229533.V6R057
100560	229533.A0A098DNS0	pp	229533.A0A0E0RU55
100561	229533.A0A098DNS0	pp	229533.A0A0E0SH88
100562	229533.A0A098DNS0	pp	229533.I1RGL3
100563	229533.A0A098DNS0	pp	229533.I1S0T4
100564	229533.A0A098DNS0	pp	229533.A0A0E0SK74
100565	229533.A0A098DNS0	pp	229533.A0A0E0SFP0
100566	229533.A0A098DNS0	pp	229533.I1RI91
100567	229533.A0A098DNS0	pp	229533.A0A1C3YLB6
100568	229533.A0A098DNS0	pp	229533.A0A1C3YLC1
100569	229533.A0A098DNS0	pp	229533.I1RDX0
100570	229533.A0A098DNS0	pp	229533.A0A1C3YHU3
100571	229533.A0A098DNS0	pp	229533.I1RQ30
100572	229533.A0A098DNS0	pp	229533.I1RUB4
100573	229533.A0A098DNS0	pp	229533.V6QVH8
100574	229533.A0A098DNS0	pp	229533.I1RMH2
100575	229533.A0A098DNS0	pp	229533.I1SAJ3
100576	229533.A0A098DNS0	pp	229533.I1RIH0
100577	229533.A0A098DNS0	pp	229533.I1RD19
100578	229533.A0A098DNS0	pp	229533.I1RY74
100579	229533.A0A098DNS0	pp	229533.I1RCG5
100580	229533.A0A098DNS0	pp	229533.I1RE53
100581	229533.A0A098DNS0	pp	229533.V6RM02
100582	229533.A0A098DNS0	pp	229533.I1RNG4
100583	229533.A0A098DNS0	pp	229533.I1RZ70
100584	229533.A0A098DNS0	pp	229533.I1RRP3
100585	229533.A0A098DNS0	pp	229533.I1RSY2
100586	229533.A0A098DNS0	pp	229533.A0A1C3YL99
100587	229533.A0A098DNS0	pp	229533.I1RZN1
100588	229533.A0A098DNS0	pp	229533.I1RXR1
100589	229533.A0A098DNS0	pp	229533.I1RMG6
100590	229533.A0A098DNS0	pp	229533.A0A1C3YLN5
100591	229533.A0A098DNS0	pp	229533.I1RW26
100592	229533.A0A098DNS0	pp	229533.I1RDJ8
100593	229533.A0A098DNS0	pp	229533.A0A0E0SMR8
100594	229533.A0A098DNS0	pp	229533.I1RZ83

100595 229533.A0A098DNS0 pp 229533.I1RP97
100596 229533.A0A098DNS0 pp 229533.Q4HXF0
100597 229533.A0A098DNS0 pp 229533.A0A1C3YMC1
100598 229533.A0A098DNS0 pp 229533.I1RBY9
100599 229533.A0A098DNS0 pp 229533.A0A0E0SPV0
100600 229533.A0A098DNS0 pp 229533.I1RSS7
100601 229533.A0A098DNS0 pp 229533.I1S4B7
100602 229533.A0A098DNS0 pp 229533.I1RWW7
100603 229533.A0A098DNS0 pp 229533.A0A1C3YL55
100604 229533.A0A098DNS0 pp 229533.I1RQG4
100605 229533.A0A098DNS0 pp 229533.I1RT75
100606 229533.A0A098DNS0 pp 229533.I1RSC0
100607 229533.A0A098DNS0 pp 229533.I1RWF9
100608 229533.A0A098DNS0 pp 229533.A0A1C3YI26
100609 229533.A0A098DNS0 pp 229533.I1RQ67
100610 229533.A0A098DNS0 pp 229533.I1RKI7
100611 229533.A0A098DNS0 pp 229533.I1S2P8
100612 229533.A0A098DNS0 pp 229533.A0A0E0S0Q5
100613 229533.A0A098DNS0 pp 229533.I1RFZ2
100614 229533.A0A098DNS0 pp 229533.I1RXA9
100615 229533.A0A098DNS0 pp 229533.A0A1C3YLE2
100616 229533.A0A098DNS0 pp 229533.I1R9W4
100617 229533.A0A098DNS0 pp 229533.I1S237
100618 229533.A0A098DNS0 pp 229533.I1RYX9
100619 229533.A0A098DNS0 pp 229533.A0A098E0A5
100620 229533.A0A098DNS0 pp 229533.I1S9C6
100621 229533.A0A098DNS0 pp 229533.I1RP28
100622 229533.A0A098DNS0 pp 229533.I1RVU7
100623 229533.A0A098DNS0 pp 229533.I1RC31
100624 229533.A0A098DNS0 pp 229533.I1RBD7
100625 229533.A0A098DNS0 pp 229533.I1RBW1
100626 229533.A0A098DNS0 pp 229533.I1S0J8
100627 229533.A0A098DNS0 pp 229533.I1RMJ3
100628 229533.A0A098DNS0 pp 229533.A0A0E0SGM4
100629 229533.A0A098DNS0 pp 229533.I1RWI5
100630 229533.A0A098DNS0 pp 229533.I1S3G4
100631 229533.A0A098DNS0 pp 229533.A0A1C3YL82
100632 229533.A0A098DNS0 pp 229533.I1RJK3
100633 229533.A0A098DNS0 pp 229533.A0A0E0S1J9
100634 229533.A0A098DNS0 pp 229533.O74638
100635 229533.A0A098DNS0 pp 229533.I1RJS9
100636 229533.A0A098DNS0 pp 229533.I1RDK0
100637 229533.A0A098DNS0 pp 229533.I1RWS4
100638 229533.A0A098DNS0 pp 229533.I1REI3
100639 229533.A0A098DNS0 pp 229533.I1RF70
100640 229533.A0A098DNS0 pp 229533.I1RJQ5
100641 229533.A0A098DNS0 pp 229533.I1S0K9
100642 229533.A0A098DNS0 pp 229533.I1RXV3
100643 229533.A0A098DNS0 pp 229533.I1RZ52
100644 229533.A0A098DNS0 pp 229533.A0A098DQK4
100645 229533.A0A098DNS0 pp 229533.I1S277
100646 229533.A0A098DNS0 pp 229533.A0A098DTI0
100647 229533.A0A098DNS0 pp 229533.I1RSK5
100648 229533.A0A098DNS0 pp 229533.I1RXB2
100649 229533.A0A098DNS0 pp 229533.I1RW43
100650 229533.A0A098DNS0 pp 229533.I1S0Y7
100651 229533.A0A098DNS0 pp 229533.I1RH91
100652 229533.A0A098DNS0 pp 229533.I1S4B6
100653 229533.A0A098DNS0 pp 229533.I1RZ86
100654 229533.A0A098DNS0 pp 229533.I1RVR2
100655 229533.A0A098DNS0 pp 229533.I1RUR8
100656 229533.A0A098DNS0 pp 229533.I1RDE7
100657 229533.A0A098DNS0 pp 229533.I1RBF3
100658 229533.A0A098DNS0 pp 229533.I1RTG7
100659 229533.A0A098DNS0 pp 229533.I1RC35
100660 229533.A0A098DNS0 pp 229533.V6RLW4
100661 229533.A0A098DNS0 pp 229533.I1RCA5
100662 229533.A0A098DNS0 pp 229533.I1RSZ7
100663 229533.I1RNF3 pp 229533.I1RRF9
100664 229533.I1RNF3 pp 229533.I1RTA3
100665 229533.Q4HZQ1 pp 229533.V6RDH3
100666 229533.Q4HZQ1 pp 229533.V6QV31
100667 229533.Q4HZQ1 pp 229533.Q4I375

100668 229533.Q4HZQ1 pp 229533.Q4I1N3
100669 229533.Q4HZQ1 pp 229533.V6RS91
100670 229533.A0A098D9U9 pp 229533.A0A098DNS0
100671 229533.I1RFY9 pp 229533.I1RSE6
100672 229533.I1RFY9 pp 229533.I1RYD8
100673 229533.A0A1C3YJT9 pp 229533.I1RUP2
100674 229533.A0A1C3YJT9 pp 229533.A0A1C3YM54
100675 229533.A0A1C3YJT9 pp 229533.I1RW06
100676 229533.A0A1C3YJT9 pp 229533.I1RNJ7
100677 229533.A0A1C3YJT9 pp 229533.I1RQG7
100678 229533.A0A1C3YJT9 pp 229533.I1RBE4
100679 229533.I1R9Y8 pp 229533.I1RMQ5
100680 229533.I1R9Y8 pp 229533.I1RLW5
100681 229533.I1R9Y8 pp 229533.I1RSQ3
100682 229533.I1R9Y8 pp 229533.I1RBS5
100683 229533.I1R9Y8 pp 229533.I1RQ21
100684 229533.I1R9Y8 pp 229533.I1RZ95
100685 229533.I1R9Y8 pp 229533.I1S0S5
100686 229533.I1R9Y8 pp 229533.I1RGX1
100687 229533.I1R9Y8 pp 229533.I1RSF1
100688 229533.I1R9Y8 pp 229533.I1RMQ3
100689 229533.I1R9Y8 pp 229533.I1S272
100690 229533.I1R9Y8 pp 229533.Q4IPZ1
100691 229533.I1R9Y8 pp 229533.I1RME4
100692 229533.I1R9Y8 pp 229533.V6RLM7
100693 229533.I1R9Y8 pp 229533.I1RRN2
100694 229533.I1R9Y8 pp 229533.I1RWH4
100695 229533.I1R9Y8 pp 229533.I1RZD3
100696 229533.I1R9Y8 pp 229533.I1RBY6
100697 229533.I1R9Y8 pp 229533.I1RFS5
100698 229533.I1R9Y8 pp 229533.I1RNJ5
100699 229533.I1R9Y8 pp 229533.Q4HZ95
100700 229533.I1R9Y8 pp 229533.I1RAN6
100701 229533.I1R9Y8 pp 229533.I1S0Z8
100702 229533.I1R9Y8 pp 229533.I1RNU7
100703 229533.I1R9Y8 pp 229533.I1S5R6
100704 229533.I1R9Y8 pp 229533.I1S1W3
100705 229533.I1R9Y8 pp 229533.I1RHW9
100706 229533.I1R9Y8 pp 229533.I1RPV0
100707 229533.I1R9Y8 pp 229533.I1RDJ8
100708 229533.I1R9Y8 pp 229533.I1RCH5
100709 229533.I1R9Y8 pp 229533.Q4I7G0
100710 229533.I1R9Y8 pp 229533.I1RZS8
100711 229533.I1R9Y8 pp 229533.Q4I375
100712 229533.I1R9Y8 pp 229533.V6RRM0
100713 229533.I1R9Y8 pp 229533.I1REG1
100714 229533.I1R9Y8 pp 229533.I1S5H2
100715 229533.I1R9Y8 pp 229533.I1RPN8
100716 229533.I1R9Y8 pp 229533.I1RZS4
100717 229533.I1R9Y8 pp 229533.I1RL79
100718 229533.I1R9Y8 pp 229533.I1RCS6
100719 229533.I1R9Y8 pp 229533.I1RRI2
100720 229533.I1R9Y8 pp 229533.I1REB8
100721 229533.I1R9Y8 pp 229533.I1RVX0
100722 229533.I1R9Y8 pp 229533.I1RZ13
100723 229533.I1R9Y8 pp 229533.I1RVY0
100724 229533.I1R9Y8 pp 229533.I1S0R9
100725 229533.I1R9Y8 pp 229533.I1RA86
100726 229533.I1R9Y8 pp 229533.I1RBA4
100727 229533.I1R9Y8 pp 229533.I1RAM2
100728 229533.I1R9Y8 pp 229533.I1RMB1
100729 229533.I1R9Y8 pp 229533.I1RZI8
100730 229533.I1R9Y8 pp 229533.I1REC7
100731 229533.I1R9Y8 pp 229533.I1S277
100732 229533.I1R9Y8 pp 229533.I1RZK1
100733 229533.I1R9Y8 pp 229533.I1RKB6
100734 229533.I1R9Y8 pp 229533.I1RL04
100735 229533.I1R9Y8 pp 229533.I1REC6
100736 229533.I1R9Y8 pp 229533.I1RU27
100737 229533.I1R9Y8 pp 229533.I1RPV3
100738 229533.I1R9Y8 pp 229533.I1RTE9
100739 229533.I1R9Y8 pp 229533.I1RPP3
100740 229533.I1R9Y8 pp 229533.I1RQG4

100741	229533.I1R9Y8	pp	229533.I1RFS3
100742	229533.I1R9Y8	pp	229533.Q4I099
100743	229533.I1R9Y8	pp	229533.I1RC55
100744	229533.I1R9Y8	pp	229533.I1RBT8
100745	229533.I1R9Y8	pp	229533.I1RBF3
100746	229533.I1R9Y8	pp	229533.I1RP28
100747	229533.I1R9Y8	pp	229533.Q4I7T5
100748	229533.I1R9Y8	pp	229533.I1RYP2
100749	229533.I1R9Y8	pp	229533.I1RPY0
100750	229533.I1R9Y8	pp	229533.I1RRT3
100751	229533.I1R9Y8	pp	229533.I1RC18
100752	229533.I1R9Y8	pp	229533.I1RTK5
100753	229533.I1R9Y8	pp	229533.I1RPD6
100754	229533.I1R9Y8	pp	229533.I1RRT8
100755	229533.I1R9Y8	pp	229533.I1RSX0
100756	229533.I1R9Y8	pp	229533.I1RY57
100757	229533.I1R9Y8	pp	229533.Q4IB65
100758	229533.I1R9Y8	pp	229533.V6R3J1
100759	229533.I1R9Y8	pp	229533.O74638
100760	229533.I1R9Y8	pp	229533.I1RKP3
100761	229533.I1R9Y8	pp	229533.I1RZH5
100762	229533.I1R9Y8	pp	229533.I1RJT3
100763	229533.I1R9Y8	pp	229533.I1RC61
100764	229533.I1R9Y8	pp	229533.I1S2V2
100765	229533.I1R9Y8	pp	229533.I1RY80
100766	229533.I1R9Y8	pp	229533.I1RG71
100767	229533.I1R9Y8	pp	229533.I1RWK9
100768	229533.I1R9Y8	pp	229533.I1RXF0
100769	229533.I1R9Y8	pp	229533.I1RPW1
100770	229533.I1R9Y8	pp	229533.I1RKP1
100771	229533.I1R9Y8	pp	229533.I1RW87
100772	229533.I1R9Y8	pp	229533.I1RBW1
100773	229533.I1R9Y8	pp	229533.I1S235
100774	229533.I1R9Y8	pp	229533.I1RUY4
100775	229533.I1R9Y8	pp	229533.I1S3G4
100776	229533.I1R9Y8	pp	229533.I1RKZ4
100777	229533.I1R9Y8	pp	229533.I1RBY7
100778	229533.I1R9Y8	pp	229533.I1S6K9
100779	229533.I1R9Y8	pp	229533.I1RT38
100780	229533.I1R9Y8	pp	229533.I1RWI5
100781	229533.I1R9Y8	pp	229533.I1RBE4
100782	229533.I1R9Y8	pp	229533.I1RSQ4
100783	229533.I1R9Y8	pp	229533.I1RDE4
100784	229533.I1R9Y8	pp	229533.I1RBL7
100785	229533.I1R9Y8	pp	229533.I1RPR9
100786	229533.I1R9Y8	pp	229533.I1RPT0
100787	229533.I1R9Y8	pp	229533.I1S821
100788	229533.I1R9Y8	pp	229533.I1S0F9
100789	229533.I1R9Y8	pp	229533.I1RBC1
100790	229533.I1R9Y8	pp	229533.I1RXZ3
100791	229533.I1R9Y8	pp	229533.I1RAB3
100792	229533.I1R9Y8	pp	229533.I1RYL5
100793	229533.I1R9Y8	pp	229533.I1RBC4
100794	229533.I1R9Y8	pp	229533.I1S8S9
100795	229533.I1R9Y8	pp	229533.I1RGG6
100796	229533.I1R9Y8	pp	229533.I1S3S5
100797	229533.I1R9Y8	pp	229533.I1RBN0
100798	229533.I1R9Y8	pp	229533.I1RDH7
100799	229533.I1R9Y8	pp	229533.I1RG14
100800	229533.I1R9Y8	pp	229533.Q4I6B0
100801	229533.I1R9Y8	pp	229533.I1RPL2
100802	229533.I1R9Y8	pp	229533.I1RP66
100803	229533.I1R9Y8	pp	229533.I1RT09
100804	229533.I1R9Y8	pp	229533.I1RQA5
100805	229533.I1R9Y8	pp	229533.I1RKK8
100806	229533.I1R9Y8	pp	229533.Q4ICM9
100807	229533.I1R9Y8	pp	229533.I1RUL3
100808	229533.I1R9Y8	pp	229533.I1RFY0
100809	229533.I1R9Y8	pp	229533.I1RQR6
100810	229533.I1R9Y8	pp	229533.I1RAR2
100811	229533.I1R9Y8	pp	229533.I1S2A1
100812	229533.I1R9Y8	pp	229533.I1RG60
100813	229533.I1R9Y8	pp	229533.I1RBU8

229533.I1R9Y8	pp	229533.I1RA94
229533.I1R9Y8	pp	229533.I1RM62
229533.I1R9Y8	pp	229533.I1RG62
229533.I1R9Y8	pp	229533.I1RY63
229533.I1R9Y8	pp	229533.I1RF94
229533.I1R9Y8	pp	229533.I1RK45
229533.I1R9Y8	pp	229533.V6QUG0
229533.I1R9Y8	pp	229533.Q4IJW4
229533.I1R9Y8	pp	229533.I1S6D7
229533.I1R9Y8	pp	229533.Q4IK03
229533.I1R9Y8	pp	229533.I1RM03
229533.I1R9Y8	pp	229533.I1RPN1
229533.I1R9Y8	pp	229533.I1RE48
229533.I1R9Y8	pp	229533.I1RDI8
229533.I1R9Y8	pp	229533.I1RQH1
229533.I1R9Y8	pp	229533.I1RPA4
229533.I1R9Y8	pp	229533.I1RRF8
229533.I1R9Y8	pp	229533.I1RJT9
229533.I1R9Y8	pp	229533.I1RDJ1
229533.I1R9Y8	pp	229533.V6R7V8
229533.I1R9Y8	pp	229533.I1RAR3
229533.I1R9Y8	pp	229533.I1S2C1
229533.I1R9Y8	pp	229533.I1RJN7
229533.I1R9Y8	pp	229533.I1RRT6
229533.I1R9Y8	pp	229533.I1RP86
229533.I1R9Y8	pp	229533.I1S1W8
229533.I1R9Y8	pp	229533.I1RBW0
229533.I1R9Y8	pp	229533.I1RTU8
229533.I1R9Y8	pp	229533.I1S7L6
229533.I1R9Y8	pp	229533.I1RM67
229533.I1R9Y8	pp	229533.I1S4S4
229533.I1R9Y8	pp	229533.I1RAM6
229533.I1R9Y8	pp	229533.I1S4S1
229533.Q4IEA5	pp	229533.V6RGI1
229533.Q4IEA5	pp	229533.V6RLW4
229533.Q4IEA5	pp	229533.Q4IQM6
229533.Q4IEA5	pp	229533.Q4IP51
229533.A0A098DPB8	pp	229533.I1RY92
229533.A0A098DPB8	pp	229533.I1S3L8
229533.A0A098DPB8	pp	229533.I1RGJ3
229533.A0A098DPB8	pp	229533.I1RFI9
229533.A0A098DPB8	pp	229533.I1RXD9
229533.A0A098DPB8	pp	229533.I1RA52
229533.A0A098DPB8	pp	229533.A0A1C3YL46
229533.A0A098DPB8	pp	229533.I1RDL0
229533.A0A098DPB8	pp	229533.I1RBF3
229533.A0A098DPB8	pp	229533.V6RUY9
229533.A0A098DPB8	pp	229533.A0A1C3YLM7
229533.A0A098DPB8	pp	229533.I1RWA3
229533.A0A098DPB8	pp	229533.I1RGY6
229533.A0A098DPB8	pp	229533.I1RZ90
229533.A0A098DPB8	pp	229533.I1RN63
229533.A0A098DPB8	pp	229533.I1RE53
229533.A0A098DPB8	pp	229533.I1S464
229533.A0A098DPB8	pp	229533.A0A1C3YN70
229533.A0A098DPB8	pp	229533.I1RT88
229533.A0A098DPB8	pp	229533.I1RF33
229533.A0A098DPB8	pp	229533.I1RY21
229533.A0A098DPB8	pp	229533.I1S5C6
229533.A0A098DPB8	pp	229533.I1RZW7
229533.A0A098DPB8	pp	229533.I1RWF9
229533.A0A098DPB8	pp	229533.I1RFJ6
229533.A0A098DPB8	pp	229533.A0A1C3YK33
229533.A0A098DPB8	pp	229533.A0A0E0S8Z0
229533.A0A098DPB8	pp	229533.I1RBT8
229533.A0A098DPB8	pp	229533.A0A1C3YJ17
229533.A0A098DPB8	pp	229533.I1RX96
229533.A0A098DPB8	pp	229533.I1S5X9
229533.A0A098DPB8	pp	229533.I1RT05
229533.A0A098DPB8	pp	229533.I1RG46
229533.A0A098DPB8	pp	229533.A0A0E0SH57
229533.A0A098DPB8	pp	229533.A0A1C3YLB6
229533.A0A098DPB8	pp	229533.A0A0E0SAR4

100887 229533.A0A098DPB8 pp 229533.I1RMQ6
100888 229533.A0A098DPB8 pp 229533.I1RYT8
100889 229533.A0A098DPB8 pp 229533.A0A1C3YJK5
100890 229533.A0A098DPB8 pp 229533.I1RFK3
100891 229533.A0A098DPB8 pp 229533.A0A1C3YNA1
100892 229533.A0A098DPB8 pp 229533.I1RZJ8
100893 229533.A0A098DPB8 pp 229533.I1RS05
100894 229533.A0A098DPB8 pp 229533.I1S0P0
100895 229533.A0A098DPB8 pp 229533.I1RHK2
100896 229533.A0A098DPB8 pp 229533.V6R7V8
100897 229533.A0A098DPB8 pp 229533.I1RYM5
100898 229533.A0A098DPB8 pp 229533.I1RDH5
100899 229533.A0A098DPB8 pp 229533.I1S0K2
100900 229533.A0A098DPB8 pp 229533.A0A1C3YKZ8
100901 229533.A0A098DPB8 pp 229533.I1RYD3
100902 229533.A0A098DPB8 pp 229533.I1RMK7
100903 229533.A0A098DPB8 pp 229533.A0A1C3YHV3
100904 229533.A0A098DPB8 pp 229533.I1RPN1
100905 229533.A0A098DPB8 pp 229533.I1RS29
100906 229533.A0A098DPB8 pp 229533.I1S287
100907 229533.A0A098DPB8 pp 229533.A0A1C3YKE0
100908 229533.A0A098DPB8 pp 229533.I1S2A1
100909 229533.A0A098DPB8 pp 229533.I1RVV7
100910 229533.A0A098DPB8 pp 229533.I1RAE8
100911 229533.A0A098DPB8 pp 229533.I1RAM9
100912 229533.A0A098DPB8 pp 229533.A0A1C3YN57
100913 229533.A0A098DPB8 pp 229533.V6RJP8
100914 229533.A0A098DPB8 pp 229533.I1RST9
100915 229533.A0A098DPB8 pp 229533.I1RUL3
100916 229533.A0A098DPB8 pp 229533.I1RFY0
100917 229533.A0A098DPB8 pp 229533.A0A1C3YKT4
100918 229533.A0A098DPB8 pp 229533.I1RCV1
100919 229533.A0A098DPB8 pp 229533.I1RJT5
100920 229533.A0A098DPB8 pp 229533.I1S267
100921 229533.A0A098D9K8 pp 229533.A0A1C3YM61
100922 229533.A0A098D9K8 pp 229533.I1RTA6
100923 229533.A0A098D9K8 pp 229533.A0A1C3YJC0
100924 229533.I1RPY0 pp 229533.I1S464
100925 229533.I1RPY0 pp 229533.I1RYI5
100926 229533.I1RPY0 pp 229533.I1RXD9
100927 229533.I1RPY0 pp 229533.I1RT88
100928 229533.I1RPY0 pp 229533.I1RWF9
100929 229533.I1RPY0 pp 229533.I1RR65
100930 229533.I1RPY0 pp 229533.I1S3L8
100931 229533.I1RPY0 pp 229533.I1RX96
100932 229533.I1RPY0 pp 229533.I1RY92
100933 229533.I1RPY0 pp 229533.I1RZ90
100934 229533.I1RPY0 pp 229533.I1RZJ8
100935 229533.I1RPY0 pp 229533.I1RWA3
100936 229533.I1RPY0 pp 229533.I1RWF4
100937 229533.I1RPY0 pp 229533.I1RWT0
100938 229533.I1RPY0 pp 229533.I1S5X9
100939 229533.I1RPY0 pp 229533.I1RS40
100940 229533.I1RPY0 pp 229533.I1RZJ3
100941 229533.I1RPY0 pp 229533.V6RN69
100942 229533.I1RPY0 pp 229533.Q4HZQ1
100943 229533.I1RPY0 pp 229533.I1RW01
100944 229533.I1RPY0 pp 229533.I1RPY8
100945 229533.I1RPY0 pp 229533.I1S0P0
100946 229533.I1RPY0 pp 229533.I1RYT8
100947 229533.I1RPY0 pp 229533.I1S0C2
100948 229533.I1RPY0 pp 229533.V6R7V8
100949 229533.I1RPY0 pp 229533.I1RS05
100950 229533.I1RPY0 pp 229533.I1RZW7
100951 229533.I1RPY0 pp 229533.I1RYD3
100952 229533.I1RPY0 pp 229533.I1S287
100953 229533.I1RPY0 pp 229533.I1S5C6
100954 229533.I1RPY0 pp 229533.V6QUG0
100955 229533.I1RPY0 pp 229533.V6RJP8
100956 229533.I1RPY0 pp 229533.I1S267
100957 229533.I1RPY0 pp 229533.I1RUL3
100958 229533.I1RPY0 pp 229533.I1S8S9
100959 229533.I1RPY0 pp 229533.Q4HVU7

100960 229533.I1RPY0 pp 229533.I1RY21
100961 229533.I1RPY0 pp 229533.I1RVV7
100962 229533.I1RPY0 pp 229533.I1RST9
100963 229533.I1RPY0 pp 229533.Q4I5G1
100964 229533.I1RPY0 pp 229533.I1RYM5
100965 229533.I1RPY0 pp 229533.I1S2A1
100966 229533.I1RPY0 pp 229533.I1RS29
100967 229533.I1RPY0 pp 229533.I1S0K2
100968 229533.I1RPY0 pp 229533.I1RT09
100969 229533.A0A0E0SEG7 pp 229533.I1RZ25
100970 229533.I1SAE8 pp 229533.V6RUB1
100971 229533.I1SAE8 pp 229533.V6QV31
100972 229533.I1RJG9 pp 229533.I1RW98
100973 229533.A0A0E0RWM1 pp 229533.Q4IPI4
100974 229533.A0A0E0RWM1 pp 229533.I1RQP1
100975 229533.A0A0E0RWM1 pp 229533.I1RTE5
100976 229533.A0A0E0RWM1 pp 229533.I1S0Z3
100977 229533.A0A0E0RWM1 pp 229533.Q4ICA8
100978 229533.A0A0E0RWM1 pp 229533.I1RRF0
100979 229533.A0A0E0RWM1 pp 229533.I1RQP3
100980 229533.A0A0E0RWM1 pp 229533.I1RT41
100981 229533.I1R9E2 pp 229533.I1RW98
100982 229533.I1R9E2 pp 229533.I1RJG9
100983 229533.I1R9E2 pp 229533.I1RY33
100984 229533.A0A0E0SEC8 pp 229533.I1RA52
100985 229533.I1RPN1 pp 229533.I1RYP2
100986 229533.I1RPN1 pp 229533.V6RM66
100987 229533.I1RPN1 pp 229533.Q4IA56
100988 229533.I1RPN1 pp 229533.I1RWI0
100989 229533.I1RPN1 pp 229533.I1S4S1
100990 229533.I1RPN1 pp 229533.V6QUG0
100991 229533.I1RPN1 pp 229533.I1RWF4
100992 229533.I1RPN1 pp 229533.I1S8F8
100993 229533.I1RPN1 pp 229533.V6R7V8
100994 229533.I1RPN1 pp 229533.V6R3J1
100995 229533.I1RPN1 pp 229533.I1RPU2
100996 229533.I1RPN1 pp 229533.Q4HZQ1
100997 229533.I1RPN1 pp 229533.I1RQ21
100998 229533.I1RPN1 pp 229533.I1RRS7
100999 229533.I1RPN1 pp 229533.I1RTA1
101000 229533.I1RPN1 pp 229533.I1RPY0
101001 229533.I1RPN1 pp 229533.I1RVT8
101002 229533.I1RPN1 pp 229533.V6RN69
101003 229533.I1RPN1 pp 229533.I1RZS4
101004 229533.I1RPN1 pp 229533.Q4I7T5
101005 229533.I1RPN1 pp 229533.I1RPR9
101006 229533.I1RPN1 pp 229533.Q4HVU7
101007 229533.I1RPN1 pp 229533.I1S0C2
101008 229533.I1RPN1 pp 229533.I1RT09
101009 229533.I1RPN1 pp 229533.I1RQA5
101010 229533.I1RPN1 pp 229533.I1S2A1
101011 229533.I1RPN1 pp 229533.I1RUQ3
101012 229533.A0A1C3YL94 pp 229533.I1RNG4
101013 229533.Q4IC11 pp 229533.Q4IPC8
101014 229533.Q4IC11 pp 229533.V6RGI1
101015 229533.Q4IC11 pp 229533.V6RLW4
101016 229533.Q4IC11 pp 229533.Q4IEA5
101017 229533.Q4IC11 pp 229533.Q4IQM6
101018 229533.Q4IC11 pp 229533.V6R382
101019 229533.Q4IC11 pp 229533.Q4IP51
101020 229533.I1RD94 pp 229533.I1S2Q0
101021 229533.I1RD94 pp 229533.I1S1K2
101022 229533.I1RD94 pp 229533.I1RK41
101023 229533.I1RD94 pp 229533.I1S3S7
101024 229533.I1RD94 pp 229533.I1RT87
101025 229533.I1RD94 pp 229533.I1RF95
101026 229533.I1RD94 pp 229533.I1RPB1
101027 229533.I1RD94 pp 229533.I1RKN7
101028 229533.I1RD94 pp 229533.I1S2J3
101029 229533.A0A1C3YKT4 pp 229533.I1RZK3
101030 229533.A0A1C3YKT4 pp 229533.I1RQC3
101031 229533.A0A1C3YKT4 pp 229533.I1RMM1
101032 229533.A0A1C3YKT4 pp 229533.I1RNB9

101033 229533.A0A1C3YKT4 pp 229533.I1RX95
101034 229533.A0A1C3YKT4 pp 229533.I1RY40
101035 229533.A0A1C3YKT4 pp 229533.I1S7X9
101036 229533.A0A1C3YKT4 pp 229533.I1RVA8
101037 229533.A0A1C3YKT4 pp 229533.I1RWZ9
101038 229533.A0A1C3YKT4 pp 229533.A0A1C3YNA1
101039 229533.A0A1C3YKT4 pp 229533.I1SAM5
101040 229533.A0A1C3YKT4 pp 229533.I1RCQ0
101041 229533.A0A1C3YKT4 pp 229533.I1S0V5
101042 229533.A0A1C3YKT4 pp 229533.I1RBS3
101043 229533.A0A1C3YKT4 pp 229533.I1S4N5
101044 229533.A0A1C3YKT4 pp 229533.I1RPY3
101045 229533.A0A1C3YKT4 pp 229533.I1RW76
101046 229533.A0A1C3YKT4 pp 229533.I1RG01
101047 229533.A0A1C3YKT4 pp 229533.I1RXT7
101048 229533.A0A1C3YKT4 pp 229533.I1RVA7
101049 229533.A0A1C3YKT4 pp 229533.I1S2W1
101050 229533.A0A1C3YKT4 pp 229533.I1RAF6
101051 229533.A0A1C3YKT4 pp 229533.I1RN70
101052 229533.A0A1C3YKT4 pp 229533.I1RYP0
101053 229533.A0A1C3YKT4 pp 229533.I1RSH3
101054 229533.A0A1C3YKT4 pp 229533.I1RQF9
101055 229533.A0A1C3YKT4 pp 229533.I1RQF7
101056 229533.A0A1C3YKT4 pp 229533.I1RX96
101057 229533.A0A1C3YKT4 pp 229533.Q4I5R9
101058 229533.A0A1C3YKT4 pp 229533.I1RMF2
101059 229533.A0A1C3YKT4 pp 229533.I1RA52
101060 229533.A0A1C3YKT4 pp 229533.I1RGC1
101061 229533.A0A1C3YKT4 pp 229533.I1RAH7
101062 229533.A0A1C3YKT4 pp 229533.I1RTQ9
101063 229533.A0A1C3YKT4 pp 229533.I1RJV7
101064 229533.A0A1C3YKT4 pp 229533.I1RP87
101065 229533.A0A1C3YKT4 pp 229533.I1RNG8
101066 229533.A0A1C3YKT4 pp 229533.I1RQ70
101067 229533.A0A1C3YKT4 pp 229533.I1S1V7
101068 229533.A0A1C3YKT4 pp 229533.I1RDH5
101069 229533.A0A1C3YKT4 pp 229533.I1RQ32
101070 229533.A0A1C3YKT4 pp 229533.I1RQH7
101071 229533.A0A1C3YKT4 pp 229533.I1RPY0
101072 229533.A0A1C3YKT4 pp 229533.I1RD45
101073 229533.A0A1C3YKT4 pp 229533.I1RQD6
101074 229533.A0A1C3YKT4 pp 229533.I1RYE4
101075 229533.A0A1C3YKT4 pp 229533.I1RZW7
101076 229533.A0A1C3YKT4 pp 229533.Q4HY20
101077 229533.A0A1C3YKT4 pp 229533.I1S1X7
101078 229533.A0A1C3YKT4 pp 229533.I1RUN9
101079 229533.A0A1C3YKT4 pp 229533.I1RAH5
101080 229533.A0A1C3YKT4 pp 229533.I1RW36
101081 229533.A0A1C3YKT4 pp 229533.I1RX87
101082 229533.A0A1C3YKT4 pp 229533.A0A1C3YLM7
101083 229533.A0A1C3YKT4 pp 229533.I1S051
101084 229533.A0A1C3YKT4 pp 229533.I1RBX3
101085 229533.A0A1C3YKT4 pp 229533.I1S830
101086 229533.A0A1C3YKT4 pp 229533.I1RBS4
101087 229533.A0A1C3YKT4 pp 229533.I1RCV3
101088 229533.A0A1C3YKT4 pp 229533.I1RDD0
101089 229533.A0A1C3YKT4 pp 229533.I1RFS8
101090 229533.A0A1C3YKT4 pp 229533.V6RXW9
101091 229533.A0A1C3YKT4 pp 229533.I1RNM3
101092 229533.A0A1C3YKT4 pp 229533.I1RTF2
101093 229533.A0A1C3YKT4 pp 229533.I1S6S3
101094 229533.A0A1C3YKT4 pp 229533.A0A1C3YL89
101095 229533.A0A1C3YKT4 pp 229533.I1RK54
101096 229533.A0A1C3YKT4 pp 229533.I1S3B7
101097 229533.A0A1C3YKT4 pp 229533.I1RRC8
101098 229533.A0A1C3YKT4 pp 229533.I1RPC6
101099 229533.A0A1C3YKT4 pp 229533.I1RWU9
101100 229533.A0A1C3YKT4 pp 229533.I1RE44
101101 229533.A0A1C3YKT4 pp 229533.I1RCH2
101102 229533.A0A1C3YKT4 pp 229533.I1RBR9
101103 229533.A0A1C3YKT4 pp 229533.I1RAK7
101104 229533.A0A1C3YKT4 pp 229533.I1RMN3
101105 229533.A0A1C3YKT4 pp 229533.I1RMK1

101106 229533.A0A1C3YKT4 pp 229533.I1RQF6
101107 229533.A0A1C3YKT4 pp 229533.I1RDJ8
101108 229533.A0A1C3YKT4 pp 229533.I1RRC9
101109 229533.A0A1C3YKT4 pp 229533.I1RIG4
101110 229533.A0A1C3YKT4 pp 229533.I1RFK0
101111 229533.A0A1C3YKT4 pp 229533.I1S9S4
101112 229533.A0A1C3YKT4 pp 229533.I1RQP7
101113 229533.A0A1C3YKT4 pp 229533.I1RM71
101114 229533.A0A1C3YKT4 pp 229533.I1S7S5
101115 229533.A0A1C3YKT4 pp 229533.I1RWB4
101116 229533.A0A1C3YKT4 pp 229533.I1RNU0
101117 229533.A0A1C3YKT4 pp 229533.I1RBF2
101118 229533.A0A1C3YKT4 pp 229533.I1S1U6
101119 229533.A0A1C3YKT4 pp 229533.I1RQC4
101120 229533.A0A1C3YKT4 pp 229533.I1RQN8
101121 229533.A0A1C3YKT4 pp 229533.I1S2B6
101122 229533.A0A1C3YKT4 pp 229533.A0A1C3YN54
101123 229533.A0A1C3YKT4 pp 229533.I1RK73
101124 229533.A0A1C3YKT4 pp 229533.I1RWR8
101125 229533.A0A1C3YKT4 pp 229533.I1RSJ0
101126 229533.A0A1C3YKT4 pp 229533.I1RPG2
101127 229533.A0A1C3YKT4 pp 229533.I1RZ84
101128 229533.A0A1C3YKT4 pp 229533.I1RD70
101129 229533.A0A1C3YKT4 pp 229533.I1RS34
101130 229533.A0A1C3YHJ8 pp 229533.I1RW06
101131 229533.A0A1C3YHJ8 pp 229533.I1RMM8
101132 229533.A0A1C3YHJ8 pp 229533.I1RBE4
101133 229533.I1RZN3 pp 229533.I1S0I2
101134 229533.I1RZN3 pp 229533.I1S0B0
101135 229533.I1R9D8 pp 229533.I1S6L6
101136 229533.I1R9D8 pp 229533.I1RD94
101137 229533.I1R9D8 pp 229533.I1S8M4
101138 229533.I1RZR8 pp 229533.Q4HYR2
101139 229533.I1RGU6 pp 229533.V6QWD8
101140 229533.I1RGU6 pp 229533.I1RZ29
101141 229533.I1RGU6 pp 229533.I1RZH8
101142 229533.I1RGU6 pp 229533.I1S6P5
101143 229533.I1RGU6 pp 229533.I1RMU5
101144 229533.I1RGU6 pp 229533.V6R6F6
101145 229533.I1RGU6 pp 229533.I1RZA7
101146 229533.I1RGU6 pp 229533.V6RUB1
101147 229533.I1RGU6 pp 229533.V6RQW4
101148 229533.I1RGU6 pp 229533.I1RMJ3
101149 229533.I1RGU6 pp 229533.I1RNB4
101150 229533.I1RGU6 pp 229533.I1RSE0
101151 229533.I1RGU6 pp 229533.I1RJU9
101152 229533.I1RGU6 pp 229533.I1RNG4
101153 229533.I1RGU6 pp 229533.I1RLM3
101154 229533.I1RGU6 pp 229533.I1SAE8
101155 229533.I1RGU6 pp 229533.I1RRK5
101156 229533.I1RGU6 pp 229533.V6RN31
101157 229533.I1RGU6 pp 229533.I1RR45
101158 229533.Q4HVU7 pp 229533.V6RN69
101159 229533.Q4HVU7 pp 229533.V6RRM0
101160 229533.Q4HVU7 pp 229533.Q4I174
101161 229533.Q4HVU7 pp 229533.Q4IB89
101162 229533.Q4HVU7 pp 229533.Q4HZ34
101163 229533.Q4HVU7 pp 229533.Q4HZQ1
101164 229533.Q4HVU7 pp 229533.V6R7V8
101165 229533.Q4HVU7 pp 229533.V6QUG0
101166 229533.Q4HVU7 pp 229533.V6QWB1
101167 229533.I1S1Z8 pp 229533.Q4IMM0
101168 229533.A0A1C3YIW3 pp 229533.I1S0B0
101169 229533.A0A1C3YIW3 pp 229533.I1RZN3
101170 229533.I1RKZ4 pp 229533.I1S4S1
101171 229533.I1RKZ4 pp 229533.I1RQJ5
101172 229533.I1RKZ4 pp 229533.Q4HZQ1
101173 229533.I1RKZ4 pp 229533.I1RMJ3
101174 229533.I1RKZ4 pp 229533.V6RLW2
101175 229533.I1RKZ4 pp 229533.I1S4Z8
101176 229533.I1RKZ4 pp 229533.I1RPN1
101177 229533.I1RKZ4 pp 229533.V6R7V8
101178 229533.I1RKZ4 pp 229533.V6QUG0

229533.I1RKZ4 pp 229533.I1RR65
229533.I1RKZ4 pp 229533.I1S2G9
229533.I1RKZ4 pp 229533.I1RQM7
229533.I1RKZ4 pp 229533.V6RN69
229533.I1RKZ4 pp 229533.I1RPY0
229533.I1RKZ4 pp 229533.Q4HVU7
229533.I1RKZ4 pp 229533.I1RQH5
229533.I1RKZ4 pp 229533.I1RT09
229533.I1RKZ4 pp 229533.I1S0C2
229533.I1RKZ4 pp 229533.Q4IQT8
229533.I1RKZ4 pp 229533.I1S2A1
229533.A0A098D9J3 pp 229533.Q4IPI4
229533.A0A098D9J3 pp 229533.I1S1V2
229533.A0A098D9J3 pp 229533.I1RYV3
229533.A0A098D9J3 pp 229533.I1RAN8
229533.A0A098D9J3 pp 229533.I1RN49
229533.A0A098D9J3 pp 229533.I1RFW1
229533.A0A098D9J3 pp 229533.I1RE51
229533.A0A098D9J3 pp 229533.I1RBJ0
229533.A0A098D9J3 pp 229533.Q4IER8
229533.A0A098D9J3 pp 229533.A0A0E0SQV5
229533.A0A098D9J3 pp 229533.I1RCW8
229533.A0A098D9J3 pp 229533.I1RQ19
229533.A0A098D9J3 pp 229533.I1RSD1
229533.A0A098D9J3 pp 229533.I1RU02
229533.A0A098D9J3 pp 229533.Q4I5R3
229533.A0A0E0SE45 pp 229533.I1RA81
229533.A0A0E0SE45 pp 229533.I1RKG1
229533.A0A0E0SE45 pp 229533.P0C431
229533.A0A098DJU9 pp 229533.I1RZH8
229533.A0A098DJU9 pp 229533.I1RMU5
229533.A0A098DJU9 pp 229533.V6QWD8
229533.A0A098DJU9 pp 229533.A0A1C3YMN0
229533.A0A098DJU9 pp 229533.I1RKM3
229533.A0A098DJU9 pp 229533.I1REW3
229533.A0A098DJU9 pp 229533.V6RN31
229533.A0A098DJU9 pp 229533.I1RR45
229533.A0A098DJU9 pp 229533.I1RZA7
229533.A0A098DJU9 pp 229533.I1RMJ3
229533.A0A098DJU9 pp 229533.I1RSE0
229533.A0A098DJU9 pp 229533.I1RNB4
229533.A0A098DJU9 pp 229533.I1RNG4
229533.A0A098DJU9 pp 229533.A0A098DMQ4
229533.A0A098DJU9 pp 229533.V6RUB1
229533.A0A098DJU9 pp 229533.I1S6P5
229533.A0A098DJU9 pp 229533.I1SAE8
229533.A0A098DJU9 pp 229533.I1RLM3
229533.A0A098DJU9 pp 229533.A0A1C3YKK8
229533.A0A098DJU9 pp 229533.I1RZ29
229533.A0A098DJU9 pp 229533.I1RRK5
229533.A0A098DJU9 pp 229533.A0A1C3YK58
229533.A0A098DJU9 pp 229533.A0A1C3YI53
229533.A0A098DJU9 pp 229533.I1RJU9
229533.A0A098DJU9 pp 229533.I1RGU6
229533.A0A098DJU9 pp 229533.A0A098E320
229533.A0A098DJU9 pp 229533.V6RQW4
229533.A0A098DJU9 pp 229533.V6R6F6
229533.A0A1C3YLB6 pp 229533.I1RST9
229533.A0A1C3YLB6 pp 229533.I1REE2
229533.A0A1C3YLB6 pp 229533.I1RHC7
229533.A0A1C3YLB6 pp 229533.V6RHX2
229533.A0A1C3YLB6 pp 229533.V6QUE6
229533.A0A1C3YLB6 pp 229533.I1RM59
229533.A0A1C3YLB6 pp 229533.I1RN14
229533.A0A1C3YLB6 pp 229533.I1RSI8
229533.A0A1C3YLB6 pp 229533.A0A1C3YMV8
229533.A0A1C3YLB6 pp 229533.V6RJP8
229533.A0A1C3YLB6 pp 229533.I1RFC3
229533.A0A1C3YLB6 pp 229533.I1RWP4
229533.A0A1C3YLB6 pp 229533.I1S8D3
229533.A0A1C3YLB6 pp 229533.I1RQ85
229533.A0A1C3YLB6 pp 229533.I1RP02
229533.A0A1C3YLB6 pp 229533.I1RXC4

101252 229533.A0A1C3YLB6 pp 229533.I1RUY5
101253 229533.A0A1C3YLB6 pp 229533.I1RK15
101254 229533.A0A1C3YLB6 pp 229533.I1RA68
101255 229533.A0A1C3YLB6 pp 229533.I1RXE6
101256 229533.A0A1C3YLB6 pp 229533.I1S267
101257 229533.A0A1C3YLB6 pp 229533.I1RZ68
101258 229533.A0A1C3YLB6 pp 229533.A0A1C3YMC4
101259 229533.A0A1C3YLB6 pp 229533.I1S5B0
101260 229533.A0A1C3YLB6 pp 229533.I1S209
101261 229533.A0A1C3YLB6 pp 229533.V6RPN0
101262 229533.A0A1C3YLB6 pp 229533.I1RMB9
101263 229533.A0A1C3YLB6 pp 229533.I1RVC7
101264 229533.A0A1C3YLB6 pp 229533.I1RIM7
101265 229533.A0A1C3YLB6 pp 229533.I1RS18
101266 229533.A0A1C3YLB6 pp 229533.I1RBE2
101267 229533.A0A1C3YLB6 pp 229533.I1R9N8
101268 229533.A0A1C3YLB6 pp 229533.I1RW13
101269 229533.A0A1C3YLB6 pp 229533.I1RCT9
101270 229533.A0A1C3YLB6 pp 229533.I1RVP5
101271 229533.A0A1C3YLB6 pp 229533.Q4IE79
101272 229533.A0A1C3YLB6 pp 229533.I1RFY0
101273 229533.A0A1C3YLB6 pp 229533.V6RCZ8
101274 229533.A0A1C3YLB6 pp 229533.I1RRN4
101275 229533.A0A1C3YLB6 pp 229533.I1RQR6
101276 229533.A0A1C3YLB6 pp 229533.I1RBE4
101277 229533.A0A1C3YLB6 pp 229533.I1REK8
101278 229533.A0A1C3YLB6 pp 229533.I1RBE7
101279 229533.A0A1C3YLB6 pp 229533.I1RW55
101280 229533.A0A1C3YLB6 pp 229533.I1RZM8
101281 229533.A0A1C3YLB6 pp 229533.Q4IQC1
101282 229533.A0A1C3YLB6 pp 229533.I1S6K9
101283 229533.A0A1C3YLB6 pp 229533.I1SAJ7
101284 229533.A0A1C3YLB6 pp 229533.Q4INS6
101285 229533.A0A1C3YLB6 pp 229533.I1RQH8
101286 229533.A0A1C3YLB6 pp 229533.I1RS29
101287 229533.A0A1C3YLB6 pp 229533.Q4IJK2
101288 229533.A0A1C3YLB6 pp 229533.I1RNG5
101289 229533.A0A1C3YLB6 pp 229533.I1S2M7
101290 229533.A0A1C3YLB6 pp 229533.I1S5R5
101291 229533.A0A1C3YLB6 pp 229533.I1RBC1
101292 229533.A0A1C3YLB6 pp 229533.I1RJY7
101293 229533.A0A1C3YLB6 pp 229533.I1RUQ3
101294 229533.A0A1C3YLB6 pp 229533.V6RWB3
101295 229533.A0A1C3YLB6 pp 229533.I1RI31
101296 229533.A0A1C3YLB6 pp 229533.I1RCL0
101297 229533.A0A1C3YLB6 pp 229533.I1REI2
101298 229533.A0A1C3YLB6 pp 229533.I1RRC4
101299 229533.A0A1C3YLB6 pp 229533.I1RNZ6
101300 229533.A0A1C3YLB6 pp 229533.I1S0K2
101301 229533.A0A1C3YLB6 pp 229533.I1RKF5
101302 229533.A0A1C3YLB6 pp 229533.A0A1C3YLC2
101303 229533.A0A1C3YLB6 pp 229533.I1RM26
101304 229533.A0A1C3YLB6 pp 229533.I1RWB0
101305 229533.A0A1C3YLB6 pp 229533.I1RAV5
101306 229533.A0A1C3YLB6 pp 229533.I1S2F7
101307 229533.A0A1C3YLB6 pp 229533.I1RY12
101308 229533.A0A1C3YLB6 pp 229533.I1RWR7
101309 229533.A0A1C3YLB6 pp 229533.I1RWL4
101310 229533.A0A1C3YLB6 pp 229533.I1RRY9
101311 229533.A0A1C3YLB6 pp 229533.I1R982
101312 229533.A0A1C3YLB6 pp 229533.I1S212
101313 229533.A0A1C3YLB6 pp 229533.A0A1C3YN86
101314 229533.A0A1C3YLB6 pp 229533.I1RQC5
101315 229533.A0A1C3YLB6 pp 229533.I1RDU0
101316 229533.A0A1C3YLB6 pp 229533.I1S3T6
101317 229533.A0A1C3YLB6 pp 229533.I1S0P0
101318 229533.A0A1C3YLB6 pp 229533.I1RVB7
101319 229533.A0A1C3YLB6 pp 229533.I1RPR9
101320 229533.A0A1C3YLB6 pp 229533.I1RE66
101321 229533.A0A1C3YLB6 pp 229533.I1S8C1
101322 229533.A0A1C3YLB6 pp 229533.I1RSR3
101323 229533.A0A1C3YLB6 pp 229533.I1S0T7
101324 229533.A0A1C3YLB6 pp 229533.I1S8S9

229533.A0A1C3YLB6 pp 229533.I1RIF7
229533.A0A1C3YLB6 pp 229533.I1RAM9
229533.A0A1C3YLB6 pp 229533.I1RFY6
229533.A0A1C3YLB6 pp 229533.I1RP38
229533.A0A1C3YLB6 pp 229533.I1RIN2
229533.A0A1C3YLB6 pp 229533.I1RJB3
229533.A0A1C3YLB6 pp 229533.I1RCM5
229533.A0A1C3YLB6 pp 229533.A0A1C3YLL3
229533.A0A1C3YLB6 pp 229533.I1RD52
229533.A0A1C3YLB6 pp 229533.I1RFV2
229533.A0A1C3YLB6 pp 229533.I1RHT5
229533.A0A1C3YLB6 pp 229533.I1RZK5
229533.A0A1C3YLB6 pp 229533.I1RRG3
229533.A0A1C3YLB6 pp 229533.I1S4S7
229533.A0A1C3YLB6 pp 229533.A0A1C3YLC3
229533.A0A1C3YLB6 pp 229533.I1RYX6
229533.A0A1C3YLB6 pp 229533.I1R9I3
229533.A0A1C3YLB6 pp 229533.I1RWG0
229533.A0A1C3YLB6 pp 229533.I1RGJ4
229533.A0A1C3YLB6 pp 229533.Q4I5R9
229533.A0A1C3YLB6 pp 229533.Q4I1Y1
229533.A0A1C3YLB6 pp 229533.I1R9A3
229533.A0A1C3YLB6 pp 229533.I1RGY4
229533.A0A1C3YLB6 pp 229533.I1S223
229533.A0A1C3YLB6 pp 229533.I1RYU6
229533.A0A1C3YLB6 pp 229533.I1RSQ7
229533.A0A1C3YLB6 pp 229533.I1RGU3
229533.A0A1C3YLB6 pp 229533.I1RDE6
229533.A0A1C3YLB6 pp 229533.I1RS05
229533.A0A1C3YLB6 pp 229533.I1RP86
229533.A0A1C3YLB6 pp 229533.I1RX46
229533.A0A1C3YLB6 pp 229533.I1RRX5
229533.A0A1C3YLB6 pp 229533.I1RIT9
229533.A0A1C3YLB6 pp 229533.A0A1C3YNA1
229533.A0A1C3YLB6 pp 229533.I1RYC7
229533.A0A1C3YLB6 pp 229533.I1RVX9
229533.A0A1C3YLB6 pp 229533.I1RW54
229533.A0A1C3YLB6 pp 229533.I1S0K1
229533.A0A1C3YLB6 pp 229533.I1RAX6
229533.A0A1C3YLB6 pp 229533.I1RA49
229533.A0A1C3YLB6 pp 229533.I1RBR2
229533.A0A1C3YLB6 pp 229533.I1RQE1
229533.A0A1C3YLB6 pp 229533.I1RZ92
229533.A0A1C3YLB6 pp 229533.I1RNA0
229533.A0A1C3YLB6 pp 229533.I1RAI4
229533.A0A1C3YLB6 pp 229533.I1RKI2
229533.A0A1C3YLB6 pp 229533.I1REJ4
229533.A0A1C3YLB6 pp 229533.V6QYF0
229533.A0A1C3YLB6 pp 229533.I1RXD7
229533.A0A1C3YLB6 pp 229533.I1RYJ6
229533.A0A1C3YLB6 pp 229533.I1RQV8
229533.A0A1C3YLB6 pp 229533.I1RPF1
229533.A0A1C3YLB6 pp 229533.I1RD01
229533.A0A1C3YLB6 pp 229533.I1RLP1
229533.A0A1C3YLB6 pp 229533.I1REV6
229533.A0A1C3YLB6 pp 229533.I1RRK9
229533.A0A1C3YLB6 pp 229533.I1RY87
229533.A0A1C3YLB6 pp 229533.A0A1C3YLR6
229533.A0A1C3YLB6 pp 229533.I1RMK7
229533.A0A1C3YLB6 pp 229533.I1RGK3
229533.A0A1C3YLB6 pp 229533.I1RQV7
229533.A0A1C3YLB6 pp 229533.I1RYT8
229533.A0A1C3YLB6 pp 229533.I1S438
229533.A0A1C3YLB6 pp 229533.I1RVD9
229533.A0A1C3YLB6 pp 229533.I1S0U0
229533.A0A1C3YLB6 pp 229533.I1S334
229533.A0A1C3YLB6 pp 229533.I1RG62
229533.A0A1C3YLB6 pp 229533.I1RNM7
229533.A0A1C3YLB6 pp 229533.I1S210
229533.A0A1C3YLB6 pp 229533.I1RBB1
229533.A0A1C3YLB6 pp 229533.I1RSC5
229533.A0A1C3YLB6 pp 229533.I1RY21
229533.A0A1C3YLB6 pp 229533.V6RCC0

101398	229533.A0A1C3YLB6	pp	229533.V6R5T7
101399	229533.A0A1C3YLB6	pp	229533.I1RRT1
101400	229533.A0A1C3YLB6	pp	229533.I1S5C6
101401	229533.A0A1C3YLB6	pp	229533.I1RS87
101402	229533.A0A1C3YLB6	pp	229533.I1S4S4
101403	229533.A0A1C3YLB6	pp	229533.I1S1P9
101404	229533.A0A1C3YLB6	pp	229533.Q4IPB3
101405	229533.A0A1C3YLB6	pp	229533.I1RFB6
101406	229533.A0A1C3YLB6	pp	229533.I1RL40
101407	229533.A0A1C3YLB6	pp	229533.A0A1C3YN55
101408	229533.A0A1C3YLB6	pp	229533.I1S2P2
101409	229533.A0A1C3YLB6	pp	229533.I1RWF4
101410	229533.A0A1C3YLB6	pp	229533.I1RRL5
101411	229533.A0A1C3YLB6	pp	229533.I1RWL3
101412	229533.A0A1C3YLB6	pp	229533.I1S0R1
101413	229533.A0A1C3YLB6	pp	229533.I1RR24
101414	229533.A0A1C3YLB6	pp	229533.I1RR05
101415	229533.A0A1C3YLB6	pp	229533.I1RRG1
101416	229533.A0A1C3YLB6	pp	229533.I1R9X3
101417	229533.A0A1C3YLB6	pp	229533.I1S1N7
101418	229533.A0A1C3YLB6	pp	229533.I1REF0
101419	229533.A0A1C3YLB6	pp	229533.I1RE01
101420	229533.A0A1C3YLB6	pp	229533.A0A1C3YMS7
101421	229533.A0A1C3YLB6	pp	229533.Q4IB89
101422	229533.A0A1C3YLB6	pp	229533.I1S1W9
101423	229533.A0A1C3YLB6	pp	229533.I1RKF4
101424	229533.A0A1C3YLB6	pp	229533.I1RBT1
101425	229533.A0A1C3YLB6	pp	229533.I1RB38
101426	229533.A0A1C3YLB6	pp	229533.I1RT05
101427	229533.A0A1C3YLB6	pp	229533.I1S4P6
101428	229533.A0A1C3YLB6	pp	229533.I1S7W5
101429	229533.A0A1C3YLB6	pp	229533.I1R9Y4
101430	229533.A0A1C3YLB6	pp	229533.I1S064
101431	229533.A0A1C3YLB6	pp	229533.A0A1C3YN26
101432	229533.A0A1C3YLB6	pp	229533.I1S0F1
101433	229533.A0A1C3YLB6	pp	229533.I1RKB7
101434	229533.A0A1C3YLB6	pp	229533.Q4IPB8
101435	229533.A0A1C3YLB6	pp	229533.I1RJ13
101436	229533.A0A1C3YLB6	pp	229533.I1RJU3
101437	229533.A0A1C3YLB6	pp	229533.I1RMQ6
101438	229533.A0A1C3YLB6	pp	229533.I1S147
101439	229533.A0A1C3YLB6	pp	229533.I1S0J1
101440	229533.A0A1C3YLB6	pp	229533.V6RLM7
101441	229533.A0A1C3YLB6	pp	229533.I1RNL5
101442	229533.A0A1C3YLB6	pp	229533.I1RPU2
101443	229533.A0A1C3YLB6	pp	229533.I1RE52
101444	229533.A0A1C3YLB6	pp	229533.I1RNL4
101445	229533.A0A1C3YLB6	pp	229533.I1RSC7
101446	229533.A0A1C3YLB6	pp	229533.I1S256
101447	229533.A0A1C3YLB6	pp	229533.I1S302
101448	229533.A0A1C3YLB6	pp	229533.I1RJ14
101449	229533.A0A1C3YLB6	pp	229533.I1S0S5
101450	229533.A0A1C3YLB6	pp	229533.I1RRN2
101451	229533.A0A1C3YLB6	pp	229533.I1S9Z4
101452	229533.A0A1C3YLB6	pp	229533.I1SAV3
101453	229533.A0A1C3YLB6	pp	229533.I1RE65
101454	229533.A0A1C3YLB6	pp	229533.I1RBC8
101455	229533.A0A1C3YLB6	pp	229533.I1RN84
101456	229533.A0A1C3YLB6	pp	229533.I1RMV0
101457	229533.A0A1C3YLB6	pp	229533.I1RSB8
101458	229533.A0A1C3YLB6	pp	229533.I1RIV2
101459	229533.A0A1C3YLB6	pp	229533.I1S5R6
101460	229533.A0A1C3YLB6	pp	229533.I1RZQ1
101461	229533.A0A1C3YLB6	pp	229533.I1RFS5
101462	229533.A0A1C3YLB6	pp	229533.I1RI95
101463	229533.A0A1C3YLB6	pp	229533.I1RSU2
101464	229533.A0A1C3YLB6	pp	229533.I1S0Q7
101465	229533.A0A1C3YLB6	pp	229533.I1S2T6
101466	229533.A0A1C3YLB6	pp	229533.I1RBS5
101467	229533.A0A1C3YLB6	pp	229533.V6RRM0
101468	229533.A0A1C3YLB6	pp	229533.V6RPQ8
101469	229533.A0A1C3YLB6	pp	229533.V6R042
101470	229533.A0A1C3YLB6	pp	229533.I1RQW4

101471	229533.A0A1C3YLB6	pp	229533.I1RBT6
101472	229533.A0A1C3YLB6	pp	229533.I1RQJ3
101473	229533.A0A1C3YLB6	pp	229533.I1RUG4
101474	229533.A0A1C3YLB6	pp	229533.I1RC54
101475	229533.A0A1C3YLB6	pp	229533.I1RSQ3
101476	229533.A0A1C3YLB6	pp	229533.I1RUQ7
101477	229533.A0A1C3YLB6	pp	229533.V6RN69
101478	229533.A0A1C3YLB6	pp	229533.Q4I624
101479	229533.A0A1C3YLB6	pp	229533.I1RBR1
101480	229533.A0A1C3YLB6	pp	229533.I1RRM3
101481	229533.A0A1C3YLB6	pp	229533.I1RHG5
101482	229533.A0A1C3YLB6	pp	229533.I1RLN1
101483	229533.A0A1C3YLB6	pp	229533.I1RNC1
101484	229533.A0A1C3YLB6	pp	229533.Q4IPH4
101485	229533.A0A1C3YLB6	pp	229533.I1RML0
101486	229533.A0A1C3YLB6	pp	229533.I1RUG3
101487	229533.A0A1C3YLB6	pp	229533.I1RTY9
101488	229533.A0A1C3YLB6	pp	229533.I1RE79
101489	229533.A0A1C3YLB6	pp	229533.I1S0T4
101490	229533.A0A1C3YLB6	pp	229533.I1S8H5
101491	229533.A0A1C3YLB6	pp	229533.I1RN50
101492	229533.A0A1C3YLB6	pp	229533.I1S0R8
101493	229533.A0A1C3YLB6	pp	229533.V6R2A1
101494	229533.A0A1C3YLB6	pp	229533.I1S266
101495	229533.A0A1C3YLB6	pp	229533.I1RP12
101496	229533.A0A1C3YLB6	pp	229533.I1RQ33
101497	229533.A0A1C3YLB6	pp	229533.I1S5X9
101498	229533.A0A1C3YLB6	pp	229533.I1RS06
101499	229533.A0A1C3YLB6	pp	229533.I1RB05
101500	229533.A0A1C3YLB6	pp	229533.V6RM02
101501	229533.A0A1C3YLB6	pp	229533.I1RUE3
101502	229533.A0A1C3YLB6	pp	229533.A0A1C3YLZ6
101503	229533.A0A1C3YLB6	pp	229533.I1RSY2
101504	229533.A0A1C3YLB6	pp	229533.I1RDJ8
101505	229533.A0A1C3YLB6	pp	229533.I1RGI8
101506	229533.A0A1C3YLB6	pp	229533.I1RCH5
101507	229533.A0A1C3YLB6	pp	229533.I1RHU8
101508	229533.A0A1C3YLB6	pp	229533.I1RE39
101509	229533.A0A1C3YLB6	pp	229533.I1RU95
101510	229533.A0A1C3YLB6	pp	229533.I1RHJ0
101511	229533.A0A1C3YLB6	pp	229533.I1RB92
101512	229533.A0A1C3YLB6	pp	229533.Q4HXF6
101513	229533.A0A1C3YLB6	pp	229533.I1S8P6
101514	229533.A0A1C3YLB6	pp	229533.I1S8H4
101515	229533.A0A1C3YLB6	pp	229533.I1RQE7
101516	229533.A0A1C3YLB6	pp	229533.I1S2C4
101517	229533.A0A1C3YLB6	pp	229533.I1RQW1
101518	229533.A0A1C3YLB6	pp	229533.I1RE89
101519	229533.A0A1C3YLB6	pp	229533.I1RKB6
101520	229533.A0A1C3YLB6	pp	229533.I1RFJ6
101521	229533.A0A1C3YLB6	pp	229533.I1S437
101522	229533.A0A1C3YLB6	pp	229533.I1RJV3
101523	229533.A0A1C3YLB6	pp	229533.I1S6V0
101524	229533.A0A1C3YLB6	pp	229533.I1RKJ8
101525	229533.A0A1C3YLB6	pp	229533.I1S1T5
101526	229533.A0A1C3YLB6	pp	229533.I1RRI2
101527	229533.A0A1C3YLB6	pp	229533.I1RDQ1
101528	229533.A0A1C3YLB6	pp	229533.I1RGY6
101529	229533.A0A1C3YLB6	pp	229533.I1RE53
101530	229533.A0A1C3YLB6	pp	229533.I1RF24
101531	229533.A0A1C3YLB6	pp	229533.I1S0X1
101532	229533.A0A1C3YLB6	pp	229533.I1RWS4
101533	229533.A0A1C3YLB6	pp	229533.I1RYR0
101534	229533.A0A1C3YLB6	pp	229533.Q4IBK5
101535	229533.A0A1C3YLB6	pp	229533.I1RN42
101536	229533.A0A1C3YLB6	pp	229533.I1RZ38
101537	229533.A0A1C3YLB6	pp	229533.I1RNG4
101538	229533.A0A1C3YLB6	pp	229533.I1S3H8
101539	229533.A0A1C3YLB6	pp	229533.I1RW26
101540	229533.A0A1C3YLB6	pp	229533.I1S5U0
101541	229533.A0A1C3YLB6	pp	229533.I1RJN9
101542	229533.A0A1C3YLB6	pp	229533.I1RSC0
101543	229533.A0A1C3YLB6	pp	229533.I1RKB2

101544 229533.A0A1C3YLB6 pp 229533.I1RMJ3
101545 229533.A0A1C3YLB6 pp 229533.I1RJD4
101546 229533.A0A1C3YLB6 pp 229533.Q4HVQ9
101547 229533.A0A1C3YLB6 pp 229533.I1RW82
101548 229533.A0A1C3YLB6 pp 229533.I1S2A6
101549 229533.A0A1C3YLB6 pp 229533.I1RC34
101550 229533.A0A1C3YLB6 pp 229533.I1RTA1
101551 229533.A0A1C3YLB6 pp 229533.I1RF33
101552 229533.A0A1C3YLB6 pp 229533.I1RVZ2
101553 229533.A0A1C3YLB6 pp 229533.I1RY62
101554 229533.A0A1C3YLB6 pp 229533.I1RC31
101555 229533.A0A1C3YLB6 pp 229533.I1RD89
101556 229533.A0A1C3YLB6 pp 229533.I1RFB0
101557 229533.A0A1C3YLB6 pp 229533.I1S3K7
101558 229533.A0A1C3YLB6 pp 229533.I1RBY9
101559 229533.A0A1C3YLB6 pp 229533.I1S464
101560 229533.A0A1C3YLB6 pp 229533.I1RYP2
101561 229533.A0A1C3YLB6 pp 229533.I1RSX0
101562 229533.A0A1C3YLB6 pp 229533.I1RYW9
101563 229533.A0A1C3YLB6 pp 229533.I1R9B2
101564 229533.A0A1C3YLB6 pp 229533.I1RSA5
101565 229533.A0A1C3YLB6 pp 229533.I1RYK0
101566 229533.A0A1C3YLB6 pp 229533.V6R3J1
101567 229533.A0A1C3YLB6 pp 229533.I1S1F3
101568 229533.A0A1C3YLB6 pp 229533.I1RWL8
101569 229533.A0A1C3YLB6 pp 229533.I1RX39
101570 229533.A0A1C3YLB6 pp 229533.I1RQ48
101571 229533.A0A1C3YLB6 pp 229533.I1RYI5
101572 229533.A0A1C3YLB6 pp 229533.I1RBE6
101573 229533.A0A1C3YLB6 pp 229533.I1RUR8
101574 229533.A0A1C3YLB6 pp 229533.I1RZN1
101575 229533.A0A1C3YLB6 pp 229533.I1RZH5
101576 229533.A0A1C3YLB6 pp 229533.I1RZB2
101577 229533.A0A1C3YLB6 pp 229533.I1R9M0
101578 229533.A0A1C3YLB6 pp 229533.I1RIJ6
101579 229533.A0A1C3YLB6 pp 229533.V6R5D2
101580 229533.A0A1C3YLB6 pp 229533.V6RIE5
101581 229533.A0A1C3YLB6 pp 229533.I1RC82
101582 229533.A0A1C3YLB6 pp 229533.I1RXD9
101583 229533.A0A1C3YLB6 pp 229533.I1RWF5
101584 229533.A0A1C3YLB6 pp 229533.I1RGA4
101585 229533.A0A1C3YLB6 pp 229533.I1RHH5
101586 229533.A0A1C3YLB6 pp 229533.I1RA52
101587 229533.A0A1C3YLB6 pp 229533.I1S7U6
101588 229533.A0A1C3YLB6 pp 229533.I1RYQ1
101589 229533.A0A1C3YLB6 pp 229533.I1RRR9
101590 229533.A0A1C3YLB6 pp 229533.I1RXF8
101591 229533.A0A1C3YLB6 pp 229533.I1RK02
101592 229533.A0A1C3YLB6 pp 229533.I1RFI4
101593 229533.A0A1C3YLB6 pp 229533.I1RFI9
101594 229533.A0A1C3YLB6 pp 229533.I1S1C3
101595 229533.A0A1C3YLB6 pp 229533.I1RAW3
101596 229533.A0A1C3YLB6 pp 229533.I1RGC3
101597 229533.A0A1C3YLB6 pp 229533.I1RPV3
101598 229533.A0A1C3YLB6 pp 229533.I1RTD5
101599 229533.A0A1C3YLB6 pp 229533.I1RR68
101600 229533.A0A1C3YLB6 pp 229533.I1RY35
101601 229533.A0A1C3YLB6 pp 229533.I1RW61
101602 229533.A0A1C3YLB6 pp 229533.I1RKD3
101603 229533.A0A1C3YLB6 pp 229533.I1RE40
101604 229533.A0A1C3YLB6 pp 229533.I1RK39
101605 229533.A0A1C3YLB6 pp 229533.I1RC29
101606 229533.A0A1C3YLB6 pp 229533.I1RWA3
101607 229533.A0A1C3YLB6 pp 229533.A0A1C3YM60
101608 229533.A0A1C3YLB6 pp 229533.V6RUY9
101609 229533.A0A1C3YLB6 pp 229533.I1RPY0
101610 229533.A0A1C3YLB6 pp 229533.I1RSZ7
101611 229533.A0A1C3YLB6 pp 229533.I1RN88
101612 229533.A0A1C3YLB6 pp 229533.I1S2J4
101613 229533.A0A1C3YLB6 pp 229533.I1RZ86
101614 229533.A0A1C3YLB6 pp 229533.I1RWI8
101615 229533.A0A1C3YLB6 pp 229533.A0A1C3YLE2
101616 229533.A0A1C3YLB6 pp 229533.I1RA11

229533.A0A1C3YLB6 pp 229533.A0A1C3YLM7
229533.A0A1C3YLB6 pp 229533.I1SAB4
229533.A0A1C3YLB6 pp 229533.I1S2H4
229533.A0A1C3YLB6 pp 229533.I1RRT8
229533.A0A1C3YLB6 pp 229533.I1RX00
229533.A0A1C3YLB6 pp 229533.I1RPE0
229533.A0A1C3YLB6 pp 229533.I1S5R1
229533.A0A1C3YLB6 pp 229533.I1RH91
229533.A0A1C3YLB6 pp 229533.I1S0L4
229533.A0A1C3YLB6 pp 229533.I1RZ39
229533.A0A1C3YLB6 pp 229533.I1RHD2
229533.A0A1C3YLB6 pp 229533.I1SAH9
229533.A0A1C3YLB6 pp 229533.I1RTG7
229533.A0A1C3YLB6 pp 229533.I1RXC2
229533.A0A1C3YLB6 pp 229533.I1RRI1
229533.A0A1C3YLB6 pp 229533.Q4IN52
229533.A0A1C3YLB6 pp 229533.I1RY92
229533.A0A1C3YLB6 pp 229533.I1RB64
229533.A0A1C3YLB6 pp 229533.I1S1L0
229533.A0A1C3YLB6 pp 229533.I1RFX2
229533.A0A1C3YLB6 pp 229533.A0A1C3YMP1
229533.A0A1C3YLB6 pp 229533.V6RCJ5
229533.A0A1C3YLB6 pp 229533.I1SB04
229533.A0A1C3YLB6 pp 229533.I1S458
229533.A0A1C3YLB6 pp 229533.I1RSQ4
229533.A0A1C3YLB6 pp 229533.I1RF49
229533.A0A1C3YLB6 pp 229533.I1RC66
229533.A0A1C3YK39 pp 229533.I1S574
229533.A0A1C3YK39 pp 229533.I1RJL3
229533.A0A1C3YK39 pp 229533.I1RNG4
229533.A0A1C3YK39 pp 229533.I1RSQ9
229533.A0A1C3YK39 pp 229533.I1RB36
229533.A0A1C3YK39 pp 229533.I1RZ29
229533.A0A1C3YK39 pp 229533.I1RAI1
229533.A0A1C3YK39 pp 229533.V6QWD8
229533.A0A1C3YK39 pp 229533.Q4IPB8
229533.A0A1C3YK39 pp 229533.I1RC61
229533.A0A1C3YK39 pp 229533.I1RNA1
229533.A0A1C3YK39 pp 229533.I1RC48
229533.A0A1C3YK39 pp 229533.I1S984
229533.A0A098D9H2 pp 229533.I1S1F3
229533.A0A098D9H2 pp 229533.A0A0E0S8Z0
229533.A0A098D9H2 pp 229533.A0A0E0SAR4
229533.I1S0C2 pp 229533.Q4HVU7
229533.I1S0C2 pp 229533.I1SA55
229533.I1S0C2 pp 229533.V6RN69
229533.I1S0C2 pp 229533.I1S4S4
229533.I1S0C2 pp 229533.Q4HZ34
229533.I1S0C2 pp 229533.I1S767
229533.I1S0C2 pp 229533.V6QWB1
229533.I1S0C2 pp 229533.Q4I174
229533.I1S0C2 pp 229533.V6QUG0
229533.I1S0C2 pp 229533.Q4HZQ1
229533.A0A1C3YIV9 pp 229533.I1S5R1
229533.I1RAN4 pp 229533.I1RMV5
229533.I1RAN4 pp 229533.I1RZG7
229533.I1RNB4 pp 229533.I1RZA7
229533.I1RNB4 pp 229533.I1RVX9
229533.I1RNB4 pp 229533.I1RQ60
229533.I1RNB4 pp 229533.I1S3G4
229533.I1RNB4 pp 229533.I1RQE1
229533.I1RNB4 pp 229533.V6RUB1
229533.I1RNB4 pp 229533.I1RP86
229533.I1RNB4 pp 229533.I1RYQ4
229533.I1RNB4 pp 229533.I1RZ29
229533.I1RNB4 pp 229533.I1RNG4
229533.I1RNB4 pp 229533.I1RY49
229533.I1RNB4 pp 229533.I1RR68
229533.I1RNB4 pp 229533.V6QWD8
229533.I1RNB4 pp 229533.I1RSQ9
229533.I1RNB4 pp 229533.I1S685
229533.I1RNB4 pp 229533.I1RSZ7
229533.I1RNB4 pp 229533.I1S1W8

101690 229533.I1RNB4 pp 229533.I1RNV4
101691 229533.I1RNB4 pp 229533.I1S5R6
101692 229533.I1RNB4 pp 229533.I1S6P5
101693 229533.I1RNB4 pp 229533.I1RNK9
101694 229533.I1RNB4 pp 229533.I1S0A0
101695 229533.I1RNB4 pp 229533.V6RM02
101696 229533.I1RNB4 pp 229533.V6RN31
101697 229533.I1RNB4 pp 229533.I1RY74
101698 229533.I1RAE8 pp 229533.I1SA55
101699 229533.I1RAE8 pp 229533.I1RFK3
101700 229533.I1RAE8 pp 229533.V6QUG0
101701 229533.I1RAE8 pp 229533.I1RY80
101702 229533.I1RAE8 pp 229533.I1RKA7
101703 229533.I1RAE8 pp 229533.I1RPY0
101704 229533.I1RAE8 pp 229533.Q4HYR2
101705 229533.I1RAE8 pp 229533.I1RSI0
101706 229533.I1RAE8 pp 229533.I1RNG4
101707 229533.I1RAE8 pp 229533.I1RVT8
101708 229533.I1RAE8 pp 229533.I1RFY0
101709 229533.I1RAE8 pp 229533.I1RE53
101710 229533.I1RAE8 pp 229533.I1RWX1
101711 229533.I1RAE8 pp 229533.I1S2A1
101712 229533.I1RAE8 pp 229533.Q4HZQ1
101713 229533.I1RAE8 pp 229533.Q4HZ34
101714 229533.I1RAE8 pp 229533.I1RSP7
101715 229533.I1RAE8 pp 229533.Q4I174
101716 229533.I1RAE8 pp 229533.I1S0C2
101717 229533.I1RAE8 pp 229533.Q4HVU7
101718 229533.I1RMN1 pp 229533.I1RVZ3
101719 229533.I1S0H2 pp 229533.I1S149
101720 229533.I1S0H2 pp 229533.I1S694
101721 229533.I1S0H2 pp 229533.I1SAI4
101722 229533.I1S0H2 pp 229533.V6R2Z2
101723 229533.I1S0H2 pp 229533.V6R5D2
101724 229533.I1S0H2 pp 229533.V6RPS5
101725 229533.I1S0H2 pp 229533.V6R756
101726 229533.I1S0H2 pp 229533.V6R0W1
101727 229533.I1S0H2 pp 229533.V6QU99
101728 229533.I1S0H2 pp 229533.V6RAM6
101729 229533.I1RBR9 pp 229533.Q4IP51
101730 229533.I1RBR9 pp 229533.I1S287
101731 229533.I1RBR9 pp 229533.I1S2W1
101732 229533.I1RBR9 pp 229533.I1RK54
101733 229533.I1RBR9 pp 229533.I1RS80
101734 229533.I1RBR9 pp 229533.I1RPU8
101735 229533.I1RBR9 pp 229533.I1RDJ8
101736 229533.I1RBR9 pp 229533.I1RBX3
101737 229533.I1RBR9 pp 229533.I1S3B7
101738 229533.A0A0E0SDY2 pp 229533.I1RQ88
101739 229533.A0A0E0SDY2 pp 229533.I1S7S9
101740 229533.A0A0E0SDY2 pp 229533.I1RGC2
101741 229533.A0A0E0SDY2 pp 229533.I1RS41
101742 229533.A0A0E0SDY2 pp 229533.I1RAI9
101743 229533.I1RUL3 pp 229533.V6R3J1
101744 229533.I1RUL3 pp 229533.V6RM66
101745 229533.I1RUL3 pp 229533.I1RYP2
101746 229533.I1RUL3 pp 229533.Q4IA56
101747 229533.I1RUL3 pp 229533.V6R7V8
101748 229533.I1RUL3 pp 229533.I1RVW7
101749 229533.I1RUL3 pp 229533.V6QUG0
101750 229533.I1RUL3 pp 229533.V6RN69
101751 229533.I1RUL3 pp 229533.Q4HVU7
101752 229533.I1RUL3 pp 229533.I1S2A1
101753 229533.I1RUL3 pp 229533.I1S4S1
101754 229533.I1RUL3 pp 229533.I1S094
101755 229533.A0A0E0RW71 pp 229533.I1RAX8
101756 229533.A0A0E0RW71 pp 229533.I1S1Z8
101757 229533.A0A0E0RW71 pp 229533.I1RS64
101758 229533.A0A0E0RW71 pp 229533.Q4I0J6
101759 229533.A0A0E0RW71 pp 229533.Q4IBX0
101760 229533.A0A0E0RW71 pp 229533.I1S148
101761 229533.A0A0E0RW71 pp 229533.A0A1C3YM21
101762 229533.A0A0E0RW71 pp 229533.A0A0E0SJN8

101763	229533.A0A0E0RW71	pp	229533.Q4IMM0
101764	229533.A0A0E0RW71	pp	229533.A0A0E0SH87
101765	229533.A0A0E0RW71	pp	229533.I1RMN1
101766	229533.A0A0E0RW71	pp	229533.I1RUQ1
101767	229533.I1RAG9	pp	229533.I1RNV4
101768	229533.I1RAG9	pp	229533.I1RK03
101769	229533.I1RAG9	pp	229533.I1RCG5
101770	229533.I1RAG9	pp	229533.I1RM26
101771	229533.I1RAG9	pp	229533.I1RBE7
101772	229533.I1RAG9	pp	229533.V6RM02
101773	229533.I1RAG9	pp	229533.I1RY74
101774	229533.I1RAG9	pp	229533.I1RNG4
101775	229533.I1RAG9	pp	229533.I1RSQ9
101776	229533.I1RAG9	pp	229533.I1RMJ3
101777	229533.I1RAG9	pp	229533.I1S5R6
101778	229533.I1RAG9	pp	229533.I1RKZ7
101779	229533.I1RAG9	pp	229533.I1RZA7
101780	229533.I1RAG9	pp	229533.I1RN84
101781	229533.I1RAG9	pp	229533.I1RYQ4
101782	229533.I1RAG9	pp	229533.I1RDE7
101783	229533.I1RAG9	pp	229533.I1RHH5
101784	229533.I1RAG9	pp	229533.I1RSZ7
101785	229533.I1RAG9	pp	229533.I1S0A0
101786	229533.I1RAG9	pp	229533.I1S3G4
101787	229533.I1RAG9	pp	229533.I1S6P5
101788	229533.I1RAG9	pp	229533.I1S1W8
101789	229533.I1RAG9	pp	229533.I1RQ60
101790	229533.I1RAG9	pp	229533.I1RZ29
101791	229533.I1RAG9	pp	229533.V6RUB1
101792	229533.I1RAG9	pp	229533.I1RVX9
101793	229533.I1RAG9	pp	229533.V6QWD8
101794	229533.I1RAG9	pp	229533.I1RY49
101795	229533.I1RAG9	pp	229533.I1RDQ2
101796	229533.I1RAG9	pp	229533.I1RMK4
101797	229533.I1RAG9	pp	229533.I1RAR3
101798	229533.I1RAG9	pp	229533.I1RP86
101799	229533.I1RAG9	pp	229533.I1RMM8
101800	229533.I1RAG9	pp	229533.I1RQE1
101801	229533.I1RAG9	pp	229533.I1RR68
101802	229533.I1RJS8	pp	229533.I1RJZ7
101803	229533.I1RJS8	pp	229533.I1RW71
101804	229533.I1RJS8	pp	229533.I1RNI2
101805	229533.I1RDJ8	pp	229533.I1RN57
101806	229533.I1RDJ8	pp	229533.I1RG11
101807	229533.I1RDJ8	pp	229533.I1RSL8
101808	229533.I1RDJ8	pp	229533.I1RQI6
101809	229533.I1RDJ8	pp	229533.Q4IB89
101810	229533.I1RDJ8	pp	229533.I1RSC7
101811	229533.I1RDJ8	pp	229533.I1RRW0
101812	229533.I1RDJ8	pp	229533.I1RZ98
101813	229533.I1RDJ8	pp	229533.V6R382
101814	229533.I1RDJ8	pp	229533.I1RW16
101815	229533.I1RDJ8	pp	229533.Q4I073
101816	229533.I1RDJ8	pp	229533.I1RK54
101817	229533.I1RDJ8	pp	229533.I1S0S5
101818	229533.I1RDJ8	pp	229533.I1RZQ1
101819	229533.I1RDJ8	pp	229533.I1RJ13
101820	229533.I1RDJ8	pp	229533.I1RY44
101821	229533.I1RDJ8	pp	229533.I1RPS0
101822	229533.I1RDJ8	pp	229533.I1S4P6
101823	229533.I1RDJ8	pp	229533.I1RNA2
101824	229533.I1RDJ8	pp	229533.I1RSP7
101825	229533.I1RDJ8	pp	229533.I1RSZ3
101826	229533.I1RDJ8	pp	229533.I1RUG4
101827	229533.I1RDJ8	pp	229533.I1RZT1
101828	229533.I1RDJ8	pp	229533.I1RMJ8
101829	229533.I1RDJ8	pp	229533.I1RPF5
101830	229533.I1RDJ8	pp	229533.I1RZP8
101831	229533.I1RDJ8	pp	229533.I1S8P6
101832	229533.I1RDJ8	pp	229533.I1RY29
101833	229533.I1RDJ8	pp	229533.I1RZK9
101834	229533.I1RDJ8	pp	229533.I1RTN6
101835	229533.I1RDJ8	pp	229533.I1RY58

101836 229533.I1RDJ8 pp 229533.I1RNU0
101837 229533.I1RDJ8 pp 229533.I1S0Q5
101838 229533.I1RDJ8 pp 229533.I1RVV5
101839 229533.I1RDJ8 pp 229533.I1RX64
101840 229533.I1RDJ8 pp 229533.I1S0D5
101841 229533.I1RDJ8 pp 229533.I1RP99
101842 229533.I1RDJ8 pp 229533.I1RSY2
101843 229533.I1RDJ8 pp 229533.I1RSR9
101844 229533.I1RDJ8 pp 229533.I1S2E9
101845 229533.I1RDJ8 pp 229533.I1RWI8
101846 229533.I1RDJ8 pp 229533.Q4IEV4
101847 229533.I1RDJ8 pp 229533.I1RDX0
101848 229533.I1RDJ8 pp 229533.I1RJV8
101849 229533.I1RDJ8 pp 229533.I1RW28
101850 229533.I1RDJ8 pp 229533.I1RPR8
101851 229533.I1RDJ8 pp 229533.I1RWK3
101852 229533.I1RDJ8 pp 229533.I1RZ86
101853 229533.I1RDJ8 pp 229533.I1RDV4
101854 229533.I1RDJ8 pp 229533.I1S6H2
101855 229533.I1RDJ8 pp 229533.I1S7X9
101856 229533.I1RDJ8 pp 229533.I1S004
101857 229533.I1RDJ8 pp 229533.I1RYQ1
101858 229533.I1RDJ8 pp 229533.I1S214
101859 229533.I1RDJ8 pp 229533.I1RKQ0
101860 229533.I1RDJ8 pp 229533.I1RQH8
101861 229533.I1RDJ8 pp 229533.I1RU61
101862 229533.I1RDJ8 pp 229533.Q4HTT1
101863 229533.I1RDJ8 pp 229533.I1RXI6
101864 229533.I1RDJ8 pp 229533.Q4INS6
101865 229533.I1RDJ8 pp 229533.I1RRM5
101866 229533.I1RDJ8 pp 229533.Q4HTT2
101867 229533.I1RDJ8 pp 229533.I1RRJ6
101868 229533.I1RDJ8 pp 229533.I1S0Q4
101869 229533.I1RDJ8 pp 229533.I1RHD2
101870 229533.I1RDJ8 pp 229533.I1S998
101871 229533.I1RDJ8 pp 229533.I1RVW0
101872 229533.I1RDJ8 pp 229533.I1S209
101873 229533.I1RDJ8 pp 229533.I1S3B7
101874 229533.I1RDJ8 pp 229533.I1RMW4
101875 229533.I1RDJ8 pp 229533.I1RFD5
101876 229533.I1RDJ8 pp 229533.I1RVZ9
101877 229533.I1RDJ8 pp 229533.I1RNE2
101878 229533.I1RDJ8 pp 229533.I1RWT6
101879 229533.I1RDJ8 pp 229533.I1RQA3
101880 229533.I1RDJ8 pp 229533.I1RN55
101881 229533.I1RDJ8 pp 229533.I1RXZ4
101882 229533.I1RDJ8 pp 229533.I1RQS4
101883 229533.I1RDJ8 pp 229533.I1RG77
101884 229533.I1RDJ8 pp 229533.I1RRY1
101885 229533.I1RDJ8 pp 229533.I1RUY6
101886 229533.I1RDJ8 pp 229533.I1RKI2
101887 229533.I1RDJ8 pp 229533.I1S2W1
101888 229533.I1RDJ8 pp 229533.I1RJK7
101889 229533.I1RDJ8 pp 229533.I1RZV9
101890 229533.I1RDJ8 pp 229533.I1RLY0
101891 229533.I1RDJ8 pp 229533.I1S287
101892 229533.I1RDJ8 pp 229533.I1RLX6
101893 229533.I1RDJ8 pp 229533.I1RLJ8
101894 229533.I1RDJ8 pp 229533.I1RPI0
101895 229533.I1RDJ8 pp 229533.Q4IM48
101896 229533.I1RDJ8 pp 229533.I1RUR0
101897 229533.I1RDJ8 pp 229533.I1RPQ1
101898 229533.I1RDJ8 pp 229533.I1RWM6
101899 229533.I1RDJ8 pp 229533.I1RS87
101900 229533.I1RDJ8 pp 229533.I1RPF4
101901 229533.I1RDJ8 pp 229533.I1S224
101902 229533.I1RDJ8 pp 229533.I1RFS6
101903 229533.I1RDJ8 pp 229533.I1RG34
101904 229533.I1RDJ8 pp 229533.I1S788
101905 229533.I1RDJ8 pp 229533.I1REY8
101906 229533.I1RDJ8 pp 229533.I1RLS4
101907 229533.I1RDJ8 pp 229533.I1S987
101908 229533.I1RDJ8 pp 229533.I1RRT1

101909 229533.I1RDJ8 pp 229533.I1RQM6
101910 229533.I1RDJ8 pp 229533.I1RIE7
101911 229533.I1RDJ8 pp 229533.I1RSK0
101912 229533.I1RDJ8 pp 229533.Q4IPN9
101913 229533.I1RDJ8 pp 229533.I1RN31
101914 229533.I1RDJ8 pp 229533.I1RZ94
101915 229533.I1RDJ8 pp 229533.I1RQQ0
101916 229533.I1S183 pp 229533.I1S5C5
101917 229533.I1S183 pp 229533.I1SAJ3
101918 229533.I1S183 pp 229533.V6RN31
101919 229533.I1S183 pp 229533.V6QU63
101920 229533.I1S183 pp 229533.V6RK40
101921 229533.I1S183 pp 229533.I1S788
101922 229533.I1S183 pp 229533.I1S3G4
101923 229533.I1S183 pp 229533.V6RQW4
101924 229533.I1S183 pp 229533.I1S209
101925 229533.I1S183 pp 229533.V6RW66
101926 229533.I1S183 pp 229533.I1S1W8
101927 229533.A0A1C3YIV8 pp 229533.I1RPU8
101928 229533.A0A1C3YIV8 pp 229533.I1RBR9
101929 229533.A0A1C3YIV8 pp 229533.I1RDJ8
101930 229533.A0A1C3YIV8 pp 229533.A0A1C3YKT4
101931 229533.A0A1C3YIV8 pp 229533.I1S287
101932 229533.I1RT09 pp 229533.V6RM02
101933 229533.I1RT09 pp 229533.I1RXS0
101934 229533.I1RT09 pp 229533.V6RN69
101935 229533.I1RT09 pp 229533.Q4HVU7
101936 229533.I1RT09 pp 229533.I1S2A1
101937 229533.I1RT09 pp 229533.V6RK96
101938 229533.I1RT09 pp 229533.I1S8R4
101939 229533.I1RT09 pp 229533.I1RUL3
101940 229533.I1RT09 pp 229533.I1RVR2
101941 229533.I1RT09 pp 229533.I1S0C2
101942 229533.I1RT09 pp 229533.I1S3L2
101943 229533.I1RT09 pp 229533.V6R7V8
101944 229533.I1RT09 pp 229533.V6R382
101945 229533.I1RT09 pp 229533.I1S0K0
101946 229533.I1RT09 pp 229533.I1S4S1
101947 229533.I1RT09 pp 229533.I1SA55
101948 229533.I1RT09 pp 229533.V6QUG0
101949 229533.I1RT09 pp 229533.I1RVX9
101950 229533.I1RT09 pp 229533.I1RZM4
101951 229533.I1RT09 pp 229533.I1S8C1
101952 229533.I1RT09 pp 229533.Q4HZQ1
101953 229533.I1RIJ2 pp 229533.I1RJT5
101954 229533.I1RIJ2 pp 229533.V6RY56
101955 229533.I1RIJ2 pp 229533.I1S2C4
101956 229533.I1RIJ2 pp 229533.I1S4D0
101957 229533.I1RIJ2 pp 229533.I1S029
101958 229533.I1RIJ2 pp 229533.I1RMM8
101959 229533.I1RIJ2 pp 229533.I1RSQ9
101960 229533.I1RIJ2 pp 229533.V6R729
101961 229533.I1RIJ2 pp 229533.V6QWD8
101962 229533.I1RIJ2 pp 229533.V6RRM0
101963 229533.I1RIJ2 pp 229533.I1RNG4
101964 229533.I1RIJ2 pp 229533.I1S1W8
101965 229533.I1RIJ2 pp 229533.I1RPU8
101966 229533.I1RIJ2 pp 229533.I1RL93
101967 229533.I1RIJ2 pp 229533.I1SAF3
101968 229533.I1RIJ2 pp 229533.I1SAI0
101969 229533.I1RIJ2 pp 229533.I1S3G4
101970 229533.I1RIJ2 pp 229533.I1RZ29
101971 229533.A0A1C3YHJ5 pp 229533.I1RML0
101972 229533.A0A1C3YHJ5 pp 229533.I1RFP9
101973 229533.A0A1C3YHJ5 pp 229533.I1S0B6
101974 229533.A0A1C3YHJ5 pp 229533.A0A1C3YKG7
101975 229533.A0A1C3YHJ5 pp 229533.I1RK90
101976 229533.A0A1C3YHJ5 pp 229533.I1S2A8
101977 229533.A0A1C3YHJ5 pp 229533.I1RAC1
101978 229533.A0A1C3YHJ5 pp 229533.Q4HZB8
101979 229533.A0A1C3YHJ5 pp 229533.I1RAN4
101980 229533.A0A1C3YHJ5 pp 229533.I1RLX7
101981 229533.A0A1C3YHJ5 pp 229533.I1RZS7

101982 229533.A0A1C3YHJ5 pp 229533.I1RJF9
101983 229533.A0A1C3YHJ5 pp 229533.Q4I5I4
101984 229533.A0A1C3YHJ5 pp 229533.I1RUK7
101985 229533.A0A1C3YHJ5 pp 229533.I1RDI9
101986 229533.I1RD63 pp 229533.I1RMR7
101987 229533.I1RD63 pp 229533.Q4I1H6
101988 229533.I1RD63 pp 229533.I1RLH5
101989 229533.I1RD63 pp 229533.I1REC4
101990 229533.I1RD63 pp 229533.I1RXT2
101991 229533.I1RD63 pp 229533.I1RRG1
101992 229533.I1RD63 pp 229533.I1RFU6
101993 229533.I1RD63 pp 229533.I1RFQ0
101994 229533.I1RD63 pp 229533.I1RJT0
101995 229533.I1RD63 pp 229533.I1RX87
101996 229533.I1RD63 pp 229533.I1S8F9
101997 229533.I1RD63 pp 229533.I1RS69
101998 229533.I1RD63 pp 229533.I1RYY6
101999 229533.I1RD63 pp 229533.I1RSZ1
102000 229533.I1RD63 pp 229533.I1REK3
102001 229533.I1RD63 pp 229533.I1RDJ8
102002 229533.I1RD63 pp 229533.I1RG69
102003 229533.I1RD63 pp 229533.I1RFV4
102004 229533.I1RD63 pp 229533.I1RG99
102005 229533.I1RD63 pp 229533.I1RW76
102006 229533.I1RD63 pp 229533.I1RKI1
102007 229533.I1RD63 pp 229533.I1RMF2
102008 229533.I1RD63 pp 229533.I1RTG7
102009 229533.I1RD63 pp 229533.I1RRX3
102010 229533.I1RD63 pp 229533.I1S5R2
102011 229533.I1RD63 pp 229533.I1RE15
102012 229533.A0A098D153 pp 229533.I1RFI9
102013 229533.A0A098D153 pp 229533.V6QUG0
102014 229533.A0A098D153 pp 229533.A0A098DAD8
102015 229533.A0A098D153 pp 229533.I1S287
102016 229533.A0A098D153 pp 229533.Q4HZQ1
102017 229533.A0A098D153 pp 229533.I1R9Y8
102018 229533.A0A098D153 pp 229533.A0A098DPB8
102019 229533.A0A098D153 pp 229533.I1RPY0
102020 229533.A0A098D153 pp 229533.V6R7V8
102021 229533.A0A098D153 pp 229533.I1RE53
102022 229533.A0A098D153 pp 229533.I1RGY6
102023 229533.A0A098D153 pp 229533.I1RF33
102024 229533.A0A098D153 pp 229533.I1RPN1
102025 229533.A0A098D153 pp 229533.I1RFY0
102026 229533.A0A098D153 pp 229533.A0A1C3YKT4
102027 229533.A0A098D153 pp 229533.Q4HVU7
102028 229533.A0A098D153 pp 229533.A0A1C3YJK5
102029 229533.A0A098D153 pp 229533.I1RKZ4
102030 229533.A0A098D153 pp 229533.A0A1C3YLB6
102031 229533.A0A098D153 pp 229533.A0A1C3YKE0
102032 229533.A0A098D153 pp 229533.I1S0C2
102033 229533.A0A098D153 pp 229533.I1RAE8
102034 229533.A0A098D153 pp 229533.I1RUL3
102035 229533.A0A098D153 pp 229533.I1RDJ8
102036 229533.A0A098D153 pp 229533.I1RT09
102037 229533.A0A098D153 pp 229533.I1S2A1
102038 229533.A0A0E0RW58 pp 229533.I1RJS8
102039 229533.A0A0E0RW58 pp 229533.I1RFR6
102040 229533.A0A0E0RW58 pp 229533.I1RRB0
102041 229533.A0A0E0RW58 pp 229533.I1S1C8
102042 229533.I1S3K8 pp 229533.V6RUB1
102043 229533.I1S3K8 pp 229533.O74638
102044 229533.I1S3K8 pp 229533.I1S4D0
102045 229533.I1S3K8 pp 229533.V6RY56
102046 229533.I1S3K8 pp 229533.V6QWD8
102047 229533.I1S3K8 pp 229533.I1S6P5
102048 229533.I1S3K8 pp 229533.V6RRM0
102049 229533.I1S3K8 pp 229533.I1S8G4
102050 229533.I1RJF1 pp 229533.I1S3F3
102051 229533.I1RJF1 pp 229533.I1RZ52
102052 229533.I1RJF1 pp 229533.I1RVX9
102053 229533.I1RJF1 pp 229533.I1RU76
102054 229533.I1RJF1 pp 229533.I1S9D4

102055	229533.I1RJF1	pp	229533.I1RPD6
102056	229533.I1RJF1	pp	229533.I1RQE0
102057	229533.I1RJF1	pp	229533.I1RY74
102058	229533.I1RJF1	pp	229533.I1RNG4
102059	229533.I1RJF1	pp	229533.I1RPW2
102060	229533.I1RJF1	pp	229533.I1RK03
102061	229533.I1RJF1	pp	229533.I1S2E2
102062	229533.I1RJF1	pp	229533.I1RM26
102063	229533.I1RJF1	pp	229533.I1RMM8
102064	229533.I1RBS0	pp	229533.I1RD22
102065	229533.I1RBS0	pp	229533.I1S7J8
102066	229533.I1RBS0	pp	229533.I1S0N4
102067	229533.I1RBS0	pp	229533.I1SA33
102068	229533.I1RBS0	pp	229533.I1RSQ3
102069	229533.I1RBS0	pp	229533.I1RXT9
102070	229533.I1RBS0	pp	229533.V6RMW6
102071	229533.I1RBS0	pp	229533.V6R042
102072	229533.I1RBS0	pp	229533.I1RE06
102073	229533.I1RBS0	pp	229533.I1S0Q7
102074	229533.I1RBS0	pp	229533.I1RRV1
102075	229533.I1RBS0	pp	229533.I1RCR8
102076	229533.I1RBS0	pp	229533.I1RNC1
102077	229533.I1RBS0	pp	229533.I1S0M2
102078	229533.I1RBS0	pp	229533.I1S8V4
102079	229533.I1RBS0	pp	229533.I1RUG3
102080	229533.I1RBS0	pp	229533.I1RSA4
102081	229533.I1RBS0	pp	229533.I1S5N3
102082	229533.I1RBS0	pp	229533.I1RRE5
102083	229533.I1RBS0	pp	229533.I1S238
102084	229533.I1RBS0	pp	229533.I1S2C0
102085	229533.I1RBS0	pp	229533.I1RJS5
102086	229533.I1RBS0	pp	229533.I1RC33
102087	229533.I1RBS0	pp	229533.I1RY44
102088	229533.I1RBS0	pp	229533.I1RCL5
102089	229533.I1RBS0	pp	229533.I1S4P6
102090	229533.I1RBS0	pp	229533.I1RJW6
102091	229533.I1RBS0	pp	229533.I1RWN5
102092	229533.I1RBS0	pp	229533.I1S503
102093	229533.I1RBS0	pp	229533.I1RTH6
102094	229533.I1RBS0	pp	229533.I1S8R3
102095	229533.I1RBS0	pp	229533.V6RIV9
102096	229533.I1RBS0	pp	229533.I1RPP4
102097	229533.I1RBS0	pp	229533.I1S2E9
102098	229533.I1RBS0	pp	229533.I1RZW8
102099	229533.I1RBS0	pp	229533.I1RS17
102100	229533.I1RBS0	pp	229533.I1RWX5
102101	229533.I1RBS0	pp	229533.I1RT75
102102	229533.I1RBS0	pp	229533.I1RRI2
102103	229533.I1RBS0	pp	229533.I1RT04
102104	229533.I1RBS0	pp	229533.V6RUY9
102105	229533.I1RBS0	pp	229533.I1S1C7
102106	229533.I1RBS0	pp	229533.V6RLM7
102107	229533.I1RBS0	pp	229533.I1RGI5
102108	229533.I1RBS0	pp	229533.I1RYW3
102109	229533.I1RBS0	pp	229533.I1RPK0
102110	229533.I1RBS0	pp	229533.I1RSH0
102111	229533.I1RBS0	pp	229533.I1S0J1
102112	229533.I1RBS0	pp	229533.I1RTD5
102113	229533.I1RBS0	pp	229533.I1RTA0
102114	229533.I1RBS0	pp	229533.Q4I624
102115	229533.I1RBS0	pp	229533.I1RW46
102116	229533.I1RBS0	pp	229533.I1RT40
102117	229533.I1RBS0	pp	229533.I1RD17
102118	229533.I1RBS0	pp	229533.I1RWN6
102119	229533.I1RBS0	pp	229533.I1RE01
102120	229533.I1RBS0	pp	229533.I1RV91
102121	229533.I1RBS0	pp	229533.I1RG90
102122	229533.I1RBS0	pp	229533.I1RT03
102123	229533.I1RBS0	pp	229533.I1RQE7
102124	229533.I1RBS0	pp	229533.I1RJV3
102125	229533.I1RBS0	pp	229533.I1RVX0
102126	229533.I1RBS0	pp	229533.I1RSK1
102127	229533.I1RBS0	pp	229533.I1S2P0

102128	229533.I1RBS0	pp	229533.I1RFL4
102129	229533.I1RBS0	pp	229533.I1RCH5
102130	229533.I1RBS0	pp	229533.I1RP28
102131	229533.I1RBS0	pp	229533.I1RRY3
102132	229533.I1RBS0	pp	229533.I1S0F6
102133	229533.I1RBS0	pp	229533.I1RN18
102134	229533.I1RBS0	pp	229533.I1RJV4
102135	229533.I1RBS0	pp	229533.I1RKX6
102136	229533.I1RBS0	pp	229533.V6R3X5
102137	229533.I1RBS0	pp	229533.I1RR73
102138	229533.I1RBS0	pp	229533.I1RN67
102139	229533.I1RBS0	pp	229533.I1RGA4
102140	229533.I1RBS0	pp	229533.I1RQG4
102141	229533.I1RBS0	pp	229533.I1RJS2
102142	229533.I1RBS0	pp	229533.V6R3J1
102143	229533.I1RBS0	pp	229533.I1RBS6
102144	229533.I1RBS0	pp	229533.I1RNN1
102145	229533.I1RBS0	pp	229533.I1RZ39
102146	229533.I1RBS0	pp	229533.I1S8P5
102147	229533.I1RBS0	pp	229533.V6RCZ8
102148	229533.I1RBS0	pp	229533.I1RFT1
102149	229533.I1RBS0	pp	229533.I1RPP3
102150	229533.I1RBS0	pp	229533.I1RYS9
102151	229533.I1RBS0	pp	229533.I1RI82
102152	229533.I1RBS0	pp	229533.I1RWK3
102153	229533.I1RBS0	pp	229533.I1S8V3
102154	229533.I1RBS0	pp	229533.I1RN63
102155	229533.I1RBS0	pp	229533.Q4I283
102156	229533.I1RBS0	pp	229533.I1RZM8
102157	229533.I1RBS0	pp	229533.I1RWI5
102158	229533.I1RBS0	pp	229533.I1RC66
102159	229533.I1RBS0	pp	229533.I1RZZ9
102160	229533.I1RBS0	pp	229533.I1S0L4
102161	229533.I1RBS0	pp	229533.I1RLP9
102162	229533.I1RBS0	pp	229533.I1RGC3
102163	229533.I1RBS0	pp	229533.I1RS20
102164	229533.I1RBS0	pp	229533.I1RRI6
102165	229533.I1RBS0	pp	229533.I1RBY4
102166	229533.I1RBS0	pp	229533.I1RPU3
102167	229533.I1RBS0	pp	229533.I1RZM1
102168	229533.I1RBS0	pp	229533.I1S504
102169	229533.I1RBS0	pp	229533.I1S2H4
102170	229533.I1RBS0	pp	229533.I1RC00
102171	229533.I1RBS0	pp	229533.I1RJZ7
102172	229533.I1RBS0	pp	229533.I1RE76
102173	229533.I1RBS0	pp	229533.I1RNQ5
102174	229533.I1RBS0	pp	229533.I1S2B8
102175	229533.I1RBS0	pp	229533.I1RRT8
102176	229533.I1RBS0	pp	229533.I1RWL2
102177	229533.I1RBS0	pp	229533.I1RLU6
102178	229533.I1RBS0	pp	229533.I1RJD6
102179	229533.I1RBS0	pp	229533.I1S5R1
102180	229533.I1RBS0	pp	229533.I1RRM5
102181	229533.I1RBS0	pp	229533.I1RE22
102182	229533.I1RBS0	pp	229533.I1RS18
102183	229533.I1RBS0	pp	229533.I1RW99
102184	229533.I1RBS0	pp	229533.I1RPM4
102185	229533.I1RBS0	pp	229533.I1RM82
102186	229533.I1RBS0	pp	229533.I1RJS4
102187	229533.I1RBS0	pp	229533.I1RQ81
102188	229533.I1RBS0	pp	229533.Q4I8P5
102189	229533.I1RBS0	pp	229533.I1S5B0
102190	229533.I1RBS0	pp	229533.I1RCR5
102191	229533.I1RBS0	pp	229533.I1RCY5
102192	229533.I1RBS0	pp	229533.I1REK8
102193	229533.I1RBS0	pp	229533.I1SA32
102194	229533.I1RBS0	pp	229533.I1RF19
102195	229533.I1RBS0	pp	229533.I1RC95
102196	229533.I1RBS0	pp	229533.I1RKK0
102197	229533.I1RBS0	pp	229533.I1RRU9
102198	229533.I1RBS0	pp	229533.I1RFT2
102199	229533.I1RBS0	pp	229533.V6QWZ7
102200	229533.I1RBS0	pp	229533.I1RWC9

102201	229533.I1RBS0	pp	229533.I1RRY9
102202	229533.I1RBS0	pp	229533.I1RRV4
102203	229533.I1RBS0	pp	229533.Q4I2J8
102204	229533.I1RBS0	pp	229533.I1RPR9
102205	229533.I1RBS0	pp	229533.I1S1J7
102206	229533.I1RBS0	pp	229533.V6RCG5
102207	229533.I1RBS0	pp	229533.I1RF71
102208	229533.I1RBS0	pp	229533.I1RMM9
102209	229533.I1RBS0	pp	229533.V6RBN2
102210	229533.I1RBS0	pp	229533.I1RVQ0
102211	229533.I1RBS0	pp	229533.I1RZZ1
102212	229533.I1RBS0	pp	229533.I1S5B5
102213	229533.I1RBS0	pp	229533.V6QW22
102214	229533.I1RBS0	pp	229533.I1RC20
102215	229533.I1RBS0	pp	229533.I1RZK5
102216	229533.I1RBS0	pp	229533.I1RJL2
102217	229533.I1RBS0	pp	229533.Q4IJK2
102218	229533.I1RBS0	pp	229533.I1RSQ7
102219	229533.I1RBS0	pp	229533.Q4IP51
102220	229533.I1RBS0	pp	229533.Q4I1Y1
102221	229533.I1RBS0	pp	229533.I1RX46
102222	229533.I1RBS0	pp	229533.V6RTJ8
102223	229533.I1RBS0	pp	229533.I1RCG4
102224	229533.I1RBS0	pp	229533.I1RW71
102225	229533.I1RBS0	pp	229533.I1RN08
102226	229533.I1RBS0	pp	229533.I1RFU8
102227	229533.I1RBS0	pp	229533.I1RY03
102228	229533.I1RBS0	pp	229533.I1RXD7
102229	229533.I1RBS0	pp	229533.I1RQH3
102230	229533.I1RBS0	pp	229533.I1RRG3
102231	229533.I1RBS0	pp	229533.I1RQ26
102232	229533.I1RBS0	pp	229533.Q4IJT5
102233	229533.I1RBS0	pp	229533.I1S0R7
102234	229533.I1RBS0	pp	229533.I1RNL0
102235	229533.I1RBS0	pp	229533.I1REF2
102236	229533.I1RBS0	pp	229533.I1RQC9
102237	229533.I1RBS0	pp	229533.I1RM67
102238	229533.I1RBS0	pp	229533.I1RR24
102239	229533.I1RBS0	pp	229533.I1RWJ8
102240	229533.I1RBS0	pp	229533.I1S0L3
102241	229533.I1RBS0	pp	229533.I1RD52
102242	229533.I1RBS0	pp	229533.I1S0X6
102243	229533.I1RBS0	pp	229533.I1RBT1
102244	229533.I1RBS0	pp	229533.I1RJU3
102245	229533.I1RBS0	pp	229533.I1RDU9
102246	229533.I1RBS0	pp	229533.I1RW25
102247	229533.I1RMY7	pp	229533.Q4IER8
102248	229533.I1RMY7	pp	229533.I1RSD1
102249	229533.I1RMY7	pp	229533.Q4I5R3
102250	229533.I1RMY7	pp	229533.I1RYV3
102251	229533.I1S0Q4	pp	229533.I1S0Q5
102252	229533.I1RM10	pp	229533.I1RY40
102253	229533.I1RM10	pp	229533.I1RZF7
102254	229533.I1RM10	pp	229533.I1RZV9
102255	229533.I1RM10	pp	229533.I1S2F3
102256	229533.I1RRQ3	pp	229533.Q4IPI1
102257	229533.I1RRQ3	pp	229533.I1RRT2
102258	229533.I1RRQ3	pp	229533.I1RWD4
102259	229533.I1RRQ3	pp	229533.I1S503
102260	229533.I1RRQ3	pp	229533.Q4IAS1
102261	229533.I1RRQ3	pp	229533.Q4I5R9
102262	229533.I1RRQ3	pp	229533.I1RS30
102263	229533.I1RRQ3	pp	229533.I1RT33
102264	229533.I1RRQ3	pp	229533.I1RS47
102265	229533.I1RRQ3	pp	229533.Q4IQW0
102266	229533.I1RRQ3	pp	229533.I1RSL9
102267	229533.I1RRQ3	pp	229533.I1S0L8
102268	229533.I1RRQ3	pp	229533.Q4HZ42
102269	229533.I1RRQ3	pp	229533.V6R283
102270	229533.I1RRQ3	pp	229533.I1RTD5
102271	229533.I1RRQ3	pp	229533.I1S6F0
102272	229533.I1RRQ3	pp	229533.I1RZJ6
102273	229533.I1RRQ3	pp	229533.I1RWH5

102274	229533.I1RRQ3	pp	229533.I1RZ99
102275	229533.I1RRQ3	pp	229533.I1RUQ2
102276	229533.I1RRQ3	pp	229533.I1RT98
102277	229533.I1RRQ3	pp	229533.Q4HZ47
102278	229533.I1RRQ3	pp	229533.I1RSQ7
102279	229533.I1RRQ3	pp	229533.I1S2E9
102280	229533.I1RRQ3	pp	229533.I1RXD7
102281	229533.I1RRQ3	pp	229533.Q4I830
102282	229533.I1RRQ3	pp	229533.I1RSV2
102283	229533.I1RRQ3	pp	229533.Q4IFI0
102284	229533.I1RRQ3	pp	229533.I1RW71
102285	229533.I1RRQ3	pp	229533.I1S2B8
102286	229533.I1RRQ3	pp	229533.Q4HXF0
102287	229533.I1RRQ3	pp	229533.I1RWJ4
102288	229533.I1RRQ3	pp	229533.I1RX46
102289	229533.I1RRQ3	pp	229533.I1S0R7
102290	229533.I1RRQ3	pp	229533.Q4I662
102291	229533.I1RRQ3	pp	229533.I1RT04
102292	229533.I1RRQ3	pp	229533.I1S9U2
102293	229533.I1RRQ3	pp	229533.I1S0F1
102294	229533.I1RRQ3	pp	229533.I1RW25
102295	229533.I1RRQ3	pp	229533.I1RY44
102296	229533.I1RRQ3	pp	229533.I1RU69
102297	229533.I1RRQ3	pp	229533.I1RW61
102298	229533.I1RRQ3	pp	229533.I1RT05
102299	229533.I1RRQ3	pp	229533.I1RWN5
102300	229533.I1RRQ3	pp	229533.I1RYT5
102301	229533.I1RRQ3	pp	229533.Q4IF76
102302	229533.I1RRQ3	pp	229533.I1RZS6
102303	229533.I1RRQ3	pp	229533.V6R227
102304	229533.I1RRQ3	pp	229533.I1RX24
102305	229533.I1RRQ3	pp	229533.I1S4T3
102306	229533.I1RRQ3	pp	229533.Q4HVW2
102307	229533.I1RRQ3	pp	229533.Q4IQK7
102308	229533.I1RRQ3	pp	229533.I1S253
102309	229533.I1RRQ3	pp	229533.V6QYF0
102310	229533.I1RRQ3	pp	229533.I1RRR7
102311	229533.I1RRQ3	pp	229533.I1RRT8
102312	229533.I1RRQ3	pp	229533.V6R3X5
102313	229533.I1RRQ3	pp	229533.I1RZF6
102314	229533.I1RRQ3	pp	229533.I1S0J1
102315	229533.I1RRQ3	pp	229533.I1S504
102316	229533.I1RRQ3	pp	229533.I1RZ39
102317	229533.I1RRQ3	pp	229533.I1S060
102318	229533.I1RRQ3	pp	229533.O74638
102319	229533.I1RRQ3	pp	229533.I1RTF4
102320	229533.I1RRQ3	pp	229533.I1S9Y1
102321	229533.I1RRQ3	pp	229533.Q4IJH1
102322	229533.I1RRQ3	pp	229533.I1RZV6
102323	229533.I1RRQ3	pp	229533.Q4IAA0
102324	229533.I1RRQ3	pp	229533.I1RT80
102325	229533.I1RRQ3	pp	229533.I1S8E2
102326	229533.I1RRQ3	pp	229533.Q4IJ56
102327	229533.I1RRQ3	pp	229533.I1S0M9
102328	229533.I1RRQ3	pp	229533.Q4IEK8
102329	229533.I1RRQ3	pp	229533.V6QUE6
102330	229533.I1RRQ3	pp	229533.Q4IBS2
102331	229533.I1RRQ3	pp	229533.I1RWB5
102332	229533.I1RRQ3	pp	229533.V6RGN5
102333	229533.I1RRQ3	pp	229533.I1S044
102334	229533.I1RRQ3	pp	229533.I1S1J6
102335	229533.I1RRQ3	pp	229533.I1S296
102336	229533.I1RRQ3	pp	229533.Q4I327
102337	229533.I1RRQ3	pp	229533.Q4HZ68
102338	229533.I1RRQ3	pp	229533.I1RRY9
102339	229533.I1RRQ3	pp	229533.I1S021
102340	229533.I1RRQ3	pp	229533.I1S558
102341	229533.I1RRQ3	pp	229533.V6RGY3
102342	229533.I1RRQ3	pp	229533.I1RWN6
102343	229533.I1RRQ3	pp	229533.I1RYB9
102344	229533.I1RRQ3	pp	229533.I1RZZ1
102345	229533.I1RRQ3	pp	229533.I1RZK6
102346	229533.I1RRQ3	pp	229533.I1RSR3

102347 229533.I1RRQ3 pp 229533.I1RSD8
102348 229533.I1RRQ3 pp 229533.I1S2E8
102349 229533.I1RRQ3 pp 229533.I1S274
102350 229533.I1RRQ3 pp 229533.Q4IN63
102351 229533.I1RRQ3 pp 229533.I1S2U2
102352 229533.I1RRQ3 pp 229533.V6R042
102353 229533.I1RRQ3 pp 229533.Q4I948
102354 229533.I1RRQ3 pp 229533.I1RT03
102355 229533.I1RRQ3 pp 229533.I1S1C7
102356 229533.I1RRQ3 pp 229533.V6RMW6
102357 229533.I1RRQ3 pp 229533.I1RVX0
102358 229533.I1RRQ3 pp 229533.I1RSI6
102359 229533.I1RRQ3 pp 229533.I1RZE5
102360 229533.I1RRQ3 pp 229533.I1RTD9
102361 229533.I1RRQ3 pp 229533.I1RSR8
102362 229533.I1RRQ3 pp 229533.I1S6E5
102363 229533.I1RRQ3 pp 229533.I1S1X9
102364 229533.I1RRQ3 pp 229533.I1RY03
102365 229533.I1RRQ3 pp 229533.I1RSS4
102366 229533.I1RRQ3 pp 229533.I1RY63
102367 229533.I1RRQ3 pp 229533.I1RTE9
102368 229533.I1RRQ3 pp 229533.I1RVZ0
102369 229533.I1RRQ3 pp 229533.I1RZK5
102370 229533.I1RRQ3 pp 229533.I1S2C0
102371 229533.I1RRQ3 pp 229533.Q4HWE2
102372 229533.I1RRQ3 pp 229533.I1RSC6
102373 229533.I1RRQ3 pp 229533.I1RTH6
102374 229533.I1RE23 pp 229533.I1RX64
102375 229533.I1RE23 pp 229533.I1RVZ9
102376 229533.I1RE23 pp 229533.I1S183
102377 229533.I1RE23 pp 229533.I1S209
102378 229533.I1RE23 pp 229533.I1RSP7
102379 229533.I1RE23 pp 229533.I1RRJ6
102380 229533.I1RE23 pp 229533.I1RSC7
102381 229533.I1RE23 pp 229533.I1S788
102382 229533.I1RE23 pp 229533.I1RRT1
102383 229533.I1RE23 pp 229533.I1RKI2
102384 229533.I1RSS8 pp 229533.Q4I7L0
102385 229533.I1RSS8 pp 229533.I1RZ94
102386 229533.I1RHR0 pp 229533.I1RSI4
102387 229533.I1RHR0 pp 229533.Q4IEA5
102388 229533.I1RHR0 pp 229533.I1RYR2
102389 229533.I1RHR0 pp 229533.I1RN57
102390 229533.I1RHR0 pp 229533.I1S209
102391 229533.I1RHR0 pp 229533.I1RKI2
102392 229533.I1RHR0 pp 229533.Q4IQM6
102393 229533.I1RHR0 pp 229533.I1RPQ1
102394 229533.I1RHR0 pp 229533.Q4IPC8
102395 229533.I1RHR0 pp 229533.I1RN55
102396 229533.I1RHR0 pp 229533.I1RMS4
102397 229533.I1RHR0 pp 229533.I1RRS7
102398 229533.I1RHR0 pp 229533.V6R382
102399 229533.I1RHR0 pp 229533.I1S461
102400 229533.I1RHR0 pp 229533.I1RSP7
102401 229533.I1RHR0 pp 229533.I1RQJ4
102402 229533.I1RHR0 pp 229533.I1RUG1
102403 229533.I1RHR0 pp 229533.I1S283
102404 229533.I1RHR0 pp 229533.I1RSA7
102405 229533.I1RHR0 pp 229533.Q4IP51
102406 229533.I1RHR0 pp 229533.V6RLW4
102407 229533.I1RHR0 pp 229533.I1RYX9
102408 229533.I1RHR0 pp 229533.I1RZP8
102409 229533.A0A0E0S0W9 pp 229533.I1RX46
102410 229533.A0A0E0S0W9 pp 229533.I1RXD7
102411 229533.A0A0E0S0W9 pp 229533.I1S0R7
102412 229533.A0A0E0S0W9 pp 229533.I1RKA0
102413 229533.A0A0E0S0W9 pp 229533.A0A1C3YIP6
102414 229533.A0A0E0S0W9 pp 229533.I1RSQ7
102415 229533.A0A0E0S0W9 pp 229533.I1RNG8
102416 229533.A0A0E0S0W9 pp 229533.I1RQC9
102417 229533.A0A0E0S0W9 pp 229533.I1RCY0
102418 229533.A0A0E0S0W9 pp 229533.I1RN65
102419 229533.A0A0E0S0W9 pp 229533.I1RR00

102420 229533.A0A0E0S0W9 pp 229533.I1RTC2
102421 229533.A0A0E0S0W9 pp 229533.A0A0E0SFN0
102422 229533.A0A0E0S0W9 pp 229533.I1REF2
102423 229533.A0A0E0S0W9 pp 229533.I1RWB3
102424 229533.A0A0E0S0W9 pp 229533.I1RWL6
102425 229533.A0A0E0S0W9 pp 229533.I1RJZ6
102426 229533.A0A0E0S0W9 pp 229533.A0A1C3YKW5
102427 229533.A0A0E0S0W9 pp 229533.V6RTJ8
102428 229533.A0A0E0S0W9 pp 229533.I1RLX6
102429 229533.A0A0E0S0W9 pp 229533.I1RN31
102430 229533.A0A0E0S0W9 pp 229533.I1RZZ1
102431 229533.A0A0E0S0W9 pp 229533.I1RW25
102432 229533.A0A0E0S0W9 pp 229533.I1RDX3
102433 229533.A0A0E0S0W9 pp 229533.A0A1C3YKY3
102434 229533.A0A0E0S0W9 pp 229533.I1RAX6
102435 229533.A0A0E0S0W9 pp 229533.I1RX84
102436 229533.A0A0E0S0W9 pp 229533.I1RQA3
102437 229533.A0A0E0S0W9 pp 229533.I1RQ70
102438 229533.A0A0E0S0W9 pp 229533.I1S0S6
102439 229533.A0A0E0S0W9 pp 229533.Q4IJK2
102440 229533.A0A0E0S0W9 pp 229533.I1RAL2
102441 229533.A0A0E0S0W9 pp 229533.I1RA20
102442 229533.A0A0E0S0W9 pp 229533.A0A0E0SA11
102443 229533.A0A0E0S0W9 pp 229533.I1S6U8
102444 229533.A0A0E0S0W9 pp 229533.I1RTQ9
102445 229533.A0A0E0S0W9 pp 229533.I1RRY9
102446 229533.A0A0E0S0W9 pp 229533.I1RKH9
102447 229533.A0A0E0S0W9 pp 229533.A0A1C3YK34
102448 229533.A0A0E0S0W9 pp 229533.A0A1C3YHX8
102449 229533.A0A0E0S0W9 pp 229533.A0A1C3YKC0
102450 229533.A0A0E0S0W9 pp 229533.I1RN43
102451 229533.A0A0E0S0W9 pp 229533.I1RD03
102452 229533.A0A0E0S0W9 pp 229533.I1RAU5
102453 229533.A0A0E0S0W9 pp 229533.P0C431
102454 229533.A0A0E0S0W9 pp 229533.I1RWT6
102455 229533.A0A0E0S0W9 pp 229533.I1RZK5
102456 229533.A0A0E0S0W9 pp 229533.A0A0E0SGT0
102457 229533.A0A0E0S0W9 pp 229533.I1RNQ5
102458 229533.A0A0E0S0W9 pp 229533.I1RNF5
102459 229533.A0A0E0S0W9 pp 229533.I1RBC3
102460 229533.A0A0E0S0W9 pp 229533.I1RM09
102461 229533.A0A0E0S0W9 pp 229533.I1RFU8
102462 229533.A0A0E0S0W9 pp 229533.I1S461
102463 229533.A0A0E0S0W9 pp 229533.I1RPP3
102464 229533.A0A0E0S0W9 pp 229533.A0A1C3YN54
102465 229533.A0A0E0S0W9 pp 229533.I1RWF7
102466 229533.A0A0E0S0W9 pp 229533.V6R283
102467 229533.A0A0E0S0W9 pp 229533.I1RXF7
102468 229533.A0A0E0S0W9 pp 229533.I1RVU9
102469 229533.A0A0E0S0W9 pp 229533.A0A1C3YIJ3
102470 229533.A0A0E0S0W9 pp 229533.I1RZE5
102471 229533.A0A0E0S0W9 pp 229533.I1RUG2
102472 229533.A0A0E0S0W9 pp 229533.I1RPU3
102473 229533.A0A0E0S0W9 pp 229533.I1RY03
102474 229533.A0A0E0S0W9 pp 229533.I1S2B8
102475 229533.A0A0E0S0W9 pp 229533.I1RAY2
102476 229533.A0A0E0S0W9 pp 229533.I1RB88
102477 229533.A0A0E0S0W9 pp 229533.I1RGA4
102478 229533.A0A0E0S0W9 pp 229533.I1S1B6
102479 229533.A0A0E0S0W9 pp 229533.I1R9M0
102480 229533.A0A0E0S0W9 pp 229533.I1RKF9
102481 229533.A0A0E0S0W9 pp 229533.I1RN67
102482 229533.A0A0E0S0W9 pp 229533.I1RC35
102483 229533.A0A0E0S0W9 pp 229533.I1RW41
102484 229533.A0A0E0S0W9 pp 229533.I1RQN9
102485 229533.A0A0E0S0W9 pp 229533.I1S0S4
102486 229533.A0A0E0S0W9 pp 229533.I1S2E9
102487 229533.A0A0E0S0W9 pp 229533.A0A1C3YI46
102488 229533.A0A0E0S0W9 pp 229533.I1RY44
102489 229533.A0A0E0S0W9 pp 229533.I1RR73
102490 229533.A0A0E0S0W9 pp 229533.I1RT04
102491 229533.A0A0E0S0W9 pp 229533.I1RTD5
102492 229533.A0A0E0S0W9 pp 229533.I1RWN5

102493	229533.A0A0E0S0W9	pp	229533.A0A1C3YHY9
102494	229533.A0A0E0S0W9	pp	229533.I1S0E4
102495	229533.A0A0E0S0W9	pp	229533.A0A1C3YIZ8
102496	229533.A0A0E0S0W9	pp	229533.V6R3X5
102497	229533.A0A0E0S0W9	pp	229533.I1RZ39
102498	229533.A0A0E0S0W9	pp	229533.I1RSU1
102499	229533.A0A0E0S0W9	pp	229533.A0A1C3YKM9
102500	229533.A0A0E0S0W9	pp	229533.V6RMW6
102501	229533.A0A0E0S0W9	pp	229533.I1RS64
102502	229533.A0A0E0S0W9	pp	229533.I1RNY2
102503	229533.A0A0E0S0W9	pp	229533.I1RHM4
102504	229533.A0A0E0S0W9	pp	229533.I1RCR8
102505	229533.A0A0E0S0W9	pp	229533.Q4IN63
102506	229533.A0A0E0S0W9	pp	229533.I1RPP4
102507	229533.A0A0E0S0W9	pp	229533.I1RW16
102508	229533.A0A0E0S0W9	pp	229533.I1RWQ0
102509	229533.A0A0E0S0W9	pp	229533.I1RNC1
102510	229533.A0A0E0S0W9	pp	229533.I1S0J1
102511	229533.A0A0E0S0W9	pp	229533.I1RAU7
102512	229533.A0A0E0S0W9	pp	229533.A0A0E0SCK7
102513	229533.A0A0E0S0W9	pp	229533.I1RNW8
102514	229533.A0A0E0S0W9	pp	229533.I1RT36
102515	229533.A0A0E0S0W9	pp	229533.I1RE44
102516	229533.A0A0E0S0W9	pp	229533.I1RD22
102517	229533.A0A0E0S0W9	pp	229533.Q4I1Q6
102518	229533.A0A0E0S0W9	pp	229533.I1RRS7
102519	229533.A0A0E0S0W9	pp	229533.I1RVX0
102520	229533.A0A0E0S0W9	pp	229533.I1RMN6
102521	229533.A0A0E0S0W9	pp	229533.I1RT03
102522	229533.A0A0E0S0W9	pp	229533.V6R042
102523	229533.A0A0E0S0W9	pp	229533.I1RAW0
102524	229533.A0A0E0S0W9	pp	229533.I1RSW5
102525	229533.A0A0E0S0W9	pp	229533.I1RSQ3
102526	229533.A0A0E0S0W9	pp	229533.I1RWN6
102527	229533.A0A0E0S0W9	pp	229533.I1R975
102528	229533.A0A0E0S0W9	pp	229533.I1RJM9
102529	229533.A0A0E0S0W9	pp	229533.I1RD70
102530	229533.A0A0E0S0W9	pp	229533.A0A1C3YHN3
102531	229533.A0A0E0S0W9	pp	229533.I1RJV3
102532	229533.A0A0E0S0W9	pp	229533.I1RTH6
102533	229533.A0A0E0S0W9	pp	229533.I1RA70
102534	229533.A0A0E0S0W9	pp	229533.I1S1C7
102535	229533.A0A1C3YJN7	pp	229533.I1RSR9
102536	229533.A0A1C3YJN7	pp	229533.I1S0D5
102537	229533.A0A1C3YJN7	pp	229533.I1RMJ8
102538	229533.A0A1C3YJN7	pp	229533.I1RDX0
102539	229533.A0A1C3YJN7	pp	229533.I1RSP7
102540	229533.A0A1C3YJN7	pp	229533.I1RQJ4
102541	229533.A0A1C3YJN7	pp	229533.I1RPS0
102542	229533.A0A1C3YJN7	pp	229533.I1RK79
102543	229533.A0A1C3YJN7	pp	229533.I1RK90
102544	229533.A0A1C3YJN7	pp	229533.I1RD19
102545	229533.A0A1C3YJN7	pp	229533.I1RG57
102546	229533.A0A1C3YJN7	pp	229533.I1RZP8
102547	229533.A0A1C3YJN7	pp	229533.I1RX64
102548	229533.A0A1C3YJN7	pp	229533.I1RTN6
102549	229533.A0A1C3YJN7	pp	229533.I1RBN9
102550	229533.A0A1C3YJN7	pp	229533.I1S4P6
102551	229533.A0A1C3YJN7	pp	229533.I1RBJ1
102552	229533.A0A1C3YJN7	pp	229533.A0A1C3YN55
102553	229533.A0A1C3YJN7	pp	229533.I1RN57
102554	229533.A0A1C3YJN7	pp	229533.V6R382
102555	229533.A0A1C3YJN7	pp	229533.I1RUG4
102556	229533.A0A1C3YJN7	pp	229533.I1RR25
102557	229533.A0A1C3YJN7	pp	229533.I1RRS7
102558	229533.A0A1C3YJN7	pp	229533.I1RDJ8
102559	229533.A0A1C3YJN7	pp	229533.I1RPM7
102560	229533.A0A1C3YJN7	pp	229533.I1RA10
102561	229533.A0A1C3YJN7	pp	229533.I1RG11
102562	229533.A0A1C3YJN7	pp	229533.I1RC54
102563	229533.A0A1C3YJN7	pp	229533.I1RCA5
102564	229533.A0A1C3YJN7	pp	229533.I1RRJ6
102565	229533.A0A1C3YJN7	pp	229533.I1RVL8

102566 229533.A0A1C3YJN7 pp 229533.I1RSC7
102567 229533.A0A1C3YJN7 pp 229533.I1RYQ1
102568 229533.A0A1C3YJN7 pp 229533.I1RSA7
102569 229533.A0A1C3YJN7 pp 229533.V6RLW4
102570 229533.A0A1C3YJN7 pp 229533.I1RBW1
102571 229533.A0A1C3YJN7 pp 229533.I1RZ63
102572 229533.A0A1C3YJN7 pp 229533.I1RSZ3
102573 229533.A0A1C3YJN7 pp 229533.I1RPF5
102574 229533.A0A1C3YJN7 pp 229533.I1S0J3
102575 229533.A0A1C3YJN7 pp 229533.I1RY29
102576 229533.A0A1C3YJN7 pp 229533.I1RMQ7
102577 229533.A0A1C3YJN7 pp 229533.I1S183
102578 229533.A0A1C3YJN7 pp 229533.I1RBC1
102579 229533.A0A1C3YJN7 pp 229533.I1RFJ2
102580 229533.A0A1C3YJN7 pp 229533.I1RSN7
102581 229533.A0A1C3YJN7 pp 229533.I1RYR2
102582 229533.A0A1C3YJN7 pp 229533.I1RE46
102583 229533.A0A1C3YJN7 pp 229533.I1RMX3
102584 229533.A0A1C3YJN7 pp 229533.I1RFQ5
102585 229533.A0A1C3YJN7 pp 229533.I1RN46
102586 229533.A0A1C3YJN7 pp 229533.I1S209
102587 229533.A0A1C3YJN7 pp 229533.I1RFL7
102588 229533.A0A1C3YJN7 pp 229533.A0A1C3YMJ3
102589 229533.A0A1C3YJN7 pp 229533.I1RE23
102590 229533.A0A1C3YJN7 pp 229533.A0A1C3YM82
102591 229533.A0A1C3YJN7 pp 229533.I1RBW0
102592 229533.A0A1C3YJN7 pp 229533.I1REN3
102593 229533.A0A1C3YJN7 pp 229533.A0A1C3YK21
102594 229533.A0A1C3YJN7 pp 229533.I1RYX9
102595 229533.A0A1C3YJN7 pp 229533.I1RCL7
102596 229533.A0A1C3YJN7 pp 229533.I1RAH4
102597 229533.A0A1C3YJN7 pp 229533.I1RFX8
102598 229533.A0A1C3YJN7 pp 229533.I1RG77
102599 229533.A0A1C3YJN7 pp 229533.A0A1C3YLR6
102600 229533.A0A1C3YJN7 pp 229533.I1RRG3
102601 229533.A0A1C3YJN7 pp 229533.I1RCQ4
102602 229533.A0A1C3YJN7 pp 229533.I1RN55
102603 229533.A0A1C3YJN7 pp 229533.A0A1C3YJT5
102604 229533.A0A1C3YJN7 pp 229533.I1RRT1
102605 229533.A0A1C3YJN7 pp 229533.I1RJL2
102606 229533.A0A1C3YJN7 pp 229533.I1RSI4
102607 229533.A0A1C3YJN7 pp 229533.I1RZ94
102608 229533.A0A1C3YJN7 pp 229533.I1RTC2
102609 229533.A0A1C3YJN7 pp 229533.I1RZB5
102610 229533.A0A1C3YJN7 pp 229533.I1RAT5
102611 229533.A0A1C3YJN7 pp 229533.I1S283
102612 229533.A0A1C3YJN7 pp 229533.I1RA12
102613 229533.A0A1C3YJN7 pp 229533.I1S788
102614 229533.A0A1C3YJN7 pp 229533.A0A1C3YML3
102615 229533.A0A1C3YJN7 pp 229533.I1RPQ1
102616 229533.A0A1C3YJN7 pp 229533.I1RKB1
102617 229533.A0A1C3YJN7 pp 229533.Q4I0J6
102618 229533.A0A1C3YJN7 pp 229533.I1RKI2
102619 229533.A0A1C3YJN7 pp 229533.V6QWX8
102620 229533.A0A1C3YJN7 pp 229533.I1RN03
102621 229533.A0A1C3YJN7 pp 229533.I1RSJ1
102622 229533.A0A1C3YJN7 pp 229533.I1RCM7
102623 229533.A0A1C3YJN7 pp 229533.I1RQC5
102624 229533.I1RZT0 pp 229533.I1S1P8
102625 229533.I1RZT0 pp 229533.V6QWD8
102626 229533.I1RZT0 pp 229533.I1S3K8
102627 229533.I1RZT0 pp 229533.I1S0Y1
102628 229533.I1RZT0 pp 229533.I1S029
102629 229533.I1RZT0 pp 229533.V6RY56
102630 229533.I1RZT0 pp 229533.I1S084
102631 229533.I1RZT0 pp 229533.I1S4D0
102632 229533.I1RZT0 pp 229533.I1S3G4
102633 229533.I1RZT0 pp 229533.O74638
102634 229533.I1RZT0 pp 229533.I1S2C4
102635 229533.I1RZT0 pp 229533.I1RZT1
102636 229533.I1RZT0 pp 229533.I1S1W8
102637 229533.I1RZT0 pp 229533.V6RRM0
102638 229533.I1RZT0 pp 229533.V6RUB1

102639	229533.I1RZT0	pp	229533.I1S8G4
102640	229533.I1RZT0	pp	229533.I1S6P5
102641	229533.I1RN67	pp	229533.I1RZJ5
102642	229533.I1RN67	pp	229533.I1RR24
102643	229533.I1RN67	pp	229533.I1S0X6
102644	229533.I1RN67	pp	229533.I1S6U8
102645	229533.I1RN67	pp	229533.I1RX46
102646	229533.I1RN67	pp	229533.I1S4S4
102647	229533.I1RN67	pp	229533.I1S0T2
102648	229533.I1RN67	pp	229533.I1RRQ1
102649	229533.I1RN67	pp	229533.I1RPH9
102650	229533.I1RN67	pp	229533.I1RWJ8
102651	229533.I1RN67	pp	229533.I1RSQ7
102652	229533.I1RN67	pp	229533.I1RW25
102653	229533.I1RN67	pp	229533.I1RRX5
102654	229533.I1RN67	pp	229533.V6QW22
102655	229533.I1RN67	pp	229533.V6QUW4
102656	229533.I1RN67	pp	229533.I1RX25
102657	229533.I1RN67	pp	229533.I1RXD7
102658	229533.I1RN67	pp	229533.I1RPN4
102659	229533.I1RN67	pp	229533.I1S0B7
102660	229533.I1RN67	pp	229533.I1RQH3
102661	229533.I1RN67	pp	229533.I1RR00
102662	229533.I1RN67	pp	229533.I1RW07
102663	229533.I1RN67	pp	229533.I1RNM7
102664	229533.I1RN67	pp	229533.V6RUT5
102665	229533.I1RN67	pp	229533.I1S3S3
102666	229533.I1RN67	pp	229533.I1RVQ0
102667	229533.I1RN67	pp	229533.I1RW71
102668	229533.I1RN67	pp	229533.I1S0R7
102669	229533.I1RN67	pp	229533.I1S4S7
102670	229533.I1RN67	pp	229533.I1S887
102671	229533.I1RN67	pp	229533.Q4IJT5
102672	229533.I1RN67	pp	229533.V6R4J1
102673	229533.I1RN67	pp	229533.I1RXU7
102674	229533.I1RN67	pp	229533.I1RRG3
102675	229533.I1RN67	pp	229533.I1RQC9
102676	229533.I1RN67	pp	229533.I1S7A8
102677	229533.I1RN67	pp	229533.I1RQ26
102678	229533.I1RN67	pp	229533.I1S0F5
102679	229533.I1RN67	pp	229533.I1RZB5
102680	229533.I1RN67	pp	229533.I1RZK5
102681	229533.I1RN67	pp	229533.I1S0L3
102682	229533.I1RN67	pp	229533.V6RTJ8
102683	229533.I1RN67	pp	229533.I1RYY5
102684	229533.I1RN67	pp	229533.I1RY03
102685	229533.I1RN67	pp	229533.I1RQH1
102686	229533.I1RN67	pp	229533.I1S1J7
102687	229533.I1RN67	pp	229533.I1RRY9
102688	229533.I1RN67	pp	229533.V6R1K4
102689	229533.I1RN67	pp	229533.I1RU29
102690	229533.I1RN67	pp	229533.I1RYS0
102691	229533.I1RN67	pp	229533.I1RT33
102692	229533.I1RN67	pp	229533.I1RZE5
102693	229533.I1RN67	pp	229533.I1RPR9
102694	229533.I1RN67	pp	229533.I1RYU0
102695	229533.I1RN67	pp	229533.I1RWB0
102696	229533.I1RN67	pp	229533.I1RWJ3
102697	229533.I1RN67	pp	229533.I1RRV4
102698	229533.I1RN67	pp	229533.Q4IJM4
102699	229533.I1RN67	pp	229533.Q4I2J8
102700	229533.I1RN67	pp	229533.I1RVR0
102701	229533.I1RN67	pp	229533.I1RZ35
102702	229533.I1RN67	pp	229533.V6RCG5
102703	229533.I1RN67	pp	229533.I1RQQ5
102704	229533.I1RN67	pp	229533.Q4I8P5
102705	229533.I1RN67	pp	229533.I1RS18
102706	229533.I1RN67	pp	229533.I1RR51
102707	229533.I1RN67	pp	229533.V6RBN2
102708	229533.I1RN67	pp	229533.I1RQ81
102709	229533.I1RN67	pp	229533.I1RTD3
102710	229533.I1RN67	pp	229533.I1RPP5
102711	229533.I1RN67	pp	229533.I1RZZ1

229533.I1RN67 pp 229533.I1RPL2
229533.I1RN67 pp 229533.I1SA32
229533.I1RN67 pp 229533.Q4IJK2
229533.I1RN67 pp 229533.I1S6K9
229533.I1RN67 pp 229533.I1RZH4
229533.I1RN67 pp 229533.V6RCZ8
229533.I1RN67 pp 229533.I1RWD7
229533.I1RN67 pp 229533.I1RZM8
229533.I1RN67 pp 229533.I1RQA3
229533.I1RN67 pp 229533.I1RWT6
229533.I1RN67 pp 229533.I1RXF0
229533.I1RN67 pp 229533.I1RXC4
229533.I1RN67 pp 229533.I1RSI0
229533.I1RN67 pp 229533.Q4I283
229533.I1RN67 pp 229533.I1S835
229533.I1RN67 pp 229533.I1RW99
229533.I1RN67 pp 229533.I1S4Q4
229533.I1RN67 pp 229533.I1RR73
229533.I1RN67 pp 229533.I1S5B0
229533.I1RN67 pp 229533.V6R3X5
229533.I1RN67 pp 229533.I1S504
229533.I1RN67 pp 229533.I1S253
229533.I1RN67 pp 229533.I1RZ39
229533.I1RN67 pp 229533.Q4ICA8
229533.I1RN67 pp 229533.I1S044
229533.I1RN67 pp 229533.I1RZM1
229533.I1RN67 pp 229533.I1RNN1
229533.I1RN67 pp 229533.I1S3K6
229533.I1RN67 pp 229533.I1RNQ5
229533.I1RN67 pp 229533.I1RRU9
229533.I1RN67 pp 229533.V6QUE6
229533.I1RN67 pp 229533.I1S8V3
229533.I1RN67 pp 229533.I1S419
229533.I1RN67 pp 229533.I1RSQ4
229533.I1RN67 pp 229533.I1RT04
229533.I1RN67 pp 229533.I1RZZ9
229533.I1RN67 pp 229533.I1RWI5
229533.I1RN67 pp 229533.I1S0M6
229533.I1RN67 pp 229533.Q4HXF0
229533.I1RN67 pp 229533.I1RR72
229533.I1RN67 pp 229533.V6RUY9
229533.I1RN67 pp 229533.I1RPM4
229533.I1RN67 pp 229533.I1RPV3
229533.I1RN67 pp 229533.I1S0L4
229533.I1RN67 pp 229533.I1S2H4
229533.I1RN67 pp 229533.I1RS20
229533.I1RN67 pp 229533.I1S6Y8
229533.I1RN67 pp 229533.I1RTD5
229533.I1RN67 pp 229533.I1RNA5
229533.I1RN67 pp 229533.I1RQ23
229533.I1RN67 pp 229533.I1RWL2
229533.I1RN67 pp 229533.V6RAC9
229533.I1RN67 pp 229533.I1RN88
229533.I1RN67 pp 229533.O74638
229533.I1RN67 pp 229533.I1RXC2
229533.I1RN67 pp 229533.I1RSZ7
229533.I1RN67 pp 229533.I1RT40
229533.I1RN67 pp 229533.I1RP27
229533.I1RN67 pp 229533.I1RV91
229533.I1RN67 pp 229533.I1RRT8
229533.I1RN67 pp 229533.I1RX39
229533.I1RN67 pp 229533.I1S7S9
229533.I1RN67 pp 229533.I1S9Y1
229533.I1RN67 pp 229533.I1RPU3
229533.I1RN67 pp 229533.I1S5R1
229533.I1RN67 pp 229533.I1RSX0
229533.I1RN67 pp 229533.I1S2B8
229533.I1RN67 pp 229533.V6R3J1
229533.I1RN67 pp 229533.I1RSV2
229533.I1RN67 pp 229533.I1RRM5
229533.I1RN67 pp 229533.I1S2E9
229533.I1RN67 pp 229533.I1S503
229533.I1RN67 pp 229533.I1RWU2

102785 229533.I1RN67 pp 229533.I1S2C0
102786 229533.I1RN67 pp 229533.I1S352
102787 229533.I1RN67 pp 229533.I1RRZ0
102788 229533.I1RN67 pp 229533.I1RQG4
102789 229533.I1RN67 pp 229533.I1RVZ2
102790 229533.I1RN67 pp 229533.I1RWF7
102791 229533.I1RN67 pp 229533.I1S8R3
102792 229533.I1RN67 pp 229533.I1RRJ8
102793 229533.I1RN67 pp 229533.V6R283
102794 229533.I1RN67 pp 229533.I1RTH6
102795 229533.I1RN67 pp 229533.I1RTA1
102796 229533.I1RN67 pp 229533.I1RZW8
102797 229533.I1RN67 pp 229533.I1S8P5
102798 229533.I1RN67 pp 229533.I1RZ79
102799 229533.I1RN67 pp 229533.I1RWK3
102800 229533.I1RN67 pp 229533.I1RPP3
102801 229533.I1RN67 pp 229533.I1RRI2
102802 229533.I1RN67 pp 229533.I1RYI5
102803 229533.I1RN67 pp 229533.I1S2G7
102804 229533.I1RN67 pp 229533.I1RRN5
102805 229533.I1RN67 pp 229533.I1RQ87
102806 229533.I1RN67 pp 229533.I1RQ65
102807 229533.I1RN67 pp 229533.I1S1F3
102808 229533.I1RN67 pp 229533.I1RSS4
102809 229533.I1RN67 pp 229533.I1RZL6
102810 229533.I1RN67 pp 229533.I1RS47
102811 229533.I1RN67 pp 229533.I1RWX5
102812 229533.I1RN67 pp 229533.I1S1C7
102813 229533.I1RN67 pp 229533.I1RXR1
102814 229533.I1RN67 pp 229533.I1RYW3
102815 229533.I1RN67 pp 229533.I1RY62
102816 229533.I1RN67 pp 229533.V6R2A1
102817 229533.I1RN67 pp 229533.I1RWN6
102818 229533.I1RN67 pp 229533.I1RT03
102819 229533.I1RN67 pp 229533.I1RP28
102820 229533.I1RN67 pp 229533.I1S0F6
102821 229533.I1RN67 pp 229533.I1RVX0
102822 229533.I1RN67 pp 229533.I1RQE7
102823 229533.I1RN67 pp 229533.I1RNG4
102824 229533.I1RN67 pp 229533.I1RXU9
102825 229533.I1RN67 pp 229533.Q4I624
102826 229533.I1RN67 pp 229533.I1S2E8
102827 229533.I1RN67 pp 229533.I1RSK1
102828 229533.I1RN67 pp 229533.I1RS17
102829 229533.I1RN67 pp 229533.V6R057
102830 229533.I1RN67 pp 229533.Q4I5V2
102831 229533.I1RN67 pp 229533.I1RPK0
102832 229533.I1RN67 pp 229533.V6RLM7
102833 229533.I1RN67 pp 229533.V6R042
102834 229533.I1RN67 pp 229533.I1S569
102835 229533.I1RN67 pp 229533.I1S0J1
102836 229533.I1RN67 pp 229533.I1S7W5
102837 229533.I1RN67 pp 229533.I1RP69
102838 229533.I1RN67 pp 229533.I1RZS7
102839 229533.I1RN67 pp 229533.I1RTA0
102840 229533.I1RN67 pp 229533.I1RRV1
102841 229533.I1RN67 pp 229533.I1RPU8
102842 229533.I1RN67 pp 229533.I1SAN9
102843 229533.I1RN67 pp 229533.V6RRH2
102844 229533.I1RN67 pp 229533.I1RW46
102845 229533.I1RN67 pp 229533.I1RNY2
102846 229533.I1RN67 pp 229533.Q4IN63
102847 229533.I1RN67 pp 229533.V6RMW6
102848 229533.I1RN67 pp 229533.I1S8V4
102849 229533.I1RN67 pp 229533.I1RUG3
102850 229533.I1RN67 pp 229533.I1S1W4
102851 229533.I1RN67 pp 229533.I1RSQ3
102852 229533.I1RN67 pp 229533.I1RXT3
102853 229533.I1RN67 pp 229533.I1RWE8
102854 229533.I1RN67 pp 229533.V6RVG3
102855 229533.I1RN67 pp 229533.I1RRE5
102856 229533.I1RN67 pp 229533.I1RNC1
102857 229533.I1RN67 pp 229533.I1RND4

102858 229533.I1RN67 pp 229533.I1S5R6
102859 229533.I1RN67 pp 229533.I1S0N4
102860 229533.I1RN67 pp 229533.I1RWH4
102861 229533.I1RN67 pp 229533.I1S1W3
102862 229533.I1RN67 pp 229533.Q4HZB8
102863 229533.I1RN67 pp 229533.I1RRN2
102864 229533.I1RN67 pp 229533.I1RY44
102865 229533.I1RN67 pp 229533.I1SA33
102866 229533.I1RN67 pp 229533.I1S1W9
102867 229533.I1RN67 pp 229533.I1S256
102868 229533.I1RN67 pp 229533.I1RPP4
102869 229533.I1RN67 pp 229533.I1RWN5
102870 229533.I1RN67 pp 229533.I1RNB8
102871 229533.I1RN67 pp 229533.V6RIV9
102872 229533.A0A1C3YN36 pp 229533.I1RUG4
102873 229533.A0A1C3YN36 pp 229533.I1RDJ8
102874 229533.A0A1C3YN36 pp 229533.I1RB87
102875 229533.A0A1C3YN36 pp 229533.I1RA10
102876 229533.A0A1C3YN36 pp 229533.I1RDH0
102877 229533.A0A1C3YN36 pp 229533.I1RRN5
102878 229533.A0A1C3YN36 pp 229533.I1RP99
102879 229533.A0A1C3YN36 pp 229533.I1RSR9
102880 229533.A0A1C3YN36 pp 229533.I1S0D5
102881 229533.A0A1C3YN36 pp 229533.I1RDX0
102882 229533.A0A1C3YN36 pp 229533.I1RPM7
102883 229533.A0A1C3YN36 pp 229533.I1S276
102884 229533.A0A1C3YN36 pp 229533.I1RMJ8
102885 229533.A0A1C3YN36 pp 229533.I1RK79
102886 229533.A0A1C3YN36 pp 229533.I1RBW1
102887 229533.A0A1C3YN36 pp 229533.I1RWJ1
102888 229533.A0A1C3YN36 pp 229533.I1RK90
102889 229533.A0A1C3YN36 pp 229533.I1RD19
102890 229533.A0A1C3YN36 pp 229533.I1RSN7
102891 229533.A0A1C3YN36 pp 229533.I1RW15
102892 229533.A0A1C3YN36 pp 229533.I1RPS0
102893 229533.A0A1C3YN36 pp 229533.I1RSP7
102894 229533.A0A1C3YN36 pp 229533.I1RYX9
102895 229533.A0A1C3YN36 pp 229533.V6RLW4
102896 229533.A0A1C3YN36 pp 229533.I1RQJ4
102897 229533.A0A1C3YN36 pp 229533.I1RPF5
102898 229533.A0A1C3YN36 pp 229533.I1RYQ1
102899 229533.A0A1C3YN36 pp 229533.I1RRJ6
102900 229533.A0A1C3YN36 pp 229533.I1RX64
102901 229533.A0A1C3YN36 pp 229533.I1RBN9
102902 229533.A0A1C3YN36 pp 229533.I1RVL8
102903 229533.A0A1C3YN36 pp 229533.I1RCA5
102904 229533.A0A1C3YN36 pp 229533.I1RBF3
102905 229533.A0A1C3YN36 pp 229533.I1RWI8
102906 229533.A0A1C3YN36 pp 229533.I1RSZ3
102907 229533.A0A1C3YN36 pp 229533.I1RZP8
102908 229533.A0A1C3YN36 pp 229533.I1RY29
102909 229533.A0A1C3YN36 pp 229533.I1RTN6
102910 229533.A0A1C3YN36 pp 229533.I1RQI6
102911 229533.A0A1C3YN36 pp 229533.I1RFX8
102912 229533.A0A1C3YN36 pp 229533.I1RG57
102913 229533.A0A1C3YN36 pp 229533.I1RPQ1
102914 229533.A0A1C3YN36 pp 229533.A0A1C3YN55
102915 229533.A0A1C3YN36 pp 229533.I1RKB1
102916 229533.A0A1C3YN36 pp 229533.I1RBW0
102917 229533.A0A1C3YN36 pp 229533.I1S4P6
102918 229533.A0A1C3YN36 pp 229533.I1RRT1
102919 229533.A0A1C3YN36 pp 229533.I1RZ94
102920 229533.A0A1C3YN36 pp 229533.I1RWM6
102921 229533.A0A1C3YN36 pp 229533.I1RKI2
102922 229533.A0A1C3YN36 pp 229533.I1RCV6
102923 229533.A0A1C3YN36 pp 229533.I1RE23
102924 229533.A0A1C3YN36 pp 229533.I1RCL7
102925 229533.A0A1C3YN36 pp 229533.I1RAT5
102926 229533.A0A1C3YN36 pp 229533.I1RN55
102927 229533.A0A1C3YN36 pp 229533.I1RUR0
102928 229533.A0A1C3YN36 pp 229533.I1S788
102929 229533.A0A1C3YN36 pp 229533.I1RG76
102930 229533.A0A1C3YN36 pp 229533.Q4IB89

102931 229533.A0A1C3YN36 pp 229533.I1RSI4
102932 229533.A0A1C3YN36 pp 229533.I1RA38
102933 229533.A0A1C3YN36 pp 229533.I1RN57
102934 229533.A0A1C3YN36 pp 229533.I1RG77
102935 229533.A0A1C3YN36 pp 229533.I1S9G7
102936 229533.A0A1C3YN36 pp 229533.I1RBC8
102937 229533.A0A1C3YN36 pp 229533.I1RC54
102938 229533.A0A1C3YN36 pp 229533.I1RTC2
102939 229533.A0A1C3YN36 pp 229533.I1RPI0
102940 229533.A0A1C3YN36 pp 229533.I1RR49
102941 229533.A0A1C3YN36 pp 229533.I1RZB5
102942 229533.A0A1C3YN36 pp 229533.I1RSC7
102943 229533.A0A1C3YN36 pp 229533.I1S209
102944 229533.A0A1C3YN36 pp 229533.I1RJL2
102945 229533.A0A1C3YN36 pp 229533.I1RC88
102946 229533.A0A1C3YN36 pp 229533.I1RA12
102947 229533.A0A1C3YN36 pp 229533.Q4INS6
102948 229533.A0A1C3YN36 pp 229533.I1RMQ7
102949 229533.A0A1C3YN36 pp 229533.I1S183
102950 229533.I1R989 pp 229533.I1RPW2
102951 229533.I1R989 pp 229533.I1RVX9
102952 229533.I1R989 pp 229533.I1S3F3
102953 229533.I1R989 pp 229533.I1S9D4
102954 229533.I1R989 pp 229533.I1RIY0
102955 229533.I1R989 pp 229533.I1RM26
102956 229533.I1R989 pp 229533.I1RU76
102957 229533.I1R989 pp 229533.I1RZ52
102958 229533.I1R989 pp 229533.I1R9K3
102959 229533.I1R989 pp 229533.I1RK03
102960 229533.I1R989 pp 229533.I1RNG4
102961 229533.I1R989 pp 229533.I1RQE0
102962 229533.I1R989 pp 229533.I1S2E2
102963 229533.I1R989 pp 229533.I1RPD6
102964 229533.I1R989 pp 229533.I1RMM8
102965 229533.I1R989 pp 229533.I1RY74
102966 229533.A0A1C3YL83 pp 229533.Q4HZ47
102967 229533.I1RNG1 pp 229533.I1RRV1
102968 229533.I1RNG1 pp 229533.I1S9F1
102969 229533.I1RNG1 pp 229533.Q4IJM4
102970 229533.I1S0F6 pp 229533.I1S7M8
102971 229533.I1S0F6 pp 229533.V6RUY9
102972 229533.I1S0F6 pp 229533.I1S0L3
102973 229533.I1S0F6 pp 229533.V6RTJ8
102974 229533.I1S0F6 pp 229533.I1S7A8
102975 229533.I1S0F6 pp 229533.I1S503
102976 229533.I1S0F6 pp 229533.I1S2E9
102977 229533.I1S0F6 pp 229533.V6QW22
102978 229533.I1S0F6 pp 229533.I1S8P5
102979 229533.I1S0F6 pp 229533.V6R1K4
102980 229533.I1S0F6 pp 229533.I1S0R7
102981 229533.I1S0F6 pp 229533.Q4IJT5
102982 229533.I1S0F6 pp 229533.I1S0X6
102983 229533.I1S0F6 pp 229533.I1S5B0
102984 229533.I1S0F6 pp 229533.V6R3X5
102985 229533.I1S0F6 pp 229533.V6RCZ8
102986 229533.I1S0F6 pp 229533.V6RMW6
102987 229533.I1S0F6 pp 229533.I1S0L4
102988 229533.I1S0F6 pp 229533.V6QYF0
102989 229533.I1S0F6 pp 229533.I1S2H4
102990 229533.I1S0F6 pp 229533.V6R042
102991 229533.I1S0F6 pp 229533.I1S2B8
102992 229533.I1S0F6 pp 229533.V6QUW4
102993 229533.I1S0F6 pp 229533.I1S504
102994 229533.I1S0F6 pp 229533.I1S0J1
102995 229533.I1S0F6 pp 229533.I1S5R1
102996 229533.I1S0F6 pp 229533.V6RCG5
102997 229533.I1S0F6 pp 229533.V6RIV9
102998 229533.I1S0F6 pp 229533.I1S1J7
102999 229533.I1S0F6 pp 229533.I1S0M6
103000 229533.I1S0F6 pp 229533.I1S274
103001 229533.I1S0F6 pp 229533.Q4I283
103002 229533.I1S0F6 pp 229533.Q4I2J8
103003 229533.I1S0F6 pp 229533.Q4IJK2

103004	229533.I1S0F6	pp	229533.Q4I624
103005	229533.I1S0F6	pp	229533.I1S8R3
103006	229533.I1S0F6	pp	229533.I1S2C4
103007	229533.I1S0F6	pp	229533.I1S1C7
103008	229533.I1S0F6	pp	229533.V6R057
103009	229533.I1RBC1	pp	229533.I1RRY1
103010	229533.I1RBC1	pp	229533.I1RPQ1
103011	229533.I1RBC1	pp	229533.I1S4S7
103012	229533.I1RBC1	pp	229533.I1RMK7
103013	229533.I1RBC1	pp	229533.I1RRT1
103014	229533.I1RBC1	pp	229533.I1RSI4
103015	229533.I1RBC1	pp	229533.I1RFX8
103016	229533.I1RBC1	pp	229533.I1S987
103017	229533.I1RBC1	pp	229533.I1RY21
103018	229533.I1RBC1	pp	229533.I1RT33
103019	229533.I1RBC1	pp	229533.I1RS05
103020	229533.I1RBC1	pp	229533.I1RBW0
103021	229533.I1RBC1	pp	229533.I1RUG0
103022	229533.I1RBC1	pp	229533.I1S4S4
103023	229533.I1RBC1	pp	229533.I1RP86
103024	229533.I1RBC1	pp	229533.I1RMQ6
103025	229533.I1RBC1	pp	229533.I1RKI2
103026	229533.I1RBC1	pp	229533.I1RHR3
103027	229533.I1RBC1	pp	229533.I1RQ81
103028	229533.I1RBC1	pp	229533.I1S0F1
103029	229533.I1RBC1	pp	229533.I1RE46
103030	229533.I1RBC1	pp	229533.I1RXB6
103031	229533.I1RBC1	pp	229533.I1S283
103032	229533.I1RBC1	pp	229533.I1RMG6
103033	229533.I1RBC1	pp	229533.I1RPD6
103034	229533.I1RBC1	pp	229533.I1RN67
103035	229533.I1RBC1	pp	229533.I1RPZ0
103036	229533.I1RBC1	pp	229533.I1RCL7
103037	229533.I1RBC1	pp	229533.I1S0K2
103038	229533.I1RBC1	pp	229533.I1RWH1
103039	229533.I1RBC1	pp	229533.I1RBE4
103040	229533.I1RBC1	pp	229533.I1RSN7
103041	229533.I1RBC1	pp	229533.I1RWI8
103042	229533.I1RBC1	pp	229533.I1RQ82
103043	229533.I1RBC1	pp	229533.I1RXA9
103044	229533.I1RBC1	pp	229533.I1RN46
103045	229533.I1RBC1	pp	229533.Q4INS6
103046	229533.I1RBC1	pp	229533.I1S461
103047	229533.I1RBC1	pp	229533.I1RXD9
103048	229533.I1RBC1	pp	229533.I1S604
103049	229533.I1RBC1	pp	229533.I1RBE6
103050	229533.I1RBC1	pp	229533.I1RFJ2
103051	229533.I1RBC1	pp	229533.I1RJH8
103052	229533.I1RBC1	pp	229533.I1RYR1
103053	229533.I1RBC1	pp	229533.I1RK49
103054	229533.I1RBC1	pp	229533.I1S209
103055	229533.I1RBC1	pp	229533.I1RZB2
103056	229533.I1RBC1	pp	229533.I1S8S9
103057	229533.I1RBC1	pp	229533.I1S9G7
103058	229533.I1RBC1	pp	229533.I1RRV4
103059	229533.I1RBC1	pp	229533.I1RN55
103060	229533.I1RBC1	pp	229533.I1RWA3
103061	229533.I1RBC1	pp	229533.I1RYX9
103062	229533.I1RBC1	pp	229533.I1RZ86
103063	229533.I1RBC1	pp	229533.I1RCA5
103064	229533.I1RBC1	pp	229533.I1RK95
103065	229533.I1RBC1	pp	229533.I1RS29
103066	229533.I1RBC1	pp	229533.I1RMG7
103067	229533.I1RBC1	pp	229533.I1RVU7
103068	229533.I1RBC1	pp	229533.I1RYI5
103069	229533.I1RBC1	pp	229533.I1RUG1
103070	229533.I1RBC1	pp	229533.I1RVW0
103071	229533.I1RBC1	pp	229533.I1RQJ4
103072	229533.I1RBC1	pp	229533.Q4IN00
103073	229533.I1RBC1	pp	229533.I1RTA1
103074	229533.I1RBC1	pp	229533.I1RKB2
103075	229533.I1RBC1	pp	229533.I1RQ30
103076	229533.I1RBC1	pp	229533.I1S998

103077 229533.I1RBC1 pp 229533.I1RZ98
103078 229533.I1RBC1 pp 229533.I1RDX0
103079 229533.I1RBC1 pp 229533.I1RSR9
103080 229533.I1RBC1 pp 229533.V6RLW4
103081 229533.I1RBC1 pp 229533.I1RVY0
103082 229533.I1RBC1 pp 229533.I1RQI6
103083 229533.I1RBC1 pp 229533.I1S0D5
103084 229533.I1RBC1 pp 229533.I1RSC7
103085 229533.I1RBC1 pp 229533.I1RFJ6
103086 229533.I1RBC1 pp 229533.I1RWI0
103087 229533.I1RBC1 pp 229533.I1RFS5
103088 229533.I1RBC1 pp 229533.I1RC54
103089 229533.I1RBC1 pp 229533.I1RRS7
103090 229533.I1RBC1 pp 229533.I1RBC8
103091 229533.I1RBC1 pp 229533.I1RSS4
103092 229533.I1RBC1 pp 229533.I1RZ39
103093 229533.I1RBC1 pp 229533.I1RBW1
103094 229533.I1RBC1 pp 229533.I1S9Y1
103095 229533.I1RBC1 pp 229533.I1RUG4
103096 229533.I1RBC1 pp 229533.I1RSY2
103097 229533.I1RBC1 pp 229533.I1RZD3
103098 229533.I1RBC1 pp 229533.I1RJH7
103099 229533.I1RBC1 pp 229533.I1RPK0
103100 229533.I1RBC1 pp 229533.O74638
103101 229533.I1RBC1 pp 229533.I1RX64
103102 229533.I1RBC1 pp 229533.I1RZP8
103103 229533.I1RBC1 pp 229533.V6R382
103104 229533.I1RBC1 pp 229533.I1RBN9
103105 229533.I1RBC1 pp 229533.I1RN57
103106 229533.I1RBC1 pp 229533.I1RSP7
103107 229533.A0A1C3YL19 pp 229533.I1RNT2
103108 229533.A0A1C3YL19 pp 229533.I1S279
103109 229533.A0A1C3YL19 pp 229533.I1RS94
103110 229533.A0A1C3YL19 pp 229533.I1RNG5
103111 229533.A0A1C3YL19 pp 229533.I1RNE4
103112 229533.I1RZZ9 pp 229533.I1S6K9
103113 229533.I1RZZ9 pp 229533.V6R227
103114 229533.I1RZZ9 pp 229533.I1S835
103115 229533.I1RZZ9 pp 229533.I1S5B0
103116 229533.I1RZZ9 pp 229533.V6RCZ8
103117 229533.I1RZZ9 pp 229533.V6RCG5
103118 229533.I1RZZ9 pp 229533.I1S504
103119 229533.I1RZZ9 pp 229533.V6RTJ8
103120 229533.I1RZZ9 pp 229533.I1S419
103121 229533.I1RZZ9 pp 229533.Q4I8P5
103122 229533.I1RZZ9 pp 229533.V6QUE6
103123 229533.I1RZZ9 pp 229533.Q4I283
103124 229533.I1RZZ9 pp 229533.Q4I2J8
103125 229533.I1RZZ9 pp 229533.I1S0T2
103126 229533.I1RZZ9 pp 229533.I1S7A8
103127 229533.I1RZZ9 pp 229533.I1S0A0
103128 229533.I1RZZ9 pp 229533.I1SA32
103129 229533.I1RZZ9 pp 229533.I1S000
103130 229533.I1RZZ9 pp 229533.I1S1J7
103131 229533.I1RZZ9 pp 229533.V6RUT5
103132 229533.I1RZZ9 pp 229533.Q4I5Z5
103133 229533.I1RZZ9 pp 229533.I1S0R7
103134 229533.I1RZZ9 pp 229533.I1S4Q4
103135 229533.I1RZZ9 pp 229533.I1S0F5
103136 229533.I1RZZ9 pp 229533.I1S0L3
103137 229533.I1RZZ9 pp 229533.I1S2U2
103138 229533.I1RZZ9 pp 229533.I1S887
103139 229533.I1RZZ9 pp 229533.Q4IJK2
103140 229533.I1RZZ9 pp 229533.V6R1K4
103141 229533.I1RZZ9 pp 229533.I1S5R6
103142 229533.I1RZZ9 pp 229533.I1S4S7
103143 229533.I1RZZ9 pp 229533.V6QW22
103144 229533.I1RZZ9 pp 229533.V6RBN2
103145 229533.I1RZZ9 pp 229533.V6RGN5
103146 229533.I1RZZ9 pp 229533.I1S0X6
103147 229533.I1RZZ9 pp 229533.I1S0N4
103148 229533.I1RZZ9 pp 229533.Q4IJT5
103149 229533.I1RZZ9 pp 229533.V6R4J1

103150 229533.I1RZZ9 pp 229533.I1S4S4
103151 229533.I1RZZ9 pp 229533.I1S8R3
103152 229533.I1RZZ9 pp 229533.I1SA33
103153 229533.I1RZZ9 pp 229533.V6QUW4
103154 229533.I1RZZ9 pp 229533.Q4I5V2
103155 229533.I1RZZ9 pp 229533.V6QYF0
103156 229533.I1RZZ9 pp 229533.I1S1C7
103157 229533.I1RZZ9 pp 229533.V6RMW6
103158 229533.I1RZZ9 pp 229533.V6R042
103159 229533.I1RZZ9 pp 229533.I1S2C0
103160 229533.I1RZZ9 pp 229533.Q4HZB8
103161 229533.I1RZZ9 pp 229533.I1S2E8
103162 229533.I1RZZ9 pp 229533.Q4I624
103163 229533.I1RZZ9 pp 229533.I1S1W3
103164 229533.I1RZZ9 pp 229533.V6RIV9
103165 229533.I1RZZ9 pp 229533.V6R2A1
103166 229533.I1RZZ9 pp 229533.I1S1W9
103167 229533.I1RZZ9 pp 229533.V6RVG3
103168 229533.I1RZZ9 pp 229533.I1S503
103169 229533.I1RZZ9 pp 229533.V6R057
103170 229533.I1RZZ9 pp 229533.I1S569
103171 229533.I1RZZ9 pp 229533.I1S1B6
103172 229533.I1RZZ9 pp 229533.I1S0J1
103173 229533.I1RZZ9 pp 229533.Q4HXF0
103174 229533.I1RZZ9 pp 229533.I1S0R2
103175 229533.I1RZZ9 pp 229533.I1S1J6
103176 229533.I1RZZ9 pp 229533.V6R3J1
103177 229533.I1RZZ9 pp 229533.V6RUY9
103178 229533.I1RZZ9 pp 229533.I1S2H4
103179 229533.I1RZZ9 pp 229533.I1S2E9
103180 229533.I1RZZ9 pp 229533.V6RRH2
103181 229533.I1RZZ9 pp 229533.I1S7W5
103182 229533.I1RZZ9 pp 229533.I1S0F6
103183 229533.I1RZZ9 pp 229533.I1S8P5
103184 229533.I1RZZ9 pp 229533.I1S2B8
103185 229533.I1RZZ9 pp 229533.I1S9Y1
103186 229533.I1RZZ9 pp 229533.I1S352
103187 229533.I1RZZ9 pp 229533.I1S0L4
103188 229533.I1RZZ9 pp 229533.I1S0M6
103189 229533.I1RZZ9 pp 229533.O74638
103190 229533.I1RZZ9 pp 229533.I1S7S9
103191 229533.I1RZZ9 pp 229533.I1S2M6
103192 229533.I1RZZ9 pp 229533.I1S5R1
103193 229533.I1RZZ9 pp 229533.I1S6Y8
103194 229533.I1RZZ9 pp 229533.V6R3X5
103195 229533.Q4IJT0 pp 229533.V6RPQ8
103196 229533.Q4IJT0 pp 229533.V6QUV7
103197 229533.A0A098DNT9 pp 229533.I1SAD2
103198 229533.A0A098DNT9 pp 229533.I1RG77
103199 229533.A0A098DNT9 pp 229533.I1RBW0
103200 229533.A0A098DNT9 pp 229533.I1RAT5
103201 229533.A0A098DNT9 pp 229533.I1RBW1
103202 229533.A0A098DNT9 pp 229533.A0A0E0SIK8
103203 229533.A0A098DNT9 pp 229533.I1RPF5
103204 229533.A0A098DNT9 pp 229533.A0A1C3YML3
103205 229533.A0A098DNT9 pp 229533.I1S209
103206 229533.I1RBB3 pp 229533.I1RC74
103207 229533.I1RBB3 pp 229533.V6RDH3
103208 229533.I1RBB3 pp 229533.I1RZQ1
103209 229533.I1RBB3 pp 229533.I1S4S7
103210 229533.I1RBB3 pp 229533.I1RZ21
103211 229533.I1RBB3 pp 229533.I1RRJ3
103212 229533.I1RBB3 pp 229533.I1RE58
103213 229533.I1RBB3 pp 229533.I1S3V2
103214 229533.I1RBB3 pp 229533.I1RSQ9
103215 229533.I1RBB3 pp 229533.I1RBT1
103216 229533.I1RBB3 pp 229533.V6QWD8
103217 229533.I1RPP3 pp 229533.I1RZV3
103218 229533.I1RPP3 pp 229533.V6RVG3
103219 229533.I1RPP3 pp 229533.I1RY44
103220 229533.I1RPP3 pp 229533.I1RSA4
103221 229533.I1RPP3 pp 229533.I1S1W3
103222 229533.I1RPP3 pp 229533.I1S016

103223 229533.I1RPP3 pp 229533.I1RUG3
103224 229533.I1RPP3 pp 229533.Q4HZB8
103225 229533.I1RPP3 pp 229533.I1RPU8
103226 229533.I1RPP3 pp 229533.I1RRM3
103227 229533.I1RPP3 pp 229533.V6RMW6
103228 229533.I1RPP3 pp 229533.I1RWN5
103229 229533.I1RPP3 pp 229533.I1S1W9
103230 229533.I1RPP3 pp 229533.I1S8V4
103231 229533.I1RPP3 pp 229533.I1RSQ3
103232 229533.I1RPP3 pp 229533.V6RIV9
103233 229533.I1RPP3 pp 229533.I1RRV1
103234 229533.I1RPP3 pp 229533.V6R042
103235 229533.I1RPP3 pp 229533.I1RPU2
103236 229533.I1RPP3 pp 229533.I1RWH4
103237 229533.I1RPP3 pp 229533.I1RVZ7
103238 229533.I1RPP3 pp 229533.I1RRR7
103239 229533.I1RPP3 pp 229533.I1S256
103240 229533.I1RPP3 pp 229533.I1RSF1
103241 229533.I1RPP3 pp 229533.I1S1W4
103242 229533.I1RPP3 pp 229533.I1RRN2
103243 229533.I1RPP3 pp 229533.I1RZW8
103244 229533.I1RPP3 pp 229533.I1RRE5
103245 229533.I1RPP3 pp 229533.I1SA33
103246 229533.I1RPP3 pp 229533.I1S5R6
103247 229533.I1RPP3 pp 229533.I1RRI2
103248 229533.I1RPP3 pp 229533.I1S0N4
103249 229533.I1RPP3 pp 229533.I1S0J1
103250 229533.I1RPP3 pp 229533.I1RZ79
103251 229533.I1RPP3 pp 229533.I1S7W5
103252 229533.I1RPP3 pp 229533.I1RTD7
103253 229533.I1RPP3 pp 229533.I1RWX5
103254 229533.I1RPP3 pp 229533.I1S1C7
103255 229533.I1RPP3 pp 229533.I1RTA0
103256 229533.I1RPP3 pp 229533.I1RYW3
103257 229533.I1RPP3 pp 229533.I1RW46
103258 229533.I1RPP3 pp 229533.I1RVF8
103259 229533.I1RPP3 pp 229533.Q4IN63
103260 229533.I1RPP3 pp 229533.I1S9Z4
103261 229533.I1RPP3 pp 229533.Q4I327
103262 229533.I1RPP3 pp 229533.I1RWB5
103263 229533.I1RPP3 pp 229533.I1S437
103264 229533.I1RPP3 pp 229533.I1RPP4
103265 229533.I1RPP3 pp 229533.I1RT03
103266 229533.I1RPP3 pp 229533.I1RSY2
103267 229533.I1RPP3 pp 229533.I1S8R3
103268 229533.I1RPP3 pp 229533.I1RS17
103269 229533.I1RPP3 pp 229533.I1S2C0
103270 229533.I1RPP3 pp 229533.I1RTH6
103271 229533.I1RPP3 pp 229533.V6R3J1
103272 229533.I1RPP3 pp 229533.V6RRH2
103273 229533.I1RPP3 pp 229533.I1RQ33
103274 229533.I1RPP3 pp 229533.I1RWQ6
103275 229533.I1RPP3 pp 229533.I1RSK1
103276 229533.I1RPP3 pp 229533.Q4I624
103277 229533.I1RPP3 pp 229533.I1RSX0
103278 229533.I1RPP3 pp 229533.V6R057
103279 229533.I1RPP3 pp 229533.I1RWN6
103280 229533.I1RPP3 pp 229533.I1S503
103281 229533.I1RPP3 pp 229533.I1S569
103282 229533.I1RPP3 pp 229533.I1RRZ0
103283 229533.I1RPP3 pp 229533.I1RQE7
103284 229533.I1RPP3 pp 229533.I1RQM7
103285 229533.I1RPP3 pp 229533.I1RXU9
103286 229533.I1RPP3 pp 229533.I1RVZ2
103287 229533.I1RPP3 pp 229533.I1S2E8
103288 229533.I1RPP3 pp 229533.I1RTD5
103289 229533.I1RPP3 pp 229533.I1RXF8
103290 229533.I1RPP3 pp 229533.I1RSD8
103291 229533.I1RPP3 pp 229533.I1RV91
103292 229533.I1RPP3 pp 229533.I1RT40
103293 229533.I1RPP3 pp 229533.I1RTA1
103294 229533.I1RPP3 pp 229533.I1RXR1
103295 229533.I1RPP3 pp 229533.V6R283

103296 229533.I1RPP3 pp 229533.I1RRN5
103297 229533.I1RPP3 pp 229533.I1RYR0
103298 229533.I1RPP3 pp 229533.I1S2E9
103299 229533.I1RPP3 pp 229533.I1RWS4
103300 229533.I1RPP3 pp 229533.I1RX22
103301 229533.I1RPP3 pp 229533.I1RT75
103302 229533.I1RPP3 pp 229533.I1RWK3
103303 229533.I1RPP3 pp 229533.V6RUY9
103304 229533.I1RPP3 pp 229533.I1RRQ3
103305 229533.I1RPP3 pp 229533.I1S0F6
103306 229533.I1RPP3 pp 229533.I1RT04
103307 229533.I1RPP3 pp 229533.I1RRJ8
103308 229533.I1RPP3 pp 229533.I1S0W4
103309 229533.I1RPP3 pp 229533.I1RQG4
103310 229533.I1RPP3 pp 229533.I1S1F3
103311 229533.I1RPP3 pp 229533.O74638
103312 229533.I1RPP3 pp 229533.I1RWL2
103313 229533.I1RPP3 pp 229533.I1S2G7
103314 229533.I1RPP3 pp 229533.I1S8P5
103315 229533.I1RPP3 pp 229533.I1S5R1
103316 229533.I1RPP3 pp 229533.I1RWF7
103317 229533.I1RPP3 pp 229533.I1RXC2
103318 229533.I1RPP3 pp 229533.I1RQ65
103319 229533.I1RPP3 pp 229533.I1RYI5
103320 229533.I1RPP3 pp 229533.I1RSA5
103321 229533.I1RPP3 pp 229533.I1RSV2
103322 229533.I1RPP3 pp 229533.I1RS47
103323 229533.I1RPP3 pp 229533.I1S8V3
103324 229533.I1RPP3 pp 229533.I1S9Y1
103325 229533.I1RPP3 pp 229533.I1RRT8
103326 229533.I1RPP3 pp 229533.I1RZV6
103327 229533.I1RPP3 pp 229533.I1S8G4
103328 229533.I1RPP3 pp 229533.I1S352
103329 229533.I1RPP3 pp 229533.I1RRI1
103330 229533.I1RPP3 pp 229533.I1RY62
103331 229533.I1RPP3 pp 229533.I1RSQ4
103332 229533.I1RPP3 pp 229533.I1S7S9
103333 229533.I1RPP3 pp 229533.I1RZZ9
103334 229533.I1RPP3 pp 229533.I1RS20
103335 229533.I1RPP3 pp 229533.I1RPU3
103336 229533.I1RPP3 pp 229533.I1S0L4
103337 229533.I1RPP3 pp 229533.I1RWI5
103338 229533.I1RPP3 pp 229533.I1S2H4
103339 229533.I1RPP3 pp 229533.I1RRM5
103340 229533.I1RPP3 pp 229533.V6R3X5
103341 229533.I1RPP3 pp 229533.I1S2B8
103342 229533.I1RPP3 pp 229533.I1S4Z8
103343 229533.I1RPP3 pp 229533.I1RR73
103344 229533.I1RPP3 pp 229533.I1S6Y8
103345 229533.I1RPP3 pp 229533.I1RPV3
103346 229533.I1RPP3 pp 229533.I1S8E2
103347 229533.I1RPP3 pp 229533.I1RQ23
103348 229533.I1RPP3 pp 229533.I1RYB9
103349 229533.I1RPP3 pp 229533.Q4I662
103350 229533.I1RPP3 pp 229533.I1RRU9
103351 229533.I1RPP3 pp 229533.I1S021
103352 229533.I1RPP3 pp 229533.I1RZ39
103353 229533.I1RPP3 pp 229533.I1RZM8
103354 229533.I1RPP3 pp 229533.I1RSZ7
103355 229533.I1RPP3 pp 229533.I1RXF0
103356 229533.I1RPP3 pp 229533.V6QUE6
103357 229533.I1RPP3 pp 229533.I1RZS6
103358 229533.I1RPP3 pp 229533.I1S0M6
103359 229533.I1RPP3 pp 229533.I1RW99
103360 229533.I1RPP3 pp 229533.I1RRV4
103361 229533.I1RPP3 pp 229533.I1RQ85
103362 229533.I1RPP3 pp 229533.V6RCG5
103363 229533.I1RPP3 pp 229533.I1RQD2
103364 229533.I1RPP3 pp 229533.I1RR51
103365 229533.I1RPP3 pp 229533.I1S5B0
103366 229533.I1RPP3 pp 229533.Q4I283
103367 229533.I1RPP3 pp 229533.I1S6K9
103368 229533.I1RPP3 pp 229533.I1RWT6

103369	229533.I1RPP3	pp	229533.V6RCZ8
103370	229533.I1RPP3	pp	229533.I1SA32
103371	229533.I1RPP3	pp	229533.I1RWB0
103372	229533.I1RPP3	pp	229533.I1RXC4
103373	229533.I1RPP3	pp	229533.I1S504
103374	229533.I1RPP3	pp	229533.I1RZF6
103375	229533.I1RPP3	pp	229533.I1RRY9
103376	229533.I1RPP3	pp	229533.Q4I8P5
103377	229533.I1RPP3	pp	229533.I1RU29
103378	229533.I1RPP3	pp	229533.I1RS18
103379	229533.I1RPP3	pp	229533.I1RWR7
103380	229533.I1RPP3	pp	229533.I1RYY5
103381	229533.I1RPP3	pp	229533.I1RQ81
103382	229533.I1RPP3	pp	229533.I1RPR9
103383	229533.I1RPP3	pp	229533.I1S0M9
103384	229533.I1RPP3	pp	229533.I1RY03
103385	229533.I1RPP3	pp	229533.I1RSR8
103386	229533.I1RPP3	pp	229533.I1RZM1
103387	229533.I1RPP3	pp	229533.I1S419
103388	229533.I1RPP3	pp	229533.I1S887
103389	229533.I1RPP3	pp	229533.I1RQQ5
103390	229533.I1RPP3	pp	229533.I1RZK5
103391	229533.I1RPP3	pp	229533.Q4IJT5
103392	229533.I1RPP3	pp	229533.V6RBN2
103393	229533.I1RPP3	pp	229533.I1RZZ1
103394	229533.I1RPP3	pp	229533.I1S1J7
103395	229533.I1RPP3	pp	229533.I1RZK6
103396	229533.I1RPP3	pp	229533.I1RRG3
103397	229533.I1RPP3	pp	229533.I1RYS0
103398	229533.I1RPP3	pp	229533.I1RWD7
103399	229533.I1RPP3	pp	229533.I1RX95
103400	229533.I1RPP3	pp	229533.I1RWH5
103401	229533.I1RPP3	pp	229533.I1RPY3
103402	229533.I1RPP3	pp	229533.Q4IJK2
103403	229533.I1RPP3	pp	229533.I1RSQ7
103404	229533.I1RPP3	pp	229533.I1RQA3
103405	229533.I1RPP3	pp	229533.I1RRX5
103406	229533.I1RPP3	pp	229533.I1RYJ6
103407	229533.I1RPP3	pp	229533.I1RT98
103408	229533.I1RPP3	pp	229533.V6QW22
103409	229533.I1RPP3	pp	229533.I1RXD7
103410	229533.I1RPP3	pp	229533.Q4IJM4
103411	229533.I1RPP3	pp	229533.I1RR00
103412	229533.I1RPP3	pp	229533.I1RQJ5
103413	229533.I1RPP3	pp	229533.I1RSI0
103414	229533.I1RPP3	pp	229533.V6RUT5
103415	229533.I1RPP3	pp	229533.I1S4Q4
103416	229533.I1RPP3	pp	229533.I1RQH3
103417	229533.I1RPP3	pp	229533.I1RZE5
103418	229533.I1RPP3	pp	229533.I1RW71
103419	229533.I1RPP3	pp	229533.Q4I2J8
103420	229533.I1RPP3	pp	229533.I1S0R7
103421	229533.I1RPP3	pp	229533.I1S4S7
103422	229533.I1RPP3	pp	229533.V6R4J1
103423	229533.I1RPP3	pp	229533.I1RVQ0
103424	229533.I1RPP3	pp	229533.I1S3S3
103425	229533.I1RPP3	pp	229533.I1RXD6
103426	229533.I1RPP3	pp	229533.I1RZ35
103427	229533.I1RPP3	pp	229533.I1RVZ0
103428	229533.I1RPP3	pp	229533.I1RX46
103429	229533.I1RPP3	pp	229533.I1RQC9
103430	229533.I1RPP3	pp	229533.I1S0X6
103431	229533.I1RPP3	pp	229533.I1S0F5
103432	229533.I1RPP3	pp	229533.I1S0T2
103433	229533.I1RPP3	pp	229533.I1S7A8
103434	229533.I1RPP3	pp	229533.I1S0L3
103435	229533.I1RPP3	pp	229533.V6R1K4
103436	229533.I1RPP3	pp	229533.V6RTJ8
103437	229533.I1RPP3	pp	229533.I1RQS9
103438	229533.I1RPP3	pp	229533.I1RT80
103439	229533.I1RPP3	pp	229533.I1RY86
103440	229533.I1RPP3	pp	229533.I1S6U8
103441	229533.I1RPP3	pp	229533.I1RZJ5

103442	229533.I1RPP3	pp	229533.I1S438
103443	229533.I1RPP3	pp	229533.I1RX25
103444	229533.I1RPP3	pp	229533.I1RPU0
103445	229533.I1RPP3	pp	229533.V6QUW4
103446	229533.I1RPP3	pp	229533.I1RWJ8
103447	229533.I1RPP3	pp	229533.I1RW25
103448	229533.I1RPP3	pp	229533.I1S4S4
103449	229533.I1RPP3	pp	229533.I1S9U2
103450	229533.I1RPP3	pp	229533.Q4IFI0
103451	229533.I1RPP3	pp	229533.I1RW07
103452	229533.I1RPP3	pp	229533.I1RPW9
103453	229533.I1RPP3	pp	229533.I1RRS8
103454	229533.I1RPP3	pp	229533.I1RR24
103455	229533.I1RWI5	pp	229533.I1S5R1
103456	229533.I1RWI5	pp	229533.I1S9Y1
103457	229533.I1RWI5	pp	229533.I1S6Y8
103458	229533.I1RWI5	pp	229533.I1RWL2
103459	229533.I1RWI5	pp	229533.I1S253
103460	229533.I1RWI5	pp	229533.I1RXC2
103461	229533.I1RWI5	pp	229533.V6R3X5
103462	229533.I1RWI5	pp	229533.I1S2H4
103463	229533.I1RWI5	pp	229533.I1S0L4
103464	229533.I1RWI5	pp	229533.I1S0F6
103465	229533.I1RWI5	pp	229533.I1S2B8
103466	229533.I1RWI5	pp	229533.I1S2G7
103467	229533.I1RWI5	pp	229533.I1RZZ9
103468	229533.I1RWI5	pp	229533.I1RXU9
103469	229533.I1RWI5	pp	229533.I1RWK3
103470	229533.I1RWI5	pp	229533.I1RWN6
103471	229533.I1RWI5	pp	229533.I1RZB2
103472	229533.I1RWI5	pp	229533.I1S1C7
103473	229533.I1RWI5	pp	229533.I1RZ39
103474	229533.I1RWI5	pp	229533.I1S8P5
103475	229533.I1RWI5	pp	229533.I1RYW3
103476	229533.I1RWI5	pp	229533.I1RYI5
103477	229533.I1RWI5	pp	229533.I1RZW8
103478	229533.I1RWI5	pp	229533.I1S2E9
103479	229533.I1RWI5	pp	229533.Q4IN00
103480	229533.I1RWI5	pp	229533.I1S503
103481	229533.I1RWI5	pp	229533.V6RRH2
103482	229533.I1RWI5	pp	229533.I1S8R3
103483	229533.I1RWI5	pp	229533.V6R057
103484	229533.I1RWI5	pp	229533.V6R3J1
103485	229533.I1RWI5	pp	229533.I1RWN5
103486	229533.I1RWI5	pp	229533.Q4I624
103487	229533.I1RWI5	pp	229533.V6RIV9
103488	229533.I1RWI5	pp	229533.V6QT89
103489	229533.I1RWI5	pp	229533.I1S2E8
103490	229533.I1RWI5	pp	229533.I1S1W9
103491	229533.I1RWI5	pp	229533.I1RXR1
103492	229533.I1RWI5	pp	229533.I1S1W4
103493	229533.I1RWI5	pp	229533.V6RY33
103494	229533.I1RWI5	pp	229533.V6R042
103495	229533.I1RWI5	pp	229533.V6RMW6
103496	229533.I1RWI5	pp	229533.I1S0J1
103497	229533.I1RWI5	pp	229533.Q4HZB8
103498	229533.I1RWI5	pp	229533.I1SA33
103499	229533.I1RWI5	pp	229533.I1RX25
103500	229533.I1RWI5	pp	229533.I1RY44
103501	229533.I1RWI5	pp	229533.I1S5R6
103502	229533.I1RWI5	pp	229533.I1S1W3
103503	229533.I1RWI5	pp	229533.I1RWJ8
103504	229533.I1RWI5	pp	229533.V6R1K4
103505	229533.I1RWI5	pp	229533.V6QUW4
103506	229533.I1RWI5	pp	229533.I1S0L3
103507	229533.I1RWI5	pp	229533.I1S0N4
103508	229533.I1RWI5	pp	229533.I1S4S4
103509	229533.I1RWI5	pp	229533.I1S0X6
103510	229533.I1RWI5	pp	229533.V6RTJ8
103511	229533.I1RWI5	pp	229533.Q4INZ9
103512	229533.I1RWI5	pp	229533.I1S0F1
103513	229533.I1RWI5	pp	229533.I1S4S7
103514	229533.I1RWI5	pp	229533.I1S0T2

103515 229533.I1RWI5 pp 229533.I1S3S3
103516 229533.I1RWI5 pp 229533.V6QW22
103517 229533.I1RWI5 pp 229533.I1S1J7
103518 229533.I1RWI5 pp 229533.I1S0R7
103519 229533.I1RWI5 pp 229533.V6RUT5
103520 229533.I1RWI5 pp 229533.I1RY03
103521 229533.I1RWI5 pp 229533.I1RX46
103522 229533.I1RWI5 pp 229533.I1RYY5
103523 229533.I1RWI5 pp 229533.I1RXD7
103524 229533.I1RWI5 pp 229533.I1RWT6
103525 229533.I1RWI5 pp 229533.I1RZB5
103526 229533.I1RWI5 pp 229533.I1RXC4
103527 229533.I1RWI5 pp 229533.I1SA32
103528 229533.I1RWI5 pp 229533.I1RZE5
103529 229533.I1RWI5 pp 229533.I1RZ35
103530 229533.I1RWI5 pp 229533.I1S887
103531 229533.I1RWI5 pp 229533.I1RZK5
103532 229533.I1RWI5 pp 229533.Q4IJK2
103533 229533.I1RWI5 pp 229533.I1S419
103534 229533.I1RWI5 pp 229533.Q4I8P5
103535 229533.I1RWI5 pp 229533.V6RBN2
103536 229533.I1RWI5 pp 229533.V6QUE6
103537 229533.I1RWI5 pp 229533.I1RZZ1
103538 229533.I1RWI5 pp 229533.I1S504
103539 229533.I1RWI5 pp 229533.I1S5B0
103540 229533.I1RWI5 pp 229533.I1RZM1
103541 229533.I1RWI5 pp 229533.I1S044
103542 229533.I1RWI5 pp 229533.Q4IJM4
103543 229533.I1RWI5 pp 229533.I1S6K9
103544 229533.I1RWI5 pp 229533.I1RZM8
103545 229533.I1RWI5 pp 229533.I1RXF0
103546 229533.I1RWI5 pp 229533.V6RCG5
103547 229533.I1RWI5 pp 229533.V6RCZ8
103548 229533.I1RPM6 pp 229533.I1S2F7
103549 229533.I1RPM6 pp 229533.I1S3T6
103550 229533.I1RPM6 pp 229533.I1RST9
103551 229533.I1RPM6 pp 229533.V6RHX2
103552 229533.I1RPM6 pp 229533.I1S0A0
103553 229533.I1RPM6 pp 229533.I1S8D3
103554 229533.I1RPM6 pp 229533.V6RWB3
103555 229533.I1RPM6 pp 229533.I1RTU0
103556 229533.I1RPM6 pp 229533.I1RQJ7
103557 229533.I1RPM6 pp 229533.I1RQE0
103558 229533.I1RPM6 pp 229533.I1RW55
103559 229533.I1RPM6 pp 229533.V6RY56
103560 229533.I1RPM6 pp 229533.I1S223
103561 229533.I1RPM6 pp 229533.V6QWD8
103562 229533.I1RPM6 pp 229533.I1RYM5
103563 229533.I1RPM6 pp 229533.I1RWL3
103564 229533.I1RPM6 pp 229533.I1RUV4
103565 229533.I1RPM6 pp 229533.I1S1W8
103566 229533.I1RPM6 pp 229533.I1RQU9
103567 229533.I1RPM6 pp 229533.V6RUB1
103568 229533.I1RPM6 pp 229533.I1RW54
103569 229533.I1RPM6 pp 229533.I1RQV8
103570 229533.I1RPM6 pp 229533.I1S210
103571 229533.I1RPM6 pp 229533.I1RPZ1
103572 229533.I1RPM6 pp 229533.I1RZA7
103573 229533.I1RPM6 pp 229533.I1RQV7
103574 229533.I1RPM6 pp 229533.I1RUQ7
103575 229533.I1RPM6 pp 229533.V6R5T7
103576 229533.I1RPM6 pp 229533.I1S2C4
103577 229533.I1RPM6 pp 229533.I1RZ38
103578 229533.I1RPM6 pp 229533.V6RN31
103579 229533.I1RPM6 pp 229533.I1S6V0
103580 229533.I1RPM6 pp 229533.V6RCC0
103581 229533.I1RPM6 pp 229533.I1RSA5
103582 229533.I1RPM6 pp 229533.I1RSQ9
103583 229533.I1RPM6 pp 229533.I1RR68
103584 229533.I1RPM6 pp 229533.I1S2B0
103585 229533.I1RPM6 pp 229533.V6RCJ5
103586 229533.I1RPM6 pp 229533.I1S3G4
103587 229533.I1RPM6 pp 229533.I1RZ29

103588 229533.I1RPM6 pp 229533.I1SAB4
103589 229533.I1RPM6 pp 229533.I1RVR7
103590 229533.I1RPM6 pp 229533.I1S1K6
103591 229533.I1RPM6 pp 229533.I1S3X7
103592 229533.I1RPM6 pp 229533.I1RVZ3
103593 229533.I1RPM6 pp 229533.I1S6P5
103594 229533.I1RXX1 pp 229533.I1RZD0
103595 229533.I1RXX1 pp 229533.I1SAS4
103596 229533.I1RXX1 pp 229533.I1RY35
103597 229533.I1RXX1 pp 229533.I1S3G4
103598 229533.I1RXX1 pp 229533.I1S2A8
103599 229533.I1RXX1 pp 229533.Q4INZ9
103600 229533.I1RXX1 pp 229533.I1SAS3
103601 229533.I1RXX1 pp 229533.V6RLM7
103602 229533.I1RXX1 pp 229533.I1RY25
103603 229533.I1RXX1 pp 229533.I1RXY7
103604 229533.I1RXX1 pp 229533.V6RM66
103605 229533.I1RXX1 pp 229533.I1RZT6
103606 229533.I1RXX1 pp 229533.V6QUV7
103607 229533.I1RXX1 pp 229533.V6RTJ8
103608 229533.I1RXX1 pp 229533.I1S130
103609 229533.I1RXX1 pp 229533.I1RZ92
103610 229533.I1RXX1 pp 229533.V6RPN0
103611 229533.I1RXX1 pp 229533.I1RZE7
103612 229533.I1RXX1 pp 229533.I1S2M5
103613 229533.I1RXX1 pp 229533.I1S1W8
103614 229533.I1RXX1 pp 229533.Q4I4A4
103615 229533.I1RXX1 pp 229533.V6RR38
103616 229533.I1RXX1 pp 229533.V6RG22
103617 229533.I1RXX1 pp 229533.P0C431
103618 229533.I1RXX1 pp 229533.I1S1Q3
103619 229533.I1RXX1 pp 229533.I1S5R5
103620 229533.I1RXX1 pp 229533.I1S267
103621 229533.I1RXX1 pp 229533.V6RH92
103622 229533.I1RXX1 pp 229533.I1RY81
103623 229533.I1RXX1 pp 229533.V6RQW4
103624 229533.A0A098DJU8 pp 229533.A0A1C3YK39
103625 229533.A0A098DJU8 pp 229533.I1R9B6
103626 229533.A0A098DJU8 pp 229533.I1RNB4
103627 229533.A0A098DJU8 pp 229533.I1RDQ2
103628 229533.A0A098DJU8 pp 229533.I1S1W8
103629 229533.A0A098DJU8 pp 229533.I1RAG9
103630 229533.A0A098DJU8 pp 229533.I1RNG4
103631 229533.A0A098DJU8 pp 229533.I1RIJ2
103632 229533.A0A098DJU8 pp 229533.I1S3K8
103633 229533.A0A098DJU8 pp 229533.I1RQ60
103634 229533.A0A098DJU8 pp 229533.I1RAR9
103635 229533.A0A098DJU8 pp 229533.I1RYQ4
103636 229533.A0A098DJU8 pp 229533.A0A1C3YJW4
103637 229533.A0A098DJU8 pp 229533.I1RZT0
103638 229533.A0A098DJU8 pp 229533.I1RY49
103639 229533.A0A098DJU8 pp 229533.I1S3G4
103640 229533.A0A098DJU8 pp 229533.I1RL76
103641 229533.A0A098DJU8 pp 229533.I1RNV4
103642 229533.A0A098DJU8 pp 229533.I1RKZ7
103643 229533.A0A098DJU8 pp 229533.A0A1C3YKX1
103644 229533.A0A098DJU8 pp 229533.I1RBB3
103645 229533.A0A098DJU8 pp 229533.A0A0E0SBC4
103646 229533.A0A098DJU8 pp 229533.I1RPM6
103647 229533.I1S8P5 pp 229533.V6RCG5
103648 229533.I1S8P5 pp 229533.Q4I283
103649 229533.I1S8P5 pp 229533.Q4IJK2
103650 229533.I1S8P5 pp 229533.Q4I5R9
103651 229533.I1S8P5 pp 229533.Q4I2J8
103652 229533.I1S8P5 pp 229533.V6RCZ8
103653 229533.I1S8P5 pp 229533.V6RBN2
103654 229533.I1S8P5 pp 229533.Q4I8P5
103655 229533.I1S8P5 pp 229533.V6RTJ8
103656 229533.I1S8P5 pp 229533.V6QUE6
103657 229533.I1S8P5 pp 229533.I1SA32
103658 229533.I1S8P5 pp 229533.Q4I5I4
103659 229533.I1S8P5 pp 229533.Q4I624
103660 229533.I1S8P5 pp 229533.Q5I2J3

229533.I1S8P5	pp	229533.V6R4J1
229533.I1S8P5	pp	229533.V6R057
229533.I1S8P5	pp	229533.Q4HZS8
229533.I1S8P5	pp	229533.V6QUV7
229533.I1S8P5	pp	229533.I1SA33
229533.I1S8P5	pp	229533.Q4IJT5
229533.I1S8P5	pp	229533.V6QW22
229533.I1S8P5	pp	229533.V6R1K4
229533.I1S8P5	pp	229533.Q4HXF6
229533.I1S8P5	pp	229533.V6RMW6
229533.I1S8P5	pp	229533.I1S8R3
229533.I1S8P5	pp	229533.V6R042
229533.I1S8P5	pp	229533.V6QX21
229533.I1S8P5	pp	229533.V6RIV9
229533.I1S8P5	pp	229533.V6R3X5
229533.I1S8P5	pp	229533.I1S9Y1
229533.I1S8P5	pp	229533.V6RRH2
229533.I1S8P5	pp	229533.V6R3J1
229533.I1RUB5	pp	229533.I1S8P5
229533.I1RUB5	pp	229533.I1S8P4
229533.I1RUB5	pp	229533.I1S6P7
229533.A0A1C3YMA1	pp	229533.V6RCG5
229533.A0A1C3YMA1	pp	229533.I1RR51
229533.A0A1C3YMA1	pp	229533.V6QUE6
229533.A0A1C3YMA1	pp	229533.I1RRU9
229533.A0A1C3YMA1	pp	229533.I1RAU5
229533.A0A1C3YMA1	pp	229533.I1RZP6
229533.A0A1C3YMA1	pp	229533.I1S5B0
229533.A0A1C3YMA1	pp	229533.I1RUY5
229533.A0A1C3YMA1	pp	229533.I1RXC4
229533.A0A1C3YMA1	pp	229533.I1RW99
229533.A0A1C3YMA1	pp	229533.I1SA32
229533.A0A1C3YMA1	pp	229533.I1RQ81
229533.A0A1C3YMA1	pp	229533.I1RS18
229533.A0A1C3YMA1	pp	229533.Q4I8P5
229533.A0A1C3YMA1	pp	229533.I1S419
229533.A0A1C3YMA1	pp	229533.I1RZM8
229533.A0A1C3YMA1	pp	229533.I1RI82
229533.A0A1C3YMA1	pp	229533.I1RNQ5
229533.A0A1C3YMA1	pp	229533.I1RLU6
229533.A0A1C3YMA1	pp	229533.V6RCZ8
229533.A0A1C3YMA1	pp	229533.I1RXD7
229533.A0A1C3YMA1	pp	229533.I1RZM1
229533.A0A1C3YMA1	pp	229533.I1RX46
229533.A0A1C3YMA1	pp	229533.I1RZK5
229533.A0A1C3YMA1	pp	229533.I1RBL7
229533.A0A1C3YMA1	pp	229533.I1S504
229533.A0A1C3YMA1	pp	229533.I1RRY9
229533.A0A1C3YMA1	pp	229533.I1S0T2
229533.A0A1C3YMA1	pp	229533.I1S0S6
229533.A0A1C3YMA1	pp	229533.I1RWT6
229533.A0A1C3YMA1	pp	229533.Q4IJK2
229533.A0A1C3YMA1	pp	229533.I1RY03
229533.A0A1C3YMA1	pp	229533.I1RQA3
229533.A0A1C3YMA1	pp	229533.I1RFU8
229533.A0A1C3YMA1	pp	229533.I1RAX6
229533.A0A1C3YMA1	pp	229533.V6RTJ8
229533.A0A1C3YMA1	pp	229533.Q4IJT5
229533.A0A1C3YMA1	pp	229533.I1RCG4
229533.A0A1C3YMA1	pp	229533.I1RZZ1
229533.A0A1C3YMA1	pp	229533.V6R042
229533.A0A1C3YMA1	pp	229533.I1S071
229533.A0A1C3YMA1	pp	229533.I1RDU0
229533.A0A1C3YMA1	pp	229533.I1RSQ3
229533.A0A1C3YMA1	pp	229533.I1REL7
229533.A0A1C3YMA1	pp	229533.I1RRG3
229533.A0A1C3YMA1	pp	229533.Q4I2J8
229533.A0A1C3YMA1	pp	229533.I1RBC9
229533.A0A1C3YMA1	pp	229533.I1SA33
229533.A0A1C3YMA1	pp	229533.I1S4S7
229533.A0A1C3YMA1	pp	229533.I1RW71
229533.A0A1C3YMA1	pp	229533.I1RF19
229533.A0A1C3YMA1	pp	229533.I1S256

103734 229533.A0A1C3YMA1 pp 229533.I1RD22
103735 229533.A0A1C3YMA1 pp 229533.I1S4Q4
103736 229533.A0A1C3YMA1 pp 229533.I1RSQ7
103737 229533.A0A1C3YMA1 pp 229533.V6QW22
103738 229533.A0A1C3YMA1 pp 229533.I1S5R6
103739 229533.A0A1C3YMA1 pp 229533.I1REK8
103740 229533.A0A1C3YMA1 pp 229533.I1RCR5
103741 229533.A0A1C3YMA1 pp 229533.I1RQC9
103742 229533.A0A1C3YMA1 pp 229533.I1S0R7
103743 229533.A0A1C3YMA1 pp 229533.I1S0N4
103744 229533.A0A1C3YMA1 pp 229533.I1RC20
103745 229533.A0A1C3YMA1 pp 229533.I1RVQ0
103746 229533.A0A1C3YMA1 pp 229533.I1RD52
103747 229533.A0A1C3YMA1 pp 229533.I1RE01
103748 229533.A0A1C3YMA1 pp 229533.A0A1C3YN30
103749 229533.A0A1C3YMA1 pp 229533.I1RW46
103750 229533.A0A1C3YMA1 pp 229533.V6R1K4
103751 229533.A0A1C3YMA1 pp 229533.I1RJU3
103752 229533.A0A1C3YMA1 pp 229533.I1RTA0
103753 229533.A0A1C3YMA1 pp 229533.I1RA06
103754 229533.A0A1C3YMA1 pp 229533.I1S0L3
103755 229533.A0A1C3YMA1 pp 229533.I1S0J1
103756 229533.A0A1C3YMA1 pp 229533.I1S4S4
103757 229533.A0A1C3YMA1 pp 229533.I1RPK0
103758 229533.A0A1C3YMA1 pp 229533.I1R9Y4
103759 229533.A0A1C3YMA1 pp 229533.I1RBT1
103760 229533.A0A1C3YMA1 pp 229533.I1RWJ8
103761 229533.A0A1C3YMA1 pp 229533.I1RS17
103762 229533.A0A1C3YMA1 pp 229533.I1S0X6
103763 229533.A0A1C3YMA1 pp 229533.I1RR24
103764 229533.A0A1C3YMA1 pp 229533.I1RPP4
103765 229533.A0A1C3YMA1 pp 229533.I1S503
103766 229533.A0A1C3YMA1 pp 229533.I1RT41
103767 229533.A0A1C3YMA1 pp 229533.I1RCS1
103768 229533.A0A1C3YMA1 pp 229533.I1S1W9
103769 229533.A0A1C3YMA1 pp 229533.I1RW25
103770 229533.A0A1C3YMA1 pp 229533.I1RBS6
103771 229533.A0A1C3YMA1 pp 229533.I1RJW6
103772 229533.A0A1C3YMA1 pp 229533.V6RIV9
103773 229533.A0A1C3YMA1 pp 229533.I1RGA4
103774 229533.A0A1C3YMA1 pp 229533.I1RUG3
103775 229533.A0A1C3YMA1 pp 229533.I1RP27
103776 229533.A0A1C3YMA1 pp 229533.I1RWN5
103777 229533.A0A1C3YMA1 pp 229533.I1RNC1
103778 229533.A0A1C3YMA1 pp 229533.I1RJV4
103779 229533.A0A1C3YMA1 pp 229533.I1RC33
103780 229533.A0A1C3YMA1 pp 229533.I1RCR8
103781 229533.A0A1C3YMA1 pp 229533.A0A1C3YMI5
103782 229533.A0A1C3YMA1 pp 229533.V6RMW6
103783 229533.A0A1C3YMA1 pp 229533.I1RRV1
103784 229533.A0A1C3YMA1 pp 229533.I1RWK3
103785 229533.A0A1C3YMA1 pp 229533.I1RN63
103786 229533.A0A1C3YMA1 pp 229533.I1RY44
103787 229533.A0A1C3YMA1 pp 229533.I1RTA1
103788 229533.A0A1C3YMA1 pp 229533.I1RVZ2
103789 229533.A0A1C3YMA1 pp 229533.I1RJV3
103790 229533.A0A1C3YMA1 pp 229533.I1RWN6
103791 229533.A0A1C3YMA1 pp 229533.I1RRE5
103792 229533.A0A1C3YMA1 pp 229533.I1RAW0
103793 229533.A0A1C3YMA1 pp 229533.I1RFT1
103794 229533.A0A1C3YMA1 pp 229533.I1S8V4
103795 229533.A0A1C3YMA1 pp 229533.I1S8P5
103796 229533.A0A1C3YMA1 pp 229533.I1RPP3
103797 229533.A0A1C3YMA1 pp 229533.I1RN18
103798 229533.A0A1C3YMA1 pp 229533.I1S1C7
103799 229533.A0A1C3YMA1 pp 229533.I1RFL4
103800 229533.A0A1C3YMA1 pp 229533.I1RCH5
103801 229533.A0A1C3YMA1 pp 229533.I1RQE7
103802 229533.A0A1C3YMA1 pp 229533.I1RYW3
103803 229533.A0A1C3YMA1 pp 229533.I1RYI5
103804 229533.A0A1C3YMA1 pp 229533.I1RN67
103805 229533.A0A1C3YMA1 pp 229533.I1RCW6
103806 229533.A0A1C3YMA1 pp 229533.I1RNG4

103807 229533.A0A1C3YMA1 pp 229533.I1RVX0
103808 229533.A0A1C3YMA1 pp 229533.I1RRI2
103809 229533.A0A1C3YMA1 pp 229533.I1S0F6
103810 229533.A0A1C3YMA1 pp 229533.I1RB92
103811 229533.A0A1C3YMA1 pp 229533.I1RT03
103812 229533.A0A1C3YMA1 pp 229533.I1RWX5
103813 229533.A0A1C3YMA1 pp 229533.I1RG90
103814 229533.A0A1C3YMA1 pp 229533.I1RP28
103815 229533.A0A1C3YMA1 pp 229533.V6RRH2
103816 229533.A0A1C3YMA1 pp 229533.I1RBS0
103817 229533.A0A1C3YMA1 pp 229533.I1RZW8
103818 229533.A0A1C3YMA1 pp 229533.I1RA70
103819 229533.A0A1C3YMA1 pp 229533.I1RC05
103820 229533.A0A1C3YMA1 pp 229533.V6R057
103821 229533.A0A1C3YMA1 pp 229533.I1RY62
103822 229533.A0A1C3YMA1 pp 229533.I1RS47
103823 229533.A0A1C3YMA1 pp 229533.I1S8R3
103824 229533.A0A1C3YMA1 pp 229533.I1RD17
103825 229533.A0A1C3YMA1 pp 229533.I1RTH6
103826 229533.A0A1C3YMA1 pp 229533.I1RAU7
103827 229533.A0A1C3YMA1 pp 229533.Q4I624
103828 229533.A0A1C3YMA1 pp 229533.I1RAB4
103829 229533.A0A1C3YMA1 pp 229533.V6R3X5
103830 229533.A0A1C3YMA1 pp 229533.I1RV91
103831 229533.A0A1C3YMA1 pp 229533.I1RSQ4
103832 229533.A0A1C3YMA1 pp 229533.I1RR73
103833 229533.A0A1C3YMA1 pp 229533.I1RT40
103834 229533.A0A1C3YMA1 pp 229533.I1RXC2
103835 229533.A0A1C3YMA1 pp 229533.I1RZZ9
103836 229533.A0A1C3YMA1 pp 229533.I1RWI5
103837 229533.A0A1C3YMA1 pp 229533.I1RTD5
103838 229533.A0A1C3YMA1 pp 229533.I1RT04
103839 229533.A0A1C3YMA1 pp 229533.I1RRT8
103840 229533.A0A1C3YMA1 pp 229533.I1S2E9
103841 229533.A0A1C3YMA1 pp 229533.I1S8V3
103842 229533.A0A1C3YMA1 pp 229533.I1RC66
103843 229533.A0A1C3YMA1 pp 229533.I1S5R1
103844 229533.A0A1C3YMA1 pp 229533.I1RA11
103845 229533.A0A1C3YMA1 pp 229533.I1RZ39
103846 229533.A0A1C3YMA1 pp 229533.I1S2H4
103847 229533.A0A1C3YMA1 pp 229533.I1RC00
103848 229533.A0A1C3YMA1 pp 229533.I1RFW3
103849 229533.A0A1C3YMA1 pp 229533.I1S0L4
103850 229533.A0A1C3YMA1 pp 229533.I1RN88
103851 229533.A0A1C3YMA1 pp 229533.I1RSZ7
103852 229533.A0A1C3YMA1 pp 229533.I1RFS3
103853 229533.A0A1C3YMA1 pp 229533.I1RPM4
103854 229533.A0A1C3YMA1 pp 229533.I1RE22
103855 229533.A0A1C3YMA1 pp 229533.I1S6Y8
103856 229533.A0A1C3YMA1 pp 229533.I1R9M0
103857 229533.A0A1C3YMA1 pp 229533.I1RRM5
103858 229533.A0A1C3YMA1 pp 229533.I1RJZ7
103859 229533.A0A1C3YMA1 pp 229533.I1S2B8
103860 229533.A0A1C3YMA1 pp 229533.I1RBY4
103861 229533.A0A1C3YKM5 pp 229533.I1RTN6
103862 229533.A0A1C3YKM5 pp 229533.I1RX64
103863 229533.A0A1C3YKM5 pp 229533.I1RVZ9
103864 229533.A0A1C3YKM5 pp 229533.I1S209
103865 229533.A0A1C3YKM5 pp 229533.I1RSY2
103866 229533.A0A1C3YKM5 pp 229533.I1RC54
103867 229533.A0A1C3YKM5 pp 229533.I1RMQ7
103868 229533.A0A1C3YKM5 pp 229533.I1RYI5
103869 229533.A0A1C3YKM5 pp 229533.A0A1C3YMJ3
103870 229533.A0A1C3YKM5 pp 229533.I1RBW1
103871 229533.A0A1C3YKM5 pp 229533.I1RCM7
103872 229533.A0A1C3YKM5 pp 229533.I1RBW0
103873 229533.A0A1C3YKM5 pp 229533.I1RKB7
103874 229533.A0A1C3YKM5 pp 229533.I1RZ94
103875 229533.A0A1C3YKM5 pp 229533.A0A1C3YML3
103876 229533.A0A1C3YKM5 pp 229533.I1RKI2
103877 229533.A0A1C3YIS7 pp 229533.I1RZZ9
103878 229533.A0A1C3YIS7 pp 229533.I1S0L4
103879 229533.A0A1C3YIS7 pp 229533.I1S0X6

103880	229533.A0A1C3YIS7	pp	229533.I1RR24
103881	229533.A0A1C3YIS7	pp	229533.I1RC00
103882	229533.A0A1C3YIS7	pp	229533.I1RQS8
103883	229533.A0A1C3YIS7	pp	229533.I1RD64
103884	229533.A0A1C3YIS7	pp	229533.A0A1C3YMA1
103885	229533.A0A1C3YIS7	pp	229533.I1RE27
103886	229533.A0A1C3YIS7	pp	229533.I1RBY4
103887	229533.A0A1C3YIS7	pp	229533.I1S2H4
103888	229533.A0A1C3YIS7	pp	229533.I1RE76
103889	229533.A0A1C3YIS7	pp	229533.I1RS20
103890	229533.A0A1C3YIS7	pp	229533.I1RW25
103891	229533.A0A1C3YIS7	pp	229533.A0A1C3YJQ9
103892	229533.A0A1C3YIS7	pp	229533.I1RRT8
103893	229533.A0A1C3YIS7	pp	229533.I1RX25
103894	229533.A0A1C3YIS7	pp	229533.I1RJG2
103895	229533.A0A1C3YIS7	pp	229533.I1S5R1
103896	229533.A0A1C3YIS7	pp	229533.I1RM06
103897	229533.A0A1C3YIS7	pp	229533.I1R9M0
103898	229533.A0A1C3YIS7	pp	229533.I1S0M6
103899	229533.A0A1C3YIS7	pp	229533.I1RJZ7
103900	229533.A0A1C3YIS7	pp	229533.O74638
103901	229533.A0A1C3YIS7	pp	229533.A0A1C3YLD8
103902	229533.A0A1C3YIS7	pp	229533.I1RRM5
103903	229533.A0A1C3YIS7	pp	229533.V6QUW4
103904	229533.A0A1C3YIS7	pp	229533.I1RW54
103905	229533.A0A1C3YIS7	pp	229533.I1RDA0
103906	229533.A0A1C3YIS7	pp	229533.I1S2B8
103907	229533.A0A1C3YIS7	pp	229533.Q4HVW2
103908	229533.A0A1C3YIS7	pp	229533.I1RMG8
103909	229533.A0A1C3YIS7	pp	229533.I1RMK3
103910	229533.A0A1C3YIS7	pp	229533.I1RA11
103911	229533.A0A1C3YIS7	pp	229533.I1RE22
103912	229533.A0A1C3YIS7	pp	229533.I1RNA0
103913	229533.A0A1C3YIS7	pp	229533.V6QYF0
103914	229533.A0A1C3YIS7	pp	229533.I1RWL2
103915	229533.A0A1C3YIS7	pp	229533.I1RR73
103916	229533.A0A1C3YIS7	pp	229533.I1RZJ5
103917	229533.A0A1C3YIS7	pp	229533.I1RRQ6
103918	229533.A0A1C3YIS7	pp	229533.V6R3X5
103919	229533.A0A1C3YIS7	pp	229533.I1RM82
103920	229533.A0A1C3YIS7	pp	229533.I1RQ23
103921	229533.A0A1C3YIS7	pp	229533.I1RWJ8
103922	229533.A0A1C3YIS7	pp	229533.I1RTF4
103923	229533.A0A1C3YIS7	pp	229533.A0A1C3YKM9
103924	229533.A0A1C3YIS7	pp	229533.I1RZ39
103925	229533.A0A1C3YIS7	pp	229533.I1S4S4
103926	229533.A0A1C3YIS7	pp	229533.I1S6Y8
103927	229533.A0A1C3YIS7	pp	229533.I1RPM4
103928	229533.A0A1C3YIS7	pp	229533.A0A1C3YJH8
103929	229533.A0A1C3YIS7	pp	229533.I1RRL9
103930	229533.A0A1C3YIS7	pp	229533.I1RJU3
103931	229533.A0A1C3YIS7	pp	229533.I1RCS1
103932	229533.A0A1C3YIS7	pp	229533.I1RSG9
103933	229533.A0A1C3YIS7	pp	229533.I1RCI9
103934	229533.A0A1C3YIS7	pp	229533.I1RW61
103935	229533.A0A1C3YIS7	pp	229533.A0A1C3YN30
103936	229533.A0A1C3YIS7	pp	229533.I1RG53
103937	229533.A0A1C3YIS7	pp	229533.I1RFH8
103938	229533.A0A1C3YIS7	pp	229533.I1RRG3
103939	229533.A0A1C3YIS7	pp	229533.I1RAM6
103940	229533.A0A1C3YIS7	pp	229533.I1RQJ5
103941	229533.A0A1C3YIS7	pp	229533.I1RC66
103942	229533.A0A1C3YIS7	pp	229533.I1RMC8
103943	229533.A0A1C3YIS7	pp	229533.I1S2M6
103944	229533.A0A1C3YIS7	pp	229533.Q4IJT5
103945	229533.A0A1C3YIS7	pp	229533.I1RPP3
103946	229533.A0A1C3YIS7	pp	229533.I1RNN1
103947	229533.A0A1C3YIS7	pp	229533.I1RWI5
103948	229533.A0A1C3YIS7	pp	229533.I1RBT1
103949	229533.A0A1C3YIS7	pp	229533.I1S8P5
103950	229533.A0A1C3YIS7	pp	229533.A0A1C3YKC0
103951	229533.A0A1C3YIS7	pp	229533.I1RWK3
103952	229533.A0A1C3YIS7	pp	229533.I1RN63

103953	229533.A0A1C3YIS7	pp	229533.V6QW22
103954	229533.A0A1C3YIS7	pp	229533.A0A1C3YM63
103955	229533.A0A1C3YIS7	pp	229533.I1S0R7
103956	229533.A0A1C3YIS7	pp	229533.I1RTD5
103957	229533.A0A1C3YIS7	pp	229533.I1RRJ8
103958	229533.A0A1C3YIS7	pp	229533.I1RLX7
103959	229533.A0A1C3YIS7	pp	229533.I1RQG4
103960	229533.A0A1C3YIS7	pp	229533.A0A1C3YL54
103961	229533.A0A1C3YIS7	pp	229533.V6R1K4
103962	229533.A0A1C3YIS7	pp	229533.Q4INZ9
103963	229533.A0A1C3YIS7	pp	229533.I1RSQ7
103964	229533.A0A1C3YIS7	pp	229533.I1RV91
103965	229533.A0A1C3YIS7	pp	229533.A0A1C3YK13
103966	229533.A0A1C3YIS7	pp	229533.Q4IEV5
103967	229533.A0A1C3YIS7	pp	229533.I1RW71
103968	229533.A0A1C3YIS7	pp	229533.I1RBC9
103969	229533.A0A1C3YIS7	pp	229533.A0A1C3YL15
103970	229533.A0A1C3YIS7	pp	229533.I1S0L3
103971	229533.A0A1C3YIS7	pp	229533.I1RCJ7
103972	229533.A0A1C3YIS7	pp	229533.I1RT40
103973	229533.A0A1C3YIS7	pp	229533.A0A1C3YKN3
103974	229533.A0A1C3YIS7	pp	229533.I1RFT1
103975	229533.A0A1C3YIS7	pp	229533.V6RTJ8
103976	229533.A0A1C3YIS7	pp	229533.I1RZZ1
103977	229533.A0A1C3YIS7	pp	229533.I1S0F5
103978	229533.A0A1C3YIS7	pp	229533.I1S0D4
103979	229533.A0A1C3YIS7	pp	229533.I1RCH5
103980	229533.A0A1C3YIS7	pp	229533.I1RD52
103981	229533.A0A1C3YIS7	pp	229533.I1S7A8
103982	229533.A0A1C3YIS7	pp	229533.I1RQQ5
103983	229533.A0A1C3YIS7	pp	229533.I1RS47
103984	229533.A0A1C3YIS7	pp	229533.I1RQE7
103985	229533.A0A1C3YIS7	pp	229533.I1RJ74
103986	229533.A0A1C3YIS7	pp	229533.I1RJS2
103987	229533.A0A1C3YIS7	pp	229533.I1RFL4
103988	229533.A0A1C3YIS7	pp	229533.I1RN18
103989	229533.A0A1C3YIS7	pp	229533.I1RJV4
103990	229533.A0A1C3YIS7	pp	229533.I1S1B6
103991	229533.A0A1C3YIS7	pp	229533.I1S6F0
103992	229533.A0A1C3YIS7	pp	229533.I1RAH0
103993	229533.A0A1C3YIS7	pp	229533.I1RQA3
103994	229533.A0A1C3YIS7	pp	229533.I1RP28
103995	229533.A0A1C3YIS7	pp	229533.I1RBS6
103996	229533.A0A1C3YIS7	pp	229533.I1RGA4
103997	229533.A0A1C3YIS7	pp	229533.I1RC82
103998	229533.A0A1C3YIS7	pp	229533.Q4IJK2
103999	229533.A0A1C3YIS7	pp	229533.I1RAX6
104000	229533.A0A1C3YIS7	pp	229533.A0A1C3YN63
104001	229533.A0A1C3YIS7	pp	229533.I1S0T2
104002	229533.A0A1C3YIS7	pp	229533.I1RBS0
104003	229533.A0A1C3YIS7	pp	229533.I1RQ72
104004	229533.A0A1C3YIS7	pp	229533.I1RAU7
104005	229533.A0A1C3YIS7	pp	229533.I1RVQ0
104006	229533.A0A1C3YIS7	pp	229533.I1RRT2
104007	229533.A0A1C3YIS7	pp	229533.I1RN67
104008	229533.A0A1C3YIS7	pp	229533.I1RRP3
104009	229533.A0A1C3YIS7	pp	229533.I1RWD7
104010	229533.A0A1C3YIS7	pp	229533.I1RWD4
104011	229533.A0A1C3YIS7	pp	229533.V6R057
104012	229533.A0A1C3YIS7	pp	229533.I1RJL4
104013	229533.A0A1C3YIS7	pp	229533.I1RX46
104014	229533.A0A1C3YIS7	pp	229533.I1S0F6
104015	229533.A0A1C3YIS7	pp	229533.Q4I624
104016	229533.A0A1C3YIS7	pp	229533.A0A1C3YMW0
104017	229533.A0A1C3YIS7	pp	229533.I1RQH3
104018	229533.A0A1C3YIS7	pp	229533.I1REK8
104019	229533.A0A1C3YIS7	pp	229533.I1RRZ0
104020	229533.A0A1C3YIS7	pp	229533.I1RXD7
104021	229533.A0A1C3YIS7	pp	229533.I1RD17
104022	229533.A0A1C3YIS7	pp	229533.I1RCR5
104023	229533.A0A1C3YIS7	pp	229533.I1RJW6
104024	229533.A0A1C3YIS7	pp	229533.I1RUQ2
104025	229533.A0A1C3YIS7	pp	229533.I1RE00

104026	229533.A0A1C3YIS7	pp	229533.I1S2E9
104027	229533.A0A1C3YIS7	pp	229533.A0A1C3YLK8
104028	229533.A0A1C3YIS7	pp	229533.I1RSI0
104029	229533.A0A1C3YIS7	pp	229533.I1S503
104030	229533.A0A1C3YIS7	pp	229533.I1RN99
104031	229533.A0A1C3YIS7	pp	229533.I1RFJ9
104032	229533.A0A1C3YIS7	pp	229533.I1RQC9
104033	229533.A0A1C3YIS7	pp	229533.A0A1C3YLF5
104034	229533.A0A1C3YIS7	pp	229533.I1RB92
104035	229533.A0A1C3YIS7	pp	229533.I1RF19
104036	229533.A0A1C3YIS7	pp	229533.I1RB41
104037	229533.A0A1C3YIS7	pp	229533.I1RDG0
104038	229533.A0A1C3YIS7	pp	229533.I1RVX0
104039	229533.A0A1C3YIS7	pp	229533.I1RT04
104040	229533.A0A1C3YIS7	pp	229533.I1RKC6
104041	229533.A0A1C3YIS7	pp	229533.I1RAZ8
104042	229533.A0A1C3YIS7	pp	229533.V6RUY9
104043	229533.A0A1C3YIS7	pp	229533.I1RT03
104044	229533.A0A1C3YIS7	pp	229533.Q4I5Z5
104045	229533.A0A1C3YIS7	pp	229533.Q4IJM4
104046	229533.A0A1C3YIS7	pp	229533.I1RY63
104047	229533.A0A1C3YIS7	pp	229533.A0A1C3YKV3
104048	229533.A0A1C3YIS7	pp	229533.Q4I2J8
104049	229533.A0A1C3YIS7	pp	229533.I1RS17
104050	229533.A0A1C3YIS7	pp	229533.I1RQ81
104051	229533.A0A1C3YIS7	pp	229533.I1SA32
104052	229533.A0A1C3YIS7	pp	229533.I1RZK5
104053	229533.A0A1C3YIS7	pp	229533.I1RSC6
104054	229533.A0A1C3YIS7	pp	229533.I1RRN5
104055	229533.A0A1C3YIS7	pp	229533.I1RTE9
104056	229533.A0A1C3YIS7	pp	229533.I1S0R2
104057	229533.A0A1C3YIS7	pp	229533.I1RAM7
104058	229533.A0A1C3YIS7	pp	229533.Q4HZB8
104059	229533.A0A1C3YIS7	pp	229533.I1RJL2
104060	229533.A0A1C3YIS7	pp	229533.I1S0J1
104061	229533.A0A1C3YIS7	pp	229533.I1RR51
104062	229533.A0A1C3YIS7	pp	229533.I1S2C0
104063	229533.A0A1C3YIS7	pp	229533.I1RA70
104064	229533.A0A1C3YIS7	pp	229533.A0A1C3YLU7
104065	229533.A0A1C3YIS7	pp	229533.I1RC72
104066	229533.A0A1C3YIS7	pp	229533.I1RFU8
104067	229533.A0A1C3YIS7	pp	229533.V6R042
104068	229533.A0A1C3YIS7	pp	229533.I1RW46
104069	229533.A0A1C3YIS7	pp	229533.I1RE01
104070	229533.A0A1C3YIS7	pp	229533.I1RTH6
104071	229533.A0A1C3YIS7	pp	229533.I1RSQ3
104072	229533.A0A1C3YIS7	pp	229533.I1RZH4
104073	229533.A0A1C3YIS7	pp	229533.V6RCG5
104074	229533.A0A1C3YIS7	pp	229533.I1S8R3
104075	229533.A0A1C3YIS7	pp	229533.I1RZS7
104076	229533.A0A1C3YIS7	pp	229533.V6RMW6
104077	229533.A0A1C3YIS7	pp	229533.I1RCG4
104078	229533.A0A1C3YIS7	pp	229533.A0A1C3YK34
104079	229533.A0A1C3YIS7	pp	229533.I1RTA0
104080	229533.A0A1C3YIS7	pp	229533.A0A1C3YLK2
104081	229533.A0A1C3YIS7	pp	229533.I1RWX5
104082	229533.A0A1C3YIS7	pp	229533.A0A1C3YKB0
104083	229533.A0A1C3YIS7	pp	229533.V6R227
104084	229533.A0A1C3YIS7	pp	229533.I1RRI2
104085	229533.A0A1C3YIS7	pp	229533.V6RCZ8
104086	229533.A0A1C3YIS7	pp	229533.I1RMG3
104087	229533.A0A1C3YIS7	pp	229533.I1RZW8
104088	229533.A0A1C3YIS7	pp	229533.I1S0C2
104089	229533.A0A1C3YIS7	pp	229533.A0A1C3YLS9
104090	229533.A0A1C3YIS7	pp	229533.Q4I283
104091	229533.A0A1C3YIS7	pp	229533.I1RSI6
104092	229533.A0A1C3YIS7	pp	229533.I1RRV1
104093	229533.A0A1C3YIS7	pp	229533.I1S1C7
104094	229533.A0A1C3YIS7	pp	229533.I1RWT6
104095	229533.A0A1C3YIS7	pp	229533.I1RYW3
104096	229533.A0A1C3YIS7	pp	229533.I1RRE5
104097	229533.A0A1C3YIS7	pp	229533.A0A1C3YKW9
104098	229533.A0A1C3YIS7	pp	229533.I1RI82

104099 229533.A0A1C3YIS7 pp 229533.I1RMD5
104100 229533.A0A1C3YIS7 pp 229533.I1RMC0
104101 229533.A0A1C3YIS7 pp 229533.I1RNC1
104102 229533.A0A1C3YIS7 pp 229533.I1RY03
104103 229533.A0A1C3YIS7 pp 229533.I1S504
104104 229533.A0A1C3YIS7 pp 229533.I1RTD9
104105 229533.A0A1C3YIS7 pp 229533.I1RXT3
104106 229533.A0A1C3YIS7 pp 229533.I1RD39
104107 229533.A0A1C3YIS7 pp 229533.I1RUG3
104108 229533.A0A1C3YIS7 pp 229533.I1RNQ5
104109 229533.A0A1C3YIS7 pp 229533.A0A1C3YK33
104110 229533.A0A1C3YIS7 pp 229533.I1RLU6
104111 229533.A0A1C3YIS7 pp 229533.I1RQ31
104112 229533.A0A1C3YIS7 pp 229533.I1RXF0
104113 229533.A0A1C3YIS7 pp 229533.I1RWN6
104114 229533.A0A1C3YIS7 pp 229533.I1RAS5
104115 229533.A0A1C3YIS7 pp 229533.I1RRY9
104116 229533.A0A1C3YIS7 pp 229533.I1RAW0
104117 229533.A0A1C3YIS7 pp 229533.Q4IEK8
104118 229533.A0A1C3YIS7 pp 229533.I1RZM8
104119 229533.A0A1C3YIS7 pp 229533.I1RSR3
104120 229533.A0A1C3YIS7 pp 229533.I1S0N4
104121 229533.A0A1C3YIS7 pp 229533.I1RCR8
104122 229533.A0A1C3YIS7 pp 229533.I1RY44
104123 229533.A0A1C3YIS7 pp 229533.I1RSK1
104124 229533.A0A1C3YIS7 pp 229533.I1RB53
104125 229533.A0A1C3YIS7 pp 229533.I1RC33
104126 229533.A0A1C3YIS7 pp 229533.I1RZM1
104127 229533.A0A1C3YIS7 pp 229533.V6RGN5
104128 229533.A0A1C3YIS7 pp 229533.I1S5B0
104129 229533.A0A1C3YIS7 pp 229533.I1RJV3
104130 229533.A0A1C3YIS7 pp 229533.I1S1J7
104131 229533.A0A1C3YIS7 pp 229533.A0A1C3YJ93
104132 229533.A0A1C3YIS7 pp 229533.V6RIV9
104133 229533.A0A1C3YIS7 pp 229533.I1RW99
104134 229533.A0A1C3YIS7 pp 229533.I1RQA2
104135 229533.A0A1C3YIS7 pp 229533.I1RPP4
104136 229533.A0A1C3YIS7 pp 229533.I1RWQ8
104137 229533.A0A1C3YIS7 pp 229533.I1RWN5
104138 229533.A0A1C3YIS7 pp 229533.I1R9X7
104139 229533.A0A1C3YIS7 pp 229533.I1RC17
104140 229533.A0A1C3YIS7 pp 229533.I1RC16
104141 229533.A0A1C3YIS7 pp 229533.I1RS18
104142 229533.A0A1C3YIS7 pp 229533.I1RRU9
104143 229533.A0A1C3YIS7 pp 229533.I1RD22
104144 229533.A0A1C3YIS7 pp 229533.I1RAU5
104145 229533.A0A1C3YIS7 pp 229533.Q4I8P5
104146 229533.A0A1C3YIS7 pp 229533.I1SA33
104147 229533.A0A1C3YIS7 pp 229533.A0A1C3YK36
104148 229533.A0A1C3YIS7 pp 229533.I1RPK0
104149 229533.A0A1C3YIS7 pp 229533.I1S419
104150 229533.A0A1C3YIS7 pp 229533.I1RGC2
104151 229533.I1S6P7 pp 229533.I1S8P4
104152 229533.I1S6P7 pp 229533.I1S8P5
104153 229533.I1RZV2 pp 229533.I1S272
104154 229533.I1RZV2 pp 229533.V6RPQ8
104155 229533.I1RZV2 pp 229533.Q4IJT0
104156 229533.I1RI56 pp 229533.I1RZ52
104157 229533.I1RI56 pp 229533.I1RPD6
104158 229533.I1RI56 pp 229533.I1S4F4
104159 229533.I1RI56 pp 229533.I1RM69
104160 229533.I1RI56 pp 229533.I1RU76
104161 229533.I1RI56 pp 229533.I1RIY0
104162 229533.I1RI56 pp 229533.I1RNG4
104163 229533.I1RI56 pp 229533.I1S2E2
104164 229533.I1RI56 pp 229533.I1S2H3
104165 229533.I1RI56 pp 229533.I1RMM8
104166 229533.I1RI56 pp 229533.I1RQE0
104167 229533.I1RI56 pp 229533.I1RPW2
104168 229533.I1RI56 pp 229533.I1RY74
104169 229533.I1RI56 pp 229533.I1RK03
104170 229533.I1RI56 pp 229533.I1S3F3
104171 229533.I1RI56 pp 229533.I1RM26

104172 229533.I1RI56 pp 229533.I1RVX9
104173 229533.I1RI56 pp 229533.I1S9D4
104174 229533.A0A1C3YMK9 pp 229533.I1S8P4
104175 229533.A0A1C3YMK9 pp 229533.I1RUB4
104176 229533.A0A1C3YMK9 pp 229533.I1S8P5
104177 229533.A0A1C3YMK9 pp 229533.I1RH32
104178 229533.A0A1C3YMK9 pp 229533.I1RUB5
104179 229533.A0A1C3YMK9 pp 229533.I1S6P7
104180 229533.A0A1C3YMK9 pp 229533.I1RUB1
104181 229533.A0A1C3YMK9 pp 229533.I1RH30
104182 229533.A0A1C3YMK9 pp 229533.A0A1C3YMS7
104183 229533.A0A1C3YMK9 pp 229533.A0A1C3YMP8
104184 229533.I1RWF7 pp 229533.V6RMW6
104185 229533.I1RWF7 pp 229533.V6R042
104186 229533.I1RWF7 pp 229533.V6R283
104187 229533.I1RWF7 pp 229533.Q4IN63
104188 229533.I1RWF7 pp 229533.I1S0J1
104189 229533.I1RWF7 pp 229533.V6R3X5
104190 229533.I1RWF7 pp 229533.I1RWN5
104191 229533.I1RWF7 pp 229533.I1RWN6
104192 229533.I1RWF7 pp 229533.I1RZS6
104193 229533.I1RWF7 pp 229533.I1S503
104194 229533.I1RWF7 pp 229533.I1S1C7
104195 229533.I1RWF7 pp 229533.Q4I662
104196 229533.I1RWF7 pp 229533.I1S296
104197 229533.I1RWF7 pp 229533.I1RZF6
104198 229533.I1RWF7 pp 229533.I1S9U2
104199 229533.I1RWF7 pp 229533.V6RTJ8
104200 229533.I1RWF7 pp 229533.Q4IFI0
104201 229533.I1RWF7 pp 229533.I1S504
104202 229533.I1RWF7 pp 229533.I1RY03
104203 229533.I1RWF7 pp 229533.V6QYF0
104204 229533.I1RWF7 pp 229533.I1RZE5
104205 229533.I1RWF7 pp 229533.I1S021
104206 229533.I1RWF7 pp 229533.I1RX46
104207 229533.I1RM69 pp 229533.I1RMM8
104208 229533.I1RM69 pp 229533.I1RPW2
104209 229533.I1RM69 pp 229533.I1S2E2
104210 229533.I1RM69 pp 229533.I1S9D4
104211 229533.I1RM69 pp 229533.I1RVX9
104212 229533.I1RM69 pp 229533.I1S3F3
104213 229533.I1RM69 pp 229533.I1RU76
104214 229533.I1RM69 pp 229533.I1S2H3
104215 229533.I1RM69 pp 229533.I1RZ52
104216 229533.I1RM69 pp 229533.I1RQE0
104217 229533.I1RM69 pp 229533.I1RY74
104218 229533.I1RM69 pp 229533.I1RNG4
104219 229533.I1RM69 pp 229533.I1RPD6
104220 229533.I1RM69 pp 229533.I1S4F4
104221 229533.A0A1C3YI14 pp 229533.I1RZ94
104222 229533.A0A1C3YI14 pp 229533.Q4I7L0
104223 229533.A0A0E0RNR8 pp 229533.I1RE13
104224 229533.A0A0E0RNR8 pp 229533.A0A1C3YI39
104225 229533.A0A0E0RNR8 pp 229533.A0A0E0SCP7
104226 229533.A0A0E0RNR8 pp 229533.A0A1C3YM91
104227 229533.A0A0E0RNR8 pp 229533.A0A1C3YI64
104228 229533.A0A0E0RNR8 pp 229533.A0A1C3YKM5
104229 229533.A0A0E0RNR8 pp 229533.I1S209
104230 229533.A0A0E0RNR8 pp 229533.I1RWI8
104231 229533.A0A0E0RNR8 pp 229533.I1S0Q4
104232 229533.A0A0E0RNR8 pp 229533.I1RSJ1
104233 229533.A0A0E0RNR8 pp 229533.I1S072
104234 229533.A0A0E0RNR8 pp 229533.I1S0Q5
104235 229533.A0A0E0RNR8 pp 229533.A0A1C3YML3
104236 229533.A0A0E0RNR8 pp 229533.I1RCF4
104237 229533.A0A0E0RNR8 pp 229533.I1RBI6
104238 229533.A0A0E0RNR8 pp 229533.I1S1Y7
104239 229533.A0A0E0RNR8 pp 229533.I1RCM7
104240 229533.A0A0E0RNR8 pp 229533.I1RZT1
104241 229533.A0A0E0RNR8 pp 229533.I1RVZ9
104242 229533.A0A0E0RNR8 pp 229533.I1RWH8
104243 229533.I1RB74 pp 229533.I1RLU1
104244 229533.I1RB74 pp 229533.I1RNP7

104245 229533.I1RB74 pp 229533.I1RRM9
104246 229533.I1RB74 pp 229533.O74638
104247 229533.I1RB74 pp 229533.I1RM82
104248 229533.I1RB74 pp 229533.I1RWL2
104249 229533.I1RB74 pp 229533.I1RFD1
104250 229533.I1RB74 pp 229533.I1RWI5
104251 229533.I1RB74 pp 229533.I1RUN8
104252 229533.I1RB74 pp 229533.I1RX38
104253 229533.I1RB74 pp 229533.I1RR73
104254 229533.I1RB74 pp 229533.V6R3X5
104255 229533.I1RB74 pp 229533.I1S2E9
104256 229533.I1RB74 pp 229533.I1RNN1
104257 229533.I1RB74 pp 229533.I1RCD4
104258 229533.I1RB74 pp 229533.I1RJ88
104259 229533.I1RB74 pp 229533.I1RC24
104260 229533.I1RB74 pp 229533.I1RZ63
104261 229533.I1RB74 pp 229533.I1RUF8
104262 229533.I1RB74 pp 229533.I1RPD6
104263 229533.I1RB74 pp 229533.I1S503
104264 229533.I1RB74 pp 229533.I1RBV5
104265 229533.I1RB74 pp 229533.I1RR95
104266 229533.I1RB74 pp 229533.I1RZ39
104267 229533.I1RB74 pp 229533.I1RN67
104268 229533.I1RB74 pp 229533.I1RP28
104269 229533.I1RB74 pp 229533.I1RPU3
104270 229533.I1RB74 pp 229533.I1RP27
104271 229533.I1RB74 pp 229533.I1S0J7
104272 229533.I1RB74 pp 229533.I1RNF4
104273 229533.I1RB74 pp 229533.I1RC97
104274 229533.I1RB74 pp 229533.I1RTD5
104275 229533.I1RB74 pp 229533.I1RQG4
104276 229533.I1RB74 pp 229533.I1RJZ7
104277 229533.I1RB74 pp 229533.I1RZP1
104278 229533.I1RB74 pp 229533.I1RV91
104279 229533.I1RB74 pp 229533.I1S2B8
104280 229533.I1RB74 pp 229533.I1RCJ7
104281 229533.I1RB74 pp 229533.I1RFL7
104282 229533.I1RB74 pp 229533.I1RVX0
104283 229533.I1RB74 pp 229533.I1RPW0
104284 229533.I1RB74 pp 229533.I1RTH6
104285 229533.I1RB74 pp 229533.I1RPP3
104286 229533.I1RB74 pp 229533.I1RD22
104287 229533.I1RB74 pp 229533.I1RSV2
104288 229533.I1RB74 pp 229533.I1RK75
104289 229533.I1RB74 pp 229533.I1RJV3
104290 229533.I1RB74 pp 229533.I1RD15
104291 229533.I1RB74 pp 229533.V6RGI1
104292 229533.I1RB74 pp 229533.I1S0N4
104293 229533.I1RB74 pp 229533.I1RQ72
104294 229533.I1RB74 pp 229533.I1RCL8
104295 229533.I1RB74 pp 229533.I1RRY3
104296 229533.I1RB74 pp 229533.I1RSK1
104297 229533.I1RB74 pp 229533.I1RT36
104298 229533.I1RB74 pp 229533.I1RY58
104299 229533.I1RB74 pp 229533.I1RMJ8
104300 229533.I1RB74 pp 229533.I1RCM3
104301 229533.I1RB74 pp 229533.I1RDJ8
104302 229533.I1RB74 pp 229533.I1RRW0
104303 229533.I1RB74 pp 229533.I1RZE4
104304 229533.I1RB74 pp 229533.I1RRP7
104305 229533.I1RB74 pp 229533.I1RWN6
104306 229533.I1RB74 pp 229533.I1S0M2
104307 229533.I1RB74 pp 229533.I1RQB4
104308 229533.I1RB74 pp 229533.I1S0E8
104309 229533.I1RB74 pp 229533.I1RZT1
104310 229533.I1RB74 pp 229533.I1RNC1
104311 229533.I1RB74 pp 229533.I1RDZ4
104312 229533.I1RB74 pp 229533.I1RQF2
104313 229533.I1RB74 pp 229533.I1RCB7
104314 229533.I1RB74 pp 229533.I1S1C7
104315 229533.I1RB74 pp 229533.I1RMQ5
104316 229533.I1RB74 pp 229533.V6RMW6
104317 229533.I1RB74 pp 229533.I1RNY9

104318	229533.I1RB74	pp	229533.I1RRN5
104319	229533.I1RB74	pp	229533.I1RY44
104320	229533.I1RB74	pp	229533.V6RVG3
104321	229533.I1RB74	pp	229533.I1RHQ8
104322	229533.I1RB74	pp	229533.I1RXU0
104323	229533.I1RB74	pp	229533.I1RT03
104324	229533.I1RB74	pp	229533.I1RRV1
104325	229533.I1RB74	pp	229533.V6RKY7
104326	229533.I1RB74	pp	229533.I1S0D1
104327	229533.I1RB74	pp	229533.I1S9Q3
104328	229533.I1RB74	pp	229533.I1S4P6
104329	229533.I1RB74	pp	229533.I1RNC0
104330	229533.I1RB74	pp	229533.I1RFQ7
104331	229533.I1RB74	pp	229533.I1RST8
104332	229533.I1RB74	pp	229533.I1RWN5
104333	229533.I1RB74	pp	229533.I1RV66
104334	229533.I1RB74	pp	229533.I1S2K7
104335	229533.I1RB74	pp	229533.I1RD18
104336	229533.I1RB74	pp	229533.I1S1W4
104337	229533.I1RB74	pp	229533.Q4HZS8
104338	229533.I1RB74	pp	229533.I1RWJ6
104339	229533.I1RB74	pp	229533.I1RDB1
104340	229533.I1RB74	pp	229533.I1RX70
104341	229533.I1RB74	pp	229533.I1RL24
104342	229533.I1RB74	pp	229533.I1RD01
104343	229533.I1RB74	pp	229533.Q4IB89
104344	229533.I1RB74	pp	229533.I1RNY8
104345	229533.I1RB74	pp	229533.I1RX46
104346	229533.I1RB74	pp	229533.I1S224
104347	229533.I1RB74	pp	229533.I1RYZ9
104348	229533.I1RB74	pp	229533.I1RYH9
104349	229533.I1RB74	pp	229533.I1RPP4
104350	229533.I1RB74	pp	229533.I1RQC9
104351	229533.I1RB74	pp	229533.I1RXH6
104352	229533.I1RB74	pp	229533.I1RQH3
104353	229533.I1RB74	pp	229533.V6QV09
104354	229533.I1RB74	pp	229533.V6RTJ8
104355	229533.I1RB74	pp	229533.Q5I2J3
104356	229533.I1RB74	pp	229533.I1RBZ9
104357	229533.I1RB74	pp	229533.I1RM49
104358	229533.I1RB74	pp	229533.I1RQ26
104359	229533.I1RB74	pp	229533.I1RZK5
104360	229533.I1RB74	pp	229533.I1RFZ3
104361	229533.I1RB74	pp	229533.I1RC20
104362	229533.I1RB74	pp	229533.I1S0D4
104363	229533.I1RB74	pp	229533.I1RW71
104364	229533.I1RB74	pp	229533.I1RZB5
104365	229533.I1RB74	pp	229533.I1RN61
104366	229533.I1RB74	pp	229533.I1RN08
104367	229533.I1RB74	pp	229533.I1RJH1
104368	229533.I1RB74	pp	229533.I1RRG3
104369	229533.I1RB74	pp	229533.I1RMM9
104370	229533.I1RB74	pp	229533.I1RFU8
104371	229533.I1RB74	pp	229533.I1RCL7
104372	229533.I1RB74	pp	229533.Q4IJK2
104373	229533.I1RB74	pp	229533.I1RSI0
104374	229533.I1RB74	pp	229533.I1RC22
104375	229533.I1RB74	pp	229533.I1RYZ0
104376	229533.I1RB74	pp	229533.I1RRY9
104377	229533.I1RB74	pp	229533.I1RY03
104378	229533.I1RB74	pp	229533.I1RUQ5
104379	229533.I1RB74	pp	229533.I1S439
104380	229533.I1RB74	pp	229533.I1RN65
104381	229533.I1RB74	pp	229533.Q4IJM4
104382	229533.I1RB74	pp	229533.I1RC95
104383	229533.I1RB74	pp	229533.I1REN3
104384	229533.I1RB74	pp	229533.I1RBY8
104385	229533.I1RB74	pp	229533.I1RFT2
104386	229533.I1RB74	pp	229533.I1RZM1
104387	229533.I1RB74	pp	229533.I1RKK0
104388	229533.I1RB74	pp	229533.I1RJK7
104389	229533.I1RB74	pp	229533.I1RC17
104390	229533.I1RB74	pp	229533.I1RZZ1

229533.I1RB74 pp 229533.I1S504
229533.I1RB74 pp 229533.I1RWT6
229533.I1RB74 pp 229533.I1RCB3
229533.I1RB74 pp 229533.I1RM09
229533.I1RB74 pp 229533.I1RZH4
229533.I1RB74 pp 229533.I1RQA3
229533.I1RB74 pp 229533.I1RMQ7
229533.I1RB74 pp 229533.I1RQ81
229533.A0A1C3YMP8 pp 229533.I1S8P5
229533.A0A1C3YMP8 pp 229533.I1RW43
229533.A0A1C3YMP8 pp 229533.I1RH32
229533.A0A1C3YMP8 pp 229533.I1RH30
229533.A0A1C3YMP8 pp 229533.I1RUB1
229533.A0A1C3YMP8 pp 229533.I1RUB4
229533.A0A1C3YMP8 pp 229533.A0A1C3YMS7
229533.A0A1C3YMP8 pp 229533.I1S6P7
229533.A0A1C3YMP8 pp 229533.I1RUB5
229533.A0A1C3YMP8 pp 229533.I1S8P4
229533.A0A098DB54 pp 229533.A0A1C3YJ56
229533.A0A098DB54 pp 229533.I1RNG4
229533.A0A098DB54 pp 229533.V6RKY8
229533.A0A098DB54 pp 229533.I1RMJ3
229533.A0A098DB54 pp 229533.I1RBF3
229533.A0A098DB54 pp 229533.I1RYP2
229533.A0A098DB54 pp 229533.A0A098DJ03
229533.A0A098DB54 pp 229533.I1S3G4
229533.A0A098DB54 pp 229533.I1RC67
229533.A0A098DB54 pp 229533.I1RCG7
229533.A0A098DB54 pp 229533.A0A1C3YN55
229533.A0A098DB54 pp 229533.I1RGX1
229533.A0A098DB54 pp 229533.A0A1C3YLL1
229533.A0A098DB54 pp 229533.A0A0E0SK74
229533.A0A098DB54 pp 229533.I1RUG4
229533.A0A098DB54 pp 229533.A0A1C3YIV0
229533.A0A098DB54 pp 229533.A0A098E375
229533.A0A098DB54 pp 229533.I1RBS5
229533.A0A098DB54 pp 229533.I1RBA4
229533.A0A098DB54 pp 229533.I1S1W8
229533.A0A098DB54 pp 229533.A0A1C3YK11
229533.A0A098DB54 pp 229533.I1RA32
229533.A0A098DB54 pp 229533.I1RJN7
229533.A0A098DB54 pp 229533.A0A098DVZ4
229533.A0A098DB54 pp 229533.A0A098DIL3
229533.A0A098DB54 pp 229533.I1RUY4
229533.A0A098DB54 pp 229533.I1RY12
229533.A0A098DB54 pp 229533.A0A1C3YKF3
229533.A0A098DB54 pp 229533.I1RXF0
229533.A0A098DB54 pp 229533.I1RVS4
229533.I1RBY4 pp 229533.I1RXC4
229533.I1RBY4 pp 229533.I1SA32
229533.I1RBY4 pp 229533.I1RQ81
229533.I1RBY4 pp 229533.I1RJ17
229533.I1RBY4 pp 229533.I1RS18
229533.I1RBY4 pp 229533.Q4I8P5
229533.I1RBY4 pp 229533.I1RLU6
229533.I1RBY4 pp 229533.I1S419
229533.I1RBY4 pp 229533.I1RZM1
229533.I1RBY4 pp 229533.I1S504
229533.I1RBY4 pp 229533.I1RG14
229533.I1RBY4 pp 229533.I1RNQ5
229533.I1RBY4 pp 229533.I1RWT6
229533.I1RBY4 pp 229533.I1RQ85
229533.I1RBY4 pp 229533.I1RGC5
229533.I1RBY4 pp 229533.V6RCZ8
229533.I1RBY4 pp 229533.Q4I283
229533.I1RBY4 pp 229533.I1RR51
229533.I1RBY4 pp 229533.V6R227
229533.I1RBY4 pp 229533.V6RCG5
229533.I1RBY4 pp 229533.V6QUE6
229533.I1RBY4 pp 229533.I1RZM8
229533.I1RBY4 pp 229533.I1RRV4
229533.I1RBY4 pp 229533.I1RGC2
229533.I1RBY4 pp 229533.I1RXF0

104464	229533.I1RBY4	pp	229533.I1RRU9
104465	229533.I1RBY4	pp	229533.I1RC17
104466	229533.I1RBY4	pp	229533.V6RPS5
104467	229533.I1RBY4	pp	229533.I1RW99
104468	229533.I1RBY4	pp	229533.I1RQ31
104469	229533.I1RBY4	pp	229533.Q4HTT2
104470	229533.I1RBY4	pp	229533.I1RI82
104471	229533.I1RBY4	pp	229533.I1RCT9
104472	229533.I1RBY4	pp	229533.I1S5B0
104473	229533.I1RBY4	pp	229533.Q4I5Z5
104474	229533.I1RBY4	pp	229533.I1RZH4
104475	229533.I1RBY4	pp	229533.I1S2U2
104476	229533.I1RBY4	pp	229533.I1S6K9
104477	229533.I1RBY4	pp	229533.I1RF19
104478	229533.I1RBY4	pp	229533.I1RPR9
104479	229533.I1RBY4	pp	229533.I1RSR3
104480	229533.I1RBY4	pp	229533.I1RFJ9
104481	229533.I1RBY4	pp	229533.I1RSI0
104482	229533.I1RBY4	pp	229533.Q4IJK2
104483	229533.I1RBY4	pp	229533.I1RQA3
104484	229533.I1RBY4	pp	229533.I1RRY9
104485	229533.I1RBY4	pp	229533.I1RQQ5
104486	229533.I1RBY4	pp	229533.I1RWR7
104487	229533.I1RBY4	pp	229533.I1S4Q4
104488	229533.I1RBY4	pp	229533.I1RU29
104489	229533.I1RBY4	pp	229533.I1RZZ1
104490	229533.I1RBY4	pp	229533.I1RE00
104491	229533.I1RBY4	pp	229533.I1RLM7
104492	229533.I1RBY4	pp	229533.I1RLX7
104493	229533.I1RBY4	pp	229533.I1REK8
104494	229533.I1RBY4	pp	229533.I1RSC6
104495	229533.I1RBY4	pp	229533.I1RCR5
104496	229533.I1RBY4	pp	229533.I1RDG0
104497	229533.I1RBY4	pp	229533.Q4IJT5
104498	229533.I1RBY4	pp	229533.I1RX95
104499	229533.I1RBY4	pp	229533.I1RK95
104500	229533.I1RBY4	pp	229533.I1RM35
104501	229533.I1RBY4	pp	229533.I1RY03
104502	229533.I1RBY4	pp	229533.I1RWD7
104503	229533.I1RBY4	pp	229533.I1REL7
104504	229533.I1RBY4	pp	229533.Q4I2J8
104505	229533.I1RBY4	pp	229533.I1RIL8
104506	229533.I1RBY4	pp	229533.I1RRG3
104507	229533.I1RBY4	pp	229533.I1RW71
104508	229533.I1RBY4	pp	229533.I1RPN4
104509	229533.I1RBY4	pp	229533.I1S887
104510	229533.I1RBY4	pp	229533.I1RCG4
104511	229533.I1RBY4	pp	229533.I1RFU8
104512	229533.I1RBY4	pp	229533.I1RZK5
104513	229533.I1RBY4	pp	229533.I1S4S7
104514	229533.I1RBY4	pp	229533.Q4IJU0
104515	229533.I1RBY4	pp	229533.I1RSQ7
104516	229533.I1RBY4	pp	229533.I1RPY3
104517	229533.I1RBY4	pp	229533.I1S1J7
104518	229533.I1RBY4	pp	229533.I1RWB0
104519	229533.I1RBY4	pp	229533.I1S0R7
104520	229533.I1RBY4	pp	229533.I1RRX5
104521	229533.I1RBY4	pp	229533.I1RQC9
104522	229533.I1RBY4	pp	229533.I1RFC0
104523	229533.I1RBY4	pp	229533.I1RI03
104524	229533.I1RBY4	pp	229533.V6QW22
104525	229533.I1RBY4	pp	229533.I1RUQ2
104526	229533.I1RBY4	pp	229533.I1RLP1
104527	229533.I1RBY4	pp	229533.I1RYS0
104528	229533.I1RBY4	pp	229533.I1RR24
104529	229533.I1RBY4	pp	229533.I1RXD7
104530	229533.I1RBY4	pp	229533.I1RZ99
104531	229533.I1RBY4	pp	229533.I1RG53
104532	229533.I1RBY4	pp	229533.I1RTA0
104533	229533.I1RBY4	pp	229533.I1RVQ0
104534	229533.I1RBY4	pp	229533.I1RWD4
104535	229533.I1RBY4	pp	229533.V6RUT5
104536	229533.I1RBY4	pp	229533.I1RX46

104537 229533.I1RBY4 pp 229533.I1RW46
104538 229533.I1RBY4 pp 229533.I1RZS7
104539 229533.I1RBY4 pp 229533.V6R4J1
104540 229533.I1RBY4 pp 229533.I1RE01
104541 229533.I1RBY4 pp 229533.I1RD52
104542 229533.I1RBY4 pp 229533.I1S0T2
104543 229533.I1RBY4 pp 229533.I1RXD6
104544 229533.I1RBY4 pp 229533.I1S0J1
104545 229533.I1RBY4 pp 229533.I1S7A8
104546 229533.I1RBY4 pp 229533.I1RC72
104547 229533.I1RBY4 pp 229533.I1S0F5
104548 229533.I1RBY4 pp 229533.I1S7W5
104549 229533.I1RBY4 pp 229533.I1RCS1
104550 229533.I1RBY4 pp 229533.V6RTJ8
104551 229533.I1RBY4 pp 229533.I1S0L3
104552 229533.I1RBY4 pp 229533.I1RS17
104553 229533.I1RBY4 pp 229533.I1RPK0
104554 229533.I1RBY4 pp 229533.V6R1K4
104555 229533.I1RBY4 pp 229533.I1RMV0
104556 229533.I1RBY4 pp 229533.V6QYF0
104557 229533.I1RBY4 pp 229533.I1RLC7
104558 229533.I1RBY4 pp 229533.I1RPP4
104559 229533.I1RBY4 pp 229533.I1RM06
104560 229533.I1RBY4 pp 229533.I1RCR8
104561 229533.I1RBY4 pp 229533.I1RL23
104562 229533.I1RBY4 pp 229533.V6QUW4
104563 229533.I1RBY4 pp 229533.Q4HZB8
104564 229533.I1RBY4 pp 229533.I1RW25
104565 229533.I1RBY4 pp 229533.I1RX25
104566 229533.I1RBY4 pp 229533.I1RUG3
104567 229533.I1RBY4 pp 229533.I1S1W9
104568 229533.I1RBY4 pp 229533.I1RW07
104569 229533.I1RBY4 pp 229533.I1RWN5
104570 229533.I1RBY4 pp 229533.I1RXT3
104571 229533.I1RBY4 pp 229533.V6RIV9
104572 229533.I1RBY4 pp 229533.I1RNC1
104573 229533.I1RBY4 pp 229533.I1S0X6
104574 229533.I1RBY4 pp 229533.I1RI02
104575 229533.I1RBY4 pp 229533.I1RRV1
104576 229533.I1RBY4 pp 229533.I1RZV3
104577 229533.I1RBY4 pp 229533.I1RMC8
104578 229533.I1RBY4 pp 229533.I1RSI6
104579 229533.I1RBY4 pp 229533.I1RJU3
104580 229533.I1RBY4 pp 229533.I1RMG3
104581 229533.I1RBY4 pp 229533.I1RPU8
104582 229533.I1RBY4 pp 229533.I1RRM3
104583 229533.I1RBY4 pp 229533.I1RNV6
104584 229533.I1RBY4 pp 229533.I1RMK3
104585 229533.I1RBY4 pp 229533.I1S1W3
104586 229533.I1RBY4 pp 229533.I1RDB1
104587 229533.I1RBY4 pp 229533.I1RSA4
104588 229533.I1RBY4 pp 229533.V6RMW6
104589 229533.I1RBY4 pp 229533.I1RSQ3
104590 229533.I1RBY4 pp 229533.I1RDA0
104591 229533.I1RBY4 pp 229533.I1S4S4
104592 229533.I1RBY4 pp 229533.I1RC33
104593 229533.I1RBY4 pp 229533.I1RY44
104594 229533.I1RBY4 pp 229533.I1RWJ8
104595 229533.I1RBY4 pp 229533.I1RRE5
104596 229533.I1RBY4 pp 229533.V6R042
104597 229533.I1RBY4 pp 229533.I1RZJ5
104598 229533.I1RBY4 pp 229533.I1RM67
104599 229533.I1RBY4 pp 229533.I1RMN6
104600 229533.I1RBY4 pp 229533.I1S8V4
104601 229533.I1RBY4 pp 229533.I1SA33
104602 229533.I1RBY4 pp 229533.I1RMG8
104603 229533.I1RBY4 pp 229533.I1RPU2
104604 229533.I1RBY4 pp 229533.I1RD22
104605 229533.I1RBY4 pp 229533.I1RT03
104606 229533.I1RBY4 pp 229533.I1RXR1
104607 229533.I1RBY4 pp 229533.I1RRN2
104608 229533.I1RBY4 pp 229533.I1S5R6
104609 229533.I1RBY4 pp 229533.I1RWE1

104610 229533.I1RBY4 pp 229533.I1RVZ7
104611 229533.I1RBY4 pp 229533.I1RWQ8
104612 229533.I1RBY4 pp 229533.I1RVX0
104613 229533.I1RBY4 pp 229533.I1RG11
104614 229533.I1RBY4 pp 229533.I1RQ87
104615 229533.I1RBY4 pp 229533.V6RRH2
104616 229533.I1RBY4 pp 229533.I1RC81
104617 229533.I1RBY4 pp 229533.I1RJV3
104618 229533.I1RBY4 pp 229533.I1RGL4
104619 229533.I1RBY4 pp 229533.I1RSK1
104620 229533.I1RBY4 pp 229533.I1S0N4
104621 229533.I1RBY4 pp 229533.I1RN18
104622 229533.I1RBY4 pp 229533.I1RCD8
104623 229533.I1RBY4 pp 229533.I1RD17
104624 229533.I1RBY4 pp 229533.I1RFL4
104625 229533.I1RBY4 pp 229533.I1RXU9
104626 229533.I1RBY4 pp 229533.Q4I624
104627 229533.I1RBY4 pp 229533.I1RWN6
104628 229533.I1RBY4 pp 229533.I1RQE7
104629 229533.I1RBY4 pp 229533.I1S569
104630 229533.I1RBY4 pp 229533.V6R057
104631 229533.I1RBY4 pp 229533.I1S2E8
104632 229533.I1RBY4 pp 229533.I1RN63
104633 229533.I1RBY4 pp 229533.I1RCH5
104634 229533.I1RBY4 pp 229533.I1RD39
104635 229533.I1RBY4 pp 229533.I1S2M6
104636 229533.I1RBY4 pp 229533.I1RWK3
104637 229533.I1RBY4 pp 229533.I1RRN5
104638 229533.I1RBY4 pp 229533.I1RQ33
104639 229533.I1RBY4 pp 229533.I1S149
104640 229533.I1RBY4 pp 229533.I1RVZ2
104641 229533.I1RBY4 pp 229533.I1RZZ9
104642 229533.I1RBY4 pp 229533.I1RQ72
104643 229533.I1RBY4 pp 229533.I1RSQ4
104644 229533.I1RBY4 pp 229533.I1RTA1
104645 229533.I1RBY4 pp 229533.I1RJN9
104646 229533.I1RBY4 pp 229533.I1S1C7
104647 229533.I1RBY4 pp 229533.I1RYW3
104648 229533.I1RBY4 pp 229533.I1RWI5
104649 229533.I1RBY4 pp 229533.I1RSX1
104650 229533.I1RBY4 pp 229533.I1RFT1
104651 229533.I1RBY4 pp 229533.I1RCW6
104652 229533.I1RBY4 pp 229533.I1S352
104653 229533.I1RBY4 pp 229533.I1RZ79
104654 229533.I1RBY4 pp 229533.I1RNN1
104655 229533.I1RBY4 pp 229533.I1RZW8
104656 229533.I1RBY4 pp 229533.I1S8V3
104657 229533.I1RBY4 pp 229533.I1RRJ8
104658 229533.I1RBY4 pp 229533.I1RQG4
104659 229533.I1RBY4 pp 229533.I1RPP3
104660 229533.I1RBY4 pp 229533.I1RC66
104661 229533.I1RBY4 pp 229533.I1RWX5
104662 229533.I1RBY4 pp 229533.I1RRI2
104663 229533.I1RBY4 pp 229533.I1RFH8
104664 229533.I1RBY4 pp 229533.I1S8P5
104665 229533.I1RBY4 pp 229533.I1RRL9
104666 229533.I1RBY4 pp 229533.I1S8R3
104667 229533.I1RBY4 pp 229533.I1RTH6
104668 229533.I1RBY4 pp 229533.I1RYI5
104669 229533.I1RBY4 pp 229533.I1RP28
104670 229533.I1RBY4 pp 229533.I1RZ39
104671 229533.I1RBY4 pp 229533.I1RCL5
104672 229533.I1RBY4 pp 229533.I1RDF7
104673 229533.I1RBY4 pp 229533.I1S0W4
104674 229533.I1RBY4 pp 229533.I1S0F6
104675 229533.I1RBY4 pp 229533.I1RN67
104676 229533.I1RBY4 pp 229533.V6R3X5
104677 229533.I1RBY4 pp 229533.I1RQ65
104678 229533.I1RBY4 pp 229533.I1RS47
104679 229533.I1RBY4 pp 229533.I1RR73
104680 229533.I1RBY4 pp 229533.I1RC05
104681 229533.I1RBY4 pp 229533.I1RM82
104682 229533.I1RBY4 pp 229533.I1RCJ7

104683	229533.I1RBY4	pp	229533.I1RJV4
104684	229533.I1RBY4	pp	229533.I1RZB2
104685	229533.I1RBY4	pp	229533.I1S9Y1
104686	229533.I1RBY4	pp	229533.I1RV91
104687	229533.I1RBY4	pp	229533.I1RFL3
104688	229533.I1RBY4	pp	229533.I1RGF5
104689	229533.I1RBY4	pp	229533.I1RRR9
104690	229533.I1RBY4	pp	229533.I1RTD5
104691	229533.I1RBY4	pp	229533.I1RQM7
104692	229533.I1RBY4	pp	229533.I1RJ74
104693	229533.I1RBY4	pp	229533.I1RW61
104694	229533.I1RBY4	pp	229533.O74638
104695	229533.I1RBY4	pp	229533.I1RSZ7
104696	229533.I1RBY4	pp	229533.I1RXC2
104697	229533.I1RBY4	pp	229533.I1RT04
104698	229533.I1RBY4	pp	229533.Q4IR18
104699	229533.I1RBY4	pp	229533.I1RRT8
104700	229533.I1RBY4	pp	229533.I1RPV3
104701	229533.I1RBY4	pp	229533.V6RUY9
104702	229533.I1RBY4	pp	229533.I1RPM4
104703	229533.I1RBY4	pp	229533.I1S6Y8
104704	229533.I1RBY4	pp	229533.I1RNA5
104705	229533.I1RBY4	pp	229533.I1S2E9
104706	229533.I1RBY4	pp	229533.I1S5R1
104707	229533.I1RBY4	pp	229533.I1RS20
104708	229533.I1RBY4	pp	229533.I1RL04
104709	229533.I1RBY4	pp	229533.I1S503
104710	229533.I1RBY4	pp	229533.I1RFS3
104711	229533.I1RBY4	pp	229533.I1RQ23
104712	229533.I1RBY4	pp	229533.I1RJW6
104713	229533.I1RBY4	pp	229533.I1RE22
104714	229533.I1RBY4	pp	229533.I1RPE0
104715	229533.I1RBY4	pp	229533.I1S2H4
104716	229533.I1RBY4	pp	229533.I1S0M6
104717	229533.I1RBY4	pp	229533.I1RRZ0
104718	229533.I1RBY4	pp	229533.I1S2B8
104719	229533.I1RBY4	pp	229533.I1RLN6
104720	229533.I1RBY4	pp	229533.V6R3J1
104721	229533.I1RBY4	pp	229533.I1RRM5
104722	229533.I1RBY4	pp	229533.I1RQS8
104723	229533.I1RBY4	pp	229533.I1RJZ7
104724	229533.I1RBY4	pp	229533.I1REC5
104725	229533.I1RBY4	pp	229533.I1RSX0
104726	229533.I1RBY4	pp	229533.I1RGF6
104727	229533.I1RBY4	pp	229533.I1RE76
104728	229533.I1RBY4	pp	229533.I1RRT2
104729	229533.I1RBY4	pp	229533.I1RPU3
104730	229533.I1RBY4	pp	229533.I1RC00
104731	229533.I1RBY4	pp	229533.I1RE27
104732	229533.I1RBY4	pp	229533.I1RRI1
104733	229533.I1RBY4	pp	229533.I1S4Z8
104734	229533.I1RBY4	pp	229533.I1RGA4
104735	229533.I1RBY4	pp	229533.I1RC82
104736	229533.I1RBY4	pp	229533.I1S0L4
104737	229533.I1RBY4	pp	229533.I1RIL3
104738	229533.A0A0E0SGX8	pp	229533.I1RUB1
104739	229533.A0A0E0SGX8	pp	229533.I1RH30
104740	229533.A0A0E0SGX8	pp	229533.I1S8P4
104741	229533.A0A0E0SGX8	pp	229533.I1RH32
104742	229533.A0A0E0SGX8	pp	229533.A0A1C3YMK9
104743	229533.A0A0E0SGX8	pp	229533.A0A1C3YMS7
104744	229533.A0A0E0SGX8	pp	229533.A0A1C3YMJ9
104745	229533.A0A0E0SGX8	pp	229533.A0A1C3YMP8
104746	229533.A0A0E0SGX8	pp	229533.I1RUB4
104747	229533.A0A0E0SGX8	pp	229533.I1S6P7
104748	229533.A0A0E0SGX8	pp	229533.I1S8P5
104749	229533.A0A0E0SGX8	pp	229533.A0A1C3YMK0
104750	229533.A0A0E0SGX8	pp	229533.I1RUB5
104751	229533.A0A0E0SGX8	pp	229533.I1RUB2
104752	229533.I1RAT5	pp	229533.I1RE23
104753	229533.I1RAT5	pp	229533.I1RSC7
104754	229533.I1RAT5	pp	229533.I1S209
104755	229533.I1RAT5	pp	229533.I1RBW1

104756	229533.I1RAT5	pp	229533.I1S183
104757	229533.I1RAT5	pp	229533.I1RZP8
104758	229533.I1RAT5	pp	229533.I1S788
104759	229533.I1RAT5	pp	229533.I1RZ94
104760	229533.I1RAT5	pp	229533.I1RX64
104761	229533.I1RAT5	pp	229533.I1RCA5
104762	229533.I1RAT5	pp	229533.I1RSP7
104763	229533.I1RAT5	pp	229533.I1RSR9
104764	229533.I1RAT5	pp	229533.I1RRT1
104765	229533.I1RAT5	pp	229533.I1RVZ9
104766	229533.I1RAT5	pp	229533.I1RRJ6
104767	229533.I1RAT5	pp	229533.I1RKI2
104768	229533.I1RAT5	pp	229533.I1RBW0
104769	229533.I1RAT5	pp	229533.I1RPF5
104770	229533.I1RAT5	pp	229533.I1RPQ1
104771	229533.I1RAT5	pp	229533.I1RSN7
104772	229533.I1RRJ8	pp	229533.I1RXD7
104773	229533.I1RRJ8	pp	229533.I1S0R7
104774	229533.I1RRJ8	pp	229533.I1RT04
104775	229533.I1RRJ8	pp	229533.I1RUQ2
104776	229533.I1RRJ8	pp	229533.I1RT40
104777	229533.I1RRJ8	pp	229533.I1RTD5
104778	229533.I1RRJ8	pp	229533.I1RV91
104779	229533.I1RRJ8	pp	229533.Q4INZ9
104780	229533.I1RRJ8	pp	229533.I1RT98
104781	229533.I1RRJ8	pp	229533.Q4IQW0
104782	229533.I1RRJ8	pp	229533.I1S0L3
104783	229533.I1RRJ8	pp	229533.V6RTJ8
104784	229533.I1RRJ8	pp	229533.I1S1B6
104785	229533.I1RRJ8	pp	229533.I1S0D4
104786	229533.I1RRJ8	pp	229533.Q4IPI1
104787	229533.I1RRJ8	pp	229533.Q4IEV5
104788	229533.I1RRJ8	pp	229533.V6R1K4
104789	229533.I1RRJ8	pp	229533.I1RRT2
104790	229533.I1RRJ8	pp	229533.Q4HZ42
104791	229533.I1RRJ8	pp	229533.I1S0T2
104792	229533.I1RRJ8	pp	229533.V6QW22
104793	229533.I1RRJ8	pp	229533.I1RT33
104794	229533.I1RRJ8	pp	229533.I1RS47
104795	229533.I1RRJ8	pp	229533.I1RSQ7
104796	229533.I1RRJ8	pp	229533.I1RX46
104797	229533.I1RRJ8	pp	229533.I1S6F0
104798	229533.I1RRJ8	pp	229533.I1S503
104799	229533.I1RRJ8	pp	229533.I1RVQ0
104800	229533.I1RRJ8	pp	229533.I1RRZ0
104801	229533.I1RRJ8	pp	229533.I1RZ99
104802	229533.I1RRJ8	pp	229533.I1RRQ3
104803	229533.I1RRJ8	pp	229533.Q4I830
104804	229533.I1RRJ8	pp	229533.I1RSX1
104805	229533.I1RRJ8	pp	229533.Q4HZ47
104806	229533.I1RRJ8	pp	229533.I1S0F6
104807	229533.I1RRJ8	pp	229533.Q4IAS1
104808	229533.I1RRJ8	pp	229533.V6R042
104809	229533.I1RRJ8	pp	229533.I1RW71
104810	229533.I1RRJ8	pp	229533.V6R283
104811	229533.I1RRJ8	pp	229533.I1RSQ3
104812	229533.I1RRJ8	pp	229533.I1S2M6
104813	229533.I1RRJ8	pp	229533.V6RMW6
104814	229533.I1RRJ8	pp	229533.I1RRP3
104815	229533.I1RRJ8	pp	229533.I1RS30
104816	229533.I1RRJ8	pp	229533.I1RWK3
104817	229533.I1RRJ8	pp	229533.I1RWD4
104818	229533.I1RRJ8	pp	229533.I1S9U2
104819	229533.I1RRJ8	pp	229533.V6RCG5
104820	229533.I1RRJ8	pp	229533.I1RXF7
104821	229533.I1RRJ8	pp	229533.I1RRM5
104822	229533.I1RRJ8	pp	229533.I1RSI6
104823	229533.I1RRJ8	pp	229533.Q4IFI0
104824	229533.I1RRJ8	pp	229533.V6RCZ8
104825	229533.I1RRJ8	pp	229533.I1S0L4
104826	229533.I1RRJ8	pp	229533.I1RX08
104827	229533.I1RRJ8	pp	229533.I1S2B8
104828	229533.I1RRJ8	pp	229533.I1RW41

104829	229533.I1RRJ8	pp	229533.I1S021
104830	229533.I1RRJ8	pp	229533.I1S2H4
104831	229533.I1RRJ8	pp	229533.I1RZS6
104832	229533.I1RRJ8	pp	229533.Q4IQK7
104833	229533.I1RRJ8	pp	229533.I1RRT8
104834	229533.I1RRJ8	pp	229533.I1S5R1
104835	229533.I1RRJ8	pp	229533.I1RRQ6
104836	229533.I1RRJ8	pp	229533.I1RYB9
104837	229533.I1RRJ8	pp	229533.V6QYF0
104838	229533.I1RRJ8	pp	229533.I1RWJ8
104839	229533.I1RRJ8	pp	229533.Q4I327
104840	229533.I1RRJ8	pp	229533.I1RX24
104841	229533.I1RRJ8	pp	229533.I1RZM8
104842	229533.I1RRJ8	pp	229533.V6R3X5
104843	229533.I1RRJ8	pp	229533.I1S6Y8
104844	229533.I1RRJ8	pp	229533.Q4I662
104845	229533.I1RRJ8	pp	229533.I1RW61
104846	229533.I1RRJ8	pp	229533.Q4HVW2
104847	229533.I1RRJ8	pp	229533.Q4IN63
104848	229533.I1RRJ8	pp	229533.I1RW99
104849	229533.I1RRJ8	pp	229533.I1RUG3
104850	229533.I1RRJ8	pp	229533.Q4IJH1
104851	229533.I1RRJ8	pp	229533.I1RUQ9
104852	229533.I1RRJ8	pp	229533.I1RTF4
104853	229533.I1RRJ8	pp	229533.V6RGN5
104854	229533.I1RRJ8	pp	229533.I1RRR7
104855	229533.I1RRJ8	pp	229533.I1S0N4
104856	229533.I1RRJ8	pp	229533.I1RT80
104857	229533.I1RRJ8	pp	229533.I1RZ39
104858	229533.I1RRJ8	pp	229533.I1S044
104859	229533.I1RRJ8	pp	229533.I1RU69
104860	229533.I1RRJ8	pp	229533.V6R227
104861	229533.I1RRJ8	pp	229533.Q4IF76
104862	229533.I1RRJ8	pp	229533.I1S5B0
104863	229533.I1RRJ8	pp	229533.I1S255
104864	229533.I1RRJ8	pp	229533.I1RT05
104865	229533.I1RRJ8	pp	229533.I1S504
104866	229533.I1RRJ8	pp	229533.I1S9U1
104867	229533.I1RRJ8	pp	229533.Q4IBS2
104868	229533.I1RRJ8	pp	229533.I1RTG6
104869	229533.I1RRJ8	pp	229533.I1RZF6
104870	229533.I1RRJ8	pp	229533.Q4HZI0
104871	229533.I1RRJ8	pp	229533.Q4I5I4
104872	229533.I1RRJ8	pp	229533.I1RW25
104873	229533.I1RRJ8	pp	229533.I1RZZ9
104874	229533.I1RRJ8	pp	229533.V6QUE6
104875	229533.I1RRJ8	pp	229533.I1RZM1
104876	229533.I1RRJ8	pp	229533.I1SA33
104877	229533.I1RRJ8	pp	229533.Q4IEK8
104878	229533.I1RRJ8	pp	229533.I1RTH6
104879	229533.I1RRJ8	pp	229533.I1RZB5
104880	229533.I1RRJ8	pp	229533.Q4I8X0
104881	229533.I1RRJ8	pp	229533.I1S8R3
104882	229533.I1RRJ8	pp	229533.Q4HZK7
104883	229533.I1RRJ8	pp	229533.Q4I8P5
104884	229533.I1RRJ8	pp	229533.I1S419
104885	229533.I1RRJ8	pp	229533.Q4IAA0
104886	229533.I1RRJ8	pp	229533.I1RZW8
104887	229533.I1RRJ8	pp	229533.V6RIV9
104888	229533.I1RRJ8	pp	229533.I1RS17
104889	229533.I1RRJ8	pp	229533.I1RWN5
104890	229533.I1RRJ8	pp	229533.I1RWB5
104891	229533.I1RRJ8	pp	229533.I1RSR8
104892	229533.I1RRJ8	pp	229533.I1RS64
104893	229533.I1RRJ8	pp	229533.I1RY03
104894	229533.I1RRJ8	pp	229533.Q4IJ56
104895	229533.I1RRJ8	pp	229533.I1S4T3
104896	229533.I1RRJ8	pp	229533.I1RTD9
104897	229533.I1RRJ8	pp	229533.I1RS18
104898	229533.I1RRJ8	pp	229533.I1SA32
104899	229533.I1RRJ8	pp	229533.I1RSC6
104900	229533.I1RRJ8	pp	229533.I1S0J1
104901	229533.I1RRJ8	pp	229533.Q4I624

229533.I1RRJ8	pp	229533.I1RSS4
229533.I1RRJ8	pp	229533.I1S1C7
229533.I1RRJ8	pp	229533.I1S247
229533.I1RRJ8	pp	229533.I1RW46
229533.I1RRJ8	pp	229533.I1RVX0
229533.I1RRJ8	pp	229533.I1RTG5
229533.I1RRJ8	pp	229533.I1S060
229533.I1RRJ8	pp	229533.I1RT03
229533.I1RRJ8	pp	229533.I1RX14
229533.I1RRJ8	pp	229533.I1S296
229533.I1RRJ8	pp	229533.Q4HZ68
229533.I1RRJ8	pp	229533.V6RGY3
229533.I1RRJ8	pp	229533.I1RSR3
229533.I1RRJ8	pp	229533.V6R057
229533.I1RRJ8	pp	229533.I1RY63
229533.I1RRJ8	pp	229533.I1S2E8
229533.I1RRJ8	pp	229533.I1RWN6
229533.I1RRJ8	pp	229533.I1S1X9
229533.I1RRJ8	pp	229533.I1RZE5
229533.I1RRJ8	pp	229533.I1RRY9
229533.I1RRJ8	pp	229533.Q4I5Z5
229533.I1RRJ8	pp	229533.I1S1J6
229533.I1RRJ8	pp	229533.I1RZK6
229533.I1RRJ8	pp	229533.I1RZZ1
229533.I1RRJ8	pp	229533.I1S2C0
229533.I1RRJ8	pp	229533.I1RTE9
229533.I1RRJ8	pp	229533.I1S2U2
229533.I1RRJ8	pp	229533.I1RZK5
229533.I1RRJ8	pp	229533.I1RSD8
229533.I1RRJ8	pp	229533.Q4IJK2
229533.I1RRJ8	pp	229533.I1RVZ0
229533.A0A0E0SKH0	pp	229533.Q4IEA7
229533.A0A0E0SKH0	pp	229533.I1RBF2
229533.A0A0E0SKH0	pp	229533.I1S286
229533.A0A0E0SKH0	pp	229533.A0A1C3YKU7
229533.A0A0E0SKH0	pp	229533.A0A1C3YID3
229533.A0A0E0SKH0	pp	229533.I1S1V4
229533.A0A0E0SKH0	pp	229533.I1RY87
229533.I1SAM9	pp	229533.Q4HZE0
229533.I1RE76	pp	229533.I1RTH6
229533.I1RE76	pp	229533.V6R057
229533.I1RE76	pp	229533.I1S8R3
229533.I1RE76	pp	229533.Q4I624
229533.I1RE76	pp	229533.I1RRI2
229533.I1RE76	pp	229533.I1RZW8
229533.I1RE76	pp	229533.I1RWX5
229533.I1RE76	pp	229533.V6RRH2
229533.I1RE76	pp	229533.I1RG90
229533.I1RE76	pp	229533.I1RT03
229533.I1RE76	pp	229533.I1S1C7
229533.I1RE76	pp	229533.I1RVX0
229533.I1RE76	pp	229533.I1RYW3
229533.I1RE76	pp	229533.I1S8V4
229533.I1RE76	pp	229533.I1REF5
229533.I1RE76	pp	229533.I1RQE7
229533.I1RE76	pp	229533.I1RRE5
229533.I1RE76	pp	229533.I1RFL4
229533.I1RE76	pp	229533.I1RN18
229533.I1RE76	pp	229533.I1RJV3
229533.I1RE76	pp	229533.I1RWN6
229533.I1RE76	pp	229533.V6R3X5
229533.I1RE76	pp	229533.I1S5R1
229533.I1RE76	pp	229533.I1RY44
229533.I1RE76	pp	229533.I1S1W9
229533.I1RE76	pp	229533.I1RSQ3
229533.I1RE76	pp	229533.V6RMW6
229533.I1RE76	pp	229533.I1RWN5
229533.I1RE76	pp	229533.I1RR73
229533.I1RE76	pp	229533.V6RIV9
229533.I1RE76	pp	229533.I1RZ39
229533.I1RE76	pp	229533.V6R042
229533.I1RE76	pp	229533.I1S8V3
229533.I1RE76	pp	229533.I1RPP4

104975	229533.I1RE76	pp	229533.I1RRV1
104976	229533.I1RE76	pp	229533.I1RFT6
104977	229533.I1RE76	pp	229533.I1RUG3
104978	229533.I1RE76	pp	229533.I1RNC1
104979	229533.I1RE76	pp	229533.I1RPK0
104980	229533.I1RE76	pp	229533.I1RZZ9
104981	229533.I1RE76	pp	229533.I1RS17
104982	229533.I1RE76	pp	229533.I1RRM5
104983	229533.I1RE76	pp	229533.I1S2B8
104984	229533.I1RE76	pp	229533.I1RWI5
104985	229533.I1RE76	pp	229533.I1RSQ4
104986	229533.I1RE76	pp	229533.I1RJZ7
104987	229533.I1RE76	pp	229533.I1S0J1
104988	229533.I1RE76	pp	229533.I1RS47
104989	229533.I1RE76	pp	229533.I1RN88
104990	229533.I1RE76	pp	229533.I1RP28
104991	229533.I1RE76	pp	229533.I1RW46
104992	229533.I1RE76	pp	229533.I1RY62
104993	229533.I1RE76	pp	229533.I1RTA0
104994	229533.I1RE76	pp	229533.I1S0N4
104995	229533.I1RE76	pp	229533.I1RPM4
104996	229533.I1RE76	pp	229533.I1S6Y8
104997	229533.I1RE76	pp	229533.I1RSZ7
104998	229533.I1RE76	pp	229533.I1RPD6
104999	229533.I1RE76	pp	229533.I1RFS3
105000	229533.I1RE76	pp	229533.I1RN67
105001	229533.I1RE76	pp	229533.I1RNG4
105002	229533.I1RE76	pp	229533.I1S0L4
105003	229533.I1RE76	pp	229533.I1S5R6
105004	229533.I1RE76	pp	229533.I1S0F6
105005	229533.I1RE76	pp	229533.I1RFW3
105006	229533.I1RE76	pp	229533.I1S2H4
105007	229533.I1RE76	pp	229533.I1S256
105008	229533.I1RE76	pp	229533.I1S8P5
105009	229533.I1RE76	pp	229533.I1RPP3
105010	229533.I1RE76	pp	229533.I1RXC2
105011	229533.I1RE76	pp	229533.I1SA33
105012	229533.I1RE76	pp	229533.I1RYI5
105013	229533.I1RE76	pp	229533.I1RZZ1
105014	229533.I1RE76	pp	229533.I1RP27
105015	229533.I1RE76	pp	229533.I1RRT8
105016	229533.I1RE76	pp	229533.Q4IJK2
105017	229533.I1RE76	pp	229533.I1RQG4
105018	229533.I1RE76	pp	229533.I1RR95
105019	229533.I1RE76	pp	229533.I1RFT1
105020	229533.I1RE76	pp	229533.I1RTA1
105021	229533.I1RE76	pp	229533.I1RJV4
105022	229533.I1RE76	pp	229533.I1RVZ2
105023	229533.I1RE76	pp	229533.I1RQA3
105024	229533.I1RE76	pp	229533.I1RWK3
105025	229533.I1RE76	pp	229533.I1RRY9
105026	229533.I1RE76	pp	229533.I1RZK5
105027	229533.I1RE76	pp	229533.I1RXG6
105028	229533.I1RE76	pp	229533.I1RGA4
105029	229533.I1RE76	pp	229533.I1RN63
105030	229533.I1RE76	pp	229533.I1RFU8
105031	229533.I1RE76	pp	229533.I1RE87
105032	229533.I1RE76	pp	229533.I1S503
105033	229533.I1RE76	pp	229533.I1RQ81
105034	229533.I1RE76	pp	229533.I1S2E9
105035	229533.I1RE76	pp	229533.Q4I963
105036	229533.I1RE76	pp	229533.I1RJW6
105037	229533.I1RE76	pp	229533.I1RY03
105038	229533.I1RE76	pp	229533.I1RXC4
105039	229533.I1RE76	pp	229533.I1SA32
105040	229533.I1RE76	pp	229533.I1RWT6
105041	229533.I1RE76	pp	229533.I1RT04
105042	229533.I1RE76	pp	229533.I1S504
105043	229533.I1RE76	pp	229533.I1RTD5
105044	229533.I1RE76	pp	229533.I1S5B0
105045	229533.I1RE76	pp	229533.I1RV91
105046	229533.I1RE76	pp	229533.I1RNQ5
105047	229533.I1RE76	pp	229533.I1RUY5

229533.I1RE76 pp 229533.I1RW99
229533.I1RE76 pp 229533.I1RT40
229533.I1RE76 pp 229533.V6QUE6
229533.I1RE76 pp 229533.I1RLU6
229533.I1RE76 pp 229533.I1RRU9
229533.I1RE76 pp 229533.I1RF19
229533.I1RE76 pp 229533.Q4ICA8
229533.I1RE76 pp 229533.I1REK8
229533.I1RE76 pp 229533.I1RZM1
229533.I1RE76 pp 229533.I1S4Q4
229533.I1RE76 pp 229533.V6RCG5
229533.I1RE76 pp 229533.I1REL7
229533.I1RE76 pp 229533.I1RR51
229533.I1RE76 pp 229533.I1S419
229533.I1RE76 pp 229533.I1RR24
229533.I1RE76 pp 229533.Q4I2J8
229533.I1RE76 pp 229533.Q4I8P5
229533.I1RE76 pp 229533.V6RCZ8
229533.I1RE76 pp 229533.I1RS18
229533.I1RE76 pp 229533.I1RI82
229533.I1RE76 pp 229533.I1S4S4
229533.I1RE76 pp 229533.I1RZM8
229533.I1RE76 pp 229533.I1S4S7
229533.I1RE76 pp 229533.I1RWJ8
229533.I1RE76 pp 229533.V6QUW4
229533.I1RE76 pp 229533.I1RW25
229533.I1RE76 pp 229533.I1S0X6
229533.I1RE76 pp 229533.I1RJU3
229533.I1RE76 pp 229533.Q4IJT5
229533.I1RE76 pp 229533.V6R1K4
229533.I1RE76 pp 229533.V6RTJ8
229533.I1RE76 pp 229533.I1RRG3
229533.I1RE76 pp 229533.I1RX46
229533.I1RE76 pp 229533.I1S0L3
229533.I1RE76 pp 229533.I1RSQ7
229533.I1RE76 pp 229533.I1RVQ0
229533.I1RE76 pp 229533.I1S0T2
229533.I1RE76 pp 229533.V6QW22
229533.I1RE76 pp 229533.I1RQC9
229533.I1RE76 pp 229533.I1RXD7
229533.I1RE76 pp 229533.I1S0R7
229533.I1RE76 pp 229533.I1RW71
229533.I1RVZ9 pp 229533.I1S072
229533.I1RVZ9 pp 229533.I1S0Q5
229533.I1RVZ9 pp 229533.I1RZ86
229533.I1RVZ9 pp 229533.I1RXV3
229533.I1RVZ9 pp 229533.Q4HXF0
229533.I1RVZ9 pp 229533.I1S0Q4
229533.I1RVZ9 pp 229533.I1RXF0
229533.I1RVZ9 pp 229533.V6RVJ3
229533.I1RVZ9 pp 229533.I1S209
229533.I1RVZ9 pp 229533.Q4I0J6
229533.I1RVZ9 pp 229533.I1S4P6
229533.I1RVZ9 pp 229533.I1RX64
229533.A0A0E0SAE0 pp 229533.I1S8P4
229533.A0A0E0SAE0 pp 229533.I1RJA2
229533.A0A0E0SAE0 pp 229533.I1RUD3
229533.A0A0E0SAE0 pp 229533.I1RW91
229533.A0A0E0SAE0 pp 229533.I1REM5
229533.A0A0E0SAE0 pp 229533.I1R994
229533.A0A0E0SAE0 pp 229533.I1RH98
229533.A0A0E0SAE0 pp 229533.I1RIZ1
229533.A0A0E0SAE0 pp 229533.I1RPW8
229533.A0A0E0SAE0 pp 229533.I1RJQ9
229533.A0A0E0SAE0 pp 229533.I1REM3
229533.A0A0E0SAE0 pp 229533.I1RN04
229533.A0A0E0SAE0 pp 229533.I1RCG6
229533.A0A0E0SAE0 pp 229533.I1RCQ5
229533.A0A0E0SAE0 pp 229533.I1REL9
229533.A0A0E0SAE0 pp 229533.I1RUB4
229533.A0A0E0SAE0 pp 229533.I1RRF7
229533.A0A0E0SAE0 pp 229533.I1RG46
229533.I1RPU3 pp 229533.I1RT03

105121 229533.I1RPU3 pp 229533.I1RRN5
105122 229533.I1RPU3 pp 229533.I1RTA0
105123 229533.I1RPU3 pp 229533.I1S0J1
105124 229533.I1RPU3 pp 229533.I1RVX0
105125 229533.I1RPU3 pp 229533.I1RQ81
105126 229533.I1RPU3 pp 229533.I1RSI0
105127 229533.I1RPU3 pp 229533.Q4IJM4
105128 229533.I1RPU3 pp 229533.I1RY44
105129 229533.I1RPU3 pp 229533.I1RZM1
105130 229533.I1RPU3 pp 229533.I1RY03
105131 229533.I1RPU3 pp 229533.I1RRI2
105132 229533.I1RPU3 pp 229533.I1RYY5
105133 229533.I1RPU3 pp 229533.I1S1C7
105134 229533.I1RPU3 pp 229533.I1RWN5
105135 229533.I1RPU3 pp 229533.V6RKY7
105136 229533.I1RPU3 pp 229533.I1RYW3
105137 229533.I1RPU3 pp 229533.V6RBN2
105138 229533.I1RPU3 pp 229533.I1RWT6
105139 229533.I1RPU3 pp 229533.I1RZE4
105140 229533.I1RPU3 pp 229533.I1RT35
105141 229533.I1RPU3 pp 229533.I1RZZ1
105142 229533.I1RPU3 pp 229533.I1RZK5
105143 229533.I1RPU3 pp 229533.I1S504
105144 229533.I1RPU3 pp 229533.I1RQA3
105145 229533.I1RPU3 pp 229533.I1RTH6
105146 229533.I1RPU3 pp 229533.I1RSK1
105147 229533.I1RPU3 pp 229533.I1RUG3
105148 229533.I1RPU3 pp 229533.I1S1W4
105149 229533.I1RPU3 pp 229533.I1RRY9
105150 229533.I1RPU3 pp 229533.Q4IJK2
105151 229533.I1RPU3 pp 229533.I1RWN6
105152 229533.I1RPU3 pp 229533.I1RRU9
105153 229533.I1RPU3 pp 229533.I1RSQ3
105154 229533.I1RPU3 pp 229533.I1S0N4
105155 229533.I1RPU3 pp 229533.I1RSI4
105156 229533.I1RPU3 pp 229533.V6R042
105157 229533.I1RPU3 pp 229533.I1S5B0
105158 229533.I1RPU3 pp 229533.V6RMW6
105159 229533.I1RPU3 pp 229533.I1RQ26
105160 229533.I1RPU3 pp 229533.I1RWK3
105161 229533.I1RPU3 pp 229533.I1RRV1
105162 229533.I1RPU3 pp 229533.I1RRJ8
105163 229533.I1RPU3 pp 229533.I1S044
105164 229533.I1RPU3 pp 229533.V6QW22
105165 229533.I1RPU3 pp 229533.I1RT04
105166 229533.I1RPU3 pp 229533.I1RTG3
105167 229533.I1RPU3 pp 229533.I1S0R7
105168 229533.I1RPU3 pp 229533.I1RW25
105169 229533.I1RPU3 pp 229533.I1RSQ7
105170 229533.I1RPU3 pp 229533.I1RX46
105171 229533.I1RPU3 pp 229533.I1RQC9
105172 229533.I1RPU3 pp 229533.I1S8P5
105173 229533.I1RPU3 pp 229533.I1RSV2
105174 229533.I1RPU3 pp 229533.I1S503
105175 229533.I1RPU3 pp 229533.I1RYH9
105176 229533.I1RPU3 pp 229533.I1RVQ0
105177 229533.I1RPU3 pp 229533.I1S2B8
105178 229533.I1RPU3 pp 229533.I1S2G7
105179 229533.I1RPU3 pp 229533.I1RWI5
105180 229533.I1RPU3 pp 229533.V6RTJ8
105181 229533.I1RPU3 pp 229533.I1RR73
105182 229533.I1RPU3 pp 229533.I1S0L3
105183 229533.I1RPU3 pp 229533.I1RZZ9
105184 229533.I1RPU3 pp 229533.I1RXU7
105185 229533.I1RPU3 pp 229533.I1RTD5
105186 229533.I1RPU3 pp 229533.I1RZ39
105187 229533.I1RPU3 pp 229533.I1RT40
105188 229533.I1RPU3 pp 229533.V6R3X5
105189 229533.I1RPU3 pp 229533.I1S2E9
105190 229533.I1RPU3 pp 229533.I1RWL2
105191 229533.I1RPU3 pp 229533.I1RXD7
105192 229533.I1RPU3 pp 229533.I1RQH3
105193 229533.I1RPU3 pp 229533.I1RRT8

105194 229533.I1RCM7 pp 229533.I1RVZ9
105195 229533.I1RCM7 pp 229533.I1RSY2
105196 229533.I1RCM7 pp 229533.I1RDH0
105197 229533.I1RCM7 pp 229533.I1S0Q4
105198 229533.I1RCM7 pp 229533.I1S0D5
105199 229533.I1RCM7 pp 229533.I1S072
105200 229533.I1RCM7 pp 229533.I1S183
105201 229533.I1RCM7 pp 229533.I1RTN6
105202 229533.I1RCM7 pp 229533.I1S209
105203 229533.I1RCM7 pp 229533.I1RSC7
105204 229533.I1RCM7 pp 229533.I1RSF1
105205 229533.I1RCM7 pp 229533.V6RG79
105206 229533.I1RCM7 pp 229533.I1RM26
105207 229533.I1RCM7 pp 229533.I1RRY1
105208 229533.I1RCM7 pp 229533.I1RQI6
105209 229533.I1RCM7 pp 229533.Q4INS6
105210 229533.I1RCM7 pp 229533.I1RVW0
105211 229533.I1RCM7 pp 229533.I1S699
105212 229533.I1RCM7 pp 229533.I1RKP1
105213 229533.I1RCM7 pp 229533.I1S9F9
105214 229533.I1RCM7 pp 229533.I1RKI2
105215 229533.I1RCM7 pp 229533.I1RVX9
105216 229533.I1RCM7 pp 229533.I1RY29
105217 229533.I1RCM7 pp 229533.I1RCM8
105218 229533.I1RCM7 pp 229533.I1S0Q5
105219 229533.I1RCM7 pp 229533.I1RXV3
105220 229533.I1RCM7 pp 229533.I1RWI8
105221 229533.A0A1C3YMJ9 pp 229533.I1RUB4
105222 229533.A0A1C3YMJ9 pp 229533.A0A1C3YMK9
105223 229533.A0A1C3YMJ9 pp 229533.I1S6P7
105224 229533.A0A1C3YMJ9 pp 229533.A0A1C3YMK0
105225 229533.A0A1C3YMJ9 pp 229533.I1RUB5
105226 229533.A0A1C3YMJ9 pp 229533.I1S8P4
105227 229533.A0A1C3YMJ9 pp 229533.A0A1C3YMP8
105228 229533.A0A1C3YMJ9 pp 229533.I1RUB1
105229 229533.A0A1C3YMJ9 pp 229533.I1RH30
105230 229533.A0A1C3YMJ9 pp 229533.I1S8P5
105231 229533.A0A1C3YMJ9 pp 229533.A0A1C3YMS7
105232 229533.A0A1C3YMJ9 pp 229533.I1RH32
105233 229533.I1RQG4 pp 229533.I1RW25
105234 229533.I1RQG4 pp 229533.I1RZ02
105235 229533.I1RQG4 pp 229533.I1RX46
105236 229533.I1RQG4 pp 229533.I1RVQ0
105237 229533.I1RQG4 pp 229533.I1RQQ8
105238 229533.I1RQG4 pp 229533.I1S0R7
105239 229533.I1RQG4 pp 229533.I1S270
105240 229533.I1RQG4 pp 229533.I1RT41
105241 229533.I1RQG4 pp 229533.V6RTJ8
105242 229533.I1RQG4 pp 229533.Q5I2J3
105243 229533.I1RQG4 pp 229533.I1S0B7
105244 229533.I1RQG4 pp 229533.I1RYZ9
105245 229533.I1RQG4 pp 229533.Q4HZS8
105246 229533.I1RQG4 pp 229533.V6QW22
105247 229533.I1RQG4 pp 229533.I1RSQ7
105248 229533.I1RQG4 pp 229533.I1S5B0
105249 229533.I1RQG4 pp 229533.I1RQQ6
105250 229533.I1RQG4 pp 229533.I1RW71
105251 229533.I1RQG4 pp 229533.I1RQH1
105252 229533.I1RQG4 pp 229533.I1S0Z3
105253 229533.I1RQG4 pp 229533.I1RYJ8
105254 229533.I1RQG4 pp 229533.V6QV09
105255 229533.I1RQG4 pp 229533.I1S0X6
105256 229533.I1RQG4 pp 229533.V6QUE6
105257 229533.I1RQG4 pp 229533.I1S235
105258 229533.I1RQG4 pp 229533.I1RW87
105259 229533.I1RQG4 pp 229533.I1RZM1
105260 229533.I1RQG4 pp 229533.I1RZB5
105261 229533.I1RQG4 pp 229533.I1RYS6
105262 229533.I1RQG4 pp 229533.Q4ICA8
105263 229533.I1RQG4 pp 229533.I1RT65
105264 229533.I1RQG4 pp 229533.Q4IAA0
105265 229533.I1RQG4 pp 229533.Q4I948
105266 229533.I1RQG4 pp 229533.I1RZH4

105267 229533.I1RQG4 pp 229533.V6RBN2
105268 229533.I1RQG4 pp 229533.I1S439
105269 229533.I1RQG4 pp 229533.I1RRY9
105270 229533.I1RQG4 pp 229533.I1RYL5
105271 229533.I1RQG4 pp 229533.I1S2G9
105272 229533.I1RQG4 pp 229533.I1RR95
105273 229533.I1RQG4 pp 229533.I1RRT8
105274 229533.I1RQG4 pp 229533.I1RXT9
105275 229533.I1RQG4 pp 229533.I1S5S8
105276 229533.I1RQG4 pp 229533.I1RZG0
105277 229533.I1RQG4 pp 229533.I1RSH0
105278 229533.I1RQG4 pp 229533.I1RWH4
105279 229533.I1RQG4 pp 229533.I1RST4
105280 229533.I1RQG4 pp 229533.I1RT04
105281 229533.I1RQG4 pp 229533.I1RTD5
105282 229533.I1RQG4 pp 229533.I1S9U6
105283 229533.I1RQG4 pp 229533.I1RRM9
105284 229533.I1RQG4 pp 229533.I1S8P5
105285 229533.I1RQG4 pp 229533.I1S2V2
105286 229533.I1RQG4 pp 229533.I1RS47
105287 229533.I1RQG4 pp 229533.I1RWK9
105288 229533.I1RQG4 pp 229533.V6R042
105289 229533.I1RQG4 pp 229533.I1RZ95
105290 229533.I1RQG4 pp 229533.I1S1V5
105291 229533.I1RQG4 pp 229533.V6RMW6
105292 229533.I1RQG4 pp 229533.I1RZZ9
105293 229533.I1RQG4 pp 229533.I1S4Z7
105294 229533.I1RQG4 pp 229533.I1RZ13
105295 229533.I1RQG4 pp 229533.I1RRV1
105296 229533.I1RQG4 pp 229533.I1S0N4
105297 229533.I1RQG4 pp 229533.V6QX21
105298 229533.I1RQG4 pp 229533.I1S1W4
105299 229533.I1RQG4 pp 229533.I1RUG3
105300 229533.I1RQG4 pp 229533.Q4I8C9
105301 229533.I1RQG4 pp 229533.V6RVG3
105302 229533.I1RQG4 pp 229533.I1RTH6
105303 229533.I1RQG4 pp 229533.I1RYW3
105304 229533.I1RQG4 pp 229533.I1RRI2
105305 229533.I1RQG4 pp 229533.V6RRH2
105306 229533.I1RQG4 pp 229533.I1S238
105307 229533.I1RQG4 pp 229533.I1RTA0
105308 229533.I1RQG4 pp 229533.I1RZE4
105309 229533.I1RQG4 pp 229533.I1S247
105310 229533.I1RQG4 pp 229533.I1RVX0
105311 229533.A0A1C3YML3 pp 229533.I1RBH9
105312 229533.A0A1C3YML3 pp 229533.I1RA36
105313 229533.A0A1C3YML3 pp 229533.I1S183
105314 229533.A0A1C3YML3 pp 229533.I1RG77
105315 229533.A0A1C3YML3 pp 229533.I1RBI6
105316 229533.A0A1C3YML3 pp 229533.A0A1C3YN36
105317 229533.A0A1C3YML3 pp 229533.I1S7M2
105318 229533.A0A1C3YML3 pp 229533.I1RMG1
105319 229533.A0A1C3YML3 pp 229533.I1RN55
105320 229533.A0A1C3YML3 pp 229533.I1S0Q4
105321 229533.A0A1C3YML3 pp 229533.I1RVZ9
105322 229533.A0A1C3YML3 pp 229533.I1RA12
105323 229533.A0A1C3YML3 pp 229533.I1RKI2
105324 229533.A0A1C3YML3 pp 229533.I1RE23
105325 229533.A0A1C3YML3 pp 229533.I1S072
105326 229533.A0A1C3YML3 pp 229533.I1RYJ6
105327 229533.A0A1C3YML3 pp 229533.Q4INS6
105328 229533.A0A1C3YML3 pp 229533.I1RUZ8
105329 229533.A0A1C3YML3 pp 229533.I1RPQ1
105330 229533.A0A1C3YML3 pp 229533.I1RZ94
105331 229533.A0A1C3YML3 pp 229533.I1S236
105332 229533.A0A1C3YML3 pp 229533.I1S7M3
105333 229533.A0A1C3YML3 pp 229533.I1RCQ6
105334 229533.A0A1C3YML3 pp 229533.I1RRY1
105335 229533.A0A1C3YML3 pp 229533.I1S209
105336 229533.A0A1C3YML3 pp 229533.I1RRT1
105337 229533.A0A1C3YML3 pp 229533.I1RRN5
105338 229533.A0A1C3YML3 pp 229533.I1RB50
105339 229533.A0A1C3YML3 pp 229533.I1RBW0

105340 229533.A0A1C3YML3 pp 229533.I1RAK6
105341 229533.A0A1C3YML3 pp 229533.I1RSY2
105342 229533.A0A1C3YML3 pp 229533.I1RDH0
105343 229533.A0A1C3YML3 pp 229533.I1RCM7
105344 229533.A0A1C3YML3 pp 229533.I1RBW1
105345 229533.A0A1C3YML3 pp 229533.I1RAT5
105346 229533.A0A1C3YML3 pp 229533.I1RA10
105347 229533.A0A1C3YML3 pp 229533.I1RX38
105348 229533.A0A1C3YML3 pp 229533.I1RG90
105349 229533.A0A1C3YML3 pp 229533.I1RDJ8
105350 229533.A0A1C3YML3 pp 229533.I1S9F9
105351 229533.A0A1C3YML3 pp 229533.I1RUG4
105352 229533.A0A1C3YML3 pp 229533.I1RTN6
105353 229533.A0A1C3YML3 pp 229533.I1RBD7
105354 229533.A0A1C3YML3 pp 229533.I1S0B1
105355 229533.A0A1C3YML3 pp 229533.I1RWS4
105356 229533.A0A1C3YML3 pp 229533.I1RZP8
105357 229533.A0A1C3YML3 pp 229533.I1RBN9
105358 229533.A0A1C3YML3 pp 229533.I1RPD6
105359 229533.A0A1C3YML3 pp 229533.I1RQI6
105360 229533.A0A1C3YML3 pp 229533.I1RSP7
105361 229533.A0A1C3YML3 pp 229533.I1RC31
105362 229533.A0A1C3YML3 pp 229533.I1RZ98
105363 229533.A0A1C3YML3 pp 229533.I1RSN7
105364 229533.A0A1C3YML3 pp 229533.I1RSC7
105365 229533.A0A1C3YML3 pp 229533.I1S274
105366 229533.A0A1C3YML3 pp 229533.I1S0Q5
105367 229533.A0A1C3YML3 pp 229533.I1RC54
105368 229533.A0A1C3YML3 pp 229533.I1RSR9
105369 229533.A0A1C3YML3 pp 229533.I1S237
105370 229533.A0A1C3YML3 pp 229533.I1RBF3
105371 229533.A0A1C3YML3 pp 229533.I1RBC8
105372 229533.A0A1C3YML3 pp 229533.I1RN57
105373 229533.A0A1C3YML3 pp 229533.I1RZ86
105374 229533.A0A1C3YML3 pp 229533.I1RSA4
105375 229533.A0A1C3YML3 pp 229533.I1RY29
105376 229533.A0A1C3YML3 pp 229533.I1RXF2
105377 229533.A0A1C3YML3 pp 229533.I1RCA5
105378 229533.A0A1C3YML3 pp 229533.I1RVW0
105379 229533.A0A1C3YML3 pp 229533.I1RRJ6
105380 229533.A0A1C3YML3 pp 229533.I1RWI8
105381 229533.A0A1C3YML3 pp 229533.I1RBV3
105382 229533.A0A1C3YML3 pp 229533.I1RX41
105383 229533.A0A1C3YML3 pp 229533.I1RPF5
105384 229533.A0A1C3YML3 pp 229533.I1RX39
105385 229533.I1RXJ1 pp 229533.Q4I7L0
105386 229533.I1RXJ1 pp 229533.I1RZ94
105387 229533.V6QU20 pp 229533.V6QYC5
105388 229533.V6QU20 pp 229533.V6RQ28
105389 229533.V6QU20 pp 229533.V6R5Q6
105390 229533.I1RH32 pp 229533.I1S8P5
105391 229533.I1RH32 pp 229533.I1RQE8
105392 229533.I1RH32 pp 229533.I1S673
105393 229533.I1RH32 pp 229533.I1S0K0
105394 229533.I1RH32 pp 229533.I1S9T6
105395 229533.I1RH32 pp 229533.I1RZM4
105396 229533.I1RH32 pp 229533.I1RSX9
105397 229533.I1RH32 pp 229533.I1RQP2
105398 229533.I1RH32 pp 229533.I1S3L2
105399 229533.I1RH32 pp 229533.I1RVR2
105400 229533.I1RH32 pp 229533.I1RXS0
105401 229533.I1RH32 pp 229533.I1S8P4
105402 229533.I1RH32 pp 229533.I1RUB4
105403 229533.I1RH32 pp 229533.I1S6P7
105404 229533.I1RH32 pp 229533.V6RK96
105405 229533.I1RH32 pp 229533.I1RUB5
105406 229533.I1RH32 pp 229533.I1S8R4
105407 229533.I1RH32 pp 229533.I1RUB1
105408 229533.I1RH32 pp 229533.I1RHZ5
105409 229533.I1RH32 pp 229533.I1RMY1
105410 229533.A0A0E0RU84 pp 229533.A0A0E0RZM5
105411 229533.A0A0E0RU84 pp 229533.I1RS17
105412 229533.A0A0E0RU84 pp 229533.I1RQ81

105413	229533.A0A0E0RU84	pp	229533.A0A1C3YKV3
105414	229533.A0A0E0RU84	pp	229533.I1RS18
105415	229533.A0A0E0RU84	pp	229533.Q4I8P5
105416	229533.A0A0E0RU84	pp	229533.I1RPP4
105417	229533.A0A0E0RU84	pp	229533.A0A1C3YK34
105418	229533.A0A0E0RU84	pp	229533.I1RTA0
105419	229533.A0A0E0RU84	pp	229533.I1RE01
105420	229533.A0A0E0RU84	pp	229533.I1RW46
105421	229533.A0A0E0RU84	pp	229533.A0A0E0S134
105422	229533.A0A0E0RU84	pp	229533.I1S0J1
105423	229533.A0A0E0RU84	pp	229533.I1SA32
105424	229533.A0A0E0RU84	pp	229533.I1S504
105425	229533.A0A0E0RU84	pp	229533.I1RRE5
105426	229533.A0A0E0RU84	pp	229533.I1RWT6
105427	229533.A0A0E0RU84	pp	229533.A0A1C3YKW9
105428	229533.A0A0E0RU84	pp	229533.A0A1C3YKB0
105429	229533.A0A0E0RU84	pp	229533.Q4I283
105430	229533.A0A0E0RU84	pp	229533.I1S419
105431	229533.A0A0E0RU84	pp	229533.V6RIV9
105432	229533.A0A0E0RU84	pp	229533.I1RWN5
105433	229533.A0A0E0RU84	pp	229533.I1RC16
105434	229533.A0A0E0RU84	pp	229533.I1RZM1
105435	229533.A0A0E0RU84	pp	229533.I1RNQ5
105436	229533.A0A0E0RU84	pp	229533.I1RLU6
105437	229533.A0A0E0RU84	pp	229533.I1RBJ5
105438	229533.A0A0E0RU84	pp	229533.I1RY44
105439	229533.A0A0E0RU84	pp	229533.I1RRV1
105440	229533.A0A0E0RU84	pp	229533.V6RCZ8
105441	229533.A0A0E0RU84	pp	229533.I1RMG3
105442	229533.A0A0E0RU84	pp	229533.A0A1C3YLS9
105443	229533.A0A0E0RU84	pp	229533.V6R042
105444	229533.A0A0E0RU84	pp	229533.I1RCR8
105445	229533.A0A0E0RU84	pp	229533.I1RNC1
105446	229533.A0A0E0RU84	pp	229533.I1RUG3
105447	229533.A0A0E0RU84	pp	229533.I1RSQ3
105448	229533.A0A0E0RU84	pp	229533.V6RCG5
105449	229533.A0A0E0RU84	pp	229533.I1RQ31
105450	229533.A0A0E0RU84	pp	229533.I1RI82
105451	229533.A0A0E0RU84	pp	229533.I1RC17
105452	229533.A0A0E0RU84	pp	229533.I1RWQ8
105453	229533.A0A0E0RU84	pp	229533.V6RMW6
105454	229533.A0A0E0RU84	pp	229533.I1RR51
105455	229533.A0A0E0RU84	pp	229533.I1R9D1
105456	229533.A0A0E0RU84	pp	229533.I1RW99
105457	229533.A0A0E0RU84	pp	229533.I1S0N4
105458	229533.A0A0E0RU84	pp	229533.A0A1C3YLU7
105459	229533.A0A0E0RU84	pp	229533.I1RZM8
105460	229533.A0A0E0RU84	pp	229533.I1RXF0
105461	229533.A0A0E0RU84	pp	229533.I1S5B0
105462	229533.A0A0E0RU84	pp	229533.I1RSI0
105463	229533.A0A0E0RU84	pp	229533.I1SA33
105464	229533.A0A0E0RU84	pp	229533.I1RWK3
105465	229533.A0A0E0RU84	pp	229533.A0A0E0SBE9
105466	229533.A0A0E0RU84	pp	229533.I1RD22
105467	229533.A0A0E0RU84	pp	229533.I1RLM7
105468	229533.A0A0E0RU84	pp	229533.I1RN63
105469	229533.A0A0E0RU84	pp	229533.A0A1C3YI73
105470	229533.A0A0E0RU84	pp	229533.I1RGL4
105471	229533.A0A0E0RU84	pp	229533.I1RAU5
105472	229533.A0A0E0RU84	pp	229533.I1RIL8
105473	229533.A0A0E0RU84	pp	229533.A0A0E0RV94
105474	229533.A0A0E0RU84	pp	229533.I1RRU9
105475	229533.A0A0E0RU84	pp	229533.I1RSC6
105476	229533.A0A0E0RU84	pp	229533.I1S2M6
105477	229533.A0A0E0RU84	pp	229533.I1RRN5
105478	229533.A0A0E0RU84	pp	229533.I1RVX0
105479	229533.A0A0E0RU84	pp	229533.I1RT03
105480	229533.A0A0E0RU84	pp	229533.I1RTH6
105481	229533.A0A0E0RU84	pp	229533.I1S8R3
105482	229533.A0A0E0RU84	pp	229533.I1RCG4
105483	229533.A0A0E0RU84	pp	229533.Q4IJT5
105484	229533.A0A0E0RU84	pp	229533.I1RA70
105485	229533.A0A0E0RU84	pp	229533.I1RD52

229533.A0A0E0RU84	pp	229533.I1RFU8
229533.A0A0E0RU84	pp	229533.I1RB92
229533.A0A0E0RU84	pp	229533.A0A0E0RVZ1
229533.A0A0E0RU84	pp	229533.I1RQA3
229533.A0A0E0RU84	pp	229533.I1RZK5
229533.A0A0E0RU84	pp	229533.I1RS47
229533.A0A0E0RU84	pp	229533.I1RY03
229533.A0A0E0RU84	pp	229533.Q4IJK2
229533.A0A0E0RU84	pp	229533.I1S0L3
229533.A0A0E0RU84	pp	229533.I1RFL4
229533.A0A0E0RU84	pp	229533.A0A1C3YHX6
229533.A0A0E0RU84	pp	229533.I1S1C7
229533.A0A0E0RU84	pp	229533.I1S0F5
229533.A0A0E0RU84	pp	229533.I1RYW3
229533.A0A0E0RU84	pp	229533.I1RN18
229533.A0A0E0RU84	pp	229533.I1RQQ5
229533.A0A0E0RU84	pp	229533.V6R1K4
229533.A0A0E0RU84	pp	229533.I1RQE7
229533.A0A0E0RU84	pp	229533.I1RZW8
229533.A0A0E0RU84	pp	229533.A0A1C3YIP5
229533.A0A0E0RU84	pp	229533.I1RWX5
229533.A0A0E0RU84	pp	229533.I1RZZ1
229533.A0A0E0RU84	pp	229533.I1S0F6
229533.A0A0E0RU84	pp	229533.I1RCH5
229533.A0A0E0RU84	pp	229533.I1RN67
229533.A0A0E0RU84	pp	229533.A0A1C3YLK2
229533.A0A0E0RU84	pp	229533.Q4I624
229533.A0A0E0RU84	pp	229533.I1RRI2
229533.A0A0E0RU84	pp	229533.I1RAW0
229533.A0A0E0RU84	pp	229533.V6R4J1
229533.A0A0E0RU84	pp	229533.I1RSR3
229533.A0A0E0RU84	pp	229533.I1RD39
229533.A0A0E0RU84	pp	229533.I1RD17
229533.A0A0E0RU84	pp	229533.I1REK8
229533.A0A0E0RU84	pp	229533.I1RZL6
229533.A0A0E0RU84	pp	229533.I1RCR5
229533.A0A0E0RU84	pp	229533.I1S569
229533.A0A0E0RU84	pp	229533.I1RU29
229533.A0A0E0RU84	pp	229533.I1RVQ0
229533.A0A0E0RU84	pp	229533.I1RWN6
229533.A0A0E0RU84	pp	229533.I1RAH0
229533.A0A0E0RU84	pp	229533.V6R057
229533.A0A0E0RU84	pp	229533.I1RRY9
229533.A0A0E0RU84	pp	229533.I1RP28
229533.A0A0E0RU84	pp	229533.I1RBS0
229533.A0A0E0RU84	pp	229533.I1RQ87
229533.A0A0E0RU84	pp	229533.I1RAU7
229533.A0A0E0RU84	pp	229533.A0A1C3YM60
229533.A0A0E0RU84	pp	229533.I1R9J6
229533.A0A0E0RU84	pp	229533.Q4I2J8
229533.A0A0E0RU84	pp	229533.I1RJV3
229533.A0A0E0RU84	pp	229533.I1RT04
229533.A0A0E0RU84	pp	229533.A0A0E0SPV2
229533.A0A0E0RU84	pp	229533.I1RI03
229533.A0A0E0RU84	pp	229533.I1S1J7
229533.A0A0E0RU84	pp	229533.I1RB41
229533.A0A0E0RU84	pp	229533.A0A1C3YIJ3
229533.A0A0E0RU84	pp	229533.I1S0R7
229533.A0A0E0RU84	pp	229533.A0A1C3YLF5
229533.A0A0E0RU84	pp	229533.I1RX46
229533.A0A0E0RU84	pp	229533.A0A1C3YIE7
229533.A0A0E0RU84	pp	229533.A0A1C3YJ93
229533.A0A0E0RU84	pp	229533.I1RSK1
229533.A0A0E0RU84	pp	229533.I1RE22
229533.A0A0E0RU84	pp	229533.I1RQC9
229533.A0A0E0RU84	pp	229533.A0A0E0SFN0
229533.A0A0E0RU84	pp	229533.A0A1C3YLZ7
229533.A0A0E0RU84	pp	229533.V6QYF0
229533.A0A0E0RU84	pp	229533.A0A1C3YKN3
229533.A0A0E0RU84	pp	229533.I1RFT1
229533.A0A0E0RU84	pp	229533.I1RNA5
229533.A0A0E0RU84	pp	229533.I1S352
229533.A0A0E0RU84	pp	229533.I1RXD7

105559 229533.A0A0E0RU84 pp 229533.A0A1C3YK13
105560 229533.A0A0E0RU84 pp 229533.I1RBC9
105561 229533.A0A0E0RU84 pp 229533.I1RUQ2
105562 229533.A0A0E0RU84 pp 229533.V6QW22
105563 229533.A0A0E0RU84 pp 229533.I1S0M6
105564 229533.A0A0E0RU84 pp 229533.I1S2E9
105565 229533.A0A0E0RU84 pp 229533.I1RCS1
105566 229533.A0A0E0RU84 pp 229533.I1S6Y8
105567 229533.A0A0E0RU84 pp 229533.I1RRJ8
105568 229533.A0A0E0RU84 pp 229533.I1RQH3
105569 229533.A0A0E0RU84 pp 229533.I1RQG4
105570 229533.A0A0E0RU84 pp 229533.I1RCJ7
105571 229533.A0A0E0RU84 pp 229533.I1RPM4
105572 229533.A0A0E0RU84 pp 229533.I1RSQ7
105573 229533.A0A0E0RU84 pp 229533.I1RT40
105574 229533.A0A0E0RU84 pp 229533.I1RG53
105575 229533.A0A0E0RU84 pp 229533.I1S8P5
105576 229533.A0A0E0RU84 pp 229533.I1RQ23
105577 229533.A0A0E0RU84 pp 229533.A0A0E0RUI4
105578 229533.A0A0E0RU84 pp 229533.I1RV91
105579 229533.A0A0E0RU84 pp 229533.I1RE27
105580 229533.A0A0E0RU84 pp 229533.I1RGF5
105581 229533.A0A0E0RU84 pp 229533.I1RW07
105582 229533.A0A0E0RU84 pp 229533.I1RPP3
105583 229533.A0A0E0RU84 pp 229533.I1RTD5
105584 229533.A0A0E0RU84 pp 229533.A0A1C3YIS7
105585 229533.A0A0E0RU84 pp 229533.A0A1C3YM63
105586 229533.A0A0E0RU84 pp 229533.I1RLX7
105587 229533.A0A0E0RU84 pp 229533.I1RI02
105588 229533.A0A0E0RU84 pp 229533.I1RGA4
105589 229533.A0A0E0RU84 pp 229533.A0A1C3YMA1
105590 229533.A0A0E0RU84 pp 229533.I1RAX6
105591 229533.A0A0E0RU84 pp 229533.I1RAM6
105592 229533.A0A0E0RU84 pp 229533.I1RG62
105593 229533.A0A0E0RU84 pp 229533.I1RBS6
105594 229533.A0A0E0RU84 pp 229533.I1RLC7
105595 229533.A0A0E0RU84 pp 229533.I1RC66
105596 229533.A0A0E0RU84 pp 229533.I1RJV4
105597 229533.A0A0E0RU84 pp 229533.I1RQS8
105598 229533.A0A0E0RU84 pp 229533.I1RRM5
105599 229533.A0A0E0RU84 pp 229533.I1S2B8
105600 229533.A0A0E0RU84 pp 229533.I1S2H4
105601 229533.A0A0E0RU84 pp 229533.V6RTJ8
105602 229533.A0A0E0RU84 pp 229533.I1RS20
105603 229533.A0A0E0RU84 pp 229533.I1R9M0
105604 229533.A0A0E0RU84 pp 229533.I1RP27
105605 229533.A0A0E0RU84 pp 229533.I1RW25
105606 229533.A0A0E0RU84 pp 229533.I1RX25
105607 229533.A0A0E0RU84 pp 229533.I1RR24
105608 229533.A0A0E0RU84 pp 229533.I1S7A8
105609 229533.A0A0E0RU84 pp 229533.I1S0L4
105610 229533.A0A0E0RU84 pp 229533.I1RJ74
105611 229533.A0A0E0RU84 pp 229533.I1RBY4
105612 229533.A0A0E0RU84 pp 229533.A0A1C3YJQ9
105613 229533.A0A0E0RU84 pp 229533.I1RIL3
105614 229533.A0A0E0RU84 pp 229533.I1RJW6
105615 229533.A0A0E0RU84 pp 229533.A0A0E0SC46
105616 229533.A0A0E0RU84 pp 229533.I1RE76
105617 229533.A0A0E0RU84 pp 229533.A0A1C3YLK8
105618 229533.A0A0E0RU84 pp 229533.I1RJU3
105619 229533.A0A0E0RU84 pp 229533.I1RRL9
105620 229533.A0A0E0RU84 pp 229533.I1RM82
105621 229533.A0A0E0RU84 pp 229533.I1RWJ8
105622 229533.A0A0E0RU84 pp 229533.I1S503
105623 229533.A0A0E0RU84 pp 229533.A0A1C3YJH8
105624 229533.A0A0E0RU84 pp 229533.I1RMG8
105625 229533.A0A0E0RU84 pp 229533.A0A1C3YN30
105626 229533.A0A0E0RU84 pp 229533.A0A1C3YKM9
105627 229533.A0A0E0RU84 pp 229533.I1RLN6
105628 229533.A0A0E0RU84 pp 229533.I1RZ39
105629 229533.A0A0E0RU84 pp 229533.I1RR73
105630 229533.A0A0E0RU84 pp 229533.V6R3X5
105631 229533.A0A0E0RU84 pp 229533.I1RA11

105632 229533.A0A0E0RU84 pp 229533.I1RWL2
105633 229533.A0A0E0RU84 pp 229533.I1RZJ5
105634 229533.A0A0E0RU84 pp 229533.I1S0T2
105635 229533.A0A0E0RU84 pp 229533.I1RRT8
105636 229533.A0A0E0RU84 pp 229533.A0A0E0SA11
105637 229533.A0A0E0RU84 pp 229533.A0A1C3YK73
105638 229533.A0A0E0RU84 pp 229533.I1S0X6
105639 229533.A0A0E0RU84 pp 229533.I1RFQ7
105640 229533.A0A0E0RU84 pp 229533.I1S5R1
105641 229533.A0A0E0RU84 pp 229533.O74638
105642 229533.A0A0E0RU84 pp 229533.I1RWI5
105643 229533.A0A0E0RU84 pp 229533.I1RBT1
105644 229533.A0A0E0RU84 pp 229533.A0A1C3YKC0
105645 229533.A0A0E0RU84 pp 229533.I1RZZ9
105646 229533.A0A0E0RU84 pp 229533.I1RMC8
105647 229533.A0A0E0RU84 pp 229533.A0A1C3YHX8
105648 229533.A0A1C3YIL9 pp 229533.I1RNM4
105649 229533.A0A1C3YIL9 pp 229533.I1RZ18
105650 229533.A0A1C3YIL9 pp 229533.Q4I0K3
105651 229533.A0A1C3YIL9 pp 229533.I1RC31
105652 229533.A0A1C3YIL9 pp 229533.I1RAH0
105653 229533.A0A1C3YIL9 pp 229533.I1RZ34
105654 229533.A0A1C3YIL9 pp 229533.I1RNK8
105655 229533.A0A1C3YIL9 pp 229533.A0A1C3YKR7
105656 229533.A0A1C3YIL9 pp 229533.I1RZM0
105657 229533.A0A1C3YIL9 pp 229533.I1RD09
105658 229533.A0A1C3YIL9 pp 229533.Q4I785
105659 229533.A0A1C3YIL9 pp 229533.Q4IB63
105660 229533.A0A1C3YIL9 pp 229533.I1RUP0
105661 229533.A0A1C3YIL9 pp 229533.I1RST0
105662 229533.A0A1C3YIL9 pp 229533.I1RZ17
105663 229533.A0A1C3YIL9 pp 229533.I1R9N7
105664 229533.A0A1C3YIL9 pp 229533.I1RMP9
105665 229533.A0A1C3YIL9 pp 229533.I1RZ13
105666 229533.A0A1C3YIL9 pp 229533.V6RPQ8
105667 229533.A0A1C3YIL9 pp 229533.Q4HZE0
105668 229533.A0A1C3YIL9 pp 229533.A0A1C3YMK7
105669 229533.A0A1C3YIL9 pp 229533.A0A1C3YLQ1
105670 229533.A0A1C3YIL9 pp 229533.V6QUV7
105671 229533.A0A1C3YIL9 pp 229533.I1RQ75
105672 229533.A0A1C3YIL9 pp 229533.I1RDF6
105673 229533.A0A1C3YIL9 pp 229533.Q4I073
105674 229533.A0A1C3YIL9 pp 229533.I1S272
105675 229533.A0A1C3YIL9 pp 229533.I1RZV2
105676 229533.A0A1C3YIL9 pp 229533.Q4IJT0
105677 229533.A0A1C3YIL9 pp 229533.I1RJL9
105678 229533.I1RE55 pp 229533.I1RRY1
105679 229533.I1RE55 pp 229533.I1RKI2
105680 229533.I1RE55 pp 229533.I1RSJ1
105681 229533.I1RE55 pp 229533.I1S1W8
105682 229533.I1RE55 pp 229533.I1RRT1
105683 229533.I1RE55 pp 229533.I1RZ94
105684 229533.I1RE55 pp 229533.Q4INS6
105685 229533.I1RE55 pp 229533.I1RUR0
105686 229533.I1RE55 pp 229533.I1RY29
105687 229533.I1RE55 pp 229533.I1S209
105688 229533.I1RE55 pp 229533.I1RPI0
105689 229533.I1RE55 pp 229533.Q4IB89
105690 229533.I1RE55 pp 229533.I1RVW0
105691 229533.I1RE55 pp 229533.I1RSD0
105692 229533.I1RE55 pp 229533.I1S523
105693 229533.I1RE55 pp 229533.I1RZ98
105694 229533.I1RE55 pp 229533.I1S3G4
105695 229533.I1RE55 pp 229533.I1RQI6
105696 229533.I1RE55 pp 229533.I1RSC7
105697 229533.I1RE55 pp 229533.I1RTN6
105698 229533.I1RE55 pp 229533.I1RSP7
105699 229533.I1RE55 pp 229533.I1RE57
105700 229533.I1RE55 pp 229533.I1S0D5
105701 229533.A0A098E4D7 pp 229533.I1RPY0
105702 229533.A0A098E4D7 pp 229533.I1RKF2
105703 229533.A0A098E4D7 pp 229533.I1RE53
105704 229533.A0A098E4D7 pp 229533.A0A1C3YM01

105705	229533.A0A098E4D7	pp	229533.I1RZ52
105706	229533.A0A098E4D7	pp	229533.I1RMG6
105707	229533.A0A098E4D7	pp	229533.I1RQ30
105708	229533.A0A098E4D7	pp	229533.I1RTI7
105709	229533.A0A098E4D7	pp	229533.I1RZ20
105710	229533.A0A098E4D7	pp	229533.O74638
105711	229533.A0A098E4D7	pp	229533.I1RWF4
105712	229533.A0A098E4D7	pp	229533.I1RQ48
105713	229533.A0A098E4D7	pp	229533.I1RCB4
105714	229533.A0A098E4D7	pp	229533.I1R9K3
105715	229533.A0A098E4D7	pp	229533.I1RYU5
105716	229533.A0A098E4D7	pp	229533.I1RGK9
105717	229533.A0A098E4D7	pp	229533.I1RMG7
105718	229533.A0A098E4D7	pp	229533.Q4HXF0
105719	229533.A0A098E4D7	pp	229533.V6RQW4
105720	229533.A0A098E4D7	pp	229533.A0A1C3YLU9
105721	229533.A0A098E4D7	pp	229533.I1RIY0
105722	229533.A0A098E4D7	pp	229533.I1S9D4
105723	229533.A0A098E4D7	pp	229533.I1S2E2
105724	229533.A0A098E4D7	pp	229533.I1RTJ2
105725	229533.I1S8P4	pp	229533.I1S8P5
105726	229533.I1R9M0	pp	229533.I1RSF1
105727	229533.I1R9M0	pp	229533.I1RPU2
105728	229533.I1R9M0	pp	229533.I1RRV1
105729	229533.I1R9M0	pp	229533.I1RMG3
105730	229533.I1R9M0	pp	229533.I1RVZ7
105731	229533.I1R9M0	pp	229533.V6RIV9
105732	229533.I1R9M0	pp	229533.I1RWN5
105733	229533.I1R9M0	pp	229533.I1RCR8
105734	229533.I1R9M0	pp	229533.V6R042
105735	229533.I1R9M0	pp	229533.Q4IN63
105736	229533.I1R9M0	pp	229533.I1S1W9
105737	229533.I1R9M0	pp	229533.V6RMW6
105738	229533.I1R9M0	pp	229533.I1RKJ4
105739	229533.I1R9M0	pp	229533.I1RRM3
105740	229533.I1R9M0	pp	229533.I1RAG7
105741	229533.I1R9M0	pp	229533.I1RVF8
105742	229533.I1R9M0	pp	229533.I1S016
105743	229533.I1R9M0	pp	229533.I1RNC1
105744	229533.I1R9M0	pp	229533.I1RXT3
105745	229533.I1R9M0	pp	229533.I1RE65
105746	229533.I1R9M0	pp	229533.I1RY44
105747	229533.I1R9M0	pp	229533.I1S5R6
105748	229533.I1R9M0	pp	229533.I1S1W3
105749	229533.I1R9M0	pp	229533.I1RC33
105750	229533.I1R9M0	pp	229533.I1RUG3
105751	229533.I1R9M0	pp	229533.I1S0N4
105752	229533.I1R9M0	pp	229533.I1RG11
105753	229533.I1R9M0	pp	229533.I1RWN6
105754	229533.I1R9M0	pp	229533.I1RB26
105755	229533.I1R9M0	pp	229533.I1RZV3
105756	229533.I1R9M0	pp	229533.I1RVQ3
105757	229533.I1R9M0	pp	229533.I1RAW0
105758	229533.I1R9M0	pp	229533.I1SA33
105759	229533.I1R9M0	pp	229533.I1RE89
105760	229533.I1R9M0	pp	229533.I1RA70
105761	229533.I1R9M0	pp	229533.I1RMN6
105762	229533.I1R9M0	pp	229533.I1RTH6
105763	229533.I1R9M0	pp	229533.I1RRN2
105764	229533.I1R9M0	pp	229533.I1S8R3
105765	229533.I1R9M0	pp	229533.I1RD22
105766	229533.I1R9M0	pp	229533.I1RCM3
105767	229533.I1R9M0	pp	229533.I1RSK1
105768	229533.I1R9M0	pp	229533.I1RFJ6
105769	229533.I1R9M0	pp	229533.I1RPK0
105770	229533.I1R9M0	pp	229533.I1S0F8
105771	229533.I1R9M0	pp	229533.I1RS17
105772	229533.I1R9M0	pp	229533.I1RJV3
105773	229533.I1R9M0	pp	229533.I1RKJ8
105774	229533.I1R9M0	pp	229533.I1RCA0
105775	229533.I1R9M0	pp	229533.I1RNB8
105776	229533.I1R9M0	pp	229533.I1S437
105777	229533.I1R9M0	pp	229533.I1RVX0

105778 229533.I1R9M0 pp 229533.I1RPP4
105779 229533.I1R9M0 pp 229533.I1RB44
105780 229533.I1R9M0 pp 229533.I1RXR1
105781 229533.I1R9M0 pp 229533.I1RT03
105782 229533.I1R9M0 pp 229533.I1S9Z4
105783 229533.I1R9M0 pp 229533.I1RMV0
105784 229533.I1R9M0 pp 229533.I1S1C7
105785 229533.I1R9M0 pp 229533.I1RRN5
105786 229533.I1R9M0 pp 229533.I1RE01
105787 229533.I1R9M0 pp 229533.I1RYW3
105788 229533.I1R9M0 pp 229533.I1RW46
105789 229533.I1R9M0 pp 229533.I1RUF7
105790 229533.I1R9M0 pp 229533.I1RB92
105791 229533.I1R9M0 pp 229533.I1RFL4
105792 229533.I1R9M0 pp 229533.I1RZ79
105793 229533.I1R9M0 pp 229533.I1RN18
105794 229533.I1R9M0 pp 229533.I1S7W5
105795 229533.I1R9M0 pp 229533.I1RRI2
105796 229533.I1R9M0 pp 229533.I1RQE7
105797 229533.I1R9M0 pp 229533.I1RCH5
105798 229533.I1R9M0 pp 229533.I1S0J1
105799 229533.I1R9M0 pp 229533.I1S569
105800 229533.I1R9M0 pp 229533.I1RRE5
105801 229533.I1R9M0 pp 229533.I1RZW8
105802 229533.I1R9M0 pp 229533.I1RXU9
105803 229533.I1R9M0 pp 229533.I1RLN6
105804 229533.I1R9M0 pp 229533.I1RG90
105805 229533.I1R9M0 pp 229533.I1RD39
105806 229533.I1R9M0 pp 229533.V6R057
105807 229533.I1R9M0 pp 229533.Q4HVQ9
105808 229533.I1R9M0 pp 229533.I1RB41
105809 229533.I1R9M0 pp 229533.Q4I624
105810 229533.I1R9M0 pp 229533.I1RC34
105811 229533.I1R9M0 pp 229533.I1RBR1
105812 229533.I1R9M0 pp 229533.I1RJW6
105813 229533.I1R9M0 pp 229533.I1RCR1
105814 229533.I1R9M0 pp 229533.I1RD17
105815 229533.I1R9M0 pp 229533.I1RAU7
105816 229533.I1R9M0 pp 229533.I1RRK3
105817 229533.I1R9M0 pp 229533.I1RML0
105818 229533.I1R9M0 pp 229533.I1RCD8
105819 229533.I1R9M0 pp 229533.I1RGL4
105820 229533.I1R9M0 pp 229533.I1RQ33
105821 229533.I1R9M0 pp 229533.I1RNA5
105822 229533.I1R9M0 pp 229533.I1S503
105823 229533.I1R9M0 pp 229533.I1S0W4
105824 229533.I1R9M0 pp 229533.I1RSX0
105825 229533.I1R9M0 pp 229533.I1RT04
105826 229533.I1R9M0 pp 229533.V6RUY9
105827 229533.I1R9M0 pp 229533.I1RN42
105828 229533.I1R9M0 pp 229533.I1S0F6
105829 229533.I1R9M0 pp 229533.I1RDF7
105830 229533.I1R9M0 pp 229533.V6R3J1
105831 229533.I1R9M0 pp 229533.I1RBS0
105832 229533.I1R9M0 pp 229533.I1S464
105833 229533.I1R9M0 pp 229533.I1S2E9
105834 229533.I1R9M0 pp 229533.I1RFL3
105835 229533.I1R9M0 pp 229533.I1RXD9
105836 229533.I1R9M0 pp 229533.I1S352
105837 229533.I1R9M0 pp 229533.I1RZ39
105838 229533.I1R9M0 pp 229533.I1RY92
105839 229533.I1R9M0 pp 229533.I1RV91
105840 229533.I1R9M0 pp 229533.I1RR73
105841 229533.I1R9M0 pp 229533.I1RSV2
105842 229533.I1R9M0 pp 229533.V6R3X5
105843 229533.I1R9M0 pp 229533.I1RCJ7
105844 229533.I1R9M0 pp 229533.I1RT40
105845 229533.I1R9M0 pp 229533.I1RFT1
105846 229533.I1R9M0 pp 229533.I1RGF5
105847 229533.I1R9M0 pp 229533.I1RYI5
105848 229533.I1R9M0 pp 229533.I1RXF8
105849 229533.I1R9M0 pp 229533.I1RWI5
105850 229533.I1R9M0 pp 229533.I1RPP3

105851 229533.I1R9M0 pp 229533.I1RTD5
105852 229533.I1R9M0 pp 229533.I1RRJ8
105853 229533.I1R9M0 pp 229533.I1RSQ4
105854 229533.I1R9M0 pp 229533.I1RZZ9
105855 229533.I1R9M0 pp 229533.I1RPH6
105856 229533.I1R9M0 pp 229533.I1S268
105857 229533.I1R9M0 pp 229533.I1RFL7
105858 229533.I1R9M0 pp 229533.I1RC66
105859 229533.I1R9M0 pp 229533.I1RNN1
105860 229533.I1R9M0 pp 229533.I1REC5
105861 229533.I1R9M0 pp 229533.I1RWK3
105862 229533.I1R9M0 pp 229533.I1RBS6
105863 229533.I1R9M0 pp 229533.I1RYR0
105864 229533.I1R9M0 pp 229533.I1S2H4
105865 229533.I1R9M0 pp 229533.I1RBC7
105866 229533.I1R9M0 pp 229533.I1RPE0
105867 229533.I1R9M0 pp 229533.I1RGA4
105868 229533.I1R9M0 pp 229533.V6R283
105869 229533.I1R9M0 pp 229533.I1RP27
105870 229533.I1R9M0 pp 229533.I1RQM7
105871 229533.I1R9M0 pp 229533.I1S0L4
105872 229533.I1R9M0 pp 229533.I1RIL3
105873 229533.I1R9M0 pp 229533.I1RJN9
105874 229533.I1R9M0 pp 229533.I1RJV4
105875 229533.I1R9M0 pp 229533.I1RTA1
105876 229533.I1R9M0 pp 229533.I1RRI1
105877 229533.I1R9M0 pp 229533.I1RRZ0
105878 229533.I1R9M0 pp 229533.I1RVZ2
105879 229533.I1R9M0 pp 229533.I1RKP1
105880 229533.I1R9M0 pp 229533.I1RS47
105881 229533.I1R9M0 pp 229533.I1S5R1
105882 229533.I1R9M0 pp 229533.I1RXC2
105883 229533.I1R9M0 pp 229533.I1RM82
105884 229533.I1R9M0 pp 229533.I1S0F7
105885 229533.I1R9M0 pp 229533.I1S9Y1
105886 229533.I1R9M0 pp 229533.I1RRT8
105887 229533.I1R9M0 pp 229533.O74638
105888 229533.I1R9M0 pp 229533.I1RA11
105889 229533.I1R9M0 pp 229533.I1RSA5
105890 229533.I1R9M0 pp 229533.I1RN67
105891 229533.I1R9M0 pp 229533.I1RWL2
105892 229533.I1R9M0 pp 229533.V6RCZ8
105893 229533.I1R9M0 pp 229533.I1RE76
105894 229533.I1R9M0 pp 229533.I1RQ81
105895 229533.I1R9M0 pp 229533.I1RGU7
105896 229533.I1R9M0 pp 229533.I1RE22
105897 229533.I1R9M0 pp 229533.I1RGC5
105898 229533.I1R9M0 pp 229533.I1RS18
105899 229533.I1R9M0 pp 229533.I1RC29
105900 229533.I1R9M0 pp 229533.I1S6K9
105901 229533.I1R9M0 pp 229533.I1S0M6
105902 229533.I1R9M0 pp 229533.Q4I8P5
105903 229533.I1R9M0 pp 229533.I1S821
105904 229533.I1R9M0 pp 229533.I1RXC4
105905 229533.I1R9M0 pp 229533.I1RSZ7
105906 229533.I1R9M0 pp 229533.I1RQ23
105907 229533.I1R9M0 pp 229533.I1RFS3
105908 229533.I1R9M0 pp 229533.I1SA32
105909 229533.I1R9M0 pp 229533.I1RR51
105910 229533.I1R9M0 pp 229533.V6RCG5
105911 229533.I1R9M0 pp 229533.I1RPV3
105912 229533.I1R9M0 pp 229533.I1S6Y8
105913 229533.I1R9M0 pp 229533.I1RJ17
105914 229533.I1R9M0 pp 229533.I1RPM4
105915 229533.I1R9M0 pp 229533.I1RL04
105916 229533.I1R9M0 pp 229533.I1RZM8
105917 229533.I1R9M0 pp 229533.I1RXF0
105918 229533.I1R9M0 pp 229533.I1S504
105919 229533.I1R9M0 pp 229533.I1RI82
105920 229533.I1R9M0 pp 229533.I1RQD2
105921 229533.I1R9M0 pp 229533.I1S4Z8
105922 229533.I1R9M0 pp 229533.I1RWT6
105923 229533.I1R9M0 pp 229533.I1RC17

229533.I1R9M0 pp 229533.I1R9Y8
229533.I1R9M0 pp 229533.I1S5B0
229533.I1R9M0 pp 229533.I1RJZ7
229533.I1R9M0 pp 229533.I1RQ85
229533.I1R9M0 pp 229533.I1RG14
229533.I1R9M0 pp 229533.I1RRM5
229533.I1R9M0 pp 229533.I1S2B8
229533.I1R9M0 pp 229533.I1RW99
229533.I1R9M0 pp 229533.I1RRV3
229533.I1R9M0 pp 229533.I1S419
229533.I1R9M0 pp 229533.I1RPU3
229533.I1R9M0 pp 229533.I1RLU6
229533.I1R9M0 pp 229533.V6QUE6
229533.I1R9M0 pp 229533.I1RBY4
229533.I1R9M0 pp 229533.I1RNQ5
229533.I1R9M0 pp 229533.I1RBJ5
229533.I1R9M0 pp 229533.I1S1B2
229533.I1R9M0 pp 229533.I1RFT2
229533.I1R9M0 pp 229533.I1S887
229533.I1R9M0 pp 229533.I1RZM1
229533.I1R9M0 pp 229533.I1RFU8
229533.I1R9M0 pp 229533.I1RNT9
229533.I1R9M0 pp 229533.I1RY03
229533.I1R9M0 pp 229533.I1RAU5
229533.I1R9M0 pp 229533.I1RS29
229533.I1R9M0 pp 229533.I1RBU8
229533.I1R9M0 pp 229533.I1RCG4
229533.I1R9M0 pp 229533.I1RBL7
229533.I1R9M0 pp 229533.I1RQA3
229533.I1R9M0 pp 229533.I1RPR9
229533.I1R9M0 pp 229533.I1RZK5
229533.I1R9M0 pp 229533.Q4IJK2
229533.I1R9M0 pp 229533.I1RCM5
229533.I1R9M0 pp 229533.I1RWR7
229533.I1R9M0 pp 229533.I1RU29
229533.I1R9M0 pp 229533.I1RGB2
229533.I1R9M0 pp 229533.I1RPY3
229533.I1R9M0 pp 229533.I1RZZ1
229533.I1R9M0 pp 229533.I1RRY9
229533.I1R9M0 pp 229533.V6R9Z0
229533.I1R9M0 pp 229533.I1S540
229533.I1R9M0 pp 229533.V6RBN2
229533.I1R9M0 pp 229533.I1RAX6
229533.I1R9M0 pp 229533.I1RJZ6
229533.I1R9M0 pp 229533.I1RWB0
229533.I1R9M0 pp 229533.I1REK8
229533.I1R9M0 pp 229533.V6RTJ8
229533.I1R9M0 pp 229533.I1RYS0
229533.I1R9M0 pp 229533.I1S7A8
229533.I1R9M0 pp 229533.I1RI03
229533.I1R9M0 pp 229533.I1RCR5
229533.I1R9M0 pp 229533.I1S1J7
229533.I1R9M0 pp 229533.I1RWJ3
229533.I1R9M0 pp 229533.I1RBR2
229533.I1R9M0 pp 229533.I1RLM7
229533.I1R9M0 pp 229533.I1RZE5
229533.I1R9M0 pp 229533.I1RSI0
229533.I1R9M0 pp 229533.I1S0T2
229533.I1R9M0 pp 229533.I1RUQ3
229533.I1R9M0 pp 229533.I1RX46
229533.I1R9M0 pp 229533.I1RF19
229533.I1R9M0 pp 229533.I1RK95
229533.I1R9M0 pp 229533.I1RMF6
229533.I1R9M0 pp 229533.I1RQC9
229533.I1R9M0 pp 229533.I1S0R7
229533.I1R9M0 pp 229533.I1RCC2
229533.I1R9M0 pp 229533.I1RD52
229533.I1R9M0 pp 229533.I1RY86
229533.I1R9M0 pp 229533.I1RA85
229533.I1R9M0 pp 229533.I1S4S7
229533.I1R9M0 pp 229533.V6R1K4
229533.I1R9M0 pp 229533.I1RTB3
229533.I1R9M0 pp 229533.V6RUT5

105997 229533.I1R9M0 pp 229533.I1RW71
105998 229533.I1R9M0 pp 229533.I1RBC9
105999 229533.I1R9M0 pp 229533.I1S0L3
106000 229533.I1R9M0 pp 229533.I1RQH3
106001 229533.I1R9M0 pp 229533.I1RR00
106002 229533.I1R9M0 pp 229533.I1RG53
106003 229533.I1R9M0 pp 229533.I1RXD7
106004 229533.I1R9M0 pp 229533.I1RN61
106005 229533.I1R9M0 pp 229533.I1S0F5
106006 229533.I1R9M0 pp 229533.I1RDU4
106007 229533.I1R9M0 pp 229533.I1RXD6
106008 229533.I1R9M0 pp 229533.V6QW22
106009 229533.I1R9M0 pp 229533.I1RLP1
106010 229533.I1R9M0 pp 229533.I1RNB2
106011 229533.I1R9M0 pp 229533.I1RC20
106012 229533.I1R9M0 pp 229533.I1RSQ7
106013 229533.I1R9M0 pp 229533.I1S0P0
106014 229533.I1R9M0 pp 229533.I1RE66
106015 229533.I1R9M0 pp 229533.I1RFC0
106016 229533.I1R9M0 pp 229533.I1RRX5
106017 229533.I1R9M0 pp 229533.V6R4J1
106018 229533.I1R9M0 pp 229533.I1RVQ0
106019 229533.I1R9M0 pp 229533.I1RX95
106020 229533.I1R9M0 pp 229533.I1RKJ5
106021 229533.I1R9M0 pp 229533.I1RJU3
106022 229533.I1R9M0 pp 229533.I1S2R0
106023 229533.I1R9M0 pp 229533.I1RBD5
106024 229533.I1R9M0 pp 229533.I1RBT1
106025 229533.I1R9M0 pp 229533.I1RIL8
106026 229533.I1R9M0 pp 229533.I1RRG3
106027 229533.I1R9M0 pp 229533.I1S0X6
106028 229533.I1R9M0 pp 229533.Q4IJT5
106029 229533.I1R9M0 pp 229533.I1R9Y4
106030 229533.I1R9M0 pp 229533.I1RE17
106031 229533.I1R9M0 pp 229533.I1S0F1
106032 229533.I1R9M0 pp 229533.I1RRQ1
106033 229533.I1R9M0 pp 229533.I1RMG8
106034 229533.I1R9M0 pp 229533.I1RMC8
106035 229533.I1R9M0 pp 229533.I1RM67
106036 229533.I1R9M0 pp 229533.I1RWJ8
106037 229533.I1R9M0 pp 229533.I1RR24
106038 229533.I1R9M0 pp 229533.I1RW54
106039 229533.I1R9M0 pp 229533.I1S4S4
106040 229533.I1R9M0 pp 229533.I1RCS1
106041 229533.I1R9M0 pp 229533.I1S438
106042 229533.I1R9M0 pp 229533.I1RFQ7
106043 229533.I1R9M0 pp 229533.I1RPA4
106044 229533.I1R9M0 pp 229533.I1RW07
106045 229533.I1R9M0 pp 229533.I1RI02
106046 229533.I1R9M0 pp 229533.I1RRS8
106047 229533.I1R9M0 pp 229533.I1RLC7
106048 229533.I1R9M0 pp 229533.I1S9I5
106049 229533.I1R9M0 pp 229533.I1RW25
106050 229533.I1R9M0 pp 229533.I1RX25
106051 229533.A0A098DJU7 pp 229533.I1RRQ1
106052 229533.A0A098DJU7 pp 229533.V6RR38
106053 229533.A0A098DJU7 pp 229533.V6RPN0
106054 229533.A0A098DJU7 pp 229533.I1RZZ3
106055 229533.A0A098DJU7 pp 229533.A0A0E0SP71
106056 229533.A0A098DJU7 pp 229533.I1RCC2
106057 229533.A0A098DJU7 pp 229533.I1RQ76
106058 229533.A0A098DJU7 pp 229533.I1S1Q3
106059 229533.A0A098DJU7 pp 229533.A0A1C3YLL7
106060 229533.A0A098DJU7 pp 229533.I1RI22
106061 229533.A0A098DJU7 pp 229533.I1RT23
106062 229533.A0A098DJU7 pp 229533.I1SAS3
106063 229533.A0A098DJU7 pp 229533.I1RQ48
106064 229533.A0A098DJU7 pp 229533.I1RNT1
106065 229533.A0A098DJU7 pp 229533.I1SAS4
106066 229533.A0A098DJU7 pp 229533.I1RR90
106067 229533.A0A098DJU7 pp 229533.I1RY35
106068 229533.A0A098DJU7 pp 229533.I1RCA0
106069 229533.A0A098DJU7 pp 229533.I1RJG1

106070	229533.A0A098DJU7	pp	229533.I1RF79
106071	229533.A0A098DJU7	pp	229533.A0A1C3YMS7
106072	229533.A0A098DJU7	pp	229533.I1RFM2
106073	229533.A0A0E0RVC1	pp	229533.I1RZ63
106074	229533.A0A0E0RVC1	pp	229533.A0A0E0SDV1
106075	229533.A0A0E0RVC1	pp	229533.A0A1C3YKK8
106076	229533.A0A0E0RVC1	pp	229533.V6R949
106077	229533.A0A0E0RVC1	pp	229533.I1RJQ5
106078	229533.A0A0E0RVC1	pp	229533.I1RZ78
106079	229533.A0A0E0RVC1	pp	229533.I1RCD4
106080	229533.A0A0E0RVC1	pp	229533.I1RC31
106081	229533.A0A0E0RVC1	pp	229533.I1RRY3
106082	229533.A0A0E0RVC1	pp	229533.I1RSW5
106083	229533.A0A0E0RVC1	pp	229533.I1S0E4
106084	229533.A0A0E0RVC1	pp	229533.A0A0E0SKE0
106085	229533.A0A0E0RVC1	pp	229533.I1RVU9
106086	229533.A0A0E0RVC1	pp	229533.I1RR45
106087	229533.A0A0E0RVC1	pp	229533.I1REW3
106088	229533.A0A0E0RVC1	pp	229533.I1RA62
106089	229533.A0A0E0RVC1	pp	229533.A0A1C3YHZ7
106090	229533.A0A0E0RVC1	pp	229533.I1S0E2
106091	229533.A0A0E0RVC1	pp	229533.I1RTE8
106092	229533.A0A0E0RVC1	pp	229533.I1RPS2
106093	229533.A0A0E0RVC1	pp	229533.I1RNU7
106094	229533.A0A0E0RVC1	pp	229533.I1RN52
106095	229533.A0A0E0RVC1	pp	229533.I1RC11
106096	229533.A0A0E0RVC1	pp	229533.I1RKB5
106097	229533.A0A0E0RVC1	pp	229533.I1S444
106098	229533.A0A0E0RVC1	pp	229533.A0A1C3YHP2
106099	229533.A0A0E0RVC1	pp	229533.A0A0E0SK74
106100	229533.A0A0E0RVC1	pp	229533.I1RP86
106101	229533.A0A0E0RVC1	pp	229533.I1RGY2
106102	229533.A0A0E0RVC1	pp	229533.I1RPT0
106103	229533.A0A0E0RVC1	pp	229533.A0A1C3YMN0
106104	229533.A0A0E0RVC1	pp	229533.A0A1C3YN26
106105	229533.A0A0E0RVC1	pp	229533.V6REE9
106106	229533.A0A0E0RVC1	pp	229533.A0A1C3YM97
106107	229533.A0A0E0RVC1	pp	229533.A0A1C3YKF3
106108	229533.A0A0E0RVC1	pp	229533.I1RAM7
106109	229533.A0A0E0RVC1	pp	229533.I1RRK5
106110	229533.A0A0E0RVC1	pp	229533.I1RUM7
106111	229533.I1RKL3	pp	229533.I1RT98
106112	229533.I1RKL3	pp	229533.Q4IPI1
106113	229533.I1RKL3	pp	229533.I1RSL9
106114	229533.I1RKL3	pp	229533.Q4IQW0
106115	229533.I1RKL3	pp	229533.I1RRT2
106116	229533.I1RKL3	pp	229533.I1RX46
106117	229533.I1RKL3	pp	229533.I1RKQ5
106118	229533.I1RKL3	pp	229533.Q4I5R9
106119	229533.I1RKL3	pp	229533.I1RQS5
106120	229533.I1RKL3	pp	229533.I1S503
106121	229533.I1RKL3	pp	229533.I1RUQ2
106122	229533.I1RKL3	pp	229533.I1RS47
106123	229533.I1RKL3	pp	229533.I1RRH5
106124	229533.I1RKL3	pp	229533.I1RXD7
106125	229533.I1RKL3	pp	229533.I1S0R7
106126	229533.I1RKL3	pp	229533.I1RNH1
106127	229533.I1RKL3	pp	229533.Q4IAS1
106128	229533.I1RKL3	pp	229533.I1S6F0
106129	229533.I1RKL3	pp	229533.I1RLV9
106130	229533.I1RKL3	pp	229533.I1RRQ3
106131	229533.I1RKL3	pp	229533.I1RSQ7
106132	229533.I1RKL3	pp	229533.I1RTI6
106133	229533.I1RKL3	pp	229533.I1RTD5
106134	229533.I1RKL3	pp	229533.I1RRJ8
106135	229533.I1RKL3	pp	229533.I1RT04
106136	229533.I1RKL3	pp	229533.I1RWD4
106137	229533.I1RKL3	pp	229533.I1RPP3
106138	229533.I1RKL3	pp	229533.I1RW26
106139	229533.I1RKL3	pp	229533.I1RQJ5
106140	229533.I1RKL3	pp	229533.I1S8I8
106141	229533.I1RKL3	pp	229533.I1RZ99
106142	229533.I1RKL3	pp	229533.I1RS30

106143	229533.I1RKL3	pp	229533.Q4INZ9
106144	229533.I1RKL3	pp	229533.I1RZJ6
106145	229533.I1RKL3	pp	229533.I1RR00
106146	229533.I1RKL3	pp	229533.V6R3X5
106147	229533.I1RKL3	pp	229533.I1RW71
106148	229533.I1RKL3	pp	229533.I1RND6
106149	229533.I1RKL3	pp	229533.I1RR73
106150	229533.I1RKL3	pp	229533.I1RPG6
106151	229533.I1RKL3	pp	229533.I1RM67
106152	229533.I1RKL3	pp	229533.Q4HVW2
106153	229533.I1RKL3	pp	229533.Q4I830
106154	229533.I1RKL3	pp	229533.I1RMK3
106155	229533.I1RKL3	pp	229533.I1S2M6
106156	229533.I1RKL3	pp	229533.O74638
106157	229533.I1RKL3	pp	229533.I1RWH5
106158	229533.I1RKL3	pp	229533.I1S8E2
106159	229533.I1RKL3	pp	229533.I1RPU0
106160	229533.I1RKL3	pp	229533.V6R283
106161	229533.I1RKL3	pp	229533.I1RZV6
106162	229533.I1RKL3	pp	229533.I1RNV6
106163	229533.I1RKL3	pp	229533.I1RT80
106164	229533.I1RKL3	pp	229533.I1RZS6
106165	229533.I1RKL3	pp	229533.I1RTF4
106166	229533.I1RKL3	pp	229533.I1RZ39
106167	229533.I1RKL3	pp	229533.I1RQ65
106168	229533.I1RKL3	pp	229533.Q4IEV5
106169	229533.I1RKL3	pp	229533.V6QYF0
106170	229533.I1RKL3	pp	229533.I1RM06
106171	229533.I1RKL3	pp	229533.Q4IJH1
106172	229533.I1RKL3	pp	229533.I1RNA0
106173	229533.I1RKL3	pp	229533.I1RQM6
106174	229533.I1RKL3	pp	229533.I1RU69
106175	229533.I1RKL3	pp	229533.I1RT33
106176	229533.I1RKL3	pp	229533.Q4HZ42
106177	229533.I1RKL3	pp	229533.I1RX24
106178	229533.I1RKL3	pp	229533.Q4IQK7
106179	229533.I1RKL3	pp	229533.Q4IF76
106180	229533.I1RKL3	pp	229533.Q4I662
106181	229533.I1RKL3	pp	229533.I1RYB9
106182	229533.I1RKL3	pp	229533.I1RT05
106183	229533.I1RKL3	pp	229533.I1RSI6
106184	229533.I1RKL3	pp	229533.I1S9U2
106185	229533.I1RKL3	pp	229533.I1RMN7
106186	229533.I1RKL3	pp	229533.I1RWJ4
106187	229533.I1RKL3	pp	229533.I1RNH6
106188	229533.I1RKL3	pp	229533.I1RYT5
106189	229533.I1RKL3	pp	229533.I1S021
106190	229533.I1RKL3	pp	229533.I1RPW9
106191	229533.I1RKL3	pp	229533.I1RYG3
106192	229533.I1RKL3	pp	229533.I1RW25
106193	229533.I1RKL3	pp	229533.I1RNY2
106194	229533.I1RKL3	pp	229533.I1RS20
106195	229533.I1RKL3	pp	229533.I1S504
106196	229533.I1RKL3	pp	229533.I1RQS8
106197	229533.I1RKL3	pp	229533.I1S2B8
106198	229533.I1RKL3	pp	229533.I1RZF6
106199	229533.I1RKL3	pp	229533.Q4I327
106200	229533.I1RKL3	pp	229533.I1RP31
106201	229533.I1RKL3	pp	229533.Q4IFI0
106202	229533.I1RKL3	pp	229533.Q4IN63
106203	229533.I1RKL3	pp	229533.Q4IEK8
106204	229533.I1RKL3	pp	229533.I1RQ31
106205	229533.I1RKL3	pp	229533.V6RMW6
106206	229533.I1RKL3	pp	229533.I1RL23
106207	229533.I1RKL3	pp	229533.I1RM96
106208	229533.I1RKL3	pp	229533.I1RNQ5
106209	229533.I1RKL3	pp	229533.V6R042
106210	229533.I1RKL3	pp	229533.I1RMC0
106211	229533.I1RKL3	pp	229533.I1RPY1
106212	229533.I1RKL3	pp	229533.I1S044
106213	229533.I1RKL3	pp	229533.V6RGN5
106214	229533.I1RKL3	pp	229533.I1RQA2
106215	229533.I1RKL3	pp	229533.I1S4T3

106216 229533.I1RKL3 pp 229533.I1RWN5
106217 229533.I1RKL3 pp 229533.I1RN99
106218 229533.I1RKL3 pp 229533.I1RQ28
106219 229533.I1RKL3 pp 229533.I1S2C0
106220 229533.I1RKL3 pp 229533.Q4IBS2
106221 229533.I1RKL3 pp 229533.I1RSS4
106222 229533.I1RKL3 pp 229533.V6QUE6
106223 229533.I1RKL3 pp 229533.Q4HWE2
106224 229533.I1RKL3 pp 229533.I1RTE9
106225 229533.I1RKL3 pp 229533.I1RMN6
106226 229533.I1RKL3 pp 229533.I1RPJ3
106227 229533.I1RKL3 pp 229533.I1RSC6
106228 229533.I1RKL3 pp 229533.I1RSG2
106229 229533.I1RKL3 pp 229533.I1RZK5
106230 229533.I1RKL3 pp 229533.I1RY63
106231 229533.I1RKL3 pp 229533.Q4IAA0
106232 229533.I1RKL3 pp 229533.I1S1X9
106233 229533.I1RKL3 pp 229533.I1RTH6
106234 229533.I1RKL3 pp 229533.I1RQ81
106235 229533.I1RKL3 pp 229533.I1RT03
106236 229533.I1RKL3 pp 229533.I1RWB5
106237 229533.I1RKL3 pp 229533.I1RM25
106238 229533.I1RKL3 pp 229533.I1RVX0
106239 229533.I1RKL3 pp 229533.I1RNU6
106240 229533.I1RKL3 pp 229533.I1RY03
106241 229533.I1RKL3 pp 229533.I1RRI2
106242 229533.I1RKL3 pp 229533.I1S2E8
106243 229533.I1RKL3 pp 229533.I1RTD9
106244 229533.I1RKL3 pp 229533.I1S0M9
106245 229533.I1RKL3 pp 229533.I1RZK6
106246 229533.I1RKL3 pp 229533.Q4IJ56
106247 229533.I1RKL3 pp 229533.I1RZZ1
106248 229533.I1RKL3 pp 229533.I1S0J1
106249 229533.I1RKL3 pp 229533.I1RSR8
106250 229533.I1RKL3 pp 229533.I1S2U2
106251 229533.I1RKL3 pp 229533.I1RX14
106252 229533.I1RKL3 pp 229533.I1S1C7
106253 229533.I1RKL3 pp 229533.V6RGY3
106254 229533.I1RKL3 pp 229533.I1RSR3
106255 229533.I1RKL3 pp 229533.I1RMQ0
106256 229533.I1RKL3 pp 229533.I1S296
106257 229533.I1RKL3 pp 229533.I1S060
106258 229533.I1RKL3 pp 229533.I1RSD8
106259 229533.I1RKL3 pp 229533.I1S1U7
106260 229533.I1RKL3 pp 229533.I1RRR7
106261 229533.I1RKL3 pp 229533.V6R227
106262 229533.I1RKL3 pp 229533.Q4HZ68
106263 229533.I1RKL3 pp 229533.I1RWN6
106264 229533.I1RKL3 pp 229533.I1RRY9
106265 229533.I1RKL3 pp 229533.I1RZE5
106266 229533.I1RKL3 pp 229533.I1RYB1
106267 229533.I1RKL3 pp 229533.I1S9U1
106268 229533.I1RKL3 pp 229533.I1S274
106269 229533.I1RKL3 pp 229533.I1RVZ0
106270 229533.I1RKL3 pp 229533.I1S0Z9
106271 229533.I1RKL3 pp 229533.I1S558
106272 229533.I1RKL3 pp 229533.I1RNK2
106273 229533.I1RUB1 pp 229533.I1S6P7
106274 229533.I1RUB1 pp 229533.I1S8P5
106275 229533.I1RUB1 pp 229533.I1RUB5
106276 229533.I1RUB1 pp 229533.I1RUB2
106277 229533.I1RUB1 pp 229533.I1RUB4
106278 229533.A0A1C3YKI7 pp 229533.I1S7X9
106279 229533.Q4IJ84 pp 229533.Q4IPI4
106280 229533.Q4IJ84 pp 229533.Q4INS6
106281 229533.I1RM74 pp 229533.I1RV65
106282 229533.I1RM74 pp 229533.I1RSH9
106283 229533.I1RM74 pp 229533.I1RP97
106284 229533.I1RM74 pp 229533.I1RNR3
106285 229533.I1RM74 pp 229533.I1RND7
106286 229533.A0A1C3YI64 pp 229533.A0A1C3YMC9
106287 229533.A0A1C3YI64 pp 229533.I1RSL8
106288 229533.A0A1C3YI64 pp 229533.I1RN57

229533.A0A1C3YI64	pp	229533.A0A1C3YN69
229533.A0A1C3YI64	pp	229533.A0A1C3YLB6
229533.A0A1C3YI64	pp	229533.I1RAP2
229533.A0A1C3YI64	pp	229533.Q4IB89
229533.A0A1C3YI64	pp	229533.I1RY44
229533.A0A1C3YI64	pp	229533.I1RBA4
229533.A0A1C3YI64	pp	229533.I1RRN5
229533.A0A1C3YI64	pp	229533.I1S4P6
229533.A0A1C3YI64	pp	229533.A0A1C3YN55
229533.A0A1C3YI64	pp	229533.I1RP99
229533.A0A1C3YI64	pp	229533.I1RDH0
229533.A0A1C3YI64	pp	229533.I1RTN6
229533.A0A1C3YI64	pp	229533.I1RZP8
229533.A0A1C3YI64	pp	229533.I1RY58
229533.A0A1C3YI64	pp	229533.I1RDJ8
229533.A0A1C3YI64	pp	229533.Q4IJ84
229533.A0A1C3YI64	pp	229533.I1RA10
229533.A0A1C3YI64	pp	229533.I1RX64
229533.A0A1C3YI64	pp	229533.I1RBN9
229533.A0A1C3YI64	pp	229533.I1RUG4
229533.A0A1C3YI64	pp	229533.I1S0N5
229533.A0A1C3YI64	pp	229533.I1RQJ4
229533.A0A1C3YI64	pp	229533.A0A1C3YM91
229533.A0A1C3YI64	pp	229533.I1RSP7
229533.A0A1C3YI64	pp	229533.I1S2E9
229533.A0A1C3YI64	pp	229533.I1RZ98
229533.A0A1C3YI64	pp	229533.I1RMJ8
229533.A0A1C3YI64	pp	229533.A0A1C3YM67
229533.A0A1C3YI64	pp	229533.I1S276
229533.A0A1C3YI64	pp	229533.I1RQI6
229533.A0A1C3YI64	pp	229533.A0A1C3YL55
229533.A0A1C3YI64	pp	229533.I1RY29
229533.A0A1C3YI64	pp	229533.I1RSC7
229533.A0A1C3YI64	pp	229533.I1RDX0
229533.A0A1C3YI64	pp	229533.I1RC54
229533.A0A1C3YI64	pp	229533.I1RPM7
229533.A0A1C3YI64	pp	229533.I1RBC8
229533.A0A1C3YI64	pp	229533.Q4HXF0
229533.A0A1C3YI64	pp	229533.I1S0D5
229533.A0A1C3YI64	pp	229533.V6R382
229533.A0A1C3YI64	pp	229533.I1RWI8
229533.A0A1C3YI64	pp	229533.I1RSR9
229533.A0A1C3YI64	pp	229533.I1RHD2
229533.A0A1C3YI64	pp	229533.I1RF02
229533.A0A1C3YI64	pp	229533.I1SAA0
229533.A0A1C3YI64	pp	229533.I1RFL7
229533.A0A1C3YI64	pp	229533.I1RYX9
229533.A0A1C3YI64	pp	229533.I1RSZ3
229533.A0A1C3YI64	pp	229533.I1RPF5
229533.A0A1C3YI64	pp	229533.A0A1C3YMJ3
229533.A0A1C3YI64	pp	229533.I1S9F9
229533.A0A1C3YI64	pp	229533.I1RB25
229533.A0A1C3YI64	pp	229533.I1RYQ1
229533.A0A1C3YI64	pp	229533.I1RJV8
229533.A0A1C3YI64	pp	229533.I1RZ86
229533.A0A1C3YI64	pp	229533.I1RCA5
229533.A0A1C3YI64	pp	229533.I1RYR2
229533.A0A1C3YI64	pp	229533.I1RSN7
229533.A0A1C3YI64	pp	229533.I1RUF8
229533.A0A1C3YI64	pp	229533.I1RVL8
229533.A0A1C3YI64	pp	229533.I1RRM8
229533.A0A1C3YI64	pp	229533.I1R9W7
229533.A0A1C3YI64	pp	229533.I1S998
229533.A0A1C3YI64	pp	229533.I1RRJ6
229533.A0A1C3YI64	pp	229533.A0A1C3YM61
229533.A0A1C3YI64	pp	229533.I1RVZ9
229533.A0A1C3YI64	pp	229533.I1S0J3
229533.A0A1C3YI64	pp	229533.A0A1C3YLQ7
229533.A0A1C3YI64	pp	229533.I1RCL7
229533.A0A1C3YI64	pp	229533.I1RSA7
229533.A0A1C3YI64	pp	229533.I1RBW1
229533.A0A1C3YI64	pp	229533.I1RE23
229533.A0A1C3YI64	pp	229533.V6RLW4

106362	229533.A0A1C3YI64	pp	229533.I1REN3
106363	229533.A0A1C3YI64	pp	229533.I1RDC0
106364	229533.A0A1C3YI64	pp	229533.I1S3G4
106365	229533.A0A1C3YI64	pp	229533.A0A1C3YJ79
106366	229533.A0A1C3YI64	pp	229533.I1RFJ2
106367	229533.A0A1C3YI64	pp	229533.I1RBC1
106368	229533.A0A1C3YI64	pp	229533.I1S523
106369	229533.A0A1C3YI64	pp	229533.I1RMQ7
106370	229533.A0A1C3YI64	pp	229533.A0A1C3YJN7
106371	229533.A0A1C3YI64	pp	229533.I1RFQ5
106372	229533.A0A1C3YI64	pp	229533.I1RWT6
106373	229533.A0A1C3YI64	pp	229533.A0A1C3YJL7
106374	229533.A0A1C3YI64	pp	229533.I1RSJ1
106375	229533.A0A1C3YI64	pp	229533.I1RA12
106376	229533.A0A1C3YI64	pp	229533.I1RQ93
106377	229533.A0A1C3YI64	pp	229533.I1RQH8
106378	229533.A0A1C3YI64	pp	229533.A0A1C3YJQ6
106379	229533.A0A1C3YI64	pp	229533.I1RKB1
106380	229533.A0A1C3YI64	pp	229533.Q4INS6
106381	229533.A0A1C3YI64	pp	229533.I1S9J6
106382	229533.A0A1C3YI64	pp	229533.I1RMS2
106383	229533.A0A1C3YI64	pp	229533.I1RPQ1
106384	229533.A0A1C3YI64	pp	229533.I1RM36
106385	229533.A0A1C3YI64	pp	229533.I1S5B0
106386	229533.A0A1C3YI64	pp	229533.I1RA38
106387	229533.A0A1C3YI64	pp	229533.I1RG77
106388	229533.A0A1C3YI64	pp	229533.Q4IQX3
106389	229533.A0A1C3YI64	pp	229533.I1RN55
106390	229533.A0A1C3YI64	pp	229533.A0A1C3YML3
106391	229533.A0A1C3YI64	pp	229533.A0A1C3YJW8
106392	229533.A0A1C3YI64	pp	229533.I1RBW0
106393	229533.A0A1C3YI64	pp	229533.I1RE55
106394	229533.A0A1C3YI64	pp	229533.A0A1C3YK82
106395	229533.A0A1C3YI64	pp	229533.I1RAH4
106396	229533.A0A1C3YI64	pp	229533.I1RQA3
106397	229533.A0A1C3YI64	pp	229533.I1S987
106398	229533.A0A1C3YI64	pp	229533.A0A1C3YJT5
106399	229533.A0A1C3YI64	pp	229533.I1RTC2
106400	229533.A0A1C3YI64	pp	229533.I1RFX8
106401	229533.A0A1C3YI64	pp	229533.I1RAL9
106402	229533.A0A1C3YI64	pp	229533.I1RCM7
106403	229533.A0A1C3YI64	pp	229533.A0A1C3YN36
106404	229533.A0A1C3YI64	pp	229533.I1RSI4
106405	229533.A0A1C3YI64	pp	229533.V6QWX8
106406	229533.A0A1C3YI64	pp	229533.I1RC98
106407	229533.A0A1C3YI64	pp	229533.I1S788
106408	229533.A0A1C3YI64	pp	229533.I1RAT5
106409	229533.A0A1C3YI64	pp	229533.I1RUR0
106410	229533.A0A1C3YI64	pp	229533.I1RZ94
106411	229533.A0A1C3YI64	pp	229533.I1S283
106412	229533.A0A1C3YI64	pp	229533.I1RD01
106413	229533.A0A1C3YI64	pp	229533.I1S1W8
106414	229533.A0A1C3YI64	pp	229533.I1RRT1
106415	229533.A0A1C3YI64	pp	229533.I1RCQ4
106416	229533.A0A1C3YI64	pp	229533.A0A1C3YLR6
106417	229533.A0A1C3YKI4	pp	229533.I1RRN5
106418	229533.A0A1C3YKI4	pp	229533.I1RDZ4
106419	229533.A0A098DTN5	pp	229533.A0A1C3YMK0
106420	229533.A0A098DTN5	pp	229533.I1RUB5
106421	229533.A0A098DTN5	pp	229533.I1S6P7
106422	229533.A0A098DTN5	pp	229533.A0A1C3YMK9
106423	229533.A0A098DTN5	pp	229533.I1RUB4
106424	229533.A0A098DTN5	pp	229533.A0A1C3YMP8
106425	229533.A0A098DTN5	pp	229533.A0A0E0SGX8
106426	229533.A0A098DTN5	pp	229533.I1RH30
106427	229533.A0A098DTN5	pp	229533.I1RUB2
106428	229533.A0A098DTN5	pp	229533.A0A0E0SAE0
106429	229533.A0A098DTN5	pp	229533.A0A1C3YMS7
106430	229533.A0A098DTN5	pp	229533.A0A1C3YMJ9
106431	229533.A0A098DTN5	pp	229533.I1RH32
106432	229533.A0A098DTN5	pp	229533.I1S8P4
106433	229533.A0A098DTN5	pp	229533.I1RUB1
106434	229533.A0A098DTN5	pp	229533.I1S8P5

106435 229533.I1RRM5 pp 229533.I1RSI6
106436 229533.I1RRM5 pp 229533.I1RRV1
106437 229533.I1RRM5 pp 229533.V6RCZ8
106438 229533.I1RRM5 pp 229533.V6RMW6
106439 229533.I1RRM5 pp 229533.I1RS17
106440 229533.I1RRM5 pp 229533.V6RCG5
106441 229533.I1RRM5 pp 229533.I1RSQ3
106442 229533.I1RRM5 pp 229533.Q4I8P5
106443 229533.I1RRM5 pp 229533.I1RW46
106444 229533.I1RRM5 pp 229533.V6R042
106445 229533.I1RRM5 pp 229533.I1RS18
106446 229533.I1RRM5 pp 229533.I1RTH6
106447 229533.I1RRM5 pp 229533.I1RUG3
106448 229533.I1RRM5 pp 229533.I1S8R3
106449 229533.I1RRM5 pp 229533.I1RXT3
106450 229533.I1RRM5 pp 229533.I1SA32
106451 229533.I1RRM5 pp 229533.I1RZM8
106452 229533.I1RRM5 pp 229533.I1RZK5
106453 229533.I1RRM5 pp 229533.I1S0J1
106454 229533.I1RRM5 pp 229533.I1RXF0
106455 229533.I1RRM5 pp 229533.I1S504
106456 229533.I1RRM5 pp 229533.I1RTE9
106457 229533.I1RRM5 pp 229533.I1S1C7
106458 229533.I1RRM5 pp 229533.Q4I283
106459 229533.I1RRM5 pp 229533.I1S5B0
106460 229533.I1RRM5 pp 229533.I1RYW3
106461 229533.I1RRM5 pp 229533.I1RWQ8
106462 229533.I1RRM5 pp 229533.I1RW99
106463 229533.I1RRM5 pp 229533.I1RY03
106464 229533.I1RRM5 pp 229533.V6R227
106465 229533.I1RRM5 pp 229533.I1RZW8
106466 229533.I1RRM5 pp 229533.I1RWX5
106467 229533.I1RRM5 pp 229533.I1RWN5
106468 229533.I1RRM5 pp 229533.I1SA33
106469 229533.I1RRM5 pp 229533.I1RWT6
106470 229533.I1RRM5 pp 229533.I1RRY9
106471 229533.I1RRM5 pp 229533.I1RWN6
106472 229533.I1RRM5 pp 229533.V6RIV9
106473 229533.I1RRM5 pp 229533.I1RU29
106474 229533.I1RRM5 pp 229533.V6R057
106475 229533.I1RRM5 pp 229533.I1RY44
106476 229533.I1RRM5 pp 229533.I1S419
106477 229533.I1RRM5 pp 229533.I1S569
106478 229533.I1RRM5 pp 229533.Q4I624
106479 229533.I1RRM5 pp 229533.I1S1J7
106480 229533.I1RRM5 pp 229533.I1RSR3
106481 229533.I1RRM5 pp 229533.Q4I2J8
106482 229533.I1RRM5 pp 229533.I1RT03
106483 229533.I1RRM5 pp 229533.I1RSI0
106484 229533.I1RRM5 pp 229533.I1RVX0
106485 229533.I1RRM5 pp 229533.I1RZM1
106486 229533.I1RRM5 pp 229533.I1RRZ0
106487 229533.I1RRM5 pp 229533.I1S0R7
106488 229533.I1RRM5 pp 229533.I1RRN5
106489 229533.I1RRM5 pp 229533.I1RSC6
106490 229533.I1RRM5 pp 229533.I1S503
106491 229533.I1RRM5 pp 229533.I1RZZ1
106492 229533.I1RRM5 pp 229533.Q4IJK2
106493 229533.I1RRM5 pp 229533.I1RT04
106494 229533.I1RRM5 pp 229533.I1RRT2
106495 229533.I1RRM5 pp 229533.V6RUY9
106496 229533.I1RRM5 pp 229533.I1RX46
106497 229533.I1RRM5 pp 229533.I1RZ39
106498 229533.I1RRM5 pp 229533.I1S2E9
106499 229533.I1RRM5 pp 229533.I1RW71
106500 229533.I1RRM5 pp 229533.I1RUQ2
106501 229533.I1RRM5 pp 229533.I1S0T2
106502 229533.I1RRM5 pp 229533.I1RZZ9
106503 229533.I1RRM5 pp 229533.I1RXD7
106504 229533.I1RRM5 pp 229533.V6R3X5
106505 229533.I1RRM5 pp 229533.I1RV91
106506 229533.I1RRM5 pp 229533.I1S352
106507 229533.I1RRM5 pp 229533.I1RT40

106508	229533.I1RRM5	pp	229533.Q4HZI0
106509	229533.I1RRM5	pp	229533.I1RSQ7
106510	229533.I1RRM5	pp	229533.I1RTD5
106511	229533.I1RRM5	pp	229533.V6QW22
106512	229533.I1RRM5	pp	229533.I1RWI5
106513	229533.I1RRM5	pp	229533.I1S0X6
106514	229533.I1RRM5	pp	229533.I1RWK3
106515	229533.I1RRM5	pp	229533.I1RS20
106516	229533.I1RRM5	pp	229533.I1RZ29
106517	229533.I1RRM5	pp	229533.I1S7A8
106518	229533.I1RRM5	pp	229533.V6RTJ8
106519	229533.I1RRM5	pp	229533.Q4IJT5
106520	229533.I1RRM5	pp	229533.I1S2M6
106521	229533.I1RRM5	pp	229533.I1S2H4
106522	229533.I1RRM5	pp	229533.I1RS47
106523	229533.I1RRM5	pp	229533.V6R1K4
106524	229533.I1RRM5	pp	229533.I1S0L4
106525	229533.I1RRM5	pp	229533.I1RW61
106526	229533.I1RRM5	pp	229533.I1RWJ8
106527	229533.I1RRM5	pp	229533.I1S0F5
106528	229533.I1RRM5	pp	229533.I1S6Y8
106529	229533.I1RRM5	pp	229533.O74638
106530	229533.I1RRM5	pp	229533.I1RW07
106531	229533.I1RRM5	pp	229533.I1RWD4
106532	229533.I1RRM5	pp	229533.I1S2B8
106533	229533.I1RRM5	pp	229533.I1S0L3
106534	229533.I1RRM5	pp	229533.I1S5R1
106535	229533.I1RRM5	pp	229533.I1RW25
106536	229533.I1RRM5	pp	229533.I1RRT8
106537	229533.I1RRM5	pp	229533.I1RX25
106538	229533.I1RRM5	pp	229533.I1S0F6
106539	229533.I1RRM5	pp	229533.V6QUW4
106540	229533.I1RRM5	pp	229533.I1RZJ5
106541	229533.I1RRM5	pp	229533.I1RVQ0
106542	229533.I1RRM5	pp	229533.I1S0M6
106543	229533.I1RRM5	pp	229533.V6QYF0
106544	229533.I1RRM5	pp	229533.V6R4J1
106545	229533.I1RM68	pp	229533.I1S9D4
106546	229533.I1RM68	pp	229533.I1RY74
106547	229533.I1RM68	pp	229533.I1S3F3
106548	229533.I1RM68	pp	229533.I1RMM8
106549	229533.I1RM68	pp	229533.I1RPW2
106550	229533.I1RM68	pp	229533.I1RPD6
106551	229533.I1RM68	pp	229533.I1S2E2
106552	229533.I1RM68	pp	229533.I1RVX9
106553	229533.I1RM68	pp	229533.I1RNG4
106554	229533.I1RM68	pp	229533.I1RU76
106555	229533.I1RM68	pp	229533.I1RQE0
106556	229533.I1RM68	pp	229533.I1RZ52
106557	229533.I1S2B8	pp	229533.I1S8V4
106558	229533.I1S2B8	pp	229533.I1S7W5
106559	229533.I1S2B8	pp	229533.V6RIV9
106560	229533.I1S2B8	pp	229533.Q4IN63
106561	229533.I1S2B8	pp	229533.I1S569
106562	229533.I1S2B8	pp	229533.I1S5R6
106563	229533.I1S2B8	pp	229533.V6RLM7
106564	229533.I1S2B8	pp	229533.I1S9Z4
106565	229533.I1S2B8	pp	229533.Q4I624
106566	229533.I1S2B8	pp	229533.V6R042
106567	229533.I1S2B8	pp	229533.I1SA33
106568	229533.I1S2B8	pp	229533.Q4I327
106569	229533.I1S2B8	pp	229533.V6R057
106570	229533.I1S2B8	pp	229533.Q4I8X0
106571	229533.I1S2B8	pp	229533.I1S2C0
106572	229533.I1S2B8	pp	229533.I1S352
106573	229533.I1S2B8	pp	229533.I1S8R3
106574	229533.I1S2B8	pp	229533.I1S437
106575	229533.I1S2B8	pp	229533.I1S2E8
106576	229533.I1S2B8	pp	229533.V6R283
106577	229533.I1S2B8	pp	229533.Q4HVQ9
106578	229533.I1S2B8	pp	229533.I1S2G7
106579	229533.I1S2B8	pp	229533.I1S6Y8
106580	229533.I1S2B8	pp	229533.I1S8P5

106581	229533.I1S2B8	pp	229533.I1S503
106582	229533.I1S2B8	pp	229533.I1S2E9
106583	229533.I1S2B8	pp	229533.I1S8V3
106584	229533.I1S2B8	pp	229533.I1S9Y1
106585	229533.I1S2B8	pp	229533.V6RUY9
106586	229533.I1S2B8	pp	229533.I1S5R1
106587	229533.I1S2B8	pp	229533.I1S2H4
106588	229533.I1S2B8	pp	229533.V6R3X5
106589	229533.I1S2B8	pp	229533.Q4I283
106590	229533.I1S2B8	pp	229533.O74638
106591	229533.I1S2B8	pp	229533.I1S4Z8
106592	229533.I1S2B8	pp	229533.I1SA32
106593	229533.I1S2B8	pp	229533.V6RCZ8
106594	229533.I1S2B8	pp	229533.I1S419
106595	229533.I1S2B8	pp	229533.I1S504
106596	229533.I1S2B8	pp	229533.I1S5B0
106597	229533.I1S2B8	pp	229533.Q4IJK2
106598	229533.I1S2B8	pp	229533.V6QUE6
106599	229533.I1S2B8	pp	229533.V6RBN2
106600	229533.I1S2B8	pp	229533.Q4I8P5
106601	229533.I1S2B8	pp	229533.I1S6K9
106602	229533.I1S2B8	pp	229533.I1S821
106603	229533.I1S2B8	pp	229533.Q4I2J8
106604	229533.I1S2B8	pp	229533.Q4IJM4
106605	229533.I1S2B8	pp	229533.V6R1K4
106606	229533.I1S2B8	pp	229533.V6RCG5
106607	229533.I1S2B8	pp	229533.V6R4J1
106608	229533.I1S2B8	pp	229533.V6RTJ8
106609	229533.I1S2B8	pp	229533.Q4IJT5
106610	229533.I1S2B8	pp	229533.V6R9Z0
106611	229533.I1S2B8	pp	229533.I1S2R0
106612	229533.I1S2B8	pp	229533.Q4IPI1
106613	229533.I1S2B8	pp	229533.I1S438
106614	229533.I1S2B8	pp	229533.I1S4S4
106615	229533.I1S2B8	pp	229533.V6QW22
106616	229533.I1S2B8	pp	229533.I1S4S7
106617	229533.I1S2B8	pp	229533.I1S7A8
106618	229533.I1S2B8	pp	229533.I1S3S3
106619	229533.I1S2B8	pp	229533.I1S9U2
106620	229533.I1S2B8	pp	229533.I1S9I5
106621	229533.A0A1C3YIH8	pp	229533.I1RKJ8
106622	229533.A0A1C3YIH8	pp	229533.A0A1C3YL89
106623	229533.A0A1C3YIH8	pp	229533.A0A1C3YKU7
106624	229533.A0A1C3YIH8	pp	229533.I1RE18
106625	229533.A0A1C3YIH8	pp	229533.I1RQH7
106626	229533.A0A1C3YIH8	pp	229533.I1RQD6
106627	229533.A0A1C3YIH8	pp	229533.I1RBF2
106628	229533.A0A1C3YIH8	pp	229533.I1S1V4
106629	229533.A0A1C3YIH8	pp	229533.I1RAH7
106630	229533.A0A1C3YIH8	pp	229533.I1RND7
106631	229533.A0A1C3YIH8	pp	229533.I1RCH2
106632	229533.A0A1C3YIH8	pp	229533.I1S287
106633	229533.A0A1C3YIH8	pp	229533.I1RCQ0
106634	229533.A0A1C3YIH8	pp	229533.I1RW76
106635	229533.A0A1C3YIH8	pp	229533.A0A1C3YKT4
106636	229533.A0A1C3YIH8	pp	229533.I1SAM5
106637	229533.A0A1C3YIH8	pp	229533.I1RNH7
106638	229533.A0A1C3YIH8	pp	229533.I1RFK0
106639	229533.A0A1C3YIH8	pp	229533.I1RZK3
106640	229533.A0A0E0SCP7	pp	229533.I1S0D5
106641	229533.A0A0E0SCP7	pp	229533.I1S183
106642	229533.A0A0E0SCP7	pp	229533.I1RSD4
106643	229533.A0A0E0SCP7	pp	229533.I1S0Q4
106644	229533.A0A0E0SCP7	pp	229533.I1RNE2
106645	229533.A0A0E0SCP7	pp	229533.A0A1C3YN55
106646	229533.A0A0E0SCP7	pp	229533.A0A1C3YL34
106647	229533.A0A0E0SCP7	pp	229533.A0A1C3YKM5
106648	229533.A0A0E0SCP7	pp	229533.I1RQI6
106649	229533.A0A0E0SCP7	pp	229533.I1RZ98
106650	229533.A0A0E0SCP7	pp	229533.I1RSC7
106651	229533.A0A0E0SCP7	pp	229533.I1RM09
106652	229533.A0A0E0SCP7	pp	229533.I1S209
106653	229533.A0A0E0SCP7	pp	229533.I1RBC8

229533.A0A0E0SCP7 pp 229533.I1RRT1
229533.A0A0E0SCP7 pp 229533.I1RXV9
229533.A0A0E0SCP7 pp 229533.I1RCQ4
229533.A0A0E0SCP7 pp 229533.I1RK47
229533.A0A0E0SCP7 pp 229533.I1RCM7
229533.A0A0E0SCP7 pp 229533.I1RAT5
229533.A0A0E0SCP7 pp 229533.A0A1C3YK21
229533.A0A0E0SCP7 pp 229533.A0A1C3YI88
229533.A0A0E0SCP7 pp 229533.I1RDH0
229533.A0A0E0SCP7 pp 229533.A0A1C3YMJ3
229533.A0A0E0SCP7 pp 229533.I1R9U7
229533.A0A0E0SCP7 pp 229533.A0A1C3YN36
229533.A0A0E0SCP7 pp 229533.I1RVZ9
229533.A0A0E0SCP7 pp 229533.I1RBI6
229533.A0A0E0SCP7 pp 229533.I1RE23
229533.A0A0E0SCP7 pp 229533.I1RRJ6
229533.A0A0E0SCP7 pp 229533.I1S0Q5
229533.A0A0E0SCP7 pp 229533.I1RW06
229533.A0A0E0SCP7 pp 229533.A0A1C3YJN7
229533.A0A0E0SCP7 pp 229533.I1RTN6
229533.A0A0E0SCP7 pp 229533.I1RY29
229533.A0A0E0SCP7 pp 229533.I1RX64
229533.A0A0E0SCP7 pp 229533.I1RKI2
229533.A0A0E0SCP7 pp 229533.A0A1C3YI64
229533.A0A0E0SCP7 pp 229533.I1REI0
229533.A0A0E0SCP7 pp 229533.I1RSJ1
229533.A0A0E0SCP7 pp 229533.A0A1C3YKE4
229533.A0A0E0SCP7 pp 229533.A0A1C3YML3
229533.I1S352 pp 229533.I1S8R3
229533.I1S352 pp 229533.V6RIV9
229533.I1S352 pp 229533.Q4IJK2
229533.I1S352 pp 229533.V6RCG5
229533.I1S352 pp 229533.Q4I1Y5
229533.I1S352 pp 229533.V6R042
229533.I1S352 pp 229533.V6RMW6
229533.I1S352 pp 229533.I1S504
229533.I1S352 pp 229533.I1S8P5
229533.I1S352 pp 229533.V6QW22
229533.I1S352 pp 229533.I1S503
229533.I1S352 pp 229533.V6R3X5
229533.I1S352 pp 229533.V6RTJ8
229533.I1RRY1 pp 229533.I1RX64
229533.I1RRY1 pp 229533.I1RSY2
229533.I1RRY1 pp 229533.I1S0D5
229533.I1RRY1 pp 229533.I1RZT1
229533.I1RRY1 pp 229533.I1RZ98
229533.I1RRY1 pp 229533.I1RUG4
229533.I1RRY1 pp 229533.I1RY58
229533.I1RRY1 pp 229533.Q4INS6
229533.I1RRY1 pp 229533.I1RSC7
229533.I1RRY1 pp 229533.I1RSZ3
229533.I1RRY1 pp 229533.I1S4P6
229533.I1RRY1 pp 229533.V6RGI1
229533.I1RRY1 pp 229533.I1RXF0
229533.I1RRY1 pp 229533.I1RYQ1
229533.I1RRY1 pp 229533.I1RXV3
229533.I1RRY1 pp 229533.I1RZ86
229533.I1RRY1 pp 229533.I1S209
229533.I1RRY1 pp 229533.I1S998
229533.I1RRY1 pp 229533.I1RVZ9
229533.I1RRY1 pp 229533.I1S5Z3
229533.I1RRY1 pp 229533.I1RWI8
229533.I1RRY1 pp 229533.I1RUR0
229533.I1RRY1 pp 229533.I1S461
229533.I1RRY1 pp 229533.I1RVX9
229533.I1RRY1 pp 229533.I1S987
229533.I1RMV7 pp 229533.I1RPP9
229533.I1RN55 pp 229533.I1S2F2
229533.I1RN55 pp 229533.I1RYQ1
229533.I1RN55 pp 229533.I1RYX9
229533.I1RN55 pp 229533.I1RQS5
229533.I1RN55 pp 229533.I1S3G4
229533.I1RN55 pp 229533.Q4IQM6

106727	229533.I1RN55	pp	229533.I1RWI8
106728	229533.I1RN55	pp	229533.I1S4P6
106729	229533.I1RN55	pp	229533.V6R382
106730	229533.I1RN55	pp	229533.I1RSN7
106731	229533.I1RN55	pp	229533.I1RYR2
106732	229533.I1RN55	pp	229533.I1RUG1
106733	229533.I1RN55	pp	229533.I1RW16
106734	229533.I1RN55	pp	229533.I1S998
106735	229533.I1RN55	pp	229533.Q4HYB8
106736	229533.I1RN55	pp	229533.I1RUG4
106737	229533.I1RN55	pp	229533.I1S0A6
106738	229533.I1RN55	pp	229533.I1RRS7
106739	229533.I1RN55	pp	229533.I1RSA7
106740	229533.I1RN55	pp	229533.I1RZP8
106741	229533.I1RN55	pp	229533.V6RLW4
106742	229533.I1RN55	pp	229533.I1RSR9
106743	229533.I1RN55	pp	229533.I1RZ86
106744	229533.I1RN55	pp	229533.I1RSL6
106745	229533.I1RN55	pp	229533.I1RTN6
106746	229533.I1RN55	pp	229533.I1RSC7
106747	229533.I1RN55	pp	229533.I1RQI6
106748	229533.I1RN55	pp	229533.I1RQJ4
106749	229533.I1RN55	pp	229533.I1RX64
106750	229533.I1RN55	pp	229533.I1RPQ1
106751	229533.I1RN55	pp	229533.Q4IC11
106752	229533.I1RN55	pp	229533.I1RNI4
106753	229533.I1RN55	pp	229533.I1RSP7
106754	229533.I1RN55	pp	229533.I1S209
106755	229533.I1RN55	pp	229533.I1RPZ0
106756	229533.I1RN55	pp	229533.Q4IP51
106757	229533.I1RN55	pp	229533.I1S987
106758	229533.I1RN55	pp	229533.I1RQ92
106759	229533.I1RN55	pp	229533.I1RZ94
106760	229533.I1RN55	pp	229533.I1RY73
106761	229533.I1RN55	pp	229533.I1S461
106762	229533.I1RN55	pp	229533.I1RQ13
106763	229533.I1RN55	pp	229533.I1S604
106764	229533.I1RN55	pp	229533.I1S1W8
106765	229533.I1RN55	pp	229533.Q4IEA5
106766	229533.I1RN55	pp	229533.I1RSI4
106767	229533.I1RN55	pp	229533.Q4IJU0
106768	229533.I1RN55	pp	229533.I1RRT1
106769	229533.I1RN55	pp	229533.I1RUG2
106770	229533.I1RN55	pp	229533.I1S283
106771	229533.I1RPP9	pp	229533.I1RZX0
106772	229533.I1RP27	pp	229533.I1RS20
106773	229533.I1RP27	pp	229533.I1RQC9
106774	229533.I1RP27	pp	229533.I1RWL2
106775	229533.I1RP27	pp	229533.I1RRT8
106776	229533.I1RP27	pp	229533.I1S0R7
106777	229533.I1RP27	pp	229533.I1RZ39
106778	229533.I1RP27	pp	229533.I1RT04
106779	229533.I1RP27	pp	229533.I1S0X5
106780	229533.I1RP27	pp	229533.I1S1W7
106781	229533.I1RP27	pp	229533.I1RXD7
106782	229533.I1RP27	pp	229533.I1RQH3
106783	229533.I1RP27	pp	229533.I1RR73
106784	229533.I1RP27	pp	229533.V6R3X5
106785	229533.I1RP27	pp	229533.I1RPW4
106786	229533.I1RP27	pp	229533.I1RWI5
106787	229533.I1RP27	pp	229533.I1S2E9
106788	229533.I1RP27	pp	229533.Q4IFI0
106789	229533.I1RP27	pp	229533.I1RPU3
106790	229533.I1RP27	pp	229533.I1S2B8
106791	229533.I1RP27	pp	229533.V6RAC9
106792	229533.I1RP27	pp	229533.I1RW25
106793	229533.I1RP27	pp	229533.I1RZZ9
106794	229533.I1RP27	pp	229533.I1RSM2
106795	229533.I1RP27	pp	229533.I1RSV2
106796	229533.I1RP27	pp	229533.I1RXU7
106797	229533.I1RP27	pp	229533.V6QYF0
106798	229533.I1RP27	pp	229533.I1RPP3
106799	229533.I1RP27	pp	229533.V6QUW4

106800	229533.I1RP27	pp	229533.I1RS47
106801	229533.I1RP27	pp	229533.I1RTD5
106802	229533.I1RP27	pp	229533.I1RQG4
106803	229533.I1RP27	pp	229533.I1RT33
106804	229533.I1RP27	pp	229533.I1S8P5
106805	229533.I1RP27	pp	229533.I1RSQ7
106806	229533.I1RP27	pp	229533.I1RWJ4
106807	229533.I1RP27	pp	229533.Q4HZ42
106808	229533.I1RP27	pp	229533.I1RRY9
106809	229533.I1RP27	pp	229533.I1RQ23
106810	229533.I1RP27	pp	229533.I1RRI3
106811	229533.I1RP27	pp	229533.V6RTJ8
106812	229533.I1RP27	pp	229533.I1RPM4
106813	229533.I1RP27	pp	229533.I1RQK8
106814	229533.I1RP27	pp	229533.I1RWN6
106815	229533.I1RP27	pp	229533.I1S2G7
106816	229533.I1RP27	pp	229533.I1S558
106817	229533.I1RP27	pp	229533.I1S503
106818	229533.I1RP27	pp	229533.I1RYS0
106819	229533.I1RP27	pp	229533.I1RP28
106820	229533.I1RP27	pp	229533.I1RX46
106821	229533.I1RP27	pp	229533.I1RSK1
106822	229533.I1RP27	pp	229533.I1RQS5
106823	229533.I1RP27	pp	229533.I1RVQ0
106824	229533.I1RP27	pp	229533.I1RW71
106825	229533.I1RP27	pp	229533.I1S504
106826	229533.I1RP27	pp	229533.I1RTH6
106827	229533.I1RP27	pp	229533.I1RQA3
106828	229533.I1RP27	pp	229533.I1S1J7
106829	229533.I1RP27	pp	229533.Q4IJK2
106830	229533.I1RP27	pp	229533.V6R227
106831	229533.I1RP27	pp	229533.I1S1W4
106832	229533.I1RP27	pp	229533.I1RZE4
106833	229533.I1RP27	pp	229533.I1RZK5
106834	229533.I1RP27	pp	229533.I1RYW3
106835	229533.I1RP27	pp	229533.I1RZZ1
106836	229533.I1RP27	pp	229533.I1S2C0
106837	229533.I1RP27	pp	229533.I1S2E8
106838	229533.I1RP27	pp	229533.I1RWT6
106839	229533.I1RP27	pp	229533.I1RQE7
106840	229533.I1RP27	pp	229533.V6RBN2
106841	229533.I1RP27	pp	229533.I1S1C7
106842	229533.I1RP27	pp	229533.I1RY03
106843	229533.I1RP27	pp	229533.I1RT35
106844	229533.I1RP27	pp	229533.I1RWN5
106845	229533.I1RP27	pp	229533.I1RYY5
106846	229533.I1RP27	pp	229533.I1S0N4
106847	229533.I1RP27	pp	229533.Q4IEK8
106848	229533.I1RP27	pp	229533.I1RY44
106849	229533.I1RP27	pp	229533.I1S5B0
106850	229533.I1RP27	pp	229533.I1RWD7
106851	229533.I1RP27	pp	229533.I1RZM1
106852	229533.I1RP27	pp	229533.I1RWX5
106853	229533.I1RP27	pp	229533.Q4ICA8
106854	229533.I1RP27	pp	229533.I1RRU9
106855	229533.I1RP27	pp	229533.I1RW99
106856	229533.I1RP27	pp	229533.Q4IJ56
106857	229533.I1RP27	pp	229533.V6RRH2
106858	229533.I1RP27	pp	229533.I1RRV1
106859	229533.I1RP27	pp	229533.I1RSI0
106860	229533.I1RP27	pp	229533.Q4I6A4
106861	229533.I1RP27	pp	229533.I1RSQ3
106862	229533.I1RP27	pp	229533.I1RQ81
106863	229533.I1RP27	pp	229533.I1RT03
106864	229533.I1RP27	pp	229533.V6R042
106865	229533.I1RP27	pp	229533.I1RVX0
106866	229533.I1RP27	pp	229533.Q4IJM4
106867	229533.I1RP27	pp	229533.I1RPP4
106868	229533.I1RP27	pp	229533.Q4IAA0
106869	229533.I1RP27	pp	229533.I1RSS4
106870	229533.I1RP27	pp	229533.V6RMW6
106871	229533.I1RP27	pp	229533.I1RWD3
106872	229533.I1RP27	pp	229533.Q4I7K4

106873	229533.I1RP27	pp	229533.I1RUG3
106874	229533.I1RP27	pp	229533.I1RQ31
106875	229533.I1RP27	pp	229533.I1RTA0
106876	229533.I1RP27	pp	229533.I1RRN5
106877	229533.I1RP27	pp	229533.I1S0J1
106878	229533.I1RP27	pp	229533.I1RWE8
106879	229533.I1S8J7	pp	229533.Q4I5R3
106880	229533.I1S8J7	pp	229533.Q4IER8
106881	229533.A0A1C3YKH9	pp	229533.I1RTI7
106882	229533.A0A1C3YKH9	pp	229533.I1RFH9
106883	229533.A0A1C3YKH7	pp	229533.I1RRZ3
106884	229533.I1RSV2	pp	229533.I1S0N4
106885	229533.I1RSV2	pp	229533.I1RWN6
106886	229533.I1RSV2	pp	229533.Q4IBS2
106887	229533.I1RSV2	pp	229533.V6R042
106888	229533.I1RSV2	pp	229533.Q4ILH3
106889	229533.I1RSV2	pp	229533.I1S044
106890	229533.I1RSV2	pp	229533.I1RYY5
106891	229533.I1RSV2	pp	229533.V6RMW6
106892	229533.I1RSV2	pp	229533.I1RVX0
106893	229533.I1RSV2	pp	229533.I1RT03
106894	229533.I1RSV2	pp	229533.Q4I6A4
106895	229533.I1RSV2	pp	229533.I1S1C7
106896	229533.I1RSV2	pp	229533.Q4IJM4
106897	229533.I1RSV2	pp	229533.I1RY03
106898	229533.I1RSV2	pp	229533.I1RY44
106899	229533.I1RSV2	pp	229533.I1RZM1
106900	229533.I1RSV2	pp	229533.I1RX46
106901	229533.I1RSV2	pp	229533.I1S2C0
106902	229533.I1RSV2	pp	229533.I1S1X9
106903	229533.I1RSV2	pp	229533.I1S0P4
106904	229533.I1RSV2	pp	229533.I1RZK5
106905	229533.I1RSV2	pp	229533.I1RWN5
106906	229533.I1RSV2	pp	229533.I1RTH6
106907	229533.I1RSV2	pp	229533.I1S503
106908	229533.I1RSV2	pp	229533.Q4I2J8
106909	229533.I1RSV2	pp	229533.I1RWT6
106910	229533.I1RSV2	pp	229533.V6RTJ8
106911	229533.I1RSV2	pp	229533.I1RWD7
106912	229533.I1RSV2	pp	229533.I1RTD5
106913	229533.I1RSV2	pp	229533.I1S504
106914	229533.I1RSV2	pp	229533.I1S1W4
106915	229533.I1RSV2	pp	229533.I1RXU7
106916	229533.I1RSV2	pp	229533.I1RXD7
106917	229533.I1RSV2	pp	229533.I1S0J1
106918	229533.I1RSV2	pp	229533.V6R057
106919	229533.I1RSV2	pp	229533.I1RTA0
106920	229533.I1RSV2	pp	229533.I1S2E9
106921	229533.I1RSV2	pp	229533.I1S2E8
106922	229533.I1RSV2	pp	229533.I1RZZ1
106923	229533.I1RSV2	pp	229533.Q4HYB8
106924	229533.I1RSV2	pp	229533.I1S0R7
106925	229533.I1RSV2	pp	229533.I1S2G7
106926	229533.I1RSV2	pp	229533.I1RZ60
106927	229533.I1RSV2	pp	229533.I1RWI5
106928	229533.I1RSV2	pp	229533.I1RT04
106929	229533.I1RSV2	pp	229533.Q4IJK2
106930	229533.I1RSV2	pp	229533.I1RX24
106931	229533.I1RSV2	pp	229533.I1S8P5
106932	229533.I1RSV2	pp	229533.I1RW71
106933	229533.I1RSV2	pp	229533.V6QUW4
106934	229533.I1RSV2	pp	229533.I1RZZ9
106935	229533.I1RSV2	pp	229533.I1RZ39
106936	229533.I1RSV2	pp	229533.V6R3X5
106937	229533.I1RSV2	pp	229533.I1RWL2
106938	229533.I1RSV2	pp	229533.I1S2B8
106939	229533.I1RSV2	pp	229533.I1RW25
106940	229533.I1RKI2	pp	229533.I1RNE2
106941	229533.I1RKI2	pp	229533.I1S283
106942	229533.I1RKI2	pp	229533.I1RQ93
106943	229533.I1RKI2	pp	229533.I1S788
106944	229533.I1RKI2	pp	229533.I1RN55
106945	229533.I1RKI2	pp	229533.I1RMS2

106946	229533.I1RKI2	pp	229533.Q4INS6
106947	229533.I1RKI2	pp	229533.I1RVZ9
106948	229533.I1RKI2	pp	229533.I1RUR0
106949	229533.I1RKI2	pp	229533.I1S461
106950	229533.I1RKI2	pp	229533.I1RQH8
106951	229533.I1RKI2	pp	229533.I1S1W8
106952	229533.I1RKI2	pp	229533.I1RX64
106953	229533.I1RKI2	pp	229533.I1RQ13
106954	229533.I1RKI2	pp	229533.I1RZ94
106955	229533.I1RKI2	pp	229533.I1RMQ7
106956	229533.I1RKI2	pp	229533.I1RMX3
106957	229533.I1RKI2	pp	229533.I1RRT1
106958	229533.I1RKI2	pp	229533.I1RTN6
106959	229533.I1RKI2	pp	229533.I1S987
106960	229533.I1RKI2	pp	229533.I1RSJ1
106961	229533.I1RKI2	pp	229533.I1RZP8
106962	229533.I1RKI2	pp	229533.I1RSP7
106963	229533.I1RKI2	pp	229533.I1RPQ1
106964	229533.I1RKI2	pp	229533.Q4IC11
106965	229533.I1RKI2	pp	229533.I1S276
106966	229533.I1RKI2	pp	229533.I1RSC7
106967	229533.I1RKI2	pp	229533.I1RQJ4
106968	229533.I1RKI2	pp	229533.I1RQI6
106969	229533.I1RKI2	pp	229533.I1RRY1
106970	229533.I1RKI2	pp	229533.I1RZ98
106971	229533.I1RKI2	pp	229533.I1RMJ8
106972	229533.I1RKI2	pp	229533.I1RSI4
106973	229533.I1RKI2	pp	229533.I1RRS7
106974	229533.I1RKI2	pp	229533.I1S3G4
106975	229533.I1RKI2	pp	229533.I1RSR9
106976	229533.I1RKI2	pp	229533.I1S0D5
106977	229533.I1RKI2	pp	229533.I1SAA0
106978	229533.I1RKI2	pp	229533.I1RP99
106979	229533.I1RKI2	pp	229533.I1RPM7
106980	229533.I1RKI2	pp	229533.V6R382
106981	229533.I1RKI2	pp	229533.I1RZ86
106982	229533.I1RKI2	pp	229533.Q4IB89
106983	229533.I1RKI2	pp	229533.I1SAD2
106984	229533.I1RKI2	pp	229533.I1RSL8
106985	229533.I1RKI2	pp	229533.I1RUG4
106986	229533.I1RKI2	pp	229533.I1RN57
106987	229533.I1RKI2	pp	229533.I1RVW0
106988	229533.I1RKI2	pp	229533.V6RLW4
106989	229533.I1RKI2	pp	229533.I1RSA7
106990	229533.I1RKI2	pp	229533.I1S998
106991	229533.I1RKI2	pp	229533.I1RKR9
106992	229533.I1RKI2	pp	229533.I1RWI8
106993	229533.I1RKI2	pp	229533.I1S4P6
106994	229533.I1RKI2	pp	229533.I1RY29
106995	229533.I1RKI2	pp	229533.I1S523
106996	229533.I1RKI2	pp	229533.I1RRJ6
106997	229533.I1RKI2	pp	229533.I1RYR2
106998	229533.I1RKI2	pp	229533.I1RSZ3
106999	229533.I1RKI2	pp	229533.I1RPF5
107000	229533.I1RKI2	pp	229533.I1RYQ1
107001	229533.I1RKI2	pp	229533.I1RSN7
107002	229533.I1RKI2	pp	229533.I1RYX9
107003	229533.I1RKI2	pp	229533.I1RUF8
107004	229533.I1RUN1	pp	229533.I1RZK3
107005	229533.I1RUN1	pp	229533.I1RWI6
107006	229533.I1RUN1	pp	229533.I1S213
107007	229533.I1RUN1	pp	229533.I1S7W2
107008	229533.I1RUN1	pp	229533.I1RUN2
107009	229533.I1RJZ7	pp	229533.I1S503
107010	229533.I1RJZ7	pp	229533.I1RSS7
107011	229533.I1RJZ7	pp	229533.I1RP27
107012	229533.I1RJZ7	pp	229533.I1RQG4
107013	229533.I1RJZ7	pp	229533.Q4IAS1
107014	229533.I1RJZ7	pp	229533.I1RRJ8
107015	229533.I1RJZ7	pp	229533.I1RT04
107016	229533.I1RJZ7	pp	229533.I1S2E9
107017	229533.I1RJZ7	pp	229533.I1RPP3
107018	229533.I1RJZ7	pp	229533.I1S8P5

107019 229533.I1RJZ7 pp 229533.Q4HXF0
107020 229533.I1RJZ7 pp 229533.I1RTD5
107021 229533.I1RJZ7 pp 229533.I1RUP6
107022 229533.I1RJZ7 pp 229533.I1RT40
107023 229533.I1RJZ7 pp 229533.I1RWK3
107024 229533.I1RJZ7 pp 229533.I1RT81
107025 229533.I1RJZ7 pp 229533.I1RSV2
107026 229533.I1RJZ7 pp 229533.I1RYI5
107027 229533.I1RJZ7 pp 229533.I1RV91
107028 229533.I1RJZ7 pp 229533.Q4I830
107029 229533.I1RJZ7 pp 229533.I1S2G7
107030 229533.I1RJZ7 pp 229533.I1RN36
107031 229533.I1RJZ7 pp 229533.I1RQ65
107032 229533.I1RJZ7 pp 229533.I1RR95
107033 229533.I1RJZ7 pp 229533.I1RW15
107034 229533.I1RJZ7 pp 229533.I1RVZ2
107035 229533.I1RJZ7 pp 229533.I1S0L8
107036 229533.I1RJZ7 pp 229533.I1RNR2
107037 229533.I1RJZ7 pp 229533.I1RTA1
107038 229533.I1RJZ7 pp 229533.I1RKI1
107039 229533.I1RJZ7 pp 229533.Q4HZ42
107040 229533.I1RJZ7 pp 229533.I1RZR2
107041 229533.I1RJZ7 pp 229533.I1RS47
107042 229533.I1RJZ7 pp 229533.I1RXT8
107043 229533.I1RJZ7 pp 229533.Q4IF76
107044 229533.I1RJZ7 pp 229533.I1RZZ9
107045 229533.I1RJZ7 pp 229533.Q4HW67
107046 229533.I1RJZ7 pp 229533.I1RWX7
107047 229533.I1RJZ7 pp 229533.I1RWI5
107048 229533.I1RJZ7 pp 229533.I1S9L6
107049 229533.I1RJZ7 pp 229533.I1S0L4
107050 229533.I1RJZ7 pp 229533.V6R283
107051 229533.I1RJZ7 pp 229533.I1RMP2
107052 229533.I1RJZ7 pp 229533.I1RS20
107053 229533.I1RJZ7 pp 229533.I1RP28
107054 229533.I1RJZ7 pp 229533.I1RKD2
107055 229533.I1RJZ7 pp 229533.I1S2F2
107056 229533.I1RJZ7 pp 229533.I1RRQ3
107057 229533.I1RJZ7 pp 229533.I1RN67
107058 229533.I1RJZ7 pp 229533.I1S0F6
107059 229533.I1RJZ7 pp 229533.I1RX24
107060 229533.I1RJZ7 pp 229533.Q4I662
107061 229533.I1RJZ7 pp 229533.I1RPM4
107062 229533.I1RJZ7 pp 229533.V6R3X5
107063 229533.I1RJZ7 pp 229533.I1RV78
107064 229533.I1RJZ7 pp 229533.I1S2H4
107065 229533.I1RJZ7 pp 229533.I1RR73
107066 229533.I1RJZ7 pp 229533.I1RSZ7
107067 229533.I1RJZ7 pp 229533.O74638
107068 229533.I1RJZ7 pp 229533.I1RR31
107069 229533.I1RJZ7 pp 229533.I1S0X5
107070 229533.I1RJZ7 pp 229533.I1RRT8
107071 229533.I1RJZ7 pp 229533.I1RXC2
107072 229533.I1RJZ7 pp 229533.I1S5R1
107073 229533.I1RJZ7 pp 229533.Q4IPI4
107074 229533.I1RJZ7 pp 229533.I1RWJ1
107075 229533.I1RJZ7 pp 229533.I1RZG0
107076 229533.I1RJZ7 pp 229533.I1RZ39
107077 229533.I1RJZ7 pp 229533.I1RVY0
107078 229533.I1RJZ7 pp 229533.I1RKR9
107079 229533.I1RJZ7 pp 229533.I1RNN1
107080 229533.I1RJZ7 pp 229533.I1RKJ6
107081 229533.I1RJZ7 pp 229533.I1RU69
107082 229533.I1RJZ7 pp 229533.I1RSL0
107083 229533.I1RJZ7 pp 229533.I1S9Y1
107084 229533.I1RJZ7 pp 229533.I1S2V2
107085 229533.I1RJZ7 pp 229533.V6RMW6
107086 229533.I1RJZ7 pp 229533.I1RRM5
107087 229533.I1RJZ7 pp 229533.I1S2B8
107088 229533.I1RJZ7 pp 229533.I1RWK9
107089 229533.I1RJZ7 pp 229533.I1RSQ3
107090 229533.I1RJZ7 pp 229533.I1SAN9
107091 229533.I1RJZ7 pp 229533.I1RZS6

107092 229533.I1RJZ7 pp 229533.I1RUG3
107093 229533.I1RJZ7 pp 229533.I1RMQ5
107094 229533.I1RJZ7 pp 229533.V6R042
107095 229533.I1RJZ7 pp 229533.I1RZ86
107096 229533.I1RJZ7 pp 229533.I1RS17
107097 229533.I1RJZ7 pp 229533.I1RRV1
107098 229533.I1RJZ7 pp 229533.I1RNC1
107099 229533.I1RJZ7 pp 229533.I1RNU6
107100 229533.I1RJZ7 pp 229533.V6RGN5
107101 229533.I1RJZ7 pp 229533.I1RPN8
107102 229533.I1RJZ7 pp 229533.I1S2C0
107103 229533.I1RJZ7 pp 229533.I1RM25
107104 229533.I1RJZ7 pp 229533.I1RS90
107105 229533.I1RJZ7 pp 229533.I1S0J1
107106 229533.I1RJZ7 pp 229533.I1RTE9
107107 229533.I1RJZ7 pp 229533.I1S8R3
107108 229533.I1RJZ7 pp 229533.I1RTA0
107109 229533.I1RJZ7 pp 229533.I1S471
107110 229533.I1RJZ7 pp 229533.I1RTH6
107111 229533.I1RJZ7 pp 229533.I1S5R6
107112 229533.I1RJZ7 pp 229533.I1RWH4
107113 229533.I1RJZ7 pp 229533.I1RZW8
107114 229533.I1RJZ7 pp 229533.Q4IB89
107115 229533.I1RJZ7 pp 229533.I1RW46
107116 229533.I1RJZ7 pp 229533.Q4IN00
107117 229533.I1RJZ7 pp 229533.I1SA33
107118 229533.I1RJZ7 pp 229533.I1RQ21
107119 229533.I1RJZ7 pp 229533.I1RMN6
107120 229533.I1RJZ7 pp 229533.I1RST4
107121 229533.I1RJZ7 pp 229533.I1RZ79
107122 229533.I1RJZ7 pp 229533.I1S1W4
107123 229533.I1RJZ7 pp 229533.I1RWE8
107124 229533.I1RJZ7 pp 229533.I1RRI2
107125 229533.I1RJZ7 pp 229533.I1RQP8
107126 229533.I1RJZ7 pp 229533.I1RY44
107127 229533.I1RJZ7 pp 229533.Q4I7K4
107128 229533.I1RJZ7 pp 229533.I1RM96
107129 229533.I1RJZ7 pp 229533.I1RWX5
107130 229533.I1RJZ7 pp 229533.I1RWD3
107131 229533.I1RJZ7 pp 229533.I1RPS0
107132 229533.I1RJZ7 pp 229533.Q4HZB8
107133 229533.I1RJZ7 pp 229533.I1RRN5
107134 229533.I1RJZ7 pp 229533.I1S1C7
107135 229533.I1RJZ7 pp 229533.I1RPP4
107136 229533.I1RJZ7 pp 229533.I1RWN5
107137 229533.I1RJZ7 pp 229533.I1S1W9
107138 229533.I1RJZ7 pp 229533.I1RYW3
107139 229533.I1RJZ7 pp 229533.V6RIV9
107140 229533.I1RJZ7 pp 229533.Q4HWE2
107141 229533.I1RJZ7 pp 229533.I1RWN6
107142 229533.I1RJZ7 pp 229533.I1S1X9
107143 229533.I1RJZ7 pp 229533.I1RNE4
107144 229533.I1RJZ7 pp 229533.I1S855
107145 229533.I1RJZ7 pp 229533.I1RRF0
107146 229533.I1RJZ7 pp 229533.I1RT03
107147 229533.I1RJZ7 pp 229533.I1RVX0
107148 229533.I1RJZ7 pp 229533.V6RRH2
107149 229533.I1RJZ7 pp 229533.I1RSQ7
107150 229533.I1RJZ7 pp 229533.I1RQE7
107151 229533.I1RJZ7 pp 229533.I1RXD7
107152 229533.I1RJZ7 pp 229533.I1S0R7
107153 229533.I1RJZ7 pp 229533.I1RME6
107154 229533.I1RJZ7 pp 229533.I1S2B7
107155 229533.I1RJZ7 pp 229533.I1RN18
107156 229533.I1RJZ7 pp 229533.I1S2E8
107157 229533.I1RJZ7 pp 229533.I1S9L5
107158 229533.I1RJZ7 pp 229533.I1RQC9
107159 229533.I1RJZ7 pp 229533.I1RPN6
107160 229533.I1RJZ7 pp 229533.I1RSM2
107161 229533.I1RJZ7 pp 229533.V6QW22
107162 229533.I1RJZ7 pp 229533.I1RSD8
107163 229533.I1RJZ7 pp 229533.I1RXU7
107164 229533.I1RJZ7 pp 229533.Q4I1Y5

107165 229533.I1RJZ7 pp 229533.I1S241
107166 229533.I1RJZ7 pp 229533.I1RTG5
107167 229533.I1RJZ7 pp 229533.I1RN61
107168 229533.I1RJZ7 pp 229533.Q4I624
107169 229533.I1RJZ7 pp 229533.Q4INZ9
107170 229533.I1RJZ7 pp 229533.I1RQJ5
107171 229533.I1RJZ7 pp 229533.I1RPN4
107172 229533.I1RJZ7 pp 229533.I1RZJ6
107173 229533.I1RJZ7 pp 229533.I1RWM6
107174 229533.I1RJZ7 pp 229533.I1RW71
107175 229533.I1RJZ7 pp 229533.I1RND2
107176 229533.I1RJZ7 pp 229533.V6RTJ8
107177 229533.I1RJZ7 pp 229533.I1RM03
107178 229533.I1RJZ7 pp 229533.V6R057
107179 229533.I1RJZ7 pp 229533.I1S4S7
107180 229533.I1RJZ7 pp 229533.I1RR49
107181 229533.I1RJZ7 pp 229533.I1RQH1
107182 229533.I1RJZ7 pp 229533.I1S0F5
107183 229533.I1RJZ7 pp 229533.Q4HZ68
107184 229533.I1RJZ7 pp 229533.I1S540
107185 229533.I1RJZ7 pp 229533.I1RNJ2
107186 229533.I1RJZ7 pp 229533.I1S296
107187 229533.I1RJZ7 pp 229533.Q4I1N3
107188 229533.I1RJZ7 pp 229533.I1S0L3
107189 229533.I1RJZ7 pp 229533.I1RMU8
107190 229533.I1RJZ7 pp 229533.I1RMW3
107191 229533.I1RJZ7 pp 229533.V6R1K4
107192 229533.I1RJZ7 pp 229533.I1RKC6
107193 229533.I1RJZ7 pp 229533.I1RVQ0
107194 229533.I1RJZ7 pp 229533.I1S3J4
107195 229533.I1RJZ7 pp 229533.I1RX46
107196 229533.I1RJZ7 pp 229533.I1RNH1
107197 229533.I1RJZ7 pp 229533.I1S854
107198 229533.I1RJZ7 pp 229533.I1RKQ5
107199 229533.I1RJZ7 pp 229533.Q5I2J3
107200 229533.I1RJZ7 pp 229533.I1RY40
107201 229533.I1RJZ7 pp 229533.I1RYH0
107202 229533.I1RJZ7 pp 229533.I1S6F0
107203 229533.I1RJZ7 pp 229533.Q4IFI0
107204 229533.I1RJZ7 pp 229533.I1RT41
107205 229533.I1RJZ7 pp 229533.Q4HVW2
107206 229533.I1RJZ7 pp 229533.I1RRN4
107207 229533.I1RJZ7 pp 229533.I1RRV4
107208 229533.I1RJZ7 pp 229533.V6RCG5
107209 229533.I1RJZ7 pp 229533.I1RT80
107210 229533.I1RJZ7 pp 229533.Q4I8P5
107211 229533.I1RJZ7 pp 229533.Q4IJH1
107212 229533.I1RJZ7 pp 229533.I1RS18
107213 229533.I1RJZ7 pp 229533.I1RQM6
107214 229533.I1RJZ7 pp 229533.I1S235
107215 229533.I1RJZ7 pp 229533.Q4IAA0
107216 229533.I1RJZ7 pp 229533.I1RT05
107217 229533.I1RJZ7 pp 229533.I1RQ81
107218 229533.I1RJZ7 pp 229533.V6RCZ8
107219 229533.I1RJZ7 pp 229533.I1RVP5
107220 229533.I1RJZ7 pp 229533.I1RP66
107221 229533.I1RJZ7 pp 229533.I1S0M9
107222 229533.I1RJZ7 pp 229533.I1RNH6
107223 229533.I1RJZ7 pp 229533.I1RPW9
107224 229533.I1RJZ7 pp 229533.I1RZM8
107225 229533.I1RJZ7 pp 229533.I1RPL2
107226 229533.I1RJZ7 pp 229533.I1RNP1
107227 229533.I1RJZ7 pp 229533.I1RN58
107228 229533.I1RJZ7 pp 229533.I1RR24
107229 229533.I1RJZ7 pp 229533.I1SA32
107230 229533.I1RJZ7 pp 229533.I1RM67
107231 229533.I1RJZ7 pp 229533.Q4IJ56
107232 229533.I1RJZ7 pp 229533.I1RMC0
107233 229533.I1RJZ7 pp 229533.I1RWJ8
107234 229533.I1RJZ7 pp 229533.I1RQ31
107235 229533.I1RJZ7 pp 229533.I1RZ94
107236 229533.I1RJZ7 pp 229533.I1RW99
107237 229533.I1RJZ7 pp 229533.I1RXC4

107238	229533.I1RJZ7	pp	229533.I1S5B0
107239	229533.I1RJZ7	pp	229533.I1RMQ4
107240	229533.I1RJZ7	pp	229533.Q4IP34
107241	229533.I1RJZ7	pp	229533.I1RWT6
107242	229533.I1RJZ7	pp	229533.V6QYF0
107243	229533.I1RJZ7	pp	229533.I1RWM4
107244	229533.I1RJZ7	pp	229533.Q4INI2
107245	229533.I1RJZ7	pp	229533.V6R227
107246	229533.I1RJZ7	pp	229533.Q4HVU7
107247	229533.I1RJZ7	pp	229533.I1S270
107248	229533.I1RJZ7	pp	229533.I1S361
107249	229533.I1RJZ7	pp	229533.V6QUW4
107250	229533.I1RJZ7	pp	229533.Q4IBS2
107251	229533.I1RJZ7	pp	229533.I1S504
107252	229533.I1RJZ7	pp	229533.I1RPW4
107253	229533.I1RJZ7	pp	229533.I1RUI6
107254	229533.I1RJZ7	pp	229533.Q4HZS8
107255	229533.I1RJZ7	pp	229533.I1RW25
107256	229533.I1RJZ7	pp	229533.I1RRU9
107257	229533.I1RJZ7	pp	229533.I1RZF6
107258	229533.I1RJZ7	pp	229533.I1S0Z3
107259	229533.I1RJZ7	pp	229533.Q4I5I4
107260	229533.I1RJZ7	pp	229533.I1RZM1
107261	229533.I1RJZ7	pp	229533.V6QUE6
107262	229533.I1RJZ7	pp	229533.I1RMB7
107263	229533.I1RJZ7	pp	229533.I1RLU6
107264	229533.I1RJZ7	pp	229533.Q4IEK8
107265	229533.I1RJZ7	pp	229533.I1RNQ5
107266	229533.I1RJZ7	pp	229533.I1RQA5
107267	229533.I1RJZ7	pp	229533.I1RSI0
107268	229533.I1RJZ7	pp	229533.I1RU02
107269	229533.I1RJZ7	pp	229533.I1S4Q4
107270	229533.I1RJZ7	pp	229533.I1RYS0
107271	229533.I1RJZ7	pp	229533.I1S0C1
107272	229533.I1RJZ7	pp	229533.I1RPW1
107273	229533.I1RJZ7	pp	229533.I1RVZ0
107274	229533.I1RJZ7	pp	229533.I1RW87
107275	229533.I1RJZ7	pp	229533.I1RQA2
107276	229533.I1RJZ7	pp	229533.I1RK95
107277	229533.I1RJZ7	pp	229533.I1RRC4
107278	229533.I1RJZ7	pp	229533.I1RZK5
107279	229533.I1RJZ7	pp	229533.I1RN65
107280	229533.I1RJZ7	pp	229533.I1RN99
107281	229533.I1RJZ7	pp	229533.Q4IER8
107282	229533.I1RJZ7	pp	229533.Q4IJM4
107283	229533.I1RJZ7	pp	229533.I1RYL5
107284	229533.I1RJZ7	pp	229533.I1RT65
107285	229533.I1RJZ7	pp	229533.Q4I7F9
107286	229533.I1RJZ7	pp	229533.I1RYY5
107287	229533.I1RJZ7	pp	229533.V6RBN2
107288	229533.I1RJZ7	pp	229533.I1RSR3
107289	229533.I1RJZ7	pp	229533.I1RY03
107290	229533.I1RJZ7	pp	229533.I1RRJ0
107291	229533.I1RJZ7	pp	229533.I1RRY9
107292	229533.I1RJZ7	pp	229533.I1RRE0
107293	229533.I1RJZ7	pp	229533.I1RZZ1
107294	229533.I1RJZ7	pp	229533.Q4I061
107295	229533.I1RJZ7	pp	229533.Q4I948
107296	229533.I1RJZ7	pp	229533.I1RWJ3
107297	229533.I1RJZ7	pp	229533.Q4IJK2
107298	229533.I1RJZ7	pp	229533.I1RQA3
107299	229533.I1RJZ7	pp	229533.I1S1J7
107300	229533.I1RJZ7	pp	229533.I1RWD7
107301	229533.I1RJZ7	pp	229533.I1RKQ9
107302	229533.I1RJZ7	pp	229533.I1RKK8
107303	229533.I1R9N5	pp	229533.I1S2D5
107304	229533.I1R9N5	pp	229533.I1S094
107305	229533.I1R9N5	pp	229533.I1RDN3
107306	229533.I1R9N5	pp	229533.I1RY49
107307	229533.I1R9N5	pp	229533.I1S334
107308	229533.I1R9N5	pp	229533.I1RLA2
107309	229533.I1R9N5	pp	229533.I1RNM7
107310	229533.I1R9N5	pp	229533.I1RRL5

107311 229533.I1R9N5 pp 229533.I1RC74
107312 229533.I1R9N5 pp 229533.I1S6H3
107313 229533.I1R9N5 pp 229533.I1RA13
107314 229533.I1R9N5 pp 229533.V6RDH3
107315 229533.I1R9N5 pp 229533.I1RDU0
107316 229533.I1R9N5 pp 229533.I1RCT7
107317 229533.I1R9N5 pp 229533.I1S8F8
107318 229533.I1R9N5 pp 229533.I1RST9
107319 229533.I1R9N5 pp 229533.I1RUY5
107320 229533.I1R9N5 pp 229533.I1RGZ3
107321 229533.I1R9N5 pp 229533.I1S3H8
107322 229533.I1R9N5 pp 229533.I1RNG4
107323 229533.I1R9N5 pp 229533.I1RZ25
107324 229533.I1R9N5 pp 229533.I1S0A5
107325 229533.I1R9N5 pp 229533.I1RKB2
107326 229533.I1R9N5 pp 229533.I1RZ38
107327 229533.I1R9N5 pp 229533.I1RPV8
107328 229533.I1R9N5 pp 229533.I1RJS1
107329 229533.I1R9N5 pp 229533.I1RZ56
107330 229533.I1RZ98 pp 229533.I1S998
107331 229533.I1RZ98 pp 229533.V6RLW4
107332 229533.I1RZ98 pp 229533.I1S0Q4
107333 229533.I1RZ98 pp 229533.I1S0D5
107334 229533.I1RZ98 pp 229533.I1S209
107335 229533.I1RZ98 pp 229533.I1S523
107336 229533.I1RZ98 pp 229533.I1S3G4
107337 229533.I1RZ98 pp 229533.I1S0Q5
107338 229533.I1RZ98 pp 229533.I1S1W8
107339 229533.I1RZ98 pp 229533.I1S788
107340 229533.I1RZ98 pp 229533.Q4INS6
107341 229533.I1RZ98 pp 229533.I1S987
107342 229533.I1RZ98 pp 229533.Q4IB89
107343 229533.A0A1C3YKN3 pp 229533.I1RSQ3
107344 229533.A0A1C3YKN3 pp 229533.I1RZK5
107345 229533.A0A1C3YKN3 pp 229533.I1RXD7
107346 229533.A0A1C3YKN3 pp 229533.I1RR73
107347 229533.A0A1C3YKN3 pp 229533.V6R3X5
107348 229533.A0A1C3YKN3 pp 229533.V6R042
107349 229533.A0A1C3YKN3 pp 229533.V6RCG5
107350 229533.A0A1C3YKN3 pp 229533.V6RMW6
107351 229533.A0A1C3YKN3 pp 229533.I1RQC9
107352 229533.A0A1C3YKN3 pp 229533.I1S2E9
107353 229533.A0A1C3YKN3 pp 229533.I1RZ39
107354 229533.A0A1C3YKN3 pp 229533.I1RA70
107355 229533.A0A1C3YKN3 pp 229533.I1RFU8
107356 229533.A0A1C3YKN3 pp 229533.V6RCZ8
107357 229533.A0A1C3YKN3 pp 229533.I1RTH6
107358 229533.A0A1C3YKN3 pp 229533.I1RJU3
107359 229533.A0A1C3YKN3 pp 229533.A0A1C3YN30
107360 229533.A0A1C3YKN3 pp 229533.I1S0R7
107361 229533.A0A1C3YKN3 pp 229533.I1RT04
107362 229533.A0A1C3YKN3 pp 229533.I1S8R3
107363 229533.A0A1C3YKN3 pp 229533.I1RRT8
107364 229533.A0A1C3YKN3 pp 229533.I1RCG4
107365 229533.A0A1C3YKN3 pp 229533.V6QW22
107366 229533.A0A1C3YKN3 pp 229533.I1S5R1
107367 229533.A0A1C3YKN3 pp 229533.I1RX46
107368 229533.A0A1C3YKN3 pp 229533.I1RB92
107369 229533.A0A1C3YKN3 pp 229533.Q4I8P5
107370 229533.A0A1C3YKN3 pp 229533.I1RAU5
107371 229533.A0A1C3YKN3 pp 229533.I1RSQ7
107372 229533.A0A1C3YKN3 pp 229533.I1RD22
107373 229533.A0A1C3YKN3 pp 229533.I1RJV3
107374 229533.A0A1C3YKN3 pp 229533.I1RT03
107375 229533.A0A1C3YKN3 pp 229533.I1RWJ8
107376 229533.A0A1C3YKN3 pp 229533.I1RA11
107377 229533.A0A1C3YKN3 pp 229533.I1RVX0
107378 229533.A0A1C3YKN3 pp 229533.A0A1C3YKV3
107379 229533.A0A1C3YKN3 pp 229533.I1RJW6
107380 229533.A0A1C3YKN3 pp 229533.A0A1C3YLK8
107381 229533.A0A1C3YKN3 pp 229533.I1RBC9
107382 229533.A0A1C3YKN3 pp 229533.I1SA33
107383 229533.A0A1C3YKN3 pp 229533.I1RE22

107384 229533.A0A1C3YKN3 pp 229533.I1RS17
107385 229533.A0A1C3YKN3 pp 229533.I1S503
107386 229533.A0A1C3YKN3 pp 229533.I1RC66
107387 229533.A0A1C3YKN3 pp 229533.I1RI82
107388 229533.A0A1C3YKN3 pp 229533.I1RNQ5
107389 229533.A0A1C3YKN3 pp 229533.I1RRI2
107390 229533.A0A1C3YKN3 pp 229533.V6RTJ8
107391 229533.A0A1C3YKN3 pp 229533.I1RBY4
107392 229533.A0A1C3YKN3 pp 229533.I1RLU6
107393 229533.A0A1C3YKN3 pp 229533.I1S0L4
107394 229533.A0A1C3YKN3 pp 229533.I1RZW8
107395 229533.A0A1C3YKN3 pp 229533.I1RTD5
107396 229533.A0A1C3YKN3 pp 229533.I1RY44
107397 229533.A0A1C3YKN3 pp 229533.I1RWN6
107398 229533.A0A1C3YKN3 pp 229533.I1RZM1
107399 229533.A0A1C3YKN3 pp 229533.I1RBT1
107400 229533.A0A1C3YKN3 pp 229533.I1S5B0
107401 229533.A0A1C3YKN3 pp 229533.I1RNC1
107402 229533.A0A1C3YKN3 pp 229533.I1RW25
107403 229533.A0A1C3YKN3 pp 229533.I1RRY9
107404 229533.A0A1C3YKN3 pp 229533.I1RZZ9
107405 229533.A0A1C3YKN3 pp 229533.I1RVQ0
107406 229533.A0A1C3YKN3 pp 229533.I1RAW0
107407 229533.A0A1C3YKN3 pp 229533.I1RWN5
107408 229533.A0A1C3YKN3 pp 229533.I1S1C7
107409 229533.A0A1C3YKN3 pp 229533.I1RYW3
107410 229533.A0A1C3YKN3 pp 229533.I1RV91
107411 229533.A0A1C3YKN3 pp 229533.I1RJV4
107412 229533.A0A1C3YKN3 pp 229533.I1RW99
107413 229533.A0A1C3YKN3 pp 229533.V6RIV9
107414 229533.A0A1C3YKN3 pp 229533.I1RBS6
107415 229533.A0A1C3YKN3 pp 229533.I1RN67
107416 229533.A0A1C3YKN3 pp 229533.I1RR24
107417 229533.A0A1C3YKN3 pp 229533.I1RUG3
107418 229533.A0A1C3YKN3 pp 229533.I1RJZ7
107419 229533.A0A1C3YKN3 pp 229533.I1RT40
107420 229533.A0A1C3YKN3 pp 229533.I1RGA4
107421 229533.A0A1C3YKN3 pp 229533.I1RZM8
107422 229533.A0A1C3YKN3 pp 229533.I1RAX6
107423 229533.A0A1C3YKN3 pp 229533.I1S0F6
107424 229533.A0A1C3YKN3 pp 229533.I1SA32
107425 229533.A0A1C3YKN3 pp 229533.I1RZZ1
107426 229533.A0A1C3YKN3 pp 229533.I1RCR8
107427 229533.A0A1C3YKN3 pp 229533.I1RY03
107428 229533.A0A1C3YKN3 pp 229533.I1S2B8
107429 229533.A0A1C3YKN3 pp 229533.I1RRM5
107430 229533.A0A1C3YKN3 pp 229533.I1RCH5
107431 229533.A0A1C3YKN3 pp 229533.I1RQE7
107432 229533.A0A1C3YKN3 pp 229533.I1RAU7
107433 229533.A0A1C3YKN3 pp 229533.I1RPM4
107434 229533.A0A1C3YKN3 pp 229533.V6R1K4
107435 229533.A0A1C3YKN3 pp 229533.I1S0L3
107436 229533.A0A1C3YKN3 pp 229533.I1S0J1
107437 229533.A0A1C3YKN3 pp 229533.I1RW46
107438 229533.A0A1C3YKN3 pp 229533.I1RD52
107439 229533.A0A1C3YKN3 pp 229533.I1RFL4
107440 229533.A0A1C3YKN3 pp 229533.I1RE01
107441 229533.A0A1C3YKN3 pp 229533.V6R057
107442 229533.A0A1C3YKN3 pp 229533.I1RWK3
107443 229533.A0A1C3YKN3 pp 229533.I1S504
107444 229533.A0A1C3YKN3 pp 229533.I1RN18
107445 229533.A0A1C3YKN3 pp 229533.Q4IJK2
107446 229533.A0A1C3YKN3 pp 229533.Q4I624
107447 229533.A0A1C3YKN3 pp 229533.I1RPP4
107448 229533.A0A1C3YKN3 pp 229533.I1REK8
107449 229533.A0A1C3YKN3 pp 229533.I1RD17
107450 229533.A0A1C3YKN3 pp 229533.I1RPP3
107451 229533.I1RZM0 pp 229533.I1SAM9
107452 229533.I1RZM0 pp 229533.I1RZS6
107453 229533.I1RZM0 pp 229533.Q4HZE0
107454 229533.I1RZM0 pp 229533.V6QX21
107455 229533.I1RZM0 pp 229533.Q4IJT0
107456 229533.I1RZM0 pp 229533.I1S0Z8

229533.I1RZM0 pp 229533.V6QUV7
229533.I1RZM0 pp 229533.I1S9Z4
229533.I1RZM0 pp 229533.Q4HY20
229533.I1RZM0 pp 229533.I1S4Z7
229533.I1RMW0 pp 229533.I1RNW8
229533.I1RMW0 pp 229533.I1RQP5
229533.I1RT19 pp 229533.I1RWM0
229533.I1RT19 pp 229533.Q4IPI1
229533.I1RT19 pp 229533.P0C431
229533.I1RT19 pp 229533.I1S4T3
229533.A0A098DE18 pp 229533.I1S044
229533.A0A098DE18 pp 229533.A0A0E0RZN1
229533.A0A098DE18 pp 229533.Q4IP34
229533.A0A098DE18 pp 229533.I1RMN6
229533.A0A098DE18 pp 229533.I1RGC2
229533.A0A098DE18 pp 229533.I1RRK8
229533.A0A098DE18 pp 229533.I1RSI6
229533.A0A098DE18 pp 229533.I1RD22
229533.A0A098DE18 pp 229533.Q4IBS2
229533.A0A098DE18 pp 229533.I1RRV1
229533.A0A098DE18 pp 229533.I1RAU5
229533.A0A098DE18 pp 229533.I1RX08
229533.A0A098DE18 pp 229533.I1RRU9
229533.A0A098DE18 pp 229533.I1RNU6
229533.A0A098DE18 pp 229533.V6R042
229533.A0A098DE18 pp 229533.I1RM25
229533.A0A098DE18 pp 229533.I1RWB5
229533.A0A098DE18 pp 229533.V6RMW6
229533.A0A098DE18 pp 229533.A0A1C3YJ29
229533.A0A098DE18 pp 229533.I1RRM7
229533.A0A098DE18 pp 229533.I1RCR8
229533.A0A098DE18 pp 229533.I1RM96
229533.A0A098DE18 pp 229533.I1RNQ5
229533.A0A098DE18 pp 229533.Q4IEK8
229533.A0A098DE18 pp 229533.Q4IJ56
229533.A0A098DE18 pp 229533.Q4IN63
229533.A0A098DE18 pp 229533.I1S0M9
229533.A0A098DE18 pp 229533.I1RC33
229533.A0A098DE18 pp 229533.I1RMC0
229533.A0A098DE18 pp 229533.I1RTH6
229533.A0A098DE18 pp 229533.I1RQ31
229533.A0A098DE18 pp 229533.I1RWD3
229533.A0A098DE18 pp 229533.A0A1C3YJG0
229533.A0A098DE18 pp 229533.I1RZB5
229533.A0A098DE18 pp 229533.I1RHM4
229533.A0A098DE18 pp 229533.I1RNH6
229533.A0A098DE18 pp 229533.I1R9X7
229533.A0A098DE18 pp 229533.I1RB27
229533.A0A098DE18 pp 229533.I1RFU8
229533.A0A098DE18 pp 229533.I1RTE9
229533.A0A098DE18 pp 229533.I1S021
229533.A0A098DE18 pp 229533.I1RK83
229533.A0A098DE18 pp 229533.Q4I7K4
229533.A0A098DE18 pp 229533.I1RMN7
229533.A0A098DE18 pp 229533.Q4IAA0
229533.A0A098DE18 pp 229533.I1S2C0
229533.A0A098DE18 pp 229533.I1RYB9
229533.A0A098DE18 pp 229533.I1RZK5
229533.A0A098DE18 pp 229533.I1RWS7
229533.A0A098DE18 pp 229533.I1RSG2
229533.A0A098DE18 pp 229533.I1RNY2
229533.A0A098DE18 pp 229533.I1RAC1
229533.A0A098DE18 pp 229533.I1S0C1
229533.A0A098DE18 pp 229533.I1S060
229533.A0A098DE18 pp 229533.Q4I327
229533.A0A098DE18 pp 229533.I1RX14
229533.A0A098DE18 pp 229533.I1S0J1
229533.A0A098DE18 pp 229533.I1RBD2
229533.A0A098DE18 pp 229533.I1RSR8
229533.A0A098DE18 pp 229533.A0A1C3YL07
229533.A0A098DE18 pp 229533.I1RY03
229533.A0A098DE18 pp 229533.I1RC72
229533.A0A098DE18 pp 229533.I1RC27

107530	229533.A0A098DE18	pp	229533.A0A0E0S621
107531	229533.A0A098DE18	pp	229533.I1S504
107532	229533.A0A098DE18	pp	229533.A0A1C3YJR3
107533	229533.A0A098DE18	pp	229533.I1S1C7
107534	229533.A0A098DE18	pp	229533.V6RGN5
107535	229533.A0A098DE18	pp	229533.I1RTD7
107536	229533.A0A098DE18	pp	229533.I1RJV3
107537	229533.A0A098DE18	pp	229533.I1RMD5
107538	229533.A0A098DE18	pp	229533.A0A0E0SJN2
107539	229533.A0A098DE18	pp	229533.I1RRI2
107540	229533.A0A098DE18	pp	229533.I1RZF6
107541	229533.A0A098DE18	pp	229533.I1S855
107542	229533.A0A098DE18	pp	229533.A0A1C3YKW9
107543	229533.A0A098DE18	pp	229533.I1RRR7
107544	229533.A0A098DE18	pp	229533.I1RC81
107545	229533.A0A098DE18	pp	229533.I1RCL3
107546	229533.A0A098DE18	pp	229533.V6R227
107547	229533.A0A098DE18	pp	229533.I1RBZ3
107548	229533.A0A098DE18	pp	229533.I1S274
107549	229533.A0A098DE18	pp	229533.I1RSC6
107550	229533.A0A098DE18	pp	229533.A0A098DRR8
107551	229533.A0A098DE18	pp	229533.I1RBL7
107552	229533.A0A098DE18	pp	229533.I1RG56
107553	229533.A0A098DE18	pp	229533.I1RPY1
107554	229533.A0A098DE18	pp	229533.I1RSS4
107555	229533.A0A098DE18	pp	229533.I1RDK7
107556	229533.A0A098DE18	pp	229533.I1RWN5
107557	229533.A0A098DE18	pp	229533.I1RAW0
107558	229533.A0A098DE18	pp	229533.Q4HWE2
107559	229533.A0A098DE18	pp	229533.I1RSR3
107560	229533.A0A098DE18	pp	229533.I1R9E6
107561	229533.A0A098DE18	pp	229533.I1RBI8
107562	229533.A0A098DE18	pp	229533.I1RA48
107563	229533.A0A098DE18	pp	229533.I1S4T3
107564	229533.A0A098DE18	pp	229533.V6RGY3
107565	229533.A0A098DE18	pp	229533.I1RVX0
107566	229533.A0A098DE18	pp	229533.I1S1U7
107567	229533.A0A098DE18	pp	229533.I1RT03
107568	229533.A0A098DE18	pp	229533.I1R9V7
107569	229533.A0A098DE18	pp	229533.I1RWN6
107570	229533.A0A098DE18	pp	229533.I1RY63
107571	229533.A0A098DE18	pp	229533.I1RNK2
107572	229533.A0A098DE18	pp	229533.I1RRY9
107573	229533.A0A098DE18	pp	229533.I1RAN5
107574	229533.A0A098DE18	pp	229533.I1S1X9
107575	229533.A0A098DE18	pp	229533.A0A0E0RP78
107576	229533.A0A098DE18	pp	229533.I1RCN5
107577	229533.A0A098DE18	pp	229533.I1RVZ0
107578	229533.A0A098DE18	pp	229533.I1RQ28
107579	229533.A0A098DE18	pp	229533.A0A1C3YIJ3
107580	229533.A0A098DE18	pp	229533.I1RQA2
107581	229533.A0A098DE18	pp	229533.I1RK95
107582	229533.A0A098DE18	pp	229533.I1S854
107583	229533.A0A098DE18	pp	229533.I1RE00
107584	229533.A0A098DE18	pp	229533.I1RDG0
107585	229533.A0A098DE18	pp	229533.I1RFJ9
107586	229533.A0A098DE18	pp	229533.A0A098DEC2
107587	229533.A0A098DE18	pp	229533.I1S0M3
107588	229533.A0A098DE18	pp	229533.I1RN99
107589	229533.A0A098DE18	pp	229533.I1RJL4
107590	229533.A0A098DE18	pp	229533.Q4IPI1
107591	229533.A0A098DE18	pp	229533.I1S2U2
107592	229533.A0A098DE18	pp	229533.I1RSD8
107593	229533.A0A098DE18	pp	229533.I1RZE5
107594	229533.A0A098DE18	pp	229533.I1RYB1
107595	229533.A0A098DE18	pp	229533.A0A1C3YMW0
107596	229533.A0A098DE18	pp	229533.I1RRT2
107597	229533.A0A098DE18	pp	229533.I1S2E8
107598	229533.A0A098DE18	pp	229533.I1RQS5
107599	229533.A0A098DE18	pp	229533.I1RT04
107600	229533.A0A098DE18	pp	229533.Q4I7F9
107601	229533.A0A098DE18	pp	229533.I1S0R7
107602	229533.A0A098DE18	pp	229533.I1RJZ6

107603	229533.A0A098DE18	pp	229533.I1RX46
107604	229533.A0A098DE18	pp	229533.I1RR00
107605	229533.A0A098DE18	pp	229533.A0A1C3YLF5
107606	229533.A0A098DE18	pp	229533.A0A0E0SFN0
107607	229533.A0A098DE18	pp	229533.I1RZK6
107608	229533.A0A098DE18	pp	229533.I1RXD7
107609	229533.A0A098DE18	pp	229533.A0A1C3YMP5
107610	229533.A0A098DE18	pp	229533.Q4I830
107611	229533.A0A098DE18	pp	229533.I1RZZ1
107612	229533.A0A098DE18	pp	229533.I1S296
107613	229533.A0A098DE18	pp	229533.I1RKL3
107614	229533.A0A098DE18	pp	229533.I1RW71
107615	229533.A0A098DE18	pp	229533.Q4HZ68
107616	229533.A0A098DE18	pp	229533.I1RRJ8
107617	229533.A0A098DE18	pp	229533.I1RAF8
107618	229533.A0A098DE18	pp	229533.I1RC06
107619	229533.A0A098DE18	pp	229533.I1S8I8
107620	229533.A0A098DE18	pp	229533.I1RF08
107621	229533.A0A098DE18	pp	229533.A0A0E0SFM9
107622	229533.A0A098DE18	pp	229533.I1RSQ7
107623	229533.A0A098DE18	pp	229533.A0A1C3YLK9
107624	229533.A0A098DE18	pp	229533.I1RT98
107625	229533.A0A098DE18	pp	229533.I1RAU7
107626	229533.A0A098DE18	pp	229533.I1RQJ5
107627	229533.A0A098DE18	pp	229533.I1RAZ8
107628	229533.A0A098DE18	pp	229533.I1RZJ6
107629	229533.A0A098DE18	pp	229533.I1RKC6
107630	229533.A0A098DE18	pp	229533.I1RZ99
107631	229533.A0A098DE18	pp	229533.A0A1C3YMK1
107632	229533.A0A098DE18	pp	229533.I1RTD5
107633	229533.A0A098DE18	pp	229533.I1RPP3
107634	229533.A0A098DE18	pp	229533.I1S0Z9
107635	229533.A0A098DE18	pp	229533.A0A098DVS9
107636	229533.A0A098DE18	pp	229533.I1RWF7
107637	229533.A0A098DE18	pp	229533.I1RSL9
107638	229533.A0A098DE18	pp	229533.I1RS30
107639	229533.A0A098DE18	pp	229533.I1RBC7
107640	229533.A0A098DE18	pp	229533.I1RFN1
107641	229533.A0A098DE18	pp	229533.I1RWH5
107642	229533.A0A098DE18	pp	229533.I1RRP3
107643	229533.A0A098DE18	pp	229533.V6R283
107644	229533.A0A098DE18	pp	229533.I1RC82
107645	229533.A0A098DE18	pp	229533.I1RGA4
107646	229533.A0A098DE18	pp	229533.I1RNH1
107647	229533.A0A098DE18	pp	229533.I1RAX6
107648	229533.A0A098DE18	pp	229533.I1RAY2
107649	229533.A0A098DE18	pp	229533.A0A0E0S6M0
107650	229533.A0A098DE18	pp	229533.Q4HW67
107651	229533.A0A098DE18	pp	229533.Q4IQW0
107652	229533.A0A098DE18	pp	229533.I1S6F0
107653	229533.A0A098DE18	pp	229533.Q4I5R9
107654	229533.A0A098DE18	pp	229533.I1RFG0
107655	229533.A0A098DE18	pp	229533.I1RRQ3
107656	229533.A0A098DE18	pp	229533.A0A1C3YKK5
107657	229533.A0A098DE18	pp	229533.I1RC03
107658	229533.A0A098DE18	pp	229533.I1RKQ5
107659	229533.A0A098DE18	pp	229533.A0A1C3YN63
107660	229533.A0A098DE18	pp	229533.A0A1C3YIY6
107661	229533.A0A098DE18	pp	229533.A0A0E0RNT7
107662	229533.A0A098DE18	pp	229533.I1S503
107663	229533.A0A098DE18	pp	229533.A0A0E0SGM4
107664	229533.A0A098DE18	pp	229533.I1RFW5
107665	229533.A0A098DE18	pp	229533.A0A0E0S7B3
107666	229533.A0A098DE18	pp	229533.I1RD56
107667	229533.A0A098DE18	pp	229533.Q4HZ42
107668	229533.A0A098DE18	pp	229533.I1RLV9
107669	229533.A0A098DE18	pp	229533.Q4IAS1
107670	229533.A0A098DE18	pp	229533.A0A0E0SAR4
107671	229533.A0A098DE18	pp	229533.I1RAV6
107672	229533.A0A098DE18	pp	229533.I1RT33
107673	229533.A0A098DE18	pp	229533.I1S8E2
107674	229533.A0A098DE18	pp	229533.I1RRH5
107675	229533.A0A098DE18	pp	229533.A0A1C3YHW7

107676 229533.A0A098DE18 pp 229533.I1RTF4
107677 229533.A0A098DE18 pp 229533.I1RS47
107678 229533.A0A098DE18 pp 229533.I1RQ65
107679 229533.A0A098DE18 pp 229533.Q4IJH1
107680 229533.A0A098DE18 pp 229533.A0A1C3YKM9
107681 229533.A0A098DE18 pp 229533.A0A1C3YIP5
107682 229533.A0A098DE18 pp 229533.A0A1C3YK72
107683 229533.A0A098DE18 pp 229533.A0A1C3YIY7
107684 229533.A0A098DE18 pp 229533.Q4HZ47
107685 229533.A0A098DE18 pp 229533.I1RZ39
107686 229533.A0A098DE18 pp 229533.I1RT80
107687 229533.A0A098DE18 pp 229533.I1S9Y1
107688 229533.A0A098DE18 pp 229533.I1RNV6
107689 229533.A0A098DE18 pp 229533.I1RPG6
107690 229533.A0A098DE18 pp 229533.I1RR73
107691 229533.A0A098DE18 pp 229533.A0A1C3YIN1
107692 229533.A0A098DE18 pp 229533.I1RM67
107693 229533.A0A098DE18 pp 229533.I1RRT8
107694 229533.A0A098DE18 pp 229533.V6R3X5
107695 229533.A0A098DE18 pp 229533.I1RC29
107696 229533.A0A098DE18 pp 229533.I1RND6
107697 229533.A0A098DE18 pp 229533.I1RNA0
107698 229533.A0A098DE18 pp 229533.Q4IQK7
107699 229533.A0A098DE18 pp 229533.I1S6U8
107700 229533.A0A098DE18 pp 229533.I1RDA0
107701 229533.A0A098DE18 pp 229533.I1RBG9
107702 229533.A0A098DE18 pp 229533.I1RKF9
107703 229533.A0A098DE18 pp 229533.I1S253
107704 229533.A0A098DE18 pp 229533.V6QYF0
107705 229533.A0A098DE18 pp 229533.I1RBN1
107706 229533.A0A098DE18 pp 229533.I1RX24
107707 229533.A0A098DE18 pp 229533.Q4HVW2
107708 229533.A0A098DE18 pp 229533.I1RMK3
107709 229533.A0A098DE18 pp 229533.I1RZS6
107710 229533.A0A098DE18 pp 229533.A0A1C3YHQ4
107711 229533.A0A098DE18 pp 229533.I1RM06
107712 229533.A0A098DE18 pp 229533.Q4IF76
107713 229533.A0A098DE18 pp 229533.I1RC32
107714 229533.A0A098DE18 pp 229533.Q4I662
107715 229533.A0A098DE18 pp 229533.I1RT05
107716 229533.A0A098DE18 pp 229533.A0A098DPR9
107717 229533.A0A098DE18 pp 229533.I1RQM6
107718 229533.A0A098DE18 pp 229533.I1RFS3
107719 229533.A0A098DE18 pp 229533.I1RG51
107720 229533.A0A098DE18 pp 229533.I1RU69
107721 229533.A0A098DE18 pp 229533.A0A1C3YHX8
107722 229533.A0A098DE18 pp 229533.I1RD64
107723 229533.A0A098DE18 pp 229533.I1RGF6
107724 229533.A0A098DE18 pp 229533.A0A0E0RUI4
107725 229533.A0A098DE18 pp 229533.I1R9G0
107726 229533.A0A098DE18 pp 229533.I1RJZ7
107727 229533.A0A098DE18 pp 229533.I1S9U2
107728 229533.A0A098DE18 pp 229533.Q4IFI0
107729 229533.A0A098DE18 pp 229533.I1RPW9
107730 229533.A0A098DE18 pp 229533.I1RA29
107731 229533.A0A098DE18 pp 229533.I1S2B8
107732 229533.A0A098DE18 pp 229533.I1RPU0
107733 229533.A0A098DE18 pp 229533.I1RL23
107734 229533.A0A098DE18 pp 229533.I1RKJ6
107735 229533.A0A098DE18 pp 229533.A0A098DXE2
107736 229533.A0A098DE18 pp 229533.I1RW25
107737 229533.A0A098DE18 pp 229533.A0A1C3YMI6
107738 229533.A0A098DE18 pp 229533.I1RZV6
107739 229533.A0A098DE18 pp 229533.I1RAC2
107740 229533.I1RKB5 pp 229533.I1S0P6
107741 229533.I1RKB5 pp 229533.V6R3J1
107742 229533.I1RKB5 pp 229533.I1S3G4
107743 229533.I1RKB5 pp 229533.I1S1W8
107744 229533.I1RKB5 pp 229533.I1RP86
107745 229533.I1RKB5 pp 229533.I1RT54
107746 229533.I1RKB5 pp 229533.I1RUM7
107747 229533.I1RKB5 pp 229533.V6RUY9
107748 229533.I1RKB5 pp 229533.V6RUG6

107749	229533.I1RKB5	pp	229533.V6R3R0
107750	229533.I1RKB5	pp	229533.I1RPT0
107751	229533.I1RKB5	pp	229533.I1RNG4
107752	229533.I1RKB5	pp	229533.I1RW43
107753	229533.I1RKB5	pp	229533.I1RZ13
107754	229533.I1RKB5	pp	229533.I1RQE0
107755	229533.I1RKB5	pp	229533.I1SAC4
107756	229533.I1RKB5	pp	229533.I1RKP3
107757	229533.I1RKB5	pp	229533.I1RZM3
107758	229533.I1RKB5	pp	229533.I1RZ78
107759	229533.I1RKB5	pp	229533.V6RLM7
107760	229533.I1RKB5	pp	229533.I1RYK0
107761	229533.I1RKB5	pp	229533.I1RPS2
107762	229533.I1RKB5	pp	229533.I1RRT3
107763	229533.I1RKB5	pp	229533.I1S272
107764	229533.I1RKB5	pp	229533.I1RNU7
107765	229533.I1RKB5	pp	229533.I1S9Z4
107766	229533.I1RKB5	pp	229533.I1RSY2
107767	229533.I1RKB5	pp	229533.I1S8J1
107768	229533.I1RBI6	pp	229533.I1RC54
107769	229533.I1RBI6	pp	229533.I1S209
107770	229533.I1RBI6	pp	229533.I1RX64
107771	229533.I1RBI6	pp	229533.I1RMQ7
107772	229533.I1RBI6	pp	229533.I1RTN6
107773	229533.I1RBI6	pp	229533.I1RBW1
107774	229533.I1RBI6	pp	229533.I1RCM7
107775	229533.I1RBI6	pp	229533.I1RSY2
107776	229533.I1RBI6	pp	229533.Q00909
107777	229533.I1RBI6	pp	229533.I1RKB7
107778	229533.I1RBI6	pp	229533.I1RBW0
107779	229533.I1RBI6	pp	229533.I1RVZ9
107780	229533.I1RBI6	pp	229533.Q4IPI4
107781	229533.I1RBI6	pp	229533.I1RKI2
107782	229533.I1RBI6	pp	229533.I1RYI5
107783	229533.I1RBI6	pp	229533.I1RZ94
107784	229533.I1RJV4	pp	229533.I1RSX1
107785	229533.I1RJV4	pp	229533.I1RW71
107786	229533.I1RJV4	pp	229533.I1S0F6
107787	229533.I1RJV4	pp	229533.I1RSQ7
107788	229533.I1RJV4	pp	229533.I1S352
107789	229533.I1RJV4	pp	229533.I1RWD4
107790	229533.I1RJV4	pp	229533.V6QW22
107791	229533.I1RJV4	pp	229533.I1RPP3
107792	229533.I1RJV4	pp	229533.I1RN67
107793	229533.I1RJV4	pp	229533.I1RRJ8
107794	229533.I1RJV4	pp	229533.I1S8P5
107795	229533.I1RJV4	pp	229533.V6R4J1
107796	229533.I1RJV4	pp	229533.I1RVQ0
107797	229533.I1RJV4	pp	229533.I1S503
107798	229533.I1RJV4	pp	229533.I1RS47
107799	229533.I1RJV4	pp	229533.I1RLN6
107800	229533.I1RJV4	pp	229533.I1S0F5
107801	229533.I1RJV4	pp	229533.I1RJW6
107802	229533.I1RJV4	pp	229533.V6R1K4
107803	229533.I1RJV4	pp	229533.I1RX46
107804	229533.I1RJV4	pp	229533.I1S0L3
107805	229533.I1RJV4	pp	229533.I1RLX7
107806	229533.I1RJV4	pp	229533.I1RRT2
107807	229533.I1RJV4	pp	229533.I1RN63
107808	229533.I1RJV4	pp	229533.I1RWK3
107809	229533.I1RJV4	pp	229533.I1S1B6
107810	229533.I1RJV4	pp	229533.V6RTJ8
107811	229533.I1RJV4	pp	229533.I1S2M6
107812	229533.I1RJV4	pp	229533.I1S7A8
107813	229533.I1RJV4	pp	229533.I1S2E9
107814	229533.I1RJV4	pp	229533.I1RQC9
107815	229533.I1RJV4	pp	229533.I1RRG3
107816	229533.I1RJV4	pp	229533.I1RT40
107817	229533.I1RJV4	pp	229533.Q4IJT5
107818	229533.I1RJV4	pp	229533.I1S0M6
107819	229533.I1RJV4	pp	229533.I1RUQ2
107820	229533.I1RJV4	pp	229533.I1RV91
107821	229533.I1RJV4	pp	229533.I1RM06

107822	229533.I1RJV4	pp	229533.I1RTD5
107823	229533.I1RJV4	pp	229533.I1RQH3
107824	229533.I1RJV4	pp	229533.I1RX04
107825	229533.I1RJV4	pp	229533.I1RXD7
107826	229533.I1RJV4	pp	229533.I1S0R7
107827	229533.I1RJV4	pp	229533.I1RWJ8
107828	229533.I1RJV4	pp	229533.I1S2B8
107829	229533.I1RJV4	pp	229533.I1RLC7
107830	229533.I1RJV4	pp	229533.I1RWL2
107831	229533.I1RJV4	pp	229533.I1RJZ7
107832	229533.I1RJV4	pp	229533.I1RRM5
107833	229533.I1RJV4	pp	229533.I1RT04
107834	229533.I1RJV4	pp	229533.V6RUY9
107835	229533.I1RJV4	pp	229533.I1RRT8
107836	229533.I1RJV4	pp	229533.I1RZJ5
107837	229533.I1RJV4	pp	229533.I1RW25
107838	229533.I1RJV4	pp	229533.I1S5R1
107839	229533.I1RJV4	pp	229533.I1RS20
107840	229533.I1RJV4	pp	229533.I1RX25
107841	229533.I1RJV4	pp	229533.I1RNA5
107842	229533.I1RJV4	pp	229533.I1S7S9
107843	229533.I1RJV4	pp	229533.I1S2H4
107844	229533.I1RJV4	pp	229533.I1RR73
107845	229533.I1RJV4	pp	229533.I1RW07
107846	229533.I1RJV4	pp	229533.I1RMK3
107847	229533.I1RJV4	pp	229533.I1RR24
107848	229533.I1RJV4	pp	229533.Q4HVW2
107849	229533.I1RJV4	pp	229533.I1RQS8
107850	229533.I1RJV4	pp	229533.I1RQ23
107851	229533.I1RJV4	pp	229533.I1RPM4
107852	229533.I1RJV4	pp	229533.I1S0L4
107853	229533.I1RJV4	pp	229533.V6QYF0
107854	229533.I1RJV4	pp	229533.Q4HZB8
107855	229533.I1RJV4	pp	229533.I1RWN5
107856	229533.I1RJV4	pp	229533.I1S0X6
107857	229533.I1RJV4	pp	229533.I1RPY1
107858	229533.I1RJV4	pp	229533.I1RZZ9
107859	229533.I1RJV4	pp	229533.I1RNA0
107860	229533.I1RJV4	pp	229533.I1RRQ6
107861	229533.I1RJV4	pp	229533.V6RIV9
107862	229533.I1RJV4	pp	229533.I1RWI5
107863	229533.I1RJV4	pp	229533.I1RZM1
107864	229533.I1RJV4	pp	229533.V6QUW4
107865	229533.I1RJV4	pp	229533.I1RMC8
107866	229533.I1RJV4	pp	229533.I1RY44
107867	229533.I1RJV4	pp	229533.I1S8V3
107868	229533.I1RJV4	pp	229533.Q4I8P5
107869	229533.I1RJV4	pp	229533.I1RS18
107870	229533.I1RJV4	pp	229533.I1RRL9
107871	229533.I1RJV4	pp	229533.I1RNV6
107872	229533.I1RJV4	pp	229533.I1RPP4
107873	229533.I1RJV4	pp	229533.Q4IEK8
107874	229533.I1RJV4	pp	229533.V6QUE6
107875	229533.I1RJV4	pp	229533.I1RZ39
107876	229533.I1RJV4	pp	229533.I1RNQ5
107877	229533.I1RJV4	pp	229533.I1RTF4
107878	229533.I1RJV4	pp	229533.I1RLU6
107879	229533.I1RJV4	pp	229533.I1S504
107880	229533.I1RJV4	pp	229533.I1SA33
107881	229533.I1RJV4	pp	229533.V6R3X5
107882	229533.I1RJV4	pp	229533.I1RWQ8
107883	229533.I1RJV4	pp	229533.I1RRE5
107884	229533.I1RJV4	pp	229533.I1RRU9
107885	229533.I1RJV4	pp	229533.I1S8V4
107886	229533.I1RJV4	pp	229533.Q4I2J8
107887	229533.I1RJV4	pp	229533.I1RWT6
107888	229533.I1RJV4	pp	229533.I1RW99
107889	229533.I1RJV4	pp	229533.Q4I5Z5
107890	229533.I1RJV4	pp	229533.I1RKC6
107891	229533.I1RJV4	pp	229533.V6RGN5
107892	229533.I1RJV4	pp	229533.I1S5B0
107893	229533.I1RJV4	pp	229533.I1RLM7
107894	229533.I1RJV4	pp	229533.I1RSI0

107895 229533.I1RJV4 pp 229533.V6R057
107896 229533.I1RJV4 pp 229533.Q4I624
107897 229533.I1RJV4 pp 229533.Q4I283
107898 229533.I1RJV4 pp 229533.V6R227
107899 229533.I1RJV4 pp 229533.I1RXF0
107900 229533.I1RJV4 pp 229533.I1S0N4
107901 229533.I1RJV4 pp 229533.I1RTA0
107902 229533.I1RJV4 pp 229533.I1RZM8
107903 229533.I1RJV4 pp 229533.I1RZH4
107904 229533.I1RJV4 pp 229533.I1RUG3
107905 229533.I1RJV4 pp 229533.I1S569
107906 229533.I1RJV4 pp 229533.I1RWD7
107907 229533.I1RJV4 pp 229533.I1RNC1
107908 229533.I1RJV4 pp 229533.I1RQ31
107909 229533.I1RJV4 pp 229533.I1RQ72
107910 229533.I1RJV4 pp 229533.I1RW46
107911 229533.I1RJV4 pp 229533.I1S0J1
107912 229533.I1RJV4 pp 229533.I1RSI6
107913 229533.I1RJV4 pp 229533.Q4IJK2
107914 229533.I1RJV4 pp 229533.I1S0R2
107915 229533.I1RJV4 pp 229533.I1RQA3
107916 229533.I1RJV4 pp 229533.V6RCZ8
107917 229533.I1RJV4 pp 229533.I1RMG3
107918 229533.I1RJV4 pp 229533.I1RN18
107919 229533.I1RJV4 pp 229533.V6RMW6
107920 229533.I1RJV4 pp 229533.I1SA32
107921 229533.I1RJV4 pp 229533.V6RCG5
107922 229533.I1RJV4 pp 229533.I1RS17
107923 229533.I1RJV4 pp 229533.I1RQE7
107924 229533.I1RJV4 pp 229533.I1RQQ5
107925 229533.I1RJV4 pp 229533.I1RSR3
107926 229533.I1RJV4 pp 229533.I1RZZ1
107927 229533.I1RJV4 pp 229533.I1RWN6
107928 229533.I1RJV4 pp 229533.I1RRN5
107929 229533.I1RJV4 pp 229533.I1RSQ3
107930 229533.I1RJV4 pp 229533.I1RRY9
107931 229533.I1RJV4 pp 229533.V6R042
107932 229533.I1RJV4 pp 229533.I1RSC6
107933 229533.I1RJV4 pp 229533.I1RVX0
107934 229533.I1RJV4 pp 229533.I1RT03
107935 229533.I1RJV4 pp 229533.I1RY03
107936 229533.I1RJV4 pp 229533.I1RN99
107937 229533.I1RJV4 pp 229533.I1RU29
107938 229533.I1RJV4 pp 229533.I1S1J7
107939 229533.I1RJV4 pp 229533.I1RTD9
107940 229533.I1RJV4 pp 229533.I1RQA2
107941 229533.I1RJV4 pp 229533.I1RYW3
107942 229533.I1RJV4 pp 229533.I1S1C7
107943 229533.I1RJV4 pp 229533.I1RRI2
107944 229533.I1RJV4 pp 229533.I1RZW8
107945 229533.I1RJV4 pp 229533.I1RMD5
107946 229533.I1RJV4 pp 229533.I1RTE9
107947 229533.I1RJV4 pp 229533.I1RTH6
107948 229533.I1RJV4 pp 229533.I1RWX5
107949 229533.I1RJV4 pp 229533.I1RZK5
107950 229533.I1RCL9 pp 229533.I1RNH7
107951 229533.I1RCL9 pp 229533.I1RFK0
107952 229533.I1RCL9 pp 229533.I1RCQ0
107953 229533.I1RCL9 pp 229533.I1SAM5
107954 229533.I1RCL9 pp 229533.Q4IN63
107955 229533.I1RCL9 pp 229533.I1RD23
107956 229533.I1RCL9 pp 229533.I1RW76
107957 229533.I1RCL9 pp 229533.I1RJZ6
107958 229533.I1RCL9 pp 229533.I1S1U6
107959 229533.I1RCL9 pp 229533.I1RQF7
107960 229533.I1RCL9 pp 229533.I1RZJ5
107961 229533.I1RCL9 pp 229533.I1RND7
107962 229533.I1RCL9 pp 229533.I1RQH7
107963 229533.I1RCL9 pp 229533.I1RJS5
107964 229533.I1RCL9 pp 229533.I1RJS4
107965 229533.I1RCL9 pp 229533.I1RPG2
107966 229533.I1RCL9 pp 229533.I1RZK3
107967 229533.I1RCL9 pp 229533.I1RE18

107968 229533.I1RCL9 pp 229533.I1S5B5
107969 229533.I1S0M6 pp 229533.I1S503
107970 229533.I1S0M6 pp 229533.I1S2E9
107971 229533.I1S0M6 pp 229533.V6QW22
107972 229533.I1S0M6 pp 229533.I1S2B8
107973 229533.I1S0M6 pp 229533.V6R3X5
107974 229533.I1S0M6 pp 229533.I1S0R7
107975 229533.I1S0M6 pp 229533.V6R1K4
107976 229533.I1S0M6 pp 229533.I1S2H4
107977 229533.I1S0M6 pp 229533.I1SA33
107978 229533.I1S0M6 pp 229533.I1S504
107979 229533.I1S0M6 pp 229533.V6RGI1
107980 229533.I1S0M6 pp 229533.V6QV09
107981 229533.I1S0M6 pp 229533.V6RTJ8
107982 229533.I1S0M6 pp 229533.V6RIV9
107983 229533.I1S0M6 pp 229533.I1S1C7
107984 229533.I1S0M6 pp 229533.V6RMW6
107985 229533.I1S0M6 pp 229533.V6R042
107986 229533.I1S0M6 pp 229533.I1S5R1
107987 229533.I1S0M6 pp 229533.I1SA32
107988 229533.I1S0M6 pp 229533.Q4I624
107989 229533.I1S0M6 pp 229533.Q4I8P5
107990 229533.I1S0M6 pp 229533.V6RCG5
107991 229533.I1S0M6 pp 229533.V6R057
107992 229533.I1S0M6 pp 229533.I1S8R3
107993 229533.I1S0M6 pp 229533.Q4IJK2
107994 229533.I1S0B2 pp 229533.I1S604
107995 229533.I1S0B2 pp 229533.Q4IR08
107996 229533.I1S0B2 pp 229533.I1S2E8
107997 229533.I1S0B2 pp 229533.I1S0D5
107998 229533.A0A1C3YM59 pp 229533.I1RPA6
107999 229533.I1RQI6 pp 229533.I1RTN6
108000 229533.I1RQI6 pp 229533.I1RZP8
108001 229533.I1RQI6 pp 229533.I1RSP7
108002 229533.I1RQI6 pp 229533.I1RQJ4
108003 229533.I1RQI6 pp 229533.I1RX64
108004 229533.I1RQI6 pp 229533.I1RSR9
108005 229533.I1RQI6 pp 229533.I1RSC7
108006 229533.I1RQI6 pp 229533.I1S0D5
108007 229533.I1RQI6 pp 229533.Q4IB89
108008 229533.I1RQI6 pp 229533.I1RZ98
108009 229533.I1RQI6 pp 229533.I1RY29
108010 229533.I1RQI6 pp 229533.I1RRJ6
108011 229533.I1RQI6 pp 229533.I1RZ86
108012 229533.I1RQI6 pp 229533.I1RUG2
108013 229533.I1RQI6 pp 229533.I1RWI8
108014 229533.I1RQI6 pp 229533.I1RSA7
108015 229533.I1RQI6 pp 229533.I1S0A0
108016 229533.I1RQI6 pp 229533.V6RLW4
108017 229533.I1RQI6 pp 229533.I1RZ94
108018 229533.I1RQI6 pp 229533.Q4INS6
108019 229533.I1RQI6 pp 229533.I1RSK0
108020 229533.I1RQI6 pp 229533.I1RSI4
108021 229533.I1RQI6 pp 229533.I1RRT1
108022 229533.I1RQI6 pp 229533.I1S209
108023 229533.I1RQI6 pp 229533.I1RSJ1
108024 229533.I1RFT1 pp 229533.V6RUY9
108025 229533.I1RFT1 pp 229533.I1RT04
108026 229533.I1RFT1 pp 229533.I1RRJ8
108027 229533.I1RFT1 pp 229533.I1RPP3
108028 229533.I1RFT1 pp 229533.Q4HXF0
108029 229533.I1RFT1 pp 229533.I1S2E9
108030 229533.I1RFT1 pp 229533.I1S8P5
108031 229533.I1RFT1 pp 229533.I1RJW6
108032 229533.I1RFT1 pp 229533.I1S503
108033 229533.I1RFT1 pp 229533.I1S5S2
108034 229533.I1RFT1 pp 229533.I1RXF2
108035 229533.I1RFT1 pp 229533.I1RRZ0
108036 229533.I1RFT1 pp 229533.I1RYI5
108037 229533.I1RFT1 pp 229533.I1RY62
108038 229533.I1RFT1 pp 229533.I1RN67
108039 229533.I1RFT1 pp 229533.Q4I5M4
108040 229533.I1RFT1 pp 229533.I1S9Y1

108041	229533.I1RFT1	pp	229533.V6R3J1
108042	229533.I1RFT1	pp	229533.Q4IQ72
108043	229533.I1RFT1	pp	229533.O74638
108044	229533.I1RFT1	pp	229533.I1RSX0
108045	229533.I1RFT1	pp	229533.I1RS47
108046	229533.I1RFT1	pp	229533.I1RRT2
108047	229533.I1RFT1	pp	229533.I1RXC2
108048	229533.I1RFT1	pp	229533.I1RGA4
108049	229533.I1RFT1	pp	229533.I1S9L6
108050	229533.I1RFT1	pp	229533.I1RRT8
108051	229533.I1RFT1	pp	229533.I1RP27
108052	229533.I1RFT1	pp	229533.I1RJV4
108053	229533.I1RFT1	pp	229533.I1S5R1
108054	229533.I1RFT1	pp	229533.I1RWK3
108055	229533.I1RFT1	pp	229533.I1RN63
108056	229533.I1RFT1	pp	229533.I1RT40
108057	229533.I1RFT1	pp	229533.I1S2H4
108058	229533.I1RFT1	pp	229533.I1RVZ2
108059	229533.I1RFT1	pp	229533.I1RTA1
108060	229533.I1RFT1	pp	229533.I1RV91
108061	229533.I1RFT1	pp	229533.I1RTD5
108062	229533.I1RFT1	pp	229533.I1RST1
108063	229533.I1RFT1	pp	229533.I1S5R6
108064	229533.I1RFT1	pp	229533.Q4I9U7
108065	229533.I1RFT1	pp	229533.I1RJF9
108066	229533.I1RFT1	pp	229533.I1RG11
108067	229533.I1RFT1	pp	229533.I1S0L4
108068	229533.I1RFT1	pp	229533.I1S4M5
108069	229533.I1RFT1	pp	229533.I1RRM5
108070	229533.I1RFT1	pp	229533.I1RZZ9
108071	229533.I1RFT1	pp	229533.I1RSQ4
108072	229533.I1RFT1	pp	229533.V6RIS2
108073	229533.I1RFT1	pp	229533.I1RJZ7
108074	229533.I1RFT1	pp	229533.I1RUG3
108075	229533.I1RFT1	pp	229533.I1RJG6
108076	229533.I1RFT1	pp	229533.I1S277
108077	229533.I1RFT1	pp	229533.I1RNC1
108078	229533.I1RFT1	pp	229533.Q4HZI0
108079	229533.I1RFT1	pp	229533.I1RX47
108080	229533.I1RFT1	pp	229533.I1RWI5
108081	229533.I1RFT1	pp	229533.I1RSZ7
108082	229533.I1RFT1	pp	229533.I1S8V3
108083	229533.I1RFT1	pp	229533.V6RMW6
108084	229533.I1RFT1	pp	229533.I1S6Y8
108085	229533.I1RFT1	pp	229533.I1RPM4
108086	229533.I1RFT1	pp	229533.I1RVN1
108087	229533.I1RFT1	pp	229533.I1RZ39
108088	229533.I1RFT1	pp	229533.I1RSQ3
108089	229533.I1RFT1	pp	229533.I1RPV3
108090	229533.I1RFT1	pp	229533.V6R042
108091	229533.I1RFT1	pp	229533.I1S524
108092	229533.I1RFT1	pp	229533.I1RPY4
108093	229533.I1RFT1	pp	229533.I1S1W9
108094	229533.I1RFT1	pp	229533.V6R3X5
108095	229533.I1RFT1	pp	229533.I1RN88
108096	229533.I1RFT1	pp	229533.I1RR73
108097	229533.I1RFT1	pp	229533.I1S2B8
108098	229533.I1RFT1	pp	229533.V6QXG1
108099	229533.I1RFT1	pp	229533.I1RT47
108100	229533.I1RFT1	pp	229533.I1RK96
108101	229533.I1RFT1	pp	229533.I1RRL0
108102	229533.I1RFT1	pp	229533.I1RSU2
108103	229533.I1RFT1	pp	229533.I1S0J1
108104	229533.I1RFT1	pp	229533.I1RWN5
108105	229533.I1RFT1	pp	229533.I1RW46
108106	229533.I1RFT1	pp	229533.I1S256
108107	229533.I1RFT1	pp	229533.I1RRN2
108108	229533.I1RFT1	pp	229533.I1RSD4
108109	229533.I1RFT1	pp	229533.V6RIV9
108110	229533.I1RFT1	pp	229533.I1RS90
108111	229533.I1RFT1	pp	229533.I1S1W3
108112	229533.I1RFT1	pp	229533.I1S056
108113	229533.I1RFT1	pp	229533.I1S7W5

108114	229533.I1RFT1	pp	229533.I1RSA4
108115	229533.I1RFT1	pp	229533.I1RRI2
108116	229533.I1RFT1	pp	229533.I1RS28
108117	229533.I1RFT1	pp	229533.I1RND5
108118	229533.I1RFT1	pp	229533.I1RNB8
108119	229533.I1RFT1	pp	229533.I1RWX5
108120	229533.I1RFT1	pp	229533.I1RS17
108121	229533.I1RFT1	pp	229533.I1RQP8
108122	229533.I1RFT1	pp	229533.I1S8R3
108123	229533.I1RFT1	pp	229533.V6RLM7
108124	229533.I1RFT1	pp	229533.I1RY44
108125	229533.I1RFT1	pp	229533.I1RPK0
108126	229533.I1RFT1	pp	229533.I1RRE5
108127	229533.I1RFT1	pp	229533.I1RG57
108128	229533.I1RFT1	pp	229533.I1RTH6
108129	229533.I1RFT1	pp	229533.I1S8V4
108130	229533.I1RFT1	pp	229533.I1RMV0
108131	229533.I1RFT1	pp	229533.I1RPP4
108132	229533.I1RFT1	pp	229533.V6RRH2
108133	229533.I1RFT1	pp	229533.I1RJV3
108134	229533.I1RFT1	pp	229533.V6RY33
108135	229533.I1RFT1	pp	229533.I1RTA0
108136	229533.I1RFT1	pp	229533.I1RXU9
108137	229533.I1RFT1	pp	229533.I1RG90
108138	229533.I1RFT1	pp	229533.I1RK92
108139	229533.I1RFT1	pp	229533.I1S0L3
108140	229533.I1RFT1	pp	229533.I1S0B1
108141	229533.I1RFT1	pp	229533.I1S1C7
108142	229533.I1RFT1	pp	229533.I1RWN6
108143	229533.I1RFT1	pp	229533.V6R057
108144	229533.I1RFT1	pp	229533.Q4I624
108145	229533.I1RFT1	pp	229533.V6R1K4
108146	229533.I1RFT1	pp	229533.I1RRQ4
108147	229533.I1RFT1	pp	229533.I1RRG3
108148	229533.I1RFT1	pp	229533.I1S2Y5
108149	229533.I1RFT1	pp	229533.I1RZW8
108150	229533.I1RFT1	pp	229533.I1RK90
108151	229533.I1RFT1	pp	229533.I1RZQ5
108152	229533.I1RFT1	pp	229533.I1S296
108153	229533.I1RFT1	pp	229533.I1RS75
108154	229533.I1RFT1	pp	229533.I1S1C6
108155	229533.I1RFT1	pp	229533.I1RN18
108156	229533.I1RFT1	pp	229533.I1RPN4
108157	229533.I1RFT1	pp	229533.I1RQE7
108158	229533.I1RFT1	pp	229533.I1RW71
108159	229533.I1RFT1	pp	229533.I1S4S7
108160	229533.I1RFT1	pp	229533.I1RXR1
108161	229533.I1RFT1	pp	229533.I1S3J4
108162	229533.I1RFT1	pp	229533.I1RSY2
108163	229533.I1RFT1	pp	229533.I1RSQ7
108164	229533.I1RFT1	pp	229533.I1RX46
108165	229533.I1RFT1	pp	229533.I1S0T2
108166	229533.I1RFT1	pp	229533.I1RT03
108167	229533.I1RFT1	pp	229533.I1RVX0
108168	229533.I1RFT1	pp	229533.I1RRX5
108169	229533.I1RFT1	pp	229533.V6RTJ8
108170	229533.I1RFT1	pp	229533.V6QUE6
108171	229533.I1RFT1	pp	229533.I1S0R7
108172	229533.I1RFT1	pp	229533.I1S9L5
108173	229533.I1RFT1	pp	229533.I1RP86
108174	229533.I1RFT1	pp	229533.V6QW22
108175	229533.I1RFT1	pp	229533.I1S4S4
108176	229533.I1RFT1	pp	229533.I1RRV4
108177	229533.I1RFT1	pp	229533.I1RQC9
108178	229533.I1RFT1	pp	229533.I1RXD7
108179	229533.I1RFT1	pp	229533.I1RQD8
108180	229533.I1RFT1	pp	229533.I1RLP1
108181	229533.I1RFT1	pp	229533.I1RWJ8
108182	229533.I1RFT1	pp	229533.I1RZ11
108183	229533.I1RFT1	pp	229533.I1S7M2
108184	229533.I1RFT1	pp	229533.I1RW99
108185	229533.I1RFT1	pp	229533.I1RR24
108186	229533.I1RFT1	pp	229533.I1RS83

229533.I1RFT1	pp	229533.I1RPW9
229533.I1RFT1	pp	229533.V6RUT5
229533.I1RFT1	pp	229533.I1S0X6
229533.I1RFT1	pp	229533.I1S5B0
229533.I1RFT1	pp	229533.I1RMW7
229533.I1RFT1	pp	229533.I1RVQ0
229533.I1RFT1	pp	229533.I1RSA8
229533.I1RFT1	pp	229533.I1RXF0
229533.I1RFT1	pp	229533.I1RQ31
229533.I1RFT1	pp	229533.I1RZM8
229533.I1RFT1	pp	229533.I1RW25
229533.I1RFT1	pp	229533.I1RJU3
229533.I1RFT1	pp	229533.I1RZG7
229533.I1RFT1	pp	229533.Q4HWB3
229533.I1RFT1	pp	229533.I1RN59
229533.I1RFT1	pp	229533.I1RXC4
229533.I1RFT1	pp	229533.I1S7M3
229533.I1RFT1	pp	229533.I1RI82
229533.I1RFT1	pp	229533.I1S6K9
229533.I1RFT1	pp	229533.I1RK72
229533.I1RFT1	pp	229533.I1RJ17
229533.I1RFT1	pp	229533.I1RPA4
229533.I1RFT1	pp	229533.I1RRX0
229533.I1RFT1	pp	229533.V6RCZ8
229533.I1RFT1	pp	229533.I1RPY5
229533.I1RFT1	pp	229533.I1RX81
229533.I1RFT1	pp	229533.V6RCG5
229533.I1RFT1	pp	229533.I1RQ81
229533.I1RFT1	pp	229533.Q4IRI9
229533.I1RFT1	pp	229533.I1RK95
229533.I1RFT1	pp	229533.I1RJV2
229533.I1RFT1	pp	229533.I1RS18
229533.I1RFT1	pp	229533.I1S419
229533.I1RFT1	pp	229533.I1RR51
229533.I1RFT1	pp	229533.I1RWX8
229533.I1RFT1	pp	229533.I1RWB0
229533.I1RFT1	pp	229533.Q4IB70
229533.I1RFT1	pp	229533.I1S1J7
229533.I1RFT1	pp	229533.I1RZM1
229533.I1RFT1	pp	229533.I1RLU6
229533.I1RFT1	pp	229533.I1RPR9
229533.I1RFT1	pp	229533.I1RNQ5
229533.I1RFT1	pp	229533.I1RPA0
229533.I1RFT1	pp	229533.I1S504
229533.I1RFT1	pp	229533.I1RY03
229533.I1RFT1	pp	229533.I1RSR3
229533.I1RFT1	pp	229533.I1RRY9
229533.I1RFT1	pp	229533.I1S887
229533.I1RFT1	pp	229533.I1RZK5
229533.I1RFT1	pp	229533.I1RFU8
229533.I1RFT1	pp	229533.I1RZZ1
229533.I1RFT1	pp	229533.Q4IJK2
229533.A0A1C3YN55	pp	229533.I1SAD2
229533.A0A1C3YN55	pp	229533.I1RZ86
229533.A0A1C3YN55	pp	229533.I1S004
229533.A0A1C3YN55	pp	229533.I1RVL8
229533.A0A1C3YN55	pp	229533.I1RN04
229533.A0A1C3YN55	pp	229533.I1RP99
229533.A0A1C3YN55	pp	229533.I1RCA5
229533.A0A1C3YN55	pp	229533.I1RRJ6
229533.A0A1C3YN55	pp	229533.I1RDC0
229533.A0A1C3YN55	pp	229533.I1RBW1
229533.A0A1C3YN55	pp	229533.I1RSS4
229533.A0A1C3YN55	pp	229533.I1RJZ7
229533.A0A1C3YN55	pp	229533.I1RBR5
229533.A0A1C3YN55	pp	229533.I1RRN5
229533.A0A1C3YN55	pp	229533.I1RA84
229533.A0A1C3YN55	pp	229533.I1RMV1
229533.A0A1C3YN55	pp	229533.I1S9Y1
229533.A0A1C3YN55	pp	229533.I1RVY0
229533.A0A1C3YN55	pp	229533.I1RDJ8
229533.A0A1C3YN55	pp	229533.I1RHD2
229533.A0A1C3YN55	pp	229533.I1RA10

108260	229533.A0A1C3YN55	pp	229533.I1RLR0
108261	229533.A0A1C3YN55	pp	229533.I1S0J3
108262	229533.A0A1C3YN55	pp	229533.I1RE30
108263	229533.A0A1C3YN55	pp	229533.I1RY58
108264	229533.A0A1C3YN55	pp	229533.I1RDH0
108265	229533.A0A1C3YN55	pp	229533.I1RFL7
108266	229533.A0A1C3YN55	pp	229533.I1R9Q0
108267	229533.A0A1C3YN55	pp	229533.I1RR61
108268	229533.A0A1C3YN55	pp	229533.I1RHL8
108269	229533.A0A1C3YN55	pp	229533.I1RC24
108270	229533.A0A1C3YN55	pp	229533.I1RZT1
108271	229533.A0A1C3YN55	pp	229533.I1RD36
108272	229533.A0A1C3YN55	pp	229533.I1RFW1
108273	229533.A0A1C3YN55	pp	229533.I1S276
108274	229533.A0A1C3YN55	pp	229533.I1RZM2
108275	229533.A0A1C3YN55	pp	229533.I1RX63
108276	229533.A0A1C3YN55	pp	229533.I1RBC7
108277	229533.A0A1C3YN55	pp	229533.I1RB25
108278	229533.A0A1C3YN55	pp	229533.I1RYQ1
108279	229533.A0A1C3YN55	pp	229533.I1RSR9
108280	229533.A0A1C3YN55	pp	229533.I1S0D5
108281	229533.A0A1C3YN55	pp	229533.I1RFZ2
108282	229533.A0A1C3YN55	pp	229533.I1S3N0
108283	229533.A0A1C3YN55	pp	229533.I1R9W7
108284	229533.A0A1C3YN55	pp	229533.I1RRM8
108285	229533.A0A1C3YN55	pp	229533.I1RK71
108286	229533.A0A1C3YN55	pp	229533.I1RPM7
108287	229533.A0A1C3YN55	pp	229533.I1RSN7
108288	229533.A0A1C3YN55	pp	229533.I1RJV8
108289	229533.A0A1C3YN55	pp	229533.I1RX64
108290	229533.A0A1C3YN55	pp	229533.V6RVG3
108291	229533.A0A1C3YN55	pp	229533.I1RUF8
108292	229533.A0A1C3YN55	pp	229533.I1RBN9
108293	229533.A0A1C3YN55	pp	229533.I1RW80
108294	229533.A0A1C3YN55	pp	229533.I1RZ42
108295	229533.A0A1C3YN55	pp	229533.Q4IJ84
108296	229533.A0A1C3YN55	pp	229533.I1RHQ5
108297	229533.A0A1C3YN55	pp	229533.I1RQZ3
108298	229533.A0A1C3YN55	pp	229533.I1RWI8
108299	229533.A0A1C3YN55	pp	229533.I1RTN6
108300	229533.A0A1C3YN55	pp	229533.I1RZP8
108301	229533.A0A1C3YN55	pp	229533.I1RMS4
108302	229533.A0A1C3YN55	pp	229533.Q4IB89
108303	229533.A0A1C3YN55	pp	229533.I1RY29
108304	229533.A0A1C3YN55	pp	229533.I1RK79
108305	229533.A0A1C3YN55	pp	229533.I1S2E9
108306	229533.A0A1C3YN55	pp	229533.I1RK90
108307	229533.A0A1C3YN55	pp	229533.I1RAU8
108308	229533.A0A1C3YN55	pp	229533.Q4HYB8
108309	229533.A0A1C3YN55	pp	229533.I1RPF5
108310	229533.A0A1C3YN55	pp	229533.I1RD19
108311	229533.A0A1C3YN55	pp	229533.I1RUP9
108312	229533.A0A1C3YN55	pp	229533.I1RSP7
108313	229533.A0A1C3YN55	pp	229533.I1RSZ3
108314	229533.A0A1C3YN55	pp	229533.I1RBJ1
108315	229533.A0A1C3YN55	pp	229533.I1R9C7
108316	229533.A0A1C3YN55	pp	229533.I1RWF3
108317	229533.A0A1C3YN55	pp	229533.I1S4P6
108318	229533.A0A1C3YN55	pp	229533.I1RN57
108319	229533.A0A1C3YN55	pp	229533.A0A1C3YN69
108320	229533.A0A1C3YN55	pp	229533.I1RRF7
108321	229533.A0A1C3YN55	pp	229533.I1RR25
108322	229533.A0A1C3YN55	pp	229533.I1RC54
108323	229533.A0A1C3YN55	pp	229533.I1RBW0
108324	229533.A0A1C3YN55	pp	229533.I1RD01
108325	229533.A0A1C3YN55	pp	229533.I1RY44
108326	229533.A0A1C3YN55	pp	229533.Q4IPC8
108327	229533.A0A1C3YN55	pp	229533.Q4I5U9
108328	229533.A0A1C3YN55	pp	229533.I1RZ94
108329	229533.A0A1C3YN55	pp	229533.I1RG11
108330	229533.A0A1C3YN55	pp	229533.I1RRT1
108331	229533.A0A1C3YN55	pp	229533.I1RSL6
108332	229533.A0A1C3YN55	pp	229533.I1RAP2

108333	229533.A0A1C3YN55	pp	229533.I1RC98
108334	229533.A0A1C3YN55	pp	229533.I1RMR1
108335	229533.A0A1C3YN55	pp	229533.I1RRG3
108336	229533.A0A1C3YN55	pp	229533.I1S0C6
108337	229533.A0A1C3YN55	pp	229533.I1RBC8
108338	229533.A0A1C3YN55	pp	229533.I1RRS7
108339	229533.A0A1C3YN55	pp	229533.I1S283
108340	229533.A0A1C3YN55	pp	229533.I1S3N1
108341	229533.A0A1C3YN55	pp	229533.I1RPQ1
108342	229533.A0A1C3YN55	pp	229533.I1RKB1
108343	229533.A0A1C3YN55	pp	229533.I1S5Z3
108344	229533.A0A1C3YN55	pp	229533.I1RUP7
108345	229533.A0A1C3YN55	pp	229533.I1RUR0
108346	229533.A0A1C3YN55	pp	229533.I1RE55
108347	229533.A0A1C3YN55	pp	229533.V6R1X4
108348	229533.A0A1C3YN55	pp	229533.I1RW90
108349	229533.A0A1C3YN55	pp	229533.I1RKQ5
108350	229533.A0A1C3YN55	pp	229533.I1RXU0
108351	229533.A0A1C3YN55	pp	229533.I1RLV1
108352	229533.A0A1C3YN55	pp	229533.I1RW91
108353	229533.A0A1C3YN55	pp	229533.I1RCB7
108354	229533.A0A1C3YN55	pp	229533.Q4IQX3
108355	229533.A0A1C3YN55	pp	229533.I1RSC7
108356	229533.A0A1C3YN55	pp	229533.I1RQM6
108357	229533.A0A1C3YN55	pp	229533.I1RP96
108358	229533.A0A1C3YN55	pp	229533.V6QWX8
108359	229533.A0A1C3YN55	pp	229533.I1RZ98
108360	229533.A0A1C3YN55	pp	229533.I1RQ42
108361	229533.A0A1C3YN55	pp	229533.I1RML9
108362	229533.A0A1C3YN55	pp	229533.I1RKI2
108363	229533.A0A1C3YN55	pp	229533.I1RT33
108364	229533.A0A1C3YN55	pp	229533.I1RAT7
108365	229533.A0A1C3YN55	pp	229533.I1RNP4
108366	229533.A0A1C3YN55	pp	229533.I1RHL7
108367	229533.A0A1C3YN55	pp	229533.I1RPW8
108368	229533.A0A1C3YN55	pp	229533.I1RVZ9
108369	229533.A0A1C3YN55	pp	229533.I1RGR8
108370	229533.A0A1C3YN55	pp	229533.I1S182
108371	229533.A0A1C3YN55	pp	229533.I1REN3
108372	229533.A0A1C3YN55	pp	229533.I1RFJ2
108373	229533.A0A1C3YN55	pp	229533.I1RFQ5
108374	229533.A0A1C3YN55	pp	229533.I1RW71
108375	229533.A0A1C3YN55	pp	229533.V6QV38
108376	229533.A0A1C3YN55	pp	229533.I1RDZ6
108377	229533.A0A1C3YN55	pp	229533.I1RBC1
108378	229533.A0A1C3YN55	pp	229533.I1RTC2
108379	229533.A0A1C3YN55	pp	229533.Q4IJU0
108380	229533.A0A1C3YN55	pp	229533.Q4IJM4
108381	229533.A0A1C3YN55	pp	229533.I1RMS2
108382	229533.A0A1C3YN55	pp	229533.I1S209
108383	229533.A0A1C3YN55	pp	229533.Q4IER8
108384	229533.A0A1C3YN55	pp	229533.I1RAR2
108385	229533.A0A1C3YN55	pp	229533.I1RZB5
108386	229533.A0A1C3YN55	pp	229533.I1RG77
108387	229533.A0A1C3YN55	pp	229533.I1RN55
108388	229533.A0A1C3YN55	pp	229533.V6QUE6
108389	229533.A0A1C3YN55	pp	229533.I1RK95
108390	229533.A0A1C3YN55	pp	229533.I1RMX3
108391	229533.A0A1C3YN55	pp	229533.I1RJL2
108392	229533.A0A1C3YN55	pp	229533.I1RYY5
108393	229533.A0A1C3YN55	pp	229533.I1RQA3
108394	229533.A0A1C3YN55	pp	229533.Q4HYI0
108395	229533.A0A1C3YN55	pp	229533.I1RM65
108396	229533.A0A1C3YN55	pp	229533.I1RAE8
108397	229533.A0A1C3YN55	pp	229533.I1RWT6
108398	229533.A0A1C3YN55	pp	229533.I1RA12
108399	229533.A0A1C3YN55	pp	229533.Q4INS6
108400	229533.A0A1C3YN55	pp	229533.I1RCG6
108401	229533.A0A1C3YN55	pp	229533.I1RQH8
108402	229533.A0A1C3YN55	pp	229533.I1RY80
108403	229533.A0A1C3YN55	pp	229533.I1RXE5
108404	229533.A0A1C3YN55	pp	229533.I1RC15
108405	229533.A0A1C3YN55	pp	229533.I1RRT0

108406	229533.A0A1C3YN55	pp	229533.I1RMQ7
108407	229533.A0A1C3YN55	pp	229533.I1RKA7
108408	229533.A0A1C3YN55	pp	229533.I1RDC9
108409	229533.I1RZS6	pp	229533.I1S9U2
108410	229533.I1RZS6	pp	229533.I1RZV6
108411	229533.I1RZS6	pp	229533.Q4IJH1
108412	229533.I1RZS6	pp	229533.I1S2B8
108413	229533.I1RZS6	pp	229533.I1S6U8
108414	229533.I1RZS6	pp	229533.I1S8E2
108415	229533.I1RZS6	pp	229533.Q4HVW2
108416	229533.I1RZS6	pp	229533.I1S253
108417	229533.I1RZS6	pp	229533.Q4IFI0
108418	229533.I1RZS6	pp	229533.Q4IAS1
108419	229533.I1RZS6	pp	229533.V6QYF0
108420	229533.I1RZS6	pp	229533.V6R3X5
108421	229533.I1RZS6	pp	229533.Q4IQW0
108422	229533.I1RZS6	pp	229533.Q4IF76
108423	229533.I1RZS6	pp	229533.I1S6F0
108424	229533.I1RZS6	pp	229533.I1S503
108425	229533.I1RZS6	pp	229533.Q4I830
108426	229533.I1RZS6	pp	229533.Q4I5R9
108427	229533.I1RZS6	pp	229533.Q4IQK7
108428	229533.I1RZS6	pp	229533.I1S1B6
108429	229533.I1RZS6	pp	229533.I1S0R7
108430	229533.I1RZS6	pp	229533.V6R283
108431	229533.I1RZS6	pp	229533.Q4I662
108432	229533.I1RZS6	pp	229533.Q4IPI1
108433	229533.I1RZS6	pp	229533.V6R042
108434	229533.I1RZS6	pp	229533.I1S274
108435	229533.I1RZS6	pp	229533.I1S1U7
108436	229533.I1RZS6	pp	229533.I1S2M6
108437	229533.I1RZS6	pp	229533.V6RMW6
108438	229533.I1RZS6	pp	229533.I1S2E9
108439	229533.I1RZS6	pp	229533.Q4IEV5
108440	229533.I1RZS6	pp	229533.Q4IN63
108441	229533.I1RZS6	pp	229533.I1S558
108442	229533.I1RZS6	pp	229533.Q4HZ42
108443	229533.I1RZS6	pp	229533.V6RGY3
108444	229533.I1RZS6	pp	229533.Q4HZK7
108445	229533.I1RZS6	pp	229533.I1S021
108446	229533.I1RZS6	pp	229533.I1S0D4
108447	229533.I1RZS6	pp	229533.I1S1X9
108448	229533.I1RZS6	pp	229533.Q4I327
108449	229533.I1RZS6	pp	229533.I1RZZ1
108450	229533.I1RZS6	pp	229533.I1S2E8
108451	229533.I1RZS6	pp	229533.I1S9U1
108452	229533.I1RZS6	pp	229533.I1S2U2
108453	229533.I1RZS6	pp	229533.I1S2C0
108454	229533.I1RZS6	pp	229533.Q4IJT0
108455	229533.I1RZS6	pp	229533.I1S1C7
108456	229533.I1RZS6	pp	229533.I1S4T3
108457	229533.I1RZS6	pp	229533.I1S044
108458	229533.I1RZS6	pp	229533.I1S504
108459	229533.I1RZS6	pp	229533.V6R227
108460	229533.I1RZS6	pp	229533.I1S296
108461	229533.I1RZS6	pp	229533.I1S0M9
108462	229533.I1RZS6	pp	229533.Q4HZ68
108463	229533.I1RZS6	pp	229533.V6RGN5
108464	229533.I1RZS6	pp	229533.Q4IEK8
108465	229533.I1RZS6	pp	229533.Q4IJ56
108466	229533.I1RZS6	pp	229533.Q4IAA0
108467	229533.I1RZS6	pp	229533.I1S060
108468	229533.I1RZS6	pp	229533.V6QUE6
108469	229533.I1RZS6	pp	229533.I1S0J1
108470	229533.I1RZS6	pp	229533.Q4IBS2
108471	229533.I1RA98	pp	229533.I1S306
108472	229533.I1RA98	pp	229533.Q4ICM0
108473	229533.I1RA98	pp	229533.I1RAI0
108474	229533.I1RA98	pp	229533.I1RR95
108475	229533.I1RA98	pp	229533.I1RRC2
108476	229533.I1RA98	pp	229533.I1RZB7
108477	229533.I1RA98	pp	229533.Q5I2J3
108478	229533.I1RA98	pp	229533.Q4HZS8

108479 229533.I1RA98 pp 229533.I1RIG3
108480 229533.I1RA98 pp 229533.I1RZK4
108481 229533.I1RA98 pp 229533.I1RLA2
108482 229533.I1RA98 pp 229533.I1RYQ0
108483 229533.I1RV70 pp 229533.I1RWX7
108484 229533.I1RV70 pp 229533.I1RZR6
108485 229533.I1RV70 pp 229533.I1S9M0
108486 229533.I1RYN9 pp 229533.I1S0X0
108487 229533.I1RYN9 pp 229533.I1S0S5
108488 229533.I1RYN9 pp 229533.I1RYP1
108489 229533.A0A1C3YJA8 pp 229533.I1S0C0
108490 229533.A0A1C3YJA8 pp 229533.A0A1C3YL40
108491 229533.A0A1C3YJA8 pp 229533.I1RYV3
108492 229533.A0A1C3YJA8 pp 229533.I1RSK0
108493 229533.A0A1C3YJA8 pp 229533.I1S5M1
108494 229533.A0A1C3YJA8 pp 229533.I1RHY7
108495 229533.A0A1C3YJA8 pp 229533.Q4IER8
108496 229533.A0A1C3YJA8 pp 229533.A0A1C3YJT5
108497 229533.A0A1C3YJA8 pp 229533.I1RMQ7
108498 229533.A0A1C3YJA8 pp 229533.I1RH33
108499 229533.A0A1C3YJA8 pp 229533.I1S0V3
108500 229533.A0A1C3YJA8 pp 229533.I1R9R7
108501 229533.A0A1C3YJA8 pp 229533.Q4I5R3
108502 229533.A0A1C3YJA8 pp 229533.I1RCI2
108503 229533.A0A1C3YJA8 pp 229533.Q4I7L0
108504 229533.A0A1C3YJA8 pp 229533.I1RBJ0
108505 229533.A0A1C3YJA8 pp 229533.I1RSD1
108506 229533.A0A1C3YJA8 pp 229533.I1RFW1
108507 229533.A0A1C3YJA8 pp 229533.I1S1W4
108508 229533.I1RBS6 pp 229533.I1REK8
108509 229533.I1RBS6 pp 229533.I1RWD7
108510 229533.I1RBS6 pp 229533.I1RCR5
108511 229533.I1RBS6 pp 229533.I1RLM7
108512 229533.I1RBS6 pp 229533.I1RSI0
108513 229533.I1RBS6 pp 229533.I1RQA3
108514 229533.I1RBS6 pp 229533.I1RFJ9
108515 229533.I1RBS6 pp 229533.Q4IJK2
108516 229533.I1RBS6 pp 229533.I1RF19
108517 229533.I1RBS6 pp 229533.I1RE00
108518 229533.I1RBS6 pp 229533.I1RSM3
108519 229533.I1RBS6 pp 229533.I1RMN3
108520 229533.I1RBS6 pp 229533.Q4I2J8
108521 229533.I1RBS6 pp 229533.I1RRY9
108522 229533.I1RBS6 pp 229533.I1RSR3
108523 229533.I1RBS6 pp 229533.I1RU29
108524 229533.I1RBS6 pp 229533.I1S1J7
108525 229533.I1RBS6 pp 229533.Q4I5Z5
108526 229533.I1RBS6 pp 229533.I1RI03
108527 229533.I1RBS6 pp 229533.I1RN99
108528 229533.I1RBS6 pp 229533.I1RZK5
108529 229533.I1RBS6 pp 229533.I1RQA2
108530 229533.I1RBS6 pp 229533.I1RSC6
108531 229533.I1RBS6 pp 229533.I1RDG0
108532 229533.I1RBS6 pp 229533.I1RQ81
108533 229533.I1RBS6 pp 229533.I1RFU8
108534 229533.I1RBS6 pp 229533.I1RZK6
108535 229533.I1RBS6 pp 229533.I1SA32
108536 229533.I1RBS6 pp 229533.I1RMD5
108537 229533.I1RBS6 pp 229533.I1RCG4
108538 229533.I1RBS6 pp 229533.I1RZZ1
108539 229533.I1RBS6 pp 229533.I1RZH4
108540 229533.I1RBS6 pp 229533.I1S5B0
108541 229533.I1RBS6 pp 229533.I1RY03
108542 229533.I1RBS6 pp 229533.I1RQQ5
108543 229533.I1RBS6 pp 229533.I1RW99
108544 229533.I1RBS6 pp 229533.I1RM06
108545 229533.I1RBS6 pp 229533.I1RTD9
108546 229533.I1RBS6 pp 229533.V6R227
108547 229533.I1RBS6 pp 229533.V6QUW4
108548 229533.I1RBS6 pp 229533.I1RC17
108549 229533.I1RBS6 pp 229533.I1RNA0
108550 229533.I1RBS6 pp 229533.V6QYF0
108551 229533.I1RBS6 pp 229533.I1RRU9

108552	229533.I1RBS6	pp	229533.Q4IBS2
108553	229533.I1RBS6	pp	229533.I1RFT2
108554	229533.I1RBS6	pp	229533.I1RGC2
108555	229533.I1RBS6	pp	229533.Q4I283
108556	229533.I1RBS6	pp	229533.I1RWT6
108557	229533.I1RBS6	pp	229533.I1RCS1
108558	229533.I1RBS6	pp	229533.I1RR24
108559	229533.I1RBS6	pp	229533.I1RZJ5
108560	229533.I1RBS6	pp	229533.I1RR51
108561	229533.I1RBS6	pp	229533.I1RMG8
108562	229533.I1RBS6	pp	229533.I1S504
108563	229533.I1RBS6	pp	229533.V6RCG5
108564	229533.I1RBS6	pp	229533.Q4IEK8
108565	229533.I1RBS6	pp	229533.V6RCZ8
108566	229533.I1RBS6	pp	229533.I1RWJ8
108567	229533.I1RBS6	pp	229533.I1RNQ5
108568	229533.I1RBS6	pp	229533.I1RI82
108569	229533.I1RBS6	pp	229533.I1RMK3
108570	229533.I1RBS6	pp	229533.I1RLU6
108571	229533.I1RBS6	pp	229533.I1RKB7
108572	229533.I1RBS6	pp	229533.I1RMC0
108573	229533.I1RBS6	pp	229533.I1RTF4
108574	229533.I1RBS6	pp	229533.I1RJU3
108575	229533.I1RBS6	pp	229533.I1RZM1
108576	229533.I1RBS6	pp	229533.I1RC16
108577	229533.I1RBS6	pp	229533.I1RQ31
108578	229533.I1RBS6	pp	229533.I1RMC8
108579	229533.I1RBS6	pp	229533.I1RXF0
108580	229533.I1RBS6	pp	229533.I1RZM8
108581	229533.I1RBS6	pp	229533.I1RBT1
108582	229533.I1RBS6	pp	229533.I1S0X6
108583	229533.I1RBS6	pp	229533.I1RI02
108584	229533.I1RBS6	pp	229533.I1RS18
108585	229533.I1RBS6	pp	229533.I1S419
108586	229533.I1RBS6	pp	229533.I1RW25
108587	229533.I1RBS6	pp	229533.I1RW07
108588	229533.I1RBS6	pp	229533.Q4I8P5
108589	229533.I1RBS6	pp	229533.V6R1K4
108590	229533.I1RBS6	pp	229533.I1S0F5
108591	229533.I1RBS6	pp	229533.I1RX25
108592	229533.I1RBS6	pp	229533.I1S6F0
108593	229533.I1RBS6	pp	229533.I1S0L3
108594	229533.I1RBS6	pp	229533.I1RZW8
108595	229533.I1RBS6	pp	229533.I1RLC7
108596	229533.I1RBS6	pp	229533.I1RD52
108597	229533.I1RBS6	pp	229533.I1S0D4
108598	229533.I1RBS6	pp	229533.V6R4J1
108599	229533.I1RBS6	pp	229533.I1RVQ0
108600	229533.I1RBS6	pp	229533.I1RYW3
108601	229533.I1RBS6	pp	229533.I1S1C7
108602	229533.I1RBS6	pp	229533.I1RWD4
108603	229533.I1RBS6	pp	229533.I1S569
108604	229533.I1RBS6	pp	229533.I1S8V4
108605	229533.I1RBS6	pp	229533.V6R057
108606	229533.I1RBS6	pp	229533.I1RG53
108607	229533.I1RBS6	pp	229533.I1RD17
108608	229533.I1RBS6	pp	229533.V6QW22
108609	229533.I1RBS6	pp	229533.I1RRE5
108610	229533.I1RBS6	pp	229533.I1RC33
108611	229533.I1RBS6	pp	229533.Q4I624
108612	229533.I1RBS6	pp	229533.I1RGL4
108613	229533.I1RBS6	pp	229533.I1RSQ7
108614	229533.I1RBS6	pp	229533.I1RY44
108615	229533.I1RBS6	pp	229533.I1RW71
108616	229533.I1RBS6	pp	229533.I1RT03
108617	229533.I1RBS6	pp	229533.I1RKC6
108618	229533.I1RBS6	pp	229533.I1RVX0
108619	229533.I1RBS6	pp	229533.I1RIL8
108620	229533.I1RBS6	pp	229533.V6RIV9
108621	229533.I1RBS6	pp	229533.I1RWN5
108622	229533.I1RBS6	pp	229533.Q4IJT5
108623	229533.I1RBS6	pp	229533.I1RLX7
108624	229533.I1RBS6	pp	229533.I1RCH5

108625	229533.I1RBS6	pp	229533.I1RRN5
108626	229533.I1RBS6	pp	229533.Q4HZB8
108627	229533.I1RBS6	pp	229533.I1RQE7
108628	229533.I1RBS6	pp	229533.I1RFL4
108629	229533.I1RBS6	pp	229533.V6RTJ8
108630	229533.I1RBS6	pp	229533.I1RPY1
108631	229533.I1RBS6	pp	229533.I1S7A8
108632	229533.I1RBS6	pp	229533.I1RD39
108633	229533.I1RBS6	pp	229533.I1RN18
108634	229533.I1RBS6	pp	229533.I1RPP4
108635	229533.I1RBS6	pp	229533.I1RWN6
108636	229533.I1RBS6	pp	229533.I1RS17
108637	229533.I1RBS6	pp	229533.I1RPK0
108638	229533.I1RBS6	pp	229533.I1S0T2
108639	229533.I1RBS6	pp	229533.I1RX46
108640	229533.I1RBS6	pp	229533.I1RJV3
108641	229533.I1RBS6	pp	229533.I1RSK1
108642	229533.I1RBS6	pp	229533.I1RNB8
108643	229533.I1RBS6	pp	229533.I1RQ87
108644	229533.I1RBS6	pp	229533.I1RTE9
108645	229533.I1RBS6	pp	229533.I1RUQ2
108646	229533.I1RBS6	pp	229533.I1S0R2
108647	229533.I1RBS6	pp	229533.I1RTH6
108648	229533.I1RBS6	pp	229533.I1S8R3
108649	229533.I1RBS6	pp	229533.I1RXD7
108650	229533.I1RBS6	pp	229533.I1RC72
108651	229533.I1RBS6	pp	229533.I1S0J1
108652	229533.I1RBS6	pp	229533.I1RE01
108653	229533.I1RBS6	pp	229533.I1RQC9
108654	229533.I1RBS6	pp	229533.I1RW46
108655	229533.I1RBS6	pp	229533.I1S0R7
108656	229533.I1RBS6	pp	229533.I1RRI2
108657	229533.I1RBS6	pp	229533.I1RWX5
108658	229533.I1RBS6	pp	229533.I1RQ72
108659	229533.I1RBS6	pp	229533.I1RZS7
108660	229533.I1RBS6	pp	229533.V6RGN5
108661	229533.I1RBS6	pp	229533.I1RTA0
108662	229533.I1RBS6	pp	229533.I1RD22
108663	229533.I1RBS6	pp	229533.I1RWH4
108664	229533.I1RBS6	pp	229533.I1RPP3
108665	229533.I1RBS6	pp	229533.I1S8P5
108666	229533.I1RBS6	pp	229533.I1SA33
108667	229533.I1RBS6	pp	229533.I1S0N4
108668	229533.I1RBS6	pp	229533.I1RRJ8
108669	229533.I1RBS6	pp	229533.I1RWQ8
108670	229533.I1RBS6	pp	229533.I1RQG4
108671	229533.I1RBS6	pp	229533.I1S352
108672	229533.I1RBS6	pp	229533.I1RQS8
108673	229533.I1RBS6	pp	229533.I1RSQ3
108674	229533.I1RBS6	pp	229533.I1RS20
108675	229533.I1RBS6	pp	229533.V6R042
108676	229533.I1RBS6	pp	229533.I1S2M6
108677	229533.I1RBS6	pp	229533.I1S2H4
108678	229533.I1RBS6	pp	229533.I1RFT1
108679	229533.I1RBS6	pp	229533.I1RMG3
108680	229533.I1RBS6	pp	229533.I1RWK3
108681	229533.I1RBS6	pp	229533.V6RMW6
108682	229533.I1RBS6	pp	229533.I1RSI6
108683	229533.I1RBS6	pp	229533.I1RRT8
108684	229533.I1RBS6	pp	229533.I1RJ74
108685	229533.I1RBS6	pp	229533.I1S5R1
108686	229533.I1RBS6	pp	229533.I1RN63
108687	229533.I1RBS6	pp	229533.I1RJS2
108688	229533.I1RBS6	pp	229533.O74638
108689	229533.I1RBS6	pp	229533.I1RRV1
108690	229533.I1RBS6	pp	229533.I1RM82
108691	229533.I1RBS6	pp	229533.I1RXT3
108692	229533.I1RBS6	pp	229533.I1RJV4
108693	229533.I1RBS6	pp	229533.I1RP27
108694	229533.I1RBS6	pp	229533.I1RNC1
108695	229533.I1RBS6	pp	229533.I1RR73
108696	229533.I1RBS6	pp	229533.I1RUG3
108697	229533.I1RBS6	pp	229533.I1RGA4

108698 229533.I1RBS6 pp 229533.I1RC82
108699 229533.I1RBS6 pp 229533.V6R3X5
108700 229533.I1RBS6 pp 229533.I1RE27
108701 229533.I1RBS6 pp 229533.I1RCR8
108702 229533.I1RBS6 pp 229533.I1RRT2
108703 229533.I1RBS6 pp 229533.I1RJL4
108704 229533.I1RBS6 pp 229533.I1RPU3
108705 229533.I1RBS6 pp 229533.I1RZ39
108706 229533.I1RBS6 pp 229533.I1RBY4
108707 229533.I1RBS6 pp 229533.I1RLN6
108708 229533.I1RBS6 pp 229533.I1RE76
108709 229533.I1RBS6 pp 229533.I1RRZ0
108710 229533.I1RBS6 pp 229533.I1RRL9
108711 229533.I1RBS6 pp 229533.I1RJW6
108712 229533.I1RBS6 pp 229533.I1S0J7
108713 229533.I1RBS6 pp 229533.I1S8V3
108714 229533.I1RBS6 pp 229533.I1RC66
108715 229533.I1RBS6 pp 229533.I1S503
108716 229533.I1RBS6 pp 229533.I1RNN1
108717 229533.I1RBS6 pp 229533.I1RJZ7
108718 229533.I1RBS6 pp 229533.I1RFH8
108719 229533.I1RBS6 pp 229533.I1S2B8
108720 229533.I1RBS6 pp 229533.I1RNA5
108721 229533.I1RBS6 pp 229533.I1S2E9
108722 229533.I1RBS6 pp 229533.I1RRM5
108723 229533.I1RBS6 pp 229533.I1RWI5
108724 229533.I1RBS6 pp 229533.I1S0M6
108725 229533.I1RBS6 pp 229533.I1RZZ9
108726 229533.I1RBS6 pp 229533.I1RT04
108727 229533.I1RBS6 pp 229533.Q4IEV5
108728 229533.I1RBS6 pp 229533.I1RE22
108729 229533.I1RBS6 pp 229533.V6RUY9
108730 229533.I1RBS6 pp 229533.I1RS47
108731 229533.I1RBS6 pp 229533.I1RQ23
108732 229533.I1RBS6 pp 229533.I1RPM4
108733 229533.I1RBS6 pp 229533.I1RGF5
108734 229533.I1RBS6 pp 229533.I1RTD5
108735 229533.I1RBS6 pp 229533.I1RP28
108736 229533.I1RBS6 pp 229533.I1RZL6
108737 229533.I1RBS6 pp 229533.I1RV91
108738 229533.I1RBS6 pp 229533.I1RN67
108739 229533.I1RBS6 pp 229533.I1S6Y8
108740 229533.I1RBS6 pp 229533.I1S0F6
108741 229533.I1RBS6 pp 229533.I1RT40
108742 229533.I1RBS6 pp 229533.I1S0L4
108743 229533.I1RBS6 pp 229533.I1RIL3
108744 229533.I1RDX0 pp 229533.I1RQ92
108745 229533.I1RDX0 pp 229533.I1RYR2
108746 229533.I1RDX0 pp 229533.I1RFZ2
108747 229533.I1RDX0 pp 229533.I1RZ94
108748 229533.I1RDX0 pp 229533.I1RQM4
108749 229533.I1RDX0 pp 229533.I1RSN7
108750 229533.I1RDX0 pp 229533.I1RRT1
108751 229533.I1RDX0 pp 229533.I1RYX9
108752 229533.I1RDX0 pp 229533.Q4IP51
108753 229533.I1RDX0 pp 229533.Q4IQM6
108754 229533.I1RDX0 pp 229533.I1S209
108755 229533.I1RDX0 pp 229533.I1RWK3
108756 229533.I1RDX0 pp 229533.I1RSI4
108757 229533.I1RDX0 pp 229533.I1RPQ1
108758 229533.I1RDX0 pp 229533.I1S604
108759 229533.I1RDX0 pp 229533.I1RMX3
108760 229533.I1RDX0 pp 229533.I1S2F2
108761 229533.I1RDX0 pp 229533.I1RYQ1
108762 229533.I1RDX0 pp 229533.I1RZ98
108763 229533.I1RDX0 pp 229533.I1S283
108764 229533.I1RDX0 pp 229533.I1RSC7
108765 229533.I1RDX0 pp 229533.Q4IMD1
108766 229533.I1RDX0 pp 229533.I1RY73
108767 229533.I1RDX0 pp 229533.I1RKI2
108768 229533.I1RDX0 pp 229533.I1S8E2
108769 229533.I1RDX0 pp 229533.I1RSJ4
108770 229533.I1RDX0 pp 229533.I1RLX6

108771 229533.I1RDX0 pp 229533.I1RZP8
108772 229533.I1RDX0 pp 229533.I1RQ81
108773 229533.I1RDX0 pp 229533.I1RNE2
108774 229533.I1RDX0 pp 229533.I1RE46
108775 229533.I1RDX0 pp 229533.I1RNI4
108776 229533.I1RDX0 pp 229533.I1RLE7
108777 229533.I1RDX0 pp 229533.I1RSP7
108778 229533.I1RDX0 pp 229533.V6R382
108779 229533.I1RDX0 pp 229533.I1RUG1
108780 229533.I1RDX0 pp 229533.I1RW16
108781 229533.I1RDX0 pp 229533.I1S0C2
108782 229533.I1RDX0 pp 229533.I1RMS4
108783 229533.I1RDX0 pp 229533.I1RQJ4
108784 229533.I1RDX0 pp 229533.I1RGC3
108785 229533.I1RDX0 pp 229533.I1RN31
108786 229533.I1RDX0 pp 229533.I1RN46
108787 229533.I1RDX0 pp 229533.Q4HYB8
108788 229533.I1RDX0 pp 229533.I1S461
108789 229533.I1RDX0 pp 229533.I1RRM5
108790 229533.I1RDX0 pp 229533.I1RN57
108791 229533.I1RDX0 pp 229533.I1RPZ0
108792 229533.I1RDX0 pp 229533.I1RFX8
108793 229533.I1RDX0 pp 229533.V6RLW4
108794 229533.I1RDX0 pp 229533.Q4IJU0
108795 229533.I1RDX0 pp 229533.I1RSR9
108796 229533.I1RDX0 pp 229533.I1RSA7
108797 229533.I1RDX0 pp 229533.I1RFJ2
108798 229533.I1RDX0 pp 229533.I1RMB7
108799 229533.I1RDX0 pp 229533.I1RG77
108800 229533.I1RDX0 pp 229533.I1RZB5
108801 229533.I1RDX0 pp 229533.I1RJV3
108802 229533.I1RDX0 pp 229533.I1RN55
108803 229533.I1RDX0 pp 229533.I1RUG2
108804 229533.I1RDX0 pp 229533.I1RZK6
108805 229533.I1RDX0 pp 229533.I1RQ13
108806 229533.A0A1C3YI40 pp 229533.I1RUR0
108807 229533.A0A1C3YI40 pp 229533.I1RZ94
108808 229533.A0A1C3YI40 pp 229533.V6RKK5
108809 229533.A0A1C3YI40 pp 229533.I1S461
108810 229533.A0A1C3YI40 pp 229533.I1RFW6
108811 229533.A0A1C3YI40 pp 229533.A0A1C3YJN7
108812 229533.A0A1C3YI40 pp 229533.I1RCA5
108813 229533.A0A1C3YI40 pp 229533.I1RFJ2
108814 229533.A0A1C3YI40 pp 229533.I1RRT1
108815 229533.A0A1C3YI40 pp 229533.Q4HTT2
108816 229533.A0A1C3YI40 pp 229533.A0A1C3YJ79
108817 229533.A0A1C3YI40 pp 229533.A0A1C3YI64
108818 229533.A0A1C3YI40 pp 229533.I1RXU0
108819 229533.A0A1C3YI40 pp 229533.I1S604
108820 229533.A0A1C3YI40 pp 229533.I1RN31
108821 229533.A0A1C3YI40 pp 229533.V6RLW4
108822 229533.A0A1C3YI40 pp 229533.I1RSC7
108823 229533.A0A1C3YI40 pp 229533.I1RFX8
108824 229533.A0A1C3YI40 pp 229533.I1RBN9
108825 229533.A0A1C3YI40 pp 229533.I1RCA9
108826 229533.A0A1C3YI40 pp 229533.I1RX64
108827 229533.A0A1C3YI40 pp 229533.I1RSA7
108828 229533.A0A1C3YI40 pp 229533.I1RY73
108829 229533.A0A1C3YI40 pp 229533.I1RBW0
108830 229533.A0A1C3YI40 pp 229533.I1RC54
108831 229533.A0A1C3YI40 pp 229533.I1RZP8
108832 229533.A0A1C3YI40 pp 229533.I1RAH4
108833 229533.A0A1C3YI40 pp 229533.I1S209
108834 229533.A0A1C3YI40 pp 229533.I1RWI8
108835 229533.A0A1C3YI40 pp 229533.I1RAP1
108836 229533.A0A1C3YI40 pp 229533.I1RRS7
108837 229533.A0A1C3YI40 pp 229533.I1RSP7
108838 229533.A0A1C3YI40 pp 229533.I1RBC8
108839 229533.A0A1C3YI40 pp 229533.I1S2E9
108840 229533.A0A1C3YI40 pp 229533.I1RE46
108841 229533.A0A1C3YI40 pp 229533.I1RW16
108842 229533.A0A1C3YI40 pp 229533.I1RQJ4
108843 229533.A0A1C3YI40 pp 229533.I1S9J6

108844 229533.A0A1C3YI40 pp 229533.A0A1C3YM67
108845 229533.A0A1C3YI40 pp 229533.I1RKI2
108846 229533.A0A1C3YI40 pp 229533.I1RMQ7
108847 229533.A0A1C3YI40 pp 229533.I1RA31
108848 229533.A0A1C3YI40 pp 229533.I1RLX6
108849 229533.A0A1C3YI40 pp 229533.I1RDX0
108850 229533.A0A1C3YI40 pp 229533.Q4IB89
108851 229533.A0A1C3YI40 pp 229533.I1RN57
108852 229533.A0A1C3YI40 pp 229533.I1RSZ3
108853 229533.A0A1C3YI40 pp 229533.I1RSR9
108854 229533.A0A1C3YI40 pp 229533.I1RM36
108855 229533.A0A1C3YI40 pp 229533.I1RN55
108856 229533.A0A1C3YI40 pp 229533.I1RG77
108857 229533.A0A1C3YI40 pp 229533.A0A1C3YJR3
108858 229533.A0A1C3YI40 pp 229533.I1RWT6
108859 229533.A0A1C3YI40 pp 229533.I1RFP4
108860 229533.A0A1C3YI40 pp 229533.Q4IQM6
108861 229533.A0A1C3YI40 pp 229533.I1RQA3
108862 229533.A0A1C3YI40 pp 229533.I1RCL7
108863 229533.A0A1C3YI40 pp 229533.I1RKB1
108864 229533.A0A1C3YI40 pp 229533.I1S4P6
108865 229533.A0A1C3YI40 pp 229533.I1RPQ1
108866 229533.A0A1C3YI40 pp 229533.A0A1C3YJP9
108867 229533.A0A1C3YI40 pp 229533.A0A1C3YN55
108868 229533.A0A1C3YI40 pp 229533.I1RA12
108869 229533.A0A1C3YI40 pp 229533.I1RYQ1
108870 229533.A0A1C3YI40 pp 229533.I1RA10
108871 229533.A0A1C3YI40 pp 229533.I1RAP2
108872 229533.A0A1C3YI40 pp 229533.A0A1C3YML3
108873 229533.A0A1C3YI40 pp 229533.I1RUG4
108874 229533.A0A1C3YI40 pp 229533.I1RY44
108875 229533.A0A1C3YI40 pp 229533.I1S0N5
108876 229533.A0A1C3YI40 pp 229533.I1RKE9
108877 229533.A0A1C3YI40 pp 229533.Q4IAS1
108878 229533.A0A1C3YI40 pp 229533.Q4IJH1
108879 229533.A0A1C3YI40 pp 229533.A0A1C3YKE4
108880 229533.A0A1C3YI40 pp 229533.A0A1C3YN36
108881 229533.A0A1C3YI40 pp 229533.A0A1C3YMJ3
108882 229533.A0A1C3YI40 pp 229533.I1RSI4
108883 229533.A0A1C3YI40 pp 229533.I1RYX9
108884 229533.A0A1C3YI40 pp 229533.I1RYR2
108885 229533.A0A1C3YI40 pp 229533.A0A1C3YIR8
108886 229533.A0A1C3YI40 pp 229533.Q4IP51
108887 229533.A0A1C3YI40 pp 229533.I1RBW1
108888 229533.A0A1C3YI40 pp 229533.I1RFL7
108889 229533.A0A1C3YI40 pp 229533.I1RQ92
108890 229533.A0A1C3YI40 pp 229533.I1RSN7
108891 229533.A0A1C3YI40 pp 229533.A0A1C3YLT1
108892 229533.I1RGA4 pp 229533.I1S5B0
108893 229533.I1RGA4 pp 229533.I1RQD2
108894 229533.I1RGA4 pp 229533.I1RZM8
108895 229533.I1RGA4 pp 229533.I1S044
108896 229533.I1RGA4 pp 229533.I1RW99
108897 229533.I1RGA4 pp 229533.I1RI82
108898 229533.I1RGA4 pp 229533.I1RNH6
108899 229533.I1RGA4 pp 229533.I1RXF0
108900 229533.I1RGA4 pp 229533.I1RYB9
108901 229533.I1RGA4 pp 229533.I1S021
108902 229533.I1RGA4 pp 229533.I1RRV3
108903 229533.I1RGA4 pp 229533.I1S6K9
108904 229533.I1RGA4 pp 229533.I1RGC5
108905 229533.I1RGA4 pp 229533.V6RCZ8
108906 229533.I1RGA4 pp 229533.V6QUE6
108907 229533.I1RGA4 pp 229533.I1RRV4
108908 229533.I1RGA4 pp 229533.I1RGC2
108909 229533.I1RGA4 pp 229533.I1S821
108910 229533.I1RGA4 pp 229533.I1RR51
108911 229533.I1RGA4 pp 229533.I1RN73
108912 229533.I1RGA4 pp 229533.I1RNQ5
108913 229533.I1RGA4 pp 229533.I1RLU6
108914 229533.I1RGA4 pp 229533.I1S1B2
108915 229533.I1RGA4 pp 229533.I1RZM1
108916 229533.I1RGA4 pp 229533.V6RCG5

229533.I1RGA4	pp	229533.Q4I8P5
229533.I1RGA4	pp	229533.I1RZF6
229533.I1RGA4	pp	229533.I1RS18
229533.I1RGA4	pp	229533.V6R9Z0
229533.I1RGA4	pp	229533.I1S504
229533.I1RGA4	pp	229533.I1S419
229533.I1RGA4	pp	229533.Q4I283
229533.I1RGA4	pp	229533.I1RZK5
229533.I1RGA4	pp	229533.I1RYS0
229533.I1RGA4	pp	229533.I1RWB0
229533.I1RGA4	pp	229533.I1S887
229533.I1RGA4	pp	229533.I1RVZ0
229533.I1RGA4	pp	229533.I1RWJ3
229533.I1RGA4	pp	229533.I1RK83
229533.I1RGA4	pp	229533.I1RWT6
229533.I1RGA4	pp	229533.I1RQ85
229533.I1RGA4	pp	229533.I1RGB1
229533.I1RGA4	pp	229533.I1S1J7
229533.I1RGA4	pp	229533.Q4I2J8
229533.I1RGA4	pp	229533.I1RM26
229533.I1RGA4	pp	229533.I1SA32
229533.I1RGA4	pp	229533.I1RXC4
229533.I1RGA4	pp	229533.I1S217
229533.I1RGA4	pp	229533.I1RPR9
229533.I1RGA4	pp	229533.I1RJ17
229533.I1RGA4	pp	229533.I1RRY9
229533.I1RGA4	pp	229533.I1RWR7
229533.I1RGA4	pp	229533.I1S0M9
229533.I1RGA4	pp	229533.I1RSR8
229533.I1RGA4	pp	229533.I1RZE5
229533.I1RGA4	pp	229533.I1RNT9
229533.I1RGA4	pp	229533.I1RSI0
229533.I1RGA4	pp	229533.I1RQ81
229533.I1RGA4	pp	229533.I1RY03
229533.I1RGA4	pp	229533.I1S0P0
229533.I1RGA4	pp	229533.Q4IJK2
229533.I1RGA4	pp	229533.I1S4Q4
229533.I1RGA4	pp	229533.I1RQA3
229533.I1RGA4	pp	229533.I1RZK6
229533.I1RGA4	pp	229533.I1RGB2
229533.I1RGA4	pp	229533.I1RVQ0
229533.I1RGA4	pp	229533.I1RXD6
229533.I1RGA4	pp	229533.Q4IPI1
229533.I1RGA4	pp	229533.I1RY86
229533.I1RGA4	pp	229533.I1S540
229533.I1RGA4	pp	229533.I1RZZ1
229533.I1RGA4	pp	229533.I1RQQ5
229533.I1RGA4	pp	229533.I1RT33
229533.I1RGA4	pp	229533.I1RJZ6
229533.I1RGA4	pp	229533.V6RBN2
229533.I1RGA4	pp	229533.V6RTJ8
229533.I1RGA4	pp	229533.I1S7A8
229533.I1RGA4	pp	229533.I1S0F5
229533.I1RGA4	pp	229533.V6R1K4
229533.I1RGA4	pp	229533.I1RNB2
229533.I1RGA4	pp	229533.I1RUQ3
229533.I1RGA4	pp	229533.I1RPY3
229533.I1RGA4	pp	229533.I1RK95
229533.I1RGA4	pp	229533.I1S0L3
229533.I1RGA4	pp	229533.I1RVX9
229533.I1RGA4	pp	229533.I1RQC9
229533.I1RGA4	pp	229533.I1RX95
229533.I1RGA4	pp	229533.I1S2R0
229533.I1RGA4	pp	229533.I1S0T2
229533.I1RGA4	pp	229533.I1RX46
229533.I1RGA4	pp	229533.I1S0R7
229533.I1RGA4	pp	229533.I1RWH5
229533.I1RGA4	pp	229533.I1RGU0
229533.I1RGA4	pp	229533.I1RRG3
229533.I1RGA4	pp	229533.I1RTB3
229533.I1RGA4	pp	229533.Q4IJT5
229533.I1RGA4	pp	229533.I1RQH3
229533.I1RGA4	pp	229533.I1RN61

108990	229533.I1RGA4	pp	229533.V6QW22
108991	229533.I1RGA4	pp	229533.V6RUT5
108992	229533.I1RGA4	pp	229533.I1S4S7
108993	229533.I1RGA4	pp	229533.I1RLP1
108994	229533.I1RGA4	pp	229533.I1RRX5
108995	229533.I1RGA4	pp	229533.I1RPN4
108996	229533.I1RGA4	pp	229533.I1RW71
108997	229533.I1RGA4	pp	229533.I1RXD7
108998	229533.I1RGA4	pp	229533.I1RSQ7
108999	229533.I1RGA4	pp	229533.I1RWJ8
109000	229533.I1RGA4	pp	229533.I1RR00
109001	229533.I1RGA4	pp	229533.I1RQJ5
109002	229533.I1RGA4	pp	229533.I1S4S4
109003	229533.I1RGA4	pp	229533.I1RPA4
109004	229533.I1RGA4	pp	229533.I1RT80
109005	229533.I1RGA4	pp	229533.I1RM67
109006	229533.I1RGA4	pp	229533.I1RPU0
109007	229533.I1RGA4	pp	229533.I1RJU3
109008	229533.I1RGA4	pp	229533.I1RR24
109009	229533.I1RGA4	pp	229533.I1S9U2
109010	229533.I1RGA4	pp	229533.I1RKJ5
109011	229533.I1RGA4	pp	229533.I1S9I5
109012	229533.I1RGA4	pp	229533.I1RW54
109013	229533.I1RGA4	pp	229533.I1S438
109014	229533.I1RGA4	pp	229533.I1RM06
109015	229533.I1RGA4	pp	229533.I1RX25
109016	229533.I1RGA4	pp	229533.I1RW25
109017	229533.I1RGA4	pp	229533.I1RMC8
109018	229533.I1RGA4	pp	229533.I1S0X6
109019	229533.I1RGA4	pp	229533.I1RSU2
109020	229533.I1RGA4	pp	229533.I1RTG6
109021	229533.I1RGA4	pp	229533.I1SA33
109022	229533.I1RGA4	pp	229533.I1RMN6
109023	229533.I1RGA4	pp	229533.Q4ICG5
109024	229533.I1RGA4	pp	229533.I1RHK2
109025	229533.I1RGA4	pp	229533.I1S0N4
109026	229533.I1RGA4	pp	229533.I1S5R6
109027	229533.I1RGA4	pp	229533.I1RRS8
109028	229533.I1RGA4	pp	229533.I1RRN2
109029	229533.I1RGA4	pp	229533.I1RNC1
109030	229533.I1RGA4	pp	229533.V6RIV9
109031	229533.I1RGA4	pp	229533.I1RXT3
109032	229533.I1RGA4	pp	229533.I1RUG3
109033	229533.I1RGA4	pp	229533.I1S1W9
109034	229533.I1RGA4	pp	229533.I1RWB5
109035	229533.I1RGA4	pp	229533.I1RS17
109036	229533.I1RGA4	pp	229533.I1S016
109037	229533.I1RGA4	pp	229533.Q4IN63
109038	229533.I1RGA4	pp	229533.I1RM96
109039	229533.I1RGA4	pp	229533.I1RY44
109040	229533.I1RGA4	pp	229533.Q4I327
109041	229533.I1RGA4	pp	229533.I1S1W3
109042	229533.I1RGA4	pp	229533.I1RNB8
109043	229533.I1RGA4	pp	229533.I1RZV3
109044	229533.I1RGA4	pp	229533.I1RPP4
109045	229533.I1RGA4	pp	229533.I1RMV0
109046	229533.I1RGA4	pp	229533.I1RKJ4
109047	229533.I1RGA4	pp	229533.I1RVF8
109048	229533.I1RGA4	pp	229533.I1RSQ3
109049	229533.I1RGA4	pp	229533.I1RRE5
109050	229533.I1RGA4	pp	229533.I1RRV1
109051	229533.I1RGA4	pp	229533.I1S9Z4
109052	229533.I1RGA4	pp	229533.I1RRR7
109053	229533.I1RGA4	pp	229533.I1RRM3
109054	229533.I1RGA4	pp	229533.I1RSF1
109055	229533.I1RGA4	pp	229533.I1RVZ7
109056	229533.I1RGA4	pp	229533.I1RVQ3
109057	229533.I1RGA4	pp	229533.I1RWN5
109058	229533.I1RGA4	pp	229533.V6RMW6
109059	229533.I1RGA4	pp	229533.I1RSK1
109060	229533.I1RGA4	pp	229533.I1RPU2
109061	229533.I1RGA4	pp	229533.I1RWX5
109062	229533.I1RGA4	pp	229533.I1RW46

109063 229533.I1RGA4 pp 229533.I1RTA0
109064 229533.I1RGA4 pp 229533.I1RJV3
109065 229533.I1RGA4 pp 229533.I1RZ79
109066 229533.I1RGA4 pp 229533.I1RRI2
109067 229533.I1RGA4 pp 229533.I1RXU9
109068 229533.I1RGA4 pp 229533.I1S7W5
109069 229533.I1RGA4 pp 229533.I1RZW8
109070 229533.I1RGA4 pp 229533.I1S0J1
109071 229533.I1RGA4 pp 229533.I1RPK0
109072 229533.I1RGA4 pp 229533.I1RWN6
109073 229533.I1RGA4 pp 229533.I1S8R3
109074 229533.I1RGA4 pp 229533.I1RTH6
109075 229533.I1RGA4 pp 229533.I1S437
109076 229533.I1RGA4 pp 229533.Q4I624
109077 229533.I1RGA4 pp 229533.I1RKJ8
109078 229533.I1RGA4 pp 229533.I1RQ33
109079 229533.I1RGA4 pp 229533.I1RSD8
109080 229533.I1RGA4 pp 229533.I1RYW3
109081 229533.I1RGA4 pp 229533.V6RRH2
109082 229533.I1RGA4 pp 229533.I1S2C0
109083 229533.I1RGA4 pp 229533.Q4IPZ1
109084 229533.I1RGA4 pp 229533.I1RML0
109085 229533.I1RGA4 pp 229533.I1RQE7
109086 229533.I1RGA4 pp 229533.V6R057
109087 229533.I1RGA4 pp 229533.I1RN18
109088 229533.I1RGA4 pp 229533.I1S0R8
109089 229533.I1RGA4 pp 229533.I1RT03
109090 229533.I1RGA4 pp 229533.I1S2E8
109091 229533.I1RGA4 pp 229533.I1RVX0
109092 229533.I1RGA4 pp 229533.I1S1C7
109093 229533.I1RGA4 pp 229533.V6R283
109094 229533.I1RGA4 pp 229533.I1RWK3
109095 229533.I1RGA4 pp 229533.I1RJW6
109096 229533.I1RGA4 pp 229533.I1RSS4
109097 229533.I1RGA4 pp 229533.I1S503
109098 229533.I1RGA4 pp 229533.I1RSY2
109099 229533.I1RGA4 pp 229533.I1RYR0
109100 229533.I1RGA4 pp 229533.I1RXR1
109101 229533.I1RGA4 pp 229533.I1RSX0
109102 229533.I1RGA4 pp 229533.I1RJH0
109103 229533.I1RGA4 pp 229533.I1RRN5
109104 229533.I1RGA4 pp 229533.V6R3J1
109105 229533.I1RGA4 pp 229533.I1RTA1
109106 229533.I1RGA4 pp 229533.Q4HVQ9
109107 229533.I1RGA4 pp 229533.I1RUF7
109108 229533.I1RGA4 pp 229533.I1RVZ2
109109 229533.I1RGA4 pp 229533.I1RJN9
109110 229533.I1RGA4 pp 229533.I1S268
109111 229533.I1RGA4 pp 229533.I1RKL3
109112 229533.I1RGA4 pp 229533.I1RSV2
109113 229533.I1RGA4 pp 229533.I1RQM7
109114 229533.I1RGA4 pp 229533.I1RP27
109115 229533.I1RGA4 pp 229533.I1RRZ0
109116 229533.I1RGA4 pp 229533.Q4HXF0
109117 229533.I1RGA4 pp 229533.I1RLV9
109118 229533.I1RGA4 pp 229533.I1RJV4
109119 229533.I1RGA4 pp 229533.I1RV91
109120 229533.I1RGA4 pp 229533.I1S8P5
109121 229533.I1RGA4 pp 229533.I1RYI5
109122 229533.I1RGA4 pp 229533.V6RUY9
109123 229533.I1RGA4 pp 229533.I1RPP3
109124 229533.I1RGA4 pp 229533.I1RRJ8
109125 229533.I1RGA4 pp 229533.I1RT40
109126 229533.I1RGA4 pp 229533.I1RT04
109127 229533.I1RGA4 pp 229533.I1S9Y1
109128 229533.I1RGA4 pp 229533.I1S2E9
109129 229533.I1RGA4 pp 229533.I1RXF8
109130 229533.I1RGA4 pp 229533.I1S0W4
109131 229533.I1RGA4 pp 229533.I1RPH6
109132 229533.I1RGA4 pp 229533.I1RZV6
109133 229533.I1RGA4 pp 229533.I1RM82
109134 229533.I1RGA4 pp 229533.I1RTD5
109135 229533.I1RGA4 pp 229533.I1RWL2

109136	229533.I1RGA4	pp	229533.I1S0F6
109137	229533.I1RGA4	pp	229533.I1RN67
109138	229533.I1RGA4	pp	229533.I1RPE0
109139	229533.I1RGA4	pp	229533.I1RKJ6
109140	229533.I1RGA4	pp	229533.I1S2H4
109141	229533.I1RGA4	pp	229533.I1S5R1
109142	229533.I1RGA4	pp	229533.I1RRQ3
109143	229533.I1RGA4	pp	229533.I1RQ65
109144	229533.I1RGA4	pp	229533.I1RRT8
109145	229533.I1RGA4	pp	229533.I1RS47
109146	229533.I1RGA4	pp	229533.I1S0L4
109147	229533.I1RGA4	pp	229533.O74638
109148	229533.I1RGA4	pp	229533.I1RRI1
109149	229533.I1RGA4	pp	229533.I1RXC2
109150	229533.I1RGA4	pp	229533.I1RSA5
109151	229533.I1RGA4	pp	229533.I1RKP1
109152	229533.I1RGA4	pp	229533.I1RR73
109153	229533.I1RGA4	pp	229533.I1RQ23
109154	229533.I1RGA4	pp	229533.I1RGB0
109155	229533.I1RGA4	pp	229533.I1RL04
109156	229533.I1RGA4	pp	229533.I1RND6
109157	229533.I1RGA4	pp	229533.I1RPM4
109158	229533.I1RGA4	pp	229533.I1S6Y8
109159	229533.I1RGA4	pp	229533.I1RWI5
109160	229533.I1RGA4	pp	229533.I1RJZ7
109161	229533.I1RGA4	pp	229533.V6R3X5
109162	229533.I1RGA4	pp	229533.I1RPV3
109163	229533.I1RGA4	pp	229533.I1S2B8
109164	229533.I1RGA4	pp	229533.I1S0M6
109165	229533.I1RGA4	pp	229533.I1RGU7
109166	229533.I1RGA4	pp	229533.I1RZZ9
109167	229533.I1RGA4	pp	229533.I1RSQ4
109168	229533.I1RGA4	pp	229533.I1RPU3
109169	229533.I1RGA4	pp	229533.I1RRM5
109170	229533.I1RGA4	pp	229533.I1RNN1
109171	229533.I1RGA4	pp	229533.I1RZS6
109172	229533.I1RGA4	pp	229533.I1S277
109173	229533.I1RGA4	pp	229533.I1RTN3
109174	229533.I1RGA4	pp	229533.I1RZ39
109175	229533.I1RGA4	pp	229533.I1S4Z8
109176	229533.I1RGA4	pp	229533.I1RSZ7
109177	229533.I1RGA4	pp	229533.I1RY92
109178	229533.I1RGA4	pp	229533.I1S8E2
109179	229533.A0A098E4A1	pp	229533.A0A1C3YME3
109180	229533.A0A098E4A1	pp	229533.I1RBR0
109181	229533.A0A098E4A1	pp	229533.A0A1C3YIF4
109182	229533.A0A098E4A1	pp	229533.I1RX09
109183	229533.A0A098E4A1	pp	229533.I1RWI6
109184	229533.A0A098E4A1	pp	229533.I1S0X0
109185	229533.A0A098E4A1	pp	229533.I1RYN9
109186	229533.A0A098E4A1	pp	229533.I1S034
109187	229533.A0A098E4A1	pp	229533.I1RCT2
109188	229533.I1RPQ6	pp	229533.I1RSN7
109189	229533.I1RPQ6	pp	229533.I1RSP7
109190	229533.I1RDH0	pp	229533.I1RTC2
109191	229533.I1RDH0	pp	229533.I1RJL3
109192	229533.I1RDH0	pp	229533.I1RFL7
109193	229533.I1RDH0	pp	229533.I1RSZ3
109194	229533.I1RDH0	pp	229533.Q4INS6
109195	229533.I1RDH0	pp	229533.I1RPF5
109196	229533.I1RDH0	pp	229533.I1RQP1
109197	229533.I1RDH0	pp	229533.Q4HYI0
109198	229533.I1RDH0	pp	229533.I1S2E9
109199	229533.I1RDH0	pp	229533.I1RY29
109200	229533.I1RDH0	pp	229533.V6RCZ8
109201	229533.I1RDH0	pp	229533.I1RKI2
109202	229533.I1RDH0	pp	229533.I1S5B0
109203	229533.I1RDH0	pp	229533.I1RY58
109204	229533.I1RDH0	pp	229533.I1S209
109205	229533.I1RDH0	pp	229533.I1RWI8
109206	229533.I1RDH0	pp	229533.I1RQA3
109207	229533.I1RDH0	pp	229533.I1RUF8
109208	229533.I1RDH0	pp	229533.I1RSJ1

229533.I1RDH0	pp	229533.I1RRN5
229533.I1RDH0	pp	229533.I1RRJ6
229533.I1RDH0	pp	229533.I1RN55
229533.I1RDH0	pp	229533.V6QWX8
229533.I1RDH0	pp	229533.Q4IQX3
229533.I1RDH0	pp	229533.I1RW90
229533.I1RDH0	pp	229533.I1RP99
229533.I1RDH0	pp	229533.I1RRM8
229533.I1RDH0	pp	229533.I1S0J3
229533.I1RDH0	pp	229533.I1RFQ5
229533.I1RDH0	pp	229533.I1RR25
229533.I1RDH0	pp	229533.I1RE55
229533.I1RDH0	pp	229533.I1RPQ1
229533.I1RDH0	pp	229533.I1RUR0
229533.I1RDH0	pp	229533.I1REN3
229533.I1RDH0	pp	229533.I1RKB1
229533.I1RDH0	pp	229533.I1RM36
229533.I1RDH0	pp	229533.I1SAA0
229533.I1RDH0	pp	229533.I1RVL8
229533.I1RDH0	pp	229533.I1RRT1
229533.I1RDH0	pp	229533.I1RUG4
229533.I1RDH0	pp	229533.I1RMQ7
229533.I1RDH0	pp	229533.I1S0N5
229533.I1RDH0	pp	229533.I1RN57
229533.I1RDH0	pp	229533.I1RZ94
229533.I1RDH0	pp	229533.Q4IB89
229533.I1RDH0	pp	229533.I1RY44
229533.I1RDH0	pp	229533.I1S9J6
229533.I1RDH0	pp	229533.I1S004
229533.I1RDH0	pp	229533.I1S072
229533.I1RDH0	pp	229533.I1RZ86
229533.I1RDH0	pp	229533.I1RSP7
229533.I1RDH0	pp	229533.I1SAD2
229533.I1RDH0	pp	229533.I1RK90
229533.I1RDH0	pp	229533.I1RTN6
229533.I1RDH0	pp	229533.I1RQ93
229533.I1RDH0	pp	229533.I1RWT6
229533.I1RDH0	pp	229533.I1RZ98
229533.I1RDH0	pp	229533.Q4IJ84
229533.I1RDH0	pp	229533.I1RQI6
229533.I1RDH0	pp	229533.I1RX64
229533.I1RDH0	pp	229533.I1RSC7
229533.I1RDH0	pp	229533.I1S0D5
229533.I1RDH0	pp	229533.I1RXU0
229533.I1RDH0	pp	229533.I1RPM7
229533.I1RDH0	pp	229533.I1RDQ9
229533.I1RDH0	pp	229533.I1RSR9
229533.I1RDH0	pp	229533.I1RMS2
229533.I1RDH0	pp	229533.I1RZT1
229533.I1RDH0	pp	229533.I1S276
229533.I1RWK3	pp	229533.V6RIV9
229533.I1RWK3	pp	229533.Q4I8P5
229533.I1RWK3	pp	229533.I1S419
229533.I1RWK3	pp	229533.I1RWN5
229533.I1RWK3	pp	229533.I1RY44
229533.I1RWK3	pp	229533.Q4IJK2
229533.I1RWK3	pp	229533.Q4HZB8
229533.I1RWK3	pp	229533.Q4I2J8
229533.I1RWK3	pp	229533.I1RZZ1
229533.I1RWK3	pp	229533.I1RZM1
229533.I1RWK3	pp	229533.I1S504
229533.I1RWK3	pp	229533.I1S569
229533.I1RWK3	pp	229533.Q4I624
229533.I1RWK3	pp	229533.V6R057
229533.I1RWK3	pp	229533.Q4I283
229533.I1RWK3	pp	229533.I1RWT6
229533.I1RWK3	pp	229533.I1RYW3
229533.I1RWK3	pp	229533.I1RZW8
229533.I1RWK3	pp	229533.I1S8R3
229533.I1RWK3	pp	229533.I1S8V4
229533.I1RWK3	pp	229533.I1RZK5
229533.I1RWK3	pp	229533.I1S1C7
229533.I1RWK3	pp	229533.I1RXT3

229533.I1RWK3 pp 229533.I1RZM8
229533.I1RWK3 pp 229533.I1RWX5
229533.I1RWK3 pp 229533.I1RY03
229533.I1RWK3 pp 229533.I1RXF0
229533.I1RWK3 pp 229533.I1S1J7
229533.I1RWK3 pp 229533.I1S0N4
229533.I1RWK3 pp 229533.I1RWQ8
229533.I1RWK3 pp 229533.I1RWN6
229533.I1RWK3 pp 229533.V6R042
229533.I1RWK3 pp 229533.I1RZH4
229533.I1RWK3 pp 229533.V6RCZ8
229533.I1RWK3 pp 229533.I1SA32
229533.I1RWK3 pp 229533.I1S5B0
229533.I1RWK3 pp 229533.I1S0J1
229533.I1RWK3 pp 229533.V6RMW6
229533.I1RWK3 pp 229533.I1S6Y8
229533.I1RWK3 pp 229533.I1S8V3
229533.I1RWK3 pp 229533.V6QUW4
229533.I1RWK3 pp 229533.V6RCG5
229533.I1RWK3 pp 229533.I1SA33
229533.I1RWK3 pp 229533.V6QYF0
229533.I1RWK3 pp 229533.I1S0M6
229533.I1RWK3 pp 229533.I1S8P5
229533.I1RWK3 pp 229533.I1RZ39
229533.I1RWK3 pp 229533.V6QW22
229533.I1RWK3 pp 229533.I1S2B8
229533.I1RWK3 pp 229533.V6R3X5
229533.I1RWK3 pp 229533.I1S0F6
229533.I1RWK3 pp 229533.I1S5R1
229533.I1RWK3 pp 229533.I1RX25
229533.I1RWK3 pp 229533.O74638
229533.I1RWK3 pp 229533.I1S7S9
229533.I1RWK3 pp 229533.V6R1K4
229533.I1RWK3 pp 229533.I1RZJ5
229533.I1RWK3 pp 229533.V6R4J1
229533.I1RWK3 pp 229533.I1RWL2
229533.I1RWK3 pp 229533.I1RZZ9
229533.I1RWK3 pp 229533.I1S0L3
229533.I1RWK3 pp 229533.I1S2H4
229533.I1RWK3 pp 229533.I1S0X6
229533.I1RWK3 pp 229533.I1S0L4
229533.I1RWK3 pp 229533.I1S0F5
229533.I1RWK3 pp 229533.I1S0T2
229533.I1RWK3 pp 229533.I1S8F0
229533.I1RWK3 pp 229533.Q4IR18
229533.I1RWK3 pp 229533.I1RX39
229533.I1RWK3 pp 229533.I1RX46
229533.I1RWK3 pp 229533.Q4IJT5
229533.I1RWK3 pp 229533.I1S0R7
229533.I1RWK3 pp 229533.I1S2M6
229533.I1RWK3 pp 229533.V6RUY9
229533.I1RWK3 pp 229533.I1S352
229533.I1RWK3 pp 229533.I1S1B6
229533.I1RWK3 pp 229533.I1S2E9
229533.I1RWK3 pp 229533.I1RXD7
229533.I1RWK3 pp 229533.V6RTJ8
229533.I1RWK3 pp 229533.I1S503
229533.I1RWK3 pp 229533.I1S7A8
229533.I1RP90 pp 229533.I1RPD9
229533.A0A1C3YII7 pp 229533.A0A1C3YKZ0
229533.A0A1C3YII7 pp 229533.I1RAU5
229533.A0A1C3YII7 pp 229533.I1RD22
229533.A0A1C3YII7 pp 229533.I1RRU9
229533.A0A1C3YII7 pp 229533.I1S290
229533.A0A1C3YII7 pp 229533.A0A1C3YK79
229533.A0A1C3YII7 pp 229533.V6RIS2
229533.A0A1C3YII7 pp 229533.I1RK78
229533.A0A1C3YII7 pp 229533.I1S3V2
229533.A0A1C3YII7 pp 229533.I1RKK9
229533.A0A1C3YII7 pp 229533.I1S471
229533.A0A1C3YII7 pp 229533.A0A1C3YIQ6
229533.A0A1C3YII7 pp 229533.I1RYB9
229533.A0A1C3YII7 pp 229533.Q4IAA0

109355 229533.A0A1C3YII7 pp 229533.I1RNB8
109356 229533.A0A1C3YII7 pp 229533.I1RUG3
109357 229533.A0A1C3YII7 pp 229533.I1RC81
109358 229533.A0A1C3YII7 pp 229533.A0A1C3YKV3
109359 229533.A0A1C3YII7 pp 229533.I1S6F0
109360 229533.A0A1C3YII7 pp 229533.I1RMC0
109361 229533.A0A1C3YII7 pp 229533.I1RN67
109362 229533.A0A1C3YII7 pp 229533.A0A1C3YIP5
109363 229533.A0A1C3YII7 pp 229533.I1RBZ3
109364 229533.A0A1C3YII7 pp 229533.I1RB53
109365 229533.A0A1C3YII7 pp 229533.I1RB91
109366 229533.A0A1C3YII7 pp 229533.V6RMW6
109367 229533.A0A1C3YII7 pp 229533.I1RE71
109368 229533.A0A1C3YII7 pp 229533.I1RK83
109369 229533.A0A1C3YII7 pp 229533.I1REF5
109370 229533.A0A1C3YII7 pp 229533.A0A1C3YL15
109371 229533.A0A1C3YII7 pp 229533.I1RLU5
109372 229533.A0A1C3YII7 pp 229533.Q4HW67
109373 229533.A0A1C3YII7 pp 229533.I1RAV4
109374 229533.A0A1C3YII7 pp 229533.V6R042
109375 229533.A0A1C3YII7 pp 229533.I1RWH5
109376 229533.A0A1C3YII7 pp 229533.I1RA66
109377 229533.A0A1C3YII7 pp 229533.I1RTH6
109378 229533.A0A1C3YII7 pp 229533.I1RA70
109379 229533.A0A1C3YII7 pp 229533.I1S6F1
109380 229533.A0A1C3YII7 pp 229533.I1S6E5
109381 229533.A0A1C3YII7 pp 229533.I1RZG9
109382 229533.A0A1C3YII7 pp 229533.I1RNE7
109383 229533.A0A1C3YII7 pp 229533.I1RIS3
109384 229533.A0A1C3YII7 pp 229533.Q4IEK8
109385 229533.A0A1C3YII7 pp 229533.I1RN48
109386 229533.A0A1C3YII7 pp 229533.I1RD17
109387 229533.A0A1C3YII7 pp 229533.I1RBB7
109388 229533.A0A1C3YII7 pp 229533.Q4HZ68
109389 229533.A0A1C3YII7 pp 229533.I1RZV6
109390 229533.A0A1C3YII7 pp 229533.I1S0X6
109391 229533.A0A1C3YII7 pp 229533.I1RT35
109392 229533.A0A1C3YII7 pp 229533.I1RN18
109393 229533.A0A1C3YII7 pp 229533.V6RIV9
109394 229533.A0A1C3YII7 pp 229533.Q4HWE2
109395 229533.A0A1C3YII7 pp 229533.I1RFL4
109396 229533.A0A1C3YII7 pp 229533.I1RMW7
109397 229533.A0A1C3YII7 pp 229533.I1RNI4
109398 229533.A0A1C3YII7 pp 229533.I1RND6
109399 229533.A0A1C3YII7 pp 229533.I1S504
109400 229533.A0A1C3YII7 pp 229533.I1RSE2
109401 229533.A0A1C3YII7 pp 229533.I1RAD1
109402 229533.A0A1C3YII7 pp 229533.I1RKF8
109403 229533.A0A1C3YII7 pp 229533.I1S503
109404 229533.A0A1C3YII7 pp 229533.Q4HVW2
109405 229533.A0A1C3YII7 pp 229533.I1RBN1
109406 229533.A0A1C3YII7 pp 229533.I1RYI6
109407 229533.A0A1C3YII7 pp 229533.I1RZS7
109408 229533.A0A1C3YII7 pp 229533.Q4IAS1
109409 229533.A0A1C3YII7 pp 229533.I1RZ39
109410 229533.A0A1C3YII7 pp 229533.I1S8E2
109411 229533.A0A1C3YII7 pp 229533.A0A1C3YL39
109412 229533.A0A1C3YII7 pp 229533.I1S289
109413 229533.A0A1C3YII7 pp 229533.I1RZ40
109414 229533.A0A1C3YII7 pp 229533.V6RTJ8
109415 229533.A0A1C3YII7 pp 229533.A0A1C3YKM9
109416 229533.A0A1C3YII7 pp 229533.A0A1C3YJQ9
109417 229533.A0A1C3YII7 pp 229533.I1RFH9
109418 229533.A0A1C3YII7 pp 229533.I1RBY4
109419 229533.A0A1C3YII7 pp 229533.I1S5S2
109420 229533.A0A1C3YII7 pp 229533.I1S524
109421 229533.A0A1C3YII7 pp 229533.I1RQC9
109422 229533.A0A1C3YII7 pp 229533.I1S9F1
109423 229533.A0A1C3YII7 pp 229533.I1RSZ1
109424 229533.A0A1C3YMZ4 pp 229533.I1S5X9
109425 229533.A0A1C3YMZ4 pp 229533.I1RS05
109426 229533.A0A1C3YMZ4 pp 229533.I1RXD9
109427 229533.A0A1C3YMZ4 pp 229533.I1RYT8

109428 229533.A0A1C3YMZ4 pp 229533.V6RJP8
109429 229533.A0A1C3YMZ4 pp 229533.I1RA52
109430 229533.A0A1C3YMZ4 pp 229533.I1RS29
109431 229533.A0A1C3YJF3 pp 229533.I1RBW1
109432 229533.A0A1C3YJF3 pp 229533.I1RSA7
109433 229533.A0A1C3YJF3 pp 229533.I1RRT1
109434 229533.A0A1C3YJF3 pp 229533.I1RBW0
109435 229533.A0A1C3YJF3 pp 229533.I1RYQ1
109436 229533.A0A1C3YJF3 pp 229533.I1S2E8
109437 229533.A0A1C3YJF3 pp 229533.I1S0D5
109438 229533.A0A1C3YJF3 pp 229533.I1S604
109439 229533.A0A1C3YJF3 pp 229533.Q4IR08
109440 229533.I1RTN6 pp 229533.V6RLW4
109441 229533.I1RTN6 pp 229533.I1S523
109442 229533.I1RTN6 pp 229533.I1S998
109443 229533.I1RTN6 pp 229533.I1RZ86
109444 229533.I1RTN6 pp 229533.I1RZP8
109445 229533.I1RTN6 pp 229533.I1S0D5
109446 229533.I1RTN6 pp 229533.I1RYQ1
109447 229533.I1RTN6 pp 229533.I1RX64
109448 229533.I1RTN6 pp 229533.I1S0Q4
109449 229533.I1RTN6 pp 229533.I1S1Y7
109450 229533.I1RTN6 pp 229533.Q4IMD1
109451 229533.I1RTN6 pp 229533.I1RUF8
109452 229533.I1RTN6 pp 229533.I1S788
109453 229533.I1RTN6 pp 229533.I1RZ98
109454 229533.I1RTN6 pp 229533.Q4HYB8
109455 229533.I1RTN6 pp 229533.I1RY29
109456 229533.I1RTN6 pp 229533.I1RUR0
109457 229533.I1RTN6 pp 229533.I1RWI8
109458 229533.I1RTN6 pp 229533.I1S0Q5
109459 229533.I1RTN6 pp 229533.I1S987
109460 229533.I1RTN6 pp 229533.Q4IB89
109461 229533.I1RTN6 pp 229533.I1S283
109462 229533.I1RTN6 pp 229533.I1RZ94
109463 229533.I1RTN6 pp 229533.I1RWB3
109464 229533.I1RTN6 pp 229533.Q4INS6
109465 229533.I1RTN6 pp 229533.I1RWM6
109466 229533.I1RTN6 pp 229533.I1S209
109467 229533.I1RC03 pp 229533.I1S2E9
109468 229533.I1RC03 pp 229533.I1S2B8
109469 229533.I1RC03 pp 229533.I1RR73
109470 229533.I1RC03 pp 229533.I1RTD5
109471 229533.I1RC03 pp 229533.I1RT04
109472 229533.I1RC03 pp 229533.V6R3X5
109473 229533.I1RC03 pp 229533.I1RND6
109474 229533.I1RC03 pp 229533.I1S253
109475 229533.I1RC03 pp 229533.Q4IQW0
109476 229533.I1RC03 pp 229533.I1RFN1
109477 229533.I1RC03 pp 229533.I1S8E2
109478 229533.I1RC03 pp 229533.I1RRI2
109479 229533.I1RC03 pp 229533.I1RSL9
109480 229533.I1RC03 pp 229533.I1RC82
109481 229533.I1RC03 pp 229533.I1RGA4
109482 229533.I1RC03 pp 229533.I1RZ39
109483 229533.I1RC03 pp 229533.I1S1C7
109484 229533.I1RC03 pp 229533.I1S1U7
109485 229533.I1RC03 pp 229533.I1RU69
109486 229533.I1RC03 pp 229533.I1RJL4
109487 229533.I1RC03 pp 229533.I1S1B6
109488 229533.I1RC03 pp 229533.Q4HZI0
109489 229533.I1RC03 pp 229533.V6RGY3
109490 229533.I1RC03 pp 229533.I1RWN6
109491 229533.I1RC03 pp 229533.I1RRT2
109492 229533.I1RC03 pp 229533.I1RD64
109493 229533.I1RC03 pp 229533.I1RJV3
109494 229533.I1RC03 pp 229533.I1RCL3
109495 229533.I1RC03 pp 229533.I1S503
109496 229533.I1RC03 pp 229533.I1RRT8
109497 229533.I1RC03 pp 229533.I1RGF6
109498 229533.I1RC03 pp 229533.I1S558
109499 229533.I1RC03 pp 229533.Q4IAS1
109500 229533.I1RC03 pp 229533.I1RKJ6

109501	229533.I1RC03	pp	229533.I1RCN5
109502	229533.I1RC03	pp	229533.I1RZV6
109503	229533.I1RC03	pp	229533.I1RLV9
109504	229533.I1RC03	pp	229533.I1RZS6
109505	229533.I1RC03	pp	229533.I1RRJ8
109506	229533.I1RC03	pp	229533.Q4HWE2
109507	229533.I1RC03	pp	229533.I1RSS4
109508	229533.I1RC03	pp	229533.I1RWF7
109509	229533.I1RC03	pp	229533.I1RPP3
109510	229533.I1RC03	pp	229533.I1RFS3
109511	229533.I1RC03	pp	229533.I1RX24
109512	229533.I1RC03	pp	229533.I1RT03
109513	229533.I1RC03	pp	229533.Q4I662
109514	229533.I1RC03	pp	229533.I1RKL3
109515	229533.I1RC03	pp	229533.Q4I830
109516	229533.I1RC03	pp	229533.I1RVX0
109517	229533.I1RC03	pp	229533.I1RYT5
109518	229533.I1RC03	pp	229533.I1S1X9
109519	229533.I1RC03	pp	229533.I1RWJ4
109520	229533.I1RC03	pp	229533.V6R283
109521	229533.I1RC03	pp	229533.I1S2E8
109522	229533.I1RC03	pp	229533.Q4HZ68
109523	229533.I1RC03	pp	229533.I1RSD8
109524	229533.I1RC03	pp	229533.Q4IEV5
109525	229533.I1RC03	pp	229533.I1RWB5
109526	229533.I1RC03	pp	229533.I1RM25
109527	229533.I1RC03	pp	229533.I1RQ65
109528	229533.I1RC03	pp	229533.Q4HZ42
109529	229533.I1RC03	pp	229533.I1S296
109530	229533.I1RC03	pp	229533.I1RS47
109531	229533.I1RC03	pp	229533.I1RKC6
109532	229533.I1RC03	pp	229533.I1RNU6
109533	229533.I1RC03	pp	229533.I1S0J1
109534	229533.I1RC03	pp	229533.I1S1J6
109535	229533.I1RC03	pp	229533.I1RC72
109536	229533.I1RC03	pp	229533.V6R042
109537	229533.I1RC03	pp	229533.I1RRQ3
109538	229533.I1RC03	pp	229533.I1RPJ3
109539	229533.I1RC03	pp	229533.I1S060
109540	229533.I1RC03	pp	229533.I1RS30
109541	229533.I1RC03	pp	229533.V6RMW6
109542	229533.I1RC03	pp	229533.I1RTE9
109543	229533.I1RC03	pp	229533.I1RRR7
109544	229533.I1RC03	pp	229533.I1RM96
109545	229533.I1RC03	pp	229533.I1RSI6
109546	229533.I1RC03	pp	229533.I1RC33
109547	229533.I1RC03	pp	229533.I1RY44
109548	229533.I1RC03	pp	229533.I1S2C0
109549	229533.I1RC03	pp	229533.I1RTH6
109550	229533.I1RC03	pp	229533.I1RNY2
109551	229533.I1RC03	pp	229533.Q4I327
109552	229533.I1RC03	pp	229533.Q4IN63
109553	229533.I1RC03	pp	229533.I1RCR8
109554	229533.I1RC03	pp	229533.I1S4T3
109555	229533.I1RC03	pp	229533.I1RPY1
109556	229533.I1RC03	pp	229533.I1RWN5
109557	229533.I1RC03	pp	229533.I1RW90
109558	229533.I1RC03	pp	229533.I1RUQ2
109559	229533.I1RC03	pp	229533.I1RSG8
109560	229533.I1RC03	pp	229533.I1RDA0
109561	229533.I1RC03	pp	229533.V6RGN5
109562	229533.I1RC03	pp	229533.I1RMK3
109563	229533.I1RC03	pp	229533.I1S6U8
109564	229533.I1RC03	pp	229533.I1RXD7
109565	229533.I1RC03	pp	229533.I1RD22
109566	229533.I1RC03	pp	229533.I1S0R7
109567	229533.I1RC03	pp	229533.I1RC27
109568	229533.I1RC03	pp	229533.Q4HVW2
109569	229533.I1RC03	pp	229533.I1RMN6
109570	229533.I1RC03	pp	229533.I1RSR8
109571	229533.I1RC03	pp	229533.I1REF2
109572	229533.I1RC03	pp	229533.Q4IJH1
109573	229533.I1RC03	pp	229533.I1RY03

109574	229533.I1RC03	pp	229533.I1RT98
109575	229533.I1RC03	pp	229533.I1RZJ6
109576	229533.I1RC03	pp	229533.I1RNV6
109577	229533.I1RC03	pp	229533.I1RTF4
109578	229533.I1RC03	pp	229533.I1RSM3
109579	229533.I1RC03	pp	229533.I1RSR3
109580	229533.I1RC03	pp	229533.Q4IPI1
109581	229533.I1RC03	pp	229533.I1RT80
109582	229533.I1RC03	pp	229533.I1RX46
109583	229533.I1RC03	pp	229533.I1RRY9
109584	229533.I1RC03	pp	229533.I1RKQ5
109585	229533.I1RC03	pp	229533.I1RQM6
109586	229533.I1RC03	pp	229533.I1RK95
109587	229533.I1RC03	pp	229533.I1RQA2
109588	229533.I1RC03	pp	229533.I1S9U2
109589	229533.I1RC03	pp	229533.I1RQ28
109590	229533.I1RC03	pp	229533.I1RDG0
109591	229533.I1RC03	pp	229533.Q4I5R9
109592	229533.I1RC03	pp	229533.I1RM67
109593	229533.I1RC03	pp	229533.I1RPW9
109594	229533.I1RC03	pp	229533.I1RN99
109595	229533.I1RC03	pp	229533.I1RSQ7
109596	229533.I1RC03	pp	229533.I1RPU0
109597	229533.I1RC03	pp	229533.I1RY63
109598	229533.I1RC03	pp	229533.I1RM06
109599	229533.I1RC03	pp	229533.I1RZ99
109600	229533.I1RC03	pp	229533.I1RNA0
109601	229533.I1RC03	pp	229533.I1RQJ5
109602	229533.I1RC03	pp	229533.I1RZK6
109603	229533.I1RC03	pp	229533.V6QYF0
109604	229533.I1RC03	pp	229533.I1RJZ6
109605	229533.I1RC03	pp	229533.I1RZZ1
109606	229533.I1RC03	pp	229533.I1RW71
109607	229533.I1RC03	pp	229533.I1RR00
109608	229533.I1RC03	pp	229533.I1RC32
109609	229533.I1RC03	pp	229533.I1RG51
109610	229533.I1RC03	pp	229533.I1RW25
109611	229533.I1RC03	pp	229533.I1RRG3
109612	229533.I1RC03	pp	229533.I1S2U2
109613	229533.I1RC03	pp	229533.Q4IFI0
109614	229533.I1RC03	pp	229533.I1RF08
109615	229533.I1RC03	pp	229533.I1RFW5
109616	229533.I1RC03	pp	229533.I1RFJ9
109617	229533.I1RC03	pp	229533.I1RWH5
109618	229533.I1RC03	pp	229533.I1RL23
109619	229533.I1RC03	pp	229533.I1RE00
109620	229533.I1RC03	pp	229533.I1RF19
109621	229533.I1RC03	pp	229533.I1RD56
109622	229533.I1RC03	pp	229533.Q4IJ56
109623	229533.I1RC03	pp	229533.I1S0D4
109624	229533.I1RC03	pp	229533.I1RT33
109625	229533.I1RC03	pp	229533.I1RZE5
109626	229533.I1RC03	pp	229533.I1S0M9
109627	229533.I1RC03	pp	229533.I1RNK2
109628	229533.I1RC03	pp	229533.I1RNH1
109629	229533.I1RC03	pp	229533.I1RVZ0
109630	229533.I1RC03	pp	229533.I1RMN7
109631	229533.I1RC03	pp	229533.I1RX14
109632	229533.I1RC03	pp	229533.I1S021
109633	229533.I1RC03	pp	229533.I1S6F0
109634	229533.I1RC03	pp	229533.V6R227
109635	229533.I1RC03	pp	229533.I1RK83
109636	229533.I1RC03	pp	229533.I1RNH6
109637	229533.I1RC03	pp	229533.I1S504
109638	229533.I1RC03	pp	229533.I1RYB9
109639	229533.I1RC03	pp	229533.I1RZK5
109640	229533.I1RC03	pp	229533.I1RMC0
109641	229533.I1RC03	pp	229533.I1RZB5
109642	229533.I1RC03	pp	229533.Q4IEK8
109643	229533.I1RC03	pp	229533.I1RZF6
109644	229533.I1RC03	pp	229533.I1RFU8
109645	229533.I1RC03	pp	229533.I1S044
109646	229533.I1RC03	pp	229533.I1RNQ5

109647	229533.I1RC03	pp	229533.I1RQ31
109648	229533.I1RC03	pp	229533.V6QUE6
109649	229533.I1RC03	pp	229533.I1RGC2
109650	229533.I1RC03	pp	229533.I1RG56
109651	229533.I1RLV9	pp	229533.I1RY03
109652	229533.I1RLV9	pp	229533.I1RMD5
109653	229533.I1RLV9	pp	229533.I1S1C7
109654	229533.I1RLV9	pp	229533.I1RRI2
109655	229533.I1RLV9	pp	229533.I1RSR8
109656	229533.I1RLV9	pp	229533.I1RTD9
109657	229533.I1RLV9	pp	229533.I1S2C0
109658	229533.I1RLV9	pp	229533.I1RSG2
109659	229533.I1RLV9	pp	229533.I1RP75
109660	229533.I1RLV9	pp	229533.I1RTE9
109661	229533.I1RLV9	pp	229533.Q4HZK7
109662	229533.I1RLV9	pp	229533.I1RQA2
109663	229533.I1RLV9	pp	229533.I1RTH6
109664	229533.I1RLV9	pp	229533.I1S212
109665	229533.I1RLV9	pp	229533.I1RZK5
109666	229533.I1RLV9	pp	229533.I1RWN6
109667	229533.I1RLV9	pp	229533.I1S0J1
109668	229533.I1RLV9	pp	229533.I1RZE5
109669	229533.I1RLV9	pp	229533.I1RRY9
109670	229533.I1RLV9	pp	229533.I1RYB1
109671	229533.I1RLV9	pp	229533.I1S0M9
109672	229533.I1RLV9	pp	229533.Q4IJ56
109673	229533.I1RLV9	pp	229533.I1RML0
109674	229533.I1RLV9	pp	229533.Q4HZ68
109675	229533.I1RLV9	pp	229533.I1RNU6
109676	229533.I1RLV9	pp	229533.I1RSR3
109677	229533.I1RLV9	pp	229533.I1RM25
109678	229533.I1RLV9	pp	229533.I1RQ31
109679	229533.I1RLV9	pp	229533.I1RSD8
109680	229533.I1RLV9	pp	229533.I1RWB5
109681	229533.I1RLV9	pp	229533.I1RZZ1
109682	229533.I1RLV9	pp	229533.Q4IN63
109683	229533.I1RLV9	pp	229533.Q4HYR2
109684	229533.I1RLV9	pp	229533.Q4IAA0
109685	229533.I1RLV9	pp	229533.I1RPY1
109686	229533.I1RLV9	pp	229533.I1RZK6
109687	229533.I1RLV9	pp	229533.I1RWN5
109688	229533.I1RLV9	pp	229533.I1S2E8
109689	229533.I1RLV9	pp	229533.I1RNY2
109690	229533.I1RLV9	pp	229533.I1RY63
109691	229533.I1RLV9	pp	229533.I1RM96
109692	229533.I1RLV9	pp	229533.Q4I327
109693	229533.I1RLV9	pp	229533.I1RYB9
109694	229533.I1RLV9	pp	229533.Q4IEK8
109695	229533.I1RLV9	pp	229533.I1RNQ5
109696	229533.I1RLV9	pp	229533.I1S021
109697	229533.I1RLV9	pp	229533.I1S1X9
109698	229533.I1RLV9	pp	229533.I1RNH6
109699	229533.I1RLV9	pp	229533.I1RZF6
109700	229533.I1RLV9	pp	229533.I1RVX0
109701	229533.I1RLV9	pp	229533.I1RX08
109702	229533.I1RLV9	pp	229533.I1RT03
109703	229533.I1RLV9	pp	229533.I1RMN7
109704	229533.I1RLV9	pp	229533.I1RSC6
109705	229533.I1RLV9	pp	229533.I1S504
109706	229533.I1RLV9	pp	229533.I1RN99
109707	229533.I1RLV9	pp	229533.I1RSI6
109708	229533.I1RLV9	pp	229533.I1S9U1
109709	229533.I1RLV9	pp	229533.I1RQ28
109710	229533.I1RLV9	pp	229533.I1RRR7
109711	229533.I1RLV9	pp	229533.I1S0Q7
109712	229533.I1RLV9	pp	229533.I1RVZ0
109713	229533.I1RLV9	pp	229533.I1S255
109714	229533.I1RLV9	pp	229533.V6R042
109715	229533.I1RLV9	pp	229533.I1RNK2
109716	229533.I1RLV9	pp	229533.V6RMW6
109717	229533.I1RLV9	pp	229533.I1S0Z9
109718	229533.I1RLV9	pp	229533.I1RMW7
109719	229533.I1RLV9	pp	229533.I1RWE1

109720 229533.I1RLV9 pp 229533.I1RT80
109721 229533.I1RLV9 pp 229533.I1RU69
109722 229533.I1RLV9 pp 229533.V6R3X5
109723 229533.I1RLV9 pp 229533.I1RMN6
109724 229533.I1RLV9 pp 229533.I1RRK8
109725 229533.I1RLV9 pp 229533.I1RND6
109726 229533.I1RLV9 pp 229533.I1RTF4
109727 229533.I1RLV9 pp 229533.Q4IBS2
109728 229533.I1RLV9 pp 229533.I1RR73
109729 229533.I1RLV9 pp 229533.Q4IJH1
109730 229533.I1RLV9 pp 229533.V6RGN5
109731 229533.I1RLV9 pp 229533.I1RZV6
109732 229533.I1RLV9 pp 229533.I1S044
109733 229533.I1RLV9 pp 229533.I1S8E2
109734 229533.I1RLV9 pp 229533.I1RX14
109735 229533.I1RLV9 pp 229533.I1RZ39
109736 229533.I1RLV9 pp 229533.Q4HVW2
109737 229533.I1RLV9 pp 229533.I1RPU0
109738 229533.I1RLV9 pp 229533.I1RM67
109739 229533.I1RLV9 pp 229533.I1RRT8
109740 229533.I1RLV9 pp 229533.I1S060
109741 229533.I1RLV9 pp 229533.I1RPG6
109742 229533.I1RLV9 pp 229533.I1RMK3
109743 229533.I1RLV9 pp 229533.I1RT98
109744 229533.I1RLV9 pp 229533.I1S8I8
109745 229533.I1RLV9 pp 229533.I1S6U8
109746 229533.I1RLV9 pp 229533.I1RW71
109747 229533.I1RLV9 pp 229533.I1RTD5
109748 229533.I1RLV9 pp 229533.I1RPW9
109749 229533.I1RLV9 pp 229533.I1RT04
109750 229533.I1RLV9 pp 229533.Q4I830
109751 229533.I1RLV9 pp 229533.I1S9U2
109752 229533.I1RLV9 pp 229533.I1S0R7
109753 229533.I1RLV9 pp 229533.Q4I662
109754 229533.I1RLV9 pp 229533.I1RPP3
109755 229533.I1RLV9 pp 229533.I1RX24
109756 229533.I1RLV9 pp 229533.I1RWF7
109757 229533.I1RLV9 pp 229533.I1RUQ2
109758 229533.I1RLV9 pp 229533.I1RXD7
109759 229533.I1RLV9 pp 229533.I1RZ99
109760 229533.I1RLV9 pp 229533.Q4IAS1
109761 229533.I1RLV9 pp 229533.V6QYF0
109762 229533.I1RLV9 pp 229533.I1RM06
109763 229533.I1RLV9 pp 229533.I1RQJ5
109764 229533.I1RLV9 pp 229533.I1RNA0
109765 229533.I1RLV9 pp 229533.I1S503
109766 229533.I1RLV9 pp 229533.I1RRJ8
109767 229533.I1RLV9 pp 229533.I1RSQ7
109768 229533.I1RLV9 pp 229533.I1RRT2
109769 229533.I1RLV9 pp 229533.I1RZS6
109770 229533.I1RLV9 pp 229533.Q4HZ47
109771 229533.I1RLV9 pp 229533.I1S2B8
109772 229533.I1RLV9 pp 229533.I1RS30
109773 229533.I1RLV9 pp 229533.Q4IFI0
109774 229533.I1RLV9 pp 229533.Q4IPI1
109775 229533.I1RLV9 pp 229533.I1RX46
109776 229533.I1RLV9 pp 229533.I1RRQ3
109777 229533.I1RLV9 pp 229533.I1RSL9
109778 229533.I1RLV9 pp 229533.I1RS47
109779 229533.I1RLV9 pp 229533.I1RNH1
109780 229533.I1RLV9 pp 229533.I1RW25
109781 229533.I1RLV9 pp 229533.I1RWH5
109782 229533.I1RLV9 pp 229533.I1RZG3
109783 229533.I1RLV9 pp 229533.Q4IQW0
109784 229533.I1RLV9 pp 229533.I1RQ65
109785 229533.I1RLV9 pp 229533.Q4HZ42
109786 229533.I1RLV9 pp 229533.Q4IEV5
109787 229533.A0A0E0SPV0 pp 229533.I1S267
109788 229533.A0A0E0SPV0 pp 229533.I1RWH1
109789 229533.A0A0E0SPV0 pp 229533.A0A1C3YJZ0
109790 229533.A0A0E0SPV0 pp 229533.I1RCN8
109791 229533.A0A0E0SPV0 pp 229533.I1RBE4
109792 229533.A0A0E0SPV0 pp 229533.I1RC61

109793 229533.A0A0E0SPV0 pp 229533.I1S835
109794 229533.A0A0E0SPV0 pp 229533.I1RA94
109795 229533.A0A0E0SPV0 pp 229533.I1RRX5
109796 229533.A0A0E0SPV0 pp 229533.A0A1C3YHV3
109797 229533.A0A0E0SPV0 pp 229533.I1RXV9
109798 229533.A0A0E0SPV0 pp 229533.I1RVZ9
109799 229533.A0A0E0SPV0 pp 229533.I1RLP1
109800 229533.A0A0E0SPV0 pp 229533.I1RXB1
109801 229533.A0A0E0SPV0 pp 229533.A0A1C3YJL7
109802 229533.A0A0E0SPV0 pp 229533.I1RAU9
109803 229533.A0A0E0SPV0 pp 229533.I1RVY2
109804 229533.A0A0E0SPV0 pp 229533.I1RT33
109805 229533.A0A0E0SPV0 pp 229533.I1RK03
109806 229533.A0A0E0SPV0 pp 229533.I1S6E5
109807 229533.A0A0E0SPV0 pp 229533.I1RCE6
109808 229533.A0A0E0SPV0 pp 229533.I1R9Y2
109809 229533.A0A0E0SPV0 pp 229533.A0A1C3YLZ8
109810 229533.A0A0E0SPV0 pp 229533.I1RFH9
109811 229533.A0A0E0SPV0 pp 229533.A0A1C3YL20
109812 229533.A0A0E0SPV0 pp 229533.I1S8E0
109813 229533.A0A0E0SPV0 pp 229533.I1RMM8
109814 229533.A0A0E0SPV0 pp 229533.V6RQW4
109815 229533.A0A0E0SPV0 pp 229533.V6RUT5
109816 229533.A0A0E0SPV0 pp 229533.I1RBC1
109817 229533.A0A0E0SPV0 pp 229533.I1R9Y4
109818 229533.A0A0E0SPV0 pp 229533.I1RB46
109819 229533.A0A0E0SPV0 pp 229533.V6R382
109820 229533.A0A0E0SPV0 pp 229533.I1RWC9
109821 229533.A0A0E0SPV0 pp 229533.I1RZB2
109822 229533.A0A0E0SPV0 pp 229533.I1RBA6
109823 229533.A0A0E0SPV0 pp 229533.I1RIY0
109824 229533.A0A0E0SPV0 pp 229533.I1RPR9
109825 229533.A0A0E0SPV0 pp 229533.V6RLM7
109826 229533.A0A0E0SPV0 pp 229533.I1RXW3
109827 229533.A0A0E0SPV0 pp 229533.I1S7W5
109828 229533.A0A0E0SPV0 pp 229533.I1RWB0
109829 229533.A0A0E0SPV0 pp 229533.I1RWW7
109830 229533.A0A0E0SPV0 pp 229533.I1RWF9
109831 229533.A0A0E0SPV0 pp 229533.I1RBF3
109832 229533.A0A0E0SPV0 pp 229533.A0A1C3YLB6
109833 229533.A0A0E0SPV0 pp 229533.V6RN69
109834 229533.A0A0E0SPV0 pp 229533.I1RF60
109835 229533.A0A0E0SPV0 pp 229533.I1RYX5
109836 229533.A0A0E0SPV0 pp 229533.I1R9W4
109837 229533.A0A0E0SPV0 pp 229533.I1RP97
109838 229533.A0A0E0SPV0 pp 229533.I1S5R6
109839 229533.A0A0E0SPV0 pp 229533.I1S2B9
109840 229533.A0A0E0SPV0 pp 229533.I1RZ52
109841 229533.A0A0E0SPV0 pp 229533.A0A1C3YI20
109842 229533.A0A0E0SPV0 pp 229533.I1RS90
109843 229533.A0A0E0SPV0 pp 229533.I1RSQ4
109844 229533.A0A0E0SPV0 pp 229533.A0A1C3YI72
109845 229533.A0A0E0SPV0 pp 229533.I1RSU2
109846 229533.A0A0E0SPV0 pp 229533.I1RWI5
109847 229533.A0A0E0SPV0 pp 229533.I1RSS4
109848 229533.A0A0E0SPV0 pp 229533.I1RMH4
109849 229533.A0A0E0SPV0 pp 229533.I1RBA4
109850 229533.A0A0E0SPV0 pp 229533.I1RY74
109851 229533.A0A0E0SPV0 pp 229533.I1RMV0
109852 229533.A0A0E0SPV0 pp 229533.A0A1C3YI50
109853 229533.A0A0E0SPV0 pp 229533.I1S0T4
109854 229533.A0A0E0SPV0 pp 229533.I1RCG5
109855 229533.A0A0E0SPV0 pp 229533.Q4I5V2
109856 229533.A0A0E0SPV0 pp 229533.I1RC81
109857 229533.A0A0E0SPV0 pp 229533.I1RKB6
109858 229533.A0A1C3YL86 pp 229533.I1RW37
109859 229533.A0A1C3YL86 pp 229533.I1RIC2
109860 229533.A0A1C3YL86 pp 229533.I1RR07
109861 229533.A0A1C3YL86 pp 229533.I1RBH7
109862 229533.I1RDP9 pp 229533.I1RP90
109863 229533.I1RDP9 pp 229533.I1RR40
109864 229533.I1RDP9 pp 229533.I1RJE5
109865 229533.I1RDP9 pp 229533.I1RPD9

109866 229533.I1RDP9 pp 229533.I1RXJ5
109867 229533.I1RDP9 pp 229533.I1RDQ0
109868 229533.I1RLN6 pp 229533.I1RR24
109869 229533.I1RLN6 pp 229533.I1S0L3
109870 229533.I1RLN6 pp 229533.I1RVQ0
109871 229533.I1RLN6 pp 229533.I1RSQ7
109872 229533.I1RLN6 pp 229533.I1RR39
109873 229533.I1RLN6 pp 229533.V6RTJ8
109874 229533.I1RLN6 pp 229533.Q4IJK2
109875 229533.I1RLN6 pp 229533.I1RW25
109876 229533.I1RLN6 pp 229533.I1RQC9
109877 229533.I1RLN6 pp 229533.I1RRY9
109878 229533.I1RLN6 pp 229533.V6QW22
109879 229533.I1RLN6 pp 229533.I1RY03
109880 229533.I1RLN6 pp 229533.I1S3A5
109881 229533.I1RLN6 pp 229533.I1RX46
109882 229533.I1RLN6 pp 229533.I1RZK5
109883 229533.I1RLN6 pp 229533.V6RCG5
109884 229533.I1RLN6 pp 229533.I1RZZ9
109885 229533.I1RLN6 pp 229533.I1RPD9
109886 229533.I1RLN6 pp 229533.I1S2B8
109887 229533.I1RLN6 pp 229533.I1RLU6
109888 229533.I1RLN6 pp 229533.I1RZ39
109889 229533.I1RLN6 pp 229533.I1RZM1
109890 229533.I1RLN6 pp 229533.I1RPM4
109891 229533.I1RLN6 pp 229533.I1RRM5
109892 229533.I1RLN6 pp 229533.V6R3X5
109893 229533.I1RLN6 pp 229533.I1RRT8
109894 229533.I1RLN6 pp 229533.I1S504
109895 229533.I1RLN6 pp 229533.I1RS18
109896 229533.I1RLN6 pp 229533.I1RR73
109897 229533.I1RLN6 pp 229533.I1S2H4
109898 229533.I1RLN6 pp 229533.I1S0L4
109899 229533.I1RLN6 pp 229533.I1RWK3
109900 229533.I1RLN6 pp 229533.I1RN67
109901 229533.I1RLN6 pp 229533.I1RT04
109902 229533.I1RLN6 pp 229533.I1RZW8
109903 229533.I1RLN6 pp 229533.I1S8P5
109904 229533.I1RLN6 pp 229533.I1S8R3
109905 229533.I1RLN6 pp 229533.I1RPP3
109906 229533.I1RLN6 pp 229533.I1S5J8
109907 229533.I1RLN6 pp 229533.I1RTH6
109908 229533.I1RLN6 pp 229533.I1S2E9
109909 229533.I1RLN6 pp 229533.I1RWN5
109910 229533.I1RLN6 pp 229533.I1S503
109911 229533.I1RLN6 pp 229533.Q4I1Y5
109912 229533.I1RLN6 pp 229533.I1RTD5
109913 229533.I1RLN6 pp 229533.I1RV91
109914 229533.I1RLN6 pp 229533.I1RY44
109915 229533.I1RLN6 pp 229533.I1RN18
109916 229533.I1RLN6 pp 229533.V6RIV9
109917 229533.I1RLN6 pp 229533.I1RTA0
109918 229533.I1RLN6 pp 229533.I1RT03
109919 229533.I1RLN6 pp 229533.I1RWN6
109920 229533.I1RLN6 pp 229533.I1S1Q6
109921 229533.I1RLN6 pp 229533.I1RVX0
109922 229533.I1RLN6 pp 229533.I1RQ75
109923 229533.I1RLN6 pp 229533.I1S1C7
109924 229533.I1RLN6 pp 229533.I1RPP4
109925 229533.I1RLN6 pp 229533.I1RNC1
109926 229533.I1RLN6 pp 229533.I1RUG3
109927 229533.I1RLN6 pp 229533.V6R042
109928 229533.I1RLN6 pp 229533.I1RSQ3
109929 229533.I1RLN6 pp 229533.V6RMW6
109930 229533.I1RWI8 pp 229533.I1S276
109931 229533.I1RWI8 pp 229533.I1RX64
109932 229533.I1RWI8 pp 229533.I1S0N5
109933 229533.I1RWI8 pp 229533.I1S0D5
109934 229533.I1RWI8 pp 229533.I1S998
109935 229533.I1RWI8 pp 229533.I1RZP8
109936 229533.I1RWI8 pp 229533.I1S9J6
109937 229533.I1RWI8 pp 229533.I1S987
109938 229533.I1RWI8 pp 229533.I1S216

109939	229533.I1RWI8	pp	229533.I1RZ94
109940	229533.I1RWI8	pp	229533.I1RWT6
109941	229533.I1RWI8	pp	229533.I1RY44
109942	229533.I1RWI8	pp	229533.I1RZ86
109943	229533.I1RWI8	pp	229533.Q4IB89
109944	229533.I1RWI8	pp	229533.I1S4P6
109945	229533.I1RWI8	pp	229533.I1S110
109946	229533.I1RWI8	pp	229533.I1RYP7
109947	229533.I1RWI8	pp	229533.I1S788
109948	229533.I1RWI8	pp	229533.I1S209
109949	229533.I1RWI8	pp	229533.I1S2H4
109950	229533.I1RWI8	pp	229533.I1S9F9
109951	229533.I1RWI8	pp	229533.Q4INS6
109952	229533.I1RWI8	pp	229533.I1S8G4
109953	229533.I1RWI8	pp	229533.I1S0J3
109954	229533.I1RWI8	pp	229533.I1RZ98
109955	229533.I1RWI8	pp	229533.I1S2E9
109956	229533.I1RWI8	pp	229533.I1RYQ1
109957	229533.I1RWI8	pp	229533.I1RX22
109958	229533.I1RWI8	pp	229533.I1RY29
109959	229533.V6R283	pp	229533.V6RGN5
109960	229533.V6R283	pp	229533.V6R3X5
109961	229533.V6R283	pp	229533.V6RGY3
109962	229533.V6R283	pp	229533.V6RTJ8
109963	229533.V6R283	pp	229533.V6RMW6
109964	229533.I1RY29	pp	229533.Q4INS6
109965	229533.I1RY29	pp	229533.I1RZ98
109966	229533.I1RY29	pp	229533.I1RYP7
109967	229533.I1RY29	pp	229533.I1S209
109968	229533.I1RY29	pp	229533.I1S072
109969	229533.I1RY29	pp	229533.I1S183
109970	229533.I1RY29	pp	229533.Q4IB89
109971	229533.I1RY29	pp	229533.I1RY44
109972	229533.I1RY29	pp	229533.I1S4P6
109973	229533.I1RY29	pp	229533.I1S8G4
109974	229533.I1RY29	pp	229533.I1S0D5
109975	229533.I1RY29	pp	229533.I1S2E9
109976	229533.I1RY29	pp	229533.I1S788
109977	229533.I1RY29	pp	229533.I1S276
109978	229533.I1RY29	pp	229533.I1S216
109979	229533.I1RY29	pp	229533.I1RZ86
109980	229533.I1RY29	pp	229533.I1RYQ1
109981	229533.I1RY29	pp	229533.I1S2H4
109982	229533.I1RY29	pp	229533.I1S0J3
109983	229533.I1RY29	pp	229533.I1RZ94
109984	229533.I1RSN7	pp	229533.I1RSR9
109985	229533.I1RSN7	pp	229533.V6RLW4
109986	229533.I1RSN7	pp	229533.I1RZ86
109987	229533.I1RSN7	pp	229533.I1RX64
109988	229533.I1RSN7	pp	229533.Q4IA56
109989	229533.I1RSN7	pp	229533.I1RSP7
109990	229533.I1RSN7	pp	229533.I1RZ94
109991	229533.I1RSN7	pp	229533.I1RZP8
109992	229533.I1RSN7	pp	229533.I1RZ98
109993	229533.I1RSN7	pp	229533.I1RYQ1
109994	229533.I1RSN7	pp	229533.I1RUG4
109995	229533.I1RSN7	pp	229533.I1S8J1
109996	229533.I1RSN7	pp	229533.I1S0A6
109997	229533.I1RSN7	pp	229533.I1S209
109998	229533.I1RSN7	pp	229533.I1S283
109999	229533.I1RSN7	pp	229533.I1RYX9
110000	229533.I1RSN7	pp	229533.I1S0P6
110001	229533.I1RSN7	pp	229533.I1S5Z3
110002	229533.I1S503	pp	229533.I1S5B0
110003	229533.I1S503	pp	229533.I1S8V4
110004	229533.I1S503	pp	229533.I1SA33
110005	229533.I1S503	pp	229533.Q4I283
110006	229533.I1S503	pp	229533.V6RKK5
110007	229533.I1S503	pp	229533.I1S569
110008	229533.I1S503	pp	229533.V6RIV9
110009	229533.I1S503	pp	229533.Q4HZB8
110010	229533.I1S503	pp	229533.V6QUE6
110011	229533.I1S503	pp	229533.V6R057

110012	229533.I1S503	pp	229533.V6RMW6
110013	229533.I1S503	pp	229533.Q4I624
110014	229533.I1S503	pp	229533.Q4I8P5
110015	229533.I1S503	pp	229533.V6R042
110016	229533.I1S503	pp	229533.I1SA32
110017	229533.I1S503	pp	229533.I1S8P5
110018	229533.I1S503	pp	229533.Q4I2J8
110019	229533.I1S503	pp	229533.V6RCZ8
110020	229533.I1S503	pp	229533.V6R4J1
110021	229533.I1S503	pp	229533.V6RCG5
110022	229533.I1S503	pp	229533.Q4IJT5
110023	229533.I1S503	pp	229533.Q4IJK2
110024	229533.I1S503	pp	229533.Q4IJM4
110025	229533.I1S503	pp	229533.V6QW22
110026	229533.I1S503	pp	229533.Q4I327
110027	229533.I1S503	pp	229533.I1S6U8
110028	229533.I1S503	pp	229533.V6RTJ8
110029	229533.I1S503	pp	229533.V6R1K4
110030	229533.I1S503	pp	229533.Q4IN63
110031	229533.I1S503	pp	229533.I1S8R3
110032	229533.I1S503	pp	229533.V6R283
110033	229533.I1S503	pp	229533.I1S7A8
110034	229533.I1S503	pp	229533.I1S8V3
110035	229533.I1S503	pp	229533.V6RUY9
110036	229533.I1S503	pp	229533.V6R3X5
110037	229533.I1S503	pp	229533.V6QUW4
110038	229533.I1S503	pp	229533.I1S9U2
110039	229533.I1S503	pp	229533.I1S5R1
110040	229533.I1RBG9	pp	229533.I1RL23
110041	229533.I1RBG9	pp	229533.I1RJZ7
110042	229533.I1RBG9	pp	229533.I1S2B8
110043	229533.I1RBG9	pp	229533.Q4IFI0
110044	229533.I1RBG9	pp	229533.I1RYT5
110045	229533.I1RBG9	pp	229533.I1RW25
110046	229533.I1RBG9	pp	229533.I1RW61
110047	229533.I1RBG9	pp	229533.I1RG51
110048	229533.I1RBG9	pp	229533.I1REX4
110049	229533.I1RBG9	pp	229533.I1RC32
110050	229533.I1RBG9	pp	229533.I1RFS3
110051	229533.I1RBG9	pp	229533.Q4I662
110052	229533.I1RBG9	pp	229533.I1RGF6
110053	229533.I1RBG9	pp	229533.I1RM67
110054	229533.I1RBG9	pp	229533.I1RNH1
110055	229533.I1RBG9	pp	229533.I1RX24
110056	229533.I1RBG9	pp	229533.I1RRH5
110057	229533.I1RBG9	pp	229533.I1RD64
110058	229533.I1RBG9	pp	229533.I1RPW9
110059	229533.I1RBG9	pp	229533.I1RNA0
110060	229533.I1RBG9	pp	229533.Q4IQK7
110061	229533.I1RBG9	pp	229533.I1RS47
110062	229533.I1RBG9	pp	229533.I1S9U2
110063	229533.I1RBG9	pp	229533.I1RC29
110064	229533.I1RBG9	pp	229533.I1RKF9
110065	229533.I1RBG9	pp	229533.I1RD56
110066	229533.I1RBG9	pp	229533.Q4HZ42
110067	229533.I1RBG9	pp	229533.Q4IF76
110068	229533.I1RBG9	pp	229533.I1RQ65
110069	229533.I1RBG9	pp	229533.I1RT05
110070	229533.I1RBG9	pp	229533.V6QYF0
110071	229533.I1RBG9	pp	229533.I1RQM6
110072	229533.I1RBG9	pp	229533.I1RWH5
110073	229533.I1RBG9	pp	229533.I1RM06
110074	229533.I1RBG9	pp	229533.I1RZS6
110075	229533.I1RBG9	pp	229533.I1RU69
110076	229533.I1RBG9	pp	229533.V6R283
110077	229533.I1RBG9	pp	229533.I1RZV6
110078	229533.I1RBG9	pp	229533.I1RTF4
110079	229533.I1RBG9	pp	229533.I1RC03
110080	229533.I1RBG9	pp	229533.I1S9Y1
110081	229533.I1RBG9	pp	229533.Q4IJH1
110082	229533.I1RBG9	pp	229533.I1RZ39
110083	229533.I1RBG9	pp	229533.I1RFW5
110084	229533.I1RBG9	pp	229533.I1RKJ6

110085	229533.I1RBG9	pp	229533.I1RPU0
110086	229533.I1RBG9	pp	229533.Q4I830
110087	229533.I1RBG9	pp	229533.I1RKL3
110088	229533.I1RBG9	pp	229533.I1RNV6
110089	229533.I1RBG9	pp	229533.I1RT80
110090	229533.I1RBG9	pp	229533.I1RRT8
110091	229533.I1RBG9	pp	229533.I1S8E2
110092	229533.I1RBG9	pp	229533.I1RW71
110093	229533.I1RBG9	pp	229533.I1S2L2
110094	229533.I1RBG9	pp	229533.I1RSL9
110095	229533.I1RBG9	pp	229533.I1S253
110096	229533.I1RBG9	pp	229533.I1RFN1
110097	229533.I1RBG9	pp	229533.I1RBN1
110098	229533.I1RBG9	pp	229533.I1RPN4
110099	229533.I1RBG9	pp	229533.I1RC82
110100	229533.I1RBG9	pp	229533.I1RDA0
110101	229533.I1RBG9	pp	229533.I1RGA4
110102	229533.I1RBG9	pp	229533.Q4IQW0
110103	229533.I1RBG9	pp	229533.Q4HVW2
110104	229533.I1RBG9	pp	229533.I1RQJ5
110105	229533.I1RBG9	pp	229533.I1RZ99
110106	229533.I1RBG9	pp	229533.I1RT98
110107	229533.I1RBG9	pp	229533.I1RMK3
110108	229533.I1RBG9	pp	229533.I1RR73
110109	229533.I1RBG9	pp	229533.I1RPP3
110110	229533.I1RBG9	pp	229533.I1RZJ6
110111	229533.I1RBG9	pp	229533.V6R3X5
110112	229533.I1RBG9	pp	229533.I1RTD5
110113	229533.I1RBG9	pp	229533.I1RRJ8
110114	229533.I1RBG9	pp	229533.Q4HXF0
110115	229533.I1RBG9	pp	229533.I1RSQ7
110116	229533.I1RBG9	pp	229533.I1RND6
110117	229533.I1RBG9	pp	229533.I1RLV9
110118	229533.I1RBG9	pp	229533.I1RT04
110119	229533.I1RBG9	pp	229533.Q4IAS1
110120	229533.I1RBG9	pp	229533.I1RWD4
110121	229533.I1RBG9	pp	229533.I1RS30
110122	229533.I1RBG9	pp	229533.I1S503
110123	229533.I1RBG9	pp	229533.I1S0R7
110124	229533.I1RBG9	pp	229533.I1S6F0
110125	229533.I1RBG9	pp	229533.I1RXD7
110126	229533.I1RBG9	pp	229533.I1RNK2
110127	229533.I1RBG9	pp	229533.Q4I5R9
110128	229533.I1RBG9	pp	229533.I1RRQ3
110129	229533.I1RBG9	pp	229533.I1RFG0
110130	229533.I1RBG9	pp	229533.I1RKQ5
110131	229533.I1RBG9	pp	229533.I1RVZ0
110132	229533.I1RBG9	pp	229533.I1RSD8
110133	229533.I1RBG9	pp	229533.I1RKC6
110134	229533.I1RBG9	pp	229533.I1RRT2
110135	229533.I1RBG9	pp	229533.I1S0Z9
110136	229533.I1RBG9	pp	229533.I1RX46
110137	229533.I1RBG9	pp	229533.I1S2U2
110138	229533.I1RBG9	pp	229533.I1RJL4
110139	229533.I1RBG9	pp	229533.I1RZE5
110140	229533.I1RBG9	pp	229533.I1RYB1
110141	229533.I1RBG9	pp	229533.I1RZK6
110142	229533.I1RBG9	pp	229533.I1RZZ1
110143	229533.I1RBG9	pp	229533.Q4IPI1
110144	229533.I1RBG9	pp	229533.I1RSI0
110145	229533.I1RBG9	pp	229533.I1RE00
110146	229533.I1RBG9	pp	229533.I1S1C7
110147	229533.I1RBG9	pp	229533.I1S2E8
110148	229533.I1RBG9	pp	229533.I1RTD7
110149	229533.I1RBG9	pp	229533.I1RJZ6
110150	229533.I1RBG9	pp	229533.I1S296
110151	229533.I1RBG9	pp	229533.I1RVX0
110152	229533.I1RBG9	pp	229533.I1RSR8
110153	229533.I1RBG9	pp	229533.Q4HZ68
110154	229533.I1RBG9	pp	229533.I1RY63
110155	229533.I1RBG9	pp	229533.I1RT03
110156	229533.I1RBG9	pp	229533.I1RC72
110157	229533.I1RBG9	pp	229533.I1RF08

110158	229533.I1RBG9	pp	229533.I1RY03
110159	229533.I1RBG9	pp	229533.Q4IJ56
110160	229533.I1RBG9	pp	229533.I1S1X9
110161	229533.I1RBG9	pp	229533.I1S0J1
110162	229533.I1RBG9	pp	229533.I1RRI2
110163	229533.I1RBG9	pp	229533.I1RSC6
110164	229533.I1RBG9	pp	229533.Q4HWE2
110165	229533.I1RBG9	pp	229533.I1RSS4
110166	229533.I1RBG9	pp	229533.I1RFU8
110167	229533.I1RBG9	pp	229533.I1RTH6
110168	229533.I1RBG9	pp	229533.I1RNU6
110169	229533.I1RBG9	pp	229533.I1S0M9
110170	229533.I1RBG9	pp	229533.I1RN99
110171	229533.I1RBG9	pp	229533.I1RM25
110172	229533.I1RBG9	pp	229533.I1RWB5
110173	229533.I1RBG9	pp	229533.I1RZK5
110174	229533.I1RBG9	pp	229533.I1RQ28
110175	229533.I1RBG9	pp	229533.Q4IAA0
110176	229533.I1RBG9	pp	229533.I1RK95
110177	229533.I1RBG9	pp	229533.I1RTE9
110178	229533.I1RBG9	pp	229533.I1RDG0
110179	229533.I1RBG9	pp	229533.I1RPJ3
110180	229533.I1RBG9	pp	229533.I1RQA2
110181	229533.I1RBG9	pp	229533.I1RCN5
110182	229533.I1RBG9	pp	229533.I1RK83
110183	229533.I1RBG9	pp	229533.I1RMN6
110184	229533.I1RBG9	pp	229533.V6QUE6
110185	229533.I1RBG9	pp	229533.I1S558
110186	229533.I1RBG9	pp	229533.I1RGC2
110187	229533.I1RBG9	pp	229533.I1S2C0
110188	229533.I1RBG9	pp	229533.Q4IBS2
110189	229533.I1RBG9	pp	229533.I1RWN5
110190	229533.I1RBG9	pp	229533.I1RD22
110191	229533.I1RBG9	pp	229533.I1RBZ3
110192	229533.I1RBG9	pp	229533.I1RZV9
110193	229533.I1RBG9	pp	229533.I1RCL3
110194	229533.I1RBG9	pp	229533.I1RJV3
110195	229533.I1RBG9	pp	229533.I1S4T3
110196	229533.I1RBG9	pp	229533.I1RBL7
110197	229533.I1RBG9	pp	229533.I1RG56
110198	229533.I1RBG9	pp	229533.I1RCK4
110199	229533.I1RBG9	pp	229533.I1RC27
110200	229533.I1RBG9	pp	229533.I1RPY1
110201	229533.I1RBG9	pp	229533.I1S274
110202	229533.I1RBG9	pp	229533.I1RBH0
110203	229533.I1RBG9	pp	229533.I1S6Y3
110204	229533.I1RBG9	pp	229533.I1RM96
110205	229533.I1RBG9	pp	229533.V6RGN5
110206	229533.I1RBG9	pp	229533.I1RWN6
110207	229533.I1RBG9	pp	229533.I1RRY9
110208	229533.I1RBG9	pp	229533.I1S044
110209	229533.I1RBG9	pp	229533.I1RNQ5
110210	229533.I1RBG9	pp	229533.Q4IEK8
110211	229533.I1RBG9	pp	229533.V6RGY3
110212	229533.I1RBG9	pp	229533.I1RMC0
110213	229533.I1RBG9	pp	229533.I1RSR3
110214	229533.I1RBG9	pp	229533.I1RZF6
110215	229533.I1RBG9	pp	229533.I1S1U7
110216	229533.I1RBG9	pp	229533.I1S504
110217	229533.I1RBG9	pp	229533.I1RQ31
110218	229533.I1RBG9	pp	229533.I1RCR8
110219	229533.I1RBG9	pp	229533.I1RNH9
110220	229533.I1RBG9	pp	229533.V6R227
110221	229533.I1RBG9	pp	229533.I1RRR7
110222	229533.I1RBG9	pp	229533.Q4IN63
110223	229533.I1RBG9	pp	229533.I1S060
110224	229533.I1RBG9	pp	229533.I1S021
110225	229533.I1RBG9	pp	229533.Q4I327
110226	229533.I1RBG9	pp	229533.Q4HYB8
110227	229533.I1RBG9	pp	229533.I1RNH6
110228	229533.I1RBG9	pp	229533.V6R042
110229	229533.I1RBG9	pp	229533.I1RYB9
110230	229533.I1RBG9	pp	229533.I1RSI6

110231 229533.I1RBG9 pp 229533.V6RMW6
110232 229533.I1S0Q5 pp 229533.Q4IB96
110233 229533.A0A1C3YN49 pp 229533.I1RJV4
110234 229533.I1RIL3 pp 229533.I1RRY9
110235 229533.I1RIL3 pp 229533.I1RZM1
110236 229533.I1RIL3 pp 229533.I1RY03
110237 229533.I1RIL3 pp 229533.I1RZK5
110238 229533.I1RIL3 pp 229533.I1RLU6
110239 229533.I1RIL3 pp 229533.V6RCG5
110240 229533.I1RIL3 pp 229533.I1S504
110241 229533.I1RIL3 pp 229533.I1S0L3
110242 229533.I1RIL3 pp 229533.I1RVQ0
110243 229533.I1RIL3 pp 229533.I1RS18
110244 229533.I1RIL3 pp 229533.Q4IJK2
110245 229533.I1RIL3 pp 229533.V6RTJ8
110246 229533.I1RIL3 pp 229533.V6RIV9
110247 229533.I1RIL3 pp 229533.I1RR39
110248 229533.I1RIL3 pp 229533.I1RJW1
110249 229533.I1RIL3 pp 229533.I1RSQ7
110250 229533.I1RIL3 pp 229533.V6QW22
110251 229533.I1RIL3 pp 229533.I1RQC9
110252 229533.I1RIL3 pp 229533.I1RWN5
110253 229533.I1RIL3 pp 229533.I1RW25
110254 229533.I1RIL3 pp 229533.I1RY44
110255 229533.I1RIL3 pp 229533.I1RX46
110256 229533.I1RIL3 pp 229533.I1RTA0
110257 229533.I1RIL3 pp 229533.I1RR24
110258 229533.I1RIL3 pp 229533.Q4I1Y5
110259 229533.I1RIL3 pp 229533.I1RN18
110260 229533.I1RIL3 pp 229533.I1RK44
110261 229533.I1RIL3 pp 229533.I1RQ75
110262 229533.I1RIL3 pp 229533.I1RPP4
110263 229533.I1RIL3 pp 229533.I1RWK3
110264 229533.I1RIL3 pp 229533.I1RPP3
110265 229533.I1RIL3 pp 229533.I1RT03
110266 229533.I1RIL3 pp 229533.I1RVX0
110267 229533.I1RIL3 pp 229533.I1RUG3
110268 229533.I1RIL3 pp 229533.I1RNC1
110269 229533.I1RIL3 pp 229533.I1RJV3
110270 229533.I1RIL3 pp 229533.I1S8P5
110271 229533.I1RIL3 pp 229533.V6R042
110272 229533.I1RIL3 pp 229533.I1RWN6
110273 229533.I1RIL3 pp 229533.I1S1C7
110274 229533.I1RIL3 pp 229533.I1RJW6
110275 229533.I1RIL3 pp 229533.I1S503
110276 229533.I1RIL3 pp 229533.I1RSQ3
110277 229533.I1RIL3 pp 229533.I1RZW8
110278 229533.I1RIL3 pp 229533.I1RJV4
110279 229533.I1RIL3 pp 229533.V6RMW6
110280 229533.I1RIL3 pp 229533.I1RTH6
110281 229533.I1RIL3 pp 229533.I1RPM4
110282 229533.I1RIL3 pp 229533.I1RV91
110283 229533.I1RIL3 pp 229533.I1RRT8
110284 229533.I1RIL3 pp 229533.I1RTD5
110285 229533.I1RIL3 pp 229533.I1RN67
110286 229533.I1RIL3 pp 229533.I1S8R3
110287 229533.I1RIL3 pp 229533.I1RT04
110288 229533.I1RIL3 pp 229533.I1S2E9
110289 229533.I1RIL3 pp 229533.I1S0L4
110290 229533.I1RIL3 pp 229533.I1S2H4
110291 229533.I1RIL3 pp 229533.I1RZZ9
110292 229533.I1RIL3 pp 229533.I1RRM5
110293 229533.I1RIL3 pp 229533.I1RR73
110294 229533.I1RIL3 pp 229533.I1S2B8
110295 229533.I1RIL3 pp 229533.I1RZ39
110296 229533.I1RIL3 pp 229533.V6R3X5
110297 229533.I1RCT2 pp 229533.I1S0Z3
110298 229533.I1RCT2 pp 229533.I1S0X0
110299 229533.I1RCT2 pp 229533.I1S275
110300 229533.I1RCT2 pp 229533.I1RWI6
110301 229533.I1RCT2 pp 229533.I1RKF9
110302 229533.I1RCT2 pp 229533.I1RRF9
110303 229533.I1RCT2 pp 229533.I1REK3

110304	229533.I1RCT2	pp	229533.Q4I5Z5
110305	229533.I1RCT2	pp	229533.I1RW71
110306	229533.I1RCT2	pp	229533.I1RKR9
110307	229533.I1RCT2	pp	229533.I1RJZ7
110308	229533.I1RCT2	pp	229533.I1RMP2
110309	229533.I1RCT2	pp	229533.I1S8E0
110310	229533.I1RCT2	pp	229533.I1RT41
110311	229533.I1RCT2	pp	229533.Q4I963
110312	229533.I1RCT2	pp	229533.I1REF2
110313	229533.I1RCT2	pp	229533.V6REE9
110314	229533.I1RCT2	pp	229533.I1RK80
110315	229533.I1RCT2	pp	229533.I1RE19
110316	229533.I1RCT2	pp	229533.I1S559
110317	229533.I1RCT2	pp	229533.V6RDH3
110318	229533.I1RCT2	pp	229533.I1RZL3
110319	229533.I1RCT2	pp	229533.I1S034
110320	229533.I1S5G0	pp	229533.Q4I7L0
110321	229533.A0A098DJN7	pp	229533.A0A1C3YN49
110322	229533.A0A098DJN7	pp	229533.I1S619
110323	229533.I1RE22	pp	229533.I1RZZ9
110324	229533.I1RE22	pp	229533.I1S0X6
110325	229533.I1RE22	pp	229533.I1RWI5
110326	229533.I1RE22	pp	229533.I1RX46
110327	229533.I1RE22	pp	229533.I1RR24
110328	229533.I1RE22	pp	229533.I1RQC9
110329	229533.I1RE22	pp	229533.I1S2H4
110330	229533.I1RE22	pp	229533.I1RMC8
110331	229533.I1RE22	pp	229533.I1S0L4
110332	229533.I1RE22	pp	229533.I1RWJ8
110333	229533.I1RE22	pp	229533.I1RJZ7
110334	229533.I1RE22	pp	229533.I1S0R7
110335	229533.I1RE22	pp	229533.I1S2B8
110336	229533.I1RE22	pp	229533.I1S5R1
110337	229533.I1RE22	pp	229533.I1RRM5
110338	229533.I1RE22	pp	229533.I1RE76
110339	229533.I1RE22	pp	229533.I1RRT8
110340	229533.I1RE22	pp	229533.I1RX25
110341	229533.I1RE22	pp	229533.V6RUY9
110342	229533.I1RE22	pp	229533.I1RW25
110343	229533.I1RE22	pp	229533.I1RT04
110344	229533.I1RE22	pp	229533.I1RZ39
110345	229533.I1RE22	pp	229533.I1RZJ5
110346	229533.I1RE22	pp	229533.I1RUQ2
110347	229533.I1RE22	pp	229533.I1RXD7
110348	229533.I1RE22	pp	229533.I1S0M6
110349	229533.I1RE22	pp	229533.V6QYF0
110350	229533.I1RE22	pp	229533.I1S2E9
110351	229533.I1RE22	pp	229533.I1RJU3
110352	229533.I1RE22	pp	229533.I1RQ23
110353	229533.I1RE22	pp	229533.I1RR73
110354	229533.I1RE22	pp	229533.V6R3X5
110355	229533.I1RE22	pp	229533.I1RPM4
110356	229533.I1RE22	pp	229533.I1RGA4
110357	229533.I1RE22	pp	229533.I1RV91
110358	229533.I1RE22	pp	229533.V6RTJ8
110359	229533.I1RE22	pp	229533.I1RT40
110360	229533.I1RE22	pp	229533.I1RFL4
110361	229533.I1RE22	pp	229533.I1RRN5
110362	229533.I1RE22	pp	229533.I1RTD5
110363	229533.I1RE22	pp	229533.I1S7A8
110364	229533.I1RE22	pp	229533.I1RS47
110365	229533.I1RE22	pp	229533.I1RN18
110366	229533.I1RE22	pp	229533.I1RQA3
110367	229533.I1RE22	pp	229533.I1RJV4
110368	229533.I1RE22	pp	229533.I1RJW6
110369	229533.I1RE22	pp	229533.V6R1K4
110370	229533.I1RE22	pp	229533.Q4IJK2
110371	229533.I1RE22	pp	229533.I1S0F5
110372	229533.I1RE22	pp	229533.I1RZZ1
110373	229533.I1RE22	pp	229533.I1S503
110374	229533.I1RE22	pp	229533.Q4IJT5
110375	229533.I1RE22	pp	229533.I1S0L3
110376	229533.I1RE22	pp	229533.I1RQQ5

110377 229533.I1RE22 pp 229533.I1RQE7
110378 229533.I1RE22 pp 229533.I1RN67
110379 229533.I1RE22 pp 229533.I1RRY9
110380 229533.I1RE22 pp 229533.I1RVQ0
110381 229533.I1RE22 pp 229533.I1S5B0
110382 229533.I1RE22 pp 229533.I1RW71
110383 229533.I1RE22 pp 229533.I1RWN6
110384 229533.I1RE22 pp 229533.I1RG53
110385 229533.I1RE22 pp 229533.I1RSR3
110386 229533.I1RE22 pp 229533.I1RW99
110387 229533.I1RE22 pp 229533.I1RJV3
110388 229533.I1RE22 pp 229533.I1S8P5
110389 229533.I1RE22 pp 229533.V6RCZ8
110390 229533.I1RE22 pp 229533.I1RSQ3
110391 229533.I1RE22 pp 229533.I1S1J7
110392 229533.I1RE22 pp 229533.I1RFU8
110393 229533.I1RE22 pp 229533.I1RPP3
110394 229533.I1RE22 pp 229533.V6R042
110395 229533.I1RE22 pp 229533.I1RRJ8
110396 229533.I1RE22 pp 229533.I1S8R3
110397 229533.I1RE22 pp 229533.I1RTH6
110398 229533.I1RE22 pp 229533.V6QW22
110399 229533.I1RE22 pp 229533.V6RCG5
110400 229533.I1RE22 pp 229533.I1RSQ7
110401 229533.I1RE22 pp 229533.V6RMW6
110402 229533.I1RE22 pp 229533.I1S2C4
110403 229533.I1RE22 pp 229533.I1RWK3
110404 229533.I1RE22 pp 229533.I1RNC1
110405 229533.I1RE22 pp 229533.I1RZK5
110406 229533.I1RE22 pp 229533.I1RYW3
110407 229533.I1RE22 pp 229533.I1RQ31
110408 229533.I1RE22 pp 229533.I1RUG3
110409 229533.I1RE22 pp 229533.I1RN63
110410 229533.I1RE22 pp 229533.I1S1C7
110411 229533.I1RE22 pp 229533.I1RZM8
110412 229533.I1RE22 pp 229533.I1RLU6
110413 229533.I1RE22 pp 229533.I1RNQ5
110414 229533.I1RE22 pp 229533.I1RXF0
110415 229533.I1RE22 pp 229533.I1RY03
110416 229533.I1RE22 pp 229533.I1RRI2
110417 229533.I1RE22 pp 229533.I1RY44
110418 229533.I1RE22 pp 229533.I1RI82
110419 229533.I1RE22 pp 229533.I1RZM1
110420 229533.I1RE22 pp 229533.V6R057
110421 229533.I1RE22 pp 229533.I1RZW8
110422 229533.I1RE22 pp 229533.I1RS17
110423 229533.I1RE22 pp 229533.I1RRE5
110424 229533.I1RE22 pp 229533.Q4I283
110425 229533.I1RE22 pp 229533.I1S504
110426 229533.I1RE22 pp 229533.I1RQ81
110427 229533.I1RE22 pp 229533.I1RPP4
110428 229533.I1RE22 pp 229533.Q4I624
110429 229533.I1RE22 pp 229533.I1REK8
110430 229533.I1RE22 pp 229533.Q4I2J8
110431 229533.I1RE22 pp 229533.I1RS18
110432 229533.I1RE22 pp 229533.I1RF19
110433 229533.I1RE22 pp 229533.I1RW46
110434 229533.I1RE22 pp 229533.I1RWT6
110435 229533.I1RE22 pp 229533.I1RT03
110436 229533.I1RE22 pp 229533.I1RWN5
110437 229533.I1RE22 pp 229533.I1RTA0
110438 229533.I1RE22 pp 229533.V6RIV9
110439 229533.I1RE22 pp 229533.I1S0J1
110440 229533.I1RE22 pp 229533.I1RVX0
110441 229533.A0A0E0SDN6 pp 229533.I1RNE7
110442 229533.A0A0E0SDN6 pp 229533.I1RBM1
110443 229533.A0A0E0SDN6 pp 229533.A0A0E0SKE0
110444 229533.A0A0E0SDN6 pp 229533.I1RVZ9
110445 229533.A0A0E0SDN6 pp 229533.I1S444
110446 229533.A0A0E0SDN6 pp 229533.I1RYI6
110447 229533.I1RXV3 pp 229533.V6QT89
110448 229533.I1RXV3 pp 229533.I1S290
110449 229533.I1RXV3 pp 229533.V6R382

110450 229533.I1RXV3 pp 229533.I1RZ92
110451 229533.I1RXV3 pp 229533.V6RG22
110452 229533.I1RXV3 pp 229533.I1S0Q5
110453 229533.I1RXV3 pp 229533.I1RYU0
110454 229533.I1RXV3 pp 229533.I1S0Q4
110455 229533.I1RXV3 pp 229533.I1RY74
110456 229533.I1RXV3 pp 229533.I1S9F1
110457 229533.I1RXV3 pp 229533.I1S0Y7
110458 229533.I1RXV3 pp 229533.I1RZ52
110459 229533.I1RXV3 pp 229533.I1S0J8
110460 229533.I1RXV3 pp 229533.O74638
110461 229533.I1RIP6 pp 229533.V6RLM7
110462 229533.I1RIP6 pp 229533.V6RVJ3
110463 229533.I1RIP6 pp 229533.I1RUY6
110464 229533.A0A0E0SC46 pp 229533.I1S0B1
110465 229533.A0A0E0SC46 pp 229533.I1SAN9
110466 229533.A0A0E0SC46 pp 229533.A0A1C3YLS9
110467 229533.A0A0E0SC46 pp 229533.V6RMW6
110468 229533.A0A0E0SC46 pp 229533.V6R042
110469 229533.A0A0E0SC46 pp 229533.I1RSQ3
110470 229533.A0A0E0SC46 pp 229533.I1RCR8
110471 229533.A0A0E0SC46 pp 229533.I1RUG3
110472 229533.A0A0E0SC46 pp 229533.I1S8R3
110473 229533.A0A0E0SC46 pp 229533.I1RTH6
110474 229533.A0A0E0SC46 pp 229533.I1RA70
110475 229533.A0A0E0SC46 pp 229533.I1RG57
110476 229533.A0A0E0SC46 pp 229533.I1RNC1
110477 229533.A0A0E0SC46 pp 229533.I1RX47
110478 229533.A0A0E0SC46 pp 229533.I1RK90
110479 229533.A0A0E0SC46 pp 229533.I1S1C7
110480 229533.A0A0E0SC46 pp 229533.I1RCN5
110481 229533.A0A0E0SC46 pp 229533.A0A1C3YK19
110482 229533.A0A0E0SC46 pp 229533.I1S855
110483 229533.A0A0E0SC46 pp 229533.I1RCW6
110484 229533.A0A0E0SC46 pp 229533.I1RJV3
110485 229533.A0A0E0SC46 pp 229533.I1S056
110486 229533.A0A0E0SC46 pp 229533.I1RZW8
110487 229533.A0A0E0SC46 pp 229533.I1RT91
110488 229533.A0A0E0SC46 pp 229533.I1RRI2
110489 229533.A0A0E0SC46 pp 229533.I1RS90
110490 229533.A0A0E0SC46 pp 229533.I1RQR3
110491 229533.A0A0E0SC46 pp 229533.I1RXR1
110492 229533.A0A0E0SC46 pp 229533.I1S5R6
110493 229533.A0A0E0SC46 pp 229533.I1RWX5
110494 229533.A0A0E0SC46 pp 229533.I1RSY2
110495 229533.A0A0E0SC46 pp 229533.I1S471
110496 229533.A0A0E0SC46 pp 229533.I1RQP8
110497 229533.A0A0E0SC46 pp 229533.I1S1X9
110498 229533.A0A0E0SC46 pp 229533.I1RXU9
110499 229533.A0A0E0SC46 pp 229533.A0A1C3YIM4
110500 229533.A0A0E0SC46 pp 229533.I1RK92
110501 229533.A0A0E0SC46 pp 229533.I1RVX0
110502 229533.A0A0E0SC46 pp 229533.I1RAW0
110503 229533.A0A0E0SC46 pp 229533.I1RG11
110504 229533.A0A0E0SC46 pp 229533.I1RT03
110505 229533.A0A0E0SC46 pp 229533.V6RIS2
110506 229533.A0A0E0SC46 pp 229533.I1RBZ8
110507 229533.A0A0E0SC46 pp 229533.I1RB92
110508 229533.A0A0E0SC46 pp 229533.A0A1C3YK33
110509 229533.A0A0E0SC46 pp 229533.I1RWN6
110510 229533.A0A0E0SC46 pp 229533.I1RZQ5
110511 229533.A0A0E0SC46 pp 229533.V6QUV7
110512 229533.A0A0E0SC46 pp 229533.I1RT47
110513 229533.A0A0E0SC46 pp 229533.I1RN18
110514 229533.A0A0E0SC46 pp 229533.I1RFL4
110515 229533.A0A0E0SC46 pp 229533.I1RN09
110516 229533.A0A0E0SC46 pp 229533.I1RC84
110517 229533.A0A0E0SC46 pp 229533.A0A1C3YN84
110518 229533.A0A0E0SC46 pp 229533.I1RQE7
110519 229533.A0A0E0SC46 pp 229533.I1RTA0
110520 229533.A0A0E0SC46 pp 229533.I1RCH5
110521 229533.A0A0E0SC46 pp 229533.A0A1C3YLC1
110522 229533.A0A0E0SC46 pp 229533.I1RSU2

110523	229533.A0A0E0SC46	pp	229533.I1RPL3
110524	229533.A0A0E0SC46	pp	229533.I1RD22
110525	229533.A0A0E0SC46	pp	229533.I1RE01
110526	229533.A0A0E0SC46	pp	229533.I1RD17
110527	229533.A0A0E0SC46	pp	229533.I1RW46
110528	229533.A0A0E0SC46	pp	229533.I1RRL0
110529	229533.A0A0E0SC46	pp	229533.I1S256
110530	229533.A0A0E0SC46	pp	229533.Q4I624
110531	229533.A0A0E0SC46	pp	229533.I1RRN2
110532	229533.A0A0E0SC46	pp	229533.I1S0J1
110533	229533.A0A0E0SC46	pp	229533.V6R057
110534	229533.A0A0E0SC46	pp	229533.I1RSD4
110535	229533.A0A0E0SC46	pp	229533.I1RNB8
110536	229533.A0A0E0SC46	pp	229533.I1RRQ4
110537	229533.A0A0E0SC46	pp	229533.I1RM25
110538	229533.A0A0E0SC46	pp	229533.I1RS28
110539	229533.A0A0E0SC46	pp	229533.A0A1C3YHK3
110540	229533.A0A0E0SC46	pp	229533.I1RFQ1
110541	229533.A0A0E0SC46	pp	229533.Q4IPH4
110542	229533.A0A0E0SC46	pp	229533.I1S7W5
110543	229533.A0A0E0SC46	pp	229533.I1RAU7
110544	229533.A0A0E0SC46	pp	229533.I1RS17
110545	229533.A0A0E0SC46	pp	229533.I1RYJ9
110546	229533.A0A0E0SC46	pp	229533.I1RPK0
110547	229533.A0A0E0SC46	pp	229533.I1RG90
110548	229533.A0A0E0SC46	pp	229533.V6RLM7
110549	229533.A0A0E0SC46	pp	229533.I1RRE5
110550	229533.A0A0E0SC46	pp	229533.V6RRH2
110551	229533.A0A0E0SC46	pp	229533.I1RBH8
110552	229533.A0A0E0SC46	pp	229533.I1S8V4
110553	229533.A0A0E0SC46	pp	229533.I1RTE8
110554	229533.A0A0E0SC46	pp	229533.A0A1C3YI20
110555	229533.A0A0E0SC46	pp	229533.Q4I7K4
110556	229533.A0A0E0SC46	pp	229533.A0A0E0SID1
110557	229533.A0A0E0SC46	pp	229533.V6RY33
110558	229533.A0A0E0SC46	pp	229533.I1RU69
110559	229533.A0A0E0SC46	pp	229533.I1RX51
110560	229533.A0A0E0SC46	pp	229533.I1RWW5
110561	229533.A0A0E0SC46	pp	229533.V6QXG1
110562	229533.A0A0E0SC46	pp	229533.I1RWD3
110563	229533.A0A0E0SC46	pp	229533.I1RC66
110564	229533.A0A0E0SC46	pp	229533.I1RMV0
110565	229533.A0A0E0SC46	pp	229533.I1S8V3
110566	229533.A0A0E0SC46	pp	229533.I1RVN1
110567	229533.A0A0E0SC46	pp	229533.I1S1W9
110568	229533.A0A0E0SC46	pp	229533.I1RPP4
110569	229533.A0A0E0SC46	pp	229533.I1S2H4
110570	229533.A0A0E0SC46	pp	229533.A0A0E0SPS2
110571	229533.A0A0E0SC46	pp	229533.I1RPY4
110572	229533.A0A0E0SC46	pp	229533.V6RIV9
110573	229533.A0A0E0SC46	pp	229533.Q4I9U7
110574	229533.A0A0E0SC46	pp	229533.I1RWN5
110575	229533.A0A0E0SC46	pp	229533.I1RA29
110576	229533.A0A0E0SC46	pp	229533.I1RN88
110577	229533.A0A0E0SC46	pp	229533.I1RST1
110578	229533.A0A0E0SC46	pp	229533.I1RSA4
110579	229533.A0A0E0SC46	pp	229533.I1S277
110580	229533.A0A0E0SC46	pp	229533.I1S0L4
110581	229533.A0A0E0SC46	pp	229533.A0A1C3YIS7
110582	229533.A0A0E0SC46	pp	229533.I1RND5
110583	229533.A0A0E0SC46	pp	229533.I1S9Y1
110584	229533.A0A0E0SC46	pp	229533.I1S1W3
110585	229533.A0A0E0SC46	pp	229533.I1RJG6
110586	229533.A0A0E0SC46	pp	229533.I1RA11
110587	229533.A0A0E0SC46	pp	229533.A0A1C3YMA1
110588	229533.A0A0E0SC46	pp	229533.I1RZ39
110589	229533.A0A0E0SC46	pp	229533.Q4I5M4
110590	229533.A0A0E0SC46	pp	229533.A0A1C3YKM9
110591	229533.A0A0E0SC46	pp	229533.V6R3X5
110592	229533.A0A0E0SC46	pp	229533.I1RRM5
110593	229533.A0A0E0SC46	pp	229533.I1RXC2
110594	229533.A0A0E0SC46	pp	229533.I1S2B8
110595	229533.A0A0E0SC46	pp	229533.I1RR73

110596	229533.A0A0E0SC46	pp	229533.O74638
110597	229533.A0A0E0SC46	pp	229533.A0A1C3YIR8
110598	229533.A0A0E0SC46	pp	229533.A0A1C3YHQ4
110599	229533.A0A0E0SC46	pp	229533.I1RSQ4
110600	229533.A0A0E0SC46	pp	229533.I1S5R1
110601	229533.A0A0E0SC46	pp	229533.I1RZZ9
110602	229533.A0A0E0SC46	pp	229533.I1RRT8
110603	229533.A0A0E0SC46	pp	229533.I1RWI5
110604	229533.A0A0E0SC46	pp	229533.I1RK96
110605	229533.A0A0E0SC46	pp	229533.A0A1C3YJD4
110606	229533.A0A0E0SC46	pp	229533.I1RJZ7
110607	229533.A0A0E0SC46	pp	229533.I1RC29
110608	229533.A0A0E0SC46	pp	229533.I1R9M0
110609	229533.A0A0E0SC46	pp	229533.Q4HZI0
110610	229533.A0A0E0SC46	pp	229533.I1RX24
110611	229533.A0A0E0SC46	pp	229533.I1S503
110612	229533.A0A0E0SC46	pp	229533.I1RMV5
110613	229533.A0A0E0SC46	pp	229533.I1RSZ7
110614	229533.A0A0E0SC46	pp	229533.I1RFS3
110615	229533.A0A0E0SC46	pp	229533.I1RE76
110616	229533.A0A0E0SC46	pp	229533.A0A1C3YI62
110617	229533.A0A0E0SC46	pp	229533.I1RJW6
110618	229533.A0A0E0SC46	pp	229533.I1RPM4
110619	229533.A0A0E0SC46	pp	229533.I1REX2
110620	229533.A0A0E0SC46	pp	229533.A0A1C3YLK8
110621	229533.A0A0E0SC46	pp	229533.I1RRZ0
110622	229533.A0A0E0SC46	pp	229533.I1RBY4
110623	229533.A0A0E0SC46	pp	229533.I1S6Y8
110624	229533.A0A0E0SC46	pp	229533.V6RUY9
110625	229533.A0A0E0SC46	pp	229533.V6R3J1
110626	229533.A0A0E0SC46	pp	229533.I1RPV3
110627	229533.A0A0E0SC46	pp	229533.A0A1C3YKM6
110628	229533.A0A0E0SC46	pp	229533.I1RSX0
110629	229533.A0A0E0SC46	pp	229533.I1RT04
110630	229533.A0A0E0SC46	pp	229533.I1S524
110631	229533.A0A0E0SC46	pp	229533.Q4HXF0
110632	229533.A0A0E0SC46	pp	229533.I1RQS5
110633	229533.A0A0E0SC46	pp	229533.I1RN67
110634	229533.A0A0E0SC46	pp	229533.I1S4M5
110635	229533.A0A0E0SC46	pp	229533.I1RND9
110636	229533.A0A0E0SC46	pp	229533.I1S5S2
110637	229533.A0A0E0SC46	pp	229533.I1RC05
110638	229533.A0A0E0SC46	pp	229533.I1RJF9
110639	229533.A0A0E0SC46	pp	229533.I1RWU2
110640	229533.A0A0E0SC46	pp	229533.I1RY62
110641	229533.A0A0E0SC46	pp	229533.I1RXF2
110642	229533.A0A0E0SC46	pp	229533.I1RG13
110643	229533.A0A0E0SC46	pp	229533.I1RCA7
110644	229533.A0A0E0SC46	pp	229533.I1RBS0
110645	229533.A0A0E0SC46	pp	229533.I1S8P5
110646	229533.A0A0E0SC46	pp	229533.I1RT40
110647	229533.A0A0E0SC46	pp	229533.I1RME9
110648	229533.A0A0E0SC46	pp	229533.I1RV91
110649	229533.A0A0E0SC46	pp	229533.I1RCG4
110650	229533.A0A0E0SC46	pp	229533.I1RPP3
110651	229533.A0A0E0SC46	pp	229533.I1RAZ3
110652	229533.A0A0E0SC46	pp	229533.A0A1C3YJI6
110653	229533.A0A0E0SC46	pp	229533.I1RFL3
110654	229533.A0A0E0SC46	pp	229533.I1RYI5
110655	229533.A0A0E0SC46	pp	229533.I1RFU8
110656	229533.A0A0E0SC46	pp	229533.Q4IBK5
110657	229533.A0A0E0SC46	pp	229533.I1RTD5
110658	229533.A0A0E0SC46	pp	229533.I1RBV3
110659	229533.A0A0E0SC46	pp	229533.I1S887
110660	229533.A0A0E0SC46	pp	229533.I1RGA4
110661	229533.A0A0E0SC46	pp	229533.A0A1C3YK68
110662	229533.A0A0E0SC46	pp	229533.I1RBC7
110663	229533.A0A0E0SC46	pp	229533.I1RQB5
110664	229533.A0A0E0SC46	pp	229533.I1RCM5
110665	229533.A0A0E0SC46	pp	229533.A0A1C3YIA6
110666	229533.A0A0E0SC46	pp	229533.I1RWK3
110667	229533.A0A0E0SC46	pp	229533.I1RBS6
110668	229533.A0A0E0SC46	pp	229533.A0A1C3YLA6

110669	229533.A0A0E0SC46	pp	229533.I1RT17
110670	229533.A0A0E0SC46	pp	229533.Q4I1Y1
110671	229533.A0A0E0SC46	pp	229533.I1RJV4
110672	229533.A0A0E0SC46	pp	229533.I1RP27
110673	229533.A0A0E0SC46	pp	229533.I1RN63
110674	229533.A0A0E0SC46	pp	229533.I1RCS2
110675	229533.A0A0E0SC46	pp	229533.I1RZK5
110676	229533.A0A0E0SC46	pp	229533.I1RVZ2
110677	229533.A0A0E0SC46	pp	229533.I1RAC1
110678	229533.A0A0E0SC46	pp	229533.V6RCZ8
110679	229533.A0A0E0SC46	pp	229533.I1RTA1
110680	229533.A0A0E0SC46	pp	229533.I1S0C1
110681	229533.A0A0E0SC46	pp	229533.I1RAF3
110682	229533.A0A0E0SC46	pp	229533.Q4IQ72
110683	229533.A0A0E0SC46	pp	229533.I1S6K9
110684	229533.A0A0E0SC46	pp	229533.I1RY03
110685	229533.A0A0E0SC46	pp	229533.A0A1C3YHX6
110686	229533.A0A0E0SC46	pp	229533.A0A1C3YHS4
110687	229533.A0A0E0SC46	pp	229533.V6RCG5
110688	229533.A0A0E0SC46	pp	229533.I1RS47
110689	229533.A0A0E0SC46	pp	229533.I1RPR9
110690	229533.A0A0E0SC46	pp	229533.I1RBL7
110691	229533.A0A0E0SC46	pp	229533.I1S9L6
110692	229533.A0A0E0SC46	pp	229533.A0A1C3YI23
110693	229533.A0A0E0SC46	pp	229533.I1RJV2
110694	229533.A0A0E0SC46	pp	229533.I1RPA0
110695	229533.A0A0E0SC46	pp	229533.I1RR51
110696	229533.A0A0E0SC46	pp	229533.I1RA36
110697	229533.A0A0E0SC46	pp	229533.A0A1C3YNC3
110698	229533.A0A0E0SC46	pp	229533.I1RWB0
110699	229533.A0A0E0SC46	pp	229533.A0A1C3YLU7
110700	229533.A0A0E0SC46	pp	229533.I1RZM8
110701	229533.A0A0E0SC46	pp	229533.I1RRY9
110702	229533.A0A0E0SC46	pp	229533.I1RXF0
110703	229533.A0A0E0SC46	pp	229533.I1S1J7
110704	229533.A0A0E0SC46	pp	229533.Q4IAA0
110705	229533.A0A0E0SC46	pp	229533.Q4IB70
110706	229533.A0A0E0SC46	pp	229533.A0A1C3YK34
110707	229533.A0A0E0SC46	pp	229533.I1RSA8
110708	229533.A0A0E0SC46	pp	229533.I1RI82
110709	229533.A0A0E0SC46	pp	229533.I1RXC4
110710	229533.A0A0E0SC46	pp	229533.I1S7M3
110711	229533.A0A0E0SC46	pp	229533.A0A1C3YKY6
110712	229533.A0A0E0SC46	pp	229533.Q4IJM4
110713	229533.A0A0E0SC46	pp	229533.I1RK72
110714	229533.A0A0E0SC46	pp	229533.I1RJ17
110715	229533.A0A0E0SC46	pp	229533.I1RK95
110716	229533.A0A0E0SC46	pp	229533.Q4IJK2
110717	229533.A0A0E0SC46	pp	229533.A0A1C3YI30
110718	229533.A0A0E0SC46	pp	229533.I1RW99
110719	229533.A0A0E0SC46	pp	229533.I1RRX0
110720	229533.A0A0E0SC46	pp	229533.Q4IP34
110721	229533.A0A0E0SC46	pp	229533.I1RPY5
110722	229533.A0A0E0SC46	pp	229533.I1RS83
110723	229533.A0A0E0SC46	pp	229533.Q4I948
110724	229533.A0A0E0SC46	pp	229533.I1S504
110725	229533.A0A0E0SC46	pp	229533.A0A1C3YJR3
110726	229533.A0A0E0SC46	pp	229533.Q4I7F9
110727	229533.A0A0E0SC46	pp	229533.A0A0E0SJQ3
110728	229533.A0A0E0SC46	pp	229533.I1S5B0
110729	229533.A0A0E0SC46	pp	229533.V6QUE6
110730	229533.A0A0E0SC46	pp	229533.I1RZZ1
110731	229533.A0A0E0SC46	pp	229533.A0A1C3YJ46
110732	229533.A0A0E0SC46	pp	229533.A0A1C3YKB0
110733	229533.A0A0E0SC46	pp	229533.A0A1C3YJ42
110734	229533.A0A0E0SC46	pp	229533.I1REK8
110735	229533.A0A0E0SC46	pp	229533.I1RRV4
110736	229533.A0A0E0SC46	pp	229533.I1RG17
110737	229533.A0A0E0SC46	pp	229533.I1RFP9
110738	229533.A0A0E0SC46	pp	229533.A0A1C3YL25
110739	229533.A0A0E0SC46	pp	229533.I1S419
110740	229533.A0A0E0SC46	pp	229533.I1S0Z9
110741	229533.A0A0E0SC46	pp	229533.I1REL7

110742	229533.A0A0E0SC46	pp	229533.I1RAU5
110743	229533.A0A0E0SC46	pp	229533.I1RZ11
110744	229533.A0A0E0SC46	pp	229533.I1S8F8
110745	229533.A0A0E0SC46	pp	229533.I1RRU9
110746	229533.A0A0E0SC46	pp	229533.I1RE00
110747	229533.A0A0E0SC46	pp	229533.A0A1C3YIJ3
110748	229533.A0A0E0SC46	pp	229533.I1RT35
110749	229533.A0A0E0SC46	pp	229533.A0A1C3YJW9
110750	229533.A0A0E0SC46	pp	229533.I1RT41
110751	229533.A0A0E0SC46	pp	229533.I1RQD8
110752	229533.A0A0E0SC46	pp	229533.I1RWX8
110753	229533.A0A0E0SC46	pp	229533.A0A1C3YIG2
110754	229533.A0A0E0SC46	pp	229533.I1RQ81
110755	229533.A0A0E0SC46	pp	229533.A0A1C3YKV3
110756	229533.A0A0E0SC46	pp	229533.I1RW25
110757	229533.A0A0E0SC46	pp	229533.I1RZM1
110758	229533.A0A0E0SC46	pp	229533.A0A1C3YJQ9
110759	229533.A0A0E0SC46	pp	229533.I1RCI4
110760	229533.A0A0E0SC46	pp	229533.Q4IRI9
110761	229533.A0A0E0SC46	pp	229533.I1RNQ5
110762	229533.A0A0E0SC46	pp	229533.I1RS18
110763	229533.A0A0E0SC46	pp	229533.I1S0R7
110764	229533.A0A0E0SC46	pp	229533.I1RLU6
110765	229533.A0A0E0SC46	pp	229533.A0A1C3YN30
110766	229533.A0A0E0SC46	pp	229533.A0A0E0SFN0
110767	229533.A0A0E0SC46	pp	229533.A0A1C3YKC0
110768	229533.A0A0E0SC46	pp	229533.I1RP86
110769	229533.A0A0E0SC46	pp	229533.I1RJU3
110770	229533.A0A0E0SC46	pp	229533.A0A0E0SLM3
110771	229533.A0A0E0SC46	pp	229533.I1RQC9
110772	229533.A0A0E0SC46	pp	229533.I1RBT1
110773	229533.A0A0E0SC46	pp	229533.I1RB50
110774	229533.A0A0E0SC46	pp	229533.A0A1C3YHX8
110775	229533.A0A0E0SC46	pp	229533.I1RN59
110776	229533.A0A0E0SC46	pp	229533.I1R9Y4
110777	229533.A0A0E0SC46	pp	229533.I1RXD7
110778	229533.A0A0E0SC46	pp	229533.I1RMW7
110779	229533.A0A0E0SC46	pp	229533.I1RLP1
110780	229533.A0A0E0SC46	pp	229533.V6RUT5
110781	229533.A0A0E0SC46	pp	229533.A0A1C3YN26
110782	229533.A0A0E0SC46	pp	229533.I1S0X6
110783	229533.A0A0E0SC46	pp	229533.I1RR24
110784	229533.A0A0E0SC46	pp	229533.I1S9M0
110785	229533.A0A0E0SC46	pp	229533.I1RME6
110786	229533.A0A0E0SC46	pp	229533.I1RPN4
110787	229533.A0A0E0SC46	pp	229533.I1S7M2
110788	229533.A0A0E0SC46	pp	229533.I1RKK4
110789	229533.A0A0E0SC46	pp	229533.A0A1C3YL11
110790	229533.A0A0E0SC46	pp	229533.I1RBC9
110791	229533.A0A0E0SC46	pp	229533.I1S4S4
110792	229533.A0A0E0SC46	pp	229533.I1RBU6
110793	229533.A0A0E0SC46	pp	229533.A0A1C3YL54
110794	229533.A0A0E0SC46	pp	229533.I1RW71
110795	229533.A0A0E0SC46	pp	229533.I1RWJ8
110796	229533.A0A0E0SC46	pp	229533.I1S4Y4
110797	229533.A0A0E0SC46	pp	229533.I1S4S7
110798	229533.A0A0E0SC46	pp	229533.I1RAX6
110799	229533.A0A0E0SC46	pp	229533.I1RAU9
110800	229533.A0A0E0SC46	pp	229533.I1RRX5
110801	229533.A0A0E0SC46	pp	229533.I1RPZ3
110802	229533.A0A0E0SC46	pp	229533.I1RAE1
110803	229533.A0A0E0SC46	pp	229533.I1RFC0
110804	229533.A0A0E0SC46	pp	229533.I1S0B6
110805	229533.A0A0E0SC46	pp	229533.I1RSQ7
110806	229533.A0A0E0SC46	pp	229533.V6RTJ8
110807	229533.A0A0E0SC46	pp	229533.I1RX81
110808	229533.A0A0E0SC46	pp	229533.I1RZG7
110809	229533.A0A0E0SC46	pp	229533.V6QW22
110810	229533.A0A0E0SC46	pp	229533.I1RPA4
110811	229533.A0A0E0SC46	pp	229533.I1RYI9
110812	229533.A0A0E0SC46	pp	229533.I1S9L5
110813	229533.A0A0E0SC46	pp	229533.I1S1C6
110814	229533.A0A0E0SC46	pp	229533.I1RCS1

229533.A0A0E0SC46	pp	229533.I1RCQ6
229533.A0A0E0SC46	pp	229533.I1RZJ1
229533.A0A0E0SC46	pp	229533.I1R9N6
229533.A0A0E0SC46	pp	229533.Q4IPB3
229533.A0A0E0SC46	pp	229533.I1S854
229533.A0A0E0SC46	pp	229533.I1S246
229533.A0A0E0SC46	pp	229533.A0A1C3YML3
229533.A0A0E0SC46	pp	229533.A0A1C3YM63
229533.A0A0E0SC46	pp	229533.I1S3J4
229533.A0A0E0SC46	pp	229533.I1RC91
229533.A0A0E0SC46	pp	229533.I1RBD5
229533.A0A0E0SC46	pp	229533.I1S2Y5
229533.A0A0E0SC46	pp	229533.I1RCI3
229533.A0A0E0SC46	pp	229533.Q4HWB3
229533.A0A0E0SC46	pp	229533.I1RRG3
229533.A0A0E0SC46	pp	229533.I1RX46
229533.A0A0E0SC46	pp	229533.I1RS75
229533.A0A0E0SC46	pp	229533.I1RAD1
229533.A0A0E0SC46	pp	229533.I1S0T2
229533.A0A0E0SC46	pp	229533.I1RCD6
229533.A0A0E0SC46	pp	229533.I1RLU5
229533.A0A0E0SC46	pp	229533.I1RA85
229533.A0A0E0SC46	pp	229533.I1RD56
229533.A0A0E0SC46	pp	229533.I1RD52
229533.A0A0E0SC46	pp	229533.I1S0L3
229533.A0A0E0SC46	pp	229533.V6R1K4
229533.A0A0E0SC46	pp	229533.I1RQC5
229533.A0A0E0SC46	pp	229533.A0A1C3YIP5
229533.A0A0E0SC46	pp	229533.I1RVQ0
229533.I1RBP6	pp	229533.I1S5X9
229533.I1RBP6	pp	229533.V6RJP8
229533.I1RBP6	pp	229533.I1RXD9
229533.I1RBP6	pp	229533.I1RYT8
229533.I1RBP6	pp	229533.I1RS29
229533.I1RBP6	pp	229533.I1RS05
229533.I1RQ23	pp	229533.I1RR73
229533.I1RQ23	pp	229533.V6QW22
229533.I1RQ23	pp	229533.I1RSQ7
229533.I1RQ23	pp	229533.V6R3X5
229533.I1RQ23	pp	229533.I1RR24
229533.I1RQ23	pp	229533.I1RZZ9
229533.I1RQ23	pp	229533.I1RWK3
229533.I1RQ23	pp	229533.I1RXF0
229533.I1RQ23	pp	229533.I1S0L4
229533.I1RQ23	pp	229533.V6R1K4
229533.I1RQ23	pp	229533.I1S0L3
229533.I1RQ23	pp	229533.I1RZM8
229533.I1RQ23	pp	229533.I1S0F6
229533.I1RQ23	pp	229533.I1RWJ8
229533.I1RQ23	pp	229533.I1S5B0
229533.I1RQ23	pp	229533.I1RVQ0
229533.I1RQ23	pp	229533.I1RUN9
229533.I1RQ23	pp	229533.I1S5R1
229533.I1RQ23	pp	229533.I1RRT8
229533.I1RQ23	pp	229533.I1RW99
229533.I1RQ23	pp	229533.I1SA33
229533.I1RQ23	pp	229533.I1RZJ5
229533.I1RQ23	pp	229533.I1RQC9
229533.I1RQ23	pp	229533.I1RS17
229533.I1RQ23	pp	229533.I1RT03
229533.I1RQ23	pp	229533.I1S0R7
229533.I1RQ23	pp	229533.Q4I8P5
229533.I1RQ23	pp	229533.I1RT04
229533.I1RQ23	pp	229533.Q4IJK2
229533.I1RQ23	pp	229533.I1RXD7
229533.I1RQ23	pp	229533.I1RVX0
229533.I1RQ23	pp	229533.I1SA32
229533.I1RQ23	pp	229533.I1S2E9
229533.I1RQ23	pp	229533.I1RW46
229533.I1RQ23	pp	229533.I1RV91
229533.I1RQ23	pp	229533.I1RZZ1
229533.I1RQ23	pp	229533.I1RT40
229533.I1RQ23	pp	229533.I1S0J1

229533.I1RQ23	pp	229533.I1RQE7
229533.I1RQ23	pp	229533.I1RTD5
229533.I1RQ23	pp	229533.V6R057
229533.I1RQ23	pp	229533.I1S504
229533.I1RQ23	pp	229533.I1RWN5
229533.I1RQ23	pp	229533.Q4I624
229533.I1RQ23	pp	229533.Q4I2J8
229533.I1RQ23	pp	229533.V6RIV9
229533.I1RQ23	pp	229533.V6RTJ8
229533.I1RQ23	pp	229533.I1RY44
229533.I1RQ23	pp	229533.I1RTH6
229533.I1RQ23	pp	229533.I1S7A8
229533.I1RQ23	pp	229533.I1RZM1
229533.I1RQ23	pp	229533.I1S503
229533.I1RQ23	pp	229533.I1RZK5
229533.I1RQ23	pp	229533.I1RX46
229533.I1RQ23	pp	229533.I1S8R3
229533.I1RQ23	pp	229533.I1RQH8
229533.I1RQ23	pp	229533.Q4IFI0
229533.I1RQ23	pp	229533.V6RCZ8
229533.I1RQ23	pp	229533.I1S1C7
229533.I1RQ23	pp	229533.I1RSQ3
229533.I1RQ23	pp	229533.V6R042
229533.I1RQ23	pp	229533.I1RY03
229533.I1RQ23	pp	229533.I1S2B8
229533.I1RQ23	pp	229533.I1RRI2
229533.I1RQ23	pp	229533.V6RMW6
229533.I1RQ23	pp	229533.I1RRM5
229533.I1RQ23	pp	229533.I1RZW8
229533.I1RQ23	pp	229533.I1RW25
229533.I1RQ23	pp	229533.I1RZ39
229533.I1RQ23	pp	229533.V6RCG5
229533.I1RQ23	pp	229533.I1RXX1
229533.I1RQ23	pp	229533.I1RWN6
229533.I1RQ23	pp	229533.I1RRY9
229533.I1RQ23	pp	229533.I1RUG3
229533.I1RIK1	pp	229533.I1S2N3
229533.I1RIK1	pp	229533.I1S2K2
229533.I1RIK1	pp	229533.I1RXM0
229533.A0A1C3YIU8	pp	229533.I1RCK4
229533.A0A1C3YIU8	pp	229533.A0A1C3YK34
229533.A0A1C3YIU8	pp	229533.A0A1C3YND3
229533.A0A1C3YIU8	pp	229533.I1REK4
229533.A0A1C3YIU8	pp	229533.I1RZV9
229533.A0A1C3YIU8	pp	229533.I1RCU7
229533.Q4I662	pp	229533.Q4IAA0
229533.Q4I662	pp	229533.Q4IJ56
229533.Q4I662	pp	229533.Q4IPI1
229533.Q4I662	pp	229533.V6QYF0
229533.Q4I662	pp	229533.Q4IFI0
229533.Q4I662	pp	229533.V6R227
229533.Q4I662	pp	229533.V6R3X5
229533.Q4I662	pp	229533.Q4IEK8
229533.Q4I662	pp	229533.Q4IJH1
229533.Q4I662	pp	229533.Q4IBS2
229533.Q4I662	pp	229533.V6RMW6
229533.Q4I662	pp	229533.Q4I830
229533.Q4I662	pp	229533.V6QUE6
229533.Q4I662	pp	229533.Q4IF76
229533.Q4I662	pp	229533.Q4IEV5
229533.Q4I662	pp	229533.V6RGY3
229533.Q4I662	pp	229533.Q4IN63
229533.Q4I662	pp	229533.V6RGN5
229533.Q4I662	pp	229533.V6R042
229533.Q4I662	pp	229533.Q4IQW0
229533.Q4I662	pp	229533.V6R283
229533.Q4I662	pp	229533.V6RY33
229533.Q4I662	pp	229533.Q4IAS1
229533.Q4I662	pp	229533.V6RIS2
229533.A0A1C3YK21	pp	229533.I1RSC7
229533.A0A1C3YK21	pp	229533.I1RPF5
229533.A0A1C3YK21	pp	229533.I1RW15
229533.A0A1C3YK21	pp	229533.I1RRJ6

110961 229533.A0A1C3YK21 pp 229533.A0A1C3YLR6
110962 229533.A0A1C3YK21 pp 229533.I1RP99
110963 229533.A0A1C3YK21 pp 229533.I1RBX4
110964 229533.A0A1C3YK21 pp 229533.I1S183
110965 229533.A0A1C3YK21 pp 229533.A0A1C3YN36
110966 229533.A0A1C3YK21 pp 229533.I1RDJ8
110967 229533.A0A1C3YK21 pp 229533.I1S788
110968 229533.A0A1C3YK21 pp 229533.I1RDH0
110969 229533.A0A1C3YK21 pp 229533.I1RYQ1
110970 229533.A0A1C3YK21 pp 229533.I1S0D5
110971 229533.A0A1C3YK21 pp 229533.I1RAT5
110972 229533.A0A1C3YK21 pp 229533.I1S209
110973 229533.A0A1C3YK21 pp 229533.I1S573
110974 229533.A0A1C3YK21 pp 229533.I1RCM7
110975 229533.A0A1C3YK21 pp 229533.I1RX64
110976 229533.A0A1C3YK21 pp 229533.I1RC54
110977 229533.A0A1C3YK21 pp 229533.A0A1C3YML3
110978 229533.A0A1C3YK21 pp 229533.I1RY29
110979 229533.A0A1C3YK21 pp 229533.Q4INS6
110980 229533.A0A1C3YK21 pp 229533.A0A1C3YL34
110981 229533.A0A1C3YK21 pp 229533.I1RWI8
110982 229533.A0A1C3YIU9 pp 229533.A0A1C3YLT9
110983 229533.A0A0E0SML0 pp 229533.I1S0H2
110984 229533.A0A0E0SML0 pp 229533.I1RIK1
110985 229533.A0A0E0SML0 pp 229533.I1RRG1
110986 229533.I1RJW6 pp 229533.I1S0L4
110987 229533.I1RJW6 pp 229533.I1RS20
110988 229533.I1RJW6 pp 229533.I1RQS8
110989 229533.I1RJW6 pp 229533.I1RR24
110990 229533.I1RJW6 pp 229533.I1S5R1
110991 229533.I1RJW6 pp 229533.I1S2H4
110992 229533.I1RJW6 pp 229533.I1RMG8
110993 229533.I1RJW6 pp 229533.O74638
110994 229533.I1RJW6 pp 229533.I1RRT8
110995 229533.I1RJW6 pp 229533.I1RMK3
110996 229533.I1RJW6 pp 229533.I1RWJ8
110997 229533.I1RJW6 pp 229533.I1RZJ5
110998 229533.I1RJW6 pp 229533.I1RRL9
110999 229533.I1RJW6 pp 229533.Q4HVW2
111000 229533.I1RJW6 pp 229533.I1RMC8
111001 229533.I1RJW6 pp 229533.I1RR73
111002 229533.I1RJW6 pp 229533.I1S8V3
111003 229533.I1RJW6 pp 229533.I1RZ39
111004 229533.I1RJW6 pp 229533.V6R3X5
111005 229533.I1RJW6 pp 229533.I1RWI5
111006 229533.I1RJW6 pp 229533.I1S0X6
111007 229533.I1RJW6 pp 229533.I1RZZ9
111008 229533.I1RJW6 pp 229533.I1RW61
111009 229533.I1RJW6 pp 229533.I1RS47
111010 229533.I1RJW6 pp 229533.I1RTF4
111011 229533.I1RJW6 pp 229533.I1RX25
111012 229533.I1RJW6 pp 229533.I1S6F0
111013 229533.I1RJW6 pp 229533.I1RW07
111014 229533.I1RJW6 pp 229533.V6RTJ8
111015 229533.I1RJW6 pp 229533.I1RW25
111016 229533.I1RJW6 pp 229533.I1RJZ7
111017 229533.I1RJW6 pp 229533.I1S7A8
111018 229533.I1RJW6 pp 229533.V6R4J1
111019 229533.I1RJW6 pp 229533.I1S0T2
111020 229533.I1RJW6 pp 229533.I1S2B8
111021 229533.I1RJW6 pp 229533.I1RVQ0
111022 229533.I1RJW6 pp 229533.I1RX46
111023 229533.I1RJW6 pp 229533.I1RWD4
111024 229533.I1RJW6 pp 229533.I1RRM5
111025 229533.I1RJW6 pp 229533.I1RRP3
111026 229533.I1RJW6 pp 229533.I1RLC7
111027 229533.I1RJW6 pp 229533.I1RM06
111028 229533.I1RJW6 pp 229533.I1RN67
111029 229533.I1RJW6 pp 229533.I1RRT2
111030 229533.I1RJW6 pp 229533.I1RLN6
111031 229533.I1RJW6 pp 229533.I1S0M6
111032 229533.I1RJW6 pp 229533.I1S503
111033 229533.I1RJW6 pp 229533.I1RRZ0

111034	229533.I1RJW6	pp	229533.I1S0F6
111035	229533.I1RJW6	pp	229533.I1RNA0
111036	229533.I1RJW6	pp	229533.I1S8P5
111037	229533.I1RJW6	pp	229533.V6QUW4
111038	229533.I1RJW6	pp	229533.V6QYF0
111039	229533.I1RJW6	pp	229533.I1RPP3
111040	229533.I1RJW6	pp	229533.I1RXD7
111041	229533.I1RJW6	pp	229533.I1RQG4
111042	229533.I1RJW6	pp	229533.I1RUQ2
111043	229533.I1RJW6	pp	229533.I1RQC9
111044	229533.I1RJW6	pp	229533.I1S2E9
111045	229533.I1RJW6	pp	229533.I1RRJ8
111046	229533.I1RJW6	pp	229533.I1S6Y8
111047	229533.I1RJW6	pp	229533.I1RQ23
111048	229533.I1RJW6	pp	229533.I1RNA5
111049	229533.I1RJW6	pp	229533.I1RPM4
111050	229533.I1RJW6	pp	229533.V6QW22
111051	229533.I1RJW6	pp	229533.I1RSQ3
111052	229533.I1RJW6	pp	229533.I1S0R7
111053	229533.I1RJW6	pp	229533.I1RSQ7
111054	229533.I1RJW6	pp	229533.V6R042
111055	229533.I1RJW6	pp	229533.I1S352
111056	229533.I1RJW6	pp	229533.I1RT04
111057	229533.I1RJW6	pp	229533.I1RW71
111058	229533.I1RJW6	pp	229533.V6RUY9
111059	229533.I1RJW6	pp	229533.V6RMW6
111060	229533.I1RJW6	pp	229533.I1S2M6
111061	229533.I1RJW6	pp	229533.V6RCG5
111062	229533.I1RJW6	pp	229533.I1RV91
111063	229533.I1RJW6	pp	229533.I1RTD5
111064	229533.I1RJW6	pp	229533.I1RMG3
111065	229533.I1RJW6	pp	229533.I1RT40
111066	229533.I1RJW6	pp	229533.Q4IJT5
111067	229533.I1RJW6	pp	229533.V6R1K4
111068	229533.I1RJW6	pp	229533.V6RCZ8
111069	229533.I1RJW6	pp	229533.I1RWK3
111070	229533.I1RJW6	pp	229533.I1RLX7
111071	229533.I1RJW6	pp	229533.I1RN63
111072	229533.I1RJW6	pp	229533.I1RRY9
111073	229533.I1RJW6	pp	229533.I1RSI6
111074	229533.I1RJW6	pp	229533.I1S0F5
111075	229533.I1RJW6	pp	229533.I1RWN6
111076	229533.I1RJW6	pp	229533.I1S0L3
111077	229533.I1RJW6	pp	229533.I1RRV1
111078	229533.I1RJW6	pp	229533.I1RNC1
111079	229533.I1RJW6	pp	229533.I1RSK1
111080	229533.I1RJW6	pp	229533.I1RXT3
111081	229533.I1RJW6	pp	229533.I1RU29
111082	229533.I1RJW6	pp	229533.I1RMC0
111083	229533.I1RJW6	pp	229533.I1RQA2
111084	229533.I1RJW6	pp	229533.I1RSR3
111085	229533.I1RJW6	pp	229533.I1S1J7
111086	229533.I1RJW6	pp	229533.I1RKC6
111087	229533.I1RJW6	pp	229533.I1RUG3
111088	229533.I1RJW6	pp	229533.I1RZK5
111089	229533.I1RJW6	pp	229533.I1RXF0
111090	229533.I1RJW6	pp	229533.I1RQ31
111091	229533.I1RJW6	pp	229533.I1RN99
111092	229533.I1RJW6	pp	229533.I1RTE9
111093	229533.I1RJW6	pp	229533.Q4I2J8
111094	229533.I1RJW6	pp	229533.I1RTH6
111095	229533.I1RJW6	pp	229533.I1RZM8
111096	229533.I1RJW6	pp	229533.V6R227
111097	229533.I1RJW6	pp	229533.I1S8R3
111098	229533.I1RJW6	pp	229533.I1RT03
111099	229533.I1RJW6	pp	229533.I1RWX5
111100	229533.I1RJW6	pp	229533.Q4I283
111101	229533.I1RJW6	pp	229533.I1RVX0
111102	229533.I1RJW6	pp	229533.I1RRI2
111103	229533.I1RJW6	pp	229533.I1RY63
111104	229533.I1RJW6	pp	229533.I1RRN5
111105	229533.I1RJW6	pp	229533.I1RZW8
111106	229533.I1RJW6	pp	229533.I1RSC6

111107 229533.I1RJW6 pp 229533.I1RYW3
111108 229533.I1RJW6 pp 229533.I1S8V4
111109 229533.I1RJW6 pp 229533.I1RWT6
111110 229533.I1RJW6 pp 229533.I1RMD5
111111 229533.I1RJW6 pp 229533.I1RZB4
111112 229533.I1RJW6 pp 229533.I1RZZ1
111113 229533.I1RJW6 pp 229533.I1S1C7
111114 229533.I1RJW6 pp 229533.I1RRE5
111115 229533.I1RJW6 pp 229533.I1RQE7
111116 229533.I1RJW6 pp 229533.I1S504
111117 229533.I1RJW6 pp 229533.I1RY03
111118 229533.I1RJW6 pp 229533.I1RWD7
111119 229533.I1RJW6 pp 229533.Q4IEK8
111120 229533.I1RJW6 pp 229533.I1RN18
111121 229533.I1RJW6 pp 229533.I1S569
111122 229533.I1RJW6 pp 229533.I1RQQ5
111123 229533.I1RJW6 pp 229533.V6R057
111124 229533.I1RJW6 pp 229533.I1RQA3
111125 229533.I1RJW6 pp 229533.I1RNQ5
111126 229533.I1RJW6 pp 229533.I1RY44
111127 229533.I1RJW6 pp 229533.I1RLU6
111128 229533.I1RJW6 pp 229533.Q4I624
111129 229533.I1RJW6 pp 229533.I1RZM1
111130 229533.I1RJW6 pp 229533.Q4IJK2
111131 229533.I1RJW6 pp 229533.I1RSI0
111132 229533.I1RJW6 pp 229533.I1RLM7
111133 229533.I1RJW6 pp 229533.V6RGN5
111134 229533.I1RJW6 pp 229533.I1RWN5
111135 229533.I1RJW6 pp 229533.I1S0N4
111136 229533.I1RJW6 pp 229533.I1S5B0
111137 229533.I1RJW6 pp 229533.I1RS17
111138 229533.I1RJW6 pp 229533.V6RIV9
111139 229533.I1RJW6 pp 229533.I1SA32
111140 229533.I1RJW6 pp 229533.I1RW99
111141 229533.I1RJW6 pp 229533.I1RQ81
111142 229533.I1RJW6 pp 229533.I1RWQ8
111143 229533.I1RJW6 pp 229533.I1S419
111144 229533.I1RJW6 pp 229533.I1RRU9
111145 229533.I1RJW6 pp 229533.I1S0R2
111146 229533.I1RJW6 pp 229533.Q4HZB8
111147 229533.I1RJW6 pp 229533.I1SA33
111148 229533.I1RJW6 pp 229533.I1RPP4
111149 229533.I1RJW6 pp 229533.I1S0J1
111150 229533.I1RJW6 pp 229533.Q4I8P5
111151 229533.I1RJW6 pp 229533.I1RS18
111152 229533.I1RJW6 pp 229533.I1RR51
111153 229533.I1RJW6 pp 229533.I1RTA0
111154 229533.I1RJW6 pp 229533.I1RW46
111155 229533.I1REZ5 pp 229533.I1RMQ7
111156 229533.I1REZ5 pp 229533.I1RFW1
111157 229533.I1REZ5 pp 229533.I1RSD1
111158 229533.I1REZ5 pp 229533.I1RYV3
111159 229533.I1REZ5 pp 229533.Q4IER8
111160 229533.I1REZ5 pp 229533.Q4I5R3
111161 229533.A0A0E0SDM0 pp 229533.I1S2N3
111162 229533.A0A0E0SDM0 pp 229533.I1RXM0
111163 229533.A0A0E0SDM0 pp 229533.I1RIK1
111164 229533.A0A0E0SDM0 pp 229533.E6PBP1
111165 229533.A0A0E0SDM0 pp 229533.A0A0E0SML0
111166 229533.A0A0E0SDM0 pp 229533.I1S2K2
111167 229533.I1R9C6 pp 229533.I1RH68
111168 229533.I1R9C6 pp 229533.I1RAN7
111169 229533.A0A1C3YLK8 pp 229533.I1RE27
111170 229533.A0A1C3YLK8 pp 229533.A0A1C3YMA1
111171 229533.A0A1C3YLK8 pp 229533.I1RFS3
111172 229533.A0A1C3YLK8 pp 229533.I1S4Z8
111173 229533.A0A1C3YLK8 pp 229533.I1RL04
111174 229533.A0A1C3YLK8 pp 229533.I1S6Y8
111175 229533.A0A1C3YLK8 pp 229533.I1RPV3
111176 229533.A0A1C3YLK8 pp 229533.I1RPM4
111177 229533.A0A1C3YLK8 pp 229533.I1RQ23
111178 229533.A0A1C3YLK8 pp 229533.I1RSZ7
111179 229533.A0A1C3YLK8 pp 229533.I1RE76

111180	229533.A0A1C3YLK8	pp	229533.I1RW61
111181	229533.A0A1C3YLK8	pp	229533.I1RBY4
111182	229533.A0A1C3YLK8	pp	229533.I1RPU3
111183	229533.A0A1C3YLK8	pp	229533.I1RKP1
111184	229533.A0A1C3YLK8	pp	229533.I1RN04
111185	229533.A0A1C3YLK8	pp	229533.I1RR72
111186	229533.A0A1C3YLK8	pp	229533.I1RRM5
111187	229533.A0A1C3YLK8	pp	229533.I1S2B8
111188	229533.A0A1C3YLK8	pp	229533.I1RRI1
111189	229533.A0A1C3YLK8	pp	229533.I1RC29
111190	229533.A0A1C3YLK8	pp	229533.I1RE22
111191	229533.A0A1C3YLK8	pp	229533.I1RGU7
111192	229533.A0A1C3YLK8	pp	229533.I1S0L4
111193	229533.A0A1C3YLK8	pp	229533.I1RA11
111194	229533.A0A1C3YLK8	pp	229533.I1RXC2
111195	229533.A0A1C3YLK8	pp	229533.I1R9M0
111196	229533.A0A1C3YLK8	pp	229533.I1RJZ7
111197	229533.A0A1C3YLK8	pp	229533.I1S2H4
111198	229533.A0A1C3YLK8	pp	229533.I1S0F6
111199	229533.A0A1C3YLK8	pp	229533.O74638
111200	229533.A0A1C3YLK8	pp	229533.I1RPE0
111201	229533.A0A1C3YLK8	pp	229533.I1RDF7
111202	229533.A0A1C3YLK8	pp	229533.I1RN67
111203	229533.A0A1C3YLK8	pp	229533.I1S0W4
111204	229533.A0A1C3YLK8	pp	229533.I1RQS8
111205	229533.A0A1C3YLK8	pp	229533.I1S5R1
111206	229533.A0A1C3YLK8	pp	229533.I1S3G4
111207	229533.A0A1C3YLK8	pp	229533.I1RSA5
111208	229533.A0A1C3YLK8	pp	229533.I1RRT8
111209	229533.A0A1C3YLK8	pp	229533.I1REC5
111210	229533.A0A1C3YLK8	pp	229533.I1SAA0
111211	229533.A0A1C3YLK8	pp	229533.I1S0F7
111212	229533.A0A1C3YLK8	pp	229533.I1RFH8
111213	229533.A0A1C3YLK8	pp	229533.I1RC66
111214	229533.A0A1C3YLK8	pp	229533.I1S1F3
111215	229533.A0A1C3YLK8	pp	229533.I1S8V3
111216	229533.A0A1C3YLK8	pp	229533.I1S9Y1
111217	229533.A0A1C3YLK8	pp	229533.I1RM82
111218	229533.A0A1C3YLK8	pp	229533.I1RSQ4
111219	229533.A0A1C3YLK8	pp	229533.I1RS47
111220	229533.A0A1C3YLK8	pp	229533.I1RW80
111221	229533.A0A1C3YLK8	pp	229533.I1RZZ9
111222	229533.A0A1C3YLK8	pp	229533.I1S2M6
111223	229533.A0A1C3YLK8	pp	229533.I1RWI5
111224	229533.A0A1C3YLK8	pp	229533.V6R3J1
111225	229533.A0A1C3YLK8	pp	229533.I1RSX0
111226	229533.A0A1C3YLK8	pp	229533.I1S5Z5
111227	229533.A0A1C3YLK8	pp	229533.I1RVZ2
111228	229533.A0A1C3YLK8	pp	229533.V6R3X5
111229	229533.A0A1C3YLK8	pp	229533.I1RJL4
111230	229533.A0A1C3YLK8	pp	229533.I1RR73
111231	229533.A0A1C3YLK8	pp	229533.I1RJN9
111232	229533.A0A1C3YLK8	pp	229533.I1RRT2
111233	229533.A0A1C3YLK8	pp	229533.I1RTA1
111234	229533.A0A1C3YLK8	pp	229533.I1RRK3
111235	229533.A0A1C3YLK8	pp	229533.I1S503
111236	229533.A0A1C3YLK8	pp	229533.I1S253
111237	229533.A0A1C3YLK8	pp	229533.I1RWK3
111238	229533.A0A1C3YLK8	pp	229533.I1RRL9
111239	229533.A0A1C3YLK8	pp	229533.I1RN63
111240	229533.A0A1C3YLK8	pp	229533.I1RC24
111241	229533.A0A1C3YLK8	pp	229533.I1RRJ8
111242	229533.A0A1C3YLK8	pp	229533.I1RJW6
111243	229533.A0A1C3YLK8	pp	229533.I1RZ39
111244	229533.A0A1C3YLK8	pp	229533.I1RC34
111245	229533.A0A1C3YLK8	pp	229533.I1RRZ0
111246	229533.A0A1C3YLK8	pp	229533.I1RC05
111247	229533.A0A1C3YLK8	pp	229533.A0A1C3YN63
111248	229533.A0A1C3YLK8	pp	229533.I1S8P5
111249	229533.A0A1C3YLK8	pp	229533.Q4HVQ9
111250	229533.A0A1C3YLK8	pp	229533.I1RBS0
111251	229533.A0A1C3YLK8	pp	229533.I1RPP3
111252	229533.A0A1C3YLK8	pp	229533.I1RJV4

111253 229533.A0A1C3YLK8 pp 229533.I1RYI5
111254 229533.A0A1C3YLK8 pp 229533.I1S9H8
111255 229533.A0A1C3YLK8 pp 229533.I1RZB2
111256 229533.A0A1C3YLK8 pp 229533.I1RFT1
111257 229533.A0A1C3YLK8 pp 229533.I1RQM7
111258 229533.A0A1C3YLK8 pp 229533.V6RUY9
111259 229533.A0A1C3YLK8 pp 229533.I1RYW3
111260 229533.A0A1C3YLK8 pp 229533.I1RJ74
111261 229533.A0A1C3YLK8 pp 229533.I1RT04
111262 229533.A0A1C3YLK8 pp 229533.I1S1C7
111263 229533.A0A1C3YLK8 pp 229533.I1RGA4
111264 229533.A0A1C3YLK8 pp 229533.I1RC82
111265 229533.A0A1C3YLK8 pp 229533.I1RML0
111266 229533.A0A1C3YLK8 pp 229533.I1RBC7
111267 229533.A0A1C3YLK8 pp 229533.I1S437
111268 229533.A0A1C3YLK8 pp 229533.I1RCD8
111269 229533.A0A1C3YLK8 pp 229533.I1S268
111270 229533.A0A1C3YLK8 pp 229533.I1RCR1
111271 229533.A0A1C3YLK8 pp 229533.I1RBS6
111272 229533.A0A1C3YLK8 pp 229533.I1RKC6
111273 229533.A0A1C3YLK8 pp 229533.I1RCW6
111274 229533.A0A1C3YLK8 pp 229533.I1RB41
111275 229533.A0A1C3YLK8 pp 229533.I1RKJ8
111276 229533.A0A1C3YLK8 pp 229533.I1RB44
111277 229533.A0A1C3YLK8 pp 229533.I1S8R3
111278 229533.A0A1C3YLK8 pp 229533.I1RTD5
111279 229533.A0A1C3YLK8 pp 229533.V6RRH2
111280 229533.A0A1C3YLK8 pp 229533.I1RXF8
111281 229533.A0A1C3YLK8 pp 229533.I1RBR1
111282 229533.A0A1C3YLK8 pp 229533.I1RT40
111283 229533.A0A1C3YLK8 pp 229533.I1S0F8
111284 229533.A0A1C3YLK8 pp 229533.I1RV91
111285 229533.A0A1C3YLK8 pp 229533.I1RQ33
111286 229533.A0A1C3YLK8 pp 229533.I1RAU7
111287 229533.A0A1C3YLK8 pp 229533.I1RA70
111288 229533.A0A1C3YLK8 pp 229533.I1RTH6
111289 229533.A0A1C3YLK8 pp 229533.I1S2E9
111290 229533.A0A1C3YLK8 pp 229533.Q4I624
111291 229533.A0A1C3YLK8 pp 229533.I1RD17
111292 229533.A0A1C3YLK8 pp 229533.I1RAP1
111293 229533.A0A1C3YLK8 pp 229533.I1RFL4
111294 229533.A0A1C3YLK8 pp 229533.I1RB92
111295 229533.A0A1C3YLK8 pp 229533.I1RZV3
111296 229533.A0A1C3YLK8 pp 229533.I1RUF7
111297 229533.A0A1C3YLK8 pp 229533.V6R057
111298 229533.A0A1C3YLK8 pp 229533.I1S1W3
111299 229533.A0A1C3YLK8 pp 229533.I1RR61
111300 229533.A0A1C3YLK8 pp 229533.I1RRN5
111301 229533.A0A1C3YLK8 pp 229533.I1RXR1
111302 229533.A0A1C3YLK8 pp 229533.I1RY44
111303 229533.A0A1C3YLK8 pp 229533.I1RCH5
111304 229533.A0A1C3YLK8 pp 229533.I1RQE7
111305 229533.A0A1C3YLK8 pp 229533.I1RC33
111306 229533.A0A1C3YLK8 pp 229533.I1RM37
111307 229533.A0A1C3YLK8 pp 229533.I1RT03
111308 229533.A0A1C3YLK8 pp 229533.I1RVX0
111309 229533.A0A1C3YLK8 pp 229533.I1RN18
111310 229533.A0A1C3YLK8 pp 229533.I1RJV3
111311 229533.A0A1C3YLK8 pp 229533.I1S016
111312 229533.A0A1C3YLK8 pp 229533.I1RAG7
111313 229533.A0A1C3YLK8 pp 229533.I1S7W5
111314 229533.A0A1C3YLK8 pp 229533.I1S1W9
111315 229533.A0A1C3YLK8 pp 229533.I1RSK1
111316 229533.A0A1C3YLK8 pp 229533.V6RIV9
111317 229533.A0A1C3YLK8 pp 229533.I1RKB6
111318 229533.A0A1C3YLK8 pp 229533.I1RTA0
111319 229533.A0A1C3YLK8 pp 229533.I1RE01
111320 229533.A0A1C3YLK8 pp 229533.I1RE89
111321 229533.A0A1C3YLK8 pp 229533.I1RWN5
111322 229533.A0A1C3YLK8 pp 229533.I1RVQ3
111323 229533.A0A1C3YLK8 pp 229533.I1RAW0
111324 229533.A0A1C3YLK8 pp 229533.I1RW46
111325 229533.A0A1C3YLK8 pp 229533.I1R9X7

111326 229533.A0A1C3YLK8 pp 229533.I1RVZ7
111327 229533.A0A1C3YLK8 pp 229533.I1RD39
111328 229533.A0A1C3YLK8 pp 229533.I1RWN6
111329 229533.A0A1C3YLK8 pp 229533.I1RPU2
111330 229533.A0A1C3YLK8 pp 229533.I1S9Z4
111331 229533.A0A1C3YLK8 pp 229533.I1RXU9
111332 229533.A0A1C3YLK8 pp 229533.I1S8V4
111333 229533.A0A1C3YLK8 pp 229533.I1RMV0
111334 229533.A0A1C3YLK8 pp 229533.I1RZW8
111335 229533.A0A1C3YLK8 pp 229533.I1RPP4
111336 229533.A0A1C3YLK8 pp 229533.I1RKR8
111337 229533.A0A1C3YLK8 pp 229533.I1RA65
111338 229533.A0A1C3YLK8 pp 229533.I1RRI2
111339 229533.A0A1C3YLK8 pp 229533.I1RZ79
111340 229533.A0A1C3YLK8 pp 229533.I1RS17
111341 229533.A0A1C3YLK8 pp 229533.I1RSF1
111342 229533.A0A1C3YLK8 pp 229533.I1RPK0
111343 229533.A0A1C3YLK8 pp 229533.I1RD22
111344 229533.A0A1C3YLK8 pp 229533.I1RRE5
111345 229533.A0A1C3YLK8 pp 229533.I1RWX5
111346 229533.A0A1C3YLK8 pp 229533.I1S0J1
111347 229533.A0A1C3YLK8 pp 229533.I1RVF8
111348 229533.A0A1C3YLK8 pp 229533.I1RRN2
111349 229533.A0A1C3YLK8 pp 229533.I1RKJ4
111350 229533.A0A1C3YLK8 pp 229533.I1SA33
111351 229533.A0A1C3YLK8 pp 229533.I1RCS1
111352 229533.A0A1C3YLK8 pp 229533.I1RCR8
111353 229533.A0A1C3YLK8 pp 229533.I1RSU2
111354 229533.A0A1C3YLK8 pp 229533.I1S438
111355 229533.A0A1C3YLK8 pp 229533.I1RB26
111356 229533.A0A1C3YLK8 pp 229533.I1RUG3
111357 229533.A0A1C3YLK8 pp 229533.I1RNC1
111358 229533.A0A1C3YLK8 pp 229533.I1RG11
111359 229533.A0A1C3YLK8 pp 229533.I1RM06
111360 229533.A0A1C3YLK8 pp 229533.V6RMW6
111361 229533.A0A1C3YLK8 pp 229533.I1S0N4
111362 229533.A0A1C3YLK8 pp 229533.V6QYF0
111363 229533.A0A1C3YLK8 pp 229533.I1RNA0
111364 229533.A0A1C3YLK8 pp 229533.I1RRM3
111365 229533.A0A1C3YLK8 pp 229533.V6RGN5
111366 229533.A0A1C3YLK8 pp 229533.I1S1W8
111367 229533.A0A1C3YLK8 pp 229533.I1RRF7
111368 229533.A0A1C3YLK8 pp 229533.V6R042
111369 229533.A0A1C3YLK8 pp 229533.I1RSQ3
111370 229533.A0A1C3YLK8 pp 229533.I1RZJ5
111371 229533.A0A1C3YLK8 pp 229533.I1RM67
111372 229533.A0A1C3YLK8 pp 229533.I1RWQ8
111373 229533.A0A1C3YLK8 pp 229533.I1RRV1
111374 229533.A0A1C3YLK8 pp 229533.A0A1C3YLS9
111375 229533.A0A1C3YLK8 pp 229533.I1RMG3
111376 229533.A0A1C3YLK8 pp 229533.I1S5R6
111377 229533.A0A1C3YLK8 pp 229533.I1RMG8
111378 229533.A0A1C3YLK8 pp 229533.I1RBT1
111379 229533.A0A1C3YLK8 pp 229533.I1RX25
111380 229533.A0A1C3YLK8 pp 229533.I1S4S4
111381 229533.A0A1C3YLK8 pp 229533.I1RMK3
111382 229533.A0A1C3YLK8 pp 229533.I1S9I5
111383 229533.A0A1C3YLK8 pp 229533.I1RKJ5
111384 229533.A0A1C3YLK8 pp 229533.I1RWJ8
111385 229533.A0A1C3YLK8 pp 229533.A0A1C3YN30
111386 229533.A0A1C3YLK8 pp 229533.I1RR24
111387 229533.A0A1C3YLK8 pp 229533.I1RW25
111388 229533.A0A1C3YLK8 pp 229533.I1RJU3
111389 229533.A0A1C3YLK8 pp 229533.I1RMC8
111390 229533.A0A1C3YLK8 pp 229533.I1RTF4
111391 229533.A0A1C3YLK8 pp 229533.I1RE17
111392 229533.A0A1C3YLK8 pp 229533.A0A1C3YN26
111393 229533.A0A1C3YLK8 pp 229533.I1RRS8
111394 229533.A0A1C3YLK8 pp 229533.I1RQ42
111395 229533.A0A1C3YLK8 pp 229533.A0A1C3YLR6
111396 229533.A0A1C3YLK8 pp 229533.I1RVQ0
111397 229533.A0A1C3YLK8 pp 229533.I1R9Y4
111398 229533.A0A1C3YLK8 pp 229533.I1RW91

111399	229533.A0A1C3YLK8	pp	229533.I1S0X6
111400	229533.A0A1C3YLK8	pp	229533.I1RXD6
111401	229533.A0A1C3YLK8	pp	229533.I1RNB2
111402	229533.A0A1C3YLK8	pp	229533.I1RXD7
111403	229533.A0A1C3YLK8	pp	229533.I1RUQ2
111404	229533.A0A1C3YLK8	pp	229533.I1RWD4
111405	229533.A0A1C3YLK8	pp	229533.I1RBD5
111406	229533.A0A1C3YLK8	pp	229533.I1RPW8
111407	229533.A0A1C3YLK8	pp	229533.I1RLP1
111408	229533.A0A1C3YLK8	pp	229533.I1RX95
111409	229533.A0A1C3YLK8	pp	229533.I1S0L3
111410	229533.A0A1C3YLK8	pp	229533.V6RUT5
111411	229533.A0A1C3YLK8	pp	229533.I1RSQ7
111412	229533.A0A1C3YLK8	pp	229533.I1RRX5
111413	229533.A0A1C3YLK8	pp	229533.V6R1K4
111414	229533.A0A1C3YLK8	pp	229533.V6QW22
111415	229533.A0A1C3YLK8	pp	229533.I1RA85
111416	229533.A0A1C3YLK8	pp	229533.I1RTB3
111417	229533.A0A1C3YLK8	pp	229533.I1S0R7
111418	229533.A0A1C3YLK8	pp	229533.I1RY86
111419	229533.A0A1C3YLK8	pp	229533.I1RD52
111420	229533.A0A1C3YLK8	pp	229533.I1RG53
111421	229533.A0A1C3YLK8	pp	229533.I1RPN4
111422	229533.A0A1C3YLK8	pp	229533.I1RW71
111423	229533.A0A1C3YLK8	pp	229533.I1RF19
111424	229533.A0A1C3YLK8	pp	229533.I1RQC9
111425	229533.A0A1C3YLK8	pp	229533.Q4IJT5
111426	229533.A0A1C3YLK8	pp	229533.I1S4Q4
111427	229533.A0A1C3YLK8	pp	229533.I1RRG3
111428	229533.A0A1C3YLK8	pp	229533.I1RBC9
111429	229533.A0A1C3YLK8	pp	229533.I1S4S7
111430	229533.A0A1C3YLK8	pp	229533.I1RSI0
111431	229533.A0A1C3YLK8	pp	229533.A0A1C3YMW0
111432	229533.A0A1C3YLK8	pp	229533.I1RLX7
111433	229533.A0A1C3YLK8	pp	229533.I1RFJ9
111434	229533.A0A1C3YLK8	pp	229533.A0A1C3YM63
111435	229533.A0A1C3YLK8	pp	229533.A0A1C3YMU9
111436	229533.A0A1C3YLK8	pp	229533.I1REL7
111437	229533.A0A1C3YLK8	pp	229533.I1RX46
111438	229533.A0A1C3YLK8	pp	229533.I1S0T2
111439	229533.A0A1C3YLK8	pp	229533.I1RZZ1
111440	229533.A0A1C3YLK8	pp	229533.Q4I2J8
111441	229533.A0A1C3YLK8	pp	229533.I1RGB2
111442	229533.A0A1C3YLK8	pp	229533.I1RAZ8
111443	229533.A0A1C3YLK8	pp	229533.I1REK8
111444	229533.A0A1C3YLK8	pp	229533.I1RKF3
111445	229533.A0A1C3YLK8	pp	229533.I1RBR2
111446	229533.A0A1C3YLK8	pp	229533.I1S7A8
111447	229533.A0A1C3YLK8	pp	229533.V6RTJ8
111448	229533.A0A1C3YLK8	pp	229533.Q4IJK2
111449	229533.A0A1C3YLK8	pp	229533.I1RCR5
111450	229533.A0A1C3YLK8	pp	229533.I1RM35
111451	229533.A0A1C3YLK8	pp	229533.A0A1C3YLX0
111452	229533.A0A1C3YLK8	pp	229533.I1RQA3
111453	229533.A0A1C3YLK8	pp	229533.I1RK95
111454	229533.A0A1C3YLK8	pp	229533.I1RAX6
111455	229533.A0A1C3YLK8	pp	229533.V6RBN2
111456	229533.A0A1C3YLK8	pp	229533.I1RPY3
111457	229533.A0A1C3YLK8	pp	229533.I1RN99
111458	229533.A0A1C3YLK8	pp	229533.I1RQQ5
111459	229533.A0A1C3YLK8	pp	229533.I1RUQ3
111460	229533.A0A1C3YLK8	pp	229533.I1RY03
111461	229533.A0A1C3YLK8	pp	229533.I1RLI4
111462	229533.A0A1C3YLK8	pp	229533.I1RWT6
111463	229533.A0A1C3YLK8	pp	229533.I1RQ85
111464	229533.A0A1C3YLK8	pp	229533.I1S1J7
111465	229533.A0A1C3YLK8	pp	229533.I1RNT9
111466	229533.A0A1C3YLK8	pp	229533.I1RYS0
111467	229533.A0A1C3YLK8	pp	229533.I1S887
111468	229533.A0A1C3YLK8	pp	229533.I1RMD5
111469	229533.A0A1C3YLK8	pp	229533.I1RWB0
111470	229533.A0A1C3YLK8	pp	229533.I1RQ93
111471	229533.A0A1C3YLK8	pp	229533.V6R9Z0

111472 229533.A0A1C3YLK8 pp 229533.Q4I283
111473 229533.A0A1C3YLK8 pp 229533.V6R227
111474 229533.A0A1C3YLK8 pp 229533.I1RCM5
111475 229533.A0A1C3YLK8 pp 229533.I1RZK5
111476 229533.A0A1C3YLK8 pp 229533.I1RSR3
111477 229533.A0A1C3YLK8 pp 229533.I1S504
111478 229533.A0A1C3YLK8 pp 229533.I1RWR7
111479 229533.A0A1C3YLK8 pp 229533.I1RBU8
111480 229533.A0A1C3YLK8 pp 229533.I1RCG4
111481 229533.A0A1C3YLK8 pp 229533.I1RZB5
111482 229533.A0A1C3YLK8 pp 229533.I1RRY9
111483 229533.A0A1C3YLK8 pp 229533.I1RPR9
111484 229533.A0A1C3YLK8 pp 229533.I1SA32
111485 229533.A0A1C3YLK8 pp 229533.I1RXC4
111486 229533.A0A1C3YLK8 pp 229533.I1RAZ3
111487 229533.A0A1C3YLK8 pp 229533.I1RBL7
111488 229533.A0A1C3YLK8 pp 229533.I1RFU8
111489 229533.A0A1C3YLK8 pp 229533.I1RZM1
111490 229533.A0A1C3YLK8 pp 229533.Q4I8P5
111491 229533.A0A1C3YLK8 pp 229533.I1RS18
111492 229533.A0A1C3YLK8 pp 229533.I1RW99
111493 229533.A0A1C3YLK8 pp 229533.I1RCG6
111494 229533.A0A1C3YLK8 pp 229533.I1RNQ5
111495 229533.A0A1C3YLK8 pp 229533.I1RLU6
111496 229533.A0A1C3YLK8 pp 229533.I1S5B0
111497 229533.A0A1C3YLK8 pp 229533.I1RC17
111498 229533.A0A1C3YLK8 pp 229533.I1S419
111499 229533.A0A1C3YLK8 pp 229533.I1RQD2
111500 229533.A0A1C3YLK8 pp 229533.I1RQ81
111501 229533.A0A1C3YLK8 pp 229533.I1S1B2
111502 229533.A0A1C3YLK8 pp 229533.I1RFI5
111503 229533.A0A1C3YLK8 pp 229533.I1RG14
111504 229533.A0A1C3YLK8 pp 229533.I1RC16
111505 229533.A0A1C3YLK8 pp 229533.I1RAU5
111506 229533.A0A1C3YLK8 pp 229533.I1RI82
111507 229533.A0A1C3YLK8 pp 229533.I1RC61
111508 229533.A0A1C3YLK8 pp 229533.V6QUE6
111509 229533.A0A1C3YLK8 pp 229533.I1RXF0
111510 229533.A0A1C3YLK8 pp 229533.I1RRV4
111511 229533.A0A1C3YLK8 pp 229533.I1RZM8
111512 229533.A0A1C3YLK8 pp 229533.I1RAR2
111513 229533.A0A1C3YLK8 pp 229533.I1RRV3
111514 229533.A0A1C3YLK8 pp 229533.I1RQ31
111515 229533.A0A1C3YLK8 pp 229533.I1RR51
111516 229533.A0A1C3YLK8 pp 229533.V6RCG5
111517 229533.A0A1C3YLK8 pp 229533.A0A1C3YLU7
111518 229533.A0A1C3YLK8 pp 229533.I1S821
111519 229533.A0A1C3YLK8 pp 229533.V6RCZ8
111520 229533.A0A1C3YLK8 pp 229533.I1RGC5
111521 229533.A0A1C3YLK8 pp 229533.I1S6K9
111522 229533.I1RBF0 pp 229533.I1RXV3
111523 229533.I1RLH3 pp 229533.I1RS05
111524 229533.I1RLH3 pp 229533.I1RS29
111525 229533.I1RLH3 pp 229533.V6RJP8
111526 229533.I1RLH3 pp 229533.I1S5X9
111527 229533.I1RLH3 pp 229533.I1RYT8
111528 229533.I1RLH3 pp 229533.I1RXD9
111529 229533.A0A0E0SMA3 pp 229533.I1SAK7
111530 229533.A0A0E0SMA3 pp 229533.I1S1Q3
111531 229533.A0A0E0SMA3 pp 229533.I1RQ76
111532 229533.A0A0E0SMA3 pp 229533.V6RR38
111533 229533.A0A0E0SMA3 pp 229533.I1RJR2
111534 229533.A0A0E0SMA3 pp 229533.V6RXE7
111535 229533.A0A0E0SMA3 pp 229533.I1RRS6
111536 229533.A0A0E0SMA3 pp 229533.I1RI22
111537 229533.A0A0E0SMA3 pp 229533.I1RT23
111538 229533.A0A0E0SMA3 pp 229533.I1RPF2
111539 229533.A0A0E0SMA3 pp 229533.A0A0E0SP71
111540 229533.A0A0E0SMA3 pp 229533.V6RPN0
111541 229533.A0A0E0SMA3 pp 229533.A0A1C3YLL7
111542 229533.A0A0E0SMA3 pp 229533.I1RZZ3
111543 229533.A0A0E0SMA3 pp 229533.I1S2M5
111544 229533.A0A0E0SMA3 pp 229533.I1RBR4

111545 229533.A0A0E0SMA3 pp 229533.I1RCA0
111546 229533.A0A0E0SMA3 pp 229533.I1RGC4
111547 229533.A0A0E0SMA3 pp 229533.I1RE80
111548 229533.A0A0E0SMA3 pp 229533.I1RFM2
111549 229533.A0A0E0SMA3 pp 229533.I1RY34
111550 229533.A0A0E0SMA3 pp 229533.I1RF79
111551 229533.A0A0E0SMA3 pp 229533.I1RJG1
111552 229533.A0A0E0SMA3 pp 229533.I1RY35
111553 229533.A0A0E0SMA3 pp 229533.I1RR90
111554 229533.A0A0E0SMA3 pp 229533.Q4HXT6
111555 229533.A0A0E0SMA3 pp 229533.I1RI23
111556 229533.A0A0E0SMA3 pp 229533.I1RNT1
111557 229533.I1S0L4 pp 229533.I1S1C7
111558 229533.I1S0L4 pp 229533.I1S7W5
111559 229533.I1S0L4 pp 229533.I1S437
111560 229533.I1S0L4 pp 229533.V6R057
111561 229533.I1S0L4 pp 229533.I1S569
111562 229533.I1S0L4 pp 229533.I1S8R3
111563 229533.I1S0L4 pp 229533.V6R042
111564 229533.I1S0L4 pp 229533.I1S2H4
111565 229533.I1S0L4 pp 229533.I1S1W3
111566 229533.I1S0L4 pp 229533.V6RMW6
111567 229533.I1S0L4 pp 229533.I1S0R2
111568 229533.I1S0L4 pp 229533.I1S1W9
111569 229533.I1S0L4 pp 229533.I1S5R1
111570 229533.I1S0L4 pp 229533.I1S3G4
111571 229533.I1S0L4 pp 229533.I1S5R6
111572 229533.I1S0L4 pp 229533.O74638
111573 229533.I1S0L4 pp 229533.I1S6Y8
111574 229533.I1S0L4 pp 229533.I1S9Y1
111575 229533.I1S0L4 pp 229533.Q4HZB8
111576 229533.I1S0L4 pp 229533.I1SA33
111577 229533.I1S0L4 pp 229533.I1S0M6
111578 229533.I1S0L4 pp 229533.V6RIV9
111579 229533.I1S0L4 pp 229533.I1S2E9
111580 229533.I1S0L4 pp 229533.I1S2B8
111581 229533.I1S0L4 pp 229533.I1S352
111582 229533.I1S0L4 pp 229533.I1S8P5
111583 229533.I1S0L4 pp 229533.I1S7S9
111584 229533.I1S0L4 pp 229533.V6R3X5
111585 229533.I1S0L4 pp 229533.Q4HXF0
111586 229533.I1S0L4 pp 229533.V6RUY9
111587 229533.I1S0L4 pp 229533.I1S2M6
111588 229533.I1S0L4 pp 229533.I1S0W4
111589 229533.I1S0L4 pp 229533.Q4I8P5
111590 229533.I1S0L4 pp 229533.V6RCG5
111591 229533.I1S0L4 pp 229533.V6R3J1
111592 229533.I1S0L4 pp 229533.I1S1J7
111593 229533.I1S0L4 pp 229533.I1S1B6
111594 229533.I1S0L4 pp 229533.I1S419
111595 229533.I1S0L4 pp 229533.I1S503
111596 229533.I1S0L4 pp 229533.Q4I283
111597 229533.I1S0L4 pp 229533.V6RCZ8
111598 229533.I1S0L4 pp 229533.I1S6K9
111599 229533.I1S0L4 pp 229533.I1SA32
111600 229533.I1S0L4 pp 229533.V6QUE6
111601 229533.I1S0L4 pp 229533.V6R227
111602 229533.I1S0L4 pp 229533.Q4IJK2
111603 229533.I1S0L4 pp 229533.I1S5B0
111604 229533.I1S0L4 pp 229533.Q4I2J8
111605 229533.I1S0L4 pp 229533.I1S4S4
111606 229533.I1S0L4 pp 229533.V6QUW4
111607 229533.I1S0L4 pp 229533.I1S1W8
111608 229533.I1S0L4 pp 229533.I1S887
111609 229533.I1S0L4 pp 229533.I1S504
111610 229533.I1S0L4 pp 229533.I1S438
111611 229533.I1S0L4 pp 229533.I1S4S7
111612 229533.I1S0L4 pp 229533.V6RUT5
111613 229533.I1S0L4 pp 229533.Q4IJT5
111614 229533.I1S0L4 pp 229533.V6QW22
111615 229533.I1S0L4 pp 229533.I1S0X6
111616 229533.I1S0L4 pp 229533.V6RTJ8
111617 229533.I1S0L4 pp 229533.V6R4J1

229533.I1S0L4 pp 229533.I1S0R7
229533.I1S0L4 pp 229533.V6R1K4
229533.I1S0L4 pp 229533.I1S7A8
229533.I1S0L4 pp 229533.I1S0T2
229533.A0A098D9E2 pp 229533.I1S209
229533.A0A098D9E2 pp 229533.I1S0H2
229533.A0A098D9E2 pp 229533.I1S072
229533.A0A098D9E2 pp 229533.I1S183
229533.A0A098D9E2 pp 229533.A0A098E4W0
229533.A0A098D9E2 pp 229533.I1RD63
229533.A0A098D9E2 pp 229533.I1RUL3
229533.A0A098D9E2 pp 229533.I1S0Q4
229533.A0A098D9E2 pp 229533.I1RE23
229533.A0A098D9E2 pp 229533.A0A1C3YJN7
229533.A0A098D9E2 pp 229533.A0A1C3YN36
229533.A0A098D9E2 pp 229533.A0A1C3YM63
229533.A0A098D9E2 pp 229533.I1RBC1
229533.A0A098D9E2 pp 229533.I1RAM7
229533.A0A098D9E2 pp 229533.A0A098DNT9
229533.A0A098D9E2 pp 229533.I1RPN1
229533.A0A098D9E2 pp 229533.A0A1C3YL10
229533.A0A098D9E2 pp 229533.I1RXX1
229533.A0A098D9E2 pp 229533.A0A1C3YKF3
229533.A0A098D9E2 pp 229533.I1RFM6
229533.A0A098D9E2 pp 229533.A0A1C3YKM5
229533.A0A098D9E2 pp 229533.I1RRG3
229533.A0A098D9E2 pp 229533.A0A0E0RSD7
229533.A0A098D9E2 pp 229533.A0A0E0RNR8
229533.A0A098D9E2 pp 229533.A0A098DB54
229533.A0A098D9E2 pp 229533.I1RAT5
229533.A0A098D9E2 pp 229533.I1RM67
229533.A0A098D9E2 pp 229533.I1RVZ9
229533.A0A098D9E2 pp 229533.I1RCM7
229533.A0A098D9E2 pp 229533.A0A1C3YML3
229533.A0A098D9E2 pp 229533.A0A1C3YN26
229533.A0A098D9E2 pp 229533.I1RE55
229533.A0A098D9E2 pp 229533.A0A0E0RVC1
229533.A0A098D9E2 pp 229533.A0A1C3YI64
229533.A0A098D9E2 pp 229533.A0A1C3YLR6
229533.A0A098D9E2 pp 229533.I1S1W8
229533.A0A098D9E2 pp 229533.A0A0E0SCP7
229533.A0A098D9E2 pp 229533.I1RRY1
229533.A0A098D9E2 pp 229533.A0A098DVZ4
229533.A0A098D9E2 pp 229533.I1RKI2
229533.A0A098D9E2 pp 229533.I1RSC7
229533.A0A098D9E2 pp 229533.I1RZ98
229533.A0A098D9E2 pp 229533.I1RT19
229533.A0A098D9E2 pp 229533.I1RBI6
229533.A0A098D9E2 pp 229533.I1RRT1
229533.A0A098D9E2 pp 229533.I1RRN5
229533.A0A098D9E2 pp 229533.I1RQI6
229533.A0A098D9E2 pp 229533.A0A1C3YN55
229533.A0A098D9E2 pp 229533.A0A098DLA7
229533.A0A098D9E2 pp 229533.I1RDX0
229533.A0A098D9E2 pp 229533.I1RRY3
229533.A0A098D9E2 pp 229533.I1RDH0
229533.A0A098D9E2 pp 229533.I1RZB2
229533.A0A098D9E2 pp 229533.I1RS41
229533.A0A098D9E2 pp 229533.I1RTN6
229533.A0A098D9E2 pp 229533.I1RZQ6
229533.A0A098D9E2 pp 229533.A0A0E0SPV0
229533.A0A098D9E2 pp 229533.I1RWI8
229533.A0A098D9E2 pp 229533.I1RY29
229533.A0A098D9E2 pp 229533.I1RSP7
229533.A0A098D9E2 pp 229533.I1RUG4
229533.A0A098D9E2 pp 229533.I1S0Q5
229533.A0A098D9E2 pp 229533.I1RSZ3
229533.A0A098D9E2 pp 229533.I1RDE7
229533.A0A098D9E2 pp 229533.I1RZ63
229533.A0A098D9E2 pp 229533.I1RXV3
229533.A0A098D9E2 pp 229533.I1RVW0
229533.A0A098D9E2 pp 229533.A0A098E4E9
229533.A0A098D9E2 pp 229533.I1RMG6

229533.A0A098D9E2 pp 229533.A0A1C3YK21
229533.A0A098D9E2 pp 229533.A0A1C3YMJ3
229533.A0A098D9E2 pp 229533.I1S3G4
229533.A0A098D9E2 pp 229533.I1RTI7
229533.A0A098D9E2 pp 229533.I1RBF3
229533.A0A098D9E2 pp 229533.I1RUN8
229533.Q4IA62 pp 229533.Q4IBX0
229533.I1RIQ3 pp 229533.I1RS05
229533.I1RIQ3 pp 229533.I1RYT8
229533.I1RIQ3 pp 229533.V6RJP8
229533.I1RIQ3 pp 229533.I1S5X9
229533.I1RIQ3 pp 229533.I1RXD9
229533.I1RIQ3 pp 229533.I1RS29
229533.A0A098DRZ0 pp 229533.A0A1C3YIN6
229533.A0A098DRZ0 pp 229533.I1RCM3
229533.A0A098DRZ0 pp 229533.I1RM82
229533.A0A098DRZ0 pp 229533.I1RJV3
229533.A0A098DRZ0 pp 229533.I1RNC1
229533.A0A098DRZ0 pp 229533.I1RSK1
229533.A0A098DRZ0 pp 229533.I1RFQ7
229533.A0A098DRZ0 pp 229533.I1RAW0
229533.A0A098DRZ0 pp 229533.I1RWN6
229533.A0A098DRZ0 pp 229533.I1RRY9
229533.A0A098DRZ0 pp 229533.I1RTD5
229533.A0A098DRZ0 pp 229533.A0A1C3YMI5
229533.A0A098DRZ0 pp 229533.I1RT04
229533.A0A098DRZ0 pp 229533.I1S0R7
229533.A0A098DRZ0 pp 229533.I1RRV1
229533.A0A098DRZ0 pp 229533.I1RY03
229533.A0A098DRZ0 pp 229533.I1S1C7
229533.A0A098DRZ0 pp 229533.A0A0E0SFN0
229533.A0A098DRZ0 pp 229533.I1RQC9
229533.A0A098DRZ0 pp 229533.I1S2E9
229533.A0A098DRZ0 pp 229533.V6RMW6
229533.A0A098DRZ0 pp 229533.I1RWI5
229533.A0A098DRZ0 pp 229533.A0A1C3YKC0
229533.A0A098DRZ0 pp 229533.A0A1C3YHX8
229533.A0A098DRZ0 pp 229533.I1RNN1
229533.A0A098DRZ0 pp 229533.I1RYY5
229533.A0A098DRZ0 pp 229533.I1RXD7
229533.A0A098DRZ0 pp 229533.I1RPU3
229533.A0A098DRZ0 pp 229533.A0A0E0RU84
229533.A0A098DRZ0 pp 229533.V6R042
229533.A0A098DRZ0 pp 229533.I1RTH6
229533.A0A098DRZ0 pp 229533.I1RB75
229533.A0A098DRZ0 pp 229533.A0A0E0RUI4
229533.A0A098DRZ0 pp 229533.A0A1C3YIS7
229533.A0A098DRZ0 pp 229533.I1RFU8
229533.A0A098DRZ0 pp 229533.I1RAU7
229533.A0A098DRZ0 pp 229533.A0A1C3YKM9
229533.A0A098DRZ0 pp 229533.I1RZ39
229533.A0A098DRZ0 pp 229533.I1RSQ3
229533.A0A098DRZ0 pp 229533.Q4IJK2
229533.A0A098DRZ0 pp 229533.I1RN67
229533.A0A098DRZ0 pp 229533.I1RA70
229533.A0A098DRZ0 pp 229533.V6R3X5
229533.A0A098DRZ0 pp 229533.I1RZZ1
229533.A0A098DRZ0 pp 229533.I1RR73
229533.A0A098DRZ0 pp 229533.I1RWN5
229533.A0A098DRZ0 pp 229533.Q4IJM4
229533.A0A098DRZ0 pp 229533.I1RSA4
229533.A0A098DRZ0 pp 229533.I1RZM1
229533.A0A098DRZ0 pp 229533.I1RZK5
229533.A0A098DRZ0 pp 229533.I1RVX0
229533.A0A098DRZ0 pp 229533.A0A0E0SA11
229533.A0A098DRZ0 pp 229533.I1S2B8
229533.A0A098DRZ0 pp 229533.I1RT03
229533.A0A098DRZ0 pp 229533.I1RBS0
229533.A0A098DRZ0 pp 229533.A0A0E0RVZ1
229533.A0A098DRZ0 pp 229533.I1R9M0
229533.A0A098DRZ0 pp 229533.A0A098DXE2
229533.A0A098DRZ0 pp 229533.I1RW25
229533.A0A098DRZ0 pp 229533.I1RY44

111764 229533.A0A098DRZ0 pp 229533.I1S503
111765 229533.A0A098DRZ0 pp 229533.I1RNQ5
111766 229533.A0A098DRZ0 pp 229533.A0A1C3YIJ3
111767 229533.A0A098DRZ0 pp 229533.I1RD22
111768 229533.A0A098DRZ0 pp 229533.I1RSI0
111769 229533.A0A098DRZ0 pp 229533.I1RLU6
111770 229533.A0A098DRZ0 pp 229533.I1RAU5
111771 229533.A0A098DRZ0 pp 229533.I1S1W4
111772 229533.A0A098DRZ0 pp 229533.I1RFT2
111773 229533.A0A098DRZ0 pp 229533.I1S504
111774 229533.A0A098DRZ0 pp 229533.I1RSV2
111775 229533.A0A098DRZ0 pp 229533.A0A1C3YKV3
111776 229533.A0A098DRZ0 pp 229533.I1RRU9
111777 229533.A0A098DRZ0 pp 229533.I1RSQ7
111778 229533.A0A098DRZ0 pp 229533.I1RX46
111779 229533.A0A098DRZ0 pp 229533.V6QW22
111780 229533.A0A098DRZ0 pp 229533.I1RC17
111781 229533.A0A098DRZ0 pp 229533.I1RGA4
111782 229533.A0A098DRZ0 pp 229533.I1S0L3
111783 229533.A0A098DRZ0 pp 229533.I1RN61
111784 229533.A0A098DRZ0 pp 229533.I1RAX6
111785 229533.A0A098DRZ0 pp 229533.I1RC20
111786 229533.A0A098DRZ0 pp 229533.I1RJV4
111787 229533.A0A098DRZ0 pp 229533.I1S2G7
111788 229533.A0A098DRZ0 pp 229533.I1S8P5
111789 229533.A0A098DRZ0 pp 229533.I1RP27
111790 229533.A0A098DRZ0 pp 229533.I1RPP3
111791 229533.A0A098DRZ0 pp 229533.I1RTA0
111792 229533.A0A098DRZ0 pp 229533.I1S0N4
111793 229533.A0A098DRZ0 pp 229533.A0A1C3YK34
111794 229533.A0A098DRZ0 pp 229533.I1RWK3
111795 229533.A0A098DRZ0 pp 229533.I1RPP4
111796 229533.A0A098DRZ0 pp 229533.I1RB74
111797 229533.A0A098DRZ0 pp 229533.V6RTJ8
111798 229533.A0A098DRZ0 pp 229533.I1S0J1
111799 229533.A0A098DRZ0 pp 229533.I1RCJ7
111800 229533.A0A098DRZ0 pp 229533.I1RQ81
111801 229533.A0A098DRZ0 pp 229533.I1RCR8
111802 229533.I1RE80 pp 229533.I1RY81
111803 229533.I1RE80 pp 229533.I1RT23
111804 229533.I1RE80 pp 229533.I1S2M5
111805 229533.I1RE80 pp 229533.I1RI22
111806 229533.I1RE80 pp 229533.V6RR38
111807 229533.I1RE80 pp 229533.I1RNT1
111808 229533.I1RE80 pp 229533.I1RJG1
111809 229533.I1RE80 pp 229533.I1RF79
111810 229533.I1RE80 pp 229533.I1RJR2
111811 229533.I1RE80 pp 229533.I1RY35
111812 229533.I1RE80 pp 229533.I1RR90
111813 229533.I1RE80 pp 229533.I1RFM2
111814 229533.I1RE80 pp 229533.I1RWF4
111815 229533.I1RE80 pp 229533.I1S1Q3
111816 229533.I1RE80 pp 229533.I1RSE9
111817 229533.I1RE80 pp 229533.I1RQ76
111818 229533.I1RE80 pp 229533.V6RPN0
111819 229533.I1RE80 pp 229533.I1RGC4
111820 229533.I1RE80 pp 229533.I1RZZ3
111821 229533.A0A0E0SDL4 pp 229533.I1RQX3
111822 229533.A0A0E0SDL4 pp 229533.I1RCJ9
111823 229533.A0A0E0SDL4 pp 229533.I1RBR0
111824 229533.A0A0E0SDL4 pp 229533.I1RWI6
111825 229533.A0A0E0SDL4 pp 229533.A0A1C3YK51
111826 229533.I1S2E9 pp 229533.V6RVG3
111827 229533.I1S2E9 pp 229533.Q4IN63
111828 229533.I1S2E9 pp 229533.V6RGI1
111829 229533.I1S2E9 pp 229533.Q4HZB8
111830 229533.I1S2E9 pp 229533.I1S569
111831 229533.I1S2E9 pp 229533.V6RKY7
111832 229533.I1S2E9 pp 229533.I1S757
111833 229533.I1S2E9 pp 229533.Q4IB89
111834 229533.I1S2E9 pp 229533.I1SA33
111835 229533.I1S2E9 pp 229533.V6R042
111836 229533.I1S2E9 pp 229533.I1SAF7

111837	229533.I1S2E9	pp	229533.I1S8R3
111838	229533.I1S2E9	pp	229533.V6RIV9
111839	229533.I1S2E9	pp	229533.V6RMW6
111840	229533.I1S2E9	pp	229533.I1S8P5
111841	229533.I1S2E9	pp	229533.I1S352
111842	229533.I1S2E9	pp	229533.I1S549
111843	229533.I1S2E9	pp	229533.I1S5R1
111844	229533.I1S2E9	pp	229533.V6R283
111845	229533.I1S2E9	pp	229533.V6RLW4
111846	229533.I1S2E9	pp	229533.Q4HTT1
111847	229533.I1S2E9	pp	229533.Q4I7L0
111848	229533.I1S2E9	pp	229533.I1S2H4
111849	229533.I1S2E9	pp	229533.I1S3H8
111850	229533.I1S2E9	pp	229533.I1S2G7
111851	229533.I1S2E9	pp	229533.Q4IJK2
111852	229533.I1S2E9	pp	229533.I1S7X9
111853	229533.I1S2E9	pp	229533.I1S4R1
111854	229533.I1S2E9	pp	229533.V6R3X5
111855	229533.I1S2E9	pp	229533.Q4IJM4
111856	229533.I1S2E9	pp	229533.Q4I283
111857	229533.I1S2E9	pp	229533.Q4I7N9
111858	229533.I1S2E9	pp	229533.I1S503
111859	229533.I1S2E9	pp	229533.I1S439
111860	229533.I1S2E9	pp	229533.Q4IR87
111861	229533.I1S2E9	pp	229533.V6RCG5
111862	229533.I1S2E9	pp	229533.Q4I2J8
111863	229533.I1S2E9	pp	229533.Q4I8P5
111864	229533.I1S2E9	pp	229533.I1S504
111865	229533.I1S2E9	pp	229533.Q4HTT2
111866	229533.I1S2E9	pp	229533.V6RCZ8
111867	229533.I1S2E9	pp	229533.V6QV38
111868	229533.I1S2E9	pp	229533.Q4I5R3
111869	229533.I1S2E9	pp	229533.Q4IER8
111870	229533.I1S2E9	pp	229533.Q4IEA7
111871	229533.I1S2E9	pp	229533.I1S604
111872	229533.I1S2E9	pp	229533.Q4ICG5
111873	229533.I1S2E9	pp	229533.V6QW22
111874	229533.I1S2E9	pp	229533.Q4INS6
111875	229533.I1S2E9	pp	229533.I1SA32
111876	229533.I1S2E9	pp	229533.V6QUW4
111877	229533.I1S2E9	pp	229533.Q4IJP1
111878	229533.I1S2E9	pp	229533.I1S5B0
111879	229533.I1S2E9	pp	229533.Q4IJT5
111880	229533.I1S2E9	pp	229533.I1S334
111881	229533.I1S2E9	pp	229533.I1S7A8
111882	229533.I1S2E9	pp	229533.V6QV09
111883	229533.I1S2E9	pp	229533.V6R4J1
111884	229533.I1S2E9	pp	229533.V6RTJ8
111885	229533.I1RGB3	pp	229533.I1RJR2
111886	229533.I1RGB3	pp	229533.I1RGS5
111887	229533.I1RGB3	pp	229533.I1S1Z9
111888	229533.I1RGB3	pp	229533.I1SAK7
111889	229533.I1RGB3	pp	229533.I1RZ29
111890	229533.I1RGB3	pp	229533.I1RYB2
111891	229533.I1RGB3	pp	229533.V6RXR0
111892	229533.I1S008	pp	229533.Q4I7L0
111893	229533.I1RC00	pp	229533.I1RMQ5
111894	229533.I1RC00	pp	229533.I1RCB7
111895	229533.I1RC00	pp	229533.I1RGF4
111896	229533.I1RC00	pp	229533.Q4IPB8
111897	229533.I1RC00	pp	229533.V6RIV9
111898	229533.I1RC00	pp	229533.I1RDY5
111899	229533.I1RC00	pp	229533.I1RQ49
111900	229533.I1RC00	pp	229533.I1RSL8
111901	229533.I1RC00	pp	229533.I1RE01
111902	229533.I1RC00	pp	229533.I1RZE1
111903	229533.I1RC00	pp	229533.V6RRH2
111904	229533.I1RC00	pp	229533.I1RTA0
111905	229533.I1RC00	pp	229533.V6R0U9
111906	229533.I1RC00	pp	229533.Q4IPH4
111907	229533.I1RC00	pp	229533.I1RD22
111908	229533.I1RC00	pp	229533.I1S0N4
111909	229533.I1RC00	pp	229533.I1S149

111910 229533.I1RC00 pp 229533.I1RG11
111911 229533.I1RC00 pp 229533.I1RPK0
111912 229533.I1RC00 pp 229533.I1RJS5
111913 229533.I1RC00 pp 229533.I1RNB8
111914 229533.I1RC00 pp 229533.I1RZT1
111915 229533.I1RC00 pp 229533.Q4HTT1
111916 229533.I1RC00 pp 229533.V6REZ4
111917 229533.I1RC00 pp 229533.I1RRU9
111918 229533.I1RC00 pp 229533.I1RIR9
111919 229533.I1RC00 pp 229533.I1RCD4
111920 229533.I1RC00 pp 229533.I1RCH5
111921 229533.I1RC00 pp 229533.I1RWX5
111922 229533.I1RC00 pp 229533.I1RGC2
111923 229533.I1RC00 pp 229533.I1S2E8
111924 229533.I1RC00 pp 229533.I1RCK4
111925 229533.I1RC00 pp 229533.V6QUE6
111926 229533.I1RC00 pp 229533.I1RQE7
111927 229533.I1RC00 pp 229533.I1RK79
111928 229533.I1RC00 pp 229533.I1RUP8
111929 229533.I1RC00 pp 229533.I1RNE4
111930 229533.I1RC00 pp 229533.I1S5B0
111931 229533.I1RC00 pp 229533.I1S044
111932 229533.I1RC00 pp 229533.I1RYW3
111933 229533.I1RC00 pp 229533.I1RTH6
111934 229533.I1RC00 pp 229533.Q4IMD1
111935 229533.I1RC00 pp 229533.Q4HXF6
111936 229533.I1RC00 pp 229533.I1RNF4
111937 229533.I1RC00 pp 229533.I1RYJ8
111938 229533.I1RC00 pp 229533.Q4IBK5
111939 229533.I1RC00 pp 229533.Q4IE79
111940 229533.I1RC00 pp 229533.I1RVA7
111941 229533.I1RC00 pp 229533.I1RJS2
111942 229533.I1RC00 pp 229533.V6RPS5
111943 229533.I1RC00 pp 229533.I1RYT7
111944 229533.I1RC00 pp 229533.V6RCZ8
111945 229533.I1RC00 pp 229533.I1S8P5
111946 229533.I1RC00 pp 229533.I1RTD5
111947 229533.I1RC00 pp 229533.I1RWF5
111948 229533.I1RC00 pp 229533.I1RPP3
111949 229533.I1RC00 pp 229533.Q4HTT2
111950 229533.I1RC00 pp 229533.I1RP28
111951 229533.I1RC00 pp 229533.I1RQG4
111952 229533.I1RC00 pp 229533.I1RMY3
111953 229533.I1RC00 pp 229533.I1RW24
111954 229533.I1RC00 pp 229533.I1RT35
111955 229533.I1RC00 pp 229533.I1RZM1
111956 229533.I1RC00 pp 229533.I1RJS4
111957 229533.I1RC00 pp 229533.I1RN67
111958 229533.I1RC00 pp 229533.I1RRE0
111959 229533.I1RC00 pp 229533.I1S0P4
111960 229533.I1RC00 pp 229533.I1S0L4
111961 229533.I1RC00 pp 229533.I1RDY1
111962 229533.I1RC00 pp 229533.I1RYP7
111963 229533.I1RC00 pp 229533.Q4I1Y1
111964 229533.I1RC00 pp 229533.I1RFW3
111965 229533.I1RC00 pp 229533.I1S5B5
111966 229533.I1RC00 pp 229533.I1RSQ4
111967 229533.I1RC00 pp 229533.I1RWI5
111968 229533.I1RC00 pp 229533.I1RTE5
111969 229533.I1RC00 pp 229533.I1RZF6
111970 229533.I1RC00 pp 229533.I1RZZ9
111971 229533.I1RC00 pp 229533.I1RQA5
111972 229533.I1RC00 pp 229533.I1RSP1
111973 229533.I1RC00 pp 229533.I1RQF6
111974 229533.I1RC00 pp 229533.I1RW36
111975 229533.I1RC00 pp 229533.Q4IER8
111976 229533.I1RC00 pp 229533.I1RZ63
111977 229533.I1RC00 pp 229533.I1RRT0
111978 229533.I1RC00 pp 229533.Q4IAA0
111979 229533.I1RC00 pp 229533.I1RZB5
111980 229533.I1RC00 pp 229533.Q4I2J8
111981 229533.I1RC00 pp 229533.I1RZV9
111982 229533.I1RC00 pp 229533.I1RC24

111983 229533.I1RC00 pp 229533.I1S2A8
111984 229533.I1RC00 pp 229533.I1RZZ1
111985 229533.I1RC00 pp 229533.I1RE76
111986 229533.I1RC00 pp 229533.I1RRY9
111987 229533.I1RC00 pp 229533.I1S1J7
111988 229533.I1RC00 pp 229533.I1RGC3
111989 229533.I1RC00 pp 229533.I1RX46
111990 229533.I1RC00 pp 229533.I1RJV7
111991 229533.I1RC00 pp 229533.I1RDG0
111992 229533.I1RC00 pp 229533.Q4I5R9
111993 229533.I1RC00 pp 229533.I1RP86
111994 229533.I1RC00 pp 229533.I1RQC5
111995 229533.I1RC00 pp 229533.I1RY40
111996 229533.I1RC00 pp 229533.V6RTJ8
111997 229533.I1RC00 pp 229533.I1RRG3
111998 229533.I1RC00 pp 229533.I1RCU7
111999 229533.I1RC00 pp 229533.I1RQH1
112000 229533.I1RC00 pp 229533.I1RNT2
112001 229533.I1RC00 pp 229533.I1RDN3
112002 229533.I1RC00 pp 229533.I1REX4
112003 229533.I1RC00 pp 229533.Q4IPB3
112004 229533.I1RC00 pp 229533.I1RYE4
112005 229533.I1RC00 pp 229533.I1RW25
112006 229533.I1RC00 pp 229533.I1S6S3
112007 229533.A0A1C3YJ72 pp 229533.I1RGS5
112008 229533.A0A1C3YJ72 pp 229533.I1RZ29
112009 229533.A0A1C3YJ72 pp 229533.V6RXR0
112010 229533.A0A1C3YJ72 pp 229533.I1SAK7
112011 229533.A0A1C3YJ72 pp 229533.I1RF82
112012 229533.A0A1C3YJ72 pp 229533.I1RGB3
112013 229533.A0A1C3YK53 pp 229533.I1RUN9
112014 229533.A0A1C3YK53 pp 229533.I1RRN6
112015 229533.A0A1C3YK53 pp 229533.I1RAZ1
112016 229533.A0A1C3YK53 pp 229533.I1RBX0
112017 229533.A0A1C3YK53 pp 229533.I1RGC1
112018 229533.A0A1C3YK53 pp 229533.I1RN70
112019 229533.A0A1C3YK53 pp 229533.I1RQ61
112020 229533.A0A1C3YK53 pp 229533.I1RSR3
112021 229533.A0A1C3YK53 pp 229533.I1RB71
112022 229533.I1RNA5 pp 229533.I1RZZ9
112023 229533.I1RNA5 pp 229533.I1RRT8
112024 229533.I1RNA5 pp 229533.V6R3X5
112025 229533.I1RNA5 pp 229533.I1S2H4
112026 229533.I1RNA5 pp 229533.I1RR73
112027 229533.I1RNA5 pp 229533.I1RZ39
112028 229533.I1RNA5 pp 229533.I1S2E9
112029 229533.I1RNA5 pp 229533.I1RWN6
112030 229533.I1RNA5 pp 229533.I1S0L4
112031 229533.I1RNA5 pp 229533.I1RPM4
112032 229533.I1RNA5 pp 229533.I1RVX0
112033 229533.I1RNA5 pp 229533.I1RT03
112034 229533.I1RNA5 pp 229533.I1RRM5
112035 229533.I1RNA5 pp 229533.I1S503
112036 229533.I1RNA5 pp 229533.I1S2B8
112037 229533.I1RNA5 pp 229533.Q4I1Y5
112038 229533.I1RNA5 pp 229533.I1RWK3
112039 229533.I1RNA5 pp 229533.I1RV91
112040 229533.I1RNA5 pp 229533.I1S8P5
112041 229533.I1RNA5 pp 229533.I1RUG3
112042 229533.I1RNA5 pp 229533.I1RPP3
112043 229533.I1RNA5 pp 229533.I1RNC1
112044 229533.I1RNA5 pp 229533.I1RT04
112045 229533.I1RNA5 pp 229533.I1RTD5
112046 229533.I1RNA5 pp 229533.V6R042
112047 229533.I1RNA5 pp 229533.I1RZW8
112048 229533.I1RNA5 pp 229533.I1S1C7
112049 229533.I1RNA5 pp 229533.I1RSQ3
112050 229533.I1RNA5 pp 229533.I1RPP4
112051 229533.I1RNA5 pp 229533.I1RTA0
112052 229533.I1RNA5 pp 229533.I1RQ75
112053 229533.I1RNA5 pp 229533.I1RTH6
112054 229533.I1RNA5 pp 229533.V6RMW6
112055 229533.I1RNA5 pp 229533.I1S8R3

112056	229533.I1RNA5	pp	229533.V6RIV9
112057	229533.I1RNA5	pp	229533.I1RQC9
112058	229533.I1RNA5	pp	229533.I1RW25
112059	229533.I1RNA5	pp	229533.I1RWN5
112060	229533.I1RNA5	pp	229533.I1RX46
112061	229533.I1RNA5	pp	229533.I1RR24
112062	229533.I1RNA5	pp	229533.I1RY44
112063	229533.I1RNA5	pp	229533.I1RR39
112064	229533.I1RNA5	pp	229533.V6RTJ8
112065	229533.I1RNA5	pp	229533.V6QW22
112066	229533.I1RNA5	pp	229533.I1RY03
112067	229533.I1RNA5	pp	229533.I1S0L3
112068	229533.I1RNA5	pp	229533.I1RZK5
112069	229533.I1RNA5	pp	229533.I1RVQ0
112070	229533.I1RNA5	pp	229533.I1RSQ7
112071	229533.I1RNA5	pp	229533.Q4IJK2
112072	229533.I1RNA5	pp	229533.I1RRY9
112073	229533.I1RNA5	pp	229533.I1RZM1
112074	229533.I1RNA5	pp	229533.I1RS18
112075	229533.I1RNA5	pp	229533.V6RCG5
112076	229533.I1RNA5	pp	229533.I1S504
112077	229533.I1RYB2	pp	229533.I1SAK7
112078	229533.I1RYB2	pp	229533.I1RZ29
112079	229533.I1RYB2	pp	229533.V6RXR0
112080	229533.I1RQ44	pp	229533.I1RY59
112081	229533.I1RQ44	pp	229533.I1RW06
112082	229533.I1RQ44	pp	229533.I1RT65
112083	229533.I1RED5	pp	229533.I1RGE7
112084	229533.I1RED5	pp	229533.V6R5G9
112085	229533.I1RED5	pp	229533.I1RL39
112086	229533.I1RED5	pp	229533.I1S071
112087	229533.I1RED5	pp	229533.I1RUP6
112088	229533.I1RED5	pp	229533.I1RWN8
112089	229533.I1RED5	pp	229533.I1RHE5
112090	229533.I1RED5	pp	229533.I1S2L3
112091	229533.I1RFS3	pp	229533.I1RTA1
112092	229533.I1RFS3	pp	229533.I1RVZ2
112093	229533.I1RFS3	pp	229533.I1RKL3
112094	229533.I1RFS3	pp	229533.I1RKB2
112095	229533.I1RFS3	pp	229533.I1RFT1
112096	229533.I1RFS3	pp	229533.I1RSX1
112097	229533.I1RFS3	pp	229533.I1RTD5
112098	229533.I1RFS3	pp	229533.I1RT40
112099	229533.I1RFS3	pp	229533.I1RZ39
112100	229533.I1RFS3	pp	229533.Q4I830
112101	229533.I1RFS3	pp	229533.V6RUY9
112102	229533.I1RFS3	pp	229533.I1RT04
112103	229533.I1RFS3	pp	229533.I1RRJ8
112104	229533.I1RFS3	pp	229533.Q4HXF0
112105	229533.I1RFS3	pp	229533.V6R3X5
112106	229533.I1RFS3	pp	229533.I1RPP3
112107	229533.I1RFS3	pp	229533.I1RND6
112108	229533.I1RFS3	pp	229533.I1RYI5
112109	229533.I1RFS3	pp	229533.I1RR73
112110	229533.I1RFS3	pp	229533.I1RN67
112111	229533.I1RFS3	pp	229533.I1S2E9
112112	229533.I1RFS3	pp	229533.I1RKJ6
112113	229533.I1RFS3	pp	229533.I1RZV6
112114	229533.I1RFS3	pp	229533.I1RS30
112115	229533.I1RFS3	pp	229533.I1RRP3
112116	229533.I1RFS3	pp	229533.I1S503
112117	229533.I1RFS3	pp	229533.I1S9Y1
112118	229533.I1RFS3	pp	229533.I1RLV9
112119	229533.I1RFS3	pp	229533.I1RNG4
112120	229533.I1RFS3	pp	229533.I1RRQ3
112121	229533.I1RFS3	pp	229533.V6R3J1
112122	229533.I1RFS3	pp	229533.Q4IAS1
112123	229533.I1RFS3	pp	229533.Q4HZ42
112124	229533.I1RFS3	pp	229533.I1RXC2
112125	229533.I1RFS3	pp	229533.I1RS47
112126	229533.I1RFS3	pp	229533.O74638
112127	229533.I1RFS3	pp	229533.I1RSX0
112128	229533.I1RFS3	pp	229533.I1RRT8

112129 229533.I1RFS3 pp 229533.I1RQ65
112130 229533.I1RFS3 pp 229533.I1RJL4
112131 229533.I1RFS3 pp 229533.I1RRT2
112132 229533.I1RFS3 pp 229533.I1S5R1
112133 229533.I1RFS3 pp 229533.I1RGA4
112134 229533.I1RFS3 pp 229533.I1RQS5
112135 229533.I1RFS3 pp 229533.Q4IF76
112136 229533.I1RFS3 pp 229533.I1RSQ4
112137 229533.I1RFS3 pp 229533.I1S3G4
112138 229533.I1RFS3 pp 229533.I1S2H4
112139 229533.I1RFS3 pp 229533.I1RZZ9
112140 229533.I1RFS3 pp 229533.I1RSL9
112141 229533.I1RFS3 pp 229533.I1RWI5
112142 229533.I1RFS3 pp 229533.Q4IQW0
112143 229533.I1RFS3 pp 229533.I1S0L4
112144 229533.I1RFS3 pp 229533.V6R283
112145 229533.I1RFS3 pp 229533.I1RU69
112146 229533.I1RFS3 pp 229533.I1RIQ7
112147 229533.I1RFS3 pp 229533.I1RSZ7
112148 229533.I1RFS3 pp 229533.I1S060
112149 229533.I1RFS3 pp 229533.I1RPV3
112150 229533.I1RFS3 pp 229533.Q4I662
112151 229533.I1RFS3 pp 229533.I1RKI1
112152 229533.I1RFS3 pp 229533.I1S8E2
112153 229533.I1RFS3 pp 229533.I1RNU6
112154 229533.I1RFS3 pp 229533.I1S0J1
112155 229533.I1RFS3 pp 229533.I1RKF9
112156 229533.I1RFS3 pp 229533.I1RM25
112157 229533.I1RFS3 pp 229533.I1RZS6
112158 229533.I1RFS3 pp 229533.I1RX24
112159 229533.I1RFS3 pp 229533.I1S277
112160 229533.I1RFS3 pp 229533.I1RWB5
112161 229533.I1RFS3 pp 229533.Q4IPI4
112162 229533.I1RFS3 pp 229533.I1RS17
112163 229533.I1RFS3 pp 229533.V6RLM7
112164 229533.I1RFS3 pp 229533.I1RPK0
112165 229533.I1RFS3 pp 229533.I1RKR9
112166 229533.I1RFS3 pp 229533.I1RVY0
112167 229533.I1RFS3 pp 229533.I1S2B8
112168 229533.I1RFS3 pp 229533.I1RTE9
112169 229533.I1RFS3 pp 229533.I1S9Z4
112170 229533.I1RFS3 pp 229533.I1RJZ7
112171 229533.I1RFS3 pp 229533.I1RPY1
112172 229533.I1RFS3 pp 229533.Q4IN63
112173 229533.I1RFS3 pp 229533.I1RJV3
112174 229533.I1RFS3 pp 229533.I1S4T3
112175 229533.I1RFS3 pp 229533.I1RLP2
112176 229533.I1RFS3 pp 229533.I1RUG3
112177 229533.I1RFS3 pp 229533.I1S274
112178 229533.I1RFS3 pp 229533.I1RGC6
112179 229533.I1RFS3 pp 229533.I1RNC1
112180 229533.I1RFS3 pp 229533.I1S1W9
112181 229533.I1RFS3 pp 229533.Q4I327
112182 229533.I1RFS3 pp 229533.I1RWN5
112183 229533.I1RFS3 pp 229533.V6RGY3
112184 229533.I1RFS3 pp 229533.I1S1W3
112185 229533.I1RFS3 pp 229533.I1RSI6
112186 229533.I1RFS3 pp 229533.I1S1U7
112187 229533.I1RFS3 pp 229533.I1RM96
112188 229533.I1RFS3 pp 229533.I1RY44
112189 229533.I1RFS3 pp 229533.I1RWN6
112190 229533.I1RFS3 pp 229533.I1RRF0
112191 229533.I1RFS3 pp 229533.V6RMW6
112192 229533.I1RFS3 pp 229533.I1S6S6
112193 229533.I1RFS3 pp 229533.V6R042
112194 229533.I1RFS3 pp 229533.I1RSD8
112195 229533.I1RFS3 pp 229533.I1S2E8
112196 229533.I1RFS3 pp 229533.I1RRR7
112197 229533.I1RFS3 pp 229533.I1RMN6
112198 229533.I1RFS3 pp 229533.I1RSQ3
112199 229533.I1RFS3 pp 229533.I1RQE7
112200 229533.I1RFS3 pp 229533.I1RST4
112201 229533.I1RFS3 pp 229533.I1S1C7

112202 229533.I1RFS3 pp 229533.Q4HWE2
112203 229533.I1RFS3 pp 229533.I1RYW3
112204 229533.I1RFS3 pp 229533.I1RRN2
112205 229533.I1RFS3 pp 229533.I1S1X9
112206 229533.I1RFS3 pp 229533.I1RRI2
112207 229533.I1RFS3 pp 229533.I1RSS4
112208 229533.I1RFS3 pp 229533.I1RG11
112209 229533.I1RFS3 pp 229533.I1RVX0
112210 229533.I1RFS3 pp 229533.I1S5R6
112211 229533.I1RFS3 pp 229533.I1RTH6
112212 229533.I1RFS3 pp 229533.I1RT03
112213 229533.I1RFS3 pp 229533.I1RKC6
112214 229533.I1RFS3 pp 229533.V6RGN5
112215 229533.I1RFS3 pp 229533.I1S2C0
112216 229533.I1RFS3 pp 229533.V6RRH2
112217 229533.I1RFS3 pp 229533.I1RW71
112218 229533.I1RFS3 pp 229533.I1RPN4
112219 229533.I1RFS3 pp 229533.Q4I624
112220 229533.I1RFS3 pp 229533.I1RML0
112221 229533.I1RFS3 pp 229533.I1S296
112222 229533.I1RFS3 pp 229533.Q4HZ68
112223 229533.I1RFS3 pp 229533.I1S4S7
112224 229533.I1RFS3 pp 229533.I1RT98
112225 229533.I1RFS3 pp 229533.V6R057
112226 229533.I1RFS3 pp 229533.I1RSQ7
112227 229533.I1RFS3 pp 229533.Q4HVW2
112228 229533.I1RFS3 pp 229533.I1RZ99
112229 229533.I1RFS3 pp 229533.I1RZJ6
112230 229533.I1RFS3 pp 229533.Q4INZ9
112231 229533.I1RFS3 pp 229533.I1RQJ5
112232 229533.I1RFS3 pp 229533.Q4HZ47
112233 229533.I1RFS3 pp 229533.I1RPU0
112234 229533.I1RFS3 pp 229533.I1RMK3
112235 229533.I1RFS3 pp 229533.I1RNH1
112236 229533.I1RFS3 pp 229533.I1RZ92
112237 229533.I1RFS3 pp 229533.I1S6F0
112238 229533.I1RFS3 pp 229533.I1S0R7
112239 229533.I1RFS3 pp 229533.I1S4S4
112240 229533.I1RFS3 pp 229533.I1RP86
112241 229533.I1RFS3 pp 229533.I1RT33
112242 229533.I1RFS3 pp 229533.I1RM67
112243 229533.I1RFS3 pp 229533.I1RR24
112244 229533.I1RFS3 pp 229533.I1RXD7
112245 229533.I1RFS3 pp 229533.I1RKQ5
112246 229533.I1RFS3 pp 229533.I1RRH5
112247 229533.I1RFS3 pp 229533.I1S9U2
112248 229533.I1RFS3 pp 229533.I1RPW9
112249 229533.I1RFS3 pp 229533.V6R1K4
112250 229533.I1RFS3 pp 229533.Q4I5R9
112251 229533.I1RFS3 pp 229533.I1RPA4
112252 229533.I1RFS3 pp 229533.I1RH48
112253 229533.I1RFS3 pp 229533.I1RT05
112254 229533.I1RFS3 pp 229533.I1RG51
112255 229533.I1RFS3 pp 229533.Q4IPI1
112256 229533.I1RFS3 pp 229533.I1RQM6
112257 229533.I1RFS3 pp 229533.V6QYF0
112258 229533.I1RFS3 pp 229533.I1RX46
112259 229533.I1RFS3 pp 229533.I1S0F1
112260 229533.I1RFS3 pp 229533.I1RQL4
112261 229533.I1RFS3 pp 229533.I1RWH5
112262 229533.I1RFS3 pp 229533.V6RTJ8
112263 229533.I1RFS3 pp 229533.I1RJU3
112264 229533.I1RFS3 pp 229533.I1RT80
112265 229533.I1RFS3 pp 229533.I1S1W8
112266 229533.I1RFS3 pp 229533.I1RNA0
112267 229533.I1RFS3 pp 229533.I1RFW5
112268 229533.I1RFS3 pp 229533.I1S270
112269 229533.I1RFS3 pp 229533.I1RNV6
112270 229533.I1RFS3 pp 229533.I1RMM8
112271 229533.I1RFS3 pp 229533.I1RRG3
112272 229533.I1RFS3 pp 229533.I1RM06
112273 229533.I1RFS3 pp 229533.I1RTF4
112274 229533.I1RFS3 pp 229533.I1RXF0

112275 229533.I1RFS3 pp 229533.I1RT41
112276 229533.I1RFS3 pp 229533.Q4IFI0
112277 229533.I1RFS3 pp 229533.Q4IJH1
112278 229533.I1RFS3 pp 229533.I1RZM8
112279 229533.I1RFS3 pp 229533.I1RQ31
112280 229533.I1RFS3 pp 229533.I1RYB9
112281 229533.I1RFS3 pp 229533.I1RW25
112282 229533.I1RFS3 pp 229533.I1RMC0
112283 229533.I1RFS3 pp 229533.Q4IJ56
112284 229533.I1RFS3 pp 229533.I1S7Q8
112285 229533.I1RFS3 pp 229533.I1S021
112286 229533.I1RFS3 pp 229533.I1RS18
112287 229533.I1RFS3 pp 229533.I1S1J7
112288 229533.I1RFS3 pp 229533.V6RPN0
112289 229533.I1RFS3 pp 229533.I1RMN7
112290 229533.I1RFS3 pp 229533.I1RNH6
112291 229533.I1RFS3 pp 229533.I1RSR3
112292 229533.I1RFS3 pp 229533.V6RCZ8
112293 229533.I1RFS3 pp 229533.Q4IJK2
112294 229533.I1RFS3 pp 229533.I1RRY9
112295 229533.I1RFS3 pp 229533.Q4IAA0
112296 229533.I1RFS3 pp 229533.I1S6K9
112297 229533.I1RFS3 pp 229533.I1S0Z3
112298 229533.I1RFS3 pp 229533.I1RG56
112299 229533.I1RFS3 pp 229533.V6RBN2
112300 229533.I1RFS3 pp 229533.I1RJZ6
112301 229533.I1RFS3 pp 229533.I1RGC2
112302 229533.I1RFS3 pp 229533.I1S5R5
112303 229533.I1RFS3 pp 229533.Q4IEK8
112304 229533.I1RFS3 pp 229533.Q4I7F9
112305 229533.I1RFS3 pp 229533.I1S504
112306 229533.I1RFS3 pp 229533.V6QUE6
112307 229533.I1RFS3 pp 229533.I1RZK6
112308 229533.I1RFS3 pp 229533.I1RZZ1
112309 229533.I1RFS3 pp 229533.I1RZF6
112310 229533.I1RFS3 pp 229533.I1RRV4
112311 229533.I1RFS3 pp 229533.Q4IBS2
112312 229533.I1RFS3 pp 229533.I1RY63
112313 229533.I1RFS3 pp 229533.I1RQ28
112314 229533.I1RFS3 pp 229533.I1S044
112315 229533.I1RFS3 pp 229533.I1RK95
112316 229533.I1RFS3 pp 229533.V6R227
112317 229533.I1RFS3 pp 229533.I1RX14
112318 229533.I1RFS3 pp 229533.I1S5B0
112319 229533.I1RFS3 pp 229533.I1RY03
112320 229533.I1RFS3 pp 229533.I1RSR8
112321 229533.I1RFS3 pp 229533.I1S0Z9
112322 229533.I1RFS3 pp 229533.I1RN99
112323 229533.I1RFS3 pp 229533.I1RT65
112324 229533.I1RFS3 pp 229533.I1RXC4
112325 229533.I1RFS3 pp 229533.I1RFU8
112326 229533.I1RFS3 pp 229533.I1RJ17
112327 229533.I1RFS3 pp 229533.I1RVZ0
112328 229533.I1RFS3 pp 229533.I1S0M9
112329 229533.I1RFS3 pp 229533.I1RK83
112330 229533.I1RFS3 pp 229533.I1RNK2
112331 229533.I1RFS3 pp 229533.I1RZK5
112332 229533.I1RFS3 pp 229533.I1S6E5
112333 229533.I1RFS3 pp 229533.I1RZE5
112334 229533.I1RFS3 pp 229533.I1RSI0
112335 229533.I1RFS3 pp 229533.I1RQA2
112336 229533.I1RFS3 pp 229533.I1RYB1
112337 229533.A0A098E442 pp 229533.I1RVB6
112338 229533.A0A098E442 pp 229533.A0A0E0SP71
112339 229533.I1RGC4 pp 229533.I1RY81
112340 229533.I1RGC4 pp 229533.I1RI22
112341 229533.I1RGC4 pp 229533.I1RRS6
112342 229533.I1RGC4 pp 229533.I1RT23
112343 229533.I1RGC4 pp 229533.I1RXX1
112344 229533.I1RGC4 pp 229533.I1S2M5
112345 229533.I1RGC4 pp 229533.I1RPF2
112346 229533.I1RGC4 pp 229533.I1S130
112347 229533.I1RGC4 pp 229533.I1S1Q3

229533.I1RGC4	pp	229533.I1RQ76
229533.I1RGC4	pp	229533.V6RPN0
229533.I1RGC4	pp	229533.V6RG22
229533.I1RGC4	pp	229533.I1SAK7
229533.I1RGC4	pp	229533.V6RXE7
229533.I1RGC4	pp	229533.I1RZD0
229533.I1RGC4	pp	229533.I1RPE5
229533.I1RGC4	pp	229533.I1RZZ3
229533.I1RGC4	pp	229533.I1RNT1
229533.I1RGC4	pp	229533.I1RJR2
229533.I1RGC4	pp	229533.V6RIE5
229533.I1RGC4	pp	229533.Q4HXT6
229533.I1RGC4	pp	229533.I1RY35
229533.I1RGC4	pp	229533.I1RR90
229533.I1RGC4	pp	229533.V6RR38
229533.I1RGC4	pp	229533.I1RY34
229533.I1RGC4	pp	229533.I1RJG1
229533.I1RGC4	pp	229533.I1RI23
229533.I1S2H4	pp	229533.I1S8P5
229533.I1S2H4	pp	229533.Q4IR18
229533.I1S2H4	pp	229533.V6RUY9
229533.I1S2H4	pp	229533.I1S503
229533.I1S2H4	pp	229533.I1S4Z8
229533.I1S2H4	pp	229533.I1S352
229533.I1S2H4	pp	229533.Q4HXF0
229533.I1S2H4	pp	229533.V6R3J1
229533.I1S2H4	pp	229533.I1S6Y8
229533.I1S2H4	pp	229533.I1S8V4
229533.I1S2H4	pp	229533.O74638
229533.I1S2H4	pp	229533.I1S5R1
229533.I1S2H4	pp	229533.V6RIV9
229533.I1S2H4	pp	229533.I1S8V3
229533.I1S2H4	pp	229533.V6RMW6
229533.I1S2H4	pp	229533.V6R042
229533.I1S2H4	pp	229533.I1S9Y1
229533.I1S2H4	pp	229533.I1S7W5
229533.I1S2H4	pp	229533.V6RRH2
229533.I1S2H4	pp	229533.I1S5R6
229533.I1S2H4	pp	229533.I1S437
229533.I1S2H4	pp	229533.V6R3X5
229533.I1S2H4	pp	229533.I1SA33
229533.I1S2H4	pp	229533.V6RGN5
229533.I1S2H4	pp	229533.V6QW22
229533.I1S2H4	pp	229533.I1S569
229533.I1S2H4	pp	229533.I1S4S7
229533.I1S2H4	pp	229533.Q4I624
229533.I1S2H4	pp	229533.I1S8R3
229533.I1S2H4	pp	229533.V6RTJ8
229533.I1S2H4	pp	229533.I1S419
229533.I1S2H4	pp	229533.V6R4J1
229533.I1S2H4	pp	229533.V6R057
229533.I1S2H4	pp	229533.V6R1K4
229533.I1S2H4	pp	229533.V6RCG5
229533.I1S2H4	pp	229533.I1S6K9
229533.I1S2H4	pp	229533.I1S438
229533.I1S2H4	pp	229533.V6RUT5
229533.I1S2H4	pp	229533.Q4IJT5
229533.I1S2H4	pp	229533.V6R227
229533.I1S2H4	pp	229533.V6QUW4
229533.I1S2H4	pp	229533.V6RCZ8
229533.I1S2H4	pp	229533.I1S504
229533.I1S2H4	pp	229533.V6QYF0
229533.I1S2H4	pp	229533.Q4I283
229533.I1S2H4	pp	229533.Q4HVW2
229533.I1S2H4	pp	229533.I1S4Q4
229533.I1S2H4	pp	229533.Q4INS6
229533.I1S2H4	pp	229533.I1S4S4
229533.I1S2H4	pp	229533.I1SA32
229533.I1S2H4	pp	229533.Q4IEK8
229533.I1S2H4	pp	229533.V6QUE6
229533.I1S2H4	pp	229533.Q4IJK2
229533.I1S2H4	pp	229533.Q4I8P5
229533.I1S2H4	pp	229533.Q4I2J8

112421	229533.I1S2H4	pp	229533.V6RBN2
112422	229533.I1S2H4	pp	229533.I1S887
112423	229533.I1S2H4	pp	229533.I1S5B0
112424	229533.A0A1C3YJT2	pp	229533.I1RT01
112425	229533.A0A1C3YJT2	pp	229533.I1RMP0
112426	229533.A0A1C3YJT2	pp	229533.I1RJK6
112427	229533.A0A1C3YJT2	pp	229533.I1RW23
112428	229533.A0A1C3YJT2	pp	229533.I1RQ88
112429	229533.A0A1C3YJT2	pp	229533.I1RZR6
112430	229533.A0A1C3YJT2	pp	229533.I1RS41
112431	229533.A0A1C3YJT1	pp	229533.I1RRK7
112432	229533.I1RD25	pp	229533.Q4I7L0
112433	229533.I1RD25	pp	229533.I1RZ94
112434	229533.I1RWR7	pp	229533.V6RTJ8
112435	229533.I1RWR7	pp	229533.I1RX46
112436	229533.I1RWR7	pp	229533.Q4HVQ9
112437	229533.I1RWR7	pp	229533.I1S0F7
112438	229533.I1RWR7	pp	229533.I1RY86
112439	229533.I1RWR7	pp	229533.I1RXD6
112440	229533.I1RWR7	pp	229533.I1RXD7
112441	229533.I1RWR7	pp	229533.I1S4S7
112442	229533.I1RWR7	pp	229533.I1RYR0
112443	229533.I1RWR7	pp	229533.I1S0W4
112444	229533.I1RWR7	pp	229533.I1RYI5
112445	229533.I1RWR7	pp	229533.I1S2R0
112446	229533.I1RWR7	pp	229533.I1S5R1
112447	229533.I1RWR7	pp	229533.I1RXF8
112448	229533.I1RWR7	pp	229533.I1RXC2
112449	229533.I1RWR7	pp	229533.I1S438
112450	229533.I1RWR7	pp	229533.I1S5R6
112451	229533.I1RWR7	pp	229533.I1S0L4
112452	229533.I1RWR7	pp	229533.I1RZ39
112453	229533.I1RWR7	pp	229533.I1RX95
112454	229533.I1RWR7	pp	229533.I1S5B0
112455	229533.I1RWR7	pp	229533.I1S4S4
112456	229533.I1RWR7	pp	229533.V6RMW6
112457	229533.I1RWR7	pp	229533.I1S4Z8
112458	229533.I1RWR7	pp	229533.V6R042
112459	229533.I1RWR7	pp	229533.I1S2H4
112460	229533.I1RWR7	pp	229533.I1S1B2
112461	229533.I1RWR7	pp	229533.I1S0F8
112462	229533.I1RWR7	pp	229533.I1S821
112463	229533.I1RWR7	pp	229533.V6R9Z0
112464	229533.I1RWR7	pp	229533.I1S9I5
112465	229533.I1RWR7	pp	229533.I1RZK5
112466	229533.I1RWR7	pp	229533.I1RZM8
112467	229533.I1RWR7	pp	229533.I1S437
112468	229533.I1RWR7	pp	229533.I1RZV3
112469	229533.I1RWR7	pp	229533.I1S2B8
112470	229533.I1RWR7	pp	229533.V6RCZ8
112471	229533.I1RWR7	pp	229533.I1S1C7
112472	229533.I1RWR7	pp	229533.I1S1W9
112473	229533.I1RWR7	pp	229533.Q4IJK2
112474	229533.I1RWR7	pp	229533.I1S0J1
112475	229533.I1RWR7	pp	229533.Q4I624
112476	229533.I1RWR7	pp	229533.I1S6K9
112477	229533.I1RWR7	pp	229533.Q4IPZ1
112478	229533.I1RWR7	pp	229533.I1S5X9
112479	229533.I1RWR7	pp	229533.I1RXC4
112480	229533.I1RT50	pp	229533.V6RIE5
112481	229533.I1RT50	pp	229533.I1RUJ5
112482	229533.I1RPM4	pp	229533.I1RWK3
112483	229533.I1RPM4	pp	229533.I1S2M6
112484	229533.I1RPM4	pp	229533.I1RQG4
112485	229533.I1RPM4	pp	229533.I1S8P5
112486	229533.I1RPM4	pp	229533.I1RPP3
112487	229533.I1RPM4	pp	229533.I1RRJ8
112488	229533.I1RPM4	pp	229533.I1RV91
112489	229533.I1RPM4	pp	229533.I1RTD5
112490	229533.I1RPM4	pp	229533.I1RT40
112491	229533.I1RPM4	pp	229533.I1S352
112492	229533.I1RPM4	pp	229533.I1RT04
112493	229533.I1RPM4	pp	229533.I1S2E9

112494 229533.I1RPM4 pp 229533.I1RZL6
112495 229533.I1RPM4 pp 229533.V6RUY9
112496 229533.I1RPM4 pp 229533.Q4IEV5
112497 229533.I1RPM4 pp 229533.I1S0F6
112498 229533.I1RPM4 pp 229533.I1RRZ0
112499 229533.I1RPM4 pp 229533.I1RRT2
112500 229533.I1RPM4 pp 229533.I1S8V3
112501 229533.I1RPM4 pp 229533.I1RS47
112502 229533.I1RPM4 pp 229533.I1S503
112503 229533.I1RPM4 pp 229533.I1RSA4
112504 229533.I1RPM4 pp 229533.I1RUG3
112505 229533.I1RPM4 pp 229533.I1RZZ9
112506 229533.I1RPM4 pp 229533.I1RWI5
112507 229533.I1RPM4 pp 229533.V6RMW6
112508 229533.I1RPM4 pp 229533.I1RSQ3
112509 229533.I1RPM4 pp 229533.V6R3X5
112510 229533.I1RPM4 pp 229533.I1RWN5
112511 229533.I1RPM4 pp 229533.I1RR73
112512 229533.I1RPM4 pp 229533.V6R042
112513 229533.I1RPM4 pp 229533.V6RIV9
112514 229533.I1RPM4 pp 229533.Q4HZB8
112515 229533.I1RPM4 pp 229533.I1RZ39
112516 229533.I1RPM4 pp 229533.I1RSI6
112517 229533.I1RPM4 pp 229533.I1RRV1
112518 229533.I1RPM4 pp 229533.I1RRL9
112519 229533.I1RPM4 pp 229533.O74638
112520 229533.I1RPM4 pp 229533.I1SA33
112521 229533.I1RPM4 pp 229533.I1S8V4
112522 229533.I1RPM4 pp 229533.I1RRT8
112523 229533.I1RPM4 pp 229533.I1RRE5
112524 229533.I1RPM4 pp 229533.V6RGN5
112525 229533.I1RPM4 pp 229533.I1S5R1
112526 229533.I1RPM4 pp 229533.I1RRI2
112527 229533.I1RPM4 pp 229533.I1RWQ8
112528 229533.I1RPM4 pp 229533.I1S0N4
112529 229533.I1RPM4 pp 229533.I1RWX5
112530 229533.I1RPM4 pp 229533.I1RZW8
112531 229533.I1RPM4 pp 229533.I1S0L4
112532 229533.I1RPM4 pp 229533.I1RUP9
112533 229533.I1RPM4 pp 229533.I1S2H4
112534 229533.I1RPM4 pp 229533.I1RS20
112535 229533.I1RPM4 pp 229533.I1RQS8
112536 229533.I1RPM4 pp 229533.I1S0J1
112537 229533.I1RPM4 pp 229533.I1S1C7
112538 229533.I1RPM4 pp 229533.I1RQ23
112539 229533.I1RPM4 pp 229533.I1RYW3
112540 229533.I1RPM4 pp 229533.I1RW46
112541 229533.I1RPM4 pp 229533.I1RTE9
112542 229533.I1RPM4 pp 229533.I1S0R2
112543 229533.I1RPM4 pp 229533.I1S0M6
112544 229533.I1RPM4 pp 229533.I1S8R3
112545 229533.I1RPM4 pp 229533.I1S6Y8
112546 229533.I1RPM4 pp 229533.I1RTH6
112547 229533.I1RPM4 pp 229533.I1RTA0
112548 229533.I1RPM4 pp 229533.I1RSK1
112549 229533.I1RPM4 pp 229533.I1S7S9
112550 229533.I1RPM4 pp 229533.I1RZS7
112551 229533.I1RPM4 pp 229533.I1RQ87
112552 229533.I1RPM4 pp 229533.I1RPP4
112553 229533.I1RPM4 pp 229533.I1S2B8
112554 229533.I1RPM4 pp 229533.I1RRM5
112555 229533.I1RPM4 pp 229533.I1RS17
112556 229533.I1RPM4 pp 229533.I1RY44
112557 229533.I1RPM4 pp 229533.Q4IJT5
112558 229533.I1RPM4 pp 229533.I1RWN6
112559 229533.I1RPM4 pp 229533.I1RQE7
112560 229533.I1RPM4 pp 229533.I1RT03
112561 229533.I1RPM4 pp 229533.I1RZE4
112562 229533.I1RPM4 pp 229533.I1RRN5
112563 229533.I1RPM4 pp 229533.I1RVX0
112564 229533.I1RPM4 pp 229533.I1S2E8
112565 229533.I1RPM4 pp 229533.I1RSQ7
112566 229533.I1RPM4 pp 229533.I1RQ26

112567 229533.I1RPM4 pp 229533.I1RZJ5
112568 229533.I1RPM4 pp 229533.V6R057
112569 229533.I1RPM4 pp 229533.V6QW22
112570 229533.I1RPM4 pp 229533.I1RW71
112571 229533.I1RPM4 pp 229533.I1S569
112572 229533.I1RPM4 pp 229533.Q4I624
112573 229533.I1RPM4 pp 229533.I1S130
112574 229533.I1RPM4 pp 229533.I1RWJ8
112575 229533.I1RPM4 pp 229533.I1S6F0
112576 229533.I1RPM4 pp 229533.I1RUQ2
112577 229533.I1RPM4 pp 229533.I1RR24
112578 229533.I1RPM4 pp 229533.I1RVQ0
112579 229533.I1RPM4 pp 229533.V6QUW4
112580 229533.I1RPM4 pp 229533.V6QYF0
112581 229533.I1RPM4 pp 229533.I1RXD7
112582 229533.I1RPM4 pp 229533.V6R4J1
112583 229533.I1RPM4 pp 229533.I1RW25
112584 229533.I1RPM4 pp 229533.I1S0R7
112585 229533.I1RPM4 pp 229533.I1RQC9
112586 229533.I1RPM4 pp 229533.V6R1K4
112587 229533.I1RPM4 pp 229533.I1RWD4
112588 229533.I1RPM4 pp 229533.I1S0T2
112589 229533.I1RPM4 pp 229533.I1RX25
112590 229533.I1RPM4 pp 229533.I1RX46
112591 229533.I1RPM4 pp 229533.I1S0D4
112592 229533.I1RPM4 pp 229533.I1S7A8
112593 229533.I1RPM4 pp 229533.I1RW07
112594 229533.I1RPM4 pp 229533.I1S0F5
112595 229533.I1RPM4 pp 229533.I1S1Z2
112596 229533.I1RPM4 pp 229533.I1S0L3
112597 229533.I1RPM4 pp 229533.V6RTJ8
112598 229533.I1RPM4 pp 229533.I1RQ31
112599 229533.I1RPM4 pp 229533.I1RRU9
112600 229533.I1RPM4 pp 229533.I1S0X6
112601 229533.I1RPM4 pp 229533.I1RXF0
112602 229533.I1RPM4 pp 229533.I1S5B0
112603 229533.I1RPM4 pp 229533.I1RZM8
112604 229533.I1RPM4 pp 229533.I1RTF4
112605 229533.I1RPM4 pp 229533.I1RR51
112606 229533.I1RPM4 pp 229533.Q4HVW2
112607 229533.I1RPM4 pp 229533.I1SA32
112608 229533.I1RPM4 pp 229533.V6RCG5
112609 229533.I1RPM4 pp 229533.I1RW99
112610 229533.I1RPM4 pp 229533.I1RY03
112611 229533.I1RPM4 pp 229533.I1RZH4
112612 229533.I1RPM4 pp 229533.I1RZK5
112613 229533.I1RPM4 pp 229533.V6RCZ8
112614 229533.I1RPM4 pp 229533.I1RTD9
112615 229533.I1RPM4 pp 229533.I1S1J7
112616 229533.I1RPM4 pp 229533.I1RS18
112617 229533.I1RPM4 pp 229533.Q4I8P5
112618 229533.I1RPM4 pp 229533.I1RQ81
112619 229533.I1RPM4 pp 229533.I1RQA2
112620 229533.I1RPM4 pp 229533.I1RU29
112621 229533.I1RPM4 pp 229533.I1RRY9
112622 229533.I1RPM4 pp 229533.Q4IEK8
112623 229533.I1RPM4 pp 229533.I1RPR9
112624 229533.I1RPM4 pp 229533.I1RSR3
112625 229533.I1RPM4 pp 229533.I1RZM1
112626 229533.I1RPM4 pp 229533.I1S419
112627 229533.I1RPM4 pp 229533.I1RZZ1
112628 229533.I1RPM4 pp 229533.Q4IJK2
112629 229533.I1RPM4 pp 229533.I1RQQ5
112630 229533.I1RPM4 pp 229533.Q4I283
112631 229533.I1RPM4 pp 229533.I1RSC6
112632 229533.I1RPM4 pp 229533.I1RQA3
112633 229533.I1RPM4 pp 229533.V6R227
112634 229533.I1RPM4 pp 229533.I1S504
112635 229533.I1RPM4 pp 229533.I1RWT6
112636 229533.I1RPM4 pp 229533.I1RWD7
112637 229533.I1RPM4 pp 229533.Q4I2J8
112638 229533.I1RPM4 pp 229533.I1RSI0
112639 229533.A0A098DAE0 pp 229533.I1S1J6

229533.A0A098DAE0	pp	229533.I1RKC6
229533.A0A098DAE0	pp	229533.I1RSD8
229533.A0A098DAE0	pp	229533.I1S2E8
229533.A0A098DAE0	pp	229533.I1S0Z9
229533.A0A098DAE0	pp	229533.I1RJZ6
229533.A0A098DAE0	pp	229533.I1RNK2
229533.A0A098DAE0	pp	229533.I1RZK6
229533.A0A098DAE0	pp	229533.I1RVZ0
229533.A0A098DAE0	pp	229533.I1RE00
229533.A0A098DAE0	pp	229533.I1RZZ1
229533.A0A098DAE0	pp	229533.A0A1C3YIJ3
229533.A0A098DAE0	pp	229533.I1RFJ9
229533.A0A098DAE0	pp	229533.I1RSS4
229533.A0A098DAE0	pp	229533.I1RSI0
229533.A0A098DAE0	pp	229533.A0A098DEC2
229533.A0A098DAE0	pp	229533.Q4HWE2
229533.A0A098DAE0	pp	229533.I1RZE5
229533.A0A098DAE0	pp	229533.A0A0E0SDE1
229533.A0A098DAE0	pp	229533.I1S0J1
229533.A0A098DAE0	pp	229533.I1RA48
229533.A0A098DAE0	pp	229533.I1RVX0
229533.A0A098DAE0	pp	229533.I1RYB1
229533.A0A098DAE0	pp	229533.I1RT03
229533.A0A098DAE0	pp	229533.I1RC72
229533.A0A098DAE0	pp	229533.I1RE37
229533.A0A098DAE0	pp	229533.A0A0E0S621
229533.A0A098DAE0	pp	229533.I1RM25
229533.A0A098DAE0	pp	229533.I1RNU6
229533.A0A098DAE0	pp	229533.I1R9V7
229533.A0A098DAE0	pp	229533.I1S1X9
229533.A0A098DAE0	pp	229533.I1S296
229533.A0A098DAE0	pp	229533.I1RY63
229533.A0A098DAE0	pp	229533.I1RWB5
229533.A0A098DAE0	pp	229533.I1RRM7
229533.A0A098DAE0	pp	229533.Q4HZ68
229533.A0A098DAE0	pp	229533.I1RQ81
229533.A0A098DAE0	pp	229533.I1RK95
229533.A0A098DAE0	pp	229533.I1RF08
229533.A0A098DAE0	pp	229533.I1RDG0
229533.A0A098DAE0	pp	229533.A0A1C3YLK9
229533.A0A098DAE0	pp	229533.I1RN99
229533.A0A098DAE0	pp	229533.I1RAU7
229533.A0A098DAE0	pp	229533.I1RAN5
229533.A0A098DAE0	pp	229533.I1S558
229533.A0A098DAE0	pp	229533.Q4IJ56
229533.A0A098DAE0	pp	229533.I1S1C7
229533.A0A098DAE0	pp	229533.I1S0M9
229533.A0A098DAE0	pp	229533.I1R9X7
229533.A0A098DAE0	pp	229533.I1RB27
229533.A0A098DAE0	pp	229533.I1RCN5
229533.A0A098DAE0	pp	229533.I1RQA2
229533.A0A098DAE0	pp	229533.I1RJV3
229533.A0A098DAE0	pp	229533.I1RRI2
229533.A0A098DAE0	pp	229533.I1RCL3
229533.A0A098DAE0	pp	229533.I1RBZ3
229533.A0A098DAE0	pp	229533.I1RTH6
229533.A0A098DAE0	pp	229533.I1RYI7
229533.A0A098DAE0	pp	229533.A0A098DRR8
229533.A0A098DAE0	pp	229533.I1RBL7
229533.A0A098DAE0	pp	229533.I1RAW0
229533.A0A098DAE0	pp	229533.I1RSR3
229533.A0A098DAE0	pp	229533.I1RX64
229533.A0A098DAE0	pp	229533.I1RRY9
229533.A0A098DAE0	pp	229533.I1RMN6
229533.A0A098DAE0	pp	229533.I1RPP7
229533.A0A098DAE0	pp	229533.I1RGC2
229533.A0A098DAE0	pp	229533.I1RFU8
229533.A0A098DAE0	pp	229533.I1RWN6
229533.A0A098DAE0	pp	229533.I1S193
229533.A0A098DAE0	pp	229533.I1RTE9
229533.A0A098DAE0	pp	229533.Q4IBS2
229533.A0A098DAE0	pp	229533.I1RSR8
229533.A0A098DAE0	pp	229533.I1RK83

112713	229533.A0A098DAE0	pp	229533.I1RY03
112714	229533.A0A098DAE0	pp	229533.I1S2C0
112715	229533.A0A098DAE0	pp	229533.A0A1C3YN89
112716	229533.A0A098DAE0	pp	229533.V6R042
112717	229533.A0A098DAE0	pp	229533.A0A0E0RQ72
112718	229533.A0A098DAE0	pp	229533.I1RD22
112719	229533.A0A098DAE0	pp	229533.I1RAU5
112720	229533.A0A098DAE0	pp	229533.I1RC27
112721	229533.A0A098DAE0	pp	229533.I1RZK5
112722	229533.A0A098DAE0	pp	229533.Q4IR08
112723	229533.A0A098DAE0	pp	229533.I1RAC1
112724	229533.A0A098DAE0	pp	229533.I1RBD2
112725	229533.A0A098DAE0	pp	229533.V6RMW6
112726	229533.A0A098DAE0	pp	229533.I1RG56
112727	229533.A0A098DAE0	pp	229533.I1RG11
112728	229533.A0A098DAE0	pp	229533.A0A1C3YMB9
112729	229533.A0A098DAE0	pp	229533.I1RPY1
112730	229533.A0A098DAE0	pp	229533.A0A1C3YJ29
112731	229533.A0A098DAE0	pp	229533.I1S0N4
112732	229533.A0A098DAE0	pp	229533.V6RGN5
112733	229533.A0A098DAE0	pp	229533.Q4IFI0
112734	229533.A0A098DAE0	pp	229533.I1RWN5
112735	229533.A0A098DAE0	pp	229533.A0A0E0SAC4
112736	229533.A0A098DAE0	pp	229533.I1S2B8
112737	229533.A0A098DAE0	pp	229533.V6RIS2
112738	229533.A0A098DAE0	pp	229533.I1RNQ5
112739	229533.A0A098DAE0	pp	229533.I1S044
112740	229533.A0A098DAE0	pp	229533.I1RM96
112741	229533.A0A098DAE0	pp	229533.I1RCR8
112742	229533.A0A098DAE0	pp	229533.Q4IN63
112743	229533.A0A098DAE0	pp	229533.A0A098DXE2
112744	229533.A0A098DAE0	pp	229533.I1RW25
112745	229533.A0A098DAE0	pp	229533.I1RAC2
112746	229533.A0A098DAE0	pp	229533.Q4IEK8
112747	229533.A0A098DAE0	pp	229533.I1RY44
112748	229533.A0A098DAE0	pp	229533.I1RYH6
112749	229533.A0A098DAE0	pp	229533.I1S504
112750	229533.A0A098DAE0	pp	229533.A0A1C3YJR3
112751	229533.A0A098DAE0	pp	229533.I1RMC0
112752	229533.A0A098DAE0	pp	229533.A0A1C3YJG0
112753	229533.A0A098DAE0	pp	229533.I1RQ31
112754	229533.A0A098DAE0	pp	229533.I1RZF6
112755	229533.A0A098DAE0	pp	229533.A0A0E0RUI4
112756	229533.A0A098DAE0	pp	229533.I1RYT5
112757	229533.A0A098DAE0	pp	229533.I1RNH6
112758	229533.A0A098DAE0	pp	229533.I1RWE1
112759	229533.A0A098DAE0	pp	229533.I1S021
112760	229533.A0A098DAE0	pp	229533.A0A098DVE3
112761	229533.A0A098DAE0	pp	229533.I1RFS3
112762	229533.A0A098DAE0	pp	229533.I1RRR7
112763	229533.A0A098DAE0	pp	229533.Q4I662
112764	229533.A0A098DAE0	pp	229533.I1RYB9
112765	229533.A0A098DAE0	pp	229533.V6R227
112766	229533.A0A098DAE0	pp	229533.Q4I327
112767	229533.A0A098DAE0	pp	229533.I1RKL0
112768	229533.A0A098DAE0	pp	229533.I1RSI6
112769	229533.A0A098DAE0	pp	229533.I1RG51
112770	229533.A0A098DAE0	pp	229533.I1RWJ4
112771	229533.A0A098DAE0	pp	229533.I1S060
112772	229533.A0A098DAE0	pp	229533.I1RA46
112773	229533.A0A098DAE0	pp	229533.I1RBG9
112774	229533.A0A098DAE0	pp	229533.V6QYF0
112775	229533.A0A098DAE0	pp	229533.I1RX24
112776	229533.A0A098DAE0	pp	229533.I1RFT1
112777	229533.A0A098DAE0	pp	229533.I1RZS6
112778	229533.A0A098DAE0	pp	229533.Q4I830
112779	229533.A0A098DAE0	pp	229533.A0A1C3YKM9
112780	229533.A0A098DAE0	pp	229533.I1R9Q0
112781	229533.A0A098DAE0	pp	229533.I1RM06
112782	229533.A0A098DAE0	pp	229533.I1RKL3
112783	229533.A0A098DAE0	pp	229533.I1RPU0
112784	229533.A0A098DAE0	pp	229533.I1RKJ6
112785	229533.A0A098DAE0	pp	229533.I1RT80

112786	229533.A0A098DAE0	pp	229533.I1RNV6
112787	229533.A0A098DAE0	pp	229533.I1RW71
112788	229533.A0A098DAE0	pp	229533.I1RR73
112789	229533.A0A098DAE0	pp	229533.I1RCL4
112790	229533.A0A098DAE0	pp	229533.I1RRJ8
112791	229533.A0A098DAE0	pp	229533.I1RZV6
112792	229533.A0A098DAE0	pp	229533.A0A0E0SFM9
112793	229533.A0A098DAE0	pp	229533.I1RM67
112794	229533.A0A098DAE0	pp	229533.A0A1C3YIN1
112795	229533.A0A098DAE0	pp	229533.I1RND6
112796	229533.A0A098DAE0	pp	229533.I1RSQ7
112797	229533.A0A098DAE0	pp	229533.V6R3X5
112798	229533.A0A098DAE0	pp	229533.A0A098E1Q1
112799	229533.A0A098DAE0	pp	229533.O74638
112800	229533.A0A098DAE0	pp	229533.I1RQJ5
112801	229533.A0A098DAE0	pp	229533.I1RD64
112802	229533.A0A098DAE0	pp	229533.A0A0E0SA11
112803	229533.A0A098DAE0	pp	229533.I1RDA0
112804	229533.A0A098DAE0	pp	229533.I1RS81
112805	229533.A0A098DAE0	pp	229533.I1RMK3
112806	229533.A0A098DAE0	pp	229533.A0A0E0RSD7
112807	229533.A0A098DAE0	pp	229533.I1S9U2
112808	229533.A0A098DAE0	pp	229533.I1RPP3
112809	229533.A0A098DAE0	pp	229533.I1RPW9
112810	229533.A0A098DAE0	pp	229533.A0A098DVS9
112811	229533.A0A098DAE0	pp	229533.I1RWD4
112812	229533.A0A098DAE0	pp	229533.I1RNH1
112813	229533.A0A098DAE0	pp	229533.Q4I5R9
112814	229533.A0A098DAE0	pp	229533.A0A0E0S6M0
112815	229533.A0A098DAE0	pp	229533.I1S6F0
112816	229533.A0A098DAE0	pp	229533.Q4IF76
112817	229533.A0A098DAE0	pp	229533.I1RKQ5
112818	229533.A0A098DAE0	pp	229533.I1RQM6
112819	229533.A0A098DAE0	pp	229533.I1RRQ3
112820	229533.A0A098DAE0	pp	229533.I1S503
112821	229533.A0A098DAE0	pp	229533.I1RFG0
112822	229533.A0A098DAE0	pp	229533.A0A1C3YHX8
112823	229533.A0A098DAE0	pp	229533.I1RU69
112824	229533.A0A098DAE0	pp	229533.I1RLV9
112825	229533.A0A098DAE0	pp	229533.A0A1C3YIY6
112826	229533.A0A098DAE0	pp	229533.I1S8E2
112827	229533.A0A098DAE0	pp	229533.A0A1C3YN63
112828	229533.A0A098DAE0	pp	229533.I1RD56
112829	229533.A0A098DAE0	pp	229533.Q4IAS1
112830	229533.A0A098DAE0	pp	229533.I1RJL4
112831	229533.A0A098DAE0	pp	229533.Q4HZ42
112832	229533.A0A098DAE0	pp	229533.Q4IPI1
112833	229533.A0A098DAE0	pp	229533.Q4IJH1
112834	229533.A0A098DAE0	pp	229533.I1RTF4
112835	229533.A0A098DAE0	pp	229533.I1RT33
112836	229533.A0A098DAE0	pp	229533.I1RRH5
112837	229533.A0A098DAE0	pp	229533.A0A1C3YMW0
112838	229533.A0A098DAE0	pp	229533.A0A1C3YHW7
112839	229533.A0A098DAE0	pp	229533.I1RS47
112840	229533.A0A098DAE0	pp	229533.I1RZ39
112841	229533.A0A098DAE0	pp	229533.I1RRT2
112842	229533.A0A098DAE0	pp	229533.I1RT04
112843	229533.A0A098DAE0	pp	229533.I1RQ65
112844	229533.A0A098DAE0	pp	229533.I1RX46
112845	229533.A0A098DAE0	pp	229533.I1RWH5
112846	229533.A0A098DAE0	pp	229533.I1RBC7
112847	229533.A0A098DAE0	pp	229533.V6R283
112848	229533.A0A098DAE0	pp	229533.I1S0R7
112849	229533.A0A098DAE0	pp	229533.I1RSL9
112850	229533.A0A098DAE0	pp	229533.A0A1C3YLF5
112851	229533.A0A098DAE0	pp	229533.I1RFN1
112852	229533.A0A098DAE0	pp	229533.A0A0E0SFN0
112853	229533.A0A098DAE0	pp	229533.I1RXD7
112854	229533.A0A098DAE0	pp	229533.I1RC82
112855	229533.A0A098DAE0	pp	229533.I1RAX6
112856	229533.A0A098DAE0	pp	229533.I1RGA4
112857	229533.A0A098DAE0	pp	229533.A0A1C3YL39
112858	229533.A0A098DAE0	pp	229533.I1RC03

112859 229533.A0A098DAE0 pp 229533.A0A1C3YKK5
112860 229533.A0A098DAE0 pp 229533.I1RAW7
112861 229533.A0A098DAE0 pp 229533.I1RFW5
112862 229533.A0A098DAE0 pp 229533.I1S2E9
112863 229533.A0A098DAE0 pp 229533.A0A098DE18
112864 229533.A0A098DAE0 pp 229533.A0A0E0S7B3
112865 229533.A0A098DAE0 pp 229533.I1RAF8
112866 229533.A0A098DAE0 pp 229533.I1RT98
112867 229533.A0A098DAE0 pp 229533.A0A1C3YMK1
112868 229533.A0A098DAE0 pp 229533.I1RTD5
112869 229533.A0A098DAE0 pp 229533.Q4IR18
112870 229533.I1RT14 pp 229533.V6RGI1
112871 229533.I1RT14 pp 229533.I1S2A8
112872 229533.I1RT14 pp 229533.V6RIE5
112873 229533.I1RT14 pp 229533.I1RUJ5
112874 229533.I1RT14 pp 229533.I1RT50
112875 229533.I1RLR4 pp 229533.I1RNG4
112876 229533.I1RJR2 pp 229533.I1RZ92
112877 229533.I1RJR2 pp 229533.I1RPF2
112878 229533.I1RJR2 pp 229533.I1S2M5
112879 229533.I1RJR2 pp 229533.I1S1Q3
112880 229533.I1RJR2 pp 229533.I1SAK7
112881 229533.I1RJR2 pp 229533.I1RXG2
112882 229533.I1RJR2 pp 229533.I1S130
112883 229533.I1RJR2 pp 229533.V6RPN0
112884 229533.I1RJR2 pp 229533.I1RZZ3
112885 229533.I1RJR2 pp 229533.V6RG22
112886 229533.I1RJR2 pp 229533.V6RR38
112887 229533.I1RJR2 pp 229533.I1RRQ1
112888 229533.I1RJR2 pp 229533.I1RQ76
112889 229533.I1RJR2 pp 229533.I1RT23
112890 229533.I1RJR2 pp 229533.I1RMA2
112891 229533.I1RJR2 pp 229533.I1RY81
112892 229533.I1RJR2 pp 229533.I1RXX1
112893 229533.I1RJR2 pp 229533.I1RQ48
112894 229533.I1RJR2 pp 229533.I1RXF8
112895 229533.I1RJR2 pp 229533.I1RNT1
112896 229533.I1RJR2 pp 229533.I1RY34
112897 229533.I1RJR2 pp 229533.I1S2P4
112898 229533.I1RJR2 pp 229533.I1RNK1
112899 229533.I1RJR2 pp 229533.I1RRA5
112900 229533.I1RJR2 pp 229533.I1RWF4
112901 229533.I1RJR2 pp 229533.I1RY35
112902 229533.I1RJR2 pp 229533.I1S1W6
112903 229533.I1RJR2 pp 229533.I1RL13
112904 229533.I1RJR2 pp 229533.Q4IPH4
112905 229533.I1RJR2 pp 229533.I1RR90
112906 229533.I1RJR2 pp 229533.I1SAS3
112907 229533.I1RJR2 pp 229533.I1SAS4
112908 229533.I1S0N4 pp 229533.V6RIV9
112909 229533.I1S0N4 pp 229533.I1S193
112910 229533.I1S0N4 pp 229533.I1S504
112911 229533.I1S0N4 pp 229533.I1S1W4
112912 229533.I1S0N4 pp 229533.I1S5B0
112913 229533.I1S0N4 pp 229533.Q4IEK8
112914 229533.I1S0N4 pp 229533.Q4IJ56
112915 229533.I1S0N4 pp 229533.Q4IJK2
112916 229533.I1S0N4 pp 229533.Q4IJM4
112917 229533.I1S0N4 pp 229533.Q4ICA8
112918 229533.I1S0N4 pp 229533.V6R042
112919 229533.I1S0N4 pp 229533.I1S1J7
112920 229533.I1S0N4 pp 229533.V6RTJ8
112921 229533.I1S0N4 pp 229533.Q4IAA0
112922 229533.I1S0N4 pp 229533.V6RMW6
112923 229533.I1S0N4 pp 229533.I1S2E8
112924 229533.I1S0N4 pp 229533.I1S1C7
112925 229533.I1S0N4 pp 229533.I1S0T2
112926 229533.I1S0N4 pp 229533.I1S2C0
112927 229533.I1S0N4 pp 229533.Q4IJT5
112928 229533.I1S0N4 pp 229533.I1S503
112929 229533.I1S0N4 pp 229533.Q4I2J8
112930 229533.I1S0N4 pp 229533.Q4IJH1
112931 229533.I1S0N4 pp 229533.I1S0R7

112932 229533.I1S0N4 pp 229533.Q4HXF0
112933 229533.I1S0N4 pp 229533.I1S2H4
112934 229533.I1S0N4 pp 229533.Q4I7L0
112935 229533.I1S0N4 pp 229533.V6R3X5
112936 229533.I1S0N4 pp 229533.I1S2B8
112937 229533.I1S0N4 pp 229533.Q4IF76
112938 229533.I1S0N4 pp 229533.Q4IFI0
112939 229533.I1S0N4 pp 229533.I1S2E9
112940 229533.I1S0N4 pp 229533.I1S0X6
112941 229533.I1S0N4 pp 229533.I1S2G7
112942 229533.I1S0N4 pp 229533.V6QUW4
112943 229533.I1S0N4 pp 229533.I1S8P5
112944 229533.A0A1C3YJT0 pp 229533.I1RBJ0
112945 229533.A0A1C3YJT0 pp 229533.Q4HTT2
112946 229533.A0A1C3YJT0 pp 229533.I1RYV3
112947 229533.A0A1C3YJT0 pp 229533.I1RQF7
112948 229533.A0A1C3YJT0 pp 229533.I1RQE5
112949 229533.A0A1C3YJT0 pp 229533.I1RFX9
112950 229533.I1RT04 pp 229533.I1RTA0
112951 229533.I1RT04 pp 229533.I1RZS7
112952 229533.I1RT04 pp 229533.I1RW46
112953 229533.I1RT04 pp 229533.I1RWB5
112954 229533.I1RT04 pp 229533.I1RUG3
112955 229533.I1RT04 pp 229533.I1S0J1
112956 229533.I1RT04 pp 229533.Q4IN63
112957 229533.I1RT04 pp 229533.I1RWN5
112958 229533.I1RT04 pp 229533.Q4HZB8
112959 229533.I1RT04 pp 229533.Q4I327
112960 229533.I1RT04 pp 229533.V6RIV9
112961 229533.I1RT04 pp 229533.V6RMW6
112962 229533.I1RT04 pp 229533.I1RY44
112963 229533.I1RT04 pp 229533.V6R042
112964 229533.I1RT04 pp 229533.I1SA33
112965 229533.I1RT04 pp 229533.V6RVG3
112966 229533.I1RT04 pp 229533.I1RWX5
112967 229533.I1RT04 pp 229533.V6RY33
112968 229533.I1RT04 pp 229533.I1RT47
112969 229533.I1RT04 pp 229533.I1S8V4
112970 229533.I1RT04 pp 229533.I1RZW8
112971 229533.I1RT04 pp 229533.V6RIS2
112972 229533.I1RT04 pp 229533.I1S1C7
112973 229533.I1RT04 pp 229533.V6RGN5
112974 229533.I1RT04 pp 229533.I1S8R3
112975 229533.I1RT04 pp 229533.I1RTH6
112976 229533.I1RT04 pp 229533.I1RYW3
112977 229533.I1RT04 pp 229533.I1RTD7
112978 229533.I1RT04 pp 229533.I1S2C0
112979 229533.I1RT04 pp 229533.I1S0N4
112980 229533.I1RT04 pp 229533.I1RWI5
112981 229533.I1RT04 pp 229533.I1S569
112982 229533.I1RT04 pp 229533.I1S2E8
112983 229533.I1RT04 pp 229533.V6R057
112984 229533.I1RT04 pp 229533.I1RWN6
112985 229533.I1RT04 pp 229533.Q4I624
112986 229533.I1RT04 pp 229533.I1RZZ9
112987 229533.I1RT04 pp 229533.I1RVX0
112988 229533.I1RT04 pp 229533.I1RV91
112989 229533.I1RT04 pp 229533.I1S8V3
112990 229533.I1RT04 pp 229533.I1RT40
112991 229533.I1RT04 pp 229533.I1S503
112992 229533.I1RT04 pp 229533.I1RZ39
112993 229533.I1RT04 pp 229533.I1RTD5
112994 229533.I1RT04 pp 229533.I1S8E2
112995 229533.I1RT04 pp 229533.I1RWK3
112996 229533.I1RT04 pp 229533.V6R283
112997 229533.I1RT04 pp 229533.V6R3X5
112998 229533.I1RT04 pp 229533.I1RZV6
112999 229533.I1RT04 pp 229533.I1S352
113000 229533.I1RT04 pp 229533.V6RUY9
113001 229533.I1RT04 pp 229533.I1RWF7
113002 229533.I1RT04 pp 229533.I1S5R1
113003 229533.I1RT04 pp 229533.I1RXF2
113004 229533.I1RT04 pp 229533.I1S8P5

113005 229533.I1RT04 pp 229533.I1S5S2
113006 229533.I1RT04 pp 229533.I1S2E9
113007 229533.I1RT04 pp 229533.O74638
113008 229533.I1RT04 pp 229533.I1S0F6
113009 229533.I1RT04 pp 229533.V6RCZ8
113010 229533.I1RT04 pp 229533.I1S2H4
113011 229533.I1RT04 pp 229533.I1RWL2
113012 229533.I1RT04 pp 229533.Q4ICA8
113013 229533.I1RT04 pp 229533.I1RZM1
113014 229533.I1RT04 pp 229533.I1RZF6
113015 229533.I1RT04 pp 229533.V6RCG5
113016 229533.I1RT04 pp 229533.I1S0L4
113017 229533.I1RT04 pp 229533.I1S504
113018 229533.I1RT04 pp 229533.V6QUE6
113019 229533.I1RT04 pp 229533.I1S6Y8
113020 229533.I1RT04 pp 229533.I1RZ11
113021 229533.I1RT04 pp 229533.I1S0M6
113022 229533.I1RT04 pp 229533.Q4I283
113023 229533.I1RT04 pp 229533.I1RZS6
113024 229533.I1RT04 pp 229533.V6R227
113025 229533.I1RT04 pp 229533.I1S044
113026 229533.I1RT04 pp 229533.I1S2B8
113027 229533.I1RT04 pp 229533.I1S5B0
113028 229533.I1RT04 pp 229533.I1S7S9
113029 229533.I1RT04 pp 229533.I1RW99
113030 229533.I1RT04 pp 229533.I1RXF0
113031 229533.I1RT04 pp 229533.I1SA32
113032 229533.I1RT04 pp 229533.I1RWT6
113033 229533.I1RT04 pp 229533.I1RZM8
113034 229533.I1RT04 pp 229533.I1RY03
113035 229533.I1RT04 pp 229533.I1S0M9
113036 229533.I1RT04 pp 229533.Q4I8P5
113037 229533.I1RT04 pp 229533.I1RZB5
113038 229533.I1RT04 pp 229533.I1RZK5
113039 229533.I1RT04 pp 229533.I1RWX8
113040 229533.I1RT04 pp 229533.I1S7M3
113041 229533.I1RT04 pp 229533.I1S419
113042 229533.I1RT04 pp 229533.I1RWJ3
113043 229533.I1RT04 pp 229533.I1S021
113044 229533.I1RT04 pp 229533.I1RW71
113045 229533.I1RT04 pp 229533.I1S1J7
113046 229533.I1RT04 pp 229533.V6QW22
113047 229533.I1RT04 pp 229533.I1S7M2
113048 229533.I1RT04 pp 229533.I1RU29
113049 229533.I1RT04 pp 229533.V6R4J1
113050 229533.I1RT04 pp 229533.Q4IJK2
113051 229533.I1RT04 pp 229533.I1RUQ2
113052 229533.I1RT04 pp 229533.I1RXD7
113053 229533.I1RT04 pp 229533.I1RZK6
113054 229533.I1RT04 pp 229533.I1RZZ1
113055 229533.I1RT04 pp 229533.I1S0T2
113056 229533.I1RT04 pp 229533.I1RVQ0
113057 229533.I1RT04 pp 229533.I1RX46
113058 229533.I1RT04 pp 229533.I1RVZ0
113059 229533.I1RT04 pp 229533.I1RT33
113060 229533.I1RT04 pp 229533.V6R1K4
113061 229533.I1RT04 pp 229533.I1S540
113062 229533.I1RT04 pp 229533.I1S0F5
113063 229533.I1RT04 pp 229533.Q4I2J8
113064 229533.I1RT04 pp 229533.V6RTJ8
113065 229533.I1RT04 pp 229533.I1S7A8
113066 229533.I1RT04 pp 229533.I1S0L3
113067 229533.I1RT04 pp 229533.I1RZE5
113068 229533.I1RT04 pp 229533.I1S0X6
113069 229533.I1RT04 pp 229533.I1RWD7
113070 229533.I1RT04 pp 229533.I1RTF4
113071 229533.I1RT04 pp 229533.I1RWH5
113072 229533.I1RT04 pp 229533.I1RW07
113073 229533.I1RT04 pp 229533.I1S6U8
113074 229533.I1RT04 pp 229533.I1RT80
113075 229533.I1RT04 pp 229533.Q4IJT5
113076 229533.I1RT04 pp 229533.I1RZJ5
113077 229533.I1RT04 pp 229533.I1RWJ8

113078	229533.I1RT04	pp	229533.I1RX81
113079	229533.I1RT04	pp	229533.V6QUW4
113080	229533.I1RT04	pp	229533.I1S9U2
113081	229533.I1RT04	pp	229533.I1RX25
113082	229533.I1RT04	pp	229533.I1RW25
113083	229533.I1RPE0	pp	229533.I1S4S7
113084	229533.I1RPE0	pp	229533.I1RTA1
113085	229533.I1RPE0	pp	229533.I1RPK0
113086	229533.I1RPE0	pp	229533.I1RXD6
113087	229533.I1RPE0	pp	229533.I1RVZ2
113088	229533.I1RPE0	pp	229533.I1RZV3
113089	229533.I1RPE0	pp	229533.I1S0J1
113090	229533.I1RPE0	pp	229533.I1RVF8
113091	229533.I1RPE0	pp	229533.I1RTD5
113092	229533.I1RPE0	pp	229533.I1RXF8
113093	229533.I1RPE0	pp	229533.I1RXC4
113094	229533.I1RPE0	pp	229533.I1RPH6
113095	229533.I1RPE0	pp	229533.I1S1W9
113096	229533.I1RPE0	pp	229533.I1S2R0
113097	229533.I1RPE0	pp	229533.I1S0W4
113098	229533.I1RPE0	pp	229533.I1RX95
113099	229533.I1RPE0	pp	229533.I1RZM8
113100	229533.I1RPE0	pp	229533.I1RSF1
113101	229533.I1RPE0	pp	229533.I1RUG3
113102	229533.I1RPE0	pp	229533.I1S0F7
113103	229533.I1RPE0	pp	229533.I1RYR0
113104	229533.I1RPE0	pp	229533.I1RSA5
113105	229533.I1RPE0	pp	229533.I1RT40
113106	229533.I1RPE0	pp	229533.I1RYI5
113107	229533.I1RPE0	pp	229533.I1RQ85
113108	229533.I1RPE0	pp	229533.I1RPP3
113109	229533.I1RPE0	pp	229533.I1RTB3
113110	229533.I1RPE0	pp	229533.I1RXD7
113111	229533.I1RPE0	pp	229533.I1RSQ7
113112	229533.I1RPE0	pp	229533.I1RRK3
113113	229533.I1RPE0	pp	229533.V6R042
113114	229533.I1RPE0	pp	229533.I1RPU2
113115	229533.I1RPE0	pp	229533.I1RSQ3
113116	229533.I1RPE0	pp	229533.V6RTJ8
113117	229533.I1RPE0	pp	229533.I1RVZ7
113118	229533.I1RPE0	pp	229533.V6RMW6
113119	229533.I1RPE0	pp	229533.I1RY86
113120	229533.I1RPE0	pp	229533.I1RQM7
113121	229533.I1RPE0	pp	229533.I1RPY3
113122	229533.I1RPE0	pp	229533.I1S5B0
113123	229533.I1RPE0	pp	229533.I1RVX0
113124	229533.I1RPE0	pp	229533.I1RRM3
113125	229533.I1RPE0	pp	229533.I1RZB2
113126	229533.I1RPE0	pp	229533.I1RQD2
113127	229533.I1RPE0	pp	229533.V6RCZ8
113128	229533.I1RPE0	pp	229533.I1S6K9
113129	229533.I1RPE0	pp	229533.I1S5R6
113130	229533.I1RPE0	pp	229533.I1RT03
113131	229533.I1RPE0	pp	229533.I1RQE7
113132	229533.I1RPE0	pp	229533.I1S821
113133	229533.I1RPE0	pp	229533.I1S9I5
113134	229533.I1RPE0	pp	229533.Q4IFI0
113135	229533.I1RPE0	pp	229533.I1RPV3
113136	229533.I1RPE0	pp	229533.I1RRN2
113137	229533.I1RPE0	pp	229533.I1RX46
113138	229533.I1RPE0	pp	229533.I1RW61
113139	229533.I1RPE0	pp	229533.I1RSZ7
113140	229533.I1RPE0	pp	229533.I1RSX0
113141	229533.I1RPE0	pp	229533.I1S2B8
113142	229533.I1RPE0	pp	229533.Q4IJK2
113143	229533.I1RPE0	pp	229533.I1S4Z8
113144	229533.I1RPE0	pp	229533.Q4HVQ9
113145	229533.I1RPE0	pp	229533.I1RQ33
113146	229533.I1RPE0	pp	229533.I1S1B2
113147	229533.I1RPE0	pp	229533.I1RUQ3
113148	229533.I1RPE0	pp	229533.I1RRS8
113149	229533.I1RPE0	pp	229533.I1RUF7
113150	229533.I1RPE0	pp	229533.I1S437

113151	229533.I1RPE0	pp	229533.I1S438
113152	229533.I1RPE0	pp	229533.Q4I624
113153	229533.I1RPE0	pp	229533.I1S4S4
113154	229533.I1RPE0	pp	229533.I1RRV3
113155	229533.I1RPE0	pp	229533.I1S1C7
113156	229533.I1RPE0	pp	229533.I1RSQ4
113157	229533.I1RPE0	pp	229533.V6R9Z0
113158	229533.I1RPE0	pp	229533.I1RWR7
113159	229533.I1RPE0	pp	229533.I1S0L4
113160	229533.I1RPE0	pp	229533.I1RZ39
113161	229533.I1RPE0	pp	229533.I1RZK5
113162	229533.I1RPE0	pp	229533.I1RRI1
113163	229533.I1RPE0	pp	229533.I1RR24
113164	229533.I1RPE0	pp	229533.I1RRY9
113165	229533.I1RPE0	pp	229533.I1S0F8
113166	229533.I1RPE0	pp	229533.I1RXC2
113167	229533.I1RPE0	pp	229533.I1S5R1
113168	229533.I1RPE0	pp	229533.I1RVQ3
113169	229533.I1RS20	pp	229533.I1RSV2
113170	229533.I1RS20	pp	229533.I1RWK3
113171	229533.I1RS20	pp	229533.I1RS47
113172	229533.I1RS20	pp	229533.I1S8P5
113173	229533.I1RS20	pp	229533.Q4HXF0
113174	229533.I1RS20	pp	229533.I1RTD5
113175	229533.I1RS20	pp	229533.I1RT40
113176	229533.I1RS20	pp	229533.I1S503
113177	229533.I1RS20	pp	229533.V6RGN5
113178	229533.I1RS20	pp	229533.I1S2E9
113179	229533.I1RS20	pp	229533.I1S0N4
113180	229533.I1RS20	pp	229533.I1RT04
113181	229533.I1RS20	pp	229533.I1S2B8
113182	229533.I1RS20	pp	229533.I1RX24
113183	229533.I1RS20	pp	229533.I1S0L4
113184	229533.I1RS20	pp	229533.I1RWI5
113185	229533.I1RS20	pp	229533.I1RT03
113186	229533.I1RS20	pp	229533.I1S2E8
113187	229533.I1RS20	pp	229533.I1RZS7
113188	229533.I1RS20	pp	229533.I1RZZ9
113189	229533.I1RS20	pp	229533.I1RWN5
113190	229533.I1RS20	pp	229533.I1S1X9
113191	229533.I1RS20	pp	229533.I1RTA0
113192	229533.I1RS20	pp	229533.V6R3X5
113193	229533.I1RS20	pp	229533.I1RTD7
113194	229533.I1RS20	pp	229533.I1RZ39
113195	229533.I1RS20	pp	229533.I1RYW3
113196	229533.I1RS20	pp	229533.V6RIV9
113197	229533.I1RS20	pp	229533.I1RUG3
113198	229533.I1RS20	pp	229533.I1RY44
113199	229533.I1RS20	pp	229533.I1S1C7
113200	229533.I1RS20	pp	229533.I1RW71
113201	229533.I1RS20	pp	229533.I1RZW8
113202	229533.I1RS20	pp	229533.I1S9Y1
113203	229533.I1RS20	pp	229533.I1RSQ3
113204	229533.I1RS20	pp	229533.V6QW22
113205	229533.I1RS20	pp	229533.V6RMW6
113206	229533.I1RS20	pp	229533.I1RWX5
113207	229533.I1RS20	pp	229533.V6R042
113208	229533.I1RS20	pp	229533.I1RVQ0
113209	229533.I1RS20	pp	229533.I1RTH6
113210	229533.I1RS20	pp	229533.I1RSQ7
113211	229533.I1RS20	pp	229533.I1S0L3
113212	229533.I1RS20	pp	229533.I1S2C0
113213	229533.I1RS20	pp	229533.I1RSM9
113214	229533.I1RS20	pp	229533.I1RW25
113215	229533.I1RS20	pp	229533.I1S0R7
113216	229533.I1RS20	pp	229533.I1RX46
113217	229533.I1RS20	pp	229533.I1RWJ8
113218	229533.I1RS20	pp	229533.I1RWN6
113219	229533.I1RS20	pp	229533.V6RTJ8
113220	229533.I1RS20	pp	229533.I1S504
113221	229533.I1RS20	pp	229533.I1RT35
113222	229533.I1RS20	pp	229533.I1RZF6
113223	229533.I1RS20	pp	229533.I1S4S4

113224	229533.I1RS20	pp	229533.Q4ICA8
113225	229533.I1RS20	pp	229533.V6RCG5
113226	229533.I1RS20	pp	229533.I1RZM1
113227	229533.I1RS20	pp	229533.Q4IEK8
113228	229533.I1RS20	pp	229533.I1RW99
113229	229533.I1RS20	pp	229533.Q4IJK2
113230	229533.I1RS20	pp	229533.I1RWD7
113231	229533.I1RS20	pp	229533.I1S5B0
113232	229533.I1RS20	pp	229533.I1S6F1
113233	229533.I1RS20	pp	229533.I1RZZ1
113234	229533.I1RS20	pp	229533.I1RY03
113235	229533.I1RS20	pp	229533.I1RSI0
113236	229533.I1RAU3	pp	229533.I1RFB6
113237	229533.I1RAU3	pp	229533.I1RT50
113238	229533.I1RAU3	pp	229533.I1RQ48
113239	229533.I1RAU3	pp	229533.I1REV6
113240	229533.I1RAU3	pp	229533.I1RWW4
113241	229533.I1RAU3	pp	229533.I1RUJ5
113242	229533.I1RAU3	pp	229533.I1S267
113243	229533.I1RAU3	pp	229533.V6RIE5
113244	229533.I1RAU3	pp	229533.I1RSV4
113245	229533.I1RAU3	pp	229533.Q4I1B1
113246	229533.I1RAU3	pp	229533.I1RP33
113247	229533.I1RAU3	pp	229533.I1RV48
113248	229533.I1RAU3	pp	229533.I1RWC2
113249	229533.I1RAU3	pp	229533.I1RT14
113250	229533.I1RRI1	pp	229533.I1RX95
113251	229533.I1RRI1	pp	229533.I1RTD5
113252	229533.I1RRI1	pp	229533.I1RT40
113253	229533.I1RRI1	pp	229533.I1RTA1
113254	229533.I1RRI1	pp	229533.I1RYR0
113255	229533.I1RRI1	pp	229533.I1RXF8
113256	229533.I1RRI1	pp	229533.I1RVZ2
113257	229533.I1RRI1	pp	229533.I1RTB3
113258	229533.I1RRI1	pp	229533.Q4HVQ9
113259	229533.I1RRI1	pp	229533.I1S4S7
113260	229533.I1RRI1	pp	229533.I1RXD7
113261	229533.I1RRI1	pp	229533.I1RYJ6
113262	229533.I1RRI1	pp	229533.I1RRK3
113263	229533.I1RRI1	pp	229533.I1RYP2
113264	229533.I1RRI1	pp	229533.I1RX46
113265	229533.I1RRI1	pp	229533.I1RYI5
113266	229533.I1RRI1	pp	229533.I1RSX0
113267	229533.I1RRI1	pp	229533.Q4IB65
113268	229533.I1RRI1	pp	229533.I1RSQ7
113269	229533.I1RRI1	pp	229533.V6RTJ8
113270	229533.I1RRI1	pp	229533.I1S0W4
113271	229533.I1RRI1	pp	229533.I1RSA5
113272	229533.I1RRI1	pp	229533.I1S0F7
113273	229533.I1RRI1	pp	229533.I1RXD6
113274	229533.I1RRI1	pp	229533.I1RT05
113275	229533.I1RRI1	pp	229533.I1RSQ4
113276	229533.I1RRI1	pp	229533.I1RZB2
113277	229533.I1RRI1	pp	229533.I1RWS4
113278	229533.I1RRI1	pp	229533.I1RY86
113279	229533.I1RRI1	pp	229533.I1S0P0
113280	229533.I1RRI1	pp	229533.I1S0F1
113281	229533.I1RRI1	pp	229533.I1S2H4
113282	229533.I1RRI1	pp	229533.I1S0L4
113283	229533.I1RRI1	pp	229533.I1RZ39
113284	229533.I1RRI1	pp	229533.I1S9I5
113285	229533.I1RRI1	pp	229533.I1RY92
113286	229533.I1RRI1	pp	229533.I1RSZ7
113287	229533.I1RRI1	pp	229533.I1S4Z8
113288	229533.I1RRI1	pp	229533.V6RCJ5
113289	229533.I1RRI1	pp	229533.I1RUG3
113290	229533.I1RRI1	pp	229533.V6R5T7
113291	229533.I1RRI1	pp	229533.I1RZM8
113292	229533.I1RRI1	pp	229533.I1RW61
113293	229533.I1RRI1	pp	229533.I1RXC2
113294	229533.I1RRI1	pp	229533.I1RVF8
113295	229533.I1RRI1	pp	229533.I1S4S4
113296	229533.I1RRI1	pp	229533.V6RCZ8

229533.I1RRI1	pp	229533.I1RWA3
229533.I1RRI1	pp	229533.I1S6K9
229533.I1RRI1	pp	229533.I1S5R1
229533.I1RRI1	pp	229533.V6QYF0
229533.I1RRI1	pp	229533.I1S821
229533.I1RRI1	pp	229533.I1S438
229533.I1RRI1	pp	229533.I1RRV3
229533.I1RRI1	pp	229533.V6RCC0
229533.I1RRI1	pp	229533.I1S2B8
229533.I1RRI1	pp	229533.V6RMW6
229533.I1RRI1	pp	229533.I1S8D3
229533.I1RRI1	pp	229533.I1RRN2
229533.I1RRI1	pp	229533.I1S5R6
229533.I1RRI1	pp	229533.I1RRM3
229533.I1RRI1	pp	229533.I1S1B2
229533.I1RRI1	pp	229533.V6R042
229533.I1RRI1	pp	229533.I1RYT8
229533.I1RRI1	pp	229533.I1RSQ3
229533.I1RRI1	pp	229533.I1S5B0
229533.I1RRI1	pp	229533.I1S1W9
229533.I1RRI1	pp	229533.I1RZK5
229533.I1RRI1	pp	229533.I1RZV3
229533.I1RRI1	pp	229533.I1RXC4
229533.I1RRI1	pp	229533.I1S0J1
229533.I1RRI1	pp	229533.V6RHX2
229533.I1RRI1	pp	229533.Q4IPB8
229533.I1RRI1	pp	229533.I1RS18
229533.I1RRI1	pp	229533.I1S6V0
229533.I1RRI1	pp	229533.I1RVX0
229533.I1RRI1	pp	229533.V6RJP8
229533.I1RRI1	pp	229533.I1RVQ3
229533.I1RRI1	pp	229533.I1RT03
229533.I1RRI1	pp	229533.V6R9Z0
229533.I1RRI1	pp	229533.I1RWR7
229533.I1RRI1	pp	229533.I1RSF1
229533.I1RRI1	pp	229533.I1RUF7
229533.I1RRI1	pp	229533.I1RRY9
229533.I1RRI1	pp	229533.I1RUQ3
229533.I1RRI1	pp	229533.I1RVZ7
229533.I1RRI1	pp	229533.I1S1C7
229533.I1RRI1	pp	229533.Q4HXF6
229533.I1RRI1	pp	229533.Q4IPZ1
229533.I1RRI1	pp	229533.I1S437
229533.I1RRI1	pp	229533.I1RSR3
229533.I1RRI1	pp	229533.I1S0F8
229533.I1RRI1	pp	229533.Q4I624
229533.I1RRI1	pp	229533.I1S5X9
229533.I1RRI1	pp	229533.Q4IPH4
229533.I1RRI1	pp	229533.Q4IJK2
229533.I1RRI1	pp	229533.V6RWB3
229533.I1RTD5	pp	229533.I1S0R7
229533.I1RTD5	pp	229533.I1RVQ0
229533.I1RTD5	pp	229533.I1RXD6
229533.I1RTD5	pp	229533.I1RX46
229533.I1RTD5	pp	229533.V6R1K4
229533.I1RTD5	pp	229533.V6R4J1
229533.I1RTD5	pp	229533.I1S0F5
229533.I1RTD5	pp	229533.I1RZJ5
229533.I1RTD5	pp	229533.I1S0L3
229533.I1RTD5	pp	229533.I1S4S4
229533.I1RTD5	pp	229533.I1S0T2
229533.I1RTD5	pp	229533.I1RWJ8
229533.I1RTD5	pp	229533.Q4IJT5
229533.I1RTD5	pp	229533.I1S7A8
229533.I1RTD5	pp	229533.I1S9U2
229533.I1RTD5	pp	229533.V6RTJ8
229533.I1RTD5	pp	229533.I1RX95
229533.I1RTD5	pp	229533.V6QW22
229533.I1RTD5	pp	229533.V6RUT5
229533.I1RTD5	pp	229533.I1S4S7
229533.I1RTD5	pp	229533.I1RW71
229533.I1RTD5	pp	229533.I1S0X6
229533.I1RTD5	pp	229533.I1S6U8

113370	229533.I1RTD5	pp	229533.I1RW99
113371	229533.I1RTD5	pp	229533.I1RXD7
113372	229533.I1RTD5	pp	229533.I1RW07
113373	229533.I1RTD5	pp	229533.I1S021
113374	229533.I1RTD5	pp	229533.I1RXF0
113375	229533.I1RTD5	pp	229533.I1S438
113376	229533.I1RTD5	pp	229533.I1S9I5
113377	229533.I1RTD5	pp	229533.V6RCG5
113378	229533.I1RTD5	pp	229533.I1RZM8
113379	229533.I1RTD5	pp	229533.I1RX25
113380	229533.I1RTD5	pp	229533.I1RW25
113381	229533.I1RTD5	pp	229533.I1S1B2
113382	229533.I1RTD5	pp	229533.V6RCZ8
113383	229533.I1RTD5	pp	229533.I1S5B0
113384	229533.I1RTD5	pp	229533.V6QUE6
113385	229533.I1RTD5	pp	229533.I1RXC4
113386	229533.I1RTD5	pp	229533.I1S6K9
113387	229533.I1RTD5	pp	229533.I1SA32
113388	229533.I1RTD5	pp	229533.I1RY03
113389	229533.I1RTD5	pp	229533.I1S821
113390	229533.I1RTD5	pp	229533.I1S887
113391	229533.I1RTD5	pp	229533.Q4I8P5
113392	229533.I1RTD5	pp	229533.I1RZK5
113393	229533.I1RTD5	pp	229533.I1S419
113394	229533.I1RTD5	pp	229533.I1S0M9
113395	229533.I1RTD5	pp	229533.I1RZM1
113396	229533.I1RTD5	pp	229533.I1RYS0
113397	229533.I1RTD5	pp	229533.I1RZE5
113398	229533.I1RTD5	pp	229533.Q4I283
113399	229533.I1RTD5	pp	229533.I1S1J7
113400	229533.I1RTD5	pp	229533.I1S4Q4
113401	229533.I1RTD5	pp	229533.I1RZ35
113402	229533.I1RTD5	pp	229533.V6R9Z0
113403	229533.I1RTD5	pp	229533.I1RVZ0
113404	229533.I1RTD5	pp	229533.I1RWB0
113405	229533.I1RTD5	pp	229533.I1RWT6
113406	229533.I1RTD5	pp	229533.I1RU29
113407	229533.I1RTD5	pp	229533.Q4I2J8
113408	229533.I1RTD5	pp	229533.I1RZF6
113409	229533.I1RTD5	pp	229533.I1RWR7
113410	229533.I1RTD5	pp	229533.I1S504
113411	229533.I1RTD5	pp	229533.I1RWD7
113412	229533.I1RTD5	pp	229533.I1RZZ1
113413	229533.I1RTD5	pp	229533.V6R283
113414	229533.I1RTD5	pp	229533.I1RWF7
113415	229533.I1RTD5	pp	229533.I1RZK6
113416	229533.I1RTD5	pp	229533.I1RYY5
113417	229533.I1RTD5	pp	229533.V6RBN2
113418	229533.I1RTD5	pp	229533.I1S8P5
113419	229533.I1RTD5	pp	229533.I1S2E9
113420	229533.I1RTD5	pp	229533.Q4IJK2
113421	229533.I1RTD5	pp	229533.V6RUY9
113422	229533.I1RTD5	pp	229533.I1S352
113423	229533.I1RTD5	pp	229533.Q4HXF0
113424	229533.I1RTD5	pp	229533.I1RYI5
113425	229533.I1RTD5	pp	229533.V6R3J1
113426	229533.I1RTD5	pp	229533.I1S0W4
113427	229533.I1RTD5	pp	229533.Q4IJM4
113428	229533.I1RTD5	pp	229533.I1S0F6
113429	229533.I1RTD5	pp	229533.I1RXC2
113430	229533.I1RTD5	pp	229533.I1S2G7
113431	229533.I1RTD5	pp	229533.I1S503
113432	229533.I1RTD5	pp	229533.O74638
113433	229533.I1RTD5	pp	229533.I1RVZ2
113434	229533.I1RTD5	pp	229533.I1RV91
113435	229533.I1RTD5	pp	229533.I1S5R1
113436	229533.I1RTD5	pp	229533.I1RWL2
113437	229533.I1RTD5	pp	229533.I1SA33
113438	229533.I1RTD5	pp	229533.I1S0L4
113439	229533.I1RTD5	pp	229533.I1RWK3
113440	229533.I1RTD5	pp	229533.I1S9Y1
113441	229533.I1RTD5	pp	229533.I1S5R6
113442	229533.I1RTD5	pp	229533.V6R3X5

113443 229533.I1RTD5 pp 229533.I1S0N4
113444 229533.I1RTD5 pp 229533.I1RZ39
113445 229533.I1RTD5 pp 229533.I1S2H4
113446 229533.I1RTD5 pp 229533.I1S2B8
113447 229533.I1RTD5 pp 229533.Q4I327
113448 229533.I1RTD5 pp 229533.I1RZZ9
113449 229533.I1RTD5 pp 229533.I1S6Y8
113450 229533.I1RTD5 pp 229533.I1RWI5
113451 229533.I1RTD5 pp 229533.I1RUG3
113452 229533.I1RTD5 pp 229533.I1S0M6
113453 229533.I1RTD5 pp 229533.I1RVQ3
113454 229533.I1RTD5 pp 229533.I1RZS6
113455 229533.I1RTD5 pp 229533.Q4IN63
113456 229533.I1RTD5 pp 229533.I1RXT3
113457 229533.I1RTD5 pp 229533.I1S1W9
113458 229533.I1RTD5 pp 229533.I1RWN6
113459 229533.I1RTD5 pp 229533.V6RMW6
113460 229533.I1RTD5 pp 229533.I1RZ79
113461 229533.I1RTD5 pp 229533.I1RXU9
113462 229533.I1RTD5 pp 229533.I1RY44
113463 229533.I1RTD5 pp 229533.V6RIV9
113464 229533.I1RTD5 pp 229533.V6R042
113465 229533.I1RTD5 pp 229533.I1RZW8
113466 229533.I1RTD5 pp 229533.I1RWN5
113467 229533.I1RTD5 pp 229533.I1RWX5
113468 229533.I1RTD5 pp 229533.I1RYW3
113469 229533.I1RTD5 pp 229533.Q4HZB8
113470 229533.I1RTD5 pp 229533.I1S1W3
113471 229533.I1RTD5 pp 229533.I1S016
113472 229533.I1RTD5 pp 229533.I1RZV3
113473 229533.I1RTD5 pp 229533.I1S1W4
113474 229533.I1RTD5 pp 229533.I1S1C7
113475 229533.I1RTD5 pp 229533.I1RTD7
113476 229533.I1RTD5 pp 229533.I1S437
113477 229533.I1RTD5 pp 229533.I1RUP9
113478 229533.I1RTD5 pp 229533.I1S7W5
113479 229533.I1RTD5 pp 229533.I1S2C0
113480 229533.I1RTD5 pp 229533.V6R057
113481 229533.I1RTD5 pp 229533.I1S0J1
113482 229533.I1RTD5 pp 229533.I1S569
113483 229533.I1RTD5 pp 229533.I1S8R3
113484 229533.I1RTD5 pp 229533.I1RW46
113485 229533.I1RTD5 pp 229533.Q4I624
113486 229533.I1RTD5 pp 229533.I1S2E8
113487 229533.I1RTD5 pp 229533.V6RRH2
113488 229533.I1RTD5 pp 229533.I1RTH6
113489 229533.I1RTD5 pp 229533.I1RVX0
113490 229533.I1RTD5 pp 229533.I1S9Z4
113491 229533.I1RTD5 pp 229533.I1RUF7
113492 229533.I1RTD5 pp 229533.I1RXR1
113493 229533.I1RQ33 pp 229533.Q4I624
113494 229533.I1RQ33 pp 229533.Q4IPH4
113495 229533.I1RQ33 pp 229533.I1RUF7
113496 229533.I1RQ33 pp 229533.Q4HXF6
113497 229533.I1RQ33 pp 229533.I1RT03
113498 229533.I1RQ33 pp 229533.I1RVX0
113499 229533.I1RQ33 pp 229533.I1RUQ3
113500 229533.I1RQ33 pp 229533.I1RSR3
113501 229533.I1RQ33 pp 229533.V6RWB3
113502 229533.I1RQ33 pp 229533.I1RQE7
113503 229533.I1RQ33 pp 229533.I1RRN2
113504 229533.I1RQ33 pp 229533.I1RWR7
113505 229533.I1RQ33 pp 229533.V6RMW6
113506 229533.I1RQ33 pp 229533.I1RRV3
113507 229533.I1RQ33 pp 229533.Q4IJK2
113508 229533.I1RQ33 pp 229533.I1RVQ3
113509 229533.I1RQ33 pp 229533.I1RZV3
113510 229533.I1RQ33 pp 229533.I1RRY9
113511 229533.I1RQ33 pp 229533.V6R9Z0
113512 229533.I1RQ33 pp 229533.I1RZM1
113513 229533.I1RQ33 pp 229533.V6RHX2
113514 229533.I1RQ33 pp 229533.V6R042
113515 229533.I1RQ33 pp 229533.I1RRM3

113516	229533.I1RQ33	pp	229533.I1S1W9
113517	229533.I1RQ33	pp	229533.I1RZK5
113518	229533.I1RQ33	pp	229533.I1RSQ3
113519	229533.I1RQ33	pp	229533.I1S821
113520	229533.I1RQ33	pp	229533.Q4I1Y1
113521	229533.I1RQ33	pp	229533.I1S6V0
113522	229533.I1RQ33	pp	229533.V6RCZ8
113523	229533.I1RQ33	pp	229533.I1RS18
113524	229533.I1RQ33	pp	229533.I1RXC4
113525	229533.I1RQ33	pp	229533.I1S0F8
113526	229533.I1RQ33	pp	229533.I1S0J1
113527	229533.I1RQ33	pp	229533.I1S6K9
113528	229533.I1RQ33	pp	229533.I1S1C7
113529	229533.I1RQ33	pp	229533.I1S437
113530	229533.I1RQ33	pp	229533.I1RVF8
113531	229533.I1RQ33	pp	229533.I1S5B0
113532	229533.I1RQ33	pp	229533.I1RUG3
113533	229533.I1RQ33	pp	229533.I1RZM8
113534	229533.I1RQ33	pp	229533.I1RQD2
113535	229533.I1RQ33	pp	229533.I1S5R6
113536	229533.I1RQ33	pp	229533.I1RVZ7
113537	229533.I1RQ33	pp	229533.I1S4Z8
113538	229533.I1RQ33	pp	229533.I1RSF1
113539	229533.I1RQ33	pp	229533.I1RQ85
113540	229533.I1RQ33	pp	229533.I1RQV7
113541	229533.I1RQ33	pp	229533.I1S277
113542	229533.I1RQ33	pp	229533.I1S8D3
113543	229533.I1RQ33	pp	229533.I1S438
113544	229533.I1RQ33	pp	229533.I1S1B2
113545	229533.I1RQ33	pp	229533.V6RCC0
113546	229533.I1RQ33	pp	229533.I1S9I5
113547	229533.I1RQ33	pp	229533.V6QYF0
113548	229533.I1RQ33	pp	229533.I1S2B8
113549	229533.I1RQ33	pp	229533.I1RZ39
113550	229533.I1RQ33	pp	229533.V6RCJ5
113551	229533.I1RQ33	pp	229533.I1S0F1
113552	229533.I1RQ33	pp	229533.Q4IPB3
113553	229533.I1RQ33	pp	229533.I1RT05
113554	229533.I1RQ33	pp	229533.Q4IFI0
113555	229533.I1RQ33	pp	229533.V6R5T7
113556	229533.I1RQ33	pp	229533.I1RSQ4
113557	229533.I1RQ33	pp	229533.I1RSZ7
113558	229533.I1RQ33	pp	229533.I1RVZ2
113559	229533.I1RQ33	pp	229533.I1RTA1
113560	229533.I1RQ33	pp	229533.I1RW61
113561	229533.I1RQ33	pp	229533.I1RRI1
113562	229533.I1RQ33	pp	229533.I1RYR0
113563	229533.I1RQ33	pp	229533.I1S0F7
113564	229533.I1RQ33	pp	229533.I1RWS4
113565	229533.I1RQ33	pp	229533.I1S0L4
113566	229533.I1RQ33	pp	229533.I1RSA5
113567	229533.I1RQ33	pp	229533.I1RX95
113568	229533.I1RQ33	pp	229533.I1RY86
113569	229533.I1RQ33	pp	229533.I1RR24
113570	229533.I1RQ33	pp	229533.I1RXD6
113571	229533.I1RQ33	pp	229533.I1S2H4
113572	229533.I1RQ33	pp	229533.I1S0W4
113573	229533.I1RQ33	pp	229533.I1RZB2
113574	229533.I1RQ33	pp	229533.I1RXC2
113575	229533.I1RQ33	pp	229533.V6RTJ8
113576	229533.I1RQ33	pp	229533.I1RQM7
113577	229533.I1RQ33	pp	229533.I1RYI5
113578	229533.I1RQ33	pp	229533.I1RSQ7
113579	229533.I1RQ33	pp	229533.I1S5R1
113580	229533.I1RQ33	pp	229533.I1RYP2
113581	229533.I1RQ33	pp	229533.I1RSX0
113582	229533.I1RQ33	pp	229533.I1S4S4
113583	229533.I1RQ33	pp	229533.I1RQV8
113584	229533.I1RQ33	pp	229533.I1RX46
113585	229533.I1RQ33	pp	229533.I1S4S7
113586	229533.I1RQ33	pp	229533.I1RRK3
113587	229533.I1RQ33	pp	229533.I1RT40
113588	229533.I1RQ33	pp	229533.I1RXD7

113589 229533.I1RQ33 pp 229533.I1RYJ6
113590 229533.I1RQ33 pp 229533.Q4HVQ9
113591 229533.I1RQ33 pp 229533.I1RTD5
113592 229533.I1RQ33 pp 229533.I1RTB3
113593 229533.I1RQ33 pp 229533.I1RXF8
113594 229533.I1RP33 pp 229533.I1RT50
113595 229533.I1RP33 pp 229533.V6RIE5
113596 229533.I1RP33 pp 229533.I1RT14
113597 229533.I1RP33 pp 229533.I1RQ48
113598 229533.I1RP33 pp 229533.I1RUJ5
113599 229533.I1RBR4 pp 229533.I1RXF8
113600 229533.I1RBR4 pp 229533.I1RY34
113601 229533.I1RBR4 pp 229533.I1RY35
113602 229533.I1RBR4 pp 229533.I1RCN2
113603 229533.I1RBR4 pp 229533.I1RF79
113604 229533.I1RBR4 pp 229533.I1RR90
113605 229533.I1RBR4 pp 229533.I1RCC2
113606 229533.I1RBR4 pp 229533.I1RJG1
113607 229533.I1RBR4 pp 229533.I1RI23
113608 229533.I1RBR4 pp 229533.I1RNK1
113609 229533.I1RBR4 pp 229533.I1RQ48
113610 229533.I1RBR4 pp 229533.I1RPF2
113611 229533.I1RBR4 pp 229533.I1RZ92
113612 229533.I1RBR4 pp 229533.I1RNT1
113613 229533.I1RBR4 pp 229533.I1RGC4
113614 229533.I1RBR4 pp 229533.I1RWF4
113615 229533.I1RBR4 pp 229533.I1S2M5
113616 229533.I1RBR4 pp 229533.I1RFM2
113617 229533.I1RBR4 pp 229533.I1S1Q3
113618 229533.I1RBR4 pp 229533.I1RRA5
113619 229533.I1RBR4 pp 229533.I1RMA2
113620 229533.I1RBR4 pp 229533.I1S1W6
113621 229533.I1RBR4 pp 229533.Q4IPH4
113622 229533.I1RBR4 pp 229533.I1RQ76
113623 229533.I1RBR4 pp 229533.I1RE80
113624 229533.I1RBR4 pp 229533.I1RCA0
113625 229533.I1RBR4 pp 229533.I1RQW1
113626 229533.I1RBR4 pp 229533.V6RG22
113627 229533.I1RBR4 pp 229533.I1S3I4
113628 229533.I1RBR4 pp 229533.I1RRQ1
113629 229533.I1RBR4 pp 229533.I1SAS4
113630 229533.I1RBR4 pp 229533.V6RPN0
113631 229533.I1RBR4 pp 229533.V6RR38
113632 229533.I1RBR4 pp 229533.I1SAS3
113633 229533.I1RBR4 pp 229533.I1RY81
113634 229533.I1RBR4 pp 229533.I1RZZ3
113635 229533.I1RBR4 pp 229533.I1S130
113636 229533.I1RBR4 pp 229533.I1RJR2
113637 229533.I1RBR4 pp 229533.I1RT23
113638 229533.I1RBR4 pp 229533.I1RP83
113639 229533.I1RBR4 pp 229533.I1RI22
113640 229533.I1RBR4 pp 229533.I1RXX1
113641 229533.I1RD64 pp 229533.I1RNK2
113642 229533.I1RD64 pp 229533.I1RVZ0
113643 229533.I1RD64 pp 229533.I1RZE5
113644 229533.I1RD64 pp 229533.I1RKC6
113645 229533.I1RD64 pp 229533.I1S1J6
113646 229533.I1RD64 pp 229533.I1RFJ9
113647 229533.I1RD64 pp 229533.I1RSI0
113648 229533.I1RD64 pp 229533.I1RF19
113649 229533.I1RD64 pp 229533.I1RF08
113650 229533.I1RD64 pp 229533.I1RE00
113651 229533.I1RD64 pp 229533.I1RFU8
113652 229533.I1RD64 pp 229533.Q4HZ68
113653 229533.I1RD64 pp 229533.I1S2U2
113654 229533.I1RD64 pp 229533.I1S296
113655 229533.I1RD64 pp 229533.I1RTH6
113656 229533.I1RD64 pp 229533.I1RZZ1
113657 229533.I1RD64 pp 229533.I1RJZ6
113658 229533.I1RD64 pp 229533.I1RSD8
113659 229533.I1RD64 pp 229533.I1S1X9
113660 229533.I1RD64 pp 229533.I1S2E8
113661 229533.I1RD64 pp 229533.I1S2C0

113662	229533.I1RD64	pp	229533.I1RTE9
113663	229533.I1RD64	pp	229533.V6QUE6
113664	229533.I1RD64	pp	229533.I1RPP4
113665	229533.I1RD64	pp	229533.I1RSS4
113666	229533.I1RD64	pp	229533.I1RPJ3
113667	229533.I1RD64	pp	229533.I1S4T3
113668	229533.I1RD64	pp	229533.I1RSC6
113669	229533.I1RD64	pp	229533.I1RMN6
113670	229533.I1RD64	pp	229533.I1RN99
113671	229533.I1RD64	pp	229533.I1RPY1
113672	229533.I1RD64	pp	229533.Q4IBS2
113673	229533.I1RD64	pp	229533.I1RG56
113674	229533.I1RD64	pp	229533.I1RDG0
113675	229533.I1RD64	pp	229533.I1RQ28
113676	229533.I1RD64	pp	229533.V6RGN5
113677	229533.I1RD64	pp	229533.I1RK95
113678	229533.I1RD64	pp	229533.I1RQA2
113679	229533.I1RD64	pp	229533.I1RG11
113680	229533.I1RD64	pp	229533.Q4IEK8
113681	229533.I1RD64	pp	229533.I1RLU6
113682	229533.I1RD64	pp	229533.I1RQ31
113683	229533.I1RD64	pp	229533.I1RNQ5
113684	229533.I1RD64	pp	229533.I1RMC0
113685	229533.I1RD64	pp	229533.I1RJV3
113686	229533.I1RD64	pp	229533.I1RM96
113687	229533.I1RD64	pp	229533.I1S274
113688	229533.I1RD64	pp	229533.I1RRE0
113689	229533.I1RD64	pp	229533.Q4IN63
113690	229533.I1RD64	pp	229533.I1RZF6
113691	229533.I1RD64	pp	229533.I1S1U7
113692	229533.I1RD64	pp	229533.Q4I327
113693	229533.I1RD64	pp	229533.I1RRY9
113694	229533.I1RD64	pp	229533.I1RRR7
113695	229533.I1RD64	pp	229533.I1RSR3
113696	229533.I1RD64	pp	229533.V6R227
113697	229533.I1RD64	pp	229533.I1RMN7
113698	229533.I1RD64	pp	229533.I1RNY2
113699	229533.I1RD64	pp	229533.I1RDY5
113700	229533.I1RD64	pp	229533.V6RGY3
113701	229533.I1RD64	pp	229533.I1S060
113702	229533.I1RD64	pp	229533.I1S021
113703	229533.I1RD64	pp	229533.I1S0J1
113704	229533.I1RD64	pp	229533.I1RTD7
113705	229533.I1RD64	pp	229533.I1RX08
113706	229533.I1RD64	pp	229533.I1RNH6
113707	229533.I1RD64	pp	229533.I1RSI6
113708	229533.I1RD64	pp	229533.I1S0M9
113709	229533.I1RD64	pp	229533.I1RRI2
113710	229533.I1RD64	pp	229533.V6RMW6
113711	229533.I1RD64	pp	229533.I1RM25
113712	229533.I1RD64	pp	229533.Q4IJ56
113713	229533.I1RD64	pp	229533.V6R042
113714	229533.I1RD64	pp	229533.I1RYT5
113715	229533.I1RD64	pp	229533.I1RW61
113716	229533.I1RD64	pp	229533.I1RNU6
113717	229533.I1RD64	pp	229533.I1RL23
113718	229533.I1RD64	pp	229533.I1RG51
113719	229533.I1RD64	pp	229533.I1RFS3
113720	229533.I1RD64	pp	229533.I1RT05
113721	229533.I1RD64	pp	229533.Q4IF76
113722	229533.I1RD64	pp	229533.I1S2B8
113723	229533.I1RD64	pp	229533.I1S0F1
113724	229533.I1RD64	pp	229533.Q4IFI0
113725	229533.I1RD64	pp	229533.Q4I662
113726	229533.I1RD64	pp	229533.I1RJZ7
113727	229533.I1RD64	pp	229533.I1RU69
113728	229533.I1RD64	pp	229533.I1REX4
113729	229533.I1RD64	pp	229533.I1RX24
113730	229533.I1RD64	pp	229533.V6R283
113731	229533.I1RD64	pp	229533.I1RNV6
113732	229533.I1RD64	pp	229533.I1RQM6
113733	229533.I1RD64	pp	229533.V6QYF0
113734	229533.I1RD64	pp	229533.I1RW71

113735 229533.I1RD64 pp 229533.I1RT80
113736 229533.I1RD64 pp 229533.I1RKF9
113737 229533.I1RD64 pp 229533.Q4I830
113738 229533.I1RD64 pp 229533.I1RKL3
113739 229533.I1RD64 pp 229533.I1RNA0
113740 229533.I1RD64 pp 229533.Q4IQK7
113741 229533.I1RD64 pp 229533.Q4IJH1
113742 229533.I1RD64 pp 229533.I1RTF4
113743 229533.I1RD64 pp 229533.I1RR00
113744 229533.I1RD64 pp 229533.I1RSX1
113745 229533.I1RD64 pp 229533.I1RMK3
113746 229533.I1RD64 pp 229533.I1RM06
113747 229533.I1RD64 pp 229533.I1RWF7
113748 229533.I1RD64 pp 229533.I1RZS6
113749 229533.I1RD64 pp 229533.I1S9Y1
113750 229533.I1RD64 pp 229533.I1RPP3
113751 229533.I1RD64 pp 229533.I1RDA0
113752 229533.I1RD64 pp 229533.Q4HVW2
113753 229533.I1RD64 pp 229533.I1RPU0
113754 229533.I1RD64 pp 229533.I1RKJ6
113755 229533.I1RD64 pp 229533.I1RZ99
113756 229533.I1RD64 pp 229533.I1RYI5
113757 229533.I1RD64 pp 229533.I1S6U8
113758 229533.I1RD64 pp 229533.I1S2A8
113759 229533.I1RD64 pp 229533.I1RRT8
113760 229533.I1RD64 pp 229533.O74638
113761 229533.I1RD64 pp 229533.I1RR73
113762 229533.I1RD64 pp 229533.I1RQJ5
113763 229533.I1RD64 pp 229533.I1RRP3
113764 229533.I1RD64 pp 229533.I1RRJ8
113765 229533.I1RD64 pp 229533.I1RM67
113766 229533.I1RD64 pp 229533.Q4IAS1
113767 229533.I1RD64 pp 229533.I1RN67
113768 229533.I1RD64 pp 229533.I1RGF6
113769 229533.I1RD64 pp 229533.I1RPW9
113770 229533.I1RD64 pp 229533.I1RLV9
113771 229533.I1RD64 pp 229533.I1RKQ5
113772 229533.I1RD64 pp 229533.I1RS30
113773 229533.I1RD64 pp 229533.I1S9U2
113774 229533.I1RD64 pp 229533.I1RX46
113775 229533.I1RD64 pp 229533.I1RFG0
113776 229533.I1RD64 pp 229533.I1RRQ3
113777 229533.I1RD64 pp 229533.I1RT98
113778 229533.I1RD64 pp 229533.I1RZJ6
113779 229533.I1RD64 pp 229533.I1S6F0
113780 229533.I1RD64 pp 229533.I1RQS5
113781 229533.I1RD64 pp 229533.I1RRT2
113782 229533.I1RD64 pp 229533.I1RTD5
113783 229533.I1RD64 pp 229533.I1RNH1
113784 229533.I1RD64 pp 229533.Q4IPI1
113785 229533.I1RD64 pp 229533.I1RS47
113786 229533.I1RD64 pp 229533.I1RQC9
113787 229533.I1RD64 pp 229533.I1S1B6
113788 229533.I1RD64 pp 229533.I1RJL4
113789 229533.I1RD64 pp 229533.I1S0R7
113790 229533.I1RD64 pp 229533.I1RGA4
113791 229533.I1RD64 pp 229533.I1RT04
113792 229533.I1RD64 pp 229533.I1RRH5
113793 229533.I1RD64 pp 229533.I1RUQ2
113794 229533.I1RD64 pp 229533.I1RT33
113795 229533.I1RD64 pp 229533.Q4HZ42
113796 229533.I1RD64 pp 229533.I1RFN1
113797 229533.I1RD64 pp 229533.I1RSL9
113798 229533.I1RD64 pp 229533.I1RQ65
113799 229533.I1RD64 pp 229533.I1RN63
113800 229533.I1RD64 pp 229533.V6RTJ8
113801 229533.I1R9Z6 pp 229533.I1RYK0
113802 229533.I1R9Z6 pp 229533.I1RJR2
113803 229533.I1R9Z6 pp 229533.I1RND4
113804 229533.I1R9Z6 pp 229533.I1S9T8
113805 229533.I1R9Z6 pp 229533.I1SAK7
113806 229533.I1R9Z6 pp 229533.I1RGF4
113807 229533.I1R9Z6 pp 229533.I1RVN0

113808 229533.I1R9Z6 pp 229533.I1RW06
113809 229533.I1R9Z6 pp 229533.I1RFV9
113810 229533.I1R9Z6 pp 229533.I1RQ76
113811 229533.I1R9Z6 pp 229533.V6RLM7
113812 229533.I1R9D1 pp 229533.Q4IJK2
113813 229533.I1R9D1 pp 229533.I1RAU5
113814 229533.I1R9D1 pp 229533.I1RRY9
113815 229533.I1R9D1 pp 229533.I1RZK5
113816 229533.I1R9D1 pp 229533.V6RCG5
113817 229533.I1R9D1 pp 229533.I1RCG4
113818 229533.I1R9D1 pp 229533.I1RFU8
113819 229533.I1R9D1 pp 229533.I1RI82
113820 229533.I1R9D1 pp 229533.I1RIE8
113821 229533.I1R9D1 pp 229533.I1S504
113822 229533.I1R9D1 pp 229533.I1RY03
113823 229533.I1R9D1 pp 229533.I1RLU6
113824 229533.I1R9D1 pp 229533.I1RR24
113825 229533.I1R9D1 pp 229533.I1RBT1
113826 229533.I1R9D1 pp 229533.I1REK8
113827 229533.I1R9D1 pp 229533.I1RZM1
113828 229533.I1R9D1 pp 229533.I1RQC9
113829 229533.I1R9D1 pp 229533.I1RW25
113830 229533.I1R9D1 pp 229533.I1RS18
113831 229533.I1R9D1 pp 229533.I1RR39
113832 229533.I1R9D1 pp 229533.V6RTJ8
113833 229533.I1R9D1 pp 229533.I1S0L3
113834 229533.I1R9D1 pp 229533.I1RTH6
113835 229533.I1R9D1 pp 229533.I1S8R3
113836 229533.I1R9D1 pp 229533.I1RJW1
113837 229533.I1R9D1 pp 229533.I1RAX6
113838 229533.I1R9D1 pp 229533.I1RVQ0
113839 229533.I1R9D1 pp 229533.I1RX46
113840 229533.I1R9D1 pp 229533.I1RZW8
113841 229533.I1R9D1 pp 229533.I1S1C7
113842 229533.I1R9D1 pp 229533.V6QW22
113843 229533.I1R9D1 pp 229533.I1RCR8
113844 229533.I1R9D1 pp 229533.I1RSQ7
113845 229533.I1R9D1 pp 229533.I1RAU7
113846 229533.I1R9D1 pp 229533.I1RY44
113847 229533.I1R9D1 pp 229533.I1RBC9
113848 229533.I1R9D1 pp 229533.Q4I1Y5
113849 229533.I1R9D1 pp 229533.I1RWN6
113850 229533.I1R9D1 pp 229533.I1RWN5
113851 229533.I1R9D1 pp 229533.I1RAW0
113852 229533.I1R9D1 pp 229533.V6RIV9
113853 229533.I1R9D1 pp 229533.I1RB92
113854 229533.I1R9D1 pp 229533.I1RD17
113855 229533.I1R9D1 pp 229533.I1RJV3
113856 229533.I1R9D1 pp 229533.I1RPP4
113857 229533.I1R9D1 pp 229533.I1RA70
113858 229533.I1R9D1 pp 229533.I1RT03
113859 229533.I1R9D1 pp 229533.I1RVX0
113860 229533.I1R9D1 pp 229533.I1RBY4
113861 229533.I1R9D1 pp 229533.I1R9M0
113862 229533.I1R9D1 pp 229533.I1RK44
113863 229533.I1R9D1 pp 229533.I1RFL4
113864 229533.I1R9D1 pp 229533.I1RN18
113865 229533.I1R9D1 pp 229533.I1S2B8
113866 229533.I1R9D1 pp 229533.I1RTA0
113867 229533.I1R9D1 pp 229533.I1S0L4
113868 229533.I1R9D1 pp 229533.I1RD22
113869 229533.I1R9D1 pp 229533.I1RQ75
113870 229533.I1R9D1 pp 229533.I1RRM5
113871 229533.I1R9D1 pp 229533.I1S2H4
113872 229533.I1R9D1 pp 229533.I1RPM4
113873 229533.I1R9D1 pp 229533.I1RRT8
113874 229533.I1R9D1 pp 229533.I1RSQ3
113875 229533.I1R9D1 pp 229533.I1RJV4
113876 229533.I1R9D1 pp 229533.V6R042
113877 229533.I1R9D1 pp 229533.I1RR73
113878 229533.I1R9D1 pp 229533.I1RBS6
113879 229533.I1R9D1 pp 229533.I1RJW6
113880 229533.I1R9D1 pp 229533.V6RMW6

113881	229533.I1R9D1	pp	229533.I1S503
113882	229533.I1R9D1	pp	229533.V6R3X5
113883	229533.I1R9D1	pp	229533.I1RZ39
113884	229533.I1R9D1	pp	229533.I1RNC1
113885	229533.I1R9D1	pp	229533.I1RUG3
113886	229533.I1R9D1	pp	229533.I1S2E9
113887	229533.I1R9D1	pp	229533.I1RZZ9
113888	229533.I1R9D1	pp	229533.I1RT04
113889	229533.I1R9D1	pp	229533.I1RTD5
113890	229533.I1R9D1	pp	229533.I1RN67
113891	229533.I1R9D1	pp	229533.I1RPP3
113892	229533.I1R9D1	pp	229533.I1RV91
113893	229533.I1R9D1	pp	229533.I1RWK3
113894	229533.I1R9D1	pp	229533.I1S8P5
113895	229533.I1RRM3	pp	229533.I1RVZ2
113896	229533.I1RRM3	pp	229533.I1RTA1
113897	229533.I1RRM3	pp	229533.I1S0W4
113898	229533.I1RRM3	pp	229533.I1S2R0
113899	229533.I1RRM3	pp	229533.I1RXD6
113900	229533.I1RRM3	pp	229533.I1RYR0
113901	229533.I1RRM3	pp	229533.I1RSA5
113902	229533.I1RRM3	pp	229533.I1RX95
113903	229533.I1RRM3	pp	229533.I1S0F7
113904	229533.I1RRM3	pp	229533.Q4HVQ9
113905	229533.I1RRM3	pp	229533.I1RY86
113906	229533.I1RRM3	pp	229533.V6RTJ8
113907	229533.I1RRM3	pp	229533.I1RYI5
113908	229533.I1RRM3	pp	229533.I1RXF8
113909	229533.I1RRM3	pp	229533.I1RTD5
113910	229533.I1RRM3	pp	229533.I1RSQ7
113911	229533.I1RRM3	pp	229533.I1S4S7
113912	229533.I1RRM3	pp	229533.I1RZB2
113913	229533.I1RRM3	pp	229533.I1S4Z8
113914	229533.I1RRM3	pp	229533.I1RT40
113915	229533.I1RRM3	pp	229533.Q4IPB3
113916	229533.I1RRM3	pp	229533.I1RSQ4
113917	229533.I1RRM3	pp	229533.I1RSZ7
113918	229533.I1RRM3	pp	229533.I1RTB3
113919	229533.I1RRM3	pp	229533.I1S438
113920	229533.I1RRM3	pp	229533.I1RW61
113921	229533.I1RRM3	pp	229533.I1RX46
113922	229533.I1RRM3	pp	229533.I1RZ39
113923	229533.I1RRM3	pp	229533.I1RXD7
113924	229533.I1RRM3	pp	229533.I1RSX0
113925	229533.I1RRM3	pp	229533.I1S9I5
113926	229533.I1RRM3	pp	229533.I1RXC2
113927	229533.I1RRM3	pp	229533.I1RZM8
113928	229533.I1RRM3	pp	229533.I1S5R1
113929	229533.I1RRM3	pp	229533.I1S0J1
113930	229533.I1RRM3	pp	229533.I1S4S4
113931	229533.I1RRM3	pp	229533.I1S2B8
113932	229533.I1RRM3	pp	229533.V6R042
113933	229533.I1RRM3	pp	229533.I1S0L4
113934	229533.I1RRM3	pp	229533.V6RMW6
113935	229533.I1RRM3	pp	229533.I1RS18
113936	229533.I1RRM3	pp	229533.I1RXC4
113937	229533.I1RRM3	pp	229533.I1RSQ3
113938	229533.I1RRM3	pp	229533.I1RZV3
113939	229533.I1RRM3	pp	229533.I1S2H4
113940	229533.I1RRM3	pp	229533.I1S1W9
113941	229533.I1RRM3	pp	229533.V6RCZ8
113942	229533.I1RRM3	pp	229533.I1RUF7
113943	229533.I1RRM3	pp	229533.I1RSF1
113944	229533.I1RRM3	pp	229533.I1RUQ3
113945	229533.I1RRM3	pp	229533.I1S821
113946	229533.I1RRM3	pp	229533.Q4HXF6
113947	229533.I1RRM3	pp	229533.I1S6K9
113948	229533.I1RRM3	pp	229533.I1RVF8
113949	229533.I1RRM3	pp	229533.I1RVX0
113950	229533.I1RRM3	pp	229533.I1RVZ7
113951	229533.I1RRM3	pp	229533.I1RT03
113952	229533.I1RRM3	pp	229533.I1RRN2
113953	229533.I1RRM3	pp	229533.Q4IPZ1

113954	229533.I1RRM3	pp	229533.I1RUG3
113955	229533.I1RRM3	pp	229533.I1S1B2
113956	229533.I1RRM3	pp	229533.I1S5B0
113957	229533.I1RRM3	pp	229533.I1RRV3
113958	229533.I1RRM3	pp	229533.I1S0F8
113959	229533.I1RRM3	pp	229533.Q4IPH4
113960	229533.I1RRM3	pp	229533.I1RZB5
113961	229533.I1RRM3	pp	229533.I1S5R6
113962	229533.I1RRM3	pp	229533.V6R9Z0
113963	229533.I1RRM3	pp	229533.I1RVQ3
113964	229533.I1RRM3	pp	229533.I1RWR7
113965	229533.I1RRM3	pp	229533.Q4I624
113966	229533.I1RRM3	pp	229533.Q4IJK2
113967	229533.I1RRM3	pp	229533.I1RRY9
113968	229533.I1RRM3	pp	229533.I1S1C7
113969	229533.I1RRM3	pp	229533.I1RZK5
113970	229533.I1RRM3	pp	229533.I1S437
113971	229533.I1RUJ5	pp	229533.V6RIE5
113972	229533.A0A098E428	pp	229533.I1R982
113973	229533.A0A098E428	pp	229533.I1RT50
113974	229533.A0A098E428	pp	229533.I1REV6
113975	229533.A0A098E428	pp	229533.I1RT14
113976	229533.A0A098E428	pp	229533.V6RGI1
113977	229533.A0A098E428	pp	229533.I1RQL4
113978	229533.A0A098E428	pp	229533.I1RQ48
113979	229533.A0A098E428	pp	229533.I1S2A8
113980	229533.A0A098E428	pp	229533.I1RAU3
113981	229533.A0A098E428	pp	229533.I1RFB6
113982	229533.A0A098E428	pp	229533.I1RP33
113983	229533.A0A098E428	pp	229533.V6RIE5
113984	229533.A0A098E428	pp	229533.A0A1C3YHL0
113985	229533.A0A098E428	pp	229533.I1RUJ5
113986	229533.I1RD22	pp	229533.I1RX25
113987	229533.I1RD22	pp	229533.I1RW25
113988	229533.I1RD22	pp	229533.I1RPA4
113989	229533.I1RD22	pp	229533.I1RRS8
113990	229533.I1RD22	pp	229533.I1RI02
113991	229533.I1RD22	pp	229533.I1RFQ7
113992	229533.I1RD22	pp	229533.I1RW07
113993	229533.I1RD22	pp	229533.I1RM67
113994	229533.I1RD22	pp	229533.I1S0X6
113995	229533.I1RD22	pp	229533.I1RW54
113996	229533.I1RD22	pp	229533.I1RMC8
113997	229533.I1RD22	pp	229533.I1RZJ5
113998	229533.I1RD22	pp	229533.V6QUW4
113999	229533.I1RD22	pp	229533.I1RKB7
114000	229533.I1RD22	pp	229533.I1RT80
114001	229533.I1RD22	pp	229533.I1S438
114002	229533.I1RD22	pp	229533.I1S1W8
114003	229533.I1RD22	pp	229533.I1RRQ1
114004	229533.I1RD22	pp	229533.I1RMG8
114005	229533.I1RD22	pp	229533.I1RJU3
114006	229533.I1RD22	pp	229533.I1RIG2
114007	229533.I1RD22	pp	229533.I1RR24
114008	229533.I1RD22	pp	229533.I1S6U8
114009	229533.I1RD22	pp	229533.I1RPW9
114010	229533.I1RD22	pp	229533.I1RLC7
114011	229533.I1RD22	pp	229533.Q4IFI0
114012	229533.I1RD22	pp	229533.I1RDU4
114013	229533.I1RD22	pp	229533.I1RXD6
114014	229533.I1RD22	pp	229533.I1S9U2
114015	229533.I1RD22	pp	229533.I1RX95
114016	229533.I1RD22	pp	229533.I1S4S4
114017	229533.I1RD22	pp	229533.I1RQ26
114018	229533.I1RD22	pp	229533.I1RWJ8
114019	229533.I1RD22	pp	229533.I1RVQ0
114020	229533.I1RD22	pp	229533.I1RPU0
114021	229533.I1RD22	pp	229533.I1RQH2
114022	229533.I1RD22	pp	229533.V6R4J1
114023	229533.I1RD22	pp	229533.I1RD52
114024	229533.I1RD22	pp	229533.I1S0T2
114025	229533.I1RD22	pp	229533.I1RY86
114026	229533.I1RD22	pp	229533.Q4IJT5

114027	229533.I1RD22	pp	229533.I1RD56
114028	229533.I1RD22	pp	229533.I1RIL8
114029	229533.I1RD22	pp	229533.I1RRG3
114030	229533.I1RD22	pp	229533.Q4IPI1
114031	229533.I1RD22	pp	229533.V6R1K4
114032	229533.I1RD22	pp	229533.I1S540
114033	229533.I1RD22	pp	229533.I1S7A8
114034	229533.I1RD22	pp	229533.I1RW71
114035	229533.I1RD22	pp	229533.I1S0F5
114036	229533.I1RD22	pp	229533.V6RTJ8
114037	229533.I1RD22	pp	229533.I1S0L3
114038	229533.I1RD22	pp	229533.I1S4S7
114039	229533.I1RD22	pp	229533.I1RPY3
114040	229533.I1RD22	pp	229533.I1RPN4
114041	229533.I1RD22	pp	229533.I1RQA3
114042	229533.I1RD22	pp	229533.Q4IJK2
114043	229533.I1RD22	pp	229533.I1RR00
114044	229533.I1RD22	pp	229533.I1RQC9
114045	229533.I1RD22	pp	229533.I1S3S3
114046	229533.I1RD22	pp	229533.I1RZZ1
114047	229533.I1RD22	pp	229533.I1S0R7
114048	229533.I1RD22	pp	229533.I1RQJ5
114049	229533.I1RD22	pp	229533.I1RQH3
114050	229533.I1RD22	pp	229533.I1RZK6
114051	229533.I1RD22	pp	229533.I1RG53
114052	229533.I1RD22	pp	229533.V6RUT5
114053	229533.I1RD22	pp	229533.I1RGB2
114054	229533.I1RD22	pp	229533.I1RSQ7
114055	229533.I1RD22	pp	229533.I1RRX5
114056	229533.I1RD22	pp	229533.I1RJZ6
114057	229533.I1RD22	pp	229533.I1RLP1
114058	229533.I1RD22	pp	229533.I1RQQ5
114059	229533.I1RD22	pp	229533.I1RFC0
114060	229533.I1RD22	pp	229533.I1RXD7
114061	229533.I1RD22	pp	229533.I1RDH4
114062	229533.I1RD22	pp	229533.V6QW22
114063	229533.I1RD22	pp	229533.V6RBN2
114064	229533.I1RD22	pp	229533.I1RX46
114065	229533.I1RD22	pp	229533.Q4IJM4
114066	229533.I1RD22	pp	229533.I1RN61
114067	229533.I1RD22	pp	229533.I1RDU0
114068	229533.I1RD22	pp	229533.I1RDG0
114069	229533.I1RD22	pp	229533.I1REN8
114070	229533.I1RD22	pp	229533.Q4I8P5
114071	229533.I1RD22	pp	229533.I1RK95
114072	229533.I1RD22	pp	229533.I1REL7
114073	229533.I1RD22	pp	229533.I1RZK5
114074	229533.I1RD22	pp	229533.I1RS18
114075	229533.I1RD22	pp	229533.Q4I2J8
114076	229533.I1RD22	pp	229533.I1S419
114077	229533.I1RD22	pp	229533.I1RK83
114078	229533.I1RD22	pp	229533.Q4IEK8
114079	229533.I1RD22	pp	229533.I1S887
114080	229533.I1RD22	pp	229533.I1RZE5
114081	229533.I1RD22	pp	229533.I1RZ35
114082	229533.I1RD22	pp	229533.I1RQA5
114083	229533.I1RD22	pp	229533.I1RWB0
114084	229533.I1RD22	pp	229533.I1RLU6
114085	229533.I1RD22	pp	229533.I1RNQ5
114086	229533.I1RD22	pp	229533.I1RYS0
114087	229533.I1RD22	pp	229533.I1RLM7
114088	229533.I1RD22	pp	229533.I1S4Q4
114089	229533.I1RD22	pp	229533.I1RF19
114090	229533.I1RD22	pp	229533.I1RSI0
114091	229533.I1RD22	pp	229533.I1RZF6
114092	229533.I1RD22	pp	229533.I1S504
114093	229533.I1RD22	pp	229533.I1RZM1
114094	229533.I1RD22	pp	229533.Q4I283
114095	229533.I1RD22	pp	229533.I1RWJ3
114096	229533.I1RD22	pp	229533.I1S1J7
114097	229533.I1RD22	pp	229533.I1RE00
114098	229533.I1RD22	pp	229533.I1REK8
114099	229533.I1RD22	pp	229533.I1RI03

114100	229533.I1RD22	pp	229533.I1S439
114101	229533.I1RD22	pp	229533.I1RWD7
114102	229533.I1RD22	pp	229533.I1RRY9
114103	229533.I1RD22	pp	229533.I1RPR9
114104	229533.I1RD22	pp	229533.I1RQ85
114105	229533.I1RD22	pp	229533.I1RY03
114106	229533.I1RD22	pp	229533.I1RG14
114107	229533.I1RD22	pp	229533.I1RWR7
114108	229533.I1RD22	pp	229533.I1RNT9
114109	229533.I1RD22	pp	229533.I1RSR8
114110	229533.I1RD22	pp	229533.I1RU29
114111	229533.I1RD22	pp	229533.I1RWT6
114112	229533.I1RD22	pp	229533.I1RYY5
114113	229533.I1RD22	pp	229533.I1RN58
114114	229533.I1RD22	pp	229533.I1RZM8
114115	229533.I1RD22	pp	229533.I1RFU8
114116	229533.I1RD22	pp	229533.V6QY25
114117	229533.I1RD22	pp	229533.I1RZH4
114118	229533.I1RD22	pp	229533.I1RXC4
114119	229533.I1RD22	pp	229533.I1RXF0
114120	229533.I1RD22	pp	229533.I1RYB9
114121	229533.I1RD22	pp	229533.I1RI82
114122	229533.I1RD22	pp	229533.I1RJ17
114123	229533.I1RD22	pp	229533.I1SA32
114124	229533.I1RD22	pp	229533.V6RCG5
114125	229533.I1RD22	pp	229533.I1RR51
114126	229533.I1RD22	pp	229533.I1S021
114127	229533.I1RD22	pp	229533.I1RNH6
114128	229533.I1RD22	pp	229533.V6QUE6
114129	229533.I1RD22	pp	229533.I1S0M9
114130	229533.I1RD22	pp	229533.I1S6K9
114131	229533.I1RD22	pp	229533.I1RGC2
114132	229533.I1RD22	pp	229533.I1RK96
114133	229533.I1RD22	pp	229533.I1RQ81
114134	229533.I1RD22	pp	229533.I1RGC5
114135	229533.I1RD22	pp	229533.I1RYI7
114136	229533.I1RD22	pp	229533.I1REE2
114137	229533.I1RD22	pp	229533.I1RE22
114138	229533.I1RD22	pp	229533.V6RCZ8
114139	229533.I1RD22	pp	229533.I1RRV4
114140	229533.I1RD22	pp	229533.I1RFT2
114141	229533.I1RD22	pp	229533.I1S0M6
114142	229533.I1RD22	pp	229533.I1RZS6
114143	229533.I1RD22	pp	229533.I1RZ39
114144	229533.I1RD22	pp	229533.I1RRU9
114145	229533.I1RD22	pp	229533.I1RDE7
114146	229533.I1RD22	pp	229533.V6R3X5
114147	229533.I1RD22	pp	229533.I1S2B8
114148	229533.I1RD22	pp	229533.I1S5B0
114149	229533.I1RD22	pp	229533.I1RQD2
114150	229533.I1RD22	pp	229533.I1RND6
114151	229533.I1RD22	pp	229533.I1RRM5
114152	229533.I1RD22	pp	229533.I1RR73
114153	229533.I1RD22	pp	229533.I1RJZ7
114154	229533.I1RD22	pp	229533.I1S044
114155	229533.I1RD22	pp	229533.I1RM82
114156	229533.I1RD22	pp	229533.I1RUY5
114157	229533.I1RD22	pp	229533.I1RE76
114158	229533.I1RD22	pp	229533.I1RW99
114159	229533.I1RD22	pp	229533.I1RPU3
114160	229533.I1RD22	pp	229533.I1S9Y1
114161	229533.I1RD22	pp	229533.I1RSZ7
114162	229533.I1RD22	pp	229533.I1RVL8
114163	229533.I1RD22	pp	229533.I1RKJ6
114164	229533.I1RD22	pp	229533.I1S7S9
114165	229533.I1RD22	pp	229533.I1RZV6
114166	229533.I1RD22	pp	229533.I1RQ23
114167	229533.I1RD22	pp	229533.O74638
114168	229533.I1RD22	pp	229533.I1S6Y8
114169	229533.I1RD22	pp	229533.I1S277
114170	229533.I1RD22	pp	229533.I1RPM4
114171	229533.I1RD22	pp	229533.I1S4Z8
114172	229533.I1RD22	pp	229533.I1RRT8

114173	229533.I1RD22	pp	229533.I1RXC2
114174	229533.I1RD22	pp	229533.I1S5R1
114175	229533.I1RD22	pp	229533.I1RZZ9
114176	229533.I1RD22	pp	229533.I1RSQ4
114177	229533.I1RD22	pp	229533.I1RPV3
114178	229533.I1RD22	pp	229533.I1RL04
114179	229533.I1RD22	pp	229533.I1RWL2
114180	229533.I1RD22	pp	229533.Q4HZI0
114181	229533.I1RD22	pp	229533.Q4I662
114182	229533.I1RD22	pp	229533.I1RS20
114183	229533.I1RD22	pp	229533.I1REC5
114184	229533.I1RD22	pp	229533.I1RFS3
114185	229533.I1RD22	pp	229533.I1RPE0
114186	229533.I1RD22	pp	229533.I1S2H4
114187	229533.I1RD22	pp	229533.I1RYR0
114188	229533.I1RD22	pp	229533.I1RWI5
114189	229533.I1RD22	pp	229533.I1RGU7
114190	229533.I1RD22	pp	229533.I1RGA6
114191	229533.I1RD22	pp	229533.I1RTA1
114192	229533.I1RD22	pp	229533.I1RNN1
114193	229533.I1RD22	pp	229533.I1RJN9
114194	229533.I1RD22	pp	229533.I1S3G4
114195	229533.I1RD22	pp	229533.I1RVZ2
114196	229533.I1RD22	pp	229533.I1RFT6
114197	229533.I1RD22	pp	229533.I1RD64
114198	229533.I1RD22	pp	229533.I1RRI1
114199	229533.I1RD22	pp	229533.I1RKL3
114200	229533.I1RD22	pp	229533.I1S0L4
114201	229533.I1RD22	pp	229533.I1RIL3
114202	229533.I1RD22	pp	229533.I1RU69
114203	229533.I1RD22	pp	229533.I1RFL3
114204	229533.I1RD22	pp	229533.I1RFT1
114205	229533.I1RD22	pp	229533.I1S352
114206	229533.I1RD22	pp	229533.I1S3T7
114207	229533.I1RD22	pp	229533.I1RT40
114208	229533.I1RD22	pp	229533.I1RKP1
114209	229533.I1RD22	pp	229533.I1RSV2
114210	229533.I1RD22	pp	229533.I1RTD5
114211	229533.I1RD22	pp	229533.I1RGF5
114212	229533.I1RD22	pp	229533.I1RPP3
114213	229533.I1RD22	pp	229533.I1S8P5
114214	229533.I1RD22	pp	229533.I1RXF8
114215	229533.I1RD22	pp	229533.I1RWK3
114216	229533.I1RD22	pp	229533.I1RNA5
114217	229533.I1RD22	pp	229533.V6R283
114218	229533.I1RD22	pp	229533.I1RWF7
114219	229533.I1RD22	pp	229533.I1RJV4
114220	229533.I1RD22	pp	229533.I1RT04
114221	229533.I1RD22	pp	229533.I1RYI5
114222	229533.I1RD22	pp	229533.I1RSD8
114223	229533.I1RD22	pp	229533.I1RN18
114224	229533.I1RD22	pp	229533.I1RRJ8
114225	229533.I1RD22	pp	229533.I1RQG4
114226	229533.I1RD22	pp	229533.V6RUY9
114227	229533.I1RD22	pp	229533.I1RFL4
114228	229533.I1RD22	pp	229533.I1RZE4
114229	229533.I1RD22	pp	229533.I1S0W4
114230	229533.I1RD22	pp	229533.I1RQE7
114231	229533.I1RD22	pp	229533.Q4HXF0
114232	229533.I1RD22	pp	229533.I1S0F6
114233	229533.I1RD22	pp	229533.I1RN67
114234	229533.I1RD22	pp	229533.I1S2E9
114235	229533.I1RD22	pp	229533.I1S2E8
114236	229533.I1RD22	pp	229533.I1RLN6
114237	229533.I1RD22	pp	229533.I1RFK2
114238	229533.I1RD22	pp	229533.I1RS06
114239	229533.I1RD22	pp	229533.I1RDF7
114240	229533.I1RD22	pp	229533.I1RRQ3
114241	229533.I1RD22	pp	229533.I1RP28
114242	229533.I1RD22	pp	229533.I1RRZ0
114243	229533.I1RD22	pp	229533.I1RSY2
114244	229533.I1RD22	pp	229533.I1S503
114245	229533.I1RD22	pp	229533.I1RS47

114246	229533.I1RD22	pp	229533.I1RJW6
114247	229533.I1RD22	pp	229533.I1RT03
114248	229533.I1RD22	pp	229533.I1RVX0
114249	229533.I1RD22	pp	229533.I1RQ65
114250	229533.I1RD22	pp	229533.I1S2G7
114251	229533.I1RD22	pp	229533.I1RRN5
114252	229533.I1RD22	pp	229533.Q4HWE2
114253	229533.I1RD22	pp	229533.V6R3J1
114254	229533.I1RD22	pp	229533.I1RSX0
114255	229533.I1RD22	pp	229533.I1RV91
114256	229533.I1RD22	pp	229533.I1RXR1
114257	229533.I1RD22	pp	229533.I1RQ33
114258	229533.I1RD22	pp	229533.I1S268
114259	229533.I1RD22	pp	229533.I1S1C7
114260	229533.I1RD22	pp	229533.V6RRH2
114261	229533.I1RD22	pp	229533.I1RGA4
114262	229533.I1RD22	pp	229533.I1RJS2
114263	229533.I1RD22	pp	229533.I1RQM7
114264	229533.I1RD22	pp	229533.I1RML0
114265	229533.I1RD22	pp	229533.I1RTD7
114266	229533.I1RD22	pp	229533.I1RGL4
114267	229533.I1RD22	pp	229533.V6R057
114268	229533.I1RD22	pp	229533.I1RYW3
114269	229533.I1RD22	pp	229533.I1RP27
114270	229533.I1RD22	pp	229533.I1S437
114271	229533.I1RD22	pp	229533.I1RJV3
114272	229533.I1RD22	pp	229533.Q4I624
114273	229533.I1RD22	pp	229533.I1S569
114274	229533.I1RD22	pp	229533.I1RXU9
114275	229533.I1RD22	pp	229533.I1RWN6
114276	229533.I1RD22	pp	229533.I1RD39
114277	229533.I1RD22	pp	229533.I1RZ79
114278	229533.I1RD22	pp	229533.I1RQJ4
114279	229533.I1RD22	pp	229533.I1RRI2
114280	229533.I1RD22	pp	229533.I1RZV3
114281	229533.I1RD22	pp	229533.I1RM96
114282	229533.I1RD22	pp	229533.I1S1U7
114283	229533.I1RD22	pp	229533.I1RW46
114284	229533.I1RD22	pp	229533.I1RE01
114285	229533.I1RD22	pp	229533.I1RWX5
114286	229533.I1RD22	pp	229533.I1RTA0
114287	229533.I1RD22	pp	229533.I1RZW8
114288	229533.I1RD22	pp	229533.I1RZS7
114289	229533.I1RD22	pp	229533.I1S1W3
114290	229533.I1RD22	pp	229533.I1S8R3
114291	229533.I1RD22	pp	229533.I1RKJ8
114292	229533.I1RD22	pp	229533.I1RRE5
114293	229533.I1RD22	pp	229533.I1RTH6
114294	229533.I1RD22	pp	229533.I1S7W5
114295	229533.I1RD22	pp	229533.I1S1W4
114296	229533.I1RD22	pp	229533.I1S0J1
114297	229533.I1RD22	pp	229533.I1RSF1
114298	229533.I1RD22	pp	229533.I1S2C0
114299	229533.I1RD22	pp	229533.I1RE89
114300	229533.I1RD22	pp	229533.I1RRR7
114301	229533.I1RD22	pp	229533.I1RTE9
114302	229533.I1RD22	pp	229533.V6RLM7
114303	229533.I1RD22	pp	229533.I1RQ21
114304	229533.I1RD22	pp	229533.I1RSK1
114305	229533.I1RD22	pp	229533.I1RPU2
114306	229533.I1RD22	pp	229533.I1RPK0
114307	229533.I1RD22	pp	229533.I1RNB8
114308	229533.I1RD22	pp	229533.I1RUG3
114309	229533.I1RD22	pp	229533.I1RVZ7
114310	229533.I1RD22	pp	229533.I1RXT3
114311	229533.I1RD22	pp	229533.I1RS17
114312	229533.I1RD22	pp	229533.I1S5R6
114313	229533.I1RD22	pp	229533.I1RWH4
114314	229533.I1RD22	pp	229533.I1RM25
114315	229533.I1RD22	pp	229533.I1RNC1
114316	229533.I1RD22	pp	229533.I1RS90
114317	229533.I1RD22	pp	229533.I1RMV0
114318	229533.I1RD22	pp	229533.Q4IN63

114319	229533.I1RD22	pp	229533.I1S9Z4
114320	229533.I1RD22	pp	229533.I1S0N4
114321	229533.I1RD22	pp	229533.V6RGN5
114322	229533.I1RD22	pp	229533.I1RPP4
114323	229533.I1RD22	pp	229533.I1RNY2
114324	229533.I1RD22	pp	229533.I1RVF8
114325	229533.I1RD22	pp	229533.I1S1W9
114326	229533.I1RD22	pp	229533.I1RWN5
114327	229533.I1RD22	pp	229533.I1RG11
114328	229533.I1RD22	pp	229533.V6RIV9
114329	229533.I1RD22	pp	229533.I1RJP4
114330	229533.I1RD22	pp	229533.I1RMG3
114331	229533.I1RD22	pp	229533.Q4HZB8
114332	229533.I1RD22	pp	229533.I1RRM3
114333	229533.I1RD22	pp	229533.I1RRV1
114334	229533.I1RD22	pp	229533.I1S016
114335	229533.I1RD22	pp	229533.V6RMW6
114336	229533.I1RD22	pp	229533.V6R042
114337	229533.I1RD22	pp	229533.I1RSQ3
114338	229533.I1RD22	pp	229533.I1RY44
114339	229533.I1RD22	pp	229533.I1SA33
114340	229533.I1RD22	pp	229533.I1RRN2
114341	229533.I1RD22	pp	229533.I1RMN6
114342	229533.I1R9C2	pp	229533.I1RNB8
114343	229533.I1R9C2	pp	229533.I1RG90
114344	229533.I1R9C2	pp	229533.I1RT35
114345	229533.I1SAK7	pp	229533.V6RXR0
114346	229533.I1RGD5	pp	229533.I1RTB8
114347	229533.I1RGD5	pp	229533.I1RZF7
114348	229533.A0A1C3YIT9	pp	229533.I1S027
114349	229533.A0A1C3YIT9	pp	229533.I1S2B8
114350	229533.I1RGF5	pp	229533.I1RZK5
114351	229533.I1RGF5	pp	229533.Q4IJK2
114352	229533.I1RGF5	pp	229533.I1RRY9
114353	229533.I1RGF5	pp	229533.I1RY03
114354	229533.I1RGF5	pp	229533.V6RCG5
114355	229533.I1RGF5	pp	229533.I1RS18
114356	229533.I1RGF5	pp	229533.I1S504
114357	229533.I1RGF5	pp	229533.I1RI82
114358	229533.I1RGF5	pp	229533.I1RR24
114359	229533.I1RGF5	pp	229533.I1RIE8
114360	229533.I1RGF5	pp	229533.I1RLU6
114361	229533.I1RGF5	pp	229533.I1RW25
114362	229533.I1RGF5	pp	229533.I1RZM1
114363	229533.I1RGF5	pp	229533.I1RR39
114364	229533.I1RGF5	pp	229533.I1RQC9
114365	229533.I1RGF5	pp	229533.I1RX46
114366	229533.I1RGF5	pp	229533.I1S8R3
114367	229533.I1RGF5	pp	229533.I1S0L3
114368	229533.I1RGF5	pp	229533.I1RVQ0
114369	229533.I1RGF5	pp	229533.Q4I1Y5
114370	229533.I1RGF5	pp	229533.I1RTH6
114371	229533.I1RGF5	pp	229533.I1RSQ7
114372	229533.I1RGF5	pp	229533.V6QW22
114373	229533.I1RGF5	pp	229533.I1RVX0
114374	229533.I1RGF5	pp	229533.V6RTJ8
114375	229533.I1RGF5	pp	229533.I1RZW8
114376	229533.I1RGF5	pp	229533.I1RJW1
114377	229533.I1RGF5	pp	229533.I1RN18
114378	229533.I1RGF5	pp	229533.I1S1C7
114379	229533.I1RGF5	pp	229533.I1RT03
114380	229533.I1RGF5	pp	229533.I1RY44
114381	229533.I1RGF5	pp	229533.I1RWN6
114382	229533.I1RGF5	pp	229533.I1RJV3
114383	229533.I1RGF5	pp	229533.I1RUG3
114384	229533.I1RGF5	pp	229533.I1RNC1
114385	229533.I1RGF5	pp	229533.V6RIV9
114386	229533.I1RGF5	pp	229533.I1S8P5
114387	229533.I1RGF5	pp	229533.I1RN67
114388	229533.I1RGF5	pp	229533.I1RWN5
114389	229533.I1RGF5	pp	229533.I1RRM5
114390	229533.I1RGF5	pp	229533.I1RPP4
114391	229533.I1RGF5	pp	229533.I1S2B8

114392	229533.I1RGF5	pp	229533.I1RPP3
114393	229533.I1RGF5	pp	229533.I1RPM4
114394	229533.I1RGF5	pp	229533.I1RK44
114395	229533.I1RGF5	pp	229533.I1RWK3
114396	229533.I1RGF5	pp	229533.I1RJV4
114397	229533.I1RGF5	pp	229533.I1S0L4
114398	229533.I1RGF5	pp	229533.I1S2H4
114399	229533.I1RGF5	pp	229533.I1RTA0
114400	229533.I1RGF5	pp	229533.I1S503
114401	229533.I1RGF5	pp	229533.I1RRT8
114402	229533.I1RGF5	pp	229533.I1RJW6
114403	229533.I1RGF5	pp	229533.I1RQ75
114404	229533.I1RGF5	pp	229533.V6RMW6
114405	229533.I1RGF5	pp	229533.I1S2E9
114406	229533.I1RGF5	pp	229533.V6R042
114407	229533.I1RGF5	pp	229533.I1RT04
114408	229533.I1RGF5	pp	229533.V6R3X5
114409	229533.I1RGF5	pp	229533.I1RZ39
114410	229533.I1RGF5	pp	229533.I1RTD5
114411	229533.I1RGF5	pp	229533.I1RR73
114412	229533.I1RGF5	pp	229533.I1RV91
114413	229533.I1RGF5	pp	229533.I1RSQ3
114414	229533.I1RGF5	pp	229533.I1RZZ9
114415	229533.I1RRT8	pp	229533.I1S419
114416	229533.I1RRT8	pp	229533.I1RWT6
114417	229533.I1RRT8	pp	229533.Q4I283
114418	229533.I1RRT8	pp	229533.V6R227
114419	229533.I1RRT8	pp	229533.I1RW99
114420	229533.I1RRT8	pp	229533.I1S504
114421	229533.I1RRT8	pp	229533.I1RZF6
114422	229533.I1RRT8	pp	229533.I1RZM8
114423	229533.I1RRT8	pp	229533.I1SA32
114424	229533.I1RRT8	pp	229533.I1RZH4
114425	229533.I1RRT8	pp	229533.I1RXF0
114426	229533.I1RRT8	pp	229533.I1RXC4
114427	229533.I1RRT8	pp	229533.Q4I8P5
114428	229533.I1RRT8	pp	229533.I1RS18
114429	229533.I1RRT8	pp	229533.V6RCG5
114430	229533.I1RRT8	pp	229533.I1S0M9
114431	229533.I1RRT8	pp	229533.I1S6K9
114432	229533.I1RRT8	pp	229533.I1RRU9
114433	229533.I1RRT8	pp	229533.I1S0Z3
114434	229533.I1RRT8	pp	229533.V6RCZ8
114435	229533.I1RRT8	pp	229533.I1RSI0
114436	229533.I1RRT8	pp	229533.I1RRV4
114437	229533.I1RRT8	pp	229533.I1RZE5
114438	229533.I1RRT8	pp	229533.I1RZM1
114439	229533.I1RRT8	pp	229533.V6QUE6
114440	229533.I1RRT8	pp	229533.I1S5B0
114441	229533.I1RRT8	pp	229533.I1S4Q4
114442	229533.I1RRT8	pp	229533.I1RT35
114443	229533.I1RRT8	pp	229533.I1RTD9
114444	229533.I1RRT8	pp	229533.I1RY03
114445	229533.I1RRT8	pp	229533.I1RVZ0
114446	229533.I1RRT8	pp	229533.Q4I2J8
114447	229533.I1RRT8	pp	229533.I1RZK6
114448	229533.I1RRT8	pp	229533.I1RZK5
114449	229533.I1RRT8	pp	229533.V6RBN2
114450	229533.I1RRT8	pp	229533.I1RZZ1
114451	229533.I1RRT8	pp	229533.I1RWD7
114452	229533.I1RRT8	pp	229533.I1S887
114453	229533.I1RRT8	pp	229533.Q4I5Z5
114454	229533.I1RRT8	pp	229533.Q4IJK2
114455	229533.I1RRT8	pp	229533.I1RZB5
114456	229533.I1RRT8	pp	229533.I1RSC6
114457	229533.I1RRT8	pp	229533.I1RY63
114458	229533.I1RRT8	pp	229533.I1RVY2
114459	229533.I1RRT8	pp	229533.I1RVQ0
114460	229533.I1RRT8	pp	229533.Q4IJT5
114461	229533.I1RRT8	pp	229533.V6R4J1
114462	229533.I1RRT8	pp	229533.I1S1J7
114463	229533.I1RRT8	pp	229533.I1RWD4
114464	229533.I1RRT8	pp	229533.Q5I2J3

114465 229533.I1RRT8 pp 229533.I1RSM3
114466 229533.I1RRT8 pp 229533.I1RXB1
114467 229533.I1RRT8 pp 229533.I1RRY9
114468 229533.I1RRT8 pp 229533.V6R1K4
114469 229533.I1RRT8 pp 229533.I1RZT6
114470 229533.I1RRT8 pp 229533.V6QW22
114471 229533.I1RRT8 pp 229533.I1RU29
114472 229533.I1RRT8 pp 229533.I1S0F5
114473 229533.I1RRT8 pp 229533.I1RSQ7
114474 229533.I1RRT8 pp 229533.I1RSR3
114475 229533.I1RRT8 pp 229533.I1S0L3
114476 229533.I1RRT8 pp 229533.I1RRX5
114477 229533.I1RRT8 pp 229533.I1RX04
114478 229533.I1RRT8 pp 229533.V6RUT5
114479 229533.I1RRT8 pp 229533.I1S0D4
114480 229533.I1RRT8 pp 229533.I1RW71
114481 229533.I1RRT8 pp 229533.I1RUQ2
114482 229533.I1RRT8 pp 229533.I1S4S7
114483 229533.I1RRT8 pp 229533.I1S0T2
114484 229533.I1RRT8 pp 229533.I1RXD7
114485 229533.I1RRT8 pp 229533.I1RT33
114486 229533.I1RRT8 pp 229533.V6RLM7
114487 229533.I1RRT8 pp 229533.I1S0R7
114488 229533.I1RRT8 pp 229533.I1S256
114489 229533.I1RRT8 pp 229533.I1RS17
114490 229533.I1RRT8 pp 229533.I1RX46
114491 229533.I1RRT8 pp 229533.I1RX25
114492 229533.I1RRT8 pp 229533.V6RTJ8
114493 229533.I1RRT8 pp 229533.I1RW25
114494 229533.I1RRT8 pp 229533.I1RWH4
114495 229533.I1RRT8 pp 229533.I1RT41
114496 229533.I1RRT8 pp 229533.I1SA33
114497 229533.I1RRT8 pp 229533.I1RSU2
114498 229533.I1RRT8 pp 229533.I1S7A8
114499 229533.I1RRT8 pp 229533.I1RY44
114500 229533.I1RRT8 pp 229533.V6QUW4
114501 229533.I1RRT8 pp 229533.I1RW07
114502 229533.I1RRT8 pp 229533.V6RGN5
114503 229533.I1RRT8 pp 229533.I1S1W3
114504 229533.I1RRT8 pp 229533.I1RZJ5
114505 229533.I1RRT8 pp 229533.I1S0N4
114506 229533.I1RRT8 pp 229533.V6QYF0
114507 229533.I1RRT8 pp 229533.I1RWN5
114508 229533.I1RRT8 pp 229533.I1RWJ8
114509 229533.I1RRT8 pp 229533.I1S7J8
114510 229533.I1RRT8 pp 229533.I1S5R6
114511 229533.I1RRT8 pp 229533.V6RIV9
114512 229533.I1RRT8 pp 229533.Q4I327
114513 229533.I1RRT8 pp 229533.I1RWQ8
114514 229533.I1RRT8 pp 229533.I1S4S4
114515 229533.I1RRT8 pp 229533.I1S0X6
114516 229533.I1RRT8 pp 229533.I1S016
114517 229533.I1RRT8 pp 229533.I1S1W9
114518 229533.I1RRT8 pp 229533.I1RTF4
114519 229533.I1RRT8 pp 229533.I1S5N3
114520 229533.I1RRT8 pp 229533.I1RT80
114521 229533.I1RRT8 pp 229533.I1S8V4
114522 229533.I1RRT8 pp 229533.I1RUG3
114523 229533.I1RRT8 pp 229533.V6QUV7
114524 229533.I1RRT8 pp 229533.Q4IN63
114525 229533.I1RRT8 pp 229533.I1S0J1
114526 229533.I1RRT8 pp 229533.I1RW46
114527 229533.I1RRT8 pp 229533.I1RSQ3
114528 229533.I1RRT8 pp 229533.V6R042
114529 229533.I1RRT8 pp 229533.I1RZS7
114530 229533.I1RRT8 pp 229533.I1RWN6
114531 229533.I1RRT8 pp 229533.I1RTA0
114532 229533.I1RRT8 pp 229533.V6RMW6
114533 229533.I1RRT8 pp 229533.V6RGY3
114534 229533.I1RRT8 pp 229533.I1RRV1
114535 229533.I1RRT8 pp 229533.I1RXU9
114536 229533.I1RRT8 pp 229533.Q4IEV5
114537 229533.I1RRT8 pp 229533.I1RWX5

114538	229533.I1RRT8	pp	229533.I1RSI6
114539	229533.I1RRT8	pp	229533.I1S1J6
114540	229533.I1RRT8	pp	229533.I1RZW8
114541	229533.I1RRT8	pp	229533.V6RRH2
114542	229533.I1RRT8	pp	229533.I1RS47
114543	229533.I1RRT8	pp	229533.I1S569
114544	229533.I1RRT8	pp	229533.I1RVZ2
114545	229533.I1RRT8	pp	229533.I1S1C7
114546	229533.I1RRT8	pp	229533.I1RTA1
114547	229533.I1RRT8	pp	229533.I1S2M6
114548	229533.I1RRT8	pp	229533.Q4I624
114549	229533.I1RRT8	pp	229533.V6R057
114550	229533.I1RRT8	pp	229533.I1RWK3
114551	229533.I1RRT8	pp	229533.I1RYW3
114552	229533.I1RRT8	pp	229533.V6R283
114553	229533.I1RRT8	pp	229533.I1S2E8
114554	229533.I1RRT8	pp	229533.I1RTE9
114555	229533.I1RRT8	pp	229533.I1RSD8
114556	229533.I1RRT8	pp	229533.I1RYI5
114557	229533.I1RRT8	pp	229533.I1S2C0
114558	229533.I1RRT8	pp	229533.I1RTH6
114559	229533.I1RRT8	pp	229533.I1RT03
114560	229533.I1RRT8	pp	229533.I1S8R3
114561	229533.I1RRT8	pp	229533.I1S8P5
114562	229533.I1RRT8	pp	229533.I1RVX0
114563	229533.I1RRT8	pp	229533.I1RSS4
114564	229533.I1RRT8	pp	229533.I1RSY2
114565	229533.I1RRT8	pp	229533.I1S352
114566	229533.I1RRT8	pp	229533.I1RXR1
114567	229533.I1RRT8	pp	229533.Q4HZK7
114568	229533.I1RRT8	pp	229533.I1RSV2
114569	229533.I1RRT8	pp	229533.I1S2P0
114570	229533.I1RRT8	pp	229533.V6RM66
114571	229533.I1RRT8	pp	229533.I1S503
114572	229533.I1RRT8	pp	229533.I1RY62
114573	229533.I1RRT8	pp	229533.I1RSK1
114574	229533.I1RRT8	pp	229533.I1RS30
114575	229533.I1RRT8	pp	229533.I1RSX0
114576	229533.I1RRT8	pp	229533.I1S0F6
114577	229533.I1RRT8	pp	229533.I1RZB2
114578	229533.I1RRT8	pp	229533.V6R3J1
114579	229533.I1RRT8	pp	229533.I1RS20
114580	229533.I1RRT8	pp	229533.I1RRZ0
114581	229533.I1RRT8	pp	229533.Q4IR18
114582	229533.I1RRT8	pp	229533.I1RTD5
114583	229533.I1RRT8	pp	229533.I1S6Y8
114584	229533.I1RRT8	pp	229533.I1RW61
114585	229533.I1RRT8	pp	229533.I1RV91
114586	229533.I1RRT8	pp	229533.I1S2H4
114587	229533.I1RRT8	pp	229533.I1RYT5
114588	229533.I1RRT8	pp	229533.I1RSZ7
114589	229533.I1RRT8	pp	229533.I1S8V3
114590	229533.I1RRT8	pp	229533.I1RZ29
114591	229533.I1RRT8	pp	229533.I1RT40
114592	229533.I1RRT8	pp	229533.I1RWI5
114593	229533.I1RRT8	pp	229533.I1RZS6
114594	229533.I1RRT8	pp	229533.I1S2E9
114595	229533.I1RRT8	pp	229533.I1RZZ9
114596	229533.I1RRT8	pp	229533.Q4HXF0
114597	229533.I1RRT8	pp	229533.I1RTG3
114598	229533.I1RRT8	pp	229533.I1S5R1
114599	229533.I1RRT8	pp	229533.I1RT04
114600	229533.I1RRT8	pp	229533.V6RUY9
114601	229533.I1RRT8	pp	229533.O74638
114602	229533.I1RRT8	pp	229533.I1RSQ4
114603	229533.I1RRT8	pp	229533.I1S2B8
114604	229533.I1RRT8	pp	229533.I1RVY0
114605	229533.I1RRT8	pp	229533.V6R3X5
114606	229533.I1RRT8	pp	229533.I1RZ39
114607	229533.I1RRT8	pp	229533.I1RXC2
114608	229533.I1RRT8	pp	229533.I1S0L4
114609	229533.I1RRT8	pp	229533.I1RTG7
114610	229533.I1RRT8	pp	229533.I1S9Y1

114611 229533.I1RP08 pp 229533.I1RU02
114612 229533.I1RP08 pp 229533.Q4IER8
114613 229533.I1RP08 pp 229533.I1RQ93
114614 229533.I1RP08 pp 229533.I1RVY0
114615 229533.I1RP08 pp 229533.I1SAA0
114616 229533.I1RP08 pp 229533.I1S1U7
114617 229533.I1RGS5 pp 229533.I1RZ29
114618 229533.I1RGS5 pp 229533.V6RXR0
114619 229533.I1RGS5 pp 229533.I1SAK7
114620 229533.A0A1C3YI53 pp 229533.A0A1C3YMC0
114621 229533.A0A1C3YI53 pp 229533.A0A1C3YKB0
114622 229533.A0A1C3YI53 pp 229533.A0A1C3YK34
114623 229533.A0A1C3YI53 pp 229533.V6RK96
114624 229533.A0A1C3YI53 pp 229533.I1S6K9
114625 229533.A0A1C3YI53 pp 229533.I1RAU5
114626 229533.A0A1C3YI53 pp 229533.Q4I2J8
114627 229533.A0A1C3YI53 pp 229533.A0A1C3YK11
114628 229533.A0A1C3YI53 pp 229533.Q4IJK2
114629 229533.A0A1C3YI53 pp 229533.I1RFJ5
114630 229533.A0A1C3YI53 pp 229533.A0A1C3YLU7
114631 229533.A0A1C3YI53 pp 229533.I1REK8
114632 229533.A0A1C3YI53 pp 229533.I1RW71
114633 229533.A0A1C3YI53 pp 229533.I1S4S4
114634 229533.A0A1C3YI53 pp 229533.I1RQE8
114635 229533.A0A1C3YI53 pp 229533.I1RGB2
114636 229533.A0A1C3YI53 pp 229533.I1RJU3
114637 229533.A0A1C3YI53 pp 229533.I1RRX5
114638 229533.A0A1C3YI53 pp 229533.I1RZM4
114639 229533.A0A1C3YI53 pp 229533.I1RPR9
114640 229533.A0A1C3YI53 pp 229533.I1R9Y4
114641 229533.A0A1C3YI53 pp 229533.I1RC49
114642 229533.A0A1C3YI53 pp 229533.I1RWC9
114643 229533.A0A1C3YI53 pp 229533.I1RBT1
114644 229533.A0A1C3YI53 pp 229533.A0A1C3YN26
114645 229533.A0A1C3YI53 pp 229533.I1RBD5
114646 229533.A0A1C3YI53 pp 229533.I1RCK9
114647 229533.A0A1C3YI53 pp 229533.I1RAU9
114648 229533.A0A1C3YI53 pp 229533.I1RRY9
114649 229533.A0A1C3YI53 pp 229533.I1RA49
114650 229533.A0A1C3YI53 pp 229533.I1RWB0
114651 229533.A0A1C3YI53 pp 229533.I1S1W9
114652 229533.A0A1C3YI53 pp 229533.V6RTJ8
114653 229533.A0A1C3YI53 pp 229533.I1S1J7
114654 229533.A0A1C3YI53 pp 229533.I1S1W3
114655 229533.A0A1C3YI53 pp 229533.I1RP86
114656 229533.A0A1C3YI53 pp 229533.I1RPK0
114657 229533.A0A1C3YI53 pp 229533.A0A1C3YKY6
114658 229533.A0A1C3YI53 pp 229533.I1S887
114659 229533.A0A1C3YI53 pp 229533.I1RLP1
114660 229533.A0A1C3YI53 pp 229533.V6RUT5
114661 229533.A0A1C3YI53 pp 229533.I1RMV0
114662 229533.A0A1C3YI53 pp 229533.A0A1C3YLC1
114663 229533.A0A1C3YI53 pp 229533.I1RCM5
114664 229533.A0A1C3YI53 pp 229533.I1RTA0
114665 229533.A0A1C3YI53 pp 229533.I1RA85
114666 229533.A0A1C3YI53 pp 229533.I1S0P0
114667 229533.A0A1C3YI53 pp 229533.I1RS90
114668 229533.A0A1C3YI53 pp 229533.I1RCZ8
114669 229533.A0A1C3YI53 pp 229533.Q4HZS8
114670 229533.A0A1C3YI53 pp 229533.I1S2B9
114671 229533.A0A1C3YI53 pp 229533.I1RTD5
114672 229533.A0A1C3YI53 pp 229533.I1RCU4
114673 229533.A0A1C3YI53 pp 229533.I1RYX5
114674 229533.A0A1C3YI53 pp 229533.I1RAE4
114675 229533.A0A1C3YI53 pp 229533.I1S5R6
114676 229533.A0A1C3YI53 pp 229533.I1RNC1
114677 229533.A0A1C3YI53 pp 229533.I1RRN2
114678 229533.A0A1C3YI53 pp 229533.I1RAW3
114679 229533.A0A1C3YI53 pp 229533.A0A1C3YMA1
114680 229533.A0A1C3YI53 pp 229533.I1RYW3
114681 229533.A0A1C3YI53 pp 229533.I1RMR9
114682 229533.A0A1C3YI53 pp 229533.I1RJZ7
114683 229533.A0A1C3YI53 pp 229533.I1RWX5

114684 229533.A0A1C3YI53 pp 229533.A0A1C3YKU8
114685 229533.A0A1C3YI53 pp 229533.I1S0N5
114686 229533.A0A1C3YI53 pp 229533.I1RE76
114687 229533.A0A1C3YI53 pp 229533.I1RRI2
114688 229533.A0A1C3YI53 pp 229533.I1RPV3
114689 229533.A0A1C3YI53 pp 229533.I1RC29
114690 229533.A0A1C3YI53 pp 229533.I1RNG4
114691 229533.A0A1C3YI53 pp 229533.I1RYH7
114692 229533.A0A1C3YI53 pp 229533.I1RN67
114693 229533.A0A1C3YI53 pp 229533.I1RN88
114694 229533.A0A1C3YI53 pp 229533.I1REC6
114695 229533.A0A1C3YI53 pp 229533.Q4I7L0
114696 229533.A0A1C3YI53 pp 229533.I1S8P5
114697 229533.A0A1C3YI53 pp 229533.I1RXR1
114698 229533.A0A1C3YI53 pp 229533.I1RIH0
114699 229533.A0A1C3YI53 pp 229533.I1RFL4
114700 229533.A0A1C3YI53 pp 229533.I1RRT8
114701 229533.A0A1C3YI53 pp 229533.I1RTA1
114702 229533.A0A1C3YI53 pp 229533.V6QVH8
114703 229533.A0A1C3YI53 pp 229533.I1RBA6
114704 229533.A0A1C3YI53 pp 229533.I1RR95
114705 229533.A0A1C3YI53 pp 229533.I1RY92
114706 229533.A0A1C3YI53 pp 229533.I1RXU9
114707 229533.A0A1C3YI53 pp 229533.A0A1C3YKM9
114708 229533.A0A1C3YI53 pp 229533.I1RSX0
114709 229533.A0A1C3YI53 pp 229533.I1RZ39
114710 229533.A0A1C3YI53 pp 229533.I1RZZ9
114711 229533.A0A1C3YI53 pp 229533.I1REC7
114712 229533.A0A1C3YI53 pp 229533.V6R3J1
114713 229533.A0A1C3YI53 pp 229533.I1RG12
114714 229533.A0A1C3YI53 pp 229533.A0A1C3YM60
114715 229533.I1RBF1 pp 229533.I1S3G4
114716 229533.I1RBF1 pp 229533.I1S1W8
114717 229533.I1RBF1 pp 229533.I1RPD6
114718 229533.I1RBF1 pp 229533.I1RRH5
114719 229533.I1RBF1 pp 229533.I1RW43
114720 229533.I1RVN0 pp 229533.I1SAK7
114721 229533.I1RV91 pp 229533.I1S0X7
114722 229533.I1RV91 pp 229533.I1RWJ6
114723 229533.I1RV91 pp 229533.I1RZ94
114724 229533.I1RV91 pp 229533.I1S8V3
114725 229533.I1RV91 pp 229533.Q4HTT1
114726 229533.I1RV91 pp 229533.I1S0M6
114727 229533.I1RV91 pp 229533.Q4ICG5
114728 229533.I1RV91 pp 229533.I1S549
114729 229533.I1RV91 pp 229533.I1S0J7
114730 229533.I1RV91 pp 229533.I1RW07
114731 229533.I1RV91 pp 229533.I1RX38
114732 229533.I1RV91 pp 229533.I1RX70
114733 229533.I1RV91 pp 229533.I1S2B8
114734 229533.I1RV91 pp 229533.I1RVY0
114735 229533.I1RV91 pp 229533.I1S0J3
114736 229533.I1RV91 pp 229533.I1RZ39
114737 229533.I1RV91 pp 229533.I1RZ63
114738 229533.I1RV91 pp 229533.I1S2H4
114739 229533.I1RV91 pp 229533.V6R3X5
114740 229533.I1RV91 pp 229533.I1S0L4
114741 229533.I1RV91 pp 229533.I1RX25
114742 229533.I1RV91 pp 229533.I1RW25
114743 229533.I1RV91 pp 229533.I1RYH9
114744 229533.I1RV91 pp 229533.I1S224
114745 229533.I1RV91 pp 229533.I1S1V5
114746 229533.I1RV91 pp 229533.I1RWI5
114747 229533.I1RV91 pp 229533.I1S0X6
114748 229533.I1RV91 pp 229533.I1RZJ5
114749 229533.I1RV91 pp 229533.I1RWK3
114750 229533.I1RV91 pp 229533.I1RZZ9
114751 229533.I1RV91 pp 229533.I1RWJ8
114752 229533.I1RV91 pp 229533.I1S5R1
114753 229533.I1RV91 pp 229533.Q4IJT5
114754 229533.I1RV91 pp 229533.I1RW71
114755 229533.I1RV91 pp 229533.I1S352
114756 229533.I1RV91 pp 229533.V6QV09

114757	229533.I1RV91	pp	229533.I1S0F6
114758	229533.I1RV91	pp	229533.I1RZ51
114759	229533.I1RV91	pp	229533.V6QW22
114760	229533.I1RV91	pp	229533.Q4IJP1
114761	229533.I1RV91	pp	229533.I1S8P5
114762	229533.I1RV91	pp	229533.V6R4J1
114763	229533.I1RV91	pp	229533.I1RZ12
114764	229533.I1RV91	pp	229533.I1RYI3
114765	229533.I1RV91	pp	229533.I1RVQ0
114766	229533.I1RV91	pp	229533.I1S0L3
114767	229533.I1RV91	pp	229533.I1RW38
114768	229533.I1RV91	pp	229533.I1S0F5
114769	229533.I1RV91	pp	229533.I1RW83
114770	229533.I1RV91	pp	229533.V6RUY9
114771	229533.I1RV91	pp	229533.I1S7X9
114772	229533.I1RV91	pp	229533.I1RYZ9
114773	229533.I1RV91	pp	229533.V6R1K4
114774	229533.I1RV91	pp	229533.I1S2G7
114775	229533.I1RV91	pp	229533.V6RTJ8
114776	229533.I1RV91	pp	229533.I1S0R7
114777	229533.I1RV91	pp	229533.I1RW76
114778	229533.I1RV91	pp	229533.I1RXD7
114779	229533.I1RV91	pp	229533.I1RY32
114780	229533.I1RV91	pp	229533.I1S503
114781	229533.I1RV91	pp	229533.I1S7A8
114782	229533.I1RV91	pp	229533.I1RX39
114783	229533.I1RV91	pp	229533.I1RWC2
114784	229533.I1RV91	pp	229533.I1S2E9
114785	229533.I1RV91	pp	229533.Q4IJK2
114786	229533.I1RV91	pp	229533.I1S268
114787	229533.I1RV91	pp	229533.I1RXU1
114788	229533.I1RV91	pp	229533.I1RX46
114789	229533.I1RV91	pp	229533.I1RY58
114790	229533.I1RV91	pp	229533.I1RZK5
114791	229533.I1RV91	pp	229533.I1RZK9
114792	229533.I1RV91	pp	229533.I1RVX0
114793	229533.I1RV91	pp	229533.I1RZZ1
114794	229533.I1RV91	pp	229533.I1RZE4
114795	229533.I1RV91	pp	229533.Q4IJM4
114796	229533.I1RV91	pp	229533.I1RYV3
114797	229533.I1RV91	pp	229533.Q4I624
114798	229533.I1RV91	pp	229533.I1RY03
114799	229533.I1RV91	pp	229533.I1S1C7
114800	229533.I1RV91	pp	229533.Q4I2J8
114801	229533.I1RV91	pp	229533.V6R057
114802	229533.I1RV91	pp	229533.I1S569
114803	229533.I1RV91	pp	229533.I1RZB5
114804	229533.I1RV91	pp	229533.I1S8R3
114805	229533.I1RV91	pp	229533.I1S757
114806	229533.I1RV91	pp	229533.I1RYW3
114807	229533.I1RV91	pp	229533.I1RXZ4
114808	229533.I1RV91	pp	229533.I1RZW8
114809	229533.I1RV91	pp	229533.I1RWX5
114810	229533.I1RV91	pp	229533.I1S0A6
114811	229533.I1RV91	pp	229533.I1RX64
114812	229533.I1RV91	pp	229533.I1RYY5
114813	229533.I1RV91	pp	229533.Q4IER8
114814	229533.I1RV91	pp	229533.I1S439
114815	229533.I1RV91	pp	229533.I1S0N8
114816	229533.I1RV91	pp	229533.I1RWN6
114817	229533.I1RV91	pp	229533.V6QV38
114818	229533.I1RV91	pp	229533.I1RW46
114819	229533.I1RV91	pp	229533.Q4I8P5
114820	229533.I1RV91	pp	229533.I1S1J7
114821	229533.I1RV91	pp	229533.Q4I5R3
114822	229533.I1RV91	pp	229533.I1RZH4
114823	229533.I1RV91	pp	229533.I1RZT1
114824	229533.I1RV91	pp	229533.I1RW84
114825	229533.I1RV91	pp	229533.V6RCZ8
114826	229533.I1RV91	pp	229533.I1S0J1
114827	229533.I1RV91	pp	229533.I1S8V4
114828	229533.I1RV91	pp	229533.I1RX09
114829	229533.I1RV91	pp	229533.Q4I283

114830	229533.I1RV91	pp	229533.I1SA32
114831	229533.I1RV91	pp	229533.I1S504
114832	229533.I1RV91	pp	229533.V6R042
114833	229533.I1RV91	pp	229533.V6RMW6
114834	229533.I1RV91	pp	229533.V6RKY7
114835	229533.I1RV91	pp	229533.I1S1W4
114836	229533.I1RV91	pp	229533.I1RWT6
114837	229533.I1RV91	pp	229533.I1RWN5
114838	229533.I1RV91	pp	229533.V6RIV9
114839	229533.I1RV91	pp	229533.V6RCG5
114840	229533.I1RV91	pp	229533.V6RGI1
114841	229533.I1RV91	pp	229533.I1RZM1
114842	229533.I1RV91	pp	229533.I1S5B0
114843	229533.I1RV91	pp	229533.I1RW99
114844	229533.I1RV91	pp	229533.I1RY44
114845	229533.I1RV91	pp	229533.I1RZM8
114846	229533.I1RV91	pp	229533.V6RVG3
114847	229533.I1RV91	pp	229533.I1RXF0
114848	229533.I1RV91	pp	229533.I1SA33
114849	229533.I1RV91	pp	229533.Q4HTT2
114850	229533.I1RV91	pp	229533.I1SAF7
114851	229533.I1RV91	pp	229533.V6QUE6
114852	229533.I1RV91	pp	229533.I1S0M2
114853	229533.I1RCZ5	pp	229533.I1RYZ0
114854	229533.I1RCZ5	pp	229533.I1RQ93
114855	229533.I1RCZ5	pp	229533.I1RD69
114856	229533.I1RCZ5	pp	229533.I1SAA0
114857	229533.I1RAF8	pp	229533.I1RS30
114858	229533.I1RAF8	pp	229533.I1RQ65
114859	229533.I1RAF8	pp	229533.Q4HZ47
114860	229533.I1RAF8	pp	229533.Q4IEV5
114861	229533.I1RAF8	pp	229533.I1RC03
114862	229533.I1RAF8	pp	229533.I1RWH5
114863	229533.I1RAF8	pp	229533.V6R283
114864	229533.I1RAF8	pp	229533.I1S2M6
114865	229533.I1RAF8	pp	229533.I1RFW5
114866	229533.I1RAF8	pp	229533.I1RW71
114867	229533.I1RAF8	pp	229533.I1RKL3
114868	229533.I1RAF8	pp	229533.Q4I830
114869	229533.I1RAF8	pp	229533.I1RR00
114870	229533.I1RAF8	pp	229533.I1RWF7
114871	229533.I1RAF8	pp	229533.I1RPP3
114872	229533.I1RAF8	pp	229533.I1RQJ5
114873	229533.I1RAF8	pp	229533.I1RZ99
114874	229533.I1RAF8	pp	229533.I1RRJ8
114875	229533.I1RAF8	pp	229533.I1RSQ7
114876	229533.I1RAF8	pp	229533.I1RAV6
114877	229533.I1RAF8	pp	229533.I1RLV9
114878	229533.I1RAF8	pp	229533.I1RKQ5
114879	229533.I1RAF8	pp	229533.Q4IAS1
114880	229533.I1RAF8	pp	229533.I1S6F0
114881	229533.I1RAF8	pp	229533.I1RFG0
114882	229533.I1RAF8	pp	229533.Q4I5R9
114883	229533.I1RAF8	pp	229533.I1RRQ3
114884	229533.I1RAF8	pp	229533.I1S503
114885	229533.I1RAF8	pp	229533.I1RAY2
114886	229533.I1RAF8	pp	229533.I1RS47
114887	229533.I1RAF8	pp	229533.I1RNH1
114888	229533.I1RAF8	pp	229533.I1RRT2
114889	229533.I1RAF8	pp	229533.I1RX24
114890	229533.I1RAF8	pp	229533.I1RRH5
114891	229533.I1RAF8	pp	229533.I1RX46
114892	229533.I1RAF8	pp	229533.I1RBG9
114893	229533.I1RAF8	pp	229533.Q4HZ42
114894	229533.I1RAF8	pp	229533.V6QYF0
114895	229533.I1RAF8	pp	229533.Q4IPI1
114896	229533.I1RAF8	pp	229533.I1RJL4
114897	229533.I1RAF8	pp	229533.I1RD56
114898	229533.I1RAF8	pp	229533.I1RBC7
114899	229533.I1RAF8	pp	229533.I1RC29
114900	229533.I1RAF8	pp	229533.I1RFN1
114901	229533.I1RAF8	pp	229533.I1S1B6
114902	229533.I1RAF8	pp	229533.I1RB71

229533.I1RAF8	pp	229533.I1RNA0
229533.I1RAF8	pp	229533.I1RC82
229533.I1RAF8	pp	229533.I1RJ74
229533.I1RAF8	pp	229533.I1RGA4
229533.I1RAF8	pp	229533.I1RT98
229533.I1RAF8	pp	229533.I1RM06
229533.I1RAF8	pp	229533.I1RAX6
229533.I1RAF8	pp	229533.I1RZJ6
229533.I1RAF8	pp	229533.I1RSL9
229533.I1RAF8	pp	229533.I1RZS6
229533.I1RAF8	pp	229533.I1RTD5
229533.I1RAF8	pp	229533.I1RZV6
229533.I1RAF8	pp	229533.I1S8E2
229533.I1RAF8	pp	229533.Q4HVW2
229533.I1RAF8	pp	229533.I1REF2
229533.I1RAF8	pp	229533.I1S9Y1
229533.I1RAF8	pp	229533.I1RT04
229533.I1RAF8	pp	229533.I1S0R7
229533.I1RAF8	pp	229533.I1RMK3
229533.I1RAF8	pp	229533.I1S6U8
229533.I1RAF8	pp	229533.I1RPU0
229533.I1RAF8	pp	229533.I1S2E9
229533.I1RAF8	pp	229533.I1RKJ6
229533.I1RAF8	pp	229533.I1RXD7
229533.I1RAF8	pp	229533.I1RRT8
229533.I1RAF8	pp	229533.I1RDA0
229533.I1RAF8	pp	229533.I1RBN1
229533.I1RAF8	pp	229533.V6R3X5
229533.I1RAF8	pp	229533.I1RM67
229533.I1RAF8	pp	229533.I1RUQ2
229533.I1RAF8	pp	229533.I1RGF6
229533.I1RAF8	pp	229533.I1RL23
229533.I1RAF8	pp	229533.I1RD64
229533.I1RAF8	pp	229533.I1S2B8
229533.I1RAF8	pp	229533.I1RND6
229533.I1RAF8	pp	229533.I1RR73
229533.I1RAF8	pp	229533.I1RQS8
229533.I1RAF8	pp	229533.I1RPW9
229533.I1RAF8	pp	229533.Q4IFI0
229533.I1RAF8	pp	229533.I1REC2
229533.I1RAF8	pp	229533.I1S4T3
229533.I1RAF8	pp	229533.I1RWN5
229533.I1RAF8	pp	229533.I1S9U2
229533.I1RAF8	pp	229533.I1RJZ7
229533.I1RAF8	pp	229533.I1RW25
229533.I1RAF8	pp	229533.I1RPY1
229533.I1RAF8	pp	229533.Q4IF76
229533.I1RAF8	pp	229533.I1RG56
229533.I1RAF8	pp	229533.I1RY44
229533.I1RAF8	pp	229533.I1RT05
229533.I1RAF8	pp	229533.I1RYT5
229533.I1RAF8	pp	229533.I1RPW4
229533.I1RAF8	pp	229533.I1RM96
229533.I1RAF8	pp	229533.I1S0F1
229533.I1RAF8	pp	229533.Q4IEK8
229533.I1RAF8	pp	229533.I1RNQ5
229533.I1RAF8	pp	229533.I1RG51
229533.I1RAF8	pp	229533.I1RU69
229533.I1RAF8	pp	229533.Q4I662
229533.I1RAF8	pp	229533.I1RAF9
229533.I1RAF8	pp	229533.I1RQM6
229533.I1RAF8	pp	229533.I1RZF6
229533.I1RAF8	pp	229533.I1RFS3
229533.I1RAF8	pp	229533.I1RT80
229533.I1RAF8	pp	229533.I1S9U1
229533.I1RAF8	pp	229533.I1RNV6
229533.I1RAF8	pp	229533.Q4IAA0
229533.I1RAF8	pp	229533.I1S504
229533.I1RAF8	pp	229533.I1RTF4
229533.I1RAF8	pp	229533.I1RHM4
229533.I1RAF8	pp	229533.V6R227
229533.I1RAF8	pp	229533.I1RB27
229533.I1RAF8	pp	229533.Q4IJH1

114976 229533.I1RAF8 pp 229533.I1RRR7
114977 229533.I1RAF8 pp 229533.V6QUE6
114978 229533.I1RAF8 pp 229533.I1RRK8
114979 229533.I1RAF8 pp 229533.I1RZ39
114980 229533.I1RAF8 pp 229533.I1RGC2
114981 229533.I1RAF8 pp 229533.I1S1J6
114982 229533.I1RAF8 pp 229533.I1S255
114983 229533.I1RAF8 pp 229533.I1S060
114984 229533.I1RAF8 pp 229533.I1RMN6
114985 229533.I1RAF8 pp 229533.I1RD22
114986 229533.I1RAF8 pp 229533.I1RKC6
114987 229533.I1RAF8 pp 229533.Q4IBS2
114988 229533.I1RAF8 pp 229533.I1S0J1
114989 229533.I1RAF8 pp 229533.I1RC72
114990 229533.I1RAF8 pp 229533.I1RZE5
114991 229533.I1RAF8 pp 229533.I1S0M9
114992 229533.I1RAF8 pp 229533.I1RAU5
114993 229533.I1RAF8 pp 229533.I1RC27
114994 229533.I1RAF8 pp 229533.I1RYB1
114995 229533.I1RAF8 pp 229533.I1RE00
114996 229533.I1RAF8 pp 229533.Q4IJ56
114997 229533.I1RAF8 pp 229533.I1S044
114998 229533.I1RAF8 pp 229533.I1RAH6
114999 229533.I1RAF8 pp 229533.I1RFJ9
115000 229533.I1RAF8 pp 229533.V6RGN5
115001 229533.I1RAF8 pp 229533.Q4HZ68
115002 229533.I1RAF8 pp 229533.I1RNU6
115003 229533.I1RAF8 pp 229533.I1RM25
115004 229533.I1RAF8 pp 229533.I1RWB5
115005 229533.I1RAF8 pp 229533.I1S296
115006 229533.I1RAF8 pp 229533.I1RG11
115007 229533.I1RAF8 pp 229533.I1RQ31
115008 229533.I1RAF8 pp 229533.I1RF08
115009 229533.I1RAF8 pp 229533.I1RQ72
115010 229533.I1RAF8 pp 229533.I1RC81
115011 229533.I1RAF8 pp 229533.I1RJV3
115012 229533.I1RAF8 pp 229533.I1RAU7
115013 229533.I1RAF8 pp 229533.I1RBL7
115014 229533.I1RAF8 pp 229533.I1RMC0
115015 229533.I1RAF8 pp 229533.I1RMQ0
115016 229533.I1RAF8 pp 229533.I1S274
115017 229533.I1RAF8 pp 229533.Q4IN63
115018 229533.I1RAF8 pp 229533.I1RWN6
115019 229533.I1RAF8 pp 229533.I1RSD8
115020 229533.I1RAF8 pp 229533.I1RCR8
115021 229533.I1RAF8 pp 229533.I1RRY9
115022 229533.I1RAF8 pp 229533.I1RNY2
115023 229533.I1RAF8 pp 229533.I1S2U2
115024 229533.I1RAF8 pp 229533.I1RSM3
115025 229533.I1RAF8 pp 229533.I1RZZ1
115026 229533.I1RAF8 pp 229533.Q4I327
115027 229533.I1RAF8 pp 229533.I1RYB9
115028 229533.I1RAF8 pp 229533.I1RAW0
115029 229533.I1RAF8 pp 229533.I1RZK6
115030 229533.I1RAF8 pp 229533.I1RSR3
115031 229533.I1RAF8 pp 229533.V6RGY3
115032 229533.I1RAF8 pp 229533.I1RNH6
115033 229533.I1RAF8 pp 229533.I1S021
115034 229533.I1RAF8 pp 229533.I1RJZ6
115035 229533.I1RAF8 pp 229533.I1S1C7
115036 229533.I1RAF8 pp 229533.I1S2E8
115037 229533.I1RAF8 pp 229533.I1RSI6
115038 229533.I1RAF8 pp 229533.I1RY03
115039 229533.I1RAF8 pp 229533.I1RY63
115040 229533.I1RAF8 pp 229533.V6RMW6
115041 229533.I1RAF8 pp 229533.I1S1X9
115042 229533.I1RAF8 pp 229533.I1RTD9
115043 229533.I1RAF8 pp 229533.V6R042
115044 229533.I1RAF8 pp 229533.I1RSR8
115045 229533.I1RAF8 pp 229533.I1RRI2
115046 229533.I1RAF8 pp 229533.I1RVX0
115047 229533.I1RAF8 pp 229533.I1RVZ0
115048 229533.I1RAF8 pp 229533.I1RT03

115049	229533.I1RAF8	pp	229533.I1S0Z9
115050	229533.I1RAF8	pp	229533.I1RNK2
115051	229533.I1RAF8	pp	229533.Q4HWE2
115052	229533.I1RAF8	pp	229533.I1RFU8
115053	229533.I1RAF8	pp	229533.I1RSC6
115054	229533.I1RAF8	pp	229533.I1RTH6
115055	229533.I1RAF8	pp	229533.I1RAZ8
115056	229533.I1RAF8	pp	229533.I1RBD2
115057	229533.I1RAF8	pp	229533.I1RZK5
115058	229533.I1RAF8	pp	229533.I1RSS4
115059	229533.I1RAF8	pp	229533.I1RN99
115060	229533.I1RAF8	pp	229533.I1RDG0
115061	229533.I1RAF8	pp	229533.I1S2C0
115062	229533.I1RAF8	pp	229533.I1RK83
115063	229533.I1RAF8	pp	229533.I1RQ28
115064	229533.I1RAF8	pp	229533.I1RK95
115065	229533.I1RAF8	pp	229533.I1RCN5
115066	229533.I1RAF8	pp	229533.I1RPJ3
115067	229533.I1RAF8	pp	229533.I1RTE9
115068	229533.I1RAF8	pp	229533.I1RQA2
115069	229533.I1RAF8	pp	229533.I1RG57
115070	229533.I1RAF8	pp	229533.I1RAN5
115071	229533.I1RAF8	pp	229533.I1RCL3
115072	229533.I1RAF8	pp	229533.I1RBZ3
115073	229533.I1RZV3	pp	229533.I1S9Z4
115074	229533.I1RZV3	pp	229533.I1S6K9
115075	229533.I1RZV3	pp	229533.I1S0J1
115076	229533.I1RZV3	pp	229533.I1S1W9
115077	229533.I1RZV3	pp	229533.I1S1B2
115078	229533.I1RZV3	pp	229533.I1S5B0
115079	229533.I1RZV3	pp	229533.I1S0F8
115080	229533.I1RZV3	pp	229533.V6RCZ8
115081	229533.I1RZV3	pp	229533.I1S5R6
115082	229533.I1RZV3	pp	229533.I1S821
115083	229533.I1RZV3	pp	229533.V6R042
115084	229533.I1RZV3	pp	229533.V6R9Z0
115085	229533.I1RZV3	pp	229533.I1S437
115086	229533.I1RZV3	pp	229533.V6RMW6
115087	229533.I1RZV3	pp	229533.I1S1C7
115088	229533.I1RZV3	pp	229533.Q4IJK2
115089	229533.I1RZV3	pp	229533.I1S0W4
115090	229533.I1RZV3	pp	229533.I1S4S7
115091	229533.I1RZV3	pp	229533.I1S2B8
115092	229533.I1RZV3	pp	229533.I1S0F7
115093	229533.I1RZV3	pp	229533.Q4I624
115094	229533.I1RZV3	pp	229533.I1S438
115095	229533.I1RZV3	pp	229533.Q4HVQ9
115096	229533.I1RZV3	pp	229533.I1S2R0
115097	229533.I1RZV3	pp	229533.V6RTJ8
115098	229533.I1RZV3	pp	229533.I1S9I5
115099	229533.I1RZV3	pp	229533.I1S4S4
115100	229533.I1RZV3	pp	229533.I1S5R1
115101	229533.I1RZV3	pp	229533.I1S2H4
115102	229533.I1RZV3	pp	229533.I1S4Z8
115103	229533.I1RZV3	pp	229533.I1S0L4
115104	229533.I1SA33	pp	229533.V6RMW6
115105	229533.I1SA33	pp	229533.Q4I2J8
115106	229533.I1SA33	pp	229533.Q4I283
115107	229533.I1SA33	pp	229533.V6RCG5
115108	229533.I1SA33	pp	229533.V6R042
115109	229533.I1SA33	pp	229533.V6RCZ8
115110	229533.I1SA33	pp	229533.V6RUY9
115111	229533.I1SA33	pp	229533.Q4I624
115112	229533.I1SA33	pp	229533.V6R1K4
115113	229533.I1SA33	pp	229533.V6RIV9
115114	229533.I1SA33	pp	229533.V6R057
115115	229533.I1SA33	pp	229533.V6QUW4
115116	229533.I1SA33	pp	229533.Q4IJT5
115117	229533.I1SA33	pp	229533.Q4IJK2
115118	229533.I1SA33	pp	229533.V6QW22
115119	229533.I1SA33	pp	229533.V6QYF0
115120	229533.I1SA33	pp	229533.Q4HYR2
115121	229533.I1SA33	pp	229533.V6RTJ8

115122 229533.I1SA33 pp 229533.V6R3X5
115123 229533.A0A1C3YIT8 pp 229533.I1RJM9
115124 229533.A0A1C3YIT8 pp 229533.I1RCP3
115125 229533.I1RQ85 pp 229533.I1RSY2
115126 229533.I1RQ85 pp 229533.I1RT03
115127 229533.I1RQ85 pp 229533.I1RVX0
115128 229533.I1RQ85 pp 229533.I1RUQ3
115129 229533.I1RQ85 pp 229533.I1RUF7
115130 229533.I1RQ85 pp 229533.I1RVQ3
115131 229533.I1RQ85 pp 229533.I1S1B2
115132 229533.I1RQ85 pp 229533.I1RRV3
115133 229533.I1RQ85 pp 229533.I1RQD2
115134 229533.I1RQ85 pp 229533.I1RRN2
115135 229533.I1RQ85 pp 229533.V6R9Z0
115136 229533.I1RQ85 pp 229533.I1RRY9
115137 229533.I1RQ85 pp 229533.I1RWR7
115138 229533.I1RQ85 pp 229533.I1S5B0
115139 229533.I1RQ85 pp 229533.I1S5R6
115140 229533.I1RQ85 pp 229533.I1S437
115141 229533.I1RQ85 pp 229533.I1S1W9
115142 229533.I1RQ85 pp 229533.I1S1C7
115143 229533.I1RQ85 pp 229533.I1RSU1
115144 229533.I1RQ85 pp 229533.I1RRI2
115145 229533.I1RQ85 pp 229533.I1RZM8
115146 229533.I1RQ85 pp 229533.I1RZV3
115147 229533.I1RQ85 pp 229533.I1RVF8
115148 229533.I1RQ85 pp 229533.I1RUG3
115149 229533.I1RQ85 pp 229533.I1S821
115150 229533.I1RQ85 pp 229533.I1RVZ7
115151 229533.I1RQ85 pp 229533.I1S0F8
115152 229533.I1RQ85 pp 229533.V6RCZ8
115153 229533.I1RQ85 pp 229533.I1RZK5
115154 229533.I1RQ85 pp 229533.Q4IPZ1
115155 229533.I1RQ85 pp 229533.I1RXC4
115156 229533.I1RQ85 pp 229533.I1RSF1
115157 229533.I1RQ85 pp 229533.I1S4S4
115158 229533.I1RQ85 pp 229533.I1RS18
115159 229533.I1RQ85 pp 229533.I1S0J1
115160 229533.I1RQ85 pp 229533.V6R042
115161 229533.I1RQ85 pp 229533.I1S6K9
115162 229533.I1RQ85 pp 229533.V6RMW6
115163 229533.I1RQ85 pp 229533.I1RSQ3
115164 229533.I1RQ85 pp 229533.Q4I624
115165 229533.I1RQ85 pp 229533.I1RXC2
115166 229533.I1RQ85 pp 229533.Q4IJK2
115167 229533.I1RQ85 pp 229533.I1S5R1
115168 229533.I1RQ85 pp 229533.I1RQE7
115169 229533.I1RQ85 pp 229533.I1RRM3
115170 229533.I1RQ85 pp 229533.I1RXF8
115171 229533.I1RQ85 pp 229533.I1S2H4
115172 229533.I1RQ85 pp 229533.I1RR24
115173 229533.I1RQ85 pp 229533.I1RTD5
115174 229533.I1RQ85 pp 229533.I1S0L4
115175 229533.I1RQ85 pp 229533.I1RW61
115176 229533.I1RQ85 pp 229533.I1RSZ7
115177 229533.I1RQ85 pp 229533.I1RRI1
115178 229533.I1RQ85 pp 229533.I1RSQ4
115179 229533.I1RQ85 pp 229533.I1S438
115180 229533.I1RQ85 pp 229533.I1RXD7
115181 229533.I1RQ85 pp 229533.I1RRK3
115182 229533.I1RQ85 pp 229533.I1RZ39
115183 229533.I1RQ85 pp 229533.I1RTB3
115184 229533.I1RQ85 pp 229533.Q4HVQ9
115185 229533.I1RQ85 pp 229533.I1RS30
115186 229533.I1RQ85 pp 229533.I1S9I5
115187 229533.I1RQ85 pp 229533.I1S0W4
115188 229533.I1RQ85 pp 229533.I1S2B8
115189 229533.I1RQ85 pp 229533.I1S8F0
115190 229533.I1RQ85 pp 229533.I1RXD6
115191 229533.I1RQ85 pp 229533.I1RSX0
115192 229533.I1RQ85 pp 229533.I1RY86
115193 229533.I1RQ85 pp 229533.I1S277
115194 229533.I1RQ85 pp 229533.I1S0F7

115195	229533.I1RQ85	pp	229533.I1RX46
115196	229533.I1RQ85	pp	229533.I1S4Z8
115197	229533.I1RQ85	pp	229533.I1RYR0
115198	229533.I1RQ85	pp	229533.I1RSA5
115199	229533.I1RQ85	pp	229533.V6RTJ8
115200	229533.I1RQ85	pp	229533.I1RT40
115201	229533.I1RQ85	pp	229533.I1RQM7
115202	229533.I1RQ85	pp	229533.I1S4S7
115203	229533.I1RQ85	pp	229533.I1S2R0
115204	229533.I1RQ85	pp	229533.I1RX95
115205	229533.I1RQ85	pp	229533.I1RYI5
115206	229533.I1RQ85	pp	229533.I1RSQ7
115207	229533.I1RQ85	pp	229533.I1RVZ2
115208	229533.I1RQ85	pp	229533.I1RTA1
115209	229533.I1RGY5	pp	229533.I1S1U7
115210	229533.I1RGY5	pp	229533.I1RVY0
115211	229533.I1RGY5	pp	229533.Q4IER8
115212	229533.I1RGY5	pp	229533.I1SAA0
115213	229533.I1RGY5	pp	229533.I1RU02
115214	229533.I1RGY5	pp	229533.I1RQ93
115215	229533.I1S5R1	pp	229533.V6QYF0
115216	229533.I1S5R1	pp	229533.I1S7A8
115217	229533.I1S5R1	pp	229533.V6RCG5
115218	229533.I1S5R1	pp	229533.V6QW22
115219	229533.I1S5R1	pp	229533.I1S9I5
115220	229533.I1S5R1	pp	229533.V6RUT5
115221	229533.I1S5R1	pp	229533.Q4IJT5
115222	229533.I1S5R1	pp	229533.Q4IJK2
115223	229533.I1S5R1	pp	229533.I1S6K9
115224	229533.I1S5R1	pp	229533.V6RCZ8
115225	229533.I1S5R1	pp	229533.V6RTJ8
115226	229533.I1S5R1	pp	229533.V6R1K4
115227	229533.I1S5R1	pp	229533.V6QUE6
115228	229533.I1S5R1	pp	229533.Q4I8P5
115229	229533.I1S5R1	pp	229533.V6RBN2
115230	229533.I1S5R1	pp	229533.I1S821
115231	229533.I1S5R1	pp	229533.I1S887
115232	229533.I1S5R1	pp	229533.I1SA32
115233	229533.I1S5R1	pp	229533.Q4IPZ1
115234	229533.I1S5R1	pp	229533.I1S9Y1
115235	229533.I1S5R1	pp	229533.V6R9Z0
115236	229533.I1S5R1	pp	229533.V6R227
115237	229533.I1S5R1	pp	229533.I1S6Y8
115238	229533.I1S5R1	pp	229533.I1S8P5
115239	229533.I1S5R1	pp	229533.V6RRH2
115240	229533.I1S5R1	pp	229533.O74638
115241	229533.I1S5R1	pp	229533.V6R3X5
115242	229533.I1S5R1	pp	229533.I1S5R2
115243	229533.I1S5R1	pp	229533.V6RUY9
115244	229533.I1S5R1	pp	229533.Q4HVQ9
115245	229533.I1S5R1	pp	229533.Q4IPH4
115246	229533.I1S5R1	pp	229533.V6R057
115247	229533.I1S5R1	pp	229533.V6R3J1
115248	229533.I1S5R1	pp	229533.Q4HXF6
115249	229533.I1S5R1	pp	229533.V6RMW6
115250	229533.I1S5R1	pp	229533.I1S7W5
115251	229533.I1S5R1	pp	229533.Q4I624
115252	229533.I1S5R1	pp	229533.V6RIV9
115253	229533.I1S5R1	pp	229533.I1S8R3
115254	229533.I1S5R1	pp	229533.I1S5R6
115255	229533.I1S5R1	pp	229533.I1SA33
115256	229533.I1S5R1	pp	229533.V6RGN5
115257	229533.I1S5R1	pp	229533.V6R042
115258	229533.A0A098DJ84	pp	229533.A0A0E0SMA3
115259	229533.A0A098DJ84	pp	229533.I1RFM2
115260	229533.A0A098DJ84	pp	229533.I1RE80
115261	229533.A0A098DJ84	pp	229533.I1RGB3
115262	229533.A0A098DJ84	pp	229533.A0A1C3YJ72
115263	229533.A0A098DJ84	pp	229533.I1RYB2
115264	229533.A0A098DJ84	pp	229533.I1RWF4
115265	229533.A0A098DJ84	pp	229533.I1RGC4
115266	229533.A0A098DJ84	pp	229533.V6RXR0
115267	229533.A0A098DJ84	pp	229533.V6RR38

115268	229533.A0A098DJ84	pp	229533.I1RR90
115269	229533.A0A098DJ84	pp	229533.I1RY35
115270	229533.A0A098DJ84	pp	229533.I1RJR2
115271	229533.A0A098DJ84	pp	229533.I1RJG1
115272	229533.A0A098DJ84	pp	229533.I1RZ29
115273	229533.A0A098DJ84	pp	229533.I1RF79
115274	229533.A0A098DJ84	pp	229533.I1RNT1
115275	229533.A0A098DJ84	pp	229533.I1S1Q3
115276	229533.A0A098DJ84	pp	229533.I1RCA0
115277	229533.A0A098DJ84	pp	229533.I1RBR4
115278	229533.A0A098DJ84	pp	229533.I1R9Z6
115279	229533.A0A098DJ84	pp	229533.I1RT23
115280	229533.A0A098DJ84	pp	229533.I1SAK7
115281	229533.A0A098DJ84	pp	229533.I1RY81
115282	229533.A0A098DJ84	pp	229533.I1RQ76
115283	229533.A0A098DJ84	pp	229533.I1RI22
115284	229533.A0A098DJ84	pp	229533.I1RGS5
115285	229533.A0A098DJ84	pp	229533.I1RVN0
115286	229533.A0A098DJ84	pp	229533.I1S2M5
115287	229533.A0A098DJ84	pp	229533.I1RZZ3
115288	229533.A0A098DJ84	pp	229533.A0A0E0SP71
115289	229533.A0A098DJ84	pp	229533.A0A1C3YLL7
115290	229533.A0A098DJ84	pp	229533.I1RF82
115291	229533.A0A098DJ84	pp	229533.V6RPN0
115292	229533.A0A0E0SKJ9	pp	229533.I1RKJ8
115293	229533.A0A0E0SKJ9	pp	229533.I1RJU3
115294	229533.A0A0E0SKJ9	pp	229533.I1RBU8
115295	229533.A0A0E0SKJ9	pp	229533.I1S0F8
115296	229533.A0A0E0SKJ9	pp	229533.I1RQE7
115297	229533.A0A0E0SKJ9	pp	229533.A0A1C3YKM9
115298	229533.A0A0E0SKJ9	pp	229533.I1RGB2
115299	229533.A0A0E0SKJ9	pp	229533.I1RZ39
115300	229533.A0A0E0SKJ9	pp	229533.I1RCH5
115301	229533.A0A0E0SKJ9	pp	229533.I1RJV3
115302	229533.A0A0E0SKJ9	pp	229533.I1RA11
115303	229533.A0A0E0SKJ9	pp	229533.Q4IJK2
115304	229533.A0A0E0SKJ9	pp	229533.I1S5R1
115305	229533.A0A0E0SKJ9	pp	229533.I1RSZ7
115306	229533.A0A0E0SKJ9	pp	229533.I1RB92
115307	229533.A0A0E0SKJ9	pp	229533.I1RXC2
115308	229533.A0A0E0SKJ9	pp	229533.I1RBY4
115309	229533.A0A0E0SKJ9	pp	229533.I1RCR1
115310	229533.A0A0E0SKJ9	pp	229533.I1RUF7
115311	229533.A0A0E0SKJ9	pp	229533.I1S2B8
115312	229533.A0A0E0SKJ9	pp	229533.I1RUQ3
115313	229533.A0A0E0SKJ9	pp	229533.I1R9M0
115314	229533.A0A0E0SKJ9	pp	229533.I1RBR1
115315	229533.A0A0E0SKJ9	pp	229533.I1RQ33
115316	229533.A0A0E0SKJ9	pp	229533.I1RVQ3
115317	229533.A0A0E0SKJ9	pp	229533.I1S438
115318	229533.A0A0E0SKJ9	pp	229533.I1RE89
115319	229533.A0A0E0SKJ9	pp	229533.V6R9Z0
115320	229533.A0A0E0SKJ9	pp	229533.A0A1C3YJH6
115321	229533.A0A0E0SKJ9	pp	229533.Q4I624
115322	229533.A0A0E0SKJ9	pp	229533.I1RVX0
115323	229533.A0A0E0SKJ9	pp	229533.I1RT03
115324	229533.A0A0E0SKJ9	pp	229533.I1REK8
115325	229533.A0A0E0SKJ9	pp	229533.I1RKP1
115326	229533.A0A0E0SKJ9	pp	229533.I1RGU7
115327	229533.A0A0E0SKJ9	pp	229533.I1RAW0
115328	229533.A0A0E0SKJ9	pp	229533.I1S0L4
115329	229533.A0A0E0SKJ9	pp	229533.Q4IPZ1
115330	229533.A0A0E0SKJ9	pp	229533.A0A1C3YLX0
115331	229533.A0A0E0SKJ9	pp	229533.A0A1C3YHX8
115332	229533.A0A0E0SKJ9	pp	229533.I1RC66
115333	229533.A0A0E0SKJ9	pp	229533.I1RNT9
115334	229533.A0A0E0SKJ9	pp	229533.I1RWR7
115335	229533.A0A0E0SKJ9	pp	229533.I1RRI1
115336	229533.A0A0E0SKJ9	pp	229533.I1S9I5
115337	229533.A0A0E0SKJ9	pp	229533.I1RRY9
115338	229533.A0A0E0SKJ9	pp	229533.I1RE17
115339	229533.A0A0E0SKJ9	pp	229533.I1RML0
115340	229533.A0A0E0SKJ9	pp	229533.A0A1C3YHX6

115341	229533.A0A0E0SKJ9	pp	229533.I1S437
115342	229533.A0A0E0SKJ9	pp	229533.I1S1C7
115343	229533.A0A0E0SKJ9	pp	229533.A0A1C3YJI5
115344	229533.A0A0E0SKJ9	pp	229533.I1RZK5
115345	229533.A0A0E0SKJ9	pp	229533.I1RPE0
115346	229533.A0A0E0SKJ9	pp	229533.I1RR24
115347	229533.A0A0E0SKJ9	pp	229533.I1REC5
115348	229533.A0A0E0SKJ9	pp	229533.I1RVZ2
115349	229533.A0A0E0SKJ9	pp	229533.Q4HVQ9
115350	229533.A0A0E0SKJ9	pp	229533.I1S0J1
115351	229533.A0A0E0SKJ9	pp	229533.I1RRV3
115352	229533.A0A0E0SKJ9	pp	229533.I1RKJ5
115353	229533.A0A0E0SKJ9	pp	229533.I1RC34
115354	229533.A0A0E0SKJ9	pp	229533.I1RJN9
115355	229533.A0A0E0SKJ9	pp	229533.I1RB26
115356	229533.A0A0E0SKJ9	pp	229533.I1RD52
115357	229533.A0A0E0SKJ9	pp	229533.I1RBR2
115358	229533.A0A0E0SKJ9	pp	229533.I1RVF8
115359	229533.A0A0E0SKJ9	pp	229533.I1RTA1
115360	229533.A0A0E0SKJ9	pp	229533.A0A1C3YJV6
115361	229533.A0A0E0SKJ9	pp	229533.A0A1C3YK34
115362	229533.A0A0E0SKJ9	pp	229533.I1RE01
115363	229533.A0A0E0SKJ9	pp	229533.I1RXF8
115364	229533.A0A0E0SKJ9	pp	229533.V6RTJ8
115365	229533.A0A0E0SKJ9	pp	229533.I1RTD5
115366	229533.A0A0E0SKJ9	pp	229533.I1RPH6
115367	229533.A0A0E0SKJ9	pp	229533.I1RKJ4
115368	229533.A0A0E0SKJ9	pp	229533.I1RPY3
115369	229533.A0A0E0SKJ9	pp	229533.I1RXC4
115370	229533.A0A0E0SKJ9	pp	229533.I1RX95
115371	229533.A0A0E0SKJ9	pp	229533.I1S2R0
115372	229533.A0A0E0SKJ9	pp	229533.I1S5B0
115373	229533.A0A0E0SKJ9	pp	229533.I1RGA4
115374	229533.A0A0E0SKJ9	pp	229533.I1RSQ7
115375	229533.A0A0E0SKJ9	pp	229533.I1RCR8
115376	229533.A0A0E0SKJ9	pp	229533.I1RAX6
115377	229533.A0A0E0SKJ9	pp	229533.I1RZM8
115378	229533.A0A0E0SKJ9	pp	229533.I1RYI5
115379	229533.A0A0E0SKJ9	pp	229533.I1RQD2
115380	229533.A0A0E0SKJ9	pp	229533.I1RPP3
115381	229533.A0A0E0SKJ9	pp	229533.I1RT40
115382	229533.A0A0E0SKJ9	pp	229533.I1RNC1
115383	229533.A0A0E0SKJ9	pp	229533.I1S5R6
115384	229533.A0A0E0SKJ9	pp	229533.I1RUG3
115385	229533.A0A0E0SKJ9	pp	229533.I1S4S7
115386	229533.A0A0E0SKJ9	pp	229533.A0A1C3YKV3
115387	229533.A0A0E0SKJ9	pp	229533.A0A1C3YJI6
115388	229533.A0A0E0SKJ9	pp	229533.I1RNB2
115389	229533.A0A0E0SKJ9	pp	229533.I1RZV3
115390	229533.A0A0E0SKJ9	pp	229533.I1RXD6
115391	229533.A0A0E0SKJ9	pp	229533.I1RXD7
115392	229533.A0A0E0SKJ9	pp	229533.V6R042
115393	229533.A0A0E0SKJ9	pp	229533.A0A1C3YJN3
115394	229533.A0A0E0SKJ9	pp	229533.I1RAG7
115395	229533.A0A0E0SKJ9	pp	229533.I1RDF7
115396	229533.A0A0E0SKJ9	pp	229533.I1RSQ3
115397	229533.A0A0E0SKJ9	pp	229533.V6RMW6
115398	229533.A0A0E0SKJ9	pp	229533.I1S0W4
115399	229533.A0A0E0SKJ9	pp	229533.I1RRM3
115400	229533.A0A0E0SKJ9	pp	229533.I1RTB3
115401	229533.A0A0E0SKJ9	pp	229533.I1S1W9
115402	229533.A0A0E0SKJ9	pp	229533.I1S0F7
115403	229533.A0A0E0SKJ9	pp	229533.I1RSA5
115404	229533.A0A0E0SKJ9	pp	229533.I1S821
115405	229533.A0A0E0SKJ9	pp	229533.I1RQ85
115406	229533.A0A0E0SKJ9	pp	229533.I1RPU2
115407	229533.A0A0E0SKJ9	pp	229533.V6RCZ8
115408	229533.A0A0E0SKJ9	pp	229533.I1RSF1
115409	229533.A0A0E0SKJ9	pp	229533.I1RY86
115410	229533.A0A0E0SKJ9	pp	229533.I1RGC5
115411	229533.A0A0E0SKJ9	pp	229533.A0A1C3YJ21
115412	229533.A0A0E0SKJ9	pp	229533.I1R9H9
115413	229533.A0A0E0SKJ9	pp	229533.I1RD22

115414 229533.A0A0E0SKJ9 pp 229533.I1S1B2
115415 229533.A0A0E0SKJ9 pp 229533.A0A1C3YLL3
115416 229533.A0A0E0SKJ9 pp 229533.A0A0E0SMQ1
115417 229533.A0A0E0SKJ9 pp 229533.I1RX46
115418 229533.A0A0E0SKJ9 pp 229533.A0A1C3YLK8
115419 229533.A0A0E0SKJ9 pp 229533.I1RRK3
115420 229533.I1RMN6 pp 229533.I1RU69
115421 229533.I1RMN6 pp 229533.I1RT05
115422 229533.I1RMN6 pp 229533.Q4IF76
115423 229533.I1RMN6 pp 229533.I1RNV6
115424 229533.I1RMN6 pp 229533.I1RT80
115425 229533.I1RMN6 pp 229533.I1S8E2
115426 229533.I1RMN6 pp 229533.I1RNR3
115427 229533.I1RMN6 pp 229533.I1RTF4
115428 229533.I1RMN6 pp 229533.I1RZ39
115429 229533.I1RMN6 pp 229533.I1RPH9
115430 229533.I1RMN6 pp 229533.Q4IJH1
115431 229533.I1RMN6 pp 229533.I1RND6
115432 229533.I1RMN6 pp 229533.V6R3X5
115433 229533.I1RMN6 pp 229533.I1S2A8
115434 229533.I1RMN6 pp 229533.Q4HVW2
115435 229533.I1RMN6 pp 229533.I1RR73
115436 229533.I1RMN6 pp 229533.I1S9U2
115437 229533.I1RMN6 pp 229533.I1S253
115438 229533.I1RMN6 pp 229533.I1S6U8
115439 229533.I1RMN6 pp 229533.I1RZV6
115440 229533.I1RMN6 pp 229533.I1RPU0
115441 229533.I1RMN6 pp 229533.I1RPW9
115442 229533.I1RMN6 pp 229533.I1S0L4
115443 229533.I1RMN6 pp 229533.I1S9Y1
115444 229533.I1RMN6 pp 229533.O74638
115445 229533.I1RMN6 pp 229533.I1RW61
115446 229533.I1RMN6 pp 229533.I1RYT5
115447 229533.I1RMN6 pp 229533.I1S1B6
115448 229533.I1RMN6 pp 229533.Q4I662
115449 229533.I1RMN6 pp 229533.I1RRT8
115450 229533.I1RMN6 pp 229533.I1RQS8
115451 229533.I1RMN6 pp 229533.V6QYF0
115452 229533.I1RMN6 pp 229533.I1RT98
115453 229533.I1RMN6 pp 229533.I1RT40
115454 229533.I1RMN6 pp 229533.I1S540
115455 229533.I1RMN6 pp 229533.Q4IQK7
115456 229533.I1RMN6 pp 229533.Q4IQW0
115457 229533.I1RMN6 pp 229533.I1RNA0
115458 229533.I1RMN6 pp 229533.Q4INZ9
115459 229533.I1RMN6 pp 229533.V6QUW4
115460 229533.I1RMN6 pp 229533.I1RWH5
115461 229533.I1RMN6 pp 229533.I1RZJ6
115462 229533.I1RMN6 pp 229533.V6RTJ8
115463 229533.I1RMN6 pp 229533.I1RTD5
115464 229533.I1RMN6 pp 229533.I1RZS6
115465 229533.I1RMN6 pp 229533.I1RX24
115466 229533.I1RMN6 pp 229533.V6RAC9
115467 229533.I1RMN6 pp 229533.V6R283
115468 229533.I1RMN6 pp 229533.I1RT04
115469 229533.I1RMN6 pp 229533.I1RWK3
115470 229533.I1RMN6 pp 229533.I1RR72
115471 229533.I1RMN6 pp 229533.I1S0R7
115472 229533.I1RMN6 pp 229533.Q4IFI0
115473 229533.I1RMN6 pp 229533.I1S2B8
115474 229533.I1RMN6 pp 229533.I1RQC9
115475 229533.I1RMN6 pp 229533.I1S2M6
115476 229533.I1RMN6 pp 229533.I1S9F8
115477 229533.I1RMN6 pp 229533.I1S2E9
115478 229533.I1RMN6 pp 229533.I1RR00
115479 229533.I1RMN6 pp 229533.I1RXD7
115480 229533.I1RMN6 pp 229533.I1RW25
115481 229533.I1RMN6 pp 229533.I1S2L2
115482 229533.I1RMN6 pp 229533.I1RUQ2
115483 229533.I1RMN6 pp 229533.I1RW71
115484 229533.I1RMN6 pp 229533.Q4I830
115485 229533.I1RMN6 pp 229533.I1S6F0
115486 229533.I1RMN6 pp 229533.Q4I5R9

115487 229533.I1RMN6 pp 229533.Q4HZ42
115488 229533.I1RMN6 pp 229533.I1RSQ7
115489 229533.I1RMN6 pp 229533.I1RRQ3
115490 229533.I1RMN6 pp 229533.I1RRJ8
115491 229533.I1RMN6 pp 229533.I1S503
115492 229533.I1RMN6 pp 229533.Q4IPI1
115493 229533.I1RMN6 pp 229533.I1RPP3
115494 229533.I1RMN6 pp 229533.Q4IAS1
115495 229533.I1RMN6 pp 229533.I1RWF7
115496 229533.I1RMN6 pp 229533.I1RS47
115497 229533.I1RMN6 pp 229533.I1RT33
115498 229533.I1RMN6 pp 229533.I1RQJ5
115499 229533.I1RMN6 pp 229533.I1RRH5
115500 229533.I1RMN6 pp 229533.I1RZ99
115501 229533.I1RMN6 pp 229533.I1RQ65
115502 229533.I1RMN6 pp 229533.I1RRT2
115503 229533.I1RMN6 pp 229533.I1RN67
115504 229533.I1RMN6 pp 229533.I1RQS5
115505 229533.I1RMN6 pp 229533.Q4IEV5
115506 229533.I1RMN6 pp 229533.I1RS30
115507 229533.I1RMN6 pp 229533.I1S9Y0
115508 229533.I1RMN6 pp 229533.I1RRP3
115509 229533.I1RMN6 pp 229533.I1RX46
115510 229533.I1RMN6 pp 229533.I1RY03
115511 229533.I1RMN6 pp 229533.I1RSR8
115512 229533.I1RMN6 pp 229533.I1RTD9
115513 229533.I1RMN6 pp 229533.I1RSL9
115514 229533.I1RMN6 pp 229533.I1S1C7
115515 229533.I1RMN6 pp 229533.Q4HZ47
115516 229533.I1RMN6 pp 229533.I1RVZ0
115517 229533.I1RMN6 pp 229533.I1RNH1
115518 229533.I1RMN6 pp 229533.I1S1J6
115519 229533.I1RMN6 pp 229533.I1RNH9
115520 229533.I1RMN6 pp 229533.I1RQA2
115521 229533.I1RMN6 pp 229533.I1RTD7
115522 229533.I1RMN6 pp 229533.I1RSI0
115523 229533.I1RMN6 pp 229533.I1RNK2
115524 229533.I1RMN6 pp 229533.I1RRI2
115525 229533.I1RMN6 pp 229533.I1RZ59
115526 229533.I1RMN6 pp 229533.I1RWJ3
115527 229533.I1RMN6 pp 229533.I1RZE5
115528 229533.I1RMN6 pp 229533.I1RTH6
115529 229533.I1RMN6 pp 229533.I1S274
115530 229533.I1RMN6 pp 229533.I1RYB1
115531 229533.I1RMN6 pp 229533.V6RGY3
115532 229533.I1RMN6 pp 229533.I1S2C0
115533 229533.I1RMN6 pp 229533.I1RSR3
115534 229533.I1RMN6 pp 229533.I1RSG8
115535 229533.I1RMN6 pp 229533.Q4HZ68
115536 229533.I1RMN6 pp 229533.I1S1U7
115537 229533.I1RMN6 pp 229533.I1S296
115538 229533.I1RMN6 pp 229533.I1RWN6
115539 229533.I1RMN6 pp 229533.I1RTE9
115540 229533.I1RMN6 pp 229533.I1RRY9
115541 229533.I1RMN6 pp 229533.Q4IN63
115542 229533.I1RMN6 pp 229533.I1RNF5
115543 229533.I1RMN6 pp 229533.I1S2U2
115544 229533.I1RMN6 pp 229533.I1RZK5
115545 229533.I1RMN6 pp 229533.I1RQ31
115546 229533.I1RMN6 pp 229533.I1RTG6
115547 229533.I1RMN6 pp 229533.I1RT94
115548 229533.I1RMN6 pp 229533.I1RNC1
115549 229533.I1RMN6 pp 229533.I1S044
115550 229533.I1RMN6 pp 229533.Q4IJK2
115551 229533.I1RMN6 pp 229533.I1RSD8
115552 229533.I1RMN6 pp 229533.I1RYB9
115553 229533.I1RMN6 pp 229533.I1RN18
115554 229533.I1RMN6 pp 229533.I1RNH6
115555 229533.I1RMN6 pp 229533.V6RGN5
115556 229533.I1RMN6 pp 229533.I1RMN7
115557 229533.I1RMN6 pp 229533.I1S021
115558 229533.I1RMN6 pp 229533.I1S2E8
115559 229533.I1RMN6 pp 229533.I1RZZ1

115560 229533.I1RMN6 pp 229533.I1S060
115561 229533.I1RMN6 pp 229533.I1RX14
115562 229533.I1RMN6 pp 229533.I1RNY2
115563 229533.I1RMN6 pp 229533.I1S0J1
115564 229533.I1RMN6 pp 229533.I1RSC6
115565 229533.I1RMN6 pp 229533.Q4I327
115566 229533.I1RMN6 pp 229533.I1RZK6
115567 229533.I1RMN6 pp 229533.I1RSI6
115568 229533.I1RMN6 pp 229533.I1RWB5
115569 229533.I1RMN6 pp 229533.I1RNU6
115570 229533.I1RMN6 pp 229533.I1RX08
115571 229533.I1RMN6 pp 229533.I1RSS4
115572 229533.I1RMN6 pp 229533.Q4IJ56
115573 229533.I1RMN6 pp 229533.I1RY63
115574 229533.I1RMN6 pp 229533.I1S0M9
115575 229533.I1RMN6 pp 229533.V6R042
115576 229533.I1RMN6 pp 229533.I1S1X9
115577 229533.I1RMN6 pp 229533.V6RMW6
115578 229533.I1RMN6 pp 229533.I1RVX0
115579 229533.I1RMN6 pp 229533.I1RPP4
115580 229533.I1RMN6 pp 229533.I1RSQ3
115581 229533.I1RMN6 pp 229533.I1RT03
115582 229533.I1RMN6 pp 229533.Q4IAA0
115583 229533.I1RMN6 pp 229533.V6QUE6
115584 229533.I1RMN6 pp 229533.I1RPY1
115585 229533.I1RMN6 pp 229533.I1RQ28
115586 229533.I1RMN6 pp 229533.Q4IBS2
115587 229533.I1RMN6 pp 229533.I1RN99
115588 229533.I1RMN6 pp 229533.I1RWN5
115589 229533.I1RMN6 pp 229533.I1S4T3
115590 229533.I1RMN6 pp 229533.I1S504
115591 229533.I1RMN6 pp 229533.I1RY44
115592 229533.I1RMN6 pp 229533.I1S0Z9
115593 229533.I1RMN6 pp 229533.I1RZM1
115594 229533.I1RMN6 pp 229533.I1RRR7
115595 229533.I1RMN6 pp 229533.I1RZF6
115596 229533.I1RMN6 pp 229533.V6R227
115597 229533.I1RMN6 pp 229533.Q4IEK8
115598 229533.I1RMN6 pp 229533.I1RNQ5
115599 229533.I1RPU2 pp 229533.I1S9I5
115600 229533.I1RPU2 pp 229533.I1S277
115601 229533.I1RPU2 pp 229533.I1S4Z8
115602 229533.I1RPU2 pp 229533.V6RCC0
115603 229533.I1RPU2 pp 229533.I1S2B8
115604 229533.I1RPU2 pp 229533.Q4IFI0
115605 229533.I1RPU2 pp 229533.I1RPV3
115606 229533.I1RPU2 pp 229533.I1RVZ2
115607 229533.I1RPU2 pp 229533.I1RSQ4
115608 229533.I1RPU2 pp 229533.I1RX95
115609 229533.I1RPU2 pp 229533.I1S438
115610 229533.I1RPU2 pp 229533.I1S2R0
115611 229533.I1RPU2 pp 229533.I1RSZ7
115612 229533.I1RPU2 pp 229533.I1RW61
115613 229533.I1RPU2 pp 229533.I1RSA5
115614 229533.I1RPU2 pp 229533.V6R5T7
115615 229533.I1RPU2 pp 229533.Q4IB65
115616 229533.I1RPU2 pp 229533.I1S0F7
115617 229533.I1RPU2 pp 229533.I1RYR0
115618 229533.I1RPU2 pp 229533.I1RRI1
115619 229533.I1RPU2 pp 229533.V6RTJ8
115620 229533.I1RPU2 pp 229533.I1S0L4
115621 229533.I1RPU2 pp 229533.I1RR24
115622 229533.I1RPU2 pp 229533.I1RQM7
115623 229533.I1RPU2 pp 229533.I1RXD6
115624 229533.I1RPU2 pp 229533.I1RW68
115625 229533.I1RPU2 pp 229533.I1RY86
115626 229533.I1RPU2 pp 229533.I1S5R1
115627 229533.I1RPU2 pp 229533.I1RZB2
115628 229533.I1RPU2 pp 229533.I1S2H4
115629 229533.I1RPU2 pp 229533.I1RX46
115630 229533.I1RPU2 pp 229533.I1S0W4
115631 229533.I1RPU2 pp 229533.I1RSX0
115632 229533.I1RPU2 pp 229533.I1RXC2

115633 229533.I1RPU2 pp 229533.I1RYI5
115634 229533.I1RPU2 pp 229533.I1S4S7
115635 229533.I1RPU2 pp 229533.I1RSQ7
115636 229533.I1RPU2 pp 229533.I1S4S4
115637 229533.I1RPU2 pp 229533.I1RYP2
115638 229533.I1RPU2 pp 229533.Q4HVQ9
115639 229533.I1RPU2 pp 229533.I1RTD5
115640 229533.I1RPU2 pp 229533.I1RRK3
115641 229533.I1RPU2 pp 229533.I1RZ39
115642 229533.I1RPU2 pp 229533.I1RTA1
115643 229533.I1RPU2 pp 229533.I1RT40
115644 229533.I1RPU2 pp 229533.I1RTB3
115645 229533.I1RPU2 pp 229533.V6RGN5
115646 229533.I1RPU2 pp 229533.I1RXD7
115647 229533.I1RPU2 pp 229533.Q4I624
115648 229533.I1RPU2 pp 229533.I1S0R7
115649 229533.I1RPU2 pp 229533.I1RQ33
115650 229533.I1RPU2 pp 229533.I1RQD2
115651 229533.I1RPU2 pp 229533.I1RPY3
115652 229533.I1RPU2 pp 229533.I1S5B0
115653 229533.I1RPU2 pp 229533.I1RUF7
115654 229533.I1RPU2 pp 229533.I1RXF8
115655 229533.I1RPU2 pp 229533.I1RRN2
115656 229533.I1RPU2 pp 229533.I1RQE7
115657 229533.I1RPU2 pp 229533.I1S5R6
115658 229533.I1RPU2 pp 229533.Q4IJK2
115659 229533.I1RPU2 pp 229533.I1RUQ3
115660 229533.I1RPU2 pp 229533.I1S8D3
115661 229533.I1RPU2 pp 229533.I1RWR7
115662 229533.I1RPU2 pp 229533.I1RVX0
115663 229533.I1RPU2 pp 229533.I1RRY9
115664 229533.I1RPU2 pp 229533.I1S1B2
115665 229533.I1RPU2 pp 229533.I1RT03
115666 229533.I1RPU2 pp 229533.I1RVQ3
115667 229533.I1RPU2 pp 229533.V6RWB3
115668 229533.I1RPU2 pp 229533.I1RRV3
115669 229533.I1RPU2 pp 229533.V6R042
115670 229533.I1RPU2 pp 229533.I1S1C7
115671 229533.I1RPU2 pp 229533.I1RSF1
115672 229533.I1RPU2 pp 229533.I1RSQ3
115673 229533.I1RPU2 pp 229533.V6R9Z0
115674 229533.I1RPU2 pp 229533.I1RRM3
115675 229533.I1RPU2 pp 229533.I1RZK5
115676 229533.I1RPU2 pp 229533.V6RMW6
115677 229533.I1RPU2 pp 229533.I1RVZ7
115678 229533.I1RPU2 pp 229533.I1RQ85
115679 229533.I1RPU2 pp 229533.V6RCZ8
115680 229533.I1RPU2 pp 229533.I1RZV3
115681 229533.I1RPU2 pp 229533.I1S6V0
115682 229533.I1RPU2 pp 229533.I1S1W9
115683 229533.I1RPU2 pp 229533.I1S6K9
115684 229533.I1RPU2 pp 229533.I1RUL3
115685 229533.I1RPU2 pp 229533.I1RS18
115686 229533.I1RPU2 pp 229533.I1S0F8
115687 229533.I1RPU2 pp 229533.I1S821
115688 229533.I1RPU2 pp 229533.I1S0J1
115689 229533.I1RPU2 pp 229533.I1S8Y0
115690 229533.I1RPU2 pp 229533.I1S8Y1
115691 229533.I1RPU2 pp 229533.I1S437
115692 229533.I1RPU2 pp 229533.I1RVF8
115693 229533.I1RPU2 pp 229533.I1RXC4
115694 229533.I1RPU2 pp 229533.I1RUG3
115695 229533.I1RPU2 pp 229533.I1RZM8
115696 229533.I1RCJ7 pp 229533.I1RRV1
115697 229533.I1RCJ7 pp 229533.V6R042
115698 229533.I1RCJ7 pp 229533.I1RFT2
115699 229533.I1RCJ7 pp 229533.V6RMW6
115700 229533.I1RCJ7 pp 229533.I1RSQ3
115701 229533.I1RCJ7 pp 229533.I1RN58
115702 229533.I1RCJ7 pp 229533.V6RCG5
115703 229533.I1RCJ7 pp 229533.I1RCR8
115704 229533.I1RCJ7 pp 229533.I1RQ31
115705 229533.I1RCJ7 pp 229533.I1S504

115706 229533.I1RCJ7 pp 229533.I1RNC1
115707 229533.I1RCJ7 pp 229533.I1RWT6
115708 229533.I1RCJ7 pp 229533.I1S1W4
115709 229533.I1RCJ7 pp 229533.I1RZM1
115710 229533.I1RCJ7 pp 229533.V6RIV9
115711 229533.I1RCJ7 pp 229533.I1RWN5
115712 229533.I1RCJ7 pp 229533.I1S0N4
115713 229533.I1RCJ7 pp 229533.I1RLU6
115714 229533.I1RCJ7 pp 229533.V6RVG3
115715 229533.I1RCJ7 pp 229533.I1RY44
115716 229533.I1RCJ7 pp 229533.I1RNQ5
115717 229533.I1RCJ7 pp 229533.I1RRU9
115718 229533.I1RCJ7 pp 229533.I1S044
115719 229533.I1RCJ7 pp 229533.I1RD22
115720 229533.I1RCJ7 pp 229533.I1RRN5
115721 229533.I1RCJ7 pp 229533.I1RPL2
115722 229533.I1RCJ7 pp 229533.I1RQ81
115723 229533.I1RCJ7 pp 229533.I1RVX0
115724 229533.I1RCJ7 pp 229533.I1RWN6
115725 229533.I1RCJ7 pp 229533.I1RT03
115726 229533.I1RCJ7 pp 229533.I1RRY9
115727 229533.I1RCJ7 pp 229533.I1RS18
115728 229533.I1RCJ7 pp 229533.Q4IAA0
115729 229533.I1RCJ7 pp 229533.I1RCM3
115730 229533.I1RCJ7 pp 229533.I1RPP4
115731 229533.I1RCJ7 pp 229533.I1RSK1
115732 229533.I1RCJ7 pp 229533.I1RJV3
115733 229533.I1RCJ7 pp 229533.I1RQA3
115734 229533.I1RCJ7 pp 229533.Q4IJM4
115735 229533.I1RCJ7 pp 229533.I1RTH6
115736 229533.I1RCJ7 pp 229533.I1RFU8
115737 229533.I1RCJ7 pp 229533.I1RZK5
115738 229533.I1RCJ7 pp 229533.I1RTA0
115739 229533.I1RCJ7 pp 229533.Q4IJK2
115740 229533.I1RCJ7 pp 229533.I1RE01
115741 229533.I1RCJ7 pp 229533.I1S2G7
115742 229533.I1RCJ7 pp 229533.I1S0J1
115743 229533.I1RCJ7 pp 229533.I1RFL4
115744 229533.I1RCJ7 pp 229533.I1RZZ1
115745 229533.I1RCJ7 pp 229533.I1S1C7
115746 229533.I1RCJ7 pp 229533.I1RY03
115747 229533.I1RCJ7 pp 229533.I1RN67
115748 229533.I1RCJ7 pp 229533.I1RGA4
115749 229533.I1RCJ7 pp 229533.V6RTJ8
115750 229533.I1RCJ7 pp 229533.I1RP28
115751 229533.I1RCJ7 pp 229533.I1RYW3
115752 229533.I1RCJ7 pp 229533.I1RSV2
115753 229533.I1RCJ7 pp 229533.I1RP27
115754 229533.I1RCJ7 pp 229533.I1RYY5
115755 229533.I1RCJ7 pp 229533.I1RQG4
115756 229533.I1RCJ7 pp 229533.I1RN61
115757 229533.I1RCJ7 pp 229533.V6RRH2
115758 229533.I1RCJ7 pp 229533.V6RKK5
115759 229533.I1RCJ7 pp 229533.I1S503
115760 229533.I1RCJ7 pp 229533.I1RX46
115761 229533.I1RCJ7 pp 229533.I1S8P5
115762 229533.I1RCJ7 pp 229533.I1RSQ7
115763 229533.I1RCJ7 pp 229533.I1RT04
115764 229533.I1RCJ7 pp 229533.I1RE00
115765 229533.I1RCJ7 pp 229533.I1RN63
115766 229533.I1RCJ7 pp 229533.I1RSI0
115767 229533.I1RCJ7 pp 229533.I1RPP3
115768 229533.I1RCJ7 pp 229533.I1S0R7
115769 229533.I1RCJ7 pp 229533.Q4IL82
115770 229533.I1RCJ7 pp 229533.I1RRB0
115771 229533.I1RCJ7 pp 229533.I1RQC9
115772 229533.I1RCJ7 pp 229533.I1S2H4
115773 229533.I1RCJ7 pp 229533.I1RWI5
115774 229533.I1RCJ7 pp 229533.I1RR24
115775 229533.I1RCJ7 pp 229533.I1RXD7
115776 229533.I1RCJ7 pp 229533.I1S0L4
115777 229533.I1RCJ7 pp 229533.I1S2E9
115778 229533.I1RCJ7 pp 229533.I1RZZ9

115779 229533.I1RCJ7 pp 229533.I1RQH3
115780 229533.I1RCJ7 pp 229533.I1RM82
115781 229533.I1RCJ7 pp 229533.I1RPW4
115782 229533.I1RCJ7 pp 229533.I1RNN1
115783 229533.I1RCJ7 pp 229533.I1S4S4
115784 229533.I1RCJ7 pp 229533.I1S2B8
115785 229533.I1RCJ7 pp 229533.I1RWL2
115786 229533.I1RCJ7 pp 229533.I1RTD5
115787 229533.I1RCJ7 pp 229533.I1RRT8
115788 229533.I1RCJ7 pp 229533.I1RXU7
115789 229533.I1RCJ7 pp 229533.I1RYH9
115790 229533.I1RCJ7 pp 229533.I1RW25
115791 229533.I1RCJ7 pp 229533.I1RPU3
115792 229533.I1RCJ7 pp 229533.I1RZ39
115793 229533.I1RCJ7 pp 229533.I1RR73
115794 229533.I1RCJ7 pp 229533.I1RFQ7
115795 229533.I1RCJ7 pp 229533.V6R3X5
115796 229533.I1S6Y6 pp 229533.Q4IR08
115797 229533.A0A1C3YJL4 pp 229533.Q4HWB3
115798 229533.A0A1C3YJL4 pp 229533.I1S092
115799 229533.A0A1C3YJL4 pp 229533.I1RA91
115800 229533.A0A1C3YJL4 pp 229533.I1RSR2
115801 229533.A0A1C3YJL4 pp 229533.I1RY38
115802 229533.A0A1C3YJL4 pp 229533.I1RS02
115803 229533.A0A1C3YJL4 pp 229533.I1RRQ9
115804 229533.A0A1C3YJL4 pp 229533.I1RC78
115805 229533.A0A1C3YJL4 pp 229533.I1S1X2
115806 229533.A0A1C3YJL4 pp 229533.I1RAZ2
115807 229533.A0A1C3YJL4 pp 229533.I1RBP9
115808 229533.I1RA11 pp 229533.I1RAU5
115809 229533.I1RA11 pp 229533.I1S1B2
115810 229533.I1RA11 pp 229533.I1RRV3
115811 229533.I1RA11 pp 229533.I1RR51
115812 229533.I1RA11 pp 229533.V6QUE6
115813 229533.I1RA11 pp 229533.I1RRY9
115814 229533.I1RA11 pp 229533.I1RWR7
115815 229533.I1RA11 pp 229533.I1RNT9
115816 229533.I1RA11 pp 229533.V6RCG5
115817 229533.I1RA11 pp 229533.I1S6K9
115818 229533.I1RA11 pp 229533.I1RBL7
115819 229533.I1RA11 pp 229533.V6RKK5
115820 229533.I1RA11 pp 229533.I1RPR9
115821 229533.I1RA11 pp 229533.I1RGB1
115822 229533.I1RA11 pp 229533.V6RCZ8
115823 229533.I1RA11 pp 229533.I1S1J7
115824 229533.I1RA11 pp 229533.I1RCR5
115825 229533.I1RA11 pp 229533.I1REK8
115826 229533.I1RA11 pp 229533.V6R9Z0
115827 229533.I1RA11 pp 229533.I1RF19
115828 229533.I1RA11 pp 229533.I1RGC5
115829 229533.I1RA11 pp 229533.I1S821
115830 229533.I1RA11 pp 229533.I1REL7
115831 229533.I1RA11 pp 229533.I1RI82
115832 229533.I1RA11 pp 229533.I1RWB0
115833 229533.I1RA11 pp 229533.Q4I2J8
115834 229533.I1RA11 pp 229533.I1RXF0
115835 229533.I1RA11 pp 229533.I1S217
115836 229533.I1RA11 pp 229533.I1RZK5
115837 229533.I1RA11 pp 229533.I1S887
115838 229533.I1RA11 pp 229533.I1RZM8
115839 229533.I1RA11 pp 229533.Q4I283
115840 229533.I1RA11 pp 229533.I1RUQ3
115841 229533.I1RA11 pp 229533.I1RFU8
115842 229533.I1RA11 pp 229533.I1RK95
115843 229533.I1RA11 pp 229533.I1RCG4
115844 229533.I1RA11 pp 229533.I1RBU8
115845 229533.I1RA11 pp 229533.I1RY03
115846 229533.I1RA11 pp 229533.I1RZZ1
115847 229533.I1RA11 pp 229533.I1RG14
115848 229533.I1RA11 pp 229533.I1RGB2
115849 229533.I1RA11 pp 229533.Q4I8P5
115850 229533.I1RA11 pp 229533.I1RQQ5
115851 229533.I1RA11 pp 229533.I1RQ85

115852	229533.I1RA11	pp	229533.I1RS18
115853	229533.I1RA11	pp	229533.I1S504
115854	229533.I1RA11	pp	229533.Q4IJK2
115855	229533.I1RA11	pp	229533.I1RQ81
115856	229533.I1RA11	pp	229533.I1RLU6
115857	229533.I1RA11	pp	229533.I1S5B0
115858	229533.I1RA11	pp	229533.I1SA32
115859	229533.I1RA11	pp	229533.I1RZM1
115860	229533.I1RA11	pp	229533.I1RNQ5
115861	229533.I1RA11	pp	229533.I1RW99
115862	229533.I1RA11	pp	229533.I1RC16
115863	229533.I1RA11	pp	229533.I1RQD2
115864	229533.I1RA11	pp	229533.I1RXC4
115865	229533.I1RA11	pp	229533.I1RR24
115866	229533.I1RA11	pp	229533.I1S419
115867	229533.I1RA11	pp	229533.I1S9I5
115868	229533.I1RA11	pp	229533.I1RLP1
115869	229533.I1RA11	pp	229533.I1RZJ5
115870	229533.I1RA11	pp	229533.I1RXD7
115871	229533.I1RA11	pp	229533.I1RQC9
115872	229533.I1RA11	pp	229533.I1S4S4
115873	229533.I1RA11	pp	229533.I1RWJ8
115874	229533.I1RA11	pp	229533.I1RX25
115875	229533.I1RA11	pp	229533.I1RW25
115876	229533.I1RA11	pp	229533.I1S0R7
115877	229533.I1RA11	pp	229533.I1RKJ5
115878	229533.I1RA11	pp	229533.I1RJU3
115879	229533.I1RA11	pp	229533.I1RMC8
115880	229533.I1RA11	pp	229533.I1RPY3
115881	229533.I1RA11	pp	229533.I1S438
115882	229533.I1RA11	pp	229533.I1RNB2
115883	229533.I1RA11	pp	229533.I1RBT1
115884	229533.I1RA11	pp	229533.I1RW54
115885	229533.I1RA11	pp	229533.I1RE17
115886	229533.I1RA11	pp	229533.V6R1K4
115887	229533.I1RA11	pp	229533.I1RRS8
115888	229533.I1RA11	pp	229533.I1S0X6
115889	229533.I1RA11	pp	229533.I1S1W8
115890	229533.I1RA11	pp	229533.I1S0F5
115891	229533.I1RA11	pp	229533.Q4IJT5
115892	229533.I1RA11	pp	229533.I1RPA4
115893	229533.I1RA11	pp	229533.I1RCS1
115894	229533.I1RA11	pp	229533.I1S7A8
115895	229533.I1RA11	pp	229533.I1RD52
115896	229533.I1RA11	pp	229533.I1S0L3
115897	229533.I1RA11	pp	229533.V6RTJ8
115898	229533.I1RA11	pp	229533.I1RBD5
115899	229533.I1RA11	pp	229533.I1RX95
115900	229533.I1RA11	pp	229533.I1RA85
115901	229533.I1RA11	pp	229533.I1RY86
115902	229533.I1RA11	pp	229533.I1RBR2
115903	229533.I1RA11	pp	229533.I1RX46
115904	229533.I1RA11	pp	229533.I1RAX6
115905	229533.I1RA11	pp	229533.I1RWN6
115906	229533.I1RA11	pp	229533.I1RXD6
115907	229533.I1RA11	pp	229533.I1RAW0
115908	229533.I1RA11	pp	229533.I1RD39
115909	229533.I1RA11	pp	229533.I1RXU9
115910	229533.I1RA11	pp	229533.I1S0T2
115911	229533.I1RA11	pp	229533.I1RGU0
115912	229533.I1RA11	pp	229533.I1RVQ0
115913	229533.I1RA11	pp	229533.V6RUT5
115914	229533.I1RA11	pp	229533.I1RJV3
115915	229533.I1RA11	pp	229533.I1RE89
115916	229533.I1RA11	pp	229533.I1RRX5
115917	229533.I1RA11	pp	229533.I1RVQ3
115918	229533.I1RA11	pp	229533.I1RTB3
115919	229533.I1RA11	pp	229533.I1SA33
115920	229533.I1RA11	pp	229533.I1RSQ7
115921	229533.I1RA11	pp	229533.I1RA70
115922	229533.I1RA11	pp	229533.I1S0F8
115923	229533.I1RA11	pp	229533.I1RBC9
115924	229533.I1RA11	pp	229533.V6QW22

115925	229533.I1RA11	pp	229533.I1S8R3
115926	229533.I1RA11	pp	229533.I1RRM3
115927	229533.I1RA11	pp	229533.V6RMW6
115928	229533.I1RA11	pp	229533.I1RW71
115929	229533.I1RA11	pp	229533.I1RSQ3
115930	229533.I1RA11	pp	229533.I1RTH6
115931	229533.I1RA11	pp	229533.I1S4S7
115932	229533.I1RA11	pp	229533.I1RRI2
115933	229533.I1RA11	pp	229533.I1RWX5
115934	229533.I1RA11	pp	229533.V6R042
115935	229533.I1RA11	pp	229533.I1RRV1
115936	229533.I1RA11	pp	229533.I1RQ33
115937	229533.I1RA11	pp	229533.I1RZW8
115938	229533.I1RA11	pp	229533.V6R057
115939	229533.I1RA11	pp	229533.I1RD17
115940	229533.I1RA11	pp	229533.I1RYW3
115941	229533.I1RA11	pp	229533.I1S1C7
115942	229533.I1RA11	pp	229533.I1RKJ4
115943	229533.I1RA11	pp	229533.Q4IPZ1
115944	229533.I1RA11	pp	229533.I1RVF8
115945	229533.I1RA11	pp	229533.I1RUG3
115946	229533.I1RA11	pp	229533.I1S437
115947	229533.I1RA11	pp	229533.I1RCR1
115948	229533.I1RA11	pp	229533.I1RBR1
115949	229533.I1RA11	pp	229533.I1RCW6
115950	229533.I1RA11	pp	229533.I1RNC1
115951	229533.I1RA11	pp	229533.I1RCR8
115952	229533.I1RA11	pp	229533.I1RUF7
115953	229533.I1RA11	pp	229533.I1RAU7
115954	229533.I1RA11	pp	229533.I1RB92
115955	229533.I1RA11	pp	229533.I1RSF1
115956	229533.I1RA11	pp	229533.I1S7W5
115957	229533.I1RA11	pp	229533.I1RT03
115958	229533.I1RA11	pp	229533.I1RPU2
115959	229533.I1RA11	pp	229533.I1S0J1
115960	229533.I1RA11	pp	229533.I1RRE5
115961	229533.I1RA11	pp	229533.I1RVX0
115962	229533.I1RA11	pp	229533.I1RW46
115963	229533.I1RA11	pp	229533.I1RXR1
115964	229533.I1RA11	pp	229533.I1RC33
115965	229533.I1RA11	pp	229533.I1RM37
115966	229533.I1RA11	pp	229533.I1RCH5
115967	229533.I1RA11	pp	229533.I1RE01
115968	229533.I1RA11	pp	229533.I1RZV3
115969	229533.I1RA11	pp	229533.I1RTA0
115970	229533.I1RA11	pp	229533.I1RQE7
115971	229533.I1RA11	pp	229533.I1RRI1
115972	229533.I1RA11	pp	229533.I1RN18
115973	229533.I1RA11	pp	229533.I1RFL4
115974	229533.I1RA11	pp	229533.I1S1W3
115975	229533.I1RA11	pp	229533.I1RB26
115976	229533.I1RA11	pp	229533.I1RKP1
115977	229533.I1RA11	pp	229533.I1RGB0
115978	229533.I1RA11	pp	229533.I1RG11
115979	229533.I1RA11	pp	229533.I1RPE0
115980	229533.I1RA11	pp	229533.I1REC5
115981	229533.I1RA11	pp	229533.I1S1W9
115982	229533.I1RA11	pp	229533.I1S5R6
115983	229533.I1RA11	pp	229533.I1S2H4
115984	229533.I1RA11	pp	229533.I1RWN5
115985	229533.I1RA11	pp	229533.I1RWQ8
115986	229533.I1RA11	pp	229533.I1S3G4
115987	229533.I1RA11	pp	229533.I1RRN2
115988	229533.I1RA11	pp	229533.V6RIV9
115989	229533.I1RA11	pp	229533.I1RAG7
115990	229533.I1RA11	pp	229533.I1RD22
115991	229533.I1RA11	pp	229533.I1RMV0
115992	229533.I1RA11	pp	229533.O74638
115993	229533.I1RA11	pp	229533.I1RE27
115994	229533.I1RA11	pp	229533.I1RPP4
115995	229533.I1RA11	pp	229533.I1RXC2
115996	229533.I1RA11	pp	229533.I1RA52
115997	229533.I1RA11	pp	229533.I1RPK0

115998 229533.I1RA11 pp 229533.I1S277
115999 229533.I1RA11 pp 229533.I1S5R1
116000 229533.I1RA11 pp 229533.I1RS17
116001 229533.I1RA11 pp 229533.I1RE76
116002 229533.I1RA11 pp 229533.I1RBY4
116003 229533.I1RA11 pp 229533.I1S9Y1
116004 229533.I1RA11 pp 229533.I1RRT8
116005 229533.I1RA11 pp 229533.V6R3J1
116006 229533.I1RA11 pp 229533.I1S2B8
116007 229533.I1RA11 pp 229533.V6R3X5
116008 229533.I1RA11 pp 229533.I1RSX0
116009 229533.I1RA11 pp 229533.I1RC34
116010 229533.I1RA11 pp 229533.I1RRM5
116011 229533.I1RA11 pp 229533.I1RJZ7
116012 229533.I1RA11 pp 229533.Q4HVQ9
116013 229533.I1RA11 pp 229533.I1S503
116014 229533.I1RA11 pp 229533.I1RR73
116015 229533.I1RA11 pp 229533.I1RZ39
116016 229533.I1RA11 pp 229533.I1S0M6
116017 229533.I1RA11 pp 229533.I1RTN3
116018 229533.I1RA11 pp 229533.I1RZZ9
116019 229533.I1RA11 pp 229533.I1RGU7
116020 229533.I1RA11 pp 229533.I1RRK3
116021 229533.I1RA11 pp 229533.I1RJW6
116022 229533.I1RA11 pp 229533.I1RSQ4
116023 229533.I1RA11 pp 229533.I1RWI5
116024 229533.I1RA11 pp 229533.I1RE22
116025 229533.I1RA11 pp 229533.V6RUY9
116026 229533.I1RA11 pp 229533.I1RQ23
116027 229533.I1RA11 pp 229533.I1RT04
116028 229533.I1RA11 pp 229533.I1RL04
116029 229533.I1RA11 pp 229533.I1S0W4
116030 229533.I1RA11 pp 229533.I1RPV3
116031 229533.I1RA11 pp 229533.Q4HXF0
116032 229533.I1RA11 pp 229533.I1RN67
116033 229533.I1RA11 pp 229533.I1RPM4
116034 229533.I1RA11 pp 229533.I1RTD5
116035 229533.I1RA11 pp 229533.I1S0F6
116036 229533.I1RA11 pp 229533.I1RV91
116037 229533.I1RA11 pp 229533.I1S6Y8
116038 229533.I1RA11 pp 229533.I1RDF7
116039 229533.I1RA11 pp 229533.I1S8P5
116040 229533.I1RA11 pp 229533.I1RFS3
116041 229533.I1RA11 pp 229533.I1RT40
116042 229533.I1RA11 pp 229533.I1RYT5
116043 229533.I1RA11 pp 229533.I1S1F3
116044 229533.I1RA11 pp 229533.I1RCJ7
116045 229533.I1RA11 pp 229533.I1RPP3
116046 229533.I1RA11 pp 229533.I1RSZ7
116047 229533.I1RA11 pp 229533.I1RSA5
116048 229533.I1RA11 pp 229533.I1RYI5
116049 229533.I1RA11 pp 229533.I1RW61
116050 229533.I1RA11 pp 229533.I1S0F7
116051 229533.I1RA11 pp 229533.I1RFT1
116052 229533.I1RA11 pp 229533.I1RRJ8
116053 229533.I1RA11 pp 229533.I1RZB2
116054 229533.I1RA11 pp 229533.I1RTA1
116055 229533.I1RA11 pp 229533.I1RJV4
116056 229533.I1RA11 pp 229533.I1RS47
116057 229533.I1RA11 pp 229533.I1RBS6
116058 229533.I1RA11 pp 229533.I1RBS0
116059 229533.I1RA11 pp 229533.I1RJN9
116060 229533.I1RA11 pp 229533.I1RC05
116061 229533.I1RA11 pp 229533.I1RGA4
116062 229533.I1RA11 pp 229533.I1RVZ2
116063 229533.I1RA11 pp 229533.I1REV8
116064 229533.I1RA11 pp 229533.I1RBC7
116065 229533.I1RA11 pp 229533.I1RN63
116066 229533.I1RA11 pp 229533.I1RWK3
116067 229533.I1RA11 pp 229533.I1RYR0
116068 229533.A0A098DJ67 pp 229533.I1RYV3
116069 229533.A0A098DJ67 pp 229533.Q4I7L0
116070 229533.A0A098DJ67 pp 229533.I1RBJ0

116071 229533.A0A098DJ67 pp 229533.Q4HTT2
116072 229533.A0A098DJ67 pp 229533.I1S4Z5
116073 229533.I1RN78 pp 229533.I1RT35
116074 229533.I1RN78 pp 229533.I1RQJ5
116075 229533.I1RN78 pp 229533.I1RNB8
116076 229533.I1RFX9 pp 229533.I1RZX0
116077 229533.I1RFX9 pp 229533.I1RYZ0
116078 229533.I1RFX9 pp 229533.I1RZ87
116079 229533.I1RFX9 pp 229533.I1RNG5
116080 229533.I1RFX9 pp 229533.I1RS71
116081 229533.I1RFX9 pp 229533.I1RZV9
116082 229533.I1RFX9 pp 229533.I1RQ93
116083 229533.I1RFX9 pp 229533.I1RJH6
116084 229533.I1RFX9 pp 229533.I1SAA0
116085 229533.I1RFX9 pp 229533.I1RWU3
116086 229533.I1RT40 pp 229533.I1S5B0
116087 229533.I1RT40 pp 229533.I1RW99
116088 229533.I1RT40 pp 229533.I1S1J7
116089 229533.I1RT40 pp 229533.I1RZM8
116090 229533.I1RT40 pp 229533.I1RWB0
116091 229533.I1RT40 pp 229533.I1RYS0
116092 229533.I1RT40 pp 229533.I1RXF0
116093 229533.I1RT40 pp 229533.V6R9Z0
116094 229533.I1RT40 pp 229533.Q4I2J8
116095 229533.I1RT40 pp 229533.V6RCG5
116096 229533.I1RT40 pp 229533.I1RWR7
116097 229533.I1RT40 pp 229533.I1S821
116098 229533.I1RT40 pp 229533.I1RY03
116099 229533.I1RT40 pp 229533.I1RZZ1
116100 229533.I1RT40 pp 229533.I1S6K9
116101 229533.I1RT40 pp 229533.Q4IJK2
116102 229533.I1RT40 pp 229533.I1S887
116103 229533.I1RT40 pp 229533.V6RCZ8
116104 229533.I1RT40 pp 229533.I1RUQ3
116105 229533.I1RT40 pp 229533.I1RZM1
116106 229533.I1RT40 pp 229533.I1S4Q4
116107 229533.I1RT40 pp 229533.I1S419
116108 229533.I1RT40 pp 229533.I1RZK5
116109 229533.I1RT40 pp 229533.V6QUE6
116110 229533.I1RT40 pp 229533.I1RWT6
116111 229533.I1RT40 pp 229533.I1S1B2
116112 229533.I1RT40 pp 229533.V6R227
116113 229533.I1RT40 pp 229533.I1RX46
116114 229533.I1RT40 pp 229533.I1RUQ2
116115 229533.I1RT40 pp 229533.I1S7A8
116116 229533.I1RT40 pp 229533.V6RTJ8
116117 229533.I1RT40 pp 229533.Q4I283
116118 229533.I1RT40 pp 229533.Q4I8P5
116119 229533.I1RT40 pp 229533.I1RXC4
116120 229533.I1RT40 pp 229533.I1RXD7
116121 229533.I1RT40 pp 229533.I1RZJ5
116122 229533.I1RT40 pp 229533.V6RUT5
116123 229533.I1RT40 pp 229533.I1RTB3
116124 229533.I1RT40 pp 229533.I1S504
116125 229533.I1RT40 pp 229533.I1SA32
116126 229533.I1RT40 pp 229533.I1S4S4
116127 229533.I1RT40 pp 229533.I1RWJ8
116128 229533.I1RT40 pp 229533.I1RX25
116129 229533.I1RT40 pp 229533.I1S0X6
116130 229533.I1RT40 pp 229533.Q4IJT5
116131 229533.I1RT40 pp 229533.I1RVQ0
116132 229533.I1RT40 pp 229533.I1S0R7
116133 229533.I1RT40 pp 229533.I1RXD6
116134 229533.I1RT40 pp 229533.I1RX95
116135 229533.I1RT40 pp 229533.I1S9I5
116136 229533.I1RT40 pp 229533.I1RWD4
116137 229533.I1RT40 pp 229533.I1RW25
116138 229533.I1RT40 pp 229533.V6QW22
116139 229533.I1RT40 pp 229533.I1S438
116140 229533.I1RT40 pp 229533.I1S2R0
116141 229533.I1RT40 pp 229533.I1S0L3
116142 229533.I1RT40 pp 229533.V6QYF0
116143 229533.I1RT40 pp 229533.I1S0F5

229533.I1RT40 pp 229533.I1S4S7
229533.I1RT40 pp 229533.V6R1K4
229533.I1RT40 pp 229533.I1RY86
229533.I1RT40 pp 229533.I1RW71
229533.I1RT40 pp 229533.I1S0T2
229533.I1RT40 pp 229533.I1RVQ3
229533.I1RT40 pp 229533.I1RZ79
229533.I1RT40 pp 229533.I1RVX0
229533.I1RT40 pp 229533.I1SA33
229533.I1RT40 pp 229533.I1RWN6
229533.I1RT40 pp 229533.I1S0N4
229533.I1RT40 pp 229533.I1S437
229533.I1RT40 pp 229533.I1RXU9
229533.I1RT40 pp 229533.I1S5R6
229533.I1RT40 pp 229533.I1RZW8
229533.I1RT40 pp 229533.I1RYW3
229533.I1RT40 pp 229533.I1RWQ8
229533.I1RT40 pp 229533.I1RWX5
229533.I1RT40 pp 229533.I1RVF8
229533.I1RT40 pp 229533.I1S1C7
229533.I1RT40 pp 229533.I1RWN5
229533.I1RT40 pp 229533.I1RXT3
229533.I1RT40 pp 229533.V6RIV9
229533.I1RT40 pp 229533.I1RTH6
229533.I1RT40 pp 229533.I1S0F8
229533.I1RT40 pp 229533.I1S1W9
229533.I1RT40 pp 229533.I1S8V4
229533.I1RT40 pp 229533.I1S8R3
229533.I1RT40 pp 229533.I1RUG3
229533.I1RT40 pp 229533.V6RRH2
229533.I1RT40 pp 229533.I1RVZ7
229533.I1RT40 pp 229533.V6R042
229533.I1RT40 pp 229533.Q4I624
229533.I1RT40 pp 229533.V6RMW6
229533.I1RT40 pp 229533.I1S7W5
229533.I1RT40 pp 229533.I1S1W3
229533.I1RT40 pp 229533.I1S0J1
229533.I1RT40 pp 229533.V6R057
229533.I1RT40 pp 229533.I1RW46
229533.I1RT40 pp 229533.I1RTA0
229533.I1RT40 pp 229533.I1RZV3
229533.I1RT40 pp 229533.I1RUF7
229533.I1RT40 pp 229533.I1RY44
229533.I1RT40 pp 229533.I1S5R1
229533.I1RT40 pp 229533.I1RXR1
229533.I1RT40 pp 229533.I1S2H4
229533.I1RT40 pp 229533.I1S0L4
229533.I1RT40 pp 229533.O74638
229533.I1RT40 pp 229533.I1S8V3
229533.I1RT40 pp 229533.I1S016
229533.I1RT40 pp 229533.I1S9Y1
229533.I1RT40 pp 229533.I1S268
229533.I1RT40 pp 229533.I1RXC2
229533.I1RT40 pp 229533.I1RWI5
229533.I1RT40 pp 229533.I1S6Y8
229533.I1RT40 pp 229533.I1RTD5
229533.I1RT40 pp 229533.Q4IR18
229533.I1RT40 pp 229533.I1RZZ9
229533.I1RT40 pp 229533.I1RV91
229533.I1RT40 pp 229533.I1RW61
229533.I1RT40 pp 229533.V6R3X5
229533.I1RT40 pp 229533.I1S2E9
229533.I1RT40 pp 229533.I1RZ39
229533.I1RT40 pp 229533.I1S0M6
229533.I1RT40 pp 229533.V6R3J1
229533.I1RT40 pp 229533.I1S2B8
229533.I1RT40 pp 229533.I1S4Z8
229533.I1RT40 pp 229533.I1S503
229533.I1RT40 pp 229533.V6RUY9
229533.I1RT40 pp 229533.Q4HVQ9
229533.I1RT40 pp 229533.I1S2M6
229533.I1RT40 pp 229533.I1S0F6
229533.I1RT40 pp 229533.I1RZB2

116217 229533.I1RT40 pp 229533.I1S0W4
116218 229533.I1RT40 pp 229533.I1RWK3
116219 229533.I1RT40 pp 229533.I1S0F7
116220 229533.I1RT40 pp 229533.I1RTA1
116221 229533.I1RT40 pp 229533.I1S8P5
116222 229533.I1RT40 pp 229533.I1RYI5
116223 229533.I1RT40 pp 229533.I1RVZ2
116224 229533.A0A098DQY4 pp 229533.I1RKV1
116225 229533.A0A098DQY4 pp 229533.I1RM41
116226 229533.A0A098DQY4 pp 229533.I1RIB9
116227 229533.A0A098DQY4 pp 229533.I1RM38
116228 229533.A0A098D9A9 pp 229533.A0A1C3YLK8
116229 229533.A0A098D9A9 pp 229533.A0A1C3YJI6
116230 229533.A0A098D9A9 pp 229533.I1RT40
116231 229533.A0A098D9A9 pp 229533.I1RXD7
116232 229533.A0A098D9A9 pp 229533.I1RD52
116233 229533.A0A098D9A9 pp 229533.I1RSA5
116234 229533.A0A098D9A9 pp 229533.I1RTA1
116235 229533.A0A098D9A9 pp 229533.I1RVZ2
116236 229533.A0A098D9A9 pp 229533.I1S4S7
116237 229533.A0A098D9A9 pp 229533.I1RYI5
116238 229533.A0A098D9A9 pp 229533.I1S5R1
116239 229533.A0A098D9A9 pp 229533.I1RWR7
116240 229533.A0A098D9A9 pp 229533.I1RAW0
116241 229533.A0A098D9A9 pp 229533.I1RA11
116242 229533.A0A098D9A9 pp 229533.A0A098DR40
116243 229533.A0A098D9A9 pp 229533.I1RR24
116244 229533.A0A098D9A9 pp 229533.I1RZK5
116245 229533.A0A098D9A9 pp 229533.I1RPE0
116246 229533.A0A098D9A9 pp 229533.I1REK8
116247 229533.A0A098D9A9 pp 229533.I1RRI1
116248 229533.A0A098D9A9 pp 229533.I1RQ33
116249 229533.A0A098D9A9 pp 229533.V6RMW6
116250 229533.A0A098D9A9 pp 229533.I1RCH5
116251 229533.A0A098D9A9 pp 229533.I1RRM3
116252 229533.A0A098D9A9 pp 229533.I1S1W9
116253 229533.A0A098D9A9 pp 229533.I1RZ39
116254 229533.A0A098D9A9 pp 229533.I1RJU3
116255 229533.A0A098D9A9 pp 229533.I1R9M0
116256 229533.A0A098D9A9 pp 229533.I1S2B8
116257 229533.A0A098D9A9 pp 229533.I1RQE7
116258 229533.A0A098D9A9 pp 229533.I1RB92
116259 229533.A0A098D9A9 pp 229533.I1RSZ7
116260 229533.A0A098D9A9 pp 229533.A0A1C3YJN3
116261 229533.A0A098D9A9 pp 229533.I1RZV3
116262 229533.A0A098D9A9 pp 229533.I1S5R6
116263 229533.A0A098D9A9 pp 229533.I1RQ85
116264 229533.A0A098D9A9 pp 229533.A0A0E0SKJ9
116265 229533.A0A098D9A9 pp 229533.I1RPU2
116266 229533.A0A098D9A9 pp 229533.I1RUG3
116267 229533.A0A098D9A9 pp 229533.I1RE01
116268 229533.A0A098D9A9 pp 229533.A0A1C3YK34
116269 229533.A0A098D9A9 pp 229533.A0A1C3YKV3
116270 229533.A0A098D9A9 pp 229533.I1RZM8
116271 229533.I1RQ65 pp 229533.I1RWE1
116272 229533.I1RQ65 pp 229533.Q4I327
116273 229533.I1RQ65 pp 229533.Q4IN63
116274 229533.I1RQ65 pp 229533.I1RRR7
116275 229533.I1RQ65 pp 229533.V6R042
116276 229533.I1RQ65 pp 229533.I1RSI6
116277 229533.I1RQ65 pp 229533.I1RTG6
116278 229533.I1RQ65 pp 229533.I1S1C7
116279 229533.I1RQ65 pp 229533.V6RMW6
116280 229533.I1RQ65 pp 229533.I1RTH6
116281 229533.I1RQ65 pp 229533.V6RGN5
116282 229533.I1RQ65 pp 229533.I1RRI2
116283 229533.I1RQ65 pp 229533.I1S0J1
116284 229533.I1RQ65 pp 229533.I1S060
116285 229533.I1RQ65 pp 229533.I1S2E8
116286 229533.I1RQ65 pp 229533.Q4HZK7
116287 229533.I1RQ65 pp 229533.I1RSD8
116288 229533.I1RQ65 pp 229533.I1RT03
116289 229533.I1RQ65 pp 229533.I1RSG2

116290	229533.I1RQ65	pp	229533.V6RLM7
116291	229533.I1RQ65	pp	229533.I1RVX0
116292	229533.I1RQ65	pp	229533.I1RTE9
116293	229533.I1RQ65	pp	229533.I1S2C0
116294	229533.I1RQ65	pp	229533.I1RWB5
116295	229533.I1RQ65	pp	229533.I1S1X9
116296	229533.I1RQ65	pp	229533.I1RSS4
116297	229533.I1RQ65	pp	229533.Q4I7K4
116298	229533.I1RQ65	pp	229533.I1RWD3
116299	229533.I1RQ65	pp	229533.Q4HWE2
116300	229533.I1RQ65	pp	229533.I1S558
116301	229533.I1RQ65	pp	229533.I1S4T3
116302	229533.I1RQ65	pp	229533.I1S855
116303	229533.I1RQ65	pp	229533.I1RWN5
116304	229533.I1RQ65	pp	229533.Q4HZ68
116305	229533.I1RQ65	pp	229533.I1S274
116306	229533.I1RQ65	pp	229533.I1S0M3
116307	229533.I1RQ65	pp	229533.I1RWN6
116308	229533.I1RQ65	pp	229533.I1RY44
116309	229533.I1RQ65	pp	229533.V6RGY3
116310	229533.I1RQ65	pp	229533.I1RU69
116311	229533.I1RQ65	pp	229533.I1RT40
116312	229533.I1RQ65	pp	229533.V6R283
116313	229533.I1RQ65	pp	229533.I1RTD5
116314	229533.I1RQ65	pp	229533.I1RZ39
116315	229533.I1RQ65	pp	229533.I1S2E9
116316	229533.I1RQ65	pp	229533.I1RT04
116317	229533.I1RQ65	pp	229533.I1S253
116318	229533.I1RQ65	pp	229533.Q4HW67
116319	229533.I1RQ65	pp	229533.I1S8E2
116320	229533.I1RQ65	pp	229533.Q4I830
116321	229533.I1RQ65	pp	229533.Q4IAS1
116322	229533.I1RQ65	pp	229533.I1RR73
116323	229533.I1RQ65	pp	229533.V6R3X5
116324	229533.I1RQ65	pp	229533.I1S503
116325	229533.I1RQ65	pp	229533.I1S9Y0
116326	229533.I1RQ65	pp	229533.I1RZV6
116327	229533.I1RQ65	pp	229533.I1RWF7
116328	229533.I1RQ65	pp	229533.I1RRT8
116329	229533.I1RQ65	pp	229533.I1RRT2
116330	229533.I1RQ65	pp	229533.I1RRJ8
116331	229533.I1RQ65	pp	229533.I1RX14
116332	229533.I1RQ65	pp	229533.I1RS30
116333	229533.I1RQ65	pp	229533.I1RSL9
116334	229533.I1RQ65	pp	229533.I1RRQ3
116335	229533.I1RQ65	pp	229533.I1S1B6
116336	229533.I1RQ65	pp	229533.Q4HZ42
116337	229533.I1RQ65	pp	229533.Q4IJ56
116338	229533.I1RQ65	pp	229533.Q4IQW0
116339	229533.I1RQ65	pp	229533.I1S021
116340	229533.I1RQ65	pp	229533.I1S0M9
116341	229533.I1RQ65	pp	229533.I1RQ81
116342	229533.I1RQ65	pp	229533.Q4I662
116343	229533.I1RQ65	pp	229533.I1RWJ4
116344	229533.I1RQ65	pp	229533.I1RX08
116345	229533.I1RQ65	pp	229533.I1RYB9
116346	229533.I1RQ65	pp	229533.Q4IAA0
116347	229533.I1RQ65	pp	229533.I1RX24
116348	229533.I1RQ65	pp	229533.Q4IEK8
116349	229533.I1RQ65	pp	229533.I1RZS6
116350	229533.I1RQ65	pp	229533.I1RR72
116351	229533.I1RQ65	pp	229533.I1RRK8
116352	229533.I1RQ65	pp	229533.I1S504
116353	229533.I1RQ65	pp	229533.V6QUE6
116354	229533.I1RQ65	pp	229533.I1RZF6
116355	229533.I1RQ65	pp	229533.I1RRU9
116356	229533.I1RQ65	pp	229533.I1S9F8
116357	229533.I1RQ65	pp	229533.Q4IBS2
116358	229533.I1RQ65	pp	229533.I1S9U1
116359	229533.I1RQ65	pp	229533.I1S2B8
116360	229533.I1RQ65	pp	229533.I1RWI5
116361	229533.I1RQ65	pp	229533.V6R227
116362	229533.I1RQ65	pp	229533.I1S255

116363 229533.I1RQ65 pp 229533.Q4IF76
116364 229533.I1RQ65 pp 229533.Q4IP34
116365 229533.I1RQ65 pp 229533.I1RTJ2
116366 229533.I1RQ65 pp 229533.I1RZJ6
116367 229533.I1RQ65 pp 229533.I1S044
116368 229533.I1RQ65 pp 229533.I1RY03
116369 229533.I1RQ65 pp 229533.I1RTD9
116370 229533.I1RQ65 pp 229533.I1S2U2
116371 229533.I1RQ65 pp 229533.I1RZK6
116372 229533.I1RQ65 pp 229533.Q4INZ9
116373 229533.I1RQ65 pp 229533.I1RZZ1
116374 229533.I1RQ65 pp 229533.I1RZB5
116375 229533.I1RQ65 pp 229533.I1RT98
116376 229533.I1RQ65 pp 229533.Q4I7F9
116377 229533.I1RQ65 pp 229533.I1S8I8
116378 229533.I1RQ65 pp 229533.I1S0C1
116379 229533.I1RQ65 pp 229533.I1RZK5
116380 229533.I1RQ65 pp 229533.I1RTI6
116381 229533.I1RQ65 pp 229533.I1S0R7
116382 229533.I1RQ65 pp 229533.I1RQA2
116383 229533.I1RQ65 pp 229533.I1RY63
116384 229533.I1RQ65 pp 229533.I1RSM3
116385 229533.I1RQ65 pp 229533.I1RUQ2
116386 229533.I1RQ65 pp 229533.I1RSC6
116387 229533.I1RQ65 pp 229533.I1RRY9
116388 229533.I1RQ65 pp 229533.I1RVZ0
116389 229533.I1RQ65 pp 229533.I1RSR3
116390 229533.I1RQ65 pp 229533.I1S854
116391 229533.I1RQ65 pp 229533.I1RXD7
116392 229533.I1RQ65 pp 229533.I1S0Z9
116393 229533.I1RQ65 pp 229533.I1RWH5
116394 229533.I1RQ65 pp 229533.Q4I5Z5
116395 229533.I1RQ65 pp 229533.I1RW71
116396 229533.I1RQ65 pp 229533.Q4I5R9
116397 229533.I1RQ65 pp 229533.I1RZ99
116398 229533.I1RQ65 pp 229533.I1RR00
116399 229533.I1RQ65 pp 229533.I1RYB1
116400 229533.I1RQ65 pp 229533.Q4IPI1
116401 229533.I1RQ65 pp 229533.I1RZE5
116402 229533.I1RQ65 pp 229533.I1RX46
116403 229533.I1RQ65 pp 229533.I1RZ59
116404 229533.I1RQ65 pp 229533.I1RQJ5
116405 229533.I1RQ65 pp 229533.I1RSR8
116406 229533.I1RQ65 pp 229533.V6RTJ8
116407 229533.I1RQ65 pp 229533.I1RSQ7
116408 229533.I1RQ65 pp 229533.Q4IJH1
116409 229533.I1RQ65 pp 229533.Q4HZ47
116410 229533.I1RQ65 pp 229533.Q4IQK7
116411 229533.I1RQ65 pp 229533.V6QYF0
116412 229533.I1RQ65 pp 229533.I1RWD4
116413 229533.I1RQ65 pp 229533.I1S6F0
116414 229533.I1RQ65 pp 229533.Q4IFI0
116415 229533.I1RQ65 pp 229533.I1RTF4
116416 229533.I1RQ65 pp 229533.I1RW25
116417 229533.I1RQ65 pp 229533.I1RT33
116418 229533.I1RQ65 pp 229533.I1RT80
116419 229533.I1RQ65 pp 229533.I1RZG3
116420 229533.I1RQ65 pp 229533.I1S6U8
116421 229533.I1RQ65 pp 229533.I1S9U2
116422 229533.I1RQ65 pp 229533.I1RT05
116423 229533.I1RQ65 pp 229533.Q4HVW2
116424 229533.A0A1C3YIT7 pp 229533.I1RMP0
116425 229533.A0A1C3YIT7 pp 229533.I1S549
116426 229533.A0A1C3YIT7 pp 229533.I1RKI0
116427 229533.A0A1C3YIT7 pp 229533.I1RJU7
116428 229533.A0A1C3YIT7 pp 229533.I1RQL4
116429 229533.A0A1C3YIT7 pp 229533.A0A1C3YJL4
116430 229533.A0A1C3YIT7 pp 229533.I1RS02
116431 229533.A0A1C3YIT7 pp 229533.I1RRN4
116432 229533.A0A1C3YIT7 pp 229533.A0A1C3YJG7
116433 229533.I1RIB9 pp 229533.I1RP66
116434 229533.I1RIB9 pp 229533.I1S9I6
116435 229533.I1RIB9 pp 229533.I1SAS4

116436	229533.I1RIB9	pp	229533.I1S6L5
116437	229533.I1RIB9	pp	229533.I1RRN5
116438	229533.I1RIB9	pp	229533.I1RPU1
116439	229533.I1RIB9	pp	229533.I1RM38
116440	229533.I1RIB9	pp	229533.I1RM41
116441	229533.I1RIB9	pp	229533.I1RME4
116442	229533.I1RIB9	pp	229533.I1RWD7
116443	229533.I1RIB9	pp	229533.I1S1J6
116444	229533.I1RIB9	pp	229533.I1RLN1
116445	229533.I1RIB9	pp	229533.I1RW71
116446	229533.I1RIB9	pp	229533.I1S2R6
116447	229533.I1RIB9	pp	229533.I1RKV1
116448	229533.I1RIB9	pp	229533.I1RYJ7
116449	229533.I1RIB9	pp	229533.I1RV58
116450	229533.I1RIB9	pp	229533.I1RJZ7
116451	229533.I1R9G2	pp	229533.I1RHD9
116452	229533.I1R9G2	pp	229533.I1RF02
116453	229533.I1R9G2	pp	229533.I1RGP4
116454	229533.I1RPM2	pp	229533.I1RZ94
116455	229533.I1RPM2	pp	229533.Q4I7L0
116456	229533.I1RWL2	pp	229533.Q4IJM4
116457	229533.I1RWL2	pp	229533.Q4IJK2
116458	229533.I1RWL2	pp	229533.I1RZZ1
116459	229533.I1RWL2	pp	229533.V6R3X5
116460	229533.I1RWL2	pp	229533.I1RWN6
116461	229533.I1RWL2	pp	229533.V6RTJ8
116462	229533.I1RWL2	pp	229533.I1RX46
116463	229533.I1RWL2	pp	229533.I1S2G7
116464	229533.I1RWL2	pp	229533.I1S0N4
116465	229533.I1RWL2	pp	229533.I1S0L3
116466	229533.I1RWL2	pp	229533.I1RZK5
116467	229533.I1RWL2	pp	229533.I1S1W4
116468	229533.I1RWL2	pp	229533.I1S503
116469	229533.I1RWL2	pp	229533.I1RZ39
116470	229533.I1RWL2	pp	229533.I1S504
116471	229533.I1RWL2	pp	229533.I1RYY5
116472	229533.I1RWL2	pp	229533.I1S2E9
116473	229533.I1RWL2	pp	229533.I1RY03
116474	229533.I1RWL2	pp	229533.V6RMW6
116475	229533.I1RWL2	pp	229533.I1RXD7
116476	229533.I1RWL2	pp	229533.I1S1C7
116477	229533.I1RWL2	pp	229533.I1RZM1
116478	229533.I1RWL2	pp	229533.I1S2B8
116479	229533.I1RWL2	pp	229533.V6R042
116480	229533.I1RWL2	pp	229533.I1RY44
116481	229533.I1RWL2	pp	229533.I1S0R7
116482	229533.I1RWL2	pp	229533.I1RWN5
116483	229533.I1RWL2	pp	229533.I1S8P5
116484	229533.I1RWL2	pp	229533.I1S0J1
116485	229533.I1RWL2	pp	229533.V6QW22
116486	229533.A0A1C3YJE2	pp	229533.I1RI22
116487	229533.A0A1C3YJE2	pp	229533.I1R9G2
116488	229533.A0A1C3YJE2	pp	229533.A0A1C3YMK4
116489	229533.A0A1C3YJE2	pp	229533.I1RQM9
116490	229533.A0A1C3YJE2	pp	229533.I1RI23
116491	229533.A0A1C3YJE2	pp	229533.I1RY34
116492	229533.A0A1C3YJE2	pp	229533.I1RHD9
116493	229533.A0A1C3YJE2	pp	229533.I1RGP4
116494	229533.A0A1C3YJE2	pp	229533.I1RPF2
116495	229533.A0A1C3YJE2	pp	229533.I1REC9
116496	229533.A0A1C3YJE2	pp	229533.I1RF02
116497	229533.A0A1C3YJE2	pp	229533.I1RCM8
116498	229533.I1RYE2	pp	229533.I1S5Z2
116499	229533.I1RYE2	pp	229533.Q4IPC8
116500	229533.I1RYE2	pp	229533.V6QV38
116501	229533.I1RYE2	pp	229533.I1SAA0
116502	229533.I1RYE2	pp	229533.I1S9Y1
116503	229533.I1RYE2	pp	229533.I1S1X4
116504	229533.I1RM41	pp	229533.I1RSI3
116505	229533.I1RM41	pp	229533.I1RST9
116506	229533.I1RM41	pp	229533.I1S968
116507	229533.I1RM41	pp	229533.I1RRW0
116508	229533.I1RM41	pp	229533.I1RWC1

116509 229533.I1RM41 pp 229533.I1RQV8
116510 229533.I1RM41 pp 229533.I1RY32
116511 229533.I1RM41 pp 229533.I1RY44
116512 229533.I1RM41 pp 229533.I1RZC3
116513 229533.I1RM41 pp 229533.I1S2E9
116514 229533.I1RM41 pp 229533.I1S969
116515 229533.I1RM41 pp 229533.V6RHX2
116516 229533.I1RM41 pp 229533.I1S267
116517 229533.I1RM41 pp 229533.I1SAB4
116518 229533.I1RM41 pp 229533.I1RYP5
116519 229533.I1RM41 pp 229533.I1S0M4
116520 229533.I1RM41 pp 229533.I1RZT1
116521 229533.I1RM41 pp 229533.I1RQV7
116522 229533.I1RM41 pp 229533.I1RS35
116523 229533.I1RM41 pp 229533.I1RZX3
116524 229533.I1RM41 pp 229533.I1S224
116525 229533.I1RM41 pp 229533.I1RQS4
116526 229533.V6RMW6 pp 229533.V6RRH2
116527 229533.V6RMW6 pp 229533.V6RUY9
116528 229533.V6RMW6 pp 229533.V6RVG3
116529 229533.V6RMW6 pp 229533.V6RTJ8
116530 229533.I1RGP4 pp 229533.I1S2N3
116531 229533.I1RGP4 pp 229533.I1RIJ1
116532 229533.I1RGP4 pp 229533.I1RXM0
116533 229533.I1RGP4 pp 229533.I1RHD9
116534 229533.I1RGP4 pp 229533.I1S2K2
116535 229533.I1RA91 pp 229533.I1RAZ2
116536 229533.I1RA91 pp 229533.I1RW63
116537 229533.I1RA91 pp 229533.I1RQ93
116538 229533.I1RA91 pp 229533.I1RB06
116539 229533.I1RA91 pp 229533.I1RD01
116540 229533.I1RA91 pp 229533.I1S1X2
116541 229533.I1RA91 pp 229533.I1RD69
116542 229533.I1RA91 pp 229533.I1RCK7
116543 229533.I1RA91 pp 229533.I1RVV9
116544 229533.I1RA91 pp 229533.I1RYR9
116545 229533.I1RA91 pp 229533.I1RYZ0
116546 229533.I1RA91 pp 229533.I1RNI7
116547 229533.I1RA91 pp 229533.I1SAA0
116548 229533.I1RA91 pp 229533.I1RFY9
116549 229533.A0A1C3YJS0 pp 229533.I1S0N7
116550 229533.A0A1C3YJS0 pp 229533.I1RNB8
116551 229533.A0A1C3YJS0 pp 229533.A0A1C3YKN3
116552 229533.A0A1C3YJS0 pp 229533.I1RN48
116553 229533.A0A1C3YJS0 pp 229533.I1RNE7
116554 229533.A0A1C3YJS0 pp 229533.I1RWD7
116555 229533.A0A1C3YJS0 pp 229533.I1RA18
116556 229533.A0A1C3YJS0 pp 229533.I1RN78
116557 229533.A0A1C3YJS0 pp 229533.I1RN58
116558 229533.A0A1C3YJS0 pp 229533.I1RAS2
116559 229533.A0A1C3YJS0 pp 229533.I1RCU3
116560 229533.A0A1C3YJS0 pp 229533.I1R9X2
116561 229533.A0A1C3YJS0 pp 229533.I1RAV4
116562 229533.A0A1C3YJS0 pp 229533.I1RG90
116563 229533.A0A1C3YJS0 pp 229533.I1RRU9
116564 229533.A0A1C3YJS0 pp 229533.I1RT35
116565 229533.A0A1C3YJR9 pp 229533.I1RWL9
116566 229533.I1RKV1 pp 229533.I1S6T1
116567 229533.I1RKV1 pp 229533.I1RM41
116568 229533.I1RKV1 pp 229533.I1RM38
116569 229533.I1RLM2 pp 229533.I1RYU0
116570 229533.I1RLM2 pp 229533.I1RTT0
116571 229533.I1RLM2 pp 229533.V6QWD8
116572 229533.I1RLM2 pp 229533.I1S3G4
116573 229533.I1RLM2 pp 229533.I1S1W8
116574 229533.I1RLM2 pp 229533.I1RXH1
116575 229533.I1RLM2 pp 229533.I1RMU5
116576 229533.I1RLM2 pp 229533.I1RSL2
116577 229533.I1RLM2 pp 229533.I1RMS5
116578 229533.I1RLM2 pp 229533.I1RSE0
116579 229533.I1RLM2 pp 229533.I1RLM3
116580 229533.A0A098DJ50 pp 229533.A0A098DQY4
116581 229533.A0A098DJ50 pp 229533.I1RIB9

116582 229533.A0A098DJ50 pp 229533.I1RM38
116583 229533.A0A098DJ50 pp 229533.I1RM41
116584 229533.A0A098DJ50 pp 229533.I1RKV1
116585 229533.I1S2G7 pp 229533.V6RTJ8
116586 229533.I1S2G7 pp 229533.Q4IJK2
116587 229533.I1S2G7 pp 229533.I1S439
116588 229533.I1S2G7 pp 229533.V6RVG3
116589 229533.I1S2G7 pp 229533.V6R3X5
116590 229533.I1S2G7 pp 229533.Q4IFI0
116591 229533.I1S2G7 pp 229533.Q4IJM4
116592 229533.I1S2G7 pp 229533.I1S504
116593 229533.I1S2G7 pp 229533.V6RGI1
116594 229533.I1S2G7 pp 229533.I1S503
116595 229533.I1S2G7 pp 229533.V6RKY7
116596 229533.I1S2G7 pp 229533.V6RMW6
116597 229533.I1RF02 pp 229533.I1RZ29
116598 229533.I1RF02 pp 229533.I1RIP9
116599 229533.I1RF02 pp 229533.I1S2F2
116600 229533.I1RF02 pp 229533.I1RV78
116601 229533.I1RF02 pp 229533.I1RVV5
116602 229533.I1RF02 pp 229533.I1RZ86
116603 229533.I1RF02 pp 229533.I1RSJ4
116604 229533.I1RF02 pp 229533.I1RHD9
116605 229533.I1RF02 pp 229533.I1RGP4
116606 229533.I1RF02 pp 229533.I1RX64
116607 229533.I1RF02 pp 229533.I1RWX7
116608 229533.I1RF02 pp 229533.I1RUG1
116609 229533.I1RF02 pp 229533.I1RW03
116610 229533.I1RF02 pp 229533.I1RFN0
116611 229533.I1RF02 pp 229533.I1RSC7
116612 229533.I1RF02 pp 229533.I1RZT1
116613 229533.I1RF02 pp 229533.I1RSL6
116614 229533.I1RF02 pp 229533.I1S0D5
116615 229533.I1RF02 pp 229533.I1RLE7
116616 229533.I1RF02 pp 229533.I1RRT1
116617 229533.I1RF02 pp 229533.I1RMB7
116618 229533.I1RF02 pp 229533.I1RWM6
116619 229533.I1RF02 pp 229533.I1RG34
116620 229533.I1RF02 pp 229533.I1RYV1
116621 229533.I1RF02 pp 229533.I1RLJ8
116622 229533.I1RF02 pp 229533.Q4INS6
116623 229533.I1RF02 pp 229533.I1RWB3
116624 229533.I1RF02 pp 229533.I1RFJ2
116625 229533.I1RF02 pp 229533.Q4I0J6
116626 229533.I1RG09 pp 229533.I1RSE0
116627 229533.I1RG09 pp 229533.I1RJU9
116628 229533.I1RG09 pp 229533.I1RLM3
116629 229533.I1RG09 pp 229533.I1RLM2
116630 229533.I1RG09 pp 229533.I1RSL2
116631 229533.I1RG09 pp 229533.I1RXH1
116632 229533.I1RG09 pp 229533.I1RMS5
116633 229533.I1RG09 pp 229533.I1RMU5
116634 229533.I1RG09 pp 229533.I1S1W8
116635 229533.I1RG09 pp 229533.I1RYU0
116636 229533.I1RG09 pp 229533.V6QWD8
116637 229533.I1RG09 pp 229533.I1RTT0
116638 229533.I1RG09 pp 229533.I1S3G4
116639 229533.I1RM82 pp 229533.I1RT40
116640 229533.I1RM82 pp 229533.I1RXU7
116641 229533.I1RM82 pp 229533.I1RTD5
116642 229533.I1RM82 pp 229533.I1RZJ6
116643 229533.I1RM82 pp 229533.I1RQG4
116644 229533.I1RM82 pp 229533.Q4HXF0
116645 229533.I1RM82 pp 229533.I1RSV2
116646 229533.I1RM82 pp 229533.I1S0R7
116647 229533.I1RM82 pp 229533.I1RRJ8
116648 229533.I1RM82 pp 229533.V6QW22
116649 229533.I1RM82 pp 229533.I1RT04
116650 229533.I1RM82 pp 229533.I1RN61
116651 229533.I1RM82 pp 229533.I1RXD7
116652 229533.I1RM82 pp 229533.I1RSQ7
116653 229533.I1RM82 pp 229533.I1RQC9
116654 229533.I1RM82 pp 229533.I1S2E9

116655 229533.I1RM82 pp 229533.I1RPP3
116656 229533.I1RM82 pp 229533.I1RNG4
116657 229533.I1RM82 pp 229533.I1RQH3
116658 229533.I1RM82 pp 229533.I1S8P5
116659 229533.I1RM82 pp 229533.I1RVQ0
116660 229533.I1RM82 pp 229533.I1RN67
116661 229533.I1RM82 pp 229533.I1S503
116662 229533.I1RM82 pp 229533.Q4HZ42
116663 229533.I1RM82 pp 229533.I1RX46
116664 229533.I1RM82 pp 229533.I1RZ39
116665 229533.I1RM82 pp 229533.I1RS47
116666 229533.I1RM82 pp 229533.V6RTJ8
116667 229533.I1RM82 pp 229533.I1S2G7
116668 229533.I1RM82 pp 229533.I1RTF4
116669 229533.I1RM82 pp 229533.I1RP27
116670 229533.I1RM82 pp 229533.I1S0X6
116671 229533.I1RM82 pp 229533.I1RWK3
116672 229533.I1RM82 pp 229533.V6R3X5
116673 229533.I1RM82 pp 229533.I1RRG3
116674 229533.I1RM82 pp 229533.I1RR73
116675 229533.I1RM82 pp 229533.I1RWI5
116676 229533.I1RM82 pp 229533.I1RNR2
116677 229533.I1RM82 pp 229533.Q4IF76
116678 229533.I1RM82 pp 229533.I1S0J1
116679 229533.I1RM82 pp 229533.I1S4S4
116680 229533.I1RM82 pp 229533.I1RZZ9
116681 229533.I1RM82 pp 229533.I1RQ81
116682 229533.I1RM82 pp 229533.I1RWL2
116683 229533.I1RM82 pp 229533.Q4IJ56
116684 229533.I1RM82 pp 229533.I1RQM6
116685 229533.I1RM82 pp 229533.I1RRT8
116686 229533.I1RM82 pp 229533.I1RS20
116687 229533.I1RM82 pp 229533.I1RNN1
116688 229533.I1RM82 pp 229533.Q4I662
116689 229533.I1RM82 pp 229533.I1RPM4
116690 229533.I1RM82 pp 229533.I1RWD3
116691 229533.I1RM82 pp 229533.I1RPU3
116692 229533.I1RM82 pp 229533.Q4I7K4
116693 229533.I1RM82 pp 229533.I1RW25
116694 229533.I1RM82 pp 229533.V6QUW4
116695 229533.I1RM82 pp 229533.I1RPW4
116696 229533.I1RM82 pp 229533.I1S0R3
116697 229533.I1RM82 pp 229533.I1S2B8
116698 229533.I1RM82 pp 229533.Q4IAA0
116699 229533.I1RM82 pp 229533.I1RNC1
116700 229533.I1RM82 pp 229533.I1RPP4
116701 229533.I1RM82 pp 229533.I1RN58
116702 229533.I1RM82 pp 229533.I1RWN5
116703 229533.I1RM82 pp 229533.I1S0R4
116704 229533.I1RM82 pp 229533.I1RZM1
116705 229533.I1RM82 pp 229533.I1RQ31
116706 229533.I1RM82 pp 229533.I1S2C0
116707 229533.I1RM82 pp 229533.I1RZK5
116708 229533.I1RM82 pp 229533.I1RRV1
116709 229533.I1RM82 pp 229533.I1RUG3
116710 229533.I1RM82 pp 229533.I1RSK1
116711 229533.I1RM82 pp 229533.Q4IEK8
116712 229533.I1RM82 pp 229533.V6R042
116713 229533.I1RM82 pp 229533.I1RNQ5
116714 229533.I1RM82 pp 229533.I1RSQ3
116715 229533.I1RM82 pp 229533.I1RWN6
116716 229533.I1RM82 pp 229533.I1RY44
116717 229533.I1RM82 pp 229533.I1RRY9
116718 229533.I1RM82 pp 229533.I1S504
116719 229533.I1RM82 pp 229533.V6RMW6
116720 229533.I1RM82 pp 229533.I1S1W4
116721 229533.I1RM82 pp 229533.Q4IJK2
116722 229533.I1RM82 pp 229533.V6QUE6
116723 229533.I1RM82 pp 229533.I1RQE7
116724 229533.I1RM82 pp 229533.I1RN26
116725 229533.I1RM82 pp 229533.I1RN18
116726 229533.I1RM82 pp 229533.I1RRU9
116727 229533.I1RM82 pp 229533.I1S2E8

116728 229533.I1RM82 pp 229533.I1S0N4
116729 229533.I1RM82 pp 229533.V6R227
116730 229533.I1RM82 pp 229533.I1RW99
116731 229533.I1RM82 pp 229533.V6QVH8
116732 229533.I1RM82 pp 229533.I1RY03
116733 229533.I1RM82 pp 229533.V6RBN2
116734 229533.I1RM82 pp 229533.I1RYW3
116735 229533.I1RM82 pp 229533.I1S5B0
116736 229533.I1RM82 pp 229533.I1S1C7
116737 229533.I1RM82 pp 229533.I1RZK6
116738 229533.I1RM82 pp 229533.I1RTA0
116739 229533.I1RM82 pp 229533.I1RYY5
116740 229533.I1RM82 pp 229533.Q4IJM4
116741 229533.I1RM82 pp 229533.I1RZZ1
116742 229533.I1RM82 pp 229533.Q4I2J8
116743 229533.I1RM82 pp 229533.I1RRI2
116744 229533.I1RM82 pp 229533.I1RZB5
116745 229533.I1RM82 pp 229533.I1RT03
116746 229533.I1RM82 pp 229533.I1RSS4
116747 229533.I1RM82 pp 229533.I1RWD7
116748 229533.I1RM82 pp 229533.I1RSI0
116749 229533.I1RM82 pp 229533.I1RVX0
116750 229533.I1RM82 pp 229533.I1RTH6
116751 229533.I1RE98 pp 229533.I1S2B4
116752 229533.I1RE98 pp 229533.I1S8Y0
116753 229533.A0A098D991 pp 229533.I1R9G2
116754 229533.A0A098D991 pp 229533.A0A1C3YJE2
116755 229533.A0A098D991 pp 229533.I1RGP4
116756 229533.A0A098D991 pp 229533.A0A1C3YMK4
116757 229533.A0A098D991 pp 229533.I1RF02
116758 229533.A0A098D991 pp 229533.I1RHD9
116759 229533.I1RZA9 pp 229533.I1SAA0
116760 229533.I1RZA9 pp 229533.Q4ICA8
116761 229533.V6R042 pp 229533.V6RCZ8
116762 229533.V6R042 pp 229533.V6RUT5
116763 229533.V6R042 pp 229533.V6RCG5
116764 229533.V6R042 pp 229533.V6R1K4
116765 229533.V6R042 pp 229533.V6RBN2
116766 229533.V6R042 pp 229533.V6RTJ8
116767 229533.V6R042 pp 229533.V6R3J1
116768 229533.V6R042 pp 229533.V6RUY9
116769 229533.V6R042 pp 229533.V6RGN5
116770 229533.V6R042 pp 229533.V6R4J1
116771 229533.V6R042 pp 229533.V6RMW6
116772 229533.V6R042 pp 229533.V6R3X5
116773 229533.V6R042 pp 229533.V6RRH2
116774 229533.V6R042 pp 229533.V6R057
116775 229533.V6R042 pp 229533.V6RIV9
116776 229533.V6R042 pp 229533.V6R283
116777 229533.A0A098DEC2 pp 229533.I1RT40
116778 229533.A0A098DEC2 pp 229533.I1RAF8
116779 229533.A0A098DEC2 pp 229533.Q4IR18
116780 229533.A0A098DEC2 pp 229533.A0A1C3YMK1
116781 229533.A0A098DEC2 pp 229533.A0A0E0SFN0
116782 229533.A0A098DEC2 pp 229533.I1S0R7
116783 229533.A0A098DEC2 pp 229533.I1RT04
116784 229533.A0A098DEC2 pp 229533.I1RUQ2
116785 229533.A0A098DEC2 pp 229533.I1RLV9
116786 229533.A0A098DEC2 pp 229533.A0A1C3YLK8
116787 229533.A0A098DEC2 pp 229533.I1RJW6
116788 229533.A0A098DEC2 pp 229533.I1RX46
116789 229533.A0A098DEC2 pp 229533.A0A1C3YMW0
116790 229533.A0A098DEC2 pp 229533.Q4IPI1
116791 229533.A0A098DEC2 pp 229533.I1RBS6
116792 229533.A0A098DEC2 pp 229533.I1RGA4
116793 229533.A0A098DEC2 pp 229533.I1RC82
116794 229533.A0A098DEC2 pp 229533.I1RRM5
116795 229533.A0A098DEC2 pp 229533.I1RJV4
116796 229533.A0A098DEC2 pp 229533.I1RN63
116797 229533.A0A098DEC2 pp 229533.Q4IFI0
116798 229533.A0A098DEC2 pp 229533.I1RC03
116799 229533.A0A098DEC2 pp 229533.I1RBY4
116800 229533.A0A098DEC2 pp 229533.A0A1C3YKK5

116801	229533.A0A098DEC2	pp	229533.A0A0E0RU84
116802	229533.A0A098DEC2	pp	229533.I1RWH5
116803	229533.A0A098DEC2	pp	229533.I1RW25
116804	229533.A0A098DEC2	pp	229533.I1RT98
116805	229533.A0A098DEC2	pp	229533.I1RFW5
116806	229533.A0A098DEC2	pp	229533.A0A1C3YIS7
116807	229533.A0A098DEC2	pp	229533.I1RBC9
116808	229533.A0A098DEC2	pp	229533.I1RKL3
116809	229533.A0A098DEC2	pp	229533.I1RQ31
116810	229533.A0A098DEC2	pp	229533.I1RD56
116811	229533.A0A098DEC2	pp	229533.I1RQ65
116812	229533.A0A098DEC2	pp	229533.I1S0L3
116813	229533.A0A098DEC2	pp	229533.I1RPP3
116814	229533.A0A098DEC2	pp	229533.I1RQJ5
116815	229533.A0A098DEC2	pp	229533.I1RSQ7
116816	229533.A0A098DEC2	pp	229533.I1RUG3
116817	229533.A0A098DEC2	pp	229533.I1RRJ8
116818	229533.A0A098DEC2	pp	229533.I1RZZ9
116819	229533.A0A098DEC2	pp	229533.A0A098DPR9
116820	229533.A0A098DEC2	pp	229533.Q4IN63
116821	229533.A0A098DEC2	pp	229533.I1RBT1
116822	229533.A0A098DEC2	pp	229533.I1RU69
116823	229533.A0A098DEC2	pp	229533.V6QW22
116824	229533.A0A098DEC2	pp	229533.I1RZM8
116825	229533.A0A098DEC2	pp	229533.I1RWD4
116826	229533.A0A098DEC2	pp	229533.I1RNV6
116827	229533.A0A098DEC2	pp	229533.I1RI82
116828	229533.A0A098DEC2	pp	229533.I1RT80
116829	229533.A0A098DEC2	pp	229533.A0A098DVS9
116830	229533.A0A098DEC2	pp	229533.A0A1C3YIP5
116831	229533.A0A098DEC2	pp	229533.I1RRQ3
116832	229533.A0A098DEC2	pp	229533.I1RYB9
116833	229533.A0A098DEC2	pp	229533.I1RVQ0
116834	229533.A0A098DEC2	pp	229533.I1S8E2
116835	229533.A0A098DEC2	pp	229533.I1RNH6
116836	229533.A0A098DEC2	pp	229533.I1RJU3
116837	229533.A0A098DEC2	pp	229533.I1RS47
116838	229533.A0A098DEC2	pp	229533.I1S021
116839	229533.A0A098DEC2	pp	229533.I1RD52
116840	229533.A0A098DEC2	pp	229533.I1RND6
116841	229533.A0A098DEC2	pp	229533.A0A1C3YIN1
116842	229533.A0A098DEC2	pp	229533.I1RX08
116843	229533.A0A098DEC2	pp	229533.I1RR73
116844	229533.A0A098DEC2	pp	229533.I1RWJ8
116845	229533.A0A098DEC2	pp	229533.I1RZV6
116846	229533.A0A098DEC2	pp	229533.I1RPY1
116847	229533.A0A098DEC2	pp	229533.I1RC33
116848	229533.A0A098DEC2	pp	229533.I1RM96
116849	229533.A0A098DEC2	pp	229533.I1RZF6
116850	229533.A0A098DEC2	pp	229533.I1RSI6
116851	229533.A0A098DEC2	pp	229533.I1RRR7
116852	229533.A0A098DEC2	pp	229533.I1RKJ6
116853	229533.A0A098DEC2	pp	229533.I1RPU0
116854	229533.A0A098DEC2	pp	229533.V6RCG5
116855	229533.A0A098DEC2	pp	229533.I1S5R1
116856	229533.A0A098DEC2	pp	229533.A0A1C3YKB0
116857	229533.A0A098DEC2	pp	229533.A0A1C3YHX6
116858	229533.A0A098DEC2	pp	229533.I1RTD9
116859	229533.A0A098DEC2	pp	229533.V6RMW6
116860	229533.A0A098DEC2	pp	229533.I1RPS0
116861	229533.A0A098DEC2	pp	229533.I1RSR8
116862	229533.A0A098DEC2	pp	229533.I1RRT8
116863	229533.A0A098DEC2	pp	229533.I1RSJ4
116864	229533.A0A098DEC2	pp	229533.V6R042
116865	229533.A0A098DEC2	pp	229533.I1RR24
116866	229533.A0A098DEC2	pp	229533.I1RGC2
116867	229533.A0A098DEC2	pp	229533.I1S2F2
116868	229533.A0A098DEC2	pp	229533.I1RRI2
116869	229533.A0A098DEC2	pp	229533.I1RMN6
116870	229533.A0A098DEC2	pp	229533.A0A1C3YLK2
116871	229533.A0A098DEC2	pp	229533.I1RYD9
116872	229533.A0A098DEC2	pp	229533.I1RPW9
116873	229533.A0A098DEC2	pp	229533.I1S044

116874	229533.A0A098DEC2	pp	229533.I1S9U2
116875	229533.A0A098DEC2	pp	229533.I1RZW8
116876	229533.A0A098DEC2	pp	229533.V6RGN5
116877	229533.A0A098DEC2	pp	229533.A0A0E0SJN2
116878	229533.A0A098DEC2	pp	229533.I1S8R3
116879	229533.A0A098DEC2	pp	229533.I1RPM4
116880	229533.A0A098DEC2	pp	229533.I1RFS3
116881	229533.A0A098DEC2	pp	229533.I1RW99
116882	229533.A0A098DEC2	pp	229533.Q4I662
116883	229533.A0A098DEC2	pp	229533.I1RCG4
116884	229533.A0A098DEC2	pp	229533.I1RW46
116885	229533.A0A098DEC2	pp	229533.I1S2C0
116886	229533.A0A098DEC2	pp	229533.I1RX24
116887	229533.A0A098DEC2	pp	229533.I1S060
116888	229533.A0A098DEC2	pp	229533.V6QYF0
116889	229533.A0A098DEC2	pp	229533.I1RK83
116890	229533.A0A098DEC2	pp	229533.I1RE01
116891	229533.A0A098DEC2	pp	229533.I1RCN5
116892	229533.A0A098DEC2	pp	229533.I1RAN5
116893	229533.A0A098DEC2	pp	229533.I1S0J1
116894	229533.A0A098DEC2	pp	229533.I1RBG9
116895	229533.A0A098DEC2	pp	229533.I1RZS6
116896	229533.A0A098DEC2	pp	229533.I1RBZ3
116897	229533.A0A098DEC2	pp	229533.I1RC81
116898	229533.A0A098DEC2	pp	229533.I1RC72
116899	229533.A0A098DEC2	pp	229533.A0A1C3YKV3
116900	229533.A0A098DEC2	pp	229533.I1RJV3
116901	229533.A0A098DEC2	pp	229533.I1RBL7
116902	229533.A0A098DEC2	pp	229533.I1RSM3
116903	229533.A0A098DEC2	pp	229533.I1RSD8
116904	229533.A0A098DEC2	pp	229533.I1RSC6
116905	229533.A0A098DEC2	pp	229533.I1RDG0
116906	229533.A0A098DEC2	pp	229533.I1RQ28
116907	229533.A0A098DEC2	pp	229533.I1S0M9
116908	229533.A0A098DEC2	pp	229533.I1RB92
116909	229533.A0A098DEC2	pp	229533.Q4I5Z5
116910	229533.A0A098DEC2	pp	229533.I1RFL4
116911	229533.A0A098DEC2	pp	229533.I1RCH5
116912	229533.A0A098DEC2	pp	229533.I1RWB5
116913	229533.A0A098DEC2	pp	229533.I1RZZ1
116914	229533.A0A098DEC2	pp	229533.I1RS17
116915	229533.A0A098DEC2	pp	229533.I1RAZ8
116916	229533.A0A098DEC2	pp	229533.I1S6E5
116917	229533.A0A098DEC2	pp	229533.I1RF19
116918	229533.A0A098DEC2	pp	229533.I1RFJ9
116919	229533.A0A098DEC2	pp	229533.I1RE00
116920	229533.A0A098DEC2	pp	229533.A0A1C3YIJ3
116921	229533.A0A098DEC2	pp	229533.I1RZK6
116922	229533.A0A098DEC2	pp	229533.I1RQE7
116923	229533.A0A098DEC2	pp	229533.I1S2E8
116924	229533.A0A098DEC2	pp	229533.I1S1X9
116925	229533.A0A098DEC2	pp	229533.I1RM25
116926	229533.A0A098DEC2	pp	229533.I1R9V7
116927	229533.A0A098DEC2	pp	229533.I1RA48
116928	229533.A0A098DEC2	pp	229533.A0A1C3YI50
116929	229533.A0A098DEC2	pp	229533.I1REK8
116930	229533.A0A098DEC2	pp	229533.I1RD17
116931	229533.A0A098DEC2	pp	229533.I1RAU7
116932	229533.A0A098DEC2	pp	229533.V6RIV9
116933	229533.I1S0D6	pp	229533.Q4I7L0
116934	229533.I1RKJ6	pp	229533.I1RRQ3
116935	229533.I1RKJ6	pp	229533.I1S6F0
116936	229533.I1RKJ6	pp	229533.I1RRP3
116937	229533.I1RKJ6	pp	229533.I1RNH1
116938	229533.I1RKJ6	pp	229533.Q4IEV5
116939	229533.I1RKJ6	pp	229533.I1RQ65
116940	229533.I1RKJ6	pp	229533.Q4HZ47
116941	229533.I1RKJ6	pp	229533.I1RRH5
116942	229533.I1RKJ6	pp	229533.I1RS47
116943	229533.I1RKJ6	pp	229533.I1S0D4
116944	229533.I1RKJ6	pp	229533.I1RT33
116945	229533.I1RKJ6	pp	229533.Q4HZ42
116946	229533.I1RKJ6	pp	229533.I1RZJ6

116947 229533.I1RKJ6 pp 229533.I1RT98
116948 229533.I1RKJ6 pp 229533.I1RTD5
116949 229533.I1RKJ6 pp 229533.I1RWH5
116950 229533.I1RKJ6 pp 229533.Q4INZ9
116951 229533.I1RKJ6 pp 229533.V6R283
116952 229533.I1RKJ6 pp 229533.I1S8I8
116953 229533.I1RKJ6 pp 229533.I1RPP3
116954 229533.I1RKJ6 pp 229533.I1RZ99
116955 229533.I1RKJ6 pp 229533.I1RXD7
116956 229533.I1RKJ6 pp 229533.I1RQJ5
116957 229533.I1RKJ6 pp 229533.I1RT04
116958 229533.I1RKJ6 pp 229533.I1RSQ7
116959 229533.I1RKJ6 pp 229533.I1RUQ2
116960 229533.I1RKJ6 pp 229533.I1RTI6
116961 229533.I1RKJ6 pp 229533.I1RRJ8
116962 229533.I1RKJ6 pp 229533.Q4IAS1
116963 229533.I1RKJ6 pp 229533.I1RKL3
116964 229533.I1RKJ6 pp 229533.I1RLV9
116965 229533.I1RKJ6 pp 229533.I1RQS5
116966 229533.I1RKJ6 pp 229533.I1RW71
116967 229533.I1RKJ6 pp 229533.I1S0R7
116968 229533.I1RKJ6 pp 229533.I1RKQ5
116969 229533.I1RKJ6 pp 229533.I1RRT2
116970 229533.I1RKJ6 pp 229533.I1S503
116971 229533.I1RKJ6 pp 229533.Q4I830
116972 229533.I1RKJ6 pp 229533.Q4I5R9
116973 229533.I1RKJ6 pp 229533.Q4IPI1
116974 229533.I1RKJ6 pp 229533.I1RR00
116975 229533.I1RKJ6 pp 229533.I1RX46
116976 229533.I1RKJ6 pp 229533.I1S0J1
116977 229533.I1RKJ6 pp 229533.Q4IQW0
116978 229533.I1RKJ6 pp 229533.I1RWJ4
116979 229533.I1RKJ6 pp 229533.I1RX14
116980 229533.I1RKJ6 pp 229533.I1RSL9
116981 229533.I1RKJ6 pp 229533.I1RT05
116982 229533.I1RKJ6 pp 229533.I1RU69
116983 229533.I1RKJ6 pp 229533.I1S060
116984 229533.I1RKJ6 pp 229533.Q4IAA0
116985 229533.I1RKJ6 pp 229533.Q4I662
116986 229533.I1RKJ6 pp 229533.I1S0M9
116987 229533.I1RKJ6 pp 229533.Q4IF76
116988 229533.I1RKJ6 pp 229533.I1RW61
116989 229533.I1RKJ6 pp 229533.I1RR72
116990 229533.I1RKJ6 pp 229533.I1RM06
116991 229533.I1RKJ6 pp 229533.Q4HVW2
116992 229533.I1RKJ6 pp 229533.I1RZS6
116993 229533.I1RKJ6 pp 229533.Q4IJ56
116994 229533.I1RKJ6 pp 229533.I1S253
116995 229533.I1RKJ6 pp 229533.I1RM25
116996 229533.I1RKJ6 pp 229533.I1RMK3
116997 229533.I1RKJ6 pp 229533.V6QYF0
116998 229533.I1RKJ6 pp 229533.I1RNU6
116999 229533.I1RKJ6 pp 229533.I1RX24
117000 229533.I1RKJ6 pp 229533.I1RWB5
117001 229533.I1RKJ6 pp 229533.I1RND6
117002 229533.I1RKJ6 pp 229533.I1RNA0
117003 229533.I1RKJ6 pp 229533.I1RNQ5
117004 229533.I1RKJ6 pp 229533.V6R3X5
117005 229533.I1RKJ6 pp 229533.I1S2B8
117006 229533.I1RKJ6 pp 229533.I1RW25
117007 229533.I1RKJ6 pp 229533.I1RM96
117008 229533.I1RKJ6 pp 229533.I1RT80
117009 229533.I1RKJ6 pp 229533.I1RR73
117010 229533.I1RKJ6 pp 229533.Q4IJH1
117011 229533.I1RKJ6 pp 229533.I1RNV6
117012 229533.I1RKJ6 pp 229533.Q4IEK8
117013 229533.I1RKJ6 pp 229533.I1RWN5
117014 229533.I1RKJ6 pp 229533.I1S4T3
117015 229533.I1RKJ6 pp 229533.Q4IFI0
117016 229533.I1RKJ6 pp 229533.I1RTF4
117017 229533.I1RKJ6 pp 229533.I1RPW4
117018 229533.I1RKJ6 pp 229533.V6RMW6
117019 229533.I1RKJ6 pp 229533.I1RZ39

117020	229533.I1RKJ6	pp	229533.I1RPY1
117021	229533.I1RKJ6	pp	229533.I1S8E2
117022	229533.I1RKJ6	pp	229533.V6R042
117023	229533.I1RKJ6	pp	229533.I1RWE1
117024	229533.I1RKJ6	pp	229533.V6R227
117025	229533.I1RKJ6	pp	229533.I1RRT8
117026	229533.I1RKJ6	pp	229533.I1RRR7
117027	229533.I1RKJ6	pp	229533.I1RSI6
117028	229533.I1RKJ6	pp	229533.I1RM67
117029	229533.I1RKJ6	pp	229533.I1RZF6
117030	229533.I1RKJ6	pp	229533.I1RPG6
117031	229533.I1RKJ6	pp	229533.Q4IBS2
117032	229533.I1RKJ6	pp	229533.I1RRK8
117033	229533.I1RKJ6	pp	229533.I1RZV6
117034	229533.I1RKJ6	pp	229533.I1S9Y1
117035	229533.I1RKJ6	pp	229533.V6QUE6
117036	229533.I1RKJ6	pp	229533.I1S504
117037	229533.I1RKJ6	pp	229533.I1RMN6
117038	229533.I1RKJ6	pp	229533.I1RPU0
117039	229533.I1RKJ6	pp	229533.Q4I327
117040	229533.I1RKJ6	pp	229533.I1RPW9
117041	229533.I1RKJ6	pp	229533.I1S044
117042	229533.I1RKJ6	pp	229533.V6RGN5
117043	229533.I1RKJ6	pp	229533.I1S9U2
117044	229533.I1RKJ6	pp	229533.I1RNY2
117045	229533.I1RKJ6	pp	229533.I1RZZ1
117046	229533.I1RKJ6	pp	229533.I1RNH6
117047	229533.I1RKJ6	pp	229533.I1RYB9
117048	229533.I1RKJ6	pp	229533.I1S021
117049	229533.I1RKJ6	pp	229533.I1RVZ0
117050	229533.I1RKJ6	pp	229533.I1S1J6
117051	229533.I1RKJ6	pp	229533.I1S0Z9
117052	229533.I1RKJ6	pp	229533.I1RNK2
117053	229533.I1RKJ6	pp	229533.I1RQ31
117054	229533.I1RKJ6	pp	229533.I1S2E8
117055	229533.I1RKJ6	pp	229533.I1RZK6
117056	229533.I1RKJ6	pp	229533.I1RMC0
117057	229533.I1RKJ6	pp	229533.I1RMQ0
117058	229533.I1RKJ6	pp	229533.I1RN99
117059	229533.I1RKJ6	pp	229533.I1RSD8
117060	229533.I1RKJ6	pp	229533.Q4HZ68
117061	229533.I1RKJ6	pp	229533.Q4IN63
117062	229533.I1RKJ6	pp	229533.I1S296
117063	229533.I1RKJ6	pp	229533.I1RQ28
117064	229533.I1RKJ6	pp	229533.I1S1X9
117065	229533.I1RKJ6	pp	229533.I1RRI2
117066	229533.I1RKJ6	pp	229533.I1RMD5
117067	229533.I1RKJ6	pp	229533.I1RVX0
117068	229533.I1RKJ6	pp	229533.I1RY63
117069	229533.I1RKJ6	pp	229533.Q4HWE2
117070	229533.I1RKJ6	pp	229533.I1RT03
117071	229533.I1RKJ6	pp	229533.I1S1C7
117072	229533.I1RKJ6	pp	229533.I1RSP7
117073	229533.I1RKJ6	pp	229533.I1RY03
117074	229533.I1RKJ6	pp	229533.I1RSR8
117075	229533.I1RKJ6	pp	229533.I1RSS4
117076	229533.I1RKJ6	pp	229533.I1RSC6
117077	229533.I1RKJ6	pp	229533.I1RZE5
117078	229533.I1RKJ6	pp	229533.I1RSG2
117079	229533.I1RKJ6	pp	229533.I1RZK5
117080	229533.I1RKJ6	pp	229533.I1S2C0
117081	229533.I1RKJ6	pp	229533.I1RYB1
117082	229533.I1RKJ6	pp	229533.I1S6E5
117083	229533.I1RKJ6	pp	229533.I1S0M3
117084	229533.I1RKJ6	pp	229533.I1RPJ3
117085	229533.I1RKJ6	pp	229533.I1S274
117086	229533.I1RKJ6	pp	229533.I1RTH6
117087	229533.I1RKJ6	pp	229533.I1RTE9
117088	229533.I1RKJ6	pp	229533.I1S558
117089	229533.I1RKJ6	pp	229533.I1RQA2
117090	229533.I1RKJ6	pp	229533.I1S1U7
117091	229533.I1RKJ6	pp	229533.I1RWN6
117092	229533.I1RKJ6	pp	229533.I1RRY9

229533.I1RKJ6 pp 229533.V6RGY3
229533.I1RKJ6 pp 229533.I1RSR3
229533.I1RE67 pp 229533.I1RQ93
229533.I1RE67 pp 229533.I1SAA0
229533.I1RE67 pp 229533.I1RYZ0
229533.A0A1C3YK73 pp 229533.I1RQC9
229533.A0A1C3YK73 pp 229533.I1RAX6
229533.A0A1C3YK73 pp 229533.I1RX46
229533.A0A1C3YK73 pp 229533.I1RJW1
229533.A0A1C3YK73 pp 229533.V6RTJ8
229533.A0A1C3YK73 pp 229533.I1RIE8
229533.A0A1C3YK73 pp 229533.I1RBC9
229533.A0A1C3YK73 pp 229533.I1RSQ7
229533.A0A1C3YK73 pp 229533.I1RI82
229533.A0A1C3YK73 pp 229533.V6RCG5
229533.A0A1C3YK73 pp 229533.V6QW22
229533.A0A1C3YK73 pp 229533.I1RVQ0
229533.A0A1C3YK73 pp 229533.I1RAU5
229533.A0A1C3YK73 pp 229533.A0A1C3YKV3
229533.A0A1C3YK73 pp 229533.I1RCG4
229533.A0A1C3YK73 pp 229533.I1RFU8
229533.A0A1C3YK73 pp 229533.I1RZK5
229533.A0A1C3YK73 pp 229533.I1RS18
229533.A0A1C3YK73 pp 229533.I1S0L3
229533.A0A1C3YK73 pp 229533.I1RR39
229533.A0A1C3YK73 pp 229533.A0A1C3YKC0
229533.A0A1C3YK73 pp 229533.I1RZM1
229533.A0A1C3YK73 pp 229533.I1RRY9
229533.A0A1C3YK73 pp 229533.Q4IJK2
229533.A0A1C3YK73 pp 229533.I1RBT1
229533.A0A1C3YK73 pp 229533.I1RLU6
229533.A0A1C3YK73 pp 229533.I1RR24
229533.A0A1C3YK73 pp 229533.I1RW25
229533.A0A1C3YK73 pp 229533.I1S504
229533.A0A1C3YK73 pp 229533.I1RI02
229533.A0A1C3YK73 pp 229533.A0A1C3YKB0
229533.A0A1C3YK73 pp 229533.I1S2E9
229533.A0A1C3YK73 pp 229533.I1REK8
229533.A0A1C3YK73 pp 229533.A0A1C3YKK9
229533.A0A1C3YK73 pp 229533.I1RY03
229533.A0A1C3YK73 pp 229533.I1RBE7
229533.A0A1C3YK73 pp 229533.I1RV91
229533.A0A1C3YK73 pp 229533.I1RRT8
229533.A0A1C3YK73 pp 229533.I1S503
229533.A0A1C3YK73 pp 229533.I1RJW6
229533.A0A1C3YK73 pp 229533.I1RTD5
229533.A0A1C3YK73 pp 229533.I1RT04
229533.A0A1C3YK73 pp 229533.I1S2H4
229533.A0A1C3YK73 pp 229533.I1RBS6
229533.A0A1C3YK73 pp 229533.I1RJV4
229533.A0A1C3YK73 pp 229533.I1RWK3
229533.A0A1C3YK73 pp 229533.I1S0L4
229533.A0A1C3YK73 pp 229533.I1RN18
229533.A0A1C3YK73 pp 229533.I1RFL4
229533.A0A1C3YK73 pp 229533.I1RVX0
229533.A0A1C3YK73 pp 229533.I1RPM4
229533.A0A1C3YK73 pp 229533.I1RPP3
229533.A0A1C3YK73 pp 229533.I1RRM5
229533.A0A1C3YK73 pp 229533.I1RN67
229533.A0A1C3YK73 pp 229533.I1S8P5
229533.A0A1C3YK73 pp 229533.I1RT03
229533.A0A1C3YK73 pp 229533.I1RB92
229533.A0A1C3YK73 pp 229533.I1R9M0
229533.A0A1C3YK73 pp 229533.I1S2B8
229533.A0A1C3YK73 pp 229533.I1RD17
229533.A0A1C3YK73 pp 229533.I1S569
229533.A0A1C3YK73 pp 229533.I1RBY4
229533.A0A1C3YK73 pp 229533.I1RZZ9
229533.A0A1C3YK73 pp 229533.I1RFI9
229533.A0A1C3YK73 pp 229533.I1RZ39
229533.A0A1C3YK73 pp 229533.Q4I1Y5
229533.A0A1C3YK73 pp 229533.I1RCR8
229533.A0A1C3YK73 pp 229533.A0A1C3YKM9

117166 229533.A0A1C3YK73 pp 229533.I1RUG3
117167 229533.A0A1C3YK73 pp 229533.I1RAU7
117168 229533.A0A1C3YK73 pp 229533.V6R3X5
117169 229533.A0A1C3YK73 pp 229533.I1RNC1
117170 229533.A0A1C3YK73 pp 229533.I1RR73
117171 229533.A0A1C3YK73 pp 229533.I1RD22
117172 229533.A0A1C3YK73 pp 229533.V6RMW6
117173 229533.A0A1C3YK73 pp 229533.I1RTA0
117174 229533.A0A1C3YK73 pp 229533.V6R042
117175 229533.A0A1C3YK73 pp 229533.I1RPP4
117176 229533.A0A1C3YK73 pp 229533.I1RK44
117177 229533.A0A1C3YK73 pp 229533.I1RSQ3
117178 229533.A0A1C3YK73 pp 229533.V6RIV9
117179 229533.A0A1C3YK73 pp 229533.I1RWN5
117180 229533.A0A1C3YK73 pp 229533.I1RQ75
117181 229533.A0A1C3YK73 pp 229533.I1RY44
117182 229533.A0A1C3YK73 pp 229533.I1S1C7
117183 229533.A0A1C3YK73 pp 229533.I1S8R3
117184 229533.A0A1C3YK73 pp 229533.I1RZW8
117185 229533.A0A1C3YK73 pp 229533.I1RTH6
117186 229533.A0A1C3YK73 pp 229533.I1RA70
117187 229533.A0A1C3YK73 pp 229533.I1RAW0
117188 229533.A0A1C3YK73 pp 229533.I1RJV3
117189 229533.A0A1C3YK73 pp 229533.I1RWN6
117190 229533.I1RX42 pp 229533.I1RX44
117191 229533.I1RA18 pp 229533.Q4IPC8
117192 229533.I1RA18 pp 229533.I1RG90
117193 229533.I1RA18 pp 229533.Q4HWE2
117194 229533.I1RA18 pp 229533.I1RWI0
117195 229533.I1RA18 pp 229533.I1RNB8
117196 229533.I1RA18 pp 229533.I1S5Z2
117197 229533.I1RA18 pp 229533.I1RA84
117198 229533.I1RA18 pp 229533.I1RBJ1
117199 229533.I1RA18 pp 229533.I1RW69
117200 229533.I1RA18 pp 229533.I1RT35
117201 229533.I1RA18 pp 229533.Q4I5W5
117202 229533.I1RA18 pp 229533.I1RDG0
117203 229533.I1RA18 pp 229533.Q4I5I4
117204 229533.I1RA18 pp 229533.I1RA46
117205 229533.I1RA18 pp 229533.I1RCU3
117206 229533.I1RAN2 pp 229533.I1RZM1
117207 229533.I1RAN2 pp 229533.I1RWT6
117208 229533.I1RAN2 pp 229533.I1RQ81
117209 229533.I1RAN2 pp 229533.I1RZH4
117210 229533.I1RAN2 pp 229533.Q4IJM4
117211 229533.I1RAN2 pp 229533.I1RN65
117212 229533.I1RAN2 pp 229533.I1RDB1
117213 229533.I1RAN2 pp 229533.I1RKK0
117214 229533.I1RAN2 pp 229533.I1RQA3
117215 229533.I1RAN2 pp 229533.I1RC17
117216 229533.I1RAN2 pp 229533.I1RX70
117217 229533.I1RAN2 pp 229533.I1RMM9
117218 229533.I1RAN2 pp 229533.I1RAU5
117219 229533.I1RAN2 pp 229533.I1RC95
117220 229533.I1RAN2 pp 229533.I1RWJ6
117221 229533.I1RAN2 pp 229533.I1REE1
117222 229533.I1RAN2 pp 229533.I1S0Z7
117223 229533.I1RAN2 pp 229533.I1RM09
117224 229533.I1RAN2 pp 229533.I1S1W4
117225 229533.I1RAN2 pp 229533.I1RN08
117226 229533.I1RAN2 pp 229533.I1RCB3
117227 229533.I1RAN2 pp 229533.I1RUQ5
117228 229533.I1RAN2 pp 229533.I1S439
117229 229533.I1RAN2 pp 229533.I1RY44
117230 229533.I1RAN2 pp 229533.I1RYZ0
117231 229533.I1RAN2 pp 229533.I1RB75
117232 229533.I1RAN2 pp 229533.I1RC22
117233 229533.I1RAN2 pp 229533.I1RBZ9
117234 229533.I1RAN2 pp 229533.V6RVG3
117235 229533.I1RAN2 pp 229533.I1RBC9
117236 229533.I1RAN2 pp 229533.V6QV09
117237 229533.I1RAN2 pp 229533.I1RAY4
117238 229533.I1RAN2 pp 229533.I1RQ26

117239 229533.I1RAN2 pp 229533.I1RAW7
117240 229533.I1RAN2 pp 229533.V6RTJ8
117241 229533.I1RAN2 pp 229533.I1RJH1
117242 229533.I1RAN2 pp 229533.I1RNC1
117243 229533.I1RAN2 pp 229533.V6RKY7
117244 229533.I1RAN2 pp 229533.I1RQB4
117245 229533.I1RAN2 pp 229533.I1S0D1
117246 229533.I1RAN2 pp 229533.I1RYZ9
117247 229533.I1RAN2 pp 229533.I1S0M2
117248 229533.I1RAN2 pp 229533.I1RNY8
117249 229533.I1RAN2 pp 229533.I1RNC0
117250 229533.I1RAN2 pp 229533.I1RQ72
117251 229533.I1RAN2 pp 229533.I1RYH9
117252 229533.I1RAN2 pp 229533.I1S2K7
117253 229533.I1RAN2 pp 229533.I1RNW8
117254 229533.I1RAN2 pp 229533.I1S1D3
117255 229533.I1RAN2 pp 229533.I1RXH6
117256 229533.I1RAN2 pp 229533.I1S224
117257 229533.I1RAN2 pp 229533.I1RB41
117258 229533.I1RAN2 pp 229533.I1RTA0
117259 229533.I1RAN2 pp 229533.I1RDZ4
117260 229533.I1RAN2 pp 229533.I1RNY9
117261 229533.I1RAN2 pp 229533.I1RPW0
117262 229533.I1RAN2 pp 229533.I1S0N4
117263 229533.I1RAN2 pp 229533.I1RL24
117264 229533.I1RAN2 pp 229533.I1RD18
117265 229533.I1RAN2 pp 229533.I1RV66
117266 229533.I1RAN2 pp 229533.I1RT36
117267 229533.I1RAN2 pp 229533.I1RHQ8
117268 229533.I1RAN2 pp 229533.I1RZE4
117269 229533.I1RAN2 pp 229533.I1RY58
117270 229533.I1RAN2 pp 229533.V6RGI1
117271 229533.I1RAN2 pp 229533.I1RC97
117272 229533.I1RAN2 pp 229533.I1RBS0
117273 229533.I1RAN2 pp 229533.I1RD22
117274 229533.I1RAN2 pp 229533.I1RSK1
117275 229533.I1RAN2 pp 229533.I1RP28
117276 229533.I1RAN2 pp 229533.I1RMQ5
117277 229533.I1RAN2 pp 229533.I1RRY3
117278 229533.I1RAN2 pp 229533.I1RRV1
117279 229533.I1RAN2 pp 229533.I1RPD6
117280 229533.I1RAN2 pp 229533.I1S8P5
117281 229533.I1RAN2 pp 229533.I1RJV3
117282 229533.I1RAN2 pp 229533.I1RRW0
117283 229533.I1RAN2 pp 229533.I1RCL5
117284 229533.I1RAN2 pp 229533.I1RYW3
117285 229533.I1RAN2 pp 229533.I1RB74
117286 229533.I1RAN2 pp 229533.I1RUN8
117287 229533.I1RAN2 pp 229533.I1RFL7
117288 229533.I1RAN2 pp 229533.I1RQG4
117289 229533.I1RAN2 pp 229533.I1RNF4
117290 229533.I1RAN2 pp 229533.I1RPM4
117291 229533.I1RAN2 pp 229533.I1RBS6
117292 229533.I1RAN2 pp 229533.I1S2E9
117293 229533.I1RAN2 pp 229533.I1S2G9
117294 229533.I1RAN2 pp 229533.I1RAS9
117295 229533.I1RAN2 pp 229533.I1RV91
117296 229533.I1RAN2 pp 229533.I1RLU1
117297 229533.I1RAN2 pp 229533.I1RBY4
117298 229533.I1RAN2 pp 229533.I1RNP7
117299 229533.I1RAN2 pp 229533.I1S549
117300 229533.I1RAN2 pp 229533.I1RRM9
117301 229533.I1RAN2 pp 229533.I1S1V5
117302 229533.A0A0E0SKT0 pp 229533.A0A1C3YMH5
117303 229533.A0A0E0SKT0 pp 229533.A0A1C3YKJ7
117304 229533.A0A0E0SKT0 pp 229533.A0A1C3YID5
117305 229533.A0A0E0SKT0 pp 229533.I1RRD2
117306 229533.I1RT95 pp 229533.I1RV70
117307 229533.I1RT95 pp 229533.I1RX47
117308 229533.I1RT95 pp 229533.I1RT96
117309 229533.A0A1C3YM51 pp 229533.I1RF42
117310 229533.I1RN28 pp 229533.Q4IPC8
117311 229533.I1RN28 pp 229533.I1RN49

117312	229533.I1RN28	pp	229533.I1S214
117313	229533.I1RN28	pp	229533.Q4IF44
117314	229533.I1RN28	pp	229533.I1RVP5
117315	229533.I1RN28	pp	229533.I1RT35
117316	229533.I1RN28	pp	229533.Q4I7X1
117317	229533.I1RN28	pp	229533.I1RR68
117318	229533.I1RN28	pp	229533.I1S8U9
117319	229533.I1RN28	pp	229533.Q4IPA4
117320	229533.I1RN28	pp	229533.I1RPY7
117321	229533.I1RN28	pp	229533.I1RQK2
117322	229533.I1RN28	pp	229533.I1RSF5
117323	229533.I1RN28	pp	229533.I1S8V0
117324	229533.I1RN28	pp	229533.I1RS03
117325	229533.I1RN28	pp	229533.I1S0N7
117326	229533.I1RN28	pp	229533.I1RNB8
117327	229533.I1RN28	pp	229533.I1S246
117328	229533.I1RN28	pp	229533.Q4HWE2
117329	229533.I1RN28	pp	229533.I1RQZ2
117330	229533.I1RN28	pp	229533.Q4IPN9
117331	229533.I1RN28	pp	229533.Q4IR09
117332	229533.I1RN28	pp	229533.Q4IAS1
117333	229533.I1RN28	pp	229533.I1RZL5
117334	229533.I1RN28	pp	229533.I1S5Z2
117335	229533.I1RN28	pp	229533.I1RWL3
117336	229533.I1RN28	pp	229533.I1S2C4
117337	229533.I1RN28	pp	229533.I1RZP2
117338	229533.I1RSQ3	pp	229533.I1RZZ9
117339	229533.I1RSQ3	pp	229533.I1RSQ4
117340	229533.I1RSQ3	pp	229533.I1RWI5
117341	229533.I1RSQ3	pp	229533.I1S5R1
117342	229533.I1RSQ3	pp	229533.I1S2H4
117343	229533.I1RSQ3	pp	229533.I1S0L4
117344	229533.I1RSQ3	pp	229533.I1RXC2
117345	229533.I1RSQ3	pp	229533.I1S6Y8
117346	229533.I1RSQ3	pp	229533.O74638
117347	229533.I1RSQ3	pp	229533.I1RSZ7
117348	229533.I1RSQ3	pp	229533.I1S0M6
117349	229533.I1RSQ3	pp	229533.I1RWL2
117350	229533.I1RSQ3	pp	229533.I1S0W4
117351	229533.I1RSQ3	pp	229533.I1S4Z8
117352	229533.I1RSQ3	pp	229533.V6R3X5
117353	229533.I1RSQ3	pp	229533.I1S2B8
117354	229533.I1RSQ3	pp	229533.I1S2E9
117355	229533.I1RSQ3	pp	229533.I1S9Y1
117356	229533.I1RSQ3	pp	229533.I1S0F6
117357	229533.I1RSQ3	pp	229533.I1RT04
117358	229533.I1RSQ3	pp	229533.I1RZ39
117359	229533.I1RSQ3	pp	229533.I1RYI5
117360	229533.I1RSQ3	pp	229533.V6RUY9
117361	229533.I1RSQ3	pp	229533.I1S352
117362	229533.I1RSQ3	pp	229533.I1RW46
117363	229533.I1RSQ3	pp	229533.I1RSV2
117364	229533.I1RSQ3	pp	229533.I1S0J1
117365	229533.I1RSQ3	pp	229533.I1RTD5
117366	229533.I1RSQ3	pp	229533.I1RTA1
117367	229533.I1RSQ3	pp	229533.I1RXF8
117368	229533.I1RSQ3	pp	229533.I1RVZ2
117369	229533.I1RSQ3	pp	229533.I1RZS7
117370	229533.I1RSQ3	pp	229533.I1RWK3
117371	229533.I1RSQ3	pp	229533.I1RV91
117372	229533.I1RSQ3	pp	229533.I1RTA0
117373	229533.I1RSQ3	pp	229533.I1RYR0
117374	229533.I1RSQ3	pp	229533.I1RVZ7
117375	229533.I1RSQ3	pp	229533.I1RT40
117376	229533.I1RSQ3	pp	229533.I1S1W3
117377	229533.I1RSQ3	pp	229533.I1RY44
117378	229533.I1RSQ3	pp	229533.V6R283
117379	229533.I1RSQ3	pp	229533.I1S8F0
117380	229533.I1RSQ3	pp	229533.I1RX39
117381	229533.I1RSQ3	pp	229533.I1S8R3
117382	229533.I1RSQ3	pp	229533.I1RTH6
117383	229533.I1RSQ3	pp	229533.I1RSX0
117384	229533.I1RSQ3	pp	229533.V6R3J1

117385 229533.I1RSQ3 pp 229533.I1RZV3
117386 229533.I1RSQ3 pp 229533.I1S503
117387 229533.I1RSQ3 pp 229533.I1RZ79
117388 229533.I1RSQ3 pp 229533.V6RIV9
117389 229533.I1RSQ3 pp 229533.I1RVX0
117390 229533.I1RSQ3 pp 229533.I1RWN5
117391 229533.I1RSQ3 pp 229533.I1RZW8
117392 229533.I1RSQ3 pp 229533.I1S437
117393 229533.I1RSQ3 pp 229533.I1S1W9
117394 229533.I1RSQ3 pp 229533.I1S016
117395 229533.I1RSQ3 pp 229533.I1S1C7
117396 229533.I1RSQ3 pp 229533.I1RT03
117397 229533.I1RSQ3 pp 229533.V6R042
117398 229533.I1RSQ3 pp 229533.I1RXR1
117399 229533.I1RSQ3 pp 229533.Q4HZB8
117400 229533.I1RSQ3 pp 229533.I1S569
117401 229533.I1RSQ3 pp 229533.I1RYW3
117402 229533.I1RSQ3 pp 229533.V6R057
117403 229533.I1RSQ3 pp 229533.V6RMW6
117404 229533.I1RSQ3 pp 229533.I1RWN6
117405 229533.I1RSQ3 pp 229533.I1RUG3
117406 229533.I1RSQ3 pp 229533.I1RXT3
117407 229533.I1RSQ3 pp 229533.I1RVF8
117408 229533.I1RSQ3 pp 229533.I1S9Z4
117409 229533.I1RSQ3 pp 229533.I1RXU9
117410 229533.I1RSQ3 pp 229533.Q4IN63
117411 229533.I1RSQ3 pp 229533.I1S7W5
117412 229533.I1RSQ3 pp 229533.Q4I624
117413 229533.I1RSQ3 pp 229533.V6R1K4
117414 229533.I1RSQ3 pp 229533.I1S0N4
117415 229533.I1RSQ3 pp 229533.I1S0F1
117416 229533.I1RSQ3 pp 229533.I1S0L3
117417 229533.I1RSQ3 pp 229533.I1S0X6
117418 229533.I1RSQ3 pp 229533.I1SA33
117419 229533.I1RSQ3 pp 229533.I1RZJ5
117420 229533.I1RSQ3 pp 229533.I1S5R6
117421 229533.I1RSQ3 pp 229533.I1RY86
117422 229533.I1RSQ3 pp 229533.I1RWJ8
117423 229533.I1RSQ3 pp 229533.I1S0F5
117424 229533.I1RSQ3 pp 229533.I1S4S4
117425 229533.I1RSQ3 pp 229533.I1S438
117426 229533.I1RSQ3 pp 229533.V6QW22
117427 229533.I1RSQ3 pp 229533.I1RXD6
117428 229533.I1RSQ3 pp 229533.V6QUW4
117429 229533.I1RSQ3 pp 229533.I1RSQ7
117430 229533.I1RSQ3 pp 229533.I1RW54
117431 229533.I1RSQ3 pp 229533.V6R4J1
117432 229533.I1RSQ3 pp 229533.I1RVQ0
117433 229533.I1RSQ3 pp 229533.I1RW07
117434 229533.I1RSQ3 pp 229533.I1RW25
117435 229533.I1RSQ3 pp 229533.V6RUT5
117436 229533.I1RSQ3 pp 229533.I1S4S7
117437 229533.I1RSQ3 pp 229533.I1S029
117438 229533.I1RSQ3 pp 229533.I1RW71
117439 229533.I1RSQ3 pp 229533.I1RX25
117440 229533.I1RSQ3 pp 229533.Q4IJT5
117441 229533.I1RSQ3 pp 229533.I1RXD7
117442 229533.I1RSQ3 pp 229533.I1RX95
117443 229533.I1RSQ3 pp 229533.V6RTJ8
117444 229533.I1RSQ3 pp 229533.I1S0R7
117445 229533.I1RSQ3 pp 229533.I1S7A8
117446 229533.I1RSQ3 pp 229533.I1RX46
117447 229533.I1RSQ3 pp 229533.Q4I283
117448 229533.I1RSQ3 pp 229533.I1RZZ1
117449 229533.I1RSQ3 pp 229533.I1RWT6
117450 229533.I1RSQ3 pp 229533.V6RBN2
117451 229533.I1RSQ3 pp 229533.I1S540
117452 229533.I1RSQ3 pp 229533.I1S504
117453 229533.I1RSQ3 pp 229533.Q4IJK2
117454 229533.I1RSQ3 pp 229533.I1RZM1
117455 229533.I1RSQ3 pp 229533.I1S0T2
117456 229533.I1RSQ3 pp 229533.I1RWR7
117457 229533.I1RSQ3 pp 229533.I1RY03

117458	229533.I1RSQ3	pp	229533.V6RY56
117459	229533.I1RSQ3	pp	229533.I1S419
117460	229533.I1RSQ3	pp	229533.I1RT35
117461	229533.I1RSQ3	pp	229533.I1RZE5
117462	229533.I1RSQ3	pp	229533.I1RU29
117463	229533.I1RSQ3	pp	229533.V6RCG5
117464	229533.I1RSQ3	pp	229533.V6RCZ8
117465	229533.I1RSQ3	pp	229533.I1RYS0
117466	229533.I1RSQ3	pp	229533.I1S1J7
117467	229533.I1RSQ3	pp	229533.I1S6K9
117468	229533.I1RSQ3	pp	229533.I1RZ35
117469	229533.I1RSQ3	pp	229533.I1S4D0
117470	229533.I1RSQ3	pp	229533.I1RWJ3
117471	229533.I1RSQ3	pp	229533.Q4I2J8
117472	229533.I1RSQ3	pp	229533.I1RZK5
117473	229533.I1RSQ3	pp	229533.I1RWB0
117474	229533.I1RSQ3	pp	229533.I1S887
117475	229533.I1RSQ3	pp	229533.I1RZM8
117476	229533.I1RSQ3	pp	229533.Q4I8P5
117477	229533.I1RSQ3	pp	229533.V6QUE6
117478	229533.I1RSQ3	pp	229533.I1SA32
117479	229533.I1RSQ3	pp	229533.I1RXF0
117480	229533.I1RSQ3	pp	229533.I1S044
117481	229533.I1RSQ3	pp	229533.I1RXC4
117482	229533.I1RSQ3	pp	229533.I1RW99
117483	229533.I1RSQ3	pp	229533.I1S5B0
117484	229533.I1RJP4	pp	229533.I1S503
117485	229533.I1RJP4	pp	229533.I1RSQ3
117486	229533.I1RJP4	pp	229533.I1RQC9
117487	229533.I1RJP4	pp	229533.V6RMW6
117488	229533.I1RJP4	pp	229533.I1S504
117489	229533.I1RJP4	pp	229533.I1RT04
117490	229533.I1RJP4	pp	229533.I1RNC1
117491	229533.I1RJP4	pp	229533.I1RNQ5
117492	229533.I1RJP4	pp	229533.I1RZM1
117493	229533.I1RJP4	pp	229533.I1RLU6
117494	229533.I1RJP4	pp	229533.I1RN67
117495	229533.I1RJP4	pp	229533.I1RRY9
117496	229533.I1RJP4	pp	229533.I1RR73
117497	229533.I1RJP4	pp	229533.I1RJV3
117498	229533.I1RJP4	pp	229533.I1RPP4
117499	229533.I1RJP4	pp	229533.I1RTH6
117500	229533.I1RJP4	pp	229533.I1S0J1
117501	229533.I1RJP4	pp	229533.I1RPP3
117502	229533.I1RJP4	pp	229533.V6RTJ8
117503	229533.I1RJP4	pp	229533.I1RVX0
117504	229533.I1RJP4	pp	229533.I1RX46
117505	229533.I1RB14	pp	229533.I1RDY3
117506	229533.I1RB14	pp	229533.I1S472
117507	229533.I1RB14	pp	229533.I1S7A3
117508	229533.I1RB14	pp	229533.I1RZP4
117509	229533.I1RB14	pp	229533.I1S087
117510	229533.I1RBZ3	pp	229533.Q4I662
117511	229533.I1RBZ3	pp	229533.I1RFS3
117512	229533.I1RBZ3	pp	229533.I1S1W8
117513	229533.I1RBZ3	pp	229533.I1RNA0
117514	229533.I1RBZ3	pp	229533.I1RND6
117515	229533.I1RBZ3	pp	229533.I1RR73
117516	229533.I1RBZ3	pp	229533.I1RQ65
117517	229533.I1RBZ3	pp	229533.I1RD56
117518	229533.I1RBZ3	pp	229533.I1RN48
117519	229533.I1RBZ3	pp	229533.I1RM06
117520	229533.I1RBZ3	pp	229533.I1RZV6
117521	229533.I1RBZ3	pp	229533.I1RZS6
117522	229533.I1RBZ3	pp	229533.Q4IEV5
117523	229533.I1RBZ3	pp	229533.Q4IFI0
117524	229533.I1RBZ3	pp	229533.I1RPU0
117525	229533.I1RBZ3	pp	229533.I1S0D4
117526	229533.I1RBZ3	pp	229533.I1RT98
117527	229533.I1RBZ3	pp	229533.I1S7S9
117528	229533.I1RBZ3	pp	229533.I1RM67
117529	229533.I1RBZ3	pp	229533.I1RKJ6
117530	229533.I1RBZ3	pp	229533.I1RFL3

117531	229533.I1RBZ3	pp	229533.I1RIS3
117532	229533.I1RBZ3	pp	229533.I1RUQ2
117533	229533.I1RBZ3	pp	229533.I1S0R7
117534	229533.I1RBZ3	pp	229533.I1S3G4
117535	229533.I1RBZ3	pp	229533.I1RKE7
117536	229533.I1RBZ3	pp	229533.I1RPW9
117537	229533.I1RBZ3	pp	229533.I1RU69
117538	229533.I1RBZ3	pp	229533.I1RLV9
117539	229533.I1RBZ3	pp	229533.I1RC03
117540	229533.I1RBZ3	pp	229533.I1RT80
117541	229533.I1RBZ3	pp	229533.I1RRT2
117542	229533.I1RBZ3	pp	229533.I1RX46
117543	229533.I1RBZ3	pp	229533.I1S8E2
117544	229533.I1RBZ3	pp	229533.Q4IPI1
117545	229533.I1RBZ3	pp	229533.I1RWH5
117546	229533.I1RBZ3	pp	229533.I1RFW5
117547	229533.I1RBZ3	pp	229533.I1RKL3
117548	229533.I1RBZ3	pp	229533.I1S6U8
117549	229533.I1RBZ3	pp	229533.I1RTG7
117550	229533.I1RBZ3	pp	229533.I1RWF7
117551	229533.I1RBZ3	pp	229533.I1RGA4
117552	229533.I1RBZ3	pp	229533.I1RM96
117553	229533.I1RBZ3	pp	229533.I1RSD8
117554	229533.I1RBZ3	pp	229533.I1RPP3
117555	229533.I1RBZ3	pp	229533.I1RQJ5
117556	229533.I1RBZ3	pp	229533.I1RZK6
117557	229533.I1RBZ3	pp	229533.I1RZZ1
117558	229533.I1RBZ3	pp	229533.I1RFC0
117559	229533.I1RBZ3	pp	229533.I1RZF6
117560	229533.I1RBZ3	pp	229533.I1RJZ6
117561	229533.I1RBZ3	pp	229533.I1RRR7
117562	229533.I1RBZ3	pp	229533.I1RS30
117563	229533.I1RBZ3	pp	229533.I1RRQ3
117564	229533.I1RBZ3	pp	229533.Q4IN63
117565	229533.I1RBZ3	pp	229533.I1S2E8
117566	229533.I1RBZ3	pp	229533.Q4HWE2
117567	229533.I1RBZ3	pp	229533.I1RK83
117568	229533.I1RBZ3	pp	229533.Q4I327
117569	229533.I1RBZ3	pp	229533.I1S2C0
117570	229533.I1RBZ3	pp	229533.I1RDG0
117571	229533.I1RBZ3	pp	229533.I1RNY2
117572	229533.I1RBZ3	pp	229533.I1RCN5
117573	229533.I1RBZ3	pp	229533.I1RQ28
117574	229533.I1RBZ3	pp	229533.I1RJV3
117575	229533.I1RBZ3	pp	229533.I1RC81
117576	229533.I1RBZ3	pp	229533.I1RYB9
117577	229533.I1RBZ3	pp	229533.I1RVZ0
117578	229533.I1RBZ3	pp	229533.I1S060
117579	229533.I1RBZ3	pp	229533.I1S6E5
117580	229533.I1RBZ3	pp	229533.I1RNH6
117581	229533.I1RBZ3	pp	229533.I1S021
117582	229533.I1RBZ3	pp	229533.I1RDY5
117583	229533.I1RBZ3	pp	229533.V6RMW6
117584	229533.I1RBZ3	pp	229533.I1RE00
117585	229533.I1RBZ3	pp	229533.I1RJ17
117586	229533.I1RBZ3	pp	229533.I1S0J1
117587	229533.I1RBZ3	pp	229533.I1RSP7
117588	229533.I1RBZ3	pp	229533.I1RPK0
117589	229533.I1RBZ3	pp	229533.I1S0M9
117590	229533.I1RBZ3	pp	229533.I1RSR8
117591	229533.I1RBZ3	pp	229533.V6R042
117592	229533.I1RBZ3	pp	229533.I1RRI2
117593	229533.I1RBZ3	pp	229533.I1RGC2
117594	229533.I1RBZ3	pp	229533.Q4IJ56
117595	229533.I1RBZ3	pp	229533.I1RMN6
117596	229533.I1RBZ3	pp	229533.I1RD22
117597	229533.I1RBZ3	pp	229533.I1RM25
117598	229533.I1RBZ3	pp	229533.I1S044
117599	229533.I1RBZ3	pp	229533.Q4HZK7
117600	229533.I1RBZ3	pp	229533.V6R382
117601	229533.I1RBZ3	pp	229533.I1S209
117602	229533.I1RBZ3	pp	229533.I1RWB5
117603	229533.I1RBZ3	pp	229533.V6RGN5

117604	229533.I1RBZ3	pp	229533.I1RZB5
117605	229533.I1REP7	pp	229533.I1RZ94
117606	229533.I1REP7	pp	229533.Q4I7L0
117607	229533.A0A1C3YIT1	pp	229533.I1RNG4
117608	229533.V6R3X5	pp	229533.V6RCG5
117609	229533.V6R3X5	pp	229533.V6RCZ8
117610	229533.V6R3X5	pp	229533.V6R4J1
117611	229533.V6R3X5	pp	229533.V6RMW6
117612	229533.V6R3X5	pp	229533.V6RIV9
117613	229533.V6R3X5	pp	229533.V6RUY9
117614	229533.V6R3X5	pp	229533.V6RTJ8
117615	229533.I1RYB9	pp	229533.I1S8E2
117616	229533.I1RYB9	pp	229533.I1RZ39
117617	229533.I1RYB9	pp	229533.I1S060
117618	229533.I1RYB9	pp	229533.V6R382
117619	229533.I1RYB9	pp	229533.I1RZV6
117620	229533.I1RYB9	pp	229533.I1S3G4
117621	229533.I1RYB9	pp	229533.I1S0J1
117622	229533.I1RYB9	pp	229533.Q4IN63
117623	229533.I1RYB9	pp	229533.I1RZS6
117624	229533.I1RYB9	pp	229533.I1S7S9
117625	229533.I1RYB9	pp	229533.Q4IEV5
117626	229533.I1RYB9	pp	229533.Q4I662
117627	229533.I1RYB9	pp	229533.Q4I327
117628	229533.I1RYB9	pp	229533.I1S2E8
117629	229533.I1RYB9	pp	229533.I1S0R7
117630	229533.I1RYB9	pp	229533.Q4IPI1
117631	229533.I1RYB9	pp	229533.V6RMW6
117632	229533.I1RYB9	pp	229533.V6R042
117633	229533.I1RYB9	pp	229533.I1S0M9
117634	229533.I1RYB9	pp	229533.I1S2C0
117635	229533.I1RYB9	pp	229533.I1S1W8
117636	229533.I1RYB9	pp	229533.I1S1X9
117637	229533.I1RYB9	pp	229533.Q4IFI0
117638	229533.I1RYB9	pp	229533.I1RZK6
117639	229533.I1RYB9	pp	229533.I1S044
117640	229533.I1RYB9	pp	229533.I1S6E5
117641	229533.I1RYB9	pp	229533.I1RZF6
117642	229533.I1RYB9	pp	229533.I1RZZ1
117643	229533.I1RYB9	pp	229533.I1S021
117644	229533.I1RYB9	pp	229533.I1RZB5
117645	229533.I1RP28	pp	229533.I1RPM4
117646	229533.I1RP28	pp	229533.I1RX24
117647	229533.I1RP28	pp	229533.I1S0L4
117648	229533.I1RP28	pp	229533.I1S2G9
117649	229533.I1RP28	pp	229533.I1RS20
117650	229533.I1RP28	pp	229533.I1S2V2
117651	229533.I1RP28	pp	229533.I1RZZ9
117652	229533.I1RP28	pp	229533.I1RWK9
117653	229533.I1RP28	pp	229533.I1S1V5
117654	229533.I1RP28	pp	229533.I1RWI5
117655	229533.I1RP28	pp	229533.I1RS47
117656	229533.I1RP28	pp	229533.I1RRT8
117657	229533.I1RP28	pp	229533.I1S0F9
117658	229533.I1RP28	pp	229533.I1RRT2
117659	229533.I1RP28	pp	229533.I1RRM9
117660	229533.I1RP28	pp	229533.I1RR95
117661	229533.I1RP28	pp	229533.I1RPP3
117662	229533.I1RP28	pp	229533.I1RUF8
117663	229533.I1RP28	pp	229533.I1RQG4
117664	229533.I1RP28	pp	229533.V6RRH2
117665	229533.I1RP28	pp	229533.I1S8P5
117666	229533.I1RP28	pp	229533.I1S5S8
117667	229533.I1RP28	pp	229533.I1S1X9
117668	229533.I1RP28	pp	229533.I1RSH0
117669	229533.I1RP28	pp	229533.I1S247
117670	229533.I1RP28	pp	229533.I1RSK1
117671	229533.I1RP28	pp	229533.I1RQF2
117672	229533.I1RP28	pp	229533.I1RTA0
117673	229533.I1RP28	pp	229533.V6RSF3
117674	229533.I1RP28	pp	229533.I1RZE4
117675	229533.I1RP28	pp	229533.I1RTH6
117676	229533.I1RP28	pp	229533.I1S0Z8

117677 229533.I1RP28 pp 229533.I1RPN8
117678 229533.I1RP28 pp 229533.I1S2C0
117679 229533.I1RP28 pp 229533.I1RXT9
117680 229533.I1RP28 pp 229533.I1S0N4
117681 229533.I1RP28 pp 229533.I1S2E8
117682 229533.I1RP28 pp 229533.I1RYW3
117683 229533.I1RP28 pp 229533.I1RQE7
117684 229533.I1RP28 pp 229533.I1S238
117685 229533.I1RP28 pp 229533.I1S0M2
117686 229533.I1RP28 pp 229533.I1RWH4
117687 229533.I1RP28 pp 229533.I1RRV1
117688 229533.I1RP28 pp 229533.I1RZ02
117689 229533.I1RP28 pp 229533.I1RZ95
117690 229533.I1RP28 pp 229533.V6RVG3
117691 229533.I1RP28 pp 229533.I1RT41
117692 229533.I1RP28 pp 229533.I1RQ21
117693 229533.I1RP28 pp 229533.I1RYH9
117694 229533.I1RP28 pp 229533.I1S9U6
117695 229533.I1RP28 pp 229533.I1RQ26
117696 229533.I1RP28 pp 229533.V6RMW6
117697 229533.I1RP28 pp 229533.I1RW25
117698 229533.I1RP28 pp 229533.V6RTJ8
117699 229533.I1RP28 pp 229533.I1RTU8
117700 229533.I1RP28 pp 229533.I1RQH1
117701 229533.I1RP28 pp 229533.I1RRF8
117702 229533.I1RP28 pp 229533.I1RUG3
117703 229533.I1RP28 pp 229533.I1RX46
117704 229533.I1RP28 pp 229533.I1RW71
117705 229533.I1RP28 pp 229533.I1RVQ0
117706 229533.I1RP28 pp 229533.I1RQQ8
117707 229533.I1RP28 pp 229533.I1RRT6
117708 229533.I1RP28 pp 229533.Q4HZS8
117709 229533.I1RP28 pp 229533.Q4ICA8
117710 229533.I1RP28 pp 229533.I1RRY9
117711 229533.I1RP28 pp 229533.I1RW87
117712 229533.I1RP28 pp 229533.I1RZM1
117713 229533.I1RP28 pp 229533.Q4I2J8
117714 229533.I1RP28 pp 229533.I1RQA5
117715 229533.I1RP28 pp 229533.I1S1J7
117716 229533.I1RP28 pp 229533.I1RSI0
117717 229533.I1RP28 pp 229533.I1RPL2
117718 229533.I1RP28 pp 229533.I1S0Z3
117719 229533.I1RP28 pp 229533.I1RYL5
117720 229533.I1RP28 pp 229533.I1RP66
117721 229533.I1RP28 pp 229533.V6RBN2
117722 229533.I1RP28 pp 229533.I1RQ81
117723 229533.I1RP28 pp 229533.V6RXW9
117724 229533.I1RP28 pp 229533.I1RQQ6
117725 229533.I1RP28 pp 229533.I1RYJ8
117726 229533.I1RP28 pp 229533.I1RZH4
117727 229533.I1RP28 pp 229533.I1S235
117728 229533.I1RP28 pp 229533.I1S5B0
117729 229533.I1RNC6 pp 229533.I1RVY0
117730 229533.I1RNC6 pp 229533.I1RSG9
117731 229533.I1RMG3 pp 229533.I1RRM5
117732 229533.I1RMG3 pp 229533.I1RW25
117733 229533.I1RMG3 pp 229533.V6RMW6
117734 229533.I1RMG3 pp 229533.I1S2E9
117735 229533.I1RMG3 pp 229533.Q4I2J8
117736 229533.I1RMG3 pp 229533.V6R042
117737 229533.I1RMG3 pp 229533.V6RCG5
117738 229533.I1RMG3 pp 229533.V6RCZ8
117739 229533.I1RMG3 pp 229533.I1RSQ3
117740 229533.I1RMG3 pp 229533.I1RXD7
117741 229533.I1RMG3 pp 229533.I1RT04
117742 229533.I1RMG3 pp 229533.I1RZW8
117743 229533.I1RMG3 pp 229533.I1RQC9
117744 229533.I1RMG3 pp 229533.I1RYL5
117745 229533.I1RMG3 pp 229533.I1S0R7
117746 229533.I1RMG3 pp 229533.I1RUG3
117747 229533.I1RMG3 pp 229533.I1RTD5
117748 229533.I1RMG3 pp 229533.I1S1C7
117749 229533.I1RMG3 pp 229533.I1RZZ9

117750 229533.I1RMG3 pp 229533.I1RNC1
117751 229533.I1RMG3 pp 229533.V6R3X5
117752 229533.I1RMG3 pp 229533.I1RY03
117753 229533.I1RMG3 pp 229533.I1RR73
117754 229533.I1RMG3 pp 229533.I1RZM8
117755 229533.I1RMG3 pp 229533.I1RTH6
117756 229533.I1RMG3 pp 229533.I1RXF0
117757 229533.I1RMG3 pp 229533.I1RWN6
117758 229533.I1RMG3 pp 229533.I1RRI2
117759 229533.I1RMG3 pp 229533.I1RRY9
117760 229533.I1RMG3 pp 229533.I1S7A8
117761 229533.I1RMG3 pp 229533.I1RT40
117762 229533.I1RMG3 pp 229533.I1S0L4
117763 229533.I1RMG3 pp 229533.I1S5B0
117764 229533.I1RMG3 pp 229533.I1SA32
117765 229533.I1RMG3 pp 229533.I1S5R1
117766 229533.I1RMG3 pp 229533.I1RR24
117767 229533.I1RMG3 pp 229533.I1RWK3
117768 229533.I1RMG3 pp 229533.I1RRT8
117769 229533.I1RMG3 pp 229533.I1RW46
117770 229533.I1RMG3 pp 229533.I1RW99
117771 229533.I1RMG3 pp 229533.Q4I283
117772 229533.I1RMG3 pp 229533.V6RTJ8
117773 229533.I1RMG3 pp 229533.I1S0L3
117774 229533.I1RMG3 pp 229533.I1RZJ5
117775 229533.I1RMG3 pp 229533.I1RX46
117776 229533.I1RMG3 pp 229533.I1S503
117777 229533.I1RMG3 pp 229533.I1RWJ8
117778 229533.I1RMG3 pp 229533.I1RT03
117779 229533.I1RMG3 pp 229533.I1SA33
117780 229533.I1RMG3 pp 229533.I1RQ41
117781 229533.I1RMG3 pp 229533.I1RY44
117782 229533.I1RMG3 pp 229533.I1RZZ1
117783 229533.I1RMG3 pp 229533.I1S504
117784 229533.I1RMG3 pp 229533.I1RRR5
117785 229533.I1RMG3 pp 229533.I1RQ23
117786 229533.I1RMG3 pp 229533.I1RNQ5
117787 229533.I1RMG3 pp 229533.I1RPM4
117788 229533.I1RMG3 pp 229533.I1RPP3
117789 229533.I1RMG3 pp 229533.I1RVQ0
117790 229533.I1RMG3 pp 229533.I1RPP4
117791 229533.I1RMG3 pp 229533.I1RQE7
117792 229533.I1RMG3 pp 229533.Q4I8P5
117793 229533.I1RMG3 pp 229533.I1RSQ7
117794 229533.I1RMG3 pp 229533.I1RZM1
117795 229533.I1RMG3 pp 229533.Q4I624
117796 229533.I1RMG3 pp 229533.I1RN18
117797 229533.I1RMG3 pp 229533.V6RIV9
117798 229533.I1RMG3 pp 229533.V6QW22
117799 229533.I1RMG3 pp 229533.I1RN67
117800 229533.I1RMG3 pp 229533.I1S0F6
117801 229533.I1RMG3 pp 229533.I1RS17
117802 229533.I1RWN4 pp 229533.I1RYH0
117803 229533.A0A1C3YMB5 pp 229533.Q4IRI9
117804 229533.A0A1C3YMB5 pp 229533.I1RGC2
117805 229533.A0A1C3YMB5 pp 229533.I1S044
117806 229533.A0A1C3YMB5 pp 229533.I1S0N7
117807 229533.A0A1C3YMB5 pp 229533.A0A1C3YMK1
117808 229533.A0A1C3YMB5 pp 229533.I1RMK1
117809 229533.A0A1C3YMB5 pp 229533.I1RJV7
117810 229533.A0A1C3YMB5 pp 229533.I1RCP7
117811 229533.A0A1C3YMB5 pp 229533.I1RE45
117812 229533.A0A1C3YMB5 pp 229533.I1RDG0
117813 229533.A0A1C3YMB5 pp 229533.I1RYW2
117814 229533.A0A1C3YMB5 pp 229533.I1S5Z2
117815 229533.A0A1C3YMB5 pp 229533.I1RRH3
117816 229533.A0A1C3YMB5 pp 229533.I1RSD4
117817 229533.A0A1C3YMB5 pp 229533.I1RN28
117818 229533.A0A1C3YMB5 pp 229533.I1RPS0
117819 229533.I1RR73 pp 229533.I1RS17
117820 229533.I1RR73 pp 229533.I1RTA0
117821 229533.I1RR73 pp 229533.I1RW46
117822 229533.I1RR73 pp 229533.I1RWB5

117823 229533.I1RR73 pp 229533.V6RVG3
117824 229533.I1RR73 pp 229533.I1RZS7
117825 229533.I1RR73 pp 229533.I1RRE5
117826 229533.I1RR73 pp 229533.I1RRV1
117827 229533.I1RR73 pp 229533.I1S0J1
117828 229533.I1RR73 pp 229533.I1RSQ3
117829 229533.I1RR73 pp 229533.I1S1W4
117830 229533.I1RR73 pp 229533.V6RMW6
117831 229533.I1RR73 pp 229533.V6R042
117832 229533.I1RR73 pp 229533.I1RRR7
117833 229533.I1RR73 pp 229533.V6R057
117834 229533.I1RR73 pp 229533.I1RWN5
117835 229533.I1RR73 pp 229533.I1S2E8
117836 229533.I1RR73 pp 229533.I1RXT3
117837 229533.I1RR73 pp 229533.V6RIV9
117838 229533.I1RR73 pp 229533.I1RUG3
117839 229533.I1RR73 pp 229533.Q4HZB8
117840 229533.I1RR73 pp 229533.I1S569
117841 229533.I1RR73 pp 229533.I1RTH6
117842 229533.I1RR73 pp 229533.I1S8R3
117843 229533.I1RR73 pp 229533.Q4I624
117844 229533.I1RR73 pp 229533.I1RY44
117845 229533.I1RR73 pp 229533.Q4I327
117846 229533.I1RR73 pp 229533.Q4IN63
117847 229533.I1RR73 pp 229533.I1RSG2
117848 229533.I1RR73 pp 229533.V6RGN5
117849 229533.I1RR73 pp 229533.I1S352
117850 229533.I1RR73 pp 229533.I1S2C0
117851 229533.I1RR73 pp 229533.I1RSK1
117852 229533.I1RR73 pp 229533.I1RSV2
117853 229533.I1RR73 pp 229533.I1RTE9
117854 229533.I1RR73 pp 229533.I1S0N4
117855 229533.I1RR73 pp 229533.I1SA33
117856 229533.I1RR73 pp 229533.I1S1C7
117857 229533.I1RR73 pp 229533.I1RYW3
117858 229533.I1RR73 pp 229533.I1RWH4
117859 229533.I1RR73 pp 229533.I1RWF7
117860 229533.I1RR73 pp 229533.I1S1X9
117861 229533.I1RR73 pp 229533.I1RTD7
117862 229533.I1RR73 pp 229533.I1S8P5
117863 229533.I1RR73 pp 229533.I1RT03
117864 229533.I1RR73 pp 229533.I1RRJ8
117865 229533.I1RR73 pp 229533.I1RRN5
117866 229533.I1RR73 pp 229533.I1RVX0
117867 229533.I1RR73 pp 229533.I1RWK3
117868 229533.I1RR73 pp 229533.I1RWX5
117869 229533.I1RR73 pp 229533.V6R283
117870 229533.I1RR73 pp 229533.I1RRI2
117871 229533.I1RR73 pp 229533.I1RS47
117872 229533.I1RR73 pp 229533.Q4HWE2
117873 229533.I1RR73 pp 229533.I1RWN6
117874 229533.I1RR73 pp 229533.I1RZW8
117875 229533.I1RR73 pp 229533.I1RSD8
117876 229533.I1RR73 pp 229533.I1RRQ3
117877 229533.I1RR73 pp 229533.I1S2H4
117878 229533.I1RR73 pp 229533.I1S2G7
117879 229533.I1RR73 pp 229533.I1S0F6
117880 229533.I1RR73 pp 229533.I1RT04
117881 229533.I1RR73 pp 229533.Q4I662
117882 229533.I1RR73 pp 229533.I1S0L4
117883 229533.I1RR73 pp 229533.I1S2E9
117884 229533.I1RR73 pp 229533.I1RV91
117885 229533.I1RR73 pp 229533.I1RRT8
117886 229533.I1RR73 pp 229533.I1RZV6
117887 229533.I1RR73 pp 229533.I1RT40
117888 229533.I1RR73 pp 229533.I1S2B8
117889 229533.I1RR73 pp 229533.I1RWL2
117890 229533.I1RR73 pp 229533.I1RRM5
117891 229533.I1RR73 pp 229533.I1S5R1
117892 229533.I1RR73 pp 229533.I1RZ39
117893 229533.I1RR73 pp 229533.I1RSL9
117894 229533.I1RR73 pp 229533.I1RTD5
117895 229533.I1RR73 pp 229533.I1S0M9

117896 229533.I1RR73 pp 229533.I1S5O3
117897 229533.I1RR73 pp 229533.I1RX24
117898 229533.I1RR73 pp 229533.Q4I8P5
117899 229533.I1RR73 pp 229533.I1S8E2
117900 229533.I1RR73 pp 229533.I1RZS6
117901 229533.I1RR73 pp 229533.I1S0M6
117902 229533.I1RR73 pp 229533.V6R3X5
117903 229533.I1RR73 pp 229533.I1RZZ9
117904 229533.I1RR73 pp 229533.I1RS18
117905 229533.I1RR73 pp 229533.I1RZM1
117906 229533.I1RR73 pp 229533.I1RWI5
117907 229533.I1RR73 pp 229533.I1RZH4
117908 229533.I1RR73 pp 229533.I1RZF6
117909 229533.I1RR73 pp 229533.I1RU69
117910 229533.I1RR73 pp 229533.I1SA32
117911 229533.I1RR73 pp 229533.I1RS20
117912 229533.I1RR73 pp 229533.V6RCZ8
117913 229533.I1RR73 pp 229533.I1RZZ1
117914 229533.I1RR73 pp 229533.I1S5O4
117915 229533.I1RR73 pp 229533.V6RCG5
117916 229533.I1RR73 pp 229533.I1RZK6
117917 229533.I1RR73 pp 229533.I1RZM8
117918 229533.I1RR73 pp 229533.Q4I283
117919 229533.I1RR73 pp 229533.V6RBN2
117920 229533.I1RR73 pp 229533.I1RXF0
117921 229533.I1RR73 pp 229533.I1RWD7
117922 229533.I1RR73 pp 229533.I1RYB9
117923 229533.I1RR73 pp 229533.I1RWT6
117924 229533.I1RR73 pp 229533.I1RVZ0
117925 229533.I1RR73 pp 229533.I1RRY9
117926 229533.I1RR73 pp 229533.I1S021
117927 229533.I1RR73 pp 229533.I1S5B0
117928 229533.I1RR73 pp 229533.I1RU29
117929 229533.I1RR73 pp 229533.I1RW99
117930 229533.I1RR73 pp 229533.I1S044
117931 229533.I1RR73 pp 229533.Q4I2J8
117932 229533.I1RR73 pp 229533.I1RW71
117933 229533.I1RR73 pp 229533.I1S0Z9
117934 229533.I1RR73 pp 229533.I1S1J7
117935 229533.I1RR73 pp 229533.I1RSQ7
117936 229533.I1RR73 pp 229533.I1RRU9
117937 229533.I1RR73 pp 229533.Q4IJM4
117938 229533.I1RR73 pp 229533.V6QW22
117939 229533.I1RR73 pp 229533.I1RZ35
117940 229533.I1RR73 pp 229533.I1RWH5
117941 229533.I1RR73 pp 229533.Q4IJK2
117942 229533.I1RR73 pp 229533.I1RYB1
117943 229533.I1RR73 pp 229533.Q4IJT5
117944 229533.I1RR73 pp 229533.I1RZE5
117945 229533.I1RR73 pp 229533.I1RZK5
117946 229533.I1RR73 pp 229533.I1RVQ0
117947 229533.I1RR73 pp 229533.I1RSI0
117948 229533.I1RR73 pp 229533.I1RY03
117949 229533.I1RR73 pp 229533.V6R4J1
117950 229533.I1RR73 pp 229533.V6R1K4
117951 229533.I1RR73 pp 229533.I1S0F5
117952 229533.I1RR73 pp 229533.I1S0R7
117953 229533.I1RR73 pp 229533.I1RXD7
117954 229533.I1RR73 pp 229533.I1S0L3
117955 229533.I1RR73 pp 229533.I1RSR8
117956 229533.I1RR73 pp 229533.I1S9U2
117957 229533.I1RR73 pp 229533.I1S8I8
117958 229533.I1RR73 pp 229533.I1RYY5
117959 229533.I1RR73 pp 229533.I1RWJ8
117960 229533.I1RR73 pp 229533.I1RZG3
117961 229533.I1RR73 pp 229533.I1S7A8
117962 229533.I1RR73 pp 229533.I1RW07
117963 229533.I1RR73 pp 229533.I1RZA5
117964 229533.I1RR73 pp 229533.I1RRQ1
117965 229533.I1RR73 pp 229533.Q4IFI0
117966 229533.I1RR73 pp 229533.I1RZJ5
117967 229533.I1RR73 pp 229533.I1RW25
117968 229533.I1RR73 pp 229533.V6RTJ8

117969	229533.I1RR73	pp	229533.I1RX46
117970	229533.I1RR73	pp	229533.I1RX25
117971	229533.I1RR73	pp	229533.Q4I5R9
117972	229533.I1RR73	pp	229533.V6QUW4
117973	229533.I1RR73	pp	229533.Q4IPI1
117974	229533.I1RR73	pp	229533.I1RT80
117975	229533.I1RR73	pp	229533.I1S0X6
117976	229533.I1RR73	pp	229533.I1S6U8
117977	229533.I1RE45	pp	229533.I1S214
117978	229533.I1RE45	pp	229533.I1RT35
117979	229533.I1RE45	pp	229533.Q4IPA4
117980	229533.I1RE45	pp	229533.I1S246
117981	229533.I1RE45	pp	229533.I1RQK2
117982	229533.I1RE45	pp	229533.I1RN49
117983	229533.I1RE45	pp	229533.I1RNB8
117984	229533.I1RE45	pp	229533.Q4IF44
117985	229533.I1RE45	pp	229533.Q4IAS1
117986	229533.I1RE45	pp	229533.I1S0N7
117987	229533.I1RE45	pp	229533.I1S8U9
117988	229533.I1RE45	pp	229533.Q4I7X1
117989	229533.I1RE45	pp	229533.Q4IPC8
117990	229533.I1RE45	pp	229533.I1RS03
117991	229533.I1RE45	pp	229533.Q4IR09
117992	229533.I1RE45	pp	229533.Q4HWE2
117993	229533.I1RE45	pp	229533.I1RPY7
117994	229533.I1RE45	pp	229533.I1S8V0
117995	229533.I1RE45	pp	229533.I1RQZ2
117996	229533.I1RE45	pp	229533.Q4IPN9
117997	229533.I1RE45	pp	229533.I1RK70
117998	229533.I1RE45	pp	229533.I1S5Z2
117999	229533.I1RE45	pp	229533.I1RZP2
118000	229533.I1RE45	pp	229533.I1RZL5
118001	229533.I1RE45	pp	229533.I1RHI1
118002	229533.I1RRV1	pp	229533.V6RIV9
118003	229533.I1RRV1	pp	229533.I1S1W9
118004	229533.I1RRV1	pp	229533.I1S0N4
118005	229533.I1RRV1	pp	229533.I1S5R6
118006	229533.I1RRV1	pp	229533.I1RWN5
118007	229533.I1RRV1	pp	229533.I1RWE8
118008	229533.I1RRV1	pp	229533.I1S1W4
118009	229533.I1RRV1	pp	229533.I1RY44
118010	229533.I1RRV1	pp	229533.I1S0R3
118011	229533.I1RRV1	pp	229533.I1RUG3
118012	229533.I1RRV1	pp	229533.I1S0J1
118013	229533.I1RRV1	pp	229533.I1RTA0
118014	229533.I1RRV1	pp	229533.I1SAN9
118015	229533.I1RRV1	pp	229533.V6RLM7
118016	229533.I1RRV1	pp	229533.V6R042
118017	229533.I1RRV1	pp	229533.V6RMW6
118018	229533.I1RRV1	pp	229533.I1RS17
118019	229533.I1RRV1	pp	229533.I1RVX0
118020	229533.I1RRV1	pp	229533.I1RSQ3
118021	229533.I1RRV1	pp	229533.I1RY11
118022	229533.I1RRV1	pp	229533.I1RT03
118023	229533.I1RRV1	pp	229533.Q4I7K4
118024	229533.I1RRV1	pp	229533.V6RRH2
118025	229533.I1RRV1	pp	229533.I1RSK1
118026	229533.I1RRV1	pp	229533.I1RWD3
118027	229533.I1RRV1	pp	229533.I1S855
118028	229533.I1RRV1	pp	229533.V6R057
118029	229533.I1RRV1	pp	229533.I1RWN6
118030	229533.I1RRV1	pp	229533.Q4I1N3
118031	229533.I1RRV1	pp	229533.I1RWK3
118032	229533.I1RRV1	pp	229533.I1RYW3
118033	229533.I1RRV1	pp	229533.I1S2G7
118034	229533.I1RRV1	pp	229533.I1S1C7
118035	229533.I1RRV1	pp	229533.Q4I624
118036	229533.I1RRV1	pp	229533.I1RZ79
118037	229533.I1RRV1	pp	229533.I1RVA8
118038	229533.I1RRV1	pp	229533.Q4I1Y5
118039	229533.I1RRV1	pp	229533.I1RWX5
118040	229533.I1RRV1	pp	229533.I1RZE4
118041	229533.I1RRV1	pp	229533.I1RTA1

118042	229533.I1RRV1	pp	229533.I1RVZ2
118043	229533.I1RRV1	pp	229533.I1S2E8
118044	229533.I1RRV1	pp	229533.I1S8P5
118045	229533.I1RRV1	pp	229533.I1RZW8
118046	229533.I1RRV1	pp	229533.I1RSV2
118047	229533.I1RRV1	pp	229533.I1RYI5
118048	229533.I1RRV1	pp	229533.I1S2E9
118049	229533.I1RRV1	pp	229533.I1RTH6
118050	229533.I1RRV1	pp	229533.I1S503
118051	229533.I1RRV1	pp	229533.I1S0R4
118052	229533.I1RRV1	pp	229533.I1RX22
118053	229533.I1RRV1	pp	229533.I1S2B8
118054	229533.I1RRV1	pp	229533.I1RTG2
118055	229533.I1RRV1	pp	229533.I1RS47
118056	229533.I1RRV1	pp	229533.I1RV91
118057	229533.I1RRV1	pp	229533.I1S0X5
118058	229533.I1RRV1	pp	229533.I1RSZ7
118059	229533.I1RRV1	pp	229533.I1RT40
118060	229533.I1RRV1	pp	229533.I1RTD5
118061	229533.I1RRV1	pp	229533.O74638
118062	229533.I1RRV1	pp	229533.I1RSQ4
118063	229533.I1RRV1	pp	229533.I1S5R1
118064	229533.I1RRV1	pp	229533.V6RUY9
118065	229533.I1RRV1	pp	229533.I1RZZ9
118066	229533.I1RRV1	pp	229533.I1RWL2
118067	229533.I1RRV1	pp	229533.I1RWI5
118068	229533.I1RRV1	pp	229533.Q4IAA0
118069	229533.I1RRV1	pp	229533.I1S8G4
118070	229533.I1RRV1	pp	229533.I1RT04
118071	229533.I1RRV1	pp	229533.I1RS20
118072	229533.I1RRV1	pp	229533.I1RZ39
118073	229533.I1RRV1	pp	229533.I1S2H4
118074	229533.I1RRV1	pp	229533.I1RZ63
118075	229533.I1RRV1	pp	229533.I1RS18
118076	229533.I1RRV1	pp	229533.V6R3X5
118077	229533.I1RRV1	pp	229533.I1S0L4
118078	229533.I1RRV1	pp	229533.I1RRV4
118079	229533.I1RRV1	pp	229533.I1S5B0
118080	229533.I1RRV1	pp	229533.I1S044
118081	229533.I1RRV1	pp	229533.I1RZZ1
118082	229533.I1RRV1	pp	229533.I1RW99
118083	229533.I1RRV1	pp	229533.I1RZM1
118084	229533.I1RRV1	pp	229533.I1S0K2
118085	229533.I1RRV1	pp	229533.I1RZK6
118086	229533.I1RRV1	pp	229533.I1RZM8
118087	229533.I1RRV1	pp	229533.Q4IJM4
118088	229533.I1RRV1	pp	229533.I1S504
118089	229533.I1RRV1	pp	229533.V6RBN2
118090	229533.I1RRV1	pp	229533.I1RWT6
118091	229533.I1RRV1	pp	229533.V6RCZ8
118092	229533.I1RRV1	pp	229533.I1RYS0
118093	229533.I1RRV1	pp	229533.I1RVQ0
118094	229533.I1RRV1	pp	229533.V6RCG5
118095	229533.I1RRV1	pp	229533.V6R1K4
118096	229533.I1RRV1	pp	229533.I1RSI0
118097	229533.I1RRV1	pp	229533.I1S1J7
118098	229533.I1RRV1	pp	229533.I1RRY9
118099	229533.I1RRV1	pp	229533.I1RWD7
118100	229533.I1RRV1	pp	229533.Q4IJK2
118101	229533.I1RRV1	pp	229533.I1RY03
118102	229533.I1RRV1	pp	229533.I1S0L3
118103	229533.I1RRV1	pp	229533.I1RSQ7
118104	229533.I1RRV1	pp	229533.I1RYY5
118105	229533.I1RRV1	pp	229533.I1RW71
118106	229533.I1RRV1	pp	229533.V6QW22
118107	229533.I1RRV1	pp	229533.I1RZK5
118108	229533.I1RRV1	pp	229533.I1RXU7
118109	229533.I1RRV1	pp	229533.I1S854
118110	229533.I1RRV1	pp	229533.I1S0C1
118111	229533.I1RRV1	pp	229533.I1S0R7
118112	229533.I1RRV1	pp	229533.I1RSM2
118113	229533.I1RRV1	pp	229533.I1RX46
118114	229533.I1RRV1	pp	229533.V6RTJ8

118115 229533.I1RRV1 pp 229533.I1RXD7
118116 229533.I1RRV1 pp 229533.I1S5S3
118117 229533.I1RRV1 pp 229533.V6QUW4
118118 229533.I1RRV1 pp 229533.I1RW25
118119 229533.I1RRV1 pp 229533.I1RY21
118120 229533.I1RPQ7 pp 229533.I1RT81
118121 229533.I1RPQ7 pp 229533.I1RYH0
118122 229533.I1RPQ7 pp 229533.I1RST4
118123 229533.A0A1C3YJR7 pp 229533.I1RWN4
118124 229533.A0A1C3YJR7 pp 229533.I1RW71
118125 229533.A0A1C3YJR7 pp 229533.I1RJZ7
118126 229533.A0A1C3YJR7 pp 229533.I1RPQ7
118127 229533.A0A1C3YJR7 pp 229533.I1RMP0
118128 229533.A0A1C3YLW0 pp 229533.I1RB73
118129 229533.A0A1C3YLW0 pp 229533.I1SAA0
118130 229533.A0A1C3YLW0 pp 229533.I1RJX2
118131 229533.A0A1C3YLW0 pp 229533.I1RQ88
118132 229533.A0A1C3YLW0 pp 229533.I1RCP3
118133 229533.A0A1C3YLW0 pp 229533.I1RQ93
118134 229533.A0A1C3YLW0 pp 229533.I1RKJ7
118135 229533.A0A1C3YLW0 pp 229533.I1RCN2
118136 229533.A0A1C3YLW0 pp 229533.I1RAI9
118137 229533.A0A1C3YLW0 pp 229533.Q4ICA8
118138 229533.I1RRX1 pp 229533.I1RZD9
118139 229533.I1RRX1 pp 229533.I1RSW5
118140 229533.I1RRX1 pp 229533.I1S4J4
118141 229533.I1RRX1 pp 229533.I1S095
118142 229533.I1RRX1 pp 229533.P0C431
118143 229533.I1RRX1 pp 229533.I1RT36
118144 229533.I1RRX1 pp 229533.I1RZP6
118145 229533.I1RRX1 pp 229533.I1S0C0
118146 229533.I1RPX0 pp 229533.I1RQ26
118147 229533.I1S5E8 pp 229533.I1S6D1
118148 229533.A0A1C3YIN1 pp 229533.Q4IF76
118149 229533.A0A1C3YIN1 pp 229533.I1RT05
118150 229533.A0A1C3YIN1 pp 229533.I1RMK3
118151 229533.A0A1C3YIN1 pp 229533.Q4HVW2
118152 229533.A0A1C3YIN1 pp 229533.I1RBN1
118153 229533.A0A1C3YIN1 pp 229533.I1RW61
118154 229533.A0A1C3YIN1 pp 229533.I1RC32
118155 229533.A0A1C3YIN1 pp 229533.I1RDA0
118156 229533.A0A1C3YIN1 pp 229533.I1S0M6
118157 229533.A0A1C3YIN1 pp 229533.I1RND6
118158 229533.A0A1C3YIN1 pp 229533.V6R3X5
118159 229533.A0A1C3YIN1 pp 229533.I1RM06
118160 229533.A0A1C3YIN1 pp 229533.I1RZS6
118161 229533.A0A1C3YIN1 pp 229533.I1RR73
118162 229533.A0A1C3YIN1 pp 229533.I1RNV6
118163 229533.A0A1C3YIN1 pp 229533.I1RX24
118164 229533.A0A1C3YIN1 pp 229533.I1RZ39
118165 229533.A0A1C3YIN1 pp 229533.I1RT80
118166 229533.A0A1C3YIN1 pp 229533.V6QYF0
118167 229533.A0A1C3YIN1 pp 229533.A0A1C3YKM9
118168 229533.A0A1C3YIN1 pp 229533.Q4IJH1
118169 229533.A0A1C3YIN1 pp 229533.I1RKF9
118170 229533.A0A1C3YIN1 pp 229533.I1RBG9
118171 229533.A0A1C3YIN1 pp 229533.I1RNA0
118172 229533.A0A1C3YIN1 pp 229533.I1RC29
118173 229533.A0A1C3YIN1 pp 229533.I1RTF4
118174 229533.A0A1C3YIN1 pp 229533.I1RRT8
118175 229533.A0A1C3YIN1 pp 229533.I1RM67
118176 229533.A0A1C3YIN1 pp 229533.I1S8E2
118177 229533.A0A1C3YIN1 pp 229533.I1RPG6
118178 229533.A0A1C3YIN1 pp 229533.A0A1C3YIY6
118179 229533.A0A1C3YIN1 pp 229533.I1RAC2
118180 229533.A0A1C3YIN1 pp 229533.I1RX46
118181 229533.A0A1C3YIN1 pp 229533.A0A1C3YMI6
118182 229533.A0A1C3YIN1 pp 229533.I1RW25
118183 229533.A0A1C3YIN1 pp 229533.I1RZV6
118184 229533.A0A1C3YIN1 pp 229533.I1RKJ6
118185 229533.A0A1C3YIN1 pp 229533.I1RQS5
118186 229533.A0A1C3YIN1 pp 229533.I1RPU0
118187 229533.A0A1C3YIN1 pp 229533.I1RL23

229533.A0A1C3YIN1	pp	229533.I1S2B8
229533.A0A1C3YIN1	pp	229533.I1RRT2
229533.A0A1C3YIN1	pp	229533.A0A1C3YMW0
229533.A0A1C3YIN1	pp	229533.Q4IFI0
229533.A0A1C3YIN1	pp	229533.I1RUF8
229533.A0A1C3YIN1	pp	229533.Q4I7L0
229533.A0A1C3YIN1	pp	229533.I1RPW9
229533.A0A1C3YIN1	pp	229533.I1RJZ7
229533.A0A1C3YIN1	pp	229533.I1R9G0
229533.A0A1C3YIN1	pp	229533.Q4IPI1
229533.A0A1C3YIN1	pp	229533.I1S9U2
229533.A0A1C3YIN1	pp	229533.I1RA87
229533.A0A1C3YIN1	pp	229533.I1RGF6
229533.A0A1C3YIN1	pp	229533.I1RJL4
229533.A0A1C3YIN1	pp	229533.I1RD64
229533.A0A1C3YIN1	pp	229533.I1RU69
229533.A0A1C3YIN1	pp	229533.Q4IAS1
229533.A0A1C3YIN1	pp	229533.I1RG51
229533.A0A1C3YIN1	pp	229533.I1RFS3
229533.A0A1C3YIN1	pp	229533.Q4I662
229533.A0A1C3YIN1	pp	229533.I1S8I8
229533.A0A1C3YIN1	pp	229533.I1RLV9
229533.A0A1C3YIN1	pp	229533.I1S503
229533.A0A1C3YIN1	pp	229533.I1RAF8
229533.A0A1C3YIN1	pp	229533.I1RC03
229533.A0A1C3YIN1	pp	229533.I1RAT7
229533.A0A1C3YIN1	pp	229533.I1RKQ5
229533.A0A1C3YIN1	pp	229533.A0A1C3YKK5
229533.A0A1C3YIN1	pp	229533.Q4I5R9
229533.A0A1C3YIN1	pp	229533.I1S2E9
229533.A0A1C3YIN1	pp	229533.I1RUQ2
229533.A0A1C3YIN1	pp	229533.I1S098
229533.A0A1C3YIN1	pp	229533.Q4IQW0
229533.A0A1C3YIN1	pp	229533.V6R283
229533.A0A1C3YIN1	pp	229533.I1RWH5
229533.A0A1C3YIN1	pp	229533.I1RAX6
229533.A0A1C3YIN1	pp	229533.I1RGA4
229533.A0A1C3YIN1	pp	229533.I1RXD7
229533.A0A1C3YIN1	pp	229533.I1RPP3
229533.A0A1C3YIN1	pp	229533.I1RC82
229533.A0A1C3YIN1	pp	229533.I1S0R7
229533.A0A1C3YIN1	pp	229533.A0A1C3YLF5
229533.A0A1C3YIN1	pp	229533.I1RB74
229533.A0A1C3YIN1	pp	229533.I1RFN1
229533.A0A1C3YIN1	pp	229533.I1RBC7
229533.A0A1C3YIN1	pp	229533.I1RZ99
229533.A0A1C3YIN1	pp	229533.I1RT04
229533.A0A1C3YIN1	pp	229533.I1RQJ5
229533.A0A1C3YIN1	pp	229533.I1RFL7
229533.A0A1C3YIN1	pp	229533.I1RSL9
229533.A0A1C3YIN1	pp	229533.A0A1C3YN63
229533.A0A1C3YIN1	pp	229533.I1RE66
229533.A0A1C3YIN1	pp	229533.I1RRQ3
229533.A0A1C3YIN1	pp	229533.I1RTD5
229533.A0A1C3YIN1	pp	229533.I1RSQ7
229533.A0A1C3YIN1	pp	229533.A0A1C3YMK1
229533.A0A1C3YIN1	pp	229533.I1RRJ8
229533.A0A1C3YIN1	pp	229533.I1REF2
229533.A0A1C3YIN1	pp	229533.I1RW71
229533.A0A1C3YIN1	pp	229533.I1RFG0
229533.A0A1C3YIN1	pp	229533.I1RZJ6
229533.A0A1C3YIN1	pp	229533.I1S6F0
229533.A0A1C3YIN1	pp	229533.I1RNH1
229533.A0A1C3YIN1	pp	229533.I1RKL3
229533.A0A1C3YIN1	pp	229533.Q4I830
229533.A0A1C3YIN1	pp	229533.I1RT98
229533.A0A1C3YIN1	pp	229533.I1RWD4
229533.A0A1C3YIN1	pp	229533.I1RBD2
229533.A0A1C3YIN1	pp	229533.I1RSG2
229533.A0A1C3YIN1	pp	229533.I1RS30
229533.A0A1C3YIN1	pp	229533.A0A1C3YMP5
229533.A0A1C3YIN1	pp	229533.I1RAC1
229533.A0A1C3YIN1	pp	229533.I1RBZ3

118261	229533.A0A1C3YIN1	pp	229533.I1R9N1
118262	229533.A0A1C3YIN1	pp	229533.I1RCL3
118263	229533.A0A1C3YIN1	pp	229533.A0A1C3YIY7
118264	229533.A0A1C3YIN1	pp	229533.A0A1C3YIP5
118265	229533.A0A1C3YIN1	pp	229533.A0A1C3YK72
118266	229533.A0A1C3YIN1	pp	229533.I1RT62
118267	229533.A0A1C3YIN1	pp	229533.I1RGC6
118268	229533.A0A1C3YIN1	pp	229533.I1RZK5
118269	229533.A0A1C3YIN1	pp	229533.I1RJV3
118270	229533.A0A1C3YIN1	pp	229533.I1RQA2
118271	229533.A0A1C3YIN1	pp	229533.I1RQ65
118272	229533.A0A1C3YIN1	pp	229533.I1RS47
118273	229533.A0A1C3YIN1	pp	229533.I1S2C0
118274	229533.A0A1C3YIN1	pp	229533.I1RCN5
118275	229533.A0A1C3YIN1	pp	229533.I1RAN5
118276	229533.A0A1C3YIN1	pp	229533.A0A1C3YIN6
118277	229533.A0A1C3YIN1	pp	229533.I1RK83
118278	229533.A0A1C3YIN1	pp	229533.I1RRY9
118279	229533.A0A1C3YIN1	pp	229533.I1RRH5
118280	229533.A0A1C3YIN1	pp	229533.Q4HZ42
118281	229533.A0A1C3YIN1	pp	229533.I1RTE9
118282	229533.A0A1C3YIN1	pp	229533.I1RWN6
118283	229533.A0A1C3YIN1	pp	229533.I1RFU8
118284	229533.A0A1C3YIN1	pp	229533.I1RTH6
118285	229533.A0A1C3YIN1	pp	229533.I1S1U7
118286	229533.A0A1C3YIN1	pp	229533.I1RSR3
118287	229533.A0A1C3YIN1	pp	229533.I1RYB1
118288	229533.A0A1C3YIN1	pp	229533.I1RFJ9
118289	229533.A0A1C3YIN1	pp	229533.I1RD56
118290	229533.A0A1C3YIN1	pp	229533.V6RGY3
118291	229533.A0A1C3YIN1	pp	229533.I1RFW5
118292	229533.A0A1C3YIN1	pp	229533.I1RAW7
118293	229533.A0A1C3YIN1	pp	229533.A0A1C3YJT5
118294	229533.A0A1C3YIN1	pp	229533.I1RSI0
118295	229533.A0A1C3YIN1	pp	229533.I1RE00
118296	229533.A0A1C3YIN1	pp	229533.I1RAW0
118297	229533.A0A1C3YIN1	pp	229533.I1RDG0
118298	229533.A0A1C3YIN1	pp	229533.A0A1C3YMB6
118299	229533.A0A1C3YIN1	pp	229533.I1RBL7
118300	229533.A0A1C3YIN1	pp	229533.I1RK95
118301	229533.A0A1C3YIN1	pp	229533.I1S274
118302	229533.A0A1C3YIN1	pp	229533.I1RQ28
118303	229533.A0A1C3YIN1	pp	229533.I1RVZ0
118304	229533.A0A1C3YIN1	pp	229533.I1RK75
118305	229533.A0A1C3YIN1	pp	229533.I1S1X9
118306	229533.A0A1C3YIN1	pp	229533.I1RY63
118307	229533.A0A1C3YIN1	pp	229533.Q4IJM4
118308	229533.A0A1C3YIN1	pp	229533.I1RYY5
118309	229533.A0A1C3YIN1	pp	229533.I1RRI2
118310	229533.A0A1C3YIN1	pp	229533.I1RNK2
118311	229533.A0A1C3YIN1	pp	229533.I1S1C7
118312	229533.A0A1C3YIN1	pp	229533.I1R9V7
118313	229533.A0A1C3YIN1	pp	229533.I1S0Z9
118314	229533.A0A1C3YIN1	pp	229533.I1RT03
118315	229533.A0A1C3YIN1	pp	229533.I1RKC6
118316	229533.A0A1C3YIN1	pp	229533.I1RSR8
118317	229533.A0A1C3YIN1	pp	229533.I1RY03
118318	229533.A0A1C3YIN1	pp	229533.I1RVX0
118319	229533.A0A1C3YIN1	pp	229533.I1S1J6
118320	229533.A0A1C3YIN1	pp	229533.I1RAZ8
118321	229533.A0A1C3YIN1	pp	229533.I1RA48
118322	229533.A0A1C3YIN1	pp	229533.I1R9E6
118323	229533.A0A1C3YIN1	pp	229533.I1RAU7
118324	229533.A0A1C3YIN1	pp	229533.Q4HWE2
118325	229533.A0A1C3YIN1	pp	229533.V6RMW6
118326	229533.A0A1C3YIN1	pp	229533.A0A1C3YLK9
118327	229533.A0A1C3YIN1	pp	229533.I1RF08
118328	229533.A0A1C3YIN1	pp	229533.I1RSC6
118329	229533.A0A1C3YIN1	pp	229533.I1RSS4
118330	229533.A0A1C3YIN1	pp	229533.V6R042
118331	229533.A0A1C3YIN1	pp	229533.Q4HZ68
118332	229533.A0A1C3YIN1	pp	229533.I1RQR3
118333	229533.A0A1C3YIN1	pp	229533.I1RSI6

118334 229533.A0A1C3YIN1 pp 229533.I1S296
118335 229533.A0A1C3YIN1 pp 229533.I1RAU5
118336 229533.A0A1C3YIN1 pp 229533.I1RB75
118337 229533.A0A1C3YIN1 pp 229533.I1RY44
118338 229533.A0A1C3YIN1 pp 229533.Q4IEK8
118339 229533.A0A1C3YIN1 pp 229533.Q4IBS2
118340 229533.A0A1C3YIN1 pp 229533.I1RD22
118341 229533.A0A1C3YIN1 pp 229533.I1RZZ1
118342 229533.A0A1C3YIN1 pp 229533.I1S193
118343 229533.A0A1C3YIN1 pp 229533.I1RNQ5
118344 229533.A0A1C3YIN1 pp 229533.I1RM96
118345 229533.A0A1C3YIN1 pp 229533.I1RZK6
118346 229533.A0A1C3YIN1 pp 229533.I1RJZ6
118347 229533.A0A1C3YIN1 pp 229533.V6QXG1
118348 229533.A0A1C3YIN1 pp 229533.I1S2E8
118349 229533.A0A1C3YIN1 pp 229533.V6QUE6
118350 229533.A0A1C3YIN1 pp 229533.I1RGC2
118351 229533.A0A1C3YIN1 pp 229533.I1RMN6
118352 229533.A0A1C3YIN1 pp 229533.I1S4T3
118353 229533.A0A1C3YIN1 pp 229533.V6RBN2
118354 229533.A0A1C3YIN1 pp 229533.I1S044
118355 229533.A0A1C3YIN1 pp 229533.I1RSD8
118356 229533.A0A1C3YIN1 pp 229533.I1RWN5
118357 229533.A0A1C3YIN1 pp 229533.V6RGN5
118358 229533.A0A1C3YIN1 pp 229533.I1S2U2
118359 229533.A0A1C3YIN1 pp 229533.I1RDK7
118360 229533.A0A1C3YIN1 pp 229533.I1RC27
118361 229533.A0A1C3YIN1 pp 229533.I1RPY1
118362 229533.A0A1C3YIN1 pp 229533.A0A1C3YIS2
118363 229533.A0A1C3YIN1 pp 229533.I1RC17
118364 229533.A0A1C3YIN1 pp 229533.I1S0E8
118365 229533.A0A1C3YIN1 pp 229533.A0A1C3YL07
118366 229533.A0A1C3YIN1 pp 229533.Q4I327
118367 229533.A0A1C3YIN1 pp 229533.I1RN99
118368 229533.A0A1C3YIN1 pp 229533.I1RG56
118369 229533.A0A1C3YIN1 pp 229533.A0A1C3YK36
118370 229533.A0A1C3YIN1 pp 229533.V6R227
118371 229533.A0A1C3YIN1 pp 229533.Q4IJ56
118372 229533.A0A1C3YIN1 pp 229533.I1RYB9
118373 229533.A0A1C3YIN1 pp 229533.I1RMN7
118374 229533.A0A1C3YIN1 pp 229533.I1RRR7
118375 229533.A0A1C3YIN1 pp 229533.I1RZF6
118376 229533.A0A1C3YIN1 pp 229533.I1RWB5
118377 229533.A0A1C3YIN1 pp 229533.I1S021
118378 229533.A0A1C3YIN1 pp 229533.I1RM25
118379 229533.A0A1C3YIN1 pp 229533.I1S504
118380 229533.A0A1C3YIN1 pp 229533.I1RNH6
118381 229533.A0A1C3YIN1 pp 229533.I1RNU6
118382 229533.A0A1C3YIN1 pp 229533.I1RC72
118383 229533.A0A1C3YIN1 pp 229533.I1RQ31
118384 229533.A0A1C3YIN1 pp 229533.A0A1C3YJG0
118385 229533.A0A1C3YIN1 pp 229533.I1RMC0
118386 229533.A0A1C3YIN1 pp 229533.I1RKA7
118387 229533.A0A1C3YIN1 pp 229533.Q4IQT8
118388 229533.A0A1C3YIN1 pp 229533.I1S0J1
118389 229533.A0A1C3YIN1 pp 229533.I1S060
118390 229533.A0A1C3YIN1 pp 229533.Q4IN63
118391 229533.A0A1C3YIN1 pp 229533.Q4IAA0
118392 229533.A0A1C3YIN1 pp 229533.I1RMQ7
118393 229533.A0A1C3YIN1 pp 229533.A0A1C3YJZ9
118394 229533.A0A1C3YIN1 pp 229533.I1RCR8
118395 229533.A0A1C3YIN1 pp 229533.I1RB27
118396 229533.A0A1C3YIN1 pp 229533.A0A1C3YJ29
118397 229533.A0A1C3YIN1 pp 229533.I1R9X7
118398 229533.A0A1C3YIN1 pp 229533.I1S0M9
118399 229533.Q4I8B6 pp 229533.Q4IMZ7
118400 229533.Q4I8B6 pp 229533.Q4IA62
118401 229533.I1SAN0 pp 229533.V6QUE6
118402 229533.I1SAN0 pp 229533.Q4I7L0
118403 229533.I1RY98 pp 229533.V6RR76
118404 229533.I1RY98 pp 229533.V6R476
118405 229533.I1RY98 pp 229533.I1S7B9
118406 229533.I1RY98 pp 229533.I1SAA0

118407 229533.I1RY98 pp 229533.I1S0Z2
118408 229533.I1RY98 pp 229533.P0C431
118409 229533.I1RY98 pp 229533.I1S0S6
118410 229533.I1RE93 pp 229533.I1RJG9
118411 229533.I1RE93 pp 229533.I1RW98
118412 229533.I1S7S9 pp 229533.V6RCG5
118413 229533.I1S7S9 pp 229533.V6RMW6
118414 229533.I1S7S9 pp 229533.I1S9T1
118415 229533.I1S7S9 pp 229533.I1S8E2
118416 229533.I1S7S9 pp 229533.V6R042
118417 229533.I1S7S9 pp 229533.V6R3X5
118418 229533.I1S7S9 pp 229533.V6QUW4
118419 229533.Q4IN63 pp 229533.V6QUW4
118420 229533.Q4IN63 pp 229533.Q4IQW0
118421 229533.Q4IN63 pp 229533.V6R283
118422 229533.Q4IN63 pp 229533.V6QYF0
118423 229533.Q4IN63 pp 229533.V6RGN5
118424 229533.Q4IN63 pp 229533.V6R227
118425 229533.Q4IN63 pp 229533.V6RGY3
118426 229533.Q4IN63 pp 229533.V6RTJ8
118427 229533.Q4IN63 pp 229533.Q4IPI1
118428 229533.Q4IN63 pp 229533.V6R3X5
118429 229533.Q4IN63 pp 229533.V6R042
118430 229533.Q4IN63 pp 229533.V6QUE6
118431 229533.Q4IN63 pp 229533.V6RMW6
118432 229533.I1RCU3 pp 229533.I1SA48
118433 229533.I1RCU3 pp 229533.I1RE14
118434 229533.I1RCU3 pp 229533.Q4I7X1
118435 229533.I1RCU3 pp 229533.I1RVZ4
118436 229533.I1RCU3 pp 229533.I1RG11
118437 229533.I1RCU3 pp 229533.Q4HWE2
118438 229533.I1RCU3 pp 229533.I1S5Z2
118439 229533.I1RCU3 pp 229533.Q4I5I4
118440 229533.I1RCU3 pp 229533.I1RZZ7
118441 229533.I1RCU3 pp 229533.I1S802
118442 229533.I1RCU3 pp 229533.I1RNB8
118443 229533.I1RCU3 pp 229533.I1RT35
118444 229533.I1RCU3 pp 229533.I1S3Z3
118445 229533.I1RCU3 pp 229533.Q4IR09
118446 229533.I1RCU3 pp 229533.I1SA47
118447 229533.I1RCU3 pp 229533.I1RWH7
118448 229533.I1RCU3 pp 229533.I1RWI0
118449 229533.I1RCU3 pp 229533.I1RTH2
118450 229533.A0A1C3YKM9 pp 229533.I1S7W5
118451 229533.A0A1C3YKM9 pp 229533.I1RW46
118452 229533.A0A1C3YKM9 pp 229533.I1S0J1
118453 229533.A0A1C3YKM9 pp 229533.I1RE01
118454 229533.A0A1C3YKM9 pp 229533.I1RZS7
118455 229533.A0A1C3YKM9 pp 229533.I1RTA0
118456 229533.A0A1C3YKM9 pp 229533.A0A1C3YLB6
118457 229533.A0A1C3YKM9 pp 229533.A0A1C3YLC1
118458 229533.A0A1C3YKM9 pp 229533.I1RPP4
118459 229533.A0A1C3YKM9 pp 229533.I1S9Z4
118460 229533.A0A1C3YKM9 pp 229533.I1RMV0
118461 229533.A0A1C3YKM9 pp 229533.I1RPK0
118462 229533.A0A1C3YKM9 pp 229533.V6RLM7
118463 229533.A0A1C3YKM9 pp 229533.I1RM96
118464 229533.A0A1C3YKM9 pp 229533.I1RS17
118465 229533.A0A1C3YKM9 pp 229533.I1RY44
118466 229533.A0A1C3YKM9 pp 229533.I1RC33
118467 229533.A0A1C3YKM9 pp 229533.I1RZV3
118468 229533.A0A1C3YKM9 pp 229533.I1S1W3
118469 229533.A0A1C3YKM9 pp 229533.I1RPU2
118470 229533.A0A1C3YKM9 pp 229533.I1RRE5
118471 229533.A0A1C3YKM9 pp 229533.I1S1W4
118472 229533.A0A1C3YKM9 pp 229533.I1RAQ5
118473 229533.A0A1C3YKM9 pp 229533.Q4I327
118474 229533.A0A1C3YKM9 pp 229533.I1RWN5
118475 229533.A0A1C3YKM9 pp 229533.I1RNY2
118476 229533.A0A1C3YKM9 pp 229533.V6RIV9
118477 229533.A0A1C3YKM9 pp 229533.I1RVF8
118478 229533.A0A1C3YKM9 pp 229533.A0A1C3YMI5
118479 229533.A0A1C3YKM9 pp 229533.I1S1W9

118480	229533.A0A1C3YKM9	pp	229533.I1RNC1
118481	229533.A0A1C3YKM9	pp	229533.I1RAG7
118482	229533.A0A1C3YKM9	pp	229533.Q4HZB8
118483	229533.A0A1C3YKM9	pp	229533.I1RSF1
118484	229533.A0A1C3YKM9	pp	229533.I1RRR7
118485	229533.A0A1C3YKM9	pp	229533.V6RRH2
118486	229533.A0A1C3YKM9	pp	229533.I1RUG3
118487	229533.A0A1C3YKM9	pp	229533.I1RBR1
118488	229533.A0A1C3YKM9	pp	229533.I1S8V4
118489	229533.A0A1C3YKM9	pp	229533.I1RG90
118490	229533.A0A1C3YKM9	pp	229533.I1RVZ7
118491	229533.A0A1C3YKM9	pp	229533.I1RB41
118492	229533.A0A1C3YKM9	pp	229533.I1RCR8
118493	229533.A0A1C3YKM9	pp	229533.Q4IN63
118494	229533.A0A1C3YKM9	pp	229533.I1R9J6
118495	229533.A0A1C3YKM9	pp	229533.I1RSQ3
118496	229533.A0A1C3YKM9	pp	229533.I1RAU7
118497	229533.A0A1C3YKM9	pp	229533.I1S0N4
118498	229533.A0A1C3YKM9	pp	229533.I1RQ33
118499	229533.A0A1C3YKM9	pp	229533.I1R9D1
118500	229533.A0A1C3YKM9	pp	229533.I1RG11
118501	229533.A0A1C3YKM9	pp	229533.I1S5R6
118502	229533.A0A1C3YKM9	pp	229533.V6R042
118503	229533.A0A1C3YKM9	pp	229533.I1RRM3
118504	229533.A0A1C3YKM9	pp	229533.I1S569
118505	229533.A0A1C3YKM9	pp	229533.I1RCH5
118506	229533.A0A1C3YKM9	pp	229533.V6R057
118507	229533.A0A1C3YKM9	pp	229533.I1S2E8
118508	229533.A0A1C3YKM9	pp	229533.I1RQE7
118509	229533.A0A1C3YKM9	pp	229533.V6RMW6
118510	229533.A0A1C3YKM9	pp	229533.Q4I624
118511	229533.A0A1C3YKM9	pp	229533.I1RD17
118512	229533.A0A1C3YKM9	pp	229533.I1RMG3
118513	229533.A0A1C3YKM9	pp	229533.I1RJP4
118514	229533.A0A1C3YKM9	pp	229533.A0A1C3YLS9
118515	229533.A0A1C3YKM9	pp	229533.I1RWX5
118516	229533.A0A1C3YKM9	pp	229533.I1RSD8
118517	229533.A0A1C3YKM9	pp	229533.I1RFL4
118518	229533.A0A1C3YKM9	pp	229533.I1RN18
118519	229533.A0A1C3YKM9	pp	229533.I1RRI2
118520	229533.A0A1C3YKM9	pp	229533.I1RRV1
118521	229533.A0A1C3YKM9	pp	229533.I1RRN2
118522	229533.A0A1C3YKM9	pp	229533.I1S256
118523	229533.A0A1C3YKM9	pp	229533.I1RT03
118524	229533.A0A1C3YKM9	pp	229533.I1RB92
118525	229533.A0A1C3YKM9	pp	229533.I1RZ79
118526	229533.A0A1C3YKM9	pp	229533.I1RVX0
118527	229533.A0A1C3YKM9	pp	229533.I1RZW8
118528	229533.A0A1C3YKM9	pp	229533.I1RD22
118529	229533.A0A1C3YKM9	pp	229533.I1RMN6
118530	229533.A0A1C3YKM9	pp	229533.I1RSY2
118531	229533.A0A1C3YKM9	pp	229533.I1RS06
118532	229533.A0A1C3YKM9	pp	229533.I1RB44
118533	229533.A0A1C3YKM9	pp	229533.I1SA33
118534	229533.A0A1C3YKM9	pp	229533.I1S437
118535	229533.A0A1C3YKM9	pp	229533.I1RXR1
118536	229533.A0A1C3YKM9	pp	229533.I1RWF7
118537	229533.A0A1C3YKM9	pp	229533.I1RYW3
118538	229533.A0A1C3YKM9	pp	229533.I1RRN5
118539	229533.A0A1C3YKM9	pp	229533.I1RML0
118540	229533.A0A1C3YKM9	pp	229533.I1RCD8
118541	229533.A0A1C3YKM9	pp	229533.I1RGL4
118542	229533.A0A1C3YKM9	pp	229533.I1RPP3
118543	229533.A0A1C3YKM9	pp	229533.I1RTD7
118544	229533.A0A1C3YKM9	pp	229533.I1S8P5
118545	229533.A0A1C3YKM9	pp	229533.I1RQG4
118546	229533.A0A1C3YKM9	pp	229533.I1RRJ8
118547	229533.A0A1C3YKM9	pp	229533.I1S1C7
118548	229533.A0A1C3YKM9	pp	229533.I1RTH6
118549	229533.A0A1C3YKM9	pp	229533.I1RWT0
118550	229533.A0A1C3YKM9	pp	229533.I1RKJ8
118551	229533.A0A1C3YKM9	pp	229533.I1S352
118552	229533.A0A1C3YKM9	pp	229533.I1S8R3

118553 229533.A0A1C3YKM9 pp 229533.I1S2C0
118554 229533.A0A1C3YKM9 pp 229533.I1RKL3
118555 229533.A0A1C3YKM9 pp 229533.I1RFT1
118556 229533.A0A1C3YKM9 pp 229533.I1RBZ3
118557 229533.A0A1C3YKM9 pp 229533.I1RA70
118558 229533.A0A1C3YKM9 pp 229533.A0A1C3YKN3
118559 229533.A0A1C3YKM9 pp 229533.I1RSK1
118560 229533.A0A1C3YKM9 pp 229533.I1RSV2
118561 229533.A0A1C3YKM9 pp 229533.I1RT40
118562 229533.A0A1C3YKM9 pp 229533.I1RJV3
118563 229533.A0A1C3YKM9 pp 229533.I1RRQ3
118564 229533.A0A1C3YKM9 pp 229533.I1RCJ7
118565 229533.A0A1C3YKM9 pp 229533.I1RCA0
118566 229533.A0A1C3YKM9 pp 229533.I1RAF8
118567 229533.A0A1C3YKM9 pp 229533.I1RCM3
118568 229533.A0A1C3YKM9 pp 229533.I1RE89
118569 229533.A0A1C3YKM9 pp 229533.I1RBS0
118570 229533.A0A1C3YKM9 pp 229533.I1RP28
118571 229533.A0A1C3YKM9 pp 229533.I1RY62
118572 229533.A0A1C3YKM9 pp 229533.I1S2E9
118573 229533.A0A1C3YKM9 pp 229533.I1RNA5
118574 229533.A0A1C3YKM9 pp 229533.I1RNG4
118575 229533.A0A1C3YKM9 pp 229533.I1RKB6
118576 229533.A0A1C3YKM9 pp 229533.I1S0W4
118577 229533.A0A1C3YKM9 pp 229533.I1RD39
118578 229533.A0A1C3YKM9 pp 229533.Q4HXF0
118579 229533.A0A1C3YKM9 pp 229533.A0A1C3YL55
118580 229533.A0A1C3YKM9 pp 229533.I1RWN6
118581 229533.A0A1C3YKM9 pp 229533.I1RN67
118582 229533.A0A1C3YKM9 pp 229533.I1RAW0
118583 229533.A0A1C3YKM9 pp 229533.A0A1C3YKY0
118584 229533.A0A1C3YKM9 pp 229533.I1RT75
118585 229533.A0A1C3YKM9 pp 229533.I1RXU9
118586 229533.A0A1C3YKM9 pp 229533.I1RTA1
118587 229533.A0A1C3YKM9 pp 229533.I1S0F6
118588 229533.A0A1C3YKM9 pp 229533.I1RDF7
118589 229533.A0A1C3YKM9 pp 229533.V6RUY9
118590 229533.A0A1C3YKM9 pp 229533.I1RQ65
118591 229533.A0A1C3YKM9 pp 229533.I1RT04
118592 229533.A0A1C3YKM9 pp 229533.I1RJN9
118593 229533.A0A1C3YKM9 pp 229533.I1RVZ2
118594 229533.A0A1C3YKM9 pp 229533.I1S1F3
118595 229533.A0A1C3YKM9 pp 229533.I1S2G7
118596 229533.A0A1C3YKM9 pp 229533.I1RSX0
118597 229533.A0A1C3YKM9 pp 229533.I1RS47
118598 229533.A0A1C3YKM9 pp 229533.A0A1C3YL82
118599 229533.A0A1C3YKM9 pp 229533.V6R3J1
118600 229533.A0A1C3YKM9 pp 229533.I1RN63
118601 229533.A0A1C3YKM9 pp 229533.I1RWK3
118602 229533.A0A1C3YKM9 pp 229533.I1RLN6
118603 229533.A0A1C3YKM9 pp 229533.I1RAB4
118604 229533.A0A1C3YKM9 pp 229533.V6R283
118605 229533.A0A1C3YKM9 pp 229533.I1RRZ0
118606 229533.A0A1C3YKM9 pp 229533.I1RLV9
118607 229533.A0A1C3YKM9 pp 229533.I1RYR0
118608 229533.A0A1C3YKM9 pp 229533.I1RXF8
118609 229533.A0A1C3YKM9 pp 229533.A0A1C3YLK8
118610 229533.A0A1C3YKM9 pp 229533.I1RJW6
118611 229533.A0A1C3YKM9 pp 229533.I1RYI5
118612 229533.A0A1C3YKM9 pp 229533.I1RC03
118613 229533.A0A1C3YKM9 pp 229533.I1RGF5
118614 229533.A0A1C3YKM9 pp 229533.I1RTD5
118615 229533.A0A1C3YKM9 pp 229533.I1RFL3
118616 229533.A0A1C3YKM9 pp 229533.I1S503
118617 229533.A0A1C3YKM9 pp 229533.I1RV91
118618 229533.A0A1C3YKM9 pp 229533.I1RB74
118619 229533.A0A1C3YKM9 pp 229533.I1RFT6
118620 229533.A0A1C3YKM9 pp 229533.I1RJS2
118621 229533.A0A1C3YKM9 pp 229533.I1RWI5
118622 229533.A0A1C3YKM9 pp 229533.I1RQM7
118623 229533.A0A1C3YKM9 pp 229533.I1RZZ9
118624 229533.A0A1C3YKM9 pp 229533.Q4I662
118625 229533.A0A1C3YKM9 pp 229533.I1RP27

118626 229533.A0A1C3YKM9 pp 229533.I1RSQ4
118627 229533.A0A1C3YKM9 pp 229533.I1RZB2
118628 229533.A0A1C3YKM9 pp 229533.I1RSZ7
118629 229533.A0A1C3YKM9 pp 229533.I1S0M6
118630 229533.A0A1C3YKM9 pp 229533.I1RJV4
118631 229533.A0A1C3YKM9 pp 229533.I1RBS6
118632 229533.A0A1C3YKM9 pp 229533.I1RR73
118633 229533.A0A1C3YKM9 pp 229533.I1RZS6
118634 229533.A0A1C3YKM9 pp 229533.V6R3X5
118635 229533.A0A1C3YKM9 pp 229533.I1RGA4
118636 229533.A0A1C3YKM9 pp 229533.I1RBC7
118637 229533.A0A1C3YKM9 pp 229533.I1S8E2
118638 229533.A0A1C3YKM9 pp 229533.I1RGU7
118639 229533.A0A1C3YKM9 pp 229533.I1RE22
118640 229533.A0A1C3YKM9 pp 229533.I1RN88
118641 229533.A0A1C3YKM9 pp 229533.I1RC29
118642 229533.A0A1C3YKM9 pp 229533.I1RQ23
118643 229533.A0A1C3YKM9 pp 229533.I1RBG9
118644 229533.A0A1C3YKM9 pp 229533.I1S5R1
118645 229533.A0A1C3YKM9 pp 229533.I1RPU3
118646 229533.A0A1C3YKM9 pp 229533.I1RL04
118647 229533.A0A1C3YKM9 pp 229533.I1RRT8
118648 229533.A0A1C3YKM9 pp 229533.I1RE76
118649 229533.A0A1C3YKM9 pp 229533.I1RPV3
118650 229533.A0A1C3YKM9 pp 229533.O74638
118651 229533.A0A1C3YKM9 pp 229533.I1R9M0
118652 229533.A0A1C3YKM9 pp 229533.I1RBY4
118653 229533.A0A1C3YKM9 pp 229533.I1RJZ7
118654 229533.A0A1C3YKM9 pp 229533.I1S6Y8
118655 229533.A0A1C3YKM9 pp 229533.I1RXC2
118656 229533.A0A1C3YKM9 pp 229533.I1S2B8
118657 229533.A0A1C3YKM9 pp 229533.I1RPM4
118658 229533.A0A1C3YKM9 pp 229533.A0A1C3YLD8
118659 229533.A0A1C3YKM9 pp 229533.I1RFS3
118660 229533.A0A1C3YKM9 pp 229533.I1RRM5
118661 229533.A0A1C3YKM9 pp 229533.I1S4Z8
118662 229533.A0A1C3YKM9 pp 229533.I1RA11
118663 229533.A0A1C3YKM9 pp 229533.I1RJ17
118664 229533.A0A1C3YKM9 pp 229533.I1RWL2
118665 229533.A0A1C3YKM9 pp 229533.I1S9Y1
118666 229533.A0A1C3YKM9 pp 229533.I1RXC4
118667 229533.A0A1C3YKM9 pp 229533.I1RM82
118668 229533.A0A1C3YKM9 pp 229533.A0A1C3YMA1
118669 229533.A0A1C3YKM9 pp 229533.I1S0A0
118670 229533.A0A1C3YKM9 pp 229533.I1RB27
118671 229533.A0A1C3YKM9 pp 229533.I1RKJ6
118672 229533.A0A1C3YKM9 pp 229533.I1RJQ5
118673 229533.A0A1C3YKM9 pp 229533.I1RA52
118674 229533.A0A1C3YKM9 pp 229533.I1RC66
118675 229533.A0A1C3YKM9 pp 229533.I1S8V3
118676 229533.A0A1C3YKM9 pp 229533.I1RS18
118677 229533.A0A1C3YKM9 pp 229533.I1RZ29
118678 229533.A0A1C3YKM9 pp 229533.I1S0L4
118679 229533.A0A1C3YKM9 pp 229533.I1RRI1
118680 229533.A0A1C3YKM9 pp 229533.Q4I8P5
118681 229533.A0A1C3YKM9 pp 229533.A0A1C3YKV3
118682 229533.A0A1C3YKM9 pp 229533.I1RNN1
118683 229533.A0A1C3YKM9 pp 229533.I1S0M9
118684 229533.A0A1C3YKM9 pp 229533.I1RIL3
118685 229533.A0A1C3YKM9 pp 229533.I1RKP1
118686 229533.A0A1C3YKM9 pp 229533.I1RC61
118687 229533.A0A1C3YKM9 pp 229533.I1RC00
118688 229533.A0A1C3YKM9 pp 229533.I1RI82
118689 229533.A0A1C3YKM9 pp 229533.I1REC5
118690 229533.A0A1C3YKM9 pp 229533.I1RQ81
118691 229533.A0A1C3YKM9 pp 229533.I1S021
118692 229533.A0A1C3YKM9 pp 229533.I1S3G4
118693 229533.A0A1C3YKM9 pp 229533.I1RLU6
118694 229533.A0A1C3YKM9 pp 229533.I1RNH6
118695 229533.A0A1C3YKM9 pp 229533.I1RNQ5
118696 229533.A0A1C3YKM9 pp 229533.I1RD64
118697 229533.A0A1C3YKM9 pp 229533.I1RPE0
118698 229533.A0A1C3YKM9 pp 229533.I1RYB9

118699	229533.A0A1C3YKM9	pp	229533.I1RXF0
118700	229533.A0A1C3YKM9	pp	229533.I1RBJ5
118701	229533.A0A1C3YKM9	pp	229533.I1RS20
118702	229533.A0A1C3YKM9	pp	229533.I1RZM1
118703	229533.A0A1C3YKM9	pp	229533.I1RC16
118704	229533.A0A1C3YKM9	pp	229533.I1S2H4
118705	229533.A0A1C3YKM9	pp	229533.I1SA32
118706	229533.A0A1C3YKM9	pp	229533.I1RZM8
118707	229533.A0A1C3YKM9	pp	229533.I1S419
118708	229533.A0A1C3YKM9	pp	229533.A0A1C3YLU7
118709	229533.A0A1C3YKM9	pp	229533.I1RR51
118710	229533.A0A1C3YKM9	pp	229533.V6RCG5
118711	229533.A0A1C3YKM9	pp	229533.I1RQD2
118712	229533.A0A1C3YKM9	pp	229533.I1S6K9
118713	229533.A0A1C3YKM9	pp	229533.I1RC17
118714	229533.A0A1C3YKM9	pp	229533.I1RZK6
118715	229533.A0A1C3YKM9	pp	229533.A0A1C3YKW9
118716	229533.A0A1C3YKM9	pp	229533.Q4I283
118717	229533.A0A1C3YKM9	pp	229533.I1RZZ1
118718	229533.A0A1C3YKM9	pp	229533.I1RBE4
118719	229533.A0A1C3YKM9	pp	229533.V6RCZ8
118720	229533.A0A1C3YKM9	pp	229533.I1RQ85
118721	229533.A0A1C3YKM9	pp	229533.I1RQQ5
118722	229533.A0A1C3YKM9	pp	229533.I1RWT6
118723	229533.A0A1C3YKM9	pp	229533.I1RGC5
118724	229533.A0A1C3YKM9	pp	229533.V6RBN2
118725	229533.A0A1C3YKM9	pp	229533.I1RG14
118726	229533.A0A1C3YKM9	pp	229533.I1RRU9
118727	229533.A0A1C3YKM9	pp	229533.I1RFT2
118728	229533.A0A1C3YKM9	pp	229533.I1RZF6
118729	229533.A0A1C3YKM9	pp	229533.I1RAU5
118730	229533.A0A1C3YKM9	pp	229533.I1RRV4
118731	229533.A0A1C3YKM9	pp	229533.I1RQA3
118732	229533.A0A1C3YKM9	pp	229533.I1RJZ6
118733	229533.A0A1C3YKM9	pp	229533.I1S504
118734	229533.A0A1C3YKM9	pp	229533.Q4IJK2
118735	229533.A0A1C3YKM9	pp	229533.I1RZ35
118736	229533.A0A1C3YKM9	pp	229533.V6QUE6
118737	229533.A0A1C3YKM9	pp	229533.I1RUY5
118738	229533.A0A1C3YKM9	pp	229533.I1RK95
118739	229533.A0A1C3YKM9	pp	229533.I1RWD7
118740	229533.A0A1C3YKM9	pp	229533.I1R9V7
118741	229533.A0A1C3YKM9	pp	229533.Q4IJM4
118742	229533.A0A1C3YKM9	pp	229533.I1S5B0
118743	229533.A0A1C3YKM9	pp	229533.I1RB75
118744	229533.A0A1C3YKM9	pp	229533.I1RM35
118745	229533.A0A1C3YKM9	pp	229533.I1S044
118746	229533.A0A1C3YKM9	pp	229533.I1RDU0
118747	229533.A0A1C3YKM9	pp	229533.I1RA48
118748	229533.A0A1C3YKM9	pp	229533.I1RCR5
118749	229533.A0A1C3YKM9	pp	229533.I1REK8
118750	229533.A0A1C3YKM9	pp	229533.I1RLM7
118751	229533.A0A1C3YKM9	pp	229533.I1RW99
118752	229533.A0A1C3YKM9	pp	229533.I1RZE5
118753	229533.A0A1C3YKM9	pp	229533.I1RYY5
118754	229533.A0A1C3YKM9	pp	229533.A0A1C3YLK2
118755	229533.A0A1C3YKM9	pp	229533.I1RSI0
118756	229533.A0A1C3YKM9	pp	229533.I1S1J7
118757	229533.A0A1C3YKM9	pp	229533.I1RSR8
118758	229533.A0A1C3YKM9	pp	229533.I1RE00
118759	229533.A0A1C3YKM9	pp	229533.I1RWB0
118760	229533.A0A1C3YKM9	pp	229533.I1S4Q4
118761	229533.A0A1C3YKM9	pp	229533.I1RNT9
118762	229533.A0A1C3YKM9	pp	229533.I1RAN5
118763	229533.A0A1C3YKM9	pp	229533.I1RRY9
118764	229533.A0A1C3YKM9	pp	229533.I1RY03
118765	229533.A0A1C3YKM9	pp	229533.I1RF19
118766	229533.A0A1C3YKM9	pp	229533.I1RVZ0
118767	229533.A0A1C3YKM9	pp	229533.I1RBC1
118768	229533.A0A1C3YKM9	pp	229533.I1RZK5
118769	229533.A0A1C3YKM9	pp	229533.I1RCM5
118770	229533.A0A1C3YKM9	pp	229533.I1RU29
118771	229533.A0A1C3YKM9	pp	229533.Q4I2J8

118772 229533.A0A1C3YKM9 pp 229533.I1RWR7
118773 229533.A0A1C3YKM9 pp 229533.I1S887
118774 229533.A0A1C3YKM9 pp 229533.I1RR00
118775 229533.A0A1C3YKM9 pp 229533.I1RBL7
118776 229533.A0A1C3YKM9 pp 229533.I1RK83
118777 229533.A0A1C3YKM9 pp 229533.I1RPR9
118778 229533.A0A1C3YKM9 pp 229533.I1RFU8
118779 229533.A0A1C3YKM9 pp 229533.I1RBU8
118780 229533.A0A1C3YKM9 pp 229533.I1RXD6
118781 229533.A0A1C3YKM9 pp 229533.I1RRG3
118782 229533.A0A1C3YKM9 pp 229533.I1RCG4
118783 229533.A0A1C3YKM9 pp 229533.V6R4J1
118784 229533.A0A1C3YKM9 pp 229533.I1RDU4
118785 229533.A0A1C3YKM9 pp 229533.I1RIL8
118786 229533.A0A1C3YKM9 pp 229533.I1RYS0
118787 229533.A0A1C3YKM9 pp 229533.I1RB53
118788 229533.A0A1C3YKM9 pp 229533.I1RFW5
118789 229533.A0A1C3YKM9 pp 229533.Q4IJT5
118790 229533.A0A1C3YKM9 pp 229533.A0A1C3YKY6
118791 229533.A0A1C3YKM9 pp 229533.I1RX95
118792 229533.A0A1C3YKM9 pp 229533.I1RVQ0
118793 229533.A0A1C3YKM9 pp 229533.I1RBD5
118794 229533.A0A1C3YKM9 pp 229533.V6R1K4
118795 229533.A0A1C3YKM9 pp 229533.I1S0F5
118796 229533.A0A1C3YKM9 pp 229533.I1RI03
118797 229533.A0A1C3YKM9 pp 229533.A0A1C3YM63
118798 229533.A0A1C3YKM9 pp 229533.I1RQJ5
118799 229533.A0A1C3YKM9 pp 229533.I1S0R7
118800 229533.A0A1C3YKM9 pp 229533.I1S0L3
118801 229533.A0A1C3YKM9 pp 229533.I1RD52
118802 229533.A0A1C3YKM9 pp 229533.I1RG53
118803 229533.A0A1C3YKM9 pp 229533.I1RC20
118804 229533.A0A1C3YKM9 pp 229533.I1S0T2
118805 229533.A0A1C3YKM9 pp 229533.I1RX46
118806 229533.A0A1C3YKM9 pp 229533.I1RN61
118807 229533.A0A1C3YKM9 pp 229533.I1RCC2
118808 229533.A0A1C3YKM9 pp 229533.A0A1C3YLL3
118809 229533.A0A1C3YKM9 pp 229533.I1RY86
118810 229533.A0A1C3YKM9 pp 229533.I1RA85
118811 229533.A0A1C3YKM9 pp 229533.Q4I5R9
118812 229533.A0A1C3YKM9 pp 229533.V6QW22
118813 229533.A0A1C3YKM9 pp 229533.I1RFC0
118814 229533.A0A1C3YKM9 pp 229533.I1RRX5
118815 229533.A0A1C3YKM9 pp 229533.A0A1C3YL54
118816 229533.A0A1C3YKM9 pp 229533.I1RPY3
118817 229533.A0A1C3YKM9 pp 229533.I1RQH3
118818 229533.A0A1C3YKM9 pp 229533.V6RTJ8
118819 229533.A0A1C3YKM9 pp 229533.I1RSQ7
118820 229533.A0A1C3YKM9 pp 229533.V6RUT5
118821 229533.A0A1C3YKM9 pp 229533.I1S4S7
118822 229533.A0A1C3YKM9 pp 229533.I1S7A8
118823 229533.A0A1C3YKM9 pp 229533.I1RW71
118824 229533.A0A1C3YKM9 pp 229533.I1RBR2
118825 229533.A0A1C3YKM9 pp 229533.I1RBC9
118826 229533.A0A1C3YKM9 pp 229533.I1RLP1
118827 229533.A0A1C3YKM9 pp 229533.I1REK4
118828 229533.A0A1C3YKM9 pp 229533.I1RXD7
118829 229533.A0A1C3YKM9 pp 229533.I1RAX6
118830 229533.A0A1C3YKM9 pp 229533.I1RQC9
118831 229533.A0A1C3YKM9 pp 229533.I1RCS1
118832 229533.A0A1C3YKM9 pp 229533.I1S6U8
118833 229533.A0A1C3YKM9 pp 229533.I1RM06
118834 229533.A0A1C3YKM9 pp 229533.I1RMQ6
118835 229533.A0A1C3YKM9 pp 229533.I1S438
118836 229533.A0A1C3YKM9 pp 229533.V6QUW4
118837 229533.A0A1C3YKM9 pp 229533.I1RW54
118838 229533.A0A1C3YKM9 pp 229533.I1S1W8
118839 229533.A0A1C3YKM9 pp 229533.I1RJU3
118840 229533.A0A1C3YKM9 pp 229533.I1RT80
118841 229533.A0A1C3YKM9 pp 229533.I1RIG2
118842 229533.A0A1C3YKM9 pp 229533.A0A1C3YN30
118843 229533.A0A1C3YKM9 pp 229533.I1RX25
118844 229533.A0A1C3YKM9 pp 229533.A0A1C3YLZ7

118845 229533.A0A1C3YKM9 pp 229533.I1RW25
118846 229533.A0A1C3YKM9 pp 229533.I1RFQ7
118847 229533.A0A1C3YKM9 pp 229533.Q4IFI0
118848 229533.A0A1C3YKM9 pp 229533.I1RMG8
118849 229533.A0A1C3YKM9 pp 229533.I1RRQ1
118850 229533.A0A1C3YKM9 pp 229533.I1RM67
118851 229533.A0A1C3YKM9 pp 229533.I1RLC7
118852 229533.A0A1C3YKM9 pp 229533.I1RZJ5
118853 229533.A0A1C3YKM9 pp 229533.A0A1C3YLR6
118854 229533.A0A1C3YKM9 pp 229533.I1RI02
118855 229533.A0A1C3YKM9 pp 229533.I1RMC8
118856 229533.A0A1C3YKM9 pp 229533.I1RW07
118857 229533.A0A1C3YKM9 pp 229533.I1RWJ8
118858 229533.A0A1C3YKM9 pp 229533.I1S4S4
118859 229533.A0A1C3YKM9 pp 229533.I1RBT1
118860 229533.A0A1C3YKM9 pp 229533.I1RPU0
118861 229533.A0A1C3YKM9 pp 229533.I1S0X6
118862 229533.A0A1C3YKM9 pp 229533.I1RPW9
118863 229533.A0A1C3YKM9 pp 229533.I1R9Y4
118864 229533.A0A1C3YKM9 pp 229533.I1S9U2
118865 229533.A0A1C3YKM9 pp 229533.I1RR24
118866 229533.A0A1C3YLT1 pp 229533.I1RRF9
118867 229533.A0A1C3YLT1 pp 229533.I1RVY0
118868 229533.A0A1C3YLT1 pp 229533.I1RAR2
118869 229533.A0A1C3YLT1 pp 229533.I1RKR6
118870 229533.A0A1C3YLT1 pp 229533.I1RB36
118871 229533.A0A1C3YLT1 pp 229533.I1RNC6
118872 229533.A0A1C3YL68 pp 229533.I1S272
118873 229533.A0A1C3YL68 pp 229533.I1RDF6
118874 229533.A0A1C3YL68 pp 229533.I1RZ13
118875 229533.A0A1C3YL68 pp 229533.V6RPQ8
118876 229533.I1RWI0 pp 229533.Q4IB50
118877 229533.I1RWI0 pp 229533.Q4I5W5
118878 229533.I1RWI0 pp 229533.I1S0Y1
118879 229533.I1RWI0 pp 229533.Q4I5I4
118880 229533.I1RWI0 pp 229533.Q4IPC8
118881 229533.I1RWI0 pp 229533.Q4IPN9
118882 229533.I1RWI0 pp 229533.I1S5Z2
118883 229533.I1RWI0 pp 229533.Q4IR09
118884 229533.I1RWI0 pp 229533.Q4HWE2
118885 229533.I1RNY2 pp 229533.I1RWN5
118886 229533.I1RNY2 pp 229533.Q4IQW0
118887 229533.I1RNY2 pp 229533.I1RRT2
118888 229533.I1RNY2 pp 229533.I1S503
118889 229533.I1RNY2 pp 229533.V6RGN5
118890 229533.I1RNY2 pp 229533.I1S0J1
118891 229533.I1RNY2 pp 229533.I1RPP4
118892 229533.I1RNY2 pp 229533.I1RQK4
118893 229533.I1RNY2 pp 229533.I1RTD5
118894 229533.I1RNY2 pp 229533.I1RT04
118895 229533.I1RNY2 pp 229533.I1RT94
118896 229533.I1RNY2 pp 229533.I1RZS6
118897 229533.I1RNY2 pp 229533.I1RQ65
118898 229533.I1RNY2 pp 229533.V6R042
118899 229533.I1RNY2 pp 229533.I1RSI6
118900 229533.I1RNY2 pp 229533.V6RMW6
118901 229533.I1RNY2 pp 229533.I1RRP3
118902 229533.I1RNY2 pp 229533.I1RYT5
118903 229533.I1RNY2 pp 229533.I1RS30
118904 229533.I1RNY2 pp 229533.Q4I662
118905 229533.I1RNY2 pp 229533.I1S253
118906 229533.I1RNY2 pp 229533.I1RW26
118907 229533.I1RNY2 pp 229533.I1RQS8
118908 229533.I1RNY2 pp 229533.I1RR73
118909 229533.I1RNY2 pp 229533.Q4IN63
118910 229533.I1RNY2 pp 229533.I1RRQ3
118911 229533.I1RNY2 pp 229533.V6R3X5
118912 229533.I1RNY2 pp 229533.I1RPP3
118913 229533.I1RNY2 pp 229533.I1RVX0
118914 229533.I1RNY2 pp 229533.V6R283
118915 229533.I1RNY2 pp 229533.I1S296
118916 229533.I1RNY2 pp 229533.I1S2M6
118917 229533.I1RNY2 pp 229533.Q4HZI0

118918 229533.I1RNY2 pp 229533.I1RT03
118919 229533.I1RNY2 pp 229533.I1RSD8
118920 229533.I1RNY2 pp 229533.I1RTF4
118921 229533.I1RNY2 pp 229533.I1RT80
118922 229533.I1RNY2 pp 229533.I1S2E8
118923 229533.I1RNY2 pp 229533.I1S1C7
118924 229533.I1RNY2 pp 229533.I1RWN6
118925 229533.I1RNY2 pp 229533.Q4HVW2
118926 229533.I1RNY2 pp 229533.I1RRI2
118927 229533.I1RNY2 pp 229533.I1RTH6
118928 229533.I1RNY2 pp 229533.I1RR00
118929 229533.I1RNY2 pp 229533.I1RZF6
118930 229533.I1RNY2 pp 229533.I1RZ99
118931 229533.I1RNY2 pp 229533.I1RT05
118932 229533.I1RNY2 pp 229533.I1RPJ3
118933 229533.I1RNY2 pp 229533.I1S540
118934 229533.I1RNY2 pp 229533.I1RTE9
118935 229533.I1RNY2 pp 229533.I1S504
118936 229533.I1RNY2 pp 229533.I1RTD7
118937 229533.I1RNY2 pp 229533.I1RQ28
118938 229533.I1RNY2 pp 229533.Q4IEK8
118939 229533.I1RNY2 pp 229533.V6RTJ8
118940 229533.I1RNY2 pp 229533.Q4IJ56
118941 229533.I1RNY2 pp 229533.I1RZK6
118942 229533.I1RNY2 pp 229533.I1RX46
118943 229533.I1RNY2 pp 229533.I1S0M9
118944 229533.I1RNY2 pp 229533.I1RQC9
118945 229533.I1RNY2 pp 229533.I1RPI0
118946 229533.I1RNY2 pp 229533.I1S0R7
118947 229533.I1RNY2 pp 229533.I1RZZ1
118948 229533.I1RNY2 pp 229533.I1RQ31
118949 229533.I1RNY2 pp 229533.I1RUQ2
118950 229533.I1RNY2 pp 229533.I1RX14
118951 229533.I1RNY2 pp 229533.I1RSM3
118952 229533.I1RNY2 pp 229533.I1S021
118953 229533.I1RNY2 pp 229533.I1RQA2
118954 229533.I1RNY2 pp 229533.Q4IFI0
118955 229533.I1RNY2 pp 229533.I1RRY9
118956 229533.I1RNY2 pp 229533.V6QYF0
118957 229533.I1RNY2 pp 229533.I1RVZ0
118958 229533.I1RNY2 pp 229533.I1RWJ3
118959 229533.I1RNY2 pp 229533.I1RZB5
118960 229533.I1RNY2 pp 229533.V6QWZ7
118961 229533.I1RNY2 pp 229533.I1RY03
118962 229533.I1RNY2 pp 229533.I1RSR8
118963 229533.I1RNY2 pp 229533.I1RPW9
118964 229533.I1RNY2 pp 229533.I1RSC6
118965 229533.I1RNY2 pp 229533.I1RY63
118966 229533.I1RNY2 pp 229533.I1RZE5
118967 229533.I1RNY2 pp 229533.I1S9U2
118968 229533.A0A0E0SA25 pp 229533.I1RW82
118969 229533.A0A0E0SA25 pp 229533.I1RW80
118970 229533.A0A0E0SA25 pp 229533.I1S0A5
118971 229533.A0A0E0SA25 pp 229533.I1RNG4
118972 229533.A0A0E0SA25 pp 229533.A0A1C3YHG7
118973 229533.A0A0E0SA25 pp 229533.I1RN26
118974 229533.A0A0E0SA25 pp 229533.I1RN04
118975 229533.A0A0E0SA25 pp 229533.I1SAT2
118976 229533.A0A0E0SA25 pp 229533.A0A1C3YLB6
118977 229533.A0A0E0SA25 pp 229533.I1RF85
118978 229533.A0A0E0SA25 pp 229533.I1RZ56
118979 229533.A0A0E0SA25 pp 229533.I1RJS1
118980 229533.A0A0E0SA25 pp 229533.I1RPV8
118981 229533.A0A0E0SA25 pp 229533.I1RHU8
118982 229533.A0A0E0SA25 pp 229533.I1RH83
118983 229533.A0A0E0SA25 pp 229533.I1RE52
118984 229533.A0A0E0SA25 pp 229533.I1RPW8
118985 229533.A0A0E0SA25 pp 229533.I1R9F2
118986 229533.A0A0E0SA25 pp 229533.I1RCU2
118987 229533.A0A0E0SA25 pp 229533.I1RDN3
118988 229533.A0A0E0SA25 pp 229533.I1RQV7
118989 229533.A0A0E0SA25 pp 229533.I1RRS6
118990 229533.A0A0E0SA25 pp 229533.I1S6H3

118991 229533.A0A0E0SA25 pp 229533.I1S5D0
118992 229533.A0A0E0SA25 pp 229533.I1RCU7
118993 229533.A0A0E0SA25 pp 229533.I1RST9
118994 229533.A0A0E0SA25 pp 229533.I1S094
118995 229533.A0A0E0SA25 pp 229533.I1RIB5
118996 229533.A0A0E0SA25 pp 229533.I1RW91
118997 229533.A0A0E0SA25 pp 229533.I1RCG6
118998 229533.A0A0E0SA25 pp 229533.I1S267
118999 229533.A0A0E0SA25 pp 229533.A0A1C3YHX6
119000 229533.A0A0E0SA25 pp 229533.I1REK8
119001 229533.A0A0E0SA25 pp 229533.I1RSY8
119002 229533.A0A0E0SA25 pp 229533.I1S659
119003 229533.A0A0E0SA25 pp 229533.I1RA13
119004 229533.I1RFY2 pp 229533.I1RYZ0
119005 229533.I1RFY2 pp 229533.I1RQ93
119006 229533.I1RFY2 pp 229533.I1SAA0
119007 229533.I1RWH5 pp 229533.I1RZZ1
119008 229533.I1RWH5 pp 229533.I1S2E8
119009 229533.I1RWH5 pp 229533.Q4I662
119010 229533.I1RWH5 pp 229533.I1S9U2
119011 229533.I1RWH5 pp 229533.I1RZK6
119012 229533.I1RWH5 pp 229533.I1S7S9
119013 229533.I1RWH5 pp 229533.I1S044
119014 229533.I1RWH5 pp 229533.I1S6E5
119015 229533.I1RWH5 pp 229533.I1S2C0
119016 229533.I1RWH5 pp 229533.Q4IPI1
119017 229533.I1RWH5 pp 229533.I1RZV6
119018 229533.I1RWH5 pp 229533.I1RX46
119019 229533.I1RWH5 pp 229533.Q4IFI0
119020 229533.I1RWH5 pp 229533.V6RMW6
119021 229533.I1RWH5 pp 229533.Q4HVW2
119022 229533.I1RWH5 pp 229533.Q4IPN9
119023 229533.I1RWH5 pp 229533.V6R042
119024 229533.I1RWH5 pp 229533.I1RZS6
119025 229533.I1RWH5 pp 229533.I1S8E2
119026 229533.I1RWH5 pp 229533.I1S060
119027 229533.I1RWH5 pp 229533.I1S1X9
119028 229533.I1RWH5 pp 229533.I1RYB9
119029 229533.I1RWH5 pp 229533.I1RX24
119030 229533.I1RWH5 pp 229533.I1S0R7
119031 229533.I1RWH5 pp 229533.Q4I327
119032 229533.I1RWH5 pp 229533.I1S021
119033 229533.I1RWH5 pp 229533.Q4IN63
119034 229533.I1RWH5 pp 229533.I1S0M9
119035 229533.I1RWH5 pp 229533.I1RZF6
119036 229533.I1RWH5 pp 229533.I1S0J1
119037 229533.I1RJM7 pp 229533.I1RSA4
119038 229533.I1RJM7 pp 229533.I1S8V4
119039 229533.I1RJM7 pp 229533.I1RN18
119040 229533.I1RJM7 pp 229533.I1RYI9
119041 229533.I1RJM7 pp 229533.I1RJW6
119042 229533.I1RJM7 pp 229533.I1S0G0
119043 229533.I1RJM7 pp 229533.I1S8V3
119044 229533.I1RCR8 pp 229533.I1RR00
119045 229533.I1RCR8 pp 229533.I1S4S7
119046 229533.I1RCR8 pp 229533.I1RSQ7
119047 229533.I1RCR8 pp 229533.I1RW71
119048 229533.I1RCR8 pp 229533.I1RRX5
119049 229533.I1RCR8 pp 229533.I1RW07
119050 229533.I1RCR8 pp 229533.I1RLC7
119051 229533.I1RCR8 pp 229533.I1RN61
119052 229533.I1RCR8 pp 229533.I1RI02
119053 229533.I1RCR8 pp 229533.V6QW22
119054 229533.I1RCR8 pp 229533.I1RQJ5
119055 229533.I1RCR8 pp 229533.I1RW25
119056 229533.I1RCR8 pp 229533.I1RX25
119057 229533.I1RCR8 pp 229533.I1RM06
119058 229533.I1RCR8 pp 229533.I1RX95
119059 229533.I1RCR8 pp 229533.I1S9I5
119060 229533.I1RCR8 pp 229533.I1RG53
119061 229533.I1RCR8 pp 229533.I1RFW5
119062 229533.I1RCR8 pp 229533.Q4IJT5
119063 229533.I1RCR8 pp 229533.I1RCS1

119064 229533.I1RCR8 pp 229533.I1RT80
119065 229533.I1RCR8 pp 229533.I1RJU3
119066 229533.I1RCR8 pp 229533.I1RIL8
119067 229533.I1RCR8 pp 229533.I1RRS8
119068 229533.I1RCR8 pp 229533.I1S6U8
119069 229533.I1RCR8 pp 229533.I1RRG3
119070 229533.I1RCR8 pp 229533.I1RQH3
119071 229533.I1RCR8 pp 229533.I1RXD7
119072 229533.I1RCR8 pp 229533.I1RKJ5
119073 229533.I1RCR8 pp 229533.I1RD52
119074 229533.I1RCR8 pp 229533.I1S0F5
119075 229533.I1RCR8 pp 229533.I1S0X6
119076 229533.I1RCR8 pp 229533.I1RMC8
119077 229533.I1RCR8 pp 229533.V6RUT5
119078 229533.I1RCR8 pp 229533.V6R1K4
119079 229533.I1RCR8 pp 229533.I1S0L3
119080 229533.I1RCR8 pp 229533.I1RTB3
119081 229533.I1RCR8 pp 229533.I1RR24
119082 229533.I1RCR8 pp 229533.I1RVQ0
119083 229533.I1RCR8 pp 229533.I1S9U2
119084 229533.I1RCR8 pp 229533.I1RNB2
119085 229533.I1RCR8 pp 229533.I1RPU0
119086 229533.I1RCR8 pp 229533.I1S4S4
119087 229533.I1RCR8 pp 229533.I1RPY3
119088 229533.I1RCR8 pp 229533.I1S7A8
119089 229533.I1RCR8 pp 229533.V6R4J1
119090 229533.I1RCR8 pp 229533.V6RTJ8
119091 229533.I1RCR8 pp 229533.I1RXD6
119092 229533.I1RCR8 pp 229533.I1RWJ8
119093 229533.I1RCR8 pp 229533.I1RFQ7
119094 229533.I1RCR8 pp 229533.I1S0R7
119095 229533.I1RCR8 pp 229533.I1RM67
119096 229533.I1RCR8 pp 229533.I1RQC9
119097 229533.I1RCR8 pp 229533.I1RX46
119098 229533.I1RCR8 pp 229533.I1S0T2
119099 229533.I1RCR8 pp 229533.I1RLP1
119100 229533.I1RCR8 pp 229533.I1RRY9
119101 229533.I1RCR8 pp 229533.V6RBN2
119102 229533.I1RCR8 pp 229533.I1RK95
119103 229533.I1RCR8 pp 229533.I1RWR7
119104 229533.I1RCR8 pp 229533.I1RU29
119105 229533.I1RCR8 pp 229533.I1RZZ1
119106 229533.I1RCR8 pp 229533.I1RQA3
119107 229533.I1RCR8 pp 229533.Q4IJK2
119108 229533.I1RCR8 pp 229533.I1RWB0
119109 229533.I1RCR8 pp 229533.V6R9Z0
119110 229533.I1RCR8 pp 229533.I1RJZ6
119111 229533.I1RCR8 pp 229533.I1RZK6
119112 229533.I1RCR8 pp 229533.I1RGB2
119113 229533.I1RCR8 pp 229533.I1S1J7
119114 229533.I1RCR8 pp 229533.Q4I2J8
119115 229533.I1RCR8 pp 229533.I1REK8
119116 229533.I1RCR8 pp 229533.I1RQQ5
119117 229533.I1RCR8 pp 229533.I1RWT6
119118 229533.I1RCR8 pp 229533.I1RYS0
119119 229533.I1RCR8 pp 229533.I1RI03
119120 229533.I1RCR8 pp 229533.I1S0M9
119121 229533.I1RCR8 pp 229533.I1RQ81
119122 229533.I1RCR8 pp 229533.I1RSI0
119123 229533.I1RCR8 pp 229533.I1RVZ0
119124 229533.I1RCR8 pp 229533.I1RF19
119125 229533.I1RCR8 pp 229533.I1RE00
119126 229533.I1RCR8 pp 229533.I1RZE5
119127 229533.I1RCR8 pp 229533.Q4I283
119128 229533.I1RCR8 pp 229533.I1S419
119129 229533.I1RCR8 pp 229533.Q4I8P5
119130 229533.I1RCR8 pp 229533.I1RFU8
119131 229533.I1RCR8 pp 229533.I1RS18
119132 229533.I1RCR8 pp 229533.I1RLM7
119133 229533.I1RCR8 pp 229533.I1S887
119134 229533.I1RCR8 pp 229533.I1RXC4
119135 229533.I1RCR8 pp 229533.I1RZM1
119136 229533.I1RCR8 pp 229533.I1RPK5

119137 229533.I1RCR8 pp 229533.I1RLU6
119138 229533.I1RCR8 pp 229533.I1RZK5
119139 229533.I1RCR8 pp 229533.I1RNQ5
119140 229533.I1RCR8 pp 229533.I1SA32
119141 229533.I1RCR8 pp 229533.I1S821
119142 229533.I1RCR8 pp 229533.I1RSR8
119143 229533.I1RCR8 pp 229533.I1RY03
119144 229533.I1RCR8 pp 229533.I1RZF6
119145 229533.I1RCR8 pp 229533.I1S504
119146 229533.I1RCR8 pp 229533.I1RPR9
119147 229533.I1RCR8 pp 229533.I1S6K9
119148 229533.I1RCR8 pp 229533.I1RG14
119149 229533.I1RCR8 pp 229533.V6RCZ8
119150 229533.I1RCR8 pp 229533.I1RE22
119151 229533.I1RCR8 pp 229533.I1RFT2
119152 229533.I1RCR8 pp 229533.I1RQ85
119153 229533.I1RCR8 pp 229533.I1RZS6
119154 229533.I1RCR8 pp 229533.I1RSQ4
119155 229533.I1RCR8 pp 229533.I1RZZ9
119156 229533.I1RCR8 pp 229533.V6RCG5
119157 229533.I1RCR8 pp 229533.I1RR51
119158 229533.I1RCR8 pp 229533.I1RSZ7
119159 229533.I1RCR8 pp 229533.I1S0M6
119160 229533.I1RCR8 pp 229533.I1RZM8
119161 229533.I1RCR8 pp 229533.I1RWI5
119162 229533.I1RCR8 pp 229533.I1RXF0
119163 229533.I1RCR8 pp 229533.I1RNN1
119164 229533.I1RCR8 pp 229533.I1RPV3
119165 229533.I1RCR8 pp 229533.I1S8V3
119166 229533.I1RCR8 pp 229533.I1S021
119167 229533.I1RCR8 pp 229533.I1S6Y8
119168 229533.I1RCR8 pp 229533.I1RPM4
119169 229533.I1RCR8 pp 229533.I1RPE0
119170 229533.I1RCR8 pp 229533.I1RS20
119171 229533.I1RCR8 pp 229533.I1RNH6
119172 229533.I1RCR8 pp 229533.I1RL04
119173 229533.I1RCR8 pp 229533.I1S2H4
119174 229533.I1RCR8 pp 229533.I1RFS3
119175 229533.I1RCR8 pp 229533.I1RI82
119176 229533.I1RCR8 pp 229533.I1RQ23
119177 229533.I1RCR8 pp 229533.I1RQD2
119178 229533.I1RCR8 pp 229533.I1RRI1
119179 229533.I1RCR8 pp 229533.I1REC5
119180 229533.I1RCR8 pp 229533.I1RW99
119181 229533.I1RCR8 pp 229533.I1S2B8
119182 229533.I1RCR8 pp 229533.I1S5B0
119183 229533.I1RCR8 pp 229533.I1RIL3
119184 229533.I1RCR8 pp 229533.I1S0L4
119185 229533.I1RCR8 pp 229533.I1RRM5
119186 229533.I1RCR8 pp 229533.I1RM82
119187 229533.I1RCR8 pp 229533.V6QUE6
119188 229533.I1RCR8 pp 229533.I1RKJ6
119189 229533.I1RCR8 pp 229533.I1RRV4
119190 229533.I1RCR8 pp 229533.I1RE76
119191 229533.I1RCR8 pp 229533.I1RJZ7
119192 229533.I1RCR8 pp 229533.I1RPU3
119193 229533.I1RCR8 pp 229533.I1S9Y1
119194 229533.I1RCR8 pp 229533.V6R3X5
119195 229533.I1RCR8 pp 229533.I1RRV3
119196 229533.I1RCR8 pp 229533.I1RZ39
119197 229533.I1RCR8 pp 229533.I1S1B2
119198 229533.I1RCR8 pp 229533.O74638
119199 229533.I1RCR8 pp 229533.I1RWL2
119200 229533.I1RCR8 pp 229533.I1RR73
119201 229533.I1RCR8 pp 229533.I1RRU9
119202 229533.I1RCR8 pp 229533.I1RJV4
119203 229533.I1RCR8 pp 229533.I1RXC2
119204 229533.I1RCR8 pp 229533.I1RTA1
119205 229533.I1RCR8 pp 229533.I1RJN9
119206 229533.I1RCR8 pp 229533.I1S5R1
119207 229533.I1RCR8 pp 229533.I1RRT8
119208 229533.I1RCR8 pp 229533.I1S503
119209 229533.I1RCR8 pp 229533.I1RP27

119210	229533.I1RCR8	pp	229533.I1RFT1
119211	229533.I1RCR8	pp	229533.I1RS47
119212	229533.I1RCR8	pp	229533.I1RJW6
119213	229533.I1RCR8	pp	229533.I1RSV2
119214	229533.I1RCR8	pp	229533.I1RLN6
119215	229533.I1RCR8	pp	229533.I1S2G7
119216	229533.I1RCR8	pp	229533.I1RSA5
119217	229533.I1RCR8	pp	229533.I1RKL3
119218	229533.I1RCR8	pp	229533.I1RQ65
119219	229533.I1RCR8	pp	229533.I1S352
119220	229533.I1RCR8	pp	229533.I1RLV9
119221	229533.I1RCR8	pp	229533.V6R3J1
119222	229533.I1RCR8	pp	229533.I1RRJ8
119223	229533.I1RCR8	pp	229533.I1RRZ0
119224	229533.I1RCR8	pp	229533.I1RDF7
119225	229533.I1RCR8	pp	229533.I1S0F6
119226	229533.I1RCR8	pp	229533.I1RSX0
119227	229533.I1RCR8	pp	229533.I1RWF7
119228	229533.I1RCR8	pp	229533.I1S8P5
119229	229533.I1RCR8	pp	229533.I1RRN5
119230	229533.I1RCR8	pp	229533.I1RN67
119231	229533.I1RCR8	pp	229533.I1S0W4
119232	229533.I1RCR8	pp	229533.I1RYI5
119233	229533.I1RCR8	pp	229533.I1RPP3
119234	229533.I1RCR8	pp	229533.I1RVX0
119235	229533.I1RCR8	pp	229533.I1RXR1
119236	229533.I1RCR8	pp	229533.I1RRQ3
119237	229533.I1RCR8	pp	229533.V6R283
119238	229533.I1RCR8	pp	229533.I1RUF7
119239	229533.I1RCR8	pp	229533.I1RT04
119240	229533.I1RCR8	pp	229533.I1RWK3
119241	229533.I1RCR8	pp	229533.I1RN18
119242	229533.I1RCR8	pp	229533.I1RSD8
119243	229533.I1RCR8	pp	229533.V6RUY9
119244	229533.I1RCR8	pp	229533.I1RVZ2
119245	229533.I1RCR8	pp	229533.Q4HXF0
119246	229533.I1RCR8	pp	229533.I1RQE7
119247	229533.I1RCR8	pp	229533.I1RFL4
119248	229533.I1RCR8	pp	229533.I1RNA5
119249	229533.I1RCR8	pp	229533.I1RJV3
119250	229533.I1RCR8	pp	229533.I1S2E9
119251	229533.I1RCR8	pp	229533.I1RSK1
119252	229533.I1RCR8	pp	229533.I1S2E8
119253	229533.I1RCR8	pp	229533.I1RS17
119254	229533.I1RCR8	pp	229533.I1RT40
119255	229533.I1RCR8	pp	229533.Q4I624
119256	229533.I1RCR8	pp	229533.I1RD17
119257	229533.I1RCR8	pp	229533.I1RPK0
119258	229533.I1RCR8	pp	229533.I1RV91
119259	229533.I1RCR8	pp	229533.V6R057
119260	229533.I1RCR8	pp	229533.I1RMV0
119261	229533.I1RCR8	pp	229533.I1RTD5
119262	229533.I1RCR8	pp	229533.I1S569
119263	229533.I1RCR8	pp	229533.I1S9Z4
119264	229533.I1RCR8	pp	229533.I1RPP4
119265	229533.I1RCR8	pp	229533.I1RGF5
119266	229533.I1RCR8	pp	229533.I1RGA4
119267	229533.I1RCR8	pp	229533.I1RTA0
119268	229533.I1RCR8	pp	229533.I1RQ33
119269	229533.I1RCR8	pp	229533.I1RW46
119270	229533.I1RCR8	pp	229533.Q4IPZ1
119271	229533.I1RCR8	pp	229533.I1RY44
119272	229533.I1RCR8	pp	229533.I1RM96
119273	229533.I1RCR8	pp	229533.I1RE01
119274	229533.I1RCR8	pp	229533.I1S0J1
119275	229533.I1RCR8	pp	229533.I1RGL4
119276	229533.I1RCR8	pp	229533.I1RRV1
119277	229533.I1RCR8	pp	229533.I1S7W5
119278	229533.I1RCR8	pp	229533.I1RRM3
119279	229533.I1RCR8	pp	229533.I1S8R3
119280	229533.I1RCR8	pp	229533.I1RTH6
119281	229533.I1RCR8	pp	229533.V6RMW6
119282	229533.I1RCR8	pp	229533.I1S2C0

229533.I1RCR8 pp 229533.I1RRE5
229533.I1RCR8 pp 229533.I1S8V4
229533.I1RCR8 pp 229533.V6R042
229533.I1RCR8 pp 229533.I1RYW3
229533.I1RCR8 pp 229533.I1RSQ3
229533.I1RCR8 pp 229533.I1RSF1
229533.I1RCR8 pp 229533.I1RPU2
229533.I1RCR8 pp 229533.I1RRR7
229533.I1RCR8 pp 229533.I1RZW8
229533.I1RCR8 pp 229533.Q4IN63
229533.I1RCR8 pp 229533.I1S1W9
229533.I1RCR8 pp 229533.I1RUG3
229533.I1RCR8 pp 229533.I1RZ79
229533.I1RCR8 pp 229533.I1RNC1
229533.I1RCR8 pp 229533.I1RRI2
229533.I1RCR8 pp 229533.V6RIV9
229533.I1RCR8 pp 229533.I1RWN5
229533.I1RCR8 pp 229533.I1RWX5
229533.I1RCR8 pp 229533.I1RZV3
229533.I1RCR8 pp 229533.I1RNY2
229533.I1RCR8 pp 229533.I1RXU9
229533.I1RCR8 pp 229533.I1RWN6
229533.I1RCR8 pp 229533.I1RKJ4
229533.I1RCR8 pp 229533.I1S1W3
229533.I1RCR8 pp 229533.Q4I327
229533.I1RCR8 pp 229533.I1RD39
229533.I1RCR8 pp 229533.I1RVQ3
229533.I1RCR8 pp 229533.I1RE89
229533.I1RCR8 pp 229533.I1S5R6
229533.I1RCR8 pp 229533.I1S0N4
229533.I1RCR8 pp 229533.I1RG11
229533.I1RCR8 pp 229533.I1RD22
229533.I1RCR8 pp 229533.I1RMN6
229533.I1RCR8 pp 229533.I1SA33
229533.I1RCR8 pp 229533.I1RRN2
229533.I1RND6 pp 229533.I1RW25
229533.I1RND6 pp 229533.I1S7S9
229533.I1RND6 pp 229533.I1RZS6
229533.I1RND6 pp 229533.I1RWI0
229533.I1RND6 pp 229533.Q4IFI0
229533.I1RND6 pp 229533.Q4I662
229533.I1RND6 pp 229533.I1RX24
229533.I1RND6 pp 229533.I1RNI4
229533.I1RND6 pp 229533.I1RPU0
229533.I1RND6 pp 229533.I1RPW9
229533.I1RND6 pp 229533.I1RR73
229533.I1RND6 pp 229533.I1RQ65
229533.I1RND6 pp 229533.I1RZV6
229533.I1RND6 pp 229533.Q4HVW2
229533.I1RND6 pp 229533.Q4HZ42
229533.I1RND6 pp 229533.I1S8E2
229533.I1RND6 pp 229533.I1RWD4
229533.I1RND6 pp 229533.I1RRQ3
229533.I1RND6 pp 229533.I1RPP3
229533.I1RND6 pp 229533.I1RT80
229533.I1RND6 pp 229533.I1RQJ5
229533.I1RND6 pp 229533.I1RWH5
229533.I1RND6 pp 229533.I1RU69
229533.I1RND6 pp 229533.I1RRI2
229533.I1RND6 pp 229533.Q4IPI1
229533.I1RND6 pp 229533.I1RT98
229533.I1RND6 pp 229533.I1RRR7
229533.I1RND6 pp 229533.I1S6E5
229533.I1RND6 pp 229533.I1RSR8
229533.I1RND6 pp 229533.I1RVZ0
229533.I1RND6 pp 229533.I1RX46
229533.I1RND6 pp 229533.I1RWB5
229533.I1RND6 pp 229533.I1RZF6
229533.I1RND6 pp 229533.Q4IAS1
229533.I1RND6 pp 229533.I1S0R7
229533.I1RND6 pp 229533.Q4IJ56
229533.I1RND6 pp 229533.Q4HWE2
229533.I1RND6 pp 229533.I1RT04

119356 229533.I1RND6 pp 229533.I1S1X9
119357 229533.I1RND6 pp 229533.I1S0J1
119358 229533.I1RND6 pp 229533.I1S2E8
119359 229533.I1RND6 pp 229533.I1S0M9
119360 229533.I1RND6 pp 229533.I1RNH6
119361 229533.I1RND6 pp 229533.I1RZK6
119362 229533.I1RND6 pp 229533.V6RMW6
119363 229533.I1RND6 pp 229533.I1RSL8
119364 229533.I1RND6 pp 229533.I1S021
119365 229533.I1RND6 pp 229533.I1RSD8
119366 229533.I1RND6 pp 229533.I1S060
119367 229533.I1RND6 pp 229533.I1RZZ1
119368 229533.I1RND6 pp 229533.Q4I327
119369 229533.I1RND6 pp 229533.V6RGN5
119370 229533.I1RND6 pp 229533.I1RYB9
119371 229533.I1RND6 pp 229533.Q4IN63
119372 229533.I1RND6 pp 229533.I1RPJ3
119373 229533.I1RND6 pp 229533.V6R042
119374 229533.I1RND6 pp 229533.I1S2C0
119375 229533.I1RND6 pp 229533.I1S044
119376 229533.A0A1C3YJ81 pp 229533.A0A1C3YJU4
119377 229533.A0A1C3YJ81 pp 229533.I1RZP4
119378 229533.A0A1C3YJ81 pp 229533.I1RB14
119379 229533.A0A1C3YJ81 pp 229533.V6RV57
119380 229533.A0A1C3YJ81 pp 229533.I1S7A3
119381 229533.A0A1C3YJ81 pp 229533.I1RH82
119382 229533.A0A1C3YJ81 pp 229533.I1RDY3
119383 229533.A0A1C3YJ81 pp 229533.I1S087
119384 229533.A0A1C3YJ81 pp 229533.I1RYH7
119385 229533.A0A1C3YJ81 pp 229533.A0A1C3YLD0
119386 229533.A0A1C3YJ81 pp 229533.I1S472
119387 229533.A0A1C3YJ81 pp 229533.I1RB01
119388 229533.I1RZ39 pp 229533.I1S0T2
119389 229533.I1RZ39 pp 229533.I1S0R7
119390 229533.I1RZ39 pp 229533.I1S4S7
119391 229533.I1RZ39 pp 229533.V6RTJ8
119392 229533.I1RZ39 pp 229533.I1S338
119393 229533.I1RZ39 pp 229533.V6RUT5
119394 229533.I1RZ39 pp 229533.V6QW22
119395 229533.I1RZ39 pp 229533.I1S2R0
119396 229533.I1RZ39 pp 229533.I1S9U2
119397 229533.I1RZ39 pp 229533.I1S0P0
119398 229533.I1RZ39 pp 229533.Q4IJT5
119399 229533.I1RZ39 pp 229533.I1S7A8
119400 229533.I1RZ39 pp 229533.I1S438
119401 229533.I1RZ39 pp 229533.I1S0F5
119402 229533.I1RZ39 pp 229533.V6R4J1
119403 229533.I1RZ39 pp 229533.I1S4S4
119404 229533.I1RZ39 pp 229533.V6R1K4
119405 229533.I1RZ39 pp 229533.I1S021
119406 229533.I1RZ39 pp 229533.I1S9I5
119407 229533.I1RZ39 pp 229533.I1SA32
119408 229533.I1RZ39 pp 229533.I1S0X6
119409 229533.I1RZ39 pp 229533.I1S1W8
119410 229533.I1RZ39 pp 229533.I1S0L3
119411 229533.I1RZ39 pp 229533.I1S5B0
119412 229533.I1RZ39 pp 229533.V6RCG5
119413 229533.I1RZ39 pp 229533.I1S1B2
119414 229533.I1RZ39 pp 229533.I1RZM8
119415 229533.I1RZ39 pp 229533.I1S0A0
119416 229533.I1RZ39 pp 229533.I1S504
119417 229533.I1RZ39 pp 229533.V6QUE6
119418 229533.I1RZ39 pp 229533.I1S6K9
119419 229533.I1RZ39 pp 229533.V6RCZ8
119420 229533.I1RZ39 pp 229533.I1RZF6
119421 229533.I1RZ39 pp 229533.V6R9Z0
119422 229533.I1RZ39 pp 229533.I1S419
119423 229533.I1RZ39 pp 229533.I1S0M9
119424 229533.I1RZ39 pp 229533.I1RZM1
119425 229533.I1RZ39 pp 229533.I1S821
119426 229533.I1RZ39 pp 229533.Q4I8P5
119427 229533.I1RZ39 pp 229533.I1S887
119428 229533.I1RZ39 pp 229533.Q4IJM4

229533.I1RZ39 pp 229533.I1S4Q4
229533.I1RZ39 pp 229533.I1RZK5
229533.I1RZ39 pp 229533.I1RZE5
229533.I1RZ39 pp 229533.I1S1J7
229533.I1RZ39 pp 229533.V6RBN2
229533.I1RZ39 pp 229533.I1RZK6
229533.I1RZ39 pp 229533.I1RZZ1
229533.I1RZ39 pp 229533.Q4I2J8
229533.I1RZ39 pp 229533.Q4HXF0
229533.I1RZ39 pp 229533.I1RZZ9
229533.I1RZ39 pp 229533.I1S0L4
229533.I1RZ39 pp 229533.I1S2E9
229533.I1RZ39 pp 229533.Q4IJK2
229533.I1RZ39 pp 229533.I1S8V3
229533.I1RZ39 pp 229533.I1S268
229533.I1RZ39 pp 229533.I1S3G4
229533.I1RZ39 pp 229533.V6R283
229533.I1RZ39 pp 229533.I1RZB2
229533.I1RZ39 pp 229533.V6RUY9
229533.I1RZ39 pp 229533.Q4HVQ9
229533.I1RZ39 pp 229533.I1S5R1
229533.I1RZ39 pp 229533.I1S2H4
229533.I1RZ39 pp 229533.V6R3J1
229533.I1RZ39 pp 229533.I1S1F3
229533.I1RZ39 pp 229533.I1S0F7
229533.I1RZ39 pp 229533.I1S8P5
229533.I1RZ39 pp 229533.I1S9Y1
229533.I1RZ39 pp 229533.I1S2G7
229533.I1RZ39 pp 229533.O74638
229533.I1RZ39 pp 229533.I1S503
229533.I1RZ39 pp 229533.I1S352
229533.I1RZ39 pp 229533.I1S0F6
229533.I1RZ39 pp 229533.I1S1W3
229533.I1RZ39 pp 229533.I1S0W4
229533.I1RZ39 pp 229533.I1S0M6
229533.I1RZ39 pp 229533.I1RZV3
229533.I1RZ39 pp 229533.I1S6Y8
229533.I1RZ39 pp 229533.I1RZS6
229533.I1RZ39 pp 229533.V6R3X5
229533.I1RZ39 pp 229533.V6R042
229533.I1RZ39 pp 229533.Q4I327
229533.I1RZ39 pp 229533.I1S1W9
229533.I1RZ39 pp 229533.I1S016
229533.I1RZ39 pp 229533.V6RIV9
229533.I1RZ39 pp 229533.V6RMW6
229533.I1RZ39 pp 229533.I1S4Z8
229533.I1RZ39 pp 229533.Q4IN63
229533.I1RZ39 pp 229533.I1SA33
229533.I1RZ39 pp 229533.I1S0N4
229533.I1RZ39 pp 229533.I1S2B8
229533.I1RZ39 pp 229533.I1S2C0
229533.I1RZ39 pp 229533.I1RZ79
229533.I1RZ39 pp 229533.I1S5R6
229533.I1RZ39 pp 229533.I1S0F8
229533.I1RZ39 pp 229533.I1S8R3
229533.I1RZ39 pp 229533.I1S9Z4
229533.I1RZ39 pp 229533.I1S7W5
229533.I1RZ39 pp 229533.I1S437
229533.I1RZ39 pp 229533.I1S569
229533.I1RZ39 pp 229533.V6RLM7
229533.I1RZ39 pp 229533.I1S256
229533.I1RZ39 pp 229533.I1RZW8
229533.I1RZ39 pp 229533.I1S8V4
229533.I1RZ39 pp 229533.I1S1C7
229533.I1RZ39 pp 229533.Q4I624
229533.I1RZ39 pp 229533.I1S0J1
229533.I1RZ39 pp 229533.V6R057
229533.I1RZ39 pp 229533.I1S1W4
229533.I1RZ39 pp 229533.V6RRH2
229533.I1RZ39 pp 229533.I1S2E8
229533.A0A1C3YM60 pp 229533.I1RB38
229533.A0A1C3YM60 pp 229533.I1S7W5
229533.A0A1C3YM60 pp 229533.I1RE52

119502 229533.A0A1C3YM60 pp 229533.I1S5X9
119503 229533.A0A1C3YM60 pp 229533.V6RJP8
119504 229533.A0A1C3YM60 pp 229533.I1RPK0
119505 229533.A0A1C3YM60 pp 229533.I1RS29
119506 229533.A0A1C3YM60 pp 229533.Q4I7K4
119507 229533.A0A1C3YM60 pp 229533.I1RAE2
119508 229533.A0A1C3YM60 pp 229533.I1RNY6
119509 229533.A0A1C3YM60 pp 229533.V6RCG5
119510 229533.A0A1C3YM60 pp 229533.I1RWD3
119511 229533.A0A1C3YM60 pp 229533.I1S0R8
119512 229533.A0A1C3YM60 pp 229533.I1S0K2
119513 229533.A0A1C3YM60 pp 229533.I1RMV0
119514 229533.A0A1C3YM60 pp 229533.I1RAE5
119515 229533.A0A1C3YM60 pp 229533.V6RMW6
119516 229533.A0A1C3YM60 pp 229533.I1R9Z5
119517 229533.A0A1C3YM60 pp 229533.I1RB92
119518 229533.A0A1C3YM60 pp 229533.Q4I2J8
119519 229533.A0A1C3YM60 pp 229533.I1S0Q7
119520 229533.A0A1C3YM60 pp 229533.I1RWA4
119521 229533.A0A1C3YM60 pp 229533.I1RYH7
119522 229533.A0A1C3YM60 pp 229533.I1RAV5
119523 229533.A0A1C3YM60 pp 229533.I1S0C1
119524 229533.A0A1C3YM60 pp 229533.I1RFJ5
119525 229533.A0A1C3YM60 pp 229533.I1REE2
119526 229533.A0A1C3YM60 pp 229533.I1RFS5
119527 229533.A0A1C3YM60 pp 229533.I1RWB0
119528 229533.A0A1C3YM60 pp 229533.I1RAE4
119529 229533.A0A1C3YM60 pp 229533.I1RP02
119530 229533.A0A1C3YM60 pp 229533.I1RAM9
119531 229533.A0A1C3YM60 pp 229533.I1RBT6
119532 229533.A0A1C3YM60 pp 229533.I1RAA2
119533 229533.A0A1C3YM60 pp 229533.I1S855
119534 229533.A0A1C3YM60 pp 229533.I1RZQ6
119535 229533.A0A1C3YM60 pp 229533.I1RP88
119536 229533.A0A1C3YM60 pp 229533.I1RE65
119537 229533.A0A1C3YM60 pp 229533.I1S5B0
119538 229533.A0A1C3YM60 pp 229533.I1RFJ6
119539 229533.A0A1C3YM60 pp 229533.I1S212
119540 229533.A0A1C3YM60 pp 229533.I1RXE6
119541 229533.A0A1C3YM60 pp 229533.I1RBS0
119542 229533.A0A1C3YM60 pp 229533.I1S8S9
119543 229533.A0A1C3YM60 pp 229533.I1S0P0
119544 229533.A0A1C3YM60 pp 229533.I1RCG4
119545 229533.A0A1C3YM60 pp 229533.I1RPR9
119546 229533.A0A1C3YM60 pp 229533.I1RX39
119547 229533.A0A1C3YM60 pp 229533.I1RBS6
119548 229533.A0A1C3YM60 pp 229533.I1RE66
119549 229533.A0A1C3YM60 pp 229533.I1RWK3
119550 229533.A0A1C3YM60 pp 229533.I1RYW9
119551 229533.A0A1C3YM60 pp 229533.I1RAE3
119552 229533.A0A1C3YM60 pp 229533.I1RBE6
119553 229533.A0A1C3YM60 pp 229533.I1RA49
119554 229533.A0A1C3YM60 pp 229533.I1RNM7
119555 229533.A0A1C3YM60 pp 229533.I1S464
119556 229533.A0A1C3YM60 pp 229533.I1RXD9
119557 229533.A0A1C3YM60 pp 229533.I1RN88
119558 229533.A0A1C3YM60 pp 229533.I1RAI4
119559 229533.A0A1C3YM60 pp 229533.I1RRX5
119560 229533.A0A1C3YM60 pp 229533.I1RJT3
119561 229533.A0A1C3YM60 pp 229533.I1RPP3
119562 229533.A0A1C3YM60 pp 229533.I1RWA3
119563 229533.A0A1C3YM60 pp 229533.I1RS05
119564 229533.A0A1C3YM60 pp 229533.I1RN67
119565 229533.A0A1C3YM60 pp 229533.I1S1U9
119566 229533.A0A1C3YM60 pp 229533.I1RLP1
119567 229533.A0A1C3YM60 pp 229533.I1S854
119568 229533.A0A1C3YM60 pp 229533.V6R3J1
119569 229533.A0A1C3YM60 pp 229533.I1RG62
119570 229533.A0A1C3YM60 pp 229533.I1RMK7
119571 229533.A0A1C3YM60 pp 229533.I1RYT8
119572 229533.A0A1C3YM60 pp 229533.I1RMR9
119573 229533.A0A1C3YM60 pp 229533.I1RAX6
119574 229533.A0A1C3YM60 pp 229533.A0A1C3YMC1

119575 229533.A0A1C3YM60 pp 229533.I1RY92
119576 229533.A0A1C3YM60 pp 229533.I1R9M0
119577 229533.A0A1C3YM60 pp 229533.I1RFI4
119578 229533.A0A1C3YM60 pp 229533.I1RUR8
119579 229533.A0A1C3YM60 pp 229533.I1RMQ6
119580 229533.A0A1C3YM60 pp 229533.I1RKB7
119581 229533.A0A1C3YM60 pp 229533.I1RA52
119582 229533.A0A1C3YM60 pp 229533.I1RR73
119583 229533.A0A1C3YM60 pp 229533.I1RGC3
119584 229533.A0A1C3YM60 pp 229533.I1RAW3
119585 229533.A0A1C3YM60 pp 229533.I1RX21
119586 229533.A0A1C3YM60 pp 229533.I1R9Y4
119587 229533.A0A1C3YM60 pp 229533.I1RY21
119588 229533.A0A1C3YM60 pp 229533.I1S3T7
119589 229533.A0A1C3YM60 pp 229533.I1RA29
119590 229533.I1RRR7 pp 229533.Q4IQW0
119591 229533.I1RRR7 pp 229533.I1RX46
119592 229533.I1RRR7 pp 229533.Q4IPI1
119593 229533.I1RRR7 pp 229533.I1RTD5
119594 229533.I1RRR7 pp 229533.I1RRT2
119595 229533.I1RRR7 pp 229533.Q4IAS1
119596 229533.I1RRR7 pp 229533.I1RT98
119597 229533.I1RRR7 pp 229533.I1RZJ6
119598 229533.I1RRR7 pp 229533.I1RUQ2
119599 229533.I1RRR7 pp 229533.V6R283
119600 229533.I1RRR7 pp 229533.I1S2E9
119601 229533.I1RRR7 pp 229533.Q4I5R9
119602 229533.I1RRR7 pp 229533.I1RXD7
119603 229533.I1RRR7 pp 229533.I1S503
119604 229533.I1RRR7 pp 229533.I1RWH5
119605 229533.I1RRR7 pp 229533.I1S0R7
119606 229533.I1RRR7 pp 229533.Q4I830
119607 229533.I1RRR7 pp 229533.I1RSQ7
119608 229533.I1RRR7 pp 229533.V6R3X5
119609 229533.I1RRR7 pp 229533.I1RT04
119610 229533.I1RRR7 pp 229533.I1RTI6
119611 229533.I1RRR7 pp 229533.I1RZ39
119612 229533.I1RRR7 pp 229533.I1RW71
119613 229533.I1RRR7 pp 229533.Q4HZ47
119614 229533.I1RRR7 pp 229533.Q4I5M4
119615 229533.I1RRR7 pp 229533.I1RRT8
119616 229533.I1RRR7 pp 229533.Q4IEV5
119617 229533.I1RRR7 pp 229533.I1RTF4
119618 229533.I1RRR7 pp 229533.I1RS30
119619 229533.I1RRR7 pp 229533.I1RZV6
119620 229533.I1RRR7 pp 229533.Q4IJH1
119621 229533.I1RRR7 pp 229533.I1S9Y1
119622 229533.I1RRR7 pp 229533.Q4IQ72
119623 229533.I1RRR7 pp 229533.I1RS47
119624 229533.I1RRR7 pp 229533.Q4I7L0
119625 229533.I1RRR7 pp 229533.I1RT33
119626 229533.I1RRR7 pp 229533.I1RT80
119627 229533.I1RRR7 pp 229533.Q4HZ42
119628 229533.I1RRR7 pp 229533.I1S9U2
119629 229533.I1RRR7 pp 229533.I1S8E2
119630 229533.I1RRR7 pp 229533.V6QUE6
119631 229533.I1RRR7 pp 229533.I1RW25
119632 229533.I1RRR7 pp 229533.Q4IFI0
119633 229533.I1RRR7 pp 229533.Q4IF76
119634 229533.I1RRR7 pp 229533.I1RU69
119635 229533.I1RRR7 pp 229533.I1S2B8
119636 229533.I1RRR7 pp 229533.V6RGN5
119637 229533.I1RRR7 pp 229533.I1S0J1
119638 229533.I1RRR7 pp 229533.I1S060
119639 229533.I1RRR7 pp 229533.I1S044
119640 229533.I1RRR7 pp 229533.I1S021
119641 229533.I1RRR7 pp 229533.Q4I662
119642 229533.I1RRR7 pp 229533.Q4I327
119643 229533.I1RRR7 pp 229533.I1S0M9
119644 229533.I1RRR7 pp 229533.I1RZS6
119645 229533.I1RRR7 pp 229533.Q4IJ56
119646 229533.I1RRR7 pp 229533.I1RYB9
119647 229533.I1RRR7 pp 229533.I1RWB5

119648 229533.I1RRR7 pp 229533.I1RWN6
119649 229533.I1RRR7 pp 229533.I1RSA8
119650 229533.I1RRR7 pp 229533.Q4IPN9
119651 229533.I1RRR7 pp 229533.I1RSR3
119652 229533.I1RRR7 pp 229533.I1RRY9
119653 229533.I1RRR7 pp 229533.I1RX24
119654 229533.I1RRR7 pp 229533.Q4IN63
119655 229533.I1RRR7 pp 229533.V6RGY3
119656 229533.I1RRR7 pp 229533.V6RMW6
119657 229533.I1RRR7 pp 229533.V6QYF0
119658 229533.I1RRR7 pp 229533.V6R042
119659 229533.I1RRR7 pp 229533.I1S1J6
119660 229533.I1RRR7 pp 229533.I1S274
119661 229533.I1RRR7 pp 229533.I1RVZ0
119662 229533.I1RRR7 pp 229533.Q4IEK8
119663 229533.I1RRR7 pp 229533.I1RSI6
119664 229533.I1RRR7 pp 229533.I1RSR8
119665 229533.I1RRR7 pp 229533.Q4HZ68
119666 229533.I1RRR7 pp 229533.I1S1C7
119667 229533.I1RRR7 pp 229533.I1RWN5
119668 229533.I1RRR7 pp 229533.I1S296
119669 229533.I1RRR7 pp 229533.I1RY44
119670 229533.I1RRR7 pp 229533.I1RZK6
119671 229533.I1RRR7 pp 229533.I1RY03
119672 229533.I1RRR7 pp 229533.I1RZZ1
119673 229533.I1RRR7 pp 229533.I1S4T3
119674 229533.I1RRR7 pp 229533.I1RTD9
119675 229533.I1RRR7 pp 229533.I1RTE9
119676 229533.I1RRR7 pp 229533.I1RZK5
119677 229533.I1RRR7 pp 229533.V6R227
119678 229533.I1RRR7 pp 229533.I1S2C0
119679 229533.I1RRR7 pp 229533.I1S2E8
119680 229533.I1RRR7 pp 229533.I1S504
119681 229533.I1RRR7 pp 229533.I1RSD8
119682 229533.I1RRR7 pp 229533.I1RZF6
119683 229533.I1RRR7 pp 229533.I1RVX0
119684 229533.I1RRR7 pp 229533.I1RTH6
119685 229533.I1RRR7 pp 229533.I1RT03
119686 229533.I1RRR7 pp 229533.I1S1X9
119687 229533.I1RRR7 pp 229533.I1RY63
119688 229533.I1RRR7 pp 229533.Q4HWE2
119689 229533.I1RRR7 pp 229533.I1RSS4
119690 229533.A0A1C3YL15 pp 229533.I1RWB5
119691 229533.A0A1C3YL15 pp 229533.I1RN28
119692 229533.A0A1C3YL15 pp 229533.I1RKK9
119693 229533.A0A1C3YL15 pp 229533.I1RZS7
119694 229533.A0A1C3YL15 pp 229533.Q4IAA0
119695 229533.A0A1C3YL15 pp 229533.I1S289
119696 229533.A0A1C3YL15 pp 229533.I1RYI6
119697 229533.A0A1C3YL15 pp 229533.I1S3V2
119698 229533.A0A1C3YL15 pp 229533.I1RDY5
119699 229533.A0A1C3YL15 pp 229533.I1RC54
119700 229533.A0A1C3YL15 pp 229533.I1RK78
119701 229533.A0A1C3YL15 pp 229533.I1RW99
119702 229533.A0A1C3YL15 pp 229533.I1S290
119703 229533.A0A1C3YL15 pp 229533.V6RIS2
119704 229533.A0A1C3YL15 pp 229533.I1S044
119705 229533.A0A1C3YL15 pp 229533.I1RGC2
119706 229533.A0A1C3YL15 pp 229533.I1S504
119707 229533.A0A1C3YL15 pp 229533.I1RD22
119708 229533.A0A1C3YL15 pp 229533.I1RDD6
119709 229533.A0A1C3YL15 pp 229533.I1RSE2
119710 229533.A0A1C3YL15 pp 229533.I1RT35
119711 229533.A0A1C3YL15 pp 229533.I1RRU9
119712 229533.A0A1C3YL15 pp 229533.I1RZM1
119713 229533.A0A1C3YL15 pp 229533.I1RA66
119714 229533.A0A1C3YL15 pp 229533.I1RC94
119715 229533.A0A1C3YL15 pp 229533.V6R042
119716 229533.A0A1C3YL15 pp 229533.I1RJW8
119717 229533.A0A1C3YL15 pp 229533.I1RD17
119718 229533.A0A1C3YL15 pp 229533.I1REK8
119719 229533.A0A1C3YL15 pp 229533.I1RAU7
119720 229533.A0A1C3YL15 pp 229533.I1RWD7

119721	229533.A0A1C3YL15	pp	229533.V6RCG5
119722	229533.A0A1C3YL15	pp	229533.V6RMW6
119723	229533.A0A1C3YL15	pp	229533.I1RNQ5
119724	229533.A0A1C3YL15	pp	229533.I1S6F1
119725	229533.A0A1C3YL15	pp	229533.I1RLU6
119726	229533.A0A1C3YL15	pp	229533.I1RS17
119727	229533.A0A1C3YL15	pp	229533.I1RNC1
119728	229533.A0A1C3YL15	pp	229533.I1RUG3
119729	229533.A0A1C3YL15	pp	229533.I1RSI0
119730	229533.A0A1C3YL15	pp	229533.I1RYB9
119731	229533.A0A1C3YL15	pp	229533.I1RJV4
119732	229533.A0A1C3YL15	pp	229533.V6RTJ8
119733	229533.A0A1C3YL15	pp	229533.I1S6E5
119734	229533.A0A1C3YL15	pp	229533.I1RML0
119735	229533.A0A1C3YL15	pp	229533.I1S503
119736	229533.A0A1C3YL15	pp	229533.I1RSS4
119737	229533.A0A1C3YL15	pp	229533.I1RTH6
119738	229533.A0A1C3YL15	pp	229533.I1RA70
119739	229533.A0A1C3YL15	pp	229533.I1RFU8
119740	229533.A0A1C3YL15	pp	229533.I1RKF8
119741	229533.A0A1C3YL15	pp	229533.I1RK83
119742	229533.A0A1C3YL15	pp	229533.I1RAD1
119743	229533.A0A1C3YL15	pp	229533.Q4HWE2
119744	229533.A0A1C3YL15	pp	229533.V6RNM4
119745	229533.A0A1C3YL15	pp	229533.I1RX46
119746	229533.A0A1C3YL15	pp	229533.I1RDG0
119747	229533.A0A1C3YL15	pp	229533.I1RKJ8
119748	229533.A0A1C3YL15	pp	229533.Q4IJK2
119749	229533.A0A1C3YL15	pp	229533.I1RT04
119750	229533.A0A1C3YL15	pp	229533.I1RQC9
119751	229533.A0A1C3YL15	pp	229533.I1REF5
119752	229533.A0A1C3YL15	pp	229533.I1RE71
119753	229533.A0A1C3YL15	pp	229533.I1RFL4
119754	229533.A0A1C3YL15	pp	229533.A0A1C3YLK2
119755	229533.A0A1C3YL15	pp	229533.I1S5S2
119756	229533.A0A1C3YL15	pp	229533.Q4I613
119757	229533.A0A1C3YL15	pp	229533.I1RN18
119758	229533.A0A1C3YL15	pp	229533.I1RT33
119759	229533.A0A1C3YL15	pp	229533.I1RQE7
119760	229533.A0A1C3YL15	pp	229533.A0A1C3YLI6
119761	229533.A0A1C3YL15	pp	229533.I1RD52
119762	229533.A0A1C3YL15	pp	229533.A0A1C3YMK1
119763	229533.A0A1C3YL15	pp	229533.I1S0L3
119764	229533.A0A1C3YL15	pp	229533.I1RN67
119765	229533.A0A1C3YL15	pp	229533.I1RB91
119766	229533.A0A1C3YL15	pp	229533.I1RXF8
119767	229533.A0A1C3YL15	pp	229533.I1RWN6
119768	229533.A0A1C3YL15	pp	229533.I1RZ40
119769	229533.A0A1C3YL15	pp	229533.I1RQ88
119770	229533.A0A1C3YL15	pp	229533.I1RS43
119771	229533.A0A1C3YL15	pp	229533.I1RRY9
119772	229533.A0A1C3YL15	pp	229533.I1RJV3
119773	229533.A0A1C3YL15	pp	229533.I1RQK8
119774	229533.A0A1C3YL15	pp	229533.I1RG53
119775	229533.A0A1C3YL15	pp	229533.I1RPP3
119776	229533.A0A1C3YL15	pp	229533.I1RE45
119777	229533.A0A1C3YL15	pp	229533.A0A1C3YM36
119778	229533.A0A1C3YL15	pp	229533.I1RFH9
119779	229533.A0A1C3YL15	pp	229533.I1S5Z2
119780	229533.A0A1C3YL15	pp	229533.I1RC81
119781	229533.A0A1C3YL15	pp	229533.I1S2B8
119782	229533.A0A1C3YL15	pp	229533.I1RWH5
119783	229533.A0A1C3YL15	pp	229533.I1RB53
119784	229533.A0A1C3YL15	pp	229533.I1RLX7
119785	229533.A0A1C3YL15	pp	229533.I1RWI0
119786	229533.A0A1C3YL15	pp	229533.I1RBZ3
119787	229533.A0A1C3YL15	pp	229533.Q4HW67
119788	229533.A0A1C3YL15	pp	229533.I1RK70
119789	229533.A0A1C3YL15	pp	229533.I1RAV4
119790	229533.A0A1C3YL15	pp	229533.I1S7S9
119791	229533.A0A1C3YL15	pp	229533.I1RW25
119792	229533.A0A1C3YL15	pp	229533.I1S9F1
119793	229533.A0A1C3YL15	pp	229533.V6QUW4

119794 229533.A0A1C3YL15 pp 229533.I1RB59
119795 229533.A0A1C3YL15 pp 229533.I1S524
119796 229533.A0A1C3YL15 pp 229533.I1R9X2
119797 229533.A0A1C3YL15 pp 229533.I1S0L4
119798 229533.A0A1C3YL15 pp 229533.I1RNE7
119799 229533.A0A1C3YL15 pp 229533.I1RZV6
119800 229533.A0A1C3YL15 pp 229533.I1RIS3
119801 229533.A0A1C3YL15 pp 229533.I1RN48
119802 229533.A0A1C3YL15 pp 229533.I1RZG9
119803 229533.A0A1C3YL15 pp 229533.I1S8E2
119804 229533.A0A1C3YL15 pp 229533.I1RND6
119805 229533.A0A1C3YL15 pp 229533.I1RR73
119806 229533.A0A1C3YL15 pp 229533.Q4IB50
119807 229533.A0A1C3YL15 pp 229533.I1RXV3
119808 229533.A0A1C3YL15 pp 229533.I1RNI4
119809 229533.A0A1C3YL15 pp 229533.I1RMW7
119810 229533.A0A1C3YL15 pp 229533.I1RZZ9
119811 229533.Q4I327 pp 229533.V6R227
119812 229533.Q4I327 pp 229533.Q4IJ56
119813 229533.Q4I327 pp 229533.V6RGN5
119814 229533.Q4I327 pp 229533.Q4IN63
119815 229533.Q4I327 pp 229533.Q4IEK8
119816 229533.Q4I327 pp 229533.Q4IAA0
119817 229533.Q4I327 pp 229533.Q4IBS2
119818 229533.Q4I327 pp 229533.Q4I5R9
119819 229533.Q4I327 pp 229533.Q4I5Z5
119820 229533.Q4I327 pp 229533.V6R283
119821 229533.Q4I327 pp 229533.V6R042
119822 229533.Q4I327 pp 229533.Q4I830
119823 229533.Q4I327 pp 229533.V6RGY3
119824 229533.Q4I327 pp 229533.Q4IPI1
119825 229533.Q4I327 pp 229533.Q4IF76
119826 229533.Q4I327 pp 229533.V6RMW6
119827 229533.Q4I327 pp 229533.V6RBN2
119828 229533.Q4I327 pp 229533.Q4IAS1
119829 229533.Q4I327 pp 229533.Q4IQW0
119830 229533.Q4I327 pp 229533.Q4IFI0
119831 229533.Q4I327 pp 229533.Q4INZ9
119832 229533.Q4I327 pp 229533.Q4IEV5
119833 229533.Q4I327 pp 229533.Q4IJH1
119834 229533.Q4I327 pp 229533.V6R3X5
119835 229533.Q4I327 pp 229533.Q4I662
119836 229533.Q4I327 pp 229533.V6QYF0
119837 229533.A0A1C3YMA5 pp 229533.I1S0W5
119838 229533.A0A1C3YMA5 pp 229533.I1R982
119839 229533.A0A1C3YMA5 pp 229533.I1RFB6
119840 229533.A0A1C3YMA5 pp 229533.I1REV6
119841 229533.A0A1C3YMA5 pp 229533.I1S0C0
119842 229533.A0A1C3YMA5 pp 229533.I1RQL4
119843 229533.A0A1C3YMA5 pp 229533.V6RGI1
119844 229533.A0A1C3YMA5 pp 229533.I1RAU3
119845 229533.A0A1C3YMA5 pp 229533.I1S2A8
119846 229533.A0A1C3YMA5 pp 229533.I1RIV2
119847 229533.A0A1C3YMA5 pp 229533.I1RBF3
119848 229533.A0A1C3YMA5 pp 229533.V6RIE5
119849 229533.A0A1C3YMA5 pp 229533.I1RMJ3
119850 229533.I1S8E2 pp 229533.Q4I662
119851 229533.I1S8E2 pp 229533.Q4IPI1
119852 229533.I1S8E2 pp 229533.Q4IAS1
119853 229533.I1S8E2 pp 229533.V6R042
119854 229533.I1S8E2 pp 229533.Q4IFI0
119855 229533.I1S8E2 pp 229533.V6RMW6
119856 229533.I1S8E2 pp 229533.Q4I5R9
119857 229533.I1S8E2 pp 229533.V6R3X5
119858 229533.I1S8E2 pp 229533.Q4IJ56
119859 229533.I1S8E2 pp 229533.Q4IEK8
119860 229533.I1S8E2 pp 229533.Q4I327
119861 229533.I1S8E2 pp 229533.Q4IN63
119862 229533.I1S8E2 pp 229533.V6QUE6
119863 229533.I1S8E2 pp 229533.V6RGN5
119864 229533.I1S8E2 pp 229533.I1S9U2
119865 229533.I1RZ92 pp 229533.I1S1Q3
119866 229533.I1RZ92 pp 229533.V6RG22

119867	229533.I1RZ92	pp	229533.I1S130
119868	229533.I1RZ92	pp	229533.I1S5R5
119869	229533.I1RZ92	pp	229533.V6RR38
119870	229533.I1RZ92	pp	229533.V6RPN0
119871	229533.I1RZ92	pp	229533.I1S1K6
119872	229533.I1RZ92	pp	229533.I1RZD0
119873	229533.I1RZ92	pp	229533.I1SAS3
119874	229533.I1RZ92	pp	229533.I1S2M5
119875	229533.I1RZ92	pp	229533.I1SAS4
119876	229533.I1RC66	pp	229533.I1S5R6
119877	229533.I1RC66	pp	229533.I1S0Z8
119878	229533.I1RC66	pp	229533.I1RG11
119879	229533.I1RC66	pp	229533.I1RCR8
119880	229533.I1RC66	pp	229533.I1RWQ8
119881	229533.I1RC66	pp	229533.I1RUG3
119882	229533.I1RC66	pp	229533.I1RNC1
119883	229533.I1RC66	pp	229533.I1RSQ3
119884	229533.I1RC66	pp	229533.I1RRV1
119885	229533.I1RC66	pp	229533.I1RMG3
119886	229533.I1RC66	pp	229533.I1RRM3
119887	229533.I1RC66	pp	229533.V6RMW6
119888	229533.I1RC66	pp	229533.V6R042
119889	229533.I1RC66	pp	229533.I1RWN5
119890	229533.I1RC66	pp	229533.I1RZS8
119891	229533.I1RC66	pp	229533.I1S1W3
119892	229533.I1RC66	pp	229533.V6RIV9
119893	229533.I1RC66	pp	229533.I1S1W9
119894	229533.I1RC66	pp	229533.I1SA33
119895	229533.I1RC66	pp	229533.I1RD22
119896	229533.I1RC66	pp	229533.I1RZV3
119897	229533.I1RC66	pp	229533.I1RRE5
119898	229533.I1RC66	pp	229533.I1RTD5
119899	229533.I1RC66	pp	229533.I1RTA0
119900	229533.I1RC66	pp	229533.I1RWH4
119901	229533.I1RC66	pp	229533.I1RPU2
119902	229533.I1RC66	pp	229533.I1RRN2
119903	229533.I1RC66	pp	229533.I1RT04
119904	229533.I1RC66	pp	229533.Q4HXF0
119905	229533.I1RC66	pp	229533.I1RW46
119906	229533.I1RC66	pp	229533.V6RUY9
119907	229533.I1RC66	pp	229533.I1S0J1
119908	229533.I1RC66	pp	229533.Q4I624
119909	229533.I1RC66	pp	229533.I1RE01
119910	229533.I1RC66	pp	229533.V6R057
119911	229533.I1RC66	pp	229533.I1S0F6
119912	229533.I1RC66	pp	229533.I1RDF7
119913	229533.I1RC66	pp	229533.I1RQ33
119914	229533.I1RC66	pp	229533.I1S7W5
119915	229533.I1RC66	pp	229533.I1RS17
119916	229533.I1RC66	pp	229533.I1S0W4
119917	229533.I1RC66	pp	229533.I1RFL4
119918	229533.I1RC66	pp	229533.I1RN67
119919	229533.I1RC66	pp	229533.I1RS47
119920	229533.I1RC66	pp	229533.I1RN18
119921	229533.I1RC66	pp	229533.I1RPP4
119922	229533.I1RC66	pp	229533.I1RPK0
119923	229533.I1RC66	pp	229533.I1RMP0
119924	229533.I1RC66	pp	229533.I1RQE7
119925	229533.I1RC66	pp	229533.I1RMV0
119926	229533.I1RC66	pp	229533.I1RYR0
119927	229533.I1RC66	pp	229533.I1RCH5
119928	229533.I1RC66	pp	229533.I1REK7
119929	229533.I1RC66	pp	229533.I1RXU9
119930	229533.I1RC66	pp	229533.I1RJV3
119931	229533.I1RC66	pp	229533.I1RN63
119932	229533.I1RC66	pp	229533.I1RW83
119933	229533.I1RC66	pp	229533.I1RXR1
119934	229533.I1RC66	pp	229533.I1RT03
119935	229533.I1RC66	pp	229533.I1RWN6
119936	229533.I1RC66	pp	229533.I1RD39
119937	229533.I1RC66	pp	229533.I1RVX0
119938	229533.I1RC66	pp	229533.I1RWK3
119939	229533.I1RC66	pp	229533.I1S437

119940 229533.I1RC66 pp 229533.I1RVZ2
119941 229533.I1RC66 pp 229533.I1RCW6
119942 229533.I1RC66 pp 229533.I1S1C7
119943 229533.I1RC66 pp 229533.I1RTA1
119944 229533.I1RC66 pp 229533.I1RSY2
119945 229533.I1RC66 pp 229533.Q4HXF6
119946 229533.I1RC66 pp 229533.I1RJN9
119947 229533.I1RC66 pp 229533.I1RJW6
119948 229533.I1RC66 pp 229533.I1RFT1
119949 229533.I1RC66 pp 229533.I1RYW3
119950 229533.I1RC66 pp 229533.I1RRJ8
119951 229533.I1RC66 pp 229533.I1RYI5
119952 229533.I1RC66 pp 229533.I1RZW8
119953 229533.I1RC66 pp 229533.I1RPP3
119954 229533.I1RC66 pp 229533.I1S503
119955 229533.I1RC66 pp 229533.I1RRI2
119956 229533.I1RC66 pp 229533.I1RWX5
119957 229533.I1RC66 pp 229533.I1RSX0
119958 229533.I1RC66 pp 229533.I1S8P5
119959 229533.I1RC66 pp 229533.V6R3J1
119960 229533.I1RC66 pp 229533.I1RTH6
119961 229533.I1RC66 pp 229533.I1RGA4
119962 229533.I1RC66 pp 229533.I1S9Y1
119963 229533.I1RC66 pp 229533.I1RRT8
119964 229533.I1RC66 pp 229533.I1S8R3
119965 229533.I1RC66 pp 229533.I1RZB2
119966 229533.I1RC66 pp 229533.I1RJV4
119967 229533.I1RC66 pp 229533.I1RME4
119968 229533.I1RC66 pp 229533.I1RZD3
119969 229533.I1RC66 pp 229533.I1RT40
119970 229533.I1RC66 pp 229533.I1S5R1
119971 229533.I1RC66 pp 229533.I1S419
119972 229533.I1RC66 pp 229533.O74638
119973 229533.I1RC66 pp 229533.I1RD17
119974 229533.I1RC66 pp 229533.I1RZM1
119975 229533.I1RC66 pp 229533.I1RPV3
119976 229533.I1RC66 pp 229533.I1RV91
119977 229533.I1RC66 pp 229533.I1RPM4
119978 229533.I1RC66 pp 229533.I1RXC2
119979 229533.I1RC66 pp 229533.I1S3G4
119980 229533.I1RC66 pp 229533.I1S6Y8
119981 229533.I1RC66 pp 229533.I1RLU6
119982 229533.I1RC66 pp 229533.I1RPE0
119983 229533.I1RC66 pp 229533.I1RL04
119984 229533.I1RC66 pp 229533.I1RNQ5
119985 229533.I1RC66 pp 229533.I1S504
119986 229533.I1RC66 pp 229533.I1RFS3
119987 229533.I1RC66 pp 229533.I1RQ23
119988 229533.I1RC66 pp 229533.I1S2H4
119989 229533.I1RC66 pp 229533.I1RQ85
119990 229533.I1RC66 pp 229533.I1RE22
119991 229533.I1RC66 pp 229533.I1S0M6
119992 229533.I1RC66 pp 229533.I1RRI1
119993 229533.I1RC66 pp 229533.V6R227
119994 229533.I1RC66 pp 229533.I1RG14
119995 229533.I1RC66 pp 229533.V6QUE6
119996 229533.I1RC66 pp 229533.I1RWI5
119997 229533.I1RC66 pp 229533.Q4I283
119998 229533.I1RC66 pp 229533.I1RQD2
119999 229533.I1RC66 pp 229533.I1RSQ4
120000 229533.I1RC66 pp 229533.I1RZZ9
120001 229533.I1RC66 pp 229533.I1RZ39
120002 229533.I1RC66 pp 229533.I1RW99
120003 229533.I1RC66 pp 229533.I1RR73
120004 229533.I1RC66 pp 229533.I1S5B0
120005 229533.I1RC66 pp 229533.I1RXC4
120006 229533.I1RC66 pp 229533.I1RZM8
120007 229533.I1RC66 pp 229533.V6R3X5
120008 229533.I1RC66 pp 229533.I1SA32
120009 229533.I1RC66 pp 229533.I1RQ81
120010 229533.I1RC66 pp 229533.I1RXF0
120011 229533.I1RC66 pp 229533.I1RI82
120012 229533.I1RC66 pp 229533.I1RJZ7

120013 229533.I1RC66 pp 229533.I1RS18
120014 229533.I1RC66 pp 229533.I1S6K9
120015 229533.I1RC66 pp 229533.Q4I8P5
120016 229533.I1RC66 pp 229533.I1S2B8
120017 229533.I1RC66 pp 229533.I1RWB0
120018 229533.I1RC66 pp 229533.I1RRM5
120019 229533.I1RC66 pp 229533.I1RE27
120020 229533.I1RC66 pp 229533.V6RCZ8
120021 229533.I1RC66 pp 229533.I1RE76
120022 229533.I1RC66 pp 229533.I1S1J7
120023 229533.I1RC66 pp 229533.I1RSZ7
120024 229533.I1RC66 pp 229533.V6RCG5
120025 229533.I1RC66 pp 229533.I1RRY9
120026 229533.I1RC66 pp 229533.I1RPR9
120027 229533.I1RC66 pp 229533.I1RR51
120028 229533.I1RC66 pp 229533.I1S235
120029 229533.I1RC66 pp 229533.I1RYT5
120030 229533.I1RC66 pp 229533.I1S0T2
120031 229533.I1RC66 pp 229533.I1RY03
120032 229533.I1RC66 pp 229533.I1RWR7
120033 229533.I1RC66 pp 229533.I1RX46
120034 229533.I1RC66 pp 229533.I1RYL5
120035 229533.I1RC66 pp 229533.I1S887
120036 229533.I1RC66 pp 229533.I1RCG4
120037 229533.I1RC66 pp 229533.I1RFU8
120038 229533.I1RC66 pp 229533.V6RTJ8
120039 229533.I1RC66 pp 229533.I1S4S7
120040 229533.I1RC66 pp 229533.Q4IJT5
120041 229533.I1RC66 pp 229533.I1S7A8
120042 229533.I1RC66 pp 229533.I1RZK5
120043 229533.I1RC66 pp 229533.I1RRT6
120044 229533.I1RC66 pp 229533.I1RYS6
120045 229533.I1RC66 pp 229533.I1RW71
120046 229533.I1RC66 pp 229533.I1RJT9
120047 229533.I1RC66 pp 229533.Q4I2J8
120048 229533.I1RC66 pp 229533.V6QW22
120049 229533.I1RC66 pp 229533.I1S0R7
120050 229533.I1RC66 pp 229533.I1RSQ7
120051 229533.I1RC66 pp 229533.I1RF19
120052 229533.I1RC66 pp 229533.I1RRX5
120053 229533.I1RC66 pp 229533.I1REL7
120054 229533.I1RC66 pp 229533.I1RCR5
120055 229533.I1RC66 pp 229533.I1RWJ8
120056 229533.I1RC66 pp 229533.I1RQC9
120057 229533.I1RC66 pp 229533.I1S4S4
120058 229533.I1RC66 pp 229533.I1RLP1
120059 229533.I1RC66 pp 229533.I1RXD7
120060 229533.I1RC66 pp 229533.I1REK8
120061 229533.I1RC66 pp 229533.Q4IJK2
120062 229533.I1RC66 pp 229533.I1RMG8
120063 229533.I1RC66 pp 229533.V6RUT5
120064 229533.I1RC66 pp 229533.I1RQQ5
120065 229533.I1RC66 pp 229533.I1RZZ1
120066 229533.I1RC66 pp 229533.I1RZJ5
120067 229533.I1RC66 pp 229533.I1RK95
120068 229533.I1RC66 pp 229533.I1RVQ0
120069 229533.I1RC66 pp 229533.I1RR24
120070 229533.I1RC66 pp 229533.I1S0X6
120071 229533.I1RC66 pp 229533.I1RCS1
120072 229533.I1RC66 pp 229533.I1RD52
120073 229533.I1RC66 pp 229533.I1S1W8
120074 229533.I1RC66 pp 229533.I1S0F5
120075 229533.I1RC66 pp 229533.Q4IPN9
120076 229533.I1RC66 pp 229533.I1RMC8
120077 229533.I1RC66 pp 229533.I1RJU3
120078 229533.I1RC66 pp 229533.I1S438
120079 229533.I1RC66 pp 229533.I1RW25
120080 229533.I1RC66 pp 229533.I1S0L3
120081 229533.I1RC66 pp 229533.V6R1K4
120082 229533.I1RC66 pp 229533.I1RX25
120083 229533.A0A098D985 pp 229533.I1RDG0
120084 229533.A0A098D985 pp 229533.A0A098DEC2
120085 229533.A0A098D985 pp 229533.I1RFJ9

120086 229533.A0A098D985 pp 229533.I1RZE5
120087 229533.A0A098D985 pp 229533.I1RA18
120088 229533.A0A098D985 pp 229533.I1RN28
120089 229533.A0A098D985 pp 229533.I1RT35
120090 229533.A0A098D985 pp 229533.I1RBZ3
120091 229533.A0A098D985 pp 229533.I1RYB9
120092 229533.A0A098D985 pp 229533.I1RWB5
120093 229533.A0A098D985 pp 229533.I1S021
120094 229533.A0A098D985 pp 229533.A0A1C3YMB5
120095 229533.A0A098D985 pp 229533.A0A1C3YM23
120096 229533.A0A098D985 pp 229533.I1RE45
120097 229533.A0A098D985 pp 229533.I1RGC2
120098 229533.A0A098D985 pp 229533.I1S9U2
120099 229533.A0A098D985 pp 229533.I1S7S9
120100 229533.A0A098D985 pp 229533.I1RCU3
120101 229533.A0A098D985 pp 229533.I1RWI0
120102 229533.A0A098D985 pp 229533.I1RWH5
120103 229533.A0A098D985 pp 229533.I1RND6
120104 229533.A0A098D985 pp 229533.A0A1C3YMW0
120105 229533.A0A098D985 pp 229533.A0A1C3YL15
120106 229533.A0A098D985 pp 229533.I1RAF8
120107 229533.A0A098D985 pp 229533.I1S8E2
120108 229533.I1RXH1 pp 229533.I1S3G4
120109 229533.I1RXH1 pp 229533.I1S5S3
120110 229533.I1RXH1 pp 229533.V6QWD8
120111 229533.I1RXH1 pp 229533.I1S1W8
120112 229533.I1RJZ4 pp 229533.I1RTB7
120113 229533.I1RJZ4 pp 229533.I1RQ81
120114 229533.I1RJZ4 pp 229533.Q4HW67
120115 229533.I1RFT6 pp 229533.I1RWD3
120116 229533.I1RFT6 pp 229533.I1RP88
120117 229533.I1RFT6 pp 229533.I1RYW3
120118 229533.I1RFT6 pp 229533.I1RNC1
120119 229533.I1RFT6 pp 229533.Q4I7K4
120120 229533.I1RFT6 pp 229533.I1S1C7
120121 229533.I1RFT6 pp 229533.I1RWN6
120122 229533.I1RFT6 pp 229533.I1S0N4
120123 229533.I1RFT6 pp 229533.I1RNB8
120124 229533.I1RFT6 pp 229533.I1RPP4
120125 229533.I1RFT6 pp 229533.I1RK92
120126 229533.I1RFT6 pp 229533.I1RSD4
120127 229533.I1RFT6 pp 229533.I1RT03
120128 229533.I1RFT6 pp 229533.I1RJV3
120129 229533.I1RFT6 pp 229533.I1RSK1
120130 229533.I1RFT6 pp 229533.I1S2E8
120131 229533.I1RFT6 pp 229533.I1RSV2
120132 229533.I1RFT6 pp 229533.I1RPP3
120133 229533.I1RFT6 pp 229533.V6R042
120134 229533.I1RFT6 pp 229533.I1RG90
120135 229533.I1RFT6 pp 229533.I1S4M5
120136 229533.I1RFT6 pp 229533.I1RSQ3
120137 229533.I1RFT6 pp 229533.V6RMW6
120138 229533.I1RFT6 pp 229533.I1S2C0
120139 229533.I1RFT6 pp 229533.I1RN67
120140 229533.I1RFT6 pp 229533.I1RTH6
120141 229533.I1RFT6 pp 229533.I1RN63
120142 229533.I1RFT6 pp 229533.I1RT04
120143 229533.I1RFT6 pp 229533.I1RNN1
120144 229533.I1RFT6 pp 229533.I1RRV1
120145 229533.I1RFT6 pp 229533.I1RS20
120146 229533.I1RFT6 pp 229533.I1RWI5
120147 229533.I1RFT6 pp 229533.I1RM82
120148 229533.I1RFT6 pp 229533.I1RP27
120149 229533.I1RFT6 pp 229533.I1S503
120150 229533.I1RFT6 pp 229533.I1RPU3
120151 229533.I1RFT6 pp 229533.I1RR73
120152 229533.I1RFT6 pp 229533.Q4IAA0
120153 229533.I1RFT6 pp 229533.I1S504
120154 229533.I1RFT6 pp 229533.I1RQ81
120155 229533.I1RFT6 pp 229533.I1RQ31
120156 229533.I1RFT6 pp 229533.I1RZM1
120157 229533.I1RFT6 pp 229533.I1RN58
120158 229533.I1RFT6 pp 229533.V6QUE6

120159 229533.I1RFT6 pp 229533.Q4IEK8
120160 229533.I1RFT6 pp 229533.I1RRU9
120161 229533.I1RFT6 pp 229533.I1RQC9
120162 229533.I1RFT6 pp 229533.I1RT35
120163 229533.I1RFT6 pp 229533.V6RTJ8
120164 229533.I1RFT6 pp 229533.I1RWD7
120165 229533.I1RFT6 pp 229533.I1RN61
120166 229533.I1RFT6 pp 229533.I1RPW4
120167 229533.I1RFT6 pp 229533.I1RQM6
120168 229533.I1RFT6 pp 229533.I1RRG3
120169 229533.A0A1C3YJC0 pp 229533.I1RB22
120170 229533.A0A1C3YJC0 pp 229533.I1RL88
120171 229533.A0A1C3YJC0 pp 229533.I1RWG1
120172 229533.A0A1C3YJC0 pp 229533.I1RNG8
120173 229533.A0A1C3YJC0 pp 229533.I1RN72
120174 229533.A0A1C3YJC0 pp 229533.I1RJM4
120175 229533.A0A1C3YJC0 pp 229533.I1RU50
120176 229533.A0A1C3YJC0 pp 229533.I1R9U3
120177 229533.A0A1C3YJC0 pp 229533.I1S4N8
120178 229533.A0A1C3YJC0 pp 229533.I1S5U9
120179 229533.A0A1C3YJC0 pp 229533.I1RKJ2
120180 229533.A0A1C3YJC0 pp 229533.I1RHA9
120181 229533.A0A1C3YJC0 pp 229533.I1S4N7
120182 229533.A0A1C3YJC0 pp 229533.I1S4P4
120183 229533.A0A1C3YJC0 pp 229533.I1S5L1
120184 229533.A0A1C3YJC0 pp 229533.I1RZP6
120185 229533.A0A1C3YJC0 pp 229533.I1S0L8
120186 229533.A0A1C3YJC0 pp 229533.I1RUP2
120187 229533.A0A1C3YJC0 pp 229533.A0A1C3YME0
120188 229533.A0A1C3YJC0 pp 229533.I1RJM9
120189 229533.A0A1C3YJC0 pp 229533.I1RE51
120190 229533.A0A1C3YJC0 pp 229533.I1RPS0
120191 229533.A0A1C3YJC0 pp 229533.I1RUU5
120192 229533.A0A1C3YJC0 pp 229533.A0A1C3YKY5
120193 229533.A0A1C3YJC0 pp 229533.A0A1C3YM54
120194 229533.A0A1C3YJC0 pp 229533.A0A1C3YLW8
120195 229533.A0A1C3YJC0 pp 229533.I1RU70
120196 229533.A0A1C3YJC0 pp 229533.I1RZP7
120197 229533.A0A1C3YJC0 pp 229533.A0A1C3YJT3
120198 229533.A0A1C3YJC0 pp 229533.I1RC43
120199 229533.A0A1C3YJC0 pp 229533.A0A1C3YM82
120200 229533.A0A1C3YJC0 pp 229533.I1RCX1
120201 229533.A0A1C3YJC0 pp 229533.I1RYM3
120202 229533.A0A1C3YJC0 pp 229533.I1R9U0
120203 229533.A0A1C3YJC0 pp 229533.I1RQG7
120204 229533.I1RYB8 pp 229533.I1RYP2
120205 229533.I1RYB8 pp 229533.I1RYL5
120206 229533.I1RYB8 pp 229533.I1S235
120207 229533.I1RYB8 pp 229533.I1RZK1
120208 229533.I1RYB8 pp 229533.I1S3G4
120209 229533.I1RYB8 pp 229533.V6QX21
120210 229533.I1RYB8 pp 229533.V6QUV7
120211 229533.I1RYB8 pp 229533.I1S1W8
120212 229533.I1RYB8 pp 229533.V6RKY8
120213 229533.I1RYB8 pp 229533.I1S4Z7
120214 229533.A0A1C3YMI5 pp 229533.I1RFQ7
120215 229533.A0A1C3YMI5 pp 229533.I1RSQ7
120216 229533.A0A1C3YMI5 pp 229533.I1RC20
120217 229533.A0A1C3YMI5 pp 229533.I1RN61
120218 229533.A0A1C3YMI5 pp 229533.I1RBC9
120219 229533.A0A1C3YMI5 pp 229533.I1RQH3
120220 229533.A0A1C3YMI5 pp 229533.I1RME6
120221 229533.A0A1C3YMI5 pp 229533.V6QUW4
120222 229533.A0A1C3YMI5 pp 229533.I1RPW4
120223 229533.A0A1C3YMI5 pp 229533.I1RXD7
120224 229533.A0A1C3YMI5 pp 229533.I1RQC9
120225 229533.A0A1C3YMI5 pp 229533.I1RW25
120226 229533.A0A1C3YMI5 pp 229533.I1RZZ1
120227 229533.A0A1C3YMI5 pp 229533.Q4IJK2
120228 229533.A0A1C3YMI5 pp 229533.I1RBT1
120229 229533.A0A1C3YMI5 pp 229533.I1RIG2
120230 229533.A0A1C3YMI5 pp 229533.I1S0R7
120231 229533.A0A1C3YMI5 pp 229533.I1R9X2

120232 229533.A0A1C3YMI5 pp 229533.I1RQA3
120233 229533.A0A1C3YMI5 pp 229533.I1RXU7
120234 229533.A0A1C3YMI5 pp 229533.I1RWD7
120235 229533.A0A1C3YMI5 pp 229533.I1RWT6
120236 229533.A0A1C3YMI5 pp 229533.I1S504
120237 229533.A0A1C3YMI5 pp 229533.V6RTJ8
120238 229533.A0A1C3YMI5 pp 229533.I1RM35
120239 229533.A0A1C3YMI5 pp 229533.I1RAX6
120240 229533.A0A1C3YMI5 pp 229533.I1RX46
120241 229533.A0A1C3YMI5 pp 229533.I1RSI0
120242 229533.A0A1C3YMI5 pp 229533.I1RZK5
120243 229533.A0A1C3YMI5 pp 229533.I1RJL2
120244 229533.A0A1C3YMI5 pp 229533.I1RNQ5
120245 229533.A0A1C3YMI5 pp 229533.I1RLU6
120246 229533.A0A1C3YMI5 pp 229533.I1RZM1
120247 229533.A0A1C3YMI5 pp 229533.Q4IJM4
120248 229533.A0A1C3YMI5 pp 229533.I1RFU8
120249 229533.A0A1C3YMI5 pp 229533.I1RN58
120250 229533.A0A1C3YMI5 pp 229533.I1RY03
120251 229533.A0A1C3YMI5 pp 229533.V6R3X5
120252 229533.A0A1C3YMI5 pp 229533.I1S044
120253 229533.A0A1C3YMI5 pp 229533.I1RRY9
120254 229533.A0A1C3YMI5 pp 229533.I1RR73
120255 229533.A0A1C3YMI5 pp 229533.I1RC17
120256 229533.A0A1C3YMI5 pp 229533.I1S1J7
120257 229533.A0A1C3YMI5 pp 229533.I1RRU9
120258 229533.A0A1C3YMI5 pp 229533.I1RZ39
120259 229533.A0A1C3YMI5 pp 229533.Q4IRI9
120260 229533.A0A1C3YMI5 pp 229533.I1RFT6
120261 229533.A0A1C3YMI5 pp 229533.I1RFT2
120262 229533.A0A1C3YMI5 pp 229533.I1RAU5
120263 229533.A0A1C3YMI5 pp 229533.I1RNN1
120264 229533.A0A1C3YMI5 pp 229533.I1RYX5
120265 229533.A0A1C3YMI5 pp 229533.I1RQ81
120266 229533.A0A1C3YMI5 pp 229533.I1RWI5
120267 229533.A0A1C3YMI5 pp 229533.I1RA52
120268 229533.A0A1C3YMI5 pp 229533.I1RAW0
120269 229533.A0A1C3YMI5 pp 229533.I1RE76
120270 229533.A0A1C3YMI5 pp 229533.I1RSK1
120271 229533.A0A1C3YMI5 pp 229533.I1RS20
120272 229533.A0A1C3YMI5 pp 229533.I1RPU3
120273 229533.A0A1C3YMI5 pp 229533.I1RJV3
120274 229533.A0A1C3YMI5 pp 229533.I1R9M0
120275 229533.A0A1C3YMI5 pp 229533.I1RWL2
120276 229533.A0A1C3YMI5 pp 229533.I1RCM3
120277 229533.A0A1C3YMI5 pp 229533.I1S2B8
120278 229533.A0A1C3YMI5 pp 229533.I1RPP4
120279 229533.A0A1C3YMI5 pp 229533.I1RM82
120280 229533.A0A1C3YMI5 pp 229533.I1RV91
120281 229533.A0A1C3YMI5 pp 229533.I1RS17
120282 229533.A0A1C3YMI5 pp 229533.I1RPP3
120283 229533.A0A1C3YMI5 pp 229533.I1RCJ7
120284 229533.A0A1C3YMI5 pp 229533.I1RYI5
120285 229533.A0A1C3YMI5 pp 229533.I1S0J1
120286 229533.A0A1C3YMI5 pp 229533.I1RWE8
120287 229533.A0A1C3YMI5 pp 229533.I1RP27
120288 229533.A0A1C3YMI5 pp 229533.I1RSV2
120289 229533.A0A1C3YMI5 pp 229533.I1RGA4
120290 229533.A0A1C3YMI5 pp 229533.I1S503
120291 229533.A0A1C3YMI5 pp 229533.I1RY44
120292 229533.A0A1C3YMI5 pp 229533.I1RWN5
120293 229533.A0A1C3YMI5 pp 229533.I1RNR2
120294 229533.A0A1C3YMI5 pp 229533.V6RMW6
120295 229533.A0A1C3YMI5 pp 229533.I1RAS2
120296 229533.A0A1C3YMI5 pp 229533.I1RN63
120297 229533.A0A1C3YMI5 pp 229533.I1RVX0
120298 229533.A0A1C3YMI5 pp 229533.I1RT03
120299 229533.A0A1C3YMI5 pp 229533.I1RBS0
120300 229533.A0A1C3YMI5 pp 229533.I1RG90
120301 229533.A0A1C3YMI5 pp 229533.I1RAU7
120302 229533.A0A1C3YMI5 pp 229533.V6R042
120303 229533.A0A1C3YMI5 pp 229533.I1RN67
120304 229533.A0A1C3YMI5 pp 229533.I1RB41

120305 229533.A0A1C3YMI5 pp 229533.I1RSQ3
120306 229533.A0A1C3YMI5 pp 229533.I1S2C0
120307 229533.A0A1C3YMI5 pp 229533.I1S2E9
120308 229533.A0A1C3YMI5 pp 229533.I1RJP4
120309 229533.A0A1C3YMI5 pp 229533.I1RA70
120310 229533.A0A1C3YMI5 pp 229533.I1RT04
120311 229533.A0A1C3YMI5 pp 229533.I1RRV1
120312 229533.A0A1C3YMI5 pp 229533.I1RTH6
120313 229533.A0A1C3YMI5 pp 229533.I1RNC1
120314 229533.A0A1C3YMI5 pp 229533.I1RYW3
120315 229533.A0A1C3YMI5 pp 229533.I1RTD5
120316 229533.A0A1C3YMI5 pp 229533.I1RCR8
120317 229533.A0A1C3YMI5 pp 229533.I1S1C7
120318 229533.A0A1C3YMI5 pp 229533.I1S0N4
120319 229533.A0A1C3YMI5 pp 229533.I1RG11
120320 229533.A0A1C3YMI5 pp 229533.I1RWN6
120321 229533.A0A1C3YMI5 pp 229533.I1RD22
120322 229533.A0A098E3Y7 pp 229533.I1RQS1
120323 229533.A0A098E3Y7 pp 229533.I1RQL4
120324 229533.A0A098E3Y7 pp 229533.A0A1C3YJG7
120325 229533.I1RF64 pp 229533.I1RKM2
120326 229533.I1RF64 pp 229533.I1RMH0
120327 229533.I1RF64 pp 229533.I1RLH5
120328 229533.I1RTA6 pp 229533.I1S5L1
120329 229533.I1RTA6 pp 229533.I1RTG9
120330 229533.I1RTA6 pp 229533.I1RUU5
120331 229533.I1RTA6 pp 229533.I1RZP7
120332 229533.I1RTA6 pp 229533.I1S0L8
120333 229533.I1RTA6 pp 229533.I1S4N7
120334 229533.I1RTA6 pp 229533.I1S4P4
120335 229533.I1RTA6 pp 229533.I1S4N8
120336 229533.I1RTA6 pp 229533.I1RWG1
120337 229533.I1RTA6 pp 229533.I1RYM3
120338 229533.I1RTA6 pp 229533.I1RUP2
120339 229533.I1RTA6 pp 229533.I1RU50
120340 229533.I1RTA6 pp 229533.I1RU70
120341 229533.I1RTA6 pp 229533.I1RZP6
120342 229533.I1RTA6 pp 229533.I1S5U9
120343 229533.I1RTA6 pp 229533.I1S1V1
120344 229533.I1RUG3 pp 229533.I1RZM8
120345 229533.I1RUG3 pp 229533.I1S5B0
120346 229533.I1RUG3 pp 229533.I1RW99
120347 229533.I1RUG3 pp 229533.I1S6K9
120348 229533.I1RUG3 pp 229533.V6RCZ8
120349 229533.I1RUG3 pp 229533.I1RXF0
120350 229533.I1RUG3 pp 229533.V6RCG5
120351 229533.I1RUG3 pp 229533.I1S887
120352 229533.I1RUG3 pp 229533.Q4I5Z5
120353 229533.I1RUG3 pp 229533.I1S419
120354 229533.I1RUG3 pp 229533.I1RZK5
120355 229533.I1RUG3 pp 229533.Q4I2J8
120356 229533.I1RUG3 pp 229533.Q4IEK8
120357 229533.I1RUG3 pp 229533.I1RZM1
120358 229533.I1RUG3 pp 229533.I1S4Q4
120359 229533.I1RUG3 pp 229533.I1S504
120360 229533.I1RUG3 pp 229533.V6R227
120361 229533.I1RUG3 pp 229533.I1RWT6
120362 229533.I1RUG3 pp 229533.I1RWD7
120363 229533.I1RUG3 pp 229533.I1S1J7
120364 229533.I1RUG3 pp 229533.Q4IJK2
120365 229533.I1RUG3 pp 229533.I1RWB0
120366 229533.I1RUG3 pp 229533.Q4I283
120367 229533.I1RUG3 pp 229533.V6RBN2
120368 229533.I1RUG3 pp 229533.I1RWR7
120369 229533.I1RUG3 pp 229533.I1RZZ1
120370 229533.I1RUG3 pp 229533.I1RYS0
120371 229533.I1RUG3 pp 229533.I1RXC4
120372 229533.I1RUG3 pp 229533.I1RY63
120373 229533.I1RUG3 pp 229533.I1RY03
120374 229533.I1RUG3 pp 229533.I1SA32
120375 229533.I1RUG3 pp 229533.I1RZH4
120376 229533.I1RUG3 pp 229533.I1RX25
120377 229533.I1RUG3 pp 229533.Q4I8P5

120378	229533.I1RUG3	pp	229533.V6QUE6
120379	229533.I1RUG3	pp	229533.I1RWJ8
120380	229533.I1RUG3	pp	229533.I1RW07
120381	229533.I1RUG3	pp	229533.I1S0R7
120382	229533.I1RUG3	pp	229533.I1S4S4
120383	229533.I1RUG3	pp	229533.I1RXD7
120384	229533.I1RUG3	pp	229533.V6QYF0
120385	229533.I1RUG3	pp	229533.I1RUQ2
120386	229533.I1RUG3	pp	229533.I1S1W8
120387	229533.I1RUG3	pp	229533.I1S0X6
120388	229533.I1RUG3	pp	229533.V6RUT5
120389	229533.I1RUG3	pp	229533.I1RZJ5
120390	229533.I1RUG3	pp	229533.I1RWD4
120391	229533.I1RUG3	pp	229533.V6QUW4
120392	229533.I1RUG3	pp	229533.V6R4J1
120393	229533.I1RUG3	pp	229533.I1S0F1
120394	229533.I1RUG3	pp	229533.I1S438
120395	229533.I1RUG3	pp	229533.I1RVQ0
120396	229533.I1RUG3	pp	229533.I1S0T2
120397	229533.I1RUG3	pp	229533.I1RW25
120398	229533.I1RUG3	pp	229533.I1RXU9
120399	229533.I1RUG3	pp	229533.I1RX46
120400	229533.I1RUG3	pp	229533.V6RTJ8
120401	229533.I1RUG3	pp	229533.I1RWN6
120402	229533.I1RUG3	pp	229533.I1S6F0
120403	229533.I1RUG3	pp	229533.I1S437
120404	229533.I1RUG3	pp	229533.I1S7A8
120405	229533.I1RUG3	pp	229533.I1RXD6
120406	229533.I1RUG3	pp	229533.I1S1C7
120407	229533.I1RUG3	pp	229533.I1RY86
120408	229533.I1RUG3	pp	229533.I1RYJ6
120409	229533.I1RUG3	pp	229533.I1S0L3
120410	229533.I1RUG3	pp	229533.I1RZ92
120411	229533.I1RUG3	pp	229533.I1RYW3
120412	229533.I1RUG3	pp	229533.I1RX04
120413	229533.I1RUG3	pp	229533.I1S0F5
120414	229533.I1RUG3	pp	229533.V6R1K4
120415	229533.I1RUG3	pp	229533.I1RZW8
120416	229533.I1RUG3	pp	229533.I1RX95
120417	229533.I1RUG3	pp	229533.V6R042
120418	229533.I1RUG3	pp	229533.I1RWX5
120419	229533.I1RUG3	pp	229533.Q4IJT5
120420	229533.I1RUG3	pp	229533.I1RZ79
120421	229533.I1RUG3	pp	229533.V6RMW6
120422	229533.I1RUG3	pp	229533.Q4HZB8
120423	229533.I1RUG3	pp	229533.I1S8R3
120424	229533.I1RUG3	pp	229533.I1S0F8
120425	229533.I1RUG3	pp	229533.V6RIV9
120426	229533.I1RUG3	pp	229533.I1S4S7
120427	229533.I1RUG3	pp	229533.I1RUG4
120428	229533.I1RUG3	pp	229533.V6RRH2
120429	229533.I1RUG3	pp	229533.I1RW71
120430	229533.I1RUG3	pp	229533.I1S1W9
120431	229533.I1RUG3	pp	229533.I1RWN5
120432	229533.I1RUG3	pp	229533.Q4I624
120433	229533.I1RUG3	pp	229533.I1S1W3
120434	229533.I1RUG3	pp	229533.V6QW22
120435	229533.I1RUG3	pp	229533.I1RXR1
120436	229533.I1RUG3	pp	229533.I1S569
120437	229533.I1RUG3	pp	229533.V6R057
120438	229533.I1RUG3	pp	229533.I1RVX0
120439	229533.I1RUG3	pp	229533.I1RZV3
120440	229533.I1RUG3	pp	229533.I1RY44
120441	229533.I1RUG3	pp	229533.I1RXC2
120442	229533.I1RUG3	pp	229533.I1S9Y1
120443	229533.I1RUG3	pp	229533.O74638
120444	229533.I1RUG3	pp	229533.I1RVZ7
120445	229533.I1RUG3	pp	229533.I1SA33
120446	229533.I1RUG3	pp	229533.I1S3G4
120447	229533.I1RUG3	pp	229533.I1RWQ8
120448	229533.I1RUG3	pp	229533.I1RW46
120449	229533.I1RUG3	pp	229533.I1S5R6
120450	229533.I1RUG3	pp	229533.I1RZS7

120451	229533.I1RUG3	pp	229533.I1S0N4
120452	229533.I1RUG3	pp	229533.I1S2H4
120453	229533.I1RUG3	pp	229533.I1S0R2
120454	229533.I1RUG3	pp	229533.I1S0J1
120455	229533.I1RUG3	pp	229533.I1S0L4
120456	229533.I1RUG3	pp	229533.I1RWI5
120457	229533.I1RUG3	pp	229533.I1S7W5
120458	229533.I1RUG3	pp	229533.V6RGN5
120459	229533.I1RUG3	pp	229533.I1S2B8
120460	229533.I1RUG3	pp	229533.I1RZZ9
120461	229533.I1RUG3	pp	229533.I1RVF8
120462	229533.I1RUG3	pp	229533.I1S7S9
120463	229533.I1RUG3	pp	229533.I1RZB2
120464	229533.I1RUG3	pp	229533.V6R3X5
120465	229533.I1RUG3	pp	229533.I1RV91
120466	229533.I1RUG3	pp	229533.I1RZ39
120467	229533.I1RUG3	pp	229533.I1S4Z8
120468	229533.I1RUG3	pp	229533.I1SB04
120469	229533.I1RUG3	pp	229533.I1RXF8
120470	229533.I1RUG3	pp	229533.V6RUY9
120471	229533.I1RUG3	pp	229533.I1RW61
120472	229533.I1RUG3	pp	229533.I1S2E9
120473	229533.I1RUG3	pp	229533.I1RVZ2
120474	229533.I1RUG3	pp	229533.I1S6Y8
120475	229533.I1RUG3	pp	229533.I1S0F6
120476	229533.I1RUG3	pp	229533.I1S0M6
120477	229533.I1RUG3	pp	229533.I1S2M6
120478	229533.I1RUG3	pp	229533.I1S352
120479	229533.I1RUG3	pp	229533.I1S503
120480	229533.I1RUG3	pp	229533.V6R3J1
120481	229533.I1RUG3	pp	229533.I1RYI5
120482	229533.I1RUG3	pp	229533.I1S0W4
120483	229533.I1RUG3	pp	229533.I1S8P5
120484	229533.I1RUG3	pp	229533.I1S1B6
120485	229533.I1RUG3	pp	229533.I1S0F7
120486	229533.I1RUG3	pp	229533.I1RWK3
120487	229533.I1RUG3	pp	229533.I1RYR0
120488	229533.I1RUG3	pp	229533.I1RWS4
120489	229533.A0A098D978	pp	229533.A0A1C3YJC0
120490	229533.A0A098D978	pp	229533.A0A1C3YM61
120491	229533.A0A098D978	pp	229533.I1RTA6
120492	229533.I1RK00	pp	229533.Q4HW67
120493	229533.I1RK00	pp	229533.I1S5K9
120494	229533.I1RK00	pp	229533.I1RRQ0
120495	229533.I1RK00	pp	229533.I1RQP8
120496	229533.I1RK00	pp	229533.I1RZ40
120497	229533.I1RRE5	pp	229533.I1RRY9
120498	229533.I1RRE5	pp	229533.I1RWJ8
120499	229533.I1RRE5	pp	229533.I1S1J7
120500	229533.I1RRE5	pp	229533.I1RW25
120501	229533.I1RRE5	pp	229533.I1S0X6
120502	229533.I1RRE5	pp	229533.V6RTJ8
120503	229533.I1RRE5	pp	229533.I1RX46
120504	229533.I1RRE5	pp	229533.I1RT65
120505	229533.I1RRE5	pp	229533.I1RZK5
120506	229533.I1RRE5	pp	229533.I1RXD7
120507	229533.I1RRE5	pp	229533.I1RY03
120508	229533.I1RRE5	pp	229533.I1RVQ0
120509	229533.I1RRE5	pp	229533.I1S0R7
120510	229533.I1RRE5	pp	229533.Q5I2J3
120511	229533.I1RRE5	pp	229533.I1RWK3
120512	229533.I1RRE5	pp	229533.I1RZZ1
120513	229533.I1RRE5	pp	229533.V6R1K4
120514	229533.I1RRE5	pp	229533.Q4IJK2
120515	229533.I1RRE5	pp	229533.I1S0L3
120516	229533.I1RRE5	pp	229533.I1RW99
120517	229533.I1RRE5	pp	229533.I1S8P5
120518	229533.I1RRE5	pp	229533.I1RZW8
120519	229533.I1RRE5	pp	229533.I1RWN6
120520	229533.I1RRE5	pp	229533.I1RSV2
120521	229533.I1RRE5	pp	229533.I1S5B0
120522	229533.I1RRE5	pp	229533.V6QW22
120523	229533.I1RRE5	pp	229533.I1RSQ7

120524 229533.I1RRE5 pp 229533.I1RRI2
120525 229533.I1RRE5 pp 229533.Q4I8P5
120526 229533.I1RRE5 pp 229533.I1S1C7
120527 229533.I1RRE5 pp 229533.I1RYW3
120528 229533.I1RRE5 pp 229533.I1RZM8
120529 229533.I1RRE5 pp 229533.I1S5R1
120530 229533.I1RRE5 pp 229533.I1RS18
120531 229533.I1RRE5 pp 229533.I1S8R3
120532 229533.I1RRE5 pp 229533.I1RRT8
120533 229533.I1RRE5 pp 229533.V6RCG5
120534 229533.I1RRE5 pp 229533.I1S0L4
120535 229533.I1RRE5 pp 229533.Q4I624
120536 229533.I1RRE5 pp 229533.I1RTH6
120537 229533.I1RRE5 pp 229533.I1RZZ9
120538 229533.I1RRE5 pp 229533.V6RCZ8
120539 229533.I1RRE5 pp 229533.I1S2H4
120540 229533.I1RRE5 pp 229533.I1RZM1
120541 229533.I1RRE5 pp 229533.V6R057
120542 229533.I1RRE5 pp 229533.I1RVX0
120543 229533.I1RRE5 pp 229533.I1RT03
120544 229533.I1RRE5 pp 229533.I1RRN5
120545 229533.I1RRE5 pp 229533.I1RZ39
120546 229533.I1RRE5 pp 229533.V6R3X5
120547 229533.I1RRE5 pp 229533.I1SA33
120548 229533.I1RRE5 pp 229533.I1RRM5
120549 229533.I1RRE5 pp 229533.I1S2B8
120550 229533.I1RRE5 pp 229533.I1RTD5
120551 229533.I1RRE5 pp 229533.I1S503
120552 229533.I1RRE5 pp 229533.I1RWT6
120553 229533.I1RRE5 pp 229533.I1RWP4
120554 229533.I1RRE5 pp 229533.V6RMW6
120555 229533.I1RRE5 pp 229533.I1RUG3
120556 229533.I1RRE5 pp 229533.I1RT40
120557 229533.I1RRE5 pp 229533.I1RSQ3
120558 229533.I1RRE5 pp 229533.I1S2E9
120559 229533.I1RRE5 pp 229533.I1S504
120560 229533.I1RRE5 pp 229533.I1RV91
120561 229533.I1RRE5 pp 229533.I1RY44
120562 229533.I1RRE5 pp 229533.V6R042
120563 229533.I1RRE5 pp 229533.I1RT04
120564 229533.I1RRE5 pp 229533.I1SA32
120565 229533.I1RRE5 pp 229533.I1RWN5
120566 229533.I1RRE5 pp 229533.I1RS47
120567 229533.I1RRE5 pp 229533.I1S0F6
120568 229533.I1RRE5 pp 229533.V6RIV9
120569 229533.I1RRE5 pp 229533.I1RW46
120570 229533.I1RRE5 pp 229533.I1S0J1
120571 229533.I1RRE5 pp 229533.I1RTA0
120572 229533.I1RRE5 pp 229533.I1RS17
120573 229533.I1S3L2 pp 229533.I1S8R2
120574 229533.I1S3L2 pp 229533.I1S9T6
120575 229533.I1S3L2 pp 229533.I1S702
120576 229533.I1S3L2 pp 229533.V6RM02
120577 229533.I1S3L2 pp 229533.I1S8C1
120578 229533.I1S3L2 pp 229533.I1S6A2
120579 229533.I1S3L2 pp 229533.V6RK96
120580 229533.I1S3L2 pp 229533.V6R382
120581 229533.I1S3L2 pp 229533.I1S568
120582 229533.I1S345 pp 229533.Q4HW67
120583 229533.I1S4Z7 pp 229533.V6RXW9
120584 229533.I1S4Z7 pp 229533.Q4I5R9
120585 229533.I1S4Z7 pp 229533.Q5I2J3
120586 229533.I1S4Z7 pp 229533.I1S9X9
120587 229533.I1S4Z7 pp 229533.Q4I1Y5
120588 229533.I1S4Z7 pp 229533.Q4IN00
120589 229533.I1S4Z7 pp 229533.I1S7B8
120590 229533.I1S4Z7 pp 229533.Q4IEA7
120591 229533.I1S4Z7 pp 229533.Q4HZS8
120592 229533.I1S4Z7 pp 229533.V6RKY8
120593 229533.I1S4Z7 pp 229533.V6QUV7
120594 229533.I1S4Z7 pp 229533.I1S8P5
120595 229533.I1S1W4 pp 229533.Q4IJM4
120596 229533.I1S1W4 pp 229533.V6QV09

120597 229533.I1S1W4 pp 229533.I1S439
120598 229533.I1S1W4 pp 229533.Q4IJK2
120599 229533.I1S1W4 pp 229533.I1S2K7
120600 229533.I1S1W4 pp 229533.V6RVG3
120601 229533.I1S1W4 pp 229533.I1S504
120602 229533.I1S1W4 pp 229533.I1S5M1
120603 229533.I1S1W4 pp 229533.I1S224
120604 229533.I1S1W4 pp 229533.V6RMW6
120605 229533.I1S1W4 pp 229533.V6RRH2
120606 229533.I1S1W4 pp 229533.I1S503
120607 229533.I1S1W4 pp 229533.V6RTJ8
120608 229533.I1S1W4 pp 229533.I1S2E9
120609 229533.I1S1W4 pp 229533.V6RKY7
120610 229533.I1S1W4 pp 229533.I1S2B8
120611 229533.I1S1W4 pp 229533.V6R3X5
120612 229533.I1S1W4 pp 229533.V6RGI1
120613 229533.I1S1W4 pp 229533.I1S549
120614 229533.I1RQH5 pp 229533.I1RR65
120615 229533.I1RQH5 pp 229533.I1S2A1
120616 229533.I1RQH5 pp 229533.I1RS29
120617 229533.I1RQH5 pp 229533.I1S8F0
120618 229533.I1RQH5 pp 229533.I1S0C2
120619 229533.I1RQH5 pp 229533.V6QWB1
120620 229533.I1RQH5 pp 229533.I1RYV4
120621 229533.I1RQH5 pp 229533.V6RN69
120622 229533.I1RQH5 pp 229533.I1RWA3
120623 229533.I1RQH5 pp 229533.V6R7V8
120624 229533.I1RQH5 pp 229533.I1RXD9
120625 229533.A0A1C3YKM8 pp 229533.I1RC49
120626 229533.A0A1C3YKM8 pp 229533.I1RS90
120627 229533.A0A1C3YKM8 pp 229533.V6RGI1
120628 229533.A0A1C3YKM8 pp 229533.I1RMV0
120629 229533.A0A1C3YKM8 pp 229533.I1RG12
120630 229533.A0A1C3YKM8 pp 229533.I1RBA6
120631 229533.A0A1C3YKM8 pp 229533.I1RWC9
120632 229533.A0A1C3YKM8 pp 229533.I1RAU9
120633 229533.A0A1C3YKM8 pp 229533.I1RCK9
120634 229533.I1RRS1 pp 229533.Q4HW67
120635 229533.I1RNN1 pp 229533.V6RIV9
120636 229533.I1RNN1 pp 229533.I1RZM1
120637 229533.I1RNN1 pp 229533.I1RWT6
120638 229533.I1RNN1 pp 229533.Q4I283
120639 229533.I1RNN1 pp 229533.I1RWN5
120640 229533.I1RNN1 pp 229533.I1S1W4
120641 229533.I1RNN1 pp 229533.I1S504
120642 229533.I1RNN1 pp 229533.I1RPP4
120643 229533.I1RNN1 pp 229533.I1RTA0
120644 229533.I1RNN1 pp 229533.I1S0J1
120645 229533.I1RNN1 pp 229533.I1RSI0
120646 229533.I1RNN1 pp 229533.I1RQ81
120647 229533.I1RNN1 pp 229533.I1RS18
120648 229533.I1RNN1 pp 229533.I1RNQ5
120649 229533.I1RNN1 pp 229533.I1RRU9
120650 229533.I1RNN1 pp 229533.V6RBN2
120651 229533.I1RNN1 pp 229533.I1S0N4
120652 229533.I1RNN1 pp 229533.I1RZZ1
120653 229533.I1RNN1 pp 229533.I1RTH6
120654 229533.I1RNN1 pp 229533.I1S5B0
120655 229533.I1RNN1 pp 229533.I1RY44
120656 229533.I1RNN1 pp 229533.I1RQE7
120657 229533.I1RNN1 pp 229533.I1RZB5
120658 229533.I1RNN1 pp 229533.Q4IJK2
120659 229533.I1RNN1 pp 229533.V6R042
120660 229533.I1RNN1 pp 229533.I1RQA3
120661 229533.I1RNN1 pp 229533.I1S0L3
120662 229533.I1RNN1 pp 229533.I1RP28
120663 229533.I1RNN1 pp 229533.I1RSQ3
120664 229533.I1RNN1 pp 229533.V6RMW6
120665 229533.I1RNN1 pp 229533.I1RVX0
120666 229533.I1RNN1 pp 229533.I1RSI6
120667 229533.I1RNN1 pp 229533.I1S2G7
120668 229533.I1RNN1 pp 229533.I1RT03
120669 229533.I1RNN1 pp 229533.I1RRV1

120670	229533.I1RNN1	pp	229533.Q4IJM4
120671	229533.I1RNN1	pp	229533.I1RWK3
120672	229533.I1RNN1	pp	229533.I1S8P5
120673	229533.I1RNN1	pp	229533.I1RSK1
120674	229533.I1RNN1	pp	229533.I1RWN6
120675	229533.I1RNN1	pp	229533.I1RPP3
120676	229533.I1RNN1	pp	229533.I1RXU7
120677	229533.I1RNN1	pp	229533.V6QW22
120678	229533.I1RNN1	pp	229533.I1RRY9
120679	229533.I1RNN1	pp	229533.I1RTD5
120680	229533.I1RNN1	pp	229533.I1RSQ7
120681	229533.I1RNN1	pp	229533.I1RQH3
120682	229533.I1RNN1	pp	229533.I1S1C7
120683	229533.I1RNN1	pp	229533.I1RXD7
120684	229533.I1RNN1	pp	229533.I1RYY5
120685	229533.I1RNN1	pp	229533.I1RSV2
120686	229533.I1RNN1	pp	229533.I1S2E9
120687	229533.I1RNN1	pp	229533.I1RW71
120688	229533.I1RNN1	pp	229533.I1RQC9
120689	229533.I1RNN1	pp	229533.I1RYW3
120690	229533.I1RNN1	pp	229533.I1RT04
120691	229533.I1RNN1	pp	229533.I1RX46
120692	229533.I1RNN1	pp	229533.I1RY03
120693	229533.I1RNN1	pp	229533.I1S0R7
120694	229533.I1RNN1	pp	229533.I1RZK5
120695	229533.I1RNN1	pp	229533.I1S503
120696	229533.I1RNN1	pp	229533.V6RTJ8
120697	229533.I1RNN1	pp	229533.I1S2H4
120698	229533.I1RNN1	pp	229533.I1RWI5
120699	229533.I1RNN1	pp	229533.I1RP27
120700	229533.I1RNN1	pp	229533.I1RPU3
120701	229533.I1RNN1	pp	229533.I1S2B8
120702	229533.I1RNN1	pp	229533.I1RZZ9
120703	229533.I1RNN1	pp	229533.I1RW25
120704	229533.I1RNN1	pp	229533.I1RWL2
120705	229533.I1RNN1	pp	229533.I1RR73
120706	229533.I1RNN1	pp	229533.I1RZ39
120707	229533.I1RNN1	pp	229533.Q4IR09
120708	229533.I1RNN1	pp	229533.V6R3X5
120709	229533.I1RXS0	pp	229533.I1S6A2
120710	229533.I1RXS0	pp	229533.I1RYI1
120711	229533.I1RXS0	pp	229533.V6RK96
120712	229533.I1RXS0	pp	229533.I1S8C1
120713	229533.I1RXS0	pp	229533.I1RZM4
120714	229533.I1RXS0	pp	229533.I1S8R2
120715	229533.I1RXS0	pp	229533.I1RXY7
120716	229533.I1RXS0	pp	229533.I1S568
120717	229533.I1RXS0	pp	229533.I1S1D7
120718	229533.I1RXS0	pp	229533.V6RM02
120719	229533.I1RXS0	pp	229533.I1S299
120720	229533.I1RXS0	pp	229533.I1S702
120721	229533.I1RXS0	pp	229533.V6R382
120722	229533.I1RXS0	pp	229533.I1S9T6
120723	229533.I1RXS0	pp	229533.I1RY72
120724	229533.Q4I8S2	pp	229533.Q4ICI6
120725	229533.I1RQ38	pp	229533.I1S4Z7
120726	229533.I1RQ38	pp	229533.I1RYP2
120727	229533.I1RQ38	pp	229533.V6RKY8
120728	229533.I1RQ38	pp	229533.I1RYB8
120729	229533.I1RQ38	pp	229533.I1RZK1
120730	229533.I1RQ38	pp	229533.V6QX21
120731	229533.I1RQ38	pp	229533.I1RQD6
120732	229533.I1RQ38	pp	229533.V6QUV7
120733	229533.I1RQ38	pp	229533.I1S3G4
120734	229533.I1RQ38	pp	229533.I1RYL5
120735	229533.I1RQ38	pp	229533.I1RXF0
120736	229533.I1RQ38	pp	229533.I1S235
120737	229533.I1RQ38	pp	229533.I1S1W8
120738	229533.I1RBB7	pp	229533.I1RPV5
120739	229533.I1RBB7	pp	229533.I1RLV3
120740	229533.I1RBB7	pp	229533.Q4HW67
120741	229533.I1RBB7	pp	229533.I1RZ40
120742	229533.I1RBB7	pp	229533.I1RD69

120743	229533.I1RBB7	pp	229533.I1RIS3
120744	229533.I1RBB7	pp	229533.I1RN48
120745	229533.I1RBB7	pp	229533.I1RSI1
120746	229533.I1RBB7	pp	229533.I1S201
120747	229533.I1RBB7	pp	229533.I1REC2
120748	229533.I1RY44	pp	229533.I1RYZ9
120749	229533.I1RY44	pp	229533.V6QW22
120750	229533.I1RY44	pp	229533.I1S7A8
120751	229533.I1RY44	pp	229533.V6RTJ8
120752	229533.I1RY44	pp	229533.I1S0R7
120753	229533.I1RY44	pp	229533.I1S0F5
120754	229533.I1RY44	pp	229533.I1RYI3
120755	229533.I1RY44	pp	229533.V6R4J1
120756	229533.I1RY44	pp	229533.V6QV09
120757	229533.I1RY44	pp	229533.I1RZ51
120758	229533.I1RY44	pp	229533.I1S1W5
120759	229533.I1RY44	pp	229533.I1RZ12
120760	229533.I1RY44	pp	229533.I1RZ94
120761	229533.I1RY44	pp	229533.Q4IJT5
120762	229533.I1RY44	pp	229533.I1S0L3
120763	229533.I1RY44	pp	229533.I1S0X6
120764	229533.I1RY44	pp	229533.I1SA32
120765	229533.I1RY44	pp	229533.V6QUW4
120766	229533.I1RY44	pp	229533.Q4IJP1
120767	229533.I1RY44	pp	229533.I1S5B0
120768	229533.I1RY44	pp	229533.Q4IEA7
120769	229533.I1RY44	pp	229533.I1S604
120770	229533.I1RY44	pp	229533.Q4I5R3
120771	229533.I1RY44	pp	229533.I1RYH9
120772	229533.I1RY44	pp	229533.I1S021
120773	229533.I1RY44	pp	229533.I1S224
120774	229533.I1RY44	pp	229533.Q4ICG5
120775	229533.I1RY44	pp	229533.I1RZH4
120776	229533.I1RY44	pp	229533.V6RCG5
120777	229533.I1RY44	pp	229533.I1S334
120778	229533.I1RY44	pp	229533.I1RZM8
120779	229533.I1RY44	pp	229533.Q4I283
120780	229533.I1RY44	pp	229533.I1S0M9
120781	229533.I1RY44	pp	229533.Q4I8P5
120782	229533.I1RY44	pp	229533.I1RZM1
120783	229533.I1RY44	pp	229533.Q4INS6
120784	229533.I1RY44	pp	229533.V6QV38
120785	229533.I1RY44	pp	229533.I1RZF6
120786	229533.I1RY44	pp	229533.I1S0V6
120787	229533.I1RY44	pp	229533.V6RCZ8
120788	229533.I1RY44	pp	229533.I1S267
120789	229533.I1RY44	pp	229533.Q4HTT2
120790	229533.I1RY44	pp	229533.I1RZK5
120791	229533.I1RY44	pp	229533.I1RZK6
120792	229533.I1RY44	pp	229533.Q4I7N9
120793	229533.I1RY44	pp	229533.I1S504
120794	229533.I1RY44	pp	229533.I1RZZ1
120795	229533.I1RY44	pp	229533.Q4I2J8
120796	229533.I1RY44	pp	229533.Q4IJK2
120797	229533.I1RY44	pp	229533.I1RYY5
120798	229533.I1RY44	pp	229533.Q4IER8
120799	229533.I1RY44	pp	229533.I1RYZ0
120800	229533.I1RY44	pp	229533.Q4IR87
120801	229533.I1RY44	pp	229533.I1S0N8
120802	229533.I1RY44	pp	229533.I1RYV3
120803	229533.I1RY44	pp	229533.I1RZB5
120804	229533.I1RY44	pp	229533.I1S1J7
120805	229533.I1RY44	pp	229533.Q4IJM4
120806	229533.I1RY44	pp	229533.I1RZP1
120807	229533.I1RY44	pp	229533.I1S098
120808	229533.I1RY44	pp	229533.I1S7X9
120809	229533.I1RY44	pp	229533.I1RZE5
120810	229533.I1RY44	pp	229533.I1S439
120811	229533.I1RY44	pp	229533.I1RZ63
120812	229533.I1RY44	pp	229533.V6R283
120813	229533.I1RY44	pp	229533.I1S074
120814	229533.I1RY44	pp	229533.I1S503
120815	229533.I1RY44	pp	229533.I1RZZ9

229533.I1RY44	pp	229533.I1S0L4
229533.I1RY44	pp	229533.I1RZ39
229533.I1RY44	pp	229533.I1S352
229533.I1RY44	pp	229533.V6R3X5
229533.I1RY44	pp	229533.Q4I7L0
229533.I1RY44	pp	229533.I1S8P5
229533.I1RY44	pp	229533.I1S5R1
229533.I1RY44	pp	229533.I1S4R1
229533.I1RY44	pp	229533.I1S3H8
229533.I1RY44	pp	229533.I1S0J3
229533.I1RY44	pp	229533.I1S0F6
229533.I1RY44	pp	229533.Q4IN63
229533.I1RY44	pp	229533.I1S0M6
229533.I1RY44	pp	229533.I1S2H4
229533.I1RY44	pp	229533.I1S1V5
229533.I1RY44	pp	229533.I1S2G7
229533.I1RY44	pp	229533.I1RZS6
229533.I1RY44	pp	229533.V6RMW6
229533.I1RY44	pp	229533.V6R042
229533.I1RY44	pp	229533.I1S549
229533.I1RY44	pp	229533.I1S0X7
229533.I1RY44	pp	229533.I1S0M2
229533.I1RY44	pp	229533.I1SA33
229533.I1RY44	pp	229533.I1S2B8
229533.I1RY44	pp	229533.I1RZC3
229533.I1RY44	pp	229533.I1S004
229533.I1RY44	pp	229533.Q4HTT1
229533.I1RY44	pp	229533.V6RLW4
229533.I1RY44	pp	229533.I1S0V7
229533.I1RY44	pp	229533.I1S0N4
229533.I1RY44	pp	229533.V6RGI1
229533.I1RY44	pp	229533.I1SAF7
229533.I1RY44	pp	229533.V6RVG3
229533.I1RY44	pp	229533.I1RYW3
229533.I1RY44	pp	229533.I1S1C7
229533.I1RY44	pp	229533.I1S0J1
229533.I1RY44	pp	229533.I1S0J7
229533.I1RY44	pp	229533.Q4IB89
229533.I1RY44	pp	229533.I1S1Y7
229533.I1RY44	pp	229533.I1S0D1
229533.I1RY44	pp	229533.Q4HZB8
229533.I1RY44	pp	229533.V6RIV9
229533.I1RY44	pp	229533.I1S1A3
229533.I1RY44	pp	229533.I1RZK9
229533.I1RY44	pp	229533.I1RZE4
229533.I1RY44	pp	229533.I1RZP8
229533.I1RY44	pp	229533.V6RKY7
229533.I1RY44	pp	229533.I1RZT1
229533.I1RY44	pp	229533.I1S2C0
229533.I1RY44	pp	229533.I1RY58
229533.I1RY44	pp	229533.I1S8R3
229533.I1RY44	pp	229533.I1S2E8
229533.I1RY44	pp	229533.I1S1W4
229533.I1RY44	pp	229533.I1RZW8
229533.I1RY44	pp	229533.I1S286
229533.I1RY44	pp	229533.I1S276
229533.I1RY44	pp	229533.I1RZX3
229533.I1RY44	pp	229533.I1RYK6
229533.I1RY44	pp	229533.I1S0E8
229533.I1RY44	pp	229533.I1S0A6
229533.I1RY44	pp	229533.I1S757
229533.I1RY44	pp	229533.I1S569
229533.I1RQP2	pp	229533.I1RSK5
229533.I1RQP2	pp	229533.I1S702
229533.I1RQP2	pp	229533.I1RR29
229533.I1RQP2	pp	229533.I1RVR2
229533.I1RQP2	pp	229533.I1S9T6
229533.I1RQP2	pp	229533.I1RSS7
229533.I1RQP2	pp	229533.I1RW10
229533.I1RQP2	pp	229533.I1RYR0
229533.I1RQP2	pp	229533.I1RUR8
229533.I1RQP2	pp	229533.I1RY72
229533.I1RQP2	pp	229533.I1S299

120889 229533.I1RQP2 pp 229533.I1RVY1
120890 229533.I1RQP2 pp 229533.I1S8R2
120891 229533.I1RQP2 pp 229533.V6R382
120892 229533.I1RQP2 pp 229533.I1RWZ4
120893 229533.I1RQP2 pp 229533.I1RXY7
120894 229533.I1RQP2 pp 229533.V6RM02
120895 229533.I1RQP2 pp 229533.I1RVX9
120896 229533.I1RQP2 pp 229533.I1S1D7
120897 229533.I1RQP2 pp 229533.I1S6A2
120898 229533.I1RQP2 pp 229533.I1S8C1
120899 229533.I1RQP2 pp 229533.I1RZM4
120900 229533.I1RQP2 pp 229533.I1RUQ1
120901 229533.I1RQP2 pp 229533.I1S568
120902 229533.I1RQP2 pp 229533.V6RK96
120903 229533.I1RQP2 pp 229533.I1RUY6
120904 229533.I1RQP2 pp 229533.I1RYI1
120905 229533.I1RQP2 pp 229533.I1RUK0
120906 229533.I1RQP2 pp 229533.I1RT09
120907 229533.I1RQP2 pp 229533.I1RZF5
120908 229533.A0A098E3U6 pp 229533.I1RW71
120909 229533.A0A098E3U6 pp 229533.I1RJZ7
120910 229533.I1S2C0 pp 229533.Q4IJH1
120911 229533.I1S2C0 pp 229533.Q4ICA8
120912 229533.I1S2C0 pp 229533.I1S6F0
120913 229533.I1S2C0 pp 229533.I1S419
120914 229533.I1S2C0 pp 229533.Q4IFI0
120915 229533.I1S2C0 pp 229533.Q4IPI1
120916 229533.I1S2C0 pp 229533.Q4IEK8
120917 229533.I1S2C0 pp 229533.Q4HZ47
120918 229533.I1S2C0 pp 229533.I1S504
120919 229533.I1S2C0 pp 229533.I1S9U2
120920 229533.I1S2C0 pp 229533.V6R227
120921 229533.I1S2C0 pp 229533.Q4I5R9
120922 229533.I1S2C0 pp 229533.V6QYF0
120923 229533.I1S2C0 pp 229533.Q4IJ56
120924 229533.I1S2C0 pp 229533.Q4INZ9
120925 229533.I1S2C0 pp 229533.Q4HVW2
120926 229533.I1S2C0 pp 229533.I1S854
120927 229533.I1S2C0 pp 229533.Q4IAA0
120928 229533.I1S2C0 pp 229533.V6RTJ8
120929 229533.I1S2C0 pp 229533.Q4IEV5
120930 229533.I1S2C0 pp 229533.V6QUE6
120931 229533.I1S2C0 pp 229533.Q4HZ42
120932 229533.I1S2C0 pp 229533.Q4IBS2
120933 229533.I1S2C0 pp 229533.I1S503
120934 229533.I1S2C0 pp 229533.I1S2E9
120935 229533.I1S2C0 pp 229533.Q4HW67
120936 229533.I1S2C0 pp 229533.Q4IAS1
120937 229533.I1S2C0 pp 229533.Q4IP34
120938 229533.I1S2C0 pp 229533.Q4IQW0
120939 229533.I1S2C0 pp 229533.Q4HXF0
120940 229533.I1S2C0 pp 229533.Q4I948
120941 229533.I1S2C0 pp 229533.I1S6Y8
120942 229533.I1S2C0 pp 229533.V6R283
120943 229533.I1S2C0 pp 229533.Q4I7F9
120944 229533.I1S2C0 pp 229533.Q4I830
120945 229533.I1S2C0 pp 229533.V6RGN5
120946 229533.I1S2C0 pp 229533.Q4I662
120947 229533.I1S2C0 pp 229533.Q4HZ68
120948 229533.I1S2C0 pp 229533.Q4I7K4
120949 229533.I1S2C0 pp 229533.O74638
120950 229533.I1S2C0 pp 229533.Q4IF76
120951 229533.I1S2C0 pp 229533.Q4IN63
120952 229533.I1S2C0 pp 229533.I1S9Y1
120953 229533.I1S2C0 pp 229533.I1S2E8
120954 229533.I1S2C0 pp 229533.Q4I327
120955 229533.I1S2C0 pp 229533.I1S8E2
120956 229533.I1S2C0 pp 229533.V6RMW6
120957 229533.I1S2C0 pp 229533.V6RGY3
120958 229533.I1S2C0 pp 229533.Q4HWE2
120959 229533.I1S2C0 pp 229533.V6R3X5
120960 229533.I1S2C0 pp 229533.I1S855
120961 229533.I1S2C0 pp 229533.V6R042

120962	229533.I1S2C0	pp	229533.I1S4T3
120963	229533.I1S2C0	pp	229533.Q4HZK7
120964	229533.I1S2C0	pp	229533.Q4HZB8
120965	229533.I1S2C0	pp	229533.Q4IN00
120966	229533.I1RHZ5	pp	229533.I1RQE8
120967	229533.I1RHZ5	pp	229533.I1S8C1
120968	229533.I1RHZ5	pp	229533.I1RZM4
120969	229533.I1RHZ5	pp	229533.I1S568
120970	229533.I1RHZ5	pp	229533.I1RWZ4
120971	229533.I1RHZ5	pp	229533.I1RY72
120972	229533.I1RHZ5	pp	229533.I1RUY6
120973	229533.I1RHZ5	pp	229533.I1RVX9
120974	229533.I1RHZ5	pp	229533.I1RW10
120975	229533.I1RHZ5	pp	229533.I1RUK0
120976	229533.I1RHZ5	pp	229533.I1RYI1
120977	229533.I1RHZ5	pp	229533.I1RUR8
120978	229533.I1RHZ5	pp	229533.I1RVR2
120979	229533.I1RHZ5	pp	229533.I1RM26
120980	229533.I1RHZ5	pp	229533.I1S702
120981	229533.I1RHZ5	pp	229533.I1RLX1
120982	229533.I1RHZ5	pp	229533.I1RN88
120983	229533.I1RHZ5	pp	229533.I1RT09
120984	229533.I1RHZ5	pp	229533.V6RK96
120985	229533.I1RHZ5	pp	229533.I1RJY7
120986	229533.I1RHZ5	pp	229533.I1RHZ7
120987	229533.I1RHZ5	pp	229533.I1S299
120988	229533.I1RHZ5	pp	229533.I1RSK5
120989	229533.I1RHZ5	pp	229533.I1S6A2
120990	229533.I1RHZ5	pp	229533.I1RR29
120991	229533.I1RHZ5	pp	229533.I1RMJ3
120992	229533.I1RHZ5	pp	229533.I1RPR1
120993	229533.I1RHZ5	pp	229533.I1RKB2
120994	229533.I1RHZ5	pp	229533.I1S9T6
120995	229533.I1RHZ5	pp	229533.I1RJK3
120996	229533.I1RHZ5	pp	229533.V6RM02
120997	229533.I1RHZ5	pp	229533.I1S1D7
120998	229533.I1RHZ5	pp	229533.V6R382
120999	229533.I1RHZ5	pp	229533.I1RLE1
121000	229533.I1RHZ5	pp	229533.I1RVY1
121001	229533.I1RHZ5	pp	229533.I1RQ18
121002	229533.I1RHZ5	pp	229533.I1S8R2
121003	229533.I1RHZ5	pp	229533.I1RSS7
121004	229533.I1RHZ5	pp	229533.I1RXY7
121005	229533.I1RHZ5	pp	229533.I1RN84
121006	229533.I1RHZ5	pp	229533.I1RIB2
121007	229533.I1RHZ5	pp	229533.I1RML4
121008	229533.I1RA70	pp	229533.I1RN63
121009	229533.I1RA70	pp	229533.I1S8P5
121010	229533.I1RA70	pp	229533.I1RRJ8
121011	229533.I1RA70	pp	229533.I1S503
121012	229533.I1RA70	pp	229533.I1RJW6
121013	229533.I1RA70	pp	229533.I1RRZ0
121014	229533.I1RA70	pp	229533.I1RPP3
121015	229533.I1RA70	pp	229533.I1RWF7
121016	229533.I1RA70	pp	229533.I1RLN6
121017	229533.I1RA70	pp	229533.I1RJV4
121018	229533.I1RA70	pp	229533.I1RB74
121019	229533.I1RA70	pp	229533.I1RYI5
121020	229533.I1RA70	pp	229533.I1RP27
121021	229533.I1RA70	pp	229533.I1RJS2
121022	229533.I1RA70	pp	229533.I1RSV2
121023	229533.I1RA70	pp	229533.I1RFT1
121024	229533.I1RA70	pp	229533.I1RGA4
121025	229533.I1RA70	pp	229533.I1S352
121026	229533.I1RA70	pp	229533.I1RBC7
121027	229533.I1RA70	pp	229533.I1RBS6
121028	229533.I1RA70	pp	229533.I1RC05
121029	229533.I1RA70	pp	229533.Q4I662
121030	229533.I1RA70	pp	229533.I1RX22
121031	229533.I1RA70	pp	229533.I1RFS3
121032	229533.I1RA70	pp	229533.I1RBS0
121033	229533.I1RA70	pp	229533.I1S6Y8
121034	229533.I1RA70	pp	229533.I1S0F6

121035	229533.I1RA70	pp	229533.I1RN67
121036	229533.I1RA70	pp	229533.I1RPM4
121037	229533.I1RA70	pp	229533.I1RPV3
121038	229533.I1RA70	pp	229533.I1RNG4
121039	229533.I1RA70	pp	229533.I1S2G7
121040	229533.I1RA70	pp	229533.I1RVZ2
121041	229533.I1RA70	pp	229533.I1RTA1
121042	229533.I1RA70	pp	229533.I1RL04
121043	229533.I1RA70	pp	229533.I1RS47
121044	229533.I1RA70	pp	229533.I1RQ23
121045	229533.I1RA70	pp	229533.V6R283
121046	229533.I1RA70	pp	229533.I1RTD5
121047	229533.I1RA70	pp	229533.I1RSZ7
121048	229533.I1RA70	pp	229533.I1RWK3
121049	229533.I1RA70	pp	229533.I1RCJ7
121050	229533.I1RA70	pp	229533.I1RGF5
121051	229533.I1RA70	pp	229533.I1S0M6
121052	229533.I1RA70	pp	229533.I1RT40
121053	229533.I1RA70	pp	229533.I1RE22
121054	229533.I1RA70	pp	229533.I1RC29
121055	229533.I1RA70	pp	229533.I1RV91
121056	229533.I1RA70	pp	229533.I1RSQ4
121057	229533.I1RA70	pp	229533.I1RFL3
121058	229533.I1RA70	pp	229533.I1RZZ9
121059	229533.I1RA70	pp	229533.I1S7S9
121060	229533.I1RA70	pp	229533.I1S2E9
121061	229533.I1RA70	pp	229533.I1RWI5
121062	229533.I1RA70	pp	229533.I1RBY4
121063	229533.I1RA70	pp	229533.I1RT04
121064	229533.I1RA70	pp	229533.I1RE76
121065	229533.I1RA70	pp	229533.V6RUY9
121066	229533.I1RA70	pp	229533.V6R3X5
121067	229533.I1RA70	pp	229533.I1RPU3
121068	229533.I1RA70	pp	229533.I1RR73
121069	229533.I1RA70	pp	229533.I1RDE7
121070	229533.I1RA70	pp	229533.I1RRM5
121071	229533.I1RA70	pp	229533.I1RZ39
121072	229533.I1RA70	pp	229533.Q4HXF0
121073	229533.I1RA70	pp	229533.I1RNA5
121074	229533.I1RA70	pp	229533.I1RVY0
121075	229533.I1RA70	pp	229533.V6R3J1
121076	229533.I1RA70	pp	229533.I1RWL2
121077	229533.I1RA70	pp	229533.I1RXC2
121078	229533.I1RA70	pp	229533.I1S2B8
121079	229533.I1RA70	pp	229533.I1RJZ7
121080	229533.I1RA70	pp	229533.O74638
121081	229533.I1RA70	pp	229533.I1S277
121082	229533.I1RA70	pp	229533.I1RSX0
121083	229533.I1RA70	pp	229533.I1RY44
121084	229533.I1RA70	pp	229533.I1RFT6
121085	229533.I1RA70	pp	229533.I1RNN1
121086	229533.I1RA70	pp	229533.I1RRT8
121087	229533.I1RA70	pp	229533.I1S5R1
121088	229533.I1RA70	pp	229533.I1RC33
121089	229533.I1RA70	pp	229533.I1RC66
121090	229533.I1RA70	pp	229533.I1RPY1
121091	229533.I1RA70	pp	229533.I1RCH5
121092	229533.I1RA70	pp	229533.Q4HZB8
121093	229533.I1RA70	pp	229533.I1RN18
121094	229533.I1RA70	pp	229533.I1RM82
121095	229533.I1RA70	pp	229533.I1S9Y1
121096	229533.I1RA70	pp	229533.I1S1W9
121097	229533.I1RA70	pp	229533.I1RB92
121098	229533.I1RA70	pp	229533.I1RFL4
121099	229533.I1RA70	pp	229533.V6RIV9
121100	229533.I1RA70	pp	229533.I1RIL3
121101	229533.I1RA70	pp	229533.I1S0L4
121102	229533.I1RA70	pp	229533.I1RRN5
121103	229533.I1RA70	pp	229533.I1RWN5
121104	229533.I1RA70	pp	229533.I1RXR1
121105	229533.I1RA70	pp	229533.I1S2H4
121106	229533.I1RA70	pp	229533.I1S1W4
121107	229533.I1RA70	pp	229533.I1RSS4

121108 229533.I1RA70 pp 229533.I1RS20
121109 229533.I1RA70 pp 229533.I1RD64
121110 229533.I1RA70 pp 229533.I1RS06
121111 229533.I1RA70 pp 229533.I1S3G4
121112 229533.I1RA70 pp 229533.I1RRE5
121113 229533.I1RA70 pp 229533.I1RNY2
121114 229533.I1RA70 pp 229533.I1RSY2
121115 229533.I1RA70 pp 229533.Q4IN63
121116 229533.I1RA70 pp 229533.I1RC00
121117 229533.I1RA70 pp 229533.I1S0J1
121118 229533.I1RA70 pp 229533.I1RVX0
121119 229533.I1RA70 pp 229533.I1RT03
121120 229533.I1RA70 pp 229533.I1S7W5
121121 229533.I1RA70 pp 229533.I1RGL4
121122 229533.I1RA70 pp 229533.I1RCR8
121123 229533.I1RA70 pp 229533.I1RUG3
121124 229533.I1RA70 pp 229533.I1RB41
121125 229533.I1RA70 pp 229533.I1RXT3
121126 229533.I1RA70 pp 229533.I1RNC1
121127 229533.I1RA70 pp 229533.I1RTA0
121128 229533.I1RA70 pp 229533.V6RRH2
121129 229533.I1RA70 pp 229533.I1RAU7
121130 229533.I1RA70 pp 229533.I1RZS7
121131 229533.I1RA70 pp 229533.I1RE01
121132 229533.I1RA70 pp 229533.I1RW46
121133 229533.I1RA70 pp 229533.Q4I624
121134 229533.I1RA70 pp 229533.V6RMW6
121135 229533.I1RA70 pp 229533.I1RPU8
121136 229533.I1RA70 pp 229533.I1RD17
121137 229533.I1RA70 pp 229533.I1RMV0
121138 229533.I1RA70 pp 229533.V6R042
121139 229533.I1RA70 pp 229533.I1RPP4
121140 229533.I1RA70 pp 229533.V6R057
121141 229533.I1RA70 pp 229533.I1RPK0
121142 229533.I1RA70 pp 229533.I1RS17
121143 229533.I1RA70 pp 229533.I1RSQ3
121144 229533.I1RA70 pp 229533.I1S569
121145 229533.I1RA70 pp 229533.I1SAN9
121146 229533.I1RA70 pp 229533.I1RRV1
121147 229533.I1RA70 pp 229533.I1RZW8
121148 229533.I1RA70 pp 229533.I1S1W3
121149 229533.I1RA70 pp 229533.I1RZ79
121150 229533.I1RA70 pp 229533.I1RJP4
121151 229533.I1RA70 pp 229533.I1RMG3
121152 229533.I1RA70 pp 229533.I1RRI2
121153 229533.I1RA70 pp 229533.I1RJV3
121154 229533.I1RA70 pp 229533.I1RCA0
121155 229533.I1RA70 pp 229533.I1RWH4
121156 229533.I1RA70 pp 229533.I1RWX5
121157 229533.I1RA70 pp 229533.I1RRN2
121158 229533.I1RA70 pp 229533.I1RD22
121159 229533.I1RA70 pp 229533.I1SA33
121160 229533.I1RA70 pp 229533.I1RSK1
121161 229533.I1RA70 pp 229533.I1S1C7
121162 229533.I1RA70 pp 229533.I1RCM3
121163 229533.I1RA70 pp 229533.I1RAW0
121164 229533.I1RA70 pp 229533.I1RWN6
121165 229533.I1RA70 pp 229533.I1RTD7
121166 229533.I1RA70 pp 229533.I1RMN6
121167 229533.I1RA70 pp 229533.I1RG11
121168 229533.I1RA70 pp 229533.I1S0N4
121169 229533.I1RA70 pp 229533.I1RYW3
121170 229533.I1RA70 pp 229533.I1RD39
121171 229533.I1RA70 pp 229533.I1RXU9
121172 229533.I1RA70 pp 229533.I1RCW6
121173 229533.I1RA70 pp 229533.I1RCL5
121174 229533.I1RA70 pp 229533.I1S2B9
121175 229533.I1RA70 pp 229533.Q4IJT5
121176 229533.I1RA70 pp 229533.I1RIL8
121177 229533.I1RA70 pp 229533.I1S8R3
121178 229533.I1RA70 pp 229533.I1S5R6
121179 229533.I1RA70 pp 229533.I1RQE7
121180 229533.I1RA70 pp 229533.I1RPA4

121181	229533.I1RA70	pp	229533.I1RBD5
121182	229533.I1RA70	pp	229533.I1RRG3
121183	229533.I1RA70	pp	229533.I1RTH6
121184	229533.I1RA70	pp	229533.I1RSQ7
121185	229533.I1RA70	pp	229533.I1RCS1
121186	229533.I1RA70	pp	229533.I1RRX5
121187	229533.I1RA70	pp	229533.I1RCE6
121188	229533.I1RA70	pp	229533.V6R1K4
121189	229533.I1RA70	pp	229533.I1RFC0
121190	229533.I1RA70	pp	229533.I1S1W8
121191	229533.I1RA70	pp	229533.I1RA85
121192	229533.I1RA70	pp	229533.V6QW22
121193	229533.I1RA70	pp	229533.V6QUW4
121194	229533.I1RA70	pp	229533.I1RD52
121195	229533.I1RA70	pp	229533.I1REK4
121196	229533.I1RA70	pp	229533.I1RW25
121197	229533.I1RA70	pp	229533.I1RXD7
121198	229533.I1RA70	pp	229533.I1RN61
121199	229533.I1RA70	pp	229533.I1RC20
121200	229533.I1RA70	pp	229533.I1RLP1
121201	229533.I1RA70	pp	229533.I1RX25
121202	229533.I1RA70	pp	229533.I1RG53
121203	229533.I1RA70	pp	229533.I1RLC7
121204	229533.I1RA70	pp	229533.V6RUT5
121205	229533.I1RA70	pp	229533.I1RR00
121206	229533.I1RA70	pp	229533.I1RW07
121207	229533.I1RA70	pp	229533.I1RME6
121208	229533.I1RA70	pp	229533.I1RPN4
121209	229533.I1RA70	pp	229533.I1RQH3
121210	229533.I1RA70	pp	229533.I1RI02
121211	229533.I1RA70	pp	229533.I1RMC8
121212	229533.I1RA70	pp	229533.I1S0X6
121213	229533.I1RA70	pp	229533.I1S0R7
121214	229533.I1RA70	pp	229533.I1RW71
121215	229533.I1RA70	pp	229533.I1RBC9
121216	229533.I1RA70	pp	229533.I1S4S7
121217	229533.I1RA70	pp	229533.I1RQS9
121218	229533.I1RA70	pp	229533.I1RP86
121219	229533.I1RA70	pp	229533.I1RQC9
121220	229533.I1RA70	pp	229533.I1RBT1
121221	229533.I1RA70	pp	229533.I1RCC2
121222	229533.I1RA70	pp	229533.I1RVQ0
121223	229533.I1RA70	pp	229533.V6R4J1
121224	229533.I1RA70	pp	229533.I1RX46
121225	229533.I1RA70	pp	229533.I1S6U8
121226	229533.I1RA70	pp	229533.I1RDU4
121227	229533.I1RA70	pp	229533.I1S0T2
121228	229533.I1RA70	pp	229533.I1RIG2
121229	229533.I1RA70	pp	229533.I1S0L3
121230	229533.I1RA70	pp	229533.I1RJU3
121231	229533.I1RA70	pp	229533.I1S0F5
121232	229533.I1RA70	pp	229533.I1S7A8
121233	229533.I1RA70	pp	229533.I1RKB7
121234	229533.I1RA70	pp	229533.I1RI82
121235	229533.I1RA70	pp	229533.I1RZJ5
121236	229533.I1RA70	pp	229533.V6RTJ8
121237	229533.I1RA70	pp	229533.I1RMG8
121238	229533.I1RA70	pp	229533.I1RM67
121239	229533.I1RA70	pp	229533.I1RRQ1
121240	229533.I1RA70	pp	229533.I1RXF0
121241	229533.I1RA70	pp	229533.I1RAX6
121242	229533.I1RA70	pp	229533.I1RZM8
121243	229533.I1RA70	pp	229533.V6RCG5
121244	229533.I1RA70	pp	229533.I1RE00
121245	229533.I1RA70	pp	229533.I1RSI0
121246	229533.I1RA70	pp	229533.I1RFQ7
121247	229533.I1RA70	pp	229533.I1RR51
121248	229533.I1RA70	pp	229533.V6RCZ8
121249	229533.I1RA70	pp	229533.I1RZE5
121250	229533.I1RA70	pp	229533.I1S4S4
121251	229533.I1RA70	pp	229533.I1RWJ8
121252	229533.I1RA70	pp	229533.I1RLM7
121253	229533.I1RA70	pp	229533.I1RR24

121254	229533.I1RA70	pp	229533.I1S6K9
121255	229533.I1RA70	pp	229533.I1RZ35
121256	229533.I1RA70	pp	229533.I1RJ17
121257	229533.I1RA70	pp	229533.I1REL7
121258	229533.I1RA70	pp	229533.I1RAU5
121259	229533.I1RA70	pp	229533.I1RFT2
121260	229533.I1RA70	pp	229533.I1RRU9
121261	229533.I1RA70	pp	229533.Q4I2J8
121262	229533.I1RA70	pp	229533.I1SA32
121263	229533.I1RA70	pp	229533.I1RMQ4
121264	229533.I1RA70	pp	229533.I1RGC2
121265	229533.I1RA70	pp	229533.I1RZH4
121266	229533.I1RA70	pp	229533.V6QUE6
121267	229533.I1RA70	pp	229533.I1RWD7
121268	229533.I1RA70	pp	229533.I1REK8
121269	229533.I1RA70	pp	229533.I1RCR5
121270	229533.I1RA70	pp	229533.I1RXC4
121271	229533.I1RA70	pp	229533.I1RRV4
121272	229533.I1RA70	pp	229533.I1RB75
121273	229533.I1RA70	pp	229533.Q4I8P5
121274	229533.I1RA70	pp	229533.I1RW99
121275	229533.I1RA70	pp	229533.I1RM35
121276	229533.I1RA70	pp	229533.I1RS18
121277	229533.I1RA70	pp	229533.I1RYY5
121278	229533.I1RA70	pp	229533.I1S044
121279	229533.I1RA70	pp	229533.I1S5B0
121280	229533.I1RA70	pp	229533.I1RQ81
121281	229533.I1RA70	pp	229533.I1RZM1
121282	229533.I1RA70	pp	229533.I1RY03
121283	229533.I1RA70	pp	229533.I1RBJ5
121284	229533.I1RA70	pp	229533.I1RC17
121285	229533.I1RA70	pp	229533.I1S887
121286	229533.I1RA70	pp	229533.I1RJZ6
121287	229533.I1RA70	pp	229533.V6RBN2
121288	229533.I1RA70	pp	229533.I1RNQ5
121289	229533.I1RA70	pp	229533.I1RLU6
121290	229533.I1RA70	pp	229533.I1RCM5
121291	229533.I1RA70	pp	229533.I1RZK5
121292	229533.I1RA70	pp	229533.I1S419
121293	229533.I1RA70	pp	229533.I1RCG4
121294	229533.I1RA70	pp	229533.I1RQQ5
121295	229533.I1RA70	pp	229533.I1RZZ1
121296	229533.I1RA70	pp	229533.I1RC16
121297	229533.I1RA70	pp	229533.I1RG14
121298	229533.I1RA70	pp	229533.I1RAZ3
121299	229533.I1RA70	pp	229533.I1RFU8
121300	229533.I1RA70	pp	229533.I1RBC4
121301	229533.I1RA70	pp	229533.Q4I283
121302	229533.I1RA70	pp	229533.I1RWT6
121303	229533.I1RA70	pp	229533.Q4IJK2
121304	229533.I1RA70	pp	229533.I1RQA3
121305	229533.I1RA70	pp	229533.I1S1J7
121306	229533.I1RA70	pp	229533.I1RDG0
121307	229533.I1RA70	pp	229533.I1RK95
121308	229533.I1RA70	pp	229533.I1RI03
121309	229533.I1RA70	pp	229533.I1S504
121310	229533.I1RA70	pp	229533.Q4IJM4
121311	229533.I1RA70	pp	229533.I1RD03
121312	229533.I1RA70	pp	229533.I1RB53
121313	229533.I1RA70	pp	229533.I1RF19
121314	229533.I1RA70	pp	229533.I1RPR9
121315	229533.I1RA70	pp	229533.I1RYS0
121316	229533.I1RA70	pp	229533.I1S4Q4
121317	229533.I1RA70	pp	229533.I1RWB0
121318	229533.I1RA70	pp	229533.I1RBL7
121319	229533.I1RA70	pp	229533.I1RU29
121320	229533.I1RA70	pp	229533.I1RRY9
121321	229533.I1S0K0	pp	229533.I1S1D7
121322	229533.I1S0K0	pp	229533.I1S8R2
121323	229533.I1S0K0	pp	229533.I1S299
121324	229533.I1S0K0	pp	229533.I1S702
121325	229533.I1S0K0	pp	229533.V6R382
121326	229533.I1S0K0	pp	229533.I1S6A2

121327 229533.I1S0K0 pp 229533.I1S568
121328 229533.I1S0K0 pp 229533.V6RM02
121329 229533.I1S0K0 pp 229533.I1S9T6
121330 229533.I1S0K0 pp 229533.I1S8C1
121331 229533.I1S0K0 pp 229533.V6RK96
121332 229533.A0A1C3YKP2 pp 229533.I1RRE9
121333 229533.A0A1C3YKP2 pp 229533.I1RYR2
121334 229533.A0A1C3YKP2 pp 229533.I1RQF7
121335 229533.A0A1C3YKP2 pp 229533.I1RCM6
121336 229533.A0A1C3YKP2 pp 229533.I1RMN2
121337 229533.I1RNC1 pp 229533.I1RW99
121338 229533.I1RNC1 pp 229533.I1S044
121339 229533.I1RNC1 pp 229533.I1S5B0
121340 229533.I1RNC1 pp 229533.I1RXF0
121341 229533.I1RNC1 pp 229533.I1RQD2
121342 229533.I1RNC1 pp 229533.I1RQ85
121343 229533.I1RNC1 pp 229533.I1RZM8
121344 229533.I1RNC1 pp 229533.Q4I283
121345 229533.I1RNC1 pp 229533.V6RCG5
121346 229533.I1RNC1 pp 229533.I1RWT6
121347 229533.I1RNC1 pp 229533.I1RR51
121348 229533.I1RNC1 pp 229533.I1S6K9
121349 229533.I1RNC1 pp 229533.I1S504
121350 229533.I1RNC1 pp 229533.I1RNQ5
121351 229533.I1RNC1 pp 229533.I1SA32
121352 229533.I1RNC1 pp 229533.V6RCZ8
121353 229533.I1RNC1 pp 229533.I1RZE5
121354 229533.I1RNC1 pp 229533.I1RXC4
121355 229533.I1RNC1 pp 229533.I1RZM1
121356 229533.I1RNC1 pp 229533.Q4ICA8
121357 229533.I1RNC1 pp 229533.I1RS18
121358 229533.I1RNC1 pp 229533.Q4I2J8
121359 229533.I1RNC1 pp 229533.I1RSI0
121360 229533.I1RNC1 pp 229533.I1S419
121361 229533.I1RNC1 pp 229533.I1RZ35
121362 229533.I1RNC1 pp 229533.I1RQ81
121363 229533.I1RNC1 pp 229533.Q4I8P5
121364 229533.I1RNC1 pp 229533.I1RRU9
121365 229533.I1RNC1 pp 229533.I1RZZ1
121366 229533.I1RNC1 pp 229533.I1RYM5
121367 229533.I1RNC1 pp 229533.V6RBN2
121368 229533.I1RNC1 pp 229533.I1RQQ5
121369 229533.I1RNC1 pp 229533.I1RYY5
121370 229533.I1RNC1 pp 229533.V6QUE6
121371 229533.I1RNC1 pp 229533.I1RWD7
121372 229533.I1RNC1 pp 229533.I1RQA3
121373 229533.I1RNC1 pp 229533.I1RRG3
121374 229533.I1RNC1 pp 229533.Q4IJT5
121375 229533.I1RNC1 pp 229533.I1RNT9
121376 229533.I1RNC1 pp 229533.Q4IJM4
121377 229533.I1RNC1 pp 229533.I1RZK5
121378 229533.I1RNC1 pp 229533.I1RQ26
121379 229533.I1RNC1 pp 229533.I1RY03
121380 229533.I1RNC1 pp 229533.I1RX95
121381 229533.I1RNC1 pp 229533.I1RYS0
121382 229533.I1RNC1 pp 229533.I1S1J7
121383 229533.I1RNC1 pp 229533.I1S887
121384 229533.I1RNC1 pp 229533.I1RWJ3
121385 229533.I1RNC1 pp 229533.I1RQA5
121386 229533.I1RNC1 pp 229533.I1RWB0
121387 229533.I1RNC1 pp 229533.V6QW22
121388 229533.I1RNC1 pp 229533.I1RXD6
121389 229533.I1RNC1 pp 229533.I1RSQ7
121390 229533.I1RNC1 pp 229533.I1RWR7
121391 229533.I1RNC1 pp 229533.I1RRX5
121392 229533.I1RNC1 pp 229533.I1RRY9
121393 229533.I1RNC1 pp 229533.I1RVQ0
121394 229533.I1RNC1 pp 229533.I1S4S7
121395 229533.I1RNC1 pp 229533.V6R4J1
121396 229533.I1RNC1 pp 229533.I1RW71
121397 229533.I1RNC1 pp 229533.I1RU29
121398 229533.I1RNC1 pp 229533.I1RR00
121399 229533.I1RNC1 pp 229533.V6R1K4

121400 229533.I1RNC1 pp 229533.I1RPR9
121401 229533.I1RNC1 pp 229533.I1S0T2
121402 229533.I1RNC1 pp 229533.I1RQH3
121403 229533.I1RNC1 pp 229533.I1S0F5
121404 229533.I1RNC1 pp 229533.I1S0L3
121405 229533.I1RNC1 pp 229533.V6RUT5
121406 229533.I1RNC1 pp 229533.I1RXD7
121407 229533.I1RNC1 pp 229533.I1RY86
121408 229533.I1RNC1 pp 229533.I1RX46
121409 229533.I1RNC1 pp 229533.I1S0R7
121410 229533.I1RNC1 pp 229533.V6RTJ8
121411 229533.I1RNC1 pp 229533.I1RQC9
121412 229533.I1RNC1 pp 229533.I1RY44
121413 229533.I1RNC1 pp 229533.I1S7A8
121414 229533.I1RNC1 pp 229533.I1RW25
121415 229533.I1RNC1 pp 229533.I1S540
121416 229533.I1RNC1 pp 229533.I1RYU3
121417 229533.I1RNC1 pp 229533.I1RZV3
121418 229533.I1RNC1 pp 229533.I1RPY3
121419 229533.I1RNC1 pp 229533.I1S1W3
121420 229533.I1RNC1 pp 229533.I1RTA0
121421 229533.I1RNC1 pp 229533.I1RX25
121422 229533.I1RNC1 pp 229533.I1RWN5
121423 229533.I1RNC1 pp 229533.I1RPP4
121424 229533.I1RNC1 pp 229533.I1RRS8
121425 229533.I1RNC1 pp 229533.I1RPA4
121426 229533.I1RNC1 pp 229533.I1S9Z4
121427 229533.I1RNC1 pp 229533.I1RW07
121428 229533.I1RNC1 pp 229533.I1S438
121429 229533.I1RNC1 pp 229533.V6RIV9
121430 229533.I1RNC1 pp 229533.I1S1W9
121431 229533.I1RNC1 pp 229533.V6QUW4
121432 229533.I1RNC1 pp 229533.V6RLM7
121433 229533.I1RNC1 pp 229533.I1RS17
121434 229533.I1RNC1 pp 229533.I1S016
121435 229533.I1RNC1 pp 229533.I1RPK0
121436 229533.I1RNC1 pp 229533.I1RRQ1
121437 229533.I1RNC1 pp 229533.I1RW54
121438 229533.I1RNC1 pp 229533.I1RSF1
121439 229533.I1RNC1 pp 229533.I1RXT9
121440 229533.I1RNC1 pp 229533.Q4HZB8
121441 229533.I1RNC1 pp 229533.I1RZJ5
121442 229533.I1RNC1 pp 229533.I1S256
121443 229533.I1RNC1 pp 229533.I1S5Z3
121444 229533.I1RNC1 pp 229533.I1RRN2
121445 229533.I1RNC1 pp 229533.I1S8V4
121446 229533.I1RNC1 pp 229533.I1RWH4
121447 229533.I1RNC1 pp 229533.I1RWJ8
121448 229533.I1RNC1 pp 229533.I1RPU2
121449 229533.I1RNC1 pp 229533.I1SA33
121450 229533.I1RNC1 pp 229533.I1RRE5
121451 229533.I1RNC1 pp 229533.I1S4S4
121452 229533.I1RNC1 pp 229533.I1S7W5
121453 229533.I1RNC1 pp 229533.I1S1W4
121454 229533.I1RNC1 pp 229533.I1S0N4
121455 229533.I1RNC1 pp 229533.I1S0X6
121456 229533.I1RNC1 pp 229533.I1RR24
121457 229533.I1RNC1 pp 229533.I1S5R6
121458 229533.I1RNC1 pp 229533.I1S0J1
121459 229533.I1RNC1 pp 229533.I1S6U8
121460 229533.I1RNC1 pp 229533.I1RS90
121461 229533.I1RNC1 pp 229533.I1RW46
121462 229533.I1RNC1 pp 229533.I1RNY2
121463 229533.I1RNC1 pp 229533.I1RSQ3
121464 229533.I1RNC1 pp 229533.I1RT03
121465 229533.I1RNC1 pp 229533.I1RSY2
121466 229533.I1RNC1 pp 229533.V6R042
121467 229533.I1RNC1 pp 229533.I1RVX0
121468 229533.I1RNC1 pp 229533.I1RXR1
121469 229533.I1RNC1 pp 229533.I1RVF8
121470 229533.I1RNC1 pp 229533.V6RMW6
121471 229533.I1RNC1 pp 229533.I1RXT3
121472 229533.I1RNC1 pp 229533.I1RRM3

121473	229533.I1RNC1	pp	229533.I1RSK1
121474	229533.I1RNC1	pp	229533.I1RUG3
121475	229533.I1RNC1	pp	229533.I1RRN5
121476	229533.I1RNC1	pp	229533.I1RRV1
121477	229533.I1RNC1	pp	229533.I1RQC0
121478	229533.I1RNC1	pp	229533.I1RTH6
121479	229533.I1RNC1	pp	229533.V6RRH2
121480	229533.I1RNC1	pp	229533.Q4IN63
121481	229533.I1RNC1	pp	229533.I1S8R3
121482	229533.I1RNC1	pp	229533.I1RWN6
121483	229533.I1RNC1	pp	229533.I1RQ33
121484	229533.I1RNC1	pp	229533.I1S569
121485	229533.I1RNC1	pp	229533.I1RXU9
121486	229533.I1RNC1	pp	229533.I1RWX5
121487	229533.I1RNC1	pp	229533.V6R057
121488	229533.I1RNC1	pp	229533.I1RP28
121489	229533.I1RNC1	pp	229533.Q4I624
121490	229533.I1RNC1	pp	229533.I1RZ79
121491	229533.I1RNC1	pp	229533.I1RY62
121492	229533.I1RNC1	pp	229533.I1RZE4
121493	229533.I1RNC1	pp	229533.I1S0W4
121494	229533.I1RNC1	pp	229533.I1RZW8
121495	229533.I1RNC1	pp	229533.I1S0F6
121496	229533.I1RNC1	pp	229533.I1RRI2
121497	229533.I1RNC1	pp	229533.I1RYK0
121498	229533.I1RNC1	pp	229533.I1S2G7
121499	229533.I1RNC1	pp	229533.I1RQE7
121500	229533.I1RNC1	pp	229533.I1RS47
121501	229533.I1RNC1	pp	229533.I1S437
121502	229533.I1RNC1	pp	229533.I1RTA1
121503	229533.I1RNC1	pp	229533.I1RYI5
121504	229533.I1RNC1	pp	229533.I1S1C7
121505	229533.I1RNC1	pp	229533.I1RYW3
121506	229533.I1RNC1	pp	229533.I1RVZ2
121507	229533.I1RNC1	pp	229533.I1S8P5
121508	229533.I1RNC1	pp	229533.I1RPP3
121509	229533.I1RNC1	pp	229533.I1RWK3
121510	229533.I1RNC1	pp	229533.V6R283
121511	229533.I1RNC1	pp	229533.I1S2E9
121512	229533.I1RNC1	pp	229533.I1RWF7
121513	229533.I1RNC1	pp	229533.I1RRJ8
121514	229533.I1RNC1	pp	229533.I1RQG4
121515	229533.I1RNC1	pp	229533.Q4HXF0
121516	229533.I1RNC1	pp	229533.I1S352
121517	229533.I1RNC1	pp	229533.I1RYR0
121518	229533.I1RNC1	pp	229533.I1RT04
121519	229533.I1RNC1	pp	229533.I1S0M6
121520	229533.I1RNC1	pp	229533.I1RT75
121521	229533.I1RNC1	pp	229533.I1S8F0
121522	229533.I1RNC1	pp	229533.I1RRT8
121523	229533.I1RNC1	pp	229533.I1RSV2
121524	229533.I1RNC1	pp	229533.I1S5R1
121525	229533.I1RNC1	pp	229533.V6RUY9
121526	229533.I1RNC1	pp	229533.I1RSX0
121527	229533.I1RNC1	pp	229533.I1S7S9
121528	229533.I1RNC1	pp	229533.I1RPU3
121529	229533.I1RNC1	pp	229533.I1S2B8
121530	229533.I1RNC1	pp	229533.V6R3J1
121531	229533.I1RNC1	pp	229533.O74638
121532	229533.I1RNC1	pp	229533.I1RRZ0
121533	229533.I1RNC1	pp	229533.I1RXC2
121534	229533.I1RNC1	pp	229533.I1S503
121535	229533.I1RNC1	pp	229533.I1RRM5
121536	229533.I1RNC1	pp	229533.I1RWL2
121537	229533.I1RNC1	pp	229533.I1S9Y1
121538	229533.I1RNC1	pp	229533.I1RYX5
121539	229533.I1RNC1	pp	229533.I1RP27
121540	229533.I1RNC1	pp	229533.I1S268
121541	229533.I1RNC1	pp	229533.I1S277
121542	229533.I1RNC1	pp	229533.I1RQ23
121543	229533.I1RNC1	pp	229533.I1RXF8
121544	229533.I1RNC1	pp	229533.I1S0L4
121545	229533.I1RNC1	pp	229533.I1RTD5

229533.I1RNC1 pp 229533.I1S6Y8
229533.I1RNC1 pp 229533.I1RRI1
229533.I1RNC1 pp 229533.I1RV91
229533.I1RNC1 pp 229533.I1RPE0
229533.I1RNC1 pp 229533.I1RPV3
229533.I1RNC1 pp 229533.I1RS20
229533.I1RNC1 pp 229533.I1RPM4
229533.I1RNC1 pp 229533.I1RSZ7
229533.I1RNC1 pp 229533.I1S2H4
229533.I1RNC1 pp 229533.I1RT40
229533.I1RNC1 pp 229533.I1RNN1
229533.I1RNC1 pp 229533.I1S8V3
229533.I1RNC1 pp 229533.I1RSQ4
229533.I1RNC1 pp 229533.I1RZZ9
229533.I1RNC1 pp 229533.I1RWI5
229533.I1RNC1 pp 229533.I1RZ39
229533.I1RNC1 pp 229533.V6R3X5
229533.I1RNC1 pp 229533.I1RR73
229533.I1RRE7 pp 229533.I1S032
229533.I1RRE7 pp 229533.I1RRN1
229533.I1RJ42 pp 229533.V6RUG6
229533.I1RJ42 pp 229533.I1RQE0
229533.I1RJ42 pp 229533.I1RWN8
229533.I1RJ42 pp 229533.I1RK45
229533.I1RJ42 pp 229533.V6RUY9
229533.I1RJ42 pp 229533.I1RKB5
229533.I1RJ42 pp 229533.I1RJ82
229533.I1RJ42 pp 229533.I1SAC4
229533.I1RJ42 pp 229533.I1RL39
229533.I1RJ42 pp 229533.I1RUM7
229533.I1RJ42 pp 229533.I1RZ78
229533.I1RJ42 pp 229533.I1RPS2
229533.A0A1C3YL57 pp 229533.I1RZB7
229533.A0A1C3YL57 pp 229533.I1RIH0
229533.A0A1C3YL57 pp 229533.I1RYQ0
229533.A0A1C3YL57 pp 229533.I1RDX8
229533.A0A1C3YL57 pp 229533.I1RSR2
229533.A0A1C3YL57 pp 229533.V6QVH8
229533.A0A1C3YL57 pp 229533.I1RR68
229533.A0A1C3YL57 pp 229533.I1RV65
229533.A0A098DT87 pp 229533.V6QX21
229533.A0A098DT87 pp 229533.A0A0E0SC50
229533.A0A098DT87 pp 229533.Q4IQK7
229533.A0A098DT87 pp 229533.I1RRI2
229533.A0A098DT87 pp 229533.I1RN64
229533.A0A098DT87 pp 229533.I1S8Q3
229533.A0A098DT87 pp 229533.I1RNG8
229533.A0A098DT87 pp 229533.I1RRE7
229533.I1RQH3 pp 229533.I1RZM1
229533.I1RQH3 pp 229533.I1RSA4
229533.I1RQH3 pp 229533.I1RSV2
229533.I1RQH3 pp 229533.I1S0J1
229533.I1RQH3 pp 229533.I1RY44
229533.I1RQH3 pp 229533.I1S0L3
229533.I1RQH3 pp 229533.I1S2G7
229533.I1RQH3 pp 229533.I1RWN5
229533.I1RQH3 pp 229533.I1S1W4
229533.I1RQH3 pp 229533.I1RWK3
229533.I1RQH3 pp 229533.I1RTA0
229533.I1RQH3 pp 229533.I1S504
229533.I1RQH3 pp 229533.I1RRU9
229533.I1RQH3 pp 229533.I1S503
229533.I1RQH3 pp 229533.V6QW22
229533.I1RQH3 pp 229533.I1RSQ7
229533.I1RQH3 pp 229533.V6RTJ8
229533.I1RQH3 pp 229533.I1RTD5
229533.I1RQH3 pp 229533.I1S8P5
229533.I1RQH3 pp 229533.I1S0N4
229533.I1RQH3 pp 229533.V6RMW6
229533.I1RQH3 pp 229533.I1RY03
229533.I1RQH3 pp 229533.I1S2E9
229533.I1RQH3 pp 229533.I1RYY5
229533.I1RQH3 pp 229533.I1RSQ3

121619	229533.I1RQH3	pp	229533.I1S1C7
121620	229533.I1RQH3	pp	229533.I1RZZ1
121621	229533.I1RQH3	pp	229533.Q4IJK2
121622	229533.I1RQH3	pp	229533.I1RXD7
121623	229533.I1RQH3	pp	229533.Q4IJM4
121624	229533.I1RQH3	pp	229533.I1RWI5
121625	229533.I1RQH3	pp	229533.V6R042
121626	229533.I1RQH3	pp	229533.I1RT03
121627	229533.I1RQH3	pp	229533.I1RRV1
121628	229533.I1RQH3	pp	229533.V6R3X5
121629	229533.I1RQH3	pp	229533.I1RR73
121630	229533.I1RQH3	pp	229533.I1S0R7
121631	229533.I1RQH3	pp	229533.I1RT04
121632	229533.I1RQH3	pp	229533.I1RZK5
121633	229533.I1RQH3	pp	229533.I1RVX0
121634	229533.I1RQH3	pp	229533.I1RTH6
121635	229533.I1RQH3	pp	229533.I1RX46
121636	229533.I1RQH3	pp	229533.I1RW25
121637	229533.I1RQH3	pp	229533.I1RZ39
121638	229533.I1RQH3	pp	229533.I1RSI0
121639	229533.I1RQH3	pp	229533.I1RWN6
121640	229533.I1RQH3	pp	229533.I1S2B8
121641	229533.I1RQH3	pp	229533.I1RSK1
121642	229533.I1RQH3	pp	229533.I1RRY9
121643	229533.I1RM96	pp	229533.I1RSR3
121644	229533.I1RM96	pp	229533.V6RGY3
121645	229533.I1RM96	pp	229533.I1RWN6
121646	229533.I1RM96	pp	229533.I1S1U7
121647	229533.I1RM96	pp	229533.I1RSD8
121648	229533.I1RM96	pp	229533.I1S2U2
121649	229533.I1RM96	pp	229533.I1RRY9
121650	229533.I1RM96	pp	229533.I1S2E8
121651	229533.I1RM96	pp	229533.V6RBN2
121652	229533.I1RM96	pp	229533.I1RMQ0
121653	229533.I1RM96	pp	229533.I1RZZ1
121654	229533.I1RM96	pp	229533.I1RZK6
121655	229533.I1RM96	pp	229533.I1RSR8
121656	229533.I1RM96	pp	229533.I1RSS4
121657	229533.I1RM96	pp	229533.I1RSC6
121658	229533.I1RM96	pp	229533.I1RVX0
121659	229533.I1RM96	pp	229533.I1RY03
121660	229533.I1RM96	pp	229533.Q4HWE2
121661	229533.I1RM96	pp	229533.I1RT03
121662	229533.I1RM96	pp	229533.I1S1C7
121663	229533.I1RM96	pp	229533.I1S1X9
121664	229533.I1RM96	pp	229533.I1RRI2
121665	229533.I1RM96	pp	229533.I1RY63
121666	229533.I1RM96	pp	229533.I1RTE9
121667	229533.I1RM96	pp	229533.I1RQ28
121668	229533.I1RM96	pp	229533.I1RTH6
121669	229533.I1RM96	pp	229533.I1RPJ3
121670	229533.I1RM96	pp	229533.I1RN99
121671	229533.I1RM96	pp	229533.I1S2C0
121672	229533.I1RM96	pp	229533.I1S0Z9
121673	229533.I1RM96	pp	229533.I1S1J6
121674	229533.I1RM96	pp	229533.I1RVZ0
121675	229533.I1RM96	pp	229533.I1RZK5
121676	229533.I1RM96	pp	229533.I1RNK2
121677	229533.I1RM96	pp	229533.I1RSI0
121678	229533.I1RM96	pp	229533.I1RNF5
121679	229533.I1RM96	pp	229533.I1S6E5
121680	229533.I1RM96	pp	229533.V6R227
121681	229533.I1RM96	pp	229533.I1S558
121682	229533.I1RM96	pp	229533.I1RYS0
121683	229533.I1RM96	pp	229533.I1RZE5
121684	229533.I1RM96	pp	229533.I1RQA2
121685	229533.I1RM96	pp	229533.I1S255
121686	229533.I1RM96	pp	229533.I1RRR7
121687	229533.I1RM96	pp	229533.Q4HZ68
121688	229533.I1RM96	pp	229533.I1S274
121689	229533.I1RM96	pp	229533.I1RYB1
121690	229533.I1RM96	pp	229533.Q4IF76
121691	229533.I1RM96	pp	229533.I1RMA8

121692	229533.I1RM96	pp	229533.I1RWE1
121693	229533.I1RM96	pp	229533.I1S296
121694	229533.I1RM96	pp	229533.I1RT05
121695	229533.I1RM96	pp	229533.I1S044
121696	229533.I1RM96	pp	229533.Q4IN63
121697	229533.I1RM96	pp	229533.V6RGN5
121698	229533.I1RM96	pp	229533.I1RU69
121699	229533.I1RM96	pp	229533.I1RMC0
121700	229533.I1RM96	pp	229533.I1RQM6
121701	229533.I1RM96	pp	229533.I1S060
121702	229533.I1RM96	pp	229533.I1RQ31
121703	229533.I1RM96	pp	229533.I1S021
121704	229533.I1RM96	pp	229533.I1S0F1
121705	229533.I1RM96	pp	229533.I1RX14
121706	229533.I1RM96	pp	229533.I1S0J1
121707	229533.I1RM96	pp	229533.I1RNH6
121708	229533.I1RM96	pp	229533.I1S8E2
121709	229533.I1RM96	pp	229533.I1RMN7
121710	229533.I1RM96	pp	229533.I1RTF4
121711	229533.I1RM96	pp	229533.I1RYB9
121712	229533.I1RM96	pp	229533.I1RNU6
121713	229533.I1RM96	pp	229533.Q4IJH1
121714	229533.I1RM96	pp	229533.Q4IJ56
121715	229533.I1RM96	pp	229533.I1RNY2
121716	229533.I1RM96	pp	229533.I1RWB5
121717	229533.I1RM96	pp	229533.I1RT80
121718	229533.I1RM96	pp	229533.I1RZ39
121719	229533.I1RM96	pp	229533.I1S0M9
121720	229533.I1RM96	pp	229533.I1RNV6
121721	229533.I1RM96	pp	229533.Q4I327
121722	229533.I1RM96	pp	229533.I1RR73
121723	229533.I1RM96	pp	229533.Q4IAA0
121724	229533.I1RM96	pp	229533.I1RSI6
121725	229533.I1RM96	pp	229533.V6R3X5
121726	229533.I1RM96	pp	229533.I1RP20
121727	229533.I1RM96	pp	229533.I1RWN5
121728	229533.I1RM96	pp	229533.I1RX08
121729	229533.I1RM96	pp	229533.I1RPY1
121730	229533.I1RM96	pp	229533.V6R042
121731	229533.I1RM96	pp	229533.I1RND6
121732	229533.I1RM96	pp	229533.I1S4T3
121733	229533.I1RM96	pp	229533.I1S253
121734	229533.I1RM96	pp	229533.I1S6U8
121735	229533.I1RM96	pp	229533.Q4IEK8
121736	229533.I1RM96	pp	229533.V6RMW6
121737	229533.I1RM96	pp	229533.I1RNQ5
121738	229533.I1RM96	pp	229533.I1RMK3
121739	229533.I1RM96	pp	229533.Q4HVW2
121740	229533.I1RM96	pp	229533.I1RMN6
121741	229533.I1RM96	pp	229533.I1S504
121742	229533.I1RM96	pp	229533.I1RY44
121743	229533.I1RM96	pp	229533.I1RPU0
121744	229533.I1RM96	pp	229533.I1S9U1
121745	229533.I1RM96	pp	229533.V6QUE6
121746	229533.I1RM96	pp	229533.Q4IBS2
121747	229533.I1RM96	pp	229533.I1S9Y1
121748	229533.I1RM96	pp	229533.I1RZV6
121749	229533.I1RM96	pp	229533.I1RP31
121750	229533.I1RM96	pp	229533.I1RRT8
121751	229533.I1RM96	pp	229533.I1RT98
121752	229533.I1RM96	pp	229533.I1S9U2
121753	229533.I1RM96	pp	229533.I1RZF6
121754	229533.I1RM96	pp	229533.I1RS20
121755	229533.I1RM96	pp	229533.I1RPW9
121756	229533.I1RM96	pp	229533.O74638
121757	229533.I1RM96	pp	229533.I1RW61
121758	229533.I1RM96	pp	229533.Q4INZ9
121759	229533.I1RM96	pp	229533.I1RYT5
121760	229533.I1RM96	pp	229533.Q4IQW0
121761	229533.I1RM96	pp	229533.I1RZJ6
121762	229533.I1RM96	pp	229533.Q4IR18
121763	229533.I1RM96	pp	229533.I1RTD5
121764	229533.I1RM96	pp	229533.V6R283

121765 229533.I1RM96 pp 229533.Q4HXF0
121766 229533.I1RM96 pp 229533.Q4I662
121767 229533.I1RM96 pp 229533.I1RZH6
121768 229533.I1RM96 pp 229533.I1RWJ4
121769 229533.I1RM96 pp 229533.I1RWH5
121770 229533.I1RM96 pp 229533.I1S0R7
121771 229533.I1RM96 pp 229533.I1RT04
121772 229533.I1RM96 pp 229533.I1RS19
121773 229533.I1RM96 pp 229533.Q4IQK7
121774 229533.I1RM96 pp 229533.Q4I830
121775 229533.I1RM96 pp 229533.I1RRJ8
121776 229533.I1RM96 pp 229533.I1RNA0
121777 229533.I1RM96 pp 229533.I1RW71
121778 229533.I1RM96 pp 229533.I1RXD7
121779 229533.I1RM96 pp 229533.I1RTI6
121780 229533.I1RM96 pp 229533.I1RSQ7
121781 229533.I1RM96 pp 229533.I1RUQ2
121782 229533.I1RM96 pp 229533.V6QYF0
121783 229533.I1RM96 pp 229533.I1RQJ5
121784 229533.I1RM96 pp 229533.I1RX24
121785 229533.I1RM96 pp 229533.I1RZ99
121786 229533.I1RM96 pp 229533.I1S503
121787 229533.I1RM96 pp 229533.I1RZS6
121788 229533.I1RM96 pp 229533.I1S2E9
121789 229533.I1RM96 pp 229533.I1RPP3
121790 229533.I1RM96 pp 229533.I1RWF7
121791 229533.I1RM96 pp 229533.Q4I5R9
121792 229533.I1RM96 pp 229533.Q4HZ47
121793 229533.I1RM96 pp 229533.Q4IFI0
121794 229533.I1RM96 pp 229533.I1RS30
121795 229533.I1RM96 pp 229533.Q4IAS1
121796 229533.I1RM96 pp 229533.I1S2B8
121797 229533.I1RM96 pp 229533.I1RRP3
121798 229533.I1RM96 pp 229533.I1RW25
121799 229533.I1RM96 pp 229533.I1RRT2
121800 229533.I1RM96 pp 229533.I1RYG3
121801 229533.I1RM96 pp 229533.Q4IPI1
121802 229533.I1RM96 pp 229533.I1S6F0
121803 229533.I1RM96 pp 229533.I1RNH1
121804 229533.I1RM96 pp 229533.I1RWD4
121805 229533.I1RM96 pp 229533.I1RX46
121806 229533.I1RM96 pp 229533.I1RQS5
121807 229533.I1RM96 pp 229533.Q4HZ42
121808 229533.I1RM96 pp 229533.I1RRQ3
121809 229533.I1RM96 pp 229533.I1RSL9
121810 229533.I1RM96 pp 229533.I1RRH5
121811 229533.I1RM96 pp 229533.I1RT33
121812 229533.I1RM96 pp 229533.I1S1B6
121813 229533.I1RM96 pp 229533.I1RQ65
121814 229533.I1RM96 pp 229533.I1RS47
121815 229533.I1RY72 pp 229533.I1S0K0
121816 229533.I1RY72 pp 229533.I1S3L2
121817 229533.I1RY72 pp 229533.I1S8R4
121818 229533.I1RY72 pp 229533.V6RM66
121819 229533.I1RN33 pp 229533.I1S767
121820 229533.I1RN33 pp 229533.Q4HZQ1
121821 229533.I1RN33 pp 229533.I1RTT0
121822 229533.I1RN33 pp 229533.I1RW11
121823 229533.I1RN33 pp 229533.I1RVW6
121824 229533.I1RN33 pp 229533.I1RZU2
121825 229533.I1RN33 pp 229533.I1RZE7
121826 229533.I1RN33 pp 229533.I1RN65
121827 229533.I1RN33 pp 229533.I1RP70
121828 229533.A0A0E0SD52 pp 229533.I1RX09
121829 229533.A0A0E0SD52 pp 229533.A0A1C3YLK5
121830 229533.I1S8R3 pp 229533.V6RGN5
121831 229533.I1S8R3 pp 229533.V6QUE6
121832 229533.I1S8R3 pp 229533.I1SA33
121833 229533.I1S8R3 pp 229533.Q4I2J8
121834 229533.I1S8R3 pp 229533.Q4IJK2
121835 229533.I1S8R3 pp 229533.V6R227
121836 229533.I1S8R3 pp 229533.V6RMW6
121837 229533.I1S8R3 pp 229533.I1S8V4

121838	229533.I1S8R3	pp	229533.Q4I283
121839	229533.I1S8R3	pp	229533.V6RCG5
121840	229533.I1S8R3	pp	229533.Q4I624
121841	229533.I1S8R3	pp	229533.V6RCZ8
121842	229533.I1S8R3	pp	229533.V6R042
121843	229533.I1S8R3	pp	229533.V6R057
121844	229533.I1S8R3	pp	229533.V6RIV9
121845	229533.I1S8R3	pp	229533.V6RTJ8
121846	229533.I1S8R3	pp	229533.I1SA32
121847	229533.I1S8R3	pp	229533.V6RUY9
121848	229533.I1S8R3	pp	229533.Q4I8P5
121849	229533.I1S8R3	pp	229533.V6R3X5
121850	229533.I1S8R3	pp	229533.V6QYF0
121851	229533.I1S8R3	pp	229533.V6R4J1
121852	229533.I1S8R3	pp	229533.V6QW22
121853	229533.I1S8R3	pp	229533.Q4IJT5
121854	229533.I1S8R3	pp	229533.I1S8V3
121855	229533.I1S8R3	pp	229533.V6R1K4
121856	229533.I1RG57	pp	229533.I1RMU1
121857	229533.I1RG57	pp	229533.I1RVK5
121858	229533.I1RG57	pp	229533.I1RK90
121859	229533.I1RG57	pp	229533.I1RQP8
121860	229533.I1RG57	pp	229533.V6RY33
121861	229533.I1RG57	pp	229533.I1RYW3
121862	229533.I1RG57	pp	229533.I1RQQ7
121863	229533.I1RG57	pp	229533.I1S0B1
121864	229533.I1RG57	pp	229533.Q4IPB8
121865	229533.I1RG57	pp	229533.I1RPY7
121866	229533.I1RG57	pp	229533.I1RG90
121867	229533.I1RG57	pp	229533.I1RYB1
121868	229533.I1RG57	pp	229533.Q4IPH4
121869	229533.I1RG57	pp	229533.V6QX58
121870	229533.I1RG57	pp	229533.I1RYJ9
121871	229533.I1RG57	pp	229533.I1RSA4
121872	229533.I1RG57	pp	229533.I1RND5
121873	229533.I1RG57	pp	229533.I1S471
121874	229533.I1RG57	pp	229533.Q4HXF6
121875	229533.I1RG57	pp	229533.I1S0Z9
121876	229533.I1RG57	pp	229533.I1S056
121877	229533.I1RG57	pp	229533.I1RWX8
121878	229533.I1RG57	pp	229533.I1RRR6
121879	229533.I1RG57	pp	229533.I1RT35
121880	229533.I1RG57	pp	229533.I1RQD8
121881	229533.I1RG57	pp	229533.I1RWH4
121882	229533.I1RG57	pp	229533.V6QXG1
121883	229533.I1RG57	pp	229533.I1S8F8
121884	229533.I1RG57	pp	229533.I1RT47
121885	229533.I1RG57	pp	229533.I1S1X9
121886	229533.I1RG57	pp	229533.I1S020
121887	229533.I1RG57	pp	229533.I1RPL3
121888	229533.I1RG57	pp	229533.I1RN09
121889	229533.I1RG57	pp	229533.I1S9L0
121890	229533.I1RG57	pp	229533.I1RX51
121891	229533.I1RG57	pp	229533.I1RZQ5
121892	229533.I1RG57	pp	229533.Q4IAA0
121893	229533.I1RG57	pp	229533.I1S7M3
121894	229533.I1RG57	pp	229533.I1RPA0
121895	229533.I1RG57	pp	229533.I1RX47
121896	229533.I1RG57	pp	229533.Q4IRI9
121897	229533.I1RG57	pp	229533.Q4IE79
121898	229533.I1RG57	pp	229533.Q4IB70
121899	229533.I1RG57	pp	229533.I1RK92
121900	229533.I1RG57	pp	229533.I1RM25
121901	229533.I1RG57	pp	229533.I1RJG6
121902	229533.I1RG57	pp	229533.I1RNB8
121903	229533.I1RG57	pp	229533.I1RMV5
121904	229533.I1RG57	pp	229533.Q4I1Y1
121905	229533.I1RG57	pp	229533.Q4IPN9
121906	229533.I1RG57	pp	229533.I1RZG7
121907	229533.I1RG57	pp	229533.I1RS28
121908	229533.I1RG57	pp	229533.I1RX24
121909	229533.I1RG57	pp	229533.I1RPY5
121910	229533.I1RG57	pp	229533.I1RPW9

229533.I1RG57	pp	229533.I1RSD4
229533.I1RG57	pp	229533.I1RRL0
229533.I1RG57	pp	229533.I1RX81
229533.I1RG57	pp	229533.I1S9M0
229533.I1RG57	pp	229533.I1RST1
229533.I1RG57	pp	229533.I1RQ13
229533.I1RG57	pp	229533.I1RK72
229533.I1RG57	pp	229533.I1S283
229533.I1RG57	pp	229533.I1RK96
229533.I1RG57	pp	229533.I1RQC5
229533.I1RG57	pp	229533.I1RN91
229533.I1RG57	pp	229533.I1S9L5
229533.I1RG57	pp	229533.I1RPY4
229533.I1RG57	pp	229533.Q4IPB3
229533.I1RG57	pp	229533.I1RWU2
229533.I1RG57	pp	229533.I1RWW5
229533.I1RG57	pp	229533.I1RUM5
229533.I1RG57	pp	229533.I1S4M5
229533.I1RG57	pp	229533.I1RN59
229533.I1RG57	pp	229533.I1RJF9
229533.I1RG57	pp	229533.I1RN03
229533.I1RG57	pp	229533.I1RU69
229533.I1RG57	pp	229533.I1RS75
229533.I1RG57	pp	229533.I1S246
229533.I1RG57	pp	229533.I1S9L6
229533.I1RG57	pp	229533.I1S2Y5
229533.I1RG57	pp	229533.I1RLU5
229533.I1RG57	pp	229533.I1RME9
229533.I1RG57	pp	229533.Q4IBK5
229533.I1RG57	pp	229533.I1RZD0
229533.I1RG57	pp	229533.I1RND9
229533.I1RG57	pp	229533.I1RT17
229533.I1RG57	pp	229533.I1RT95
229533.I1RG57	pp	229533.I1RPZ3
229533.I1RG57	pp	229533.I1S0N7
229533.I1RG57	pp	229533.I1RLU0
229533.I1RG57	pp	229533.I1RSL9
229533.I1RG57	pp	229533.Q4I5R9
229533.I1RG57	pp	229533.I1S1C6
229533.I1RG57	pp	229533.I1RXF2
229533.I1RG57	pp	229533.I1S7M2
229533.V6RIV9	pp	229533.V6RMW6
229533.V6RIV9	pp	229533.V6RTJ8
229533.V6RIV9	pp	229533.V6RUY9
229533.V6RIV9	pp	229533.V6RVG3
229533.A0A1C3YJ36	pp	229533.O74638
229533.A0A1C3YJ36	pp	229533.I1RBS0
229533.A0A1C3YJ36	pp	229533.I1RDJ7
229533.A0A1C3YJ36	pp	229533.I1RY57
229533.A0A1C3YJ36	pp	229533.I1REA8
229533.A0A1C3YJ36	pp	229533.V6R949
229533.A0A1C3YJ36	pp	229533.A0A1C3YN25
229533.A0A1C3YJ36	pp	229533.I1S3G4
229533.A0A1C3YJ36	pp	229533.I1RE76
229533.A0A1C3YJ36	pp	229533.I1RPD6
229533.A0A1C3YJ36	pp	229533.I1RY72
229533.A0A1C3YJ36	pp	229533.A0A1C3YMA1
229533.A0A1C3YJ36	pp	229533.I1RUE7
229533.A0A1C3YJ36	pp	229533.I1RN67
229533.A0A1C3YJ36	pp	229533.I1RCB9
229533.A0A1C3YJ36	pp	229533.I1RNG4
229533.A0A1C3YJ36	pp	229533.I1REV2
229533.A0A1C3YJ36	pp	229533.I1RP53
229533.A0A1C3YJ36	pp	229533.I1RXG6
229533.A0A1C3YJ36	pp	229533.I1RMJ3
229533.A0A1C3YJ36	pp	229533.I1S4F4
229533.A0A1C3YJ36	pp	229533.I1RPW2
229533.A0A1C3YJ36	pp	229533.A0A1C3YL82
229533.A0A1C3YJ36	pp	229533.A0A1C3YJ47
229533.A0A1C3YJ36	pp	229533.I1R9B8
229533.A0A1C3YJ36	pp	229533.I1RHX4
229533.A0A1C3YJ36	pp	229533.I1S5R6
229533.A0A1C3YJ36	pp	229533.I1S2E8

121984	229533.A0A1C3YJ36	pp	229533.I1RW06
121985	229533.A0A1C3YJ36	pp	229533.I1RQX9
121986	229533.A0A1C3YJ36	pp	229533.I1S2H3
121987	229533.A0A1C3YJ36	pp	229533.I1REX4
121988	229533.A0A1C3YJ36	pp	229533.I1RA28
121989	229533.A0A1C3YJ36	pp	229533.I1S1W8
121990	229533.A0A1C3YJ36	pp	229533.A0A1C3YJI4
121991	229533.A0A1C3YJ36	pp	229533.I1RVX9
121992	229533.A0A1C3YJ36	pp	229533.I1RZQ1
121993	229533.A0A1C3YJ36	pp	229533.I1S0R7
121994	229533.A0A1C3YJ36	pp	229533.I1RXY7
121995	229533.A0A1C3YJ36	pp	229533.I1RA06
121996	229533.A0A1C3YJ36	pp	229533.A0A1C3YLE9
121997	229533.A0A1C3YJ36	pp	229533.I1S1J7
121998	229533.A0A1C3YJ36	pp	229533.I1RN80
121999	229533.A0A1C3YJ36	pp	229533.I1RE00
122000	229533.A0A1C3YJ36	pp	229533.I1REB0
122001	229533.A0A1C3YJ36	pp	229533.I1RM26
122002	229533.A0A1C3YJ36	pp	229533.I1RQE0
122003	229533.A0A1C3YJ36	pp	229533.A0A1C3YKF3
122004	229533.A0A1C3YJ36	pp	229533.I1S0A0
122005	229533.A0A1C3YJ36	pp	229533.I1RA94
122006	229533.A0A1C3YJ36	pp	229533.I1RFV9
122007	229533.I1RTW1	pp	229533.I1S1F9
122008	229533.I1RTW1	pp	229533.I1S1G1
122009	229533.I1RTW1	pp	229533.I1SAS4
122010	229533.I1RTW1	pp	229533.I1S1G0
122011	229533.I1RUJ3	pp	229533.O74638
122012	229533.I1RXD7	pp	229533.I1S021
122013	229533.I1RXD7	pp	229533.I1RXF0
122014	229533.I1RXD7	pp	229533.I1S6K9
122015	229533.I1RXD7	pp	229533.I1S0M9
122016	229533.I1RXD7	pp	229533.V6RCZ8
122017	229533.I1RXD7	pp	229533.I1RZM8
122018	229533.I1RXD7	pp	229533.V6RCG5
122019	229533.I1RXD7	pp	229533.Q4I8P5
122020	229533.I1RXD7	pp	229533.I1S821
122021	229533.I1RXD7	pp	229533.I1S419
122022	229533.I1RXD7	pp	229533.I1S1B2
122023	229533.I1RXD7	pp	229533.I1RZM1
122024	229533.I1RXD7	pp	229533.I1RZK5
122025	229533.I1RXD7	pp	229533.I1S1J7
122026	229533.I1RXD7	pp	229533.Q4I283
122027	229533.I1RXD7	pp	229533.V6QUE6
122028	229533.I1RXD7	pp	229533.I1S887
122029	229533.I1RXD7	pp	229533.I1RYS0
122030	229533.I1RXD7	pp	229533.I1SA32
122031	229533.I1RXD7	pp	229533.I1RZZ1
122032	229533.I1RXD7	pp	229533.I1RZF6
122033	229533.I1RXD7	pp	229533.V6R9Z0
122034	229533.I1RXD7	pp	229533.I1S5B0
122035	229533.I1RXD7	pp	229533.V6R227
122036	229533.I1RXD7	pp	229533.I1S504
122037	229533.I1RXD7	pp	229533.I1S0R7
122038	229533.I1RXD7	pp	229533.V6RBN2
122039	229533.I1RXD7	pp	229533.I1RZK6
122040	229533.I1RXD7	pp	229533.V6RUT5
122041	229533.I1RXD7	pp	229533.I1RZE5
122042	229533.I1RXD7	pp	229533.I1S7A8
122043	229533.I1RXD7	pp	229533.I1RY03
122044	229533.I1RXD7	pp	229533.Q4IJK2
122045	229533.I1RXD7	pp	229533.I1S0T2
122046	229533.I1RXD7	pp	229533.V6RTJ8
122047	229533.I1RXD7	pp	229533.Q4I2J8
122048	229533.I1RXD7	pp	229533.Q4IJT5
122049	229533.I1RXD7	pp	229533.I1S4S7
122050	229533.I1RXD7	pp	229533.I1S4Q4
122051	229533.I1RXD7	pp	229533.V6R1K4
122052	229533.I1RXD7	pp	229533.I1S0L3
122053	229533.I1RXD7	pp	229533.Q4I327
122054	229533.I1RXD7	pp	229533.I1RY86
122055	229533.I1RXD7	pp	229533.V6QW22
122056	229533.I1RXD7	pp	229533.I1S0F5

122057 229533.I1RXD7 pp 229533.Q4IN63
122058 229533.I1RXD7 pp 229533.I1S9Z4
122059 229533.I1RXD7 pp 229533.I1RZJ5
122060 229533.I1RXD7 pp 229533.I1S4S4
122061 229533.I1RXD7 pp 229533.I1S0X6
122062 229533.I1RXD7 pp 229533.I1RY44
122063 229533.I1RXD7 pp 229533.V6R042
122064 229533.I1RXD7 pp 229533.I1RZV3
122065 229533.I1RXD7 pp 229533.I1S9U2
122066 229533.I1RXD7 pp 229533.V6RMW6
122067 229533.I1RXD7 pp 229533.I1S438
122068 229533.I1RXD7 pp 229533.Q4IFI0
122069 229533.I1RXD7 pp 229533.I1S1W3
122070 229533.I1RXD7 pp 229533.I1SA33
122071 229533.I1RXD7 pp 229533.I1S9I5
122072 229533.I1RXD7 pp 229533.I1S256
122073 229533.I1RXD7 pp 229533.I1S1C7
122074 229533.I1RXD7 pp 229533.V6RIV9
122075 229533.I1RXD7 pp 229533.I1S1W9
122076 229533.I1RXD7 pp 229533.I1RYW3
122077 229533.I1RXD7 pp 229533.V6RGN5
122078 229533.I1RXD7 pp 229533.I1RZ79
122079 229533.I1RXD7 pp 229533.I1S437
122080 229533.I1RXD7 pp 229533.I1S7W5
122081 229533.I1RXD7 pp 229533.I1S0N4
122082 229533.I1RXD7 pp 229533.I1S016
122083 229533.I1RXD7 pp 229533.I1S5R6
122084 229533.I1RXD7 pp 229533.I1RZW8
122085 229533.I1RXD7 pp 229533.I1S0J1
122086 229533.I1RXD7 pp 229533.I1RXR1
122087 229533.I1RXD7 pp 229533.I1S2C0
122088 229533.I1RXD7 pp 229533.I1S0F8
122089 229533.I1RXD7 pp 229533.I1RXU9
122090 229533.I1RXD7 pp 229533.V6R057
122091 229533.I1RXD7 pp 229533.V6RRH2
122092 229533.I1RXD7 pp 229533.I1S8R3
122093 229533.I1RXD7 pp 229533.V6RUY9
122094 229533.I1RXD7 pp 229533.Q4HVQ9
122095 229533.I1RXD7 pp 229533.V6R3X5
122096 229533.I1RXD7 pp 229533.I1RXF8
122097 229533.I1RXD7 pp 229533.V6R3J1
122098 229533.I1RXD7 pp 229533.Q4I624
122099 229533.I1RXD7 pp 229533.I1S2E8
122100 229533.I1RXD7 pp 229533.I1RZ39
122101 229533.I1RXD7 pp 229533.I1RZB2
122102 229533.I1RXD7 pp 229533.O74638
122103 229533.I1RXD7 pp 229533.I1S503
122104 229533.I1RXD7 pp 229533.I1S2E9
122105 229533.I1RXD7 pp 229533.I1S5R1
122106 229533.I1RXD7 pp 229533.V6R283
122107 229533.I1RXD7 pp 229533.I1RYI5
122108 229533.I1RXD7 pp 229533.I1S268
122109 229533.I1RXD7 pp 229533.I1RYP2
122110 229533.I1RXD7 pp 229533.I1S0L4
122111 229533.I1RXD7 pp 229533.I1S2H4
122112 229533.I1RXD7 pp 229533.I1S0W4
122113 229533.I1RXD7 pp 229533.I1S9Y1
122114 229533.I1RXD7 pp 229533.I1RY62
122115 229533.I1RXD7 pp 229533.I1RYR0
122116 229533.I1RXD7 pp 229533.I1S0F7
122117 229533.I1RXD7 pp 229533.I1S6Y8
122118 229533.I1RXD7 pp 229533.I1S2B8
122119 229533.I1RXD7 pp 229533.I1S0F6
122120 229533.I1RXD7 pp 229533.I1S8P5
122121 229533.I1RXD7 pp 229533.I1RZS6
122122 229533.I1RXD7 pp 229533.I1S277
122123 229533.I1RXD7 pp 229533.I1S0M6
122124 229533.I1RXD7 pp 229533.I1RZZ9
122125 229533.I1RXD7 pp 229533.I1S4Z8
122126 229533.A0A0E0SGH2 pp 229533.V6R476
122127 229533.A0A0E0SGH2 pp 229533.V6QUU6
122128 229533.A0A0E0SGH2 pp 229533.A0A1C3YJJ1
122129 229533.A0A0E0SGH2 pp 229533.A0A1C3YN54

122130 229533.A0A0E0SGH2 pp 229533.I1RN81
122131 229533.A0A0E0SGH2 pp 229533.I1RM71
122132 229533.A0A0E0SGH2 pp 229533.I1RBF2
122133 229533.A0A0E0SGH2 pp 229533.I1RTQ9
122134 229533.A0A0E0SGH2 pp 229533.I1RQ70
122135 229533.A0A0E0SGH2 pp 229533.I1RZF7
122136 229533.A0A0E0SGH2 pp 229533.I1RD39
122137 229533.A0A0E0SGH2 pp 229533.I1RAH7
122138 229533.A0A0E0SGH2 pp 229533.I1RT41
122139 229533.A0A0E0SGH2 pp 229533.I1RWR8
122140 229533.A0A0E0SGH2 pp 229533.I1RQF7
122141 229533.A0A0E0SGH2 pp 229533.I1RMJ1
122142 229533.A0A0E0SGH2 pp 229533.I1RW76
122143 229533.A0A0E0SGH2 pp 229533.I1RYW8
122144 229533.A0A0E0SGH2 pp 229533.I1RTC1
122145 229533.A0A0E0SGH2 pp 229533.I1RZ60
122146 229533.A0A0E0SGH2 pp 229533.I1RTG5
122147 229533.A0A0E0SGH2 pp 229533.I1RX84
122148 229533.A0A0E0SGH2 pp 229533.I1RCQ0
122149 229533.A0A0E0SGH2 pp 229533.Q4ILI9
122150 229533.A0A0E0SGH2 pp 229533.I1RCE7
122151 229533.A0A0E0SGH2 pp 229533.I1RA25
122152 229533.A0A0E0SGH2 pp 229533.I1RCN6
122153 229533.A0A0E0SGH2 pp 229533.I1S254
122154 229533.A0A0E0SGH2 pp 229533.A0A0E0SNH8
122155 229533.A0A0E0SGH2 pp 229533.A0A1C3YHZ2
122156 229533.A0A0E0SGH2 pp 229533.A0A1C3YID2
122157 229533.A0A0E0SGH2 pp 229533.Q4HYZ2
122158 229533.A0A0E0SGH2 pp 229533.Q4I877
122159 229533.A0A0E0SGH2 pp 229533.A0A1C3YLS7
122160 229533.A0A0E0SGH2 pp 229533.I1RZK3
122161 229533.A0A0E0SGH2 pp 229533.V6RR76
122162 229533.A0A0E0SGH2 pp 229533.A0A1C3YL28
122163 229533.A0A0E0SGH2 pp 229533.I1RKR7
122164 229533.A0A1C3YI46 pp 229533.I1RA20
122165 229533.A0A1C3YI46 pp 229533.I1RTB7
122166 229533.A0A1C3YI46 pp 229533.I1RDE0
122167 229533.A0A1C3YI46 pp 229533.A0A1C3YK43
122168 229533.A0A1C3YI46 pp 229533.I1RH68
122169 229533.A0A1C3YI46 pp 229533.I1S0S6
122170 229533.A0A1C3YI46 pp 229533.I1RQL4
122171 229533.A0A1C3YI46 pp 229533.I1RLX6
122172 229533.A0A1C3YI46 pp 229533.I1RWL6
122173 229533.A0A1C3YI46 pp 229533.P0C431
122174 229533.A0A1C3YI46 pp 229533.I1S050
122175 229533.A0A1C3YI46 pp 229533.I1RKK2
122176 229533.A0A1C3YI46 pp 229533.A0A1C3YIZ8
122177 229533.A0A1C3YI46 pp 229533.I1RE19
122178 229533.A0A1C3YI46 pp 229533.I1RMP2
122179 229533.A0A1C3YI46 pp 229533.I1RQN9
122180 229533.A0A1C3YI46 pp 229533.I1RJV8
122181 229533.A0A1C3YI46 pp 229533.I1RCS6
122182 229533.A0A1C3YI46 pp 229533.I1RRM8
122183 229533.A0A1C3YI46 pp 229533.I1RPU5
122184 229533.I1RTH6 pp 229533.V6QW22
122185 229533.I1RTH6 pp 229533.Q4IJT5
122186 229533.I1RTH6 pp 229533.I1S0T2
122187 229533.I1RTH6 pp 229533.I1RW71
122188 229533.I1RTH6 pp 229533.I1RXD7
122189 229533.I1RTH6 pp 229533.V6R4J1
122190 229533.I1RTH6 pp 229533.I1RX46
122191 229533.I1RTH6 pp 229533.Q4I5R9
122192 229533.I1RTH6 pp 229533.I1RVQ0
122193 229533.I1RTH6 pp 229533.Q4IPI1
122194 229533.I1RTH6 pp 229533.V6R1K4
122195 229533.I1RTH6 pp 229533.I1S0R7
122196 229533.I1RTH6 pp 229533.V6RTJ8
122197 229533.I1RTH6 pp 229533.I1RX25
122198 229533.I1RTH6 pp 229533.I1RW25
122199 229533.I1RTH6 pp 229533.Q4IFI0
122200 229533.I1RTH6 pp 229533.I1S7A8
122201 229533.I1RTH6 pp 229533.I1S0L3
122202 229533.I1RTH6 pp 229533.I1RW07

122203	229533.I1RTH6	pp	229533.I1S0F5
122204	229533.I1RTH6	pp	229533.I1RZM8
122205	229533.I1RTH6	pp	229533.Q4I283
122206	229533.I1RTH6	pp	229533.I1RXU7
122207	229533.I1RTH6	pp	229533.I1RZJ5
122208	229533.I1RTH6	pp	229533.I1RWT6
122209	229533.I1RTH6	pp	229533.V6QUW4
122210	229533.I1RTH6	pp	229533.I1RZF6
122211	229533.I1RTH6	pp	229533.I1RXF0
122212	229533.I1RTH6	pp	229533.I1S504
122213	229533.I1RTH6	pp	229533.I1RWJ8
122214	229533.I1RTH6	pp	229533.V6RCG5
122215	229533.I1RTH6	pp	229533.I1SA32
122216	229533.I1RTH6	pp	229533.P0C431
122217	229533.I1RTH6	pp	229533.I1S9U2
122218	229533.I1RTH6	pp	229533.I1S0X6
122219	229533.I1RTH6	pp	229533.V6RCZ8
122220	229533.I1RTH6	pp	229533.I1RZE5
122221	229533.I1RTH6	pp	229533.I1S6U8
122222	229533.I1RTH6	pp	229533.I1RZH4
122223	229533.I1RTH6	pp	229533.I1S0M9
122224	229533.I1RTH6	pp	229533.I1RVZ0
122225	229533.I1RTH6	pp	229533.Q4I8P5
122226	229533.I1RTH6	pp	229533.I1S419
122227	229533.I1RTH6	pp	229533.I1RZM1
122228	229533.I1RTH6	pp	229533.V6QUE6
122229	229533.I1RTH6	pp	229533.Q4I2J8
122230	229533.I1RTH6	pp	229533.I1RZ35
122231	229533.I1RTH6	pp	229533.Q4IJM4
122232	229533.I1RTH6	pp	229533.I1S044
122233	229533.I1RTH6	pp	229533.I1RWD7
122234	229533.I1RTH6	pp	229533.I1S5B0
122235	229533.I1RTH6	pp	229533.I1RZK6
122236	229533.I1RTH6	pp	229533.V6RBN2
122237	229533.I1RTH6	pp	229533.I1RW99
122238	229533.I1RTH6	pp	229533.I1RZZ1
122239	229533.I1RTH6	pp	229533.I1S021
122240	229533.I1RTH6	pp	229533.I1RU29
122241	229533.I1RTH6	pp	229533.I1S1J7
122242	229533.I1RTH6	pp	229533.Q4IJK2
122243	229533.I1RTH6	pp	229533.I1RYY5
122244	229533.I1RTH6	pp	229533.I1RY03
122245	229533.I1RTH6	pp	229533.V6R3J1
122246	229533.I1RTH6	pp	229533.I1S0F6
122247	229533.I1RTH6	pp	229533.I1RWK3
122248	229533.I1RTH6	pp	229533.I1S0V6
122249	229533.I1RTH6	pp	229533.I1S2G7
122250	229533.I1RTH6	pp	229533.Q4I1T9
122251	229533.I1RTH6	pp	229533.I1S503
122252	229533.I1RTH6	pp	229533.I1RWI5
122253	229533.I1RTH6	pp	229533.V6R283
122254	229533.I1RTH6	pp	229533.I1RZK5
122255	229533.I1RTH6	pp	229533.I1S2E9
122256	229533.I1RTH6	pp	229533.I1RV91
122257	229533.I1RTH6	pp	229533.I1RWF7
122258	229533.I1RTH6	pp	229533.V6R3X5
122259	229533.I1RTH6	pp	229533.I1RZZ9
122260	229533.I1RTH6	pp	229533.V6RUY9
122261	229533.I1RTH6	pp	229533.I1RZ39
122262	229533.I1RTH6	pp	229533.I1S8P5
122263	229533.I1RTH6	pp	229533.I1S8E2
122264	229533.I1RTH6	pp	229533.I1S7S9
122265	229533.I1RTH6	pp	229533.I1S352
122266	229533.I1RTH6	pp	229533.I1RY44
122267	229533.I1RTH6	pp	229533.I1S8F0
122268	229533.I1RTH6	pp	229533.I1RYX5
122269	229533.I1RTH6	pp	229533.Q4I662
122270	229533.I1RTH6	pp	229533.I1S2B8
122271	229533.I1RTH6	pp	229533.V6RIV9
122272	229533.I1RTH6	pp	229533.I1S0V7
122273	229533.I1RTH6	pp	229533.I1S6Y8
122274	229533.I1RTH6	pp	229533.I1RWN5
122275	229533.I1RTH6	pp	229533.I1S0M6

122276 229533.I1RTH6 pp 229533.I1RZS6
122277 229533.I1RTH6 pp 229533.I1SA33
122278 229533.I1RTH6 pp 229533.I1RWH4
122279 229533.I1RTH6 pp 229533.I1S5R1
122280 229533.I1RTH6 pp 229533.Q4HZB8
122281 229533.I1RTH6 pp 229533.I1S0N4
122282 229533.I1RTH6 pp 229533.I1RWL2
122283 229533.I1RTH6 pp 229533.I1S8V4
122284 229533.I1RTH6 pp 229533.I1RZV6
122285 229533.I1RTH6 pp 229533.I1S1W4
122286 229533.I1RTH6 pp 229533.Q4I327
122287 229533.I1RTH6 pp 229533.I1RW46
122288 229533.I1RTH6 pp 229533.I1S0L4
122289 229533.I1RTH6 pp 229533.I1S0J1
122290 229533.I1RTH6 pp 229533.I1S2H4
122291 229533.I1RTH6 pp 229533.I1RUG3
122292 229533.I1RTH6 pp 229533.I1RZS7
122293 229533.I1RTH6 pp 229533.V6R042
122294 229533.I1RTH6 pp 229533.Q4IN63
122295 229533.I1RTH6 pp 229533.I1RWX5
122296 229533.I1RTH6 pp 229533.Q4ILH3
122297 229533.I1RTH6 pp 229533.I1S8V3
122298 229533.I1RTH6 pp 229533.I1S569
122299 229533.I1RTH6 pp 229533.V6RMW6
122300 229533.I1RTH6 pp 229533.I1RYW3
122301 229533.I1RTH6 pp 229533.I1S1C7
122302 229533.I1RTH6 pp 229533.I1RZW8
122303 229533.I1RTH6 pp 229533.V6QVH8
122304 229533.I1RTH6 pp 229533.Q4I624
122305 229533.I1RTH6 pp 229533.V6R057
122306 229533.I1RTH6 pp 229533.I1S8R3
122307 229533.I1RTH6 pp 229533.I1S2C0
122308 229533.I1RTH6 pp 229533.I1RWN6
122309 229533.I1RTH6 pp 229533.I1RVX0
122310 229533.I1RTH6 pp 229533.I1S2E8
122311 229533.A0A098DQM6 pp 229533.I1S0K0
122312 229533.A0A098DQM6 pp 229533.I1RHZ5
122313 229533.A0A098DQM6 pp 229533.I1RC48
122314 229533.A0A098DQM6 pp 229533.I1RXS0
122315 229533.A0A098DQM6 pp 229533.I1RQP2
122316 229533.A0A098DQM6 pp 229533.I1S3L2
122317 229533.A0A098DQM6 pp 229533.A0A1C3YL10
122318 229533.A0A098DQM6 pp 229533.I1RNG4
122319 229533.A0A098DQM6 pp 229533.V6RM66
122320 229533.A0A098DQM6 pp 229533.I1S8R4
122321 229533.I1RBU1 pp 229533.I1RCN8
122322 229533.I1RBU1 pp 229533.I1RMU9
122323 229533.I1RBU1 pp 229533.I1RZ89
122324 229533.I1RBU1 pp 229533.I1RPP5
122325 229533.I1RBU1 pp 229533.I1S0R9
122326 229533.I1RBU1 pp 229533.I1RMW4
122327 229533.I1RBU1 pp 229533.I1RC18
122328 229533.I1RBU1 pp 229533.I1RVN1
122329 229533.I1RBU1 pp 229533.Q4I1Q6
122330 229533.I1RBU1 pp 229533.I1REX4
122331 229533.I1RLE3 pp 229533.I1RTV7
122332 229533.I1RLE3 pp 229533.I1RTW1
122333 229533.I1RLE3 pp 229533.I1RTN1
122334 229533.I1RLE3 pp 229533.I1S1G0
122335 229533.I1RLE3 pp 229533.I1SAS4
122336 229533.I1RLE3 pp 229533.I1RQW2
122337 229533.I1RLE3 pp 229533.I1S1F9
122338 229533.I1RLE3 pp 229533.I1RLN1
122339 229533.I1RLE3 pp 229533.I1S1G1
122340 229533.I1RHA2 pp 229533.I1RJX2
122341 229533.A0A098DPS3 pp 229533.I1RPM4
122342 229533.A0A098DPS3 pp 229533.I1RJZ7
122343 229533.A0A098DPS3 pp 229533.I1RRM5
122344 229533.A0A098DPS3 pp 229533.I1RE76
122345 229533.A0A098DPS3 pp 229533.I1RBY4
122346 229533.A0A098DPS3 pp 229533.I1RC46
122347 229533.A0A098DPS3 pp 229533.A0A1C3YN63
122348 229533.A0A098DPS3 pp 229533.I1RP74

122349	229533.A0A098DPS3	pp	229533.I1RAY2
122350	229533.A0A098DPS3	pp	229533.I1RS47
122351	229533.A0A098DPS3	pp	229533.A0A1C3YHG7
122352	229533.A0A098DPS3	pp	229533.A0A1C3YIS7
122353	229533.A0A098DPS3	pp	229533.I1RZZ9
122354	229533.A0A098DPS3	pp	229533.A0A1C3YKM9
122355	229533.A0A098DPS3	pp	229533.I1RR73
122356	229533.A0A098DPS3	pp	229533.V6R3X5
122357	229533.A0A098DPS3	pp	229533.I1S0V4
122358	229533.A0A098DPS3	pp	229533.I1RYT5
122359	229533.A0A098DPS3	pp	229533.I1RTD5
122360	229533.A0A098DPS3	pp	229533.A0A1C3YIE7
122361	229533.A0A098DPS3	pp	229533.Q4HXF0
122362	229533.A0A098DPS3	pp	229533.I1RRT8
122363	229533.A0A098DPS3	pp	229533.I1RZW8
122364	229533.A0A098DPS3	pp	229533.I1RA70
122365	229533.A0A098DPS3	pp	229533.I1RRM9
122366	229533.A0A098DPS3	pp	229533.I1RS20
122367	229533.A0A098DPS3	pp	229533.I1RT04
122368	229533.A0A098DPS3	pp	229533.I1RTH6
122369	229533.A0A098DPS3	pp	229533.I1RJW6
122370	229533.A0A098DPS3	pp	229533.I1RL82
122371	229533.A0A098DPS3	pp	229533.I1RZP8
122372	229533.A0A098DPS3	pp	229533.I1S0L4
122373	229533.A0A098DPS3	pp	229533.I1S503
122374	229533.A0A098DPS3	pp	229533.I1S2C0
122375	229533.A0A098DPS3	pp	229533.I1RRT2
122376	229533.A0A098DPS3	pp	229533.I1RWK3
122377	229533.A0A098DPS3	pp	229533.I1RJV3
122378	229533.A0A098DPS3	pp	229533.I1RC03
122379	229533.A0A098DPS3	pp	229533.I1RBS6
122380	229533.A0A098DPS3	pp	229533.I1RF85
122381	229533.A0A098DPS3	pp	229533.I1RJV4
122382	229533.A0A098DPS3	pp	229533.I1RFL4
122383	229533.A0A098DPS3	pp	229533.I1RKL3
122384	229533.A0A098DPS3	pp	229533.I1RB74
122385	229533.A0A098DPS3	pp	229533.I1RWN6
122386	229533.A0A098DPS3	pp	229533.I1RPP3
122387	229533.A0A098DPS3	pp	229533.I1RN18
122388	229533.A0A098DPS3	pp	229533.I1RE01
122389	229533.A0A098DPS3	pp	229533.I1S0E8
122390	229533.A0A098DPS3	pp	229533.I1RC72
122391	229533.A0A098DPS3	pp	229533.I1RQG4
122392	229533.A0A098DPS3	pp	229533.I1RS17
122393	229533.A0A098DPS3	pp	229533.I1RN67
122394	229533.A0A098DPS3	pp	229533.I1RJL3
122395	229533.A0A098DPS3	pp	229533.I1RC28
122396	229533.A0A098DPS3	pp	229533.I1RQE7
122397	229533.A0A098DPS3	pp	229533.I1RPP4
122398	229533.A0A098DPS3	pp	229533.I1RVX0
122399	229533.A0A098DPS3	pp	229533.I1RP28
122400	229533.A0A098DPS3	pp	229533.I1RHM4
122401	229533.A0A098DPS3	pp	229533.V6RMW6
122402	229533.A0A098DPS3	pp	229533.I1RT03
122403	229533.A0A098DPS3	pp	229533.V6RIV9
122404	229533.A0A098DPS3	pp	229533.I1RCU1
122405	229533.A0A098DPS3	pp	229533.V6RE46
122406	229533.A0A098DPS3	pp	229533.I1RSS4
122407	229533.A0A098DPS3	pp	229533.A0A1C3YIK9
122408	229533.A0A098DPS3	pp	229533.I1RD22
122409	229533.A0A098DPS3	pp	229533.I1S7T4
122410	229533.A0A098DPS3	pp	229533.I1RWN5
122411	229533.A0A098DPS3	pp	229533.I1RG51
122412	229533.A0A098DPS3	pp	229533.V6QUW4
122413	229533.A0A098DPS3	pp	229533.I1RB92
122414	229533.A0A098DPS3	pp	229533.V6RRH2
122415	229533.A0A098DPS3	pp	229533.V6RKY7
122416	229533.A0A098DPS3	pp	229533.A0A0E0RP23
122417	229533.A0A098DPS3	pp	229533.I1RKC6
122418	229533.A0A098DPS3	pp	229533.I1RD17
122419	229533.A0A098DPS3	pp	229533.V6QYF0
122420	229533.A0A098DPS3	pp	229533.V6RVG3
122421	229533.A0A098DPS3	pp	229533.A0A098DXE2

122422	229533.A0A098DPS3	pp	229533.Q4IFI0
122423	229533.A0A098DPS3	pp	229533.I1RAU7
122424	229533.A0A098DPS3	pp	229533.I1S1C7
122425	229533.A0A098DPS3	pp	229533.I1RNC1
122426	229533.A0A098DPS3	pp	229533.I1RWM7
122427	229533.A0A098DPS3	pp	229533.I1RRI2
122428	229533.A0A098DPS3	pp	229533.I1RAC2
122429	229533.A0A098DPS3	pp	229533.I1RW25
122430	229533.A0A098DPS3	pp	229533.I1RUG3
122431	229533.A0A098DPS3	pp	229533.A0A0E0RUI4
122432	229533.A0A098DPS3	pp	229533.A0A1C3YKC0
122433	229533.A0A098DPS3	pp	229533.I1RW71
122434	229533.A0A098DPS3	pp	229533.I1RRV1
122435	229533.A0A098DPS3	pp	229533.I1RBC9
122436	229533.A0A098DPS3	pp	229533.V6QW22
122437	229533.A0A098DPS3	pp	229533.I1RBT1
122438	229533.A0A098DPS3	pp	229533.I1RQM6
122439	229533.A0A098DPS3	pp	229533.I1RMQ5
122440	229533.A0A098DPS3	pp	229533.A0A1C3YHX8
122441	229533.A0A098DPS3	pp	229533.I1RDB1
122442	229533.A0A098DPS3	pp	229533.I1RT80
122443	229533.A0A098DPS3	pp	229533.I1RSQ7
122444	229533.A0A098DPS3	pp	229533.V6R042
122445	229533.A0A098DPS3	pp	229533.A0A1C3YIP5
122446	229533.A0A098DPS3	pp	229533.I1RVQ0
122447	229533.A0A098DPS3	pp	229533.I1RZS5
122448	229533.A0A098DPS3	pp	229533.I1RSQ3
122449	229533.A0A098DPS3	pp	229533.A0A098DSZ8
122450	229533.A0A098DPS3	pp	229533.A0A0E0RSD7
122451	229533.A0A098DPS3	pp	229533.I1RQ26
122452	229533.A0A098DPS3	pp	229533.I1RCU2
122453	229533.A0A098DPS3	pp	229533.I1S0L3
122454	229533.A0A098DPS3	pp	229533.I1RQC9
122455	229533.A0A098DPS3	pp	229533.V6RCG5
122456	229533.A0A098DPS3	pp	229533.A0A0E0SFN0
122457	229533.A0A098DPS3	pp	229533.I1RRK8
122458	229533.A0A098DPS3	pp	229533.I1S0R7
122459	229533.A0A098DPS3	pp	229533.I1S0K3
122460	229533.A0A098DPS3	pp	229533.A0A0E0S7B3
122461	229533.A0A098DPS3	pp	229533.I1RB75
122462	229533.A0A098DPS3	pp	229533.V6QUE6
122463	229533.A0A098DPS3	pp	229533.A0A0E0RV94
122464	229533.A0A098DPS3	pp	229533.I1REK8
122465	229533.A0A098DPS3	pp	229533.I1RAU5
122466	229533.A0A098DPS3	pp	229533.A0A1C3YHX6
122467	229533.A0A098DPS3	pp	229533.I1RY03
122468	229533.A0A098DPS3	pp	229533.A0A1C3YL64
122469	229533.A0A098DPS3	pp	229533.I1RX46
122470	229533.A0A098DPS3	pp	229533.I1S5B0
122471	229533.A0A098DPS3	pp	229533.I1RAX6
122472	229533.A0A098DPS3	pp	229533.V6RTJ8
122473	229533.A0A098DPS3	pp	229533.I1RFU8
122474	229533.A0A098DPS3	pp	229533.I1RZB5
122475	229533.A0A098DPS3	pp	229533.I1RCG4
122476	229533.A0A098DPS3	pp	229533.I1RJB9
122477	229533.A0A098DPS3	pp	229533.I1RW99
122478	229533.A0A098DPS3	pp	229533.I1RAX0
122479	229533.A0A098DPS3	pp	229533.I1RRY9
122480	229533.A0A098DPS3	pp	229533.I1RPP5
122481	229533.A0A098DPS3	pp	229533.I1RZK6
122482	229533.A0A098DPS3	pp	229533.A0A0E0SJS1
122483	229533.A0A098DPS3	pp	229533.I1RAM7
122484	229533.A0A098DPS3	pp	229533.I1RPL2
122485	229533.A0A098DPS3	pp	229533.A0A1C3YKV3
122486	229533.A0A098DPS3	pp	229533.I1RQ81
122487	229533.A0A098DPS3	pp	229533.I1RZZ1
122488	229533.A0A098DPS3	pp	229533.V6RBN2
122489	229533.A0A098DPS3	pp	229533.I1RG56
122490	229533.A0A098DPS3	pp	229533.I1RUI2
122491	229533.A0A098DPS3	pp	229533.I1RNQ5
122492	229533.A0A098DPS3	pp	229533.I1RLU6
122493	229533.A0A098DPS3	pp	229533.I1RZM1
122494	229533.A0A098DPS3	pp	229533.I1RE00

122495	229533.A0A098DPS3	pp	229533.A0A1C3YIJ3
122496	229533.A0A098DPS3	pp	229533.I1RSI0
122497	229533.A0A098DPS3	pp	229533.A0A0E0SMM8
122498	229533.A0A098DPS3	pp	229533.I1RT93
122499	229533.A0A098DPS3	pp	229533.I1S504
122500	229533.A0A098DPS3	pp	229533.A0A1C3YKB0
122501	229533.A0A098DPS3	pp	229533.A0A1C3YLE3
122502	229533.I1S050	pp	229533.I1S0S6
122503	229533.I1S050	pp	229533.Q4I5U9
122504	229533.I1S050	pp	229533.I1SAT1
122505	229533.I1RCH2	pp	229533.I1S8F8
122506	229533.I1RCH2	pp	229533.I1RRC5
122507	229533.I1RCH2	pp	229533.I1RQ32
122508	229533.I1RCH2	pp	229533.I1RQF7
122509	229533.I1RCH2	pp	229533.Q4I7X1
122510	229533.I1RCH2	pp	229533.I1RJS4
122511	229533.I1RCH2	pp	229533.I1RQD6
122512	229533.I1RCH2	pp	229533.I1RND7
122513	229533.I1RCH2	pp	229533.I1S1V4
122514	229533.I1RCH2	pp	229533.I1RQH7
122515	229533.I1RCH2	pp	229533.I1RPG2
122516	229533.I1RCH2	pp	229533.I1RNH7
122517	229533.I1RCH2	pp	229533.I1RE18
122518	229533.I1RCH2	pp	229533.I1SAM5
122519	229533.I1RCH2	pp	229533.I1RUP6
122520	229533.I1RCH2	pp	229533.I1S5B5
122521	229533.I1RCH2	pp	229533.I1S2B6
122522	229533.I1RCH2	pp	229533.I1RKJ8
122523	229533.I1RCH2	pp	229533.I1RZK3
122524	229533.I1RCH2	pp	229533.I1RJS5
122525	229533.I1RCH2	pp	229533.I1REJ0
122526	229533.I1RCH2	pp	229533.I1RWB4
122527	229533.I1RCH2	pp	229533.I1S287
122528	229533.I1RCH2	pp	229533.I1RXF8
122529	229533.I1RCH2	pp	229533.I1RW76
122530	229533.I1RCH2	pp	229533.I1RCQ0
122531	229533.I1RCH2	pp	229533.I1RFK0
122532	229533.A0A0E0SFN0	pp	229533.I1RA48
122533	229533.A0A0E0SFN0	pp	229533.I1R9V7
122534	229533.A0A0E0SFN0	pp	229533.I1RK95
122535	229533.A0A0E0SFN0	pp	229533.I1RDG0
122536	229533.A0A0E0SFN0	pp	229533.I1RN99
122537	229533.A0A0E0SFN0	pp	229533.I1RQA3
122538	229533.A0A0E0SFN0	pp	229533.Q4IJK2
122539	229533.A0A0E0SFN0	pp	229533.V6RBN2
122540	229533.A0A0E0SFN0	pp	229533.A0A0E0SPV2
122541	229533.A0A0E0SFN0	pp	229533.I1RI03
122542	229533.A0A0E0SFN0	pp	229533.A0A1C3YJ93
122543	229533.A0A0E0SFN0	pp	229533.I1RQQ5
122544	229533.A0A0E0SFN0	pp	229533.I1RJZ6
122545	229533.A0A0E0SFN0	pp	229533.I1S1J7
122546	229533.A0A0E0SFN0	pp	229533.Q4I2J8
122547	229533.A0A0E0SFN0	pp	229533.I1RZK6
122548	229533.A0A0E0SFN0	pp	229533.A0A1C3YKY6
122549	229533.A0A0E0SFN0	pp	229533.I1RB53
122550	229533.A0A0E0SFN0	pp	229533.I1RZZ1
122551	229533.A0A0E0SFN0	pp	229533.I1REL7
122552	229533.A0A0E0SFN0	pp	229533.I1RYS0
122553	229533.A0A0E0SFN0	pp	229533.I1RVZ0
122554	229533.A0A0E0SFN0	pp	229533.I1RBC4
122555	229533.A0A0E0SFN0	pp	229533.I1RQ81
122556	229533.A0A0E0SFN0	pp	229533.I1REK8
122557	229533.A0A0E0SFN0	pp	229533.I1RCR5
122558	229533.A0A0E0SFN0	pp	229533.I1RWD7
122559	229533.A0A0E0SFN0	pp	229533.I1S4Q4
122560	229533.A0A0E0SFN0	pp	229533.I1S0M9
122561	229533.A0A0E0SFN0	pp	229533.A0A1C3YIJ3
122562	229533.A0A0E0SFN0	pp	229533.I1RE00
122563	229533.A0A0E0SFN0	pp	229533.A0A1C3YKV3
122564	229533.A0A0E0SFN0	pp	229533.I1RAZ8
122565	229533.A0A0E0SFN0	pp	229533.Q4I5Z5
122566	229533.A0A0E0SFN0	pp	229533.I1RSI0
122567	229533.A0A0E0SFN0	pp	229533.I1RS18

122568	229533.A0A0E0SFN0	pp	229533.I1RFJ9
122569	229533.A0A0E0SFN0	pp	229533.I1RB27
122570	229533.A0A0E0SFN0	pp	229533.I1RNQ5
122571	229533.A0A0E0SFN0	pp	229533.I1RLU6
122572	229533.A0A0E0SFN0	pp	229533.Q4IEK8
122573	229533.A0A0E0SFN0	pp	229533.I1RF19
122574	229533.A0A0E0SFN0	pp	229533.I1RLM7
122575	229533.A0A0E0SFN0	pp	229533.I1RZE5
122576	229533.A0A0E0SFN0	pp	229533.I1RCG4
122577	229533.A0A0E0SFN0	pp	229533.Q4I8P5
122578	229533.A0A0E0SFN0	pp	229533.A0A1C3YK34
122579	229533.A0A0E0SFN0	pp	229533.I1RFU8
122580	229533.A0A0E0SFN0	pp	229533.I1S887
122581	229533.A0A0E0SFN0	pp	229533.A0A1C3YJN3
122582	229533.A0A0E0SFN0	pp	229533.I1RBJ5
122583	229533.A0A0E0SFN0	pp	229533.I1RXC4
122584	229533.A0A0E0SFN0	pp	229533.A0A1C3YJW9
122585	229533.A0A0E0SFN0	pp	229533.I1RCM5
122586	229533.A0A0E0SFN0	pp	229533.I1RZK5
122587	229533.A0A0E0SFN0	pp	229533.I1SA32
122588	229533.A0A0E0SFN0	pp	229533.I1RSR8
122589	229533.A0A0E0SFN0	pp	229533.V6RCZ8
122590	229533.A0A0E0SFN0	pp	229533.I1RJ17
122591	229533.A0A0E0SFN0	pp	229533.I1S6K9
122592	229533.A0A0E0SFN0	pp	229533.V6RCG5
122593	229533.A0A0E0SFN0	pp	229533.I1RY03
122594	229533.A0A0E0SFN0	pp	229533.I1RTD9
122595	229533.A0A0E0SFN0	pp	229533.I1S504
122596	229533.A0A0E0SFN0	pp	229533.A0A1C3YHX6
122597	229533.A0A0E0SFN0	pp	229533.I1RMD5
122598	229533.A0A0E0SFN0	pp	229533.A0A1C3YJR3
122599	229533.A0A0E0SFN0	pp	229533.I1RR51
122600	229533.A0A0E0SFN0	pp	229533.A0A1C3YHS4
122601	229533.A0A0E0SFN0	pp	229533.A0A1C3YLK2
122602	229533.A0A0E0SFN0	pp	229533.I1RZF6
122603	229533.A0A0E0SFN0	pp	229533.I1RPR9
122604	229533.A0A0E0SFN0	pp	229533.A0A1C3YLU7
122605	229533.A0A0E0SFN0	pp	229533.A0A1C3YI23
122606	229533.A0A0E0SFN0	pp	229533.I1RZM8
122607	229533.A0A0E0SFN0	pp	229533.I1RBL7
122608	229533.A0A0E0SFN0	pp	229533.I1RWT6
122609	229533.A0A0E0SFN0	pp	229533.I1RSR3
122610	229533.A0A0E0SFN0	pp	229533.A0A1C3YKW9
122611	229533.A0A0E0SFN0	pp	229533.I1RWR7
122612	229533.A0A0E0SFN0	pp	229533.I1RXF0
122613	229533.A0A0E0SFN0	pp	229533.I1RQ85
122614	229533.A0A0E0SFN0	pp	229533.I1RMC0
122615	229533.A0A0E0SFN0	pp	229533.I1RG14
122616	229533.A0A0E0SFN0	pp	229533.I1RU29
122617	229533.A0A0E0SFN0	pp	229533.A0A1C3YKB0
122618	229533.A0A0E0SFN0	pp	229533.I1RRY9
122619	229533.A0A0E0SFN0	pp	229533.I1RQ31
122620	229533.A0A0E0SFN0	pp	229533.I1RNH6
122621	229533.A0A0E0SFN0	pp	229533.I1S021
122622	229533.A0A0E0SFN0	pp	229533.I1RI82
122623	229533.A0A0E0SFN0	pp	229533.V6R227
122624	229533.A0A0E0SFN0	pp	229533.A0A1C3YI30
122625	229533.A0A0E0SFN0	pp	229533.I1RC17
122626	229533.A0A0E0SFN0	pp	229533.I1RYJ8
122627	229533.A0A0E0SFN0	pp	229533.I1RAN5
122628	229533.A0A0E0SFN0	pp	229533.I1RWB0
122629	229533.A0A0E0SFN0	pp	229533.Q4I283
122630	229533.A0A0E0SFN0	pp	229533.I1RQA2
122631	229533.A0A0E0SFN0	pp	229533.I1RM06
122632	229533.A0A0E0SFN0	pp	229533.I1RQD2
122633	229533.A0A0E0SFN0	pp	229533.I1S419
122634	229533.A0A0E0SFN0	pp	229533.I1RW99
122635	229533.A0A0E0SFN0	pp	229533.I1RC16
122636	229533.A0A0E0SFN0	pp	229533.I1S438
122637	229533.A0A0E0SFN0	pp	229533.A0A0E0SMM8
122638	229533.A0A0E0SFN0	pp	229533.A0A1C3YJV6
122639	229533.A0A0E0SFN0	pp	229533.I1S5B0
122640	229533.A0A0E0SFN0	pp	229533.I1RCS1

122641	229533.A0A0E0SFN0	pp	229533.I1RZM1
122642	229533.A0A0E0SFN0	pp	229533.I1RPA4
122643	229533.A0A0E0SFN0	pp	229533.I1RRV4
122644	229533.A0A0E0SFN0	pp	229533.I1RMC8
122645	229533.A0A0E0SFN0	pp	229533.A0A1C3YHX8
122646	229533.A0A0E0SFN0	pp	229533.V6QUE6
122647	229533.A0A0E0SFN0	pp	229533.I1RAM6
122648	229533.A0A0E0SFN0	pp	229533.I1RW07
122649	229533.A0A0E0SFN0	pp	229533.I1RR24
122650	229533.A0A0E0SFN0	pp	229533.I1RI02
122651	229533.A0A0E0SFN0	pp	229533.I1RAU5
122652	229533.A0A0E0SFN0	pp	229533.I1RFT2
122653	229533.A0A0E0SFN0	pp	229533.I1RRU9
122654	229533.A0A0E0SFN0	pp	229533.I1S9U2
122655	229533.A0A0E0SFN0	pp	229533.I1RNA0
122656	229533.A0A0E0SFN0	pp	229533.I1RLC7
122657	229533.A0A0E0SFN0	pp	229533.I1RW25
122658	229533.A0A0E0SFN0	pp	229533.I1RPU0
122659	229533.A0A0E0SFN0	pp	229533.I1RX25
122660	229533.A0A0E0SFN0	pp	229533.A0A1C3YLZ7
122661	229533.A0A0E0SFN0	pp	229533.V6QYF0
122662	229533.A0A0E0SFN0	pp	229533.I1RWJ8
122663	229533.A0A0E0SFN0	pp	229533.V6QUW4
122664	229533.A0A0E0SFN0	pp	229533.A0A1C3YJQ9
122665	229533.A0A0E0SFN0	pp	229533.I1RJU3
122666	229533.A0A0E0SFN0	pp	229533.I1S4S4
122667	229533.A0A0E0SFN0	pp	229533.A0A1C3YJH8
122668	229533.A0A0E0SFN0	pp	229533.A0A1C3YN30
122669	229533.A0A0E0SFN0	pp	229533.I1RTF4
122670	229533.A0A0E0SFN0	pp	229533.I1RMG8
122671	229533.A0A0E0SFN0	pp	229533.I1RT80
122672	229533.A0A0E0SFN0	pp	229533.I1RZJ5
122673	229533.A0A0E0SFN0	pp	229533.Q4HVW2
122674	229533.A0A0E0SFN0	pp	229533.I1RM67
122675	229533.A0A0E0SFN0	pp	229533.I1RA85
122676	229533.A0A0E0SFN0	pp	229533.I1RT33
122677	229533.A0A0E0SFN0	pp	229533.I1RFQ7
122678	229533.A0A0E0SFN0	pp	229533.I1RD52
122679	229533.A0A0E0SFN0	pp	229533.I1RMK3
122680	229533.A0A0E0SFN0	pp	229533.I1RPN4
122681	229533.A0A0E0SFN0	pp	229533.I1S0X6
122682	229533.A0A0E0SFN0	pp	229533.I1S0L3
122683	229533.A0A0E0SFN0	pp	229533.A0A1C3YK13
122684	229533.A0A0E0SFN0	pp	229533.I1S0F5
122685	229533.A0A0E0SFN0	pp	229533.I1R9Y4
122686	229533.A0A0E0SFN0	pp	229533.I1S4S7
122687	229533.A0A0E0SFN0	pp	229533.I1RBT1
122688	229533.A0A0E0SFN0	pp	229533.I1RBC9
122689	229533.A0A0E0SFN0	pp	229533.V6R1K4
122690	229533.A0A0E0SFN0	pp	229533.A0A1C3YKC0
122691	229533.A0A0E0SFN0	pp	229533.I1S0T2
122692	229533.A0A0E0SFN0	pp	229533.I1RW71
122693	229533.A0A0E0SFN0	pp	229533.I1RX46
122694	229533.A0A0E0SFN0	pp	229533.A0A1C3YIP5
122695	229533.A0A0E0SFN0	pp	229533.I1RWD4
122696	229533.A0A0E0SFN0	pp	229533.V6QW22
122697	229533.A0A0E0SFN0	pp	229533.V6R4J1
122698	229533.A0A0E0SFN0	pp	229533.I1RXR1
122699	229533.A0A0E0SFN0	pp	229533.I1RN61
122700	229533.A0A0E0SFN0	pp	229533.I1RSS4
122701	229533.A0A0E0SFN0	pp	229533.I1RC20
122702	229533.A0A0E0SFN0	pp	229533.I1RVQ0
122703	229533.A0A0E0SFN0	pp	229533.I1RRN5
122704	229533.A0A0E0SFN0	pp	229533.I1RRX5
122705	229533.A0A0E0SFN0	pp	229533.I1RVX0
122706	229533.A0A0E0SFN0	pp	229533.I1S6F0
122707	229533.A0A0E0SFN0	pp	229533.I1RFC0
122708	229533.A0A0E0SFN0	pp	229533.I1RG53
122709	229533.A0A0E0SFN0	pp	229533.I1RAH0
122710	229533.A0A0E0SFN0	pp	229533.I1S0R7
122711	229533.A0A0E0SFN0	pp	229533.I1RT03
122712	229533.A0A0E0SFN0	pp	229533.I1RQJ5
122713	229533.A0A0E0SFN0	pp	229533.I1RSY2

229533.A0A0E0SFN0	pp	229533.A0A1C3YM63
229533.A0A0E0SFN0	pp	229533.I1RQC9
229533.A0A0E0SFN0	pp	229533.I1RLP1
229533.A0A0E0SFN0	pp	229533.I1RUQ2
229533.A0A0E0SFN0	pp	229533.I1RB92
229533.A0A0E0SFN0	pp	229533.I1RFL4
229533.A0A0E0SFN0	pp	229533.I1RSD8
229533.A0A0E0SFN0	pp	229533.I1RLX7
229533.A0A0E0SFN0	pp	229533.I1RN18
229533.A0A0E0SFN0	pp	229533.I1RBD5
229533.A0A0E0SFN0	pp	229533.V6RUT5
229533.A0A0E0SFN0	pp	229533.I1S2E8
229533.A0A0E0SFN0	pp	229533.I1RIL8
229533.A0A0E0SFN0	pp	229533.I1RQH3
229533.A0A0E0SFN0	pp	229533.I1RRG3
229533.A0A0E0SFN0	pp	229533.I1RQE7
229533.A0A0E0SFN0	pp	229533.A0A1C3YL54
229533.A0A0E0SFN0	pp	229533.I1RAX6
229533.A0A0E0SFN0	pp	229533.I1RBR2
229533.A0A0E0SFN0	pp	229533.Q4IJT5
229533.A0A0E0SFN0	pp	229533.I1RCH5
229533.A0A0E0SFN0	pp	229533.V6RTJ8
229533.A0A0E0SFN0	pp	229533.I1RFW5
229533.A0A0E0SFN0	pp	229533.I1S7A8
229533.A0A0E0SFN0	pp	229533.I1RD17
229533.A0A0E0SFN0	pp	229533.Q4I624
229533.A0A0E0SFN0	pp	229533.I1RTH6
229533.A0A0E0SFN0	pp	229533.I1S569
229533.A0A0E0SFN0	pp	229533.I1S8R3
229533.A0A0E0SFN0	pp	229533.I1RA70
229533.A0A0E0SFN0	pp	229533.V6R057
229533.A0A0E0SFN0	pp	229533.A0A1C3YMW0
229533.A0A0E0SFN0	pp	229533.I1RTE9
229533.A0A0E0SFN0	pp	229533.I1R9X7
229533.A0A0E0SFN0	pp	229533.I1RPP4
229533.A0A0E0SFN0	pp	229533.I1RQ33
229533.A0A0E0SFN0	pp	229533.I1S2C0
229533.A0A0E0SFN0	pp	229533.I1RAU7
229533.A0A0E0SFN0	pp	229533.I1RKC6
229533.A0A0E0SFN0	pp	229533.I1S9Z4
229533.A0A0E0SFN0	pp	229533.I1RKJ8
229533.A0A0E0SFN0	pp	229533.I1RCW6
229533.A0A0E0SFN0	pp	229533.I1RMV0
229533.A0A0E0SFN0	pp	229533.I1RZS7
229533.A0A0E0SFN0	pp	229533.I1R9J6
229533.A0A0E0SFN0	pp	229533.I1RB41
229533.A0A0E0SFN0	pp	229533.I1RBR1
229533.A0A0E0SFN0	pp	229533.V6RRH2
229533.A0A0E0SFN0	pp	229533.I1S437
229533.A0A0E0SFN0	pp	229533.I1RML0
229533.A0A0E0SFN0	pp	229533.A0A1C3YI73
229533.A0A0E0SFN0	pp	229533.I1RGL4
229533.A0A0E0SFN0	pp	229533.A0A1C3YLC1
229533.A0A0E0SFN0	pp	229533.I1RTA0
229533.A0A0E0SFN0	pp	229533.A0A1C3YLB6
229533.A0A0E0SFN0	pp	229533.I1RE01
229533.A0A0E0SFN0	pp	229533.I1RW46
229533.A0A0E0SFN0	pp	229533.I1S1C7
229533.A0A0E0SFN0	pp	229533.I1S0J1
229533.A0A0E0SFN0	pp	229533.I1RMG3
229533.A0A0E0SFN0	pp	229533.I1RYW3
229533.A0A0E0SFN0	pp	229533.A0A1C3YLS9
229533.A0A0E0SFN0	pp	229533.V6R042
229533.A0A0E0SFN0	pp	229533.I1RC72
229533.A0A0E0SFN0	pp	229533.I1RZW8
229533.A0A0E0SFN0	pp	229533.I1S7W5
229533.A0A0E0SFN0	pp	229533.I1RSQ3
229533.A0A0E0SFN0	pp	229533.I1RWX5
229533.A0A0E0SFN0	pp	229533.V6RMW6
229533.A0A0E0SFN0	pp	229533.I1RRE5
229533.A0A0E0SFN0	pp	229533.I1RZ79
229533.A0A0E0SFN0	pp	229533.I1RPU2
229533.A0A0E0SFN0	pp	229533.I1RRR7

122787 229533.A0A0E0SFN0 pp 229533.I1RRI2
122788 229533.A0A0E0SFN0 pp 229533.I1RPY1
122789 229533.A0A0E0SFN0 pp 229533.I1RRM3
122790 229533.A0A0E0SFN0 pp 229533.I1RCR8
122791 229533.A0A0E0SFN0 pp 229533.I1RXU9
122792 229533.A0A0E0SFN0 pp 229533.A0A1C3YKY0
122793 229533.A0A0E0SFN0 pp 229533.Q4IN63
122794 229533.A0A0E0SFN0 pp 229533.V6RIV9
122795 229533.A0A0E0SFN0 pp 229533.I1RAW0
122796 229533.A0A0E0SFN0 pp 229533.I1RD39
122797 229533.A0A0E0SFN0 pp 229533.A0A1C3YK33
122798 229533.A0A0E0SFN0 pp 229533.I1RWN5
122799 229533.A0A0E0SFN0 pp 229533.I1RWN6
122800 229533.A0A0E0SFN0 pp 229533.I1RNC1
122801 229533.A0A0E0SFN0 pp 229533.I1RCM3
122802 229533.A0A0E0SFN0 pp 229533.I1RE89
122803 229533.A0A0E0SFN0 pp 229533.I1RUG3
122804 229533.A0A0E0SFN0 pp 229533.I1S1W9
122805 229533.A0A0E0SFN0 pp 229533.I1RJV3
122806 229533.A0A0E0SFN0 pp 229533.I1S1W3
122807 229533.A0A0E0SFN0 pp 229533.A0A0E0SKJ9
122808 229533.A0A0E0SFN0 pp 229533.A0A1C3YMI5
122809 229533.A0A0E0SFN0 pp 229533.Q4I327
122810 229533.A0A0E0SFN0 pp 229533.I1RWQ8
122811 229533.A0A0E0SFN0 pp 229533.I1RSK1
122812 229533.A0A0E0SFN0 pp 229533.I1S5R6
122813 229533.A0A0E0SFN0 pp 229533.I1RS17
122814 229533.A0A0E0SFN0 pp 229533.I1RZV3
122815 229533.A0A0E0SFN0 pp 229533.I1RM96
122816 229533.A0A0E0SFN0 pp 229533.I1R9D1
122817 229533.A0A0E0SFN0 pp 229533.I1RPK0
122818 229533.A0A0E0SFN0 pp 229533.I1RG11
122819 229533.A0A0E0SFN0 pp 229533.I1RC33
122820 229533.A0A0E0SFN0 pp 229533.I1RY44
122821 229533.A0A0E0SFN0 pp 229533.I1S0N4
122822 229533.A0A0E0SFN0 pp 229533.I1RSZ7
122823 229533.A0A0E0SFN0 pp 229533.I1RFS3
122824 229533.A0A0E0SFN0 pp 229533.V6RGN5
122825 229533.A0A0E0SFN0 pp 229533.I1RSI6
122826 229533.A0A0E0SFN0 pp 229533.I1RPM4
122827 229533.A0A0E0SFN0 pp 229533.I1RRV1
122828 229533.A0A0E0SFN0 pp 229533.I1SA33
122829 229533.A0A0E0SFN0 pp 229533.I1S6Y8
122830 229533.A0A0E0SFN0 pp 229533.I1RMN6
122831 229533.A0A0E0SFN0 pp 229533.I1RNN1
122832 229533.A0A0E0SFN0 pp 229533.I1RPV3
122833 229533.A0A0E0SFN0 pp 229533.I1RFH8
122834 229533.A0A0E0SFN0 pp 229533.A0A1C3YIK9
122835 229533.A0A0E0SFN0 pp 229533.I1RD22
122836 229533.A0A0E0SFN0 pp 229533.I1RC66
122837 229533.A0A0E0SFN0 pp 229533.I1RL04
122838 229533.A0A0E0SFN0 pp 229533.I1RQS8
122839 229533.A0A0E0SFN0 pp 229533.I1RWH4
122840 229533.A0A0E0SFN0 pp 229533.I1RN88
122841 229533.A0A0E0SFN0 pp 229533.I1S256
122842 229533.A0A0E0SFN0 pp 229533.A0A0E0SPS2
122843 229533.A0A0E0SFN0 pp 229533.I1S2H4
122844 229533.A0A0E0SFN0 pp 229533.I1RQ23
122845 229533.A0A0E0SFN0 pp 229533.I1RS20
122846 229533.A0A0E0SFN0 pp 229533.I1RPE0
122847 229533.A0A0E0SFN0 pp 229533.I1S0L4
122848 229533.A0A0E0SFN0 pp 229533.I1RRN2
122849 229533.A0A0E0SFN0 pp 229533.I1RC29
122850 229533.A0A0E0SFN0 pp 229533.I1RE22
122851 229533.A0A0E0SFN0 pp 229533.I1RBG9
122852 229533.A0A0E0SFN0 pp 229533.I1S277
122853 229533.A0A0E0SFN0 pp 229533.A0A1C3YJD4
122854 229533.A0A0E0SFN0 pp 229533.I1RE27
122855 229533.A0A0E0SFN0 pp 229533.I1RIL3
122856 229533.A0A0E0SFN0 pp 229533.A0A1C3YIS7
122857 229533.A0A0E0SFN0 pp 229533.A0A1C3YMA1
122858 229533.A0A0E0SFN0 pp 229533.I1RZS6
122859 229533.A0A0E0SFN0 pp 229533.I1RRI1

122860	229533.A0A0E0SFN0	pp	229533.I1RJZ7
122861	229533.A0A0E0SFN0	pp	229533.I1RKJ6
122862	229533.A0A0E0SFN0	pp	229533.I1RM82
122863	229533.A0A0E0SFN0	pp	229533.I1RRM5
122864	229533.A0A0E0SFN0	pp	229533.I1S0M6
122865	229533.A0A0E0SFN0	pp	229533.I1S9Y1
122866	229533.A0A0E0SFN0	pp	229533.I1S2B8
122867	229533.A0A0E0SFN0	pp	229533.I1S2M6
122868	229533.A0A0E0SFN0	pp	229533.I1R9M0
122869	229533.A0A0E0SFN0	pp	229533.A0A1C3YLD8
122870	229533.A0A0E0SFN0	pp	229533.I1RWL2
122871	229533.A0A0E0SFN0	pp	229533.I1RJN9
122872	229533.A0A0E0SFN0	pp	229533.I1RPU3
122873	229533.A0A0E0SFN0	pp	229533.I1RBY4
122874	229533.A0A0E0SFN0	pp	229533.I1RTA1
122875	229533.A0A0E0SFN0	pp	229533.I1RA11
122876	229533.A0A0E0SFN0	pp	229533.I1RRT8
122877	229533.A0A0E0SFN0	pp	229533.I1RE76
122878	229533.A0A0E0SFN0	pp	229533.I1S5R1
122879	229533.A0A0E0SFN0	pp	229533.I1RRL9
122880	229533.A0A0E0SFN0	pp	229533.A0A1C3YKM9
122881	229533.A0A0E0SFN0	pp	229533.I1RS47
122882	229533.A0A0E0SFN0	pp	229533.I1RXC2
122883	229533.A0A0E0SFN0	pp	229533.I1RZ39
122884	229533.A0A0E0SFN0	pp	229533.O74638
122885	229533.A0A0E0SFN0	pp	229533.I1RQ65
122886	229533.A0A0E0SFN0	pp	229533.I1RR73
122887	229533.A0A0E0SFN0	pp	229533.I1RDF7
122888	229533.A0A0E0SFN0	pp	229533.A0A1C3YIN1
122889	229533.A0A0E0SFN0	pp	229533.I1S0F6
122890	229533.A0A0E0SFN0	pp	229533.I1RN67
122891	229533.A0A0E0SFN0	pp	229533.I1RSV2
122892	229533.A0A0E0SFN0	pp	229533.V6R3X5
122893	229533.A0A0E0SFN0	pp	229533.A0A1C3YKN3
122894	229533.A0A0E0SFN0	pp	229533.A0A1C3YK73
122895	229533.A0A0E0SFN0	pp	229533.I1RFT1
122896	229533.A0A0E0SFN0	pp	229533.A0A1C3YIR8
122897	229533.A0A0E0SFN0	pp	229533.I1S352
122898	229533.A0A0E0SFN0	pp	229533.I1S0W4
122899	229533.A0A0E0SFN0	pp	229533.I1RWI5
122900	229533.A0A0E0SFN0	pp	229533.I1RC05
122901	229533.A0A0E0SFN0	pp	229533.I1RY62
122902	229533.A0A0E0SFN0	pp	229533.I1RKL3
122903	229533.A0A0E0SFN0	pp	229533.I1RSQ4
122904	229533.A0A0E0SFN0	pp	229533.I1RRJ8
122905	229533.A0A0E0SFN0	pp	229533.I1RQG4
122906	229533.A0A0E0SFN0	pp	229533.I1RRQ3
122907	229533.A0A0E0SFN0	pp	229533.I1RZZ9
122908	229533.A0A0E0SFN0	pp	229533.A0A1C3YN63
122909	229533.A0A0E0SFN0	pp	229533.A0A1C3YI20
122910	229533.A0A0E0SFN0	pp	229533.I1RBS0
122911	229533.A0A0E0SFN0	pp	229533.I1RT04
122912	229533.A0A0E0SFN0	pp	229533.I1RYI5
122913	229533.A0A0E0SFN0	pp	229533.I1RBS6
122914	229533.A0A0E0SFN0	pp	229533.I1RPP3
122915	229533.A0A0E0SFN0	pp	229533.I1RJV4
122916	229533.A0A0E0SFN0	pp	229533.I1S8P5
122917	229533.A0A0E0SFN0	pp	229533.V6RUY9
122918	229533.A0A0E0SFN0	pp	229533.I1RZB2
122919	229533.A0A0E0SFN0	pp	229533.A0A1C3YLF5
122920	229533.A0A0E0SFN0	pp	229533.A0A1C3YIE7
122921	229533.A0A0E0SFN0	pp	229533.I1RNA5
122922	229533.A0A0E0SFN0	pp	229533.I1RYR0
122923	229533.A0A0E0SFN0	pp	229533.I1RP27
122924	229533.A0A0E0SFN0	pp	229533.I1RJ74
122925	229533.A0A0E0SFN0	pp	229533.V6R283
122926	229533.A0A0E0SFN0	pp	229533.I1S2E9
122927	229533.A0A0E0SFN0	pp	229533.I1RJW6
122928	229533.A0A0E0SFN0	pp	229533.I1RAF8
122929	229533.A0A0E0SFN0	pp	229533.A0A1C3YLK8
122930	229533.A0A0E0SFN0	pp	229533.I1RC03
122931	229533.A0A0E0SFN0	pp	229533.I1RWK3
122932	229533.A0A0E0SFN0	pp	229533.I1RCJ7

122933	229533.A0A0E0SFN0	pp	229533.I1RT40
122934	229533.A0A0E0SFN0	pp	229533.I1S503
122935	229533.A0A0E0SFN0	pp	229533.I1RFL3
122936	229533.A0A0E0SFN0	pp	229533.A0A1C3YJI6
122937	229533.A0A0E0SFN0	pp	229533.I1RN63
122938	229533.A0A0E0SFN0	pp	229533.I1RLV9
122939	229533.A0A0E0SFN0	pp	229533.I1RVZ2
122940	229533.A0A0E0SFN0	pp	229533.I1RV91
122941	229533.A0A0E0SFN0	pp	229533.I1RRZ0
122942	229533.A0A0E0SFN0	pp	229533.I1RGF5
122943	229533.A0A0E0SFN0	pp	229533.I1RXF8
122944	229533.A0A0E0SFN0	pp	229533.I1RLN6
122945	229533.A0A0E0SFN0	pp	229533.I1RSX0
122946	229533.A0A0E0SFN0	pp	229533.I1RTD5
122947	229533.A0A0E0SFN0	pp	229533.I1RJL4
122948	229533.A0A0E0SFN0	pp	229533.I1RBC7
122949	229533.A0A0E0SFN0	pp	229533.I1RC82
122950	229533.A0A0E0SFN0	pp	229533.I1RGA4
122951	229533.A0A0E0SFN0	pp	229533.V6R3J1
122952	229533.A0A0E0SFN0	pp	229533.I1RRT2
122953	229533.I1S8R4	pp	229533.V6RK96
122954	229533.I1S8R4	pp	229533.V6R382
122955	229533.I1S8R4	pp	229533.I1S9T6
122956	229533.I1S8R4	pp	229533.V6RM02
122957	229533.I1RQC9	pp	229533.I1RTA0
122958	229533.I1RQC9	pp	229533.I1S0N4
122959	229533.I1RQC9	pp	229533.I1S0J1
122960	229533.I1RQC9	pp	229533.I1RW46
122961	229533.I1RQC9	pp	229533.I1RZS7
122962	229533.I1RQC9	pp	229533.I1RWN5
122963	229533.I1RQC9	pp	229533.Q4HZB8
122964	229533.I1RQC9	pp	229533.I1RS17
122965	229533.I1RQC9	pp	229533.Q4IN63
122966	229533.I1RQC9	pp	229533.V6RIV9
122967	229533.I1RQC9	pp	229533.I1RUG3
122968	229533.I1RQC9	pp	229533.I1RY44
122969	229533.I1RQC9	pp	229533.V6RVG3
122970	229533.I1RQC9	pp	229533.I1RRV1
122971	229533.I1RQC9	pp	229533.I1S1W4
122972	229533.I1RQC9	pp	229533.I1RXT3
122973	229533.I1RQC9	pp	229533.V6RMW6
122974	229533.I1RQC9	pp	229533.I1RRE5
122975	229533.I1RQC9	pp	229533.I1RYW3
122976	229533.I1RQC9	pp	229533.I1RSQ3
122977	229533.I1RQC9	pp	229533.I1S1C7
122978	229533.I1RQC9	pp	229533.I1SA33
122979	229533.I1RQC9	pp	229533.I1RZW8
122980	229533.I1RQC9	pp	229533.V6R042
122981	229533.I1RQC9	pp	229533.I1RQP8
122982	229533.I1RQC9	pp	229533.I1RRI2
122983	229533.I1RQC9	pp	229533.I1RSK1
122984	229533.I1RQC9	pp	229533.I1RWX5
122985	229533.I1RQC9	pp	229533.I1RWH4
122986	229533.I1RQC9	pp	229533.I1RWK3
122987	229533.I1RQC9	pp	229533.I1S8R3
122988	229533.I1RQC9	pp	229533.V6R283
122989	229533.I1RQC9	pp	229533.I1RTH6
122990	229533.I1RQC9	pp	229533.I1RWN6
122991	229533.I1RQC9	pp	229533.I1RSV2
122992	229533.I1RQC9	pp	229533.I1RRN5
122993	229533.I1RQC9	pp	229533.I1RQE7
122994	229533.I1RQC9	pp	229533.I1RT03
122995	229533.I1RQC9	pp	229533.I1S352
122996	229533.I1RQC9	pp	229533.I1RWL2
122997	229533.I1RQC9	pp	229533.I1RVX0
122998	229533.I1RQC9	pp	229533.I1RRT8
122999	229533.I1RQC9	pp	229533.I1S5R1
123000	229533.I1RQC9	pp	229533.I1RRJ8
123001	229533.I1RQC9	pp	229533.I1RQG4
123002	229533.I1RQC9	pp	229533.I1RS20
123003	229533.I1RQC9	pp	229533.Q4I624
123004	229533.I1RQC9	pp	229533.I1RWF7
123005	229533.I1RQC9	pp	229533.I1S8P5

123006 229533.I1RQC9 pp 229533.V6R057
123007 229533.I1RQC9 pp 229533.I1S569
123008 229533.I1RQC9 pp 229533.I1S0F6
123009 229533.I1RQC9 pp 229533.I1S2H4
123010 229533.I1RQC9 pp 229533.I1RS47
123011 229533.I1RQC9 pp 229533.I1RT40
123012 229533.I1RQC9 pp 229533.I1S0L4
123013 229533.I1RQC9 pp 229533.I1S2G7
123014 229533.I1RQC9 pp 229533.I1S0M6
123015 229533.I1RQC9 pp 229533.I1RTD5
123016 229533.I1RQC9 pp 229533.I1S2B8
123017 229533.I1RQC9 pp 229533.I1RV91
123018 229533.I1RQC9 pp 229533.I1RWI5
123019 229533.I1RQC9 pp 229533.I1RRM5
123020 229533.I1RQC9 pp 229533.I1RZZ9
123021 229533.I1RQC9 pp 229533.I1S2E9
123022 229533.I1RQC9 pp 229533.I1RT04
123023 229533.I1RQC9 pp 229533.I1RZ39
123024 229533.I1RQC9 pp 229533.I1S503
123025 229533.I1RQC9 pp 229533.I1S7S9
123026 229533.I1RQC9 pp 229533.I1RXF0
123027 229533.I1RQC9 pp 229533.V6R3X5
123028 229533.I1RQC9 pp 229533.I1RZM8
123029 229533.I1RQC9 pp 229533.I1RRU9
123030 229533.I1RQC9 pp 229533.I1RR73
123031 229533.I1RQC9 pp 229533.I1S5B0
123032 229533.I1RQC9 pp 229533.V6RCZ8
123033 229533.I1RQC9 pp 229533.I1RW99
123034 229533.I1RQC9 pp 229533.V6RCG5
123035 229533.I1RQC9 pp 229533.I1S7A8
123036 229533.I1RQC9 pp 229533.I1S540
123037 229533.I1RQC9 pp 229533.I1S044
123038 229533.I1RQC9 pp 229533.V6RTJ8
123039 229533.I1RQC9 pp 229533.I1RRY9
123040 229533.I1RQC9 pp 229533.I1RZH4
123041 229533.I1RQC9 pp 229533.I1S1J7
123042 229533.I1RQC9 pp 229533.I1RU29
123043 229533.I1RQC9 pp 229533.I1SA32
123044 229533.I1RQC9 pp 229533.Q4IJT5
123045 229533.I1RQC9 pp 229533.I1RR00
123046 229533.I1RQC9 pp 229533.Q4I8P5
123047 229533.I1RQC9 pp 229533.I1RSQ7
123048 229533.I1RQC9 pp 229533.Q4IJK2
123049 229533.I1RQC9 pp 229533.I1RW71
123050 229533.I1RQC9 pp 229533.V6RBN2
123051 229533.I1RQC9 pp 229533.I1RZM1
123052 229533.I1RQC9 pp 229533.I1RS18
123053 229533.I1RQC9 pp 229533.V6QW22
123054 229533.I1RQC9 pp 229533.I1S504
123055 229533.I1RQC9 pp 229533.V6R4J1
123056 229533.I1RQC9 pp 229533.I1RVQ0
123057 229533.I1RQC9 pp 229533.I1RZZ1
123058 229533.I1RQC9 pp 229533.Q4I283
123059 229533.I1RQC9 pp 229533.Q4IJM4
123060 229533.I1RQC9 pp 229533.I1RWT6
123061 229533.I1RQC9 pp 229533.Q4I2J8
123062 229533.I1RQC9 pp 229533.I1S0F5
123063 229533.I1RQC9 pp 229533.V6R1K4
123064 229533.I1RQC9 pp 229533.I1S0L3
123065 229533.I1RQC9 pp 229533.I1RSI0
123066 229533.I1RQC9 pp 229533.I1RYY5
123067 229533.I1RQC9 pp 229533.I1RZE5
123068 229533.I1RQC9 pp 229533.I1RY03
123069 229533.I1RQC9 pp 229533.I1RZK5
123070 229533.I1RQC9 pp 229533.I1RWD7
123071 229533.I1RQC9 pp 229533.I1S6U8
123072 229533.I1RQC9 pp 229533.I1S0R7
123073 229533.I1RQC9 pp 229533.I1S0X6
123074 229533.I1RQC9 pp 229533.I1RR24
123075 229533.I1RQC9 pp 229533.I1RXD7
123076 229533.I1RQC9 pp 229533.I1RWJ3
123077 229533.I1RQC9 pp 229533.I1RWJ8
123078 229533.I1RQC9 pp 229533.I1RQH3

123079 229533.I1RQC9 pp 229533.I1RX46
123080 229533.I1RQC9 pp 229533.V6QUW4
123081 229533.I1RQC9 pp 229533.I1RZJ5
123082 229533.I1RQC9 pp 229533.I1RRQ1
123083 229533.I1RQC9 pp 229533.I1RX25
123084 229533.I1RQC9 pp 229533.I1RW25
123085 229533.I1RQC9 pp 229533.I1RW07
123086 229533.I1RBS7 pp 229533.I1RS50
123087 229533.I1RBS7 pp 229533.I1S9K9
123088 229533.I1RBS7 pp 229533.V6RM66
123089 229533.I1RBS7 pp 229533.I1RMK7
123090 229533.I1RBS7 pp 229533.I1RFJ1
123091 229533.A0A098E3Q0 pp 229533.I1RHM4
123092 229533.A0A098E3Q0 pp 229533.A0A1C3YI46
123093 229533.A0A098E3Q0 pp 229533.I1RE19
123094 229533.A0A098E3Q0 pp 229533.I1RQN9
123095 229533.A0A098E3Q0 pp 229533.I1RMP2
123096 229533.A0A098E3Q0 pp 229533.I1S050
123097 229533.A0A098E3Q0 pp 229533.A0A0E0S0W9
123098 229533.A0A098E3Q0 pp 229533.I1S0S6
123099 229533.A0A098E3Q0 pp 229533.I1RQL4
123100 229533.A0A098D8Z6 pp 229533.I1S3L2
123101 229533.A0A098D8Z6 pp 229533.I1RQH5
123102 229533.A0A098D8Z6 pp 229533.I1RXS0
123103 229533.A0A098D8Z6 pp 229533.I1RQP2
123104 229533.A0A098D8Z6 pp 229533.I1RHZ5
123105 229533.A0A098D8Z6 pp 229533.I1S0K0
123106 229533.A0A098D8Z6 pp 229533.I1RJ42
123107 229533.A0A098D8Z6 pp 229533.A0A1C3YIP5
123108 229533.A0A098D8Z6 pp 229533.I1RC48
123109 229533.A0A098D8Z6 pp 229533.I1RY72
123110 229533.A0A098D8Z6 pp 229533.A0A1C3YKM2
123111 229533.A0A098D8Z6 pp 229533.I1RQ01
123112 229533.A0A098D8Z6 pp 229533.A0A1C3YJ36
123113 229533.A0A098D8Z6 pp 229533.A0A0E0SGH2
123114 229533.A0A098D8Z6 pp 229533.A0A098DQM6
123115 229533.A0A098D8Z6 pp 229533.I1RNG4
123116 229533.A0A098D8Z6 pp 229533.I1S2C4
123117 229533.A0A098D8Z6 pp 229533.I1S8R4
123118 229533.A0A098D8Z6 pp 229533.I1RBS7
123119 229533.I1R9B3 pp 229533.I1RWZ0
123120 229533.I1R9B3 pp 229533.I1RM91
123121 229533.I1RZZ4 pp 229533.I1S2L3
123122 229533.I1RSQ2 pp 229533.V6QX21
123123 229533.I1RSQ2 pp 229533.V6RKY8
123124 229533.I1RSQ2 pp 229533.I1RZK1
123125 229533.I1RSQ2 pp 229533.I1S4Z7
123126 229533.I1RSQ2 pp 229533.V6QUV7
123127 229533.I1RSQ2 pp 229533.I1S3G4
123128 229533.I1RSQ2 pp 229533.I1S235
123129 229533.I1RSQ2 pp 229533.I1RYL5
123130 229533.I1RSQ2 pp 229533.I1RYP2
123131 229533.I1RSQ2 pp 229533.I1S1W8
123132 229533.I1S0R7 pp 229533.I1S9U2
123133 229533.I1S0R7 pp 229533.I1S0X6
123134 229533.I1S0R7 pp 229533.V6QYF0
123135 229533.I1S0R7 pp 229533.Q4IQK7
123136 229533.I1S0R7 pp 229533.Q4IPI1
123137 229533.I1S0R7 pp 229533.I1S7A8
123138 229533.I1S0R7 pp 229533.Q4IJH1
123139 229533.I1S0R7 pp 229533.Q4HZ47
123140 229533.I1S0R7 pp 229533.I1S0T2
123141 229533.I1S0R7 pp 229533.I1S540
123142 229533.I1S0R7 pp 229533.I1S7M2
123143 229533.I1S0R7 pp 229533.I1S6F0
123144 229533.I1S0R7 pp 229533.Q4INZ9
123145 229533.I1S0R7 pp 229533.V6R1K4
123146 229533.I1S0R7 pp 229533.Q4IFI0
123147 229533.I1S0R7 pp 229533.V6RTJ8
123148 229533.I1S0R7 pp 229533.Q4IJT5
123149 229533.I1S0R7 pp 229533.I1S5B0
123150 229533.I1S0R7 pp 229533.V6QW22
123151 229533.I1S0R7 pp 229533.Q4I2J8

123152	229533.I1S0R7	pp	229533.I1S1J7
123153	229533.I1S0R7	pp	229533.Q4IJK2
123154	229533.I1S0R7	pp	229533.Q4I283
123155	229533.I1S0R7	pp	229533.Q4I6B0
123156	229533.I1S0R7	pp	229533.I1S7M3
123157	229533.I1S0R7	pp	229533.Q4I5Z5
123158	229533.I1S0R7	pp	229533.Q4I948
123159	229533.I1S0R7	pp	229533.V6RCG5
123160	229533.I1S0R7	pp	229533.V6QUE6
123161	229533.I1S0R7	pp	229533.I1SA32
123162	229533.I1S0R7	pp	229533.V6RCZ8
123163	229533.I1S0R7	pp	229533.V6R227
123164	229533.I1S0R7	pp	229533.Q4IJ56
123165	229533.I1S0R7	pp	229533.Q4IEK8
123166	229533.I1S0R7	pp	229533.I1S419
123167	229533.I1S0R7	pp	229533.I1S2G9
123168	229533.I1S0R7	pp	229533.Q4ICA8
123169	229533.I1S0R7	pp	229533.I1S504
123170	229533.I1S0R7	pp	229533.O74638
123171	229533.I1S0R7	pp	229533.V6RUY9
123172	229533.I1S0R7	pp	229533.Q4IF76
123173	229533.I1S0R7	pp	229533.Q4I8P5
123174	229533.I1S0R7	pp	229533.I1S8V3
123175	229533.I1S0R7	pp	229533.V6R3X5
123176	229533.I1S0R7	pp	229533.I1S2E9
123177	229533.I1S0R7	pp	229533.I1S2H4
123178	229533.I1S0R7	pp	229533.I1S8E2
123179	229533.I1S0R7	pp	229533.I1S5R1
123180	229533.I1S0R7	pp	229533.I1S2B8
123181	229533.I1S0R7	pp	229533.Q4HZ42
123182	229533.I1S0R7	pp	229533.I1S5S2
123183	229533.I1S0R7	pp	229533.I1S253
123184	229533.I1S0R7	pp	229533.I1S524
123185	229533.I1S0R7	pp	229533.I1S1B6
123186	229533.I1S0R7	pp	229533.V6RIS2
123187	229533.I1S0R7	pp	229533.I1S4M5
123188	229533.I1S0R7	pp	229533.I1S503
123189	229533.I1S0R7	pp	229533.V6R283
123190	229533.I1S0R7	pp	229533.I1S6Y8
123191	229533.I1S0R7	pp	229533.V6RGN5
123192	229533.I1S0R7	pp	229533.I1S2C0
123193	229533.I1S0R7	pp	229533.Q4IN63
123194	229533.I1S0R7	pp	229533.Q4IQW0
123195	229533.I1S0R7	pp	229533.I1S8P5
123196	229533.I1S0R7	pp	229533.Q4IPZ1
123197	229533.I1S0R7	pp	229533.I1S1C7
123198	229533.I1S0R7	pp	229533.I1S1U7
123199	229533.I1S0R7	pp	229533.I1S1J6
123200	229533.I1S0R7	pp	229533.V6RGY3
123201	229533.I1S0R7	pp	229533.V6R057
123202	229533.I1S0R7	pp	229533.V6RMW6
123203	229533.I1S0R7	pp	229533.Q4HZK7
123204	229533.I1S0R7	pp	229533.Q4I327
123205	229533.I1S0R7	pp	229533.Q4I624
123206	229533.I1S0R7	pp	229533.V6RXB3
123207	229533.I1S0R7	pp	229533.V6R042
123208	229533.I1S0R7	pp	229533.V6QVH8
123209	229533.I1S0R7	pp	229533.V6RIV9
123210	229533.I1S0R7	pp	229533.I1S2E8
123211	229533.I1S0R7	pp	229533.I1S8R3
123212	229533.I1S0R7	pp	229533.V6RVG3
123213	229533.I1S0R7	pp	229533.I1SA33
123214	229533.I1S0R7	pp	229533.I1S8V4
123215	229533.I1S0R7	pp	229533.I1S1X9
123216	229533.I1S0R7	pp	229533.V6RY33
123217	229533.I1S0R7	pp	229533.Q4I8X0
123218	229533.V6QVV9	pp	229533.V6RE46
123219	229533.I1RWN5	pp	229533.V6R283
123220	229533.I1RWN5	pp	229533.I1S2G7
123221	229533.I1RWN5	pp	229533.I1S503
123222	229533.I1RWN5	pp	229533.I1S8P5
123223	229533.I1RWN5	pp	229533.I1S352
123224	229533.I1RWN5	pp	229533.I1RZS6

123225	229533.I1RWN5	pp	229533.I1RZZ9
123226	229533.I1RWN5	pp	229533.I1S0F6
123227	229533.I1RWN5	pp	229533.I1S8V3
123228	229533.I1RWN5	pp	229533.I1S2E9
123229	229533.I1RWN5	pp	229533.Q4IN63
123230	229533.I1RWN5	pp	229533.I1S2B8
123231	229533.I1RWN5	pp	229533.V6R3X5
123232	229533.I1RWN5	pp	229533.I1S0M6
123233	229533.I1RWN5	pp	229533.V6RUY9
123234	229533.I1RWN5	pp	229533.I1S0L4
123235	229533.I1RWN5	pp	229533.I1S5R1
123236	229533.I1RWN5	pp	229533.I1S0N4
123237	229533.I1RWN5	pp	229533.I1RYW3
123238	229533.I1RWN5	pp	229533.I1S2H4
123239	229533.I1RWN5	pp	229533.I1RZ39
123240	229533.I1RWN5	pp	229533.I1S8V4
123241	229533.I1RWN5	pp	229533.I1S2E8
123242	229533.I1RWN5	pp	229533.I1S0J1
123243	229533.I1RWN5	pp	229533.I1S1C7
123244	229533.I1RWN5	pp	229533.I1SA33
123245	229533.I1RWN5	pp	229533.Q4I624
123246	229533.I1RWN5	pp	229533.I1RY44
123247	229533.I1RWN5	pp	229533.V6R057
123248	229533.I1RWN5	pp	229533.I1RWN6
123249	229533.I1RWN5	pp	229533.I1S1W4
123250	229533.I1RWN5	pp	229533.V6QW22
123251	229533.I1RWN5	pp	229533.I1S0F5
123252	229533.I1RWN5	pp	229533.I1S569
123253	229533.I1RWN5	pp	229533.V6RMW6
123254	229533.I1RWN5	pp	229533.Q4IJT5
123255	229533.I1RWN5	pp	229533.V6RIV9
123256	229533.I1RWN5	pp	229533.V6R042
123257	229533.I1RWN5	pp	229533.V6R4J1
123258	229533.I1RWN5	pp	229533.I1S2C0
123259	229533.I1RWN5	pp	229533.I1S0L3
123260	229533.I1RWN5	pp	229533.V6R1K4
123261	229533.I1RWN5	pp	229533.I1RWX5
123262	229533.I1RWN5	pp	229533.I1RXD7
123263	229533.I1RWN5	pp	229533.I1S0X6
123264	229533.I1RWN5	pp	229533.I1S8R3
123265	229533.I1RWN5	pp	229533.I1RZW8
123266	229533.I1RWN5	pp	229533.Q4I327
123267	229533.I1RWN5	pp	229533.I1S9U2
123268	229533.I1RWN5	pp	229533.I1S021
123269	229533.I1RWN5	pp	229533.I1S7A8
123270	229533.I1RWN5	pp	229533.I1S0R7
123271	229533.I1RWN5	pp	229533.V6RCZ8
123272	229533.I1RWN5	pp	229533.I1S0M9
123273	229533.I1RWN5	pp	229533.I1RZM8
123274	229533.I1RWN5	pp	229533.Q4I8P5
123275	229533.I1RWN5	pp	229533.V6RTJ8
123276	229533.I1RWN5	pp	229533.I1RXF0
123277	229533.I1RWN5	pp	229533.I1S5B0
123278	229533.I1RWN5	pp	229533.I1RWT6
123279	229533.I1RWN5	pp	229533.V6QUE6
123280	229533.I1RWN5	pp	229533.I1RX46
123281	229533.I1RWN5	pp	229533.I1SA32
123282	229533.I1RWN5	pp	229533.I1RX25
123283	229533.I1RWN5	pp	229533.Q4I283
123284	229533.I1RWN5	pp	229533.I1RZK5
123285	229533.I1RWN5	pp	229533.I1RZZ1
123286	229533.I1RWN5	pp	229533.Q4IJM4
123287	229533.I1RWN5	pp	229533.I1S6U8
123288	229533.I1RWN5	pp	229533.I1RZF6
123289	229533.I1RWN5	pp	229533.I1RY03
123290	229533.I1RWN5	pp	229533.I1S504
123291	229533.I1RWN5	pp	229533.I1RZK6
123292	229533.I1RWN5	pp	229533.I1RYY5
123293	229533.I1RWN5	pp	229533.Q4I2J8
123294	229533.I1RWN5	pp	229533.I1S1J7
123295	229533.I1RWN5	pp	229533.V6RCG5
123296	229533.I1RWN5	pp	229533.Q4IJK2
123297	229533.I1RWN5	pp	229533.I1RZM1

123298	229533.I1RWN5	pp	229533.I1RZE5
123299	229533.I1RUT8	pp	229533.I1SAK0
123300	229533.I1RUT8	pp	229533.V6QTX7
123301	229533.I1RUT8	pp	229533.V6RM09
123302	229533.I1RUT8	pp	229533.V6REA4
123303	229533.I1RUT8	pp	229533.I1S2L3
123304	229533.I1RUT8	pp	229533.V6R556
123305	229533.I1RWX5	pp	229533.Q4I1Y1
123306	229533.I1RWX5	pp	229533.I1RZM1
123307	229533.I1RWX5	pp	229533.I1RY03
123308	229533.I1RWX5	pp	229533.I1RXC1
123309	229533.I1RWX5	pp	229533.I1RZK5
123310	229533.I1RWX5	pp	229533.I1S5B0
123311	229533.I1RWX5	pp	229533.I1RZF6
123312	229533.I1RWX5	pp	229533.I1RZM8
123313	229533.I1RWX5	pp	229533.Q4IJK2
123314	229533.I1RWX5	pp	229533.I1S0Z3
123315	229533.I1RWX5	pp	229533.I1RZZ1
123316	229533.I1RWX5	pp	229533.I1S1J7
123317	229533.I1RWX5	pp	229533.I1S0X6
123318	229533.I1RWX5	pp	229533.I1S504
123319	229533.I1RWX5	pp	229533.V6RCZ8
123320	229533.I1RWX5	pp	229533.I1RXG2
123321	229533.I1RWX5	pp	229533.I1S8R3
123322	229533.I1RWX5	pp	229533.I1S1W8
123323	229533.I1RWX5	pp	229533.I1S0L3
123324	229533.I1RWX5	pp	229533.Q4IJT5
123325	229533.I1RWX5	pp	229533.Q4HZS8
123326	229533.I1RWX5	pp	229533.I1S0R7
123327	229533.I1RWX5	pp	229533.I1S282
123328	229533.I1RWX5	pp	229533.I1RXD7
123329	229533.I1RWX5	pp	229533.V6QW22
123330	229533.I1RWX5	pp	229533.V6RIV9
123331	229533.I1RWX5	pp	229533.I1RX46
123332	229533.I1RWX5	pp	229533.I1S569
123333	229533.I1RWX5	pp	229533.I1S5N3
123334	229533.I1RWX5	pp	229533.Q4I624
123335	229533.I1RWX5	pp	229533.I1RY44
123336	229533.I1RWX5	pp	229533.V6RTJ8
123337	229533.I1RWX5	pp	229533.V6RLM7
123338	229533.I1RWX5	pp	229533.I1RYW3
123339	229533.I1RWX5	pp	229533.I1S0J1
123340	229533.I1RWX5	pp	229533.V6RS91
123341	229533.I1RWX5	pp	229533.I1S1C7
123342	229533.I1RWX5	pp	229533.I1S2B8
123343	229533.I1RWX5	pp	229533.I1S7J8
123344	229533.I1RWX5	pp	229533.Q4IPI4
123345	229533.I1RWX5	pp	229533.V6R3X5
123346	229533.I1RWX5	pp	229533.I1S0N4
123347	229533.I1RWX5	pp	229533.I1S2P0
123348	229533.I1RWX5	pp	229533.I1S1F3
123349	229533.I1RWX5	pp	229533.V6R042
123350	229533.I1RWX5	pp	229533.I1RYI5
123351	229533.I1RWX5	pp	229533.I1S5R1
123352	229533.I1RWX5	pp	229533.I1S2P4
123353	229533.I1RWX5	pp	229533.I1S8P5
123354	229533.I1RWX5	pp	229533.I1S503
123355	229533.I1RWX5	pp	229533.I1S3G4
123356	229533.I1RWX5	pp	229533.V6RMW6
123357	229533.I1RWX5	pp	229533.I1S2H4
123358	229533.I1RWX5	pp	229533.I1RZ39
123359	229533.I1RWX5	pp	229533.I1S2E9
123360	229533.I1RWX5	pp	229533.V6R3J1
123361	229533.I1RWX5	pp	229533.I1S0L4
123362	229533.I1RWX5	pp	229533.I1RZZ9
123363	229533.I1R9B0	pp	229533.I1RUY5
123364	229533.I1R9B0	pp	229533.I1RMT6
123365	229533.I1R9B0	pp	229533.I1RSA5
123366	229533.I1R9B0	pp	229533.I1RDU0
123367	229533.Q4IPZ1	pp	229533.V6QWP4
123368	229533.Q4IPZ1	pp	229533.V6RM66
123369	229533.Q4HZB8	pp	229533.Q4IPH4
123370	229533.Q4HZB8	pp	229533.V6RMW6

123371 229533.Q4HZB8 pp 229533.Q4IPB8
123372 229533.Q4HZB8 pp 229533.V6RIV9
123373 229533.Q4HZB8 pp 229533.V6R042
123374 229533.Q4HZB8 pp 229533.Q4IMD1
123375 229533.Q4HZB8 pp 229533.V6R227
123376 229533.Q4HZB8 pp 229533.Q4I665
123377 229533.Q4HZB8 pp 229533.V6RDZ4
123378 229533.Q4HZB8 pp 229533.Q4IJ84
123379 229533.Q4HZB8 pp 229533.V6R3X5
123380 229533.Q4HZB8 pp 229533.Q4I5R9
123381 229533.Q4HZB8 pp 229533.V6QW22
123382 229533.Q4HZB8 pp 229533.V6RTJ8
123383 229533.Q4HZB8 pp 229533.V6RCG5
123384 229533.Q4HZB8 pp 229533.Q4IPB3
123385 229533.Q4HZB8 pp 229533.Q4IQX3
123386 229533.Q4HZB8 pp 229533.Q4INI2
123387 229533.Q4HZB8 pp 229533.V6QUW4
123388 229533.A0A1C3YM39 pp 229533.Q4I2W2
123389 229533.I1RZW8 pp 229533.I1S1J7
123390 229533.I1RZW8 pp 229533.I1S8R3
123391 229533.I1RZW8 pp 229533.I1S504
123392 229533.I1RZW8 pp 229533.Q4I283
123393 229533.I1RZW8 pp 229533.V6R057
123394 229533.I1RZW8 pp 229533.I1S1C7
123395 229533.I1RZW8 pp 229533.Q4I624
123396 229533.I1RZW8 pp 229533.Q4I2J8
123397 229533.I1RZW8 pp 229533.Q4HZB8
123398 229533.I1RZW8 pp 229533.I1S569
123399 229533.I1RZW8 pp 229533.Q4IEK8
123400 229533.I1RZW8 pp 229533.V6R227
123401 229533.I1RZW8 pp 229533.I1S419
123402 229533.I1RZW8 pp 229533.I1RZZ1
123403 229533.I1RZW8 pp 229533.V6RIV9
123404 229533.I1RZW8 pp 229533.Q4I8P5
123405 229533.I1RZW8 pp 229533.Q4IJK2
123406 229533.I1RZW8 pp 229533.I1S0J1
123407 229533.I1RZW8 pp 229533.I1S5B0
123408 229533.I1RZW8 pp 229533.I1S0F5
123409 229533.I1RZW8 pp 229533.V6RCG5
123410 229533.I1RZW8 pp 229533.I1SA32
123411 229533.I1RZW8 pp 229533.V6RCZ8
123412 229533.I1RZW8 pp 229533.I1SA33
123413 229533.I1RZW8 pp 229533.V6R1K4
123414 229533.I1RZW8 pp 229533.I1S0L3
123415 229533.I1RZW8 pp 229533.V6RGN5
123416 229533.I1RZW8 pp 229533.V6RMW6
123417 229533.I1RZW8 pp 229533.I1S2B8
123418 229533.I1RZW8 pp 229533.I1S2H4
123419 229533.I1RZW8 pp 229533.V6R4J1
123420 229533.I1RZW8 pp 229533.V6R042
123421 229533.I1RZW8 pp 229533.I1S0F6
123422 229533.I1RZW8 pp 229533.I1S6Y8
123423 229533.I1RZW8 pp 229533.I1S0L4
123424 229533.I1RZW8 pp 229533.V6QUW4
123425 229533.I1RZW8 pp 229533.I1S5R1
123426 229533.I1RZW8 pp 229533.V6QYF0
123427 229533.I1RZW8 pp 229533.I1S6F0
123428 229533.I1RZW8 pp 229533.I1S0M6
123429 229533.I1RZW8 pp 229533.I1S8P5
123430 229533.I1RZW8 pp 229533.V6R3X5
123431 229533.I1RZW8 pp 229533.I1S352
123432 229533.I1RZW8 pp 229533.V6QW22
123433 229533.I1RZW8 pp 229533.I1S2M6
123434 229533.I1RZW8 pp 229533.V6RTJ8
123435 229533.I1RZW8 pp 229533.Q4IJT5
123436 229533.I1RZW8 pp 229533.Q4HVW2
123437 229533.I1RZW8 pp 229533.I1S7A8
123438 229533.I1RZW8 pp 229533.I1S0X6
123439 229533.I1RZW8 pp 229533.I1S0T2
123440 229533.I1RZW8 pp 229533.I1S0R7
123441 229533.I1RZW8 pp 229533.I1S2E9
123442 229533.I1RZW8 pp 229533.I1RZZ9
123443 229533.I1RZW8 pp 229533.I1S503

123444 229533.I1RBM1 pp 229533.I1RCR9
123445 229533.I1RBM1 pp 229533.I1RJV4
123446 229533.I1RPP4 pp 229533.Q4I2J8
123447 229533.I1RPP4 pp 229533.I1RSI0
123448 229533.I1RPP4 pp 229533.I1RZ35
123449 229533.I1RPP4 pp 229533.Q4I624
123450 229533.I1RPP4 pp 229533.I1RZE5
123451 229533.I1RPP4 pp 229533.Q4IPZ1
123452 229533.I1RPP4 pp 229533.I1RT03
123453 229533.I1RPP4 pp 229533.V6R057
123454 229533.I1RPP4 pp 229533.I1S569
123455 229533.I1RPP4 pp 229533.I1RVX0
123456 229533.I1RPP4 pp 229533.I1RWD7
123457 229533.I1RPP4 pp 229533.Q4IJM4
123458 229533.I1RPP4 pp 229533.Q4IJK2
123459 229533.I1RPP4 pp 229533.I1RQA3
123460 229533.I1RPP4 pp 229533.I1S1J7
123461 229533.I1RPP4 pp 229533.I1RQQ5
123462 229533.I1RPP4 pp 229533.I1RQE7
123463 229533.I1RPP4 pp 229533.I1RSK1
123464 229533.I1RPP4 pp 229533.V6RBN2
123465 229533.I1RPP4 pp 229533.I1RYY5
123466 229533.I1RPP4 pp 229533.I1RWT5
123467 229533.I1RPP4 pp 229533.I1RZK6
123468 229533.I1RPP4 pp 229533.I1RRN5
123469 229533.I1RPP4 pp 229533.I1RRY9
123470 229533.I1RPP4 pp 229533.I1RZZ1
123471 229533.I1RPP4 pp 229533.I1RWN6
123472 229533.I1RPP4 pp 229533.I1RWX5
123473 229533.I1RPP4 pp 229533.I1S0N4
123474 229533.I1RPP4 pp 229533.I1RU29
123475 229533.I1RPP4 pp 229533.I1RRI2
123476 229533.I1RPP4 pp 229533.I1RY03
123477 229533.I1RPP4 pp 229533.I1S8R3
123478 229533.I1RPP4 pp 229533.I1RTH6
123479 229533.I1RPP4 pp 229533.I1RTD7
123480 229533.I1RPP4 pp 229533.I1S5B0
123481 229533.I1RPP4 pp 229533.I1RYW3
123482 229533.I1RPP4 pp 229533.I1RZK5
123483 229533.I1RPP4 pp 229533.I1S044
123484 229533.I1RPP4 pp 229533.I1S1C7
123485 229533.I1RPP4 pp 229533.I1RXF0
123486 229533.I1RPP4 pp 229533.Q4HZB8
123487 229533.I1RPP4 pp 229533.I1RWN5
123488 229533.I1RPP4 pp 229533.I1RZM8
123489 229533.I1RPP4 pp 229533.I1RZW8
123490 229533.I1RPP4 pp 229533.Q4IN63
123491 229533.I1RPP4 pp 229533.V6RIV9
123492 229533.I1RPP4 pp 229533.I1RZM1
123493 229533.I1RPP4 pp 229533.I1RZS7
123494 229533.I1RPP4 pp 229533.I1RXT3
123495 229533.I1RPP4 pp 229533.I1RTA0
123496 229533.I1RPP4 pp 229533.I1RUG3
123497 229533.I1RPP4 pp 229533.I1RW46
123498 229533.I1RPP4 pp 229533.V6RVG3
123499 229533.I1RPP4 pp 229533.I1RRV1
123500 229533.I1RPP4 pp 229533.I1RZH4
123501 229533.I1RPP4 pp 229533.V6RCZ8
123502 229533.I1RPP4 pp 229533.I1RY44
123503 229533.I1RPP4 pp 229533.I1S504
123504 229533.I1RPP4 pp 229533.I1RSQ3
123505 229533.I1RPP4 pp 229533.I1RRE5
123506 229533.I1RPP4 pp 229533.I1S1W4
123507 229533.I1RPP4 pp 229533.I1S0J1
123508 229533.I1RPP4 pp 229533.V6RCG5
123509 229533.I1RPP4 pp 229533.I1SA32
123510 229533.I1RPP4 pp 229533.V6R042
123511 229533.I1RPP4 pp 229533.I1RS17
123512 229533.I1RPP4 pp 229533.I1RWT6
123513 229533.I1RPP4 pp 229533.I1RQ81
123514 229533.I1RPP4 pp 229533.V6RMW6
123515 229533.I1RPP4 pp 229533.Q4I283
123516 229533.I1RPP4 pp 229533.I1RRM5

123517	229533.I1RPP4	pp	229533.I1S2B8
123518	229533.I1RPP4	pp	229533.I1RS18
123519	229533.I1RPP4	pp	229533.I1RQ23
123520	229533.I1RPP4	pp	229533.Q4I8P5
123521	229533.I1RPP4	pp	229533.I1SA33
123522	229533.I1RPP4	pp	229533.I1RX25
123523	229533.I1RPP4	pp	229533.V6QUW4
123524	229533.I1RPP4	pp	229533.I1S0M6
123525	229533.I1RPP4	pp	229533.I1RRU9
123526	229533.I1RPP4	pp	229533.I1RW25
123527	229533.I1RPP4	pp	229533.I1RYJ8
123528	229533.I1RPP4	pp	229533.I1RPU3
123529	229533.I1RPP4	pp	229533.I1RW99
123530	229533.I1RPP4	pp	229533.I1RWJ8
123531	229533.I1RPP4	pp	229533.I1RW07
123532	229533.I1RPP4	pp	229533.I1RZJ5
123533	229533.I1RPP4	pp	229533.I1RS47
123534	229533.I1RPP4	pp	229533.I1RRQ1
123535	229533.I1RPP4	pp	229533.I1S0X6
123536	229533.I1RPP4	pp	229533.I1S0F5
123537	229533.I1RPP4	pp	229533.I1RWI5
123538	229533.I1RPP4	pp	229533.I1RWL2
123539	229533.I1RPP4	pp	229533.I1S2G7
123540	229533.I1RPP4	pp	229533.I1RZZ9
123541	229533.I1RPP4	pp	229533.I1S0L3
123542	229533.I1RPP4	pp	229533.I1S5R1
123543	229533.I1RPP4	pp	229533.I1RRT8
123544	229533.I1RPP4	pp	229533.V6R1K4
123545	229533.I1RPP4	pp	229533.V6R283
123546	229533.I1RPP4	pp	229533.I1RS20
123547	229533.I1RPP4	pp	229533.I1RZ39
123548	229533.I1RPP4	pp	229533.I1RR24
123549	229533.I1RPP4	pp	229533.I1S2H4
123550	229533.I1RPP4	pp	229533.I1RWK3
123551	229533.I1RPP4	pp	229533.I1S0L4
123552	229533.I1RPP4	pp	229533.I1RR73
123553	229533.I1RPP4	pp	229533.I1S0Y1
123554	229533.I1RPP4	pp	229533.V6R3X5
123555	229533.I1RPP4	pp	229533.Q4IJT5
123556	229533.I1RPP4	pp	229533.I1S0F6
123557	229533.I1RPP4	pp	229533.I1RR00
123558	229533.I1RPP4	pp	229533.I1S6U8
123559	229533.I1RPP4	pp	229533.I1S352
123560	229533.I1RPP4	pp	229533.I1RSV2
123561	229533.I1RPP4	pp	229533.I1RVQ0
123562	229533.I1RPP4	pp	229533.I1RW71
123563	229533.I1RPP4	pp	229533.V6R4J1
123564	229533.I1RPP4	pp	229533.V6QW22
123565	229533.I1RPP4	pp	229533.I1RRJ8
123566	229533.I1RPP4	pp	229533.I1RQG4
123567	229533.I1RPP4	pp	229533.I1S8P5
123568	229533.I1RPP4	pp	229533.I1RSQ7
123569	229533.I1RPP4	pp	229533.I1RX46
123570	229533.I1RPP4	pp	229533.I1S503
123571	229533.I1RPP4	pp	229533.I1RWF7
123572	229533.I1RPP4	pp	229533.I1RT40
123573	229533.I1RPP4	pp	229533.I1S7A8
123574	229533.I1RPP4	pp	229533.V6RTJ8
123575	229533.I1RPP4	pp	229533.I1RTD5
123576	229533.I1RPP4	pp	229533.I1RV91
123577	229533.I1RPP4	pp	229533.I1RQC9
123578	229533.I1RPP4	pp	229533.I1RT04
123579	229533.I1RPP4	pp	229533.I1S0R7
123580	229533.I1RPP4	pp	229533.I1RQH3
123581	229533.I1RPP4	pp	229533.I1S2E9
123582	229533.I1RPP4	pp	229533.I1RXD7
123583	229533.I1RRW5	pp	229533.V6R1X4
123584	229533.A0A0E0SCQ6	pp	229533.A0A0E0SP71
123585	229533.I1RH76	pp	229533.V6RB81
123586	229533.I1RH76	pp	229533.I1RUA7
123587	229533.I1RH76	pp	229533.I1RIJ2
123588	229533.I1RH76	pp	229533.I1RL93
123589	229533.I1RH76	pp	229533.I1RQE8

123590 229533.I1RH76 pp 229533.I1RZM4
123591 229533.I1RH76 pp 229533.V6RK96
123592 229533.I1RH76 pp 229533.I1SAI0
123593 229533.I1RH76 pp 229533.I1RHS7
123594 229533.I1RH76 pp 229533.V6QVL9
123595 229533.I1RH76 pp 229533.V6QZS3
123596 229533.I1S1C7 pp 229533.I1S1J7
123597 229533.I1S1C7 pp 229533.Q4I8P5
123598 229533.I1S1C7 pp 229533.V6RCG5
123599 229533.I1S1C7 pp 229533.I1SA32
123600 229533.I1S1C7 pp 229533.Q4IJK2
123601 229533.I1S1C7 pp 229533.I1S504
123602 229533.I1S1C7 pp 229533.V6RBN2
123603 229533.I1S1C7 pp 229533.I1S419
123604 229533.I1S1C7 pp 229533.Q4I2J8
123605 229533.I1S1C7 pp 229533.V6RCZ8
123606 229533.I1S1C7 pp 229533.I1S887
123607 229533.I1S1C7 pp 229533.Q4I283
123608 229533.I1S1C7 pp 229533.I1S6K9
123609 229533.I1S1C7 pp 229533.V6QUE6
123610 229533.I1S1C7 pp 229533.I1S4S4
123611 229533.I1S1C7 pp 229533.I1S5B0
123612 229533.I1S1C7 pp 229533.I1S9U2
123613 229533.I1S1C7 pp 229533.V6R4J1
123614 229533.I1S1C7 pp 229533.I1S6U8
123615 229533.I1S1C7 pp 229533.I1S4S7
123616 229533.I1S1C7 pp 229533.I1S2E8
123617 229533.I1S1C7 pp 229533.V6QUW4
123618 229533.I1S1C7 pp 229533.V6QW22
123619 229533.I1S1C7 pp 229533.V6RUT5
123620 229533.I1S1C7 pp 229533.I1S7A8
123621 229533.I1S1C7 pp 229533.Q4I624
123622 229533.I1S1C7 pp 229533.V6R1K4
123623 229533.I1S1C7 pp 229533.V6RTJ8
123624 229533.I1S1C7 pp 229533.Q4IJT5
123625 229533.I1S1C7 pp 229533.V6R057
123626 229533.I1S1C7 pp 229533.I1S1W3
123627 229533.I1S1C7 pp 229533.I1S569
123628 229533.I1S1C7 pp 229533.I1S8V4
123629 229533.I1S1C7 pp 229533.I1S8V3
123630 229533.I1S1C7 pp 229533.I1S5R1
123631 229533.I1S1C7 pp 229533.I1S8R3
123632 229533.I1S1C7 pp 229533.V6RIV9
123633 229533.I1S1C7 pp 229533.I1S2C0
123634 229533.I1S1C7 pp 229533.I1S2H4
123635 229533.I1S1C7 pp 229533.Q4HZB8
123636 229533.I1S1C7 pp 229533.I1S1W9
123637 229533.I1S1C7 pp 229533.I1S9Y1
123638 229533.I1S1C7 pp 229533.I1S7W5
123639 229533.I1S1C7 pp 229533.I1S9Z4
123640 229533.I1S1C7 pp 229533.O74638
123641 229533.I1S1C7 pp 229533.V6RMW6
123642 229533.I1S1C7 pp 229533.I1S352
123643 229533.I1S1C7 pp 229533.V6R042
123644 229533.I1S1C7 pp 229533.I1SA33
123645 229533.I1S1C7 pp 229533.I1S8P5
123646 229533.I1S1C7 pp 229533.I1S2B8
123647 229533.I1S1C7 pp 229533.V6R283
123648 229533.I1S1C7 pp 229533.Q4IN63
123649 229533.I1S1C7 pp 229533.I1S6Y8
123650 229533.I1S1C7 pp 229533.Q4I327
123651 229533.I1S1C7 pp 229533.Q4I662
123652 229533.I1S1C7 pp 229533.I1S5R6
123653 229533.I1S1C7 pp 229533.I1S2E9
123654 229533.I1S1C7 pp 229533.I1S503
123655 229533.I1S1C7 pp 229533.I1S2G7
123656 229533.I1S1C7 pp 229533.V6R3X5
123657 229533.I1S1C7 pp 229533.Q4HXF0
123658 229533.I1S1C7 pp 229533.V6RUY9
123659 229533.I1S1C7 pp 229533.V6R3J1
123660 229533.I1RJG2 pp 229533.I1S927
123661 229533.V6R1K4 pp 229533.V6R2A1
123662 229533.V6R1K4 pp 229533.V6RRH2

123663 229533.V6R1K4 pp 229533.V6RIV9
123664 229533.V6R1K4 pp 229533.V6RMW6
123665 229533.V6R1K4 pp 229533.V6RCG5
123666 229533.V6R1K4 pp 229533.V6RCZ8
123667 229533.V6R1K4 pp 229533.V6R3X5
123668 229533.V6R1K4 pp 229533.V6RY56
123669 229533.V6R1K4 pp 229533.V6RBN2
123670 229533.V6R1K4 pp 229533.V6RVJ3
123671 229533.V6R1K4 pp 229533.V6RTJ8
123672 229533.I1RME1 pp 229533.I1RTN1
123673 229533.I1RME1 pp 229533.I1RTN8
123674 229533.I1RME1 pp 229533.I1RME2
123675 229533.I1RME1 pp 229533.I1S984
123676 229533.I1RME1 pp 229533.I1RUT8
123677 229533.I1RME1 pp 229533.I1RNA1
123678 229533.I1RME1 pp 229533.I1S574
123679 229533.I1RME1 pp 229533.I1RY27
123680 229533.I1RME1 pp 229533.I1SAK0
123681 229533.I1RME1 pp 229533.I1S2L3
123682 229533.I1RME1 pp 229533.I1RQW2
123683 229533.I1RUG5 pp 229533.Q4I7L0
123684 229533.I1RYW3 pp 229533.Q4HWB3
123685 229533.I1RYW3 pp 229533.Q5I2J3
123686 229533.I1RYW3 pp 229533.Q4HZS8
123687 229533.I1RYW3 pp 229533.V6QW22
123688 229533.I1RYW3 pp 229533.I1S4S7
123689 229533.I1RYW3 pp 229533.V6QUW4
123690 229533.I1RYW3 pp 229533.I1RZG7
123691 229533.I1RYW3 pp 229533.I1S4S4
123692 229533.I1RYW3 pp 229533.I1S0T2
123693 229533.I1RYW3 pp 229533.Q4IJH1
123694 229533.I1RYW3 pp 229533.I1S9L5
123695 229533.I1RYW3 pp 229533.I1S3J4
123696 229533.I1RYW3 pp 229533.I1RZM4
123697 229533.I1RYW3 pp 229533.Q4IJT5
123698 229533.I1RYW3 pp 229533.I1S2Y5
123699 229533.I1RYW3 pp 229533.V6RTJ8
123700 229533.I1RYW3 pp 229533.I1S0X6
123701 229533.I1RYW3 pp 229533.I1S0L3
123702 229533.I1RYW3 pp 229533.V6RUT5
123703 229533.I1RYW3 pp 229533.I1S8F8
123704 229533.I1RYW3 pp 229533.I1S0R7
123705 229533.I1RYW3 pp 229533.I1S1C6
123706 229533.I1RYW3 pp 229533.V6R1K4
123707 229533.I1RYW3 pp 229533.I1RZM1
123708 229533.I1RYW3 pp 229533.Q4INZ9
123709 229533.I1RYW3 pp 229533.I1S0Z3
123710 229533.I1RYW3 pp 229533.I1S7M2
123711 229533.I1RYW3 pp 229533.Q4IJK2
123712 229533.I1RYW3 pp 229533.I1RZZ1
123713 229533.I1RYW3 pp 229533.Q4IB70
123714 229533.I1RYW3 pp 229533.Q4IRI9
123715 229533.I1RYW3 pp 229533.I1S1J7
123716 229533.I1RYW3 pp 229533.Q4I061
123717 229533.I1RYW3 pp 229533.Q4I2J8
123718 229533.I1RYW3 pp 229533.Q4HTT2
123719 229533.I1RYW3 pp 229533.Q4IAA0
123720 229533.I1RYW3 pp 229533.Q4I8P5
123721 229533.I1RYW3 pp 229533.I1RZ11
123722 229533.I1RYW3 pp 229533.I1S887
123723 229533.I1RYW3 pp 229533.V6RCZ8
123724 229533.I1RYW3 pp 229533.I1S6K9
123725 229533.I1RYW3 pp 229533.I1S504
123726 229533.I1RYW3 pp 229533.I1RZK5
123727 229533.I1RYW3 pp 229533.V6RK96
123728 229533.I1RYW3 pp 229533.I1SA32
123729 229533.I1RYW3 pp 229533.I1S5B0
123730 229533.I1RYW3 pp 229533.V6QUE6
123731 229533.I1RYW3 pp 229533.I1S419
123732 229533.I1RYW3 pp 229533.V6RCG5
123733 229533.I1RYW3 pp 229533.I1RZM8
123734 229533.I1RYW3 pp 229533.Q4IE79
123735 229533.I1RYW3 pp 229533.O74638

123736 229533.I1RYW3 pp 229533.I1S5R1
123737 229533.I1RYW3 pp 229533.I1S097
123738 229533.I1RYW3 pp 229533.I1S4Z7
123739 229533.I1RYW3 pp 229533.I1S524
123740 229533.I1RYW3 pp 229533.I1S277
123741 229533.I1RYW3 pp 229533.V6R3X5
123742 229533.I1RYW3 pp 229533.I1S6Y8
123743 229533.I1RYW3 pp 229533.I1RZ39
123744 229533.I1RYW3 pp 229533.I1S7M3
123745 229533.I1RYW3 pp 229533.Q4IPI4
123746 229533.I1RYW3 pp 229533.I1S0L4
123747 229533.I1RYW3 pp 229533.I1S4Z8
123748 229533.I1RYW3 pp 229533.I1S2H4
123749 229533.I1RYW3 pp 229533.Q4IF76
123750 229533.I1RYW3 pp 229533.I1S8V3
123751 229533.I1RYW3 pp 229533.I1S2B8
123752 229533.I1RYW3 pp 229533.Q4HZI0
123753 229533.I1RYW3 pp 229533.Q4I9U7
123754 229533.I1RYW3 pp 229533.I1RZZ9
123755 229533.I1RYW3 pp 229533.Q4IQ72
123756 229533.I1RYW3 pp 229533.Q4I5M4
123757 229533.I1RYW3 pp 229533.I1S9L6
123758 229533.I1RYW3 pp 229533.I1S9Y1
123759 229533.I1RYW3 pp 229533.Q4HXF0
123760 229533.I1RYW3 pp 229533.V6RUY9
123761 229533.I1RYW3 pp 229533.I1S2E9
123762 229533.I1RYW3 pp 229533.I1S0F6
123763 229533.I1RYW3 pp 229533.I1S8P5
123764 229533.I1RYW3 pp 229533.I1S4M5
123765 229533.I1RYW3 pp 229533.I1S5S2
123766 229533.I1RYW3 pp 229533.V6R057
123767 229533.I1RYW3 pp 229533.V6QXG1
123768 229533.I1RYW3 pp 229533.I1S2P0
123769 229533.I1RYW3 pp 229533.Q4I624
123770 229533.I1RYW3 pp 229533.I1S503
123771 229533.I1RYW3 pp 229533.V6RRH2
123772 229533.I1RYW3 pp 229533.I1S8R3
123773 229533.I1RYW3 pp 229533.I1RZD3
123774 229533.I1RYW3 pp 229533.V6R3J1
123775 229533.I1RYW3 pp 229533.I1S1W9
123776 229533.I1RYW3 pp 229533.I1S1W3
123777 229533.I1RYW3 pp 229533.V6QX21
123778 229533.I1RYW3 pp 229533.I1S7W5
123779 229533.I1RYW3 pp 229533.I1S0B1
123780 229533.I1RYW3 pp 229533.V6RLM7
123781 229533.I1RYW3 pp 229533.I1S0J1
123782 229533.I1RYW3 pp 229533.I1S056
123783 229533.I1RYW3 pp 229533.I1RZW8
123784 229533.I1RYW3 pp 229533.I1RZQ5
123785 229533.I1RYW3 pp 229533.I1S1C7
123786 229533.I1RYW3 pp 229533.V6QUV7
123787 229533.I1RYW3 pp 229533.I1S7J8
123788 229533.I1RYW3 pp 229533.I1S5N3
123789 229533.I1RYW3 pp 229533.I1S0N4
123790 229533.I1RYW3 pp 229533.I1S5R6
123791 229533.I1RYW3 pp 229533.I1S8V4
123792 229533.I1RYW3 pp 229533.I1S256
123793 229533.I1RYW3 pp 229533.V6R042
123794 229533.I1RYW3 pp 229533.V6RY33
123795 229533.I1RYW3 pp 229533.I1RZS8
123796 229533.I1RYW3 pp 229533.V6RIS2
123797 229533.I1RYW3 pp 229533.I1SAN9
123798 229533.I1RYW3 pp 229533.V6RMW6
123799 229533.I1RYW3 pp 229533.I1SA33
123800 229533.I1RYW3 pp 229533.V6RIV9
123801 229533.A0A1C3YM38 pp 229533.I1RJS2
123802 229533.A0A1C3YM38 pp 229533.I1RN81
123803 229533.A0A1C3YM38 pp 229533.V6QUU6
123804 229533.A0A1C3YM38 pp 229533.I1RMR5
123805 229533.A0A1C3YM38 pp 229533.V6RR76
123806 229533.A0A1C3YM38 pp 229533.I1REK3
123807 229533.I1RPQ9 pp 229533.I1RWN8
123808 229533.I1RPQ9 pp 229533.I1S071

123809	229533.I1RPQ9	pp	229533.I1S2L3
123810	229533.I1RYR3	pp	229533.I1RZA7
123811	229533.I1RYR3	pp	229533.V6RUB1
123812	229533.I1RYR3	pp	229533.Q4HYR2
123813	229533.I1RYR3	pp	229533.I1S9T6
123814	229533.I1RYR3	pp	229533.I1S6P5
123815	229533.I1RYR3	pp	229533.I1SA33
123816	229533.I1RS72	pp	229533.I1RT47
123817	229533.I1RS72	pp	229533.I1RT35
123818	229533.I1RS72	pp	229533.I1S9M0
123819	229533.I1RAU7	pp	229533.I1S6F0
123820	229533.I1RAU7	pp	229533.I1RDU4
123821	229533.I1RAU7	pp	229533.I1RD52
123822	229533.I1RAU7	pp	229533.I1RVQ0
123823	229533.I1RAU7	pp	229533.V6R4J1
123824	229533.I1RAU7	pp	229533.I1RD56
123825	229533.I1RAU7	pp	229533.V6R1K4
123826	229533.I1RAU7	pp	229533.I1RR00
123827	229533.I1RAU7	pp	229533.I1RG53
123828	229533.I1RAU7	pp	229533.I1RQJ5
123829	229533.I1RAU7	pp	229533.I1S0L3
123830	229533.I1RAU7	pp	229533.I1RSQ7
123831	229533.I1RAU7	pp	229533.I1S0F5
123832	229533.I1RAU7	pp	229533.I1RN61
123833	229533.I1RAU7	pp	229533.V6QW22
123834	229533.I1RAU7	pp	229533.I1RC20
123835	229533.I1RAU7	pp	229533.I1RSI4
123836	229533.I1RAU7	pp	229533.I1RWH5
123837	229533.I1RAU7	pp	229533.I1RX46
123838	229533.I1RAU7	pp	229533.I1RFW5
123839	229533.I1RAU7	pp	229533.I1S854
123840	229533.I1RAU7	pp	229533.I1RIL8
123841	229533.I1RAU7	pp	229533.I1S0T2
123842	229533.I1RAU7	pp	229533.Q4IJT5
123843	229533.I1RAU7	pp	229533.I1RBC9
123844	229533.I1RAU7	pp	229533.Q4IPI1
123845	229533.I1RAU7	pp	229533.I1RLC7
123846	229533.I1RAU7	pp	229533.I1RW71
123847	229533.I1RAU7	pp	229533.I1RM67
123848	229533.I1RAU7	pp	229533.I1RZJ5
123849	229533.I1RAU7	pp	229533.I1RAX6
123850	229533.I1RAU7	pp	229533.I1RCC2
123851	229533.I1RAU7	pp	229533.Q4IFI0
123852	229533.I1RAU7	pp	229533.I1S7A8
123853	229533.I1RAU7	pp	229533.I1RX25
123854	229533.I1RAU7	pp	229533.I1RRQ1
123855	229533.I1RAU7	pp	229533.I1RMG8
123856	229533.I1RAU7	pp	229533.I1RPA7
123857	229533.I1RAU7	pp	229533.I1RT98
123858	229533.I1RAU7	pp	229533.V6RTJ8
123859	229533.I1RAU7	pp	229533.I1RR24
123860	229533.I1RAU7	pp	229533.I1RW25
123861	229533.I1RAU7	pp	229533.I1RPW9
123862	229533.I1RAU7	pp	229533.I1RI02
123863	229533.I1RAU7	pp	229533.I1S8I8
123864	229533.I1RAU7	pp	229533.I1RC11
123865	229533.I1RAU7	pp	229533.I1RW07
123866	229533.I1RAU7	pp	229533.I1RXU7
123867	229533.I1RAU7	pp	229533.I1S9U2
123868	229533.I1RAU7	pp	229533.I1RCS1
123869	229533.I1RAU7	pp	229533.I1RQC9
123870	229533.I1RAU7	pp	229533.V6QUW4
123871	229533.I1RAU7	pp	229533.I1RBT1
123872	229533.I1RAU7	pp	229533.I1S0X6
123873	229533.I1RAU7	pp	229533.I1RM06
123874	229533.I1RAU7	pp	229533.I1S0R7
123875	229533.I1RAU7	pp	229533.I1RWJ8
123876	229533.I1RAU7	pp	229533.I1RMC8
123877	229533.I1RAU7	pp	229533.I1RPU0
123878	229533.I1RAU7	pp	229533.I1RQH3
123879	229533.I1RAU7	pp	229533.I1RDB1
123880	229533.I1RAU7	pp	229533.I1RT80
123881	229533.I1RAU7	pp	229533.I1RN58

123882	229533.I1RAU7	pp	229533.I1RXD7
123883	229533.I1RAU7	pp	229533.I1RFQ7
123884	229533.I1RAU7	pp	229533.I1RXF0
123885	229533.I1RAU7	pp	229533.I1RIG2
123886	229533.I1RAU7	pp	229533.I1S504
123887	229533.I1RAU7	pp	229533.Q4IJH1
123888	229533.I1RAU7	pp	229533.I1RZM8
123889	229533.I1RAU7	pp	229533.I1RJU3
123890	229533.I1RAU7	pp	229533.Q4I283
123891	229533.I1RAU7	pp	229533.I1RYB9
123892	229533.I1RAU7	pp	229533.I1RI82
123893	229533.I1RAU7	pp	229533.I1RWT6
123894	229533.I1RAU7	pp	229533.Q4HVW2
123895	229533.I1RAU7	pp	229533.I1RBN1
123896	229533.I1RAU7	pp	229533.I1SA32
123897	229533.I1RAU7	pp	229533.I1S021
123898	229533.I1RAU7	pp	229533.I1RZH4
123899	229533.I1RAU7	pp	229533.V6RCZ8
123900	229533.I1RAU7	pp	229533.I1RNH6
123901	229533.I1RAU7	pp	229533.I1RR51
123902	229533.I1RAU7	pp	229533.I1S6U8
123903	229533.I1RAU7	pp	229533.I1RDK7
123904	229533.I1RAU7	pp	229533.I1S9T2
123905	229533.I1RAU7	pp	229533.I1S419
123906	229533.I1RAU7	pp	229533.V6RCG5
123907	229533.I1RAU7	pp	229533.I1RBJ5
123908	229533.I1RAU7	pp	229533.I1S0M9
123909	229533.I1RAU7	pp	229533.Q4IEK8
123910	229533.I1RAU7	pp	229533.Q4IJ56
123911	229533.I1RAU7	pp	229533.I1RVZ0
123912	229533.I1RAU7	pp	229533.I1RLU6
123913	229533.I1RAU7	pp	229533.I1RQ81
123914	229533.I1RAU7	pp	229533.Q4I2J8
123915	229533.I1RAU7	pp	229533.I1RZ35
123916	229533.I1RAU7	pp	229533.Q4IAA0
123917	229533.I1RAU7	pp	229533.I1S6F1
123918	229533.I1RAU7	pp	229533.I1S0Z9
123919	229533.I1RAU7	pp	229533.I1RNQ5
123920	229533.I1RAU7	pp	229533.I1RZM1
123921	229533.I1RAU7	pp	229533.I1RB27
123922	229533.I1RAU7	pp	229533.Q4I8P5
123923	229533.I1RAU7	pp	229533.I1RGC2
123924	229533.I1RAU7	pp	229533.I1RS18
123925	229533.I1RAU7	pp	229533.I1RZE5
123926	229533.I1RAU7	pp	229533.I1RYB1
123927	229533.I1RAU7	pp	229533.I1RZF6
123928	229533.I1RAU7	pp	229533.I1RKL3
123929	229533.I1RAU7	pp	229533.I1RLM7
123930	229533.I1RAU7	pp	229533.I1RRU9
123931	229533.I1RAU7	pp	229533.I1RFT2
123932	229533.I1RAU7	pp	229533.I1RF19
123933	229533.I1RAU7	pp	229533.I1RC17
123934	229533.I1RAU7	pp	229533.Q4IBS2
123935	229533.I1RAU7	pp	229533.Q4I830
123936	229533.I1RAU7	pp	229533.I1RSI0
123937	229533.I1RAU7	pp	229533.I1RE00
123938	229533.I1RAU7	pp	229533.Q4IP34
123939	229533.I1RAU7	pp	229533.I1RF08
123940	229533.I1RAU7	pp	229533.I1S352
123941	229533.I1RAU7	pp	229533.I1REK8
123942	229533.I1RAU7	pp	229533.I1RFT1
123943	229533.I1RAU7	pp	229533.I1RCR5
123944	229533.I1RAU7	pp	229533.I1RSV2
123945	229533.I1RAU7	pp	229533.I1S044
123946	229533.I1RAU7	pp	229533.I1S5B0
123947	229533.I1RAU7	pp	229533.I1RWD7
123948	229533.I1RAU7	pp	229533.I1RB75
123949	229533.I1RAU7	pp	229533.I1RW99
123950	229533.I1RAU7	pp	229533.I1RWF7
123951	229533.I1RAU7	pp	229533.Q4IJK2
123952	229533.I1RAU7	pp	229533.I1RMC0
123953	229533.I1RAU7	pp	229533.I1RQA3
123954	229533.I1RAU7	pp	229533.I1RPP3

123955 229533.I1RAU7 pp 229533.I1RB74
123956 229533.I1RAU7 pp 229533.I1S8P5
123957 229533.I1RAU7 pp 229533.I1RZZ1
123958 229533.I1RAU7 pp 229533.I1RBC4
123959 229533.I1RAU7 pp 229533.I1RCG4
123960 229533.I1RAU7 pp 229533.I1RZK6
123961 229533.I1RAU7 pp 229533.I1S0C1
123962 229533.I1RAU7 pp 229533.I1RQG4
123963 229533.I1RAU7 pp 229533.I1RZK5
123964 229533.I1RAU7 pp 229533.I1RRJ8
123965 229533.I1RAU7 pp 229533.Q4IAS1
123966 229533.I1RAU7 pp 229533.I1RJZ6
123967 229533.I1RAU7 pp 229533.I1RQQ5
123968 229533.I1RAU7 pp 229533.I1RK83
123969 229533.I1RAU7 pp 229533.I1RLN6
123970 229533.I1RAU7 pp 229533.Q4I7F9
123971 229533.I1RAU7 pp 229533.I1RRZ0
123972 229533.I1RAU7 pp 229533.I1RN67
123973 229533.I1RAU7 pp 229533.I1RLV9
123974 229533.I1RAU7 pp 229533.I1S0F6
123975 229533.I1RAU7 pp 229533.V6RBN2
123976 229533.I1RAU7 pp 229533.I1RBS0
123977 229533.I1RAU7 pp 229533.I1RRQ3
123978 229533.I1RAU7 pp 229533.Q4IJM4
123979 229533.I1RAU7 pp 229533.I1RFG0
123980 229533.I1RAU7 pp 229533.I1S503
123981 229533.I1RAU7 pp 229533.I1RD03
123982 229533.I1RAU7 pp 229533.I1RDG0
123983 229533.I1RAU7 pp 229533.I1RJW6
123984 229533.I1RAU7 pp 229533.I1RBS6
123985 229533.I1RAU7 pp 229533.Q4HZ42
123986 229533.I1RAU7 pp 229533.I1RS47
123987 229533.I1RAU7 pp 229533.I1RGA4
123988 229533.I1RAU7 pp 229533.I1RB53
123989 229533.I1RAU7 pp 229533.I1RQ65
123990 229533.I1RAU7 pp 229533.I1S1J7
123991 229533.I1RAU7 pp 229533.I1RSL9
123992 229533.I1RAU7 pp 229533.I1RJS2
123993 229533.I1RAU7 pp 229533.I1RBL7
123994 229533.I1RAU7 pp 229533.I1RI03
123995 229533.I1RAU7 pp 229533.I1S2G7
123996 229533.I1RAU7 pp 229533.I1RRY9
123997 229533.I1RAU7 pp 229533.I1RU29
123998 229533.I1RAU7 pp 229533.I1RP27
123999 229533.I1RAU7 pp 229533.I1RN63
124000 229533.I1RAU7 pp 229533.I1RY03
124001 229533.I1RAU7 pp 229533.I1RWK3
124002 229533.I1RAU7 pp 229533.I1S0L8
124003 229533.I1RAU7 pp 229533.I1RC03
124004 229533.I1RAU7 pp 229533.I1RJV4
124005 229533.I1RAU7 pp 229533.I1RV91
124006 229533.I1RAU7 pp 229533.Q4HW67
124007 229533.I1RAU7 pp 229533.I1RSR8
124008 229533.I1RAU7 pp 229533.I1RT40
124009 229533.I1RAU7 pp 229533.V6R283
124010 229533.I1RAU7 pp 229533.I1RYY5
124011 229533.I1RAU7 pp 229533.I1RFU8
124012 229533.I1RAU7 pp 229533.I1RCJ7
124013 229533.I1RAU7 pp 229533.I1RM82
124014 229533.I1RAU7 pp 229533.V6RUY9
124015 229533.I1RAU7 pp 229533.I1RTD5
124016 229533.I1RAU7 pp 229533.I1RZV6
124017 229533.I1RAU7 pp 229533.I1RT04
124018 229533.I1RAU7 pp 229533.I1RGF5
124019 229533.I1RAU7 pp 229533.I1S2E9
124020 229533.I1RAU7 pp 229533.I1RNA5
124021 229533.I1RAU7 pp 229533.I1S5R1
124022 229533.I1RAU7 pp 229533.I1RKJ6
124023 229533.I1RAU7 pp 229533.I1RC33
124024 229533.I1RAU7 pp 229533.I1RY44
124025 229533.I1RAU7 pp 229533.I1S2B8
124026 229533.I1RAU7 pp 229533.I1RJU5
124027 229533.I1RAU7 pp 229533.I1RM96

124028 229533.I1RAU7 pp 229533.I1RRT8
124029 229533.I1RAU7 pp 229533.I1RC00
124030 229533.I1RAU7 pp 229533.I1RWL2
124031 229533.I1RAU7 pp 229533.I1RPG6
124032 229533.I1RAU7 pp 229533.I1RRM5
124033 229533.I1RAU7 pp 229533.I1RJZ7
124034 229533.I1RAU7 pp 229533.I1RS20
124035 229533.I1RAU7 pp 229533.I1RBY4
124036 229533.I1RAU7 pp 229533.I1RE76
124037 229533.I1RAU7 pp 229533.I1S7S9
124038 229533.I1RAU7 pp 229533.V6RVG3
124039 229533.I1RAU7 pp 229533.I1RPU3
124040 229533.I1RAU7 pp 229533.I1RRE5
124041 229533.I1RAU7 pp 229533.I1RD64
124042 229533.I1RAU7 pp 229533.I1S2H4
124043 229533.I1RAU7 pp 229533.I1S1W4
124044 229533.I1RAU7 pp 229533.I1RIL3
124045 229533.I1RAU7 pp 229533.I1RQ23
124046 229533.I1RAU7 pp 229533.I1S0L4
124047 229533.I1RAU7 pp 229533.I1RRR7
124048 229533.I1RAU7 pp 229533.I1RZZ9
124049 229533.I1RAU7 pp 229533.I1S8V4
124050 229533.I1RAU7 pp 229533.I1RPM4
124051 229533.I1RAU7 pp 229533.I1S6Y8
124052 229533.I1RAU7 pp 229533.I1RWI5
124053 229533.I1RAU7 pp 229533.Q4I662
124054 229533.I1RAU7 pp 229533.I1RW46
124055 229533.I1RAU7 pp 229533.I1RFS3
124056 229533.I1RAU7 pp 229533.I1S060
124057 229533.I1RAU7 pp 229533.I1RC66
124058 229533.I1RAU7 pp 229533.Q4IF76
124059 229533.I1RAU7 pp 229533.I1S8V3
124060 229533.I1RAU7 pp 229533.I1RE01
124061 229533.I1RAU7 pp 229533.I1RX24
124062 229533.I1RAU7 pp 229533.I1RTG3
124063 229533.I1RAU7 pp 229533.I1RTA0
124064 229533.I1RAU7 pp 229533.I1RZS7
124065 229533.I1RAU7 pp 229533.I1RU69
124066 229533.I1RAU7 pp 229533.I1RNN1
124067 229533.I1RAU7 pp 229533.I1RBG9
124068 229533.I1RAU7 pp 229533.I1RFT6
124069 229533.I1RAU7 pp 229533.I1S0M6
124070 229533.I1RAU7 pp 229533.I1RE22
124071 229533.I1RAU7 pp 229533.I1S0J1
124072 229533.I1RAU7 pp 229533.I1RPK0
124073 229533.I1RAU7 pp 229533.I1S8E2
124074 229533.I1RAU7 pp 229533.I1RZ39
124075 229533.I1RAU7 pp 229533.I1RWB5
124076 229533.I1RAU7 pp 229533.I1RZS6
124077 229533.I1RAU7 pp 229533.I1RS17
124078 229533.I1RAU7 pp 229533.V6RGN5
124079 229533.I1RAU7 pp 229533.I1RND6
124080 229533.I1RAU7 pp 229533.I1RM25
124081 229533.I1RAU7 pp 229533.I1S0N4
124082 229533.I1RAU7 pp 229533.V6R3X5
124083 229533.I1RAU7 pp 229533.I1RUG3
124084 229533.I1RAU7 pp 229533.I1RPP4
124085 229533.I1RAU7 pp 229533.Q4I7K4
124086 229533.I1RAU7 pp 229533.I1RR73
124087 229533.I1RAU7 pp 229533.I1RWN5
124088 229533.I1RAU7 pp 229533.Q4HZB8
124089 229533.I1RAU7 pp 229533.I1RXT3
124090 229533.I1RAU7 pp 229533.V6RIV9
124091 229533.I1RAU7 pp 229533.I1RWD3
124092 229533.I1RAU7 pp 229533.I1RNC1
124093 229533.I1RAU7 pp 229533.I1RN18
124094 229533.I1RAU7 pp 229533.I1RMN6
124095 229533.I1RAU7 pp 229533.Q4IN63
124096 229533.I1RAU7 pp 229533.I1RSD8
124097 229533.I1RAU7 pp 229533.I1RCR8
124098 229533.I1RAU7 pp 229533.I1SA33
124099 229533.I1RAU7 pp 229533.I1RFL4
124100 229533.I1RAU7 pp 229533.Q4I327

124101	229533.I1RAU7	pp	229533.I1RQE7
124102	229533.I1RAU7	pp	229533.I1RWH4
124103	229533.I1RAU7	pp	229533.I1RCH5
124104	229533.I1RAU7	pp	229533.I1RD22
124105	229533.I1RAU7	pp	229533.I1RJP4
124106	229533.I1RAU7	pp	229533.I1RNY2
124107	229533.I1RAU7	pp	229533.I1RYW3
124108	229533.I1RAU7	pp	229533.I1S2E8
124109	229533.I1RAU7	pp	229533.I1RP88
124110	229533.I1RAU7	pp	229533.I1S1X9
124111	229533.I1RAU7	pp	229533.I1RMG3
124112	229533.I1RAU7	pp	229533.V6RMW6
124113	229533.I1RAU7	pp	229533.I1RTD7
124114	229533.I1RAU7	pp	229533.I1RRV1
124115	229533.I1RAU7	pp	229533.I1RSQ3
124116	229533.I1RAU7	pp	229533.I1RT03
124117	229533.I1RAU7	pp	229533.I1RRN5
124118	229533.I1RAU7	pp	229533.I1RVX0
124119	229533.I1RAU7	pp	229533.I1S1C7
124120	229533.I1RAU7	pp	229533.V6R042
124121	229533.I1RAU7	pp	229533.I1RG90
124122	229533.I1RAU7	pp	229533.Q4HWE2
124123	229533.I1RAU7	pp	229533.I1RB41
124124	229533.I1RAU7	pp	229533.I1RPS0
124125	229533.I1RAU7	pp	229533.I1RRI2
124126	229533.I1RAU7	pp	229533.I1RGL4
124127	229533.I1RAU7	pp	229533.I1RB92
124128	229533.I1RAU7	pp	229533.I1RCM3
124129	229533.I1RAU7	pp	229533.V6R057
124130	229533.I1RAU7	pp	229533.I1RCN5
124131	229533.I1RAU7	pp	229533.I1RE71
124132	229533.I1RAU7	pp	229533.I1RWX5
124133	229533.I1RAU7	pp	229533.I1RZW8
124134	229533.I1RAU7	pp	229533.I1S569
124135	229533.I1RAU7	pp	229533.I1RBZ3
124136	229533.I1RAU7	pp	229533.I1RCA0
124137	229533.I1RAU7	pp	229533.I1S855
124138	229533.I1RAU7	pp	229533.I1RSK1
124139	229533.I1RAU7	pp	229533.I1S8R3
124140	229533.I1RAU7	pp	229533.Q4HZ68
124141	229533.I1RAU7	pp	229533.I1RTH6
124142	229533.I1RAU7	pp	229533.I1RD17
124143	229533.I1RAU7	pp	229533.Q4I624
124144	229533.I1RAU7	pp	229533.I1RWN6
124145	229533.I1RAU7	pp	229533.I1RSG2
124146	229533.I1RAU7	pp	229533.I1RJV3
124147	229533.I1RAU7	pp	229533.I1S2C0
124148	229533.I1RAU7	pp	229533.I1RD39
124149	229533.I1RAU7	pp	229533.I1RAW0
124150	229533.I1RAU7	pp	229533.I1RTE9
124151	229533.I1S9T6	pp	229533.Q4HYR2
124152	229533.I1S9T6	pp	229533.V6RK96
124153	229533.I1RS17	pp	229533.I1RY44
124154	229533.I1RS17	pp	229533.V6RIV9
124155	229533.I1RS17	pp	229533.I1RWN5
124156	229533.I1RS17	pp	229533.I1RSA4
124157	229533.I1RS17	pp	229533.I1RWE8
124158	229533.I1RS17	pp	229533.I1S2C5
124159	229533.I1RS17	pp	229533.Q4HZB8
124160	229533.I1RS17	pp	229533.I1S0R2
124161	229533.I1RS17	pp	229533.I1RUG3
124162	229533.I1RS17	pp	229533.I1RSQ3
124163	229533.I1RS17	pp	229533.I1S6H1
124164	229533.I1RS17	pp	229533.I1S0J1
124165	229533.I1RS17	pp	229533.I1S1C7
124166	229533.I1RS17	pp	229533.I1RW46
124167	229533.I1RS17	pp	229533.V6R042
124168	229533.I1RS17	pp	229533.V6RMW6
124169	229533.I1RS17	pp	229533.I1RTA0
124170	229533.I1RS17	pp	229533.I1RY62
124171	229533.I1RS17	pp	229533.I1RSI6
124172	229533.I1RS17	pp	229533.I1RYW3
124173	229533.I1RS17	pp	229533.I1RTN8

124174 229533.I1RS17 pp 229533.I1S0F6
124175 229533.I1RS17 pp 229533.I1S2C4
124176 229533.I1RS17 pp 229533.I1S256
124177 229533.I1RS17 pp 229533.I1RTE9
124178 229533.I1RS17 pp 229533.I1S190
124179 229533.I1RS17 pp 229533.I1RYR6
124180 229533.I1RS17 pp 229533.I1RW26
124181 229533.I1RS17 pp 229533.V6RGN5
124182 229533.I1RS17 pp 229533.I1RTH6
124183 229533.I1RS17 pp 229533.I1SA33
124184 229533.I1RS17 pp 229533.I1S5G3
124185 229533.I1RS17 pp 229533.I1RT03
124186 229533.I1RS17 pp 229533.I1RS47
124187 229533.I1RS17 pp 229533.I1S8R3
124188 229533.I1RS17 pp 229533.I1S9H4
124189 229533.I1RS17 pp 229533.V6RRH2
124190 229533.I1RS17 pp 229533.I1RWN6
124191 229533.I1RS17 pp 229533.I1RVX0
124192 229533.I1RS17 pp 229533.I1S2M6
124193 229533.I1RS17 pp 229533.I1RTD5
124194 229533.I1RS17 pp 229533.I1RT40
124195 229533.I1RS17 pp 229533.Q4I624
124196 229533.I1RS17 pp 229533.I1RV91
124197 229533.I1RS17 pp 229533.I1RWX5
124198 229533.I1RS17 pp 229533.I1S2E9
124199 229533.I1RS17 pp 229533.I1RWK3
124200 229533.I1RS17 pp 229533.I1RZW8
124201 229533.I1RS17 pp 229533.I1SAJ0
124202 229533.I1RS17 pp 229533.Q4HXF0
124203 229533.I1RS17 pp 229533.I1S8P5
124204 229533.I1RS17 pp 229533.I1RTA9
124205 229533.I1RS17 pp 229533.I1S0M6
124206 229533.I1RS17 pp 229533.I1S2L3
124207 229533.I1RS17 pp 229533.I1S1B6
124208 229533.I1RS17 pp 229533.I1S8F0
124209 229533.I1RS17 pp 229533.I1RT04
124210 229533.I1RS17 pp 229533.I1RTG3
124211 229533.I1RS17 pp 229533.I1S503
124212 229533.I1RS17 pp 229533.I1RSX1
124213 229533.I1RS17 pp 229533.I1S2B8
124214 229533.I1RS17 pp 229533.I1RZH4
124215 229533.I1RS17 pp 229533.I1S7S9
124216 229533.I1RS17 pp 229533.I1S1C9
124217 229533.I1RS17 pp 229533.I1RWI5
124218 229533.I1RS17 pp 229533.I1RS18
124219 229533.I1RS17 pp 229533.I1S6B4
124220 229533.I1RS17 pp 229533.I1RZZ9
124221 229533.I1RS17 pp 229533.Q4I8P5
124222 229533.I1RS17 pp 229533.I1RXR5
124223 229533.I1RS17 pp 229533.I1S5B0
124224 229533.I1RS17 pp 229533.V6R3X5
124225 229533.I1RS17 pp 229533.I1RSQ4
124226 229533.I1RS17 pp 229533.I1RV18
124227 229533.I1RS17 pp 229533.Q4IEK8
124228 229533.I1RS17 pp 229533.I1RW99
124229 229533.I1RS17 pp 229533.I1S076
124230 229533.I1RS17 pp 229533.I1RZ39
124231 229533.I1RS17 pp 229533.I1RZM1
124232 229533.I1RS17 pp 229533.V6R227
124233 229533.I1RS17 pp 229533.I1RZZ1
124234 229533.I1RS17 pp 229533.I1RTJ7
124235 229533.I1RS17 pp 229533.Q4IJK2
124236 229533.I1RS17 pp 229533.V6RBN2
124237 229533.I1RS17 pp 229533.Q4I283
124238 229533.I1RS17 pp 229533.O74638
124239 229533.I1RS17 pp 229533.I1S5R1
124240 229533.I1RS17 pp 229533.I1RSI0
124241 229533.I1RS17 pp 229533.I1S0L4
124242 229533.I1RS17 pp 229533.I1S3G4
124243 229533.I1RS17 pp 229533.I1S4Q4
124244 229533.I1RS17 pp 229533.I1RWT6
124245 229533.I1RS17 pp 229533.I1S6E5
124246 229533.I1RS17 pp 229533.I1RS20

124247 229533.I1RS17 pp 229533.I1S5O4
124248 229533.I1RS17 pp 229533.Q4I2J8
124249 229533.I1RS17 pp 229533.I1RXF0
124250 229533.I1RS17 pp 229533.I1SA32
124251 229533.I1RS17 pp 229533.I1RZM8
124252 229533.I1RS17 pp 229533.I1S2H4
124253 229533.I1RS17 pp 229533.I1RWD7
124254 229533.I1RS17 pp 229533.V6RCG5
124255 229533.I1RS17 pp 229533.Q4I5Z5
124256 229533.I1RS17 pp 229533.I1S3S3
124257 229533.I1RS17 pp 229533.I1S0R7
124258 229533.I1RS17 pp 229533.I1RYB5
124259 229533.I1RS17 pp 229533.I1RX46
124260 229533.I1RS17 pp 229533.V6RCZ8
124261 229533.I1RS17 pp 229533.I1RY03
124262 229533.I1RS17 pp 229533.V6QW22
124263 229533.I1RS17 pp 229533.I1RYE6
124264 229533.I1RS17 pp 229533.I1RSQ7
124265 229533.I1RS17 pp 229533.V6RTJ8
124266 229533.I1RS17 pp 229533.I1RW71
124267 229533.I1RS17 pp 229533.I1RZK5
124268 229533.I1RS17 pp 229533.I1S084
124269 229533.I1RS17 pp 229533.I1RVQ0
124270 229533.I1RS17 pp 229533.I1S7A8
124271 229533.I1RS17 pp 229533.I1S1J7
124272 229533.I1RS17 pp 229533.I1RWD4
124273 229533.I1RS17 pp 229533.V6QUW4
124274 229533.I1RS17 pp 229533.I1S6T4
124275 229533.I1RS17 pp 229533.I1S1W8
124276 229533.I1RS17 pp 229533.I1RSR3
124277 229533.I1RS17 pp 229533.I1S0F5
124278 229533.I1RS17 pp 229533.Q4IJT5
124279 229533.I1RS17 pp 229533.I1S1P8
124280 229533.I1RS17 pp 229533.I1S0X6
124281 229533.I1RS17 pp 229533.I1S0L3
124282 229533.I1RS17 pp 229533.I1RW25
124283 229533.I1RS17 pp 229533.I1RX04
124284 229533.I1RS17 pp 229533.V6QYF0
124285 229533.I1RS17 pp 229533.I1RZJ5
124286 229533.I1RS17 pp 229533.I1RUQ2
124287 229533.I1RS17 pp 229533.I1RTF4
124288 229533.I1RS17 pp 229533.I1RX25
124289 229533.I1RS17 pp 229533.I1RWJ8
124290 229533.I1RS17 pp 229533.I1S4S4
124291 229533.I1RS17 pp 229533.I1RXD7
124292 229533.A0A0E0RW05 pp 229533.I1RT47
124293 229533.A0A0E0RW05 pp 229533.Q4IP34
124294 229533.A0A0E0RW05 pp 229533.I1RSD4
124295 229533.A0A0E0RW05 pp 229533.A0A1C3YHK3
124296 229533.A0A0E0RW05 pp 229533.I1RM25
124297 229533.A0A0E0RW05 pp 229533.I1RNU6
124298 229533.A0A0E0RW05 pp 229533.I1RND5
124299 229533.A0A0E0RW05 pp 229533.I1RAC1
124300 229533.A0A0E0RW05 pp 229533.I1RC84
124301 229533.A0A0E0RW05 pp 229533.I1RSA4
124302 229533.A0A0E0RW05 pp 229533.I1RCN5
124303 229533.A0A0E0RW05 pp 229533.I1RA36
124304 229533.A0A0E0RW05 pp 229533.I1RVT9
124305 229533.A0A0E0RW05 pp 229533.I1RK92
124306 229533.A0A0E0RW05 pp 229533.A0A1C3YM79
124307 229533.A0A0E0RW05 pp 229533.A0A1C3YI62
124308 229533.A0A0E0RW05 pp 229533.I1S1C6
124309 229533.A0A0E0RW05 pp 229533.I1RPA0
124310 229533.A0A0E0RW05 pp 229533.I1RSL9
124311 229533.A0A0E0RW05 pp 229533.I1RBH8
124312 229533.A0A0E0RW05 pp 229533.Q4I7F9
124313 229533.A0A0E0RW05 pp 229533.I1RU69
124314 229533.A0A0E0RW05 pp 229533.I1S4M5
124315 229533.A0A0E0RW05 pp 229533.I1S1X9
124316 229533.A0A0E0RW05 pp 229533.A0A1C3YNC3
124317 229533.A0A0E0RW05 pp 229533.I1RD56
124318 229533.A0A0E0RW05 pp 229533.I1RYB1
124319 229533.A0A0E0RW05 pp 229533.A0A1C3YHQ4

124320 229533.A0A0E0RW05 pp 229533.I1RB50
124321 229533.A0A0E0RW05 pp 229533.A0A1C3YL11
124322 229533.A0A0E0RW05 pp 229533.I1RPW9
124323 229533.A0A0E0RW05 pp 229533.A0A1C3YHW7
124324 229533.A0A0E0RW05 pp 229533.I1S0B1
124325 229533.A0A0E0RW05 pp 229533.I1RCQ6
124326 229533.A0A0E0RW05 pp 229533.A0A0E0RZU7
124327 229533.A0A0E0RW05 pp 229533.I1S0Z9
124328 229533.A0A0E0RW05 pp 229533.I1S0M6
124329 229533.A0A0E0RW05 pp 229533.I1RG90
124330 229533.A0A0E0RW05 pp 229533.I1RX81
124331 229533.A0A0E0RW05 pp 229533.I1S7M3
124332 229533.A0A0E0RW05 pp 229533.I1RX24
124333 229533.A0A0E0RW05 pp 229533.I1RMV5
124334 229533.A0A0E0RW05 pp 229533.I1RBV3
124335 229533.A0A0E0RW05 pp 229533.A0A1C3YJ29
124336 229533.A0A0E0RW05 pp 229533.I1RS72
124337 229533.A0A0E0RW05 pp 229533.I1S7M2
124338 229533.A0A0E0RW05 pp 229533.I1RXF2
124339 229533.I1S0L3 pp 229533.I1S0L4
124340 229533.I1S0L3 pp 229533.I1S2H4
124341 229533.I1S0L3 pp 229533.I1S6Y8
124342 229533.I1S0L3 pp 229533.O74638
124343 229533.I1S0L3 pp 229533.I1S5R1
124344 229533.I1S0L3 pp 229533.I1S8V3
124345 229533.I1S0L3 pp 229533.I1S4Z7
124346 229533.I1S0L3 pp 229533.V6RUY9
124347 229533.I1S0L3 pp 229533.I1S0M6
124348 229533.I1S0L3 pp 229533.I1S2E9
124349 229533.I1S0L3 pp 229533.I1S8F0
124350 229533.I1S0L3 pp 229533.I1S352
124351 229533.I1S0L3 pp 229533.I1S7S9
124352 229533.I1S0L3 pp 229533.I1S2B8
124353 229533.I1S0L3 pp 229533.V6R3X5
124354 229533.I1S0L3 pp 229533.I1S1B6
124355 229533.I1S0L3 pp 229533.Q4I624
124356 229533.I1S0L3 pp 229533.I1S1C7
124357 229533.I1S0L3 pp 229533.I1S503
124358 229533.I1S0L3 pp 229533.Q4IEV5
124359 229533.I1S0L3 pp 229533.I1S2E8
124360 229533.I1S0L3 pp 229533.V6R057
124361 229533.I1S0L3 pp 229533.I1S8P5
124362 229533.I1S0L3 pp 229533.I1S569
124363 229533.I1S0L3 pp 229533.I1S2M6
124364 229533.I1S0L3 pp 229533.I1S2C0
124365 229533.I1S0L3 pp 229533.V6QX21
124366 229533.I1S0L3 pp 229533.V6RMW6
124367 229533.I1S0L3 pp 229533.I1S0M2
124368 229533.I1S0L3 pp 229533.I1S8R3
124369 229533.I1S0L3 pp 229533.I1SA33
124370 229533.I1S0L3 pp 229533.I1S0N4
124371 229533.I1S0L3 pp 229533.I1S0T2
124372 229533.I1S0L3 pp 229533.I1S0R2
124373 229533.I1S0L3 pp 229533.V6RGN5
124374 229533.I1S0L3 pp 229533.V6R042
124375 229533.I1S0L3 pp 229533.I1S0R7
124376 229533.I1S0L3 pp 229533.Q4HZB8
124377 229533.I1S0L3 pp 229533.Q4HVW2
124378 229533.I1S0L3 pp 229533.V6QYF0
124379 229533.I1S0L3 pp 229533.I1S7A8
124380 229533.I1S0L3 pp 229533.I1S0X6
124381 229533.I1S0L3 pp 229533.V6QW22
124382 229533.I1S0L3 pp 229533.V6QUW4
124383 229533.I1S0L3 pp 229533.V6RTJ8
124384 229533.I1S0L3 pp 229533.V6RIV9
124385 229533.I1S0L3 pp 229533.Q4IJT5
124386 229533.I1S0L3 pp 229533.Q4I8P5
124387 229533.I1S0L3 pp 229533.I1S6F0
124388 229533.I1S0L3 pp 229533.Q4I5Z5
124389 229533.I1S0L3 pp 229533.I1S8V4
124390 229533.I1S0L3 pp 229533.Q4I2J8
124391 229533.I1S0L3 pp 229533.V6R4J1
124392 229533.I1S0L3 pp 229533.V6R1K4

124393	229533.I1S0L3	pp	229533.I1S419
124394	229533.I1S0L3	pp	229533.I1S1J7
124395	229533.I1S0L3	pp	229533.Q4IEK8
124396	229533.I1S0L3	pp	229533.V6RCG5
124397	229533.I1S0L3	pp	229533.Q4I283
124398	229533.I1S0L3	pp	229533.I1SA32
124399	229533.I1S0L3	pp	229533.Q4IJK2
124400	229533.I1S0L3	pp	229533.V6RCZ8
124401	229533.I1S0L3	pp	229533.I1S5B0
124402	229533.I1S0L3	pp	229533.V6R227
124403	229533.I1S0L3	pp	229533.I1S504
124404	229533.A0A0E0RVM5	pp	229533.I1RNG4
124405	229533.I1SAK0	pp	229533.V6REA4
124406	229533.I1SAK0	pp	229533.V6RM09
124407	229533.I1SAK0	pp	229533.V6R556
124408	229533.I1SAK0	pp	229533.V6QTX7
124409	229533.V6R057	pp	229533.V6RTJ8
124410	229533.V6R057	pp	229533.V6RRH2
124411	229533.V6R057	pp	229533.V6RCG5
124412	229533.V6R057	pp	229533.V6RCZ8
124413	229533.V6R057	pp	229533.V6RBN2
124414	229533.V6R057	pp	229533.V6RIV9
124415	229533.V6R057	pp	229533.V6R3X5
124416	229533.V6R057	pp	229533.V6R1K4
124417	229533.V6R057	pp	229533.V6R2A1
124418	229533.V6R057	pp	229533.V6RMW6
124419	229533.I1RDU6	pp	229533.I1S0B0
124420	229533.I1RDU6	pp	229533.I1RZA7
124421	229533.I1RDU6	pp	229533.V6RUB1
124422	229533.I1RDU6	pp	229533.I1S6P5
124423	229533.I1RDU6	pp	229533.I1RFD4
124424	229533.I1RDU6	pp	229533.I1SA33
124425	229533.I1RDU6	pp	229533.Q4HYR2
124426	229533.I1RDU6	pp	229533.I1RWM4
124427	229533.I1RDU6	pp	229533.I1S9T6
124428	229533.I1S0F5	pp	229533.V6R042
124429	229533.I1S0F5	pp	229533.I1SA33
124430	229533.I1S0F5	pp	229533.I1S0J1
124431	229533.I1S0F5	pp	229533.V6RMW6
124432	229533.I1S0F5	pp	229533.V6R3X5
124433	229533.I1S0F5	pp	229533.Q4I624
124434	229533.I1S0F5	pp	229533.V6RIV9
124435	229533.I1S0F5	pp	229533.I1S8R3
124436	229533.I1S0F5	pp	229533.I1S1C7
124437	229533.I1S0F5	pp	229533.I1S0L4
124438	229533.I1S0F5	pp	229533.V6R057
124439	229533.I1S0F5	pp	229533.V6RCZ8
124440	229533.I1S0F5	pp	229533.Q4IJK2
124441	229533.I1S0F5	pp	229533.I1S2G9
124442	229533.I1S0F5	pp	229533.V6RCG5
124443	229533.I1S0F5	pp	229533.V6QUW4
124444	229533.I1S0F5	pp	229533.I1S0X7
124445	229533.I1S0F5	pp	229533.I1S0R7
124446	229533.I1S0F5	pp	229533.I1S5R1
124447	229533.I1S0F5	pp	229533.I1S5B0
124448	229533.I1S0F5	pp	229533.V6RTJ8
124449	229533.I1S0F5	pp	229533.I1S2E9
124450	229533.I1S0F5	pp	229533.Q4I8P5
124451	229533.I1S0F5	pp	229533.I1S2B8
124452	229533.I1S0F5	pp	229533.I1SA32
124453	229533.I1S0F5	pp	229533.V6RXW9
124454	229533.I1S0F5	pp	229533.I1S0L3
124455	229533.I1S0F5	pp	229533.I1S0F6
124456	229533.I1S0F5	pp	229533.V6QW22
124457	229533.I1S0F5	pp	229533.I1S503
124458	229533.I1S0F5	pp	229533.I1S504
124459	229533.I1S0F5	pp	229533.V6R1K4
124460	229533.I1S0J1	pp	229533.V6R227
124461	229533.I1S0J1	pp	229533.I1S419
124462	229533.I1S0J1	pp	229533.I1S887
124463	229533.I1S0J1	pp	229533.Q4IEK8
124464	229533.I1S0J1	pp	229533.I1S1J7
124465	229533.I1S0J1	pp	229533.Q4I2J8

124466	229533.I1S0J1	pp	229533.Q4IJ56
124467	229533.I1S0J1	pp	229533.V6QUE6
124468	229533.I1S0J1	pp	229533.I1S4Q4
124469	229533.I1S0J1	pp	229533.I1S5B0
124470	229533.I1S0J1	pp	229533.Q4IJK2
124471	229533.I1S0J1	pp	229533.Q4IJH1
124472	229533.I1S0J1	pp	229533.Q4I8P5
124473	229533.I1S0J1	pp	229533.I1S6K9
124474	229533.I1S0J1	pp	229533.I1S0M9
124475	229533.I1S0J1	pp	229533.V6RBN2
124476	229533.I1S0J1	pp	229533.Q4IBS2
124477	229533.I1S0J1	pp	229533.V6RCZ8
124478	229533.I1S0J1	pp	229533.Q4HVW2
124479	229533.I1S0J1	pp	229533.I1SA32
124480	229533.I1S0J1	pp	229533.Q4IAA0
124481	229533.I1S0J1	pp	229533.I1S6U8
124482	229533.I1S0J1	pp	229533.I1S504
124483	229533.I1S0J1	pp	229533.Q4IFI0
124484	229533.I1S0J1	pp	229533.V6RCG5
124485	229533.I1S0J1	pp	229533.I1S9U2
124486	229533.I1S0J1	pp	229533.I1S0X6
124487	229533.I1S0J1	pp	229533.Q4HZK7
124488	229533.I1S0J1	pp	229533.I1S438
124489	229533.I1S0J1	pp	229533.I1S8R3
124490	229533.I1S0J1	pp	229533.I1S4S4
124491	229533.I1S0J1	pp	229533.V6QYF0
124492	229533.I1S0J1	pp	229533.I1S0R7
124493	229533.I1S0J1	pp	229533.I1S0L3
124494	229533.I1S0J1	pp	229533.V6R1K4
124495	229533.I1S0J1	pp	229533.I1S2C0
124496	229533.I1S0J1	pp	229533.V6RUT5
124497	229533.I1S0J1	pp	229533.I1S540
124498	229533.I1S0J1	pp	229533.I1S7A8
124499	229533.I1S0J1	pp	229533.I1S6F0
124500	229533.I1S0J1	pp	229533.V6RTJ8
124501	229533.I1S0J1	pp	229533.I1S437
124502	229533.I1S0J1	pp	229533.Q4HZ47
124503	229533.I1S0J1	pp	229533.I1S0T2
124504	229533.I1S0J1	pp	229533.Q4I624
124505	229533.I1S0J1	pp	229533.V6QW22
124506	229533.I1S0J1	pp	229533.Q4IPI1
124507	229533.I1S0J1	pp	229533.I1S1C7
124508	229533.I1S0J1	pp	229533.I1S4S7
124509	229533.I1S0J1	pp	229533.Q4HZ68
124510	229533.I1S0J1	pp	229533.I1S1X9
124511	229533.I1S0J1	pp	229533.V6R057
124512	229533.I1S0J1	pp	229533.Q4HWE2
124513	229533.I1S0J1	pp	229533.Q4IJT5
124514	229533.I1S0J1	pp	229533.V6RRH2
124515	229533.I1S0J1	pp	229533.I1S5R6
124516	229533.I1S0J1	pp	229533.I1S274
124517	229533.I1S0J1	pp	229533.I1S2E8
124518	229533.I1S0J1	pp	229533.V6RGY3
124519	229533.I1S0J1	pp	229533.I1S0N4
124520	229533.I1S0J1	pp	229533.I1S2H4
124521	229533.I1S0J1	pp	229533.V6RMW6
124522	229533.I1S0J1	pp	229533.I1S0M3
124523	229533.I1S0J1	pp	229533.I1S7W5
124524	229533.I1S0J1	pp	229533.V6RGN5
124525	229533.I1S0J1	pp	229533.I1SA33
124526	229533.I1S0J1	pp	229533.I1S9Y1
124527	229533.I1S0J1	pp	229533.I1S256
124528	229533.I1S0J1	pp	229533.Q4IN63
124529	229533.I1S0J1	pp	229533.I1S0L4
124530	229533.I1S0J1	pp	229533.O74638
124531	229533.I1S0J1	pp	229533.I1S1B6
124532	229533.I1S0J1	pp	229533.Q4I327
124533	229533.I1S0J1	pp	229533.I1S5R1
124534	229533.I1S0J1	pp	229533.V6R042
124535	229533.I1S0J1	pp	229533.Q4IQW0
124536	229533.I1S0J1	pp	229533.I1S1W3
124537	229533.I1S0J1	pp	229533.V6R3X5
124538	229533.I1S0J1	pp	229533.I1S8E2

124539 229533.I1S0J1 pp 229533.V6RIV9
124540 229533.I1S0J1 pp 229533.I1S1W9
124541 229533.I1S0J1 pp 229533.Q4HZ42
124542 229533.I1S0J1 pp 229533.Q4IAS1
124543 229533.I1S0J1 pp 229533.I1S2G7
124544 229533.I1S0J1 pp 229533.I1S503
124545 229533.I1S0J1 pp 229533.I1S253
124546 229533.I1S0J1 pp 229533.Q4IEV5
124547 229533.I1S0J1 pp 229533.I1S9Y0
124548 229533.I1S0J1 pp 229533.Q4IF76
124549 229533.I1S0J1 pp 229533.V6R3J1
124550 229533.I1S0J1 pp 229533.I1S2B8
124551 229533.I1S0J1 pp 229533.Q4HXF0
124552 229533.I1S0J1 pp 229533.Q4I662
124553 229533.I1S0J1 pp 229533.Q4I830
124554 229533.I1S0J1 pp 229533.I1S0W4
124555 229533.I1S0J1 pp 229533.I1S0M6
124556 229533.I1S0J1 pp 229533.I1S6Y8
124557 229533.I1S0J1 pp 229533.I1S2E9
124558 229533.I1S0J1 pp 229533.V6R283
124559 229533.I1S0J1 pp 229533.Q4I613
124560 229533.I1S0J1 pp 229533.V6RUY9
124561 229533.I1S0J1 pp 229533.I1S8P5
124562 229533.A0A1C3YIE0 pp 229533.I1R9K2
124563 229533.A0A1C3YIE0 pp 229533.I1RSG0
124564 229533.A0A1C3YIE0 pp 229533.I1R9M7
124565 229533.A0A1C3YIE0 pp 229533.I1RFD4
124566 229533.A0A1C3YIE0 pp 229533.A0A1C3YJM7
124567 229533.A0A1C3YIE0 pp 229533.I1RSX9
124568 229533.A0A1C3YIE0 pp 229533.I1RXS0
124569 229533.A0A1C3YIE0 pp 229533.Q4HYR2
124570 229533.A0A1C3YIE0 pp 229533.A0A1C3YJU5
124571 229533.A0A1C3YIE0 pp 229533.I1S6A2
124572 229533.A0A1C3YIE0 pp 229533.I1RHZ5
124573 229533.A0A1C3YIE0 pp 229533.A0A1C3YJL9
124574 229533.A0A1C3YIE0 pp 229533.I1RZM4
124575 229533.A0A1C3YIE0 pp 229533.I1RZC3
124576 229533.A0A1C3YIE0 pp 229533.I1RWM4
124577 229533.A0A1C3YIE0 pp 229533.I1RAG3
124578 229533.A0A1C3YIE0 pp 229533.I1S8R4
124579 229533.A0A1C3YIE0 pp 229533.I1RW10
124580 229533.A0A1C3YIE0 pp 229533.I1RVY1
124581 229533.A0A1C3YIE0 pp 229533.I1RM66
124582 229533.A0A1C3YIE0 pp 229533.I1RMY1
124583 229533.A0A1C3YIE0 pp 229533.I1RQP2
124584 229533.A0A1C3YIE0 pp 229533.I1RH30
124585 229533.A0A1C3YIE0 pp 229533.I1RHA8
124586 229533.A0A1C3YIE0 pp 229533.I1RHL9
124587 229533.A0A1C3YIE0 pp 229533.I1RVR2
124588 229533.A0A1C3YIE0 pp 229533.I1RC48
124589 229533.A0A1C3YIE0 pp 229533.A0A1C3YJ22
124590 229533.A0A1C3YIE0 pp 229533.I1RDV8
124591 229533.A0A1C3YIE0 pp 229533.V6RK96
124592 229533.A0A1C3YIE0 pp 229533.I1S0K0
124593 229533.A0A1C3YIE0 pp 229533.A0A1C3YJT8
124594 229533.A0A1C3YIE0 pp 229533.I1S9T6
124595 229533.A0A1C3YIE0 pp 229533.I1S267
124596 229533.A0A1C3YIE0 pp 229533.I1RF16
124597 229533.A0A1C3YIE0 pp 229533.I1RAX2
124598 229533.A0A1C3YIE0 pp 229533.I1RY32
124599 229533.A0A1C3YIE0 pp 229533.I1RDU6
124600 229533.A0A1C3YIE0 pp 229533.I1S3L2
124601 229533.A0A1C3YIE0 pp 229533.I1RJR8
124602 229533.A0A1C3YIE0 pp 229533.I1S673
124603 229533.A0A1C3YIE0 pp 229533.A0A1C3YKN4
124604 229533.A0A1C3YIE0 pp 229533.I1RC71
124605 229533.A0A1C3YIE0 pp 229533.I1RYR3
124606 229533.A0A1C3YIE0 pp 229533.I1RQE8
124607 229533.A0A1C3YIE0 pp 229533.I1RR29
124608 229533.A0A1C3YIE0 pp 229533.I1RIL0
124609 229533.A0A1C3YIE0 pp 229533.I1RH32
124610 229533.A0A1C3YIE0 pp 229533.I1S749
124611 229533.I1R9A9 pp 229533.Q4HTT2

124612	229533.I1R9A9	pp	229533.I1RYV3
124613	229533.I1R9A9	pp	229533.Q4I7L0
124614	229533.I1R9A9	pp	229533.I1RBJ0
124615	229533.I1R9A9	pp	229533.I1S4Z5
124616	229533.I1RE01	pp	229533.I1RY63
124617	229533.I1RE01	pp	229533.I1RY03
124618	229533.I1RE01	pp	229533.I1RNT9
124619	229533.I1RE01	pp	229533.I1S0K2
124620	229533.I1RE01	pp	229533.I1RK95
124621	229533.I1RE01	pp	229533.I1RMD5
124622	229533.I1RE01	pp	229533.V6RBN2
124623	229533.I1RE01	pp	229533.I1RQQ5
124624	229533.I1RE01	pp	229533.I1RWR7
124625	229533.I1RE01	pp	229533.I1RSR3
124626	229533.I1RE01	pp	229533.I1RZZ1
124627	229533.I1RE01	pp	229533.I1RN99
124628	229533.I1RE01	pp	229533.I1RSM3
124629	229533.I1RE01	pp	229533.Q4I283
124630	229533.I1RE01	pp	229533.I1RRY9
124631	229533.I1RE01	pp	229533.I1RPR9
124632	229533.I1RE01	pp	229533.V6R227
124633	229533.I1RE01	pp	229533.Q4IJK2
124634	229533.I1RE01	pp	229533.I1REK8
124635	229533.I1RE01	pp	229533.I1RQA3
124636	229533.I1RE01	pp	229533.I1RM35
124637	229533.I1RE01	pp	229533.I1S504
124638	229533.I1RE01	pp	229533.I1S1J7
124639	229533.I1RE01	pp	229533.I1RZM1
124640	229533.I1RE01	pp	229533.I1S6E5
124641	229533.I1RE01	pp	229533.I1RYS0
124642	229533.I1RE01	pp	229533.I1RWB0
124643	229533.I1RE01	pp	229533.I1RNQ5
124644	229533.I1RE01	pp	229533.I1RLU6
124645	229533.I1RE01	pp	229533.I1RF19
124646	229533.I1RE01	pp	229533.I1RQA2
124647	229533.I1RE01	pp	229533.Q4IEK8
124648	229533.I1RE01	pp	229533.Q4I8P5
124649	229533.I1RE01	pp	229533.I1RFJ9
124650	229533.I1RE01	pp	229533.I1REL7
124651	229533.I1RE01	pp	229533.I1RSI0
124652	229533.I1RE01	pp	229533.I1S419
124653	229533.I1RE01	pp	229533.I1RS18
124654	229533.I1RE01	pp	229533.I1RQ81
124655	229533.I1RE01	pp	229533.Q4I2J8
124656	229533.I1RE01	pp	229533.I1RR51
124657	229533.I1RE01	pp	229533.V6RCG5
124658	229533.I1RE01	pp	229533.I1S887
124659	229533.I1RE01	pp	229533.I1SA32
124660	229533.I1RE01	pp	229533.I1RZK5
124661	229533.I1RE01	pp	229533.I1RZH4
124662	229533.I1RE01	pp	229533.I1RGC5
124663	229533.I1RE01	pp	229533.I1RXC4
124664	229533.I1RE01	pp	229533.I1S6K9
124665	229533.I1RE01	pp	229533.V6RCZ8
124666	229533.I1RE01	pp	229533.I1RFU8
124667	229533.I1RE01	pp	229533.I1RG14
124668	229533.I1RE01	pp	229533.I1RI82
124669	229533.I1RE01	pp	229533.I1RWT6
124670	229533.I1RE01	pp	229533.I1RRV4
124671	229533.I1RE01	pp	229533.I1RQ85
124672	229533.I1RE01	pp	229533.I1S438
124673	229533.I1RE01	pp	229533.I1RXF0
124674	229533.I1RE01	pp	229533.I1RQ31
124675	229533.I1RE01	pp	229533.I1RK15
124676	229533.I1RE01	pp	229533.I1RW99
124677	229533.I1RE01	pp	229533.I1RM06
124678	229533.I1RE01	pp	229533.I1RMC0
124679	229533.I1RE01	pp	229533.I1RTF4
124680	229533.I1RE01	pp	229533.I1RNA0
124681	229533.I1RE01	pp	229533.V6QYF0
124682	229533.I1RE01	pp	229533.I1RMC8
124683	229533.I1RE01	pp	229533.I1S5B0
124684	229533.I1RE01	pp	229533.I1RR24

124685 229533.I1RE01 pp 229533.I1RE17
124686 229533.I1RE01 pp 229533.I1S0X6
124687 229533.I1RE01 pp 229533.I1RRU9
124688 229533.I1RE01 pp 229533.V6QUW4
124689 229533.I1RE01 pp 229533.I1RQD2
124690 229533.I1RE01 pp 229533.V6QUE6
124691 229533.I1RE01 pp 229533.I1RGC2
124692 229533.I1RE01 pp 229533.I1RMG8
124693 229533.I1RE01 pp 229533.I1RZJ5
124694 229533.I1RE01 pp 229533.I1RPA4
124695 229533.I1RE01 pp 229533.V6R1K4
124696 229533.I1RE01 pp 229533.I1S4S4
124697 229533.I1RE01 pp 229533.I1RX25
124698 229533.I1RE01 pp 229533.I1RWJ8
124699 229533.I1RE01 pp 229533.I1RW25
124700 229533.I1RE01 pp 229533.I1RVQ0
124701 229533.I1RE01 pp 229533.I1RY86
124702 229533.I1RE01 pp 229533.I1RYT8
124703 229533.I1RE01 pp 229533.I1RRX5
124704 229533.I1RE01 pp 229533.I1RSQ7
124705 229533.I1RE01 pp 229533.I1RXD6
124706 229533.I1RE01 pp 229533.V6QW22
124707 229533.I1RE01 pp 229533.Q4HVW2
124708 229533.I1RE01 pp 229533.I1RKJ5
124709 229533.I1RE01 pp 229533.I1RMK3
124710 229533.I1RE01 pp 229533.I1S6F0
124711 229533.I1RE01 pp 229533.I1RPN4
124712 229533.I1RE01 pp 229533.I1RWD4
124713 229533.I1RE01 pp 229533.I1RG53
124714 229533.I1RE01 pp 229533.I1RXD7
124715 229533.I1RE01 pp 229533.I1RJU3
124716 229533.I1RE01 pp 229533.I1S4S7
124717 229533.I1RE01 pp 229533.I1RW71
124718 229533.I1RE01 pp 229533.I1S0T2
124719 229533.I1RE01 pp 229533.I1RRN5
124720 229533.I1RE01 pp 229533.I1RLP1
124721 229533.I1RE01 pp 229533.I1RUQ2
124722 229533.I1RE01 pp 229533.Q4IJT5
124723 229533.I1RE01 pp 229533.V6RUT5
124724 229533.I1RE01 pp 229533.I1RRG3
124725 229533.I1RE01 pp 229533.I1RXR1
124726 229533.I1RE01 pp 229533.I1RSY2
124727 229533.I1RE01 pp 229533.I1S0R7
124728 229533.I1RE01 pp 229533.I1RVX0
124729 229533.I1RE01 pp 229533.I1RLX7
124730 229533.I1RE01 pp 229533.I1RX95
124731 229533.I1RE01 pp 229533.I1S3S3
124732 229533.I1RE01 pp 229533.I1RQC9
124733 229533.I1RE01 pp 229533.I1RT03
124734 229533.I1RE01 pp 229533.I1S0L3
124735 229533.I1RE01 pp 229533.V6R2A1
124736 229533.I1RE01 pp 229533.I1S7A8
124737 229533.I1RE01 pp 229533.I1RPY3
124738 229533.I1RE01 pp 229533.I1RQE7
124739 229533.I1RE01 pp 229533.I1S0F5
124740 229533.I1RE01 pp 229533.V6RTJ8
124741 229533.I1RE01 pp 229533.I1S0J1
124742 229533.I1RE01 pp 229533.I1RN18
124743 229533.I1RE01 pp 229533.I1S0R2
124744 229533.I1RE01 pp 229533.I1RQ33
124745 229533.I1RE01 pp 229533.I1RFL4
124746 229533.I1RE01 pp 229533.I1RTE9
124747 229533.I1RE01 pp 229533.I1RX46
124748 229533.I1RE01 pp 229533.I1RTA0
124749 229533.I1RE01 pp 229533.I1S7W5
124750 229533.I1RE01 pp 229533.Q4I624
124751 229533.I1RE01 pp 229533.I1RKJ8
124752 229533.I1RE01 pp 229533.V6R057
124753 229533.I1RE01 pp 229533.I1RZS7
124754 229533.I1RE01 pp 229533.I1RML0
124755 229533.I1RE01 pp 229533.I1S8R3
124756 229533.I1RE01 pp 229533.I1RKC6
124757 229533.I1RE01 pp 229533.I1S0F8

124758	229533.I1RE01	pp	229533.I1RW46
124759	229533.I1RE01	pp	229533.I1RPU2
124760	229533.I1RE01	pp	229533.I1RTH6
124761	229533.I1RE01	pp	229533.I1RVZ7
124762	229533.I1RE01	pp	229533.I1RZW8
124763	229533.I1RE01	pp	229533.V6RRH2
124764	229533.I1RE01	pp	229533.I1S2C0
124765	229533.I1RE01	pp	229533.I1RRI2
124766	229533.I1RE01	pp	229533.I1S8V4
124767	229533.I1RE01	pp	229533.I1RZ79
124768	229533.I1RE01	pp	229533.I1RVF8
124769	229533.I1RE01	pp	229533.I1RRE5
124770	229533.I1RE01	pp	229533.I1RUG3
124771	229533.I1RE01	pp	229533.I1RWX5
124772	229533.I1RE01	pp	229533.I1RNC1
124773	229533.I1RE01	pp	229533.I1RYW3
124774	229533.I1RE01	pp	229533.V6RVG3
124775	229533.I1RE01	pp	229533.I1S1C7
124776	229533.I1RE01	pp	229533.I1RXT3
124777	229533.I1RE01	pp	229533.I1RZV3
124778	229533.I1RE01	pp	229533.I1S437
124779	229533.I1RE01	pp	229533.I1RG11
124780	229533.I1RE01	pp	229533.I1RWN6
124781	229533.I1RE01	pp	229533.I1S1W3
124782	229533.I1RE01	pp	229533.I1RY44
124783	229533.I1RE01	pp	229533.I1RXU9
124784	229533.I1RE01	pp	229533.I1RPY1
124785	229533.I1RE01	pp	229533.I1S0N4
124786	229533.I1RE01	pp	229533.Q4HZB8
124787	229533.I1RE01	pp	229533.V6RGN5
124788	229533.I1RE01	pp	229533.I1RJV3
124789	229533.I1RE01	pp	229533.I1S274
124790	229533.I1RE01	pp	229533.I1RWQ8
124791	229533.I1RE01	pp	229533.I1S1W9
124792	229533.I1RE01	pp	229533.I1S5R6
124793	229533.I1RE01	pp	229533.I1RE89
124794	229533.I1RE01	pp	229533.I1RMV0
124795	229533.I1RE01	pp	229533.V6RIV9
124796	229533.I1RE01	pp	229533.I1RS17
124797	229533.I1RE01	pp	229533.I1RRN2
124798	229533.I1RE01	pp	229533.I1RPP4
124799	229533.I1RE01	pp	229533.I1SA33
124800	229533.I1RE01	pp	229533.V6RMW6
124801	229533.I1RE01	pp	229533.I1RWN5
124802	229533.I1RE01	pp	229533.I1S0M6
124803	229533.I1RE01	pp	229533.I1RPK0
124804	229533.I1RE01	pp	229533.I1RE22
124805	229533.I1RE01	pp	229533.I1S0L4
124806	229533.I1RE01	pp	229533.I1RRM3
124807	229533.I1RE01	pp	229533.V6R042
124808	229533.I1RE01	pp	229533.I1S2H4
124809	229533.I1RE01	pp	229533.I1RGU7
124810	229533.I1RE01	pp	229533.I1RL04
124811	229533.I1RE01	pp	229533.I1RFS3
124812	229533.I1RE01	pp	229533.I1RSQ3
124813	229533.I1RE01	pp	229533.I1RPE0
124814	229533.I1RE01	pp	229533.I1RS20
124815	229533.I1RE01	pp	229533.I1S6Y8
124816	229533.I1RE01	pp	229533.I1RSI6
124817	229533.I1RE01	pp	229533.I1RRV1
124818	229533.I1RE01	pp	229533.I1RPM4
124819	229533.I1RE01	pp	229533.I1RQS8
124820	229533.I1RE01	pp	229533.I1RMG3
124821	229533.I1RE01	pp	229533.I1REC5
124822	229533.I1RE01	pp	229533.I1RXC2
124823	229533.I1RE01	pp	229533.I1S4Z8
124824	229533.I1RE01	pp	229533.I1RQ23
124825	229533.I1RE01	pp	229533.I1RPV3
124826	229533.I1RE01	pp	229533.I1RSZ7
124827	229533.I1RE01	pp	229533.O74638
124828	229533.I1RE01	pp	229533.I1RW61
124829	229533.I1RE01	pp	229533.I1S5R1
124830	229533.I1RE01	pp	229533.I1S277

124831	229533.I1RE01	pp	229533.I1RPU3
124832	229533.I1RE01	pp	229533.I1RRT8
124833	229533.I1RE01	pp	229533.I1RE76
124834	229533.I1RE01	pp	229533.I1S9Y1
124835	229533.I1RE01	pp	229533.I1RE27
124836	229533.I1RE01	pp	229533.I1S2B8
124837	229533.I1RE01	pp	229533.I1RRM5
124838	229533.I1RE01	pp	229533.I1RS47
124839	229533.I1RE01	pp	229533.I1RM82
124840	229533.I1RE01	pp	229533.I1RRJ8
124841	229533.I1RE01	pp	229533.I1RP28
124842	229533.I1RE01	pp	229533.I1S0F6
124843	229533.I1RE01	pp	229533.I1RJZ7
124844	229533.I1RE01	pp	229533.I1RRP3
124845	229533.I1RE01	pp	229533.V6R3X5
124846	229533.I1RE01	pp	229533.I1RQG4
124847	229533.I1RE01	pp	229533.I1RR73
124848	229533.I1RE01	pp	229533.I1RN67
124849	229533.I1RE01	pp	229533.I1S8P5
124850	229533.I1RE01	pp	229533.I1RPP3
124851	229533.I1RE01	pp	229533.I1RRL9
124852	229533.I1RE01	pp	229533.I1RZ39
124853	229533.I1RE01	pp	229533.I1S0W4
124854	229533.I1RE01	pp	229533.I1RYI5
124855	229533.I1RE01	pp	229533.I1RNN1
124856	229533.I1RE01	pp	229533.I1RFH8
124857	229533.I1RE01	pp	229533.I1S2E9
124858	229533.I1RE01	pp	229533.I1RYP2
124859	229533.I1RE01	pp	229533.I1S8V3
124860	229533.I1RE01	pp	229533.I1S2M6
124861	229533.I1RE01	pp	229533.I1RFT1
124862	229533.I1RE01	pp	229533.V6RUY9
124863	229533.I1RE01	pp	229533.I1RVZ2
124864	229533.I1RE01	pp	229533.I1RJN9
124865	229533.I1RE01	pp	229533.I1RT04
124866	229533.I1RE01	pp	229533.I1RSQ4
124867	229533.I1RE01	pp	229533.I1RGA6
124868	229533.I1RE01	pp	229533.I1RTA1
124869	229533.I1RE01	pp	229533.Q4HXF0
124870	229533.I1RE01	pp	229533.I1RZZ9
124871	229533.I1RE01	pp	229533.I1RYR0
124872	229533.I1RE01	pp	229533.I1RWI5
124873	229533.I1RE01	pp	229533.I1RTD5
124874	229533.I1RE01	pp	229533.I1RRI1
124875	229533.I1RE01	pp	229533.I1RXF8
124876	229533.I1RE01	pp	229533.I1RN63
124877	229533.I1RE01	pp	229533.I1RWK3
124878	229533.I1RE01	pp	229533.I1RT40
124879	229533.I1RE01	pp	229533.I1RV91
124880	229533.I1RE01	pp	229533.I1RJW6
124881	229533.I1RE01	pp	229533.I1S503
124882	229533.I1RE01	pp	229533.I1RJV4
124883	229533.I1RE01	pp	229533.I1S0F7
124884	229533.I1RE01	pp	229533.I1RZB2
124885	229533.I1RE01	pp	229533.Q4IEV5
124886	229533.I1RE01	pp	229533.I1RRZ0
124887	229533.I1RE01	pp	229533.I1RQM7
124888	229533.I1RE01	pp	229533.I1RJS2
124889	229533.I1RE01	pp	229533.I1RJ74
124890	229533.I1RE01	pp	229533.V6R3J1
124891	229533.I1RE01	pp	229533.I1RGA4
124892	229533.I1RE01	pp	229533.I1RRT2
124893	229533.I1RE01	pp	229533.I1RSX0
124894	229533.I1RE01	pp	229533.I1RJL4
124895	229533.I1RE01	pp	229533.I1RRK3
124896	229533.V6QU65	pp	229533.V6RXR0
124897	229533.V6QU65	pp	229533.V6RHD0
124898	229533.V6QU65	pp	229533.V6RDH3
124899	229533.V6QU65	pp	229533.V6RK96
124900	229533.I1RAA2	pp	229533.I1RAI4
124901	229533.I1RAA2	pp	229533.V6R1X4
124902	229533.I1RAA2	pp	229533.I1RZ43
124903	229533.I1RAA2	pp	229533.I1RRX5

124904 229533.I1RAA2 pp 229533.I1RMR9
124905 229533.I1RAA2 pp 229533.I1RYH7
124906 229533.I1RAA2 pp 229533.I1RFJ5
124907 229533.I1RAA2 pp 229533.V6R3J1
124908 229533.I1RAA2 pp 229533.I1RAE3
124909 229533.I1RAA2 pp 229533.I1RAE4
124910 229533.I1RAA2 pp 229533.I1RRH6
124911 229533.I1RAA2 pp 229533.I1RAW3
124912 229533.I1RZ26 pp 229533.V6R756
124913 229533.I1RZ26 pp 229533.V6R5D2
124914 229533.I1RZ26 pp 229533.I1SAI4
124915 229533.I1RZ26 pp 229533.V6QU99
124916 229533.I1RZ26 pp 229533.V6R2Z2
124917 229533.I1RZ26 pp 229533.V6R0W1
124918 229533.I1RZ26 pp 229533.V6RAM6
124919 229533.I1S569 pp 229533.V6QW22
124920 229533.I1S569 pp 229533.V6RTJ8
124921 229533.I1S569 pp 229533.Q4I1Y5
124922 229533.I1S569 pp 229533.Q4IJK2
124923 229533.I1S569 pp 229533.V6RCG5
124924 229533.I1S569 pp 229533.V6R042
124925 229533.I1S569 pp 229533.V6R3X5
124926 229533.I1S569 pp 229533.V6RMW6
124927 229533.I1S569 pp 229533.V6RIV9
124928 229533.I1S569 pp 229533.I1S8R3
124929 229533.I1S569 pp 229533.I1S8P5
124930 229533.I1RUQ3 pp 229533.I1S3T7
124931 229533.I1RUQ3 pp 229533.I1RYI5
124932 229533.I1RUQ3 pp 229533.I1S299
124933 229533.I1RUQ3 pp 229533.I1S5R1
124934 229533.I1RUQ3 pp 229533.I1RUR8
124935 229533.I1RUQ3 pp 229533.I1RVZ2
124936 229533.I1RUQ3 pp 229533.I1RVR2
124937 229533.I1RUQ3 pp 229533.I1S2B8
124938 229533.I1RUQ3 pp 229533.I1RYW9
124939 229533.I1RUQ3 pp 229533.I1S0W4
124940 229533.I1RUQ3 pp 229533.I1RY92
124941 229533.I1RUQ3 pp 229533.I1RZ39
124942 229533.I1RUQ3 pp 229533.I1S0A5
124943 229533.I1RUQ3 pp 229533.I1RVX8
124944 229533.I1RUQ3 pp 229533.I1S5R6
124945 229533.I1RUQ3 pp 229533.V6RRM0
124946 229533.I1RUQ3 pp 229533.V6RLM7
124947 229533.I1RUQ3 pp 229533.I1S1W9
124948 229533.I1RUQ3 pp 229533.V6RGI1
124949 229533.I1RUQ3 pp 229533.V6RAC9
124950 229533.I1RUQ3 pp 229533.I1S599
124951 229533.I1RUQ3 pp 229533.I1RYC8
124952 229533.I1RUQ3 pp 229533.V6R3J1
124953 229533.I1RUQ3 pp 229533.I1RYA0
124954 229533.I1RUQ3 pp 229533.V6RN69
124955 229533.I1RUQ3 pp 229533.I1S8R2
124956 229533.I1RUQ3 pp 229533.I1RZV3
124957 229533.I1RUQ3 pp 229533.I1S0V7
124958 229533.I1RUQ3 pp 229533.I1RVB2
124959 229533.I1RUQ3 pp 229533.V6QUG0
124960 229533.I1RUQ3 pp 229533.I1RZM8
124961 229533.I1RUQ3 pp 229533.Q4HVU7
124962 229533.I1RUQ3 pp 229533.I1SAF0
124963 229533.I1RUQ3 pp 229533.I1S4S7
124964 229533.I1RUQ3 pp 229533.I1RWM4
124965 229533.I1RUQ3 pp 229533.I1RY21
124966 229533.I1RUQ3 pp 229533.I1S2A1
124967 229533.I1RUQ3 pp 229533.I1S423
124968 229533.I1RUQ3 pp 229533.I1RXD7
124969 229533.I1RUQ3 pp 229533.Q4IJK2
124970 229533.I1RUQ3 pp 229533.I1RYX6
124971 229533.I1RUQ3 pp 229533.Q4HYR2
124972 229533.I1RUQ3 pp 229533.I1S0P0
124973 229533.I1RUQ3 pp 229533.I1RUY5
124974 229533.I1RUQ3 pp 229533.I1S0V6
124975 229533.I1RUQ3 pp 229533.I1S0K2
124976 229533.I1RUQ3 pp 229533.I1S8S9

124977 229533.I1RUQ3 pp 229533.I1RWR7
124978 229533.I1RUQ3 pp 229533.I1RZK5
124979 229533.I1RUQ3 pp 229533.I1S8F8
124980 229533.I1RUQ3 pp 229533.I1RYA2
124981 229533.I1RW46 pp 229533.V6R042
124982 229533.I1RW46 pp 229533.V6RGN5
124983 229533.I1RW46 pp 229533.I1SA33
124984 229533.I1RW46 pp 229533.I1RZW8
124985 229533.I1RW46 pp 229533.V6RMW6
124986 229533.I1RW46 pp 229533.I1RWN6
124987 229533.I1RW46 pp 229533.I1S1C7
124988 229533.I1RW46 pp 229533.I1RYW3
124989 229533.I1RW46 pp 229533.V6RIV9
124990 229533.I1RW46 pp 229533.I1RWN5
124991 229533.I1RW46 pp 229533.I1RY44
124992 229533.I1RW46 pp 229533.V6R057
124993 229533.I1RW46 pp 229533.I1S503
124994 229533.I1RW46 pp 229533.I1S8R3
124995 229533.I1RW46 pp 229533.I1S2E9
124996 229533.I1RW46 pp 229533.I1S0J1
124997 229533.I1RW46 pp 229533.V6RUY9
124998 229533.I1RW46 pp 229533.Q4I624
124999 229533.I1RW46 pp 229533.I1S5B0
125000 229533.I1RW46 pp 229533.I1S0F6
125001 229533.I1RW46 pp 229533.I1RW99
125002 229533.I1RW46 pp 229533.I1S0L4
125003 229533.I1RW46 pp 229533.I1RZM8
125004 229533.I1RW46 pp 229533.I1RXF0
125005 229533.I1RW46 pp 229533.I1RWI5
125006 229533.I1RW46 pp 229533.I1S2M6
125007 229533.I1RW46 pp 229533.I1S2H4
125008 229533.I1RW46 pp 229533.V6RCZ8
125009 229533.I1RW46 pp 229533.I1RWK3
125010 229533.I1RW46 pp 229533.V6RCG5
125011 229533.I1RW46 pp 229533.V6R3X5
125012 229533.I1RW46 pp 229533.I1RZZ9
125013 229533.I1RW46 pp 229533.Q4IEK8
125014 229533.I1RW46 pp 229533.I1RZ39
125015 229533.I1RW46 pp 229533.I1S5R1
125016 229533.I1RW46 pp 229533.I1S2B8
125017 229533.I1RW46 pp 229533.I1RZM1
125018 229533.I1RW46 pp 229533.O74638
125019 229533.I1RW46 pp 229533.I1S0M6
125020 229533.I1RW46 pp 229533.I1RXD7
125021 229533.I1RW46 pp 229533.Q4I283
125022 229533.I1RW46 pp 229533.I1S0R7
125023 229533.I1RW46 pp 229533.V6R227
125024 229533.I1RW46 pp 229533.I1RZK5
125025 229533.I1RW46 pp 229533.I1S6F0
125026 229533.I1RW46 pp 229533.I1RY03
125027 229533.I1RW46 pp 229533.Q4I2J8
125028 229533.I1RW46 pp 229533.I1RWD4
125029 229533.I1RW46 pp 229533.I1S504
125030 229533.I1RW46 pp 229533.V6R1K4
125031 229533.I1RW46 pp 229533.I1RWT6
125032 229533.I1RW46 pp 229533.Q4IJK2
125033 229533.I1RW46 pp 229533.I1S0F5
125034 229533.I1RW46 pp 229533.I1RZZ1
125035 229533.I1RW46 pp 229533.I1S0L3
125036 229533.I1RW46 pp 229533.I1SA32
125037 229533.I1RW46 pp 229533.Q4IJT5
125038 229533.I1RW46 pp 229533.I1RY63
125039 229533.I1RW46 pp 229533.Q4I8P5
125040 229533.I1RW46 pp 229533.I1S1J7
125041 229533.I1RW46 pp 229533.V6RTJ8
125042 229533.I1RW46 pp 229533.I1RX46
125043 229533.I1RW46 pp 229533.I1RW71
125044 229533.I1RW46 pp 229533.V6QW22
125045 229533.I1RW46 pp 229533.I1RX25
125046 229533.I1RW46 pp 229533.I1RZJ5
125047 229533.I1RW46 pp 229533.I1S7A8
125048 229533.I1RW46 pp 229533.I1S0X6
125049 229533.I1RW46 pp 229533.V6QYF0

125050	229533.I1RW46	pp	229533.I1RWJ8
125051	229533.I1R9K2	pp	229533.I1RDU6
125052	229533.I1R9K2	pp	229533.I1RIL0
125053	229533.I1R9K2	pp	229533.I1RM66
125054	229533.I1R9K2	pp	229533.I1RYR3
125055	229533.I1R9K2	pp	229533.I1S9T6
125056	229533.I1R9K2	pp	229533.Q4HYR2
125057	229533.I1R9K2	pp	229533.I1RJ27
125058	229533.I1R9K2	pp	229533.I1RFD4
125059	229533.I1R9K2	pp	229533.I1REW3
125060	229533.I1R9K2	pp	229533.I1RR45
125061	229533.I1R9K2	pp	229533.I1R9M7
125062	229533.I1R9K2	pp	229533.I1RH30
125063	229533.I1R9K2	pp	229533.I1RWM4
125064	229533.I1R9K2	pp	229533.I1RRK5
125065	229533.I1R9K2	pp	229533.I1SA33
125066	229533.I1RTA0	pp	229533.I1S8R3
125067	229533.I1RTA0	pp	229533.V6RIV9
125068	229533.I1RTA0	pp	229533.I1RWN5
125069	229533.I1RTA0	pp	229533.I1RTH6
125070	229533.I1RTA0	pp	229533.I1S1C7
125071	229533.I1RTA0	pp	229533.V6QX21
125072	229533.I1RTA0	pp	229533.I1RY44
125073	229533.I1RTA0	pp	229533.V6R3X5
125074	229533.I1RTA0	pp	229533.I1RWN6
125075	229533.I1RTA0	pp	229533.I1RZ39
125076	229533.I1RTA0	pp	229533.V6QUV7
125077	229533.I1RTA0	pp	229533.I1RYW3
125078	229533.I1RTA0	pp	229533.I1RVX0
125079	229533.I1RTA0	pp	229533.I1RZZ9
125080	229533.I1RTA0	pp	229533.I1RZW8
125081	229533.I1RTA0	pp	229533.Q4HXF6
125082	229533.I1RTA0	pp	229533.I1RZ79
125083	229533.I1RTA0	pp	229533.I1RWI5
125084	229533.I1RTA0	pp	229533.I1RZ29
125085	229533.I1RTA0	pp	229533.I1RWX5
125086	229533.I1RTA0	pp	229533.I1S2H4
125087	229533.I1RTA0	pp	229533.V6RMW6
125088	229533.I1RTA0	pp	229533.I1S2E8
125089	229533.I1RTA0	pp	229533.I1RYN4
125090	229533.I1RTA0	pp	229533.V6R042
125091	229533.I1RTA0	pp	229533.I1RZE4
125092	229533.I1RTA0	pp	229533.Q4I624
125093	229533.I1RTA0	pp	229533.I1S0L4
125094	229533.I1RTA0	pp	229533.I1S0N4
125095	229533.I1RTA0	pp	229533.I1RUG3
125096	229533.I1RTA0	pp	229533.I1S9Y1
125097	229533.I1RTA0	pp	229533.I1RWL2
125098	229533.I1RTA0	pp	229533.I1SA33
125099	229533.I1RTA0	pp	229533.V6R057
125100	229533.I1RTA0	pp	229533.I1S5R1
125101	229533.I1RTA0	pp	229533.I1S569
125102	229533.I1RTA0	pp	229533.V6RRH2
125103	229533.I1RTA0	pp	229533.I1S0J1
125104	229533.I1RTA0	pp	229533.I1S4Z7
125105	229533.I1RTA0	pp	229533.I1S2B8
125106	229533.I1RTA0	pp	229533.I1RTG3
125107	229533.I1RTA0	pp	229533.I1RWE8
125108	229533.I1RTA0	pp	229533.I1RZK5
125109	229533.I1RTA0	pp	229533.I1S503
125110	229533.I1RTA0	pp	229533.I1RYL5
125111	229533.I1RTA0	pp	229533.I1S2E9
125112	229533.I1RTA0	pp	229533.I1RY03
125113	229533.I1RTA0	pp	229533.V6R3J1
125114	229533.I1RTA0	pp	229533.I1RU29
125115	229533.I1RTA0	pp	229533.I1RV91
125116	229533.I1RTA0	pp	229533.I1S352
125117	229533.I1RTA0	pp	229533.I1RTD5
125118	229533.I1RTA0	pp	229533.I1RYS0
125119	229533.I1RTA0	pp	229533.I1RWD7
125120	229533.I1RTA0	pp	229533.I1S1J7
125121	229533.I1RTA0	pp	229533.I1RWK3
125122	229533.I1RTA0	pp	229533.Q4I2J8

125123	229533.I1RTA0	pp	229533.I1S8P5
125124	229533.I1RTA0	pp	229533.Q4IJK2
125125	229533.I1RTA0	pp	229533.I1RTA1
125126	229533.I1RTA0	pp	229533.I1S0F6
125127	229533.I1RTA0	pp	229533.V6RBN2
125128	229533.I1RTA0	pp	229533.V6RCZ8
125129	229533.I1RTA0	pp	229533.I1RZZ1
125130	229533.I1RTA0	pp	229533.Q4I8P5
125131	229533.I1RTA0	pp	229533.V6RCG5
125132	229533.I1RTA0	pp	229533.I1S0R7
125133	229533.I1RTA0	pp	229533.I1RZM8
125134	229533.I1RTA0	pp	229533.I1S504
125135	229533.I1RTA0	pp	229533.I1RW99
125136	229533.I1RTA0	pp	229533.I1SA32
125137	229533.I1RTA0	pp	229533.I1S5B0
125138	229533.I1RTA0	pp	229533.I1RXD7
125139	229533.I1RTA0	pp	229533.I1S3S3
125140	229533.I1RTA0	pp	229533.V6QUE6
125141	229533.I1RTA0	pp	229533.V6RTJ8
125142	229533.I1RTA0	pp	229533.Q4I5I4
125143	229533.I1RTA0	pp	229533.Q4I283
125144	229533.I1RTA0	pp	229533.Q4I5R9
125145	229533.I1RTA0	pp	229533.I1RX46
125146	229533.I1RTA0	pp	229533.I1S0X6
125147	229533.I1RTA0	pp	229533.I1RZM1
125148	229533.I1RTA0	pp	229533.V6QW22
125149	229533.I1RTA0	pp	229533.I1RW71
125150	229533.I1RTA0	pp	229533.I1RW25
125151	229533.I1RTA0	pp	229533.I1RW07
125152	229533.I1RTA0	pp	229533.Q4HZS8
125153	229533.I1RTA0	pp	229533.V6R1K4
125154	229533.I1RTA0	pp	229533.I1S0L3
125155	229533.I1RTA0	pp	229533.Q4IJT5
125156	229533.I1RTA0	pp	229533.I1RVQ0
125157	229533.I1RTA0	pp	229533.Q5I2J3
125158	229533.I1RTA0	pp	229533.V6R4J1
125159	229533.I1RIL0	pp	229533.I1S9T6
125160	229533.I1RIL0	pp	229533.I1RWM4
125161	229533.I1RIL0	pp	229533.I1RZA7
125162	229533.I1RIL0	pp	229533.I1S6P5
125163	229533.I1RIL0	pp	229533.V6RUB1
125164	229533.I1RIL0	pp	229533.Q4HYR2
125165	229533.I1RIL0	pp	229533.I1SA33
125166	229533.I1RPW1	pp	229533.V6RSF3
125167	229533.I1RPW1	pp	229533.V6RN31
125168	229533.I1RPW1	pp	229533.I1SAJ3
125169	229533.I1RPW1	pp	229533.I1RW69
125170	229533.I1RPW1	pp	229533.I1RR05
125171	229533.I1RPW1	pp	229533.I1S5C5
125172	229533.I1RPW1	pp	229533.I1S183
125173	229533.I1RPW1	pp	229533.I1RVY2
125174	229533.I1RPW1	pp	229533.I1RQE0
125175	229533.I1RPW1	pp	229533.V6RQW4
125176	229533.I1RPW1	pp	229533.V6RLM7
125177	229533.I1RPW1	pp	229533.I1RQ12
125178	229533.I1RPW1	pp	229533.I1S0A0
125179	229533.I1RPW1	pp	229533.V6QVH8
125180	229533.I1RPW1	pp	229533.I1RYU0
125181	229533.I1RPW1	pp	229533.I1S0I3
125182	229533.I1RPW1	pp	229533.I1RYI7
125183	229533.I1RPW1	pp	229533.I1RY49
125184	229533.I1RPW1	pp	229533.I1RZQ1
125185	229533.I1RPW1	pp	229533.I1RPW2
125186	229533.I1RPW1	pp	229533.I1S1W8
125187	229533.I1RPW1	pp	229533.I1RVJ6
125188	229533.I1RPW1	pp	229533.I1RYJ7
125189	229533.I1RPW1	pp	229533.I1RW43
125190	229533.I1RPW1	pp	229533.I1S3G4
125191	229533.I1RPW1	pp	229533.I1S9Y1
125192	229533.I1RPW1	pp	229533.I1RZ29
125193	229533.I1RPW1	pp	229533.I1RW71
125194	229533.I1RPW1	pp	229533.I1RYX6
125195	229533.I1RPW1	pp	229533.I1RSQ4

125196	229533.I1RPW1	pp	229533.I1RVX9
125197	229533.I1RPW1	pp	229533.I1RXB1
125198	229533.I1RPW1	pp	229533.I1RRG3
125199	229533.I1RD52	pp	229533.I1RFY6
125200	229533.I1RD52	pp	229533.I1RY86
125201	229533.I1RD52	pp	229533.I1RT33
125202	229533.I1RD52	pp	229533.V6R1K4
125203	229533.I1RD52	pp	229533.I1RPN1
125204	229533.I1RD52	pp	229533.Q4IJT5
125205	229533.I1RD52	pp	229533.I1RRG3
125206	229533.I1RD52	pp	229533.V6RTJ8
125207	229533.I1RD52	pp	229533.I1S0L3
125208	229533.I1RD52	pp	229533.I1S0F5
125209	229533.I1RD52	pp	229533.I1RWD4
125210	229533.I1RD52	pp	229533.I1S6F0
125211	229533.I1RD52	pp	229533.I1S7A8
125212	229533.I1RD52	pp	229533.I1S0T2
125213	229533.I1RD52	pp	229533.I1S854
125214	229533.I1RD52	pp	229533.I1S4S7
125215	229533.I1RD52	pp	229533.I1RXD6
125216	229533.I1RD52	pp	229533.I1RVQ0
125217	229533.I1RD52	pp	229533.I1RQC9
125218	229533.I1RD52	pp	229533.I1S0R7
125219	229533.I1RD52	pp	229533.I1RX46
125220	229533.I1RD52	pp	229533.I1RPY3
125221	229533.I1RD52	pp	229533.I1RW71
125222	229533.I1RD52	pp	229533.I1RTB3
125223	229533.I1RD52	pp	229533.I1RG53
125224	229533.I1RD52	pp	229533.I1RRS8
125225	229533.I1RD52	pp	229533.V6QW22
125226	229533.I1RD52	pp	229533.Q4IFI0
125227	229533.I1RD52	pp	229533.V6RUT5
125228	229533.I1RD52	pp	229533.I1RXD7
125229	229533.I1RD52	pp	229533.I1RSQ7
125230	229533.I1RD52	pp	229533.I1RRX5
125231	229533.I1RD52	pp	229533.I1RW25
125232	229533.I1RD52	pp	229533.I1RLP1
125233	229533.I1RD52	pp	229533.I1RX25
125234	229533.I1RD52	pp	229533.I1RX95
125235	229533.I1RD52	pp	229533.I1RFC0
125236	229533.I1RD52	pp	229533.I1S9I5
125237	229533.I1RD52	pp	229533.I1RUQ2
125238	229533.I1RD52	pp	229533.I1RW54
125239	229533.I1RD52	pp	229533.I1RYJ6
125240	229533.I1RD52	pp	229533.I1RE17
125241	229533.I1RD52	pp	229533.I1RNB2
125242	229533.I1RD52	pp	229533.I1RNA0
125243	229533.I1RD52	pp	229533.I1S0F1
125244	229533.I1RD52	pp	229533.I1RZF6
125245	229533.I1RD52	pp	229533.I1RMC8
125246	229533.I1RD52	pp	229533.V6QYF0
125247	229533.I1RD52	pp	229533.I1RG51
125248	229533.I1RD52	pp	229533.I1RR24
125249	229533.I1RD52	pp	229533.I1S504
125250	229533.I1RD52	pp	229533.I1RPF1
125251	229533.I1RD52	pp	229533.I1RWJ8
125252	229533.I1RD52	pp	229533.I1S4S4
125253	229533.I1RD52	pp	229533.V6R227
125254	229533.I1RD52	pp	229533.I1RZJ5
125255	229533.I1RD52	pp	229533.I1RM06
125256	229533.I1RD52	pp	229533.I1RMG8
125257	229533.I1RD52	pp	229533.Q4I283
125258	229533.I1RD52	pp	229533.I1RM67
125259	229533.I1RD52	pp	229533.I1S438
125260	229533.I1RD52	pp	229533.I1RTF4
125261	229533.I1RD52	pp	229533.I1RPA4
125262	229533.I1RD52	pp	229533.I1RWT6
125263	229533.I1RD52	pp	229533.I1RXC4
125264	229533.I1RD52	pp	229533.Q4IJH1
125265	229533.I1RD52	pp	229533.I1SA32
125266	229533.I1RD52	pp	229533.I1RG14
125267	229533.I1RD52	pp	229533.I1RQ85
125268	229533.I1RD52	pp	229533.I1RJ17

125269 229533.I1RD52 pp 229533.I1RT80
125270 229533.I1RD52 pp 229533.I1RQD2
125271 229533.I1RD52 pp 229533.I1RJU3
125272 229533.I1RD52 pp 229533.I1S419
125273 229533.I1RD52 pp 229533.I1RNQ5
125274 229533.I1RD52 pp 229533.I1RKJ5
125275 229533.I1RD52 pp 229533.I1RG56
125276 229533.I1RD52 pp 229533.Q4HVW2
125277 229533.I1RD52 pp 229533.I1RDA0
125278 229533.I1RD52 pp 229533.I1RLU6
125279 229533.I1RD52 pp 229533.I1S5B0
125280 229533.I1RD52 pp 229533.I1RMK3
125281 229533.I1RD52 pp 229533.Q4IEK8
125282 229533.I1RD52 pp 229533.I1S0X6
125283 229533.I1RD52 pp 229533.Q4IP34
125284 229533.I1RD52 pp 229533.I1RT05
125285 229533.I1RD52 pp 229533.I1RW99
125286 229533.I1RD52 pp 229533.Q4IJ56
125287 229533.I1RD52 pp 229533.I1RZM1
125288 229533.I1RD52 pp 229533.I1RRV3
125289 229533.I1RD52 pp 229533.V6RCG5
125290 229533.I1RD52 pp 229533.I1RMC0
125291 229533.I1RD52 pp 229533.I1RRV4
125292 229533.I1RD52 pp 229533.V6QUE6
125293 229533.I1RD52 pp 229533.I1RQ81
125294 229533.I1RD52 pp 229533.I1RRU9
125295 229533.I1RD52 pp 229533.Q4IAA0
125296 229533.I1RD52 pp 229533.I1RQ31
125297 229533.I1RD52 pp 229533.I1RS18
125298 229533.I1RD52 pp 229533.Q4IBS2
125299 229533.I1RD52 pp 229533.I1RXF0
125300 229533.I1RD52 pp 229533.I1RZM8
125301 229533.I1RD52 pp 229533.Q4I8P5
125302 229533.I1RD52 pp 229533.I1RN99
125303 229533.I1RD52 pp 229533.I1S1B2
125304 229533.I1RD52 pp 229533.I1RI82
125305 229533.I1RD52 pp 229533.I1RGC5
125306 229533.I1RD52 pp 229533.I1REK8
125307 229533.I1RD52 pp 229533.I1RF08
125308 229533.I1RD52 pp 229533.I1RUQ3
125309 229533.I1RD52 pp 229533.Q4I5Z5
125310 229533.I1RD52 pp 229533.I1RQA3
125311 229533.I1RD52 pp 229533.I1RK95
125312 229533.I1RD52 pp 229533.V6RCZ8
125313 229533.I1RD52 pp 229533.I1S6K9
125314 229533.I1RD52 pp 229533.Q4I2J8
125315 229533.I1RD52 pp 229533.I1REL7
125316 229533.I1RD52 pp 229533.I1S821
125317 229533.I1RD52 pp 229533.Q4IJK2
125318 229533.I1RD52 pp 229533.I1RZE5
125319 229533.I1RD52 pp 229533.I1RGB2
125320 229533.I1RD52 pp 229533.I1RZZ1
125321 229533.I1RD52 pp 229533.I1RR51
125322 229533.I1RD52 pp 229533.I1RSI0
125323 229533.I1RD52 pp 229533.I1RE00
125324 229533.I1RD52 pp 229533.I1RY62
125325 229533.I1RD52 pp 229533.V6RBN2
125326 229533.I1RD52 pp 229533.I1RF19
125327 229533.I1RD52 pp 229533.Q4I7F9
125328 229533.I1RD52 pp 229533.I1RFJ9
125329 229533.I1RD52 pp 229533.Q4I830
125330 229533.I1RD52 pp 229533.I1RZK5
125331 229533.I1RD52 pp 229533.I1RY63
125332 229533.I1RD52 pp 229533.I1RYP2
125333 229533.I1RD52 pp 229533.I1RQQ5
125334 229533.I1RD52 pp 229533.I1RWR7
125335 229533.I1RD52 pp 229533.I1RPR9
125336 229533.I1RD52 pp 229533.I1RFT1
125337 229533.I1RD52 pp 229533.I1RYI5
125338 229533.I1RD52 pp 229533.I1RQA5
125339 229533.I1RD52 pp 229533.I1S0C1
125340 229533.I1RD52 pp 229533.I1RRY9
125341 229533.I1RD52 pp 229533.I1S887

125342 229533.I1RD52 pp 229533.I1RQA2
125343 229533.I1RD52 pp 229533.I1RSR3
125344 229533.I1RD52 pp 229533.I1S8P5
125345 229533.I1RD52 pp 229533.V6R9Z0
125346 229533.I1RD52 pp 229533.I1RMD5
125347 229533.I1RD52 pp 229533.I1RPP3
125348 229533.I1RD52 pp 229533.I1RNT9
125349 229533.I1RD52 pp 229533.I1RRJ8
125350 229533.I1RD52 pp 229533.I1RWB0
125351 229533.I1RD52 pp 229533.I1RY03
125352 229533.I1RD52 pp 229533.I1RWK3
125353 229533.I1RD52 pp 229533.I1RN63
125354 229533.I1RD52 pp 229533.I1RS47
125355 229533.I1RD52 pp 229533.I1RPT0
125356 229533.I1RD52 pp 229533.I1RWS4
125357 229533.I1RD52 pp 229533.I1RYS0
125358 229533.I1RD52 pp 229533.I1RYR0
125359 229533.I1RD52 pp 229533.I1S1J7
125360 229533.I1RD52 pp 229533.Q4HZ42
125361 229533.I1RD52 pp 229533.I1RFU8
125362 229533.I1RD52 pp 229533.I1RRZ0
125363 229533.I1RD52 pp 229533.I1S0F7
125364 229533.I1RD52 pp 229533.Q4IAS1
125365 229533.I1RD52 pp 229533.Q4HW67
125366 229533.I1RD52 pp 229533.I1RN67
125367 229533.I1RD52 pp 229533.I1RSA5
125368 229533.I1RD52 pp 229533.I1RJN9
125369 229533.I1RD52 pp 229533.I1RRP3
125370 229533.I1RD52 pp 229533.I1S0W4
125371 229533.I1RD52 pp 229533.I1RTA1
125372 229533.I1RD52 pp 229533.I1RVZ2
125373 229533.I1RD52 pp 229533.Q4HVQ9
125374 229533.I1RD52 pp 229533.I1RRK3
125375 229533.I1RD52 pp 229533.I1RJW6
125376 229533.I1RD52 pp 229533.I1RNG4
125377 229533.I1RD52 pp 229533.I1S503
125378 229533.I1RD52 pp 229533.I1RDF7
125379 229533.I1RD52 pp 229533.I1S2M6
125380 229533.I1RD52 pp 229533.I1S268
125381 229533.I1RD52 pp 229533.I1S0F6
125382 229533.I1RD52 pp 229533.I1RGA4
125383 229533.I1RD52 pp 229533.I1RFG0
125384 229533.I1RD52 pp 229533.I1RRT2
125385 229533.I1RD52 pp 229533.I1RQM7
125386 229533.I1RD52 pp 229533.I1RJZ7
125387 229533.I1RD52 pp 229533.I1RSX0
125388 229533.I1RD52 pp 229533.I1RJ74
125389 229533.I1RD52 pp 229533.I1RJL4
125390 229533.I1RD52 pp 229533.I1RJV4
125391 229533.I1RD52 pp 229533.V6R3J1
125392 229533.I1RD52 pp 229533.I1RRM5
125393 229533.I1RD52 pp 229533.I1RE76
125394 229533.I1RD52 pp 229533.I1RKG2
125395 229533.I1RD52 pp 229533.I1S2B8
125396 229533.I1RD52 pp 229533.I1RT04
125397 229533.I1RD52 pp 229533.I1RZB2
125398 229533.I1RD52 pp 229533.I1RGU7
125399 229533.I1RD52 pp 229533.V6RUY9
125400 229533.I1RD52 pp 229533.I1RKD3
125401 229533.I1RD52 pp 229533.I1RE22
125402 229533.I1RD52 pp 229533.I1RPE0
125403 229533.I1RD52 pp 229533.I1S2H4
125404 229533.I1RD52 pp 229533.I1S2E9
125405 229533.I1RD52 pp 229533.I1S0M6
125406 229533.I1RD52 pp 229533.I1RV91
125407 229533.I1RD52 pp 229533.I1RS20
125408 229533.I1RD52 pp 229533.I1RFL3
125409 229533.I1RD52 pp 229533.I1S0L4
125410 229533.I1RD52 pp 229533.I1RSZ7
125411 229533.I1RD52 pp 229533.I1RW61
125412 229533.I1RD52 pp 229533.I1RN88
125413 229533.I1RD52 pp 229533.I1RT40
125414 229533.I1RD52 pp 229533.I1RXF8

125415	229533.I1RD52	pp	229533.Q4I7L0
125416	229533.I1RD52	pp	229533.I1RQ23
125417	229533.I1RD52	pp	229533.I1RTD5
125418	229533.I1RD52	pp	229533.Q4I662
125419	229533.I1RD52	pp	229533.I1RRI1
125420	229533.I1RD52	pp	229533.I1RFS3
125421	229533.I1RD52	pp	229533.I1RKP1
125422	229533.I1RD52	pp	229533.I1S277
125423	229533.I1RD52	pp	229533.I1S6Y8
125424	229533.I1RD52	pp	229533.I1S9Y1
125425	229533.I1RD52	pp	229533.I1RPM4
125426	229533.I1RD52	pp	229533.I1RPV3
125427	229533.I1RD52	pp	229533.I1RRT8
125428	229533.I1RD52	pp	229533.I1S5R1
125429	229533.I1RD52	pp	229533.I1S4Z8
125430	229533.I1RD52	pp	229533.I1REC5
125431	229533.I1RD52	pp	229533.I1RE27
125432	229533.I1RD52	pp	229533.I1RL04
125433	229533.I1RD52	pp	229533.I1RQS8
125434	229533.I1RD52	pp	229533.I1S8V3
125435	229533.I1RD52	pp	229533.I1RXC2
125436	229533.I1RD52	pp	229533.I1RRE5
125437	229533.I1RD52	pp	229533.O74638
125438	229533.I1RD52	pp	229533.I1RWD3
125439	229533.I1RD52	pp	229533.I1RQ21
125440	229533.I1RD52	pp	229533.I1RMV0
125441	229533.I1RD52	pp	229533.Q4I7K4
125442	229533.I1RD52	pp	229533.I1RSF1
125443	229533.I1RD52	pp	229533.I1RZG9
125444	229533.I1RD52	pp	229533.I1RPU2
125445	229533.I1RD52	pp	229533.I1RZ39
125446	229533.I1RD52	pp	229533.I1RE01
125447	229533.I1RD52	pp	229533.I1RRL9
125448	229533.I1RD52	pp	229533.I1RVZ7
125449	229533.I1RD52	pp	229533.I1RR73
125450	229533.I1RD52	pp	229533.I1RTA0
125451	229533.I1RD52	pp	229533.I1RW46
125452	229533.I1RD52	pp	229533.V6R3X5
125453	229533.I1RD52	pp	229533.I1S8V4
125454	229533.I1RD52	pp	229533.I1RWI5
125455	229533.I1RD52	pp	229533.V6RIV9
125456	229533.I1RD52	pp	229533.I1S7W5
125457	229533.I1RD52	pp	229533.I1RWN5
125458	229533.I1RD52	pp	229533.I1RSQ4
125459	229533.I1RD52	pp	229533.I1S0R2
125460	229533.I1RD52	pp	229533.I1S0J1
125461	229533.I1RD52	pp	229533.I1S1W9
125462	229533.I1RD52	pp	229533.I1RWQ8
125463	229533.I1RD52	pp	229533.I1RZZ9
125464	229533.I1RD52	pp	229533.I1RNL5
125465	229533.I1RD52	pp	229533.Q4IF76
125466	229533.I1RD52	pp	229533.I1RM37
125467	229533.I1RD52	pp	229533.I1S016
125468	229533.I1RD52	pp	229533.I1RY44
125469	229533.I1RD52	pp	229533.I1S5R6
125470	229533.I1RD52	pp	229533.I1S0N4
125471	229533.I1RD52	pp	229533.I1RFH8
125472	229533.I1RD52	pp	229533.I1S1W3
125473	229533.I1RD52	pp	229533.V6RGN5
125474	229533.I1RD52	pp	229533.I1RSQ3
125475	229533.I1RD52	pp	229533.V6R042
125476	229533.I1RD52	pp	229533.I1RG11
125477	229533.I1RD52	pp	229533.I1RZV3
125478	229533.I1RD52	pp	229533.I1S256
125479	229533.I1RD52	pp	229533.V6RMW6
125480	229533.I1RD52	pp	229533.I1SA33
125481	229533.I1RD52	pp	229533.I1RPK0
125482	229533.I1RD52	pp	229533.I1RRM3
125483	229533.I1RD52	pp	229533.V6RLM7
125484	229533.I1RD52	pp	229533.I1RRN2
125485	229533.I1RD52	pp	229533.I1RKG3
125486	229533.I1RD52	pp	229533.I1RSI6
125487	229533.I1RD52	pp	229533.I1RNC1

125488 229533.I1RD52 pp 229533.I1RMG3
125489 229533.I1RD52 pp 229533.I1RPP4
125490 229533.I1RD52 pp 229533.I1RXT3
125491 229533.I1RD52 pp 229533.I1RS17
125492 229533.I1RD52 pp 229533.I1RRV1
125493 229533.I1RD52 pp 229533.I1RVX0
125494 229533.I1RD52 pp 229533.I1RE79
125495 229533.I1RD52 pp 229533.I1RUG3
125496 229533.I1RD52 pp 229533.I1RTH6
125497 229533.I1RD52 pp 229533.I1RKJ4
125498 229533.I1RD52 pp 229533.I1S8R3
125499 229533.I1RD52 pp 229533.I1RT03
125500 229533.I1RD52 pp 229533.I1RVF8
125501 229533.I1RD52 pp 229533.I1RSY2
125502 229533.I1RD52 pp 229533.I1S0F8
125503 229533.I1RD52 pp 229533.I1RXR1
125504 229533.I1RD52 pp 229533.I1RSS4
125505 229533.I1RD52 pp 229533.I1RKJ8
125506 229533.I1RD52 pp 229533.I1RRN5
125507 229533.I1RD52 pp 229533.Q4HZ68
125508 229533.I1RD52 pp 229533.V6R057
125509 229533.I1RD52 pp 229533.I1RTE9
125510 229533.I1RD52 pp 229533.I1RNU7
125511 229533.I1RD52 pp 229533.I1S2C0
125512 229533.I1RD52 pp 229533.I1RUF7
125513 229533.I1RD52 pp 229533.I1S437
125514 229533.I1RD52 pp 229533.Q4I624
125515 229533.I1RD52 pp 229533.Q4HXF6
125516 229533.I1RD52 pp 229533.I1RFL4
125517 229533.I1RD52 pp 229533.I1S1C7
125518 229533.I1RD52 pp 229533.I1S2E8
125519 229533.I1RD52 pp 229533.I1RN18
125520 229533.I1RD52 pp 229533.Q4IPH4
125521 229533.I1RD52 pp 229533.I1RQE7
125522 229533.I1RD52 pp 229533.I1RG90
125523 229533.I1RD52 pp 229533.I1RQ33
125524 229533.I1RD52 pp 229533.I1RXU9
125525 229533.I1RD52 pp 229533.I1RYW3
125526 229533.I1RD52 pp 229533.I1RZ79
125527 229533.I1RD52 pp 229533.I1RWX5
125528 229533.I1RD52 pp 229533.V6RRH2
125529 229533.I1RD52 pp 229533.I1RKC6
125530 229533.I1RD52 pp 229533.I1RVQ3
125531 229533.I1RD52 pp 229533.I1RWN6
125532 229533.I1RD52 pp 229533.I1RML0
125533 229533.I1RD52 pp 229533.I1RRI2
125534 229533.I1RD52 pp 229533.I1RJV3
125535 229533.I1RD52 pp 229533.I1RZW8
125536 229533.I1RD52 pp 229533.I1RE89
125537 229533.I1RD52 pp 229533.I1S855
125538 229533.Q4I624 pp 229533.Q4IJK2
125539 229533.Q4I624 pp 229533.V6R1K4
125540 229533.Q4I624 pp 229533.V6RCZ8
125541 229533.Q4I624 pp 229533.Q4I8P5
125542 229533.Q4I624 pp 229533.Q4IP34
125543 229533.Q4I624 pp 229533.V6QUE6
125544 229533.Q4I624 pp 229533.V6RTJ8
125545 229533.Q4I624 pp 229533.V6QW22
125546 229533.Q4I624 pp 229533.Q4IPN9
125547 229533.Q4I624 pp 229533.V6RCG5
125548 229533.Q4I624 pp 229533.V6R227
125549 229533.Q4I624 pp 229533.V6R057
125550 229533.Q4I624 pp 229533.V6R3J1
125551 229533.Q4I624 pp 229533.Q4IJT5
125552 229533.Q4I624 pp 229533.V6RUT5
125553 229533.Q4I624 pp 229533.V6RIV9
125554 229533.Q4I624 pp 229533.V6R3X5
125555 229533.Q4I624 pp 229533.V6RMW6
125556 229533.Q4I624 pp 229533.V6RUY9
125557 229533.Q4I624 pp 229533.V6R042
125558 229533.I1S599 pp 229533.Q4HVQ9
125559 229533.I1RM66 pp 229533.I1RZA7
125560 229533.I1RM66 pp 229533.I1S6P5

125561	229533.I1RM66	pp	229533.I1SA33
125562	229533.I1RM66	pp	229533.V6RUB1
125563	229533.I1RM66	pp	229533.I1S9T6
125564	229533.I1RM66	pp	229533.Q4HYR2
125565	229533.I1RM66	pp	229533.I1RWM4
125566	229533.I1RD17	pp	229533.I1RDA0
125567	229533.I1RD17	pp	229533.Q4HVW2
125568	229533.I1RD17	pp	229533.I1RJU3
125569	229533.I1RD17	pp	229533.I1RMK3
125570	229533.I1RD17	pp	229533.I1S0X6
125571	229533.I1RD17	pp	229533.I1RTF4
125572	229533.I1RD17	pp	229533.I1RMC8
125573	229533.I1RD17	pp	229533.I1RI02
125574	229533.I1RD17	pp	229533.I1S0R7
125575	229533.I1RD17	pp	229533.I1RW07
125576	229533.I1RD17	pp	229533.I1RQC9
125577	229533.I1RD17	pp	229533.I1RW25
125578	229533.I1RD17	pp	229533.I1RLC7
125579	229533.I1RD17	pp	229533.I1RX25
125580	229533.I1RD17	pp	229533.I1S0L3
125581	229533.I1RD17	pp	229533.I1S0F5
125582	229533.I1RD17	pp	229533.I1RNA0
125583	229533.I1RD17	pp	229533.I1RM06
125584	229533.I1RD17	pp	229533.V6R1K4
125585	229533.I1RD17	pp	229533.I1RRQ6
125586	229533.I1RD17	pp	229533.V6QUW4
125587	229533.I1RD17	pp	229533.V6QYF0
125588	229533.I1RD17	pp	229533.I1RD52
125589	229533.I1RD17	pp	229533.I1RMG8
125590	229533.I1RD17	pp	229533.V6R4J1
125591	229533.I1RD17	pp	229533.I1RR24
125592	229533.I1RD17	pp	229533.I1RVQ0
125593	229533.I1RD17	pp	229533.I1RZJ5
125594	229533.I1RD17	pp	229533.I1S6F0
125595	229533.I1RD17	pp	229533.V6RTJ8
125596	229533.I1RD17	pp	229533.I1RM67
125597	229533.I1RD17	pp	229533.I1RWJ8
125598	229533.I1RD17	pp	229533.I1RT62
125599	229533.I1RD17	pp	229533.I1S7A8
125600	229533.I1RD17	pp	229533.I1S0T2
125601	229533.I1RD17	pp	229533.I1RZK5
125602	229533.I1RD17	pp	229533.I1RXD7
125603	229533.I1RD17	pp	229533.I1RWD4
125604	229533.I1RD17	pp	229533.I1RX46
125605	229533.I1RD17	pp	229533.I1RY03
125606	229533.I1RD17	pp	229533.V6QW22
125607	229533.I1RD17	pp	229533.I1RFU8
125608	229533.I1RD17	pp	229533.I1RSQ7
125609	229533.I1RD17	pp	229533.I1RTD9
125610	229533.I1RD17	pp	229533.I1RG53
125611	229533.I1RD17	pp	229533.I1RMD5
125612	229533.I1RD17	pp	229533.I1RUQ2
125613	229533.I1RD17	pp	229533.I1RWD7
125614	229533.I1RD17	pp	229533.I1RW71
125615	229533.I1RD17	pp	229533.V6RCZ8
125616	229533.I1RD17	pp	229533.I1RIL8
125617	229533.I1RD17	pp	229533.I1REK8
125618	229533.I1RD17	pp	229533.I1RI82
125619	229533.I1RD17	pp	229533.I1RSI0
125620	229533.I1RD17	pp	229533.I1RFJ9
125621	229533.I1RD17	pp	229533.Q4IJT5
125622	229533.I1RD17	pp	229533.I1RZM8
125623	229533.I1RD17	pp	229533.I1RXF0
125624	229533.I1RD17	pp	229533.I1RLX7
125625	229533.I1RD17	pp	229533.I1RF19
125626	229533.I1RD17	pp	229533.I1RMC0
125627	229533.I1RD17	pp	229533.I1RLM7
125628	229533.I1RD17	pp	229533.Q4I5Z5
125629	229533.I1RD17	pp	229533.I1RQ31
125630	229533.I1RD17	pp	229533.I1RU29
125631	229533.I1RD17	pp	229533.I1RSR3
125632	229533.I1RD17	pp	229533.I1RWT6
125633	229533.I1RD17	pp	229533.Q4I2J8

125634	229533.I1RD17	pp	229533.I1RGB6
125635	229533.I1RD17	pp	229533.I1RRY9
125636	229533.I1RD17	pp	229533.I1RDG0
125637	229533.I1RD17	pp	229533.V6R227
125638	229533.I1RD17	pp	229533.I1RN99
125639	229533.I1RD17	pp	229533.I1RI03
125640	229533.I1RD17	pp	229533.I1S1J7
125641	229533.I1RD17	pp	229533.I1RSC6
125642	229533.I1RD17	pp	229533.I1RQQ5
125643	229533.I1RD17	pp	229533.I1RY63
125644	229533.I1RD17	pp	229533.I1RQA2
125645	229533.I1RD17	pp	229533.I1RRT8
125646	229533.I1RD17	pp	229533.I1S504
125647	229533.I1RD17	pp	229533.Q4I283
125648	229533.I1RD17	pp	229533.I1RQA3
125649	229533.I1RD17	pp	229533.I1RZZ1
125650	229533.I1RD17	pp	229533.I1RZM1
125651	229533.I1RD17	pp	229533.I1S5R1
125652	229533.I1RD17	pp	229533.Q4IJK2
125653	229533.I1RD17	pp	229533.I1RLU6
125654	229533.I1RD17	pp	229533.I1RNQ5
125655	229533.I1RD17	pp	229533.O74638
125656	229533.I1RD17	pp	229533.I1RR73
125657	229533.I1RD17	pp	229533.I1RW99
125658	229533.I1RD17	pp	229533.V6R3X5
125659	229533.I1RD17	pp	229533.I1S5B0
125660	229533.I1RD17	pp	229533.Q4IEK8
125661	229533.I1RD17	pp	229533.I1RRU9
125662	229533.I1RD17	pp	229533.I1RRL9
125663	229533.I1RD17	pp	229533.I1S419
125664	229533.I1RD17	pp	229533.V6RCG5
125665	229533.I1RD17	pp	229533.I1RGC2
125666	229533.I1RD17	pp	229533.Q4I8P5
125667	229533.I1RD17	pp	229533.I1RS18
125668	229533.I1RD17	pp	229533.I1RZ39
125669	229533.I1RD17	pp	229533.I1RQ81
125670	229533.I1RD17	pp	229533.I1RR51
125671	229533.I1RD17	pp	229533.I1RFH8
125672	229533.I1RD17	pp	229533.I1RW61
125673	229533.I1RD17	pp	229533.I1S8V3
125674	229533.I1RD17	pp	229533.I1RZH4
125675	229533.I1RD17	pp	229533.I1SA32
125676	229533.I1RD17	pp	229533.I1RWI5
125677	229533.I1RD17	pp	229533.I1RP27
125678	229533.I1RD17	pp	229533.I1RZZ9
125679	229533.I1RD17	pp	229533.I1RJV4
125680	229533.I1RD17	pp	229533.I1RJS2
125681	229533.I1RD17	pp	229533.I1RE27
125682	229533.I1RD17	pp	229533.I1RPU3
125683	229533.I1RD17	pp	229533.I1S0L4
125684	229533.I1RD17	pp	229533.I1RJ74
125685	229533.I1RD17	pp	229533.I1RE76
125686	229533.I1RD17	pp	229533.I1RIL3
125687	229533.I1RD17	pp	229533.I1RGA4
125688	229533.I1RD17	pp	229533.I1RJL4
125689	229533.I1RD17	pp	229533.I1RQS8
125690	229533.I1RD17	pp	229533.I1S2H4
125691	229533.I1RD17	pp	229533.I1RJZ7
125692	229533.I1RD17	pp	229533.I1S8F0
125693	229533.I1RD17	pp	229533.I1RS20
125694	229533.I1RD17	pp	229533.I1RRT2
125695	229533.I1RD17	pp	229533.I1RRM5
125696	229533.I1RD17	pp	229533.I1RJW6
125697	229533.I1RD17	pp	229533.I1S2B8
125698	229533.I1RD17	pp	229533.I1S0M6
125699	229533.I1RD17	pp	229533.I1S503
125700	229533.I1RD17	pp	229533.I1RJM7
125701	229533.I1RD17	pp	229533.I1RE22
125702	229533.I1RD17	pp	229533.I1RWK3
125703	229533.I1RD17	pp	229533.I1RN63
125704	229533.I1RD17	pp	229533.I1RPM4
125705	229533.I1RD17	pp	229533.I1RRZ0
125706	229533.I1RD17	pp	229533.I1RD39

125707	229533.I1RD17	pp	229533.I1RLN6
125708	229533.I1RD17	pp	229533.I1S2E9
125709	229533.I1RD17	pp	229533.I1S6Y8
125710	229533.I1RD17	pp	229533.I1RJV3
125711	229533.I1RD17	pp	229533.I1RWN6
125712	229533.I1RD17	pp	229533.I1RQ23
125713	229533.I1RD17	pp	229533.I1RT04
125714	229533.I1RD17	pp	229533.I1RTE9
125715	229533.I1RD17	pp	229533.V6RUY9
125716	229533.I1RD17	pp	229533.I1RGC6
125717	229533.I1RD17	pp	229533.I1RTH6
125718	229533.I1RD17	pp	229533.I1RNA5
125719	229533.I1RD17	pp	229533.I1RT03
125720	229533.I1RD17	pp	229533.I1RFL4
125721	229533.I1RD17	pp	229533.I1RQE7
125722	229533.I1RD17	pp	229533.I1S8R3
125723	229533.I1RD17	pp	229533.I1RGF5
125724	229533.I1RD17	pp	229533.I1RTD5
125725	229533.I1RD17	pp	229533.I1RZW8
125726	229533.I1RD17	pp	229533.I1RT40
125727	229533.I1RD17	pp	229533.I1RN18
125728	229533.I1RD17	pp	229533.I1RV91
125729	229533.I1RD17	pp	229533.I1S0N4
125730	229533.I1RD17	pp	229533.I1RRI2
125731	229533.I1RD17	pp	229533.I1RS47
125732	229533.I1RD17	pp	229533.I1RWX5
125733	229533.I1RD17	pp	229533.I1S1C7
125734	229533.I1RD17	pp	229533.I1S0F6
125735	229533.I1RD17	pp	229533.V6RGN5
125736	229533.I1RD17	pp	229533.I1RD22
125737	229533.I1RD17	pp	229533.I1RWQ8
125738	229533.I1RD17	pp	229533.I1RYW3
125739	229533.I1RD17	pp	229533.I1RN67
125740	229533.I1RD17	pp	229533.I1RRP3
125741	229533.I1RD17	pp	229533.Q4I624
125742	229533.I1RD17	pp	229533.I1RRJ8
125743	229533.I1RD17	pp	229533.I1RWH4
125744	229533.I1RD17	pp	229533.I1S8P5
125745	229533.I1RD17	pp	229533.I1SA33
125746	229533.I1RD17	pp	229533.V6R042
125747	229533.I1RD17	pp	229533.I1S569
125748	229533.I1RD17	pp	229533.V6R057
125749	229533.I1RD17	pp	229533.I1RPP3
125750	229533.I1RD17	pp	229533.I1RFT1
125751	229533.I1RD17	pp	229533.I1RGL4
125752	229533.I1RD17	pp	229533.I1RSQ3
125753	229533.I1RD17	pp	229533.I1S352
125754	229533.I1RD17	pp	229533.I1RG90
125755	229533.I1RD17	pp	229533.I1S2M6
125756	229533.I1RD17	pp	229533.I1RKC6
125757	229533.I1RD17	pp	229533.I1RRN5
125758	229533.I1RD17	pp	229533.I1RVX0
125759	229533.I1RD17	pp	229533.V6RMW6
125760	229533.I1RD17	pp	229533.I1RS17
125761	229533.I1RD17	pp	229533.I1RSI6
125762	229533.I1RD17	pp	229533.I1RMG3
125763	229533.I1RD17	pp	229533.I1RRV1
125764	229533.I1RD17	pp	229533.I1RNB8
125765	229533.I1RD17	pp	229533.I1RXT3
125766	229533.I1RD17	pp	229533.I1S0J1
125767	229533.I1RD17	pp	229533.I1S0R2
125768	229533.I1RD17	pp	229533.I1RZS7
125769	229533.I1RD17	pp	229533.I1RNC1
125770	229533.I1RD17	pp	229533.I1S8V4
125771	229533.I1RD17	pp	229533.I1RUG3
125772	229533.I1RD17	pp	229533.I1RTA0
125773	229533.I1RD17	pp	229533.I1RW46
125774	229533.I1RD17	pp	229533.I1RRE5
125775	229533.I1RD17	pp	229533.I1RE01
125776	229533.I1RD17	pp	229533.Q4HZB8
125777	229533.I1RD17	pp	229533.I1RY44
125778	229533.I1RD17	pp	229533.I1S272
125779	229533.I1RD17	pp	229533.V6RIV9

125780 229533.I1RD17 pp 229533.I1RWN5
125781 229533.I1RD17 pp 229533.I1RPP4
125782 229533.A0A098DHF2 pp 229533.I1RC74
125783 229533.A0A098DHF2 pp 229533.I1RQE8
125784 229533.A0A098DHF2 pp 229533.I1RYQ4
125785 229533.A0A098DHF2 pp 229533.I1RY12
125786 229533.A0A098DHF2 pp 229533.I1RY49
125787 229533.A0A098DHF2 pp 229533.I1RQM4
125788 229533.A0A098DHF2 pp 229533.V6QU65
125789 229533.A0A098DHF2 pp 229533.I1S1W8
125790 229533.A0A098DHF2 pp 229533.I1S241
125791 229533.A0A098DHF2 pp 229533.V6RDH3
125792 229533.A0A098DHF2 pp 229533.I1RZM4
125793 229533.A0A098DHF2 pp 229533.I1RG66
125794 229533.A0A098DHF2 pp 229533.I1S3G4
125795 229533.A0A098DHF2 pp 229533.I1RHU0
125796 229533.A0A098DHF2 pp 229533.I1R9B6
125797 229533.A0A098DHF2 pp 229533.I1RW69
125798 229533.A0A098DHF2 pp 229533.A0A1C3YJU0
125799 229533.A0A098DHF2 pp 229533.I1RF42
125800 229533.A0A098DHF2 pp 229533.I1RQE1
125801 229533.A0A098DHF2 pp 229533.I1RE58
125802 229533.A0A098DHF2 pp 229533.A0A1C3YJW4
125803 229533.A0A098DHF2 pp 229533.I1RH91
125804 229533.A0A098DHF2 pp 229533.I1RZK8
125805 229533.A0A098DHF2 pp 229533.A0A1C3YKN4
125806 229533.A0A098DHF2 pp 229533.I1S673
125807 229533.A0A098DHF2 pp 229533.I1RBE7
125808 229533.A0A098DHF2 pp 229533.I1RYM5
125809 229533.A0A098DHF2 pp 229533.V6RK96
125810 229533.A0A098DHF2 pp 229533.I1RX59
125811 229533.A0A098DHF2 pp 229533.I1RNG4
125812 229533.A0A098DHF2 pp 229533.I1RJS0
125813 229533.A0A098DHF2 pp 229533.V6RHD0
125814 229533.A0A098DHF2 pp 229533.I1RMY1
125815 229533.A0A098DHF2 pp 229533.I1RRF5
125816 229533.A0A098DHF2 pp 229533.A0A098DTS1
125817 229533.A0A098DHF2 pp 229533.V6RXR0
125818 229533.A0A098DHF2 pp 229533.A0A1C3YJA1
125819 229533.A0A098DHF2 pp 229533.I1RAH3
125820 229533.A0A098DHF2 pp 229533.A0A1C3YKL7
125821 229533.A0A098DHF2 pp 229533.I1RBY9
125822 229533.A0A098DHF2 pp 229533.A0A1C3YJU5
125823 229533.A0A098DHF2 pp 229533.I1RWF5
125824 229533.A0A098DHF2 pp 229533.I1S0I3
125825 229533.A0A098DHF2 pp 229533.I1RNV4
125826 229533.A0A098DHF2 pp 229533.I1RXY7
125827 229533.A0A098DHF2 pp 229533.I1RUP5
125828 229533.A0A098DHF2 pp 229533.A0A0E0SID1
125829 229533.A0A098DHF2 pp 229533.A0A0E0SH57
125830 229533.A0A098DHF2 pp 229533.I1S147
125831 229533.A0A098DHF2 pp 229533.I1RKZ7
125832 229533.A0A098D6T7 pp 229533.Q4IPX8
125833 229533.A0A098D6T7 pp 229533.I1RKQ5
125834 229533.A0A098D6T7 pp 229533.A0A1C3YJR8
125835 229533.A0A098D6T7 pp 229533.I1RWC2
125836 229533.A0A098D6T7 pp 229533.I1RB36
125837 229533.A0A098D6T7 pp 229533.A0A1C3YMW0
125838 229533.A0A098D6T7 pp 229533.I1RX46
125839 229533.A0A098D6T7 pp 229533.I1RM49
125840 229533.A0A098D6T7 pp 229533.I1RAX6
125841 229533.A0A098D6T7 pp 229533.I1RUQ6
125842 229533.A0A098D6T7 pp 229533.I1RYZ9
125843 229533.A0A098D6T7 pp 229533.I1RW71
125844 229533.A0A098D6T7 pp 229533.A0A1C3YJI0
125845 229533.A0A098D6T7 pp 229533.V6RTJ8
125846 229533.A0A098D6T7 pp 229533.I1S7A8
125847 229533.A0A098D6T7 pp 229533.I1RC20
125848 229533.A0A098D6T7 pp 229533.V6QW22
125849 229533.A0A098D6T7 pp 229533.A0A1C3YL54
125850 229533.A0A098D6T7 pp 229533.A0A0E0RQK8
125851 229533.A0A098D6T7 pp 229533.I1RN61
125852 229533.A0A098D6T7 pp 229533.A0A1C3YM63

229533.A0A098D6T7	pp	229533.I1RFZ3
229533.A0A098D6T7	pp	229533.I1RLX7
229533.A0A098D6T7	pp	229533.A0A098DT74
229533.A0A098D6T7	pp	229533.I1RTG0
229533.A0A098D6T7	pp	229533.I1RZ12
229533.A0A098D6T7	pp	229533.I1S0R7
229533.A0A098D6T7	pp	229533.I1RSQ7
229533.A0A098D6T7	pp	229533.I1RQ26
229533.A0A098D6T7	pp	229533.I1RQC9
229533.A0A098D6T7	pp	229533.I1RFS6
229533.A0A098D6T7	pp	229533.A0A0E0SFN0
229533.A0A098D6T7	pp	229533.A0A1C3YI31
229533.A0A098D6T7	pp	229533.I1RAW7
229533.A0A098D6T7	pp	229533.I1RRG3
229533.A0A098D6T7	pp	229533.I1RG53
229533.A0A098D6T7	pp	229533.I1RXD7
229533.A0A098D6T7	pp	229533.I1RW38
229533.A0A098D6T7	pp	229533.A0A1C3YIL0
229533.A0A098D6T7	pp	229533.A0A1C3YIP5
229533.A0A098D6T7	pp	229533.I1RNH1
229533.A0A098D6T7	pp	229533.V6R4J1
229533.A0A098D6T7	pp	229533.I1RIL8
229533.A0A098D6T7	pp	229533.Q4IJT5
229533.A0A098D6T7	pp	229533.I1RZ51
229533.A0A098D6T7	pp	229533.I1RN81
229533.A0A098D6T7	pp	229533.A0A0E0SPD4
229533.A0A098D6T7	pp	229533.I1RSI4
229533.A0A098D6T7	pp	229533.I1RRT1
229533.A0A098D6T7	pp	229533.A0A098DYZ0
229533.A0A098D6T7	pp	229533.I1RVQ0
229533.A0A098D6T7	pp	229533.I1RC91
229533.A0A098D6T7	pp	229533.V6QV09
229533.A0A098D6T7	pp	229533.A0A1C3YLZ7
229533.A0A098D6T7	pp	229533.I1RE66
229533.A0A098D6T7	pp	229533.I1RD52
229533.A0A098D6T7	pp	229533.Q4IJP1
229533.A0A098D6T7	pp	229533.I1S0F5
229533.A0A098D6T7	pp	229533.A0A1C3YK00
229533.A0A098D6T7	pp	229533.A0A1C3YK13
229533.A0A098D6T7	pp	229533.I1RBC9
229533.A0A098D6T7	pp	229533.I1RJU3
229533.A0A098D6T7	pp	229533.V6QUW4
229533.A0A098D6T7	pp	229533.A0A1C3YN30
229533.A0A098D6T7	pp	229533.A0A098E4R5
229533.A0A098D6T7	pp	229533.I1RX70
229533.A0A098D6T7	pp	229533.I1RYI3
229533.A0A098D6T7	pp	229533.I1RZ94
229533.A0A098D6T7	pp	229533.I1RD01
229533.A0A098D6T7	pp	229533.I1S0L3
229533.A0A098D6T7	pp	229533.A0A0E0SAC4
229533.A0A098D6T7	pp	229533.I1RDB1
229533.A0A098D6T7	pp	229533.A0A098DXS1
229533.A0A098D6T7	pp	229533.I1RLC7
229533.A0A098D6T7	pp	229533.V6R1K4
229533.A0A098D6T7	pp	229533.I1RG62
229533.A0A098D6T7	pp	229533.A0A098DXE2
229533.A0A098D6T7	pp	229533.A0A0E0SA11
229533.A0A098D6T7	pp	229533.I1RIH8
229533.A0A098D6T7	pp	229533.I1RRX6
229533.A0A098D6T7	pp	229533.I1RWJ8
229533.A0A098D6T7	pp	229533.I1RX25
229533.A0A098D6T7	pp	229533.A0A1C3YJX0
229533.A0A098D6T7	pp	229533.I1RQA9
229533.A0A098D6T7	pp	229533.I1RV66
229533.A0A098D6T7	pp	229533.I1RW25
229533.A0A098D6T7	pp	229533.A0A1C3YJH8
229533.A0A098D6T7	pp	229533.A0A1C3YJV7
229533.A0A098D6T7	pp	229533.A0A098DSW2
229533.A0A098D6T7	pp	229533.A0A098DR40
229533.A0A098D6T7	pp	229533.A0A1C3YKF2
229533.A0A098D6T7	pp	229533.I1RZJ5
229533.A0A098D6T7	pp	229533.I1S0X6
229533.A0A098D6T7	pp	229533.I1S224

229533.A0A098D6T7	pp	229533.A0A1C3YKC0
229533.A0A098D6T7	pp	229533.A0A1C3YJQ9
229533.A0A098D6T7	pp	229533.I1RBT1
229533.A0A098D6T7	pp	229533.I1RMC8
229533.A0A098D6T7	pp	229533.I1RBW0
229533.A0A098D6T7	pp	229533.A0A1C3YHX8
229533.A0A098D6T7	pp	229533.I1RWJ6
229533.A0A098D6T7	pp	229533.I1RR94
229533.A0A098D6T7	pp	229533.A0A098E0E1
229533.A0A098D6T7	pp	229533.I1RYH9
229533.A0A098D6T7	pp	229533.I1RD18
229533.A0A098D6T7	pp	229533.I1RAU6
229533.A0A098D6T7	pp	229533.I1RR24
229533.A0A098D6T7	pp	229533.A0A0E0RSD7
229533.A0A098D6T7	pp	229533.Q4ICG5
229533.A0A098D6T7	pp	229533.I1RD69
229533.A0A098D6T7	pp	229533.I1RW99
229533.A0A098D6T7	pp	229533.A0A1C3YLR4
229533.A0A098D6T7	pp	229533.I1RN89
229533.A0A098D6T7	pp	229533.I1RCT9
229533.A0A098D6T7	pp	229533.A0A1C3YN58
229533.A0A098D6T7	pp	229533.I1S5B0
229533.A0A098D6T7	pp	229533.I1RL24
229533.A0A098D6T7	pp	229533.I1RNC0
229533.A0A098D6T7	pp	229533.A0A0E0RUI4
229533.A0A098D6T7	pp	229533.I1RW07
229533.A0A098D6T7	pp	229533.A0A1C3YK34
229533.A0A098D6T7	pp	229533.I1RMQ7
229533.A0A098D6T7	pp	229533.I1RN31
229533.A0A098D6T7	pp	229533.A0A0E0SQV5
229533.A0A098D6T7	pp	229533.I1RDC9
229533.A0A098D6T7	pp	229533.I1RI02
229533.A0A098D6T7	pp	229533.A0A1C3YJR3
229533.A0A098D6T7	pp	229533.A0A1C3YJZ9
229533.A0A098D6T7	pp	229533.I1RXF0
229533.A0A098D6T7	pp	229533.I1RZM8
229533.A0A098D6T7	pp	229533.I1RS07
229533.A0A098D6T7	pp	229533.I1RZH4
229533.A0A098D6T7	pp	229533.Q4I5R3
229533.A0A098D6T7	pp	229533.I1S504
229533.A0A098D6T7	pp	229533.I1RCW8
229533.A0A098D6T7	pp	229533.I1SA32
229533.A0A098D6T7	pp	229533.I1RCB3
229533.A0A098D6T7	pp	229533.I1RWT6
229533.A0A098D6T7	pp	229533.I1RM09
229533.A0A098D6T7	pp	229533.I1RQ19
229533.A0A098D6T7	pp	229533.I1REE1
229533.A0A098D6T7	pp	229533.A0A1C3YKB0
229533.A0A098D6T7	pp	229533.A0A1C3YIG3
229533.A0A098D6T7	pp	229533.I1RI82
229533.A0A098D6T7	pp	229533.Q4HTT2
229533.A0A098D6T7	pp	229533.I1RME5
229533.A0A098D6T7	pp	229533.I1RAM7
229533.A0A098D6T7	pp	229533.I1RX09
229533.A0A098D6T7	pp	229533.A0A0E0RZM5
229533.A0A098D6T7	pp	229533.A0A1C3YKV3
229533.A0A098D6T7	pp	229533.I1RS18
229533.A0A098D6T7	pp	229533.Q4I283
229533.A0A098D6T7	pp	229533.I1RUQ5
229533.A0A098D6T7	pp	229533.I1RC22
229533.A0A098D6T7	pp	229533.Q4I8P5
229533.A0A098D6T7	pp	229533.V6RCZ8
229533.A0A098D6T7	pp	229533.I1RY03
229533.A0A098D6T7	pp	229533.A0A1C3YIF4
229533.A0A098D6T7	pp	229533.A0A1C3YHX6
229533.A0A098D6T7	pp	229533.V6RCG5
229533.A0A098D6T7	pp	229533.A0A098DNE7
229533.A0A098D6T7	pp	229533.I1RZF2
229533.A0A098D6T7	pp	229533.A0A1C3YLU7
229533.A0A098D6T7	pp	229533.I1RYY5
229533.A0A098D6T7	pp	229533.A0A1C3YK75
229533.A0A098D6T7	pp	229533.A0A1C3YI23
229533.A0A098D6T7	pp	229533.V6QUE6

125999 229533.A0A098D6T7 pp 229533.I1RJT1
126000 229533.A0A098D6T7 pp 229533.V6RR76
126001 229533.A0A098D6T7 pp 229533.A0A1C3YLK2
126002 229533.A0A098D6T7 pp 229533.I1RST9
126003 229533.A0A098D6T7 pp 229533.I1RZB5
126004 229533.A0A098D6T7 pp 229533.I1RMB2
126005 229533.A0A098D6T7 pp 229533.A0A1C3YIJ4
126006 229533.A0A098D6T7 pp 229533.I1RAU5
126007 229533.A0A098D6T7 pp 229533.I1RN08
126008 229533.A0A098D6T7 pp 229533.I1RC95
126009 229533.A0A098D6T7 pp 229533.A0A0E0RV94
126010 229533.A0A098D6T7 pp 229533.I1RC17
126011 229533.A0A098D6T7 pp 229533.I1RMR7
126012 229533.A0A098D6T7 pp 229533.I1RKK0
126013 229533.A0A098D6T7 pp 229533.I1RLT6
126014 229533.A0A098D6T7 pp 229533.I1RZM1
126015 229533.A0A098D6T7 pp 229533.I1RKE9
126016 229533.A0A098D6T7 pp 229533.I1RXZ4
126017 229533.A0A098D6T7 pp 229533.I1RNQ5
126018 229533.A0A098D6T7 pp 229533.I1RCG4
126019 229533.A0A098D6T7 pp 229533.I1RLU6
126020 229533.A0A098D6T7 pp 229533.I1RFU8
126021 229533.A0A098D6T7 pp 229533.Q4I2J8
126022 229533.A0A098D6T7 pp 229533.I1RBJ5
126023 229533.A0A098D6T7 pp 229533.Q4IER8
126024 229533.A0A098D6T7 pp 229533.I1RMS2
126025 229533.A0A098D6T7 pp 229533.I1RJL2
126026 229533.A0A098D6T7 pp 229533.A0A1C3YK01
126027 229533.A0A098D6T7 pp 229533.I1RQS4
126028 229533.A0A098D6T7 pp 229533.I1S0N8
126029 229533.A0A098D6T7 pp 229533.I1RZK5
126030 229533.A0A098D6T7 pp 229533.I1RMM9
126031 229533.A0A098D6T7 pp 229533.I1RFQ5
126032 229533.A0A098D6T7 pp 229533.I1RFJ9
126033 229533.A0A098D6T7 pp 229533.A0A0E0RMM5
126034 229533.A0A098D6T7 pp 229533.A0A1C3YIJ3
126035 229533.A0A098D6T7 pp 229533.I1RF19
126036 229533.A0A098D6T7 pp 229533.I1RLM7
126037 229533.A0A098D6T7 pp 229533.I1RI03
126038 229533.A0A098D6T7 pp 229533.A0A0E0SPV2
126039 229533.A0A098D6T7 pp 229533.A0A1C3YJ93
126040 229533.A0A098D6T7 pp 229533.I1S1J7
126041 229533.A0A098D6T7 pp 229533.I1RU02
126042 229533.A0A098D6T7 pp 229533.I1RQE9
126043 229533.A0A098D6T7 pp 229533.I1RB75
126044 229533.A0A098D6T7 pp 229533.A0A0E0SQG9
126045 229533.A0A098D6T7 pp 229533.I1RCR5
126046 229533.A0A098D6T7 pp 229533.A0A1C3YLK8
126047 229533.A0A098D6T7 pp 229533.I1REK8
126048 229533.A0A098D6T7 pp 229533.I1RJW6
126049 229533.A0A098D6T7 pp 229533.A0A0E0RSG3
126050 229533.A0A098D6T7 pp 229533.I1RJK7
126051 229533.A0A098D6T7 pp 229533.I1R9X6
126052 229533.A0A098D6T7 pp 229533.V6QV38
126053 229533.A0A098D6T7 pp 229533.I1RYV3
126054 229533.A0A098D6T7 pp 229533.I1S503
126055 229533.A0A098D6T7 pp 229533.A0A098E2M3
126056 229533.A0A098D6T7 pp 229533.I1S439
126057 229533.A0A098D6T7 pp 229533.A0A098DYT7
126058 229533.A0A098D6T7 pp 229533.I1RS38
126059 229533.A0A098D6T7 pp 229533.I1RLN6
126060 229533.A0A098D6T7 pp 229533.I1RRY9
126061 229533.A0A098D6T7 pp 229533.I1RDV9
126062 229533.A0A098D6T7 pp 229533.I1RRZ0
126063 229533.A0A098D6T7 pp 229533.I1RT40
126064 229533.A0A098D6T7 pp 229533.I1RA87
126065 229533.A0A098D6T7 pp 229533.I1RU29
126066 229533.A0A098D6T7 pp 229533.A0A1C3YKE4
126067 229533.A0A098D6T7 pp 229533.I1RY32
126068 229533.A0A098D6T7 pp 229533.A0A1C3YKY3
126069 229533.A0A098D6T7 pp 229533.A0A1C3YLX2
126070 229533.A0A098D6T7 pp 229533.I1RXU1
126071 229533.A0A098D6T7 pp 229533.I1RQA3

126072	229533.A0A098D6T7	pp	229533.I1RPU7
126073	229533.A0A098D6T7	pp	229533.I1RRT2
126074	229533.A0A098D6T7	pp	229533.A0A0E0SB24
126075	229533.A0A098D6T7	pp	229533.Q4IJK2
126076	229533.A0A098D6T7	pp	229533.A0A098DMX4
126077	229533.A0A098D6T7	pp	229533.I1RGF5
126078	229533.A0A098D6T7	pp	229533.I1RBC7
126079	229533.A0A098D6T7	pp	229533.I1RQ88
126080	229533.A0A098D6T7	pp	229533.I1RGA4
126081	229533.A0A098D6T7	pp	229533.I1RTD5
126082	229533.A0A098D6T7	pp	229533.I1RQQ5
126083	229533.A0A098D6T7	pp	229533.I1RBC4
126084	229533.A0A098D6T7	pp	229533.I1RX39
126085	229533.A0A098D6T7	pp	229533.I1RZZ1
126086	229533.A0A098D6T7	pp	229533.I1RSZ3
126087	229533.A0A098D6T7	pp	229533.I1RBS6
126088	229533.A0A098D6T7	pp	229533.I1RW76
126089	229533.A0A098D6T7	pp	229533.I1S268
126090	229533.A0A098D6T7	pp	229533.A0A1C3YKJ6
126091	229533.A0A098D6T7	pp	229533.V6RUY9
126092	229533.A0A098D6T7	pp	229533.I1RN65
126093	229533.A0A098D6T7	pp	229533.I1S1B6
126094	229533.A0A098D6T7	pp	229533.A0A1C3YKT6
126095	229533.A0A098D6T7	pp	229533.A0A1C3YJF1
126096	229533.A0A098D6T7	pp	229533.Q4IJM4
126097	229533.A0A098D6T7	pp	229533.I1RT04
126098	229533.A0A098D6T7	pp	229533.I1RJV4
126099	229533.A0A098D6T7	pp	229533.A0A1C3YLF5
126100	229533.A0A098D6T7	pp	229533.I1RJ74
126101	229533.A0A098D6T7	pp	229533.I1RBV5
126102	229533.A0A098D6T7	pp	229533.I1RNA5
126103	229533.A0A098D6T7	pp	229533.I1RK95
126104	229533.A0A098D6T7	pp	229533.A0A0E0RVZ1
126105	229533.A0A098D6T7	pp	229533.A0A1C3YIB8
126106	229533.A0A098D6T7	pp	229533.I1S7X9
126107	229533.A0A098D6T7	pp	229533.I1RW83
126108	229533.A0A098D6T7	pp	229533.A0A1C3YKN3
126109	229533.A0A098D6T7	pp	229533.I1S2E9
126110	229533.A0A098D6T7	pp	229533.I1RWK3
126111	229533.A0A098D6T7	pp	229533.I1RX38
126112	229533.A0A098D6T7	pp	229533.I1S352
126113	229533.A0A098D6T7	pp	229533.I1RN63
126114	229533.A0A098D6T7	pp	229533.I1RRJ8
126115	229533.A0A098D6T7	pp	229533.A0A1C3YMJ3
126116	229533.A0A098D6T7	pp	229533.I1RW24
126117	229533.A0A098D6T7	pp	229533.I1RQS8
126118	229533.A0A098D6T7	pp	229533.I1S2M6
126119	229533.A0A098D6T7	pp	229533.I1S2H4
126120	229533.A0A098D6T7	pp	229533.I1RFL7
126121	229533.A0A098D6T7	pp	229533.I1RFW1
126122	229533.A0A098D6T7	pp	229533.I1RB74
126123	229533.A0A098D6T7	pp	229533.I1RPP3
126124	229533.A0A098D6T7	pp	229533.I1RFT1
126125	229533.A0A098D6T7	pp	229533.A0A1C3YIR8
126126	229533.A0A098D6T7	pp	229533.I1RBW1
126127	229533.A0A098D6T7	pp	229533.I1S8P5
126128	229533.A0A098D6T7	pp	229533.I1RWI5
126129	229533.A0A098D6T7	pp	229533.I1S0F6
126130	229533.A0A098D6T7	pp	229533.I1S0L4
126131	229533.A0A098D6T7	pp	229533.I1RZZ9
126132	229533.A0A098D6T7	pp	229533.Q4I7L0
126133	229533.A0A098D6T7	pp	229533.I1RN67
126134	229533.A0A098D6T7	pp	229533.I1RU69
126135	229533.A0A098D6T7	pp	229533.A0A1C3YIN0
126136	229533.A0A098D6T7	pp	229533.I1RTB2
126137	229533.A0A098D6T7	pp	229533.A0A098D8H0
126138	229533.A0A098D6T7	pp	229533.A0A0E0SC46
126139	229533.A0A098D6T7	pp	229533.I1RIL3
126140	229533.A0A098D6T7	pp	229533.I1RC66
126141	229533.A0A098D6T7	pp	229533.A0A0E0S0W9
126142	229533.A0A098D6T7	pp	229533.A0A1C3YJG7
126143	229533.A0A098D6T7	pp	229533.I1RBS0
126144	229533.A0A098D6T7	pp	229533.I1RC97

126145 229533.A0A098D6T7 pp 229533.A0A1C3YI39
126146 229533.A0A098D6T7 pp 229533.I1RE15
126147 229533.A0A098D6T7 pp 229533.A0A098DVE3
126148 229533.A0A098D6T7 pp 229533.I1RPM4
126149 229533.A0A098D6T7 pp 229533.I1S8V3
126150 229533.A0A098D6T7 pp 229533.I1RUN8
126151 229533.A0A098D6T7 pp 229533.I1RBU5
126152 229533.A0A098D6T7 pp 229533.A0A098E4E9
126153 229533.A0A098D6T7 pp 229533.I1RQ23
126154 229533.A0A098D6T7 pp 229533.I1RZ39
126155 229533.A0A098D6T7 pp 229533.I1RE22
126156 229533.A0A098D6T7 pp 229533.I1S0J3
126157 229533.A0A098D6T7 pp 229533.I1RS47
126158 229533.A0A098D6T7 pp 229533.A0A1C3YKM9
126159 229533.A0A098D6T7 pp 229533.I1RGF1
126160 229533.A0A098D6T7 pp 229533.A0A1C3YK57
126161 229533.A0A098D6T7 pp 229533.Q4HTT1
126162 229533.A0A098D6T7 pp 229533.I1S2G7
126163 229533.A0A098D6T7 pp 229533.I1RR73
126164 229533.A0A098D6T7 pp 229533.I1RMP0
126165 229533.A0A098D6T7 pp 229533.I1RNF4
126166 229533.A0A098D6T7 pp 229533.V6R3X5
126167 229533.A0A098D6T7 pp 229533.A0A0E0RP92
126168 229533.A0A098D6T7 pp 229533.A0A1C3YKN4
126169 229533.A0A098D6T7 pp 229533.I1REX8
126170 229533.A0A098D6T7 pp 229533.I1RC71
126171 229533.A0A098D6T7 pp 229533.I1RAN2
126172 229533.A0A098D6T7 pp 229533.A0A1C3YK73
126173 229533.A0A098D6T7 pp 229533.I1S549
126174 229533.A0A098D6T7 pp 229533.V6R042
126175 229533.A0A098D6T7 pp 229533.I1RZ63
126176 229533.A0A098D6T7 pp 229533.A0A1C3YM91
126177 229533.A0A098D6T7 pp 229533.V6RMW6
126178 229533.A0A098D6T7 pp 229533.I1S1V5
126179 229533.A0A098D6T7 pp 229533.I1RM82
126180 229533.A0A098D6T7 pp 229533.A0A1C3YJ12
126181 229533.A0A098D6T7 pp 229533.A0A1C3YK17
126182 229533.A0A098D6T7 pp 229533.I1SA33
126183 229533.A0A098D6T7 pp 229533.A0A1C3YJZ2
126184 229533.A0A098D6T7 pp 229533.I1S0M6
126185 229533.A0A098D6T7 pp 229533.I1RNP7
126186 229533.A0A098D6T7 pp 229533.I1S0X7
126187 229533.A0A098D6T7 pp 229533.I1RD22
126188 229533.A0A098D6T7 pp 229533.I1RLU1
126189 229533.A0A098D6T7 pp 229533.I1RWH4
126190 229533.A0A098D6T7 pp 229533.I1RA11
126191 229533.A0A098D6T7 pp 229533.I1S0J7
126192 229533.A0A098D6T7 pp 229533.I1S5R1
126193 229533.A0A098D6T7 pp 229533.I1RJZ7
126194 229533.A0A098D6T7 pp 229533.I1S2B8
126195 229533.A0A098D6T7 pp 229533.I1SAF7
126196 229533.A0A098D6T7 pp 229533.I1RRT8
126197 229533.A0A098D6T7 pp 229533.I1R9M0
126198 229533.A0A098D6T7 pp 229533.A0A0E0RU84
126199 229533.A0A098D6T7 pp 229533.V6RGI1
126200 229533.A0A098D6T7 pp 229533.I1RVY0
126201 229533.A0A098D6T7 pp 229533.I1RE76
126202 229533.A0A098D6T7 pp 229533.I1RBY4
126203 229533.A0A098D6T7 pp 229533.I1RRM5
126204 229533.A0A098D6T7 pp 229533.I1RT36
126205 229533.A0A098D6T7 pp 229533.I1RPX5
126206 229533.A0A098D6T7 pp 229533.A0A1C3YMQ5
126207 229533.A0A098D6T7 pp 229533.I1RE65
126208 229533.A0A098D6T7 pp 229533.A0A1C3YMC9
126209 229533.A0A098D6T7 pp 229533.A0A1C3YK43
126210 229533.A0A098D6T7 pp 229533.I1RE27
126211 229533.A0A098D6T7 pp 229533.A0A1C3YL52
126212 229533.A0A098D6T7 pp 229533.A0A1C3YIS7
126213 229533.A0A098D6T7 pp 229533.I1RPP4
126214 229533.A0A098D6T7 pp 229533.A0A1C3YHL0
126215 229533.A0A098D6T7 pp 229533.A0A1C3YMA1
126216 229533.A0A098D6T7 pp 229533.I1RG11
126217 229533.A0A098D6T7 pp 229533.I1RW16

229533.A0A098D6T7 pp 229533.I1S0M2
229533.A0A098D6T7 pp 229533.A0A098DX13
229533.A0A098D6T7 pp 229533.V6RKY7
229533.A0A098D6T7 pp 229533.I1R9D1
229533.A0A098D6T7 pp 229533.I1RSI3
229533.A0A098D6T7 pp 229533.I1RSD1
229533.A0A098D6T7 pp 229533.I1RCR8
229533.A0A098D6T7 pp 229533.I1RTA0
229533.A0A098D6T7 pp 229533.I1RQB4
229533.A0A098D6T7 pp 229533.I1RW46
229533.A0A098D6T7 pp 229533.I1RNC1
229533.A0A098D6T7 pp 229533.A0A1C3YI28
229533.A0A098D6T7 pp 229533.I1RA43
229533.A0A098D6T7 pp 229533.I1RWN5
229533.A0A098D6T7 pp 229533.V6RIV9
229533.A0A098D6T7 pp 229533.I1RE01
229533.A0A098D6T7 pp 229533.I1S0J1
229533.A0A098D6T7 pp 229533.I1RUG3
229533.A0A098D6T7 pp 229533.I1RJT0
229533.A0A098D6T7 pp 229533.I1RRW0
229533.A0A098D6T7 pp 229533.A0A1C3YMI5
229533.A0A098D6T7 pp 229533.I1RNY8
229533.A0A098D6T7 pp 229533.A0A1C3YKS0
229533.A0A098D6T7 pp 229533.A0A1C3YN69
229533.A0A098D6T7 pp 229533.I1RMA4
229533.A0A098D6T7 pp 229533.I1R9Y9
229533.A0A098D6T7 pp 229533.I1RS17
229533.A0A098D6T7 pp 229533.I1RRV1
229533.A0A098D6T7 pp 229533.V6RVG3
229533.A0A098D6T7 pp 229533.I1RMG3
229533.A0A098D6T7 pp 229533.I1RCB7
229533.A0A098D6T7 pp 229533.I1RA56
229533.A0A098D6T7 pp 229533.I1RC33
229533.A0A098D6T7 pp 229533.I1RSQ3
229533.A0A098D6T7 pp 229533.I1RY44
229533.A0A098D6T7 pp 229533.A0A098DF98
229533.A0A098D6T7 pp 229533.I1RTH6
229533.A0A098D6T7 pp 229533.I1RQE7
229533.A0A098D6T7 pp 229533.I1S1W4
229533.A0A098D6T7 pp 229533.I1RY58
229533.A0A098D6T7 pp 229533.A0A1C3YLN8
229533.A0A098D6T7 pp 229533.A0A1C3YHP8
229533.A0A098D6T7 pp 229533.I1S8R3
229533.A0A098D6T7 pp 229533.A0A0E0RS85
229533.A0A098D6T7 pp 229533.I1RRE5
229533.A0A098D6T7 pp 229533.I1RZE4
229533.A0A098D6T7 pp 229533.I1S8V4
229533.A0A098D6T7 pp 229533.I1RA70
229533.A0A098D6T7 pp 229533.I1RCH5
229533.A0A098D6T7 pp 229533.I1RSG8
229533.A0A098D6T7 pp 229533.I1RQK5
229533.A0A098D6T7 pp 229533.I1RRN5
229533.A0A098D6T7 pp 229533.I1RDC5
229533.A0A098D6T7 pp 229533.I1RX64
229533.A0A098D6T7 pp 229533.I1RT03
229533.A0A098D6T7 pp 229533.I1RZK9
229533.A0A098D6T7 pp 229533.I1RSP7
229533.A0A098D6T7 pp 229533.I1RPW0
229533.A0A098D6T7 pp 229533.I1S1Y7
229533.A0A098D6T7 pp 229533.A0A1C3YIN6
229533.A0A098D6T7 pp 229533.I1RDQ9
229533.A0A098D6T7 pp 229533.I1RVX0
229533.A0A098D6T7 pp 229533.I1RJV3
229533.A0A098D6T7 pp 229533.I1RK79
229533.A0A098D6T7 pp 229533.I1RB92
229533.A0A098D6T7 pp 229533.A0A0E0SBC4
229533.A0A098D6T7 pp 229533.I1RRY3
229533.A0A098D6T7 pp 229533.I1RYW3
229533.A0A098D6T7 pp 229533.I1RDZ4
229533.A0A098D6T7 pp 229533.I1R9J6
229533.A0A098D6T7 pp 229533.I1S1C7
229533.A0A098D6T7 pp 229533.I1RGC6
229533.A0A098D6T7 pp 229533.I1RK75

126291 229533.A0A098D6T7 pp 229533.I1RTN0
126292 229533.A0A098D6T7 pp 229533.A0A1C3YM29
126293 229533.A0A098D6T7 pp 229533.I1RB41
126294 229533.A0A098D6T7 pp 229533.I1RZW8
126295 229533.A0A098D6T7 pp 229533.I1RW84
126296 229533.A0A098D6T7 pp 229533.I1RG90
126297 229533.A0A098D6T7 pp 229533.I1R9Y7
126298 229533.A0A098D6T7 pp 229533.I1RS41
126299 229533.A0A098D6T7 pp 229533.I1RW32
126300 229533.A0A098D6T7 pp 229533.I1RGL4
126301 229533.A0A098D6T7 pp 229533.A0A1C3YI73
126302 229533.A0A098D6T7 pp 229533.I1RWX5
126303 229533.A0A098D6T7 pp 229533.I1RZT1
126304 229533.A0A098D6T7 pp 229533.I1RD17
126305 229533.A0A098D6T7 pp 229533.I1RAW0
126306 229533.A0A098D6T7 pp 229533.I1RRI2
126307 229533.A0A098D6T7 pp 229533.Q4I624
126308 229533.A0A098D6T7 pp 229533.I1RMJ8
126309 229533.A0A098D6T7 pp 229533.I1S569
126310 229533.A0A098D6T7 pp 229533.I1RD39
126311 229533.A0A098D6T7 pp 229533.I1RBJ0
126312 229533.A0A098D6T7 pp 229533.I1S0A6
126313 229533.A0A098D6T7 pp 229533.I1RWN6
126314 229533.A0A098D6T7 pp 229533.A0A1C3YK33
126315 229533.A0A098D6T7 pp 229533.I1S757
126316 229533.A0A098D6T7 pp 229533.I1RFL4
126317 229533.A0A098D6T7 pp 229533.I1RN18
126318 229533.A0A098D6T7 pp 229533.V6R057
126319 229533.A0A098D6T7 pp 229533.I1RR25
126320 229533.A0A098D6T7 pp 229533.I1RAU7
126321 229533.A0A098D6T7 pp 229533.I1RQ72
126322 229533.A0A1C3YJ22 pp 229533.I1S6P5
126323 229533.A0A1C3YJ22 pp 229533.I1S9T6
126324 229533.A0A1C3YJ22 pp 229533.I1RZA7
126325 229533.A0A1C3YJ22 pp 229533.V6RUB1
126326 229533.A0A1C3YJ22 pp 229533.A0A1C3YKN4
126327 229533.A0A1C3YJ22 pp 229533.I1R9M7
126328 229533.A0A1C3YJ22 pp 229533.Q4HYR2
126329 229533.A0A1C3YJ22 pp 229533.I1R9K2
126330 229533.A0A1C3YJ22 pp 229533.I1RFD4
126331 229533.A0A1C3YJ22 pp 229533.I1SA33
126332 229533.A0A1C3YJ22 pp 229533.I1RWM4
126333 229533.A0A1C3YJ22 pp 229533.A0A1C3YK58
126334 229533.I1RDU4 pp 229533.I1S2B8
126335 229533.I1RDU4 pp 229533.I1RR73
126336 229533.I1RDU4 pp 229533.I1RN67
126337 229533.I1RDU4 pp 229533.I1RK44
126338 229533.I1RDU4 pp 229533.Q4I1Y5
126339 229533.I1RDU4 pp 229533.I1RZ39
126340 229533.I1RDU4 pp 229533.I1RPP4
126341 229533.I1RDU4 pp 229533.I1RWN5
126342 229533.I1RDU4 pp 229533.I1RQC9
126343 229533.I1RDU4 pp 229533.I1RTD5
126344 229533.I1RDU4 pp 229533.I1RJW1
126345 229533.I1RDU4 pp 229533.I1RNC1
126346 229533.I1RDU4 pp 229533.I1RWN6
126347 229533.I1RDU4 pp 229533.Q4IJK2
126348 229533.I1RDU4 pp 229533.I1RTH6
126349 229533.I1RDU4 pp 229533.I1RHT5
126350 229533.I1RDU4 pp 229533.I1RSQ3
126351 229533.I1RDU4 pp 229533.V6RMW6
126352 229533.I1RDU4 pp 229533.I1RRY9
126353 229533.I1RDU4 pp 229533.I1RR39
126354 229533.I1RDU4 pp 229533.I1RIE8
126355 229533.I1RDU4 pp 229533.I1S267
126356 229533.I1RDU4 pp 229533.I1RW55
126357 229533.I1RYA2 pp 229533.I1S299
126358 229533.I1RYA2 pp 229533.I1S3G4
126359 229533.I1RYA2 pp 229533.I1S2Q4
126360 229533.I1RYA2 pp 229533.Q4HVQ9
126361 229533.I1RYA2 pp 229533.I1S1B8
126362 229533.I1RYA2 pp 229533.I1S1W8
126363 229533.I1RYA2 pp 229533.V6RRM0

126364 229533.I1RYA2 pp 229533.I1RYX6
126365 229533.I1RYA2 pp 229533.I1S8R2
126366 229533.I1RYA2 pp 229533.I1S8F8
126367 229533.I1RYA2 pp 229533.I1S8C1
126368 229533.I1RYA2 pp 229533.I1S4G3
126369 229533.A0A1C3YI73 pp 229533.I1RPP4
126370 229533.A0A1C3YI73 pp 229533.I1S272
126371 229533.A0A1C3YI73 pp 229533.A0A1C3YKV3
126372 229533.A0A1C3YI73 pp 229533.Q4I8P5
126373 229533.A0A1C3YI73 pp 229533.I1RS17
126374 229533.A0A1C3YI73 pp 229533.I1SA32
126375 229533.A0A1C3YI73 pp 229533.I1RW46
126376 229533.A0A1C3YI73 pp 229533.I1RE01
126377 229533.A0A1C3YI73 pp 229533.A0A1C3YK34
126378 229533.A0A1C3YI73 pp 229533.I1S0N4
126379 229533.A0A1C3YI73 pp 229533.I1S5B0
126380 229533.A0A1C3YI73 pp 229533.I1RUN9
126381 229533.A0A1C3YI73 pp 229533.I1RW99
126382 229533.A0A1C3YI73 pp 229533.I1R9U9
126383 229533.A0A1C3YI73 pp 229533.V6RPQ8
126384 229533.A0A1C3YI73 pp 229533.I1RDF6
126385 229533.A0A1C3YI73 pp 229533.I1RD22
126386 229533.A0A1C3YI73 pp 229533.I1RAU5
126387 229533.A0A1C3YI73 pp 229533.I1RQ75
126388 229533.A0A1C3YI73 pp 229533.I1SA33
126389 229533.A0A1C3YI73 pp 229533.V6R042
126390 229533.A0A1C3YI73 pp 229533.A0A1C3YKB0
126391 229533.A0A1C3YI73 pp 229533.I1RJL9
126392 229533.A0A1C3YI73 pp 229533.I1RV63
126393 229533.A0A1C3YI73 pp 229533.A0A1C3YLH6
126394 229533.A0A1C3YI73 pp 229533.V6RMW6
126395 229533.A0A1C3YI73 pp 229533.I1S504
126396 229533.A0A1C3YI73 pp 229533.V6RCG5
126397 229533.A0A1C3YI73 pp 229533.I1RNQ5
126398 229533.A0A1C3YI73 pp 229533.I1RZM1
126399 229533.A0A1C3YI73 pp 229533.Q4HZE0
126400 229533.A0A1C3YI73 pp 229533.V6RCZ8
126401 229533.A0A1C3YI73 pp 229533.V6RIV9
126402 229533.A0A1C3YI73 pp 229533.I1RI82
126403 229533.A0A1C3YI73 pp 229533.I1RB92
126404 229533.A0A1C3YI73 pp 229533.I1RNC1
126405 229533.A0A1C3YI73 pp 229533.I1RA48
126406 229533.A0A1C3YI73 pp 229533.I1RUG3
126407 229533.A0A1C3YI73 pp 229533.I1RXF0
126408 229533.A0A1C3YI73 pp 229533.I1RCH5
126409 229533.A0A1C3YI73 pp 229533.I1S0F6
126410 229533.A0A1C3YI73 pp 229533.I1RZZ1
126411 229533.A0A1C3YI73 pp 229533.I1RPP3
126412 229533.A0A1C3YI73 pp 229533.A0A1C3YIP5
126413 229533.A0A1C3YI73 pp 229533.I1RZM8
126414 229533.A0A1C3YI73 pp 229533.I1RQE7
126415 229533.A0A1C3YI73 pp 229533.V6QW22
126416 229533.A0A1C3YI73 pp 229533.I1RAU7
126417 229533.A0A1C3YI73 pp 229533.I1RFL4
126418 229533.A0A1C3YI73 pp 229533.I1RN18
126419 229533.A0A1C3YI73 pp 229533.I1RBC9
126420 229533.A0A1C3YI73 pp 229533.I1RSQ7
126421 229533.A0A1C3YI73 pp 229533.A0A1C3YKN3
126422 229533.A0A1C3YI73 pp 229533.I1REK8
126423 229533.A0A1C3YI73 pp 229533.Q4IJT0
126424 229533.A0A1C3YI73 pp 229533.I1RD17
126425 229533.A0A1C3YI73 pp 229533.Q4I624
126426 229533.A0A1C3YI73 pp 229533.I1RD39
126427 229533.A0A1C3YI73 pp 229533.I1RWK3
126428 229533.A0A1C3YI73 pp 229533.A0A1C3YLP4
126429 229533.A0A1C3YI73 pp 229533.I1RRV9
126430 229533.A0A1C3YI73 pp 229533.A0A1C3YIJ3
126431 229533.A0A1C3YI73 pp 229533.V6RTJ8
126432 229533.A0A1C3YI73 pp 229533.I1RZV2
126433 229533.A0A1C3YI73 pp 229533.I1RRM5
126434 229533.A0A1C3YI73 pp 229533.V6QUV7
126435 229533.A0A1C3YI73 pp 229533.I1RJV4
126436 229533.A0A1C3YI73 pp 229533.I1RNM4

126437 229533.A0A1C3YI73 pp 229533.Q4IB63
126438 229533.A0A1C3YI73 pp 229533.I1RRY9
126439 229533.A0A1C3YI73 pp 229533.I1S2B8
126440 229533.A0A1C3YI73 pp 229533.I1RWN6
126441 229533.A0A1C3YI73 pp 229533.I1SAM9
126442 229533.A0A1C3YI73 pp 229533.I1RAW0
126443 229533.A0A1C3YI73 pp 229533.I1R9N7
126444 229533.A0A1C3YI73 pp 229533.I1RBS6
126445 229533.A0A1C3YI73 pp 229533.I1RJV3
126446 229533.A0A1C3YI73 pp 229533.A0A1C3YIL9
126447 229533.A0A1C3YI73 pp 229533.A0A1C3YID9
126448 229533.A0A1C3YI73 pp 229533.I1RSM8
126449 229533.A0A1C3YI73 pp 229533.I1RA70
126450 229533.A0A1C3YI73 pp 229533.I1RE22
126451 229533.A0A1C3YI73 pp 229533.I1RX46
126452 229533.A0A1C3YI73 pp 229533.I1RGA4
126453 229533.A0A1C3YI73 pp 229533.I1S4Z7
126454 229533.A0A1C3YI73 pp 229533.V6QX21
126455 229533.A0A1C3YI73 pp 229533.I1RFU8
126456 229533.A0A1C3YI73 pp 229533.I1RMM6
126457 229533.A0A1C3YI73 pp 229533.I1RPM4
126458 229533.A0A1C3YI73 pp 229533.I1RJW6
126459 229533.A0A1C3YI73 pp 229533.I1RTH6
126460 229533.A0A1C3YI73 pp 229533.A0A1C3YLK8
126461 229533.A0A1C3YI73 pp 229533.I1RXD7
126462 229533.A0A1C3YI73 pp 229533.I1S0L4
126463 229533.A0A1C3YI73 pp 229533.I1RZM0
126464 229533.A0A1C3YI73 pp 229533.I1RCG4
126465 229533.A0A1C3YI73 pp 229533.I1S2E9
126466 229533.A0A1C3YI73 pp 229533.I1RQC9
126467 229533.A0A1C3YI73 pp 229533.I1S8R3
126468 229533.A0A1C3YI73 pp 229533.I1RR24
126469 229533.A0A1C3YI73 pp 229533.I1RRI2
126470 229533.A0A1C3YI73 pp 229533.I1RT04
126471 229533.A0A1C3YI73 pp 229533.I1S5R1
126472 229533.A0A1C3YI73 pp 229533.I1RZW8
126473 229533.A0A1C3YI73 pp 229533.I1S0R7
126474 229533.A0A1C3YI73 pp 229533.I1RYL5
126475 229533.A0A1C3YI73 pp 229533.I1RRT8
126476 229533.A0A1C3YI73 pp 229533.I1RA11
126477 229533.A0A1C3YI73 pp 229533.I1RWJ8
126478 229533.A0A1C3YI73 pp 229533.I1RRC6
126479 229533.A0A1C3YI73 pp 229533.I1RUP0
126480 229533.A0A1C3YI73 pp 229533.I1RYW3
126481 229533.A0A1C3YI73 pp 229533.I1RV91
126482 229533.A0A1C3YI73 pp 229533.I1S0L3
126483 229533.A0A1C3YI73 pp 229533.I1RR73
126484 229533.A0A1C3YI73 pp 229533.V6R3X5
126485 229533.A0A1C3YI73 pp 229533.I1RT40
126486 229533.A0A1C3YI73 pp 229533.I1RAH0
126487 229533.A0A1C3YI73 pp 229533.I1RD52
126488 229533.A0A1C3YI73 pp 229533.I1RZ13
126489 229533.A0A1C3YI73 pp 229533.I1RZ34
126490 229533.A0A1C3YI73 pp 229533.A0A1C3YKM9
126491 229533.A0A1C3YI73 pp 229533.I1RVQ0
126492 229533.A0A1C3YI73 pp 229533.I1RJU3
126493 229533.A0A1C3YI73 pp 229533.A0A1C3YIS7
126494 229533.A0A1C3YI73 pp 229533.I1RN67
126495 229533.A0A1C3YI73 pp 229533.I1RBY4
126496 229533.A0A1C3YI73 pp 229533.I1RX25
126497 229533.A0A1C3YI73 pp 229533.I1RC66
126498 229533.A0A1C3YI73 pp 229533.I1RBT1
126499 229533.A0A1C3YI73 pp 229533.A0A1C3YKC0
126500 229533.A0A1C3YI73 pp 229533.I1RW25
126501 229533.A0A1C3YI73 pp 229533.I1RGC1
126502 229533.A0A1C3YI73 pp 229533.I1RZZ9
126503 229533.I1RH30 pp 229533.I1SA33
126504 229533.I1RH30 pp 229533.I1RUB4
126505 229533.I1RH30 pp 229533.I1S6P7
126506 229533.I1RH30 pp 229533.I1RWM4
126507 229533.I1RH30 pp 229533.I1RZA7
126508 229533.I1RH30 pp 229533.Q4HYR2
126509 229533.I1RH30 pp 229533.I1RUB5

126510	229533.I1RH30	pp	229533.I1S8P4
126511	229533.I1RH30	pp	229533.I1RUB1
126512	229533.I1RH30	pp	229533.I1S8P5
126513	229533.I1RH30	pp	229533.I1S9T6
126514	229533.I1RH30	pp	229533.V6RUB1
126515	229533.I1RH30	pp	229533.I1RH32
126516	229533.I1RH30	pp	229533.I1S6P5
126517	229533.I1RVA9	pp	229533.I1S3U8
126518	229533.I1RVQ0	pp	229533.I1S0R7
126519	229533.I1RVQ0	pp	229533.I1RX46
126520	229533.I1RVQ0	pp	229533.I1S352
126521	229533.I1RVQ0	pp	229533.I1RW71
126522	229533.I1RVQ0	pp	229533.I1S0T2
126523	229533.I1RVQ0	pp	229533.I1RXD7
126524	229533.I1RVQ0	pp	229533.I1S2E9
126525	229533.I1RVQ0	pp	229533.V6QW22
126526	229533.I1RVQ0	pp	229533.I1S8P5
126527	229533.I1RVQ0	pp	229533.I1S7A8
126528	229533.I1RVQ0	pp	229533.I1RW07
126529	229533.I1RVQ0	pp	229533.V6RTJ8
126530	229533.I1RVQ0	pp	229533.I1RWK3
126531	229533.I1RVQ0	pp	229533.Q4IJT5
126532	229533.I1RVQ0	pp	229533.I1S503
126533	229533.I1RVQ0	pp	229533.V6R1K4
126534	229533.I1RVQ0	pp	229533.I1S2M6
126535	229533.I1RVQ0	pp	229533.I1RVY0
126536	229533.I1RVQ0	pp	229533.I1S2B8
126537	229533.I1RVQ0	pp	229533.I1S0F6
126538	229533.I1RVQ0	pp	229533.I1RWD4
126539	229533.I1RVQ0	pp	229533.I1S0F5
126540	229533.I1RVQ0	pp	229533.I1S6F0
126541	229533.I1RVQ0	pp	229533.I1S2H4
126542	229533.I1RVQ0	pp	229533.I1RW25
126543	229533.I1RVQ0	pp	229533.I1S0L3
126544	229533.I1RVQ0	pp	229533.I1RX25
126545	229533.I1RVQ0	pp	229533.V6R4J1
126546	229533.I1RVQ0	pp	229533.I1RZL6
126547	229533.I1RVQ0	pp	229533.Q4HVW2
126548	229533.I1RVQ0	pp	229533.I1RZ39
126549	229533.I1RVQ0	pp	229533.O74638
126550	229533.I1RVQ0	pp	229533.I1RWJ8
126551	229533.I1RVQ0	pp	229533.I1S0L4
126552	229533.I1RVQ0	pp	229533.I1RZJ5
126553	229533.I1RVQ0	pp	229533.I1S5R1
126554	229533.I1RVQ0	pp	229533.V6RCZ8
126555	229533.I1RVQ0	pp	229533.I1RZZ9
126556	229533.I1RVQ0	pp	229533.V6R3X5
126557	229533.I1RVQ0	pp	229533.I1S0X6
126558	229533.I1RVQ0	pp	229533.I1RWI5
126559	229533.I1RVQ0	pp	229533.V6RMW6
126560	229533.I1RVQ0	pp	229533.V6RCG5
126561	229533.I1RVQ0	pp	229533.V6QYF0
126562	229533.I1RVQ0	pp	229533.V6QUW4
126563	229533.I1RVQ0	pp	229533.I1RW61
126564	229533.I1RVQ0	pp	229533.I1S0M6
126565	229533.I1RVQ0	pp	229533.V6R042
126566	229533.I1RVQ0	pp	229533.I1S0J1
126567	229533.I1RVQ0	pp	229533.I1SA32
126568	229533.I1RVQ0	pp	229533.Q4I283
126569	229533.I1RVQ0	pp	229533.I1S504
126570	229533.I1RVQ0	pp	229533.I1S0R2
126571	229533.I1RVQ0	pp	229533.I1S5B0
126572	229533.I1RVQ0	pp	229533.I1S6Y8
126573	229533.I1RVQ0	pp	229533.I1RZM8
126574	229533.I1RVQ0	pp	229533.V6RGN5
126575	229533.I1RVQ0	pp	229533.I1RXT3
126576	229533.I1RVQ0	pp	229533.I1S0N4
126577	229533.I1RVQ0	pp	229533.I1RWT6
126578	229533.I1RVQ0	pp	229533.V6R227
126579	229533.I1RVQ0	pp	229533.I1RXF0
126580	229533.I1RVQ0	pp	229533.I1RW99
126581	229533.I1RVQ0	pp	229533.I1RWN5
126582	229533.I1RVQ0	pp	229533.Q4I8P5

126583	229533.I1RVQ0	pp	229533.I1SA33
126584	229533.I1RVQ0	pp	229533.Q4HZB8
126585	229533.I1RVQ0	pp	229533.I1RWQ8
126586	229533.I1RVQ0	pp	229533.V6RIV9
126587	229533.I1RVQ0	pp	229533.I1RY03
126588	229533.I1RVQ0	pp	229533.I1RZH4
126589	229533.I1RVQ0	pp	229533.I1S419
126590	229533.I1RVQ0	pp	229533.I1RZS7
126591	229533.I1RVQ0	pp	229533.I1RW46
126592	229533.I1RVQ0	pp	229533.Q4IEK8
126593	229533.I1RVQ0	pp	229533.I1RWX5
126594	229533.I1RVQ0	pp	229533.I1RY44
126595	229533.I1RVQ0	pp	229533.I1RZW8
126596	229533.I1RVQ0	pp	229533.Q4IJK2
126597	229533.I1RVQ0	pp	229533.I1RZM1
126598	229533.I1RVQ0	pp	229533.I1S2E8
126599	229533.I1RVQ0	pp	229533.I1RZZ1
126600	229533.I1RVQ0	pp	229533.V6R057
126601	229533.I1RVQ0	pp	229533.I1S8R3
126602	229533.I1RVQ0	pp	229533.I1RWN6
126603	229533.I1RVQ0	pp	229533.I1S1J7
126604	229533.I1RVQ0	pp	229533.I1S569
126605	229533.I1RVQ0	pp	229533.I1S1C7
126606	229533.I1RVQ0	pp	229533.I1RZK5
126607	229533.I1RVQ0	pp	229533.Q4I624
126608	229533.I1RVQ0	pp	229533.I1RWD7
126609	229533.I1RVQ0	pp	229533.I1RYW3
126610	229533.I1RVQ0	pp	229533.I1RY63
126611	229533.I1RVQ0	pp	229533.I1RVX0
126612	229533.I1RVQ0	pp	229533.Q4I2J8
126613	229533.I1RKZ2	pp	229533.Q4HVQ9
126614	229533.I1RKZ2	pp	229533.I1RWZ3
126615	229533.I1RKZ2	pp	229533.I1RNG4
126616	229533.I1RKZ2	pp	229533.I1S1B8
126617	229533.I1RKZ2	pp	229533.I1RTY0
126618	229533.I1RKZ2	pp	229533.I1S3G4
126619	229533.I1RKZ2	pp	229533.I1RQE1
126620	229533.I1RKZ2	pp	229533.I1S1W8
126621	229533.I1RKZ2	pp	229533.I1RYX6
126622	229533.I1RKZ2	pp	229533.I1S2Q4
126623	229533.I1RKZ2	pp	229533.I1S4G3
126624	229533.I1RFD4	pp	229533.Q4HYR2
126625	229533.I1RFD4	pp	229533.I1RIL0
126626	229533.I1RFD4	pp	229533.I1RM66
126627	229533.I1RFD4	pp	229533.I1RVR2
126628	229533.I1RFD4	pp	229533.I1RMM8
126629	229533.I1RFD4	pp	229533.I1RH30
126630	229533.I1RFD4	pp	229533.I1RWM4
126631	229533.I1RFD4	pp	229533.I1RJ27
126632	229533.I1RFD4	pp	229533.I1S9T6
126633	229533.I1RFD4	pp	229533.I1RZ26
126634	229533.I1RFD4	pp	229533.I1RYR3
126635	229533.I1RFD4	pp	229533.I1SA33
126636	229533.I1RFD4	pp	229533.V6RM02
126637	229533.I1RFD4	pp	229533.V6RUY9
126638	229533.I1R9Z5	pp	229533.I1RWA3
126639	229533.I1R9Z5	pp	229533.I1RAI4
126640	229533.I1R9Z5	pp	229533.I1RG62
126641	229533.I1R9Z5	pp	229533.I1RAW3
126642	229533.I1R9Z5	pp	229533.I1RMR9
126643	229533.I1R9Z5	pp	229533.I1RYT8
126644	229533.I1R9Z5	pp	229533.I1RLP1
126645	229533.I1R9Z5	pp	229533.I1RS05
126646	229533.I1R9Z5	pp	229533.I1RXD9
126647	229533.I1R9Z5	pp	229533.I1RX39
126648	229533.I1R9Z5	pp	229533.I1RSC0
126649	229533.I1R9Z5	pp	229533.I1RA49
126650	229533.I1R9Z5	pp	229533.I1S8F0
126651	229533.I1R9Z5	pp	229533.V6R3J1
126652	229533.I1R9Z5	pp	229533.I1RPR9
126653	229533.I1R9Z5	pp	229533.I1S464
126654	229533.I1R9Z5	pp	229533.I1RRX5
126655	229533.I1R9Z5	pp	229533.I1RWB0

229533.I1R9Z5	pp	229533.I1RE66
229533.I1R9Z5	pp	229533.I1RAE3
229533.I1R9Z5	pp	229533.I1S5X9
229533.I1R9Z5	pp	229533.I1RS29
229533.I1R9Z5	pp	229533.I1RBT6
229533.I1R9Z5	pp	229533.I1S0R8
229533.I1R9Z5	pp	229533.I1RYH7
229533.I1R9Z5	pp	229533.I1RFJ5
229533.I1R9Z5	pp	229533.I1RSQ3
229533.I1R9Z5	pp	229533.I1S0Q7
229533.I1R9Z5	pp	229533.I1RE65
229533.I1R9Z5	pp	229533.I1RAE4
229533.I1R9Z5	pp	229533.Q4I7K4
229533.I1R9Z5	pp	229533.I1RMV0
229533.I1R9Z5	pp	229533.I1RWD3
229533.I1R9Z5	pp	229533.I1S7W5
229533.I1R9Z5	pp	229533.V6RJP8
229533.I1RIE1	pp	229533.I1S3U8
229533.I1RIE1	pp	229533.I1RU48
229533.I1RIE1	pp	229533.I1RVA9
229533.I1RCI9	pp	229533.I1RQP7
229533.I1RCI9	pp	229533.I1RYP0
229533.I1RCI9	pp	229533.I1RCV3
229533.I1RCI9	pp	229533.I1RGD1
229533.I1RCI9	pp	229533.I1RX87
229533.I1RGG7	pp	229533.I1RZT1
229533.I1RGG7	pp	229533.V6RK96
229533.I1RGG7	pp	229533.I1S0I3
229533.I1RGG7	pp	229533.I1RY49
229533.I1RGG7	pp	229533.I1RH91
229533.I1RGG7	pp	229533.I1S3G4
229533.I1RGG7	pp	229533.I1S1W8
229533.I1RGG7	pp	229533.I1RNG4
229533.I1RGG7	pp	229533.I1RQE8
229533.I1RGG7	pp	229533.I1RZM4
229533.I1RGG7	pp	229533.I1RQE1
229533.I1RGG7	pp	229533.I1RYX6
229533.Q4HYR2	pp	229533.Q4I2D7
229533.Q4HYR2	pp	229533.V6QVV9
229533.Q4HYR2	pp	229533.Q4IBS9
229533.Q4HYR2	pp	229533.V6RE46
229533.Q4HYR2	pp	229533.V6RV57
229533.Q4HYR2	pp	229533.V6RLM7
229533.I1RGL4	pp	229533.I1RY03
229533.I1RGL4	pp	229533.I1RZK5
229533.I1RGL4	pp	229533.I1RJW1
229533.I1RGL4	pp	229533.I1RRY9
229533.I1RGL4	pp	229533.V6RTJ8
229533.I1RGL4	pp	229533.Q4IJK2
229533.I1RGL4	pp	229533.V6RCG5
229533.I1RGL4	pp	229533.I1RX46
229533.I1RGL4	pp	229533.I1RIE8
229533.I1RGL4	pp	229533.V6QW22
229533.I1RGL4	pp	229533.I1RZM1
229533.I1RGL4	pp	229533.I1RLU6
229533.I1RGL4	pp	229533.I1RSQ7
229533.I1RGL4	pp	229533.I1RR39
229533.I1RGL4	pp	229533.I1RS18
229533.I1RGL4	pp	229533.I1RI82
229533.I1RGL4	pp	229533.I1RR24
229533.I1RGL4	pp	229533.I1S504
229533.I1RGL4	pp	229533.I1RVQ0
229533.I1RGL4	pp	229533.I1S0L3
229533.I1RGL4	pp	229533.I1RW25
229533.I1RGL4	pp	229533.I1RPP4
229533.I1RGL4	pp	229533.I1RTH6
229533.I1RGL4	pp	229533.I1RQC9
229533.I1RGL4	pp	229533.I1RK44
229533.I1RGL4	pp	229533.I1RTA0
229533.I1RGL4	pp	229533.I1S8R3
229533.I1RGL4	pp	229533.I1RJV3
229533.I1RGL4	pp	229533.I1RVX0
229533.I1RGL4	pp	229533.V6RIV9

126729 229533.I1RGL4 pp 229533.I1S1C7
126730 229533.I1RGL4 pp 229533.I1RT03
126731 229533.I1RGL4 pp 229533.I1RN18
126732 229533.I1RGL4 pp 229533.I1RWN5
126733 229533.I1RGL4 pp 229533.V6R042
126734 229533.I1RGL4 pp 229533.Q4I1Y5
126735 229533.I1RGL4 pp 229533.I1RZW8
126736 229533.I1RGL4 pp 229533.I1RWN6
126737 229533.I1RGL4 pp 229533.I1RZ39
126738 229533.I1RGL4 pp 229533.I1RY44
126739 229533.I1RGL4 pp 229533.I1RR73
126740 229533.I1RGL4 pp 229533.I1RPM4
126741 229533.I1RGL4 pp 229533.V6RMW6
126742 229533.I1RGL4 pp 229533.I1RSQ3
126743 229533.I1RGL4 pp 229533.I1RRM5
126744 229533.I1RGL4 pp 229533.V6R3X5
126745 229533.I1RGL4 pp 229533.I1RZZ9
126746 229533.I1RGL4 pp 229533.I1RNC1
126747 229533.I1RGL4 pp 229533.I1S2H4
126748 229533.I1RGL4 pp 229533.I1S2B8
126749 229533.I1RGL4 pp 229533.I1RQ75
126750 229533.I1RGL4 pp 229533.I1RUG3
126751 229533.I1RGL4 pp 229533.I1RT04
126752 229533.I1RGL4 pp 229533.I1S2E9
126753 229533.I1RGL4 pp 229533.I1RRT8
126754 229533.I1RGL4 pp 229533.I1S0L4
126755 229533.I1RGL4 pp 229533.I1RV91
126756 229533.I1RGL4 pp 229533.I1S8P5
126757 229533.I1RGL4 pp 229533.I1RWK3
126758 229533.I1RGL4 pp 229533.I1RPP3
126759 229533.I1RGL4 pp 229533.I1RTD5
126760 229533.I1RGL4 pp 229533.I1RN67
126761 229533.I1RGL4 pp 229533.I1RJW6
126762 229533.I1RGL4 pp 229533.I1RJV4
126763 229533.I1RGL4 pp 229533.I1S503
126764 229533.A0A0E0SCN4 pp 229533.I1RQL4
126765 229533.A0A0E0SCN4 pp 229533.A0A1C3YJG7
126766 229533.A0A1C3YKX1 pp 229533.I1RH91
126767 229533.A0A1C3YKX1 pp 229533.I1S2Q4
126768 229533.A0A1C3YKX1 pp 229533.I1S3G4
126769 229533.A0A1C3YKX1 pp 229533.I1RNG4
126770 229533.A0A1C3YKX1 pp 229533.I1RNB4
126771 229533.A0A1C3YKX1 pp 229533.I1RZ83
126772 229533.A0A1C3YKX1 pp 229533.I1RSQ9
126773 229533.A0A1C3YKX1 pp 229533.I1RUR8
126774 229533.A0A1C3YKX1 pp 229533.I1RJJ7
126775 229533.A0A1C3YKX1 pp 229533.I1RWF5
126776 229533.A0A1C3YKX1 pp 229533.I1RBY9
126777 229533.A0A1C3YKX1 pp 229533.I1RGC3
126778 229533.A0A1C3YKX1 pp 229533.I1RY74
126779 229533.A0A1C3YKX1 pp 229533.I1RWZ3
126780 229533.A0A1C3YKX1 pp 229533.I1RXY7
126781 229533.A0A1C3YKX1 pp 229533.I1RY49
126782 229533.A0A1C3YKX1 pp 229533.V6QWD8
126783 229533.A0A1C3YKX1 pp 229533.A0A1C3YMF1
126784 229533.A0A1C3YKX1 pp 229533.I1RAV0
126785 229533.A0A1C3YKX1 pp 229533.I1RAR3
126786 229533.A0A1C3YKX1 pp 229533.I1S1W8
126787 229533.A0A1C3YKX1 pp 229533.I1RAJ6
126788 229533.A0A1C3YKX1 pp 229533.A0A1C3YN04
126789 229533.A0A1C3YKX1 pp 229533.I1RBE7
126790 229533.A0A1C3YKX1 pp 229533.I1RAG9
126791 229533.A0A1C3YKX1 pp 229533.I1RYX6
126792 229533.A0A1C3YKX1 pp 229533.I1RMK4
126793 229533.A0A1C3YKX1 pp 229533.I1RG66
126794 229533.A0A1C3YKX1 pp 229533.I1RY12
126795 229533.A0A1C3YKX1 pp 229533.A0A1C3YKY4
126796 229533.A0A1C3YKX1 pp 229533.I1RTY0
126797 229533.A0A1C3YKX1 pp 229533.I1RQE1
126798 229533.A0A1C3YKX1 pp 229533.I1RA94
126799 229533.A0A1C3YKX1 pp 229533.I1S1B8
126800 229533.A0A1C3YKX1 pp 229533.I1RVX9
126801 229533.A0A1C3YKX1 pp 229533.I1RM26

126802 229533.A0A1C3YKX1 pp 229533.I1S4G3
126803 229533.I1RU48 pp 229533.I1S3U8
126804 229533.I1RU48 pp 229533.I1RVA9
126805 229533.I1R9M7 pp 229533.I1RUQ3
126806 229533.I1R9M7 pp 229533.I1RA13
126807 229533.I1R9M7 pp 229533.Q4HYR2
126808 229533.I1R9M7 pp 229533.I1RWM4
126809 229533.I1R9M7 pp 229533.V6RV57
126810 229533.I1R9M7 pp 229533.V6QVV9
126811 229533.I1R9M7 pp 229533.I1RXE6
126812 229533.I1R9M7 pp 229533.I1SA33
126813 229533.I1R9M7 pp 229533.I1RZ25
126814 229533.I1R9M7 pp 229533.I1S0V7
126815 229533.I1R9M7 pp 229533.I1RC34
126816 229533.I1R9M7 pp 229533.Q4HVQ9
126817 229533.I1R9M7 pp 229533.I1RH30
126818 229533.I1R9M7 pp 229533.I1S0Q7
126819 229533.I1R9M7 pp 229533.I1RZ56
126820 229533.I1R9M7 pp 229533.I1RLV9
126821 229533.I1R9M7 pp 229533.I1S9T6
126822 229533.I1R9M7 pp 229533.I1RBY9
126823 229533.I1R9M7 pp 229533.I1RPV8
126824 229533.I1R9M7 pp 229533.I1S6H3
126825 229533.I1R9M7 pp 229533.I1RYX6
126826 229533.I1R9M7 pp 229533.I1S267
126827 229533.I1R9M7 pp 229533.I1RDU6
126828 229533.I1R9M7 pp 229533.I1RYR3
126829 229533.I1R9M7 pp 229533.I1RH91
126830 229533.I1R9M7 pp 229533.I1RF85
126831 229533.I1R9M7 pp 229533.I1RJS1
126832 229533.I1R9M7 pp 229533.I1RMW7
126833 229533.I1R9M7 pp 229533.I1S0V6
126834 229533.I1R9M7 pp 229533.I1RCM5
126835 229533.I1R9M7 pp 229533.I1RF24
126836 229533.I1R9M7 pp 229533.I1S0A5
126837 229533.I1R9M7 pp 229533.I1RFI4
126838 229533.I1R9M7 pp 229533.I1S094
126839 229533.I1R9M7 pp 229533.I1RW71
126840 229533.I1R9M7 pp 229533.I1RFD4
126841 229533.I1R9M7 pp 229533.I1RIL0
126842 229533.I1R9M7 pp 229533.I1RDN3
126843 229533.I1R9M7 pp 229533.I1RNM7
126844 229533.I1R9M7 pp 229533.I1RCU2
126845 229533.I1R9M7 pp 229533.I1RM66
126846 229533.I1R9M7 pp 229533.I1R9N6
126847 229533.I1RB41 pp 229533.I1RZZ1
126848 229533.I1RB41 pp 229533.I1RCG4
126849 229533.I1RB41 pp 229533.I1RQA3
126850 229533.I1RB41 pp 229533.I1REK8
126851 229533.I1RB41 pp 229533.I1RN08
126852 229533.I1RB41 pp 229533.I1S439
126853 229533.I1RB41 pp 229533.I1RFU8
126854 229533.I1RB41 pp 229533.I1RB75
126855 229533.I1RB41 pp 229533.Q4IR87
126856 229533.I1RB41 pp 229533.I1RS38
126857 229533.I1RB41 pp 229533.I1RZK5
126858 229533.I1RB41 pp 229533.I1RHB0
126859 229533.I1RB41 pp 229533.I1RY03
126860 229533.I1RB41 pp 229533.I1RUQ5
126861 229533.I1RB41 pp 229533.I1RRY9
126862 229533.I1RB41 pp 229533.I1RSI0
126863 229533.I1RB41 pp 229533.I1RC22
126864 229533.I1RB41 pp 229533.I1RMS2
126865 229533.I1RB41 pp 229533.Q4HTT2
126866 229533.I1RB41 pp 229533.I1S267
126867 229533.I1RB41 pp 229533.I1RMM9
126868 229533.I1RB41 pp 229533.Q4IJK2
126869 229533.I1RB41 pp 229533.V6RCZ8
126870 229533.I1RB41 pp 229533.I1S0N8
126871 229533.I1RB41 pp 229533.Q4I8P5
126872 229533.I1RB41 pp 229533.V6RR76
126873 229533.I1RB41 pp 229533.V6RCG5
126874 229533.I1RB41 pp 229533.I1RLU6

126875	229533.I1RB41	pp	229533.I1RZM8
126876	229533.I1RB41	pp	229533.I1RJU3
126877	229533.I1RB41	pp	229533.I1RNQ5
126878	229533.I1RB41	pp	229533.I1RS18
126879	229533.I1RB41	pp	229533.I1RMQ7
126880	229533.I1RB41	pp	229533.I1RI82
126881	229533.I1RB41	pp	229533.I1RCB3
126882	229533.I1RB41	pp	229533.I1RDB1
126883	229533.I1RB41	pp	229533.I1RPU4
126884	229533.I1RB41	pp	229533.I1S0X6
126885	229533.I1RB41	pp	229533.I1RX70
126886	229533.I1RB41	pp	229533.I1RX09
126887	229533.I1RB41	pp	229533.I1RKK0
126888	229533.I1RB41	pp	229533.I1SA32
126889	229533.I1RB41	pp	229533.I1RMR7
126890	229533.I1RB41	pp	229533.I1S5B0
126891	229533.I1RB41	pp	229533.I1RW99
126892	229533.I1RB41	pp	229533.I1RBT1
126893	229533.I1RB41	pp	229533.I1S635
126894	229533.I1RB41	pp	229533.I1S504
126895	229533.I1RB41	pp	229533.I1RFT2
126896	229533.I1RB41	pp	229533.I1RWT6
126897	229533.I1RB41	pp	229533.I1S1W5
126898	229533.I1RB41	pp	229533.I1RQ81
126899	229533.I1RB41	pp	229533.I1RC95
126900	229533.I1RB41	pp	229533.I1RWJ8
126901	229533.I1RB41	pp	229533.I1RR24
126902	229533.I1RB41	pp	229533.I1S1B1
126903	229533.I1RB41	pp	229533.I1RLT6
126904	229533.I1RB41	pp	229533.I1RX46
126905	229533.I1RB41	pp	229533.V6RTJ8
126906	229533.I1RB41	pp	229533.I1RZM1
126907	229533.I1RB41	pp	229533.I1RV66
126908	229533.I1RB41	pp	229533.I1RBC9
126909	229533.I1RB41	pp	229533.I1RW25
126910	229533.I1RB41	pp	229533.I1RFQ7
126911	229533.I1RB41	pp	229533.I1RBW0
126912	229533.I1RB41	pp	229533.I1RL24
126913	229533.I1RB41	pp	229533.I1RW71
126914	229533.I1RB41	pp	229533.I1RMR5
126915	229533.I1RB41	pp	229533.I1RYH9
126916	229533.I1RB41	pp	229533.I1S0R7
126917	229533.I1RB41	pp	229533.I1RSQ7
126918	229533.I1RB41	pp	229533.I1RD69
126919	229533.I1RB41	pp	229533.I1RN81
126920	229533.I1RB41	pp	229533.V6QW22
126921	229533.I1RB41	pp	229533.I1RN61
126922	229533.I1RB41	pp	229533.I1RRT1
126923	229533.I1RB41	pp	229533.I1RQC9
126924	229533.I1RB41	pp	229533.I1RYZ9
126925	229533.I1RB41	pp	229533.I1RXD7
126926	229533.I1RB41	pp	229533.I1RC91
126927	229533.I1RB41	pp	229533.I1RFZ3
126928	229533.I1RB41	pp	229533.Q4ICG5
126929	229533.I1RB41	pp	229533.I1RW38
126930	229533.I1RB41	pp	229533.I1RD52
126931	229533.I1RB41	pp	229533.I1RQ26
126932	229533.I1RB41	pp	229533.V6QV09
126933	229533.I1RB41	pp	229533.I1RWN6
126934	229533.I1RB41	pp	229533.I1RBJ0
126935	229533.I1RB41	pp	229533.I1RFL4
126936	229533.I1RB41	pp	229533.I1RJV3
126937	229533.I1RB41	pp	229533.I1RYI3
126938	229533.I1RB41	pp	229533.I1S0L3
126939	229533.I1RB41	pp	229533.I1RW84
126940	229533.I1RB41	pp	229533.I1RVQ0
126941	229533.I1RB41	pp	229533.I1RRY3
126942	229533.I1RB41	pp	229533.I1RQE7
126943	229533.I1RB41	pp	229533.I1RY58
126944	229533.I1RB41	pp	229533.I1RRN5
126945	229533.I1RB41	pp	229533.I1RDH0
126946	229533.I1RB41	pp	229533.I1RT03
126947	229533.I1RB41	pp	229533.I1S8R3

126948 229533.I1RB41 pp 229533.I1RZE4
126949 229533.I1RB41 pp 229533.I1RPW0
126950 229533.I1RB41 pp 229533.I1RCH5
126951 229533.I1RB41 pp 229533.I1RD17
126952 229533.I1RB41 pp 229533.I1RVX0
126953 229533.I1RB41 pp 229533.I1RTH6
126954 229533.I1RB41 pp 229533.I1RJT8
126955 229533.I1RB41 pp 229533.I1S1C7
126956 229533.I1RB41 pp 229533.I1RW32
126957 229533.I1RB41 pp 229533.I1RD15
126958 229533.I1RB41 pp 229533.I1RRV1
126959 229533.I1RB41 pp 229533.I1RB92
126960 229533.I1RB41 pp 229533.I1S0E8
126961 229533.I1RB41 pp 229533.V6RMW6
126962 229533.I1RB41 pp 229533.I1RN18
126963 229533.I1RB41 pp 229533.I1RZW8
126964 229533.I1RB41 pp 229533.V6R042
126965 229533.I1RB41 pp 229533.I1RSQ3
126966 229533.I1RB41 pp 229533.I1RRI2
126967 229533.I1RB41 pp 229533.I1RQB4
126968 229533.I1RB41 pp 229533.I1RW46
126969 229533.I1RB41 pp 229533.I1RPP4
126970 229533.I1RB41 pp 229533.I1RZ91
126971 229533.I1RB41 pp 229533.I1RCR8
126972 229533.I1RB41 pp 229533.I1S0J1
126973 229533.I1RB41 pp 229533.I1RE01
126974 229533.I1RB41 pp 229533.I1S0M2
126975 229533.I1RB41 pp 229533.I1RUG3
126976 229533.I1RB41 pp 229533.I1RZ39
126977 229533.I1RB41 pp 229533.I1RGF1
126978 229533.I1RB41 pp 229533.I1RNC1
126979 229533.I1RB41 pp 229533.I1RNY8
126980 229533.I1RB41 pp 229533.I1REN4
126981 229533.I1RB41 pp 229533.I1S1W4
126982 229533.I1RB41 pp 229533.I1RRE5
126983 229533.I1RB41 pp 229533.V6RGI1
126984 229533.I1RB41 pp 229533.V6R3X5
126985 229533.I1RB41 pp 229533.I1RNR3
126986 229533.I1RB41 pp 229533.I1S9S6
126987 229533.I1RB41 pp 229533.V6RKY7
126988 229533.I1RB41 pp 229533.I1RZ63
126989 229533.I1RB41 pp 229533.I1RWN5
126990 229533.I1RB41 pp 229533.I1RR73
126991 229533.I1RB41 pp 229533.I1RC71
126992 229533.I1RB41 pp 229533.V6RIV9
126993 229533.I1RB41 pp 229533.I1SA33
126994 229533.I1RB41 pp 229533.V6RVG3
126995 229533.I1RB41 pp 229533.I1RD22
126996 229533.I1RB41 pp 229533.I1RWI5
126997 229533.I1RB41 pp 229533.I1RZZ9
126998 229533.I1RB41 pp 229533.I1RLU1
126999 229533.I1RB41 pp 229533.I1RU69
127000 229533.I1RB41 pp 229533.I1RY44
127001 229533.I1RB41 pp 229533.I1RNP7
127002 229533.I1RB41 pp 229533.I1RUN8
127003 229533.I1RB41 pp 229533.I1S5R1
127004 229533.I1RB41 pp 229533.I1S2H4
127005 229533.I1RB41 pp 229533.I1S2B8
127006 229533.I1RB41 pp 229533.I1RGD1
127007 229533.I1RB41 pp 229533.I1RX38
127008 229533.I1RB41 pp 229533.I1RE22
127009 229533.I1RB41 pp 229533.I1RRT8
127010 229533.I1RB41 pp 229533.I1RMF5
127011 229533.I1RB41 pp 229533.I1RRM5
127012 229533.I1RB41 pp 229533.I1RPK4
127013 229533.I1RB41 pp 229533.I1S0J7
127014 229533.I1RB41 pp 229533.I1RBW1
127015 229533.I1RB41 pp 229533.Q4I7L0
127016 229533.I1RB41 pp 229533.I1RJZ7
127017 229533.I1RB41 pp 229533.I1S549
127018 229533.I1RB41 pp 229533.I1S0M6
127019 229533.I1RB41 pp 229533.I1RBY4
127020 229533.I1RB41 pp 229533.I1RT40

127021 229533.I1RB41 pp 229533.I1RPU3
127022 229533.I1RB41 pp 229533.I1RT04
127023 229533.I1RB41 pp 229533.I1RCJ7
127024 229533.I1RB41 pp 229533.I1RE15
127025 229533.I1RB41 pp 229533.I1S0X7
127026 229533.I1RB41 pp 229533.I1RBV5
127027 229533.I1RB41 pp 229533.I1RV91
127028 229533.I1RB41 pp 229533.I1RTD5
127029 229533.I1RB41 pp 229533.I1RB59
127030 229533.I1RB41 pp 229533.I1S2E9
127031 229533.I1RB41 pp 229533.I1RW76
127032 229533.I1RB41 pp 229533.I1S098
127033 229533.I1RB41 pp 229533.I1RSZ3
127034 229533.I1RB41 pp 229533.I1RPM4
127035 229533.I1RB41 pp 229533.I1RY29
127036 229533.I1RB41 pp 229533.I1RBS6
127037 229533.I1RB41 pp 229533.I1RT28
127038 229533.I1RB41 pp 229533.I1RGA4
127039 229533.I1RB41 pp 229533.I1RFL7
127040 229533.I1RB41 pp 229533.I1RP27
127041 229533.I1RB41 pp 229533.I1RJV4
127042 229533.I1RB41 pp 229533.I1RPP3
127043 229533.I1RB41 pp 229533.I1S503
127044 229533.I1RB41 pp 229533.I1S7X9
127045 229533.I1RB41 pp 229533.I1RB74
127046 229533.I1RB41 pp 229533.I1RW83
127047 229533.I1RB41 pp 229533.I1RXU1
127048 229533.I1RB41 pp 229533.I1RSV2
127049 229533.I1RB41 pp 229533.I1RJW6
127050 229533.I1RB41 pp 229533.I1RZP1
127051 229533.I1RB41 pp 229533.I1RWK3
127052 229533.I1RB41 pp 229533.I1RNF4
127053 229533.I1RB41 pp 229533.I1RC97
127054 229533.I1RB41 pp 229533.I1RMP0
127055 229533.I1RB41 pp 229533.I1REF9
127056 229533.I1RB41 pp 229533.I1RN67
127057 229533.I1RB41 pp 229533.I1S0F6
127058 229533.A0A098DJ01 pp 229533.I1RYR3
127059 229533.A0A098DJ01 pp 229533.A0A1C3YKN4
127060 229533.A0A098DJ01 pp 229533.I1S9T6
127061 229533.A0A098DJ01 pp 229533.V6RUY9
127062 229533.A0A098DJ01 pp 229533.I1RJ27
127063 229533.A0A098DJ01 pp 229533.I1RVR2
127064 229533.A0A098DJ01 pp 229533.I1RDU6
127065 229533.A0A098DJ01 pp 229533.I1SA33
127066 229533.A0A098DJ01 pp 229533.A0A1C3YIE0
127067 229533.A0A098DJ01 pp 229533.V6RM02
127068 229533.A0A098DJ01 pp 229533.I1RZ26
127069 229533.A0A098DJ01 pp 229533.A0A1C3YK33
127070 229533.A0A098DJ01 pp 229533.I1R9K2
127071 229533.A0A098DJ01 pp 229533.I1RIL0
127072 229533.A0A098DJ01 pp 229533.A0A098DVZ4
127073 229533.A0A098DJ01 pp 229533.A0A1C3YL54
127074 229533.A0A098DJ01 pp 229533.A0A098DVI8
127075 229533.A0A098DJ01 pp 229533.I1RM66
127076 229533.A0A098DJ01 pp 229533.A0A1C3YN26
127077 229533.A0A098DJ01 pp 229533.I1RMM8
127078 229533.A0A098DJ01 pp 229533.A0A1C3YJ22
127079 229533.A0A098DJ01 pp 229533.I1RWM4
127080 229533.A0A098DJ01 pp 229533.I1RH30
127081 229533.A0A098DJ01 pp 229533.I1RFD4
127082 229533.A0A098DJ01 pp 229533.Q4HYR2
127083 229533.A0A098DJ01 pp 229533.I1R9M7
127084 229533.I1RDB3 pp 229533.V6RK96
127085 229533.I1RDB3 pp 229533.V6QVL9
127086 229533.I1RDB3 pp 229533.I1RQE8
127087 229533.I1RDB3 pp 229533.I1RHS7
127088 229533.I1RDB3 pp 229533.V6QZS3
127089 229533.I1RDB3 pp 229533.I1RIJ2
127090 229533.I1RDB3 pp 229533.V6RB81
127091 229533.I1RDB3 pp 229533.I1RUA7
127092 229533.I1RDB3 pp 229533.I1RL93
127093 229533.I1RDB3 pp 229533.I1RZM4

127094	229533.I1RDB3	pp	229533.I1SAI0
127095	229533.I1RRG9	pp	229533.I1RSC5
127096	229533.I1RGH6	pp	229533.I1RNG4
127097	229533.I1RGH6	pp	229533.I1RIE1
127098	229533.I1RGH6	pp	229533.I1RH65
127099	229533.I1RGH6	pp	229533.I1S3U8
127100	229533.I1RGH6	pp	229533.I1RU48
127101	229533.I1RWN6	pp	229533.I1RZF6
127102	229533.I1RWN6	pp	229533.I1S504
127103	229533.I1RWN6	pp	229533.I1RZM1
127104	229533.I1RWN6	pp	229533.I1RWT6
127105	229533.I1RWN6	pp	229533.I1S0F5
127106	229533.I1RWN6	pp	229533.I1RZH4
127107	229533.I1RWN6	pp	229533.Q4I283
127108	229533.I1RWN6	pp	229533.V6R4J1
127109	229533.I1RWN6	pp	229533.V6R1K4
127110	229533.I1RWN6	pp	229533.Q4IJT5
127111	229533.I1RWN6	pp	229533.I1S0M9
127112	229533.I1RWN6	pp	229533.Q4I8P5
127113	229533.I1RWN6	pp	229533.I1S0L3
127114	229533.I1RWN6	pp	229533.I1SA32
127115	229533.I1RWN6	pp	229533.I1RX25
127116	229533.I1RWN6	pp	229533.I1S7A8
127117	229533.I1RWN6	pp	229533.V6QW22
127118	229533.I1RWN6	pp	229533.I1RX46
127119	229533.I1RWN6	pp	229533.V6QUE6
127120	229533.I1RWN6	pp	229533.V6RTJ8
127121	229533.I1RWN6	pp	229533.I1RZM8
127122	229533.I1RWN6	pp	229533.I1RZJ5
127123	229533.I1RWN6	pp	229533.V6QUW4
127124	229533.I1RWN6	pp	229533.I1S5B0
127125	229533.I1RWN6	pp	229533.I1RXF0
127126	229533.I1RWN6	pp	229533.I1RYB9
127127	229533.I1RWN6	pp	229533.I1RXD7
127128	229533.I1RWN6	pp	229533.I1S021
127129	229533.I1RWN6	pp	229533.I1S0R7
127130	229533.I1RWN6	pp	229533.I1S9U2
127131	229533.I1RWN6	pp	229533.V6RCZ8
127132	229533.I1RWN6	pp	229533.Q4IJK2
127133	229533.I1RWN6	pp	229533.I1S0X6
127134	229533.I1RWN6	pp	229533.I1RZE5
127135	229533.I1RWN6	pp	229533.I1S6U8
127136	229533.I1RWN6	pp	229533.Q4I2J8
127137	229533.I1RWN6	pp	229533.I1RZK6
127138	229533.I1RWN6	pp	229533.I1RZK5
127139	229533.I1RWN6	pp	229533.V6RCG5
127140	229533.I1RWN6	pp	229533.I1RZZ1
127141	229533.I1RWN6	pp	229533.V6RUY9
127142	229533.I1RWN6	pp	229533.I1S2B8
127143	229533.I1RWN6	pp	229533.I1S0F6
127144	229533.I1RWN6	pp	229533.Q4IJM4
127145	229533.I1RWN6	pp	229533.I1S2E9
127146	229533.I1RWN6	pp	229533.V6RBN2
127147	229533.I1RWN6	pp	229533.I1RZS6
127148	229533.I1RWN6	pp	229533.V6R283
127149	229533.I1RWN6	pp	229533.I1S2G7
127150	229533.I1RWN6	pp	229533.I1S0M6
127151	229533.I1RWN6	pp	229533.I1S1J7
127152	229533.I1RWN6	pp	229533.I1S352
127153	229533.I1RWN6	pp	229533.I1RYY5
127154	229533.I1RWN6	pp	229533.I1S8P5
127155	229533.I1RWN6	pp	229533.I1S5R1
127156	229533.I1RWN6	pp	229533.I1RY03
127157	229533.I1RWN6	pp	229533.I1S2H4
127158	229533.I1RWN6	pp	229533.I1RZZ9
127159	229533.I1RWN6	pp	229533.I1S503
127160	229533.I1RWN6	pp	229533.I1S8E2
127161	229533.I1RWN6	pp	229533.I1S0L4
127162	229533.I1RWN6	pp	229533.I1RZ39
127163	229533.I1RWN6	pp	229533.I1S1C7
127164	229533.I1RWN6	pp	229533.V6RIV9
127165	229533.I1RWN6	pp	229533.I1RX39
127166	229533.I1RWN6	pp	229533.I1S8V3

229533.I1RWN6 pp 229533.V6R3X5
229533.I1RWN6 pp 229533.Q4HZB8
229533.I1RWN6 pp 229533.I1RWX5
229533.I1RWN6 pp 229533.I1RY44
229533.I1RWN6 pp 229533.Q4IN63
229533.I1RWN6 pp 229533.Q4I327
229533.I1RWN6 pp 229533.I1RYW3
229533.I1RWN6 pp 229533.I1S1W4
229533.I1RWN6 pp 229533.V6R042
229533.I1RWN6 pp 229533.I1S8R3
229533.I1RWN6 pp 229533.I1RZW8
229533.I1RWN6 pp 229533.V6RMW6
229533.I1RWN6 pp 229533.I1RZS7
229533.I1RWN6 pp 229533.I1S569
229533.I1RWN6 pp 229533.I1S2C0
229533.I1RWN6 pp 229533.I1S8V4
229533.I1RWN6 pp 229533.V6R057
229533.I1RWN6 pp 229533.I1S2E8
229533.I1RWN6 pp 229533.I1S0N4
229533.I1RWN6 pp 229533.I1S0J1
229533.I1RWN6 pp 229533.Q4I624
229533.I1RWN6 pp 229533.I1SA33
229533.I1S099 pp 229533.V6QU65
229533.A0A0E0SCF4 pp 229533.A0A0E0SMR8
229533.I1S2L1 pp 229533.V6R9L8
229533.I1S2L1 pp 229533.I1S368
229533.V6R4J1 pp 229533.V6RMW6
229533.V6R4J1 pp 229533.V6RIV9
229533.V6R4J1 pp 229533.V6RCG5
229533.V6R4J1 pp 229533.V6RTJ8
229533.I1RL49 pp 229533.I1RN84
229533.I1RL49 pp 229533.I1RSS7
229533.I1RL49 pp 229533.I1RMJ3
229533.I1RL49 pp 229533.V6RM02
229533.I1RLN4 pp 229533.I1RMK7
229533.I1R999 pp 229533.I1RJZ7
229533.I1R999 pp 229533.I1RW71
229533.I1R9J6 pp 229533.I1RZZ9
229533.I1R9J6 pp 229533.V6R3X5
229533.I1R9J6 pp 229533.I1RR73
229533.I1R9J6 pp 229533.I1RRT8
229533.I1R9J6 pp 229533.I1RZ39
229533.I1R9J6 pp 229533.I1S0L4
229533.I1R9J6 pp 229533.I1S2H4
229533.I1R9J6 pp 229533.I1RPM4
229533.I1R9J6 pp 229533.I1RTD5
229533.I1R9J6 pp 229533.I1RZW8
229533.I1R9J6 pp 229533.I1RBY4
229533.I1R9J6 pp 229533.I1R9M0
229533.I1R9J6 pp 229533.I1RV91
229533.I1R9J6 pp 229533.I1S2E9
229533.I1R9J6 pp 229533.I1S1C7
229533.I1R9J6 pp 229533.I1RA70
229533.I1R9J6 pp 229533.I1RRM5
229533.I1R9J6 pp 229533.I1S8R3
229533.I1R9J6 pp 229533.I1RT04
229533.I1R9J6 pp 229533.I1S503
229533.I1R9J6 pp 229533.I1S2B8
229533.I1R9J6 pp 229533.I1RTH6
229533.I1R9J6 pp 229533.I1RNC1
229533.I1R9J6 pp 229533.I1RJV3
229533.I1R9J6 pp 229533.I1RCR8
229533.I1R9J6 pp 229533.I1RWN6
229533.I1R9J6 pp 229533.I1RJW6
229533.I1R9J6 pp 229533.V6RMW6
229533.I1R9J6 pp 229533.I1RJV4
229533.I1R9J6 pp 229533.I1RBS6
229533.I1R9J6 pp 229533.I1RAW0
229533.I1R9J6 pp 229533.I1RWK3
229533.I1R9J6 pp 229533.I1RN18
229533.I1R9J6 pp 229533.I1RPP3
229533.I1R9J6 pp 229533.V6R042
229533.I1R9J6 pp 229533.I1RFL4

127240	229533.I1R9J6	pp	229533.I1S8P5
127241	229533.I1R9J6	pp	229533.I1RB92
127242	229533.I1R9J6	pp	229533.I1RSQ3
127243	229533.I1R9J6	pp	229533.I1RD22
127244	229533.I1R9J6	pp	229533.I1RVX0
127245	229533.I1R9J6	pp	229533.I1RN67
127246	229533.I1R9J6	pp	229533.I1RT03
127247	229533.I1R9J6	pp	229533.I1RAU7
127248	229533.I1R9J6	pp	229533.I1RQ75
127249	229533.I1R9J6	pp	229533.I1RSQ7
127250	229533.I1R9J6	pp	229533.I1RK44
127251	229533.I1R9J6	pp	229533.Q4I1Y5
127252	229533.I1R9J6	pp	229533.V6QW22
127253	229533.I1R9J6	pp	229533.I1RTA0
127254	229533.I1R9J6	pp	229533.I1RD17
127255	229533.I1R9J6	pp	229533.I1RBC9
127256	229533.I1R9J6	pp	229533.I1RPP4
127257	229533.I1R9J6	pp	229533.I1RVQ0
127258	229533.I1R9J6	pp	229533.I1RUG3
127259	229533.I1R9J6	pp	229533.I1RR24
127260	229533.I1R9J6	pp	229533.I1RY44
127261	229533.I1R9J6	pp	229533.I1RW25
127262	229533.I1R9J6	pp	229533.I1RR39
127263	229533.I1R9J6	pp	229533.V6RIV9
127264	229533.I1R9J6	pp	229533.I1S0L3
127265	229533.I1R9J6	pp	229533.I1RY03
127266	229533.I1R9J6	pp	229533.I1RQC9
127267	229533.I1R9J6	pp	229533.I1RX46
127268	229533.I1R9J6	pp	229533.I1RWN5
127269	229533.I1R9J6	pp	229533.I1RBT1
127270	229533.I1R9J6	pp	229533.I1RZK5
127271	229533.I1R9J6	pp	229533.I1RJW1
127272	229533.I1R9J6	pp	229533.I1REK8
127273	229533.I1R9J6	pp	229533.V6RTJ8
127274	229533.I1R9J6	pp	229533.I1RFU8
127275	229533.I1R9J6	pp	229533.I1RCG4
127276	229533.I1R9J6	pp	229533.I1RI82
127277	229533.I1R9J6	pp	229533.I1RRY9
127278	229533.I1R9J6	pp	229533.I1RIE8
127279	229533.I1R9J6	pp	229533.I1RAX6
127280	229533.I1R9J6	pp	229533.Q4IJK2
127281	229533.I1R9J6	pp	229533.I1RZM1
127282	229533.I1R9J6	pp	229533.V6RCG5
127283	229533.I1R9J6	pp	229533.I1S504
127284	229533.I1R9J6	pp	229533.I1RAU5
127285	229533.I1R9J6	pp	229533.I1RS18
127286	229533.I1R9J6	pp	229533.I1RLU6
127287	229533.I1RC12	pp	229533.I1RC90
127288	229533.I1RC12	pp	229533.I1RYN7
127289	229533.I1RC12	pp	229533.I1S1V2
127290	229533.I1RB38	pp	229533.I1RE52
127291	229533.I1RB38	pp	229533.I1RYM5
127292	229533.I1RB38	pp	229533.I1RXE6
127293	229533.I1RB38	pp	229533.Q4I5G1
127294	229533.I1RB38	pp	229533.I1RBT6
127295	229533.I1RB38	pp	229533.I1S2A1
127296	229533.I1RB38	pp	229533.I1RNJ5
127297	229533.I1RB38	pp	229533.I1RLZ6
127298	229533.I1RB38	pp	229533.Q4IPZ1
127299	229533.I1RB38	pp	229533.I1RP02
127300	229533.I1RB38	pp	229533.V6RRM0
127301	229533.I1RB38	pp	229533.V6RM02
127302	229533.I1RB38	pp	229533.V6RF62
127303	229533.I1RB38	pp	229533.I1RG33
127304	229533.I1RB38	pp	229533.I1RE65
127305	229533.I1RB38	pp	229533.I1RGI4
127306	229533.I1RB38	pp	229533.I1S0K2
127307	229533.I1RB38	pp	229533.I1S0R8
127308	229533.I1RB38	pp	229533.Q4IN52
127309	229533.I1RB38	pp	229533.I1RQR6
127310	229533.I1RB38	pp	229533.I1RFS5
127311	229533.I1RB38	pp	229533.I1REE2
127312	229533.I1RB38	pp	229533.I1S8R2

127313	229533.I1RB38	pp	229533.I1RYA0
127314	229533.I1RB38	pp	229533.I1S0Q7
127315	229533.I1RB38	pp	229533.I1S8E9
127316	229533.I1RB38	pp	229533.I1RB86
127317	229533.I1RB38	pp	229533.I1S5X9
127318	229533.I1RB38	pp	229533.I1RN84
127319	229533.I1RB38	pp	229533.I1RPR1
127320	229533.I1RB38	pp	229533.I1S5C6
127321	229533.I1RB38	pp	229533.I1RY21
127322	229533.I1RB38	pp	229533.I1RPY8
127323	229533.I1RB38	pp	229533.I1RS29
127324	229533.I1RB38	pp	229533.I1RY92
127325	229533.I1RB38	pp	229533.I1RC86
127326	229533.I1RB38	pp	229533.V6R3V4
127327	229533.I1RB38	pp	229533.I1RVZ3
127328	229533.I1RB38	pp	229533.I1RFJ6
127329	229533.I1RB38	pp	229533.I1RFI4
127330	229533.I1RB38	pp	229533.I1RKB7
127331	229533.I1RB38	pp	229533.I1RMQ6
127332	229533.I1RB38	pp	229533.I1RQY8
127333	229533.I1RB38	pp	229533.I1S4S1
127334	229533.I1RB38	pp	229533.I1RZ43
127335	229533.I1RB38	pp	229533.V6QUG0
127336	229533.I1RB38	pp	229533.I1S0B8
127337	229533.I1RB38	pp	229533.Q4IB65
127338	229533.I1RB38	pp	229533.I1S458
127339	229533.I1RB38	pp	229533.I1S9E7
127340	229533.I1RB38	pp	229533.I1S8S9
127341	229533.I1RB38	pp	229533.I1RG62
127342	229533.I1RB38	pp	229533.I1RG66
127343	229533.I1RB38	pp	229533.V6RZD4
127344	229533.I1RB38	pp	229533.I1RYT8
127345	229533.I1RB38	pp	229533.V6RJP8
127346	229533.I1RB38	pp	229533.I1RYC8
127347	229533.I1RB38	pp	229533.I1RYW9
127348	229533.I1RB38	pp	229533.I1RL81
127349	229533.I1RB38	pp	229533.I1RUR8
127350	229533.I1RB38	pp	229533.I1S8F8
127351	229533.I1RB38	pp	229533.I1RQU9
127352	229533.I1RB38	pp	229533.Q4IJW4
127353	229533.I1RB38	pp	229533.I1RMK7
127354	229533.I1RB38	pp	229533.I1RBE6
127355	229533.I1RB38	pp	229533.I1S6P5
127356	229533.I1RB38	pp	229533.I1RE66
127357	229533.I1RB38	pp	229533.I1RX39
127358	229533.I1RB38	pp	229533.I1RWA3
127359	229533.I1RB38	pp	229533.V6RUB1
127360	229533.I1RB38	pp	229533.I1RNM7
127361	229533.I1RB38	pp	229533.I1S0P0
127362	229533.I1RB38	pp	229533.I1S3T7
127363	229533.I1RB38	pp	229533.I1RS05
127364	229533.I1RB38	pp	229533.I1RRX5
127365	229533.I1RB38	pp	229533.I1RXD9
127366	229533.I1RB38	pp	229533.I1RX98
127367	229533.I1RB38	pp	229533.I1S299
127368	229533.I1RB38	pp	229533.I1S464
127369	229533.I1RB38	pp	229533.I1RZA7
127370	229533.I1RB38	pp	229533.I1RKB2
127371	229533.I1RB38	pp	229533.I1S4S5
127372	229533.I1RB92	pp	229533.I1RXR1
127373	229533.I1RB92	pp	229533.I1RT03
127374	229533.I1RB92	pp	229533.I1RVX0
127375	229533.I1RB92	pp	229533.Q4HXF6
127376	229533.I1RB92	pp	229533.I1RTH6
127377	229533.I1RB92	pp	229533.I1RRN5
127378	229533.I1RB92	pp	229533.I1RFL4
127379	229533.I1RB92	pp	229533.I1RMV0
127380	229533.I1RB92	pp	229533.I1RCH5
127381	229533.I1RB92	pp	229533.I1RN18
127382	229533.I1RB92	pp	229533.I1S0F8
127383	229533.I1RB92	pp	229533.I1RW46
127384	229533.I1RB92	pp	229533.I1RE01
127385	229533.I1RB92	pp	229533.I1RQE7

127386 229533.I1RB92 pp 229533.I1RZS7
127387 229533.I1RB92 pp 229533.I1S8R3
127388 229533.I1RB92 pp 229533.Q4HZK7
127389 229533.I1RB92 pp 229533.I1RTA0
127390 229533.I1RB92 pp 229533.I1S569
127391 229533.I1RB92 pp 229533.I1RCL5
127392 229533.I1RB92 pp 229533.I1S7W5
127393 229533.I1RB92 pp 229533.V6R057
127394 229533.I1RB92 pp 229533.I1RD17
127395 229533.I1RB92 pp 229533.I1RTE9
127396 229533.I1RB92 pp 229533.I1S1C7
127397 229533.I1RB92 pp 229533.Q4I624
127398 229533.I1RB92 pp 229533.I1S0R2
127399 229533.I1RB92 pp 229533.I1RQ72
127400 229533.I1RB92 pp 229533.I1RYW3
127401 229533.I1RB92 pp 229533.I1S0J1
127402 229533.I1RB92 pp 229533.I1RC72
127403 229533.I1RB92 pp 229533.I1RQ33
127404 229533.I1RB92 pp 229533.I1RSF1
127405 229533.I1RB92 pp 229533.I1RRE5
127406 229533.I1RB92 pp 229533.I1RWX5
127407 229533.I1RB92 pp 229533.I1RGL4
127408 229533.I1RB92 pp 229533.I1RZ79
127409 229533.I1RB92 pp 229533.I1RKC6
127410 229533.I1RB92 pp 229533.I1S8V4
127411 229533.I1RB92 pp 229533.V6RMW6
127412 229533.I1RB92 pp 229533.I1RXT3
127413 229533.I1RB92 pp 229533.I1RRI2
127414 229533.I1RB92 pp 229533.I1RPU2
127415 229533.I1RB92 pp 229533.I1RNC1
127416 229533.I1RB92 pp 229533.I1RWN5
127417 229533.I1RB92 pp 229533.I1RZW8
127418 229533.I1RB92 pp 229533.V6RIV9
127419 229533.I1RB92 pp 229533.I1RXU9
127420 229533.I1RB92 pp 229533.I1RUG3
127421 229533.I1RB92 pp 229533.I1RD39
127422 229533.I1RB92 pp 229533.I1S1W9
127423 229533.I1RB92 pp 229533.I1RWN6
127424 229533.I1RB92 pp 229533.I1S5R6
127425 229533.I1RB92 pp 229533.I1RCR8
127426 229533.I1RB92 pp 229533.I1RWQ8
127427 229533.I1RB92 pp 229533.I1RE89
127428 229533.I1RB92 pp 229533.I1RC33
127429 229533.I1RB92 pp 229533.Q4HZB8
127430 229533.I1RB92 pp 229533.I1RSK1
127431 229533.I1RB92 pp 229533.V6RGN5
127432 229533.I1RB92 pp 229533.I1RY44
127433 229533.I1RB92 pp 229533.I1RJV3
127434 229533.I1RB92 pp 229533.I1S1W3
127435 229533.I1RB92 pp 229533.I1RPK0
127436 229533.I1RB92 pp 229533.I1RG11
127437 229533.I1RB92 pp 229533.I1S0N4
127438 229533.I1RB92 pp 229533.I1RS17
127439 229533.I1RB92 pp 229533.I1RNB8
127440 229533.I1RB92 pp 229533.V6RVG3
127441 229533.I1RB92 pp 229533.I1RZV3
127442 229533.I1RB92 pp 229533.I1RPP4
127443 229533.I1RB92 pp 229533.I1SA33
127444 229533.I1RB92 pp 229533.I1RQS8
127445 229533.I1RB92 pp 229533.I1RC00
127446 229533.I1RB92 pp 229533.I1RXT9
127447 229533.I1RB92 pp 229533.I1RS20
127448 229533.I1RB92 pp 229533.I1RD22
127449 229533.I1RB92 pp 229533.I1RRN2
127450 229533.I1RB92 pp 229533.I1RMG3
127451 229533.I1RB92 pp 229533.I1RWH4
127452 229533.I1RB92 pp 229533.I1RRV1
127453 229533.I1RB92 pp 229533.I1RPE0
127454 229533.I1RB92 pp 229533.I1RSI6
127455 229533.I1RB92 pp 229533.I1RE22
127456 229533.I1RB92 pp 229533.I1S2H4
127457 229533.I1RB92 pp 229533.I1RC29
127458 229533.I1RB92 pp 229533.I1RSQ3

127459 229533.I1RB92 pp 229533.I1S0M6
127460 229533.I1RB92 pp 229533.I1RW61
127461 229533.I1RB92 pp 229533.I1S0L4
127462 229533.I1RB92 pp 229533.V6R042
127463 229533.I1RB92 pp 229533.I1RSZ7
127464 229533.I1RB92 pp 229533.I1RIL3
127465 229533.I1RB92 pp 229533.I1RRI1
127466 229533.I1RB92 pp 229533.I1RQ23
127467 229533.I1RB92 pp 229533.I1RRM3
127468 229533.I1RB92 pp 229533.I1S0F7
127469 229533.I1RB92 pp 229533.I1RKP1
127470 229533.I1RB92 pp 229533.I1RPM4
127471 229533.I1RB92 pp 229533.I1RSA5
127472 229533.I1RB92 pp 229533.I1S6Y8
127473 229533.I1RB92 pp 229533.I1RM82
127474 229533.I1RB92 pp 229533.I1RPV3
127475 229533.I1RB92 pp 229533.I1S9Y1
127476 229533.I1RB92 pp 229533.I1RN67
127477 229533.I1RB92 pp 229533.I1S0W4
127478 229533.I1RB92 pp 229533.I1RRT8
127479 229533.I1RB92 pp 229533.I1RL04
127480 229533.I1RB92 pp 229533.I1RFS3
127481 229533.I1RB92 pp 229533.I1RDF7
127482 229533.I1RB92 pp 229533.I1RE27
127483 229533.I1RB92 pp 229533.I1S5R1
127484 229533.I1RB92 pp 229533.I1RBS0
127485 229533.I1RB92 pp 229533.I1RJZ7
127486 229533.I1RB92 pp 229533.I1S0F6
127487 229533.I1RB92 pp 229533.I1RNA5
127488 229533.I1RB92 pp 229533.I1S2B8
127489 229533.I1RB92 pp 229533.O74638
127490 229533.I1RB92 pp 229533.I1RRM5
127491 229533.I1RB92 pp 229533.I1RXC2
127492 229533.I1RB92 pp 229533.I1S352
127493 229533.I1RB92 pp 229533.I1RPU3
127494 229533.I1RB92 pp 229533.Q4HXF0
127495 229533.I1RB92 pp 229533.I1RFT1
127496 229533.I1RB92 pp 229533.V6RUY9
127497 229533.I1RB92 pp 229533.I1RT04
127498 229533.I1RB92 pp 229533.I1RE76
127499 229533.I1RB92 pp 229533.I1RBY4
127500 229533.I1RB92 pp 229533.I1RRL9
127501 229533.I1RB92 pp 229533.I1RZ39
127502 229533.I1RB92 pp 229533.I1RYI5
127503 229533.I1RB92 pp 229533.I1S8P5
127504 229533.I1RB92 pp 229533.I1RV91
127505 229533.I1RB92 pp 229533.I1S2E9
127506 229533.I1RB92 pp 229533.I1RPP3
127507 229533.I1RB92 pp 229533.I1RR73
127508 229533.I1RB92 pp 229533.I1RQG4
127509 229533.I1RB92 pp 229533.V6R3X5
127510 229533.I1RB92 pp 229533.I1RT40
127511 229533.I1RB92 pp 229533.I1RRJ8
127512 229533.I1RB92 pp 229533.I1RWI5
127513 229533.I1RB92 pp 229533.I1RCJ7
127514 229533.I1RB92 pp 229533.I1RGF5
127515 229533.I1RB92 pp 229533.I1RZZ9
127516 229533.I1RB92 pp 229533.I1RBS6
127517 229533.I1RB92 pp 229533.I1RTD5
127518 229533.I1RB92 pp 229533.I1RWK3
127519 229533.I1RB92 pp 229533.I1RSQ4
127520 229533.I1RB92 pp 229533.I1S8V3
127521 229533.I1RB92 pp 229533.I1RN63
127522 229533.I1RB92 pp 229533.I1RGA4
127523 229533.I1RB92 pp 229533.I1RBC7
127524 229533.I1RB92 pp 229533.I1RJ74
127525 229533.I1RB92 pp 229533.I1RWS4
127526 229533.I1RB92 pp 229533.I1RC82
127527 229533.I1RB92 pp 229533.I1RC66
127528 229533.I1RB92 pp 229533.Q4IBK5
127529 229533.I1RB92 pp 229533.I1RJV4
127530 229533.I1RB92 pp 229533.I1RNN1
127531 229533.I1RB92 pp 229533.I1RFH8

127532	229533.I1RB92	pp	229533.I1RLN6
127533	229533.I1RB92	pp	229533.I1RZB2
127534	229533.I1RB92	pp	229533.I1RTA1
127535	229533.I1RB92	pp	229533.I1RJN9
127536	229533.I1RB92	pp	229533.I1RZH4
127537	229533.I1RB92	pp	229533.I1REC5
127538	229533.I1RB92	pp	229533.I1RVZ2
127539	229533.I1RB92	pp	229533.I1RRZ0
127540	229533.I1RB92	pp	229533.I1S503
127541	229533.I1RB92	pp	229533.I1RJW6
127542	229533.I1RB92	pp	229533.I1SA32
127543	229533.I1RB92	pp	229533.I1S504
127544	229533.I1RB92	pp	229533.I1RRK3
127545	229533.I1RB92	pp	229533.I1S2M6
127546	229533.I1RB92	pp	229533.I1RRT2
127547	229533.I1RB92	pp	229533.I1RS47
127548	229533.I1RB92	pp	229533.I1RBU8
127549	229533.I1RB92	pp	229533.V6R227
127550	229533.I1RB92	pp	229533.I1RCG4
127551	229533.I1RB92	pp	229533.I1RJL4
127552	229533.I1RB92	pp	229533.I1RCM5
127553	229533.I1RB92	pp	229533.Q4I283
127554	229533.I1RB92	pp	229533.V6R3J1
127555	229533.I1RB92	pp	229533.I1RSX0
127556	229533.I1RB92	pp	229533.I1S887
127557	229533.I1RB92	pp	229533.I1RZK5
127558	229533.I1RB92	pp	229533.I1RY63
127559	229533.I1RB92	pp	229533.I1RMD5
127560	229533.I1RB92	pp	229533.I1RSC6
127561	229533.I1RB92	pp	229533.I1RQ85
127562	229533.I1RB92	pp	229533.I1RWT6
127563	229533.I1RB92	pp	229533.I1RN99
127564	229533.I1RB92	pp	229533.I1RBL7
127565	229533.I1RB92	pp	229533.I1RY03
127566	229533.I1RB92	pp	229533.I1RG14
127567	229533.I1RB92	pp	229533.I1S419
127568	229533.I1RB92	pp	229533.I1RK95
127569	229533.I1RB92	pp	229533.I1RC16
127570	229533.I1RB92	pp	229533.I1RDG0
127571	229533.I1RB92	pp	229533.I1RPR9
127572	229533.I1RB92	pp	229533.I1RRY9
127573	229533.I1RB92	pp	229533.Q4IEK8
127574	229533.I1RB92	pp	229533.I1RQA3
127575	229533.I1RB92	pp	229533.I1RWR7
127576	229533.I1RB92	pp	229533.I1RNQ5
127577	229533.I1RB92	pp	229533.Q4IJK2
127578	229533.I1RB92	pp	229533.I1RBC4
127579	229533.I1RB92	pp	229533.I1RLU6
127580	229533.I1RB92	pp	229533.I1RBJ5
127581	229533.I1RB92	pp	229533.I1RU29
127582	229533.I1RB92	pp	229533.I1RZZ1
127583	229533.I1RB92	pp	229533.I1RZM1
127584	229533.I1RB92	pp	229533.I1RQQ5
127585	229533.I1RB92	pp	229533.I1RSR3
127586	229533.I1RB92	pp	229533.V6RBN2
127587	229533.I1RB92	pp	229533.I1RQA2
127588	229533.I1RB92	pp	229533.I1RGC5
127589	229533.I1RB92	pp	229533.I1S6K9
127590	229533.I1RB92	pp	229533.I1RWB0
127591	229533.I1RB92	pp	229533.I1RCR5
127592	229533.I1RB92	pp	229533.I1REK8
127593	229533.I1RB92	pp	229533.V6RCZ8
127594	229533.I1RB92	pp	229533.I1RWD7
127595	229533.I1RB92	pp	229533.I1RYS0
127596	229533.I1RB92	pp	229533.I1RR51
127597	229533.I1RB92	pp	229533.I1RI03
127598	229533.I1RB92	pp	229533.Q4I2J8
127599	229533.I1RB92	pp	229533.V6RCG5
127600	229533.I1RB92	pp	229533.I1RLM7
127601	229533.I1RB92	pp	229533.I1S1J7
127602	229533.I1RB92	pp	229533.I1RFJ9
127603	229533.I1RB92	pp	229533.I1RQ81
127604	229533.I1RB92	pp	229533.I1RQ31

127605 229533.I1RB92 pp 229533.I1RMC0
127606 229533.I1RB92 pp 229533.I1RS18
127607 229533.I1RB92 pp 229533.I1RSI0
127608 229533.I1RB92 pp 229533.I1RF19
127609 229533.I1RB92 pp 229533.I1RZM8
127610 229533.I1RB92 pp 229533.I1RCV1
127611 229533.I1RB92 pp 229533.Q4I8P5
127612 229533.I1RB92 pp 229533.I1RXF0
127613 229533.I1RB92 pp 229533.I1RFU8
127614 229533.I1RB92 pp 229533.I1RBC9
127615 229533.I1RB92 pp 229533.I1RI82
127616 229533.I1RB92 pp 229533.I1RW71
127617 229533.I1RB92 pp 229533.I1RXC4
127618 229533.I1RB92 pp 229533.I1RG53
127619 229533.I1RB92 pp 229533.I1RC17
127620 229533.I1RB92 pp 229533.I1S5B0
127621 229533.I1RB92 pp 229533.V6QW22
127622 229533.I1RB92 pp 229533.I1RW07
127623 229533.I1RB92 pp 229533.I1RW25
127624 229533.I1RB92 pp 229533.I1RW99
127625 229533.I1RB92 pp 229533.I1RLC7
127626 229533.I1RB92 pp 229533.I1RSQ7
127627 229533.I1RB92 pp 229533.I1RX25
127628 229533.I1RB92 pp 229533.I1RX95
127629 229533.I1RB92 pp 229533.I1RRV4
127630 229533.I1RB92 pp 229533.I1RRX5
127631 229533.I1RB92 pp 229533.V6QUE6
127632 229533.I1RB92 pp 229533.I1RDB1
127633 229533.I1RB92 pp 229533.I1RTF4
127634 229533.I1RB92 pp 229533.I1RGC2
127635 229533.I1RB92 pp 229533.I1RBD5
127636 229533.I1RB92 pp 229533.I1RKJ5
127637 229533.I1RB92 pp 229533.I1RLX7
127638 229533.I1RB92 pp 229533.I1RJU3
127639 229533.I1RB92 pp 229533.I1RMK3
127640 229533.I1RB92 pp 229533.Q4HVW2
127641 229533.I1RB92 pp 229533.I1RRU9
127642 229533.I1RB92 pp 229533.I1RIL8
127643 229533.I1RB92 pp 229533.I1RFY6
127644 229533.I1RB92 pp 229533.Q4IJT5
127645 229533.I1RB92 pp 229533.I1RRG3
127646 229533.I1RB92 pp 229533.V6QUW4
127647 229533.I1RB92 pp 229533.I1RD52
127648 229533.I1RB92 pp 229533.I1RBT1
127649 229533.I1RB92 pp 229533.I1RNA0
127650 229533.I1RB92 pp 229533.V6QYF0
127651 229533.I1RB92 pp 229533.I1S0X6
127652 229533.I1RB92 pp 229533.I1RRQ6
127653 229533.I1RB92 pp 229533.I1RT05
127654 229533.I1RB92 pp 229533.V6R1K4
127655 229533.I1RB92 pp 229533.I1RE17
127656 229533.I1RB92 pp 229533.I1RM06
127657 229533.I1RB92 pp 229533.I1S0F5
127658 229533.I1RB92 pp 229533.I1RPF1
127659 229533.I1RB92 pp 229533.I1S0F1
127660 229533.I1RB92 pp 229533.I1RWJ8
127661 229533.I1RB92 pp 229533.I1S0L3
127662 229533.I1RB92 pp 229533.I1RMC8
127663 229533.I1RB92 pp 229533.I1RWD4
127664 229533.I1RB92 pp 229533.I1S6F0
127665 229533.I1RB92 pp 229533.I1S4S4
127666 229533.I1RB92 pp 229533.I1RI02
127667 229533.I1RB92 pp 229533.I1RCS1
127668 229533.I1RB92 pp 229533.I1RM67
127669 229533.I1RB92 pp 229533.V6R4J1
127670 229533.I1RB92 pp 229533.I1RMG8
127671 229533.I1RB92 pp 229533.I1RZJ5
127672 229533.I1RB92 pp 229533.I1RVQ0
127673 229533.I1RB92 pp 229533.I1RRS8
127674 229533.I1RB92 pp 229533.I1RQC9
127675 229533.I1RB92 pp 229533.I1RYJ6
127676 229533.I1RB92 pp 229533.I1S0R7
127677 229533.I1RB92 pp 229533.I1S4S7

127678 229533.I1RB92 pp 229533.I1RPY3
127679 229533.I1RB92 pp 229533.V6RTJ8
127680 229533.I1RB92 pp 229533.I1RLP1
127681 229533.I1RB92 pp 229533.V6RUT5
127682 229533.I1RB92 pp 229533.I1RUQ2
127683 229533.I1RB92 pp 229533.I1S7A8
127684 229533.I1RB92 pp 229533.I1RX46
127685 229533.I1RB92 pp 229533.I1S0T2
127686 229533.I1RB92 pp 229533.I1RXD7
127687 229533.I1RD39 pp 229533.V6RCG5
127688 229533.I1RD39 pp 229533.V6RMW6
127689 229533.I1RD39 pp 229533.V6RIV9
127690 229533.I1RD39 pp 229533.I1RSQ3
127691 229533.I1RD39 pp 229533.I1RPP4
127692 229533.I1RD39 pp 229533.I1RS18
127693 229533.I1RD39 pp 229533.V6R042
127694 229533.I1RD39 pp 229533.Q4I8P5
127695 229533.I1RD39 pp 229533.I1RMG3
127696 229533.I1RD39 pp 229533.I1RS17
127697 229533.I1RD39 pp 229533.V6RCZ8
127698 229533.I1RD39 pp 229533.I1RI82
127699 229533.I1RD39 pp 229533.I1SA32
127700 229533.I1RD39 pp 229533.I1RZM8
127701 229533.I1RD39 pp 229533.I1RUG3
127702 229533.I1RD39 pp 229533.I1RNC1
127703 229533.I1RD39 pp 229533.I1S0J1
127704 229533.I1RD39 pp 229533.Q4HY20
127705 229533.I1RD39 pp 229533.I1RXF0
127706 229533.I1RD39 pp 229533.I1RE01
127707 229533.I1RD39 pp 229533.I1RW46
127708 229533.I1RD39 pp 229533.I1RW99
127709 229533.I1RD39 pp 229533.I1RWT6
127710 229533.I1RD39 pp 229533.I1S5B0
127711 229533.I1RD39 pp 229533.I1RY44
127712 229533.I1RD39 pp 229533.I1RVN2
127713 229533.I1RD39 pp 229533.I1S504
127714 229533.I1RD39 pp 229533.I1RT03
127715 229533.I1RD39 pp 229533.I1SA33
127716 229533.I1RD39 pp 229533.I1RLU6
127717 229533.I1RD39 pp 229533.I1RNQ5
127718 229533.I1RD39 pp 229533.I1RNL7
127719 229533.I1RD39 pp 229533.I1RZM1
127720 229533.I1RD39 pp 229533.I1RVX0
127721 229533.I1RD39 pp 229533.I1RE44
127722 229533.I1RD39 pp 229533.I1RZK5
127723 229533.I1RD39 pp 229533.I1RTF5
127724 229533.I1RD39 pp 229533.I1RTB8
127725 229533.I1RD39 pp 229533.I1RZZ1
127726 229533.I1RD39 pp 229533.I1RWN5
127727 229533.I1RD39 pp 229533.I1RFU8
127728 229533.I1RD39 pp 229533.I1RQE7
127729 229533.I1RD39 pp 229533.I1RWF4
127730 229533.I1RD39 pp 229533.I1S0L3
127731 229533.I1RD39 pp 229533.Q4I7N9
127732 229533.I1RD39 pp 229533.I1RLZ4
127733 229533.I1RD39 pp 229533.I1S8R3
127734 229533.I1RD39 pp 229533.I1RD52
127735 229533.I1RD39 pp 229533.I1RVQ0
127736 229533.I1RD39 pp 229533.I1RN18
127737 229533.I1RD39 pp 229533.I1RTH6
127738 229533.I1RD39 pp 229533.I1RRI2
127739 229533.I1RD39 pp 229533.I1RFL4
127740 229533.I1RD39 pp 229533.I1RYL5
127741 229533.I1RD39 pp 229533.Q4IJK2
127742 229533.I1RD39 pp 229533.I1RKI8
127743 229533.I1RD39 pp 229533.I1RMJ1
127744 229533.I1RD39 pp 229533.I1RZW8
127745 229533.I1RD39 pp 229533.I1RWZ9
127746 229533.I1RD39 pp 229533.I1RQA3
127747 229533.I1RD39 pp 229533.I1RQN8
127748 229533.I1RD39 pp 229533.I1S1C7
127749 229533.I1RD39 pp 229533.I1RN67
127750 229533.I1RD39 pp 229533.I1RY40

127751 229533.I1RD39 pp 229533.V6R057
127752 229533.I1RD39 pp 229533.I1RY03
127753 229533.I1RD39 pp 229533.I1S0F6
127754 229533.I1RD39 pp 229533.I1REK8
127755 229533.I1RD39 pp 229533.I1REK9
127756 229533.I1RD39 pp 229533.I1S2E9
127757 229533.I1RD39 pp 229533.Q4I624
127758 229533.I1RD39 pp 229533.I1RT28
127759 229533.I1RD39 pp 229533.I1RRY9
127760 229533.I1RD39 pp 229533.I1RXD7
127761 229533.I1RD39 pp 229533.I1RWN6
127762 229533.I1RD39 pp 229533.I1RSI0
127763 229533.I1RD39 pp 229533.I1RZV5
127764 229533.I1RD39 pp 229533.I1RW25
127765 229533.I1RD39 pp 229533.V6QUV7
127766 229533.I1RD39 pp 229533.I1RFL6
127767 229533.I1RD39 pp 229533.I1RZF7
127768 229533.I1RD39 pp 229533.I1S0R7
127769 229533.I1RD39 pp 229533.I1RQC9
127770 229533.I1RD39 pp 229533.I1RJV3
127771 229533.I1RD39 pp 229533.I1RX25
127772 229533.I1RD39 pp 229533.I1RT04
127773 229533.I1RD39 pp 229533.I1RFL8
127774 229533.I1RD39 pp 229533.I1RPP3
127775 229533.I1RD39 pp 229533.I1S2B8
127776 229533.I1RD39 pp 229533.I1RRR5
127777 229533.I1RD39 pp 229533.I1RUP0
127778 229533.I1RD39 pp 229533.I1RG53
127779 229533.I1RD39 pp 229533.I1RRM5
127780 229533.I1RD39 pp 229533.I1RSQ7
127781 229533.I1RD39 pp 229533.I1RW76
127782 229533.I1RD39 pp 229533.I1RFV4
127783 229533.I1RD39 pp 229533.V6R3X5
127784 229533.I1RD39 pp 229533.I1RTD5
127785 229533.I1RD39 pp 229533.I1RFW0
127786 229533.I1RD39 pp 229533.V6QW22
127787 229533.I1RD39 pp 229533.I1RRC6
127788 229533.I1RD39 pp 229533.I1S0Y1
127789 229533.I1RD39 pp 229533.I1RV91
127790 229533.I1RD39 pp 229533.I1RR73
127791 229533.I1RD39 pp 229533.I1RGS6
127792 229533.I1RD39 pp 229533.I1RT40
127793 229533.I1RD39 pp 229533.I1RZ39
127794 229533.I1RD39 pp 229533.I1S7A8
127795 229533.I1RD39 pp 229533.I1RZP1
127796 229533.I1RD39 pp 229533.I1RJU3
127797 229533.I1RD39 pp 229533.I1RRL9
127798 229533.I1RD39 pp 229533.I1RWK3
127799 229533.I1RD39 pp 229533.V6RTJ8
127800 229533.I1RD39 pp 229533.V6R1K4
127801 229533.I1RD39 pp 229533.I1RZZ9
127802 229533.I1RD39 pp 229533.I1S0X6
127803 229533.I1RD39 pp 229533.I1RJV4
127804 229533.I1RD39 pp 229533.I1RJH8
127805 229533.I1RD39 pp 229533.I1RQ41
127806 229533.I1RD39 pp 229533.I1S051
127807 229533.I1RD39 pp 229533.I1RX46
127808 229533.I1RD39 pp 229533.I1S0L4
127809 229533.I1RD39 pp 229533.I1RGA4
127810 229533.I1RD39 pp 229533.I1S503
127811 229533.I1RD39 pp 229533.I1RR24
127812 229533.I1RD39 pp 229533.I1RS20
127813 229533.I1RD39 pp 229533.I1RJW6
127814 229533.I1RD39 pp 229533.I1RZM0
127815 229533.I1RD39 pp 229533.I1RQ23
127816 229533.I1RD39 pp 229533.I1RPM4
127817 229533.I1RD39 pp 229533.I1RE22
127818 229533.I1RD39 pp 229533.I1RX87
127819 229533.I1RD39 pp 229533.I1S2H4
127820 229533.I1RD39 pp 229533.I1RRT8
127821 229533.I1RD39 pp 229533.I1RYX4
127822 229533.I1RD39 pp 229533.I1RJH7
127823 229533.I1RD39 pp 229533.I1RMG8

127824	229533.I1RD39	pp	229533.I1S5R1
127825	229533.I1RD39	pp	229533.I1RWJ8
127826	229533.I1RAW0	pp	229533.I1RCM3
127827	229533.I1RAW0	pp	229533.I1RQ33
127828	229533.I1RAW0	pp	229533.Q4I624
127829	229533.I1RAW0	pp	229533.I1RD17
127830	229533.I1RAW0	pp	229533.V6R057
127831	229533.I1RAW0	pp	229533.I1RE89
127832	229533.I1RAW0	pp	229533.I1S569
127833	229533.I1RAW0	pp	229533.I1RVQ3
127834	229533.I1RAW0	pp	229533.I1RZV3
127835	229533.I1RAW0	pp	229533.I1S2C0
127836	229533.I1RAW0	pp	229533.I1RKJ8
127837	229533.I1RAW0	pp	229533.I1RCD8
127838	229533.I1RAW0	pp	229533.I1S0F8
127839	229533.I1RAW0	pp	229533.I1RGL4
127840	229533.I1RAW0	pp	229533.I1RCR1
127841	229533.I1RAW0	pp	229533.I1RML0
127842	229533.I1RAW0	pp	229533.I1S1W3
127843	229533.I1RAW0	pp	229533.V6RRH2
127844	229533.I1RAW0	pp	229533.I1S8R3
127845	229533.I1RAW0	pp	229533.I1RY44
127846	229533.I1RAW0	pp	229533.I1RBR1
127847	229533.I1RAW0	pp	229533.I1RTH6
127848	229533.I1RAW0	pp	229533.I1RZW8
127849	229533.I1RAW0	pp	229533.I1RB41
127850	229533.I1RAW0	pp	229533.I1RC33
127851	229533.I1RAW0	pp	229533.I1RB92
127852	229533.I1RAW0	pp	229533.I1RUF7
127853	229533.I1RAW0	pp	229533.I1RRI2
127854	229533.I1RAW0	pp	229533.I1S016
127855	229533.I1RAW0	pp	229533.I1RM96
127856	229533.I1RAW0	pp	229533.I1RRN5
127857	229533.I1RAW0	pp	229533.I1S1W9
127858	229533.I1RAW0	pp	229533.I1RS06
127859	229533.I1RAW0	pp	229533.I1RXR1
127860	229533.I1RAW0	pp	229533.I1RZ79
127861	229533.I1RAW0	pp	229533.I1RWX5
127862	229533.I1RAW0	pp	229533.V6RIV9
127863	229533.I1RAW0	pp	229533.I1RWN5
127864	229533.I1RAW0	pp	229533.I1RYW3
127865	229533.I1RAW0	pp	229533.I1RT03
127866	229533.I1RAW0	pp	229533.I1S2E8
127867	229533.I1RAW0	pp	229533.I1RVX0
127868	229533.I1RAW0	pp	229533.I1RQE7
127869	229533.I1RAW0	pp	229533.I1S9Z4
127870	229533.I1RAW0	pp	229533.I1S1C7
127871	229533.I1RAW0	pp	229533.I1RMV0
127872	229533.I1RAW0	pp	229533.I1RPP4
127873	229533.I1RAW0	pp	229533.I1RB44
127874	229533.I1RAW0	pp	229533.I1RN18
127875	229533.I1RAW0	pp	229533.I1RS17
127876	229533.I1RAW0	pp	229533.I1RCH5
127877	229533.I1RAW0	pp	229533.I1RSD8
127878	229533.I1RAW0	pp	229533.I1RNU6
127879	229533.I1RAW0	pp	229533.I1S437
127880	229533.I1RAW0	pp	229533.V6RLM7
127881	229533.I1RAW0	pp	229533.I1RCW6
127882	229533.I1RAW0	pp	229533.I1RVZ7
127883	229533.I1RAW0	pp	229533.I1RFL4
127884	229533.I1RAW0	pp	229533.I1RWN6
127885	229533.I1RAW0	pp	229533.I1RD39
127886	229533.I1RAW0	pp	229533.I1RPK0
127887	229533.I1RAW0	pp	229533.I1RPU2
127888	229533.I1RAW0	pp	229533.I1RXU9
127889	229533.I1RAW0	pp	229533.I1S0J1
127890	229533.I1RAW0	pp	229533.I1RRR7
127891	229533.I1RAW0	pp	229533.I1RSF1
127892	229533.I1RAW0	pp	229533.I1RCA0
127893	229533.I1RAW0	pp	229533.I1RJV3
127894	229533.I1RAW0	pp	229533.I1RBZ3
127895	229533.I1RAW0	pp	229533.I1S7W5
127896	229533.I1RAW0	pp	229533.I1S1W4

127897 229533.I1RAW0 pp 229533.I1RSK1
127898 229533.I1RAW0 pp 229533.I1RB26
127899 229533.I1RAW0 pp 229533.I1RTA0
127900 229533.I1RAW0 pp 229533.I1RW46
127901 229533.I1RAW0 pp 229533.I1RG11
127902 229533.I1RAW0 pp 229533.I1S0N4
127903 229533.I1RAW0 pp 229533.I1RRE5
127904 229533.I1RAW0 pp 229533.I1RUG3
127905 229533.I1RAW0 pp 229533.I1RE01
127906 229533.I1RAW0 pp 229533.I1RS90
127907 229533.I1RAW0 pp 229533.I1RL04
127908 229533.I1RAW0 pp 229533.I1RNC1
127909 229533.I1RAW0 pp 229533.I1RPM4
127910 229533.I1RAW0 pp 229533.I1RPV3
127911 229533.I1RAW0 pp 229533.I1S6Y8
127912 229533.I1RAW0 pp 229533.I1RRN2
127913 229533.I1RAW0 pp 229533.I1S5R6
127914 229533.I1RAW0 pp 229533.I1SA33
127915 229533.I1RAW0 pp 229533.I1RXT3
127916 229533.I1RAW0 pp 229533.I1RMN6
127917 229533.I1RAW0 pp 229533.I1RQ23
127918 229533.I1RAW0 pp 229533.I1S4Z8
127919 229533.I1RAW0 pp 229533.I1RFS3
127920 229533.I1RAW0 pp 229533.I1RSZ7
127921 229533.I1RAW0 pp 229533.I1RVL8
127922 229533.I1RAW0 pp 229533.V6RMW6
127923 229533.I1RAW0 pp 229533.I1S3T7
127924 229533.I1RAW0 pp 229533.I1RRM3
127925 229533.I1RAW0 pp 229533.I1RE76
127926 229533.I1RAW0 pp 229533.I1RSQ3
127927 229533.I1RAW0 pp 229533.I1S277
127928 229533.I1RAW0 pp 229533.I1RKP1
127929 229533.I1RAW0 pp 229533.I1RBY4
127930 229533.I1RAW0 pp 229533.I1RPU3
127931 229533.I1RAW0 pp 229533.V6R042
127932 229533.I1RAW0 pp 229533.I1RRI1
127933 229533.I1RAW0 pp 229533.I1RRV1
127934 229533.I1RAW0 pp 229533.I1S2B8
127935 229533.I1RAW0 pp 229533.I1RIL3
127936 229533.I1RAW0 pp 229533.I1S0L4
127937 229533.I1RAW0 pp 229533.I1RVF8
127938 229533.I1RAW0 pp 229533.I1RKJ4
127939 229533.I1RAW0 pp 229533.I1RRM5
127940 229533.I1RAW0 pp 229533.I1RPE0
127941 229533.I1RAW0 pp 229533.Q4IN63
127942 229533.I1RAW0 pp 229533.I1RS20
127943 229533.I1RAW0 pp 229533.I1RJZ7
127944 229533.I1RAW0 pp 229533.I1S2H4
127945 229533.I1RAW0 pp 229533.I1RZS6
127946 229533.I1RAW0 pp 229533.I1S0M6
127947 229533.I1RAW0 pp 229533.I1RCR8
127948 229533.I1RAW0 pp 229533.I1S3G4
127949 229533.I1RAW0 pp 229533.I1RD64
127950 229533.I1RAW0 pp 229533.I1RRQ3
127951 229533.I1RAW0 pp 229533.I1RBG9
127952 229533.I1RAW0 pp 229533.I1S0F6
127953 229533.I1RAW0 pp 229533.I1RK96
127954 229533.I1RAW0 pp 229533.I1RWL2
127955 229533.I1RAW0 pp 229533.I1REC5
127956 229533.I1RAW0 pp 229533.I1RC29
127957 229533.I1RAW0 pp 229533.O74638
127958 229533.I1RAW0 pp 229533.I1RDF7
127959 229533.I1RAW0 pp 229533.I1RE22
127960 229533.I1RAW0 pp 229533.I1RGU7
127961 229533.I1RAW0 pp 229533.I1S0W4
127962 229533.I1RAW0 pp 229533.I1RXC2
127963 229533.I1RAW0 pp 229533.I1RN67
127964 229533.I1RAW0 pp 229533.I1RR73
127965 229533.I1RAW0 pp 229533.I1S5R1
127966 229533.I1RAW0 pp 229533.I1RRJ8
127967 229533.I1RAW0 pp 229533.I1RDE7
127968 229533.I1RAW0 pp 229533.I1RZ39
127969 229533.I1RAW0 pp 229533.I1S8P5

127970 229533.I1RAW0 pp 229533.I1RNN1
127971 229533.I1RAW0 pp 229533.I1RPP3
127972 229533.I1RAW0 pp 229533.I1RRT8
127973 229533.I1RAW0 pp 229533.I1RC66
127974 229533.I1RAW0 pp 229533.I1RB74
127975 229533.I1RAW0 pp 229533.I1RKJ6
127976 229533.I1RAW0 pp 229533.I1S9Y1
127977 229533.I1RAW0 pp 229533.I1RYI5
127978 229533.I1RAW0 pp 229533.I1RFT1
127979 229533.I1RAW0 pp 229533.I1RZZ9
127980 229533.I1RAW0 pp 229533.I1RM82
127981 229533.I1RAW0 pp 229533.I1RSV2
127982 229533.I1RAW0 pp 229533.I1RYP2
127983 229533.I1RAW0 pp 229533.I1RSQ4
127984 229533.I1RAW0 pp 229533.Q4HZI0
127985 229533.I1RAW0 pp 229533.I1RZV6
127986 229533.I1RAW0 pp 229533.I1RKL3
127987 229533.I1RAW0 pp 229533.I1RND6
127988 229533.I1RAW0 pp 229533.V6R3X5
127989 229533.I1RAW0 pp 229533.I1S352
127990 229533.I1RAW0 pp 229533.I1S2G7
127991 229533.I1RAW0 pp 229533.I1RWI5
127992 229533.I1RAW0 pp 229533.I1RQM7
127993 229533.I1RAW0 pp 229533.I1RVZ2
127994 229533.I1RAW0 pp 229533.I1RGA4
127995 229533.I1RAW0 pp 229533.I1RSA5
127996 229533.I1RAW0 pp 229533.I1RP27
127997 229533.I1RAW0 pp 229533.I1RJN9
127998 229533.I1RAW0 pp 229533.I1S0F7
127999 229533.I1RAW0 pp 229533.I1RTA1
128000 229533.I1RAW0 pp 229533.I1RBC7
128001 229533.I1RAW0 pp 229533.I1RC03
128002 229533.I1RAW0 pp 229533.V6R283
128003 229533.I1RAW0 pp 229533.I1RQ65
128004 229533.I1RAW0 pp 229533.I1RBS6
128005 229533.I1RAW0 pp 229533.I1RS47
128006 229533.I1RAW0 pp 229533.I1RYR0
128007 229533.I1RAW0 pp 229533.V6R3J1
128008 229533.I1RAW0 pp 229533.I1RWK3
128009 229533.I1RAW0 pp 229533.I1RC05
128010 229533.I1RAW0 pp 229533.I1S503
128011 229533.I1RAW0 pp 229533.I1RSX0
128012 229533.I1RAW0 pp 229533.I1RBS0
128013 229533.I1RAW0 pp 229533.I1RJV4
128014 229533.I1RAW0 pp 229533.I1RT40
128015 229533.I1RAW0 pp 229533.I1RJZ6
128016 229533.I1RAW0 pp 229533.V6RBN2
128017 229533.I1RAW0 pp 229533.I1RRK3
128018 229533.I1RAW0 pp 229533.I1RV91
128019 229533.I1RAW0 pp 229533.I1RJW6
128020 229533.I1RAW0 pp 229533.I1RCJ7
128021 229533.I1RAW0 pp 229533.Q4HVQ9
128022 229533.I1RAW0 pp 229533.I1RZZ1
128023 229533.I1RAW0 pp 229533.I1RC34
128024 229533.I1RAW0 pp 229533.I1RLN6
128025 229533.I1RAW0 pp 229533.I1RGB2
128026 229533.I1RAW0 pp 229533.I1RZK6
128027 229533.I1RAW0 pp 229533.I1RFL3
128028 229533.I1RAW0 pp 229533.I1RWD7
128029 229533.I1RAW0 pp 229533.I1RRZ0
128030 229533.I1RAW0 pp 229533.I1S2E9
128031 229533.I1RAW0 pp 229533.I1RCR5
128032 229533.I1RAW0 pp 229533.V6RUY9
128033 229533.I1RAW0 pp 229533.Q4IJK2
128034 229533.I1RAW0 pp 229533.I1RB75
128035 229533.I1RAW0 pp 229533.I1RQA3
128036 229533.I1RAW0 pp 229533.I1REK8
128037 229533.I1RAW0 pp 229533.I1RT04
128038 229533.I1RAW0 pp 229533.I1RF19
128039 229533.I1RAW0 pp 229533.I1RRY9
128040 229533.I1RAW0 pp 229533.I1RU29
128041 229533.I1RAW0 pp 229533.I1RSI0
128042 229533.I1RAW0 pp 229533.Q4HXF0

128043	229533.I1RAW0	pp	229533.I1RE00
128044	229533.I1RAW0	pp	229533.I1S4Q4
128045	229533.I1RAW0	pp	229533.I1RNA5
128046	229533.I1RAW0	pp	229533.I1RPR9
128047	229533.I1RAW0	pp	229533.I1RWR7
128048	229533.I1RAW0	pp	229533.I1RPH6
128049	229533.I1RAW0	pp	229533.I1RZE5
128050	229533.I1RAW0	pp	229533.I1RXF8
128051	229533.I1RAW0	pp	229533.I1RBL7
128052	229533.I1RAW0	pp	229533.I1RTD5
128053	229533.I1RAW0	pp	229533.I1RLM7
128054	229533.I1RAW0	pp	229533.I1REL7
128055	229533.I1RAW0	pp	229533.I1RGF5
128056	229533.I1RAW0	pp	229533.I1RWJ3
128057	229533.I1RAW0	pp	229533.Q4I2J8
128058	229533.I1RAW0	pp	229533.I1S1J7
128059	229533.I1RAW0	pp	229533.I1RAZ8
128060	229533.I1RAW0	pp	229533.I1RCM5
128061	229533.I1RAW0	pp	229533.I1RDG0
128062	229533.I1RAW0	pp	229533.I1RK95
128063	229533.I1RAW0	pp	229533.I1RZK5
128064	229533.I1RAW0	pp	229533.I1RUQ3
128065	229533.I1RAW0	pp	229533.I1RI03
128066	229533.I1RAW0	pp	229533.I1RBU8
128067	229533.I1RAW0	pp	229533.I1RCG4
128068	229533.I1RAW0	pp	229533.I1RD03
128069	229533.I1RAW0	pp	229533.I1RDU0
128070	229533.I1RAW0	pp	229533.I1RYS0
128071	229533.I1RAW0	pp	229533.I1RZB5
128072	229533.I1RAW0	pp	229533.I1RB53
128073	229533.I1RAW0	pp	229533.Q4IJM4
128074	229533.I1RAW0	pp	229533.I1RWB0
128075	229533.I1RAW0	pp	229533.I1RFU8
128076	229533.I1RAW0	pp	229533.I1RAZ3
128077	229533.I1RAW0	pp	229533.I1S1B2
128078	229533.I1RAW0	pp	229533.V6QY25
128079	229533.I1RAW0	pp	229533.I1RQD2
128080	229533.I1RAW0	pp	229533.V6R9Z0
128081	229533.I1RAW0	pp	229533.I1RY03
128082	229533.I1RAW0	pp	229533.I1RYY5
128083	229533.I1RAW0	pp	229533.I1RK83
128084	229533.I1RAW0	pp	229533.I1RFT2
128085	229533.I1RAW0	pp	229533.V6QUE6
128086	229533.I1RAW0	pp	229533.I1RSR8
128087	229533.I1RAW0	pp	229533.I1S887
128088	229533.I1RAW0	pp	229533.I1RGC2
128089	229533.I1RAW0	pp	229533.I1RBJ5
128090	229533.I1RAW0	pp	229533.I1RYI7
128091	229533.I1RAW0	pp	229533.I1RNT9
128092	229533.I1RAW0	pp	229533.I1RNQ5
128093	229533.I1RAW0	pp	229533.I1RQ85
128094	229533.I1RAW0	pp	229533.I1RG14
128095	229533.I1RAW0	pp	229533.I1RRV4
128096	229533.I1RAW0	pp	229533.I1REE2
128097	229533.I1RAW0	pp	229533.I1RWT6
128098	229533.I1RAW0	pp	229533.I1RLU6
128099	229533.I1RAW0	pp	229533.I1RRV3
128100	229533.I1RAW0	pp	229533.I1S504
128101	229533.I1RAW0	pp	229533.V6RCG5
128102	229533.I1RAW0	pp	229533.I1S419
128103	229533.I1RAW0	pp	229533.I1RR51
128104	229533.I1RAW0	pp	229533.Q4I8P5
128105	229533.I1RAW0	pp	229533.I1RZF6
128106	229533.I1RAW0	pp	229533.I1RS18
128107	229533.I1RAW0	pp	229533.I1S821
128108	229533.I1RAW0	pp	229533.I1RZM1
128109	229533.I1RAW0	pp	229533.I1S6K9
128110	229533.I1RAW0	pp	229533.I1RQ81
128111	229533.I1RAW0	pp	229533.I1RGC5
128112	229533.I1RAW0	pp	229533.I1S0M9
128113	229533.I1RAW0	pp	229533.I1RW07
128114	229533.I1RAW0	pp	229533.I1RRS8
128115	229533.I1RAW0	pp	229533.I1RJ17

128116 229533.I1RAW0 pp 229533.I1SA32
128117 229533.I1RAW0 pp 229533.I1RYB9
128118 229533.I1RAW0 pp 229533.V6RCZ8
128119 229533.I1RAW0 pp 229533.I1RX25
128120 229533.I1RAW0 pp 229533.I1RI02
128121 229533.I1RAW0 pp 229533.I1RNH6
128122 229533.I1RAW0 pp 229533.I1RXC4
128123 229533.I1RAW0 pp 229533.I1S9I5
128124 229533.I1RAW0 pp 229533.I1S021
128125 229533.I1RAW0 pp 229533.I1RC61
128126 229533.I1RAW0 pp 229533.I1RW99
128127 229533.I1RAW0 pp 229533.I1S5B0
128128 229533.I1RAW0 pp 229533.I1RW25
128129 229533.I1RAW0 pp 229533.I1RXF0
128130 229533.I1RAW0 pp 229533.I1RLC7
128131 229533.I1RAW0 pp 229533.I1RI82
128132 229533.I1RAW0 pp 229533.I1RZM8
128133 229533.I1RAW0 pp 229533.I1S438
128134 229533.I1RAW0 pp 229533.I1S044
128135 229533.I1RAW0 pp 229533.I1RM06
128136 229533.I1RAW0 pp 229533.I1RUY5
128137 229533.I1RAW0 pp 229533.I1RPA4
128138 229533.I1RAW0 pp 229533.I1RC17
128139 229533.I1RAW0 pp 229533.I1S1W8
128140 229533.I1RAW0 pp 229533.I1RCS1
128141 229533.I1RAW0 pp 229533.I1RMC8
128142 229533.I1RAW0 pp 229533.I1RKB7
128143 229533.I1RAW0 pp 229533.I1RE17
128144 229533.I1RAW0 pp 229533.I1RR24
128145 229533.I1RAW0 pp 229533.I1S0X6
128146 229533.I1RAW0 pp 229533.I1RG53
128147 229533.I1RAW0 pp 229533.I1RM67
128148 229533.I1RAW0 pp 229533.I1RBT1
128149 229533.I1RAW0 pp 229533.I1RFQ7
128150 229533.I1RAW0 pp 229533.I1RRQ1
128151 229533.I1RAW0 pp 229533.I1S0F5
128152 229533.I1RAW0 pp 229533.I1RPU0
128153 229533.I1RAW0 pp 229533.I1S4S4
128154 229533.I1RAW0 pp 229533.I1RPN4
128155 229533.I1RAW0 pp 229533.I1RR00
128156 229533.I1RAW0 pp 229533.I1RWJ8
128157 229533.I1RAW0 pp 229533.I1RW71
128158 229533.I1RAW0 pp 229533.I1S0L3
128159 229533.I1RAW0 pp 229533.I1RBC9
128160 229533.I1RAW0 pp 229533.V6R1K4
128161 229533.I1RAW0 pp 229533.I1RKJ5
128162 229533.I1RAW0 pp 229533.I1S4S7
128163 229533.I1RAW0 pp 229533.I1RY86
128164 229533.I1RAW0 pp 229533.Q4IJT5
128165 229533.I1RAW0 pp 229533.I1RIG2
128166 229533.I1RAW0 pp 229533.I1RD52
128167 229533.I1RAW0 pp 229533.I1RJU3
128168 229533.I1RAW0 pp 229533.I1RVQ0
128169 229533.I1RAW0 pp 229533.I1RRG3
128170 229533.I1RAW0 pp 229533.I1RT80
128171 229533.I1RAW0 pp 229533.I1RIL8
128172 229533.I1RAW0 pp 229533.I1RBD5
128173 229533.I1RAW0 pp 229533.I1S2R0
128174 229533.I1RAW0 pp 229533.V6R4J1
128175 229533.I1RAW0 pp 229533.I1RXD6
128176 229533.I1RAW0 pp 229533.I1RLP1
128177 229533.I1RAW0 pp 229533.I1RX95
128178 229533.I1RAW0 pp 229533.I1RXD7
128179 229533.I1RAW0 pp 229533.I1RDU4
128180 229533.I1RAW0 pp 229533.I1RQH3
128181 229533.I1RAW0 pp 229533.I1RBR2
128182 229533.I1RAW0 pp 229533.I1REN8
128183 229533.I1RAW0 pp 229533.I1S7A8
128184 229533.I1RAW0 pp 229533.I1RRX5
128185 229533.I1RAW0 pp 229533.I1RTB3
128186 229533.I1RAW0 pp 229533.V6RUT5
128187 229533.I1RAW0 pp 229533.I1RFC0
128188 229533.I1RAW0 pp 229533.V6RTJ8

128189	229533.I1RAW0	pp	229533.I1RC20
128190	229533.I1RAW0	pp	229533.I1RSQ7
128191	229533.I1RAW0	pp	229533.I1S540
128192	229533.I1RAW0	pp	229533.I1RAX6
128193	229533.I1RAW0	pp	229533.V6QW22
128194	229533.I1RAW0	pp	229533.I1S0R7
128195	229533.I1RAW0	pp	229533.I1RQC9
128196	229533.I1RAW0	pp	229533.I1RN61
128197	229533.I1RAW0	pp	229533.I1RNB2
128198	229533.I1RAW0	pp	229533.I1RPY3
128199	229533.I1RAW0	pp	229533.I1RQJ5
128200	229533.I1RAW0	pp	229533.Q4IPI1
128201	229533.I1RAW0	pp	229533.I1S0T2
128202	229533.I1RAW0	pp	229533.I1RCC2
128203	229533.I1RAW0	pp	229533.I1RX46
128204	229533.I1RPI4	pp	229533.I1S0Y2
128205	229533.I1RPI4	pp	229533.I1S0I3
128206	229533.I1RPI4	pp	229533.I1S3G4
128207	229533.I1RPI4	pp	229533.I1RZJ8
128208	229533.I1RPI4	pp	229533.I1RTE9
128209	229533.I1RPI4	pp	229533.V6RK96
128210	229533.I1RPI4	pp	229533.I1RYH1
128211	229533.I1RPI4	pp	229533.I1RUP5
128212	229533.I1RPI4	pp	229533.I1RZM4
128213	229533.I1RPI4	pp	229533.I1RXX1
128214	229533.I1RPI4	pp	229533.Q4I785
128215	229533.I1RPI4	pp	229533.I1S1W8
128216	229533.I1RPI4	pp	229533.Q4HXT5
128217	229533.I1RPI4	pp	229533.I1RQE1
128218	229533.I1RPI4	pp	229533.I1RY49
128219	229533.I1RPI4	pp	229533.I1RT00
128220	229533.I1RPI4	pp	229533.I1RQE8
128221	229533.A0A1C3YK95	pp	229533.I1RE88
128222	229533.A0A1C3YK95	pp	229533.I1RTP2
128223	229533.A0A098DRR8	pp	229533.V6RAC9
128224	229533.A0A098DRR8	pp	229533.I1RTD5
128225	229533.A0A098DRR8	pp	229533.Q4INZ9
128226	229533.A0A098DRR8	pp	229533.I1RZJ6
128227	229533.A0A098DRR8	pp	229533.I1RAF8
128228	229533.A0A098DRR8	pp	229533.I1RUQ2
128229	229533.A0A098DRR8	pp	229533.A0A0E0SMR8
128230	229533.A0A098DRR8	pp	229533.I1RXD7
128231	229533.A0A098DRR8	pp	229533.I1S2E9
128232	229533.A0A098DRR8	pp	229533.I1RQC9
128233	229533.A0A098DRR8	pp	229533.A0A1C3YLF5
128234	229533.A0A098DRR8	pp	229533.A0A0E0SFN0
128235	229533.A0A098DRR8	pp	229533.I1S0R7
128236	229533.A0A098DRR8	pp	229533.I1RBC7
128237	229533.A0A098DRR8	pp	229533.I1RT04
128238	229533.A0A098DRR8	pp	229533.I1RGA4
128239	229533.A0A098DRR8	pp	229533.I1RAX6
128240	229533.A0A098DRR8	pp	229533.I1RC82
128241	229533.A0A098DRR8	pp	229533.A0A0E0S7B3
128242	229533.A0A098DRR8	pp	229533.A0A0E0SGM4
128243	229533.A0A098DRR8	pp	229533.I1S9Y0
128244	229533.A0A098DRR8	pp	229533.I1RFW5
128245	229533.A0A098DRR8	pp	229533.I1RX46
128246	229533.A0A098DRR8	pp	229533.I1RQH2
128247	229533.A0A098DRR8	pp	229533.I1RRT2
128248	229533.A0A098DRR8	pp	229533.V6R283
128249	229533.A0A098DRR8	pp	229533.I1RQS5
128250	229533.A0A098DRR8	pp	229533.I1RJL4
128251	229533.A0A098DRR8	pp	229533.A0A1C3YMW0
128252	229533.A0A098DRR8	pp	229533.I1RC03
128253	229533.A0A098DRR8	pp	229533.Q4IPI1
128254	229533.A0A098DRR8	pp	229533.I1RQJ5
128255	229533.A0A098DRR8	pp	229533.Q4IAS1
128256	229533.A0A098DRR8	pp	229533.I1RZ99
128257	229533.A0A098DRR8	pp	229533.I1RLV9
128258	229533.A0A098DRR8	pp	229533.I1RPP3
128259	229533.A0A098DRR8	pp	229533.I1RKQ5
128260	229533.A0A098DRR8	pp	229533.I1RWF7
128261	229533.A0A098DRR8	pp	229533.A0A0E0SFM9

128262	229533.A0A098DRR8	pp	229533.A0A0E0RNT7
128263	229533.A0A098DRR8	pp	229533.I1S503
128264	229533.A0A098DRR8	pp	229533.I1RRJ8
128265	229533.A0A098DRR8	pp	229533.V6RTJ8
128266	229533.A0A098DRR8	pp	229533.I1RSQ7
128267	229533.A0A098DRR8	pp	229533.Q4IQW0
128268	229533.A0A098DRR8	pp	229533.Q4I830
128269	229533.A0A098DRR8	pp	229533.I1RT33
128270	229533.A0A098DRR8	pp	229533.I1S540
128271	229533.A0A098DRR8	pp	229533.I1RW71
128272	229533.A0A098DRR8	pp	229533.I1RKL3
128273	229533.A0A098DRR8	pp	229533.I1S1B6
128274	229533.A0A098DRR8	pp	229533.I1RR00
128275	229533.A0A098DRR8	pp	229533.Q4HZ42
128276	229533.A0A098DRR8	pp	229533.I1RFN1
128277	229533.A0A098DRR8	pp	229533.I1RRQ3
128278	229533.A0A098DRR8	pp	229533.I1RM06
128279	229533.A0A098DRR8	pp	229533.I1RZS6
128280	229533.A0A098DRR8	pp	229533.I1RU69
128281	229533.A0A098DRR8	pp	229533.I1S6F0
128282	229533.A0A098DRR8	pp	229533.A0A1C3YHX8
128283	229533.A0A098DRR8	pp	229533.A0A1C3YN63
128284	229533.A0A098DRR8	pp	229533.I1RBT1
128285	229533.A0A098DRR8	pp	229533.A0A0E0S0W9
128286	229533.A0A098DRR8	pp	229533.A0A1C3YKC0
128287	229533.A0A098DRR8	pp	229533.I1RT05
128288	229533.A0A098DRR8	pp	229533.I1RK81
128289	229533.A0A098DRR8	pp	229533.I1RNH1
128290	229533.A0A098DRR8	pp	229533.I1RX24
128291	229533.A0A098DRR8	pp	229533.A0A1C3YK72
128292	229533.A0A098DRR8	pp	229533.A0A098DVS9
128293	229533.A0A098DRR8	pp	229533.V6QUW4
128294	229533.A0A098DRR8	pp	229533.I1S253
128295	229533.A0A098DRR8	pp	229533.I1RS30
128296	229533.A0A098DRR8	pp	229533.I1RDA0
128297	229533.A0A098DRR8	pp	229533.I1RN67
128298	229533.A0A098DRR8	pp	229533.I1RBN1
128299	229533.A0A098DRR8	pp	229533.I1S6U8
128300	229533.A0A098DRR8	pp	229533.I1RC29
128301	229533.A0A098DRR8	pp	229533.I1RNA0
128302	229533.A0A098DRR8	pp	229533.I1RRP3
128303	229533.A0A098DRR8	pp	229533.I1RA02
128304	229533.A0A098DRR8	pp	229533.V6QYF0
128305	229533.A0A098DRR8	pp	229533.A0A1C3YIP5
128306	229533.A0A098DRR8	pp	229533.A0A0E0SA11
128307	229533.A0A098DRR8	pp	229533.I1RMK3
128308	229533.A0A098DRR8	pp	229533.I1REN8
128309	229533.A0A098DRR8	pp	229533.Q4HVW2
128310	229533.A0A098DRR8	pp	229533.I1RBG9
128311	229533.A0A098DRR8	pp	229533.I1RAC2
128312	229533.A0A098DRR8	pp	229533.Q4IEV5
128313	229533.A0A098DRR8	pp	229533.I1RQK4
128314	229533.A0A098DRR8	pp	229533.I1RW25
128315	229533.A0A098DRR8	pp	229533.I1R9M0
128316	229533.A0A098DRR8	pp	229533.I1S9F8
128317	229533.A0A098DRR8	pp	229533.A0A1C3YIN1
128318	229533.A0A098DRR8	pp	229533.I1RR73
128319	229533.A0A098DRR8	pp	229533.V6R3X5
128320	229533.A0A098DRR8	pp	229533.I1RQ65
128321	229533.A0A098DRR8	pp	229533.I1S2A8
128322	229533.A0A098DRR8	pp	229533.I1RD56
128323	229533.A0A098DRR8	pp	229533.A0A098DXE2
128324	229533.A0A098DRR8	pp	229533.I1RJZ7
128325	229533.A0A098DRR8	pp	229533.I1RMN6
128326	229533.A0A098DRR8	pp	229533.A0A1C3YKM9
128327	229533.A0A098DRR8	pp	229533.I1RPH9
128328	229533.A0A098DRR8	pp	229533.I1RZ39
128329	229533.A0A098DRR8	pp	229533.Q4IFI0
128330	229533.A0A098DRR8	pp	229533.V6QUE6
128331	229533.A0A098DRR8	pp	229533.I1S2B8
128332	229533.A0A098DRR8	pp	229533.I1RTF4
128333	229533.A0A098DRR8	pp	229533.V6RGN5
128334	229533.A0A098DRR8	pp	229533.A0A0E0RZN1

128335 229533.A0A098DRR8 pp 229533.Q4IJH1
128336 229533.A0A098DRR8 pp 229533.I1RG11
128337 229533.A0A098DRR8 pp 229533.I1RNV6
128338 229533.A0A098DRR8 pp 229533.A0A0E0RUI4
128339 229533.A0A098DRR8 pp 229533.I1RNY2
128340 229533.A0A098DRR8 pp 229533.I1RTG6
128341 229533.A0A098DRR8 pp 229533.Q4I327
128342 229533.A0A098DRR8 pp 229533.I1RC27
128343 229533.A0A098DRR8 pp 229533.I1RT80
128344 229533.A0A098DRR8 pp 229533.A0A1C3YL07
128345 229533.A0A098DRR8 pp 229533.A0A0E0S621
128346 229533.A0A098DRR8 pp 229533.I1RMN7
128347 229533.A0A098DRR8 pp 229533.I1S0J1
128348 229533.A0A098DRR8 pp 229533.I1RNH6
128349 229533.A0A098DRR8 pp 229533.I1RC72
128350 229533.A0A098DRR8 pp 229533.I1S021
128351 229533.A0A098DRR8 pp 229533.I1S060
128352 229533.A0A098DRR8 pp 229533.I1RRT8
128353 229533.A0A098DRR8 pp 229533.I1RNC1
128354 229533.A0A098DRR8 pp 229533.I1RX14
128355 229533.A0A098DRR8 pp 229533.I1RMC0
128356 229533.A0A098DRR8 pp 229533.I1RM67
128357 229533.A0A098DRR8 pp 229533.A0A1C3YK34
128358 229533.A0A098DRR8 pp 229533.A0A0E0SJS1
128359 229533.A0A098DRR8 pp 229533.I1S9Y1
128360 229533.A0A098DRR8 pp 229533.I1RKJ6
128361 229533.A0A098DRR8 pp 229533.Q4IAA0
128362 229533.A0A098DRR8 pp 229533.I1RPU0
128363 229533.A0A098DRR8 pp 229533.I1RQ31
128364 229533.A0A098DRR8 pp 229533.I1RPP4
128365 229533.A0A098DRR8 pp 229533.A0A1C3YJG0
128366 229533.A0A098DRR8 pp 229533.I1RCR8
128367 229533.A0A098DRR8 pp 229533.I1RB27
128368 229533.A0A098DRR8 pp 229533.I1RPW9
128369 229533.A0A098DRR8 pp 229533.Q4IN63
128370 229533.A0A098DRR8 pp 229533.I1R9X7
128371 229533.A0A098DRR8 pp 229533.I1RSQ3
128372 229533.A0A098DRR8 pp 229533.V6R042
128373 229533.A0A098DRR8 pp 229533.I1S9U2
128374 229533.A0A098DRR8 pp 229533.A0A0E0RSD7
128375 229533.A0A098DRR8 pp 229533.I1RD64
128376 229533.A0A098DRR8 pp 229533.I1RUN8
128377 229533.A0A098DRR8 pp 229533.Q4IJ56
128378 229533.A0A098DRR8 pp 229533.I1RWJ4
128379 229533.A0A098DRR8 pp 229533.V6RMW6
128380 229533.A0A098DRR8 pp 229533.I1S0M9
128381 229533.A0A098DRR8 pp 229533.I1RX08
128382 229533.A0A098DRR8 pp 229533.I1RG51
128383 229533.A0A098DRR8 pp 229533.A0A098DVE3
128384 229533.A0A098DRR8 pp 229533.I1RNU6
128385 229533.A0A098DRR8 pp 229533.Q4I662
128386 229533.A0A098DRR8 pp 229533.I1RM25
128387 229533.A0A098DRR8 pp 229533.I1RFS3
128388 229533.A0A098DRR8 pp 229533.I1RSI6
128389 229533.A0A098DRR8 pp 229533.I1RAU5
128390 229533.A0A098DRR8 pp 229533.I1RD22
128391 229533.A0A098DRR8 pp 229533.Q4IEK8
128392 229533.A0A098DRR8 pp 229533.Q4IBS2
128393 229533.A0A098DRR8 pp 229533.I1RM96
128394 229533.A0A098DRR8 pp 229533.I1RW61
128395 229533.A0A098DRR8 pp 229533.I1RNQ5
128396 229533.A0A098DRR8 pp 229533.I1RXZ9
128397 229533.A0A098DRR8 pp 229533.I1RC32
128398 229533.A0A098DRR8 pp 229533.I1RLU6
128399 229533.A0A098DRR8 pp 229533.I1RRI2
128400 229533.A0A098DRR8 pp 229533.I1RC33
128401 229533.A0A098DRR8 pp 229533.V6RBN2
128402 229533.A0A098DRR8 pp 229533.A0A0E0SJN2
128403 229533.A0A098DRR8 pp 229533.I1S1C7
128404 229533.A0A098DRR8 pp 229533.I1RY44
128405 229533.A0A098DRR8 pp 229533.I1RTD7
128406 229533.A0A098DRR8 pp 229533.I1S2E8
128407 229533.A0A098DRR8 pp 229533.I1RZM1

128408 229533.A0A098DRR8 pp 229533.I1RJZ6
128409 229533.A0A098DRR8 pp 229533.I1RSD8
128410 229533.A0A098DRR8 pp 229533.I1RSR8
128411 229533.A0A098DRR8 pp 229533.I1RWN5
128412 229533.A0A098DRR8 pp 229533.I1RY03
128413 229533.A0A098DRR8 pp 229533.I1RZK5
128414 229533.A0A098DRR8 pp 229533.I1RNF5
128415 229533.A0A098DRR8 pp 229533.I1RQA3
128416 229533.A0A098DRR8 pp 229533.Q4IJK2
128417 229533.A0A098DRR8 pp 229533.I1S4T3
128418 229533.A0A098DRR8 pp 229533.I1RN99
128419 229533.A0A098DRR8 pp 229533.I1RTE9
128420 229533.A0A098DRR8 pp 229533.I1RS64
128421 229533.A0A098DRR8 pp 229533.I1S2C0
128422 229533.A0A098DRR8 pp 229533.I1RQ28
128423 229533.A0A098DRR8 pp 229533.I1RG56
128424 229533.A0A098DRR8 pp 229533.A0A1C3YK36
128425 229533.A0A098DRR8 pp 229533.I1RK95
128426 229533.A0A098DRR8 pp 229533.I1RA70
128427 229533.A0A098DRR8 pp 229533.I1RDG0
128428 229533.A0A098DRR8 pp 229533.I1RPY1
128429 229533.A0A098DRR8 pp 229533.I1RT03
128430 229533.A0A098DRR8 pp 229533.I1RSG8
128431 229533.A0A098DRR8 pp 229533.I1RRR7
128432 229533.A0A098DRR8 pp 229533.I1RFU8
128433 229533.A0A098DRR8 pp 229533.I1RTH6
128434 229533.A0A098DRR8 pp 229533.I1R9V7
128435 229533.A0A098DRR8 pp 229533.I1RVX0
128436 229533.A0A098DRR8 pp 229533.V6R227
128437 229533.A0A098DRR8 pp 229533.I1S1X9
128438 229533.A0A098DRR8 pp 229533.I1RBZ3
128439 229533.A0A098DRR8 pp 229533.A0A1C3YKW9
128440 229533.A0A098DRR8 pp 229533.I1RCL3
128441 229533.A0A098DRR8 pp 229533.I1RD03
128442 229533.A0A098DRR8 pp 229533.I1RSS4
128443 229533.A0A098DRR8 pp 229533.I1RWT6
128444 229533.A0A098DRR8 pp 229533.I1RSC6
128445 229533.A0A098DRR8 pp 229533.I1RJV3
128446 229533.A0A098DRR8 pp 229533.I1RZF6
128447 229533.A0A098DRR8 pp 229533.A0A1C3YJR3
128448 229533.A0A098DRR8 pp 229533.I1RWJ3
128449 229533.A0A098DRR8 pp 229533.I1RA48
128450 229533.A0A098DRR8 pp 229533.I1RZE5
128451 229533.A0A098DRR8 pp 229533.I1S9U1
128452 229533.A0A098DRR8 pp 229533.I1RFJ9
128453 229533.A0A098DRR8 pp 229533.A0A1C3YIJ3
128454 229533.A0A098DRR8 pp 229533.I1RE00
128455 229533.A0A098DRR8 pp 229533.I1RF19
128456 229533.A0A098DRR8 pp 229533.I1RNK2
128457 229533.A0A098DRR8 pp 229533.I1RVZ0
128458 229533.A0A098DRR8 pp 229533.I1RQA2
128459 229533.A0A098DRR8 pp 229533.I1RCN5
128460 229533.A0A098DRR8 pp 229533.I1S558
128461 229533.A0A098DRR8 pp 229533.A0A0E0RP78
128462 229533.A0A098DRR8 pp 229533.I1RWN6
128463 229533.A0A098DRR8 pp 229533.I1S504
128464 229533.A0A098DRR8 pp 229533.I1RRY9
128465 229533.A0A098DRR8 pp 229533.I1RAZ8
128466 229533.A0A098DRR8 pp 229533.I1S1J6
128467 229533.A0A098DRR8 pp 229533.I1RKC6
128468 229533.A0A098DRR8 pp 229533.I1RAW0
128469 229533.A0A098DRR8 pp 229533.V6RGY3
128470 229533.A0A098DRR8 pp 229533.I1RSR3
128471 229533.A0A098DRR8 pp 229533.I1RZ35
128472 229533.A0A098DRR8 pp 229533.I1S1U7
128473 229533.A0A098DRR8 pp 229533.I1RBL7
128474 229533.A0A098DRR8 pp 229533.I1S274
128475 229533.A0A098DRR8 pp 229533.I1RAU7
128476 229533.A0A098DRR8 pp 229533.A0A1C3YLK9
128477 229533.A0A098DRR8 pp 229533.I1RCY5
128478 229533.A0A098DRR8 pp 229533.I1RF08
128479 229533.A0A098DRR8 pp 229533.I1RC06
128480 229533.A0A098DRR8 pp 229533.I1S296

128481 229533.A0A098DRR8 pp 229533.I1RK01
128482 229533.A0A098DRR8 pp 229533.I1RZK6
128483 229533.A0A098DRR8 pp 229533.Q4HZ68
128484 229533.A0A098DRR8 pp 229533.I1RZZ1
128485 229533.I1RPG8 pp 229533.I1RRC7
128486 229533.I1RPG8 pp 229533.I1RTP2
128487 229533.I1RPG8 pp 229533.Q4ICM0
128488 229533.I1RSK1 pp 229533.I1S5B0
128489 229533.I1RSK1 pp 229533.I1RW99
128490 229533.I1RSK1 pp 229533.I1RX46
128491 229533.I1RSK1 pp 229533.V6RTJ8
128492 229533.I1RSK1 pp 229533.I1S0N4
128493 229533.I1RSK1 pp 229533.V6RMW6
128494 229533.I1RSK1 pp 229533.I1S2E9
128495 229533.I1RSK1 pp 229533.I1S503
128496 229533.I1RSK1 pp 229533.I1RWT6
128497 229533.I1RSK1 pp 229533.I1RXD7
128498 229533.I1RSK1 pp 229533.I1RT04
128499 229533.I1RSK1 pp 229533.I1RSQ3
128500 229533.I1RSK1 pp 229533.V6R042
128501 229533.I1RSK1 pp 229533.I1S0R7
128502 229533.I1RSK1 pp 229533.I1S1W4
128503 229533.I1RSK1 pp 229533.I1S2G7
128504 229533.I1RSK1 pp 229533.I1S504
128505 229533.I1RSK1 pp 229533.I1RTD5
128506 229533.I1RSK1 pp 229533.I1RT40
128507 229533.I1RSK1 pp 229533.I1S0M2
128508 229533.I1RSK1 pp 229533.I1RVQ0
128509 229533.I1RSK1 pp 229533.I1RZM1
128510 229533.I1RSK1 pp 229533.Q4ICA8
128511 229533.I1RSK1 pp 229533.I1RUG3
128512 229533.I1RSK1 pp 229533.I1RYY5
128513 229533.I1RSK1 pp 229533.I1RTA0
128514 229533.I1RSK1 pp 229533.I1RY03
128515 229533.I1RSK1 pp 229533.I1RWK3
128516 229533.I1RSK1 pp 229533.I1RY44
128517 229533.I1RSK1 pp 229533.I1RWN6
128518 229533.I1RSK1 pp 229533.I1S1C7
128519 229533.I1RSK1 pp 229533.V6RIV9
128520 229533.I1RSK1 pp 229533.I1RWN5
128521 229533.I1RSK1 pp 229533.I1RZZ9
128522 229533.I1RSK1 pp 229533.I1RYW3
128523 229533.I1RSK1 pp 229533.I1RSQ7
128524 229533.I1RSK1 pp 229533.I1S2H4
128525 229533.I1RSK1 pp 229533.I1S8P5
128526 229533.I1RSK1 pp 229533.I1RWI5
128527 229533.I1RSK1 pp 229533.I1RWL2
128528 229533.I1RSK1 pp 229533.I1RSV2
128529 229533.I1RSK1 pp 229533.V6R3X5
128530 229533.I1RSK1 pp 229533.I1RZK5
128531 229533.I1RSK1 pp 229533.I1RW25
128532 229533.I1RSK1 pp 229533.I1S0J1
128533 229533.I1RSK1 pp 229533.Q4I2J8
128534 229533.I1RSK1 pp 229533.I1RTH6
128535 229533.I1RSK1 pp 229533.I1RYH9
128536 229533.I1RSK1 pp 229533.I1RZ39
128537 229533.I1RSK1 pp 229533.V6RBN2
128538 229533.I1RSK1 pp 229533.I1RZE4
128539 229533.I1RSK1 pp 229533.I1S2B8
128540 229533.I1RSK1 pp 229533.I1RZZ1
128541 229533.I1RSK1 pp 229533.I1RVX0
128542 229533.I1RSK1 pp 229533.Q4IJK2
128543 229533.I1RSK1 pp 229533.Q4IJM4
128544 229533.I1RSK1 pp 229533.I1RT03
128545 229533.I1RD53 pp 229533.I1RX17
128546 229533.I1RD53 pp 229533.I1RSV9
128547 229533.I1RD53 pp 229533.I1S1F3
128548 229533.A0A098DVS9 pp 229533.I1RMN6
128549 229533.A0A098DVS9 pp 229533.I1RGC2
128550 229533.A0A098DVS9 pp 229533.V6QUE6
128551 229533.A0A098DVS9 pp 229533.V6RGN5
128552 229533.A0A098DVS9 pp 229533.I1S044
128553 229533.A0A098DVS9 pp 229533.I1RUN9

128554 229533.A0A098DVS9 pp 229533.I1RG11
128555 229533.A0A098DVS9 pp 229533.I1RTG6
128556 229533.A0A098DVS9 pp 229533.I1RC27
128557 229533.A0A098DVS9 pp 229533.A0A0E0S621
128558 229533.A0A098DVS9 pp 229533.Q4I327
128559 229533.A0A098DVS9 pp 229533.I1S0J1
128560 229533.A0A098DVS9 pp 229533.I1RC72
128561 229533.A0A098DVS9 pp 229533.I1RNH6
128562 229533.A0A098DVS9 pp 229533.I1S021
128563 229533.A0A098DVS9 pp 229533.I1S060
128564 229533.A0A098DVS9 pp 229533.I1RYB9
128565 229533.A0A098DVS9 pp 229533.I1RX14
128566 229533.A0A098DVS9 pp 229533.I1RMC0
128567 229533.A0A098DVS9 pp 229533.Q4IAA0
128568 229533.A0A098DVS9 pp 229533.I1RQ31
128569 229533.A0A098DVS9 pp 229533.I1RB27
128570 229533.A0A098DVS9 pp 229533.A0A1C3YK36
128571 229533.A0A098DVS9 pp 229533.I1R9X7
128572 229533.A0A098DVS9 pp 229533.I1RCR8
128573 229533.A0A098DVS9 pp 229533.Q4IN63
128574 229533.A0A098DVS9 pp 229533.A0A1C3YJ29
128575 229533.A0A098DVS9 pp 229533.V6R042
128576 229533.A0A098DVS9 pp 229533.V6R227
128577 229533.A0A098DVS9 pp 229533.I1RRR7
128578 229533.A0A098DVS9 pp 229533.I1RZF6
128579 229533.A0A098DVS9 pp 229533.Q4IJ56
128580 229533.A0A098DVS9 pp 229533.A0A1C3YJR3
128581 229533.A0A098DVS9 pp 229533.I1S504
128582 229533.A0A098DVS9 pp 229533.V6RMW6
128583 229533.A0A098DVS9 pp 229533.I1S0M9
128584 229533.A0A098DVS9 pp 229533.I1RSI6
128585 229533.A0A098DVS9 pp 229533.I1RWB5
128586 229533.A0A098DVS9 pp 229533.A0A0E0RV94
128587 229533.A0A098DVS9 pp 229533.I1RAU5
128588 229533.A0A098DVS9 pp 229533.I1RNU6
128589 229533.A0A098DVS9 pp 229533.I1RRI2
128590 229533.A0A098DVS9 pp 229533.I1RD22
128591 229533.A0A098DVS9 pp 229533.I1RM25
128592 229533.A0A098DVS9 pp 229533.Q4IBS2
128593 229533.A0A098DVS9 pp 229533.I1S1C7
128594 229533.A0A098DVS9 pp 229533.Q4IEK8
128595 229533.A0A098DVS9 pp 229533.I1RMD5
128596 229533.A0A098DVS9 pp 229533.I1RQA2
128597 229533.A0A098DVS9 pp 229533.I1RM96
128598 229533.A0A098DVS9 pp 229533.I1RCN5
128599 229533.A0A098DVS9 pp 229533.I1RAN5
128600 229533.A0A098DVS9 pp 229533.I1RNE4
128601 229533.A0A098DVS9 pp 229533.I1RNQ5
128602 229533.A0A098DVS9 pp 229533.I1RSR8
128603 229533.A0A098DVS9 pp 229533.I1RWN6
128604 229533.A0A098DVS9 pp 229533.I1RLU6
128605 229533.A0A098DVS9 pp 229533.I1RRY9
128606 229533.A0A098DVS9 pp 229533.I1RY03
128607 229533.A0A098DVS9 pp 229533.I1RY44
128608 229533.A0A098DVS9 pp 229533.I1RAC1
128609 229533.A0A098DVS9 pp 229533.I1RZK5
128610 229533.A0A098DVS9 pp 229533.I1RAW0
128611 229533.A0A098DVS9 pp 229533.V6RGY3
128612 229533.A0A098DVS9 pp 229533.I1RWN5
128613 229533.A0A098DVS9 pp 229533.I1RSR3
128614 229533.A0A098DVS9 pp 229533.I1RBL7
128615 229533.A0A098DVS9 pp 229533.I1RRE0
128616 229533.A0A098DVS9 pp 229533.I1RPJ3
128617 229533.A0A098DVS9 pp 229533.I1RTE9
128618 229533.A0A098DVS9 pp 229533.I1S2C0
128619 229533.A0A098DVS9 pp 229533.I1S4T3
128620 229533.A0A098DVS9 pp 229533.I1RK83
128621 229533.A0A098DVS9 pp 229533.I1RZK6
128622 229533.A0A098DVS9 pp 229533.I1RG56
128623 229533.A0A098DVS9 pp 229533.I1RZZ1
128624 229533.A0A098DVS9 pp 229533.I1RFU8
128625 229533.A0A098DVS9 pp 229533.I1RTH6
128626 229533.A0A098DVS9 pp 229533.I1RYB1

229533.A0A098DVS9	pp	229533.I1RZB5
229533.A0A098DVS9	pp	229533.I1S2E8
229533.A0A098DVS9	pp	229533.I1RSI0
229533.A0A098DVS9	pp	229533.A0A1C3YIJ3
229533.A0A098DVS9	pp	229533.I1RJZ6
229533.A0A098DVS9	pp	229533.I1RBZ3
229533.A0A098DVS9	pp	229533.I1RE00
229533.A0A098DVS9	pp	229533.I1RCL3
229533.A0A098DVS9	pp	229533.I1RLP2
229533.A0A098DVS9	pp	229533.I1RSD8
229533.A0A098DVS9	pp	229533.I1RNK2
229533.A0A098DVS9	pp	229533.I1RN99
229533.A0A098DVS9	pp	229533.I1RK95
229533.A0A098DVS9	pp	229533.I1RJV3
229533.A0A098DVS9	pp	229533.I1RVZ0
229533.A0A098DVS9	pp	229533.I1RX46
229533.A0A098DVS9	pp	229533.I1RAZ8
229533.A0A098DVS9	pp	229533.I1RDG0
229533.A0A098DVS9	pp	229533.I1RT03
229533.A0A098DVS9	pp	229533.I1R9V7
229533.A0A098DVS9	pp	229533.I1RVX0
229533.A0A098DVS9	pp	229533.I1RRT2
229533.A0A098DVS9	pp	229533.I1RKC6
229533.A0A098DVS9	pp	229533.I1RQS5
229533.A0A098DVS9	pp	229533.I1RY63
229533.A0A098DVS9	pp	229533.I1S0Z9
229533.A0A098DVS9	pp	229533.I1RAU7
229533.A0A098DVS9	pp	229533.I1S1X9
229533.A0A098DVS9	pp	229533.I1RJL4
229533.A0A098DVS9	pp	229533.I1RF08
229533.A0A098DVS9	pp	229533.Q4IPI1
229533.A0A098DVS9	pp	229533.I1RC06
229533.A0A098DVS9	pp	229533.I1RSS4
229533.A0A098DVS9	pp	229533.I1RA48
229533.A0A098DVS9	pp	229533.Q4HWE2
229533.A0A098DVS9	pp	229533.Q4IAS1
229533.A0A098DVS9	pp	229533.Q4HZ68
229533.A0A098DVS9	pp	229533.I1RLV9
229533.A0A098DVS9	pp	229533.Q4IR18
229533.A0A098DVS9	pp	229533.Q4I5R9
229533.A0A098DVS9	pp	229533.I1RSX1
229533.A0A098DVS9	pp	229533.A0A1C3YMK1
229533.A0A098DVS9	pp	229533.I1RW71
229533.A0A098DVS9	pp	229533.I1RTD5
229533.A0A098DVS9	pp	229533.I1S503
229533.A0A098DVS9	pp	229533.A0A0E0RNT7
229533.A0A098DVS9	pp	229533.Q4IQW0
229533.A0A098DVS9	pp	229533.I1RKL3
229533.A0A098DVS9	pp	229533.I1RPN4
229533.A0A098DVS9	pp	229533.Q4INZ9
229533.A0A098DVS9	pp	229533.I1RSV2
229533.A0A098DVS9	pp	229533.I1RZJ6
229533.A0A098DVS9	pp	229533.A0A1C3YL39
229533.A0A098DVS9	pp	229533.A0A1C3YM43
229533.A0A098DVS9	pp	229533.I1RFN1
229533.A0A098DVS9	pp	229533.I1RFG0
229533.A0A098DVS9	pp	229533.I1RRQ3
229533.A0A098DVS9	pp	229533.I1RT98
229533.A0A098DVS9	pp	229533.A0A0E0S6M0
229533.A0A098DVS9	pp	229533.I1S6F0
229533.A0A098DVS9	pp	229533.I1RSL9
229533.A0A098DVS9	pp	229533.I1RBC7
229533.A0A098DVS9	pp	229533.I1RGA4
229533.A0A098DVS9	pp	229533.I1RAF8
229533.A0A098DVS9	pp	229533.I1RAX6
229533.A0A098DVS9	pp	229533.I1RC82
229533.A0A098DVS9	pp	229533.A0A1C3YN63
229533.A0A098DVS9	pp	229533.I1RNH1
229533.A0A098DVS9	pp	229533.I1RXD7
229533.A0A098DVS9	pp	229533.A0A1C3YK72
229533.A0A098DVS9	pp	229533.Q4HZ47
229533.A0A098DVS9	pp	229533.Q4HYQ4
229533.A0A098DVS9	pp	229533.I1S2E9

128700	229533.A0A098DVS9	pp	229533.I1RFW5
128701	229533.A0A098DVS9	pp	229533.A0A1C3YLF5
128702	229533.A0A098DVS9	pp	229533.A0A0E0SFN0
128703	229533.A0A098DVS9	pp	229533.I1RWD4
128704	229533.A0A098DVS9	pp	229533.V6R283
128705	229533.A0A098DVS9	pp	229533.A0A1C3YIP5
128706	229533.A0A098DVS9	pp	229533.I1RQ65
128707	229533.A0A098DVS9	pp	229533.Q4HXF0
128708	229533.A0A098DVS9	pp	229533.I1RQ48
128709	229533.A0A098DVS9	pp	229533.I1RWH5
128710	229533.A0A098DVS9	pp	229533.A0A1C3YKK5
128711	229533.A0A098DVS9	pp	229533.I1S0R7
128712	229533.A0A098DVS9	pp	229533.I1RC03
128713	229533.A0A098DVS9	pp	229533.A0A1C3YHW7
128714	229533.A0A098DVS9	pp	229533.I1RRH5
128715	229533.A0A098DVS9	pp	229533.I1RTI6
128716	229533.A0A098DVS9	pp	229533.I1RS47
128717	229533.A0A098DVS9	pp	229533.I1RQJ5
128718	229533.A0A098DVS9	pp	229533.I1RT04
128719	229533.A0A098DVS9	pp	229533.I1RDA0
128720	229533.A0A098DVS9	pp	229533.I1RPP3
128721	229533.A0A098DVS9	pp	229533.I1RBN1
128722	229533.A0A098DVS9	pp	229533.A0A0E0SA11
128723	229533.A0A098DVS9	pp	229533.A0A0E0SFM9
128724	229533.A0A098DVS9	pp	229533.I1RRJ8
128725	229533.A0A098DVS9	pp	229533.I1RSQ7
128726	229533.A0A098DVS9	pp	229533.I1RD56
128727	229533.A0A098DVS9	pp	229533.I1RT33
128728	229533.A0A098DVS9	pp	229533.Q4HVW2
128729	229533.A0A098DVS9	pp	229533.Q4HZ42
128730	229533.A0A098DVS9	pp	229533.A0A1C3YIN1
128731	229533.A0A098DVS9	pp	229533.Q4I830
128732	229533.A0A098DVS9	pp	229533.I1RR73
128733	229533.A0A098DVS9	pp	229533.I1RNT2
128734	229533.A0A098DVS9	pp	229533.I1S0F1
128735	229533.A0A098DVS9	pp	229533.V6R3X5
128736	229533.A0A098DVS9	pp	229533.I1RQM6
128737	229533.A0A098DVS9	pp	229533.I1RND6
128738	229533.A0A098DVS9	pp	229533.A0A1C3YKM9
128739	229533.A0A098DVS9	pp	229533.I1RU69
128740	229533.A0A098DVS9	pp	229533.I1RZ39
128741	229533.A0A098DVS9	pp	229533.A0A1C3YHX8
128742	229533.A0A098DVS9	pp	229533.Q4IF76
128743	229533.A0A098DVS9	pp	229533.Q4IJH1
128744	229533.A0A098DVS9	pp	229533.I1RG51
128745	229533.A0A098DVS9	pp	229533.I1RNV6
128746	229533.A0A098DVS9	pp	229533.I1REX4
128747	229533.A0A098DVS9	pp	229533.Q4I662
128748	229533.A0A098DVS9	pp	229533.I1RT80
128749	229533.A0A098DVS9	pp	229533.A0A1C3YIY6
128750	229533.A0A098DVS9	pp	229533.I1RFS3
128751	229533.A0A098DVS9	pp	229533.I1RW61
128752	229533.A0A098DVS9	pp	229533.I1S8E2
128753	229533.A0A098DVS9	pp	229533.I1RFQ7
128754	229533.A0A098DVS9	pp	229533.I1RM06
128755	229533.A0A098DVS9	pp	229533.I1RZS6
128756	229533.A0A098DVS9	pp	229533.O74638
128757	229533.A0A098DVS9	pp	229533.I1RX24
128758	229533.A0A098DVS9	pp	229533.I1RM67
128759	229533.A0A098DVS9	pp	229533.V6QYF0
128760	229533.A0A098DVS9	pp	229533.I1S9Y1
128761	229533.A0A098DVS9	pp	229533.I1RBG9
128762	229533.A0A098DVS9	pp	229533.I1RAC2
128763	229533.A0A098DVS9	pp	229533.I1RZV6
128764	229533.A0A098DVS9	pp	229533.A0A1C3YHT9
128765	229533.A0A098DVS9	pp	229533.I1RW25
128766	229533.A0A098DVS9	pp	229533.I1RKJ6
128767	229533.A0A098DVS9	pp	229533.A0A098DXE2
128768	229533.A0A098DVS9	pp	229533.Q4IFI0
128769	229533.A0A098DVS9	pp	229533.I1RPU0
128770	229533.A0A098DVS9	pp	229533.I1S0L4
128771	229533.A0A098DVS9	pp	229533.I1RJZ7
128772	229533.A0A098DVS9	pp	229533.I1S2B8

128773 229533.A0A098DVS9 pp 229533.I1RPW9
128774 229533.A0A098DVS9 pp 229533.A0A1C3YIS7
128775 229533.A0A098DVS9 pp 229533.I1S9U2
128776 229533.A0A098DVS9 pp 229533.I1RD64
128777 229533.A0A098DVS9 pp 229533.A0A0E0RUI4
128778 229533.A0A1C3YIP5 pp 229533.I1RAX6
128779 229533.A0A1C3YIP5 pp 229533.V6RTJ8
128780 229533.A0A1C3YIP5 pp 229533.I1RX46
128781 229533.A0A1C3YIP5 pp 229533.A0A1C3YJJ2
128782 229533.A0A1C3YIP5 pp 229533.Q4IPI1
128783 229533.A0A1C3YIP5 pp 229533.I1S0T2
128784 229533.A0A1C3YIP5 pp 229533.A0A1C3YMW0
128785 229533.A0A1C3YIP5 pp 229533.Q4I5R9
128786 229533.A0A1C3YIP5 pp 229533.I1RG53
128787 229533.A0A1C3YIP5 pp 229533.I1RKQ5
128788 229533.A0A1C3YIP5 pp 229533.I1RSQ7
128789 229533.A0A1C3YIP5 pp 229533.I1RFC0
128790 229533.A0A1C3YIP5 pp 229533.V6QW22
128791 229533.A0A1C3YIP5 pp 229533.I1RRX5
128792 229533.A0A1C3YIP5 pp 229533.I1RBC9
128793 229533.A0A1C3YIP5 pp 229533.I1RW71
128794 229533.A0A1C3YIP5 pp 229533.A0A1C3YK13
128795 229533.A0A1C3YIP5 pp 229533.V6RUT5
128796 229533.A0A1C3YIP5 pp 229533.I1RXD7
128797 229533.A0A1C3YIP5 pp 229533.I1S4S7
128798 229533.A0A1C3YIP5 pp 229533.I1RLP1
128799 229533.A0A1C3YIP5 pp 229533.I1RPN4
128800 229533.A0A1C3YIP5 pp 229533.I1RUQ2
128801 229533.A0A1C3YIP5 pp 229533.I1RQC9
128802 229533.A0A1C3YIP5 pp 229533.I1S0R7
128803 229533.A0A1C3YIP5 pp 229533.Q4HYQ4
128804 229533.A0A1C3YIP5 pp 229533.Q4IJT5
128805 229533.A0A1C3YIP5 pp 229533.A0A1C3YJL7
128806 229533.A0A1C3YIP5 pp 229533.I1RT98
128807 229533.A0A1C3YIP5 pp 229533.I1RFW5
128808 229533.A0A1C3YIP5 pp 229533.I1RRG3
128809 229533.A0A1C3YIP5 pp 229533.A0A1C3YL54
128810 229533.A0A1C3YIP5 pp 229533.I1RIL8
128811 229533.A0A1C3YIP5 pp 229533.Q4INZ9
128812 229533.A0A1C3YIP5 pp 229533.I1RBD5
128813 229533.A0A1C3YIP5 pp 229533.I1S7A8
128814 229533.A0A1C3YIP5 pp 229533.I1RAH0
128815 229533.A0A1C3YIP5 pp 229533.I1RLX7
128816 229533.A0A1C3YIP5 pp 229533.A0A1C3YM63
128817 229533.A0A1C3YIP5 pp 229533.I1S6F0
128818 229533.A0A1C3YIP5 pp 229533.I1RPU0
128819 229533.A0A1C3YIP5 pp 229533.I1RVQ0
128820 229533.A0A1C3YIP5 pp 229533.V6R1K4
128821 229533.A0A1C3YIP5 pp 229533.I1RM06
128822 229533.A0A1C3YIP5 pp 229533.V6R4J1
128823 229533.A0A1C3YIP5 pp 229533.V6QUW4
128824 229533.A0A1C3YIP5 pp 229533.I1S0D4
128825 229533.A0A1C3YIP5 pp 229533.I1RNH1
128826 229533.A0A1C3YIP5 pp 229533.I1RRQ6
128827 229533.A0A1C3YIP5 pp 229533.I1S0L3
128828 229533.A0A1C3YIP5 pp 229533.V6QYF0
128829 229533.A0A1C3YIP5 pp 229533.I1RWD4
128830 229533.A0A1C3YIP5 pp 229533.I1RD52
128831 229533.A0A1C3YIP5 pp 229533.I1S0F5
128832 229533.A0A1C3YIP5 pp 229533.A0A1C3YK72
128833 229533.A0A1C3YIP5 pp 229533.Q4HVW2
128834 229533.A0A1C3YIP5 pp 229533.I1RNA0
128835 229533.A0A1C3YIP5 pp 229533.I1RA85
128836 229533.A0A1C3YIP5 pp 229533.Q4IQK7
128837 229533.A0A1C3YIP5 pp 229533.I1RMK3
128838 229533.A0A1C3YIP5 pp 229533.I1RBN1
128839 229533.A0A1C3YIP5 pp 229533.I1RT33
128840 229533.A0A1C3YIP5 pp 229533.I1RDA0
128841 229533.A0A1C3YIP5 pp 229533.A0A1C3YLZ7
128842 229533.A0A1C3YIP5 pp 229533.I1RG51
128843 229533.A0A1C3YIP5 pp 229533.I1S021
128844 229533.A0A1C3YIP5 pp 229533.I1RT80
128845 229533.A0A1C3YIP5 pp 229533.I1RNH6

128846 229533.A0A1C3YIP5 pp 229533.I1RQ31
128847 229533.A0A1C3YIP5 pp 229533.I1RNV6
128848 229533.A0A1C3YIP5 pp 229533.I1RCS1
128849 229533.A0A1C3YIP5 pp 229533.I1RTF4
128850 229533.A0A1C3YIP5 pp 229533.Q4IJH1
128851 229533.A0A1C3YIP5 pp 229533.I1RMC0
128852 229533.A0A1C3YIP5 pp 229533.I1RC32
128853 229533.A0A1C3YIP5 pp 229533.I1RI02
128854 229533.A0A1C3YIP5 pp 229533.A0A1C3YLR6
128855 229533.A0A1C3YIP5 pp 229533.I1RXF0
128856 229533.A0A1C3YIP5 pp 229533.A0A1C3YN30
128857 229533.A0A1C3YIP5 pp 229533.I1RJU3
128858 229533.A0A1C3YIP5 pp 229533.I1RZM8
128859 229533.A0A1C3YIP5 pp 229533.I1RW07
128860 229533.A0A1C3YIP5 pp 229533.A0A1C3YJQ9
128861 229533.A0A1C3YIP5 pp 229533.A0A1C3YJZ9
128862 229533.A0A1C3YIP5 pp 229533.I1RW25
128863 229533.A0A1C3YIP5 pp 229533.I1RAC2
128864 229533.A0A1C3YIP5 pp 229533.I1S0F1
128865 229533.A0A1C3YIP5 pp 229533.I1S5B0
128866 229533.A0A1C3YIP5 pp 229533.I1RMC8
128867 229533.A0A1C3YIP5 pp 229533.I1RQM6
128868 229533.A0A1C3YIP5 pp 229533.I1RX25
128869 229533.A0A1C3YIP5 pp 229533.I1RW99
128870 229533.A0A1C3YIP5 pp 229533.I1RC17
128871 229533.A0A1C3YIP5 pp 229533.I1RLC7
128872 229533.A0A1C3YIP5 pp 229533.I1RN51
128873 229533.A0A1C3YIP5 pp 229533.I1RBT1
128874 229533.A0A1C3YIP5 pp 229533.I1R9Y4
128875 229533.A0A1C3YIP5 pp 229533.A0A1C3YKC0
128876 229533.A0A1C3YIP5 pp 229533.I1RRU9
128877 229533.A0A1C3YIP5 pp 229533.I1S0X6
128878 229533.A0A1C3YIP5 pp 229533.Q4IFI0
128879 229533.A0A1C3YIP5 pp 229533.I1S9U2
128880 229533.A0A1C3YIP5 pp 229533.I1RR51
128881 229533.A0A1C3YIP5 pp 229533.Q4IBS2
128882 229533.A0A1C3YIP5 pp 229533.A0A1C3YLU7
128883 229533.A0A1C3YIP5 pp 229533.I1RAU5
128884 229533.A0A1C3YIP5 pp 229533.I1RGC2
128885 229533.A0A1C3YIP5 pp 229533.V6RCG5
128886 229533.A0A1C3YIP5 pp 229533.I1RZJ5
128887 229533.A0A1C3YIP5 pp 229533.I1RR24
128888 229533.A0A1C3YIP5 pp 229533.V6QUE6
128889 229533.A0A1C3YIP5 pp 229533.I1RM67
128890 229533.A0A1C3YIP5 pp 229533.V6RCZ8
128891 229533.A0A1C3YIP5 pp 229533.I1RYI7
128892 229533.A0A1C3YIP5 pp 229533.I1RMG8
128893 229533.A0A1C3YIP5 pp 229533.I1S4S4
128894 229533.A0A1C3YIP5 pp 229533.A0A1C3YJH8
128895 229533.A0A1C3YIP5 pp 229533.Q4IAA0
128896 229533.A0A1C3YIP5 pp 229533.I1RWJ8
128897 229533.A0A1C3YIP5 pp 229533.I1S6K9
128898 229533.A0A1C3YIP5 pp 229533.A0A1C3YJW9
128899 229533.A0A1C3YIP5 pp 229533.Q4I8P5
128900 229533.A0A1C3YIP5 pp 229533.A0A1C3YK34
128901 229533.A0A1C3YIP5 pp 229533.I1RI82
128902 229533.A0A1C3YIP5 pp 229533.I1RF19
128903 229533.A0A1C3YIP5 pp 229533.I1RS18
128904 229533.A0A1C3YIP5 pp 229533.I1RB27
128905 229533.A0A1C3YIP5 pp 229533.Q4I283
128906 229533.A0A1C3YIP5 pp 229533.I1S0M9
128907 229533.A0A1C3YIP5 pp 229533.I1S4Q4
128908 229533.A0A1C3YIP5 pp 229533.A0A1C3YKV3
128909 229533.A0A1C3YIP5 pp 229533.I1RE00
128910 229533.A0A1C3YIP5 pp 229533.I1RSI0
128911 229533.A0A1C3YIP5 pp 229533.I1RFJ9
128912 229533.A0A1C3YIP5 pp 229533.A0A1C3YKB0
128913 229533.A0A1C3YIP5 pp 229533.V6R227
128914 229533.A0A1C3YIP5 pp 229533.I1RG14
128915 229533.A0A1C3YIP5 pp 229533.Q4IJ56
128916 229533.A0A1C3YIP5 pp 229533.I1RNK2
128917 229533.A0A1C3YIP5 pp 229533.I1RQ81
128918 229533.A0A1C3YIP5 pp 229533.I1RWT6

128919	229533.A0A1C3YIP5	pp	229533.Q4I5Z5
128920	229533.A0A1C3YIP5	pp	229533.I1RJ17
128921	229533.A0A1C3YIP5	pp	229533.Q4I2J8
128922	229533.A0A1C3YIP5	pp	229533.I1RZF6
128923	229533.A0A1C3YIP5	pp	229533.A0A1C3YKW9
128924	229533.A0A1C3YIP5	pp	229533.I1SA32
128925	229533.A0A1C3YIP5	pp	229533.I1S504
128926	229533.A0A1C3YIP5	pp	229533.I1S2E9
128927	229533.A0A1C3YIP5	pp	229533.I1RAZ8
128928	229533.A0A1C3YIP5	pp	229533.I1RZH4
128929	229533.A0A1C3YIP5	pp	229533.I1RNA5
128930	229533.A0A1C3YIP5	pp	229533.I1RBL7
128931	229533.A0A1C3YIP5	pp	229533.I1RXC4
128932	229533.A0A1C3YIP5	pp	229533.A0A1C3YLF5
128933	229533.A0A1C3YIP5	pp	229533.A0A1C3YJR3
128934	229533.A0A1C3YIP5	pp	229533.I1RT04
128935	229533.A0A1C3YIP5	pp	229533.I1RBJ5
128936	229533.A0A1C3YIP5	pp	229533.I1RPR9
128937	229533.A0A1C3YIP5	pp	229533.V6RUY9
128938	229533.A0A1C3YIP5	pp	229533.A0A1C3YKY6
128939	229533.A0A1C3YIP5	pp	229533.I1RLU6
128940	229533.A0A1C3YIP5	pp	229533.I1RNQ5
128941	229533.A0A1C3YIP5	pp	229533.I1RB53
128942	229533.A0A1C3YIP5	pp	229533.A0A1C3YL55
128943	229533.A0A1C3YIP5	pp	229533.Q4IEK8
128944	229533.A0A1C3YIP5	pp	229533.I1RYS0
128945	229533.A0A1C3YIP5	pp	229533.Q4HXF0
128946	229533.A0A1C3YIP5	pp	229533.Q4IR18
128947	229533.A0A1C3YIP5	pp	229533.A0A1C3YJ93
128948	229533.A0A1C3YIP5	pp	229533.I1RZM1
128949	229533.A0A1C3YIP5	pp	229533.I1S1J7
128950	229533.A0A1C3YIP5	pp	229533.I1RI03
128951	229533.A0A1C3YIP5	pp	229533.I1RC16
128952	229533.A0A1C3YIP5	pp	229533.I1RTD5
128953	229533.A0A1C3YIP5	pp	229533.I1RGF5
128954	229533.A0A1C3YIP5	pp	229533.A0A1C3YJI6
128955	229533.A0A1C3YIP5	pp	229533.A0A1C3YMK1
128956	229533.A0A1C3YIP5	pp	229533.A0A1C3YK36
128957	229533.A0A1C3YIP5	pp	229533.I1RG56
128958	229533.A0A1C3YIP5	pp	229533.I1RNF5
128959	229533.A0A1C3YIP5	pp	229533.I1S419
128960	229533.A0A1C3YIP5	pp	229533.I1RQA2
128961	229533.A0A1C3YIP5	pp	229533.I1RZK5
128962	229533.A0A1C3YIP5	pp	229533.I1RWB0
128963	229533.A0A1C3YIP5	pp	229533.I1RV91
128964	229533.A0A1C3YIP5	pp	229533.I1RFL3
128965	229533.A0A1C3YIP5	pp	229533.I1RT40
128966	229533.A0A1C3YIP5	pp	229533.I1RN99
128967	229533.A0A1C3YIP5	pp	229533.I1RCM5
128968	229533.A0A1C3YIP5	pp	229533.I1S887
128969	229533.A0A1C3YIP5	pp	229533.I1RDG0
128970	229533.A0A1C3YIP5	pp	229533.I1RQ28
128971	229533.A0A1C3YIP5	pp	229533.I1RP27
128972	229533.A0A1C3YIP5	pp	229533.I1RFU8
128973	229533.A0A1C3YIP5	pp	229533.I1RCG4
128974	229533.A0A1C3YIP5	pp	229533.I1RK95
128975	229533.A0A1C3YIP5	pp	229533.I1RJS2
128976	229533.A0A1C3YIP5	pp	229533.I1RJ74
128977	229533.A0A1C3YIP5	pp	229533.I1RZB2
128978	229533.A0A1C3YIP5	pp	229533.I1RY63
128979	229533.A0A1C3YIP5	pp	229533.I1RZB5
128980	229533.A0A1C3YIP5	pp	229533.I1R9V7
128981	229533.A0A1C3YIP5	pp	229533.A0A1C3YLK2
128982	229533.A0A1C3YIP5	pp	229533.I1S1B6
128983	229533.A0A1C3YIP5	pp	229533.I1RJV4
128984	229533.A0A1C3YIP5	pp	229533.I1RMD5
128985	229533.A0A1C3YIP5	pp	229533.I1RSC6
128986	229533.A0A1C3YIP5	pp	229533.I1RTD9
128987	229533.A0A1C3YIP5	pp	229533.I1RY03
128988	229533.A0A1C3YIP5	pp	229533.I1RBS6
128989	229533.A0A1C3YIP5	pp	229533.I1RZZ1
128990	229533.A0A1C3YIP5	pp	229533.I1RGA4
128991	229533.A0A1C3YIP5	pp	229533.I1RC82

128992	229533.A0A1C3YIP5	pp	229533.I1RSR8
128993	229533.A0A1C3YIP5	pp	229533.I1RZK6
128994	229533.A0A1C3YIP5	pp	229533.I1RBC7
128995	229533.A0A1C3YIP5	pp	229533.I1RU29
128996	229533.A0A1C3YIP5	pp	229533.I1RFN1
128997	229533.A0A1C3YIP5	pp	229533.I1RJZ6
128998	229533.A0A1C3YIP5	pp	229533.I1RQS5
128999	229533.A0A1C3YIP5	pp	229533.I1RRT2
129000	229533.A0A1C3YIP5	pp	229533.I1RRY9
129001	229533.A0A1C3YIP5	pp	229533.I1RSM3
129002	229533.A0A1C3YIP5	pp	229533.I1RSR3
129003	229533.A0A1C3YIP5	pp	229533.V6RBN2
129004	229533.A0A1C3YIP5	pp	229533.V6R3J1
129005	229533.A0A1C3YIP5	pp	229533.I1RQQ5
129006	229533.A0A1C3YIP5	pp	229533.I1RPP3
129007	229533.A0A1C3YIP5	pp	229533.I1RSX0
129008	229533.A0A1C3YIP5	pp	229533.Q4IJK2
129009	229533.A0A1C3YIP5	pp	229533.I1S8P5
129010	229533.A0A1C3YIP5	pp	229533.I1RQA3
129011	229533.A0A1C3YIP5	pp	229533.I1RJL4
129012	229533.A0A1C3YIP5	pp	229533.I1RLV9
129013	229533.A0A1C3YIP5	pp	229533.I1RM35
129014	229533.A0A1C3YIP5	pp	229533.I1RRZ0
129015	229533.A0A1C3YIP5	pp	229533.I1RYI5
129016	229533.A0A1C3YIP5	pp	229533.I1RF08
129017	229533.A0A1C3YIP5	pp	229533.Q4IAS1
129018	229533.A0A1C3YIP5	pp	229533.I1REK8
129019	229533.A0A1C3YIP5	pp	229533.I1RRJ8
129020	229533.A0A1C3YIP5	pp	229533.I1RCR5
129021	229533.A0A1C3YIP5	pp	229533.I1S352
129022	229533.A0A1C3YIP5	pp	229533.I1RLM7
129023	229533.A0A1C3YIP5	pp	229533.I1RLN6
129024	229533.A0A1C3YIP5	pp	229533.I1S503
129025	229533.A0A1C3YIP5	pp	229533.Q4I830
129026	229533.A0A1C3YIP5	pp	229533.A0A1C3YKN3
129027	229533.A0A1C3YIP5	pp	229533.I1RZ39
129028	229533.A0A1C3YIP5	pp	229533.I1RFT1
129029	229533.A0A1C3YIP5	pp	229533.I1RTA1
129030	229533.A0A1C3YIP5	pp	229533.I1RJW6
129031	229533.A0A1C3YIP5	pp	229533.I1S2M6
129032	229533.A0A1C3YIP5	pp	229533.A0A1C3YLK8
129033	229533.A0A1C3YIP5	pp	229533.I1RC29
129034	229533.A0A1C3YIP5	pp	229533.I1RRL9
129035	229533.A0A1C3YIP5	pp	229533.I1RQ23
129036	229533.A0A1C3YIP5	pp	229533.Q4I662
129037	229533.A0A1C3YIP5	pp	229533.I1RFH8
129038	229533.A0A1C3YIP5	pp	229533.I1RC66
129039	229533.A0A1C3YIP5	pp	229533.I1RVZ2
129040	229533.A0A1C3YIP5	pp	229533.I1RFS3
129041	229533.A0A1C3YIP5	pp	229533.I1RN63
129042	229533.A0A1C3YIP5	pp	229533.I1RWK3
129043	229533.A0A1C3YIP5	pp	229533.I1RNN1
129044	229533.A0A1C3YIP5	pp	229533.I1RPV3
129045	229533.A0A1C3YIP5	pp	229533.I1RSQ4
129046	229533.A0A1C3YIP5	pp	229533.I1S6Y8
129047	229533.A0A1C3YIP5	pp	229533.I1RPM4
129048	229533.A0A1C3YIP5	pp	229533.V6R283
129049	229533.A0A1C3YIP5	pp	229533.I1RZZ9
129050	229533.A0A1C3YIP5	pp	229533.I1RWI5
129051	229533.A0A1C3YIP5	pp	229533.I1RQ65
129052	229533.A0A1C3YIP5	pp	229533.I1RL04
129053	229533.A0A1C3YIP5	pp	229533.A0A1C3YIR8
129054	229533.A0A1C3YIP5	pp	229533.I1RW61
129055	229533.A0A1C3YIP5	pp	229533.I1RSZ7
129056	229533.A0A1C3YIP5	pp	229533.Q4IEV5
129057	229533.A0A1C3YIP5	pp	229533.Q4IF76
129058	229533.A0A1C3YIP5	pp	229533.I1RIL3
129059	229533.A0A1C3YIP5	pp	229533.I1RS47
129060	229533.A0A1C3YIP5	pp	229533.Q4HZ42
129061	229533.A0A1C3YIP5	pp	229533.A0A1C3YMA1
129062	229533.A0A1C3YIP5	pp	229533.I1RE27
129063	229533.A0A1C3YIP5	pp	229533.A0A1C3YIS7
129064	229533.A0A1C3YIP5	pp	229533.I1S0L4

129065 229533.A0A1C3YIP5 pp 229533.I1RC00
129066 229533.A0A1C3YIP5 pp 229533.I1S2H4
129067 229533.A0A1C3YIP5 pp 229533.I1RBY4
129068 229533.A0A1C3YIP5 pp 229533.A0A1C3YN63
129069 229533.A0A1C3YIP5 pp 229533.I1RP28
129070 229533.A0A1C3YIP5 pp 229533.I1RPU3
129071 229533.A0A1C3YIP5 pp 229533.I1RE76
129072 229533.A0A1C3YIP5 pp 229533.I1RS20
129073 229533.A0A1C3YIP5 pp 229533.I1R9M0
129074 229533.A0A1C3YIP5 pp 229533.I1RBS0
129075 229533.A0A1C3YIP5 pp 229533.I1RRM5
129076 229533.A0A1C3YIP5 pp 229533.I1RFG0
129077 229533.A0A1C3YIP5 pp 229533.I1RQS8
129078 229533.A0A1C3YIP5 pp 229533.I1RD64
129079 229533.A0A1C3YIP5 pp 229533.I1RRQ3
129080 229533.A0A1C3YIP5 pp 229533.I1RS30
129081 229533.A0A1C3YIP5 pp 229533.I1S2B8
129082 229533.A0A1C3YIP5 pp 229533.I1RJZ7
129083 229533.A0A1C3YIP5 pp 229533.I1RXC2
129084 229533.A0A1C3YIP5 pp 229533.V6RMW6
129085 229533.A0A1C3YIP5 pp 229533.I1RN67
129086 229533.A0A1C3YIP5 pp 229533.O74638
129087 229533.A0A1C3YIP5 pp 229533.I1RRP3
129088 229533.A0A1C3YIP5 pp 229533.I1S0F6
129089 229533.A0A1C3YIP5 pp 229533.I1RRT8
129090 229533.A0A1C3YIP5 pp 229533.I1RW26
129091 229533.A0A1C3YIP5 pp 229533.V6R042
129092 229533.A0A1C3YIP5 pp 229533.I1S5R1
129093 229533.A0A1C3YIP5 pp 229533.I1RSQ3
129094 229533.A0A1C3YIP5 pp 229533.A0A1C3YK73
129095 229533.A0A1C3YIP5 pp 229533.I1RA11
129096 229533.A0A1C3YIP5 pp 229533.I1S253
129097 229533.A0A1C3YIP5 pp 229533.A0A1C3YLD8
129098 229533.A0A1C3YIP5 pp 229533.I1RMG3
129099 229533.A0A1C3YIP5 pp 229533.A0A1C3YLS9
129100 229533.A0A1C3YIP5 pp 229533.I1RM82
129101 229533.A0A1C3YIP5 pp 229533.I1RRV1
129102 229533.A0A1C3YIP5 pp 229533.I1S9Y1
129103 229533.A0A1C3YIP5 pp 229533.V6R3X5
129104 229533.A0A1C3YIP5 pp 229533.I1RR73
129105 229533.A0A1C3YIP5 pp 229533.I1RKJ6
129106 229533.A0A1C3YIP5 pp 229533.A0A1C3YKM9
129107 229533.A0A1C3YIP5 pp 229533.I1RSI6
129108 229533.A0A1C3YIP5 pp 229533.I1RUQ9
129109 229533.A0A1C3YIP5 pp 229533.I1S0M6
129110 229533.A0A1C3YIP5 pp 229533.Q4I327
129111 229533.A0A1C3YIP5 pp 229533.I1RRR7
129112 229533.A0A1C3YIP5 pp 229533.I1RR72
129113 229533.A0A1C3YIP5 pp 229533.I1RUG3
129114 229533.A0A1C3YIP5 pp 229533.I1RZS6
129115 229533.A0A1C3YIP5 pp 229533.I1RRE5
129116 229533.A0A1C3YIP5 pp 229533.I1RWE8
129117 229533.A0A1C3YIP5 pp 229533.I1RX24
129118 229533.A0A1C3YIP5 pp 229533.I1RC33
129119 229533.A0A1C3YIP5 pp 229533.I1RBG9
129120 229533.A0A1C3YIP5 pp 229533.I1RXT3
129121 229533.A0A1C3YIP5 pp 229533.A0A1C3YJD4
129122 229533.A0A1C3YIP5 pp 229533.I1RE22
129123 229533.A0A1C3YIP5 pp 229533.I1RY44
129124 229533.A0A1C3YIP5 pp 229533.I1RNC1
129125 229533.A0A1C3YIP5 pp 229533.Q4IN63
129126 229533.A0A1C3YIP5 pp 229533.I1RXU9
129127 229533.A0A1C3YIP5 pp 229533.I1RM96
129128 229533.A0A1C3YIP5 pp 229533.A0A1C3YKY0
129129 229533.A0A1C3YIP5 pp 229533.I1RCL3
129130 229533.A0A1C3YIP5 pp 229533.I1RSK1
129131 229533.A0A1C3YIP5 pp 229533.I1S1W3
129132 229533.A0A1C3YIP5 pp 229533.I1RC81
129133 229533.A0A1C3YIP5 pp 229533.I1RCR8
129134 229533.A0A1C3YIP5 pp 229533.I1RFK3
129135 229533.A0A1C3YIP5 pp 229533.I1RJV3
129136 229533.A0A1C3YIP5 pp 229533.I1RB92
129137 229533.A0A1C3YIP5 pp 229533.I1S1W9

129138 229533.A0A1C3YIP5 pp 229533.I1S1X9
129139 229533.A0A1C3YIP5 pp 229533.I1S0N4
129140 229533.A0A1C3YIP5 pp 229533.V6RGN5
129141 229533.A0A1C3YIP5 pp 229533.V6RIV9
129142 229533.A0A1C3YIP5 pp 229533.I1RWN5
129143 229533.A0A1C3YIP5 pp 229533.I1RVX0
129144 229533.A0A1C3YIP5 pp 229533.I1R9D1
129145 229533.A0A1C3YIP5 pp 229533.I1RT03
129146 229533.A0A1C3YIP5 pp 229533.I1RC27
129147 229533.A0A1C3YIP5 pp 229533.I1RG11
129148 229533.A0A1C3YIP5 pp 229533.I1RWQ8
129149 229533.A0A1C3YIP5 pp 229533.I1RPY1
129150 229533.A0A1C3YIP5 pp 229533.Q4HZB8
129151 229533.A0A1C3YIP5 pp 229533.I1RRN5
129152 229533.A0A1C3YIP5 pp 229533.I1S5R6
129153 229533.A0A1C3YIP5 pp 229533.I1RXR1
129154 229533.A0A1C3YIP5 pp 229533.I1S2C0
129155 229533.A0A1C3YIP5 pp 229533.I1RRN2
129156 229533.A0A1C3YIP5 pp 229533.I1RSS4
129157 229533.A0A1C3YIP5 pp 229533.I1RPJ3
129158 229533.A0A1C3YIP5 pp 229533.I1RD22
129159 229533.A0A1C3YIP5 pp 229533.I1RCH5
129160 229533.A0A1C3YIP5 pp 229533.I1SA33
129161 229533.A0A1C3YIP5 pp 229533.I1RTE9
129162 229533.A0A1C3YIP5 pp 229533.I1RA70
129163 229533.A0A1C3YIP5 pp 229533.I1S2E8
129164 229533.A0A1C3YIP5 pp 229533.I1S8R3
129165 229533.A0A1C3YIP5 pp 229533.I1RMN6
129166 229533.A0A1C3YIP5 pp 229533.I1RMV0
129167 229533.A0A1C3YIP5 pp 229533.I1RQE7
129168 229533.A0A1C3YIP5 pp 229533.I1RN18
129169 229533.A0A1C3YIP5 pp 229533.I1RTH6
129170 229533.A0A1C3YIP5 pp 229533.I1RFL4
129171 229533.A0A1C3YIP5 pp 229533.I1RPP4
129172 229533.A0A1C3YIP5 pp 229533.I1R9X7
129173 229533.A0A1C3YIP5 pp 229533.V6RLM7
129174 229533.A0A1C3YIP5 pp 229533.I1RSD8
129175 229533.A0A1C3YIP5 pp 229533.I1RZ79
129176 229533.A0A1C3YIP5 pp 229533.I1RRI2
129177 229533.A0A1C3YIP5 pp 229533.I1RQ72
129178 229533.A0A1C3YIP5 pp 229533.I1RPK0
129179 229533.A0A1C3YIP5 pp 229533.I1RAU7
129180 229533.A0A1C3YIP5 pp 229533.I1RNB8
129181 229533.A0A1C3YIP5 pp 229533.V6R057
129182 229533.A0A1C3YIP5 pp 229533.I1RS17
129183 229533.A0A1C3YIP5 pp 229533.I1RNU6
129184 229533.A0A1C3YIP5 pp 229533.I1RQJ4
129185 229533.A0A1C3YIP5 pp 229533.I1RWX5
129186 229533.A0A1C3YIP5 pp 229533.I1RZW8
129187 229533.A0A1C3YIP5 pp 229533.I1S296
129188 229533.A0A1C3YIP5 pp 229533.I1S7W5
129189 229533.A0A1C3YIP5 pp 229533.Q4HZ68
129190 229533.A0A1C3YIP5 pp 229533.I1S0J1
129191 229533.A0A1C3YIP5 pp 229533.I1RC72
129192 229533.A0A1C3YIP5 pp 229533.I1S569
129193 229533.A0A1C3YIP5 pp 229533.I1RD17
129194 229533.A0A1C3YIP5 pp 229533.I1RYW3
129195 229533.A0A1C3YIP5 pp 229533.I1S1C7
129196 229533.A0A1C3YIP5 pp 229533.I1S0R2
129197 229533.A0A1C3YIP5 pp 229533.I1RE01
129198 229533.A0A1C3YIP5 pp 229533.I1RW46
129199 229533.A0A1C3YIP5 pp 229533.Q4I624
129200 229533.A0A1C3YIP5 pp 229533.I1RGL4
129201 229533.A0A1C3YIP5 pp 229533.A0A1C3YK33
129202 229533.A0A1C3YIP5 pp 229533.I1RB41
129203 229533.A0A1C3YIP5 pp 229533.I1RWN6
129204 229533.A0A1C3YIP5 pp 229533.I1RDX0
129205 229533.A0A1C3YIP5 pp 229533.I1RD39
129206 229533.A0A1C3YIP5 pp 229533.I1RTA0
129207 229533.A0A1C3YIP5 pp 229533.A0A1C3YLC1
129208 229533.A0A1C3YIP5 pp 229533.I1RZS7
129209 229533.A0A1C3YIP5 pp 229533.V6RRH2
129210 229533.A0A1C3YIP5 pp 229533.I1S1U7

129211 229533.A0A1C3YIP5 pp 229533.I1RAW0
129212 229533.A0A1C3YIP5 pp 229533.I1R9J6
129213 229533.A0A1C3YIP5 pp 229533.I1RKC6
129214 229533.A0A1C3YIP5 pp 229533.I1S1J6
129215 229533.I1S360 pp 229533.V6R9L8
129216 229533.I1S360 pp 229533.I1S368
129217 229533.I1RG53 pp 229533.I1RT04
129218 229533.I1RG53 pp 229533.I1RV91
129219 229533.I1RG53 pp 229533.I1RXD7
129220 229533.I1RG53 pp 229533.I1S2E9
129221 229533.I1RG53 pp 229533.I1RTD5
129222 229533.I1RG53 pp 229533.I1RGA4
129223 229533.I1RG53 pp 229533.I1RT40
129224 229533.I1RG53 pp 229533.I1RJV4
129225 229533.I1RG53 pp 229533.V6RTJ8
129226 229533.I1RG53 pp 229533.I1RX46
129227 229533.I1RG53 pp 229533.I1RJW6
129228 229533.I1RG53 pp 229533.I1S503
129229 229533.I1RG53 pp 229533.I1RPP3
129230 229533.I1RG53 pp 229533.I1RZ39
129231 229533.I1RG53 pp 229533.I1RJV8
129232 229533.I1RG53 pp 229533.I1RJU3
129233 229533.I1RG53 pp 229533.V6QW22
129234 229533.I1RG53 pp 229533.V6R3X5
129235 229533.I1RG53 pp 229533.I1RR73
129236 229533.I1RG53 pp 229533.I1RSQ7
129237 229533.I1RG53 pp 229533.I1RLX7
129238 229533.I1RG53 pp 229533.I1RZZ9
129239 229533.I1RG53 pp 229533.I1RWK3
129240 229533.I1RG53 pp 229533.V6R1K4
129241 229533.I1RG53 pp 229533.I1S0X6
129242 229533.I1RG53 pp 229533.I1RS20
129243 229533.I1RG53 pp 229533.I1RR24
129244 229533.I1RG53 pp 229533.I1S0L3
129245 229533.I1RG53 pp 229533.I1S0L4
129246 229533.I1RG53 pp 229533.I1RWR8
129247 229533.I1RG53 pp 229533.I1RN67
129248 229533.I1RG53 pp 229533.I1RZZ1
129249 229533.I1RG53 pp 229533.I1S0F6
129250 229533.I1RG53 pp 229533.I1S4S4
129251 229533.I1RG53 pp 229533.I1RWJ8
129252 229533.I1RG53 pp 229533.I1RVQ0
129253 229533.I1RG53 pp 229533.V6R057
129254 229533.I1RG53 pp 229533.I1RTH6
129255 229533.I1RG53 pp 229533.I1RQE7
129256 229533.I1RG53 pp 229533.I1S5R1
129257 229533.I1RG53 pp 229533.I1RWD7
129258 229533.I1RG53 pp 229533.I1RSI0
129259 229533.I1RG53 pp 229533.I1S8R3
129260 229533.I1RG53 pp 229533.I1RRT8
129261 229533.I1RG53 pp 229533.V6RCZ8
129262 229533.I1RG53 pp 229533.V6QUW4
129263 229533.I1RG53 pp 229533.I1RZK5
129264 229533.I1RG53 pp 229533.I1RPM4
129265 229533.I1RG53 pp 229533.I1S1C7
129266 229533.I1RG53 pp 229533.V6R042
129267 229533.I1RG53 pp 229533.I1RSQ3
129268 229533.I1RG53 pp 229533.I1RY03
129269 229533.I1RG53 pp 229533.I1RWX5
129270 229533.I1RG53 pp 229533.I1RRM5
129271 229533.I1RG53 pp 229533.I1S2B8
129272 229533.I1RG53 pp 229533.V6RCG5
129273 229533.I1RG53 pp 229533.I1RQP8
129274 229533.I1RG53 pp 229533.I1RW25
129275 229533.I1RG53 pp 229533.I1S7S9
129276 229533.I1RG53 pp 229533.I1RRI2
129277 229533.I1RG53 pp 229533.I1RZW8
129278 229533.I1RG53 pp 229533.I1RNC1
129279 229533.I1RG53 pp 229533.V6RMW6
129280 229533.I1RG53 pp 229533.I1RRY9
129281 229533.I1RG53 pp 229533.I1RQC9
129282 229533.I1RG53 pp 229533.I1RWN6
129283 229533.I1RG53 pp 229533.I1S0R7

129284	229533.I1RG53	pp	229533.I1RUG3
129285	229533.I1RG53	pp	229533.I1RJV3
129286	229533.I1RG53	pp	229533.I1RI82
129287	229533.I1RG53	pp	229533.I1RZM8
129288	229533.I1RG53	pp	229533.I1RWQ8
129289	229533.I1RG53	pp	229533.I1RT03
129290	229533.I1RG53	pp	229533.I1RN18
129291	229533.I1RG53	pp	229533.I1RVX0
129292	229533.I1RG53	pp	229533.I1S5B0
129293	229533.I1RG53	pp	229533.I1SA33
129294	229533.I1RG53	pp	229533.Q4IJK2
129295	229533.I1RG53	pp	229533.I1RW99
129296	229533.I1RG53	pp	229533.I1RS17
129297	229533.I1RG53	pp	229533.I1RGC2
129298	229533.I1RG53	pp	229533.I1RW46
129299	229533.I1RG53	pp	229533.I1RPP4
129300	229533.I1RG53	pp	229533.Q4I8P5
129301	229533.I1RG53	pp	229533.I1SA32
129302	229533.I1RG53	pp	229533.I1S0J1
129303	229533.I1RG53	pp	229533.I1RZS7
129304	229533.I1RG53	pp	229533.I1RWN5
129305	229533.I1RG53	pp	229533.I1S504
129306	229533.I1RG53	pp	229533.I1RLU6
129307	229533.I1RG53	pp	229533.V6RIV9
129308	229533.I1RG53	pp	229533.I1RNQ5
129309	229533.I1RG53	pp	229533.I1RZM1
129310	229533.I1RG53	pp	229533.I1RY44
129311	229533.I1RNY6	pp	229533.I1S3G4
129312	229533.I1RNY6	pp	229533.I1S8F8
129313	229533.I1RNY6	pp	229533.I1S2Q4
129314	229533.I1RNY6	pp	229533.I1S4S5
129315	229533.I1RNY6	pp	229533.I1RRX5
129316	229533.I1RNY6	pp	229533.I1RYX6
129317	229533.I1RNY6	pp	229533.I1S6P5
129318	229533.I1RNY6	pp	229533.I1S3T7
129319	229533.I1RNY6	pp	229533.V6R3V4
129320	229533.I1RNY6	pp	229533.I1S8C1
129321	229533.I1RNY6	pp	229533.I1RUR8
129322	229533.I1RNY6	pp	229533.I1RY21
129323	229533.I1RNY6	pp	229533.I1RVZ3
129324	229533.I1RNY6	pp	229533.I1S1W8
129325	229533.I1RNY6	pp	229533.I1S0V6
129326	229533.I1RNY6	pp	229533.Q4HVQ9
129327	229533.I1RNY6	pp	229533.I1S8S9
129328	229533.I1RNY6	pp	229533.I1RQE1
129329	229533.I1RNY6	pp	229533.V6R3J1
129330	229533.I1RNY6	pp	229533.I1S1B8
129331	229533.I1RNY6	pp	229533.V6RUB1
129332	229533.I1RNY6	pp	229533.I1RTY0
129333	229533.I1RNY6	pp	229533.I1RXV9
129334	229533.I1RNY6	pp	229533.I1RYA2
129335	229533.I1RNY6	pp	229533.I1RZA7
129336	229533.I1RNY6	pp	229533.I1RUQ3
129337	229533.I1RNY6	pp	229533.I1S4G3
129338	229533.I1RNY6	pp	229533.I1S299
129339	229533.I1RNY6	pp	229533.V6RZD4
129340	229533.I1RNY6	pp	229533.I1RWZ3
129341	229533.I1RNY6	pp	229533.I1RYK0
129342	229533.I1RNY6	pp	229533.I1RYW9
129343	229533.I1RNY6	pp	229533.V6RF62
129344	229533.I1RNY6	pp	229533.I1S8R2
129345	229533.I1RNY6	pp	229533.I1S0V7
129346	229533.I1RNY6	pp	229533.V6RRM0
129347	229533.I1RHB4	pp	229533.I1RW43
129348	229533.I1RHB4	pp	229533.I1RY49
129349	229533.I1RVX0	pp	229533.I1S4S4
129350	229533.I1RVX0	pp	229533.I1RWJ8
129351	229533.I1RVX0	pp	229533.I1S9U2
129352	229533.I1RVX0	pp	229533.I1RW07
129353	229533.I1RVX0	pp	229533.I1RW25
129354	229533.I1RVX0	pp	229533.I1S6U8
129355	229533.I1RVX0	pp	229533.I1RY86
129356	229533.I1RVX0	pp	229533.I1RX25

129357 229533.I1RVX0 pp 229533.I1S0F5
129358 229533.I1RVX0 pp 229533.I1S0X6
129359 229533.I1RVX0 pp 229533.V6R4J1
129360 229533.I1RVX0 pp 229533.I1RYH9
129361 229533.I1RVX0 pp 229533.I1S0L3
129362 229533.I1RVX0 pp 229533.I1S9I5
129363 229533.I1RVX0 pp 229533.V6QW22
129364 229533.I1RVX0 pp 229533.I1RXD6
129365 229533.I1RVX0 pp 229533.I1S4Q4
129366 229533.I1RVX0 pp 229533.I1S438
129367 229533.I1RVX0 pp 229533.V6R1K4
129368 229533.I1RVX0 pp 229533.I1RW71
129369 229533.I1RVX0 pp 229533.I1S0T2
129370 229533.I1RVX0 pp 229533.I1S4S7
129371 229533.I1RVX0 pp 229533.I1RX46
129372 229533.I1RVX0 pp 229533.I1RZE5
129373 229533.I1RVX0 pp 229533.I1RXD7
129374 229533.I1RVX0 pp 229533.Q4IJT5
129375 229533.I1RVX0 pp 229533.Q4I2J8
129376 229533.I1RVX0 pp 229533.V6RUT5
129377 229533.I1RVX0 pp 229533.I1RZ35
129378 229533.I1RVX0 pp 229533.I1RX95
129379 229533.I1RVX0 pp 229533.V6RTJ8
129380 229533.I1RVX0 pp 229533.I1RVZ0
129381 229533.I1RVX0 pp 229533.Q4IJM4
129382 229533.I1RVX0 pp 229533.I1S7A8
129383 229533.I1RVX0 pp 229533.I1S3S3
129384 229533.I1RVX0 pp 229533.I1S1J7
129385 229533.I1RVX0 pp 229533.V6RBN2
129386 229533.I1RVX0 pp 229533.I1S0R7
129387 229533.I1RVX0 pp 229533.I1RYS0
129388 229533.I1RVX0 pp 229533.I1RYJ6
129389 229533.I1RVX0 pp 229533.I1RZZ1
129390 229533.I1RVX0 pp 229533.I1RWT6
129391 229533.I1RVX0 pp 229533.I1RZK6
129392 229533.I1RVX0 pp 229533.V6R9Z0
129393 229533.I1RVX0 pp 229533.I1RWB0
129394 229533.I1RVX0 pp 229533.Q4IJK2
129395 229533.I1RVX0 pp 229533.I1S887
129396 229533.I1RVX0 pp 229533.Q4I283
129397 229533.I1RVX0 pp 229533.I1S1B2
129398 229533.I1RVX0 pp 229533.I1S504
129399 229533.I1RVX0 pp 229533.I1RWR7
129400 229533.I1RVX0 pp 229533.I1RYY5
129401 229533.I1RVX0 pp 229533.I1RZF6
129402 229533.I1RVX0 pp 229533.I1RZK5
129403 229533.I1RVX0 pp 229533.I1RZM1
129404 229533.I1RVX0 pp 229533.V6QUE6
129405 229533.I1RVX0 pp 229533.I1S419
129406 229533.I1RVX0 pp 229533.I1RY03
129407 229533.I1RVX0 pp 229533.V6RCG5
129408 229533.I1RVX0 pp 229533.I1S821
129409 229533.I1RVX0 pp 229533.I1S2G9
129410 229533.I1RVX0 pp 229533.I1S0L4
129411 229533.I1RVX0 pp 229533.I1S6K9
129412 229533.I1RVX0 pp 229533.Q4I8P5
129413 229533.I1RVX0 pp 229533.I1RWL2
129414 229533.I1RVX0 pp 229533.I1S0M9
129415 229533.I1RVX0 pp 229533.I1S2H4
129416 229533.I1RVX0 pp 229533.I1S021
129417 229533.I1RVX0 pp 229533.V6RCZ8
129418 229533.I1RVX0 pp 229533.I1S5R1
129419 229533.I1RVX0 pp 229533.I1RXF0
129420 229533.I1RVX0 pp 229533.I1SA32
129421 229533.I1RVX0 pp 229533.I1RY57
129422 229533.I1RVX0 pp 229533.I1RZM8
129423 229533.I1RVX0 pp 229533.I1RXC4
129424 229533.I1RVX0 pp 229533.I1RXC2
129425 229533.I1RVX0 pp 229533.O74638
129426 229533.I1RVX0 pp 229533.I1RW99
129427 229533.I1RVX0 pp 229533.I1S5B0
129428 229533.I1RVX0 pp 229533.I1S4Z8
129429 229533.I1RVX0 pp 229533.I1S2B8

129430 229533.I1RVX0 pp 229533.I1S9Y1
129431 229533.I1RVX0 pp 229533.I1S1F3
129432 229533.I1RVX0 pp 229533.I1RZS6
129433 229533.I1RVX0 pp 229533.I1RY62
129434 229533.I1RVX0 pp 229533.V6R3X5
129435 229533.I1RVX0 pp 229533.I1S0M6
129436 229533.I1RVX0 pp 229533.I1S0F6
129437 229533.I1RVX0 pp 229533.I1RX00
129438 229533.I1RVX0 pp 229533.I1S0W4
129439 229533.I1RVX0 pp 229533.I1RZ39
129440 229533.I1RVX0 pp 229533.I1RYI5
129441 229533.I1RVX0 pp 229533.I1S8V3
129442 229533.I1RVX0 pp 229533.I1S6Y8
129443 229533.I1RVX0 pp 229533.Q4I662
129444 229533.I1RVX0 pp 229533.I1S8P5
129445 229533.I1RVX0 pp 229533.I1RZZ9
129446 229533.I1RVX0 pp 229533.I1RWI5
129447 229533.I1RVX0 pp 229533.I1RWF7
129448 229533.I1RVX0 pp 229533.I1S464
129449 229533.I1RVX0 pp 229533.I1S2G7
129450 229533.I1RVX0 pp 229533.V6R283
129451 229533.I1RVX0 pp 229533.I1S352
129452 229533.I1RVX0 pp 229533.I1RYR0
129453 229533.I1RVX0 pp 229533.Q4I624
129454 229533.I1RVX0 pp 229533.I1RVZ2
129455 229533.I1RVX0 pp 229533.I1RWK3
129456 229533.I1RVX0 pp 229533.I1RWQ6
129457 229533.I1RVX0 pp 229533.I1S569
129458 229533.I1RVX0 pp 229533.I1RWS4
129459 229533.I1RVX0 pp 229533.I1RZW8
129460 229533.I1RVX0 pp 229533.V6R3J1
129461 229533.I1RVX0 pp 229533.Q4IPZ1
129462 229533.I1RVX0 pp 229533.I1S503
129463 229533.I1RVX0 pp 229533.V6R057
129464 229533.I1RVX0 pp 229533.I1S2E9
129465 229533.I1RVX0 pp 229533.I1RZ79
129466 229533.I1RVX0 pp 229533.I1RWX5
129467 229533.I1RVX0 pp 229533.V6RRH2
129468 229533.I1RVX0 pp 229533.I1S0R9
129469 229533.I1RVX0 pp 229533.I1S437
129470 229533.I1RVX0 pp 229533.I1RXR1
129471 229533.I1RVX0 pp 229533.V6RUY9
129472 229533.I1RVX0 pp 229533.I1S2E8
129473 229533.I1RVX0 pp 229533.I1S1C7
129474 229533.I1RVX0 pp 229533.I1RXF8
129475 229533.I1RVX0 pp 229533.Q4I327
129476 229533.I1RVX0 pp 229533.I1RWN6
129477 229533.I1RVX0 pp 229533.I1RYW3
129478 229533.I1RVX0 pp 229533.I1S2C0
129479 229533.I1RVX0 pp 229533.I1RXU9
129480 229533.I1RVX0 pp 229533.I1RVZ7
129481 229533.I1RVX0 pp 229533.Q4IN63
129482 229533.I1RVX0 pp 229533.I1S8V4
129483 229533.I1RVX0 pp 229533.I1S9Z4
129484 229533.I1RVX0 pp 229533.I1S8R3
129485 229533.I1RVX0 pp 229533.I1RZV3
129486 229533.I1RVX0 pp 229533.I1S1W3
129487 229533.I1RVX0 pp 229533.I1S1W4
129488 229533.I1RVX0 pp 229533.I1RW46
129489 229533.I1RVX0 pp 229533.I1S7W5
129490 229533.I1RVX0 pp 229533.I1S0J1
129491 229533.I1RVX0 pp 229533.I1RY44
129492 229533.I1RVX0 pp 229533.I1S0N4
129493 229533.I1RVX0 pp 229533.I1S256
129494 229533.I1RVX0 pp 229533.I1S5R6
129495 229533.I1RVX0 pp 229533.I1S016
129496 229533.I1RVX0 pp 229533.V6RIV9
129497 229533.I1RVX0 pp 229533.I1RWN5
129498 229533.I1RVX0 pp 229533.I1SA33
129499 229533.I1RVX0 pp 229533.V6R042
129500 229533.I1RVX0 pp 229533.I1S1W9
129501 229533.I1RVX0 pp 229533.V6RMW6
129502 229533.I1RUS5 pp 229533.I1S3G4

129503	229533.I1RUS5	pp	229533.I1RYX6
129504	229533.I1RUS5	pp	229533.I1S1W8
129505	229533.I1RUS5	pp	229533.Q4HVQ9
129506	229533.I1RUS5	pp	229533.I1S2Q4
129507	229533.I1RUS5	pp	229533.I1S1B8
129508	229533.I1RUS5	pp	229533.I1S4G3
129509	229533.I1RUS5	pp	229533.I1RWZ3
129510	229533.I1RQJ5	pp	229533.I1RW25
129511	229533.I1RQJ5	pp	229533.I1S2B8
129512	229533.I1RQJ5	pp	229533.Q4IFI0
129513	229533.I1RQJ5	pp	229533.I1RR72
129514	229533.I1RQJ5	pp	229533.I1RVL8
129515	229533.I1RQJ5	pp	229533.V6QYF0
129516	229533.I1RQJ5	pp	229533.I1RZS6
129517	229533.I1RQJ5	pp	229533.Q4I662
129518	229533.I1RQJ5	pp	229533.I1RTF4
129519	229533.I1RQJ5	pp	229533.I1RZV6
129520	229533.I1RQJ5	pp	229533.I1RX24
129521	229533.I1RQJ5	pp	229533.I1S0L3
129522	229533.I1RQJ5	pp	229533.I1S9Y1
129523	229533.I1RQJ5	pp	229533.I1RT33
129524	229533.I1RQJ5	pp	229533.O74638
129525	229533.I1RQJ5	pp	229533.Q4IJH1
129526	229533.I1RQJ5	pp	229533.I1RWH5
129527	229533.I1RQJ5	pp	229533.V6R283
129528	229533.I1RQJ5	pp	229533.I1RZ39
129529	229533.I1RQJ5	pp	229533.I1S8E2
129530	229533.I1RQJ5	pp	229533.I1S9U2
129531	229533.I1RQJ5	pp	229533.I1RT80
129532	229533.I1RQJ5	pp	229533.Q4HZI0
129533	229533.I1RQJ5	pp	229533.Q4I830
129534	229533.I1RQJ5	pp	229533.Q4HVW2
129535	229533.I1RQJ5	pp	229533.I1S253
129536	229533.I1RQJ5	pp	229533.I1RW71
129537	229533.I1RQJ5	pp	229533.I1RZ99
129538	229533.I1RQJ5	pp	229533.I1RR73
129539	229533.I1RQJ5	pp	229533.Q4IF76
129540	229533.I1RQJ5	pp	229533.I1RU69
129541	229533.I1RQJ5	pp	229533.I1RRJ8
129542	229533.I1RQJ5	pp	229533.Q4IPI1
129543	229533.I1RQJ5	pp	229533.V6R3X5
129544	229533.I1RQJ5	pp	229533.I1RSQ7
129545	229533.I1RQJ5	pp	229533.I1RSL9
129546	229533.I1RQJ5	pp	229533.Q4IAS1
129547	229533.I1RQJ5	pp	229533.I1RS30
129548	229533.I1RQJ5	pp	229533.I1S6F0
129549	229533.I1RQJ5	pp	229533.I1S503
129550	229533.I1RQJ5	pp	229533.Q4I5R9
129551	229533.I1RQJ5	pp	229533.I1RRQ3
129552	229533.I1RQJ5	pp	229533.I1RT98
129553	229533.I1RQJ5	pp	229533.I1RRH5
129554	229533.I1RQJ5	pp	229533.I1RRT2
129555	229533.I1RQJ5	pp	229533.Q4INZ9
129556	229533.I1RQJ5	pp	229533.V6RAC9
129557	229533.I1RQJ5	pp	229533.I1RS47
129558	229533.I1RQJ5	pp	229533.I1S9Y0
129559	229533.I1RQJ5	pp	229533.I1RQS5
129560	229533.I1RQJ5	pp	229533.Q4HZ42
129561	229533.I1RQJ5	pp	229533.I1RZE5
129562	229533.I1RQJ5	pp	229533.I1RX46
129563	229533.I1RQJ5	pp	229533.I1RTD5
129564	229533.I1RQJ5	pp	229533.I1RYB1
129565	229533.I1RQJ5	pp	229533.Q4HXF0
129566	229533.I1RQJ5	pp	229533.I1RZ59
129567	229533.I1RQJ5	pp	229533.I1S274
129568	229533.I1RQJ5	pp	229533.I1RT04
129569	229533.I1RQJ5	pp	229533.I1RSM3
129570	229533.I1RQJ5	pp	229533.Q4HZ68
129571	229533.I1RQJ5	pp	229533.I1S0R7
129572	229533.I1RQJ5	pp	229533.I1RWN6
129573	229533.I1RQJ5	pp	229533.I1RXD7
129574	229533.I1RQJ5	pp	229533.I1S2E9
129575	229533.I1RQJ5	pp	229533.I1RSD8

129576	229533.I1RQJ5	pp	229533.I1RRY9
129577	229533.I1RQJ5	pp	229533.I1RVZ0
129578	229533.I1RQJ5	pp	229533.V6RGY3
129579	229533.I1RQJ5	pp	229533.I1RZK6
129580	229533.I1RQJ5	pp	229533.I1RZZ1
129581	229533.I1RQJ5	pp	229533.I1RSR3
129582	229533.I1RQJ5	pp	229533.I1S1C7
129583	229533.I1RQJ5	pp	229533.I1S2E8
129584	229533.I1RQJ5	pp	229533.I1S1J6
129585	229533.I1RQJ5	pp	229533.I1RVX0
129586	229533.I1RQJ5	pp	229533.I1RSR8
129587	229533.I1RQJ5	pp	229533.I1S0Z9
129588	229533.I1RQJ5	pp	229533.I1RT03
129589	229533.I1RQJ5	pp	229533.I1RTE9
129590	229533.I1RQJ5	pp	229533.I1RTD9
129591	229533.I1RQJ5	pp	229533.I1RY03
129592	229533.I1RQJ5	pp	229533.I1RY63
129593	229533.I1RQJ5	pp	229533.I1S1X9
129594	229533.I1RQJ5	pp	229533.I1RRI2
129595	229533.I1RQJ5	pp	229533.I1S2C0
129596	229533.I1RQJ5	pp	229533.I1RSS4
129597	229533.I1RQJ5	pp	229533.I1RWN5
129598	229533.I1RQJ5	pp	229533.V6QY25
129599	229533.I1RQJ5	pp	229533.I1RSG8
129600	229533.I1RQJ5	pp	229533.I1S4T3
129601	229533.I1RQJ5	pp	229533.I1S0M9
129602	229533.I1RQJ5	pp	229533.Q4IEK8
129603	229533.I1RQJ5	pp	229533.Q4HWE2
129604	229533.I1RQJ5	pp	229533.I1RWB5
129605	229533.I1RQJ5	pp	229533.I1RY44
129606	229533.I1RQJ5	pp	229533.I1RZF6
129607	229533.I1RQJ5	pp	229533.I1RZK5
129608	229533.I1RQJ5	pp	229533.I1RTH6
129609	229533.I1RQJ5	pp	229533.V6QUE6
129610	229533.I1RQJ5	pp	229533.I1RRK8
129611	229533.I1RQJ5	pp	229533.I1RSI6
129612	229533.I1RQJ5	pp	229533.Q4IBS2
129613	229533.I1RQJ5	pp	229533.I1S504
129614	229533.I1RQJ5	pp	229533.V6R042
129615	229533.I1RQJ5	pp	229533.V6R227
129616	229533.I1RQJ5	pp	229533.I1RRV1
129617	229533.I1RQJ5	pp	229533.V6RGN5
129618	229533.I1RQJ5	pp	229533.V6RMW6
129619	229533.I1RQJ5	pp	229533.I1RRR7
129620	229533.I1RQJ5	pp	229533.I1S044
129621	229533.I1RQJ5	pp	229533.I1RWE1
129622	229533.I1RQJ5	pp	229533.Q4IN63
129623	229533.I1RQJ5	pp	229533.Q4IQT8
129624	229533.I1RQJ5	pp	229533.I1S060
129625	229533.I1RQJ5	pp	229533.Q4IJ56
129626	229533.I1RQJ5	pp	229533.Q4I327
129627	229533.I1RQJ5	pp	229533.I1S0J1
129628	229533.I1RQJ5	pp	229533.I1RYB9
129629	229533.I1RQJ5	pp	229533.I1S021
129630	229533.I1RKZ7	pp	229533.I1RNV4
129631	229533.I1RKZ7	pp	229533.I1RWF5
129632	229533.I1RKZ7	pp	229533.I1RWZ3
129633	229533.I1RKZ7	pp	229533.I1S147
129634	229533.I1RKZ7	pp	229533.I1S024
129635	229533.I1RKZ7	pp	229533.I1RXY7
129636	229533.I1RKZ7	pp	229533.I1RY74
129637	229533.I1RKZ7	pp	229533.I1RW43
129638	229533.I1RKZ7	pp	229533.I1S0I3
129639	229533.I1RKZ7	pp	229533.I1RZ83
129640	229533.I1RKZ7	pp	229533.I1S2Q4
129641	229533.I1RKZ7	pp	229533.I1RNG4
129642	229533.I1RKZ7	pp	229533.I1S3G4
129643	229533.I1RKZ7	pp	229533.I1S4G3
129644	229533.I1RKZ7	pp	229533.I1RUR8
129645	229533.I1RKZ7	pp	229533.V6RHD0
129646	229533.I1RKZ7	pp	229533.I1RX59
129647	229533.I1RKZ7	pp	229533.I1RYQ4
129648	229533.I1RKZ7	pp	229533.I1RZK8

129649 229533.I1RKZ7 pp 229533.I1RM26
129650 229533.I1RKZ7 pp 229533.I1RSQ9
129651 229533.I1RKZ7 pp 229533.I1RNB4
129652 229533.I1RKZ7 pp 229533.I1RY12
129653 229533.I1RKZ7 pp 229533.V6RXR0
129654 229533.I1RKZ7 pp 229533.V6QU65
129655 229533.I1RKZ7 pp 229533.I1RY49
129656 229533.I1RKZ7 pp 229533.I1S1W8
129657 229533.I1RKZ7 pp 229533.V6QWD8
129658 229533.I1RKZ7 pp 229533.I1S1B8
129659 229533.I1RKZ7 pp 229533.I1RMK4
129660 229533.I1RKZ7 pp 229533.I1RVX9
129661 229533.I1RKZ7 pp 229533.I1RTY0
129662 229533.I1RKZ7 pp 229533.I1RQE1
129663 229533.I1RKZ7 pp 229533.I1RYX6
129664 229533.I1RKZ7 pp 229533.I1RZY1
129665 229533.A0A1C3YJ69 pp 229533.I1RE88
129666 229533.A0A1C3YJ69 pp 229533.I1RTP2
129667 229533.A0A1C3YJ69 pp 229533.A0A1C3YK95
129668 229533.A0A1C3YJ69 pp 229533.I1RWX5
129669 229533.I1RT03 pp 229533.I1RV91
129670 229533.I1RT03 pp 229533.I1RTD5
129671 229533.I1RT03 pp 229533.I1RT40
129672 229533.I1RT03 pp 229533.I1RT04
129673 229533.I1RT03 pp 229533.I1S352
129674 229533.I1RT03 pp 229533.I1RVZ2
129675 229533.I1RT03 pp 229533.I1S8P5
129676 229533.I1RT03 pp 229533.I1RWF7
129677 229533.I1RT03 pp 229533.V6RUY9
129678 229533.I1RT03 pp 229533.I1S2E9
129679 229533.I1RT03 pp 229533.Q4HXF0
129680 229533.I1RT03 pp 229533.I1S0F6
129681 229533.I1RT03 pp 229533.I1S0W4
129682 229533.I1RT03 pp 229533.I1RYI5
129683 229533.I1RT03 pp 229533.I1S2G7
129684 229533.I1RT03 pp 229533.V6R3J1
129685 229533.I1RT03 pp 229533.I1S503
129686 229533.I1RT03 pp 229533.I1RWK3
129687 229533.I1RT03 pp 229533.I1RZZ9
129688 229533.I1RT03 pp 229533.V6R3X5
129689 229533.I1RT03 pp 229533.I1RWI5
129690 229533.I1RT03 pp 229533.V6R283
129691 229533.I1RT03 pp 229533.I1S9Y1
129692 229533.I1RT03 pp 229533.I1RTA1
129693 229533.I1RT03 pp 229533.I1S8V3
129694 229533.I1RT03 pp 229533.I1RZ39
129695 229533.I1RT03 pp 229533.I1S9Z4
129696 229533.I1RT03 pp 229533.I1S1W9
129697 229533.I1RT03 pp 229533.I1RUG3
129698 229533.I1RT03 pp 229533.I1RXC2
129699 229533.I1RT03 pp 229533.I1S5R1
129700 229533.I1RT03 pp 229533.V6RIV9
129701 229533.I1RT03 pp 229533.O74638
129702 229533.I1RT03 pp 229533.Q4IN63
129703 229533.I1RT03 pp 229533.I1RWN5
129704 229533.I1RT03 pp 229533.Q4HZB8
129705 229533.I1RT03 pp 229533.Q4I327
129706 229533.I1RT03 pp 229533.I1RWL2
129707 229533.I1RT03 pp 229533.V6RMW6
129708 229533.I1RT03 pp 229533.I1RY44
129709 229533.I1RT03 pp 229533.I1S2H4
129710 229533.I1RT03 pp 229533.V6R042
129711 229533.I1RT03 pp 229533.I1S0L4
129712 229533.I1RT03 pp 229533.I1S1W3
129713 229533.I1RT03 pp 229533.Q4I662
129714 229533.I1RT03 pp 229533.I1RZV3
129715 229533.I1RT03 pp 229533.I1S5R6
129716 229533.I1RT03 pp 229533.I1S6Y8
129717 229533.I1RT03 pp 229533.I1SA33
129718 229533.I1RT03 pp 229533.I1S0M6
129719 229533.I1RT03 pp 229533.I1S8V4
129720 229533.I1RT03 pp 229533.I1RZS6
129721 229533.I1RT03 pp 229533.I1S0N4

129722	229533.I1RT03	pp	229533.I1S1C7
129723	229533.I1RT03	pp	229533.I1RYW3
129724	229533.I1RT03	pp	229533.I1RTA0
129725	229533.I1RT03	pp	229533.I1RW46
129726	229533.I1RT03	pp	229533.I1RZ79
129727	229533.I1RT03	pp	229533.I1S2B8
129728	229533.I1RT03	pp	229533.V6R057
129729	229533.I1RT03	pp	229533.I1RZS7
129730	229533.I1RT03	pp	229533.I1RWX5
129731	229533.I1RT03	pp	229533.I1S569
129732	229533.I1RT03	pp	229533.I1RZW8
129733	229533.I1RT03	pp	229533.I1S7W5
129734	229533.I1RT03	pp	229533.I1S0J1
129735	229533.I1RT03	pp	229533.I1S8R3
129736	229533.I1RT03	pp	229533.I1RTH6
129737	229533.I1RT03	pp	229533.I1RX95
129738	229533.I1RT03	pp	229533.Q4I624
129739	229533.I1RT03	pp	229533.I1S0R7
129740	229533.I1RT03	pp	229533.Q4IJT5
129741	229533.I1RT03	pp	229533.I1S2C0
129742	229533.I1RT03	pp	229533.I1RXU9
129743	229533.I1RT03	pp	229533.I1RW71
129744	229533.I1RT03	pp	229533.I1S4S7
129745	229533.I1RT03	pp	229533.I1RWN6
129746	229533.I1RT03	pp	229533.V6RUT5
129747	229533.I1RT03	pp	229533.I1S2E8
129748	229533.I1RT03	pp	229533.V6QW22
129749	229533.I1RT03	pp	229533.I1RXD7
129750	229533.I1RT03	pp	229533.I1RVX0
129751	229533.I1RT03	pp	229533.I1RX46
129752	229533.I1RT03	pp	229533.V6R4J1
129753	229533.I1RT03	pp	229533.I1S0T2
129754	229533.I1RT03	pp	229533.I1RVQ0
129755	229533.I1RT03	pp	229533.I1RT80
129756	229533.I1RT03	pp	229533.I1S7A8
129757	229533.I1RT03	pp	229533.I1RXR1
129758	229533.I1RT03	pp	229533.V6R1K4
129759	229533.I1RT03	pp	229533.I1S6U8
129760	229533.I1RT03	pp	229533.I1S4S4
129761	229533.I1RT03	pp	229533.I1S0L3
129762	229533.I1RT03	pp	229533.V6RTJ8
129763	229533.I1RT03	pp	229533.I1S0F5
129764	229533.I1RT03	pp	229533.I1RWJ8
129765	229533.I1RT03	pp	229533.I1RW25
129766	229533.I1RT03	pp	229533.I1S0X6
129767	229533.I1RT03	pp	229533.I1RX25
129768	229533.I1RT03	pp	229533.V6QUW4
129769	229533.I1RT03	pp	229533.I1RW07
129770	229533.I1RT03	pp	229533.I1S9U2
129771	229533.I1RT03	pp	229533.I1SA32
129772	229533.I1RT03	pp	229533.I1RZM8
129773	229533.I1RT03	pp	229533.I1RXC4
129774	229533.I1RT03	pp	229533.I1S0M9
129775	229533.I1RT03	pp	229533.I1RXF0
129776	229533.I1RT03	pp	229533.I1S887
129777	229533.I1RT03	pp	229533.I1S021
129778	229533.I1RT03	pp	229533.I1RZK5
129779	229533.I1RT03	pp	229533.I1S419
129780	229533.I1RT03	pp	229533.V6RCZ8
129781	229533.I1RT03	pp	229533.Q4I8P5
129782	229533.I1RT03	pp	229533.I1RYS0
129783	229533.I1RT03	pp	229533.I1RWB0
129784	229533.I1RT03	pp	229533.I1RZM1
129785	229533.I1RT03	pp	229533.I1S1J7
129786	229533.I1RT03	pp	229533.I1S6K9
129787	229533.I1RT03	pp	229533.I1S504
129788	229533.I1RT03	pp	229533.I1RZF6
129789	229533.I1RT03	pp	229533.V6RCG5
129790	229533.I1RT03	pp	229533.V6QUE6
129791	229533.I1RT03	pp	229533.I1RWR7
129792	229533.I1RT03	pp	229533.I1S5B0
129793	229533.I1RT03	pp	229533.Q4IJK2
129794	229533.I1RT03	pp	229533.I1RU29

129795 229533.I1RT03 pp 229533.I1RW99
129796 229533.I1RT03 pp 229533.I1RZZ1
129797 229533.I1RT03 pp 229533.Q4I283
129798 229533.I1RT03 pp 229533.I1RWT6
129799 229533.I1RT03 pp 229533.I1RZK6
129800 229533.I1RT03 pp 229533.I1RVZ0
129801 229533.I1RT03 pp 229533.V6RBN2
129802 229533.I1RT03 pp 229533.I1RY03
129803 229533.I1RT03 pp 229533.I1RZE5
129804 229533.I1RT03 pp 229533.Q4I2J8
129805 229533.A0A098DYP8 pp 229533.I1RT51
129806 229533.A0A098DYP8 pp 229533.I1RG66
129807 229533.A0A098DYP8 pp 229533.I1S4G3
129808 229533.A0A098DYP8 pp 229533.I1RBE7
129809 229533.A0A098DYP8 pp 229533.A0A1C3YI08
129810 229533.A0A098DYP8 pp 229533.A0A1C3YN04
129811 229533.A0A098DYP8 pp 229533.I1RYX6
129812 229533.A0A098DYP8 pp 229533.I1RTY0
129813 229533.A0A098DYP8 pp 229533.I1S1W8
129814 229533.A0A098DYP8 pp 229533.I1RBY9
129815 229533.A0A098DYP8 pp 229533.A0A1C3YMF1
129816 229533.A0A098DYP8 pp 229533.I1RAV0
129817 229533.A0A098DYP8 pp 229533.I1RQE1
129818 229533.A0A098DYP8 pp 229533.Q4HVQ9
129819 229533.A0A098DYP8 pp 229533.I1S1B8
129820 229533.A0A098DYP8 pp 229533.I1RWZ3
129821 229533.A0A098DYP8 pp 229533.A0A1C3YKL7
129822 229533.A0A098DYP8 pp 229533.I1RJJ7
129823 229533.A0A098DYP8 pp 229533.I1RNG4
129824 229533.A0A098DYP8 pp 229533.I1RH91
129825 229533.A0A098DYP8 pp 229533.I1S2Q4
129826 229533.A0A098DYP8 pp 229533.I1S3G4
129827 229533.I1R9P1 pp 229533.I1RE88
129828 229533.I1R9P1 pp 229533.I1RTP2
129829 229533.I1R9P1 pp 229533.I1RPG8
129830 229533.I1R9P1 pp 229533.Q4ICM0
129831 229533.I1RSQ7 pp 229533.I1RZW8
129832 229533.I1RSQ7 pp 229533.V6RMW6
129833 229533.I1RSQ7 pp 229533.I1RUG3
129834 229533.I1RSQ7 pp 229533.I1RWH4
129835 229533.I1RSQ7 pp 229533.V6R042
129836 229533.I1RSQ7 pp 229533.I1RZ79
129837 229533.I1RSQ7 pp 229533.I1RWX5
129838 229533.I1RSQ7 pp 229533.I1S256
129839 229533.I1RSQ7 pp 229533.I1S1C7
129840 229533.I1RSQ7 pp 229533.I1S2E8
129841 229533.I1RSQ7 pp 229533.I1RYW3
129842 229533.I1RSQ7 pp 229533.I1RSS4
129843 229533.I1RSQ7 pp 229533.I1RXR1
129844 229533.I1RSQ7 pp 229533.I1SA33
129845 229533.I1RSQ7 pp 229533.I1RSY2
129846 229533.I1RSQ7 pp 229533.I1S0N4
129847 229533.I1RSQ7 pp 229533.I1RVX0
129848 229533.I1RSQ7 pp 229533.V6RGN5
129849 229533.I1RSQ7 pp 229533.I1S437
129850 229533.I1RSQ7 pp 229533.I1RT03
129851 229533.I1RSQ7 pp 229533.I1S2C0
129852 229533.I1RSQ7 pp 229533.I1RWQ8
129853 229533.I1RSQ7 pp 229533.I1S5R6
129854 229533.I1RSQ7 pp 229533.I1RTE9
129855 229533.I1RSQ7 pp 229533.I1S7W5
129856 229533.I1RSQ7 pp 229533.I1S0J1
129857 229533.I1RSQ7 pp 229533.V6RRH2
129858 229533.I1RSQ7 pp 229533.I1RTH6
129859 229533.I1RSQ7 pp 229533.Q4I624
129860 229533.I1RSQ7 pp 229533.I1S8R3
129861 229533.I1RSQ7 pp 229533.I1RWN6
129862 229533.I1RSQ7 pp 229533.I1RTA0
129863 229533.I1RSQ7 pp 229533.I1RZS7
129864 229533.I1RSQ7 pp 229533.I1RXU9
129865 229533.I1RSQ7 pp 229533.V6R057
129866 229533.I1RSQ7 pp 229533.Q4I327
129867 229533.I1RSQ7 pp 229533.I1RW46

129868	229533.I1RSQ7	pp	229533.I1S569
129869	229533.I1RSQ7	pp	229533.I1RZV3
129870	229533.I1RSQ7	pp	229533.I1S9Z4
129871	229533.I1RSQ7	pp	229533.I1RZ39
129872	229533.I1RSQ7	pp	229533.Q4IN63
129873	229533.I1RSQ7	pp	229533.I1S1W3
129874	229533.I1RSQ7	pp	229533.I1S2B8
129875	229533.I1RSQ7	pp	229533.I1RY44
129876	229533.I1RSQ7	pp	229533.I1RWL2
129877	229533.I1RSQ7	pp	229533.I1RXC2
129878	229533.I1RSQ7	pp	229533.I1S1W9
129879	229533.I1RSQ7	pp	229533.V6RIV9
129880	229533.I1RSQ7	pp	229533.O74638
129881	229533.I1RSQ7	pp	229533.I1S277
129882	229533.I1RSQ7	pp	229533.I1S5R1
129883	229533.I1RSQ7	pp	229533.I1RWN5
129884	229533.I1RSQ7	pp	229533.I1RTD5
129885	229533.I1RSQ7	pp	229533.I1S9Y1
129886	229533.I1RSQ7	pp	229533.I1RXF8
129887	229533.I1RSQ7	pp	229533.I1RWI5
129888	229533.I1RSQ7	pp	229533.I1RT40
129889	229533.I1RSQ7	pp	229533.I1RZZ9
129890	229533.I1RSQ7	pp	229533.I1S2H4
129891	229533.I1RSQ7	pp	229533.I1RV91
129892	229533.I1RSQ7	pp	229533.I1S0L4
129893	229533.I1RSQ7	pp	229533.I1S2E9
129894	229533.I1RSQ7	pp	229533.V6R3X5
129895	229533.I1RSQ7	pp	229533.V6RUY9
129896	229533.I1RSQ7	pp	229533.I1S6Y8
129897	229533.I1RSQ7	pp	229533.I1S2M6
129898	229533.I1RSQ7	pp	229533.I1RT04
129899	229533.I1RSQ7	pp	229533.I1RVZ2
129900	229533.I1RSQ7	pp	229533.I1RSZ7
129901	229533.I1RSQ7	pp	229533.I1RYR0
129902	229533.I1RSQ7	pp	229533.I1RTA1
129903	229533.I1RSQ7	pp	229533.I1S0M6
129904	229533.I1RSQ7	pp	229533.V6R3J1
129905	229533.I1RSQ7	pp	229533.I1RZS6
129906	229533.I1RSQ7	pp	229533.V6R283
129907	229533.I1RSQ7	pp	229533.I1RTD9
129908	229533.I1RSQ7	pp	229533.I1RSX0
129909	229533.I1RSQ7	pp	229533.I1RWK3
129910	229533.I1RSQ7	pp	229533.I1S503
129911	229533.I1RSQ7	pp	229533.I1RSR8
129912	229533.I1RSQ7	pp	229533.I1RY03
129913	229533.I1RSQ7	pp	229533.I1S8P5
129914	229533.I1RSQ7	pp	229533.I1RYI5
129915	229533.I1RSQ7	pp	229533.I1S887
129916	229533.I1RSQ7	pp	229533.I1RZB2
129917	229533.I1RSQ7	pp	229533.I1RSV2
129918	229533.I1RSQ7	pp	229533.V6RBN2
129919	229533.I1RSQ7	pp	229533.I1S1J7
129920	229533.I1RSQ7	pp	229533.I1RZZ1
129921	229533.I1RSQ7	pp	229533.I1RZK5
129922	229533.I1RSQ7	pp	229533.I1RY62
129923	229533.I1RSQ7	pp	229533.I1RZK6
129924	229533.I1RSQ7	pp	229533.I1S352
129925	229533.I1RSQ7	pp	229533.I1RYS0
129926	229533.I1RSQ7	pp	229533.I1S0F6
129927	229533.I1RSQ7	pp	229533.I1RSR3
129928	229533.I1RSQ7	pp	229533.Q4IJK2
129929	229533.I1RSQ7	pp	229533.I1RWB0
129930	229533.I1RSQ7	pp	229533.I1RXF0
129931	229533.I1RSQ7	pp	229533.I1S4Q4
129932	229533.I1RSQ7	pp	229533.I1S0W4
129933	229533.I1RSQ7	pp	229533.I1RZE5
129934	229533.I1RSQ7	pp	229533.I1RU29
129935	229533.I1RSQ7	pp	229533.Q4I5Z5
129936	229533.I1RSQ7	pp	229533.I1RZM8
129937	229533.I1RSQ7	pp	229533.I1RWR7
129938	229533.I1RSQ7	pp	229533.I1RZM1
129939	229533.I1RSQ7	pp	229533.I1S0M9
129940	229533.I1RSQ7	pp	229533.V6RCG5

129941	229533.I1RSQ7	pp	229533.Q4IEK8
129942	229533.I1RSQ7	pp	229533.Q4I2J8
129943	229533.I1RSQ7	pp	229533.I1S6K9
129944	229533.I1RSQ7	pp	229533.I1RVZ0
129945	229533.I1RSQ7	pp	229533.V6RCZ8
129946	229533.I1RSQ7	pp	229533.I1RWT6
129947	229533.I1RSQ7	pp	229533.I1S419
129948	229533.I1RSQ7	pp	229533.I1RWD7
129949	229533.I1RSQ7	pp	229533.I1S021
129950	229533.I1RSQ7	pp	229533.V6QUE6
129951	229533.I1RSQ7	pp	229533.I1S5B0
129952	229533.I1RSQ7	pp	229533.I1RT80
129953	229533.I1RSQ7	pp	229533.I1RW99
129954	229533.I1RSQ7	pp	229533.I1RYJ8
129955	229533.I1RSQ7	pp	229533.Q4I283
129956	229533.I1RSQ7	pp	229533.I1RTF4
129957	229533.I1RSQ7	pp	229533.I1SA32
129958	229533.I1RSQ7	pp	229533.I1RZJ5
129959	229533.I1RSQ7	pp	229533.I1S504
129960	229533.I1RSQ7	pp	229533.I1S4S4
129961	229533.I1RSQ7	pp	229533.V6R227
129962	229533.I1RSQ7	pp	229533.Q4I8P5
129963	229533.I1RSQ7	pp	229533.I1RWJ8
129964	229533.I1RSQ7	pp	229533.I1RXC4
129965	229533.I1RSQ7	pp	229533.I1S9U2
129966	229533.I1RSQ7	pp	229533.I1RZF6
129967	229533.I1RSQ7	pp	229533.I1S438
129968	229533.I1RSQ7	pp	229533.V6QW22
129969	229533.I1RSQ7	pp	229533.I1S0X6
129970	229533.I1RSQ7	pp	229533.V6QYF0
129971	229533.I1RSQ7	pp	229533.Q4HVW2
129972	229533.I1RSQ7	pp	229533.I1RW71
129973	229533.I1RSQ7	pp	229533.V6RUT5
129974	229533.I1RSQ7	pp	229533.I1RUQ2
129975	229533.I1RSQ7	pp	229533.I1RVQ0
129976	229533.I1RSQ7	pp	229533.V6QUW4
129977	229533.I1RSQ7	pp	229533.I1S4S7
129978	229533.I1RSQ7	pp	229533.I1S0R7
129979	229533.I1RSQ7	pp	229533.V6R4J1
129980	229533.I1RSQ7	pp	229533.I1RX25
129981	229533.I1RSQ7	pp	229533.I1RX46
129982	229533.I1RSQ7	pp	229533.I1RW25
129983	229533.I1RSQ7	pp	229533.I1RW07
129984	229533.I1RSQ7	pp	229533.I1S6F0
129985	229533.I1RSQ7	pp	229533.I1S0T2
129986	229533.I1RSQ7	pp	229533.V6RTJ8
129987	229533.I1RSQ7	pp	229533.I1S7A8
129988	229533.I1RSQ7	pp	229533.I1RWD4
129989	229533.I1RSQ7	pp	229533.I1S0F5
129990	229533.I1RSQ7	pp	229533.I1S0L3
129991	229533.I1RSQ7	pp	229533.I1RT33
129992	229533.I1RSQ7	pp	229533.Q4IJT5
129993	229533.I1RSQ7	pp	229533.V6R1K4
129994	229533.I1RYA8	pp	229533.I1S1B8
129995	229533.I1RYA8	pp	229533.I1S3G4
129996	229533.I1RYA8	pp	229533.I1S2Q4
129997	229533.I1RYA8	pp	229533.I1RYX6
129998	229533.I1RYA8	pp	229533.I1S4G3
129999	229533.I1RYA8	pp	229533.Q4HVQ9
130000	229533.I1RYA8	pp	229533.I1S1W8
130001	229533.I1RRN5	pp	229533.I1RZM1
130002	229533.I1RRN5	pp	229533.I1S267
130003	229533.I1RRN5	pp	229533.I1SA32
130004	229533.I1RRN5	pp	229533.I1RW99
130005	229533.I1RRN5	pp	229533.I1RS18
130006	229533.I1RRN5	pp	229533.Q4I8P5
130007	229533.I1RRN5	pp	229533.I1RZM8
130008	229533.I1RRN5	pp	229533.Q4IJM4
130009	229533.I1RRN5	pp	229533.I1S5B0
130010	229533.I1RRN5	pp	229533.I1RW25
130011	229533.I1RRN5	pp	229533.I1RRY9
130012	229533.I1RRN5	pp	229533.Q4INS6
130013	229533.I1RRN5	pp	229533.I1RY03

130014 229533.I1RRN5 pp 229533.Q4HTT2
130015 229533.I1RRN5 pp 229533.I1S7L1
130016 229533.I1RRN5 pp 229533.I1RUQ5
130017 229533.I1RRN5 pp 229533.I1RYH9
130018 229533.I1RRN5 pp 229533.I1S0X6
130019 229533.I1RRN5 pp 229533.I1RRT1
130020 229533.I1RRN5 pp 229533.I1RT03
130021 229533.I1RRN5 pp 229533.I1RZE4
130022 229533.I1RRN5 pp 229533.I1RYZ9
130023 229533.I1RRN5 pp 229533.I1RX70
130024 229533.I1RRN5 pp 229533.I1RWJ8
130025 229533.I1RRN5 pp 229533.I1RVQ0
130026 229533.I1RRN5 pp 229533.I1RW84
130027 229533.I1RRN5 pp 229533.I1RSJ1
130028 229533.I1RRN5 pp 229533.I1S276
130029 229533.I1RRN5 pp 229533.I1S0D5
130030 229533.I1RRN5 pp 229533.I1RSQ7
130031 229533.I1RRN5 pp 229533.I1RRW5
130032 229533.I1RRN5 pp 229533.V6QV09
130033 229533.I1RRN5 pp 229533.I1RY58
130034 229533.I1RRN5 pp 229533.I1S0E8
130035 229533.I1RRN5 pp 229533.V6QW22
130036 229533.I1RRN5 pp 229533.I1RZW8
130037 229533.I1RRN5 pp 229533.I1RWN6
130038 229533.I1RRN5 pp 229533.I1RXU0
130039 229533.I1RRN5 pp 229533.I1RY44
130040 229533.I1RRN5 pp 229533.I1S8R3
130041 229533.I1RRN5 pp 229533.I1RTN6
130042 229533.I1RRN5 pp 229533.I1S0J1
130043 229533.I1RRN5 pp 229533.V6RIV9
130044 229533.I1RRN5 pp 229533.I1RZP8
130045 229533.I1RRN5 pp 229533.I1S2B8
130046 229533.I1RRN5 pp 229533.I1RRV1
130047 229533.I1RRN5 pp 229533.Q4IPI4
130048 229533.I1RRN5 pp 229533.I1RUG3
130049 229533.I1RRN5 pp 229533.I1RVL8
130050 229533.I1RRN5 pp 229533.V6RMW6
130051 229533.I1RRN5 pp 229533.I1S2H4
130052 229533.I1RRN5 pp 229533.I1RUN8
130053 229533.I1RRN5 pp 229533.I1S5R1
130054 229533.I1RRN5 pp 229533.I1S7X9
130055 229533.I1RRN5 pp 229533.I1SAD2
130056 229533.I1RRN5 pp 229533.I1RSQ3
130057 229533.I1RRN5 pp 229533.I1RW83
130058 229533.I1RRN5 pp 229533.I1S004
130059 229533.I1RRN5 pp 229533.I1S0X7
130060 229533.I1RRN5 pp 229533.I1RU69
130061 229533.I1RRN5 pp 229533.I1RWK3
130062 229533.I1RRN5 pp 229533.I1RSI0
130063 229533.I1RRN5 pp 229533.I1RSV2
130064 229533.I1RRN5 pp 229533.I1S0J3
130065 229533.I1RRN5 pp 229533.V6R3X5
130066 229533.I1RRN5 pp 229533.I1RUF8
130067 229533.I1RRN5 pp 229533.I1RT40
130068 229533.I1RRN5 pp 229533.I1S0F6
130069 229533.I1RRN5 pp 229533.I1S439
130070 229533.I1RRN5 pp 229533.I1S098
130071 229533.I1RRN5 pp 229533.Q4IJK2
130072 229533.I1RRN5 pp 229533.I1RSZ3
130073 229533.I1RRN5 pp 229533.I1RYW8
130074 229533.I1RRN5 pp 229533.I1RZK5
130075 229533.I1RRN5 pp 229533.I1RZZ1
130076 229533.I1RRN5 pp 229533.I1RT28
130077 229533.I1RRN5 pp 229533.Q4ICA8
130078 229533.I1RRN5 pp 229533.I1S0N8
130079 229533.I1RRN5 pp 229533.I1S504
130080 229533.I1RRN5 pp 229533.Q4IR87
130081 229533.I1RRN5 pp 229533.V6RCZ8
130082 229533.I1RRN5 pp 229533.I1RWT6
130083 229533.I1RRN5 pp 229533.Q4IEA7
130084 229533.I1RRN5 pp 229533.V6RCG5
130085 229533.I1RRN5 pp 229533.I1RYY5
130086 229533.I1RRN5 pp 229533.I1RW71

130087 229533.I1RRN5 pp 229533.I1RSS1
130088 229533.I1RRN5 pp 229533.I1RTC2
130089 229533.I1RRN5 pp 229533.I1RZ94
130090 229533.I1RRN5 pp 229533.I1RXH6
130091 229533.I1RRN5 pp 229533.I1S224
130092 229533.I1RRN5 pp 229533.I1RV66
130093 229533.I1RRN5 pp 229533.I1RYI9
130094 229533.I1RRN5 pp 229533.I1S0L3
130095 229533.I1RRN5 pp 229533.I1S1C7
130096 229533.I1RRN5 pp 229533.I1RX46
130097 229533.I1RRN5 pp 229533.I1RXD7
130098 229533.I1RRN5 pp 229533.V6RTJ8
130099 229533.I1RRN5 pp 229533.I1S0R7
130100 229533.I1RRN5 pp 229533.V6RKY7
130101 229533.I1RRN5 pp 229533.I1RTH6
130102 229533.I1RRN5 pp 229533.I1RVX0
130103 229533.I1RRN5 pp 229533.V6R057
130104 229533.I1RRN5 pp 229533.I1RWN5
130105 229533.I1RRN5 pp 229533.I1S286
130106 229533.I1RRN5 pp 229533.Q4HTT1
130107 229533.I1RRN5 pp 229533.I1S1W4
130108 229533.I1RRN5 pp 229533.I1S549
130109 229533.I1RRN5 pp 229533.V6RVG3
130110 229533.I1RRN5 pp 229533.I1S9S6
130111 229533.I1RRN5 pp 229533.I1S0M6
130112 229533.I1RRN5 pp 229533.I1RZT1
130113 229533.I1RRN5 pp 229533.I1RRT8
130114 229533.I1RRN5 pp 229533.I1RW46
130115 229533.I1RRN5 pp 229533.I1RX38
130116 229533.I1RRN5 pp 229533.V6RGI1
130117 229533.I1RRN5 pp 229533.I1S2K7
130118 229533.I1RRN5 pp 229533.I1S4Z5
130119 229533.I1RRN5 pp 229533.V6R042
130120 229533.I1RRN5 pp 229533.I1RRW0
130121 229533.I1RRN5 pp 229533.I1SA33
130122 229533.I1RRN5 pp 229533.Q4I7L0
130123 229533.I1RRN5 pp 229533.I1RZZ9
130124 229533.I1RRN5 pp 229533.I1S1J2
130125 229533.I1RRN5 pp 229533.I1RWI8
130126 229533.I1RRN5 pp 229533.I1RT04
130127 229533.I1RRN5 pp 229533.I1RW82
130128 229533.I1RRN5 pp 229533.I1S2E9
130129 229533.I1RRN5 pp 229533.I1RWI5
130130 229533.I1RRN5 pp 229533.I1RZ39
130131 229533.I1RRN5 pp 229533.I1S503
130132 229533.I1RRN5 pp 229533.I1RZP1
130133 229533.I1RRN5 pp 229533.I1RZ63
130134 229533.I1RRN5 pp 229533.I1RY29
130135 229533.I1RRN5 pp 229533.I1RXU1
130136 229533.I1RRN5 pp 229533.I1RTD5
130137 229533.I1RRN5 pp 229533.I1RV91
130138 229533.I1RYK1 pp 229533.I1S1W8
130139 229533.I1RYK1 pp 229533.I1S3G4
130140 229533.I1RYK1 pp 229533.I1S2Q4
130141 229533.I1RYK1 pp 229533.I1S4G3
130142 229533.I1RYK1 pp 229533.I1RYX6
130143 229533.I1RYK1 pp 229533.I1S1B8
130144 229533.I1RYK1 pp 229533.Q4HVQ9
130145 229533.V6QW22 pp 229533.V6RTJ8
130146 229533.V6QW22 pp 229533.V6R4J1
130147 229533.V6QW22 pp 229533.V6R042
130148 229533.V6QW22 pp 229533.V6RIV9
130149 229533.V6QW22 pp 229533.V6R1K4
130150 229533.V6QW22 pp 229533.V6RCG5
130151 229533.V6QW22 pp 229533.V6RMW6
130152 229533.V6QW22 pp 229533.V6QYF0
130153 229533.V6QW22 pp 229533.V6R057
130154 229533.V6QW22 pp 229533.V6RCZ8
130155 229533.V6QW22 pp 229533.V6RGN5
130156 229533.V6QW22 pp 229533.V6R3X5
130157 229533.V6QW22 pp 229533.V6R227
130158 229533.I1RHC8 pp 229533.V6RRM0
130159 229533.I1RHC8 pp 229533.I1RNG4

130160	229533.I1RHC8	pp	229533.Q4HVQ9
130161	229533.I1RHC8	pp	229533.I1RN84
130162	229533.I1RHC8	pp	229533.I1S299
130163	229533.I1RHC8	pp	229533.I1S8R2
130164	229533.I1RHC8	pp	229533.I1RWZ3
130165	229533.I1RHC8	pp	229533.I1RTY0
130166	229533.I1RHC8	pp	229533.I1RKB2
130167	229533.I1RHC8	pp	229533.I1S1W8
130168	229533.I1RHC8	pp	229533.I1S2Q4
130169	229533.I1RHC8	pp	229533.I1RQE1
130170	229533.I1RHC8	pp	229533.I1RNY6
130171	229533.I1RHC8	pp	229533.I1S1B8
130172	229533.I1RHC8	pp	229533.I1S3G4
130173	229533.I1RHC8	pp	229533.I1RYX6
130174	229533.I1RHC8	pp	229533.I1RUR8
130175	229533.I1RHC8	pp	229533.I1RJY7
130176	229533.I1RHC8	pp	229533.I1S8F8
130177	229533.I1RHC8	pp	229533.I1RJJ7
130178	229533.I1RHC8	pp	229533.I1RN88
130179	229533.I1RHC8	pp	229533.I1RRX5
130180	229533.I1RHC8	pp	229533.I1S8C1
130181	229533.I1RHC8	pp	229533.I1S4G3
130182	229533.I1RHC8	pp	229533.I1RUQ3
130183	229533.I1RHS0	pp	229533.I1RHS6
130184	229533.I1RHS0	pp	229533.I1RKT9
130185	229533.I1RHS0	pp	229533.I1S3U8
130186	229533.I1RHS0	pp	229533.I1RHS5
130187	229533.I1RCA0	pp	229533.I1RQC9
130188	229533.I1RCA0	pp	229533.I1RGL6
130189	229533.I1RCA0	pp	229533.I1RJR2
130190	229533.I1RCA0	pp	229533.I1RR39
130191	229533.I1RCA0	pp	229533.I1S2M5
130192	229533.I1RCA0	pp	229533.V6RR38
130193	229533.I1RCA0	pp	229533.I1RQ76
130194	229533.I1RCA0	pp	229533.I1RGK2
130195	229533.I1RCA0	pp	229533.I1RZ92
130196	229533.I1RCA0	pp	229533.I1REM2
130197	229533.I1RCA0	pp	229533.I1S130
130198	229533.I1RCA0	pp	229533.I1RJW1
130199	229533.I1RCA0	pp	229533.I1RJS0
130200	229533.I1RCA0	pp	229533.I1RPE5
130201	229533.I1RCA0	pp	229533.I1RT23
130202	229533.I1RCA0	pp	229533.V6RPN0
130203	229533.I1RCA0	pp	229533.I1RRY9
130204	229533.I1RCA0	pp	229533.I1S1Q3
130205	229533.I1RCA0	pp	229533.I1RZZ3
130206	229533.I1RCA0	pp	229533.V6RG22
130207	229533.I1RCA0	pp	229533.I1S2C2
130208	229533.I1RCA0	pp	229533.I1RY65
130209	229533.I1RCA0	pp	229533.I1REL9
130210	229533.I1RCA0	pp	229533.I1RIE8
130211	229533.I1RCA0	pp	229533.I1RNX0
130212	229533.I1RCA0	pp	229533.I1RXX1
130213	229533.I1RCA0	pp	229533.I1RJG1
130214	229533.I1RCA0	pp	229533.I1RI22
130215	229533.I1RCA0	pp	229533.I1RZ39
130216	229533.I1RCA0	pp	229533.Q4IJK2
130217	229533.I1RCA0	pp	229533.I1RNT1
130218	229533.I1RCA0	pp	229533.I1RTD5
130219	229533.I1RCA0	pp	229533.I1RF79
130220	229533.I1RCA0	pp	229533.V6RIE5
130221	229533.I1RCA0	pp	229533.I1RY35
130222	229533.I1RCA0	pp	229533.I1RR90
130223	229533.I1RCA0	pp	229533.I1RY81
130224	229533.I1RCA0	pp	229533.I1S2B8
130225	229533.I1RCA0	pp	229533.I1RIZ8
130226	229533.I1RCA0	pp	229533.I1RFM2
130227	229533.I1RCA0	pp	229533.I1S5K2
130228	229533.I1RCA0	pp	229533.I1RNC1
130229	229533.I1RCA0	pp	229533.I1RN67
130230	229533.I1RCA0	pp	229533.I1RD22
130231	229533.I1RCA0	pp	229533.I1RSQ3
130232	229533.I1RCA0	pp	229533.I1RR73

130233 229533.I1RCA0 pp 229533.V6RMW6
130234 229533.I1RCA0 pp 229533.I1RPP4
130235 229533.I1RCA0 pp 229533.I1RE80
130236 229533.I1RCA0 pp 229533.I1RK44
130237 229533.I1RCA0 pp 229533.I1SAS4
130238 229533.I1RCA0 pp 229533.I1S1W6
130239 229533.I1RCA0 pp 229533.I1RGC4
130240 229533.I1RCA0 pp 229533.I1RWN6
130241 229533.I1RCA0 pp 229533.I1RWN5
130242 229533.I1RCA0 pp 229533.I1RUP5
130243 229533.I1RCA0 pp 229533.I1RTH6
130244 229533.I1RCA0 pp 229533.Q4I1Y5
130245 229533.I1RCA0 pp 229533.I1RSX9
130246 229533.I1RCA0 pp 229533.I1SAS3
130247 229533.I1SAF0 pp 229533.Q4HVQ9
130248 229533.A0A1C3YLM8 pp 229533.I1RKT9
130249 229533.A0A1C3YLM8 pp 229533.I1RHS5
130250 229533.A0A1C3YLM8 pp 229533.I1RHS6
130251 229533.A0A1C3YLM8 pp 229533.I1RHS0
130252 229533.A0A1C3YLM8 pp 229533.I1S3U8
130253 229533.I1RCH5 pp 229533.I1S1W9
130254 229533.I1RCH5 pp 229533.V6RIV9
130255 229533.I1RCH5 pp 229533.I1RWN5
130256 229533.I1RCH5 pp 229533.I1RRE5
130257 229533.I1RCH5 pp 229533.I1RZV3
130258 229533.I1RCH5 pp 229533.I1S9S6
130259 229533.I1RCH5 pp 229533.I1S8V4
130260 229533.I1RCH5 pp 229533.I1S5R6
130261 229533.I1RCH5 pp 229533.V6RGI1
130262 229533.I1RCH5 pp 229533.I1RVZ7
130263 229533.I1RCH5 pp 229533.I1S1W3
130264 229533.I1RCH5 pp 229533.I1RM37
130265 229533.I1RCH5 pp 229533.I1RY44
130266 229533.I1RCH5 pp 229533.I1RPU2
130267 229533.I1RCH5 pp 229533.I1RSF1
130268 229533.I1RCH5 pp 229533.I1RG11
130269 229533.I1RCH5 pp 229533.I1RWQ8
130270 229533.I1RCH5 pp 229533.I1RS17
130271 229533.I1RCH5 pp 229533.I1SA33
130272 229533.I1RCH5 pp 229533.I1RSU2
130273 229533.I1RCH5 pp 229533.I1RQ21
130274 229533.I1RCH5 pp 229533.V6RLM7
130275 229533.I1RCH5 pp 229533.I1RD22
130276 229533.I1RCH5 pp 229533.I1RPK0
130277 229533.I1RCH5 pp 229533.I1S016
130278 229533.I1RCH5 pp 229533.I1RMV0
130279 229533.I1RCH5 pp 229533.I1RFV3
130280 229533.I1RCH5 pp 229533.I1RRN2
130281 229533.I1RCH5 pp 229533.I1S256
130282 229533.I1RCH5 pp 229533.I1RUG3
130283 229533.I1RCH5 pp 229533.I1RNC1
130284 229533.I1RCH5 pp 229533.I1RTA0
130285 229533.I1RCH5 pp 229533.I1RPP4
130286 229533.I1RCH5 pp 229533.Q4IQT8
130287 229533.I1RCH5 pp 229533.I1RXT3
130288 229533.I1RCH5 pp 229533.I1RW46
130289 229533.I1RCH5 pp 229533.I1RTN8
130290 229533.I1RCH5 pp 229533.I1RRV1
130291 229533.I1RCH5 pp 229533.I1RE01
130292 229533.I1RCH5 pp 229533.I1RKJ4
130293 229533.I1RCH5 pp 229533.I1RVF8
130294 229533.I1RCH5 pp 229533.I1RMG3
130295 229533.I1RCH5 pp 229533.I1SAN9
130296 229533.I1RCH5 pp 229533.I1S7W5
130297 229533.I1RCH5 pp 229533.I1S0J1
130298 229533.I1RCH5 pp 229533.Q4I624
130299 229533.I1RCH5 pp 229533.I1RRM3
130300 229533.I1RCH5 pp 229533.V6RMW6
130301 229533.I1RCH5 pp 229533.I1RD17
130302 229533.I1RCH5 pp 229533.V6R057
130303 229533.I1RCH5 pp 229533.I1RML0
130304 229533.I1RCH5 pp 229533.I1RXR1
130305 229533.I1RCH5 pp 229533.I1RRN5

130306	229533.I1RCH5	pp	229533.I1RPL4
130307	229533.I1RCH5	pp	229533.I1RQ33
130308	229533.I1RCH5	pp	229533.V6R042
130309	229533.I1RCH5	pp	229533.I1RSQ3
130310	229533.I1RCH5	pp	229533.I1RE79
130311	229533.I1RCH5	pp	229533.I1RCR1
130312	229533.I1RCH5	pp	229533.I1RT03
130313	229533.I1RCH5	pp	229533.I1RCR8
130314	229533.I1RCH5	pp	229533.I1RSY2
130315	229533.I1RCH5	pp	229533.I1RVX0
130316	229533.I1RCH5	pp	229533.I1RE89
130317	229533.I1RCH5	pp	229533.V6RRH2
130318	229533.I1RCH5	pp	229533.I1RVQ3
130319	229533.I1RCH5	pp	229533.I1RZD3
130320	229533.I1RCH5	pp	229533.I1RUF7
130321	229533.I1RCH5	pp	229533.I1RJV3
130322	229533.I1RCH5	pp	229533.Q4HXF6
130323	229533.I1RCH5	pp	229533.I1RN18
130324	229533.I1RCH5	pp	229533.I1RS47
130325	229533.I1RCH5	pp	229533.Q4IPZ1
130326	229533.I1RCH5	pp	229533.I1S8R3
130327	229533.I1RCH5	pp	229533.I1S0F8
130328	229533.I1RCH5	pp	229533.I1RFL4
130329	229533.I1RCH5	pp	229533.I1S5X9
130330	229533.I1RCH5	pp	229533.I1S9H4
130331	229533.I1RCH5	pp	229533.I1S1F3
130332	229533.I1RCH5	pp	229533.I1RTH6
130333	229533.I1RCH5	pp	229533.I1RQE7
130334	229533.I1RCH5	pp	229533.I1RZJ3
130335	229533.I1RCH5	pp	229533.I1RXU9
130336	229533.I1RCH5	pp	229533.I1RWN6
130337	229533.I1RCH5	pp	229533.I1RSA5
130338	229533.I1RCH5	pp	229533.I1RKJ8
130339	229533.I1RCH5	pp	229533.I1RD39
130340	229533.I1RCH5	pp	229533.I1RKB6
130341	229533.I1RCH5	pp	229533.I1S0F7
130342	229533.I1RCH5	pp	229533.I1S190
130343	229533.I1RCH5	pp	229533.I1RYW3
130344	229533.I1RCH5	pp	229533.I1S0F6
130345	229533.I1RCH5	pp	229533.I1S1C7
130346	229533.I1RCH5	pp	229533.I1S8P5
130347	229533.I1RCH5	pp	229533.I1RPP3
130348	229533.I1RCH5	pp	229533.I1RYI5
130349	229533.I1RCH5	pp	229533.I1RDF7
130350	229533.I1RCH5	pp	229533.I1RYR0
130351	229533.I1RCH5	pp	229533.I1S0W4
130352	229533.I1RCH5	pp	229533.I1RCW6
130353	229533.I1RCH5	pp	229533.I1S437
130354	229533.I1RCH5	pp	229533.I1RN67
130355	229533.I1RCH5	pp	229533.I1REV8
130356	229533.I1RCH5	pp	229533.I1RY62
130357	229533.I1RCH5	pp	229533.I1RZW8
130358	229533.I1RCH5	pp	229533.I1RFT1
130359	229533.I1RCH5	pp	229533.I1RWK3
130360	229533.I1RCH5	pp	229533.I1RZ79
130361	229533.I1RCH5	pp	229533.I1RN63
130362	229533.I1RCH5	pp	229533.I1RRI2
130363	229533.I1RCH5	pp	229533.I1RWX5
130364	229533.I1RCH5	pp	229533.I1S2M6
130365	229533.I1RCH5	pp	229533.I1S2L3
130366	229533.I1RCH5	pp	229533.I1RVZ2
130367	229533.I1RCH5	pp	229533.I1S464
130368	229533.I1RCH5	pp	229533.V6R3J1
130369	229533.I1RCH5	pp	229533.I1RFL7
130370	229533.I1RCH5	pp	229533.I1RTA1
130371	229533.I1RCH5	pp	229533.I1RTK5
130372	229533.I1RCH5	pp	229533.I1RJN9
130373	229533.I1RCH5	pp	229533.I1RRJ8
130374	229533.I1RCH5	pp	229533.I1RSZ7
130375	229533.I1RCH5	pp	229533.I1RGA4
130376	229533.I1RCH5	pp	229533.I1RW61
130377	229533.I1RCH5	pp	229533.I1S268
130378	229533.I1RCH5	pp	229533.I1RSX0

130379	229533.I1RCH5	pp	229533.V6RUY9
130380	229533.I1RCH5	pp	229533.I1RJV4
130381	229533.I1RCH5	pp	229533.I1RT75
130382	229533.I1RCH5	pp	229533.Q4I099
130383	229533.I1RCH5	pp	229533.I1RZB2
130384	229533.I1RCH5	pp	229533.I1RT04
130385	229533.I1RCH5	pp	229533.I1RQM7
130386	229533.I1RCH5	pp	229533.I1RL04
130387	229533.I1RCH5	pp	229533.I1RJ74
130388	229533.I1RCH5	pp	229533.I1RJS2
130389	229533.I1RCH5	pp	229533.Q4HXF0
130390	229533.I1RCH5	pp	229533.I1S503
130391	229533.I1RCH5	pp	229533.I1S6Y8
130392	229533.I1RCH5	pp	229533.I1RPV3
130393	229533.I1RCH5	pp	229533.I1RPM4
130394	229533.I1RCH5	pp	229533.I1S2E9
130395	229533.I1RCH5	pp	229533.I1RT40
130396	229533.I1RCH5	pp	229533.I1RFS3
130397	229533.I1RCH5	pp	229533.I1RRK3
130398	229533.I1RCH5	pp	229533.I1RJW6
130399	229533.I1RCH5	pp	229533.I1RV91
130400	229533.I1RCH5	pp	229533.I1RFL3
130401	229533.I1RCH5	pp	229533.I1RQ23
130402	229533.I1RCH5	pp	229533.Q4HVQ9
130403	229533.I1RCH5	pp	229533.I1RTD5
130404	229533.I1RCH5	pp	229533.I1RRZ0
130405	229533.I1RCH5	pp	229533.I1RN88
130406	229533.I1RCH5	pp	229533.I1S5R1
130407	229533.I1RCH5	pp	229533.I1RPE0
130408	229533.I1RCH5	pp	229533.I1S6B4
130409	229533.I1RCH5	pp	229533.I1RXF8
130410	229533.I1RCH5	pp	229533.I1RS20
130411	229533.I1RCH5	pp	229533.I1S2H4
130412	229533.I1RCH5	pp	229533.I1RY57
130413	229533.I1RCH5	pp	229533.I1RRT8
130414	229533.I1RCH5	pp	229533.I1S3G4
130415	229533.I1RCH5	pp	229533.I1S277
130416	229533.I1RCH5	pp	229533.I1RQS8
130417	229533.I1RCH5	pp	229533.I1RFI4
130418	229533.I1RCH5	pp	229533.I1RRL9
130419	229533.I1RCH5	pp	229533.I1RE27
130420	229533.I1RCH5	pp	229533.I1S4Z8
130421	229533.I1RCH5	pp	229533.I1RY92
130422	229533.I1RCH5	pp	229533.I1RHE5
130423	229533.I1RCH5	pp	229533.I1REC5
130424	229533.I1RCH5	pp	229533.I1S2B8
130425	229533.I1RCH5	pp	229533.I1RKP1
130426	229533.I1RCH5	pp	229533.I1RZ39
130427	229533.I1RCH5	pp	229533.I1RRM5
130428	229533.I1RCH5	pp	229533.V6R3X5
130429	229533.I1RCH5	pp	229533.I1RGB0
130430	229533.I1RCH5	pp	229533.I1RR73
130431	229533.I1RCH5	pp	229533.I1RRI1
130432	229533.I1RCH5	pp	229533.I1RE76
130433	229533.I1RCH5	pp	229533.I1RJZ7
130434	229533.I1RCH5	pp	229533.I1S2G9
130435	229533.I1RCH5	pp	229533.I1REX8
130436	229533.I1RCH5	pp	229533.I1S0L4
130437	229533.I1RCH5	pp	229533.I1RE22
130438	229533.I1RCH5	pp	229533.I1RSQ4
130439	229533.I1RCH5	pp	229533.I1RZZ9
130440	229533.I1RCH5	pp	229533.I1RTN3
130441	229533.I1RCH5	pp	229533.I1RGU7
130442	229533.I1RCH5	pp	229533.I1RKD3
130443	229533.I1RCH5	pp	229533.I1S0M6
130444	229533.I1RCH5	pp	229533.I1RWI5
130445	229533.I1RCH5	pp	229533.I1S9Y1
130446	229533.I1RCH5	pp	229533.I1RT38
130447	229533.I1RCH5	pp	229533.O74638
130448	229533.I1RCH5	pp	229533.I1RXC2
130449	229533.I1RCH5	pp	229533.I1S8V3
130450	229533.I1RCH5	pp	229533.I1RJ17
130451	229533.I1RCH5	pp	229533.I1RFH8

229533.I1RCH5	pp	229533.I1RWT6
229533.I1RCH5	pp	229533.I1SA32
229533.I1RCH5	pp	229533.V6RJP8
229533.I1RCH5	pp	229533.I1S504
229533.I1RCH5	pp	229533.I1S5B0
229533.I1RCH5	pp	229533.I1RQD2
229533.I1RCH5	pp	229533.I1RW99
229533.I1RCH5	pp	229533.I1RUY5
229533.I1RCH5	pp	229533.V6QUE6
229533.I1RCH5	pp	229533.Q4I283
229533.I1RCH5	pp	229533.I1RQ85
229533.I1RCH5	pp	229533.I1RG14
229533.I1RCH5	pp	229533.I1RR51
229533.I1RCH5	pp	229533.V6R227
229533.I1RCH5	pp	229533.I1RKZ4
229533.I1RCH5	pp	229533.I1S419
229533.I1RCH5	pp	229533.I1RFY0
229533.I1RCH5	pp	229533.I1RRV4
229533.I1RCH5	pp	229533.I1RZM1
229533.I1RCH5	pp	229533.I1RXF0
229533.I1RCH5	pp	229533.I1RNQ5
229533.I1RCH5	pp	229533.I1RZM8
229533.I1RCH5	pp	229533.I1RRV3
229533.I1RCH5	pp	229533.I1RLU6
229533.I1RCH5	pp	229533.I1S821
229533.I1RCH5	pp	229533.I1S1B2
229533.I1RCH5	pp	229533.I1RI82
229533.I1RCH5	pp	229533.I1RQ31
229533.I1RCH5	pp	229533.I1REK8
229533.I1RCH5	pp	229533.I1RQ81
229533.I1RCH5	pp	229533.I1RCR5
229533.I1RCH5	pp	229533.Q4I8P5
229533.I1RCH5	pp	229533.I1RGC5
229533.I1RCH5	pp	229533.I1S6K9
229533.I1RCH5	pp	229533.Q4I2J8
229533.I1RCH5	pp	229533.I1REL7
229533.I1RCH5	pp	229533.V6RCZ8
229533.I1RCH5	pp	229533.I1RS18
229533.I1RCH5	pp	229533.I1S217
229533.I1RCH5	pp	229533.V6RCG5
229533.I1RCH5	pp	229533.I1RXC4
229533.I1RCH5	pp	229533.I1S1C9
229533.I1RCH5	pp	229533.I1RF19
229533.I1RCH5	pp	229533.I1RDU0
229533.I1RCH5	pp	229533.I1RD52
229533.I1RCH5	pp	229533.I1RY86
229533.I1RCH5	pp	229533.I1RK95
229533.I1RCH5	pp	229533.I1RFJ9
229533.I1RCH5	pp	229533.I1RSI0
229533.I1RCH5	pp	229533.I1S0F5
229533.I1RCH5	pp	229533.I1S4Q4
229533.I1RCH5	pp	229533.I1RUQ3
229533.I1RCH5	pp	229533.V6R1K4
229533.I1RCH5	pp	229533.I1S0L3
229533.I1RCH5	pp	229533.Q4IJK2
229533.I1RCH5	pp	229533.I1RQA3
229533.I1RCH5	pp	229533.I1RWR7
229533.I1RCH5	pp	229533.I1RRY9
229533.I1RCH5	pp	229533.I1RWB0
229533.I1RCH5	pp	229533.I1RQQ5
229533.I1RCH5	pp	229533.V6RBN2
229533.I1RCH5	pp	229533.I1RWD4
229533.I1RCH5	pp	229533.I1RVQ0
229533.I1RCH5	pp	229533.V6R9Z0
229533.I1RCH5	pp	229533.I1RZZ1
229533.I1RCH5	pp	229533.I1S0P0
229533.I1RCH5	pp	229533.I1RGB2
229533.I1RCH5	pp	229533.I1S1J7
229533.I1RCH5	pp	229533.I1RK56
229533.I1RCH5	pp	229533.I1RXD6
229533.I1RCH5	pp	229533.I1RCM6
229533.I1RCH5	pp	229533.I1RYS0
229533.I1RCH5	pp	229533.I1RZK6

130525	229533.I1RCH5	pp	229533.I1RFU8
130526	229533.I1RCH5	pp	229533.I1RZB5
130527	229533.I1RCH5	pp	229533.I1RPR9
130528	229533.I1RCH5	pp	229533.I1RPN4
130529	229533.I1RCH5	pp	229533.I1RW71
130530	229533.I1RCH5	pp	229533.I1S887
130531	229533.I1RCH5	pp	229533.I1RGB1
130532	229533.I1RCH5	pp	229533.I1S4S7
130533	229533.I1RCH5	pp	229533.I1RHW8
130534	229533.I1RCH5	pp	229533.I1RCM5
130535	229533.I1RCH5	pp	229533.I1RZK5
130536	229533.I1RCH5	pp	229533.I1RSR3
130537	229533.I1RCH5	pp	229533.I1RRX5
130538	229533.I1RCH5	pp	229533.I1RY03
130539	229533.I1RCH5	pp	229533.I1RFC0
130540	229533.I1RCH5	pp	229533.I1RG53
130541	229533.I1RCH5	pp	229533.I1RX95
130542	229533.I1RCH5	pp	229533.I1RSQ7
130543	229533.I1RCH5	pp	229533.Q4IJT5
130544	229533.I1RCH5	pp	229533.V6QW22
130545	229533.I1RCH5	pp	229533.I1RNT9
130546	229533.I1RCH5	pp	229533.I1S8S9
130547	229533.I1RCH5	pp	229533.I1RGU0
130548	229533.I1RCH5	pp	229533.I1RYB5
130549	229533.I1RCH5	pp	229533.I1RQJ5
130550	229533.I1RCH5	pp	229533.I1S6T4
130551	229533.I1RCH5	pp	229533.I1RYT8
130552	229533.I1RCH5	pp	229533.I1RRG3
130553	229533.I1RCH5	pp	229533.I1RQF7
130554	229533.I1RCH5	pp	229533.I1S7A8
130555	229533.I1RCH5	pp	229533.I1RQC9
130556	229533.I1RCH5	pp	229533.I1S0R7
130557	229533.I1RCH5	pp	229533.Q4IFI0
130558	229533.I1RCH5	pp	229533.I1RUQ2
130559	229533.I1RCH5	pp	229533.I1RLP1
130560	229533.I1RCH5	pp	229533.V6RTJ8
130561	229533.I1RCH5	pp	229533.I1S9I5
130562	229533.I1RCH5	pp	229533.I1RXD7
130563	229533.I1RCH5	pp	229533.I1RX25
130564	229533.I1RCH5	pp	229533.I1RX46
130565	229533.I1RCH5	pp	229533.I1RME6
130566	229533.I1RCH5	pp	229533.I1RW25
130567	229533.I1RCH5	pp	229533.I1RTB3
130568	229533.I1RCH5	pp	229533.V6RUT5
130569	229533.I1RCH5	pp	229533.I1S0T2
130570	229533.I1RCH5	pp	229533.I1RR24
130571	229533.I1RCH5	pp	229533.I1S1W8
130572	229533.I1RCH5	pp	229533.V6QYF0
130573	229533.I1RCH5	pp	229533.I1RPY3
130574	229533.I1RCH5	pp	229533.I1RNB2
130575	229533.I1RCH5	pp	229533.I1RWJ8
130576	229533.I1RCH5	pp	229533.I1S4S4
130577	229533.I1RCH5	pp	229533.I1RN89
130578	229533.I1RCH5	pp	229533.I1RRS8
130579	229533.I1RCH5	pp	229533.I1RY21
130580	229533.I1RCH5	pp	229533.I1RW54
130581	229533.I1RCH5	pp	229533.I1RDW0
130582	229533.I1RCH5	pp	229533.I1RKJ5
130583	229533.I1RCH5	pp	229533.I1S438
130584	229533.I1RCH5	pp	229533.I1RM67
130585	229533.I1RCH5	pp	229533.I1S0X6
130586	229533.I1RCH5	pp	229533.I1RZJ5
130587	229533.I1RCH5	pp	229533.I1RE17
130588	229533.I1RCH5	pp	229533.I1RPA4
130589	229533.I1RCH5	pp	229533.I1RMC8
130590	229533.I1RCH5	pp	229533.I1RJU3
130591	229533.I1RCH5	pp	229533.I1RMG8
130592	229533.I1RCH5	pp	229533.I1RCS1
130593	229533.I1RHS5	pp	229533.I1RKT9
130594	229533.I1RHS5	pp	229533.I1S3U8
130595	229533.I1RJV3	pp	229533.I1RSS4
130596	229533.I1RJV3	pp	229533.I1RT03
130597	229533.I1RJV3	pp	229533.I1RRN5

130598 229533.I1RJV3 pp 229533.I1RXR1
130599 229533.I1RJV3 pp 229533.I1RSY2
130600 229533.I1RJV3 pp 229533.I1RPP4
130601 229533.I1RJV3 pp 229533.I1RVX0
130602 229533.I1RJV3 pp 229533.I1RMV0
130603 229533.I1RJV3 pp 229533.V6RM02
130604 229533.I1RJV3 pp 229533.V6RRH2
130605 229533.I1RJV3 pp 229533.I1RKC6
130606 229533.I1RJV3 pp 229533.I1S9Z4
130607 229533.I1RJV3 pp 229533.V6RIV9
130608 229533.I1RJV3 pp 229533.I1RWN5
130609 229533.I1RJV3 pp 229533.Q4HZB8
130610 229533.I1RJV3 pp 229533.Q4I624
130611 229533.I1RJV3 pp 229533.I1RML0
130612 229533.I1RJV3 pp 229533.I1S1W3
130613 229533.I1RJV3 pp 229533.I1S1W9
130614 229533.I1RJV3 pp 229533.I1S569
130615 229533.I1RJV3 pp 229533.I1RZV3
130616 229533.I1RJV3 pp 229533.I1RSD8
130617 229533.I1RJV3 pp 229533.V6R057
130618 229533.I1RJV3 pp 229533.I1RN18
130619 229533.I1RJV3 pp 229533.I1RQ33
130620 229533.I1RJV3 pp 229533.I1RM96
130621 229533.I1RJV3 pp 229533.I1RQE7
130622 229533.I1RJV3 pp 229533.I1S437
130623 229533.I1RJV3 pp 229533.I1RTD7
130624 229533.I1RJV3 pp 229533.I1S2E8
130625 229533.I1RJV3 pp 229533.I1RYW3
130626 229533.I1RJV3 pp 229533.I1S1W4
130627 229533.I1RJV3 pp 229533.I1RY44
130628 229533.I1RJV3 pp 229533.I1RTE9
130629 229533.I1RJV3 pp 229533.I1RRE5
130630 229533.I1RJV3 pp 229533.I1S8R3
130631 229533.I1RJV3 pp 229533.I1S2C0
130632 229533.I1RJV3 pp 229533.I1S1C7
130633 229533.I1RJV3 pp 229533.I1RPU2
130634 229533.I1RJV3 pp 229533.I1RRR7
130635 229533.I1RJV3 pp 229533.I1RZW8
130636 229533.I1RJV3 pp 229533.I1RWX5
130637 229533.I1RJV3 pp 229533.Q4IN63
130638 229533.I1RJV3 pp 229533.I1RKJ8
130639 229533.I1RJV3 pp 229533.I1RNC1
130640 229533.I1RJV3 pp 229533.I1RQJ4
130641 229533.I1RJV3 pp 229533.I1RSK1
130642 229533.I1RJV3 pp 229533.I1RXT3
130643 229533.I1RJV3 pp 229533.I1RXU9
130644 229533.I1RJV3 pp 229533.I1RZ79
130645 229533.I1RJV3 pp 229533.I1RWN6
130646 229533.I1RJV3 pp 229533.I1RRI2
130647 229533.I1RJV3 pp 229533.I1RTH6
130648 229533.I1RJV3 pp 229533.I1RUG3
130649 229533.I1RJV3 pp 229533.I1RZS7
130650 229533.I1RJV3 pp 229533.V6RMW6
130651 229533.I1RJV3 pp 229533.I1RTA0
130652 229533.I1RJV3 pp 229533.I1RNY2
130653 229533.I1RJV3 pp 229533.I1RW46
130654 229533.I1RJV3 pp 229533.Q4I327
130655 229533.I1RJV3 pp 229533.I1SA33
130656 229533.I1RJV3 pp 229533.I1S0J1
130657 229533.I1RJV3 pp 229533.I1RMN6
130658 229533.I1RJV3 pp 229533.I1RRV1
130659 229533.I1RJV3 pp 229533.I1RRN2
130660 229533.I1RJV3 pp 229533.I1RSQ3
130661 229533.I1RJV3 pp 229533.I1S7W5
130662 229533.I1RJV3 pp 229533.I1RMG3
130663 229533.I1RJV3 pp 229533.I1S5R6
130664 229533.I1RJV3 pp 229533.I1RS17
130665 229533.I1RJV3 pp 229533.I1S256
130666 229533.I1RJV3 pp 229533.I1RPK0
130667 229533.I1RJV3 pp 229533.V6R042
130668 229533.I1RJV3 pp 229533.I1S7S9
130669 229533.I1RJV3 pp 229533.I1RPE0
130670 229533.I1RJV3 pp 229533.I1RRM3

130671	229533.I1RJV3	pp	229533.V6RGN5
130672	229533.I1RJV3	pp	229533.I1RS20
130673	229533.I1RJV3	pp	229533.I1RWQ8
130674	229533.I1RJV3	pp	229533.I1S2H4
130675	229533.I1RJV3	pp	229533.I1RPU3
130676	229533.I1RJV3	pp	229533.I1S0L4
130677	229533.I1RJV3	pp	229533.I1S0N4
130678	229533.I1RJV3	pp	229533.I1S277
130679	229533.I1RJV3	pp	229533.I1RL04
130680	229533.I1RJV3	pp	229533.I1RRI1
130681	229533.I1RJV3	pp	229533.I1RSZ7
130682	229533.I1RJV3	pp	229533.Q4IF76
130683	229533.I1RJV3	pp	229533.I1RPV3
130684	229533.I1RJV3	pp	229533.I1S6Y8
130685	229533.I1RJV3	pp	229533.I1RWI5
130686	229533.I1RJV3	pp	229533.V6R283
130687	229533.I1RJV3	pp	229533.I1RN63
130688	229533.I1RJV3	pp	229533.I1RPM4
130689	229533.I1RJV3	pp	229533.I1RYR0
130690	229533.I1RJV3	pp	229533.Q4I662
130691	229533.I1RJV3	pp	229533.I1RZZ9
130692	229533.I1RJV3	pp	229533.I1RSQ4
130693	229533.I1RJV3	pp	229533.I1RN88
130694	229533.I1RJV3	pp	229533.I1RWK3
130695	229533.I1RJV3	pp	229533.I1S8E2
130696	229533.I1RJV3	pp	229533.I1RVZ2
130697	229533.I1RJV3	pp	229533.I1RNN1
130698	229533.I1RJV3	pp	229533.I1RTA1
130699	229533.I1RJV3	pp	229533.I1RQ23
130700	229533.I1RJV3	pp	229533.I1RZ39
130701	229533.I1RJV3	pp	229533.I1RSV2
130702	229533.I1RJV3	pp	229533.I1RZS6
130703	229533.I1RJV3	pp	229533.I1S352
130704	229533.I1RJV3	pp	229533.I1S0M6
130705	229533.I1RJV3	pp	229533.I1RR73
130706	229533.I1RJV3	pp	229533.I1RND6
130707	229533.I1RJV3	pp	229533.I1RJZ7
130708	229533.I1RJV3	pp	229533.I1S2B8
130709	229533.I1RJV3	pp	229533.V6R3X5
130710	229533.I1RJV3	pp	229533.I1RKL3
130711	229533.I1RJV3	pp	229533.I1RRJ8
130712	229533.I1RJV3	pp	229533.I1RRM5
130713	229533.I1RJV3	pp	229533.I1RQG4
130714	229533.I1RJV3	pp	229533.I1S9Y1
130715	229533.I1RJV3	pp	229533.I1RKJ6
130716	229533.I1RJV3	pp	229533.I1RQ65
130717	229533.I1RJV3	pp	229533.I1RYI5
130718	229533.I1RJV3	pp	229533.I1RS47
130719	229533.I1RJV3	pp	229533.I1RV91
130720	229533.I1RJV3	pp	229533.I1RT40
130721	229533.I1RJV3	pp	229533.I1RWF7
130722	229533.I1RJV3	pp	229533.I1RZV6
130723	229533.I1RJV3	pp	229533.I1RM82
130724	229533.I1RJV3	pp	229533.I1RXF8
130725	229533.I1RJV3	pp	229533.I1RWL2
130726	229533.I1RJV3	pp	229533.I1S8P5
130727	229533.I1RJV3	pp	229533.I1S5R1
130728	229533.I1RJV3	pp	229533.I1RTD5
130729	229533.I1RJV3	pp	229533.I1RPP3
130730	229533.I1RJV3	pp	229533.I1RRT8
130731	229533.I1RJV3	pp	229533.I1S0F6
130732	229533.I1RJV3	pp	229533.I1RT04
130733	229533.I1RJV3	pp	229533.I1S0W4
130734	229533.I1RJV3	pp	229533.V6RUY9
130735	229533.I1RJV3	pp	229533.I1RXC2
130736	229533.I1RJV3	pp	229533.I1RN67
130737	229533.I1RJV3	pp	229533.O74638
130738	229533.I1RJV3	pp	229533.I1RY62
130739	229533.I1RJV3	pp	229533.I1RR68
130740	229533.I1RJV3	pp	229533.I1RQ81
130741	229533.I1RJV3	pp	229533.I1RRQ3
130742	229533.I1RJV3	pp	229533.I1RNA5
130743	229533.I1RJV3	pp	229533.I1RP28

130744	229533.I1RJV3	pp	229533.I1S2E9
130745	229533.I1RJV3	pp	229533.I1S0M9
130746	229533.I1RJV3	pp	229533.I1S503
130747	229533.I1RJV3	pp	229533.I1RJW6
130748	229533.I1RJV3	pp	229533.Q4I8P5
130749	229533.I1RJV3	pp	229533.I1RQA3
130750	229533.I1RJV3	pp	229533.I1RS18
130751	229533.I1RJV3	pp	229533.I1RRZ0
130752	229533.I1RJV3	pp	229533.Q4IJK2
130753	229533.I1RJV3	pp	229533.I1RLN6
130754	229533.I1RJV3	pp	229533.I1RQQ5
130755	229533.I1RJV3	pp	229533.I1S419
130756	229533.I1RJV3	pp	229533.I1RQN9
130757	229533.I1RJV3	pp	229533.I1RLV9
130758	229533.I1RJV3	pp	229533.I1RT35
130759	229533.I1RJV3	pp	229533.I1RZM1
130760	229533.I1RJV3	pp	229533.I1RSX0
130761	229533.I1RJV3	pp	229533.V6RBN2
130762	229533.I1RJV3	pp	229533.Q4ICA8
130763	229533.I1RJV3	pp	229533.I1RJZ6
130764	229533.I1RJV3	pp	229533.I1RRT2
130765	229533.I1RJV3	pp	229533.I1RZK6
130766	229533.I1RJV3	pp	229533.V6R3J1
130767	229533.I1RJV3	pp	229533.I1RNQ5
130768	229533.I1RJV3	pp	229533.I1RZZ1
130769	229533.I1RJV3	pp	229533.I1RLU6
130770	229533.I1RJV3	pp	229533.I1RK95
130771	229533.I1RJV3	pp	229533.I1RZB2
130772	229533.I1RJV3	pp	229533.I1RN99
130773	229533.I1RJV3	pp	229533.I1S504
130774	229533.I1RJV3	pp	229533.I1RJV4
130775	229533.I1RJV3	pp	229533.I1RMD5
130776	229533.I1RJV3	pp	229533.I1RP27
130777	229533.I1RJV3	pp	229533.I1RZF6
130778	229533.I1RJV3	pp	229533.I1S887
130779	229533.I1RJV3	pp	229533.Q4I2J8
130780	229533.I1RJV3	pp	229533.I1RZ35
130781	229533.I1RJV3	pp	229533.I1RWT6
130782	229533.I1RJV3	pp	229533.I1RQ85
130783	229533.I1RJV3	pp	229533.I1RVZ0
130784	229533.I1RJV3	pp	229533.I1RZK5
130785	229533.I1RJV3	pp	229533.V6R227
130786	229533.I1RJV3	pp	229533.I1RSI0
130787	229533.I1RJV3	pp	229533.I1RWB0
130788	229533.I1RJV3	pp	229533.I1S4Q4
130789	229533.I1RJV3	pp	229533.I1S1J7
130790	229533.I1RJV3	pp	229533.Q4I283
130791	229533.I1RJV3	pp	229533.I1RPR9
130792	229533.I1RJV3	pp	229533.I1RLM7
130793	229533.I1RJV3	pp	229533.I1RYS0
130794	229533.I1RJV3	pp	229533.I1RZE5
130795	229533.I1RJV3	pp	229533.I1RXF0
130796	229533.I1RJV3	pp	229533.I1RWD7
130797	229533.I1RJV3	pp	229533.I1RXX1
130798	229533.I1RJV3	pp	229533.I1RZM8
130799	229533.I1RJV3	pp	229533.I1RSR8
130800	229533.I1RJV3	pp	229533.I1RN58
130801	229533.I1RJV3	pp	229533.I1RRY9
130802	229533.I1RJV3	pp	229533.I1S021
130803	229533.I1RJV3	pp	229533.I1RQ31
130804	229533.I1RJV3	pp	229533.I1RY03
130805	229533.I1RJV3	pp	229533.I1RWR7
130806	229533.I1RJV3	pp	229533.I1RU29
130807	229533.I1RJV3	pp	229533.P0C431
130808	229533.I1RJV3	pp	229533.I1RNH6
130809	229533.I1RJV3	pp	229533.I1RXC4
130810	229533.I1RJV3	pp	229533.I1S6K9
130811	229533.I1RJV3	pp	229533.I1RYB9
130812	229533.I1RJV3	pp	229533.I1SA32
130813	229533.I1RJV3	pp	229533.V6RCG5
130814	229533.I1RJV3	pp	229533.I1RZH4
130815	229533.I1RJV3	pp	229533.I1S438
130816	229533.I1RJV3	pp	229533.V6RCZ8

130817	229533.I1RJV3	pp	229533.I1RR51
130818	229533.I1RJV3	pp	229533.Q4IFI0
130819	229533.I1RJV3	pp	229533.I1RRV4
130820	229533.I1RJV3	pp	229533.I1RWH5
130821	229533.I1RJV3	pp	229533.V6QUE6
130822	229533.I1RJV3	pp	229533.I1RPW9
130823	229533.I1RJV3	pp	229533.I1RX25
130824	229533.I1RJV3	pp	229533.I1RLC7
130825	229533.I1RJV3	pp	229533.I1RRU9
130826	229533.I1RJV3	pp	229533.I1RRG3
130827	229533.I1RJV3	pp	229533.I1RQ26
130828	229533.I1RJV3	pp	229533.I1RW25
130829	229533.I1RJV3	pp	229533.Q4IJT5
130830	229533.I1RJV3	pp	229533.I1RYJ8
130831	229533.I1RJV3	pp	229533.I1RW99
130832	229533.I1RJV3	pp	229533.I1RW07
130833	229533.I1RJV3	pp	229533.I1RQD2
130834	229533.I1RJV3	pp	229533.I1RR00
130835	229533.I1RJV3	pp	229533.I1RPN4
130836	229533.I1RJV3	pp	229533.I1RMC8
130837	229533.I1RJV3	pp	229533.I1S5B0
130838	229533.I1RJV3	pp	229533.I1S0X6
130839	229533.I1RJV3	pp	229533.I1S044
130840	229533.I1RJV3	pp	229533.I1S4S7
130841	229533.I1RJV3	pp	229533.Q4IJH1
130842	229533.I1RJV3	pp	229533.I1RPA4
130843	229533.I1RJV3	pp	229533.I1RW71
130844	229533.I1RJV3	pp	229533.I1RNA0
130845	229533.I1RJV3	pp	229533.I1RTF4
130846	229533.I1RJV3	pp	229533.I1RN61
130847	229533.I1RJV3	pp	229533.V6QW22
130848	229533.I1RJV3	pp	229533.I1S6U8
130849	229533.I1RJV3	pp	229533.I1RT80
130850	229533.I1RJV3	pp	229533.I1RRX5
130851	229533.I1RJV3	pp	229533.V6QUW4
130852	229533.I1RJV3	pp	229533.I1RPU0
130853	229533.I1RJV3	pp	229533.I1RMK3
130854	229533.I1RJV3	pp	229533.I1RM06
130855	229533.I1RJV3	pp	229533.I1RQJ5
130856	229533.I1RJV3	pp	229533.I1S0R7
130857	229533.I1RJV3	pp	229533.I1RSQ7
130858	229533.I1RJV3	pp	229533.I1RWJ8
130859	229533.I1RJV3	pp	229533.Q5I2J3
130860	229533.I1RJV3	pp	229533.I1RUQ2
130861	229533.I1RJV3	pp	229533.I1S4S4
130862	229533.I1RJV3	pp	229533.I1RQC9
130863	229533.I1RJV3	pp	229533.I1RLP1
130864	229533.I1RJV3	pp	229533.V6R4J1
130865	229533.I1RJV3	pp	229533.I1RMG8
130866	229533.I1RJV3	pp	229533.I1RM67
130867	229533.I1RJV3	pp	229533.I1RVQ0
130868	229533.I1RJV3	pp	229533.I1RT33
130869	229533.I1RJV3	pp	229533.I1RZJ5
130870	229533.I1RJV3	pp	229533.I1RQH3
130871	229533.I1RJV3	pp	229533.I1S0F5
130872	229533.I1RJV3	pp	229533.I1RTB7
130873	229533.I1RJV3	pp	229533.V6RUT5
130874	229533.I1RJV3	pp	229533.I1S9U2
130875	229533.I1RJV3	pp	229533.I1RR24
130876	229533.I1RJV3	pp	229533.I1S0T2
130877	229533.I1RJV3	pp	229533.I1S0L3
130878	229533.I1RJV3	pp	229533.Q4IPI1
130879	229533.I1RJV3	pp	229533.I1S0S6
130880	229533.I1RJV3	pp	229533.I1RX46
130881	229533.I1RJV3	pp	229533.V6R1K4
130882	229533.I1RJV3	pp	229533.I1S7A8
130883	229533.I1RJV3	pp	229533.V6RTJ8
130884	229533.I1RJB0	pp	229533.I1S4G3
130885	229533.I1RJB0	pp	229533.I1RQE1
130886	229533.I1RJB0	pp	229533.I1RUQ3
130887	229533.I1RJB0	pp	229533.I1RWZ3
130888	229533.I1RJB0	pp	229533.I1S1B8
130889	229533.I1RJB0	pp	229533.I1RNG4

130890 229533.I1RJB0 pp 229533.Q4HVQ9
130891 229533.I1RJB0 pp 229533.I1RTY0
130892 229533.I1RJB0 pp 229533.I1S2Q4
130893 229533.I1RJB0 pp 229533.I1RJJ7
130894 229533.I1RJB0 pp 229533.I1S3G4
130895 229533.I1RJB0 pp 229533.I1RYX6
130896 229533.I1RJB0 pp 229533.I1S1W8
130897 229533.I1RKT9 pp 229533.I1RQ70
130898 229533.I1RKT9 pp 229533.I1S3U8
130899 229533.I1RKT9 pp 229533.I1RWI6
130900 229533.I1RKT9 pp 229533.I1RW76
130901 229533.I1RKT9 pp 229533.I1RZ92
130902 229533.A0A1C3YHF1 pp 229533.A0A1C3YLB6
130903 229533.A0A1C3YHF1 pp 229533.I1RNG4
130904 229533.A0A1C3YIN6 pp 229533.I1RZM1
130905 229533.A0A1C3YIN6 pp 229533.Q4ICA8
130906 229533.A0A1C3YIN6 pp 229533.Q4HYI0
130907 229533.A0A1C3YIN6 pp 229533.I1RQ81
130908 229533.A0A1C3YIN6 pp 229533.I1RMQ7
130909 229533.A0A1C3YIN6 pp 229533.I1RC17
130910 229533.A0A1C3YIN6 pp 229533.V6RDP1
130911 229533.A0A1C3YIN6 pp 229533.A0A1C3YK34
130912 229533.A0A1C3YIN6 pp 229533.I1RKK0
130913 229533.A0A1C3YIN6 pp 229533.I1RRT0
130914 229533.A0A1C3YIN6 pp 229533.I1RAM7
130915 229533.A0A1C3YIN6 pp 229533.I1RSI0
130916 229533.A0A1C3YIN6 pp 229533.I1RZB5
130917 229533.A0A1C3YIN6 pp 229533.I1RN08
130918 229533.A0A1C3YIN6 pp 229533.I1RFU8
130919 229533.A0A1C3YIN6 pp 229533.A0A1C3YIS2
130920 229533.A0A1C3YIN6 pp 229533.I1RFT2
130921 229533.A0A1C3YIN6 pp 229533.I1RAU5
130922 229533.A0A1C3YIN6 pp 229533.I1RC95
130923 229533.A0A1C3YIN6 pp 229533.Q4IJK2
130924 229533.A0A1C3YIN6 pp 229533.I1RQA3
130925 229533.A0A1C3YIN6 pp 229533.I1S504
130926 229533.A0A1C3YIN6 pp 229533.I1REN3
130927 229533.A0A1C3YIN6 pp 229533.I1RJL2
130928 229533.A0A1C3YIN6 pp 229533.I1RWT6
130929 229533.A0A1C3YIN6 pp 229533.I1RZK5
130930 229533.A0A1C3YIN6 pp 229533.A0A1C3YKF2
130931 229533.A0A1C3YIN6 pp 229533.I1RY03
130932 229533.A0A1C3YIN6 pp 229533.I1RFQ7
130933 229533.A0A1C3YIN6 pp 229533.I1S439
130934 229533.A0A1C3YIN6 pp 229533.I1RFQ5
130935 229533.A0A1C3YIN6 pp 229533.I1RUQ5
130936 229533.A0A1C3YIN6 pp 229533.I1RA43
130937 229533.A0A1C3YIN6 pp 229533.I1RPP4
130938 229533.A0A1C3YIN6 pp 229533.I1RC22
130939 229533.A0A1C3YIN6 pp 229533.I1RAT7
130940 229533.A0A1C3YIN6 pp 229533.I1RQH3
130941 229533.A0A1C3YIN6 pp 229533.I1R9Y9
130942 229533.A0A1C3YIN6 pp 229533.I1RYY5
130943 229533.A0A1C3YIN6 pp 229533.I1RHB0
130944 229533.A0A1C3YIN6 pp 229533.I1S1W4
130945 229533.A0A1C3YIN6 pp 229533.I1RAX6
130946 229533.A0A1C3YIN6 pp 229533.I1RM49
130947 229533.A0A1C3YIN6 pp 229533.I1RRY9
130948 229533.A0A1C3YIN6 pp 229533.I1RYZ9
130949 229533.A0A1C3YIN6 pp 229533.I1RMM9
130950 229533.A0A1C3YIN6 pp 229533.V6RKY7
130951 229533.A0A1C3YIN6 pp 229533.I1RKB1
130952 229533.A0A1C3YIN6 pp 229533.V6RTJ8
130953 229533.A0A1C3YIN6 pp 229533.A0A1C3YN55
130954 229533.A0A1C3YIN6 pp 229533.I1RW71
130955 229533.A0A1C3YIN6 pp 229533.I1RWN5
130956 229533.A0A1C3YIN6 pp 229533.I1RC20
130957 229533.A0A1C3YIN6 pp 229533.I1RN61
130958 229533.A0A1C3YIN6 pp 229533.I1RQ26
130959 229533.A0A1C3YIN6 pp 229533.I1RQ13
130960 229533.A0A1C3YIN6 pp 229533.I1RCU8
130961 229533.A0A1C3YIN6 pp 229533.V6RVG3
130962 229533.A0A1C3YIN6 pp 229533.I1RL24

130963	229533.A0A1C3YIN6	pp	229533.A0A1C3YJT5
130964	229533.A0A1C3YIN6	pp	229533.I1RY44
130965	229533.A0A1C3YIN6	pp	229533.I1RRG3
130966	229533.A0A1C3YIN6	pp	229533.V6QV09
130967	229533.A0A1C3YIN6	pp	229533.I1RRV1
130968	229533.A0A1C3YIN6	pp	229533.I1RYH9
130969	229533.A0A1C3YIN6	pp	229533.I1RJH1
130970	229533.A0A1C3YIN6	pp	229533.V6RMW6
130971	229533.A0A1C3YIN6	pp	229533.I1RDB1
130972	229533.A0A1C3YIN6	pp	229533.I1RCB7
130973	229533.A0A1C3YIN6	pp	229533.I1RT62
130974	229533.A0A1C3YIN6	pp	229533.I1RQC9
130975	229533.A0A1C3YIN6	pp	229533.I1RX70
130976	229533.A0A1C3YIN6	pp	229533.I1RW90
130977	229533.A0A1C3YIN6	pp	229533.I1RXU0
130978	229533.A0A1C3YIN6	pp	229533.I1R9Y4
130979	229533.A0A1C3YIN6	pp	229533.V6RGI1
130980	229533.A0A1C3YIN6	pp	229533.I1RG11
130981	229533.A0A1C3YIN6	pp	229533.I1S0M2
130982	229533.A0A1C3YIN6	pp	229533.A0A1C3YKC0
130983	229533.A0A1C3YIN6	pp	229533.A0A1C3YK57
130984	229533.A0A1C3YIN6	pp	229533.A0A1C3YL67
130985	229533.A0A1C3YIN6	pp	229533.I1RNC1
130986	229533.A0A1C3YIN6	pp	229533.I1RVL8
130987	229533.A0A1C3YIN6	pp	229533.I1S0N4
130988	229533.A0A1C3YIN6	pp	229533.A0A1C3YJC3
130989	229533.A0A1C3YIN6	pp	229533.I1RGC6
130990	229533.A0A1C3YIN6	pp	229533.I1RUN8
130991	229533.A0A1C3YIN6	pp	229533.I1RSK1
130992	229533.A0A1C3YIN6	pp	229533.I1RD22
130993	229533.A0A1C3YIN6	pp	229533.Q4IPI4
130994	229533.A0A1C3YIN6	pp	229533.I1RRN5
130995	229533.A0A1C3YIN6	pp	229533.I1RFJ6
130996	229533.A0A1C3YIN6	pp	229533.I1RSV2
130997	229533.A0A1C3YIN6	pp	229533.I1RVX0
130998	229533.A0A1C3YIN6	pp	229533.I1S2B8
130999	229533.A0A1C3YIN6	pp	229533.I1RFL7
131000	229533.A0A1C3YIN6	pp	229533.I1S0J7
131001	229533.A0A1C3YIN6	pp	229533.I1RJZ7
131002	229533.A0A1C3YIN6	pp	229533.I1RDZ4
131003	229533.A0A1C3YIN6	pp	229533.I1S0E8
131004	229533.A0A1C3YIN6	pp	229533.I1RPP3
131005	229533.A0A1C3YIN6	pp	229533.I1RZE4
131006	229533.A0A1C3YIN6	pp	229533.I1RR25
131007	229533.A0A1C3YIN6	pp	229533.I1RPU3
131008	229533.A0A1C3YIN6	pp	229533.I1RC24
131009	229533.A0A1C3YIN6	pp	229533.I1RC97
131010	229533.A0A1C3YIN6	pp	229533.I1RAU7
131011	229533.A0A1C3YIN6	pp	229533.I1RN67
131012	229533.A0A1C3YIN6	pp	229533.A0A1C3YKM9
131013	229533.A0A1C3YIN6	pp	229533.I1RD15
131014	229533.A0A1C3YIN6	pp	229533.A0A1C3YM61
131015	229533.A0A1C3YIN6	pp	229533.I1RZ39
131016	229533.A0A1C3YIN6	pp	229533.I1RK75
131017	229533.A0A1C3YIN6	pp	229533.I1R9N1
131018	229533.A0A1C3YIN6	pp	229533.I1S0J3
131019	229533.A0A1C3YIN6	pp	229533.I1R9Y7
131020	229533.A0A1C3YIN6	pp	229533.I1S2E9
131021	229533.A0A1C3YIN6	pp	229533.I1RTH6
131022	229533.A0A1C3YIN6	pp	229533.I1RA70
131023	229533.A0A1C3YIN6	pp	229533.I1RK79
131024	229533.A0A1C3YIN6	pp	229533.I1S098
131025	229533.A0A1C3YIN6	pp	229533.V6R3X5
131026	229533.A0A1C3YIN6	pp	229533.I1RCJ7
131027	229533.A0A1C3YIN6	pp	229533.I1RR73
131028	229533.A0A1C3YIN6	pp	229533.I1RAW0
131029	229533.A0A1C3YIN6	pp	229533.I1RZ63
131030	229533.A0A1C3YIN6	pp	229533.I1RRF0
131031	229533.A0A1C3YIN6	pp	229533.I1RV91
131032	229533.A0A1C3YIN6	pp	229533.I1RTD5
131033	229533.A0A1C3YIN6	pp	229533.I1RWN6
131034	229533.A0A1C3YIN6	pp	229533.I1RAN2
131035	229533.A0A1C3YIN6	pp	229533.I1RCM3

131036 229533.A0A1C3YIN6 pp 229533.A0A1C3YIR8
131037 229533.A0A1C3YIN6 pp 229533.I1RPM7
131038 229533.A0A1C3YIN6 pp 229533.I1RWI5
131039 229533.A0A1C3YIN6 pp 229533.I1RSZ3
131040 229533.A0A1C3YIN6 pp 229533.I1RP27
131041 229533.A0A1C3YIN6 pp 229533.I1RNN1
131042 229533.A0A1C3YIN6 pp 229533.I1RCU9
131043 229533.A0A1C3YIN6 pp 229533.Q4I7L0
131044 229533.A0A1C3YIN6 pp 229533.I1RM82
131045 229533.A0A1C3YIN6 pp 229533.I1S503
131046 229533.A0A1C3YIN6 pp 229533.I1RUF8
131047 229533.A0A1C3YIN6 pp 229533.I1RWL2
131048 229533.A0A1C3YIN6 pp 229533.I1RNP7
131049 229533.I1RQ60 pp 229533.I1S4G3
131050 229533.I1RQ60 pp 229533.I1RWF5
131051 229533.I1RQ60 pp 229533.I1RXY7
131052 229533.I1RQ60 pp 229533.I1RVX9
131053 229533.I1RQ60 pp 229533.I1RSQ9
131054 229533.I1RQ60 pp 229533.I1S2Q4
131055 229533.I1RQ60 pp 229533.I1RTY0
131056 229533.I1RQ60 pp 229533.I1RZ83
131057 229533.I1RQ60 pp 229533.I1RY74
131058 229533.I1RQ60 pp 229533.I1RWZ3
131059 229533.I1RQ60 pp 229533.I1RQE1
131060 229533.I1RQ60 pp 229533.I1S3G4
131061 229533.I1RQ60 pp 229533.I1S1B8
131062 229533.I1RQ60 pp 229533.I1RY12
131063 229533.I1RQ60 pp 229533.I1RYX6
131064 229533.I1RQ60 pp 229533.I1RUR8
131065 229533.I1RQ60 pp 229533.I1RY49
131066 229533.I1RQ60 pp 229533.V6QWD8
131067 229533.I1RQ60 pp 229533.I1S1W8
131068 229533.I1RHS6 pp 229533.I1RKT9
131069 229533.I1RHS6 pp 229533.I1S3U8
131070 229533.I1RZE4 pp 229533.I1S1D3
131071 229533.I1RZE4 pp 229533.I1S2K7
131072 229533.I1RZE4 pp 229533.I1S0D1
131073 229533.I1RZE4 pp 229533.V6RGI1
131074 229533.I1RZE4 pp 229533.I1S0M2
131075 229533.I1RZE4 pp 229533.V6RKY7
131076 229533.I1RZE4 pp 229533.V6RVG3
131077 229533.I1RZE4 pp 229533.I1S1W4
131078 229533.I1RZE4 pp 229533.I1RZM1
131079 229533.I1RZE4 pp 229533.V6RDP1
131080 229533.I1RZE4 pp 229533.I1S2G7
131081 229533.I1RZE4 pp 229533.I1S8P5
131082 229533.I1RZE4 pp 229533.I1S0N4
131083 229533.I1RZE4 pp 229533.Q4IJM4
131084 229533.I1RZE4 pp 229533.I1S0Z7
131085 229533.I1RZE4 pp 229533.I1S039
131086 229533.I1RZE4 pp 229533.V6QV09
131087 229533.I1RZE4 pp 229533.I1S439
131088 229533.I1RZE4 pp 229533.I1S2E9
131089 229533.I1RZE4 pp 229533.I1S224
131090 229533.I1RZE4 pp 229533.I1RZH4
131091 229533.I1RZE4 pp 229533.V6RTJ8
131092 229533.A0A098DWX8 pp 229533.V6RGI1
131093 229533.A0A098DWX8 pp 229533.I1RHS6
131094 229533.A0A098DWX8 pp 229533.I1RHS5
131095 229533.A0A098DWX8 pp 229533.I1RKT9
131096 229533.A0A098DWX8 pp 229533.I1R9Y7
131097 229533.A0A098DWX8 pp 229533.I1RYZ9
131098 229533.A0A098DWX8 pp 229533.A0A1C3YLM8
131099 229533.A0A098DWX8 pp 229533.A0A098E0E1
131100 229533.A0A098DWX8 pp 229533.I1RHS0
131101 229533.A0A098DWX8 pp 229533.I1RFL7
131102 229533.A0A098DWX8 pp 229533.I1RL24
131103 229533.A0A098DWX8 pp 229533.I1S3U8
131104 229533.A0A098DWX8 pp 229533.V6QV09
131105 229533.A0A098DWX8 pp 229533.A0A1C3YHL0
131106 229533.I1RN61 pp 229533.I1RSM2
131107 229533.I1RN61 pp 229533.I1RV91
131108 229533.I1RN61 pp 229533.I1RXU7

131109	229533.I1RN61	pp	229533.I1RTD5
131110	229533.I1RN61	pp	229533.I1RXD7
131111	229533.I1RN61	pp	229533.I1S2E9
131112	229533.I1RN61	pp	229533.I1RQH3
131113	229533.I1RN61	pp	229533.V6R3X5
131114	229533.I1RN61	pp	229533.I1RWI5
131115	229533.I1RN61	pp	229533.I1RZ39
131116	229533.I1RN61	pp	229533.I1RR73
131117	229533.I1RN61	pp	229533.I1S0R7
131118	229533.I1RN61	pp	229533.I1RQC9
131119	229533.I1RN61	pp	229533.I1RT04
131120	229533.I1RN61	pp	229533.I1RX46
131121	229533.I1RN61	pp	229533.I1RS20
131122	229533.I1RN61	pp	229533.I1RWL2
131123	229533.I1RN61	pp	229533.V6QUW4
131124	229533.I1RN61	pp	229533.I1S0B7
131125	229533.I1RN61	pp	229533.I1S503
131126	229533.I1RN61	pp	229533.Q4IAS1
131127	229533.I1RN61	pp	229533.I1S854
131128	229533.I1RN61	pp	229533.I1S2B8
131129	229533.I1RN61	pp	229533.I1RPU3
131130	229533.I1RN61	pp	229533.I1RW25
131131	229533.I1RN61	pp	229533.I1RNR2
131132	229533.I1RN61	pp	229533.V6RTJ8
131133	229533.I1RN61	pp	229533.I1RP27
131134	229533.I1RN61	pp	229533.I1S0X5
131135	229533.I1RN61	pp	229533.I1RPW4
131136	229533.I1RN61	pp	229533.I1RNN1
131137	229533.I1RN61	pp	229533.I1RSQ7
131138	229533.I1RN61	pp	229533.I1RN63
131139	229533.I1RN61	pp	229533.I1RRU9
131140	229533.I1RN61	pp	229533.I1RN67
131141	229533.I1RN61	pp	229533.I1RPP3
131142	229533.I1RN61	pp	229533.I1S8P5
131143	229533.I1RN61	pp	229533.I1S2G7
131144	229533.I1RN61	pp	229533.I1RSV2
131145	229533.I1RN61	pp	229533.Q4HZ42
131146	229533.I1RN61	pp	229533.I1S044
131147	229533.I1RN61	pp	229533.I1RYY5
131148	229533.I1RN61	pp	229533.I1RW71
131149	229533.I1RN61	pp	229533.I1S0N4
131150	229533.I1RN61	pp	229533.I1RZS4
131151	229533.I1RN61	pp	229533.I1S0J1
131152	229533.I1RN61	pp	229533.I1RY03
131153	229533.I1RN61	pp	229533.I1RTA0
131154	229533.I1RN61	pp	229533.I1RZZ1
131155	229533.I1RN61	pp	229533.Q4I7K4
131156	229533.I1RN61	pp	229533.I1RZK6
131157	229533.I1RN61	pp	229533.I1S1C7
131158	229533.I1RN61	pp	229533.I1RYW3
131159	229533.I1RN61	pp	229533.Q4IJK2
131160	229533.I1RN61	pp	229533.I1RQA3
131161	229533.I1RN61	pp	229533.I1S0C1
131162	229533.I1RN61	pp	229533.I1RWD3
131163	229533.I1RN61	pp	229533.Q4IJM4
131164	229533.I1RN61	pp	229533.Q4IAA0
131165	229533.I1RN61	pp	229533.I1RRN5
131166	229533.I1RN61	pp	229533.I1RPP4
131167	229533.I1RN61	pp	229533.I1RZK5
131168	229533.I1RN61	pp	229533.I1S855
131169	229533.I1RN61	pp	229533.I1RQ81
131170	229533.I1RN61	pp	229533.I1RTH6
131171	229533.I1RN61	pp	229533.I1RVX0
131172	229533.I1RN61	pp	229533.I1RZM1
131173	229533.I1RN61	pp	229533.I1RT03
131174	229533.I1RN61	pp	229533.I1RSK1
131175	229533.I1RN61	pp	229533.I1RSI0
131176	229533.I1RN61	pp	229533.I1RRY9
131177	229533.I1RN61	pp	229533.I1RWN6
131178	229533.I1RN61	pp	229533.I1RY44
131179	229533.I1RN61	pp	229533.I1RQ31
131180	229533.I1RN61	pp	229533.Q4IEK8
131181	229533.I1RN61	pp	229533.I1RWD7

131182	229533.I1RN61	pp	229533.I1RNC1
131183	229533.I1RN61	pp	229533.I1RNQ5
131184	229533.I1RN61	pp	229533.I1RWT6
131185	229533.I1RN61	pp	229533.I1RWN5
131186	229533.I1RN61	pp	229533.V6RMW6
131187	229533.I1RN61	pp	229533.I1S1W4
131188	229533.I1RN61	pp	229533.V6R042
131189	229533.I1RN61	pp	229533.I1RSQ3
131190	229533.I1RN61	pp	229533.I1RWE8
131191	229533.I1RN61	pp	229533.I1RRV1
131192	229533.I1RN61	pp	229533.I1S504
131193	229533.I1RN61	pp	229533.I1SAN9
131194	229533.I1RC20	pp	229533.I1S1C7
131195	229533.I1RC20	pp	229533.I1RG90
131196	229533.I1RC20	pp	229533.I1RYW3
131197	229533.I1RC20	pp	229533.I1S2C0
131198	229533.I1RC20	pp	229533.I1RTH6
131199	229533.I1RC20	pp	229533.I1S238
131200	229533.I1RC20	pp	229533.I1RJV3
131201	229533.I1RC20	pp	229533.I1RWN5
131202	229533.I1RC20	pp	229533.I1RCR8
131203	229533.I1RC20	pp	229533.I1S1W4
131204	229533.I1RC20	pp	229533.I1RSK1
131205	229533.I1RC20	pp	229533.I1RNC1
131206	229533.I1RC20	pp	229533.I1RWN6
131207	229533.I1RC20	pp	229533.I1RCM3
131208	229533.I1RC20	pp	229533.V6R042
131209	229533.I1RC20	pp	229533.I1S0J1
131210	229533.I1RC20	pp	229533.I1RVX0
131211	229533.I1RC20	pp	229533.I1RPP4
131212	229533.I1RC20	pp	229533.I1RC28
131213	229533.I1RC20	pp	229533.I1RT03
131214	229533.I1RC20	pp	229533.I1RNN1
131215	229533.I1RC20	pp	229533.I1RSQ3
131216	229533.I1RC20	pp	229533.I1S1W3
131217	229533.I1RC20	pp	229533.I1RNB8
131218	229533.I1RC20	pp	229533.I1RWI5
131219	229533.I1RC20	pp	229533.V6RMW6
131220	229533.I1RC20	pp	229533.Q4IBU4
131221	229533.I1RC20	pp	229533.I1RR73
131222	229533.I1RC20	pp	229533.I1RJP4
131223	229533.I1RC20	pp	229533.I1RRV1
131224	229533.I1RC20	pp	229533.I1RD22
131225	229533.I1RC20	pp	229533.I1RY44
131226	229533.I1RC20	pp	229533.I1RZ39
131227	229533.I1RC20	pp	229533.V6R3X5
131228	229533.I1RC20	pp	229533.I1RCI8
131229	229533.I1RC20	pp	229533.I1S0N4
131230	229533.I1RC20	pp	229533.I1RCJ7
131231	229533.I1RC20	pp	229533.I1RTD5
131232	229533.I1RC20	pp	229533.I1RV91
131233	229533.I1RC20	pp	229533.I1RPU3
131234	229533.I1RC20	pp	229533.I1RM82
131235	229533.I1RC20	pp	229533.I1RWL2
131236	229533.I1RC20	pp	229533.I1RE76
131237	229533.I1RC20	pp	229533.I1S2E9
131238	229533.I1RC20	pp	229533.I1S2B8
131239	229533.I1RC20	pp	229533.I1RS20
131240	229533.I1RC20	pp	229533.I1RCZ9
131241	229533.I1RC20	pp	229533.I1RT04
131242	229533.I1RC20	pp	229533.I1S503
131243	229533.I1RC20	pp	229533.I1RN63
131244	229533.I1RC20	pp	229533.I1RYI5
131245	229533.I1RC20	pp	229533.I1RP27
131246	229533.I1RC20	pp	229533.I1RJL2
131247	229533.I1RC20	pp	229533.I1RY03
131248	229533.I1RC20	pp	229533.I1RZZ1
131249	229533.I1RC20	pp	229533.I1RGA4
131250	229533.I1RC20	pp	229533.I1RZK5
131251	229533.I1RC20	pp	229533.I1RPP3
131252	229533.I1RC20	pp	229533.I1RSV2
131253	229533.I1RC20	pp	229533.I1RN67
131254	229533.I1RC20	pp	229533.I1RS30

131255 229533.I1RC20 pp 229533.I1RFU8
131256 229533.I1RC20 pp 229533.I1S1J7
131257 229533.I1RC20 pp 229533.Q4IJK2
131258 229533.I1RC20 pp 229533.I1RRY9
131259 229533.I1RC20 pp 229533.Q4IJM4
131260 229533.I1RC20 pp 229533.I1RN61
131261 229533.I1RC20 pp 229533.I1RQ81
131262 229533.I1RC20 pp 229533.I1RSI0
131263 229533.I1RC20 pp 229533.I1RSQ7
131264 229533.I1RC20 pp 229533.I1RZM1
131265 229533.I1RC20 pp 229533.I1RLU5
131266 229533.I1RC20 pp 229533.I1RS83
131267 229533.I1RC20 pp 229533.I1RXD7
131268 229533.I1RC20 pp 229533.I1RFT2
131269 229533.I1RC20 pp 229533.I1RCS1
131270 229533.I1RC20 pp 229533.I1RW25
131271 229533.I1RC20 pp 229533.I1RNQ5
131272 229533.I1RC20 pp 229533.I1RQH3
131273 229533.I1RC20 pp 229533.I1RYH9
131274 229533.I1RC20 pp 229533.I1RLU6
131275 229533.I1RC20 pp 229533.I1S0R7
131276 229533.I1RC20 pp 229533.I1RQC9
131277 229533.I1RC20 pp 229533.I1RIG2
131278 229533.I1RC20 pp 229533.I1RTF4
131279 229533.I1RC20 pp 229533.I1RX46
131280 229533.I1RC20 pp 229533.I1RFQ7
131281 229533.I1RC20 pp 229533.I1S504
131282 229533.I1RC20 pp 229533.V6RTJ8
131283 229533.A0A1C3YJW4 pp 229533.A0A1C3YMK0
131284 229533.A0A1C3YJW4 pp 229533.I1RIJ5
131285 229533.A0A1C3YJW4 pp 229533.I1RXY7
131286 229533.A0A1C3YJW4 pp 229533.I1RF53
131287 229533.A0A1C3YJW4 pp 229533.A0A1C3YKW4
131288 229533.A0A1C3YJW4 pp 229533.I1RGG7
131289 229533.A0A1C3YJW4 pp 229533.I1SAJ3
131290 229533.A0A1C3YJW4 pp 229533.I1RBJ1
131291 229533.A0A1C3YJW4 pp 229533.V6RN31
131292 229533.A0A1C3YJW4 pp 229533.I1RL76
131293 229533.A0A1C3YJW4 pp 229533.I1RE52
131294 229533.A0A1C3YJW4 pp 229533.V6RRH2
131295 229533.A0A1C3YJW4 pp 229533.I1S3G4
131296 229533.A0A1C3YJW4 pp 229533.I1S9C6
131297 229533.A0A1C3YJW4 pp 229533.I1RZ29
131298 229533.A0A1C3YJW4 pp 229533.I1RAN1
131299 229533.A0A1C3YJW4 pp 229533.I1RSQ4
131300 229533.A0A1C3YJW4 pp 229533.I1S0I3
131301 229533.A0A1C3YJW4 pp 229533.I1RZ70
131302 229533.A0A1C3YJW4 pp 229533.I1S253
131303 229533.A0A1C3YJW4 pp 229533.I1RDE7
131304 229533.A0A1C3YJW4 pp 229533.V6RN69
131305 229533.A0A1C3YJW4 pp 229533.Q4IPC8
131306 229533.A0A1C3YJW4 pp 229533.I1RSX4
131307 229533.A0A1C3YJW4 pp 229533.I1S4B6
131308 229533.A0A1C3YJW4 pp 229533.I1RPD6
131309 229533.A0A1C3YJW4 pp 229533.I1RH91
131310 229533.A0A1C3YJW4 pp 229533.I1RSK5
131311 229533.A0A1C3YJW4 pp 229533.I1RAH6
131312 229533.A0A1C3YJW4 pp 229533.I1RNB4
131313 229533.A0A1C3YJW4 pp 229533.I1RW43
131314 229533.A0A1C3YJW4 pp 229533.I1S3X7
131315 229533.A0A1C3YJW4 pp 229533.V6RHD0
131316 229533.A0A1C3YJW4 pp 229533.I1RAZ3
131317 229533.A0A1C3YJW4 pp 229533.I1RM54
131318 229533.A0A1C3YJW4 pp 229533.I1RNG4
131319 229533.A0A1C3YJW4 pp 229533.A0A1C3YLI1
131320 229533.A0A1C3YJW4 pp 229533.I1RSQ9
131321 229533.A0A1C3YJW4 pp 229533.V6RGB5
131322 229533.A0A1C3YJW4 pp 229533.I1RK03
131323 229533.A0A1C3YJW4 pp 229533.V6RXR0
131324 229533.A0A1C3YJW4 pp 229533.A0A1C3YKN4
131325 229533.A0A1C3YJW4 pp 229533.I1S673
131326 229533.A0A1C3YJW4 pp 229533.I1RZ52
131327 229533.A0A1C3YJW4 pp 229533.I1RM26

131328 229533.A0A1C3YJW4 pp 229533.I1RBE7
131329 229533.A0A1C3YJW4 pp 229533.I1RVZ3
131330 229533.A0A1C3YJW4 pp 229533.I1S3A5
131331 229533.A0A1C3YJW4 pp 229533.I1S5L5
131332 229533.A0A1C3YJW4 pp 229533.I1RVZ2
131333 229533.A0A1C3YJW4 pp 229533.I1RVR2
131334 229533.A0A1C3YJW4 pp 229533.I1RBF3
131335 229533.A0A1C3YJW4 pp 229533.I1RTU0
131336 229533.A0A1C3YJW4 pp 229533.I1RR72
131337 229533.A0A1C3YJW4 pp 229533.A0A1C3YL82
131338 229533.A0A1C3YJW4 pp 229533.I1RCR3
131339 229533.A0A1C3YJW4 pp 229533.I1S4B7
131340 229533.A0A1C3YJW4 pp 229533.Q4I5I4
131341 229533.A0A1C3YJW4 pp 229533.I1RIY0
131342 229533.A0A1C3YJW4 pp 229533.I1RC83
131343 229533.A0A1C3YJW4 pp 229533.I1RBY9
131344 229533.A0A1C3YJW4 pp 229533.A0A1C3YKL7
131345 229533.A0A1C3YJW4 pp 229533.V6QUE6
131346 229533.A0A1C3YJW4 pp 229533.I1RNG7
131347 229533.A0A1C3YJW4 pp 229533.A0A1C3YLA6
131348 229533.A0A1C3YJW4 pp 229533.I1RA90
131349 229533.A0A1C3YJW4 pp 229533.I1RRV4
131350 229533.A0A1C3YJW4 pp 229533.I1RU76
131351 229533.A0A1C3YJW4 pp 229533.A0A1C3YK58
131352 229533.A0A1C3YJW4 pp 229533.A0A1C3YLN6
131353 229533.A0A1C3YJW4 pp 229533.V6QU65
131354 229533.A0A1C3YJW4 pp 229533.I1RJF2
131355 229533.A0A1C3YJW4 pp 229533.I1S0A0
131356 229533.A0A1C3YJW4 pp 229533.I1RMY1
131357 229533.A0A1C3YJW4 pp 229533.I1RD81
131358 229533.A0A1C3YJW4 pp 229533.I1RG66
131359 229533.A0A1C3YJW4 pp 229533.I1RW69
131360 229533.A0A1C3YJW4 pp 229533.A0A1C3YK68
131361 229533.A0A1C3YJW4 pp 229533.A0A1C3YNB6
131362 229533.A0A1C3YJW4 pp 229533.I1RF38
131363 229533.A0A1C3YJW4 pp 229533.I1RVX9
131364 229533.A0A1C3YJW4 pp 229533.I1RAR3
131365 229533.A0A1C3YJW4 pp 229533.A0A1C3YN26
131366 229533.A0A1C3YJW4 pp 229533.I1RY49
131367 229533.A0A1C3YJW4 pp 229533.V6QWD8
131368 229533.A0A1C3YJW4 pp 229533.I1RQE1
131369 229533.A0A1C3YJW4 pp 229533.I1RMH9
131370 229533.A0A1C3YJW4 pp 229533.I1RPW2
131371 229533.A0A1C3YJW4 pp 229533.I1RP86
131372 229533.A0A1C3YJW4 pp 229533.I1RMQ6
131373 229533.A0A1C3YJW4 pp 229533.I1RMM8
131374 229533.A0A1C3YJW4 pp 229533.I1RUV4
131375 229533.A0A1C3YJW4 pp 229533.I1RPE5
131376 229533.A0A1C3YJW4 pp 229533.I1RMJ4
131377 229533.A0A1C3YJW4 pp 229533.A0A1C3YLR6
131378 229533.A0A1C3YJW4 pp 229533.I1RAG9
131379 229533.A0A1C3YJW4 pp 229533.I1RPI4
131380 229533.A0A1C3YJW4 pp 229533.A0A1C3YLV0
131381 229533.A0A1C3YJW4 pp 229533.I1RYM6
131382 229533.A0A1C3YJW4 pp 229533.I1RTG4
131383 229533.A0A1C3YJW4 pp 229533.I1RYX6
131384 229533.A0A1C3YJW4 pp 229533.I1RR12
131385 229533.A0A1C3YJW4 pp 229533.I1S1W8
131386 229533.A0A1C3YJW4 pp 229533.I1RH36
131387 229533.A0A1C3YJW4 pp 229533.I1RPE7
131388 229533.A0A098DIZ1 pp 229533.I1RHS0
131389 229533.A0A098DIZ1 pp 229533.A0A1C3YLM8
131390 229533.A0A098DIZ1 pp 229533.I1RHS5
131391 229533.A0A098DIZ1 pp 229533.I1RKT9
131392 229533.A0A098DIZ1 pp 229533.I1RHS6
131393 229533.A0A098DIZ1 pp 229533.A0A098DWX8
131394 229533.A0A098DIZ1 pp 229533.I1S3U8
131395 229533.I1S2E8 pp 229533.I1S6Y6
131396 229533.I1S2E8 pp 229533.Q4IN63
131397 229533.I1S2E8 pp 229533.Q4I327
131398 229533.I1S2E8 pp 229533.Q4HZ42
131399 229533.I1S2E8 pp 229533.I1S8U9
131400 229533.I1S2E8 pp 229533.Q4I830

131401	229533.I1S2E8	pp	229533.I1S8P5
131402	229533.I1S2E8	pp	229533.V6R042
131403	229533.I1S2E8	pp	229533.I1S2E9
131404	229533.I1S2E8	pp	229533.V6R283
131405	229533.I1S2E8	pp	229533.Q4HXF0
131406	229533.I1S2E8	pp	229533.Q4IQW0
131407	229533.I1S2E8	pp	229533.I1S8E2
131408	229533.I1S2E8	pp	229533.V6RGN5
131409	229533.I1S2E8	pp	229533.Q4IAS1
131410	229533.I1S2E8	pp	229533.V6RMW6
131411	229533.I1S2E8	pp	229533.Q4IF76
131412	229533.I1S2E8	pp	229533.I1S503
131413	229533.I1S2E8	pp	229533.I1S9Y1
131414	229533.I1S2E8	pp	229533.I1S8V0
131415	229533.I1S2E8	pp	229533.Q4HWE2
131416	229533.I1S2E8	pp	229533.V6RIS2
131417	229533.I1S2E8	pp	229533.V6R3X5
131418	229533.I1S2E8	pp	229533.Q4HZ68
131419	229533.I1S2E8	pp	229533.Q4HZK7
131420	229533.I1S2E8	pp	229533.O74638
131421	229533.I1S2E8	pp	229533.V6RRH2
131422	229533.I1S2E8	pp	229533.V6RIV9
131423	229533.I1S2E8	pp	229533.V6QW22
131424	229533.I1S2E8	pp	229533.Q4IAA0
131425	229533.I1S2E8	pp	229533.I1S6F0
131426	229533.I1S2E8	pp	229533.Q4IJ56
131427	229533.I1S2E8	pp	229533.V6RGY3
131428	229533.I1S2E8	pp	229533.V6R057
131429	229533.I1S2E8	pp	229533.Q4IQK7
131430	229533.I1S2E8	pp	229533.Q4IEK8
131431	229533.I1S2E8	pp	229533.V6QYF0
131432	229533.I1S2E8	pp	229533.Q4ICA8
131433	229533.I1S2E8	pp	229533.V6R1K4
131434	229533.I1S2E8	pp	229533.Q4IFI0
131435	229533.I1S2E8	pp	229533.V6R227
131436	229533.I1S2E8	pp	229533.Q4INZ9
131437	229533.I1S2E8	pp	229533.I1S504
131438	229533.I1S2E8	pp	229533.Q4I5R9
131439	229533.I1S2E8	pp	229533.V6RCG5
131440	229533.I1S2E8	pp	229533.I1S5B0
131441	229533.I1S2E8	pp	229533.Q4I948
131442	229533.I1S2E8	pp	229533.Q4IJH1
131443	229533.I1S2E8	pp	229533.Q4HVW2
131444	229533.I1S2E8	pp	229533.Q4IPI1
131445	229533.I1S2E8	pp	229533.V6RBN2
131446	229533.I1S2E8	pp	229533.Q4IBS2
131447	229533.I1S2E8	pp	229533.I1S4Q4
131448	229533.I1S2E8	pp	229533.I1S9U2
131449	229533.I1S2E8	pp	229533.V6QUE6
131450	229533.I1S2E8	pp	229533.Q4IR08
131451	229533.I1RVB2	pp	229533.I1S2Q4
131452	229533.I1RVB2	pp	229533.I1S3G4
131453	229533.I1RVB2	pp	229533.I1RYX6
131454	229533.I1RVB2	pp	229533.Q4HVQ9
131455	229533.I1RVB2	pp	229533.I1S1W8
131456	229533.I1RVB2	pp	229533.I1RWZ3
131457	229533.I1RVB2	pp	229533.I1S1B8
131458	229533.I1RVB2	pp	229533.I1S4G3
131459	229533.I1RCM3	pp	229533.I1S2B8
131460	229533.I1RCM3	pp	229533.I1RW25
131461	229533.I1RCM3	pp	229533.I1RPU3
131462	229533.I1RCM3	pp	229533.V6QUW4
131463	229533.I1RCM3	pp	229533.I1RT03
131464	229533.I1RCM3	pp	229533.Q4INI2
131465	229533.I1RCM3	pp	229533.I1RVX0
131466	229533.I1RCM3	pp	229533.I1RN67
131467	229533.I1RCM3	pp	229533.I1RQ23
131468	229533.I1RCM3	pp	229533.I1RN61
131469	229533.I1RCM3	pp	229533.Q4IJM4
131470	229533.I1RCM3	pp	229533.I1RS20
131471	229533.I1RCM3	pp	229533.I1RPP3
131472	229533.I1RCM3	pp	229533.I1RSQ7
131473	229533.I1RCM3	pp	229533.V6RBN2

131474	229533.I1RCM3	pp	229533.I1RWL2
131475	229533.I1RCM3	pp	229533.I1RFQ7
131476	229533.I1RCM3	pp	229533.I1RSV2
131477	229533.I1RCM3	pp	229533.I1RZZ1
131478	229533.I1RCM3	pp	229533.I1RKK4
131479	229533.I1RCM3	pp	229533.I1RN63
131480	229533.I1RCM3	pp	229533.I1S0B6
131481	229533.I1RCM3	pp	229533.I1S4S4
131482	229533.I1RCM3	pp	229533.I1RM82
131483	229533.I1RCM3	pp	229533.I1RRY9
131484	229533.I1RCM3	pp	229533.Q4IJK2
131485	229533.I1RCM3	pp	229533.V6RTJ8
131486	229533.I1RCM3	pp	229533.I1RWN6
131487	229533.I1RCM3	pp	229533.I1RP27
131488	229533.I1RCM3	pp	229533.I1RR73
131489	229533.I1RCM3	pp	229533.V6R3X5
131490	229533.I1RCM3	pp	229533.I1RXX1
131491	229533.I1RCM3	pp	229533.I1RJV3
131492	229533.I1RCM3	pp	229533.I1RGA4
131493	229533.I1RCM3	pp	229533.I1RIG2
131494	229533.I1RCM3	pp	229533.I1RSK1
131495	229533.I1RCM3	pp	229533.I1RZ39
131496	229533.I1RCM3	pp	229533.I1RX46
131497	229533.I1RCM3	pp	229533.I1RMC8
131498	229533.I1RCM3	pp	229533.I1S503
131499	229533.I1RCM3	pp	229533.I1RZK5
131500	229533.I1RCM3	pp	229533.I1RNN1
131501	229533.I1RCM3	pp	229533.I1RCM5
131502	229533.I1RCM3	pp	229533.I1RTH6
131503	229533.I1RCM3	pp	229533.I1RFT6
131504	229533.I1RCM3	pp	229533.I1RXD7
131505	229533.I1RCM3	pp	229533.I1S2E9
131506	229533.I1RCM3	pp	229533.I1RWI5
131507	229533.I1RCM3	pp	229533.I1RYY5
131508	229533.I1RCM3	pp	229533.I1S2G7
131509	229533.I1RCM3	pp	229533.I1RFU8
131510	229533.I1RCM3	pp	229533.I1RY03
131511	229533.I1RCM3	pp	229533.I1S0R7
131512	229533.I1RCM3	pp	229533.I1RQH3
131513	229533.I1RCM3	pp	229533.I1S1C7
131514	229533.I1RCM3	pp	229533.I1S0J1
131515	229533.I1RCM3	pp	229533.I1S0N4
131516	229533.I1RCM3	pp	229533.I1RT04
131517	229533.I1RCM3	pp	229533.I1S504
131518	229533.I1RCM3	pp	229533.I1RDF6
131519	229533.I1RCM3	pp	229533.I1RTD5
131520	229533.I1RCM3	pp	229533.I1RQC9
131521	229533.I1RCM3	pp	229533.I1RD22
131522	229533.I1RCM3	pp	229533.I1S1W4
131523	229533.I1RCM3	pp	229533.I1RXU7
131524	229533.I1RCM3	pp	229533.I1RFT2
131525	229533.I1RCM3	pp	229533.I1RRU9
131526	229533.I1RCM3	pp	229533.I1RZM1
131527	229533.I1RCM3	pp	229533.I1RWD7
131528	229533.I1RCM3	pp	229533.I1RSQ3
131529	229533.I1RCM3	pp	229533.I1RLU6
131530	229533.I1RCM3	pp	229533.I1RNQ5
131531	229533.I1RCM3	pp	229533.V6RMW6
131532	229533.I1RCM3	pp	229533.V6R042
131533	229533.I1RCM3	pp	229533.I1RSI0
131534	229533.I1RCM3	pp	229533.I1RWN5
131535	229533.I1RCM3	pp	229533.I1RRV1
131536	229533.I1RCM3	pp	229533.I1RY44
131537	229533.I1RCM3	pp	229533.I1RQ81
131538	229533.I1RCM3	pp	229533.I1RPP4
131539	229533.I1RCM3	pp	229533.I1RNC1
131540	229533.I1RCM3	pp	229533.I1RN58
131541	229533.I1RCM3	pp	229533.I1RCR8
131542	229533.I1RHV0	pp	229533.I1RZM1
131543	229533.I1RHV0	pp	229533.I1RJS0
131544	229533.I1RHV0	pp	229533.I1RUP5
131545	229533.I1RHV0	pp	229533.I1RPI4
131546	229533.I1RW71	pp	229533.I1RWX5

131547	229533.I1RW71	pp	229533.Q4IN00
131548	229533.I1RW71	pp	229533.I1RZ79
131549	229533.I1RW71	pp	229533.I1S8R3
131550	229533.I1RW71	pp	229533.I1RZW8
131551	229533.I1RW71	pp	229533.I1S1X9
131552	229533.I1RW71	pp	229533.Q4I1N3
131553	229533.I1RW71	pp	229533.I1S2C0
131554	229533.I1RW71	pp	229533.V6RRH2
131555	229533.I1RW71	pp	229533.Q4HZ68
131556	229533.I1RW71	pp	229533.I1S855
131557	229533.I1RW71	pp	229533.I1RWN6
131558	229533.I1RW71	pp	229533.I1S296
131559	229533.I1RW71	pp	229533.I1S274
131560	229533.I1RW71	pp	229533.I1S1C7
131561	229533.I1RW71	pp	229533.V6RGY3
131562	229533.I1RW71	pp	229533.V6R057
131563	229533.I1RW71	pp	229533.Q4I624
131564	229533.I1RW71	pp	229533.I1S1U7
131565	229533.I1RW71	pp	229533.I1RYW3
131566	229533.I1RW71	pp	229533.I1S0J1
131567	229533.I1RW71	pp	229533.I1S2E8
131568	229533.I1RW71	pp	229533.I1SAN9
131569	229533.I1RW71	pp	229533.Q4I1Y5
131570	229533.I1RW71	pp	229533.V6RGY0
131571	229533.I1RW71	pp	229533.I1RWD3
131572	229533.I1RW71	pp	229533.V6R042
131573	229533.I1RW71	pp	229533.I1RZZ9
131574	229533.I1RW71	pp	229533.Q4I7K4
131575	229533.I1RW71	pp	229533.Q4IN63
131576	229533.I1RW71	pp	229533.Q4IF76
131577	229533.I1RW71	pp	229533.V6RMW6
131578	229533.I1RW71	pp	229533.V6RIV9
131579	229533.I1RW71	pp	229533.I1RWN5
131580	229533.I1RW71	pp	229533.I1SA33
131581	229533.I1RW71	pp	229533.I1RWH4
131582	229533.I1RW71	pp	229533.I1S1W9
131583	229533.I1RW71	pp	229533.I1RZR2
131584	229533.I1RW71	pp	229533.I1S471
131585	229533.I1RW71	pp	229533.I1S016
131586	229533.I1RW71	pp	229533.I1RZ39
131587	229533.I1RW71	pp	229533.Q4HZB8
131588	229533.I1RW71	pp	229533.I1RWJ1
131589	229533.I1RW71	pp	229533.V6RGN5
131590	229533.I1RW71	pp	229533.I1S5R6
131591	229533.I1RW71	pp	229533.I1RY44
131592	229533.I1RW71	pp	229533.I1RWE8
131593	229533.I1RW71	pp	229533.I1S7J8
131594	229533.I1RW71	pp	229533.I1S253
131595	229533.I1RW71	pp	229533.I1S1W4
131596	229533.I1RW71	pp	229533.V6R3X5
131597	229533.I1RW71	pp	229533.Q4IB89
131598	229533.I1RW71	pp	229533.I1RWK9
131599	229533.I1RW71	pp	229533.I1S060
131600	229533.I1RW71	pp	229533.I1RWI5
131601	229533.I1RW71	pp	229533.I1S2V2
131602	229533.I1RW71	pp	229533.I1S5N3
131603	229533.I1RW71	pp	229533.Q4I662
131604	229533.I1RW71	pp	229533.I1S9Y1
131605	229533.I1RW71	pp	229533.I1RWX7
131606	229533.I1RW71	pp	229533.I1RZG0
131607	229533.I1RW71	pp	229533.I1RWK3
131608	229533.I1RW71	pp	229533.I1RZS6
131609	229533.I1RW71	pp	229533.I1RX24
131610	229533.I1RW71	pp	229533.V6R283
131611	229533.I1RW71	pp	229533.I1S9F8
131612	229533.I1RW71	pp	229533.I1S5R1
131613	229533.I1RW71	pp	229533.I1RXT8
131614	229533.I1RW71	pp	229533.Q4HW67
131615	229533.I1RW71	pp	229533.I1RXC2
131616	229533.I1RW71	pp	229533.I1RY90
131617	229533.I1RW71	pp	229533.O74638
131618	229533.I1RW71	pp	229533.I1S2B8
131619	229533.I1RW71	pp	229533.I1S2F2

131620 229533.I1RW71 pp 229533.I1S2M6
131621 229533.I1RW71 pp 229533.I1S2H4
131622 229533.I1RW71 pp 229533.Q4IPI4
131623 229533.I1RW71 pp 229533.Q4I830
131624 229533.I1RW71 pp 229533.I1S0L4
131625 229533.I1RW71 pp 229533.I1RYI5
131626 229533.I1RW71 pp 229533.I1S0X5
131627 229533.I1RW71 pp 229533.Q4HXF0
131628 229533.I1RW71 pp 229533.I1S8P5
131629 229533.I1RW71 pp 229533.I1S0F6
131630 229533.I1RW71 pp 229533.I1S2E9
131631 229533.I1RW71 pp 229533.I1S2G7
131632 229533.I1RW71 pp 229533.Q4IAS1
131633 229533.I1RW71 pp 229533.I1S2P0
131634 229533.I1RW71 pp 229533.I1RY03
131635 229533.I1RW71 pp 229533.I1S503
131636 229533.I1RW71 pp 229533.I1RYY5
131637 229533.I1RW71 pp 229533.Q4HZ42
131638 229533.I1RW71 pp 229533.I1RYL5
131639 229533.I1RW71 pp 229533.I1S9L6
131640 229533.I1RW71 pp 229533.I1RZB5
131641 229533.I1RW71 pp 229533.Q4IER8
131642 229533.I1RW71 pp 229533.I1S0L8
131643 229533.I1RW71 pp 229533.I1RWM4
131644 229533.I1RW71 pp 229533.Q4I948
131645 229533.I1RW71 pp 229533.I1S5B0
131646 229533.I1RW71 pp 229533.I1S044
131647 229533.I1RW71 pp 229533.I1RZK5
131648 229533.I1RW71 pp 229533.I1RZZ1
131649 229533.I1RW71 pp 229533.I1S0C1
131650 229533.I1RW71 pp 229533.Q4IJK2
131651 229533.I1RW71 pp 229533.Q4I7F9
131652 229533.I1RW71 pp 229533.I1RW87
131653 229533.I1RW71 pp 229533.I1RW99
131654 229533.I1RW71 pp 229533.I1RYS0
131655 229533.I1RW71 pp 229533.Q4IP34
131656 229533.I1RW71 pp 229533.I1RXC4
131657 229533.I1RW71 pp 229533.Q4IJM4
131658 229533.I1RW71 pp 229533.I1S1J7
131659 229533.I1RW71 pp 229533.I1SA32
131660 229533.I1RW71 pp 229533.I1RWJ3
131661 229533.I1RW71 pp 229533.Q4I061
131662 229533.I1RW71 pp 229533.I1S4Q4
131663 229533.I1RW71 pp 229533.I1RX09
131664 229533.I1RW71 pp 229533.I1RZF6
131665 229533.I1RW71 pp 229533.Q4IJ56
131666 229533.I1RW71 pp 229533.I1RWD7
131667 229533.I1RW71 pp 229533.I1RZM8
131668 229533.I1RW71 pp 229533.I1S0M9
131669 229533.I1RW71 pp 229533.Q4IAA0
131670 229533.I1RW71 pp 229533.I1S021
131671 229533.I1RW71 pp 229533.I1S504
131672 229533.I1RW71 pp 229533.I1S0P4
131673 229533.I1RW71 pp 229533.Q4I8P5
131674 229533.I1RW71 pp 229533.I1S361
131675 229533.I1RW71 pp 229533.I1S235
131676 229533.I1RW71 pp 229533.V6RCZ8
131677 229533.I1RW71 pp 229533.I1RWT6
131678 229533.I1RW71 pp 229533.V6RCG5
131679 229533.I1RW71 pp 229533.V6R227
131680 229533.I1RW71 pp 229533.I1S0F1
131681 229533.I1RW71 pp 229533.V6QUE6
131682 229533.I1RW71 pp 229533.Q4IEK8
131683 229533.I1RW71 pp 229533.V6RG06
131684 229533.I1RW71 pp 229533.Q4I5I4
131685 229533.I1RW71 pp 229533.I1S0Z3
131686 229533.I1RW71 pp 229533.I1RZM1
131687 229533.I1RW71 pp 229533.Q4IJH1
131688 229533.I1RW71 pp 229533.Q4HVW2
131689 229533.I1RW71 pp 229533.I1RXZ9
131690 229533.I1RW71 pp 229533.Q4IBS2
131691 229533.I1RW71 pp 229533.Q4INZ9
131692 229533.I1RW71 pp 229533.I1S9U2

131693	229533.I1RW71	pp	229533.Q4HZS8
131694	229533.I1RW71	pp	229533.I1RZJ6
131695	229533.I1RW71	pp	229533.I1RWJ8
131696	229533.I1RW71	pp	229533.I1RYN7
131697	229533.I1RW71	pp	229533.I1S270
131698	229533.I1RW71	pp	229533.I1S540
131699	229533.I1RW71	pp	229533.I1RXU7
131700	229533.I1RW71	pp	229533.V6RTJ8
131701	229533.I1RW71	pp	229533.I1RWM6
131702	229533.I1RW71	pp	229533.V6QUW4
131703	229533.I1RW71	pp	229533.I1S0R7
131704	229533.I1RW71	pp	229533.I1S4S7
131705	229533.I1RW71	pp	229533.I1RXD7
131706	229533.I1RW71	pp	229533.Q4IQK7
131707	229533.I1RW71	pp	229533.V6QYF0
131708	229533.I1RW71	pp	229533.I1S854
131709	229533.I1RW71	pp	229533.I1RZY1
131710	229533.I1RW71	pp	229533.Q4IFI0
131711	229533.I1RW71	pp	229533.I1S2L2
131712	229533.I1RW71	pp	229533.I1S6F0
131713	229533.I1RW71	pp	229533.Q4I5R9
131714	229533.I1RW71	pp	229533.I1S241
131715	229533.I1RW71	pp	229533.I1RYH0
131716	229533.I1RW71	pp	229533.V6QW22
131717	229533.I1RW71	pp	229533.V6R1K4
131718	229533.I1RW71	pp	229533.I1S9L5
131719	229533.I1RW71	pp	229533.I1RX46
131720	229533.I1RW71	pp	229533.I1RY40
131721	229533.I1RW71	pp	229533.I1S0L3
131722	229533.I1RW71	pp	229533.I1S2B7
131723	229533.I1RW71	pp	229533.Q5I2J3
131724	229533.I1RW71	pp	229533.I1RYQ3
131725	229533.I1RQE7	pp	229533.I1RRV4
131726	229533.I1RQE7	pp	229533.I1RRU9
131727	229533.I1RQE7	pp	229533.I1S419
131728	229533.I1RQE7	pp	229533.I1RWT6
131729	229533.I1RQE7	pp	229533.V6QUE6
131730	229533.I1RQE7	pp	229533.Q4IP34
131731	229533.I1RQE7	pp	229533.I1RW99
131732	229533.I1RQE7	pp	229533.V6R227
131733	229533.I1RQE7	pp	229533.I1S504
131734	229533.I1RQE7	pp	229533.Q4I283
131735	229533.I1RQE7	pp	229533.I1S5B0
131736	229533.I1RQE7	pp	229533.I1RZM8
131737	229533.I1RQE7	pp	229533.I1RXF0
131738	229533.I1RQE7	pp	229533.I1SA32
131739	229533.I1RQE7	pp	229533.I1RZF6
131740	229533.I1RQE7	pp	229533.V6RCG5
131741	229533.I1RQE7	pp	229533.I1RXC4
131742	229533.I1RQE7	pp	229533.V6RCZ8
131743	229533.I1RQE7	pp	229533.I1RR51
131744	229533.I1RQE7	pp	229533.I1S6K9
131745	229533.I1RQE7	pp	229533.I1RZH4
131746	229533.I1RQE7	pp	229533.I1RZM1
131747	229533.I1RQE7	pp	229533.Q4I8P5
131748	229533.I1RQE7	pp	229533.I1RS18
131749	229533.I1RQE7	pp	229533.Q4IBS2
131750	229533.I1RQE7	pp	229533.Q4IAA0
131751	229533.I1RQE7	pp	229533.Q4IEK8
131752	229533.I1RQE7	pp	229533.I1RYS0
131753	229533.I1RQE7	pp	229533.Q4IJ56
131754	229533.I1RQE7	pp	229533.I1RSR3
131755	229533.I1RQE7	pp	229533.I1RWB0
131756	229533.I1RQE7	pp	229533.I1RZE5
131757	229533.I1RQE7	pp	229533.I1RX46
131758	229533.I1RQE7	pp	229533.Q4I5Z5
131759	229533.I1RQE7	pp	229533.I1S1J7
131760	229533.I1RQE7	pp	229533.I1RSM3
131761	229533.I1RQE7	pp	229533.Q4I5R9
131762	229533.I1RQE7	pp	229533.I1RRY9
131763	229533.I1RQE7	pp	229533.Q4I2J8
131764	229533.I1RQE7	pp	229533.I1RWR7
131765	229533.I1RQE7	pp	229533.I1RTD9

131766	229533.I1RQE7	pp	229533.I1S854
131767	229533.I1RQE7	pp	229533.I1RWD7
131768	229533.I1RQE7	pp	229533.V6RBN2
131769	229533.I1RQE7	pp	229533.V6RTJ8
131770	229533.I1RQE7	pp	229533.I1S887
131771	229533.I1RQE7	pp	229533.I1RY03
131772	229533.I1RQE7	pp	229533.I1S7A8
131773	229533.I1RQE7	pp	229533.I1RZK5
131774	229533.I1RQE7	pp	229533.I1RQQ5
131775	229533.I1RQE7	pp	229533.Q4I7F9
131776	229533.I1RQE7	pp	229533.I1RZZ1
131777	229533.I1RQE7	pp	229533.I1RX04
131778	229533.I1RQE7	pp	229533.I1RSI0
131779	229533.I1RQE7	pp	229533.I1S0C1
131780	229533.I1RQE7	pp	229533.I1RYJ6
131781	229533.I1RQE7	pp	229533.I1RUQ2
131782	229533.I1RQE7	pp	229533.Q4IJK2
131783	229533.I1RQE7	pp	229533.I1RXD7
131784	229533.I1RQE7	pp	229533.I1RY63
131785	229533.I1RQE7	pp	229533.I1S0F5
131786	229533.I1RQE7	pp	229533.I1S0T2
131787	229533.I1RQE7	pp	229533.V6RUT5
131788	229533.I1RQE7	pp	229533.I1S0D4
131789	229533.I1RQE7	pp	229533.I1RWJ8
131790	229533.I1RQE7	pp	229533.V6R1K4
131791	229533.I1RQE7	pp	229533.I1S0R7
131792	229533.I1RQE7	pp	229533.I1RVQ0
131793	229533.I1RQE7	pp	229533.I1RY86
131794	229533.I1RQE7	pp	229533.I1S6F0
131795	229533.I1RQE7	pp	229533.I1S4S4
131796	229533.I1RQE7	pp	229533.I1RXD6
131797	229533.I1RQE7	pp	229533.I1RRG3
131798	229533.I1RQE7	pp	229533.I1RR24
131799	229533.I1RQE7	pp	229533.I1RT33
131800	229533.I1RQE7	pp	229533.I1S0F1
131801	229533.I1RQE7	pp	229533.Q4IJT5
131802	229533.I1RQE7	pp	229533.I1S0L3
131803	229533.I1RQE7	pp	229533.I1RT05
131804	229533.I1RQE7	pp	229533.I1RWD4
131805	229533.I1RQE7	pp	229533.V6QW22
131806	229533.I1RQE7	pp	229533.I1S0X6
131807	229533.I1RQE7	pp	229533.I1RX95
131808	229533.I1RQE7	pp	229533.Q4HVW2
131809	229533.I1RQE7	pp	229533.I1S256
131810	229533.I1RQE7	pp	229533.I1RRX5
131811	229533.I1RQE7	pp	229533.I1SA33
131812	229533.I1RQE7	pp	229533.I1RRN2
131813	229533.I1RQE7	pp	229533.I1S4S7
131814	229533.I1RQE7	pp	229533.Q4IJH1
131815	229533.I1RQE7	pp	229533.I1RSQ7
131816	229533.I1RQE7	pp	229533.I1RTF4
131817	229533.I1RQE7	pp	229533.I1S0N4
131818	229533.I1RQE7	pp	229533.I1RT80
131819	229533.I1RQE7	pp	229533.I1RW71
131820	229533.I1RQE7	pp	229533.V6RGN5
131821	229533.I1RQE7	pp	229533.I1RUN9
131822	229533.I1RQE7	pp	229533.I1RX25
131823	229533.I1RQE7	pp	229533.I1RZJ5
131824	229533.I1RQE7	pp	229533.Q4HZB8
131825	229533.I1RQE7	pp	229533.I1RWQ8
131826	229533.I1RQE7	pp	229533.I1S5R6
131827	229533.I1RQE7	pp	229533.V6RIV9
131828	229533.I1RQE7	pp	229533.I1RW25
131829	229533.I1RQE7	pp	229533.I1RWN5
131830	229533.I1RQE7	pp	229533.I1RVF8
131831	229533.I1RQE7	pp	229533.Q4IFI0
131832	229533.I1RQE7	pp	229533.I1S1W9
131833	229533.I1RQE7	pp	229533.I1S438
131834	229533.I1RQE7	pp	229533.I1RXT3
131835	229533.I1RQE7	pp	229533.I1RVZ7
131836	229533.I1RQE7	pp	229533.V6QYF0
131837	229533.I1RQE7	pp	229533.V6R042
131838	229533.I1RQE7	pp	229533.I1RUG3

229533.I1RQE7	pp	229533.I1RRQ6
229533.I1RQE7	pp	229533.V6QUW4
229533.I1RQE7	pp	229533.I1S8V4
229533.I1RQE7	pp	229533.I1RRE5
229533.I1RQE7	pp	229533.I1RSQ3
229533.I1RQE7	pp	229533.I1RSF1
229533.I1RQE7	pp	229533.I1S0R2
229533.I1RQE7	pp	229533.I1RSD4
229533.I1RQE7	pp	229533.I1RW54
229533.I1RQE7	pp	229533.I1RWH4
229533.I1RQE7	pp	229533.V6RMW6
229533.I1RQE7	pp	229533.I1S0J1
229533.I1RQE7	pp	229533.I1RSI6
229533.I1RQE7	pp	229533.I1RRM3
229533.I1RQE7	pp	229533.I1RRV1
229533.I1RQE7	pp	229533.I1S7W5
229533.I1RQE7	pp	229533.V6RLM7
229533.I1RQE7	pp	229533.I1S1W3
229533.I1RQE7	pp	229533.I1S855
229533.I1RQE7	pp	229533.I1RTA0
229533.I1RQE7	pp	229533.I1RWN6
229533.I1RQE7	pp	229533.I1RZS7
229533.I1RQE7	pp	229533.I1RZV3
229533.I1RQE7	pp	229533.I1RW46
229533.I1RQE7	pp	229533.I1RXU9
229533.I1RQE7	pp	229533.I1RY44
229533.I1RQE7	pp	229533.I1RZW8
229533.I1RQE7	pp	229533.Q4I7K4
229533.I1RQE7	pp	229533.I1RWX5
229533.I1RQE7	pp	229533.I1RWD3
229533.I1RQE7	pp	229533.I1RTH6
229533.I1RQE7	pp	229533.I1RRI2
229533.I1RQE7	pp	229533.I1S016
229533.I1RQE7	pp	229533.I1RZ79
229533.I1RQE7	pp	229533.I1RS17
229533.I1RQE7	pp	229533.I1S437
229533.I1RQE7	pp	229533.I1S8R3
229533.I1RQE7	pp	229533.I1RXR1
229533.I1RQE7	pp	229533.I1RYW3
229533.I1RQE7	pp	229533.I1RSS4
229533.I1RQE7	pp	229533.I1S0F8
229533.I1RQE7	pp	229533.I1RVX0
229533.I1RQE7	pp	229533.I1RRN5
229533.I1RQE7	pp	229533.V6RRH2
229533.I1RQE7	pp	229533.I1S1J6
229533.I1RQE7	pp	229533.I1RT03
229533.I1RQE7	pp	229533.I1RSY2
229533.I1RQE7	pp	229533.Q4I624
229533.I1RQE7	pp	229533.I1S1C7
229533.I1RQE7	pp	229533.I1RSX0
229533.I1RQE7	pp	229533.I1RTE9
229533.I1RQE7	pp	229533.I1S2C0
229533.I1RQE7	pp	229533.Q4HZ68
229533.I1RQE7	pp	229533.V6R3J1
229533.I1RQE7	pp	229533.I1S2E9
229533.I1RQE7	pp	229533.I1RRT2
229533.I1RQE7	pp	229533.V6R057
229533.I1RQE7	pp	229533.I1S503
229533.I1RQE7	pp	229533.I1RRK3
229533.I1RQE7	pp	229533.I1RWK3
229533.I1RQE7	pp	229533.V6RUY9
229533.I1RQE7	pp	229533.I1S2E8
229533.I1RQE7	pp	229533.I1RRZ0
229533.I1RQE7	pp	229533.I1RWS4
229533.I1RQE7	pp	229533.I1RT04
229533.I1RQE7	pp	229533.I1RY62
229533.I1RQE7	pp	229533.Q4IAS1
229533.I1RQE7	pp	229533.I1S0F6
229533.I1RQE7	pp	229533.I1RRJ8
229533.I1RQE7	pp	229533.I1RZB2
229533.I1RQE7	pp	229533.I1RQM7
229533.I1RQE7	pp	229533.I1RRP3
229533.I1RQE7	pp	229533.I1RQG4

131912	229533.I1RQE7	pp	229533.I1RYI5
131913	229533.I1RQE7	pp	229533.I1S1B6
131914	229533.I1RQE7	pp	229533.I1RXF8
131915	229533.I1RQE7	pp	229533.I1S8P5
131916	229533.I1RQE7	pp	229533.I1S0W4
131917	229533.I1RQE7	pp	229533.I1RSX1
131918	229533.I1RQE7	pp	229533.I1RTD5
131919	229533.I1RQE7	pp	229533.I1S0F7
131920	229533.I1RQE7	pp	229533.Q4IEV5
131921	229533.I1RQE7	pp	229533.Q4I830
131922	229533.I1RQE7	pp	229533.I1RV91
131923	229533.I1RQE7	pp	229533.I1RT40
131924	229533.I1RQE7	pp	229533.I1S5R1
131925	229533.I1RQE7	pp	229533.I1RRT8
131926	229533.I1RQE7	pp	229533.I1S8V3
131927	229533.I1RQE7	pp	229533.Q4HZ42
131928	229533.I1RQE7	pp	229533.I1RXC2
131929	229533.I1RQE7	pp	229533.I1RS47
131930	229533.I1RQE7	pp	229533.O74638
131931	229533.I1RQE7	pp	229533.Q4IF76
131932	229533.I1RQE7	pp	229533.I1RVZ2
131933	229533.I1RQE7	pp	229533.I1RWI5
131934	229533.I1RQE7	pp	229533.I1S9Y1
131935	229533.I1RQE7	pp	229533.I1S2M6
131936	229533.I1RQE7	pp	229533.I1RSQ4
131937	229533.I1RQE7	pp	229533.I1RTA1
131938	229533.I1RQE7	pp	229533.I1RYR0
131939	229533.I1RQE7	pp	229533.I1S0L4
131940	229533.I1RQE7	pp	229533.I1RZZ9
131941	229533.I1RQE7	pp	229533.Q4HW67
131942	229533.I1RQE7	pp	229533.I1RR73
131943	229533.I1RQE7	pp	229533.I1RRI1
131944	229533.I1RQE7	pp	229533.Q4I7L0
131945	229533.I1RQE7	pp	229533.V6R3X5
131946	229533.I1RQE7	pp	229533.I1S2H4
131947	229533.I1RQE7	pp	229533.I1RQS8
131948	229533.I1RQE7	pp	229533.I1S277
131949	229533.I1RQE7	pp	229533.Q4I662
131950	229533.I1RQE7	pp	229533.I1RZ39
131951	229533.I1RQE7	pp	229533.I1RRL9
131952	229533.I1RQE7	pp	229533.I1RS20
131953	229533.I1RQE7	pp	229533.I1S7S9
131954	229533.I1RQE7	pp	229533.I1RW61
131955	229533.I1RQE7	pp	229533.I1RRM5
131956	229533.I1RQE7	pp	229533.I1S6Y8
131957	229533.I1RQE7	pp	229533.I1S4Z8
131958	229533.I1RQE7	pp	229533.I1RYT5
131959	229533.I1RQE7	pp	229533.I1S2B8
131960	229533.I1RQE7	pp	229533.I1S0M6
131961	229533.I1RQE7	pp	229533.I1RSZ7
131962	229533.I1RQE7	pp	229533.I1RZ86
131963	229533.I1RY49	pp	229533.I1RZA7
131964	229533.I1RY49	pp	229533.I1RYP2
131965	229533.I1RY49	pp	229533.I1RYQ4
131966	229533.I1RY49	pp	229533.V6RUB1
131967	229533.I1RY49	pp	229533.I1RYK0
131968	229533.I1RY49	pp	229533.I1RZT0
131969	229533.I1RY49	pp	229533.I1S4B6
131970	229533.I1RY49	pp	229533.I1S241
131971	229533.I1RY49	pp	229533.I1S4B7
131972	229533.I1RY49	pp	229533.I1S5L5
131973	229533.I1RY49	pp	229533.I1S1D4
131974	229533.I1RY49	pp	229533.I1S6P5
131975	229533.I1RY49	pp	229533.V6QWD8
131976	229533.I1RY49	pp	229533.I1RZ29
131977	229533.I1RY49	pp	229533.I1RYI5
131978	229533.I1RY49	pp	229533.I1S1W8
131979	229533.I1RY49	pp	229533.I1RYX6
131980	229533.I1RY49	pp	229533.V6RHD0
131981	229533.I1RY49	pp	229533.I1S3G4
131982	229533.I1RY49	pp	229533.I1RYM6
131983	229533.I1RY49	pp	229533.I1S3X7
131984	229533.I1RY49	pp	229533.V6QU65

131985 229533.I1RY49 pp 229533.I1S9C6
131986 229533.I1RY49 pp 229533.I1S3K8
131987 229533.I1RY49 pp 229533.I1S9Y1
131988 229533.I1RY49 pp 229533.I1RZK8
131989 229533.I1RY49 pp 229533.V6RDH3
131990 229533.I1RY49 pp 229533.I1SAJ3
131991 229533.I1RY49 pp 229533.V6RN69
131992 229533.I1RY49 pp 229533.I1S183
131993 229533.I1RY49 pp 229533.V6RXR0
131994 229533.I1RY49 pp 229533.I1RYL5
131995 229533.I1RY49 pp 229533.I1S332
131996 229533.I1RY49 pp 229533.I1S0I3
131997 229533.I1RY49 pp 229533.I1S0A0
131998 229533.I1RY49 pp 229533.I1RZ70
131999 229533.I1RY49 pp 229533.V6RQW4
132000 229533.I1RBC9 pp 229533.I1S1C7
132001 229533.I1RBC9 pp 229533.I1RZW8
132002 229533.I1RBC9 pp 229533.I1RWX5
132003 229533.I1RBC9 pp 229533.I1RYW3
132004 229533.I1RBC9 pp 229533.I1RFL4
132005 229533.I1RBC9 pp 229533.I1RRI2
132006 229533.I1RBC9 pp 229533.I1RQE7
132007 229533.I1RBC9 pp 229533.I1RTH6
132008 229533.I1RBC9 pp 229533.I1RN18
132009 229533.I1RBC9 pp 229533.I1RCH5
132010 229533.I1RBC9 pp 229533.I1RTE9
132011 229533.I1RBC9 pp 229533.I1S8R3
132012 229533.I1RBC9 pp 229533.I1RC33
132013 229533.I1RBC9 pp 229533.I1RRN5
132014 229533.I1RBC9 pp 229533.I1RVX0
132015 229533.I1RBC9 pp 229533.I1RY44
132016 229533.I1RBC9 pp 229533.I1RQ87
132017 229533.I1RBC9 pp 229533.I1RRE5
132018 229533.I1RBC9 pp 229533.I1RT03
132019 229533.I1RBC9 pp 229533.I1RJV3
132020 229533.I1RBC9 pp 229533.I1RSK1
132021 229533.I1RBC9 pp 229533.I1S8V4
132022 229533.I1RBC9 pp 229533.I1RG90
132023 229533.I1RBC9 pp 229533.I1RKC6
132024 229533.I1RBC9 pp 229533.I1RD39
132025 229533.I1RBC9 pp 229533.I1RCR8
132026 229533.I1RBC9 pp 229533.I1RGL4
132027 229533.I1RBC9 pp 229533.I1RWN6
132028 229533.I1RBC9 pp 229533.I1RD17
132029 229533.I1RBC9 pp 229533.I1RXT3
132030 229533.I1RBC9 pp 229533.I1RNC1
132031 229533.I1RBC9 pp 229533.I1RZS7
132032 229533.I1RBC9 pp 229533.I1RTA0
132033 229533.I1RBC9 pp 229533.Q4I624
132034 229533.I1RBC9 pp 229533.I1RSI6
132035 229533.I1RBC9 pp 229533.I1RUG3
132036 229533.I1RBC9 pp 229533.I1RW46
132037 229533.I1RBC9 pp 229533.I1RE01
132038 229533.I1RBC9 pp 229533.I1RRV1
132039 229533.I1RBC9 pp 229533.I1S569
132040 229533.I1RBC9 pp 229533.I1S0J1
132041 229533.I1RBC9 pp 229533.V6R057
132042 229533.I1RBC9 pp 229533.V6R042
132043 229533.I1RBC9 pp 229533.I1RMG3
132044 229533.I1RBC9 pp 229533.I1RS17
132045 229533.I1RBC9 pp 229533.I1RC72
132046 229533.I1RBC9 pp 229533.I1RSQ3
132047 229533.I1RBC9 pp 229533.I1RQ72
132048 229533.I1RBC9 pp 229533.I1RPK0
132049 229533.I1RBC9 pp 229533.V6RMW6
132050 229533.I1RBC9 pp 229533.I1RPP4
132051 229533.I1RBC9 pp 229533.I1S0M6
132052 229533.I1RBC9 pp 229533.I1RPY1
132053 229533.I1RBC9 pp 229533.I1RJZ7
132054 229533.I1RBC9 pp 229533.I1RRM5
132055 229533.I1RBC9 pp 229533.I1SA33
132056 229533.I1RBC9 pp 229533.Q4HZB8
132057 229533.I1RBC9 pp 229533.I1RBD0

132058	229533.I1RBC9	pp	229533.V6RIV9
132059	229533.I1RBC9	pp	229533.I1RD22
132060	229533.I1RBC9	pp	229533.I1S2B8
132061	229533.I1RBC9	pp	229533.I1RWN5
132062	229533.I1RBC9	pp	229533.I1RWQ8
132063	229533.I1RBC9	pp	229533.I1RPU3
132064	229533.I1RBC9	pp	229533.I1S0N4
132065	229533.I1RBC9	pp	229533.I1RE76
132066	229533.I1RBC9	pp	229533.I1RBY4
132067	229533.I1RBC9	pp	229533.I1RE27
132068	229533.I1RBC9	pp	229533.I1S2H4
132069	229533.I1RBC9	pp	229533.I1RWI5
132070	229533.I1RBC9	pp	229533.V6RGN5
132071	229533.I1RBC9	pp	229533.I1RS20
132072	229533.I1RBC9	pp	229533.I1RW61
132073	229533.I1RBC9	pp	229533.I1RZZ9
132074	229533.I1RBC9	pp	229533.I1S0L4
132075	229533.I1RBC9	pp	229533.I1RIL3
132076	229533.I1RBC9	pp	229533.I1RPM4
132077	229533.I1RBC9	pp	229533.I1S6Y8
132078	229533.I1RBC9	pp	229533.I1RFH8
132079	229533.I1RBC9	pp	229533.I1RWK3
132080	229533.I1RBC9	pp	229533.I1RQ23
132081	229533.I1RBC9	pp	229533.I1RN63
132082	229533.I1RBC9	pp	229533.I1S8V3
132083	229533.I1RBC9	pp	229533.I1S2M6
132084	229533.I1RBC9	pp	229533.I1RC66
132085	229533.I1RBC9	pp	229533.I1RNA5
132086	229533.I1RBC9	pp	229533.I1RE22
132087	229533.I1RBC9	pp	229533.I1RRL9
132088	229533.I1RBC9	pp	229533.I1RZ39
132089	229533.I1RBC9	pp	229533.I1S352
132090	229533.I1RBC9	pp	229533.I1RFT1
132091	229533.I1RBC9	pp	229533.I1RR73
132092	229533.I1RBC9	pp	229533.I1S2E9
132093	229533.I1RBC9	pp	229533.I1RJW6
132094	229533.I1RBC9	pp	229533.I1RRJ8
132095	229533.I1RBC9	pp	229533.I1RQG4
132096	229533.I1RBC9	pp	229533.V6R3X5
132097	229533.I1RBC9	pp	229533.I1RRZ0
132098	229533.I1RBC9	pp	229533.I1S503
132099	229533.I1RBC9	pp	229533.I1RM82
132100	229533.I1RBC9	pp	229533.I1S8P5
132101	229533.I1RBC9	pp	229533.I1RRT8
132102	229533.I1RBC9	pp	229533.I1RPP3
132103	229533.I1RBC9	pp	229533.I1RLN6
132104	229533.I1RBC9	pp	229533.I1RRT2
132105	229533.I1RBC9	pp	229533.I1S0F6
132106	229533.I1RBC9	pp	229533.I1RJL4
132107	229533.I1RBC9	pp	229533.I1RN67
132108	229533.I1RBC9	pp	229533.I1S5R1
132109	229533.I1RBC9	pp	229533.I1RGA4
132110	229533.I1RBC9	pp	229533.I1RRP3
132111	229533.I1RBC9	pp	229533.I1RC82
132112	229533.I1RBC9	pp	229533.I1RBS6
132113	229533.I1RBC9	pp	229533.I1RJV4
132114	229533.I1RBC9	pp	229533.O74638
132115	229533.I1RBC9	pp	229533.I1RQS8
132116	229533.I1RBC9	pp	229533.I1RZL6
132117	229533.I1RBC9	pp	229533.I1RJ74
132118	229533.I1RBC9	pp	229533.I1RBS0
132119	229533.I1RBC9	pp	229533.I1RS47
132120	229533.I1RBC9	pp	229533.I1RWD7
132121	229533.I1RBC9	pp	229533.I1RQA3
132122	229533.I1RBC9	pp	229533.I1RJS2
132123	229533.I1RBC9	pp	229533.I1RCJ7
132124	229533.I1RBC9	pp	229533.Q4IJK2
132125	229533.I1RBC9	pp	229533.I1RMD5
132126	229533.I1RBC9	pp	229533.I1RT40
132127	229533.I1RBC9	pp	229533.I1RY03
132128	229533.I1RBC9	pp	229533.I1RCG4
132129	229533.I1RBC9	pp	229533.I1RQQ5
132130	229533.I1RBC9	pp	229533.I1RV91

132131	229533.I1RBC9	pp	229533.I1RFU8
132132	229533.I1RBC9	pp	229533.I1RGF5
132133	229533.I1RBC9	pp	229533.I1RZK5
132134	229533.I1RBC9	pp	229533.I1RTD5
132135	229533.I1RBC9	pp	229533.I1RZZ1
132136	229533.I1RBC9	pp	229533.I1RT04
132137	229533.I1RBC9	pp	229533.I1RQA2
132138	229533.I1RBC9	pp	229533.I1RSC6
132139	229533.I1RBC9	pp	229533.I1RY63
132140	229533.I1RBC9	pp	229533.V6RUY9
132141	229533.I1RBC9	pp	229533.I1RN99
132142	229533.I1RBC9	pp	229533.I1RI03
132143	229533.I1RBC9	pp	229533.I1RDG0
132144	229533.I1RBC9	pp	229533.I1RBJ5
132145	229533.I1RBC9	pp	229533.I1S504
132146	229533.I1RBC9	pp	229533.I1S1J7
132147	229533.I1RBC9	pp	229533.I1RSR3
132148	229533.I1RBC9	pp	229533.Q4I2J8
132149	229533.I1RBC9	pp	229533.I1RU29
132150	229533.I1RBC9	pp	229533.I1RSI0
132151	229533.I1RBC9	pp	229533.I1RWT6
132152	229533.I1RBC9	pp	229533.I1RFJ9
132153	229533.I1RBC9	pp	229533.I1RRY9
132154	229533.I1RBC9	pp	229533.V6R227
132155	229533.I1RBC9	pp	229533.I1RLM7
132156	229533.I1RBC9	pp	229533.I1RF19
132157	229533.I1RBC9	pp	229533.I1REK8
132158	229533.I1RBC9	pp	229533.I1RZH4
132159	229533.I1RBC9	pp	229533.Q4I283
132160	229533.I1RBC9	pp	229533.I1SA32
132161	229533.I1RBC9	pp	229533.I1RW07
132162	229533.I1RBC9	pp	229533.I1RCR5
132163	229533.I1RBC9	pp	229533.I1RI02
132164	229533.I1RBC9	pp	229533.I1RQ81
132165	229533.I1RBC9	pp	229533.I1RXF0
132166	229533.I1RBC9	pp	229533.I1RZM8
132167	229533.I1RBC9	pp	229533.I1RS18
132168	229533.I1RBC9	pp	229533.I1S0X6
132169	229533.I1RBC9	pp	229533.I1RBT1
132170	229533.I1RBC9	pp	229533.I1RMC0
132171	229533.I1RBC9	pp	229533.I1RMC8
132172	229533.I1RBC9	pp	229533.Q4I8P5
132173	229533.I1RBC9	pp	229533.I1RI82
132174	229533.I1RBC9	pp	229533.I1S4S1
132175	229533.I1RBC9	pp	229533.I1RQ31
132176	229533.I1RBC9	pp	229533.V6RCZ8
132177	229533.I1RBC9	pp	229533.I1RJU3
132178	229533.I1RBC9	pp	229533.I1S419
132179	229533.I1RBC9	pp	229533.I1RTF4
132180	229533.I1RBC9	pp	229533.I1RC16
132181	229533.I1RBC9	pp	229533.I1RNV6
132182	229533.I1RBC9	pp	229533.I1RZM1
132183	229533.I1RBC9	pp	229533.V6RCG5
132184	229533.I1RBC9	pp	229533.I1RDA0
132185	229533.I1RBC9	pp	229533.I1RR51
132186	229533.I1RBC9	pp	229533.I1RNQ5
132187	229533.I1RBC9	pp	229533.I1RGC2
132188	229533.I1RBC9	pp	229533.I1RLU6
132189	229533.I1RBC9	pp	229533.I1RMK3
132190	229533.I1RBC9	pp	229533.I1RRU9
132191	229533.I1RBC9	pp	229533.Q4HVW2
132192	229533.I1RBC9	pp	229533.I1RWJ8
132193	229533.I1RBC9	pp	229533.I1RC17
132194	229533.I1RBC9	pp	229533.Q4IEK8
132195	229533.I1RBC9	pp	229533.I1RVQ0
132196	229533.I1RBC9	pp	229533.Q4IR08
132197	229533.I1RBC9	pp	229533.I1RZJ5
132198	229533.I1RBC9	pp	229533.I1RW99
132199	229533.I1RBC9	pp	229533.I1RMG8
132200	229533.I1RBC9	pp	229533.I1RD52
132201	229533.I1RBC9	pp	229533.I1RM67
132202	229533.I1RBC9	pp	229533.I1S6F0
132203	229533.I1RBC9	pp	229533.I1S5B0

132204 229533.I1RBC9 pp 229533.I1RR24
132205 229533.I1RBC9 pp 229533.I1RNA0
132206 229533.I1RBC9 pp 229533.I1S0L3
132207 229533.I1RBC9 pp 229533.I1RCS1
132208 229533.I1RBC9 pp 229533.I1S0F5
132209 229533.I1RBC9 pp 229533.I1RRG3
132210 229533.I1RBC9 pp 229533.I1RLX7
132211 229533.I1RBC9 pp 229533.V6QYF0
132212 229533.I1RBC9 pp 229533.I1RIL8
132213 229533.I1RBC9 pp 229533.V6QUW4
132214 229533.I1RBC9 pp 229533.V6R1K4
132215 229533.I1RBC9 pp 229533.I1RRQ6
132216 229533.I1RBC9 pp 229533.I1S0R7
132217 229533.I1RBC9 pp 229533.I1RW71
132218 229533.I1RBC9 pp 229533.Q4IJT5
132219 229533.I1RBC9 pp 229533.I1RM06
132220 229533.I1RBC9 pp 229533.I1RQC9
132221 229533.I1RBC9 pp 229533.I1RXD7
132222 229533.I1RBC9 pp 229533.I1RLC7
132223 229533.I1RBC9 pp 229533.V6QW22
132224 229533.I1RBC9 pp 229533.I1RW25
132225 229533.I1RBC9 pp 229533.I1RUQ2
132226 229533.I1RBC9 pp 229533.I1RC20
132227 229533.I1RBC9 pp 229533.I1RSQ7
132228 229533.I1RBC9 pp 229533.I1RX25
132229 229533.I1RBC9 pp 229533.I1RWD4
132230 229533.I1RBC9 pp 229533.I1RG53
132231 229533.I1RBC9 pp 229533.I1S0T2
132232 229533.I1RBC9 pp 229533.V6R4J1
132233 229533.I1RBC9 pp 229533.V6RTJ8
132234 229533.I1RBC9 pp 229533.I1RX46
132235 229533.I1RBC9 pp 229533.I1S7A8
132236 229533.I1RDQ2 pp 229533.I1RYX6
132237 229533.I1RDQ2 pp 229533.I1RWF5
132238 229533.I1RDQ2 pp 229533.I1RUR8
132239 229533.I1RDQ2 pp 229533.I1RVX9
132240 229533.I1RDQ2 pp 229533.I1RMK4
132241 229533.I1RDQ2 pp 229533.I1RNB4
132242 229533.I1RDQ2 pp 229533.I1RGC3
132243 229533.I1RDQ2 pp 229533.I1RTY0
132244 229533.I1RDQ2 pp 229533.I1RJJ7
132245 229533.I1RDQ2 pp 229533.I1RZ83
132246 229533.I1RDQ2 pp 229533.I1RNG4
132247 229533.I1RDQ2 pp 229533.I1S3G4
132248 229533.I1RDQ2 pp 229533.I1S1B8
132249 229533.I1RDQ2 pp 229533.I1S2Q4
132250 229533.I1RDQ2 pp 229533.I1RQE1
132251 229533.I1RDQ2 pp 229533.I1S1W8
132252 229533.I1RDQ2 pp 229533.I1RH91
132253 229533.I1RDQ2 pp 229533.I1RSQ9
132254 229533.I1RDQ2 pp 229533.I1RM26
132255 229533.I1RDQ2 pp 229533.I1RG66
132256 229533.I1RDQ2 pp 229533.V6QWD8
132257 229533.I1RDQ2 pp 229533.I1RY49
132258 229533.I1RDQ2 pp 229533.I1RXY7
132259 229533.I1RDQ2 pp 229533.I1RY74
132260 229533.I1RDQ2 pp 229533.I1RY12
132261 229533.I1RDQ2 pp 229533.I1S4G3
132262 229533.I1RDQ2 pp 229533.I1RWZ3
132263 229533.I1RQC1 pp 229533.I1RX74
132264 229533.I1RQC1 pp 229533.I1RSV8
132265 229533.A0A1C3YK13 pp 229533.I1RE76
132266 229533.A0A1C3YK13 pp 229533.I1RJZ7
132267 229533.A0A1C3YK13 pp 229533.I1RBY4
132268 229533.A0A1C3YK13 pp 229533.I1RRM5
132269 229533.A0A1C3YK13 pp 229533.I1S2B8
132270 229533.A0A1C3YK13 pp 229533.I1RJG6
132271 229533.A0A1C3YK13 pp 229533.I1R9M0
132272 229533.A0A1C3YK13 pp 229533.I1S4Z8
132273 229533.A0A1C3YK13 pp 229533.A0A1C3YMA1
132274 229533.A0A1C3YK13 pp 229533.I1RFS3
132275 229533.A0A1C3YK13 pp 229533.I1S277
132276 229533.A0A1C3YK13 pp 229533.I1RL04

132277 229533.A0A1C3YK13 pp 229533.I1RPM4
132278 229533.A0A1C3YK13 pp 229533.I1S0L4
132279 229533.A0A1C3YK13 pp 229533.I1S6Y8
132280 229533.A0A1C3YK13 pp 229533.I1RPV3
132281 229533.A0A1C3YK13 pp 229533.I1RN88
132282 229533.A0A1C3YK13 pp 229533.I1S524
132283 229533.A0A1C3YK13 pp 229533.Q4I9U7
132284 229533.A0A1C3YK13 pp 229533.I1RST1
132285 229533.A0A1C3YK13 pp 229533.I1RPY4
132286 229533.A0A1C3YK13 pp 229533.I1RSZ7
132287 229533.A0A1C3YK13 pp 229533.I1S2H4
132288 229533.A0A1C3YK13 pp 229533.I1RVN1
132289 229533.A0A1C3YK13 pp 229533.I1RC29
132290 229533.A0A1C3YK13 pp 229533.I1RK96
132291 229533.A0A1C3YK13 pp 229533.I1RVZ2
132292 229533.A0A1C3YK13 pp 229533.I1RTA1
132293 229533.A0A1C3YK13 pp 229533.I1RWK3
132294 229533.A0A1C3YK13 pp 229533.I1RC66
132295 229533.A0A1C3YK13 pp 229533.I1RGA6
132296 229533.A0A1C3YK13 pp 229533.I1S8V3
132297 229533.A0A1C3YK13 pp 229533.Q4I5M4
132298 229533.A0A1C3YK13 pp 229533.I1RWI5
132299 229533.A0A1C3YK13 pp 229533.I1RA11
132300 229533.A0A1C3YK13 pp 229533.I1RN63
132301 229533.A0A1C3YK13 pp 229533.I1RCS2
132302 229533.A0A1C3YK13 pp 229533.I1RCA7
132303 229533.A0A1C3YK13 pp 229533.I1S5R1
132304 229533.A0A1C3YK13 pp 229533.I1RYI5
132305 229533.A0A1C3YK13 pp 229533.Q4HZI0
132306 229533.A0A1C3YK13 pp 229533.I1RSQ4
132307 229533.A0A1C3YK13 pp 229533.I1RRT8
132308 229533.A0A1C3YK13 pp 229533.I1RPP3
132309 229533.A0A1C3YK13 pp 229533.I1RXC2
132310 229533.A0A1C3YK13 pp 229533.I1RZZ9
132311 229533.A0A1C3YK13 pp 229533.I1S8P5
132312 229533.A0A1C3YK13 pp 229533.O74638
132313 229533.A0A1C3YK13 pp 229533.I1RR73
132314 229533.A0A1C3YK13 pp 229533.I1S4M5
132315 229533.A0A1C3YK13 pp 229533.V6R3X5
132316 229533.A0A1C3YK13 pp 229533.I1S9Y1
132317 229533.A0A1C3YK13 pp 229533.I1RJF9
132318 229533.A0A1C3YK13 pp 229533.I1RSX0
132319 229533.A0A1C3YK13 pp 229533.I1RZ39
132320 229533.A0A1C3YK13 pp 229533.I1RT40
132321 229533.A0A1C3YK13 pp 229533.V6R3J1
132322 229533.A0A1C3YK13 pp 229533.I1RFL3
132323 229533.A0A1C3YK13 pp 229533.A0A1C3YKM9
132324 229533.A0A1C3YK13 pp 229533.I1RC05
132325 229533.A0A1C3YK13 pp 229533.I1RY62
132326 229533.A0A1C3YK13 pp 229533.I1RJW6
132327 229533.A0A1C3YK13 pp 229533.I1RV91
132328 229533.A0A1C3YK13 pp 229533.A0A1C3YLK8
132329 229533.A0A1C3YK13 pp 229533.I1S503
132330 229533.A0A1C3YK13 pp 229533.I1RN67
132331 229533.A0A1C3YK13 pp 229533.I1RBS0
132332 229533.A0A1C3YK13 pp 229533.I1S5S2
132333 229533.A0A1C3YK13 pp 229533.A0A1C3YNC3
132334 229533.A0A1C3YK13 pp 229533.I1RRZ0
132335 229533.A0A1C3YK13 pp 229533.Q4IQ72
132336 229533.A0A1C3YK13 pp 229533.I1RXF2
132337 229533.A0A1C3YK13 pp 229533.I1RJV4
132338 229533.A0A1C3YK13 pp 229533.Q4HXF0
132339 229533.A0A1C3YK13 pp 229533.I1S9L6
132340 229533.A0A1C3YK13 pp 229533.I1RS47
132341 229533.A0A1C3YK13 pp 229533.I1RZB2
132342 229533.A0A1C3YK13 pp 229533.V6RUY9
132343 229533.A0A1C3YK13 pp 229533.I1RP27
132344 229533.A0A1C3YK13 pp 229533.I1RQM7
132345 229533.A0A1C3YK13 pp 229533.I1RZV3
132346 229533.A0A1C3YK13 pp 229533.V6RIV9
132347 229533.A0A1C3YK13 pp 229533.I1RT04
132348 229533.A0A1C3YK13 pp 229533.I1RBC7
132349 229533.A0A1C3YK13 pp 229533.I1RGA4

229533.A0A1C3YK13	pp	229533.I1RBS6
229533.A0A1C3YK13	pp	229533.I1RZD3
229533.A0A1C3YK13	pp	229533.I1RCD8
229533.A0A1C3YK13	pp	229533.A0A1C3YLA6
229533.A0A1C3YK13	pp	229533.I1RTD5
229533.A0A1C3YK13	pp	229533.I1RBV3
229533.A0A1C3YK13	pp	229533.I1RWN5
229533.A0A1C3YK13	pp	229533.I1S0B1
229533.A0A1C3YK13	pp	229533.I1RG90
229533.A0A1C3YK13	pp	229533.V6QXG1
229533.A0A1C3YK13	pp	229533.I1RVX0
229533.A0A1C3YK13	pp	229533.I1RT03
229533.A0A1C3YK13	pp	229533.I1S1W9
229533.A0A1C3YK13	pp	229533.V6RRH2
229533.A0A1C3YK13	pp	229533.I1RSY2
229533.A0A1C3YK13	pp	229533.I1RQ33
229533.A0A1C3YK13	pp	229533.I1RJV3
229533.A0A1C3YK13	pp	229533.I1RAU7
229533.A0A1C3YK13	pp	229533.I1RD17
229533.A0A1C3YK13	pp	229533.I1RVZ7
229533.A0A1C3YK13	pp	229533.I1RC84
229533.A0A1C3YK13	pp	229533.I1S8V4
229533.A0A1C3YK13	pp	229533.A0A1C3YN84
229533.A0A1C3YK13	pp	229533.I1RE89
229533.A0A1C3YK13	pp	229533.I1RAW0
229533.A0A1C3YK13	pp	229533.Q4I624
229533.A0A1C3YK13	pp	229533.A0A1C3YK33
229533.A0A1C3YK13	pp	229533.I1RWN6
229533.A0A1C3YK13	pp	229533.I1RRQ4
229533.A0A1C3YK13	pp	229533.I1RQ21
229533.A0A1C3YK13	pp	229533.V6RY33
229533.A0A1C3YK13	pp	229533.I1RXU9
229533.A0A1C3YK13	pp	229533.I1RK92
229533.A0A1C3YK13	pp	229533.V6R057
229533.A0A1C3YK13	pp	229533.I1RZW8
229533.A0A1C3YK13	pp	229533.I1RRE5
229533.A0A1C3YK13	pp	229533.I1RBH8
229533.A0A1C3YK13	pp	229533.I1RQE7
229533.A0A1C3YK13	pp	229533.I1RSD4
229533.A0A1C3YK13	pp	229533.I1RCH5
229533.A0A1C3YK13	pp	229533.I1RWX5
229533.A0A1C3YK13	pp	229533.I1S0J1
229533.A0A1C3YK13	pp	229533.I1RZQ5
229533.A0A1C3YK13	pp	229533.I1RQP8
229533.A0A1C3YK13	pp	229533.I1RRI2
229533.A0A1C3YK13	pp	229533.I1RFL4
229533.A0A1C3YK13	pp	229533.I1RS28
229533.A0A1C3YK13	pp	229533.I1RN18
229533.A0A1C3YK13	pp	229533.I1RCW6
229533.A0A1C3YK13	pp	229533.I1RFQ1
229533.A0A1C3YK13	pp	229533.I1RB92
229533.A0A1C3YK13	pp	229533.I1RB44
229533.A0A1C3YK13	pp	229533.I1S7W5
229533.A0A1C3YK13	pp	229533.I1RK90
229533.A0A1C3YK13	pp	229533.I1RXR1
229533.A0A1C3YK13	pp	229533.I1RTA0
229533.A0A1C3YK13	pp	229533.I1RG11
229533.A0A1C3YK13	pp	229533.I1S1C7
229533.A0A1C3YK13	pp	229533.I1RG57
229533.A0A1C3YK13	pp	229533.I1RTH6
229533.A0A1C3YK13	pp	229533.I1RE01
229533.A0A1C3YK13	pp	229533.A0A1C3YLC1
229533.A0A1C3YK13	pp	229533.I1RW46
229533.A0A1C3YK13	pp	229533.I1RRL0
229533.A0A1C3YK13	pp	229533.I1RPP4
229533.A0A1C3YK13	pp	229533.V6RIS2
229533.A0A1C3YK13	pp	229533.I1RMV0
229533.A0A1C3YK13	pp	229533.I1S8R3
229533.A0A1C3YK13	pp	229533.I1RA70
229533.A0A1C3YK13	pp	229533.I1S056
229533.A0A1C3YK13	pp	229533.I1RS17
229533.A0A1C3YK13	pp	229533.I1RS90
229533.A0A1C3YK13	pp	229533.I1RPK0

229533.A0A1C3YK13	pp	229533.I1RNB8
229533.A0A1C3YK13	pp	229533.I1RSA4
229533.A0A1C3YK13	pp	229533.I1RND5
229533.A0A1C3YK13	pp	229533.I1S1W3
229533.A0A1C3YK13	pp	229533.I1S5R6
229533.A0A1C3YK13	pp	229533.V6RLM7
229533.A0A1C3YK13	pp	229533.I1RX47
229533.A0A1C3YK13	pp	229533.I1RD22
229533.A0A1C3YK13	pp	229533.I1RCR8
229533.A0A1C3YK13	pp	229533.I1RNC1
229533.A0A1C3YK13	pp	229533.I1S256
229533.A0A1C3YK13	pp	229533.I1RUG3
229533.A0A1C3YK13	pp	229533.I1RVQ0
229533.A0A1C3YK13	pp	229533.I1S0L3
229533.A0A1C3YK13	pp	229533.I1RRN2
229533.A0A1C3YK13	pp	229533.V6R042
229533.A0A1C3YK13	pp	229533.I1RT47
229533.A0A1C3YK13	pp	229533.I1RSU2
229533.A0A1C3YK13	pp	229533.V6R1K4
229533.A0A1C3YK13	pp	229533.I1RA85
229533.A0A1C3YK13	pp	229533.I1RSQ3
229533.A0A1C3YK13	pp	229533.I1RD52
229533.A0A1C3YK13	pp	229533.V6RMW6
229533.A0A1C3YK13	pp	229533.I1RRG3
229533.A0A1C3YK13	pp	229533.I1RZG7
229533.A0A1C3YK13	pp	229533.I1S2Y5
229533.A0A1C3YK13	pp	229533.I1RRM3
229533.A0A1C3YK13	pp	229533.A0A1C3YLS9
229533.A0A1C3YK13	pp	229533.I1RS75
229533.A0A1C3YK13	pp	229533.I1RCS1
229533.A0A1C3YK13	pp	229533.I1RPA4
229533.A0A1C3YK13	pp	229533.I1RBD5
229533.A0A1C3YK13	pp	229533.A0A1C3YM63
229533.A0A1C3YK13	pp	229533.I1RW25
229533.A0A1C3YK13	pp	229533.V6QW22
229533.A0A1C3YK13	pp	229533.I1RX81
229533.A0A1C3YK13	pp	229533.I1RCI4
229533.A0A1C3YK13	pp	229533.I1RRS8
229533.A0A1C3YK13	pp	229533.I1S9L5
229533.A0A1C3YK13	pp	229533.I1RCI3
229533.A0A1C3YK13	pp	229533.A0A1C3YL11
229533.A0A1C3YK13	pp	229533.I1RWJ8
229533.A0A1C3YK13	pp	229533.I1S4S4
229533.A0A1C3YK13	pp	229533.Q4HWB3
229533.A0A1C3YK13	pp	229533.I1RRX5
229533.A0A1C3YK13	pp	229533.I1RFC0
229533.A0A1C3YK13	pp	229533.I1R9N6
229533.A0A1C3YK13	pp	229533.I1RSQ7
229533.A0A1C3YK13	pp	229533.I1RR24
229533.A0A1C3YK13	pp	229533.I1RCQ6
229533.A0A1C3YK13	pp	229533.I1RPN4
229533.A0A1C3YK13	pp	229533.I1RN59
229533.A0A1C3YK13	pp	229533.I1S4S7
229533.A0A1C3YK13	pp	229533.A0A1C3YN26
229533.A0A1C3YK13	pp	229533.I1S1C6
229533.A0A1C3YK13	pp	229533.I1S0X6
229533.A0A1C3YK13	pp	229533.I1RMW7
229533.A0A1C3YK13	pp	229533.I1R9Y4
229533.A0A1C3YK13	pp	229533.A0A1C3YKC0
229533.A0A1C3YK13	pp	229533.I1RBC9
229533.A0A1C3YK13	pp	229533.I1RBT1
229533.A0A1C3YK13	pp	229533.I1RW71
229533.A0A1C3YK13	pp	229533.I1S3J4
229533.A0A1C3YK13	pp	229533.I1S0T2
229533.A0A1C3YK13	pp	229533.V6RTJ8
229533.A0A1C3YK13	pp	229533.I1RB50
229533.A0A1C3YK13	pp	229533.I1RJU3
229533.A0A1C3YK13	pp	229533.I1RAU9
229533.A0A1C3YK13	pp	229533.I1RAX6
229533.A0A1C3YK13	pp	229533.A0A1C3YN30
229533.A0A1C3YK13	pp	229533.A0A1C3YL54
229533.A0A1C3YK13	pp	229533.I1RZZ1
229533.A0A1C3YK13	pp	229533.I1RX46

132496 229533.A0A1C3YK13 pp 229533.I1RXD7
132497 229533.A0A1C3YK13 pp 229533.Q4IJK2
132498 229533.A0A1C3YK13 pp 229533.I1RLP1
132499 229533.A0A1C3YK13 pp 229533.I1RAD1
132500 229533.A0A1C3YK13 pp 229533.I1RZK5
132501 229533.A0A1C3YK13 pp 229533.A0A1C3YK68
132502 229533.A0A1C3YK13 pp 229533.I1RK95
132503 229533.A0A1C3YK13 pp 229533.I1RCG4
132504 229533.A0A1C3YK13 pp 229533.V6RUT5
132505 229533.A0A1C3YK13 pp 229533.Q4IB70
132506 229533.A0A1C3YK13 pp 229533.I1RFU8
132507 229533.A0A1C3YK13 pp 229533.I1RAZ3
132508 229533.A0A1C3YK13 pp 229533.I1RZM1
132509 229533.A0A1C3YK13 pp 229533.I1S1J7
132510 229533.A0A1C3YK13 pp 229533.A0A1C3YKY6
132511 229533.A0A1C3YK13 pp 229533.I1S7M2
132512 229533.A0A1C3YK13 pp 229533.I1RNQ5
132513 229533.A0A1C3YK13 pp 229533.I1S0R7
132514 229533.A0A1C3YK13 pp 229533.I1RQC9
132515 229533.A0A1C3YK13 pp 229533.I1RLU6
132516 229533.A0A1C3YK13 pp 229533.I1S419
132517 229533.A0A1C3YK13 pp 229533.I1RWB0
132518 229533.A0A1C3YK13 pp 229533.I1REL7
132519 229533.A0A1C3YK13 pp 229533.I1RP86
132520 229533.A0A1C3YK13 pp 229533.I1RA36
132521 229533.A0A1C3YK13 pp 229533.I1RRY9
132522 229533.A0A1C3YK13 pp 229533.I1REK8
132523 229533.A0A1C3YK13 pp 229533.I1RT35
132524 229533.A0A1C3YK13 pp 229533.I1RWX8
132525 229533.A0A1C3YK13 pp 229533.I1RPR9
132526 229533.A0A1C3YK13 pp 229533.I1RPA0
132527 229533.A0A1C3YK13 pp 229533.I1RZ11
132528 229533.A0A1C3YK13 pp 229533.I1RQ85
132529 229533.A0A1C3YK13 pp 229533.I1RQD8
132530 229533.A0A1C3YK13 pp 229533.I1RBL7
132531 229533.A0A1C3YK13 pp 229533.A0A1C3YKB0
132532 229533.A0A1C3YK13 pp 229533.I1RY03
132533 229533.A0A1C3YK13 pp 229533.I1RRV4
132534 229533.A0A1C3YK13 pp 229533.I1S504
132535 229533.A0A1C3YK13 pp 229533.I1RQA5
132536 229533.A0A1C3YK13 pp 229533.V6QUE6
132537 229533.A0A1C3YK13 pp 229533.I1RPY5
132538 229533.A0A1C3YK13 pp 229533.I1RW99
132539 229533.A0A1C3YK13 pp 229533.I1S887
132540 229533.A0A1C3YK13 pp 229533.I1RCM5
132541 229533.A0A1C3YK13 pp 229533.I1RJ17
132542 229533.A0A1C3YK13 pp 229533.I1RS83
132543 229533.A0A1C3YK13 pp 229533.I1RRX0
132544 229533.A0A1C3YK13 pp 229533.A0A1C3YK34
132545 229533.A0A1C3YK13 pp 229533.I1RXC4
132546 229533.A0A1C3YK13 pp 229533.I1RK72
132547 229533.A0A1C3YK13 pp 229533.I1S5B0
132548 229533.A0A1C3YK13 pp 229533.I1S7M3
132549 229533.A0A1C3YK13 pp 229533.I1RS18
132550 229533.A0A1C3YK13 pp 229533.Q4IRI9
132551 229533.A0A1C3YK13 pp 229533.I1RI82
132552 229533.A0A1C3YK13 pp 229533.I1RXF0
132553 229533.A0A1C3YK13 pp 229533.I1RQ81
132554 229533.A0A1C3YK13 pp 229533.A0A1C3YKV3
132555 229533.A0A1C3YK13 pp 229533.I1RZM8
132556 229533.A0A1C3YK13 pp 229533.I1RAU5
132557 229533.A0A1C3YK13 pp 229533.I1RSA8
132558 229533.A0A1C3YK13 pp 229533.I1RR51
132559 229533.A0A1C3YK13 pp 229533.V6RCG5
132560 229533.A0A1C3YK13 pp 229533.A0A1C3YLU7
132561 229533.A0A1C3YK13 pp 229533.I1RJV2
132562 229533.A0A1C3YK13 pp 229533.I1S6K9
132563 229533.A0A1C3YK13 pp 229533.V6RCZ8
132564 229533.I1R978 pp 229533.I1SAT1
132565 229533.I1RR00 pp 229533.V6QYF0
132566 229533.I1RR00 pp 229533.I1RW25
132567 229533.I1RR00 pp 229533.I1RW06
132568 229533.I1RR00 pp 229533.I1RZZ1

132569 229533.I1RR00 pp 229533.Q4HVW2
132570 229533.I1RR00 pp 229533.I1RVX9
132571 229533.I1RR00 pp 229533.Q4IJH1
132572 229533.I1RR00 pp 229533.V6RBN2
132573 229533.I1RR00 pp 229533.I1S6U8
132574 229533.I1RR00 pp 229533.I1RTF4
132575 229533.I1RR00 pp 229533.I1RT80
132576 229533.I1RR00 pp 229533.I1RZE5
132577 229533.I1RR00 pp 229533.I1RZF6
132578 229533.I1RR00 pp 229533.Q4IJK2
132579 229533.I1RR00 pp 229533.Q4HYC1
132580 229533.I1RR00 pp 229533.I1S504
132581 229533.I1RR00 pp 229533.I1S9U2
132582 229533.I1RR00 pp 229533.I1RZM1
132583 229533.I1RR00 pp 229533.I1RVZ0
132584 229533.I1RR00 pp 229533.I1RXD7
132585 229533.I1RR00 pp 229533.I1RZK5
132586 229533.I1RR00 pp 229533.I1RZ35
132587 229533.I1RR00 pp 229533.I1RY03
132588 229533.I1RR00 pp 229533.I1S0R7
132589 229533.I1RR00 pp 229533.Q4I7X1
132590 229533.I1RR00 pp 229533.I1RRY9
132591 229533.I1RR00 pp 229533.I1S021
132592 229533.I1RR00 pp 229533.Q4IBS2
132593 229533.I1RR00 pp 229533.V6RTJ8
132594 229533.I1RR00 pp 229533.I1RT98
132595 229533.I1RR00 pp 229533.I1RSR3
132596 229533.I1RR00 pp 229533.I1RSM3
132597 229533.I1RR00 pp 229533.I1S540
132598 229533.I1RR00 pp 229533.I1RWJ3
132599 229533.I1RR00 pp 229533.I1RWJ4
132600 229533.I1RR00 pp 229533.Q4IPI1
132601 229533.I1RR00 pp 229533.I1S2B8
132602 229533.I1RR00 pp 229533.I1RX46
132603 229533.I1RR00 pp 229533.I1RSH4
132604 229533.I1RR00 pp 229533.V6R3X5
132605 229533.I1RR00 pp 229533.Q4IJ56
132606 229533.I1RR00 pp 229533.I1RR73
132607 229533.I1RR00 pp 229533.I1RWF7
132608 229533.I1RR00 pp 229533.I1RZ39
132609 229533.I1RR00 pp 229533.V6R283
132610 229533.I1RR00 pp 229533.I1S2E8
132611 229533.I1RR00 pp 229533.I1RWI5
132612 229533.I1RR00 pp 229533.I1S2C0
132613 229533.I1RR00 pp 229533.Q4HZ42
132614 229533.I1RR00 pp 229533.I1RWN5
132615 229533.I1RR00 pp 229533.I1RTE9
132616 229533.I1RR00 pp 229533.I1RTD5
132617 229533.I1RR00 pp 229533.I1RT04
132618 229533.I1RR00 pp 229533.I1RTH6
132619 229533.I1RR00 pp 229533.I1S1C7
132620 229533.I1RR00 pp 229533.V6RMW6
132621 229533.I1RR00 pp 229533.I1RRI2
132622 229533.I1RR00 pp 229533.Q4IQW0
132623 229533.I1RR00 pp 229533.I1RWN6
132624 229533.I1RR00 pp 229533.I1RSQ3
132625 229533.I1RR00 pp 229533.Q4IAS1
132626 229533.I1RR00 pp 229533.V6R042
132627 229533.I1RR00 pp 229533.I1S503
132628 229533.I1RR00 pp 229533.I1S0J1
132629 229533.I1RR00 pp 229533.I1S558
132630 229533.I1RR00 pp 229533.I1RVX0
132631 229533.I1RR00 pp 229533.Q4I327
132632 229533.I1RR00 pp 229533.I1RWF3
132633 229533.I1RR00 pp 229533.Q4IN63
132634 229533.I1RR00 pp 229533.I1S4T3
132635 229533.I1RR00 pp 229533.I1RTG6
132636 229533.I1RR00 pp 229533.I1RT03
132637 229533.I1RMU0 pp 229533.I1RYR9
132638 229533.I1S056 pp 229533.Q4IBK5
132639 229533.I1S056 pp 229533.I1S246
132640 229533.I1S056 pp 229533.I1S0Z9
132641 229533.I1S056 pp 229533.Q4IPH4

132642 229533.I1S056 pp 229533.Q4IPB8
132643 229533.I1S056 pp 229533.Q4IAA0
132644 229533.I1S056 pp 229533.Q4HXF6
132645 229533.I1S056 pp 229533.Q4IPB3
132646 229533.I1S056 pp 229533.I1S1C6
132647 229533.I1S056 pp 229533.Q4IE79
132648 229533.I1S056 pp 229533.Q4IB70
132649 229533.I1S056 pp 229533.Q4I1Y1
132650 229533.I1S056 pp 229533.Q4I5R9
132651 229533.I1S056 pp 229533.I1S9L0
132652 229533.A0A1C3YKS8 pp 229533.I1RPR8
132653 229533.A0A1C3YKS8 pp 229533.I1RPF4
132654 229533.A0A1C3YKS8 pp 229533.I1RW28
132655 229533.A0A1C3YKS8 pp 229533.Q4IEV4
132656 229533.A0A1C3YHC8 pp 229533.I1RK86
132657 229533.A0A1C3YHC8 pp 229533.A0A1C3YJG7
132658 229533.A0A1C3YHC8 pp 229533.I1RQS5
132659 229533.A0A1C3YHC8 pp 229533.I1RHV0
132660 229533.A0A1C3YHC8 pp 229533.I1S039
132661 229533.A0A1C3YHC8 pp 229533.I1S1W5
132662 229533.A0A1C3YHC8 pp 229533.Q4IQW0
132663 229533.A0A1C3YHC8 pp 229533.A0A1C3YM29
132664 229533.A0A1C3YHC8 pp 229533.I1RBW6
132665 229533.A0A1C3YHC8 pp 229533.I1RYB7
132666 229533.A0A1C3YHC8 pp 229533.I1RMV6
132667 229533.A0A1C3YHC8 pp 229533.I1RZM1
132668 229533.A0A0E0RV94 pp 229533.I1RNQ5
132669 229533.A0A0E0RV94 pp 229533.I1RZM1
132670 229533.A0A0E0RV94 pp 229533.A0A0E0SMM8
132671 229533.A0A0E0RV94 pp 229533.I1RZF6
132672 229533.A0A0E0RV94 pp 229533.I1S504
132673 229533.A0A0E0RV94 pp 229533.A0A1C3YKB0
132674 229533.A0A0E0RV94 pp 229533.A0A1C3YK34
132675 229533.A0A0E0RV94 pp 229533.A0A0E0SJS1
132676 229533.A0A0E0RV94 pp 229533.I1SA32
132677 229533.A0A0E0RV94 pp 229533.A0A1C3YKV3
132678 229533.A0A0E0RV94 pp 229533.I1RQ81
132679 229533.A0A0E0RV94 pp 229533.Q4I8P5
132680 229533.A0A0E0RV94 pp 229533.I1RB27
132681 229533.A0A0E0RV94 pp 229533.I1RAU5
132682 229533.A0A0E0RV94 pp 229533.A0A1C3YIJ3
132683 229533.A0A0E0RV94 pp 229533.I1S5B0
132684 229533.A0A0E0RV94 pp 229533.I1REK8
132685 229533.A0A0E0RV94 pp 229533.I1RW99
132686 229533.A0A0E0RV94 pp 229533.Q4IJK2
132687 229533.A0A0E0RV94 pp 229533.I1RZM8
132688 229533.A0A0E0RV94 pp 229533.I1RBC4
132689 229533.A0A0E0RV94 pp 229533.I1RK95
132690 229533.A0A0E0RV94 pp 229533.V6RCG5
132691 229533.A0A0E0RV94 pp 229533.I1RYS0
132692 229533.A0A0E0RV94 pp 229533.I1RBL7
132693 229533.A0A0E0RV94 pp 229533.I1RW25
132694 229533.A0A0E0RV94 pp 229533.I1RRY9
132695 229533.A0A0E0RV94 pp 229533.I1RW54
132696 229533.A0A0E0RV94 pp 229533.A0A1C3YHX6
132697 229533.A0A0E0RV94 pp 229533.I1RGD2
132698 229533.A0A0E0RV94 pp 229533.I1RCH5
132699 229533.A0A0E0RV94 pp 229533.I1RY03
132700 229533.A0A0E0RV94 pp 229533.I1RQE7
132701 229533.A0A0E0RV94 pp 229533.V6QUW4
132702 229533.A0A0E0RV94 pp 229533.I1RFU8
132703 229533.A0A0E0RV94 pp 229533.I1RT03
132704 229533.A0A0E0RV94 pp 229533.I1RCG4
132705 229533.A0A0E0RV94 pp 229533.I1RWJ8
132706 229533.A0A0E0RV94 pp 229533.I1RVX0
132707 229533.A0A0E0RV94 pp 229533.I1RR24
132708 229533.A0A0E0RV94 pp 229533.A0A1C3YKS6
132709 229533.A0A0E0RV94 pp 229533.I1S027
132710 229533.A0A0E0RV94 pp 229533.A0A1C3YKC0
132711 229533.A0A0E0RV94 pp 229533.V6R042
132712 229533.A0A0E0RV94 pp 229533.A0A1C3YHX8
132713 229533.A0A0E0RV94 pp 229533.I1RB92
132714 229533.A0A0E0RV94 pp 229533.I1RJU3

132715	229533.A0A0E0RV94	pp	229533.I1RCL3
132716	229533.A0A0E0RV94	pp	229533.I1S2B8
132717	229533.A0A0E0RV94	pp	229533.A0A0E0SA11
132718	229533.A0A0E0RV94	pp	229533.A0A1C3YL93
132719	229533.A0A0E0RV94	pp	229533.A0A1C3YIP5
132720	229533.A0A0E0RV94	pp	229533.I1RJV3
132721	229533.A0A0E0RV94	pp	229533.I1RWN6
132722	229533.A0A0E0RV94	pp	229533.I1RRM5
132723	229533.A0A0E0RV94	pp	229533.I1RVQ0
132724	229533.A0A0E0RV94	pp	229533.I1RD52
132725	229533.A0A0E0RV94	pp	229533.I1RJZ7
132726	229533.A0A0E0RV94	pp	229533.I1RAW0
132727	229533.A0A0E0RV94	pp	229533.I1RYW3
132728	229533.A0A0E0RV94	pp	229533.I1RE76
132729	229533.A0A0E0RV94	pp	229533.I1RBY4
132730	229533.A0A0E0RV94	pp	229533.I1S0L3
132731	229533.A0A0E0RV94	pp	229533.A0A1C3YIS7
132732	229533.A0A0E0RV94	pp	229533.I1RBC9
132733	229533.A0A0E0RV94	pp	229533.I1S1C7
132734	229533.A0A0E0RV94	pp	229533.I1RPM4
132735	229533.A0A0E0RV94	pp	229533.I1RFS3
132736	229533.A0A0E0RV94	pp	229533.I1RW71
132737	229533.A0A0E0RV94	pp	229533.I1S5R1
132738	229533.A0A0E0RV94	pp	229533.I1RNE4
132739	229533.A0A0E0RV94	pp	229533.I1RQJ5
132740	229533.A0A0E0RV94	pp	229533.I1RRI2
132741	229533.A0A0E0RV94	pp	229533.I1RQP8
132742	229533.A0A0E0RV94	pp	229533.I1RRT8
132743	229533.A0A0E0RV94	pp	229533.I1RSQ7
132744	229533.A0A0E0RV94	pp	229533.I1S0L4
132745	229533.A0A0E0RV94	pp	229533.I1RZW8
132746	229533.A0A0E0RV94	pp	229533.V6QW22
132747	229533.A0A0E0RV94	pp	229533.I1RA70
132748	229533.A0A0E0RV94	pp	229533.I1RTH6
132749	229533.A0A0E0RV94	pp	229533.I1RZZ9
132750	229533.A0A0E0RV94	pp	229533.I1RX46
132751	229533.A0A0E0RV94	pp	229533.I1RZ39
132752	229533.A0A0E0RV94	pp	229533.I1RAX6
132753	229533.A0A0E0RV94	pp	229533.A0A1C3YKM9
132754	229533.A0A0E0RV94	pp	229533.V6R3X5
132755	229533.A0A0E0RV94	pp	229533.V6RTJ8
132756	229533.A0A0E0RV94	pp	229533.I1RWN5
132757	229533.A0A0E0RV94	pp	229533.V6RIV9
132758	229533.A0A0E0RV94	pp	229533.I1RZJ6
132759	229533.A0A0E0RV94	pp	229533.A0A0E0SFN0
132760	229533.A0A0E0RV94	pp	229533.I1RR73
132761	229533.A0A0E0RV94	pp	229533.I1RQC9
132762	229533.A0A0E0RV94	pp	229533.I1RN67
132763	229533.A0A0E0RV94	pp	229533.I1RY44
132764	229533.A0A0E0RV94	pp	229533.I1RME6
132765	229533.A0A0E0RV94	pp	229533.A0A1C3YI73
132766	229533.A0A0E0RV94	pp	229533.I1RM96
132767	229533.A0A0E0RV94	pp	229533.I1RW26
132768	229533.A0A0E0RV94	pp	229533.I1RD17
132769	229533.A0A0E0RV94	pp	229533.I1RW46
132770	229533.A0A0E0RV94	pp	229533.I1RAU7
132771	229533.A0A0E0RV94	pp	229533.I1S0F6
132772	229533.A0A0E0RV94	pp	229533.I1RE01
132773	229533.A0A0E0RV94	pp	229533.I1RN18
132774	229533.A0A0E0RV94	pp	229533.I1RS17
132775	229533.A0A0E0RV94	pp	229533.I1RFL4
132776	229533.A0A0E0RV94	pp	229533.I1RC28
132777	229533.A0A0E0RV94	pp	229533.I1RWK3
132778	229533.A0A0E0RV94	pp	229533.I1RBS6
132779	229533.A0A0E0RV94	pp	229533.I1RGA4
132780	229533.A0A0E0RV94	pp	229533.I1RPP4
132781	229533.A0A0E0RV94	pp	229533.I1RNR2
132782	229533.A0A0E0RV94	pp	229533.I1RQM7
132783	229533.A0A0E0RV94	pp	229533.I1RAH9
132784	229533.A0A0E0RV94	pp	229533.I1SA33
132785	229533.A0A0E0RV94	pp	229533.I1RJV4
132786	229533.A0A0E0RV94	pp	229533.I1RWH4
132787	229533.A0A0E0RV94	pp	229533.A0A1C3YKN3

132788 229533.A0A0E0RV94 pp 229533.I1RPP3
132789 229533.A0A0E0RV94 pp 229533.I1RT40
132790 229533.A0A0E0RV94 pp 229533.I1RCA7
132791 229533.A0A0E0RV94 pp 229533.I1RD22
132792 229533.A0A0E0RV94 pp 229533.I1RTD5
132793 229533.A0A0E0RV94 pp 229533.A0A0E0SQI9
132794 229533.A0A0E0RV94 pp 229533.I1RUG3
132795 229533.A0A0E0RV94 pp 229533.I1RTH2
132796 229533.A0A0E0RV94 pp 229533.I1RT04
132797 229533.A0A0E0RV94 pp 229533.I1S503
132798 229533.A0A0E0RV94 pp 229533.I1RNC1
132799 229533.A0A0E0RV94 pp 229533.I1RRV1
132800 229533.A0A0E0RV94 pp 229533.I1S2E9
132801 229533.A0A0E0RV94 pp 229533.V6RMW6
132802 229533.A0A0E0RV94 pp 229533.A0A1C3YLK8
132803 229533.A0A0E0RV94 pp 229533.I1RJW6
132804 229533.I1S6M5 pp 229533.Q4HVQ9
132805 229533.I1S8J4 pp 229533.Q4I0C5
132806 229533.I1RFT2 pp 229533.I1RVX0
132807 229533.I1RFT2 pp 229533.Q4IJM4
132808 229533.I1RFT2 pp 229533.I1RRN5
132809 229533.I1RFT2 pp 229533.Q4IJK2
132810 229533.I1RFT2 pp 229533.I1RT03
132811 229533.I1RFT2 pp 229533.I1RZZ1
132812 229533.I1RFT2 pp 229533.I1RQA3
132813 229533.I1RFT2 pp 229533.I1RZ93
132814 229533.I1RFT2 pp 229533.V6RBN2
132815 229533.I1RFT2 pp 229533.I1RKT9
132816 229533.I1RFT2 pp 229533.I1RSK1
132817 229533.I1RFT2 pp 229533.I1RJV3
132818 229533.I1RFT2 pp 229533.I1RQ81
132819 229533.I1RFT2 pp 229533.V6RRH2
132820 229533.I1RFT2 pp 229533.I1RFU8
132821 229533.I1RFT2 pp 229533.I1RPP4
132822 229533.I1RFT2 pp 229533.I1RSI0
132823 229533.I1RFT2 pp 229533.I1S2B8
132824 229533.I1RFT2 pp 229533.I1S0J1
132825 229533.I1RFT2 pp 229533.I1RTA0
132826 229533.I1RFT2 pp 229533.I1S1W4
132827 229533.I1RFT2 pp 229533.I1RTH6
132828 229533.I1RFT2 pp 229533.I1RW25
132829 229533.I1RFT2 pp 229533.I1RPU3
132830 229533.I1RFT2 pp 229533.I1RZK5
132831 229533.I1RFT2 pp 229533.I1RYW3
132832 229533.I1RFT2 pp 229533.I1RWT6
132833 229533.I1RFT2 pp 229533.I1S504
132834 229533.I1RFT2 pp 229533.I1RM18
132835 229533.I1RFT2 pp 229533.V6R3X5
132836 229533.I1RFT2 pp 229533.I1RZ39
132837 229533.I1RFT2 pp 229533.I1S1C7
132838 229533.I1RFT2 pp 229533.I1RY03
132839 229533.I1RFT2 pp 229533.I1RY44
132840 229533.I1RFT2 pp 229533.I1RWN5
132841 229533.I1RFT2 pp 229533.I1RR73
132842 229533.I1RFT2 pp 229533.I1RYY5
132843 229533.I1RFT2 pp 229533.I1RNQ5
132844 229533.I1RFT2 pp 229533.I1RLU6
132845 229533.I1RFT2 pp 229533.I1RRY9
132846 229533.I1RFT2 pp 229533.I1RZM1
132847 229533.I1RFT2 pp 229533.I1RWI5
132848 229533.I1RFT2 pp 229533.I1RZZ9
132849 229533.I1RFT2 pp 229533.I1RFT6
132850 229533.I1RFT2 pp 229533.I1RWN6
132851 229533.I1RFT2 pp 229533.V6RMW6
132852 229533.I1RFT2 pp 229533.I1RRV1
132853 229533.I1RFT2 pp 229533.I1RQC9
132854 229533.I1RFT2 pp 229533.I1RT04
132855 229533.I1RFT2 pp 229533.I1RZD2
132856 229533.I1RFT2 pp 229533.I1RNN1
132857 229533.I1RFT2 pp 229533.V6R042
132858 229533.I1RFT2 pp 229533.I1RM82
132859 229533.I1RFT2 pp 229533.I1RWL2
132860 229533.I1RFT2 pp 229533.I1S0R7

132861	229533.I1RFT2	pp	229533.I1RSQ3
132862	229533.I1RFT2	pp	229533.I1RSV2
132863	229533.I1RFT2	pp	229533.I1RNC1
132864	229533.I1RFT2	pp	229533.I1RQH3
132865	229533.I1RFT2	pp	229533.I1RN58
132866	229533.I1RFT2	pp	229533.I1S2E9
132867	229533.I1RFT2	pp	229533.I1RPP3
132868	229533.I1RFT2	pp	229533.I1RR93
132869	229533.I1RFT2	pp	229533.I1S8P5
132870	229533.I1RFT2	pp	229533.I1S044
132871	229533.I1RFT2	pp	229533.I1RXD7
132872	229533.I1RFT2	pp	229533.I1S0N4
132873	229533.I1RFT2	pp	229533.I1RXU7
132874	229533.I1RFT2	pp	229533.I1RN61
132875	229533.I1RFT2	pp	229533.I1RSQ7
132876	229533.I1RFT2	pp	229533.I1RTD5
132877	229533.I1RFT2	pp	229533.I1RQG4
132878	229533.I1RFT2	pp	229533.I1RRU9
132879	229533.I1RFT2	pp	229533.I1RGA4
132880	229533.I1RFT2	pp	229533.I1S2G7
132881	229533.I1RFT2	pp	229533.I1RP27
132882	229533.I1RFT2	pp	229533.I1RP28
132883	229533.I1RFT2	pp	229533.I1RN67
132884	229533.I1RFT2	pp	229533.V6RTJ8
132885	229533.I1RFT2	pp	229533.I1RX46
132886	229533.I1RFT2	pp	229533.I1S503
132887	229533.I1RRU9	pp	229533.Q4HXF6
132888	229533.I1RRU9	pp	229533.Q4I1Y5
132889	229533.I1RRU9	pp	229533.I1RVX0
132890	229533.I1RRU9	pp	229533.I1S1X9
132891	229533.I1RRU9	pp	229533.V6RIS2
132892	229533.I1RRU9	pp	229533.I1S0N4
132893	229533.I1RRU9	pp	229533.I1RSS4
132894	229533.I1RRU9	pp	229533.I1S3V2
132895	229533.I1RRU9	pp	229533.I1RSK1
132896	229533.I1RRU9	pp	229533.I1S471
132897	229533.I1RRU9	pp	229533.V6R042
132898	229533.I1RRU9	pp	229533.I1S1C7
132899	229533.I1RRU9	pp	229533.I1RSL9
132900	229533.I1RRU9	pp	229533.I1SAN9
132901	229533.I1RRU9	pp	229533.I1RTH6
132902	229533.I1RRU9	pp	229533.I1RTD5
132903	229533.I1RRU9	pp	229533.I1RZ40
132904	229533.I1RRU9	pp	229533.V6R223
132905	229533.I1RRU9	pp	229533.Q4I7K4
132906	229533.I1RRU9	pp	229533.I1RWN5
132907	229533.I1RRU9	pp	229533.I1RST1
132908	229533.I1RRU9	pp	229533.I1S2E9
132909	229533.I1RRU9	pp	229533.Q4HZB8
132910	229533.I1RRU9	pp	229533.I1RZZ9
132911	229533.I1RRU9	pp	229533.I1RT04
132912	229533.I1RRU9	pp	229533.V6RY33
132913	229533.I1RRU9	pp	229533.I1RTA0
132914	229533.I1RRU9	pp	229533.Q4HXF0
132915	229533.I1RRU9	pp	229533.I1RS94
132916	229533.I1RRU9	pp	229533.I1RZS7
132917	229533.I1RRU9	pp	229533.I1RWI5
132918	229533.I1RRU9	pp	229533.I1RWT6
132919	229533.I1RRU9	pp	229533.I1S8P5
132920	229533.I1RRU9	pp	229533.I1RX24
132921	229533.I1RRU9	pp	229533.I1S504
132922	229533.I1RRU9	pp	229533.I1S503
132923	229533.I1RRU9	pp	229533.I1RWD7
132924	229533.I1RRU9	pp	229533.I1RX46
132925	229533.I1RRU9	pp	229533.I1RSI0
132926	229533.I1RRU9	pp	229533.I1RTF4
132927	229533.I1RRU9	pp	229533.V6RTJ8
132928	229533.I1RRU9	pp	229533.Q4IAA0
132929	229533.I1RRU9	pp	229533.I1RXU7
132930	229533.I1RRU9	pp	229533.I1RWN6
132931	229533.I1RRU9	pp	229533.I1S0L3
132932	229533.I1RRU9	pp	229533.V6RIV9
132933	229533.I1RRU9	pp	229533.I1RXF6

132934	229533.I1RRU9	pp	229533.I1RWX5
132935	229533.I1RRU9	pp	229533.I1RSD4
132936	229533.I1RRU9	pp	229533.I1RWE8
132937	229533.I1RRU9	pp	229533.Q4IJT5
132938	229533.I1RRU9	pp	229533.I1RYW3
132939	229533.I1RRU9	pp	229533.I1RRX5
132940	229533.I1RRU9	pp	229533.I1S289
132941	229533.I1RRU9	pp	229533.V6RNM4
132942	229533.I1RRU9	pp	229533.I1RWL2
132943	229533.I1RRU9	pp	229533.I1RW71
132944	229533.I1RRU9	pp	229533.I1RWD3
132945	229533.I1RRU9	pp	229533.I1RT03
132946	229533.I1RRU9	pp	229533.I1S2C0
132947	229533.I1RRU9	pp	229533.I1S0L4
132948	229533.I1RRU9	pp	229533.I1RWH4
132949	229533.I1RRU9	pp	229533.I1S855
132950	229533.I1RRU9	pp	229533.I1RUG3
132951	229533.I1RRU9	pp	229533.I1S2H4
132952	229533.I1RRU9	pp	229533.I1RSV2
132953	229533.I1RRU9	pp	229533.V6RMW6
132954	229533.I1RRU9	pp	229533.I1S1J7
132955	229533.I1RRU9	pp	229533.I1RW36
132956	229533.I1RRU9	pp	229533.I1RRY9
132957	229533.I1RRU9	pp	229533.I1RS20
132958	229533.I1RRU9	pp	229533.I1RRV1
132959	229533.I1RRU9	pp	229533.I1RY03
132960	229533.I1RRU9	pp	229533.I1RSQ3
132961	229533.I1RRU9	pp	229533.I1RYB1
132962	229533.I1RRU9	pp	229533.V6R3X5
132963	229533.I1RRU9	pp	229533.I1S0Z9
132964	229533.I1RRU9	pp	229533.I1RY44
132965	229533.I1RRU9	pp	229533.I1S0X5
132966	229533.I1RRU9	pp	229533.I1RW99
132967	229533.I1RRU9	pp	229533.Q4I2J8
132968	229533.I1RRU9	pp	229533.I1S5B0
132969	229533.I1RRU9	pp	229533.I1S0X6
132970	229533.I1RRU9	pp	229533.I1RZM1
132971	229533.I1RRU9	pp	229533.I1RW25
132972	229533.I1RRU9	pp	229533.I1S6S3
132973	229533.I1RRU9	pp	229533.I1RT35
132974	229533.I1RRU9	pp	229533.I1RSM2
132975	229533.I1RRU9	pp	229533.Q4IPI1
132976	229533.I1RRU9	pp	229533.V6R227
132977	229533.I1RRU9	pp	229533.I1S246
132978	229533.I1RRU9	pp	229533.I1RS18
132979	229533.I1RRU9	pp	229533.I1RSQ7
132980	229533.I1RRU9	pp	229533.I1RVQ0
132981	229533.I1RN18	pp	229533.I1RQC9
132982	229533.I1RN18	pp	229533.I1RX04
132983	229533.I1RN18	pp	229533.I1RTD5
132984	229533.I1RN18	pp	229533.I1RRT8
132985	229533.I1RN18	pp	229533.I1RT40
132986	229533.I1RN18	pp	229533.I1S5R1
132987	229533.I1RN18	pp	229533.V6R3X5
132988	229533.I1RN18	pp	229533.I1RWJ8
132989	229533.I1RN18	pp	229533.I1RV91
132990	229533.I1RN18	pp	229533.I1RR73
132991	229533.I1RN18	pp	229533.I1S7A8
132992	229533.I1RN18	pp	229533.V6RTJ8
132993	229533.I1RN18	pp	229533.V6QYF0
132994	229533.I1RN18	pp	229533.I1S0M6
132995	229533.I1RN18	pp	229533.I1RZ39
132996	229533.I1RN18	pp	229533.I1RRL9
132997	229533.I1RN18	pp	229533.V6QUW4
132998	229533.I1RN18	pp	229533.I1RX46
132999	229533.I1RN18	pp	229533.I1S1B6
133000	229533.I1RN18	pp	229533.I1RRT2
133001	229533.I1RN18	pp	229533.I1S6Y8
133002	229533.I1RN18	pp	229533.I1S0X6
133003	229533.I1RN18	pp	229533.I1S0T2
133004	229533.I1RN18	pp	229533.I1RZZ9
133005	229533.I1RN18	pp	229533.I1RPM4
133006	229533.I1RN18	pp	229533.I1S0L4

133007 229533.I1RN18 pp 229533.I1RWI5
133008 229533.I1RN18 pp 229533.I1S503
133009 229533.I1RN18 pp 229533.I1RRF8
133010 229533.I1RN18 pp 229533.I1RQ23
133011 229533.I1RN18 pp 229533.I1RRZ0
133012 229533.I1RN18 pp 229533.I1RS20
133013 229533.I1RN18 pp 229533.I1RW07
133014 229533.I1RN18 pp 229533.I1RX25
133015 229533.I1RN18 pp 229533.I1S2H4
133016 229533.I1RN18 pp 229533.I1RRJ8
133017 229533.I1RN18 pp 229533.I1RW25
133018 229533.I1RN18 pp 229533.I1RSQ7
133019 229533.I1RN18 pp 229533.I1S7S9
133020 229533.I1RN18 pp 229533.I1RR24
133021 229533.I1RN18 pp 229533.I1RQS8
133022 229533.I1RN18 pp 229533.I1S2B8
133023 229533.I1RN18 pp 229533.V6QW22
133024 229533.I1RN18 pp 229533.I1RRM9
133025 229533.I1RN18 pp 229533.I1RPP3
133026 229533.I1RN18 pp 229533.I1RZJ5
133027 229533.I1RN18 pp 229533.I1S8P5
133028 229533.I1RN18 pp 229533.I1RRM5
133029 229533.I1RN18 pp 229533.O74638
133030 229533.I1RN18 pp 229533.I1RW71
133031 229533.I1RN18 pp 229533.I1S2E9
133032 229533.I1RN18 pp 229533.I1S0L3
133033 229533.I1RN18 pp 229533.I1RXD7
133034 229533.I1RN18 pp 229533.I1RUQ2
133035 229533.I1RN18 pp 229533.I1S0R7
133036 229533.I1RN18 pp 229533.V6R1K4
133037 229533.I1RN18 pp 229533.I1S352
133038 229533.I1RN18 pp 229533.I1RT04
133039 229533.I1RN18 pp 229533.I1S2M6
133040 229533.I1RN18 pp 229533.I1RVQ0
133041 229533.I1RN18 pp 229533.Q4IJT5
133042 229533.I1RN18 pp 229533.I1RS47
133043 229533.I1RN18 pp 229533.I1RWK3
133044 229533.I1RN18 pp 229533.I1RNA5
133045 229533.I1RN18 pp 229533.V6R4J1
133046 229533.I1RN18 pp 229533.I1RN63
133047 229533.I1RN18 pp 229533.V6RCZ8
133048 229533.I1RN18 pp 229533.I1S0F5
133049 229533.I1RN18 pp 229533.I1S0F6
133050 229533.I1RN18 pp 229533.I1RZM8
133051 229533.I1RN18 pp 229533.I1RZK1
133052 229533.I1RN18 pp 229533.I1RXF0
133053 229533.I1RN18 pp 229533.I1RN67
133054 229533.I1RN18 pp 229533.I1RZK5
133055 229533.I1RN18 pp 229533.I1RRN5
133056 229533.I1RN18 pp 229533.I1RQ31
133057 229533.I1RN18 pp 229533.I1RSC6
133058 229533.I1RN18 pp 229533.I1RUG3
133059 229533.I1RN18 pp 229533.I1RT03
133060 229533.I1RN18 pp 229533.I1S8R3
133061 229533.I1RN18 pp 229533.I1RNC1
133062 229533.I1RN18 pp 229533.I1RW99
133063 229533.I1RN18 pp 229533.I1RTH6
133064 229533.I1RN18 pp 229533.I1RZW8
133065 229533.I1RN18 pp 229533.I1S5B0
133066 229533.I1RN18 pp 229533.I1RVX0
133067 229533.I1RN18 pp 229533.I1RRI2
133068 229533.I1RN18 pp 229533.I1RQQ5
133069 229533.I1RN18 pp 229533.I1RQE7
133070 229533.I1RN18 pp 229533.I1RWQ8
133071 229533.I1RN18 pp 229533.I1RWX5
133072 229533.I1RN18 pp 229533.Q4I8P5
133073 229533.I1RN18 pp 229533.I1RZZ1
133074 229533.I1RN18 pp 229533.I1SA33
133075 229533.I1RN18 pp 229533.I1RYW3
133076 229533.I1RN18 pp 229533.Q4IJK2
133077 229533.I1RN18 pp 229533.I1RY03
133078 229533.I1RN18 pp 229533.I1S1C7
133079 229533.I1RN18 pp 229533.I1RQA3

133080	229533.I1RN18	pp	229533.I1RPP4
133081	229533.I1RN18	pp	229533.I1RQ72
133082	229533.I1RN18	pp	229533.I1RS18
133083	229533.I1RN18	pp	229533.Q4I624
133084	229533.I1RN18	pp	229533.I1RSR3
133085	229533.I1RN18	pp	229533.I1RQ81
133086	229533.I1RN18	pp	229533.I1RWN6
133087	229533.I1RN18	pp	229533.I1RRY9
133088	229533.I1RN18	pp	229533.V6R057
133089	229533.I1RN18	pp	229533.I1S0J1
133090	229533.I1RN18	pp	229533.I1RS17
133091	229533.I1RN18	pp	229533.I1S569
133092	229533.I1RN18	pp	229533.I1RU29
133093	229533.I1RN18	pp	229533.I1S0R2
133094	229533.I1RN18	pp	229533.I1SA32
133095	229533.I1RN18	pp	229533.I1RSM3
133096	229533.I1RN18	pp	229533.I1S1J7
133097	229533.I1RN18	pp	229533.I1RTA0
133098	229533.I1RN18	pp	229533.I1RSI0
133099	229533.I1RN18	pp	229533.I1RZS7
133100	229533.I1RN18	pp	229533.I1S504
133101	229533.I1RN18	pp	229533.Q4I2J8
133102	229533.I1RN18	pp	229533.I1RZH4
133103	229533.I1RN18	pp	229533.I1RYH7
133104	229533.I1RN18	pp	229533.I1RZM1
133105	229533.I1RN18	pp	229533.V6RCG5
133106	229533.I1RN18	pp	229533.I1RRE5
133107	229533.I1RN18	pp	229533.V6RMW6
133108	229533.I1RN18	pp	229533.I1RWT6
133109	229533.I1RN18	pp	229533.I1RW46
133110	229533.I1RN18	pp	229533.I1RSA4
133111	229533.I1RN18	pp	229533.Q4I283
133112	229533.I1RN18	pp	229533.V6R042
133113	229533.I1RN18	pp	229533.I1RY44
133114	229533.I1RN18	pp	229533.I1RSQ3
133115	229533.I1RN18	pp	229533.Q4HZB8
133116	229533.I1RN18	pp	229533.I1RNQ5
133117	229533.I1RN18	pp	229533.V6R227
133118	229533.I1RN18	pp	229533.I1S419
133119	229533.I1RN18	pp	229533.I1RR51
133120	229533.I1RN18	pp	229533.V6RIV9
133121	229533.I1RN18	pp	229533.I1RWN5
133122	229533.I1RN18	pp	229533.I1RRV1
133123	229533.V6RHD0	pp	229533.V6RXR0
133124	229533.V6RHD0	pp	229533.V6RK96
133125	229533.I1RFL4	pp	229533.I1S2C0
133126	229533.I1RFL4	pp	229533.I1S8R3
133127	229533.I1RFL4	pp	229533.I1RTH6
133128	229533.I1RFL4	pp	229533.I1RTE9
133129	229533.I1RFL4	pp	229533.I1RZ79
133130	229533.I1RFL4	pp	229533.I1RRI2
133131	229533.I1RFL4	pp	229533.I1RXU9
133132	229533.I1RFL4	pp	229533.I1RWX5
133133	229533.I1RFL4	pp	229533.I1RZW8
133134	229533.I1RFL4	pp	229533.I1RYW3
133135	229533.I1RFL4	pp	229533.I1RJV3
133136	229533.I1RFL4	pp	229533.I1RSK1
133137	229533.I1RFL4	pp	229533.I1S1C7
133138	229533.I1RFL4	pp	229533.I1RSY2
133139	229533.I1RFL4	pp	229533.I1RGL4
133140	229533.I1RFL4	pp	229533.I1RVX0
133141	229533.I1RFL4	pp	229533.I1RWN6
133142	229533.I1RFL4	pp	229533.I1RT03
133143	229533.I1RFL4	pp	229533.I1RRN5
133144	229533.I1RFL4	pp	229533.V6RRH2
133145	229533.I1RFL4	pp	229533.I1S1J6
133146	229533.I1RFL4	pp	229533.I1RKC6
133147	229533.I1RFL4	pp	229533.I1RMV0
133148	229533.I1RFL4	pp	229533.I1SA33
133149	229533.I1RFL4	pp	229533.I1RXR1
133150	229533.I1RFL4	pp	229533.I1RPP4
133151	229533.I1RFL4	pp	229533.V6RMW6
133152	229533.I1RFL4	pp	229533.I1RQE7

133153 229533.I1RFL4 pp 229533.V6R042
133154 229533.I1RFL4 pp 229533.I1RN18
133155 229533.I1RFL4 pp 229533.I1RNB8
133156 229533.I1RFL4 pp 229533.I1RPK0
133157 229533.I1RFL4 pp 229533.I1RQ72
133158 229533.I1RFL4 pp 229533.I1RSQ3
133159 229533.I1RFL4 pp 229533.V6R057
133160 229533.I1RFL4 pp 229533.I1RS17
133161 229533.I1RFL4 pp 229533.I1S7W5
133162 229533.I1RFL4 pp 229533.I1RMG3
133163 229533.I1RFL4 pp 229533.I1RRV1
133164 229533.I1RFL4 pp 229533.I1S296
133165 229533.I1RFL4 pp 229533.I1S0J1
133166 229533.I1RFL4 pp 229533.I1S569
133167 229533.I1RFL4 pp 229533.I1RSI6
133168 229533.I1RFL4 pp 229533.I1SAN9
133169 229533.I1RFL4 pp 229533.I1S0R2
133170 229533.I1RFL4 pp 229533.I1RUG3
133171 229533.I1RFL4 pp 229533.I1RTA0
133172 229533.I1RFL4 pp 229533.I1RW46
133173 229533.I1RFL4 pp 229533.Q4I624
133174 229533.I1RFL4 pp 229533.I1RXT3
133175 229533.I1RFL4 pp 229533.V6R3X5
133176 229533.I1RFL4 pp 229533.I1RZS7
133177 229533.I1RFL4 pp 229533.I1RR73
133178 229533.I1RFL4 pp 229533.I1RNC1
133179 229533.I1RFL4 pp 229533.I1RZ39
133180 229533.I1RFL4 pp 229533.I1S8V4
133181 229533.I1RFL4 pp 229533.I1S0N4
133182 229533.I1RFL4 pp 229533.I1RRE5
133183 229533.I1RFL4 pp 229533.I1RRL9
133184 229533.I1RFL4 pp 229533.V6RGN5
133185 229533.I1RFL4 pp 229533.I1RG11
133186 229533.I1RFL4 pp 229533.I1RY44
133187 229533.I1RFL4 pp 229533.I1S5R6
133188 229533.I1RFL4 pp 229533.I1RWQ8
133189 229533.I1RFL4 pp 229533.I1S8V3
133190 229533.I1RFL4 pp 229533.V6RVG3
133191 229533.I1RFL4 pp 229533.I1RZZ9
133192 229533.I1RFL4 pp 229533.I1RSQ4
133193 229533.I1RFL4 pp 229533.I1RWH4
133194 229533.I1RFL4 pp 229533.I1RRN2
133195 229533.I1RFL4 pp 229533.V6QXG1
133196 229533.I1RFL4 pp 229533.I1S1W3
133197 229533.I1RFL4 pp 229533.I1RWI5
133198 229533.I1RFL4 pp 229533.I1S2E9
133199 229533.I1RFL4 pp 229533.I1RNA5
133200 229533.I1RFL4 pp 229533.I1RIL3
133201 229533.I1RFL4 pp 229533.I1S0L4
133202 229533.I1RFL4 pp 229533.I1S1W9
133203 229533.I1RFL4 pp 229533.I1S2H4
133204 229533.I1RFL4 pp 229533.V6RIV9
133205 229533.I1RFL4 pp 229533.I1RR68
133206 229533.I1RFL4 pp 229533.I1RWN5
133207 229533.I1RFL4 pp 229533.I1RS20
133208 229533.I1RFL4 pp 229533.V6RUY9
133209 229533.I1RFL4 pp 229533.I1RT04
133210 229533.I1RFL4 pp 229533.Q4HZB8
133211 229533.I1RFL4 pp 229533.I1S3G4
133212 229533.I1RFL4 pp 229533.I1RQS8
133213 229533.I1RFL4 pp 229533.I1RT75
133214 229533.I1RFL4 pp 229533.I1S9Y1
133215 229533.I1RFL4 pp 229533.I1RXC2
133216 229533.I1RFL4 pp 229533.I1RTD5
133217 229533.I1RFL4 pp 229533.I1S0M6
133218 229533.I1RFL4 pp 229533.I1RM82
133219 229533.I1RFL4 pp 229533.O74638
133220 229533.I1RFL4 pp 229533.I1RGF5
133221 229533.I1RFL4 pp 229533.I1RV91
133222 229533.I1RFL4 pp 229533.I1RJS2
133223 229533.I1RFL4 pp 229533.I1RQ23
133224 229533.I1RFL4 pp 229533.I1RT40
133225 229533.I1RFL4 pp 229533.I1RN88

133226	229533.I1RFL4	pp	229533.I1S5R1
133227	229533.I1RFL4	pp	229533.I1RZB2
133228	229533.I1RFL4	pp	229533.I1RFS3
133229	229533.I1RFL4	pp	229533.I1RJ74
133230	229533.I1RFL4	pp	229533.I1RJV4
133231	229533.I1RFL4	pp	229533.I1RL04
133232	229533.I1RFL4	pp	229533.I1RRT8
133233	229533.I1RFL4	pp	229533.I1RPV3
133234	229533.I1RFL4	pp	229533.I1RRJ8
133235	229533.I1RFL4	pp	229533.I1RQG4
133236	229533.I1RFL4	pp	229533.I1RPM4
133237	229533.I1RFL4	pp	229533.I1S1B6
133238	229533.I1RFL4	pp	229533.I1S6Y8
133239	229533.I1RFL4	pp	229533.I1S352
133240	229533.I1RFL4	pp	229533.I1S268
133241	229533.I1RFL4	pp	229533.I1RGA4
133242	229533.I1RFL4	pp	229533.I1RSZ7
133243	229533.I1RFL4	pp	229533.I1RFT1
133244	229533.I1RFL4	pp	229533.I1RSX1
133245	229533.I1RFL4	pp	229533.I1RRT2
133246	229533.I1RFL4	pp	229533.I1RW61
133247	229533.I1RFL4	pp	229533.I1RTA1
133248	229533.I1RFL4	pp	229533.V6R3J1
133249	229533.I1RFL4	pp	229533.I1S7S9
133250	229533.I1RFL4	pp	229533.I1S277
133251	229533.I1RFL4	pp	229533.I1RJL4
133252	229533.I1RFL4	pp	229533.I1RSX0
133253	229533.I1RFL4	pp	229533.I1S2M6
133254	229533.I1RFL4	pp	229533.I1RRM5
133255	229533.I1RFL4	pp	229533.I1RRZ0
133256	229533.I1RFL4	pp	229533.I1RVZ2
133257	229533.I1RFL4	pp	229533.I1RLN6
133258	229533.I1RFL4	pp	229533.I1RN63
133259	229533.I1RFL4	pp	229533.I1RWK3
133260	229533.I1RFL4	pp	229533.I1S503
133261	229533.I1RFL4	pp	229533.I1S2B8
133262	229533.I1RFL4	pp	229533.I1RS47
133263	229533.I1RFL4	pp	229533.I1RJW6
133264	229533.I1RFL4	pp	229533.I1RJZ7
133265	229533.I1RFL4	pp	229533.V6RBN2
133266	229533.I1RFL4	pp	229533.I1S8P5
133267	229533.I1RFL4	pp	229533.I1RN67
133268	229533.I1RFL4	pp	229533.I1RRP3
133269	229533.I1RFL4	pp	229533.I1RQQ5
133270	229533.I1RFL4	pp	229533.Q4IJK2
133271	229533.I1RFL4	pp	229533.I1RW26
133272	229533.I1RFL4	pp	229533.I1RPP3
133273	229533.I1RFL4	pp	229533.I1RYI5
133274	229533.I1RFL4	pp	229533.I1RQA3
133275	229533.I1RFL4	pp	229533.I1RMD5
133276	229533.I1RFL4	pp	229533.I1S0F6
133277	229533.I1RFL4	pp	229533.I1RFU8
133278	229533.I1RFL4	pp	229533.I1RZK5
133279	229533.I1RFL4	pp	229533.I1RLM7
133280	229533.I1RFL4	pp	229533.I1RWD7
133281	229533.I1RFL4	pp	229533.I1RTD9
133282	229533.I1RFL4	pp	229533.I1S4Q4
133283	229533.I1RFL4	pp	229533.I1RU29
133284	229533.I1RFL4	pp	229533.I1RY03
133285	229533.I1RFL4	pp	229533.I1S887
133286	229533.I1RFL4	pp	229533.I1RRU9
133287	229533.I1RFL4	pp	229533.Q4I5Z5
133288	229533.I1RFL4	pp	229533.I1RSI0
133289	229533.I1RFL4	pp	229533.I1RGC2
133290	229533.I1RFL4	pp	229533.I1RRY9
133291	229533.I1RFL4	pp	229533.V6QUE6
133292	229533.I1RFL4	pp	229533.I1RSR3
133293	229533.I1RFL4	pp	229533.Q4I2J8
133294	229533.I1RFL4	pp	229533.I1RPR9
133295	229533.I1RFL4	pp	229533.I1RYS0
133296	229533.I1RFL4	pp	229533.I1RR51
133297	229533.I1RFL4	pp	229533.I1S1J7
133298	229533.I1RFL4	pp	229533.I1RRV4

133299 229533.I1RFL4 pp 229533.V6RCZ8
133300 229533.I1RFL4 pp 229533.V6RCG5
133301 229533.I1RFL4 pp 229533.Q4I8P5
133302 229533.I1RFL4 pp 229533.I1RS18
133303 229533.I1RFL4 pp 229533.I1RQA2
133304 229533.I1RFL4 pp 229533.I1RI03
133305 229533.I1RFL4 pp 229533.I1RWB0
133306 229533.I1RFL4 pp 229533.I1RQ81
133307 229533.I1RFL4 pp 229533.I1RI82
133308 229533.I1RFL4 pp 229533.I1S6K9
133309 229533.I1RFL4 pp 229533.I1RJ17
133310 229533.I1RFL4 pp 229533.I1RMQ4
133311 229533.I1RFL4 pp 229533.I1RK95
133312 229533.I1RFL4 pp 229533.I1RN99
133313 229533.I1RFL4 pp 229533.I1RQ31
133314 229533.I1RFL4 pp 229533.I1RY63
133315 229533.I1RFL4 pp 229533.I1RZM8
133316 229533.I1RFL4 pp 229533.I1RMC0
133317 229533.I1RFL4 pp 229533.I1SA32
133318 229533.I1RFL4 pp 229533.I1RZH4
133319 229533.I1RFL4 pp 229533.I1RZZ1
133320 229533.I1RFL4 pp 229533.I1RSC6
133321 229533.I1RFL4 pp 229533.I1RXF0
133322 229533.I1RFL4 pp 229533.I1RZJ5
133323 229533.I1RFL4 pp 229533.I1RXC4
133324 229533.I1RFL4 pp 229533.I1S044
133325 229533.I1RFL4 pp 229533.I1S5B0
133326 229533.I1RFL4 pp 229533.I1RMG8
133327 229533.I1RFL4 pp 229533.I1S4S4
133328 229533.I1RFL4 pp 229533.I1RW99
133329 229533.I1RFL4 pp 229533.I1RGB6
133330 229533.I1RFL4 pp 229533.Q4I283
133331 229533.I1RFL4 pp 229533.I1RLU6
133332 229533.I1RFL4 pp 229533.I1RWJ8
133333 229533.I1RFL4 pp 229533.I1S504
133334 229533.I1RFL4 pp 229533.V6R227
133335 229533.I1RFL4 pp 229533.V6QUW4
133336 229533.I1RFL4 pp 229533.I1RM06
133337 229533.I1RFL4 pp 229533.I1RG14
133338 229533.I1RFL4 pp 229533.I1RNQ5
133339 229533.I1RFL4 pp 229533.I1RWT6
133340 229533.I1RFL4 pp 229533.Q4IEK8
133341 229533.I1RFL4 pp 229533.I1RRQ6
133342 229533.I1RFL4 pp 229533.I1RZM1
133343 229533.I1RFL4 pp 229533.V6RUT5
133344 229533.I1RFL4 pp 229533.V6QYF0
133345 229533.I1RFL4 pp 229533.I1RNA0
133346 229533.I1RFL4 pp 229533.I1RT35
133347 229533.I1RFL4 pp 229533.I1S419
133348 229533.I1RFL4 pp 229533.I1RME6
133349 229533.I1RFL4 pp 229533.I1S1W8
133350 229533.I1RFL4 pp 229533.Q4HVW2
133351 229533.I1RFL4 pp 229533.I1RUQ2
133352 229533.I1RFL4 pp 229533.I1RXD7
133353 229533.I1RFL4 pp 229533.I1RLP1
133354 229533.I1RFL4 pp 229533.I1S0B7
133355 229533.I1RFL4 pp 229533.I1RQC9
133356 229533.I1RFL4 pp 229533.I1RMK3
133357 229533.I1RFL4 pp 229533.I1RNV6
133358 229533.I1RFL4 pp 229533.I1RI02
133359 229533.I1RFL4 pp 229533.I1RTF4
133360 229533.I1RFL4 pp 229533.I1RPA4
133361 229533.I1RFL4 pp 229533.I1RJU3
133362 229533.I1RFL4 pp 229533.I1S0R7
133363 229533.I1RFL4 pp 229533.I1RX04
133364 229533.I1RFL4 pp 229533.I1RMC8
133365 229533.I1RFL4 pp 229533.I1RW07
133366 229533.I1RFL4 pp 229533.I1RX25
133367 229533.I1RFL4 pp 229533.I1S0X6
133368 229533.I1RFL4 pp 229533.I1RRS8
133369 229533.I1RFL4 pp 229533.I1RR24
133370 229533.I1RFL4 pp 229533.I1S7A8
133371 229533.I1RFL4 pp 229533.I1RFX8

133372 229533.I1RFL4 pp 229533.I1RW25
133373 229533.I1RFL4 pp 229533.V6RTJ8
133374 229533.I1RFL4 pp 229533.I1RX46
133375 229533.I1RFL4 pp 229533.Q4IJT5
133376 229533.I1RFL4 pp 229533.I1RLC7
133377 229533.I1RFL4 pp 229533.I1RG53
133378 229533.I1RFL4 pp 229533.I1S0T2
133379 229533.I1RFL4 pp 229533.I1RIL8
133380 229533.I1RFL4 pp 229533.I1RRG3
133381 229533.I1RFL4 pp 229533.I1RRX5
133382 229533.I1RFL4 pp 229533.I1RSQ7
133383 229533.I1RFL4 pp 229533.V6R1K4
133384 229533.I1RFL4 pp 229533.I1RLX7
133385 229533.I1RFL4 pp 229533.I1S0L3
133386 229533.I1RFL4 pp 229533.V6QW22
133387 229533.I1RFL4 pp 229533.I1S4S7
133388 229533.I1RFL4 pp 229533.I1RW71
133389 229533.I1RFL4 pp 229533.I1S0F5
133390 229533.I1RFL4 pp 229533.I1RPN4
133391 229533.I1RFL4 pp 229533.I1RVQ0
133392 229533.I1RFL4 pp 229533.I1S6F0
133393 229533.I1RFL4 pp 229533.I1RWD4
133394 229533.I1RFL4 pp 229533.V6R4J1
133395 229533.A0A1C3YHA4 pp 229533.I1S8J4
133396 229533.A0A1C3YHA4 pp 229533.I1RNG4
133397 229533.A0A1C3YHA4 pp 229533.A0A1C3YLB6
133398 229533.A0A1C3YHA4 pp 229533.I1RZK5
133399 229533.A0A1C3YHA4 pp 229533.Q4I0C5
133400 229533.A0A1C3YHA4 pp 229533.I1RXV1
133401 229533.I1RRF5 pp 229533.I1S1W8
133402 229533.I1RRF5 pp 229533.I1S2Q4
133403 229533.I1RRF5 pp 229533.V6RXR0
133404 229533.I1RRF5 pp 229533.Q4IA56
133405 229533.I1RRF5 pp 229533.I1S3G4
133406 229533.I1RRF5 pp 229533.I1RYX6
133407 229533.I1RRF5 pp 229533.V6RHD0
133408 229533.I1RRF5 pp 229533.I1S1B8
133409 229533.I1RRF5 pp 229533.Q4I7L0
133410 229533.I1RRF5 pp 229533.I1S4G3
133411 229533.I1RRF5 pp 229533.Q4HVQ9
133412 229533.I1RRF5 pp 229533.I1RTY0
133413 229533.I1RRF5 pp 229533.I1RUQ3
133414 229533.I1RRF5 pp 229533.I1RWZ3
133415 229533.I1RRF5 pp 229533.V6QU65
133416 229533.I1RGC2 pp 229533.Q4IRI9
133417 229533.I1RGC2 pp 229533.I1S002
133418 229533.I1RGC2 pp 229533.Q4IE79
133419 229533.I1RGC2 pp 229533.I1RNQ5
133420 229533.I1RGC2 pp 229533.I1S0M9
133421 229533.I1RGC2 pp 229533.I1RV79
133422 229533.I1RGC2 pp 229533.I1RLU6
133423 229533.I1RGC2 pp 229533.I1RT35
133424 229533.I1RGC2 pp 229533.I1RZM1
133425 229533.I1RGC2 pp 229533.I1RQ31
133426 229533.I1RGC2 pp 229533.I1RZV9
133427 229533.I1RGC2 pp 229533.I1S9L0
133428 229533.I1RGC2 pp 229533.V6RCG5
133429 229533.I1RGC2 pp 229533.I1RZF6
133430 229533.I1RGC2 pp 229533.I1RRU9
133431 229533.I1RGC2 pp 229533.I1RRY9
133432 229533.I1RGC2 pp 229533.I1RI82
133433 229533.I1RGC2 pp 229533.Q4IBS2
133434 229533.I1RGC2 pp 229533.I1RQX6
133435 229533.I1RGC2 pp 229533.I1RW99
133436 229533.I1RGC2 pp 229533.I1RNH6
133437 229533.I1RGC2 pp 229533.I1RLX7
133438 229533.I1RGC2 pp 229533.I1RWH5
133439 229533.I1RGC2 pp 229533.I1RQJ5
133440 229533.I1RGC2 pp 229533.I1S021
133441 229533.I1RGC2 pp 229533.I1S246
133442 229533.I1RGC2 pp 229533.I1RYJ8
133443 229533.I1RGC2 pp 229533.I1RYB9
133444 229533.I1RGC2 pp 229533.I1S9T1

133445 229533.I1RGC2 pp 229533.I1RZZ1
133446 229533.I1RGC2 pp 229533.I1RZK6
133447 229533.I1RGC2 pp 229533.V6QUW4
133448 229533.I1RGC2 pp 229533.V6QW22
133449 229533.I1RGC2 pp 229533.I1RW71
133450 229533.I1RGC2 pp 229533.I1S0L3
133451 229533.I1RGC2 pp 229533.Q4I7F9
133452 229533.I1RGC2 pp 229533.I1RVQ0
133453 229533.I1RGC2 pp 229533.I1RVX1
133454 229533.I1RGC2 pp 229533.I1RW25
133455 229533.I1RGC2 pp 229533.I1RT98
133456 229533.I1RGC2 pp 229533.I1RRC6
133457 229533.I1RGC2 pp 229533.I1RNT2
133458 229533.I1RGC2 pp 229533.I1RIC5
133459 229533.I1RGC2 pp 229533.I1RSI0
133460 229533.I1RGC2 pp 229533.Q4IJK2
133461 229533.I1RGC2 pp 229533.I1RT80
133462 229533.I1RGC2 pp 229533.I1RQC9
133463 229533.I1RGC2 pp 229533.I1S1K9
133464 229533.I1RGC2 pp 229533.I1S0R7
133465 229533.I1RGC2 pp 229533.I1S6E5
133466 229533.I1RGC2 pp 229533.I1RX46
133467 229533.I1RGC2 pp 229533.I1RIS3
133468 229533.I1RGC2 pp 229533.I1S0Z9
133469 229533.I1RGC2 pp 229533.I1RSR8
133470 229533.I1RGC2 pp 229533.Q4IPI1
133471 229533.I1RGC2 pp 229533.I1RKF8
133472 229533.I1RGC2 pp 229533.I1RWJ8
133473 229533.I1RGC2 pp 229533.I1RK83
133474 229533.I1RGC2 pp 229533.V6RTJ8
133475 229533.I1RGC2 pp 229533.I1RM96
133476 229533.I1RGC2 pp 229533.I1RPU0
133477 229533.I1RGC2 pp 229533.V6RIV9
133478 229533.I1RGC2 pp 229533.I1S0J1
133479 229533.I1RGC2 pp 229533.I1RRH3
133480 229533.I1RGC2 pp 229533.I1RZW8
133481 229533.I1RGC2 pp 229533.I1S289
133482 229533.I1RGC2 pp 229533.I1RW46
133483 229533.I1RGC2 pp 229533.V6RY33
133484 229533.I1RGC2 pp 229533.I1RRR7
133485 229533.I1RGC2 pp 229533.I1S060
133486 229533.I1RGC2 pp 229533.I1RNE4
133487 229533.I1RGC2 pp 229533.I1S2C0
133488 229533.I1RGC2 pp 229533.V6RNM4
133489 229533.I1RGC2 pp 229533.I1RZS7
133490 229533.I1RGC2 pp 229533.I1RPP4
133491 229533.I1RGC2 pp 229533.I1RUG3
133492 229533.I1RGC2 pp 229533.I1RNC1
133493 229533.I1RGC2 pp 229533.V6R042
133494 229533.I1RGC2 pp 229533.I1RM25
133495 229533.I1RGC2 pp 229533.I1S8R3
133496 229533.I1RGC2 pp 229533.I1RTH6
133497 229533.I1RGC2 pp 229533.I1RWB5
133498 229533.I1RGC2 pp 229533.I1RS17
133499 229533.I1RGC2 pp 229533.I1RJV3
133500 229533.I1RGC2 pp 229533.V6RMW6
133501 229533.I1RGC2 pp 229533.I1RLV9
133502 229533.I1RGC2 pp 229533.I1RYW2
133503 229533.I1RGC2 pp 229533.I1RXF6
133504 229533.I1RGC2 pp 229533.I1RMN6
133505 229533.I1RGC2 pp 229533.I1RJW6
133506 229533.I1RGC2 pp 229533.I1RJV4
133507 229533.I1RGC2 pp 229533.I1RQE7
133508 229533.I1RGC2 pp 229533.I1S2E8
133509 229533.I1RGC2 pp 229533.I1RT17
133510 229533.I1RGC2 pp 229533.I1RWK3
133511 229533.I1RGC2 pp 229533.I1RSL9
133512 229533.I1RGC2 pp 229533.Q4HW67
133513 229533.I1RGC2 pp 229533.I1RSD8
133514 229533.I1RGC2 pp 229533.I1RPM4
133515 229533.I1RGC2 pp 229533.I1S1X9
133516 229533.I1RGC2 pp 229533.I1RN18
133517 229533.I1RGC2 pp 229533.I1RRJ8

133518 229533.I1RGC2 pp 229533.I1RPP3
133519 229533.I1RGC2 pp 229533.Q4HWE2
133520 229533.I1RGC2 pp 229533.I1RZS6
133521 229533.I1RGC2 pp 229533.I1RWU2
133522 229533.I1RGC2 pp 229533.V6R0U9
133523 229533.I1RGC2 pp 229533.V6REZ4
133524 229533.I1RGC2 pp 229533.I1RX24
133525 229533.I1RGC2 pp 229533.I1RRQ3
133526 229533.I1RGC2 pp 229533.I1RKL3
133527 229533.I1RGC2 pp 229533.I1RN67
133528 229533.I1RGC2 pp 229533.I1S2R6
133529 229533.I1RGC2 pp 229533.I1S7S9
133530 229533.I1RGC2 pp 229533.I1RQ65
133531 229533.I1RGC2 pp 229533.I1S0V1
133532 229533.I1RGC2 pp 229533.I1RU69
133533 229533.I1RGC2 pp 229533.I1RV82
133534 229533.I1RGC2 pp 229533.I1RZZ9
133535 229533.I1RGC2 pp 229533.Q4I613
133536 229533.I1RGC2 pp 229533.I1RR73
133537 229533.I1RGC2 pp 229533.I1RND6
133538 229533.I1RGC2 pp 229533.I1RT04
133539 229533.I1RGC2 pp 229533.I1RZV6
133540 229533.I1RGC2 pp 229533.I1S8E2
133541 229533.I1RGC2 pp 229533.I1RKJ6
133542 229533.I1RGC2 pp 229533.I1RN48
133543 229533.I1RGC2 pp 229533.I1S0L4
133544 229533.I1RGC2 pp 229533.I1RST1
133545 229533.A0A0E0SMT9 pp 229533.A0A1C3YKL7
133546 229533.A0A0E0SMT9 pp 229533.A0A1C3YLA6
133547 229533.A0A0E0SMT9 pp 229533.I1R9W4
133548 229533.A0A0E0SMT9 pp 229533.I1RY29
133549 229533.A0A0E0SMT9 pp 229533.A0A1C3YN25
133550 229533.A0A0E0SMT9 pp 229533.I1RWI8
133551 229533.A0A0E0SMT9 pp 229533.I1RDE7
133552 229533.A0A0E0SMT9 pp 229533.A0A1C3YHG7
133553 229533.A0A0E0SMT9 pp 229533.I1RPD6
133554 229533.A0A0E0SMT9 pp 229533.I1RW43
133555 229533.A0A0E0SMT9 pp 229533.I1RNG4
133556 229533.A0A0E0SMT9 pp 229533.I1RKB2
133557 229533.A0A0E0SMT9 pp 229533.A0A1C3YKK9
133558 229533.A0A0E0SMT9 pp 229533.A0A1C3YJW4
133559 229533.A0A0E0SMT9 pp 229533.I1S3G4
133560 229533.A0A0E0SMT9 pp 229533.I1S0J3
133561 229533.A0A0E0SMT9 pp 229533.I1RZ29
133562 229533.A0A0E0SMT9 pp 229533.I1RDH0
133563 229533.A0A0E0SMT9 pp 229533.I1S4P6
133564 229533.A0A0E0SMT9 pp 229533.I1RRN5
133565 229533.A0A0E0SMT9 pp 229533.A0A1C3YN55
133566 229533.A0A0E0SMT9 pp 229533.A0A1C3YK97
133567 229533.A0A0E0SMT9 pp 229533.A0A1C3YN83
133568 229533.A0A0E0SMT9 pp 229533.I1RF85
133569 229533.A0A0E0SMT9 pp 229533.A0A1C3YMC9
133570 229533.A0A0E0SMT9 pp 229533.I1SAJ3
133571 229533.A0A0E0SMT9 pp 229533.A0A1C3YI71
133572 229533.A0A0E0SMT9 pp 229533.I1RE86
133573 229533.A0A0E0SMT9 pp 229533.V6RLM7
133574 229533.A0A0E0SMT9 pp 229533.A0A1C3YJL7
133575 229533.A0A0E0SMT9 pp 229533.I1RNQ1
133576 229533.A0A0E0SMT9 pp 229533.V6RN31
133577 229533.A0A0E0SMT9 pp 229533.I1RQ75
133578 229533.A0A0E0SMT9 pp 229533.I1S5C5
133579 229533.A0A0E0SMT9 pp 229533.A0A1C3YLR6
133580 229533.A0A0E0SMT9 pp 229533.A0A1C3YI64
133581 229533.A0A0E0SMT9 pp 229533.I1RZ94
133582 229533.A0A0E0SMT9 pp 229533.V6RRM0
133583 229533.A0A0E0SMT9 pp 229533.I1S1W8
133584 229533.A0A0E0SMT9 pp 229533.I1RTN6
133585 229533.A0A0E0SMT9 pp 229533.I1RZQ1
133586 229533.A0A0E0SMT9 pp 229533.I1R9Y7
133587 229533.A0A0E0SMT9 pp 229533.A0A0E0SQ99
133588 229533.A0A0E0SMT9 pp 229533.I1RCU2
133589 229533.A0A0E0SMT9 pp 229533.Q4I1Y5
133590 229533.A0A0E0SMT9 pp 229533.A0A1C3YK76

133591	229533.A0A0E0SMT9	pp	229533.I1RAR3
133592	229533.A0A0E0SMT9	pp	229533.I1RY49
133593	229533.A0A0E0SMT9	pp	229533.I1RVX9
133594	229533.A0A0E0SMT9	pp	229533.I1RW69
133595	229533.A0A0E0SMT9	pp	229533.I1RWM6
133596	229533.A0A0E0SMT9	pp	229533.I1RPW2
133597	229533.A0A0E0SMT9	pp	229533.I1S183
133598	229533.A0A0E0SMT9	pp	229533.A0A1C3YJW8
133599	229533.A0A0E0SMT9	pp	229533.I1RYI7
133600	229533.A0A0E0SMT9	pp	229533.I1RG66
133601	229533.A0A0E0SMT9	pp	229533.I1RIE8
133602	229533.A0A0E0SMT9	pp	229533.I1RJL9
133603	229533.A0A0E0SMT9	pp	229533.A0A1C3YJQ6
133604	229533.A0A0E0SMT9	pp	229533.I1RA90
133605	229533.A0A0E0SMT9	pp	229533.V6RQW4
133606	229533.A0A0E0SMT9	pp	229533.I1RM26
133607	229533.A0A0E0SMT9	pp	229533.I1RFQ5
133608	229533.A0A0E0SMT9	pp	229533.I1RBE7
133609	229533.A0A0E0SMT9	pp	229533.I1RMS2
133610	229533.A0A0E0SMT9	pp	229533.I1RPW1
133611	229533.A0A1C3YLU7	pp	229533.I1RF71
133612	229533.A0A1C3YLU7	pp	229533.I1S1J7
133613	229533.A0A1C3YLU7	pp	229533.I1RAN5
133614	229533.A0A1C3YLU7	pp	229533.I1RRY9
133615	229533.A0A1C3YLU7	pp	229533.I1RGB2
133616	229533.A0A1C3YLU7	pp	229533.A0A1C3YME9
133617	229533.A0A1C3YLU7	pp	229533.I1RZZ1
133618	229533.A0A1C3YLU7	pp	229533.I1RQA3
133619	229533.A0A1C3YLU7	pp	229533.I1RCY5
133620	229533.A0A1C3YLU7	pp	229533.Q4IJK2
133621	229533.A0A1C3YLU7	pp	229533.I1REK8
133622	229533.A0A1C3YLU7	pp	229533.I1RCR5
133623	229533.A0A1C3YLU7	pp	229533.I1RY03
133624	229533.A0A1C3YLU7	pp	229533.I1RI82
133625	229533.A0A1C3YLU7	pp	229533.I1RCM5
133626	229533.A0A1C3YLU7	pp	229533.I1RZK5
133627	229533.A0A1C3YLU7	pp	229533.I1RRU9
133628	229533.A0A1C3YLU7	pp	229533.I1RZM8
133629	229533.A0A1C3YLU7	pp	229533.I1RBL8
133630	229533.A0A1C3YLU7	pp	229533.I1RFU8
133631	229533.A0A1C3YLU7	pp	229533.I1RCG4
133632	229533.A0A1C3YLU7	pp	229533.I1RAU5
133633	229533.A0A1C3YLU7	pp	229533.V6RCZ8
133634	229533.A0A1C3YLU7	pp	229533.I1RRV4
133635	229533.A0A1C3YLU7	pp	229533.Q4IJT5
133636	229533.A0A1C3YLU7	pp	229533.I1RWJ8
133637	229533.A0A1C3YLU7	pp	229533.I1S5B0
133638	229533.A0A1C3YLU7	pp	229533.I1RG53
133639	229533.A0A1C3YLU7	pp	229533.I1RR24
133640	229533.A0A1C3YLU7	pp	229533.V6QW22
133641	229533.A0A1C3YLU7	pp	229533.I1RW99
133642	229533.A0A1C3YLU7	pp	229533.I1RNL0
133643	229533.A0A1C3YLU7	pp	229533.I1RSQ7
133644	229533.A0A1C3YLU7	pp	229533.I1RS18
133645	229533.A0A1C3YLU7	pp	229533.I1RQ81
133646	229533.A0A1C3YLU7	pp	229533.I1RW71
133647	229533.A0A1C3YLU7	pp	229533.I1RDU9
133648	229533.A0A1C3YLU7	pp	229533.I1RNQ5
133649	229533.A0A1C3YLU7	pp	229533.I1RBC9
133650	229533.A0A1C3YLU7	pp	229533.I1RPN4
133651	229533.A0A1C3YLU7	pp	229533.I1RLU6
133652	229533.A0A1C3YLU7	pp	229533.I1RN64
133653	229533.A0A1C3YLU7	pp	229533.I1S1W8
133654	229533.A0A1C3YLU7	pp	229533.I1RVQ0
133655	229533.A0A1C3YLU7	pp	229533.I1S0L3
133656	229533.A0A1C3YLU7	pp	229533.I1RZM1
133657	229533.A0A1C3YLU7	pp	229533.I1RD52
133658	229533.A0A1C3YLU7	pp	229533.I1RRI2
133659	229533.A0A1C3YLU7	pp	229533.I1S1C7
133660	229533.A0A1C3YLU7	pp	229533.I1RYW3
133661	229533.A0A1C3YLU7	pp	229533.I1RW25
133662	229533.A0A1C3YLU7	pp	229533.I1RT41
133663	229533.A0A1C3YLU7	pp	229533.I1RME6

133664 229533.A0A1C3YLU7 pp 229533.V6RS91
133665 229533.A0A1C3YLU7 pp 229533.I1S0Z3
133666 229533.A0A1C3YLU7 pp 229533.I1RXD7
133667 229533.A0A1C3YLU7 pp 229533.I1RWT6
133668 229533.A0A1C3YLU7 pp 229533.I1RA70
133669 229533.A0A1C3YLU7 pp 229533.I1RTH6
133670 229533.A0A1C3YLU7 pp 229533.I1S8R3
133671 229533.A0A1C3YLU7 pp 229533.I1S0R7
133672 229533.A0A1C3YLU7 pp 229533.I1RQC9
133673 229533.A0A1C3YLU7 pp 229533.I1RZF6
133674 229533.A0A1C3YLU7 pp 229533.I1RX46
133675 229533.A0A1C3YLU7 pp 229533.I1S504
133676 229533.A0A1C3YLU7 pp 229533.I1RJV3
133677 229533.A0A1C3YLU7 pp 229533.A0A1C3YN26
133678 229533.A0A1C3YLU7 pp 229533.I1REC7
133679 229533.A0A1C3YLU7 pp 229533.V6RTJ8
133680 229533.A0A1C3YLU7 pp 229533.I1RBT1
133681 229533.A0A1C3YLU7 pp 229533.I1RWN6
133682 229533.A0A1C3YLU7 pp 229533.I1RAW0
133683 229533.A0A1C3YLU7 pp 229533.I1RCH5
133684 229533.A0A1C3YLU7 pp 229533.I1S0X6
133685 229533.A0A1C3YLU7 pp 229533.I1RQE7
133686 229533.A0A1C3YLU7 pp 229533.I1RFL4
133687 229533.A0A1C3YLU7 pp 229533.I1RQL4
133688 229533.A0A1C3YLU7 pp 229533.I1RN18
133689 229533.A0A1C3YLU7 pp 229533.I1RAX6
133690 229533.A0A1C3YLU7 pp 229533.I1RJU3
133691 229533.A0A1C3YLU7 pp 229533.Q4I624
133692 229533.A0A1C3YLU7 pp 229533.I1RAU7
133693 229533.A0A1C3YLU7 pp 229533.I1RB92
133694 229533.A0A1C3YLU7 pp 229533.I1RVX0
133695 229533.A0A1C3YLU7 pp 229533.I1RT03
133696 229533.A0A1C3YLU7 pp 229533.V6RIV9
133697 229533.A0A1C3YLU7 pp 229533.I1RD17
133698 229533.A0A1C3YLU7 pp 229533.I1RWN5
133699 229533.A0A1C3YLU7 pp 229533.I1RE76
133700 229533.A0A1C3YLU7 pp 229533.I1RNC1
133701 229533.A0A1C3YLU7 pp 229533.I1RBY4
133702 229533.A0A1C3YLU7 pp 229533.I1RUG3
133703 229533.A0A1C3YLU7 pp 229533.I1RCR8
133704 229533.A0A1C3YLU7 pp 229533.I1S5N3
133705 229533.A0A1C3YLU7 pp 229533.I1RJZ7
133706 229533.A0A1C3YLU7 pp 229533.I1RC66
133707 229533.A0A1C3YLU7 pp 229533.V6R042
133708 229533.A0A1C3YLU7 pp 229533.I1RRM5
133709 229533.A0A1C3YLU7 pp 229533.I1RZZ9
133710 229533.A0A1C3YLU7 pp 229533.V6RMW6
133711 229533.A0A1C3YLU7 pp 229533.I1RR73
133712 229533.A0A1C3YLU7 pp 229533.I1S2B8
133713 229533.A0A1C3YLU7 pp 229533.I1RVY0
133714 229533.A0A1C3YLU7 pp 229533.I1RKX6
133715 229533.A0A1C3YLU7 pp 229533.Q4IPI4
133716 229533.A0A1C3YLU7 pp 229533.A0A1C3YMA1
133717 229533.A0A1C3YLU7 pp 229533.I1RRV1
133718 229533.A0A1C3YLU7 pp 229533.I1RD22
133719 229533.A0A1C3YLU7 pp 229533.I1S0N4
133720 229533.A0A1C3YLU7 pp 229533.V6R3X5
133721 229533.A0A1C3YLU7 pp 229533.I1RTD5
133722 229533.A0A1C3YLU7 pp 229533.I1RG11
133723 229533.A0A1C3YLU7 pp 229533.I1S7J8
133724 229533.A0A1C3YLU7 pp 229533.I1RZ39
133725 229533.A0A1C3YLU7 pp 229533.I1RV91
133726 229533.A0A1C3YLU7 pp 229533.I1S5R1
133727 229533.A0A1C3YLU7 pp 229533.I1RS90
133728 229533.A0A1C3YLU7 pp 229533.I1RT40
133729 229533.A0A1C3YLU7 pp 229533.I1RRT8
133730 229533.A0A1C3YLU7 pp 229533.I1S0J1
133731 229533.A0A1C3YLU7 pp 229533.I1RA11
133732 229533.A0A1C3YLU7 pp 229533.I1S2E9
133733 229533.A0A1C3YLU7 pp 229533.I1RT04
133734 229533.A0A1C3YLU7 pp 229533.V6R3J1
133735 229533.A0A1C3YLU7 pp 229533.I1RTA0
133736 229533.A0A1C3YLU7 pp 229533.I1RE01

133737 229533.A0A1C3YLU7 pp 229533.I1S0L4
133738 229533.A0A1C3YLU7 pp 229533.I1RC00
133739 229533.A0A1C3YLU7 pp 229533.V6RLM7
133740 229533.A0A1C3YLU7 pp 229533.I1RPP4
133741 229533.A0A1C3YLU7 pp 229533.I1RJW6
133742 229533.A0A1C3YLU7 pp 229533.I1S3G4
133743 229533.A0A1C3YLU7 pp 229533.I1RPK0
133744 229533.A0A1C3YLU7 pp 229533.I1S503
133745 229533.A0A1C3YLU7 pp 229533.I1S2H4
133746 229533.A0A1C3YLU7 pp 229533.I1RS17
133747 229533.A0A1C3YLU7 pp 229533.I1RLP9
133748 229533.A0A1C3YLU7 pp 229533.I1RP27
133749 229533.A0A1C3YLU7 pp 229533.I1RJV4
133750 229533.A0A1C3YLU7 pp 229533.I1RE19
133751 229533.A0A1C3YLU7 pp 229533.I1RS20
133752 229533.A0A1C3YLU7 pp 229533.I1S282
133753 229533.A0A1C3YLU7 pp 229533.I1RY44
133754 229533.A0A1C3YLU7 pp 229533.I1RBS6
133755 229533.A0A1C3YLU7 pp 229533.I1RRI6
133756 229533.A0A1C3YLU7 pp 229533.I1RGA4
133757 229533.A0A1C3YLU7 pp 229533.I1RWK3
133758 229533.A0A1C3YLU7 pp 229533.I1REC6
133759 229533.A0A1C3YLU7 pp 229533.I1S8P5
133760 229533.A0A1C3YLU7 pp 229533.I1S1F3
133761 229533.A0A1C3YLU7 pp 229533.I1RPM4
133762 229533.A0A1C3YLU7 pp 229533.I1RJD6
133763 229533.A0A1C3YLU7 pp 229533.I1RC29
133764 229533.A0A1C3YLU7 pp 229533.I1RPP3
133765 229533.A0A1C3YLU7 pp 229533.I1RFT1
133766 229533.A0A1C3YLU7 pp 229533.I1RBS0
133767 229533.A0A1C3YLU7 pp 229533.I1RAB4
133768 229533.A0A1C3YLU7 pp 229533.I1S2P0
133769 229533.A0A1C3YLU7 pp 229533.I1RN67
133770 229533.Q4IJT5 pp 229533.V6QW22
133771 229533.Q4IJT5 pp 229533.V6RMW6
133772 229533.Q4IJT5 pp 229533.V6RIV9
133773 229533.Q4IJT5 pp 229533.V6RCZ8
133774 229533.Q4IJT5 pp 229533.V6RTJ8
133775 229533.Q4IJT5 pp 229533.V6RCG5
133776 229533.Q4IJT5 pp 229533.V6R3X5
133777 229533.Q4IJT5 pp 229533.V6R042
133778 229533.I1RCR6 pp 229533.I1RJJ7
133779 229533.I1RCR6 pp 229533.I1RN84
133780 229533.I1RCR6 pp 229533.I1S2Q4
133781 229533.I1RCR6 pp 229533.I1RN88
133782 229533.I1RCR6 pp 229533.I1S3G4
133783 229533.I1RCR6 pp 229533.I1S8R2
133784 229533.I1RCR6 pp 229533.I1RF85
133785 229533.I1RCR6 pp 229533.I1RH91
133786 229533.I1RCR6 pp 229533.I1RNG4
133787 229533.I1RCR6 pp 229533.I1RWZ3
133788 229533.I1RCR6 pp 229533.V6RLM7
133789 229533.I1RCR6 pp 229533.I1RG66
133790 229533.I1RCR6 pp 229533.I1RD81
133791 229533.I1RCR6 pp 229533.I1S8F8
133792 229533.I1RCR6 pp 229533.I1S299
133793 229533.I1RCR6 pp 229533.I1RKB2
133794 229533.I1RCR6 pp 229533.I1RNY6
133795 229533.I1RCR6 pp 229533.V6RRM0
133796 229533.I1RCR6 pp 229533.Q4HVQ9
133797 229533.I1RCR6 pp 229533.I1RJY7
133798 229533.I1RCR6 pp 229533.I1S4G3
133799 229533.I1RCR6 pp 229533.I1RUQ3
133800 229533.I1RCR6 pp 229533.I1RGC3
133801 229533.I1RCR6 pp 229533.I1RCV1
133802 229533.I1RCR6 pp 229533.I1RYX6
133803 229533.I1RCR6 pp 229533.I1RUR8
133804 229533.I1RCR6 pp 229533.I1RRX5
133805 229533.I1RCR6 pp 229533.I1S8C1
133806 229533.I1RCR6 pp 229533.I1RQE1
133807 229533.I1RCR6 pp 229533.I1RCU2
133808 229533.I1RCR6 pp 229533.I1S1B8
133809 229533.I1RCR6 pp 229533.I1S1W8

229533.I1RCR6	pp	229533.V6QWD8
229533.I1RCR6	pp	229533.I1RTY0
229533.I1RCR6	pp	229533.I1S2D5
229533.I1RSD8	pp	229533.Q4IEK8
229533.I1RSD8	pp	229533.I1S4T3
229533.I1RSD8	pp	229533.I1RWN5
229533.I1RSD8	pp	229533.V6QUE6
229533.I1RSD8	pp	229533.I1RZF6
229533.I1RSD8	pp	229533.I1RY44
229533.I1RSD8	pp	229533.Q4IBS2
229533.I1RSD8	pp	229533.I1S9U1
229533.I1RSD8	pp	229533.V6R227
229533.I1RSD8	pp	229533.V6RGN5
229533.I1RSD8	pp	229533.I1S504
229533.I1RSD8	pp	229533.I1RX14
229533.I1RSD8	pp	229533.I1S060
229533.I1RSD8	pp	229533.I1S044
229533.I1RSD8	pp	229533.I1S0J1
229533.I1RSD8	pp	229533.Q4IN63
229533.I1RSD8	pp	229533.V6RGY3
229533.I1RSD8	pp	229533.Q4IJ56
229533.I1RSD8	pp	229533.Q4I327
229533.I1RSD8	pp	229533.I1RYB9
229533.I1RSD8	pp	229533.I1S0M9
229533.I1RSD8	pp	229533.I1S021
229533.I1RSD8	pp	229533.I1RWB5
229533.I1RSD8	pp	229533.I1RSR3
229533.I1RSD8	pp	229533.I1RX08
229533.I1RSD8	pp	229533.I1S1C7
229533.I1RSD8	pp	229533.I1S1U7
229533.I1RSD8	pp	229533.V6R042
229533.I1RSD8	pp	229533.I1RSI6
229533.I1RSD8	pp	229533.Q4IAA0
229533.I1RSD8	pp	229533.I1S2U2
229533.I1RSD8	pp	229533.I1RSR8
229533.I1RSD8	pp	229533.I1RZK6
229533.I1RSD8	pp	229533.V6RMW6
229533.I1RSD8	pp	229533.I1RY03
229533.I1RSD8	pp	229533.I1RZE5
229533.I1RSD8	pp	229533.I1RVZ0
229533.I1RSD8	pp	229533.I1RZZ1
229533.I1RSD8	pp	229533.I1S2E8
229533.I1RSD8	pp	229533.I1RSG8
229533.I1RSD8	pp	229533.I1RVX0
229533.I1RSD8	pp	229533.I1RSI0
229533.I1RSD8	pp	229533.I1RTH6
229533.I1RSD8	pp	229533.I1RT03
229533.I1RSD8	pp	229533.I1RZK5
229533.I1RSD8	pp	229533.Q4HZ68
229533.I1RSD8	pp	229533.I1RY63
229533.I1RSD8	pp	229533.I1RTE9
229533.I1RSD8	pp	229533.I1S1X9
229533.I1RSD8	pp	229533.I1S2C0
229533.I1RSD8	pp	229533.I1S296
229533.I1RSD8	pp	229533.I1RSS4
229533.I1RSD8	pp	229533.V6R283
229533.I1RSD8	pp	229533.Q4HZ47
229533.I1RSD8	pp	229533.I1RWD4
229533.I1RSD8	pp	229533.I1RWH5
229533.I1RSD8	pp	229533.I1S2M6
229533.I1RSD8	pp	229533.I1S6F0
229533.I1RSD8	pp	229533.Q4HWE2
229533.I1RSD8	pp	229533.Q4HZ42
229533.I1RSD8	pp	229533.Q4I830
229533.I1RSD8	pp	229533.I1S274
229533.I1RSD8	pp	229533.I1RT33
229533.I1RSD8	pp	229533.I1RSM3
229533.I1RSD8	pp	229533.I1RZ99
229533.I1RSD8	pp	229533.I1RWN6
229533.I1RSD8	pp	229533.I1RW71
229533.I1RSD8	pp	229533.I1RZV6
229533.I1RSD8	pp	229533.V6RAC9
229533.I1RSD8	pp	229533.I1RZS6

133883 229533.I1RSD8 pp 229533.I1RTD5
133884 229533.I1RSD8 pp 229533.I1RSQ7
133885 229533.I1RSD8 pp 229533.I1S9Y1
133886 229533.I1RSD8 pp 229533.Q4I5R9
133887 229533.I1RSD8 pp 229533.Q4IAS1
133888 229533.I1RSD8 pp 229533.I1RT04
133889 229533.I1RSD8 pp 229533.O74638
133890 229533.I1RSD8 pp 229533.Q4HXF0
133891 229533.I1RSD8 pp 229533.I1S9U2
133892 229533.I1RSD8 pp 229533.I1S503
133893 229533.I1RSD8 pp 229533.I1RX46
133894 229533.I1RSD8 pp 229533.Q4IF76
133895 229533.I1RSD8 pp 229533.I1S0R7
133896 229533.I1RSD8 pp 229533.I1RT05
133897 229533.I1RSD8 pp 229533.Q4IPI1
133898 229533.I1RSD8 pp 229533.I1RTI6
133899 229533.I1RSD8 pp 229533.I1S0F1
133900 229533.I1RSD8 pp 229533.I1RXD7
133901 229533.I1RSD8 pp 229533.I1RSL9
133902 229533.I1RSD8 pp 229533.I1S2E9
133903 229533.I1RSD8 pp 229533.I1S1B6
133904 229533.I1RSD8 pp 229533.I1RTF4
133905 229533.I1RSD8 pp 229533.Q4IFI0
133906 229533.I1RSD8 pp 229533.I1RU69
133907 229533.I1RSD8 pp 229533.Q4IQW0
133908 229533.I1RSD8 pp 229533.Q4IJH1
133909 229533.I1RSD8 pp 229533.I1RW25
133910 229533.I1RSD8 pp 229533.I1RT98
133911 229533.I1RSD8 pp 229533.I1S2B8
133912 229533.I1RSD8 pp 229533.I1RW61
133913 229533.I1RSD8 pp 229533.I1RZJ6
133914 229533.I1RSD8 pp 229533.I1RZ39
133915 229533.I1RSD8 pp 229533.I1RYT5
133916 229533.I1RSD8 pp 229533.I1RT80
133917 229533.I1RSD8 pp 229533.Q4I662
133918 229533.I1RSD8 pp 229533.I1S253
133919 229533.I1RSD8 pp 229533.I1S8E2
133920 229533.I1RSD8 pp 229533.V6R3X5
133921 229533.I1RSD8 pp 229533.I1RX24
133922 229533.I1RSD8 pp 229533.Q4HVW2
133923 229533.I1RSD8 pp 229533.V6QYF0
133924 229533.I1RSD8 pp 229533.Q4IQK7
133925 229533.A0A0E0SQX4 pp 229533.I1RNW8
133926 229533.A0A0E0SQX4 pp 229533.I1RQP5
133927 229533.I1RD67 pp 229533.I1RWZ3
133928 229533.I1RD67 pp 229533.I1RG66
133929 229533.I1RD67 pp 229533.I1S4G3
133930 229533.I1RD67 pp 229533.I1S1B8
133931 229533.I1RD67 pp 229533.Q4HVQ9
133932 229533.I1RD67 pp 229533.I1RQE1
133933 229533.I1RD67 pp 229533.I1RNG4
133934 229533.I1RD67 pp 229533.I1RTY0
133935 229533.I1RD67 pp 229533.I1S1W8
133936 229533.I1RD67 pp 229533.I1RYX6
133937 229533.I1RD67 pp 229533.I1RH91
133938 229533.I1RD67 pp 229533.I1S3G4
133939 229533.I1RD67 pp 229533.I1RJJ7
133940 229533.I1RD67 pp 229533.I1S2Q4
133941 229533.I1RFW5 pp 229533.I1RQ31
133942 229533.I1RFW5 pp 229533.V6RMW6
133943 229533.I1RFW5 pp 229533.I1RM96
133944 229533.I1RFW5 pp 229533.V6R042
133945 229533.I1RFW5 pp 229533.I1RNQ5
133946 229533.I1RFW5 pp 229533.I1RPY1
133947 229533.I1RFW5 pp 229533.I1RSI6
133948 229533.I1RFW5 pp 229533.I1RWN5
133949 229533.I1RFW5 pp 229533.I1S4T3
133950 229533.I1RFW5 pp 229533.I1RRR7
133951 229533.I1RFW5 pp 229533.I1S0M3
133952 229533.I1RFW5 pp 229533.Q4I5Z5
133953 229533.I1RFW5 pp 229533.I1RZE5
133954 229533.I1RFW5 pp 229533.I1S504
133955 229533.I1RFW5 pp 229533.I1RKC6

133956	229533.I1RFW5	pp	229533.I1S0J1
133957	229533.I1RFW5	pp	229533.I1RZF6
133958	229533.I1RFW5	pp	229533.I1RX14
133959	229533.I1RFW5	pp	229533.I1RVZ0
133960	229533.I1RFW5	pp	229533.I1RJZ6
133961	229533.I1RFW5	pp	229533.I1RNK2
133962	229533.I1RFW5	pp	229533.I1RNU6
133963	229533.I1RFW5	pp	229533.I1S2E8
133964	229533.I1RFW5	pp	229533.I1RWB5
133965	229533.I1RFW5	pp	229533.I1RM25
133966	229533.I1RFW5	pp	229533.I1RZZ1
133967	229533.I1RFW5	pp	229533.I1S0M9
133968	229533.I1RFW5	pp	229533.I1RMQ0
133969	229533.I1RFW5	pp	229533.I1S267
133970	229533.I1RFW5	pp	229533.I1RZK6
133971	229533.I1RFW5	pp	229533.I1RSD8
133972	229533.I1RFW5	pp	229533.I1RMN6
133973	229533.I1RFW5	pp	229533.Q4IJ56
133974	229533.I1RFW5	pp	229533.V6RGN5
133975	229533.I1RFW5	pp	229533.I1RQ28
133976	229533.I1RFW5	pp	229533.I1S1X9
133977	229533.I1RFW5	pp	229533.I1RSC6
133978	229533.I1RFW5	pp	229533.I1RYB9
133979	229533.I1RFW5	pp	229533.I1RVX0
133980	229533.I1RFW5	pp	229533.I1RNH6
133981	229533.I1RFW5	pp	229533.I1S021
133982	229533.I1RFW5	pp	229533.I1RT03
133983	229533.I1RFW5	pp	229533.I1RRY9
133984	229533.I1RFW5	pp	229533.I1RJV3
133985	229533.I1RFW5	pp	229533.I1RRI2
133986	229533.I1RFW5	pp	229533.I1RNY2
133987	229533.I1RFW5	pp	229533.I1RQA2
133988	229533.I1RFW5	pp	229533.I1RY03
133989	229533.I1RFW5	pp	229533.Q4I327
133990	229533.I1RFW5	pp	229533.Q4IN63
133991	229533.I1RFW5	pp	229533.I1RSR3
133992	229533.I1RFW5	pp	229533.I1S1C7
133993	229533.I1RFW5	pp	229533.I1RSR8
133994	229533.I1RFW5	pp	229533.V6RGY3
133995	229533.I1RFW5	pp	229533.I1RX24
133996	229533.I1RFW5	pp	229533.I1RWN6
133997	229533.I1RFW5	pp	229533.I1RNA0
133998	229533.I1RFW5	pp	229533.I1RMD5
133999	229533.I1RFW5	pp	229533.I1RM67
134000	229533.I1RFW5	pp	229533.I1RK83
134001	229533.I1RFW5	pp	229533.I1RJL4
134002	229533.I1RFW5	pp	229533.I1RNH9
134003	229533.I1RFW5	pp	229533.I1RX46
134004	229533.I1RFW5	pp	229533.I1RKJ6
134005	229533.I1RFW5	pp	229533.I1RPU0
134006	229533.I1RFW5	pp	229533.I1S2C0
134007	229533.I1RFW5	pp	229533.I1RZV6
134008	229533.I1RFW5	pp	229533.I1RKQ5
134009	229533.I1RFW5	pp	229533.I1S503
134010	229533.I1RFW5	pp	229533.I1RTE9
134011	229533.I1RFW5	pp	229533.I1RZK5
134012	229533.I1RFW5	pp	229533.Q4I5R9
134013	229533.I1RFW5	pp	229533.I1S9U2
134014	229533.I1RFW5	pp	229533.I1RPW9
134015	229533.I1RFW5	pp	229533.Q4HZK7
134016	229533.I1RFW5	pp	229533.V6R3X5
134017	229533.I1RFW5	pp	229533.I1RLV9
134018	229533.I1RFW5	pp	229533.I1RU69
134019	229533.I1RFW5	pp	229533.Q4IQW0
134020	229533.I1RFW5	pp	229533.I1RSG8
134021	229533.I1RFW5	pp	229533.I1RTH6
134022	229533.I1RFW5	pp	229533.I1RNH1
134023	229533.I1RFW5	pp	229533.I1RGA4
134024	229533.I1RFW5	pp	229533.I1RR73
134025	229533.I1RFW5	pp	229533.I1RND6
134026	229533.I1RFW5	pp	229533.I1RTD5
134027	229533.I1RFW5	pp	229533.I1RRQ3
134028	229533.I1RFW5	pp	229533.I1RS30

134029	229533.I1RFW5	pp	229533.Q4HVW2
134030	229533.I1RFW5	pp	229533.I1S0D4
134031	229533.I1RFW5	pp	229533.I1RT98
134032	229533.I1RFW5	pp	229533.I1S6U8
134033	229533.I1RFW5	pp	229533.I1RXD7
134034	229533.I1RFW5	pp	229533.I1RMK3
134035	229533.I1RFW5	pp	229533.I1RUQ2
134036	229533.I1RFW5	pp	229533.Q4IEV5
134037	229533.I1RFW5	pp	229533.I1RQ65
134038	229533.I1RFW5	pp	229533.Q4HZ42
134039	229533.I1RFW5	pp	229533.I1RT04
134040	229533.I1RFW5	pp	229533.I1S8E2
134041	229533.I1RFW5	pp	229533.I1S253
134042	229533.I1RFW5	pp	229533.I1S0R7
134043	229533.I1RFW5	pp	229533.I1RWH5
134044	229533.I1RFW5	pp	229533.I1RSQ7
134045	229533.I1RFW5	pp	229533.I1RW25
134046	229533.I1RFW5	pp	229533.I1S2B8
134047	229533.I1RFW5	pp	229533.I1RT80
134048	229533.I1RFW5	pp	229533.I1RWF7
134049	229533.I1RFW5	pp	229533.I1RNV6
134050	229533.I1RFW5	pp	229533.I1RRJ8
134051	229533.I1RFW5	pp	229533.I1RTF4
134052	229533.I1RFW5	pp	229533.I1RZ39
134053	229533.I1RFW5	pp	229533.I1RPP3
134054	229533.I1RFW5	pp	229533.I1RZ99
134055	229533.I1RFW5	pp	229533.Q4IFI0
134056	229533.I1RFW5	pp	229533.I1RKL3
134057	229533.I1RFW5	pp	229533.Q4I662
134058	229533.I1RFW5	pp	229533.I1RQJ5
134059	229533.I1RFW5	pp	229533.I1RM06
134060	229533.I1RFW5	pp	229533.V6QYF0
134061	229533.I1RFW5	pp	229533.I1RZS6
134062	229533.I1RIL8	pp	229533.I1RK44
134063	229533.I1RIL8	pp	229533.I1RPP4
134064	229533.I1RIL8	pp	229533.I1RY44
134065	229533.I1RIL8	pp	229533.I1RTA0
134066	229533.I1RIL8	pp	229533.I1RWN5
134067	229533.I1RIL8	pp	229533.V6RIV9
134068	229533.I1RIL8	pp	229533.V6R042
134069	229533.I1RIL8	pp	229533.I1RNC1
134070	229533.I1RIL8	pp	229533.I1RUG3
134071	229533.I1RIL8	pp	229533.I1RSQ3
134072	229533.I1RIL8	pp	229533.I1RVX0
134073	229533.I1RIL8	pp	229533.I1RN18
134074	229533.I1RIL8	pp	229533.V6RMW6
134075	229533.I1RIL8	pp	229533.I1RT03
134076	229533.I1RIL8	pp	229533.I1RQ75
134077	229533.I1RIL8	pp	229533.I1S8P5
134078	229533.I1RIL8	pp	229533.Q4I1Y5
134079	229533.I1RIL8	pp	229533.I1RRM5
134080	229533.I1RIL8	pp	229533.I1S1C7
134081	229533.I1RIL8	pp	229533.I1RZW8
134082	229533.I1RIL8	pp	229533.I1RN67
134083	229533.I1RIL8	pp	229533.I1RPP3
134084	229533.I1RIL8	pp	229533.I1RTH6
134085	229533.I1RIL8	pp	229533.I1S2B8
134086	229533.I1RIL8	pp	229533.I1RTD5
134087	229533.I1RIL8	pp	229533.I1S8R3
134088	229533.I1RIL8	pp	229533.I1RV91
134089	229533.I1RIL8	pp	229533.I1RZZ9
134090	229533.I1RIL8	pp	229533.I1S2E9
134091	229533.I1RIL8	pp	229533.I1RR73
134092	229533.I1RIL8	pp	229533.I1RWN6
134093	229533.I1RIL8	pp	229533.I1RT04
134094	229533.I1RIL8	pp	229533.I1RJV3
134095	229533.I1RIL8	pp	229533.I1RWK3
134096	229533.I1RIL8	pp	229533.I1RJW6
134097	229533.I1RIL8	pp	229533.V6R3X5
134098	229533.I1RIL8	pp	229533.I1S503
134099	229533.I1RIL8	pp	229533.I1RZ39
134100	229533.I1RIL8	pp	229533.I1RZK5
134101	229533.I1RIL8	pp	229533.I1RY03

134102	229533.I1RIL8	pp	229533.I1RRT8
134103	229533.I1RIL8	pp	229533.I1RPM4
134104	229533.I1RIL8	pp	229533.I1S0L4
134105	229533.I1RIL8	pp	229533.I1RJV4
134106	229533.I1RIL8	pp	229533.I1RRY9
134107	229533.I1RIL8	pp	229533.I1RZM1
134108	229533.I1RIL8	pp	229533.I1RLU6
134109	229533.I1RIL8	pp	229533.I1S2H4
134110	229533.I1RIL8	pp	229533.V6QW22
134111	229533.I1RIL8	pp	229533.I1S504
134112	229533.I1RIL8	pp	229533.I1RSQ7
134113	229533.I1RIL8	pp	229533.I1RS18
134114	229533.I1RIL8	pp	229533.I1RVQ0
134115	229533.I1RIL8	pp	229533.I1RQC9
134116	229533.I1RIL8	pp	229533.Q4IJK2
134117	229533.I1RIL8	pp	229533.V6RCG5
134118	229533.I1RIL8	pp	229533.I1RX46
134119	229533.I1RIL8	pp	229533.I1RR39
134120	229533.I1RIL8	pp	229533.V6RTJ8
134121	229533.I1RIL8	pp	229533.I1S0L3
134122	229533.I1RIL8	pp	229533.I1RR24
134123	229533.I1RIL8	pp	229533.I1RJW1
134124	229533.I1RIL8	pp	229533.I1RW25
134125	229533.I1SAY2	pp	229533.Q4IER8
134126	229533.I1SAY2	pp	229533.V6RGI1
134127	229533.I1SAY2	pp	229533.Q4IMD1
134128	229533.I1SAY2	pp	229533.Q4IB50
134129	229533.V6RCG5	pp	229533.V6RIV9
134130	229533.V6RCG5	pp	229533.V6RMW6
134131	229533.V6RCG5	pp	229533.V6RTJ8
134132	229533.V6RCG5	pp	229533.V6RCZ8
134133	229533.V6RCG5	pp	229533.V6RGN5
134134	229533.V6RCG5	pp	229533.V6RIS2
134135	229533.I1RIA1	pp	229533.I1RZ29
134136	229533.I1RIA1	pp	229533.I1RLQ5
134137	229533.I1RIA1	pp	229533.V6QWD8
134138	229533.I1RIA1	pp	229533.I1RMU5
134139	229533.I1RIA1	pp	229533.I1S3X8
134140	229533.I1RIA1	pp	229533.I1S6P5
134141	229533.I1RIA1	pp	229533.I1RJ03
134142	229533.I1RIA1	pp	229533.I1S2V3
134143	229533.I1RIA1	pp	229533.I1RNP4
134144	229533.I1RIA1	pp	229533.V6RUB1
134145	229533.I1RIA1	pp	229533.V6R5G9
134146	229533.I1RIA1	pp	229533.I1RKU0
134147	229533.I1RIA1	pp	229533.I1RIA3
134148	229533.I1RIA1	pp	229533.I1RZA7
134149	229533.I1RIA1	pp	229533.I1S3N0
134150	229533.I1RYW9	pp	229533.I1S2Q4
134151	229533.I1RYW9	pp	229533.I1S3G4
134152	229533.I1RYW9	pp	229533.V6RZD4
134153	229533.I1RYW9	pp	229533.I1S6P5
134154	229533.I1RYW9	pp	229533.V6RUB1
134155	229533.I1RYW9	pp	229533.V6R3J1
134156	229533.I1RYW9	pp	229533.Q4HVQ9
134157	229533.I1RYW9	pp	229533.I1S299
134158	229533.I1RYW9	pp	229533.I1RZA7
134159	229533.I1RYW9	pp	229533.I1S8S9
134160	229533.I1RYW9	pp	229533.V6RRM0
134161	229533.I1RYW9	pp	229533.V6RF62
134162	229533.I1RYW9	pp	229533.I1S0V7
134163	229533.I1RYW9	pp	229533.I1S8R2
134164	229533.I1RYW9	pp	229533.V6R3V4
134165	229533.I1RYW9	pp	229533.I1RYX6
134166	229533.I1RYW9	pp	229533.I1S8F8
134167	229533.I1RYW9	pp	229533.I1S8C1
134168	229533.I1RYW9	pp	229533.I1S1W8
134169	229533.I1RYW9	pp	229533.I1S0V6
134170	229533.I1RYW9	pp	229533.I1S4G3
134171	229533.I1RYW9	pp	229533.I1S4S5
134172	229533.I1RYW9	pp	229533.I1S1B8
134173	229533.I1S7A8	pp	229533.Q4IJK2
134174	229533.I1S7A8	pp	229533.Q4I624

134175	229533.I1S7A8	pp	229533.V6R057
134176	229533.I1S7A8	pp	229533.V6R042
134177	229533.I1S7A8	pp	229533.I1S8R3
134178	229533.I1S7A8	pp	229533.V6RCZ8
134179	229533.I1S7A8	pp	229533.V6RMW6
134180	229533.I1S7A8	pp	229533.V6RCG5
134181	229533.I1S7A8	pp	229533.V6R3X5
134182	229533.I1S7A8	pp	229533.Q4I8P5
134183	229533.I1S7A8	pp	229533.I1SA33
134184	229533.I1S7A8	pp	229533.V6RIV9
134185	229533.I1S7A8	pp	229533.V6QW22
134186	229533.I1S7A8	pp	229533.V6R1K4
134187	229533.I1S7A8	pp	229533.I1SA32
134188	229533.I1S7A8	pp	229533.V6RTJ8
134189	229533.I1RIH9	pp	229533.I1RLN6
134190	229533.I1RIH9	pp	229533.I1RNG4
134191	229533.I1RIH9	pp	229533.I1RPD9
134192	229533.I1RA80	pp	229533.I1RZ25
134193	229533.I1RA80	pp	229533.I1RQV8
134194	229533.I1RA80	pp	229533.I1RYP5
134195	229533.I1RA80	pp	229533.I1S969
134196	229533.I1RA80	pp	229533.I1RF24
134197	229533.I1RA80	pp	229533.I1S968
134198	229533.I1RA80	pp	229533.V6RHX2
134199	229533.I1RA80	pp	229533.I1RK55
134200	229533.V6RCZ8	pp	229533.V6RIV9
134201	229533.V6RCZ8	pp	229533.V6RMW6
134202	229533.V6RCZ8	pp	229533.V6RTJ8
134203	229533.V6RCZ8	pp	229533.V6RRH2
134204	229533.V6RCZ8	pp	229533.V6RUY9
134205	229533.V6RCZ8	pp	229533.V6RUT5
134206	229533.I1RFS5	pp	229533.I1RKB7
134207	229533.I1RFS5	pp	229533.I1RS05
134208	229533.I1RFS5	pp	229533.I1RMQ6
134209	229533.I1RFS5	pp	229533.I1S2C1
134210	229533.I1RFS5	pp	229533.I1RNM7
134211	229533.I1RFS5	pp	229533.I1RY21
134212	229533.I1RFS5	pp	229533.I1RQ26
134213	229533.I1RFS5	pp	229533.I1RMK7
134214	229533.I1RFS5	pp	229533.I1RPN1
134215	229533.I1RFS5	pp	229533.I1RRX5
134216	229533.I1RFS5	pp	229533.I1RG62
134217	229533.I1RFS5	pp	229533.I1S8F8
134218	229533.I1RFS5	pp	229533.I1RYT8
134219	229533.I1RFS5	pp	229533.I1RSE2
134220	229533.I1RFS5	pp	229533.I1RYN2
134221	229533.I1RFS5	pp	229533.I1S0P0
134222	229533.I1RFS5	pp	229533.I1RQR6
134223	229533.I1RFS5	pp	229533.I1RRS8
134224	229533.I1RFS5	pp	229533.I1RFV2
134225	229533.I1RFS5	pp	229533.V6RJP8
134226	229533.I1RFS5	pp	229533.I1S212
134227	229533.I1RFS5	pp	229533.I1RXE6
134228	229533.I1RFS5	pp	229533.V6RM66
134229	229533.I1RFS5	pp	229533.I1RP02
134230	229533.I1RFS5	pp	229533.I1RS29
134231	229533.I1RFS5	pp	229533.I1S464
134232	229533.I1RFS5	pp	229533.V6R3J1
134233	229533.I1RFS5	pp	229533.Q4I7T5
134234	229533.I1RFS5	pp	229533.I1RZN1
134235	229533.I1RFS5	pp	229533.I1RX39
134236	229533.I1RFS5	pp	229533.I1RG33
134237	229533.I1RFS5	pp	229533.I1S0K2
134238	229533.I1RFS5	pp	229533.I1RYE5
134239	229533.I1RFS5	pp	229533.Q4I099
134240	229533.I1RFS5	pp	229533.I1RQ82
134241	229533.I1RFS5	pp	229533.I1S8S9
134242	229533.I1RFS5	pp	229533.I1RL81
134243	229533.I1RFS5	pp	229533.Q4IB65
134244	229533.I1RFS5	pp	229533.I1RWA3
134245	229533.I1RFS5	pp	229533.I1RN08
134246	229533.I1RFS5	pp	229533.I1S1F3
134247	229533.I1RFS5	pp	229533.I1RUL3

134248 229533.I1RFS5 pp 229533.I1RY72
134249 229533.I1RFS5 pp 229533.I1RQA5
134250 229533.I1RFS5 pp 229533.I1RSQ4
134251 229533.I1RFS5 pp 229533.I1RT38
134252 229533.I1RFS5 pp 229533.I1RZ43
134253 229533.I1RFS5 pp 229533.I1RXD9
134254 229533.I1RFS5 pp 229533.I1RVN1
134255 229533.I1RFS5 pp 229533.I1RJT3
134256 229533.I1RFS5 pp 229533.I1RZH5
134257 229533.I1RFS5 pp 229533.I1RY92
134258 229533.I1RFS5 pp 229533.Q4I375
134259 229533.I1RFS5 pp 229533.I1RPK0
134260 229533.I1RFS5 pp 229533.I1RS50
134261 229533.I1RFS5 pp 229533.I1RYA0
134262 229533.I1RFS5 pp 229533.I1RY57
134263 229533.I1RFS5 pp 229533.I1RM37
134264 229533.I1RFS5 pp 229533.I1RYC8
134265 229533.I1RFS5 pp 229533.I1S0R8
134266 229533.I1RFS5 pp 229533.I1S5X9
134267 229533.I1RFS5 pp 229533.Q4HZ95
134268 229533.I1RFS5 pp 229533.I1RZ95
134269 229533.I1RFS5 pp 229533.I1S0Q7
134270 229533.I1RFS5 pp 229533.I1S0R9
134271 229533.I1RFS5 pp 229533.I1RWT0
134272 229533.I1RFS5 pp 229533.I1RNJ5
134273 229533.I1RRK7 pp 229533.I1RYX6
134274 229533.I1RRK7 pp 229533.I1RS94
134275 229533.I1RRK7 pp 229533.I1S0I3
134276 229533.A0A098DQ41 pp 229533.I1RSQ7
134277 229533.A0A098DQ41 pp 229533.I1RBW0
134278 229533.A0A098DQ41 pp 229533.I1RBC9
134279 229533.A0A098DQ41 pp 229533.I1RW71
134280 229533.A0A098DQ41 pp 229533.I1RYH9
134281 229533.A0A098DQ41 pp 229533.I1RX46
134282 229533.A0A098DQ41 pp 229533.I1RW25
134283 229533.A0A098DQ41 pp 229533.I1RFX8
134284 229533.A0A098DQ41 pp 229533.A0A0E0SAC4
134285 229533.A0A098DQ41 pp 229533.A0A098DVZ4
134286 229533.A0A098DQ41 pp 229533.A0A0E0SL81
134287 229533.A0A098DQ41 pp 229533.V6RTJ8
134288 229533.A0A098DQ41 pp 229533.I1RT41
134289 229533.A0A098DQ41 pp 229533.A0A1C3YN12
134290 229533.A0A098DQ41 pp 229533.A0A1C3YKJ7
134291 229533.A0A098DQ41 pp 229533.I1RM03
134292 229533.A0A098DQ41 pp 229533.I1RDI8
134293 229533.A0A098DQ41 pp 229533.I1RQH1
134294 229533.A0A098DQ41 pp 229533.I1RQQ8
134295 229533.A0A098DQ41 pp 229533.I1RZ02
134296 229533.A0A098DQ41 pp 229533.I1RJT9
134297 229533.A0A098DQ41 pp 229533.I1RNY7
134298 229533.A0A098DQ41 pp 229533.A0A1C3YIA8
134299 229533.A0A098DQ41 pp 229533.I1RRT6
134300 229533.A0A098DQ41 pp 229533.A0A0E0RSD7
134301 229533.A0A098DQ41 pp 229533.I1RMB3
134302 229533.A0A098DQ41 pp 229533.A0A1C3YN26
134303 229533.A0A098DQ41 pp 229533.Q4HZS8
134304 229533.A0A098DQ41 pp 229533.I1RAM6
134305 229533.A0A098DQ41 pp 229533.I1RDB1
134306 229533.A0A098DQ41 pp 229533.I1RAW7
134307 229533.A0A098DQ41 pp 229533.I1RQ26
134308 229533.A0A098DQ41 pp 229533.I1RE00
134309 229533.A0A098DQ41 pp 229533.I1RSI0
134310 229533.A0A098DQ41 pp 229533.I1RYS6
134311 229533.A0A098DQ41 pp 229533.A0A1C3YK11
134312 229533.A0A098DQ41 pp 229533.I1RQ81
134313 229533.A0A098DQ41 pp 229533.I1RYL5
134314 229533.A0A098DQ41 pp 229533.A0A1C3YHS4
134315 229533.A0A098DQ41 pp 229533.A0A1C3YHX6
134316 229533.A0A098DQ41 pp 229533.I1RFT2
134317 229533.A0A098DQ41 pp 229533.I1S439
134318 229533.A0A098DQ41 pp 229533.I1RQA5
134319 229533.A0A098DQ41 pp 229533.I1RDG9
134320 229533.A0A098DQ41 pp 229533.I1RM35

134321	229533.A0A098DQ41	pp	229533.I1RB75
134322	229533.A0A098DQ41	pp	229533.I1RAU5
134323	229533.A0A098DQ41	pp	229533.I1RN08
134324	229533.A0A098DQ41	pp	229533.I1S5B0
134325	229533.A0A098DQ41	pp	229533.I1RBC4
134326	229533.A0A098DQ41	pp	229533.A0A098DRS5
134327	229533.A0A098DQ41	pp	229533.I1RKK8
134328	229533.A0A098DQ41	pp	229533.I1RZM1
134329	229533.A0A098DQ41	pp	229533.I1RW87
134330	229533.A0A098DQ41	pp	229533.I1S0Z3
134331	229533.A0A098DQ41	pp	229533.I1RMM9
134332	229533.A0A098DQ41	pp	229533.I1RYJ8
134333	229533.A0A098DQ41	pp	229533.Q4ICA8
134334	229533.A0A098DQ41	pp	229533.I1RKK0
134335	229533.A0A098DQ41	pp	229533.I1RAM7
134336	229533.A0A098DQ41	pp	229533.I1RQQ6
134337	229533.A0A098DQ41	pp	229533.I1RPM4
134338	229533.A0A098DQ41	pp	229533.I1RZH4
134339	229533.A0A098DQ41	pp	229533.I1RB59
134340	229533.A0A098DQ41	pp	229533.I1RRT8
134341	229533.A0A098DQ41	pp	229533.I1S235
134342	229533.A0A098DQ41	pp	229533.I1RMQ7
134343	229533.A0A098DQ41	pp	229533.I1RBE4
134344	229533.A0A098DQ41	pp	229533.I1RE76
134345	229533.A0A098DQ41	pp	229533.I1RJZ7
134346	229533.A0A098DQ41	pp	229533.I1RRM9
134347	229533.A0A098DQ41	pp	229533.Q4IAA0
134348	229533.A0A098DQ41	pp	229533.I1RWK9
134349	229533.A0A098DQ41	pp	229533.A0A1C3YIK4
134350	229533.A0A098DQ41	pp	229533.I1RVY0
134351	229533.A0A098DQ41	pp	229533.I1RPL2
134352	229533.A0A098DQ41	pp	229533.I1S2V2
134353	229533.A0A098DQ41	pp	229533.A0A1C3YKV3
134354	229533.A0A098DQ41	pp	229533.I1S1V5
134355	229533.A0A098DQ41	pp	229533.I1R9Y8
134356	229533.A0A098DQ41	pp	229533.A0A1C3YIS7
134357	229533.A0A098DQ41	pp	229533.I1RKR9
134358	229533.A0A098DQ41	pp	229533.I1RP66
134359	229533.A0A098DQ41	pp	229533.I1REC6
134360	229533.A0A098DQ41	pp	229533.I1RAN2
134361	229533.A0A098DQ41	pp	229533.A0A1C3YK08
134362	229533.A0A098DQ41	pp	229533.I1RCQ3
134363	229533.A0A098DQ41	pp	229533.I1RP27
134364	229533.A0A098DQ41	pp	229533.I1RP28
134365	229533.A0A098DQ41	pp	229533.I1RJS2
134366	229533.A0A098DQ41	pp	229533.I1S2G9
134367	229533.A0A098DQ41	pp	229533.I1RN67
134368	229533.A0A098DQ41	pp	229533.I1RBW1
134369	229533.A0A098DQ41	pp	229533.I1RR95
134370	229533.A0A098DQ41	pp	229533.I1RNG4
134371	229533.A0A098DQ41	pp	229533.Q4IQM6
134372	229533.A0A098DQ41	pp	229533.I1RC00
134373	229533.A0A098DQ41	pp	229533.A0A0E0SBC0
134374	229533.A0A098DQ41	pp	229533.I1RS20
134375	229533.A0A098DQ41	pp	229533.I1RS47
134376	229533.A0A098DQ41	pp	229533.I1RBS6
134377	229533.A0A098DQ41	pp	229533.A0A1C3YI20
134378	229533.A0A098DQ41	pp	229533.I1RKI0
134379	229533.A0A098DQ41	pp	229533.I1RB74
134380	229533.A0A098DQ41	pp	229533.I1RZZ9
134381	229533.A0A098DQ41	pp	229533.I1RCJ7
134382	229533.A0A098DQ41	pp	229533.I1S5S8
134383	229533.A0A098DQ41	pp	229533.I1RPP3
134384	229533.A0A098DQ41	pp	229533.I1RSH0
134385	229533.A0A098DQ41	pp	229533.I1S8P5
134386	229533.A0A098DQ41	pp	229533.I1RXT9
134387	229533.A0A098DQ41	pp	229533.I1RME4
134388	229533.A0A098DQ41	pp	229533.I1RQG4
134389	229533.A0A098DQ41	pp	229533.I1RZD3
134390	229533.A0A098DQ41	pp	229533.I1RCL5
134391	229533.A0A098DQ41	pp	229533.A0A0E0RV61
134392	229533.A0A098DQ41	pp	229533.I1RD22
134393	229533.A0A098DQ41	pp	229533.I1RWH4

134394 229533.A0A098DQ41 pp 229533.V6RRH2
134395 229533.A0A098DQ41 pp 229533.I1S238
134396 229533.A0A098DQ41 pp 229533.A0A098E4F8
134397 229533.A0A098DQ41 pp 229533.I1RAU7
134398 229533.A0A098DQ41 pp 229533.I1RZE4
134399 229533.A0A098DQ41 pp 229533.I1S0N4
134400 229533.A0A098DQ41 pp 229533.I1RPN8
134401 229533.A0A098DQ41 pp 229533.I1RTH6
134402 229533.A0A098DQ41 pp 229533.A0A0E0SIF3
134403 229533.A0A098DQ41 pp 229533.I1RNC1
134404 229533.A0A098DQ41 pp 229533.I1RUG3
134405 229533.A0A098DQ41 pp 229533.I1RQF2
134406 229533.A0A098DQ41 pp 229533.I1RJT8
134407 229533.A0A098DQ41 pp 229533.V6RKY7
134408 229533.A0A098DQ41 pp 229533.I1RJV3
134409 229533.A0A098DQ41 pp 229533.I1RSR9
134410 229533.A0A098DQ41 pp 229533.I1S0M2
134411 229533.A0A098DQ41 pp 229533.I1RQ21
134412 229533.A0A098DQ41 pp 229533.A0A0E0RS85
134413 229533.A0A098DQ41 pp 229533.I1S9U6
134414 229533.A0A098DQ41 pp 229533.I1REC7
134415 229533.A0A098DQ41 pp 229533.I1RSL8
134416 229533.A0A098DQ41 pp 229533.I1RIG5
134417 229533.A0A098DQ41 pp 229533.I1RE01
134418 229533.A0A098DQ41 pp 229533.I1RYW3
134419 229533.A0A098DQ41 pp 229533.I1RMQ5
134420 229533.A0A098DQ41 pp 229533.I1RJS3
134421 229533.A0A098DQ41 pp 229533.I1RBS5
134422 229533.A0A098DQ41 pp 229533.A0A0E0SK74
134423 229533.A0A098DQ41 pp 229533.I1RZ95
134424 229533.A0A098DQ41 pp 229533.I1RA86
134425 229533.A0A098DQ41 pp 229533.I1RTA0
134426 229533.A0A098DQ41 pp 229533.I1RRV1
134427 229533.I1R9W4 pp 229533.I1RUP5
134428 229533.I1R9W4 pp 229533.I1RRZ6
134429 229533.I1R9W4 pp 229533.I1S064
134430 229533.I1R9W4 pp 229533.I1RA90
134431 229533.I1R9W4 pp 229533.I1S9Z4
134432 229533.I1R9W4 pp 229533.I1RD81
134433 229533.I1R9W4 pp 229533.I1RXB6
134434 229533.I1R9W4 pp 229533.V6RLM7
134435 229533.I1R9W4 pp 229533.I1RCB4
134436 229533.I1R9W4 pp 229533.I1RJS0
134437 229533.I1R9W4 pp 229533.I1RFY0
134438 229533.I1R9W4 pp 229533.I1RSU2
134439 229533.I1R9W4 pp 229533.I1RA94
134440 229533.I1R9W4 pp 229533.V6RN69
134441 229533.I1R9W4 pp 229533.I1RC61
134442 229533.I1R9W4 pp 229533.V6RPQ8
134443 229533.I1R9W4 pp 229533.I1RCN8
134444 229533.I1R9W4 pp 229533.I1S4P6
134445 229533.I1R9W4 pp 229533.I1RE79
134446 229533.I1R9W4 pp 229533.I1RVP5
134447 229533.I1R9W4 pp 229533.I1RXY7
134448 229533.I1R9W4 pp 229533.I1RXF0
134449 229533.I1R9W4 pp 229533.I1RG66
134450 229533.I1R9W4 pp 229533.I1RGL3
134451 229533.I1R9W4 pp 229533.I1S2C4
134452 229533.I1R9W4 pp 229533.I1RK03
134453 229533.I1R9W4 pp 229533.I1RHG5
134454 229533.I1R9W4 pp 229533.I1RM26
134455 229533.I1R9W4 pp 229533.I1RMW4
134456 229533.I1R9W4 pp 229533.I1RSX9
134457 229533.I1R9W4 pp 229533.I1RBE7
134458 229533.I1R9W4 pp 229533.I1RPD6
134459 229533.I1R9W4 pp 229533.I1RE53
134460 229533.I1R9W4 pp 229533.I1RZ83
134461 229533.I1R9W4 pp 229533.I1RT09
134462 229533.I1R9W4 pp 229533.I1RY74
134463 229533.I1R9W4 pp 229533.I1RJU6
134464 229533.I1R9W4 pp 229533.I1RZ20
134465 229533.I1R9W4 pp 229533.I1RWF9
134466 229533.I1R9W4 pp 229533.I1S2A1

134467 229533.I1R9W4 pp 229533.I1RC83
134468 229533.I1R9W4 pp 229533.I1RSS7
134469 229533.I1R9W4 pp 229533.I1RAE8
134470 229533.I1R9W4 pp 229533.I1RYU0
134471 229533.I1R9W4 pp 229533.I1RVX9
134472 229533.I1R9W4 pp 229533.I1RDH6
134473 229533.I1R9W4 pp 229533.I1S8E0
134474 229533.I1R9W4 pp 229533.I1S0C2
134475 229533.I1R9W4 pp 229533.I1RZ92
134476 229533.I1R9W4 pp 229533.I1RMK4
134477 229533.I1R9W4 pp 229533.I1RR68
134478 229533.I1R9W4 pp 229533.I1RMM8
134479 229533.I1R9W4 pp 229533.I1RC14
134480 229533.I1R9W4 pp 229533.I1RP97
134481 229533.I1R9W4 pp 229533.I1RGZ3
134482 229533.I1R9W4 pp 229533.I1RYU5
134483 229533.I1R9W4 pp 229533.I1RAJ6
134484 229533.V6RTJ8 pp 229533.V6RUT5
134485 229533.V6RTJ8 pp 229533.V6RWB3
134486 229533.V6RTJ8 pp 229533.V6RUY9
134487 229533.I1RF91 pp 229533.I1RLN6
134488 229533.I1RF91 pp 229533.I1RXF2
134489 229533.I1RF91 pp 229533.I1S7M3
134490 229533.I1RF91 pp 229533.I1S7M2
134491 229533.I1RF91 pp 229533.I1S0B1
134492 229533.I1RF91 pp 229533.I1S5J8
134493 229533.I1RF91 pp 229533.I1RST1
134494 229533.I1RF91 pp 229533.I1RG90
134495 229533.I1RF91 pp 229533.I1RSA4
134496 229533.I1RF91 pp 229533.I1S4S7
134497 229533.I1RF91 pp 229533.I1RPD9
134498 229533.I1RF91 pp 229533.I1RIH9
134499 229533.I1S259 pp 229533.I1SB04
134500 229533.I1S259 pp 229533.Q4IER8
134501 229533.I1S259 pp 229533.I1S4Z5
134502 229533.I1S259 pp 229533.Q4IB50
134503 229533.I1S259 pp 229533.Q4I7L0
134504 229533.I1S259 pp 229533.Q4HTT2
134505 229533.A0A1C3YM63 pp 229533.I1RA94
134506 229533.A0A1C3YM63 pp 229533.I1S5B0
134507 229533.A0A1C3YM63 pp 229533.I1S835
134508 229533.A0A1C3YM63 pp 229533.I1RW99
134509 229533.A0A1C3YM63 pp 229533.I1RRV4
134510 229533.A0A1C3YM63 pp 229533.I1RAU5
134511 229533.A0A1C3YM63 pp 229533.V6QUE6
134512 229533.A0A1C3YM63 pp 229533.V6RCG5
134513 229533.A0A1C3YM63 pp 229533.I1RBE4
134514 229533.A0A1C3YM63 pp 229533.V6RCZ8
134515 229533.A0A1C3YM63 pp 229533.I1RC61
134516 229533.A0A1C3YM63 pp 229533.I1RI82
134517 229533.A0A1C3YM63 pp 229533.I1RZM8
134518 229533.A0A1C3YM63 pp 229533.I1RXF0
134519 229533.A0A1C3YM63 pp 229533.Q4I283
134520 229533.A0A1C3YM63 pp 229533.I1RWT6
134521 229533.A0A1C3YM63 pp 229533.I1S504
134522 229533.A0A1C3YM63 pp 229533.I1RG14
134523 229533.A0A1C3YM63 pp 229533.I1RLU6
134524 229533.A0A1C3YM63 pp 229533.I1RNQ5
134525 229533.A0A1C3YM63 pp 229533.I1RCN8
134526 229533.A0A1C3YM63 pp 229533.I1RZM1
134527 229533.A0A1C3YM63 pp 229533.Q4I2J8
134528 229533.A0A1C3YM63 pp 229533.I1RC16
134529 229533.A0A1C3YM63 pp 229533.I1RK95
134530 229533.A0A1C3YM63 pp 229533.I1RS18
134531 229533.A0A1C3YM63 pp 229533.I1RZZ1
134532 229533.A0A1C3YM63 pp 229533.Q4I8P5
134533 229533.A0A1C3YM63 pp 229533.I1RXB6
134534 229533.A0A1C3YM63 pp 229533.V6RBN2
134535 229533.A0A1C3YM63 pp 229533.I1RQA3
134536 229533.A0A1C3YM63 pp 229533.V6RK96
134537 229533.A0A1C3YM63 pp 229533.I1RXC4
134538 229533.A0A1C3YM63 pp 229533.I1SA32
134539 229533.A0A1C3YM63 pp 229533.I1RVZ9

134540 229533.A0A1C3YM63 pp 229533.I1RMQ4
134541 229533.A0A1C3YM63 pp 229533.I1RD63
134542 229533.A0A1C3YM63 pp 229533.Q4IJK2
134543 229533.A0A1C3YM63 pp 229533.I1RQ41
134544 229533.A0A1C3YM63 pp 229533.I1RRY9
134545 229533.A0A1C3YM63 pp 229533.V6RTJ8
134546 229533.A0A1C3YM63 pp 229533.I1RZK5
134547 229533.A0A1C3YM63 pp 229533.I1S0L3
134548 229533.A0A1C3YM63 pp 229533.I1RD52
134549 229533.A0A1C3YM63 pp 229533.I1RFU8
134550 229533.A0A1C3YM63 pp 229533.I1S7A8
134551 229533.A0A1C3YM63 pp 229533.I1RT33
134552 229533.A0A1C3YM63 pp 229533.I1RAZ3
134553 229533.A0A1C3YM63 pp 229533.I1RYL5
134554 229533.A0A1C3YM63 pp 229533.I1RCG4
134555 229533.A0A1C3YM63 pp 229533.I1RAX6
134556 229533.A0A1C3YM63 pp 229533.I1RX46
134557 229533.A0A1C3YM63 pp 229533.I1RME6
134558 229533.A0A1C3YM63 pp 229533.I1RVQ0
134559 229533.A0A1C3YM63 pp 229533.I1RY03
134560 229533.A0A1C3YM63 pp 229533.I1RRR5
134561 229533.A0A1C3YM63 pp 229533.I1RXD7
134562 229533.A0A1C3YM63 pp 229533.I1RM35
134563 229533.A0A1C3YM63 pp 229533.V6QW22
134564 229533.A0A1C3YM63 pp 229533.I1RQC9
134565 229533.A0A1C3YM63 pp 229533.I1REK8
134566 229533.A0A1C3YM63 pp 229533.I1RCR5
134567 229533.A0A1C3YM63 pp 229533.I1RSQ7
134568 229533.A0A1C3YM63 pp 229533.I1S4Q4
134569 229533.A0A1C3YM63 pp 229533.I1RZM4
134570 229533.A0A1C3YM63 pp 229533.I1S0R7
134571 229533.A0A1C3YM63 pp 229533.I1RRC6
134572 229533.A0A1C3YM63 pp 229533.V6R1K4
134573 229533.A0A1C3YM63 pp 229533.A0A1C3YN30
134574 229533.A0A1C3YM63 pp 229533.I1RBC1
134575 229533.A0A1C3YM63 pp 229533.I1S4S7
134576 229533.A0A1C3YM63 pp 229533.A0A1C3YN26
134577 229533.A0A1C3YM63 pp 229533.I1RBC9
134578 229533.A0A1C3YM63 pp 229533.I1RBT1
134579 229533.A0A1C3YM63 pp 229533.I1RFL6
134580 229533.A0A1C3YM63 pp 229533.I1RPN4
134581 229533.A0A1C3YM63 pp 229533.I1RBW0
134582 229533.A0A1C3YM63 pp 229533.I1RMG3
134583 229533.A0A1C3YM63 pp 229533.I1RW25
134584 229533.A0A1C3YM63 pp 229533.I1RTC7
134585 229533.A0A1C3YM63 pp 229533.I1SAN9
134586 229533.A0A1C3YM63 pp 229533.I1S7L6
134587 229533.A0A1C3YM63 pp 229533.I1RR24
134588 229533.A0A1C3YM63 pp 229533.I1RRV1
134589 229533.A0A1C3YM63 pp 229533.I1RX25
134590 229533.A0A1C3YM63 pp 229533.I1RMG8
134591 229533.A0A1C3YM63 pp 229533.I1RM67
134592 229533.A0A1C3YM63 pp 229533.I1RNC1
134593 229533.A0A1C3YM63 pp 229533.I1RFL8
134594 229533.A0A1C3YM63 pp 229533.I1RUG3
134595 229533.A0A1C3YM63 pp 229533.I1RZJ5
134596 229533.A0A1C3YM63 pp 229533.I1RCR8
134597 229533.A0A1C3YM63 pp 229533.I1S1W8
134598 229533.A0A1C3YM63 pp 229533.I1RY44
134599 229533.A0A1C3YM63 pp 229533.I1S0B7
134600 229533.A0A1C3YM63 pp 229533.I1RPA4
134601 229533.A0A1C3YM63 pp 229533.I1RWJ8
134602 229533.A0A1C3YM63 pp 229533.I1RWN5
134603 229533.A0A1C3YM63 pp 229533.I1S4S4
134604 229533.A0A1C3YM63 pp 229533.I1S5R6
134605 229533.A0A1C3YM63 pp 229533.V6RIV9
134606 229533.A0A1C3YM63 pp 229533.I1RCL4
134607 229533.A0A1C3YM63 pp 229533.I1RQE8
134608 229533.A0A1C3YM63 pp 229533.I1SA33
134609 229533.A0A1C3YM63 pp 229533.I1RD22
134610 229533.A0A1C3YM63 pp 229533.I1S1W9
134611 229533.A0A1C3YM63 pp 229533.I1RJU3
134612 229533.A0A1C3YM63 pp 229533.I1RKB6

134613	229533.A0A1C3YM63	pp	229533.I1RPP4
134614	229533.A0A1C3YM63	pp	229533.I1RSQ3
134615	229533.A0A1C3YM63	pp	229533.I1RS17
134616	229533.A0A1C3YM63	pp	229533.I1S0J1
134617	229533.A0A1C3YM63	pp	229533.I1RA70
134618	229533.A0A1C3YM63	pp	229533.V6R042
134619	229533.A0A1C3YM63	pp	229533.I1RTH6
134620	229533.A0A1C3YM63	pp	229533.I1RW46
134621	229533.A0A1C3YM63	pp	229533.V6RMW6
134622	229533.A0A1C3YM63	pp	229533.I1S8R3
134623	229533.A0A1C3YM63	pp	229533.I1RE01
134624	229533.A0A1C3YM63	pp	229533.I1RVZ2
134625	229533.A0A1C3YM63	pp	229533.I1RRI2
134626	229533.A0A1C3YM63	pp	229533.I1RD39
134627	229533.A0A1C3YM63	pp	229533.I1RWN6
134628	229533.A0A1C3YM63	pp	229533.I1RAW0
134629	229533.A0A1C3YM63	pp	229533.I1RZW8
134630	229533.A0A1C3YM63	pp	229533.Q4I5V2
134631	229533.A0A1C3YM63	pp	229533.I1RZP1
134632	229533.A0A1C3YM63	pp	229533.I1S1C7
134633	229533.A0A1C3YM63	pp	229533.I1RN63
134634	229533.A0A1C3YM63	pp	229533.I1RWK3
134635	229533.A0A1C3YM63	pp	229533.I1RJV3
134636	229533.A0A1C3YM63	pp	229533.I1RAU7
134637	229533.A0A1C3YM63	pp	229533.I1RYW3
134638	229533.A0A1C3YM63	pp	229533.I1RZB2
134639	229533.A0A1C3YM63	pp	229533.I1RJV4
134640	229533.A0A1C3YM63	pp	229533.I1RBW1
134641	229533.A0A1C3YM63	pp	229533.I1S0L4
134642	229533.A0A1C3YM63	pp	229533.V6R057
134643	229533.A0A1C3YM63	pp	229533.I1S3G4
134644	229533.A0A1C3YM63	pp	229533.I1RBS6
134645	229533.A0A1C3YM63	pp	229533.I1RD17
134646	229533.A0A1C3YM63	pp	229533.I1S2H4
134647	229533.A0A1C3YM63	pp	229533.I1RGA4
134648	229533.A0A1C3YM63	pp	229533.Q4I624
134649	229533.A0A1C3YM63	pp	229533.I1RJW6
134650	229533.A0A1C3YM63	pp	229533.I1S503
134651	229533.A0A1C3YM63	pp	229533.I1RRT8
134652	229533.A0A1C3YM63	pp	229533.I1RB92
134653	229533.A0A1C3YM63	pp	229533.I1RNL7
134654	229533.A0A1C3YM63	pp	229533.I1S5R1
134655	229533.A0A1C3YM63	pp	229533.I1RT28
134656	229533.A0A1C3YM63	pp	229533.I1S2E9
134657	229533.A0A1C3YM63	pp	229533.I1RT03
134658	229533.A0A1C3YM63	pp	229533.O74638
134659	229533.A0A1C3YM63	pp	229533.I1RVX0
134660	229533.A0A1C3YM63	pp	229533.I1RT04
134661	229533.A0A1C3YM63	pp	229533.I1RXC2
134662	229533.A0A1C3YM63	pp	229533.I1RSS4
134663	229533.A0A1C3YM63	pp	229533.I1RWF5
134664	229533.A0A1C3YM63	pp	229533.I1RA11
134665	229533.A0A1C3YM63	pp	229533.I1RBA4
134666	229533.A0A1C3YM63	pp	229533.I1RCH5
134667	229533.A0A1C3YM63	pp	229533.I1RTD5
134668	229533.A0A1C3YM63	pp	229533.I1RQE7
134669	229533.A0A1C3YM63	pp	229533.I1S9Y1
134670	229533.A0A1C3YM63	pp	229533.I1RR73
134671	229533.A0A1C3YM63	pp	229533.I1RV91
134672	229533.A0A1C3YM63	pp	229533.I1RT40
134673	229533.A0A1C3YM63	pp	229533.I1RFL4
134674	229533.A0A1C3YM63	pp	229533.V6R3X5
134675	229533.A0A1C3YM63	pp	229533.I1RN18
134676	229533.A0A1C3YM63	pp	229533.I1RZ39
134677	229533.A0A1C3YM63	pp	229533.I1R9M0
134678	229533.A0A1C3YM63	pp	229533.I1RGC1
134679	229533.A0A1C3YM63	pp	229533.I1RC66
134680	229533.A0A1C3YM63	pp	229533.I1RN67
134681	229533.A0A1C3YM63	pp	229533.I1RC31
134682	229533.A0A1C3YM63	pp	229533.I1S2B8
134683	229533.A0A1C3YM63	pp	229533.I1S0F6
134684	229533.A0A1C3YM63	pp	229533.I1RRM5
134685	229533.A0A1C3YM63	pp	229533.I1RQD6

134686 229533.A0A1C3YM63 pp 229533.I1RZZ9
134687 229533.A0A1C3YM63 pp 229533.I1RBF3
134688 229533.A0A1C3YM63 pp 229533.I1RE22
134689 229533.A0A1C3YM63 pp 229533.I1RYI5
134690 229533.A0A1C3YM63 pp 229533.I1RSQ4
134691 229533.A0A1C3YM63 pp 229533.I1RPP3
134692 229533.A0A1C3YM63 pp 229533.I1RFT1
134693 229533.A0A1C3YM63 pp 229533.I1RQ23
134694 229533.A0A1C3YM63 pp 229533.I1RGC3
134695 229533.A0A1C3YM63 pp 229533.I1RE27
134696 229533.A0A1C3YM63 pp 229533.I1R9Y8
134697 229533.A0A1C3YM63 pp 229533.I1RPM4
134698 229533.A0A1C3YM63 pp 229533.I1S277
134699 229533.A0A1C3YM63 pp 229533.I1RTA1
134700 229533.A0A1C3YM63 pp 229533.I1RSZ7
134701 229533.A0A1C3YM63 pp 229533.I1RL04
134702 229533.A0A1C3YM63 pp 229533.I1RBY4
134703 229533.A0A098DDV4 pp 229533.A0A1C3YJI5
134704 229533.A0A098DDV4 pp 229533.I1RH91
134705 229533.A0A098DDV4 pp 229533.I1S3G4
134706 229533.A0A098DDV4 pp 229533.I1S2Q4
134707 229533.A0A098DDV4 pp 229533.I1RJJ7
134708 229533.A0A098DDV4 pp 229533.V6RXR0
134709 229533.A0A098DDV4 pp 229533.I1RBY9
134710 229533.A0A098DDV4 pp 229533.Q4HVQ9
134711 229533.A0A098DDV4 pp 229533.A0A1C3YKL7
134712 229533.A0A098DDV4 pp 229533.I1RRF5
134713 229533.A0A098DDV4 pp 229533.V6RHD0
134714 229533.A0A098DDV4 pp 229533.I1RNG4
134715 229533.A0A098DDV4 pp 229533.V6QU65
134716 229533.A0A098DDV4 pp 229533.A0A098DUS9
134717 229533.A0A098DDV4 pp 229533.I1RWZ3
134718 229533.A0A098DDV4 pp 229533.A0A098DEV0
134719 229533.A0A098DDV4 pp 229533.A0A098DHF2
134720 229533.A0A098DDV4 pp 229533.A0A1C3YMF1
134721 229533.A0A098DDV4 pp 229533.I1RG66
134722 229533.A0A098DDV4 pp 229533.A0A1C3YIP7
134723 229533.A0A098DDV4 pp 229533.I1RAV0
134724 229533.A0A098DDV4 pp 229533.A0A1C3YI08
134725 229533.A0A098DDV4 pp 229533.I1RUQ3
134726 229533.A0A098DDV4 pp 229533.I1S1W8
134727 229533.A0A098DDV4 pp 229533.I1S4G3
134728 229533.A0A098DDV4 pp 229533.I1RAY1
134729 229533.A0A098DDV4 pp 229533.I1RTY0
134730 229533.A0A098DDV4 pp 229533.I1RBE7
134731 229533.A0A098DDV4 pp 229533.I1S1B8
134732 229533.A0A098DDV4 pp 229533.I1RQE1
134733 229533.A0A098DDV4 pp 229533.A0A1C3YN04
134734 229533.A0A098DDV4 pp 229533.I1RYX6
134735 229533.A0A1C3YN84 pp 229533.I1RFT1
134736 229533.A0A1C3YN84 pp 229533.I1RCN4
134737 229533.A0A1C3YN84 pp 229533.I1S4T6
134738 229533.A0A1C3YN84 pp 229533.I1RBE2
134739 229533.A0A1C3YN84 pp 229533.I1RX84
134740 229533.A0A1C3YN84 pp 229533.I1RCR5
134741 229533.A0A1C3YN84 pp 229533.V6RQR4
134742 229533.A0A1C3YN84 pp 229533.I1RYW3
134743 229533.A0A1C3YN84 pp 229533.I1RN64
134744 229533.A0A1C3YN84 pp 229533.I1RTQ9
134745 229533.A0A1C3YN84 pp 229533.I1RT91
134746 229533.I1RAX6 pp 229533.I1RYI5
134747 229533.I1RAX6 pp 229533.I1RC03
134748 229533.I1RAX6 pp 229533.I1S8P5
134749 229533.I1RAX6 pp 229533.I1RB74
134750 229533.I1RAX6 pp 229533.I1RWF7
134751 229533.I1RAX6 pp 229533.I1RRJ8
134752 229533.I1RAX6 pp 229533.I1RQG4
134753 229533.I1RAX6 pp 229533.I1RPP3
134754 229533.I1RAX6 pp 229533.I1S352
134755 229533.I1RAX6 pp 229533.V6RUY9
134756 229533.I1RAX6 pp 229533.I1RSV2
134757 229533.I1RAX6 pp 229533.I1RSX0
134758 229533.I1RAX6 pp 229533.I1RKL3

134759 229533.I1RAX6 pp 229533.I1RFT1
134760 229533.I1RAX6 pp 229533.I1RLV9
134761 229533.I1RAX6 pp 229533.V6R3J1
134762 229533.I1RAX6 pp 229533.I1RLN6
134763 229533.I1RAX6 pp 229533.I1RRQ3
134764 229533.I1RAX6 pp 229533.I1RN67
134765 229533.I1RAX6 pp 229533.I1RBS0
134766 229533.I1RAX6 pp 229533.I1RJW6
134767 229533.I1RAX6 pp 229533.I1S503
134768 229533.I1RAX6 pp 229533.I1RDF7
134769 229533.I1RAX6 pp 229533.I1S0W4
134770 229533.I1RAX6 pp 229533.I1S0F6
134771 229533.I1RAX6 pp 229533.I1RP27
134772 229533.I1RAX6 pp 229533.I1RQ65
134773 229533.I1RAX6 pp 229533.I1RJV4
134774 229533.I1RAX6 pp 229533.I1RBS6
134775 229533.I1RAX6 pp 229533.I1S2G7
134776 229533.I1RAX6 pp 229533.I1RGA4
134777 229533.I1RAX6 pp 229533.I1RBC7
134778 229533.I1RAX6 pp 229533.I1RQ23
134779 229533.I1RAX6 pp 229533.I1RS47
134780 229533.I1RAX6 pp 229533.I1RGF5
134781 229533.I1RAX6 pp 229533.I1RJN9
134782 229533.I1RAX6 pp 229533.I1RTA1
134783 229533.I1RAX6 pp 229533.Q4I662
134784 229533.I1RAX6 pp 229533.I1RV91
134785 229533.I1RAX6 pp 229533.I1RTD5
134786 229533.I1RAX6 pp 229533.I1RFS3
134787 229533.I1RAX6 pp 229533.I1RVZ2
134788 229533.I1RAX6 pp 229533.I1RPV3
134789 229533.I1RAX6 pp 229533.I1RN63
134790 229533.I1RAX6 pp 229533.I1RCJ7
134791 229533.I1RAX6 pp 229533.I1S2E9
134792 229533.I1RAX6 pp 229533.I1RWK3
134793 229533.I1RAX6 pp 229533.I1RT40
134794 229533.I1RAX6 pp 229533.V6R283
134795 229533.I1RAX6 pp 229533.I1S6Y8
134796 229533.I1RAX6 pp 229533.I1RL04
134797 229533.I1RAX6 pp 229533.I1RPM4
134798 229533.I1RAX6 pp 229533.I1RKG2
134799 229533.I1RAX6 pp 229533.I1RNA5
134800 229533.I1RAX6 pp 229533.I1RSZ7
134801 229533.I1RAX6 pp 229533.I1S2H4
134802 229533.I1RAX6 pp 229533.I1RPE0
134803 229533.I1RAX6 pp 229533.I1RT04
134804 229533.I1RAX6 pp 229533.I1RJZ7
134805 229533.I1RAX6 pp 229533.I1S0M6
134806 229533.I1RAX6 pp 229533.I1RS20
134807 229533.I1RAX6 pp 229533.I1RRM5
134808 229533.I1RAX6 pp 229533.I1RZS6
134809 229533.I1RAX6 pp 229533.I1S7W5
134810 229533.I1RAX6 pp 229533.I1RE22
134811 229533.I1RAX6 pp 229533.I1S2B8
134812 229533.I1RAX6 pp 229533.I1RPU3
134813 229533.I1RAX6 pp 229533.I1S0J1
134814 229533.I1RAX6 pp 229533.I1RBG9
134815 229533.I1RAX6 pp 229533.I1RBY4
134816 229533.I1RAX6 pp 229533.I1RW46
134817 229533.I1RAX6 pp 229533.I1RE01
134818 229533.I1RAX6 pp 229533.I1RE76
134819 229533.I1RAX6 pp 229533.I1S8V3
134820 229533.I1RAX6 pp 229533.I1RNC1
134821 229533.I1RAX6 pp 229533.I1RC66
134822 229533.I1RAX6 pp 229533.I1RTA0
134823 229533.I1RAX6 pp 229533.I1RMV0
134824 229533.I1RAX6 pp 229533.I1RFT6
134825 229533.I1RAX6 pp 229533.I1RPP4
134826 229533.I1RAX6 pp 229533.I1RUG3
134827 229533.I1RAX6 pp 229533.I1RNN1
134828 229533.I1RAX6 pp 229533.I1RPK0
134829 229533.I1RAX6 pp 229533.I1RCR8
134830 229533.I1RAX6 pp 229533.I1RWI5
134831 229533.I1RAX6 pp 229533.I1RKG3

134832	229533.I1RAX6	pp	229533.I1RS17
134833	229533.I1RAX6	pp	229533.I1RE79
134834	229533.I1RAX6	pp	229533.I1RZZ9
134835	229533.I1RAX6	pp	229533.I1RSQ4
134836	229533.I1RAX6	pp	229533.I1RNB8
134837	229533.I1RAX6	pp	229533.Q4IN63
134838	229533.I1RAX6	pp	229533.I1RR73
134839	229533.I1RAX6	pp	229533.V6R042
134840	229533.I1RAX6	pp	229533.I1RY44
134841	229533.I1RAX6	pp	229533.I1RM96
134842	229533.I1RAX6	pp	229533.I1RSQ3
134843	229533.I1RAX6	pp	229533.V6R3X5
134844	229533.I1RAX6	pp	229533.I1RSA4
134845	229533.I1RAX6	pp	229533.I1RZ39
134846	229533.I1RAX6	pp	229533.I1RC33
134847	229533.I1RAX6	pp	229533.I1S1W3
134848	229533.I1RAX6	pp	229533.I1RRT8
134849	229533.I1RAX6	pp	229533.V6RMW6
134850	229533.I1RAX6	pp	229533.I1S5R1
134851	229533.I1RAX6	pp	229533.I1RRM3
134852	229533.I1RAX6	pp	229533.I1RXC2
134853	229533.I1RAX6	pp	229533.I1RMG3
134854	229533.I1RAX6	pp	229533.I1RJP4
134855	229533.I1RAX6	pp	229533.I1RRV1
134856	229533.I1RAX6	pp	229533.V6RIV9
134857	229533.I1RAX6	pp	229533.I1RZV3
134858	229533.I1RAX6	pp	229533.I1RRN2
134859	229533.I1RAX6	pp	229533.O74638
134860	229533.I1RAX6	pp	229533.I1RWN5
134861	229533.I1RAX6	pp	229533.I1RD22
134862	229533.I1RAX6	pp	229533.I1RWL2
134863	229533.I1RAX6	pp	229533.I1SA33
134864	229533.I1RAX6	pp	229533.I1S9Y1
134865	229533.I1RAX6	pp	229533.I1RM82
134866	229533.I1RAX6	pp	229533.I1RMN6
134867	229533.I1RAX6	pp	229533.I1S1W9
134868	229533.I1RAX6	pp	229533.I1S0N4
134869	229533.I1RAX6	pp	229533.I1RRR7
134870	229533.I1RAX6	pp	229533.I1RKJ6
134871	229533.I1RAX6	pp	229533.I1RSF1
134872	229533.I1RAX6	pp	229533.I1RG11
134873	229533.I1RAX6	pp	229533.I1S0L4
134874	229533.I1RAX6	pp	229533.I1S8V4
134875	229533.I1RAX6	pp	229533.I1RPU2
134876	229533.I1RAX6	pp	229533.I1RRI1
134877	229533.I1RAX6	pp	229533.I1RIL3
134878	229533.I1RAX6	pp	229533.I1S5R6
134879	229533.I1RAX6	pp	229533.I1RKP1
134880	229533.I1RAX6	pp	229533.I1REC5
134881	229533.I1RAX6	pp	229533.I1RCH5
134882	229533.I1RAX6	pp	229533.I1S2E8
134883	229533.I1RAX6	pp	229533.I1RRE5
134884	229533.I1RAX6	pp	229533.I1RQE7
134885	229533.I1RAX6	pp	229533.I1S1W4
134886	229533.I1RAX6	pp	229533.I1RD64
134887	229533.I1RAX6	pp	229533.I1RNY2
134888	229533.I1RAX6	pp	229533.I1RFL4
134889	229533.I1RAX6	pp	229533.I1RRN5
134890	229533.I1RAX6	pp	229533.I1RGL4
134891	229533.I1RAX6	pp	229533.Q4I327
134892	229533.I1RAX6	pp	229533.I1RB41
134893	229533.I1RAX6	pp	229533.I1RSD8
134894	229533.I1RAX6	pp	229533.I1RG90
134895	229533.I1RAX6	pp	229533.I1RN18
134896	229533.I1RAX6	pp	229533.I1RB92
134897	229533.I1RAX6	pp	229533.V6RRH2
134898	229533.I1RAX6	pp	229533.I1RCM3
134899	229533.I1RAX6	pp	229533.I1RQ33
134900	229533.I1RAX6	pp	229533.I1RWN6
134901	229533.I1RAX6	pp	229533.I1RVX0
134902	229533.I1RAX6	pp	229533.I1RD39
134903	229533.I1RAX6	pp	229533.I1RT03
134904	229533.I1RAX6	pp	229533.V6R057

134905	229533.I1RAX6	pp	229533.I1S569
134906	229533.I1RAX6	pp	229533.I1RXR1
134907	229533.I1RAX6	pp	229533.I1RXU9
134908	229533.I1RAX6	pp	229533.Q4I624
134909	229533.I1RAX6	pp	229533.I1RRG3
134910	229533.I1RAX6	pp	229533.I1RIL8
134911	229533.I1RAX6	pp	229533.I1RD17
134912	229533.I1RAX6	pp	229533.I1RWX5
134913	229533.I1RAX6	pp	229533.V6R1K4
134914	229533.I1RAX6	pp	229533.I1S0L3
134915	229533.I1RAX6	pp	229533.Q4IJT5
134916	229533.I1RAX6	pp	229533.I1RRI2
134917	229533.I1RAX6	pp	229533.I1RFW5
134918	229533.I1RAX6	pp	229533.I1RX95
134919	229533.I1RAX6	pp	229533.I1RZW8
134920	229533.I1RAX6	pp	229533.I1RD52
134921	229533.I1RAX6	pp	229533.I1S0F5
134922	229533.I1RAX6	pp	229533.I1RG53
134923	229533.I1RAX6	pp	229533.I1RBD5
134924	229533.I1RAX6	pp	229533.I1RYW3
134925	229533.I1RAX6	pp	229533.I1RPY3
134926	229533.I1RAX6	pp	229533.I1S1C7
134927	229533.I1RAX6	pp	229533.I1RN61
134928	229533.I1RAX6	pp	229533.V6RUT5
134929	229533.I1RAX6	pp	229533.I1RQJ5
134930	229533.I1RAX6	pp	229533.I1RTD7
134931	229533.I1RAX6	pp	229533.V6QW22
134932	229533.I1RAX6	pp	229533.I1RC20
134933	229533.I1RAX6	pp	229533.I1RQH3
134934	229533.I1RAX6	pp	229533.I1S2C0
134935	229533.I1RAX6	pp	229533.I1RSQ7
134936	229533.I1RAX6	pp	229533.I1RLP1
134937	229533.I1RAX6	pp	229533.I1RXD7
134938	229533.I1RAX6	pp	229533.I1RRX5
134939	229533.I1RAX6	pp	229533.I1RTH6
134940	229533.I1RAX6	pp	229533.I1S8R3
134941	229533.I1RAX6	pp	229533.I1RQC9
134942	229533.I1RAX6	pp	229533.I1S4S7
134943	229533.I1RAX6	pp	229533.I1RSK1
134944	229533.I1RAX6	pp	229533.I1RJV3
134945	229533.I1RAX6	pp	229533.I1S0R7
134946	229533.I1RAX6	pp	229533.I1S0T2
134947	229533.I1RAX6	pp	229533.I1RW71
134948	229533.I1RAX6	pp	229533.I1RKB7
134949	229533.I1RAX6	pp	229533.I1RBC9
134950	229533.I1RAX6	pp	229533.I1RR00
134951	229533.I1RAX6	pp	229533.I1RT80
134952	229533.I1RAX6	pp	229533.I1RJU3
134953	229533.I1RAX6	pp	229533.I1RX46
134954	229533.I1RAX6	pp	229533.V6RTJ8
134955	229533.I1RAX6	pp	229533.V6R4J1
134956	229533.I1RAX6	pp	229533.I1RFQ7
134957	229533.I1RAX6	pp	229533.I1RVQ0
134958	229533.I1RAX6	pp	229533.I1RIG2
134959	229533.I1RAX6	pp	229533.I1RMG8
134960	229533.I1RAX6	pp	229533.I1RCS1
134961	229533.I1RAX6	pp	229533.I1S7A8
134962	229533.I1RAX6	pp	229533.I1RM06
134963	229533.I1RAX6	pp	229533.I1RZJ5
134964	229533.I1RAX6	pp	229533.I1RM67
134965	229533.I1RAX6	pp	229533.Q4I283
134966	229533.I1RAX6	pp	229533.I1RWT6
134967	229533.I1RAX6	pp	229533.V6QUW4
134968	229533.I1RAX6	pp	229533.I1S4S4
134969	229533.I1RAX6	pp	229533.I1RWJ8
134970	229533.I1RAX6	pp	229533.I1RW25
134971	229533.I1RAX6	pp	229533.I1RQ85
134972	229533.I1RAX6	pp	229533.I1RX25
134973	229533.I1RAX6	pp	229533.I1RG14
134974	229533.I1RAX6	pp	229533.I1RPU0
134975	229533.I1RAX6	pp	229533.I1RZF6
134976	229533.I1RAX6	pp	229533.I1RLC7
134977	229533.I1RAX6	pp	229533.I1S9U2

134978 229533.I1RAX6 pp 229533.I1RI02
134979 229533.I1RAX6 pp 229533.I1S504
134980 229533.I1RAX6 pp 229533.I1RW07
134981 229533.I1RAX6 pp 229533.I1RI82
134982 229533.I1RAX6 pp 229533.I1RR24
134983 229533.I1RAX6 pp 229533.I1S021
134984 229533.I1RAX6 pp 229533.I1RMC8
134985 229533.I1RAX6 pp 229533.I1SA32
134986 229533.I1RAX6 pp 229533.I1RNH6
134987 229533.I1RAX6 pp 229533.I1RBT1
134988 229533.I1RAX6 pp 229533.I1S0X6
134989 229533.I1RAX6 pp 229533.I1RZM8
134990 229533.I1RAX6 pp 229533.I1RXC4
134991 229533.I1RAX6 pp 229533.I1RB27
134992 229533.I1RAX6 pp 229533.Q4I8P5
134993 229533.I1RAX6 pp 229533.I1RS18
134994 229533.I1RAX6 pp 229533.I1S6U8
134995 229533.I1RAX6 pp 229533.I1RXF0
134996 229533.I1RAX6 pp 229533.I1S0M9
134997 229533.I1RAX6 pp 229533.I1RR51
134998 229533.I1RAX6 pp 229533.V6RCG5
134999 229533.I1RAX6 pp 229533.I1RQQ5
135000 229533.I1RAX6 pp 229533.I1RQ81
135001 229533.I1RAX6 pp 229533.V6RBN2
135002 229533.I1RAX6 pp 229533.I1RJZ6
135003 229533.I1RAX6 pp 229533.V6RCZ8
135004 229533.I1RAX6 pp 229533.I1RGC5
135005 229533.I1RAX6 pp 229533.I1RLU6
135006 229533.I1RAX6 pp 229533.I1RNQ5
135007 229533.I1RAX6 pp 229533.I1RBE4
135008 229533.I1RAX6 pp 229533.I1S6K9
135009 229533.I1RAX6 pp 229533.I1RQA3
135010 229533.I1RAX6 pp 229533.I1RZM1
135011 229533.I1RAX6 pp 229533.I1RBJ5
135012 229533.I1RAX6 pp 229533.I1RRU9
135013 229533.I1RAX6 pp 229533.Q4IJK2
135014 229533.I1RAX6 pp 229533.I1S419
135015 229533.I1RAX6 pp 229533.I1RFT2
135016 229533.I1RAX6 pp 229533.V6QUE6
135017 229533.I1RAX6 pp 229533.I1RK95
135018 229533.I1RAX6 pp 229533.I1RDU0
135019 229533.I1RAX6 pp 229533.Q4IJM4
135020 229533.I1RAX6 pp 229533.I1S5B0
135021 229533.I1RAX6 pp 229533.I1RUY5
135022 229533.I1RAX6 pp 229533.I1RLM7
135023 229533.I1RAX6 pp 229533.I1RW99
135024 229533.I1RAX6 pp 229533.I1RE00
135025 229533.I1RAX6 pp 229533.I1RZE5
135026 229533.I1RAX6 pp 229533.I1RSI0
135027 229533.I1RAX6 pp 229533.I1RC17
135028 229533.I1RAX6 pp 229533.I1RZZ1
135029 229533.I1RAX6 pp 229533.I1RZK6
135030 229533.I1RAX6 pp 229533.I1RF19
135031 229533.I1RAX6 pp 229533.I1S887
135032 229533.I1RAX6 pp 229533.I1RZ35
135033 229533.I1RAX6 pp 229533.I1RVZ0
135034 229533.I1RAX6 pp 229533.Q4I2J8
135035 229533.I1RAX6 pp 229533.I1RFU8
135036 229533.I1RAX6 pp 229533.I1RBU8
135037 229533.I1RAX6 pp 229533.I1RCG4
135038 229533.I1RAX6 pp 229533.I1RB75
135039 229533.I1RAX6 pp 229533.I1RI03
135040 229533.I1RAX6 pp 229533.I1RCR5
135041 229533.I1RAX6 pp 229533.I1REK8
135042 229533.I1RAX6 pp 229533.I1RYY5
135043 229533.I1RAX6 pp 229533.I1S1J7
135044 229533.I1RAX6 pp 229533.I1RWR7
135045 229533.I1RAX6 pp 229533.I1RWB0
135046 229533.I1RAX6 pp 229533.I1RSR8
135047 229533.I1RAX6 pp 229533.I1RY03
135048 229533.I1RAX6 pp 229533.I1RRY9
135049 229533.I1RAX6 pp 229533.I1RU29
135050 229533.I1RAX6 pp 229533.I1RPR9

135051	229533.I1RAX6	pp	229533.I1RBL7
135052	229533.I1RPW9	pp	229533.I1RYB9
135053	229533.I1RPW9	pp	229533.I1RQ31
135054	229533.I1RPW9	pp	229533.I1S021
135055	229533.I1RPW9	pp	229533.Q4IAA0
135056	229533.I1RPW9	pp	229533.Q4IRI9
135057	229533.I1RPW9	pp	229533.Q4IBS2
135058	229533.I1RPW9	pp	229533.I1RVZ0
135059	229533.I1RPW9	pp	229533.Q4IJ56
135060	229533.I1RPW9	pp	229533.I1S0M9
135061	229533.I1RPW9	pp	229533.I1RZJ6
135062	229533.I1RPW9	pp	229533.I1RX14
135063	229533.I1RPW9	pp	229533.I1S504
135064	229533.I1RPW9	pp	229533.Q4IPI1
135065	229533.I1RPW9	pp	229533.V6R227
135066	229533.I1RPW9	pp	229533.I1RSR3
135067	229533.I1RPW9	pp	229533.I1RSR8
135068	229533.I1RPW9	pp	229533.I1RX46
135069	229533.I1RPW9	pp	229533.Q4I5R9
135070	229533.I1RPW9	pp	229533.I1RZF6
135071	229533.I1RPW9	pp	229533.I1S1C6
135072	229533.I1RPW9	pp	229533.I1RRY9
135073	229533.I1RPW9	pp	229533.Q4IEK8
135074	229533.I1RPW9	pp	229533.I1RZ99
135075	229533.I1RPW9	pp	229533.Q4IFI0
135076	229533.I1RPW9	pp	229533.I1RQA2
135077	229533.I1RPW9	pp	229533.I1RQ28
135078	229533.I1RPW9	pp	229533.V6RMW6
135079	229533.I1RPW9	pp	229533.I1RR00
135080	229533.I1RPW9	pp	229533.I1RZZ1
135081	229533.I1RPW9	pp	229533.I1RQJ5
135082	229533.I1RPW9	pp	229533.I1RW71
135083	229533.I1RPW9	pp	229533.I1RZK6
135084	229533.I1RPW9	pp	229533.V6R042
135085	229533.I1RPW9	pp	229533.I1RSI6
135086	229533.I1RPW9	pp	229533.I1S6F0
135087	229533.I1RPW9	pp	229533.Q4I327
135088	229533.I1RPW9	pp	229533.I1RX47
135089	229533.I1RPW9	pp	229533.I1RWH5
135090	229533.I1RPW9	pp	229533.Q4HVW2
135091	229533.I1RPW9	pp	229533.I1RRH5
135092	229533.I1RPW9	pp	229533.Q4HZ47
135093	229533.I1RPW9	pp	229533.Q4IN63
135094	229533.I1RPW9	pp	229533.I1RX51
135095	229533.I1RPW9	pp	229533.I1RT80
135096	229533.I1RPW9	pp	229533.I1RPY1
135097	229533.I1RPW9	pp	229533.I1S6U8
135098	229533.I1RPW9	pp	229533.V6RGN5
135099	229533.I1RPW9	pp	229533.Q4IJH1
135100	229533.I1RPW9	pp	229533.I1S4T3
135101	229533.I1RPW9	pp	229533.I1RT47
135102	229533.I1RPW9	pp	229533.I1S2C0
135103	229533.I1RPW9	pp	229533.I1RT05
135104	229533.I1RPW9	pp	229533.I1RTE9
135105	229533.I1RPW9	pp	229533.I1RTF4
135106	229533.I1RPW9	pp	229533.I1S0J1
135107	229533.I1RPW9	pp	229533.I1RWB5
135108	229533.I1RPW9	pp	229533.I1S9U2
135109	229533.I1RPW9	pp	229533.I1RSD4
135110	229533.I1RPW9	pp	229533.I1RTH6
135111	229533.I1RPW9	pp	229533.I1RX81
135112	229533.I1RPW9	pp	229533.I1RRI2
135113	229533.I1RPW9	pp	229533.V6QYF0
135114	229533.I1RPW9	pp	229533.I1RRL0
135115	229533.I1RPW9	pp	229533.I1RTD7
135116	229533.I1RPW9	pp	229533.I1RS28
135117	229533.I1RPW9	pp	229533.I1RZG7
135118	229533.I1RPW9	pp	229533.I1S060
135119	229533.I1RPW9	pp	229533.I1RRR7
135120	229533.I1RPW9	pp	229533.Q4ICG5
135121	229533.I1RPW9	pp	229533.I1RXF6
135122	229533.I1RPW9	pp	229533.I1S274
135123	229533.I1RPW9	pp	229533.I1RSA4

229533.I1RPW9 pp 229533.I1RWN6
229533.I1RPW9 pp 229533.I1RQ65
229533.I1RPW9 pp 229533.I1RQ48
229533.I1RPW9 pp 229533.I1RSL9
229533.I1RPW9 pp 229533.I1S1X9
229533.I1RPW9 pp 229533.Q4IQW0
229533.I1RPW9 pp 229533.I1RRQ3
229533.I1RPW9 pp 229533.I1S2E8
229533.I1RPW9 pp 229533.Q4HZ42
229533.I1RPW9 pp 229533.I1RSD8
229533.I1RPW9 pp 229533.Q4IAS1
229533.I1RPW9 pp 229533.I1S296
229533.I1RPW9 pp 229533.V6R3X5
229533.I1RPW9 pp 229533.I1RRJ8
229533.I1RPW9 pp 229533.I1RWF7
229533.I1RPW9 pp 229533.I1S8E2
229533.I1RPW9 pp 229533.I1RR73
229533.I1RPW9 pp 229533.Q4HZ68
229533.I1RPW9 pp 229533.I1S4M5
229533.I1RPW9 pp 229533.Q4IF76
229533.I1RPW9 pp 229533.I1RU69
229533.I1RPW9 pp 229533.I1RWU2
229533.I1RPW9 pp 229533.V6R283
229533.I1RPW9 pp 229533.Q4HW67
229533.I1RPW9 pp 229533.Q4I830
229533.I1RPW9 pp 229533.I1RZV6
229533.I1RPW9 pp 229533.I1RST1
229533.I1RPW9 pp 229533.Q4I662
229533.I1RPW9 pp 229533.I1RX24
229533.I1RPW9 pp 229533.I1RZS6
229533.A0A098D8J9 pp 229533.I1S1F3
229533.A0A098D8J9 pp 229533.I1RMQ6
229533.A0A098D8J9 pp 229533.I1RAM9
229533.A0A098D8J9 pp 229533.A0A0E0S8Z0
229533.I1S9U2 pp 229533.V6RGY3
229533.I1S9U2 pp 229533.Q4IEK8
229533.I1S9U2 pp 229533.Q4IJ56
229533.I1S9U2 pp 229533.V6RGN5
229533.I1S9U2 pp 229533.V6QUE6
229533.I1S9U2 pp 229533.V6R042
229533.I1S9U2 pp 229533.Q4IN63
229533.I1S9U2 pp 229533.Q4IBS2
229533.I1S9U2 pp 229533.V6RMW6
229533.I1S9U2 pp 229533.Q4HZK7
229533.I1S9U2 pp 229533.Q4IJH1
229533.I1S9U2 pp 229533.Q4I327
229533.I1S9U2 pp 229533.Q4IFI0
229533.I1S9U2 pp 229533.Q4IAA0
229533.I1S9U2 pp 229533.Q4I8X0
229533.I1S9U2 pp 229533.Q4HZ68
229533.I1S9U2 pp 229533.V6QYF0
229533.I1S9U2 pp 229533.V6R3X5
229533.I1S9U2 pp 229533.Q4HZ42
229533.I1S9U2 pp 229533.V6R227
229533.I1S9U2 pp 229533.Q4HVW2
229533.I1S9U2 pp 229533.Q4IQW0
229533.I1S9U2 pp 229533.Q4HZ47
229533.I1S9U2 pp 229533.Q4I662
229533.I1S9U2 pp 229533.Q4IEV5
229533.I1S9U2 pp 229533.Q4IF76
229533.I1S9U2 pp 229533.V6R283
229533.I1S9U2 pp 229533.Q4IPI1
229533.I1S9U2 pp 229533.Q4IAS1
229533.I1S9U2 pp 229533.Q4I830
229533.I1S9U2 pp 229533.Q4I5R9
229533.A0A098DIV1 pp 229533.Q4HXF0
229533.A0A098DIV1 pp 229533.A0A1C3YJW4
229533.A0A098DIV1 pp 229533.I1RIA1
229533.A0A098DIV1 pp 229533.I1RIH9
229533.A0A098DIV1 pp 229533.I1RJT5
229533.A0A098DIV1 pp 229533.A0A1C3YKZ8
229533.A0A098DIV1 pp 229533.V6R5G9
229533.A0A098DIV1 pp 229533.I1RNG4

135197 229533.A0A098DIV1 pp 229533.I1RF91
135198 229533.A0A098DIV1 pp 229533.I1RE53
135199 229533.A0A098DIV1 pp 229533.I1S5J8
135200 229533.A0A098DIV1 pp 229533.I1RLN6
135201 229533.A0A098DIV1 pp 229533.V6RQW4
135202 229533.A0A098DIV1 pp 229533.I1RM55
135203 229533.A0A098DIV1 pp 229533.I1RCV1
135204 229533.I1RSA7 pp 229533.Q4IQM6
135205 229533.I1RSA7 pp 229533.I1RSN7
135206 229533.I1RSA7 pp 229533.I1RWM6
135207 229533.I1RSA7 pp 229533.Q4IEA5
135208 229533.I1RSA7 pp 229533.I1RYR2
135209 229533.I1RSA7 pp 229533.I1RSI4
135210 229533.I1RSA7 pp 229533.Q4IR08
135211 229533.I1RSA7 pp 229533.I1RYQ1
135212 229533.I1RSA7 pp 229533.I1RYX9
135213 229533.I1RSA7 pp 229533.I1S6Y6
135214 229533.I1RSA7 pp 229533.Q4IP51
135215 229533.I1RSA7 pp 229533.I1S604
135216 229533.I1RSA7 pp 229533.I1S209
135217 229533.I1RSA7 pp 229533.I1RUG2
135218 229533.I1RSA7 pp 229533.I1RZ94
135219 229533.I1RSA7 pp 229533.I1RY73
135220 229533.I1RSA7 pp 229533.I1RSC7
135221 229533.I1RSA7 pp 229533.I1RW16
135222 229533.I1RSA7 pp 229533.I1RSR9
135223 229533.I1RSA7 pp 229533.I1S0B2
135224 229533.I1RSA7 pp 229533.I1S283
135225 229533.I1RSA7 pp 229533.I1RSP7
135226 229533.I1RSA7 pp 229533.V6RLW4
135227 229533.I1RSA7 pp 229533.I1RZP8
135228 229533.I1RSA7 pp 229533.Q4IC11
135229 229533.I1RSA7 pp 229533.I1RTN6
135230 229533.I1RI82 pp 229533.I1RJV3
135231 229533.I1RI82 pp 229533.I1RRY9
135232 229533.I1RI82 pp 229533.I1S1J7
135233 229533.I1RI82 pp 229533.I1RU29
135234 229533.I1RI82 pp 229533.I1RSR3
135235 229533.I1RI82 pp 229533.I1RN18
135236 229533.I1RI82 pp 229533.I1RWN6
135237 229533.I1RI82 pp 229533.I1RQA3
135238 229533.I1RI82 pp 229533.I1RYW3
135239 229533.I1RI82 pp 229533.Q4IJK2
135240 229533.I1RI82 pp 229533.I1RMD5
135241 229533.I1RI82 pp 229533.I1S1C7
135242 229533.I1RI82 pp 229533.I1RZZ1
135243 229533.I1RI82 pp 229533.I1RWX5
135244 229533.I1RI82 pp 229533.I1RY03
135245 229533.I1RI82 pp 229533.I1RRI2
135246 229533.I1RI82 pp 229533.I1RQE7
135247 229533.I1RI82 pp 229533.I1RVX0
135248 229533.I1RI82 pp 229533.I1RQQ5
135249 229533.I1RI82 pp 229533.I1RZW8
135250 229533.I1RI82 pp 229533.I1RUG3
135251 229533.I1RI82 pp 229533.I1RTH6
135252 229533.I1RI82 pp 229533.I1RNC1
135253 229533.I1RI82 pp 229533.I1RZK5
135254 229533.I1RI82 pp 229533.I1RT03
135255 229533.I1RI82 pp 229533.I1RZM8
135256 229533.I1RI82 pp 229533.I1RQ31
135257 229533.I1RI82 pp 229533.I1RRN5
135258 229533.I1RI82 pp 229533.I1RN99
135259 229533.I1RI82 pp 229533.I1RQA2
135260 229533.I1RI82 pp 229533.I1RTE9
135261 229533.I1RI82 pp 229533.I1RXF0
135262 229533.I1RI82 pp 229533.I1RKC6
135263 229533.I1RI82 pp 229533.Q4I2J8
135264 229533.I1RI82 pp 229533.V6RCZ8
135265 229533.I1RI82 pp 229533.I1RS18
135266 229533.I1RI82 pp 229533.I1RLM7
135267 229533.I1RI82 pp 229533.V6RIV9
135268 229533.I1RI82 pp 229533.Q4I8P5
135269 229533.I1RI82 pp 229533.I1S569

135270 229533.I1RI82 pp 229533.V6R042
135271 229533.I1RI82 pp 229533.I1RWN5
135272 229533.I1RI82 pp 229533.I1RSQ3
135273 229533.I1RI82 pp 229533.V6RMW6
135274 229533.I1RI82 pp 229533.I1RLU6
135275 229533.I1RI82 pp 229533.I1RNQ5
135276 229533.I1RI82 pp 229533.V6R057
135277 229533.I1RI82 pp 229533.Q4I624
135278 229533.I1RI82 pp 229533.V6RCG5
135279 229533.I1RI82 pp 229533.I1RT40
135280 229533.I1RI82 pp 229533.I1SA33
135281 229533.I1RI82 pp 229533.I1RY44
135282 229533.I1RI82 pp 229533.I1RTD5
135283 229533.I1RI82 pp 229533.I1RRE5
135284 229533.I1RI82 pp 229533.I1RZM1
135285 229533.I1RI82 pp 229533.I1RQC9
135286 229533.I1RI82 pp 229533.V6QUE6
135287 229533.I1RI82 pp 229533.I1RNA5
135288 229533.I1RI82 pp 229533.V6RGN5
135289 229533.I1RI82 pp 229533.I1S504
135290 229533.I1RI82 pp 229533.I1S5B0
135291 229533.I1RI82 pp 229533.V6RUY9
135292 229533.I1RI82 pp 229533.V6R227
135293 229533.I1RI82 pp 229533.I1RT04
135294 229533.I1RI82 pp 229533.I1RW99
135295 229533.I1RI82 pp 229533.Q4I283
135296 229533.I1RI82 pp 229533.I1RW46
135297 229533.I1RI82 pp 229533.I1S0R7
135298 229533.I1RI82 pp 229533.I1RXD7
135299 229533.I1RI82 pp 229533.I1RWT6
135300 229533.I1RI82 pp 229533.I1RTA0
135301 229533.I1RI82 pp 229533.I1S8V4
135302 229533.I1RI82 pp 229533.I1SA32
135303 229533.I1RI82 pp 229533.I1S0J1
135304 229533.I1RI82 pp 229533.I1RUQ2
135305 229533.I1RI82 pp 229533.I1RWI5
135306 229533.I1RI82 pp 229533.I1RS17
135307 229533.I1RI82 pp 229533.I1RLN6
135308 229533.I1RI82 pp 229533.I1S2E9
135309 229533.I1RI82 pp 229533.I1RPP4
135310 229533.I1RI82 pp 229533.I1RZZ9
135311 229533.I1RI82 pp 229533.I1S0X6
135312 229533.I1RI82 pp 229533.I1RJW6
135313 229533.I1RI82 pp 229533.I1RZJ5
135314 229533.I1RI82 pp 229533.I1S8V3
135315 229533.I1RI82 pp 229533.I1RR24
135316 229533.I1RI82 pp 229533.I1S503
135317 229533.I1RI82 pp 229533.I1S2H4
135318 229533.I1RI82 pp 229533.I1RRT2
135319 229533.I1RI82 pp 229533.I1RTF4
135320 229533.I1RI82 pp 229533.I1RMC8
135321 229533.I1RI82 pp 229533.I1RX46
135322 229533.I1RI82 pp 229533.I1RJL4
135323 229533.I1RI82 pp 229533.I1RIL3
135324 229533.I1RI82 pp 229533.I1S0L4
135325 229533.I1RI82 pp 229533.I1RZ39
135326 229533.I1RI82 pp 229533.I1RQ23
135327 229533.I1RI82 pp 229533.I1RMK3
135328 229533.I1RI82 pp 229533.I1RJU3
135329 229533.I1RI82 pp 229533.V6RTJ8
135330 229533.I1RI82 pp 229533.I1S7A8
135331 229533.I1RI82 pp 229533.I1RNA0
135332 229533.I1RI82 pp 229533.I1RPM4
135333 229533.I1RI82 pp 229533.I1RN63
135334 229533.I1RI82 pp 229533.I1RR73
135335 229533.I1RI82 pp 229533.I1RWK3
135336 229533.I1RI82 pp 229533.V6R3X5
135337 229533.I1RI82 pp 229533.V6QYF0
135338 229533.I1RI82 pp 229533.I1RWJ8
135339 229533.I1RI82 pp 229533.I1RRG3
135340 229533.I1RI82 pp 229533.I1RM06
135341 229533.I1RI82 pp 229533.I1S0M6
135342 229533.I1RI82 pp 229533.I1S5R1

135343	229533.I1RI82	pp	229533.I1RRM5
135344	229533.I1RI82	pp	229533.I1RIL8
135345	229533.I1RI82	pp	229533.I1RJZ7
135346	229533.I1RI82	pp	229533.Q4IJT5
135347	229533.I1RI82	pp	229533.I1S0F6
135348	229533.I1RI82	pp	229533.I1RRT8
135349	229533.I1RI82	pp	229533.I1S352
135350	229533.I1RI82	pp	229533.I1RLC7
135351	229533.I1RI82	pp	229533.I1RW71
135352	229533.I1RI82	pp	229533.I1S2B8
135353	229533.I1RI82	pp	229533.V6R4J1
135354	229533.I1RI82	pp	229533.I1RX25
135355	229533.I1RI82	pp	229533.I1RW25
135356	229533.I1RI82	pp	229533.I1RVQ0
135357	229533.I1RI82	pp	229533.I1RW07
135358	229533.I1RI82	pp	229533.I1S8P5
135359	229533.I1RI82	pp	229533.V6R1K4
135360	229533.I1RI82	pp	229533.I1RS47
135361	229533.I1RI82	pp	229533.I1RPP3
135362	229533.I1RI82	pp	229533.I1RV91
135363	229533.I1RI82	pp	229533.I1S0L3
135364	229533.I1RI82	pp	229533.I1RRJ8
135365	229533.I1RI82	pp	229533.I1S0F5
135366	229533.I1RI82	pp	229533.V6QW22
135367	229533.I1RI82	pp	229533.I1RWD4
135368	229533.I1RI82	pp	229533.I1RSQ7
135369	229533.I1RI82	pp	229533.I1RN67
135370	229533.I1RX46	pp	229533.I1RYB9
135371	229533.I1RX46	pp	229533.I1RXF0
135372	229533.I1RX46	pp	229533.I1RZM8
135373	229533.I1RX46	pp	229533.I1S021
135374	229533.I1RX46	pp	229533.I1S6K9
135375	229533.I1RX46	pp	229533.I1RZM1
135376	229533.I1RX46	pp	229533.I1RZF6
135377	229533.I1RX46	pp	229533.V6RCG5
135378	229533.I1RX46	pp	229533.V6RCZ8
135379	229533.I1RX46	pp	229533.Q4I8P5
135380	229533.I1RX46	pp	229533.V6QUE6
135381	229533.I1RX46	pp	229533.I1SA32
135382	229533.I1RX46	pp	229533.Q4IEK8
135383	229533.I1RX46	pp	229533.I1RXC4
135384	229533.I1RX46	pp	229533.Q4IAA0
135385	229533.I1RX46	pp	229533.Q4IJ56
135386	229533.I1RX46	pp	229533.Q4I283
135387	229533.I1RX46	pp	229533.I1S419
135388	229533.I1RX46	pp	229533.I1S044
135389	229533.I1RX46	pp	229533.I1S5B0
135390	229533.I1RX46	pp	229533.I1S504
135391	229533.I1RX46	pp	229533.I1S887
135392	229533.I1RX46	pp	229533.I1S1J7
135393	229533.I1RX46	pp	229533.I1S0M9
135394	229533.I1RX46	pp	229533.I1RY03
135395	229533.I1RX46	pp	229533.I1RZK5
135396	229533.I1RX46	pp	229533.I1RZB5
135397	229533.I1RX46	pp	229533.I1RYS0
135398	229533.I1RX46	pp	229533.I1RZK6
135399	229533.I1RX46	pp	229533.I1S8I8
135400	229533.I1RX46	pp	229533.Q4IJK2
135401	229533.I1RX46	pp	229533.V6RUT5
135402	229533.I1RX46	pp	229533.I1S0R7
135403	229533.I1RX46	pp	229533.I1RZZ1
135404	229533.I1RX46	pp	229533.I1S4Q4
135405	229533.I1RX46	pp	229533.I1RXD7
135406	229533.I1RX46	pp	229533.Q4IPI1
135407	229533.I1RX46	pp	229533.Q4I2J8
135408	229533.I1RX46	pp	229533.V6R4J1
135409	229533.I1RX46	pp	229533.I1RZE5
135410	229533.I1RX46	pp	229533.I1RZ35
135411	229533.I1RX46	pp	229533.V6RTJ8
135412	229533.I1RX46	pp	229533.I1RX95
135413	229533.I1RX46	pp	229533.I1S0T2
135414	229533.I1RX46	pp	229533.I1S0L3
135415	229533.I1RX46	pp	229533.V6RBN2

135416	229533.I1RX46	pp	229533.I1S7A8
135417	229533.I1RX46	pp	229533.Q4IJT5
135418	229533.I1RX46	pp	229533.I1S4S7
135419	229533.I1RX46	pp	229533.V6QW22
135420	229533.I1RX46	pp	229533.I1S0F5
135421	229533.I1RX46	pp	229533.V6R1K4
135422	229533.I1RX46	pp	229533.I1S4S4
135423	229533.I1RX46	pp	229533.I1S9U2
135424	229533.I1RX46	pp	229533.I1S438
135425	229533.I1RX46	pp	229533.I1S540
135426	229533.I1RX46	pp	229533.I1RZJ5
135427	229533.I1RX46	pp	229533.I1SA33
135428	229533.I1RX46	pp	229533.I1S0X6
135429	229533.I1RX46	pp	229533.V6QUW4
135430	229533.I1RX46	pp	229533.I1S256
135431	229533.I1RX46	pp	229533.I1S1W3
135432	229533.I1RX46	pp	229533.I1RY44
135433	229533.I1RX46	pp	229533.V6RGN5
135434	229533.I1RX46	pp	229533.I1S5R6
135435	229533.I1RX46	pp	229533.I1RZV3
135436	229533.I1RX46	pp	229533.I1S6U8
135437	229533.I1RX46	pp	229533.I1S0N4
135438	229533.I1RX46	pp	229533.Q4HZB8
135439	229533.I1RX46	pp	229533.Q4IFI0
135440	229533.I1RX46	pp	229533.Q4I327
135441	229533.I1RX46	pp	229533.Q4HVW2
135442	229533.I1RX46	pp	229533.Q4IJH1
135443	229533.I1RX46	pp	229533.I1S1W9
135444	229533.I1RX46	pp	229533.I1RXU9
135445	229533.I1RX46	pp	229533.V6RIV9
135446	229533.I1RX46	pp	229533.Q4IN63
135447	229533.I1RX46	pp	229533.Q4I5V2
135448	229533.I1RX46	pp	229533.I1RZW8
135449	229533.I1RX46	pp	229533.I1S0J1
135450	229533.I1RX46	pp	229533.I1RZ79
135451	229533.I1RX46	pp	229533.I1S1W4
135452	229533.I1RX46	pp	229533.I1RXT3
135453	229533.I1RX46	pp	229533.V6R042
135454	229533.I1RX46	pp	229533.I1S7W5
135455	229533.I1RX46	pp	229533.I1S8R3
135456	229533.I1RX46	pp	229533.I1S060
135457	229533.I1RX46	pp	229533.I1S437
135458	229533.I1RX46	pp	229533.V6RMW6
135459	229533.I1RX46	pp	229533.I1RYI5
135460	229533.I1RX46	pp	229533.I1S2C0
135461	229533.I1RX46	pp	229533.I1S1C7
135462	229533.I1RX46	pp	229533.V6RRH2
135463	229533.I1RX46	pp	229533.I1RYW3
135464	229533.I1RX46	pp	229533.V6R057
135465	229533.I1RX46	pp	229533.V6R3J1
135466	229533.I1RX46	pp	229533.I1S352
135467	229533.I1RX46	pp	229533.Q4I624
135468	229533.I1RX46	pp	229533.I1S8P5
135469	229533.I1RX46	pp	229533.I1S569
135470	229533.I1RX46	pp	229533.I1S2E8
135471	229533.I1RX46	pp	229533.I1S5R1
135472	229533.I1RX46	pp	229533.I1RXR1
135473	229533.I1RX46	pp	229533.I1S503
135474	229533.I1RX46	pp	229533.Q4IAS1
135475	229533.I1RX46	pp	229533.I1RZZ9
135476	229533.I1RX46	pp	229533.O74638
135477	229533.I1RX46	pp	229533.I1S2E9
135478	229533.I1RX46	pp	229533.I1RXC2
135479	229533.I1RX46	pp	229533.Q4HXF0
135480	229533.I1RX46	pp	229533.I1RZV6
135481	229533.I1RX46	pp	229533.I1RY62
135482	229533.I1RX46	pp	229533.I1S0F6
135483	229533.I1RX46	pp	229533.V6R3X5
135484	229533.I1RX46	pp	229533.I1S8E2
135485	229533.I1RX46	pp	229533.V6RUY9
135486	229533.I1RX46	pp	229533.I1S2B8
135487	229533.I1RX46	pp	229533.Q4I662
135488	229533.I1RX46	pp	229533.I1S0W4

135489 229533.I1RX46 pp 229533.I1RZ39
135490 229533.I1RX46 pp 229533.I1S9Y1
135491 229533.I1RX46 pp 229533.I1S6Y8
135492 229533.I1RX46 pp 229533.I1S2H4
135493 229533.I1RX46 pp 229533.I1S0L4
135494 229533.I1RX46 pp 229533.I1RYR0
135495 229533.I1RX46 pp 229533.V6R283
135496 229533.I1RX46 pp 229533.I1RZS6
135497 229533.I1RX46 pp 229533.I1S0M6
135498 229533.I1RX46 pp 229533.Q4IF76
135499 229533.Q4IQM6 pp 229533.V6RLW4
135500 229533.Q4IQM6 pp 229533.V6RGI1
135501 229533.I1RNH6 pp 229533.I1RZS6
135502 229533.I1RNH6 pp 229533.I1S2B8
135503 229533.I1RNH6 pp 229533.I1S8E2
135504 229533.I1RNH6 pp 229533.I1RW25
135505 229533.I1RNH6 pp 229533.I1RX24
135506 229533.I1RNH6 pp 229533.Q4IFI0
135507 229533.I1RNH6 pp 229533.V6QYF0
135508 229533.I1RNH6 pp 229533.I1RTF4
135509 229533.I1RNH6 pp 229533.I1RT80
135510 229533.I1RNH6 pp 229533.I1RWH5
135511 229533.I1RNH6 pp 229533.Q4I662
135512 229533.I1RNH6 pp 229533.I1RRG3
135513 229533.I1RNH6 pp 229533.I1RZ39
135514 229533.I1RNH6 pp 229533.I1S9U2
135515 229533.I1RNH6 pp 229533.Q4IJH1
135516 229533.I1RNH6 pp 229533.I1RPW9
135517 229533.I1RNH6 pp 229533.V6R283
135518 229533.I1RNH6 pp 229533.I1RQS8
135519 229533.I1RNH6 pp 229533.I1RU69
135520 229533.I1RNH6 pp 229533.Q4IQW0
135521 229533.I1RNH6 pp 229533.I1RSL9
135522 229533.I1RNH6 pp 229533.I1RPG6
135523 229533.I1RNH6 pp 229533.Q4IF76
135524 229533.I1RNH6 pp 229533.I1RT05
135525 229533.I1RNH6 pp 229533.I1RPU0
135526 229533.I1RNH6 pp 229533.Q4IPI1
135527 229533.I1RNH6 pp 229533.I1RZV6
135528 229533.I1RNH6 pp 229533.I1S1B6
135529 229533.I1RNH6 pp 229533.I1S9Y1
135530 229533.I1RNH6 pp 229533.Q4HZ42
135531 229533.I1RNH6 pp 229533.I1RQ65
135532 229533.I1RNH6 pp 229533.I1RRT2
135533 229533.I1RNH6 pp 229533.I1S6F0
135534 229533.I1RNH6 pp 229533.I1S9Y0
135535 229533.I1RNH6 pp 229533.V6R3X5
135536 229533.I1RNH6 pp 229533.I1RQS5
135537 229533.I1RNH6 pp 229533.I1RS30
135538 229533.I1RNH6 pp 229533.I1RR73
135539 229533.I1RNH6 pp 229533.I1RRQ3
135540 229533.I1RNH6 pp 229533.Q4HVW2
135541 229533.I1RNH6 pp 229533.I1RX46
135542 229533.I1RNH6 pp 229533.I1RSQ7
135543 229533.I1RNH6 pp 229533.Q4HZ47
135544 229533.I1RNH6 pp 229533.I1S503
135545 229533.I1RNH6 pp 229533.Q4I5R9
135546 229533.I1RNH6 pp 229533.I1RRJ8
135547 229533.I1RNH6 pp 229533.I1S6U8
135548 229533.I1RNH6 pp 229533.Q4IAS1
135549 229533.I1RNH6 pp 229533.I1S253
135550 229533.I1RNH6 pp 229533.I1RPP3
135551 229533.I1RNH6 pp 229533.I1S296
135552 229533.I1RNH6 pp 229533.I1RWF7
135553 229533.I1RNH6 pp 229533.Q4HZ68
135554 229533.I1RNH6 pp 229533.I1RXD7
135555 229533.I1RNH6 pp 229533.I1RZ99
135556 229533.I1RNH6 pp 229533.I1RT04
135557 229533.I1RNH6 pp 229533.I1RUQ2
135558 229533.I1RNH6 pp 229533.I1RR00
135559 229533.I1RNH6 pp 229533.I1S0M3
135560 229533.I1RNH6 pp 229533.I1RQJ5
135561 229533.I1RNH6 pp 229533.I1RZ59

135562 229533.I1RNH6 pp 229533.I1S0R7
135563 229533.I1RNH6 pp 229533.I1S2L2
135564 229533.I1RNH6 pp 229533.V6RAC9
135565 229533.I1RNH6 pp 229533.I1RTD5
135566 229533.I1RNH6 pp 229533.Q4IP51
135567 229533.I1RNH6 pp 229533.I1RZE5
135568 229533.I1RNH6 pp 229533.I1RW71
135569 229533.I1RNH6 pp 229533.Q4I830
135570 229533.I1RNH6 pp 229533.I1S8I8
135571 229533.I1RNH6 pp 229533.I1RYB1
135572 229533.I1RNH6 pp 229533.I1S2M6
135573 229533.I1RNH6 pp 229533.I1S0Z9
135574 229533.I1RNH6 pp 229533.Q4INZ9
135575 229533.I1RNH6 pp 229533.I1RVZ0
135576 229533.I1RNH6 pp 229533.I1RT98
135577 229533.I1RNH6 pp 229533.I1RZJ6
135578 229533.I1RNH6 pp 229533.Q4IAA0
135579 229533.I1RNH6 pp 229533.I1RNK2
135580 229533.I1RNH6 pp 229533.I1RQA2
135581 229533.I1RNH6 pp 229533.I1RQ28
135582 229533.I1RNH6 pp 229533.I1RWB5
135583 229533.I1RNH6 pp 229533.Q4HWE2
135584 229533.I1RNH6 pp 229533.I1RNU6
135585 229533.I1RNH6 pp 229533.I1RTE9
135586 229533.I1RNH6 pp 229533.I1RSC6
135587 229533.I1RNH6 pp 229533.I1S2C0
135588 229533.I1RNH6 pp 229533.I1RY63
135589 229533.I1RNH6 pp 229533.I1S0M9
135590 229533.I1RNH6 pp 229533.I1S1X9
135591 229533.I1RNH6 pp 229533.I1RZK5
135592 229533.I1RNH6 pp 229533.Q4IJ56
135593 229533.I1RNH6 pp 229533.I1RVX0
135594 229533.I1RNH6 pp 229533.I1S0J1
135595 229533.I1RNH6 pp 229533.I1RSG2
135596 229533.I1RNH6 pp 229533.I1RTH6
135597 229533.I1RNH6 pp 229533.I1RX14
135598 229533.I1RNH6 pp 229533.I1S060
135599 229533.I1RNH6 pp 229533.I1RRI2
135600 229533.I1RNH6 pp 229533.I1RT03
135601 229533.I1RNH6 pp 229533.I1RZZ1
135602 229533.I1RNH6 pp 229533.I1S044
135603 229533.I1RNH6 pp 229533.I1S2E8
135604 229533.I1RNH6 pp 229533.I1RY03
135605 229533.I1RNH6 pp 229533.V6RGN5
135606 229533.I1RNH6 pp 229533.I1RSR8
135607 229533.I1RNH6 pp 229533.I1RTD9
135608 229533.I1RNH6 pp 229533.I1RZK6
135609 229533.I1RNH6 pp 229533.V6QUE6
135610 229533.I1RNH6 pp 229533.I1S2U2
135611 229533.I1RNH6 pp 229533.Q4IBS2
135612 229533.I1RNH6 pp 229533.I1RSD8
135613 229533.I1RNH6 pp 229533.I1S1C7
135614 229533.I1RNH6 pp 229533.I1RSR3
135615 229533.I1RNH6 pp 229533.V6RMW6
135616 229533.I1RNH6 pp 229533.I1RRR7
135617 229533.I1RNH6 pp 229533.V6RGY3
135618 229533.I1RNH6 pp 229533.V6R227
135619 229533.I1RNH6 pp 229533.V6R042
135620 229533.I1RNH6 pp 229533.I1RWN6
135621 229533.I1RNH6 pp 229533.I1S504
135622 229533.I1RNH6 pp 229533.I1RRY9
135623 229533.I1RNH6 pp 229533.I1S9U1
135624 229533.I1RNH6 pp 229533.I1RSI6
135625 229533.I1RNH6 pp 229533.I1RYB9
135626 229533.I1RNH6 pp 229533.I1RX08
135627 229533.I1RNH6 pp 229533.I1RZF6
135628 229533.I1RNH6 pp 229533.I1S021
135629 229533.I1RNH6 pp 229533.I1S274
135630 229533.I1RNH6 pp 229533.I1RNY2
135631 229533.I1RNH6 pp 229533.Q4IEK8
135632 229533.I1RNH6 pp 229533.I1RNQ5
135633 229533.I1RNH6 pp 229533.I1S4T3
135634 229533.I1RNH6 pp 229533.I1RPY1

135635 229533.I1RNH6 pp 229533.Q4I327
135636 229533.I1RNH6 pp 229533.Q4IN63
135637 229533.I1RNH6 pp 229533.I1RWN5
135638 229533.I1RNH6 pp 229533.I1RQ31
135639 229533.I1RLQ8 pp 229533.I1S2N3
135640 229533.I1RLQ8 pp 229533.I1S2K2
135641 229533.I1RLQ8 pp 229533.I1RXM0
135642 229533.Q4I6A4 pp 229533.Q4IR08
135643 229533.Q4I6A4 pp 229533.Q4I7L0
135644 229533.Q4I6A4 pp 229533.Q4IER8
135645 229533.Q4I6A4 pp 229533.Q4IJ84
135646 229533.Q4I6A4 pp 229533.Q4IEV4
135647 229533.I1RCC2 pp 229533.I1REM2
135648 229533.I1RCC2 pp 229533.I1S2M5
135649 229533.I1RCC2 pp 229533.I1RQ76
135650 229533.I1RCC2 pp 229533.I1S1Q3
135651 229533.I1RCC2 pp 229533.I1RJR2
135652 229533.I1RCC2 pp 229533.V6RR38
135653 229533.I1RCC2 pp 229533.I1RQC9
135654 229533.I1RCC2 pp 229533.Q4IJK2
135655 229533.I1RCC2 pp 229533.I1RJW1
135656 229533.I1RCC2 pp 229533.I1RR39
135657 229533.I1RCC2 pp 229533.I1RGK2
135658 229533.I1RCC2 pp 229533.I1RT23
135659 229533.I1RCC2 pp 229533.I1RNX0
135660 229533.I1RCC2 pp 229533.I1RRY9
135661 229533.I1RCC2 pp 229533.I1RY65
135662 229533.I1RCC2 pp 229533.I1RIE8
135663 229533.I1RCC2 pp 229533.I1REL9
135664 229533.I1RCC2 pp 229533.V6RPN0
135665 229533.I1RCC2 pp 229533.I1RR73
135666 229533.I1RCC2 pp 229533.I1S2C2
135667 229533.I1RCC2 pp 229533.I1S2B8
135668 229533.I1RCC2 pp 229533.I1RJS0
135669 229533.I1RCC2 pp 229533.Q4I1Y5
135670 229533.I1RCC2 pp 229533.I1RY35
135671 229533.I1RCC2 pp 229533.I1RSX9
135672 229533.I1RCC2 pp 229533.I1RZ39
135673 229533.I1RCC2 pp 229533.I1RTH6
135674 229533.I1RCC2 pp 229533.I1RJG1
135675 229533.I1RCC2 pp 229533.I1S5K2
135676 229533.I1RCC2 pp 229533.I1RN67
135677 229533.I1RCC2 pp 229533.I1SAS3
135678 229533.I1RCC2 pp 229533.I1RWN5
135679 229533.I1RCC2 pp 229533.I1RPP4
135680 229533.I1RCC2 pp 229533.I1RTD5
135681 229533.I1RCC2 pp 229533.I1RIZ8
135682 229533.I1RCC2 pp 229533.I1RUP5
135683 229533.I1RCC2 pp 229533.I1SAS4
135684 229533.I1RCC2 pp 229533.I1RWN6
135685 229533.I1RCC2 pp 229533.I1RNT1
135686 229533.I1RCC2 pp 229533.I1RNC1
135687 229533.I1RCC2 pp 229533.I1RFM2
135688 229533.I1RCC2 pp 229533.I1RK44
135689 229533.I1RCC2 pp 229533.V6RMW6
135690 229533.I1RCC2 pp 229533.I1RD22
135691 229533.I1RCC2 pp 229533.I1RSQ3
135692 229533.A0A098DDX5 pp 229533.A0A1C3YIP6
135693 229533.A0A098DDX5 pp 229533.I1RHU0
135694 229533.A0A098DDX5 pp 229533.A0A1C3YKT9
135695 229533.A0A098DDX5 pp 229533.I1RSG9
135696 229533.A0A098DDX5 pp 229533.A0A098DTU2
135697 229533.A0A098DDX5 pp 229533.I1S270
135698 229533.A0A098DDX5 pp 229533.A0A1C3YIQ1
135699 229533.A0A098DDX5 pp 229533.I1RT41
135700 229533.A0A098DDX5 pp 229533.I1RRF9
135701 229533.A0A098DDX5 pp 229533.I1RXB8
135702 229533.A0A098DDX5 pp 229533.I1RN81
135703 229533.A0A098DDX5 pp 229533.A0A1C3YIM8
135704 229533.A0A098DDX5 pp 229533.I1S275
135705 229533.A0A098DDX5 pp 229533.I1R9N6
135706 229533.A0A098DDX5 pp 229533.I1RA34
135707 229533.A0A098DDX5 pp 229533.I1RAL2

135708 229533.A0A098DDX5 pp 229533.I1RKR9
135709 229533.A0A098DDX5 pp 229533.A0A1C3YL06
135710 229533.A0A098DDX5 pp 229533.I1S0Z3
135711 229533.A0A098DDX5 pp 229533.I1S045
135712 229533.A0A098DDX5 pp 229533.I1RE19
135713 229533.A0A098DDX5 pp 229533.A0A1C3YJP1
135714 229533.A0A098DDX5 pp 229533.I1RT81
135715 229533.A0A098DDX5 pp 229533.V6RR76
135716 229533.A0A098DDX5 pp 229533.A0A1C3YM61
135717 229533.A0A098DDX5 pp 229533.I1RMY3
135718 229533.A0A098DDX5 pp 229533.I1RMP2
135719 229533.A0A098DDX5 pp 229533.Q4I963
135720 229533.A0A098DDX5 pp 229533.I1RSZ6
135721 229533.A0A098DDX5 pp 229533.I1RZL3
135722 229533.A0A098DDX5 pp 229533.I1RTE0
135723 229533.A0A098DDX5 pp 229533.I1RA31
135724 229533.A0A098DDX5 pp 229533.A0A1C3YJJ1
135725 229533.A0A098DDX5 pp 229533.I1RTA3
135726 229533.A0A098DDX5 pp 229533.I1RMW3
135727 229533.A0A098DDX5 pp 229533.I1RD10
135728 229533.A0A098DDX5 pp 229533.I1RSL8
135729 229533.A0A098DDX5 pp 229533.I1RTG5
135730 229533.A0A1C3YIB6 pp 229533.I1RVF8
135731 229533.A0A1C3YIB6 pp 229533.I1S444
135732 229533.A0A1C3YIB6 pp 229533.I1RYZ4
135733 229533.A0A1C3YIB6 pp 229533.I1RGU7
135734 229533.A0A1C3YIB6 pp 229533.I1RNA0
135735 229533.A0A1C3YIB6 pp 229533.A0A1C3YIN1
135736 229533.A0A1C3YJH8 pp 229533.I1RAU7
135737 229533.A0A1C3YJH8 pp 229533.I1RD17
135738 229533.A0A1C3YJH8 pp 229533.I1RCH5
135739 229533.A0A1C3YJH8 pp 229533.I1RZE4
135740 229533.A0A1C3YJH8 pp 229533.I1RDH0
135741 229533.A0A1C3YJH8 pp 229533.I1RY58
135742 229533.A0A1C3YJH8 pp 229533.I1RFL4
135743 229533.A0A1C3YJH8 pp 229533.I1RQE7
135744 229533.A0A1C3YJH8 pp 229533.I1RN18
135745 229533.A0A1C3YJH8 pp 229533.I1RTH6
135746 229533.A0A1C3YJH8 pp 229533.I1RB92
135747 229533.A0A1C3YJH8 pp 229533.I1S0E8
135748 229533.A0A1C3YJH8 pp 229533.I1S8R3
135749 229533.A0A1C3YJH8 pp 229533.I1RPW0
135750 229533.A0A1C3YJH8 pp 229533.I1RJT8
135751 229533.A0A1C3YJH8 pp 229533.I1RY44
135752 229533.A0A1C3YJH8 pp 229533.V6RVG3
135753 229533.A0A1C3YJH8 pp 229533.I1RVX0
135754 229533.A0A1C3YJH8 pp 229533.V6RIV9
135755 229533.A0A1C3YJH8 pp 229533.I1RT03
135756 229533.A0A1C3YJH8 pp 229533.I1RRN5
135757 229533.A0A1C3YJH8 pp 229533.I1RWN5
135758 229533.A0A1C3YJH8 pp 229533.I1R9Y7
135759 229533.A0A1C3YJH8 pp 229533.I1RTN0
135760 229533.A0A1C3YJH8 pp 229533.I1RRY3
135761 229533.A0A1C3YJH8 pp 229533.V6RKY7
135762 229533.A0A1C3YJH8 pp 229533.I1RD15
135763 229533.A0A1C3YJH8 pp 229533.A0A1C3YM29
135764 229533.A0A1C3YJH8 pp 229533.I1RW84
135765 229533.A0A1C3YJH8 pp 229533.I1RBJ0
135766 229533.A0A1C3YJH8 pp 229533.I1RJV3
135767 229533.A0A1C3YJH8 pp 229533.I1RWN6
135768 229533.A0A1C3YJH8 pp 229533.A0A1C3YL52
135769 229533.A0A1C3YJH8 pp 229533.I1RAW0
135770 229533.A0A1C3YJH8 pp 229533.I1RRI2
135771 229533.A0A1C3YJH8 pp 229533.I1RA56
135772 229533.A0A1C3YJH8 pp 229533.A0A1C3YMC9
135773 229533.A0A1C3YJH8 pp 229533.I1RRE5
135774 229533.A0A1C3YJH8 pp 229533.I1RZW8
135775 229533.A0A1C3YJH8 pp 229533.I1RD22
135776 229533.A0A1C3YJH8 pp 229533.I1RW32
135777 229533.A0A1C3YJH8 pp 229533.I1S1W4
135778 229533.A0A1C3YJH8 pp 229533.A0A1C3YLN8
135779 229533.A0A1C3YJH8 pp 229533.I1SA33
135780 229533.A0A1C3YJH8 pp 229533.I1RNY8

229533.A0A1C3YJH8	pp	229533.I1S1C7
229533.A0A1C3YJH8	pp	229533.I1RA70
229533.A0A1C3YJH8	pp	229533.I1S9S6
229533.A0A1C3YJH8	pp	229533.I1RQB4
229533.A0A1C3YJH8	pp	229533.I1R9Y9
229533.A0A1C3YJH8	pp	229533.V6RGI1
229533.A0A1C3YJH8	pp	229533.I1S0J1
229533.A0A1C3YJH8	pp	229533.I1RE01
229533.A0A1C3YJH8	pp	229533.A0A1C3YMI5
229533.A0A1C3YJH8	pp	229533.I1RZ91
229533.A0A1C3YJH8	pp	229533.I1RW46
229533.A0A1C3YJH8	pp	229533.I1RNC1
229533.A0A1C3YJH8	pp	229533.V6R042
229533.A0A1C3YJH8	pp	229533.I1RUG3
229533.A0A1C3YJH8	pp	229533.I1RPP4
229533.A0A1C3YJH8	pp	229533.Q4I7L0
229533.A0A1C3YJH8	pp	229533.I1RSQ3
229533.A0A1C3YJH8	pp	229533.V6RMW6
229533.A0A1C3YJH8	pp	229533.I1S0M2
229533.A0A1C3YJH8	pp	229533.I1RPK4
229533.A0A1C3YJH8	pp	229533.I1RCR8
229533.A0A1C3YJH8	pp	229533.I1RX38
229533.A0A1C3YJH8	pp	229533.I1RBW1
229533.A0A1C3YJH8	pp	229533.I1RRV1
229533.A0A1C3YJH8	pp	229533.I1RUN8
229533.A0A1C3YJH8	pp	229533.I1RPM4
229533.A0A1C3YJH8	pp	229533.I1RPU3
229533.A0A1C3YJH8	pp	229533.I1S2H4
229533.A0A1C3YJH8	pp	229533.I1RB59
229533.A0A1C3YJH8	pp	229533.I1R9M0
229533.A0A1C3YJH8	pp	229533.I1RBY4
229533.A0A1C3YJH8	pp	229533.I1RE15
229533.A0A1C3YJH8	pp	229533.I1RGD1
229533.A0A1C3YJH8	pp	229533.I1RJZ7
229533.A0A1C3YJH8	pp	229533.I1RU69
229533.A0A1C3YJH8	pp	229533.I1S0J7
229533.A0A1C3YJH8	pp	229533.I1S0X7
229533.A0A1C3YJH8	pp	229533.I1RWI5
229533.A0A1C3YJH8	pp	229533.I1RRM5
229533.A0A1C3YJH8	pp	229533.I1S0M6
229533.A0A1C3YJH8	pp	229533.I1RD33
229533.A0A1C3YJH8	pp	229533.I1S2B8
229533.A0A1C3YJH8	pp	229533.I1RZZ9
229533.A0A1C3YJH8	pp	229533.I1S549
229533.A0A1C3YJH8	pp	229533.I1RPX5
229533.A0A1C3YJH8	pp	229533.I1RMF5
229533.A0A1C3YJH8	pp	229533.I1RC71
229533.A0A1C3YJH8	pp	229533.I1RAN2
229533.A0A1C3YJH8	pp	229533.I1RZ63
229533.A0A1C3YJH8	pp	229533.I1RW83
229533.A0A1C3YJH8	pp	229533.I1RE22
229533.A0A1C3YJH8	pp	229533.I1S7X9
229533.A0A1C3YJH8	pp	229533.I1RR73
229533.A0A1C3YJH8	pp	229533.V6R3X5
229533.A0A1C3YJH8	pp	229533.I1S5R1
229533.A0A1C3YJH8	pp	229533.I1RRT8
229533.A0A1C3YJH8	pp	229533.I1RB74
229533.A0A1C3YJH8	pp	229533.I1RNP7
229533.A0A1C3YJH8	pp	229533.I1RPP3
229533.A0A1C3YJH8	pp	229533.I1RNR3
229533.A0A1C3YJH8	pp	229533.A0A1C3YK17
229533.A0A1C3YJH8	pp	229533.I1RSV2
229533.A0A1C3YJH8	pp	229533.I1RFL7
229533.A0A1C3YJH8	pp	229533.I1RZ39
229533.A0A1C3YJH8	pp	229533.I1RLU1
229533.A0A1C3YJH8	pp	229533.I1R9N1
229533.A0A1C3YJH8	pp	229533.A0A1C3YKM9
229533.A0A1C3YJH8	pp	229533.I1RXU1
229533.A0A1C3YJH8	pp	229533.I1REN4
229533.A0A1C3YJH8	pp	229533.I1RN67
229533.A0A1C3YJH8	pp	229533.I1RGF1
229533.A0A1C3YJH8	pp	229533.I1RJW6
229533.A0A1C3YJH8	pp	229533.I1REF9

135854 229533.A0A1C3YJH8 pp 229533.I1S503
135855 229533.A0A1C3YJH8 pp 229533.A0A1C3YLK8
135856 229533.A0A1C3YJH8 pp 229533.I1RV91
135857 229533.A0A1C3YJH8 pp 229533.I1RCJ7
135858 229533.A0A1C3YJH8 pp 229533.I1RT40
135859 229533.A0A1C3YJH8 pp 229533.I1S0F6
135860 229533.A0A1C3YJH8 pp 229533.I1RP27
135861 229533.A0A1C3YJH8 pp 229533.I1RNF4
135862 229533.A0A1C3YJH8 pp 229533.I1RY29
135863 229533.A0A1C3YJH8 pp 229533.I1RJV4
135864 229533.A0A1C3YJH8 pp 229533.I1RMP0
135865 229533.A0A1C3YJH8 pp 229533.I1RT28
135866 229533.A0A1C3YJH8 pp 229533.A0A1C3YKT6
135867 229533.A0A1C3YJH8 pp 229533.I1S098
135868 229533.A0A1C3YJH8 pp 229533.I1RBS6
135869 229533.A0A1C3YJH8 pp 229533.I1RGA4
135870 229533.A0A1C3YJH8 pp 229533.I1RC97
135871 229533.A0A1C3YJH8 pp 229533.I1RW76
135872 229533.A0A1C3YJH8 pp 229533.I1RSZ3
135873 229533.A0A1C3YJH8 pp 229533.I1S2E9
135874 229533.A0A1C3YJH8 pp 229533.I1RBV5
135875 229533.A0A1C3YJH8 pp 229533.I1RZP1
135876 229533.A0A1C3YJH8 pp 229533.I1RT04
135877 229533.A0A1C3YJH8 pp 229533.I1RTD5
135878 229533.A0A1C3YJH8 pp 229533.I1RC22
135879 229533.A0A1C3YJH8 pp 229533.I1RWK3
135880 229533.A0A1C3YJH8 pp 229533.I1RUQ5
135881 229533.A0A1C3YJH8 pp 229533.I1RSI0
135882 229533.A0A1C3YJH8 pp 229533.Q4IR87
135883 229533.A0A1C3YJH8 pp 229533.I1RZZ1
135884 229533.A0A1C3YJH8 pp 229533.I1RY03
135885 229533.A0A1C3YJH8 pp 229533.I1RQA3
135886 229533.A0A1C3YJH8 pp 229533.I1S439
135887 229533.A0A1C3YJH8 pp 229533.I1RZK5
135888 229533.A0A1C3YJH8 pp 229533.Q4IJK2
135889 229533.A0A1C3YJH8 pp 229533.I1RB75
135890 229533.A0A1C3YJH8 pp 229533.I1S0N8
135891 229533.A0A1C3YJH8 pp 229533.I1REK8
135892 229533.A0A1C3YJH8 pp 229533.I1S1B1
135893 229533.A0A1C3YJH8 pp 229533.A0A1C3YJW1
135894 229533.A0A1C3YJH8 pp 229533.I1RMM9
135895 229533.A0A1C3YJH8 pp 229533.I1RFU8
135896 229533.A0A1C3YJH8 pp 229533.I1RN08
135897 229533.A0A1C3YJH8 pp 229533.I1RMS2
135898 229533.A0A1C3YJH8 pp 229533.I1RNQ5
135899 229533.A0A1C3YJH8 pp 229533.I1RCG4
135900 229533.A0A1C3YJH8 pp 229533.A0A1C3YKB0
135901 229533.A0A1C3YJH8 pp 229533.I1RWT6
135902 229533.A0A1C3YJH8 pp 229533.I1S504
135903 229533.A0A1C3YJH8 pp 229533.I1RRY9
135904 229533.A0A1C3YJH8 pp 229533.I1RHB0
135905 229533.A0A1C3YJH8 pp 229533.I1RLU6
135906 229533.A0A1C3YJH8 pp 229533.I1SA32
135907 229533.A0A1C3YJH8 pp 229533.I1RZM1
135908 229533.A0A1C3YJH8 pp 229533.I1RS38
135909 229533.A0A1C3YJH8 pp 229533.I1RAM7
135910 229533.A0A1C3YJH8 pp 229533.V6RCZ8
135911 229533.A0A1C3YJH8 pp 229533.I1RLT6
135912 229533.A0A1C3YJH8 pp 229533.I1RFT2
135913 229533.A0A1C3YJH8 pp 229533.I1RX09
135914 229533.A0A1C3YJH8 pp 229533.A0A1C3YK34
135915 229533.A0A1C3YJH8 pp 229533.I1RMQ7
135916 229533.A0A1C3YJH8 pp 229533.I1RAU5
135917 229533.A0A1C3YJH8 pp 229533.I1S5B0
135918 229533.A0A1C3YJH8 pp 229533.I1RAU6
135919 229533.A0A1C3YJH8 pp 229533.Q4HTT2
135920 229533.A0A1C3YJH8 pp 229533.I1RS18
135921 229533.A0A1C3YJH8 pp 229533.I1RBW0
135922 229533.A0A1C3YJH8 pp 229533.I1RW99
135923 229533.A0A1C3YJH8 pp 229533.V6RR76
135924 229533.A0A1C3YJH8 pp 229533.I1RYH9
135925 229533.A0A1C3YJH8 pp 229533.I1RMR7
135926 229533.A0A1C3YJH8 pp 229533.Q4I8P5

135927 229533.A0A1C3YJH8 pp 229533.A0A1C3YJQ9
135928 229533.A0A1C3YJH8 pp 229533.I1RKK0
135929 229533.A0A1C3YJH8 pp 229533.I1RW25
135930 229533.A0A1C3YJH8 pp 229533.A0A1C3YK51
135931 229533.A0A1C3YJH8 pp 229533.I1RPU4
135932 229533.A0A1C3YJH8 pp 229533.I1RI82
135933 229533.A0A1C3YJH8 pp 229533.I1RCB3
135934 229533.A0A1C3YJH8 pp 229533.Q4ICG5
135935 229533.A0A1C3YJH8 pp 229533.I1S267
135936 229533.A0A1C3YJH8 pp 229533.A0A1C3YKV3
135937 229533.A0A1C3YJH8 pp 229533.I1RQ81
135938 229533.A0A1C3YJH8 pp 229533.I1RC91
135939 229533.A0A1C3YJH8 pp 229533.I1RZM8
135940 229533.A0A1C3YJH8 pp 229533.V6RCG5
135941 229533.A0A1C3YJH8 pp 229533.I1RC95
135942 229533.A0A1C3YJH8 pp 229533.I1RL24
135943 229533.A0A1C3YJH8 pp 229533.I1RRT1
135944 229533.A0A1C3YJH8 pp 229533.I1RN81
135945 229533.A0A1C3YJH8 pp 229533.I1RFQ7
135946 229533.A0A1C3YJH8 pp 229533.A0A1C3YJV7
135947 229533.A0A1C3YJH8 pp 229533.I1RD69
135948 229533.A0A1C3YJH8 pp 229533.A0A1C3YKF2
135949 229533.A0A1C3YJH8 pp 229533.I1RV66
135950 229533.A0A1C3YJH8 pp 229533.I1S635
135951 229533.A0A1C3YJH8 pp 229533.I1RBT1
135952 229533.A0A1C3YJH8 pp 229533.I1RMR5
135953 229533.A0A1C3YJH8 pp 229533.A0A1C3YJT5
135954 229533.A0A1C3YJH8 pp 229533.I1RWJ8
135955 229533.A0A1C3YJH8 pp 229533.I1S0X6
135956 229533.A0A1C3YJH8 pp 229533.I1RQ26
135957 229533.A0A1C3YJH8 pp 229533.I1RR24
135958 229533.A0A1C3YJH8 pp 229533.I1RFZ3
135959 229533.A0A1C3YJH8 pp 229533.I1RJI1
135960 229533.A0A1C3YJH8 pp 229533.I1RX70
135961 229533.A0A1C3YJH8 pp 229533.I1S1W5
135962 229533.A0A1C3YJH8 pp 229533.A0A1C3YKC0
135963 229533.A0A1C3YJH8 pp 229533.I1RW38
135964 229533.A0A1C3YJH8 pp 229533.I1RDB1
135965 229533.A0A1C3YJH8 pp 229533.I1RJU3
135966 229533.A0A1C3YJH8 pp 229533.I1RVQ0
135967 229533.A0A1C3YJH8 pp 229533.V6QW22
135968 229533.A0A1C3YJH8 pp 229533.I1RN61
135969 229533.A0A1C3YJH8 pp 229533.A0A1C3YJX0
135970 229533.A0A1C3YJH8 pp 229533.I1RSQ7
135971 229533.A0A1C3YJH8 pp 229533.I1RYI3
135972 229533.A0A1C3YJH8 pp 229533.A0A1C3YK00
135973 229533.A0A1C3YJH8 pp 229533.I1S0L3
135974 229533.A0A1C3YJH8 pp 229533.I1RD52
135975 229533.A0A1C3YJH8 pp 229533.I1RW71
135976 229533.A0A1C3YJH8 pp 229533.I1RBC9
135977 229533.A0A1C3YJH8 pp 229533.V6QV09
135978 229533.A0A1C3YJH8 pp 229533.I1RB36
135979 229533.A0A1C3YJH8 pp 229533.I1RX46
135980 229533.A0A1C3YJH8 pp 229533.A0A1C3YJR8
135981 229533.A0A1C3YJH8 pp 229533.I1RYZ9
135982 229533.A0A1C3YJH8 pp 229533.I1RAT7
135983 229533.A0A1C3YJH8 pp 229533.I1RAX6
135984 229533.A0A1C3YJH8 pp 229533.V6RTJ8
135985 229533.A0A1C3YJH8 pp 229533.I1RXD7
135986 229533.A0A1C3YJH8 pp 229533.I1S0R7
135987 229533.A0A1C3YJH8 pp 229533.I1RQC9
135988 229533.A0A098DTE3 pp 229533.A0A1C3YI19
135989 229533.A0A098DTE3 pp 229533.I1RZ78
135990 229533.A0A098DTE3 pp 229533.I1RLP9
135991 229533.A0A098DTE3 pp 229533.I1RZ63
135992 229533.A0A098DTE3 pp 229533.I1RF64
135993 229533.A0A098DTE3 pp 229533.I1S5N3
135994 229533.A0A098DTE3 pp 229533.I1RTA0
135995 229533.A0A098DTE3 pp 229533.I1S8P5
135996 229533.A0A098DTE3 pp 229533.I1RVF8
135997 229533.A0A098DTE3 pp 229533.I1S444
135998 229533.A0A098DTE3 pp 229533.A0A1C3YKF3
135999 229533.I1S021 pp 229533.V6RGY3

136000 229533.I1S021 pp 229533.I1S060
136001 229533.I1S021 pp 229533.I1S2E8
136002 229533.I1S021 pp 229533.Q4HZ68
136003 229533.I1S021 pp 229533.I1S296
136004 229533.I1S021 pp 229533.I1S0J1
136005 229533.I1S021 pp 229533.I1S2U2
136006 229533.I1S021 pp 229533.I1S0M9
136007 229533.I1S021 pp 229533.I1S1X9
136008 229533.I1S021 pp 229533.I1S4T3
136009 229533.I1S021 pp 229533.Q4IJ56
136010 229533.I1S021 pp 229533.I1S1C7
136011 229533.I1S021 pp 229533.Q4HWE2
136012 229533.I1S021 pp 229533.Q4IEK8
136013 229533.I1S021 pp 229533.Q4IBS2
136014 229533.I1S021 pp 229533.I1S2C0
136015 229533.I1S021 pp 229533.Q4HZK7
136016 229533.I1S021 pp 229533.V6RMW6
136017 229533.I1S021 pp 229533.I1S274
136018 229533.I1S021 pp 229533.Q4I5I4
136019 229533.I1S021 pp 229533.I1S9U1
136020 229533.I1S021 pp 229533.I1S558
136021 229533.I1S021 pp 229533.I1S504
136022 229533.I1S021 pp 229533.Q4I662
136023 229533.I1S021 pp 229533.I1S8E2
136024 229533.I1S021 pp 229533.Q4IJH1
136025 229533.I1S021 pp 229533.V6RGN5
136026 229533.I1S021 pp 229533.V6R227
136027 229533.I1S021 pp 229533.I1S044
136028 229533.I1S021 pp 229533.I1S6U8
136029 229533.I1S021 pp 229533.V6QYF0
136030 229533.I1S021 pp 229533.Q4I327
136031 229533.I1S021 pp 229533.V6R3X5
136032 229533.I1S021 pp 229533.Q4IN63
136033 229533.I1S021 pp 229533.I1S9U2
136034 229533.I1S021 pp 229533.I1S2B8
136035 229533.I1S021 pp 229533.Q4IFI0
136036 229533.I1S021 pp 229533.Q4HVW2
136037 229533.I1S021 pp 229533.V6R042
136038 229533.I1S021 pp 229533.I1S503
136039 229533.I1S021 pp 229533.Q4I830
136040 229533.I1S021 pp 229533.Q4IAS1
136041 229533.I1S021 pp 229533.Q4I5R9
136042 229533.I1S021 pp 229533.Q4IF76
136043 229533.I1S021 pp 229533.V6R283
136044 229533.I1S021 pp 229533.I1S6F0
136045 229533.I1S021 pp 229533.I1S1B6
136046 229533.I1S021 pp 229533.Q4HZ42
136047 229533.I1S021 pp 229533.Q4HZ47
136048 229533.I1S021 pp 229533.Q4IPI1
136049 229533.I1S021 pp 229533.I1S0R7
136050 229533.I1S021 pp 229533.Q4IQW0
136051 229533.I1S021 pp 229533.I1S2E9
136052 229533.A0A1C3YHM3 pp 229533.I1RN57
136053 229533.A0A1C3YHM3 pp 229533.A0A1C3YN69
136054 229533.A0A1C3YHM3 pp 229533.Q4IB89
136055 229533.A0A1C3YHM3 pp 229533.I1RC54
136056 229533.A0A1C3YHM3 pp 229533.I1RBC8
136057 229533.A0A1C3YHM3 pp 229533.I1RRS7
136058 229533.A0A1C3YHM3 pp 229533.A0A1C3YL34
136059 229533.A0A1C3YHM3 pp 229533.I1RZ98
136060 229533.A0A1C3YHM3 pp 229533.I1RQI6
136061 229533.A0A1C3YHM3 pp 229533.I1RSC7
136062 229533.A0A1C3YHM3 pp 229533.I1RUG4
136063 229533.A0A1C3YHM3 pp 229533.I1RP99
136064 229533.A0A1C3YHM3 pp 229533.I1RDJ8
136065 229533.A0A1C3YHM3 pp 229533.I1RD19
136066 229533.A0A1C3YHM3 pp 229533.I1RA10
136067 229533.A0A1C3YHM3 pp 229533.I1RMJ8
136068 229533.A0A1C3YHM3 pp 229533.I1RYR2
136069 229533.A0A1C3YHM3 pp 229533.I1RDX0
136070 229533.A0A1C3YHM3 pp 229533.I1RZT1
136071 229533.A0A1C3YHM3 pp 229533.I1RW24
136072 229533.A0A1C3YHM3 pp 229533.I1S0D5

136073 229533.A0A1C3YHM3 pp 229533.I1S4P6
136074 229533.A0A1C3YHM3 pp 229533.I1RYX9
136075 229533.A0A1C3YHM3 pp 229533.I1RCA5
136076 229533.A0A1C3YHM3 pp 229533.I1RSR9
136077 229533.A0A1C3YHM3 pp 229533.I1RYQ1
136078 229533.A0A1C3YHM3 pp 229533.A0A1C3YHN3
136079 229533.A0A1C3YHM3 pp 229533.I1RTN6
136080 229533.A0A1C3YHM3 pp 229533.A0A1C3YI39
136081 229533.A0A1C3YHM3 pp 229533.I1RBW1
136082 229533.A0A1C3YHM3 pp 229533.I1RDC0
136083 229533.A0A1C3YHM3 pp 229533.I1RJV8
136084 229533.A0A1C3YHM3 pp 229533.I1R9W7
136085 229533.A0A1C3YHM3 pp 229533.A0A1C3YLQ7
136086 229533.A0A1C3YHM3 pp 229533.I1RA12
136087 229533.A0A1C3YHM3 pp 229533.I1S209
136088 229533.A0A1C3YHM3 pp 229533.I1RZP8
136089 229533.A0A1C3YHM3 pp 229533.I1RVV5
136090 229533.A0A1C3YHM3 pp 229533.Q4IJ84
136091 229533.A0A1C3YHM3 pp 229533.A0A1C3YL55
136092 229533.A0A1C3YHM3 pp 229533.I1RYR1
136093 229533.A0A1C3YHM3 pp 229533.I1RWI8
136094 229533.A0A1C3YHM3 pp 229533.I1RMX3
136095 229533.A0A1C3YHM3 pp 229533.I1RX64
136096 229533.A0A1C3YHM3 pp 229533.I1RRJ6
136097 229533.A0A1C3YHM3 pp 229533.I1RBN9
136098 229533.A0A1C3YHM3 pp 229533.I1RSP7
136099 229533.A0A1C3YHM3 pp 229533.I1RVW0
136100 229533.A0A1C3YHM3 pp 229533.I1RQH8
136101 229533.A0A1C3YHM3 pp 229533.I1RBC1
136102 229533.A0A1C3YHM3 pp 229533.I1S523
136103 229533.A0A1C3YHM3 pp 229533.I1RFJ2
136104 229533.A0A1C3YHM3 pp 229533.I1RG77
136105 229533.A0A1C3YHM3 pp 229533.I1RN55
136106 229533.A0A1C3YHM3 pp 229533.Q4INS6
136107 229533.A0A1C3YHM3 pp 229533.I1RCB3
136108 229533.A0A1C3YHM3 pp 229533.I1RZ86
136109 229533.A0A1C3YHM3 pp 229533.I1S461
136110 229533.A0A1C3YHM3 pp 229533.A0A1C3YK82
136111 229533.A0A1C3YHM3 pp 229533.I1RS81
136112 229533.A0A1C3YHM3 pp 229533.I1RWM6
136113 229533.A0A1C3YHM3 pp 229533.A0A1C3YJ79
136114 229533.A0A1C3YHM3 pp 229533.I1RD01
136115 229533.A0A1C3YHM3 pp 229533.I1RPQ1
136116 229533.A0A1C3YHM3 pp 229533.I1RCQ4
136117 229533.A0A1C3YHM3 pp 229533.I1S788
136118 229533.A0A1C3YHM3 pp 229533.I1RRY1
136119 229533.A0A1C3YHM3 pp 229533.I1RE55
136120 229533.A0A1C3YHM3 pp 229533.I1RKI2
136121 229533.A0A1C3YHM3 pp 229533.A0A1C3YLR6
136122 229533.A0A1C3YHM3 pp 229533.I1S5Z3
136123 229533.A0A1C3YHM3 pp 229533.I1RUR0
136124 229533.A0A1C3YHM3 pp 229533.A0A1C3YI64
136125 229533.A0A1C3YHM3 pp 229533.I1RBW0
136126 229533.A0A1C3YHM3 pp 229533.I1RFX8
136127 229533.A0A1C3YHM3 pp 229533.I1S283
136128 229533.A0A1C3YHM3 pp 229533.I1RZ94
136129 229533.A0A1C3YHM3 pp 229533.I1RDJ1
136130 229533.I1RR24 pp 229533.V6R4J1
136131 229533.I1RR24 pp 229533.I1RW71
136132 229533.I1RR24 pp 229533.I1S4S7
136133 229533.I1RR24 pp 229533.I1RVQ0
136134 229533.I1RR24 pp 229533.I1RSQ7
136135 229533.I1RR24 pp 229533.I1RRX5
136136 229533.I1RR24 pp 229533.V6QW22
136137 229533.I1RR24 pp 229533.I1RX95
136138 229533.I1RR24 pp 229533.I1S0R7
136139 229533.I1RR24 pp 229533.I1RRG3
136140 229533.I1RR24 pp 229533.I1RTB3
136141 229533.I1RR24 pp 229533.V6R1K4
136142 229533.I1RR24 pp 229533.Q4IJT5
136143 229533.I1RR24 pp 229533.I1S0F5
136144 229533.I1RR24 pp 229533.V6RUT5
136145 229533.I1RR24 pp 229533.I1RUQ2

136146	229533.I1RR24	pp	229533.I1S0L3
136147	229533.I1RR24	pp	229533.I1RYJ6
136148	229533.I1RR24	pp	229533.V6RTJ8
136149	229533.I1RR24	pp	229533.I1RXD7
136150	229533.I1RR24	pp	229533.I1RRS8
136151	229533.I1RR24	pp	229533.I1RXD6
136152	229533.I1RR24	pp	229533.I1RWD4
136153	229533.I1RR24	pp	229533.I1RW07
136154	229533.I1RR24	pp	229533.I1S7A8
136155	229533.I1RR24	pp	229533.I1S504
136156	229533.I1RR24	pp	229533.I1S0T2
136157	229533.I1RR24	pp	229533.I1S6F0
136158	229533.I1RR24	pp	229533.I1RX25
136159	229533.I1RR24	pp	229533.I1RX46
136160	229533.I1RR24	pp	229533.I1S9I5
136161	229533.I1RR24	pp	229533.I1RWJ8
136162	229533.I1RR24	pp	229533.V6R227
136163	229533.I1RR24	pp	229533.V6QYF0
136164	229533.I1RR24	pp	229533.I1RW25
136165	229533.I1RR24	pp	229533.Q4I283
136166	229533.I1RR24	pp	229533.I1RZJ5
136167	229533.I1RR24	pp	229533.I1S438
136168	229533.I1RR24	pp	229533.I1S4S4
136169	229533.I1RR24	pp	229533.I1RTF4
136170	229533.I1RR24	pp	229533.I1RWT6
136171	229533.I1RR24	pp	229533.I1RT05
136172	229533.I1RR24	pp	229533.I1S419
136173	229533.I1RR24	pp	229533.I1S5B0
136174	229533.I1RR24	pp	229533.I1S0X6
136175	229533.I1RR24	pp	229533.I1RW99
136176	229533.I1RR24	pp	229533.Q4IEK8
136177	229533.I1RR24	pp	229533.I1RRV4
136178	229533.I1RR24	pp	229533.I1RRV3
136179	229533.I1RR24	pp	229533.I1RS18
136180	229533.I1RR24	pp	229533.I1S0F1
136181	229533.I1RR24	pp	229533.I1RZM1
136182	229533.I1RR24	pp	229533.Q4I2J8
136183	229533.I1RR24	pp	229533.V6QUE6
136184	229533.I1RR24	pp	229533.Q4I8P5
136185	229533.I1RR24	pp	229533.I1RSI0
136186	229533.I1RR24	pp	229533.I1RRU9
136187	229533.I1RR24	pp	229533.I1RY63
136188	229533.I1RR24	pp	229533.I1S1B2
136189	229533.I1RR24	pp	229533.I1RXC4
136190	229533.I1RR24	pp	229533.I1SA32
136191	229533.I1RR24	pp	229533.V6RCZ8
136192	229533.I1RR24	pp	229533.I1S6K9
136193	229533.I1RR24	pp	229533.I1RYI5
136194	229533.I1RR24	pp	229533.Q4IJK2
136195	229533.I1RR24	pp	229533.I1RRJ8
136196	229533.I1RR24	pp	229533.I1S8P5
136197	229533.I1RR24	pp	229533.I1RZZ1
136198	229533.I1RR24	pp	229533.V6RBN2
136199	229533.I1RR24	pp	229533.I1RR51
136200	229533.I1RR24	pp	229533.I1S821
136201	229533.I1RR24	pp	229533.I1RWK3
136202	229533.I1RR24	pp	229533.V6RCG5
136203	229533.I1RR24	pp	229533.I1RU29
136204	229533.I1RR24	pp	229533.I1RWS4
136205	229533.I1RR24	pp	229533.I1RRY9
136206	229533.I1RR24	pp	229533.I1RWR7
136207	229533.I1RR24	pp	229533.I1RXF0
136208	229533.I1RR24	pp	229533.Q4IBK5
136209	229533.I1RR24	pp	229533.I1RSR3
136210	229533.I1RR24	pp	229533.I1RZM8
136211	229533.I1RR24	pp	229533.I1RVZ2
136212	229533.I1RR24	pp	229533.I1RTA1
136213	229533.I1RR24	pp	229533.I1S1J7
136214	229533.I1RR24	pp	229533.I1RZK5
136215	229533.I1RR24	pp	229533.I1S2M6
136216	229533.I1RR24	pp	229533.V6R9Z0
136217	229533.I1RR24	pp	229533.I1S887
136218	229533.I1RR24	pp	229533.I1RWB0

136219 229533.I1RR24 pp 229533.I1RRZ0
136220 229533.I1RR24 pp 229533.I1RYS0
136221 229533.I1RR24 pp 229533.I1RZB2
136222 229533.I1RR24 pp 229533.I1RS47
136223 229533.I1RR24 pp 229533.I1RY03
136224 229533.I1RR24 pp 229533.I1S503
136225 229533.I1RR24 pp 229533.I1RSA5
136226 229533.I1RR24 pp 229533.I1S6Y8
136227 229533.I1RR24 pp 229533.I1RRT2
136228 229533.I1RR24 pp 229533.I1RS20
136229 229533.I1RR24 pp 229533.I1RRK3
136230 229533.I1RR24 pp 229533.I1S0W4
136231 229533.I1RR24 pp 229533.I1S2H4
136232 229533.I1RR24 pp 229533.I1S0F7
136233 229533.I1RR24 pp 229533.I1S0L4
136234 229533.I1RR24 pp 229533.I1RSX0
136235 229533.I1RR24 pp 229533.I1RRI1
136236 229533.I1RR24 pp 229533.I1S0F6
136237 229533.I1RR24 pp 229533.V6R3J1
136238 229533.I1RR24 pp 229533.I1S9Y1
136239 229533.I1RR24 pp 229533.Q4HXF0
136240 229533.I1RR24 pp 229533.I1S352
136241 229533.I1RR24 pp 229533.I1RRE5
136242 229533.I1RR24 pp 229533.V6RUY9
136243 229533.I1RR24 pp 229533.I1RT04
136244 229533.I1RR24 pp 229533.I1RSF1
136245 229533.I1RR24 pp 229533.I1S5R1
136246 229533.I1RR24 pp 229533.I1S8V4
136247 229533.I1RR24 pp 229533.I1S2E9
136248 229533.I1RR24 pp 229533.I1RRT8
136249 229533.I1RR24 pp 229533.O74638
136250 229533.I1RR24 pp 229533.I1RWN5
136251 229533.I1RR24 pp 229533.I1RXC2
136252 229533.I1RR24 pp 229533.I1RV91
136253 229533.I1RR24 pp 229533.V6RIV9
136254 229533.I1RR24 pp 229533.I1S1W9
136255 229533.I1RR24 pp 229533.I1RZ39
136256 229533.I1RR24 pp 229533.I1RT40
136257 229533.I1RR24 pp 229533.I1RRL9
136258 229533.I1RR24 pp 229533.I1RTD5
136259 229533.I1RR24 pp 229533.I1RR73
136260 229533.I1RR24 pp 229533.I1RY44
136261 229533.I1RR24 pp 229533.I1S2B8
136262 229533.I1RR24 pp 229533.I1S0M6
136263 229533.I1RR24 pp 229533.I1RRM5
136264 229533.I1RR24 pp 229533.I1S1W3
136265 229533.I1RR24 pp 229533.I1RW61
136266 229533.I1RR24 pp 229533.V6R3X5
136267 229533.I1RR24 pp 229533.I1RS17
136268 229533.I1RR24 pp 229533.I1RWI5
136269 229533.I1RR24 pp 229533.I1RZV3
136270 229533.I1RR24 pp 229533.V6RGN5
136271 229533.I1RR24 pp 229533.I1RZZ9
136272 229533.I1RR24 pp 229533.I1RSZ7
136273 229533.I1RR24 pp 229533.I1RSQ4
136274 229533.I1RR24 pp 229533.I1RW46
136275 229533.I1RR24 pp 229533.I1S0N4
136276 229533.I1RR24 pp 229533.I1S7W5
136277 229533.I1RR24 pp 229533.I1RTA0
136278 229533.I1RR24 pp 229533.I1RTH6
136279 229533.I1RR24 pp 229533.I1SA33
136280 229533.I1RR24 pp 229533.I1S8V3
136281 229533.I1RR24 pp 229533.I1RRN2
136282 229533.I1RR24 pp 229533.I1S0J1
136283 229533.I1RR24 pp 229533.I1RSQ3
136284 229533.I1RR24 pp 229533.I1RRV1
136285 229533.I1RR24 pp 229533.I1S0F8
136286 229533.I1RR24 pp 229533.Q4HZK7
136287 229533.I1RR24 pp 229533.I1RWQ8
136288 229533.I1RR24 pp 229533.I1S5R6
136289 229533.I1RR24 pp 229533.V6R042
136290 229533.I1RR24 pp 229533.I1RT03
136291 229533.I1RR24 pp 229533.I1RVX0

229533.I1RR24	pp	229533.I1RRM3
229533.I1RR24	pp	229533.I1S8R3
229533.I1RR24	pp	229533.V6RMW6
229533.I1RR24	pp	229533.I1RXR1
229533.I1RR24	pp	229533.I1RTE9
229533.I1RR24	pp	229533.I1RXT3
229533.I1RR24	pp	229533.I1S437
229533.I1RR24	pp	229533.I1RUF7
229533.I1RR24	pp	229533.I1RYW3
229533.I1RR24	pp	229533.I1RRN5
229533.I1RR24	pp	229533.I1S1C7
229533.I1RR24	pp	229533.I1RUG3
229533.I1RR24	pp	229533.Q4HXF6
229533.I1RR24	pp	229533.I1RWX5
229533.I1RR24	pp	229533.I1S569
229533.I1RR24	pp	229533.I1RRI2
229533.I1RR24	pp	229533.V6R057
229533.I1RR24	pp	229533.I1RXU9
229533.I1RR24	pp	229533.I1RZ79
229533.I1RR24	pp	229533.I1RWN6
229533.I1RR24	pp	229533.Q4I624
229533.I1RR24	pp	229533.I1RZW8
229533.I1RR24	pp	229533.I1RVQ3
229533.I1RIU7	pp	229533.I1RXM0
229533.I1RIU7	pp	229533.I1S2N3
229533.I1RIU7	pp	229533.I1S2K2
229533.I1RLV8	pp	229533.I1RRQ2
229533.I1RLV8	pp	229533.I1RMB6
229533.A0A0E0RSD7	pp	229533.I1S5R1
229533.A0A0E0RSD7	pp	229533.I1S4Z8
229533.A0A0E0RSD7	pp	229533.A0A1C3YIS7
229533.A0A0E0RSD7	pp	229533.A0A0E0RU84
229533.A0A0E0RSD7	pp	229533.I1RBY4
229533.A0A0E0RSD7	pp	229533.I1R9M0
229533.A0A0E0RSD7	pp	229533.I1RRT8
229533.A0A0E0RSD7	pp	229533.O74638
229533.A0A0E0RSD7	pp	229533.I1RXC2
229533.A0A0E0RSD7	pp	229533.I1RJZ7
229533.A0A0E0RSD7	pp	229533.I1S2B8
229533.A0A0E0RSD7	pp	229533.I1RRM9
229533.A0A0E0RSD7	pp	229533.I1RA11
229533.A0A0E0RSD7	pp	229533.I1S9Y1
229533.A0A0E0RSD7	pp	229533.I1RRM5
229533.A0A0E0RSD7	pp	229533.I1RR73
229533.A0A0E0RSD7	pp	229533.I1RM82
229533.A0A0E0RSD7	pp	229533.I1RE22
229533.A0A0E0RSD7	pp	229533.V6R3X5
229533.A0A0E0RSD7	pp	229533.A0A1C3YJD4
229533.A0A0E0RSD7	pp	229533.A0A1C3YKK9
229533.A0A0E0RSD7	pp	229533.I1RYI5
229533.A0A0E0RSD7	pp	229533.I1RL04
229533.A0A0E0RSD7	pp	229533.I1S8P5
229533.A0A0E0RSD7	pp	229533.I1RZ39
229533.A0A0E0RSD7	pp	229533.I1RPV3
229533.A0A0E0RSD7	pp	229533.A0A1C3YKM9
229533.A0A0E0RSD7	pp	229533.I1RVN1
229533.A0A0E0RSD7	pp	229533.I1RPM4
229533.A0A0E0RSD7	pp	229533.I1RPP3
229533.A0A0E0RSD7	pp	229533.I1RFS3
229533.A0A0E0RSD7	pp	229533.I1RFT1
229533.A0A0E0RSD7	pp	229533.I1RSV2
229533.A0A0E0RSD7	pp	229533.I1RC66
229533.A0A0E0RSD7	pp	229533.I1RFT6
229533.A0A0E0RSD7	pp	229533.I1RSZ7
229533.A0A0E0RSD7	pp	229533.A0A1C3YI20
229533.A0A0E0RSD7	pp	229533.I1RWI5
229533.A0A0E0RSD7	pp	229533.I1RZZ9
229533.A0A0E0RSD7	pp	229533.I1RTA1
229533.A0A0E0RSD7	pp	229533.Q4HXF0
229533.A0A0E0RSD7	pp	229533.I1RJN9
229533.A0A0E0RSD7	pp	229533.A0A1C3YL55
229533.A0A0E0RSD7	pp	229533.I1RVZ2
229533.A0A0E0RSD7	pp	229533.I1RSQ4

136365	229533.A0A0E0RSD7	pp	229533.I1RT75
136366	229533.A0A0E0RSD7	pp	229533.Q4IF76
136367	229533.A0A0E0RSD7	pp	229533.I1RN63
136368	229533.A0A0E0RSD7	pp	229533.I1RT04
136369	229533.A0A0E0RSD7	pp	229533.I1RWK3
136370	229533.A0A0E0RSD7	pp	229533.V6R283
136371	229533.A0A0E0RSD7	pp	229533.A0A1C3YIR8
136372	229533.A0A0E0RSD7	pp	229533.I1S0L4
136373	229533.A0A0E0RSD7	pp	229533.V6RAC9
136374	229533.A0A0E0RSD7	pp	229533.I1RRI1
136375	229533.A0A0E0RSD7	pp	229533.I1RXF8
136376	229533.A0A0E0RSD7	pp	229533.A0A0E0SC46
136377	229533.A0A0E0RSD7	pp	229533.A0A1C3YJW4
136378	229533.A0A0E0RSD7	pp	229533.A0A1C3YJK3
136379	229533.A0A0E0RSD7	pp	229533.I1RTD5
136380	229533.A0A0E0RSD7	pp	229533.I1RP28
136381	229533.A0A0E0RSD7	pp	229533.I1S3G4
136382	229533.A0A0E0RSD7	pp	229533.I1RFL3
136383	229533.A0A0E0RSD7	pp	229533.I1RPE0
136384	229533.A0A0E0RSD7	pp	229533.I1RC05
136385	229533.A0A0E0RSD7	pp	229533.I1RC31
136386	229533.A0A0E0RSD7	pp	229533.I1RV91
136387	229533.A0A0E0RSD7	pp	229533.I1RNG4
136388	229533.A0A0E0RSD7	pp	229533.A0A1C3YJI6
136389	229533.A0A0E0RSD7	pp	229533.I1RS43
136390	229533.A0A0E0RSD7	pp	229533.I1RS20
136391	229533.A0A0E0RSD7	pp	229533.I1RT40
136392	229533.A0A0E0RSD7	pp	229533.I1S2H4
136393	229533.A0A0E0RSD7	pp	229533.I1RCJ7
136394	229533.A0A0E0RSD7	pp	229533.I1RN67
136395	229533.A0A0E0RSD7	pp	229533.I1RQM7
136396	229533.A0A0E0RSD7	pp	229533.I1S0F6
136397	229533.A0A0E0RSD7	pp	229533.I1RPP4
136398	229533.A0A0E0RSD7	pp	229533.I1RP27
136399	229533.A0A0E0RSD7	pp	229533.I1RWD3
136400	229533.A0A0E0RSD7	pp	229533.Q4I7K4
136401	229533.A0A0E0RSD7	pp	229533.I1S2E9
136402	229533.A0A0E0RSD7	pp	229533.I1RZB2
136403	229533.A0A0E0RSD7	pp	229533.I1RQ33
136404	229533.A0A0E0RSD7	pp	229533.I1RJV4
136405	229533.A0A0E0RSD7	pp	229533.I1RBS6
136406	229533.A0A0E0RSD7	pp	229533.I1RPK0
136407	229533.A0A0E0RSD7	pp	229533.Q4HZ68
136408	229533.A0A0E0RSD7	pp	229533.A0A1C3YLA6
136409	229533.A0A0E0RSD7	pp	229533.I1RBC7
136410	229533.A0A0E0RSD7	pp	229533.V6R057
136411	229533.A0A0E0RSD7	pp	229533.V6RLM7
136412	229533.A0A0E0RSD7	pp	229533.I1RSL9
136413	229533.A0A0E0RSD7	pp	229533.I1RS17
136414	229533.A0A0E0RSD7	pp	229533.I1RGA4
136415	229533.A0A0E0RSD7	pp	229533.A0A0E0SPV0
136416	229533.A0A0E0RSD7	pp	229533.A0A0E0SH88
136417	229533.A0A0E0RSD7	pp	229533.Q4I624
136418	229533.A0A0E0RSD7	pp	229533.I1S0J1
136419	229533.A0A0E0RSD7	pp	229533.I1RSX0
136420	229533.A0A0E0RSD7	pp	229533.I1RW46
136421	229533.A0A0E0RSD7	pp	229533.I1RD17
136422	229533.A0A0E0RSD7	pp	229533.I1RCD8
136423	229533.A0A0E0RSD7	pp	229533.V6R3J1
136424	229533.A0A0E0RSD7	pp	229533.I1RML0
136425	229533.A0A0E0RSD7	pp	229533.V6RRH2
136426	229533.A0A0E0RSD7	pp	229533.I1RE01
136427	229533.A0A0E0RSD7	pp	229533.A0A1C3YLB6
136428	229533.A0A0E0RSD7	pp	229533.I1RTA0
136429	229533.A0A0E0RSD7	pp	229533.Q4IAS1
136430	229533.A0A0E0RSD7	pp	229533.A0A1C3YLK8
136431	229533.A0A0E0RSD7	pp	229533.I1RVZ7
136432	229533.A0A0E0RSD7	pp	229533.I1RPU2
136433	229533.A0A0E0RSD7	pp	229533.I1RKJ8
136434	229533.A0A0E0RSD7	pp	229533.I1RJW6
136435	229533.A0A0E0RSD7	pp	229533.I1RA70
136436	229533.A0A0E0RSD7	pp	229533.I1RTH6
136437	229533.A0A0E0RSD7	pp	229533.I1RRE5

136438 229533.A0A0E0RSD7 pp 229533.I1S503
136439 229533.A0A0E0RSD7 pp 229533.I1RY44
136440 229533.A0A0E0RSD7 pp 229533.I1S8R3
136441 229533.A0A0E0RSD7 pp 229533.I1RAS2
136442 229533.A0A0E0RSD7 pp 229533.I1RB92
136443 229533.A0A0E0RSD7 pp 229533.I1RRI2
136444 229533.A0A0E0RSD7 pp 229533.I1RJW8
136445 229533.A0A0E0RSD7 pp 229533.I1RT03
136446 229533.A0A0E0RSD7 pp 229533.I1RZV3
136447 229533.A0A0E0RSD7 pp 229533.I1RZ79
136448 229533.A0A0E0RSD7 pp 229533.I1RVX0
136449 229533.A0A0E0RSD7 pp 229533.I1RZW8
136450 229533.A0A0E0RSD7 pp 229533.I1RWN5
136451 229533.A0A0E0RSD7 pp 229533.I1RSS4
136452 229533.A0A0E0RSD7 pp 229533.V6RIV9
136453 229533.A0A0E0RSD7 pp 229533.I1RCH5
136454 229533.A0A0E0RSD7 pp 229533.I1RB44
136455 229533.A0A0E0RSD7 pp 229533.I1RYW3
136456 229533.A0A0E0RSD7 pp 229533.I1RQE7
136457 229533.A0A0E0RSD7 pp 229533.I1S1W9
136458 229533.A0A0E0RSD7 pp 229533.I1RFL4
136459 229533.A0A0E0RSD7 pp 229533.I1RN18
136460 229533.A0A0E0RSD7 pp 229533.I1S1C7
136461 229533.A0A0E0RSD7 pp 229533.I1RAU7
136462 229533.A0A0E0RSD7 pp 229533.I1RC94
136463 229533.A0A0E0RSD7 pp 229533.I1RWN6
136464 229533.A0A0E0RSD7 pp 229533.I1RMQ5
136465 229533.A0A0E0RSD7 pp 229533.I1RSQ3
136466 229533.A0A0E0RSD7 pp 229533.I1SAN9
136467 229533.A0A0E0RSD7 pp 229533.V6R042
136468 229533.A0A0E0RSD7 pp 229533.I1RFT8
136469 229533.A0A0E0RSD7 pp 229533.I1RAW0
136470 229533.A0A0E0RSD7 pp 229533.I1RRV1
136471 229533.A0A0E0RSD7 pp 229533.A0A0E0SD83
136472 229533.A0A0E0RSD7 pp 229533.A0A1C3YKY0
136473 229533.A0A0E0RSD7 pp 229533.A0A1C3YMI5
136474 229533.A0A0E0RSD7 pp 229533.I1RCL3
136475 229533.A0A0E0RSD7 pp 229533.I1RSK1
136476 229533.A0A0E0RSD7 pp 229533.I1RRM3
136477 229533.A0A0E0RSD7 pp 229533.I1RNC1
136478 229533.A0A0E0RSD7 pp 229533.V6RMW6
136479 229533.A0A0E0RSD7 pp 229533.I1RJV3
136480 229533.A0A0E0RSD7 pp 229533.I1RUG3
136481 229533.A0A0E0RSD7 pp 229533.I1RE89
136482 229533.A0A0E0RSD7 pp 229533.I1RCR8
136483 229533.A0A0E0RSD7 pp 229533.I1RCM3
136484 229533.A0A0E0RSD7 pp 229533.I1RFC0
136485 229533.A0A0E0RSD7 pp 229533.I1S0N4
136486 229533.A0A0E0RSD7 pp 229533.I1RRX5
136487 229533.A0A0E0RSD7 pp 229533.A0A0E0RUI4
136488 229533.A0A0E0RSD7 pp 229533.I1RW25
136489 229533.A0A0E0RSD7 pp 229533.I1RG11
136490 229533.A0A0E0RSD7 pp 229533.A0A0E0SA11
136491 229533.A0A0E0RSD7 pp 229533.I1RSQ7
136492 229533.A0A0E0RSD7 pp 229533.I1S5R6
136493 229533.A0A0E0RSD7 pp 229533.I1S4S7
136494 229533.A0A0E0RSD7 pp 229533.I1RC27
136495 229533.A0A0E0RSD7 pp 229533.Q4HVW2
136496 229533.A0A0E0RSD7 pp 229533.I1RS90
136497 229533.A0A0E0RSD7 pp 229533.A0A1C3YK13
136498 229533.A0A0E0RSD7 pp 229533.I1RW71
136499 229533.A0A0E0RSD7 pp 229533.I1RRN2
136500 229533.A0A0E0RSD7 pp 229533.I1RJU3
136501 229533.A0A0E0RSD7 pp 229533.I1RBC9
136502 229533.A0A0E0RSD7 pp 229533.I1RD22
136503 229533.A0A0E0RSD7 pp 229533.I1RPN4
136504 229533.A0A0E0RSD7 pp 229533.I1RIG2
136505 229533.A0A0E0RSD7 pp 229533.A0A1C3YIK9
136506 229533.A0A0E0RSD7 pp 229533.A0A1C3YN30
136507 229533.A0A0E0RSD7 pp 229533.A0A1C3YN26
136508 229533.A0A0E0RSD7 pp 229533.I1RAM6
136509 229533.A0A0E0RSD7 pp 229533.A0A0E0SBU8
136510 229533.A0A0E0RSD7 pp 229533.A0A1C3YM63

136511	229533.A0A0E0RSD7	pp	229533.V6R1K4
136512	229533.A0A0E0RSD7	pp	229533.I1SA33
136513	229533.A0A0E0RSD7	pp	229533.I1S0L3
136514	229533.A0A0E0RSD7	pp	229533.A0A1C3YHX8
136515	229533.A0A0E0RSD7	pp	229533.A0A1C3YKC0
136516	229533.A0A0E0RSD7	pp	229533.I1RBT1
136517	229533.A0A0E0RSD7	pp	229533.I1S0X6
136518	229533.A0A0E0RSD7	pp	229533.V6QUW4
136519	229533.A0A0E0RSD7	pp	229533.I1R9Y4
136520	229533.A0A0E0RSD7	pp	229533.I1RPW9
136521	229533.A0A0E0RSD7	pp	229533.I1RD52
136522	229533.A0A0E0RSD7	pp	229533.I1RT33
136523	229533.A0A0E0RSD7	pp	229533.I1S1W8
136524	229533.A0A0E0RSD7	pp	229533.I1REX4
136525	229533.A0A0E0RSD7	pp	229533.I1RQK8
136526	229533.A0A0E0RSD7	pp	229533.I1R9X2
136527	229533.A0A0E0RSD7	pp	229533.A0A1C3YLR6
136528	229533.A0A0E0RSD7	pp	229533.I1RR24
136529	229533.A0A0E0RSD7	pp	229533.I1RFQ7
136530	229533.A0A0E0RSD7	pp	229533.I1RVQ0
136531	229533.A0A0E0RSD7	pp	229533.I1RPW4
136532	229533.A0A0E0RSD7	pp	229533.A0A1C3YIP5
136533	229533.A0A0E0RSD7	pp	229533.I1RME6
136534	229533.A0A0E0RSD7	pp	229533.I1RM67
136535	229533.A0A0E0RSD7	pp	229533.I1RQ81
136536	229533.A0A0E0RSD7	pp	229533.I1RWJ8
136537	229533.A0A0E0RSD7	pp	229533.I1RXD7
136538	229533.A0A0E0RSD7	pp	229533.I1RMQ4
136539	229533.A0A0E0RSD7	pp	229533.I1RQC9
136540	229533.A0A0E0RSD7	pp	229533.A0A1C3YK11
136541	229533.A0A0E0RSD7	pp	229533.I1S4S4
136542	229533.A0A0E0RSD7	pp	229533.I1SA32
136543	229533.A0A0E0RSD7	pp	229533.I1RCL4
136544	229533.A0A0E0RSD7	pp	229533.I1RP86
136545	229533.A0A0E0RSD7	pp	229533.I1RJ17
136546	229533.A0A0E0RSD7	pp	229533.A0A0E0SFN0
136547	229533.A0A0E0RSD7	pp	229533.I1RQE1
136548	229533.A0A0E0RSD7	pp	229533.I1RC20
136549	229533.A0A0E0RSD7	pp	229533.I1RN61
136550	229533.A0A0E0RSD7	pp	229533.I1RXC4
136551	229533.A0A0E0RSD7	pp	229533.V6QW22
136552	229533.A0A0E0RSD7	pp	229533.I1S0R7
136553	229533.A0A0E0RSD7	pp	229533.A0A0E0S134
136554	229533.A0A0E0RSD7	pp	229533.A0A1C3YK34
136555	229533.A0A0E0RSD7	pp	229533.A0A0E0SJS1
136556	229533.A0A0E0RSD7	pp	229533.I1RXU7
136557	229533.A0A0E0RSD7	pp	229533.A0A1C3YKB0
136558	229533.A0A0E0RSD7	pp	229533.V6RTJ8
136559	229533.A0A0E0RSD7	pp	229533.I1RQ85
136560	229533.A0A0E0RSD7	pp	229533.A0A0E0S035
136561	229533.A0A0E0RSD7	pp	229533.I1RSM2
136562	229533.A0A0E0RSD7	pp	229533.I1RWT6
136563	229533.A0A0E0RSD7	pp	229533.I1RQH1
136564	229533.A0A0E0RSD7	pp	229533.I1S4Q4
136565	229533.A0A0E0RSD7	pp	229533.I1RAZ8
136566	229533.A0A0E0RSD7	pp	229533.A0A1C3YJR3
136567	229533.A0A0E0RSD7	pp	229533.I1RBC1
136568	229533.A0A0E0RSD7	pp	229533.I1REL7
136569	229533.A0A0E0RSD7	pp	229533.I1RZK5
136570	229533.A0A0E0RSD7	pp	229533.I1RAX6
136571	229533.A0A0E0RSD7	pp	229533.A0A1C3YJJ2
136572	229533.A0A0E0RSD7	pp	229533.I1RX46
136573	229533.A0A0E0RSD7	pp	229533.I1S504
136574	229533.A0A0E0RSD7	pp	229533.I1RLU6
136575	229533.A0A0E0RSD7	pp	229533.I1RK95
136576	229533.A0A0E0RSD7	pp	229533.I1RZZ1
136577	229533.A0A0E0RSD7	pp	229533.A0A1C3YK68
136578	229533.A0A0E0RSD7	pp	229533.A0A0E0RWW0
136579	229533.A0A0E0RSD7	pp	229533.I1RCV1
136580	229533.A0A0E0RSD7	pp	229533.I1RNQ5
136581	229533.A0A0E0RSD7	pp	229533.I1RFU8
136582	229533.A0A0E0RSD7	pp	229533.A0A1C3YJN3
136583	229533.A0A0E0RSD7	pp	229533.V6RBN2

136584 229533.A0A0E0RSD7 pp 229533.I1RAZ3
136585 229533.A0A0E0RSD7 pp 229533.I1RCG4
136586 229533.A0A0E0RSD7 pp 229533.A0A1C3YLK2
136587 229533.A0A0E0RSD7 pp 229533.I1RZM1
136588 229533.A0A0E0RSD7 pp 229533.A0A1C3YJ29
136589 229533.A0A0E0RSD7 pp 229533.A0A1C3YHS4
136590 229533.A0A0E0RSD7 pp 229533.A0A1C3YHX6
136591 229533.A0A0E0RSD7 pp 229533.I1RY03
136592 229533.A0A0E0RSD7 pp 229533.I1RRY9
136593 229533.A0A0E0RSD7 pp 229533.I1RWH8
136594 229533.A0A0E0RSD7 pp 229533.A0A0E0S9Y6
136595 229533.A0A0E0RSD7 pp 229533.I1RQA3
136596 229533.A0A0E0RSD7 pp 229533.V6RCG5
136597 229533.A0A0E0RSD7 pp 229533.Q4IJK2
136598 229533.A0A0E0RSD7 pp 229533.I1RBL7
136599 229533.A0A0E0RSD7 pp 229533.I1RB53
136600 229533.A0A0E0RSD7 pp 229533.I1S6K9
136601 229533.A0A0E0RSD7 pp 229533.A0A1C3YKY6
136602 229533.A0A0E0RSD7 pp 229533.I1RBE4
136603 229533.A0A0E0RSD7 pp 229533.I1RXD0
136604 229533.A0A0E0RSD7 pp 229533.I1RQA2
136605 229533.A0A0E0RSD7 pp 229533.I1RWD7
136606 229533.A0A0E0RSD7 pp 229533.I1RM35
136607 229533.A0A0E0RSD7 pp 229533.V6RCZ8
136608 229533.A0A0E0RSD7 pp 229533.A0A1C3YJW9
136609 229533.A0A0E0RSD7 pp 229533.I1RCR5
136610 229533.A0A0E0RSD7 pp 229533.I1RI82
136611 229533.A0A0E0RSD7 pp 229533.I1REK8
136612 229533.A0A0E0RSD7 pp 229533.I1RZE5
136613 229533.A0A0E0RSD7 pp 229533.I1RS18
136614 229533.A0A0E0RSD7 pp 229533.I1RXF0
136615 229533.A0A0E0RSD7 pp 229533.Q4I8P5
136616 229533.A0A0E0RSD7 pp 229533.I1RSI0
136617 229533.A0A0E0RSD7 pp 229533.I1RZM8
136618 229533.A0A0E0RSD7 pp 229533.I1RE00
136619 229533.A0A0E0RSD7 pp 229533.A0A1C3YIJ3
136620 229533.A0A0E0RSD7 pp 229533.I1RN58
136621 229533.A0A0E0RSD7 pp 229533.A0A1C3YKV3
136622 229533.A0A0E0RSD7 pp 229533.I1RW99
136623 229533.A0A0E0RSD7 pp 229533.I1S5B0
136624 229533.A0A0E0RSD7 pp 229533.I1RQD2
136625 229533.A0A0E0RSD7 pp 229533.I1RA94
136626 229533.A0A0E0RSD7 pp 229533.A0A1C3YI30
136627 229533.A0A0E0RSD7 pp 229533.I1RRU9
136628 229533.A0A0E0RSD7 pp 229533.I1RAU5
136629 229533.A0A0E0RSD7 pp 229533.I1RRV4
136630 229533.A0A0E0RSD7 pp 229533.V6QUE6
136631 229533.I1RPL0 pp 229533.I1S2N3
136632 229533.I1RPL0 pp 229533.I1S2K2
136633 229533.I1RPL0 pp 229533.I1RXM0
136634 229533.I1RXI6 pp 229533.I1RYX9
136635 229533.I1RXI6 pp 229533.I1S4P6
136636 229533.I1RXI6 pp 229533.V6RLW4
136637 229533.I1RXI6 pp 229533.Q4IB89
136638 229533.I1RXI6 pp 229533.Q4INS6
136639 229533.I1RXI6 pp 229533.O74638
136640 229533.I1RXI6 pp 229533.I1RY73
136641 229533.I1RXI6 pp 229533.Q4IR08
136642 229533.A0A098D5E5 pp 229533.I1RN81
136643 229533.A0A098D5E5 pp 229533.A0A0E0SPD4
136644 229533.A0A098D5E5 pp 229533.I1RRT1
136645 229533.A0A098D5E5 pp 229533.I1RC91
136646 229533.A0A098D5E5 pp 229533.Q4ICG5
136647 229533.A0A098D5E5 pp 229533.A0A1C3YLR4
136648 229533.A0A098D5E5 pp 229533.A0A098E0E1
136649 229533.A0A098D5E5 pp 229533.A0A1C3YK00
136650 229533.A0A098D5E5 pp 229533.A0A1C3YK13
136651 229533.A0A098D5E5 pp 229533.I1RW71
136652 229533.A0A098D5E5 pp 229533.I1RN89
136653 229533.A0A098D5E5 pp 229533.A0A1C3YLZ7
136654 229533.A0A098D5E5 pp 229533.V6QUW4
136655 229533.A0A098D5E5 pp 229533.I1RNC0
136656 229533.A0A098D5E5 pp 229533.I1RBC9

136657	229533.A0A098D5E5	pp	229533.A0A1C3YN58
136658	229533.A0A098D5E5	pp	229533.I1RC20
136659	229533.A0A098D5E5	pp	229533.A0A0E0RUI4
136660	229533.A0A098D5E5	pp	229533.I1RZ94
136661	229533.A0A098D5E5	pp	229533.I1RN61
136662	229533.A0A098D5E5	pp	229533.I1RN31
136663	229533.A0A098D5E5	pp	229533.V6QW22
136664	229533.A0A098D5E5	pp	229533.I1RW07
136665	229533.A0A098D5E5	pp	229533.I1RZ12
136666	229533.A0A098D5E5	pp	229533.I1RI02
136667	229533.A0A098D5E5	pp	229533.A0A0E0SAC4
136668	229533.A0A098D5E5	pp	229533.I1RD01
136669	229533.A0A098D5E5	pp	229533.I1RD69
136670	229533.A0A098D5E5	pp	229533.I1RLC7
136671	229533.A0A098D5E5	pp	229533.I1RSQ7
136672	229533.A0A098D5E5	pp	229533.I1RG62
136673	229533.A0A098D5E5	pp	229533.A0A098DXE2
136674	229533.A0A098D5E5	pp	229533.I1RRX6
136675	229533.A0A098D5E5	pp	229533.I1RG53
136676	229533.A0A098D5E5	pp	229533.I1RL24
136677	229533.A0A098D5E5	pp	229533.I1RX25
136678	229533.A0A098D5E5	pp	229533.A0A098E4R5
136679	229533.A0A098D5E5	pp	229533.I1RDB1
136680	229533.A0A098D5E5	pp	229533.A0A098DXS1
136681	229533.A0A098D5E5	pp	229533.I1RW38
136682	229533.A0A098D5E5	pp	229533.I1RW25
136683	229533.A0A098D5E5	pp	229533.A0A1C3YIL0
136684	229533.A0A098D5E5	pp	229533.A0A1C3YM63
136685	229533.A0A098D5E5	pp	229533.I1RFZ3
136686	229533.A0A098D5E5	pp	229533.I1RLX7
136687	229533.A0A098D5E5	pp	229533.A0A0E0SA11
136688	229533.A0A098D5E5	pp	229533.I1S224
136689	229533.A0A098D5E5	pp	229533.I1RQA9
136690	229533.A0A098D5E5	pp	229533.A0A1C3YJQ9
136691	229533.A0A098D5E5	pp	229533.I1S0X6
136692	229533.A0A098D5E5	pp	229533.I1RQ26
136693	229533.A0A098D5E5	pp	229533.I1RBW0
136694	229533.A0A098D5E5	pp	229533.I1RTG0
136695	229533.A0A098D5E5	pp	229533.A0A1C3YKC0
136696	229533.A0A098D5E5	pp	229533.I1RYH9
136697	229533.A0A098D5E5	pp	229533.I1RBT1
136698	229533.A0A098D5E5	pp	229533.I1RMC8
136699	229533.A0A098D5E5	pp	229533.I1RFS6
136700	229533.A0A098D5E5	pp	229533.I1RJU3
136701	229533.A0A098D5E5	pp	229533.A0A1C3YI31
136702	229533.A0A098D5E5	pp	229533.A0A1C3YN30
136703	229533.A0A098D5E5	pp	229533.I1RAW7
136704	229533.A0A098D5E5	pp	229533.I1RIL8
136705	229533.A0A098D5E5	pp	229533.I1RRG3
136706	229533.A0A098D5E5	pp	229533.A0A1C3YHX8
136707	229533.A0A098D5E5	pp	229533.I1RWJ6
136708	229533.A0A098D5E5	pp	229533.I1RX70
136709	229533.A0A098D5E5	pp	229533.I1RD18
136710	229533.A0A098D5E5	pp	229533.Q4IJT5
136711	229533.A0A098D5E5	pp	229533.A0A0E0RSD7
136712	229533.A0A098D5E5	pp	229533.I1RR24
136713	229533.A0A098D5E5	pp	229533.I1RNH1
136714	229533.A0A098D5E5	pp	229533.I1RIH8
136715	229533.A0A098D5E5	pp	229533.I1RWJ8
136716	229533.A0A098D5E5	pp	229533.I1RZ51
136717	229533.A0A098D5E5	pp	229533.V6R4J1
136718	229533.A0A098D5E5	pp	229533.A0A098DYZ0
136719	229533.A0A098D5E5	pp	229533.I1RSI4
136720	229533.A0A098D5E5	pp	229533.I1RV66
136721	229533.A0A098D5E5	pp	229533.I1RVQ0
136722	229533.A0A098D5E5	pp	229533.A0A1C3YJH8
136723	229533.A0A098D5E5	pp	229533.A0A098DSW2
136724	229533.A0A098D5E5	pp	229533.A0A1C3YJV7
136725	229533.A0A098D5E5	pp	229533.A0A098DR40
136726	229533.A0A098D5E5	pp	229533.A0A1C3YKF2
136727	229533.A0A098D5E5	pp	229533.I1RZJ5
136728	229533.A0A098D5E5	pp	229533.V6QV09
136729	229533.A0A098D5E5	pp	229533.I1RD52

136730	229533.A0A098D5E5	pp	229533.I1RE66
136731	229533.A0A098D5E5	pp	229533.Q4IJP1
136732	229533.A0A098D5E5	pp	229533.I1RR94
136733	229533.A0A098D5E5	pp	229533.I1S0R7
136734	229533.A0A098D5E5	pp	229533.A0A1C3YJR8
136735	229533.A0A098D5E5	pp	229533.I1RWC2
136736	229533.A0A098D5E5	pp	229533.I1S0F5
136737	229533.A0A098D5E5	pp	229533.I1RQC9
136738	229533.A0A098D5E5	pp	229533.A0A0E0SFN0
136739	229533.A0A098D5E5	pp	229533.I1RYI3
136740	229533.A0A098D5E5	pp	229533.I1RB36
136741	229533.A0A098D5E5	pp	229533.I1RXD7
136742	229533.A0A098D5E5	pp	229533.A0A1C3YMW0
136743	229533.A0A098D5E5	pp	229533.I1RX46
136744	229533.A0A098D5E5	pp	229533.I1S0L3
136745	229533.A0A098D5E5	pp	229533.A0A1C3YL54
136746	229533.A0A098D5E5	pp	229533.A0A0E0RQK8
136747	229533.A0A098D5E5	pp	229533.A0A098DT74
136748	229533.A0A098D5E5	pp	229533.V6R1K4
136749	229533.A0A098D5E5	pp	229533.I1RM49
136750	229533.A0A098D5E5	pp	229533.I1RN65
136751	229533.A0A098D5E5	pp	229533.Q4IJM4
136752	229533.A0A098D5E5	pp	229533.I1RAX6
136753	229533.A0A098D5E5	pp	229533.A0A1C3YJX0
136754	229533.A0A098D5E5	pp	229533.A0A1C3YIP5
136755	229533.A0A098D5E5	pp	229533.I1RUQ6
136756	229533.A0A098D5E5	pp	229533.A0A1C3YIJ3
136757	229533.A0A098D5E5	pp	229533.A0A0E0RMM5
136758	229533.A0A098D5E5	pp	229533.I1RF19
136759	229533.A0A098D5E5	pp	229533.I1RLM7
136760	229533.A0A098D5E5	pp	229533.A0A1C3YJF1
136761	229533.A0A098D5E5	pp	229533.I1RK95
136762	229533.A0A098D5E5	pp	229533.A0A0E0RVZ1
136763	229533.A0A098D5E5	pp	229533.I1RYZ9
136764	229533.A0A098D5E5	pp	229533.A0A1C3YIB8
136765	229533.A0A098D5E5	pp	229533.A0A1C3YJI0
136766	229533.A0A098D5E5	pp	229533.I1RZB5
136767	229533.A0A098D5E5	pp	229533.I1RN08
136768	229533.A0A098D5E5	pp	229533.V6RTJ8
136769	229533.A0A098D5E5	pp	229533.I1RXZ4
136770	229533.A0A098D5E5	pp	229533.A0A1C3YKY3
136771	229533.A0A098D5E5	pp	229533.I1S7A8
136772	229533.A0A098D5E5	pp	229533.A0A1C3YLX2
136773	229533.A0A098D5E5	pp	229533.I1RQA3
136774	229533.A0A098D5E5	pp	229533.Q4IPX8
136775	229533.A0A098D5E5	pp	229533.Q4IJK2
136776	229533.A0A098D5E5	pp	229533.I1RCG4
136777	229533.A0A098D5E5	pp	229533.I1RFU8
136778	229533.A0A098D5E5	pp	229533.I1RKQ5
136779	229533.A0A098D5E5	pp	229533.I1RQQ5
136780	229533.A0A098D5E5	pp	229533.Q4IER8
136781	229533.A0A098D5E5	pp	229533.I1RRY9
136782	229533.A0A098D5E5	pp	229533.I1RJL2
136783	229533.A0A098D5E5	pp	229533.I1RBC4
136784	229533.A0A098D5E5	pp	229533.I1RZZ1
136785	229533.A0A098D5E5	pp	229533.I1RU29
136786	229533.A0A098D5E5	pp	229533.I1RB75
136787	229533.A0A098D5E5	pp	229533.I1RZK5
136788	229533.A0A098D5E5	pp	229533.I1RMS2
136789	229533.A0A098D5E5	pp	229533.I1RU02
136790	229533.A0A098D5E5	pp	229533.I1RCR5
136791	229533.A0A098D5E5	pp	229533.I1REK8
136792	229533.A0A098D5E5	pp	229533.I1S0N8
136793	229533.A0A098D5E5	pp	229533.A0A0E0RSG3
136794	229533.A0A098D5E5	pp	229533.I1RJK7
136795	229533.A0A098D5E5	pp	229533.I1RMM9
136796	229533.A0A098D5E5	pp	229533.I1RYV3
136797	229533.A0A098D5E5	pp	229533.I1RI03
136798	229533.A0A098D5E5	pp	229533.I1RUQ5
136799	229533.A0A098D5E5	pp	229533.A0A0E0SPV2
136800	229533.A0A098D5E5	pp	229533.I1S439
136801	229533.A0A098D5E5	pp	229533.I1RC22
136802	229533.A0A098D5E5	pp	229533.I1S1J7

136803	229533.A0A098D5E5	pp	229533.I1RY03
136804	229533.A0A098D5E5	pp	229533.I1RDV9
136805	229533.A0A098D5E5	pp	229533.A0A1C3YJ93
136806	229533.A0A098D5E5	pp	229533.Q4I2J8
136807	229533.A0A098D5E5	pp	229533.A0A1C3YIF4
136808	229533.A0A098D5E5	pp	229533.I1RQE9
136809	229533.A0A098D5E5	pp	229533.A0A1C3YHX6
136810	229533.A0A098D5E5	pp	229533.A0A0E0RZM5
136811	229533.A0A098D5E5	pp	229533.A0A098DNE7
136812	229533.A0A098D5E5	pp	229533.I1RYY5
136813	229533.A0A098D5E5	pp	229533.A0A1C3YKV3
136814	229533.A0A098D5E5	pp	229533.I1RJT1
136815	229533.A0A098D5E5	pp	229533.A0A1C3YLK2
136816	229533.A0A098D5E5	pp	229533.I1RQS4
136817	229533.A0A098D5E5	pp	229533.A0A1C3YK01
136818	229533.A0A098D5E5	pp	229533.I1RFQ5
136819	229533.A0A098D5E5	pp	229533.I1RFJ9
136820	229533.A0A098D5E5	pp	229533.I1RS18
136821	229533.A0A098D5E5	pp	229533.Q4I283
136822	229533.A0A098D5E5	pp	229533.V6QV38
136823	229533.A0A098D5E5	pp	229533.A0A098DYT7
136824	229533.A0A098D5E5	pp	229533.I1RS38
136825	229533.A0A098D5E5	pp	229533.Q4I8P5
136826	229533.A0A098D5E5	pp	229533.I1RC17
136827	229533.A0A098D5E5	pp	229533.I1RMR7
136828	229533.A0A098D5E5	pp	229533.I1RMB2
136829	229533.A0A098D5E5	pp	229533.A0A1C3YIJ4
136830	229533.A0A098D5E5	pp	229533.I1RLT6
136831	229533.A0A098D5E5	pp	229533.V6RR76
136832	229533.A0A098D5E5	pp	229533.A0A1C3YK34
136833	229533.A0A098D5E5	pp	229533.I1RZM1
136834	229533.A0A098D5E5	pp	229533.I1RKK0
136835	229533.A0A098D5E5	pp	229533.I1RMQ7
136836	229533.A0A098D5E5	pp	229533.I1RW99
136837	229533.A0A098D5E5	pp	229533.I1RLU6
136838	229533.A0A098D5E5	pp	229533.I1RDC9
136839	229533.A0A098D5E5	pp	229533.I1RKE9
136840	229533.A0A098D5E5	pp	229533.I1RAU6
136841	229533.A0A098D5E5	pp	229533.I1RCT9
136842	229533.A0A098D5E5	pp	229533.I1RS07
136843	229533.A0A098D5E5	pp	229533.I1S5B0
136844	229533.A0A098D5E5	pp	229533.I1RNQ5
136845	229533.A0A098D5E5	pp	229533.I1RBJ5
136846	229533.A0A098D5E5	pp	229533.I1RZH4
136847	229533.A0A098D5E5	pp	229533.A0A0E0SQV5
136848	229533.A0A098D5E5	pp	229533.Q4HTT2
136849	229533.A0A098D5E5	pp	229533.I1RME5
136850	229533.A0A098D5E5	pp	229533.V6QUE6
136851	229533.A0A098D5E5	pp	229533.I1RST9
136852	229533.A0A098D5E5	pp	229533.I1RAU5
136853	229533.A0A098D5E5	pp	229533.Q4I5R3
136854	229533.A0A098D5E5	pp	229533.I1RC95
136855	229533.A0A098D5E5	pp	229533.A0A0E0RV94
136856	229533.A0A098D5E5	pp	229533.I1RE22
136857	229533.A0A098D5E5	pp	229533.A0A1C3YK57
136858	229533.A0A098D5E5	pp	229533.V6RCZ8
136859	229533.A0A098D5E5	pp	229533.I1SA32
136860	229533.A0A098D5E5	pp	229533.V6RCG5
136861	229533.A0A098D5E5	pp	229533.I1RX09
136862	229533.A0A098D5E5	pp	229533.I1RAM7
136863	229533.A0A098D5E5	pp	229533.Q4HTT1
136864	229533.A0A098D5E5	pp	229533.A0A1C3YLU7
136865	229533.A0A098D5E5	pp	229533.A0A1C3YK75
136866	229533.A0A098D5E5	pp	229533.A0A1C3YI23
136867	229533.A0A098D5E5	pp	229533.A0A1C3YJZ9
136868	229533.A0A098D5E5	pp	229533.I1RXF0
136869	229533.A0A098D5E5	pp	229533.A0A1C3YJR3
136870	229533.A0A098D5E5	pp	229533.I1RZM8
136871	229533.A0A098D5E5	pp	229533.I1S504
136872	229533.A0A098D5E5	pp	229533.I1RCW8
136873	229533.A0A098D5E5	pp	229533.I1S549
136874	229533.A0A098D5E5	pp	229533.I1RWT6
136875	229533.A0A098D5E5	pp	229533.I1RCB3

136876	229533.A0A098D5E5	pp	229533.I1RQ19
136877	229533.A0A098D5E5	pp	229533.I1RM09
136878	229533.A0A098D5E5	pp	229533.A0A1C3YKB0
136879	229533.A0A098D5E5	pp	229533.A0A1C3YJZ2
136880	229533.A0A098D5E5	pp	229533.I1S0M6
136881	229533.A0A098D5E5	pp	229533.I1REE1
136882	229533.A0A098D5E5	pp	229533.A0A1C3YIG3
136883	229533.A0A098D5E5	pp	229533.I1RLU1
136884	229533.A0A098D5E5	pp	229533.I1RI82
136885	229533.A0A098D5E5	pp	229533.I1RA11
136886	229533.A0A098D5E5	pp	229533.I1RR73
136887	229533.A0A098D5E5	pp	229533.I1S0X7
136888	229533.A0A098D5E5	pp	229533.I1S5R1
136889	229533.A0A098D5E5	pp	229533.A0A1C3YI39
136890	229533.A0A098D5E5	pp	229533.I1RE15
136891	229533.A0A098D5E5	pp	229533.V6R3X5
136892	229533.A0A098D5E5	pp	229533.I1RRT8
136893	229533.A0A098D5E5	pp	229533.I1REX8
136894	229533.A0A098D5E5	pp	229533.A0A098DVE3
136895	229533.A0A098D5E5	pp	229533.I1RPM4
136896	229533.A0A098D5E5	pp	229533.I1RFT1
136897	229533.A0A098D5E5	pp	229533.A0A1C3YKN3
136898	229533.A0A098D5E5	pp	229533.I1RUN8
136899	229533.A0A098D5E5	pp	229533.I1RQ23
136900	229533.A0A098D5E5	pp	229533.I1RAN2
136901	229533.A0A098D5E5	pp	229533.A0A1C3YK73
136902	229533.A0A098D5E5	pp	229533.I1RZ63
136903	229533.A0A098D5E5	pp	229533.I1S352
136904	229533.A0A098D5E5	pp	229533.I1RC71
136905	229533.A0A098D5E5	pp	229533.I1RPX5
136906	229533.A0A098D5E5	pp	229533.I1RRJ8
136907	229533.A0A098D5E5	pp	229533.A0A1C3YMQ5
136908	229533.A0A098D5E5	pp	229533.I1RE27
136909	229533.A0A098D5E5	pp	229533.A0A1C3YIR8
136910	229533.A0A098D5E5	pp	229533.I1RWI5
136911	229533.A0A098D5E5	pp	229533.I1RW24
136912	229533.A0A098D5E5	pp	229533.I1RZZ9
136913	229533.A0A098D5E5	pp	229533.A0A1C3YIS7
136914	229533.A0A098D5E5	pp	229533.A0A1C3YHL0
136915	229533.A0A098D5E5	pp	229533.I1RFL7
136916	229533.A0A098D5E5	pp	229533.A0A1C3YMA1
136917	229533.A0A098D5E5	pp	229533.I1RB74
136918	229533.A0A098D5E5	pp	229533.I1S0J7
136919	229533.A0A098D5E5	pp	229533.I1RU69
136920	229533.A0A098D5E5	pp	229533.A0A1C3YIN0
136921	229533.A0A098D5E5	pp	229533.A0A098D8H0
136922	229533.A0A098D5E5	pp	229533.I1RC66
136923	229533.A0A098D5E5	pp	229533.I1RJZ7
136924	229533.A0A098D5E5	pp	229533.I1S8P5
136925	229533.A0A098D5E5	pp	229533.I1S2B8
136926	229533.A0A098D5E5	pp	229533.I1S8V3
136927	229533.A0A098D5E5	pp	229533.I1RPP3
136928	229533.A0A098D5E5	pp	229533.I1RX38
136929	229533.A0A098D5E5	pp	229533.I1RVY0
136930	229533.A0A098D5E5	pp	229533.I1RW83
136931	229533.A0A098D5E5	pp	229533.I1RRM5
136932	229533.A0A098D5E5	pp	229533.I1RQS8
136933	229533.A0A098D5E5	pp	229533.I1S7X9
136934	229533.A0A098D5E5	pp	229533.I1S2H4
136935	229533.A0A098D5E5	pp	229533.I1R9M0
136936	229533.A0A098D5E5	pp	229533.A0A0E0RU84
136937	229533.A0A098D5E5	pp	229533.I1RE76
136938	229533.A0A098D5E5	pp	229533.I1RN63
136939	229533.A0A098D5E5	pp	229533.I1RBY4
136940	229533.A0A098D5E5	pp	229533.I1RBU5
136941	229533.A0A098D5E5	pp	229533.I1RWK3
136942	229533.A0A098D5E5	pp	229533.I1RZ39
136943	229533.A0A098D5E5	pp	229533.A0A1C3YMJ3
136944	229533.A0A098D5E5	pp	229533.I1S0J3
136945	229533.A0A098D5E5	pp	229533.I1RBW1
136946	229533.A0A098D5E5	pp	229533.I1S0L4
136947	229533.A0A098D5E5	pp	229533.I1S2M6
136948	229533.A0A098D5E5	pp	229533.I1RFW1

136949	229533.A0A098D5E5	pp	229533.Q4I7L0
136950	229533.A0A098D5E5	pp	229533.A0A1C3YKM9
136951	229533.A0A098D5E5	pp	229533.I1RGF1
136952	229533.A0A098D5E5	pp	229533.I1RC97
136953	229533.A0A098D5E5	pp	229533.I1RFL4
136954	229533.A0A098D5E5	pp	229533.I1RN18
136955	229533.A0A098D5E5	pp	229533.A0A0E0SC46
136956	229533.A0A098D5E5	pp	229533.I1RIL3
136957	229533.A0A098D5E5	pp	229533.A0A098E4E9
136958	229533.A0A098D5E5	pp	229533.I1S1V5
136959	229533.A0A098D5E5	pp	229533.I1RM82
136960	229533.A0A098D5E5	pp	229533.A0A1C3YK17
136961	229533.A0A098D5E5	pp	229533.I1RNP7
136962	229533.A0A098D5E5	pp	229533.I1RQE7
136963	229533.A0A098D5E5	pp	229533.I1RY58
136964	229533.A0A098D5E5	pp	229533.A0A1C3YHP8
136965	229533.A0A098D5E5	pp	229533.I1RS47
136966	229533.A0A098D5E5	pp	229533.I1RZE4
136967	229533.A0A098D5E5	pp	229533.I1RCH5
136968	229533.A0A098D5E5	pp	229533.I1RNF4
136969	229533.A0A098D5E5	pp	229533.I1S2G7
136970	229533.A0A098D5E5	pp	229533.A0A1C3YKN4
136971	229533.A0A098D5E5	pp	229533.A0A0E0RP92
136972	229533.A0A098D5E5	pp	229533.I1S0F6
136973	229533.A0A098D5E5	pp	229533.Q4I624
136974	229533.A0A098D5E5	pp	229533.I1RMP0
136975	229533.A0A098D5E5	pp	229533.A0A098DMX4
136976	229533.A0A098D5E5	pp	229533.I1RD17
136977	229533.A0A098D5E5	pp	229533.I1RBC7
136978	229533.A0A098D5E5	pp	229533.I1RN67
136979	229533.A0A098D5E5	pp	229533.I1S569
136980	229533.A0A098D5E5	pp	229533.I1RGA4
136981	229533.A0A098D5E5	pp	229533.I1S0A6
136982	229533.A0A098D5E5	pp	229533.I1RTB2
136983	229533.A0A098D5E5	pp	229533.I1RX39
136984	229533.A0A098D5E5	pp	229533.A0A0E0S0W9
136985	229533.A0A098D5E5	pp	229533.A0A1C3YJG7
136986	229533.A0A098D5E5	pp	229533.I1RBS0
136987	229533.A0A098D5E5	pp	229533.V6RUY9
136988	229533.A0A098D5E5	pp	229533.I1S757
136989	229533.A0A098D5E5	pp	229533.I1S268
136990	229533.A0A098D5E5	pp	229533.I1RBS6
136991	229533.A0A098D5E5	pp	229533.V6R057
136992	229533.A0A098D5E5	pp	229533.A0A1C3YKT6
136993	229533.A0A098D5E5	pp	229533.I1RR25
136994	229533.A0A098D5E5	pp	229533.I1RT04
136995	229533.A0A098D5E5	pp	229533.A0A1C3YLF5
136996	229533.A0A098D5E5	pp	229533.I1S1B6
136997	229533.A0A098D5E5	pp	229533.I1RBV5
136998	229533.A0A098D5E5	pp	229533.I1RNA5
136999	229533.A0A098D5E5	pp	229533.I1RJV4
137000	229533.A0A098D5E5	pp	229533.I1RAU7
137001	229533.A0A098D5E5	pp	229533.I1RQ72
137002	229533.A0A098D5E5	pp	229533.I1RJ74
137003	229533.A0A098D5E5	pp	229533.A0A1C3YLK8
137004	229533.A0A098D5E5	pp	229533.I1R9J6
137005	229533.A0A098D5E5	pp	229533.I1R9X6
137006	229533.A0A098D5E5	pp	229533.A0A1C3YM29
137007	229533.A0A098D5E5	pp	229533.I1RK75
137008	229533.A0A098D5E5	pp	229533.I1S2E9
137009	229533.A0A098D5E5	pp	229533.I1RJW6
137010	229533.A0A098D5E5	pp	229533.A0A0E0SQG9
137011	229533.A0A098D5E5	pp	229533.I1RT40
137012	229533.A0A098D5E5	pp	229533.I1RG90
137013	229533.A0A098D5E5	pp	229533.I1RB41
137014	229533.A0A098D5E5	pp	229533.I1S503
137015	229533.A0A098D5E5	pp	229533.I1R9Y7
137016	229533.A0A098D5E5	pp	229533.A0A098E2M3
137017	229533.A0A098D5E5	pp	229533.I1RY32
137018	229533.A0A098D5E5	pp	229533.I1RGL4
137019	229533.A0A098D5E5	pp	229533.I1RLN6
137020	229533.A0A098D5E5	pp	229533.A0A1C3YI73
137021	229533.A0A098D5E5	pp	229533.A0A098D6T7

137022	229533.A0A098D5E5	pp	229533.I1RTH6
137023	229533.A0A098D5E5	pp	229533.I1RPU7
137024	229533.A0A098D5E5	pp	229533.I1RRZ0
137025	229533.A0A098D5E5	pp	229533.I1RA87
137026	229533.A0A098D5E5	pp	229533.A0A0E0SB24
137027	229533.A0A098D5E5	pp	229533.I1RGF5
137028	229533.A0A098D5E5	pp	229533.A0A1C3YKE4
137029	229533.A0A098D5E5	pp	229533.I1S8R3
137030	229533.A0A098D5E5	pp	229533.I1RQ88
137031	229533.A0A098D5E5	pp	229533.I1RXU1
137032	229533.A0A098D5E5	pp	229533.I1RTD5
137033	229533.A0A098D5E5	pp	229533.I1RRT2
137034	229533.A0A098D5E5	pp	229533.I1RA70
137035	229533.A0A098D5E5	pp	229533.I1RSG8
137036	229533.A0A098D5E5	pp	229533.I1RSZ3
137037	229533.A0A098D5E5	pp	229533.I1RRN5
137038	229533.A0A098D5E5	pp	229533.I1RW76
137039	229533.A0A098D5E5	pp	229533.I1RX64
137040	229533.A0A098D5E5	pp	229533.I1RT03
137041	229533.A0A098D5E5	pp	229533.I1RZK9
137042	229533.A0A098D5E5	pp	229533.A0A1C3YKJ6
137043	229533.A0A098D5E5	pp	229533.I1RPP4
137044	229533.A0A098D5E5	pp	229533.I1RPW0
137045	229533.A0A098D5E5	pp	229533.A0A098DX13
137046	229533.A0A098D5E5	pp	229533.I1RW16
137047	229533.A0A098D5E5	pp	229533.I1RSP7
137048	229533.A0A098D5E5	pp	229533.I1RVX0
137049	229533.A0A098D5E5	pp	229533.I1S1Y7
137050	229533.A0A098D5E5	pp	229533.I1RDZ4
137051	229533.A0A098D5E5	pp	229533.I1RSD1
137052	229533.A0A098D5E5	pp	229533.I1RB92
137053	229533.A0A098D5E5	pp	229533.I1RTA0
137054	229533.A0A098D5E5	pp	229533.I1RK79
137055	229533.A0A098D5E5	pp	229533.I1RCB7
137056	229533.A0A098D5E5	pp	229533.I1RYW3
137057	229533.A0A098D5E5	pp	229533.I1RW46
137058	229533.A0A098D5E5	pp	229533.I1RSQ3
137059	229533.A0A098D5E5	pp	229533.I1RE01
137060	229533.A0A098D5E5	pp	229533.I1RA43
137061	229533.A0A098D5E5	pp	229533.I1S1C7
137062	229533.A0A098D5E5	pp	229533.V6R042
137063	229533.A0A098D5E5	pp	229533.I1RZW8
137064	229533.A0A098D5E5	pp	229533.I1S0J1
137065	229533.A0A098D5E5	pp	229533.A0A1C3YM91
137066	229533.A0A098D5E5	pp	229533.I1RJT0
137067	229533.A0A098D5E5	pp	229533.I1RNY8
137068	229533.A0A098D5E5	pp	229533.I1RW32
137069	229533.A0A098D5E5	pp	229533.V6RMW6
137070	229533.A0A098D5E5	pp	229533.A0A1C3YJ12
137071	229533.A0A098D5E5	pp	229533.I1RWX5
137072	229533.A0A098D5E5	pp	229533.A0A1C3YN69
137073	229533.A0A098D5E5	pp	229533.I1RCR8
137074	229533.A0A098D5E5	pp	229533.I1R9Y9
137075	229533.A0A098D5E5	pp	229533.I1S1W4
137076	229533.A0A098D5E5	pp	229533.A0A1C3YLN8
137077	229533.A0A098D5E5	pp	229533.A0A0E0RS85
137078	229533.A0A098D5E5	pp	229533.I1RRE5
137079	229533.A0A098D5E5	pp	229533.A0A098DF98
137080	229533.A0A098D5E5	pp	229533.I1RRI2
137081	229533.A0A098D5E5	pp	229533.I1RZT1
137082	229533.A0A098D5E5	pp	229533.I1S0M2
137083	229533.A0A098D5E5	pp	229533.I1RWT5
137084	229533.A0A098D5E5	pp	229533.I1S8V4
137085	229533.A0A098D5E5	pp	229533.I1RQB4
137086	229533.A0A098D5E5	pp	229533.I1RNC1
137087	229533.A0A098D5E5	pp	229533.A0A098D635
137088	229533.A0A098D5E5	pp	229533.I1RQK5
137089	229533.A0A098D5E5	pp	229533.A0A1C3YI28
137090	229533.A0A098D5E5	pp	229533.I1RAW0
137091	229533.A0A098D5E5	pp	229533.I1RMJ8
137092	229533.A0A098D5E5	pp	229533.I1RDC5
137093	229533.A0A098D5E5	pp	229533.I1RUG3
137094	229533.A0A098D5E5	pp	229533.V6RKY7

137095 229533.A0A098D5E5 pp 229533.I1RD39
137096 229533.A0A098D5E5 pp 229533.I1RRW0
137097 229533.A0A098D5E5 pp 229533.I1RWN6
137098 229533.A0A098D5E5 pp 229533.A0A1C3YMI5
137099 229533.A0A098D5E5 pp 229533.A0A1C3YKS0
137100 229533.A0A098D5E5 pp 229533.I1RBJ0
137101 229533.A0A098D5E5 pp 229533.I1RMA4
137102 229533.A0A098D5E5 pp 229533.V6RGI1
137103 229533.A0A098D5E5 pp 229533.A0A1C3YK33
137104 229533.A0A098D5E5 pp 229533.A0A1C3YIN6
137105 229533.A0A098D5E5 pp 229533.I1RDQ9
137106 229533.A0A098D5E5 pp 229533.I1RWN5
137107 229533.A0A098D5E5 pp 229533.I1RE65
137108 229533.A0A098D5E5 pp 229533.V6RIV9
137109 229533.A0A098D5E5 pp 229533.I1RJV3
137110 229533.A0A098D5E5 pp 229533.A0A0E0SBC4
137111 229533.A0A098D5E5 pp 229533.I1RTN0
137112 229533.A0A098D5E5 pp 229533.I1RRY3
137113 229533.A0A098D5E5 pp 229533.I1RT36
137114 229533.A0A098D5E5 pp 229533.I1RGC6
137115 229533.A0A098D5E5 pp 229533.I1RG11
137116 229533.A0A098D5E5 pp 229533.I1RW84
137117 229533.A0A098D5E5 pp 229533.I1R9D1
137118 229533.A0A098D5E5 pp 229533.I1RSI3
137119 229533.A0A098D5E5 pp 229533.I1RS41
137120 229533.A0A098D5E5 pp 229533.I1RS17
137121 229533.A0A098D5E5 pp 229533.V6RVG3
137122 229533.A0A098D5E5 pp 229533.I1SA33
137123 229533.A0A098D5E5 pp 229533.I1RY44
137124 229533.A0A098D5E5 pp 229533.I1RD22
137125 229533.A0A098D5E5 pp 229533.I1RC33
137126 229533.A0A098D5E5 pp 229533.I1RWH4
137127 229533.A0A098D5E5 pp 229533.I1RRV1
137128 229533.A0A098D5E5 pp 229533.I1RMG3
137129 229533.A0A098D5E5 pp 229533.I1RA56
137130 229533.A0A098D5E5 pp 229533.A0A1C3YMC9
137131 229533.A0A098D5E5 pp 229533.A0A1C3YK43
137132 229533.A0A098D5E5 pp 229533.A0A1C3YL52
137133 229533.A0A098D5E5 pp 229533.I1SAF7
137134 229533.I1RVW0 pp 229533.I1RWM6
137135 229533.I1RVW0 pp 229533.I1RVX9
137136 229533.I1RVW0 pp 229533.I1S987
137137 229533.I1RVW0 pp 229533.I1S5Z3
137138 229533.I1RVW0 pp 229533.I1S283
137139 229533.I1RVW0 pp 229533.Q4INS6
137140 229533.I1RVW0 pp 229533.I1RXF0
137141 229533.I1RVW0 pp 229533.I1RVZ9
137142 229533.I1RVW0 pp 229533.I1S461
137143 229533.I1RVW0 pp 229533.I1S209
137144 229533.I1RVW0 pp 229533.I1S4P6
137145 229533.I1RVW0 pp 229533.I1RZ86
137146 229533.I1RVW0 pp 229533.I1RXV3
137147 229533.I1RVW0 pp 229533.Q4IJ84
137148 229533.I1RVW0 pp 229533.I1RWI8
137149 229533.I1RVW0 pp 229533.I1RZ98
137150 229533.I1RVW0 pp 229533.I1S998
137151 229533.I1RVW0 pp 229533.I1RX64
137152 229533.I1RVW0 pp 229533.I1S0D5
137153 229533.I1RVW0 pp 229533.V6RGI1
137154 229533.I1RVW0 pp 229533.I1RYQ1
137155 229533.I1RVW0 pp 229533.I1RY58
137156 229533.I1RVW0 pp 229533.I1RZT1
137157 229533.I1RFQ7 pp 229533.I1RPP4
137158 229533.I1RFQ7 pp 229533.Q4IAA0
137159 229533.I1RFQ7 pp 229533.I1RMQ5
137160 229533.I1RFQ7 pp 229533.I1S2C0
137161 229533.I1RFQ7 pp 229533.I1RFU8
137162 229533.I1RFQ7 pp 229533.I1RTH6
137163 229533.I1RFQ7 pp 229533.I1RTE9
137164 229533.I1RFQ7 pp 229533.V6R042
137165 229533.I1RFQ7 pp 229533.V6RMW6
137166 229533.I1RFQ7 pp 229533.I1RSQ3
137167 229533.I1RFQ7 pp 229533.Q4IJ56

137168	229533.I1RFQ7	pp	229533.I1RQ81
137169	229533.I1RFQ7	pp	229533.I1RYY5
137170	229533.I1RFQ7	pp	229533.Q4I6A4
137171	229533.I1RFQ7	pp	229533.I1RMQ4
137172	229533.I1RFQ7	pp	229533.I1RRV1
137173	229533.I1RFQ7	pp	229533.I1S0J1
137174	229533.I1RFQ7	pp	229533.I1S1C7
137175	229533.I1RFQ7	pp	229533.I1RYW3
137176	229533.I1RFQ7	pp	229533.I1RTA0
137177	229533.I1RFQ7	pp	229533.I1RQ31
137178	229533.I1RFQ7	pp	229533.I1RMC0
137179	229533.I1RFQ7	pp	229533.I1RY03
137180	229533.I1RFQ7	pp	229533.I1RNC1
137181	229533.I1RFQ7	pp	229533.V6R227
137182	229533.I1RFQ7	pp	229533.I1RWN6
137183	229533.I1RFQ7	pp	229533.I1RRY9
137184	229533.I1RFQ7	pp	229533.I1RWT6
137185	229533.I1RFQ7	pp	229533.I1RN26
137186	229533.I1RFQ7	pp	229533.I1RN58
137187	229533.I1RFQ7	pp	229533.Q4IN63
137188	229533.I1RFQ7	pp	229533.I1S1W4
137189	229533.I1RFQ7	pp	229533.I1RSK1
137190	229533.I1RFQ7	pp	229533.I1S0N4
137191	229533.I1RFQ7	pp	229533.I1RYS0
137192	229533.I1RFQ7	pp	229533.I1S044
137193	229533.I1RFQ7	pp	229533.I1RFT2
137194	229533.I1RFQ7	pp	229533.I1RJV3
137195	229533.I1RFQ7	pp	229533.I1S504
137196	229533.I1RFQ7	pp	229533.I1RWE8
137197	229533.I1RFQ7	pp	229533.I1RQA2
137198	229533.I1RFQ7	pp	229533.I1RY44
137199	229533.I1RFQ7	pp	229533.I1RI34
137200	229533.I1RFQ7	pp	229533.I1RRU9
137201	229533.I1RFQ7	pp	229533.I1RK95
137202	229533.I1RFQ7	pp	229533.I1RNQ5
137203	229533.I1RFQ7	pp	229533.Q4IBS2
137204	229533.I1RFQ7	pp	229533.I1RLU6
137205	229533.I1RFQ7	pp	229533.I1S1X9
137206	229533.I1RFQ7	pp	229533.I1S2E8
137207	229533.I1RFQ7	pp	229533.Q4IJM4
137208	229533.I1RFQ7	pp	229533.I1RZZ1
137209	229533.I1RFQ7	pp	229533.V6RBN2
137210	229533.I1RFQ7	pp	229533.I1RVX0
137211	229533.I1RFQ7	pp	229533.Q4IEK8
137212	229533.I1RFQ7	pp	229533.I1RT03
137213	229533.I1RFQ7	pp	229533.I1RZM1
137214	229533.I1RFQ7	pp	229533.I1RSD8
137215	229533.I1RFQ7	pp	229533.I1RRN5
137216	229533.I1RFQ7	pp	229533.I1RWN5
137217	229533.I1RFQ7	pp	229533.I1RSS4
137218	229533.I1RFQ7	pp	229533.I1RZK5
137219	229533.I1RFQ7	pp	229533.Q4IJK2
137220	229533.I1RFQ7	pp	229533.I1RP27
137221	229533.I1RFQ7	pp	229533.I1RT33
137222	229533.I1RFQ7	pp	229533.I1RQA3
137223	229533.I1RFQ7	pp	229533.I1RP28
137224	229533.I1RFQ7	pp	229533.I1S6F0
137225	229533.I1RFQ7	pp	229533.Q4IQW0
137226	229533.I1RFQ7	pp	229533.I1RSI0
137227	229533.I1RFQ7	pp	229533.I1RWD7
137228	229533.I1RFQ7	pp	229533.V6RTJ8
137229	229533.I1RFQ7	pp	229533.I1RN67
137230	229533.I1RFQ7	pp	229533.I1RGA4
137231	229533.I1RFQ7	pp	229533.V6R3X5
137232	229533.I1RFQ7	pp	229533.I1RQH3
137233	229533.I1RFQ7	pp	229533.I1RX46
137234	229533.I1RFQ7	pp	229533.I1RR73
137235	229533.I1RFQ7	pp	229533.I1S2E9
137236	229533.I1RFQ7	pp	229533.I1RXD7
137237	229533.I1RFQ7	pp	229533.Q4IJH1
137238	229533.I1RFQ7	pp	229533.I1RT04
137239	229533.I1RFQ7	pp	229533.I1S503
137240	229533.I1RFQ7	pp	229533.I1RQC9

137241 229533.I1RFQ7 pp 229533.I1S8P5
137242 229533.I1RFQ7 pp 229533.I1RFT6
137243 229533.I1RFQ7 pp 229533.I1RZ39
137244 229533.I1RFQ7 pp 229533.I1RPP3
137245 229533.I1RFQ7 pp 229533.I1S0R7
137246 229533.I1RFQ7 pp 229533.I1RQJ5
137247 229533.I1RFQ7 pp 229533.Q4HXF0
137248 229533.I1RFQ7 pp 229533.I1RNN1
137249 229533.I1RFQ7 pp 229533.I1RSQ7
137250 229533.I1RFQ7 pp 229533.I1RXU7
137251 229533.I1RFQ7 pp 229533.I1RZZ9
137252 229533.I1RFQ7 pp 229533.I1RN61
137253 229533.I1RFQ7 pp 229533.I1RWI5
137254 229533.I1RFQ7 pp 229533.I1RQG4
137255 229533.I1RFQ7 pp 229533.Q4IF76
137256 229533.I1RFQ7 pp 229533.I1RTD5
137257 229533.I1RFQ7 pp 229533.I1RS20
137258 229533.I1RFQ7 pp 229533.I1RW71
137259 229533.I1RFQ7 pp 229533.Q4INZ9
137260 229533.I1RFQ7 pp 229533.V6QUW4
137261 229533.I1RFQ7 pp 229533.I1S5R1
137262 229533.I1RFQ7 pp 229533.Q4INI2
137263 229533.I1RFQ7 pp 229533.I1RSV2
137264 229533.I1RFQ7 pp 229533.I1RN63
137265 229533.I1RFQ7 pp 229533.I1S2G7
137266 229533.I1RFQ7 pp 229533.I1RM82
137267 229533.I1RFQ7 pp 229533.I1RWL2
137268 229533.I1RFQ7 pp 229533.I1RS47
137269 229533.I1RFQ7 pp 229533.I1RX24
137270 229533.I1RFQ7 pp 229533.I1RW25
137271 229533.I1RFQ7 pp 229533.V6QYF0
137272 229533.I1RFQ7 pp 229533.I1RPU3
137273 229533.I1RFQ7 pp 229533.I1RJZ7
137274 229533.I1RFQ7 pp 229533.I1S2B8
137275 229533.A0A098DT69 pp 229533.I1RLM3
137276 229533.A0A098DT69 pp 229533.I1RJU9
137277 229533.A0A098DT69 pp 229533.A0A0E0SP71
137278 229533.I1S2K0 pp 229533.I1SAJ7
137279 229533.I1S2K0 pp 229533.I1SAJ8
137280 229533.I1RSK0 pp 229533.I1S0D5
137281 229533.I1RSK0 pp 229533.Q4IB89
137282 229533.I1RSK0 pp 229533.I1RUG2
137283 229533.I1RSK0 pp 229533.I1RZH4
137284 229533.I1RSK0 pp 229533.I1S4P6
137285 229533.I1RSK0 pp 229533.I1RY58
137286 229533.I1RSK0 pp 229533.V6RLW4
137287 229533.I1RSK0 pp 229533.Q4INS6
137288 229533.I1RSK0 pp 229533.I1S050
137289 229533.I1RSK0 pp 229533.I1RZT1
137290 229533.I1RSK0 pp 229533.I1RTN6
137291 229533.I1RSK0 pp 229533.O74638
137292 229533.I1RSK0 pp 229533.Q4IR08
137293 229533.I1RSK0 pp 229533.I1RYX9
137294 229533.I1RSK0 pp 229533.I1RY73
137295 229533.I1RWJ8 pp 229533.I1RX46
137296 229533.I1RWJ8 pp 229533.I1RXD7
137297 229533.I1RWJ8 pp 229533.I1S0R7
137298 229533.I1RWJ8 pp 229533.Q4IEK8
137299 229533.I1RWJ8 pp 229533.I1S7A8
137300 229533.I1RWJ8 pp 229533.Q4I8P5
137301 229533.I1RWJ8 pp 229533.V6QW22
137302 229533.I1RWJ8 pp 229533.V6RTJ8
137303 229533.I1RWJ8 pp 229533.Q4IJT5
137304 229533.I1RWJ8 pp 229533.I1RZM1
137305 229533.I1RWJ8 pp 229533.I1S6F0
137306 229533.I1RWJ8 pp 229533.V6QUW4
137307 229533.I1RWJ8 pp 229533.V6R1K4
137308 229533.I1RWJ8 pp 229533.I1S504
137309 229533.I1RWJ8 pp 229533.V6QYF0
137310 229533.I1RWJ8 pp 229533.I1S0L3
137311 229533.I1RWJ8 pp 229533.I1RX25
137312 229533.I1RWJ8 pp 229533.I1S0F5
137313 229533.I1RWJ8 pp 229533.Q4I283

137314 229533.I1RWJ8 pp 229533.I1RXF0
137315 229533.I1RWJ8 pp 229533.I1S0X6
137316 229533.I1RWJ8 pp 229533.V6R227
137317 229533.I1RWJ8 pp 229533.I1RWT6
137318 229533.I1RWJ8 pp 229533.I1RZJ5
137319 229533.I1RWJ8 pp 229533.I1RY63
137320 229533.I1RWJ8 pp 229533.I1RY03
137321 229533.I1RWJ8 pp 229533.I1RZM8
137322 229533.I1RWJ8 pp 229533.I1RZK5
137323 229533.I1RWJ8 pp 229533.Q4I2J8
137324 229533.I1RWJ8 pp 229533.V6RCZ8
137325 229533.I1RWJ8 pp 229533.V6RCG5
137326 229533.I1RWJ8 pp 229533.I1S1J7
137327 229533.I1RWJ8 pp 229533.I1S5B0
137328 229533.I1RWJ8 pp 229533.V6RUY9
137329 229533.I1RWJ8 pp 229533.Q4IJK2
137330 229533.I1RWJ8 pp 229533.I1RZZ1
137331 229533.I1RWJ8 pp 229533.I1S503
137332 229533.I1RWJ8 pp 229533.I1S2E9
137333 229533.I1RWJ8 pp 229533.I1S2M6
137334 229533.I1RWJ8 pp 229533.I1SA32
137335 229533.I1RWJ8 pp 229533.I1RWK3
137336 229533.I1RWJ8 pp 229533.I1S0F6
137337 229533.I1RWJ8 pp 229533.I1RWQ8
137338 229533.I1RWJ8 pp 229533.I1S0L4
137339 229533.I1RWJ8 pp 229533.V6RGN5
137340 229533.I1RWJ8 pp 229533.I1S2H4
137341 229533.I1RWJ8 pp 229533.I1S0J1
137342 229533.I1RWJ8 pp 229533.I1RZZ9
137343 229533.I1RWJ8 pp 229533.I1S0M6
137344 229533.I1RWJ8 pp 229533.V6R3X5
137345 229533.I1RWJ8 pp 229533.I1RZ39
137346 229533.I1RWJ8 pp 229533.I1RWN5
137347 229533.I1RWJ8 pp 229533.I1S2B8
137348 229533.I1RWJ8 pp 229533.V6RIV9
137349 229533.I1RWJ8 pp 229533.I1S7S9
137350 229533.I1RWJ8 pp 229533.O74638
137351 229533.I1RWJ8 pp 229533.I1S5R1
137352 229533.I1RWJ8 pp 229533.V6RMW6
137353 229533.I1RWJ8 pp 229533.Q4HZB8
137354 229533.I1RWJ8 pp 229533.V6R042
137355 229533.I1RWJ8 pp 229533.I1S8R3
137356 229533.I1RWJ8 pp 229533.I1RY44
137357 229533.I1RWJ8 pp 229533.I1SA33
137358 229533.I1RWJ8 pp 229533.I1S1C7
137359 229533.I1RWJ8 pp 229533.V6R057
137360 229533.I1RWJ8 pp 229533.I1RYW3
137361 229533.I1RWJ8 pp 229533.I1RWN6
137362 229533.I1RWJ8 pp 229533.I1RZW8
137363 229533.I1RWJ8 pp 229533.Q4I624
137364 229533.I1RWJ8 pp 229533.I1RWX5
137365 229533.I1RRS7 pp 229533.Q4IEA5
137366 229533.I1RRS7 pp 229533.I1RYR2
137367 229533.I1RRS7 pp 229533.I1RYX9
137368 229533.I1RRS7 pp 229533.Q4IP51
137369 229533.I1RRS7 pp 229533.I1RSI4
137370 229533.I1RRS7 pp 229533.I1RRY1
137371 229533.I1RRS7 pp 229533.I1RS81
137372 229533.I1RRS7 pp 229533.Q4IB89
137373 229533.I1RRS7 pp 229533.V6RLW4
137374 229533.I1RRS7 pp 229533.Q4IQM6
137375 229533.I1RRS7 pp 229533.I1RWM6
137376 229533.I1RRS7 pp 229533.Q4IA62
137377 229533.I1RRS7 pp 229533.I1RYQ1
137378 229533.I1RRS7 pp 229533.Q4IJ56
137379 229533.I1RRS7 pp 229533.I1S987
137380 229533.I1RRS7 pp 229533.I1RVW0
137381 229533.I1RRS7 pp 229533.I1RSA7
137382 229533.I1RRS7 pp 229533.I1RUG4
137383 229533.I1RRS7 pp 229533.I1RT36
137384 229533.I1RRS7 pp 229533.Q4IC11
137385 229533.I1RRS7 pp 229533.I1S461
137386 229533.I1RRS7 pp 229533.I1RUG2

137387	229533.I1RRS7	pp	229533.V6RGI1
137388	229533.I1RRS7	pp	229533.Q4I1Q6
137389	229533.I1RRS7	pp	229533.I1S998
137390	229533.I1RRS7	pp	229533.I1S209
137391	229533.I1RRS7	pp	229533.I1S0D5
137392	229533.I1RRS7	pp	229533.I1RY73
137393	229533.I1RRS7	pp	229533.I1RRT1
137394	229533.I1RRS7	pp	229533.I1RZ94
137395	229533.I1RRS7	pp	229533.I1RSC7
137396	229533.I1RRS7	pp	229533.Q4INS6
137397	229533.I1RRS7	pp	229533.I1S283
137398	229533.I1RRS7	pp	229533.I1RZV9
137399	229533.I1RRS7	pp	229533.I1RX64
137400	229533.I1RRS7	pp	229533.Q4IJU0
137401	229533.I1RRS7	pp	229533.I1RSP7
137402	229533.I1RRS7	pp	229533.I1RZP8
137403	229533.I1RRS7	pp	229533.I1RUL3
137404	229533.I1RX10	pp	229533.Q4I1N3
137405	229533.I1RAW3	pp	229533.I1RRX5
137406	229533.I1RAW3	pp	229533.I1S464
137407	229533.I1RAW3	pp	229533.I1S8F0
137408	229533.I1RAW3	pp	229533.I1RE66
137409	229533.I1RAW3	pp	229533.V6R3J1
137410	229533.I1RAW3	pp	229533.I1S0P0
137411	229533.I1RAW3	pp	229533.I1RSQ3
137412	229533.I1RAW3	pp	229533.Q4IPI1
137413	229533.I1RAW3	pp	229533.I1S0Q7
137414	229533.I1RAW3	pp	229533.I1RBE6
137415	229533.I1RAW3	pp	229533.Q4IB65
137416	229533.I1RAW3	pp	229533.I1RXD9
137417	229533.I1RAW3	pp	229533.I1RX39
137418	229533.I1RAW3	pp	229533.V6R1X4
137419	229533.I1RAW3	pp	229533.I1RXE6
137420	229533.I1RAW3	pp	229533.I1RNM7
137421	229533.I1RAW3	pp	229533.I1RLP1
137422	229533.I1RAW3	pp	229533.I1RS05
137423	229533.I1RAW3	pp	229533.I1RWA3
137424	229533.I1RAW3	pp	229533.I1RRH6
137425	229533.I1RAW3	pp	229533.I1RYT8
137426	229533.I1RAW3	pp	229533.I1RE65
137427	229533.I1RAW3	pp	229533.I1RP02
137428	229533.I1RAW3	pp	229533.I1RKB7
137429	229533.I1RAW3	pp	229533.I1RJT3
137430	229533.I1RAW3	pp	229533.I1RMR9
137431	229533.I1RAW3	pp	229533.I1S0R8
137432	229533.I1RAW3	pp	229533.I1RXZ3
137433	229533.I1RAW3	pp	229533.I1RMQ6
137434	229533.I1RAW3	pp	229533.I1RG62
137435	229533.I1RAW3	pp	229533.I1RY92
137436	229533.I1RAW3	pp	229533.I1S0K2
137437	229533.I1RAW3	pp	229533.Q4I6B0
137438	229533.I1RAW3	pp	229533.I1S7W5
137439	229533.I1RAW3	pp	229533.I1RFJ6
137440	229533.I1RAW3	pp	229533.I1RS29
137441	229533.I1RAW3	pp	229533.I1S212
137442	229533.I1RAW3	pp	229533.I1RB38
137443	229533.I1RAW3	pp	229533.I1RWB0
137444	229533.I1RAW3	pp	229533.I1RPR9
137445	229533.I1RAW3	pp	229533.I1RMV0
137446	229533.I1RAW3	pp	229533.V6QU63
137447	229533.I1RAW3	pp	229533.I1S5X9
137448	229533.I1RAW3	pp	229533.V6RJP8
137449	229533.I1RAW3	pp	229533.Q4I948
137450	229533.I1RAW3	pp	229533.I1RFJ5
137451	229533.I1RAW3	pp	229533.I1RYH7
137452	229533.I1RAW3	pp	229533.I1RBT6
137453	229533.I1RAW3	pp	229533.Q4IPZ1
137454	229533.I1RAW3	pp	229533.I1RZ43
137455	229533.I1RAW3	pp	229533.I1S8S9
137456	229533.I1RZJ5	pp	229533.I1RZW8
137457	229533.I1RZJ5	pp	229533.I1S8R3
137458	229533.I1RZJ5	pp	229533.I1RZM1
137459	229533.I1RZJ5	pp	229533.Q4I8P5

137460	229533.I1RZJ5	pp	229533.I1S0L4
137461	229533.I1RZJ5	pp	229533.V6RIV9
137462	229533.I1RZJ5	pp	229533.I1SA32
137463	229533.I1RZJ5	pp	229533.I1S5R1
137464	229533.I1RZJ5	pp	229533.V6R3X5
137465	229533.I1RZJ5	pp	229533.I1S7A8
137466	229533.I1RZJ5	pp	229533.I1RZM8
137467	229533.I1RZJ5	pp	229533.I1SA33
137468	229533.I1RZJ5	pp	229533.I1RZZ9
137469	229533.I1RZJ5	pp	229533.I1S5B0
137470	229533.I1RZJ5	pp	229533.V6RTJ8
137471	229533.I1RZJ5	pp	229533.I1S0L3
137472	229533.I1RZJ5	pp	229533.I1S0R7
137473	229533.I1RZJ5	pp	229533.I1S0F6
137474	229533.I1RZJ5	pp	229533.V6RCG5
137475	229533.I1RZJ5	pp	229533.Q4I624
137476	229533.I1RZJ5	pp	229533.I1RZZ1
137477	229533.I1RZJ5	pp	229533.V6RMW6
137478	229533.I1RZJ5	pp	229533.V6QW22
137479	229533.I1RZJ5	pp	229533.V6R042
137480	229533.I1RZJ5	pp	229533.V6RCZ8
137481	229533.I1RDF7	pp	229533.I1RJU3
137482	229533.I1RDF7	pp	229533.I1RZ39
137483	229533.I1RDF7	pp	229533.Q4I624
137484	229533.I1RDF7	pp	229533.V6R9Z0
137485	229533.I1RDF7	pp	229533.I1REK8
137486	229533.I1RDF7	pp	229533.I1RQ33
137487	229533.I1RDF7	pp	229533.I1RVQ3
137488	229533.I1RDF7	pp	229533.I1S2B8
137489	229533.I1RDF7	pp	229533.I1RZK5
137490	229533.I1RDF7	pp	229533.I1RJV3
137491	229533.I1RDF7	pp	229533.I1RE89
137492	229533.I1RDF7	pp	229533.I1RKJ5
137493	229533.I1RDF7	pp	229533.I1S5R1
137494	229533.I1RDF7	pp	229533.I1S9I5
137495	229533.I1RDF7	pp	229533.I1RKJ8
137496	229533.I1RDF7	pp	229533.I1RML0
137497	229533.I1RDF7	pp	229533.I1RNT9
137498	229533.I1RDF7	pp	229533.I1RT03
137499	229533.I1RDF7	pp	229533.I1RXC2
137500	229533.I1RDF7	pp	229533.I1S437
137501	229533.I1RDF7	pp	229533.I1S821
137502	229533.I1RDF7	pp	229533.I1S1C7
137503	229533.I1RDF7	pp	229533.I1RVX0
137504	229533.I1RDF7	pp	229533.V6RCZ8
137505	229533.I1RDF7	pp	229533.I1RGC5
137506	229533.I1RDF7	pp	229533.I1RUQ3
137507	229533.I1RDF7	pp	229533.I1RGU7
137508	229533.I1RDF7	pp	229533.I1RPE0
137509	229533.I1RDF7	pp	229533.I1RUF7
137510	229533.I1RDF7	pp	229533.I1RSQ3
137511	229533.I1RDF7	pp	229533.I1REC5
137512	229533.I1RDF7	pp	229533.I1RXD7
137513	229533.I1RDF7	pp	229533.I1RR24
137514	229533.I1RDF7	pp	229533.I1S438
137515	229533.I1RDF7	pp	229533.I1RKP1
137516	229533.I1RDF7	pp	229533.V6R042
137517	229533.I1RDF7	pp	229533.Q4IJK2
137518	229533.I1RDF7	pp	229533.I1RRY9
137519	229533.I1RDF7	pp	229533.I1S0L4
137520	229533.I1RDF7	pp	229533.I1RRM3
137521	229533.I1RDF7	pp	229533.I1RSZ7
137522	229533.I1RDF7	pp	229533.I1RQE7
137523	229533.I1RDF7	pp	229533.I1RRI1
137524	229533.I1RDF7	pp	229533.V6RMW6
137525	229533.I1RDF7	pp	229533.I1RGB2
137526	229533.I1RDF7	pp	229533.I1S1W9
137527	229533.I1RDF7	pp	229533.I1RWR7
137528	229533.I1RDF7	pp	229533.I1RT40
137529	229533.I1RDF7	pp	229533.I1RTB3
137530	229533.I1RDF7	pp	229533.I1S0W4
137531	229533.I1RDF7	pp	229533.I1RX46
137532	229533.I1RDF7	pp	229533.I1S4S7

137533 229533.I1RDF7 pp 229533.I1RZV3
137534 229533.I1RDF7 pp 229533.I1RNC1
137535 229533.I1RDF7 pp 229533.I1RZM8
137536 229533.I1RDF7 pp 229533.I1RNB2
137537 229533.I1RDF7 pp 229533.I1RSQ7
137538 229533.I1RDF7 pp 229533.I1RUG3
137539 229533.I1RDF7 pp 229533.I1RXD6
137540 229533.I1RDF7 pp 229533.Q4I7T5
137541 229533.I1RDF7 pp 229533.I1RVF8
137542 229533.I1RDF7 pp 229533.I1RPP3
137543 229533.I1RDF7 pp 229533.I1RYI5
137544 229533.I1RDF7 pp 229533.I1RX95
137545 229533.I1RDF7 pp 229533.I1RXF8
137546 229533.I1RDF7 pp 229533.I1RXC4
137547 229533.I1RDF7 pp 229533.I1RPH6
137548 229533.I1RDF7 pp 229533.I1RE01
137549 229533.I1RDF7 pp 229533.I1RPY3
137550 229533.I1RDF7 pp 229533.I1S2R0
137551 229533.I1RDF7 pp 229533.I1RTD5
137552 229533.I1RDF7 pp 229533.I1RVZ2
137553 229533.I1RDF7 pp 229533.I1RKJ4
137554 229533.I1RDF7 pp 229533.I1S5R6
137555 229533.I1RDF7 pp 229533.I1RGA4
137556 229533.I1RDF7 pp 229533.I1S0J1
137557 229533.I1RDF7 pp 229533.I1RQD2
137558 229533.I1RDF7 pp 229533.I1RJN9
137559 229533.I1RDF7 pp 229533.I1RTA1
137560 229533.I1RDF7 pp 229533.V6RTJ8
137561 229533.I1RDF7 pp 229533.I1RY86
137562 229533.I1RDF7 pp 229533.I1RSA5
137563 229533.I1RDF7 pp 229533.I1RQ85
137564 229533.I1RDF7 pp 229533.Q4HVQ9
137565 229533.I1RDF7 pp 229533.I1RPU2
137566 229533.I1RDF7 pp 229533.I1RSF1
137567 229533.I1RDF7 pp 229533.I1RRV3
137568 229533.I1RDF7 pp 229533.I1S1B2
137569 229533.A0A1C3YL77 pp 229533.Q4IB50
137570 229533.A0A1C3YL77 pp 229533.I1RCW8
137571 229533.A0A1C3YL77 pp 229533.I1RMK8
137572 229533.A0A1C3YL77 pp 229533.V6RGI1
137573 229533.A0A1C3YL77 pp 229533.I1RFW1
137574 229533.A0A1C3YL77 pp 229533.I1RU02
137575 229533.A0A1C3YL77 pp 229533.Q4IMD1
137576 229533.A0A1C3YL77 pp 229533.Q4IER8
137577 229533.I1RZM8 pp 229533.I1SA32
137578 229533.I1RZM8 pp 229533.I1S504
137579 229533.I1RZM8 pp 229533.V6R227
137580 229533.I1RZM8 pp 229533.Q4I8P5
137581 229533.I1RZM8 pp 229533.V6RCZ8
137582 229533.I1RZM8 pp 229533.I1S419
137583 229533.I1RZM8 pp 229533.V6QUE6
137584 229533.I1RZM8 pp 229533.Q4I283
137585 229533.I1RZM8 pp 229533.Q4IJK2
137586 229533.I1RZM8 pp 229533.I1S6K9
137587 229533.I1RZM8 pp 229533.I1S821
137588 229533.I1RZM8 pp 229533.V6RCG5
137589 229533.I1RZM8 pp 229533.I1S1B2
137590 229533.I1RZM8 pp 229533.I1S5B0
137591 229533.I1RZM8 pp 229533.I1S4S7
137592 229533.I1RZM8 pp 229533.Q4IJT5
137593 229533.I1RZM8 pp 229533.I1RZZ1
137594 229533.I1RZM8 pp 229533.I1S0P0
137595 229533.I1RZM8 pp 229533.I1S887
137596 229533.I1RZM8 pp 229533.V6RBN2
137597 229533.I1RZM8 pp 229533.I1S0K2
137598 229533.I1RZM8 pp 229533.V6QW22
137599 229533.I1RZM8 pp 229533.V6R1K4
137600 229533.I1RZM8 pp 229533.V6RUT5
137601 229533.I1RZM8 pp 229533.I1S0L3
137602 229533.I1RZM8 pp 229533.V6R9Z0
137603 229533.I1RZM8 pp 229533.Q4I2J8
137604 229533.I1RZM8 pp 229533.I1S1J7
137605 229533.I1RZM8 pp 229533.Q4IFI0

137606	229533.I1RZM8	pp	229533.I1S0F5
137607	229533.I1RZM8	pp	229533.V6RTJ8
137608	229533.I1RZM8	pp	229533.I1S2R0
137609	229533.I1RZM8	pp	229533.I1S0T2
137610	229533.I1RZM8	pp	229533.I1S0X6
137611	229533.I1RZM8	pp	229533.I1S7W5
137612	229533.I1RZM8	pp	229533.I1S0R7
137613	229533.I1RZM8	pp	229533.I1S9I5
137614	229533.I1RZM8	pp	229533.I1S7A8
137615	229533.I1RZM8	pp	229533.I1S4S4
137616	229533.I1RZM8	pp	229533.I1S438
137617	229533.I1RZM8	pp	229533.V6R2A1
137618	229533.I1RZM8	pp	229533.I1S0J1
137619	229533.I1RZM8	pp	229533.V6QYF0
137620	229533.I1RZM8	pp	229533.V6RIV9
137621	229533.I1RZM8	pp	229533.I1S016
137622	229533.I1RZM8	pp	229533.V6RRH2
137623	229533.I1RZM8	pp	229533.V6R042
137624	229533.I1RZM8	pp	229533.I1S1W9
137625	229533.I1RZM8	pp	229533.I1S8V4
137626	229533.I1RZM8	pp	229533.V6RMW6
137627	229533.I1RZM8	pp	229533.I1S8R3
137628	229533.I1RZM8	pp	229533.V6R057
137629	229533.I1RZM8	pp	229533.I1S1W3
137630	229533.I1RZM8	pp	229533.I1RZV3
137631	229533.I1RZM8	pp	229533.I1S1C7
137632	229533.I1RZM8	pp	229533.I1S0F8
137633	229533.I1RZM8	pp	229533.I1S5R6
137634	229533.I1RZM8	pp	229533.Q4I624
137635	229533.I1RZM8	pp	229533.I1SA33
137636	229533.I1RZM8	pp	229533.I1S437
137637	229533.I1RZM8	pp	229533.V6RGN5
137638	229533.I1RZM8	pp	229533.I1S2E9
137639	229533.I1RZM8	pp	229533.I1S2C0
137640	229533.I1RZM8	pp	229533.I1S2C4
137641	229533.I1RZM8	pp	229533.I1RZW8
137642	229533.I1RZM8	pp	229533.I1S0W4
137643	229533.I1RZM8	pp	229533.I1S503
137644	229533.I1RZM8	pp	229533.I1S8P5
137645	229533.I1RZM8	pp	229533.I1S274
137646	229533.I1RZM8	pp	229533.Q4HVQ9
137647	229533.I1RZM8	pp	229533.I1S277
137648	229533.I1RZM8	pp	229533.V6R3J1
137649	229533.I1RZM8	pp	229533.I1S0F7
137650	229533.I1RZM8	pp	229533.I1S2M6
137651	229533.I1RZM8	pp	229533.I1S2B8
137652	229533.I1RZM8	pp	229533.I1S0F6
137653	229533.I1RZM8	pp	229533.Q4HXF0
137654	229533.I1RZM8	pp	229533.I1RZZ9
137655	229533.I1RZM8	pp	229533.I1S8V3
137656	229533.I1RZM8	pp	229533.I1S268
137657	229533.I1RZM8	pp	229533.I1S4Z8
137658	229533.I1RZM8	pp	229533.V6R3X5
137659	229533.I1RZM8	pp	229533.I1S5R1
137660	229533.I1RZM8	pp	229533.I1S6Y8
137661	229533.I1RZM8	pp	229533.I1S0M6
137662	229533.I1RZM8	pp	229533.V6RUY9
137663	229533.I1RZM8	pp	229533.I1S0L4
137664	229533.I1RZM8	pp	229533.I1S9Y1
137665	229533.I1RZM8	pp	229533.O74638
137666	229533.I1RZM8	pp	229533.I1S2H4
137667	229533.A0A098DT21	pp	229533.V6QXN0
137668	229533.A0A098DT21	pp	229533.A0A1C3YJ89
137669	229533.A0A098DT21	pp	229533.I1S053
137670	229533.A0A098DT21	pp	229533.V6RIV7
137671	229533.A0A098DT21	pp	229533.I1RMD6
137672	229533.A0A1C3YND0	pp	229533.I1RNG4
137673	229533.I1RYR2	pp	229533.V6RLW4
137674	229533.I1RYR2	pp	229533.I1S987
137675	229533.I1RYR2	pp	229533.I1S604
137676	229533.I1RYR2	pp	229533.Q4IC11
137677	229533.I1RYR2	pp	229533.I1S209
137678	229533.I1RYR2	pp	229533.Q4I5R9

137679	229533.I1RYR2	pp	229533.I1S998
137680	229533.I1RYR2	pp	229533.Q4INS6
137681	229533.I1RYR2	pp	229533.I1S461
137682	229533.I1RYR2	pp	229533.V6R382
137683	229533.I1RYR2	pp	229533.Q4HYB8
137684	229533.I1RYR2	pp	229533.I1RZP8
137685	229533.I1RYR2	pp	229533.Q4IQM6
137686	229533.I1RYR2	pp	229533.I1S2F2
137687	229533.I1RYR2	pp	229533.I1RYX9
137688	229533.I1RYR2	pp	229533.I1RZ94
137689	229533.I1RYR2	pp	229533.Q4IEA5
137690	229533.I1RYR2	pp	229533.I1S283
137691	229533.I1RYR2	pp	229533.Q4IP51
137692	229533.I1RYR2	pp	229533.Q4IMM0
137693	229533.I1RBR1	pp	229533.I1RC34
137694	229533.I1RBR1	pp	229533.I1RRV3
137695	229533.I1RBR1	pp	229533.I1S5B0
137696	229533.I1RBR1	pp	229533.I1RBR2
137697	229533.I1RBR1	pp	229533.V6RTJ8
137698	229533.I1RBR1	pp	229533.I1RQD2
137699	229533.I1RBR1	pp	229533.I1S5R6
137700	229533.I1RBR1	pp	229533.I1RGA4
137701	229533.I1RBR1	pp	229533.I1RXF8
137702	229533.I1RBR1	pp	229533.I1RVF8
137703	229533.I1RBR1	pp	229533.I1RKJ4
137704	229533.I1RBR1	pp	229533.I1S1W9
137705	229533.I1RBR1	pp	229533.I1RTD5
137706	229533.I1RBR1	pp	229533.I1RQ85
137707	229533.I1RBR1	pp	229533.I1RCR8
137708	229533.I1RBR1	pp	229533.I1RSA5
137709	229533.I1RBR1	pp	229533.I1RT40
137710	229533.I1RBR1	pp	229533.I1RZM8
137711	229533.I1RBR1	pp	229533.I1RY86
137712	229533.I1RBR1	pp	229533.I1RPU2
137713	229533.I1RBR1	pp	229533.I1RSF1
137714	229533.I1RBR1	pp	229533.I1RUG3
137715	229533.I1RBR1	pp	229533.I1RD52
137716	229533.I1RBR1	pp	229533.I1RNC1
137717	229533.I1RBR1	pp	229533.I1RJN9
137718	229533.I1RBR1	pp	229533.I1RVZ2
137719	229533.I1RBR1	pp	229533.V6R042
137720	229533.I1RBR1	pp	229533.I1RNB2
137721	229533.I1RBR1	pp	229533.I1RXD7
137722	229533.I1RBR1	pp	229533.I1RYR0
137723	229533.I1RBR1	pp	229533.I1RSQ3
137724	229533.I1RBR1	pp	229533.I1RTA1
137725	229533.I1RBR1	pp	229533.I1RE01
137726	229533.I1RBR1	pp	229533.I1RXC4
137727	229533.I1RBR1	pp	229533.I1RX95
137728	229533.I1RBR1	pp	229533.V6RMW6
137729	229533.I1RBR1	pp	229533.I1RRM3
137730	229533.I1RBR1	pp	229533.I1RYI5
137731	229533.I1RBR1	pp	229533.I1RTB3
137732	229533.I1RBR1	pp	229533.I1RSQ7
137733	229533.I1RBR1	pp	229533.I1RD22
137734	229533.I1RBR1	pp	229533.I1S821
137735	229533.I1RBR1	pp	229533.I1RSX0
137736	229533.I1RBR1	pp	229533.V6RCZ8
137737	229533.I1RBR1	pp	229533.I1RPP3
137738	229533.I1RBR1	pp	229533.I1S4S7
137739	229533.I1RBR1	pp	229533.I1S6K9
137740	229533.I1RBR1	pp	229533.I1RPK0
137741	229533.I1RBR1	pp	229533.I1RZV3
137742	229533.I1RBR1	pp	229533.I1S1B2
137743	229533.I1RBR1	pp	229533.I1S5R1
137744	229533.I1RBR1	pp	229533.I1RJV3
137745	229533.I1RBR1	pp	229533.I1RRN2
137746	229533.I1RBR1	pp	229533.I1RXC2
137747	229533.I1RBR1	pp	229533.I1RM37
137748	229533.I1RBR1	pp	229533.I1RCH5
137749	229533.I1RBR1	pp	229533.Q4IJK2
137750	229533.I1RBR1	pp	229533.I1RXD6
137751	229533.I1RBR1	pp	229533.I1RSZ7

229533.I1RBR1	pp	229533.I1RDF7
229533.I1RBR1	pp	229533.I1S4S4
229533.I1RBR1	pp	229533.I1S0W4
229533.I1RBR1	pp	229533.I1RUF7
229533.I1RBR1	pp	229533.I1RVQ3
229533.I1RBR1	pp	229533.I1REC5
229533.I1RBR1	pp	229533.I1RR24
229533.I1RBR1	pp	229533.I1S438
229533.I1RBR1	pp	229533.I1RE89
229533.I1RBR1	pp	229533.V6R9Z0
229533.I1RBR1	pp	229533.I1RC66
229533.I1RBR1	pp	229533.I1RNT9
229533.I1RBR1	pp	229533.I1S0L4
229533.I1RBR1	pp	229533.I1S437
229533.I1RBR1	pp	229533.I1RVX0
229533.I1RBR1	pp	229533.I1RZK5
229533.I1RBR1	pp	229533.I1RGU7
229533.I1RBR1	pp	229533.I1RWR7
229533.I1RBR1	pp	229533.I1RPE0
229533.I1RBR1	pp	229533.I1RKJ8
229533.I1RBR1	pp	229533.I1RRI1
229533.I1RBR1	pp	229533.I1RZ39
229533.I1RBR1	pp	229533.I1RJU3
229533.I1RBR1	pp	229533.I1RCR1
229533.I1RBR1	pp	229533.Q4IPZ1
229533.I1RBR1	pp	229533.I1RML0
229533.I1RBR1	pp	229533.I1RQ33
229533.I1RBR1	pp	229533.I1REK8
229533.I1RBR1	pp	229533.I1S2B8
229533.I1RBR1	pp	229533.I1RQE7
229533.I1RBR1	pp	229533.Q4I624
229533.I1RBR1	pp	229533.I1RGB2
229533.I1RBR1	pp	229533.I1RPV3
229533.I1RBR1	pp	229533.I1RL04
229533.I1RPU0	pp	229533.I1RZJ6
229533.I1RPU0	pp	229533.I1RX46
229533.I1RPU0	pp	229533.Q4IPI1
229533.I1RPU0	pp	229533.I1RZ99
229533.I1RPU0	pp	229533.I1S9Y0
229533.I1RPU0	pp	229533.I1RQJ5
229533.I1RPU0	pp	229533.I1RT98
229533.I1RPU0	pp	229533.I1RQS5
229533.I1RPU0	pp	229533.I1RW71
229533.I1RPU0	pp	229533.I1RRT2
229533.I1RPU0	pp	229533.I1RUQ2
229533.I1RPU0	pp	229533.I1RXD7
229533.I1RPU0	pp	229533.I1S503
229533.I1RPU0	pp	229533.Q4I5R9
229533.I1RPU0	pp	229533.I1S0R7
229533.I1RPU0	pp	229533.I1RRT8
229533.I1RPU0	pp	229533.I1RT04
229533.I1RPU0	pp	229533.Q4IAS1
229533.I1RPU0	pp	229533.I1S9Y1
229533.I1RPU0	pp	229533.I1RZV6
229533.I1RPU0	pp	229533.I1RRQ3
229533.I1RPU0	pp	229533.I1S9U2
229533.I1RPU0	pp	229533.Q4IQW0
229533.I1RPU0	pp	229533.I1S6F0
229533.I1RPU0	pp	229533.I1S0L4
229533.I1RPU0	pp	229533.I1RWD4
229533.I1RPU0	pp	229533.I1RSL9
229533.I1RPU0	pp	229533.I1RTD5
229533.I1RPU0	pp	229533.I1RS30
229533.I1RPU0	pp	229533.Q4HZ47
229533.I1RPU0	pp	229533.I1RTF4
229533.I1RPU0	pp	229533.I1RPW9
229533.I1RPU0	pp	229533.I1RW25
229533.I1RPU0	pp	229533.I1RQ65
229533.I1RPU0	pp	229533.I1RU69
229533.I1RPU0	pp	229533.I1S2B8
229533.I1RPU0	pp	229533.V6R3X5
229533.I1RPU0	pp	229533.I1RS47
229533.I1RPU0	pp	229533.I1RR73

137825 229533.I1RPU0 pp 229533.Q4HZ42
137826 229533.I1RPU0 pp 229533.Q4I662
137827 229533.I1RPU0 pp 229533.I1S253
137828 229533.I1RPU0 pp 229533.Q4IFI0
137829 229533.I1RPU0 pp 229533.I1S8E2
137830 229533.I1RPU0 pp 229533.V6R283
137831 229533.I1RPU0 pp 229533.I1RT80
137832 229533.I1RPU0 pp 229533.I1RWJ4
137833 229533.I1RPU0 pp 229533.I1RWH5
137834 229533.I1RPU0 pp 229533.I1RZ39
137835 229533.I1RPU0 pp 229533.I1RSQ7
137836 229533.I1RPU0 pp 229533.I1RZS6
137837 229533.I1RPU0 pp 229533.I1RYB9
137838 229533.I1RPU0 pp 229533.I1RRJ8
137839 229533.I1RPU0 pp 229533.I1RX24
137840 229533.I1RPU0 pp 229533.Q4IJH1
137841 229533.I1RPU0 pp 229533.V6QYF0
137842 229533.I1RPU0 pp 229533.Q4IAA0
137843 229533.I1RPU0 pp 229533.Q4IBS2
137844 229533.I1RPU0 pp 229533.I1S021
137845 229533.I1RPU0 pp 229533.Q4I327
137846 229533.I1RPU0 pp 229533.V6QUE6
137847 229533.I1RPU0 pp 229533.I1S0M9
137848 229533.I1RPU0 pp 229533.Q4IN63
137849 229533.I1RPU0 pp 229533.I1RWB5
137850 229533.I1RPU0 pp 229533.I1RQ31
137851 229533.I1RPU0 pp 229533.V6RGN5
137852 229533.I1RPU0 pp 229533.I1S044
137853 229533.I1RPU0 pp 229533.I1S558
137854 229533.I1RPU0 pp 229533.Q4IJ56
137855 229533.I1RPU0 pp 229533.I1RQA2
137856 229533.I1RPU0 pp 229533.I1RYB1
137857 229533.I1RPU0 pp 229533.V6RMW6
137858 229533.I1RPU0 pp 229533.V6R042
137859 229533.I1RPU0 pp 229533.I1S0Z9
137860 229533.I1RPU0 pp 229533.I1RZE5
137861 229533.I1RPU0 pp 229533.I1RSH5
137862 229533.I1RPU0 pp 229533.V6RGY3
137863 229533.I1RPU0 pp 229533.Q4HZ68
137864 229533.I1RPU0 pp 229533.I1RSR3
137865 229533.I1RPU0 pp 229533.I1RVZ0
137866 229533.I1RPU0 pp 229533.Q4IEK8
137867 229533.I1RPU0 pp 229533.I1RRY9
137868 229533.I1RPU0 pp 229533.I1RSI6
137869 229533.I1RPU0 pp 229533.I1S296
137870 229533.I1RPU0 pp 229533.I1RWN6
137871 229533.I1RPU0 pp 229533.I1RPY1
137872 229533.I1RPU0 pp 229533.I1RZZ1
137873 229533.I1RPU0 pp 229533.I1S2E8
137874 229533.I1RPU0 pp 229533.I1S274
137875 229533.I1RPU0 pp 229533.I1RWN5
137876 229533.I1RPU0 pp 229533.I1S4T3
137877 229533.I1RPU0 pp 229533.I1RZK6
137878 229533.I1RPU0 pp 229533.I1RWE1
137879 229533.I1RPU0 pp 229533.V6R227
137880 229533.I1RPU0 pp 229533.I1S2U2
137881 229533.I1RPU0 pp 229533.I1RRI2
137882 229533.I1RPU0 pp 229533.I1RRR7
137883 229533.I1RPU0 pp 229533.I1RSR8
137884 229533.I1RPU0 pp 229533.I1RY03
137885 229533.I1RPU0 pp 229533.I1RSD8
137886 229533.I1RPU0 pp 229533.I1S504
137887 229533.I1RPU0 pp 229533.Q4HWE2
137888 229533.I1RPU0 pp 229533.I1S1C7
137889 229533.I1RPU0 pp 229533.I1RQ28
137890 229533.I1RPU0 pp 229533.I1S0J1
137891 229533.I1RPU0 pp 229533.I1S2C0
137892 229533.I1RPU0 pp 229533.I1RZF6
137893 229533.I1RPU0 pp 229533.I1RTE9
137894 229533.I1RPU0 pp 229533.I1RSS4
137895 229533.I1RPU0 pp 229533.I1RZK5
137896 229533.I1RPU0 pp 229533.I1RX14
137897 229533.I1RPU0 pp 229533.I1RSC6

137898	229533.I1RPU0	pp	229533.I1RTH6
137899	229533.I1RPU0	pp	229533.I1S1X9
137900	229533.I1RPU0	pp	229533.I1S060
137901	229533.I1RPU0	pp	229533.I1RSG8
137902	229533.I1RPU0	pp	229533.I1RY63
137903	229533.I1RPU0	pp	229533.I1RZ59
137904	229533.I1RPU0	pp	229533.I1RVX0
137905	229533.I1RPU0	pp	229533.I1RT03
137906	229533.I1RAL9	pp	229533.I1RS81
137907	229533.I1RAL9	pp	229533.I1RYR1
137908	229533.I1RAL9	pp	229533.I1RWM6
137909	229533.I1RAL9	pp	229533.I1S209
137910	229533.I1RAL9	pp	229533.I1RPQ1
137911	229533.I1RAL9	pp	229533.I1RMX3
137912	229533.I1RAL9	pp	229533.I1RE55
137913	229533.I1RAL9	pp	229533.Q4INS6
137914	229533.I1RAL9	pp	229533.I1RRY1
137915	229533.I1RAL9	pp	229533.I1RDJ8
137916	229533.I1RAL9	pp	229533.I1RKI2
137917	229533.I1RAL9	pp	229533.I1RSJ1
137918	229533.I1RAL9	pp	229533.I1RQH8
137919	229533.I1RAL9	pp	229533.I1RCB3
137920	229533.I1RAL9	pp	229533.I1S461
137921	229533.I1RAL9	pp	229533.I1RZT1
137922	229533.I1RAL9	pp	229533.I1RFX8
137923	229533.I1RAL9	pp	229533.I1RMJ8
137924	229533.I1RAL9	pp	229533.I1RBC1
137925	229533.I1RAL9	pp	229533.I1RBW0
137926	229533.I1RAL9	pp	229533.I1RDX0
137927	229533.I1RAL9	pp	229533.I1RFJ2
137928	229533.I1RAL9	pp	229533.I1RZ94
137929	229533.I1RAL9	pp	229533.I1RG77
137930	229533.I1RAL9	pp	229533.I1RSR9
137931	229533.I1RAL9	pp	229533.I1RDJ1
137932	229533.I1RAL9	pp	229533.I1RN55
137933	229533.I1RAL9	pp	229533.I1RD01
137934	229533.I1RAL9	pp	229533.I1S0D5
137935	229533.I1RAL9	pp	229533.I1RUR0
137936	229533.I1RAL9	pp	229533.I1RCQ4
137937	229533.I1RAL9	pp	229533.I1S4P6
137938	229533.I1RAL9	pp	229533.I1S788
137939	229533.I1RAL9	pp	229533.Q4IJ84
137940	229533.I1RAL9	pp	229533.I1RBN9
137941	229533.I1RAL9	pp	229533.I1S283
137942	229533.I1RAL9	pp	229533.I1S5Z3
137943	229533.I1RAL9	pp	229533.Q4IB89
137944	229533.I1RAL9	pp	229533.I1RN57
137945	229533.I1RAL9	pp	229533.I1RX64
137946	229533.I1RAL9	pp	229533.I1RTN6
137947	229533.I1RAL9	pp	229533.I1RVW0
137948	229533.I1RAL9	pp	229533.I1RZP8
137949	229533.I1RAL9	pp	229533.I1RC54
137950	229533.I1RAL9	pp	229533.I1RRS7
137951	229533.I1RAL9	pp	229533.I1RBC8
137952	229533.I1RAL9	pp	229533.I1RSP7
137953	229533.I1RAL9	pp	229533.I1RRJ6
137954	229533.I1RAL9	pp	229533.I1S523
137955	229533.I1RAL9	pp	229533.I1RQI6
137956	229533.I1RAL9	pp	229533.I1RZ98
137957	229533.I1RAL9	pp	229533.I1RD19
137958	229533.I1RAL9	pp	229533.I1RSD0
137959	229533.I1RAL9	pp	229533.I1RYR2
137960	229533.I1RAL9	pp	229533.I1RSC7
137961	229533.I1RAL9	pp	229533.I1RZ86
137962	229533.I1RAL9	pp	229533.I1RYX9
137963	229533.I1RAL9	pp	229533.I1RUG4
137964	229533.I1RAL9	pp	229533.I1RBW1
137965	229533.I1RAL9	pp	229533.I1RCA5
137966	229533.I1RAL9	pp	229533.I1RW24
137967	229533.I1RAL9	pp	229533.I1RDC0
137968	229533.I1RAL9	pp	229533.I1RYQ1
137969	229533.I1RAL9	pp	229533.I1RP99
137970	229533.I1RAL9	pp	229533.I1RJV8

137971 229533.I1RAL9 pp 229533.I1RWI8
137972 229533.I1RAL9 pp 229533.I1RVV5
137973 229533.I1S2J3 pp 229533.I1SAJ7
137974 229533.I1S2J3 pp 229533.I1SAJ8
137975 229533.I1S2J3 pp 229533.I1S2K0
137976 229533.I1S2J3 pp 229533.I1S2J8
137977 229533.I1S2J3 pp 229533.I1S2J6
137978 229533.I1S2J3 pp 229533.I1S2J5
137979 229533.I1RXF0 pp 229533.V6R3X5
137980 229533.I1RXF0 pp 229533.I1RZ39
137981 229533.I1RXF0 pp 229533.I1RZZ9
137982 229533.I1RXF0 pp 229533.I1RZM0
137983 229533.I1RXF0 pp 229533.V6R3J1
137984 229533.I1RXF0 pp 229533.I1RYI5
137985 229533.I1RXF0 pp 229533.I1S0L4
137986 229533.I1RXF0 pp 229533.I1RZP1
137987 229533.I1RXF0 pp 229533.I1S503
137988 229533.I1RXF0 pp 229533.I1S3G4
137989 229533.I1RXF0 pp 229533.I1S4Z7
137990 229533.I1RXF0 pp 229533.I1S1F3
137991 229533.I1RXF0 pp 229533.I1RYN4
137992 229533.I1RXF0 pp 229533.I1S0F6
137993 229533.I1RXF0 pp 229533.I1S7W5
137994 229533.I1RXF0 pp 229533.I1S6Y8
137995 229533.I1RXF0 pp 229533.I1S5R1
137996 229533.I1RXF0 pp 229533.Q4HXF0
137997 229533.I1RXF0 pp 229533.I1S2H4
137998 229533.I1RXF0 pp 229533.O74638
137999 229533.I1RXF0 pp 229533.I1S1W3
138000 229533.I1RXF0 pp 229533.I1RZ52
138001 229533.I1RXF0 pp 229533.I1S277
138002 229533.I1RXF0 pp 229533.I1S0J1
138003 229533.I1RXF0 pp 229533.V6RIV9
138004 229533.I1RXF0 pp 229533.V6RLM7
138005 229533.I1RXF0 pp 229533.I1S1W9
138006 229533.I1RXF0 pp 229533.I1S2B8
138007 229533.I1RXF0 pp 229533.I1S9Y1
138008 229533.I1RXF0 pp 229533.I1SA33
138009 229533.I1RXF0 pp 229533.I1RXR1
138010 229533.I1RXF0 pp 229533.I1RYW3
138011 229533.I1RXF0 pp 229533.Q4I1Y5
138012 229533.I1RXF0 pp 229533.V6R042
138013 229533.I1RXF0 pp 229533.V6R2A1
138014 229533.I1RXF0 pp 229533.V6QUV7
138015 229533.I1RXF0 pp 229533.V6RN69
138016 229533.I1RXF0 pp 229533.I1S1C7
138017 229533.I1RXF0 pp 229533.V6R057
138018 229533.I1RXF0 pp 229533.V6RMW6
138019 229533.I1RXF0 pp 229533.I1S5R6
138020 229533.I1RXF0 pp 229533.Q4I5V2
138021 229533.I1RXF0 pp 229533.V6RPQ8
138022 229533.I1RXF0 pp 229533.Q4I624
138023 229533.I1RXF0 pp 229533.I1RXU9
138024 229533.I1RXF0 pp 229533.V6QX21
138025 229533.I1RXF0 pp 229533.I1RZW8
138026 229533.I1RXF0 pp 229533.Q4HY20
138027 229533.I1RXF0 pp 229533.V6QW22
138028 229533.I1RXF0 pp 229533.I1S8R3
138029 229533.I1RXF0 pp 229533.V6RRF6
138030 229533.I1RXF0 pp 229533.V6RTJ8
138031 229533.I1RXF0 pp 229533.I1RZM1
138032 229533.I1RXF0 pp 229533.I1S504
138033 229533.I1RXF0 pp 229533.I1S0R7
138034 229533.I1RXF0 pp 229533.I1S4S7
138035 229533.I1RXF0 pp 229533.I1S0T2
138036 229533.I1RXF0 pp 229533.I1S0L3
138037 229533.I1RXF0 pp 229533.I1S1W8
138038 229533.I1RXF0 pp 229533.I1S419
138039 229533.I1RXF0 pp 229533.V6R1K4
138040 229533.I1RXF0 pp 229533.I1S235
138041 229533.I1RXF0 pp 229533.V6RCZ8
138042 229533.I1RXF0 pp 229533.I1S0B7
138043 229533.I1RXF0 pp 229533.V6RUT5

138044	229533.I1RXF0	pp	229533.V6RCG5
138045	229533.I1RXF0	pp	229533.I1RY40
138046	229533.I1RXF0	pp	229533.I1RZM8
138047	229533.I1RXF0	pp	229533.V6RBN2
138048	229533.I1RXF0	pp	229533.I1RZZ1
138049	229533.I1RXF0	pp	229533.I1S6K9
138050	229533.I1RXF0	pp	229533.I1S7L6
138051	229533.I1RXF0	pp	229533.I1S4S4
138052	229533.I1RXF0	pp	229533.I1S835
138053	229533.I1RXF0	pp	229533.I1S4Q4
138054	229533.I1RXF0	pp	229533.I1RZJ5
138055	229533.I1RXF0	pp	229533.Q4IJK2
138056	229533.I1RXF0	pp	229533.I1S5B0
138057	229533.I1RXF0	pp	229533.I1S887
138058	229533.I1RXF0	pp	229533.V6QUE6
138059	229533.I1RXF0	pp	229533.I1SA32
138060	229533.I1RXF0	pp	229533.Q4I8P5
138061	229533.I1RXF0	pp	229533.I1RZK5
138062	229533.I1RXF0	pp	229533.I1RYL5
138063	229533.I1RXF0	pp	229533.I1RY03
138064	229533.I1RXF0	pp	229533.I1RZF7
138065	229533.I1RKQ0	pp	229533.I1S4P6
138066	229533.I1RKQ0	pp	229533.Q4IB89
138067	229533.I1RKQ0	pp	229533.I1RZH4
138068	229533.I1RKQ0	pp	229533.I1RSC7
138069	229533.I1RKQ0	pp	229533.I1RQI6
138070	229533.I1RKQ0	pp	229533.Q4IR08
138071	229533.I1RKQ0	pp	229533.Q4INS6
138072	229533.I1RKQ0	pp	229533.I1RY58
138073	229533.I1RKQ0	pp	229533.I1RY73
138074	229533.I1RKQ0	pp	229533.I1S0D5
138075	229533.I1RKQ0	pp	229533.I1RUG2
138076	229533.I1RKQ0	pp	229533.I1RYX9
138077	229533.I1RKQ0	pp	229533.I1RMJ8
138078	229533.I1RKQ0	pp	229533.I1RN46
138079	229533.I1RKQ0	pp	229533.O74638
138080	229533.I1RKQ0	pp	229533.V6RLW4
138081	229533.I1RKQ0	pp	229533.I1RZT1
138082	229533.I1RKQ0	pp	229533.I1RPP0
138083	229533.I1RKQ0	pp	229533.I1RTN6
138084	229533.I1S0W4	pp	229533.V6RCZ8
138085	229533.I1S0W4	pp	229533.I1S4S7
138086	229533.I1S0W4	pp	229533.I1S1W9
138087	229533.I1S0W4	pp	229533.V6R042
138088	229533.I1S0W4	pp	229533.V6RMW6
138089	229533.I1S0W4	pp	229533.I1S9I5
138090	229533.I1S0W4	pp	229533.I1S5R6
138091	229533.I1S0W4	pp	229533.I1S438
138092	229533.I1S0W4	pp	229533.V6RTJ8
138093	229533.I1S0W4	pp	229533.I1S821
138094	229533.I1S0W4	pp	229533.Q4HVQ9
138095	229533.I1S0W4	pp	229533.Q4IJK2
138096	229533.I1S0W4	pp	229533.I1S437
138097	229533.I1S0W4	pp	229533.I1S5R1
138098	229533.I1S0W4	pp	229533.I1S1B2
138099	229533.I1S0W4	pp	229533.I1S2B8
138100	229533.I1S0W4	pp	229533.Q4I624
138101	229533.I1S0W4	pp	229533.V6R9Z0
138102	229533.I1S0W4	pp	229533.I1S1C7
138103	229533.I1RM67	pp	229533.I1S0B7
138104	229533.I1RM67	pp	229533.I1S1W8
138105	229533.I1RM67	pp	229533.V6QUW4
138106	229533.I1RM67	pp	229533.I1RMK7
138107	229533.I1RM67	pp	229533.I1RNA0
138108	229533.I1RM67	pp	229533.Q4IQK7
138109	229533.I1RM67	pp	229533.I1RQM6
138110	229533.I1RM67	pp	229533.I1RRQ6
138111	229533.I1RM67	pp	229533.V6QYF0
138112	229533.I1RM67	pp	229533.Q4IFI0
138113	229533.I1RM67	pp	229533.I1S0R7
138114	229533.I1RM67	pp	229533.I1RW25
138115	229533.I1RM67	pp	229533.I1RMT4
138116	229533.I1RM67	pp	229533.I1RT05

138117	229533.I1RM67	pp	229533.I1RZ02
138118	229533.I1RM67	pp	229533.I1S6U8
138119	229533.I1RM67	pp	229533.I1S338
138120	229533.I1RM67	pp	229533.I1RMK3
138121	229533.I1RM67	pp	229533.I1S0F1
138122	229533.I1RM67	pp	229533.I1RX46
138123	229533.I1RM67	pp	229533.Q4HVW2
138124	229533.I1RM67	pp	229533.Q4IQX3
138125	229533.I1RM67	pp	229533.Q4IPI1
138126	229533.I1RM67	pp	229533.I1RSQ7
138127	229533.I1RM67	pp	229533.I1RZ99
138128	229533.I1RM67	pp	229533.I1RTF4
138129	229533.I1RM67	pp	229533.Q4I5R9
138130	229533.I1RM67	pp	229533.I1RW71
138131	229533.I1RM67	pp	229533.I1S2L2
138132	229533.I1RM67	pp	229533.Q4IJH1
138133	229533.I1RM67	pp	229533.I1S6F0
138134	229533.I1RM67	pp	229533.V6RTJ8
138135	229533.I1RM67	pp	229533.I1RPN4
138136	229533.I1RM67	pp	229533.I1RT80
138137	229533.I1RM67	pp	229533.I1RPI5
138138	229533.I1RM67	pp	229533.I1RNV6
138139	229533.I1RM67	pp	229533.I1RZK6
138140	229533.I1RM67	pp	229533.I1RNH1
138141	229533.I1RM67	pp	229533.I1RZZ1
138142	229533.I1RM67	pp	229533.I1S4S4
138143	229533.I1RM67	pp	229533.I1RWD4
138144	229533.I1RM67	pp	229533.I1S0D4
138145	229533.I1RM67	pp	229533.I1RPW9
138146	229533.I1RM67	pp	229533.I1RPU0
138147	229533.I1RM67	pp	229533.Q4IJP1
138148	229533.I1RM67	pp	229533.V6RBN2
138149	229533.I1RM67	pp	229533.I1RR24
138150	229533.I1RM67	pp	229533.I1RMQ0
138151	229533.I1RM67	pp	229533.I1S9U2
138152	229533.I1RM67	pp	229533.I1RYJ6
138153	229533.I1RM67	pp	229533.Q4IJK2
138154	229533.I1RM67	pp	229533.I1RT33
138155	229533.I1RM67	pp	229533.I1RN99
138156	229533.I1RM67	pp	229533.I1RWH5
138157	229533.I1RM67	pp	229533.I1RQ28
138158	229533.I1RM67	pp	229533.I1RZJ6
138159	229533.I1RM67	pp	229533.I1RQJ5
138160	229533.I1RM67	pp	229533.I1RY63
138161	229533.I1RM67	pp	229533.I1RZE5
138162	229533.I1RM67	pp	229533.I1RMK4
138163	229533.I1RM67	pp	229533.I1RUQ2
138164	229533.I1RM67	pp	229533.I1S4Q4
138165	229533.I1RM67	pp	229533.I1RZF6
138166	229533.I1RM67	pp	229533.I1RXD7
138167	229533.I1RM67	pp	229533.I1RSC6
138168	229533.I1RM67	pp	229533.I1S6E5
138169	229533.I1RM67	pp	229533.V6QWZ7
138170	229533.I1RM67	pp	229533.V6RQW4
138171	229533.I1RM67	pp	229533.I1RQA2
138172	229533.I1RM67	pp	229533.I1S504
138173	229533.I1RM67	pp	229533.I1RNH6
138174	229533.I1RM67	pp	229533.I1RMN7
138175	229533.I1RM67	pp	229533.I1RRY9
138176	229533.I1RM67	pp	229533.I1RVZ0
138177	229533.I1RM67	pp	229533.I1RSR3
138178	229533.I1RM67	pp	229533.I1RNK2
138179	229533.I1RM67	pp	229533.I1S021
138180	229533.I1RM67	pp	229533.Q4I5Z5
138181	229533.I1RM67	pp	229533.I1S0A0
138182	229533.I1RM67	pp	229533.I1RYB9
138183	229533.I1RM67	pp	229533.I1RX14
138184	229533.I1RM67	pp	229533.I1RSR8
138185	229533.I1RM67	pp	229533.I1RMD5
138186	229533.I1RM67	pp	229533.I1RMC0
138187	229533.I1RM67	pp	229533.I1RY03
138188	229533.I1RM67	pp	229533.I1RZH4
138189	229533.I1RM67	pp	229533.Q4IAA0

229533.I1RM67	pp	229533.I1RXF0
229533.I1RM67	pp	229533.I1RQ31
229533.I1RM67	pp	229533.I1RS18
229533.I1RM67	pp	229533.I1RZK5
229533.I1RM67	pp	229533.V6RCZ8
229533.I1RM67	pp	229533.I1RMW4
229533.I1RM67	pp	229533.I1RNF5
229533.I1RM67	pp	229533.I1RRV4
229533.I1RM67	pp	229533.I1RXB6
229533.I1RM67	pp	229533.Q4IBS2
229533.I1RM67	pp	229533.I1RR72
229533.I1RM67	pp	229533.Q4IJ56
229533.I1RM67	pp	229533.I1RZB5
229533.I1RM67	pp	229533.I1S0M9
229533.I1RM67	pp	229533.V6QUE6
229533.I1RM67	pp	229533.Q4IEK8
229533.I1RM67	pp	229533.I1RZS6
229533.I1RM67	pp	229533.I1S5B0
229533.I1RM67	pp	229533.I1RNQ5
229533.I1RM67	pp	229533.I1S835
229533.I1RM67	pp	229533.I1RX24
229533.I1RM67	pp	229533.I1S2B8
229533.I1RM67	pp	229533.I1RQD2
229533.I1RM67	pp	229533.I1RU69
229533.I1RM67	pp	229533.V6R227
229533.I1RM67	pp	229533.I1RRM5
229533.I1RM67	pp	229533.I1RRQ3
229533.I1RM67	pp	229533.O74638
229533.I1RM67	pp	229533.Q4I662
229533.I1RM67	pp	229533.I1RWI5
229533.I1RM67	pp	229533.I1RN67
229533.I1RM67	pp	229533.I1RSQ4
229533.I1RM67	pp	229533.Q4I5M4
229533.I1RM67	pp	229533.I1RZZ9
229533.I1RM67	pp	229533.I1S9Y1
229533.I1RM67	pp	229533.I1RRP3
229533.I1RM67	pp	229533.I1RS30
229533.I1RM67	pp	229533.Q4IF76
229533.I1RM67	pp	229533.I1RW26
229533.I1RM67	pp	229533.Q4IEV5
229533.I1RM67	pp	229533.I1S253
229533.I1RM67	pp	229533.I1RZV6
229533.I1RM67	pp	229533.I1SAE3
229533.I1RM67	pp	229533.I1RQ65
229533.I1RM67	pp	229533.I1S3G4
229533.I1RM67	pp	229533.V6R3X5
229533.I1RM67	pp	229533.I1RR73
229533.I1RM67	pp	229533.I1RTA1
229533.I1RM67	pp	229533.I1RND6
229533.I1RM67	pp	229533.I1S2H4
229533.I1RM67	pp	229533.I1RZ39
229533.I1RM67	pp	229533.Q4IQ72
229533.I1RM67	pp	229533.I1RS47
229533.I1RM67	pp	229533.I1RTG7
229533.I1RM67	pp	229533.I1RWK3
229533.I1RM67	pp	229533.V6RAC9
229533.I1RM67	pp	229533.Q4HZ42
229533.I1RM67	pp	229533.I1RWS4
229533.I1RM67	pp	229533.I1RRT8
229533.I1RM67	pp	229533.V6R283
229533.I1RM67	pp	229533.I1S8E2
229533.I1RM67	pp	229533.I1RTD5
229533.I1RM67	pp	229533.I1RYI5
229533.I1RM67	pp	229533.Q4IQW0
229533.I1RM67	pp	229533.I1RPP3
229533.I1RM67	pp	229533.I1S503
229533.I1RM67	pp	229533.Q4HXF0
229533.I1RM67	pp	229533.I1RWF7
229533.I1RM67	pp	229533.I1RT40
229533.I1RM67	pp	229533.I1RRJ8
229533.I1RM67	pp	229533.I1RZB2
229533.I1RM67	pp	229533.I1RWF9
229533.I1RM67	pp	229533.I1S1B6

138263	229533.I1RM67	pp	229533.I1RT75
138264	229533.I1RM67	pp	229533.I1RRM8
138265	229533.I1RM67	pp	229533.I1RT04
138266	229533.I1RM67	pp	229533.I1S0I3
138267	229533.I1RM67	pp	229533.I1RRT2
138268	229533.I1RM67	pp	229533.Q4I830
138269	229533.I1RM67	pp	229533.I1S2E8
138270	229533.I1RM67	pp	229533.I1RPP4
138271	229533.I1RM67	pp	229533.I1RQE7
138272	229533.I1RM67	pp	229533.I1RTK5
138273	229533.I1RM67	pp	229533.I1RQS5
138274	229533.I1RM67	pp	229533.I1RSD8
138275	229533.I1RM67	pp	229533.I1RSS7
138276	229533.I1RM67	pp	229533.I1S9Z4
138277	229533.I1RM67	pp	229533.V6R382
138278	229533.I1RM67	pp	229533.I1RT03
138279	229533.I1RM67	pp	229533.Q4IAS1
138280	229533.I1RM67	pp	229533.Q4I8X0
138281	229533.I1RM67	pp	229533.I1S1X9
138282	229533.I1RM67	pp	229533.I1RVX0
138283	229533.I1RM67	pp	229533.I1RRI2
138284	229533.I1RM67	pp	229533.I1RSY2
138285	229533.I1RM67	pp	229533.I1RSS4
138286	229533.I1RM67	pp	229533.I1S1C7
138287	229533.I1RM67	pp	229533.V6RLM7
138288	229533.I1RM67	pp	229533.I1RNU6
138289	229533.I1RM67	pp	229533.I1S247
138290	229533.I1RM67	pp	229533.I1RWB5
138291	229533.I1RM67	pp	229533.Q4HZ68
138292	229533.I1RM67	pp	229533.I1RTD7
138293	229533.I1RM67	pp	229533.I1RM96
138294	229533.I1RM67	pp	229533.Q4IN63
138295	229533.I1RM67	pp	229533.Q4I665
138296	229533.I1RM67	pp	229533.I1RTE9
138297	229533.I1RM67	pp	229533.I1RPJ3
138298	229533.I1RM67	pp	229533.I1RSQ3
138299	229533.I1RM67	pp	229533.I1S2C0
138300	229533.I1RM67	pp	229533.I1RZV3
138301	229533.I1RM67	pp	229533.V6RMW6
138302	229533.I1RM67	pp	229533.I1RWN5
138303	229533.I1RM67	pp	229533.V6R042
138304	229533.I1RM67	pp	229533.I1RTH6
138305	229533.I1RM67	pp	229533.V6RIV9
138306	229533.I1RM67	pp	229533.I1RSG8
138307	229533.I1RM67	pp	229533.I1RSI6
138308	229533.I1RM67	pp	229533.Q4HZK7
138309	229533.I1RM67	pp	229533.I1RPY1
138310	229533.I1RM67	pp	229533.I1RMN6
138311	229533.I1RM67	pp	229533.I1S1W9
138312	229533.I1RM67	pp	229533.I1RRR7
138313	229533.I1RM67	pp	229533.Q4I5V2
138314	229533.I1RM67	pp	229533.V6RGN5
138315	229533.I1RM67	pp	229533.I1RUQ9
138316	229533.I1RM67	pp	229533.V6RDZ4
138317	229533.I1RM67	pp	229533.I1RWN6
138318	229533.I1RM67	pp	229533.I1S5R6
138319	229533.I1RM67	pp	229533.I1RTG6
138320	229533.I1RM67	pp	229533.I1S274
138321	229533.I1RM67	pp	229533.I1RNY2
138322	229533.I1RM67	pp	229533.I1S0S0
138323	229533.I1RM67	pp	229533.Q4I327
138324	229533.I1RM67	pp	229533.I1S0J1
138325	229533.I1RM67	pp	229533.I1RNC1
138326	229533.I1RXT0	pp	229533.I1RYW9
138327	229533.I1RXT0	pp	229533.V6R3J1
138328	229533.I1S2J8	pp	229533.I1SAJ7
138329	229533.I1S2J8	pp	229533.I1SAJ8
138330	229533.I1S2J8	pp	229533.I1S2K0
138331	229533.I1RMX3	pp	229533.I1S998
138332	229533.I1RMX3	pp	229533.I1S283
138333	229533.I1RMX3	pp	229533.I1RSA7
138334	229533.I1RMX3	pp	229533.I1RUG1
138335	229533.I1RMX3	pp	229533.I1RVW0

229533.I1RMX3 pp 229533.I1S0D5
229533.I1RMX3 pp 229533.I1S987
229533.I1RMX3 pp 229533.I1RPQ1
229533.I1RMX3 pp 229533.V6RLW4
229533.I1RMX3 pp 229533.I1RSR9
229533.I1RMX3 pp 229533.I1RZ94
229533.I1RMX3 pp 229533.I1RZP8
229533.I1RMX3 pp 229533.I1RWM6
229533.I1RMX3 pp 229533.I1RYR2
229533.I1RMX3 pp 229533.I1RX64
229533.I1RMX3 pp 229533.I1RYX9
229533.I1RMX3 pp 229533.I1RSN7
229533.I1RMX3 pp 229533.I1RRT1
229533.I1RMX3 pp 229533.I1S209
229533.I1RMX3 pp 229533.I1RSP7
229533.I1RMX3 pp 229533.I1RQJ4
229533.I1RMX3 pp 229533.I1RRS7
229533.I1RMX3 pp 229533.I1S461
229533.I1RMX3 pp 229533.I1RN46
229533.I1RMX3 pp 229533.I1S2F2
229533.I1RMX3 pp 229533.I1RSI4
229533.I1RMX3 pp 229533.I1RY73
229533.I1RMX3 pp 229533.V6RKK5
229533.I1RMX3 pp 229533.I1RSC7
229533.I1RMX3 pp 229533.I1RPZ0
229533.I1RMX3 pp 229533.I1RUG4
229533.I1RMX3 pp 229533.I1RWB3
229533.I1RMX3 pp 229533.I1RN57
229533.I1RMX3 pp 229533.I1RN55
229533.I1S6U8 pp 229533.V6RTJ8
229533.I1S6U8 pp 229533.Q4IFI0
229533.I1S6U8 pp 229533.V6QYF0
229533.I1S6U8 pp 229533.I1S9U2
229533.I1S6U8 pp 229533.V6R042
229533.I1S6U8 pp 229533.V6R283
229533.I1S6U8 pp 229533.V6RMW6
229533.I1S6U8 pp 229533.Q4I662
229533.I1S6U8 pp 229533.V6R3X5
229533.I1S6U8 pp 229533.Q4IN63
229533.I1RJV8 pp 229533.I1S0D5
229533.I1RJV8 pp 229533.I1RQJ4
229533.I1RJV8 pp 229533.I1RX64
229533.I1RJV8 pp 229533.I1RSR9
229533.I1RJV8 pp 229533.I1RSK0
229533.I1RJV8 pp 229533.I1S461
229533.I1RJV8 pp 229533.I1S987
229533.I1RJV8 pp 229533.I1RZ86
229533.I1RJV8 pp 229533.I1RUG4
229533.I1RJV8 pp 229533.I1RU61
229533.I1RJV8 pp 229533.I1RUG2
229533.I1RJV8 pp 229533.I1RMJ8
229533.I1RJV8 pp 229533.I1RRT1
229533.I1RJV8 pp 229533.I1RXV9
229533.I1RJV8 pp 229533.I1RZ94
229533.I1RJV8 pp 229533.I1RKK2
229533.I1RJV8 pp 229533.I1RYQ1
229533.I1RJV8 pp 229533.I1S4P6
229533.I1RJV8 pp 229533.I1S209
229533.I1RJV8 pp 229533.I1RMD3
229533.I1RJV8 pp 229533.I1RWM6
229533.I1RJV8 pp 229533.Q4IR08
229533.I1RJV8 pp 229533.I1RPQ1
229533.I1RJV8 pp 229533.I1RMX3
229533.I1RJV8 pp 229533.Q4IB89
229533.I1RJV8 pp 229533.I1RRS7
229533.I1RJV8 pp 229533.I1RRM8
229533.I1RJV8 pp 229533.I1S788
229533.I1RJV8 pp 229533.I1RUR0
229533.I1RJV8 pp 229533.I1RQH8
229533.I1RJV8 pp 229533.I1RQ67
229533.I1RJV8 pp 229533.I1RKI2
229533.I1RJV8 pp 229533.I1S998
229533.I1RJV8 pp 229533.I1RY29

138409 229533.I1RJV8 pp 229533.Q4INS6
138410 229533.I1RJV8 pp 229533.I1RVV5
138411 229533.I1RJV8 pp 229533.I1RWI8
138412 229533.I1RJV8 pp 229533.I1RSC7
138413 229533.I1RJV8 pp 229533.I1RRJ6
138414 229533.I1RJV8 pp 229533.I1RZ19
138415 229533.I1RJV8 pp 229533.I1RXI6
138416 229533.I1RJV8 pp 229533.I1RYX9
138417 229533.I1RJV8 pp 229533.I1RKQ0
138418 229533.A0A098DR40 pp 229533.O74638
138419 229533.A0A098DR40 pp 229533.I1RRT8
138420 229533.A0A098DR40 pp 229533.I1S6Y8
138421 229533.A0A098DR40 pp 229533.I1S5R1
138422 229533.A0A098DR40 pp 229533.I1RPM4
138423 229533.A0A098DR40 pp 229533.I1RL04
138424 229533.A0A098DR40 pp 229533.I1REC5
138425 229533.A0A098DR40 pp 229533.I1S2H4
138426 229533.A0A098DR40 pp 229533.I1RGU7
138427 229533.A0A098DR40 pp 229533.A0A1C3YJD4
138428 229533.A0A098DR40 pp 229533.I1RE22
138429 229533.A0A098DR40 pp 229533.I1RPE0
138430 229533.A0A098DR40 pp 229533.A0A0E0SPS2
138431 229533.A0A098DR40 pp 229533.I1S3G4
138432 229533.A0A098DR40 pp 229533.I1S0M6
138433 229533.A0A098DR40 pp 229533.I1RTN3
138434 229533.A0A098DR40 pp 229533.A0A0E0SC46
138435 229533.A0A098DR40 pp 229533.I1RRI1
138436 229533.A0A098DR40 pp 229533.I1R9M0
138437 229533.A0A098DR40 pp 229533.I1RGB0
138438 229533.A0A098DR40 pp 229533.I1RRM5
138439 229533.A0A098DR40 pp 229533.I1RKP1
138440 229533.A0A098DR40 pp 229533.I1REV8
138441 229533.A0A098DR40 pp 229533.I1S2B8
138442 229533.A0A098DR40 pp 229533.I1RN63
138443 229533.A0A098DR40 pp 229533.I1RJZ7
138444 229533.A0A098DR40 pp 229533.I1RWK3
138445 229533.A0A098DR40 pp 229533.I1RBY4
138446 229533.A0A098DR40 pp 229533.I1RE76
138447 229533.A0A098DR40 pp 229533.A0A0E0RU84
138448 229533.A0A098DR40 pp 229533.I1RYR0
138449 229533.A0A098DR40 pp 229533.I1RA52
138450 229533.A0A098DR40 pp 229533.I1RJN9
138451 229533.A0A098DR40 pp 229533.I1S277
138452 229533.A0A098DR40 pp 229533.I1RTA1
138453 229533.A0A098DR40 pp 229533.A0A1C3YMA1
138454 229533.A0A098DR40 pp 229533.A0A1C3YIS7
138455 229533.A0A098DR40 pp 229533.I1RVZ2
138456 229533.A0A098DR40 pp 229533.I1RE27
138457 229533.A0A098DR40 pp 229533.A0A1C3YKN3
138458 229533.A0A098DR40 pp 229533.I1RSQ4
138459 229533.A0A098DR40 pp 229533.I1RFT1
138460 229533.A0A098DR40 pp 229533.I1S8P5
138461 229533.A0A098DR40 pp 229533.I1RZZ9
138462 229533.A0A098DR40 pp 229533.I1RPP3
138463 229533.A0A098DR40 pp 229533.I1RWI5
138464 229533.A0A098DR40 pp 229533.A0A1C3YIR8
138465 229533.A0A098DR40 pp 229533.I1RYI5
138466 229533.A0A098DR40 pp 229533.A0A1C3YI20
138467 229533.A0A098DR40 pp 229533.A0A1C3YKM9
138468 229533.A0A098DR40 pp 229533.I1RRJ8
138469 229533.A0A098DR40 pp 229533.I1RSZ7
138470 229533.A0A098DR40 pp 229533.I1RZ39
138471 229533.A0A098DR40 pp 229533.I1RYT5
138472 229533.A0A098DR40 pp 229533.A0A1C3YJI5
138473 229533.A0A098DR40 pp 229533.I1RN67
138474 229533.A0A098DR40 pp 229533.I1S0W4
138475 229533.A0A098DR40 pp 229533.I1RW61
138476 229533.A0A098DR40 pp 229533.V6R3X5
138477 229533.A0A098DR40 pp 229533.I1RQ23
138478 229533.A0A098DR40 pp 229533.I1S0F6
138479 229533.A0A098DR40 pp 229533.I1RR73
138480 229533.A0A098DR40 pp 229533.I1RDF7
138481 229533.A0A098DR40 pp 229533.I1RBS0

138482	229533.A0A098DR40	pp	229533.I1RFS3
138483	229533.A0A098DR40	pp	229533.I1RPV3
138484	229533.A0A098DR40	pp	229533.I1S9Y1
138485	229533.A0A098DR40	pp	229533.I1RJV3
138486	229533.A0A098DR40	pp	229533.I1RXU9
138487	229533.A0A098DR40	pp	229533.I1RC05
138488	229533.A0A098DR40	pp	229533.I1RS47
138489	229533.A0A098DR40	pp	229533.I1S0F7
138490	229533.A0A098DR40	pp	229533.I1RSA5
138491	229533.A0A098DR40	pp	229533.I1RXC2
138492	229533.A0A098DR40	pp	229533.I1S1F3
138493	229533.A0A098DR40	pp	229533.A0A1C3YK33
138494	229533.A0A098DR40	pp	229533.I1RWN6
138495	229533.A0A098DR40	pp	229533.I1RQE7
138496	229533.A0A098DR40	pp	229533.I1RD39
138497	229533.A0A098DR40	pp	229533.A0A1C3YJI6
138498	229533.A0A098DR40	pp	229533.I1RVX0
138499	229533.A0A098DR40	pp	229533.I1RV91
138500	229533.A0A098DR40	pp	229533.I1RAW0
138501	229533.A0A098DR40	pp	229533.I1RE01
138502	229533.A0A098DR40	pp	229533.I1RW46
138503	229533.A0A098DR40	pp	229533.I1RCJ7
138504	229533.A0A098DR40	pp	229533.I1RT03
138505	229533.A0A098DR40	pp	229533.I1RT40
138506	229533.A0A098DR40	pp	229533.A0A1C3YLC1
138507	229533.A0A098DR40	pp	229533.I1RXR1
138508	229533.A0A098DR40	pp	229533.I1RTA0
138509	229533.A0A098DR40	pp	229533.I1RTD5
138510	229533.A0A098DR40	pp	229533.I1RB92
138511	229533.A0A098DR40	pp	229533.I1RBR1
138512	229533.A0A098DR40	pp	229533.A0A1C3YLF5
138513	229533.A0A098DR40	pp	229533.A0A098E3M9
138514	229533.A0A098DR40	pp	229533.A0A1C3YLB6
138515	229533.A0A098DR40	pp	229533.A0A1C3YI73
138516	229533.A0A098DR40	pp	229533.I1S7W5
138517	229533.A0A098DR40	pp	229533.I1S0J1
138518	229533.A0A098DR40	pp	229533.A0A1C3YJ21
138519	229533.A0A098DR40	pp	229533.I1RT04
138520	229533.A0A098DR40	pp	229533.V6RUY9
138521	229533.A0A098DR40	pp	229533.Q4IPZ1
138522	229533.A0A098DR40	pp	229533.Q4HXF0
138523	229533.A0A098DR40	pp	229533.I1RPK0
138524	229533.A0A098DR40	pp	229533.V6R057
138525	229533.A0A098DR40	pp	229533.I1RS17
138526	229533.A0A098DR40	pp	229533.I1RD17
138527	229533.A0A098DR40	pp	229533.I1RAU7
138528	229533.A0A098DR40	pp	229533.I1RC34
138529	229533.A0A098DR40	pp	229533.Q4HVQ9
138530	229533.A0A098DR40	pp	229533.I1RMV0
138531	229533.A0A098DR40	pp	229533.I1RQ33
138532	229533.A0A098DR40	pp	229533.I1RPP4
138533	229533.A0A098DR40	pp	229533.I1RRK3
138534	229533.A0A098DR40	pp	229533.I1S1C7
138535	229533.A0A098DR40	pp	229533.I1S503
138536	229533.A0A098DR40	pp	229533.I1RYW3
138537	229533.A0A098DR40	pp	229533.I1S1W9
138538	229533.A0A098DR40	pp	229533.I1RCW6
138539	229533.A0A098DR40	pp	229533.I1RJW6
138540	229533.A0A098DR40	pp	229533.A0A1C3YLK8
138541	229533.A0A098DR40	pp	229533.V6R3J1
138542	229533.A0A098DR40	pp	229533.V6RIV9
138543	229533.A0A098DR40	pp	229533.I1S437
138544	229533.A0A098DR40	pp	229533.I1RWN5
138545	229533.A0A098DR40	pp	229533.I1RRI2
138546	229533.A0A098DR40	pp	229533.I1RSX0
138547	229533.A0A098DR40	pp	229533.I1RAG7
138548	229533.A0A098DR40	pp	229533.I1RWX5
138549	229533.A0A098DR40	pp	229533.I1RC33
138550	229533.A0A098DR40	pp	229533.I1RBS6
138551	229533.A0A098DR40	pp	229533.I1RGA4
138552	229533.A0A098DR40	pp	229533.I1RBC7
138553	229533.A0A098DR40	pp	229533.I1RZW8
138554	229533.A0A098DR40	pp	229533.I1RZB2

138555	229533.A0A098DR40	pp	229533.I1RA70
138556	229533.A0A098DR40	pp	229533.I1RJV4
138557	229533.A0A098DR40	pp	229533.I1RZV3
138558	229533.A0A098DR40	pp	229533.I1S8R3
138559	229533.A0A098DR40	pp	229533.I1S1W3
138560	229533.A0A098DR40	pp	229533.I1RRE5
138561	229533.A0A098DR40	pp	229533.A0A1C3YM05
138562	229533.A0A098DR40	pp	229533.I1RN18
138563	229533.A0A098DR40	pp	229533.I1S0F8
138564	229533.A0A098DR40	pp	229533.I1RFL4
138565	229533.A0A098DR40	pp	229533.I1RCH5
138566	229533.A0A098DR40	pp	229533.A0A1C3YKB9
138567	229533.A0A098DR40	pp	229533.I1RTH6
138568	229533.A0A098DR40	pp	229533.I1RSF1
138569	229533.A0A098DR40	pp	229533.A0A0E0S8Z0
138570	229533.A0A098DR40	pp	229533.I1RPU2
138571	229533.A0A098DR40	pp	229533.I1RUG3
138572	229533.A0A098DR40	pp	229533.I1RE89
138573	229533.A0A098DR40	pp	229533.I1RNC1
138574	229533.A0A098DR40	pp	229533.I1RCR8
138575	229533.A0A098DR40	pp	229533.A0A0E0SKJ9
138576	229533.A0A098DR40	pp	229533.I1RRX5
138577	229533.A0A098DR40	pp	229533.I1RX25
138578	229533.A0A098DR40	pp	229533.A0A0E0RUI4
138579	229533.A0A098DR40	pp	229533.V6QW22
138580	229533.A0A098DR40	pp	229533.A0A0E0RPG8
138581	229533.A0A098DR40	pp	229533.I1RVF8
138582	229533.A0A098DR40	pp	229533.I1RRS8
138583	229533.A0A098DR40	pp	229533.A0A1C3YLS9
138584	229533.A0A098DR40	pp	229533.I1RBT1
138585	229533.A0A098DR40	pp	229533.I1RRV1
138586	229533.A0A098DR40	pp	229533.A0A1C3YKC0
138587	229533.A0A098DR40	pp	229533.A0A1C3YIP5
138588	229533.A0A098DR40	pp	229533.I1RXD6
138589	229533.A0A098DR40	pp	229533.I1R9Y4
138590	229533.A0A098DR40	pp	229533.V6RMW6
138591	229533.A0A098DR40	pp	229533.I1RE17
138592	229533.A0A098DR40	pp	229533.I1RRM3
138593	229533.A0A098DR40	pp	229533.I1RVQ0
138594	229533.A0A098DR40	pp	229533.I1S0X6
138595	229533.A0A098DR40	pp	229533.I1RD52
138596	229533.A0A098DR40	pp	229533.A0A1C3YHX8
138597	229533.A0A098DR40	pp	229533.V6R042
138598	229533.A0A098DR40	pp	229533.I1RA85
138599	229533.A0A098DR40	pp	229533.I1RMC8
138600	229533.A0A098DR40	pp	229533.I1RY86
138601	229533.A0A098DR40	pp	229533.A0A0E0SAR4
138602	229533.A0A098DR40	pp	229533.A0A1C3YN30
138603	229533.A0A098DR40	pp	229533.V6R1K4
138604	229533.A0A098DR40	pp	229533.I1RSQ3
138605	229533.A0A098DR40	pp	229533.I1RJU3
138606	229533.A0A098DR40	pp	229533.I1SA33
138607	229533.A0A098DR40	pp	229533.I1S0L3
138608	229533.A0A098DR40	pp	229533.I1RKJ5
138609	229533.A0A098DR40	pp	229533.I1S0F5
138610	229533.A0A098DR40	pp	229533.I1RRN2
138611	229533.A0A098DR40	pp	229533.I1S4S4
138612	229533.A0A098DR40	pp	229533.I1RD22
138613	229533.A0A098DR40	pp	229533.I1RWQ8
138614	229533.A0A098DR40	pp	229533.A0A1C3YL54
138615	229533.A0A098DR40	pp	229533.I1RWJ8
138616	229533.A0A098DR40	pp	229533.I1RPY3
138617	229533.A0A098DR40	pp	229533.I1S5R6
138618	229533.A0A098DR40	pp	229533.A0A0E0SFN0
138619	229533.A0A098DR40	pp	229533.I1RQC9
138620	229533.A0A098DR40	pp	229533.I1RZJ5
138621	229533.A0A098DR40	pp	229533.A0A0E0RSD7
138622	229533.A0A098DR40	pp	229533.I1S0R7
138623	229533.A0A098DR40	pp	229533.I1RB26
138624	229533.A0A098DR40	pp	229533.I1RR24
138625	229533.A0A098DR40	pp	229533.I1RG11
138626	229533.A0A098DR40	pp	229533.A0A098DSB7
138627	229533.A0A098DR40	pp	229533.I1RBD5

138628 229533.A0A098DR40 pp 229533.V6RUT5
138629 229533.A0A098DR40 pp 229533.I1RPA4
138630 229533.A0A098DR40 pp 229533.I1RCS1
138631 229533.A0A098DR40 pp 229533.I1RX95
138632 229533.A0A098DR40 pp 229533.I1RW54
138633 229533.A0A098DR40 pp 229533.I1RXD7
138634 229533.A0A098DR40 pp 229533.A0A1C3YM63
138635 229533.A0A098DR40 pp 229533.I1RLP1
138636 229533.A0A098DR40 pp 229533.I1S1W8
138637 229533.A0A098DR40 pp 229533.Q4IJT5
138638 229533.A0A098DR40 pp 229533.I1S438
138639 229533.A0A098DR40 pp 229533.I1RW71
138640 229533.A0A098DR40 pp 229533.I1RBC9
138641 229533.A0A098DR40 pp 229533.A0A098DXE2
138642 229533.A0A098DR40 pp 229533.A0A1C3YJQ9
138643 229533.A0A098DR40 pp 229533.I1RX46
138644 229533.A0A098DR40 pp 229533.A0A1C3YK13
138645 229533.A0A098DR40 pp 229533.I1S0T2
138646 229533.A0A098DR40 pp 229533.I1RW25
138647 229533.A0A098DR40 pp 229533.I1S4S7
138648 229533.A0A098DR40 pp 229533.I1S887
138649 229533.A0A098DR40 pp 229533.I1RAX6
138650 229533.A0A098DR40 pp 229533.I1RGU0
138651 229533.A0A098DR40 pp 229533.I1S7A8
138652 229533.A0A098DR40 pp 229533.I1RWB0
138653 229533.A0A098DR40 pp 229533.I1RSQ7
138654 229533.A0A098DR40 pp 229533.A0A1C3YKY6
138655 229533.A0A098DR40 pp 229533.I1S1J7
138656 229533.A0A098DR40 pp 229533.V6RTJ8
138657 229533.A0A098DR40 pp 229533.Q4I283
138658 229533.A0A098DR40 pp 229533.A0A1C3YKB0
138659 229533.A0A098DR40 pp 229533.I1RBR2
138660 229533.A0A098DR40 pp 229533.Q4IJK2
138661 229533.A0A098DR40 pp 229533.A0A1C3YJ93
138662 229533.A0A098DR40 pp 229533.I1RBL7
138663 229533.A0A098DR40 pp 229533.I1RG14
138664 229533.A0A098DR40 pp 229533.I1RPR9
138665 229533.A0A098DR40 pp 229533.I1RQ85
138666 229533.A0A098DR40 pp 229533.I1RZZ1
138667 229533.A0A098DR40 pp 229533.I1RGB1
138668 229533.A0A098DR40 pp 229533.I1RWR7
138669 229533.A0A098DR40 pp 229533.I1RXF0
138670 229533.A0A098DR40 pp 229533.I1RRY9
138671 229533.A0A098DR40 pp 229533.I1RQQ5
138672 229533.A0A098DR40 pp 229533.I1RZM8
138673 229533.A0A098DR40 pp 229533.I1RUQ3
138674 229533.A0A098DR40 pp 229533.A0A0E0S134
138675 229533.A0A098DR40 pp 229533.I1RXC4
138676 229533.A0A098DR40 pp 229533.I1RI82
138677 229533.A0A098DR40 pp 229533.A0A1C3YK34
138678 229533.A0A098DR40 pp 229533.I1SA32
138679 229533.A0A098DR40 pp 229533.I1RK95
138680 229533.A0A098DR40 pp 229533.I1S217
138681 229533.A0A098DR40 pp 229533.V6RCZ8
138682 229533.A0A098DR40 pp 229533.I1RGC5
138683 229533.A0A098DR40 pp 229533.I1REL7
138684 229533.A0A098DR40 pp 229533.Q4I2J8
138685 229533.A0A098DR40 pp 229533.A0A1C3YKV3
138686 229533.A0A098DR40 pp 229533.I1RQ81
138687 229533.A0A098DR40 pp 229533.I1S6K9
138688 229533.A0A098DR40 pp 229533.A0A1C3YIK4
138689 229533.A0A098DR40 pp 229533.A0A1C3YJW9
138690 229533.A0A098DR40 pp 229533.Q4I8P5
138691 229533.A0A098DR40 pp 229533.I1RF19
138692 229533.A0A098DR40 pp 229533.A0A1C3YIJ3
138693 229533.A0A098DR40 pp 229533.I1RR51
138694 229533.A0A098DR40 pp 229533.I1REK8
138695 229533.A0A098DR40 pp 229533.A0A1C3YLU7
138696 229533.A0A098DR40 pp 229533.I1RCR5
138697 229533.A0A098DR40 pp 229533.V6RKK5
138698 229533.A0A098DR40 pp 229533.I1RS18
138699 229533.A0A098DR40 pp 229533.I1RC16
138700 229533.A0A098DR40 pp 229533.I1S419

138701 229533.A0A098DR40 pp 229533.V6RCG5
138702 229533.A0A098DR40 pp 229533.A0A1C3YJN3
138703 229533.A0A098DR40 pp 229533.I1RLU6
138704 229533.A0A098DR40 pp 229533.A0A1C3YHX6
138705 229533.A0A098DR40 pp 229533.I1RY03
138706 229533.A0A098DR40 pp 229533.V6QUE6
138707 229533.A0A098DR40 pp 229533.I1RNT9
138708 229533.A0A098DR40 pp 229533.I1RAU5
138709 229533.A0A098DR40 pp 229533.I1RNQ5
138710 229533.A0A098DR40 pp 229533.I1RZM1
138711 229533.A0A098DR40 pp 229533.I1RFU8
138712 229533.A0A098DR40 pp 229533.I1RQD2
138713 229533.A0A098DR40 pp 229533.A0A098E4W0
138714 229533.A0A098DR40 pp 229533.I1RBU8
138715 229533.A0A098DR40 pp 229533.A0A1C3YI30
138716 229533.A0A098DR40 pp 229533.I1RCG4
138717 229533.A0A098DR40 pp 229533.I1RZK5
138718 229533.A0A098DR40 pp 229533.I1S504
138719 229533.A0A098DR40 pp 229533.I1S5B0
138720 229533.A0A098DR40 pp 229533.A0A1C3YJV6
138721 229533.A0A098DR40 pp 229533.A0A1C3YJR3
138722 229533.A0A098DR40 pp 229533.I1RW99
138723 229533.I1S437 pp 229533.V6R042
138724 229533.I1S437 pp 229533.V6RTJ8
138725 229533.I1S437 pp 229533.I1S821
138726 229533.I1S437 pp 229533.I1S5B0
138727 229533.I1S437 pp 229533.I1S5R6
138728 229533.I1S437 pp 229533.V6RMW6
138729 229533.I1S437 pp 229533.Q4I624
138730 229533.I1S437 pp 229533.V6RCZ8
138731 229533.I1S437 pp 229533.I1S4S4
138732 229533.I1S437 pp 229533.I1S4S7
138733 229533.I1S437 pp 229533.I1S5R1
138734 229533.I1S437 pp 229533.I1S6K9
138735 229533.I1S437 pp 229533.Q4IJK2
138736 229533.A0A0E0RU38 pp 229533.I1RRT1
138737 229533.A0A0E0RU38 pp 229533.A0A1C3YKZ8
138738 229533.A0A0E0RU38 pp 229533.I1RDE6
138739 229533.A0A0E0RU38 pp 229533.I1RQL4
138740 229533.A0A0E0RU38 pp 229533.I1RQ28
138741 229533.A0A0E0RU38 pp 229533.I1RJT5
138742 229533.A0A0E0RU38 pp 229533.I1RM09
138743 229533.A0A0E0RU38 pp 229533.I1RB73
138744 229533.A0A0E0RU38 pp 229533.I1RJZ7
138745 229533.A0A0E0RU38 pp 229533.A0A1C3YLX5
138746 229533.A0A0E0RU38 pp 229533.A0A1C3YM09
138747 229533.A0A0E0RU38 pp 229533.Q4IMD1
138748 229533.I1RMG8 pp 229533.V6RIV9
138749 229533.I1RMG8 pp 229533.I1RN67
138750 229533.I1RMG8 pp 229533.I1RPP4
138751 229533.I1RMG8 pp 229533.V6QW22
138752 229533.I1RMG8 pp 229533.I1RSQ7
138753 229533.I1RMG8 pp 229533.I1RW46
138754 229533.I1RMG8 pp 229533.I1RWK3
138755 229533.I1RMG8 pp 229533.I1RS17
138756 229533.I1RMG8 pp 229533.I1S7A8
138757 229533.I1RMG8 pp 229533.I1RW99
138758 229533.I1RMG8 pp 229533.I1RX46
138759 229533.I1RMG8 pp 229533.V6RTJ8
138760 229533.I1RMG8 pp 229533.I1S5B0
138761 229533.I1RMG8 pp 229533.Q4I624
138762 229533.I1RMG8 pp 229533.V6RCG5
138763 229533.I1RMG8 pp 229533.I1S503
138764 229533.I1RMG8 pp 229533.I1RRM5
138765 229533.I1RMG8 pp 229533.I1RW25
138766 229533.I1RMG8 pp 229533.V6R042
138767 229533.I1RMG8 pp 229533.I1S0L3
138768 229533.I1RMG8 pp 229533.V6RMW6
138769 229533.I1RMG8 pp 229533.I1RPM4
138770 229533.I1RMG8 pp 229533.I1S504
138771 229533.I1RMG8 pp 229533.Q4I2J8
138772 229533.I1RMG8 pp 229533.I1RVQ0
138773 229533.I1RMG8 pp 229533.I1RXD7

138774 229533.I1RMG8 pp 229533.I1RZZ1
138775 229533.I1RMG8 pp 229533.I1RQE7
138776 229533.I1RMG8 pp 229533.I1RSQ3
138777 229533.I1RMG8 pp 229533.I1RT04
138778 229533.I1RMG8 pp 229533.I1RQ23
138779 229533.I1RMG8 pp 229533.I1RZM1
138780 229533.I1RMG8 pp 229533.I1S0R7
138781 229533.I1RMG8 pp 229533.I1RNQ5
138782 229533.I1RMG8 pp 229533.I1RRY9
138783 229533.I1RMG8 pp 229533.I1RTH6
138784 229533.I1RMG8 pp 229533.I1RN18
138785 229533.I1RMG8 pp 229533.I1RQC9
138786 229533.I1RMG8 pp 229533.I1RZW8
138787 229533.I1RMG8 pp 229533.V6RCZ8
138788 229533.I1RMG8 pp 229533.I1RRI2
138789 229533.I1RMG8 pp 229533.I1S0L4
138790 229533.I1RMG8 pp 229533.I1RY03
138791 229533.I1RMG8 pp 229533.I1RTD5
138792 229533.I1RMG8 pp 229533.I1RR24
138793 229533.I1RMG8 pp 229533.I1RZJ5
138794 229533.I1RMG8 pp 229533.I1RXF0
138795 229533.I1RMG8 pp 229533.I1RZM8
138796 229533.I1RMG8 pp 229533.I1RZZ9
138797 229533.I1RMG8 pp 229533.I1S5R1
138798 229533.I1RMG8 pp 229533.I1RUG3
138799 229533.I1RMG8 pp 229533.I1RT40
138800 229533.I1RMG8 pp 229533.I1RRT8
138801 229533.I1RMG8 pp 229533.V6R3X5
138802 229533.I1RMG8 pp 229533.I1RWJ8
138803 229533.I1RMG8 pp 229533.I1RNC1
138804 229533.I1RMG8 pp 229533.I1RR73
138805 229533.I1S209 pp 229533.I1SAA0
138806 229533.I1S209 pp 229533.I1SAD2
138807 229533.I1S209 pp 229533.V6RLW4
138808 229533.I1S209 pp 229533.Q4IB89
138809 229533.I1S209 pp 229533.I1S523
138810 229533.I1S209 pp 229533.I1S9F9
138811 229533.I1S209 pp 229533.I1S998
138812 229533.I1S209 pp 229533.V6R382
138813 229533.I1S209 pp 229533.I1S276
138814 229533.I1S209 pp 229533.Q4IC11
138815 229533.I1S209 pp 229533.I1S788
138816 229533.I1S209 pp 229533.I1S4P6
138817 229533.I1S209 pp 229533.I1S3G4
138818 229533.I1S209 pp 229533.Q4IJU0
138819 229533.I1S209 pp 229533.Q4INS6
138820 229533.I1S209 pp 229533.I1S987
138821 229533.I1S209 pp 229533.I1S283
138822 229533.I1S209 pp 229533.I1S461
138823 229533.A0A1C3YJL2 pp 229533.I1RA62
138824 229533.A0A1C3YJL2 pp 229533.I1RGU7
138825 229533.I1RRQ1 pp 229533.I1RTD5
138826 229533.I1RRQ1 pp 229533.I1RSQ3
138827 229533.I1RRQ1 pp 229533.I1SAS4
138828 229533.I1RRQ1 pp 229533.I1S5K2
138829 229533.I1RRQ1 pp 229533.I1RTH6
138830 229533.I1RRQ1 pp 229533.V6RMW6
138831 229533.I1RRQ1 pp 229533.I1S8D2
138832 229533.I1RRQ1 pp 229533.I1SAS3
138833 229533.I1RRQ1 pp 229533.I1RZ39
138834 229533.I1RRQ1 pp 229533.I1RWN5
138835 229533.I1RRQ1 pp 229533.I1S2B8
138836 229533.I1RRQ1 pp 229533.I1RUP5
138837 229533.I1RRQ1 pp 229533.I1RSX9
138838 229533.I1RRQ1 pp 229533.I1RY35
138839 229533.I1RRQ1 pp 229533.Q4I2W2
138840 229533.I1RRQ1 pp 229533.I1S2C2
138841 229533.I1RRQ1 pp 229533.I1RWN6
138842 229533.I1RRQ1 pp 229533.I1RY65
138843 229533.I1RRQ1 pp 229533.Q4I1Y5
138844 229533.I1RRQ1 pp 229533.I1S2M5
138845 229533.I1RRQ1 pp 229533.V6RPN0
138846 229533.I1RRQ1 pp 229533.V6RR38

229533.I1RRQ1 pp 229533.I1S1Q3
229533.I1RRQ1 pp 229533.Q4IJK2
229533.I1RRQ1 pp 229533.I1RRY9
229533.I1RRQ1 pp 229533.I1RT23
229533.A0A0E0SA11 pp 229533.I1RK79
229533.A0A0E0SA11 pp 229533.I1RAW0
229533.A0A0E0SA11 pp 229533.I1RMJ8
229533.A0A0E0SA11 pp 229533.I1RWN6
229533.A0A0E0SA11 pp 229533.I1S2C0
229533.A0A0E0SA11 pp 229533.I1RBJ0
229533.A0A0E0SA11 pp 229533.I1S276
229533.A0A0E0SA11 pp 229533.I1RZP8
229533.A0A0E0SA11 pp 229533.I1RRP7
229533.A0A0E0SA11 pp 229533.I1RZK9
229533.A0A0E0SA11 pp 229533.I1RD39
229533.A0A0E0SA11 pp 229533.I1RD70
229533.A0A0E0SA11 pp 229533.I1S8R3
229533.A0A0E0SA11 pp 229533.I1RWT5
229533.A0A0E0SA11 pp 229533.I1RTH6
229533.A0A0E0SA11 pp 229533.I1RZT1
229533.A0A0E0SA11 pp 229533.I1RCA9
229533.A0A0E0SA11 pp 229533.I1RA70
229533.A0A0E0SA11 pp 229533.I1RPM7
229533.A0A0E0SA11 pp 229533.I1RJV3
229533.A0A0E0SA11 pp 229533.I1RW84
229533.A0A0E0SA11 pp 229533.I1RS41
229533.A0A0E0SA11 pp 229533.I1RT03
229533.A0A0E0SA11 pp 229533.I1RPW0
229533.A0A0E0SA11 pp 229533.I1RVX0
229533.A0A0E0SA11 pp 229533.I1RX64
229533.A0A0E0SA11 pp 229533.I1RJT8
229533.A0A0E0SA11 pp 229533.I1RQE7
229533.A0A0E0SA11 pp 229533.I1RSK1
229533.A0A0E0SA11 pp 229533.I1RW32
229533.A0A0E0SA11 pp 229533.I1RGC6
229533.A0A0E0SA11 pp 229533.I1S2E8
229533.A0A0E0SA11 pp 229533.I1RZW8
229533.A0A0E0SA11 pp 229533.I1RDH0
229533.A0A0E0SA11 pp 229533.I1RRY3
229533.A0A0E0SA11 pp 229533.I1RRI2
229533.A0A0E0SA11 pp 229533.I1RTN0
229533.A0A0E0SA11 pp 229533.I1REC7
229533.A0A0E0SA11 pp 229533.I1RY58
229533.A0A0E0SA11 pp 229533.I1RWX5
229533.A0A0E0SA11 pp 229533.I1RCH5
229533.A0A0E0SA11 pp 229533.I1RA10
229533.A0A0E0SA11 pp 229533.I1RDJ8
229533.A0A0E0SA11 pp 229533.I1RDQ9
229533.A0A0E0SA11 pp 229533.I1RCM3
229533.A0A0E0SA11 pp 229533.I1S1Y7
229533.A0A0E0SA11 pp 229533.A0A1C3YIN6
229533.A0A0E0SA11 pp 229533.A0A1C3YHP8
229533.A0A0E0SA11 pp 229533.I1RZE4
229533.A0A0E0SA11 pp 229533.I1RSR9
229533.A0A0E0SA11 pp 229533.I1RPS0
229533.A0A0E0SA11 pp 229533.I1S286
229533.A0A0E0SA11 pp 229533.I1S0E8
229533.A0A0E0SA11 pp 229533.I1RYW3
229533.A0A0E0SA11 pp 229533.I1RN18
229533.A0A0E0SA11 pp 229533.I1RSD8
229533.A0A0E0SA11 pp 229533.I1RB92
229533.A0A0E0SA11 pp 229533.I1RDZ4
229533.A0A0E0SA11 pp 229533.I1RAS2
229533.A0A0E0SA11 pp 229533.I1S1C7
229533.A0A0E0SA11 pp 229533.I1RRN5
229533.A0A0E0SA11 pp 229533.I1R9J6
229533.A0A0E0SA11 pp 229533.I1RYK6
229533.A0A0E0SA11 pp 229533.I1RD15
229533.A0A0E0SA11 pp 229533.I1R9D1
229533.A0A0E0SA11 pp 229533.I1RB41
229533.A0A0E0SA11 pp 229533.I1RFL4
229533.A0A0E0SA11 pp 229533.I1RSI3
229533.A0A0E0SA11 pp 229533.A0A1C3YM29

138920	229533.A0A0E0SA11	pp	229533.A0A1C3YM91
138921	229533.A0A0E0SA11	pp	229533.I1S0N4
138922	229533.A0A0E0SA11	pp	229533.I1RR25
138923	229533.A0A0E0SA11	pp	229533.I1RAU7
138924	229533.A0A0E0SA11	pp	229533.I1RXF5
138925	229533.A0A0E0SA11	pp	229533.I1RQ72
138926	229533.A0A0E0SA11	pp	229533.V6RGI1
138927	229533.A0A0E0SA11	pp	229533.I1RT36
138928	229533.A0A0E0SA11	pp	229533.I1RD17
138929	229533.A0A0E0SA11	pp	229533.I1S0A6
138930	229533.A0A0E0SA11	pp	229533.V6RMW6
138931	229533.A0A0E0SA11	pp	229533.I1RCB7
138932	229533.A0A0E0SA11	pp	229533.I1S757
138933	229533.A0A0E0SA11	pp	229533.I1RVN2
138934	229533.A0A0E0SA11	pp	229533.I1RSQ3
138935	229533.A0A0E0SA11	pp	229533.I1RWH4
138936	229533.A0A0E0SA11	pp	229533.I1S0V7
138937	229533.A0A0E0SA11	pp	229533.I1S569
138938	229533.A0A0E0SA11	pp	229533.V6R042
138939	229533.A0A0E0SA11	pp	229533.I1RZX3
138940	229533.A0A0E0SA11	pp	229533.I1RD22
138941	229533.A0A0E0SA11	pp	229533.I1RXU0
138942	229533.A0A0E0SA11	pp	229533.I1RBM3
138943	229533.A0A0E0SA11	pp	229533.I1SAF7
138944	229533.A0A0E0SA11	pp	229533.I1R9Y7
138945	229533.A0A0E0SA11	pp	229533.I1RNW8
138946	229533.A0A0E0SA11	pp	229533.I1RRV1
138947	229533.A0A0E0SA11	pp	229533.I1RGL4
138948	229533.A0A0E0SA11	pp	229533.I1RM41
138949	229533.A0A0E0SA11	pp	229533.I1RMN6
138950	229533.A0A0E0SA11	pp	229533.I1RZC3
138951	229533.A0A0E0SA11	pp	229533.I1SA33
138952	229533.A0A0E0SA11	pp	229533.I1RMG3
138953	229533.A0A0E0SA11	pp	229533.I1RSD1
138954	229533.A0A0E0SA11	pp	229533.I1RMA4
138955	229533.A0A0E0SA11	pp	229533.A0A1C3YJC3
138956	229533.A0A0E0SA11	pp	229533.A0A1C3YL52
138957	229533.A0A0E0SA11	pp	229533.I1RW16
138958	229533.A0A0E0SA11	pp	229533.I1RTA0
138959	229533.A0A0E0SA11	pp	229533.I1RRW0
138960	229533.A0A0E0SA11	pp	229533.I1RW46
138961	229533.A0A0E0SA11	pp	229533.A0A1C3YMI5
138962	229533.A0A0E0SA11	pp	229533.I1S1A3
138963	229533.A0A0E0SA11	pp	229533.I1RPP4
138964	229533.A0A0E0SA11	pp	229533.Q4IB89
138965	229533.A0A0E0SA11	pp	229533.A0A1C3YKS0
138966	229533.A0A0E0SA11	pp	229533.I1RA43
138967	229533.A0A0E0SA11	pp	229533.I1RE01
138968	229533.A0A0E0SA11	pp	229533.I1RS17
138969	229533.A0A0E0SA11	pp	229533.I1RQB4
138970	229533.A0A0E0SA11	pp	229533.I1RSH9
138971	229533.A0A0E0SA11	pp	229533.I1RQK5
138972	229533.A0A0E0SA11	pp	229533.I1RRR7
138973	229533.A0A0E0SA11	pp	229533.Q4IN63
138974	229533.A0A0E0SA11	pp	229533.I1RDC5
138975	229533.A0A0E0SA11	pp	229533.I1RCR8
138976	229533.A0A0E0SA11	pp	229533.I1S1W4
138977	229533.A0A0E0SA11	pp	229533.A0A1C3YMC9
138978	229533.A0A0E0SA11	pp	229533.A0A1C3YK43
138979	229533.A0A0E0SA11	pp	229533.I1S0M2
138980	229533.A0A0E0SA11	pp	229533.I1RA56
138981	229533.A0A0E0SA11	pp	229533.I1S0D1
138982	229533.A0A0E0SA11	pp	229533.A0A1C3YLN8
138983	229533.A0A0E0SA11	pp	229533.I1RAV0
138984	229533.A0A0E0SA11	pp	229533.I1RRE5
138985	229533.A0A0E0SA11	pp	229533.V6RVG3
138986	229533.A0A0E0SA11	pp	229533.I1RUG3
138987	229533.A0A0E0SA11	pp	229533.I1S0J1
138988	229533.A0A0E0SA11	pp	229533.I1RNC1
138989	229533.A0A0E0SA11	pp	229533.I1RM96
138990	229533.A0A0E0SA11	pp	229533.Q4HZB8
138991	229533.A0A0E0SA11	pp	229533.I1RJT0
138992	229533.A0A0E0SA11	pp	229533.A0A1C3YN69

138993 229533.A0A0E0SA11 pp 229533.I1R9Y9
138994 229533.A0A0E0SA11 pp 229533.A0A1C3YI28
138995 229533.A0A0E0SA11 pp 229533.I1RNY8
138996 229533.A0A0E0SA11 pp 229533.I1RG11
138997 229533.A0A0E0SA11 pp 229533.V6R3X5
138998 229533.A0A0E0SA11 pp 229533.I1RIL3
138999 229533.A0A0E0SA11 pp 229533.V6RKY7
139000 229533.A0A0E0SA11 pp 229533.A0A1C3YIN1
139001 229533.A0A0E0SA11 pp 229533.A0A1C3YI10
139002 229533.A0A0E0SA11 pp 229533.I1S0L4
139003 229533.A0A0E0SA11 pp 229533.I1RR73
139004 229533.A0A0E0SA11 pp 229533.I1RS20
139005 229533.A0A0E0SA11 pp 229533.A0A1C3YJ90
139006 229533.A0A0E0SA11 pp 229533.A0A1C3YK73
139007 229533.A0A0E0SA11 pp 229533.I1RZ63
139008 229533.A0A0E0SA11 pp 229533.I1S2H4
139009 229533.A0A0E0SA11 pp 229533.I1RFW0
139010 229533.A0A0E0SA11 pp 229533.I1RX38
139011 229533.A0A0E0SA11 pp 229533.I1RAN2
139012 229533.A0A0E0SA11 pp 229533.I1RC24
139013 229533.A0A0E0SA11 pp 229533.I1RBU5
139014 229533.A0A0E0SA11 pp 229533.I1RGF1
139015 229533.A0A0E0SA11 pp 229533.I1RWN5
139016 229533.A0A0E0SA11 pp 229533.A0A1C3YN55
139017 229533.A0A0E0SA11 pp 229533.I1S4R1
139018 229533.A0A0E0SA11 pp 229533.V6RIV9
139019 229533.A0A0E0SA11 pp 229533.I1S0J3
139020 229533.A0A0E0SA11 pp 229533.I1RLU1
139021 229533.A0A0E0SA11 pp 229533.I1RZ39
139022 229533.A0A0E0SA11 pp 229533.I1RWL2
139023 229533.A0A0E0SA11 pp 229533.A0A1C3YK17
139024 229533.A0A0E0SA11 pp 229533.I1REN4
139025 229533.A0A0E0SA11 pp 229533.I1RNP7
139026 229533.A0A0E0SA11 pp 229533.A0A1C3YKM9
139027 229533.A0A0E0SA11 pp 229533.I1RH91
139028 229533.A0A0E0SA11 pp 229533.I1RNN1
139029 229533.A0A0E0SA11 pp 229533.I1S5R1
139030 229533.A0A0E0SA11 pp 229533.I1RAN3
139031 229533.A0A0E0SA11 pp 229533.Q4HTT1
139032 229533.A0A0E0SA11 pp 229533.I1RU69
139033 229533.A0A0E0SA11 pp 229533.I1RRT8
139034 229533.A0A0E0SA11 pp 229533.A0A1C3YIN0
139035 229533.A0A0E0SA11 pp 229533.I1RC71
139036 229533.A0A0E0SA11 pp 229533.I1RPM4
139037 229533.A0A0E0SA11 pp 229533.I1S1V5
139038 229533.A0A0E0SA11 pp 229533.I1RFS3
139039 229533.A0A0E0SA11 pp 229533.A0A1C3YIR8
139040 229533.A0A0E0SA11 pp 229533.I1RZZ9
139041 229533.A0A0E0SA11 pp 229533.I1RM82
139042 229533.A0A0E0SA11 pp 229533.I1RWI5
139043 229533.A0A0E0SA11 pp 229533.I1RCA5
139044 229533.A0A0E0SA11 pp 229533.I1RQ23
139045 229533.A0A0E0SA11 pp 229533.I1S004
139046 229533.A0A0E0SA11 pp 229533.I1RUN8
139047 229533.A0A0E0SA11 pp 229533.I1REC6
139048 229533.A0A0E0SA11 pp 229533.I1S549
139049 229533.A0A0E0SA11 pp 229533.A0A1C3YI39
139050 229533.A0A0E0SA11 pp 229533.I1RVL8
139051 229533.A0A0E0SA11 pp 229533.I1RPK4
139052 229533.A0A0E0SA11 pp 229533.I1RBW1
139053 229533.A0A0E0SA11 pp 229533.I1RZS6
139054 229533.A0A0E0SA11 pp 229533.Q4I7L0
139055 229533.A0A0E0SA11 pp 229533.I1RE15
139056 229533.A0A0E0SA11 pp 229533.A0A1C3YIS7
139057 229533.A0A0E0SA11 pp 229533.I1S0M6
139058 229533.A0A0E0SA11 pp 229533.A0A1C3YMQ5
139059 229533.A0A0E0SA11 pp 229533.A0A1C3YMA1
139060 229533.A0A0E0SA11 pp 229533.A0A1C3YHL0
139061 229533.A0A0E0SA11 pp 229533.I1RPX5
139062 229533.A0A0E0SA11 pp 229533.I1RE76
139063 229533.A0A0E0SA11 pp 229533.I1RBY4
139064 229533.A0A0E0SA11 pp 229533.I1S0J7
139065 229533.A0A0E0SA11 pp 229533.V6RLW4

139066	229533.A0A0E0SA11	pp	229533.I1S0X7
139067	229533.A0A0E0SA11	pp	229533.I1RJZ7
139068	229533.A0A0E0SA11	pp	229533.I1RPU3
139069	229533.A0A0E0SA11	pp	229533.I1RE22
139070	229533.A0A0E0SA11	pp	229533.I1S2B8
139071	229533.A0A0E0SA11	pp	229533.I1R9M0
139072	229533.A0A0E0SA11	pp	229533.I1RFT1
139073	229533.A0A0E0SA11	pp	229533.I1S098
139074	229533.A0A0E0SA11	pp	229533.A0A1C3YKN3
139075	229533.A0A0E0SA11	pp	229533.I1RY29
139076	229533.A0A0E0SA11	pp	229533.I1RRM5
139077	229533.A0A0E0SA11	pp	229533.I1RSV2
139078	229533.A0A0E0SA11	pp	229533.I1RAF8
139079	229533.A0A0E0SA11	pp	229533.I1RV91
139080	229533.A0A0E0SA11	pp	229533.I1RT28
139081	229533.A0A0E0SA11	pp	229533.I1S352
139082	229533.A0A0E0SA11	pp	229533.A0A0E0SQG9
139083	229533.A0A0E0SA11	pp	229533.A0A0E0SQJ6
139084	229533.A0A0E0SA11	pp	229533.I1RWI8
139085	229533.A0A0E0SA11	pp	229533.A0A0E0SB24
139086	229533.A0A0E0SA11	pp	229533.I1RPF5
139087	229533.A0A0E0SA11	pp	229533.I1RZP1
139088	229533.A0A0E0SA11	pp	229533.I1RPU7
139089	229533.A0A0E0SA11	pp	229533.I1RFW1
139090	229533.A0A0E0SA11	pp	229533.I1RT04
139091	229533.A0A0E0SA11	pp	229533.A0A0E0SLZ3
139092	229533.A0A0E0SA11	pp	229533.I1RC03
139093	229533.A0A0E0SA11	pp	229533.I1RJV4
139094	229533.A0A0E0SA11	pp	229533.I1RP27
139095	229533.A0A0E0SA11	pp	229533.I1RBV5
139096	229533.A0A0E0SA11	pp	229533.I1S074
139097	229533.A0A0E0SA11	pp	229533.I1RW83
139098	229533.A0A0E0SA11	pp	229533.I1RNA5
139099	229533.A0A0E0SA11	pp	229533.I1RGA4
139100	229533.A0A0E0SA11	pp	229533.I1RBS6
139101	229533.A0A0E0SA11	pp	229533.I1S7X9
139102	229533.A0A0E0SA11	pp	229533.A0A1C3YKT6
139103	229533.A0A0E0SA11	pp	229533.I1RUF8
139104	229533.A0A0E0SA11	pp	229533.A0A1C3YKE4
139105	229533.A0A0E0SA11	pp	229533.I1RQ88
139106	229533.A0A0E0SA11	pp	229533.I1RTD5
139107	229533.A0A0E0SA11	pp	229533.V6R283
139108	229533.A0A0E0SA11	pp	229533.I1RGF5
139109	229533.A0A0E0SA11	pp	229533.I1RXU1
139110	229533.A0A0E0SA11	pp	229533.A0A1C3YLK5
139111	229533.A0A0E0SA11	pp	229533.I1S503
139112	229533.A0A0E0SA11	pp	229533.A0A0E0SNM6
139113	229533.A0A0E0SA11	pp	229533.I1RRR9
139114	229533.A0A0E0SA11	pp	229533.I1RWK3
139115	229533.A0A0E0SA11	pp	229533.I1RSZ3
139116	229533.A0A0E0SA11	pp	229533.I1RFZ2
139117	229533.A0A0E0SA11	pp	229533.I1RW76
139118	229533.A0A0E0SA11	pp	229533.A0A1C3YLK8
139119	229533.A0A0E0SA11	pp	229533.I1RJW6
139120	229533.A0A0E0SA11	pp	229533.I1R9X6
139121	229533.A0A0E0SA11	pp	229533.I1S2G7
139122	229533.A0A0E0SA11	pp	229533.I1RLN6
139123	229533.A0A0E0SA11	pp	229533.A0A1C3YKJ6
139124	229533.A0A0E0SA11	pp	229533.I1RMP0
139125	229533.A0A0E0SA11	pp	229533.I1RY32
139126	229533.A0A0E0SA11	pp	229533.I1RW24
139127	229533.A0A0E0SA11	pp	229533.I1RNF4
139128	229533.A0A0E0SA11	pp	229533.I1RT40
139129	229533.A0A0E0SA11	pp	229533.I1RFL7
139130	229533.A0A0E0SA11	pp	229533.I1RQ65
139131	229533.A0A0E0SA11	pp	229533.A0A1C3YM82
139132	229533.A0A0E0SA11	pp	229533.I1S8P5
139133	229533.A0A0E0SA11	pp	229533.I1RPP3
139134	229533.A0A0E0SA11	pp	229533.I1RCJ7
139135	229533.A0A0E0SA11	pp	229533.A0A1C3YLK2
139136	229533.A0A0E0SA11	pp	229533.I1RYY5
139137	229533.A0A0E0SA11	pp	229533.A0A1C3YKN4
139138	229533.A0A0E0SA11	pp	229533.I1RC97

139139 229533.A0A0E0SA11 pp 229533.I1RSG0
139140 229533.A0A0E0SA11 pp 229533.I1RB74
139141 229533.A0A0E0SA11 pp 229533.I1RTB2
139142 229533.A0A0E0SA11 pp 229533.I1RY03
139143 229533.A0A0E0SA11 pp 229533.I1RS47
139144 229533.A0A0E0SA11 pp 229533.A0A1C3YJG7
139145 229533.A0A0E0SA11 pp 229533.I1S0V6
139146 229533.A0A0E0SA11 pp 229533.I1RBS0
139147 229533.A0A0E0SA11 pp 229533.I1RA48
139148 229533.A0A0E0SA11 pp 229533.I1RRQ3
139149 229533.A0A0E0SA11 pp 229533.I1RG77
139150 229533.A0A0E0SA11 pp 229533.I1RC22
139151 229533.A0A0E0SA11 pp 229533.I1RUQ5
139152 229533.A0A0E0SA11 pp 229533.I1S0F6
139153 229533.A0A0E0SA11 pp 229533.I1RN65
139154 229533.A0A0E0SA11 pp 229533.I1S3H8
139155 229533.A0A0E0SA11 pp 229533.I1RSR8
139156 229533.A0A0E0SA11 pp 229533.Q4IJM4
139157 229533.A0A0E0SA11 pp 229533.A0A1C3YJF1
139158 229533.A0A0E0SA11 pp 229533.A0A1C3YIF4
139159 229533.A0A0E0SA11 pp 229533.A0A1C3YHX6
139160 229533.A0A0E0SA11 pp 229533.I1RS38
139161 229533.A0A0E0SA11 pp 229533.A0A1C3YM61
139162 229533.A0A0E0SA11 pp 229533.I1RN67
139163 229533.A0A0E0SA11 pp 229533.I1RJZ6
139164 229533.A0A0E0SA11 pp 229533.I1R9N1
139165 229533.A0A0E0SA11 pp 229533.I1RHB0
139166 229533.A0A0E0SA11 pp 229533.I1RRY9
139167 229533.A0A0E0SA11 pp 229533.I1RJL2
139168 229533.A0A0E0SA11 pp 229533.I1RQQ5
139169 229533.A0A0E0SA11 pp 229533.I1RU02
139170 229533.A0A0E0SA11 pp 229533.I1RU29
139171 229533.A0A0E0SA11 pp 229533.Q4I7N9
139172 229533.A0A0E0SA11 pp 229533.V6QV38
139173 229533.A0A0E0SA11 pp 229533.I1RBC4
139174 229533.A0A0E0SA11 pp 229533.A0A1C3YJ93
139175 229533.A0A0E0SA11 pp 229533.I1RZK5
139176 229533.A0A0E0SA11 pp 229533.I1RZZ1
139177 229533.A0A0E0SA11 pp 229533.I1RXZ4
139178 229533.A0A0E0SA11 pp 229533.I1S1J7
139179 229533.A0A0E0SA11 pp 229533.I1RZK6
139180 229533.A0A0E0SA11 pp 229533.I1RI03
139181 229533.A0A0E0SA11 pp 229533.I1RCG4
139182 229533.A0A0E0SA11 pp 229533.I1RCM6
139183 229533.A0A0E0SA11 pp 229533.I1RZB5
139184 229533.A0A0E0SA11 pp 229533.I1RN08
139185 229533.A0A0E0SA11 pp 229533.A0A0E0SPV2
139186 229533.A0A0E0SA11 pp 229533.I1RQE9
139187 229533.A0A0E0SA11 pp 229533.I1RB53
139188 229533.A0A0E0SA11 pp 229533.Q4IJK2
139189 229533.A0A0E0SA11 pp 229533.I1RMS2
139190 229533.A0A0E0SA11 pp 229533.A0A1C3YI44
139191 229533.A0A0E0SA11 pp 229533.I1RFU8
139192 229533.A0A0E0SA11 pp 229533.A0A1C3YLX2
139193 229533.A0A0E0SA11 pp 229533.I1RMM9
139194 229533.A0A0E0SA11 pp 229533.A0A1C3YKY3
139195 229533.A0A0E0SA11 pp 229533.I1S0N8
139196 229533.A0A0E0SA11 pp 229533.Q4IER8
139197 229533.A0A0E0SA11 pp 229533.I1RQA3
139198 229533.A0A0E0SA11 pp 229533.I1RCL7
139199 229533.A0A0E0SA11 pp 229533.A0A1C3YJW1
139200 229533.A0A0E0SA11 pp 229533.I1RJT1
139201 229533.A0A0E0SA11 pp 229533.I1RYV3
139202 229533.A0A0E0SA11 pp 229533.A0A1C3YIB8
139203 229533.A0A0E0SA11 pp 229533.I1S267
139204 229533.A0A0E0SA11 pp 229533.I1S439
139205 229533.A0A0E0SA11 pp 229533.Q4I5R3
139206 229533.A0A0E0SA11 pp 229533.I1RLM7
139207 229533.A0A0E0SA11 pp 229533.I1RDV9
139208 229533.A0A0E0SA11 pp 229533.Q4I2J8
139209 229533.A0A0E0SA11 pp 229533.I1REN3
139210 229533.A0A0E0SA11 pp 229533.I1REK8
139211 229533.A0A0E0SA11 pp 229533.I1RSZ4

139212	229533.A0A0E0SA11	pp	229533.Q4IR87
139213	229533.A0A0E0SA11	pp	229533.I1RB75
139214	229533.A0A0E0SA11	pp	229533.I1RX09
139215	229533.A0A0E0SA11	pp	229533.I1RJK7
139216	229533.A0A0E0SA11	pp	229533.I1RAM7
139217	229533.A0A0E0SA11	pp	229533.I1RFQ5
139218	229533.A0A0E0SA11	pp	229533.I1RSI0
139219	229533.A0A0E0SA11	pp	229533.I1RYZ0
139220	229533.A0A0E0SA11	pp	229533.I1SA32
139221	229533.A0A0E0SA11	pp	229533.I1RE00
139222	229533.A0A0E0SA11	pp	229533.A0A1C3YIJ3
139223	229533.A0A0E0SA11	pp	229533.Q4IEA7
139224	229533.A0A0E0SA11	pp	229533.I1RFJ2
139225	229533.A0A0E0SA11	pp	229533.A0A1C3YK01
139226	229533.A0A0E0SA11	pp	229533.I1RQS4
139227	229533.A0A0E0SA11	pp	229533.I1RZE5
139228	229533.A0A0E0SA11	pp	229533.V6RCG5
139229	229533.A0A0E0SA11	pp	229533.I1RDC9
139230	229533.A0A0E0SA11	pp	229533.A0A1C3YK34
139231	229533.A0A0E0SA11	pp	229533.I1RMQ7
139232	229533.A0A0E0SA11	pp	229533.I1RW99
139233	229533.A0A0E0SA11	pp	229533.I1RAU6
139234	229533.A0A0E0SA11	pp	229533.I1RRT0
139235	229533.A0A0E0SA11	pp	229533.A0A1C3YI23
139236	229533.A0A0E0SA11	pp	229533.A0A1C3YLU7
139237	229533.A0A0E0SA11	pp	229533.I1RCT9
139238	229533.A0A0E0SA11	pp	229533.A0A1C3YK75
139239	229533.A0A0E0SA11	pp	229533.Q4HTT2
139240	229533.A0A0E0SA11	pp	229533.I1RZH4
139241	229533.A0A0E0SA11	pp	229533.I1S5B0
139242	229533.A0A0E0SA11	pp	229533.I1RS07
139243	229533.A0A0E0SA11	pp	229533.I1RC17
139244	229533.A0A0E0SA11	pp	229533.I1RME5
139245	229533.A0A0E0SA11	pp	229533.I1RWT6
139246	229533.A0A0E0SA11	pp	229533.I1RPU4
139247	229533.A0A0E0SA11	pp	229533.I1RMR7
139248	229533.A0A0E0SA11	pp	229533.Q4I283
139249	229533.A0A0E0SA11	pp	229533.Q4INS6
139250	229533.A0A0E0SA11	pp	229533.I1RKK0
139251	229533.A0A0E0SA11	pp	229533.I1RAU5
139252	229533.A0A0E0SA11	pp	229533.V6RCZ8
139253	229533.A0A0E0SA11	pp	229533.I1RRU9
139254	229533.A0A0E0SA11	pp	229533.I1RFT2
139255	229533.A0A0E0SA11	pp	229533.I1RQ19
139256	229533.A0A0E0SA11	pp	229533.I1RC95
139257	229533.A0A0E0SA11	pp	229533.I1S021
139258	229533.A0A0E0SA11	pp	229533.A0A1C3YKB0
139259	229533.A0A0E0SA11	pp	229533.I1RCW8
139260	229533.A0A0E0SA11	pp	229533.I1RST9
139261	229533.A0A0E0SA11	pp	229533.I1REE1
139262	229533.A0A0E0SA11	pp	229533.A0A1C3YIG3
139263	229533.A0A0E0SA11	pp	229533.I1RI82
139264	229533.A0A0E0SA11	pp	229533.I1S504
139265	229533.A0A0E0SA11	pp	229533.Q4I8P5
139266	229533.A0A0E0SA11	pp	229533.I1RZF6
139267	229533.A0A0E0SA11	pp	229533.I1RM09
139268	229533.A0A0E0SA11	pp	229533.I1S604
139269	229533.A0A0E0SA11	pp	229533.A0A1C3YJZ9
139270	229533.A0A0E0SA11	pp	229533.I1RN58
139271	229533.A0A0E0SA11	pp	229533.I1RS18
139272	229533.A0A0E0SA11	pp	229533.I1RZM8
139273	229533.A0A0E0SA11	pp	229533.I1RLT6
139274	229533.A0A0E0SA11	pp	229533.I1RA12
139275	229533.A0A0E0SA11	pp	229533.I1RQ81
139276	229533.A0A0E0SA11	pp	229533.I1RZM1
139277	229533.A0A0E0SA11	pp	229533.A0A1C3YKV3
139278	229533.A0A0E0SA11	pp	229533.I1RCB3
139279	229533.A0A0E0SA11	pp	229533.A0A1C3YK37
139280	229533.A0A0E0SA11	pp	229533.I1RBJ5
139281	229533.A0A0E0SA11	pp	229533.I1S0M9
139282	229533.A0A0E0SA11	pp	229533.A0A0E0SQV5
139283	229533.A0A0E0SA11	pp	229533.I1RLU6
139284	229533.A0A0E0SA11	pp	229533.I1RNQ5

139285 229533.A0A0E0SA11 pp 229533.I1S1W5
139286 229533.A0A0E0SA11 pp 229533.I1RR24
139287 229533.A0A0E0SA11 pp 229533.I1RSS1
139288 229533.A0A0E0SA11 pp 229533.I1RD18
139289 229533.A0A0E0SA11 pp 229533.I1RKE9
139290 229533.A0A0E0SA11 pp 229533.I1RRT1
139291 229533.A0A0E0SA11 pp 229533.I1RMB2
139292 229533.A0A0E0SA11 pp 229533.A0A1C3YKF2
139293 229533.A0A0E0SA11 pp 229533.I1RC91
139294 229533.A0A0E0SA11 pp 229533.A0A1C3YJV7
139295 229533.A0A0E0SA11 pp 229533.A0A1C3YIJ4
139296 229533.A0A0E0SA11 pp 229533.I1RR94
139297 229533.A0A0E0SA11 pp 229533.I1RL24
139298 229533.A0A0E0SA11 pp 229533.I1RFQ7
139299 229533.A0A0E0SA11 pp 229533.I1RIH8
139300 229533.A0A0E0SA11 pp 229533.A0A1C3YJH8
139301 229533.A0A0E0SA11 pp 229533.I1RWJ8
139302 229533.A0A0E0SA11 pp 229533.I1RJU3
139303 229533.A0A0E0SA11 pp 229533.I1RD69
139304 229533.A0A0E0SA11 pp 229533.I1RDB1
139305 229533.A0A0E0SA11 pp 229533.A0A1C3YK51
139306 229533.A0A0E0SA11 pp 229533.I1RV66
139307 229533.A0A0E0SA11 pp 229533.I1RT80
139308 229533.A0A0E0SA11 pp 229533.I1RW07
139309 229533.A0A0E0SA11 pp 229533.I1RX70
139310 229533.A0A0E0SA11 pp 229533.I1RN31
139311 229533.A0A0E0SA11 pp 229533.A0A1C3YHX8
139312 229533.A0A0E0SA11 pp 229533.I1RMC8
139313 229533.A0A0E0SA11 pp 229533.I1RI02
139314 229533.A0A0E0SA11 pp 229533.I1RZ94
139315 229533.A0A0E0SA11 pp 229533.A0A1C3YLR4
139316 229533.A0A0E0SA11 pp 229533.A0A1C3YI64
139317 229533.A0A0E0SA11 pp 229533.I1RWJ6
139318 229533.A0A0E0SA11 pp 229533.A0A1C3YI02
139319 229533.A0A0E0SA11 pp 229533.I1RD01
139320 229533.A0A0E0SA11 pp 229533.I1RQ13
139321 229533.A0A0E0SA11 pp 229533.I1RQA9
139322 229533.A0A0E0SA11 pp 229533.Q4ICG5
139323 229533.A0A0E0SA11 pp 229533.I1S0X6
139324 229533.A0A0E0SA11 pp 229533.A0A1C3YN58
139325 229533.A0A0E0SA11 pp 229533.A0A1C3YLZ7
139326 229533.A0A0E0SA11 pp 229533.V6QUW4
139327 229533.A0A0E0SA11 pp 229533.I1RNC0
139328 229533.A0A0E0SA11 pp 229533.A0A1C3YKC0
139329 229533.A0A0E0SA11 pp 229533.I1RBT1
139330 229533.A0A0E0SA11 pp 229533.A0A1C3YL96
139331 229533.A0A0E0SA11 pp 229533.I1RW25
139332 229533.A0A0E0SA11 pp 229533.I1RJR8
139333 229533.A0A0E0SA11 pp 229533.I1RRL5
139334 229533.A0A0E0SA11 pp 229533.I1RRX6
139335 229533.A0A0E0SA11 pp 229533.A0A1C3YJQ9
139336 229533.A0A0E0SA11 pp 229533.I1RBW0
139337 229533.A0A0E0SA11 pp 229533.I1RAH4
139338 229533.A0A0E0SA11 pp 229533.A0A1C3YI40
139339 229533.A0A0E0SA11 pp 229533.A0A0E0SPD4
139340 229533.A0A0E0SA11 pp 229533.I1RFX8
139341 229533.A0A0E0SA11 pp 229533.I1RWC2
139342 229533.A0A0E0SA11 pp 229533.I1RYH9
139343 229533.A0A0E0SA11 pp 229533.I1RB36
139344 229533.A0A0E0SA11 pp 229533.I1RXD7
139345 229533.A0A0E0SA11 pp 229533.I1RQH3
139346 229533.A0A0E0SA11 pp 229533.I1RX46
139347 229533.A0A0E0SA11 pp 229533.I1S224
139348 229533.A0A0E0SA11 pp 229533.A0A1C3YJR8
139349 229533.A0A0E0SA11 pp 229533.I1RZ12
139350 229533.A0A0E0SA11 pp 229533.I1RLC7
139351 229533.A0A0E0SA11 pp 229533.I1RAT7
139352 229533.A0A0E0SA11 pp 229533.A0A0E0SAC4
139353 229533.A0A0E0SA11 pp 229533.I1RSQ7
139354 229533.A0A0E0SA11 pp 229533.I1S0R7
139355 229533.A0A0E0SA11 pp 229533.I1S334
139356 229533.A0A0E0SA11 pp 229533.A0A1C3YKW5
139357 229533.A0A0E0SA11 pp 229533.A0A0E0SFN0

139358 229533.A0A0E0SA11 pp 229533.I1RQC9
139359 229533.A0A0E0SA11 pp 229533.I1RC20
139360 229533.A0A0E0SA11 pp 229533.I1RN61
139361 229533.A0A0E0SA11 pp 229533.I1RFZ3
139362 229533.A0A0E0SA11 pp 229533.V6QW22
139363 229533.A0A0E0SA11 pp 229533.A0A1C3YM63
139364 229533.A0A0E0SA11 pp 229533.A0A1C3YI31
139365 229533.A0A0E0SA11 pp 229533.I1RQ26
139366 229533.A0A0E0SA11 pp 229533.I1RW38
139367 229533.A0A0E0SA11 pp 229533.I1RTG0
139368 229533.A0A0E0SA11 pp 229533.A0A1C3YIL0
139369 229533.A0A0E0SA11 pp 229533.I1RQJ5
139370 229533.A0A0E0SA11 pp 229533.I1RD47
139371 229533.A0A0E0SA11 pp 229533.I1RYZ9
139372 229533.A0A0E0SA11 pp 229533.I1RFS6
139373 229533.A0A0E0SA11 pp 229533.I1RUQ6
139374 229533.A0A0E0SA11 pp 229533.I1RG53
139375 229533.A0A0E0SA11 pp 229533.I1RBC9
139376 229533.A0A0E0SA11 pp 229533.I1S0F5
139377 229533.A0A0E0SA11 pp 229533.I1RW71
139378 229533.A0A0E0SA11 pp 229533.I1RPQ1
139379 229533.A0A0E0SA11 pp 229533.I1RQ92
139380 229533.A0A0E0SA11 pp 229533.I1S7A8
139381 229533.A0A0E0SA11 pp 229533.I1RTC2
139382 229533.A0A0E0SA11 pp 229533.I1RYI3
139383 229533.A0A0E0SA11 pp 229533.A0A1C3YK00
139384 229533.A0A0E0SA11 pp 229533.A0A1C3YJT5
139385 229533.A0A0E0SA11 pp 229533.Q4IJT5
139386 229533.A0A0E0SA11 pp 229533.I1RKB1
139387 229533.A0A0E0SA11 pp 229533.I1S0L3
139388 229533.A0A0E0SA11 pp 229533.A0A1C3YJI0
139389 229533.A0A0E0SA11 pp 229533.A0A1C3YJX0
139390 229533.A0A0E0SA11 pp 229533.V6RTJ8
139391 229533.A0A0E0SA11 pp 229533.I1RAW7
139392 229533.A0A0E0SA11 pp 229533.I1RQ80
139393 229533.A0A0E0SA11 pp 229533.Q4IJP1
139394 229533.A0A0E0SA11 pp 229533.I1RRG3
139395 229533.A0A0E0SA11 pp 229533.I1RAX6
139396 229533.A0A0E0SA11 pp 229533.I1RD52
139397 229533.A0A0E0SA11 pp 229533.I1RIL8
139398 229533.A0A0E0SA11 pp 229533.I1RVQ0
139399 229533.A0A0E0SA11 pp 229533.I1RSI4
139400 229533.A0A0E0SA11 pp 229533.A0A1C3YIP6
139401 229533.A0A0E0SA11 pp 229533.V6QV09
139402 229533.A0A0E0SA11 pp 229533.V6R4J1
139403 229533.A0A0E0SA11 pp 229533.A0A1C3YIP5
139404 229533.A0A0E0SA11 pp 229533.I1RZ51
139405 229533.A0A0E0SA11 pp 229533.I1RT62
139406 229533.A0A0E0SA11 pp 229533.I1RBZ9
139407 229533.I1R9W7 pp 229533.I1RA40
139408 229533.I1R9W7 pp 229533.I1RHD2
139409 229533.I1R9W7 pp 229533.I1RF70
139410 229533.I1R9W7 pp 229533.I1S998
139411 229533.I1R9W7 pp 229533.I1S987
139412 229533.I1R9W7 pp 229533.I1RD01
139413 229533.I1R9W7 pp 229533.I1RZ86
139414 229533.I1R9W7 pp 229533.I1RSK0
139415 229533.I1R9W7 pp 229533.I1RZ94
139416 229533.I1R9W7 pp 229533.I1RRT1
139417 229533.I1R9W7 pp 229533.I1RYQ1
139418 229533.I1R9W7 pp 229533.I1RCA5
139419 229533.I1R9W7 pp 229533.I1RPQ1
139420 229533.I1R9W7 pp 229533.I1RQ67
139421 229533.I1R9W7 pp 229533.I1RWI8
139422 229533.I1R9W7 pp 229533.I1RJV8
139423 229533.I1R9W7 pp 229533.I1RUG4
139424 229533.I1R9W7 pp 229533.I1RKI2
139425 229533.I1R9W7 pp 229533.I1S461
139426 229533.I1R9W7 pp 229533.I1RY29
139427 229533.I1R9W7 pp 229533.I1RG77
139428 229533.I1R9W7 pp 229533.I1RXI6
139429 229533.I1R9W7 pp 229533.I1RKQ0
139430 229533.I1R9W7 pp 229533.I1RPL3

139431 229533.I1R9W7 pp 229533.I1RDJ8
139432 229533.I1R9W7 pp 229533.I1RAL9
139433 229533.I1R9W7 pp 229533.I1RDX0
139434 229533.I1R9W7 pp 229533.I1RA10
139435 229533.I1R9W7 pp 229533.I1RC54
139436 229533.I1R9W7 pp 229533.I1RMJ8
139437 229533.I1R9W7 pp 229533.I1S209
139438 229533.I1R9W7 pp 229533.I1RSR9
139439 229533.I1R9W7 pp 229533.I1RBC8
139440 229533.I1R9W7 pp 229533.I1S0D5
139441 229533.I1R9W7 pp 229533.Q4INS6
139442 229533.I1R9W7 pp 229533.I1RBN9
139443 229533.I1R9W7 pp 229533.I1RD19
139444 229533.I1R9W7 pp 229533.I1RJX7
139445 229533.I1R9W7 pp 229533.I1RSC7
139446 229533.I1R9W7 pp 229533.I1RU61
139447 229533.I1R9W7 pp 229533.I1RA12
139448 229533.I1R9W7 pp 229533.Q4IB89
139449 229533.I1S8A6 pp 229533.Q4IFI0
139450 229533.I1S8A6 pp 229533.Q4ICG8
139451 229533.I1S8A6 pp 229533.Q4I4A4
139452 229533.I1RT80 pp 229533.V6RAC9
139453 229533.I1RT80 pp 229533.Q4HZ42
139454 229533.I1RT80 pp 229533.I1RZJ6
139455 229533.I1RT80 pp 229533.I1RXD7
139456 229533.I1RT80 pp 229533.Q4HXF0
139457 229533.I1RT80 pp 229533.I1RT98
139458 229533.I1RT80 pp 229533.I1S2E9
139459 229533.I1RT80 pp 229533.Q4IQW0
139460 229533.I1RT80 pp 229533.I1S6F0
139461 229533.I1RT80 pp 229533.I1S1B6
139462 229533.I1RT80 pp 229533.I1RTI6
139463 229533.I1RT80 pp 229533.Q4HZ47
139464 229533.I1RT80 pp 229533.I1RWD4
139465 229533.I1RT80 pp 229533.I1RTD5
139466 229533.I1RT80 pp 229533.I1S0R7
139467 229533.I1RT80 pp 229533.V6R3X5
139468 229533.I1RT80 pp 229533.Q4IPI1
139469 229533.I1RT80 pp 229533.O74638
139470 229533.I1RT80 pp 229533.I1S253
139471 229533.I1RT80 pp 229533.I1RX46
139472 229533.I1RT80 pp 229533.I1S6U8
139473 229533.I1RT80 pp 229533.I1S9Y1
139474 229533.I1RT80 pp 229533.I1S503
139475 229533.I1RT80 pp 229533.Q4HVW2
139476 229533.I1RT80 pp 229533.Q4I5R9
139477 229533.I1RT80 pp 229533.Q4IAS1
139478 229533.I1RT80 pp 229533.I1RZS6
139479 229533.I1RT80 pp 229533.I1RZV6
139480 229533.I1RT80 pp 229533.I1RZ99
139481 229533.I1RT80 pp 229533.I1S8E2
139482 229533.I1RT80 pp 229533.Q4IQK7
139483 229533.I1RT80 pp 229533.I1RWF7
139484 229533.I1RT80 pp 229533.I1RZ39
139485 229533.I1RT80 pp 229533.Q4I830
139486 229533.I1RT80 pp 229533.Q4IJH1
139487 229533.I1RT80 pp 229533.V6QYF0
139488 229533.I1RT80 pp 229533.I1RW71
139489 229533.I1RT80 pp 229533.I1RX24
139490 229533.I1RT80 pp 229533.I1RTF4
139491 229533.I1RT80 pp 229533.I1RU69
139492 229533.I1RT80 pp 229533.Q4I662
139493 229533.I1RT80 pp 229533.I1S9U2
139494 229533.I1RT80 pp 229533.Q4IF76
139495 229533.I1RT80 pp 229533.I1RWH5
139496 229533.I1RT80 pp 229533.V6R283
139497 229533.I1RT80 pp 229533.I1RYT5
139498 229533.I1RT80 pp 229533.I1RW61
139499 229533.I1RT80 pp 229533.I1S021
139500 229533.I1RT80 pp 229533.I1RYG3
139501 229533.I1RT80 pp 229533.I1RYB9
139502 229533.I1RT80 pp 229533.I1RW25
139503 229533.I1RT80 pp 229533.I1S504

139504	229533.I1RT80	pp	229533.Q4IEK8
139505	229533.I1RT80	pp	229533.I1RZF6
139506	229533.I1RT80	pp	229533.Q4IFI0
139507	229533.I1RT80	pp	229533.I1RY44
139508	229533.I1RT80	pp	229533.Q4I327
139509	229533.I1RT80	pp	229533.Q4IN63
139510	229533.I1RT80	pp	229533.V6R042
139511	229533.I1RT80	pp	229533.I1S2B8
139512	229533.I1RT80	pp	229533.V6RGN5
139513	229533.I1RT80	pp	229533.I1RWN5
139514	229533.I1RT80	pp	229533.V6RMW6
139515	229533.I1RT80	pp	229533.I1S0N4
139516	229533.I1RT80	pp	229533.I1S4T3
139517	229533.I1RT80	pp	229533.I1S044
139518	229533.I1RT80	pp	229533.Q4HWE2
139519	229533.I1RT80	pp	229533.I1RTE9
139520	229533.I1RT80	pp	229533.I1RTG6
139521	229533.I1RT80	pp	229533.I1S2C0
139522	229533.I1RT80	pp	229533.I1RVX0
139523	229533.I1RT80	pp	229533.I1RTH6
139524	229533.I1RT80	pp	229533.I1RZK5
139525	229533.I1RT80	pp	229533.I1RY63
139526	229533.I1RT80	pp	229533.Q4IBS2
139527	229533.I1RT80	pp	229533.I1S1X9
139528	229533.I1RT80	pp	229533.I1RZK6
139529	229533.I1RT80	pp	229533.V6QUE6
139530	229533.I1RT80	pp	229533.I1S2E8
139531	229533.I1RT80	pp	229533.I1RWB5
139532	229533.I1RT80	pp	229533.I1RTD9
139533	229533.I1RT80	pp	229533.Q4IJ56
139534	229533.I1RT80	pp	229533.I1RZZ1
139535	229533.I1RT80	pp	229533.I1RY03
139536	229533.I1RT80	pp	229533.I1S0M9
139537	229533.I1RT80	pp	229533.I1S1C7
139538	229533.I1RT80	pp	229533.I1S2U2
139539	229533.I1RT80	pp	229533.I1S296
139540	229533.I1RT80	pp	229533.I1S1U7
139541	229533.I1RT80	pp	229533.V6RGY3
139542	229533.I1RT80	pp	229533.Q4HZ68
139543	229533.I1RT80	pp	229533.I1S0J1
139544	229533.I1RT80	pp	229533.I1RZE5
139545	229533.I1RT80	pp	229533.I1RWN6
139546	229533.I1RT80	pp	229533.I1S060
139547	229533.I1RT80	pp	229533.I1RVZ0
139548	229533.I1RT80	pp	229533.I1RX14
139549	229533.I1RT80	pp	229533.V6R227
139550	229533.I1RT80	pp	229533.I1S274
139551	229533.I1RU61	pp	229533.Q4IR08
139552	229533.I1RU61	pp	229533.Q4IB89
139553	229533.I1RU61	pp	229533.Q4INS6
139554	229533.I1RU61	pp	229533.I1RYX9
139555	229533.I1RU61	pp	229533.I1RY73
139556	229533.I1RU61	pp	229533.I1S4P6
139557	229533.I1RU61	pp	229533.I1RUG2
139558	229533.I1RU61	pp	229533.O74638
139559	229533.I1RU61	pp	229533.V6RLW4
139560	229533.I1RNJ5	pp	229533.Q4HZ95
139561	229533.I1RNJ5	pp	229533.Q4IPZ1
139562	229533.I1RNJ5	pp	229533.Q4I6B0
139563	229533.I1RNJ5	pp	229533.Q4IJW4
139564	229533.I1RNJ5	pp	229533.Q4IB65
139565	229533.I1RNJ5	pp	229533.I1RPP5
139566	229533.I1RNJ5	pp	229533.I1RT38
139567	229533.I1RNJ5	pp	229533.I1S8A6
139568	229533.I1RNJ5	pp	229533.I1RXZ3
139569	229533.I1RNJ5	pp	229533.Q4I7T5
139570	229533.I1RNJ5	pp	229533.I1RUG0
139571	229533.I1RNJ5	pp	229533.I1S5X9
139572	229533.I1RNJ5	pp	229533.I1RZH5
139573	229533.I1RNJ5	pp	229533.I1RT99
139574	229533.I1RNJ5	pp	229533.I1RPX9
139575	229533.I1RNJ5	pp	229533.Q4IK03
139576	229533.I1RNJ5	pp	229533.I1RQ21

139577 229533.I1RNJ5 pp 229533.I1RYT8
139578 229533.I1RNJ5 pp 229533.I1RSY2
139579 229533.I1RNJ5 pp 229533.Q4I099
139580 229533.I1RNJ5 pp 229533.I1S0R5
139581 229533.I1RNJ5 pp 229533.I1RZS4
139582 229533.I1RNJ5 pp 229533.V6RJP8
139583 229533.I1RNJ5 pp 229533.I1RPR9
139584 229533.I1RNJ5 pp 229533.I1RQA5
139585 229533.I1RNJ5 pp 229533.I1RQR6
139586 229533.I1RNJ5 pp 229533.I1S0Q7
139587 229533.I1RNJ5 pp 229533.Q4I375
139588 229533.I1RNJ5 pp 229533.Q4ICM9
139589 229533.I1RNJ5 pp 229533.I1RPY8
139590 229533.I1SAJ7 pp 229533.I1SAJ8
139591 229533.Q4I283 pp 229533.Q4I2J8
139592 229533.Q4I283 pp 229533.V6RCG5
139593 229533.Q4I283 pp 229533.V6RCZ8
139594 229533.Q4I283 pp 229533.Q4IJT5
139595 229533.Q4I283 pp 229533.Q4I8P5
139596 229533.Q4I283 pp 229533.V6R042
139597 229533.Q4I283 pp 229533.V6RTJ8
139598 229533.Q4I283 pp 229533.V6RMW6
139599 229533.Q4I283 pp 229533.V6R3X5
139600 229533.Q4I283 pp 229533.V6RNM4
139601 229533.Q4I283 pp 229533.Q4I624
139602 229533.Q4I283 pp 229533.V6QW22
139603 229533.Q4I283 pp 229533.V6RIV9
139604 229533.A0A098E1Q1 pp 229533.I1S5B6
139605 229533.A0A098E1Q1 pp 229533.I1S209
139606 229533.A0A098E1Q1 pp 229533.Q4IR08
139607 229533.A0A098E1Q1 pp 229533.I1RBW1
139608 229533.A0A098E1Q1 pp 229533.I1RSA7
139609 229533.A0A098E1Q1 pp 229533.I1S604
139610 229533.A0A098E1Q1 pp 229533.A0A1C3YHM3
139611 229533.A0A098E1Q1 pp 229533.I1RRJ6
139612 229533.A0A098E1Q1 pp 229533.I1RYQ1
139613 229533.A0A098E1Q1 pp 229533.Q4INS6
139614 229533.A0A098E1Q1 pp 229533.I1RVW0
139615 229533.A0A098E1Q1 pp 229533.Q4I5R3
139616 229533.A0A098E1Q1 pp 229533.A0A0E0SNM6
139617 229533.A0A098E1Q1 pp 229533.A0A1C3YL55
139618 229533.A0A098E1Q1 pp 229533.A0A098E4E9
139619 229533.A0A098E1Q1 pp 229533.I1RRS7
139620 229533.A0A098E1Q1 pp 229533.A0A0E0RW49
139621 229533.A0A098E1Q1 pp 229533.I1RDJ8
139622 229533.A0A098E1Q1 pp 229533.I1RB97
139623 229533.A0A098E1Q1 pp 229533.I1S2E8
139624 229533.A0A098E1Q1 pp 229533.I1RC54
139625 229533.A0A098E1Q1 pp 229533.I1RSC7
139626 229533.A0A098E1Q1 pp 229533.I1RSS4
139627 229533.A0A098E1Q1 pp 229533.I1RBD2
139628 229533.A0A098E1Q1 pp 229533.A0A1C3YL34
139629 229533.A0A098E1Q1 pp 229533.A0A1C3YJN7
139630 229533.A0A098E1Q1 pp 229533.I1RZZ1
139631 229533.A0A098E1Q1 pp 229533.I1RAL9
139632 229533.A0A098E1Q1 pp 229533.I1S788
139633 229533.A0A098E1Q1 pp 229533.I1S4P6
139634 229533.A0A098E1Q1 pp 229533.I1RBW0
139635 229533.A0A098E1Q1 pp 229533.I1RRT1
139636 229533.A0A098E1Q1 pp 229533.I1RG77
139637 229533.A0A098E1Q1 pp 229533.I1S0D5
139638 229533.A0A098E3Y1 pp 229533.A0A1C3YHI3
139639 229533.A0A098E3Y1 pp 229533.I1RGU7
139640 229533.A0A098E3Y1 pp 229533.I1RA62
139641 229533.A0A1C3YLW7 pp 229533.I1RFW1
139642 229533.A0A1C3YLW7 pp 229533.I1RA87
139643 229533.A0A1C3YLW7 pp 229533.I1RN49
139644 229533.A0A1C3YLW7 pp 229533.I1RCW8
139645 229533.A0A1C3YLW7 pp 229533.I1RQ19
139646 229533.A0A1C3YLW7 pp 229533.Q4IPI4
139647 229533.A0A1C3YLW7 pp 229533.I1RYV3
139648 229533.A0A1C3YLW7 pp 229533.I1RBJ0
139649 229533.A0A1C3YLW7 pp 229533.I1RSD1

139650 229533.A0A1C3YLW7 pp 229533.Q4I5R3
139651 229533.A0A1C3YLW7 pp 229533.I1S1V2
139652 229533.A0A1C3YLW7 pp 229533.Q4IER8
139653 229533.A0A1C3YLW7 pp 229533.I1RYT5
139654 229533.A0A1C3YLW7 pp 229533.I1RU02
139655 229533.A0A1C3YLW7 pp 229533.I1RE51
139656 229533.A0A1C3YLW7 pp 229533.I1RAN8
139657 229533.A0A1C3YLW7 pp 229533.Q4HTT1
139658 229533.I1RKB7 pp 229533.I1RWT0
139659 229533.I1RKB7 pp 229533.I1RRY9
139660 229533.I1RKB7 pp 229533.I1RP38
139661 229533.I1RKB7 pp 229533.I1S5U0
139662 229533.I1RKB7 pp 229533.I1RPR9
139663 229533.I1RKB7 pp 229533.I1RQS2
139664 229533.I1RKB7 pp 229533.I1RU95
139665 229533.I1RKB7 pp 229533.I1S266
139666 229533.I1RKB7 pp 229533.I1S8S9
139667 229533.I1RKB7 pp 229533.I1S212
139668 229533.I1RKB7 pp 229533.Q4I5G1
139669 229533.I1RKB7 pp 229533.Q4I2J8
139670 229533.I1RKB7 pp 229533.I1RZ43
139671 229533.I1RKB7 pp 229533.I1RZM9
139672 229533.I1RKB7 pp 229533.V6R042
139673 229533.I1RKB7 pp 229533.I1RMI1
139674 229533.I1RKB7 pp 229533.I1RLN1
139675 229533.I1RKB7 pp 229533.I1S0Q7
139676 229533.I1RKB7 pp 229533.I1RZK5
139677 229533.I1RKB7 pp 229533.I1S0R8
139678 229533.I1RKB7 pp 229533.I1RWA3
139679 229533.I1RKB7 pp 229533.I1RMK7
139680 229533.I1RKB7 pp 229533.I1S0K2
139681 229533.I1RKB7 pp 229533.I1S0U0
139682 229533.I1RKB7 pp 229533.I1RNM7
139683 229533.I1RKB7 pp 229533.I1S5X9
139684 229533.I1RKB7 pp 229533.I1RYA0
139685 229533.I1RKB7 pp 229533.I1RPP5
139686 229533.I1RKB7 pp 229533.I1RKQ6
139687 229533.I1RKB7 pp 229533.I1RZ68
139688 229533.I1RKB7 pp 229533.I1RS29
139689 229533.I1RKB7 pp 229533.V6RTJ8
139690 229533.I1RKB7 pp 229533.I1RL71
139691 229533.I1RKB7 pp 229533.I1RYC7
139692 229533.I1RKB7 pp 229533.I1S504
139693 229533.I1RKB7 pp 229533.I1RW13
139694 229533.I1RKB7 pp 229533.I1RXE6
139695 229533.I1RKB7 pp 229533.I1RX39
139696 229533.I1RKB7 pp 229533.I1RP02
139697 229533.I1RKB7 pp 229533.I1RRH6
139698 229533.I1RKB7 pp 229533.V6RJP8
139699 229533.I1RKB7 pp 229533.I1RLU6
139700 229533.I1RKB7 pp 229533.I1RTL0
139701 229533.I1RKB7 pp 229533.Q4IBU4
139702 229533.I1RKB7 pp 229533.I1S0L4
139703 229533.I1RKB7 pp 229533.V6R3J1
139704 229533.I1RKB7 pp 229533.I1RX17
139705 229533.I1RKB7 pp 229533.I1RY21
139706 229533.I1RKB7 pp 229533.I1RYC8
139707 229533.I1RKB7 pp 229533.I1RY92
139708 229533.I1RKB7 pp 229533.I1S503
139709 229533.I1RKB7 pp 229533.I1S8F8
139710 229533.I1RKB7 pp 229533.I1RMQ6
139711 229533.I1RKB7 pp 229533.I1S0P0
139712 229533.I1RKB7 pp 229533.I1RRX5
139713 229533.I1RKB7 pp 229533.I1RSQ4
139714 229533.I1RKB7 pp 229533.I1RN67
139715 229533.I1RKB7 pp 229533.I1RYT8
139716 229533.I1RKB7 pp 229533.I1RYN2
139717 229533.I1RKB7 pp 229533.I1RX21
139718 229533.I1RKB7 pp 229533.I1RTD5
139719 229533.I1RKB7 pp 229533.I1RS05
139720 229533.I1RKB7 pp 229533.I1RZH5
139721 229533.I1RKB7 pp 229533.I1RYK0
139722 229533.I1RKB7 pp 229533.I1RXD9

139723	229533.I1RKB7	pp	229533.I1RUR8
139724	229533.I1RKB7	pp	229533.I1S464
139725	229533.I1RKB7	pp	229533.I1RRK9
139726	229533.I1RKB7	pp	229533.I1S0T7
139727	229533.I1RKB7	pp	229533.I1RWG0
139728	229533.I1RKB7	pp	229533.I1RL81
139729	229533.I1RKB7	pp	229533.I1S1F3
139730	229533.I1S2J6	pp	229533.I1S2K0
139731	229533.I1S2J6	pp	229533.I1SAJ7
139732	229533.I1S2J6	pp	229533.I1S2J8
139733	229533.I1S2J6	pp	229533.I1SAJ8
139734	229533.I1RCC5	pp	229533.I1RYW9
139735	229533.I1RCC5	pp	229533.I1RRX5
139736	229533.I1RCC5	pp	229533.I1RMQ5
139737	229533.I1RCC5	pp	229533.I1RMQ6
139738	229533.I1RCC5	pp	229533.V6R3J1
139739	229533.I1RCC5	pp	229533.I1RPP5
139740	229533.I1S283	pp	229533.I1S998
139741	229533.I1S283	pp	229533.I1S987
139742	229533.I1S283	pp	229533.Q4HYB8
139743	229533.I1S283	pp	229533.Q4IC11
139744	229533.I1S283	pp	229533.I1S4P6
139745	229533.I1S283	pp	229533.V6RLW4
139746	229533.I1S283	pp	229533.I1S2F2
139747	229533.I1S283	pp	229533.V6R382
139748	229533.I1S283	pp	229533.Q4IEA5
139749	229533.I1S283	pp	229533.Q4I0J6
139750	229533.I1S283	pp	229533.Q4IJU0
139751	229533.I1S283	pp	229533.Q4IP51
139752	229533.I1S283	pp	229533.I1S461
139753	229533.I1S283	pp	229533.V6RGI1
139754	229533.I1S283	pp	229533.I1S9J6
139755	229533.I1S283	pp	229533.I1S604
139756	229533.I1S283	pp	229533.Q4IQM6
139757	229533.I1S283	pp	229533.Q4IBX0
139758	229533.I1S1J7	pp	229533.I1S256
139759	229533.I1S1J7	pp	229533.I1S8V4
139760	229533.I1S1J7	pp	229533.I1S1W9
139761	229533.I1S1J7	pp	229533.I1SA33
139762	229533.I1S1J7	pp	229533.V6RIV9
139763	229533.I1S1J7	pp	229533.Q4I624
139764	229533.I1S1J7	pp	229533.V6R057
139765	229533.I1S1J7	pp	229533.I1S8R3
139766	229533.I1S1J7	pp	229533.I1S5R6
139767	229533.I1S1J7	pp	229533.V6RMW6
139768	229533.I1S1J7	pp	229533.V6RRH2
139769	229533.I1S1J7	pp	229533.I1S2E9
139770	229533.I1S1J7	pp	229533.I1S5R1
139771	229533.I1S1J7	pp	229533.I1S2H4
139772	229533.I1S1J7	pp	229533.I1S8P5
139773	229533.I1S1J7	pp	229533.V6R042
139774	229533.I1S1J7	pp	229533.I1S5B0
139775	229533.I1S1J7	pp	229533.I1S2B8
139776	229533.I1S1J7	pp	229533.I1S6Y8
139777	229533.I1S1J7	pp	229533.I1S503
139778	229533.I1S1J7	pp	229533.I1SA32
139779	229533.I1S1J7	pp	229533.Q4IJK2
139780	229533.I1S1J7	pp	229533.V6R3X5
139781	229533.I1S1J7	pp	229533.I1S504
139782	229533.I1S1J7	pp	229533.Q4I8P5
139783	229533.I1S1J7	pp	229533.I1S8V3
139784	229533.I1S1J7	pp	229533.I1S419
139785	229533.I1S1J7	pp	229533.V6RCZ8
139786	229533.I1S1J7	pp	229533.I1S4Q4
139787	229533.I1S1J7	pp	229533.I1S4S7
139788	229533.I1S1J7	pp	229533.Q4IJT5
139789	229533.I1S1J7	pp	229533.Q4I2J8
139790	229533.I1S1J7	pp	229533.V6R1K4
139791	229533.I1S1J7	pp	229533.V6RTJ8
139792	229533.I1S1J7	pp	229533.V6RCG5
139793	229533.I1S1J7	pp	229533.V6QW22
139794	229533.I1S1J7	pp	229533.V6QUE6
139795	229533.I1S1J7	pp	229533.I1S4S4

139796	229533.Q4I9U7	pp	229533.Q4IQ72
139797	229533.I1RLK2	pp	229533.I1RNX0
139798	229533.I1RLK2	pp	229533.I1SAS1
139799	229533.I1RLK2	pp	229533.I1S341
139800	229533.A0A1C3YN30	pp	229533.I1S2B8
139801	229533.A0A1C3YN30	pp	229533.I1RJZ7
139802	229533.A0A1C3YN30	pp	229533.I1RRM5
139803	229533.A0A1C3YN30	pp	229533.I1R9M0
139804	229533.A0A1C3YN30	pp	229533.I1RBY4
139805	229533.A0A1C3YN30	pp	229533.I1RE27
139806	229533.A0A1C3YN30	pp	229533.I1RE76
139807	229533.A0A1C3YN30	pp	229533.I1S8G4
139808	229533.A0A1C3YN30	pp	229533.I1RXR5
139809	229533.A0A1C3YN30	pp	229533.I1RSQ4
139810	229533.A0A1C3YN30	pp	229533.I1RZZ9
139811	229533.A0A1C3YN30	pp	229533.I1RWI5
139812	229533.A0A1C3YN30	pp	229533.I1RC66
139813	229533.A0A1C3YN30	pp	229533.I1S076
139814	229533.A0A1C3YN30	pp	229533.I1RGA6
139815	229533.A0A1C3YN30	pp	229533.I1RZ39
139816	229533.A0A1C3YN30	pp	229533.I1RPM4
139817	229533.A0A1C3YN30	pp	229533.V6R3X5
139818	229533.A0A1C3YN30	pp	229533.I1RL04
139819	229533.A0A1C3YN30	pp	229533.I1RR73
139820	229533.A0A1C3YN30	pp	229533.I1RJC7
139821	229533.A0A1C3YN30	pp	229533.O74638
139822	229533.A0A1C3YN30	pp	229533.I1RA11
139823	229533.A0A1C3YN30	pp	229533.I1RKY2
139824	229533.A0A1C3YN30	pp	229533.I1RN63
139825	229533.A0A1C3YN30	pp	229533.I1RQ23
139826	229533.A0A1C3YN30	pp	229533.I1RFS3
139827	229533.A0A1C3YN30	pp	229533.I1RWK3
139828	229533.A0A1C3YN30	pp	229533.I1RRT8
139829	229533.A0A1C3YN30	pp	229533.I1S5R1
139830	229533.A0A1C3YN30	pp	229533.I1R9G4
139831	229533.A0A1C3YN30	pp	229533.I1RN88
139832	229533.A0A1C3YN30	pp	229533.I1RFT1
139833	229533.A0A1C3YN30	pp	229533.I1RTG3
139834	229533.A0A1C3YN30	pp	229533.I1RE22
139835	229533.A0A1C3YN30	pp	229533.I1RRJ8
139836	229533.A0A1C3YN30	pp	229533.I1S2H4
139837	229533.A0A1C3YN30	pp	229533.V6RVJ3
139838	229533.A0A1C3YN30	pp	229533.I1RPP3
139839	229533.A0A1C3YN30	pp	229533.I1S3G4
139840	229533.A0A1C3YN30	pp	229533.I1S8P5
139841	229533.A0A1C3YN30	pp	229533.I1S0M6
139842	229533.A0A1C3YN30	pp	229533.I1S0L4
139843	229533.A0A1C3YN30	pp	229533.I1S0F6
139844	229533.A0A1C3YN30	pp	229533.I1S503
139845	229533.A0A1C3YN30	pp	229533.I1RN67
139846	229533.A0A1C3YN30	pp	229533.I1SAJ0
139847	229533.A0A1C3YN30	pp	229533.I1RCW6
139848	229533.A0A1C3YN30	pp	229533.I1RJW6
139849	229533.A0A1C3YN30	pp	229533.I1RGA4
139850	229533.A0A1C3YN30	pp	229533.I1RY62
139851	229533.A0A1C3YN30	pp	229533.I1RBS6
139852	229533.A0A1C3YN30	pp	229533.I1RZW8
139853	229533.A0A1C3YN30	pp	229533.I1RRI2
139854	229533.A0A1C3YN30	pp	229533.I1RX22
139855	229533.A0A1C3YN30	pp	229533.I1RJV4
139856	229533.A0A1C3YN30	pp	229533.I1RN18
139857	229533.A0A1C3YN30	pp	229533.I1RFL4
139858	229533.A0A1C3YN30	pp	229533.I1RS47
139859	229533.A0A1C3YN30	pp	229533.I1RQE7
139860	229533.A0A1C3YN30	pp	229533.I1S8R3
139861	229533.A0A1C3YN30	pp	229533.I1RTH6
139862	229533.A0A1C3YN30	pp	229533.I1S5G3
139863	229533.A0A1C3YN30	pp	229533.I1S2E8
139864	229533.A0A1C3YN30	pp	229533.I1RT40
139865	229533.A0A1C3YN30	pp	229533.I1RYR6
139866	229533.A0A1C3YN30	pp	229533.I1RCH5
139867	229533.A0A1C3YN30	pp	229533.I1RV91
139868	229533.A0A1C3YN30	pp	229533.I1RA70

139869 229533.A0A1C3YN30 pp 229533.V6R2A1
139870 229533.A0A1C3YN30 pp 229533.I1S2C4
139871 229533.A0A1C3YN30 pp 229533.I1RTD5
139872 229533.A0A1C3YN30 pp 229533.I1RT03
139873 229533.A0A1C3YN30 pp 229533.I1RT04
139874 229533.A0A1C3YN30 pp 229533.I1RJV3
139875 229533.A0A1C3YN30 pp 229533.I1RAW0
139876 229533.A0A1C3YN30 pp 229533.I1RWN6
139877 229533.A0A1C3YN30 pp 229533.I1RI34
139878 229533.A0A1C3YN30 pp 229533.I1RVX0
139879 229533.A0A1C3YN30 pp 229533.Q4HXF0
139880 229533.A0A1C3YN30 pp 229533.I1RB92
139881 229533.A0A1C3YN30 pp 229533.I1RRP7
139882 229533.A0A1C3YN30 pp 229533.V6RRH2
139883 229533.A0A1C3YN30 pp 229533.I1RD39
139884 229533.A0A1C3YN30 pp 229533.I1RTA9
139885 229533.A0A1C3YN30 pp 229533.I1RWE8
139886 229533.A0A1C3YN30 pp 229533.I1RTA0
139887 229533.A0A1C3YN30 pp 229533.Q4I624
139888 229533.A0A1C3YN30 pp 229533.I1RW46
139889 229533.A0A1C3YN30 pp 229533.I1RE01
139890 229533.A0A1C3YN30 pp 229533.I1S2C5
139891 229533.A0A1C3YN30 pp 229533.I1RD17
139892 229533.A0A1C3YN30 pp 229533.V6R057
139893 229533.A0A1C3YN30 pp 229533.I1RMI8
139894 229533.A0A1C3YN30 pp 229533.I1S0J1
139895 229533.A0A1C3YN30 pp 229533.I1S064
139896 229533.A0A1C3YN30 pp 229533.I1RRE5
139897 229533.A0A1C3YN30 pp 229533.I1RCR8
139898 229533.A0A1C3YN30 pp 229533.I1RAR1
139899 229533.A0A1C3YN30 pp 229533.I1S216
139900 229533.A0A1C3YN30 pp 229533.I1RD80
139901 229533.A0A1C3YN30 pp 229533.I1RUG3
139902 229533.A0A1C3YN30 pp 229533.I1RPK0
139903 229533.A0A1C3YN30 pp 229533.I1RAU7
139904 229533.A0A1C3YN30 pp 229533.I1S1C7
139905 229533.A0A1C3YN30 pp 229533.I1RPP4
139906 229533.A0A1C3YN30 pp 229533.I1R9X7
139907 229533.A0A1C3YN30 pp 229533.I1S6H1
139908 229533.A0A1C3YN30 pp 229533.I1RHT3
139909 229533.A0A1C3YN30 pp 229533.I1RNC1
139910 229533.A0A1C3YN30 pp 229533.I1RYW3
139911 229533.A0A1C3YN30 pp 229533.I1RB44
139912 229533.A0A1C3YN30 pp 229533.I1RWN5
139913 229533.A0A1C3YN30 pp 229533.V6RIV9
139914 229533.A0A1C3YN30 pp 229533.I1RRV1
139915 229533.A0A1C3YN30 pp 229533.I1RPN4
139916 229533.A0A1C3YN30 pp 229533.I1RPU8
139917 229533.A0A1C3YN30 pp 229533.I1RW71
139918 229533.A0A1C3YN30 pp 229533.I1RR00
139919 229533.A0A1C3YN30 pp 229533.I1S1W8
139920 229533.A0A1C3YN30 pp 229533.V6RMW6
139921 229533.A0A1C3YN30 pp 229533.I1RBC9
139922 229533.A0A1C3YN30 pp 229533.I1RM06
139923 229533.A0A1C3YN30 pp 229533.I1RSQ3
139924 229533.A0A1C3YN30 pp 229533.I1RSQ7
139925 229533.A0A1C3YN30 pp 229533.I1RW25
139926 229533.A0A1C3YN30 pp 229533.V6QW22
139927 229533.A0A1C3YN30 pp 229533.V6R042
139928 229533.A0A1C3YN30 pp 229533.I1RX25
139929 229533.A0A1C3YN30 pp 229533.I1S0X6
139930 229533.A0A1C3YN30 pp 229533.I1RB39
139931 229533.A0A1C3YN30 pp 229533.I1RG53
139932 229533.A0A1C3YN30 pp 229533.I1RWD4
139933 229533.A0A1C3YN30 pp 229533.I1RD22
139934 229533.A0A1C3YN30 pp 229533.I1SA33
139935 229533.A0A1C3YN30 pp 229533.I1RVQ0
139936 229533.A0A1C3YN30 pp 229533.I1RD52
139937 229533.A0A1C3YN30 pp 229533.I1RBT1
139938 229533.A0A1C3YN30 pp 229533.I1RQS9
139939 229533.A0A1C3YN30 pp 229533.I1S256
139940 229533.A0A1C3YN30 pp 229533.I1RMC8
139941 229533.A0A1C3YN30 pp 229533.I1S0F5

139942	229533.A0A1C3YN30	pp	229533.I1RJU3
139943	229533.A0A1C3YN30	pp	229533.I1RV23
139944	229533.A0A1C3YN30	pp	229533.I1S1P8
139945	229533.A0A1C3YN30	pp	229533.I1S0B7
139946	229533.A0A1C3YN30	pp	229533.I1RT80
139947	229533.A0A1C3YN30	pp	229533.I1RAR9
139948	229533.A0A1C3YN30	pp	229533.I1S0L3
139949	229533.A0A1C3YN30	pp	229533.I1RCL4
139950	229533.A0A1C3YN30	pp	229533.I1RC39
139951	229533.A0A1C3YN30	pp	229533.I1S4S4
139952	229533.A0A1C3YN30	pp	229533.Q4I8P5
139953	229533.A0A1C3YN30	pp	229533.V6R1K4
139954	229533.A0A1C3YN30	pp	229533.I1RWJ8
139955	229533.A0A1C3YN30	pp	229533.I1RS18
139956	229533.A0A1C3YN30	pp	229533.I1S0R7
139957	229533.A0A1C3YN30	pp	229533.I1RHD1
139958	229533.A0A1C3YN30	pp	229533.I1RF82
139959	229533.A0A1C3YN30	pp	229533.I1RCN8
139960	229533.A0A1C3YN30	pp	229533.I1S3S3
139961	229533.A0A1C3YN30	pp	229533.I1RZM1
139962	229533.A0A1C3YN30	pp	229533.I1RR24
139963	229533.A0A1C3YN30	pp	229533.I1RX46
139964	229533.A0A1C3YN30	pp	229533.I1RQC9
139965	229533.A0A1C3YN30	pp	229533.I1RUQ2
139966	229533.A0A1C3YN30	pp	229533.I1RLU6
139967	229533.A0A1C3YN30	pp	229533.I1RAX6
139968	229533.A0A1C3YN30	pp	229533.I1RNQ5
139969	229533.A0A1C3YN30	pp	229533.I1S7A8
139970	229533.A0A1C3YN30	pp	229533.I1S084
139971	229533.A0A1C3YN30	pp	229533.I1RXD7
139972	229533.A0A1C3YN30	pp	229533.I1S504
139973	229533.A0A1C3YN30	pp	229533.I1RM35
139974	229533.A0A1C3YN30	pp	229533.V6RY56
139975	229533.A0A1C3YN30	pp	229533.V6RTJ8
139976	229533.A0A1C3YN30	pp	229533.I1RK15
139977	229533.A0A1C3YN30	pp	229533.I1RWD7
139978	229533.A0A1C3YN30	pp	229533.Q4IJK2
139979	229533.A0A1C3YN30	pp	229533.I1S4D0
139980	229533.A0A1C3YN30	pp	229533.I1RY03
139981	229533.A0A1C3YN30	pp	229533.I1RXF0
139982	229533.A0A1C3YN30	pp	229533.V6RBN2
139983	229533.A0A1C3YN30	pp	229533.I1RZZ1
139984	229533.A0A1C3YN30	pp	229533.I1RYE6
139985	229533.A0A1C3YN30	pp	229533.I1RCG4
139986	229533.A0A1C3YN30	pp	229533.I1RZM8
139987	229533.A0A1C3YN30	pp	229533.I1RFU8
139988	229533.A0A1C3YN30	pp	229533.I1REX0
139989	229533.A0A1C3YN30	pp	229533.Q4I2J8
139990	229533.A0A1C3YN30	pp	229533.I1RQ31
139991	229533.A0A1C3YN30	pp	229533.I1RAZ8
139992	229533.A0A1C3YN30	pp	229533.I1S4Q4
139993	229533.A0A1C3YN30	pp	229533.I1RCV1
139994	229533.A0A1C3YN30	pp	229533.I1RAZ3
139995	229533.A0A1C3YN30	pp	229533.I1RZK5
139996	229533.A0A1C3YN30	pp	229533.I1RAN5
139997	229533.A0A1C3YN30	pp	229533.I1S1J7
139998	229533.A0A1C3YN30	pp	229533.I1RI82
139999	229533.A0A1C3YN30	pp	229533.I1RTJ7
140000	229533.A0A1C3YN30	pp	229533.I1RTD3
140001	229533.A0A1C3YN30	pp	229533.I1REK8
140002	229533.A0A1C3YN30	pp	229533.I1RCR5
140003	229533.A0A1C3YN30	pp	229533.I1RBL7
140004	229533.A0A1C3YN30	pp	229533.I1RSR3
140005	229533.A0A1C3YN30	pp	229533.I1RRY9
140006	229533.A0A1C3YN30	pp	229533.V6RCZ8
140007	229533.A0A1C3YN30	pp	229533.I1RJK0
140008	229533.A0A1C3YN30	pp	229533.V6RCG5
140009	229533.A0A1C3YN30	pp	229533.I1RRV4
140010	229533.A0A1C3YN30	pp	229533.I1RJT5
140011	229533.A0A1C3YN30	pp	229533.I1RMQ4
140012	229533.A0A1C3YN30	pp	229533.I1RAU5
140013	229533.A0A1C3YN30	pp	229533.I1RV18
140014	229533.A0A1C3YN30	pp	229533.I1SA32

140015 229533.A0A1C3YN30 pp 229533.I1RBI1
140016 229533.A0A1C3YN30 pp 229533.I1RW99
140017 229533.A0A1C3YN30 pp 229533.I1S5B0
140018 229533.A0A1C3YN30 pp 229533.I1RXB6
140019 229533.I1RZ78 pp 229533.I1S8J1
140020 229533.I1RZ78 pp 229533.V6RUG6
140021 229533.I1RZ78 pp 229533.I1S0P6
140022 229533.I1RZ78 pp 229533.I1S6S8
140023 229533.I1RZ78 pp 229533.I1S272
140024 229533.I1RZ78 pp 229533.I1S444
140025 229533.I1RZ78 pp 229533.I1RZP8
140026 229533.I1RZ78 pp 229533.V6RUY9
140027 229533.I1RZ78 pp 229533.V6R3R0
140028 229533.I1RZ78 pp 229533.I1SAC4
140029 229533.I1RZ78 pp 229533.Q4IPX8
140030 229533.I1RZ78 pp 229533.I1S3F7
140031 229533.I1RZ78 pp 229533.I1RZM3
140032 229533.A0A1C3YKS5 pp 229533.I1RL06
140033 229533.A0A1C3YKS5 pp 229533.I1S2J8
140034 229533.A0A1C3YKS5 pp 229533.I1S2J6
140035 229533.A0A1C3YKS5 pp 229533.I1S2J3
140036 229533.A0A1C3YKS5 pp 229533.I1RWZ0
140037 229533.A0A1C3YKS5 pp 229533.I1SAJ7
140038 229533.A0A1C3YKS5 pp 229533.I1S2J5
140039 229533.A0A1C3YKB0 pp 229533.I1RY03
140040 229533.A0A1C3YKB0 pp 229533.I1RNT9
140041 229533.A0A1C3YKB0 pp 229533.I1RWR7
140042 229533.A0A1C3YKB0 pp 229533.I1RU29
140043 229533.A0A1C3YKB0 pp 229533.I1RRY9
140044 229533.A0A1C3YKB0 pp 229533.I1RSR3
140045 229533.A0A1C3YKB0 pp 229533.I1RPR9
140046 229533.A0A1C3YKB0 pp 229533.I1RBL7
140047 229533.A0A1C3YKB0 pp 229533.I1RCM5
140048 229533.A0A1C3YKB0 pp 229533.I1RZK5
140049 229533.A0A1C3YKB0 pp 229533.I1RYS0
140050 229533.A0A1C3YKB0 pp 229533.I1RB53
140051 229533.A0A1C3YKB0 pp 229533.Q4I1Y1
140052 229533.A0A1C3YKB0 pp 229533.I1S1J7
140053 229533.A0A1C3YKB0 pp 229533.I1S887
140054 229533.A0A1C3YKB0 pp 229533.I1RI03
140055 229533.A0A1C3YKB0 pp 229533.I1RAZ3
140056 229533.A0A1C3YKB0 pp 229533.I1RQA2
140057 229533.A0A1C3YKB0 pp 229533.I1RFU8
140058 229533.A0A1C3YKB0 pp 229533.I1RBB3
140059 229533.A0A1C3YKB0 pp 229533.I1RCG4
140060 229533.A0A1C3YKB0 pp 229533.I1RBU8
140061 229533.A0A1C3YKB0 pp 229533.I1RMD5
140062 229533.A0A1C3YKB0 pp 229533.A0A1C3YLK2
140063 229533.A0A1C3YKB0 pp 229533.I1RWB0
140064 229533.A0A1C3YKB0 pp 229533.I1RN99
140065 229533.A0A1C3YKB0 pp 229533.I1RTD9
140066 229533.A0A1C3YKB0 pp 229533.I1RAU5
140067 229533.A0A1C3YKB0 pp 229533.I1RGC2
140068 229533.A0A1C3YKB0 pp 229533.I1RDG0
140069 229533.A0A1C3YKB0 pp 229533.I1RY63
140070 229533.A0A1C3YKB0 pp 229533.I1RK95
140071 229533.A0A1C3YKB0 pp 229533.I1REK8
140072 229533.A0A1C3YKB0 pp 229533.I1RCR5
140073 229533.A0A1C3YKB0 pp 229533.V6QUE6
140074 229533.A0A1C3YKB0 pp 229533.I1RLM7
140075 229533.A0A1C3YKB0 pp 229533.I1S6E5
140076 229533.A0A1C3YKB0 pp 229533.I1RZZ1
140077 229533.A0A1C3YKB0 pp 229533.I1RBC4
140078 229533.A0A1C3YKB0 pp 229533.Q4IAA0
140079 229533.A0A1C3YKB0 pp 229533.I1RQQ5
140080 229533.A0A1C3YKB0 pp 229533.Q4IJK2
140081 229533.A0A1C3YKB0 pp 229533.I1RFJ9
140082 229533.A0A1C3YKB0 pp 229533.I1RF19
140083 229533.A0A1C3YKB0 pp 229533.I1RSI0
140084 229533.A0A1C3YKB0 pp 229533.Q4I8P5
140085 229533.A0A1C3YKB0 pp 229533.I1RS18
140086 229533.A0A1C3YKB0 pp 229533.Q4I2J8
140087 229533.A0A1C3YKB0 pp 229533.I1RQA3

140088	229533.A0A1C3YKB0	pp	229533.A0A1C3YKV3
140089	229533.A0A1C3YKB0	pp	229533.I1RQ81
140090	229533.A0A1C3YKB0	pp	229533.I1RWD7
140091	229533.A0A1C3YKB0	pp	229533.I1RAZ8
140092	229533.A0A1C3YKB0	pp	229533.I1RR51
140093	229533.A0A1C3YKB0	pp	229533.I1RT35
140094	229533.A0A1C3YKB0	pp	229533.A0A1C3YLU7
140095	229533.A0A1C3YKB0	pp	229533.I1SA32
140096	229533.A0A1C3YKB0	pp	229533.I1S419
140097	229533.A0A1C3YKB0	pp	229533.I1RZH4
140098	229533.A0A1C3YKB0	pp	229533.V6RCG5
140099	229533.A0A1C3YKB0	pp	229533.V6RCZ8
140100	229533.A0A1C3YKB0	pp	229533.I1S6K9
140101	229533.A0A1C3YKB0	pp	229533.I1RMK3
140102	229533.A0A1C3YKB0	pp	229533.Q4HVW2
140103	229533.A0A1C3YKB0	pp	229533.I1RXC4
140104	229533.A0A1C3YKB0	pp	229533.I1RDA0
140105	229533.A0A1C3YKB0	pp	229533.I1RI82
140106	229533.A0A1C3YKB0	pp	229533.I1RTF4
140107	229533.A0A1C3YKB0	pp	229533.I1RQ31
140108	229533.A0A1C3YKB0	pp	229533.Q4I283
140109	229533.A0A1C3YKB0	pp	229533.I1RKB7
140110	229533.A0A1C3YKB0	pp	229533.Q4IE79
140111	229533.A0A1C3YKB0	pp	229533.A0A1C3YN30
140112	229533.A0A1C3YKB0	pp	229533.I1RJU3
140113	229533.A0A1C3YKB0	pp	229533.I1S0F1
140114	229533.A0A1C3YKB0	pp	229533.V6R227
140115	229533.A0A1C3YKB0	pp	229533.I1RMC0
140116	229533.A0A1C3YKB0	pp	229533.I1RGB6
140117	229533.A0A1C3YKB0	pp	229533.I1RG14
140118	229533.A0A1C3YKB0	pp	229533.I1RXF0
140119	229533.A0A1C3YKB0	pp	229533.I1RZM8
140120	229533.A0A1C3YKB0	pp	229533.I1RMC8
140121	229533.A0A1C3YKB0	pp	229533.A0A1C3YKC0
140122	229533.A0A1C3YKB0	pp	229533.I1RWT6
140123	229533.A0A1C3YKB0	pp	229533.I1RBT1
140124	229533.A0A1C3YKB0	pp	229533.A0A1C3YKW9
140125	229533.A0A1C3YKB0	pp	229533.I1R9Y4
140126	229533.A0A1C3YKB0	pp	229533.I1S5B0
140127	229533.A0A1C3YKB0	pp	229533.I1RT05
140128	229533.A0A1C3YKB0	pp	229533.I1RQ85
140129	229533.A0A1C3YKB0	pp	229533.I1RW99
140130	229533.A0A1C3YKB0	pp	229533.I1S0X6
140131	229533.A0A1C3YKB0	pp	229533.I1S504
140132	229533.A0A1C3YKB0	pp	229533.I1RBJ5
140133	229533.A0A1C3YKB0	pp	229533.I1RR24
140134	229533.A0A1C3YKB0	pp	229533.I1RQD2
140135	229533.A0A1C3YKB0	pp	229533.I1RC17
140136	229533.A0A1C3YKB0	pp	229533.I1RNQ5
140137	229533.A0A1C3YKB0	pp	229533.I1RZJ5
140138	229533.A0A1C3YKB0	pp	229533.I1RRU9
140139	229533.A0A1C3YKB0	pp	229533.I1RLU6
140140	229533.A0A1C3YKB0	pp	229533.I1RMG8
140141	229533.A0A1C3YKB0	pp	229533.V6QUW4
140142	229533.A0A1C3YKB0	pp	229533.I1S4S4
140143	229533.A0A1C3YKB0	pp	229533.Q4IEK8
140144	229533.A0A1C3YKB0	pp	229533.V6QYF0
140145	229533.A0A1C3YKB0	pp	229533.I1RZM1
140146	229533.A0A1C3YKB0	pp	229533.I1RWJ8
140147	229533.A0A1C3YKB0	pp	229533.I1S438
140148	229533.A0A1C3YKB0	pp	229533.I1RRQ6
140149	229533.A0A1C3YKB0	pp	229533.A0A1C3YL54
140150	229533.A0A1C3YKB0	pp	229533.I1S0R7
140151	229533.A0A1C3YKB0	pp	229533.I1RNA0
140152	229533.A0A1C3YKB0	pp	229533.I1RPF1
140153	229533.A0A1C3YKB0	pp	229533.A0A1C3YLZ7
140154	229533.A0A1C3YKB0	pp	229533.I1RM06
140155	229533.A0A1C3YKB0	pp	229533.Q4IPB3
140156	229533.A0A1C3YKB0	pp	229533.I1RYJ6
140157	229533.A0A1C3YKB0	pp	229533.I1RFY6
140158	229533.A0A1C3YKB0	pp	229533.I1RPA4
140159	229533.A0A1C3YKB0	pp	229533.I1RLX7
140160	229533.A0A1C3YKB0	pp	229533.A0A1C3YM63

140161	229533.A0A1C3YKB0	pp	229533.I1S7A8
140162	229533.A0A1C3YKB0	pp	229533.V6R1K4
140163	229533.A0A1C3YKB0	pp	229533.I1S0L3
140164	229533.A0A1C3YKB0	pp	229533.I1RCS1
140165	229533.A0A1C3YKB0	pp	229533.V6RTJ8
140166	229533.A0A1C3YKB0	pp	229533.I1RNM7
140167	229533.A0A1C3YKB0	pp	229533.I1RBR2
140168	229533.A0A1C3YKB0	pp	229533.I1RI02
140169	229533.A0A1C3YKB0	pp	229533.I1RAX6
140170	229533.A0A1C3YKB0	pp	229533.I1S0F5
140171	229533.A0A1C3YKB0	pp	229533.I1RD52
140172	229533.A0A1C3YKB0	pp	229533.I1RW07
140173	229533.A0A1C3YKB0	pp	229533.I1RX25
140174	229533.A0A1C3YKB0	pp	229533.I1RX46
140175	229533.A0A1C3YKB0	pp	229533.I1RW25
140176	229533.A0A1C3YKB0	pp	229533.I1RA85
140177	229533.A0A1C3YKB0	pp	229533.I1S0T2
140178	229533.A0A1C3YKB0	pp	229533.I1RY86
140179	229533.A0A1C3YKB0	pp	229533.A0A1C3YMW0
140180	229533.A0A1C3YKB0	pp	229533.I1RAH0
140181	229533.A0A1C3YKB0	pp	229533.V6RUT5
140182	229533.A0A1C3YKB0	pp	229533.I1RLC7
140183	229533.A0A1C3YKB0	pp	229533.I1RXD6
140184	229533.A0A1C3YKB0	pp	229533.Q4I5R9
140185	229533.A0A1C3YKB0	pp	229533.I1RG53
140186	229533.A0A1C3YKB0	pp	229533.I1S6F0
140187	229533.A0A1C3YKB0	pp	229533.I1RXD7
140188	229533.A0A1C3YKB0	pp	229533.I1RVQ0
140189	229533.A0A1C3YKB0	pp	229533.V6R4J1
140190	229533.A0A1C3YKB0	pp	229533.I1RRX5
140191	229533.A0A1C3YKB0	pp	229533.I1RUQ2
140192	229533.A0A1C3YKB0	pp	229533.I1RSQ7
140193	229533.A0A1C3YKB0	pp	229533.I1RQC9
140194	229533.A0A1C3YKB0	pp	229533.I1RLP1
140195	229533.A0A1C3YKB0	pp	229533.I1RWD4
140196	229533.A0A1C3YKB0	pp	229533.V6QW22
140197	229533.A0A1C3YKB0	pp	229533.I1RKJ8
140198	229533.A0A1C3YKB0	pp	229533.I1RQC5
140199	229533.A0A1C3YKB0	pp	229533.I1RAW0
140200	229533.A0A1C3YKB0	pp	229533.I1RXU9
140201	229533.A0A1C3YKB0	pp	229533.I1RBC9
140202	229533.A0A1C3YKB0	pp	229533.I1RW71
140203	229533.A0A1C3YKB0	pp	229533.I1RSK1
140204	229533.A0A1C3YKB0	pp	229533.I1RC81
140205	229533.A0A1C3YKB0	pp	229533.I1RA70
140206	229533.A0A1C3YKB0	pp	229533.I1RTE9
140207	229533.A0A1C3YKB0	pp	229533.I1S4S7
140208	229533.A0A1C3YKB0	pp	229533.I1S8R3
140209	229533.A0A1C3YKB0	pp	229533.I1RJV3
140210	229533.A0A1C3YKB0	pp	229533.I1RTH6
140211	229533.A0A1C3YKB0	pp	229533.I1RE89
140212	229533.A0A1C3YKB0	pp	229533.Q4IJT5
140213	229533.A0A1C3YKB0	pp	229533.I1RIL8
140214	229533.A0A1C3YKB0	pp	229533.Q4HXF6
140215	229533.A0A1C3YKB0	pp	229533.I1RRI2
140216	229533.A0A1C3YKB0	pp	229533.I1RB92
140217	229533.A0A1C3YKB0	pp	229533.I1RVX0
140218	229533.A0A1C3YKB0	pp	229533.I1RRG3
140219	229533.A0A1C3YKB0	pp	229533.I1RZ79
140220	229533.A0A1C3YKB0	pp	229533.I1RBD5
140221	229533.A0A1C3YKB0	pp	229533.I1RT03
140222	229533.A0A1C3YKB0	pp	229533.I1RWX5
140223	229533.A0A1C3YKB0	pp	229533.I1S569
140224	229533.A0A1C3YKB0	pp	229533.I1RRN5
140225	229533.A0A1C3YKB0	pp	229533.I1RYW3
140226	229533.A0A1C3YKB0	pp	229533.I1RZW8
140227	229533.A0A1C3YKB0	pp	229533.I1RXR1
140228	229533.A0A1C3YKB0	pp	229533.Q4I624
140229	229533.A0A1C3YKB0	pp	229533.I1RD17
140230	229533.A0A1C3YKB0	pp	229533.I1RCH5
140231	229533.A0A1C3YKB0	pp	229533.I1RQE7
140232	229533.A0A1C3YKB0	pp	229533.I1S1C7
140233	229533.A0A1C3YKB0	pp	229533.I1S437

140234	229533.A0A1C3YKB0	pp	229533.I1RGL4
140235	229533.A0A1C3YKB0	pp	229533.I1RN18
140236	229533.A0A1C3YKB0	pp	229533.I1RML0
140237	229533.A0A1C3YKB0	pp	229533.I1RRC8
140238	229533.A0A1C3YKB0	pp	229533.I1RFL4
140239	229533.A0A1C3YKB0	pp	229533.Q4IPH4
140240	229533.A0A1C3YKB0	pp	229533.I1RB44
140241	229533.A0A1C3YKB0	pp	229533.I1RWN6
140242	229533.A0A1C3YKB0	pp	229533.I1RCD8
140243	229533.A0A1C3YKB0	pp	229533.I1RB41
140244	229533.A0A1C3YKB0	pp	229533.I1RD39
140245	229533.A0A1C3YKB0	pp	229533.I1RAU7
140246	229533.A0A1C3YKB0	pp	229533.I1RY44
140247	229533.A0A1C3YKB0	pp	229533.I1RQ33
140248	229533.A0A1C3YKB0	pp	229533.V6RRH2
140249	229533.A0A1C3YKB0	pp	229533.I1RBR1
140250	229533.A0A1C3YKB0	pp	229533.V6R057
140251	229533.A0A1C3YKB0	pp	229533.I1R9J6
140252	229533.A0A1C3YKB0	pp	229533.I1RC33
140253	229533.A0A1C3YKB0	pp	229533.I1RRN2
140254	229533.A0A1C3YKB0	pp	229533.I1RKC6
140255	229533.A0A1C3YKB0	pp	229533.I1RPU8
140256	229533.A0A1C3YKB0	pp	229533.I1RZV3
140257	229533.A0A1C3YKB0	pp	229533.I1RWH4
140258	229533.A0A1C3YKB0	pp	229533.I1S1W3
140259	229533.A0A1C3YKB0	pp	229533.I1RD22
140260	229533.A0A1C3YKB0	pp	229533.V6RMW6
140261	229533.A0A1C3YKB0	pp	229533.I1RRM3
140262	229533.A0A1C3YKB0	pp	229533.I1SA33
140263	229533.A0A1C3YKB0	pp	229533.I1S1W9
140264	229533.A0A1C3YKB0	pp	229533.V6RIV9
140265	229533.A0A1C3YKB0	pp	229533.I1RPP4
140266	229533.A0A1C3YKB0	pp	229533.I1RMV0
140267	229533.A0A1C3YKB0	pp	229533.V6R042
140268	229533.A0A1C3YKB0	pp	229533.I1R9X7
140269	229533.A0A1C3YKB0	pp	229533.A0A1C3YKP5
140270	229533.A0A1C3YKB0	pp	229533.I1RSQ3
140271	229533.A0A1C3YKB0	pp	229533.I1RPK0
140272	229533.A0A1C3YKB0	pp	229533.A0A1C3YLS9
140273	229533.A0A1C3YKB0	pp	229533.I1RWN5
140274	229533.A0A1C3YKB0	pp	229533.V6RLM7
140275	229533.A0A1C3YKB0	pp	229533.I1RMG3
140276	229533.A0A1C3YKB0	pp	229533.I1RPY1
140277	229533.A0A1C3YKB0	pp	229533.I1RKG3
140278	229533.A0A1C3YKB0	pp	229533.I1RRV1
140279	229533.A0A1C3YKB0	pp	229533.I1RS17
140280	229533.A0A1C3YKB0	pp	229533.I1S7W5
140281	229533.A0A1C3YKB0	pp	229533.I1S016
140282	229533.A0A1C3YKB0	pp	229533.Q4HZB8
140283	229533.A0A1C3YKB0	pp	229533.I1RSI6
140284	229533.A0A1C3YKB0	pp	229533.I1S253
140285	229533.A0A1C3YKB0	pp	229533.V6R3X5
140286	229533.A0A1C3YKB0	pp	229533.A0A1C3YKS0
140287	229533.A0A1C3YKB0	pp	229533.I1S0J1
140288	229533.A0A1C3YKB0	pp	229533.I1RVF8
140289	229533.A0A1C3YKB0	pp	229533.I1S0R2
140290	229533.A0A1C3YKB0	pp	229533.I1RC72
140291	229533.A0A1C3YKB0	pp	229533.I1RUG3
140292	229533.A0A1C3YKB0	pp	229533.I1RR73
140293	229533.A0A1C3YKB0	pp	229533.I1RE01
140294	229533.A0A1C3YKB0	pp	229533.I1RNC1
140295	229533.A0A1C3YKB0	pp	229533.I1RH91
140296	229533.A0A1C3YKB0	pp	229533.I1RZ39
140297	229533.A0A1C3YKB0	pp	229533.I1RXT3
140298	229533.A0A1C3YKB0	pp	229533.I1RW46
140299	229533.A0A1C3YKB0	pp	229533.I1RTA0
140300	229533.A0A1C3YKB0	pp	229533.I1RE79
140301	229533.A0A1C3YKB0	pp	229533.I1RCR8
140302	229533.A0A1C3YKB0	pp	229533.A0A1C3YKM9
140303	229533.A0A1C3YKB0	pp	229533.I1RRL9
140304	229533.A0A1C3YKB0	pp	229533.A0A1C3YLC1
140305	229533.A0A1C3YKB0	pp	229533.I1RZS7
140306	229533.A0A1C3YKB0	pp	229533.A0A1C3YLB6

140307 229533.A0A1C3YKB0 pp 229533.I1RFH8
140308 229533.A0A1C3YKB0 pp 229533.I1RC66
140309 229533.A0A1C3YKB0 pp 229533.I1S8V3
140310 229533.A0A1C3YKB0 pp 229533.I1S0N4
140311 229533.A0A1C3YKB0 pp 229533.I1RSF1
140312 229533.A0A1C3YKB0 pp 229533.I1RVZ7
140313 229533.A0A1C3YKB0 pp 229533.I1RPU2
140314 229533.A0A1C3YKB0 pp 229533.V6RGN5
140315 229533.A0A1C3YKB0 pp 229533.I1RNN1
140316 229533.A0A1C3YKB0 pp 229533.I1RG11
140317 229533.A0A1C3YKB0 pp 229533.I1R9D1
140318 229533.A0A1C3YKB0 pp 229533.I1RSQ4
140319 229533.A0A1C3YKB0 pp 229533.I1S8V4
140320 229533.A0A1C3YKB0 pp 229533.I1RZZ9
140321 229533.A0A1C3YKB0 pp 229533.I1RWI5
140322 229533.A0A1C3YKB0 pp 229533.I1RWQ8
140323 229533.A0A1C3YKB0 pp 229533.Q4IPB8
140324 229533.A0A1C3YKB0 pp 229533.I1S5R6
140325 229533.A0A1C3YKB0 pp 229533.I1RIL3
140326 229533.A0A1C3YKB0 pp 229533.I1RRE5
140327 229533.A0A1C3YKB0 pp 229533.I1RRI1
140328 229533.A0A1C3YKB0 pp 229533.I1S277
140329 229533.A0A1C3YKB0 pp 229533.I1RRT2
140330 229533.A0A1C3YKB0 pp 229533.I1S0L4
140331 229533.A0A1C3YKB0 pp 229533.I1RE76
140332 229533.A0A1C3YKB0 pp 229533.I1RBY9
140333 229533.A0A1C3YKB0 pp 229533.I1RKP1
140334 229533.A0A1C3YKB0 pp 229533.I1RBY4
140335 229533.A0A1C3YKB0 pp 229533.V6R3J1
140336 229533.A0A1C3YKB0 pp 229533.I1S7S9
140337 229533.A0A1C3YKB0 pp 229533.I1S2H4
140338 229533.A0A1C3YKB0 pp 229533.I1RSX0
140339 229533.A0A1C3YKB0 pp 229533.I1R9M0
140340 229533.A0A1C3YKB0 pp 229533.I1RPE0
140341 229533.A0A1C3YKB0 pp 229533.I1RRM5
140342 229533.A0A1C3YKB0 pp 229533.I1RJL4
140343 229533.A0A1C3YKB0 pp 229533.I1RRZ0
140344 229533.A0A1C3YKB0 pp 229533.I1RS20
140345 229533.A0A1C3YKB0 pp 229533.I1RQS8
140346 229533.A0A1C3YKB0 pp 229533.I1S2B8
140347 229533.A0A1C3YKB0 pp 229533.I1RLN6
140348 229533.A0A1C3YKB0 pp 229533.I1RJZ7
140349 229533.A0A1C3YKB0 pp 229533.I1RJW6
140350 229533.A0A1C3YKB0 pp 229533.I1S503
140351 229533.A0A1C3YKB0 pp 229533.I1RXC2
140352 229533.A0A1C3YKB0 pp 229533.O74638
140353 229533.A0A1C3YKB0 pp 229533.I1S5R1
140354 229533.A0A1C3YKB0 pp 229533.V6RLW4
140355 229533.A0A1C3YKB0 pp 229533.A0A1C3YLK8
140356 229533.A0A1C3YKB0 pp 229533.I1RPP3
140357 229533.A0A1C3YKB0 pp 229533.I1S8P5
140358 229533.A0A1C3YKB0 pp 229533.I1S2E9
140359 229533.A0A1C3YKB0 pp 229533.I1RNA5
140360 229533.A0A1C3YKB0 pp 229533.I1RRT8
140361 229533.A0A1C3YKB0 pp 229533.I1RYI5
140362 229533.A0A1C3YKB0 pp 229533.A0A1C3YLF5
140363 229533.A0A1C3YKB0 pp 229533.I1RKG2
140364 229533.A0A1C3YKB0 pp 229533.I1RRJ8
140365 229533.A0A1C3YKB0 pp 229533.A0A1C3YLD8
140366 229533.A0A1C3YKB0 pp 229533.I1RA11
140367 229533.A0A1C3YKB0 pp 229533.I1S9Y1
140368 229533.A0A1C3YKB0 pp 229533.V6RUY9
140369 229533.A0A1C3YKB0 pp 229533.I1RQG4
140370 229533.A0A1C3YKB0 pp 229533.I1RM82
140371 229533.A0A1C3YKB0 pp 229533.I1RT04
140372 229533.A0A1C3YKB0 pp 229533.I1S352
140373 229533.A0A1C3YKB0 pp 229533.I1S0M6
140374 229533.A0A1C3YKB0 pp 229533.A0A1C3YL55
140375 229533.A0A1C3YKB0 pp 229533.I1RR72
140376 229533.A0A1C3YKB0 pp 229533.I1RSX1
140377 229533.A0A1C3YKB0 pp 229533.I1RFT1
140378 229533.A0A1C3YKB0 pp 229533.Q4IR18
140379 229533.A0A1C3YKB0 pp 229533.A0A1C3YKN3

229533.A0A1C3YKB0 pp 229533.I1RGU7
229533.A0A1C3YKB0 pp 229533.I1RC29
229533.A0A1C3YKB0 pp 229533.I1RTD5
229533.A0A1C3YKB0 pp 229533.I1RE22
229533.A0A1C3YKB0 pp 229533.I1RQ23
229533.A0A1C3YKB0 pp 229533.I1RTA1
229533.A0A1C3YKB0 pp 229533.I1RJN9
229533.A0A1C3YKB0 pp 229533.I1RXF8
229533.A0A1C3YKB0 pp 229533.I1S2M6
229533.A0A1C3YKB0 pp 229533.I1RFS3
229533.A0A1C3YKB0 pp 229533.I1RGF5
229533.A0A1C3YKB0 pp 229533.I1RL04
229533.A0A1C3YKB0 pp 229533.I1RV91
229533.A0A1C3YKB0 pp 229533.I1RVZ2
229533.A0A1C3YKB0 pp 229533.I1S6Y8
229533.A0A1C3YKB0 pp 229533.I1RCJ7
229533.A0A1C3YKB0 pp 229533.I1RWS4
229533.A0A1C3YKB0 pp 229533.I1RPM4
229533.A0A1C3YKB0 pp 229533.I1RT40
229533.A0A1C3YKB0 pp 229533.I1RWK3
229533.A0A1C3YKB0 pp 229533.I1RPV3
229533.A0A1C3YKB0 pp 229533.I1RSZ7
229533.A0A1C3YKB0 pp 229533.I1RQM7
229533.A0A1C3YKB0 pp 229533.I1RN63
229533.A0A1C3YKB0 pp 229533.I1RJ74
229533.A0A1C3YKB0 pp 229533.I1RJV4
229533.A0A1C3YKB0 pp 229533.Q4IBK5
229533.A0A1C3YKB0 pp 229533.I1RYR0
229533.A0A1C3YKB0 pp 229533.I1RW61
229533.A0A1C3YKB0 pp 229533.I1RZB2
229533.A0A1C3YKB0 pp 229533.A0A1C3YMA1
229533.A0A1C3YKB0 pp 229533.I1S4Z8
229533.A0A1C3YKB0 pp 229533.A0A1C3YL15
229533.A0A1C3YKB0 pp 229533.I1RS47
229533.A0A1C3YKB0 pp 229533.I1S268
229533.A0A1C3YKB0 pp 229533.I1RE27
229533.A0A1C3YKB0 pp 229533.I1RBS6
229533.A0A1C3YKB0 pp 229533.I1RRP3
229533.A0A1C3YKB0 pp 229533.A0A1C3YLA6
229533.A0A1C3YKB0 pp 229533.I1RC82
229533.A0A1C3YKB0 pp 229533.I1RGA4
229533.A0A1C3YKB0 pp 229533.I1S0F6
229533.A0A1C3YKB0 pp 229533.I1RN67
229533.A0A1C3YKB0 pp 229533.I1RAB4
229533.A0A1C3YKB0 pp 229533.A0A1C3YN63
229533.A0A1C3YKB0 pp 229533.I1RBC7
229533.A0A1C3YKB0 pp 229533.I1RBS0
229533.I1RAS5 pp 229533.I1S4Z7
229533.I1RAS5 pp 229533.I1RKK7
229533.I1RAS5 pp 229533.I1RW54
229533.I1RAS5 pp 229533.Q4INI2
229533.I1RAS5 pp 229533.I1S270
229533.I1RAS5 pp 229533.I1RQ32
229533.I1RAS5 pp 229533.I1RY50
229533.I1RAS5 pp 229533.I1RN81
229533.I1RAS5 pp 229533.I1RKR9
229533.I1RAS5 pp 229533.Q4HZS8
229533.I1RAS5 pp 229533.Q4IPI4
229533.I1RAS5 pp 229533.I1RCD3
229533.I1RAS5 pp 229533.I1RQF7
229533.I1RAS5 pp 229533.I1RT41
229533.I1RAS5 pp 229533.I1S7W2
229533.I1RAS5 pp 229533.I1RKI1
229533.I1RAS5 pp 229533.I1S8E0
229533.I1RAS5 pp 229533.I1S2G9
229533.I1RAS5 pp 229533.V6QUU6
229533.I1RAS5 pp 229533.V6R476
229533.I1RAS5 pp 229533.I1RRF0
229533.I1RAS5 pp 229533.I1RWB4
229533.I1RAS5 pp 229533.I1RZL3
229533.I1RAS5 pp 229533.I1RZD6
229533.I1RAS5 pp 229533.V6RLW2
229533.I1RAS5 pp 229533.I1RQJ5

140453	229533.I1RAS5	pp	229533.I1S247
140454	229533.I1RAS5	pp	229533.I1RCT2
140455	229533.I1RAS5	pp	229533.V6RR76
140456	229533.I1RAS5	pp	229533.I1S254
140457	229533.I1RAS5	pp	229533.I1RB89
140458	229533.I1RAS5	pp	229533.I1RQR3
140459	229533.I1RAS5	pp	229533.I1RXS3
140460	229533.I1RAS5	pp	229533.I1RQM8
140461	229533.I1RAS5	pp	229533.Q4IQT8
140462	229533.I1RAS5	pp	229533.I1RAU9
140463	229533.I1RAS5	pp	229533.I1S2A1
140464	229533.I1RAS5	pp	229533.V6RXW9
140465	229533.I1RAS5	pp	229533.I1RR95
140466	229533.I1RAS5	pp	229533.I1RBB0
140467	229533.I1RAS5	pp	229533.I1RR61
140468	229533.I1RAS5	pp	229533.I1RX69
140469	229533.I1RAS5	pp	229533.I1S0N5
140470	229533.I1RAS5	pp	229533.I1S0Z3
140471	229533.I1RAS5	pp	229533.I1S559
140472	229533.I1RAS5	pp	229533.I1RX68
140473	229533.I1RAS5	pp	229533.I1RSD7
140474	229533.I1RAS5	pp	229533.Q4HZE0
140475	229533.I1RAS5	pp	229533.I1RBR0
140476	229533.I1RAS5	pp	229533.V6QX21
140477	229533.I1RAS5	pp	229533.I1RKZ4
140478	229533.I1RAS5	pp	229533.I1RMQ5
140479	229533.I1RAS5	pp	229533.I1RQ64
140480	229533.I1RAS5	pp	229533.I1RQP5
140481	229533.I1RAS5	pp	229533.Q4ILI9
140482	229533.I1RAS5	pp	229533.I1RWI6
140483	229533.I1RAS5	pp	229533.I1RT65
140484	229533.I1RAS5	pp	229533.I1RST4
140485	229533.I1RRD2	pp	229533.I1RW57
140486	229533.I1RIG2	pp	229533.I1RSI0
140487	229533.I1RIG2	pp	229533.I1RTH6
140488	229533.I1RIG2	pp	229533.I1RRY9
140489	229533.I1RIG2	pp	229533.I1RR73
140490	229533.I1RIG2	pp	229533.I1RJV3
140491	229533.I1RIG2	pp	229533.I1RWN6
140492	229533.I1RIG2	pp	229533.I1RPP4
140493	229533.I1RIG2	pp	229533.I1S4S4
140494	229533.I1RIG2	pp	229533.I1RPP3
140495	229533.I1RIG2	pp	229533.I1S0A0
140496	229533.I1RIG2	pp	229533.I1RLU6
140497	229533.I1RIG2	pp	229533.I1RN61
140498	229533.I1RIG2	pp	229533.I1RZM1
140499	229533.I1RIG2	pp	229533.I1RNQ5
140500	229533.I1RIG2	pp	229533.I1RWN5
140501	229533.I1RIG2	pp	229533.I1RN67
140502	229533.I1RIG2	pp	229533.I1RSQ3
140503	229533.I1RIG2	pp	229533.V6RMW6
140504	229533.I1RIG2	pp	229533.I1RQC9
140505	229533.I1RIG2	pp	229533.I1RJP4
140506	229533.I1RIG2	pp	229533.I1RT04
140507	229533.I1RIG2	pp	229533.V6R042
140508	229533.I1RIG2	pp	229533.V6RTJ8
140509	229533.I1RIG2	pp	229533.I1RTD5
140510	229533.I1RIG2	pp	229533.I1RNC1
140511	229533.I1RIG2	pp	229533.I1RX46
140512	229533.A0A0E0RVW9	pp	229533.I1S5P3
140513	229533.A0A0E0RVW9	pp	229533.I1RRD2
140514	229533.A0A0E0RVW9	pp	229533.A0A0E0RXI2
140515	229533.I1S0D5	pp	229533.I1S283
140516	229533.I1S0D5	pp	229533.I1S788
140517	229533.I1S0D5	pp	229533.I1S7X9
140518	229533.I1S0D5	pp	229533.I1S209
140519	229533.I1S0D5	pp	229533.I1S998
140520	229533.I1S0D5	pp	229533.Q4IEV4
140521	229533.I1S0D5	pp	229533.I1S987
140522	229533.I1S0D5	pp	229533.Q4IR08
140523	229533.I1S0D5	pp	229533.I1S461
140524	229533.I1S0D5	pp	229533.Q4INS6
140525	229533.I1S0D5	pp	229533.V6RSF3

140526 229533.I1S0D5 pp 229533.I1S6Y6
140527 229533.I1S0D5 pp 229533.Q4IJ84
140528 229533.I1S0D5 pp 229533.I1S4P6
140529 229533.I1S0D5 pp 229533.Q4IB89
140530 229533.A0A1C3YIU6 pp 229533.A0A1C3YLE3
140531 229533.A0A1C3YIU6 pp 229533.I1RA49
140532 229533.A0A1C3YIU6 pp 229533.V6R3J1
140533 229533.A0A1C3YIU6 pp 229533.A0A1C3YLB0
140534 229533.A0A1C3YIU6 pp 229533.I1RYW9
140535 229533.A0A1C3YIU6 pp 229533.I1RRX5
140536 229533.A0A1C3YIU6 pp 229533.I1R9Y4
140537 229533.A0A1C3YIU6 pp 229533.I1RAM6
140538 229533.A0A1C3YIU6 pp 229533.I1RMQ6
140539 229533.A0A1C3YIU6 pp 229533.I1RPP5
140540 229533.A0A1C3YIU6 pp 229533.I1RMQ5
140541 229533.A0A1C3YIU6 pp 229533.I1RAI4
140542 229533.A0A1C3YIU6 pp 229533.I1RAV5
140543 229533.A0A1C3YKW9 pp 229533.I1RUQ2
140544 229533.A0A1C3YKW9 pp 229533.Q4IAS1
140545 229533.A0A1C3YKW9 pp 229533.I1RKQ5
140546 229533.A0A1C3YKW9 pp 229533.I1RLV9
140547 229533.A0A1C3YKW9 pp 229533.I1RJW6
140548 229533.A0A1C3YKW9 pp 229533.I1RX46
140549 229533.A0A1C3YKW9 pp 229533.I1RRT2
140550 229533.A0A1C3YKW9 pp 229533.I1RQS5
140551 229533.A0A1C3YKW9 pp 229533.I1RE87
140552 229533.A0A1C3YKW9 pp 229533.A0A1C3YMW0
140553 229533.A0A1C3YKW9 pp 229533.I1RJL4
140554 229533.A0A1C3YKW9 pp 229533.Q4IPI1
140555 229533.A0A1C3YKW9 pp 229533.I1RBS6
140556 229533.A0A1C3YKW9 pp 229533.A0A1C3YM43
140557 229533.A0A1C3YKW9 pp 229533.I1RC82
140558 229533.A0A1C3YKW9 pp 229533.I1RAX6
140559 229533.A0A1C3YKW9 pp 229533.I1RFN1
140560 229533.A0A1C3YKW9 pp 229533.Q4IQW0
140561 229533.A0A1C3YKW9 pp 229533.I1RJV4
140562 229533.A0A1C3YKW9 pp 229533.V6RTJ8
140563 229533.A0A1C3YKW9 pp 229533.I1RWK3
140564 229533.A0A1C3YKW9 pp 229533.I1RC03
140565 229533.A0A1C3YKW9 pp 229533.I1RFW5
140566 229533.A0A1C3YKW9 pp 229533.V6R283
140567 229533.A0A1C3YKW9 pp 229533.I1RBC9
140568 229533.A0A1C3YKW9 pp 229533.Q4INZ9
140569 229533.A0A1C3YKW9 pp 229533.I1RT98
140570 229533.A0A1C3YKW9 pp 229533.I1RKL3
140571 229533.A0A1C3YKW9 pp 229533.I1RR00
140572 229533.A0A1C3YKW9 pp 229533.I1RT40
140573 229533.A0A1C3YKW9 pp 229533.I1RAF8
140574 229533.A0A1C3YKW9 pp 229533.I1RWF7
140575 229533.A0A1C3YKW9 pp 229533.I1RPP3
140576 229533.A0A1C3YKW9 pp 229533.I1RTD5
140577 229533.A0A1C3YKW9 pp 229533.I1RQC9
140578 229533.A0A1C3YKW9 pp 229533.I1RQJ5
140579 229533.A0A1C3YKW9 pp 229533.I1RSQ7
140580 229533.A0A1C3YKW9 pp 229533.V6QYF0
140581 229533.A0A1C3YKW9 pp 229533.I1RRJ8
140582 229533.A0A1C3YKW9 pp 229533.I1S0R7
140583 229533.A0A1C3YKW9 pp 229533.I1RT04
140584 229533.A0A1C3YKW9 pp 229533.I1RNH1
140585 229533.A0A1C3YKW9 pp 229533.I1RNA0
140586 229533.A0A1C3YKW9 pp 229533.I1RBG9
140587 229533.A0A1C3YKW9 pp 229533.V6QW22
140588 229533.A0A1C3YKW9 pp 229533.I1RR72
140589 229533.A0A1C3YKW9 pp 229533.I1RRP3
140590 229533.A0A1C3YKW9 pp 229533.I1RS47
140591 229533.A0A1C3YKW9 pp 229533.Q4HZ42
140592 229533.A0A1C3YKW9 pp 229533.I1RM06
140593 229533.A0A1C3YKW9 pp 229533.I1RZS6
140594 229533.A0A1C3YKW9 pp 229533.I1RN67
140595 229533.A0A1C3YKW9 pp 229533.I1RS30
140596 229533.A0A1C3YKW9 pp 229533.I1S2B8
140597 229533.A0A1C3YKW9 pp 229533.I1RRM5
140598 229533.A0A1C3YKW9 pp 229533.Q4HZ47

140599	229533.A0A1C3YKW9	pp	229533.I1RQ65
140600	229533.A0A1C3YKW9	pp	229533.A0A1C3YN63
140601	229533.A0A1C3YKW9	pp	229533.Q4IFI0
140602	229533.A0A1C3YKW9	pp	229533.I1S0F5
140603	229533.A0A1C3YKW9	pp	229533.I1RRQ3
140604	229533.A0A1C3YKW9	pp	229533.I1RBY4
140605	229533.A0A1C3YKW9	pp	229533.I1S6F0
140606	229533.A0A1C3YKW9	pp	229533.I1S0L3
140607	229533.A0A1C3YKW9	pp	229533.I1RZZ9
140608	229533.A0A1C3YKW9	pp	229533.I1RAC2
140609	229533.A0A1C3YKW9	pp	229533.I1RVQ0
140610	229533.A0A1C3YKW9	pp	229533.I1RW25
140611	229533.A0A1C3YKW9	pp	229533.I1RQK4
140612	229533.A0A1C3YKW9	pp	229533.I1RBT1
140613	229533.A0A1C3YKW9	pp	229533.I1S0J1
140614	229533.A0A1C3YKW9	pp	229533.I1RT80
140615	229533.A0A1C3YKW9	pp	229533.I1RPH9
140616	229533.A0A1C3YKW9	pp	229533.I1S0M9
140617	229533.A0A1C3YKW9	pp	229533.I1S8E2
140618	229533.A0A1C3YKW9	pp	229533.I1RTF4
140619	229533.A0A1C3YKW9	pp	229533.I1RSW2
140620	229533.A0A1C3YKW9	pp	229533.I1RUG3
140621	229533.A0A1C3YKW9	pp	229533.I1RQ31
140622	229533.A0A1C3YKW9	pp	229533.Q4IJ56
140623	229533.A0A1C3YKW9	pp	229533.I1RMK3
140624	229533.A0A1C3YKW9	pp	229533.I1RWB5
140625	229533.A0A1C3YKW9	pp	229533.I1RMC0
140626	229533.A0A1C3YKW9	pp	229533.I1RDA0
140627	229533.A0A1C3YKW9	pp	229533.I1RNU6
140628	229533.A0A1C3YKW9	pp	229533.I1RNC1
140629	229533.A0A1C3YKW9	pp	229533.I1RPP4
140630	229533.A0A1C3YKW9	pp	229533.I1S6U8
140631	229533.A0A1C3YKW9	pp	229533.I1RB27
140632	229533.A0A1C3YKW9	pp	229533.I1RND6
140633	229533.A0A1C3YKW9	pp	229533.V6R3X5
140634	229533.A0A1C3YKW9	pp	229533.I1RS18
140635	229533.A0A1C3YKW9	pp	229533.Q4I327
140636	229533.A0A1C3YKW9	pp	229533.I1R9X7
140637	229533.A0A1C3YKW9	pp	229533.I1RR73
140638	229533.A0A1C3YKW9	pp	229533.I1RBI8
140639	229533.A0A1C3YKW9	pp	229533.I1RWJ8
140640	229533.A0A1C3YKW9	pp	229533.I1RNY2
140641	229533.A0A1C3YKW9	pp	229533.V6RIV9
140642	229533.A0A1C3YKW9	pp	229533.I1RMN7
140643	229533.A0A1C3YKW9	pp	229533.I1S021
140644	229533.A0A1C3YKW9	pp	229533.I1RKJ6
140645	229533.A0A1C3YKW9	pp	229533.I1RPU0
140646	229533.A0A1C3YKW9	pp	229533.I1RPY1
140647	229533.A0A1C3YKW9	pp	229533.I1RNH6
140648	229533.A0A1C3YKW9	pp	229533.I1RG56
140649	229533.A0A1C3YKW9	pp	229533.V6RMW6
140650	229533.A0A1C3YKW9	pp	229533.I1RSI6
140651	229533.A0A1C3YKW9	pp	229533.I1RRT8
140652	229533.A0A1C3YKW9	pp	229533.I1RM67
140653	229533.A0A1C3YKW9	pp	229533.I1S2H4
140654	229533.A0A1C3YKW9	pp	229533.Q4IEK8
140655	229533.A0A1C3YKW9	pp	229533.I1RLU6
140656	229533.A0A1C3YKW9	pp	229533.V6RCG5
140657	229533.A0A1C3YKW9	pp	229533.I1RD64
140658	229533.A0A1C3YKW9	pp	229533.I1RNQ5
140659	229533.A0A1C3YKW9	pp	229533.I1S9U2
140660	229533.A0A1C3YKW9	pp	229533.I1RM96
140661	229533.A0A1C3YKW9	pp	229533.I1RG51
140662	229533.A0A1C3YKW9	pp	229533.I1RSQ3
140663	229533.A0A1C3YKW9	pp	229533.I1RZM1
140664	229533.A0A1C3YKW9	pp	229533.I1RPM4
140665	229533.A0A1C3YKW9	pp	229533.I1RZF6
140666	229533.A0A1C3YKW9	pp	229533.V6R042
140667	229533.A0A1C3YKW9	pp	229533.V6QUE6
140668	229533.A0A1C3YKW9	pp	229533.I1RBZ3
140669	229533.A0A1C3YKW9	pp	229533.I1S9U1
140670	229533.A0A1C3YKW9	pp	229533.I1RCL3
140671	229533.A0A1C3YKW9	pp	229533.I1RJV3

140672 229533.A0A1C3YKW9 pp 229533.I1RMN6
140673 229533.A0A1C3YKW9 pp 229533.I1RRY9
140674 229533.A0A1C3YKW9 pp 229533.I1RAU5
140675 229533.A0A1C3YKW9 pp 229533.V6R227
140676 229533.A0A1C3YKW9 pp 229533.I1RAW0
140677 229533.A0A1C3YKW9 pp 229533.I1RD22
140678 229533.A0A1C3YKW9 pp 229533.I1RN18
140679 229533.A0A1C3YKW9 pp 229533.I1RC27
140680 229533.A0A1C3YKW9 pp 229533.I1RRR7
140681 229533.A0A1C3YKW9 pp 229533.I1RFL4
140682 229533.A0A1C3YKW9 pp 229533.I1RTG6
140683 229533.A0A1C3YKW9 pp 229533.I1RMD5
140684 229533.A0A1C3YKW9 pp 229533.I1RZZ1
140685 229533.A0A1C3YKW9 pp 229533.I1RTD7
140686 229533.A0A1C3YKW9 pp 229533.I1S5B0
140687 229533.A0A1C3YKW9 pp 229533.V6RGN5
140688 229533.A0A1C3YKW9 pp 229533.I1RQE7
140689 229533.A0A1C3YKW9 pp 229533.I1S2E8
140690 229533.A0A1C3YKW9 pp 229533.I1RK79
140691 229533.A0A1C3YKW9 pp 229533.I1RTD9
140692 229533.A0A1C3YKW9 pp 229533.I1R9V7
140693 229533.A0A1C3YKW9 pp 229533.I1RW99
140694 229533.A0A1C3YKW9 pp 229533.I1RA48
140695 229533.A0A1C3YKW9 pp 229533.I1RY03
140696 229533.A0A1C3YKW9 pp 229533.I1RX14
140697 229533.A0A1C3YKW9 pp 229533.I1RSR8
140698 229533.A0A1C3YKW9 pp 229533.I1RSS4
140699 229533.A0A1C3YKW9 pp 229533.I1S060
140700 229533.A0A1C3YKW9 pp 229533.I1RSC6
140701 229533.A0A1C3YKW9 pp 229533.I1RN99
140702 229533.A0A1C3YKW9 pp 229533.I1RRI2
140703 229533.A0A1C3YKW9 pp 229533.I1RE01
140704 229533.A0A1C3YKW9 pp 229533.I1RFU8
140705 229533.A0A1C3YKW9 pp 229533.I1RK95
140706 229533.A0A1C3YKW9 pp 229533.I1RC72
140707 229533.A0A1C3YKW9 pp 229533.I1RA70
140708 229533.A0A1C3YKW9 pp 229533.I1RQ28
140709 229533.A0A1C3YKW9 pp 229533.Q4HZK7
140710 229533.A0A1C3YKW9 pp 229533.I1RB92
140711 229533.A0A1C3YKW9 pp 229533.I1RVZ0
140712 229533.A0A1C3YKW9 pp 229533.I1RCG4
140713 229533.A0A1C3YKW9 pp 229533.I1RZB5
140714 229533.A0A1C3YKW9 pp 229533.I1RNK2
140715 229533.A0A1C3YKW9 pp 229533.Q4I5Z5
140716 229533.A0A1C3YKW9 pp 229533.I1RTH6
140717 229533.A0A1C3YKW9 pp 229533.I1S2C0
140718 229533.A0A1C3YKW9 pp 229533.I1RKC6
140719 229533.A0A1C3YKW9 pp 229533.I1RAZ8
140720 229533.A0A1C3YKW9 pp 229533.I1RZE5
140721 229533.A0A1C3YKW9 pp 229533.I1S0M3
140722 229533.A0A1C3YKW9 pp 229533.I1RJL2
140723 229533.A0A1C3YKW9 pp 229533.I1RTE9
140724 229533.A0A1C3YKW9 pp 229533.I1RPJ3
140725 229533.A0A1C3YKW9 pp 229533.I1RE00
140726 229533.A0A1C3YKW9 pp 229533.I1RFJ9
140727 229533.A0A1C3YKW9 pp 229533.I1RQA2
140728 229533.A0A1C3YKW9 pp 229533.I1RC06
140729 229533.A0A1C3YKW9 pp 229533.I1RD17
140730 229533.A0A1C3YKW9 pp 229533.I1REK8
140731 229533.A0A1C3YKW9 pp 229533.I1RAU7
140732 229533.I1RN57 pp 229533.I1RQI6
140733 229533.I1RN57 pp 229533.I1RSC7
140734 229533.I1RN57 pp 229533.I1S4P6
140735 229533.I1RN57 pp 229533.I1RSL6
140736 229533.I1RN57 pp 229533.I1RRS7
140737 229533.I1RN57 pp 229533.I1RW16
140738 229533.I1RN57 pp 229533.I1RQJ4
140739 229533.I1RN57 pp 229533.Q4HYB8
140740 229533.I1RN57 pp 229533.I1RTN6
140741 229533.I1RN57 pp 229533.I1RSP7
140742 229533.I1RN57 pp 229533.V6R382
140743 229533.I1RN57 pp 229533.I1RYQ1
140744 229533.I1RN57 pp 229533.I1RWI8

140745	229533.I1RN57	pp	229533.I1RZ86
140746	229533.I1RN57	pp	229533.I1RZP8
140747	229533.I1RN57	pp	229533.Q4IQM6
140748	229533.I1RN57	pp	229533.I1RYX9
140749	229533.I1RN57	pp	229533.I1RX64
140750	229533.I1RN57	pp	229533.I1RSG8
140751	229533.I1RN57	pp	229533.I1RSN7
140752	229533.I1RN57	pp	229533.I1RSA7
140753	229533.I1RN57	pp	229533.I1S0A6
140754	229533.I1RN57	pp	229533.V6RLW4
140755	229533.I1RN57	pp	229533.I1RSR9
140756	229533.I1RN57	pp	229533.I1S2F2
140757	229533.I1RN57	pp	229533.I1RYR2
140758	229533.I1RN57	pp	229533.I1RUG4
140759	229533.I1RN57	pp	229533.I1S3G4
140760	229533.I1RN57	pp	229533.I1S998
140761	229533.I1RN57	pp	229533.I1RY73
140762	229533.I1RN57	pp	229533.Q4IP51
140763	229533.I1RN57	pp	229533.I1RUG1
140764	229533.I1RN57	pp	229533.Q4IJU0
140765	229533.I1RN57	pp	229533.I1RUG2
140766	229533.I1RN57	pp	229533.I1RNI4
140767	229533.I1RN57	pp	229533.I1S604
140768	229533.I1RN57	pp	229533.I1RQ92
140769	229533.I1RN57	pp	229533.I1S461
140770	229533.I1RN57	pp	229533.I1S209
140771	229533.I1RN57	pp	229533.I1S283
140772	229533.I1RN57	pp	229533.I1RPZ0
140773	229533.I1RN57	pp	229533.I1RRT1
140774	229533.I1RN57	pp	229533.I1RSI4
140775	229533.I1RN57	pp	229533.I1RPQ1
140776	229533.I1RN57	pp	229533.I1RZ94
140777	229533.I1RN57	pp	229533.I1S1W8
140778	229533.I1RN57	pp	229533.I1RQ13
140779	229533.I1RN57	pp	229533.Q4IEA5
140780	229533.I1RN57	pp	229533.Q4IC11
140781	229533.I1RN57	pp	229533.I1S987
140782	229533.I1RXK9	pp	229533.I1SA55
140783	229533.I1RMS0	pp	229533.I1RZR2
140784	229533.I1RZF6	pp	229533.Q4IFI0
140785	229533.I1RZF6	pp	229533.Q4IAS1
140786	229533.I1RZF6	pp	229533.I1S2B8
140787	229533.I1RZF6	pp	229533.I1S1B6
140788	229533.I1RZF6	pp	229533.Q4IQW0
140789	229533.I1RZF6	pp	229533.Q4IQK7
140790	229533.I1RZF6	pp	229533.I1S503
140791	229533.I1RZF6	pp	229533.Q4HZ42
140792	229533.I1RZF6	pp	229533.Q4I5R9
140793	229533.I1RZF6	pp	229533.I1S6F0
140794	229533.I1RZF6	pp	229533.V6R283
140795	229533.I1RZF6	pp	229533.I1RZS6
140796	229533.I1RZF6	pp	229533.I1S0R7
140797	229533.I1RZF6	pp	229533.V6QYF0
140798	229533.I1RZF6	pp	229533.Q4I830
140799	229533.I1RZF6	pp	229533.Q4IPI1
140800	229533.I1RZF6	pp	229533.I1S9U2
140801	229533.I1RZF6	pp	229533.Q4IJH1
140802	229533.I1RZF6	pp	229533.Q4INZ9
140803	229533.I1RZF6	pp	229533.Q4I662
140804	229533.I1RZF6	pp	229533.I1S2E9
140805	229533.I1RZF6	pp	229533.I1S9Y1
140806	229533.I1RZF6	pp	229533.I1S6U8
140807	229533.I1RZF6	pp	229533.I1S8E2
140808	229533.I1RZF6	pp	229533.I1RZV6
140809	229533.I1RZF6	pp	229533.I1RZJ6
140810	229533.I1RZF6	pp	229533.Q4IF76
140811	229533.I1RZF6	pp	229533.Q4IEK8
140812	229533.I1RZF6	pp	229533.Q4IJ56
140813	229533.I1RZF6	pp	229533.V6R042
140814	229533.I1RZF6	pp	229533.Q4HVW2
140815	229533.I1RZF6	pp	229533.V6R3X5
140816	229533.I1RZF6	pp	229533.Q4IAA0
140817	229533.I1RZF6	pp	229533.I1S504

140818 229533.I1RZF6 pp 229533.Q4IN63
140819 229533.I1RZF6 pp 229533.V6RMW6
140820 229533.I1RZF6 pp 229533.I1S0M9
140821 229533.I1RZF6 pp 229533.Q4HYB8
140822 229533.I1RZF6 pp 229533.I1S4T3
140823 229533.I1RZF6 pp 229533.V6R227
140824 229533.I1RZF6 pp 229533.I1S060
140825 229533.I1RZF6 pp 229533.Q4I327
140826 229533.I1RZF6 pp 229533.Q4HZ68
140827 229533.I1RZF6 pp 229533.V6RGN5
140828 229533.I1RZF6 pp 229533.I1RZK6
140829 229533.I1RZF6 pp 229533.I1S021
140830 229533.I1RZF6 pp 229533.I1S2U2
140831 229533.I1RZF6 pp 229533.I1S0J1
140832 229533.I1RZF6 pp 229533.I1S044
140833 229533.I1RZF6 pp 229533.I1S0Z9
140834 229533.I1RZF6 pp 229533.Q4IBS2
140835 229533.I1RZF6 pp 229533.I1S296
140836 229533.I1RZF6 pp 229533.I1RZZ1
140837 229533.I1RZF6 pp 229533.V6RGY3
140838 229533.I1RZF6 pp 229533.Q4HWE2
140839 229533.I1RZF6 pp 229533.I1S2E8
140840 229533.I1RZF6 pp 229533.I1S1X9
140841 229533.I1RZF6 pp 229533.I1S274
140842 229533.I1RZF6 pp 229533.I1S1C7
140843 229533.I1RZF6 pp 229533.I1S1U7
140844 229533.I1RZF6 pp 229533.I1RZK5
140845 229533.I1RZF6 pp 229533.I1S2C0
140846 229533.I1RJN9 pp 229533.I1RT40
140847 229533.I1RJN9 pp 229533.I1RQM7
140848 229533.I1RJN9 pp 229533.I1RX46
140849 229533.I1RJN9 pp 229533.V6RTJ8
140850 229533.I1RJN9 pp 229533.I1RPP3
140851 229533.I1RJN9 pp 229533.I1RSX0
140852 229533.I1RJN9 pp 229533.Q4HVQ9
140853 229533.I1RJN9 pp 229533.I1RXD7
140854 229533.I1RJN9 pp 229533.I1RYI5
140855 229533.I1RJN9 pp 229533.I1RTB3
140856 229533.I1RJN9 pp 229533.I1S4S7
140857 229533.I1RJN9 pp 229533.I1RSQ7
140858 229533.I1RJN9 pp 229533.I1RR24
140859 229533.I1RJN9 pp 229533.I1RPY3
140860 229533.I1RJN9 pp 229533.I1RTD5
140861 229533.I1RJN9 pp 229533.I1RNB2
140862 229533.I1RJN9 pp 229533.I1RTA1
140863 229533.I1RJN9 pp 229533.I1RXF8
140864 229533.I1RJN9 pp 229533.I1S2H4
140865 229533.I1RJN9 pp 229533.I1RRG3
140866 229533.I1RJN9 pp 229533.I1RPE0
140867 229533.I1RJN9 pp 229533.I1RVZ2
140868 229533.I1RJN9 pp 229533.I1RYR0
140869 229533.I1RJN9 pp 229533.I1RX95
140870 229533.I1RJN9 pp 229533.I1RXC2
140871 229533.I1RJN9 pp 229533.I1RSA5
140872 229533.I1RJN9 pp 229533.I1RY86
140873 229533.I1RJN9 pp 229533.I1S9I5
140874 229533.I1RJN9 pp 229533.I1S0W4
140875 229533.I1RJN9 pp 229533.I1S5R1
140876 229533.I1RJN9 pp 229533.I1RXD6
140877 229533.I1RJN9 pp 229533.I1S4S4
140878 229533.I1RJN9 pp 229533.V6RMW6
140879 229533.I1RJN9 pp 229533.I1S2B8
140880 229533.I1RJN9 pp 229533.I1S438
140881 229533.I1RJN9 pp 229533.I1RKJ5
140882 229533.I1RJN9 pp 229533.V6R042
140883 229533.I1RJN9 pp 229533.I1RZ39
140884 229533.I1RJN9 pp 229533.I1RRM3
140885 229533.I1RJN9 pp 229533.I1RPV3
140886 229533.I1RJN9 pp 229533.I1RMC8
140887 229533.I1RJN9 pp 229533.I1RJU3
140888 229533.I1RJN9 pp 229533.I1RSQ3
140889 229533.I1RJN9 pp 229533.I1RL04
140890 229533.I1RJN9 pp 229533.V6RCZ8

140891	229533.I1RJN9	pp	229533.I1RSQ4
140892	229533.I1RJN9	pp	229533.I1RRI1
140893	229533.I1RJN9	pp	229533.I1S6K9
140894	229533.I1RJN9	pp	229533.I1S0L4
140895	229533.I1RJN9	pp	229533.I1RSZ7
140896	229533.I1RJN9	pp	229533.I1RW61
140897	229533.I1RJN9	pp	229533.I1RKP1
140898	229533.I1RJN9	pp	229533.I1S5B0
140899	229533.I1RJN9	pp	229533.I1S967
140900	229533.I1RJN9	pp	229533.I1RKJ4
140901	229533.I1RJN9	pp	229533.I1S4Z8
140902	229533.I1RJN9	pp	229533.I1S821
140903	229533.I1RJN9	pp	229533.I1RM37
140904	229533.I1RJN9	pp	229533.I1RVF8
140905	229533.I1RJN9	pp	229533.I1RUG3
140906	229533.I1RJN9	pp	229533.I1RQD2
140907	229533.I1RJN9	pp	229533.I1S5R6
140908	229533.I1RJN9	pp	229533.I1RZV3
140909	229533.I1RJN9	pp	229533.I1RZM8
140910	229533.I1RJN9	pp	229533.I1RNC1
140911	229533.I1RJN9	pp	229533.I1RRN2
140912	229533.I1RJN9	pp	229533.I1RJV3
140913	229533.I1RJN9	pp	229533.I1RRV3
140914	229533.I1RJN9	pp	229533.I1S1B2
140915	229533.I1RJN9	pp	229533.I1S1W9
140916	229533.I1RJN9	pp	229533.I1RKJ8
140917	229533.I1RJN9	pp	229533.V6R9Z0
140918	229533.I1RJN9	pp	229533.I1RVQ3
140919	229533.I1RJN9	pp	229533.I1RS18
140920	229533.I1RJN9	pp	229533.I1RUF7
140921	229533.I1RJN9	pp	229533.I1S0J1
140922	229533.I1RJN9	pp	229533.I1RPK0
140923	229533.I1RJN9	pp	229533.I1RZK5
140924	229533.I1RJN9	pp	229533.I1RUQ3
140925	229533.I1RJN9	pp	229533.I1RVX0
140926	229533.I1RJN9	pp	229533.I1RXC4
140927	229533.I1RJN9	pp	229533.I1RT03
140928	229533.I1RJN9	pp	229533.I1S1C7
140929	229533.I1RJN9	pp	229533.I1RPU2
140930	229533.I1RJN9	pp	229533.I1RQE7
140931	229533.I1RJN9	pp	229533.I1RSF1
140932	229533.I1RJN9	pp	229533.I1S437
140933	229533.I1RJN9	pp	229533.I1RQ85
140934	229533.I1RJN9	pp	229533.I1RVZ7
140935	229533.I1RJN9	pp	229533.I1RNT9
140936	229533.I1RJN9	pp	229533.I1S5X9
140937	229533.I1RJN9	pp	229533.Q4IJK2
140938	229533.I1RJN9	pp	229533.I1S966
140939	229533.I1RJN9	pp	229533.Q4IPZ1
140940	229533.I1RJN9	pp	229533.I1RWR7
140941	229533.I1RJN9	pp	229533.I1RML0
140942	229533.I1RJN9	pp	229533.I1RRY9
140943	229533.I1RJN9	pp	229533.I1RQ33
140944	229533.I1RJN9	pp	229533.Q4I624
140945	229533.I1RD19	pp	229533.I1RZH4
140946	229533.I1RD19	pp	229533.I1RMQ7
140947	229533.I1RD19	pp	229533.V6REE9
140948	229533.I1RD19	pp	229533.Q4IMM0
140949	229533.I1RD19	pp	229533.I1RE46
140950	229533.I1RD19	pp	229533.I1RPZ0
140951	229533.I1RD19	pp	229533.Q4IR08
140952	229533.I1RD19	pp	229533.I1RQ92
140953	229533.I1RD19	pp	229533.I1S209
140954	229533.I1RD19	pp	229533.I1RQ80
140955	229533.I1RD19	pp	229533.I1RSI4
140956	229533.I1RD19	pp	229533.I1S604
140957	229533.I1RD19	pp	229533.I1RPQ1
140958	229533.I1RD19	pp	229533.I1RM36
140959	229533.I1RD19	pp	229533.I1RY73
140960	229533.I1RD19	pp	229533.I1RLX6
140961	229533.I1RD19	pp	229533.I1RFX8
140962	229533.I1RD19	pp	229533.I1RT41
140963	229533.I1RD19	pp	229533.Q4INS6

140964 229533.I1RD19 pp 229533.Q4IBX0
140965 229533.I1RD19 pp 229533.I1RRT1
140966 229533.I1RD19 pp 229533.I1RN31
140967 229533.I1RD19 pp 229533.I1RQ13
140968 229533.I1RD19 pp 229533.I1RFJ2
140969 229533.I1RD19 pp 229533.I1S461
140970 229533.I1RD19 pp 229533.I1RN46
140971 229533.I1RD19 pp 229533.I1RZ94
140972 229533.I1RD19 pp 229533.Q4IB89
140973 229533.I1RD19 pp 229533.I1RG77
140974 229533.I1RD19 pp 229533.I1RSK0
140975 229533.I1RD19 pp 229533.I1S283
140976 229533.I1RD19 pp 229533.V6R382
140977 229533.I1RD19 pp 229533.I1RTN6
140978 229533.I1RD19 pp 229533.I1RUG2
140979 229533.I1RD19 pp 229533.I1RUR0
140980 229533.I1RD19 pp 229533.I1RRS7
140981 229533.I1RD19 pp 229533.I1S788
140982 229533.I1RD19 pp 229533.I1RW16
140983 229533.I1RD19 pp 229533.I1S4P6
140984 229533.I1RD19 pp 229533.I1RYR2
140985 229533.I1RD19 pp 229533.I1RSC7
140986 229533.I1RD19 pp 229533.I1RFZ2
140987 229533.I1RD19 pp 229533.I1RFN0
140988 229533.I1RD19 pp 229533.I1RDJ8
140989 229533.I1RD19 pp 229533.I1RKQ0
140990 229533.I1RD19 pp 229533.I1RYX9
140991 229533.I1RD19 pp 229533.I1RJV8
140992 229533.I1RD19 pp 229533.I1RSR9
140993 229533.I1RD19 pp 229533.Q4IQM6
140994 229533.I1RD19 pp 229533.I1S0D5
140995 229533.I1RD19 pp 229533.I1RD70
140996 229533.I1RD19 pp 229533.I1RYQ1
140997 229533.I1RD19 pp 229533.I1RSP7
140998 229533.I1RD19 pp 229533.I1RMJ8
140999 229533.I1RD19 pp 229533.I1RVV5
141000 229533.I1RD19 pp 229533.I1RPF5
141001 229533.I1RD19 pp 229533.I1RZ86
141002 229533.I1RD19 pp 229533.V6RLW4
141003 229533.I1RD19 pp 229533.I1RRJ6
141004 229533.A0A1C3YJ93 pp 229533.I1RRI2
141005 229533.A0A1C3YJ93 pp 229533.I1RWX5
141006 229533.A0A1C3YJ93 pp 229533.I1RIB9
141007 229533.A0A1C3YJ93 pp 229533.I1RZW8
141008 229533.A0A1C3YJ93 pp 229533.I1S1J6
141009 229533.A0A1C3YJ93 pp 229533.I1RYW3
141010 229533.A0A1C3YJ93 pp 229533.I1S1C7
141011 229533.A0A1C3YJ93 pp 229533.I1RY03
141012 229533.A0A1C3YJ93 pp 229533.I1RAU7
141013 229533.A0A1C3YJ93 pp 229533.Q4I2J8
141014 229533.A0A1C3YJ93 pp 229533.I1RZK5
141015 229533.A0A1C3YJ93 pp 229533.Q4I624
141016 229533.A0A1C3YJ93 pp 229533.I1RA70
141017 229533.A0A1C3YJ93 pp 229533.I1RD17
141018 229533.A0A1C3YJ93 pp 229533.I1REK8
141019 229533.A0A1C3YJ93 pp 229533.I1RCH5
141020 229533.A0A1C3YJ93 pp 229533.I1RFU8
141021 229533.A0A1C3YJ93 pp 229533.I1RZZ1
141022 229533.A0A1C3YJ93 pp 229533.I1RTH6
141023 229533.A0A1C3YJ93 pp 229533.I1RCG4
141024 229533.A0A1C3YJ93 pp 229533.I1RQE7
141025 229533.A0A1C3YJ93 pp 229533.I1S8R3
141026 229533.A0A1C3YJ93 pp 229533.I1RFL4
141027 229533.A0A1C3YJ93 pp 229533.I1RLU6
141028 229533.A0A1C3YJ93 pp 229533.I1RN18
141029 229533.A0A1C3YJ93 pp 229533.I1RNQ5
141030 229533.A0A1C3YJ93 pp 229533.Q4IJK2
141031 229533.A0A1C3YJ93 pp 229533.I1RY44
141032 229533.A0A1C3YJ93 pp 229533.I1RB92
141033 229533.A0A1C3YJ93 pp 229533.I1RZM1
141034 229533.A0A1C3YJ93 pp 229533.I1RT03
141035 229533.A0A1C3YJ93 pp 229533.I1RVX0
141036 229533.A0A1C3YJ93 pp 229533.V6RIV9

141037 229533.A0A1C3YJ93 pp 229533.I1RWN5
141038 229533.A0A1C3YJ93 pp 229533.I1RJV3
141039 229533.A0A1C3YJ93 pp 229533.I1S1J7
141040 229533.A0A1C3YJ93 pp 229533.I1S6L5
141041 229533.A0A1C3YJ93 pp 229533.A0A1C3YKB0
141042 229533.A0A1C3YJ93 pp 229533.Q4I283
141043 229533.A0A1C3YJ93 pp 229533.I1RWN6
141044 229533.A0A1C3YJ93 pp 229533.I1RRY9
141045 229533.A0A1C3YJ93 pp 229533.I1RAW0
141046 229533.A0A1C3YJ93 pp 229533.I1S9I6
141047 229533.A0A1C3YJ93 pp 229533.I1S5B0
141048 229533.A0A1C3YJ93 pp 229533.I1S504
141049 229533.A0A1C3YJ93 pp 229533.I1RW99
141050 229533.A0A1C3YJ93 pp 229533.I1S0L4
141051 229533.A0A1C3YJ93 pp 229533.I1SA32
141052 229533.A0A1C3YJ93 pp 229533.I1RI82
141053 229533.A0A1C3YJ93 pp 229533.I1RR24
141054 229533.A0A1C3YJ93 pp 229533.I1S2H4
141055 229533.A0A1C3YJ93 pp 229533.I1RNC1
141056 229533.A0A1C3YJ93 pp 229533.I1S0J1
141057 229533.A0A1C3YJ93 pp 229533.I1RUG3
141058 229533.A0A1C3YJ93 pp 229533.I1RCR8
141059 229533.A0A1C3YJ93 pp 229533.I1RW46
141060 229533.A0A1C3YJ93 pp 229533.I1RE01
141061 229533.A0A1C3YJ93 pp 229533.I1RC66
141062 229533.A0A1C3YJ93 pp 229533.I1RZM8
141063 229533.A0A1C3YJ93 pp 229533.A0A1C3YKC0
141064 229533.A0A1C3YJ93 pp 229533.I1RBT1
141065 229533.A0A1C3YJ93 pp 229533.A0A1C3YK34
141066 229533.A0A1C3YJ93 pp 229533.A0A1C3YLU7
141067 229533.A0A1C3YJ93 pp 229533.I1RTA0
141068 229533.A0A1C3YJ93 pp 229533.I1RZZ9
141069 229533.A0A1C3YJ93 pp 229533.I1RPP4
141070 229533.A0A1C3YJ93 pp 229533.I1RS18
141071 229533.A0A1C3YJ93 pp 229533.I1RSQ3
141072 229533.A0A1C3YJ93 pp 229533.I1S0X6
141073 229533.A0A1C3YJ93 pp 229533.I1RR73
141074 229533.A0A1C3YJ93 pp 229533.V6R3X5
141075 229533.A0A1C3YJ93 pp 229533.V6R042
141076 229533.A0A1C3YJ93 pp 229533.Q4I8P5
141077 229533.A0A1C3YJ93 pp 229533.A0A1C3YKV3
141078 229533.A0A1C3YJ93 pp 229533.I1RS17
141079 229533.A0A1C3YJ93 pp 229533.V6RMW6
141080 229533.A0A1C3YJ93 pp 229533.I1RP66
141081 229533.A0A1C3YJ93 pp 229533.I1RZ39
141082 229533.A0A1C3YJ93 pp 229533.A0A1C3YKM9
141083 229533.A0A1C3YJ93 pp 229533.V6RCG5
141084 229533.A0A1C3YJ93 pp 229533.I1RJU3
141085 229533.A0A1C3YJ93 pp 229533.I1RBS0
141086 229533.A0A1C3YJ93 pp 229533.I1S2R6
141087 229533.A0A1C3YJ93 pp 229533.I1RRU9
141088 229533.A0A1C3YJ93 pp 229533.I1RAU5
141089 229533.A0A1C3YJ93 pp 229533.V6RCZ8
141090 229533.A0A1C3YJ93 pp 229533.I1RD22
141091 229533.A0A1C3YJ93 pp 229533.I1SA33
141092 229533.A0A1C3YJ93 pp 229533.I1RE76
141093 229533.A0A1C3YJ93 pp 229533.A0A1C3YJQ9
141094 229533.A0A1C3YJ93 pp 229533.I1RVQ0
141095 229533.A0A1C3YJ93 pp 229533.I1RBY4
141096 229533.A0A1C3YJ93 pp 229533.I1S0N4
141097 229533.A0A1C3YJ93 pp 229533.I1RX98
141098 229533.A0A1C3YJ93 pp 229533.I1RW25
141099 229533.A0A1C3YJ93 pp 229533.I1R9M0
141100 229533.A0A1C3YJ93 pp 229533.I1S0F6
141101 229533.A0A1C3YJ93 pp 229533.I1RN67
141102 229533.A0A1C3YJ93 pp 229533.A0A1C3YLK8
141103 229533.A0A1C3YJ93 pp 229533.I1RJW6
141104 229533.A0A1C3YJ93 pp 229533.I1S503
141105 229533.A0A1C3YJ93 pp 229533.I1S2B8
141106 229533.A0A1C3YJ93 pp 229533.I1S0L3
141107 229533.A0A1C3YJ93 pp 229533.I1RRM5
141108 229533.A0A1C3YJ93 pp 229533.A0A1C3YMA1
141109 229533.A0A1C3YJ93 pp 229533.V6RTJ8

229533.A0A1C3YJ93	pp	229533.I1RJV4
229533.A0A1C3YJ93	pp	229533.I1RBS6
229533.A0A1C3YJ93	pp	229533.I1RV58
229533.A0A1C3YJ93	pp	229533.I1RD52
229533.A0A1C3YJ93	pp	229533.I1RWK3
229533.A0A1C3YJ93	pp	229533.I1S8P5
229533.A0A1C3YJ93	pp	229533.I1RPM4
229533.A0A1C3YJ93	pp	229533.I1RAX6
229533.A0A1C3YJ93	pp	229533.I1RGA4
229533.A0A1C3YJ93	pp	229533.I1RTD5
229533.A0A1C3YJ93	pp	229533.I1RE22
229533.A0A1C3YJ93	pp	229533.I1RPP3
229533.A0A1C3YJ93	pp	229533.I1S5R1
229533.A0A1C3YJ93	pp	229533.I1RV91
229533.A0A1C3YJ93	pp	229533.V6QW22
229533.A0A1C3YJ93	pp	229533.I1RRT8
229533.A0A1C3YJ93	pp	229533.I1RT40
229533.A0A1C3YJ93	pp	229533.I1RXD7
229533.A0A1C3YJ93	pp	229533.I1RA11
229533.A0A1C3YJ93	pp	229533.I1RSQ7
229533.A0A1C3YJ93	pp	229533.I1RWJ8
229533.A0A1C3YJ93	pp	229533.I1RX46
229533.A0A1C3YJ93	pp	229533.I1RBC9
229533.A0A1C3YJ93	pp	229533.I1S2E9
229533.A0A1C3YJ93	pp	229533.I1RQC9
229533.A0A1C3YJ93	pp	229533.I1RT04
229533.A0A1C3YJ93	pp	229533.I1S0R7
229533.A0A1C3YKA7	pp	229533.I1RCW8
229533.A0A1C3YKA7	pp	229533.I1RU02
229533.A0A1C3YKA7	pp	229533.Q4IER8
229533.A0A1C3YKA7	pp	229533.I1RMK8
229533.A0A1C3YKA7	pp	229533.V6RGI1
229533.A0A1C3YKA7	pp	229533.Q4IB50
229533.A0A1C3YKA7	pp	229533.Q4IMD1
229533.A0A1C3YKA7	pp	229533.I1RFW1
229533.A0A0E0SPV2	pp	229533.V6R3X5
229533.A0A0E0SPV2	pp	229533.I1RR73
229533.A0A0E0SPV2	pp	229533.I1RBY4
229533.A0A0E0SPV2	pp	229533.I1R9M0
229533.A0A0E0SPV2	pp	229533.I1RRM5
229533.A0A0E0SPV2	pp	229533.I1S2B8
229533.A0A0E0SPV2	pp	229533.A0A1C3YKM9
229533.A0A0E0SPV2	pp	229533.I1RZ39
229533.A0A0E0SPV2	pp	229533.I1RZZ9
229533.A0A0E0SPV2	pp	229533.I1S2E9
229533.A0A0E0SPV2	pp	229533.I1S0L4
229533.A0A0E0SPV2	pp	229533.I1S2H4
229533.A0A0E0SPV2	pp	229533.I1RT04
229533.A0A0E0SPV2	pp	229533.I1RTD5
229533.A0A0E0SPV2	pp	229533.I1RV91
229533.A0A0E0SPV2	pp	229533.I1RRT8
229533.A0A0E0SPV2	pp	229533.A0A1C3YKK9
229533.A0A0E0SPV2	pp	229533.I1RPM4
229533.A0A0E0SPV2	pp	229533.I1RJV4
229533.A0A0E0SPV2	pp	229533.I1RBS6
229533.A0A0E0SPV2	pp	229533.I1S503
229533.A0A0E0SPV2	pp	229533.I1RJW6
229533.A0A0E0SPV2	pp	229533.I1RWK3
229533.A0A0E0SPV2	pp	229533.I1RN67
229533.A0A0E0SPV2	pp	229533.Q4I1Y5
229533.A0A0E0SPV2	pp	229533.I1RA70
229533.A0A0E0SPV2	pp	229533.I1RD17
229533.A0A0E0SPV2	pp	229533.I1RPP3
229533.A0A0E0SPV2	pp	229533.I1S8R3
229533.A0A0E0SPV2	pp	229533.I1S8P5
229533.A0A0E0SPV2	pp	229533.V6RMW6
229533.A0A0E0SPV2	pp	229533.I1RJW1
229533.A0A0E0SPV2	pp	229533.I1RTH6
229533.A0A0E0SPV2	pp	229533.I1RZW8
229533.A0A0E0SPV2	pp	229533.V6R042
229533.A0A0E0SPV2	pp	229533.I1S1C7
229533.A0A0E0SPV2	pp	229533.V6RTJ8
229533.A0A0E0SPV2	pp	229533.I1RSQ3

141183 229533.A0A0E0SPV2 pp 229533.I1RAX6
141184 229533.A0A0E0SPV2 pp 229533.I1RX46
141185 229533.A0A0E0SPV2 pp 229533.I1RWN6
141186 229533.A0A0E0SPV2 pp 229533.I1RUG3
141187 229533.A0A0E0SPV2 pp 229533.I1RAW0
141188 229533.A0A0E0SPV2 pp 229533.I1RJV3
141189 229533.A0A0E0SPV2 pp 229533.I1RSQ7
141190 229533.A0A0E0SPV2 pp 229533.I1RNC1
141191 229533.A0A0E0SPV2 pp 229533.I1RCR8
141192 229533.A0A0E0SPV2 pp 229533.V6QW22
141193 229533.A0A0E0SPV2 pp 229533.I1RB92
141194 229533.A0A0E0SPV2 pp 229533.I1RVX0
141195 229533.A0A0E0SPV2 pp 229533.I1RD22
141196 229533.A0A0E0SPV2 pp 229533.I1RQ75
141197 229533.A0A0E0SPV2 pp 229533.I1RT03
141198 229533.A0A0E0SPV2 pp 229533.I1RBC9
141199 229533.A0A0E0SPV2 pp 229533.I1RN18
141200 229533.A0A0E0SPV2 pp 229533.A0A0E0SQ99
141201 229533.A0A0E0SPV2 pp 229533.I1RR39
141202 229533.A0A0E0SPV2 pp 229533.I1RPP4
141203 229533.A0A0E0SPV2 pp 229533.I1S0L3
141204 229533.A0A0E0SPV2 pp 229533.I1RFL4
141205 229533.A0A0E0SPV2 pp 229533.I1RK44
141206 229533.A0A0E0SPV2 pp 229533.I1RAU7
141207 229533.A0A0E0SPV2 pp 229533.I1RVQ0
141208 229533.A0A0E0SPV2 pp 229533.I1RTA0
141209 229533.A0A0E0SPV2 pp 229533.A0A1C3YIP5
141210 229533.A0A0E0SPV2 pp 229533.I1RZK5
141211 229533.A0A0E0SPV2 pp 229533.I1RFU8
141212 229533.A0A0E0SPV2 pp 229533.I1RWN5
141213 229533.A0A0E0SPV2 pp 229533.A0A1C3YHX8
141214 229533.A0A0E0SPV2 pp 229533.I1RBT1
141215 229533.A0A0E0SPV2 pp 229533.A0A1C3YKC0
141216 229533.A0A0E0SPV2 pp 229533.I1RR24
141217 229533.A0A0E0SPV2 pp 229533.I1RY44
141218 229533.A0A0E0SPV2 pp 229533.I1RW25
141219 229533.A0A0E0SPV2 pp 229533.I1RQC9
141220 229533.A0A0E0SPV2 pp 229533.I1RCG4
141221 229533.A0A0E0SPV2 pp 229533.A0A1C3YHX6
141222 229533.A0A0E0SPV2 pp 229533.I1RY03
141223 229533.A0A0E0SPV2 pp 229533.V6RIV9
141224 229533.A0A0E0SPV2 pp 229533.I1RRY9
141225 229533.A0A0E0SPV2 pp 229533.A0A1C3YI71
141226 229533.A0A0E0SPV2 pp 229533.I1RAU5
141227 229533.A0A0E0SPV2 pp 229533.I1RS18
141228 229533.A0A0E0SPV2 pp 229533.A0A1C3YKV3
141229 229533.A0A0E0SPV2 pp 229533.A0A1C3YKB0
141230 229533.A0A0E0SPV2 pp 229533.Q4IJK2
141231 229533.A0A0E0SPV2 pp 229533.I1S504
141232 229533.A0A0E0SPV2 pp 229533.I1REK8
141233 229533.A0A0E0SPV2 pp 229533.I1RLU6
141234 229533.A0A0E0SPV2 pp 229533.A0A1C3YIJ3
141235 229533.A0A0E0SPV2 pp 229533.V6RCG5
141236 229533.A0A0E0SPV2 pp 229533.I1RZM1
141237 229533.A0A0E0SPV2 pp 229533.I1RI82
141238 229533.A0A0E0SPV2 pp 229533.I1RIE8
141239 229533.I1RZP8 pp 229533.Q4IB89
141240 229533.I1RZP8 pp 229533.I1S209
141241 229533.I1RZP8 pp 229533.Q4IQM6
141242 229533.I1RZP8 pp 229533.I1S2E9
141243 229533.I1RZP8 pp 229533.V6R382
141244 229533.I1RZP8 pp 229533.Q4IEA5
141245 229533.I1RZP8 pp 229533.V6RGI1
141246 229533.I1RZP8 pp 229533.I1S604
141247 229533.I1RZP8 pp 229533.Q4INS6
141248 229533.I1RZP8 pp 229533.I1S461
141249 229533.I1RZP8 pp 229533.V6RE46
141250 229533.I1RZP8 pp 229533.V6RLW4
141251 229533.I1RZP8 pp 229533.Q4IP51
141252 229533.I1RZP8 pp 229533.Q4IC11
141253 229533.I1RZP8 pp 229533.I1S7T4
141254 229533.I1RZP8 pp 229533.I1RZS5
141255 229533.I1RZP8 pp 229533.I1S0A6

141256	229533.I1RZP8	pp	229533.I1S283
141257	229533.I1RI03	pp	229533.V6RCG5
141258	229533.I1RI03	pp	229533.I1RI82
141259	229533.I1RI03	pp	229533.I1RZM1
141260	229533.I1RI03	pp	229533.I1RY03
141261	229533.I1RI03	pp	229533.I1RRY9
141262	229533.I1RI03	pp	229533.I1RZK5
141263	229533.I1RI03	pp	229533.I1RLU6
141264	229533.I1RI03	pp	229533.Q4IJK2
141265	229533.I1RI03	pp	229533.I1S0L3
141266	229533.I1RI03	pp	229533.I1S504
141267	229533.I1RI03	pp	229533.I1RR24
141268	229533.I1RI03	pp	229533.I1RR39
141269	229533.I1RI03	pp	229533.I1RS18
141270	229533.I1RI03	pp	229533.V6QW22
141271	229533.I1RI03	pp	229533.I1RIE8
141272	229533.I1RI03	pp	229533.I1RT03
141273	229533.I1RI03	pp	229533.I1RN18
141274	229533.I1RI03	pp	229533.I1RW25
141275	229533.I1RI03	pp	229533.I1RSQ7
141276	229533.I1RI03	pp	229533.I1RVX0
141277	229533.I1RI03	pp	229533.I1RX46
141278	229533.I1RI03	pp	229533.I1RWN6
141279	229533.I1RI03	pp	229533.I1RJV3
141280	229533.I1RI03	pp	229533.I1RJW1
141281	229533.I1RI03	pp	229533.V6RTJ8
141282	229533.I1RI03	pp	229533.I1RQ75
141283	229533.I1RI03	pp	229533.I1RQC9
141284	229533.I1RI03	pp	229533.I1S1C7
141285	229533.I1RI03	pp	229533.I1RNC1
141286	229533.I1RI03	pp	229533.I1RZW8
141287	229533.I1RI03	pp	229533.I1RUG3
141288	229533.I1RI03	pp	229533.I1RPP4
141289	229533.I1RI03	pp	229533.I1RVQ0
141290	229533.I1RI03	pp	229533.I1RSQ3
141291	229533.I1RI03	pp	229533.I1RTH6
141292	229533.I1RI03	pp	229533.I1S8R3
141293	229533.I1RI03	pp	229533.I1RRT8
141294	229533.I1RI03	pp	229533.Q4I1Y5
141295	229533.I1RI03	pp	229533.V6R042
141296	229533.I1RI03	pp	229533.I1S0L4
141297	229533.I1RI03	pp	229533.I1S2H4
141298	229533.I1RI03	pp	229533.V6RMW6
141299	229533.I1RI03	pp	229533.I1RZZ9
141300	229533.I1RI03	pp	229533.I1RJW6
141301	229533.I1RI03	pp	229533.I1RWN5
141302	229533.I1RI03	pp	229533.V6RIV9
141303	229533.I1RI03	pp	229533.I1S503
141304	229533.I1RI03	pp	229533.I1RY44
141305	229533.I1RI03	pp	229533.I1RJV4
141306	229533.I1RI03	pp	229533.I1RZ39
141307	229533.I1RI03	pp	229533.V6R3X5
141308	229533.I1RI03	pp	229533.I1RV91
141309	229533.I1RI03	pp	229533.I1RR73
141310	229533.I1RI03	pp	229533.I1RTD5
141311	229533.I1RI03	pp	229533.I1RTA0
141312	229533.I1RI03	pp	229533.I1S2E9
141313	229533.I1RI03	pp	229533.I1RK44
141314	229533.I1RI03	pp	229533.I1RT04
141315	229533.I1RI03	pp	229533.I1RRM5
141316	229533.I1RI03	pp	229533.I1S8P5
141317	229533.I1RI03	pp	229533.I1S2B8
141318	229533.I1RI03	pp	229533.I1RN67
141319	229533.I1RI03	pp	229533.I1RPM4
141320	229533.I1RI03	pp	229533.I1RPP3
141321	229533.I1RI03	pp	229533.I1RWK3
141322	229533.I1RBN9	pp	229533.I1RQ67
141323	229533.I1RBN9	pp	229533.I1RJV8
141324	229533.I1RBN9	pp	229533.I1RKQ0
141325	229533.I1RBN9	pp	229533.I1RYQ1
141326	229533.I1RBN9	pp	229533.I1RSZ3
141327	229533.I1RBN9	pp	229533.I1RY29
141328	229533.I1RBN9	pp	229533.I1RYX9

141329	229533.I1RBN9	pp	229533.I1RFL7
141330	229533.I1RBN9	pp	229533.I1RHD2
141331	229533.I1RBN9	pp	229533.I1RXA9
141332	229533.I1RBN9	pp	229533.I1RWI8
141333	229533.I1RBN9	pp	229533.I1RVV5
141334	229533.I1RBN9	pp	229533.I1RXB2
141335	229533.I1RBN9	pp	229533.I1RSN7
141336	229533.I1RBN9	pp	229533.I1RSA7
141337	229533.I1RBN9	pp	229533.I1RBW1
141338	229533.I1RBN9	pp	229533.I1RRS7
141339	229533.I1RBN9	pp	229533.I1RUR0
141340	229533.I1RBN9	pp	229533.I1RSC7
141341	229533.I1RBN9	pp	229533.I1RC54
141342	229533.I1RBN9	pp	229533.V6RLW4
141343	229533.I1RBN9	pp	229533.I1S283
141344	229533.I1RBN9	pp	229533.I1S998
141345	229533.I1RBN9	pp	229533.I1S4P6
141346	229533.I1RBN9	pp	229533.I1RRJ6
141347	229533.I1RBN9	pp	229533.I1S788
141348	229533.I1RBN9	pp	229533.Q4IB89
141349	229533.I1RBN9	pp	229533.I1RN57
141350	229533.I1RBN9	pp	229533.I1RF70
141351	229533.I1RBN9	pp	229533.I1RCI4
141352	229533.I1RBN9	pp	229533.I1RCA5
141353	229533.I1RBN9	pp	229533.I1RBW0
141354	229533.I1RBN9	pp	229533.I1RDV4
141355	229533.I1RBN9	pp	229533.I1RFX8
141356	229533.I1RBN9	pp	229533.I1S987
141357	229533.I1RBN9	pp	229533.I1RMS4
141358	229533.I1RBN9	pp	229533.I1S0D5
141359	229533.I1RBN9	pp	229533.I1RRT1
141360	229533.I1RBN9	pp	229533.I1RD01
141361	229533.I1RBN9	pp	229533.I1RZ86
141362	229533.I1RBN9	pp	229533.I1RX64
141363	229533.I1RBN9	pp	229533.I1RSR9
141364	229533.I1RBN9	pp	229533.I1RSK0
141365	229533.I1RBN9	pp	229533.I1RDX0
141366	229533.I1RBN9	pp	229533.I1RMJ8
141367	229533.I1RBN9	pp	229533.I1RZ94
141368	229533.I1RBN9	pp	229533.I1RQJ4
141369	229533.I1RBN9	pp	229533.I1RUG4
141370	229533.I1RBN9	pp	229533.I1RQ13
141371	229533.I1RBN9	pp	229533.I1RDJ8
141372	229533.I1RBN9	pp	229533.I1RD19
141373	229533.I1RBN9	pp	229533.I1RDH0
141374	229533.I1RBN9	pp	229533.I1RMX3
141375	229533.I1RBN9	pp	229533.I1RPQ1
141376	229533.I1RBN9	pp	229533.I1RZP8
141377	229533.I1RBN9	pp	229533.I1RSP7
141378	229533.I1RBN9	pp	229533.I1RWM6
141379	229533.I1RBN9	pp	229533.I1S209
141380	229533.I1RBN9	pp	229533.I1RMQ7
141381	229533.I1RBN9	pp	229533.I1RKI2
141382	229533.I1RBN9	pp	229533.I1RBP0
141383	229533.I1RBN9	pp	229533.I1RCB3
141384	229533.I1RBN9	pp	229533.I1RQH8
141385	229533.I1RBN9	pp	229533.I1RE46
141386	229533.I1RBN9	pp	229533.Q4INS6
141387	229533.I1RBN9	pp	229533.I1RQ92
141388	229533.I1RBN9	pp	229533.I1RFJ2
141389	229533.I1RBN9	pp	229533.I1RLJ8
141390	229533.I1RBN9	pp	229533.I1RSI4
141391	229533.I1RBN9	pp	229533.I1RG34
141392	229533.I1RBN9	pp	229533.I1RCL7
141393	229533.I1RBN9	pp	229533.I1S461
141394	229533.I1RBN9	pp	229533.I1RG77
141395	229533.I1RVM5	pp	229533.I1RW06
141396	229533.I1RYR0	pp	229533.V6R042
141397	229533.I1RYR0	pp	229533.I1RZ39
141398	229533.I1RYR0	pp	229533.Q4I624
141399	229533.I1RYR0	pp	229533.I1RZM8
141400	229533.I1RYR0	pp	229533.V6RMW6
141401	229533.I1RYR0	pp	229533.I1S5B0

229533.I1RYR0	pp	229533.I1S4S4
229533.I1RYR0	pp	229533.I1S437
229533.I1RYR0	pp	229533.I1S6K9
229533.I1RYR0	pp	229533.V6RCZ8
229533.I1RYR0	pp	229533.I1S5R1
229533.I1RYR0	pp	229533.I1RZF5
229533.I1RYR0	pp	229533.Q4IJK2
229533.I1RYR0	pp	229533.I1S0J1
229533.I1RYR0	pp	229533.I1S0L4
229533.I1RYR0	pp	229533.I1S5R6
229533.I1RYR0	pp	229533.I1S4S7
229533.I1RYR0	pp	229533.V6RTJ8
229533.I1RYR0	pp	229533.I1S2H4
229533.I1RYR0	pp	229533.I1RZV3
229533.I1RYR0	pp	229533.I1S2B8
229533.I1RPG1	pp	229533.I1RTE0
229533.I1RPG1	pp	229533.I1RSL8
229533.I1RPG1	pp	229533.I1RTA3
229533.I1RPG1	pp	229533.I1RUE2
229533.I1RPG1	pp	229533.I1RZH6
229533.I1RPG1	pp	229533.I1RSG9
229533.I1RPG1	pp	229533.I1RTG5
229533.I1RPG1	pp	229533.I1RXB8
229533.I1RPG1	pp	229533.I1RYV4
229533.I1RPG1	pp	229533.I1RSZ6
229533.I1RPG1	pp	229533.I1RRF9
229533.I1RPG1	pp	229533.I1S270
229533.I1RKM2	pp	229533.I1RZ78
229533.I1RKM2	pp	229533.I1RLP9
229533.I1RKM2	pp	229533.I1S8P5
229533.I1RKM2	pp	229533.I1RTA0
229533.I1RKM2	pp	229533.I1S444
229533.I1RKM2	pp	229533.I1RVF8
229533.I1RKM2	pp	229533.I1S5N3
229533.I1RJU3	pp	229533.V6RLM7
229533.I1RJU3	pp	229533.I1RKG3
229533.I1RJU3	pp	229533.I1S1W3
229533.I1RJU3	pp	229533.I1RZV3
229533.I1RJU3	pp	229533.I1RPK0
229533.I1RJU3	pp	229533.I1RM37
229533.I1RJU3	pp	229533.I1RVF8
229533.I1RJU3	pp	229533.I1RY44
229533.I1RJU3	pp	229533.I1S016
229533.I1RJU3	pp	229533.I1RKJ4
229533.I1RJU3	pp	229533.I1RTN8
229533.I1RJU3	pp	229533.I1S1W9
229533.I1RJU3	pp	229533.I1RUG3
229533.I1RJU3	pp	229533.I1RSU1
229533.I1RJU3	pp	229533.I1SA33
229533.I1RJU3	pp	229533.I1RXT3
229533.I1RJU3	pp	229533.I1RWN5
229533.I1RJU3	pp	229533.I1RNC1
229533.I1RJU3	pp	229533.V6RGN5
229533.I1RJU3	pp	229533.V6RIV9
229533.I1RJU3	pp	229533.I1RRM3
229533.I1RJU3	pp	229533.I1RVZ7
229533.I1RJU3	pp	229533.I1S5R6
229533.I1RJU3	pp	229533.V6RMW6
229533.I1RJU3	pp	229533.I1RPU2
229533.I1RJU3	pp	229533.I1RSQ3
229533.I1RJU3	pp	229533.I1S0J1
229533.I1RJU3	pp	229533.I1RWQ8
229533.I1RJU3	pp	229533.I1RSF1
229533.I1RJU3	pp	229533.V6QT89
229533.I1RJU3	pp	229533.I1S7W5
229533.I1RJU3	pp	229533.V6R042
229533.I1RJU3	pp	229533.I1RRE5
229533.I1RJU3	pp	229533.I1RRV1
229533.I1RJU3	pp	229533.I1RTA0
229533.I1RJU3	pp	229533.I1RZW8
229533.I1RJU3	pp	229533.I1RSI6
229533.I1RJU3	pp	229533.I1RRN2
229533.I1RJU3	pp	229533.I1RW46

141475 229533.I1RJU3 pp 229533.I1RMG3
141476 229533.I1RJU3 pp 229533.I1RZ79
141477 229533.I1RJU3 pp 229533.I1RRI2
141478 229533.I1RJU3 pp 229533.I1S256
141479 229533.I1RJU3 pp 229533.I1RPP4
141480 229533.I1RJU3 pp 229533.I1RMV0
141481 229533.I1RJU3 pp 229533.I1RXF8
141482 229533.I1RJU3 pp 229533.I1RWX5
141483 229533.I1RJU3 pp 229533.I1RS17
141484 229533.I1RJU3 pp 229533.I1S1C7
141485 229533.I1RJU3 pp 229533.I1RYW3
141486 229533.I1RJU3 pp 229533.I1RUF7
141487 229533.I1RJU3 pp 229533.Q4IR18
141488 229533.I1RJU3 pp 229533.Q4HXF6
141489 229533.I1RJU3 pp 229533.I1RRN5
141490 229533.I1RJU3 pp 229533.I1S437
141491 229533.I1RJU3 pp 229533.I1RT40
141492 229533.I1RJU3 pp 229533.I1RSS4
141493 229533.I1RJU3 pp 229533.I1RV91
141494 229533.I1RJU3 pp 229533.I1RXD9
141495 229533.I1RJU3 pp 229533.I1RTE9
141496 229533.I1RJU3 pp 229533.I1RXR1
141497 229533.I1RJU3 pp 229533.I1RSY2
141498 229533.I1RJU3 pp 229533.I1S0F8
141499 229533.I1RJU3 pp 229533.I1RKJ8
141500 229533.I1RJU3 pp 229533.I1RT03
141501 229533.I1RJU3 pp 229533.I1S2E9
141502 229533.I1RJU3 pp 229533.V6RUY9
141503 229533.I1RJU3 pp 229533.I1S8R3
141504 229533.I1RJU3 pp 229533.I1RVX0
141505 229533.I1RJU3 pp 229533.I1RT04
141506 229533.I1RJU3 pp 229533.I1RML0
141507 229533.I1RJU3 pp 229533.I1RTH6
141508 229533.I1RJU3 pp 229533.V6RRH2
141509 229533.I1RJU3 pp 229533.I1RKC6
141510 229533.I1RJU3 pp 229533.I1RKG2
141511 229533.I1RJU3 pp 229533.Q4HXF0
141512 229533.I1RJU3 pp 229533.I1RJV3
141513 229533.I1RJU3 pp 229533.I1RSX0
141514 229533.I1RJU3 pp 229533.I1RPL4
141515 229533.I1RJU3 pp 229533.I1RVQ3
141516 229533.I1RJU3 pp 229533.I1RWN6
141517 229533.I1RJU3 pp 229533.I1RQ33
141518 229533.I1RJU3 pp 229533.I1RQE7
141519 229533.I1RJU3 pp 229533.I1RXU9
141520 229533.I1RJU3 pp 229533.I1RRT2
141521 229533.I1RJU3 pp 229533.Q4I624
141522 229533.I1RJU3 pp 229533.I1S503
141523 229533.I1RJU3 pp 229533.Q4IPH4
141524 229533.I1RJU3 pp 229533.I1RRK3
141525 229533.I1RJU3 pp 229533.I1RN18
141526 229533.I1RJU3 pp 229533.I1S2M6
141527 229533.I1RJU3 pp 229533.I1S268
141528 229533.I1RJU3 pp 229533.I1RJW6
141529 229533.I1RJU3 pp 229533.I1RTD5
141530 229533.I1RJU3 pp 229533.Q4HVQ9
141531 229533.I1RJU3 pp 229533.V6R057
141532 229533.I1RJU3 pp 229533.O74638
141533 229533.I1RJU3 pp 229533.I1RRZ0
141534 229533.I1RJU3 pp 229533.I1RTA1
141535 229533.I1RJU3 pp 229533.I1RZB2
141536 229533.I1RJU3 pp 229533.I1RVZ2
141537 229533.I1RJU3 pp 229533.I1RKB2
141538 229533.I1RJU3 pp 229533.I1RJV4
141539 229533.I1RJU3 pp 229533.I1RXC2
141540 229533.I1RJU3 pp 229533.I1S5R1
141541 229533.I1RJU3 pp 229533.I1RYR0
141542 229533.I1RJU3 pp 229533.I1RWS4
141543 229533.I1RJU3 pp 229533.I1RRT8
141544 229533.I1RJU3 pp 229533.I1RQM7
141545 229533.I1RJU3 pp 229533.I1S9Y1
141546 229533.I1RJU3 pp 229533.I1RN63
141547 229533.I1RJU3 pp 229533.I1S0W4

141548 229533.I1RJU3 pp 229533.I1RKP1
141549 229533.I1RJU3 pp 229533.I1RRP3
141550 229533.I1RJU3 pp 229533.I1RRJ8
141551 229533.I1RJU3 pp 229533.I1RN67
141552 229533.I1RJU3 pp 229533.I1RWK3
141553 229533.I1RJU3 pp 229533.I1S9H4
141554 229533.I1RJU3 pp 229533.I1RRI1
141555 229533.I1RJU3 pp 229533.I1S8P5
141556 229533.I1RJU3 pp 229533.I1RSA5
141557 229533.I1RJU3 pp 229533.I1S0L4
141558 229533.I1RJU3 pp 229533.I1RS20
141559 229533.I1RJU3 pp 229533.I1RYI5
141560 229533.I1RJU3 pp 229533.I1RPP3
141561 229533.I1RJU3 pp 229533.I1S2H4
141562 229533.I1RJU3 pp 229533.I1S0F7
141563 229533.I1RJU3 pp 229533.I1RPE0
141564 229533.I1RJU3 pp 229533.I1S190
141565 229533.I1RJU3 pp 229533.I1RYP2
141566 229533.I1RJU3 pp 229533.I1RS47
141567 229533.I1RJU3 pp 229533.I1RQS8
141568 229533.I1RJU3 pp 229533.I1S2L3
141569 229533.I1RJU3 pp 229533.I1S464
141570 229533.I1RJU3 pp 229533.I1RPV3
141571 229533.I1RJU3 pp 229533.I1RL04
141572 229533.I1RJU3 pp 229533.I1RY62
141573 229533.I1RJU3 pp 229533.I1RZZ9
141574 229533.I1RJU3 pp 229533.I1S6Y8
141575 229533.I1RJU3 pp 229533.I1RSQ4
141576 229533.I1RJU3 pp 229533.I1RPM4
141577 229533.I1RJU3 pp 229533.V6RCG5
141578 229533.I1RJU3 pp 229533.I1RWI5
141579 229533.I1RJU3 pp 229533.I1S0F6
141580 229533.I1RJU3 pp 229533.I1RN88
141581 229533.I1RJU3 pp 229533.I1RQ23
141582 229533.I1RJU3 pp 229533.I1RR73
141583 229533.I1RJU3 pp 229533.I1RSZ7
141584 229533.I1RJU3 pp 229533.V6R3X5
141585 229533.I1RJU3 pp 229533.I1S821
141586 229533.I1RJU3 pp 229533.I1RW61
141587 229533.I1RJU3 pp 229533.I1RR51
141588 229533.I1RJU3 pp 229533.I1RTD3
141589 229533.I1RJU3 pp 229533.I1RRL9
141590 229533.I1RJU3 pp 229533.I1S0M6
141591 229533.I1RJU3 pp 229533.I1RZ39
141592 229533.I1RJU3 pp 229533.I1RTN3
141593 229533.I1RJU3 pp 229533.V6RCZ8
141594 229533.I1RJU3 pp 229533.I1RXC4
141595 229533.I1RJU3 pp 229533.I1S6K9
141596 229533.I1RJU3 pp 229533.I1S2B8
141597 229533.I1RJU3 pp 229533.I1RKD3
141598 229533.I1RJU3 pp 229533.I1S1B2
141599 229533.I1RJU3 pp 229533.I1RRM5
141600 229533.I1RJU3 pp 229533.Q4I8P5
141601 229533.I1RJU3 pp 229533.I1RJZ7
141602 229533.I1RJU3 pp 229533.V6QUE6
141603 229533.I1RJU3 pp 229533.I1RS18
141604 229533.I1RJU3 pp 229533.I1S4Z8
141605 229533.I1RJU3 pp 229533.I1RQ81
141606 229533.I1RJU3 pp 229533.I1RRV3
141607 229533.I1RJU3 pp 229533.I1RZM1
141608 229533.I1RJU3 pp 229533.I1RRV4
141609 229533.I1RJU3 pp 229533.I1S277
141610 229533.I1RJU3 pp 229533.I1RLU6
141611 229533.I1RJU3 pp 229533.I1S6B4
141612 229533.I1RJU3 pp 229533.I1RNQ5
141613 229533.I1RJU3 pp 229533.I1RW99
141614 229533.I1RJU3 pp 229533.I1S419
141615 229533.I1RJU3 pp 229533.I1RXF0
141616 229533.I1RJU3 pp 229533.I1S5B0
141617 229533.I1RJU3 pp 229533.I1RQ85
141618 229533.I1RJU3 pp 229533.I1RQD2
141619 229533.I1RJU3 pp 229533.I1RZM8
141620 229533.I1RJU3 pp 229533.I1RQ31

141621 229533.I1RJU3 pp 229533.I1SA32
141622 229533.I1RJU3 pp 229533.I1RMD5
141623 229533.I1RJU3 pp 229533.I1RNT9
141624 229533.I1RJU3 pp 229533.I1RWT6
141625 229533.I1RJU3 pp 229533.I1S1C9
141626 229533.I1RJU3 pp 229533.I1RMQ4
141627 229533.I1RJU3 pp 229533.I1RYB5
141628 229533.I1RJU3 pp 229533.I1S0K2
141629 229533.I1RJU3 pp 229533.Q4I283
141630 229533.I1RJU3 pp 229533.V6R227
141631 229533.I1RJU3 pp 229533.I1S887
141632 229533.I1RJU3 pp 229533.I1RQQ5
141633 229533.I1RJU3 pp 229533.I1S504
141634 229533.I1RJU3 pp 229533.V6RBN2
141635 229533.I1RJU3 pp 229533.I1RZK5
141636 229533.I1RJU3 pp 229533.I1S1J7
141637 229533.I1RJU3 pp 229533.I1RZZ1
141638 229533.I1RJU3 pp 229533.I1RWB0
141639 229533.I1RJU3 pp 229533.I1RY03
141640 229533.I1RJU3 pp 229533.I1RYS0
141641 229533.I1RJU3 pp 229533.I1RQA3
141642 229533.I1RJU3 pp 229533.I1RWD7
141643 229533.I1RJU3 pp 229533.Q4IJK2
141644 229533.I1RJU3 pp 229533.I1RYU0
141645 229533.I1RJU3 pp 229533.I1RPY3
141646 229533.I1RJU3 pp 229533.I1RK95
141647 229533.I1RJU3 pp 229533.I1RNB2
141648 229533.I1RJU3 pp 229533.I1RS29
141649 229533.I1RJU3 pp 229533.I1RQA2
141650 229533.I1RJU3 pp 229533.I1RYJ6
141651 229533.I1RJU3 pp 229533.V6R9Z0
141652 229533.I1RJU3 pp 229533.I1RUQ2
141653 229533.I1RJU3 pp 229533.I1RUQ3
141654 229533.I1RJU3 pp 229533.I1RLP1
141655 229533.I1RJU3 pp 229533.I1RSR3
141656 229533.I1RJU3 pp 229533.I1RN99
141657 229533.I1RJU3 pp 229533.I1RXD7
141658 229533.I1RJU3 pp 229533.I1RY63
141659 229533.I1RJU3 pp 229533.I1RRY9
141660 229533.I1RJU3 pp 229533.I1S4Q4
141661 229533.I1RJU3 pp 229533.I1RSI0
141662 229533.I1RJU3 pp 229533.V6RUT5
141663 229533.I1RJU3 pp 229533.I1RPR9
141664 229533.I1RJU3 pp 229533.I1RWR7
141665 229533.I1RJU3 pp 229533.I1RTB3
141666 229533.I1RJU3 pp 229533.Q4I2J8
141667 229533.I1RJU3 pp 229533.I1S7A8
141668 229533.I1RJU3 pp 229533.V6RTJ8
141669 229533.I1RJU3 pp 229533.I1S217
141670 229533.I1RJU3 pp 229533.Q4IJT5
141671 229533.I1RJU3 pp 229533.I1S0R7
141672 229533.I1RJU3 pp 229533.I1S3S3
141673 229533.I1RJU3 pp 229533.I1S6T4
141674 229533.I1RJU3 pp 229533.I1RWD4
141675 229533.I1RJU3 pp 229533.I1RP86
141676 229533.I1RJU3 pp 229533.I1RRG3
141677 229533.I1RJU3 pp 229533.I1RQC9
141678 229533.I1RJU3 pp 229533.I1S0L3
141679 229533.I1RJU3 pp 229533.I1RX46
141680 229533.I1RJU3 pp 229533.I1S0F5
141681 229533.I1RJU3 pp 229533.I1RX95
141682 229533.I1RJU3 pp 229533.I1RLX7
141683 229533.I1RJU3 pp 229533.V6R1K4
141684 229533.I1RJU3 pp 229533.I1RRX5
141685 229533.I1RJU3 pp 229533.I1S0T2
141686 229533.I1RJU3 pp 229533.I1RT33
141687 229533.I1RJU3 pp 229533.I1S4S4
141688 229533.I1RJU3 pp 229533.I1RSQ7
141689 229533.I1RJU3 pp 229533.I1RWJ8
141690 229533.I1RJU3 pp 229533.I1RY86
141691 229533.I1RJU3 pp 229533.V6QW22
141692 229533.I1RJU3 pp 229533.I1RR24
141693 229533.I1RJU3 pp 229533.I1RMC8

141694 229533.I1RJU3 pp 229533.I1RNM7
141695 229533.I1RJU3 pp 229533.I1RPN4
141696 229533.I1RJU3 pp 229533.I1S0F1
141697 229533.I1RJU3 pp 229533.I1RT05
141698 229533.I1RJU3 pp 229533.I1RPA4
141699 229533.I1RJU3 pp 229533.I1RW71
141700 229533.I1RJU3 pp 229533.I1S4S7
141701 229533.I1RJU3 pp 229533.I1S0X6
141702 229533.I1RJU3 pp 229533.I1RM06
141703 229533.I1RJU3 pp 229533.I1RVQ0
141704 229533.I1RJU3 pp 229533.I1S438
141705 229533.I1RJU3 pp 229533.I1RXD6
141706 229533.I1RJU3 pp 229533.I1RKJ5
141707 229533.I1RJU3 pp 229533.I1RMK3
141708 229533.I1RJU3 pp 229533.I1RX25
141709 229533.I1RJU3 pp 229533.I1RPF1
141710 229533.I1RJU3 pp 229533.V6QYF0
141711 229533.I1RJU3 pp 229533.I1RTF4
141712 229533.I1RJU3 pp 229533.I1RMQ6
141713 229533.I1RJU3 pp 229533.I1RW25
141714 229533.I1RJU3 pp 229533.I1RMG8
141715 229533.I1RJU3 pp 229533.I1RZJ5
141716 229533.I1RJU3 pp 229533.I1RNA0
141717 229533.I1RJU3 pp 229533.Q4IFI0
141718 229533.I1RJU3 pp 229533.I1S9I5
141719 229533.I1RJU3 pp 229533.I1RRS8
141720 229533.I1RZK5 pp 229533.I1S9U2
141721 229533.I1RZK5 pp 229533.Q4IJT5
141722 229533.I1RZK5 pp 229533.I1S0R7
141723 229533.I1RZK5 pp 229533.V6RUT5
141724 229533.I1RZK5 pp 229533.I1S2R0
141725 229533.I1RZK5 pp 229533.I1S0X6
141726 229533.I1RZK5 pp 229533.V6QW22
141727 229533.I1RZK5 pp 229533.I1S9I5
141728 229533.I1RZK5 pp 229533.I1S0T2
141729 229533.I1RZK5 pp 229533.I1S4S7
141730 229533.I1RZK5 pp 229533.I1S419
141731 229533.I1RZK5 pp 229533.I1S7A8
141732 229533.I1RZK5 pp 229533.I1S4S4
141733 229533.I1RZK5 pp 229533.V6RTJ8
141734 229533.I1RZK5 pp 229533.V6RBN2
141735 229533.I1RZK5 pp 229533.I1RZZ1
141736 229533.I1RZK5 pp 229533.Q4I283
141737 229533.I1RZK5 pp 229533.V6R4J1
141738 229533.I1RZK5 pp 229533.I1S0F5
141739 229533.I1RZK5 pp 229533.I1S0L3
141740 229533.I1RZK5 pp 229533.V6R1K4
141741 229533.I1RZK5 pp 229533.I1S504
141742 229533.I1RZK5 pp 229533.I1S1J7
141743 229533.I1RZK5 pp 229533.I1RZK6
141744 229533.I1RZK5 pp 229533.Q4IJK2
141745 229533.I1RZK5 pp 229533.I1RZM8
141746 229533.I1RZK5 pp 229533.Q4IJM4
141747 229533.I1RZK5 pp 229533.I1S021
141748 229533.I1RZK5 pp 229533.I1SA32
141749 229533.I1RZK5 pp 229533.I1S8J4
141750 229533.I1RZK5 pp 229533.Q4I2J8
141751 229533.I1RZK5 pp 229533.I1S821
141752 229533.I1RZK5 pp 229533.I1S887
141753 229533.I1RZK5 pp 229533.Q4I0C5
141754 229533.I1RZK5 pp 229533.V6RCG5
141755 229533.I1RZK5 pp 229533.Q4I8P5
141756 229533.I1RZK5 pp 229533.I1RZM1
141757 229533.I1RZK5 pp 229533.I1S8V3
141758 229533.I1RZK5 pp 229533.V6RCZ8
141759 229533.I1RZK5 pp 229533.I1S0M9
141760 229533.I1RZK5 pp 229533.I1S6K9
141761 229533.I1RZK5 pp 229533.I1S8P5
141762 229533.I1RZK5 pp 229533.V6R3X5
141763 229533.I1RZK5 pp 229533.I1RZZ9
141764 229533.I1RZK5 pp 229533.V6R283
141765 229533.I1RZK5 pp 229533.I1S1B2
141766 229533.I1RZK5 pp 229533.I1S5B0

141767 229533.I1RZK5 pp 229533.I1S0F6
141768 229533.I1RZK5 pp 229533.I1S5R1
141769 229533.I1RZK5 pp 229533.V6QUE6
141770 229533.I1RZK5 pp 229533.O74638
141771 229533.I1RZK5 pp 229533.I1S352
141772 229533.I1RZK5 pp 229533.I1S0W4
141773 229533.I1RZK5 pp 229533.I1RZS6
141774 229533.I1RZK5 pp 229533.I1S2G7
141775 229533.I1RZK5 pp 229533.I1S6Y8
141776 229533.I1RZK5 pp 229533.I1S9Y1
141777 229533.I1RZK5 pp 229533.I1S0F7
141778 229533.I1RZK5 pp 229533.I1S2B8
141779 229533.I1RZK5 pp 229533.I1S0L4
141780 229533.I1RZK5 pp 229533.I1S0M6
141781 229533.I1RZK5 pp 229533.I1S2H4
141782 229533.I1RZK5 pp 229533.V6R3J1
141783 229533.I1RZK5 pp 229533.V6RRH2
141784 229533.I1RZK5 pp 229533.I1S1W9
141785 229533.I1RZK5 pp 229533.I1S2E9
141786 229533.I1RZK5 pp 229533.V6RUY9
141787 229533.I1RZK5 pp 229533.I1S016
141788 229533.I1RZK5 pp 229533.V6RIV9
141789 229533.I1RZK5 pp 229533.Q4HVQ9
141790 229533.I1RZK5 pp 229533.Q4I624
141791 229533.I1RZK5 pp 229533.Q4IPH4
141792 229533.I1RZK5 pp 229533.I1S503
141793 229533.I1RZK5 pp 229533.I1S1W4
141794 229533.I1RZK5 pp 229533.I1S2E8
141795 229533.I1RZK5 pp 229533.I1S8V4
141796 229533.I1RZK5 pp 229533.V6R057
141797 229533.I1RZK5 pp 229533.I1S268
141798 229533.I1RZK5 pp 229533.I1RZW8
141799 229533.I1RZK5 pp 229533.I1S1C7
141800 229533.I1RZK5 pp 229533.I1S2C0
141801 229533.I1RZK5 pp 229533.Q4HXF6
141802 229533.I1RZK5 pp 229533.I1S569
141803 229533.I1RZK5 pp 229533.Q4I327
141804 229533.I1RZK5 pp 229533.Q4IN63
141805 229533.I1RZK5 pp 229533.I1RZV3
141806 229533.I1RZK5 pp 229533.I1S0F8
141807 229533.I1RZK5 pp 229533.I1S0J1
141808 229533.I1RZK5 pp 229533.I1S7W5
141809 229533.I1RZK5 pp 229533.V6R042
141810 229533.I1RZK5 pp 229533.I1S5R6
141811 229533.I1RZK5 pp 229533.V6RMW6
141812 229533.I1RZK5 pp 229533.I1S8R3
141813 229533.I1RZK5 pp 229533.I1S1W3
141814 229533.I1RZK5 pp 229533.I1SA33
141815 229533.I1RZK5 pp 229533.I1S0N4
141816 229533.I1RLJ1 pp 229533.V6RN31
141817 229533.A0A098DJU6 pp 229533.Q4IJ84
141818 229533.A0A098DJU6 pp 229533.I1RBN9
141819 229533.A0A098DJU6 pp 229533.I1RZP8
141820 229533.A0A098DJU6 pp 229533.I1S0D5
141821 229533.A0A098DJU6 pp 229533.I1RDX0
141822 229533.A0A098DJU6 pp 229533.I1RG77
141823 229533.A0A098DJU6 pp 229533.I1RN55
141824 229533.A0A098DJU6 pp 229533.A0A0E0RW49
141825 229533.A0A098DJU6 pp 229533.I1RQI6
141826 229533.A0A098DJU6 pp 229533.I1S461
141827 229533.A0A098DJU6 pp 229533.A0A098E1Q1
141828 229533.A0A098DJU6 pp 229533.I1RBW1
141829 229533.A0A098DJU6 pp 229533.I1RSC7
141830 229533.A0A098DJU6 pp 229533.Q4INS6
141831 229533.A0A098DJU6 pp 229533.I1RKB5
141832 229533.A0A098DJU6 pp 229533.I1RRS7
141833 229533.A0A098DJU6 pp 229533.I1RZ78
141834 229533.A0A098DJU6 pp 229533.A0A0E0SQG9
141835 229533.A0A098DJU6 pp 229533.I1R9W7
141836 229533.A0A098DJU6 pp 229533.I1RRT3
141837 229533.A0A098DJU6 pp 229533.I1RC54
141838 229533.A0A098DJU6 pp 229533.I1RJV8
141839 229533.A0A098DJU6 pp 229533.I1S209

141840 229533.A0A098DJU6 pp 229533.A0A1C3YI40
141841 229533.A0A098DJU6 pp 229533.I1RPQ6
141842 229533.A0A098DJU6 pp 229533.I1RYQ1
141843 229533.A0A098DJU6 pp 229533.I1RN57
141844 229533.A0A098DJU6 pp 229533.A0A1C3YMJ3
141845 229533.A0A098DJU6 pp 229533.A0A1C3YHM3
141846 229533.A0A098DJU6 pp 229533.I1RSA7
141847 229533.A0A098DJU6 pp 229533.A0A1C3YN69
141848 229533.A0A098DJU6 pp 229533.I1RAL9
141849 229533.A0A098DJU6 pp 229533.I1RSP7
141850 229533.A0A098DJU6 pp 229533.A0A098DR13
141851 229533.A0A098DJU6 pp 229533.I1RBW0
141852 229533.A0A098DJU6 pp 229533.I1RSN7
141853 229533.A0A098DJU6 pp 229533.I1RVW0
141854 229533.A0A098DJU6 pp 229533.A0A098E4E9
141855 229533.I1RAM6 pp 229533.I1RPU3
141856 229533.I1RAM6 pp 229533.I1S1V5
141857 229533.I1RAM6 pp 229533.I1RJZ7
141858 229533.I1RAM6 pp 229533.I1RBY4
141859 229533.I1RAM6 pp 229533.I1RKI0
141860 229533.I1RAM6 pp 229533.I1S2G9
141861 229533.I1RAM6 pp 229533.I1S0F9
141862 229533.I1RAM6 pp 229533.I1RZZ9
141863 229533.I1RAM6 pp 229533.I1RCQ3
141864 229533.I1RAM6 pp 229533.I1RC00
141865 229533.I1RAM6 pp 229533.I1RAN2
141866 229533.I1RAM6 pp 229533.I1RS20
141867 229533.I1RAM6 pp 229533.I1RR73
141868 229533.I1RAM6 pp 229533.I1RB74
141869 229533.I1RAM6 pp 229533.I1RRT8
141870 229533.I1RAM6 pp 229533.I1S8P5
141871 229533.I1RAM6 pp 229533.I1RRM9
141872 229533.I1RAM6 pp 229533.I1RPP3
141873 229533.I1RAM6 pp 229533.I1RM82
141874 229533.I1RAM6 pp 229533.I1RQG4
141875 229533.I1RAM6 pp 229533.I1REC6
141876 229533.I1RAM6 pp 229533.I1S2V2
141877 229533.I1RAM6 pp 229533.I1RCC5
141878 229533.I1RAM6 pp 229533.I1RBS0
141879 229533.I1RAM6 pp 229533.I1RP28
141880 229533.I1RAM6 pp 229533.I1RNG4
141881 229533.I1RAM6 pp 229533.I1RPM4
141882 229533.I1RAM6 pp 229533.I1RWK9
141883 229533.I1RAM6 pp 229533.I1RCJ7
141884 229533.I1RAM6 pp 229533.I1RME4
141885 229533.I1RAM6 pp 229533.I1RN67
141886 229533.I1RAM6 pp 229533.V6RRH2
141887 229533.I1RAM6 pp 229533.I1RS47
141888 229533.I1RAM6 pp 229533.I1S5S8
141889 229533.I1RAM6 pp 229533.I1RSH0
141890 229533.I1RAM6 pp 229533.I1RC97
141891 229533.I1RAM6 pp 229533.I1RAU7
141892 229533.I1RAM6 pp 229533.I1RYW3
141893 229533.I1RAM6 pp 229533.I1RT04
141894 229533.I1RAM6 pp 229533.I1RUG3
141895 229533.I1RAM6 pp 229533.I1S0M2
141896 229533.I1RAM6 pp 229533.I1RJS2
141897 229533.I1RAM6 pp 229533.I1S238
141898 229533.I1RAM6 pp 229533.I1RCL5
141899 229533.I1RAM6 pp 229533.I1RMQ5
141900 229533.I1RAM6 pp 229533.I1S9U6
141901 229533.I1RAM6 pp 229533.I1RTH6
141902 229533.I1RAM6 pp 229533.I1RP27
141903 229533.I1RAM6 pp 229533.I1RBS6
141904 229533.I1RAM6 pp 229533.I1RBS5
141905 229533.I1RAM6 pp 229533.I1RSK1
141906 229533.I1RAM6 pp 229533.V6RMW6
141907 229533.I1RAM6 pp 229533.I1RZE4
141908 229533.I1RAM6 pp 229533.I1RJV3
141909 229533.I1RAM6 pp 229533.I1RZ95
141910 229533.I1RAM6 pp 229533.I1RFL4
141911 229533.I1RAM6 pp 229533.I1REC7
141912 229533.I1RAM6 pp 229533.I1RQF2

141913	229533.I1RAM6	pp	229533.I1REG1
141914	229533.I1RAM6	pp	229533.I1RIG5
141915	229533.I1RAM6	pp	229533.I1RRV1
141916	229533.I1RAM6	pp	229533.I1RN61
141917	229533.I1RAM6	pp	229533.I1RJS3
141918	229533.I1RAM6	pp	229533.I1RXT9
141919	229533.I1RAM6	pp	229533.I1RSQ7
141920	229533.I1RAM6	pp	229533.I1RMQ3
141921	229533.I1RAM6	pp	229533.I1RD22
141922	229533.I1RAM6	pp	229533.I1RBC9
141923	229533.I1RAM6	pp	229533.I1RWH4
141924	229533.I1RAM6	pp	229533.I1RTA0
141925	229533.I1RAM6	pp	229533.I1RQ21
141926	229533.I1RAM6	pp	229533.I1RW71
141927	229533.I1RAM6	pp	229533.I1RNC1
141928	229533.I1RAM6	pp	229533.I1S0N4
141929	229533.I1RAM6	pp	229533.I1S0Z8
141930	229533.I1RAM6	pp	229533.I1RVQ0
141931	229533.I1RAM6	pp	229533.I1RT41
141932	229533.I1RAM6	pp	229533.I1S0L3
141933	229533.I1RAM6	pp	229533.I1RPN8
141934	229533.I1RAM6	pp	229533.I1RF94
141935	229533.I1RAM6	pp	229533.I1RQQ8
141936	229533.I1RAM6	pp	229533.I1RTU8
141937	229533.I1RAM6	pp	229533.I1RNY7
141938	229533.I1RAM6	pp	229533.I1RZ02
141939	229533.I1RAM6	pp	229533.I1RDI8
141940	229533.I1RAM6	pp	229533.I1RYH9
141941	229533.I1RAM6	pp	229533.I1RW25
141942	229533.I1RAM6	pp	229533.I1RJT9
141943	229533.I1RAM6	pp	229533.I1RDB1
141944	229533.I1RAM6	pp	229533.V6QWB1
141945	229533.I1RAM6	pp	229533.I1RQC9
141946	229533.I1RAM6	pp	229533.I1RRT6
141947	229533.I1RAM6	pp	229533.I1RAW7
141948	229533.I1RAM6	pp	229533.I1RQ26
141949	229533.I1RAM6	pp	229533.I1RX46
141950	229533.I1RAM6	pp	229533.V6RTJ8
141951	229533.I1RAM6	pp	229533.I1RM03
141952	229533.I1RAM6	pp	229533.I1RQH1
141953	229533.I1RAM6	pp	229533.I1RXT0
141954	229533.I1RAM6	pp	229533.I1RQA5
141955	229533.I1RAM6	pp	229533.I1RDG9
141956	229533.I1RAM6	pp	229533.I1RZB5
141957	229533.I1RAM6	pp	229533.I1RBC4
141958	229533.I1RAM6	pp	229533.I1RAX6
141959	229533.I1RAM6	pp	229533.I1S0Z3
141960	229533.I1RAM6	pp	229533.I1RZZ1
141961	229533.I1RAM6	pp	229533.I1RZM1
141962	229533.I1RAM6	pp	229533.I1RN08
141963	229533.I1RAM6	pp	229533.I1RCG4
141964	229533.I1RAM6	pp	229533.I1RW87
141965	229533.I1RAM6	pp	229533.Q4ICA8
141966	229533.I1RAM6	pp	229533.V6RBN2
141967	229533.I1RAM6	pp	229533.I1RKK8
141968	229533.I1RAM6	pp	229533.I1RG60
141969	229533.I1RAM6	pp	229533.I1RRY9
141970	229533.I1RAM6	pp	229533.I1RMM9
141971	229533.I1RAM6	pp	229533.I1RQQ6
141972	229533.I1RAM6	pp	229533.I1RAM7
141973	229533.I1RAM6	pp	229533.I1S439
141974	229533.I1RAM6	pp	229533.I1RZH4
141975	229533.I1RAM6	pp	229533.I1S235
141976	229533.I1RAM6	pp	229533.I1RM35
141977	229533.I1RAM6	pp	229533.I1RPL2
141978	229533.I1RAM6	pp	229533.I1RBE4
141979	229533.I1RAM6	pp	229533.I1RB75
141980	229533.I1RAM6	pp	229533.I1RC95
141981	229533.I1RAM6	pp	229533.I1RAU5
141982	229533.I1RAM6	pp	229533.I1RYL5
141983	229533.I1RAM6	pp	229533.I1RQ81
141984	229533.I1RAM6	pp	229533.I1RP66
141985	229533.I1RAM6	pp	229533.I1S5B0

141986 229533.I1RAM6 pp 229533.I1RC22
141987 229533.I1RAM6 pp 229533.I1RW99
141988 229533.I1RAM6 pp 229533.I1RKK0
141989 229533.I1RAM6 pp 229533.I1RYJ8
141990 229533.I1S461 pp 229533.V6R382
141991 229533.I1S461 pp 229533.Q4IP51
141992 229533.I1S461 pp 229533.Q4IA62
141993 229533.I1S461 pp 229533.Q4IC11
141994 229533.I1S461 pp 229533.I1S998
141995 229533.I1S461 pp 229533.Q4I5V3
141996 229533.I1S461 pp 229533.V6RGI1
141997 229533.I1S461 pp 229533.V6RLW4
141998 229533.I1S461 pp 229533.Q4IEA5
141999 229533.I1S461 pp 229533.I1S987
142000 229533.I1S461 pp 229533.Q4INW6
142001 229533.I1S461 pp 229533.Q4IQM6
142002 229533.A0A1C3YHX8 pp 229533.I1RLP1
142003 229533.A0A1C3YHX8 pp 229533.I1RXD7
142004 229533.A0A1C3YHX8 pp 229533.A0A1C3YL54
142005 229533.A0A1C3YHX8 pp 229533.I1RPY3
142006 229533.A0A1C3YHX8 pp 229533.I1RAX6
142007 229533.A0A1C3YHX8 pp 229533.I1RIL8
142008 229533.A0A1C3YHX8 pp 229533.V6RTJ8
142009 229533.A0A1C3YHX8 pp 229533.I1RRG3
142010 229533.A0A1C3YHX8 pp 229533.I1RFW5
142011 229533.A0A1C3YHX8 pp 229533.I1S7A8
142012 229533.A0A1C3YHX8 pp 229533.Q4IJT5
142013 229533.A0A1C3YHX8 pp 229533.I1S0T2
142014 229533.A0A1C3YHX8 pp 229533.I1RD52
142015 229533.A0A1C3YHX8 pp 229533.I1RX46
142016 229533.A0A1C3YHX8 pp 229533.A0A1C3YLL3
142017 229533.A0A1C3YHX8 pp 229533.I1S4S7
142018 229533.A0A1C3YHX8 pp 229533.A0A1C3YK13
142019 229533.A0A1C3YHX8 pp 229533.I1RA85
142020 229533.A0A1C3YHX8 pp 229533.V6R1K4
142021 229533.A0A1C3YHX8 pp 229533.I1RBC9
142022 229533.A0A1C3YHX8 pp 229533.I1RW71
142023 229533.A0A1C3YHX8 pp 229533.I1S0F5
142024 229533.A0A1C3YHX8 pp 229533.I1RR00
142025 229533.A0A1C3YHX8 pp 229533.I1S0L3
142026 229533.A0A1C3YHX8 pp 229533.I1RQJ5
142027 229533.A0A1C3YHX8 pp 229533.I1RG53
142028 229533.A0A1C3YHX8 pp 229533.A0A1C3YIP5
142029 229533.A0A1C3YHX8 pp 229533.V6R4J1
142030 229533.A0A1C3YHX8 pp 229533.I1RC20
142031 229533.A0A1C3YHX8 pp 229533.I1RQC9
142032 229533.A0A1C3YHX8 pp 229533.I1RN61
142033 229533.A0A1C3YHX8 pp 229533.I1RVQ0
142034 229533.A0A1C3YHX8 pp 229533.I1RKB7
142035 229533.A0A1C3YHX8 pp 229533.I1S0R7
142036 229533.A0A1C3YHX8 pp 229533.V6QW22
142037 229533.A0A1C3YHX8 pp 229533.I1RQH3
142038 229533.A0A1C3YHX8 pp 229533.V6RUT5
142039 229533.A0A1C3YHX8 pp 229533.I1RT80
142040 229533.A0A1C3YHX8 pp 229533.I1RRX5
142041 229533.A0A1C3YHX8 pp 229533.A0A1C3YIY6
142042 229533.A0A1C3YHX8 pp 229533.I1RJU3
142043 229533.A0A1C3YHX8 pp 229533.I1RSQ7
142044 229533.A0A1C3YHX8 pp 229533.A0A1C3YLU7
142045 229533.A0A1C3YHX8 pp 229533.I1RR51
142046 229533.A0A1C3YHX8 pp 229533.I1RIG2
142047 229533.A0A1C3YHX8 pp 229533.A0A1C3YN30
142048 229533.A0A1C3YHX8 pp 229533.I1RX95
142049 229533.A0A1C3YHX8 pp 229533.I1S6U8
142050 229533.A0A1C3YHX8 pp 229533.I1RBD5
142051 229533.A0A1C3YHX8 pp 229533.A0A1C3YI23
142052 229533.A0A1C3YHX8 pp 229533.V6RCG5
142053 229533.A0A1C3YHX8 pp 229533.A0A1C3YM63
142054 229533.A0A1C3YHX8 pp 229533.A0A1C3YKC0
142055 229533.A0A1C3YHX8 pp 229533.I1RBT1
142056 229533.A0A1C3YHX8 pp 229533.I1RPU0
142057 229533.A0A1C3YHX8 pp 229533.I1RXF0
142058 229533.A0A1C3YHX8 pp 229533.I1S0X6

142059 229533.A0A1C3YHX8 pp 229533.I1RFQ7
142060 229533.A0A1C3YHX8 pp 229533.I1R9Y4
142061 229533.A0A1C3YHX8 pp 229533.I1RZM8
142062 229533.A0A1C3YHX8 pp 229533.I1RMG8
142063 229533.A0A1C3YHX8 pp 229533.I1RI82
142064 229533.A0A1C3YHX8 pp 229533.I1RAM6
142065 229533.A0A1C3YHX8 pp 229533.I1RMC8
142066 229533.A0A1C3YHX8 pp 229533.I1RM67
142067 229533.A0A1C3YHX8 pp 229533.I1RR24
142068 229533.A0A1C3YHX8 pp 229533.I1S021
142069 229533.A0A1C3YHX8 pp 229533.I1RZJ5
142070 229533.A0A1C3YHX8 pp 229533.I1RNH6
142071 229533.A0A1C3YHX8 pp 229533.A0A1C3YI30
142072 229533.A0A1C3YHX8 pp 229533.I1S9U2
142073 229533.A0A1C3YHX8 pp 229533.V6QUW4
142074 229533.A0A1C3YHX8 pp 229533.I1RWJ8
142075 229533.A0A1C3YHX8 pp 229533.I1RC17
142076 229533.A0A1C3YHX8 pp 229533.A0A1C3YLZ7
142077 229533.A0A1C3YHX8 pp 229533.I1RUY5
142078 229533.A0A1C3YHX8 pp 229533.A0A1C3YJH8
142079 229533.A0A1C3YHX8 pp 229533.I1RM06
142080 229533.A0A1C3YHX8 pp 229533.I1S4S4
142081 229533.A0A1C3YHX8 pp 229533.A0A1C3YJV6
142082 229533.A0A1C3YHX8 pp 229533.I1S5B0
142083 229533.A0A1C3YHX8 pp 229533.I1RCS1
142084 229533.A0A1C3YHX8 pp 229533.I1RI02
142085 229533.A0A1C3YHX8 pp 229533.I1RW99
142086 229533.A0A1C3YHX8 pp 229533.I1RZF6
142087 229533.A0A1C3YHX8 pp 229533.I1RW07
142088 229533.A0A1C3YHX8 pp 229533.A0A1C3YJR3
142089 229533.A0A1C3YHX8 pp 229533.I1S504
142090 229533.A0A1C3YHX8 pp 229533.V6QUE6
142091 229533.A0A1C3YHX8 pp 229533.I1RLC7
142092 229533.A0A1C3YHX8 pp 229533.A0A1C3YJQ9
142093 229533.A0A1C3YHX8 pp 229533.I1RRU9
142094 229533.A0A1C3YHX8 pp 229533.I1RX25
142095 229533.A0A1C3YHX8 pp 229533.I1RFT2
142096 229533.A0A1C3YHX8 pp 229533.I1RW25
142097 229533.A0A1C3YHX8 pp 229533.A0A1C3YKB0
142098 229533.A0A1C3YHX8 pp 229533.Q4I283
142099 229533.A0A1C3YHX8 pp 229533.I1RAU5
142100 229533.A0A1C3YHX8 pp 229533.A0A1C3YKV3
142101 229533.A0A1C3YHX8 pp 229533.I1S0M9
142102 229533.A0A1C3YHX8 pp 229533.I1S6K9
142103 229533.A0A1C3YHX8 pp 229533.I1RBE4
142104 229533.A0A1C3YHX8 pp 229533.I1RQ81
142105 229533.A0A1C3YHX8 pp 229533.I1RB27
142106 229533.A0A1C3YHX8 pp 229533.I1RS18
142107 229533.A0A1C3YHX8 pp 229533.A0A1C3YKW9
142108 229533.A0A1C3YHX8 pp 229533.V6RCZ8
142109 229533.A0A1C3YHX8 pp 229533.I1RQ85
142110 229533.A0A1C3YHX8 pp 229533.I1RGC5
142111 229533.A0A1C3YHX8 pp 229533.I1RWT6
142112 229533.A0A1C3YHX8 pp 229533.Q4I8P5
142113 229533.A0A1C3YHX8 pp 229533.A0A1C3YJW9
142114 229533.A0A1C3YHX8 pp 229533.I1RXC4
142115 229533.A0A1C3YHX8 pp 229533.I1RG14
142116 229533.A0A1C3YHX8 pp 229533.I1S419
142117 229533.A0A1C3YHX8 pp 229533.I1RWB0
142118 229533.A0A1C3YHX8 pp 229533.I1RLU6
142119 229533.A0A1C3YHX8 pp 229533.I1RNQ5
142120 229533.A0A1C3YHX8 pp 229533.A0A1C3YK34
142121 229533.A0A1C3YHX8 pp 229533.I1SA32
142122 229533.A0A1C3YHX8 pp 229533.I1RAN5
142123 229533.A0A1C3YHX8 pp 229533.A0A1C3YIJ3
142124 229533.A0A1C3YHX8 pp 229533.A0A1C3YKY6
142125 229533.A0A1C3YHX8 pp 229533.I1RF19
142126 229533.A0A1C3YHX8 pp 229533.A0A1C3YJ21
142127 229533.A0A1C3YHX8 pp 229533.I1RBJ5
142128 229533.A0A1C3YHX8 pp 229533.A0A1C3YJN3
142129 229533.A0A1C3YHX8 pp 229533.I1RI03
142130 229533.A0A1C3YHX8 pp 229533.I1RZM1
142131 229533.A0A1C3YHX8 pp 229533.A0A1C3YIE7

142132 229533.A0A1C3YHX8 pp 229533.A0A1C3YJ93
142133 229533.A0A1C3YHX8 pp 229533.I1RKG2
142134 229533.A0A1C3YHX8 pp 229533.I1RNA5
142135 229533.A0A1C3YHX8 pp 229533.I1RFU8
142136 229533.A0A1C3YHX8 pp 229533.I1S1J7
142137 229533.A0A1C3YHX8 pp 229533.I1RBU8
142138 229533.A0A1C3YHX8 pp 229533.A0A1C3YM60
142139 229533.A0A1C3YHX8 pp 229533.I1R9V7
142140 229533.A0A1C3YHX8 pp 229533.V6RUY9
142141 229533.A0A1C3YHX8 pp 229533.I1RCG4
142142 229533.A0A1C3YHX8 pp 229533.I1S887
142143 229533.A0A1C3YHX8 pp 229533.Q4IJM4
142144 229533.A0A1C3YHX8 pp 229533.I1RSR8
142145 229533.A0A1C3YHX8 pp 229533.I1RT04
142146 229533.A0A1C3YHX8 pp 229533.I1RDU0
142147 229533.A0A1C3YHX8 pp 229533.I1S2E9
142148 229533.A0A1C3YHX8 pp 229533.I1RA48
142149 229533.A0A1C3YHX8 pp 229533.I1RY03
142150 229533.A0A1C3YHX8 pp 229533.I1RK95
142151 229533.A0A1C3YHX8 pp 229533.I1RYY5
142152 229533.A0A1C3YHX8 pp 229533.I1RV91
142153 229533.A0A1C3YHX8 pp 229533.I1RQA3
142154 229533.A0A1C3YHX8 pp 229533.A0A1C3YJI6
142155 229533.A0A1C3YHX8 pp 229533.I1RT40
142156 229533.A0A1C3YHX8 pp 229533.A0A1C3YLK2
142157 229533.A0A1C3YHX8 pp 229533.I1RBL7
142158 229533.A0A1C3YHX8 pp 229533.Q4IJK2
142159 229533.A0A1C3YHX8 pp 229533.I1RPR9
142160 229533.A0A1C3YHX8 pp 229533.I1RRY9
142161 229533.A0A1C3YHX8 pp 229533.I1RCJ7
142162 229533.A0A1C3YHX8 pp 229533.I1RAF8
142163 229533.A0A1C3YHX8 pp 229533.I1RGF5
142164 229533.A0A1C3YHX8 pp 229533.I1RZK6
142165 229533.A0A1C3YHX8 pp 229533.I1RTD5
142166 229533.A0A1C3YHX8 pp 229533.I1RU29
142167 229533.A0A1C3YHX8 pp 229533.I1RZZ1
142168 229533.A0A1C3YHX8 pp 229533.I1RWR7
142169 229533.A0A1C3YHX8 pp 229533.A0A1C3YLA6
142170 229533.A0A1C3YHX8 pp 229533.I1RBS6
142171 229533.A0A1C3YHX8 pp 229533.I1RBC7
142172 229533.A0A1C3YHX8 pp 229533.I1RQQ5
142173 229533.A0A1C3YHX8 pp 229533.I1RGA4
142174 229533.A0A1C3YHX8 pp 229533.I1RP27
142175 229533.A0A1C3YHX8 pp 229533.I1RB74
142176 229533.A0A1C3YHX8 pp 229533.I1RWF7
142177 229533.A0A1C3YHX8 pp 229533.V6RBN2
142178 229533.A0A1C3YHX8 pp 229533.I1RPP3
142179 229533.A0A1C3YHX8 pp 229533.I1RJV4
142180 229533.A0A1C3YHX8 pp 229533.I1RJZ6
142181 229533.A0A1C3YHX8 pp 229533.I1RLN6
142182 229533.A0A1C3YHX8 pp 229533.I1RB75
142183 229533.A0A1C3YHX8 pp 229533.I1S8P5
142184 229533.A0A1C3YHX8 pp 229533.I1RLV9
142185 229533.A0A1C3YHX8 pp 229533.I1RCR5
142186 229533.A0A1C3YHX8 pp 229533.A0A1C3YLK8
142187 229533.A0A1C3YHX8 pp 229533.I1REK8
142188 229533.A0A1C3YHX8 pp 229533.I1RJW6
142189 229533.A0A1C3YHX8 pp 229533.I1S503
142190 229533.A0A1C3YHX8 pp 229533.I1RQG4
142191 229533.A0A1C3YHX8 pp 229533.I1RVZ0
142192 229533.A0A1C3YHX8 pp 229533.I1RRJ8
142193 229533.A0A1C3YHX8 pp 229533.I1RN63
142194 229533.A0A1C3YHX8 pp 229533.I1RSX0
142195 229533.A0A1C3YHX8 pp 229533.Q4I2J8
142196 229533.A0A1C3YHX8 pp 229533.V6R3J1
142197 229533.A0A1C3YHX8 pp 229533.I1RWK3
142198 229533.A0A1C3YHX8 pp 229533.I1RZ35
142199 229533.A0A1C3YHX8 pp 229533.V6R283
142200 229533.A0A1C3YHX8 pp 229533.I1RLM7
142201 229533.A0A1C3YHX8 pp 229533.I1S352
142202 229533.A0A1C3YHX8 pp 229533.I1RC03
142203 229533.A0A1C3YHX8 pp 229533.I1RZE5
142204 229533.A0A1C3YHX8 pp 229533.I1RTA1

229533.A0A1C3YHX8	pp	229533.I1RKL3
229533.A0A1C3YHX8	pp	229533.I1RFT1
229533.A0A1C3YHX8	pp	229533.I1RJN9
229533.A0A1C3YHX8	pp	229533.I1RSI0
229533.A0A1C3YHX8	pp	229533.I1RVZ2
229533.A0A1C3YHX8	pp	229533.I1RS47
229533.A0A1C3YHX8	pp	229533.A0A1C3YKN3
229533.A0A1C3YHX8	pp	229533.I1RSV2
229533.A0A1C3YHX8	pp	229533.I1RYI5
229533.A0A1C3YHX8	pp	229533.I1RE00
229533.A0A1C3YHX8	pp	229533.A0A1C3YMA1
229533.A0A1C3YHX8	pp	229533.A0A1C3YIR8
229533.A0A1C3YHX8	pp	229533.A0A1C3YIS7
229533.A0A1C3YHX8	pp	229533.I1RC66
229533.A0A1C3YHX8	pp	229533.I1R9M0
229533.A0A1C3YHX8	pp	229533.I1RQ65
229533.A0A1C3YHX8	pp	229533.I1S8V3
229533.A0A1C3YHX8	pp	229533.I1RJZ7
229533.A0A1C3YHX8	pp	229533.I1RNN1
229533.A0A1C3YHX8	pp	229533.I1S2G7
229533.A0A1C3YHX8	pp	229533.I1S2B8
229533.A0A1C3YHX8	pp	229533.I1S0W4
229533.A0A1C3YHX8	pp	229533.A0A1C3YI20
229533.A0A1C3YHX8	pp	229533.I1RFT6
229533.A0A1C3YHX8	pp	229533.I1REC5
229533.A0A1C3YHX8	pp	229533.I1RRM5
229533.A0A1C3YHX8	pp	229533.I1RD64
229533.A0A1C3YHX8	pp	229533.I1RS20
229533.A0A1C3YHX8	pp	229533.I1RN67
229533.A0A1C3YHX8	pp	229533.I1RPU3
229533.A0A1C3YHX8	pp	229533.I1RDF7
229533.A0A1C3YHX8	pp	229533.I1RPE0
229533.A0A1C3YHX8	pp	229533.I1S0F6
229533.A0A1C3YHX8	pp	229533.I1RE76
229533.A0A1C3YHX8	pp	229533.I1RBY4
229533.A0A1C3YHX8	pp	229533.I1RRQ3
229533.A0A1C3YHX8	pp	229533.I1S2H4
229533.A0A1C3YHX8	pp	229533.I1RMG3
229533.A0A1C3YHX8	pp	229533.I1S0L4
229533.A0A1C3YHX8	pp	229533.I1RBS0
229533.A0A1C3YHX8	pp	229533.I1RJP4
229533.A0A1C3YHX8	pp	229533.I1RZ39
229533.A0A1C3YHX8	pp	229533.A0A1C3YLS9
229533.A0A1C3YHX8	pp	229533.I1RRI1
229533.A0A1C3YHX8	pp	229533.I1RRV1
229533.A0A1C3YHX8	pp	229533.A0A1C3YKM9
229533.A0A1C3YHX8	pp	229533.A0A1C3YK73
229533.A0A1C3YHX8	pp	229533.A0A1C3YIN1
229533.A0A1C3YHX8	pp	229533.I1RR73
229533.A0A1C3YHX8	pp	229533.I1RIL3
229533.A0A1C3YHX8	pp	229533.I1RSQ3
229533.A0A1C3YHX8	pp	229533.I1RKP1
229533.A0A1C3YHX8	pp	229533.I1S9Y1
229533.A0A1C3YHX8	pp	229533.V6R3X5
229533.A0A1C3YHX8	pp	229533.V6R042
229533.A0A1C3YHX8	pp	229533.I1RWI5
229533.A0A1C3YHX8	pp	229533.I1RM82
229533.A0A1C3YHX8	pp	229533.I1RRM3
229533.A0A1C3YHX8	pp	229533.I1RKJ6
229533.A0A1C3YHX8	pp	229533.I1S5R1
229533.A0A1C3YHX8	pp	229533.I1RZZ9
229533.A0A1C3YHX8	pp	229533.V6RMW6
229533.A0A1C3YHX8	pp	229533.I1RSQ4
229533.A0A1C3YHX8	pp	229533.I1RPK0
229533.A0A1C3YHX8	pp	229533.I1RS17
229533.A0A1C3YHX8	pp	229533.I1RRT8
229533.A0A1C3YHX8	pp	229533.O74638
229533.A0A1C3YHX8	pp	229533.I1RNC1
229533.A0A1C3YHX8	pp	229533.I1RXC2
229533.A0A1C3YHX8	pp	229533.I1RNB8
229533.A0A1C3YHX8	pp	229533.I1RUG3
229533.A0A1C3YHX8	pp	229533.I1RCR8
229533.A0A1C3YHX8	pp	229533.I1RPP4

229533.A0A1C3YHX8	pp	229533.I1RWL2
229533.A0A1C3YHX8	pp	229533.I1RE79
229533.A0A1C3YHX8	pp	229533.I1RA11
229533.A0A1C3YHX8	pp	229533.I1RE22
229533.A0A1C3YHX8	pp	229533.I1RMV0
229533.A0A1C3YHX8	pp	229533.I1RW46
229533.A0A1C3YHX8	pp	229533.Q4IN63
229533.A0A1C3YHX8	pp	229533.I1RE01
229533.A0A1C3YHX8	pp	229533.A0A1C3YLB6
229533.A0A1C3YHX8	pp	229533.I1RTA0
229533.A0A1C3YHX8	pp	229533.A0A1C3YJD4
229533.A0A1C3YHX8	pp	229533.I1RBG9
229533.A0A1C3YHX8	pp	229533.I1S0M6
229533.A0A1C3YHX8	pp	229533.Q4I327
229533.A0A1C3YHX8	pp	229533.I1RZS6
229533.A0A1C3YHX8	pp	229533.I1RNY2
229533.A0A1C3YHX8	pp	229533.A0A1C3YLC1
229533.A0A1C3YHX8	pp	229533.I1S7W5
229533.A0A1C3YHX8	pp	229533.A0A1C3YMI5
229533.A0A1C3YHX8	pp	229533.I1RSZ7
229533.A0A1C3YHX8	pp	229533.I1RQ23
229533.A0A1C3YHX8	pp	229533.I1S0J1
229533.A0A1C3YHX8	pp	229533.I1S5R6
229533.A0A1C3YHX8	pp	229533.I1RRE5
229533.A0A1C3YHX8	pp	229533.I1RL04
229533.A0A1C3YHX8	pp	229533.I1S0N4
229533.A0A1C3YHX8	pp	229533.I1RPV3
229533.A0A1C3YHX8	pp	229533.I1RG11
229533.A0A1C3YHX8	pp	229533.I1S1W4
229533.A0A1C3YHX8	pp	229533.I1RPM4
229533.A0A1C3YHX8	pp	229533.I1R9D1
229533.A0A1C3YHX8	pp	229533.I1RRR7
229533.A0A1C3YHX8	pp	229533.I1S6Y8
229533.A0A1C3YHX8	pp	229533.I1RMN6
229533.A0A1C3YHX8	pp	229533.I1RFS3
229533.A0A1C3YHX8	pp	229533.I1RSF1
229533.A0A1C3YHX8	pp	229533.I1SA33
229533.A0A1C3YHX8	pp	229533.I1S8V4
229533.A0A1C3YHX8	pp	229533.Q4I662
229533.A0A1C3YHX8	pp	229533.I1RRN2
229533.A0A1C3YHX8	pp	229533.I1RD22
229533.A0A1C3YHX8	pp	229533.I1S2C0
229533.A0A1C3YHX8	pp	229533.I1RPU2
229533.A0A1C3YHX8	pp	229533.I1RWN5
229533.A0A1C3YHX8	pp	229533.I1RKG3
229533.A0A1C3YHX8	pp	229533.I1RCH5
229533.A0A1C3YHX8	pp	229533.V6RIV9
229533.A0A1C3YHX8	pp	229533.I1RYW3
229533.A0A1C3YHX8	pp	229533.I1RQE7
229533.A0A1C3YHX8	pp	229533.I1RTD7
229533.A0A1C3YHX8	pp	229533.I1S1C7
229533.A0A1C3YHX8	pp	229533.I1RWX5
229533.A0A1C3YHX8	pp	229533.I1S1W9
229533.A0A1C3YHX8	pp	229533.I1S2E8
229533.A0A1C3YHX8	pp	229533.I1RM96
229533.A0A1C3YHX8	pp	229533.I1RC33
229533.A0A1C3YHX8	pp	229533.I1RRI2
229533.A0A1C3YHX8	pp	229533.I1S569
229533.A0A1C3YHX8	pp	229533.I1RZW8
229533.A0A1C3YHX8	pp	229533.I1RXU9
229533.A0A1C3YHX8	pp	229533.I1RY44
229533.A0A1C3YHX8	pp	229533.I1S1W3
229533.A0A1C3YHX8	pp	229533.V6R057
229533.A0A1C3YHX8	pp	229533.I1RD17
229533.A0A1C3YHX8	pp	229533.I1RSA4
229533.A0A1C3YHX8	pp	229533.I1RZV3
229533.A0A1C3YHX8	pp	229533.I1RD39
229533.A0A1C3YHX8	pp	229533.I1RWN6
229533.A0A1C3YHX8	pp	229533.A0A1C3YK33
229533.A0A1C3YHX8	pp	229533.Q4I624
229533.A0A1C3YHX8	pp	229533.I1RAW0
229533.A0A1C3YHX8	pp	229533.I1RAU7
229533.A0A1C3YHX8	pp	229533.I1RCM3

142351 229533.A0A1C3YHX8 pp 229533.A0A1C3YIN6
142352 229533.A0A1C3YHX8 pp 229533.I1RSK1
142353 229533.A0A1C3YHX8 pp 229533.I1RQ33
142354 229533.A0A1C3YHX8 pp 229533.I1RJV3
142355 229533.A0A1C3YHX8 pp 229533.I1RT03
142356 229533.A0A1C3YHX8 pp 229533.I1RA70
142357 229533.A0A1C3YHX8 pp 229533.I1RTH6
142358 229533.A0A1C3YHX8 pp 229533.V6RRH2
142359 229533.A0A1C3YHX8 pp 229533.I1S8R3
142360 229533.A0A1C3YHX8 pp 229533.I1RG90
142361 229533.A0A1C3YHX8 pp 229533.I1RVX0
142362 229533.A0A1C3YHX8 pp 229533.I1RB41
142363 229533.A0A1C3YHX8 pp 229533.I1R9J6
142364 229533.A0A1C3YHX8 pp 229533.I1RXR1
142365 229533.A0A1C3YHX8 pp 229533.I1RGL4
142366 229533.A0A1C3YHX8 pp 229533.A0A1C3YI73
142367 229533.A0A1C3YHX8 pp 229533.I1RRN5
142368 229533.A0A1C3YHX8 pp 229533.I1RB92
142369 229533.A0A1C3YHX8 pp 229533.I1RSD8
142370 229533.A0A1C3YHX8 pp 229533.I1RFL4
142371 229533.A0A1C3YHX8 pp 229533.I1RN18
142372 229533.I1RCC7 pp 229533.I1RML8
142373 229533.I1RCC7 pp 229533.I1RVA8
142374 229533.I1RCC7 pp 229533.I1RWU9
142375 229533.I1RUG4 pp 229533.I1RUR0
142376 229533.I1RUG4 pp 229533.I1RW90
142377 229533.I1RUG4 pp 229533.I1S283
142378 229533.I1RUG4 pp 229533.I1S5Z3
142379 229533.I1RUG4 pp 229533.I1RW91
142380 229533.I1RUG4 pp 229533.I1S461
142381 229533.I1RUG4 pp 229533.V6QWX8
142382 229533.I1RUG4 pp 229533.Q4INS6
142383 229533.I1RUG4 pp 229533.Q4IJU0
142384 229533.I1RUG4 pp 229533.I1RWB3
142385 229533.I1RUG4 pp 229533.Q4HYI0
142386 229533.I1RUG4 pp 229533.I1RZ94
142387 229533.I1RUG4 pp 229533.I1RYQ1
142388 229533.I1RUG4 pp 229533.I1RVZ9
142389 229533.I1RUG4 pp 229533.I1S209
142390 229533.I1RUG4 pp 229533.I1RXE5
142391 229533.I1RUG4 pp 229533.I1RY80
142392 229533.I1RUG4 pp 229533.I1RW80
142393 229533.I1RUG4 pp 229533.V6QV38
142394 229533.I1RUG4 pp 229533.Q4IBS2
142395 229533.I1RUG4 pp 229533.I1RZB5
142396 229533.I1RUG4 pp 229533.I1RY29
142397 229533.I1RUG4 pp 229533.V6QUE6
142398 229533.I1RUG4 pp 229533.I1RWI8
142399 229533.I1RUG4 pp 229533.I1RVW0
142400 229533.I1RUG4 pp 229533.I1RZ86
142401 229533.I1RUG4 pp 229533.I1S9Y1
142402 229533.I1RUG4 pp 229533.I1RVY0
142403 229533.I1RUG4 pp 229533.Q4I5U9
142404 229533.I1RUG4 pp 229533.I1RVL8
142405 229533.I1RUG4 pp 229533.Q4IL82
142406 229533.I1RUG4 pp 229533.I1S4P6
142407 229533.I1RUG4 pp 229533.Q4IPC8
142408 229533.I1RUG4 pp 229533.Q4IB89
142409 229533.I1RUG4 pp 229533.I1RWF3
142410 229533.I1RUG4 pp 229533.I1RXU0
142411 229533.I1RUG4 pp 229533.I1RZP8
142412 229533.I1RUG4 pp 229533.I1S0D5
142413 229533.I1RUG4 pp 229533.I1S276
142414 229533.I1RUG4 pp 229533.I1RY58
142415 229533.I1RUG4 pp 229533.I1RX64
142416 229533.A0A1C3YKC4 pp 229533.I1RB50
142417 229533.A0A1C3YKC4 pp 229533.I1RCC7
142418 229533.I1RAU8 pp 229533.I1RLX4
142419 229533.I1RAU8 pp 229533.I1S461
142420 229533.I1RAU8 pp 229533.I1RG77
142421 229533.I1RAU8 pp 229533.I1RUG4
142422 229533.I1RAU8 pp 229533.I1RDX0
142423 229533.I1RAU8 pp 229533.I1RN55

142424 229533.I1RAU8 pp 229533.I1RSR9
142425 229533.I1RAU8 pp 229533.I1RSP7
142426 229533.I1RAU8 pp 229533.I1RX64
142427 229533.I1RAU8 pp 229533.I1RQJ4
142428 229533.I1RAU8 pp 229533.I1RSN7
142429 229533.I1RAU8 pp 229533.I1RBN9
142430 229533.I1RAU8 pp 229533.I1RWM6
142431 229533.I1RAU8 pp 229533.I1RFZ2
142432 229533.I1RAU8 pp 229533.I1RJV8
142433 229533.I1RAU8 pp 229533.I1RSA7
142434 229533.I1RAU8 pp 229533.I1RBW0
142435 229533.I1RAU8 pp 229533.I1RPQ1
142436 229533.I1RAU8 pp 229533.I1RZ94
142437 229533.I1RAU8 pp 229533.I1RCA5
142438 229533.I1RAU8 pp 229533.I1RN57
142439 229533.I1RAU8 pp 229533.I1S283
142440 229533.I1RAU8 pp 229533.I1RBW1
142441 229533.I1RAU8 pp 229533.I1RRS7
142442 229533.I1RAU8 pp 229533.I1RMX3
142443 229533.I1RAU8 pp 229533.I1RMS4
142444 229533.I1RMC8 pp 229533.I1RT04
142445 229533.I1RMC8 pp 229533.I1RT40
142446 229533.I1RMC8 pp 229533.I1RTD5
142447 229533.I1RMC8 pp 229533.I1RQC9
142448 229533.I1RMC8 pp 229533.I1S0F6
142449 229533.I1RMC8 pp 229533.I1RV91
142450 229533.I1RMC8 pp 229533.I1RZM8
142451 229533.I1RMC8 pp 229533.I1RN67
142452 229533.I1RMC8 pp 229533.I1RUG3
142453 229533.I1RMC8 pp 229533.I1RW99
142454 229533.I1RMC8 pp 229533.I1RWN5
142455 229533.I1RMC8 pp 229533.I1RNC1
142456 229533.I1RMC8 pp 229533.V6R3X5
142457 229533.I1RMC8 pp 229533.I1S5B0
142458 229533.I1RMC8 pp 229533.V6RIV9
142459 229533.I1RMC8 pp 229533.I1RR73
142460 229533.I1RMC8 pp 229533.V6RTJ8
142461 229533.I1RMC8 pp 229533.I1S8R3
142462 229533.I1RMC8 pp 229533.I1RX46
142463 229533.I1RMC8 pp 229533.I1RZK5
142464 229533.I1RMC8 pp 229533.I1SA33
142465 229533.I1RMC8 pp 229533.I1RTH6
142466 229533.I1RMC8 pp 229533.I1S503
142467 229533.I1RMC8 pp 229533.Q4I8P5
142468 229533.I1RMC8 pp 229533.I1RZ39
142469 229533.I1RMC8 pp 229533.I1RY03
142470 229533.I1RMC8 pp 229533.I1RSQ7
142471 229533.I1RMC8 pp 229533.I1RZW8
142472 229533.I1RMC8 pp 229533.I1RPP3
142473 229533.I1RMC8 pp 229533.V6QW22
142474 229533.I1RMC8 pp 229533.I1RZZ9
142475 229533.I1RMC8 pp 229533.I1RPP4
142476 229533.I1RMC8 pp 229533.I1S1C7
142477 229533.I1RMC8 pp 229533.I1RWN6
142478 229533.I1RMC8 pp 229533.I1RS17
142479 229533.I1RMC8 pp 229533.I1RRY9
142480 229533.I1RMC8 pp 229533.I1S2E9
142481 229533.I1RMC8 pp 229533.I1S0J1
142482 229533.I1RMC8 pp 229533.V6RMW6
142483 229533.I1RMC8 pp 229533.I1RS20
142484 229533.I1RMC8 pp 229533.I1S0L4
142485 229533.I1RMC8 pp 229533.V6RCG5
142486 229533.I1RMC8 pp 229533.I1RR24
142487 229533.I1RMC8 pp 229533.I1SA32
142488 229533.I1RMC8 pp 229533.I1S5R1
142489 229533.I1RMC8 pp 229533.I1RSQ3
142490 229533.I1RMC8 pp 229533.I1RW46
142491 229533.I1RMC8 pp 229533.I1RRT8
142492 229533.I1RMC8 pp 229533.I1RWK3
142493 229533.I1RMC8 pp 229533.I1RXD7
142494 229533.I1RMC8 pp 229533.I1S0L3
142495 229533.I1RMC8 pp 229533.V6R042
142496 229533.I1RMC8 pp 229533.I1RT03

142497 229533.I1RMC8 pp 229533.V6R1K4
142498 229533.I1RMC8 pp 229533.I1RWJ8
142499 229533.I1RMC8 pp 229533.Q4I624
142500 229533.I1RMC8 pp 229533.I1RVX0
142501 229533.I1RMC8 pp 229533.I1RPM4
142502 229533.I1RMC8 pp 229533.V6R057
142503 229533.I1RMC8 pp 229533.I1S504
142504 229533.I1RMC8 pp 229533.I1RW25
142505 229533.I1RMC8 pp 229533.I1RQE7
142506 229533.I1RMC8 pp 229533.I1RS47
142507 229533.I1RMC8 pp 229533.I1RSI0
142508 229533.I1RMC8 pp 229533.I1RVQ0
142509 229533.I1RMC8 pp 229533.I1RN18
142510 229533.I1RMC8 pp 229533.I1RZM1
142511 229533.I1RMC8 pp 229533.Q4IJK2
142512 229533.I1RMC8 pp 229533.I1RRM5
142513 229533.I1RMC8 pp 229533.I1RY44
142514 229533.I1RMC8 pp 229533.I1S2B8
142515 229533.I1RMC8 pp 229533.I1RNQ5
142516 229533.I1RMC8 pp 229533.I1S5X9
142517 229533.A0A1C3YKP6 pp 229533.A0A1C3YM95
142518 229533.A0A1C3YKP6 pp 229533.I1RMU9
142519 229533.I1RFU8 pp 229533.Q4IJM4
142520 229533.I1RFU8 pp 229533.I1RI03
142521 229533.I1RFU8 pp 229533.I1RZZ1
142522 229533.I1RFU8 pp 229533.I1RZK6
142523 229533.I1RFU8 pp 229533.V6RBN2
142524 229533.I1RFU8 pp 229533.I1S1J7
142525 229533.I1RFU8 pp 229533.I1RS18
142526 229533.I1RFU8 pp 229533.I1RQQ5
142527 229533.I1RFU8 pp 229533.I1RJZ6
142528 229533.I1RFU8 pp 229533.Q4I8P5
142529 229533.I1RFU8 pp 229533.I1RQA3
142530 229533.I1RFU8 pp 229533.I1S0M9
142531 229533.I1RFU8 pp 229533.I1RWD7
142532 229533.I1RFU8 pp 229533.I1RQ81
142533 229533.I1RFU8 pp 229533.Q4IJK2
142534 229533.I1RFU8 pp 229533.I1SA32
142535 229533.I1RFU8 pp 229533.I1RZE5
142536 229533.I1RFU8 pp 229533.I1RLM7
142537 229533.I1RFU8 pp 229533.I1RSI0
142538 229533.I1RFU8 pp 229533.Q4I283
142539 229533.I1RFU8 pp 229533.Q4I2J8
142540 229533.I1RFU8 pp 229533.I1RVZ0
142541 229533.I1RFU8 pp 229533.I1RWT6
142542 229533.I1RFU8 pp 229533.I1RZK5
142543 229533.I1RFU8 pp 229533.I1S504
142544 229533.I1RFU8 pp 229533.I1RZF6
142545 229533.I1RFU8 pp 229533.I1RYY5
142546 229533.I1RFU8 pp 229533.I1RNQ5
142547 229533.I1RFU8 pp 229533.I1RRU9
142548 229533.I1RFU8 pp 229533.I1RW99
142549 229533.I1RFU8 pp 229533.I1RLU6
142550 229533.I1RFU8 pp 229533.I1RZM1
142551 229533.I1RFU8 pp 229533.I1RY03
142552 229533.I1RFU8 pp 229533.I1RSR8
142553 229533.I1RFU8 pp 229533.I1RU29
142554 229533.I1RFU8 pp 229533.V6QUW4
142555 229533.I1RFU8 pp 229533.I1RI02
142556 229533.I1RFU8 pp 229533.V6RCG5
142557 229533.I1RFU8 pp 229533.I1RRY9
142558 229533.I1RFU8 pp 229533.V6RCZ8
142559 229533.I1RFU8 pp 229533.I1RX25
142560 229533.I1RFU8 pp 229533.I1RW07
142561 229533.I1RFU8 pp 229533.I1RI82
142562 229533.I1RFU8 pp 229533.I1RMG8
142563 229533.I1RFU8 pp 229533.I1RW25
142564 229533.I1RFU8 pp 229533.I1RZJ5
142565 229533.I1RFU8 pp 229533.I1RNH6
142566 229533.I1RFU8 pp 229533.I1RM67
142567 229533.I1RFU8 pp 229533.I1S021
142568 229533.I1RFU8 pp 229533.I1RLC7
142569 229533.I1RFU8 pp 229533.I1S6U8

142570 229533.I1RFU8 pp 229533.I1RWJ8
142571 229533.I1RFU8 pp 229533.I1RG53
142572 229533.I1RFU8 pp 229533.I1RPU0
142573 229533.I1RFU8 pp 229533.I1RZM8
142574 229533.I1RFU8 pp 229533.I1RXF0
142575 229533.I1RFU8 pp 229533.I1RJU3
142576 229533.I1RFU8 pp 229533.I1RT80
142577 229533.I1RFU8 pp 229533.I1RQJ5
142578 229533.I1RFU8 pp 229533.I1RIG2
142579 229533.I1RFU8 pp 229533.I1RN61
142580 229533.I1RFU8 pp 229533.V6QW22
142581 229533.I1RFU8 pp 229533.I1S5B0
142582 229533.I1RFU8 pp 229533.I1RMC8
142583 229533.I1RFU8 pp 229533.I1S0X6
142584 229533.I1RFU8 pp 229533.I1RSQ7
142585 229533.I1RFU8 pp 229533.I1S9U2
142586 229533.I1RFU8 pp 229533.I1RX46
142587 229533.I1RFU8 pp 229533.I1RR00
142588 229533.I1RFU8 pp 229533.I1RW71
142589 229533.I1RFU8 pp 229533.I1RVX0
142590 229533.I1RFU8 pp 229533.I1RT03
142591 229533.I1RFU8 pp 229533.I1RSK1
142592 229533.I1RFU8 pp 229533.I1RR24
142593 229533.I1RFU8 pp 229533.I1RRN5
142594 229533.I1RFU8 pp 229533.I1RIL8
142595 229533.I1RFU8 pp 229533.I1S2E8
142596 229533.I1RFU8 pp 229533.Q4IJT5
142597 229533.I1RFU8 pp 229533.I1RJV3
142598 229533.I1RFU8 pp 229533.I1RS17
142599 229533.I1RFU8 pp 229533.I1RFW5
142600 229533.I1RFU8 pp 229533.I1RPP4
142601 229533.I1RFU8 pp 229533.V6R1K4
142602 229533.I1RFU8 pp 229533.I1RQE7
142603 229533.I1RFU8 pp 229533.I1S0L3
142604 229533.I1RFU8 pp 229533.I1S0J1
142605 229533.I1RFU8 pp 229533.I1RSD8
142606 229533.I1RFU8 pp 229533.I1RW46
142607 229533.I1RFU8 pp 229533.I1RTA0
142608 229533.I1RFU8 pp 229533.I1RN18
142609 229533.I1RFU8 pp 229533.I1S569
142610 229533.I1RFU8 pp 229533.I1S0F5
142611 229533.I1RFU8 pp 229533.V6R057
142612 229533.I1RFU8 pp 229533.V6R4J1
142613 229533.I1RFU8 pp 229533.I1RRR7
142614 229533.I1RFU8 pp 229533.I1RRE5
142615 229533.I1RFU8 pp 229533.I1RVQ0
142616 229533.I1RFU8 pp 229533.I1S8V4
142617 229533.I1RFU8 pp 229533.Q4I624
142618 229533.I1RFU8 pp 229533.I1RQH3
142619 229533.I1RFU8 pp 229533.I1S1W4
142620 229533.I1RFU8 pp 229533.I1RXD7
142621 229533.I1RFU8 pp 229533.I1RGL4
142622 229533.I1RFU8 pp 229533.I1RQC9
142623 229533.I1RFU8 pp 229533.I1S0R7
142624 229533.I1RFU8 pp 229533.I1RG90
142625 229533.I1RFU8 pp 229533.I1S2C0
142626 229533.I1RFU8 pp 229533.I1RM96
142627 229533.I1RFU8 pp 229533.V6RVG3
142628 229533.I1RFU8 pp 229533.I1RY44
142629 229533.I1RFU8 pp 229533.I1RTH6
142630 229533.I1RFU8 pp 229533.I1S8R3
142631 229533.I1RFU8 pp 229533.I1S7A8
142632 229533.I1RFU8 pp 229533.V6RTJ8
142633 229533.I1RFU8 pp 229533.I1RWX5
142634 229533.I1RFU8 pp 229533.V6RIV9
142635 229533.I1RFU8 pp 229533.I1RRI2
142636 229533.I1RFU8 pp 229533.I1RWN5
142637 229533.I1RFU8 pp 229533.I1RIL3
142638 229533.I1RFU8 pp 229533.V6R042
142639 229533.I1RFU8 pp 229533.I1RZW8
142640 229533.I1RFU8 pp 229533.I1S2H4
142641 229533.I1RFU8 pp 229533.I1RS20
142642 229533.I1RFU8 pp 229533.I1RSQ3

142643 229533.I1RFU8 pp 229533.V6RMW6
142644 229533.I1RFU8 pp 229533.I1RTD7
142645 229533.I1RFU8 pp 229533.I1S5R1
142646 229533.I1RFU8 pp 229533.I1RMG3
142647 229533.I1RFU8 pp 229533.I1RRT8
142648 229533.I1RFU8 pp 229533.I1RYW3
142649 229533.I1RFU8 pp 229533.I1S1C7
142650 229533.I1RFU8 pp 229533.I1RWL2
142651 229533.I1RFU8 pp 229533.Q4I327
142652 229533.I1RFU8 pp 229533.I1RM82
142653 229533.I1RFU8 pp 229533.I1RRV1
142654 229533.I1RFU8 pp 229533.I1RNY2
142655 229533.I1RFU8 pp 229533.I1RKJ6
142656 229533.I1RFU8 pp 229533.I1RWN6
142657 229533.I1RFU8 pp 229533.I1SA33
142658 229533.I1RFU8 pp 229533.I1RPP3
142659 229533.I1RFU8 pp 229533.I1RWF7
142660 229533.I1RFU8 pp 229533.I1S8P5
142661 229533.I1RFU8 pp 229533.I1RMN6
142662 229533.I1RFU8 pp 229533.I1RNC1
142663 229533.I1RFU8 pp 229533.I1RUG3
142664 229533.I1RFU8 pp 229533.I1S0M6
142665 229533.I1RFU8 pp 229533.I1RZS6
142666 229533.I1RFU8 pp 229533.I1RRJ8
142667 229533.I1RFU8 pp 229533.I1RQ23
142668 229533.I1RFU8 pp 229533.I1S352
142669 229533.I1RFU8 pp 229533.Q4IN63
142670 229533.I1RFU8 pp 229533.I1RKL3
142671 229533.I1RFU8 pp 229533.I1RSV2
142672 229533.I1RFU8 pp 229533.I1S0N4
142673 229533.I1RFU8 pp 229533.I1RPM4
142674 229533.I1RFU8 pp 229533.I1RWK3
142675 229533.I1RFU8 pp 229533.I1RPU3
142676 229533.I1RFU8 pp 229533.I1RN63
142677 229533.I1RFU8 pp 229533.I1RT40
142678 229533.I1RFU8 pp 229533.I1RJV4
142679 229533.I1RFU8 pp 229533.V6R283
142680 229533.I1RFU8 pp 229533.I1RP27
142681 229533.I1RFU8 pp 229533.I1RQ65
142682 229533.I1RFU8 pp 229533.I1S7S9
142683 229533.I1RFU8 pp 229533.I1RRZ0
142684 229533.I1RFU8 pp 229533.I1RGA4
142685 229533.I1RFU8 pp 229533.I1RJZ7
142686 229533.I1RFU8 pp 229533.I1S2G7
142687 229533.I1RFU8 pp 229533.I1RRM5
142688 229533.I1RFU8 pp 229533.I1RLV9
142689 229533.I1RFU8 pp 229533.I1RLN6
142690 229533.I1RFU8 pp 229533.I1RS47
142691 229533.I1RFU8 pp 229533.I1S503
142692 229533.I1RFU8 pp 229533.I1S2B8
142693 229533.I1RFU8 pp 229533.I1RJW6
142694 229533.I1RFU8 pp 229533.I1RR73
142695 229533.I1RFU8 pp 229533.I1RN67
142696 229533.I1RFU8 pp 229533.I1RRQ3
142697 229533.I1RFU8 pp 229533.V6R3X5
142698 229533.I1RFU8 pp 229533.I1S0F6
142699 229533.I1RFU8 pp 229533.I1RNA5
142700 229533.I1RFU8 pp 229533.I1S2E9
142701 229533.I1RFU8 pp 229533.I1RZ39
142702 229533.I1RFU8 pp 229533.I1S8V3
142703 229533.I1RFU8 pp 229533.I1RNN1
142704 229533.I1RFU8 pp 229533.V6RUY9
142705 229533.I1RFU8 pp 229533.I1RWI5
142706 229533.I1RFU8 pp 229533.I1RT04
142707 229533.I1RFU8 pp 229533.I1RZZ9
142708 229533.I1RFU8 pp 229533.I1RGF5
142709 229533.I1RFU8 pp 229533.I1RTD5
142710 229533.I1RFU8 pp 229533.I1S0L4
142711 229533.I1RFU8 pp 229533.I1RV91
142712 229533.A0A1C3YLM4 pp 229533.P0C431
142713 229533.A0A1C3YLM4 pp 229533.I1RRF9
142714 229533.A0A1C3YLM4 pp 229533.I1RQL4
142715 229533.A0A1C3YLM4 pp 229533.I1RSK0

142716	229533.A0A1C3YLM4	pp	229533.I1RMP2
142717	229533.A0A1C3YLM4	pp	229533.I1RE19
142718	229533.I1RKB0	pp	229533.I1RLV8
142719	229533.I1RBR2	pp	229533.I1RC66
142720	229533.I1RBR2	pp	229533.I1RVF8
142721	229533.I1RBR2	pp	229533.I1RUG3
142722	229533.I1RBR2	pp	229533.I1S437
142723	229533.I1RBR2	pp	229533.I1RXF8
142724	229533.I1RBR2	pp	229533.I1RKJ8
142725	229533.I1RBR2	pp	229533.I1RNC1
142726	229533.I1RBR2	pp	229533.I1RNT9
142727	229533.I1RBR2	pp	229533.I1RZM8
142728	229533.I1RBR2	pp	229533.I1RCH5
142729	229533.I1RBR2	pp	229533.I1RZ39
142730	229533.I1RBR2	pp	229533.I1RZK5
142731	229533.I1RBR2	pp	229533.I1RGB2
142732	229533.I1RBR2	pp	229533.I1RPV3
142733	229533.I1RBR2	pp	229533.I1RT40
142734	229533.I1RBR2	pp	229533.I1RCR8
142735	229533.I1RBR2	pp	229533.V6RMW6
142736	229533.I1RBR2	pp	229533.I1RL04
142737	229533.I1RBR2	pp	229533.I1RQE7
142738	229533.I1RBR2	pp	229533.I1RJU3
142739	229533.I1RBR2	pp	229533.I1RXC2
142740	229533.I1RBR2	pp	229533.I1RRM3
142741	229533.I1RBR2	pp	229533.I1RSZ7
142742	229533.I1RBR2	pp	229533.I1S5R1
142743	229533.I1RBR2	pp	229533.I1RJV3
142744	229533.I1RBR2	pp	229533.I1RXD7
142745	229533.I1RBR2	pp	229533.V6R042
142746	229533.I1RBR2	pp	229533.Q4IJK2
142747	229533.I1RBR2	pp	229533.I1S4S4
142748	229533.I1RBR2	pp	229533.I1RSQ3
142749	229533.I1RBR2	pp	229533.I1RE89
142750	229533.I1RBR2	pp	229533.I1S438
142751	229533.I1RBR2	pp	229533.I1RUF7
142752	229533.I1RBR2	pp	229533.I1RWR7
142753	229533.I1RBR2	pp	229533.I1RGU7
142754	229533.I1RBR2	pp	229533.I1RRI1
142755	229533.I1RBR2	pp	229533.V6RCZ8
142756	229533.I1RBR2	pp	229533.I1S0L4
142757	229533.I1RBR2	pp	229533.I1RVX0
142758	229533.I1RBR2	pp	229533.I1S6K9
142759	229533.I1RBR2	pp	229533.I1RR24
142760	229533.I1RBR2	pp	229533.I1RML0
142761	229533.I1RBR2	pp	229533.I1RRN2
142762	229533.I1RBR2	pp	229533.I1REC5
142763	229533.I1RBR2	pp	229533.I1RPE0
142764	229533.I1RBR2	pp	229533.Q4IPZ1
142765	229533.I1RBR2	pp	229533.I1RSX0
142766	229533.I1RBR2	pp	229533.I1RD22
142767	229533.I1RBR2	pp	229533.I1S2B8
142768	229533.I1RBR2	pp	229533.I1S1B2
142769	229533.I1RBR2	pp	229533.I1S5B0
142770	229533.I1RBR2	pp	229533.I1RQ33
142771	229533.I1RBR2	pp	229533.I1RC34
142772	229533.I1RBR2	pp	229533.V6RTJ8
142773	229533.I1RBR2	pp	229533.I1REK8
142774	229533.I1RBR2	pp	229533.Q4I624
142775	229533.I1RBR2	pp	229533.I1RVZ2
142776	229533.I1RBR2	pp	229533.I1RQD2
142777	229533.I1RBR2	pp	229533.I1RXC4
142778	229533.I1RBR2	pp	229533.I1S5R6
142779	229533.I1RBR2	pp	229533.I1RE01
142780	229533.I1RBR2	pp	229533.I1RJN9
142781	229533.I1RBR2	pp	229533.I1RYR0
142782	229533.I1RBR2	pp	229533.I1RGA4
142783	229533.I1RBR2	pp	229533.I1RPP3
142784	229533.I1RBR2	pp	229533.I1RTA1
142785	229533.I1RBR2	pp	229533.I1RPK0
142786	229533.I1RBR2	pp	229533.I1RYI5
142787	229533.I1RBR2	pp	229533.I1RSQ7
142788	229533.I1RBR2	pp	229533.I1RXD6

142789	229533.I1RBR2	pp	229533.I1RZV3
142790	229533.I1RBR2	pp	229533.I1S4S7
142791	229533.I1RBR2	pp	229533.I1S1W9
142792	229533.I1RBR2	pp	229533.I1S0W4
142793	229533.I1RBR2	pp	229533.I1RPU2
142794	229533.I1RBR2	pp	229533.I1RDF7
142795	229533.I1RBR2	pp	229533.I1RSF1
142796	229533.I1RBR2	pp	229533.I1RD52
142797	229533.I1RBR2	pp	229533.I1RY86
142798	229533.I1RBR2	pp	229533.I1RQ85
142799	229533.I1S2I5	pp	229533.Q4IPX8
142800	229533.A0A1C3YI61	pp	229533.I1S8D1
142801	229533.A0A1C3YI61	pp	229533.I1RLV8
142802	229533.A0A1C3YI61	pp	229533.I1RCQ5
142803	229533.A0A1C3YI61	pp	229533.I1RKB0
142804	229533.A0A1C3YI61	pp	229533.I1RRQ2
142805	229533.I1RBT1	pp	229533.I1RMG3
142806	229533.I1RBT1	pp	229533.I1RP20
142807	229533.I1RBT1	pp	229533.I1RSI6
142808	229533.I1RBT1	pp	229533.I1RRV1
142809	229533.I1RBT1	pp	229533.V6R042
142810	229533.I1RBT1	pp	229533.I1RS17
142811	229533.I1RBT1	pp	229533.I1RSQ3
142812	229533.I1RBT1	pp	229533.I1RPP4
142813	229533.I1RBT1	pp	229533.V6RMW6
142814	229533.I1RBT1	pp	229533.I1RNC1
142815	229533.I1RBT1	pp	229533.I1RPU8
142816	229533.I1RBT1	pp	229533.I1RMV0
142817	229533.I1RBT1	pp	229533.I1RUG3
142818	229533.I1RBT1	pp	229533.I1RE01
142819	229533.I1RBT1	pp	229533.I1RW46
142820	229533.I1RBT1	pp	229533.I1RTA0
142821	229533.I1RBT1	pp	229533.I1RCR8
142822	229533.I1RBT1	pp	229533.I1RWQ8
142823	229533.I1RBT1	pp	229533.I1S7W5
142824	229533.I1RBT1	pp	229533.I1S5R6
142825	229533.I1RBT1	pp	229533.I1S0J1
142826	229533.I1RBT1	pp	229533.V6RRM0
142827	229533.I1RBT1	pp	229533.I1S0R2
142828	229533.I1RBT1	pp	229533.I1RRE5
142829	229533.I1RBT1	pp	229533.I1RC72
142830	229533.I1RBT1	pp	229533.I1S8V4
142831	229533.I1RBT1	pp	229533.V6RGN5
142832	229533.I1RBT1	pp	229533.I1RE33
142833	229533.I1RBT1	pp	229533.I1RWN5
142834	229533.I1RBT1	pp	229533.I1RG90
142835	229533.I1RBT1	pp	229533.I1RG11
142836	229533.I1RBT1	pp	229533.I1SA33
142837	229533.I1RBT1	pp	229533.I1RKC6
142838	229533.I1RBT1	pp	229533.V6RIV9
142839	229533.I1RBT1	pp	229533.I1RGL4
142840	229533.I1RBT1	pp	229533.I1RNA5
142841	229533.I1RBT1	pp	229533.I1S1W9
142842	229533.I1RBT1	pp	229533.V6RUY9
142843	229533.I1RBT1	pp	229533.I1RRN2
142844	229533.I1RBT1	pp	229533.I1RT04
142845	229533.I1RBT1	pp	229533.I1RD22
142846	229533.I1RBT1	pp	229533.Q4HZB8
142847	229533.I1RBT1	pp	229533.I1S4P6
142848	229533.I1RBT1	pp	229533.I1RZW8
142849	229533.I1RBT1	pp	229533.I1RXU9
142850	229533.I1RBT1	pp	229533.I1RD39
142851	229533.I1RBT1	pp	229533.I1S2E9
142852	229533.I1RBT1	pp	229533.I1RV91
142853	229533.I1RBT1	pp	229533.I1RFL3
142854	229533.I1RBT1	pp	229533.I1RC33
142855	229533.I1RBT1	pp	229533.I1RY44
142856	229533.I1RBT1	pp	229533.I1RWN6
142857	229533.I1RBT1	pp	229533.I1S1W3
142858	229533.I1RBT1	pp	229533.I1RT40
142859	229533.I1RBT1	pp	229533.I1RJV3
142860	229533.I1RBT1	pp	229533.I1RGF5
142861	229533.I1RBT1	pp	229533.I1RC81

142862 229533.I1RBT1 pp 229533.I1RTD5
142863 229533.I1RBT1 pp 229533.I1RT03
142864 229533.I1RBT1 pp 229533.I1RSA4
142865 229533.I1RBT1 pp 229533.I1RVX0
142866 229533.I1RBT1 pp 229533.I1S268
142867 229533.I1RBT1 pp 229533.I1RX39
142868 229533.I1RBT1 pp 229533.I1RTH6
142869 229533.I1RBT1 pp 229533.I1RSS4
142870 229533.I1RBT1 pp 229533.I1S8R3
142871 229533.I1RBT1 pp 229533.I1RXR1
142872 229533.I1RBT1 pp 229533.I1S1B6
142873 229533.I1RBT1 pp 229533.I1RTE9
142874 229533.I1RBT1 pp 229533.I1RGA4
142875 229533.I1RBT1 pp 229533.I1RRN5
142876 229533.I1RBT1 pp 229533.I1RJ74
142877 229533.I1RBT1 pp 229533.I1RC82
142878 229533.I1RBT1 pp 229533.I1RYW3
142879 229533.I1RBT1 pp 229533.I1S4S8
142880 229533.I1RBT1 pp 229533.I1RJS2
142881 229533.I1RBT1 pp 229533.I1S1C7
142882 229533.I1RBT1 pp 229533.I1RJV4
142883 229533.I1RBT1 pp 229533.I1RFL4
142884 229533.I1RBT1 pp 229533.I1RZB2
142885 229533.I1RBT1 pp 229533.I1RN18
142886 229533.I1RBT1 pp 229533.I1RLN6
142887 229533.I1RBT1 pp 229533.I1RCH5
142888 229533.I1RBT1 pp 229533.I1RQE7
142889 229533.I1RBT1 pp 229533.I1RWX5
142890 229533.I1RBT1 pp 229533.I1S0R8
142891 229533.I1RBT1 pp 229533.I1RRZ0
142892 229533.I1RBT1 pp 229533.I1RZ79
142893 229533.I1RBT1 pp 229533.I1RRI2
142894 229533.I1RBT1 pp 229533.I1S569
142895 229533.I1RBT1 pp 229533.I1RJW6
142896 229533.I1RBT1 pp 229533.I1S503
142897 229533.I1RBT1 pp 229533.I1RYI5
142898 229533.I1RBT1 pp 229533.V6R057
142899 229533.I1RBT1 pp 229533.I1S8F0
142900 229533.I1RBT1 pp 229533.I1S8P5
142901 229533.I1RBT1 pp 229533.I1RBT6
142902 229533.I1RBT1 pp 229533.Q4I624
142903 229533.I1RBT1 pp 229533.I1RRT2
142904 229533.I1RBT1 pp 229533.I1RJL4
142905 229533.I1RBT1 pp 229533.I1RPP3
142906 229533.I1RBT1 pp 229533.I1RD17
142907 229533.I1RBT1 pp 229533.I1RSX0
142908 229533.I1RBT1 pp 229533.V6R3J1
142909 229533.I1RBT1 pp 229533.I1RRJ8
142910 229533.I1RBT1 pp 229533.I1RWK3
142911 229533.I1RBT1 pp 229533.I1RC66
142912 229533.I1RBT1 pp 229533.I1S352
142913 229533.I1RBT1 pp 229533.I1S8V3
142914 229533.I1RBT1 pp 229533.I1RFH8
142915 229533.I1RBT1 pp 229533.I1RN63
142916 229533.I1RBT1 pp 229533.I1RFT1
142917 229533.I1RBT1 pp 229533.I1RTA1
142918 229533.I1RBT1 pp 229533.I1RQS8
142919 229533.I1RBT1 pp 229533.I1RE27
142920 229533.I1RBT1 pp 229533.I1RVZ2
142921 229533.I1RBT1 pp 229533.I1RJZ7
142922 229533.I1RBT1 pp 229533.I1RS20
142923 229533.I1RBT1 pp 229533.I1S2M6
142924 229533.I1RBT1 pp 229533.I1RRM5
142925 229533.I1RBT1 pp 229533.I1S2B8
142926 229533.I1RBT1 pp 229533.I1RRP3
142927 229533.I1RBT1 pp 229533.I1RS47
142928 229533.I1RBT1 pp 229533.I1S2H4
142929 229533.I1RBT1 pp 229533.I1S0L4
142930 229533.I1RBT1 pp 229533.I1RBY4
142931 229533.I1RBT1 pp 229533.I1RE76
142932 229533.I1RBT1 pp 229533.I1RN67
142933 229533.I1RBT1 pp 229533.I1S6K9
142934 229533.I1RBT1 pp 229533.I1S0F6

142935	229533.I1RBT1	pp	229533.I1RIL3
142936	229533.I1RBT1	pp	229533.I1RW26
142937	229533.I1RBT1	pp	229533.I1S9Y1
142938	229533.I1RBT1	pp	229533.I1RM82
142939	229533.I1RBT1	pp	229533.V6RCZ8
142940	229533.I1RBT1	pp	229533.V6RCG5
142941	229533.I1RBT1	pp	229533.I1RR51
142942	229533.I1RBT1	pp	229533.I1S5R1
142943	229533.I1RBT1	pp	229533.I1RZ39
142944	229533.I1RBT1	pp	229533.I1RRL9
142945	229533.I1RBT1	pp	229533.I1RRT8
142946	229533.I1RBT1	pp	229533.I1RMC0
142947	229533.I1RBT1	pp	229533.I1RR73
142948	229533.I1RBT1	pp	229533.V6R3X5
142949	229533.I1RBT1	pp	229533.I1RQ31
142950	229533.I1RBT1	pp	229533.I1RZM8
142951	229533.I1RBT1	pp	229533.O74638
142952	229533.I1RBT1	pp	229533.I1RWI5
142953	229533.I1RBT1	pp	229533.I1RXF0
142954	229533.I1RBT1	pp	229533.I1RZZ9
142955	229533.I1RBT1	pp	229533.I1RXC2
142956	229533.I1RBT1	pp	229533.I1RSQ4
142957	229533.I1RBT1	pp	229533.I1RE22
142958	229533.I1RBT1	pp	229533.I1RC17
142959	229533.I1RBT1	pp	229533.I1RI82
142960	229533.I1RBT1	pp	229533.I1S0M6
142961	229533.I1RBT1	pp	229533.I1RZH4
142962	229533.I1RBT1	pp	229533.I1RW61
142963	229533.I1RBT1	pp	229533.I1S5B0
142964	229533.I1RBT1	pp	229533.I1RW99
142965	229533.I1RBT1	pp	229533.I1SA32
142966	229533.I1RBT1	pp	229533.I1RQ23
142967	229533.I1RBT1	pp	229533.I1RSZ7
142968	229533.I1RBT1	pp	229533.I1RRV4
142969	229533.I1RBT1	pp	229533.V6QUE6
142970	229533.I1RBT1	pp	229533.I1RL04
142971	229533.I1RBT1	pp	229533.I1RJ17
142972	229533.I1RBT1	pp	229533.I1S504
142973	229533.I1RBT1	pp	229533.I1RGC2
142974	229533.I1RBT1	pp	229533.I1RQ81
142975	229533.I1RBT1	pp	229533.I1S6Y8
142976	229533.I1RBT1	pp	229533.V6R227
142977	229533.I1RBT1	pp	229533.I1RS18
142978	229533.I1RBT1	pp	229533.I1RGB6
142979	229533.I1RBT1	pp	229533.I1RPM4
142980	229533.I1RBT1	pp	229533.Q4I283
142981	229533.I1RBT1	pp	229533.I1RPV3
142982	229533.I1RBT1	pp	229533.I1RXC4
142983	229533.I1RBT1	pp	229533.Q4I8P5
142984	229533.I1RBT1	pp	229533.I1RFS3
142985	229533.I1RBT1	pp	229533.I1RLP1
142986	229533.I1RBT1	pp	229533.I1RWT6
142987	229533.I1RBT1	pp	229533.I1RUQ2
142988	229533.I1RBT1	pp	229533.I1RXD7
142989	229533.I1RBT1	pp	229533.I1RSR3
142990	229533.I1RBT1	pp	229533.I1RG14
142991	229533.I1RBT1	pp	229533.I1RQA2
142992	229533.I1RBT1	pp	229533.I1RWB0
142993	229533.I1RBT1	pp	229533.I1S419
142994	229533.I1RBT1	pp	229533.V6RTJ8
142995	229533.I1RBT1	pp	229533.I1RC16
142996	229533.I1RBT1	pp	229533.I1RYS0
142997	229533.I1RBT1	pp	229533.Q4IEK8
142998	229533.I1RBT1	pp	229533.I1S1J7
142999	229533.I1RBT1	pp	229533.I1S7A8
143000	229533.I1RBT1	pp	229533.I1RI03
143001	229533.I1RBT1	pp	229533.I1S0T2
143002	229533.I1RBT1	pp	229533.I1RLU6
143003	229533.I1RBT1	pp	229533.I1RNQ5
143004	229533.I1RBT1	pp	229533.I1RX46
143005	229533.I1RBT1	pp	229533.I1RY63
143006	229533.I1RBT1	pp	229533.I1RY16
143007	229533.I1RBT1	pp	229533.I1RSC6

143008 229533.I1RBT1 pp 229533.I1RZM1
143009 229533.I1RBT1 pp 229533.I1RCG4
143010 229533.I1RBT1 pp 229533.I1RZK5
143011 229533.I1RBT1 pp 229533.I1RFU8
143012 229533.I1RBT1 pp 229533.I1S4S7
143013 229533.I1RBT1 pp 229533.I1RN99
143014 229533.I1RBT1 pp 229533.I1RDG0
143015 229533.I1RBT1 pp 229533.I1RK95
143016 229533.I1RBT1 pp 229533.I1S887
143017 229533.I1RBT1 pp 229533.I1RQA3
143018 229533.I1RBT1 pp 229533.I1RG53
143019 229533.I1RBT1 pp 229533.I1RW71
143020 229533.I1RBT1 pp 229533.I1RMD5
143021 229533.I1RBT1 pp 229533.Q4IJK2
143022 229533.I1RBT1 pp 229533.I1RZZ1
143023 229533.I1RBT1 pp 229533.I1RC20
143024 229533.I1RBT1 pp 229533.I1RTD9
143025 229533.I1RBT1 pp 229533.I1RQQ5
143026 229533.I1RBT1 pp 229533.I1RY03
143027 229533.I1RBT1 pp 229533.V6QW22
143028 229533.I1RBT1 pp 229533.I1RPR9
143029 229533.I1RBT1 pp 229533.I1RRX5
143030 229533.I1RBT1 pp 229533.I1RSM3
143031 229533.I1RBT1 pp 229533.I1RFC0
143032 229533.I1RBT1 pp 229533.V6RBN2
143033 229533.I1RBT1 pp 229533.I1RWC9
143034 229533.I1RBT1 pp 229533.I1RCR5
143035 229533.I1RBT1 pp 229533.I1RSQ7
143036 229533.I1RBT1 pp 229533.I1RLX7
143037 229533.I1RBT1 pp 229533.I1REK8
143038 229533.I1RBT1 pp 229533.I1RWD7
143039 229533.I1RBT1 pp 229533.I1RRY9
143040 229533.I1RBT1 pp 229533.I1RU29
143041 229533.I1RBT1 pp 229533.Q4I2J8
143042 229533.I1RBT1 pp 229533.I1RIL8
143043 229533.I1RBT1 pp 229533.I1RRG3
143044 229533.I1RBT1 pp 229533.I1RLM7
143045 229533.I1RBT1 pp 229533.I1RWD4
143046 229533.I1RBT1 pp 229533.I1S6F0
143047 229533.I1RBT1 pp 229533.Q4IJT5
143048 229533.I1RBT1 pp 229533.I1RFJ9
143049 229533.I1RBT1 pp 229533.V6R4J1
143050 229533.I1RBT1 pp 229533.I1RSI0
143051 229533.I1RBT1 pp 229533.I1RD52
143052 229533.I1RBT1 pp 229533.I1RT33
143053 229533.I1RBT1 pp 229533.I1RF19
143054 229533.I1RBT1 pp 229533.I1RVQ0
143055 229533.I1RBT1 pp 229533.I1RQC9
143056 229533.I1RBT1 pp 229533.I1RQ14
143057 229533.I1RBT1 pp 229533.I1S0R7
143058 229533.I1RBT1 pp 229533.V6R1K4
143059 229533.I1RBT1 pp 229533.I1RTF4
143060 229533.I1RBT1 pp 229533.I1RNV6
143061 229533.I1RBT1 pp 229533.V6RUT5
143062 229533.I1RBT1 pp 229533.I1RDA0
143063 229533.I1RBT1 pp 229533.I1S0F5
143064 229533.I1RBT1 pp 229533.I1RJU3
143065 229533.I1RBT1 pp 229533.I1RRS8
143066 229533.I1RBT1 pp 229533.I1S0L3
143067 229533.I1RBT1 pp 229533.I1RI02
143068 229533.I1RBT1 pp 229533.I1RMK3
143069 229533.I1RBT1 pp 229533.I1RW07
143070 229533.I1RBT1 pp 229533.I1RLC7
143071 229533.I1RBT1 pp 229533.Q4HVW2
143072 229533.I1RBT1 pp 229533.I1S0X6
143073 229533.I1RBT1 pp 229533.I1RW25
143074 229533.I1RBT1 pp 229533.I1RMC8
143075 229533.I1RBT1 pp 229533.I1RX25
143076 229533.I1RBT1 pp 229533.I1RWJ8
143077 229533.I1RBT1 pp 229533.I1RR24
143078 229533.I1RBT1 pp 229533.I1RZJ5
143079 229533.I1RBT1 pp 229533.I1S4S4
143080 229533.I1RBT1 pp 229533.I1RMG8

143081 229533.I1RBT1 pp 229533.I1RNA0
143082 229533.I1RBT1 pp 229533.V6QYF0
143083 229533.I1RBT1 pp 229533.V6QUW4
143084 229533.I1RBT1 pp 229533.I1RCS1
143085 229533.I1RBT1 pp 229533.I1RM06
143086 229533.I1RUG2 pp 229533.Q4IQM6
143087 229533.I1RUG2 pp 229533.I1S283
143088 229533.I1RUG2 pp 229533.Q4IEA5
143089 229533.I1RUG2 pp 229533.I1RYH9
143090 229533.I1RUG2 pp 229533.I1RZ94
143091 229533.I1RUG2 pp 229533.I1RYX9
143092 229533.I1RUG2 pp 229533.I1RZP8
143093 229533.I1RUG2 pp 229533.I1RW16
143094 229533.I1RUG2 pp 229533.I1RX64
143095 229533.I1RUG2 pp 229533.V6RLW4
143096 229533.I1RUG2 pp 229533.I1RYR2
143097 229533.I1RUG2 pp 229533.I1S461
143098 229533.I1RUG2 pp 229533.I1RXI6
143099 229533.I1RUG2 pp 229533.I1RY73
143100 229533.I1RUG2 pp 229533.Q4I0J6
143101 229533.I1RUG2 pp 229533.I1S604
143102 229533.I1S504 pp 229533.Q4IN63
143103 229533.I1S504 pp 229533.I1S8V4
143104 229533.I1S504 pp 229533.I1S5B0
143105 229533.I1S504 pp 229533.V6RCZ8
143106 229533.I1S504 pp 229533.Q4I327
143107 229533.I1S504 pp 229533.I1SA33
143108 229533.I1S504 pp 229533.V6RMW6
143109 229533.I1S504 pp 229533.Q4HZB8
143110 229533.I1S504 pp 229533.Q4I283
143111 229533.I1S504 pp 229533.V6R042
143112 229533.I1S504 pp 229533.Q4I8P5
143113 229533.I1S504 pp 229533.Q4IJM4
143114 229533.I1S504 pp 229533.V6QUE6
143115 229533.I1S504 pp 229533.V6RIV9
143116 229533.I1S504 pp 229533.V6RCG5
143117 229533.I1S504 pp 229533.V6R1K4
143118 229533.I1S504 pp 229533.Q4IJK2
143119 229533.I1S504 pp 229533.V6R4J1
143120 229533.I1S504 pp 229533.I1S8R3
143121 229533.I1S504 pp 229533.I1SA32
143122 229533.I1S504 pp 229533.V6RTJ8
143123 229533.I1S504 pp 229533.V6QW22
143124 229533.I1S504 pp 229533.V6R3X5
143125 229533.I1S504 pp 229533.I1S569
143126 229533.I1S504 pp 229533.I1S7A8
143127 229533.I1S504 pp 229533.Q4I624
143128 229533.I1S504 pp 229533.Q4IJT5
143129 229533.I1S504 pp 229533.I1S8E2
143130 229533.I1S504 pp 229533.I1S6U8
143131 229533.I1S504 pp 229533.I1S8P5
143132 229533.I1S504 pp 229533.V6R057
143133 229533.I1S504 pp 229533.V6R283
143134 229533.I1S504 pp 229533.V6RUY9
143135 229533.I1S504 pp 229533.I1S5R1
143136 229533.I1S504 pp 229533.Q4I2J8
143137 229533.I1S504 pp 229533.I1S8V3
143138 229533.I1S504 pp 229533.I1S9U2
143139 229533.I1S504 pp 229533.V6QUW4
143140 229533.I1RG77 pp 229533.I1RQ13
143141 229533.I1RG77 pp 229533.I1S283
143142 229533.I1RG77 pp 229533.I1S5R2
143143 229533.I1RG77 pp 229533.I1RZ94
143144 229533.I1RG77 pp 229533.I1RV66
143145 229533.I1RG77 pp 229533.I1RRT1
143146 229533.I1RG77 pp 229533.I1RZ86
143147 229533.I1RG77 pp 229533.I1RUG1
143148 229533.I1RG77 pp 229533.I1RUR0
143149 229533.I1RG77 pp 229533.I1RYX9
143150 229533.I1RG77 pp 229533.I1S5Z3
143151 229533.I1RG77 pp 229533.I1RVW0
143152 229533.I1RG77 pp 229533.I1S998
143153 229533.I1RG77 pp 229533.I1RZP8

229533.I1RG77 pp 229533.I1RX64
229533.I1RG77 pp 229533.I1RYR2
229533.I1RG77 pp 229533.I1RSI4
229533.I1RG77 pp 229533.I1RTN6
229533.I1RG77 pp 229533.V6RLW4
229533.I1RG77 pp 229533.I1RSN7
229533.I1RG77 pp 229533.I1RSP7
229533.I1RG77 pp 229533.I1RQJ4
229533.I1RG77 pp 229533.I1S987
229533.I1RG77 pp 229533.I1RSA7
229533.I1RG77 pp 229533.I1RWI8
229533.I1RG77 pp 229533.I1RMB7
229533.I1RG77 pp 229533.I1S2E9
229533.I1RG77 pp 229533.I1RZT1
229533.I1RG77 pp 229533.I1RKI2
229533.I1RG77 pp 229533.I1S2F2
229533.I1RG77 pp 229533.I1RSR9
229533.I1RG77 pp 229533.I1S0D5
229533.I1RG77 pp 229533.I1RM36
229533.I1RG77 pp 229533.I1RYQ1
229533.I1RG77 pp 229533.I1RKE9
229533.I1RG77 pp 229533.I1RMS4
229533.I1RG77 pp 229533.I1RKB1
229533.I1RG77 pp 229533.I1RPQ1
229533.I1RG77 pp 229533.I1RY44
229533.I1RG77 pp 229533.I1RZS7
229533.I1RG77 pp 229533.I1RMQ7
229533.I1RG77 pp 229533.I1RJV8
229533.I1RG77 pp 229533.Q4INS6
229533.I1RG77 pp 229533.Q4IB89
229533.I1RG77 pp 229533.I1RQH8
229533.I1RG77 pp 229533.I1RN55
229533.I1RG77 pp 229533.I1RRY1
229533.I1RG77 pp 229533.I1RN57
229533.I1RG77 pp 229533.I1RQB4
229533.I1RG77 pp 229533.I1RUG4
229533.I1RG77 pp 229533.I1RQA3
229533.I1RG77 pp 229533.I1RSC7
229533.I1RG77 pp 229533.I1RWT6
229533.I1RG77 pp 229533.I1S209
229533.I1RG77 pp 229533.I1RSU7
229533.I1RG77 pp 229533.I1S0A6
229533.I1RG77 pp 229533.I1S016
229533.I1RG77 pp 229533.I1RRS7
229533.I1RG77 pp 229533.I1RMX3
229533.I1RG77 pp 229533.I1S461
229533.I1RG77 pp 229533.I1S4P6
229533.A0A098D8H1 pp 229533.I1RSA7
229533.A0A098D8H1 pp 229533.I1RAH4
229533.A0A098D8H1 pp 229533.V6RLW4
229533.A0A098D8H1 pp 229533.Q4IQM6
229533.A0A098D8H1 pp 229533.A0A098DSW9
229533.A0A098D8H1 pp 229533.A0A098DDX5
229533.A0A098D8H1 pp 229533.I1RVV5
229533.A0A098D8H1 pp 229533.I1RFX8
229533.A0A098D8H1 pp 229533.A0A1C3YHM3
229533.A0A098D8H1 pp 229533.I1RLV8
229533.A0A098D8H1 pp 229533.A0A1C3YJL7
229533.A0A098D8H1 pp 229533.I1RXI6
229533.A0A098D8H1 pp 229533.I1RVW0
229533.A0A098D8H1 pp 229533.I1RSK0
229533.A0A098D8H1 pp 229533.I1RRS7
229533.A0A098D8H1 pp 229533.A0A098E4E9
229533.A0A098D8H1 pp 229533.I1RYR2
229533.A0A098D8H1 pp 229533.I1RAL9
229533.A0A098D8H1 pp 229533.I1RKQ0
229533.A0A098D8H1 pp 229533.I1RAH9
229533.A0A098D8H1 pp 229533.I1RMX3
229533.A0A098D8H1 pp 229533.I1RJV8
229533.A0A098D8H1 pp 229533.A0A0E0RU38
229533.A0A098D8H1 pp 229533.I1S209
229533.A0A098D8H1 pp 229533.I1RYX9
229533.A0A098D8H1 pp 229533.I1R9W7

143227 229533.A0A098D8H1 pp 229533.I1RU61
143228 229533.A0A098D8H1 pp 229533.I1RSC7
143229 229533.A0A098D8H1 pp 229533.A0A098E1Q1
143230 229533.A0A098D8H1 pp 229533.I1S283
143231 229533.A0A098D8H1 pp 229533.Q4IB89
143232 229533.A0A098D8H1 pp 229533.A0A098DDT3
143233 229533.A0A098D8H1 pp 229533.A0A1C3YI10
143234 229533.A0A098D8H1 pp 229533.I1RE46
143235 229533.A0A098D8H1 pp 229533.I1RAS5
143236 229533.A0A098D8H1 pp 229533.I1S0D5
143237 229533.A0A098D8H1 pp 229533.I1RN57
143238 229533.A0A098D8H1 pp 229533.I1RMJ8
143239 229533.A0A098D8H1 pp 229533.I1RD19
143240 229533.A0A098D8H1 pp 229533.A0A1C3YHN3
143241 229533.A0A098D8H1 pp 229533.I1RZP8
143242 229533.A0A098D8H1 pp 229533.I1RBN9
143243 229533.A0A098D8H1 pp 229533.I1RPG1
143244 229533.A0A098D8H1 pp 229533.I1RFJ2
143245 229533.A0A098D8H1 pp 229533.A0A098DJU6
143246 229533.A0A098D8H1 pp 229533.I1S461
143247 229533.A0A098D8H1 pp 229533.I1RUG4
143248 229533.A0A098D8H1 pp 229533.A0A0E0RVF4
143249 229533.A0A098D8H1 pp 229533.I1RCL7
143250 229533.A0A098D8H1 pp 229533.A0A1C3YLM4
143251 229533.A0A098D8H1 pp 229533.I1RUG2
143252 229533.A0A098D8H1 pp 229533.I1RG77
143253 229533.I1RX41 pp 229533.I1RZT1
143254 229533.I1RX41 pp 229533.I1S4Z7
143255 229533.I1RX41 pp 229533.I1RYE2
143256 229533.I1RX41 pp 229533.I1RY71
143257 229533.I1RX41 pp 229533.I1RX42
143258 229533.A0A098E4W0 pp 229533.I1RVQ0
143259 229533.A0A098E4W0 pp 229533.A0A1C3YIP5
143260 229533.A0A098E4W0 pp 229533.V6R1K4
143261 229533.A0A098E4W0 pp 229533.I1S0L3
143262 229533.A0A098E4W0 pp 229533.I1RD52
143263 229533.A0A098E4W0 pp 229533.I1RT33
143264 229533.A0A098E4W0 pp 229533.I1RRS8
143265 229533.A0A098E4W0 pp 229533.A0A0E0RUI4
143266 229533.A0A098E4W0 pp 229533.A0A1C3YM63
143267 229533.A0A098E4W0 pp 229533.I1RN61
143268 229533.A0A098E4W0 pp 229533.I1S438
143269 229533.A0A098E4W0 pp 229533.V6QW22
143270 229533.A0A098E4W0 pp 229533.I1S1W8
143271 229533.A0A098E4W0 pp 229533.I1RC20
143272 229533.A0A098E4W0 pp 229533.I1RFQ7
143273 229533.A0A098E4W0 pp 229533.I1RSQ7
143274 229533.A0A098E4W0 pp 229533.I1RM67
143275 229533.A0A098E4W0 pp 229533.I1RFC0
143276 229533.A0A098E4W0 pp 229533.A0A1C3YK11
143277 229533.A0A098E4W0 pp 229533.I1RRX5
143278 229533.A0A098E4W0 pp 229533.I1RWJ8
143279 229533.A0A098E4W0 pp 229533.A0A1C3YK13
143280 229533.A0A098E4W0 pp 229533.I1S4S7
143281 229533.A0A098E4W0 pp 229533.I1RW25
143282 229533.A0A098E4W0 pp 229533.I1S4S4
143283 229533.A0A098E4W0 pp 229533.I1RCL4
143284 229533.A0A098E4W0 pp 229533.A0A1C3YLR6
143285 229533.A0A098E4W0 pp 229533.I1RW71
143286 229533.A0A098E4W0 pp 229533.I1RR24
143287 229533.A0A098E4W0 pp 229533.I1RBC9
143288 229533.A0A098E4W0 pp 229533.A0A1C3YN26
143289 229533.A0A098E4W0 pp 229533.I1RPN4
143290 229533.A0A098E4W0 pp 229533.A0A1C3YHX8
143291 229533.A0A098E4W0 pp 229533.I1S504
143292 229533.A0A098E4W0 pp 229533.I1RBT1
143293 229533.A0A098E4W0 pp 229533.A0A1C3YJJ2
143294 229533.A0A098E4W0 pp 229533.A0A1C3YKC0
143295 229533.A0A098E4W0 pp 229533.A0A1C3YJR3
143296 229533.A0A098E4W0 pp 229533.I1RBI1
143297 229533.A0A098E4W0 pp 229533.A0A1C3YIG9
143298 229533.A0A098E4W0 pp 229533.I1RX46
143299 229533.A0A098E4W0 pp 229533.I1S0X6

143300 229533.A0A098E4W0 pp 229533.V6RTJ8
143301 229533.A0A098E4W0 pp 229533.I1RMQ4
143302 229533.A0A098E4W0 pp 229533.I1R9Y4
143303 229533.A0A098E4W0 pp 229533.I1SA32
143304 229533.A0A098E4W0 pp 229533.I1RJU3
143305 229533.A0A098E4W0 pp 229533.I1RIG2
143306 229533.A0A098E4W0 pp 229533.I1RJ17
143307 229533.A0A098E4W0 pp 229533.A0A1C3YN30
143308 229533.A0A098E4W0 pp 229533.I1RXC4
143309 229533.A0A098E4W0 pp 229533.I1RAX6
143310 229533.A0A098E4W0 pp 229533.I1RK95
143311 229533.A0A098E4W0 pp 229533.A0A0E0S035
143312 229533.A0A098E4W0 pp 229533.I1RME6
143313 229533.A0A098E4W0 pp 229533.A0A1C3YKY6
143314 229533.A0A098E4W0 pp 229533.A0A0E0S134
143315 229533.A0A098E4W0 pp 229533.A0A1C3YK34
143316 229533.A0A098E4W0 pp 229533.I1RS18
143317 229533.A0A098E4W0 pp 229533.Q4I8P5
143318 229533.A0A098E4W0 pp 229533.I1RXD7
143319 229533.A0A098E4W0 pp 229533.I1RQC9
143320 229533.A0A098E4W0 pp 229533.A0A0E0SFN0
143321 229533.A0A098E4W0 pp 229533.I1RXD0
143322 229533.A0A098E4W0 pp 229533.I1RP86
143323 229533.A0A098E4W0 pp 229533.A0A1C3YKV3
143324 229533.A0A098E4W0 pp 229533.I1RQ81
143325 229533.A0A098E4W0 pp 229533.I1S0R7
143326 229533.A0A098E4W0 pp 229533.I1RWR7
143327 229533.A0A098E4W0 pp 229533.I1RQE1
143328 229533.A0A098E4W0 pp 229533.I1RAU5
143329 229533.A0A098E4W0 pp 229533.I1RRV4
143330 229533.A0A098E4W0 pp 229533.I1RRY9
143331 229533.A0A098E4W0 pp 229533.I1RE59
143332 229533.A0A098E4W0 pp 229533.I1RKB3
143333 229533.A0A098E4W0 pp 229533.A0A1C3YIJ3
143334 229533.A0A098E4W0 pp 229533.I1S4Q4
143335 229533.A0A098E4W0 pp 229533.I1RBL7
143336 229533.A0A098E4W0 pp 229533.V6QUE6
143337 229533.A0A098E4W0 pp 229533.A0A1C3YHS4
143338 229533.A0A098E4W0 pp 229533.A0A1C3YHX6
143339 229533.A0A098E4W0 pp 229533.I1S5B0
143340 229533.A0A098E4W0 pp 229533.I1RSI0
143341 229533.A0A098E4W0 pp 229533.I1RY03
143342 229533.A0A098E4W0 pp 229533.I1RBC1
143343 229533.A0A098E4W0 pp 229533.I1RAZ8
143344 229533.A0A098E4W0 pp 229533.I1RWD7
143345 229533.A0A098E4W0 pp 229533.I1RA94
143346 229533.A0A098E4W0 pp 229533.I1RZK5
143347 229533.A0A098E4W0 pp 229533.I1RW99
143348 229533.A0A098E4W0 pp 229533.A0A1C3YK68
143349 229533.A0A098E4W0 pp 229533.I1RQD2
143350 229533.A0A098E4W0 pp 229533.I1REK8
143351 229533.A0A098E4W0 pp 229533.I1RQA5
143352 229533.A0A098E4W0 pp 229533.I1RCR5
143353 229533.A0A098E4W0 pp 229533.A0A0E0RWW0
143354 229533.A0A098E4W0 pp 229533.I1RZZ1
143355 229533.A0A098E4W0 pp 229533.I1RCV1
143356 229533.A0A098E4W0 pp 229533.A0A1C3YI30
143357 229533.A0A098E4W0 pp 229533.I1RI82
143358 229533.A0A098E4W0 pp 229533.I1RAZ3
143359 229533.A0A098E4W0 pp 229533.V6RBN2
143360 229533.A0A098E4W0 pp 229533.I1RZM8
143361 229533.A0A098E4W0 pp 229533.I1RFU8
143362 229533.A0A098E4W0 pp 229533.I1RCG4
143363 229533.A0A098E4W0 pp 229533.I1RXF0
143364 229533.A0A098E4W0 pp 229533.I1RBU8
143365 229533.A0A098E4W0 pp 229533.I1RWH8
143366 229533.A0A098E4W0 pp 229533.I1RLU6
143367 229533.A0A098E4W0 pp 229533.Q4IJK2
143368 229533.A0A098E4W0 pp 229533.V6RCG5
143369 229533.A0A098E4W0 pp 229533.I1RNQ5
143370 229533.A0A098E4W0 pp 229533.A0A1C3YJN3
143371 229533.A0A098E4W0 pp 229533.V6RCZ8
143372 229533.A0A098E4W0 pp 229533.I1RBY4

229533.A0A098E4W0	pp	229533.I1RZM1
229533.A0A098E4W0	pp	229533.I1R9M0
229533.A0A098E4W0	pp	229533.A0A1C3YKB0
229533.A0A098E4W0	pp	229533.I1RJZ7
229533.A0A098E4W0	pp	229533.I1RBE4
229533.A0A098E4W0	pp	229533.I1S6K9
229533.A0A098E4W0	pp	229533.I1RQ85
229533.A0A098E4W0	pp	229533.A0A1C3YJW9
229533.A0A098E4W0	pp	229533.I1RSV2
229533.A0A098E4W0	pp	229533.I1S3G4
229533.A0A098E4W0	pp	229533.I1RRM5
229533.A0A098E4W0	pp	229533.I1RFT1
229533.A0A098E4W0	pp	229533.A0A0E0SPV0
229533.A0A098E4W0	pp	229533.I1S2B8
229533.A0A098E4W0	pp	229533.I1S2H4
229533.A0A098E4W0	pp	229533.I1RSX0
229533.A0A098E4W0	pp	229533.I1RPE0
229533.A0A098E4W0	pp	229533.I1S4Z8
229533.A0A098E4W0	pp	229533.V6R3J1
229533.A0A098E4W0	pp	229533.A0A1C3YIS7
229533.A0A098E4W0	pp	229533.I1RGA6
229533.A0A098E4W0	pp	229533.I1RVN1
229533.A0A098E4W0	pp	229533.I1RFS3
229533.A0A098E4W0	pp	229533.I1RJW6
229533.A0A098E4W0	pp	229533.I1RPV3
229533.A0A098E4W0	pp	229533.A0A1C3YLK8
229533.A0A098E4W0	pp	229533.I1RC66
229533.A0A098E4W0	pp	229533.A0A1C3YI20
229533.A0A098E4W0	pp	229533.I1RWI5
229533.A0A098E4W0	pp	229533.I1RPM4
229533.A0A098E4W0	pp	229533.I1RL04
229533.A0A098E4W0	pp	229533.I1S503
229533.A0A098E4W0	pp	229533.I1RSZ7
229533.A0A098E4W0	pp	229533.I1RSQ4
229533.A0A098E4W0	pp	229533.I1RP27
229533.A0A098E4W0	pp	229533.I1RZZ9
229533.A0A098E4W0	pp	229533.I1RTN3
229533.A0A098E4W0	pp	229533.I1RQM7
229533.A0A098E4W0	pp	229533.I1RE22
229533.A0A098E4W0	pp	229533.A0A1C3YIR8
229533.A0A098E4W0	pp	229533.I1RZB2
229533.A0A098E4W0	pp	229533.I1RR73
229533.A0A098E4W0	pp	229533.A0A1C3YJD4
229533.A0A098E4W0	pp	229533.I1RRT8
229533.A0A098E4W0	pp	229533.I1RJV4
229533.A0A098E4W0	pp	229533.V6R3X5
229533.A0A098E4W0	pp	229533.A0A1C3YLA6
229533.A0A098E4W0	pp	229533.I1S5R1
229533.A0A098E4W0	pp	229533.I1RBS6
229533.A0A098E4W0	pp	229533.I1RBC7
229533.A0A098E4W0	pp	229533.A0A1C3YKM9
229533.A0A098E4W0	pp	229533.I1RZ39
229533.A0A098E4W0	pp	229533.I1RXC2
229533.A0A098E4W0	pp	229533.I1RGA4
229533.A0A098E4W0	pp	229533.O74638
229533.A0A098E4W0	pp	229533.I1RC31
229533.A0A098E4W0	pp	229533.I1RN67
229533.A0A098E4W0	pp	229533.V6RAC9
229533.A0A098E4W0	pp	229533.I1RNG4
229533.A0A098E4W0	pp	229533.I1RA11
229533.A0A098E4W0	pp	229533.I1RM82
229533.A0A098E4W0	pp	229533.I1RXF8
229533.A0A098E4W0	pp	229533.I1S9Y1
229533.A0A098E4W0	pp	229533.I1S0F6
229533.A0A098E4W0	pp	229533.I1RTD5
229533.A0A098E4W0	pp	229533.I1RJN9
229533.A0A098E4W0	pp	229533.I1RFL3
229533.A0A098E4W0	pp	229533.I1S0L4
229533.A0A098E4W0	pp	229533.A0A1C3YJI6
229533.A0A098E4W0	pp	229533.I1RTA1
229533.A0A098E4W0	pp	229533.I1RVZ2
229533.A0A098E4W0	pp	229533.A0A0E0SC46
229533.A0A098E4W0	pp	229533.I1RT40

143446 229533.A0A098E4W0 pp 229533.I1RRI1
143447 229533.A0A098E4W0 pp 229533.A0A1C3YJ21
143448 229533.A0A098E4W0 pp 229533.A0A1C3YL55
143449 229533.A0A098E4W0 pp 229533.I1RN63
143450 229533.A0A098E4W0 pp 229533.I1RWK3
143451 229533.A0A098E4W0 pp 229533.I1RKP1
143452 229533.A0A098E4W0 pp 229533.A0A1C3YJW4
143453 229533.A0A098E4W0 pp 229533.A0A1C3YJK3
143454 229533.A0A098E4W0 pp 229533.I1RCH5
143455 229533.A0A098E4W0 pp 229533.Q4HXF0
143456 229533.A0A098E4W0 pp 229533.I1RQE7
143457 229533.A0A098E4W0 pp 229533.I1RYR0
143458 229533.A0A098E4W0 pp 229533.I1RT04
143459 229533.A0A098E4W0 pp 229533.I1RYI5
143460 229533.A0A098E4W0 pp 229533.I1RFL4
143461 229533.A0A098E4W0 pp 229533.I1RN18
143462 229533.A0A098E4W0 pp 229533.I1RT75
143463 229533.A0A098E4W0 pp 229533.I1RB92
143464 229533.A0A098E4W0 pp 229533.I1RPP3
143465 229533.A0A098E4W0 pp 229533.I1RML0
143466 229533.A0A098E4W0 pp 229533.I1RZV3
143467 229533.A0A098E4W0 pp 229533.I1RVX0
143468 229533.A0A098E4W0 pp 229533.I1RCD8
143469 229533.A0A098E4W0 pp 229533.I1RT03
143470 229533.A0A098E4W0 pp 229533.I1RSS4
143471 229533.A0A098E4W0 pp 229533.V6RIV9
143472 229533.A0A098E4W0 pp 229533.V6RRH2
143473 229533.A0A098E4W0 pp 229533.I1RAU7
143474 229533.A0A098E4W0 pp 229533.I1RWN5
143475 229533.A0A098E4W0 pp 229533.I1RJV3
143476 229533.A0A098E4W0 pp 229533.I1RQ33
143477 229533.A0A098E4W0 pp 229533.I1RE89
143478 229533.A0A098E4W0 pp 229533.I1RCM3
143479 229533.A0A098E4W0 pp 229533.I1RWN6
143480 229533.A0A098E4W0 pp 229533.V6R057
143481 229533.A0A098E4W0 pp 229533.I1S1W9
143482 229533.A0A098E4W0 pp 229533.I1RAW0
143483 229533.A0A098E4W0 pp 229533.I1RFT8
143484 229533.A0A098E4W0 pp 229533.Q4I624
143485 229533.A0A098E4W0 pp 229533.A0A0E0SD83
143486 229533.A0A098E4W0 pp 229533.A0A1C3YKY0
143487 229533.A0A098E4W0 pp 229533.I1RSF1
143488 229533.A0A098E4W0 pp 229533.I1RD17
143489 229533.A0A098E4W0 pp 229533.I1RPU2
143490 229533.A0A098E4W0 pp 229533.I1RRI2
143491 229533.A0A098E4W0 pp 229533.A0A0E0SH88
143492 229533.A0A098E4W0 pp 229533.A0A1C3YMI5
143493 229533.A0A098E4W0 pp 229533.I1RZW8
143494 229533.A0A098E4W0 pp 229533.I1RVZ7
143495 229533.A0A098E4W0 pp 229533.I1RNC1
143496 229533.A0A098E4W0 pp 229533.I1S0J1
143497 229533.A0A098E4W0 pp 229533.I1S437
143498 229533.A0A098E4W0 pp 229533.I1RUG3
143499 229533.A0A098E4W0 pp 229533.I1RB44
143500 229533.A0A098E4W0 pp 229533.I1RE01
143501 229533.A0A098E4W0 pp 229533.I1RCR8
143502 229533.A0A098E4W0 pp 229533.I1RW46
143503 229533.A0A098E4W0 pp 229533.I1RE79
143504 229533.A0A098E4W0 pp 229533.A0A1C3YLB6
143505 229533.A0A098E4W0 pp 229533.I1RPP4
143506 229533.A0A098E4W0 pp 229533.I1S1C7
143507 229533.A0A098E4W0 pp 229533.V6R042
143508 229533.A0A098E4W0 pp 229533.I1RYW3
143509 229533.A0A098E4W0 pp 229533.V6RLM7
143510 229533.A0A098E4W0 pp 229533.I1RSQ3
143511 229533.A0A098E4W0 pp 229533.I1RPK0
143512 229533.A0A098E4W0 pp 229533.I1RKJ8
143513 229533.A0A098E4W0 pp 229533.V6RMW6
143514 229533.A0A098E4W0 pp 229533.I1RA70
143515 229533.A0A098E4W0 pp 229533.I1RTH6
143516 229533.A0A098E4W0 pp 229533.I1RS17
143517 229533.A0A098E4W0 pp 229533.I1RRM3
143518 229533.A0A098E4W0 pp 229533.I1RRN2

143519	229533.A0A098E4W0	pp	229533.I1RD22
143520	229533.A0A098E4W0	pp	229533.I1S8R3
143521	229533.A0A098E4W0	pp	229533.I1SAN9
143522	229533.A0A098E4W0	pp	229533.I1SA33
143523	229533.A0A098E4W0	pp	229533.I1RRV1
143524	229533.A0A098E4W0	pp	229533.I1S0N4
143525	229533.A0A098E4W0	pp	229533.I1RG11
143526	229533.A0A098E4W0	pp	229533.I1RC27
143527	229533.A0A098E4W0	pp	229533.I1S5R6
143528	229533.A0A1C3YI19	pp	229533.I1RRT3
143529	229533.A0A1C3YI19	pp	229533.I1RCU8
143530	229533.A0A1C3YI19	pp	229533.I1RYL5
143531	229533.A0A1C3YI19	pp	229533.I1RZ78
143532	229533.A0A1C3YI19	pp	229533.I1R9N7
143533	229533.A0A1C3YI19	pp	229533.Q4I1Y5
143534	229533.A0A1C3YI19	pp	229533.I1RNM4
143535	229533.A0A1C3YI19	pp	229533.I1RKM2
143536	229533.A0A1C3YI19	pp	229533.I1S9S4
143537	229533.A0A1C3YI19	pp	229533.I1RUP0
143538	229533.A0A1C3YI19	pp	229533.I1RML4
143539	229533.A0A1C3YI19	pp	229533.I1RAH0
143540	229533.A0A1C3YI19	pp	229533.I1RA62
143541	229533.A0A1C3YI19	pp	229533.I1RHU0
143542	229533.A0A1C3YI19	pp	229533.I1S444
143543	229533.A0A1C3YI19	pp	229533.I1RZF7
143544	229533.A0A1C3YI19	pp	229533.I1RCU9
143545	229533.A0A1C3YI19	pp	229533.I1RIE8
143546	229533.A0A1C3YI19	pp	229533.A0A1C3YKR7
143547	229533.A0A1C3YI19	pp	229533.I1RGU7
143548	229533.A0A1C3YI19	pp	229533.V6QUV7
143549	229533.A0A1C3YI19	pp	229533.I1RXF0
143550	229533.A0A1C3YI19	pp	229533.I1RBH7
143551	229533.A0A1C3YI19	pp	229533.I1RVF8
143552	229533.A0A1C3YI19	pp	229533.I1RD80
143553	229533.A0A1C3YI19	pp	229533.I1RVS4
143554	229533.I1RCG4	pp	229533.I1RWK3
143555	229533.I1RCG4	pp	229533.I1RN63
143556	229533.I1RCG4	pp	229533.I1S2M6
143557	229533.I1RCG4	pp	229533.I1RLX7
143558	229533.I1RCG4	pp	229533.I1RIL8
143559	229533.I1RCG4	pp	229533.I1RV91
143560	229533.I1RCG4	pp	229533.Q4IJT5
143561	229533.I1RCG4	pp	229533.Q4IR18
143562	229533.I1RCG4	pp	229533.I1RW71
143563	229533.I1RCG4	pp	229533.I1RT40
143564	229533.I1RCG4	pp	229533.I1S352
143565	229533.I1RCG4	pp	229533.I1RTD5
143566	229533.I1RCG4	pp	229533.I1RNA5
143567	229533.I1RCG4	pp	229533.I1RFT1
143568	229533.I1RCG4	pp	229533.I1RGF5
143569	229533.I1RCG4	pp	229533.I1RQC9
143570	229533.I1RCG4	pp	229533.I1RPP3
143571	229533.I1RCG4	pp	229533.I1S8P5
143572	229533.I1RCG4	pp	229533.I1RG53
143573	229533.I1RCG4	pp	229533.I1RT04
143574	229533.I1RCG4	pp	229533.I1S0R7
143575	229533.I1RCG4	pp	229533.V6RUY9
143576	229533.I1RCG4	pp	229533.I1RSQ7
143577	229533.I1RCG4	pp	229533.I1S2E9
143578	229533.I1RCG4	pp	229533.I1RRJ8
143579	229533.I1RCG4	pp	229533.V6QW22
143580	229533.I1RCG4	pp	229533.I1RQG4
143581	229533.I1RCG4	pp	229533.I1RXD7
143582	229533.I1RCG4	pp	229533.I1RRP3
143583	229533.I1RCG4	pp	229533.I1RN67
143584	229533.I1RCG4	pp	229533.I1RUQ2
143585	229533.I1RCG4	pp	229533.I1RLN6
143586	229533.I1RCG4	pp	229533.I1RWD4
143587	229533.I1RCG4	pp	229533.I1RRZ0
143588	229533.I1RCG4	pp	229533.I1S0F6
143589	229533.I1RCG4	pp	229533.I1S503
143590	229533.I1RCG4	pp	229533.I1S6F0
143591	229533.I1RCG4	pp	229533.I1RJW6

143592 229533.I1RCG4 pp 229533.I1RRT2
143593 229533.I1RCG4 pp 229533.I1RVQ0
143594 229533.I1RCG4 pp 229533.I1RPM4
143595 229533.I1RCG4 pp 229533.I1RX46
143596 229533.I1RCG4 pp 229533.I1S0T2
143597 229533.I1RCG4 pp 229533.I1S6Y8
143598 229533.I1RCG4 pp 229533.V6QUW4
143599 229533.I1RCG4 pp 229533.V6R4J1
143600 229533.I1RCG4 pp 229533.I1RJL4
143601 229533.I1RCG4 pp 229533.I1RS47
143602 229533.I1RCG4 pp 229533.I1S7A8
143603 229533.I1RCG4 pp 229533.I1RGA4
143604 229533.I1RCG4 pp 229533.I1RJ74
143605 229533.I1RCG4 pp 229533.I1RD52
143606 229533.I1RCG4 pp 229533.I1RNA0
143607 229533.I1RCG4 pp 229533.V6QYF0
143608 229533.I1RCG4 pp 229533.V6R1K4
143609 229533.I1RCG4 pp 229533.I1RJS2
143610 229533.I1RCG4 pp 229533.V6RTJ8
143611 229533.I1RCG4 pp 229533.I1RE22
143612 229533.I1RCG4 pp 229533.I1RJV4
143613 229533.I1RCG4 pp 229533.I1S0L3
143614 229533.I1RCG4 pp 229533.I1RM06
143615 229533.I1RCG4 pp 229533.I1S0M6
143616 229533.I1RCG4 pp 229533.I1S0F5
143617 229533.I1RCG4 pp 229533.I1RCS1
143618 229533.I1RCG4 pp 229533.I1RW61
143619 229533.I1RCG4 pp 229533.I1RLC7
143620 229533.I1RCG4 pp 229533.I1RQ23
143621 229533.I1RCG4 pp 229533.I1RQ81
143622 229533.I1RCG4 pp 229533.I1S2B8
143623 229533.I1RCG4 pp 229533.I1RRM5
143624 229533.I1RCG4 pp 229533.I1RS17
143625 229533.I1RCG4 pp 229533.I1RJU3
143626 229533.I1RCG4 pp 229533.I1RJZ7
143627 229533.I1RCG4 pp 229533.I1RPP4
143628 229533.I1RCG4 pp 229533.I1RMK3
143629 229533.I1RCG4 pp 229533.I1RE76
143630 229533.I1RCG4 pp 229533.I1RW25
143631 229533.I1RCG4 pp 229533.Q4I8P5
143632 229533.I1RCG4 pp 229533.V6R3X5
143633 229533.I1RCG4 pp 229533.I1RR73
143634 229533.I1RCG4 pp 229533.I1RWJ8
143635 229533.I1RCG4 pp 229533.I1RS18
143636 229533.I1RCG4 pp 229533.I1RI02
143637 229533.I1RCG4 pp 229533.I1RX25
143638 229533.I1RCG4 pp 229533.O74638
143639 229533.I1RCG4 pp 229533.V6RIV9
143640 229533.I1RCG4 pp 229533.I1RW07
143641 229533.I1RCG4 pp 229533.I1RRT8
143642 229533.I1RCG4 pp 229533.I1RE27
143643 229533.I1RCG4 pp 229533.I1RWN5
143644 229533.I1RCG4 pp 229533.Q4HZB8
143645 229533.I1RCG4 pp 229533.I1RZZ9
143646 229533.I1RCG4 pp 229533.I1S419
143647 229533.I1RCG4 pp 229533.I1RWI5
143648 229533.I1RCG4 pp 229533.I1S5R1
143649 229533.I1RCG4 pp 229533.I1S0X6
143650 229533.I1RCG4 pp 229533.I1RY44
143651 229533.I1RCG4 pp 229533.I1RZJ5
143652 229533.I1RCG4 pp 229533.I1RMG8
143653 229533.I1RCG4 pp 229533.I1RFH8
143654 229533.I1RCG4 pp 229533.I1RMC8
143655 229533.I1RCG4 pp 229533.I1RNQ5
143656 229533.I1RCG4 pp 229533.I1RR24
143657 229533.I1RCG4 pp 229533.I1RTF4
143658 229533.I1RCG4 pp 229533.I1S2H4
143659 229533.I1RCG4 pp 229533.I1RLU6
143660 229533.I1RCG4 pp 229533.I1RQS8
143661 229533.I1RCG4 pp 229533.I1RS20
143662 229533.I1RCG4 pp 229533.Q4IEK8
143663 229533.I1RCG4 pp 229533.I1RZ39
143664 229533.I1RCG4 pp 229533.I1RRL9

143665	229533.I1RCG4	pp	229533.I1RIL3
143666	229533.I1RCG4	pp	229533.I1S0L4
143667	229533.I1RCG4	pp	229533.V6RCG5
143668	229533.I1RCG4	pp	229533.I1RZH4
143669	229533.I1RCG4	pp	229533.I1RZM1
143670	229533.I1RCG4	pp	229533.V6R042
143671	229533.I1RCG4	pp	229533.V6RMW6
143672	229533.I1RCG4	pp	229533.I1RRE5
143673	229533.I1RCG4	pp	229533.I1RE01
143674	229533.I1RCG4	pp	229533.I1RW46
143675	229533.I1RCG4	pp	229533.I1RSQ3
143676	229533.I1RCG4	pp	229533.I1S504
143677	229533.I1RCG4	pp	229533.I1RTA0
143678	229533.I1RCG4	pp	229533.I1SA33
143679	229533.I1RCG4	pp	229533.I1RZS7
143680	229533.I1RCG4	pp	229533.I1RGC2
143681	229533.I1RCG4	pp	229533.I1RRU9
143682	229533.I1RCG4	pp	229533.I1SA32
143683	229533.I1RCG4	pp	229533.Q4I283
143684	229533.I1RCG4	pp	229533.I1S0J1
143685	229533.I1RCG4	pp	229533.V6R227
143686	229533.I1RCG4	pp	229533.I1RWT6
143687	229533.I1RCG4	pp	229533.I1RD22
143688	229533.I1RCG4	pp	229533.I1RWQ8
143689	229533.I1RCG4	pp	229533.I1S0R2
143690	229533.I1RCG4	pp	229533.I1RQ31
143691	229533.I1RCG4	pp	229533.I1S5B0
143692	229533.I1RCG4	pp	229533.I1RUG3
143693	229533.I1RCG4	pp	229533.I1RPK0
143694	229533.I1RCG4	pp	229533.I1RXT3
143695	229533.I1RCG4	pp	229533.I1S0N4
143696	229533.I1RCG4	pp	229533.I1RFJ9
143697	229533.I1RCG4	pp	229533.V6RGN5
143698	229533.I1RCG4	pp	229533.I1RNC1
143699	229533.I1RCG4	pp	229533.I1RSI0
143700	229533.I1RCG4	pp	229533.V6R057
143701	229533.I1RCG4	pp	229533.I1RW99
143702	229533.I1RCG4	pp	229533.I1RN18
143703	229533.I1RCG4	pp	229533.I1RMC0
143704	229533.I1RCG4	pp	229533.I1S569
143705	229533.I1RCG4	pp	229533.I1RXF0
143706	229533.I1RCG4	pp	229533.Q4IJK2
143707	229533.I1RCG4	pp	229533.I1RFL4
143708	229533.I1RCG4	pp	229533.I1RD17
143709	229533.I1RCG4	pp	229533.I1REK8
143710	229533.I1RCG4	pp	229533.I1RCR8
143711	229533.I1RCG4	pp	229533.I1RZM8
143712	229533.I1RCG4	pp	229533.Q4I624
143713	229533.I1RCG4	pp	229533.I1RI82
143714	229533.I1RCG4	pp	229533.I1RZZ1
143715	229533.I1RCG4	pp	229533.I1RQA3
143716	229533.I1RCG4	pp	229533.I1RCR5
143717	229533.I1RCG4	pp	229533.I1RCH5
143718	229533.I1RCG4	pp	229533.I1RMD5
143719	229533.I1RCG4	pp	229533.I1S1C7
143720	229533.I1RCG4	pp	229533.V6RCZ8
143721	229533.I1RCG4	pp	229533.I1RMG3
143722	229533.I1RCG4	pp	229533.I1RQE7
143723	229533.I1RCG4	pp	229533.I1RQQ5
143724	229533.I1RCG4	pp	229533.I1RRV1
143725	229533.I1RCG4	pp	229533.I1RYW3
143726	229533.I1RCG4	pp	229533.I1RSI6
143727	229533.I1RCG4	pp	229533.I1RY03
143728	229533.I1RCG4	pp	229533.I1RY63
143729	229533.I1RCG4	pp	229533.I1RTD9
143730	229533.I1RCG4	pp	229533.I1S0Q7
143731	229533.I1RCG4	pp	229533.I1RVX0
143732	229533.I1RCG4	pp	229533.I1RWX5
143733	229533.I1RCG4	pp	229533.I1RRI2
143734	229533.I1RCG4	pp	229533.I1RR51
143735	229533.I1RCG4	pp	229533.I1RT03
143736	229533.I1RCG4	pp	229533.I1RRN5
143737	229533.I1RCG4	pp	229533.I1RZW8

229533.I1RCG4 pp 229533.I1RU29
229533.I1RCG4 pp 229533.I1RSC6
229533.I1RCG4 pp 229533.I1RN99
229533.I1RCG4 pp 229533.I1RFU8
229533.I1RCG4 pp 229533.Q4I2J8
229533.I1RCG4 pp 229533.I1RWN6
229533.I1RCG4 pp 229533.I1RDG0
229533.I1RCG4 pp 229533.I1RRY9
229533.I1RCG4 pp 229533.I1RD39
229533.I1RCG4 pp 229533.I1S8R3
229533.I1RCG4 pp 229533.I1RKC6
229533.I1RCG4 pp 229533.I1RTH6
229533.I1RCG4 pp 229533.I1RLM7
229533.I1RCG4 pp 229533.I1RSR3
229533.I1RCG4 pp 229533.I1RGL4
229533.I1RCG4 pp 229533.I1RTE9
229533.I1RCG4 pp 229533.I1RF19
229533.I1RCG4 pp 229533.I1RZK5
229533.I1RCG4 pp 229533.I1RSK1
229533.I1RCG4 pp 229533.I1RQA2
229533.I1RCG4 pp 229533.I1RI03
229533.I1RCG4 pp 229533.I1S1J7
229533.I1RCG4 pp 229533.I1RJV3
229533.Q4HZI0 pp 229533.Q4IQ72
229533.Q4HZI0 pp 229533.Q4I5M4
229533.Q4HZI0 pp 229533.V6RGN5
229533.Q4HZI0 pp 229533.V6QY25
229533.Q4HZI0 pp 229533.Q4I9U7
229533.A0A1C3YM89 pp 229533.I1S8Y5
229533.A0A1C3YM89 pp 229533.I1RQL1
229533.A0A1C3YM89 pp 229533.I1RK32
229533.A0A1C3YM89 pp 229533.I1S0Z2
229533.A0A1C3YM89 pp 229533.I1RWF9
229533.A0A1C3YM89 pp 229533.I1RY71
229533.A0A1C3YM89 pp 229533.I1RX41
229533.A0A1C3YM89 pp 229533.I1RCP3
229533.A0A1C3YM89 pp 229533.A0A1C3YN92
229533.A0A1C3YKC0 pp 229533.I1RNN1
229533.A0A1C3YKC0 pp 229533.I1RC66
229533.A0A1C3YKC0 pp 229533.I1RAM6
229533.A0A1C3YKC0 pp 229533.I1S0X6
229533.A0A1C3YKC0 pp 229533.I1RPM4
229533.A0A1C3YKC0 pp 229533.I1RBT1
229533.A0A1C3YKC0 pp 229533.I1RWI5
229533.A0A1C3YKC0 pp 229533.I1RQ23
229533.A0A1C3YKC0 pp 229533.I1S0M6
229533.A0A1C3YKC0 pp 229533.I1RR73
229533.A0A1C3YKC0 pp 229533.I1RZZ9
229533.A0A1C3YKC0 pp 229533.I1RE22
229533.A0A1C3YKC0 pp 229533.A0A1C3YLZ7
229533.A0A1C3YKC0 pp 229533.V6R3X5
229533.A0A1C3YKC0 pp 229533.V6QUW4
229533.A0A1C3YKC0 pp 229533.I1RW25
229533.A0A1C3YKC0 pp 229533.I1RJU3
229533.A0A1C3YKC0 pp 229533.I1RMG8
229533.A0A1C3YKC0 pp 229533.I1RX25
229533.A0A1C3YKC0 pp 229533.I1RIG2
229533.A0A1C3YKC0 pp 229533.I1RWL2
229533.A0A1C3YKC0 pp 229533.A0A1C3YKM9
229533.A0A1C3YKC0 pp 229533.A0A1C3YN30
229533.A0A1C3YKC0 pp 229533.I1RA11
229533.A0A1C3YKC0 pp 229533.I1RZJ5
229533.A0A1C3YKC0 pp 229533.I1RBY4
229533.A0A1C3YKC0 pp 229533.I1RPU3
229533.A0A1C3YKC0 pp 229533.I1RZ39
229533.A0A1C3YKC0 pp 229533.I1RE76
229533.A0A1C3YKC0 pp 229533.I1RRM5
229533.A0A1C3YKC0 pp 229533.I1RJZ7
229533.A0A1C3YKC0 pp 229533.I1RRT8
229533.A0A1C3YKC0 pp 229533.V6R4J1
229533.A0A1C3YKC0 pp 229533.I1RLC7
229533.A0A1C3YKC0 pp 229533.I1S5R1
229533.A0A1C3YKC0 pp 229533.I1RVQ0

143811	229533.A0A1C3YKC0	pp	229533.I1S2B8
143812	229533.A0A1C3YKC0	pp	229533.I1RBS0
143813	229533.A0A1C3YKC0	pp	229533.I1RFQ7
143814	229533.A0A1C3YKC0	pp	229533.I1RM82
143815	229533.A0A1C3YKC0	pp	229533.I1RWJ8
143816	229533.A0A1C3YKC0	pp	229533.I1S0F6
143817	229533.A0A1C3YKC0	pp	229533.I1R9M0
143818	229533.A0A1C3YKC0	pp	229533.I1RW07
143819	229533.A0A1C3YKC0	pp	229533.I1RN67
143820	229533.A0A1C3YKC0	pp	229533.A0A1C3YMA1
143821	229533.A0A1C3YKC0	pp	229533.I1S0L4
143822	229533.A0A1C3YKC0	pp	229533.I1RI02
143823	229533.A0A1C3YKC0	pp	229533.I1RIL3
143824	229533.A0A1C3YKC0	pp	229533.I1S2H4
143825	229533.A0A1C3YKC0	pp	229533.I1S0L3
143826	229533.A0A1C3YKC0	pp	229533.A0A1C3YM63
143827	229533.A0A1C3YKC0	pp	229533.V6R283
143828	229533.A0A1C3YKC0	pp	229533.I1RWK3
143829	229533.A0A1C3YKC0	pp	229533.I1S2G7
143830	229533.A0A1C3YKC0	pp	229533.I1RS20
143831	229533.A0A1C3YKC0	pp	229533.I1S0F5
143832	229533.A0A1C3YKC0	pp	229533.I1RMC8
143833	229533.A0A1C3YKC0	pp	229533.I1RR24
143834	229533.A0A1C3YKC0	pp	229533.I1RN61
143835	229533.A0A1C3YKC0	pp	229533.V6R1K4
143836	229533.A0A1C3YKC0	pp	229533.I1RFT6
143837	229533.A0A1C3YKC0	pp	229533.V6QW22
143838	229533.A0A1C3YKC0	pp	229533.A0A1C3YL15
143839	229533.A0A1C3YKC0	pp	229533.I1RTD5
143840	229533.A0A1C3YKC0	pp	229533.I1RT40
143841	229533.A0A1C3YKC0	pp	229533.I1RCJ7
143842	229533.A0A1C3YKC0	pp	229533.I1RRJ8
143843	229533.A0A1C3YKC0	pp	229533.I1RC20
143844	229533.A0A1C3YKC0	pp	229533.I1RQG4
143845	229533.A0A1C3YKC0	pp	229533.I1RQ65
143846	229533.A0A1C3YKC0	pp	229533.I1RD52
143847	229533.A0A1C3YKC0	pp	229533.I1RV91
143848	229533.A0A1C3YKC0	pp	229533.I1RSQ7
143849	229533.A0A1C3YKC0	pp	229533.I1RXD7
143850	229533.A0A1C3YKC0	pp	229533.I1RG53
143851	229533.A0A1C3YKC0	pp	229533.I1RS47
143852	229533.A0A1C3YKC0	pp	229533.I1RIL8
143853	229533.A0A1C3YKC0	pp	229533.I1RB74
143854	229533.A0A1C3YKC0	pp	229533.I1S8P5
143855	229533.A0A1C3YKC0	pp	229533.I1RPP3
143856	229533.A0A1C3YKC0	pp	229533.I1RQH3
143857	229533.A0A1C3YKC0	pp	229533.I1S2E9
143858	229533.A0A1C3YKC0	pp	229533.Q4IJT5
143859	229533.A0A1C3YKC0	pp	229533.I1RT04
143860	229533.A0A1C3YKC0	pp	229533.I1RQE7
143861	229533.A0A1C3YKC0	pp	229533.I1RSV2
143862	229533.A0A1C3YKC0	pp	229533.I1RR00
143863	229533.A0A1C3YKC0	pp	229533.I1S0R7
143864	229533.A0A1C3YKC0	pp	229533.I1RQC9
143865	229533.A0A1C3YKC0	pp	229533.I1RJZ6
143866	229533.A0A1C3YKC0	pp	229533.A0A1C3YKN3
143867	229533.A0A1C3YKC0	pp	229533.I1RFT1
143868	229533.A0A1C3YKC0	pp	229533.I1S352
143869	229533.A0A1C3YKC0	pp	229533.I1RZZ1
143870	229533.A0A1C3YKC0	pp	229533.I1RNA5
143871	229533.A0A1C3YKC0	pp	229533.I1RCH5
143872	229533.A0A1C3YKC0	pp	229533.I1RBC9
143873	229533.A0A1C3YKC0	pp	229533.I1RE00
143874	229533.A0A1C3YKC0	pp	229533.I1RQA3
143875	229533.A0A1C3YKC0	pp	229533.I1RW71
143876	229533.A0A1C3YKC0	pp	229533.I1RSI0
143877	229533.A0A1C3YKC0	pp	229533.I1RX46
143878	229533.A0A1C3YKC0	pp	229533.I1RLM7
143879	229533.A0A1C3YKC0	pp	229533.Q4IJK2
143880	229533.A0A1C3YKC0	pp	229533.I1RFL4
143881	229533.A0A1C3YKC0	pp	229533.I1RZE5
143882	229533.A0A1C3YKC0	pp	229533.I1RGL4
143883	229533.A0A1C3YKC0	pp	229533.I1RJW6

143884 229533.A0A1C3YKC0 pp 229533.I1RN18
143885 229533.A0A1C3YKC0 pp 229533.A0A1C3YLK8
143886 229533.A0A1C3YKC0 pp 229533.I1S503
143887 229533.A0A1C3YKC0 pp 229533.I1RZ35
143888 229533.A0A1C3YKC0 pp 229533.I1RDG0
143889 229533.A0A1C3YKC0 pp 229533.I1RB92
143890 229533.A0A1C3YKC0 pp 229533.I1R9J6
143891 229533.A0A1C3YKC0 pp 229533.Q4I2J8
143892 229533.A0A1C3YKC0 pp 229533.I1RRN5
143893 229533.A0A1C3YKC0 pp 229533.I1RLN6
143894 229533.A0A1C3YKC0 pp 229533.I1RJV4
143895 229533.A0A1C3YKC0 pp 229533.V6RTJ8
143896 229533.A0A1C3YKC0 pp 229533.I1RG90
143897 229533.A0A1C3YKC0 pp 229533.I1RVX0
143898 229533.A0A1C3YKC0 pp 229533.I1RB41
143899 229533.A0A1C3YKC0 pp 229533.I1RT03
143900 229533.A0A1C3YKC0 pp 229533.I1RWD7
143901 229533.A0A1C3YKC0 pp 229533.I1RAU7
143902 229533.A0A1C3YKC0 pp 229533.I1S7A8
143903 229533.A0A1C3YKC0 pp 229533.Q4IJM4
143904 229533.A0A1C3YKC0 pp 229533.I1RB75
143905 229533.A0A1C3YKC0 pp 229533.I1RP27
143906 229533.A0A1C3YKC0 pp 229533.I1RD17
143907 229533.A0A1C3YKC0 pp 229533.I1RJV3
143908 229533.A0A1C3YKC0 pp 229533.Q4I624
143909 229533.A0A1C3YKC0 pp 229533.I1REK8
143910 229533.A0A1C3YKC0 pp 229533.I1RGA4
143911 229533.A0A1C3YKC0 pp 229533.I1RAX6
143912 229533.A0A1C3YKC0 pp 229533.I1RBS6
143913 229533.A0A1C3YKC0 pp 229533.I1RI03
143914 229533.A0A1C3YKC0 pp 229533.A0A1C3YMK1
143915 229533.A0A1C3YKC0 pp 229533.I1RGF5
143916 229533.A0A1C3YKC0 pp 229533.I1RWN6
143917 229533.A0A1C3YKC0 pp 229533.I1S1J7
143918 229533.A0A1C3YKC0 pp 229533.I1RCR5
143919 229533.A0A1C3YKC0 pp 229533.I1S569
143920 229533.A0A1C3YKC0 pp 229533.I1RB53
143921 229533.A0A1C3YKC0 pp 229533.I1RWJ3
143922 229533.A0A1C3YKC0 pp 229533.V6R057
143923 229533.A0A1C3YKC0 pp 229533.I1RRY9
143924 229533.A0A1C3YKC0 pp 229533.I1RPR9
143925 229533.A0A1C3YKC0 pp 229533.I1RSK1
143926 229533.A0A1C3YKC0 pp 229533.I1RCM3
143927 229533.A0A1C3YKC0 pp 229533.I1RQQ5
143928 229533.A0A1C3YKC0 pp 229533.I1RZW8
143929 229533.A0A1C3YKC0 pp 229533.I1RAW0
143930 229533.A0A1C3YKC0 pp 229533.I1RD39
143931 229533.A0A1C3YKC0 pp 229533.I1RWX5
143932 229533.A0A1C3YKC0 pp 229533.Q4I283
143933 229533.A0A1C3YKC0 pp 229533.I1RU29
143934 229533.A0A1C3YKC0 pp 229533.I1RYY5
143935 229533.A0A1C3YKC0 pp 229533.I1R9D1
143936 229533.A0A1C3YKC0 pp 229533.I1RW99
143937 229533.A0A1C3YKC0 pp 229533.I1RRI2
143938 229533.A0A1C3YKC0 pp 229533.A0A1C3YLK2
143939 229533.A0A1C3YKC0 pp 229533.I1RY03
143940 229533.A0A1C3YKC0 pp 229533.I1S0N4
143941 229533.A0A1C3YKC0 pp 229533.I1S504
143942 229533.A0A1C3YKC0 pp 229533.I1S1W4
143943 229533.A0A1C3YKC0 pp 229533.I1S1C7
143944 229533.A0A1C3YKC0 pp 229533.I1RYW3
143945 229533.A0A1C3YKC0 pp 229533.I1S044
143946 229533.A0A1C3YKC0 pp 229533.I1RRE5
143947 229533.A0A1C3YKC0 pp 229533.I1S0J1
143948 229533.A0A1C3YKC0 pp 229533.I1RC17
143949 229533.A0A1C3YKC0 pp 229533.I1S5B0
143950 229533.A0A1C3YKC0 pp 229533.I1SA32
143951 229533.A0A1C3YKC0 pp 229533.I1RG57
143952 229533.A0A1C3YKC0 pp 229533.I1RZK5
143953 229533.A0A1C3YKC0 pp 229533.A0A1C3YMI5
143954 229533.A0A1C3YKC0 pp 229533.I1RI82
143955 229533.A0A1C3YKC0 pp 229533.I1RZS7
143956 229533.A0A1C3YKC0 pp 229533.I1RTA0

143957	229533.A0A1C3YKC0	pp	229533.I1RCR8
143958	229533.A0A1C3YKC0	pp	229533.I1RTH6
143959	229533.A0A1C3YKC0	pp	229533.I1RXF0
143960	229533.A0A1C3YKC0	pp	229533.I1S8R3
143961	229533.A0A1C3YKC0	pp	229533.I1RCG4
143962	229533.A0A1C3YKC0	pp	229533.I1RE01
143963	229533.A0A1C3YKC0	pp	229533.I1RZM8
143964	229533.A0A1C3YKC0	pp	229533.I1RZH4
143965	229533.A0A1C3YKC0	pp	229533.I1RW46
143966	229533.A0A1C3YKC0	pp	229533.I1RA70
143967	229533.A0A1C3YKC0	pp	229533.I1RFU8
143968	229533.A0A1C3YKC0	pp	229533.Q4IN63
143969	229533.A0A1C3YKC0	pp	229533.I1RZM1
143970	229533.A0A1C3YKC0	pp	229533.I1S0C9
143971	229533.A0A1C3YKC0	pp	229533.I1RXT3
143972	229533.A0A1C3YKC0	pp	229533.I1RS18
143973	229533.A0A1C3YKC0	pp	229533.Q4I8P5
143974	229533.A0A1C3YKC0	pp	229533.I1RNC1
143975	229533.A0A1C3YKC0	pp	229533.I1RPP4
143976	229533.A0A1C3YKC0	pp	229533.I1RLU6
143977	229533.A0A1C3YKC0	pp	229533.I1RNQ5
143978	229533.A0A1C3YKC0	pp	229533.I1RUG3
143979	229533.A0A1C3YKC0	pp	229533.I1RY44
143980	229533.A0A1C3YKC0	pp	229533.I1RBJ5
143981	229533.A0A1C3YKC0	pp	229533.I1RS17
143982	229533.A0A1C3YKC0	pp	229533.I1RQ81
143983	229533.A0A1C3YKC0	pp	229533.A0A1C3YKV3
143984	229533.A0A1C3YKC0	pp	229533.V6R042
143985	229533.A0A1C3YKC0	pp	229533.Q4HZB8
143986	229533.A0A1C3YKC0	pp	229533.I1RSQ3
143987	229533.A0A1C3YKC0	pp	229533.I1RD22
143988	229533.A0A1C3YKC0	pp	229533.V6RIV9
143989	229533.A0A1C3YKC0	pp	229533.I1RAU5
143990	229533.A0A1C3YKC0	pp	229533.I1RRU9
143991	229533.A0A1C3YKC0	pp	229533.I1RFT2
143992	229533.A0A1C3YKC0	pp	229533.V6RMW6
143993	229533.A0A1C3YKC0	pp	229533.I1SA33
143994	229533.A0A1C3YKC0	pp	229533.I1RWN5
143995	229533.A0A1C3YKC0	pp	229533.I1RC16
143996	229533.A0A1C3YKC0	pp	229533.V6RCG5
143997	229533.A0A1C3YKC0	pp	229533.A0A1C3YLU7
143998	229533.A0A1C3YKC0	pp	229533.I1RMN6
143999	229533.A0A1C3YKC0	pp	229533.I1RRV1
144000	229533.A0A1C3YKC0	pp	229533.I1RWT6
144001	229533.A0A1C3YKC0	pp	229533.A0A1C3YKW9
144002	229533.A0A1C3YKC0	pp	229533.I1RGC2
144003	229533.A0A1C3YKC0	pp	229533.I1RMG3
144004	229533.A0A1C3YKC0	pp	229533.V6RCZ8
144005	229533.A0A1C3YKC0	pp	229533.I1RJP4
144006	229533.A0A1C3YLK2	pp	229533.Q4I8P5
144007	229533.A0A1C3YLK2	pp	229533.I1RCL0
144008	229533.A0A1C3YLK2	pp	229533.I1RSI0
144009	229533.A0A1C3YLK2	pp	229533.I1RAU7
144010	229533.A0A1C3YLK2	pp	229533.V6R057
144011	229533.A0A1C3YLK2	pp	229533.I1RWB5
144012	229533.A0A1C3YLK2	pp	229533.I1RS17
144013	229533.A0A1C3YLK2	pp	229533.I1REK8
144014	229533.A0A1C3YLK2	pp	229533.I1RD17
144015	229533.A0A1C3YLK2	pp	229533.I1RAU5
144016	229533.A0A1C3YLK2	pp	229533.I1RD22
144017	229533.A0A1C3YLK2	pp	229533.I1RGC2
144018	229533.A0A1C3YLK2	pp	229533.I1SA33
144019	229533.A0A1C3YLK2	pp	229533.I1RCH5
144020	229533.A0A1C3YLK2	pp	229533.I1RZZ1
144021	229533.A0A1C3YLK2	pp	229533.I1S6E5
144022	229533.A0A1C3YLK2	pp	229533.I1RQE7
144023	229533.A0A1C3YLK2	pp	229533.I1S044
144024	229533.A0A1C3YLK2	pp	229533.A0A1C3YN30
144025	229533.A0A1C3YLK2	pp	229533.I1RN18
144026	229533.A0A1C3YLK2	pp	229533.I1S8E2
144027	229533.A0A1C3YLK2	pp	229533.Q4IJK2
144028	229533.A0A1C3YLK2	pp	229533.I1RFL4
144029	229533.A0A1C3YLK2	pp	229533.I1S5B0

144030	229533.A0A1C3YLK2	pp	229533.I1RJU3
144031	229533.A0A1C3YLK2	pp	229533.I1RVQ0
144032	229533.A0A1C3YLK2	pp	229533.I1RW99
144033	229533.A0A1C3YLK2	pp	229533.I1RWQ8
144034	229533.A0A1C3YLK2	pp	229533.I1RDG0
144035	229533.A0A1C3YLK2	pp	229533.I1RB92
144036	229533.A0A1C3YLK2	pp	229533.I1RN67
144037	229533.A0A1C3YLK2	pp	229533.I1RI82
144038	229533.A0A1C3YLK2	pp	229533.I1RVX0
144039	229533.A0A1C3YLK2	pp	229533.I1RT03
144040	229533.A0A1C3YLK2	pp	229533.I1S0F6
144041	229533.A0A1C3YLK2	pp	229533.I1RBZ3
144042	229533.A0A1C3YLK2	pp	229533.I1RYB9
144043	229533.A0A1C3YLK2	pp	229533.V6R1K4
144044	229533.A0A1C3YLK2	pp	229533.I1RUG3
144045	229533.A0A1C3YLK2	pp	229533.I1RNC1
144046	229533.A0A1C3YLK2	pp	229533.I1RC81
144047	229533.A0A1C3YLK2	pp	229533.I1S0L3
144048	229533.A0A1C3YLK2	pp	229533.I1RJV3
144049	229533.A0A1C3YLK2	pp	229533.I1RWN6
144050	229533.A0A1C3YLK2	pp	229533.I1RRY9
144051	229533.A0A1C3YLK2	pp	229533.I1S265
144052	229533.A0A1C3YLK2	pp	229533.I1RZM8
144053	229533.A0A1C3YLK2	pp	229533.I1RCR8
144054	229533.A0A1C3YLK2	pp	229533.I1RD52
144055	229533.A0A1C3YLK2	pp	229533.I1RD39
144056	229533.A0A1C3YLK2	pp	229533.V6RCG5
144057	229533.A0A1C3YLK2	pp	229533.I1RAW0
144058	229533.A0A1C3YLK2	pp	229533.I1RWK3
144059	229533.A0A1C3YLK2	pp	229533.V6RMW6
144060	229533.A0A1C3YLK2	pp	229533.I1RE71
144061	229533.A0A1C3YLK2	pp	229533.I1RZW8
144062	229533.A0A1C3YLK2	pp	229533.I1RN63
144063	229533.A0A1C3YLK2	pp	229533.I1S1C7
144064	229533.A0A1C3YLK2	pp	229533.I1RLX7
144065	229533.A0A1C3YLK2	pp	229533.V6R042
144066	229533.A0A1C3YLK2	pp	229533.I1RWH5
144067	229533.A0A1C3YLK2	pp	229533.I1RSQ3
144068	229533.A0A1C3YLK2	pp	229533.I1RY03
144069	229533.A0A1C3YLK2	pp	229533.I1RPP3
144070	229533.A0A1C3YLK2	pp	229533.I1RBY4
144071	229533.A0A1C3YLK2	pp	229533.I1RG53
144072	229533.A0A1C3YLK2	pp	229533.I1RZK5
144073	229533.A0A1C3YLK2	pp	229533.I1RK83
144074	229533.A0A1C3YLK2	pp	229533.I1RW25
144075	229533.A0A1C3YLK2	pp	229533.I1S7S9
144076	229533.A0A1C3YLK2	pp	229533.I1RSQ7
144077	229533.A0A1C3YLK2	pp	229533.I1RFU8
144078	229533.A0A1C3YLK2	pp	229533.I1RA70
144079	229533.A0A1C3YLK2	pp	229533.I1R9M0
144080	229533.A0A1C3YLK2	pp	229533.I1S8R3
144081	229533.A0A1C3YLK2	pp	229533.V6QW22
144082	229533.A0A1C3YLK2	pp	229533.I1RRM5
144083	229533.A0A1C3YLK2	pp	229533.I1RCG4
144084	229533.A0A1C3YLK2	pp	229533.I1RC20
144085	229533.A0A1C3YLK2	pp	229533.I1RTH6
144086	229533.A0A1C3YLK2	pp	229533.I1S2B8
144087	229533.A0A1C3YLK2	pp	229533.I1RPM4
144088	229533.A0A1C3YLK2	pp	229533.V6RM66
144089	229533.A0A1C3YLK2	pp	229533.I1RY44
144090	229533.A0A1C3YLK2	pp	229533.I1RBC9
144091	229533.A0A1C3YLK2	pp	229533.I1RCS1
144092	229533.A0A1C3YLK2	pp	229533.I1RNQ5
144093	229533.A0A1C3YLK2	pp	229533.I1RE22
144094	229533.A0A1C3YLK2	pp	229533.I1RX46
144095	229533.A0A1C3YLK2	pp	229533.I1S5R1
144096	229533.A0A1C3YLK2	pp	229533.I1RRT8
144097	229533.A0A1C3YLK2	pp	229533.I1RLU6
144098	229533.A0A1C3YLK2	pp	229533.I1RZM1
144099	229533.A0A1C3YLK2	pp	229533.I1RJW6
144100	229533.A0A1C3YLK2	pp	229533.I1S503
144101	229533.A0A1C3YLK2	pp	229533.V6RIV9
144102	229533.A0A1C3YLK2	pp	229533.A0A1C3YLK8

144103 229533.A0A1C3YLK2 pp 229533.I1RZV6
144104 229533.A0A1C3YLK2 pp 229533.I1S4S4
144105 229533.A0A1C3YLK2 pp 229533.I1RWN5
144106 229533.A0A1C3YLK2 pp 229533.V6RTJ8
144107 229533.A0A1C3YLK2 pp 229533.I1RRE5
144108 229533.A0A1C3YLK2 pp 229533.I1RJV4
144109 229533.A0A1C3YLK2 pp 229533.I1RWJ8
144110 229533.A0A1C3YLK2 pp 229533.I1RBS6
144111 229533.A0A1C3YLK2 pp 229533.I1RGA4
144112 229533.A0A1C3YLK2 pp 229533.I1S504
144113 229533.A0A1C3YLK2 pp 229533.I1S0L4
144114 229533.A0A1C3YLK2 pp 229533.I1RAX6
144115 229533.A0A1C3YLK2 pp 229533.I1RR24
144116 229533.A0A1C3YLK2 pp 229533.I1SA32
144117 229533.A0A1C3YLK2 pp 229533.I1RTD5
144118 229533.A0A1C3YLK2 pp 229533.A0A1C3YMK1
144119 229533.A0A1C3YLK2 pp 229533.I1S0J1
144120 229533.A0A1C3YLK2 pp 229533.I1RS20
144121 229533.A0A1C3YLK2 pp 229533.I1RV91
144122 229533.A0A1C3YLK2 pp 229533.I1RE01
144123 229533.A0A1C3YLK2 pp 229533.I1RT40
144124 229533.A0A1C3YLK2 pp 229533.I1RZZ9
144125 229533.A0A1C3YLK2 pp 229533.I1RBT1
144126 229533.A0A1C3YLK2 pp 229533.V6R3X5
144127 229533.A0A1C3YLK2 pp 229533.I1RZS7
144128 229533.A0A1C3YLK2 pp 229533.I1RW46
144129 229533.A0A1C3YLK2 pp 229533.I1S2E9
144130 229533.A0A1C3YLK2 pp 229533.I1RPP4
144131 229533.A0A1C3YLK2 pp 229533.I1RXD7
144132 229533.A0A1C3YLK2 pp 229533.I1RND6
144133 229533.A0A1C3YLK2 pp 229533.I1RR73
144134 229533.A0A1C3YLK2 pp 229533.I1RQC9
144135 229533.A0A1C3YLK2 pp 229533.I1RCN7
144136 229533.A0A1C3YLK2 pp 229533.I1RZ39
144137 229533.A0A1C3YLK2 pp 229533.I1RT04
144138 229533.A0A1C3YLK2 pp 229533.I1S0R7
144139 229533.I1RAY4 pp 229533.I1RB74
144140 229533.I1RAY4 pp 229533.I1RFW0
144141 229533.I1RAY4 pp 229533.I1RGF1
144142 229533.I1RAY4 pp 229533.I1S7X9
144143 229533.I1RAY4 pp 229533.I1RS47
144144 229533.I1RAY4 pp 229533.I1RZC3
144145 229533.I1RAY4 pp 229533.I1RQD6
144146 229533.I1RAY4 pp 229533.I1RX64
144147 229533.I1RAY4 pp 229533.I1S248
144148 229533.I1RAY4 pp 229533.I1RUN8
144149 229533.I1RAY4 pp 229533.I1S0X7
144150 229533.I1RAY4 pp 229533.I1RMA7
144151 229533.I1RAY4 pp 229533.I1RNY8
144152 229533.I1RAY4 pp 229533.I1S286
144153 229533.I1RAY4 pp 229533.I1RQB4
144154 229533.I1RAY4 pp 229533.I1RC46
144155 229533.I1RAY4 pp 229533.I1S1W4
144156 229533.I1RAY4 pp 229533.V6RKY7
144157 229533.I1RAY4 pp 229533.I1RRN5
144158 229533.I1RAY4 pp 229533.I1RY32
144159 229533.I1RAY4 pp 229533.V6RVG3
144160 229533.I1RAY4 pp 229533.I1RDB1
144161 229533.I1RAY4 pp 229533.I1S247
144162 229533.I1RAY4 pp 229533.I1RZE4
144163 229533.I1RAY4 pp 229533.I1RX87
144164 229533.I1RAY4 pp 229533.I1RPA6
144165 229533.I1RAY4 pp 229533.I1RV66
144166 229533.I1RAY4 pp 229533.I1RWT6
144167 229533.I1RAY4 pp 229533.I1RMB2
144168 229533.I1RAY4 pp 229533.I1RQA3
144169 229533.I1RAY4 pp 229533.I1RMM9
144170 229533.I1RAY4 pp 229533.I1RX77
144171 229533.I1RAY4 pp 229533.I1RB75
144172 229533.I1RAY4 pp 229533.I1RCB3
144173 229533.I1RAY4 pp 229533.I1RX04
144174 229533.I1RAY4 pp 229533.I1S439
144175 229533.I1RAY4 pp 229533.I1RQL4

144176	229533.I1RAY4	pp	229533.I1RCD1
144177	229533.I1RAY4	pp	229533.I1S278
144178	229533.I1RAY4	pp	229533.I1RC22
144179	229533.I1RAY4	pp	229533.I1RKK0
144180	229533.I1RAY4	pp	229533.Q4IEA7
144181	229533.I1RAY4	pp	229533.I1RUQ5
144182	229533.I1RAY4	pp	229533.I1RSS1
144183	229533.I1RAY4	pp	229533.I1RYL5
144184	229533.I1S438	pp	229533.Q4IJK2
144185	229533.I1S438	pp	229533.I1S5B0
144186	229533.I1S438	pp	229533.I1S5R6
144187	229533.I1S438	pp	229533.Q4I624
144188	229533.I1S438	pp	229533.I1S5R1
144189	229533.I1S438	pp	229533.V6RCZ8
144190	229533.I1S438	pp	229533.V6R042
144191	229533.I1S438	pp	229533.V6RMW6
144192	229533.I1S438	pp	229533.I1S821
144193	229533.I1S438	pp	229533.I1S4S7
144194	229533.I1S438	pp	229533.I1S4S4
144195	229533.I1S438	pp	229533.I1S6K9
144196	229533.I1S438	pp	229533.V6RTJ8
144197	229533.I1RYY5	pp	229533.Q4HYI0
144198	229533.I1RYY5	pp	229533.I1RZB5
144199	229533.I1RYY5	pp	229533.V6RTJ8
144200	229533.I1RYY5	pp	229533.Q4ICA8
144201	229533.I1RYY5	pp	229533.I1S439
144202	229533.I1RYY5	pp	229533.I1RZK5
144203	229533.I1RYY5	pp	229533.I1RZM1
144204	229533.I1RYY5	pp	229533.V6QV09
144205	229533.I1RYY5	pp	229533.Q4I7L0
144206	229533.I1RYY5	pp	229533.I1RYZ9
144207	229533.I1RYY5	pp	229533.Q4IJK2
144208	229533.I1RYY5	pp	229533.I1S504
144209	229533.I1RYY5	pp	229533.I1RZ39
144210	229533.I1RYY5	pp	229533.I1RZ63
144211	229533.I1RYY5	pp	229533.V6R3X5
144212	229533.I1RYY5	pp	229533.I1S0K2
144213	229533.I1RYY5	pp	229533.Q4IJM4
144214	229533.I1RYY5	pp	229533.I1S0J3
144215	229533.I1RYY5	pp	229533.I1S0J7
144216	229533.I1RYY5	pp	229533.V6RGI1
144217	229533.I1RYY5	pp	229533.I1S2B8
144218	229533.I1RYY5	pp	229533.I1S0E8
144219	229533.I1RYY5	pp	229533.I1RZE4
144220	229533.I1RYY5	pp	229533.I1S2E9
144221	229533.I1RYY5	pp	229533.V6RVG3
144222	229533.I1RYY5	pp	229533.I1S503
144223	229533.I1RYY5	pp	229533.I1S0N4
144224	229533.I1RYY5	pp	229533.V6RMW6
144225	229533.I1RYY5	pp	229533.V6RKY7
144226	229533.I1RA10	pp	229533.I1RKE9
144227	229533.I1RA10	pp	229533.I1RFJ2
144228	229533.I1RA10	pp	229533.I1RY44
144229	229533.I1RA10	pp	229533.I1S016
144230	229533.I1RA10	pp	229533.I1RTN6
144231	229533.I1RA10	pp	229533.I1RZP8
144232	229533.I1RA10	pp	229533.I1S4P6
144233	229533.I1RA10	pp	229533.I1RX64
144234	229533.I1RA10	pp	229533.I1RG77
144235	229533.I1RA10	pp	229533.I1RN55
144236	229533.I1RA10	pp	229533.I1RAX3
144237	229533.I1RA10	pp	229533.I1RQH8
144238	229533.I1RA10	pp	229533.I1RBN9
144239	229533.I1RA10	pp	229533.Q4INS6
144240	229533.I1RA10	pp	229533.I1RDH0
144241	229533.I1RA10	pp	229533.I1RQJ4
144242	229533.I1RA10	pp	229533.I1RDJ8
144243	229533.I1RA10	pp	229533.I1RQA3
144244	229533.I1RA10	pp	229533.I1RSP7
144245	229533.I1RA10	pp	229533.I1RCL7
144246	229533.I1RA10	pp	229533.I1RSC7
144247	229533.I1RA10	pp	229533.I1RAU8
144248	229533.I1RA10	pp	229533.I1RQ72

144249	229533.I1RA10	pp	229533.I1RQB4
144250	229533.I1RA10	pp	229533.I1RDX0
144251	229533.I1RA10	pp	229533.V6RN69
144252	229533.I1RA10	pp	229533.I1S461
144253	229533.I1RA10	pp	229533.I1RUG4
144254	229533.I1RA10	pp	229533.I1S0D5
144255	229533.I1RA10	pp	229533.I1RSR9
144256	229533.I1RA10	pp	229533.I1S0A6
144257	229533.I1RA10	pp	229533.I1RMS4
144258	229533.I1RA10	pp	229533.I1S209
144259	229533.I1RA10	pp	229533.I1RC54
144260	229533.I1RA10	pp	229533.I1RN57
144261	229533.I1RA10	pp	229533.I1RBC8
144262	229533.I1RA10	pp	229533.I1RSA7
144263	229533.I1RA10	pp	229533.I1RRS7
144264	229533.I1RA10	pp	229533.I1RMQ7
144265	229533.I1RA10	pp	229533.I1RZ94
144266	229533.I1RA10	pp	229533.I1RMX3
144267	229533.I1RA10	pp	229533.I1RMB7
144268	229533.I1RA10	pp	229533.I1RB00
144269	229533.I1RA10	pp	229533.Q4IB89
144270	229533.I1RA10	pp	229533.I1RAH4
144271	229533.I1RA10	pp	229533.I1RBW0
144272	229533.I1RA10	pp	229533.V6RLW4
144273	229533.I1RA10	pp	229533.I1RZ86
144274	229533.I1RA10	pp	229533.I1RD01
144275	229533.I1RA10	pp	229533.Q4HYB8
144276	229533.I1RA10	pp	229533.I1RQ13
144277	229533.I1RA10	pp	229533.I1RWT6
144278	229533.I1RA10	pp	229533.I1S987
144279	229533.I1RA10	pp	229533.I1RFX8
144280	229533.I1RA10	pp	229533.I1RSD0
144281	229533.I1RA10	pp	229533.I1RCA5
144282	229533.I1RA10	pp	229533.I1RA12
144283	229533.I1RA10	pp	229533.I1RBU5
144284	229533.I1RA10	pp	229533.I1RNF4
144285	229533.I1RA10	pp	229533.I1RRT1
144286	229533.I1RA10	pp	229533.I1RBW1
144287	229533.I1RA10	pp	229533.I1RRJ6
144288	229533.I1RA10	pp	229533.I1S2F2
144289	229533.I1RA10	pp	229533.I1RAT5
144290	229533.I1RA10	pp	229533.I1RYR2
144291	229533.I1RA10	pp	229533.I1S283
144292	229533.I1RA10	pp	229533.I1S998
144293	229533.I1RA10	pp	229533.I1RVW0
144294	229533.I1RA10	pp	229533.I1RSN7
144295	229533.I1RA10	pp	229533.I1RV66
144296	229533.I1RA10	pp	229533.I1S2E9
144297	229533.I1RA10	pp	229533.I1RKI2
144298	229533.I1RA10	pp	229533.I1RFL7
144299	229533.I1RA10	pp	229533.I1RYX9
144300	229533.I1RA10	pp	229533.I1RWI8
144301	229533.I1RA10	pp	229533.I1RSJ1
144302	229533.I1RA10	pp	229533.I1RPN1
144303	229533.I1RA10	pp	229533.I1RSI4
144304	229533.I1RA10	pp	229533.I1RAL9
144305	229533.I1RA10	pp	229533.I1RPQ1
144306	229533.I1RA10	pp	229533.I1RYQ1
144307	229533.I1RA10	pp	229533.I1RKB1
144308	229533.I1RA10	pp	229533.I1RJV8
144309	229533.I1RA10	pp	229533.I1RRY1
144310	229533.I1RBJ5	pp	229533.I1S2H4
144311	229533.I1RBJ5	pp	229533.I1RRT8
144312	229533.I1RBJ5	pp	229533.I1RZ39
144313	229533.I1RBJ5	pp	229533.I1S0L4
144314	229533.I1RBJ5	pp	229533.I1RZZ9
144315	229533.I1RBJ5	pp	229533.I1RN18
144316	229533.I1RBJ5	pp	229533.V6R3X5
144317	229533.I1RBJ5	pp	229533.Q4I1Y5
144318	229533.I1RBJ5	pp	229533.I1RR73
144319	229533.I1RBJ5	pp	229533.I1RFL4
144320	229533.I1RBJ5	pp	229533.I1S2B8
144321	229533.I1RBJ5	pp	229533.I1RRM5

144322 229533.I1RBJ5 pp 229533.I1RVX0
144323 229533.I1RBJ5 pp 229533.I1RN67
144324 229533.I1RBJ5 pp 229533.I1RWK3
144325 229533.I1RBJ5 pp 229533.I1RT03
144326 229533.I1RBJ5 pp 229533.I1RQ75
144327 229533.I1RBJ5 pp 229533.I1RBY4
144328 229533.I1RBJ5 pp 229533.I1S8P5
144329 229533.I1RBJ5 pp 229533.I1RPM4
144330 229533.I1RBJ5 pp 229533.I1RD22
144331 229533.I1RBJ5 pp 229533.I1S503
144332 229533.I1RBJ5 pp 229533.I1RUG3
144333 229533.I1RBJ5 pp 229533.I1RCR8
144334 229533.I1RBJ5 pp 229533.I1RJW6
144335 229533.I1RBJ5 pp 229533.I1RPP3
144336 229533.I1RBJ5 pp 229533.I1RJV3
144337 229533.I1RBJ5 pp 229533.I1RBS6
144338 229533.I1RBJ5 pp 229533.I1RJV4
144339 229533.I1RBJ5 pp 229533.I1RWN6
144340 229533.I1RBJ5 pp 229533.I1S1C7
144341 229533.I1RBJ5 pp 229533.I1RNC1
144342 229533.I1RBJ5 pp 229533.I1RTD5
144343 229533.I1RBJ5 pp 229533.I1RV91
144344 229533.I1RBJ5 pp 229533.I1S2E9
144345 229533.I1RBJ5 pp 229533.V6RMW6
144346 229533.I1RBJ5 pp 229533.I1RT04
144347 229533.I1RBJ5 pp 229533.I1S8R3
144348 229533.I1RBJ5 pp 229533.V6R042
144349 229533.I1RBJ5 pp 229533.I1RZW8
144350 229533.I1RBJ5 pp 229533.I1RSQ3
144351 229533.I1RBJ5 pp 229533.I1RTH6
144352 229533.I1RBJ5 pp 229533.I1RD17
144353 229533.I1RBJ5 pp 229533.I1RJW1
144354 229533.I1RBJ5 pp 229533.V6RTJ8
144355 229533.I1RBJ5 pp 229533.V6RIV9
144356 229533.I1RBJ5 pp 229533.I1RPP4
144357 229533.I1RBJ5 pp 229533.I1RK44
144358 229533.I1RBJ5 pp 229533.I1RQC9
144359 229533.I1RBJ5 pp 229533.I1RR24
144360 229533.I1RBJ5 pp 229533.I1S0L3
144361 229533.I1RBJ5 pp 229533.I1RVQ0
144362 229533.I1RBJ5 pp 229533.I1RWN5
144363 229533.I1RBJ5 pp 229533.I1RW25
144364 229533.I1RBJ5 pp 229533.I1RY44
144365 229533.I1RBJ5 pp 229533.I1RBT1
144366 229533.I1RBJ5 pp 229533.I1RR39
144367 229533.I1RBJ5 pp 229533.I1RX46
144368 229533.I1RBJ5 pp 229533.V6QW22
144369 229533.I1RBJ5 pp 229533.I1RSQ7
144370 229533.I1RBJ5 pp 229533.I1RTA0
144371 229533.I1RBJ5 pp 229533.I1RCG4
144372 229533.I1RBJ5 pp 229533.I1RRY9
144373 229533.I1RBJ5 pp 229533.I1RY03
144374 229533.I1RBJ5 pp 229533.V6RCG5
144375 229533.I1RBJ5 pp 229533.I1RFU8
144376 229533.I1RBJ5 pp 229533.I1REK8
144377 229533.I1RBJ5 pp 229533.I1RZK5
144378 229533.I1RBJ5 pp 229533.I1RZM1
144379 229533.I1RBJ5 pp 229533.I1S504
144380 229533.I1RBJ5 pp 229533.I1RS18
144381 229533.I1RBJ5 pp 229533.I1RLU6
144382 229533.I1RBJ5 pp 229533.I1RIE8
144383 229533.I1RBJ5 pp 229533.Q4IJK2
144384 229533.I1RBJ5 pp 229533.I1RI82
144385 229533.I1RCS0 pp 229533.I1RDG0
144386 229533.A0A1C3YJV6 pp 229533.I1RB92
144387 229533.A0A1C3YJV6 pp 229533.I1RSZ7
144388 229533.A0A1C3YJV6 pp 229533.I1S438
144389 229533.A0A1C3YJV6 pp 229533.I1RUQ3
144390 229533.A0A1C3YJV6 pp 229533.I1RGU7
144391 229533.A0A1C3YJV6 pp 229533.I1RUF7
144392 229533.A0A1C3YJV6 pp 229533.I1RT03
144393 229533.A0A1C3YJV6 pp 229533.I1S9I5
144394 229533.A0A1C3YJV6 pp 229533.I1RBY4

144395 229533.A0A1C3YJV6 pp 229533.I1RVX0
144396 229533.A0A1C3YJV6 pp 229533.I1RA11
144397 229533.A0A1C3YJV6 pp 229533.I1RML0
144398 229533.A0A1C3YJV6 pp 229533.I1RXC2
144399 229533.A0A1C3YJV6 pp 229533.I1RJV3
144400 229533.A0A1C3YJV6 pp 229533.I1S2B8
144401 229533.A0A1C3YJV6 pp 229533.I1RCR1
144402 229533.A0A1C3YJV6 pp 229533.I1R9M0
144403 229533.A0A1C3YJV6 pp 229533.I1S5R1
144404 229533.A0A1C3YJV6 pp 229533.I1RBR1
144405 229533.A0A1C3YJV6 pp 229533.V6R9Z0
144406 229533.A0A1C3YJV6 pp 229533.I1RE89
144407 229533.A0A1C3YJV6 pp 229533.I1RQ33
144408 229533.A0A1C3YJV6 pp 229533.I1RVQ3
144409 229533.A0A1C3YJV6 pp 229533.Q4I624
144410 229533.A0A1C3YJV6 pp 229533.I1RAW0
144411 229533.A0A1C3YJV6 pp 229533.I1REK8
144412 229533.A0A1C3YJV6 pp 229533.A0A1C3YLX0
144413 229533.A0A1C3YJV6 pp 229533.I1RKP1
144414 229533.A0A1C3YJV6 pp 229533.I1RQE7
144415 229533.A0A1C3YJV6 pp 229533.I1RRI1
144416 229533.A0A1C3YJV6 pp 229533.I1RCH5
144417 229533.A0A1C3YJV6 pp 229533.I1RRY9
144418 229533.A0A1C3YJV6 pp 229533.I1RWR7
144419 229533.A0A1C3YJV6 pp 229533.I1RGB2
144420 229533.A0A1C3YJV6 pp 229533.I1S0L4
144421 229533.A0A1C3YJV6 pp 229533.I1RBR2
144422 229533.A0A1C3YJV6 pp 229533.Q4IJK2
144423 229533.A0A1C3YJV6 pp 229533.I1RB26
144424 229533.A0A1C3YJV6 pp 229533.A0A1C3YLL3
144425 229533.A0A1C3YJV6 pp 229533.V6RTJ8
144426 229533.A0A1C3YJV6 pp 229533.I1RY86
144427 229533.A0A1C3YJV6 pp 229533.I1RPE0
144428 229533.A0A1C3YJV6 pp 229533.I1REC5
144429 229533.A0A1C3YJV6 pp 229533.I1RD52
144430 229533.A0A1C3YJV6 pp 229533.I1RAX6
144431 229533.A0A1C3YJV6 pp 229533.I1RR24
144432 229533.A0A1C3YJV6 pp 229533.I1RGA4
144433 229533.A0A1C3YJV6 pp 229533.I1S0J1
144434 229533.A0A1C3YJV6 pp 229533.I1RC66
144435 229533.A0A1C3YJV6 pp 229533.I1S5R6
144436 229533.A0A1C3YJV6 pp 229533.I1RNT9
144437 229533.A0A1C3YJV6 pp 229533.I1RVZ2
144438 229533.A0A1C3YJV6 pp 229533.I1RQD2
144439 229533.A0A1C3YJV6 pp 229533.I1S1C7
144440 229533.A0A1C3YJV6 pp 229533.I1RTA1
144441 229533.A0A1C3YJV6 pp 229533.I1RPH6
144442 229533.A0A1C3YJV6 pp 229533.I1RJN9
144443 229533.A0A1C3YJV6 pp 229533.I1S437
144444 229533.A0A1C3YJV6 pp 229533.A0A1C3YK34
144445 229533.A0A1C3YJV6 pp 229533.I1RKJ8
144446 229533.A0A1C3YJV6 pp 229533.I1RXF8
144447 229533.A0A1C3YJV6 pp 229533.I1RTD5
144448 229533.A0A1C3YJV6 pp 229533.I1S2R0
144449 229533.A0A1C3YJV6 pp 229533.I1RVF8
144450 229533.A0A1C3YJV6 pp 229533.I1RZK5
144451 229533.A0A1C3YJV6 pp 229533.I1RKJ5
144452 229533.A0A1C3YJV6 pp 229533.I1RX95
144453 229533.A0A1C3YJV6 pp 229533.I1RE01
144454 229533.A0A1C3YJV6 pp 229533.I1RJU3
144455 229533.A0A1C3YJV6 pp 229533.I1RBU8
144456 229533.A0A1C3YJV6 pp 229533.I1RPY3
144457 229533.A0A1C3YJV6 pp 229533.I1RKJ4
144458 229533.A0A1C3YJV6 pp 229533.I1RT40
144459 229533.A0A1C3YJV6 pp 229533.I1RXC4
144460 229533.A0A1C3YJV6 pp 229533.I1RZ39
144461 229533.A0A1C3YJV6 pp 229533.I1RSQ7
144462 229533.A0A1C3YJV6 pp 229533.I1RCR8
144463 229533.A0A1C3YJV6 pp 229533.A0A1C3YKM9
144464 229533.A0A1C3YJV6 pp 229533.I1RPP3
144465 229533.A0A1C3YJV6 pp 229533.I1RZM8
144466 229533.A0A1C3YJV6 pp 229533.I1RYI5
144467 229533.A0A1C3YJV6 pp 229533.I1RZV3

144468 229533.A0A1C3YJV6 pp 229533.I1RXD6
144469 229533.A0A1C3YJV6 pp 229533.I1RNB2
144470 229533.A0A1C3YJV6 pp 229533.I1RDF7
144471 229533.A0A1C3YJV6 pp 229533.A0A1C3YKV3
144472 229533.A0A1C3YJV6 pp 229533.I1RAG7
144473 229533.A0A1C3YJV6 pp 229533.I1RUG3
144474 229533.A0A1C3YJV6 pp 229533.I1RNC1
144475 229533.A0A1C3YJV6 pp 229533.I1S4S7
144476 229533.A0A1C3YJV6 pp 229533.I1RRM3
144477 229533.A0A1C3YJV6 pp 229533.I1S1W9
144478 229533.A0A1C3YJV6 pp 229533.I1S0W4
144479 229533.A0A1C3YJV6 pp 229533.I1S1B2
144480 229533.A0A1C3YJV6 pp 229533.I1R9H9
144481 229533.A0A1C3YJV6 pp 229533.V6RMW6
144482 229533.A0A1C3YJV6 pp 229533.I1RSQ3
144483 229533.A0A1C3YJV6 pp 229533.I1RD22
144484 229533.A0A1C3YJV6 pp 229533.I1RX46
144485 229533.A0A1C3YJV6 pp 229533.A0A1C3YLK8
144486 229533.A0A1C3YJV6 pp 229533.I1RC34
144487 229533.A0A1C3YJV6 pp 229533.I1RPU2
144488 229533.A0A1C3YJV6 pp 229533.V6R042
144489 229533.A0A1C3YJV6 pp 229533.I1RQ85
144490 229533.A0A1C3YJV6 pp 229533.I1RSA5
144491 229533.A0A1C3YJV6 pp 229533.I1RRV3
144492 229533.A0A1C3YJV6 pp 229533.I1RSF1
144493 229533.A0A1C3YJV6 pp 229533.V6RCZ8
144494 229533.A0A1C3YJV6 pp 229533.I1RTB3
144495 229533.A0A1C3YJV6 pp 229533.I1RXD7
144496 229533.A0A1C3YJV6 pp 229533.I1S821
144497 229533.A0A1C3YJV6 pp 229533.Q4HVQ9
144498 229533.A0A1C3YJV6 pp 229533.I1RGC5
144499 229533.I1S0X6 pp 229533.V6RCG5
144500 229533.I1S0X6 pp 229533.V6RCZ8
144501 229533.I1S0X6 pp 229533.Q4I283
144502 229533.I1S0X6 pp 229533.V6QUE6
144503 229533.I1S0X6 pp 229533.V6RMW6
144504 229533.I1S0X6 pp 229533.V6R042
144505 229533.I1S0X6 pp 229533.V6RIV9
144506 229533.I1S0X6 pp 229533.I1S504
144507 229533.I1S0X6 pp 229533.I1S1C7
144508 229533.I1S0X6 pp 229533.I1S5B0
144509 229533.I1S0X6 pp 229533.I1SA33
144510 229533.I1S0X6 pp 229533.Q4IJK2
144511 229533.I1S0X6 pp 229533.Q4I624
144512 229533.I1S0X6 pp 229533.I1S1J7
144513 229533.I1S0X6 pp 229533.I1S8R3
144514 229533.I1S0X6 pp 229533.Q4I2J8
144515 229533.I1S0X6 pp 229533.I1SA32
144516 229533.I1S0X6 pp 229533.V6R057
144517 229533.I1S0X6 pp 229533.I1S2E9
144518 229533.I1S0X6 pp 229533.V6R3X5
144519 229533.I1S0X6 pp 229533.Q4I8P5
144520 229533.I1S0X6 pp 229533.V6RTJ8
144521 229533.I1S0X6 pp 229533.Q4IJT5
144522 229533.I1S0X6 pp 229533.I1S5R1
144523 229533.I1S0X6 pp 229533.I1S8P5
144524 229533.I1S0X6 pp 229533.I1S2H4
144525 229533.I1S0X6 pp 229533.I1S503
144526 229533.I1S0X6 pp 229533.V6QW22
144527 229533.I1S0X6 pp 229533.V6R1K4
144528 229533.I1S0X6 pp 229533.V6QUW4
144529 229533.I1S0X6 pp 229533.I1S2B8
144530 229533.I1RNQ5 pp 229533.I1S0F6
144531 229533.I1RNQ5 pp 229533.I1RWK3
144532 229533.I1RNQ5 pp 229533.I1RS47
144533 229533.I1RNQ5 pp 229533.I1RQ65
144534 229533.I1RNQ5 pp 229533.I1S8P5
144535 229533.I1RNQ5 pp 229533.I1RWF7
144536 229533.I1RNQ5 pp 229533.V6R283
144537 229533.I1RNQ5 pp 229533.I1RPP3
144538 229533.I1RNQ5 pp 229533.I1RQN9
144539 229533.I1RNQ5 pp 229533.I1S503
144540 229533.I1RNQ5 pp 229533.I1RRJ8

144541 229533.I1RNQ5 pp 229533.I1RRZ0
144542 229533.I1RNQ5 pp 229533.I1RRT8
144543 229533.I1RNQ5 pp 229533.I1RP27
144544 229533.I1RNQ5 pp 229533.I1RSV2
144545 229533.I1RNQ5 pp 229533.I1S5R1
144546 229533.I1RNQ5 pp 229533.I1RTD5
144547 229533.I1RNQ5 pp 229533.I1RT40
144548 229533.I1RNQ5 pp 229533.I1RWL2
144549 229533.I1RNQ5 pp 229533.I1RV91
144550 229533.I1RNQ5 pp 229533.I1RS20
144551 229533.I1RNQ5 pp 229533.I1S2E9
144552 229533.I1RNQ5 pp 229533.I1S0L4
144553 229533.I1RNQ5 pp 229533.I1S2B8
144554 229533.I1RNQ5 pp 229533.I1RT04
144555 229533.I1RNQ5 pp 229533.V6RUY9
144556 229533.I1RNQ5 pp 229533.I1S8V3
144557 229533.I1RNQ5 pp 229533.I1S2H4
144558 229533.I1RNQ5 pp 229533.I1RQ23
144559 229533.I1RNQ5 pp 229533.I1RRM5
144560 229533.I1RNQ5 pp 229533.I1RWI5
144561 229533.I1RNQ5 pp 229533.I1RPM4
144562 229533.I1RNQ5 pp 229533.I1RRQ3
144563 229533.I1RNQ5 pp 229533.I1RR73
144564 229533.I1RNQ5 pp 229533.I1S8V4
144565 229533.I1RNQ5 pp 229533.I1RZZ9
144566 229533.I1RNQ5 pp 229533.I1SA33
144567 229533.I1RNQ5 pp 229533.I1RRE5
144568 229533.I1RNQ5 pp 229533.I1RZS6
144569 229533.I1RNQ5 pp 229533.I1S1W4
144570 229533.I1RNQ5 pp 229533.V6R3X5
144571 229533.I1RNQ5 pp 229533.I1S0J1
144572 229533.I1RNQ5 pp 229533.I1RT47
144573 229533.I1RNQ5 pp 229533.I1RZ39
144574 229533.I1RNQ5 pp 229533.I1RW46
144575 229533.I1RNQ5 pp 229533.I1RPU3
144576 229533.I1RNQ5 pp 229533.I1S0N4
144577 229533.I1RNQ5 pp 229533.I1RNY2
144578 229533.I1RNQ5 pp 229533.I1RQE7
144579 229533.I1RNQ5 pp 229533.I1S2E8
144580 229533.I1RNQ5 pp 229533.Q4I327
144581 229533.I1RNQ5 pp 229533.I1RTA0
144582 229533.I1RNQ5 pp 229533.I1RUG3
144583 229533.I1RNQ5 pp 229533.I1RSD8
144584 229533.I1RNQ5 pp 229533.I1RPP4
144585 229533.I1RNQ5 pp 229533.Q4IN63
144586 229533.I1RNQ5 pp 229533.I1RS17
144587 229533.I1RNQ5 pp 229533.I1RVX0
144588 229533.I1RNQ5 pp 229533.I1RSK1
144589 229533.I1RNQ5 pp 229533.I1RT03
144590 229533.I1RNQ5 pp 229533.V6R042
144591 229533.I1RNQ5 pp 229533.I1RWN6
144592 229533.I1RNQ5 pp 229533.I1RSQ3
144593 229533.I1RNQ5 pp 229533.I1RWX5
144594 229533.I1RNQ5 pp 229533.I1RRN5
144595 229533.I1RNQ5 pp 229533.V6RMW6
144596 229533.I1RNQ5 pp 229533.I1RRI2
144597 229533.I1RNQ5 pp 229533.I1RX46
144598 229533.I1RNQ5 pp 229533.I1S7A8
144599 229533.I1RNQ5 pp 229533.I1RZW8
144600 229533.I1RNQ5 pp 229533.V6RTJ8
144601 229533.I1RNQ5 pp 229533.I1S1C7
144602 229533.I1RNQ5 pp 229533.I1RRV1
144603 229533.I1RNQ5 pp 229533.I1RWN5
144604 229533.I1RNQ5 pp 229533.I1S0S6
144605 229533.I1RNQ5 pp 229533.I1RY44
144606 229533.I1RNQ5 pp 229533.I1RQH3
144607 229533.I1RNQ5 pp 229533.V6RIV9
144608 229533.I1RNQ5 pp 229533.I1RYW3
144609 229533.I1RNQ5 pp 229533.I1RXD7
144610 229533.I1RNQ5 pp 229533.I1RTD7
144611 229533.I1RNQ5 pp 229533.I1RQC9
144612 229533.I1RNQ5 pp 229533.Q4HZB8
144613 229533.I1RNQ5 pp 229533.I1S2C0

229533.I1RNQ5	pp	229533.I1RVQ0
229533.I1RNQ5	pp	229533.I1S0R7
229533.I1RNQ5	pp	229533.I1RTH6
229533.I1RNQ5	pp	229533.I1S8R3
229533.I1RNQ5	pp	229533.I1RRR7
229533.I1RNQ5	pp	229533.V6R057
229533.I1RNQ5	pp	229533.V6R1K4
229533.I1RNQ5	pp	229533.I1S5B0
229533.I1RNQ5	pp	229533.I1S044
229533.I1RNQ5	pp	229533.I1S0L3
229533.I1RNQ5	pp	229533.I1S0F5
229533.I1RNQ5	pp	229533.Q4I624
229533.I1RNQ5	pp	229533.I1RW99
229533.I1RNQ5	pp	229533.I1RTB7
229533.I1RNQ5	pp	229533.I1RSR8
229533.I1RNQ5	pp	229533.I1S021
229533.I1RNQ5	pp	229533.I1RY03
229533.I1RNQ5	pp	229533.I1RZM8
229533.I1RNQ5	pp	229533.I1RRG3
229533.I1RNQ5	pp	229533.Q4IJT5
229533.I1RNQ5	pp	229533.P0C431
229533.I1RNQ5	pp	229533.V6RCG5
229533.I1RNQ5	pp	229533.I1RQJ5
229533.I1RNQ5	pp	229533.I1RZK5
229533.I1RNQ5	pp	229533.V6QW22
229533.I1RNQ5	pp	229533.V6RCZ8
229533.I1RNQ5	pp	229533.I1RZE5
229533.I1RNQ5	pp	229533.I1RSQ7
229533.I1RNQ5	pp	229533.I1RSI0
229533.I1RNQ5	pp	229533.Q4I283
229533.I1RNQ5	pp	229533.I1RVZ0
229533.I1RNQ5	pp	229533.I1RZM1
229533.I1RNQ5	pp	229533.I1RR00
229533.I1RNQ5	pp	229533.Q4I2J8
229533.I1RNQ5	pp	229533.I1RW71
229533.I1RNQ5	pp	229533.I1RZJ5
229533.I1RNQ5	pp	229533.I1RZ35
229533.I1RNQ5	pp	229533.I1RWT6
229533.I1RNQ5	pp	229533.I1RWJ8
229533.I1RNQ5	pp	229533.I1RWD7
229533.I1RNQ5	pp	229533.I1S504
229533.I1RNQ5	pp	229533.I1RZK6
229533.I1RNQ5	pp	229533.I1RZF6
229533.I1RNQ5	pp	229533.I1S9U2
229533.I1RNQ5	pp	229533.I1RPU0
229533.I1RNQ5	pp	229533.I1RR24
229533.I1RNQ5	pp	229533.I1RQQ5
229533.I1RNQ5	pp	229533.I1RZZ1
229533.I1RNQ5	pp	229533.I1RS18
229533.I1RNQ5	pp	229533.I1SA32
229533.I1RNQ5	pp	229533.I1S0X6
229533.I1RNQ5	pp	229533.Q4I8P5
229533.I1RNQ5	pp	229533.V6RBN2
229533.I1RNQ5	pp	229533.I1RW25
229533.I1RNQ5	pp	229533.I1RQA3
229533.I1RNQ5	pp	229533.I1S6U8
229533.I1RNQ5	pp	229533.I1RQ81
229533.I1RNQ5	pp	229533.Q4IJK2
229533.I1RNQ5	pp	229533.I1S0M9
229533.I1RNQ5	pp	229533.V6QUW4
229533.I1RNQ5	pp	229533.I1RX25
229533.I1RNQ5	pp	229533.I1RRU9
229533.I1RNQ5	pp	229533.I1RRY9
229533.I1RNQ5	pp	229533.I1S1J7
229533.I1RQD2	pp	229533.I1RXC2
229533.I1RQD2	pp	229533.I1S2B8
229533.I1RQD2	pp	229533.I1S4Z8
229533.I1RQD2	pp	229533.I1S5R1
229533.I1RQD2	pp	229533.I1RZ39
229533.I1RQD2	pp	229533.I1S438
229533.I1RQD2	pp	229533.I1RSZ7
229533.I1RQD2	pp	229533.I1RSQ4
229533.I1RQD2	pp	229533.I1S4S4

229533.I1RQD2 pp 229533.I1RRI1
229533.I1RQD2 pp 229533.I1S0W4
229533.I1RQD2 pp 229533.I1RYI5
229533.I1RQD2 pp 229533.I1RTB3
229533.I1RQD2 pp 229533.I1S0L4
229533.I1RQD2 pp 229533.I1RR24
229533.I1RQD2 pp 229533.I1RXD7
229533.I1RQD2 pp 229533.I1RTD5
229533.I1RQD2 pp 229533.I1RQE7
229533.I1RQD2 pp 229533.I1RSQ7
229533.I1RQD2 pp 229533.Q4IJK2
229533.I1RQD2 pp 229533.I1RXF8
229533.I1RQD2 pp 229533.I1S4S7
229533.I1RQD2 pp 229533.Q4I624
229533.I1RQD2 pp 229533.I1RTA1
229533.I1RQD2 pp 229533.I1RQM7
229533.I1RQD2 pp 229533.I1RVZ2
229533.I1RQD2 pp 229533.I1RT40
229533.I1RQD2 pp 229533.Q4IPZ1
229533.I1RQD2 pp 229533.I1RX95
229533.I1RQD2 pp 229533.I1S0T4
229533.I1RQD2 pp 229533.I1RYR0
229533.I1RQD2 pp 229533.V6RTJ8
229533.I1RQD2 pp 229533.I1RZK5
229533.I1RQD2 pp 229533.I1RSA5
229533.I1RQD2 pp 229533.I1RSX0
229533.I1RQD2 pp 229533.I1RVX0
229533.I1RQD2 pp 229533.I1RY86
229533.I1RQD2 pp 229533.I1RUF7
229533.I1RQD2 pp 229533.I1RXD6
229533.I1RQD2 pp 229533.I1S437
229533.I1RQD2 pp 229533.I1RRM3
229533.I1RQD2 pp 229533.I1S1W9
229533.I1RQD2 pp 229533.I1RWR7
229533.I1RQD2 pp 229533.I1RVQ3
229533.I1RQD2 pp 229533.V6R9Z0
229533.I1RQD2 pp 229533.V6RMW6
229533.I1RQD2 pp 229533.I1RSQ3
229533.I1RQD2 pp 229533.I1S9Z4
229533.I1RQD2 pp 229533.I1S6K9
229533.I1RQD2 pp 229533.I1RXC4
229533.I1RQD2 pp 229533.V6R042
229533.I1RQD2 pp 229533.I1RZV3
229533.I1RQD2 pp 229533.I1RVZ7
229533.I1RQD2 pp 229533.I1RSF1
229533.I1RQD2 pp 229533.I1S821
229533.I1RQD2 pp 229533.V6RCZ8
229533.I1RQD2 pp 229533.I1RZM8
229533.I1RQD2 pp 229533.I1RUG3
229533.I1RQD2 pp 229533.I1RVF8
229533.I1RQD2 pp 229533.I1RRN2
229533.I1RQD2 pp 229533.I1S5R6
229533.I1RQD2 pp 229533.I1S5B0
229533.I1RQD2 pp 229533.I1RRV3
229533.I1RQD2 pp 229533.I1S1B2
229533.A0A1C3YHX6 pp 229533.I1RW07
229533.A0A1C3YHX6 pp 229533.V6QYF0
229533.A0A1C3YHX6 pp 229533.I1RI02
229533.A0A1C3YHX6 pp 229533.I1RRQ6
229533.A0A1C3YHX6 pp 229533.I1RQ42
229533.A0A1C3YHX6 pp 229533.I1RNA0
229533.A0A1C3YHX6 pp 229533.A0A1C3YLR6
229533.A0A1C3YHX6 pp 229533.A0A1C3YLZ7
229533.A0A1C3YHX6 pp 229533.I1RLC7
229533.A0A1C3YHX6 pp 229533.I1RW25
229533.A0A1C3YHX6 pp 229533.I1RR24
229533.A0A1C3YHX6 pp 229533.I1S1W8
229533.A0A1C3YHX6 pp 229533.I1S4S4
229533.A0A1C3YHX6 pp 229533.I1RX25
229533.A0A1C3YHX6 pp 229533.V6QUW4
229533.A0A1C3YHX6 pp 229533.A0A1C3YJQ9
229533.A0A1C3YHX6 pp 229533.A0A1C3YN30
229533.A0A1C3YHX6 pp 229533.I1RJU3

144760 229533.A0A1C3YHX6 pp 229533.A0A1C3YJH8
144761 229533.A0A1C3YHX6 pp 229533.A0A1C3YK11
144762 229533.A0A1C3YHX6 pp 229533.I1RWJ8
144763 229533.A0A1C3YHX6 pp 229533.I1RZ94
144764 229533.A0A1C3YHX6 pp 229533.I1RZJ5
144765 229533.A0A1C3YHX6 pp 229533.I1RTF4
144766 229533.A0A1C3YHX6 pp 229533.I1RM06
144767 229533.A0A1C3YHX6 pp 229533.I1RMK3
144768 229533.A0A1C3YHX6 pp 229533.I1RGK3
144769 229533.A0A1C3YHX6 pp 229533.I1R9Y4
144770 229533.A0A1C3YHX6 pp 229533.I1RM67
144771 229533.A0A1C3YHX6 pp 229533.I1RCS1
144772 229533.A0A1C3YHX6 pp 229533.I1RMG8
144773 229533.A0A1C3YHX6 pp 229533.I1RW91
144774 229533.A0A1C3YHX6 pp 229533.I1S0X6
144775 229533.A0A1C3YHX6 pp 229533.I1RPN4
144776 229533.A0A1C3YHX6 pp 229533.V6R1K4
144777 229533.A0A1C3YHX6 pp 229533.I1RW71
144778 229533.A0A1C3YHX6 pp 229533.A0A1C3YIP5
144779 229533.A0A1C3YHX6 pp 229533.I1RBT1
144780 229533.A0A1C3YHX6 pp 229533.A0A1C3YKC0
144781 229533.A0A1C3YHX6 pp 229533.V6QWX8
144782 229533.A0A1C3YHX6 pp 229533.I1RBC9
144783 229533.A0A1C3YHX6 pp 229533.I1RWD4
144784 229533.A0A1C3YHX6 pp 229533.A0A1C3YK13
144785 229533.A0A1C3YHX6 pp 229533.I1S4S7
144786 229533.A0A1C3YHX6 pp 229533.I1RVQ0
144787 229533.A0A1C3YHX6 pp 229533.I1RSQ7
144788 229533.A0A1C3YHX6 pp 229533.I1RRX5
144789 229533.A0A1C3YHX6 pp 229533.A0A1C3YHX8
144790 229533.A0A1C3YHX6 pp 229533.I1RMC8
144791 229533.A0A1C3YHX6 pp 229533.I1RAM6
144792 229533.A0A1C3YHX6 pp 229533.V6R4J1
144793 229533.A0A1C3YHX6 pp 229533.A0A1C3YN26
144794 229533.A0A1C3YHX6 pp 229533.I1RAH0
144795 229533.A0A1C3YHX6 pp 229533.V6RTJ8
144796 229533.A0A1C3YHX6 pp 229533.V6QW22
144797 229533.A0A1C3YHX6 pp 229533.I1S6F0
144798 229533.A0A1C3YHX6 pp 229533.I1RG53
144799 229533.A0A1C3YHX6 pp 229533.I1RLX7
144800 229533.A0A1C3YHX6 pp 229533.A0A1C3YMW0
144801 229533.A0A1C3YHX6 pp 229533.A0A1C3YMU9
144802 229533.A0A1C3YHX6 pp 229533.I1RX46
144803 229533.A0A1C3YHX6 pp 229533.I1S0R7
144804 229533.A0A1C3YHX6 pp 229533.I1S0T2
144805 229533.A0A1C3YHX6 pp 229533.I1RIF7
144806 229533.A0A1C3YHX6 pp 229533.I1S0K1
144807 229533.A0A1C3YHX6 pp 229533.A0A1C3YM63
144808 229533.A0A1C3YHX6 pp 229533.I1RBD5
144809 229533.A0A1C3YHX6 pp 229533.I1RH75
144810 229533.A0A1C3YHX6 pp 229533.I1RNG5
144811 229533.A0A1C3YHX6 pp 229533.I1RX95
144812 229533.A0A1C3YHX6 pp 229533.I1RQC9
144813 229533.A0A1C3YHX6 pp 229533.Q4IJT5
144814 229533.A0A1C3YHX6 pp 229533.I1RSC6
144815 229533.A0A1C3YHX6 pp 229533.I1RDG0
144816 229533.A0A1C3YHX6 pp 229533.I1RXD7
144817 229533.A0A1C3YHX6 pp 229533.I1RK95
144818 229533.A0A1C3YHX6 pp 229533.I1RRG3
144819 229533.A0A1C3YHX6 pp 229533.I1RLP1
144820 229533.A0A1C3YHX6 pp 229533.I1RIL8
144821 229533.A0A1C3YHX6 pp 229533.I1RN99
144822 229533.A0A1C3YHX6 pp 229533.Q4IJK2
144823 229533.A0A1C3YHX6 pp 229533.I1RUQ2
144824 229533.A0A1C3YHX6 pp 229533.I1RQA3
144825 229533.A0A1C3YHX6 pp 229533.I1RA85
144826 229533.A0A1C3YHX6 pp 229533.I1RD52
144827 229533.A0A1C3YHX6 pp 229533.A0A1C3YIB4
144828 229533.A0A1C3YHX6 pp 229533.I1RA06
144829 229533.A0A1C3YHX6 pp 229533.V6RUT5
144830 229533.A0A1C3YHX6 pp 229533.I1RPW8
144831 229533.A0A1C3YHX6 pp 229533.A0A1C3YL54
144832 229533.A0A1C3YHX6 pp 229533.I1S0L3

144833 229533.A0A1C3YHX6 pp 229533.V6RBN2
144834 229533.A0A1C3YHX6 pp 229533.I1RPY3
144835 229533.A0A1C3YHX6 pp 229533.I1RQQ5
144836 229533.A0A1C3YHX6 pp 229533.I1RAX6
144837 229533.A0A1C3YHX6 pp 229533.I1RZZ1
144838 229533.A0A1C3YHX6 pp 229533.I1REK8
144839 229533.A0A1C3YHX6 pp 229533.I1RCR5
144840 229533.A0A1C3YHX6 pp 229533.I1RKF3
144841 229533.A0A1C3YHX6 pp 229533.I1RWD7
144842 229533.A0A1C3YHX6 pp 229533.Q4I5Z5
144843 229533.A0A1C3YHX6 pp 229533.I1S7A8
144844 229533.A0A1C3YHX6 pp 229533.Q4I2J8
144845 229533.A0A1C3YHX6 pp 229533.I1RBL7
144846 229533.A0A1C3YHX6 pp 229533.I1S504
144847 229533.A0A1C3YHX6 pp 229533.I1RSR3
144848 229533.A0A1C3YHX6 pp 229533.I1REL7
144849 229533.A0A1C3YHX6 pp 229533.I1RAZ8
144850 229533.A0A1C3YHX6 pp 229533.I1RWR7
144851 229533.A0A1C3YHX6 pp 229533.A0A1C3YJR3
144852 229533.A0A1C3YHX6 pp 229533.I1RG14
144853 229533.A0A1C3YHX6 pp 229533.I1RWT6
144854 229533.A0A1C3YHX6 pp 229533.I1RF19
144855 229533.A0A1C3YHX6 pp 229533.A0A1C3YIJ3
144856 229533.A0A1C3YHX6 pp 229533.I1S4Q4
144857 229533.A0A1C3YHX6 pp 229533.I1RU29
144858 229533.A0A1C3YHX6 pp 229533.I1RSI0
144859 229533.A0A1C3YHX6 pp 229533.I1RFJ9
144860 229533.A0A1C3YHX6 pp 229533.I1RQ85
144861 229533.A0A1C3YHX6 pp 229533.A0A1C3YKW9
144862 229533.A0A1C3YHX6 pp 229533.I1RRY9
144863 229533.A0A1C3YHX6 pp 229533.Q4I283
144864 229533.A0A1C3YHX6 pp 229533.I1RED2
144865 229533.A0A1C3YHX6 pp 229533.I1RLM7
144866 229533.A0A1C3YHX6 pp 229533.I1RWB0
144867 229533.A0A1C3YHX6 pp 229533.I1RCG4
144868 229533.A0A1C3YHX6 pp 229533.I1RZB5
144869 229533.A0A1C3YHX6 pp 229533.I1RMM9
144870 229533.A0A1C3YHX6 pp 229533.A0A1C3YKB0
144871 229533.A0A1C3YHX6 pp 229533.V6R227
144872 229533.A0A1C3YHX6 pp 229533.I1RQA2
144873 229533.A0A1C3YHX6 pp 229533.I1RAZ3
144874 229533.A0A1C3YHX6 pp 229533.I1RFU8
144875 229533.A0A1C3YHX6 pp 229533.I1RJL2
144876 229533.A0A1C3YHX6 pp 229533.A0A1C3YK36
144877 229533.A0A1C3YHX6 pp 229533.I1S1J7
144878 229533.A0A1C3YHX6 pp 229533.I1S419
144879 229533.A0A1C3YHX6 pp 229533.A0A1C3YJ93
144880 229533.A0A1C3YHX6 pp 229533.I1RQ93
144881 229533.A0A1C3YHX6 pp 229533.I1RI03
144882 229533.A0A1C3YHX6 pp 229533.I1S887
144883 229533.A0A1C3YHX6 pp 229533.A0A1C3YKY6
144884 229533.A0A1C3YHX6 pp 229533.I1RC16
144885 229533.A0A1C3YHX6 pp 229533.I1RZM1
144886 229533.A0A1C3YHX6 pp 229533.I1RYS0
144887 229533.A0A1C3YHX6 pp 229533.A0A1C3YK68
144888 229533.A0A1C3YHX6 pp 229533.I1RCM5
144889 229533.A0A1C3YHX6 pp 229533.I1RZK5
144890 229533.A0A1C3YHX6 pp 229533.I1RCG6
144891 229533.A0A1C3YHX6 pp 229533.A0A1C3YJN3
144892 229533.A0A1C3YHX6 pp 229533.I1RQ81
144893 229533.A0A1C3YHX6 pp 229533.I1RBJ5
144894 229533.A0A1C3YHX6 pp 229533.I1RNQ5
144895 229533.A0A1C3YHX6 pp 229533.I1RPL2
144896 229533.A0A1C3YHX6 pp 229533.I1RY03
144897 229533.A0A1C3YHX6 pp 229533.A0A1C3YKV3
144898 229533.A0A1C3YHX6 pp 229533.I1RLU6
144899 229533.A0A1C3YHX6 pp 229533.A0A1C3YJ83
144900 229533.A0A1C3YHX6 pp 229533.Q4IEK8
144901 229533.A0A1C3YHX6 pp 229533.I1RLI4
144902 229533.A0A1C3YHX6 pp 229533.I1RTD9
144903 229533.A0A1C3YHX6 pp 229533.Q4I8P5
144904 229533.A0A1C3YHX6 pp 229533.I1RMD5
144905 229533.A0A1C3YHX6 pp 229533.I1RS18

144906	229533.A0A1C3YHX6	pp	229533.A0A1C3YJW9
144907	229533.A0A1C3YHX6	pp	229533.I1RGC5
144908	229533.A0A1C3YHX6	pp	229533.V6RCZ8
144909	229533.A0A1C3YHX6	pp	229533.A0A1C3YIK4
144910	229533.A0A1C3YHX6	pp	229533.A0A1C3YLK2
144911	229533.A0A1C3YHX6	pp	229533.I1RC15
144912	229533.A0A1C3YHX6	pp	229533.I1S6K9
144913	229533.A0A1C3YHX6	pp	229533.I1RPR9
144914	229533.A0A1C3YHX6	pp	229533.A0A1C3YK34
144915	229533.A0A1C3YHX6	pp	229533.I1RC17
144916	229533.A0A1C3YHX6	pp	229533.V6RCG5
144917	229533.A0A1C3YHX6	pp	229533.I1RXC4
144918	229533.A0A1C3YHX6	pp	229533.A0A1C3YI23
144919	229533.A0A1C3YHX6	pp	229533.I1RR51
144920	229533.A0A1C3YHX6	pp	229533.A0A1C3YI30
144921	229533.A0A1C3YHX6	pp	229533.I1RFI5
144922	229533.A0A1C3YHX6	pp	229533.I1RW99
144923	229533.A0A1C3YHX6	pp	229533.I1SA32
144924	229533.A0A1C3YHX6	pp	229533.I1S5B0
144925	229533.A0A1C3YHX6	pp	229533.A0A1C3YLU7
144926	229533.A0A1C3YHX6	pp	229533.I1RNN1
144927	229533.A0A1C3YHX6	pp	229533.I1RCH4
144928	229533.A0A1C3YHX6	pp	229533.I1RFH8
144929	229533.A0A1C3YHX6	pp	229533.A0A1C3YJV6
144930	229533.A0A1C3YHX6	pp	229533.I1RGC2
144931	229533.A0A1C3YHX6	pp	229533.V6QUE6
144932	229533.A0A1C3YHX6	pp	229533.I1RZM8
144933	229533.A0A1C3YHX6	pp	229533.I1RXF0
144934	229533.A0A1C3YHX6	pp	229533.I1S8V3
144935	229533.A0A1C3YHX6	pp	229533.I1RC66
144936	229533.A0A1C3YHX6	pp	229533.I1RQ31
144937	229533.A0A1C3YHX6	pp	229533.I1RMC0
144938	229533.A0A1C3YHX6	pp	229533.I1RRV4
144939	229533.A0A1C3YHX6	pp	229533.I1S2H4
144940	229533.A0A1C3YHX6	pp	229533.I1RAR2
144941	229533.A0A1C3YHX6	pp	229533.I1RAU5
144942	229533.A0A1C3YHX6	pp	229533.I1RI82
144943	229533.A0A1C3YHX6	pp	229533.I1RPE0
144944	229533.A0A1C3YHX6	pp	229533.I1RC61
144945	229533.A0A1C3YHX6	pp	229533.I1RRU9
144946	229533.A0A1C3YHX6	pp	229533.I1RS20
144947	229533.A0A1C3YHX6	pp	229533.A0A1C3YJD4
144948	229533.A0A1C3YHX6	pp	229533.I1RQ23
144949	229533.A0A1C3YHX6	pp	229533.A0A1C3YIS7
144950	229533.A0A1C3YHX6	pp	229533.I1RE27
144951	229533.A0A1C3YHX6	pp	229533.I1RED3
144952	229533.A0A1C3YHX6	pp	229533.I1RC29
144953	229533.A0A1C3YHX6	pp	229533.I1RE22
144954	229533.A0A1C3YHX6	pp	229533.I1RQS8
144955	229533.A0A1C3YHX6	pp	229533.I1RN04
144956	229533.A0A1C3YHX6	pp	229533.I1RR72
144957	229533.A0A1C3YHX6	pp	229533.A0A1C3YMA1
144958	229533.A0A1C3YHX6	pp	229533.I1RRM5
144959	229533.A0A1C3YHX6	pp	229533.I1S3G4
144960	229533.A0A1C3YHX6	pp	229533.I1RVY0
144961	229533.A0A1C3YHX6	pp	229533.I1REC5
144962	229533.A0A1C3YHX6	pp	229533.I1RSZ7
144963	229533.A0A1C3YHX6	pp	229533.I1RIL3
144964	229533.A0A1C3YHX6	pp	229533.I1S2B8
144965	229533.A0A1C3YHX6	pp	229533.I1RW61
144966	229533.A0A1C3YHX6	pp	229533.I1RFS3
144967	229533.A0A1C3YHX6	pp	229533.I1RJZ7
144968	229533.A0A1C3YHX6	pp	229533.I1SAH9
144969	229533.A0A1C3YHX6	pp	229533.I1R9M0
144970	229533.A0A1C3YHX6	pp	229533.I1RBY4
144971	229533.A0A1C3YHX6	pp	229533.I1S6Y8
144972	229533.A0A1C3YHX6	pp	229533.I1RRI1
144973	229533.A0A1C3YHX6	pp	229533.I1RPM4
144974	229533.A0A1C3YHX6	pp	229533.I1RE76
144975	229533.A0A1C3YHX6	pp	229533.I1RPU3
144976	229533.A0A1C3YHX6	pp	229533.I1S0L4
144977	229533.A0A1C3YHX6	pp	229533.I1RRL9
144978	229533.A0A1C3YHX6	pp	229533.I1RC24

144979 229533.A0A1C3YHX6 pp 229533.I1RPV3
144980 229533.A0A1C3YHX6 pp 229533.I1RL04
144981 229533.A0A1C3YHX6 pp 229533.I1SAA0
144982 229533.A0A1C3YHX6 pp 229533.I1RWK3
144983 229533.A0A1C3YHX6 pp 229533.A0A1C3YKM9
144984 229533.A0A1C3YHX6 pp 229533.I1RZ39
144985 229533.A0A1C3YHX6 pp 229533.I1RM82
144986 229533.A0A1C3YHX6 pp 229533.V6R3X5
144987 229533.A0A1C3YHX6 pp 229533.I1RN63
144988 229533.A0A1C3YHX6 pp 229533.I1S9Y1
144989 229533.A0A1C3YHX6 pp 229533.I1S2M6
144990 229533.A0A1C3YHX6 pp 229533.I1RA11
144991 229533.A0A1C3YHX6 pp 229533.A0A1C3YLD8
144992 229533.A0A1C3YHX6 pp 229533.I1RR73
144993 229533.A0A1C3YHX6 pp 229533.A0A1C3YK73
144994 229533.A0A1C3YHX6 pp 229533.I1S5Z5
144995 229533.A0A1C3YHX6 pp 229533.I1RXC2
144996 229533.A0A1C3YHX6 pp 229533.I1RVZ2
144997 229533.A0A1C3YHX6 pp 229533.I1S253
144998 229533.A0A1C3YHX6 pp 229533.A0A1C3YIR8
144999 229533.A0A1C3YHX6 pp 229533.O74638
145000 229533.A0A1C3YHX6 pp 229533.I1RSQ4
145001 229533.A0A1C3YHX6 pp 229533.I1RJN9
145002 229533.A0A1C3YHX6 pp 229533.I1RTA1
145003 229533.A0A1C3YHX6 pp 229533.I1RZZ9
145004 229533.A0A1C3YHX6 pp 229533.I1RRT8
145005 229533.A0A1C3YHX6 pp 229533.I1RWI5
145006 229533.A0A1C3YHX6 pp 229533.I1S5R1
145007 229533.A0A1C3YHX6 pp 229533.A0A1C3YKN3
145008 229533.A0A1C3YHX6 pp 229533.A0A1C3YI20
145009 229533.A0A1C3YHX6 pp 229533.I1RFT1
145010 229533.A0A1C3YHX6 pp 229533.I1RT40
145011 229533.A0A1C3YHX6 pp 229533.I1RS47
145012 229533.A0A1C3YHX6 pp 229533.I1S1F3
145013 229533.A0A1C3YHX6 pp 229533.I1S352
145014 229533.A0A1C3YHX6 pp 229533.A0A1C3YL15
145015 229533.A0A1C3YHX6 pp 229533.A0A1C3YJI6
145016 229533.A0A1C3YHX6 pp 229533.I1RV91
145017 229533.A0A1C3YHX6 pp 229533.I1RRJ8
145018 229533.A0A1C3YHX6 pp 229533.I1RTD5
145019 229533.A0A1C3YHX6 pp 229533.I1S0F6
145020 229533.A0A1C3YHX6 pp 229533.I1RQG4
145021 229533.A0A1C3YHX6 pp 229533.I1RDF7
145022 229533.A0A1C3YHX6 pp 229533.A0A1C3YLP8
145023 229533.A0A1C3YHX6 pp 229533.I1RN67
145024 229533.A0A1C3YHX6 pp 229533.I1S0W4
145025 229533.A0A1C3YHX6 pp 229533.I1RGF5
145026 229533.A0A1C3YHX6 pp 229533.I1RXF8
145027 229533.A0A1C3YHX6 pp 229533.I1S8P5
145028 229533.A0A1C3YHX6 pp 229533.I1RSZ3
145029 229533.A0A1C3YHX6 pp 229533.I1RPP3
145030 229533.A0A1C3YHX6 pp 229533.I1RC05
145031 229533.A0A1C3YHX6 pp 229533.I1RP28
145032 229533.A0A1C3YHX6 pp 229533.A0A1C3YN63
145033 229533.A0A1C3YHX6 pp 229533.I1RC82
145034 229533.A0A1C3YHX6 pp 229533.I1RGA4
145035 229533.A0A1C3YHX6 pp 229533.I1RBC7
145036 229533.A0A1C3YHX6 pp 229533.I1RYI5
145037 229533.A0A1C3YHX6 pp 229533.I1RBS6
145038 229533.A0A1C3YHX6 pp 229533.I1RJV4
145039 229533.A0A1C3YHX6 pp 229533.I1RBS0
145040 229533.A0A1C3YHX6 pp 229533.I1S9H8
145041 229533.A0A1C3YHX6 pp 229533.I1RVX0
145042 229533.A0A1C3YHX6 pp 229533.I1RE30
145043 229533.A0A1C3YHX6 pp 229533.I1RZB2
145044 229533.A0A1C3YHX6 pp 229533.I1RT03
145045 229533.A0A1C3YHX6 pp 229533.I1RP27
145046 229533.A0A1C3YHX6 pp 229533.I1RT04
145047 229533.A0A1C3YHX6 pp 229533.I1RB92
145048 229533.A0A1C3YHX6 pp 229533.V6RUY9
145049 229533.A0A1C3YHX6 pp 229533.I1RJ74
145050 229533.A0A1C3YHX6 pp 229533.I1RN18
145051 229533.A0A1C3YHX6 pp 229533.I1S503

229533.A0A1C3YHX6	pp	229533.I1RFL4
229533.A0A1C3YHX6	pp	229533.I1RJW6
229533.A0A1C3YHX6	pp	229533.I1RRZ0
229533.A0A1C3YHX6	pp	229533.A0A1C3YL55
229533.A0A1C3YHX6	pp	229533.A0A1C3YM60
229533.A0A1C3YHX6	pp	229533.I1RNA5
229533.A0A1C3YHX6	pp	229533.A0A1C3YLF5
229533.A0A1C3YHX6	pp	229533.I1RR61
229533.A0A1C3YHX6	pp	229533.A0A1C3YIE7
229533.A0A1C3YHX6	pp	229533.I1RAP1
229533.A0A1C3YHX6	pp	229533.I1S2E9
229533.A0A1C3YHX6	pp	229533.I1RLN6
229533.A0A1C3YHX6	pp	229533.I1RQE7
229533.A0A1C3YHX6	pp	229533.V6R3J1
229533.A0A1C3YHX6	pp	229533.I1RCH5
229533.A0A1C3YHX6	pp	229533.I1RW80
229533.A0A1C3YHX6	pp	229533.I1RSX0
229533.A0A1C3YHX6	pp	229533.I1S0X1
229533.A0A1C3YHX6	pp	229533.I1RA70
229533.A0A1C3YHX6	pp	229533.Q4I624
229533.A0A1C3YHX6	pp	229533.I1RTE9
229533.A0A1C3YHX6	pp	229533.I1RD17
229533.A0A1C3YHX6	pp	229533.I1RKJ8
229533.A0A1C3YHX6	pp	229533.I1RCL5
229533.A0A1C3YHX6	pp	229533.I1RJL4
229533.A0A1C3YHX6	pp	229533.I1RRT2
229533.A0A1C3YHX6	pp	229533.V6R057
229533.A0A1C3YHX6	pp	229533.I1RRN5
229533.A0A1C3YHX6	pp	229533.I1RXR1
229533.A0A1C3YHX6	pp	229533.I1S1C7
229533.A0A1C3YHX6	pp	229533.I1S569
229533.A0A1C3YHX6	pp	229533.I1RYW3
229533.A0A1C3YHX6	pp	229533.I1RR25
229533.A0A1C3YHX6	pp	229533.A0A1C3YMF9
229533.A0A1C3YHX6	pp	229533.I1RS17
229533.A0A1C3YHX6	pp	229533.I1RCW6
229533.A0A1C3YHX6	pp	229533.I1RK79
229533.A0A1C3YHX6	pp	229533.I1RP12
229533.A0A1C3YHX6	pp	229533.I1RZW8
229533.A0A1C3YHX6	pp	229533.I1RPK0
229533.A0A1C3YHX6	pp	229533.I1RQ33
229533.A0A1C3YHX6	pp	229533.I1R9X7
229533.A0A1C3YHX6	pp	229533.I1S9Z4
229533.A0A1C3YHX6	pp	229533.I1RZ79
229533.A0A1C3YHX6	pp	229533.I1RTY9
229533.A0A1C3YHX6	pp	229533.I1RAU7
229533.A0A1C3YHX6	pp	229533.I1RRI2
229533.A0A1C3YHX6	pp	229533.I1R9J6
229533.A0A1C3YHX6	pp	229533.I1RMV0
229533.A0A1C3YHX6	pp	229533.I1RKC6
229533.A0A1C3YHX6	pp	229533.I1RWX5
229533.A0A1C3YHX6	pp	229533.I1RPP4
229533.A0A1C3YHX6	pp	229533.I1RB41
229533.A0A1C3YHX6	pp	229533.A0A1C3YLC1
229533.A0A1C3YHX6	pp	229533.I1RTA0
229533.A0A1C3YHX6	pp	229533.I1RXU9
229533.A0A1C3YHX6	pp	229533.V6RRH2
229533.A0A1C3YHX6	pp	229533.A0A1C3YKY0
229533.A0A1C3YHX6	pp	229533.A0A1C3YI73
229533.A0A1C3YHX6	pp	229533.I1RE01
229533.A0A1C3YHX6	pp	229533.I1RW46
229533.A0A1C3YHX6	pp	229533.I1RAW0
229533.A0A1C3YHX6	pp	229533.I1RQW1
229533.A0A1C3YHX6	pp	229533.I1RGL4
229533.A0A1C3YHX6	pp	229533.A0A1C3YK33
229533.A0A1C3YHX6	pp	229533.I1RML0
229533.A0A1C3YHX6	pp	229533.I1S0J1
229533.A0A1C3YHX6	pp	229533.I1RC72
229533.A0A1C3YHX6	pp	229533.I1RWN6
229533.A0A1C3YHX6	pp	229533.I1RD39
229533.A0A1C3YHX6	pp	229533.I1S8R3
229533.A0A1C3YHX6	pp	229533.I1RKB6
229533.A0A1C3YHX6	pp	229533.I1RE89

145125 229533.A0A1C3YHX6 pp 229533.I1S0R2
145126 229533.A0A1C3YHX6 pp 229533.I1RTH6
145127 229533.A0A1C3YHX6 pp 229533.I1S7W5
145128 229533.A0A1C3YHX6 pp 229533.I1RRE5
145129 229533.A0A1C3YHX6 pp 229533.V6RIV9
145130 229533.A0A1C3YHX6 pp 229533.I1RUE3
145131 229533.A0A1C3YHX6 pp 229533.I1RJV3
145132 229533.A0A1C3YHX6 pp 229533.I1RSK1
145133 229533.A0A1C3YHX6 pp 229533.I1RPU2
145134 229533.A0A1C3YHX6 pp 229533.I1RKR8
145135 229533.A0A1C3YHX6 pp 229533.I1S8V4
145136 229533.A0A1C3YHX6 pp 229533.I1RWN5
145137 229533.A0A1C3YHX6 pp 229533.I1RA65
145138 229533.A0A1C3YHX6 pp 229533.I1RZV3
145139 229533.A0A1C3YHX6 pp 229533.Q4HZB8
145140 229533.A0A1C3YHX6 pp 229533.I1S1W3
145141 229533.A0A1C3YHX6 pp 229533.I1S016
145142 229533.A0A1C3YHX6 pp 229533.I1RY44
145143 229533.A0A1C3YHX6 pp 229533.I1RC33
145144 229533.A0A1C3YHX6 pp 229533.I1RRV1
145145 229533.A0A1C3YHX6 pp 229533.I1R9X3
145146 229533.A0A1C3YHX6 pp 229533.I1S1W9
145147 229533.A0A1C3YHX6 pp 229533.I1RSI6
145148 229533.A0A1C3YHX6 pp 229533.A0A1C3YLS9
145149 229533.A0A1C3YHX6 pp 229533.I1RD22
145150 229533.A0A1C3YHX6 pp 229533.I1RMG3
145151 229533.A0A1C3YHX6 pp 229533.I1RRN2
145152 229533.A0A1C3YHX6 pp 229533.V6RMW6
145153 229533.A0A1C3YHX6 pp 229533.I1RRM3
145154 229533.A0A1C3YHX6 pp 229533.V6R042
145155 229533.A0A1C3YHX6 pp 229533.I1RSQ3
145156 229533.A0A1C3YHX6 pp 229533.I1RCR8
145157 229533.A0A1C3YHX6 pp 229533.I1RUG3
145158 229533.A0A1C3YHX6 pp 229533.I1RNC1
145159 229533.A0A1C3YHX6 pp 229533.I1RXT3
145160 229533.A0A1C3YHX6 pp 229533.A0A1C3YKS0
145161 229533.A0A1C3YHX6 pp 229533.A0A1C3YMI5
145162 229533.A0A1C3YHX6 pp 229533.I1RRF7
145163 229533.A0A1C3YHX6 pp 229533.I1RWQ8
145164 229533.A0A1C3YHX6 pp 229533.I1S5R6
145165 229533.A0A1C3YHX6 pp 229533.I1R9D1
145166 229533.A0A1C3YHX6 pp 229533.I1RG11
145167 229533.A0A1C3YHX6 pp 229533.I1S0N4
145168 229533.A0A1C3YHX6 pp 229533.V6RGN5
145169 229533.A0A1C3YHX6 pp 229533.I1SA33
145170 229533.A0A1C3YHX6 pp 229533.A0A1C3YIK9
145171 229533.A0A1C3YHX6 pp 229533.I1RSU2
145172 229533.A0A098DH49 pp 229533.V6RVG3
145173 229533.A0A098DH49 pp 229533.I1S286
145174 229533.A0A098DH49 pp 229533.I1RZE4
145175 229533.A0A098DH49 pp 229533.I1S247
145176 229533.A0A098DH49 pp 229533.V6RKY7
145177 229533.A0A098DH49 pp 229533.I1RRN5
145178 229533.A0A098DH49 pp 229533.I1S1W4
145179 229533.A0A098DH49 pp 229533.I1RQB4
145180 229533.A0A098DH49 pp 229533.I1RQD6
145181 229533.A0A098DH49 pp 229533.I1RAN2
145182 229533.A0A098DH49 pp 229533.I1RFW0
145183 229533.A0A098DH49 pp 229533.I1RZC3
145184 229533.A0A098DH49 pp 229533.I1RGF1
145185 229533.A0A098DH49 pp 229533.I1RMA7
145186 229533.A0A098DH49 pp 229533.I1RX64
145187 229533.A0A098DH49 pp 229533.I1RNY8
145188 229533.A0A098DH49 pp 229533.I1S248
145189 229533.A0A098DH49 pp 229533.I1R9Y9
145190 229533.A0A098DH49 pp 229533.I1RUN8
145191 229533.A0A098DH49 pp 229533.I1RC46
145192 229533.A0A098DH49 pp 229533.I1RYL5
145193 229533.A0A098DH49 pp 229533.I1RC22
145194 229533.A0A098DH49 pp 229533.I1RUQ5
145195 229533.A0A098DH49 pp 229533.I1RMM9
145196 229533.A0A098DH49 pp 229533.I1RY32
145197 229533.A0A098DH49 pp 229533.I1RAX2

229533.A0A098DH49 pp 229533.I1S7X9
229533.A0A098DH49 pp 229533.I1S0X7
229533.A0A098DH49 pp 229533.I1RB74
229533.A0A098DH49 pp 229533.I1RS47
229533.A0A098DH49 pp 229533.I1RQA3
229533.A0A098DH49 pp 229533.I1RKK0
229533.A0A098DH49 pp 229533.I1S439
229533.A0A098DH49 pp 229533.Q4IEA7
229533.A0A098DH49 pp 229533.I1RB75
229533.A0A098DH49 pp 229533.I1RAM7
229533.A0A098DH49 pp 229533.A0A0E0SAC4
229533.A0A098DH49 pp 229533.I1RPA6
229533.A0A098DH49 pp 229533.I1S278
229533.A0A098DH49 pp 229533.I1RSS1
229533.A0A098DH49 pp 229533.A0A1C3YIG3
229533.A0A098DH49 pp 229533.I1RCB3
229533.A0A098DH49 pp 229533.A0A098DT74
229533.A0A098DH49 pp 229533.I1RX04
229533.A0A098DH49 pp 229533.I1RMB2
229533.A0A098DH49 pp 229533.I1RX77
229533.A0A098DH49 pp 229533.I1RWT6
229533.A0A098DH49 pp 229533.I1RDB1
229533.A0A098DH49 pp 229533.I1RQL4
229533.A0A098DH49 pp 229533.I1RCD1
229533.A0A098DH49 pp 229533.I1RV66
229533.A0A098DH49 pp 229533.I1RX87
229533.A0A098DH49 pp 229533.A0A0E0RR40
229533.I1RTV7 pp 229533.I1RTW1
229533.I1RTV7 pp 229533.I1S1G0
229533.I1RTV7 pp 229533.I1S1G1
229533.I1RTV7 pp 229533.I1S1F9
229533.I1RI02 pp 229533.Q4IJK2
229533.I1RI02 pp 229533.I1RRY9
229533.I1RI02 pp 229533.I1RVQ0
229533.I1RI02 pp 229533.I1S504
229533.I1RI02 pp 229533.V6QW22
229533.I1RI02 pp 229533.I1RZM1
229533.I1RI02 pp 229533.I1RZK5
229533.I1RI02 pp 229533.I1RLU6
229533.I1RI02 pp 229533.I1RSQ7
229533.I1RI02 pp 229533.I1RY03
229533.I1RI02 pp 229533.I1S0L3
229533.I1RI02 pp 229533.I1RS18
229533.I1RI02 pp 229533.V6RCG5
229533.I1RI02 pp 229533.V6RTJ8
229533.I1RI02 pp 229533.I1RX46
229533.I1RI02 pp 229533.I1RI82
229533.I1RI02 pp 229533.I1RJW1
229533.I1RI02 pp 229533.I1RIE8
229533.I1RI02 pp 229533.I1RQ75
229533.I1RI02 pp 229533.I1RR39
229533.I1RI02 pp 229533.I1RR24
229533.I1RI02 pp 229533.I1RQC9
229533.I1RI02 pp 229533.I1RY44
229533.I1RI02 pp 229533.I1RW07
229533.I1RI02 pp 229533.V6R042
229533.I1RI02 pp 229533.V6RMW6
229533.I1RI02 pp 229533.I1RW25
229533.I1RI02 pp 229533.I1RSQ3
229533.I1RI02 pp 229533.I1RTH6
229533.I1RI02 pp 229533.V6RIV9
229533.I1RI02 pp 229533.I1RNC1
229533.I1RI02 pp 229533.I1RWN5
229533.I1RI02 pp 229533.I1S8R3
229533.I1RI02 pp 229533.I1RUG3
229533.I1RI02 pp 229533.I1RZW8
229533.I1RI02 pp 229533.I1RPP4
229533.I1RI02 pp 229533.I1RK44
229533.I1RI02 pp 229533.Q4I1Y5
229533.I1RI02 pp 229533.I1RTA0
229533.I1RI02 pp 229533.I1S569
229533.I1RI02 pp 229533.I1S1C7
229533.I1RI02 pp 229533.I1RT04

145271 229533.I1RI02 pp 229533.I1RVX0
145272 229533.I1RI02 pp 229533.I1RN67
145273 229533.I1RI02 pp 229533.I1RTD5
145274 229533.I1RI02 pp 229533.I1S8P5
145275 229533.I1RI02 pp 229533.I1RV91
145276 229533.I1RI02 pp 229533.I1RPP3
145277 229533.I1RI02 pp 229533.I1RT03
145278 229533.I1RI02 pp 229533.I1RWN6
145279 229533.I1RI02 pp 229533.I1RWK3
145280 229533.I1RI02 pp 229533.I1RN18
145281 229533.I1RI02 pp 229533.I1RJV4
145282 229533.I1RI02 pp 229533.I1RRM5
145283 229533.I1RI02 pp 229533.I1S2B8
145284 229533.I1RI02 pp 229533.I1RJW6
145285 229533.I1RI02 pp 229533.I1RJV3
145286 229533.I1RI02 pp 229533.I1S503
145287 229533.I1RI02 pp 229533.I1RPM4
145288 229533.I1RI02 pp 229533.I1RR73
145289 229533.I1RI02 pp 229533.V6R3X5
145290 229533.I1RI02 pp 229533.I1S2E9
145291 229533.I1RI02 pp 229533.I1S2H4
145292 229533.I1RI02 pp 229533.I1S0L4
145293 229533.I1RI02 pp 229533.I1RZ39
145294 229533.I1RI02 pp 229533.I1RRT8
145295 229533.I1RI02 pp 229533.I1RZZ9
145296 229533.I1S0D1 pp 229533.V6RKY7
145297 229533.I1S0D1 pp 229533.V6RVG3
145298 229533.I1S0D1 pp 229533.I1S3G4
145299 229533.I1S0D1 pp 229533.I1S439
145300 229533.I1S0D1 pp 229533.I1S1W4
145301 229533.I1S0D1 pp 229533.I1S0M2
145302 229533.I1S0D1 pp 229533.I1S2E9
145303 229533.I1S0D1 pp 229533.I1S267
145304 229533.I1S0D1 pp 229533.I1S1W8
145305 229533.I1S0D1 pp 229533.I1S0Z7
145306 229533.A0A1C3YN92 pp 229533.I1SA27
145307 229533.A0A1C3YN92 pp 229533.I1RQL1
145308 229533.A0A1C3YN92 pp 229533.I1RDZ3
145309 229533.A0A1C3YN92 pp 229533.I1RJA7
145310 229533.A0A1C3YN92 pp 229533.I1RY71
145311 229533.A0A1C3YN92 pp 229533.I1RX41
145312 229533.A0A1C3YN92 pp 229533.I1RCP3
145313 229533.A0A1C3YN92 pp 229533.I1RPT0
145314 229533.A0A1C3YN92 pp 229533.I1REG4
145315 229533.A0A1C3YN92 pp 229533.I1RIN3
145316 229533.A0A1C3YN92 pp 229533.I1RMP5
145317 229533.I1RUX6 pp 229533.Q4IMM0
145318 229533.I1RUX6 pp 229533.Q4IBX0
145319 229533.I1RUG1 pp 229533.I1S1C8
145320 229533.I1RUG1 pp 229533.Q4IBX0
145321 229533.I1RUG1 pp 229533.V6RLW4
145322 229533.I1RUG1 pp 229533.I1S2F2
145323 229533.I1RUG1 pp 229533.I1S1D3
145324 229533.I1RUG1 pp 229533.Q4IQM6
145325 229533.I1RUG1 pp 229533.I1S283
145326 229533.I1RUG1 pp 229533.I1RYX9
145327 229533.I1RUG1 pp 229533.I1RWM6
145328 229533.I1RUG1 pp 229533.Q4IC11
145329 229533.I1RY03 pp 229533.I1RZZ9
145330 229533.I1RY03 pp 229533.I1S6U8
145331 229533.I1RY03 pp 229533.I1S0X6
145332 229533.I1RY03 pp 229533.I1S2H4
145333 229533.I1RY03 pp 229533.I1S8V3
145334 229533.I1RY03 pp 229533.V6QUW4
145335 229533.I1RY03 pp 229533.I1S9U2
145336 229533.I1RY03 pp 229533.I1S0L4
145337 229533.I1RY03 pp 229533.I1RZ39
145338 229533.I1RY03 pp 229533.I1S5R1
145339 229533.I1RY03 pp 229533.Q4I662
145340 229533.I1RY03 pp 229533.I1RZS6
145341 229533.I1RY03 pp 229533.I1RZJ5
145342 229533.I1RY03 pp 229533.V6QW22
145343 229533.I1RY03 pp 229533.I1S352

145344 229533.I1RY03 pp 229533.I1S2E9
145345 229533.I1RY03 pp 229533.I1S8P5
145346 229533.I1RY03 pp 229533.I1S0M6
145347 229533.I1RY03 pp 229533.I1S2B8
145348 229533.I1RY03 pp 229533.I1S0F6
145349 229533.I1RY03 pp 229533.V6R283
145350 229533.I1RY03 pp 229533.I1S2C0
145351 229533.I1RY03 pp 229533.V6RUY9
145352 229533.I1RY03 pp 229533.Q4IJT5
145353 229533.I1RY03 pp 229533.I1S0R7
145354 229533.I1RY03 pp 229533.I1S7A8
145355 229533.I1RY03 pp 229533.I1RZK5
145356 229533.I1RY03 pp 229533.I1S0F5
145357 229533.I1RY03 pp 229533.V6RTJ8
145358 229533.I1RY03 pp 229533.I1S0L3
145359 229533.I1RY03 pp 229533.I1S1J7
145360 229533.I1RY03 pp 229533.I1RZW8
145361 229533.I1RY03 pp 229533.I1S8R3
145362 229533.I1RY03 pp 229533.I1S503
145363 229533.I1RY03 pp 229533.I1S2G7
145364 229533.I1RY03 pp 229533.Q4IJM4
145365 229533.I1RY03 pp 229533.V6R4J1
145366 229533.I1RY03 pp 229533.I1S2E8
145367 229533.I1RY03 pp 229533.V6R1K4
145368 229533.I1RY03 pp 229533.I1RYY5
145369 229533.I1RY03 pp 229533.I1S1C7
145370 229533.I1RY03 pp 229533.Q4I327
145371 229533.I1RY03 pp 229533.I1RZK6
145372 229533.I1RY03 pp 229533.Q4I624
145373 229533.I1RY03 pp 229533.I1RYW3
145374 229533.I1RY03 pp 229533.Q4IJK2
145375 229533.I1RY03 pp 229533.I1SA32
145376 229533.I1RY03 pp 229533.I1S8V4
145377 229533.I1RY03 pp 229533.I1RZZ1
145378 229533.I1RY03 pp 229533.I1RZM8
145379 229533.I1RY03 pp 229533.I1S569
145380 229533.I1RY03 pp 229533.I1S0N4
145381 229533.I1RY03 pp 229533.I1S5B0
145382 229533.I1RY03 pp 229533.Q4I283
145383 229533.I1RY03 pp 229533.Q4IN63
145384 229533.I1RY03 pp 229533.V6R057
145385 229533.I1RY03 pp 229533.I1RZE5
145386 229533.I1RY03 pp 229533.I1SA33
145387 229533.I1RY03 pp 229533.V6R042
145388 229533.I1RY03 pp 229533.V6QUE6
145389 229533.I1RY03 pp 229533.Q4I2J8
145390 229533.I1RY03 pp 229533.I1S504
145391 229533.I1RY03 pp 229533.I1RZM1
145392 229533.I1RY03 pp 229533.Q4I8P5
145393 229533.I1RY03 pp 229533.I1S1W4
145394 229533.I1RY03 pp 229533.V6RMW6
145395 229533.I1RY03 pp 229533.I1RZF6
145396 229533.I1RY03 pp 229533.V6RCZ8
145397 229533.I1RY03 pp 229533.V6RIV9
145398 229533.I1RY03 pp 229533.I1S0J1
145399 229533.I1RY03 pp 229533.V6RCG5
145400 229533.I1RY03 pp 229533.I1S0M9
145401 229533.I1RY03 pp 229533.I1RY44
145402 229533.I1RY03 pp 229533.I1S021
145403 229533.A0A1C3YJX1 pp 229533.I1RGW0
145404 229533.A0A1C3YJX1 pp 229533.A0A1C3YK01
145405 229533.A0A1C3YJX1 pp 229533.A0A1C3YJZ2
145406 229533.A0A1C3YJX1 pp 229533.I1RWU3
145407 229533.A0A1C3YJX1 pp 229533.I1RKR6
145408 229533.A0A1C3YJX1 pp 229533.V6QV38
145409 229533.A0A1C3YJX1 pp 229533.I1RB36
145410 229533.A0A1C3YJX1 pp 229533.I1RXE5
145411 229533.A0A1C3YJX1 pp 229533.I1RMA5
145412 229533.A0A1C3YJX1 pp 229533.I1RWX8
145413 229533.A0A1C3YJX1 pp 229533.I1RDC9
145414 229533.I1RSR8 pp 229533.I1RTD5
145415 229533.I1RSR8 pp 229533.I1S8I8
145416 229533.I1RSR8 pp 229533.I1RZJ6

145417	229533.I1RSR8	pp	229533.I1RT98
145418	229533.I1RSR8	pp	229533.I1RZS6
145419	229533.I1RSR8	pp	229533.I1RUQ2
145420	229533.I1RSR8	pp	229533.I1RXD7
145421	229533.I1RSR8	pp	229533.I1RX24
145422	229533.I1RSR8	pp	229533.I1S2E9
145423	229533.I1RSR8	pp	229533.V6QYF0
145424	229533.I1RSR8	pp	229533.I1S0R7
145425	229533.I1RSR8	pp	229533.I1RT04
145426	229533.I1RSR8	pp	229533.V6R283
145427	229533.I1RSR8	pp	229533.I1RWH5
145428	229533.I1RSR8	pp	229533.I1RX46
145429	229533.I1RSR8	pp	229533.I1S6F0
145430	229533.I1RSR8	pp	229533.I1RW25
145431	229533.I1RSR8	pp	229533.Q4IAS1
145432	229533.I1RSR8	pp	229533.Q4IFI0
145433	229533.I1RSR8	pp	229533.Q4IPI1
145434	229533.I1RSR8	pp	229533.I1S9U2
145435	229533.I1RSR8	pp	229533.I1S503
145436	229533.I1RSR8	pp	229533.I1S2B8
145437	229533.I1RSR8	pp	229533.I1S0J1
145438	229533.I1RSR8	pp	229533.I1RU69
145439	229533.I1RSR8	pp	229533.Q4I662
145440	229533.I1RSR8	pp	229533.Q4HVW2
145441	229533.I1RSR8	pp	229533.Q4IQW0
145442	229533.I1RSR8	pp	229533.V6R3X5
145443	229533.I1RSR8	pp	229533.I1RX14
145444	229533.I1RSR8	pp	229533.I1S060
145445	229533.I1RSR8	pp	229533.I1RTF4
145446	229533.I1RSR8	pp	229533.Q4IAA0
145447	229533.I1RSR8	pp	229533.Q4IJH1
145448	229533.I1RSR8	pp	229533.Q4IJ56
145449	229533.I1RSR8	pp	229533.Q4IN63
145450	229533.I1RSR8	pp	229533.I1RZ39
145451	229533.I1RSR8	pp	229533.V6R042
145452	229533.I1RSR8	pp	229533.I1S0M9
145453	229533.I1RSR8	pp	229533.I1RT80
145454	229533.I1RSR8	pp	229533.V6RMW6
145455	229533.I1RSR8	pp	229533.V6RGN5
145456	229533.I1RSR8	pp	229533.Q4IEK8
145457	229533.I1RSR8	pp	229533.I1RWB5
145458	229533.I1RSR8	pp	229533.I1RY44
145459	229533.I1RSR8	pp	229533.I1S044
145460	229533.I1RSR8	pp	229533.I1S8E2
145461	229533.I1RSR8	pp	229533.I1RWN5
145462	229533.I1RSR8	pp	229533.I1RZV6
145463	229533.I1RSR8	pp	229533.I1RZK6
145464	229533.I1RSR8	pp	229533.I1S0Z9
145465	229533.I1RSR8	pp	229533.I1RTG6
145466	229533.I1RSR8	pp	229533.I1RZF6
145467	229533.I1RSR8	pp	229533.I1S255
145468	229533.I1RSR8	pp	229533.I1RWE1
145469	229533.I1RSR8	pp	229533.I1S2E8
145470	229533.I1RSR8	pp	229533.I1RZZ1
145471	229533.I1RSR8	pp	229533.I1S504
145472	229533.I1RSR8	pp	229533.I1RVX0
145473	229533.I1RSR8	pp	229533.Q4HZ68
145474	229533.I1RSR8	pp	229533.I1RT03
145475	229533.I1RSR8	pp	229533.Q4I327
145476	229533.I1RSR8	pp	229533.I1S1C7
145477	229533.I1RSR8	pp	229533.I1S1X9
145478	229533.I1RSR8	pp	229533.I1RTD7
145479	229533.I1RSR8	pp	229533.I1RYB9
145480	229533.I1RSR8	pp	229533.I1RY03
145481	229533.I1RSR8	pp	229533.I1S021
145482	229533.I1RSR8	pp	229533.I1RZE5
145483	229533.I1RSR8	pp	229533.I1RZK5
145484	229533.I1RSR8	pp	229533.Q4HWE2
145485	229533.I1RSR8	pp	229533.I1RYB1
145486	229533.I1RSR8	pp	229533.I1RTE9
145487	229533.I1RSR8	pp	229533.I1RTH6
145488	229533.I1RSR8	pp	229533.I1RVZ0
145489	229533.I1RSR8	pp	229533.I1S2C0

145490 229533.I1RSR8 pp 229533.I1RWN6
145491 229533.I1RSR8 pp 229533.I1RTK6
145492 229533.I1RG94 pp 229533.I1S0S6
145493 229533.I1RG94 pp 229533.P0C431
145494 229533.I1RG94 pp 229533.Q4IBX0
145495 229533.I1RG94 pp 229533.Q4IMM0
145496 229533.I1RG94 pp 229533.I1RH68
145497 229533.I1RG94 pp 229533.I1RQN9
145498 229533.I1RG94 pp 229533.I1RTB7
145499 229533.I1RY71 pp 229533.I1RYJ8
145500 229533.I1RY71 pp 229533.I1S8Y5
145501 229533.I1RY71 pp 229533.I1S0Z2
145502 229533.A0A098E321 pp 229533.I1RGK9
145503 229533.A0A098E321 pp 229533.I1RTJ2
145504 229533.I1RJ40 pp 229533.I1RWX8
145505 229533.I1RJ40 pp 229533.I1RXE5
145506 229533.I1RJ40 pp 229533.V6QV38
145507 229533.I1RJ40 pp 229533.I1RMA5
145508 229533.I1RJ40 pp 229533.I1RWU3
145509 229533.I1RJ40 pp 229533.I1RKR6
145510 229533.I1RLU6 pp 229533.Q4I2J8
145511 229533.I1RLU6 pp 229533.Q4IPZ1
145512 229533.I1RLU6 pp 229533.I1RSI0
145513 229533.I1RLU6 pp 229533.I1RZ35
145514 229533.I1RLU6 pp 229533.Q4I624
145515 229533.I1RLU6 pp 229533.I1RZK5
145516 229533.I1RLU6 pp 229533.I1RZE5
145517 229533.I1RLU6 pp 229533.Q4HZB8
145518 229533.I1RLU6 pp 229533.I1RQE7
145519 229533.I1RLU6 pp 229533.I1RWN5
145520 229533.I1RLU6 pp 229533.V6R057
145521 229533.I1RLU6 pp 229533.I1RQQ5
145522 229533.I1RLU6 pp 229533.I1RZZ1
145523 229533.I1RLU6 pp 229533.I1S2E8
145524 229533.I1RLU6 pp 229533.I1S569
145525 229533.I1RLU6 pp 229533.I1RRN5
145526 229533.I1RLU6 pp 229533.V6RIV9
145527 229533.I1RLU6 pp 229533.Q4IJK2
145528 229533.I1RLU6 pp 229533.I1RZM1
145529 229533.I1RLU6 pp 229533.I1RWD7
145530 229533.I1RLU6 pp 229533.I1RVX0
145531 229533.I1RLU6 pp 229533.I1RSA4
145532 229533.I1RLU6 pp 229533.I1RT03
145533 229533.I1RLU6 pp 229533.I1RY44
145534 229533.I1RLU6 pp 229533.I1RQA3
145535 229533.I1RLU6 pp 229533.I1RNQ5
145536 229533.I1RLU6 pp 229533.I1S1J7
145537 229533.I1RLU6 pp 229533.I1RN18
145538 229533.I1RLU6 pp 229533.I1RW99
145539 229533.I1RLU6 pp 229533.I1RRY9
145540 229533.I1RLU6 pp 229533.I1RSK1
145541 229533.I1RLU6 pp 229533.I1S5B0
145542 229533.I1RLU6 pp 229533.I1S504
145543 229533.I1RLU6 pp 229533.I1RU29
145544 229533.I1RLU6 pp 229533.I1RRE5
145545 229533.I1RLU6 pp 229533.I1RWN6
145546 229533.I1RLU6 pp 229533.I1S0N4
145547 229533.I1RLU6 pp 229533.I1S044
145548 229533.I1RLU6 pp 229533.I1RY03
145549 229533.I1RLU6 pp 229533.Q4IN63
145550 229533.I1RLU6 pp 229533.I1RWT6
145551 229533.I1RLU6 pp 229533.I1RTD7
145552 229533.I1RLU6 pp 229533.I1RYW3
145553 229533.I1RLU6 pp 229533.I1RXF0
145554 229533.I1RLU6 pp 229533.I1RZM8
145555 229533.I1RLU6 pp 229533.I1S1C7
145556 229533.I1RLU6 pp 229533.Q4I283
145557 229533.I1RLU6 pp 229533.I1RUG3
145558 229533.I1RLU6 pp 229533.I1RTA0
145559 229533.I1RLU6 pp 229533.I1RRI2
145560 229533.I1RLU6 pp 229533.I1RW46
145561 229533.I1RLU6 pp 229533.I1RZW8
145562 229533.I1RLU6 pp 229533.I1RNY2

145563 229533.I1RLU6 pp 229533.I1RNC1
145564 229533.I1RLU6 pp 229533.I1RWX5
145565 229533.I1RLU6 pp 229533.I1RRV1
145566 229533.I1RLU6 pp 229533.I1S0J1
145567 229533.I1RLU6 pp 229533.I1RTH6
145568 229533.I1RLU6 pp 229533.I1SA32
145569 229533.I1RLU6 pp 229533.I1S8R3
145570 229533.I1RLU6 pp 229533.V6R9P0
145571 229533.I1RLU6 pp 229533.V6RCZ8
145572 229533.I1RLU6 pp 229533.I1RMG3
145573 229533.I1RLU6 pp 229533.I1S0M6
145574 229533.I1RLU6 pp 229533.V6RMW6
145575 229533.I1RLU6 pp 229533.I1RS17
145576 229533.I1RLU6 pp 229533.I1RWJ8
145577 229533.I1RLU6 pp 229533.I1RQ81
145578 229533.I1RLU6 pp 229533.Q4I8P5
145579 229533.I1RLU6 pp 229533.I1RM82
145580 229533.I1RLU6 pp 229533.V6RCG5
145581 229533.I1RLU6 pp 229533.I1RZJ5
145582 229533.I1RLU6 pp 229533.I1RM67
145583 229533.I1RLU6 pp 229533.V6R042
145584 229533.I1RLU6 pp 229533.I1RS18
145585 229533.I1RLU6 pp 229533.I1RSQ3
145586 229533.I1RLU6 pp 229533.I1RWL2
145587 229533.I1RLU6 pp 229533.I1S2B8
145588 229533.I1RLU6 pp 229533.I1RPP4
145589 229533.I1RLU6 pp 229533.I1RMG8
145590 229533.I1RLU6 pp 229533.I1RMN6
145591 229533.I1RLU6 pp 229533.I1SA33
145592 229533.I1RLU6 pp 229533.I1RRM5
145593 229533.I1RLU6 pp 229533.I1S5R1
145594 229533.I1RLU6 pp 229533.I1RRU9
145595 229533.I1RLU6 pp 229533.I1RRT8
145596 229533.I1RLU6 pp 229533.I1RX25
145597 229533.I1RLU6 pp 229533.I1RW25
145598 229533.I1RLU6 pp 229533.I1RS20
145599 229533.I1RLU6 pp 229533.I1RW07
145600 229533.I1RLU6 pp 229533.I1RPU3
145601 229533.I1RLU6 pp 229533.I1S0F5
145602 229533.I1RLU6 pp 229533.I1S0L3
145603 229533.I1RLU6 pp 229533.I1RR24
145604 229533.I1RLU6 pp 229533.I1S2H4
145605 229533.I1RLU6 pp 229533.I1RPM4
145606 229533.I1RLU6 pp 229533.I1S0L4
145607 229533.I1RLU6 pp 229533.V6QUW4
145608 229533.I1RLU6 pp 229533.I1RQ23
145609 229533.I1RLU6 pp 229533.I1RQ65
145610 229533.I1RLU6 pp 229533.I1RZZ9
145611 229533.I1RLU6 pp 229533.V6R1K4
145612 229533.I1RLU6 pp 229533.I1S0X6
145613 229533.I1RLU6 pp 229533.I1RWI5
145614 229533.I1RLU6 pp 229533.V6R283
145615 229533.I1RLU6 pp 229533.I1RT04
145616 229533.I1RLU6 pp 229533.I1RWK3
145617 229533.I1RLU6 pp 229533.I1S0R7
145618 229533.I1RLU6 pp 229533.I1RMC8
145619 229533.I1RLU6 pp 229533.I1RNN1
145620 229533.I1RLU6 pp 229533.I1RNA5
145621 229533.I1RLU6 pp 229533.I1S2G5
145622 229533.I1RLU6 pp 229533.Q4IJT5
145623 229533.I1RLU6 pp 229533.I1RSV2
145624 229533.I1RLU6 pp 229533.I1RQC9
145625 229533.I1RLU6 pp 229533.I1RR00
145626 229533.I1RLU6 pp 229533.I1RZ39
145627 229533.I1RLU6 pp 229533.I1S2E9
145628 229533.I1RLU6 pp 229533.I1RXD7
145629 229533.I1RLU6 pp 229533.V6R3X5
145630 229533.I1RLU6 pp 229533.I1RR73
145631 229533.I1RLU6 pp 229533.I1RQH3
145632 229533.I1RLU6 pp 229533.I1RW71
145633 229533.I1RLU6 pp 229533.I1RSQ7
145634 229533.I1RLU6 pp 229533.I1S6U8
145635 229533.I1RLU6 pp 229533.I1S352

229533.I1RLU6 pp 229533.I1S0F6
229533.I1RLU6 pp 229533.I1RN61
229533.I1RLU6 pp 229533.I1RVQ0
229533.I1RLU6 pp 229533.V6QW22
229533.I1RLU6 pp 229533.I1RN67
229533.I1RLU6 pp 229533.I1RPP3
229533.I1RLU6 pp 229533.V6R4J1
229533.I1RLU6 pp 229533.I1RWF7
229533.I1RLU6 pp 229533.I1S8P5
229533.I1RLU6 pp 229533.I1RS47
229533.I1RLU6 pp 229533.I1S8F0
229533.I1RLU6 pp 229533.I1RX46
229533.I1RLU6 pp 229533.I1S503
229533.I1RLU6 pp 229533.I1RP27
229533.I1RLU6 pp 229533.I1S7A8
229533.I1RLU6 pp 229533.I1RX39
229533.I1RLU6 pp 229533.I1RT40
229533.I1RLU6 pp 229533.V6RTJ8
229533.I1RLU6 pp 229533.I1RTD5
229533.I1RLU6 pp 229533.I1RV91
229533.I1S1F9 pp 229533.I1S1G0
229533.I1S1F9 pp 229533.I1S1G1
229533.A0A0E0S1U8 pp 229533.Q4IB89
229533.A0A0E0S1U8 pp 229533.I1RJT0
229533.A0A0E0S1U8 pp 229533.I1S4N7
229533.A0A0E0S1U8 pp 229533.I1RY44
229533.A0A0E0S1U8 pp 229533.I1RX50
229533.A0A0E0S1U8 pp 229533.I1RC71
229533.A0A0E0S1U8 pp 229533.Q4IBU4
229533.A0A0E0S1U8 pp 229533.I1RZ63
229533.A0A0E0S1U8 pp 229533.I1RW37
229533.A0A0E0S1U8 pp 229533.I1RFL7
229533.A0A0E0S1U8 pp 229533.I1RC18
229533.A0A0E0S1U8 pp 229533.I1S248
229533.A0A0E0S1U8 pp 229533.A0A1C3YJG7
229533.A0A0E0S1U8 pp 229533.I1S4N8
229533.A0A0E0S1U8 pp 229533.I1S1X7
229533.A0A0E0S1U8 pp 229533.I1RR07
229533.A0A0E0S1U8 pp 229533.I1RFN8
229533.A0A0E0S1U8 pp 229533.I1RD23
229533.A0A0E0S1U8 pp 229533.A0A1C3YME0
229533.A0A0E0S1U8 pp 229533.A0A1C3YKM2
229533.A0A0E0S1U8 pp 229533.I1S074
229533.A0A0E0S1U8 pp 229533.I1S0P7
229533.A0A0E0S1U8 pp 229533.I1S2E9
229533.A0A0E0S1U8 pp 229533.I1RMF2
229533.A0A0E0S1U8 pp 229533.A0A1C3YHP8
229533.A0A0E0S1U8 pp 229533.A0A1C3YLI6
229533.A0A0E0S1U8 pp 229533.I1RB41
229533.A0A0E0S1U8 pp 229533.I1RK75
229533.A0A0E0S1U8 pp 229533.I1RXV4
229533.A0A0E0S1U8 pp 229533.I1RMV6
229533.A0A0E0S1U8 pp 229533.I1RQ01
229533.A0A0E0S1U8 pp 229533.I1RWT6
229533.A0A0E0S1U8 pp 229533.A0A1C3YK57
229533.A0A0E0S1U8 pp 229533.I1S0J7
229533.A0A0E0S1U8 pp 229533.I1S278
229533.A0A0E0S1U8 pp 229533.I1RMK1
229533.A0A0E0S1U8 pp 229533.I1RQA3
229533.A0A0E0S1U8 pp 229533.I1RC22
229533.A0A0E0S1U8 pp 229533.I1RJT1
229533.A0A0E0S1U8 pp 229533.I1S039
229533.A0A0E0S1U8 pp 229533.I1RGD8
229533.A0A0E0S1U8 pp 229533.V6QV09
229533.A0A0E0S1U8 pp 229533.I1S9X9
229533.A0A0E0S1U8 pp 229533.A0A0E0SA11
229533.A0A0E0S1U8 pp 229533.I1RFS8
229533.A0A0E0S1U8 pp 229533.I1RKA1
229533.A0A0E0S1U8 pp 229533.A0A1C3YJH8
229533.A0A0E0S1U8 pp 229533.A0A0E0SC50
229533.A0A0E0S1U8 pp 229533.I1RNG8
229533.A0A0E0S1U8 pp 229533.I1RM49
229533.A0A0E0S1U8 pp 229533.I1RYZ9

229533.A0A0E0S1U8 pp 229533.I1RL24
229533.I1RW07 pp 229533.I1RY44
229533.I1RW07 pp 229533.Q4I1Y5
229533.I1RW07 pp 229533.I1RWN5
229533.I1RW07 pp 229533.V6RIV9
229533.I1RW07 pp 229533.I1S2B8
229533.I1RW07 pp 229533.V6R042
229533.I1RW07 pp 229533.V6RMW6
229533.I1RW07 pp 229533.I1RZW8
229533.I1RW07 pp 229533.I1S569
229533.I1RW07 pp 229533.I1RWK3
229533.I1RW07 pp 229533.I1RZZ9
229533.I1RW07 pp 229533.I1S1C7
229533.I1RW07 pp 229533.I1S8R3
229533.I1RW07 pp 229533.V6R3X5
229533.I1RW07 pp 229533.I1RZ39
229533.I1RW07 pp 229533.I1RWN6
229533.I1RW07 pp 229533.I1S8P5
229533.I1RW07 pp 229533.I1S2H4
229533.I1RW07 pp 229533.I1S2E9
229533.I1RW07 pp 229533.I1S0L4
229533.I1RW07 pp 229533.I1S504
229533.I1RW07 pp 229533.I1RZM1
229533.I1RW07 pp 229533.I1RY03
229533.I1RW07 pp 229533.V6RCG5
229533.I1RW07 pp 229533.I1S503
229533.I1RW07 pp 229533.Q4IJK2
229533.I1RW07 pp 229533.I1RZK5
229533.I1RW07 pp 229533.I1S0L3
229533.I1RW07 pp 229533.V6QW22
229533.I1RW07 pp 229533.I1RX46
229533.I1RW07 pp 229533.I1RW25
229533.I1RW07 pp 229533.V6RTJ8
229533.A0A1C3YIB0 pp 229533.V6RDP1
229533.A0A1C3YIB0 pp 229533.A0A1C3YN26
229533.A0A1C3YIB0 pp 229533.I1RWM4
229533.A0A1C3YIB0 pp 229533.I1RCU9
229533.A0A1C3YIB0 pp 229533.I1RDB1
229533.A0A1C3YIB0 pp 229533.V6RRM0
229533.A0A1C3YIB0 pp 229533.I1RNM7
229533.A0A1C3YIB0 pp 229533.I1RHJ2
229533.A0A1C3YIB0 pp 229533.I1RP86
229533.A0A1C3YIB0 pp 229533.I1RST8
229533.A0A1C3YIB0 pp 229533.I1RAM7
229533.A0A1C3YIB0 pp 229533.I1RCU8
229533.A0A1C3YIB0 pp 229533.I1S3L1
229533.A0A1C3YIB0 pp 229533.I1RCG5
229533.A0A1C3YIB0 pp 229533.I1RCU2
229533.A0A1C3YIB0 pp 229533.I1RF85
229533.A0A1C3YIB0 pp 229533.I1RKB2
229533.A0A1C3YIB0 pp 229533.V6RLM7
229533.A0A1C3YIB0 pp 229533.I1RHP8
229533.A0A1C3YIB0 pp 229533.I1RYK0
229533.A0A1C3YIB0 pp 229533.I1RYA0
229533.A0A1C3YIB0 pp 229533.I1RYC8
229533.A0A1C3YIB0 pp 229533.I1RG33
229533.A0A1C3YIB0 pp 229533.I1RNG4
229533.A0A1C3YIB0 pp 229533.I1S8F8
229533.A0A1C3YIB0 pp 229533.I1RTI3
229533.A0A1C3YIB0 pp 229533.I1RL81
229533.A0A1C3YIB0 pp 229533.A0A1C3YJZ0
229533.A0A1C3YIB0 pp 229533.A0A1C3YII1
229533.A0A1C3YIB0 pp 229533.V6RQW4
229533.A0A098DIJ2 pp 229533.I1S1W5
229533.A0A098DIJ2 pp 229533.I1RZT1
229533.A0A098DIJ2 pp 229533.A0A0E0S1U8
229533.A0A098DIJ2 pp 229533.I1RSD1
229533.A0A0E0SMM8 pp 229533.I1S021
229533.A0A0E0SMM8 pp 229533.I1RNH6
229533.A0A0E0SMM8 pp 229533.I1RZM8
229533.A0A0E0SMM8 pp 229533.I1RNC1
229533.A0A0E0SMM8 pp 229533.I1RUG3
229533.A0A0E0SMM8 pp 229533.I1RQ31

229533.A0A0E0SMM8	pp	229533.I1RSQ3
229533.A0A0E0SMM8	pp	229533.V6RIV9
229533.A0A0E0SMM8	pp	229533.A0A1C3YKB0
229533.A0A0E0SMM8	pp	229533.V6R042
229533.A0A0E0SMM8	pp	229533.V6RMW6
229533.A0A0E0SMM8	pp	229533.V6RCG5
229533.A0A0E0SMM8	pp	229533.I1S504
229533.A0A0E0SMM8	pp	229533.I1RZW8
229533.A0A0E0SMM8	pp	229533.I1RAU5
229533.A0A0E0SMM8	pp	229533.I1RD22
229533.A0A0E0SMM8	pp	229533.I1RW99
229533.A0A0E0SMM8	pp	229533.I1RQP8
229533.A0A0E0SMM8	pp	229533.I1RRI2
229533.A0A0E0SMM8	pp	229533.I1S5B0
229533.A0A0E0SMM8	pp	229533.I1RY03
229533.A0A0E0SMM8	pp	229533.A0A1C3YHX6
229533.A0A0E0SMM8	pp	229533.I1S1C7
229533.A0A0E0SMM8	pp	229533.I1RC72
229533.A0A0E0SMM8	pp	229533.A0A1C3YK34
229533.A0A0E0SMM8	pp	229533.I1RE01
229533.A0A0E0SMM8	pp	229533.I1RPP4
229533.A0A0E0SMM8	pp	229533.I1RTH6
229533.A0A0E0SMM8	pp	229533.I1RA70
229533.A0A0E0SMM8	pp	229533.I1RS17
229533.A0A0E0SMM8	pp	229533.I1RNU6
229533.A0A0E0SMM8	pp	229533.I1RFU8
229533.A0A0E0SMM8	pp	229533.I1RJV3
229533.A0A0E0SMM8	pp	229533.I1RCL3
229533.A0A0E0SMM8	pp	229533.I1RQ81
229533.A0A0E0SMM8	pp	229533.A0A1C3YKV3
229533.A0A0E0SMM8	pp	229533.I1RZM1
229533.A0A0E0SMM8	pp	229533.I1RGC6
229533.A0A0E0SMM8	pp	229533.I1RSR3
229533.A0A0E0SMM8	pp	229533.I1RAW0
229533.A0A0E0SMM8	pp	229533.I1RWN6
229533.A0A0E0SMM8	pp	229533.I1RRY9
229533.A0A0E0SMM8	pp	229533.I1RQE7
229533.A0A0E0SMM8	pp	229533.I1RM96
229533.A0A0E0SMM8	pp	229533.I1RLU6
229533.A0A0E0SMM8	pp	229533.I1RNQ5
229533.A0A0E0SMM8	pp	229533.V6QW22
229533.A0A0E0SMM8	pp	229533.I1RC28
229533.A0A0E0SMM8	pp	229533.I1RT03
229533.A0A0E0SMM8	pp	229533.Q4IJK2
229533.A0A0E0SMM8	pp	229533.A0A1C3YIJ3
229533.A0A0E0SMM8	pp	229533.I1RRJ8
229533.A0A0E0SMM8	pp	229533.I1RSQ7
229533.A0A0E0SMM8	pp	229533.I1RFL4
229533.A0A0E0SMM8	pp	229533.V6QVD5
229533.A0A0E0SMM8	pp	229533.I1RN18
229533.A0A0E0SMM8	pp	229533.I1RCL0
229533.A0A0E0SMM8	pp	229533.I1RPP3
229533.A0A0E0SMM8	pp	229533.I1RAU7
229533.A0A0E0SMM8	pp	229533.I1RB92
229533.A0A0E0SMM8	pp	229533.I1REK8
229533.A0A0E0SMM8	pp	229533.I1RW71
229533.A0A0E0SMM8	pp	229533.I1RRT8
229533.A0A0E0SMM8	pp	229533.I1RD17
229533.A0A0E0SMM8	pp	229533.I1RBC9
229533.A0A0E0SMM8	pp	229533.I1RVQ0
229533.A0A0E0SMM8	pp	229533.I1S5R1
229533.A0A0E0SMM8	pp	229533.I1RTD5
229533.A0A0E0SMM8	pp	229533.I1S9U2
229533.A0A0E0SMM8	pp	229533.A0A1C3YIP5
229533.A0A0E0SMM8	pp	229533.I1RWF5
229533.A0A0E0SMM8	pp	229533.I1RN67
229533.A0A0E0SMM8	pp	229533.I1RS30
229533.A0A0E0SMM8	pp	229533.I1S0R7
229533.A0A0E0SMM8	pp	229533.I1S0L3
229533.A0A0E0SMM8	pp	229533.I1S0L4
229533.A0A0E0SMM8	pp	229533.I1RT04
229533.A0A0E0SMM8	pp	229533.I1RS20
229533.A0A0E0SMM8	pp	229533.I1RPM4

145855	229533.A0A0E0SMM8	pp	229533.I1RQC9
145856	229533.A0A0E0SMM8	pp	229533.I1RQ65
145857	229533.A0A0E0SMM8	pp	229533.V6QYF0
145858	229533.A0A0E0SMM8	pp	229533.I1RX46
145859	229533.A0A0E0SMM8	pp	229533.V6QUW4
145860	229533.A0A0E0SMM8	pp	229533.I1RNJ7
145861	229533.A0A0E0SMM8	pp	229533.I1S503
145862	229533.A0A0E0SMM8	pp	229533.A0A1C3YHX8
145863	229533.A0A0E0SMM8	pp	229533.I1RBT1
145864	229533.A0A0E0SMM8	pp	229533.I1RJV4
145865	229533.A0A0E0SMM8	pp	229533.I1RY84
145866	229533.A0A0E0SMM8	pp	229533.I1RW25
145867	229533.A0A0E0SMM8	pp	229533.A0A1C3YKC0
145868	229533.A0A0E0SMM8	pp	229533.I1RZZ9
145869	229533.A0A0E0SMM8	pp	229533.I1RR73
145870	229533.A0A0E0SMM8	pp	229533.V6RTJ8
145871	229533.A0A0E0SMM8	pp	229533.I1RAX6
145872	229533.A0A0E0SMM8	pp	229533.V6R3X5
145873	229533.A0A0E0SMM8	pp	229533.I1RAC2
145874	229533.A0A0E0SMM8	pp	229533.I1RC82
145875	229533.A0A0E0SMM8	pp	229533.I1RBY4
145876	229533.A0A0E0SMM8	pp	229533.I1RBS6
145877	229533.A0A0E0SMM8	pp	229533.I1RMK3
145878	229533.A0A0E0SMM8	pp	229533.I1RE76
145879	229533.A0A0E0SMM8	pp	229533.I1RJZ7
145880	229533.A0A0E0SMM8	pp	229533.I1RWK3
145881	229533.A0A0E0SMM8	pp	229533.I1S2B8
145882	229533.A0A0E0SMM8	pp	229533.A0A1C3YKM9
145883	229533.A0A0E0SMM8	pp	229533.I1RTF4
145884	229533.A0A0E0SMM8	pp	229533.I1RRM5
145885	229533.A0A0E0SMM8	pp	229533.A0A1C3YIS7
145886	229533.I1RNP1	pp	229533.I1S7X9
145887	229533.I1RNP1	pp	229533.V6RB81
145888	229533.I1RNP1	pp	229533.Q4I061
145889	229533.I1RNP1	pp	229533.I1S0K5
145890	229533.I1RNP1	pp	229533.Q4I6S5
145891	229533.I1RNP1	pp	229533.I1S0G3
145892	229533.I1RQL1	pp	229533.I1RUM9
145893	229533.I1RQL1	pp	229533.I1RY71
145894	229533.I1RQL1	pp	229533.I1S0Z2
145895	229533.I1RQL1	pp	229533.I1RX41
145896	229533.I1RQL1	pp	229533.I1S8Y5
145897	229533.A0A1C3YIS5	pp	229533.I1RNU7
145898	229533.A0A1C3YIS5	pp	229533.I1RPU8
145899	229533.A0A1C3YIS5	pp	229533.I1RPT0
145900	229533.A0A0E0RUI4	pp	229533.I1RA70
145901	229533.A0A0E0RUI4	pp	229533.I1S2C0
145902	229533.A0A0E0RUI4	pp	229533.V6RMW6
145903	229533.A0A0E0RUI4	pp	229533.I1S1C7
145904	229533.A0A0E0RUI4	pp	229533.I1RTD7
145905	229533.A0A0E0RUI4	pp	229533.I1RSQ3
145906	229533.A0A0E0RUI4	pp	229533.V6R042
145907	229533.A0A0E0RUI4	pp	229533.I1RYW3
145908	229533.A0A0E0RUI4	pp	229533.Q4IN63
145909	229533.A0A0E0RUI4	pp	229533.I1RZW8
145910	229533.A0A0E0RUI4	pp	229533.I1RRI2
145911	229533.A0A0E0RUI4	pp	229533.I1RCR8
145912	229533.A0A0E0RUI4	pp	229533.I1RUG3
145913	229533.A0A0E0RUI4	pp	229533.I1RWX5
145914	229533.A0A0E0RUI4	pp	229533.I1RD17
145915	229533.A0A0E0RUI4	pp	229533.I1RNC1
145916	229533.A0A0E0RUI4	pp	229533.Q4I624
145917	229533.A0A0E0RUI4	pp	229533.A0A1C3YMI5
145918	229533.A0A0E0RUI4	pp	229533.I1RNY2
145919	229533.A0A0E0RUI4	pp	229533.V6R057
145920	229533.A0A0E0RUI4	pp	229533.Q4I327
145921	229533.A0A0E0RUI4	pp	229533.I1S569
145922	229533.A0A0E0RUI4	pp	229533.I1RAU7
145923	229533.A0A0E0RUI4	pp	229533.I1S1W4
145924	229533.A0A0E0RUI4	pp	229533.I1R9J6
145925	229533.A0A0E0RUI4	pp	229533.I1RB41
145926	229533.A0A0E0RUI4	pp	229533.I1RRE5
145927	229533.A0A0E0RUI4	pp	229533.I1RG90

145928 229533.A0A0E0RUI4 pp 229533.A0A1C3YI73
145929 229533.A0A0E0RUI4 pp 229533.I1S8V4
145930 229533.A0A0E0RUI4 pp 229533.I1RGL4
145931 229533.A0A0E0RUI4 pp 229533.I1RRN5
145932 229533.A0A0E0RUI4 pp 229533.I1RRR7
145933 229533.A0A0E0RUI4 pp 229533.I1RT03
145934 229533.A0A0E0RUI4 pp 229533.I1RVX0
145935 229533.A0A0E0RUI4 pp 229533.I1RWN5
145936 229533.A0A0E0RUI4 pp 229533.I1RB92
145937 229533.A0A0E0RUI4 pp 229533.V6RIV9
145938 229533.A0A0E0RUI4 pp 229533.I1RAW0
145939 229533.A0A0E0RUI4 pp 229533.I1RWN6
145940 229533.A0A0E0RUI4 pp 229533.I1RN18
145941 229533.A0A0E0RUI4 pp 229533.A0A1C3YK33
145942 229533.A0A0E0RUI4 pp 229533.I1RSD8
145943 229533.A0A0E0RUI4 pp 229533.V6RVG3
145944 229533.A0A0E0RUI4 pp 229533.I1RD39
145945 229533.A0A0E0RUI4 pp 229533.I1RFL4
145946 229533.A0A0E0RUI4 pp 229533.I1RCM3
145947 229533.A0A0E0RUI4 pp 229533.A0A1C3YIN6
145948 229533.A0A0E0RUI4 pp 229533.I1RJV3
145949 229533.A0A0E0RUI4 pp 229533.I1RC33
145950 229533.A0A0E0RUI4 pp 229533.I1RQE7
145951 229533.A0A0E0RUI4 pp 229533.I1RY44
145952 229533.A0A0E0RUI4 pp 229533.A0A0E0SBC4
145953 229533.A0A0E0RUI4 pp 229533.I1RSK1
145954 229533.A0A0E0RUI4 pp 229533.I1S2E8
145955 229533.A0A0E0RUI4 pp 229533.I1S8R3
145956 229533.A0A0E0RUI4 pp 229533.I1RM96
145957 229533.A0A0E0RUI4 pp 229533.I1RCH5
145958 229533.A0A0E0RUI4 pp 229533.I1RS17
145959 229533.A0A0E0RUI4 pp 229533.I1R9D1
145960 229533.A0A0E0RUI4 pp 229533.I1RTH6
145961 229533.A0A0E0RUI4 pp 229533.I1S0N4
145962 229533.A0A0E0RUI4 pp 229533.I1RR73
145963 229533.A0A0E0RUI4 pp 229533.I1RMN6
145964 229533.A0A0E0RUI4 pp 229533.A0A1C3YK73
145965 229533.A0A0E0RUI4 pp 229533.I1RZZ9
145966 229533.A0A0E0RUI4 pp 229533.I1RPP4
145967 229533.A0A0E0RUI4 pp 229533.I1RTA0
145968 229533.A0A0E0RUI4 pp 229533.I1SA33
145969 229533.A0A0E0RUI4 pp 229533.I1RW46
145970 229533.A0A0E0RUI4 pp 229533.I1RD22
145971 229533.A0A0E0RUI4 pp 229533.I1RWI5
145972 229533.A0A0E0RUI4 pp 229533.I1RE01
145973 229533.A0A0E0RUI4 pp 229533.I1RRV1
145974 229533.A0A0E0RUI4 pp 229533.I1S0J1
145975 229533.A0A0E0RUI4 pp 229533.I1RNN1
145976 229533.A0A0E0RUI4 pp 229533.I1RMG3
145977 229533.A0A0E0RUI4 pp 229533.I1S8V3
145978 229533.A0A0E0RUI4 pp 229533.I1RC66
145979 229533.A0A0E0RUI4 pp 229533.I1RS20
145980 229533.A0A0E0RUI4 pp 229533.I1S2H4
145981 229533.A0A0E0RUI4 pp 229533.V6RUY9
145982 229533.A0A0E0RUI4 pp 229533.I1RNA5
145983 229533.A0A0E0RUI4 pp 229533.A0A1C3YIE7
145984 229533.A0A0E0RUI4 pp 229533.I1RD64
145985 229533.A0A0E0RUI4 pp 229533.I1RIL3
145986 229533.A0A0E0RUI4 pp 229533.A0A0E0SC46
145987 229533.A0A0E0RUI4 pp 229533.I1S0L4
145988 229533.A0A0E0RUI4 pp 229533.A0A1C3YIS7
145989 229533.A0A0E0RUI4 pp 229533.A0A1C3YMA1
145990 229533.A0A0E0RUI4 pp 229533.I1S2B8
145991 229533.A0A0E0RUI4 pp 229533.I1S2E9
145992 229533.A0A0E0RUI4 pp 229533.I1RT40
145993 229533.A0A0E0RUI4 pp 229533.I1RRM5
145994 229533.A0A0E0RUI4 pp 229533.I1RKJ6
145995 229533.A0A0E0RUI4 pp 229533.I1RAF8
145996 229533.A0A0E0RUI4 pp 229533.I1RM82
145997 229533.A0A0E0RUI4 pp 229533.I1RJZ7
145998 229533.A0A0E0RUI4 pp 229533.I1RCJ7
145999 229533.A0A0E0RUI4 pp 229533.I1R9M0
146000 229533.A0A0E0RUI4 pp 229533.I1S7S9

146001 229533.A0A0E0RUI4 pp 229533.I1RWL2
146002 229533.A0A0E0RUI4 pp 229533.I1RV91
146003 229533.A0A0E0RUI4 pp 229533.I1RA11
146004 229533.A0A0E0RUI4 pp 229533.A0A1C3YLD8
146005 229533.A0A0E0RUI4 pp 229533.I1RTD5
146006 229533.A0A0E0RUI4 pp 229533.I1RRT8
146007 229533.A0A0E0RUI4 pp 229533.I1S5R1
146008 229533.A0A0E0RUI4 pp 229533.I1RBY4
146009 229533.A0A0E0RUI4 pp 229533.I1RE76
146010 229533.A0A0E0RUI4 pp 229533.I1RPU3
146011 229533.A0A0E0RUI4 pp 229533.I1RBG9
146012 229533.A0A0E0RUI4 pp 229533.I1RGF5
146013 229533.A0A0E0RUI4 pp 229533.I1RE22
146014 229533.A0A0E0RUI4 pp 229533.A0A1C3YKM9
146015 229533.A0A0E0RUI4 pp 229533.I1RZ39
146016 229533.A0A0E0RUI4 pp 229533.I1RS47
146017 229533.A0A0E0RUI4 pp 229533.V6R3X5
146018 229533.A0A0E0RUI4 pp 229533.I1S2G7
146019 229533.A0A0E0RUI4 pp 229533.I1RZS6
146020 229533.A0A0E0RUI4 pp 229533.A0A1C3YL15
146021 229533.A0A0E0RUI4 pp 229533.A0A1C3YIN1
146022 229533.A0A0E0RUI4 pp 229533.I1S0M6
146023 229533.A0A0E0RUI4 pp 229533.I1RQ65
146024 229533.A0A0E0RUI4 pp 229533.I1RPM4
146025 229533.A0A0E0RUI4 pp 229533.I1RCG4
146026 229533.A0A0E0RUI4 pp 229533.I1RZK5
146027 229533.A0A0E0RUI4 pp 229533.I1S0F6
146028 229533.A0A0E0RUI4 pp 229533.I1RY03
146029 229533.A0A0E0RUI4 pp 229533.I1RFS3
146030 229533.A0A0E0RUI4 pp 229533.I1RN67
146031 229533.A0A0E0RUI4 pp 229533.A0A0E0S0W9
146032 229533.A0A0E0RUI4 pp 229533.I1RQ23
146033 229533.A0A0E0RUI4 pp 229533.I1RSR8
146034 229533.A0A0E0RUI4 pp 229533.I1RBS0
146035 229533.A0A0E0RUI4 pp 229533.A0A1C3YHX6
146036 229533.A0A0E0RUI4 pp 229533.I1RGA4
146037 229533.A0A0E0RUI4 pp 229533.I1RRQ3
146038 229533.A0A0E0RUI4 pp 229533.I1RBS6
146039 229533.A0A0E0RUI4 pp 229533.A0A1C3YLK2
146040 229533.A0A0E0RUI4 pp 229533.A0A1C3YKN3
146041 229533.A0A0E0RUI4 pp 229533.I1RYY5
146042 229533.A0A0E0RUI4 pp 229533.A0A1C3YLA6
146043 229533.A0A0E0RUI4 pp 229533.I1RJV4
146044 229533.A0A0E0RUI4 pp 229533.I1RFT1
146045 229533.A0A0E0RUI4 pp 229533.I1RP27
146046 229533.A0A0E0RUI4 pp 229533.I1RCE0
146047 229533.A0A0E0RUI4 pp 229533.I1RCR5
146048 229533.A0A0E0RUI4 pp 229533.I1RSV2
146049 229533.A0A0E0RUI4 pp 229533.I1REK8
146050 229533.A0A0E0RUI4 pp 229533.I1S503
146051 229533.A0A0E0RUI4 pp 229533.I1RKL3
146052 229533.A0A0E0RUI4 pp 229533.A0A1C3YLK8
146053 229533.A0A0E0RUI4 pp 229533.I1RJW6
146054 229533.A0A0E0RUI4 pp 229533.I1RB75
146055 229533.A0A0E0RUI4 pp 229533.I1RWD7
146056 229533.A0A0E0RUI4 pp 229533.I1S352
146057 229533.A0A0E0RUI4 pp 229533.Q4I2J8
146058 229533.A0A0E0RUI4 pp 229533.I1RLN6
146059 229533.A0A0E0RUI4 pp 229533.I1RLV9
146060 229533.A0A0E0RUI4 pp 229533.I1RRJ8
146061 229533.A0A0E0RUI4 pp 229533.I1RVZ0
146062 229533.A0A0E0RUI4 pp 229533.I1RRZ0
146063 229533.A0A0E0RUI4 pp 229533.I1RPP3
146064 229533.A0A0E0RUI4 pp 229533.I1RT04
146065 229533.A0A0E0RUI4 pp 229533.I1RF19
146066 229533.A0A0E0RUI4 pp 229533.I1RSI0
146067 229533.A0A0E0RUI4 pp 229533.I1S8P5
146068 229533.A0A0E0RUI4 pp 229533.A0A1C3YIJ3
146069 229533.A0A0E0RUI4 pp 229533.I1RE00
146070 229533.A0A0E0RUI4 pp 229533.I1RAU5
146071 229533.A0A0E0RUI4 pp 229533.A0A0E0RV94
146072 229533.A0A0E0RUI4 pp 229533.I1RFT2
146073 229533.A0A0E0RUI4 pp 229533.I1RWF7

146074 229533.A0A0E0RUI4 pp 229533.I1RRU9
146075 229533.A0A0E0RUI4 pp 229533.I1RZE5
146076 229533.A0A0E0RUI4 pp 229533.I1RB74
146077 229533.A0A0E0RUI4 pp 229533.I1RC03
146078 229533.A0A0E0RUI4 pp 229533.I1RLM7
146079 229533.A0A0E0RUI4 pp 229533.I1RA48
146080 229533.A0A0E0RUI4 pp 229533.V6R283
146081 229533.A0A0E0RUI4 pp 229533.Q4IJM4
146082 229533.A0A0E0RUI4 pp 229533.V6RCZ8
146083 229533.A0A0E0RUI4 pp 229533.I1RWK3
146084 229533.A0A0E0RUI4 pp 229533.V6RCG5
146085 229533.A0A0E0RUI4 pp 229533.I1R9V7
146086 229533.A0A0E0RUI4 pp 229533.A0A0E0RVZ1
146087 229533.A0A0E0RUI4 pp 229533.I1RN63
146088 229533.A0A0E0RUI4 pp 229533.Q4IJK2
146089 229533.A0A0E0RUI4 pp 229533.I1RBL7
146090 229533.A0A0E0RUI4 pp 229533.I1RQA3
146091 229533.A0A0E0RUI4 pp 229533.A0A1C3YLU7
146092 229533.A0A0E0RUI4 pp 229533.I1RZM8
146093 229533.A0A0E0RUI4 pp 229533.I1RRY9
146094 229533.A0A0E0RUI4 pp 229533.I1RXF0
146095 229533.A0A0E0RUI4 pp 229533.I1RJZ6
146096 229533.A0A0E0RUI4 pp 229533.I1RU29
146097 229533.A0A0E0RUI4 pp 229533.I1RAN5
146098 229533.A0A0E0RUI4 pp 229533.I1RQQ5
146099 229533.A0A0E0RUI4 pp 229533.A0A1C3YJ93
146100 229533.A0A0E0RUI4 pp 229533.I1RNH6
146101 229533.A0A0E0RUI4 pp 229533.I1S021
146102 229533.A0A0E0RUI4 pp 229533.V6RBN2
146103 229533.A0A0E0RUI4 pp 229533.I1S1J7
146104 229533.A0A0E0RUI4 pp 229533.A0A0E0SPV2
146105 229533.A0A0E0RUI4 pp 229533.I1RZZ1
146106 229533.A0A0E0RUI4 pp 229533.I1RI82
146107 229533.A0A0E0RUI4 pp 229533.I1RI03
146108 229533.A0A0E0RUI4 pp 229533.I1S504
146109 229533.A0A0E0RUI4 pp 229533.I1RZK6
146110 229533.A0A0E0RUI4 pp 229533.I1RX46
146111 229533.A0A0E0RUI4 pp 229533.I1RC17
146112 229533.A0A0E0RUI4 pp 229533.I1RW99
146113 229533.A0A0E0RUI4 pp 229533.I1S0R7
146114 229533.A0A0E0RUI4 pp 229533.A0A0E0SFN0
146115 229533.A0A0E0RUI4 pp 229533.I1S5B0
146116 229533.A0A0E0RUI4 pp 229533.I1RZF6
146117 229533.A0A0E0RUI4 pp 229533.I1RQC9
146118 229533.A0A0E0RUI4 pp 229533.A0A1C3YKW9
146119 229533.A0A0E0RUI4 pp 229533.I1RWT6
146120 229533.A0A0E0RUI4 pp 229533.I1RXD7
146121 229533.A0A0E0RUI4 pp 229533.Q4I283
146122 229533.A0A0E0RUI4 pp 229533.A0A1C3YJH8
146123 229533.A0A0E0RUI4 pp 229533.I1RWJ8
146124 229533.A0A0E0RUI4 pp 229533.I1RQH3
146125 229533.A0A0E0RUI4 pp 229533.A0A1C3YL54
146126 229533.A0A0E0RUI4 pp 229533.I1RM67
146127 229533.A0A0E0RUI4 pp 229533.I1RD52
146128 229533.A0A0E0RUI4 pp 229533.I1RZJ5
146129 229533.A0A0E0RUI4 pp 229533.I1S0F5
146130 229533.A0A0E0RUI4 pp 229533.I1RMG8
146131 229533.A0A0E0RUI4 pp 229533.I1RFQ7
146132 229533.A0A0E0RUI4 pp 229533.A0A1C3YKB0
146133 229533.A0A0E0RUI4 pp 229533.A0A0E0SMM8
146134 229533.A0A0E0RUI4 pp 229533.I1RZM1
146135 229533.A0A0E0RUI4 pp 229533.I1S0L3
146136 229533.A0A0E0RUI4 pp 229533.I1RBJ5
146137 229533.A0A0E0RUI4 pp 229533.I1RNQ5
146138 229533.A0A0E0RUI4 pp 229533.A0A1C3YN30
146139 229533.A0A0E0RUI4 pp 229533.I1RIG2
146140 229533.A0A0E0RUI4 pp 229533.I1RJU3
146141 229533.A0A0E0RUI4 pp 229533.V6R1K4
146142 229533.A0A0E0RUI4 pp 229533.A0A1C3YIP5
146143 229533.A0A0E0RUI4 pp 229533.I1RT80
146144 229533.A0A0E0RUI4 pp 229533.I1RLU6
146145 229533.A0A0E0RUI4 pp 229533.I1RVQ0
146146 229533.A0A0E0RUI4 pp 229533.I1RQ81

146147 229533.A0A0E0RUI4 pp 229533.I1RDB1
146148 229533.A0A0E0RUI4 pp 229533.A0A0E0RZM5
146149 229533.A0A0E0RUI4 pp 229533.I1S0M9
146150 229533.A0A0E0RUI4 pp 229533.I1S6U8
146151 229533.A0A0E0RUI4 pp 229533.V6R4J1
146152 229533.A0A0E0RUI4 pp 229533.A0A0E0SA11
146153 229533.A0A0E0RUI4 pp 229533.I1S0X6
146154 229533.A0A0E0RUI4 pp 229533.A0A1C3YKV3
146155 229533.A0A0E0RUI4 pp 229533.I1RR00
146156 229533.A0A0E0RUI4 pp 229533.I1RBC9
146157 229533.A0A0E0RUI4 pp 229533.Q4I8P5
146158 229533.A0A0E0RUI4 pp 229533.I1RBT1
146159 229533.A0A0E0RUI4 pp 229533.A0A1C3YKC0
146160 229533.A0A0E0RUI4 pp 229533.A0A1C3YHX8
146161 229533.A0A0E0RUI4 pp 229533.I1RW71
146162 229533.A0A0E0RUI4 pp 229533.I1RMC8
146163 229533.A0A0E0RUI4 pp 229533.A0A1C3YK13
146164 229533.A0A0E0RUI4 pp 229533.I1RB27
146165 229533.A0A0E0RUI4 pp 229533.I1RW07
146166 229533.A0A0E0RUI4 pp 229533.I1RSQ7
146167 229533.A0A0E0RUI4 pp 229533.I1RI02
146168 229533.A0A0E0RUI4 pp 229533.I1RS18
146169 229533.A0A0E0RUI4 pp 229533.I1RC20
146170 229533.A0A0E0RUI4 pp 229533.A0A1C3YK34
146171 229533.A0A0E0RUI4 pp 229533.A0A0E0SJS1
146172 229533.A0A0E0RUI4 pp 229533.I1RAM7
146173 229533.A0A0E0RUI4 pp 229533.I1SA32
146174 229533.A0A0E0RUI4 pp 229533.I1RLC7
146175 229533.A0A0E0RUI4 pp 229533.I1RW25
146176 229533.A0A0E0RUI4 pp 229533.V6QW22
146177 229533.A0A0E0RUI4 pp 229533.I1RX25
146178 229533.A0A0E0RUI4 pp 229533.I1RN61
146179 229533.A0A0E0RUI4 pp 229533.A0A1C3YJQ9
146180 229533.A0A0E0RUI4 pp 229533.A0A1C3YLZ7
146181 229533.A0A0E0RUI4 pp 229533.I1RQJ5
146182 229533.A0A0E0RUI4 pp 229533.I1RR24
146183 229533.A0A0E0RUI4 pp 229533.I1S9U2
146184 229533.A0A0E0RUI4 pp 229533.V6QUW4
146185 229533.A0A0E0RUI4 pp 229533.I1RAX6
146186 229533.A0A0E0RUI4 pp 229533.I1RG53
146187 229533.A0A0E0RUI4 pp 229533.I1S7A8
146188 229533.A0A0E0RUI4 pp 229533.A0A1C3YM63
146189 229533.A0A0E0RUI4 pp 229533.I1RPU0
146190 229533.A0A0E0RUI4 pp 229533.I1RFW5
146191 229533.A0A0E0RUI4 pp 229533.V6RTJ8
146192 229533.A0A0E0RUI4 pp 229533.Q4IJT5
146193 229533.A0A0E0RUI4 pp 229533.I1RIL8
146194 229533.I1RXD5 pp 229533.Q4IMM0
146195 229533.I1RXD5 pp 229533.P0C431
146196 229533.I1RXD5 pp 229533.I1S0S6
146197 229533.I1RXD5 pp 229533.Q4IBX0
146198 229533.I1S2K7 pp 229533.I1S7X9
146199 229533.I1S2K7 pp 229533.I1S439
146200 229533.I1S2K7 pp 229533.Q4IEA7
146201 229533.I1S2K7 pp 229533.V6RVG3
146202 229533.I1S2K7 pp 229533.V6RKY7
146203 229533.I1RZS3 pp 229533.I1S0S2
146204 229533.I1RZS3 pp 229533.I1S0Y7
146205 229533.A0A0E0S2K0 pp 229533.I1RTI3
146206 229533.A0A0E0S2K0 pp 229533.I1REC7
146207 229533.A0A0E0S2K0 pp 229533.A0A0E0SAK0
146208 229533.A0A0E0S2K0 pp 229533.I1RB77
146209 229533.A0A0E0S2K0 pp 229533.I1RXJ0
146210 229533.I1RZM1 pp 229533.Q4I2J8
146211 229533.I1RZM1 pp 229533.Q4I283
146212 229533.I1RZM1 pp 229533.V6RBN2
146213 229533.I1RZM1 pp 229533.I1S439
146214 229533.I1RZM1 pp 229533.I1RZZ1
146215 229533.I1RZM1 pp 229533.I1S5B0
146216 229533.I1RZM1 pp 229533.V6RCG5
146217 229533.I1RZM1 pp 229533.I1SA32
146218 229533.I1RZM1 pp 229533.Q4IJM4
146219 229533.I1RZM1 pp 229533.I1S504

229533.I1RZM1 pp 229533.I1RZM8
229533.I1RZM1 pp 229533.Q4IJK2
229533.I1RZM1 pp 229533.I1S1J7
229533.I1RZM1 pp 229533.V6RCZ8
229533.I1RZM1 pp 229533.I1S044
229533.I1RZM1 pp 229533.Q4I8P5
229533.I1RZM1 pp 229533.P0C431
229533.I1RZM1 pp 229533.V6R4J1
229533.I1RZM1 pp 229533.Q4IN00
229533.I1RZM1 pp 229533.I1S0X6
229533.I1RZM1 pp 229533.Q4IJT5
229533.I1RZM1 pp 229533.V6QUW4
229533.I1RZM1 pp 229533.I1RZW8
229533.I1RZM1 pp 229533.I1S0L3
229533.I1RZM1 pp 229533.I1RZQ6
229533.I1RZM1 pp 229533.V6QW22
229533.I1RZM1 pp 229533.V6R1K4
229533.I1RZM1 pp 229533.I1S0T2
229533.I1RZM1 pp 229533.V6RTJ8
229533.I1RZM1 pp 229533.I1S1C7
229533.I1RZM1 pp 229533.I1S0R7
229533.I1RZM1 pp 229533.Q4I5R9
229533.I1RZM1 pp 229533.I1S0F5
229533.I1RZM1 pp 229533.Q4IPH4
229533.I1RZM1 pp 229533.I1S0S6
229533.I1RZM1 pp 229533.I1S8R3
229533.I1RZM1 pp 229533.V6RIV9
229533.I1RZM1 pp 229533.I1S7A8
229533.I1RZM1 pp 229533.I1S1W4
229533.I1RZM1 pp 229533.V6R057
229533.I1RZM1 pp 229533.I1S0J1
229533.I1RZM1 pp 229533.Q4INZ9
229533.I1RZM1 pp 229533.Q4HZB8
229533.I1RZM1 pp 229533.I1S569
229533.I1RZM1 pp 229533.Q4I624
229533.I1RZM1 pp 229533.Q4IN63
229533.I1RZM1 pp 229533.I1RZZ9
229533.I1RZM1 pp 229533.I1SA33
229533.I1RZM1 pp 229533.I1S0N4
229533.I1RZM1 pp 229533.I1S0M6
229533.I1RZM1 pp 229533.V6RMW6
229533.I1RZM1 pp 229533.I1S2B8
229533.I1RZM1 pp 229533.V6R3X5
229533.I1RZM1 pp 229533.V6R042
229533.I1RZM1 pp 229533.I1RZM2
229533.I1RZM1 pp 229533.I1S7S9
229533.I1RZM1 pp 229533.I1S0L4
229533.I1RZM1 pp 229533.I1S0F6
229533.I1RZM1 pp 229533.I1S2G7
229533.I1RZM1 pp 229533.I1S5R1
229533.I1RZM1 pp 229533.I1S8P5
229533.I1RZM1 pp 229533.I1S2H4
229533.I1RZM1 pp 229533.I1S352
229533.I1RZM1 pp 229533.V6R283
229533.I1RZM1 pp 229533.Q4I7L0
229533.I1RZM1 pp 229533.V6R3J1
229533.I1RZM1 pp 229533.I1S503
229533.I1RZM1 pp 229533.I1S2E9
229533.A0A1C3YLI5 pp 229533.I1RQ72
229533.I1RJW4 pp 229533.I1RWB5
229533.I1RJW4 pp 229533.I1RPK0
229533.I1RJW4 pp 229533.I1RST8
229533.I1RJW4 pp 229533.I1RZQ6
229533.I1RJW4 pp 229533.V6RDP1
229533.A0A1C3YJL3 pp 229533.I1RX22
229533.A0A1C3YJL3 pp 229533.I1R9R2
229533.A0A1C3YJL3 pp 229533.Q4I8B6
229533.A0A1C3YJL3 pp 229533.I1S7B9
229533.A0A1C3YJL3 pp 229533.I1RW03
229533.A0A1C3YJL3 pp 229533.I1RDH3
229533.A0A1C3YJL3 pp 229533.A0A1C3YLS9
229533.I1RB03 pp 229533.I1S097
229533.I1RB03 pp 229533.I1RBY9

146293 229533.I1RB03 pp 229533.I1RB77
146294 229533.I1RB03 pp 229533.I1S2L0
146295 229533.A0A1C3YJQ9 pp 229533.I1RT03
146296 229533.A0A1C3YJQ9 pp 229533.I1RB92
146297 229533.A0A1C3YJQ9 pp 229533.I1RVX0
146298 229533.A0A1C3YJQ9 pp 229533.I1RRN5
146299 229533.A0A1C3YJQ9 pp 229533.I1RAU7
146300 229533.A0A1C3YJQ9 pp 229533.I1RB41
146301 229533.A0A1C3YJQ9 pp 229533.V6R057
146302 229533.A0A1C3YJQ9 pp 229533.Q4I624
146303 229533.A0A1C3YJQ9 pp 229533.I1RNC1
146304 229533.A0A1C3YJQ9 pp 229533.I1RD17
146305 229533.A0A1C3YJQ9 pp 229533.I1RUG3
146306 229533.A0A1C3YJQ9 pp 229533.I1RCR8
146307 229533.A0A1C3YJQ9 pp 229533.V6R042
146308 229533.A0A1C3YJQ9 pp 229533.I1RSQ3
146309 229533.A0A1C3YJQ9 pp 229533.I1RR73
146310 229533.A0A1C3YJQ9 pp 229533.V6RMW6
146311 229533.A0A1C3YJQ9 pp 229533.I1RZ39
146312 229533.A0A1C3YJQ9 pp 229533.V6R3X5
146313 229533.A0A1C3YJQ9 pp 229533.I1RD22
146314 229533.A0A1C3YJQ9 pp 229533.I1SA33
146315 229533.A0A1C3YJQ9 pp 229533.I1S5R1
146316 229533.A0A1C3YJQ9 pp 229533.A0A1C3YKM9
146317 229533.A0A1C3YJQ9 pp 229533.I1RRT8
146318 229533.A0A1C3YJQ9 pp 229533.I1S0N4
146319 229533.A0A1C3YJQ9 pp 229533.I1RA11
146320 229533.A0A1C3YJQ9 pp 229533.I1S0J1
146321 229533.A0A1C3YJQ9 pp 229533.I1RM82
146322 229533.A0A1C3YJQ9 pp 229533.I1S0L4
146323 229533.A0A1C3YJQ9 pp 229533.I1RE01
146324 229533.A0A1C3YJQ9 pp 229533.I1RW46
146325 229533.A0A1C3YJQ9 pp 229533.I1S2H4
146326 229533.A0A1C3YJQ9 pp 229533.I1RPM4
146327 229533.A0A1C3YJQ9 pp 229533.I1RTA0
146328 229533.A0A1C3YJQ9 pp 229533.I1RZS7
146329 229533.A0A1C3YJQ9 pp 229533.I1RPP4
146330 229533.A0A1C3YJQ9 pp 229533.Q4I662
146331 229533.A0A1C3YJQ9 pp 229533.I1RE22
146332 229533.A0A1C3YJQ9 pp 229533.I1RRI2
146333 229533.A0A1C3YJQ9 pp 229533.I1RS17
146334 229533.A0A1C3YJQ9 pp 229533.I1RZW8
146335 229533.A0A1C3YJQ9 pp 229533.I1RE76
146336 229533.A0A1C3YJQ9 pp 229533.I1RY44
146337 229533.A0A1C3YJQ9 pp 229533.I1RBY4
146338 229533.A0A1C3YJQ9 pp 229533.I1R9M0
146339 229533.A0A1C3YJQ9 pp 229533.I1S2B8
146340 229533.A0A1C3YJQ9 pp 229533.I1RYW3
146341 229533.A0A1C3YJQ9 pp 229533.I1RSA4
146342 229533.A0A1C3YJQ9 pp 229533.I1S1C7
146343 229533.A0A1C3YJQ9 pp 229533.I1RRM5
146344 229533.A0A1C3YJQ9 pp 229533.I1RWN5
146345 229533.A0A1C3YJQ9 pp 229533.I1RCL5
146346 229533.A0A1C3YJQ9 pp 229533.V6RIV9
146347 229533.A0A1C3YJQ9 pp 229533.A0A1C3YMA1
146348 229533.A0A1C3YJQ9 pp 229533.I1RA70
146349 229533.A0A1C3YJQ9 pp 229533.I1RTD5
146350 229533.A0A1C3YJQ9 pp 229533.I1RTH6
146351 229533.A0A1C3YJQ9 pp 229533.I1RT40
146352 229533.A0A1C3YJQ9 pp 229533.I1RV91
146353 229533.A0A1C3YJQ9 pp 229533.I1S8R3
146354 229533.A0A1C3YJQ9 pp 229533.I1RPY1
146355 229533.A0A1C3YJQ9 pp 229533.I1S2E9
146356 229533.A0A1C3YJQ9 pp 229533.I1RJV3
146357 229533.A0A1C3YJQ9 pp 229533.I1RRE5
146358 229533.A0A1C3YJQ9 pp 229533.I1RWN6
146359 229533.A0A1C3YJQ9 pp 229533.I1RT04
146360 229533.A0A1C3YJQ9 pp 229533.I1RC66
146361 229533.A0A1C3YJQ9 pp 229533.I1RAW0
146362 229533.A0A1C3YJQ9 pp 229533.V6R3J1
146363 229533.A0A1C3YJQ9 pp 229533.I1RCH5
146364 229533.A0A1C3YJQ9 pp 229533.I1RWI5
146365 229533.A0A1C3YJQ9 pp 229533.I1RZZ9

146366	229533.A0A1C3YJQ9	pp	229533.I1RQE7
146367	229533.A0A1C3YJQ9	pp	229533.I1RFL4
146368	229533.A0A1C3YJQ9	pp	229533.A0A1C3YLK8
146369	229533.A0A1C3YJQ9	pp	229533.I1RJW6
146370	229533.A0A1C3YJQ9	pp	229533.I1S503
146371	229533.A0A1C3YJQ9	pp	229533.I1RN18
146372	229533.A0A1C3YJQ9	pp	229533.I1RSQ4
146373	229533.A0A1C3YJQ9	pp	229533.Q4IJK2
146374	229533.A0A1C3YJQ9	pp	229533.I1RJV4
146375	229533.A0A1C3YJQ9	pp	229533.I1RBS6
146376	229533.A0A1C3YJQ9	pp	229533.I1RJS2
146377	229533.A0A1C3YJQ9	pp	229533.I1RDG0
146378	229533.A0A1C3YJQ9	pp	229533.A0A1C3YKC0
146379	229533.A0A1C3YJQ9	pp	229533.I1RBT1
146380	229533.A0A1C3YJQ9	pp	229533.I1RGA4
146381	229533.A0A1C3YJQ9	pp	229533.I1S0X6
146382	229533.A0A1C3YJQ9	pp	229533.Q4I2J8
146383	229533.A0A1C3YJQ9	pp	229533.I1RCR5
146384	229533.A0A1C3YJQ9	pp	229533.I1RWK3
146385	229533.A0A1C3YJQ9	pp	229533.I1REK8
146386	229533.A0A1C3YJQ9	pp	229533.I1RKB7
146387	229533.A0A1C3YJQ9	pp	229533.I1RJU3
146388	229533.A0A1C3YJQ9	pp	229533.I1RPP3
146389	229533.A0A1C3YJQ9	pp	229533.A0A1C3YN30
146390	229533.A0A1C3YJQ9	pp	229533.I1RWJ8
146391	229533.A0A1C3YJQ9	pp	229533.I1RZM8
146392	229533.A0A1C3YJQ9	pp	229533.I1RI82
146393	229533.A0A1C3YJQ9	pp	229533.I1S8P5
146394	229533.A0A1C3YJQ9	pp	229533.V6RCG5
146395	229533.A0A1C3YJQ9	pp	229533.I1RR24
146396	229533.A0A1C3YJQ9	pp	229533.I1RFT1
146397	229533.A0A1C3YJQ9	pp	229533.I1RBS0
146398	229533.A0A1C3YJQ9	pp	229533.V6RCZ8
146399	229533.A0A1C3YJQ9	pp	229533.I1RN67
146400	229533.A0A1C3YJQ9	pp	229533.I1RCQ6
146401	229533.A0A1C3YJQ9	pp	229533.V6QUW4
146402	229533.A0A1C3YJQ9	pp	229533.I1S0F6
146403	229533.A0A1C3YJQ9	pp	229533.I1RAU5
146404	229533.A0A1C3YJQ9	pp	229533.I1RRU9
146405	229533.A0A1C3YJQ9	pp	229533.I1S5B0
146406	229533.A0A1C3YJQ9	pp	229533.I1RW99
146407	229533.A0A1C3YJQ9	pp	229533.I1RG62
146408	229533.A0A1C3YJQ9	pp	229533.I1RW25
146409	229533.A0A1C3YJQ9	pp	229533.I1RXD7
146410	229533.A0A1C3YJQ9	pp	229533.I1RQC9
146411	229533.A0A1C3YJQ9	pp	229533.I1S0R7
146412	229533.A0A1C3YJQ9	pp	229533.Q4IEV5
146413	229533.A0A1C3YJQ9	pp	229533.I1RC17
146414	229533.A0A1C3YJQ9	pp	229533.I1RY03
146415	229533.A0A1C3YJQ9	pp	229533.I1RX46
146416	229533.A0A1C3YJQ9	pp	229533.I1SA32
146417	229533.A0A1C3YJQ9	pp	229533.I1RZK5
146418	229533.A0A1C3YJQ9	pp	229533.V6RTJ8
146419	229533.A0A1C3YJQ9	pp	229533.I1RFU8
146420	229533.A0A1C3YJQ9	pp	229533.I1RZH4
146421	229533.A0A1C3YJQ9	pp	229533.I1RCG4
146422	229533.A0A1C3YJQ9	pp	229533.A0A1C3YK34
146423	229533.A0A1C3YJQ9	pp	229533.I1RW01
146424	229533.A0A1C3YJQ9	pp	229533.I1RS18
146425	229533.A0A1C3YJQ9	pp	229533.I1S1J7
146426	229533.A0A1C3YJQ9	pp	229533.I1RAX6
146427	229533.A0A1C3YJQ9	pp	229533.I1RSM3
146428	229533.A0A1C3YJQ9	pp	229533.Q4I8P5
146429	229533.A0A1C3YJQ9	pp	229533.A0A1C3YKV3
146430	229533.A0A1C3YJQ9	pp	229533.I1RLU6
146431	229533.A0A1C3YJQ9	pp	229533.I1RRY9
146432	229533.A0A1C3YJQ9	pp	229533.Q4IJT5
146433	229533.A0A1C3YJQ9	pp	229533.V6QW22
146434	229533.A0A1C3YJQ9	pp	229533.I1RNQ5
146435	229533.A0A1C3YJQ9	pp	229533.I1RZM1
146436	229533.A0A1C3YJQ9	pp	229533.I1RWR7
146437	229533.A0A1C3YJQ9	pp	229533.I1RA36
146438	229533.A0A1C3YJQ9	pp	229533.A0A1C3YKB0

146439	229533.A0A1C3YJQ9	pp	229533.I1RSQ7
146440	229533.A0A1C3YJQ9	pp	229533.Q4I283
146441	229533.A0A1C3YJQ9	pp	229533.A0A1C3YK13
146442	229533.A0A1C3YJQ9	pp	229533.I1RZZ1
146443	229533.A0A1C3YJQ9	pp	229533.I1RVQ0
146444	229533.A0A1C3YJQ9	pp	229533.I1RBC9
146445	229533.A0A1C3YJQ9	pp	229533.I1RQQ5
146446	229533.A0A1C3YJQ9	pp	229533.V6R1K4
146447	229533.A0A1C3YJQ9	pp	229533.I1RWT6
146448	229533.A0A1C3YJQ9	pp	229533.I1RQA3
146449	229533.A0A1C3YJQ9	pp	229533.I1S504
146450	229533.A0A1C3YJQ9	pp	229533.I1S0L3
146451	229533.A0A1C3YJQ9	pp	229533.I1RD52
146452	229533.A0A1C3YJQ9	pp	229533.I1S0D4
146453	229533.A0A098DJL5	pp	229533.I1RCU8
146454	229533.A0A098DJL5	pp	229533.I1RST8
146455	229533.A0A098DJL5	pp	229533.I1RMD3
146456	229533.A0A098DJL5	pp	229533.A0A1C3YJZ0
146457	229533.A0A098DJL5	pp	229533.I1RAM7
146458	229533.A0A098DJL5	pp	229533.V6RDP1
146459	229533.A0A098DJL5	pp	229533.I1RDB1
146460	229533.A0A098DJL5	pp	229533.I1RCU9
146461	229533.A0A098DJL5	pp	229533.A0A1C3YIB0
146462	229533.A0A098DJL5	pp	229533.I1RHJ2
146463	229533.A0A098DLD4	pp	229533.I1RJZ7
146464	229533.A0A098DLD4	pp	229533.I1RB77
146465	229533.A0A098DLD4	pp	229533.A0A0E0S2K0
146466	229533.A0A098DLD4	pp	229533.A0A098DMK7
146467	229533.A0A098DLD4	pp	229533.I1RW71
146468	229533.I1RWD7	pp	229533.V6RTJ8
146469	229533.I1RWD7	pp	229533.I1RWH4
146470	229533.I1RWD7	pp	229533.I1RX46
146471	229533.I1RWD7	pp	229533.I1S8P5
146472	229533.I1RWD7	pp	229533.V6R042
146473	229533.I1RWD7	pp	229533.V6QW22
146474	229533.I1RWD7	pp	229533.V6R1K4
146475	229533.I1RWD7	pp	229533.I1RWK3
146476	229533.I1RWD7	pp	229533.I1RXU7
146477	229533.I1RWD7	pp	229533.V6RMW6
146478	229533.I1RWD7	pp	229533.I1S0L4
146479	229533.I1RWD7	pp	229533.V6RCG5
146480	229533.I1RWD7	pp	229533.I1RYJ7
146481	229533.I1RWD7	pp	229533.I1S0L3
146482	229533.I1RWD7	pp	229533.I1RZM1
146483	229533.I1RWD7	pp	229533.I1S4S4
146484	229533.I1RWD7	pp	229533.I1RZZ9
146485	229533.I1RWD7	pp	229533.I1RZ39
146486	229533.I1RWD7	pp	229533.I1S5B0
146487	229533.I1RWD7	pp	229533.V6RIV9
146488	229533.I1RWD7	pp	229533.Q4IAA0
146489	229533.I1RWD7	pp	229533.I1RYW3
146490	229533.I1RWD7	pp	229533.V6RSF3
146491	229533.I1RWD7	pp	229533.V6R057
146492	229533.I1RWD7	pp	229533.I1RZS7
146493	229533.I1RWD7	pp	229533.I1S6F1
146494	229533.A0A098DID0	pp	229533.A0A098DMK7
146495	229533.A0A098DID0	pp	229533.I1RB77
146496	229533.A0A098DID0	pp	229533.A0A0E0S2K0
146497	229533.A0A098DID0	pp	229533.I1RB03
146498	229533.A0A098DID0	pp	229533.A0A098DLD4
146499	229533.I1R9R2	pp	229533.I1RX22
146500	229533.I1R9R2	pp	229533.I1RW03
146501	229533.A0A1C3YM02	pp	229533.I1RUZ8
146502	229533.A0A1C3YM02	pp	229533.A0A1C3YMG8
146503	229533.A0A1C3YM02	pp	229533.I1S8F2
146504	229533.A0A1C3YM02	pp	229533.I1RII1
146505	229533.A0A1C3YM02	pp	229533.I1RXV4
146506	229533.A0A1C3YM02	pp	229533.I1S0F0
146507	229533.A0A1C3YM02	pp	229533.I1RB73
146508	229533.A0A1C3YM02	pp	229533.I1RW52
146509	229533.A0A1C3YM02	pp	229533.I1SB04
146510	229533.A0A1C3YM02	pp	229533.I1RJY5
146511	229533.A0A1C3YM02	pp	229533.I1RP30

146512	229533.A0A1C3YM02	pp	229533.I1S8V4
146513	229533.A0A1C3YM02	pp	229533.I1S0G3
146514	229533.A0A1C3YM02	pp	229533.I1RBD7
146515	229533.A0A1C3YM02	pp	229533.I1RAK6
146516	229533.A0A1C3YM02	pp	229533.Q4I7L0
146517	229533.A0A1C3YM02	pp	229533.I1RYE2
146518	229533.A0A1C3YM02	pp	229533.A0A1C3YN58
146519	229533.A0A1C3YM02	pp	229533.I1RCN2
146520	229533.A0A1C3YM02	pp	229533.I1S201
146521	229533.A0A1C3YM02	pp	229533.I1RVA8
146522	229533.I1RW25	pp	229533.I1RWN5
146523	229533.I1RW25	pp	229533.Q4HZB8
146524	229533.I1RW25	pp	229533.I1RY44
146525	229533.I1RW25	pp	229533.Q4I624
146526	229533.I1RW25	pp	229533.V6RIV9
146527	229533.I1RW25	pp	229533.I1S2C0
146528	229533.I1RW25	pp	229533.V6R057
146529	229533.I1RW25	pp	229533.I1S8R3
146530	229533.I1RW25	pp	229533.I1S0J1
146531	229533.I1RW25	pp	229533.I1RZW8
146532	229533.I1RW25	pp	229533.I1RWB5
146533	229533.I1RW25	pp	229533.I1S2E8
146534	229533.I1RW25	pp	229533.I1RWX5
146535	229533.I1RW25	pp	229533.I1S569
146536	229533.I1RW25	pp	229533.I1RZS7
146537	229533.I1RW25	pp	229533.I1S0R2
146538	229533.I1RW25	pp	229533.I1RWN6
146539	229533.I1RW25	pp	229533.I1RW46
146540	229533.I1RW25	pp	229533.I1S1C7
146541	229533.I1RW25	pp	229533.V6RIS2
146542	229533.I1RW25	pp	229533.I1RWQ8
146543	229533.I1RW25	pp	229533.I1RYW3
146544	229533.I1RW25	pp	229533.V6RGN5
146545	229533.I1RW25	pp	229533.I1S8V4
146546	229533.I1RW25	pp	229533.V6RMW6
146547	229533.I1RW25	pp	229533.V6RY33
146548	229533.I1RW25	pp	229533.I1SA33
146549	229533.I1RW25	pp	229533.I1RZV6
146550	229533.I1RW25	pp	229533.I1RZS6
146551	229533.I1RW25	pp	229533.I1S0N4
146552	229533.I1RW25	pp	229533.I1S0M6
146553	229533.I1RW25	pp	229533.V6R042
146554	229533.I1RW25	pp	229533.I1S6Y8
146555	229533.I1RW25	pp	229533.V6R3X5
146556	229533.I1RW25	pp	229533.Q4I327
146557	229533.I1RW25	pp	229533.I1S0L4
146558	229533.I1RW25	pp	229533.Q4IN63
146559	229533.I1RW25	pp	229533.I1S8E2
146560	229533.I1RW25	pp	229533.I1RZ39
146561	229533.I1RW25	pp	229533.I1RXT3
146562	229533.I1RW25	pp	229533.I1RWL2
146563	229533.I1RW25	pp	229533.I1S8V3
146564	229533.I1RW25	pp	229533.I1S2H4
146565	229533.I1RW25	pp	229533.I1RWI5
146566	229533.I1RW25	pp	229533.I1RZZ9
146567	229533.I1RW25	pp	229533.I1S7S9
146568	229533.I1RW25	pp	229533.Q4IEV5
146569	229533.I1RW25	pp	229533.O74638
146570	229533.I1RW25	pp	229533.I1S503
146571	229533.I1RW25	pp	229533.I1RXC2
146572	229533.I1RW25	pp	229533.I1S2B8
146573	229533.I1RW25	pp	229533.I1S8P5
146574	229533.I1RW25	pp	229533.I1S5R1
146575	229533.I1RW25	pp	229533.I1S0F6
146576	229533.I1RW25	pp	229533.I1RZK5
146577	229533.I1RW25	pp	229533.I1S2E9
146578	229533.I1RW25	pp	229533.I1RZB5
146579	229533.I1RW25	pp	229533.I1S352
146580	229533.I1RW25	pp	229533.I1S5S2
146581	229533.I1RW25	pp	229533.I1RY03
146582	229533.I1RW25	pp	229533.I1RXF2
146583	229533.I1RW25	pp	229533.I1S2M6
146584	229533.I1RW25	pp	229533.I1RWT6

146585 229533.I1RW25 pp 229533.V6RUY9
146586 229533.I1RW25 pp 229533.I1RWD7
146587 229533.I1RW25 pp 229533.Q4IR18
146588 229533.I1RW25 pp 229533.V6R283
146589 229533.I1RW25 pp 229533.Q4I283
146590 229533.I1RW25 pp 229533.Q4IQW0
146591 229533.I1RW25 pp 229533.I1RWK3
146592 229533.I1RW25 pp 229533.I1RZE5
146593 229533.I1RW25 pp 229533.I1S1B6
146594 229533.I1RW25 pp 229533.Q4I5Z5
146595 229533.I1RW25 pp 229533.V6R227
146596 229533.I1RW25 pp 229533.Q4I2J8
146597 229533.I1RW25 pp 229533.I1SA32
146598 229533.I1RW25 pp 229533.I1S504
146599 229533.I1RW25 pp 229533.I1S0M9
146600 229533.I1RW25 pp 229533.I1RZK6
146601 229533.I1RW25 pp 229533.I1RZF6
146602 229533.I1RW25 pp 229533.Q4ICA8
146603 229533.I1RW25 pp 229533.I1RZH4
146604 229533.I1RW25 pp 229533.Q4IJK2
146605 229533.I1RW25 pp 229533.I1RW99
146606 229533.I1RW25 pp 229533.I1RZM1
146607 229533.I1RW25 pp 229533.I1RXC4
146608 229533.I1RW25 pp 229533.I1RWJ3
146609 229533.I1RW25 pp 229533.I1S5B0
146610 229533.I1RW25 pp 229533.I1S7M3
146611 229533.I1RW25 pp 229533.Q4IEK8
146612 229533.I1RW25 pp 229533.I1S1J7
146613 229533.I1RW25 pp 229533.I1RZ11
146614 229533.I1RW25 pp 229533.I1S419
146615 229533.I1RW25 pp 229533.I1S044
146616 229533.I1RW25 pp 229533.V6QUE6
146617 229533.I1RW25 pp 229533.I1RWX8
146618 229533.I1RW25 pp 229533.Q4I8P5
146619 229533.I1RW25 pp 229533.I1RXF0
146620 229533.I1RW25 pp 229533.I1RZM8
146621 229533.I1RW25 pp 229533.I1S0F5
146622 229533.I1RW25 pp 229533.V6RCG5
146623 229533.I1RW25 pp 229533.I1RX25
146624 229533.I1RW25 pp 229533.V6QYF0
146625 229533.I1RW25 pp 229533.V6RCZ8
146626 229533.I1RW25 pp 229533.V6QUW4
146627 229533.I1RW25 pp 229533.I1RX81
146628 229533.I1RW25 pp 229533.I1S0L3
146629 229533.I1RW25 pp 229533.I1RWH5
146630 229533.I1RW25 pp 229533.V6R1K4
146631 229533.I1RW25 pp 229533.I1S021
146632 229533.I1RW25 pp 229533.I1S9U2
146633 229533.I1RW25 pp 229533.V6R4J1
146634 229533.I1RW25 pp 229533.I1RZJ5
146635 229533.I1RW25 pp 229533.I1S6F0
146636 229533.I1RW25 pp 229533.I1S7A8
146637 229533.I1RW25 pp 229533.I1RWJ8
146638 229533.I1RW25 pp 229533.I1RWD4
146639 229533.I1RW25 pp 229533.V6RTJ8
146640 229533.I1RW25 pp 229533.I1S540
146641 229533.I1RW25 pp 229533.I1S0X6
146642 229533.I1RW25 pp 229533.I1RW71
146643 229533.I1RW25 pp 229533.I1RX46
146644 229533.I1RW25 pp 229533.V6QW22
146645 229533.I1RW25 pp 229533.Q4IJT5
146646 229533.I1RW25 pp 229533.I1RXD7
146647 229533.I1RW25 pp 229533.I1S0T2
146648 229533.I1RW25 pp 229533.I1S7M2
146649 229533.A0A1C3YJ47 pp 229533.A0A1C3YKN4
146650 229533.A0A1C3YJ47 pp 229533.I1RNG4
146651 229533.A0A1C3YJ47 pp 229533.I1RYW9
146652 229533.A0A1C3YJ47 pp 229533.I1RKB2
146653 229533.A0A1C3YJ47 pp 229533.I1RZA7
146654 229533.A0A1C3YJ47 pp 229533.I1S0V7
146655 229533.A0A1C3YJ47 pp 229533.I1RIJ5
146656 229533.A0A1C3YJ47 pp 229533.V6RUB1
146657 229533.A0A1C3YJ47 pp 229533.I1RF85

229533.A0A1C3YJ47	pp	229533.I1RD17
229533.A0A1C3YJ47	pp	229533.A0A1C3YKW4
229533.A0A1C3YJ47	pp	229533.V6RG22
229533.A0A1C3YJ47	pp	229533.I1RFI4
229533.A0A1C3YJ47	pp	229533.I1RVZ3
229533.A0A1C3YJ47	pp	229533.I1S6P5
229533.A0A1C3YJ47	pp	229533.I1S2C4
229533.A0A1C3YJ47	pp	229533.I1RNY6
229533.A0A1C3YJ47	pp	229533.I1RHS0
229533.A0A1C3YJ47	pp	229533.A0A1C3YK58
229533.A0A1C3YJ47	pp	229533.I1RPR1
229533.A0A1C3YJ47	pp	229533.I1RXV3
229533.A0A1C3YJ47	pp	229533.A0A1C3YM86
229533.A0A1C3YJ47	pp	229533.I1RSX2
229533.A0A1C3YJ47	pp	229533.I1RYU0
229533.A0A1C3YJ47	pp	229533.A0A1C3YKZ8
229533.A0A1C3YJ47	pp	229533.I1RCV1
229533.A0A1C3YJ47	pp	229533.I1RCU2
229533.A0A1C3YJ47	pp	229533.I1RJT5
229533.I1REK8	pp	229533.I1S016
229533.I1REK8	pp	229533.V6RIV9
229533.I1REK8	pp	229533.I1RWN5
229533.I1REK8	pp	229533.I1S1W9
229533.I1REK8	pp	229533.I1RWH4
229533.I1REK8	pp	229533.I1SA33
229533.I1REK8	pp	229533.I1RPY1
229533.I1REK8	pp	229533.I1RZV3
229533.I1REK8	pp	229533.I1S1W3
229533.I1REK8	pp	229533.I1S256
229533.I1REK8	pp	229533.I1RRN2
229533.I1REK8	pp	229533.I1RY44
229533.I1REK8	pp	229533.I1RTN8
229533.I1REK8	pp	229533.Q4HZB8
229533.I1REK8	pp	229533.I1RVZ7
229533.I1REK8	pp	229533.I1RRE5
229533.I1REK8	pp	229533.I1RKC6
229533.I1REK8	pp	229533.I1RWQ8
229533.I1REK8	pp	229533.V6RRH2
229533.I1REK8	pp	229533.I1RPU2
229533.I1REK8	pp	229533.I1RSF1
229533.I1REK8	pp	229533.I1S5R6
229533.I1REK8	pp	229533.I1RML0
229533.I1REK8	pp	229533.I1RGL4
229533.I1REK8	pp	229533.I1RTA0
229533.I1REK8	pp	229533.I1S0N4
229533.I1REK8	pp	229533.I1RW46
229533.I1REK8	pp	229533.I1RG11
229533.I1REK8	pp	229533.I1S0J1
229533.I1REK8	pp	229533.Q4I624
229533.I1REK8	pp	229533.I1RUG3
229533.I1REK8	pp	229533.V6RGN5
229533.I1REK8	pp	229533.V6R057
229533.I1REK8	pp	229533.I1S7W5
229533.I1REK8	pp	229533.I1S569
229533.I1REK8	pp	229533.I1RXT3
229533.I1REK8	pp	229533.I1RQ33
229533.I1REK8	pp	229533.I1RS17
229533.I1REK8	pp	229533.I1RPK0
229533.I1REK8	pp	229533.I1RNC1
229533.I1REK8	pp	229533.I1RVF8
229533.I1REK8	pp	229533.V6RLM7
229533.I1REK8	pp	229533.I1RN18
229533.I1REK8	pp	229533.I1RPL4
229533.I1REK8	pp	229533.I1RFL4
229533.I1REK8	pp	229533.I1RRV1
229533.I1REK8	pp	229533.I1RMV0
229533.I1REK8	pp	229533.I1RSI6
229533.I1REK8	pp	229533.I1RKG3
229533.I1REK8	pp	229533.I1RPP4
229533.I1REK8	pp	229533.I1RMG3
229533.I1REK8	pp	229533.I1RQE7
229533.I1REK8	pp	229533.V6RMW6
229533.I1REK8	pp	229533.I1RRN5

146731 229533.I1REK8 pp 229533.I1RN67
146732 229533.I1REK8 pp 229533.I1RRP3
146733 229533.I1REK8 pp 229533.I1RXR1
146734 229533.I1REK8 pp 229533.I1RSS4
146735 229533.I1REK8 pp 229533.V6R042
146736 229533.I1REK8 pp 229533.I1RRM3
146737 229533.I1REK8 pp 229533.I1RSY2
146738 229533.I1REK8 pp 229533.I1S0W4
146739 229533.I1REK8 pp 229533.I1RVX0
146740 229533.I1REK8 pp 229533.I1RY62
146741 229533.I1REK8 pp 229533.I1RSQ3
146742 229533.I1REK8 pp 229533.I1RS47
146743 229533.I1REK8 pp 229533.I1RT03
146744 229533.I1REK8 pp 229533.I1RRZ0
146745 229533.I1REK8 pp 229533.I1S0F7
146746 229533.I1REK8 pp 229533.I1S9H4
146747 229533.I1REK8 pp 229533.I1RJL4
146748 229533.I1REK8 pp 229533.I1RLN6
146749 229533.I1REK8 pp 229533.I1RSX0
146750 229533.I1REK8 pp 229533.I1RRT2
146751 229533.I1REK8 pp 229533.I1S190
146752 229533.I1REK8 pp 229533.I1RJV3
146753 229533.I1REK8 pp 229533.I1RYR0
146754 229533.I1REK8 pp 229533.I1RXU9
146755 229533.I1REK8 pp 229533.I1RWN6
146756 229533.I1REK8 pp 229533.I1RGA4
146757 229533.I1REK8 pp 229533.I1S1B6
146758 229533.I1REK8 pp 229533.I1REV8
146759 229533.I1REK8 pp 229533.I1RWS4
146760 229533.I1REK8 pp 229533.I1RJV4
146761 229533.I1REK8 pp 229533.I1RWK3
146762 229533.I1REK8 pp 229533.I1RZ84
146763 229533.I1REK8 pp 229533.I1RJ74
146764 229533.I1REK8 pp 229533.I1RZB2
146765 229533.I1REK8 pp 229533.I1RYW3
146766 229533.I1REK8 pp 229533.I1RN63
146767 229533.I1REK8 pp 229533.I1S2M6
146768 229533.I1REK8 pp 229533.I1RQM7
146769 229533.I1REK8 pp 229533.I1RVZ2
146770 229533.I1REK8 pp 229533.I1S1C7
146771 229533.I1REK8 pp 229533.I1S437
146772 229533.I1REK8 pp 229533.I1RJS2
146773 229533.I1REK8 pp 229533.I1RT40
146774 229533.I1REK8 pp 229533.I1RJN9
146775 229533.I1REK8 pp 229533.I1RZW8
146776 229533.I1REK8 pp 229533.I1RV91
146777 229533.I1REK8 pp 229533.I1RTA1
146778 229533.I1REK8 pp 229533.I1RKB2
146779 229533.I1REK8 pp 229533.I1RXF8
146780 229533.I1REK8 pp 229533.I1RTD5
146781 229533.I1REK8 pp 229533.I1RRI2
146782 229533.I1REK8 pp 229533.I1RZ79
146783 229533.I1REK8 pp 229533.I1RFT1
146784 229533.I1REK8 pp 229533.I1RSV2
146785 229533.I1REK8 pp 229533.Q4IR18
146786 229533.I1REK8 pp 229533.I1RGF5
146787 229533.I1REK8 pp 229533.I1S352
146788 229533.I1REK8 pp 229533.I1RWX5
146789 229533.I1REK8 pp 229533.V6RUY9
146790 229533.I1REK8 pp 229533.I1S8R3
146791 229533.I1REK8 pp 229533.I1S2L3
146792 229533.I1REK8 pp 229533.I1RSX1
146793 229533.I1REK8 pp 229533.I1RRJ8
146794 229533.I1REK8 pp 229533.I1S0F8
146795 229533.I1REK8 pp 229533.I1RTH6
146796 229533.I1REK8 pp 229533.I1RT04
146797 229533.I1REK8 pp 229533.Q4HXF0
146798 229533.I1REK8 pp 229533.I1RPP3
146799 229533.I1REK8 pp 229533.I1RTE9
146800 229533.I1REK8 pp 229533.I1RNA5
146801 229533.I1REK8 pp 229533.I1RKG2
146802 229533.I1REK8 pp 229533.I1S8P5
146803 229533.I1REK8 pp 229533.I1RYI5

146804 229533.I1REK8 pp 229533.I1RKJ8
146805 229533.I1REK8 pp 229533.I1S2E9
146806 229533.I1REK8 pp 229533.I1RRK3
146807 229533.I1REK8 pp 229533.I1RRM5
146808 229533.I1REK8 pp 229533.I1S0F6
146809 229533.I1REK8 pp 229533.I1S2B8
146810 229533.I1REK8 pp 229533.I1RJZ7
146811 229533.I1REK8 pp 229533.I1RJW6
146812 229533.I1REK8 pp 229533.I1S503
146813 229533.I1REK8 pp 229533.I1S6B4
146814 229533.I1REK8 pp 229533.I1RVY0
146815 229533.I1REK8 pp 229533.I1S277
146816 229533.I1REK8 pp 229533.I1RXC2
146817 229533.I1REK8 pp 229533.O74638
146818 229533.I1REK8 pp 229533.I1S8F8
146819 229533.I1REK8 pp 229533.I1RT35
146820 229533.I1REK8 pp 229533.I1S4Z8
146821 229533.I1REK8 pp 229533.I1S5R1
146822 229533.I1REK8 pp 229533.I1RZM1
146823 229533.I1REK8 pp 229533.I1RRT8
146824 229533.I1REK8 pp 229533.I1RSZ7
146825 229533.I1REK8 pp 229533.I1RNQ5
146826 229533.I1REK8 pp 229533.I1RW61
146827 229533.I1REK8 pp 229533.I1S2H4
146828 229533.I1REK8 pp 229533.I1RFS3
146829 229533.I1REK8 pp 229533.I1RLU6
146830 229533.I1REK8 pp 229533.I1RS20
146831 229533.I1REK8 pp 229533.Q4IEK8
146832 229533.I1REK8 pp 229533.I1RL04
146833 229533.I1REK8 pp 229533.I1S504
146834 229533.I1REK8 pp 229533.I1RPM4
146835 229533.I1REK8 pp 229533.I1RQS8
146836 229533.I1REK8 pp 229533.I1RPE0
146837 229533.I1REK8 pp 229533.I1S6Y8
146838 229533.I1REK8 pp 229533.I1RGB0
146839 229533.I1REK8 pp 229533.I1RPV3
146840 229533.I1REK8 pp 229533.I1RG14
146841 229533.I1REK8 pp 229533.I1RWT6
146842 229533.I1REK8 pp 229533.I1RKP1
146843 229533.I1REK8 pp 229533.I1RIL3
146844 229533.I1REK8 pp 229533.I1RQ23
146845 229533.I1REK8 pp 229533.I1RQ85
146846 229533.I1REK8 pp 229533.I1RRI1
146847 229533.I1REK8 pp 229533.I1RGU7
146848 229533.I1REK8 pp 229533.I1RN88
146849 229533.I1REK8 pp 229533.I1S0L4
146850 229533.I1REK8 pp 229533.Q4I283
146851 229533.I1REK8 pp 229533.I1S0M6
146852 229533.I1REK8 pp 229533.I1RTN3
146853 229533.I1REK8 pp 229533.I1RSQ4
146854 229533.I1REK8 pp 229533.I1RZZ9
146855 229533.I1REK8 pp 229533.I1RWI5
146856 229533.I1REK8 pp 229533.V6R227
146857 229533.I1REK8 pp 229533.I1S9Y1
146858 229533.I1REK8 pp 229533.I1RGB6
146859 229533.I1REK8 pp 229533.I1RM82
146860 229533.I1REK8 pp 229533.I1S1C9
146861 229533.I1REK8 pp 229533.I1RFH8
146862 229533.I1REK8 pp 229533.I1RRL9
146863 229533.I1REK8 pp 229533.I1RXC4
146864 229533.I1REK8 pp 229533.I1RGA6
146865 229533.I1REK8 pp 229533.I1RMQ4
146866 229533.I1REK8 pp 229533.I1RQD2
146867 229533.I1REK8 pp 229533.I1RZ39
146868 229533.I1REK8 pp 229533.I1RHE5
146869 229533.I1REK8 pp 229533.I1RW99
146870 229533.I1REK8 pp 229533.I1SA32
146871 229533.I1REK8 pp 229533.I1S5B0
146872 229533.I1REK8 pp 229533.V6R3X5
146873 229533.I1REK8 pp 229533.I1RXF0
146874 229533.I1REK8 pp 229533.I1RQ81
146875 229533.I1REK8 pp 229533.I1RF82
146876 229533.I1REK8 pp 229533.Q4I8P5

146877	229533.I1REK8	pp	229533.I1RR73
146878	229533.I1REK8	pp	229533.I1S419
146879	229533.I1REK8	pp	229533.I1RS18
146880	229533.I1REK8	pp	229533.I1RZM8
146881	229533.I1REK8	pp	229533.I1RGC2
146882	229533.I1REK8	pp	229533.I1RQ31
146883	229533.I1REK8	pp	229533.I1RSR3
146884	229533.I1REK8	pp	229533.I1RMC0
146885	229533.I1REK8	pp	229533.V6QUE6
146886	229533.I1REK8	pp	229533.I1RRV4
146887	229533.I1REK8	pp	229533.I1RU29
146888	229533.I1REK8	pp	229533.I1RI82
146889	229533.I1REK8	pp	229533.I1RWR7
146890	229533.I1REK8	pp	229533.I1RTD3
146891	229533.I1REK8	pp	229533.I1RRU9
146892	229533.I1REK8	pp	229533.I1RFT2
146893	229533.I1REK8	pp	229533.I1RGC5
146894	229533.I1REK8	pp	229533.V6RCZ8
146895	229533.I1REK8	pp	229533.I1RTD9
146896	229533.I1REK8	pp	229533.I1RRY9
146897	229533.I1REK8	pp	229533.Q4IJK2
146898	229533.I1REK8	pp	229533.I1RWD7
146899	229533.I1REK8	pp	229533.I1RNT9
146900	229533.I1REK8	pp	229533.I1RY03
146901	229533.I1REK8	pp	229533.I1S6K9
146902	229533.I1REK8	pp	229533.I1RQA3
146903	229533.I1REK8	pp	229533.V6RCG5
146904	229533.I1REK8	pp	229533.I1RYB5
146905	229533.I1REK8	pp	229533.I1RR51
146906	229533.I1REK8	pp	229533.V6RBN2
146907	229533.I1REK8	pp	229533.I1RQQ5
146908	229533.I1REK8	pp	229533.I1RMD5
146909	229533.I1REK8	pp	229533.I1RFU8
146910	229533.I1REK8	pp	229533.I1RZZ1
146911	229533.I1REK8	pp	229533.I1S887
146912	229533.I1REK8	pp	229533.I1S217
146913	229533.I1REK8	pp	229533.I1RZK5
146914	229533.I1REK8	pp	229533.I1REL7
146915	229533.I1REK8	pp	229533.I1RY63
146916	229533.I1REK8	pp	229533.I1RWD4
146917	229533.I1REK8	pp	229533.I1RVQ0
146918	229533.I1REK8	pp	229533.I1RK95
146919	229533.I1REK8	pp	229533.Q4I2J8
146920	229533.I1REK8	pp	229533.I1RN65
146921	229533.I1REK8	pp	229533.V6R4J1
146922	229533.I1REK8	pp	229533.I1RN99
146923	229533.I1REK8	pp	229533.I1RYU0
146924	229533.I1REK8	pp	229533.I1RF19
146925	229533.I1REK8	pp	229533.I1RXD6
146926	229533.I1REK8	pp	229533.I1S4Q4
146927	229533.I1REK8	pp	229533.I1RFJ9
146928	229533.I1REK8	pp	229533.I1RWB0
146929	229533.I1REK8	pp	229533.I1S6F0
146930	229533.I1REK8	pp	229533.I1RQA2
146931	229533.I1REK8	pp	229533.I1S1J7
146932	229533.I1REK8	pp	229533.I1RSI0
146933	229533.I1REK8	pp	229533.I1RT33
146934	229533.I1REK8	pp	229533.I1RI03
146935	229533.I1REK8	pp	229533.I1RY86
146936	229533.I1REK8	pp	229533.I1RLM7
146937	229533.I1REK8	pp	229533.I1RYS0
146938	229533.I1REK8	pp	229533.I1S0L3
146939	229533.I1REK8	pp	229533.I1RFY6
146940	229533.I1REK8	pp	229533.V6R1K4
146941	229533.I1REK8	pp	229533.I1RPR9
146942	229533.I1REK8	pp	229533.I1RGB1
146943	229533.I1REK8	pp	229533.I1RLX7
146944	229533.I1REK8	pp	229533.I1S0F5
146945	229533.I1REK8	pp	229533.I1RX95
146946	229533.I1REK8	pp	229533.I1S7A8
146947	229533.I1REK8	pp	229533.Q4IJT5
146948	229533.I1REK8	pp	229533.V6QYF0
146949	229533.I1REK8	pp	229533.V6RTJ8

146950 229533.I1REK8 pp 229533.I1RIL8
146951 229533.I1REK8 pp 229533.I1RRQ6
146952 229533.I1REK8 pp 229533.I1RYJ6
146953 229533.I1REK8 pp 229533.I1RNA0
146954 229533.I1REK8 pp 229533.I1RRG3
146955 229533.I1REK8 pp 229533.I1S6T4
146956 229533.I1REK8 pp 229533.I1RPY3
146957 229533.I1REK8 pp 229533.I1S3S3
146958 229533.I1REK8 pp 229533.I1RPN4
146959 229533.I1REK8 pp 229533.V6QUW4
146960 229533.I1REK8 pp 229533.I1S0R7
146961 229533.I1REK8 pp 229533.I1RW71
146962 229533.I1REK8 pp 229533.I1RP86
146963 229533.I1REK8 pp 229533.I1S4S7
146964 229533.I1REK8 pp 229533.I1RQC9
146965 229533.I1REK8 pp 229533.I1S438
146966 229533.I1REK8 pp 229533.I1RXD7
146967 229533.I1REK8 pp 229533.I1RHW8
146968 229533.I1REK8 pp 229533.I1RM06
146969 229533.I1REK8 pp 229533.I1RPF1
146970 229533.I1REK8 pp 229533.I1RUQ2
146971 229533.I1REK8 pp 229533.I1RLP1
146972 229533.I1REK8 pp 229533.I1RRX5
146973 229533.I1REK8 pp 229533.I1S4S4
146974 229533.I1REK8 pp 229533.V6RUT5
146975 229533.I1REK8 pp 229533.I1RWJ8
146976 229533.I1REK8 pp 229533.I1RSQ7
146977 229533.I1REK8 pp 229533.I1RZJ5
146978 229533.I1REK8 pp 229533.V6QW22
146979 229533.I1REK8 pp 229533.I1RX25
146980 229533.I1REK8 pp 229533.I1RLC7
146981 229533.I1REK8 pp 229533.I1RGU0
146982 229533.I1REK8 pp 229533.I1RMG8
146983 229533.I1REK8 pp 229533.I1RS66
146984 229533.I1REK8 pp 229533.I1RG53
146985 229533.I1REK8 pp 229533.I1RX46
146986 229533.I1REK8 pp 229533.I1RW25
146987 229533.I1REK8 pp 229533.I1RR24
146988 229533.I1REK8 pp 229533.I1RW07
146989 229533.I1REK8 pp 229533.I1RRS8
146990 229533.I1REK8 pp 229533.I1S0T2
146991 229533.I1REK8 pp 229533.I1S0X6
146992 229533.I1REK8 pp 229533.I1RT05
146993 229533.I1REK8 pp 229533.I1RI02
146994 229533.I1REK8 pp 229533.I1RMQ6
146995 229533.I1REK8 pp 229533.I1RMC8
146996 229533.I1REK8 pp 229533.I1RPA4
146997 229533.I1REK8 pp 229533.I1RTF4
146998 229533.I1REK8 pp 229533.Q4HVW2
146999 229533.I1REK8 pp 229533.I1RMK3
147000 229533.I1REK8 pp 229533.I1S0F1
147001 229533.I1REK8 pp 229533.I1RKJ5
147002 229533.A0A1C3YN62 pp 229533.I1RAE1
147003 229533.A0A1C3YN62 pp 229533.I1RA94
147004 229533.A0A1C3YN62 pp 229533.I1RM67
147005 229533.A0A1C3YN62 pp 229533.I1RQ86
147006 229533.A0A1C3YN62 pp 229533.I1RYI7
147007 229533.I1RX25 pp 229533.I1S0F6
147008 229533.I1RX25 pp 229533.I1S2B8
147009 229533.I1RX25 pp 229533.I1SA33
147010 229533.I1RX25 pp 229533.I1S0L3
147011 229533.I1RX25 pp 229533.V6R1K4
147012 229533.I1RX25 pp 229533.I1S5B0
147013 229533.I1RX25 pp 229533.I1RXF0
147014 229533.I1RX25 pp 229533.Q4IB65
147015 229533.I1RX25 pp 229533.V6QW22
147016 229533.I1RX25 pp 229533.I1S8F0
147017 229533.I1RX25 pp 229533.I1RZJ5
147018 229533.I1RX25 pp 229533.I1RZM8
147019 229533.I1RX25 pp 229533.I1RX46
147020 229533.I1RX25 pp 229533.V6RMW6
147021 229533.I1RX25 pp 229533.V6R042
147022 229533.I1RX25 pp 229533.V6RCZ8

147023	229533.I1RX25	pp	229533.I1S5R1
147024	229533.I1RX25	pp	229533.V6RCG5
147025	229533.I1RX25	pp	229533.I1S503
147026	229533.I1RX25	pp	229533.I1RZM1
147027	229533.I1RX25	pp	229533.V6RTJ8
147028	229533.I1RX25	pp	229533.I1S0L4
147029	229533.I1RX25	pp	229533.I1S7A8
147030	229533.I1RX25	pp	229533.I1S2H4
147031	229533.I1RX25	pp	229533.V6R3X5
147032	229533.I1RX25	pp	229533.I1RZ89
147033	229533.I1RX25	pp	229533.I1S504
147034	229533.I1RX25	pp	229533.V6RIV9
147035	229533.I1RX25	pp	229533.I1RZZ9
147036	229533.I1RX25	pp	229533.I1RXD7
147037	229533.I1RX25	pp	229533.Q4I8P5
147038	229533.I1RX25	pp	229533.I1S0J1
147039	229533.I1RX25	pp	229533.I1SA32
147040	229533.I1RX25	pp	229533.I1RZ39
147041	229533.I1RX25	pp	229533.I1S0R7
147042	229533.I1RX25	pp	229533.I1RZK5
147043	229533.I1RX25	pp	229533.I1S8R3
147044	229533.I1RX25	pp	229533.I1RZW8
147045	229533.I1RX25	pp	229533.I1RY03
147046	229533.I1RX25	pp	229533.I1S1C7
147047	229533.I1RX25	pp	229533.I1RZZ1
147048	229533.I1RX25	pp	229533.Q4IJK2
147049	229533.I1RX25	pp	229533.Q4IPZ1
147050	229533.I1RX25	pp	229533.Q4I624
147051	229533.I1RX25	pp	229533.V6R057
147052	229533.I1S0W5	pp	229533.P0C431
147053	229533.I1S0W5	pp	229533.Q4IMM0
147054	229533.I1S0W5	pp	229533.Q4IBX0
147055	229533.I1RSM9	pp	229533.I1RWD7
147056	229533.I1RYI1	pp	229533.I1RZF5
147057	229533.I1RYI1	pp	229533.I1S0K0
147058	229533.I1RYI1	pp	229533.I1S437
147059	229533.I1RYI1	pp	229533.I1RYR0
147060	229533.I1RYI1	pp	229533.I1S3L2
147061	229533.I1RYI1	pp	229533.Q4I0J6
147062	229533.I1RYI1	pp	229533.I1S438
147063	229533.I1RYI1	pp	229533.I1S8R4
147064	229533.I1RYI1	pp	229533.Q4IBX0
147065	229533.A0A1C3YIS4	pp	229533.A0A1C3YJL3
147066	229533.A0A1C3YIS4	pp	229533.I1RX22
147067	229533.A0A1C3YIS4	pp	229533.I1RY25
147068	229533.A0A1C3YIS4	pp	229533.I1R9R2
147069	229533.A0A1C3YIS4	pp	229533.I1S7B9
147070	229533.A0A1C3YIS4	pp	229533.I1RW03
147071	229533.A0A1C3YIS4	pp	229533.I1RY98
147072	229533.A0A1C3YIS4	pp	229533.I1RTE7
147073	229533.A0A1C3YIS4	pp	229533.I1RYJ8
147074	229533.Q4I8P5	pp	229533.V6RIV9
147075	229533.Q4I8P5	pp	229533.V6R042
147076	229533.Q4I8P5	pp	229533.V6RUY9
147077	229533.Q4I8P5	pp	229533.Q4IJK2
147078	229533.Q4I8P5	pp	229533.V6QW22
147079	229533.Q4I8P5	pp	229533.V6RCG5
147080	229533.Q4I8P5	pp	229533.V6RMW6
147081	229533.Q4I8P5	pp	229533.V6RCZ8
147082	229533.Q4I8P5	pp	229533.Q4IJT5
147083	229533.Q4I8P5	pp	229533.V6QYF0
147084	229533.Q4I8P5	pp	229533.V6QUW4
147085	229533.Q4I8P5	pp	229533.V6R057
147086	229533.Q4I8P5	pp	229533.V6RTJ8
147087	229533.Q4I8P5	pp	229533.V6R1K4
147088	229533.Q4I8P5	pp	229533.V6R3X5
147089	229533.I1RHJ1	pp	229533.I1RHJ2
147090	229533.I1RHJ1	pp	229533.I1RWB5
147091	229533.I1RHJ1	pp	229533.I1RZQ6
147092	229533.I1RHJ1	pp	229533.V6RDP1
147093	229533.I1RHJ1	pp	229533.I1RPK0
147094	229533.I1RHJ1	pp	229533.P0C431
147095	229533.I1RHJ1	pp	229533.I1RRR6

147096 229533.I1RHJ1 pp 229533.I1RST8
147097 229533.I1RUC6 pp 229533.Q4IMM0
147098 229533.I1RUC6 pp 229533.Q4IBX0
147099 229533.A0A098DI91 pp 229533.I1RNG4
147100 229533.A0A098DXE2 pp 229533.A0A1C3YLF5
147101 229533.A0A098DXE2 pp 229533.I1RNA5
147102 229533.A0A098DXE2 pp 229533.I1RT04
147103 229533.A0A098DXE2 pp 229533.I1S2E9
147104 229533.A0A098DXE2 pp 229533.I1RV91
147105 229533.A0A098DXE2 pp 229533.I1RAF8
147106 229533.A0A098DXE2 pp 229533.I1RCJ7
147107 229533.A0A098DXE2 pp 229533.A0A1C3YKN3
147108 229533.A0A098DXE2 pp 229533.I1RFT1
147109 229533.A0A098DXE2 pp 229533.I1RT40
147110 229533.A0A098DXE2 pp 229533.I1RB74
147111 229533.A0A098DXE2 pp 229533.I1S8P5
147112 229533.A0A098DXE2 pp 229533.I1RGF5
147113 229533.A0A098DXE2 pp 229533.A0A1C3YMK1
147114 229533.A0A098DXE2 pp 229533.I1RPP3
147115 229533.A0A098DXE2 pp 229533.I1RTD5
147116 229533.A0A098DXE2 pp 229533.I1RWF7
147117 229533.A0A098DXE2 pp 229533.I1RX39
147118 229533.A0A098DXE2 pp 229533.I1RBS6
147119 229533.A0A098DXE2 pp 229533.I1RRJ8
147120 229533.A0A098DXE2 pp 229533.I1RKJ6
147121 229533.A0A098DXE2 pp 229533.I1RGA4
147122 229533.A0A098DXE2 pp 229533.I1S1V5
147123 229533.A0A098DXE2 pp 229533.I1RWK3
147124 229533.A0A098DXE2 pp 229533.I1RP27
147125 229533.A0A098DXE2 pp 229533.V6R283
147126 229533.A0A098DXE2 pp 229533.I1RJV4
147127 229533.A0A098DXE2 pp 229533.I1RC03
147128 229533.A0A098DXE2 pp 229533.I1RLV9
147129 229533.A0A098DXE2 pp 229533.I1RS47
147130 229533.A0A098DXE2 pp 229533.I1RRT8
147131 229533.A0A098DXE2 pp 229533.I1RLN6
147132 229533.A0A098DXE2 pp 229533.I1S5R1
147133 229533.A0A098DXE2 pp 229533.I1RJW6
147134 229533.A0A098DXE2 pp 229533.A0A1C3YLK8
147135 229533.A0A098DXE2 pp 229533.A0A1C3YLD8
147136 229533.A0A098DXE2 pp 229533.I1RA11
147137 229533.A0A098DXE2 pp 229533.I1RWL2
147138 229533.A0A098DXE2 pp 229533.A0A1C3YL15
147139 229533.A0A098DXE2 pp 229533.I1RQ65
147140 229533.A0A098DXE2 pp 229533.I1S503
147141 229533.A0A098DXE2 pp 229533.A0A1C3YII7
147142 229533.A0A098DXE2 pp 229533.I1S2G7
147143 229533.A0A098DXE2 pp 229533.I1S352
147144 229533.A0A098DXE2 pp 229533.I1RFZ2
147145 229533.A0A098DXE2 pp 229533.I1RN67
147146 229533.A0A098DXE2 pp 229533.I1RE22
147147 229533.A0A098DXE2 pp 229533.I1RKL3
147148 229533.A0A098DXE2 pp 229533.I1RBG9
147149 229533.A0A098DXE2 pp 229533.I1S0M6
147150 229533.A0A098DXE2 pp 229533.I1S0F6
147151 229533.A0A098DXE2 pp 229533.I1RSV2
147152 229533.A0A098DXE2 pp 229533.I1RRQ3
147153 229533.A0A098DXE2 pp 229533.I1RZS6
147154 229533.A0A098DXE2 pp 229533.I1RMN6
147155 229533.A0A098DXE2 pp 229533.I1RQ23
147156 229533.A0A098DXE2 pp 229533.I1RBS0
147157 229533.A0A098DXE2 pp 229533.A0A0E0S0W9
147158 229533.A0A098DXE2 pp 229533.I1RXT9
147159 229533.A0A098DXE2 pp 229533.A0A1C3YKM9
147160 229533.A0A098DXE2 pp 229533.I1RZ39
147161 229533.A0A098DXE2 pp 229533.I1S8E2
147162 229533.A0A098DXE2 pp 229533.Q4I662
147163 229533.A0A098DXE2 pp 229533.I1RD22
147164 229533.A0A098DXE2 pp 229533.I1RFS3
147165 229533.A0A098DXE2 pp 229533.I1S6Y8
147166 229533.A0A098DXE2 pp 229533.A0A1C3YK73
147167 229533.A0A098DXE2 pp 229533.I1RS17
147168 229533.A0A098DXE2 pp 229533.I1RR73

147169 229533.A0A098DXE2 pp 229533.I1RPM4
147170 229533.A0A098DXE2 pp 229533.A0A1C3YIN1
147171 229533.A0A098DXE2 pp 229533.I1RWB5
147172 229533.A0A098DXE2 pp 229533.V6R3X5
147173 229533.A0A098DXE2 pp 229533.I1RNB8
147174 229533.A0A098DXE2 pp 229533.A0A0E0SKJ2
147175 229533.A0A098DXE2 pp 229533.I1RND6
147176 229533.A0A098DXE2 pp 229533.I1RPP4
147177 229533.A0A098DXE2 pp 229533.A0A1C3YMA1
147178 229533.A0A098DXE2 pp 229533.A0A1C3YIS7
147179 229533.A0A098DXE2 pp 229533.I1RWI5
147180 229533.A0A098DXE2 pp 229533.I1RE01
147181 229533.A0A098DXE2 pp 229533.I1RZZ9
147182 229533.A0A098DXE2 pp 229533.I1R9M0
147183 229533.A0A098DXE2 pp 229533.I1RJZ7
147184 229533.A0A098DXE2 pp 229533.I1RW46
147185 229533.A0A098DXE2 pp 229533.I1RRM5
147186 229533.A0A098DXE2 pp 229533.I1S2B8
147187 229533.A0A098DXE2 pp 229533.I1RC66
147188 229533.A0A098DXE2 pp 229533.I1RFT6
147189 229533.A0A098DXE2 pp 229533.I1RZS7
147190 229533.A0A098DXE2 pp 229533.I1RNN1
147191 229533.A0A098DXE2 pp 229533.I1RPU3
147192 229533.A0A098DXE2 pp 229533.I1RTA0
147193 229533.A0A098DXE2 pp 229533.A0A0E0RU84
147194 229533.A0A098DXE2 pp 229533.I1RBY4
147195 229533.A0A098DXE2 pp 229533.I1RC00
147196 229533.A0A098DXE2 pp 229533.I1RD64
147197 229533.A0A098DXE2 pp 229533.I1S0J1
147198 229533.A0A098DXE2 pp 229533.I1S2H4
147199 229533.A0A098DXE2 pp 229533.I1RE76
147200 229533.A0A098DXE2 pp 229533.I1RMG3
147201 229533.A0A098DXE2 pp 229533.I1RRE5
147202 229533.A0A098DXE2 pp 229533.A0A0E0RS85
147203 229533.A0A098DXE2 pp 229533.I1RS20
147204 229533.A0A098DXE2 pp 229533.I1S0L4
147205 229533.A0A098DXE2 pp 229533.A0A1C3YLS9
147206 229533.A0A098DXE2 pp 229533.I1RJP4
147207 229533.A0A098DXE2 pp 229533.I1RRV1
147208 229533.A0A098DXE2 pp 229533.I1S1W4
147209 229533.A0A098DXE2 pp 229533.V6R042
147210 229533.A0A098DXE2 pp 229533.I1RIL3
147211 229533.A0A098DXE2 pp 229533.I1RRR7
147212 229533.A0A098DXE2 pp 229533.A0A0E0SC46
147213 229533.A0A098DXE2 pp 229533.I1RZV6
147214 229533.A0A098DXE2 pp 229533.I1RSQ3
147215 229533.A0A098DXE2 pp 229533.V6RIV9
147216 229533.A0A098DXE2 pp 229533.V6RMW6
147217 229533.A0A098DXE2 pp 229533.I1RM82
147218 229533.A0A098DXE2 pp 229533.I1RWN6
147219 229533.A0A098DXE2 pp 229533.I1RAW0
147220 229533.A0A098DXE2 pp 229533.I1RWN5
147221 229533.A0A098DXE2 pp 229533.A0A1C3YIN6
147222 229533.A0A098DXE2 pp 229533.I1RCM3
147223 229533.A0A098DXE2 pp 229533.I1RSK1
147224 229533.A0A098DXE2 pp 229533.I1RNC1
147225 229533.A0A098DXE2 pp 229533.Q4HZB8
147226 229533.A0A098DXE2 pp 229533.I1RUG3
147227 229533.A0A098DXE2 pp 229533.I1RJV3
147228 229533.A0A098DXE2 pp 229533.I1RM96
147229 229533.A0A098DXE2 pp 229533.I1RVX0
147230 229533.A0A098DXE2 pp 229533.I1RT03
147231 229533.A0A098DXE2 pp 229533.I1RRN5
147232 229533.A0A098DXE2 pp 229533.I1RCR8
147233 229533.A0A098DXE2 pp 229533.Q4IN63
147234 229533.A0A098DXE2 pp 229533.I1RNY2
147235 229533.A0A098DXE2 pp 229533.I1RY44
147236 229533.A0A098DXE2 pp 229533.Q4I327
147237 229533.A0A098DXE2 pp 229533.I1RB92
147238 229533.A0A098DXE2 pp 229533.I1RA70
147239 229533.A0A098DXE2 pp 229533.I1RFL4
147240 229533.A0A098DXE2 pp 229533.I1RSD8
147241 229533.A0A098DXE2 pp 229533.I1RTH6

147242 229533.A0A098DXE2 pp 229533.A0A1C3YMI5
147243 229533.A0A098DXE2 pp 229533.I1S0N4
147244 229533.A0A098DXE2 pp 229533.I1R9D1
147245 229533.A0A098DXE2 pp 229533.I1SA33
147246 229533.A0A098DXE2 pp 229533.I1RN18
147247 229533.A0A098DXE2 pp 229533.I1RCH5
147248 229533.A0A098DXE2 pp 229533.I1S2E8
147249 229533.A0A098DXE2 pp 229533.I1RQE7
147250 229533.A0A098DXE2 pp 229533.I1S8R3
147251 229533.A0A098DXE2 pp 229533.I1S569
147252 229533.A0A098DXE2 pp 229533.I1S0T2
147253 229533.A0A098DXE2 pp 229533.I1S2C0
147254 229533.A0A098DXE2 pp 229533.I1RX46
147255 229533.A0A098DXE2 pp 229533.V6R057
147256 229533.A0A098DXE2 pp 229533.I1S1C7
147257 229533.A0A098DXE2 pp 229533.Q4I624
147258 229533.A0A098DXE2 pp 229533.A0A1C3YK13
147259 229533.A0A098DXE2 pp 229533.I1RW71
147260 229533.A0A098DXE2 pp 229533.I1RYW3
147261 229533.A0A098DXE2 pp 229533.I1RBC9
147262 229533.A0A098DXE2 pp 229533.I1RR00
147263 229533.A0A098DXE2 pp 229533.I1RWX5
147264 229533.A0A098DXE2 pp 229533.I1RD17
147265 229533.A0A098DXE2 pp 229533.I1RAU7
147266 229533.A0A098DXE2 pp 229533.I1RB41
147267 229533.A0A098DXE2 pp 229533.I1RG53
147268 229533.A0A098DXE2 pp 229533.I1RQJ5
147269 229533.A0A098DXE2 pp 229533.I1RRI2
147270 229533.A0A098DXE2 pp 229533.V6QW22
147271 229533.A0A098DXE2 pp 229533.I1R9J6
147272 229533.A0A098DXE2 pp 229533.A0A1C3YI73
147273 229533.A0A098DXE2 pp 229533.I1RGL4
147274 229533.A0A098DXE2 pp 229533.I1RZW8
147275 229533.A0A098DXE2 pp 229533.I1RQC9
147276 229533.A0A098DXE2 pp 229533.I1RD39
147277 229533.A0A098DXE2 pp 229533.I1RN61
147278 229533.A0A098DXE2 pp 229533.I1RC20
147279 229533.A0A098DXE2 pp 229533.A0A0E0SFN0
147280 229533.A0A098DXE2 pp 229533.I1S0R7
147281 229533.A0A098DXE2 pp 229533.I1RYB9
147282 229533.A0A098DXE2 pp 229533.I1RQH3
147283 229533.A0A098DXE2 pp 229533.I1RSQ7
147284 229533.A0A098DXE2 pp 229533.I1RC17
147285 229533.A0A098DXE2 pp 229533.A0A1C3YM63
147286 229533.A0A098DXE2 pp 229533.I1RXD7
147287 229533.A0A098DXE2 pp 229533.I1S044
147288 229533.A0A098DXE2 pp 229533.A0A1C3YIA8
147289 229533.A0A098DXE2 pp 229533.I1RAX6
147290 229533.A0A098DXE2 pp 229533.V6RTJ8
147291 229533.A0A098DXE2 pp 229533.I1RIL8
147292 229533.A0A098DXE2 pp 229533.I1S5B0
147293 229533.A0A098DXE2 pp 229533.I1RQ80
147294 229533.A0A098DXE2 pp 229533.I1RW99
147295 229533.A0A098DXE2 pp 229533.I1RAW7
147296 229533.A0A098DXE2 pp 229533.Q4IJT5
147297 229533.A0A098DXE2 pp 229533.I1S7A8
147298 229533.A0A098DXE2 pp 229533.I1RGC2
147299 229533.A0A098DXE2 pp 229533.A0A0E0RV94
147300 229533.A0A098DXE2 pp 229533.I1RFT2
147301 229533.A0A098DXE2 pp 229533.A0A1C3YHX8
147302 229533.A0A098DXE2 pp 229533.I1RR24
147303 229533.A0A098DXE2 pp 229533.I1RFW5
147304 229533.A0A098DXE2 pp 229533.I1RRU9
147305 229533.A0A098DXE2 pp 229533.I1RD52
147306 229533.A0A098DXE2 pp 229533.V6R1K4
147307 229533.A0A098DXE2 pp 229533.A0A0E0RSD7
147308 229533.A0A098DXE2 pp 229533.I1RPW9
147309 229533.A0A098DXE2 pp 229533.I1S9U2
147310 229533.A0A098DXE2 pp 229533.I1RAU5
147311 229533.A0A098DXE2 pp 229533.I1RWJ8
147312 229533.A0A098DXE2 pp 229533.I1S0M9
147313 229533.A0A098DXE2 pp 229533.I1S0L3
147314 229533.A0A098DXE2 pp 229533.A0A1C3YKV3

147315	229533.A0A098DXE2	pp	229533.A0A1C3YJH8
147316	229533.A0A098DXE2	pp	229533.A0A0E0RZM5
147317	229533.A0A098DXE2	pp	229533.I1S0F5
147318	229533.A0A098DXE2	pp	229533.I1RPU0
147319	229533.A0A098DXE2	pp	229533.I1RQ81
147320	229533.A0A098DXE2	pp	229533.I1RFQ7
147321	229533.A0A098DXE2	pp	229533.I1RMG8
147322	229533.A0A098DXE2	pp	229533.A0A1C3YIP5
147323	229533.A0A098DXE2	pp	229533.I1RS18
147324	229533.A0A098DXE2	pp	229533.I1REN8
147325	229533.A0A098DXE2	pp	229533.I1RB27
147326	229533.A0A098DXE2	pp	229533.I1RZJ5
147327	229533.A0A098DXE2	pp	229533.V6R4J1
147328	229533.A0A098DXE2	pp	229533.I1RM67
147329	229533.A0A098DXE2	pp	229533.Q4I8P5
147330	229533.A0A098DXE2	pp	229533.V6QUW4
147331	229533.A0A098DXE2	pp	229533.I1RVQ0
147332	229533.A0A098DXE2	pp	229533.A0A0E0SNZ9
147333	229533.A0A098DXE2	pp	229533.I1RZH4
147334	229533.A0A098DXE2	pp	229533.I1RT80
147335	229533.A0A098DXE2	pp	229533.A0A0E0SJS1
147336	229533.A0A098DXE2	pp	229533.I1RMQ6
147337	229533.A0A098DXE2	pp	229533.I1RJU3
147338	229533.A0A098DXE2	pp	229533.A0A1C3YLZ7
147339	229533.A0A098DXE2	pp	229533.I1RM06
147340	229533.A0A098DXE2	pp	229533.I1RCS1
147341	229533.A0A098DXE2	pp	229533.A0A1C3YK34
147342	229533.A0A098DXE2	pp	229533.I1RYH6
147343	229533.A0A098DXE2	pp	229533.A0A1C3YIY6
147344	229533.A0A098DXE2	pp	229533.A0A1C3YN30
147345	229533.A0A098DXE2	pp	229533.I1RIG2
147346	229533.A0A098DXE2	pp	229533.A0A0E0RUI4
147347	229533.A0A098DXE2	pp	229533.I1RI02
147348	229533.A0A098DXE2	pp	229533.I1SA32
147349	229533.A0A098DXE2	pp	229533.I1RW07
147350	229533.A0A098DXE2	pp	229533.I1RLC7
147351	229533.A0A098DXE2	pp	229533.A0A1C3YJQ9
147352	229533.A0A098DXE2	pp	229533.I1RW25
147353	229533.A0A098DXE2	pp	229533.I1S6U8
147354	229533.A0A098DXE2	pp	229533.A0A0E0SA11
147355	229533.A0A098DXE2	pp	229533.I1RBT1
147356	229533.A0A098DXE2	pp	229533.A0A1C3YKC0
147357	229533.A0A098DXE2	pp	229533.I1S0X6
147358	229533.A0A098DXE2	pp	229533.I1RZF6
147359	229533.A0A098DXE2	pp	229533.I1RMC8
147360	229533.A0A098DXE2	pp	229533.I1RX25
147361	229533.A0A098DXE2	pp	229533.I1S504
147362	229533.A0A098DXE2	pp	229533.V6RCZ8
147363	229533.A0A098DXE2	pp	229533.I1RR51
147364	229533.A0A098DXE2	pp	229533.A0A1C3YLK2
147365	229533.A0A098DXE2	pp	229533.I1RBL7
147366	229533.A0A098DXE2	pp	229533.I1RU29
147367	229533.A0A098DXE2	pp	229533.I1RRY9
147368	229533.A0A098DXE2	pp	229533.I1RAN5
147369	229533.A0A098DXE2	pp	229533.A0A1C3YKB0
147370	229533.A0A098DXE2	pp	229533.I1RD03
147371	229533.A0A098DXE2	pp	229533.A0A0E0SPV2
147372	229533.A0A098DXE2	pp	229533.I1RI03
147373	229533.A0A098DXE2	pp	229533.I1S1J7
147374	229533.A0A098DXE2	pp	229533.A0A1C3YLU7
147375	229533.A0A098DXE2	pp	229533.V6RCG5
147376	229533.A0A098DXE2	pp	229533.Q4I283
147377	229533.A0A098DXE2	pp	229533.I1RXF0
147378	229533.A0A098DXE2	pp	229533.A0A1C3YJ93
147379	229533.A0A098DXE2	pp	229533.I1RWT6
147380	229533.A0A098DXE2	pp	229533.I1R9V7
147381	229533.A0A098DXE2	pp	229533.A0A1C3YKW9
147382	229533.A0A098DXE2	pp	229533.I1RZM8
147383	229533.A0A098DXE2	pp	229533.I1RI82
147384	229533.A0A098DXE2	pp	229533.Q4IJM4
147385	229533.A0A098DXE2	pp	229533.I1S419
147386	229533.A0A098DXE2	pp	229533.I1RA48
147387	229533.A0A098DXE2	pp	229533.A0A0E0RVZ1

229533.A0A098DXE2	pp	229533.I1RDG0
229533.A0A098DXE2	pp	229533.I1RNH6
229533.A0A098DXE2	pp	229533.I1RNQ5
229533.A0A098DXE2	pp	229533.I1S021
229533.A0A098DXE2	pp	229533.I1RQA3
229533.A0A098DXE2	pp	229533.I1RLU6
229533.A0A098DXE2	pp	229533.I1RBJ5
229533.A0A098DXE2	pp	229533.Q4IJK2
229533.A0A098DXE2	pp	229533.A0A0E0SMM8
229533.A0A098DXE2	pp	229533.I1RZK6
229533.A0A098DXE2	pp	229533.I1RZM1
229533.A0A098DXE2	pp	229533.A0A098E4W0
229533.A0A098DXE2	pp	229533.I1RFU8
229533.A0A098DXE2	pp	229533.I1RZZ1
229533.A0A098DXE2	pp	229533.I1RCG4
229533.A0A098DXE2	pp	229533.I1RQQ5
229533.A0A098DXE2	pp	229533.I1RZK5
229533.A0A098DXE2	pp	229533.A0A1C3YHX6
229533.A0A098DXE2	pp	229533.I1RJZ6
229533.A0A098DXE2	pp	229533.I1RB75
229533.A0A098DXE2	pp	229533.I1RSR8
229533.A0A098DXE2	pp	229533.I1REK8
229533.A0A098DXE2	pp	229533.I1RCR5
229533.A0A098DXE2	pp	229533.I1RY03
229533.A0A098DXE2	pp	229533.I1RWD7
229533.A0A098DXE2	pp	229533.I1RVZ0
229533.A0A098DXE2	pp	229533.I1RYY5
229533.A0A098DXE2	pp	229533.I1RZ35
229533.A0A098DXE2	pp	229533.Q4I2J8
229533.A0A098DXE2	pp	229533.I1RLM7
229533.A0A098DXE2	pp	229533.I1RZE5
229533.A0A098DXE2	pp	229533.A0A1C3YIJ3
229533.A0A098DXE2	pp	229533.I1RE00
229533.A0A098DXE2	pp	229533.I1RSI0
229533.A0A098E4P3	pp	229533.I1RDB1
229533.A0A098E4P3	pp	229533.I1RX70
229533.A0A098E4P3	pp	229533.I1RYH9
229533.A0A098E4P3	pp	229533.I1S1W4
229533.A0A098E4P3	pp	229533.I1RAM7
229533.A0A098E4P3	pp	229533.A0A0E0SAC4
229533.A0A098E4P3	pp	229533.A0A1C3YHL0
229533.A0A098E4P3	pp	229533.A0A0E0SD93
229533.A0A098E4P3	pp	229533.I1R9Y9
229533.A0A098E4P3	pp	229533.I1RNY8
229533.A0A098E4P3	pp	229533.Q4IEA7
229533.A0A098E4P3	pp	229533.I1RUN8
229533.A0A098E4P3	pp	229533.A0A1C3YJI8
229533.A0A098E4P3	pp	229533.I1RL24
229533.A0A098E4P3	pp	229533.I1S549
229533.A0A098E4P3	pp	229533.I1RYZ9
229533.A0A098E4P3	pp	229533.I1RAN3
229533.A0A098E4P3	pp	229533.I1RC95
229533.A0A098E4P3	pp	229533.I1RA43
229533.A0A098E4P3	pp	229533.I1S0D1
229533.A0A098E4P3	pp	229533.I1S2E9
229533.A0A098E4P3	pp	229533.I1RRY3
229533.A0A098E4P3	pp	229533.I1RT36
229533.A0A098E4P3	pp	229533.I1RBZ9
229533.A0A098E4P3	pp	229533.A0A0E0RP92
229533.A0A098E4P3	pp	229533.I1RKK0
229533.A0A098E4P3	pp	229533.I1RC17
229533.A0A098E4P3	pp	229533.V6RGI1
229533.A0A098E4P3	pp	229533.I1RMM9
229533.A0A098E4P3	pp	229533.I1S286
229533.A0A098E4P3	pp	229533.A0A1C3YL35
229533.A0A098E4P3	pp	229533.I1RC97
229533.A0A098E4P3	pp	229533.I1RAB6
229533.A0A098E4P3	pp	229533.I1RM09
229533.A0A098E4P3	pp	229533.V6QV09
229533.A0A098E4P3	pp	229533.I1RQI6
229533.A0A098E4P3	pp	229533.I1RC22
229533.A0A098E4P3	pp	229533.I1RQ26
229533.A0A098E4P3	pp	229533.I1RUQ5

147461 229533.A0A098E4P3 pp 229533.A0A1C3YI28
147462 229533.A0A098E4P3 pp 229533.I1S0M2
147463 229533.A0A098E4P3 pp 229533.A0A1C3YIL0
147464 229533.A0A098E4P3 pp 229533.I1RB74
147465 229533.A0A098E4P3 pp 229533.I1RN08
147466 229533.A0A098E4P3 pp 229533.A0A1C3YM29
147467 229533.A0A098E4P3 pp 229533.I1S439
147468 229533.A0A098E4P3 pp 229533.A0A1C3YJQ6
147469 229533.A0A098E4P3 pp 229533.I1RY44
147470 229533.A0A098E4P3 pp 229533.Q4I0K3
147471 229533.A0A098E4P3 pp 229533.V6RVG3
147472 229533.A0A098E4P3 pp 229533.A0A1C3YKF2
147473 229533.A0A098E4P3 pp 229533.I1RB75
147474 229533.A0A098E4P3 pp 229533.I1RZE4
147475 229533.A0A098E4P3 pp 229533.I1RNP7
147476 229533.A0A098E4P3 pp 229533.I1RY09
147477 229533.A0A098E4P3 pp 229533.I1RGD1
147478 229533.A0A098E4P3 pp 229533.A0A1C3YJ90
147479 229533.A0A098E4P3 pp 229533.I1RQA3
147480 229533.A0A098E4P3 pp 229533.V6RKY7
147481 229533.A0A098E4P3 pp 229533.I1RAN2
147482 229533.A0A098E4P3 pp 229533.I1RNY9
147483 229533.A0A098E4P3 pp 229533.A0A0E0SA11
147484 229533.A0A098E4P3 pp 229533.I1RWT6
147485 229533.A0A098E4P3 pp 229533.I1RDZ4
147486 229533.A0A098E4P3 pp 229533.I1RN65
147487 229533.A0A098E4P3 pp 229533.I1RRN5
147488 229533.I1RHS9 pp 229533.I1RKT7
147489 229533.I1RHS9 pp 229533.I1RIY8
147490 229533.I1RY39 pp 229533.I1S694
147491 229533.I1RY39 pp 229533.I1RZA8
147492 229533.I1RLC7 pp 229533.I1RX46
147493 229533.I1RLC7 pp 229533.I1RQC9
147494 229533.I1RLC7 pp 229533.V6QW22
147495 229533.I1RLC7 pp 229533.V6RTJ8
147496 229533.I1RLC7 pp 229533.I1RVQ0
147497 229533.I1RLC7 pp 229533.I1RS18
147498 229533.I1RLC7 pp 229533.I1RSQ7
147499 229533.I1RLC7 pp 229533.I1RLU6
147500 229533.I1RLC7 pp 229533.I1S0L3
147501 229533.I1RLC7 pp 229533.I1RR39
147502 229533.I1RLC7 pp 229533.I1S504
147503 229533.I1RLC7 pp 229533.I1RZM1
147504 229533.I1RLC7 pp 229533.I1RW25
147505 229533.I1RLC7 pp 229533.I1RR24
147506 229533.I1RLC7 pp 229533.Q4IJK2
147507 229533.I1RLC7 pp 229533.I1RY03
147508 229533.I1RLC7 pp 229533.I1RTD5
147509 229533.I1RLC7 pp 229533.I1RV91
147510 229533.I1RLC7 pp 229533.V6RCG5
147511 229533.I1RLC7 pp 229533.I1RZK5
147512 229533.I1RLC7 pp 229533.I1RTA0
147513 229533.I1RLC7 pp 229533.I1RT04
147514 229533.I1RLC7 pp 229533.I1RRY9
147515 229533.I1RLC7 pp 229533.I1RPP4
147516 229533.I1RLC7 pp 229533.I1RWK3
147517 229533.I1RLC7 pp 229533.I1S2E9
147518 229533.I1RLC7 pp 229533.I1S503
147519 229533.I1RLC7 pp 229533.I1RPP3
147520 229533.I1RLC7 pp 229533.I1S8P5
147521 229533.I1RLC7 pp 229533.I1RPM4
147522 229533.I1RLC7 pp 229533.I1RWN5
147523 229533.I1RLC7 pp 229533.I1S2B8
147524 229533.I1RLC7 pp 229533.I1RN67
147525 229533.I1RLC7 pp 229533.V6RIV9
147526 229533.I1RLC7 pp 229533.I1RRM5
147527 229533.I1RLC7 pp 229533.I1RY44
147528 229533.I1RLC7 pp 229533.I1RZ39
147529 229533.I1RLC7 pp 229533.I1S1C7
147530 229533.I1RLC7 pp 229533.I1RZZ9
147531 229533.I1RLC7 pp 229533.I1RUG3
147532 229533.I1RLC7 pp 229533.I1RNC1
147533 229533.I1RLC7 pp 229533.I1RR73

147534	229533.I1RLC7	pp	229533.I1RSQ3
147535	229533.I1RLC7	pp	229533.I1RZW8
147536	229533.I1RLC7	pp	229533.I1RRT8
147537	229533.I1RLC7	pp	229533.I1RTH6
147538	229533.I1RLC7	pp	229533.V6R3X5
147539	229533.I1RLC7	pp	229533.V6RMW6
147540	229533.I1RLC7	pp	229533.I1S8R3
147541	229533.I1RLC7	pp	229533.V6R042
147542	229533.I1RLC7	pp	229533.I1RWN6
147543	229533.I1RLC7	pp	229533.I1S2H4
147544	229533.I1RLC7	pp	229533.I1RQ75
147545	229533.I1RLC7	pp	229533.I1RN18
147546	229533.I1RLC7	pp	229533.I1S0L4
147547	229533.I1RLC7	pp	229533.I1RVX0
147548	229533.I1RLC7	pp	229533.Q4I1Y5
147549	229533.I1RLC7	pp	229533.I1RT03
147550	229533.I1RAV9	pp	229533.I1RD61
147551	229533.I1RAV9	pp	229533.I1S438
147552	229533.I1RAV9	pp	229533.I1RZF5
147553	229533.I1RAV9	pp	229533.I1S437
147554	229533.I1RAV9	pp	229533.I1RQP1
147555	229533.I1RAV9	pp	229533.I1RYI1
147556	229533.I1RAV9	pp	229533.I1RYR0
147557	229533.I1RAV9	pp	229533.I1RUQ1
147558	229533.I1RAV9	pp	229533.I1RQP2
147559	229533.I1RHY7	pp	229533.V6RU70
147560	229533.I1RHY7	pp	229533.V6RMM8
147561	229533.I1RHY7	pp	229533.I1RIR9
147562	229533.I1RHY7	pp	229533.I1S1W4
147563	229533.I1RHY7	pp	229533.I1RKL9
147564	229533.I1RHY7	pp	229533.I1S0V3
147565	229533.I1RHY7	pp	229533.I1RZZ4
147566	229533.I1RHY7	pp	229533.I1S2L3
147567	229533.I1RHY7	pp	229533.I1RJZ7
147568	229533.I1RHY7	pp	229533.I1RW71
147569	229533.I1RHY7	pp	229533.I1S5M1
147570	229533.I1RHR6	pp	229533.I1RWX8
147571	229533.I1RHR6	pp	229533.V6QV38
147572	229533.I1RHR6	pp	229533.I1RXE5
147573	229533.I1RHR6	pp	229533.I1RMA5
147574	229533.I1RHR6	pp	229533.I1RKR6
147575	229533.I1RHR6	pp	229533.I1RWU3
147576	229533.I1RCR5	pp	229533.I1RN67
147577	229533.I1RCR5	pp	229533.I1S9L6
147578	229533.I1RCR5	pp	229533.I1RY62
147579	229533.I1RCR5	pp	229533.I1RS47
147580	229533.I1RCR5	pp	229533.Q4IQ72
147581	229533.I1RCR5	pp	229533.I1S5S2
147582	229533.I1RCR5	pp	229533.I1RXF2
147583	229533.I1RCR5	pp	229533.I1RRZ0
147584	229533.I1RCR5	pp	229533.I1RZZ9
147585	229533.I1RCR5	pp	229533.Q4HZI0
147586	229533.I1RCR5	pp	229533.I1RSQ4
147587	229533.I1RCR5	pp	229533.I1S503
147588	229533.I1RCR5	pp	229533.I1RWI5
147589	229533.I1RCR5	pp	229533.V6R3J1
147590	229533.I1RCR5	pp	229533.I1RJW6
147591	229533.I1RCR5	pp	229533.I1RSX0
147592	229533.I1RCR5	pp	229533.I1S8V3
147593	229533.I1RCR5	pp	229533.I1RQM7
147594	229533.I1RCR5	pp	229533.I1RGA4
147595	229533.I1RCR5	pp	229533.I1RVN1
147596	229533.I1RCR5	pp	229533.I1RP27
147597	229533.I1RCR5	pp	229533.V6R3X5
147598	229533.I1RCR5	pp	229533.I1RZ39
147599	229533.I1RCR5	pp	229533.I1RV91
147600	229533.I1RCR5	pp	229533.I1RJV4
147601	229533.I1RCR5	pp	229533.I1RN63
147602	229533.I1RCR5	pp	229533.I1RCS2
147603	229533.I1RCR5	pp	229533.I1RR73
147604	229533.I1RCR5	pp	229533.I1RT40
147605	229533.I1RCR5	pp	229533.I1S9Y1
147606	229533.I1RCR5	pp	229533.I1RFL3

147607 229533.I1RCR5 pp 229533.O74638
147608 229533.I1RCR5 pp 229533.I1RWK3
147609 229533.I1RCR5 pp 229533.I1RT04
147610 229533.I1RCR5 pp 229533.I1RTD5
147611 229533.I1RCR5 pp 229533.I1RTA1
147612 229533.I1RCR5 pp 229533.I1RXC2
147613 229533.I1RCR5 pp 229533.V6RUY9
147614 229533.I1RCR5 pp 229533.I1RVZ2
147615 229533.I1RCR5 pp 229533.Q4HXF0
147616 229533.I1RCR5 pp 229533.I1RJF9
147617 229533.I1RCR5 pp 229533.I1S5R1
147618 229533.I1RCR5 pp 229533.I1RRT8
147619 229533.I1RCR5 pp 229533.I1S4M5
147620 229533.I1RCR5 pp 229533.I1S8P5
147621 229533.I1RCR5 pp 229533.I1RCR8
147622 229533.I1RCR5 pp 229533.Q4I5M4
147623 229533.I1RCR5 pp 229533.I1RPP3
147624 229533.I1RCR5 pp 229533.I1RX47
147625 229533.I1RCR5 pp 229533.I1S2H4
147626 229533.I1RCR5 pp 229533.I1RRM3
147627 229533.I1RCR5 pp 229533.V6RMW6
147628 229533.I1RCR5 pp 229533.I1RMV0
147629 229533.I1RCR5 pp 229533.I1RST1
147630 229533.I1RCR5 pp 229533.I1RYI5
147631 229533.I1RCR5 pp 229533.I1S1W9
147632 229533.I1RCR5 pp 229533.I1RPP4
147633 229533.I1RCR5 pp 229533.Q4I9U7
147634 229533.I1RCR5 pp 229533.I1RSQ3
147635 229533.I1RCR5 pp 229533.V6R042
147636 229533.I1RCR5 pp 229533.V6QXG1
147637 229533.I1RCR5 pp 229533.I1S0L4
147638 229533.I1RCR5 pp 229533.I1RSU2
147639 229533.I1RCR5 pp 229533.I1RT47
147640 229533.I1RCR5 pp 229533.I1RWN5
147641 229533.I1RCR5 pp 229533.V6RIV9
147642 229533.I1RCR5 pp 229533.I1RSZ7
147643 229533.I1RCR5 pp 229533.I1RRN2
147644 229533.I1RCR5 pp 229533.I1RZV3
147645 229533.I1RCR5 pp 229533.I1S256
147646 229533.I1RCR5 pp 229533.I1RPY4
147647 229533.I1RCR5 pp 229533.I1RD22
147648 229533.I1RCR5 pp 229533.I1S524
147649 229533.I1RCR5 pp 229533.I1RPV3
147650 229533.I1RCR5 pp 229533.I1S5R6
147651 229533.I1RCR5 pp 229533.I1RN88
147652 229533.I1RCR5 pp 229533.I1S056
147653 229533.I1RCR5 pp 229533.I1S1W3
147654 229533.I1RCR5 pp 229533.I1RS90
147655 229533.I1RCR5 pp 229533.I1RND5
147656 229533.I1RCR5 pp 229533.I1S6Y8
147657 229533.I1RCR5 pp 229533.I1RSA4
147658 229533.I1RCR5 pp 229533.I1RPM4
147659 229533.I1RCR5 pp 229533.V6RIS2
147660 229533.I1RCR5 pp 229533.I1RRE5
147661 229533.I1RCR5 pp 229533.I1RG11
147662 229533.I1RCR5 pp 229533.I1RL04
147663 229533.I1RCR5 pp 229533.I1RRL0
147664 229533.I1RCR5 pp 229533.I1RFS3
147665 229533.I1RCR5 pp 229533.I1RQ21
147666 229533.I1RCR5 pp 229533.V6RY33
147667 229533.I1RCR5 pp 229533.I1RK96
147668 229533.I1RCR5 pp 229533.I1S2B8
147669 229533.I1RCR5 pp 229533.I1RE01
147670 229533.I1RCR5 pp 229533.I1RW46
147671 229533.I1RCR5 pp 229533.I1S8V4
147672 229533.I1RCR5 pp 229533.I1RTA0
147673 229533.I1RCR5 pp 229533.I1RVZ7
147674 229533.I1RCR5 pp 229533.I1RRM5
147675 229533.I1RCR5 pp 229533.I1RJZ7
147676 229533.I1RCR5 pp 229533.I1RE76
147677 229533.I1RCR5 pp 229533.I1RFQ1
147678 229533.I1RCR5 pp 229533.I1S7W5
147679 229533.I1RCR5 pp 229533.I1RS28

147680	229533.I1RCR5	pp	229533.I1S277
147681	229533.I1RCR5	pp	229533.I1S4Z8
147682	229533.I1RCR5	pp	229533.I1S0J1
147683	229533.I1RCR5	pp	229533.I1S1C7
147684	229533.I1RCR5	pp	229533.I1RK90
147685	229533.I1RCR5	pp	229533.I1RCW6
147686	229533.I1RCR5	pp	229533.I1RJG6
147687	229533.I1RCR5	pp	229533.V6RLM7
147688	229533.I1RCR5	pp	229533.I1RSD4
147689	229533.I1RCR5	pp	229533.I1RRI2
147690	229533.I1RCR5	pp	229533.I1RUG3
147691	229533.I1RCR5	pp	229533.I1RPK0
147692	229533.I1RCR5	pp	229533.I1RNC1
147693	229533.I1RCR5	pp	229533.I1RWX5
147694	229533.I1RCR5	pp	229533.I1RQP8
147695	229533.I1RCR5	pp	229533.I1RNB8
147696	229533.I1RCR5	pp	229533.I1RZW8
147697	229533.I1RCR5	pp	229533.I1RRQ4
147698	229533.I1RCR5	pp	229533.V6R057
147699	229533.I1RCR5	pp	229533.I1RS17
147700	229533.I1RCR5	pp	229533.I1S2Y5
147701	229533.I1RCR5	pp	229533.I1S8R3
147702	229533.I1RCR5	pp	229533.I1RS75
147703	229533.I1RCR5	pp	229533.Q4I624
147704	229533.I1RCR5	pp	229533.I1RTH6
147705	229533.I1RCR5	pp	229533.I1RRG3
147706	229533.I1RCR5	pp	229533.I1RD17
147707	229533.I1RCR5	pp	229533.I1RG57
147708	229533.I1RCR5	pp	229533.I1RW71
147709	229533.I1RCR5	pp	229533.I1RE89
147710	229533.I1RCR5	pp	229533.I1RQ33
147711	229533.I1RCR5	pp	229533.I1RP86
147712	229533.I1RCR5	pp	229533.I1RJV3
147713	229533.I1RCR5	pp	229533.I1RQC9
147714	229533.I1RCR5	pp	229533.I1RK92
147715	229533.I1RCR5	pp	229533.I1S4S7
147716	229533.I1RCR5	pp	229533.I1RXU9
147717	229533.I1RCR5	pp	229533.I1S0R7
147718	229533.I1RCR5	pp	229533.I1RPN4
147719	229533.I1RCR5	pp	229533.I1RN18
147720	229533.I1RCR5	pp	229533.I1RWN6
147721	229533.I1RCR5	pp	229533.I1S7M2
147722	229533.I1RCR5	pp	229533.I1RSQ7
147723	229533.I1RCR5	pp	229533.I1RFC0
147724	229533.I1RCR5	pp	229533.I1S9L5
147725	229533.I1RCR5	pp	229533.I1RFL4
147726	229533.I1RCR5	pp	229533.I1RRX5
147727	229533.I1RCR5	pp	229533.V6RUT5
147728	229533.I1RCR5	pp	229533.I1RZQ5
147729	229533.I1RCR5	pp	229533.V6QW22
147730	229533.I1RCR5	pp	229533.I1RQE7
147731	229533.I1RCR5	pp	229533.I1RLP1
147732	229533.I1RCR5	pp	229533.I1RXD7
147733	229533.I1RCR5	pp	229533.I1RT03
147734	229533.I1RCR5	pp	229533.I1RSY2
147735	229533.I1RCR5	pp	229533.I1S1C6
147736	229533.I1RCR5	pp	229533.I1S6F0
147737	229533.I1RCR5	pp	229533.I1RX46
147738	229533.I1RCR5	pp	229533.I1RVQ0
147739	229533.I1RCR5	pp	229533.I1RVX0
147740	229533.I1RCR5	pp	229533.V6RRH2
147741	229533.I1RCR5	pp	229533.I1RD52
147742	229533.I1RCR5	pp	229533.I1RXR1
147743	229533.I1RCR5	pp	229533.I1S0T2
147744	229533.I1RCR5	pp	229533.V6R1K4
147745	229533.I1RCR5	pp	229533.I1S3J4
147746	229533.I1RCR5	pp	229533.I1S0L3
147747	229533.I1RCR5	pp	229533.I1RG90
147748	229533.I1RCR5	pp	229533.I1S0X6
147749	229533.I1RCR5	pp	229533.I1RMW7
147750	229533.I1RCR5	pp	229533.I1S0B1
147751	229533.I1RCR5	pp	229533.V6RTJ8
147752	229533.I1RCR5	pp	229533.I1RW25

147753 229533.I1RCR5 pp 229533.I1RK72
147754 229533.I1RCR5 pp 229533.Q4HWB3
147755 229533.I1RCR5 pp 229533.I1RN59
147756 229533.I1RCR5 pp 229533.I1RXC4
147757 229533.I1RCR5 pp 229533.I1S4S4
147758 229533.I1RCR5 pp 229533.I1RJU3
147759 229533.I1RCR5 pp 229533.I1RSA8
147760 229533.I1RCR5 pp 229533.I1RMC0
147761 229533.I1RCR5 pp 229533.I1RWJ8
147762 229533.I1RCR5 pp 229533.I1RRX0
147763 229533.I1RCR5 pp 229533.I1RZM8
147764 229533.I1RCR5 pp 229533.I1RPY5
147765 229533.I1RCR5 pp 229533.I1RR24
147766 229533.I1RCR5 pp 229533.I1RJ17
147767 229533.I1RCR5 pp 229533.I1RXF0
147768 229533.I1RCR5 pp 229533.I1RPA4
147769 229533.I1RCR5 pp 229533.I1S7M3
147770 229533.I1RCR5 pp 229533.I1RXB6
147771 229533.I1RCR5 pp 229533.I1RI82
147772 229533.I1RCR5 pp 229533.I1RQ81
147773 229533.I1RCR5 pp 229533.I1RZG7
147774 229533.I1RCR5 pp 229533.I1RCS1
147775 229533.I1RCR5 pp 229533.Q4IRI9
147776 229533.I1RCR5 pp 229533.I1S6K9
147777 229533.I1RCR5 pp 229533.I1RX81
147778 229533.I1RCR5 pp 229533.V6RCZ8
147779 229533.I1RCR5 pp 229533.I1RS18
147780 229533.I1RCR5 pp 229533.I1RZK5
147781 229533.I1RCR5 pp 229533.I1RJV2
147782 229533.I1RCR5 pp 229533.I1RWX8
147783 229533.I1RCR5 pp 229533.I1RT35
147784 229533.I1RCR5 pp 229533.I1RR51
147785 229533.I1RCR5 pp 229533.I1S419
147786 229533.I1RCR5 pp 229533.V6RCG5
147787 229533.I1RCR5 pp 229533.I1S887
147788 229533.I1RCR5 pp 229533.I1RLU6
147789 229533.I1RCR5 pp 229533.I1RWB0
147790 229533.I1RCR5 pp 229533.I1RQA5
147791 229533.I1RCR5 pp 229533.I1RNQ5
147792 229533.I1RCR5 pp 229533.V6QUE6
147793 229533.I1RCR5 pp 229533.I1RZM1
147794 229533.I1RCR5 pp 229533.I1RRV4
147795 229533.I1RCR5 pp 229533.I1S1J7
147796 229533.I1RCR5 pp 229533.I1RPA0
147797 229533.I1RCR5 pp 229533.I1RQD8
147798 229533.I1RCR5 pp 229533.Q4IB70
147799 229533.I1RCR5 pp 229533.I1S5B0
147800 229533.I1RCR5 pp 229533.I1RZ11
147801 229533.I1RCR5 pp 229533.I1RPR9
147802 229533.I1RCR5 pp 229533.I1S504
147803 229533.I1RCR5 pp 229533.I1RS83
147804 229533.I1RCR5 pp 229533.I1RRY9
147805 229533.I1RCR5 pp 229533.Q4IJK2
147806 229533.I1RCR5 pp 229533.I1RQ85
147807 229533.I1RCR5 pp 229533.I1RY03
147808 229533.I1RCR5 pp 229533.I1RW99
147809 229533.I1RCR5 pp 229533.I1REL7
147810 229533.I1RCR5 pp 229533.I1RK95
147811 229533.I1RCR5 pp 229533.I1RFU8
147812 229533.I1RCR5 pp 229533.I1REK8
147813 229533.I1RCR5 pp 229533.I1RZZ1
147814 229533.I1RHE5 pp 229533.I1RTN1
147815 229533.I1RHE5 pp 229533.I1RZZ4
147816 229533.I1RHE5 pp 229533.I1RUT8
147817 229533.I1RHE5 pp 229533.I1RIR9
147818 229533.I1RHE5 pp 229533.V6RMM8
147819 229533.I1RHE5 pp 229533.I1RTN8
147820 229533.I1RHE5 pp 229533.V6RU70
147821 229533.I1RHE5 pp 229533.I1RS17
147822 229533.I1RHE5 pp 229533.I1RJ14
147823 229533.I1RHE5 pp 229533.I1RJU3
147824 229533.I1RHE5 pp 229533.I1RME1
147825 229533.I1RHE5 pp 229533.I1RHY7

147826 229533.I1RHE5 pp 229533.Q4HY20
147827 229533.I1RHE5 pp 229533.I1S2L3
147828 229533.I1RHE5 pp 229533.V6RIV9
147829 229533.I1RHE5 pp 229533.I1RHW8
147830 229533.I1RHE5 pp 229533.I1RKL9
147831 229533.I1RHE5 pp 229533.I1RPQ9
147832 229533.I1RHE5 pp 229533.I1RQW2
147833 229533.I1RHE5 pp 229533.I1RZX4
147834 229533.I1RHE5 pp 229533.I1SAK0
147835 229533.I1RQD1 pp 229533.I1RRN5
147836 229533.I1RQD1 pp 229533.I1RZT1
147837 229533.I1RQD1 pp 229533.I1RWT6
147838 229533.I1RQD1 pp 229533.I1S0J7
147839 229533.I1RQD1 pp 229533.I1RZ63
147840 229533.I1RQD1 pp 229533.I1RWX7
147841 229533.I1RB75 pp 229533.I1RNC0
147842 229533.I1RB75 pp 229533.I1RL24
147843 229533.I1RB75 pp 229533.I1RYH9
147844 229533.I1RB75 pp 229533.I1RD01
147845 229533.I1RB75 pp 229533.I1S224
147846 229533.I1RB75 pp 229533.I1RXH6
147847 229533.I1RB75 pp 229533.I1RST8
147848 229533.I1RB75 pp 229533.I1S9Q3
147849 229533.I1RB75 pp 229533.I1RN65
147850 229533.I1RB75 pp 229533.I1RD18
147851 229533.I1RB75 pp 229533.Q4IJM4
147852 229533.I1RB75 pp 229533.I1RV66
147853 229533.I1RB75 pp 229533.I1RFQ7
147854 229533.I1RB75 pp 229533.I1RC20
147855 229533.I1RB75 pp 229533.I1RX70
147856 229533.I1RB75 pp 229533.I1RCL7
147857 229533.I1RB75 pp 229533.I1RZZ1
147858 229533.I1RB75 pp 229533.I1RDB1
147859 229533.I1RB75 pp 229533.I1RN61
147860 229533.I1RB75 pp 229533.I1RW71
147861 229533.I1RB75 pp 229533.I1RQA3
147862 229533.I1RB75 pp 229533.I1RJH1
147863 229533.I1RB75 pp 229533.I1RWJ6
147864 229533.I1RB75 pp 229533.Q4IJK2
147865 229533.I1RB75 pp 229533.I1RBZ9
147866 229533.I1RB75 pp 229533.V6QV09
147867 229533.I1RB75 pp 229533.I1S439
147868 229533.I1RB75 pp 229533.I1RQC9
147869 229533.I1RB75 pp 229533.I1RQH3
147870 229533.I1RB75 pp 229533.I1REN3
147871 229533.I1RB75 pp 229533.I1RBY8
147872 229533.I1RB75 pp 229533.V6RTJ8
147873 229533.I1RB75 pp 229533.I1RRG3
147874 229533.I1RB75 pp 229533.I1RQ26
147875 229533.I1RB75 pp 229533.I1S0D4
147876 229533.I1RB75 pp 229533.I1RSI0
147877 229533.I1RB75 pp 229533.I1RFZ3
147878 229533.I1RB75 pp 229533.I1RYZ0
147879 229533.I1RB75 pp 229533.I1RYZ9
147880 229533.I1RB75 pp 229533.I1RFU8
147881 229533.I1RB75 pp 229533.I1RM49
147882 229533.I1RB75 pp 229533.I1RZK5
147883 229533.I1RB75 pp 229533.I1RX46
147884 229533.I1RB75 pp 229533.I1RN08
147885 229533.I1RB75 pp 229533.I1RZB5
147886 229533.I1RB75 pp 229533.I1RQ81
147887 229533.I1RB75 pp 229533.I1RPU3
147888 229533.I1RB75 pp 229533.I1RZH4
147889 229533.I1RB75 pp 229533.I1S0J7
147890 229533.I1RB75 pp 229533.I1RUQ5
147891 229533.I1RB75 pp 229533.I1RY03
147892 229533.I1RB75 pp 229533.I1RJZ7
147893 229533.I1RB75 pp 229533.I1RC22
147894 229533.I1RB75 pp 229533.I1RMQ7
147895 229533.I1RB75 pp 229533.I1S2B8
147896 229533.I1RB75 pp 229533.I1RWT6
147897 229533.I1RB75 pp 229533.I1RZ63
147898 229533.I1RB75 pp 229533.I1RRY9

147899 229533.I1RB75 pp 229533.I1RFD1
147900 229533.I1RB75 pp 229533.I1S504
147901 229533.I1RB75 pp 229533.I1RMM9
147902 229533.I1RB75 pp 229533.I1RKK0
147903 229533.I1RB75 pp 229533.I1RR73
147904 229533.I1RB75 pp 229533.I1RC95
147905 229533.I1RB75 pp 229533.I1RC17
147906 229533.I1RB75 pp 229533.I1RZM1
147907 229533.I1RB75 pp 229533.V6R3X5
147908 229533.I1RB75 pp 229533.I1RM09
147909 229533.I1RB75 pp 229533.I1RFT2
147910 229533.I1RB75 pp 229533.I1RUN8
147911 229533.I1RB75 pp 229533.I1RZ39
147912 229533.I1RB75 pp 229533.I1RP27
147913 229533.I1RB75 pp 229533.I1RCB3
147914 229533.I1RB75 pp 229533.I1RJ88
147915 229533.I1RB75 pp 229533.I1RNN1
147916 229533.I1RB75 pp 229533.I1RX38
147917 229533.I1RB75 pp 229533.I1RC24
147918 229533.I1RB75 pp 229533.I1RNP7
147919 229533.I1RB75 pp 229533.I1RUF8
147920 229533.I1RB75 pp 229533.I1RNF4
147921 229533.I1RB75 pp 229533.I1RWI5
147922 229533.I1RB75 pp 229533.I1S503
147923 229533.I1RB75 pp 229533.I1RDZ4
147924 229533.I1RB75 pp 229533.I1RLU1
147925 229533.I1RB75 pp 229533.I1RRM9
147926 229533.I1RB75 pp 229533.I1RNY9
147927 229533.I1RB75 pp 229533.I1RC97
147928 229533.I1RB75 pp 229533.I1RWL2
147929 229533.I1RB75 pp 229533.I1RP28
147930 229533.I1RB75 pp 229533.I1RPD6
147931 229533.I1RB75 pp 229533.I1RM82
147932 229533.I1RB75 pp 229533.I1RPP3
147933 229533.I1RB75 pp 229533.I1RT03
147934 229533.I1RB75 pp 229533.I1RVX0
147935 229533.I1RB75 pp 229533.I1RN67
147936 229533.I1RB75 pp 229533.I1RPW0
147937 229533.I1RB75 pp 229533.I1S2E9
147938 229533.I1RB75 pp 229533.I1RQG4
147939 229533.I1RB75 pp 229533.I1RBV5
147940 229533.I1RB75 pp 229533.I1RRN5
147941 229533.I1RB75 pp 229533.I1RFL7
147942 229533.I1RB75 pp 229533.I1RSV2
147943 229533.I1RB75 pp 229533.I1RTD5
147944 229533.I1RB75 pp 229533.I1RV91
147945 229533.I1RB75 pp 229533.I1RHQ8
147946 229533.I1RB75 pp 229533.I1RZP1
147947 229533.I1RB75 pp 229533.I1RCJ7
147948 229533.I1RB75 pp 229533.I1RZE4
147949 229533.I1RB75 pp 229533.I1RDJ8
147950 229533.I1RB75 pp 229533.I1RK75
147951 229533.I1RB75 pp 229533.I1S1W4
147952 229533.I1RB75 pp 229533.I1RY44
147953 229533.I1RB75 pp 229533.I1RD15
147954 229533.I1RB75 pp 229533.I1RCL8
147955 229533.I1RB75 pp 229533.I1RY58
147956 229533.I1RB75 pp 229533.I1S1C7
147957 229533.I1RB75 pp 229533.I1RTH6
147958 229533.I1RB75 pp 229533.I1RQF2
147959 229533.I1RB75 pp 229533.V6RVG3
147960 229533.I1RB75 pp 229533.I1S0E8
147961 229533.I1RB75 pp 229533.I1RWN5
147962 229533.I1RB75 pp 229533.I1RRP7
147963 229533.I1RB75 pp 229533.I1RQB7
147964 229533.I1RB75 pp 229533.I1RMJ8
147965 229533.I1RB75 pp 229533.I1RWN6
147966 229533.I1RB75 pp 229533.I1RZT1
147967 229533.I1RB75 pp 229533.I1RQ72
147968 229533.I1RB75 pp 229533.I1S4P6
147969 229533.I1RB75 pp 229533.V6RKY7
147970 229533.I1RB75 pp 229533.I1RRY3
147971 229533.I1RB75 pp 229533.I1RT36

147972	229533.I1RB75	pp	229533.I1RJV3
147973	229533.I1RB75	pp	229533.I1RSK1
147974	229533.I1RB75	pp	229533.I1RMQ5
147975	229533.I1RB75	pp	229533.V6RGI1
147976	229533.I1RB75	pp	229533.I1RPP4
147977	229533.I1RB75	pp	229533.I1RCM3
147978	229533.I1RB75	pp	229533.I1RXU0
147979	229533.I1RB75	pp	229533.V6RMW6
147980	229533.I1RB75	pp	229533.I1RD22
147981	229533.I1RB75	pp	229533.I1RCB7
147982	229533.I1RB75	pp	229533.I1RNY8
147983	229533.I1RB75	pp	229533.I1S0D1
147984	229533.I1RB75	pp	229533.I1S2K7
147985	229533.I1RB75	pp	229533.I1RRV1
147986	229533.I1RB75	pp	229533.I1RRW0
147987	229533.I1RB75	pp	229533.I1S0M2
147988	229533.I1RB75	pp	229533.Q4IB89
147989	229533.I1RB75	pp	229533.I1RNC1
147990	229533.I1RB75	pp	229533.I1S0N4
147991	229533.I1RB75	pp	229533.I1RQB4
147992	229533.V6R6F0	pp	229533.V6RDP1
147993	229533.I1RS18	pp	229533.I1RW99
147994	229533.I1RS18	pp	229533.V6QUE6
147995	229533.I1RS18	pp	229533.I1RZM8
147996	229533.I1RS18	pp	229533.I1RXF0
147997	229533.I1RS18	pp	229533.I1S5B0
147998	229533.I1RS18	pp	229533.I1S6K9
147999	229533.I1RS18	pp	229533.V6RCG5
148000	229533.I1RS18	pp	229533.I1SA32
148001	229533.I1RS18	pp	229533.Q4INS6
148002	229533.I1RS18	pp	229533.V6RCZ8
148003	229533.I1RS18	pp	229533.I1RZM1
148004	229533.I1RS18	pp	229533.Q4I8P5
148005	229533.I1RS18	pp	229533.I1RXC4
148006	229533.I1RS18	pp	229533.I1S419
148007	229533.I1RS18	pp	229533.Q4IEK8
148008	229533.I1RS18	pp	229533.I1RYP7
148009	229533.I1RS18	pp	229533.I1RYS0
148010	229533.I1RS18	pp	229533.I1S1J7
148011	229533.I1RS18	pp	229533.I1RSR3
148012	229533.I1RS18	pp	229533.I1RWB0
148013	229533.I1RS18	pp	229533.I1RWT6
148014	229533.I1RS18	pp	229533.V6R227
148015	229533.I1RS18	pp	229533.I1RU29
148016	229533.I1RS18	pp	229533.I1RTD9
148017	229533.I1RS18	pp	229533.I1RWR7
148018	229533.I1RS18	pp	229533.Q4I283
148019	229533.I1RS18	pp	229533.I1RXD7
148020	229533.I1RS18	pp	229533.I1RY03
148021	229533.I1RS18	pp	229533.I1RUQ2
148022	229533.I1RS18	pp	229533.I1S504
148023	229533.I1RS18	pp	229533.I1S887
148024	229533.I1RS18	pp	229533.I1RZK5
148025	229533.I1RS18	pp	229533.I1RSI0
148026	229533.I1RS18	pp	229533.V6RUT5
148027	229533.I1RS18	pp	229533.I1S4Q4
148028	229533.I1RS18	pp	229533.I1S438
148029	229533.I1RS18	pp	229533.I1S0R7
148030	229533.I1RS18	pp	229533.Q4I2J8
148031	229533.I1RS18	pp	229533.V6R4J1
148032	229533.I1RS18	pp	229533.Q4I5Z5
148033	229533.I1RS18	pp	229533.I1RVQ0
148034	229533.I1RS18	pp	229533.V6QYF0
148035	229533.I1RS18	pp	229533.I1SA33
148036	229533.I1RS18	pp	229533.I1RWD4
148037	229533.I1RS18	pp	229533.V6QUW4
148038	229533.I1RS18	pp	229533.I1S0L3
148039	229533.I1RS18	pp	229533.V6RBN2
148040	229533.I1RS18	pp	229533.V6R1K4
148041	229533.I1RS18	pp	229533.V6RGN5
148042	229533.I1RS18	pp	229533.I1RZZ1
148043	229533.I1RS18	pp	229533.I1RT33
148044	229533.I1RS18	pp	229533.Q4IJK2

148045	229533.I1RS18	pp	229533.I1S0N4
148046	229533.I1RS18	pp	229533.I1S5R6
148047	229533.I1RS18	pp	229533.I1S0T2
148048	229533.I1RS18	pp	229533.I1RX46
148049	229533.I1RS18	pp	229533.I1RUG3
148050	229533.I1RS18	pp	229533.Q4IJT5
148051	229533.I1RS18	pp	229533.I1RSQ3
148052	229533.I1RS18	pp	229533.V6RTJ8
148053	229533.I1RS18	pp	229533.V6QW22
148054	229533.I1RS18	pp	229533.I1RWJ8
148055	229533.I1RS18	pp	229533.I1RSQ7
148056	229533.I1RS18	pp	229533.V6RMW6
148057	229533.I1RS18	pp	229533.I1S4S7
148058	229533.I1RS18	pp	229533.V6R042
148059	229533.I1RS18	pp	229533.I1S0X6
148060	229533.I1RS18	pp	229533.I1RW71
148061	229533.I1RS18	pp	229533.I1S4S4
148062	229533.I1RS18	pp	229533.I1S8V4
148063	229533.I1RS18	pp	229533.I1S1W3
148064	229533.I1RS18	pp	229533.I1RVZ7
148065	229533.I1RS18	pp	229533.Q4HVW2
148066	229533.I1RS18	pp	229533.I1RZV3
148067	229533.I1RS18	pp	229533.I1RY44
148068	229533.I1RS18	pp	229533.I1RX25
148069	229533.I1RS18	pp	229533.I1RTF4
148070	229533.I1RS18	pp	229533.Q4HZB8
148071	229533.I1RS18	pp	229533.I1S0J1
148072	229533.I1RS18	pp	229533.I1RW07
148073	229533.I1RS18	pp	229533.I1S7W5
148074	229533.I1RS18	pp	229533.I1RW25
148075	229533.I1RS18	pp	229533.I1RW46
148076	229533.I1RS18	pp	229533.I1RWN5
148077	229533.I1RS18	pp	229533.I1RTA0
148078	229533.I1RS18	pp	229533.I1RSK1
148079	229533.I1RS18	pp	229533.V6RIV9
148080	229533.I1RS18	pp	229533.I1RWN6
148081	229533.I1RS18	pp	229533.I1S1C7
148082	229533.I1RS18	pp	229533.I1S1W9
148083	229533.I1RS18	pp	229533.I1RXU9
148084	229533.I1RS18	pp	229533.I1RZB2
148085	229533.I1RS18	pp	229533.I1RTE9
148086	229533.I1RS18	pp	229533.I1RTH6
148087	229533.I1RS18	pp	229533.I1RTD5
148088	229533.I1RS18	pp	229533.V6RRH2
148089	229533.I1RS18	pp	229533.I1RZW8
148090	229533.I1RS18	pp	229533.I1S8R3
148091	229533.I1RS18	pp	229533.I1RT40
148092	229533.I1RS18	pp	229533.I1RWX5
148093	229533.I1RS18	pp	229533.Q4IR18
148094	229533.I1RS18	pp	229533.I1RZ79
148095	229533.I1RS18	pp	229533.Q4I624
148096	229533.I1RS18	pp	229533.I1S437
148097	229533.I1RS18	pp	229533.I1RV91
148098	229533.I1RS18	pp	229533.I1S569
148099	229533.I1RS18	pp	229533.I1RY29
148100	229533.I1RS18	pp	229533.V6R057
148101	229533.I1RS18	pp	229533.I1S2E9
148102	229533.I1RS18	pp	229533.Q4HXF0
148103	229533.I1RS18	pp	229533.I1RYW3
148104	229533.I1RS18	pp	229533.I1RXR1
148105	229533.I1RS18	pp	229533.I1RSS4
148106	229533.I1RS18	pp	229533.I1RWK3
148107	229533.I1RS18	pp	229533.I1RWI8
148108	229533.I1RS18	pp	229533.I1RT03
148109	229533.I1RS18	pp	229533.I1RW24
148110	229533.I1RS18	pp	229533.I1RYI5
148111	229533.I1RS18	pp	229533.I1RT75
148112	229533.I1RS18	pp	229533.I1RYK0
148113	229533.I1RS18	pp	229533.I1RVX0
148114	229533.I1RS18	pp	229533.V6RUY9
148115	229533.I1RS18	pp	229533.I1S8P5
148116	229533.I1RS18	pp	229533.I1RT04
148117	229533.I1RS18	pp	229533.I1RSY2

148118	229533.I1RS18	pp	229533.I1RSX0
148119	229533.I1RS18	pp	229533.I1S0F6
148120	229533.I1RS18	pp	229533.I1S503
148121	229533.I1RS18	pp	229533.I1S352
148122	229533.I1RS18	pp	229533.V6R3J1
148123	229533.I1RS18	pp	229533.I1RVZ2
148124	229533.I1RS18	pp	229533.I1S5R1
148125	229533.I1RS18	pp	229533.I1RS47
148126	229533.I1RS18	pp	229533.O74638
148127	229533.I1RS18	pp	229533.I1RTA1
148128	229533.I1RS18	pp	229533.I1RZ39
148129	229533.I1RS18	pp	229533.I1RXC2
148130	229533.I1RS18	pp	229533.I1RYR0
148131	229533.I1RS18	pp	229533.I1S0L4
148132	229533.I1RS18	pp	229533.I1S2B8
148133	229533.I1RS18	pp	229533.I1S9Y1
148134	229533.I1RS18	pp	229533.I1S8V3
148135	229533.I1RS18	pp	229533.I1S4Z8
148136	229533.I1RS18	pp	229533.I1RSZ7
148137	229533.I1RS18	pp	229533.I1RWI5
148138	229533.I1RS18	pp	229533.I1S6Y8
148139	229533.I1RS18	pp	229533.I1RSQ4
148140	229533.I1RS18	pp	229533.I1S0M6
148141	229533.I1RS18	pp	229533.I1RZZ9
148142	229533.I1RS18	pp	229533.V6R3X5
148143	229533.I1RD61	pp	229533.I1RQP2
148144	229533.I1RD61	pp	229533.I1RUG2
148145	229533.I1RD61	pp	229533.I1RYR0
148146	229533.I1RD61	pp	229533.I1S438
148147	229533.I1RD61	pp	229533.I1RYI1
148148	229533.I1RD61	pp	229533.I1RUQ1
148149	229533.I1RD61	pp	229533.I1S437
148150	229533.I1RD61	pp	229533.Q4IBX0
148151	229533.I1RD61	pp	229533.I1RQP1
148152	229533.I1RD61	pp	229533.I1RZF5
148153	229533.I1RD61	pp	229533.I1RY73
148154	229533.I1RUA4	pp	229533.Q4IMM0
148155	229533.I1RUA4	pp	229533.Q4IBX0
148156	229533.I1RUA4	pp	229533.P0C431
148157	229533.I1RUA4	pp	229533.I1S0S6
148158	229533.I1S2L3	pp	229533.I1SAK0
148159	229533.I1S2L3	pp	229533.V6RIV9
148160	229533.I1S2L3	pp	229533.V6RMM8
148161	229533.I1S2L3	pp	229533.Q4HY20
148162	229533.I1S2L3	pp	229533.V6RU70
148163	229533.I1RM06	pp	229533.Q4HZ68
148164	229533.I1RM06	pp	229533.V6R057
148165	229533.I1RM06	pp	229533.I1S1X9
148166	229533.I1RM06	pp	229533.I1S296
148167	229533.I1RM06	pp	229533.I1RSC6
148168	229533.I1RM06	pp	229533.I1RNK2
148169	229533.I1RM06	pp	229533.I1RSS4
148170	229533.I1RM06	pp	229533.I1RVZ0
148171	229533.I1RM06	pp	229533.I1RTE9
148172	229533.I1RM06	pp	229533.I1RPJ3
148173	229533.I1RM06	pp	229533.I1S1J6
148174	229533.I1RM06	pp	229533.I1RN99
148175	229533.I1RM06	pp	229533.Q4I5Z5
148176	229533.I1RM06	pp	229533.I1S2C0
148177	229533.I1RM06	pp	229533.I1RQ28
148178	229533.I1RM06	pp	229533.I1RZE5
148179	229533.I1RM06	pp	229533.I1RSD8
148180	229533.I1RM06	pp	229533.I1S1C7
148181	229533.I1RM06	pp	229533.I1RMQ0
148182	229533.I1RM06	pp	229533.I1RNH9
148183	229533.I1RM06	pp	229533.I1S0M3
148184	229533.I1RM06	pp	229533.I1S2U2
148185	229533.I1RM06	pp	229533.I1RZ59
148186	229533.I1RM06	pp	229533.I1RMD5
148187	229533.I1RM06	pp	229533.I1RTD7
148188	229533.I1RM06	pp	229533.I1RT03
148189	229533.I1RM06	pp	229533.I1RSR8
148190	229533.I1RM06	pp	229533.I1RZK6

148191	229533.I1RM06	pp	229533.I1RY03
148192	229533.I1RM06	pp	229533.I1RVX0
148193	229533.I1RM06	pp	229533.I1RZZ1
148194	229533.I1RM06	pp	229533.I1RTD9
148195	229533.I1RM06	pp	229533.I1RQE7
148196	229533.I1RM06	pp	229533.I1RS18
148197	229533.I1RM06	pp	229533.V6RBN2
148198	229533.I1RM06	pp	229533.I1RY63
148199	229533.I1RM06	pp	229533.I1RRI2
148200	229533.I1RM06	pp	229533.I1S060
148201	229533.I1RM06	pp	229533.I1S2E8
148202	229533.I1RM06	pp	229533.I1S274
148203	229533.I1RM06	pp	229533.I1RZW8
148204	229533.I1RM06	pp	229533.I1RWN6
148205	229533.I1RM06	pp	229533.I1RW46
148206	229533.I1RM06	pp	229533.I1RZF6
148207	229533.I1RM06	pp	229533.I1RRY9
148208	229533.I1RM06	pp	229533.I1S504
148209	229533.I1RM06	pp	229533.I1RX14
148210	229533.I1RM06	pp	229533.I1S0J1
148211	229533.I1RM06	pp	229533.V6R227
148212	229533.I1RM06	pp	229533.I1RSR3
148213	229533.I1RM06	pp	229533.V6RGY3
148214	229533.I1RM06	pp	229533.V6RGN5
148215	229533.I1RM06	pp	229533.I1RRR7
148216	229533.I1RM06	pp	229533.I1RMN6
148217	229533.I1RM06	pp	229533.I1RW99
148218	229533.I1RM06	pp	229533.I1S1U7
148219	229533.I1RM06	pp	229533.I1RWN5
148220	229533.I1RM06	pp	229533.I1RQA2
148221	229533.I1RM06	pp	229533.V6QUE6
148222	229533.I1RM06	pp	229533.V6RIV9
148223	229533.I1RM06	pp	229533.Q4IBS2
148224	229533.I1RM06	pp	229533.I1RRU9
148225	229533.I1RM06	pp	229533.I1RTH6
148226	229533.I1RM06	pp	229533.I1RZB5
148227	229533.I1RM06	pp	229533.I1S4T3
148228	229533.I1RM06	pp	229533.I1RMX3
148229	229533.I1RM06	pp	229533.Q4IEK8
148230	229533.I1RM06	pp	229533.I1RPY1
148231	229533.I1RM06	pp	229533.I1RM96
148232	229533.I1RM06	pp	229533.I1RX08
148233	229533.I1RM06	pp	229533.I1S8R3
148234	229533.I1RM06	pp	229533.Q4IJ56
148235	229533.I1RM06	pp	229533.Q4HZK7
148236	229533.I1RM06	pp	229533.I1RZK5
148237	229533.I1RM06	pp	229533.V6R042
148238	229533.I1RM06	pp	229533.I1RSI6
148239	229533.I1RM06	pp	229533.I1RWJ8
148240	229533.I1RM06	pp	229533.I1S0M9
148241	229533.I1RM06	pp	229533.V6RCG5
148242	229533.I1RM06	pp	229533.I1RZV6
148243	229533.I1RM06	pp	229533.I1RS17
148244	229533.I1RM06	pp	229533.I1RPU0
148245	229533.I1RM06	pp	229533.V6RMW6
148246	229533.I1RM06	pp	229533.I1RWB5
148247	229533.I1RM06	pp	229533.I1RRT8
148248	229533.I1RM06	pp	229533.I1RNU6
148249	229533.I1RM06	pp	229533.I1RMC0
148250	229533.I1RM06	pp	229533.I1S5R1
148251	229533.I1RM06	pp	229533.Q4IAA0
148252	229533.I1RM06	pp	229533.O74638
148253	229533.I1RM06	pp	229533.I1RUG3
148254	229533.I1RM06	pp	229533.Q4IFI0
148255	229533.I1RM06	pp	229533.I1S2B8
148256	229533.I1RM06	pp	229533.I1RZM8
148257	229533.I1RM06	pp	229533.I1RQ31
148258	229533.I1RM06	pp	229533.I1RRM5
148259	229533.I1RM06	pp	229533.I1RM67
148260	229533.I1RM06	pp	229533.I1RNY2
148261	229533.I1RM06	pp	229533.I1RZ39
148262	229533.I1RM06	pp	229533.Q4IN63
148263	229533.I1RM06	pp	229533.I1RX24

148264	229533.I1RM06	pp	229533.I1RTF4
148265	229533.I1RM06	pp	229533.I1RW25
148266	229533.I1RM06	pp	229533.Q4I327
148267	229533.I1RM06	pp	229533.Q4IQK7
148268	229533.I1RM06	pp	229533.I1RNV6
148269	229533.I1RM06	pp	229533.I1RMN7
148270	229533.I1RM06	pp	229533.Q4IJH1
148271	229533.I1RM06	pp	229533.V6QYF0
148272	229533.I1RM06	pp	229533.I1RNA0
148273	229533.I1RM06	pp	229533.I1RNH6
148274	229533.I1RM06	pp	229533.I1RRQ6
148275	229533.I1RM06	pp	229533.I1RT80
148276	229533.I1RM06	pp	229533.I1RWD4
148277	229533.I1RM06	pp	229533.I1S8E2
148278	229533.I1RM06	pp	229533.I1S021
148279	229533.I1RM06	pp	229533.I1S253
148280	229533.I1RM06	pp	229533.I1RZS6
148281	229533.I1RM06	pp	229533.I1RS30
148282	229533.I1RM06	pp	229533.I1RRP3
148283	229533.I1RM06	pp	229533.I1RW61
148284	229533.I1RM06	pp	229533.I1RYT5
148285	229533.I1RM06	pp	229533.I1RRQ3
148286	229533.I1RM06	pp	229533.I1RMK3
148287	229533.I1RM06	pp	229533.I1S6F0
148288	229533.I1RM06	pp	229533.I1RPM4
148289	229533.I1RM06	pp	229533.Q4HVW2
148290	229533.I1RM06	pp	229533.I1RNH1
148291	229533.I1RM06	pp	229533.I1RR73
148292	229533.I1RM06	pp	229533.V6R3X5
148293	229533.I1RM06	pp	229533.Q4I662
148294	229533.I1RM06	pp	229533.I1RVQ0
148295	229533.I1RM06	pp	229533.Q4I830
148296	229533.I1RM06	pp	229533.I1RND6
148297	229533.I1RM06	pp	229533.I1RR24
148298	229533.I1RM06	pp	229533.I1S9U2
148299	229533.I1RM06	pp	229533.I1RPW9
148300	229533.I1RM06	pp	229533.I1RW71
148301	229533.I1RM06	pp	229533.I1RZZ9
148302	229533.I1RM06	pp	229533.I1RSX1
148303	229533.I1RM06	pp	229533.I1S9Y0
148304	229533.I1RM06	pp	229533.Q4IF76
148305	229533.I1RM06	pp	229533.I1S503
148306	229533.I1RM06	pp	229533.I1RT05
148307	229533.I1RM06	pp	229533.I1RQJ5
148308	229533.I1RM06	pp	229533.I1RPP3
148309	229533.I1RM06	pp	229533.I1RQM6
148310	229533.I1RM06	pp	229533.I1RZ99
148311	229533.I1RM06	pp	229533.I1RRT2
148312	229533.I1RM06	pp	229533.I1RS47
148313	229533.I1RM06	pp	229533.I1RX46
148314	229533.I1RM06	pp	229533.I1RT33
148315	229533.I1RM06	pp	229533.V6QW22
148316	229533.I1RM06	pp	229533.I1RQS5
148317	229533.I1RM06	pp	229533.I1RSQ7
148318	229533.I1RM06	pp	229533.I1RRJ8
148319	229533.I1RM06	pp	229533.I1S0R7
148320	229533.I1RM06	pp	229533.Q4HZ42
148321	229533.I1RM06	pp	229533.Q4IPI1
148322	229533.I1RM06	pp	229533.V6R1K4
148323	229533.I1RM06	pp	229533.I1RN63
148324	229533.I1RM06	pp	229533.Q4IEV5
148325	229533.I1RM06	pp	229533.I1RT04
148326	229533.I1RM06	pp	229533.V6R283
148327	229533.I1RM06	pp	229533.I1RQ65
148328	229533.I1RM06	pp	229533.I1RWH5
148329	229533.I1RM06	pp	229533.I1RUQ2
148330	229533.I1RM06	pp	229533.Q4INZ9
148331	229533.I1RM06	pp	229533.I1RXD7
148332	229533.I1RM06	pp	229533.Q4IAS1
148333	229533.I1RM06	pp	229533.I1S0L3
148334	229533.I1RM06	pp	229533.Q4IQW0
148335	229533.I1RM06	pp	229533.I1RTD5
148336	229533.I1RM06	pp	229533.I1RZJ6

148337 229533.I1RKT7 pp 229533.I1RNJ7
148338 229533.I1RKT7 pp 229533.I1RUG0
148339 229533.I1RB27 pp 229533.I1RK95
148340 229533.I1RB27 pp 229533.I1S2U2
148341 229533.I1RB27 pp 229533.Q4I061
148342 229533.I1RB27 pp 229533.I1RRY9
148343 229533.I1RB27 pp 229533.I1RN99
148344 229533.I1RB27 pp 229533.I1RVZ0
148345 229533.I1RB27 pp 229533.I1RMQ0
148346 229533.I1RB27 pp 229533.Q4I948
148347 229533.I1RB27 pp 229533.I1RX14
148348 229533.I1RB27 pp 229533.I1RNK2
148349 229533.I1RB27 pp 229533.I1RZZ1
148350 229533.I1RB27 pp 229533.I1RJZ6
148351 229533.I1RB27 pp 229533.I1RSI0
148352 229533.I1RB27 pp 229533.I1RFJ9
148353 229533.I1RB27 pp 229533.I1RQ81
148354 229533.I1RB27 pp 229533.I1RK72
148355 229533.I1RB27 pp 229533.I1RZK6
148356 229533.I1RB27 pp 229533.I1RE00
148357 229533.I1RB27 pp 229533.Q4IJ56
148358 229533.I1RB27 pp 229533.I1S0M9
148359 229533.I1RB27 pp 229533.I1RZE5
148360 229533.I1RB27 pp 229533.I1RF08
148361 229533.I1RB27 pp 229533.I1RY63
148362 229533.I1RB27 pp 229533.I1RG77
148363 229533.I1RB27 pp 229533.Q4IAA0
148364 229533.I1RB27 pp 229533.I1RKQ9
148365 229533.I1RB27 pp 229533.I1RY03
148366 229533.I1RB27 pp 229533.I1RDG0
148367 229533.I1RB27 pp 229533.I1RQ28
148368 229533.I1RB27 pp 229533.I1RG56
148369 229533.I1RB27 pp 229533.I1RSR8
148370 229533.I1RB27 pp 229533.I1RS19
148371 229533.I1RB27 pp 229533.I1RFU8
148372 229533.I1RB27 pp 229533.Q4IEK8
148373 229533.I1RB27 pp 229533.I1RNQ5
148374 229533.I1RB27 pp 229533.I1RG51
148375 229533.I1RB27 pp 229533.I1RK83
148376 229533.I1RB27 pp 229533.I1RBD2
148377 229533.I1RB27 pp 229533.Q4IQK7
148378 229533.I1RB27 pp 229533.V6QYF0
148379 229533.I1RB27 pp 229533.I1RZK5
148380 229533.I1RB27 pp 229533.I1S504
148381 229533.I1RB27 pp 229533.V6R227
148382 229533.I1RB27 pp 229533.I1RZF6
148383 229533.I1RB27 pp 229533.I1RQA2
148384 229533.I1RB27 pp 229533.I1RRE0
148385 229533.I1RB27 pp 229533.I1RM06
148386 229533.I1RB27 pp 229533.I1RNA0
148387 229533.I1RB27 pp 229533.I1RN58
148388 229533.I1RB27 pp 229533.I1RBL7
148389 229533.I1RB27 pp 229533.I1RL23
148390 229533.I1RB27 pp 229533.Q4IFI0
148391 229533.I1RB27 pp 229533.I1RSR3
148392 229533.I1RB27 pp 229533.I1RQ31
148393 229533.I1RB27 pp 229533.I1RMC0
148394 229533.I1RB27 pp 229533.I1RW25
148395 229533.I1RB27 pp 229533.I1RYB9
148396 229533.I1RB27 pp 229533.I1S9U2
148397 229533.I1RB27 pp 229533.I1RQM6
148398 229533.I1RB27 pp 229533.I1RT05
148399 229533.I1RB27 pp 229533.I1RPW9
148400 229533.I1RB27 pp 229533.I1RWH5
148401 229533.I1RB27 pp 229533.I1RNH6
148402 229533.I1RB27 pp 229533.I1RMN7
148403 229533.I1RB27 pp 229533.I1RFW5
148404 229533.I1RB27 pp 229533.I1RNV6
148405 229533.I1RB27 pp 229533.I1RR00
148406 229533.I1RB27 pp 229533.I1RT80
148407 229533.I1RB27 pp 229533.I1S021
148408 229533.I1RB27 pp 229533.I1RTF4
148409 229533.I1RB27 pp 229533.I1RW71

229533.I1RB27	pp	229533.I1RGC2
229533.I1RB27	pp	229533.V6QUE6
229533.I1RB27	pp	229533.Q4IJH1
229533.I1RB27	pp	229533.I1RMK3
229533.I1RB27	pp	229533.I1RSQ7
229533.I1RB27	pp	229533.Q4IBS2
229533.I1RB27	pp	229533.Q4HVW2
229533.I1RB27	pp	229533.I1S044
229533.I1RB27	pp	229533.I1RQJ5
229533.I1RB27	pp	229533.I1RNH1
229533.I1RB27	pp	229533.I1RZ99
229533.I1RB27	pp	229533.I1RDA0
229533.I1RB27	pp	229533.I1RC32
229533.I1RB27	pp	229533.I1REX4
229533.I1RB27	pp	229533.I1S6U8
229533.I1RB27	pp	229533.I1S6F0
229533.I1RB27	pp	229533.I1RBN1
229533.I1RB27	pp	229533.I1RSD8
229533.I1RB27	pp	229533.I1RPU0
229533.I1RB27	pp	229533.I1RD56
229533.I1RB27	pp	229533.I1RT33
229533.I1RB27	pp	229533.I1S2E8
229533.I1RB27	pp	229533.I1RSS4
229533.I1RB27	pp	229533.I1RRH5
229533.I1RB27	pp	229533.I1RM67
229533.I1RB27	pp	229533.I1RBZ3
229533.I1RB27	pp	229533.I1S1X9
229533.I1RB27	pp	229533.Q4INZ9
229533.I1RB27	pp	229533.I1RCL3
229533.I1RB27	pp	229533.I1S274
229533.I1RB27	pp	229533.I1RT03
229533.I1RB27	pp	229533.I1RZJ6
229533.I1RB27	pp	229533.V6RGY3
229533.I1RB27	pp	229533.I1RT98
229533.I1RB27	pp	229533.I1RVX0
229533.I1RB27	pp	229533.I1RKC6
229533.I1RB27	pp	229533.I1S1U7
229533.I1RB27	pp	229533.I1S1J6
229533.I1RB27	pp	229533.I1S0R7
229533.I1RB27	pp	229533.I1S060
229533.I1RB27	pp	229533.I1RWN6
229533.I1RB27	pp	229533.I1RXD7
229533.I1RB27	pp	229533.V6R057
229533.I1RB27	pp	229533.I1RKQ5
229533.I1RB27	pp	229533.Q4HZ68
229533.I1RB27	pp	229533.I1S0J1
229533.I1RB27	pp	229533.I1S296
229533.I1RB27	pp	229533.Q4I5R9
229533.I1RB27	pp	229533.I1RC72
229533.I1RB27	pp	229533.I1S0R2
229533.I1RB27	pp	229533.I1S1C7
229533.I1RB27	pp	229533.I1RWB5
229533.I1RB27	pp	229533.I1RNB8
229533.I1RB27	pp	229533.Q4IPI1
229533.I1RB27	pp	229533.I1RX46
229533.I1RB27	pp	229533.I1RNE4
229533.I1RB27	pp	229533.I1RTD7
229533.I1RB27	pp	229533.I1RC27
229533.I1RB27	pp	229533.I1RNU6
229533.I1RB27	pp	229533.I1RRI2
229533.I1RB27	pp	229533.I1RM25
229533.I1RB27	pp	229533.I1RG11
229533.I1RB27	pp	229533.I1S4T3
229533.I1RB27	pp	229533.V6RGN5
229533.I1RB27	pp	229533.I1RPY1
229533.I1RB27	pp	229533.I1RTH6
229533.I1RB27	pp	229533.I1RWN5
229533.I1RB27	pp	229533.I1RM96
229533.I1RB27	pp	229533.I1RYT5
229533.I1RB27	pp	229533.I1RX64
229533.I1RB27	pp	229533.I1RP31
229533.I1RB27	pp	229533.I1RRR7
229533.I1RB27	pp	229533.I1RW61

148483 229533.I1RB27 pp 229533.I1RTE9
148484 229533.I1RB27 pp 229533.I1S2C0
148485 229533.I1RB27 pp 229533.I1RPJ3
148486 229533.I1RB27 pp 229533.Q4I662
148487 229533.I1RB27 pp 229533.I1RFS3
148488 229533.I1RB27 pp 229533.Q4IN63
148489 229533.I1RB27 pp 229533.I1RWJ4
148490 229533.I1RB27 pp 229533.I1S558
148491 229533.I1RB27 pp 229533.I1RKF9
148492 229533.I1RB27 pp 229533.I1RCR8
148493 229533.I1RB27 pp 229533.I1RC81
148494 229533.I1RB27 pp 229533.I1RCN5
148495 229533.I1RB27 pp 229533.I1RBG9
148496 229533.I1RB27 pp 229533.I1RNY2
148497 229533.I1RB27 pp 229533.I1RJV3
148498 229533.I1RB27 pp 229533.I1RC29
148499 229533.I1RB27 pp 229533.I1S9Y1
148500 229533.I1RB27 pp 229533.I1RX24
148501 229533.I1RB27 pp 229533.I1RSI6
148502 229533.I1RB27 pp 229533.Q4I327
148503 229533.I1RB27 pp 229533.I1RZS6
148504 229533.I1RB27 pp 229533.I1RR72
148505 229533.I1RB27 pp 229533.O74638
148506 229533.I1RB27 pp 229533.I1RZV6
148507 229533.I1RB27 pp 229533.I1S2B8
148508 229533.I1RB27 pp 229533.V6RMW6
148509 229533.I1RB27 pp 229533.I1RRT8
148510 229533.I1RB27 pp 229533.V6R042
148511 229533.I1RB27 pp 229533.I1RJZ7
148512 229533.I1RB27 pp 229533.I1S2F2
148513 229533.I1RB27 pp 229533.I1RMN6
148514 229533.I1RB27 pp 229533.I1RQK4
148515 229533.I1RB27 pp 229533.I1RD64
148516 229533.I1RB27 pp 229533.Q4IR18
148517 229533.I1RB27 pp 229533.I1RYG3
148518 229533.I1RB27 pp 229533.I1RGF6
148519 229533.I1RB27 pp 229533.I1RC03
148520 229533.I1RB27 pp 229533.I1RN63
148521 229533.I1RB27 pp 229533.I1RT75
148522 229533.I1RB27 pp 229533.V6R283
148523 229533.I1RB27 pp 229533.Q4IF76
148524 229533.I1RB27 pp 229533.I1RT04
148525 229533.I1RB27 pp 229533.I1RU69
148526 229533.I1RB27 pp 229533.Q4HXF0
148527 229533.I1RB27 pp 229533.I1RKL3
148528 229533.I1RB27 pp 229533.I1RHP9
148529 229533.I1RB27 pp 229533.I1S8E2
148530 229533.I1RB27 pp 229533.Q4I830
148531 229533.I1RB27 pp 229533.I1RSX1
148532 229533.I1RB27 pp 229533.Q4IJ11
148533 229533.I1RB27 pp 229533.I1RRJ8
148534 229533.I1RB27 pp 229533.I1RZ39
148535 229533.I1RB27 pp 229533.I1RQG4
148536 229533.I1RB27 pp 229533.V6R3X5
148537 229533.I1RB27 pp 229533.Q4IAS1
148538 229533.I1RB27 pp 229533.I1S503
148539 229533.I1RB27 pp 229533.I1RND6
148540 229533.I1RB27 pp 229533.I1RPP3
148541 229533.I1RB27 pp 229533.I1RR73
148542 229533.I1RB27 pp 229533.I1RWF7
148543 229533.I1RB27 pp 229533.I1RLV9
148544 229533.I1RB27 pp 229533.I1RVA7
148545 229533.I1RB27 pp 229533.I1RW26
148546 229533.I1RB27 pp 229533.I1RJL4
148547 229533.I1RB27 pp 229533.I1RRP3
148548 229533.I1RB27 pp 229533.I1S253
148549 229533.I1RB27 pp 229533.I1RKJ6
148550 229533.I1RB27 pp 229533.I1RFG0
148551 229533.I1RB27 pp 229533.I1RE87
148552 229533.I1RB27 pp 229533.I1RQS5
148553 229533.I1RB27 pp 229533.I1RRQ3
148554 229533.I1RB27 pp 229533.I1RRT2
148555 229533.I1RB27 pp 229533.I1RGA4

229533.I1RB27 pp 229533.Q4HZ42
229533.I1RB27 pp 229533.I1RFN1
229533.I1RB27 pp 229533.I1RC82
229533.I1RB27 pp 229533.I1RS47
229533.I1RB27 pp 229533.Q4IQW0
229533.I1RB27 pp 229533.I1RQ65
229533.I1RB27 pp 229533.I1RBC7
229533.I1RB27 pp 229533.I1RTD5
229533.I1S0M9 pp 229533.V6R227
229533.I1S0M9 pp 229533.V6R042
229533.I1S0M9 pp 229533.V6RGN5
229533.I1S0M9 pp 229533.V6RMW6
229533.I1S0M9 pp 229533.I1S504
229533.I1S0M9 pp 229533.Q4HWE2
229533.I1S0M9 pp 229533.I1S503
229533.I1S0M9 pp 229533.Q4I327
229533.I1S0M9 pp 229533.I1S1X9
229533.I1S0M9 pp 229533.Q4IN63
229533.I1S0M9 pp 229533.Q4IJ56
229533.I1S0M9 pp 229533.Q4I5R9
229533.I1S0M9 pp 229533.Q4IPI1
229533.I1S0M9 pp 229533.V6RGY3
229533.I1S0M9 pp 229533.I1S2E8
229533.I1S0M9 pp 229533.I1S0Z9
229533.I1S0M9 pp 229533.Q4IEV5
229533.I1S0M9 pp 229533.I1S1C7
229533.I1S0M9 pp 229533.I1S2C0
229533.I1S0M9 pp 229533.I1S0R7
229533.I1S0M9 pp 229533.I1S2E9
229533.I1S0M9 pp 229533.I1S2B8
229533.I1S0M9 pp 229533.I1S9U2
229533.I1S0M9 pp 229533.Q4IFI0
229533.I1S0M9 pp 229533.Q4IQW0
229533.I1S0M9 pp 229533.Q4INZ9
229533.I1S0M9 pp 229533.Q4I662
229533.I1S0M9 pp 229533.V6R3X5
229533.I1S0M9 pp 229533.I1S8E2
229533.I1S0M9 pp 229533.Q4HVW2
229533.A0A1C3YK66 pp 229533.I1RWX7
229533.A0A1C3YK66 pp 229533.I1RZ63
229533.A0A1C3YK66 pp 229533.I1RWT6
229533.A0A1C3YK66 pp 229533.A0A1C3YM91
229533.A0A1C3YK66 pp 229533.I1RC22
229533.A0A1C3YK66 pp 229533.I1RYE4
229533.A0A1C3YK66 pp 229533.I1S0J7
229533.A0A1C3YK66 pp 229533.I1RZT1
229533.A0A1C3YK66 pp 229533.I1RRN5
229533.A0A1C3YK66 pp 229533.I1RJK7
229533.A0A1C3YK66 pp 229533.I1RQA3
229533.A0A1C3YK66 pp 229533.A0A1C3YM29
229533.I1RMU4 pp 229533.Q4IMM0
229533.I1RMU4 pp 229533.Q4IBX0
229533.I1RLD7 pp 229533.I1RNM7
229533.I1RLD7 pp 229533.I1RWM4
229533.I1RLD7 pp 229533.I1RWY4
229533.I1RZE5 pp 229533.I1S296
229533.I1RZE5 pp 229533.I1RZK6
229533.I1RZE5 pp 229533.I1RZK5
229533.I1RZE5 pp 229533.I1S2C0
229533.I1RZE5 pp 229533.I1S1X9
229533.I1RZE5 pp 229533.I1RZZ1
229533.I1RZE5 pp 229533.I1S021
229533.I1RZE5 pp 229533.I1S9U2
229533.I1RZE5 pp 229533.I1S2A8
229533.I1RZE5 pp 229533.I1S1C7
229533.I1RZE5 pp 229533.Q4IJK2
229533.I1RZE5 pp 229533.I1S504
229533.I1RZE5 pp 229533.Q4I327
229533.I1RZE5 pp 229533.Q4IEK8
229533.I1RZE5 pp 229533.I1S0M9
229533.I1RZE5 pp 229533.I1RZF6
229533.I1RZE5 pp 229533.I1RZM1
229533.I1RZE5 pp 229533.I1S6U8

229533.I1RZE5	pp	229533.Q4IJ56
229533.I1RZE5	pp	229533.V6R3X5
229533.I1RZE5	pp	229533.Q4HVW2
229533.I1RZE5	pp	229533.Q4IN63
229533.I1RZE5	pp	229533.Q4IFI0
229533.I1RZE5	pp	229533.Q4IJH1
229533.I1RZE5	pp	229533.V6RGN5
229533.I1RZE5	pp	229533.I1S2B8
229533.I1RZE5	pp	229533.I1S0J1
229533.I1RZE5	pp	229533.I1S8E2
229533.I1RZE5	pp	229533.Q4IF76
229533.I1RZE5	pp	229533.V6QYF0
229533.I1RZE5	pp	229533.I1RZZ9
229533.I1RZE5	pp	229533.I1RZS6
229533.I1RZE5	pp	229533.V6RMW6
229533.I1RZE5	pp	229533.Q4I662
229533.I1RZE5	pp	229533.V6R283
229533.I1RZE5	pp	229533.V6R042
229533.I1RZE5	pp	229533.V6QUW4
229533.I1RZE5	pp	229533.Q4HZ42
229533.I1RZE5	pp	229533.V6RTJ8
229533.I1RZE5	pp	229533.I1S540
229533.I1RZE5	pp	229533.Q4IAS1
229533.I1RZE5	pp	229533.Q4IQW0
229533.I1RZE5	pp	229533.Q4IPI1
229533.I1RZE5	pp	229533.I1S503
229533.I1RZE5	pp	229533.I1S2E9
229533.I1RZE5	pp	229533.I1S0R7
229533.I1RNY9	pp	229533.V6RKY7
229533.I1RNY9	pp	229533.I1S1W4
229533.I1RNY9	pp	229533.V6RVG3
229533.I1RNY9	pp	229533.I1RUN8
229533.I1RNY9	pp	229533.I1S439
229533.I1RNY9	pp	229533.I1RYH9
229533.I1RNY9	pp	229533.I1RRY3
229533.I1RNY9	pp	229533.I1RRN5
229533.I1RNY9	pp	229533.I1RZE4
229533.I1RNY9	pp	229533.I1RX70
229533.I1RNY9	pp	229533.I1RUQ5
229533.A0A098DI90	pp	229533.I1RHS9
229533.A0A098DI90	pp	229533.I1RHY7
229533.A0A098DI90	pp	229533.I1RHE5
229533.A0A098DI90	pp	229533.I1RJU3
229533.A0A098DI90	pp	229533.V6RIV9
229533.A0A098DI90	pp	229533.I1S2L3
229533.A0A098DI90	pp	229533.I1RKT7
229533.A0A098DI90	pp	229533.A0A1C3YKV3
229533.A0A098DI90	pp	229533.I1RS17
229533.A0A098DI90	pp	229533.I1REK8
229533.A0A098DI90	pp	229533.I1RCH5
229533.I1RTD1	pp	229533.V6RR76
229533.I1RTD1	pp	229533.I1RZ91
229533.I1RLM7	pp	229533.I1RW25
229533.I1RLM7	pp	229533.I1RSQ7
229533.I1RLM7	pp	229533.I1RR24
229533.I1RLM7	pp	229533.V6QW22
229533.I1RLM7	pp	229533.I1RX46
229533.I1RLM7	pp	229533.I1RPM4
229533.I1RLM7	pp	229533.I1RVQ0
229533.I1RLM7	pp	229533.V6RTJ8
229533.I1RLM7	pp	229533.I1RR39
229533.I1RLM7	pp	229533.Q4IJK2
229533.I1RLM7	pp	229533.I1RRM5
229533.I1RLM7	pp	229533.I1S2B8
229533.I1RLM7	pp	229533.I1RY03
229533.I1RLM7	pp	229533.I1RZK5
229533.I1RLM7	pp	229533.I1RZZ9
229533.I1RLM7	pp	229533.I1S0L3
229533.I1RLM7	pp	229533.I1RZ39
229533.I1RLM7	pp	229533.I1RRY9
229533.I1RLM7	pp	229533.I1RQC9
229533.I1RLM7	pp	229533.I1RLU6
229533.I1RLM7	pp	229533.I1RS18

148702 229533.I1RLM7 pp 229533.I1S8P5
148703 229533.I1RLM7 pp 229533.I1RPP3
148704 229533.I1RLM7 pp 229533.V6R3X5
148705 229533.I1RLM7 pp 229533.I1RRT8
148706 229533.I1RLM7 pp 229533.I1RR73
148707 229533.I1RLM7 pp 229533.I1RZM1
148708 229533.I1RLM7 pp 229533.I1S504
148709 229533.I1RLM7 pp 229533.I1RN67
148710 229533.I1RLM7 pp 229533.I1S2H4
148711 229533.I1RLM7 pp 229533.I1RTD5
148712 229533.I1RLM7 pp 229533.I1RV91
148713 229533.I1RLM7 pp 229533.I1RT04
148714 229533.I1RLM7 pp 229533.I1S0L4
148715 229533.I1RLM7 pp 229533.V6RCG5
148716 229533.I1RLM7 pp 229533.I1S2E9
148717 229533.I1RLM7 pp 229533.I1RPP4
148718 229533.I1RLM7 pp 229533.I1RVX0
148719 229533.I1RLM7 pp 229533.V6RIV9
148720 229533.I1RLM7 pp 229533.I1RWK3
148721 229533.I1RLM7 pp 229533.I1RWN5
148722 229533.I1RLM7 pp 229533.I1RT03
148723 229533.I1RLM7 pp 229533.I1S503
148724 229533.I1RLM7 pp 229533.I1RN18
148725 229533.I1RLM7 pp 229533.Q4I1Y5
148726 229533.I1RLM7 pp 229533.I1S1C7
148727 229533.I1RLM7 pp 229533.I1RUG3
148728 229533.I1RLM7 pp 229533.I1RY44
148729 229533.I1RLM7 pp 229533.I1S8R3
148730 229533.I1RLM7 pp 229533.I1RZW8
148731 229533.I1RLM7 pp 229533.I1RNC1
148732 229533.I1RLM7 pp 229533.I1RTH6
148733 229533.I1RLM7 pp 229533.V6RMW6
148734 229533.I1RLM7 pp 229533.I1RSQ3
148735 229533.I1RLM7 pp 229533.I1RWN6
148736 229533.I1RLM7 pp 229533.V6R042
148737 229533.I1RLM7 pp 229533.I1RQ75
148738 229533.I1RLM7 pp 229533.I1RTA0
148739 229533.I1RLD4 pp 229533.I1RNM7
148740 229533.I1RLD4 pp 229533.I1RWM4
148741 229533.I1RHQ8 pp 229533.Q4IEA7
148742 229533.I1RHQ8 pp 229533.I1RSS1
148743 229533.I1RHQ8 pp 229533.I1RMM9
148744 229533.I1RHQ8 pp 229533.I1RKK0
148745 229533.I1RHQ8 pp 229533.I1S1W4
148746 229533.I1RHQ8 pp 229533.I1S278
148747 229533.I1RHQ8 pp 229533.V6RVG3
148748 229533.I1RHQ8 pp 229533.I1RUQ5
148749 229533.I1RHQ8 pp 229533.I1RWT6
148750 229533.I1RHQ8 pp 229533.I1RYL5
148751 229533.I1RHQ8 pp 229533.I1S439
148752 229533.I1RHQ8 pp 229533.I1RMB2
148753 229533.I1RHQ8 pp 229533.V6RKY7
148754 229533.I1RHQ8 pp 229533.I1RX77
148755 229533.I1RHQ8 pp 229533.I1RNY8
148756 229533.I1RHQ8 pp 229533.I1RQL4
148757 229533.I1RHQ8 pp 229533.I1RQA3
148758 229533.I1RHQ8 pp 229533.I1S286
148759 229533.I1RHQ8 pp 229533.I1RX64
148760 229533.I1RHQ8 pp 229533.I1RX87
148761 229533.I1RHQ8 pp 229533.I1RX04
148762 229533.I1RHQ8 pp 229533.I1RMA7
148763 229533.I1RHQ8 pp 229533.I1RPA6
148764 229533.I1RHQ8 pp 229533.I1RRN5
148765 229533.I1RHQ8 pp 229533.I1RZC3
148766 229533.I1RHQ8 pp 229533.I1RV66
148767 229533.I1RHQ8 pp 229533.I1RQB4
148768 229533.I1RHQ8 pp 229533.I1S247
148769 229533.I1RHQ8 pp 229533.I1RZE4
148770 229533.I1RHQ8 pp 229533.I1RS47
148771 229533.I1RHQ8 pp 229533.I1RY32
148772 229533.I1RHQ8 pp 229533.I1RQD6
148773 229533.I1RHQ8 pp 229533.I1S248
148774 229533.I1RHQ8 pp 229533.I1S7X9

148775	229533.I1RHQ8	pp	229533.I1RUN8
148776	229533.I1RHQ8	pp	229533.I1S0X7
148777	229533.V6QUW4	pp	229533.V6RMW6
148778	229533.V6QUW4	pp	229533.V6R3X5
148779	229533.V6QUW4	pp	229533.V6R042
148780	229533.V6QUW4	pp	229533.V6RCG5
148781	229533.V6QUW4	pp	229533.V6R283
148782	229533.V6QUW4	pp	229533.V6RIV9
148783	229533.V6QUW4	pp	229533.V6RTJ8
148784	229533.V6QUW4	pp	229533.V6QW22
148785	229533.A0A098DI57	pp	229533.A0A0E0RU85
148786	229533.A0A098DI57	pp	229533.A0A1C3YJP4
148787	229533.A0A098DI57	pp	229533.I1RFI4
148788	229533.A0A098DI57	pp	229533.I1S147
148789	229533.A0A1C3YLZ7	pp	229533.I1RPM4
148790	229533.A0A1C3YLZ7	pp	229533.I1R9M0
148791	229533.A0A1C3YLZ7	pp	229533.I1RRM5
148792	229533.A0A1C3YLZ7	pp	229533.I1RBY4
148793	229533.A0A1C3YLZ7	pp	229533.I1S2B8
148794	229533.A0A1C3YLZ7	pp	229533.I1S1C7
148795	229533.A0A1C3YLZ7	pp	229533.I1RZ39
148796	229533.A0A1C3YLZ7	pp	229533.I1RR73
148797	229533.A0A1C3YLZ7	pp	229533.I1RZZ9
148798	229533.A0A1C3YLZ7	pp	229533.V6R3X5
148799	229533.A0A1C3YLZ7	pp	229533.I1RZW8
148800	229533.A0A1C3YLZ7	pp	229533.I1RWN6
148801	229533.A0A1C3YLZ7	pp	229533.I1S2H4
148802	229533.A0A1C3YLZ7	pp	229533.I1S0L4
148803	229533.A0A1C3YLZ7	pp	229533.I1RTD5
148804	229533.A0A1C3YLZ7	pp	229533.I1RJV3
148805	229533.A0A1C3YLZ7	pp	229533.I1RAW0
148806	229533.A0A1C3YLZ7	pp	229533.I1RRT8
148807	229533.A0A1C3YLZ7	pp	229533.I1RBS6
148808	229533.A0A1C3YLZ7	pp	229533.I1RJW6
148809	229533.A0A1C3YLZ7	pp	229533.I1RJV4
148810	229533.A0A1C3YLZ7	pp	229533.I1RPP4
148811	229533.A0A1C3YLZ7	pp	229533.I1RTA0
148812	229533.A0A1C3YLZ7	pp	229533.I1RK44
148813	229533.A0A1C3YLZ7	pp	229533.I1S8P5
148814	229533.A0A1C3YLZ7	pp	229533.I1S503
148815	229533.A0A1C3YLZ7	pp	229533.I1RPP3
148816	229533.A0A1C3YLZ7	pp	229533.V6RIV9
148817	229533.A0A1C3YLZ7	pp	229533.I1RVX0
148818	229533.A0A1C3YLZ7	pp	229533.I1RWK3
148819	229533.A0A1C3YLZ7	pp	229533.I1RWN5
148820	229533.A0A1C3YLZ7	pp	229533.I1RN67
148821	229533.A0A1C3YLZ7	pp	229533.I1RT03
148822	229533.A0A1C3YLZ7	pp	229533.I1RB92
148823	229533.A0A1C3YLZ7	pp	229533.I1RFL4
148824	229533.A0A1C3YLZ7	pp	229533.I1RT04
148825	229533.A0A1C3YLZ7	pp	229533.I1RY44
148826	229533.A0A1C3YLZ7	pp	229533.V6R042
148827	229533.A0A1C3YLZ7	pp	229533.I1RN18
148828	229533.A0A1C3YLZ7	pp	229533.I1RD17
148829	229533.A0A1C3YLZ7	pp	229533.I1S2E9
148830	229533.A0A1C3YLZ7	pp	229533.I1RAU7
148831	229533.A0A1C3YLZ7	pp	229533.Q4I1Y5
148832	229533.A0A1C3YLZ7	pp	229533.I1RV91
148833	229533.A0A1C3YLZ7	pp	229533.I1RSQ3
148834	229533.A0A1C3YLZ7	pp	229533.V6RMW6
148835	229533.A0A1C3YLZ7	pp	229533.I1RA70
148836	229533.A0A1C3YLZ7	pp	229533.V6QW22
148837	229533.A0A1C3YLZ7	pp	229533.I1RTH6
148838	229533.A0A1C3YLZ7	pp	229533.I1S8R3
148839	229533.A0A1C3YLZ7	pp	229533.I1RNC1
148840	229533.A0A1C3YLZ7	pp	229533.I1RSQ7
148841	229533.A0A1C3YLZ7	pp	229533.I1RUG3
148842	229533.A0A1C3YLZ7	pp	229533.I1RCR8
148843	229533.A0A1C3YLZ7	pp	229533.I1RR39
148844	229533.A0A1C3YLZ7	pp	229533.I1RQ75
148845	229533.A0A1C3YLZ7	pp	229533.Q4IJK2
148846	229533.A0A1C3YLZ7	pp	229533.I1RD22
148847	229533.A0A1C3YLZ7	pp	229533.I1S0L3

148848	229533.A0A1C3YLZ7	pp	229533.I1RVQ0
148849	229533.A0A1C3YLZ7	pp	229533.I1RW25
148850	229533.A0A1C3YLZ7	pp	229533.I1RBT1
148851	229533.A0A1C3YLZ7	pp	229533.I1RR24
148852	229533.A0A1C3YLZ7	pp	229533.I1RFU8
148853	229533.A0A1C3YLZ7	pp	229533.I1REK8
148854	229533.A0A1C3YLZ7	pp	229533.I1RQC9
148855	229533.A0A1C3YLZ7	pp	229533.I1RCG4
148856	229533.A0A1C3YLZ7	pp	229533.I1RBC9
148857	229533.A0A1C3YLZ7	pp	229533.I1RAX6
148858	229533.A0A1C3YLZ7	pp	229533.I1S504
148859	229533.A0A1C3YLZ7	pp	229533.I1RZK5
148860	229533.A0A1C3YLZ7	pp	229533.I1RY03
148861	229533.A0A1C3YLZ7	pp	229533.V6RTJ8
148862	229533.A0A1C3YLZ7	pp	229533.I1RLU6
148863	229533.A0A1C3YLZ7	pp	229533.I1RJW1
148864	229533.A0A1C3YLZ7	pp	229533.I1RS18
148865	229533.A0A1C3YLZ7	pp	229533.I1RRY9
148866	229533.A0A1C3YLZ7	pp	229533.I1RZM1
148867	229533.A0A1C3YLZ7	pp	229533.I1RX46
148868	229533.A0A1C3YLZ7	pp	229533.I1RIE8
148869	229533.A0A1C3YLZ7	pp	229533.V6RCG5
148870	229533.A0A1C3YLZ7	pp	229533.I1RI82
148871	229533.A0A1C3YLZ7	pp	229533.I1RAU5
148872	229533.A0A1C3YMN9	pp	229533.Q4IBX0
148873	229533.A0A1C3YMN9	pp	229533.Q4IMM0
148874	229533.A0A1C3YIJ3	pp	229533.I1S503
148875	229533.A0A1C3YIJ3	pp	229533.I1RJW6
148876	229533.A0A1C3YIJ3	pp	229533.I1RRT2
148877	229533.A0A1C3YIJ3	pp	229533.A0A1C3YLK8
148878	229533.A0A1C3YIJ3	pp	229533.I1RJL4
148879	229533.A0A1C3YIJ3	pp	229533.I1RBS6
148880	229533.A0A1C3YIJ3	pp	229533.I1RGA4
148881	229533.A0A1C3YIJ3	pp	229533.A0A1C3YM43
148882	229533.A0A1C3YIJ3	pp	229533.I1RP27
148883	229533.A0A1C3YIJ3	pp	229533.I1RV91
148884	229533.A0A1C3YIJ3	pp	229533.A0A1C3YN63
148885	229533.A0A1C3YIJ3	pp	229533.I1RJV4
148886	229533.A0A1C3YIJ3	pp	229533.I1RCJ7
148887	229533.A0A1C3YIJ3	pp	229533.I1RBS0
148888	229533.A0A1C3YIJ3	pp	229533.I1RAF8
148889	229533.A0A1C3YIJ3	pp	229533.I1RWK3
148890	229533.A0A1C3YIJ3	pp	229533.I1RN63
148891	229533.A0A1C3YIJ3	pp	229533.I1RRQ3
148892	229533.A0A1C3YIJ3	pp	229533.I1RS47
148893	229533.A0A1C3YIJ3	pp	229533.I1RT40
148894	229533.A0A1C3YIJ3	pp	229533.I1RC03
148895	229533.A0A1C3YIJ3	pp	229533.I1RTD5
148896	229533.A0A1C3YIJ3	pp	229533.V6R283
148897	229533.A0A1C3YIJ3	pp	229533.A0A1C3YKK5
148898	229533.A0A1C3YIJ3	pp	229533.I1RGF5
148899	229533.A0A1C3YIJ3	pp	229533.A0A1C3YMK1
148900	229533.A0A1C3YIJ3	pp	229533.I1RQ65
148901	229533.A0A1C3YIJ3	pp	229533.I1RKL3
148902	229533.A0A1C3YIJ3	pp	229533.I1RNA5
148903	229533.A0A1C3YIJ3	pp	229533.A0A1C3YL15
148904	229533.A0A1C3YIJ3	pp	229533.I1RZZ9
148905	229533.A0A1C3YIJ3	pp	229533.A0A1C3YLF5
148906	229533.A0A1C3YIJ3	pp	229533.I1RWI5
148907	229533.A0A1C3YIJ3	pp	229533.I1S352
148908	229533.A0A1C3YIJ3	pp	229533.V6RUY9
148909	229533.A0A1C3YIJ3	pp	229533.I1RSV2
148910	229533.A0A1C3YIJ3	pp	229533.I1RFT1
148911	229533.A0A1C3YIJ3	pp	229533.A0A1C3YIR8
148912	229533.A0A1C3YIJ3	pp	229533.I1RXF2
148913	229533.A0A1C3YIJ3	pp	229533.I1RC66
148914	229533.A0A1C3YIJ3	pp	229533.I1S8V3
148915	229533.A0A1C3YIJ3	pp	229533.A0A1C3YKN3
148916	229533.A0A1C3YIJ3	pp	229533.I1S2E9
148917	229533.A0A1C3YIJ3	pp	229533.I1S8P5
148918	229533.A0A1C3YIJ3	pp	229533.I1RPP3
148919	229533.A0A1C3YIJ3	pp	229533.I1RWF7
148920	229533.A0A1C3YIJ3	pp	229533.I1RNN1

148921 229533.A0A1C3YIJ3 pp 229533.I1RFT6
148922 229533.A0A1C3YIJ3 pp 229533.I1S5S2
148923 229533.A0A1C3YIJ3 pp 229533.I1RZ39
148924 229533.A0A1C3YIJ3 pp 229533.I1RRJ8
148925 229533.A0A1C3YIJ3 pp 229533.I1RRZ0
148926 229533.A0A1C3YIJ3 pp 229533.A0A1C3YKM9
148927 229533.A0A1C3YIJ3 pp 229533.I1S8E2
148928 229533.A0A1C3YIJ3 pp 229533.I1RQG4
148929 229533.A0A1C3YIJ3 pp 229533.I1RLV9
148930 229533.A0A1C3YIJ3 pp 229533.I1RN67
148931 229533.A0A1C3YIJ3 pp 229533.I1RLN6
148932 229533.A0A1C3YIJ3 pp 229533.I1RND6
148933 229533.A0A1C3YIJ3 pp 229533.A0A1C3YK73
148934 229533.A0A1C3YIJ3 pp 229533.V6R3X5
148935 229533.A0A1C3YIJ3 pp 229533.I1S0F6
148936 229533.A0A1C3YIJ3 pp 229533.I1RS20
148937 229533.A0A1C3YIJ3 pp 229533.I1S2H4
148938 229533.A0A1C3YIJ3 pp 229533.Q4I327
148939 229533.A0A1C3YIJ3 pp 229533.I1RR73
148940 229533.A0A1C3YIJ3 pp 229533.I1RIL3
148941 229533.A0A1C3YIJ3 pp 229533.I1RD64
148942 229533.A0A1C3YIJ3 pp 229533.A0A1C3YIN1
148943 229533.A0A1C3YIJ3 pp 229533.I1RZV6
148944 229533.A0A1C3YIJ3 pp 229533.A0A1C3YMI5
148945 229533.A0A1C3YIJ3 pp 229533.I1RNY2
148946 229533.A0A1C3YIJ3 pp 229533.I1S0L4
148947 229533.A0A1C3YIJ3 pp 229533.A0A1C3YLS9
148948 229533.A0A1C3YIJ3 pp 229533.I1RM82
148949 229533.A0A1C3YIJ3 pp 229533.I1RJP4
148950 229533.A0A1C3YIJ3 pp 229533.I1RKJ6
148951 229533.A0A1C3YIJ3 pp 229533.I1RQ23
148952 229533.A0A1C3YIJ3 pp 229533.I1RMG3
148953 229533.A0A1C3YIJ3 pp 229533.O74638
148954 229533.A0A1C3YIJ3 pp 229533.I1RFS3
148955 229533.A0A1C3YIJ3 pp 229533.I1RPM4
148956 229533.A0A1C3YIJ3 pp 229533.I1S5R1
148957 229533.A0A1C3YIJ3 pp 229533.I1RRV1
148958 229533.A0A1C3YIJ3 pp 229533.I1S6Y8
148959 229533.A0A1C3YIJ3 pp 229533.I1RRT8
148960 229533.A0A1C3YIJ3 pp 229533.I1RBG9
148961 229533.A0A1C3YIJ3 pp 229533.I1RSI6
148962 229533.A0A1C3YIJ3 pp 229533.V6RMW6
148963 229533.A0A1C3YIJ3 pp 229533.I1RWL2
148964 229533.A0A1C3YIJ3 pp 229533.I1RE22
148965 229533.A0A1C3YIJ3 pp 229533.I1RK96
148966 229533.A0A1C3YIJ3 pp 229533.I1RA11
148967 229533.A0A1C3YIJ3 pp 229533.A0A1C3YLD8
148968 229533.A0A1C3YIJ3 pp 229533.I1S0M6
148969 229533.A0A1C3YIJ3 pp 229533.V6R042
148970 229533.A0A1C3YIJ3 pp 229533.I1RSQ3
148971 229533.A0A1C3YIJ3 pp 229533.I1R9X7
148972 229533.A0A1C3YIJ3 pp 229533.I1R9M0
148973 229533.A0A1C3YIJ3 pp 229533.I1RZS6
148974 229533.A0A1C3YIJ3 pp 229533.V6RIV9
148975 229533.A0A1C3YIJ3 pp 229533.I1RT47
148976 229533.A0A1C3YIJ3 pp 229533.I1SA33
148977 229533.A0A1C3YIJ3 pp 229533.I1RRM5
148978 229533.A0A1C3YIJ3 pp 229533.I1RWN5
148979 229533.A0A1C3YIJ3 pp 229533.I1S2B8
148980 229533.A0A1C3YIJ3 pp 229533.I1RMN6
148981 229533.A0A1C3YIJ3 pp 229533.I1RPY1
148982 229533.A0A1C3YIJ3 pp 229533.I1RJZ7
148983 229533.A0A1C3YIJ3 pp 229533.I1RD22
148984 229533.A0A1C3YIJ3 pp 229533.Q4HZB8
148985 229533.A0A1C3YIJ3 pp 229533.I1RE76
148986 229533.A0A1C3YIJ3 pp 229533.I1RBY4
148987 229533.A0A1C3YIJ3 pp 229533.I1RPU3
148988 229533.A0A1C3YIJ3 pp 229533.V6RGN5
148989 229533.A0A1C3YIJ3 pp 229533.I1S0N4
148990 229533.A0A1C3YIJ3 pp 229533.V6RIS2
148991 229533.A0A1C3YIJ3 pp 229533.I1RC33
148992 229533.A0A1C3YIJ3 pp 229533.I1RM96
148993 229533.A0A1C3YIJ3 pp 229533.I1RY44

148994	229533.A0A1C3YIJ3	pp	229533.I1S7S9
148995	229533.A0A1C3YIJ3	pp	229533.A0A1C3YMA1
148996	229533.A0A1C3YIJ3	pp	229533.V6RVG3
148997	229533.A0A1C3YIJ3	pp	229533.A0A1C3YIS7
148998	229533.A0A1C3YIJ3	pp	229533.I1RE27
148999	229533.A0A1C3YIJ3	pp	229533.I1R9D1
149000	229533.A0A1C3YIJ3	pp	229533.I1RW46
149001	229533.A0A1C3YIJ3	pp	229533.I1RE01
149002	229533.A0A1C3YIJ3	pp	229533.I1RTA0
149003	229533.A0A1C3YIJ3	pp	229533.I1RZS7
149004	229533.A0A1C3YIJ3	pp	229533.I1RRE5
149005	229533.A0A1C3YIJ3	pp	229533.I1RSA4
149006	229533.A0A1C3YIJ3	pp	229533.V6RY33
149007	229533.A0A1C3YIJ3	pp	229533.I1RUG3
149008	229533.A0A1C3YIJ3	pp	229533.I1RRR7
149009	229533.A0A1C3YIJ3	pp	229533.I1RNC1
149010	229533.A0A1C3YIJ3	pp	229533.I1S8V4
149011	229533.A0A1C3YIJ3	pp	229533.I1S0J1
149012	229533.A0A1C3YIJ3	pp	229533.Q4IN63
149013	229533.A0A1C3YIJ3	pp	229533.I1RC72
149014	229533.A0A1C3YIJ3	pp	229533.I1RPK0
149015	229533.A0A1C3YIJ3	pp	229533.I1RCR8
149016	229533.A0A1C3YIJ3	pp	229533.I1RWB5
149017	229533.A0A1C3YIJ3	pp	229533.I1RNB8
149018	229533.A0A1C3YIJ3	pp	229533.I1RTD7
149019	229533.A0A1C3YIJ3	pp	229533.I1RYW3
149020	229533.A0A1C3YIJ3	pp	229533.I1S1C7
149021	229533.A0A1C3YIJ3	pp	229533.I1S7A8
149022	229533.A0A1C3YIJ3	pp	229533.I1RRI2
149023	229533.A0A1C3YIJ3	pp	229533.I1RS17
149024	229533.A0A1C3YIJ3	pp	229533.I1RCM3
149025	229533.A0A1C3YIJ3	pp	229533.I1RPP4
149026	229533.A0A1C3YIJ3	pp	229533.V6RTJ8
149027	229533.A0A1C3YIJ3	pp	229533.I1RSK1
149028	229533.A0A1C3YIJ3	pp	229533.I1RWX5
149029	229533.A0A1C3YIJ3	pp	229533.I1RJV3
149030	229533.A0A1C3YIJ3	pp	229533.I1RZW8
149031	229533.A0A1C3YIJ3	pp	229533.I1RWH5
149032	229533.A0A1C3YIJ3	pp	229533.I1RA70
149033	229533.A0A1C3YIJ3	pp	229533.A0A1C3YM63
149034	229533.A0A1C3YIJ3	pp	229533.I1S8R3
149035	229533.A0A1C3YIJ3	pp	229533.Q4IJT5
149036	229533.A0A1C3YIJ3	pp	229533.I1RWN6
149037	229533.A0A1C3YIJ3	pp	229533.A0A1C3YK33
149038	229533.A0A1C3YIJ3	pp	229533.I1RFW5
149039	229533.A0A1C3YIJ3	pp	229533.I1RTH6
149040	229533.A0A1C3YIJ3	pp	229533.I1RD39
149041	229533.A0A1C3YIJ3	pp	229533.I1RIL8
149042	229533.A0A1C3YIJ3	pp	229533.I1RBC9
149043	229533.A0A1C3YIJ3	pp	229533.I1S2C0
149044	229533.A0A1C3YIJ3	pp	229533.I1RN18
149045	229533.A0A1C3YIJ3	pp	229533.I1RAW0
149046	229533.A0A1C3YIJ3	pp	229533.I1RW71
149047	229533.A0A1C3YIJ3	pp	229533.I1RFL4
149048	229533.A0A1C3YIJ3	pp	229533.V6R057
149049	229533.A0A1C3YIJ3	pp	229533.A0A1C3YK13
149050	229533.A0A1C3YIJ3	pp	229533.I1RSD8
149051	229533.A0A1C3YIJ3	pp	229533.I1RR00
149052	229533.A0A1C3YIJ3	pp	229533.I1RCH5
149053	229533.A0A1C3YIJ3	pp	229533.I1S569
149054	229533.A0A1C3YIJ3	pp	229533.I1RG53
149055	229533.A0A1C3YIJ3	pp	229533.I1S2E8
149056	229533.A0A1C3YIJ3	pp	229533.I1RQE7
149057	229533.A0A1C3YIJ3	pp	229533.I1RQJ5
149058	229533.A0A1C3YIJ3	pp	229533.I1RD17
149059	229533.A0A1C3YIJ3	pp	229533.Q4I624
149060	229533.A0A1C3YIJ3	pp	229533.I1RAU7
149061	229533.A0A1C3YIJ3	pp	229533.I1RSQ7
149062	229533.A0A1C3YIJ3	pp	229533.A0A1C3YL54
149063	229533.A0A1C3YIJ3	pp	229533.I1RVX0
149064	229533.A0A1C3YIJ3	pp	229533.V6QW22
149065	229533.A0A1C3YIJ3	pp	229533.I1RT03
149066	229533.A0A1C3YIJ3	pp	229533.I1RRN5

149067	229533.A0A1C3YIJ3	pp	229533.I1RQC9
149068	229533.A0A1C3YIJ3	pp	229533.I1RQH3
149069	229533.A0A1C3YIJ3	pp	229533.I1RN61
149070	229533.A0A1C3YIJ3	pp	229533.I1RC20
149071	229533.A0A1C3YIJ3	pp	229533.I1RSS4
149072	229533.A0A1C3YIJ3	pp	229533.I1S7M2
149073	229533.A0A1C3YIJ3	pp	229533.A0A1C3YIP5
149074	229533.A0A1C3YIJ3	pp	229533.I1REN8
149075	229533.A0A1C3YIJ3	pp	229533.I1RRW2
149076	229533.A0A1C3YIJ3	pp	229533.I1RXD7
149077	229533.A0A1C3YIJ3	pp	229533.I1RAS2
149078	229533.A0A1C3YIJ3	pp	229533.I1RVQ0
149079	229533.A0A1C3YIJ3	pp	229533.I1RUQ2
149080	229533.A0A1C3YIJ3	pp	229533.I1RB41
149081	229533.A0A1C3YIJ3	pp	229533.I1RB92
149082	229533.A0A1C3YIJ3	pp	229533.I1RG90
149083	229533.A0A1C3YIJ3	pp	229533.V6R4J1
149084	229533.A0A1C3YIJ3	pp	229533.I1RX46
149085	229533.A0A1C3YIJ3	pp	229533.I1R9J6
149086	229533.A0A1C3YIJ3	pp	229533.I1RD52
149087	229533.A0A1C3YIJ3	pp	229533.I1RAD1
149088	229533.A0A1C3YIJ3	pp	229533.I1S540
149089	229533.A0A1C3YIJ3	pp	229533.I1S0T2
149090	229533.A0A1C3YIJ3	pp	229533.I1RKC6
149091	229533.A0A1C3YIJ3	pp	229533.I1RGL4
149092	229533.A0A1C3YIJ3	pp	229533.I1RT33
149093	229533.A0A1C3YIJ3	pp	229533.I1RNA0
149094	229533.A0A1C3YIJ3	pp	229533.A0A1C3YLZ7
149095	229533.A0A1C3YIJ3	pp	229533.I1RAX6
149096	229533.A0A1C3YIJ3	pp	229533.I1RM06
149097	229533.A0A1C3YIJ3	pp	229533.I1RMR1
149098	229533.A0A1C3YIJ3	pp	229533.I1RLC7
149099	229533.A0A1C3YIJ3	pp	229533.V6R1K4
149100	229533.A0A1C3YIJ3	pp	229533.I1RAT0
149101	229533.A0A1C3YIJ3	pp	229533.A0A1C3YJQ9
149102	229533.A0A1C3YIJ3	pp	229533.I1RX25
149103	229533.A0A1C3YIJ3	pp	229533.I1S0X6
149104	229533.A0A1C3YIJ3	pp	229533.I1RW25
149105	229533.A0A1C3YIJ3	pp	229533.I1S0L3
149106	229533.A0A1C3YIJ3	pp	229533.I1RAM6
149107	229533.A0A1C3YIJ3	pp	229533.I1RQM6
149108	229533.A0A1C3YIJ3	pp	229533.I1RMC8
149109	229533.A0A1C3YIJ3	pp	229533.I1RCI3
149110	229533.A0A1C3YIJ3	pp	229533.I1RI02
149111	229533.A0A1C3YIJ3	pp	229533.A0A1C3YKC0
149112	229533.A0A1C3YIJ3	pp	229533.I1S0F5
149113	229533.A0A1C3YIJ3	pp	229533.I1RW07
149114	229533.A0A1C3YIJ3	pp	229533.I1RT80
149115	229533.A0A1C3YIJ3	pp	229533.I1RTF4
149116	229533.A0A1C3YIJ3	pp	229533.I1RBT1
149117	229533.A0A1C3YIJ3	pp	229533.I1RQ31
149118	229533.A0A1C3YIJ3	pp	229533.I1RMW7
149119	229533.A0A1C3YIJ3	pp	229533.A0A1C3YN30
149120	229533.A0A1C3YIJ3	pp	229533.I1RN58
149121	229533.A0A1C3YIJ3	pp	229533.I1RAU5
149122	229533.A0A1C3YIJ3	pp	229533.I1RIG2
149123	229533.A0A1C3YIJ3	pp	229533.I1RC17
149124	229533.A0A1C3YIJ3	pp	229533.I1RXF0
149125	229533.A0A1C3YIJ3	pp	229533.I1RZM8
149126	229533.A0A1C3YIJ3	pp	229533.I1RJU3
149127	229533.A0A1C3YIJ3	pp	229533.I1S044
149128	229533.A0A1C3YIJ3	pp	229533.I1RMK3
149129	229533.A0A1C3YIJ3	pp	229533.I1S6U8
149130	229533.A0A1C3YIJ3	pp	229533.I1RI82
149131	229533.A0A1C3YIJ3	pp	229533.I1S7M3
149132	229533.A0A1C3YIJ3	pp	229533.I1RS83
149133	229533.A0A1C3YIJ3	pp	229533.I1RB50
149134	229533.A0A1C3YIJ3	pp	229533.I1S5B0
149135	229533.A0A1C3YIJ3	pp	229533.I1RNH6
149136	229533.A0A1C3YIJ3	pp	229533.A0A1C3YJH8
149137	229533.A0A1C3YIJ3	pp	229533.I1RWJ8
149138	229533.A0A1C3YIJ3	pp	229533.I1RW99
149139	229533.A0A1C3YIJ3	pp	229533.I1S021

149140	229533.A0A1C3YIJ3	pp	229533.I1RPU0
149141	229533.A0A1C3YIJ3	pp	229533.A0A1C3YK34
149142	229533.A0A1C3YIJ3	pp	229533.V6RCZ8
149143	229533.A0A1C3YIJ3	pp	229533.I1RZJ5
149144	229533.A0A1C3YIJ3	pp	229533.I1RFQ7
149145	229533.A0A1C3YIJ3	pp	229533.I1SA32
149146	229533.A0A1C3YIJ3	pp	229533.A0A1C3YKV3
149147	229533.A0A1C3YIJ3	pp	229533.I1RR51
149148	229533.A0A1C3YIJ3	pp	229533.I1RM67
149149	229533.A0A1C3YIJ3	pp	229533.I1S0M9
149150	229533.A0A1C3YIJ3	pp	229533.A0A1C3YLU7
149151	229533.A0A1C3YIJ3	pp	229533.I1RMG8
149152	229533.A0A1C3YIJ3	pp	229533.V6RCG5
149153	229533.A0A1C3YIJ3	pp	229533.I1RR24
149154	229533.A0A1C3YIJ3	pp	229533.I1RQ81
149155	229533.A0A1C3YIJ3	pp	229533.I1RGC2
149156	229533.A0A1C3YIJ3	pp	229533.I1S9U2
149157	229533.A0A1C3YIJ3	pp	229533.Q4I8P5
149158	229533.A0A1C3YIJ3	pp	229533.V6QUE6
149159	229533.A0A1C3YIJ3	pp	229533.I1RS18
149160	229533.A0A1C3YIJ3	pp	229533.I1RCS1
149161	229533.A0A1C3YIJ3	pp	229533.I1RX81
149162	229533.A0A1C3YIJ3	pp	229533.I1RFT2
149163	229533.A0A1C3YIJ3	pp	229533.I1RZ11
149164	229533.A0A1C3YIJ3	pp	229533.I1RB27
149165	229533.A0A1C3YIJ3	pp	229533.V6QUW4
149166	229533.A0A1C3YIJ3	pp	229533.I1RWX8
149167	229533.A0A1C3YIJ3	pp	229533.I1RRU9
149168	229533.A0A1C3YIJ3	pp	229533.I1RWT6
149169	229533.A0A1C3YIJ3	pp	229533.I1S419
149170	229533.A0A1C3YIJ3	pp	229533.I1RQD8
149171	229533.A0A1C3YIJ3	pp	229533.I1RZZ1
149172	229533.A0A1C3YIJ3	pp	229533.I1RBJ5
149173	229533.A0A1C3YIJ3	pp	229533.A0A1C3YKW9
149174	229533.A0A1C3YIJ3	pp	229533.I1RMD5
149175	229533.A0A1C3YIJ3	pp	229533.I1RY03
149176	229533.A0A1C3YIJ3	pp	229533.I1RNQ5
149177	229533.A0A1C3YIJ3	pp	229533.I1RLU6
149178	229533.A0A1C3YIJ3	pp	229533.I1RZK6
149179	229533.A0A1C3YIJ3	pp	229533.I1RSR8
149180	229533.A0A1C3YIJ3	pp	229533.I1RJZ6
149181	229533.A0A1C3YIJ3	pp	229533.Q4ICA8
149182	229533.A0A1C3YIJ3	pp	229533.A0A1C3YLK2
149183	229533.A0A1C3YIJ3	pp	229533.I1RQQ5
149184	229533.A0A1C3YIJ3	pp	229533.I1RFU8
149185	229533.A0A1C3YIJ3	pp	229533.I1RZM1
149186	229533.A0A1C3YIJ3	pp	229533.I1R9V7
149187	229533.A0A1C3YIJ3	pp	229533.I1RN99
149188	229533.A0A1C3YIJ3	pp	229533.I1RA48
149189	229533.A0A1C3YIJ3	pp	229533.I1RCG4
149190	229533.A0A1C3YIJ3	pp	229533.I1RZB5
149191	229533.A0A1C3YIJ3	pp	229533.I1RZK5
149192	229533.A0A1C3YIJ3	pp	229533.I1RZF6
149193	229533.A0A1C3YIJ3	pp	229533.I1RAN5
149194	229533.A0A1C3YIJ3	pp	229533.I1RD03
149195	229533.A0A1C3YIJ3	pp	229533.A0A1C3YJ93
149196	229533.A0A1C3YIJ3	pp	229533.I1RB53
149197	229533.A0A1C3YIJ3	pp	229533.I1S1J7
149198	229533.A0A1C3YIJ3	pp	229533.I1RWJ3
149199	229533.A0A1C3YIJ3	pp	229533.I1RVZ0
149200	229533.A0A1C3YIJ3	pp	229533.I1RDG0
149201	229533.A0A1C3YIJ3	pp	229533.Q4I2J8
149202	229533.A0A1C3YIJ3	pp	229533.I1RAZ8
149203	229533.A0A1C3YIJ3	pp	229533.I1S504
149204	229533.A0A1C3YIJ3	pp	229533.I1RZE5
149205	229533.A0A1C3YIJ3	pp	229533.I1RLM7
149206	229533.A0A1C3YIJ3	pp	229533.I1RF19
149207	229533.A0A1C3YIJ3	pp	229533.Q4I283
149208	229533.A0A1C3YIJ3	pp	229533.I1RI03
149209	229533.A0A1C3YIJ3	pp	229533.I1RBL7
149210	229533.A0A1C3YIJ3	pp	229533.A0A1C3YKB0
149211	229533.A0A1C3YIJ3	pp	229533.I1RSI0
149212	229533.A0A1C3YIJ3	pp	229533.I1RE00

229533.A0A1C3YIJ3 pp 229533.I1REK8
229533.A0A1C3YIJ3 pp 229533.I1RCR5
229533.A0A1C3YIJ3 pp 229533.I1RU29
229533.A0A1C3YIJ3 pp 229533.I1RPA0
229533.A0A1C3YIJ3 pp 229533.I1RRY9
229533.A0A1C3YIJ3 pp 229533.V6R227
229533.A0A1C3YIJ3 pp 229533.Q4IJK2
229533.A0A1C3YIJ3 pp 229533.I1RMQ0
229533.A0A1C3YIJ3 pp 229533.I1RWD7
229533.A0A1C3YIJ3 pp 229533.I1RQA3
229533.I1RE00 pp 229533.I1RW06
229533.I1RE00 pp 229533.I1RW25
229533.I1RE00 pp 229533.Q4HVW2
229533.I1RE00 pp 229533.I1S6U8
229533.I1RE00 pp 229533.I1RK81
229533.I1RE00 pp 229533.Q4IFI0
229533.I1RE00 pp 229533.I1RM06
229533.I1RE00 pp 229533.V6QYF0
229533.I1RE00 pp 229533.Q4IJH1
229533.I1RE00 pp 229533.I1RNA0
229533.I1RE00 pp 229533.I1RG51
229533.I1RE00 pp 229533.I1RTF4
229533.I1RE00 pp 229533.I1RJU3
229533.I1RE00 pp 229533.I1RT80
229533.I1RE00 pp 229533.I1RNV6
229533.I1RE00 pp 229533.I1REX4
229533.I1RE00 pp 229533.I1RPW9
229533.I1RE00 pp 229533.Q4IPB3
229533.I1RE00 pp 229533.I1RQM6
229533.I1RE00 pp 229533.I1S9U2
229533.I1RE00 pp 229533.I1S6F0
229533.I1RE00 pp 229533.I1RT33
229533.I1RE00 pp 229533.V6RTJ8
229533.I1RE00 pp 229533.I1RFQ7
229533.I1RE00 pp 229533.I1S0B6
229533.I1RE00 pp 229533.I1RX46
229533.I1RE00 pp 229533.I1RNH1
229533.I1RE00 pp 229533.I1RKQ5
229533.I1RE00 pp 229533.Q4I5R9
229533.I1RE00 pp 229533.I1RVQ0
229533.I1RE00 pp 229533.I1RM67
229533.I1RE00 pp 229533.I1RQC5
229533.I1RE00 pp 229533.I1RXD7
229533.I1RE00 pp 229533.I1RPU0
229533.I1RE00 pp 229533.V6QW22
229533.I1RE00 pp 229533.I1RQJ5
229533.I1RE00 pp 229533.I1RZK6
229533.I1RE00 pp 229533.I1RY63
229533.I1RE00 pp 229533.I1S0R7
229533.I1RE00 pp 229533.I1RZZ1
229533.I1RE00 pp 229533.I1RSQ7
229533.I1RE00 pp 229533.I1RXU7
229533.I1RE00 pp 229533.I1RW71
229533.I1RE00 pp 229533.I1RT98
229533.I1RE00 pp 229533.I1RMQ0
229533.I1RE00 pp 229533.I1RRG3
229533.I1RE00 pp 229533.I1RJZ6
229533.I1RE00 pp 229533.Q4I948
229533.I1RE00 pp 229533.I1RFW5
229533.I1RE00 pp 229533.Q4INZ9
229533.I1RE00 pp 229533.I1RWH5
229533.I1RE00 pp 229533.I1RKQ9
229533.I1RE00 pp 229533.I1S246
229533.I1RE00 pp 229533.I1RF08
229533.I1RE00 pp 229533.I1RRY9
229533.I1RE00 pp 229533.I1RSR3
229533.I1RE00 pp 229533.I1RYB1
229533.I1RE00 pp 229533.I1REK8
229533.I1RE00 pp 229533.I1RY03
229533.I1RE00 pp 229533.I1RQA2
229533.I1RE00 pp 229533.I1RZK5
229533.I1RE00 pp 229533.I1RSI0
229533.I1RE00 pp 229533.V6R227

149286	229533.I1RE00	pp	229533.Q4I1Y1
149287	229533.I1RE00	pp	229533.I1RSH4
149288	229533.I1RE00	pp	229533.I1RVZ0
149289	229533.I1RE00	pp	229533.I1RZF6
149290	229533.I1RE00	pp	229533.I1RK95
149291	229533.I1RE00	pp	229533.I1RFU8
149292	229533.I1RE00	pp	229533.I1RK83
149293	229533.I1RE00	pp	229533.I1RQ28
149294	229533.I1RE00	pp	229533.I1RZB5
149295	229533.I1RE00	pp	229533.I1RNH6
149296	229533.I1RE00	pp	229533.I1S504
149297	229533.I1RE00	pp	229533.I1RNQ5
149298	229533.I1RE00	pp	229533.I1RSR8
149299	229533.I1RE00	pp	229533.I1S9Y1
149300	229533.I1RE00	pp	229533.I1S021
149301	229533.I1RE00	pp	229533.I1RLU6
149302	229533.I1RE00	pp	229533.I1RM82
149303	229533.I1RE00	pp	229533.Q4IEK8
149304	229533.I1RE00	pp	229533.I1RYB9
149305	229533.I1RE00	pp	229533.I1RZM1
149306	229533.I1RE00	pp	229533.Q4IE79
149307	229533.I1RE00	pp	229533.I1RKJ6
149308	229533.I1RE00	pp	229533.I1RJS4
149309	229533.I1RE00	pp	229533.I1RMC0
149310	229533.I1RE00	pp	229533.Q4ICA8
149311	229533.I1RE00	pp	229533.I1RQ31
149312	229533.I1RE00	pp	229533.I1RR73
149313	229533.I1RE00	pp	229533.I1RG56
149314	229533.I1RE00	pp	229533.I1S0P4
149315	229533.I1RE00	pp	229533.I1RN58
149316	229533.I1RE00	pp	229533.I1S2A8
149317	229533.I1RE00	pp	229533.I1RND6
149318	229533.I1RE00	pp	229533.Q4IAA0
149319	229533.I1RE00	pp	229533.I1RE76
149320	229533.I1RE00	pp	229533.V6R3X5
149321	229533.I1RE00	pp	229533.I1RS18
149322	229533.I1RE00	pp	229533.I1RJZ7
149323	229533.I1RE00	pp	229533.I1S2B8
149324	229533.I1RE00	pp	229533.Q4IJ56
149325	229533.I1RE00	pp	229533.I1RZ39
149326	229533.I1RE00	pp	229533.I1RR72
149327	229533.I1RE00	pp	229533.I1S0M9
149328	229533.I1RE00	pp	229533.I1S8E2
149329	229533.I1RE00	pp	229533.I1RZS6
149330	229533.I1RE00	pp	229533.I1RFT6
149331	229533.I1RE00	pp	229533.I1RX24
149332	229533.I1RE00	pp	229533.I1RWI5
149333	229533.I1RE00	pp	229533.I1RQ81
149334	229533.I1RE00	pp	229533.I1RZZ9
149335	229533.I1RE00	pp	229533.I1RFS3
149336	229533.I1RE00	pp	229533.I1RMQ4
149337	229533.I1RE00	pp	229533.I1S044
149338	229533.I1RE00	pp	229533.Q4IF76
149339	229533.I1RE00	pp	229533.I1RW99
149340	229533.I1RE00	pp	229533.I1RPM4
149341	229533.I1RE00	pp	229533.I1S5B0
149342	229533.I1RE00	pp	229533.I1RQ65
149343	229533.I1RE00	pp	229533.I1RS47
149344	229533.I1RE00	pp	229533.I1RRU9
149345	229533.I1RE00	pp	229533.Q4HZ42
149346	229533.I1RE00	pp	229533.I1S0L4
149347	229533.I1RE00	pp	229533.I1RRQ3
149348	229533.I1RE00	pp	229533.I1RGC2
149349	229533.I1RE00	pp	229533.V6QUE6
149350	229533.I1RE00	pp	229533.I1RFG0
149351	229533.I1RE00	pp	229533.I1RS20
149352	229533.I1RE00	pp	229533.I1S2H4
149353	229533.I1RE00	pp	229533.I1RP28
149354	229533.I1RE00	pp	229533.V6RCZ8
149355	229533.I1RE00	pp	229533.I1RN67
149356	229533.I1RE00	pp	229533.I1RRP3
149357	229533.I1RE00	pp	229533.I1RRT8
149358	229533.I1RE00	pp	229533.O74638

149359	229533.I1RE00	pp	229533.V6R283
149360	229533.I1RE00	pp	229533.I1RN63
149361	229533.I1RE00	pp	229533.I1RZV6
149362	229533.I1RE00	pp	229533.I1S8P5
149363	229533.I1RE00	pp	229533.I1RWF7
149364	229533.I1RE00	pp	229533.I1RPP3
149365	229533.I1RE00	pp	229533.Q4IBK5
149366	229533.I1RE00	pp	229533.I1RP27
149367	229533.I1RE00	pp	229533.I1RRJ8
149368	229533.I1RE00	pp	229533.I1S1J6
149369	229533.I1RE00	pp	229533.I1RQG4
149370	229533.I1RE00	pp	229533.I1RKC6
149371	229533.I1RE00	pp	229533.I1RQS5
149372	229533.I1RE00	pp	229533.I1RFN1
149373	229533.I1RE00	pp	229533.I1RGA4
149374	229533.I1RE00	pp	229533.I1RRT2
149375	229533.I1RE00	pp	229533.Q4I830
149376	229533.I1RE00	pp	229533.Q4HXF6
149377	229533.I1RE00	pp	229533.I1RKL3
149378	229533.I1RE00	pp	229533.I1RVX0
149379	229533.I1RE00	pp	229533.I1RLV9
149380	229533.I1RE00	pp	229533.I1RJL4
149381	229533.I1RE00	pp	229533.I1RT03
149382	229533.I1RE00	pp	229533.I1RSV2
149383	229533.I1RE00	pp	229533.I1S1X9
149384	229533.I1RE00	pp	229533.Q4IAS1
149385	229533.I1RE00	pp	229533.I1RSS4
149386	229533.I1RE00	pp	229533.I1RRI2
149387	229533.I1RE00	pp	229533.I1S2E9
149388	229533.I1RE00	pp	229533.I1RTD7
149389	229533.I1RE00	pp	229533.I1S503
149390	229533.I1RE00	pp	229533.Q4HXF0
149391	229533.I1RE00	pp	229533.I1RYW3
149392	229533.I1RE00	pp	229533.Q4HWE2
149393	229533.I1RE00	pp	229533.I1S1C7
149394	229533.I1RE00	pp	229533.I1S2E8
149395	229533.I1RE00	pp	229533.I1RRR7
149396	229533.I1RE00	pp	229533.I1RQE7
149397	229533.I1RE00	pp	229533.I1RT04
149398	229533.I1RE00	pp	229533.Q4IPB8
149399	229533.I1RE00	pp	229533.I1RTD5
149400	229533.I1RE00	pp	229533.I1RSD8
149401	229533.I1RE00	pp	229533.I1RT40
149402	229533.I1RE00	pp	229533.I1RWN6
149403	229533.I1RE00	pp	229533.I1S1U7
149404	229533.I1RE00	pp	229533.I1RRE5
149405	229533.I1RE00	pp	229533.Q4IPH4
149406	229533.I1RE00	pp	229533.I1RM96
149407	229533.I1RE00	pp	229533.V6RGY3
149408	229533.I1RE00	pp	229533.Q4HZ68
149409	229533.I1RE00	pp	229533.I1RGC6
149410	229533.I1RE00	pp	229533.I1RY44
149411	229533.I1RE00	pp	229533.I1RJV3
149412	229533.I1RE00	pp	229533.V6R057
149413	229533.I1RE00	pp	229533.I1RSA4
149414	229533.I1RE00	pp	229533.I1RJS5
149415	229533.I1RE00	pp	229533.I1RMN6
149416	229533.I1RE00	pp	229533.V6RIV9
149417	229533.I1RE00	pp	229533.V6R042
149418	229533.I1RE00	pp	229533.I1RTE9
149419	229533.I1RE00	pp	229533.I1RPP4
149420	229533.I1RE00	pp	229533.I1RWN5
149421	229533.I1RE00	pp	229533.I1S2C0
149422	229533.I1RE00	pp	229533.V6RMW6
149423	229533.I1RE00	pp	229533.I1RSG8
149424	229533.I1RE00	pp	229533.I1RWD3
149425	229533.I1RE00	pp	229533.Q4I8X0
149426	229533.I1RE00	pp	229533.I1RSI6
149427	229533.I1RE00	pp	229533.Q4I327
149428	229533.I1RE00	pp	229533.I1RTH6
149429	229533.I1RE00	pp	229533.Q4I7K4
149430	229533.I1RE00	pp	229533.I1RWB5
149431	229533.I1RE00	pp	229533.I1S0J1

149432 229533.I1RE00 pp 229533.I1RNY2
149433 229533.I1RE00 pp 229533.I1RE01
149434 229533.I1RE00 pp 229533.I1RNC1
149435 229533.I1RE00 pp 229533.I1RTA0
149436 229533.I1RE00 pp 229533.I1S060
149437 229533.I1RE00 pp 229533.Q4IN63
149438 229533.I1RE00 pp 229533.V6RGN5
149439 229533.I1RE00 pp 229533.I1RG11
149440 229533.I1RE00 pp 229533.I1S0N4
149441 229533.I1RZ15 pp 229533.V6QVV9
149442 229533.I1RZ15 pp 229533.I1RZ25
149443 229533.A0A0E0SDV1 pp 229533.I1RC31
149444 229533.A0A0E0SDV1 pp 229533.I1RYK0
149445 229533.A0A0E0SDV1 pp 229533.I1RF73
149446 229533.A0A0E0SDV1 pp 229533.I1RA62
149447 229533.A0A0E0SDV1 pp 229533.A0A1C3YM97
149448 229533.A0A0E0SDV1 pp 229533.I1S166
149449 229533.A0A0E0SDV1 pp 229533.I1RVD8
149450 229533.A0A0E0SDV1 pp 229533.A0A1C3YN26
149451 229533.A0A0E0SDV1 pp 229533.I1RP86
149452 229533.A0A0E0SDV1 pp 229533.A0A1C3YL54
149453 229533.A0A0E0SDV1 pp 229533.I1RCG5
149454 229533.I1RRM9 pp 229533.I1RWH4
149455 229533.I1RRM9 pp 229533.I1RRT2
149456 229533.I1RRM9 pp 229533.I1RS20
149457 229533.I1RRM9 pp 229533.I1RS47
149458 229533.I1RRM9 pp 229533.I1RSH0
149459 229533.I1RRM9 pp 229533.I1S0M2
149460 229533.I1RRM9 pp 229533.I1S0N4
149461 229533.I1RRM9 pp 229533.I1RWB4
149462 229533.I1RRM9 pp 229533.Q4I7L0
149463 229533.I1RRM9 pp 229533.V6R283
149464 229533.I1RRM9 pp 229533.I1RT41
149465 229533.I1RRM9 pp 229533.I1S2G9
149466 229533.I1RRM9 pp 229533.I1RRV1
149467 229533.I1RRM9 pp 229533.I1RYS6
149468 229533.I1RRM9 pp 229533.I1RYW3
149469 229533.I1RRM9 pp 229533.I1RSI0
149470 229533.I1RRM9 pp 229533.I1RYH9
149471 229533.I1RRM9 pp 229533.I1S238
149472 229533.I1RRM9 pp 229533.Q4I948
149473 229533.I1RRM9 pp 229533.I1S2C0
149474 229533.I1RRM9 pp 229533.Q4HZS8
149475 229533.I1RRM9 pp 229533.I1S0Z3
149476 229533.I1RRM9 pp 229533.V6RTJ8
149477 229533.I1RRM9 pp 229533.I1RYL5
149478 229533.I1RRM9 pp 229533.I1RZH4
149479 229533.I1RRM9 pp 229533.V6RBN2
149480 229533.I1RSI0 pp 229533.I1S0L4
149481 229533.I1RSI0 pp 229533.I1S2H4
149482 229533.I1RSI0 pp 229533.O74638
149483 229533.I1RSI0 pp 229533.I1RWI5
149484 229533.I1RSI0 pp 229533.I1RZZ9
149485 229533.I1RSI0 pp 229533.I1S2B8
149486 229533.I1RSI0 pp 229533.V6R3X5
149487 229533.I1RSI0 pp 229533.I1RZ39
149488 229533.I1RSI0 pp 229533.I1RWX1
149489 229533.I1RSI0 pp 229533.I1RTD5
149490 229533.I1RSI0 pp 229533.I1RT40
149491 229533.I1RSI0 pp 229533.I1RXK9
149492 229533.I1RSI0 pp 229533.I1RT04
149493 229533.I1RSI0 pp 229533.I1RX24
149494 229533.I1RSI0 pp 229533.I1RWL2
149495 229533.I1RSI0 pp 229533.I1S503
149496 229533.I1RSI0 pp 229533.I1S2E9
149497 229533.I1RSI0 pp 229533.I1RSK1
149498 229533.I1RSI0 pp 229533.V6RRM0
149499 229533.I1RSI0 pp 229533.I1RWN6
149500 229533.I1RSI0 pp 229533.I1S7X9
149501 229533.I1RSI0 pp 229533.I1RWX2
149502 229533.I1RSI0 pp 229533.I1S2G7
149503 229533.I1RSI0 pp 229533.V6RN69
149504 229533.I1RSI0 pp 229533.I1S1C7

149505	229533.I1RSI0	pp	229533.I1RWQ8
149506	229533.I1RSI0	pp	229533.I1RYW3
149507	229533.I1RSI0	pp	229533.V6R283
149508	229533.I1RSI0	pp	229533.I1RZH6
149509	229533.I1RSI0	pp	229533.I1RWK3
149510	229533.I1RSI0	pp	229533.I1S0C2
149511	229533.I1RSI0	pp	229533.I1S0N4
149512	229533.I1RSI0	pp	229533.V6RGN5
149513	229533.I1RSI0	pp	229533.I1RSV2
149514	229533.I1RSI0	pp	229533.I1RZW8
149515	229533.I1RSI0	pp	229533.I1RUG3
149516	229533.I1RSI0	pp	229533.I1RZ79
149517	229533.I1RSI0	pp	229533.I1S4S4
149518	229533.I1RSI0	pp	229533.V6RMW6
149519	229533.I1RSI0	pp	229533.I1RTH6
149520	229533.I1RSI0	pp	229533.I1RSQ3
149521	229533.I1RSI0	pp	229533.I1RWJ8
149522	229533.I1RSI0	pp	229533.I1S0X6
149523	229533.I1RSI0	pp	229533.V6R042
149524	229533.I1RSI0	pp	229533.I1RWN5
149525	229533.I1RSI0	pp	229533.I1S4S1
149526	229533.I1RSI0	pp	229533.I1S2C0
149527	229533.I1RSI0	pp	229533.I1RW25
149528	229533.I1RSI0	pp	229533.V6RIV9
149529	229533.I1RSI0	pp	229533.V6R057
149530	229533.I1RSI0	pp	229533.V6QUG0
149531	229533.I1RSI0	pp	229533.I1S1X9
149532	229533.I1RSI0	pp	229533.I1S1W4
149533	229533.I1RSI0	pp	229533.I1RT03
149534	229533.I1RSI0	pp	229533.I1RY44
149535	229533.I1RSI0	pp	229533.V6QYF0
149536	229533.I1RSI0	pp	229533.I1RVX0
149537	229533.I1RSI0	pp	229533.I1SA55
149538	229533.I1RSI0	pp	229533.I1RX46
149539	229533.I1RSI0	pp	229533.I1RZS7
149540	229533.I1RSI0	pp	229533.V6RTJ8
149541	229533.I1RSI0	pp	229533.V6QUW4
149542	229533.I1RSI0	pp	229533.I1S0J1
149543	229533.I1RSI0	pp	229533.I1S0R7
149544	229533.I1RSI0	pp	229533.Q4HVU7
149545	229533.I1RSI0	pp	229533.I1RY03
149546	229533.I1RSI0	pp	229533.I1RYY5
149547	229533.I1RSI0	pp	229533.I1RW99
149548	229533.I1RSI0	pp	229533.I1S5B0
149549	229533.I1RSI0	pp	229533.I1RSP1
149550	229533.I1RSI0	pp	229533.I1RXD7
149551	229533.I1RSI0	pp	229533.I1RZK5
149552	229533.I1RSI0	pp	229533.V6RCZ8
149553	229533.I1RSI0	pp	229533.I1RVQ0
149554	229533.I1RSI0	pp	229533.I1RZE5
149555	229533.I1RSI0	pp	229533.I1S6F0
149556	229533.I1RSI0	pp	229533.I1RZM1
149557	229533.I1RSI0	pp	229533.I1S0L3
149558	229533.I1RSI0	pp	229533.V6RCG5
149559	229533.I1RSI0	pp	229533.V6QWB1
149560	229533.I1RSI0	pp	229533.Q4IJK2
149561	229533.I1RSI0	pp	229533.I1RWD7
149562	229533.I1RSI0	pp	229533.Q4IEK8
149563	229533.I1RSI0	pp	229533.I1RZZ1
149564	229533.I1RSI0	pp	229533.I1RSQ7
149565	229533.I1RSI0	pp	229533.I1S504
149566	229533.I1RSI0	pp	229533.I1RW71
149567	229533.I1RSI0	pp	229533.I1RZF6
149568	229533.I1RSI0	pp	229533.Q4IJM4
149569	229533.I1RSI0	pp	229533.I1RZK6
149570	229533.I1RSI0	pp	229533.I1RWT6
149571	229533.I1RSI0	pp	229533.V6QW22
149572	229533.I1RSI0	pp	229533.I1RYS0
149573	229533.I1RSI0	pp	229533.I1S2A1
149574	229533.I1RSI0	pp	229533.I1RXB6
149575	229533.A0A1C3YKV3	pp	229533.I1RWD4
149576	229533.A0A1C3YKV3	pp	229533.Q5I2J3
149577	229533.A0A1C3YKV3	pp	229533.I1RVQ0

149578 229533.A0A1C3YKV3 pp 229533.V6R4J1
149579 229533.A0A1C3YKV3 pp 229533.I1RXD6
149580 229533.A0A1C3YKV3 pp 229533.I1RAH0
149581 229533.A0A1C3YKV3 pp 229533.I1RA85
149582 229533.A0A1C3YKV3 pp 229533.I1RY86
149583 229533.A0A1C3YKV3 pp 229533.I1S0D4
149584 229533.A0A1C3YKV3 pp 229533.I1RD52
149585 229533.A0A1C3YKV3 pp 229533.I1S0L3
149586 229533.A0A1C3YKV3 pp 229533.I1S0F5
149587 229533.A0A1C3YKV3 pp 229533.I1RLX7
149588 229533.A0A1C3YKV3 pp 229533.V6R1K4
149589 229533.A0A1C3YKV3 pp 229533.A0A1C3YM63
149590 229533.A0A1C3YKV3 pp 229533.I1RBD5
149591 229533.A0A1C3YKV3 pp 229533.Q4IJT5
149592 229533.A0A1C3YKV3 pp 229533.I1RX95
149593 229533.A0A1C3YKV3 pp 229533.I1RIL8
149594 229533.A0A1C3YKV3 pp 229533.I1RRG3
149595 229533.A0A1C3YKV3 pp 229533.I1S6T4
149596 229533.A0A1C3YKV3 pp 229533.I1RPN4
149597 229533.A0A1C3YKV3 pp 229533.I1RW71
149598 229533.A0A1C3YKV3 pp 229533.I1RHW8
149599 229533.A0A1C3YKV3 pp 229533.I1RBC9
149600 229533.A0A1C3YKV3 pp 229533.I1S4S7
149601 229533.A0A1C3YKV3 pp 229533.I1RRX5
149602 229533.A0A1C3YKV3 pp 229533.I1RSQ7
149603 229533.A0A1C3YKV3 pp 229533.I1RG53
149604 229533.A0A1C3YKV3 pp 229533.A0A1C3YMW0
149605 229533.A0A1C3YKV3 pp 229533.V6QW22
149606 229533.A0A1C3YKV3 pp 229533.I1RFC0
149607 229533.A0A1C3YKV3 pp 229533.I1RXD7
149608 229533.A0A1C3YKV3 pp 229533.I1RX46
149609 229533.A0A1C3YKV3 pp 229533.I1RUQ2
149610 229533.A0A1C3YKV3 pp 229533.I1RLP1
149611 229533.A0A1C3YKV3 pp 229533.I1S0T2
149612 229533.A0A1C3YKV3 pp 229533.V6QYF0
149613 229533.A0A1C3YKV3 pp 229533.V6RUT5
149614 229533.A0A1C3YKV3 pp 229533.I1RAX6
149615 229533.A0A1C3YKV3 pp 229533.I1RBR2
149616 229533.A0A1C3YKV3 pp 229533.I1RME6
149617 229533.A0A1C3YKV3 pp 229533.I1RQH3
149618 229533.A0A1C3YKV3 pp 229533.I1S7A8
149619 229533.A0A1C3YKV3 pp 229533.I1RLC7
149620 229533.A0A1C3YKV3 pp 229533.A0A1C3YLZ7
149621 229533.A0A1C3YKV3 pp 229533.V6RTJ8
149622 229533.A0A1C3YKV3 pp 229533.I1S1W8
149623 229533.A0A1C3YKV3 pp 229533.A0A1C3YL54
149624 229533.A0A1C3YKV3 pp 229533.I1RW54
149625 229533.A0A1C3YKV3 pp 229533.I1RPY3
149626 229533.A0A1C3YKV3 pp 229533.I1RW25
149627 229533.A0A1C3YKV3 pp 229533.I1RX25
149628 229533.A0A1C3YKV3 pp 229533.V6QUW4
149629 229533.A0A1C3YKV3 pp 229533.I1S438
149630 229533.A0A1C3YKV3 pp 229533.I1S0R7
149631 229533.A0A1C3YKV3 pp 229533.I1RN89
149632 229533.A0A1C3YKV3 pp 229533.I1RW07
149633 229533.A0A1C3YKV3 pp 229533.I1RI02
149634 229533.A0A1C3YKV3 pp 229533.I1RQC9
149635 229533.A0A1C3YKV3 pp 229533.I1RM06
149636 229533.A0A1C3YKV3 pp 229533.I1RMK3
149637 229533.A0A1C3YKV3 pp 229533.A0A1C3YLR6
149638 229533.A0A1C3YKV3 pp 229533.I1RCL4
149639 229533.A0A1C3YKV3 pp 229533.I1RKJ5
149640 229533.A0A1C3YKV3 pp 229533.I1RPA4
149641 229533.A0A1C3YKV3 pp 229533.I1RCS1
149642 229533.A0A1C3YKV3 pp 229533.I1S4S4
149643 229533.A0A1C3YKV3 pp 229533.I1S419
149644 229533.A0A1C3YKV3 pp 229533.I1RWJ8
149645 229533.A0A1C3YKV3 pp 229533.I1SA32
149646 229533.A0A1C3YKV3 pp 229533.I1RC16
149647 229533.A0A1C3YKV3 pp 229533.I1RZJ5
149648 229533.A0A1C3YKV3 pp 229533.I1RZM1
149649 229533.A0A1C3YKV3 pp 229533.I1RM67
149650 229533.A0A1C3YKV3 pp 229533.I1RBJ5

149651 229533.A0A1C3YKV3 pp 229533.I1RQ81
149652 229533.A0A1C3YKV3 pp 229533.I1RMG8
149653 229533.A0A1C3YKV3 pp 229533.I1RR24
149654 229533.A0A1C3YKV3 pp 229533.I1RNQ5
149655 229533.A0A1C3YKV3 pp 229533.Q4I8P5
149656 229533.A0A1C3YKV3 pp 229533.I1RLU6
149657 229533.A0A1C3YKV3 pp 229533.I1RE17
149658 229533.A0A1C3YKV3 pp 229533.I1RS18
149659 229533.A0A1C3YKV3 pp 229533.I1R9Y4
149660 229533.A0A1C3YKV3 pp 229533.I1S504
149661 229533.A0A1C3YKV3 pp 229533.I1RG14
149662 229533.A0A1C3YKV3 pp 229533.I1S0X6
149663 229533.A0A1C3YKV3 pp 229533.V6QUE6
149664 229533.A0A1C3YKV3 pp 229533.I1RBT1
149665 229533.A0A1C3YKV3 pp 229533.I1RMC8
149666 229533.A0A1C3YKV3 pp 229533.I1RWT6
149667 229533.A0A1C3YKV3 pp 229533.A0A1C3YKW9
149668 229533.A0A1C3YKV3 pp 229533.I1RGC2
149669 229533.A0A1C3YKV3 pp 229533.I1RAU5
149670 229533.A0A1C3YKV3 pp 229533.I1RRV4
149671 229533.A0A1C3YKV3 pp 229533.I1RRU9
149672 229533.A0A1C3YKV3 pp 229533.I1RAM6
149673 229533.A0A1C3YKV3 pp 229533.A0A1C3YN30
149674 229533.A0A1C3YKV3 pp 229533.A0A1C3YN26
149675 229533.A0A1C3YKV3 pp 229533.I1RJU3
149676 229533.A0A1C3YKV3 pp 229533.A0A1C3YL89
149677 229533.A0A1C3YKV3 pp 229533.I1RDB1
149678 229533.A0A1C3YKV3 pp 229533.I1RQ85
149679 229533.A0A1C3YKV3 pp 229533.I1RI82
149680 229533.A0A1C3YKV3 pp 229533.I1RC17
149681 229533.A0A1C3YKV3 pp 229533.Q4I283
149682 229533.A0A1C3YKV3 pp 229533.I1RQD2
149683 229533.A0A1C3YKV3 pp 229533.I1RW99
149684 229533.A0A1C3YKV3 pp 229533.I1S5B0
149685 229533.A0A1C3YKV3 pp 229533.V6RCZ8
149686 229533.A0A1C3YKV3 pp 229533.I1RGC5
149687 229533.A0A1C3YKV3 pp 229533.V6R227
149688 229533.A0A1C3YKV3 pp 229533.I1S6K9
149689 229533.A0A1C3YKV3 pp 229533.I1S1C9
149690 229533.A0A1C3YKV3 pp 229533.I1RUY5
149691 229533.A0A1C3YKV3 pp 229533.I1RXF0
149692 229533.A0A1C3YKV3 pp 229533.I1RZH4
149693 229533.A0A1C3YKV3 pp 229533.V6RCG5
149694 229533.A0A1C3YKV3 pp 229533.I1RR51
149695 229533.A0A1C3YKV3 pp 229533.I1RZM8
149696 229533.A0A1C3YKV3 pp 229533.I1RAM7
149697 229533.A0A1C3YKV3 pp 229533.I1RXC4
149698 229533.A0A1C3YKV3 pp 229533.I1RQ31
149699 229533.A0A1C3YKV3 pp 229533.A0A1C3YLU7
149700 229533.A0A1C3YKV3 pp 229533.I1RJ17
149701 229533.A0A1C3YKV3 pp 229533.I1REL7
149702 229533.A0A1C3YKV3 pp 229533.Q4I2J8
149703 229533.A0A1C3YKV3 pp 229533.V6RBN2
149704 229533.A0A1C3YKV3 pp 229533.I1RQQ5
149705 229533.A0A1C3YKV3 pp 229533.I1RSM3
149706 229533.A0A1C3YKV3 pp 229533.I1RY03
149707 229533.A0A1C3YKV3 pp 229533.I1RAZ8
149708 229533.A0A1C3YKV3 pp 229533.I1RNT9
149709 229533.A0A1C3YKV3 pp 229533.I1RZZ1
149710 229533.A0A1C3YKV3 pp 229533.I1RCM6
149711 229533.A0A1C3YKV3 pp 229533.I1RSC6
149712 229533.A0A1C3YKV3 pp 229533.I1S4Q4
149713 229533.A0A1C3YKV3 pp 229533.I1RF19
149714 229533.A0A1C3YKV3 pp 229533.I1RYB5
149715 229533.A0A1C3YKV3 pp 229533.I1RDU0
149716 229533.A0A1C3YKV3 pp 229533.I1RE00
149717 229533.A0A1C3YKV3 pp 229533.I1RDG0
149718 229533.A0A1C3YKV3 pp 229533.V6REE9
149719 229533.A0A1C3YKV3 pp 229533.I1RSI0
149720 229533.A0A1C3YKV3 pp 229533.I1RK95
149721 229533.A0A1C3YKV3 pp 229533.A0A1C3YLK2
149722 229533.A0A1C3YKV3 pp 229533.I1RCG4
149723 229533.A0A1C3YKV3 pp 229533.I1RBU8

149724	229533.A0A1C3YKV3	pp	229533.I1RFJ9
149725	229533.A0A1C3YKV3	pp	229533.I1RLM7
149726	229533.A0A1C3YKV3	pp	229533.I1RAZ3
149727	229533.A0A1C3YKV3	pp	229533.I1RWB0
149728	229533.A0A1C3YKV3	pp	229533.I1REK8
149729	229533.A0A1C3YKV3	pp	229533.I1RFU8
149730	229533.A0A1C3YKV3	pp	229533.I1S1J7
149731	229533.A0A1C3YKV3	pp	229533.I1RWD7
149732	229533.A0A1C3YKV3	pp	229533.I1RCR5
149733	229533.A0A1C3YKV3	pp	229533.I1S887
149734	229533.A0A1C3YKV3	pp	229533.Q4IJK2
149735	229533.A0A1C3YKV3	pp	229533.I1RI03
149736	229533.A0A1C3YKV3	pp	229533.I1RCM5
149737	229533.A0A1C3YKV3	pp	229533.A0A1C3YKY6
149738	229533.A0A1C3YKV3	pp	229533.I1RB53
149739	229533.A0A1C3YKV3	pp	229533.I1RZK5
149740	229533.A0A1C3YKV3	pp	229533.I1RQA3
149741	229533.A0A1C3YKV3	pp	229533.I1S0F7
149742	229533.A0A1C3YKV3	pp	229533.I1RYS0
149743	229533.A0A1C3YKV3	pp	229533.I1RPR9
149744	229533.A0A1C3YKV3	pp	229533.I1S0F6
149745	229533.A0A1C3YKV3	pp	229533.I1RDF7
149746	229533.A0A1C3YKV3	pp	229533.I1S1F3
149747	229533.A0A1C3YKV3	pp	229533.I1RSR3
149748	229533.A0A1C3YKV3	pp	229533.I1RBL7
149749	229533.A0A1C3YKV3	pp	229533.I1RN67
149750	229533.A0A1C3YKV3	pp	229533.I1RWR7
149751	229533.A0A1C3YKV3	pp	229533.I1S190
149752	229533.A0A1C3YKV3	pp	229533.A0A1C3YL15
149753	229533.A0A1C3YKV3	pp	229533.I1S0W4
149754	229533.A0A1C3YKV3	pp	229533.I1RU29
149755	229533.A0A1C3YKV3	pp	229533.Q4IEV5
149756	229533.A0A1C3YKV3	pp	229533.I1RY62
149757	229533.A0A1C3YKV3	pp	229533.I1RRY9
149758	229533.A0A1C3YKV3	pp	229533.I1RYR0
149759	229533.A0A1C3YKV3	pp	229533.I1RC05
149760	229533.A0A1C3YKV3	pp	229533.I1RRT2
149761	229533.A0A1C3YKV3	pp	229533.I1RZL6
149762	229533.A0A1C3YKV3	pp	229533.I1RWK3
149763	229533.A0A1C3YKV3	pp	229533.I1RC82
149764	229533.A0A1C3YKV3	pp	229533.I1RP28
149765	229533.A0A1C3YKV3	pp	229533.I1RN63
149766	229533.A0A1C3YKV3	pp	229533.I1RGA4
149767	229533.A0A1C3YKV3	pp	229533.I1S2M6
149768	229533.A0A1C3YKV3	pp	229533.I1RBC7
149769	229533.A0A1C3YKV3	pp	229533.A0A1C3YLA6
149770	229533.A0A1C3YKV3	pp	229533.I1RJV4
149771	229533.A0A1C3YKV3	pp	229533.I1RBS6
149772	229533.A0A1C3YKV3	pp	229533.I1RBS0
149773	229533.A0A1C3YKV3	pp	229533.I1RZB2
149774	229533.A0A1C3YKV3	pp	229533.I1RS47
149775	229533.A0A1C3YKV3	pp	229533.I1RVZ2
149776	229533.A0A1C3YKV3	pp	229533.I1RJN9
149777	229533.A0A1C3YKV3	pp	229533.I1RTA1
149778	229533.A0A1C3YKV3	pp	229533.I1RJ74
149779	229533.A0A1C3YKV3	pp	229533.I1RP27
149780	229533.A0A1C3YKV3	pp	229533.I1S9H4
149781	229533.A0A1C3YKV3	pp	229533.I1RJS2
149782	229533.A0A1C3YKV3	pp	229533.I1RBY4
149783	229533.A0A1C3YKV3	pp	229533.I1RFT1
149784	229533.A0A1C3YKV3	pp	229533.I1S299
149785	229533.A0A1C3YKV3	pp	229533.I1RE76
149786	229533.A0A1C3YKV3	pp	229533.I1RT40
149787	229533.A0A1C3YKV3	pp	229533.I1RCJ7
149788	229533.A0A1C3YKV3	pp	229533.I1S352
149789	229533.A0A1C3YKV3	pp	229533.I1RPU3
149790	229533.A0A1C3YKV3	pp	229533.I1RV91
149791	229533.A0A1C3YKV3	pp	229533.I1S2L3
149792	229533.A0A1C3YKV3	pp	229533.I1S277
149793	229533.A0A1C3YKV3	pp	229533.I1RFL3
149794	229533.A0A1C3YKV3	pp	229533.I1RFL7
149795	229533.A0A1C3YKV3	pp	229533.I1RTD5
149796	229533.A0A1C3YKV3	pp	229533.I1S6B4

149797	229533.A0A1C3YKV3	pp	229533.I1RGF5
149798	229533.A0A1C3YKV3	pp	229533.I1RRJ8
149799	229533.A0A1C3YKV3	pp	229533.I1RA52
149800	229533.A0A1C3YKV3	pp	229533.I1R9Y8
149801	229533.A0A1C3YKV3	pp	229533.I1RQG4
149802	229533.A0A1C3YKV3	pp	229533.I1RXF8
149803	229533.A0A1C3YKV3	pp	229533.I1RE27
149804	229533.A0A1C3YKV3	pp	229533.A0A1C3YMA1
149805	229533.A0A1C3YKV3	pp	229533.I1S8P5
149806	229533.A0A1C3YKV3	pp	229533.I1RPP3
149807	229533.A0A1C3YKV3	pp	229533.I1RT04
149808	229533.A0A1C3YKV3	pp	229533.I1RSZ7
149809	229533.A0A1C3YKV3	pp	229533.I1RW61
149810	229533.A0A1C3YKV3	pp	229533.V6RUY9
149811	229533.A0A1C3YKV3	pp	229533.I1RYI5
149812	229533.A0A1C3YKV3	pp	229533.I1RT75
149813	229533.A0A1C3YKV3	pp	229533.A0A1C3YL55
149814	229533.A0A1C3YKV3	pp	229533.I1RFS3
149815	229533.A0A1C3YKV3	pp	229533.Q4HXF0
149816	229533.A0A1C3YKV3	pp	229533.I1RRK3
149817	229533.A0A1C3YKV3	pp	229533.I1S503
149818	229533.A0A1C3YKV3	pp	229533.I1S6Y8
149819	229533.A0A1C3YKV3	pp	229533.I1RPM4
149820	229533.A0A1C3YKV3	pp	229533.I1RJW6
149821	229533.A0A1C3YKV3	pp	229533.I1RPV3
149822	229533.A0A1C3YKV3	pp	229533.I1RNA5
149823	229533.A0A1C3YKV3	pp	229533.A0A1C3YLF5
149824	229533.A0A1C3YKV3	pp	229533.A0A1C3YLK8
149825	229533.A0A1C3YKV3	pp	229533.I1S2E9
149826	229533.A0A1C3YKV3	pp	229533.I1RRZ0
149827	229533.A0A1C3YKV3	pp	229533.I1RL04
149828	229533.A0A1C3YKV3	pp	229533.I1RRM5
149829	229533.A0A1C3YKV3	pp	229533.I1RQ23
149830	229533.A0A1C3YKV3	pp	229533.I1RLN6
149831	229533.A0A1C3YKV3	pp	229533.V6R3J1
149832	229533.A0A1C3YKV3	pp	229533.I1RVY0
149833	229533.A0A1C3YKV3	pp	229533.I1RCD4
149834	229533.A0A1C3YKV3	pp	229533.I1RN88
149835	229533.A0A1C3YKV3	pp	229533.I1S2B8
149836	229533.A0A1C3YKV3	pp	229533.I1RSX0
149837	229533.A0A1C3YKV3	pp	229533.I1RE22
149838	229533.A0A1C3YKV3	pp	229533.I1RC29
149839	229533.A0A1C3YKV3	pp	229533.I1RM82
149840	229533.A0A1C3YKV3	pp	229533.I1RJZ7
149841	229533.A0A1C3YKV3	pp	229533.I1R9M0
149842	229533.A0A1C3YKV3	pp	229533.I1RGU7
149843	229533.A0A1C3YKV3	pp	229533.I1RSF1
149844	229533.A0A1C3YKV3	pp	229533.I1S9Y1
149845	229533.A0A1C3YKV3	pp	229533.I1RA11
149846	229533.A0A1C3YKV3	pp	229533.I1S0M6
149847	229533.A0A1C3YKV3	pp	229533.I1RWL2
149848	229533.A0A1C3YKV3	pp	229533.A0A1C3YLC1
149849	229533.A0A1C3YKV3	pp	229533.I1S8V3
149850	229533.A0A1C3YKV3	pp	229533.I1RTA0
149851	229533.A0A1C3YKV3	pp	229533.A0A1C3YLB6
149852	229533.A0A1C3YKV3	pp	229533.A0A1C3YLD8
149853	229533.A0A1C3YKV3	pp	229533.I1RXC2
149854	229533.A0A1C3YKV3	pp	229533.I1RC66
149855	229533.A0A1C3YKV3	pp	229533.I1RZS7
149856	229533.A0A1C3YKV3	pp	229533.I1RRL9
149857	229533.A0A1C3YKV3	pp	229533.O74638
149858	229533.A0A1C3YKV3	pp	229533.I1RE01
149859	229533.A0A1C3YKV3	pp	229533.I1RHE5
149860	229533.A0A1C3YKV3	pp	229533.I1RRT8
149861	229533.A0A1C3YKV3	pp	229533.I1S0J1
149862	229533.A0A1C3YKV3	pp	229533.I1RW46
149863	229533.A0A1C3YKV3	pp	229533.I1RZ39
149864	229533.A0A1C3YKV3	pp	229533.I1RC72
149865	229533.A0A1C3YKV3	pp	229533.V6R3X5
149866	229533.A0A1C3YKV3	pp	229533.I1S5R1
149867	229533.A0A1C3YKV3	pp	229533.I1S2H4
149868	229533.A0A1C3YKV3	pp	229533.I1S0R2
149869	229533.A0A1C3YKV3	pp	229533.I1S7W5

149870 229533.A0A1C3YKV3 pp 229533.I1RR73
149871 229533.A0A1C3YKV3 pp 229533.I1REX8
149872 229533.A0A1C3YKV3 pp 229533.I1RS17
149873 229533.A0A1C3YKV3 pp 229533.I1RS20
149874 229533.A0A1C3YKV3 pp 229533.I1RPE0
149875 229533.A0A1C3YKV3 pp 229533.Q4HZB8
149876 229533.A0A1C3YKV3 pp 229533.I1RQS8
149877 229533.A0A1C3YKV3 pp 229533.I1S3G4
149878 229533.A0A1C3YKV3 pp 229533.V6RLM7
149879 229533.A0A1C3YKV3 pp 229533.I1S1W9
149880 229533.A0A1C3YKV3 pp 229533.I1REC5
149881 229533.A0A1C3YKV3 pp 229533.I1RPK0
149882 229533.A0A1C3YKV3 pp 229533.I1RKP1
149883 229533.A0A1C3YKV3 pp 229533.I1RMV0
149884 229533.A0A1C3YKV3 pp 229533.V6RIV9
149885 229533.A0A1C3YKV3 pp 229533.I1SA33
149886 229533.A0A1C3YKV3 pp 229533.I1RPP4
149887 229533.A0A1C3YKV3 pp 229533.I1RSU2
149888 229533.A0A1C3YKV3 pp 229533.I1RIL3
149889 229533.A0A1C3YKV3 pp 229533.I1S0L4
149890 229533.A0A1C3YKV3 pp 229533.I1RRI1
149891 229533.A0A1C3YKV3 pp 229533.I1RWN5
149892 229533.A0A1C3YKV3 pp 229533.I1RSQ4
149893 229533.A0A1C3YKV3 pp 229533.I1RZV3
149894 229533.A0A1C3YKV3 pp 229533.I1RD22
149895 229533.A0A1C3YKV3 pp 229533.I1S256
149896 229533.A0A1C3YKV3 pp 229533.I1RZZ9
149897 229533.A0A1C3YKV3 pp 229533.V6RVG3
149898 229533.A0A1C3YKV3 pp 229533.I1RWI5
149899 229533.A0A1C3YKV3 pp 229533.I1RNN1
149900 229533.A0A1C3YKV3 pp 229533.I1RRN2
149901 229533.A0A1C3YKV3 pp 229533.I1RTN8
149902 229533.A0A1C3YKV3 pp 229533.I1S1W3
149903 229533.A0A1C3YKV3 pp 229533.I1RFH8
149904 229533.A0A1C3YKV3 pp 229533.I1S5R6
149905 229533.A0A1C3YKV3 pp 229533.I1RY44
149906 229533.A0A1C3YKV3 pp 229533.I1RC33
149907 229533.A0A1C3YKV3 pp 229533.I1S9S6
149908 229533.A0A1C3YKV3 pp 229533.I1RRE5
149909 229533.A0A1C3YKV3 pp 229533.I1R9D1
149910 229533.A0A1C3YKV3 pp 229533.V6RGI1
149911 229533.A0A1C3YKV3 pp 229533.I1RG11
149912 229533.A0A1C3YKV3 pp 229533.I1S0N4
149913 229533.A0A1C3YKV3 pp 229533.I1RPU2
149914 229533.A0A1C3YKV3 pp 229533.I1RWQ8
149915 229533.A0A1C3YKV3 pp 229533.I1RCR8
149916 229533.A0A1C3YKV3 pp 229533.I1RUG3
149917 229533.A0A1C3YKV3 pp 229533.I1S8V4
149918 229533.A0A1C3YKV3 pp 229533.I1RQ87
149919 229533.A0A1C3YKV3 pp 229533.I1RML0
149920 229533.A0A1C3YKV3 pp 229533.I1RJV3
149921 229533.A0A1C3YKV3 pp 229533.I1RC81
149922 229533.A0A1C3YKV3 pp 229533.Q4I624
149923 229533.A0A1C3YKV3 pp 229533.I1RNC1
149924 229533.A0A1C3YKV3 pp 229533.I1RXT3
149925 229533.A0A1C3YKV3 pp 229533.I1RD17
149926 229533.A0A1C3YKV3 pp 229533.I1RVF8
149927 229533.A0A1C3YKV3 pp 229533.I1RXU9
149928 229533.A0A1C3YKV3 pp 229533.A0A1C3YKY0
149929 229533.A0A1C3YKV3 pp 229533.V6R057
149930 229533.A0A1C3YKV3 pp 229533.A0A1C3YLE1
149931 229533.A0A1C3YKV3 pp 229533.I1S569
149932 229533.A0A1C3YKV3 pp 229533.I1RRV1
149933 229533.A0A1C3YKV3 pp 229533.I1SAN9
149934 229533.A0A1C3YKV3 pp 229533.I1RAW0
149935 229533.A0A1C3YKV3 pp 229533.I1RTE6
149936 229533.A0A1C3YKV3 pp 229533.I1RWN6
149937 229533.A0A1C3YKV3 pp 229533.I1RD39
149938 229533.A0A1C3YKV3 pp 229533.I1RQ33
149939 229533.A0A1C3YKV3 pp 229533.I1RPL4
149940 229533.A0A1C3YKV3 pp 229533.I1S1C7
149941 229533.A0A1C3YKV3 pp 229533.I1RAU7
149942 229533.A0A1C3YKV3 pp 229533.I1RN18

229533.A0A1C3YKV3	pp	229533.I1RSI6
229533.A0A1C3YKV3	pp	229533.A0A1C3YLS9
229533.A0A1C3YKV3	pp	229533.I1RMG3
229533.A0A1C3YKV3	pp	229533.I1RFL4
229533.A0A1C3YKV3	pp	229533.I1RYW3
229533.A0A1C3YKV3	pp	229533.I1RCW6
229533.A0A1C3YKV3	pp	229533.I1S437
229533.A0A1C3YKV3	pp	229533.V6QVH8
229533.A0A1C3YKV3	pp	229533.V6RMW6
229533.A0A1C3YKV3	pp	229533.I1RRM3
229533.A0A1C3YKV3	pp	229533.I1RQE7
229533.A0A1C3YKV3	pp	229533.V6R042
229533.A0A1C3YKV3	pp	229533.I1RZW8
229533.A0A1C3YKV3	pp	229533.I1RRN5
229533.A0A1C3YKV3	pp	229533.I1RSQ3
229533.A0A1C3YKV3	pp	229533.I1RXR1
229533.A0A1C3YKV3	pp	229533.I1RZ79
229533.A0A1C3YKV3	pp	229533.I1RRI2
229533.A0A1C3YKV3	pp	229533.I1R9J6
229533.A0A1C3YKV3	pp	229533.I1RB41
229533.A0A1C3YKV3	pp	229533.I1RSY2
229533.A0A1C3YKV3	pp	229533.I1RBR1
229533.A0A1C3YKV3	pp	229533.V6RRH2
229533.A0A1C3YKV3	pp	229533.I1RVX0
229533.A0A1C3YKV3	pp	229533.I1RWX5
229533.A0A1C3YKV3	pp	229533.I1S8R3
229533.A0A1C3YKV3	pp	229533.I1RT03
229533.A0A1C3YKV3	pp	229533.I1RB92
229533.A0A1C3YKV3	pp	229533.I1RGL4
229533.A0A1C3YKV3	pp	229533.I1S0F8
229533.A0A1C3YKV3	pp	229533.I1RIH0
229533.A0A1C3YKV3	pp	229533.I1RKB6
229533.A0A1C3YKV3	pp	229533.I1RTH6
229533.A0A1C3YKV3	pp	229533.I1RZJ3
229533.A0A1C3YKV3	pp	229533.I1RSR9
229533.A0A1C3YKV3	pp	229533.I1RE89
229533.A0A1C3YKV3	pp	229533.I1RA70
229533.A0A1C3YKV3	pp	229533.I1RTE9
229533.A0A1C3YKV3	pp	229533.I1RKJ8
229533.A0A1C3YHX4	pp	229533.I1RSU7
229533.A0A1C3YHX4	pp	229533.I1S0F0
229533.A0A1C3YHX4	pp	229533.A0A1C3YIL3
229533.A0A1C3YHX4	pp	229533.I1S4Z5
229533.A0A1C3YHX4	pp	229533.I1RPF4
229533.A0A1C3YHX4	pp	229533.Q4I5V3
229533.A0A1C3YHX4	pp	229533.I1RPR8
229533.A0A1C3YHX4	pp	229533.I1RZU9
229533.A0A1C3YHX4	pp	229533.I1RBZ8
229533.A0A1C3YHX4	pp	229533.A0A1C3YLG0
229533.A0A1C3YHX4	pp	229533.I1RX64
229533.A0A1C3YHX4	pp	229533.I1SB04
229533.A0A1C3YHX4	pp	229533.Q4IER8
229533.A0A1C3YHX4	pp	229533.I1R9Y1
229533.A0A1C3YHX4	pp	229533.I1RWD0
229533.A0A1C3YHX4	pp	229533.I1RT41
229533.A0A1C3YHX4	pp	229533.Q4IPI4
229533.A0A1C3YHX4	pp	229533.A0A1C3YK01
229533.A0A1C3YHX4	pp	229533.I1RCA8
229533.A0A1C3YHX4	pp	229533.Q4HY90
229533.A0A1C3YHX4	pp	229533.I1RBH2
229533.A0A1C3YHX4	pp	229533.Q4HTT1
229533.A0A1C3YHX4	pp	229533.A0A1C3YKS8
229533.A0A1C3YHX4	pp	229533.Q4I948
229533.A0A1C3YHX4	pp	229533.A0A1C3YKP8
229533.A0A1C3YHX4	pp	229533.I1RFN9
229533.A0A1C3YHX4	pp	229533.I1RAV2
229533.A0A1C3YHX4	pp	229533.I1RZF3
229533.A0A1C3YHX4	pp	229533.Q4IAK7
229533.A0A1C3YHX4	pp	229533.A0A1C3YIR3
229533.A0A1C3YHX4	pp	229533.Q4IMD1
229533.A0A1C3YHX4	pp	229533.Q4I7S1
229533.A0A1C3YHX4	pp	229533.I1RW28
229533.A0A1C3YHX4	pp	229533.I1S0Z3

150016	229533.A0A1C3YHX4	pp	229533.I1RQA8
150017	229533.A0A1C3YHX4	pp	229533.Q4IEV4
150018	229533.A0A1C3YHX4	pp	229533.Q4ICA8
150019	229533.A0A1C3YHX4	pp	229533.A0A1C3YM79
150020	229533.A0A1C3YHX4	pp	229533.Q4IB96
150021	229533.A0A1C3YHX4	pp	229533.I1RRH5
150022	229533.A0A1C3YHX4	pp	229533.I1RFW1
150023	229533.A0A1C3YHX4	pp	229533.A0A1C3YKV4
150024	229533.A0A1C3YHX4	pp	229533.A0A1C3YK21
150025	229533.A0A1C3YIS0	pp	229533.I1RTI7
150026	229533.A0A1C3YIS0	pp	229533.I1RFH9
150027	229533.I1R9R7	pp	229533.I1RTM8
150028	229533.I1R9R7	pp	229533.I1RPW0
150029	229533.I1R9R7	pp	229533.I1RBV5
150030	229533.I1R9R7	pp	229533.I1RQ88
150031	229533.I1R9R7	pp	229533.I1RK79
150032	229533.I1R9R7	pp	229533.I1RAI9
150033	229533.I1R9R7	pp	229533.I1RBJ0
150034	229533.I1R9R7	pp	229533.I1RX38
150035	229533.I1R9R7	pp	229533.Q4ICA8
150036	229533.I1R9R7	pp	229533.I1RG11
150037	229533.I1R9R7	pp	229533.I1RZB5
150038	229533.I1R9R7	pp	229533.I1RPU4
150039	229533.I1R9R7	pp	229533.I1RRT0
150040	229533.I1R9R7	pp	229533.I1RJL2
150041	229533.I1R9R7	pp	229533.I1S193
150042	229533.I1R9R7	pp	229533.I1REG5
150043	229533.I1S5B0	pp	229533.V6RUY9
150044	229533.I1S5B0	pp	229533.V6R3J1
150045	229533.I1S5B0	pp	229533.I1SAA0
150046	229533.I1S5B0	pp	229533.O74638
150047	229533.I1S5B0	pp	229533.V6RMW6
150048	229533.I1S5B0	pp	229533.V6R3X5
150049	229533.I1S5B0	pp	229533.I1S8P5
150050	229533.I1S5B0	pp	229533.I1S9Y1
150051	229533.I1S5B0	pp	229533.Q4HXF0
150052	229533.I1S5B0	pp	229533.I1S8V3
150053	229533.I1S5B0	pp	229533.I1S6Y8
150054	229533.I1S5B0	pp	229533.I1S5R6
150055	229533.I1S5B0	pp	229533.V6R042
150056	229533.I1S5B0	pp	229533.I1S5R1
150057	229533.I1S5B0	pp	229533.I1S8V4
150058	229533.I1S5B0	pp	229533.I1S7A8
150059	229533.I1S5B0	pp	229533.I1S7W5
150060	229533.I1S5B0	pp	229533.Q4I624
150061	229533.I1S5B0	pp	229533.I1SA33
150062	229533.I1S5B0	pp	229533.V6RUT5
150063	229533.I1S5B0	pp	229533.V6RTJ8
150064	229533.I1S5B0	pp	229533.I1S8R3
150065	229533.I1S5B0	pp	229533.V6R057
150066	229533.I1S5B0	pp	229533.V6RIV9
150067	229533.I1S5B0	pp	229533.V6RRH2
150068	229533.I1S5B0	pp	229533.Q4IJT5
150069	229533.I1S5B0	pp	229533.V6R1K4
150070	229533.I1S5B0	pp	229533.I1S6K9
150071	229533.I1S5B0	pp	229533.V6QW22
150072	229533.I1S5B0	pp	229533.V6QYF0
150073	229533.I1S5B0	pp	229533.V6RCZ8
150074	229533.I1S5B0	pp	229533.Q4I8P5
150075	229533.I1S5B0	pp	229533.Q4IQK7
150076	229533.I1S5B0	pp	229533.V6QWX8
150077	229533.I1S5B0	pp	229533.V6QUW4
150078	229533.I1S5B0	pp	229533.V6RBN2
150079	229533.I1S5B0	pp	229533.V6QUE6
150080	229533.I1S5B0	pp	229533.I1SA32
150081	229533.I1S5B0	pp	229533.V6RCG5
150082	229533.I1S5B0	pp	229533.Q4IJK2
150083	229533.I1S5B0	pp	229533.Q4IAA0
150084	229533.I1S5B0	pp	229533.Q4I5Z5
150085	229533.I1S5B0	pp	229533.Q4I283
150086	229533.I1S5B0	pp	229533.I1S887
150087	229533.I1S5B0	pp	229533.Q4I2J8
150088	229533.I1RQ81	pp	229533.I1RWN5

150089 229533.I1RQ81 pp 229533.I1S0N4
150090 229533.I1RQ81 pp 229533.V6RIV9
150091 229533.I1RQ81 pp 229533.I1SA33
150092 229533.I1RQ81 pp 229533.V6RGN5
150093 229533.I1RQ81 pp 229533.I1S0J1
150094 229533.I1RQ81 pp 229533.I1S1U7
150095 229533.I1RQ81 pp 229533.I1RTA0
150096 229533.I1RQ81 pp 229533.I1RSK1
150097 229533.I1RQ81 pp 229533.I1RWN6
150098 229533.I1RQ81 pp 229533.V6RMW6
150099 229533.I1RQ81 pp 229533.Q4I5V2
150100 229533.I1RQ81 pp 229533.I1RSQ3
150101 229533.I1RQ81 pp 229533.V6R042
150102 229533.I1RQ81 pp 229533.I1RRV1
150103 229533.I1RQ81 pp 229533.I1S2C0
150104 229533.I1RQ81 pp 229533.I1RTH6
150105 229533.I1RQ81 pp 229533.I1S0R4
150106 229533.I1RQ81 pp 229533.I1SAN9
150107 229533.I1RQ81 pp 229533.V6RSF3
150108 229533.I1RQ81 pp 229533.I1S0R3
150109 229533.I1RQ81 pp 229533.I1S0M2
150110 229533.I1RQ81 pp 229533.I1RZ79
150111 229533.I1RQ81 pp 229533.I1RYW3
150112 229533.I1RQ81 pp 229533.Q4I1Y5
150113 229533.I1RQ81 pp 229533.I1RUP5
150114 229533.I1RQ81 pp 229533.I1RUG3
150115 229533.I1RQ81 pp 229533.I1RQJ4
150116 229533.I1RQ81 pp 229533.I1RRI2
150117 229533.I1RQ81 pp 229533.I1S1W4
150118 229533.I1RQ81 pp 229533.I1RWX5
150119 229533.I1RQ81 pp 229533.Q4I624
150120 229533.I1RQ81 pp 229533.V6RRH2
150121 229533.I1RQ81 pp 229533.I1RY44
150122 229533.I1RQ81 pp 229533.I1S1C7
150123 229533.I1RQ81 pp 229533.I1RT40
150124 229533.I1RQ81 pp 229533.I1RRN5
150125 229533.I1RQ81 pp 229533.V6R3X5
150126 229533.I1RQ81 pp 229533.I1RVX0
150127 229533.I1RQ81 pp 229533.I1RR73
150128 229533.I1RQ81 pp 229533.I1RT03
150129 229533.I1RQ81 pp 229533.I1S2G7
150130 229533.I1RQ81 pp 229533.I1RZ39
150131 229533.I1RQ81 pp 229533.I1RQD6
150132 229533.I1RQ81 pp 229533.I1S0F6
150133 229533.I1RQ81 pp 229533.I1RS47
150134 229533.I1RQ81 pp 229533.I1S2E8
150135 229533.I1RQ81 pp 229533.I1RZZ9
150136 229533.I1RQ81 pp 229533.I1RQE7
150137 229533.I1RQ81 pp 229533.I1RRJ8
150138 229533.I1RQ81 pp 229533.I1RWI5
150139 229533.I1RQ81 pp 229533.I1RQF2
150140 229533.I1RQ81 pp 229533.I1RYX5
150141 229533.I1RQ81 pp 229533.I1RQG4
150142 229533.I1RQ81 pp 229533.I1S8P5
150143 229533.I1RQ81 pp 229533.I1S0X5
150144 229533.I1RQ81 pp 229533.I1RX50
150145 229533.I1RQ81 pp 229533.I1RSV2
150146 229533.I1RQ81 pp 229533.I1RSD8
150147 229533.I1RQ81 pp 229533.I1RWK3
150148 229533.I1RQ81 pp 229533.I1RRM5
150149 229533.I1RQ81 pp 229533.I1S0L8
150150 229533.I1RQ81 pp 229533.I1S0L4
150151 229533.I1RQ81 pp 229533.Q4IQW0
150152 229533.I1RQ81 pp 229533.I1S2B8
150153 229533.I1RQ81 pp 229533.I1RRT2
150154 229533.I1RQ81 pp 229533.I1RVY0
150155 229533.I1RQ81 pp 229533.I1RS20
150156 229533.I1RQ81 pp 229533.I1S503
150157 229533.I1RQ81 pp 229533.I1S2H4
150158 229533.I1RQ81 pp 229533.V6RLW4
150159 229533.I1RQ81 pp 229533.I1RWL2
150160 229533.I1RQ81 pp 229533.I1RRM9
150161 229533.I1RQ81 pp 229533.I1RW99

150162 229533.I1RQ81 pp 229533.I1S2E9
150163 229533.I1RQ81 pp 229533.I1S835
150164 229533.I1RQ81 pp 229533.I1RRT8
150165 229533.I1RQ81 pp 229533.I1RT04
150166 229533.I1RQ81 pp 229533.I1S044
150167 229533.I1RQ81 pp 229533.I1RTD5
150168 229533.I1RQ81 pp 229533.I1RRU9
150169 229533.I1RQ81 pp 229533.I1RRY9
150170 229533.I1RQ81 pp 229533.I1S5R1
150171 229533.I1RQ81 pp 229533.V6RBN2
150172 229533.I1RQ81 pp 229533.I1S1J7
150173 229533.I1RQ81 pp 229533.I1S5B0
150174 229533.I1RQ81 pp 229533.I1RYS0
150175 229533.I1RQ81 pp 229533.V6QUE6
150176 229533.I1RQ81 pp 229533.I1RZK5
150177 229533.I1RQ81 pp 229533.I1RZZ1
150178 229533.I1RQ81 pp 229533.V6RCG5
150179 229533.I1RQ81 pp 229533.I1RZB5
150180 229533.I1RQ81 pp 229533.I1RZM8
150181 229533.I1RQ81 pp 229533.Q4IJK2
150182 229533.I1RQ81 pp 229533.I1RQA3
150183 229533.I1RQ81 pp 229533.I1RWT6
150184 229533.I1RQ81 pp 229533.I1RYL5
150185 229533.I1RQ81 pp 229533.V6RTJ8
150186 229533.I1RQ81 pp 229533.I1S2B6
150187 229533.I1RQ81 pp 229533.I1RYY5
150188 229533.I1RQ81 pp 229533.I1S504
150189 229533.I1RQ81 pp 229533.I1RQH1
150190 229533.I1RQ81 pp 229533.I1RX46
150191 229533.I1RQ81 pp 229533.I1RZM1
150192 229533.I1RQ81 pp 229533.Q4IEK8
150193 229533.I1RQ81 pp 229533.I1RXD7
150194 229533.I1RQ81 pp 229533.I1RY03
150195 229533.I1RQ81 pp 229533.I1RQH3
150196 229533.I1RQ81 pp 229533.Q4I8P5
150197 229533.I1RQ81 pp 229533.I1RWD7
150198 229533.I1RQ81 pp 229533.I1S0R7
150199 229533.I1RQ81 pp 229533.I1S4Q4
150200 229533.I1RQ81 pp 229533.I1SA32
150201 229533.I1RQ81 pp 229533.I1RS18
150202 229533.I1RQ81 pp 229533.I1RSI0
150203 229533.I1RQ81 pp 229533.I1RZE5
150204 229533.I1RQ81 pp 229533.I1RSM2
150205 229533.I1RQ81 pp 229533.I1RQC9
150206 229533.I1RQ81 pp 229533.I1RR24
150207 229533.I1RQ81 pp 229533.I1RXU7
150208 229533.I1RQ81 pp 229533.I1S0L3
150209 229533.I1RQ81 pp 229533.I1RYJ7
150210 229533.I1RQ81 pp 229533.Q4IJM4
150211 229533.I1RQ81 pp 229533.I1S0X6
150212 229533.I1RQ81 pp 229533.I1RTF4
150213 229533.I1RQ81 pp 229533.I1RVQ0
150214 229533.I1RQ81 pp 229533.I1RR00
150215 229533.I1RQ81 pp 229533.I1RSQ7
150216 229533.I1RQ81 pp 229533.V6QUW4
150217 229533.I1RQ81 pp 229533.I1RW71
150218 229533.I1RQ81 pp 229533.I1RW25
150219 229533.I1RQ81 pp 229533.I1RYH9
150220 229533.A0A098DST9 pp 229533.Q4HY90
150221 229533.A0A098DST9 pp 229533.A0A1C3YHX4
150222 229533.A0A098DST9 pp 229533.I1RXE5
150223 229533.A0A098DST9 pp 229533.A0A1C3YK01
150224 229533.A0A098DST9 pp 229533.I1RZG7
150225 229533.I1RXG9 pp 229533.Q4IBX0
150226 229533.I1RXG9 pp 229533.Q4IMM0
150227 229533.A0A1C3YHH7 pp 229533.I1RRT0
150228 229533.A0A1C3YHH7 pp 229533.Q4ICA8
150229 229533.A0A1C3YHH7 pp 229533.I1RG11
150230 229533.A0A1C3YHH7 pp 229533.I1RPW0
150231 229533.A0A1C3YHH7 pp 229533.A0A1C3YK37
150232 229533.A0A1C3YHH7 pp 229533.I1S193
150233 229533.A0A1C3YHH7 pp 229533.I1REG5
150234 229533.A0A1C3YHH7 pp 229533.I1RPU4

150235 229533.A0A1C3YHH7 pp 229533.I1RAI9
150236 229533.A0A1C3YHH7 pp 229533.I1RJL2
150237 229533.A0A1C3YHH7 pp 229533.I1RK79
150238 229533.A0A1C3YHH7 pp 229533.I1RBJ0
150239 229533.A0A1C3YHH7 pp 229533.I1RTM8
150240 229533.A0A1C3YHH7 pp 229533.I1RZB5
150241 229533.A0A1C3YHH7 pp 229533.A0A1C3YKJ6
150242 229533.A0A1C3YHH7 pp 229533.I1RQ88
150243 229533.A0A1C3YHH7 pp 229533.I1RBV5
150244 229533.A0A1C3YHH7 pp 229533.A0A1C3YIH7
150245 229533.A0A1C3YHH7 pp 229533.A0A1C3YIR8
150246 229533.A0A1C3YHH7 pp 229533.A0A1C3YJA8
150247 229533.A0A1C3YHH7 pp 229533.I1RX38
150248 229533.I1RVZ0 pp 229533.I1RZV6
150249 229533.I1RVZ0 pp 229533.I1S9U2
150250 229533.I1RVZ0 pp 229533.Q4HVW2
150251 229533.I1RVZ0 pp 229533.V6R3X5
150252 229533.I1RVZ0 pp 229533.I1S9Y1
150253 229533.I1RVZ0 pp 229533.I1S6U8
150254 229533.I1RVZ0 pp 229533.Q4IQW0
150255 229533.I1RVZ0 pp 229533.I1S1B6
150256 229533.I1RVZ0 pp 229533.I1S8E2
150257 229533.I1RVZ0 pp 229533.I1S253
150258 229533.I1RVZ0 pp 229533.I1RZ39
150259 229533.I1RVZ0 pp 229533.Q4IJH1
150260 229533.I1RVZ0 pp 229533.Q4IPI1
150261 229533.I1RVZ0 pp 229533.I1RX46
150262 229533.I1RVZ0 pp 229533.Q4IF76
150263 229533.I1RVZ0 pp 229533.I1RZG3
150264 229533.I1RVZ0 pp 229533.I1S503
150265 229533.I1RVZ0 pp 229533.Q4I5R9
150266 229533.I1RVZ0 pp 229533.I1S0R7
150267 229533.I1RVZ0 pp 229533.I1RXD7
150268 229533.I1RVZ0 pp 229533.Q4IAS1
150269 229533.I1RVZ0 pp 229533.I1S2B8
150270 229533.I1RVZ0 pp 229533.I1RW25
150271 229533.I1RVZ0 pp 229533.Q4IR18
150272 229533.I1RVZ0 pp 229533.I1RZS6
150273 229533.I1RVZ0 pp 229533.Q4IFI0
150274 229533.I1RVZ0 pp 229533.V6QYF0
150275 229533.I1RVZ0 pp 229533.I1RZJ6
150276 229533.I1RVZ0 pp 229533.I1RX08
150277 229533.I1RVZ0 pp 229533.Q4IQK7
150278 229533.I1RVZ0 pp 229533.I1RYB9
150279 229533.I1RVZ0 pp 229533.I1S6F0
150280 229533.I1RVZ0 pp 229533.I1S021
150281 229533.I1RVZ0 pp 229533.Q4HZ42
150282 229533.I1RVZ0 pp 229533.Q4I662
150283 229533.I1RVZ0 pp 229533.Q4I327
150284 229533.I1RVZ0 pp 229533.I1RX24
150285 229533.I1RVZ0 pp 229533.Q4HZ47
150286 229533.I1RVZ0 pp 229533.I1RWF7
150287 229533.I1RVZ0 pp 229533.I1S296
150288 229533.I1RVZ0 pp 229533.Q4IN63
150289 229533.I1RVZ0 pp 229533.I1S1C7
150290 229533.I1RVZ0 pp 229533.V6R227
150291 229533.I1RVZ0 pp 229533.I1RW71
150292 229533.I1RVZ0 pp 229533.I1RZ99
150293 229533.I1RVZ0 pp 229533.Q4HZ68
150294 229533.I1RVZ0 pp 229533.Q4I830
150295 229533.I1RVZ0 pp 229533.I1S6E5
150296 229533.I1RVZ0 pp 229533.I1S0M3
150297 229533.I1RVZ0 pp 229533.I1S9U1
150298 229533.I1RVZ0 pp 229533.I1RWH5
150299 229533.I1RVZ0 pp 229533.I1S504
150300 229533.I1RVZ0 pp 229533.I1RZH6
150301 229533.I1RVZ0 pp 229533.I1RZE5
150302 229533.I1RVZ0 pp 229533.I1RZF6
150303 229533.I1RVZ0 pp 229533.Q4IEK8
150304 229533.I1RVZ0 pp 229533.V6R283
150305 229533.I1RVZ0 pp 229533.I1S1X9
150306 229533.I1RVZ0 pp 229533.I1S4T3
150307 229533.I1RVZ0 pp 229533.I1S2E8

150308	229533.I1RVZ0	pp	229533.V6RGY3
150309	229533.I1RVZ0	pp	229533.I1RWN6
150310	229533.I1RVZ0	pp	229533.I1RZZ1
150311	229533.I1RVZ0	pp	229533.I1RZK6
150312	229533.I1RVZ0	pp	229533.I1S274
150313	229533.I1RVZ0	pp	229533.I1RWN5
150314	229533.I1RVZ0	pp	229533.I1S2C0
150315	229533.I1RVZ0	pp	229533.I1S2U2
150316	229533.I1RVZ0	pp	229533.Q4IAA0
150317	229533.I1RVZ0	pp	229533.I1RZK5
150318	229533.I1RVZ0	pp	229533.I1RWB5
150319	229533.I1RVZ0	pp	229533.Q4IBS2
150320	229533.I1RVZ0	pp	229533.I1S0M9
150321	229533.I1RVZ0	pp	229533.V6RGN5
150322	229533.I1RVZ0	pp	229533.V6RMW6
150323	229533.I1RVZ0	pp	229533.I1RY03
150324	229533.I1RVZ0	pp	229533.Q4IJ56
150325	229533.I1RVZ0	pp	229533.V6R042
150326	229533.I1RVZ0	pp	229533.I1S0J1
150327	229533.I1RVZ0	pp	229533.I1S060
150328	229533.I1RVZ0	pp	229533.I1RX14
150329	229533.I1RCL8	pp	229533.I1S1W4
150330	229533.I1RCL8	pp	229533.V6RVG3
150331	229533.I1RCL8	pp	229533.V6RKY7
150332	229533.I1RCL8	pp	229533.I1RUQ5
150333	229533.I1RCL8	pp	229533.I1RDZ4
150334	229533.I1RCL8	pp	229533.I1RJ33
150335	229533.I1RCL8	pp	229533.I1RQB4
150336	229533.I1RCL8	pp	229533.I1RMM9
150337	229533.I1RCL8	pp	229533.I1S0D1
150338	229533.I1RCL8	pp	229533.I1RZE4
150339	229533.I1RCL8	pp	229533.V6R6F6
150340	229533.I1RCL8	pp	229533.I1RNY9
150341	229533.I1RCL8	pp	229533.I1RFL7
150342	229533.I1RCL8	pp	229533.I1RYH9
150343	229533.I1RCL8	pp	229533.I1SAG3
150344	229533.I1RCL8	pp	229533.I1RUN8
150345	229533.I1RCL8	pp	229533.I1S439
150346	229533.I1RCL8	pp	229533.I1RDB1
150347	229533.I1RCL8	pp	229533.I1RJH1
150348	229533.I1RCL8	pp	229533.I1SA77
150349	229533.I1RCL8	pp	229533.I1S1W5
150350	229533.I1RCL8	pp	229533.I1RX70
150351	229533.I1RW99	pp	229533.I1S2M6
150352	229533.I1RW99	pp	229533.I1RZZ9
150353	229533.I1RW99	pp	229533.I1RWK3
150354	229533.I1RW99	pp	229533.I1RWI5
150355	229533.I1RW99	pp	229533.I1RZ39
150356	229533.I1RW99	pp	229533.I1S8P5
150357	229533.I1RW99	pp	229533.V6R3X5
150358	229533.I1RW99	pp	229533.I1S0F6
150359	229533.I1RW99	pp	229533.O74638
150360	229533.I1RW99	pp	229533.I1RZL6
150361	229533.I1RW99	pp	229533.Q4IEV5
150362	229533.I1RW99	pp	229533.I1S5R1
150363	229533.I1RW99	pp	229533.I1S2E9
150364	229533.I1RW99	pp	229533.I1S0L4
150365	229533.I1RW99	pp	229533.I1RWN5
150366	229533.I1RW99	pp	229533.V6RIV9
150367	229533.I1RW99	pp	229533.I1S0J1
150368	229533.I1RW99	pp	229533.I1S2H4
150369	229533.I1RW99	pp	229533.I1S8V4
150370	229533.I1RW99	pp	229533.V6RUY9
150371	229533.I1RW99	pp	229533.I1RZS7
150372	229533.I1RW99	pp	229533.I1S503
150373	229533.I1RW99	pp	229533.I1S0R2
150374	229533.I1RW99	pp	229533.V6R042
150375	229533.I1RW99	pp	229533.I1S6Y8
150376	229533.I1RW99	pp	229533.V6RMW6
150377	229533.I1RW99	pp	229533.I1RY44
150378	229533.I1RW99	pp	229533.I1S0M6
150379	229533.I1RW99	pp	229533.V6RGN5
150380	229533.I1RW99	pp	229533.I1S2B8

150381 229533.I1RW99 pp 229533.I1SA33
150382 229533.I1RW99 pp 229533.Q4I624
150383 229533.I1RW99 pp 229533.I1S8V3
150384 229533.I1RW99 pp 229533.Q4HZB8
150385 229533.I1RW99 pp 229533.I1S6F0
150386 229533.I1RW99 pp 229533.I1S0N4
150387 229533.I1RW99 pp 229533.I1RWD4
150388 229533.I1RW99 pp 229533.V6R057
150389 229533.I1RW99 pp 229533.I1RWQ8
150390 229533.I1RW99 pp 229533.I1S0D4
150391 229533.I1RW99 pp 229533.I1S2E8
150392 229533.I1RW99 pp 229533.I1RZW8
150393 229533.I1RW99 pp 229533.I1RWX5
150394 229533.I1RW99 pp 229533.I1RZH4
150395 229533.I1RW99 pp 229533.Q4I8P5
150396 229533.I1RW99 pp 229533.I1S1C7
150397 229533.I1RW99 pp 229533.I1S0F5
150398 229533.I1RW99 pp 229533.I1RYW3
150399 229533.I1RW99 pp 229533.I1RZM1
150400 229533.I1RW99 pp 229533.I1S0L3
150401 229533.I1RW99 pp 229533.I1S8R3
150402 229533.I1RW99 pp 229533.V6R1K4
150403 229533.I1RW99 pp 229533.I1S419
150404 229533.I1RW99 pp 229533.I1RXD7
150405 229533.I1RW99 pp 229533.Q4IEK8
150406 229533.I1RW99 pp 229533.Q4IJT5
150407 229533.I1RW99 pp 229533.I1S0R7
150408 229533.I1RW99 pp 229533.I1RWN6
150409 229533.I1RW99 pp 229533.I1RX46
150410 229533.I1RW99 pp 229533.I1RWT6
150411 229533.I1RW99 pp 229533.I1S0T2
150412 229533.I1RW99 pp 229533.V6QW22
150413 229533.I1RW99 pp 229533.Q4I283
150414 229533.I1RW99 pp 229533.V6R227
150415 229533.I1RW99 pp 229533.I1S7A8
150416 229533.I1RW99 pp 229533.V6QYF0
150417 229533.I1RW99 pp 229533.V6RTJ8
150418 229533.I1RW99 pp 229533.V6QUW4
150419 229533.I1RW99 pp 229533.I1RZZ1
150420 229533.I1RW99 pp 229533.I1S504
150421 229533.I1RW99 pp 229533.I1RX25
150422 229533.I1RW99 pp 229533.I1S0X6
150423 229533.I1RW99 pp 229533.I1RZM8
150424 229533.I1RW99 pp 229533.I1RXF0
150425 229533.I1RW99 pp 229533.Q4IJK2
150426 229533.I1RW99 pp 229533.Q4HVW2
150427 229533.I1RW99 pp 229533.Q4I2J8
150428 229533.I1RW99 pp 229533.V6RCG5
150429 229533.I1RW99 pp 229533.I1RZJ5
150430 229533.I1RW99 pp 229533.I1RWD7
150431 229533.I1RW99 pp 229533.I1RWJ8
150432 229533.I1RW99 pp 229533.V6RCZ8
150433 229533.I1RW99 pp 229533.I1RY03
150434 229533.I1RW99 pp 229533.I1SA32
150435 229533.I1RW99 pp 229533.I1RZK5
150436 229533.I1RW99 pp 229533.I1S5B0
150437 229533.I1RW99 pp 229533.Q4IBS2
150438 229533.I1RW99 pp 229533.I1S1J7
150439 229533.I1RWT1 pp 229533.I1RYR9
150440 229533.I1RWT1 pp 229533.I1S5B2
150441 229533.I1RWT1 pp 229533.I1RX38
150442 229533.I1RWT1 pp 229533.I1RYW7
150443 229533.I1RWT1 pp 229533.I1RZB5
150444 229533.I1RWT1 pp 229533.I1RYX3
150445 229533.I1RWT1 pp 229533.I1RX63
150446 229533.I1RWT1 pp 229533.Q4ICA8
150447 229533.Q4I2J8 pp 229533.Q4IJT5
150448 229533.Q4I2J8 pp 229533.V6QW22
150449 229533.Q4I2J8 pp 229533.V6RMW6
150450 229533.Q4I2J8 pp 229533.V6RTJ8
150451 229533.Q4I2J8 pp 229533.V6R042
150452 229533.Q4I2J8 pp 229533.V6RCG5
150453 229533.Q4I2J8 pp 229533.V6R1K4

229533.Q4I2J8 pp 229533.Q4I8P5
229533.Q4I2J8 pp 229533.Q4IJK2
229533.Q4I2J8 pp 229533.V6RIV9
229533.Q4I2J8 pp 229533.Q4I624
229533.Q4I2J8 pp 229533.V6R057
229533.Q4I2J8 pp 229533.V6R3X5
229533.Q4I2J8 pp 229533.V6QUV7
229533.I1RUE4 pp 229533.P0C431
229533.I1RUE4 pp 229533.Q4IBX0
229533.I1RUE4 pp 229533.I1S0S6
229533.I1RUE4 pp 229533.Q4IMM0
229533.A0A0E0RTX3 pp 229533.I1RCU9
229533.A0A0E0RTX3 pp 229533.I1RPK0
229533.A0A0E0RTX3 pp 229533.I1RWB5
229533.A0A0E0RTX3 pp 229533.I1RDB1
229533.A0A0E0RTX3 pp 229533.I1RHJ2
229533.A0A0E0RTX3 pp 229533.A0A1C3YII1
229533.A0A0E0RTX3 pp 229533.I1RZQ6
229533.A0A0E0RTX3 pp 229533.I1RCU8
229533.A0A0E0RTX3 pp 229533.I1RST8
229533.A0A0E0RTX3 pp 229533.V6RDP1
229533.A0A0E0RTX3 pp 229533.I1RRR6
229533.A0A0E0RTX3 pp 229533.I1RAM7
229533.A0A1C3YM12 pp 229533.I1RNG4
229533.I1S1Y9 pp 229533.I1S7W5
229533.I1S1Y9 pp 229533.I1S2B9
229533.A0A0E0RZM5 pp 229533.I1RD22
229533.A0A0E0RZM5 pp 229533.I1RQ75
229533.A0A0E0RZM5 pp 229533.I1RUG3
229533.A0A0E0RZM5 pp 229533.I1RNC1
229533.A0A0E0RZM5 pp 229533.I1RCR8
229533.A0A0E0RZM5 pp 229533.V6RMW6
229533.A0A0E0RZM5 pp 229533.I1RY44
229533.A0A0E0RZM5 pp 229533.I1RJV3
229533.A0A0E0RZM5 pp 229533.V6R042
229533.A0A0E0RZM5 pp 229533.I1RWN6
229533.A0A0E0RZM5 pp 229533.I1RSQ3
229533.A0A0E0RZM5 pp 229533.V6RIV9
229533.A0A0E0RZM5 pp 229533.I1RAU7
229533.A0A0E0RZM5 pp 229533.I1RWN5
229533.A0A0E0RZM5 pp 229533.I1RAW0
229533.A0A0E0RZM5 pp 229533.I1RZW8
229533.A0A0E0RZM5 pp 229533.Q4I1Y5
229533.A0A0E0RZM5 pp 229533.I1S1C7
229533.A0A0E0RZM5 pp 229533.I1RD17
229533.A0A0E0RZM5 pp 229533.I1RK44
229533.A0A0E0RZM5 pp 229533.I1RN18
229533.A0A0E0RZM5 pp 229533.I1RA70
229533.A0A0E0RZM5 pp 229533.I1S8R3
229533.A0A0E0RZM5 pp 229533.I1RTA0
229533.A0A0E0RZM5 pp 229533.I1RFL4
229533.A0A0E0RZM5 pp 229533.I1RPP4
229533.A0A0E0RZM5 pp 229533.I1RB92
229533.A0A0E0RZM5 pp 229533.I1RTH6
229533.A0A0E0RZM5 pp 229533.I1RVX0
229533.A0A0E0RZM5 pp 229533.I1RN67
229533.A0A0E0RZM5 pp 229533.I1RWK3
229533.A0A0E0RZM5 pp 229533.I1RJV4
229533.A0A0E0RZM5 pp 229533.I1RBS6
229533.A0A0E0RZM5 pp 229533.I1RTD5
229533.A0A0E0RZM5 pp 229533.I1RT03
229533.A0A0E0RZM5 pp 229533.I1S0L4
229533.A0A0E0RZM5 pp 229533.I1RV91
229533.A0A0E0RZM5 pp 229533.I1RPP3
229533.A0A0E0RZM5 pp 229533.I1S2H4
229533.A0A0E0RZM5 pp 229533.I1S8P5
229533.A0A0E0RZM5 pp 229533.I1S2E9
229533.A0A0E0RZM5 pp 229533.I1RZZ9
229533.A0A0E0RZM5 pp 229533.I1S503
229533.A0A0E0RZM5 pp 229533.I1RJW6
229533.A0A0E0RZM5 pp 229533.I1RT04
229533.A0A0E0RZM5 pp 229533.I1RBY4
229533.A0A0E0RZM5 pp 229533.V6R3X5

150527 229533.A0A0E0RZM5 pp 229533.I1R9M0
150528 229533.A0A0E0RZM5 pp 229533.I1RRM5
150529 229533.A0A0E0RZM5 pp 229533.A0A1C3YHX6
150530 229533.A0A0E0RZM5 pp 229533.I1RY03
150531 229533.A0A0E0RZM5 pp 229533.I1RR73
150532 229533.A0A0E0RZM5 pp 229533.I1RZK5
150533 229533.A0A0E0RZM5 pp 229533.I1S2B8
150534 229533.A0A0E0RZM5 pp 229533.I1RZ39
150535 229533.A0A0E0RZM5 pp 229533.A0A1C3YKM9
150536 229533.A0A0E0RZM5 pp 229533.I1RPM4
150537 229533.A0A0E0RZM5 pp 229533.I1RFU8
150538 229533.A0A0E0RZM5 pp 229533.I1RLU6
150539 229533.A0A0E0RZM5 pp 229533.I1RCG4
150540 229533.A0A0E0RZM5 pp 229533.I1RVQ0
150541 229533.A0A0E0RZM5 pp 229533.I1RRT8
150542 229533.A0A0E0RZM5 pp 229533.I1RZM1
150543 229533.A0A0E0RZM5 pp 229533.A0A1C3YKK9
150544 229533.A0A0E0RZM5 pp 229533.A0A1C3YKB0
150545 229533.A0A0E0RZM5 pp 229533.A0A1C3YIP5
150546 229533.A0A0E0RZM5 pp 229533.I1RR39
150547 229533.A0A0E0RZM5 pp 229533.I1RI82
150548 229533.A0A0E0RZM5 pp 229533.I1RIE8
150549 229533.A0A0E0RZM5 pp 229533.I1S504
150550 229533.A0A0E0RZM5 pp 229533.V6RCG5
150551 229533.A0A0E0RZM5 pp 229533.I1S0L3
150552 229533.A0A0E0RZM5 pp 229533.I1RS18
150553 229533.A0A0E0RZM5 pp 229533.A0A1C3YKV3
150554 229533.A0A0E0RZM5 pp 229533.A0A0E0SQ99
150555 229533.A0A0E0RZM5 pp 229533.I1RSQ7
150556 229533.A0A0E0RZM5 pp 229533.A0A1C3YIJ3
150557 229533.A0A0E0RZM5 pp 229533.I1REK8
150558 229533.A0A0E0RZM5 pp 229533.I1RAU5
150559 229533.A0A0E0RZM5 pp 229533.Q4IJK2
150560 229533.A0A0E0RZM5 pp 229533.V6QW22
150561 229533.A0A0E0RZM5 pp 229533.I1RAX6
150562 229533.A0A0E0RZM5 pp 229533.A0A1C3YI71
150563 229533.A0A0E0RZM5 pp 229533.A0A0E0SFN0
150564 229533.A0A0E0RZM5 pp 229533.I1RBC9
150565 229533.A0A0E0RZM5 pp 229533.I1RQC9
150566 229533.A0A0E0RZM5 pp 229533.I1RX46
150567 229533.A0A0E0RZM5 pp 229533.I1RW25
150568 229533.A0A0E0RZM5 pp 229533.I1RRY9
150569 229533.A0A0E0RZM5 pp 229533.I1RJW1
150570 229533.A0A0E0RZM5 pp 229533.I1RR24
150571 229533.A0A0E0RZM5 pp 229533.V6RTJ8
150572 229533.A0A0E0RZM5 pp 229533.A0A1C3YHX8
150573 229533.A0A0E0RZM5 pp 229533.A0A1C3YKC0
150574 229533.A0A0E0RZM5 pp 229533.I1RBT1
150575 229533.A0A0E0RZM5 pp 229533.A0A0E0SA11
150576 229533.I1S3L1 pp 229533.V6RDP1
150577 229533.I1RK56 pp 229533.I1RPR9
150578 229533.I1RC17 pp 229533.I1S8R3
150579 229533.I1RC17 pp 229533.I1RRI2
150580 229533.I1RC17 pp 229533.I1RWT5
150581 229533.I1RC17 pp 229533.I1RZW8
150582 229533.I1RC17 pp 229533.I1RJT8
150583 229533.I1RC17 pp 229533.I1RW84
150584 229533.I1RC17 pp 229533.I1RT03
150585 229533.I1RC17 pp 229533.I1RQC0
150586 229533.I1RC17 pp 229533.I1RRN5
150587 229533.I1RC17 pp 229533.I1RWN6
150588 229533.I1RC17 pp 229533.V6RIV9
150589 229533.I1RC17 pp 229533.I1RN18
150590 229533.I1RC17 pp 229533.I1RY44
150591 229533.I1RC17 pp 229533.I1S5X9
150592 229533.I1RC17 pp 229533.V6RMW6
150593 229533.I1RC17 pp 229533.I1RFL4
150594 229533.I1RC17 pp 229533.I1RC24
150595 229533.I1RC17 pp 229533.I1RSQ3
150596 229533.I1RC17 pp 229533.I1RZE4
150597 229533.I1RC17 pp 229533.I1S0E8
150598 229533.I1RC17 pp 229533.V6R3X5
150599 229533.I1RC17 pp 229533.I1RCB7

150600 229533.I1RC17 pp 229533.I1RUG3
150601 229533.I1RC17 pp 229533.I1RU69
150602 229533.I1RC17 pp 229533.I1RQE7
150603 229533.I1RC17 pp 229533.I1RQB4
150604 229533.I1RC17 pp 229533.I1SAF7
150605 229533.I1RC17 pp 229533.I1RY58
150606 229533.I1RC17 pp 229533.I1RD22
150607 229533.I1RC17 pp 229533.I1S2H4
150608 229533.I1RC17 pp 229533.I1RCQ3
150609 229533.I1RC17 pp 229533.I1RJT0
150610 229533.I1RC17 pp 229533.I1S0J1
150611 229533.I1RC17 pp 229533.I1RNY8
150612 229533.I1RC17 pp 229533.I1RK75
150613 229533.I1RC17 pp 229533.I1RG90
150614 229533.I1RC17 pp 229533.I1RE22
150615 229533.I1RC17 pp 229533.I1RRV1
150616 229533.I1RC17 pp 229533.I1RPK4
150617 229533.I1RC17 pp 229533.I1RRE5
150618 229533.I1RC17 pp 229533.I1S5R1
150619 229533.I1RC17 pp 229533.I1S0X7
150620 229533.I1RC17 pp 229533.I1RJ88
150621 229533.I1RC17 pp 229533.I1RT40
150622 229533.I1RC17 pp 229533.I1RPC2
150623 229533.I1RC17 pp 229533.I1RWL2
150624 229533.I1RC17 pp 229533.I1RUN8
150625 229533.I1RC17 pp 229533.I1RSZ3
150626 229533.I1RC17 pp 229533.I1RNP7
150627 229533.I1RC17 pp 229533.I1RCJ7
150628 229533.I1RC17 pp 229533.I1RPM4
150629 229533.I1RC17 pp 229533.I1RUF8
150630 229533.I1RC17 pp 229533.I1RSV2
150631 229533.I1RC17 pp 229533.I1RFZ2
150632 229533.I1RC17 pp 229533.I1S2B8
150633 229533.I1RC17 pp 229533.I1RPP3
150634 229533.I1RC17 pp 229533.I1RRM5
150635 229533.I1RC17 pp 229533.I1RN67
150636 229533.I1RC17 pp 229533.I1RPU3
150637 229533.I1RC17 pp 229533.I1S0F6
150638 229533.I1RC17 pp 229533.I1RW83
150639 229533.I1RC17 pp 229533.I1RWK3
150640 229533.I1RC17 pp 229533.I1RN08
150641 229533.I1RC17 pp 229533.I1RY03
150642 229533.I1RC17 pp 229533.I1S098
150643 229533.I1RC17 pp 229533.I1RT28
150644 229533.I1RC17 pp 229533.I1RC22
150645 229533.I1RC17 pp 229533.I1S7X9
150646 229533.I1RC17 pp 229533.I1S5B0
150647 229533.I1RC17 pp 229533.I1RUQ5
150648 229533.I1RC17 pp 229533.I1S2G7
150649 229533.I1RC17 pp 229533.I1RW99
150650 229533.I1RC17 pp 229533.I1RQA3
150651 229533.I1RC17 pp 229533.I1RWD5
150652 229533.I1RC17 pp 229533.I1RYZ0
150653 229533.I1RC17 pp 229533.I1RRY9
150654 229533.I1RC17 pp 229533.I1S267
150655 229533.I1RC17 pp 229533.I1RC95
150656 229533.I1RC17 pp 229533.I1RHB0
150657 229533.I1RC17 pp 229533.I1RQS4
150658 229533.I1RC17 pp 229533.I1RQ81
150659 229533.I1RC17 pp 229533.I1RMS2
150660 229533.I1RC17 pp 229533.Q4HTT2
150661 229533.I1RC17 pp 229533.Q4I8P5
150662 229533.I1RC17 pp 229533.I1RZM8
150663 229533.I1RC17 pp 229533.Q4IJM4
150664 229533.I1RC17 pp 229533.I1RCB3
150665 229533.I1RC17 pp 229533.I1RS18
150666 229533.I1RC17 pp 229533.I1SA32
150667 229533.I1RC17 pp 229533.I1RX70
150668 229533.I1RC17 pp 229533.I1REE1
150669 229533.I1RC17 pp 229533.I1RWX8
150670 229533.I1RC17 pp 229533.I1RJU3
150671 229533.I1RC17 pp 229533.I1RI82
150672 229533.I1RC17 pp 229533.I1RKK0

150673 229533.I1RC17 pp 229533.I1RNQ5
150674 229533.I1RC17 pp 229533.I1RP02
150675 229533.I1RC17 pp 229533.I1RMR7
150676 229533.I1RC17 pp 229533.I1RSQ7
150677 229533.I1RC17 pp 229533.I1S0X6
150678 229533.I1RC17 pp 229533.Q4ICG5
150679 229533.I1RC17 pp 229533.I1RZM1
150680 229533.I1RC17 pp 229533.I1RWJ6
150681 229533.I1RC17 pp 229533.I1S1W5
150682 229533.I1RC17 pp 229533.V6QW22
150683 229533.I1RC17 pp 229533.I1RN61
150684 229533.I1RC17 pp 229533.I1RWJ8
150685 229533.I1RC17 pp 229533.I1RYH9
150686 229533.I1RC17 pp 229533.I1RIH8
150687 229533.I1RC17 pp 229533.I1RQ26
150688 229533.I1RC17 pp 229533.I1RW25
150689 229533.I1RC17 pp 229533.V6QV09
150690 229533.I1RC17 pp 229533.I1RZJ5
150691 229533.I1RC17 pp 229533.I1RM49
150692 229533.I1RC17 pp 229533.I1RYZ9
150693 229533.I1RC17 pp 229533.I1RZ51
150694 229533.I1RC17 pp 229533.I1RL24
150695 229533.I1RC17 pp 229533.I1RQ13
150696 229533.I1RC17 pp 229533.I1RUQ6
150697 229533.I1RC17 pp 229533.I1RD69
150698 229533.I1RC17 pp 229533.I1RTH6
150699 229533.I1RC17 pp 229533.I1RVQ0
150700 229533.I1RC17 pp 229533.I1RRT1
150701 229533.I1RC17 pp 229533.Q4ICM0
150702 229533.I1RC17 pp 229533.I1RCL5
150703 229533.I1RC17 pp 229533.I1RZT1
150704 229533.I1RC17 pp 229533.I1RZK9
150705 229533.I1RC17 pp 229533.I1S1C7
150706 229533.I1RC17 pp 229533.I1RRY3
150707 229533.I1RC17 pp 229533.I1S1W4
150708 229533.I1RC17 pp 229533.I1RGC6
150709 229533.I1RC17 pp 229533.I1RWN5
150710 229533.I1RC17 pp 229533.V6RKY7
150711 229533.I1RC17 pp 229533.I1RTN0
150712 229533.I1RC17 pp 229533.V6R042
150713 229533.I1RC17 pp 229533.I1RCR8
150714 229533.I1RC17 pp 229533.I1RJV3
150715 229533.I1RC17 pp 229533.I1RNC1
150716 229533.I1RC17 pp 229533.V6RVG3
150717 229533.I1RC17 pp 229533.I1RVX0
150718 229533.I1RC17 pp 229533.I1RSA4
150719 229533.I1RC17 pp 229533.I1RPW0
150720 229533.I1RC17 pp 229533.I1S0M2
150721 229533.I1RC17 pp 229533.I1RDZ4
150722 229533.I1RC17 pp 229533.I1RGF1
150723 229533.I1RC17 pp 229533.I1RRW0
150724 229533.I1RC17 pp 229533.I1RCL8
150725 229533.I1RC17 pp 229533.I1RCH5
150726 229533.I1RC17 pp 229533.I1RZ39
150727 229533.I1RC17 pp 229533.I1RDH0
150728 229533.I1RC17 pp 229533.V6RGI1
150729 229533.I1RC17 pp 229533.I1S757
150730 229533.I1RC17 pp 229533.I1RZ63
150731 229533.I1RC17 pp 229533.I1S9S6
150732 229533.I1RC17 pp 229533.I1REX8
150733 229533.I1RC17 pp 229533.I1RD17
150734 229533.I1RC17 pp 229533.I1RR73
150735 229533.I1RC17 pp 229533.I1RD15
150736 229533.I1RC17 pp 229533.I1S0N4
150737 229533.I1RC17 pp 229533.I1RRT8
150738 229533.I1RC17 pp 229533.I1RLU1
150739 229533.I1RC17 pp 229533.I1SA33
150740 229533.I1RC17 pp 229533.Q4HTT1
150741 229533.I1RC17 pp 229533.I1RZZ9
150742 229533.I1RC17 pp 229533.I1RC71
150743 229533.I1RC17 pp 229533.I1S0D1
150744 229533.I1RC17 pp 229533.I1RWI5
150745 229533.I1RC17 pp 229533.I1S0M6

150746	229533.I1RC17	pp	229533.I1RGD1
150747	229533.I1RC17	pp	229533.I1RPP4
150748	229533.I1RC17	pp	229533.I1RW46
150749	229533.I1RC17	pp	229533.I1S549
150750	229533.I1RC17	pp	229533.I1RSD1
150751	229533.I1RC17	pp	229533.I1RE01
150752	229533.I1RC17	pp	229533.I1S4R1
150753	229533.I1RC17	pp	229533.I1RX38
150754	229533.I1RC17	pp	229533.I1RT04
150755	229533.I1RC17	pp	229533.I1RJZ7
150756	229533.I1RC17	pp	229533.I1RTD5
150757	229533.I1RC17	pp	229533.I1RY29
150758	229533.I1RC17	pp	229533.I1RP27
150759	229533.I1RC17	pp	229533.I1RGA4
150760	229533.I1RC17	pp	229533.Q4I7L0
150761	229533.I1RC17	pp	229533.I1S2E9
150762	229533.I1RC17	pp	229533.I1RV91
150763	229533.I1RC17	pp	229533.I1RJV4
150764	229533.I1RC17	pp	229533.I1RPU7
150765	229533.I1RC17	pp	229533.Q4IJK2
150766	229533.I1RC17	pp	229533.I1RJW6
150767	229533.I1RC17	pp	229533.I1S503
150768	229533.I1RC17	pp	229533.I1RZZ1
150769	229533.I1RC17	pp	229533.I1RMP0
150770	229533.I1RC17	pp	229533.I1RFL7
150771	229533.I1RC17	pp	229533.I1RJK7
150772	229533.I1RC17	pp	229533.I1RJM7
150773	229533.I1RC17	pp	229533.I1REK8
150774	229533.I1RC17	pp	229533.I1RCG4
150775	229533.I1RC17	pp	229533.I1RZP1
150776	229533.I1RC17	pp	229533.I1RFU8
150777	229533.I1RC17	pp	229533.I1S439
150778	229533.I1RC17	pp	229533.I1RJL2
150779	229533.I1RC17	pp	229533.I1RZK5
150780	229533.I1RC17	pp	229533.I1RIU0
150781	229533.I1RC17	pp	229533.I1RYY5
150782	229533.I1RC17	pp	229533.Q4IR87
150783	229533.I1RC17	pp	229533.I1RC97
150784	229533.I1RC17	pp	229533.I1RFT2
150785	229533.I1RC17	pp	229533.I1RMM9
150786	229533.I1RC17	pp	229533.I1RSI0
150787	229533.I1RC17	pp	229533.I1RCK4
150788	229533.I1RC17	pp	229533.I1RRT0
150789	229533.I1RC17	pp	229533.I1S0N8
150790	229533.I1RC17	pp	229533.V6RG22
150791	229533.I1RC17	pp	229533.I1RMQ7
150792	229533.I1RC17	pp	229533.V6RCZ8
150793	229533.I1RC17	pp	229533.I1RME5
150794	229533.I1RC17	pp	229533.I1S224
150795	229533.I1RC17	pp	229533.I1RN89
150796	229533.I1RC17	pp	229533.V6RCG5
150797	229533.I1RC17	pp	229533.I1S504
150798	229533.I1RC17	pp	229533.I1RQC9
150799	229533.I1RC17	pp	229533.I1RWT6
150800	229533.I1RC17	pp	229533.I1RYU3
150801	229533.I1RC17	pp	229533.I1S0R7
150802	229533.I1RC17	pp	229533.I1RLU6
150803	229533.I1RC17	pp	229533.I1RPU4
150804	229533.I1RC17	pp	229533.I1RLT6
150805	229533.I1RC17	pp	229533.I1RQA9
150806	229533.I1RC17	pp	229533.I1RQH3
150807	229533.I1RC17	pp	229533.I1RDB1
150808	229533.I1RC17	pp	229533.I1RR24
150809	229533.I1RC17	pp	229533.I1RV66
150810	229533.I1RC17	pp	229533.V6RTJ8
150811	229533.I1RC17	pp	229533.I1RXD7
150812	229533.I1RC17	pp	229533.I1RFQ7
150813	229533.I1RC17	pp	229533.I1RR94
150814	229533.I1RC17	pp	229533.I1RX46
150815	229533.I1RC17	pp	229533.I1RYI9
150816	229533.I1RC17	pp	229533.I1RY84
150817	229533.I1RC17	pp	229533.I1RTC2
150818	229533.I1RC17	pp	229533.I1RC20

150819 229533.I1RC17 pp 229533.I1RW71
150820 229533.I1RC17 pp 229533.I1RS56
150821 229533.I1RC17 pp 229533.I1RFZ3
150822 229533.I1RC17 pp 229533.I1RRG3
150823 229533.I1RC17 pp 229533.I1RSI4
150824 229533.I1RC17 pp 229533.I1RJH1
150825 229533.I1RC17 pp 229533.I1S0F5
150826 229533.I1RC17 pp 229533.I1S0L3
150827 229533.I1RC17 pp 229533.I1RD52
150828 229533.I1RC17 pp 229533.I1RT62
150829 229533.A0A1C3YIU5 pp 229533.I1RZB5
150830 229533.A0A1C3YIU5 pp 229533.I1REG5
150831 229533.A0A1C3YIU5 pp 229533.I1RJL2
150832 229533.A0A1C3YIU5 pp 229533.I1RPU4
150833 229533.A0A1C3YIU5 pp 229533.A0A1C3YK37
150834 229533.A0A1C3YIU5 pp 229533.I1RK79
150835 229533.A0A1C3YIU5 pp 229533.I1RBJ0
150836 229533.A0A1C3YIU5 pp 229533.Q4ICA8
150837 229533.A0A1C3YIU5 pp 229533.I1RAI9
150838 229533.A0A1C3YIU5 pp 229533.I1RG11
150839 229533.A0A1C3YIU5 pp 229533.I1RPW0
150840 229533.A0A1C3YIU5 pp 229533.I1RX38
150841 229533.A0A1C3YIU5 pp 229533.I1RRT0
150842 229533.A0A1C3YIU5 pp 229533.I1RBV5
150843 229533.A0A1C3YIU5 pp 229533.A0A1C3YKJ6
150844 229533.A0A1C3YIU5 pp 229533.I1RQ88
150845 229533.A0A1C3YIU5 pp 229533.I1RTM8
150846 229533.I1S0E2 pp 229533.Q4HTT2
150847 229533.I1S0E2 pp 229533.I1S0K7
150848 229533.I1S0E2 pp 229533.I1S4T3
150849 229533.I1S0E2 pp 229533.I1S537
150850 229533.A0A1C3YK03 pp 229533.I1RG53
150851 229533.A0A1C3YK03 pp 229533.I1RCA7
150852 229533.A0A1C3YK03 pp 229533.I1RKG3
150853 229533.A0A1C3YK03 pp 229533.I1S9L5
150854 229533.A0A1C3YK03 pp 229533.I1RCI4
150855 229533.A0A1C3YK03 pp 229533.I1S9L6
150856 229533.A0A1C3YK03 pp 229533.I1RN03
150857 229533.A0A1C3YK03 pp 229533.I1RQP8
150858 229533.A0A1C3YK03 pp 229533.I1RNC1
150859 229533.A0A1C3YK03 pp 229533.I1RG57
150860 229533.A0A1C3YK03 pp 229533.I1RWQ8
150861 229533.A0A1C3YK03 pp 229533.I1RKG2
150862 229533.A0A1C3YK03 pp 229533.I1RFI5
150863 229533.I1SA32 pp 229533.Q4I2J8
150864 229533.I1SA32 pp 229533.I1SA33
150865 229533.I1SA32 pp 229533.V6R042
150866 229533.I1SA32 pp 229533.V6RCG5
150867 229533.I1SA32 pp 229533.V6R057
150868 229533.I1SA32 pp 229533.Q4I624
150869 229533.I1SA32 pp 229533.Q4IJK2
150870 229533.I1SA32 pp 229533.Q4I283
150871 229533.I1SA32 pp 229533.V6RMW6
150872 229533.I1SA32 pp 229533.V6R1K4
150873 229533.I1SA32 pp 229533.V6RIV9
150874 229533.I1SA32 pp 229533.V6R3X5
150875 229533.I1SA32 pp 229533.V6QYF0
150876 229533.I1SA32 pp 229533.V6RUY9
150877 229533.I1SA32 pp 229533.V6RCZ8
150878 229533.I1SA32 pp 229533.V6QUW4
150879 229533.I1SA32 pp 229533.Q4IJT5
150880 229533.I1SA32 pp 229533.V6QW22
150881 229533.I1SA32 pp 229533.V6RTJ8
150882 229533.I1RX84 pp 229533.Q4IN00
150883 229533.I1RX84 pp 229533.I1S1V7
150884 229533.I1RX84 pp 229533.Q4I877
150885 229533.I1RX84 pp 229533.I1RYW8
150886 229533.I1RX84 pp 229533.Q4HYZ2
150887 229533.I1RX84 pp 229533.I1RZ60
150888 229533.I1RX84 pp 229533.Q4ILI9
150889 229533.I1RTE8 pp 229533.V6R949
150890 229533.I1RZK6 pp 229533.Q4HXF0
150891 229533.I1RZK6 pp 229533.I1S503

229533.I1RZK6 pp 229533.Q4HZ47
229533.I1RZK6 pp 229533.Q4IPI1
229533.I1RZK6 pp 229533.I1S8I8
229533.I1RZK6 pp 229533.I1S0R7
229533.I1RZK6 pp 229533.Q4IAS1
229533.I1RZK6 pp 229533.I1S2E9
229533.I1RZK6 pp 229533.I1S021
229533.I1RZK6 pp 229533.I1S8E2
229533.I1RZK6 pp 229533.Q4I327
229533.I1RZK6 pp 229533.V6R283
229533.I1RZK6 pp 229533.I1S6F0
229533.I1RZK6 pp 229533.V6RGN5
229533.I1RZK6 pp 229533.I1S1Z9
229533.I1RZK6 pp 229533.I1RZS6
229533.I1RZK6 pp 229533.Q4IJH1
229533.I1RZK6 pp 229533.Q4I662
229533.I1RZK6 pp 229533.V6QUE6
229533.I1RZK6 pp 229533.I1S2B8
229533.I1RZK6 pp 229533.Q4IF76
229533.I1RZK6 pp 229533.I1S044
229533.I1RZK6 pp 229533.V6R3X5
229533.I1RZK6 pp 229533.Q4IFI0
229533.I1RZK6 pp 229533.V6R042
229533.I1RZK6 pp 229533.Q4IJ56
229533.I1RZK6 pp 229533.V6RMW6
229533.I1RZK6 pp 229533.Q4IBS2
229533.I1RZK6 pp 229533.I1S9U2
229533.I1RZK6 pp 229533.Q4IN63
229533.I1RZK6 pp 229533.Q4I8X0
229533.I1RZK6 pp 229533.I1S2G9
229533.I1RZK6 pp 229533.I1S0M9
229533.I1RZK6 pp 229533.I1RZV6
229533.I1RZK6 pp 229533.I1S060
229533.I1RZK6 pp 229533.V6RGY3
229533.I1RZK6 pp 229533.V6R227
229533.I1RZK6 pp 229533.Q4IAA0
229533.I1RZK6 pp 229533.I1S504
229533.I1RZK6 pp 229533.I1S274
229533.I1RZK6 pp 229533.Q4HWE2
229533.I1RZK6 pp 229533.I1S0J1
229533.I1RZK6 pp 229533.I1S1X9
229533.I1RZK6 pp 229533.Q4IQT8
229533.I1RZK6 pp 229533.Q4IEK8
229533.I1RZK6 pp 229533.V6RBN2
229533.I1RZK6 pp 229533.I1S2E8
229533.I1RZK6 pp 229533.I1S2C0
229533.I1RZK6 pp 229533.I1RZZ1
229533.I1RZK6 pp 229533.I1S1C7
229533.I1RZK6 pp 229533.I1S0Z9
229533.I1RZK6 pp 229533.Q4IPZ1
229533.I1RFW0 pp 229533.I1RPA6
229533.I1RFW0 pp 229533.I1RXH6
229533.I1RFW0 pp 229533.I1RMM6
229533.I1RFW0 pp 229533.I1S0X7
229533.I1RFW0 pp 229533.I1RTC7
229533.I1RFW0 pp 229533.I1RST8
229533.I1RFW0 pp 229533.I1RNF4
229533.I1RFW0 pp 229533.I1RTG0
229533.I1RFW0 pp 229533.I1RV66
229533.I1RFW0 pp 229533.I1RPK4
229533.I1RFW0 pp 229533.Q4IEA7
229533.I1RFW0 pp 229533.I1RVA8
229533.I1RFW0 pp 229533.I1S2K7
229533.I1RFW0 pp 229533.I1RPH1
229533.I1RFW0 pp 229533.I1S0Z6
229533.I1RFW0 pp 229533.I1RSW4
229533.I1RFW0 pp 229533.I1RN27
229533.I1RFW0 pp 229533.I1RGF1
229533.I1RFW0 pp 229533.I1RVN2
229533.I1RFW0 pp 229533.I1RNK8
229533.I1RFW0 pp 229533.I1RWT6
229533.I1RFW0 pp 229533.I1RX77
229533.I1RFW0 pp 229533.V6RPQ8

150965	229533.I1RFW0	pp	229533.I1RWK0
150966	229533.I1RFW0	pp	229533.I1RMB2
150967	229533.I1RFW0	pp	229533.Q4I785
150968	229533.I1RFW0	pp	229533.I1RKB3
150969	229533.I1RFW0	pp	229533.I1RSI3
150970	229533.I1RFW0	pp	229533.I1S235
150971	229533.I1RFW0	pp	229533.I1S2E9
150972	229533.I1RFW0	pp	229533.I1RQ75
150973	229533.I1RFW0	pp	229533.I1RYL5
150974	229533.I1RFW0	pp	229533.I1RY44
150975	229533.I1RFW0	pp	229533.I1RZK4
150976	229533.I1RFW0	pp	229533.I1RSS1
150977	229533.I1RFW0	pp	229533.I1RXF0
150978	229533.I1RFW0	pp	229533.I1S296
150979	229533.I1RFW0	pp	229533.I1RLU2
150980	229533.I1RFW0	pp	229533.I1RQB4
150981	229533.I1RFW0	pp	229533.Q4IR87
150982	229533.I1RFW0	pp	229533.I1RHQ8
150983	229533.I1RFW0	pp	229533.I1RQ72
150984	229533.I1RFW0	pp	229533.I1RQA3
150985	229533.I1RFW0	pp	229533.I1RRN5
150986	229533.I1RFW0	pp	229533.V6RRF6
150987	229533.I1RFW0	pp	229533.V6QUV7
150988	229533.I1RFW0	pp	229533.I1S286
150989	229533.I1RFW0	pp	229533.I1S2B6
150990	229533.I1RJZ6	pp	229533.I1RY03
150991	229533.I1RJZ6	pp	229533.I1RSR8
150992	229533.I1RJZ6	pp	229533.I1RRI2
150993	229533.I1RJZ6	pp	229533.I1RMD5
150994	229533.I1RJZ6	pp	229533.I1RTD7
150995	229533.I1RJZ6	pp	229533.I1RPJ3
150996	229533.I1RJZ6	pp	229533.I1S5B5
150997	229533.I1RJZ6	pp	229533.I1S1C7
150998	229533.I1RJZ6	pp	229533.I1S2C0
150999	229533.I1RJZ6	pp	229533.I1RK83
151000	229533.I1RJZ6	pp	229533.I1RTE9
151001	229533.I1RJZ6	pp	229533.I1RTH6
151002	229533.I1RJZ6	pp	229533.I1RZK5
151003	229533.I1RJZ6	pp	229533.I1RVZ0
151004	229533.I1RJZ6	pp	229533.Q4HZK7
151005	229533.I1RJZ6	pp	229533.I1RZB5
151006	229533.I1RJZ6	pp	229533.I1RWJ3
151007	229533.I1RJZ6	pp	229533.I1RYV3
151008	229533.I1RJZ6	pp	229533.I1S2B6
151009	229533.I1RJZ6	pp	229533.I1RQA2
151010	229533.I1RJZ6	pp	229533.Q4HZ68
151011	229533.I1RJZ6	pp	229533.I1RPP4
151012	229533.I1RJZ6	pp	229533.I1RNH6
151013	229533.I1RJZ6	pp	229533.I1S060
151014	229533.I1RJZ6	pp	229533.I1RRY9
151015	229533.I1RJZ6	pp	229533.I1S021
151016	229533.I1RJZ6	pp	229533.V6RGY3
151017	229533.I1RJZ6	pp	229533.I1RYB9
151018	229533.I1RJZ6	pp	229533.I1RM25
151019	229533.I1RJZ6	pp	229533.I1S2E8
151020	229533.I1RJZ6	pp	229533.I1RWN6
151021	229533.I1RJZ6	pp	229533.I1RWB5
151022	229533.I1RJZ6	pp	229533.I1S0M9
151023	229533.I1RJZ6	pp	229533.Q4I327
151024	229533.I1RJZ6	pp	229533.Q4IJ56
151025	229533.I1RJZ6	pp	229533.I1RZK6
151026	229533.I1RJZ6	pp	229533.I1RZM1
151027	229533.I1RJZ6	pp	229533.Q4IJK2
151028	229533.I1RJZ6	pp	229533.I1RNY2
151029	229533.I1RJZ6	pp	229533.I1RZZ1
151030	229533.I1RJZ6	pp	229533.I1RLU6
151031	229533.I1RJZ6	pp	229533.I1RNC1
151032	229533.I1RJZ6	pp	229533.I1RNQ5
151033	229533.I1RJZ6	pp	229533.I1RM96
151034	229533.I1RJZ6	pp	229533.I1RQ31
151035	229533.I1RJZ6	pp	229533.I1RND7
151036	229533.I1RJZ6	pp	229533.I1RSD8
151037	229533.I1RJZ6	pp	229533.Q4IEK8

151038 229533.I1RJZ6 pp 229533.I1RSQ3
151039 229533.I1RJZ6 pp 229533.I1RSS4
151040 229533.I1RJZ6 pp 229533.V6R042
151041 229533.I1RJZ6 pp 229533.I1RPY1
151042 229533.I1RJZ6 pp 229533.I1RY44
151043 229533.I1RJZ6 pp 229533.V6RMW6
151044 229533.I1RJZ6 pp 229533.I1RT03
151045 229533.I1RJZ6 pp 229533.I1RVX0
151046 229533.I1RJZ6 pp 229533.I1RSI6
151047 229533.I1RJZ6 pp 229533.I1RWN5
151048 229533.I1RJZ6 pp 229533.I1S1X9
151049 229533.I1RJZ6 pp 229533.I1RMN6
151050 229533.I1RJZ6 pp 229533.I1S4T3
151051 229533.I1RJZ6 pp 229533.I1RRR7
151052 229533.I1RJZ6 pp 229533.I1RKC6
151053 229533.I1RJZ6 pp 229533.I1RZE5
151054 229533.I1RJZ6 pp 229533.Q4I5Z5
151055 229533.I1RJZ6 pp 229533.V6R227
151056 229533.I1RJZ6 pp 229533.V6QUE6
151057 229533.I1RJZ6 pp 229533.I1RR73
151058 229533.I1RJZ6 pp 229533.V6RGN5
151059 229533.I1RJZ6 pp 229533.I1RSH4
151060 229533.I1RJZ6 pp 229533.I1RTG6
151061 229533.I1RJZ6 pp 229533.I1RUQ9
151062 229533.I1RJZ6 pp 229533.V6R3X5
151063 229533.I1RJZ6 pp 229533.I1RND6
151064 229533.I1RJZ6 pp 229533.I1S504
151065 229533.I1RJZ6 pp 229533.I1S6U8
151066 229533.I1RJZ6 pp 229533.I1S0J1
151067 229533.I1RJZ6 pp 229533.I1RQM6
151068 229533.I1RJZ6 pp 229533.Q4HXF0
151069 229533.I1RJZ6 pp 229533.I1RZF6
151070 229533.I1RJZ6 pp 229533.I1S8E2
151071 229533.I1RJZ6 pp 229533.I1RT04
151072 229533.I1RJZ6 pp 229533.I1RMK3
151073 229533.I1RJZ6 pp 229533.I1S0R7
151074 229533.I1RJZ6 pp 229533.I1RU69
151075 229533.I1RJZ6 pp 229533.I1RQC9
151076 229533.I1RJZ6 pp 229533.Q4IJH1
151077 229533.I1RJZ6 pp 229533.Q4IF76
151078 229533.I1RJZ6 pp 229533.Q4IPI1
151079 229533.I1RJZ6 pp 229533.I1RZS6
151080 229533.I1RJZ6 pp 229533.I1RTF4
151081 229533.I1RJZ6 pp 229533.I1RZ39
151082 229533.I1RJZ6 pp 229533.I1RM06
151083 229533.I1RJZ6 pp 229533.I1RNA0
151084 229533.I1RJZ6 pp 229533.I1RT80
151085 229533.I1RJZ6 pp 229533.I1RRT2
151086 229533.I1RJZ6 pp 229533.I1RM67
151087 229533.I1RJZ6 pp 229533.V6QYF0
151088 229533.I1RJZ6 pp 229533.I1RX46
151089 229533.I1RJZ6 pp 229533.I1RRT8
151090 229533.I1RJZ6 pp 229533.I1RKQ5
151091 229533.I1RJZ6 pp 229533.I1S503
151092 229533.I1RJZ6 pp 229533.I1RX24
151093 229533.I1RJZ6 pp 229533.I1RPU0
151094 229533.I1RJZ6 pp 229533.I1RLV9
151095 229533.I1RJZ6 pp 229533.I1S1V4
151096 229533.I1RJZ6 pp 229533.I1RW25
151097 229533.I1RJZ6 pp 229533.Q4IFI0
151098 229533.I1RJZ6 pp 229533.I1RKJ6
151099 229533.I1RJZ6 pp 229533.I1RZV6
151100 229533.I1RJZ6 pp 229533.I1S2B8
151101 229533.I1RJZ6 pp 229533.Q4IAS1
151102 229533.I1RJZ6 pp 229533.I1RPW9
151103 229533.I1RJZ6 pp 229533.V6RTJ8
151104 229533.I1RJZ6 pp 229533.I1S9U2
151105 229533.I1RJZ6 pp 229533.Q4IQW0
151106 229533.I1RJZ6 pp 229533.I1RTD5
151107 229533.I1RJZ6 pp 229533.I1S540
151108 229533.I1RJZ6 pp 229533.I1RQH7
151109 229533.I1RJZ6 pp 229533.I1RXD7
151110 229533.I1RJZ6 pp 229533.I1RWH5

151111	229533.I1RJZ6	pp	229533.I1RUQ2
151112	229533.I1RJZ6	pp	229533.Q4I662
151113	229533.I1RJZ6	pp	229533.V6R283
151114	229533.I1RJZ6	pp	229533.I1S2E9
151115	229533.I1RJZ6	pp	229533.I1RW71
151116	229533.I1RJZ6	pp	229533.I1RRJ8
151117	229533.I1RJZ6	pp	229533.I1RKL3
151118	229533.I1RJZ6	pp	229533.I1RRQ3
151119	229533.I1RJZ6	pp	229533.I1RZK3
151120	229533.I1RJZ6	pp	229533.I1RSQ7
151121	229533.I1RJZ6	pp	229533.I1RN67
151122	229533.I1RJZ6	pp	229533.I1SAM5
151123	229533.I1RJZ6	pp	229533.I1RPP3
151124	229533.I1RJZ6	pp	229533.I1RS30
151125	229533.I1RJZ6	pp	229533.I1RQJ5
151126	229533.I1RJZ6	pp	229533.I1RS47
151127	229533.I1RJZ6	pp	229533.I1RWF7
151128	229533.I1RJZ6	pp	229533.I1RQ65
151129	229533.I1RJZ6	pp	229533.I1RR00
151130	229533.I1RMF5	pp	229533.I1RRR6
151131	229533.I1RMF5	pp	229533.I1S0M2
151132	229533.I1RMF5	pp	229533.V6RVG3
151133	229533.I1RMF5	pp	229533.I1RN08
151134	229533.I1RMF5	pp	229533.I1S439
151135	229533.I1RMF5	pp	229533.I1RQ26
151136	229533.I1RMF5	pp	229533.I1RMM9
151137	229533.I1RMF5	pp	229533.I1RRY3
151138	229533.I1RMF5	pp	229533.I1RYH9
151139	229533.I1RMF5	pp	229533.I1RUN8
151140	229533.I1RBP9	pp	229533.I1RRL3
151141	229533.I1RBP9	pp	229533.I1RSR2
151142	229533.I1RBP9	pp	229533.I1RDX8
151143	229533.I1RBP9	pp	229533.I1RS64
151144	229533.I1RBP9	pp	229533.I1RRQ9
151145	229533.I1RBP9	pp	229533.I1RDE9
151146	229533.I1RBP9	pp	229533.I1RD23
151147	229533.I1RBP9	pp	229533.I1RYR9
151148	229533.I1RDG0	pp	229533.I1RZB5
151149	229533.I1RDG0	pp	229533.Q4I1Y1
151150	229533.I1RDG0	pp	229533.I1RZZ1
151151	229533.I1RDG0	pp	229533.I1RK83
151152	229533.I1RDG0	pp	229533.I1RSR8
151153	229533.I1RDG0	pp	229533.I1RZK6
151154	229533.I1RDG0	pp	229533.I1RFP9
151155	229533.I1RDG0	pp	229533.I1RYJ8
151156	229533.I1RDG0	pp	229533.I1RW99
151157	229533.I1RDG0	pp	229533.I1S050
151158	229533.I1RDG0	pp	229533.I1S5B0
151159	229533.I1RDG0	pp	229533.I1REK8
151160	229533.I1RDG0	pp	229533.I1RF08
151161	229533.I1RDG0	pp	229533.I1RS83
151162	229533.I1RDG0	pp	229533.V6QUE6
151163	229533.I1RDG0	pp	229533.Q4I5Z5
151164	229533.I1RDG0	pp	229533.I1S0Z9
151165	229533.I1RDG0	pp	229533.I1RVZ0
151166	229533.I1RDG0	pp	229533.Q4IBS2
151167	229533.I1RDG0	pp	229533.I1RG17
151168	229533.I1RDG0	pp	229533.V6RCG5
151169	229533.I1RDG0	pp	229533.I1RRE0
151170	229533.I1RDG0	pp	229533.I1S6E5
151171	229533.I1RDG0	pp	229533.I1RNK2
151172	229533.I1RDG0	pp	229533.I1RSR3
151173	229533.I1RDG0	pp	229533.I1RRY9
151174	229533.I1RDG0	pp	229533.I1RQ31
151175	229533.I1RDG0	pp	229533.I1RSA8
151176	229533.I1RDG0	pp	229533.I1RFJ9
151177	229533.I1RDG0	pp	229533.I1RF19
151178	229533.I1RDG0	pp	229533.I1RE00
151179	229533.I1RDG0	pp	229533.I1RSM3
151180	229533.I1RDG0	pp	229533.I1RSI0
151181	229533.I1RDG0	pp	229533.I1S9L0
151182	229533.I1RDG0	pp	229533.Q4IE79
151183	229533.I1RDG0	pp	229533.I1RMC0

151184 229533.I1RDG0 pp 229533.I1RMH5
151185 229533.I1RDG0 pp 229533.I1RYB1
151186 229533.I1RDG0 pp 229533.I1RQA2
151187 229533.I1RDG0 pp 229533.I1RZE5
151188 229533.I1RDG0 pp 229533.I1RYB9
151189 229533.I1RDG0 pp 229533.I1RK95
151190 229533.I1RDG0 pp 229533.Q4IB70
151191 229533.I1RDG0 pp 229533.I1S021
151192 229533.I1RDG0 pp 229533.I1RN99
151193 229533.I1RDG0 pp 229533.I1RQ28
151194 229533.I1RDG0 pp 229533.I1RI82
151195 229533.I1RDG0 pp 229533.Q4IJK2
151196 229533.I1RDG0 pp 229533.I1RNH6
151197 229533.I1RDG0 pp 229533.I1RZV9
151198 229533.I1RDG0 pp 229533.V6R227
151199 229533.I1RDG0 pp 229533.I1RZF6
151200 229533.I1RDG0 pp 229533.Q4IRI9
151201 229533.I1RDG0 pp 229533.I1RJZ6
151202 229533.I1RDG0 pp 229533.I1S0P4
151203 229533.I1RDG0 pp 229533.Q4IFI0
151204 229533.I1RDG0 pp 229533.I1RG56
151205 229533.I1RDG0 pp 229533.I1RT35
151206 229533.I1RDG0 pp 229533.I1RW25
151207 229533.I1RDG0 pp 229533.Q4IAA0
151208 229533.I1RDG0 pp 229533.V6QYF0
151209 229533.I1RDG0 pp 229533.I1RX14
151210 229533.I1RDG0 pp 229533.Q4IEK8
151211 229533.I1RDG0 pp 229533.I1RZM1
151212 229533.I1RDG0 pp 229533.I1RR24
151213 229533.I1RDG0 pp 229533.I1S9U2
151214 229533.I1RDG0 pp 229533.I1RM06
151215 229533.I1RDG0 pp 229533.I1RNA0
151216 229533.I1RDG0 pp 229533.I1RPW9
151217 229533.I1RDG0 pp 229533.I1REX4
151218 229533.I1RDG0 pp 229533.I1RNQ5
151219 229533.I1RDG0 pp 229533.I1RLU6
151220 229533.I1RDG0 pp 229533.I1RG51
151221 229533.I1RDG0 pp 229533.I1S0M9
151222 229533.I1RDG0 pp 229533.Q4IPB3
151223 229533.I1RDG0 pp 229533.I1RPU0
151224 229533.I1RDG0 pp 229533.Q4I5R9
151225 229533.I1RDG0 pp 229533.I1RWJ8
151226 229533.I1RDG0 pp 229533.I1RFQ7
151227 229533.I1RDG0 pp 229533.I1RN28
151228 229533.I1RDG0 pp 229533.I1RM67
151229 229533.I1RDG0 pp 229533.I1RKQ5
151230 229533.I1RDG0 pp 229533.I1RNV6
151231 229533.I1RDG0 pp 229533.Q4IJ56
151232 229533.I1RDG0 pp 229533.Q4IPI1
151233 229533.I1RDG0 pp 229533.I1S0D4
151234 229533.I1RDG0 pp 229533.I1S0L3
151235 229533.I1RDG0 pp 229533.I1RT80
151236 229533.I1RDG0 pp 229533.I1RX46
151237 229533.I1RDG0 pp 229533.I1RWD4
151238 229533.I1RDG0 pp 229533.I1S0R7
151239 229533.I1RDG0 pp 229533.Q4IJH1
151240 229533.I1RDG0 pp 229533.I1RQC9
151241 229533.I1RDG0 pp 229533.I1RTF4
151242 229533.I1RDG0 pp 229533.I1RNH1
151243 229533.I1RDG0 pp 229533.I1RVQ0
151244 229533.I1RDG0 pp 229533.I1RMK3
151245 229533.I1RDG0 pp 229533.I1RZJ6
151246 229533.I1RDG0 pp 229533.Q4HVW2
151247 229533.I1RDG0 pp 229533.I1RUQ2
151248 229533.I1RDG0 pp 229533.I1S6F0
151249 229533.I1RDG0 pp 229533.Q4INZ9
151250 229533.I1RDG0 pp 229533.I1S0X6
151251 229533.I1RDG0 pp 229533.I1RT05
151252 229533.I1RDG0 pp 229533.I1RW71
151253 229533.I1RDG0 pp 229533.I1RRH5
151254 229533.I1RDG0 pp 229533.I1RKK4
151255 229533.I1RDG0 pp 229533.I1RT98
151256 229533.I1RDG0 pp 229533.I1RNT2

151257	229533.I1RDG0	pp	229533.I1RK19
151258	229533.I1RDG0	pp	229533.I1RNE4
151259	229533.I1RDG0	pp	229533.I1RSQ7
151260	229533.I1RDG0	pp	229533.V6QW22
151261	229533.I1RDG0	pp	229533.I1RRH3
151262	229533.I1RDG0	pp	229533.I1RZW8
151263	229533.I1RDG0	pp	229533.V6R042
151264	229533.I1RDG0	pp	229533.Q4IN63
151265	229533.I1RDG0	pp	229533.I1RZ99
151266	229533.I1RDG0	pp	229533.I1S246
151267	229533.I1RDG0	pp	229533.I1RQJ5
151268	229533.I1RDG0	pp	229533.I1RUG3
151269	229533.I1RDG0	pp	229533.I1RRI2
151270	229533.I1RDG0	pp	229533.V6REZ4
151271	229533.I1RDG0	pp	229533.I1RWH5
151272	229533.I1RDG0	pp	229533.I1S296
151273	229533.I1RDG0	pp	229533.I1RNC1
151274	229533.I1RDG0	pp	229533.I1S274
151275	229533.I1RDG0	pp	229533.I1RFW5
151276	229533.I1RDG0	pp	229533.I1RX47
151277	229533.I1RDG0	pp	229533.Q4HZ68
151278	229533.I1RDG0	pp	229533.Q4I327
151279	229533.I1RDG0	pp	229533.I1S1U7
151280	229533.I1RDG0	pp	229533.V6RGY3
151281	229533.I1RDG0	pp	229533.I1RKC6
151282	229533.I1RDG0	pp	229533.V6R0U9
151283	229533.I1RDG0	pp	229533.Q4IPB8
151284	229533.I1RDG0	pp	229533.I1RYW2
151285	229533.I1RDG0	pp	229533.I1RML0
151286	229533.I1RDG0	pp	229533.Q4HWE2
151287	229533.I1RDG0	pp	229533.V6RY33
151288	229533.I1RDG0	pp	229533.I1RRR7
151289	229533.I1RDG0	pp	229533.I1S558
151290	229533.I1RDG0	pp	229533.Q4HZK7
151291	229533.I1RDG0	pp	229533.I1RJV3
151292	229533.I1RDG0	pp	229533.I1RX51
151293	229533.I1RDG0	pp	229533.I1S1X9
151294	229533.I1RDG0	pp	229533.V6QXG1
151295	229533.I1RDG0	pp	229533.I1RSS4
151296	229533.I1RDG0	pp	229533.I1S8R3
151297	229533.I1RDG0	pp	229533.Q4HXF6
151298	229533.I1RDG0	pp	229533.I1S2C0
151299	229533.I1RDG0	pp	229533.I1S4T3
151300	229533.I1RDG0	pp	229533.I1RTH6
151301	229533.I1RDG0	pp	229533.V6RIV9
151302	229533.I1RDG0	pp	229533.I1RSD8
151303	229533.I1RDG0	pp	229533.I1RN18
151304	229533.I1RDG0	pp	229533.I1RTE9
151305	229533.I1RDG0	pp	229533.I1RM96
151306	229533.I1RDG0	pp	229533.I1RNB8
151307	229533.I1RDG0	pp	229533.I1RFL4
151308	229533.I1RDG0	pp	229533.I1RQE7
151309	229533.I1RDG0	pp	229533.V6RNM4
151310	229533.I1RDG0	pp	229533.I1RWB5
151311	229533.I1RDG0	pp	229533.I1S2E8
151312	229533.I1RDG0	pp	229533.I1RG11
151313	229533.I1RDG0	pp	229533.I1RZV6
151314	229533.I1RDG0	pp	229533.I1RM25
151315	229533.I1RDG0	pp	229533.I1RS17
151316	229533.I1RDG0	pp	229533.V6RGN5
151317	229533.I1RDG0	pp	229533.I1RRT8
151318	229533.I1RDG0	pp	229533.I1S8E2
151319	229533.I1RDG0	pp	229533.I1RMN6
151320	229533.I1RDG0	pp	229533.I1RND6
151321	229533.I1RDG0	pp	229533.I1RNU6
151322	229533.I1RDG0	pp	229533.I1RSI6
151323	229533.I1RDG0	pp	229533.I1RN09
151324	229533.I1RDG0	pp	229533.I1RPP4
151325	229533.I1RDG0	pp	229533.I1RR73
151326	229533.I1RDG0	pp	229533.I1RZS7
151327	229533.I1RDG0	pp	229533.V6RMW6
151328	229533.I1RDG0	pp	229533.I1RW46
151329	229533.I1RDG0	pp	229533.Q4IF76

151330 229533.I1RDG0 pp 229533.I1S060
151331 229533.I1RDG0 pp 229533.Q4IQW0
151332 229533.I1RDG0 pp 229533.I1RZZ9
151333 229533.I1RDG0 pp 229533.I1RU69
151334 229533.I1RDG0 pp 229533.I1RFZ8
151335 229533.I1RDG0 pp 229533.I1RE01
151336 229533.I1RDG0 pp 229533.I1S0J1
151337 229533.I1RDG0 pp 229533.I1RJW6
151338 229533.I1RDG0 pp 229533.I1RE45
151339 229533.I1RDG0 pp 229533.Q4IAS1
151340 229533.I1RDG0 pp 229533.I1RDY5
151341 229533.I1RDG0 pp 229533.I1RLV9
151342 229533.I1RDG0 pp 229533.I1RYT7
151343 229533.I1RDG0 pp 229533.I1RST1
151344 229533.I1RDG0 pp 229533.I1RJZ7
151345 229533.I1RDG0 pp 229533.I1RRM5
151346 229533.I1RDG0 pp 229533.I1RKJ6
151347 229533.I1RDG0 pp 229533.I1S0L4
151348 229533.I1RDG0 pp 229533.I1RWI0
151349 229533.I1RDG0 pp 229533.I1S7S9
151350 229533.I1RDG0 pp 229533.I1S0N7
151351 229533.I1RDG0 pp 229533.I1RJL4
151352 229533.I1RDG0 pp 229533.I1S9Y0
151353 229533.I1RDG0 pp 229533.I1S9Y1
151354 229533.I1RDG0 pp 229533.I1RZS6
151355 229533.I1RDG0 pp 229533.I1RX24
151356 229533.I1RDG0 pp 229533.I1RKL3
151357 229533.I1RDG0 pp 229533.I1RWU2
151358 229533.I1RDG0 pp 229533.I1RT04
151359 229533.I1RDG0 pp 229533.I1RRT2
151360 229533.I1RDG0 pp 229533.I1RTS2
151361 229533.I1RDG0 pp 229533.Q4IR18
151362 229533.I1RDG0 pp 229533.I1RSX1
151363 229533.I1RDG0 pp 229533.Q4I830
151364 229533.I1RDG0 pp 229533.I1RPM4
151365 229533.I1RDG0 pp 229533.Q4I662
151366 229533.I1RDG0 pp 229533.Q4HZ42
151367 229533.I1RDG0 pp 229533.I1RFS3
151368 229533.I1RDG0 pp 229533.I1RS47
151369 229533.I1RDG0 pp 229533.I1RRJ8
151370 229533.I1RDG0 pp 229533.I1RS30
151371 229533.I1RDG0 pp 229533.I1RPP3
151372 229533.I1RDG0 pp 229533.I1RQ65
151373 229533.I1RDG0 pp 229533.I1RGA4
151374 229533.I1RDG0 pp 229533.Q4IBK5
151375 229533.I1RDG0 pp 229533.I1RWJ4
151376 229533.I1RDG0 pp 229533.I1RSL9
151377 229533.I1RDG0 pp 229533.I1RFN1
151378 229533.I1RDG0 pp 229533.I1S214
151379 229533.I1RDG0 pp 229533.I1RN67
151380 229533.I1RDG0 pp 229533.V6R283
151381 229533.I1RDG0 pp 229533.I1RN63
151382 229533.I1RDG0 pp 229533.I1RT17
151383 229533.I1RDG0 pp 229533.I1S1B6
151384 229533.I1RDG0 pp 229533.I1RWK3
151385 229533.I1RDG0 pp 229533.I1RRQ3
151386 229533.I1RDG0 pp 229533.I1RJV4
151387 229533.I1RDG0 pp 229533.I1RFG0
151388 229533.I1RDG0 pp 229533.I1RSV2
151389 229533.A0A0E0RVZ1 pp 229533.I1RQC9
151390 229533.A0A0E0RVZ1 pp 229533.A0A0E0SFN0
151391 229533.A0A0E0RVZ1 pp 229533.I1S0R7
151392 229533.A0A0E0RVZ1 pp 229533.I1RQH3
151393 229533.A0A0E0RVZ1 pp 229533.I1RAT7
151394 229533.A0A0E0RVZ1 pp 229533.I1RXU7
151395 229533.A0A0E0RVZ1 pp 229533.I1RM49
151396 229533.A0A0E0RVZ1 pp 229533.I1RAX6
151397 229533.A0A0E0RVZ1 pp 229533.V6RTJ8
151398 229533.A0A0E0RVZ1 pp 229533.I1RYZ9
151399 229533.A0A0E0RVZ1 pp 229533.I1RS18
151400 229533.A0A0E0RVZ1 pp 229533.A0A1C3YK34
151401 229533.A0A0E0RVZ1 pp 229533.I1RX46
151402 229533.A0A0E0RVZ1 pp 229533.I1RMQ7

151403 229533.A0A0E0RVZ1 pp 229533.I1RAM7
151404 229533.A0A0E0RVZ1 pp 229533.I1RBC9
151405 229533.A0A0E0RVZ1 pp 229533.I1RW71
151406 229533.A0A0E0RVZ1 pp 229533.A0A1C3YKF2
151407 229533.A0A0E0RVZ1 pp 229533.I1RL24
151408 229533.A0A0E0RVZ1 pp 229533.I1RN61
151409 229533.A0A0E0RVZ1 pp 229533.V6QW22
151410 229533.A0A0E0RVZ1 pp 229533.I1S504
151411 229533.A0A0E0RVZ1 pp 229533.I1RSQ7
151412 229533.A0A0E0RVZ1 pp 229533.I1RWP4
151413 229533.A0A0E0RVZ1 pp 229533.A0A0E0SAC4
151414 229533.A0A0E0RVZ1 pp 229533.I1RYH9
151415 229533.A0A0E0RVZ1 pp 229533.I1RQ26
151416 229533.A0A0E0RVZ1 pp 229533.I1RWT6
151417 229533.A0A0E0RVZ1 pp 229533.I1RJH1
151418 229533.A0A0E0RVZ1 pp 229533.V6QV09
151419 229533.A0A0E0RVZ1 pp 229533.I1RW25
151420 229533.A0A0E0RVZ1 pp 229533.I1S8F8
151421 229533.A0A0E0RVZ1 pp 229533.I1RN58
151422 229533.A0A0E0RVZ1 pp 229533.I1RLU6
151423 229533.A0A0E0RVZ1 pp 229533.I1RRG3
151424 229533.A0A0E0RVZ1 pp 229533.A0A1C3YJT5
151425 229533.A0A0E0RVZ1 pp 229533.I1S0L3
151426 229533.A0A0E0RVZ1 pp 229533.I1RKK0
151427 229533.A0A0E0RVZ1 pp 229533.I1S5B0
151428 229533.A0A0E0RVZ1 pp 229533.I1RNQ5
151429 229533.A0A0E0RVZ1 pp 229533.I1RW99
151430 229533.A0A0E0RVZ1 pp 229533.A0A1C3YIP5
151431 229533.A0A0E0RVZ1 pp 229533.I1RZM1
151432 229533.A0A0E0RVZ1 pp 229533.I1RVQ0
151433 229533.A0A0E0RVZ1 pp 229533.I1RFU8
151434 229533.A0A0E0RVZ1 pp 229533.A0A1C3YIP6
151435 229533.A0A0E0RVZ1 pp 229533.I1RX70
151436 229533.A0A0E0RVZ1 pp 229533.V6QUE6
151437 229533.A0A0E0RVZ1 pp 229533.I1RZB5
151438 229533.A0A0E0RVZ1 pp 229533.I1RC95
151439 229533.A0A0E0RVZ1 pp 229533.I1RRU9
151440 229533.A0A0E0RVZ1 pp 229533.I1RDB1
151441 229533.A0A0E0RVZ1 pp 229533.I1RN08
151442 229533.A0A0E0RVZ1 pp 229533.A0A0E0SA11
151443 229533.A0A0E0RVZ1 pp 229533.A0A1C3YKC0
151444 229533.A0A0E0RVZ1 pp 229533.I1RCG4
151445 229533.A0A0E0RVZ1 pp 229533.I1RAM6
151446 229533.A0A0E0RVZ1 pp 229533.I1RFT2
151447 229533.A0A0E0RVZ1 pp 229533.I1RAU5
151448 229533.A0A0E0RVZ1 pp 229533.A0A1C3YKV3
151449 229533.A0A0E0RVZ1 pp 229533.A0A1C3YHX8
151450 229533.A0A0E0RVZ1 pp 229533.I1RR24
151451 229533.A0A0E0RVZ1 pp 229533.I1RZK5
151452 229533.A0A0E0RVZ1 pp 229533.I1RFQ7
151453 229533.A0A0E0RVZ1 pp 229533.I1RQ81
151454 229533.A0A0E0RVZ1 pp 229533.A0A1C3YHX6
151455 229533.A0A0E0RVZ1 pp 229533.I1RC22
151456 229533.A0A0E0RVZ1 pp 229533.Q4IJM4
151457 229533.A0A0E0RVZ1 pp 229533.I1S503
151458 229533.A0A0E0RVZ1 pp 229533.I1RUQ5
151459 229533.A0A0E0RVZ1 pp 229533.I1RSV2
151460 229533.A0A0E0RVZ1 pp 229533.I1RK95
151461 229533.A0A0E0RVZ1 pp 229533.I1RY03
151462 229533.A0A0E0RVZ1 pp 229533.A0A0E0S9Y6
151463 229533.A0A0E0RVZ1 pp 229533.I1RQA3
151464 229533.A0A0E0RVZ1 pp 229533.I1RB74
151465 229533.A0A0E0RVZ1 pp 229533.I1S8P5
151466 229533.A0A0E0RVZ1 pp 229533.I1RYY5
151467 229533.A0A0E0RVZ1 pp 229533.I1RPP3
151468 229533.A0A0E0RVZ1 pp 229533.Q4IJK2
151469 229533.A0A0E0RVZ1 pp 229533.I1RRY9
151470 229533.A0A0E0RVZ1 pp 229533.I1RB75
151471 229533.A0A0E0RVZ1 pp 229533.I1S439
151472 229533.A0A0E0RVZ1 pp 229533.I1RQG4
151473 229533.A0A0E0RVZ1 pp 229533.I1RWN5
151474 229533.A0A0E0RVZ1 pp 229533.I1RFL7
151475 229533.A0A0E0RVZ1 pp 229533.I1RC97

151476	229533.A0A0E0RVZ1	pp	229533.Q4IB65
151477	229533.A0A0E0RVZ1	pp	229533.I1RSI0
151478	229533.A0A0E0RVZ1	pp	229533.A0A1C3YIJ3
151479	229533.A0A0E0RVZ1	pp	229533.I1RT04
151480	229533.A0A0E0RVZ1	pp	229533.V6RIV9
151481	229533.A0A0E0RVZ1	pp	229533.I1S2E9
151482	229533.A0A0E0RVZ1	pp	229533.I1RV91
151483	229533.A0A0E0RVZ1	pp	229533.I1RCJ7
151484	229533.A0A0E0RVZ1	pp	229533.I1RTD5
151485	229533.A0A0E0RVZ1	pp	229533.I1R9N1
151486	229533.A0A0E0RVZ1	pp	229533.I1RN67
151487	229533.A0A0E0RVZ1	pp	229533.I1RBS0
151488	229533.A0A0E0RVZ1	pp	229533.I1RZ39
151489	229533.A0A0E0RVZ1	pp	229533.V6RKY7
151490	229533.A0A0E0RVZ1	pp	229533.A0A1C3YKM9
151491	229533.A0A0E0RVZ1	pp	229533.I1RAN2
151492	229533.A0A0E0RVZ1	pp	229533.I1RBS6
151493	229533.A0A0E0RVZ1	pp	229533.I1RGA4
151494	229533.A0A0E0RVZ1	pp	229533.I1RY44
151495	229533.A0A0E0RVZ1	pp	229533.I1RP27
151496	229533.A0A0E0RVZ1	pp	229533.I1RZZ9
151497	229533.A0A0E0RVZ1	pp	229533.I1RZ63
151498	229533.A0A0E0RVZ1	pp	229533.A0A1C3YIN1
151499	229533.A0A0E0RVZ1	pp	229533.V6RVG3
151500	229533.A0A0E0RVZ1	pp	229533.I1RR73
151501	229533.A0A0E0RVZ1	pp	229533.I1RA70
151502	229533.A0A0E0RVZ1	pp	229533.V6R3X5
151503	229533.A0A0E0RVZ1	pp	229533.I1RNN1
151504	229533.A0A0E0RVZ1	pp	229533.I1RTH6
151505	229533.A0A0E0RVZ1	pp	229533.I1RFT6
151506	229533.A0A0E0RVZ1	pp	229533.I1RCL5
151507	229533.A0A0E0RVZ1	pp	229533.I1RWI5
151508	229533.A0A0E0RVZ1	pp	229533.I1RS20
151509	229533.A0A0E0RVZ1	pp	229533.I1RK79
151510	229533.A0A0E0RVZ1	pp	229533.I1S2H4
151511	229533.A0A0E0RVZ1	pp	229533.I1RRV1
151512	229533.A0A0E0RVZ1	pp	229533.I1RYW3
151513	229533.A0A0E0RVZ1	pp	229533.I1RSQ3
151514	229533.A0A0E0RVZ1	pp	229533.I1S0L4
151515	229533.A0A0E0RVZ1	pp	229533.I1S1C7
151516	229533.A0A0E0RVZ1	pp	229533.I1RM82
151517	229533.A0A0E0RVZ1	pp	229533.V6R042
151518	229533.A0A0E0RVZ1	pp	229533.I1RRI2
151519	229533.A0A0E0RVZ1	pp	229533.V6RMW6
151520	229533.A0A0E0RVZ1	pp	229533.I1RRT8
151521	229533.A0A0E0RVZ1	pp	229533.I1RWN6
151522	229533.A0A0E0RVZ1	pp	229533.I1RNP7
151523	229533.A0A0E0RVZ1	pp	229533.I1RWL2
151524	229533.A0A0E0RVZ1	pp	229533.I1RC29
151525	229533.A0A0E0RVZ1	pp	229533.I1RNC1
151526	229533.A0A0E0RVZ1	pp	229533.I1RAW0
151527	229533.A0A0E0RVZ1	pp	229533.I1RUG3
151528	229533.A0A0E0RVZ1	pp	229533.A0A1C3YK57
151529	229533.A0A0E0RVZ1	pp	229533.I1RCM3
151530	229533.A0A0E0RVZ1	pp	229533.I1RUN8
151531	229533.A0A0E0RVZ1	pp	229533.I1RCR8
151532	229533.A0A0E0RVZ1	pp	229533.A0A1C3YMI5
151533	229533.A0A0E0RVZ1	pp	229533.I1RSK1
151534	229533.A0A0E0RVZ1	pp	229533.V6RGI1
151535	229533.A0A0E0RVZ1	pp	229533.I1RPM4
151536	229533.A0A0E0RVZ1	pp	229533.I1RJV3
151537	229533.A0A0E0RVZ1	pp	229533.A0A1C3YHL0
151538	229533.A0A0E0RVZ1	pp	229533.A0A1C3YIS7
151539	229533.A0A0E0RVZ1	pp	229533.I1S0N4
151540	229533.A0A0E0RVZ1	pp	229533.I1S0J7
151541	229533.A0A0E0RVZ1	pp	229533.I1RT03
151542	229533.A0A0E0RVZ1	pp	229533.I1RJZ7
151543	229533.A0A0E0RVZ1	pp	229533.I1S2B8
151544	229533.A0A0E0RVZ1	pp	229533.I1RD22
151545	229533.A0A0E0RVZ1	pp	229533.I1RVX0
151546	229533.A0A0E0RVZ1	pp	229533.I1RPP4
151547	229533.A0A0E0RVZ1	pp	229533.I1RAS2
151548	229533.A0A0E0RVZ1	pp	229533.I1RE01

151549 229533.A0A0E0RVZ1 pp 229533.I1RB92
151550 229533.A0A0E0RVZ1 pp 229533.I1RTA0
151551 229533.A0A0E0RVZ1 pp 229533.I1RPU3
151552 229533.A0A0E0RVZ1 pp 229533.I1RDZ4
151553 229533.A0A0E0RVZ1 pp 229533.I1RBY4
151554 229533.A0A0E0RVZ1 pp 229533.I1R9Y9
151555 229533.A0A0E0RVZ1 pp 229533.I1RN18
151556 229533.A0A0E0RVZ1 pp 229533.I1RC72
151557 229533.A0A0E0RVZ1 pp 229533.I1S0J1
151558 229533.A0A0E0RVZ1 pp 229533.A0A1C3YHP8
151559 229533.A0A0E0RVZ1 pp 229533.I1RZE4
151560 229533.A0A0E0RVZ1 pp 229533.I1S0E8
151561 229533.A0A0E0RVZ1 pp 229533.I1RD17
151562 229533.A0A0E0RVZ1 pp 229533.I1RAU7
151563 229533.A0A0E0RVZ1 pp 229533.I1RK75
151564 229533.A0A0E0RVZ1 pp 229533.I1S8A6
151565 229533.A0A0E0RVZ1 pp 229533.I1RD15
151566 229533.A0A0E0RVZ1 pp 229533.I1R9Y7
151567 229533.I1RH74 pp 229533.I1RZT1
151568 229533.A0A1C3YL28 pp 229533.Q4IN00
151569 229533.A0A1C3YL28 pp 229533.A0A1C3YN54
151570 229533.A0A1C3YL28 pp 229533.I1RTG5
151571 229533.A0A1C3YL28 pp 229533.I1RTC1
151572 229533.A0A1C3YL28 pp 229533.I1RRB0
151573 229533.A0A1C3YL28 pp 229533.I1RWB4
151574 229533.A0A1C3YL28 pp 229533.I1RQ32
151575 229533.A0A1C3YL28 pp 229533.I1RMJ1
151576 229533.A0A1C3YL28 pp 229533.I1RQ70
151577 229533.A0A1C3YL28 pp 229533.I1RTQ9
151578 229533.A0A1C3YL28 pp 229533.I1RYW8
151579 229533.A0A1C3YL28 pp 229533.I1RMG2
151580 229533.A0A1C3YL28 pp 229533.I1RCE7
151581 229533.A0A1C3YL28 pp 229533.Q4ILI9
151582 229533.A0A1C3YL28 pp 229533.I1RTM8
151583 229533.A0A1C3YL28 pp 229533.A0A1C3YM61
151584 229533.A0A1C3YL28 pp 229533.I1REG5
151585 229533.A0A1C3YL28 pp 229533.A0A1C3YLS7
151586 229533.A0A1C3YL28 pp 229533.Q4I877
151587 229533.A0A1C3YL28 pp 229533.I1RPK5
151588 229533.A0A1C3YL28 pp 229533.Q4HYZ2
151589 229533.Q4IJM4 pp 229533.V6QWB1
151590 229533.Q4IJM4 pp 229533.V6RVG3
151591 229533.Q4IJM4 pp 229533.V6RDP1
151592 229533.Q4IJM4 pp 229533.V6QV09
151593 229533.Q4IJM4 pp 229533.V6RKY7
151594 229533.Q4IJM4 pp 229533.V6R3X5
151595 229533.Q4IJM4 pp 229533.Q4IPI4
151596 229533.Q4IJM4 pp 229533.V6RGI1
151597 229533.Q4IJM4 pp 229533.V6RTJ8
151598 229533.Q4IJM4 pp 229533.V6RMW6
151599 229533.A0A098DI12 pp 229533.I1R9R7
151600 229533.A0A098DI12 pp 229533.I1RY32
151601 229533.A0A098DI12 pp 229533.I1RC71
151602 229533.A0A098DI12 pp 229533.A0A1C3YHH7
151603 229533.A0A098DI12 pp 229533.I1RJR8
151604 229533.A0A098DI12 pp 229533.A0A1C3YIH7
151605 229533.A0A098DI12 pp 229533.I1RWT1
151606 229533.A0A098DI12 pp 229533.I1RZC3
151607 229533.A0A098DI12 pp 229533.A0A098DJG8
151608 229533.A0A098DI12 pp 229533.A0A1C3YIU5
151609 229533.A0A098DI12 pp 229533.I1RX84
151610 229533.A0A098DI12 pp 229533.I1RST9
151611 229533.A0A098DI12 pp 229533.A0A0E0SNH8
151612 229533.A0A098DI12 pp 229533.I1RQ48
151613 229533.A0A098DI12 pp 229533.I1RPK5
151614 229533.A0A098DI12 pp 229533.I1RVB8
151615 229533.A0A098DI12 pp 229533.I1S267
151616 229533.A0A098DI12 pp 229533.A0A1C3YL28
151617 229533.A0A098DI12 pp 229533.I1REG5
151618 229533.A0A098DB19 pp 229533.A0A1C3YKM9
151619 229533.A0A098DB19 pp 229533.I1S0N3
151620 229533.A0A098DB19 pp 229533.I1RA70
151621 229533.A0A098DB19 pp 229533.I1RCG4

151622	229533.A0A098DB19	pp	229533.I1RTH6
151623	229533.A0A098DB19	pp	229533.V6R3X5
151624	229533.A0A098DB19	pp	229533.I1RR73
151625	229533.A0A098DB19	pp	229533.I1RZZ9
151626	229533.A0A098DB19	pp	229533.I1S1C7
151627	229533.A0A098DB19	pp	229533.A0A1C3YHX6
151628	229533.A0A098DB19	pp	229533.I1RBT1
151629	229533.A0A098DB19	pp	229533.A0A1C3YKC0
151630	229533.A0A098DB19	pp	229533.I1RY03
151631	229533.A0A098DB19	pp	229533.I1S0X6
151632	229533.A0A098DB19	pp	229533.A0A1C3YHX8
151633	229533.A0A098DB19	pp	229533.I1RZW8
151634	229533.A0A098DB19	pp	229533.A0A0E0RUI4
151635	229533.A0A098DB19	pp	229533.I1RD17
151636	229533.A0A098DB19	pp	229533.I1REK8
151637	229533.A0A098DB19	pp	229533.I1RAU7
151638	229533.A0A098DB19	pp	229533.A0A1C3YIS7
151639	229533.A0A098DB19	pp	229533.A0A098DXE2
151640	229533.A0A098DB19	pp	229533.I1RRM5
151641	229533.A0A098DB19	pp	229533.I1RBY4
151642	229533.A0A098DB19	pp	229533.A0A1C3YJQ9
151643	229533.A0A098DB19	pp	229533.A0A0E0RU84
151644	229533.A0A098DB19	pp	229533.I1RW25
151645	229533.A0A098DB19	pp	229533.A0A1C3YIJ3
151646	229533.A0A098DB19	pp	229533.I1RT03
151647	229533.A0A098DB19	pp	229533.I1RB92
151648	229533.A0A098DB19	pp	229533.I1RN18
151649	229533.A0A098DB19	pp	229533.I1RFL4
151650	229533.A0A098DB19	pp	229533.I1RPM4
151651	229533.A0A098DB19	pp	229533.I1RQE7
151652	229533.A0A098DB19	pp	229533.I1RBS6
151653	229533.A0A098DB19	pp	229533.I1RAX6
151654	229533.A0A098DB19	pp	229533.I1S5B0
151655	229533.A0A098DB19	pp	229533.I1S504
151656	229533.A0A098DB19	pp	229533.V6RTJ8
151657	229533.A0A098DB19	pp	229533.I1RJV4
151658	229533.A0A098DB19	pp	229533.I1RW99
151659	229533.A0A098DB19	pp	229533.A0A1C3YKB0
151660	229533.A0A098DB19	pp	229533.I1S0L3
151661	229533.A0A098DB19	pp	229533.I1RN67
151662	229533.A0A098DB19	pp	229533.V6RIV9
151663	229533.A0A098DB19	pp	229533.A0A1C3YIP5
151664	229533.A0A098DB19	pp	229533.I1RM07
151665	229533.A0A098DB19	pp	229533.I1S503
151666	229533.A0A098DB19	pp	229533.I1S0F6
151667	229533.A0A098DB19	pp	229533.I1RNQ5
151668	229533.A0A098DB19	pp	229533.I1RLU6
151669	229533.A0A098DB19	pp	229533.I1RVQ0
151670	229533.A0A098DB19	pp	229533.I1RJW6
151671	229533.A0A098DB19	pp	229533.I1RX46
151672	229533.A0A098DB19	pp	229533.I1RD22
151673	229533.A0A098DB19	pp	229533.I1RAU5
151674	229533.A0A098DB19	pp	229533.A0A0E0SFN0
151675	229533.A0A098DB19	pp	229533.I1RQC9
151676	229533.A0A098DB19	pp	229533.I1RZM1
151677	229533.A0A098DB19	pp	229533.I1RZL6
151678	229533.A0A098DB19	pp	229533.I1RT04
151679	229533.A0A098DB19	pp	229533.V6RCG5
151680	229533.A0A098DB19	pp	229533.A0A1C3YKV3
151681	229533.A0A098DB19	pp	229533.I1RWN6
151682	229533.A0A098DB19	pp	229533.I1RBC9
151683	229533.A0A098DB19	pp	229533.I1RS17
151684	229533.A0A098DB19	pp	229533.I1RRY9
151685	229533.A0A098DB19	pp	229533.I1RPP3
151686	229533.A0A098DB19	pp	229533.V6RMW6
151687	229533.A0A098DB19	pp	229533.I1S0L4
151688	229533.A0A098DB19	pp	229533.I1S2E9
151689	229533.A0A098DB19	pp	229533.I1RPP4
151690	229533.A0A098DB19	pp	229533.I1RSQ7
151691	229533.A0A098DB19	pp	229533.I1RWJ8
151692	229533.A0A098DB19	pp	229533.V6QW22
151693	229533.A0A098DB19	pp	229533.V6R042
151694	229533.A0A098DB19	pp	229533.I1RJV3

151695 229533.A0A098DB19 pp 229533.I1RSQ3
151696 229533.A0A098DB19 pp 229533.I1RFU8
151697 229533.A0A098DB19 pp 229533.I1RE01
151698 229533.A0A098DB19 pp 229533.I1RUG3
151699 229533.A0A098DB19 pp 229533.I1RWK3
151700 229533.A0A098DB19 pp 229533.I1RNC1
151701 229533.I1RRL3 pp 229533.I1RS64
151702 229533.A0A1C3YK34 pp 229533.I1RB75
151703 229533.A0A1C3YK34 pp 229533.I1REK8
151704 229533.A0A1C3YK34 pp 229533.I1RM35
151705 229533.A0A1C3YK34 pp 229533.A0A1C3YLX0
151706 229533.A0A1C3YK34 pp 229533.I1RSI0
151707 229533.A0A1C3YK34 pp 229533.I1RF19
151708 229533.A0A1C3YK34 pp 229533.I1S4Q4
151709 229533.A0A1C3YK34 pp 229533.I1RZE5
151710 229533.A0A1C3YK34 pp 229533.Q4I2J8
151711 229533.A0A1C3YK34 pp 229533.I1RRY9
151712 229533.A0A1C3YK34 pp 229533.I1RWR7
151713 229533.A0A1C3YK34 pp 229533.I1RBL7
151714 229533.A0A1C3YK34 pp 229533.I1RXX1
151715 229533.A0A1C3YK34 pp 229533.I1REL7
151716 229533.A0A1C3YK34 pp 229533.I1RDG0
151717 229533.A0A1C3YK34 pp 229533.I1RK95
151718 229533.A0A1C3YK34 pp 229533.I1RUQ3
151719 229533.A0A1C3YK34 pp 229533.I1RWJ3
151720 229533.A0A1C3YK34 pp 229533.I1S1J7
151721 229533.A0A1C3YK34 pp 229533.A0A1C3YKH8
151722 229533.A0A1C3YK34 pp 229533.I1RD03
151723 229533.A0A1C3YK34 pp 229533.Q4IJM4
151724 229533.A0A1C3YK34 pp 229533.I1RYS0
151725 229533.A0A1C3YK34 pp 229533.I1RJZ6
151726 229533.A0A1C3YK34 pp 229533.I1RQQ5
151727 229533.A0A1C3YK34 pp 229533.I1RB53
151728 229533.A0A1C3YK34 pp 229533.A0A1C3YKY6
151729 229533.A0A1C3YK34 pp 229533.V6RBN2
151730 229533.A0A1C3YK34 pp 229533.I1RCR5
151731 229533.A0A1C3YK34 pp 229533.I1RZZ1
151732 229533.A0A1C3YK34 pp 229533.A0A1C3YLK2
151733 229533.A0A1C3YK34 pp 229533.I1RYY5
151734 229533.A0A1C3YK34 pp 229533.I1RY03
151735 229533.A0A1C3YK34 pp 229533.I1RWB0
151736 229533.A0A1C3YK34 pp 229533.I1RGB2
151737 229533.A0A1C3YK34 pp 229533.Q4IJK2
151738 229533.A0A1C3YK34 pp 229533.I1RQA3
151739 229533.A0A1C3YK34 pp 229533.V6R9Z0
151740 229533.A0A1C3YK34 pp 229533.I1RNT9
151741 229533.A0A1C3YK34 pp 229533.I1RG14
151742 229533.A0A1C3YK34 pp 229533.I1RK15
151743 229533.A0A1C3YK34 pp 229533.I1S887
151744 229533.A0A1C3YK34 pp 229533.A0A1C3YK68
151745 229533.A0A1C3YK34 pp 229533.A0A1C3YKV3
151746 229533.A0A1C3YK34 pp 229533.I1RQ85
151747 229533.A0A1C3YK34 pp 229533.I1SA32
151748 229533.A0A1C3YK34 pp 229533.I1RZK5
151749 229533.A0A1C3YK34 pp 229533.I1RWT6
151750 229533.A0A1C3YK34 pp 229533.I1RCG4
151751 229533.A0A1C3YK34 pp 229533.I1RCM5
151752 229533.A0A1C3YK34 pp 229533.Q4I283
151753 229533.A0A1C3YK34 pp 229533.I1RXC4
151754 229533.A0A1C3YK34 pp 229533.I1RFU8
151755 229533.A0A1C3YK34 pp 229533.I1RBU8
151756 229533.A0A1C3YK34 pp 229533.I1RW99
151757 229533.A0A1C3YK34 pp 229533.I1RAZ3
151758 229533.A0A1C3YK34 pp 229533.I1S5B0
151759 229533.A0A1C3YK34 pp 229533.A0A1C3YKB0
151760 229533.A0A1C3YK34 pp 229533.I1RTD3
151761 229533.A0A1C3YK34 pp 229533.I1RC17
151762 229533.A0A1C3YK34 pp 229533.I1RA90
151763 229533.A0A1C3YK34 pp 229533.I1RQD2
151764 229533.A0A1C3YK34 pp 229533.I1S504
151765 229533.A0A1C3YK34 pp 229533.I1S6K9
151766 229533.A0A1C3YK34 pp 229533.I1RAU5
151767 229533.A0A1C3YK34 pp 229533.I1RZM1

151768	229533.A0A1C3YK34	pp	229533.I1RBE4
151769	229533.A0A1C3YK34	pp	229533.I1S1B2
151770	229533.A0A1C3YK34	pp	229533.I1RNQ5
151771	229533.A0A1C3YK34	pp	229533.I1RLU6
151772	229533.A0A1C3YK34	pp	229533.I1RFT2
151773	229533.A0A1C3YK34	pp	229533.V6RCZ8
151774	229533.A0A1C3YK34	pp	229533.I1RGC5
151775	229533.A0A1C3YK34	pp	229533.V6QUE6
151776	229533.A0A1C3YK34	pp	229533.I1RF82
151777	229533.A0A1C3YK34	pp	229533.I1RC61
151778	229533.A0A1C3YK34	pp	229533.I1S419
151779	229533.A0A1C3YK34	pp	229533.I1RRV4
151780	229533.A0A1C3YK34	pp	229533.I1RI82
151781	229533.A0A1C3YK34	pp	229533.A0A1C3YK36
151782	229533.A0A1C3YK34	pp	229533.I1RRV3
151783	229533.A0A1C3YK34	pp	229533.Q4I8P5
151784	229533.A0A1C3YK34	pp	229533.I1RXF0
151785	229533.A0A1C3YK34	pp	229533.V6RCG5
151786	229533.A0A1C3YK34	pp	229533.I1RS18
151787	229533.A0A1C3YK34	pp	229533.I1RZM8
151788	229533.A0A1C3YK34	pp	229533.I1RQ81
151789	229533.A0A1C3YK34	pp	229533.A0A1C3YLU7
151790	229533.A0A1C3YK34	pp	229533.I1RR51
151791	229533.A0A1C3YK34	pp	229533.I1RRS8
151792	229533.A0A1C3YK34	pp	229533.I1RB39
151793	229533.A0A1C3YK34	pp	229533.I1S821
151794	229533.A0A1C3YK34	pp	229533.I1S4S4
151795	229533.A0A1C3YK34	pp	229533.A0A1C3YLR6
151796	229533.A0A1C3YK34	pp	229533.I1S9I5
151797	229533.A0A1C3YK34	pp	229533.I1RXD6
151798	229533.A0A1C3YK34	pp	229533.I1RX25
151799	229533.A0A1C3YK34	pp	229533.I1RWJ8
151800	229533.A0A1C3YK34	pp	229533.I1RKJ5
151801	229533.A0A1C3YK34	pp	229533.I1RIG2
151802	229533.A0A1C3YK34	pp	229533.I1RRX5
151803	229533.A0A1C3YK34	pp	229533.I1RW25
151804	229533.A0A1C3YK34	pp	229533.I1RSQ7
151805	229533.A0A1C3YK34	pp	229533.A0A1C3YN30
151806	229533.A0A1C3YK34	pp	229533.I1RMQ6
151807	229533.A0A1C3YK34	pp	229533.I1RJU3
151808	229533.A0A1C3YK34	pp	229533.I1S438
151809	229533.A0A1C3YK34	pp	229533.I1RMC8
151810	229533.A0A1C3YK34	pp	229533.I1R9Y4
151811	229533.A0A1C3YK34	pp	229533.A0A1C3YN26
151812	229533.A0A1C3YK34	pp	229533.I1RE17
151813	229533.A0A1C3YK34	pp	229533.I1S0X6
151814	229533.A0A1C3YK34	pp	229533.I1RN61
151815	229533.A0A1C3YK34	pp	229533.I1RC20
151816	229533.A0A1C3YK34	pp	229533.V6QW22
151817	229533.A0A1C3YK34	pp	229533.I1S1W8
151818	229533.A0A1C3YK34	pp	229533.I1RPA4
151819	229533.A0A1C3YK34	pp	229533.I1RG53
151820	229533.A0A1C3YK34	pp	229533.I1RPN4
151821	229533.A0A1C3YK34	pp	229533.A0A1C3YKC0
151822	229533.A0A1C3YK34	pp	229533.I1RCS1
151823	229533.A0A1C3YK34	pp	229533.I1RBT1
151824	229533.A0A1C3YK34	pp	229533.I1S0F5
151825	229533.A0A1C3YK34	pp	229533.I1RR00
151826	229533.A0A1C3YK34	pp	229533.I1RBC9
151827	229533.A0A1C3YK34	pp	229533.I1RR24
151828	229533.A0A1C3YK34	pp	229533.I1RW71
151829	229533.A0A1C3YK34	pp	229533.I1S0L3
151830	229533.A0A1C3YK34	pp	229533.V6R1K4
151831	229533.A0A1C3YK34	pp	229533.I1RM67
151832	229533.A0A1C3YK34	pp	229533.I1RFQ7
151833	229533.A0A1C3YK34	pp	229533.I1S4S7
151834	229533.A0A1C3YK34	pp	229533.I1RT33
151835	229533.A0A1C3YK34	pp	229533.Q4IJT5
151836	229533.A0A1C3YK34	pp	229533.I1S540
151837	229533.A0A1C3YK34	pp	229533.I1RA85
151838	229533.A0A1C3YK34	pp	229533.I1RRG3
151839	229533.A0A1C3YK34	pp	229533.I1RX46
151840	229533.A0A1C3YK34	pp	229533.A0A1C3YM63

151841	229533.A0A1C3YK34	pp	229533.I1RBD5
151842	229533.A0A1C3YK34	pp	229533.A0A1C3YLL3
151843	229533.A0A1C3YK34	pp	229533.I1S2R0
151844	229533.A0A1C3YK34	pp	229533.I1RY86
151845	229533.A0A1C3YK34	pp	229533.I1RD52
151846	229533.A0A1C3YK34	pp	229533.I1S0T2
151847	229533.A0A1C3YK34	pp	229533.I1RVQ0
151848	229533.A0A1C3YK34	pp	229533.I1RD17
151849	229533.A0A1C3YK34	pp	229533.I1RX95
151850	229533.A0A1C3YK34	pp	229533.I1RBR2
151851	229533.A0A1C3YK34	pp	229533.I1REK4
151852	229533.A0A1C3YK34	pp	229533.Q4I624
151853	229533.A0A1C3YK34	pp	229533.I1RLP1
151854	229533.A0A1C3YK34	pp	229533.I1RXD7
151855	229533.A0A1C3YK34	pp	229533.V6R057
151856	229533.A0A1C3YK34	pp	229533.I1S7A8
151857	229533.A0A1C3YK34	pp	229533.I1RQH3
151858	229533.A0A1C3YK34	pp	229533.I1RCD8
151859	229533.A0A1C3YK34	pp	229533.I1RAX6
151860	229533.A0A1C3YK34	pp	229533.I1RML0
151861	229533.A0A1C3YK34	pp	229533.V6RUT5
151862	229533.A0A1C3YK34	pp	229533.V6RRH2
151863	229533.A0A1C3YK34	pp	229533.I1RCR1
151864	229533.A0A1C3YK34	pp	229533.I1RYW3
151865	229533.A0A1C3YK34	pp	229533.I1RTB3
151866	229533.A0A1C3YK34	pp	229533.I1RBR1
151867	229533.A0A1C3YK34	pp	229533.I1S1C7
151868	229533.A0A1C3YK34	pp	229533.I1S3S3
151869	229533.A0A1C3YK34	pp	229533.I1RB41
151870	229533.A0A1C3YK34	pp	229533.I1RUF7
151871	229533.A0A1C3YK34	pp	229533.I1RB92
151872	229533.A0A1C3YK34	pp	229533.I1RB44
151873	229533.A0A1C3YK34	pp	229533.I1S437
151874	229533.A0A1C3YK34	pp	229533.I1RCW6
151875	229533.A0A1C3YK34	pp	229533.I1S0R7
151876	229533.A0A1C3YK34	pp	229533.I1RRN5
151877	229533.A0A1C3YK34	pp	229533.I1RQC9
151878	229533.A0A1C3YK34	pp	229533.I1S8V4
151879	229533.A0A1C3YK34	pp	229533.I1RSS4
151880	229533.A0A1C3YK34	pp	229533.I1RPY3
151881	229533.A0A1C3YK34	pp	229533.I1RVZ7
151882	229533.A0A1C3YK34	pp	229533.I1RNB2
151883	229533.A0A1C3YK34	pp	229533.I1RXR1
151884	229533.A0A1C3YK34	pp	229533.A0A1C3YL54
151885	229533.A0A1C3YK34	pp	229533.I1RSY2
151886	229533.A0A1C3YK34	pp	229533.I1RQ33
151887	229533.A0A1C3YK34	pp	229533.I1RPU2
151888	229533.A0A1C3YK34	pp	229533.I1RSF1
151889	229533.A0A1C3YK34	pp	229533.V6R2A1
151890	229533.A0A1C3YK34	pp	229533.I1RT03
151891	229533.A0A1C3YK34	pp	229533.I1RAU7
151892	229533.A0A1C3YK34	pp	229533.I1S1W4
151893	229533.A0A1C3YK34	pp	229533.I1RWN6
151894	229533.A0A1C3YK34	pp	229533.I1RD39
151895	229533.A0A1C3YK34	pp	229533.I1RWE8
151896	229533.A0A1C3YK34	pp	229533.I1RVX0
151897	229533.A0A1C3YK34	pp	229533.A0A1C3YKY0
151898	229533.A0A1C3YK34	pp	229533.I1RRE5
151899	229533.A0A1C3YK34	pp	229533.I1RQE7
151900	229533.A0A1C3YK34	pp	229533.I1RXU9
151901	229533.A0A1C3YK34	pp	229533.I1RZV3
151902	229533.A0A1C3YK34	pp	229533.I1RJV3
151903	229533.A0A1C3YK34	pp	229533.I1RCH5
151904	229533.A0A1C3YK34	pp	229533.I1RN18
151905	229533.A0A1C3YK34	pp	229533.I1RSK1
151906	229533.A0A1C3YK34	pp	229533.I1RCM3
151907	229533.A0A1C3YK34	pp	229533.I1RFL4
151908	229533.A0A1C3YK34	pp	229533.I1S1W3
151909	229533.A0A1C3YK34	pp	229533.I1RAW0
151910	229533.A0A1C3YK34	pp	229533.I1S0J1
151911	229533.A0A1C3YK34	pp	229533.I1RE89
151912	229533.A0A1C3YK34	pp	229533.I1RC33
151913	229533.A0A1C3YK34	pp	229533.I1RVQ3

151914 229533.A0A1C3YK34 pp 229533.I1S7W5
151915 229533.A0A1C3YK34 pp 229533.I1RY44
151916 229533.A0A1C3YK34 pp 229533.I1RKJ8
151917 229533.A0A1C3YK34 pp 229533.I1RTA0
151918 229533.A0A1C3YK34 pp 229533.I1S0F8
151919 229533.A0A1C3YK34 pp 229533.I1S016
151920 229533.A0A1C3YK34 pp 229533.I1RAG7
151921 229533.A0A1C3YK34 pp 229533.I1S1W9
151922 229533.A0A1C3YK34 pp 229533.I1RW46
151923 229533.A0A1C3YK34 pp 229533.A0A1C3YLC1
151924 229533.A0A1C3YK34 pp 229533.I1S8R3
151925 229533.A0A1C3YK34 pp 229533.I1RE01
151926 229533.A0A1C3YK34 pp 229533.I1RTH6
151927 229533.A0A1C3YK34 pp 229533.I1RB26
151928 229533.A0A1C3YK34 pp 229533.I1RG11
151929 229533.A0A1C3YK34 pp 229533.I1RA70
151930 229533.A0A1C3YK34 pp 229533.I1RWN5
151931 229533.A0A1C3YK34 pp 229533.I1S0N4
151932 229533.A0A1C3YK34 pp 229533.I1RZW8
151933 229533.A0A1C3YK34 pp 229533.V6RIV9
151934 229533.A0A1C3YK34 pp 229533.I1RRI2
151935 229533.A0A1C3YK34 pp 229533.I1RS90
151936 229533.A0A1C3YK34 pp 229533.I1S5R6
151937 229533.A0A1C3YK34 pp 229533.I1S9Z4
151938 229533.A0A1C3YK34 pp 229533.I1RPP4
151939 229533.A0A1C3YK34 pp 229533.I1RZ79
151940 229533.A0A1C3YK34 pp 229533.I1RD22
151941 229533.A0A1C3YK34 pp 229533.I1RRN2
151942 229533.A0A1C3YK34 pp 229533.I1RMN6
151943 229533.A0A1C3YK34 pp 229533.I1RWX5
151944 229533.A0A1C3YK34 pp 229533.I1RS17
151945 229533.A0A1C3YK34 pp 229533.I1RVF8
151946 229533.A0A1C3YK34 pp 229533.I1SA33
151947 229533.A0A1C3YK34 pp 229533.I1RPK0
151948 229533.A0A1C3YK34 pp 229533.A0A1C3YMI5
151949 229533.A0A1C3YK34 pp 229533.A0A1C3YKS0
151950 229533.A0A1C3YK34 pp 229533.V6RLM7
151951 229533.A0A1C3YK34 pp 229533.I1RSU2
151952 229533.A0A1C3YK34 pp 229533.I1RKJ4
151953 229533.A0A1C3YK34 pp 229533.I1RNL5
151954 229533.A0A1C3YK34 pp 229533.Q4IN63
151955 229533.A0A1C3YK34 pp 229533.I1RRM3
151956 229533.A0A1C3YK34 pp 229533.V6RMW6
151957 229533.A0A1C3YK34 pp 229533.I1RSQ3
151958 229533.A0A1C3YK34 pp 229533.I1RC29
151959 229533.A0A1C3YK34 pp 229533.I1RE22
151960 229533.A0A1C3YK34 pp 229533.I1RGU7
151961 229533.A0A1C3YK34 pp 229533.I1RCR8
151962 229533.A0A1C3YK34 pp 229533.I1RPV3
151963 229533.A0A1C3YK34 pp 229533.I1RL04
151964 229533.A0A1C3YK34 pp 229533.V6R042
151965 229533.A0A1C3YK34 pp 229533.I1RUG3
151966 229533.A0A1C3YK34 pp 229533.I1RPM4
151967 229533.A0A1C3YK34 pp 229533.I1RRV1
151968 229533.A0A1C3YK34 pp 229533.I1RXT3
151969 229533.A0A1C3YK34 pp 229533.I1S6Y8
151970 229533.A0A1C3YK34 pp 229533.I1RNC1
151971 229533.A0A1C3YK34 pp 229533.A0A1C3YLS9
151972 229533.A0A1C3YK34 pp 229533.A0A1C3YMA1
151973 229533.A0A1C3YK34 pp 229533.I1RRT8
151974 229533.A0A1C3YK34 pp 229533.I1RFS3
151975 229533.A0A1C3YK34 pp 229533.I1S9Y1
151976 229533.A0A1C3YK34 pp 229533.I1RQ23
151977 229533.A0A1C3YK34 pp 229533.I1S4Z8
151978 229533.A0A1C3YK34 pp 229533.I1RKP1
151979 229533.A0A1C3YK34 pp 229533.I1RSZ7
151980 229533.A0A1C3YK34 pp 229533.I1RM82
151981 229533.A0A1C3YK34 pp 229533.I1S277
151982 229533.A0A1C3YK34 pp 229533.I1RA52
151983 229533.A0A1C3YK34 pp 229533.V6R3X5
151984 229533.A0A1C3YK34 pp 229533.I1RR73
151985 229533.A0A1C3YK34 pp 229533.I1RZ39
151986 229533.A0A1C3YK34 pp 229533.I1S0L4

151987 229533.A0A1C3YK34 pp 229533.I1RRI1
151988 229533.A0A1C3YK34 pp 229533.I1RS20
151989 229533.A0A1C3YK34 pp 229533.I1RQK4
151990 229533.A0A1C3YK34 pp 229533.I1RPE0
151991 229533.A0A1C3YK34 pp 229533.I1RE76
151992 229533.A0A1C3YK34 pp 229533.I1S2H4
151993 229533.A0A1C3YK34 pp 229533.A0A1C3YKM9
151994 229533.A0A1C3YK34 pp 229533.I1RBY4
151995 229533.A0A1C3YK34 pp 229533.I1RNN1
151996 229533.A0A1C3YK34 pp 229533.I1RC66
151997 229533.A0A1C3YK34 pp 229533.I1RPU3
151998 229533.A0A1C3YK34 pp 229533.I1S3G4
151999 229533.A0A1C3YK34 pp 229533.I1S8V3
152000 229533.A0A1C3YK34 pp 229533.I1S2B8
152001 229533.A0A1C3YK34 pp 229533.I1REC5
152002 229533.A0A1C3YK34 pp 229533.I1RVN1
152003 229533.A0A1C3YK34 pp 229533.I1RWL2
152004 229533.A0A1C3YK34 pp 229533.I1RRM5
152005 229533.A0A1C3YK34 pp 229533.I1RGA6
152006 229533.A0A1C3YK34 pp 229533.I1RA11
152007 229533.A0A1C3YK34 pp 229533.I1RZZ9
152008 229533.A0A1C3YK34 pp 229533.I1RJZ7
152009 229533.A0A1C3YK34 pp 229533.O74638
152010 229533.A0A1C3YK34 pp 229533.I1RXC2
152011 229533.A0A1C3YK34 pp 229533.I1R9M0
152012 229533.A0A1C3YK34 pp 229533.I1RSQ4
152013 229533.A0A1C3YK34 pp 229533.I1S0M6
152014 229533.A0A1C3YK34 pp 229533.I1RC05
152015 229533.A0A1C3YK34 pp 229533.I1RWI5
152016 229533.A0A1C3YK34 pp 229533.I1S5R1
152017 229533.A0A1C3YK34 pp 229533.I1RBS0
152018 229533.A0A1C3YK34 pp 229533.I1RSA5
152019 229533.A0A1C3YK34 pp 229533.I1S2G7
152020 229533.A0A1C3YK34 pp 229533.I1S0F6
152021 229533.A0A1C3YK34 pp 229533.I1RJV4
152022 229533.A0A1C3YK34 pp 229533.I1RQM7
152023 229533.A0A1C3YK34 pp 229533.I1RDF7
152024 229533.A0A1C3YK34 pp 229533.I1RP27
152025 229533.A0A1C3YK34 pp 229533.I1S0F7
152026 229533.A0A1C3YK34 pp 229533.I1RQ65
152027 229533.A0A1C3YK34 pp 229533.I1S0W4
152028 229533.A0A1C3YK34 pp 229533.I1RGA4
152029 229533.A0A1C3YK34 pp 229533.I1RN67
152030 229533.A0A1C3YK34 pp 229533.I1RBS6
152031 229533.A0A1C3YK34 pp 229533.I1RBC7
152032 229533.A0A1C3YK34 pp 229533.A0A1C3YKN4
152033 229533.A0A1C3YK34 pp 229533.I1RAB4
152034 229533.A0A1C3YK34 pp 229533.I1RS47
152035 229533.A0A1C3YK34 pp 229533.I1RRJ8
152036 229533.A0A1C3YK34 pp 229533.I1S268
152037 229533.A0A1C3YK34 pp 229533.I1S8P5
152038 229533.A0A1C3YK34 pp 229533.I1RPP3
152039 229533.A0A1C3YK34 pp 229533.V6R3J1
152040 229533.A0A1C3YK34 pp 229533.I1RSX0
152041 229533.A0A1C3YK34 pp 229533.I1RT40
152042 229533.A0A1C3YK34 pp 229533.I1RCJ7
152043 229533.A0A1C3YK34 pp 229533.I1RYI5
152044 229533.A0A1C3YK34 pp 229533.I1RV91
152045 229533.A0A1C3YK34 pp 229533.I1R9H9
152046 229533.A0A1C3YK34 pp 229533.I1S503
152047 229533.A0A1C3YK34 pp 229533.I1RB74
152048 229533.A0A1C3YK34 pp 229533.I1RFT1
152049 229533.A0A1C3YK34 pp 229533.A0A1C3YKN3
152050 229533.A0A1C3YK34 pp 229533.I1RSV2
152051 229533.A0A1C3YK34 pp 229533.I1RRK3
152052 229533.A0A1C3YK34 pp 229533.A0A1C3YLK8
152053 229533.A0A1C3YK34 pp 229533.I1RJW6
152054 229533.A0A1C3YK34 pp 229533.I1RC34
152055 229533.A0A1C3YK34 pp 229533.I1RVZ2
152056 229533.A0A1C3YK34 pp 229533.Q4HVQ9
152057 229533.A0A1C3YK34 pp 229533.I1RTA1
152058 229533.A0A1C3YK34 pp 229533.I1RRZ0
152059 229533.A0A1C3YK34 pp 229533.I1RJN9

152060 229533.A0A1C3YK34 pp 229533.I1S2E9
152061 229533.A0A1C3YK34 pp 229533.V6R283
152062 229533.A0A1C3YK34 pp 229533.I1RYR0
152063 229533.A0A1C3YK34 pp 229533.V6RUY9
152064 229533.A0A1C3YK34 pp 229533.I1RN63
152065 229533.A0A1C3YK34 pp 229533.I1RT04
152066 229533.A0A1C3YK34 pp 229533.I1RWK3
152067 229533.A0A1C3YK34 pp 229533.Q4HXF0
152068 229533.A0A1C3YK34 pp 229533.A0A1C3YL55
152069 229533.A0A1C3YK34 pp 229533.I1RPH6
152070 229533.A0A1C3YK34 pp 229533.I1RXF8
152071 229533.A0A1C3YK34 pp 229533.I1RTD5
152072 229533.I1RU29 pp 229533.V6RTJ8
152073 229533.I1RU29 pp 229533.V6RCG5
152074 229533.I1RU29 pp 229533.I1RX46
152075 229533.I1RU29 pp 229533.V6QW22
152076 229533.I1RU29 pp 229533.I1S0L3
152077 229533.I1RU29 pp 229533.I1RVQ0
152078 229533.I1RU29 pp 229533.I1RZM1
152079 229533.I1RU29 pp 229533.I1RZK5
152080 229533.I1RU29 pp 229533.I1S504
152081 229533.I1RU29 pp 229533.I1RY03
152082 229533.I1RU29 pp 229533.I1RW25
152083 229533.I1RU29 pp 229533.Q4IJK2
152084 229533.I1RU29 pp 229533.I1S2H4
152085 229533.I1RU29 pp 229533.I1RUG3
152086 229533.I1RU29 pp 229533.I1S503
152087 229533.I1RU29 pp 229533.I1RY44
152088 229533.I1RU29 pp 229533.V6R3X5
152089 229533.I1RU29 pp 229533.V6RIV9
152090 229533.I1RU29 pp 229533.I1RWN5
152091 229533.I1RU29 pp 229533.I1RZZ9
152092 229533.I1RU29 pp 229533.I1S2E9
152093 229533.I1RU29 pp 229533.I1RZ39
152094 229533.I1RU29 pp 229533.I1S8R3
152095 229533.I1RU29 pp 229533.I1RWN6
152096 229533.I1RU29 pp 229533.I1RV91
152097 229533.I1RU29 pp 229533.I1S8P5
152098 229533.I1RU29 pp 229533.I1S2B8
152099 229533.I1RU29 pp 229533.I1RZW8
152100 229533.I1RU29 pp 229533.I1RWK3
152101 229533.I1RU29 pp 229533.V6R042
152102 229533.I1RU29 pp 229533.I1S0L4
152103 229533.I1RU29 pp 229533.V6RMW6
152104 229533.I1RU29 pp 229533.I1S1C7
152105 229533.I1RU29 pp 229533.I1RVX0
152106 229533.I1RU29 pp 229533.Q4I1Y5
152107 229533.V6RBN2 pp 229533.V6RG79
152108 229533.V6RBN2 pp 229533.V6RTJ8
152109 229533.V6RBN2 pp 229533.V6RMW6
152110 229533.V6RBN2 pp 229533.V6RQW4
152111 229533.V6RBN2 pp 229533.V6RCZ8
152112 229533.V6RBN2 pp 229533.V6RRH2
152113 229533.A0A1C3YJI8 pp 229533.I1RKK0
152114 229533.A0A1C3YJI8 pp 229533.I1RC95
152115 229533.A0A1C3YJI8 pp 229533.I1RTA0
152116 229533.A0A1C3YJI8 pp 229533.I1RQ26
152117 229533.A0A1C3YJI8 pp 229533.I1S8P5
152118 229533.A0A1C3YJI8 pp 229533.I1RA43
152119 229533.A0A1C3YJI8 pp 229533.V6RVG3
152120 229533.A0A1C3YJI8 pp 229533.I1RPD6
152121 229533.A0A1C3YJI8 pp 229533.I1RC97
152122 229533.A0A1C3YJI8 pp 229533.I1S1W4
152123 229533.A0A1C3YJI8 pp 229533.Q4IJP1
152124 229533.A0A1C3YJI8 pp 229533.I1RC22
152125 229533.A0A1C3YJI8 pp 229533.I1RMM9
152126 229533.A0A1C3YJI8 pp 229533.I1RAN2
152127 229533.A0A1C3YJI8 pp 229533.A0A1C3YKF2
152128 229533.A0A1C3YJI8 pp 229533.I1RNP7
152129 229533.A0A1C3YJI8 pp 229533.I1RPM4
152130 229533.A0A1C3YJI8 pp 229533.I1RRY3
152131 229533.A0A1C3YJI8 pp 229533.I1RUN8
152132 229533.A0A1C3YJI8 pp 229533.I1RZE4

152133 229533.A0A1C3YJI8 pp 229533.I1RYH9
152134 229533.A0A1C3YJI8 pp 229533.I1RZ94
152135 229533.A0A1C3YJI8 pp 229533.I1S0M2
152136 229533.A0A1C3YJI8 pp 229533.I1S439
152137 229533.A0A1C3YJI8 pp 229533.I1RSX9
152138 229533.I1RRY9 pp 229533.I1S1J7
152139 229533.I1RRY9 pp 229533.I1RWR7
152140 229533.I1RRY9 pp 229533.I1RU29
152141 229533.I1RRY9 pp 229533.I1RWB0
152142 229533.I1RRY9 pp 229533.I1RYS0
152143 229533.I1RRY9 pp 229533.I1RWD7
152144 229533.I1RRY9 pp 229533.I1S4Q4
152145 229533.I1RRY9 pp 229533.I1S887
152146 229533.I1RRY9 pp 229533.I1RZK5
152147 229533.I1RRY9 pp 229533.I1RZE5
152148 229533.I1RRY9 pp 229533.I1RSI0
152149 229533.I1RRY9 pp 229533.I1RZ35
152150 229533.I1RRY9 pp 229533.I1RY03
152151 229533.I1RRY9 pp 229533.I1RYY5
152152 229533.I1RRY9 pp 229533.I1RVZ0
152153 229533.I1RRY9 pp 229533.Q4I2J8
152154 229533.I1RRY9 pp 229533.I1S419
152155 229533.I1RRY9 pp 229533.V6QUE6
152156 229533.I1RRY9 pp 229533.I1RSR8
152157 229533.I1RRY9 pp 229533.V6RBN2
152158 229533.I1RRY9 pp 229533.V6RCG5
152159 229533.I1RRY9 pp 229533.Q4IJM4
152160 229533.I1RRY9 pp 229533.V6RCZ8
152161 229533.I1RRY9 pp 229533.I1S8F8
152162 229533.I1RRY9 pp 229533.I1S6K9
152163 229533.I1RRY9 pp 229533.I1RZZ1
152164 229533.I1RRY9 pp 229533.Q4I8P5
152165 229533.I1RRY9 pp 229533.I1S0M9
152166 229533.I1RRY9 pp 229533.I1RZK6
152167 229533.I1RRY9 pp 229533.I1RS18
152168 229533.I1RRY9 pp 229533.I1SA32
152169 229533.I1RRY9 pp 229533.I1S021
152170 229533.I1RRY9 pp 229533.I1RW99
152171 229533.I1RRY9 pp 229533.I1RZM8
152172 229533.I1RRY9 pp 229533.Q4IJK2
152173 229533.I1RRY9 pp 229533.I1S9U2
152174 229533.I1RRY9 pp 229533.I1RXF0
152175 229533.I1RRY9 pp 229533.I1RXC4
152176 229533.I1RRY9 pp 229533.I1S5B0
152177 229533.I1RRY9 pp 229533.I1RZJ5
152178 229533.I1RRY9 pp 229533.I1S0X6
152179 229533.I1RRY9 pp 229533.I1RWT6
152180 229533.I1RRY9 pp 229533.I1RT80
152181 229533.I1RRY9 pp 229533.Q4I283
152182 229533.I1RRY9 pp 229533.I1S4S4
152183 229533.I1RRY9 pp 229533.I1RW07
152184 229533.I1RRY9 pp 229533.I1RX25
152185 229533.I1RRY9 pp 229533.I1RWJ8
152186 229533.I1RRY9 pp 229533.I1RZF6
152187 229533.I1RRY9 pp 229533.I1RW25
152188 229533.I1RRY9 pp 229533.I1S504
152189 229533.I1RRY9 pp 229533.I1RZM1
152190 229533.I1RRY9 pp 229533.I1S6U8
152191 229533.I1RRY9 pp 229533.V6QUW4
152192 229533.I1RRY9 pp 229533.I1S7A8
152193 229533.I1RRY9 pp 229533.I1RSK1
152194 229533.I1RRY9 pp 229533.I1RVQ0
152195 229533.I1RRY9 pp 229533.V6RTJ8
152196 229533.I1RRY9 pp 229533.I1RT33
152197 229533.I1RRY9 pp 229533.V6R4J1
152198 229533.I1RRY9 pp 229533.I1RX46
152199 229533.I1RRY9 pp 229533.I1S2C0
152200 229533.I1RRY9 pp 229533.I1S8R3
152201 229533.I1RRY9 pp 229533.I1S0T2
152202 229533.I1RRY9 pp 229533.I1RXD7
152203 229533.I1RRY9 pp 229533.I1RTH6
152204 229533.I1RRY9 pp 229533.I1RSQ7
152205 229533.I1RRY9 pp 229533.I1RZW8

152206 229533.I1RRY9 pp 229533.V6RUT5
152207 229533.I1RRY9 pp 229533.V6QW22
152208 229533.I1RRY9 pp 229533.I1RZ79
152209 229533.I1RRY9 pp 229533.I1RW71
152210 229533.I1RRY9 pp 229533.I1S0L3
152211 229533.I1RRY9 pp 229533.I1S4S7
152212 229533.I1RRY9 pp 229533.I1S0R7
152213 229533.I1RRY9 pp 229533.Q4IJT5
152214 229533.I1RRY9 pp 229533.I1RTD7
152215 229533.I1RRY9 pp 229533.I1RWX5
152216 229533.I1RRY9 pp 229533.I1S0F5
152217 229533.I1RRY9 pp 229533.I1RYW3
152218 229533.I1RRY9 pp 229533.I1S1C7
152219 229533.I1RRY9 pp 229533.V6R1K4
152220 229533.I1RRY9 pp 229533.I1RX95
152221 229533.I1RRY9 pp 229533.I1RSQ3
152222 229533.I1RRY9 pp 229533.Q4I624
152223 229533.I1RRY9 pp 229533.I1RWN6
152224 229533.I1RRY9 pp 229533.Q4I327
152225 229533.I1RRY9 pp 229533.I1RXU9
152226 229533.I1RRY9 pp 229533.I1S569
152227 229533.I1RRY9 pp 229533.V6RRH2
152228 229533.I1RRY9 pp 229533.V6R057
152229 229533.I1RRY9 pp 229533.I1RXR1
152230 229533.I1RRY9 pp 229533.I1RUG3
152231 229533.I1RRY9 pp 229533.Q4IN63
152232 229533.I1RRY9 pp 229533.I1RSS4
152233 229533.I1RRY9 pp 229533.I1RS17
152234 229533.I1RRY9 pp 229533.I1RUP9
152235 229533.I1RRY9 pp 229533.I1RVX0
152236 229533.I1RRY9 pp 229533.I1S0J1
152237 229533.I1RRY9 pp 229533.I1RSF1
152238 229533.I1RRY9 pp 229533.I1RT03
152239 229533.I1RRY9 pp 229533.I1RXT3
152240 229533.I1RRY9 pp 229533.I1S7W5
152241 229533.I1RRY9 pp 229533.I1S1W4
152242 229533.I1RRY9 pp 229533.I1RSD8
152243 229533.I1RRY9 pp 229533.I1RTA0
152244 229533.I1RRY9 pp 229533.I1S2E8
152245 229533.I1RRY9 pp 229533.V6RVG3
152246 229533.I1RRY9 pp 229533.I1S0N4
152247 229533.I1RRY9 pp 229533.I1RW46
152248 229533.I1RRY9 pp 229533.I1S5R6
152249 229533.I1RRY9 pp 229533.I1S2G9
152250 229533.I1RRY9 pp 229533.I1RWH4
152251 229533.I1RRY9 pp 229533.I1RZV3
152252 229533.I1RRY9 pp 229533.I1S0L4
152253 229533.I1RRY9 pp 229533.I1S1W3
152254 229533.I1RRY9 pp 229533.I1RS20
152255 229533.I1RRY9 pp 229533.I1S2H4
152256 229533.I1RRY9 pp 229533.I1RY44
152257 229533.I1RRY9 pp 229533.I1SA33
152258 229533.I1RRY9 pp 229533.V6RMW6
152259 229533.I1RRY9 pp 229533.I1RWL2
152260 229533.I1RRY9 pp 229533.Q4HZB8
152261 229533.I1RRY9 pp 229533.I1S016
152262 229533.I1RRY9 pp 229533.O74638
152263 229533.I1RRY9 pp 229533.I1RXC2
152264 229533.I1RRY9 pp 229533.I1S1W9
152265 229533.I1RRY9 pp 229533.V6R042
152266 229533.I1RRY9 pp 229533.I1RZV6
152267 229533.I1RRY9 pp 229533.I1RSQ4
152268 229533.I1RRY9 pp 229533.I1S5R1
152269 229533.I1RRY9 pp 229533.I1RWN5
152270 229533.I1RRY9 pp 229533.I1RZZ9
152271 229533.I1RRY9 pp 229533.V6RIV9
152272 229533.I1RRY9 pp 229533.I1RWI5
152273 229533.I1RRY9 pp 229533.I1S9Y1
152274 229533.I1RRY9 pp 229533.I1S7S9
152275 229533.I1RRY9 pp 229533.I1S9Z4
152276 229533.I1RRY9 pp 229533.V6R3X5
152277 229533.I1RRY9 pp 229533.I1S8E2
152278 229533.I1RRY9 pp 229533.I1S2B8

152279 229533.I1RRY9 pp 229533.V6R3J1
152280 229533.I1RRY9 pp 229533.I1RSX0
152281 229533.I1RRY9 pp 229533.I1RVY0
152282 229533.I1RRY9 pp 229533.I1RZS6
152283 229533.I1RRY9 pp 229533.I1RZ39
152284 229533.I1RRY9 pp 229533.I1S503
152285 229533.I1RRY9 pp 229533.I1S0M6
152286 229533.I1RRY9 pp 229533.I1RRZ0
152287 229533.I1RRY9 pp 229533.I1S0F6
152288 229533.I1RRY9 pp 229533.I1S0W4
152289 229533.I1RRY9 pp 229533.I1S2E9
152290 229533.I1RRY9 pp 229533.V6RUY9
152291 229533.I1RRY9 pp 229533.I1S8P5
152292 229533.I1RRY9 pp 229533.I1S6Y8
152293 229533.I1RRY9 pp 229533.I1RWF7
152294 229533.I1RRY9 pp 229533.I1RT04
152295 229533.I1RRY9 pp 229533.I1RSZ7
152296 229533.I1RRY9 pp 229533.I1RSV2
152297 229533.I1RRY9 pp 229533.I1RYI5
152298 229533.I1RRY9 pp 229533.I1S352
152299 229533.I1RRY9 pp 229533.I1RT40
152300 229533.I1RRY9 pp 229533.Q4HXF0
152301 229533.I1RRY9 pp 229533.I1RSA5
152302 229533.I1RRY9 pp 229533.I1RV91
152303 229533.I1RRY9 pp 229533.I1RVZ2
152304 229533.I1RRY9 pp 229533.I1RTA1
152305 229533.I1RRY9 pp 229533.I1S2G7
152306 229533.I1RRY9 pp 229533.I1RWK3
152307 229533.I1RRY9 pp 229533.I1RS47
152308 229533.I1RRY9 pp 229533.V6R283
152309 229533.A0A1C3YN81 pp 229533.I1RK33
152310 229533.A0A1C3YN81 pp 229533.I1RFS7
152311 229533.A0A1C3YN81 pp 229533.I1RQD6
152312 229533.A0A1C3YN81 pp 229533.I1RD45
152313 229533.A0A1C3YN81 pp 229533.I1RFV4
152314 229533.A0A1C3YN81 pp 229533.I1RT38
152315 229533.A0A1C3YN81 pp 229533.I1S2B6
152316 229533.A0A1C3YN81 pp 229533.I1RTQ9
152317 229533.A0A1C3YN81 pp 229533.I1RMF2
152318 229533.A0A1C3YN81 pp 229533.Q4I6S5
152319 229533.A0A1C3YN81 pp 229533.I1RNP1
152320 229533.A0A1C3YN81 pp 229533.I1RPU5
152321 229533.A0A1C3YN81 pp 229533.I1S6S4
152322 229533.A0A1C3YN81 pp 229533.I1SAM5
152323 229533.A0A1C3YN81 pp 229533.I1RKE1
152324 229533.A0A1C3YN81 pp 229533.I1S0K5
152325 229533.A0A1C3YN81 pp 229533.I1RNA3
152326 229533.A0A1C3YN81 pp 229533.I1RPA7
152327 229533.A0A1C3YN81 pp 229533.I1RN72
152328 229533.A0A1C3YN81 pp 229533.I1RFK0
152329 229533.A0A1C3YN81 pp 229533.I1RYU1
152330 229533.A0A1C3YN81 pp 229533.I1S830
152331 229533.A0A1C3YN81 pp 229533.I1S1V4
152332 229533.A0A1C3YN81 pp 229533.I1S287
152333 229533.A0A1C3YN81 pp 229533.I1RG99
152334 229533.A0A1C3YN81 pp 229533.I1RRK8
152335 229533.A0A1C3YN81 pp 229533.I1RXF5
152336 229533.A0A1C3YN81 pp 229533.I1RE03
152337 229533.A0A1C3YN81 pp 229533.I1RW76
152338 229533.A0A1C3YN81 pp 229533.I1S278
152339 229533.A0A1C3YN81 pp 229533.I1RQP7
152340 229533.A0A1C3YN81 pp 229533.I1S1D0
152341 229533.I1RMD3 pp 229533.I1RTI3
152342 229533.I1RMD3 pp 229533.V6RRM0
152343 229533.I1RMD3 pp 229533.I1RYA0
152344 229533.I1RMD3 pp 229533.I1RMD4
152345 229533.I1RMD3 pp 229533.V6RDP1
152346 229533.I1RMD3 pp 229533.V6RY56
152347 229533.I1RMD3 pp 229533.I1RV23
152348 229533.I1RMD3 pp 229533.I1RWM4
152349 229533.I1RMD3 pp 229533.I1S4D0
152350 229533.I1RMD3 pp 229533.V6RQW4
152351 229533.I1RMD3 pp 229533.V6RLM7

152352	229533.I1RMD3	pp	229533.I1S8F8
152353	229533.I1RMD3	pp	229533.I1RYC8
152354	229533.I1RMD3	pp	229533.I1RY16
152355	229533.I1RMD3	pp	229533.I1RNM7
152356	229533.I1RMD3	pp	229533.I1RST8
152357	229533.I1RMD3	pp	229533.I1RRM8
152358	229533.I1RMD3	pp	229533.I1S3L1
152359	229533.I1RMD3	pp	229533.I1RYK0
152360	229533.I1RMD3	pp	229533.I1RNG4
152361	229533.I1RMD3	pp	229533.I1RP86
152362	229533.I1RRX0	pp	229533.Q4IQ72
152363	229533.I1RRX0	pp	229533.Q4I5M4
152364	229533.I1RRX0	pp	229533.Q4HXF0
152365	229533.I1RRX0	pp	229533.I1RYW3
152366	229533.I1RRX0	pp	229533.Q4HZI0
152367	229533.I1RRX0	pp	229533.Q4I9U7
152368	229533.I1RRX0	pp	229533.Q4HWB3
152369	229533.I1RC04	pp	229533.I1RQM2
152370	229533.I1RC04	pp	229533.I1RML5
152371	229533.I1RC04	pp	229533.I1RDG1
152372	229533.I1RQQ5	pp	229533.I1RZK5
152373	229533.I1RQQ5	pp	229533.Q4I2J8
152374	229533.I1RQQ5	pp	229533.I1RZZ1
152375	229533.I1RQQ5	pp	229533.I1RRY9
152376	229533.I1RQQ5	pp	229533.I1RY03
152377	229533.I1RQQ5	pp	229533.I1SA32
152378	229533.I1RQQ5	pp	229533.Q4I8P5
152379	229533.I1RQQ5	pp	229533.I1RS18
152380	229533.I1RQQ5	pp	229533.V6RCG5
152381	229533.I1RQQ5	pp	229533.I1RZM1
152382	229533.I1RQQ5	pp	229533.I1S504
152383	229533.I1RQQ5	pp	229533.V6RCZ8
152384	229533.I1RQQ5	pp	229533.Q4I624
152385	229533.I1RQQ5	pp	229533.I1RTH6
152386	229533.I1RQQ5	pp	229533.I1RZM8
152387	229533.I1RQQ5	pp	229533.V6REZ4
152388	229533.I1RQQ5	pp	229533.I1S2B8
152389	229533.I1RQQ5	pp	229533.I1RRM5
152390	229533.I1RQQ5	pp	229533.I1RW99
152391	229533.I1RQQ5	pp	229533.I1RR73
152392	229533.I1RQQ5	pp	229533.I1S8R3
152393	229533.I1RQQ5	pp	229533.I1RW25
152394	229533.I1RQQ5	pp	229533.I1RZW8
152395	229533.I1RQQ5	pp	229533.I1S5B0
152396	229533.I1RQQ5	pp	229533.I1RZZ9
152397	229533.I1RQQ5	pp	229533.I1RRI2
152398	229533.I1RQQ5	pp	229533.V6R3X5
152399	229533.I1RQQ5	pp	229533.I1S1C7
152400	229533.I1RQQ5	pp	229533.I1S0X6
152401	229533.I1RQQ5	pp	229533.I1RWN6
152402	229533.I1RQQ5	pp	229533.I1S0L4
152403	229533.I1RQQ5	pp	229533.I1RR24
152404	229533.I1RQQ5	pp	229533.I1RS17
152405	229533.I1RQQ5	pp	229533.I1RWJ8
152406	229533.I1RQQ5	pp	229533.I1S2H4
152407	229533.I1RQQ5	pp	229533.I1RRT8
152408	229533.I1RQQ5	pp	229533.V6QW22
152409	229533.I1RQQ5	pp	229533.I1RSQ7
152410	229533.I1RQQ5	pp	229533.O74638
152411	229533.I1RQQ5	pp	229533.I1RTA0
152412	229533.I1RQQ5	pp	229533.I1S8P5
152413	229533.I1RQQ5	pp	229533.I1S0L3
152414	229533.I1RQQ5	pp	229533.I1RW46
152415	229533.I1RQQ5	pp	229533.I1RWK3
152416	229533.I1RQQ5	pp	229533.I1RY44
152417	229533.I1RQQ5	pp	229533.I1RXD7
152418	229533.I1RQQ5	pp	229533.V6RIV9
152419	229533.I1RQQ5	pp	229533.I1RVQ0
152420	229533.I1RQQ5	pp	229533.I1S2E9
152421	229533.I1RQQ5	pp	229533.I1RWN5
152422	229533.I1RQQ5	pp	229533.I1S0F6
152423	229533.I1RQQ5	pp	229533.V6RTJ8
152424	229533.I1RQQ5	pp	229533.I1S0R7

152425 229533.I1RQQ5 pp 229533.I1RT04
152426 229533.I1RQQ5 pp 229533.I1S236
152427 229533.I1RQQ5 pp 229533.V6R042
152428 229533.I1RQQ5 pp 229533.I1RT40
152429 229533.I1RQQ5 pp 229533.I1RSQ3
152430 229533.I1RQQ5 pp 229533.I1RTD5
152431 229533.I1RQQ5 pp 229533.V6RMW6
152432 229533.I1RQQ5 pp 229533.I1RX46
152433 229533.I1RQQ5 pp 229533.I1RUG3
152434 229533.I1RQQ5 pp 229533.I1RV91
152435 229533.I1RQQ5 pp 229533.I1RVX0
152436 229533.I1RQQ5 pp 229533.I1RT03
152437 229533.I1RQQ5 pp 229533.I1S503
152438 229533.I1RQQ5 pp 229533.I1RYT7
152439 229533.I1RQQ5 pp 229533.I1SA33
152440 229533.A0A1C3YN39 pp 229533.I1RYL5
152441 229533.A0A1C3YN39 pp 229533.I1S278
152442 229533.A0A1C3YN39 pp 229533.I1RC22
152443 229533.A0A1C3YN39 pp 229533.I1RCB3
152444 229533.A0A1C3YN39 pp 229533.I1RUQ5
152445 229533.A0A1C3YN39 pp 229533.I1RKK0
152446 229533.A0A1C3YN39 pp 229533.Q4IEA7
152447 229533.A0A1C3YN39 pp 229533.I1RMM9
152448 229533.A0A1C3YN39 pp 229533.I1RQA3
152449 229533.A0A1C3YN39 pp 229533.I1RV66
152450 229533.A0A1C3YN39 pp 229533.I1RX87
152451 229533.A0A1C3YN39 pp 229533.I1RAM7
152452 229533.A0A1C3YN39 pp 229533.I1S439
152453 229533.A0A1C3YN39 pp 229533.I1RSS1
152454 229533.A0A1C3YN39 pp 229533.I1RPA6
152455 229533.A0A1C3YN39 pp 229533.I1RB75
152456 229533.A0A1C3YN39 pp 229533.I1RMB2
152457 229533.A0A1C3YN39 pp 229533.I1RX04
152458 229533.A0A1C3YN39 pp 229533.I1RWT6
152459 229533.A0A1C3YN39 pp 229533.I1RMA7
152460 229533.A0A1C3YN39 pp 229533.I1RX64
152461 229533.A0A1C3YN39 pp 229533.I1RX77
152462 229533.A0A1C3YN39 pp 229533.I1RQL4
152463 229533.A0A1C3YN39 pp 229533.I1RDB1
152464 229533.A0A1C3YN39 pp 229533.I1S286
152465 229533.A0A1C3YN39 pp 229533.I1RZE4
152466 229533.A0A1C3YN39 pp 229533.I1S247
152467 229533.A0A1C3YN39 pp 229533.I1RCD1
152468 229533.A0A1C3YN39 pp 229533.I1S1W4
152469 229533.A0A1C3YN39 pp 229533.I1RRN5
152470 229533.A0A1C3YN39 pp 229533.I1RAN2
152471 229533.A0A1C3YN39 pp 229533.I1RQD6
152472 229533.A0A1C3YN39 pp 229533.I1RQB4
152473 229533.A0A1C3YN39 pp 229533.I1RZC3
152474 229533.A0A1C3YN39 pp 229533.I1RGF1
152475 229533.A0A1C3YN39 pp 229533.I1RFW0
152476 229533.A0A1C3YN39 pp 229533.I1S248
152477 229533.A0A1C3YN39 pp 229533.I1RNY8
152478 229533.A0A1C3YN39 pp 229533.V6RVG3
152479 229533.A0A1C3YN39 pp 229533.I1R9Y9
152480 229533.A0A1C3YN39 pp 229533.I1RUN8
152481 229533.A0A1C3YN39 pp 229533.I1S0X7
152482 229533.A0A1C3YN39 pp 229533.I1RC46
152483 229533.A0A1C3YN39 pp 229533.V6RKY7
152484 229533.A0A1C3YN39 pp 229533.I1RY32
152485 229533.A0A1C3YN39 pp 229533.I1RAX2
152486 229533.A0A1C3YN39 pp 229533.I1RB74
152487 229533.A0A1C3YN39 pp 229533.I1S7X9
152488 229533.A0A1C3YN39 pp 229533.I1RS47
152489 229533.A0A098DI08 pp 229533.I1RT35
152490 229533.A0A098DI08 pp 229533.I1RNB8
152491 229533.A0A098DI08 pp 229533.I1RG90
152492 229533.I1RBL7 pp 229533.I1RFL4
152493 229533.I1RBL7 pp 229533.I1S2C0
152494 229533.I1RBL7 pp 229533.I1RTE9
152495 229533.I1RBL7 pp 229533.I1RSD8
152496 229533.I1RBL7 pp 229533.I1RTH6
152497 229533.I1RBL7 pp 229533.V6R057

152498 229533.I1RBL7 pp 229533.I1REV3
152499 229533.I1RBL7 pp 229533.Q4HZ68
152500 229533.I1RBL7 pp 229533.I1RRI2
152501 229533.I1RBL7 pp 229533.I1S296
152502 229533.I1RBL7 pp 229533.I1RYW3
152503 229533.I1RBL7 pp 229533.Q4I624
152504 229533.I1RBL7 pp 229533.V6RMW6
152505 229533.I1RBL7 pp 229533.V6RRH2
152506 229533.I1RBL7 pp 229533.I1RKC6
152507 229533.I1RBL7 pp 229533.I1RRF0
152508 229533.I1RBL7 pp 229533.V6R042
152509 229533.I1RBL7 pp 229533.I1S1C7
152510 229533.I1RBL7 pp 229533.I1RSQ3
152511 229533.I1RBL7 pp 229533.I1RWN6
152512 229533.I1RBL7 pp 229533.Q4I327
152513 229533.I1RBL7 pp 229533.V6RGY3
152514 229533.I1RBL7 pp 229533.I1RSI6
152515 229533.I1RBL7 pp 229533.I1RCL3
152516 229533.I1RBL7 pp 229533.I1RUG3
152517 229533.I1RBL7 pp 229533.I1S274
152518 229533.I1RBL7 pp 229533.I1RC81
152519 229533.I1RBL7 pp 229533.I1RBZ3
152520 229533.I1RBL7 pp 229533.I1RNC1
152521 229533.I1RBL7 pp 229533.I1RJV3
152522 229533.I1RBL7 pp 229533.I1RCN5
152523 229533.I1RBL7 pp 229533.Q4IN63
152524 229533.I1RBL7 pp 229533.I1RVX0
152525 229533.I1RBL7 pp 229533.I1S1X9
152526 229533.I1RBL7 pp 229533.V6RGN5
152527 229533.I1RBL7 pp 229533.I1RCR8
152528 229533.I1RBL7 pp 229533.I1RU69
152529 229533.I1RBL7 pp 229533.I1RT03
152530 229533.I1RBL7 pp 229533.I1RG11
152531 229533.I1RBL7 pp 229533.I1RSQ4
152532 229533.I1RBL7 pp 229533.I1RC27
152533 229533.I1RBL7 pp 229533.Q4HWE2
152534 229533.I1RBL7 pp 229533.I1RSS4
152535 229533.I1RBL7 pp 229533.I1S5R6
152536 229533.I1RBL7 pp 229533.I1RZZ9
152537 229533.I1RBL7 pp 229533.I1RCH5
152538 229533.I1RBL7 pp 229533.I1RRN2
152539 229533.I1RBL7 pp 229533.I1RWI5
152540 229533.I1RBL7 pp 229533.Q4IF76
152541 229533.I1RBL7 pp 229533.I1S2E8
152542 229533.I1RBL7 pp 229533.I1RST4
152543 229533.I1RBL7 pp 229533.I1RD22
152544 229533.I1RBL7 pp 229533.I1S9Z4
152545 229533.I1RBL7 pp 229533.I1RQE7
152546 229533.I1RBL7 pp 229533.I1RMN6
152547 229533.I1RBL7 pp 229533.I1RPK0
152548 229533.I1RBL7 pp 229533.I1S0L4
152549 229533.I1RBL7 pp 229533.I1RIQ7
152550 229533.I1RBL7 pp 229533.I1S2H4
152551 229533.I1RBL7 pp 229533.V6RLM7
152552 229533.I1RBL7 pp 229533.I1RVY0
152553 229533.I1RBL7 pp 229533.I1S2B8
152554 229533.I1RBL7 pp 229533.I1RM25
152555 229533.I1RBL7 pp 229533.I1RNU6
152556 229533.I1RBL7 pp 229533.I1S3G4
152557 229533.I1RBL7 pp 229533.I1RD64
152558 229533.I1RBL7 pp 229533.I1RWB5
152559 229533.I1RBL7 pp 229533.I1RJZ7
152560 229533.I1RBL7 pp 229533.I1RS17
152561 229533.I1RBL7 pp 229533.V6RUY9
152562 229533.I1RBL7 pp 229533.I1RXC2
152563 229533.I1RBL7 pp 229533.I1RT04
152564 229533.I1RBL7 pp 229533.I1S0J1
152565 229533.I1RBL7 pp 229533.O74638
152566 229533.I1RBL7 pp 229533.I1S5R1
152567 229533.I1RBL7 pp 229533.Q4HXF0
152568 229533.I1RBL7 pp 229533.I1RRT8
152569 229533.I1RBL7 pp 229533.I1RC72
152570 229533.I1RBL7 pp 229533.I1RE01

152571	229533.I1RBL7	pp	229533.I1S060
152572	229533.I1RBL7	pp	229533.I1RFL3
152573	229533.I1RBL7	pp	229533.I1RTD5
152574	229533.I1RBL7	pp	229533.I1RZV6
152575	229533.I1RBL7	pp	229533.I1RT40
152576	229533.I1RBL7	pp	229533.I1RC82
152577	229533.I1RBL7	pp	229533.I1RRR7
152578	229533.I1RBL7	pp	229533.I1S9Y1
152579	229533.I1RBL7	pp	229533.I1S6S6
152580	229533.I1RBL7	pp	229533.I1RSL9
152581	229533.I1RBL7	pp	229533.I1RGA4
152582	229533.I1RBL7	pp	229533.I1RM96
152583	229533.I1RBL7	pp	229533.I1RKJ6
152584	229533.I1RBL7	pp	229533.I1RFN1
152585	229533.I1RBL7	pp	229533.I1RZS6
152586	229533.I1RBL7	pp	229533.I1RX24
152587	229533.I1RBL7	pp	229533.I1S4T3
152588	229533.I1RBL7	pp	229533.I1RRT2
152589	229533.I1RBL7	pp	229533.I1RQS5
152590	229533.I1RBL7	pp	229533.I1S1W9
152591	229533.I1RBL7	pp	229533.I1RKF9
152592	229533.I1RBL7	pp	229533.V6R3J1
152593	229533.I1RBL7	pp	229533.I1RC29
152594	229533.I1RBL7	pp	229533.Q4I662
152595	229533.I1RBL7	pp	229533.I1RSX0
152596	229533.I1RBL7	pp	229533.I1RPY1
152597	229533.I1RBL7	pp	229533.I1RWN5
152598	229533.I1RBL7	pp	229533.I1RPV3
152599	229533.I1RBL7	pp	229533.I1RJL4
152600	229533.I1RBL7	pp	229533.I1RLV9
152601	229533.I1RBL7	pp	229533.I1RND6
152602	229533.I1RBL7	pp	229533.I1RSZ7
152603	229533.I1RBL7	pp	229533.I1RKR9
152604	229533.I1RBL7	pp	229533.V6R3X5
152605	229533.I1RBL7	pp	229533.Q4IAS1
152606	229533.I1RBL7	pp	229533.I1S503
152607	229533.I1RBL7	pp	229533.Q4IPI4
152608	229533.I1RBL7	pp	229533.I1RZ39
152609	229533.I1RBL7	pp	229533.I1RR73
152610	229533.I1RBL7	pp	229533.I1RPP3
152611	229533.I1RBL7	pp	229533.I1RYI5
152612	229533.I1RBL7	pp	229533.I1S8E2
152613	229533.I1RBL7	pp	229533.I1S277
152614	229533.I1RBL7	pp	229533.I1RC66
152615	229533.I1RBL7	pp	229533.I1RRJ8
152616	229533.I1RBL7	pp	229533.I1RBY4
152617	229533.I1RBL7	pp	229533.I1RKL3
152618	229533.I1RBL7	pp	229533.I1RE76
152619	229533.I1RBL7	pp	229533.I1RKB2
152620	229533.I1RBL7	pp	229533.Q4I830
152621	229533.I1RBL7	pp	229533.I1RKI1
152622	229533.I1RBL7	pp	229533.I1RFT1
152623	229533.I1RBL7	pp	229533.I1RNK2
152624	229533.I1RBL7	pp	229533.I1S0Z9
152625	229533.I1RBL7	pp	229533.I1RTA1
152626	229533.I1RBL7	pp	229533.I1RVZ2
152627	229533.I1RBL7	pp	229533.V6R283
152628	229533.I1RBL7	pp	229533.I1RSR8
152629	229533.I1RBL7	pp	229533.I1RC03
152630	229533.I1RBL7	pp	229533.I1RRY9
152631	229533.I1RBL7	pp	229533.I1S5R5
152632	229533.I1RBL7	pp	229533.V6RCZ8
152633	229533.I1RBL7	pp	229533.I1RSR3
152634	229533.I1RBL7	pp	229533.I1RQ65
152635	229533.I1RBL7	pp	229533.I1S1J7
152636	229533.I1RBL7	pp	229533.I1S6K9
152637	229533.I1RBL7	pp	229533.I1RS47
152638	229533.I1RBL7	pp	229533.I1RN99
152639	229533.I1RBL7	pp	229533.I1RQA2
152640	229533.I1RBL7	pp	229533.I1RRQ3
152641	229533.I1RBL7	pp	229533.Q4HZ42
152642	229533.I1RBL7	pp	229533.I1RYB9
152643	229533.I1RBL7	pp	229533.I1RNH6

152644	229533.I1RBL7	pp	229533.I1RK95
152645	229533.I1RBL7	pp	229533.I1RDG0
152646	229533.I1RBL7	pp	229533.I1RFG0
152647	229533.I1RBL7	pp	229533.I1RQ28
152648	229533.I1RBL7	pp	229533.I1RN67
152649	229533.I1RBL7	pp	229533.I1RRP3
152650	229533.I1RBL7	pp	229533.I1S021
152651	229533.I1RBL7	pp	229533.I1RMN7
152652	229533.I1RBL7	pp	229533.I1RZZ1
152653	229533.I1RBL7	pp	229533.I1RY63
152654	229533.I1RBL7	pp	229533.I1RQ31
152655	229533.I1RBL7	pp	229533.I1RNG4
152656	229533.I1RBL7	pp	229533.I1RZK5
152657	229533.I1RBL7	pp	229533.I1RCM5
152658	229533.I1RBL7	pp	229533.I1RMC0
152659	229533.I1RBL7	pp	229533.I1RZK6
152660	229533.I1RBL7	pp	229533.I1RZM8
152661	229533.I1RBL7	pp	229533.I1RK83
152662	229533.I1RBL7	pp	229533.I1RFU8
152663	229533.I1RBL7	pp	229533.I1RJZ6
152664	229533.I1RBL7	pp	229533.I1RXF0
152665	229533.I1RBL7	pp	229533.I1RT65
152666	229533.I1RBL7	pp	229533.V6RBN2
152667	229533.I1RBL7	pp	229533.I1RY03
152668	229533.I1RBL7	pp	229533.I1S044
152669	229533.I1RBL7	pp	229533.Q4I7F9
152670	229533.I1RBL7	pp	229533.Q4IJK2
152671	229533.I1RBL7	pp	229533.I1RXC4
152672	229533.I1RBL7	pp	229533.Q4IBS2
152673	229533.I1RBL7	pp	229533.I1S5B0
152674	229533.I1RBL7	pp	229533.I1RF08
152675	229533.I1RBL7	pp	229533.V6R227
152676	229533.I1RBL7	pp	229533.I1RCR5
152677	229533.I1RBL7	pp	229533.I1REK8
152678	229533.I1RBL7	pp	229533.V6QUE6
152679	229533.I1RBL7	pp	229533.I1RZE5
152680	229533.I1RBL7	pp	229533.I1RGC2
152681	229533.I1RBL7	pp	229533.I1RYB1
152682	229533.I1RBL7	pp	229533.I1RZF6
152683	229533.I1RBL7	pp	229533.I1RRV4
152684	229533.I1RBL7	pp	229533.I1S6E5
152685	229533.I1RBL7	pp	229533.I1S504
152686	229533.I1RBL7	pp	229533.Q4IEK8
152687	229533.I1RBL7	pp	229533.I1RE00
152688	229533.I1RBL7	pp	229533.I1RFJ9
152689	229533.I1RBL7	pp	229533.V6RPN0
152690	229533.I1RBL7	pp	229533.I1S0Z3
152691	229533.I1RBL7	pp	229533.Q4IAA0
152692	229533.I1RBL7	pp	229533.I1RVZ0
152693	229533.I1RBL7	pp	229533.I1RSI0
152694	229533.I1RBL7	pp	229533.I1RS18
152695	229533.I1RBL7	pp	229533.I1RG56
152696	229533.I1RBL7	pp	229533.I1S0M9
152697	229533.I1RBL7	pp	229533.Q4HVW2
152698	229533.I1RBL7	pp	229533.I1RT98
152699	229533.I1RBL7	pp	229533.Q4INZ9
152700	229533.I1RBL7	pp	229533.I1RMK3
152701	229533.I1RBL7	pp	229533.I1RZJ6
152702	229533.I1RBL7	pp	229533.Q4IJ56
152703	229533.I1RBL7	pp	229533.I1RDA0
152704	229533.I1RBL7	pp	229533.I1RJ17
152705	229533.I1RBL7	pp	229533.I1RBN1
152706	229533.I1RBL7	pp	229533.I1RMM8
152707	229533.I1RBL7	pp	229533.I1RT80
152708	229533.I1RBL7	pp	229533.I1RM67
152709	229533.I1RBL7	pp	229533.I1RZ92
152710	229533.I1RBL7	pp	229533.I1RQL4
152711	229533.I1RBL7	pp	229533.Q4IJH1
152712	229533.I1RBL7	pp	229533.I1RTF4
152713	229533.I1RBL7	pp	229533.I1S4S4
152714	229533.I1RBL7	pp	229533.V6RTJ8
152715	229533.I1RBL7	pp	229533.I1RJU3
152716	229533.I1RBL7	pp	229533.I1RX46

152717	229533.I1RBL7	pp	229533.I1RPU0
152718	229533.I1RBL7	pp	229533.Q4IPI1
152719	229533.I1RBL7	pp	229533.I1RM06
152720	229533.I1RBL7	pp	229533.I1RBT1
152721	229533.I1RBL7	pp	229533.I1S0F1
152722	229533.I1RBL7	pp	229533.I1S270
152723	229533.I1RBL7	pp	229533.I1RT05
152724	229533.I1RBL7	pp	229533.Q4I5R9
152725	229533.I1RBL7	pp	229533.I1RH48
152726	229533.I1RBL7	pp	229533.I1RKQ5
152727	229533.I1RBL7	pp	229533.V6QYF0
152728	229533.I1RBL7	pp	229533.I1S9U2
152729	229533.I1RBL7	pp	229533.I1RPW9
152730	229533.I1RBL7	pp	229533.I1S1W8
152731	229533.I1RBL7	pp	229533.I1RQJ5
152732	229533.I1RBL7	pp	229533.I1RFC0
152733	229533.I1RBL7	pp	229533.I1RR24
152734	229533.I1RBL7	pp	229533.I1RG51
152735	229533.I1RBL7	pp	229533.I1RSQ7
152736	229533.I1RBL7	pp	229533.I1RPA4
152737	229533.I1RBL7	pp	229533.I1RW71
152738	229533.I1RBL7	pp	229533.I1S6F0
152739	229533.I1RBL7	pp	229533.I1S4S7
152740	229533.I1RBL7	pp	229533.I1S7Q8
152741	229533.I1RBL7	pp	229533.I1RNH1
152742	229533.I1RBL7	pp	229533.I1RW25
152743	229533.I1RBL7	pp	229533.I1RPN4
152744	229533.I1RBL7	pp	229533.Q4IFI0
152745	229533.I1RBL7	pp	229533.I1RFW5
152746	229533.I1RBL7	pp	229533.I1RRG3
152747	229533.I1RBL7	pp	229533.I1RT41
152748	229533.I1RBL7	pp	229533.V6R1K4
152749	229533.I1RBL7	pp	229533.I1RWH5
152750	229533.I1RBL7	pp	229533.I1RD52
152751	229533.I1RBL7	pp	229533.I1RXD7
152752	229533.I1RBL7	pp	229533.I1RD56
152753	229533.I1RBL7	pp	229533.I1RT33
152754	229533.I1RBL7	pp	229533.I1RP86
152755	229533.I1RBL7	pp	229533.I1S0R7
152756	229533.V6QVS3	pp	229533.V6RDP1
152757	229533.I1RVC8	pp	229533.I1SAG3
152758	229533.I1RVC8	pp	229533.I1SA77
152759	229533.I1RCV0	pp	229533.I1RM94
152760	229533.I1RCV0	pp	229533.I1RLY6
152761	229533.I1RCV0	pp	229533.I1S0P4
152762	229533.I1RCV0	pp	229533.V6RJ21
152763	229533.I1RCV0	pp	229533.I1RZU7
152764	229533.I1RCV0	pp	229533.I1RQ06
152765	229533.I1RCV0	pp	229533.I1RSE4
152766	229533.I1RCV0	pp	229533.I1RNS2
152767	229533.I1R9V7	pp	229533.I1RPW9
152768	229533.I1R9V7	pp	229533.I1S9U2
152769	229533.I1R9V7	pp	229533.I1RD64
152770	229533.I1R9V7	pp	229533.I1RGF6
152771	229533.I1R9V7	pp	229533.I1RS20
152772	229533.I1R9V7	pp	229533.I1RRT8
152773	229533.I1R9V7	pp	229533.I1RM67
152774	229533.I1R9V7	pp	229533.O74638
152775	229533.I1R9V7	pp	229533.I1RZV6
152776	229533.I1R9V7	pp	229533.I1S9Y1
152777	229533.I1R9V7	pp	229533.I1RKJ6
152778	229533.I1R9V7	pp	229533.I1RPU0
152779	229533.I1R9V7	pp	229533.I1S253
152780	229533.I1R9V7	pp	229533.I1RDA0
152781	229533.I1R9V7	pp	229533.Q4HVW2
152782	229533.I1R9V7	pp	229533.I1RBN1
152783	229533.I1R9V7	pp	229533.I1RU69
152784	229533.I1R9V7	pp	229533.I1RT05
152785	229533.I1R9V7	pp	229533.I1RR73
152786	229533.I1R9V7	pp	229533.I1RMK3
152787	229533.I1R9V7	pp	229533.I1RND6
152788	229533.I1R9V7	pp	229533.V6R3X5
152789	229533.I1R9V7	pp	229533.I1RC82

152790	229533.I1R9V7	pp	229533.I1RFN1
152791	229533.I1R9V7	pp	229533.Q4IF76
152792	229533.I1R9V7	pp	229533.I1RAX6
152793	229533.I1R9V7	pp	229533.Q4IJH1
152794	229533.I1R9V7	pp	229533.I1RAC2
152795	229533.I1R9V7	pp	229533.I1RGA4
152796	229533.I1R9V7	pp	229533.I1RQS5
152797	229533.I1R9V7	pp	229533.I1RRT2
152798	229533.I1R9V7	pp	229533.I1RW25
152799	229533.I1R9V7	pp	229533.I1RL23
152800	229533.I1R9V7	pp	229533.I1RTF4
152801	229533.I1R9V7	pp	229533.I1RX46
152802	229533.I1R9V7	pp	229533.I1RJZ7
152803	229533.I1R9V7	pp	229533.I1RZ39
152804	229533.I1R9V7	pp	229533.I1RT80
152805	229533.I1R9V7	pp	229533.Q4IPI1
152806	229533.I1R9V7	pp	229533.I1RJL4
152807	229533.I1R9V7	pp	229533.Q4IFI0
152808	229533.I1R9V7	pp	229533.I1RLV9
152809	229533.I1R9V7	pp	229533.I1S2B8
152810	229533.I1R9V7	pp	229533.I1RNV6
152811	229533.I1R9V7	pp	229533.I1S8E2
152812	229533.I1R9V7	pp	229533.Q4IAS1
152813	229533.I1R9V7	pp	229533.I1RB71
152814	229533.I1R9V7	pp	229533.I1RM06
152815	229533.I1R9V7	pp	229533.I1RAV6
152816	229533.I1R9V7	pp	229533.Q4I5R9
152817	229533.I1R9V7	pp	229533.I1RZS6
152818	229533.I1R9V7	pp	229533.I1S0F1
152819	229533.I1R9V7	pp	229533.Q4HZ47
152820	229533.I1R9V7	pp	229533.I1RX24
152821	229533.I1R9V7	pp	229533.I1RC29
152822	229533.I1R9V7	pp	229533.I1RKQ5
152823	229533.I1R9V7	pp	229533.I1S503
152824	229533.I1R9V7	pp	229533.I1RS30
152825	229533.I1R9V7	pp	229533.I1RWD4
152826	229533.I1R9V7	pp	229533.I1RNA0
152827	229533.I1R9V7	pp	229533.I1RQJ5
152828	229533.I1R9V7	pp	229533.I1RXD7
152829	229533.I1R9V7	pp	229533.I1RUQ2
152830	229533.I1R9V7	pp	229533.Q4IQK7
152831	229533.I1R9V7	pp	229533.I1RBG9
152832	229533.I1R9V7	pp	229533.I1RT04
152833	229533.I1R9V7	pp	229533.I1RPP3
152834	229533.I1R9V7	pp	229533.I1RZ99
152835	229533.I1R9V7	pp	229533.V6QYF0
152836	229533.I1R9V7	pp	229533.I1RRJ8
152837	229533.I1R9V7	pp	229533.I1RKF9
152838	229533.I1R9V7	pp	229533.I1S0R7
152839	229533.I1R9V7	pp	229533.I1RG51
152840	229533.I1R9V7	pp	229533.I1REF2
152841	229533.I1R9V7	pp	229533.I1RSX1
152842	229533.I1R9V7	pp	229533.I1RSQ7
152843	229533.I1R9V7	pp	229533.I1RTD5
152844	229533.I1R9V7	pp	229533.I1RWJ4
152845	229533.I1R9V7	pp	229533.I1REX4
152846	229533.I1R9V7	pp	229533.I1RT98
152847	229533.I1R9V7	pp	229533.Q4I830
152848	229533.I1R9V7	pp	229533.I1RFS3
152849	229533.I1R9V7	pp	229533.I1RZJ6
152850	229533.I1R9V7	pp	229533.I1RKL3
152851	229533.I1R9V7	pp	229533.I1RAF8
152852	229533.I1R9V7	pp	229533.Q4I662
152853	229533.I1R9V7	pp	229533.I1RRH5
152854	229533.I1R9V7	pp	229533.I1RQ65
152855	229533.I1R9V7	pp	229533.I1RW71
152856	229533.I1R9V7	pp	229533.I1RW61
152857	229533.I1R9V7	pp	229533.I1RC32
152858	229533.I1R9V7	pp	229533.I1RFW5
152859	229533.I1R9V7	pp	229533.I1RWH5
152860	229533.I1R9V7	pp	229533.V6R283
152861	229533.I1R9V7	pp	229533.I1RD56
152862	229533.I1R9V7	pp	229533.I1RS47

152863	229533.I1R9V7	pp	229533.Q4IQW0
152864	229533.I1R9V7	pp	229533.Q4HZ42
152865	229533.I1R9V7	pp	229533.I1RC03
152866	229533.I1R9V7	pp	229533.I1RBC7
152867	229533.I1R9V7	pp	229533.I1RRY9
152868	229533.I1R9V7	pp	229533.I1RT33
152869	229533.I1R9V7	pp	229533.I1S6F0
152870	229533.I1R9V7	pp	229533.I1RSL9
152871	229533.I1R9V7	pp	229533.I1RWN6
152872	229533.I1R9V7	pp	229533.I1RQA2
152873	229533.I1R9V7	pp	229533.I1S1U7
152874	229533.I1R9V7	pp	229533.I1RAN5
152875	229533.I1R9V7	pp	229533.I1RRQ3
152876	229533.I1R9V7	pp	229533.I1S558
152877	229533.I1R9V7	pp	229533.I1RNH1
152878	229533.I1R9V7	pp	229533.I1RFG0
152879	229533.I1R9V7	pp	229533.I1RAW0
152880	229533.I1R9V7	pp	229533.I1RSR3
152881	229533.I1R9V7	pp	229533.V6RGY3
152882	229533.I1R9V7	pp	229533.I1RZK5
152883	229533.I1R9V7	pp	229533.I1RAC1
152884	229533.I1R9V7	pp	229533.I1RQ28
152885	229533.I1R9V7	pp	229533.I1RBL7
152886	229533.I1R9V7	pp	229533.I1RTE9
152887	229533.I1R9V7	pp	229533.I1S274
152888	229533.I1R9V7	pp	229533.I1RDG0
152889	229533.I1R9V7	pp	229533.I1RPJ3
152890	229533.I1R9V7	pp	229533.I1RVX0
152891	229533.I1R9V7	pp	229533.I1RT03
152892	229533.I1R9V7	pp	229533.I1RK83
152893	229533.I1R9V7	pp	229533.I1RCL3
152894	229533.I1R9V7	pp	229533.I1S1X9
152895	229533.I1R9V7	pp	229533.I1RBZ3
152896	229533.I1R9V7	pp	229533.I1RJV3
152897	229533.I1R9V7	pp	229533.I1RC81
152898	229533.I1R9V7	pp	229533.I1S2C0
152899	229533.I1R9V7	pp	229533.I1RFU8
152900	229533.I1R9V7	pp	229533.I1RY63
152901	229533.I1R9V7	pp	229533.I1RTH6
152902	229533.I1R9V7	pp	229533.I1RCN5
152903	229533.I1R9V7	pp	229533.I1RYB9
152904	229533.I1R9V7	pp	229533.I1RSC6
152905	229533.I1R9V7	pp	229533.I1RZB5
152906	229533.I1R9V7	pp	229533.I1RSS4
152907	229533.I1R9V7	pp	229533.Q4HWE2
152908	229533.I1R9V7	pp	229533.Q4HZK7
152909	229533.I1R9V7	pp	229533.I1RQ31
152910	229533.I1R9V7	pp	229533.I1RMC0
152911	229533.I1R9V7	pp	229533.I1RCR8
152912	229533.I1R9V7	pp	229533.Q4IN63
152913	229533.I1R9V7	pp	229533.I1RZK6
152914	229533.I1R9V7	pp	229533.I1RA48
152915	229533.I1R9V7	pp	229533.I1RZZ1
152916	229533.I1R9V7	pp	229533.I1RRI2
152917	229533.I1R9V7	pp	229533.I1S2E8
152918	229533.I1R9V7	pp	229533.I1RTD7
152919	229533.I1R9V7	pp	229533.I1RRR7
152920	229533.I1R9V7	pp	229533.V6R227
152921	229533.I1R9V7	pp	229533.I1S1C7
152922	229533.I1R9V7	pp	229533.I1RJZ6
152923	229533.I1R9V7	pp	229533.I1RSD8
152924	229533.I1R9V7	pp	229533.I1RSR8
152925	229533.I1R9V7	pp	229533.I1RZF6
152926	229533.I1R9V7	pp	229533.I1S2U2
152927	229533.I1R9V7	pp	229533.I1RY03
152928	229533.I1R9V7	pp	229533.I1S504
152929	229533.I1R9V7	pp	229533.I1RNQ5
152930	229533.I1R9V7	pp	229533.I1RAU7
152931	229533.I1R9V7	pp	229533.I1RM96
152932	229533.I1R9V7	pp	229533.V6RGN5
152933	229533.I1R9V7	pp	229533.I1S296
152934	229533.I1R9V7	pp	229533.I1S044
152935	229533.I1R9V7	pp	229533.I1RC27

152936	229533.I1R9V7	pp	229533.Q4IBS2
152937	229533.I1R9V7	pp	229533.Q4HZ68
152938	229533.I1R9V7	pp	229533.Q4IEK8
152939	229533.I1R9V7	pp	229533.I1RWN5
152940	229533.I1R9V7	pp	229533.I1RF08
152941	229533.I1R9V7	pp	229533.I1RAU5
152942	229533.I1R9V7	pp	229533.I1RD22
152943	229533.I1R9V7	pp	229533.I1S4T3
152944	229533.I1R9V7	pp	229533.I1RZE5
152945	229533.I1R9V7	pp	229533.I1RMN6
152946	229533.I1R9V7	pp	229533.I1RG56
152947	229533.I1R9V7	pp	229533.I1RGC2
152948	229533.I1R9V7	pp	229533.I1RYB1
152949	229533.I1R9V7	pp	229533.I1RPY1
152950	229533.I1R9V7	pp	229533.V6QUE6
152951	229533.I1R9V7	pp	229533.I1RE00
152952	229533.I1R9V7	pp	229533.Q4IAA0
152953	229533.I1R9V7	pp	229533.V6R042
152954	229533.I1R9V7	pp	229533.I1RSI0
152955	229533.I1R9V7	pp	229533.I1RFJ9
152956	229533.I1R9V7	pp	229533.I1R9X7
152957	229533.I1R9V7	pp	229533.Q4IJ56
152958	229533.I1R9V7	pp	229533.I1RB27
152959	229533.I1R9V7	pp	229533.V6RMW6
152960	229533.I1R9V7	pp	229533.I1S6E5
152961	229533.I1R9V7	pp	229533.I1RX08
152962	229533.I1R9V7	pp	229533.I1RNK2
152963	229533.I1R9V7	pp	229533.I1S0M9
152964	229533.I1R9V7	pp	229533.I1RVZ0
152965	229533.I1R9V7	pp	229533.I1RSI6
152966	229533.I1R9V7	pp	229533.I1RM25
152967	229533.I1R9V7	pp	229533.I1RKC6
152968	229533.I1R9V7	pp	229533.Q4I327
152969	229533.I1R9V7	pp	229533.I1RAZ8
152970	229533.I1R9V7	pp	229533.I1RNU6
152971	229533.I1R9V7	pp	229533.I1RWB5
152972	229533.I1R9V7	pp	229533.I1S0Z9
152973	229533.I1R9V7	pp	229533.I1RNY2
152974	229533.I1R9V7	pp	229533.I1S0J1
152975	229533.I1R9V7	pp	229533.I1RN99
152976	229533.I1R9V7	pp	229533.I1RNH6
152977	229533.I1R9V7	pp	229533.I1S021
152978	229533.I1R9V7	pp	229533.I1RC72
152979	229533.I1R9V7	pp	229533.I1RK95
152980	229533.I1R9V7	pp	229533.I1RMN7
152981	229533.I1R9V7	pp	229533.I1RX14
152982	229533.I1R9V7	pp	229533.I1S060
152983	229533.I1RJ88	pp	229533.V6RVG3
152984	229533.I1RJ88	pp	229533.I1RX70
152985	229533.I1RJ88	pp	229533.V6RKY7
152986	229533.I1RJ88	pp	229533.I1RZE4
152987	229533.I1RJ88	pp	229533.I1S1W4
152988	229533.I1RJ88	pp	229533.I1S0D1
152989	229533.I1RJ88	pp	229533.I1S439
152990	229533.I1RJ88	pp	229533.I1RUQ5
152991	229533.I1RJ88	pp	229533.I1RUN8
152992	229533.I1RJ88	pp	229533.I1RYH9
152993	229533.A0A098DSL2	pp	229533.I1RT35
152994	229533.A0A098DSL2	pp	229533.I1RG90
152995	229533.A0A098DSL2	pp	229533.I1RNB8
152996	229533.I1RA48	pp	229533.I1RZ99
152997	229533.I1RA48	pp	229533.I1RQJ5
152998	229533.I1RA48	pp	229533.I1RPP3
152999	229533.I1RA48	pp	229533.I1RWF7
153000	229533.I1RA48	pp	229533.I1S1B6
153001	229533.I1RA48	pp	229533.I1RRJ8
153002	229533.I1RA48	pp	229533.I1RSQ7
153003	229533.I1RA48	pp	229533.I1RS47
153004	229533.I1RA48	pp	229533.I1RSL9
153005	229533.I1RA48	pp	229533.I1RFN1
153006	229533.I1RA48	pp	229533.I1RD56
153007	229533.I1RA48	pp	229533.I1RC82
153008	229533.I1RA48	pp	229533.Q4HZ42

153009 229533.I1RA48 pp 229533.Q4I830
153010 229533.I1RA48 pp 229533.I1RGA4
153011 229533.I1RA48 pp 229533.I1S6F0
153012 229533.I1RA48 pp 229533.I1RKL3
153013 229533.I1RA48 pp 229533.I1RAX6
153014 229533.I1RA48 pp 229533.I1RW71
153015 229533.I1RA48 pp 229533.I1RRT2
153016 229533.I1RA48 pp 229533.I1RFG0
153017 229533.I1RA48 pp 229533.I1RX46
153018 229533.I1RA48 pp 229533.I1RJL4
153019 229533.I1RA48 pp 229533.I1RFW5
153020 229533.I1RA48 pp 229533.I1RRQ3
153021 229533.I1RA48 pp 229533.I1RNH1
153022 229533.I1RA48 pp 229533.I1RC05
153023 229533.I1RA48 pp 229533.I1RWH5
153024 229533.I1RA48 pp 229533.I1RS30
153025 229533.I1RA48 pp 229533.Q4IPI1
153026 229533.I1RA48 pp 229533.I1RLV9
153027 229533.I1RA48 pp 229533.V6R283
153028 229533.I1RA48 pp 229533.I1RRP3
153029 229533.I1RA48 pp 229533.I1RC03
153030 229533.I1RA48 pp 229533.Q4IAS1
153031 229533.I1RA48 pp 229533.I1RXD7
153032 229533.I1RA48 pp 229533.I1RQ65
153033 229533.I1RA48 pp 229533.I1S503
153034 229533.I1RA48 pp 229533.I1RRH5
153035 229533.I1RA48 pp 229533.I1RUQ2
153036 229533.I1RA48 pp 229533.I1REX4
153037 229533.I1RA48 pp 229533.I1S2E9
153038 229533.I1RA48 pp 229533.Q4I5R9
153039 229533.I1RA48 pp 229533.I1RT04
153040 229533.I1RA48 pp 229533.Q4I662
153041 229533.I1RA48 pp 229533.I1RKQ5
153042 229533.I1RA48 pp 229533.I1S0R7
153043 229533.I1RA48 pp 229533.I1RFS3
153044 229533.I1RA48 pp 229533.I1RM06
153045 229533.I1RA48 pp 229533.I1RB71
153046 229533.I1RA48 pp 229533.I1RX24
153047 229533.I1RA48 pp 229533.I1RZS6
153048 229533.I1RA48 pp 229533.I1REF2
153049 229533.I1RA48 pp 229533.I1RTD5
153050 229533.I1RA48 pp 229533.I1RT98
153051 229533.I1RA48 pp 229533.I1RYT5
153052 229533.I1RA48 pp 229533.I1RAC2
153053 229533.I1RA48 pp 229533.I1RZJ6
153054 229533.I1RA48 pp 229533.I1RW25
153055 229533.I1RA48 pp 229533.I1RNA0
153056 229533.I1RA48 pp 229533.I1RBG9
153057 229533.I1RA48 pp 229533.V6QYF0
153058 229533.I1RA48 pp 229533.I1RL23
153059 229533.I1RA48 pp 229533.I1RAF8
153060 229533.I1RA48 pp 229533.I1S8I8
153061 229533.I1RA48 pp 229533.I1RJZ7
153062 229533.I1RA48 pp 229533.I1RG51
153063 229533.I1RA48 pp 229533.I1RB27
153064 229533.I1RA48 pp 229533.I1RZ39
153065 229533.I1RA48 pp 229533.Q4IFI0
153066 229533.I1RA48 pp 229533.Q4IJ56
153067 229533.I1RA48 pp 229533.I1RT80
153068 229533.I1RA48 pp 229533.I1S2B8
153069 229533.I1RA48 pp 229533.I1S0M9
153070 229533.I1RA48 pp 229533.I1RNV6
153071 229533.I1RA48 pp 229533.I1RNU6
153072 229533.I1RA48 pp 229533.I1S6U8
153073 229533.I1RA48 pp 229533.I1S8E2
153074 229533.I1RA48 pp 229533.I1RM25
153075 229533.I1RA48 pp 229533.I1RBN1
153076 229533.I1RA48 pp 229533.I1RWB5
153077 229533.I1RA48 pp 229533.I1RU69
153078 229533.I1RA48 pp 229533.I1RDA0
153079 229533.I1RA48 pp 229533.I1S253
153080 229533.I1RA48 pp 229533.Q4IF76
153081 229533.I1RA48 pp 229533.I1RC72

153082 229533.I1RA48 pp 229533.Q4HVW2
153083 229533.I1RA48 pp 229533.I1S0J1
153084 229533.I1RA48 pp 229533.I1S060
153085 229533.I1RA48 pp 229533.I1RT05
153086 229533.I1RA48 pp 229533.I1RPW9
153087 229533.I1RA48 pp 229533.I1RMK3
153088 229533.I1RA48 pp 229533.I1RX14
153089 229533.I1RA48 pp 229533.I1RR73
153090 229533.I1RA48 pp 229533.I1S9U2
153091 229533.I1RA48 pp 229533.I1RRR7
153092 229533.I1RA48 pp 229533.I1RGF6
153093 229533.I1RA48 pp 229533.V6R3X5
153094 229533.I1RA48 pp 229533.V6R227
153095 229533.I1RA48 pp 229533.I1RD64
153096 229533.I1RA48 pp 229533.I1RND6
153097 229533.I1RA48 pp 229533.I1RWE1
153098 229533.I1RA48 pp 229533.I1RTF4
153099 229533.I1RA48 pp 229533.I1RRT8
153100 229533.I1RA48 pp 229533.I1RPG6
153101 229533.I1RA48 pp 229533.I1RM67
153102 229533.I1RA48 pp 229533.I1RZF6
153103 229533.I1RA48 pp 229533.I1RZV6
153104 229533.I1RA48 pp 229533.Q4IJH1
153105 229533.I1RA48 pp 229533.I1S504
153106 229533.I1RA48 pp 229533.I1S9U1
153107 229533.I1RA48 pp 229533.I1RDK7
153108 229533.I1RA48 pp 229533.I1S9Y1
153109 229533.I1RA48 pp 229533.V6R042
153110 229533.I1RA48 pp 229533.I1RKJ6
153111 229533.I1RA48 pp 229533.I1RPU0
153112 229533.I1RA48 pp 229533.I1RPP4
153113 229533.I1RA48 pp 229533.I1RM96
153114 229533.I1RA48 pp 229533.V6RMW6
153115 229533.I1RA48 pp 229533.I1RNQ5
153116 229533.I1RA48 pp 229533.Q4IEK8
153117 229533.I1RA48 pp 229533.Q4IAA0
153118 229533.I1RA48 pp 229533.I1S6E5
153119 229533.I1RA48 pp 229533.I1RX08
153120 229533.I1RA48 pp 229533.I1RY44
153121 229533.I1RA48 pp 229533.V6RGY0
153122 229533.I1RA48 pp 229533.I1RSI6
153123 229533.I1RA48 pp 229533.Q4ICA8
153124 229533.I1RA48 pp 229533.I1RWN5
153125 229533.I1RA48 pp 229533.I1RNK2
153126 229533.I1RA48 pp 229533.I1RVZ0
153127 229533.I1RA48 pp 229533.I1RNY2
153128 229533.I1RA48 pp 229533.I1RAZ8
153129 229533.I1RA48 pp 229533.I1S4T3
153130 229533.I1RA48 pp 229533.I1RG56
153131 229533.I1RA48 pp 229533.Q4I327
153132 229533.I1RA48 pp 229533.I1RPY1
153133 229533.I1RA48 pp 229533.I1RKC6
153134 229533.I1RA48 pp 229533.I1S021
153135 229533.I1RA48 pp 229533.I1S1J6
153136 229533.I1RA48 pp 229533.I1S0Z9
153137 229533.I1RA48 pp 229533.I1RNH6
153138 229533.I1RA48 pp 229533.I1RMN7
153139 229533.I1RA48 pp 229533.I1RN99
153140 229533.I1RA48 pp 229533.I1RQ28
153141 229533.I1RA48 pp 229533.I1RK95
153142 229533.I1RA48 pp 229533.I1RVX0
153143 229533.I1RA48 pp 229533.I1RDG0
153144 229533.I1RA48 pp 229533.I1REL7
153145 229533.I1RA48 pp 229533.I1RZK5
153146 229533.I1RA48 pp 229533.I1RYB9
153147 229533.I1RA48 pp 229533.I1RSG2
153148 229533.I1RA48 pp 229533.I1RAC1
153149 229533.I1RA48 pp 229533.I1RNF5
153150 229533.I1RA48 pp 229533.I1RMC0
153151 229533.I1RA48 pp 229533.I1RT03
153152 229533.I1RA48 pp 229533.I1RY63
153153 229533.I1RA48 pp 229533.I1RG57
153154 229533.I1RA48 pp 229533.I1RTE9

153155 229533.I1RA48 pp 229533.I1RQ31
153156 229533.I1RA48 pp 229533.I1RCR8
153157 229533.I1RA48 pp 229533.I1S1X9
153158 229533.I1RA48 pp 229533.I1RK83
153159 229533.I1RA48 pp 229533.I1RSC6
153160 229533.I1RA48 pp 229533.I1S2C0
153161 229533.I1RA48 pp 229533.Q4IN63
153162 229533.I1RA48 pp 229533.I1RTH6
153163 229533.I1RA48 pp 229533.I1RFU8
153164 229533.I1RA48 pp 229533.I1RSS4
153165 229533.I1RA48 pp 229533.V6RGN5
153166 229533.I1RA48 pp 229533.I1RRI2
153167 229533.I1RA48 pp 229533.Q4HWE2
153168 229533.I1RA48 pp 229533.I1S044
153169 229533.I1RA48 pp 229533.I1RZK6
153170 229533.I1RA48 pp 229533.I1RD22
153171 229533.I1RA48 pp 229533.I1RC27
153172 229533.I1RA48 pp 229533.I1S1C7
153173 229533.I1RA48 pp 229533.I1RZZ1
153174 229533.I1RA48 pp 229533.I1S2E8
153175 229533.I1RA48 pp 229533.Q4IBS2
153176 229533.I1RA48 pp 229533.I1RAU5
153177 229533.I1RA48 pp 229533.I1RJZ6
153178 229533.I1RA48 pp 229533.I1RMN6
153179 229533.I1RA48 pp 229533.I1RSP7
153180 229533.I1RA48 pp 229533.I1RSR8
153181 229533.I1RA48 pp 229533.I1RY03
153182 229533.I1RA48 pp 229533.V6QUE6
153183 229533.I1RA48 pp 229533.I1RSD8
153184 229533.I1RA48 pp 229533.I1RMQ0
153185 229533.I1RA48 pp 229533.I1S2U2
153186 229533.I1RA48 pp 229533.I1RGC2
153187 229533.I1RA48 pp 229533.I1RTD9
153188 229533.I1RA48 pp 229533.I1RBZ3
153189 229533.I1RA48 pp 229533.I1RWN6
153190 229533.I1RA48 pp 229533.I1RJV3
153191 229533.I1RA48 pp 229533.I1RAU7
153192 229533.I1RA48 pp 229533.Q4HZ68
153193 229533.I1RA48 pp 229533.I1RC81
153194 229533.I1RA48 pp 229533.I1RCN5
153195 229533.I1RA48 pp 229533.I1RRY9
153196 229533.I1RA48 pp 229533.V6RGY3
153197 229533.I1RA48 pp 229533.I1RF08
153198 229533.I1RA48 pp 229533.I1RZE5
153199 229533.I1RA48 pp 229533.I1RQA2
153200 229533.I1RA48 pp 229533.I1RAN5
153201 229533.I1RA48 pp 229533.I1RSR3
153202 229533.I1RA48 pp 229533.I1RE00
153203 229533.I1RA48 pp 229533.I1RYB1
153204 229533.I1RA48 pp 229533.I1RSI0
153205 229533.I1RA48 pp 229533.I1RAW0
153206 229533.I1RA48 pp 229533.I1S274
153207 229533.I1RA48 pp 229533.I1RBL7
153208 229533.I1RA48 pp 229533.I1RCL3
153209 229533.I1RA48 pp 229533.I1RFJ9
153210 229533.I1RXV1 pp 229533.I1RZK5
153211 229533.I1RXV1 pp 229533.I1RZQ6
153212 229533.I1RXV1 pp 229533.Q4I0C5
153213 229533.I1RXV1 pp 229533.V6RDP1
153214 229533.I1RXV1 pp 229533.I1S8J4
153215 229533.I1RZZ1 pp 229533.Q4IEK8
153216 229533.I1RZZ1 pp 229533.I1S504
153217 229533.I1RZZ1 pp 229533.Q4ICA8
153218 229533.I1RZZ1 pp 229533.I1SA32
153219 229533.I1RZZ1 pp 229533.I1S5B0
153220 229533.I1RZZ1 pp 229533.I1S044
153221 229533.I1RZZ1 pp 229533.V6R227
153222 229533.I1RZZ1 pp 229533.Q4I283
153223 229533.I1RZZ1 pp 229533.Q4IR08
153224 229533.I1RZZ1 pp 229533.I1S021
153225 229533.I1RZZ1 pp 229533.I1S7M3
153226 229533.I1RZZ1 pp 229533.Q4I8P5
153227 229533.I1RZZ1 pp 229533.Q4IJ56

229533.I1RZZ1 pp 229533.I1S0M9
229533.I1RZZ1 pp 229533.V6RCG5
229533.I1RZZ1 pp 229533.Q4I2J8
229533.I1RZZ1 pp 229533.Q4IAA0
229533.I1RZZ1 pp 229533.V6QUE6
229533.I1RZZ1 pp 229533.V6RCZ8
229533.I1RZZ1 pp 229533.I1S0F5
229533.I1RZZ1 pp 229533.I1S0L3
229533.I1RZZ1 pp 229533.Q4I948
229533.I1RZZ1 pp 229533.Q4IBS2
229533.I1RZZ1 pp 229533.I1S419
229533.I1RZZ1 pp 229533.I1S0T2
229533.I1RZZ1 pp 229533.V6R1K4
229533.I1RZZ1 pp 229533.I1S540
229533.I1RZZ1 pp 229533.Q4IJT5
229533.I1RZZ1 pp 229533.I1S4Y4
229533.I1RZZ1 pp 229533.Q4IJK2
229533.I1RZZ1 pp 229533.V6QW22
229533.I1RZZ1 pp 229533.I1S7A8
229533.I1RZZ1 pp 229533.Q4I7F9
229533.I1RZZ1 pp 229533.V6RTJ8
229533.I1RZZ1 pp 229533.I1S1J7
229533.I1RZZ1 pp 229533.I1S0B6
229533.I1RZZ1 pp 229533.I1S0R7
229533.I1RZZ1 pp 229533.I1S9U2
229533.I1RZZ1 pp 229533.Q4I5R9
229533.I1RZZ1 pp 229533.I1S6F0
229533.I1RZZ1 pp 229533.Q4INZ9
229533.I1RZZ1 pp 229533.V6R042
229533.I1RZZ1 pp 229533.V6RMW6
229533.I1RZZ1 pp 229533.I1S0X6
229533.I1RZZ1 pp 229533.I1S8U9
229533.I1RZZ1 pp 229533.I1S7M2
229533.I1RZZ1 pp 229533.Q4IPZ1
229533.I1RZZ1 pp 229533.I1SA33
229533.I1RZZ1 pp 229533.Q4IJH1
229533.I1RZZ1 pp 229533.Q4IFI0
229533.I1RZZ1 pp 229533.V6RIV9
229533.I1RZZ1 pp 229533.I1S8V4
229533.I1RZZ1 pp 229533.V6RY33
229533.I1RZZ1 pp 229533.V6RIS2
229533.I1RZZ1 pp 229533.I1S0B1
229533.I1RZZ1 pp 229533.I1S471
229533.I1RZZ1 pp 229533.Q4I624
229533.I1RZZ1 pp 229533.V6RGN5
229533.I1RZZ1 pp 229533.Q4IPH4
229533.I1RZZ1 pp 229533.I1S0N4
229533.I1RZZ1 pp 229533.I1S0J1
229533.I1RZZ1 pp 229533.V6R057
229533.I1RZZ1 pp 229533.I1S2E8
229533.I1RZZ1 pp 229533.Q4IN63
229533.I1RZZ1 pp 229533.I1S1C7
229533.I1RZZ1 pp 229533.I1S8R3
229533.I1RZZ1 pp 229533.I1S8V0
229533.I1RZZ1 pp 229533.V6R283
229533.I1RZZ1 pp 229533.I1S4M5
229533.I1RZZ1 pp 229533.I1S0F6
229533.I1RZZ1 pp 229533.V6QVH8
229533.I1RZZ1 pp 229533.I1S2C0
229533.I1RZZ1 pp 229533.Q4I327
229533.I1RZZ1 pp 229533.Q4IPA4
229533.I1RZZ1 pp 229533.Q4I830
229533.I1RZZ1 pp 229533.I1S1X9
229533.I1RZZ1 pp 229533.I1S2G7
229533.I1RZZ1 pp 229533.Q4HWE2
229533.I1RZZ1 pp 229533.Q4HXF0
229533.I1RZZ1 pp 229533.I1S2H4
229533.I1RZZ1 pp 229533.I1S8P5
229533.I1RZZ1 pp 229533.I1S1U7
229533.I1RZZ1 pp 229533.I1S503
229533.I1RZZ1 pp 229533.I1S5S2
229533.I1RZZ1 pp 229533.I1RZZ9
229533.I1RZZ1 pp 229533.I1S2E9

153301 229533.I1RZZ1 pp 229533.V6RUY9
153302 229533.I1RZZ1 pp 229533.I1S0L4
153303 229533.I1RZZ1 pp 229533.I1S6Y8
153304 229533.I1RZZ1 pp 229533.I1S5R1
153305 229533.I1RZZ1 pp 229533.O74638
153306 229533.I1RZZ1 pp 229533.I1S2B8
153307 229533.I1RZZ1 pp 229533.I1S524
153308 229533.I1RZZ1 pp 229533.I1S8V3
153309 229533.I1RZZ1 pp 229533.I1S8E2
153310 229533.I1RZZ1 pp 229533.V6R3X5
153311 229533.A0A0E0SJQ3 pp 229533.Q4I5M4
153312 229533.A0A0E0SJQ3 pp 229533.Q4I9U7
153313 229533.A0A0E0SJQ3 pp 229533.I1RCR5
153314 229533.A0A0E0SJQ3 pp 229533.Q4HZI0
153315 229533.A0A0E0SJQ3 pp 229533.Q4HWB3
153316 229533.A0A0E0SJQ3 pp 229533.I1RYW3
153317 229533.A0A0E0SJQ3 pp 229533.I1RJV7
153318 229533.A0A0E0SJQ3 pp 229533.I1RRX0
153319 229533.A0A0E0SJQ3 pp 229533.I1RBD2
153320 229533.A0A0E0SJQ3 pp 229533.I1RFT1
153321 229533.A0A0E0SJQ3 pp 229533.A0A1C3YK13
153322 229533.A0A0E0SJQ3 pp 229533.Q4IQ72
153323 229533.A0A1C3YHU9 pp 229533.I1RM94
153324 229533.A0A1C3YHU9 pp 229533.V6RJ21
153325 229533.A0A1C3YHU9 pp 229533.I1RCV0
153326 229533.A0A1C3YHU9 pp 229533.I1RLY6
153327 229533.A0A1C3YHU9 pp 229533.A0A1C3YN78
153328 229533.A0A1C3YHU9 pp 229533.I1RNS2
153329 229533.A0A1C3YHU9 pp 229533.I1RCI0
153330 229533.A0A1C3YHU9 pp 229533.A0A1C3YII1
153331 229533.A0A1C3YHU9 pp 229533.I1RCJ4
153332 229533.A0A1C3YHU9 pp 229533.I1RSE4
153333 229533.A0A1C3YHU9 pp 229533.I1S0P4
153334 229533.A0A1C3YHU9 pp 229533.I1RQ06
153335 229533.I1S3F3 pp 229533.I1SA33
153336 229533.I1S3F3 pp 229533.I1S7M8
153337 229533.I1RI72 pp 229533.I1RZQ6
153338 229533.I1RBT5 pp 229533.I1RFQ0
153339 229533.I1RBT5 pp 229533.I1RC97
153340 229533.A0A098DHS8 pp 229533.I1RCV0
153341 229533.A0A098DHS8 pp 229533.A0A1C3YN78
153342 229533.A0A098DHS8 pp 229533.A0A1C3YII1
153343 229533.A0A098DHS8 pp 229533.A0A0E0RQT8
153344 229533.A0A098DHS8 pp 229533.A0A1C3YHU9
153345 229533.Q4IJK2 pp 229533.V6RTJ8
153346 229533.Q4IJK2 pp 229533.V6QW22
153347 229533.Q4IJK2 pp 229533.V6R4J1
153348 229533.Q4IJK2 pp 229533.Q4IJM4
153349 229533.Q4IJK2 pp 229533.Q4IJT5
153350 229533.Q4IJK2 pp 229533.V6RUT5
153351 229533.Q4IJK2 pp 229533.V6RCG5
153352 229533.Q4IJK2 pp 229533.V6RCZ8
153353 229533.Q4IJK2 pp 229533.V6R9Z0
153354 229533.Q4IJK2 pp 229533.V6R1K4
153355 229533.Q4IJK2 pp 229533.V6RBN2
153356 229533.Q4IJK2 pp 229533.V6R3J1
153357 229533.Q4IJK2 pp 229533.V6R057
153358 229533.Q4IJK2 pp 229533.Q4IN63
153359 229533.Q4IJK2 pp 229533.V6RRH2
153360 229533.Q4IJK2 pp 229533.V6RUY9
153361 229533.Q4IJK2 pp 229533.V6QUE6
153362 229533.Q4IJK2 pp 229533.V6R283
153363 229533.Q4IJK2 pp 229533.V6R042
153364 229533.Q4IJK2 pp 229533.V6RLM7
153365 229533.Q4IJK2 pp 229533.V6R3X5
153366 229533.Q4IJK2 pp 229533.V6RMW6
153367 229533.Q4IJK2 pp 229533.V6RIS2
153368 229533.Q4IJK2 pp 229533.V6RIV9
153369 229533.A0A1C3YM07 pp 229533.I1RI72
153370 229533.A0A1C3YM07 pp 229533.I1RZQ6
153371 229533.A0A1C3YM07 pp 229533.I1RE53
153372 229533.A0A098D110 pp 229533.I1RBS0
153373 229533.A0A098D110 pp 229533.I1RRQ3

153374	229533.A0A098D110	pp	229533.A0A0E0S0W9
153375	229533.A0A098D110	pp	229533.I1RN67
153376	229533.A0A098D110	pp	229533.I1RNG4
153377	229533.A0A098D110	pp	229533.I1S0F6
153378	229533.A0A098D110	pp	229533.I1RZZ9
153379	229533.A0A098D110	pp	229533.I1RPP3
153380	229533.A0A098D110	pp	229533.I1RWI5
153381	229533.A0A098D110	pp	229533.I1S8P5
153382	229533.A0A098D110	pp	229533.A0A1C3YMA1
153383	229533.A0A098D110	pp	229533.A0A1C3YIS7
153384	229533.A0A098D110	pp	229533.I1RWF7
153385	229533.A0A098D110	pp	229533.I1RB74
153386	229533.A0A098D110	pp	229533.I1RBY4
153387	229533.A0A098D110	pp	229533.I1RRJ8
153388	229533.A0A098D110	pp	229533.I1RE76
153389	229533.A0A098D110	pp	229533.I1RPU3
153390	229533.A0A098D110	pp	229533.I1RQG4
153391	229533.A0A098D110	pp	229533.A0A0E0RU84
153392	229533.A0A098D110	pp	229533.I1R9M0
153393	229533.A0A098D110	pp	229533.I1RKL3
153394	229533.A0A098D110	pp	229533.I1RRM5
153395	229533.A0A098D110	pp	229533.I1S2B8
153396	229533.A0A098D110	pp	229533.I1S352
153397	229533.A0A098D110	pp	229533.I1RP27
153398	229533.A0A098D110	pp	229533.I1RSV2
153399	229533.A0A098D110	pp	229533.I1RJZ7
153400	229533.A0A098D110	pp	229533.A0A1C3YKN3
153401	229533.A0A098D110	pp	229533.A0A098DE18
153402	229533.A0A098D110	pp	229533.I1RJV4
153403	229533.A0A098D110	pp	229533.I1S0M6
153404	229533.A0A098D110	pp	229533.I1RFT1
153405	229533.A0A098D110	pp	229533.I1RZS6
153406	229533.A0A098D110	pp	229533.I1RBS6
153407	229533.A0A098D110	pp	229533.I1RGA4
153408	229533.A0A098D110	pp	229533.I1RWK3
153409	229533.A0A098D110	pp	229533.A0A1C3YII7
153410	229533.A0A098D110	pp	229533.I1RC03
153411	229533.A0A098D110	pp	229533.I1RLV9
153412	229533.A0A098D110	pp	229533.I1RLN6
153413	229533.A0A098D110	pp	229533.V6R283
153414	229533.A0A098D110	pp	229533.I1S503
153415	229533.A0A098D110	pp	229533.I1RBG9
153416	229533.A0A098D110	pp	229533.I1RIL3
153417	229533.A0A098D110	pp	229533.I1RE22
153418	229533.A0A098D110	pp	229533.A0A0E0SC46
153419	229533.A0A098D110	pp	229533.I1RQ23
153420	229533.A0A098D110	pp	229533.Q4I662
153421	229533.A0A098D110	pp	229533.I1RJW6
153422	229533.A0A098D110	pp	229533.A0A1C3YLK8
153423	229533.A0A098D110	pp	229533.I1S0L4
153424	229533.A0A098D110	pp	229533.A0A098DRZ0
153425	229533.A0A098D110	pp	229533.I1S2E9
153426	229533.A0A098D110	pp	229533.I1RC00
153427	229533.A0A098D110	pp	229533.I1RNA5
153428	229533.A0A098D110	pp	229533.I1RFS3
153429	229533.A0A098D110	pp	229533.I1S2H4
153430	229533.A0A098D110	pp	229533.I1RPM4
153431	229533.A0A098D110	pp	229533.A0A098DAE0
153432	229533.A0A098D110	pp	229533.I1S0N4
153433	229533.A0A098D110	pp	229533.I1RT04
153434	229533.A0A098D110	pp	229533.I1RS20
153435	229533.A0A098D110	pp	229533.I1RTD5
153436	229533.A0A098D110	pp	229533.I1RD64
153437	229533.A0A098D110	pp	229533.I1R9D1
153438	229533.A0A098D110	pp	229533.I1RD22
153439	229533.A0A098D110	pp	229533.I1RGF5
153440	229533.A0A098D110	pp	229533.I1RRT8
153441	229533.A0A098D110	pp	229533.A0A1C3YI53
153442	229533.A0A098D110	pp	229533.I1RV91
153443	229533.A0A098D110	pp	229533.I1RAF8
153444	229533.A0A098D110	pp	229533.I1SA33
153445	229533.A0A098D110	pp	229533.I1S5R1
153446	229533.A0A098D110	pp	229533.I1RMN6

153447	229533.A0A098D110	pp	229533.I1RCJ7
153448	229533.A0A098D110	pp	229533.I1RA11
153449	229533.A0A098D110	pp	229533.I1RT40
153450	229533.A0A098D110	pp	229533.I1RQ65
153451	229533.A0A098D110	pp	229533.I1RMQ5
153452	229533.A0A098D110	pp	229533.I1RWL2
153453	229533.A0A098D110	pp	229533.V6RMW6
153454	229533.A0A098D110	pp	229533.A0A1C3YLD8
153455	229533.A0A098D110	pp	229533.I1S2G7
153456	229533.A0A098D110	pp	229533.I1RM82
153457	229533.A0A098D110	pp	229533.I1RS47
153458	229533.A0A098D110	pp	229533.V6R042
153459	229533.A0A098D110	pp	229533.I1RKJ6
153460	229533.A0A098D110	pp	229533.A0A1C3YK73
153461	229533.A0A098D110	pp	229533.I1RAN2
153462	229533.A0A098D110	pp	229533.I1RSQ3
153463	229533.A0A098D110	pp	229533.I1RJP4
153464	229533.A0A098D110	pp	229533.V6R3X5
153465	229533.A0A098D110	pp	229533.I1RP28
153466	229533.A0A098D110	pp	229533.I1RMG3
153467	229533.A0A098D110	pp	229533.I1RR73
153468	229533.A0A098D110	pp	229533.I1RRV1
153469	229533.A0A098D110	pp	229533.A0A1C3YIN1
153470	229533.A0A098D110	pp	229533.Q4IN63
153471	229533.A0A098D110	pp	229533.A0A1C3YKM9
153472	229533.A0A098D110	pp	229533.I1RNY2
153473	229533.A0A098D110	pp	229533.I1RCR8
153474	229533.A0A098D110	pp	229533.I1RZ39
153475	229533.A0A098D110	pp	229533.I1RRR7
153476	229533.A0A098D110	pp	229533.Q4I327
153477	229533.A0A098D110	pp	229533.I1RC66
153478	229533.A0A098D110	pp	229533.I1RFT6
153479	229533.A0A098D110	pp	229533.A0A1C3YMI5
153480	229533.A0A098D110	pp	229533.I1RUG3
153481	229533.A0A098D110	pp	229533.I1RRE5
153482	229533.A0A098D110	pp	229533.I1S1W4
153483	229533.A0A098D110	pp	229533.I1RNN1
153484	229533.A0A098D110	pp	229533.I1RY44
153485	229533.A0A098D110	pp	229533.I1S2C0
153486	229533.A0A098D110	pp	229533.I1RA70
153487	229533.A0A098D110	pp	229533.I1RNC1
153488	229533.A0A098D110	pp	229533.I1RQH3
153489	229533.A0A098D110	pp	229533.I1RM96
153490	229533.A0A098D110	pp	229533.I1S8R3
153491	229533.A0A098D110	pp	229533.V6RIV9
153492	229533.A0A098D110	pp	229533.I1RXD7
153493	229533.A0A098D110	pp	229533.I1RTH6
153494	229533.A0A098D110	pp	229533.A0A098DPS3
153495	229533.A0A098D110	pp	229533.A0A0E0SFN0
153496	229533.A0A098D110	pp	229533.I1RQC9
153497	229533.A0A098D110	pp	229533.I1RRI2
153498	229533.A0A098D110	pp	229533.I1S0R7
153499	229533.A0A098D110	pp	229533.I1RWN5
153500	229533.A0A098D110	pp	229533.I1RWX5
153501	229533.A0A098D110	pp	229533.Q4HZB8
153502	229533.A0A098D110	pp	229533.I1RZW8
153503	229533.A0A098D110	pp	229533.I1RPP4
153504	229533.A0A098D110	pp	229533.I1S1C7
153505	229533.A0A098D110	pp	229533.V6R1K4
153506	229533.A0A098D110	pp	229533.I1RPK0
153507	229533.A0A098D110	pp	229533.I1RYW3
153508	229533.A0A098D110	pp	229533.I1RAU7
153509	229533.A0A098D110	pp	229533.I1RS17
153510	229533.A0A098D110	pp	229533.I1S0L3
153511	229533.A0A098D110	pp	229533.V6R057
153512	229533.A0A098D110	pp	229533.I1S0F5
153513	229533.A0A098D110	pp	229533.I1S0J1
153514	229533.A0A098D110	pp	229533.I1RE01
153515	229533.A0A098D110	pp	229533.I1S569
153516	229533.A0A098D110	pp	229533.I1RW46
153517	229533.A0A098D110	pp	229533.I1RTA0
153518	229533.A0A098D110	pp	229533.I1RD52
153519	229533.A0A098D110	pp	229533.Q4I624

153520	229533.A0A098D110	pp	229533.I1RD17
153521	229533.A0A098D110	pp	229533.A0A098D6T7
153522	229533.A0A098D110	pp	229533.I1RDU4
153523	229533.A0A098D110	pp	229533.A0A1C3YI73
153524	229533.A0A098D110	pp	229533.I1RVQ0
153525	229533.A0A098D110	pp	229533.I1RZS7
153526	229533.A0A098D110	pp	229533.I1RGL4
153527	229533.A0A098D110	pp	229533.I1RB41
153528	229533.A0A098D110	pp	229533.I1RWN6
153529	229533.A0A098D110	pp	229533.V6R4J1
153530	229533.A0A098D110	pp	229533.I1R9J6
153531	229533.A0A098D110	pp	229533.I1RB92
153532	229533.A0A098D110	pp	229533.I1RD39
153533	229533.A0A098D110	pp	229533.I1RAW0
153534	229533.A0A098D110	pp	229533.A0A098DRR8
153535	229533.A0A098D110	pp	229533.I1RSK1
153536	229533.A0A098D110	pp	229533.A0A098DVS9
153537	229533.A0A098D110	pp	229533.A0A1C3YIP5
153538	229533.A0A098D110	pp	229533.I1RG53
153539	229533.A0A098D110	pp	229533.I1RVX0
153540	229533.A0A098D110	pp	229533.I1RQJ5
153541	229533.A0A098D110	pp	229533.I1RT03
153542	229533.A0A098D110	pp	229533.I1RSQ7
153543	229533.A0A098D110	pp	229533.I1RRN5
153544	229533.A0A098D110	pp	229533.V6QW22
153545	229533.A0A098D110	pp	229533.I1RCA0
153546	229533.A0A098D110	pp	229533.I1RCH5
153547	229533.A0A098D110	pp	229533.I1RJV3
153548	229533.A0A098D110	pp	229533.A0A1C3YIN6
153549	229533.A0A098D110	pp	229533.I1RZE4
153550	229533.A0A098D110	pp	229533.I1RN61
153551	229533.A0A098D110	pp	229533.I1RC20
153552	229533.A0A098D110	pp	229533.I1S2E8
153553	229533.A0A098D110	pp	229533.I1RCM3
153554	229533.A0A098D110	pp	229533.I1RW71
153555	229533.A0A098D110	pp	229533.I1RQE7
153556	229533.A0A098D110	pp	229533.I1RBC9
153557	229533.A0A098D110	pp	229533.A0A1C3YK13
153558	229533.A0A098D110	pp	229533.I1RR00
153559	229533.A0A098D110	pp	229533.A0A0E0RV94
153560	229533.A0A098D110	pp	229533.I1RFT2
153561	229533.A0A098D110	pp	229533.I1RRU9
153562	229533.A0A098D110	pp	229533.I1RN18
153563	229533.A0A098D110	pp	229533.I1RFL4
153564	229533.A0A098D110	pp	229533.I1RGC2
153565	229533.A0A098D110	pp	229533.A0A1C3YLU7
153566	229533.A0A098D110	pp	229533.Q4IJT5
153567	229533.A0A098D110	pp	229533.I1RSD8
153568	229533.A0A098D110	pp	229533.I1RFW5
153569	229533.A0A098D110	pp	229533.I1RIL8
153570	229533.A0A098D110	pp	229533.V6RCG5
153571	229533.A0A098D110	pp	229533.I1S7A8
153572	229533.A0A098D110	pp	229533.V6RCZ8
153573	229533.A0A098D110	pp	229533.A0A098DQ41
153574	229533.A0A098D110	pp	229533.V6RTJ8
153575	229533.A0A098D110	pp	229533.A0A1C3YM63
153576	229533.A0A098D110	pp	229533.I1RAX6
153577	229533.A0A098D110	pp	229533.I1RPW9
153578	229533.A0A098D110	pp	229533.I1S9U2
153579	229533.A0A098D110	pp	229533.I1RI82
153580	229533.A0A098D110	pp	229533.I1RX46
153581	229533.A0A098D110	pp	229533.I1RNH6
153582	229533.A0A098D110	pp	229533.I1RCC2
153583	229533.A0A098D110	pp	229533.A0A1C3YJH8
153584	229533.A0A098D110	pp	229533.I1S021
153585	229533.A0A098D110	pp	229533.I1RR24
153586	229533.A0A098D110	pp	229533.A0A0E0RSD7
153587	229533.A0A098D110	pp	229533.A0A098D5E5
153588	229533.A0A098D110	pp	229533.I1RFQ7
153589	229533.A0A098D110	pp	229533.I1RWJ8
153590	229533.A0A098D110	pp	229533.I1RZJ5
153591	229533.A0A098D110	pp	229533.I1RZM8
153592	229533.A0A098D110	pp	229533.I1RPU0

153593	229533.A0A098D110	pp	229533.I1RXF0
153594	229533.A0A098D110	pp	229533.I1RM67
153595	229533.A0A098D110	pp	229533.I1S6U8
153596	229533.A0A098D110	pp	229533.A0A098DR40
153597	229533.A0A098D110	pp	229533.I1RMG8
153598	229533.A0A098D110	pp	229533.I1RRQ1
153599	229533.A0A098D110	pp	229533.A0A0E0SA11
153600	229533.A0A098D110	pp	229533.I1RT80
153601	229533.A0A098D110	pp	229533.Q4I283
153602	229533.A0A098D110	pp	229533.I1RKB7
153603	229533.A0A098D110	pp	229533.I1S1J7
153604	229533.A0A098D110	pp	229533.A0A1C3YN30
153605	229533.A0A098D110	pp	229533.A0A1C3YKB0
153606	229533.A0A098D110	pp	229533.I1RWT6
153607	229533.A0A098D110	pp	229533.I1RIG2
153608	229533.A0A098D110	pp	229533.A0A1C3YKW9
153609	229533.A0A098D110	pp	229533.I1RZF6
153610	229533.A0A098D110	pp	229533.A0A1C3YJ93
153611	229533.A0A098D110	pp	229533.A0A0E0SPV2
153612	229533.A0A098D110	pp	229533.I1RI03
153613	229533.A0A098D110	pp	229533.I1RAN5
153614	229533.A0A098D110	pp	229533.I1RJU3
153615	229533.A0A098D110	pp	229533.I1RZK5
153616	229533.A0A098D110	pp	229533.I1RAM6
153617	229533.A0A098D110	pp	229533.A0A1C3YHX8
153618	229533.A0A098D110	pp	229533.I1RMC8
153619	229533.A0A098D110	pp	229533.I1RFU8
153620	229533.A0A098D110	pp	229533.I1RBT1
153621	229533.A0A098D110	pp	229533.I1S504
153622	229533.A0A098D110	pp	229533.A0A098E4W0
153623	229533.A0A098D110	pp	229533.I1RCG4
153624	229533.A0A098D110	pp	229533.A0A1C3YKC0
153625	229533.A0A098D110	pp	229533.A0A1C3YLK2
153626	229533.A0A098D110	pp	229533.I1RYY5
153627	229533.A0A098D110	pp	229533.I1RBJ5
153628	229533.A0A098D110	pp	229533.I1S0X6
153629	229533.A0A098D110	pp	229533.I1RNQ5
153630	229533.A0A098D110	pp	229533.A0A1C3YHX6
153631	229533.A0A098D110	pp	229533.I1RI02
153632	229533.A0A098D110	pp	229533.I1RY03
153633	229533.A0A098D110	pp	229533.I1RSR8
153634	229533.A0A098D110	pp	229533.I1RLU6
153635	229533.A0A098D110	pp	229533.I1RW07
153636	229533.A0A098D110	pp	229533.A0A0E0SMM8
153637	229533.A0A098D110	pp	229533.A0A0E0RUI4
153638	229533.A0A098D110	pp	229533.I1RZM1
153639	229533.A0A098D110	pp	229533.A0A1C3YJQ9
153640	229533.A0A098D110	pp	229533.I1RWD7
153641	229533.A0A098D110	pp	229533.I1RW25
153642	229533.A0A098D110	pp	229533.I1REK8
153643	229533.A0A098D110	pp	229533.I1RX25
153644	229533.A0A098D110	pp	229533.Q4I8P5
153645	229533.A0A098D110	pp	229533.A0A098DXE2
153646	229533.A0A098D110	pp	229533.I1RLC7
153647	229533.A0A098D110	pp	229533.I1RCR5
153648	229533.A0A098D110	pp	229533.I1RB75
153649	229533.A0A098D110	pp	229533.I1RS18
153650	229533.A0A098D110	pp	229533.I1RM06
153651	229533.A0A098D110	pp	229533.I1RB27
153652	229533.A0A098D110	pp	229533.I1S0M9
153653	229533.A0A098D110	pp	229533.I1RZE5
153654	229533.A0A098D110	pp	229533.I1RLM7
153655	229533.A0A098D110	pp	229533.V6QUW4
153656	229533.A0A098D110	pp	229533.A0A1C3YLZ7
153657	229533.A0A098D110	pp	229533.A0A1C3YIJ3
153658	229533.A0A098D110	pp	229533.I1RE00
153659	229533.A0A098D110	pp	229533.I1RSI0
153660	229533.A0A098D110	pp	229533.A0A1C3YKV3
153661	229533.A0A098D110	pp	229533.I1S5B0
153662	229533.A0A098D110	pp	229533.I1RQ81
153663	229533.A0A098D110	pp	229533.I1RVZ0
153664	229533.A0A098D110	pp	229533.I1RW99
153665	229533.A0A098D110	pp	229533.Q4I2J8

229533.A0A098D110 pp 229533.A0A0E0RZM5
229533.A0A098D110 pp 229533.I1RC17
229533.A0A098D110 pp 229533.I1SA32
229533.A0A098D110 pp 229533.I1RZK6
229533.A0A098D110 pp 229533.I1RJZ6
229533.A0A098D110 pp 229533.I1RDG0
229533.A0A098D110 pp 229533.A0A0E0RVZ1
229533.A0A098D110 pp 229533.Q4IJM4
229533.A0A098D110 pp 229533.A0A098DB19
229533.A0A098D110 pp 229533.A0A1C3YK34
229533.A0A098D110 pp 229533.I1RU29
229533.A0A098D110 pp 229533.V6RBN2
229533.A0A098D110 pp 229533.I1RRY9
229533.A0A098D110 pp 229533.I1RQQ5
229533.A0A098D110 pp 229533.I1RBL7
229533.A0A098D110 pp 229533.I1R9V7
229533.A0A098D110 pp 229533.I1RA48
229533.A0A098D110 pp 229533.I1RZZ1
229533.A0A098D110 pp 229533.Q4IJK2
229533.A0A098D110 pp 229533.I1RQA3
229533.A0A1C3YJ46 pp 229533.I1S7M8
229533.A0A1C3YJ46 pp 229533.I1RRT8
229533.A0A1C3YJ46 pp 229533.A0A1C3YLE2
229533.A0A1C3YJ46 pp 229533.A0A1C3YLP8
229533.A0A1C3YJ46 pp 229533.I1RJZ7
229533.A0A1C3YJ46 pp 229533.I1RFT1
229533.A0A1C3YJ46 pp 229533.I1RYW3
229533.A0A1C3YJ46 pp 229533.I1RMJ3
229533.A0A1C3YJ46 pp 229533.I1RFL4
229533.A0A1C3YJ46 pp 229533.I1RA62
229533.A0A1C3YJ46 pp 229533.I1RN84
229533.A0A1C3YJ46 pp 229533.I1RGK9
229533.A0A1C3YJ46 pp 229533.V6QW22
229533.A0A1C3YJ46 pp 229533.I1RS10
229533.A0A1C3YJ46 pp 229533.A0A1C3YK13
229533.A0A1C3YJ46 pp 229533.I1RCR5
229533.A0A1C3YJ46 pp 229533.I1RTJ2
229533.A0A1C3YJ46 pp 229533.I1RK67
229533.A0A1C3YJ46 pp 229533.I1RA50
229533.I1RLU0 pp 229533.Q4INW6
229533.I1RLU0 pp 229533.I1RQC5
229533.I1RLU0 pp 229533.I1RZG7
229533.I1RLU0 pp 229533.I1RZQ5
229533.I1RLU0 pp 229533.V6QXG1
229533.I1RLU0 pp 229533.Q4ICI6
229533.I1RLU0 pp 229533.I1RPY4
229533.I1RLU0 pp 229533.I1S524
229533.I1RLU0 pp 229533.I1S9L0
229533.I1RE71 pp 229533.I1RYJ9
229533.I1RE71 pp 229533.Q4HWE2
229533.I1RE71 pp 229533.I1S6F1
229533.I1RE71 pp 229533.I1RWD7
229533.I1RE71 pp 229533.I1RGC2
229533.I1RE71 pp 229533.I1RSI0
229533.I1RE71 pp 229533.I1S471
229533.I1RE71 pp 229533.I1RRU9
229533.I1RE71 pp 229533.I1RPU3
229533.I1RE71 pp 229533.Q4IP34
229533.I1RE71 pp 229533.I1RS20
229533.I1RE71 pp 229533.I1RNB8
229533.I1RE71 pp 229533.Q4IAA0
229533.I1RE71 pp 229533.I1RZS7
229533.I1RE71 pp 229533.I1RT35
229533.I1RE71 pp 229533.I1S7S9
229533.I1RE71 pp 229533.I1RN48
229533.I1RE71 pp 229533.I1S0N7
229533.I1RE71 pp 229533.I1S4S4
229533.I1RE71 pp 229533.I1RPS0
229533.I1RE71 pp 229533.I1RG53
229533.I1RE71 pp 229533.Q4HW67
229533.I1RE71 pp 229533.I1RIS3
229533.I1RE71 pp 229533.I1RNE7
229533.I1RE71 pp 229533.Q4HZ42

153739 229533.I1RE71 pp 229533.I1RQK2
153740 229533.I1RE71 pp 229533.I1RLU5
153741 229533.A0A1C3YKZ8 pp 229533.I1RIJ2
153742 229533.A0A1C3YKZ8 pp 229533.I1S029
153743 229533.A0A1C3YKZ8 pp 229533.V6R1K4
153744 229533.A0A1C3YKZ8 pp 229533.I1RC39
153745 229533.A0A1C3YKZ8 pp 229533.A0A1C3YM68
153746 229533.A0A1C3YKZ8 pp 229533.I1S273
153747 229533.A0A1C3YKZ8 pp 229533.I1RC11
153748 229533.A0A1C3YKZ8 pp 229533.V6RAM6
153749 229533.A0A1C3YKZ8 pp 229533.A0A1C3YLR6
153750 229533.A0A1C3YKZ8 pp 229533.I1S3K8
153751 229533.A0A1C3YKZ8 pp 229533.I1RQE1
153752 229533.A0A1C3YKZ8 pp 229533.I1RP86
153753 229533.A0A1C3YKZ8 pp 229533.A0A1C3YN26
153754 229533.A0A1C3YKZ8 pp 229533.I1RVX9
153755 229533.A0A1C3YKZ8 pp 229533.I1RMT4
153756 229533.A0A1C3YKZ8 pp 229533.I1RMU7
153757 229533.A0A1C3YKZ8 pp 229533.I1RY49
153758 229533.A0A1C3YKZ8 pp 229533.V6R0W1
153759 229533.A0A1C3YKZ8 pp 229533.I1RZY1
153760 229533.A0A1C3YKZ8 pp 229533.V6QU99
153761 229533.A0A1C3YKZ8 pp 229533.I1RAR9
153762 229533.A0A1C3YKZ8 pp 229533.I1RAU9
153763 229533.A0A1C3YKZ8 pp 229533.I1RPW2
153764 229533.A0A1C3YKZ8 pp 229533.A0A1C3YN30
153765 229533.A0A1C3YKZ8 pp 229533.I1RYQ4
153766 229533.A0A1C3YKZ8 pp 229533.I1RCU2
153767 229533.A0A1C3YKZ8 pp 229533.I1RGW2
153768 229533.A0A1C3YKZ8 pp 229533.I1RCY0
153769 229533.A0A1C3YKZ8 pp 229533.I1RQX9
153770 229533.A0A1C3YKZ8 pp 229533.I1RWL3
153771 229533.A0A1C3YKZ8 pp 229533.I1RXH3
153772 229533.A0A1C3YKZ8 pp 229533.I1S8C1
153773 229533.A0A1C3YKZ8 pp 229533.I1SAI4
153774 229533.A0A1C3YKZ8 pp 229533.I1RFK7
153775 229533.A0A1C3YKZ8 pp 229533.I1RS52
153776 229533.A0A1C3YKZ8 pp 229533.A0A1C3YNB6
153777 229533.A0A1C3YKZ8 pp 229533.A0A1C3YLV0
153778 229533.A0A1C3YKZ8 pp 229533.I1RJT5
153779 229533.A0A1C3YKZ8 pp 229533.I1RM30
153780 229533.A0A1C3YKZ8 pp 229533.I1RMD3
153781 229533.A0A1C3YKZ8 pp 229533.I1RF38
153782 229533.A0A1C3YKZ8 pp 229533.I1RA90
153783 229533.A0A1C3YKZ8 pp 229533.I1RYU5
153784 229533.A0A1C3YKZ8 pp 229533.I1RVV7
153785 229533.A0A1C3YKZ8 pp 229533.I1RZH4
153786 229533.A0A1C3YKZ8 pp 229533.I1S0A0
153787 229533.A0A1C3YKZ8 pp 229533.I1RW54
153788 229533.A0A1C3YKZ8 pp 229533.I1S4D0
153789 229533.A0A1C3YKZ8 pp 229533.I1RCV1
153790 229533.A0A1C3YKZ8 pp 229533.I1RNY6
153791 229533.A0A1C3YKZ8 pp 229533.I1RC83
153792 229533.A0A1C3YKZ8 pp 229533.Q4I0M9
153793 229533.A0A1C3YKZ8 pp 229533.I1RY12
153794 229533.A0A1C3YKZ8 pp 229533.I1RVR0
153795 229533.A0A1C3YKZ8 pp 229533.I1RD81
153796 229533.A0A1C3YKZ8 pp 229533.I1RC48
153797 229533.A0A1C3YKZ8 pp 229533.I1RPR1
153798 229533.A0A1C3YKZ8 pp 229533.I1S3K6
153799 229533.A0A1C3YKZ8 pp 229533.I1RRM1
153800 229533.A0A1C3YKZ8 pp 229533.I1RAD3
153801 229533.A0A1C3YKZ8 pp 229533.I1RWF5
153802 229533.A0A1C3YKZ8 pp 229533.V6R398
153803 229533.A0A1C3YKZ8 pp 229533.I1S3A5
153804 229533.A0A1C3YKZ8 pp 229533.I1RMJ3
153805 229533.A0A1C3YKZ8 pp 229533.A0A1C3YLE2
153806 229533.A0A1C3YKZ8 pp 229533.A0A1C3YLX8
153807 229533.A0A1C3YKZ8 pp 229533.I1RKB2
153808 229533.A0A1C3YKZ8 pp 229533.I1RNH4
153809 229533.A0A1C3YKZ8 pp 229533.A0A1C3YL82
153810 229533.A0A1C3YKZ8 pp 229533.I1S024
153811 229533.A0A1C3YKZ8 pp 229533.A0A1C3YM86

153812 229533.A0A1C3YKZ8 pp 229533.V6RQW4
153813 229533.A0A1C3YKZ8 pp 229533.I1RZA7
153814 229533.A0A1C3YKZ8 pp 229533.Q4HXF0
153815 229533.A0A1C3YKZ8 pp 229533.I1RR68
153816 229533.A0A1C3YKZ8 pp 229533.I1RSX4
153817 229533.A0A1C3YKZ8 pp 229533.I1RJY7
153818 229533.A0A1C3YKZ8 pp 229533.V6RUB1
153819 229533.A0A1C3YKZ8 pp 229533.I1RM26
153820 229533.A0A1C3YKZ8 pp 229533.V6RY56
153821 229533.A0A1C3YKZ8 pp 229533.I1RQE0
153822 229533.A0A1C3YKZ8 pp 229533.V6R2Z2
153823 229533.A0A1C3YKZ8 pp 229533.I1RE53
153824 229533.A0A1C3YKZ8 pp 229533.V6R3J1
153825 229533.A0A1C3YKZ8 pp 229533.I1RNG7
153826 229533.A0A1C3YKZ8 pp 229533.I1RE63
153827 229533.A0A1C3YKZ8 pp 229533.I1S0V6
153828 229533.A0A1C3YKZ8 pp 229533.I1RYW9
153829 229533.A0A1C3YKZ8 pp 229533.I1RVR2
153830 229533.A0A1C3YKZ8 pp 229533.I1RUR8
153831 229533.A0A1C3YKZ8 pp 229533.I1RZ20
153832 229533.A0A1C3YKZ8 pp 229533.I1S6P5
153833 229533.A0A1C3YKZ8 pp 229533.I1RVZ3
153834 229533.A0A1C3YKZ8 pp 229533.I1RGC3
153835 229533.A0A1C3YKZ8 pp 229533.I1S5L5
153836 229533.A0A1C3YKZ8 pp 229533.I1RFI4
153837 229533.A0A1C3YKZ8 pp 229533.O74638
153838 229533.A0A1C3YKZ8 pp 229533.I1RZT0
153839 229533.A0A1C3YKZ8 pp 229533.I1RPY0
153840 229533.A0A1C3YKZ8 pp 229533.I1S0H5
153841 229533.A0A1C3YKZ8 pp 229533.I1RV23
153842 229533.A0A1C3YKZ8 pp 229533.I1RM54
153843 229533.A0A1C3YKZ8 pp 229533.V6R5D2
153844 229533.A0A1C3YKZ8 pp 229533.I1RAN1
153845 229533.A0A1C3YKZ8 pp 229533.I1RBA4
153846 229533.A0A1C3YKZ8 pp 229533.I1RZG0
153847 229533.A0A1C3YKZ8 pp 229533.A0A1C3YNB1
153848 229533.A0A1C3YKZ8 pp 229533.I1RHJ2
153849 229533.A0A1C3YKZ8 pp 229533.I1RDJ7
153850 229533.A0A1C3YKZ8 pp 229533.I1RKP1
153851 229533.A0A1C3YKZ8 pp 229533.I1RD80
153852 229533.A0A1C3YKZ8 pp 229533.V6RM02
153853 229533.A0A1C3YKZ8 pp 229533.I1RP69
153854 229533.A0A1C3YKZ8 pp 229533.I1S064
153855 229533.A0A1C3YKZ8 pp 229533.V6RLM7
153856 229533.A0A1C3YKZ8 pp 229533.I1RDT5
153857 229533.A0A1C3YKZ8 pp 229533.V6R756
153858 229533.A0A1C3YKZ8 pp 229533.I1RF53
153859 229533.A0A1C3YKZ8 pp 229533.I1RPU8
153860 229533.A0A1C3YKZ8 pp 229533.Q4INW6
153861 229533.A0A1C3YKZ8 pp 229533.I1RND2
153862 229533.A0A1C3YKZ8 pp 229533.I1RN84
153863 229533.A0A1C3YKZ8 pp 229533.I1RF85
153864 229533.A0A1C3YKZ8 pp 229533.I1R9B8
153865 229533.A0A1C3YKZ8 pp 229533.I1RLT3
153866 229533.A0A1C3YKZ8 pp 229533.I1S0V7
153867 229533.A0A1C3YKZ8 pp 229533.I1RCG5
153868 229533.A0A1C3YKZ8 pp 229533.I1RM55
153869 229533.A0A1C3YKZ8 pp 229533.I1RFK3
153870 229533.A0A1C3YKZ8 pp 229533.I1RCB4
153871 229533.A0A1C3YKZ8 pp 229533.I1RZQ6
153872 229533.A0A1C3YKZ8 pp 229533.I1S2C4
153873 229533.A0A1C3YKZ8 pp 229533.A0A1C3YM09
153874 229533.A0A098E2T0 pp 229533.A0A098E5C4
153875 229533.A0A098E2T0 pp 229533.I1S182
153876 229533.I1RRG3 pp 229533.I1S0J3
153877 229533.I1RRG3 pp 229533.I1RZ39
153878 229533.I1RRG3 pp 229533.V6R3X5
153879 229533.I1RRG3 pp 229533.Q4IB50
153880 229533.I1RRG3 pp 229533.I1S253
153881 229533.I1RRG3 pp 229533.I1RZZ9
153882 229533.I1RRG3 pp 229533.I1RWI5
153883 229533.I1RRG3 pp 229533.I1RRT8
153884 229533.I1RRG3 pp 229533.I1S5R1

153885	229533.I1RRG3	pp	229533.I1RSQ4
153886	229533.I1RRG3	pp	229533.O74638
153887	229533.I1RRG3	pp	229533.Q4IF76
153888	229533.I1RRG3	pp	229533.Q4IL82
153889	229533.I1RRG3	pp	229533.I1RXZ3
153890	229533.I1RRG3	pp	229533.I1RXC2
153891	229533.I1RRG3	pp	229533.Q4HTT1
153892	229533.I1RRG3	pp	229533.I1S2H4
153893	229533.I1RRG3	pp	229533.I1S0R5
153894	229533.I1RRG3	pp	229533.I1RRI1
153895	229533.I1RRG3	pp	229533.I1RYE2
153896	229533.I1RRG3	pp	229533.I1S2B8
153897	229533.I1RRG3	pp	229533.I1S9Y1
153898	229533.I1RRG3	pp	229533.I1RVY0
153899	229533.I1RRG3	pp	229533.I1RSZ7
153900	229533.I1RRG3	pp	229533.V6RUY9
153901	229533.I1RRG3	pp	229533.Q4I099
153902	229533.I1RRG3	pp	229533.Q4IJ11
153903	229533.I1RRG3	pp	229533.I1RTH2
153904	229533.I1RRG3	pp	229533.I1RVL8
153905	229533.I1RRG3	pp	229533.I1RXF2
153906	229533.I1RRG3	pp	229533.Q4IAK7
153907	229533.I1RRG3	pp	229533.I1RX63
153908	229533.I1RRG3	pp	229533.Q4IMD1
153909	229533.I1RRG3	pp	229533.I1S2E9
153910	229533.I1RRG3	pp	229533.I1RZI4
153911	229533.I1RRG3	pp	229533.Q4I5W5
153912	229533.I1RRG3	pp	229533.I1RV91
153913	229533.I1RRG3	pp	229533.I1S2G7
153914	229533.I1RRG3	pp	229533.I1RT40
153915	229533.I1RRG3	pp	229533.I1RRM8
153916	229533.I1RRG3	pp	229533.I1RS30
153917	229533.I1RRG3	pp	229533.I1RS43
153918	229533.I1RRG3	pp	229533.I1RW80
153919	229533.I1RRG3	pp	229533.I1RSZ3
153920	229533.I1RRG3	pp	229533.I1RSX0
153921	229533.I1RRG3	pp	229533.I1RYI5
153922	229533.I1RRG3	pp	229533.I1S3N0
153923	229533.I1RRG3	pp	229533.Q4IB96
153924	229533.I1RRG3	pp	229533.I1RRJ8
153925	229533.I1RRG3	pp	229533.I1RTD5
153926	229533.I1RRG3	pp	229533.I1RTA1
153927	229533.I1RRG3	pp	229533.I1S214
153928	229533.I1RRG3	pp	229533.I1S2E8
153929	229533.I1RRG3	pp	229533.I1RZM2
153930	229533.I1RRG3	pp	229533.I1S0N5
153931	229533.I1RRG3	pp	229533.I1RSG8
153932	229533.I1RRG3	pp	229533.I1RUG4
153933	229533.I1RRG3	pp	229533.I1RTB2
153934	229533.I1RRG3	pp	229533.I1RWN6
153935	229533.I1RRG3	pp	229533.I1S8V0
153936	229533.I1RRG3	pp	229533.I1RVZ2
153937	229533.I1RRG3	pp	229533.I1S8R3
153938	229533.I1RRG3	pp	229533.Q4IJ84
153939	229533.I1RRG3	pp	229533.I1RZU4
153940	229533.I1RRG3	pp	229533.V6RRH2
153941	229533.I1RRG3	pp	229533.I1S276
153942	229533.I1RRG3	pp	229533.I1S5C5
153943	229533.I1RRG3	pp	229533.I1S2C4
153944	229533.I1RRG3	pp	229533.I1S0B1
153945	229533.I1RRG3	pp	229533.Q4IPZ1
153946	229533.I1RRG3	pp	229533.I1RS41
153947	229533.I1RRG3	pp	229533.I1RVX0
153948	229533.I1RRG3	pp	229533.I1RZQ6
153949	229533.I1RRG3	pp	229533.I1RT03
153950	229533.I1RRG3	pp	229533.I1RTD7
153951	229533.I1RRG3	pp	229533.V6RM02
153952	229533.I1RRG3	pp	229533.I1S3N1
153953	229533.I1RRG3	pp	229533.I1RYW3
153954	229533.I1RRG3	pp	229533.I1S8U9
153955	229533.I1RRG3	pp	229533.Q4I7S1
153956	229533.I1RRG3	pp	229533.I1RS06
153957	229533.I1RRG3	pp	229533.I1S1C7

229533.I1RRG3	pp	229533.I1RRI2
229533.I1RRG3	pp	229533.I1RW23
229533.I1RRG3	pp	229533.I1RSS4
229533.I1RRG3	pp	229533.I1RSQ3
229533.I1RRG3	pp	229533.I1RXU0
229533.I1RRG3	pp	229533.I1S5R6
229533.I1RRG3	pp	229533.I1S1W4
229533.I1RRG3	pp	229533.I1S0J1
229533.I1RRG3	pp	229533.V6RGN5
229533.I1RRG3	pp	229533.V6RRM0
229533.I1RRG3	pp	229533.V6R042
229533.I1RRG3	pp	229533.V6RMW6
229533.I1RRG3	pp	229533.I1RRN6
229533.I1RRG3	pp	229533.Q4IPC8
229533.I1RRG3	pp	229533.I1RUG3
229533.I1RRG3	pp	229533.I1RWN5
229533.I1RRG3	pp	229533.I1RSU2
229533.I1RRG3	pp	229533.I1S1W9
229533.I1RRG3	pp	229533.I1RWF3
229533.I1RRG3	pp	229533.I1S0C6
229533.I1RRG3	pp	229533.I1S0F1
229533.I1RRG3	pp	229533.Q4I5U9
229533.I1RRG3	pp	229533.V6QWX8
229533.I1RRG3	pp	229533.I1RY44
229533.I1RRG3	pp	229533.I1RZ42
229533.I1RRG3	pp	229533.I1RU52
229533.I1RRG3	pp	229533.I1RRN2
229533.I1RRG3	pp	229533.I1RWL3
229533.I1RRG3	pp	229533.I1RSA4
229533.I1RRG3	pp	229533.I1RS66
229533.I1RRG3	pp	229533.Q4HYB8
229533.I1RRG3	pp	229533.Q4IJH1
229533.I1RRG3	pp	229533.I1RUR0
229533.I1RRG3	pp	229533.I1RUG0
229533.I1RRG3	pp	229533.Q4IQT8
229533.I1RRG3	pp	229533.I1RUP9
229533.I1RRG3	pp	229533.I1RW90
229533.I1RRG3	pp	229533.I1S0R7
229533.I1RRG3	pp	229533.I1RVJ1
229533.I1RRG3	pp	229533.V6R7V8
229533.I1RRG3	pp	229533.I1RW91
229533.I1RRG3	pp	229533.I1S0X6
229533.I1RRG3	pp	229533.I1S7M2
229533.I1RRG3	pp	229533.Q4IPN9
229533.I1RRG3	pp	229533.V6RTJ8
229533.I1RRG3	pp	229533.I1RYY5
229533.I1RRG3	pp	229533.I1RXD7
229533.I1RRG3	pp	229533.I1S338
229533.I1RRG3	pp	229533.I1S9J6
229533.I1RRG3	pp	229533.I1RX46
229533.I1RRG3	pp	229533.I1S201
229533.I1RRG3	pp	229533.Q4HYQ4
229533.I1RRG3	pp	229533.Q4IQX3
229533.I1RRG3	pp	229533.I1S4S7
229533.I1RRG3	pp	229533.I1S182
229533.I1RRG3	pp	229533.I1RXX1
229533.I1RRG3	pp	229533.I1RZR6
229533.I1RRG3	pp	229533.I1RTC2
229533.I1RRG3	pp	229533.V6QV38
229533.I1RRG3	pp	229533.Q4IER8
229533.I1RRG3	pp	229533.I1RRY9
229533.I1RRG3	pp	229533.I1RZK6
229533.I1RRG3	pp	229533.I1RZL5
229533.I1RRG3	pp	229533.I1RSQ7
229533.I1RRG3	pp	229533.I1RZK5
229533.I1RRG3	pp	229533.I1RZB5
229533.I1RRG3	pp	229533.I1RZU9
229533.I1RRG3	pp	229533.I1RZZ1
229533.I1RRG3	pp	229533.I1RU02
229533.I1RRG3	pp	229533.I1S6K9
229533.I1RRG3	pp	229533.I1S5Z2
229533.I1RRG3	pp	229533.Q4IJU0
229533.I1RRG3	pp	229533.I1RY03

154031 229533.I1RRG3 pp 229533.I1S1J7
154032 229533.I1RRG3 pp 229533.I1RYS4
154033 229533.I1RRG3 pp 229533.Q4HYI0
154034 229533.I1RRG3 pp 229533.V6RCZ8
154035 229533.I1RRG3 pp 229533.V6QUE6
154036 229533.I1RRG3 pp 229533.Q4IJM4
154037 229533.I1RRG3 pp 229533.I1S5B0
154038 229533.I1RRG3 pp 229533.Q4HTT2
154039 229533.I1RRG3 pp 229533.Q4I5I4
154040 229533.I1RRG3 pp 229533.I1S2A1
154041 229533.I1RRG3 pp 229533.Q4IJK2
154042 229533.I1RRG3 pp 229533.I1RZM8
154043 229533.I1RRG3 pp 229533.I1RWT6
154044 229533.I1RRG3 pp 229533.I1S0F0
154045 229533.I1RRG3 pp 229533.I1S7M3
154046 229533.I1RRG3 pp 229533.I1RXC4
154047 229533.I1RRG3 pp 229533.I1RWX8
154048 229533.I1RRG3 pp 229533.I1RS18
154049 229533.I1RRG3 pp 229533.I1RRT0
154050 229533.I1RRG3 pp 229533.I1RT01
154051 229533.I1RRG3 pp 229533.Q4IEK8
154052 229533.I1RRG3 pp 229533.I1RX14
154053 229533.I1RRG3 pp 229533.I1RT99
154054 229533.I1RRG3 pp 229533.I1S255
154055 229533.I1RRG3 pp 229533.I1RSI1
154056 229533.I1RRG3 pp 229533.I1S9U1
154057 229533.I1RRG3 pp 229533.I1RXE5
154058 229533.I1RNH3 pp 229533.I1RTB7
154059 229533.I1RNH3 pp 229533.I1S0S6
154060 229533.I1RNH3 pp 229533.I1RX09
154061 229533.I1RNH3 pp 229533.P0C431
154062 229533.I1RNH3 pp 229533.I1RQN9
154063 229533.A0A1C3YJZ0 pp 229533.A0A1C3YKY6
154064 229533.A0A1C3YJZ0 pp 229533.A0A1C3YLC2
154065 229533.A0A1C3YJZ0 pp 229533.I1REI2
154066 229533.A0A1C3YJZ0 pp 229533.I1RMD3
154067 229533.A0A1C3YJZ0 pp 229533.I1RDU0
154068 229533.A0A1C3YJZ0 pp 229533.I1RED2
154069 229533.A0A1C3YJZ0 pp 229533.I1RRC4
154070 229533.A0A1C3YJZ0 pp 229533.I1RXX0
154071 229533.A0A1C3YJZ0 pp 229533.I1RUM7
154072 229533.A0A1C3YJZ0 pp 229533.I1RC11
154073 229533.A0A1C3YJZ0 pp 229533.I1RDB1
154074 229533.A0A1C3YJZ0 pp 229533.I1RCU8
154075 229533.A0A1C3YJZ0 pp 229533.I1RST8
154076 229533.A0A1C3YJZ0 pp 229533.I1RCE6
154077 229533.A0A1C3YJZ0 pp 229533.I1RAR3
154078 229533.A0A1C3YJZ0 pp 229533.A0A1C3YN26
154079 229533.A0A1C3YJZ0 pp 229533.I1RFV2
154080 229533.A0A1C3YJZ0 pp 229533.I1RSX2
154081 229533.A0A1C3YJZ0 pp 229533.I1R9Y2
154082 229533.A0A1C3YJZ0 pp 229533.I1RW69
154083 229533.A0A1C3YJZ0 pp 229533.A0A1C3YK34
154084 229533.A0A1C3YJZ0 pp 229533.I1S0R1
154085 229533.A0A1C3YJZ0 pp 229533.Q4IR09
154086 229533.A0A1C3YJZ0 pp 229533.I1RPZ1
154087 229533.A0A1C3YJZ0 pp 229533.I1RAM7
154088 229533.A0A1C3YJZ0 pp 229533.I1S0A0
154089 229533.A0A1C3YJZ0 pp 229533.A0A1C3YLC3
154090 229533.A0A1C3YJZ0 pp 229533.I1RHP8
154091 229533.A0A1C3YJZ0 pp 229533.I1S4S4
154092 229533.A0A1C3YJZ0 pp 229533.I1RRX5
154093 229533.A0A1C3YJZ0 pp 229533.V6RDP1
154094 229533.A0A1C3YJZ0 pp 229533.I1RY21
154095 229533.A0A1C3YJZ0 pp 229533.V6RTJ8
154096 229533.A0A1C3YJZ0 pp 229533.I1RUY5
154097 229533.A0A1C3YJZ0 pp 229533.I1RM67
154098 229533.A0A1C3YJZ0 pp 229533.I1RRN4
154099 229533.A0A1C3YJZ0 pp 229533.A0A1C3YML3
154100 229533.A0A1C3YJZ0 pp 229533.I1RTP2
154101 229533.A0A1C3YJZ0 pp 229533.I1RP86
154102 229533.A0A1C3YJZ0 pp 229533.I1RCG5
154103 229533.A0A1C3YJZ0 pp 229533.I1RPK0

154104 229533.A0A1C3YJZ0 pp 229533.A0A1C3YN55
154105 229533.A0A1C3YJZ0 pp 229533.I1RSQ4
154106 229533.A0A1C3YJZ0 pp 229533.I1RUG4
154107 229533.A0A1C3YJZ0 pp 229533.V6RLM7
154108 229533.A0A1C3YJZ0 pp 229533.I1RVN1
154109 229533.A0A1C3YJZ0 pp 229533.I1S2B0
154110 229533.A0A1C3YJZ0 pp 229533.V6R382
154111 229533.A0A1C3YJZ0 pp 229533.I1RPS2
154112 229533.A0A1C3YJZ0 pp 229533.I1RNG4
154113 229533.A0A1C3YJZ0 pp 229533.I1RCF3
154114 229533.A0A1C3YJZ0 pp 229533.I1S8X0
154115 229533.A0A1C3YJZ0 pp 229533.I1RC67
154116 229533.A0A1C3YJZ0 pp 229533.I1RTI3
154117 229533.A0A1C3YJZ0 pp 229533.I1S9Z4
154118 229533.A0A1C3YJZ0 pp 229533.A0A1C3YKW4
154119 229533.A0A1C3YJZ0 pp 229533.I1RAN1
154120 229533.A0A1C3YJZ0 pp 229533.A0A1C3YLB6
154121 229533.A0A1C3YJZ0 pp 229533.I1RHJ2
154122 229533.A0A1C3YJZ0 pp 229533.I1RYK0
154123 229533.A0A1C3YJZ0 pp 229533.I1RFK3
154124 229533.A0A1C3YJZ0 pp 229533.I1RA62
154125 229533.A0A1C3YJZ0 pp 229533.A0A1C3YLA6
154126 229533.A0A1C3YJZ0 pp 229533.I1RZN1
154127 229533.A0A1C3YJZ0 pp 229533.A0A1C3YLB2
154128 229533.A0A1C3YJZ0 pp 229533.I1RSA3
154129 229533.A0A1C3YJZ0 pp 229533.I1RCU9
154130 229533.A0A1C3YJZ0 pp 229533.I1RSU2
154131 229533.A0A1C3YJZ0 pp 229533.A0A1C3YK08
154132 229533.A0A1C3YJZ0 pp 229533.I1RZ95
154133 229533.A0A1C3YJZ0 pp 229533.I1RPX7
154134 229533.A0A1C3YJZ0 pp 229533.O74638
154135 229533.A0A1C3YJZ0 pp 229533.I1RK02
154136 229533.A0A1C3YJZ0 pp 229533.I1S3L1
154137 229533.A0A1C3YJZ0 pp 229533.I1RTG7
154138 229533.A0A1C3YJZ0 pp 229533.I1REI3
154139 229533.A0A1C3YJZ0 pp 229533.I1RR68
154140 229533.A0A1C3YL48 pp 229533.I1RSC7
154141 229533.A0A1C3YL48 pp 229533.I1RBA4
154142 229533.A0A1C3YL48 pp 229533.I1S209
154143 229533.A0A1C3YL48 pp 229533.I1RVZ9
154144 229533.A0A1C3YL48 pp 229533.I1RDJ8
154145 229533.I1RFQ3 pp 229533.Q4I7L0
154146 229533.I1RST9 pp 229533.I1S5J9
154147 229533.I1RST9 pp 229533.I1RXX0
154148 229533.I1RST9 pp 229533.V6RHX2
154149 229533.I1RST9 pp 229533.I1RWT6
154150 229533.I1RST9 pp 229533.I1S267
154151 229533.I1RST9 pp 229533.I1RSX2
154152 229533.I1RST9 pp 229533.I1S8H9
154153 229533.I1RST9 pp 229533.I1RW55
154154 229533.I1RST9 pp 229533.I1S2F7
154155 229533.I1RST9 pp 229533.I1S3B2
154156 229533.I1RST9 pp 229533.I1RVP5
154157 229533.I1RST9 pp 229533.I1RWM4
154158 229533.I1RST9 pp 229533.I1S8D3
154159 229533.I1RST9 pp 229533.V6RWB3
154160 229533.I1RST9 pp 229533.I1RXH2
154161 229533.I1RST9 pp 229533.I1S4I7
154162 229533.I1RST9 pp 229533.V6R3V4
154163 229533.I1RST9 pp 229533.I1RY81
154164 229533.I1RST9 pp 229533.I1S4I8
154165 229533.I1RST9 pp 229533.I1RY12
154166 229533.I1RST9 pp 229533.I1S659
154167 229533.I1RST9 pp 229533.I1RYM5
154168 229533.I1RST9 pp 229533.I1S4S5
154169 229533.I1RST9 pp 229533.I1RYD3
154170 229533.I1RST9 pp 229533.I1RWD2
154171 229533.I1RST9 pp 229533.I1S0V6
154172 229533.I1RST9 pp 229533.I1S3M4
154173 229533.I1RST9 pp 229533.V6QVV9
154174 229533.I1RST9 pp 229533.I1S969
154175 229533.I1RST9 pp 229533.I1S223
154176 229533.I1RST9 pp 229533.V6QU99

154177	229533.I1RST9	pp	229533.I1S000
154178	229533.I1RST9	pp	229533.I1S8C1
154179	229533.I1RST9	pp	229533.I1S3T6
154180	229533.I1RST9	pp	229533.V6QVH2
154181	229533.I1RST9	pp	229533.I1RYX6
154182	229533.I1RST9	pp	229533.V6R5T7
154183	229533.I1RST9	pp	229533.I1RYP5
154184	229533.I1RST9	pp	229533.I1SAI4
154185	229533.I1RST9	pp	229533.I1RYU6
154186	229533.I1RST9	pp	229533.I1RVI8
154187	229533.I1RST9	pp	229533.I1RZ15
154188	229533.I1RST9	pp	229533.I1S0K1
154189	229533.I1RST9	pp	229533.I1S5D0
154190	229533.I1RST9	pp	229533.I1RW54
154191	229533.I1RST9	pp	229533.I1S094
154192	229533.I1RST9	pp	229533.I1S210
154193	229533.I1RST9	pp	229533.V6RAM6
154194	229533.I1RST9	pp	229533.V6RCC0
154195	229533.I1RST9	pp	229533.V6R1D2
154196	229533.I1RST9	pp	229533.I1S2F6
154197	229533.I1RST9	pp	229533.I1S224
154198	229533.I1RST9	pp	229533.I1RY16
154199	229533.I1RST9	pp	229533.V6RH92
154200	229533.I1RST9	pp	229533.I1RVF2
154201	229533.I1RST9	pp	229533.V6R0W1
154202	229533.I1RST9	pp	229533.V6R9L8
154203	229533.I1RST9	pp	229533.I1S6H3
154204	229533.I1RST9	pp	229533.I1RY44
154205	229533.I1RST9	pp	229533.I1SAT2
154206	229533.I1RST9	pp	229533.I1S0D1
154207	229533.I1RST9	pp	229533.I1S9Z4
154208	229533.I1RST9	pp	229533.I1RZ56
154209	229533.I1RST9	pp	229533.I1S0V7
154210	229533.I1RST9	pp	229533.I1S2Z7
154211	229533.I1RST9	pp	229533.I1RZC3
154212	229533.I1RST9	pp	229533.I1RX18
154213	229533.I1RST9	pp	229533.V6RM02
154214	229533.I1RST9	pp	229533.V6RF62
154215	229533.I1RST9	pp	229533.V6R2Z2
154216	229533.I1RST9	pp	229533.I1RZX3
154217	229533.I1RST9	pp	229533.I1RSU2
154218	229533.I1RST9	pp	229533.I1S2C4
154219	229533.I1RST9	pp	229533.I1RTY9
154220	229533.I1RST9	pp	229533.I1S6V0
154221	229533.I1RST9	pp	229533.I1RZQ6
154222	229533.I1RST9	pp	229533.I1RW82
154223	229533.I1RST9	pp	229533.I1RTM8
154224	229533.I1RST9	pp	229533.I1S0X1
154225	229533.I1RST9	pp	229533.I1S0A5
154226	229533.I1RST9	pp	229533.I1S968
154227	229533.I1RST9	pp	229533.I1RUE3
154228	229533.I1RST9	pp	229533.V6RZD4
154229	229533.I1RST9	pp	229533.V6R756
154230	229533.I1RST9	pp	229533.I1RVX8
154231	229533.I1RST9	pp	229533.I1RY32
154232	229533.I1RST9	pp	229533.I1RZV5
154233	229533.I1RST9	pp	229533.I1RV91
154234	229533.I1RST9	pp	229533.I1RWF5
154235	229533.I1RST9	pp	229533.I1RZ38
154236	229533.I1RST9	pp	229533.I1RZA8
154237	229533.I1RST9	pp	229533.I1S694
154238	229533.I1RST9	pp	229533.I1S2E9
154239	229533.I1RST9	pp	229533.V6R5D2
154240	229533.I1RST9	pp	229533.I1RZ25
154241	229533.I1RST9	pp	229533.I1RUR8
154242	229533.I1RST9	pp	229533.V6RIE5
154243	229533.I1RST9	pp	229533.V6QZ48
154244	229533.I1RST9	pp	229533.V6RCJ5
154245	229533.I1RST9	pp	229533.I1RW64
154246	229533.I1RST9	pp	229533.I1SAB4
154247	229533.I1RST9	pp	229533.I1SAH9
154248	229533.I1S6S9	pp	229533.P0C431
154249	229533.I1RT82	pp	229533.I1RWF5

154250	229533.I1RT82	pp	229533.I1S8C1
154251	229533.I1RT82	pp	229533.I1RY12
154252	229533.V6QUE6	pp	229533.V6QWX8
154253	229533.V6QUE6	pp	229533.V6RTJ8
154254	229533.V6QUE6	pp	229533.V6RUT5
154255	229533.V6QUE6	pp	229533.V6R227
154256	229533.V6QUE6	pp	229533.V6R3X5
154257	229533.V6QUE6	pp	229533.V6RCZ8
154258	229533.V6QUE6	pp	229533.V6QYF0
154259	229533.V6QUE6	pp	229533.V6QV38
154260	229533.V6QUE6	pp	229533.V6RBN2
154261	229533.V6QUE6	pp	229533.V6R283
154262	229533.V6QUE6	pp	229533.V6RRH2
154263	229533.V6QUE6	pp	229533.V6R2A1
154264	229533.V6QUE6	pp	229533.V6R3J1
154265	229533.V6QUE6	pp	229533.V6RUY9
154266	229533.V6QUE6	pp	229533.V6RGN5
154267	229533.V6QUE6	pp	229533.V6RMW6
154268	229533.V6QUE6	pp	229533.V6RLM7
154269	229533.V6QUE6	pp	229533.V6R042
154270	229533.I1RIR0	pp	229533.I1S439
154271	229533.I1RIR0	pp	229533.I1RYH9
154272	229533.I1RIR0	pp	229533.I1RYZ0
154273	229533.A0A1C3YL45	pp	229533.I1RTI9
154274	229533.A0A1C3YL45	pp	229533.I1RNG4
154275	229533.I1RMU7	pp	229533.I1S0E4
154276	229533.I1RMU7	pp	229533.I1S9Y1
154277	229533.I1RMU7	pp	229533.I1RSW5
154278	229533.I1RMU7	pp	229533.I1RVU9
154279	229533.I1RMU7	pp	229533.I1RTI3
154280	229533.I1RMU7	pp	229533.I1RTP2
154281	229533.I1RMU7	pp	229533.Q4HTS9
154282	229533.I1RMU7	pp	229533.I1RPU8
154283	229533.I1RMU7	pp	229533.I1RNG4
154284	229533.I1RMU7	pp	229533.I1S029
154285	229533.I1RMU7	pp	229533.I1RV85
154286	229533.I1RMU7	pp	229533.V6QUE6
154287	229533.I1RMU7	pp	229533.I1RRG3
154288	229533.I1RMU7	pp	229533.I1RVX9
154289	229533.I1RMU7	pp	229533.I1RST9
154290	229533.I1RMU7	pp	229533.Q4I8Q4
154291	229533.I1RMU7	pp	229533.V6RY56
154292	229533.I1RMU7	pp	229533.I1S4D0
154293	229533.I1RI98	pp	229533.I1RW06
154294	229533.I1RI98	pp	229533.I1RMM8
154295	229533.A0A098DSD2	pp	229533.I1RMM8
154296	229533.A0A098DSD2	pp	229533.I1RPW2
154297	229533.A0A098DSD2	pp	229533.I1S3F3
154298	229533.A0A098DSD2	pp	229533.I1RVX9
154299	229533.A0A098DSD2	pp	229533.I1S9D4
154300	229533.A0A098DSD2	pp	229533.I1RIY0
154301	229533.A0A098DSD2	pp	229533.I1RU76
154302	229533.A0A098DSD2	pp	229533.I1RM26
154303	229533.A0A098DSD2	pp	229533.I1RK03
154304	229533.A0A098DSD2	pp	229533.I1RQE0
154305	229533.A0A098DSD2	pp	229533.A0A1C3YIV7
154306	229533.A0A098DSD2	pp	229533.I1S2E2
154307	229533.A0A098DSD2	pp	229533.A0A1C3YM01
154308	229533.A0A098DSD2	pp	229533.I1RZ52
154309	229533.A0A098DSD2	pp	229533.A0A1C3YLU9
154310	229533.A0A098DSD2	pp	229533.I1R9K3
154311	229533.A0A098DSD2	pp	229533.I1RNG4
154312	229533.A0A098DSD2	pp	229533.I1RPD6
154313	229533.A0A098DSD2	pp	229533.I1RY74
154314	229533.I1RCV1	pp	229533.I1RLT3
154315	229533.I1RCV1	pp	229533.I1RF85
154316	229533.I1RCV1	pp	229533.I1RM55
154317	229533.I1RCV1	pp	229533.I1S2C4
154318	229533.I1RCV1	pp	229533.V6R756
154319	229533.I1RCV1	pp	229533.I1RUE2
154320	229533.I1RCV1	pp	229533.I1RZQ6
154321	229533.I1RCV1	pp	229533.I1S0V7
154322	229533.I1RCV1	pp	229533.I1RDT5

154323 229533.I1RCV1 pp 229533.I1RE79
154324 229533.I1RCV1 pp 229533.I1RPU8
154325 229533.I1RCV1 pp 229533.I1RSL5
154326 229533.I1RCV1 pp 229533.I1RP69
154327 229533.I1RCV1 pp 229533.I1RSY2
154328 229533.I1RCV1 pp 229533.I1RHC8
154329 229533.I1RCV1 pp 229533.V6RLM7
154330 229533.I1RCV1 pp 229533.V6RM02
154331 229533.I1RCV1 pp 229533.I1RVZ3
154332 229533.I1RCV1 pp 229533.I1RFI4
154333 229533.I1RCV1 pp 229533.I1RD80
154334 229533.I1RCV1 pp 229533.I1RGC3
154335 229533.I1RCV1 pp 229533.O74638
154336 229533.I1RCV1 pp 229533.I1RV23
154337 229533.I1RCV1 pp 229533.V6R5D2
154338 229533.I1RCV1 pp 229533.I1RM54
154339 229533.I1RCV1 pp 229533.I1RW43
154340 229533.I1RCV1 pp 229533.I1RFK3
154341 229533.I1RCV1 pp 229533.I1RSX4
154342 229533.I1RCV1 pp 229533.I1RG11
154343 229533.I1RCV1 pp 229533.I1RZT0
154344 229533.I1RCV1 pp 229533.I1S0H5
154345 229533.I1RCV1 pp 229533.I1RTA9
154346 229533.I1RCV1 pp 229533.I1RSQ4
154347 229533.I1RCV1 pp 229533.I1RR68
154348 229533.I1RCV1 pp 229533.I1RPY0
154349 229533.I1RCV1 pp 229533.Q4HXF0
154350 229533.I1RCV1 pp 229533.I1RVR2
154351 229533.I1RCV1 pp 229533.I1RYW9
154352 229533.I1RCV1 pp 229533.I1RZ20
154353 229533.I1RCV1 pp 229533.I1S3G4
154354 229533.I1RCV1 pp 229533.I1RDJ7
154355 229533.I1RCV1 pp 229533.I1RWF5
154356 229533.I1RCV1 pp 229533.I1S024
154357 229533.I1RCV1 pp 229533.I1RNG4
154358 229533.I1RCV1 pp 229533.I1RE53
154359 229533.I1RCV1 pp 229533.I1RPM6
154360 229533.I1RCV1 pp 229533.I1RUY5
154361 229533.I1RCV1 pp 229533.V6R2Z2
154362 229533.I1RCV1 pp 229533.V6RY56
154363 229533.I1RCV1 pp 229533.I1S3K6
154364 229533.I1RCV1 pp 229533.I1RYK0
154365 229533.I1RCV1 pp 229533.I1RPR1
154366 229533.I1RCV1 pp 229533.I1RM26
154367 229533.I1RCV1 pp 229533.I1RNY6
154368 229533.I1RCV1 pp 229533.I1RKB2
154369 229533.I1RCV1 pp 229533.V6RQW4
154370 229533.I1RCV1 pp 229533.I1S3A5
154371 229533.I1RCV1 pp 229533.I1S4S4
154372 229533.I1RCV1 pp 229533.I1RDU0
154373 229533.I1RCV1 pp 229533.I1RXH3
154374 229533.I1RCV1 pp 229533.I1S4D0
154375 229533.I1RCV1 pp 229533.V6R0W1
154376 229533.I1RCV1 pp 229533.I1S3K8
154377 229533.I1RCV1 pp 229533.I1RPW2
154378 229533.I1RCV1 pp 229533.I1S273
154379 229533.I1RCV1 pp 229533.I1RYA2
154380 229533.I1RCV1 pp 229533.V6RBN2
154381 229533.I1RCV1 pp 229533.I1RMU7
154382 229533.I1RCV1 pp 229533.I1RRS8
154383 229533.I1RCV1 pp 229533.I1RY49
154384 229533.I1RCV1 pp 229533.I1RQA5
154385 229533.I1RCV1 pp 229533.I1S0V6
154386 229533.I1RCV1 pp 229533.I1RYX6
154387 229533.I1RCV1 pp 229533.I1RW54
154388 229533.I1RCV1 pp 229533.I1S029
154389 229533.I1RCV1 pp 229533.V6R1K4
154390 229533.I1RCV1 pp 229533.I1RWP4
154391 229533.I1RCV1 pp 229533.I1RMD3
154392 229533.I1RCV1 pp 229533.I1RG66
154393 229533.I1RCV1 pp 229533.I1S1W8
154394 229533.I1RCV1 pp 229533.I1RIJ2
154395 229533.I1RCV1 pp 229533.I1RYU5

229533.I1RCV1	pp	229533.I1RG14
229533.I1RCV1	pp	229533.I1RVR0
229533.I1RCV1	pp	229533.I1S1P8
229533.I1RCV1	pp	229533.I1RJT5
229533.I1RCV1	pp	229533.I1RFK7
229533.I1RCV1	pp	229533.I1S0A0
229533.I1RCV1	pp	229533.I1RYD3
229533.I1RCV1	pp	229533.I1SAI4
229533.I1RCV1	pp	229533.I1RM30
229533.I1RCV1	pp	229533.I1RQE1
229533.I1RCV1	pp	229533.I1S084
229533.I1RCV1	pp	229533.V6QU99
229533.I1RCV1	pp	229533.I1RQX9
229533.I1RCV1	pp	229533.V6RAM6
229533.I1RCV1	pp	229533.I1RZY1
229533.I1RCV1	pp	229533.I1RVX9
229533.I1RCV1	pp	229533.I1RWL3
229533.A0A098DGP5	pp	229533.V6RVG3
229533.A0A098DGP5	pp	229533.V6RKY7
229533.A0A098DGP5	pp	229533.I1S1W4
229533.A0A098DGP5	pp	229533.I1RQB4
229533.A0A098DGP5	pp	229533.I1RZC3
229533.A0A098DGP5	pp	229533.I1RZE4
229533.A0A098DGP5	pp	229533.I1RRN5
229533.A0A098DGP5	pp	229533.I1S247
229533.A0A098DGP5	pp	229533.I1RNY8
229533.A0A098DGP5	pp	229533.I1RMA7
229533.A0A098DGP5	pp	229533.I1R9Y9
229533.A0A098DGP5	pp	229533.I1RX64
229533.A0A098DGP5	pp	229533.I1RAX2
229533.A0A098DGP5	pp	229533.I1RUN8
229533.A0A098DGP5	pp	229533.Q4IEA7
229533.A0A098DGP5	pp	229533.I1S0X7
229533.A0A098DGP5	pp	229533.I1S286
229533.A0A098DGP5	pp	229533.I1RAM7
229533.A0A098DGP5	pp	229533.I1S7X9
229533.A0A098DGP5	pp	229533.I1RSS1
229533.A0A098DGP5	pp	229533.I1RMB2
229533.A0A098DGP5	pp	229533.I1RC46
229533.A0A098DGP5	pp	229533.I1RQD6
229533.A0A098DGP5	pp	229533.I1RB74
229533.A0A098DGP5	pp	229533.I1RWT6
229533.A0A098DGP5	pp	229533.I1RS47
229533.A0A098DGP5	pp	229533.I1RFW0
229533.A0A098DGP5	pp	229533.I1S278
229533.A0A098DGP5	pp	229533.I1RAN2
229533.A0A098DGP5	pp	229533.A0A1C3YIG3
229533.A0A098DGP5	pp	229533.I1RY32
229533.A0A098DGP5	pp	229533.I1RGF1
229533.A0A098DGP5	pp	229533.I1RCB3
229533.A0A098DGP5	pp	229533.I1S248
229533.A0A098DGP5	pp	229533.I1RKK0
229533.A0A098DGP5	pp	229533.I1RDB1
229533.A0A098DGP5	pp	229533.I1RV66
229533.A0A098DGP5	pp	229533.I1S439
229533.A0A098DGP5	pp	229533.I1RQA3
229533.A0A098DGP5	pp	229533.A0A098DT74
229533.A0A098DGP5	pp	229533.I1RB75
229533.A0A098DGP5	pp	229533.I1RX87
229533.A0A098DGP5	pp	229533.I1RX04
229533.A0A098DGP5	pp	229533.I1RYL5
229533.A0A098DGP5	pp	229533.I1RX77
229533.A0A098DGP5	pp	229533.I1RUQ5
229533.A0A098DGP5	pp	229533.I1RCD1
229533.A0A098DGP5	pp	229533.I1RC22
229533.A0A098DGP5	pp	229533.I1RMM9
229533.A0A098DGP5	pp	229533.A0A0E0RR40
229533.A0A098DGP5	pp	229533.I1RQL4
229533.A0A098DGP5	pp	229533.A0A0E0SAC4
229533.A0A098DGP5	pp	229533.I1RPA6
229533.A0A098DGB7	pp	229533.I1RWM4
229533.A0A098DGB7	pp	229533.A0A0E0RQT8
229533.A0A098DGB7	pp	229533.Q4I8Q4

154469 229533.A0A098DGB7 pp 229533.I1RTP2
154470 229533.A0A098DGB7 pp 229533.I1REI4
154471 229533.A0A098DGB7 pp 229533.I1RTI3
154472 229533.A0A098DGB7 pp 229533.I1RV85
154473 229533.A0A098DGB7 pp 229533.I1RDZ5
154474 229533.A0A098DGB7 pp 229533.A0A0E0S5J3
154475 229533.A0A098DGB7 pp 229533.A0A1C3YJJ7
154476 229533.A0A098DGB7 pp 229533.I1RMU7
154477 229533.A0A098DGB7 pp 229533.I1REB5
154478 229533.A0A098DGB7 pp 229533.I1RE88
154479 229533.A0A098DGB7 pp 229533.A0A098DIZ7
154480 229533.A0A0E0SJS1 pp 229533.V6QUW4
154481 229533.A0A0E0SJS1 pp 229533.I1RPM4
154482 229533.A0A0E0SJS1 pp 229533.I1S7T4
154483 229533.A0A0E0SJS1 pp 229533.I1RZZ9
154484 229533.A0A0E0SJS1 pp 229533.A0A1C3YIS7
154485 229533.A0A0E0SJS1 pp 229533.I1RJZ7
154486 229533.A0A0E0SJS1 pp 229533.A0A1C3YHX8
154487 229533.A0A0E0SJS1 pp 229533.I1S0L4
154488 229533.A0A0E0SJS1 pp 229533.I1RZS5
154489 229533.A0A0E0SJS1 pp 229533.I1RRM5
154490 229533.A0A0E0SJS1 pp 229533.I1RW25
154491 229533.A0A0E0SJS1 pp 229533.I1RBY4
154492 229533.A0A0E0SJS1 pp 229533.I1RRT8
154493 229533.A0A0E0SJS1 pp 229533.I1RW71
154494 229533.A0A0E0SJS1 pp 229533.I1RE76
154495 229533.A0A0E0SJS1 pp 229533.A0A1C3YKM9
154496 229533.A0A0E0SJS1 pp 229533.I1RDB1
154497 229533.A0A0E0SJS1 pp 229533.I1RR73
154498 229533.A0A0E0SJS1 pp 229533.I1RBC9
154499 229533.A0A0E0SJS1 pp 229533.V6QW22
154500 229533.A0A0E0SJS1 pp 229533.I1RJV3
154501 229533.A0A0E0SJS1 pp 229533.V6R3X5
154502 229533.A0A0E0SJS1 pp 229533.I1RYS0
154503 229533.A0A0E0SJS1 pp 229533.I1RPP3
154504 229533.A0A0E0SJS1 pp 229533.I1S0L3
154505 229533.A0A0E0SJS1 pp 229533.I1RBT1
154506 229533.A0A0E0SJS1 pp 229533.A0A1C3YKC0
154507 229533.A0A0E0SJS1 pp 229533.I1RS17
154508 229533.A0A0E0SJS1 pp 229533.I1RN67
154509 229533.A0A0E0SJS1 pp 229533.I1RVQ0
154510 229533.A0A0E0SJS1 pp 229533.A0A1C3YIP5
154511 229533.A0A0E0SJS1 pp 229533.Q4IJ56
154512 229533.A0A0E0SJS1 pp 229533.I1RC28
154513 229533.A0A0E0SJS1 pp 229533.A0A1C3YKV3
154514 229533.A0A0E0SJS1 pp 229533.I1RRQ3
154515 229533.A0A0E0SJS1 pp 229533.I1RPP5
154516 229533.A0A0E0SJS1 pp 229533.I1RPP4
154517 229533.A0A0E0SJS1 pp 229533.I1RFL4
154518 229533.A0A0E0SJS1 pp 229533.I1RT04
154519 229533.A0A0E0SJS1 pp 229533.I1RE01
154520 229533.A0A0E0SJS1 pp 229533.I1S0R7
154521 229533.A0A0E0SJS1 pp 229533.I1RN18
154522 229533.A0A0E0SJS1 pp 229533.I1RQE7
154523 229533.A0A0E0SJS1 pp 229533.I1S504
154524 229533.A0A0E0SJS1 pp 229533.I1RCH5
154525 229533.A0A0E0SJS1 pp 229533.I1RD17
154526 229533.A0A0E0SJS1 pp 229533.I1RQC9
154527 229533.A0A0E0SJS1 pp 229533.I1RT93
154528 229533.A0A0E0SJS1 pp 229533.I1RGA4
154529 229533.A0A0E0SJS1 pp 229533.I1REK8
154530 229533.A0A0E0SJS1 pp 229533.A0A1C3YKB0
154531 229533.A0A0E0SJS1 pp 229533.I1RAU7
154532 229533.A0A0E0SJS1 pp 229533.I1RAZ8
154533 229533.A0A0E0SJS1 pp 229533.A0A1C3YIJ3
154534 229533.A0A0E0SJS1 pp 229533.I1RTH6
154535 229533.A0A0E0SJS1 pp 229533.I1RAX6
154536 229533.A0A0E0SJS1 pp 229533.I1RBS6
154537 229533.A0A0E0SJS1 pp 229533.I1RJV4
154538 229533.A0A0E0SJS1 pp 229533.V6RIV9
154539 229533.A0A0E0SJS1 pp 229533.A0A0E0SMM8
154540 229533.A0A0E0SJS1 pp 229533.V6RTJ8
154541 229533.A0A0E0SJS1 pp 229533.I1RZB5

229533.A0A0E0SJS1 pp 229533.I1RZM1
229533.A0A0E0SJS1 pp 229533.I1RJW6
229533.A0A0E0SJS1 pp 229533.I1RX46
229533.A0A0E0SJS1 pp 229533.I1RCG4
229533.A0A0E0SJS1 pp 229533.I1RA70
229533.A0A0E0SJS1 pp 229533.I1RFU8
229533.A0A0E0SJS1 pp 229533.V6RVG3
229533.A0A0E0SJS1 pp 229533.I1RZP8
229533.A0A0E0SJS1 pp 229533.I1RSS4
229533.A0A0E0SJS1 pp 229533.I1RNQ5
229533.A0A0E0SJS1 pp 229533.I1RVX0
229533.A0A0E0SJS1 pp 229533.I1RY03
229533.A0A0E0SJS1 pp 229533.I1RRV1
229533.A0A0E0SJS1 pp 229533.A0A1C3YHX6
229533.A0A0E0SJS1 pp 229533.I1RZW8
229533.A0A0E0SJS1 pp 229533.I1RB92
229533.A0A0E0SJS1 pp 229533.I1S0E8
229533.A0A0E0SJS1 pp 229533.V6R042
229533.A0A0E0SJS1 pp 229533.I1RRI2
229533.A0A0E0SJS1 pp 229533.I1RSQ3
229533.A0A0E0SJS1 pp 229533.I1RWN6
229533.A0A0E0SJS1 pp 229533.V6RCG5
229533.A0A0E0SJS1 pp 229533.I1RRY9
229533.A0A0E0SJS1 pp 229533.I1RNC1
229533.A0A0E0SJS1 pp 229533.V6RMW6
229533.A0A0E0SJS1 pp 229533.I1RUG3
229533.A0A0E0SJS1 pp 229533.A0A1C3YLE3
229533.A0A0E0SJS1 pp 229533.I1RS90
229533.A0A0E0SJS1 pp 229533.I1RW99
229533.A0A0E0SJS1 pp 229533.I1RD22
229533.I1RM26 pp 229533.I1RWS4
229533.I1RM26 pp 229533.V6RM66
229533.I1RM26 pp 229533.I1S4B7
229533.I1RM26 pp 229533.O74638
229533.I1RM26 pp 229533.I1S299
229533.I1RM26 pp 229533.I1RXG6
229533.I1RM26 pp 229533.I1S9Y1
229533.I1RM26 pp 229533.I1RZN1
229533.I1RM26 pp 229533.I1RNG7
229533.I1RM26 pp 229533.I1RW76
229533.I1RM26 pp 229533.I1S9C6
229533.I1RM26 pp 229533.I1RNW6
229533.I1RM26 pp 229533.I1RPD6
229533.I1RM26 pp 229533.I1RXA9
229533.I1RM26 pp 229533.I1RNB4
229533.I1RM26 pp 229533.I1S3G4
229533.I1RM26 pp 229533.I1RXB2
229533.I1RM26 pp 229533.I1RNG4
229533.I1RM26 pp 229533.I1RMG6
229533.I1RM26 pp 229533.I1RM69
229533.I1RM26 pp 229533.I1RZZ9
229533.I1RM26 pp 229533.I1RZ29
229533.I1RM26 pp 229533.I1S4F4
229533.I1RM26 pp 229533.I1S5L5
229533.I1RM26 pp 229533.I1RZ20
229533.I1RM26 pp 229533.V6R3J1
229533.I1RM26 pp 229533.I1S4B6
229533.I1RM26 pp 229533.I1RX72
229533.I1RM26 pp 229533.I1RW43
229533.I1RM26 pp 229533.I1RSK5
229533.I1RM26 pp 229533.I1S3X7
229533.I1RM26 pp 229533.I1RZ52
229533.I1RM26 pp 229533.I1SAJ3
229533.I1RM26 pp 229533.I1S6P6
229533.I1RM26 pp 229533.I1RNV4
229533.I1RM26 pp 229533.I1RVW0
229533.I1RM26 pp 229533.V6RN31
229533.I1RM26 pp 229533.I1RVZ3
229533.I1RM26 pp 229533.I1SAN9
229533.I1RM26 pp 229533.I1RZQ6
229533.I1RM26 pp 229533.I1S0S5
229533.I1RM26 pp 229533.I1S132
229533.I1RM26 pp 229533.I1RZ21

154615 229533.I1RM26 pp 229533.I1RZQ1
154616 229533.I1RM26 pp 229533.I1RVR2
154617 229533.I1RM26 pp 229533.I1RSZ7
154618 229533.I1RM26 pp 229533.I1RZ86
154619 229533.I1RM26 pp 229533.I1S8R4
154620 229533.I1RM26 pp 229533.I1S2C4
154621 229533.I1RM26 pp 229533.I1S685
154622 229533.I1RM26 pp 229533.V6RSF3
154623 229533.I1RM26 pp 229533.I1RUP5
154624 229533.I1RM26 pp 229533.I1RM37
154625 229533.I1RM26 pp 229533.I1RNB5
154626 229533.I1RM26 pp 229533.I1S3V2
154627 229533.I1RM26 pp 229533.I1S147
154628 229533.I1RM26 pp 229533.I1RNW2
154629 229533.I1RM26 pp 229533.I1S684
154630 229533.I1RM26 pp 229533.I1RXY7
154631 229533.I1RM26 pp 229533.V6R9T8
154632 229533.I1RM26 pp 229533.I1RSX9
154633 229533.I1RM26 pp 229533.I1RSL5
154634 229533.I1RM26 pp 229533.I1RWM0
154635 229533.I1RM26 pp 229533.I1S9Z4
154636 229533.I1RM26 pp 229533.I1RM68
154637 229533.I1RM26 pp 229533.V6RLM7
154638 229533.I1RM26 pp 229533.V6RS91
154639 229533.I1RM26 pp 229533.V6R042
154640 229533.I1RM26 pp 229533.I1RS77
154641 229533.I1RM26 pp 229533.I1S8R2
154642 229533.I1RM26 pp 229533.I1RQ30
154643 229533.I1RM26 pp 229533.I1RPK0
154644 229533.I1RM26 pp 229533.I1RMK4
154645 229533.I1RM26 pp 229533.I1S2H3
154646 229533.I1RM26 pp 229533.I1RYJ7
154647 229533.I1RM26 pp 229533.I1RYJ6
154648 229533.I1RM26 pp 229533.I1RY74
154649 229533.I1RM26 pp 229533.I1RME6
154650 229533.I1RM26 pp 229533.I1RWL3
154651 229533.I1RM26 pp 229533.I1S0I3
154652 229533.I1RM26 pp 229533.I1RZ70
154653 229533.I1RM26 pp 229533.I1RQE1
154654 229533.I1RM26 pp 229533.I1RSY2
154655 229533.I1RM26 pp 229533.I1S3L2
154656 229533.I1RM26 pp 229533.I1RP86
154657 229533.I1RM26 pp 229533.I1RRY1
154658 229533.I1RM26 pp 229533.I1RUV4
154659 229533.I1RM26 pp 229533.I1RQ60
154660 229533.I1RM26 pp 229533.I1RYM6
154661 229533.I1RM26 pp 229533.I1RYX6
154662 229533.I1RM26 pp 229533.I1RXY4
154663 229533.I1RM26 pp 229533.I1RY49
154664 229533.I1RM26 pp 229533.I1S1D4
154665 229533.I1RM26 pp 229533.I1RMH9
154666 229533.I1RM26 pp 229533.I1RQ14
154667 229533.I1RM26 pp 229533.I1RMU7
154668 229533.I1RM26 pp 229533.I1RPW2
154669 229533.I1RM26 pp 229533.I1RPE7
154670 229533.I1RM26 pp 229533.I1RZM4
154671 229533.I1RM26 pp 229533.I1S8E0
154672 229533.I1RM26 pp 229533.I1S0K0
154673 229533.I1RM26 pp 229533.I1RQE8
154674 229533.I1RM26 pp 229533.I1S241
154675 229533.I1RM26 pp 229533.I1RVX9
154676 229533.I1RM26 pp 229533.I1RYQ4
154677 229533.I1RM26 pp 229533.I1RNW1
154678 229533.I1RM26 pp 229533.I1S686
154679 229533.I1RM26 pp 229533.I1RMM8
154680 229533.I1RM26 pp 229533.V6RGB5
154681 229533.I1RM26 pp 229533.I1RTG4
154682 229533.I1RM26 pp 229533.I1S4Q4
154683 229533.I1RM26 pp 229533.I1RR00
154684 229533.I1RM26 pp 229533.I1S1W8
154685 229533.I1RM26 pp 229533.I1RYU5
154686 229533.I1RM26 pp 229533.V6RQW4
154687 229533.I1RM26 pp 229533.I1RRS8

154688 229533.I1RM26 pp 229533.I1S029
154689 229533.I1RM26 pp 229533.I1S019
154690 229533.I1RM26 pp 229533.I1RQP2
154691 229533.I1RM26 pp 229533.I1RMJ4
154692 229533.I1RM26 pp 229533.I1RUI6
154693 229533.I1RM26 pp 229533.V6R9Z3
154694 229533.I1RM26 pp 229533.I1S4D0
154695 229533.I1RM26 pp 229533.I1RWM4
154696 229533.I1RM26 pp 229533.I1RQE0
154697 229533.I1RM26 pp 229533.I1RPR1
154698 229533.I1RM26 pp 229533.I1S0K2
154699 229533.I1RM26 pp 229533.V6RY56
154700 229533.I1RM26 pp 229533.I1S2C8
154701 229533.I1RM26 pp 229533.I1RT09
154702 229533.I1RM26 pp 229533.I1S0A0
154703 229533.I1RM26 pp 229533.I1RXS0
154704 229533.I1RM26 pp 229533.V6RK96
154705 229533.I1RM26 pp 229533.I1S2M8
154706 229533.I1RM26 pp 229533.I1RZK8
154707 229533.I1RM26 pp 229533.I1RX59
154708 229533.I1RM26 pp 229533.I1RMG7
154709 229533.I1RM26 pp 229533.I1RW69
154710 229533.I1RM26 pp 229533.I1RNH4
154711 229533.I1RM26 pp 229533.I1RTU0
154712 229533.I1RM26 pp 229533.I1S183
154713 229533.I1RM26 pp 229533.I1RRM1
154714 229533.I1RM26 pp 229533.I1RPW1
154715 229533.I1RM26 pp 229533.I1RN80
154716 229533.I1RM26 pp 229533.I1RMW4
154717 229533.I1RM26 pp 229533.I1RU76
154718 229533.I1S128 pp 229533.Q4IEV5
154719 229533.I1S128 pp 229533.I1S8S9
154720 229533.I1S128 pp 229533.Q4I5G1
154721 229533.I1RSS1 pp 229533.I1RY44
154722 229533.I1RSS1 pp 229533.I1RXF0
154723 229533.I1RSS1 pp 229533.I1RVN2
154724 229533.I1RSS1 pp 229533.I1RWT6
154725 229533.I1RSS1 pp 229533.I1RZK4
154726 229533.I1RSS1 pp 229533.Q4IEA7
154727 229533.I1RSS1 pp 229533.I1S2K7
154728 229533.I1RSS1 pp 229533.I1RWK0
154729 229533.I1RSS1 pp 229533.V6QUV7
154730 229533.I1RSS1 pp 229533.I1S286
154731 229533.I1RSS1 pp 229533.V6RRF6
154732 229533.I1RSS1 pp 229533.I1RYL5
154733 229533.I1RSS1 pp 229533.I1S296
154734 229533.I1RSS1 pp 229533.Q4I785
154735 229533.I1RSS1 pp 229533.I1S2B6
154736 229533.I1RSS1 pp 229533.I1S2E9
154737 229533.I1RSS1 pp 229533.I1RVA8
154738 229533.I1RSS1 pp 229533.I1RX77
154739 229533.I1RSS1 pp 229533.Q4IR87
154740 229533.I1RSS1 pp 229533.I1RV66
154741 229533.I1RSS1 pp 229533.I1RTG0
154742 229533.I1RSS1 pp 229533.I1S0X7
154743 229533.I1RSS1 pp 229533.I1RST8
154744 229533.I1RSS1 pp 229533.I1RXH6
154745 229533.A0A0E0RV96 pp 229533.A0A0E0SC46
154746 229533.A0A0E0RV96 pp 229533.I1RAE1
154747 229533.A0A0E0RV96 pp 229533.I1S0B6
154748 229533.A0A0E0RV96 pp 229533.I1RUG0
154749 229533.A0A0E0RV96 pp 229533.I1RMW7
154750 229533.A0A0E0RV96 pp 229533.I1RCI3
154751 229533.A0A0E0RV96 pp 229533.I1RK96
154752 229533.A0A0E0RV96 pp 229533.I1S524
154753 229533.A0A0E0RV96 pp 229533.I1RFN1
154754 229533.A0A0E0RV96 pp 229533.I1RBT5
154755 229533.A0A0E0RV96 pp 229533.I1S1C6
154756 229533.A0A0E0RV96 pp 229533.I1RLU0
154757 229533.A0A0E0RV96 pp 229533.I1RAD1
154758 229533.A0A0E0RV96 pp 229533.I1RML9
154759 229533.A0A0E0RV96 pp 229533.I1RCA7
154760 229533.A0A0E0RV96 pp 229533.A0A0E0S1T0

229533.A0A0E0RV96	pp	229533.I1RCS2
229533.A0A0E0RV96	pp	229533.I1RAC1
229533.A0A0E0RV96	pp	229533.I1S5S2
229533.A0A0E0RV96	pp	229533.I1RJV2
229533.A0A0E0RV96	pp	229533.A0A1C3YKS0
229533.A0A0E0RV96	pp	229533.I1RRQ4
229533.A0A0E0RV96	pp	229533.V6QVD5
229533.A0A0E0RV96	pp	229533.V6RY33
229533.A0A0E0RV96	pp	229533.I1RB27
229533.A0A0E0RV96	pp	229533.Q4I7F9
229533.A0A0E0RV96	pp	229533.I1RZZ1
229533.A0A0E0RV96	pp	229533.V6RIS2
229533.A0A0E0RV96	pp	229533.I1RQD8
229533.I1RFH2	pp	229533.I1S4Q7
229533.I1RFH2	pp	229533.I1RMN2
229533.I1RFH2	pp	229533.I1RQF7
229533.I1RFH2	pp	229533.I1S4Z5
229533.I1RFH2	pp	229533.I1RZ18
229533.I1R9X4	pp	229533.V6RN69
229533.I1R9X4	pp	229533.I1RKZ4
229533.I1R9X4	pp	229533.I1RFY0
229533.I1R9X4	pp	229533.V6R7V8
229533.I1R9X4	pp	229533.I1RPA4
229533.I1R9X4	pp	229533.I1RPY0
229533.I1R9X4	pp	229533.V6QUG0
229533.I1R9X4	pp	229533.I1S4S1
229533.I1R9X4	pp	229533.I1RR65
229533.I1R9X4	pp	229533.I1S8E9
229533.I1R9X4	pp	229533.I1RFL7
229533.I1R9X4	pp	229533.I1S2A1
229533.I1R9X4	pp	229533.I1RT09
229533.I1R9X4	pp	229533.V6QWB1
229533.I1R9X4	pp	229533.Q4IB65
229533.I1R9X4	pp	229533.I1RQH5
229533.I1R9X4	pp	229533.I1RS29
229533.I1R9X4	pp	229533.I1RT75
229533.I1R9X4	pp	229533.I1RSH0
229533.I1R9X4	pp	229533.I1S0C2
229533.I1R9X4	pp	229533.I1RAB6
229533.I1R9X4	pp	229533.I1RYI9
229533.I1S029	pp	229533.I1S2C4
229533.I1S029	pp	229533.I1S4F4
229533.I1S029	pp	229533.I1S3G4
229533.I1S029	pp	229533.I1S2H3
229533.I1S029	pp	229533.Q4HXF0
229533.I1S029	pp	229533.I1S3K8
229533.I1S029	pp	229533.I1S1W8
229533.I1S029	pp	229533.I1S3K6
229533.I1S029	pp	229533.O74638
229533.I1S029	pp	229533.V6RQW4
229533.I1S1B2	pp	229533.I1S5R6
229533.I1S1B2	pp	229533.Q4IJK2
229533.I1S1B2	pp	229533.I1S5R1
229533.I1S1B2	pp	229533.I1S1W9
229533.I1S1B2	pp	229533.I1S821
229533.I1S1B2	pp	229533.V6RCZ8
229533.I1S1B2	pp	229533.I1S2B8
229533.I1S1B2	pp	229533.V6R9Z0
229533.I1S1B2	pp	229533.I1S4S7
229533.I1S4D0	pp	229533.V6R1K4
229533.I1S4D0	pp	229533.V6RQW4
229533.I1S4D0	pp	229533.V6QU99
229533.I1S4D0	pp	229533.V6RAM6
229533.I1S4D0	pp	229533.Q4HXF0
229533.I1S4D0	pp	229533.I1S4F4
229533.I1S4D0	pp	229533.V6R2Z2
229533.I1S4D0	pp	229533.V6RY56
229533.I1S4D0	pp	229533.I1SAI4
229533.I1S4D0	pp	229533.V6RM02
229533.I1S4D0	pp	229533.O74638
229533.I1S4D0	pp	229533.V6R5D2
229533.I1S4D0	pp	229533.V6R0W1
229533.I1S4D0	pp	229533.V6R756

229533.I1RDW0	pp	229533.I1RPR9
229533.I1RBN6	pp	229533.I1S4Q7
229533.I1RBN6	pp	229533.I1RQF7
229533.I1RBN6	pp	229533.I1RMN2
229533.I1RBN6	pp	229533.I1RDA8
229533.I1RBN6	pp	229533.I1RFH2
229533.A0A0E0RQT8	pp	229533.A0A1C3YJ07
229533.A0A0E0RQT8	pp	229533.I1RM77
229533.A0A0E0RQT8	pp	229533.A0A1C3YHY5
229533.A0A0E0RQT8	pp	229533.I1RBN0
229533.A0A0E0RQT8	pp	229533.I1RDK7
229533.A0A0E0RQT8	pp	229533.V6RDP1
229533.A0A0E0RQT8	pp	229533.I1RC37
229533.A0A0E0RQT8	pp	229533.I1RRN4
229533.A0A0E0RQT8	pp	229533.I1RHC6
229533.A0A0E0RQT8	pp	229533.I1RMG4
229533.A0A0E0RQT8	pp	229533.A0A1C3YMX3
229533.A0A0E0RQT8	pp	229533.I1RS10
229533.A0A0E0RQT8	pp	229533.Q4I8Q4
229533.A0A0E0RQT8	pp	229533.I1RRC4
229533.A0A0E0RQT8	pp	229533.I1RFV2
229533.A0A0E0RQT8	pp	229533.I1RDX7
229533.A0A0E0RQT8	pp	229533.I1RK45
229533.A0A0E0RQT8	pp	229533.A0A1C3YI43
229533.A0A0E0RQT8	pp	229533.I1RCY0
229533.A0A0E0RQT8	pp	229533.I1RM23
229533.A0A0E0RQT8	pp	229533.A0A1C3YI65
229533.A0A0E0RQT8	pp	229533.A0A1C3YLC3
229533.A0A0E0RQT8	pp	229533.I1RN31
229533.A0A0E0RQT8	pp	229533.I1RX67
229533.A0A0E0RQT8	pp	229533.I1RCJ4
229533.A0A0E0RQT8	pp	229533.I1RKT7
229533.A0A0E0RQT8	pp	229533.I1RE79
229533.A0A0E0RQT8	pp	229533.A0A0E0SE71
229533.A0A0E0RQT8	pp	229533.A0A0E0SNH8
229533.A0A0E0RQT8	pp	229533.A0A1C3YKJ5
229533.A0A0E0RQT8	pp	229533.I1RTI3
229533.A0A0E0RQT8	pp	229533.V6RJ21
229533.A0A0E0RQT8	pp	229533.I1RVS1
229533.A0A0E0RQT8	pp	229533.I1RW16
229533.A0A0E0RQT8	pp	229533.I1S0R1
229533.A0A0E0RQT8	pp	229533.I1RCV6
229533.A0A0E0RQT8	pp	229533.I1RMF9
229533.A0A0E0RQT8	pp	229533.A0A0E0SAK0
229533.A0A0E0RQT8	pp	229533.A0A1C3YII1
229533.A0A0E0RQT8	pp	229533.A0A1C3YN78
229533.A0A0E0RQT8	pp	229533.A0A0E0S5J3
229533.A0A0E0RQT8	pp	229533.I1S7X9
229533.A0A0E0RQT8	pp	229533.I1RB87
229533.A0A0E0RQT8	pp	229533.I1RRH2
229533.A0A0E0RQT8	pp	229533.I1RWN0
229533.A0A0E0RQT8	pp	229533.A0A0E0S2K0
229533.A0A0E0RQT8	pp	229533.I1RW15
229533.A0A0E0RQT8	pp	229533.I1RCV0
229533.A0A0E0RQT8	pp	229533.I1RAF3
229533.A0A0E0RQT8	pp	229533.I1RN67
229533.A0A0E0RQT8	pp	229533.I1RAM7
229533.A0A0E0RQT8	pp	229533.A0A1C3YJK9
229533.A0A0E0RQT8	pp	229533.I1RPX7
229533.A0A0E0RQT8	pp	229533.I1RZN1
229533.A0A0E0RQT8	pp	229533.A0A1C3YLR9
229533.A0A0E0RQT8	pp	229533.I1RWJ1
229533.A0A0E0RQT8	pp	229533.I1RHF8
229533.A0A0E0RQT8	pp	229533.I1RMD3
229533.A0A0E0RQT8	pp	229533.I1REI3
229533.A0A0E0RQT8	pp	229533.A0A1C3YJZ0
229533.A0A0E0RQT8	pp	229533.I1RC88
229533.A0A0E0RQT8	pp	229533.I1RFF2
229533.A0A0E0RQT8	pp	229533.I1RCU9
229533.A0A0E0RQT8	pp	229533.I1RM94
229533.A0A0E0RQT8	pp	229533.I1RE36
229533.A0A0E0RQT8	pp	229533.I1S0P4
229533.A0A0E0RQT8	pp	229533.I1RZP2

229533.A0A0E0RQT8 pp 229533.I1RST8
229533.A0A0E0RQT8 pp 229533.I1REI4
229533.A0A0E0RQT8 pp 229533.I1REI2
229533.A0A0E0RQT8 pp 229533.I1RXX1
229533.A0A0E0RQT8 pp 229533.I1RKA9
229533.A0A0E0RQT8 pp 229533.I1S111
229533.A0A0E0RQT8 pp 229533.I1RCI0
229533.A0A0E0RQT8 pp 229533.I1RPD3
229533.A0A0E0RQT8 pp 229533.I1RCJ2
229533.A0A0E0RQT8 pp 229533.I1RR49
229533.A0A0E0RQT8 pp 229533.I1RLX6
229533.A0A0E0RQT8 pp 229533.I1RZU7
229533.A0A0E0RQT8 pp 229533.I1S1Z9
229533.A0A0E0RQT8 pp 229533.I1RZT6
229533.A0A0E0RQT8 pp 229533.A0A0E0RRE1
229533.A0A0E0RQT8 pp 229533.I1RJ78
229533.A0A0E0RQT8 pp 229533.I1RNS2
229533.A0A0E0RQT8 pp 229533.I1RLY6
229533.A0A0E0RQT8 pp 229533.A0A1C3YHU9
229533.A0A0E0RQT8 pp 229533.I1RCU8
229533.A0A0E0RQT8 pp 229533.I1RMU7
229533.A0A0E0RQT8 pp 229533.A0A1C3YN48
229533.A0A0E0RQT8 pp 229533.A0A1C3YN32
229533.A0A0E0RQT8 pp 229533.I1RDB1
229533.A0A0E0RQT8 pp 229533.I1RG76
229533.A0A0E0RQT8 pp 229533.A0A0E0RSR0
229533.A0A0E0RQT8 pp 229533.Q4HXF6
229533.A0A0E0RQT8 pp 229533.I1RM11
229533.A0A0E0RQT8 pp 229533.A0A1C3YIX3
229533.A0A0E0RQT8 pp 229533.I1RQN8
229533.A0A0E0RQT8 pp 229533.I1RHJ2
229533.A0A0E0RQT8 pp 229533.A0A1C3YIB0
229533.A0A0E0RQT8 pp 229533.I1RER6
229533.A0A0E0RQT8 pp 229533.A0A1C3YLQ4
229533.A0A0E0RQT8 pp 229533.A0A1C3YJX9
229533.A0A0E0RQT8 pp 229533.I1RZK9
229533.A0A0E0RQT8 pp 229533.I1RT76
229533.A0A0E0RQT8 pp 229533.I1RX64
229533.A0A0E0RQT8 pp 229533.I1RQ06
229533.A0A0E0RQT8 pp 229533.I1RZQ6
229533.A0A0E0RQT8 pp 229533.I1RBC8
229533.A0A0E0RQT8 pp 229533.I1S6H4
229533.A0A0E0RQT8 pp 229533.I1S274
229533.A0A0E0RQT8 pp 229533.A0A1C3YLB2
229533.A0A0E0RQT8 pp 229533.I1RWV4
229533.A0A0E0RQT8 pp 229533.I1RNG4
229533.A0A0E0RQT8 pp 229533.I1RV85
229533.A0A0E0RQT8 pp 229533.I1S8X0
229533.A0A0E0RQT8 pp 229533.A0A0E0RRE0
229533.A0A0E0RQT8 pp 229533.A0A1C3YIZ4
229533.I1RPF1 pp 229533.I1S5R1
229533.I1RPF1 pp 229533.I1RSC7
229533.I1RPF1 pp 229533.I1RW61
229533.I1RPF1 pp 229533.I1RRI1
229533.I1RPF1 pp 229533.I1RUG3
229533.I1RPF1 pp 229533.I1RQE7
229533.I1RPF1 pp 229533.V6RCZ8
229533.I1RPF1 pp 229533.I1RQ33
229533.I1RPF1 pp 229533.I1RZB2
229533.I1RPF1 pp 229533.I1S5B0
229533.I1RPF1 pp 229533.I1S0F1
229533.I1RPF1 pp 229533.I1RQG5
229533.I1RPF1 pp 229533.I1RR24
229533.I1RPF1 pp 229533.I1RT05
229533.I1RJJ4 pp 229533.I1RW06
229533.I1RJJ4 pp 229533.I1RMM8
229533.I1RF71 pp 229533.V6QUE6
229533.I1RF71 pp 229533.I1RYW3
229533.I1RF71 pp 229533.I1RWX5
229533.I1RF71 pp 229533.I1RW71
229533.I1RF71 pp 229533.I1RRT8
229533.I1RIS3 pp 229533.I1S3V2
229533.I1RIS3 pp 229533.Q4HWE2

229533.I1RIS3 pp 229533.V6RIS2
229533.I1RIS3 pp 229533.I1S289
229533.I1RIS3 pp 229533.I1RZS7
229533.I1RIS3 pp 229533.I1RWB5
229533.I1RIS3 pp 229533.I1RND6
229533.I1RIS3 pp 229533.I1RXV3
229533.I1RIS3 pp 229533.O74638
229533.I1RIS3 pp 229533.I1S8E2
229533.I1RIS3 pp 229533.I1RKE7
229533.I1RIS3 pp 229533.I1RZV6
229533.I1RIS3 pp 229533.I1RZ40
229533.I1RIS3 pp 229533.I1RZG9
229533.I1RIS3 pp 229533.Q4HW67
229533.I1RIS3 pp 229533.I1S524
229533.I1RIS3 pp 229533.I1RNI4
229533.I1RIS3 pp 229533.I1RK83
229533.I1RIS3 pp 229533.Q4I613
229533.I1RIS3 pp 229533.I1S5S2
229533.I1RIS3 pp 229533.I1RMW7
229533.I1RIS3 pp 229533.I1S6E5
229533.I1RIS3 pp 229533.I1S6F1
229533.I1RIS3 pp 229533.I1RNE7
229533.I1RIS3 pp 229533.I1RWH5
229533.I1RIS3 pp 229533.I1RKF8
229533.I1RIS3 pp 229533.I1RYB9
229533.I1RIS3 pp 229533.I1RYI6
229533.I1RIS3 pp 229533.I1RRU9
229533.I1RVX9 pp 229533.I1S241
229533.I1RVX9 pp 229533.I1RYX6
229533.I1RVX9 pp 229533.I1RZM4
229533.I1RVX9 pp 229533.I1RYJ6
229533.I1RVX9 pp 229533.I1S1D4
229533.I1RVX9 pp 229533.I1RYM6
229533.I1RVX9 pp 229533.I1RWL3
229533.I1RVX9 pp 229533.I1S1W8
229533.I1RVX9 pp 229533.V6RS91
229533.I1RVX9 pp 229533.I1RYU5
229533.I1RVX9 pp 229533.I1S3L2
229533.I1RVX9 pp 229533.I1RXY4
229533.I1RVX9 pp 229533.I1RYJ7
229533.I1RVX9 pp 229533.I1S029
229533.I1RVX9 pp 229533.I1S686
229533.I1RVX9 pp 229533.I1S183
229533.I1RVX9 pp 229533.I1RZK8
229533.I1RVX9 pp 229533.I1RYQ4
229533.I1RVX9 pp 229533.I1S0K0
229533.I1RVX9 pp 229533.I1RY49
229533.I1RVX9 pp 229533.I1RW69
229533.I1RVX9 pp 229533.I1RWM4
229533.I1RVX9 pp 229533.I1S8E0
229533.I1RVX9 pp 229533.I1S019
229533.I1RVX9 pp 229533.V6RK96
229533.I1RVX9 pp 229533.I1RX59
229533.I1RVX9 pp 229533.I1S0A0
229533.I1RVX9 pp 229533.I1S4Q4
229533.I1RVX9 pp 229533.I1S2M8
229533.I1RVX9 pp 229533.V6RGB5
229533.I1RVX9 pp 229533.I1S4D0
229533.I1RVX9 pp 229533.I1S2C8
229533.I1RVX9 pp 229533.I1S0K2
229533.I1RVX9 pp 229533.I1RXS0
229533.I1RVX9 pp 229533.V6RQW4
229533.I1RVX9 pp 229533.I1S4F4
229533.I1RVX9 pp 229533.I1RWS4
229533.I1RVX9 pp 229533.V6RM66
229533.I1RVX9 pp 229533.I1RZ20
229533.I1RVX9 pp 229533.V6RY56
229533.I1RVX9 pp 229533.V6R9Z3
229533.I1RVX9 pp 229533.I1S4B7
229533.I1RVX9 pp 229533.I1S299
229533.I1RVX9 pp 229533.V6R3J1
229533.I1RVX9 pp 229533.I1S5L5
229533.I1RVX9 pp 229533.I1RX72

155053 229533.I1RVX9 pp 229533.I1S0S5
155054 229533.I1RVX9 pp 229533.I1RXA9
155055 229533.I1RVX9 pp 229533.I1RW76
155056 229533.I1RVX9 pp 229533.I1SAN9
155057 229533.I1RVX9 pp 229533.I1RZ86
155058 229533.I1RVX9 pp 229533.I1RZN1
155059 229533.I1RVX9 pp 229533.I1RXG6
155060 229533.I1RVX9 pp 229533.I1RZ21
155061 229533.I1RVX9 pp 229533.I1RVZ3
155062 229533.I1RVX9 pp 229533.I1RZQ1
155063 229533.I1RVX9 pp 229533.I1RZZ9
155064 229533.I1RVX9 pp 229533.I1RZ52
155065 229533.I1RVX9 pp 229533.I1S8R4
155066 229533.I1RVX9 pp 229533.I1S3X7
155067 229533.I1RVX9 pp 229533.V6R042
155068 229533.I1RVX9 pp 229533.I1RZ29
155069 229533.I1RVX9 pp 229533.I1S3G4
155070 229533.I1RVX9 pp 229533.I1S3V2
155071 229533.I1RVX9 pp 229533.V6R9T8
155072 229533.I1RVX9 pp 229533.I1S9C6
155073 229533.I1RVX9 pp 229533.I1S6P6
155074 229533.I1RVX9 pp 229533.I1RXB2
155075 229533.I1RVX9 pp 229533.I1S685
155076 229533.I1RVX9 pp 229533.I1S4B6
155077 229533.I1RVX9 pp 229533.I1S9Y1
155078 229533.I1RVX9 pp 229533.I1RW43
155079 229533.I1RVX9 pp 229533.O74638
155080 229533.I1RVX9 pp 229533.I1S684
155081 229533.I1RVX9 pp 229533.I1S147
155082 229533.I1RVX9 pp 229533.I1S8R2
155083 229533.I1RVX9 pp 229533.I1RZ70
155084 229533.I1RVX9 pp 229533.V6RLM7
155085 229533.I1RVX9 pp 229533.I1RY74
155086 229533.I1RVX9 pp 229533.V6RSF3
155087 229533.I1RVX9 pp 229533.I1RXY7
155088 229533.I1RVX9 pp 229533.I1S9Z4
155089 229533.I1RVX9 pp 229533.I1S2C4
155090 229533.I1RVX9 pp 229533.I1RZQ6
155091 229533.I1RVX9 pp 229533.I1S0I3
155092 229533.I1RVX9 pp 229533.V6RN31
155093 229533.I1RVX9 pp 229533.I1S132
155094 229533.I1RVX9 pp 229533.I1RWM0
155095 229533.I1RVX9 pp 229533.I1S2H3
155096 229533.I1RVX9 pp 229533.I1SAJ3
155097 229533.I1RN48 pp 229533.I1S8E2
155098 229533.I1RN48 pp 229533.I1RXV3
155099 229533.I1RN48 pp 229533.I1RND6
155100 229533.I1RN48 pp 229533.I1RZG9
155101 229533.I1RN48 pp 229533.I1S5S2
155102 229533.I1RN48 pp 229533.I1RZV6
155103 229533.I1RN48 pp 229533.I1S524
155104 229533.I1RN48 pp 229533.Q4I613
155105 229533.I1RN48 pp 229533.O74638
155106 229533.I1RN48 pp 229533.I1RZ40
155107 229533.I1RN48 pp 229533.I1S3V2
155108 229533.I1RN48 pp 229533.I1RWB5
155109 229533.I1RN48 pp 229533.V6RIS2
155110 229533.I1RN48 pp 229533.Q4HW67
155111 229533.I1RN48 pp 229533.I1RNI4
155112 229533.I1RN48 pp 229533.I1RZS7
155113 229533.I1RN48 pp 229533.I1RWH5
155114 229533.I1RN48 pp 229533.I1RNE7
155115 229533.I1RN48 pp 229533.I1S289
155116 229533.I1RN48 pp 229533.I1RRU9
155117 229533.I1RN48 pp 229533.I1RYB9
155118 229533.I1RN48 pp 229533.Q4HWE2
155119 229533.I1RN48 pp 229533.I1S6E5
155120 229533.I1RN48 pp 229533.I1S6F1
155121 229533.I1RN48 pp 229533.I1RYI6
155122 229533.Q4IR87 pp 229533.V6QZS3
155123 229533.Q4IR87 pp 229533.V6QVL9
155124 229533.A0A1C3YJR3 pp 229533.A0A1C3YL39
155125 229533.A0A1C3YJR3 pp 229533.I1RFN1

155126	229533.A0A1C3YJR3	pp	229533.I1RGA4
155127	229533.A0A1C3YJR3	pp	229533.I1RBC7
155128	229533.A0A1C3YJR3	pp	229533.I1RC82
155129	229533.A0A1C3YJR3	pp	229533.A0A1C3YM43
155130	229533.A0A1C3YJR3	pp	229533.I1S1B6
155131	229533.A0A1C3YJR3	pp	229533.A0A1C3YLA6
155132	229533.A0A1C3YJR3	pp	229533.I1RJL4
155133	229533.A0A1C3YJR3	pp	229533.I1RSX0
155134	229533.A0A1C3YJR3	pp	229533.I1RW80
155135	229533.A0A1C3YJR3	pp	229533.A0A1C3YKE4
155136	229533.A0A1C3YJR3	pp	229533.V6R3J1
155137	229533.A0A1C3YJR3	pp	229533.Q4IMD1
155138	229533.A0A1C3YJR3	pp	229533.I1RRT2
155139	229533.A0A1C3YJR3	pp	229533.I1RQS5
155140	229533.A0A1C3YJR3	pp	229533.A0A1C3YN63
155141	229533.A0A1C3YJR3	pp	229533.I1RJL3
155142	229533.A0A1C3YJR3	pp	229533.I1R9N1
155143	229533.A0A1C3YJR3	pp	229533.Q4I5W5
155144	229533.A0A1C3YJR3	pp	229533.I1RNG4
155145	229533.A0A1C3YJR3	pp	229533.I1RRM8
155146	229533.A0A1C3YJR3	pp	229533.A0A1C3YLK8
155147	229533.A0A1C3YJR3	pp	229533.I1RS43
155148	229533.A0A1C3YJR3	pp	229533.I1RTH2
155149	229533.A0A1C3YJR3	pp	229533.I1RRP3
155150	229533.A0A1C3YJR3	pp	229533.I1S0L4
155151	229533.A0A1C3YJR3	pp	229533.Q4IAS1
155152	229533.A0A1C3YJR3	pp	229533.I1RS30
155153	229533.A0A1C3YJR3	pp	229533.I1S3G4
155154	229533.A0A1C3YJR3	pp	229533.Q4IJ11
155155	229533.A0A1C3YJR3	pp	229533.I1RN67
155156	229533.A0A1C3YJR3	pp	229533.I1RRJ8
155157	229533.A0A1C3YJR3	pp	229533.I1RD64
155158	229533.A0A1C3YJR3	pp	229533.I1RFL7
155159	229533.A0A1C3YJR3	pp	229533.A0A1C3YM67
155160	229533.A0A1C3YJR3	pp	229533.Q4HXF0
155161	229533.A0A1C3YJR3	pp	229533.I1RS20
155162	229533.A0A1C3YJR3	pp	229533.I1RYK0
155163	229533.A0A1C3YJR3	pp	229533.I1S2H4
155164	229533.A0A1C3YJR3	pp	229533.I1RGF6
155165	229533.A0A1C3YJR3	pp	229533.A0A1C3YLF5
155166	229533.A0A1C3YJR3	pp	229533.I1RYI5
155167	229533.A0A1C3YJR3	pp	229533.A0A1C3YMK1
155168	229533.A0A1C3YJR3	pp	229533.I1RQ88
155169	229533.A0A1C3YJR3	pp	229533.I1RTD5
155170	229533.A0A1C3YJR3	pp	229533.I1S5R1
155171	229533.A0A1C3YJR3	pp	229533.I1RA11
155172	229533.A0A1C3YJR3	pp	229533.I1S8P5
155173	229533.A0A1C3YJR3	pp	229533.I1RRT8
155174	229533.A0A1C3YJR3	pp	229533.I1RPP3
155175	229533.A0A1C3YJR3	pp	229533.I1RSZ3
155176	229533.A0A1C3YJR3	pp	229533.A0A1C3YLP8
155177	229533.A0A1C3YJR3	pp	229533.V6RAC9
155178	229533.A0A1C3YJR3	pp	229533.I1RFT1
155179	229533.A0A1C3YJR3	pp	229533.O74638
155180	229533.A0A1C3YJR3	pp	229533.I1RT40
155181	229533.A0A1C3YJR3	pp	229533.I1RSV2
155182	229533.A0A1C3YJR3	pp	229533.I1RAF8
155183	229533.A0A1C3YJR3	pp	229533.I1RXC2
155184	229533.A0A1C3YJR3	pp	229533.I1RFL3
155185	229533.A0A1C3YJR3	pp	229533.I1RV91
155186	229533.A0A1C3YJR3	pp	229533.I1S9Y1
155187	229533.A0A1C3YJR3	pp	229533.I1RKJ6
155188	229533.A0A1C3YJR3	pp	229533.Q4I830
155189	229533.A0A1C3YJR3	pp	229533.I1RKL3
155190	229533.A0A1C3YJR3	pp	229533.I1S1F3
155191	229533.A0A1C3YJR3	pp	229533.I1S253
155192	229533.A0A1C3YJR3	pp	229533.I1RVZ2
155193	229533.A0A1C3YJR3	pp	229533.I1RB25
155194	229533.A0A1C3YJR3	pp	229533.I1RTG7
155195	229533.A0A1C3YJR3	pp	229533.I1S2G7
155196	229533.A0A1C3YJR3	pp	229533.I1RFW1
155197	229533.A0A1C3YJR3	pp	229533.I1RC24
155198	229533.A0A1C3YJR3	pp	229533.I1RZ39

229533.A0A1C3YJR3	pp	229533.I1RKB2
229533.A0A1C3YJR3	pp	229533.Q4HZ42
229533.A0A1C3YJR3	pp	229533.I1RTA1
229533.A0A1C3YJR3	pp	229533.I1S0J3
229533.A0A1C3YJR3	pp	229533.I1RS47
229533.A0A1C3YJR3	pp	229533.I1RZM2
229533.A0A1C3YJR3	pp	229533.A0A1C3YKM9
229533.A0A1C3YJR3	pp	229533.I1RVN1
229533.A0A1C3YJR3	pp	229533.I1RFT6
229533.A0A1C3YJR3	pp	229533.I1RC05
229533.A0A1C3YJR3	pp	229533.I1RRQ3
229533.A0A1C3YJR3	pp	229533.I1RE30
229533.A0A1C3YJR3	pp	229533.V6R283
229533.A0A1C3YJR3	pp	229533.I1RFG0
229533.A0A1C3YJR3	pp	229533.A0A1C3YKX5
229533.A0A1C3YJR3	pp	229533.I1RC66
229533.A0A1C3YJR3	pp	229533.I1S6Y8
229533.A0A1C3YJR3	pp	229533.Q4IF76
229533.A0A1C3YJR3	pp	229533.I1RC03
229533.A0A1C3YJR3	pp	229533.I1RPV3
229533.A0A1C3YJR3	pp	229533.I1RWI5
229533.A0A1C3YJR3	pp	229533.I1RRV1
229533.A0A1C3YJR3	pp	229533.A0A1C3YLS9
229533.A0A1C3YJR3	pp	229533.I1RZZ9
229533.A0A1C3YJR3	pp	229533.I1RFS3
229533.A0A1C3YJR3	pp	229533.I1RCR8
229533.A0A1C3YJR3	pp	229533.I1RSQ4
229533.A0A1C3YJR3	pp	229533.Q4I099
229533.A0A1C3YJR3	pp	229533.I1RVL8
229533.A0A1C3YJR3	pp	229533.Q4IN63
229533.A0A1C3YJR3	pp	229533.I1RSZ7
229533.A0A1C3YJR3	pp	229533.I1RNC1
229533.A0A1C3YJR3	pp	229533.I1RNQ6
229533.A0A1C3YJR3	pp	229533.I1SAN0
229533.A0A1C3YJR3	pp	229533.V6RGN5
229533.A0A1C3YJR3	pp	229533.I1RRR7
229533.A0A1C3YJR3	pp	229533.I1RUG3
229533.A0A1C3YJR3	pp	229533.Q4IPC8
229533.A0A1C3YJR3	pp	229533.I1RA52
229533.A0A1C3YJR3	pp	229533.I1RVW0
229533.A0A1C3YJR3	pp	229533.I1RBJ1
229533.A0A1C3YJR3	pp	229533.I1RAQ5
229533.A0A1C3YJR3	pp	229533.I1RRF7
229533.A0A1C3YJR3	pp	229533.I1S1W4
229533.A0A1C3YJR3	pp	229533.I1RBY4
229533.A0A1C3YJR3	pp	229533.I1RA84
229533.A0A1C3YJR3	pp	229533.I1S0R5
229533.A0A1C3YJR3	pp	229533.I1RRN6
229533.A0A1C3YJR3	pp	229533.I1S5R6
229533.A0A1C3YJR3	pp	229533.I1RT36
229533.A0A1C3YJR3	pp	229533.I1RE65
229533.A0A1C3YJR3	pp	229533.I1S2B8
229533.A0A1C3YJR3	pp	229533.I1RJZ7
229533.A0A1C3YJR3	pp	229533.V6QT89
229533.A0A1C3YJR3	pp	229533.I1RVY0
229533.A0A1C3YJR3	pp	229533.I1RC27
229533.A0A1C3YJR3	pp	229533.I1R9M0
229533.A0A1C3YJR3	pp	229533.I1RD22
229533.A0A1C3YJR3	pp	229533.I1RRN2
229533.A0A1C3YJR3	pp	229533.I1RMN6
229533.A0A1C3YJR3	pp	229533.I1RJW8
229533.A0A1C3YJR3	pp	229533.I1RSU2
229533.A0A1C3YJR3	pp	229533.I1RBR5
229533.A0A1C3YJR3	pp	229533.I1RM96
229533.A0A1C3YJR3	pp	229533.I1RZS6
229533.A0A1C3YJR3	pp	229533.I1RC33
229533.A0A1C3YJR3	pp	229533.I1RN04
229533.A0A1C3YJR3	pp	229533.I1RAP2
229533.A0A1C3YJR3	pp	229533.I1RCB7
229533.A0A1C3YJR3	pp	229533.I1RSQ3
229533.A0A1C3YJR3	pp	229533.V6R042
229533.A0A1C3YJR3	pp	229533.Q4I5U9
229533.A0A1C3YJR3	pp	229533.I1RR72

155272	229533.A0A1C3YJR3	pp	229533.A0A1C3YJZ2
155273	229533.A0A1C3YJR3	pp	229533.I1RC29
155274	229533.A0A1C3YJR3	pp	229533.I1RX24
155275	229533.A0A1C3YJR3	pp	229533.I1RBG9
155276	229533.A0A1C3YJR3	pp	229533.I1RRM3
155277	229533.A0A1C3YJR3	pp	229533.Q4HZB8
155278	229533.A0A1C3YJR3	pp	229533.I1RFP4
155279	229533.A0A1C3YJR3	pp	229533.I1RPU8
155280	229533.A0A1C3YJR3	pp	229533.Q4HTT1
155281	229533.A0A1C3YJR3	pp	229533.A0A1C3YN55
155282	229533.A0A1C3YJR3	pp	229533.I1RWF3
155283	229533.A0A1C3YJR3	pp	229533.V6RMW6
155284	229533.A0A1C3YJR3	pp	229533.I1RUG4
155285	229533.A0A1C3YJR3	pp	229533.Q4I624
155286	229533.A0A1C3YJR3	pp	229533.I1S1W9
155287	229533.A0A1C3YJR3	pp	229533.I1RXU0
155288	229533.A0A1C3YJR3	pp	229533.I1RSI6
155289	229533.A0A1C3YJR3	pp	229533.I1R9X7
155290	229533.A0A1C3YJR3	pp	229533.I1RNB8
155291	229533.A0A1C3YJR3	pp	229533.I1RNU6
155292	229533.A0A1C3YJR3	pp	229533.I1S0N5
155293	229533.A0A1C3YJR3	pp	229533.I1S296
155294	229533.A0A1C3YJR3	pp	229533.Q4HZ68
155295	229533.A0A1C3YJR3	pp	229533.I1S4T3
155296	229533.A0A1C3YJR3	pp	229533.I1RPK0
155297	229533.A0A1C3YJR3	pp	229533.I1RC81
155298	229533.A0A1C3YJR3	pp	229533.I1RK71
155299	229533.A0A1C3YJR3	pp	229533.I1RSD4
155300	229533.A0A1C3YJR3	pp	229533.I1S0J1
155301	229533.A0A1C3YJR3	pp	229533.V6R057
155302	229533.A0A1C3YJR3	pp	229533.I1RCL3
155303	229533.A0A1C3YJR3	pp	229533.I1RPM7
155304	229533.A0A1C3YJR3	pp	229533.I1RAI9
155305	229533.A0A1C3YJR3	pp	229533.I1RCN5
155306	229533.A0A1C3YJR3	pp	229533.I1S1J6
155307	229533.A0A1C3YJR3	pp	229533.I1RC72
155308	229533.A0A1C3YJR3	pp	229533.I1RUP9
155309	229533.A0A1C3YJR3	pp	229533.I1RTE9
155310	229533.A0A1C3YJR3	pp	229533.I1RTA0
155311	229533.A0A1C3YJR3	pp	229533.I1RKC6
155312	229533.A0A1C3YJR3	pp	229533.I1S060
155313	229533.A0A1C3YJR3	pp	229533.I1S2C0
155314	229533.A0A1C3YJR3	pp	229533.I1S2C4
155315	229533.A0A1C3YJR3	pp	229533.I1RE01
155316	229533.A0A1C3YJR3	pp	229533.Q4HYB8
155317	229533.A0A1C3YJR3	pp	229533.I1RJY3
155318	229533.A0A1C3YJR3	pp	229533.V6RRH2
155319	229533.A0A1C3YJR3	pp	229533.I1RA70
155320	229533.A0A1C3YJR3	pp	229533.Q4IQT8
155321	229533.A0A1C3YJR3	pp	229533.I1RSG8
155322	229533.A0A1C3YJR3	pp	229533.I1RAW0
155323	229533.A0A1C3YJR3	pp	229533.I1S1U7
155324	229533.A0A1C3YJR3	pp	229533.V6RGY3
155325	229533.A0A1C3YJR3	pp	229533.I1RB92
155326	229533.A0A1C3YJR3	pp	229533.I1RT03
155327	229533.A0A1C3YJR3	pp	229533.I1RSS4
155328	229533.A0A1C3YJR3	pp	229533.I1RVX0
155329	229533.A0A1C3YJR3	pp	229533.Q4IN00
155330	229533.A0A1C3YJR3	pp	229533.I1S276
155331	229533.A0A1C3YJR3	pp	229533.I1S1X9
155332	229533.A0A1C3YJR3	pp	229533.I1RRI2
155333	229533.A0A1C3YJR3	pp	229533.I1RK79
155334	229533.A0A1C3YJR3	pp	229533.I1RQE7
155335	229533.A0A1C3YJR3	pp	229533.I1S274
155336	229533.A0A1C3YJR3	pp	229533.I1RD38
155337	229533.A0A1C3YJR3	pp	229533.I1S2E8
155338	229533.A0A1C3YJR3	pp	229533.V6QVH8
155339	229533.A0A1C3YJR3	pp	229533.I1RCW6
155340	229533.A0A1C3YJR3	pp	229533.A0A1C3YKY0
155341	229533.A0A1C3YJR3	pp	229533.I1RJV3
155342	229533.A0A1C3YJR3	pp	229533.I1RW23
155343	229533.A0A1C3YJR3	pp	229533.I1RZQ6
155344	229533.A0A1C3YJR3	pp	229533.I1RYW3

229533.A0A1C3YJR3	pp	229533.I1RFL4
229533.A0A1C3YJR3	pp	229533.V6RTJ8
229533.A0A1C3YJR3	pp	229533.I1RUP7
229533.A0A1C3YJR3	pp	229533.I1RR61
229533.A0A1C3YJR3	pp	229533.I1RDJ8
229533.A0A1C3YJR3	pp	229533.I1S0E8
229533.A0A1C3YJR3	pp	229533.I1RCH5
229533.A0A1C3YJR3	pp	229533.I1S1C7
229533.A0A1C3YJR3	pp	229533.I1RMV1
229533.A0A1C3YJR3	pp	229533.I1RSD8
229533.A0A1C3YJR3	pp	229533.I1RR25
229533.A0A1C3YJR3	pp	229533.Q4INZ9
229533.A0A1C3YJR3	pp	229533.I1RKB1
229533.A0A1C3YJR3	pp	229533.I1RM36
229533.A0A1C3YJR3	pp	229533.Q4HYQ4
229533.A0A1C3YJR3	pp	229533.I1RAX6
229533.A0A1C3YJR3	pp	229533.A0A1C3YMW0
229533.A0A1C3YJR3	pp	229533.I1RPW8
229533.A0A1C3YJR3	pp	229533.I1RRY1
229533.A0A1C3YJR3	pp	229533.I1RT33
229533.A0A1C3YJR3	pp	229533.I1RSI4
229533.A0A1C3YJR3	pp	229533.I1RRH5
229533.A0A1C3YJR3	pp	229533.I1RD52
229533.A0A1C3YJR3	pp	229533.Q4IPI1
229533.A0A1C3YJR3	pp	229533.I1RWL3
229533.A0A1C3YJR3	pp	229533.I1S0T2
229533.A0A1C3YJR3	pp	229533.A0A1C3YK88
229533.A0A1C3YJR3	pp	229533.I1RNH1
229533.A0A1C3YJR3	pp	229533.I1S9U2
229533.A0A1C3YJR3	pp	229533.I1RZR6
229533.A0A1C3YJR3	pp	229533.I1RE66
229533.A0A1C3YJR3	pp	229533.I1RX46
229533.A0A1C3YJR3	pp	229533.I1RPW9
229533.A0A1C3YJR3	pp	229533.I1RC98
229533.A0A1C3YJR3	pp	229533.Q4IQX3
229533.A0A1C3YJR3	pp	229533.I1S6F0
229533.A0A1C3YJR3	pp	229533.I1RR24
229533.A0A1C3YJR3	pp	229533.I1RBW4
229533.A0A1C3YJR3	pp	229533.I1RKQ5
229533.A0A1C3YJR3	pp	229533.I1RM67
229533.A0A1C3YJR3	pp	229533.I1RRX5
229533.A0A1C3YJR3	pp	229533.I1RFQ7
229533.A0A1C3YJR3	pp	229533.I1RAV6
229533.A0A1C3YJR3	pp	229533.I1RXD7
229533.A0A1C3YJR3	pp	229533.I1RFC0
229533.A0A1C3YJR3	pp	229533.I1RSQ7
229533.A0A1C3YJR3	pp	229533.I1RS66
229533.A0A1C3YJR3	pp	229533.I1RP96
229533.A0A1C3YJR3	pp	229533.I1RPU0
229533.A0A1C3YJR3	pp	229533.I1RQJ5
229533.A0A1C3YJR3	pp	229533.I1RML9
229533.A0A1C3YJR3	pp	229533.I1RCL4
229533.A0A1C3YJR3	pp	229533.I1RZ99
229533.A0A1C3YJR3	pp	229533.I1S4S4
229533.A0A1C3YJR3	pp	229533.I1S0R7
229533.A0A1C3YJR3	pp	229533.Q4HTT2
229533.A0A1C3YJR3	pp	229533.I1RPN4
229533.A0A1C3YJR3	pp	229533.I1S4S7
229533.A0A1C3YJR3	pp	229533.I1RMU7
229533.A0A1C3YJR3	pp	229533.A0A1C3YJW9
229533.A0A1C3YJR3	pp	229533.I1RW90
229533.A0A1C3YJR3	pp	229533.A0A1C3YK13
229533.A0A1C3YJR3	pp	229533.I1S6K9
229533.A0A1C3YJR3	pp	229533.I1RDA0
229533.A0A1C3YJR3	pp	229533.I1RW71
229533.A0A1C3YJR3	pp	229533.I1RTC2
229533.A0A1C3YJR3	pp	229533.I1RBN1
229533.A0A1C3YJR3	pp	229533.I1RBE4
229533.A0A1C3YJR3	pp	229533.I1RX08
229533.A0A1C3YJR3	pp	229533.V6RCZ8
229533.A0A1C3YJR3	pp	229533.I1RMK3
229533.A0A1C3YJR3	pp	229533.I1RRG3
229533.A0A1C3YJR3	pp	229533.Q4HVW2

155418	229533.A0A1C3YJR3	pp	229533.I1RNA0
155419	229533.A0A1C3YJR3	pp	229533.A0A1C3YJT5
155420	229533.A0A1C3YJR3	pp	229533.I1RJU3
155421	229533.A0A1C3YJR3	pp	229533.Q4HYI0
155422	229533.A0A1C3YJR3	pp	229533.I1RUG0
155423	229533.A0A1C3YJR3	pp	229533.V6QYF0
155424	229533.A0A1C3YJR3	pp	229533.I1REX4
155425	229533.A0A1C3YJR3	pp	229533.I1RNH6
155426	229533.A0A1C3YJR3	pp	229533.Q4IJH1
155427	229533.A0A1C3YJR3	pp	229533.I1S021
155428	229533.A0A1C3YJR3	pp	229533.I1RJI3
155429	229533.A0A1C3YJR3	pp	229533.I1RE14
155430	229533.A0A1C3YJR3	pp	229533.I1RZM8
155431	229533.A0A1C3YJR3	pp	229533.I1RTF4
155432	229533.A0A1C3YJR3	pp	229533.I1RNV6
155433	229533.A0A1C3YJR3	pp	229533.I1RXF0
155434	229533.A0A1C3YJR3	pp	229533.I1RT80
155435	229533.A0A1C3YJR3	pp	229533.I1RG51
155436	229533.A0A1C3YJR3	pp	229533.I1RNM7
155437	229533.A0A1C3YJR3	pp	229533.I1RC32
155438	229533.A0A1C3YJR3	pp	229533.I1RQM6
155439	229533.A0A1C3YJR3	pp	229533.I1RMC0
155440	229533.A0A1C3YJR3	pp	229533.I1RQ31
155441	229533.A0A1C3YJR3	pp	229533.A0A1C3YKW9
155442	229533.A0A1C3YJR3	pp	229533.I1RUY5
155443	229533.A0A1C3YJR3	pp	229533.I1RCS1
155444	229533.A0A1C3YJR3	pp	229533.V6QWX8
155445	229533.A0A1C3YJR3	pp	229533.I1S5B0
155446	229533.A0A1C3YJR3	pp	229533.I1RT05
155447	229533.A0A1C3YJR3	pp	229533.I1S255
155448	229533.A0A1C3YJR3	pp	229533.V6R227
155449	229533.A0A1C3YJR3	pp	229533.I1S044
155450	229533.A0A1C3YJR3	pp	229533.I1R9Y4
155451	229533.A0A1C3YJR3	pp	229533.I1RW91
155452	229533.A0A1C3YJR3	pp	229533.Q4IBS2
155453	229533.A0A1C3YJR3	pp	229533.A0A1C3YKB0
155454	229533.A0A1C3YJR3	pp	229533.I1RBT1
155455	229533.A0A1C3YJR3	pp	229533.I1S9U1
155456	229533.A0A1C3YJR3	pp	229533.A0A1C3YK51
155457	229533.A0A1C3YJR3	pp	229533.A0A1C3YLR6
155458	229533.A0A1C3YJR3	pp	229533.I1RVZ4
155459	229533.A0A1C3YJR3	pp	229533.I1RCW8
155460	229533.A0A1C3YJR3	pp	229533.I1RQ42
155461	229533.A0A1C3YJR3	pp	229533.Q4I5I4
155462	229533.A0A1C3YJR3	pp	229533.I1R9G0
155463	229533.A0A1C3YJR3	pp	229533.I1RZF6
155464	229533.A0A1C3YJR3	pp	229533.I1RAC2
155465	229533.A0A1C3YJR3	pp	229533.I1S0Z3
155466	229533.A0A1C3YJR3	pp	229533.I1RT41
155467	229533.A0A1C3YJR3	pp	229533.I1RYI7
155468	229533.A0A1C3YJR3	pp	229533.I1RAR2
155469	229533.A0A1C3YJR3	pp	229533.I1RRV4
155470	229533.A0A1C3YJR3	pp	229533.I1RXE5
155471	229533.A0A1C3YJR3	pp	229533.I1RCG6
155472	229533.A0A1C3YJR3	pp	229533.Q4IFI0
155473	229533.A0A1C3YJR3	pp	229533.Q4IEK8
155474	229533.A0A1C3YJR3	pp	229533.I1RL23
155475	229533.A0A1C3YJR3	pp	229533.I1RGC2
155476	229533.A0A1C3YJR3	pp	229533.V6QUE6
155477	229533.A0A1C3YJR3	pp	229533.I1RM06
155478	229533.A0A1C3YJR3	pp	229533.I1RB71
155479	229533.A0A1C3YJR3	pp	229533.I1RT01
155480	229533.A0A1C3YJR3	pp	229533.I1S419
155481	229533.A0A1C3YJR3	pp	229533.I1RR51
155482	229533.A0A1C3YJR3	pp	229533.I1RB27
155483	229533.A0A1C3YJR3	pp	229533.I1S1W8
155484	229533.A0A1C3YJR3	pp	229533.I1RAN5
155485	229533.A0A1C3YJR3	pp	229533.I1RS18
155486	229533.A0A1C3YJR3	pp	229533.I1RT99
155487	229533.A0A1C3YJR3	pp	229533.I1RG56
155488	229533.A0A1C3YJR3	pp	229533.I1RQA2
155489	229533.A0A1C3YJR3	pp	229533.I1RT35
155490	229533.A0A1C3YJR3	pp	229533.Q4IJ56

155491 229533.A0A1C3YJR3 pp 229533.I1RC15
155492 229533.A0A1C3YJR3 pp 229533.I1RJL2
155493 229533.A0A1C3YJR3 pp 229533.I1RCM5
155494 229533.A0A1C3YJR3 pp 229533.I1RMQ4
155495 229533.A0A1C3YJR3 pp 229533.A0A1C3YKV3
155496 229533.A0A1C3YJR3 pp 229533.I1RC16
155497 229533.A0A1C3YJR3 pp 229533.I1RFJ9
155498 229533.A0A1C3YJR3 pp 229533.I1RSI0
155499 229533.A0A1C3YJR3 pp 229533.I1RZK5
155500 229533.A0A1C3YJR3 pp 229533.I1RJ17
155501 229533.A0A1C3YJR3 pp 229533.I1RU02
155502 229533.A0A1C3YJR3 pp 229533.I1RE00
155503 229533.A0A1C3YJR3 pp 229533.A0A1C3YK34
155504 229533.A0A1C3YJR3 pp 229533.I1RBD2
155505 229533.A0A1C3YJR3 pp 229533.I1S4Q4
155506 229533.A0A1C3YJR3 pp 229533.A0A1C3YK68
155507 229533.A0A1C3YJR3 pp 229533.I1RMQ7
155508 229533.A0A1C3YJR3 pp 229533.I1RDC9
155509 229533.A0A1C3YJR3 pp 229533.I1RXC4
155510 229533.A0A1C3YJR3 pp 229533.I1RZB5
155511 229533.A0A1C3YJR3 pp 229533.I1RF19
155512 229533.A0A1C3YJR3 pp 229533.I1RAZ8
155513 229533.A0A1C3YJR3 pp 229533.Q4IJU0
155514 229533.A0A1C3YJR3 pp 229533.I1S0N3
155515 229533.A0A1C3YJR3 pp 229533.I1RRT0
155516 229533.A0A1C3YJR3 pp 229533.I1RJY2
155517 229533.A0A1C3YJR3 pp 229533.I1RSR3
155518 229533.A0A1C3YJR3 pp 229533.Q4IER8
155519 229533.A0A1C3YJR3 pp 229533.I1REL7
155520 229533.A0A1C3YJR3 pp 229533.I1RVZ0
155521 229533.A0A1C3YJR3 pp 229533.I1RAZ3
155522 229533.A0A1C3YJR3 pp 229533.I1RK95
155523 229533.A0A1C3YJR3 pp 229533.I1RFQ5
155524 229533.A0A1C3YJR3 pp 229533.I1RRY9
155525 229533.A0A1C3YJR3 pp 229533.I1RRE0
155526 229533.A0A1C3YJR3 pp 229533.V6QV38
155527 229533.A0A1C3YJR3 pp 229533.I1S9J6
155528 229533.A0A1C3YJR3 pp 229533.I1RDG0
155529 229533.A0A1C3YJR3 pp 229533.I1RN17
155530 229533.A0A1C3YJR3 pp 229533.I1RKQ9
155531 229533.A0A1C3YJR3 pp 229533.I1RN99
155532 229533.A0A1C3YJR3 pp 229533.A0A1C3YKR8
155533 229533.A0A1C3YJR3 pp 229533.I1RBL7
155534 229533.A0A1C3YJR3 pp 229533.I1RDU0
155535 229533.A0A1C3YJR3 pp 229533.I1S5Z2
155536 229533.A0A1C3YJR3 pp 229533.I1RA48
155537 229533.A0A1C3YJR3 pp 229533.I1R9V7
155538 229533.A0A1C3YJR3 pp 229533.V6RBN2
155539 229533.A0A1C3YJR3 pp 229533.I1RWD7
155540 229533.A0A1C3YJR3 pp 229533.I1REN3
155541 229533.A0A1C3YJR3 pp 229533.I1RCR5
155542 229533.A0A1C3YJR3 pp 229533.I1REK8
155543 229533.A0A1C3YJR3 pp 229533.A0A1C3YKY6
155544 229533.A0A1C3YJR3 pp 229533.I1RJZ6
155545 229533.A0A1C3YJR3 pp 229533.I1RC06
155546 229533.A0A1C3YJR3 pp 229533.I1RF08
155547 229533.A0A1C3YJR3 pp 229533.I1RZK6
155548 229533.A0A1C3YJR3 pp 229533.Q4IJK2
155549 229533.A0A1C3YJR3 pp 229533.I1RBE7
155550 229533.A0A1C3YJR3 pp 229533.I1S2U2
155551 229533.I1S5B2 pp 229533.I1S605
155552 229533.I1S5B2 pp 229533.I1S5B3
155553 229533.I1RW52 pp 229533.I1SB04
155554 229533.A0A0E0RV05 pp 229533.I1S4T3
155555 229533.A0A0E0RV05 pp 229533.A0A1C3YMF9
155556 229533.A0A0E0RV05 pp 229533.I1RDA8
155557 229533.A0A0E0RV05 pp 229533.I1RJS4
155558 229533.A0A0E0RV05 pp 229533.I1RBZ1
155559 229533.A0A0E0RV05 pp 229533.I1RBQ0
155560 229533.A0A0E0RV05 pp 229533.A0A1C3YHV5
155561 229533.A0A0E0RV05 pp 229533.I1S8W6
155562 229533.A0A0E0RV05 pp 229533.A0A0E0SMG8
155563 229533.A0A0E0RV05 pp 229533.I1RB17

155564 229533.A0A0E0RV05 pp 229533.I1RDY1
155565 229533.A0A0E0RV05 pp 229533.I1RRH5
155566 229533.A0A0E0RV05 pp 229533.A0A1C3YID5
155567 229533.A0A0E0RV05 pp 229533.I1S0Z1
155568 229533.A0A0E0RV05 pp 229533.A0A1C3YKJ7
155569 229533.I1RJT5 pp 229533.V6RV57
155570 229533.I1RJT5 pp 229533.I1S024
155571 229533.I1RJT5 pp 229533.I1RNG7
155572 229533.I1RJT5 pp 229533.I1RWF5
155573 229533.I1RJT5 pp 229533.Q4HXF0
155574 229533.I1RJT5 pp 229533.I1RMJ3
155575 229533.I1RJT5 pp 229533.I1RSX4
155576 229533.I1RJT5 pp 229533.I1RZA7
155577 229533.I1RJT5 pp 229533.I1RKB2
155578 229533.I1RJT5 pp 229533.I1RZT0
155579 229533.I1RJT5 pp 229533.V6R2Z2
155580 229533.I1RJT5 pp 229533.I1RR68
155581 229533.I1RJT5 pp 229533.V6RUB1
155582 229533.I1RJT5 pp 229533.O74638
155583 229533.I1RJT5 pp 229533.I1RZ20
155584 229533.I1RJT5 pp 229533.I1RVZ3
155585 229533.I1RJT5 pp 229533.I1RYW9
155586 229533.I1RJT5 pp 229533.I1RN84
155587 229533.I1RJT5 pp 229533.I1RPU8
155588 229533.I1RJT5 pp 229533.I1RVR2
155589 229533.I1RJT5 pp 229533.I1S5L5
155590 229533.I1RJT5 pp 229533.I1S6P5
155591 229533.I1RJT5 pp 229533.I1S0V7
155592 229533.I1RJT5 pp 229533.I1S0H5
155593 229533.I1RJT5 pp 229533.I1RPY0
155594 229533.I1RJT5 pp 229533.I1S064
155595 229533.I1RJT5 pp 229533.I1RZQ6
155596 229533.I1RJT5 pp 229533.I1RV23
155597 229533.I1RJT5 pp 229533.I1S2C4
155598 229533.I1RJT5 pp 229533.I1RM54
155599 229533.I1RJT5 pp 229533.V6RLM7
155600 229533.I1RJT5 pp 229533.V6R756
155601 229533.I1RJT5 pp 229533.V6R5D2
155602 229533.I1RJT5 pp 229533.I1RWL3
155603 229533.I1RJT5 pp 229533.I1RM55
155604 229533.I1RJT5 pp 229533.I1RUR8
155605 229533.I1RJT5 pp 229533.I1SAI4
155606 229533.I1RJT5 pp 229533.I1RQX9
155607 229533.I1RJT5 pp 229533.I1RP69
155608 229533.I1RJT5 pp 229533.I1RLT3
155609 229533.I1RJT5 pp 229533.V6RM02
155610 229533.I1RJT5 pp 229533.I1S8C1
155611 229533.I1RJT5 pp 229533.I1RYU5
155612 229533.I1RJT5 pp 229533.I1RND2
155613 229533.I1RJT5 pp 229533.V6RAM6
155614 229533.I1RJT5 pp 229533.I1RW54
155615 229533.I1RJT5 pp 229533.I1S029
155616 229533.I1RJT5 pp 229533.I1RQE1
155617 229533.I1RJT5 pp 229533.V6R1K4
155618 229533.I1RJT5 pp 229533.I1RP86
155619 229533.I1RJT5 pp 229533.I1S273
155620 229533.I1RJT5 pp 229533.I1RMU7
155621 229533.I1RJT5 pp 229533.I1RY49
155622 229533.I1RJT5 pp 229533.I1RVX9
155623 229533.I1RJT5 pp 229533.V6R0W1
155624 229533.I1RJT5 pp 229533.I1S3K8
155625 229533.I1RJT5 pp 229533.I1RZY1
155626 229533.I1RJT5 pp 229533.I1RPW2
155627 229533.I1RJT5 pp 229533.V6QU99
155628 229533.I1RJT5 pp 229533.I1RVV7
155629 229533.I1RJT5 pp 229533.I1S4D0
155630 229533.I1RJT5 pp 229533.I1RWM4
155631 229533.I1RJT5 pp 229533.I1RPR1
155632 229533.I1RJT5 pp 229533.I1RXH3
155633 229533.I1RJT5 pp 229533.I1RNY6
155634 229533.I1RJT5 pp 229533.I1S3K6
155635 229533.I1RJT5 pp 229533.I1S0A0
155636 229533.I1RJT5 pp 229533.I1RM30

155637 229533.I1RJT5 pp 229533.I1RMD3
155638 229533.I1RJT5 pp 229533.I1RVR0
155639 229533.I1RJT5 pp 229533.I1RRM1
155640 229533.I1RJT5 pp 229533.I1RY12
155641 229533.I1RJT5 pp 229533.I1S0V6
155642 229533.I1RJT5 pp 229533.I1RNH4
155643 229533.I1RJT5 pp 229533.I1S3A5
155644 229533.I1RJT5 pp 229533.V6R398
155645 229533.I1RJT5 pp 229533.I1RJY7
155646 229533.I1RJT5 pp 229533.V6RQW4
155647 229533.I1RJT5 pp 229533.I1RQE0
155648 229533.I1RJT5 pp 229533.V6RY56
155649 229533.I1RJT5 pp 229533.I1RM26
155650 229533.I1S0Z7 pp 229533.I1S1W4
155651 229533.I1S0Z7 pp 229533.I1S439
155652 229533.I1S0Z7 pp 229533.Q4IBU4
155653 229533.I1S0Z7 pp 229533.V6RVG3
155654 229533.I1S9Y1 pp 229533.Q4HZ42
155655 229533.I1S9Y1 pp 229533.V6R3J1
155656 229533.I1S9Y1 pp 229533.Q4HXF0
155657 229533.I1S9Y1 pp 229533.Q4IMD1
155658 229533.I1S9Y1 pp 229533.V6RUY9
155659 229533.I1S9Y1 pp 229533.V6RGN5
155660 229533.I1S9Y1 pp 229533.I1SAN0
155661 229533.I1S9Y1 pp 229533.V6RRH2
155662 229533.I1S9Y1 pp 229533.V6R283
155663 229533.I1S9Y1 pp 229533.O74638
155664 229533.I1S9Y1 pp 229533.V6RMW6
155665 229533.I1S9Y1 pp 229533.V6RTJ8
155666 229533.I1S9Y1 pp 229533.V6R042
155667 229533.I1S9Y1 pp 229533.Q4I624
155668 229533.I1S9Y1 pp 229533.Q4IQT8
155669 229533.I1S9Y1 pp 229533.V6QYF0
155670 229533.I1S9Y1 pp 229533.Q4HVW2
155671 229533.I1S9Y1 pp 229533.V6QUE6
155672 229533.I1S9Y1 pp 229533.V6RUT5
155673 229533.I1S9Y1 pp 229533.V6QWX8
155674 229533.I1S9Y1 pp 229533.V6RCZ8
155675 229533.I1S9Y1 pp 229533.V6RQW4
155676 229533.I1S9Y1 pp 229533.Q4IJK2
155677 229533.I1S9Y1 pp 229533.V6RBN2
155678 229533.I1S9Y1 pp 229533.Q4HTT2
155679 229533.I1S9Y1 pp 229533.V6R227
155680 229533.A0A098DHF8 pp 229533.I1RD70
155681 229533.A0A098DHF8 pp 229533.A0A1C3YIP6
155682 229533.I1REI4 pp 229533.I1RTI3
155683 229533.I1REI4 pp 229533.I1RWM4
155684 229533.I1REI4 pp 229533.Q4I8Q4
155685 229533.I1REI4 pp 229533.I1RV85
155686 229533.I1REI4 pp 229533.I1RTP2
155687 229533.I1REI4 pp 229533.I1RMU7
155688 229533.I1RJY5 pp 229533.I1RKK0
155689 229533.I1RJY5 pp 229533.I1S201
155690 229533.I1RJY5 pp 229533.I1RX25
155691 229533.I1RYS0 pp 229533.I1S1W9
155692 229533.I1RYS0 pp 229533.I1S5R1
155693 229533.I1RYS0 pp 229533.V6R042
155694 229533.I1RYS0 pp 229533.I1S2H4
155695 229533.I1RYS0 pp 229533.V6R057
155696 229533.I1RYS0 pp 229533.I1S0J1
155697 229533.I1RYS0 pp 229533.I1S5R6
155698 229533.I1RYS0 pp 229533.V6RMW6
155699 229533.I1RYS0 pp 229533.I1S1C7
155700 229533.I1RYS0 pp 229533.I1RZZ9
155701 229533.I1RYS0 pp 229533.I1RYW3
155702 229533.I1RYS0 pp 229533.I1RZ39
155703 229533.I1RYS0 pp 229533.Q4HVU7
155704 229533.I1RYS0 pp 229533.I1S4S7
155705 229533.I1RYS0 pp 229533.V6RTJ8
155706 229533.I1RYS0 pp 229533.I1S2B8
155707 229533.I1RYS0 pp 229533.Q4IJK2
155708 229533.I1RYS0 pp 229533.I1S8P5
155709 229533.I1RYS0 pp 229533.I1RZM8

155710	229533.I1RK95	pp	229533.Q4HVW2
155711	229533.I1RK95	pp	229533.Q4IJH1
155712	229533.I1RK95	pp	229533.I1RQM6
155713	229533.I1RK95	pp	229533.I1RT80
155714	229533.I1RK95	pp	229533.V6QWX8
155715	229533.I1RK95	pp	229533.I1RTF4
155716	229533.I1RK95	pp	229533.I1RT05
155717	229533.I1RK95	pp	229533.I1RMM8
155718	229533.I1RK95	pp	229533.I1RNT2
155719	229533.I1RK95	pp	229533.I1RNV6
155720	229533.I1RK95	pp	229533.Q4IFI0
155721	229533.I1RK95	pp	229533.I1RY49
155722	229533.I1RK95	pp	229533.I1RM67
155723	229533.I1RK95	pp	229533.I1RMU7
155724	229533.I1RK95	pp	229533.I1RT41
155725	229533.I1RK95	pp	229533.I1S9U2
155726	229533.I1RK95	pp	229533.I1RPU0
155727	229533.I1RK95	pp	229533.I1RW90
155728	229533.I1RK95	pp	229533.I1RL23
155729	229533.I1RK95	pp	229533.I1RRG3
155730	229533.I1RK95	pp	229533.I1RPW9
155731	229533.I1RK95	pp	229533.I1RYJ6
155732	229533.I1RK95	pp	229533.I1RN61
155733	229533.I1RK95	pp	229533.I1RZJ6
155734	229533.I1RK95	pp	229533.I1RR24
155735	229533.I1RK95	pp	229533.I1S0B7
155736	229533.I1RK95	pp	229533.Q4INZ9
155737	229533.I1RK95	pp	229533.I1RXD7
155738	229533.I1RK95	pp	229533.I1RS19
155739	229533.I1RK95	pp	229533.I1RRX5
155740	229533.I1RK95	pp	229533.I1RZ94
155741	229533.I1RK95	pp	229533.V6RUT5
155742	229533.I1RK95	pp	229533.I1RSQ7
155743	229533.I1RK95	pp	229533.I1RM06
155744	229533.I1RK95	pp	229533.I1RZ99
155745	229533.I1RK95	pp	229533.I1S0R7
155746	229533.I1RK95	pp	229533.I1RNA0
155747	229533.I1RK95	pp	229533.I1RQJ5
155748	229533.I1RK95	pp	229533.Q4IQX3
155749	229533.I1RK95	pp	229533.I1RP86
155750	229533.I1RK95	pp	229533.I1RPN4
155751	229533.I1RK95	pp	229533.I1S4S7
155752	229533.I1RK95	pp	229533.V6QYF0
155753	229533.I1RK95	pp	229533.I1RSI0
155754	229533.I1RK95	pp	229533.I1RTC2
155755	229533.I1RK95	pp	229533.I1S4Q4
155756	229533.I1RK95	pp	229533.I1RVX9
155757	229533.I1RK95	pp	229533.I1RW71
155758	229533.I1RK95	pp	229533.I1RX46
155759	229533.I1RK95	pp	229533.I1RNH1
155760	229533.I1RK95	pp	229533.V6RQW4
155761	229533.I1RK95	pp	229533.I1S854
155762	229533.I1RK95	pp	229533.I1RKQ5
155763	229533.I1RK95	pp	229533.I1RZE5
155764	229533.I1RK95	pp	229533.I1RM26
155765	229533.I1RK95	pp	229533.I1RUP7
155766	229533.I1RK95	pp	229533.I1S6F0
155767	229533.I1RK95	pp	229533.I1RNK2
155768	229533.I1RK95	pp	229533.I1RT33
155769	229533.I1RK95	pp	229533.I1RKB1
155770	229533.I1RK95	pp	229533.I1RRH5
155771	229533.I1RK95	pp	229533.V6RTJ8
155772	229533.I1RK95	pp	229533.I1S9J6
155773	229533.I1RK95	pp	229533.I1RKQ9
155774	229533.I1RK95	pp	229533.I1RVZ0
155775	229533.I1RK95	pp	229533.I1RM36
155776	229533.I1RK95	pp	229533.I1RN17
155777	229533.I1RK95	pp	229533.I1RZB5
155778	229533.I1RK95	pp	229533.I1S0C1
155779	229533.I1RK95	pp	229533.I1S887
155780	229533.I1RK95	pp	229533.I1RZK5
155781	229533.I1RK95	pp	229533.I1S021
155782	229533.I1RK95	pp	229533.I1RWD7

155783	229533.I1RK95	pp	229533.I1RMN7
155784	229533.I1RK95	pp	229533.I1RNH6
155785	229533.I1RK95	pp	229533.I1RPW1
155786	229533.I1RK95	pp	229533.V6QUE6
155787	229533.I1RK95	pp	229533.I1RQA2
155788	229533.I1RK95	pp	229533.I1S5B0
155789	229533.I1RK95	pp	229533.I1RXF0
155790	229533.I1RK95	pp	229533.I1RSR3
155791	229533.I1RK95	pp	229533.I1RRY9
155792	229533.I1RK95	pp	229533.I1RZM8
155793	229533.I1RK95	pp	229533.I1RMC0
155794	229533.I1RK95	pp	229533.I1RRE0
155795	229533.I1RK95	pp	229533.I1RMQ4
155796	229533.I1RK95	pp	229533.I1RMA8
155797	229533.I1RK95	pp	229533.I1RQ31
155798	229533.I1RK95	pp	229533.I1RXC4
155799	229533.I1RK95	pp	229533.V6RBN2
155800	229533.I1RK95	pp	229533.I1RS18
155801	229533.I1RK95	pp	229533.Q4IJK2
155802	229533.I1RK95	pp	229533.I1RQ81
155803	229533.I1RK95	pp	229533.Q4HTT2
155804	229533.I1RK95	pp	229533.I1S6K9
155805	229533.I1RK95	pp	229533.Q4IJ56
155806	229533.I1RK95	pp	229533.V6RCZ8
155807	229533.I1RK95	pp	229533.I1S0Z3
155808	229533.I1RK95	pp	229533.I1S2U2
155809	229533.I1RK95	pp	229533.Q4IBS2
155810	229533.I1RK95	pp	229533.I1RN99
155811	229533.I1RK95	pp	229533.I1RMG7
155812	229533.I1RK95	pp	229533.I1RSC6
155813	229533.I1RK95	pp	229533.Q4I5I4
155814	229533.I1RK95	pp	229533.Q4IEK8
155815	229533.I1RK95	pp	229533.I1RYT5
155816	229533.I1RK95	pp	229533.I1S255
155817	229533.I1RK95	pp	229533.I1RYI7
155818	229533.I1RK95	pp	229533.I1RRV4
155819	229533.I1RK95	pp	229533.I1RSZ7
155820	229533.I1RK95	pp	229533.I1RZS6
155821	229533.I1RK95	pp	229533.Q4IF76
155822	229533.I1RK95	pp	229533.V6R227
155823	229533.I1RK95	pp	229533.I1RYE2
155824	229533.I1RK95	pp	229533.I1S9U1
155825	229533.I1RK95	pp	229533.I1RSQ4
155826	229533.I1RK95	pp	229533.I1RZF6
155827	229533.I1RK95	pp	229533.I1RWI5
155828	229533.I1RK95	pp	229533.I1RZ86
155829	229533.I1RK95	pp	229533.I1RU69
155830	229533.I1RK95	pp	229533.I1RR72
155831	229533.I1RK95	pp	229533.I1RQS5
155832	229533.I1RK95	pp	229533.I1RZZ9
155833	229533.I1RK95	pp	229533.I1RX24
155834	229533.I1RK95	pp	229533.I1RKF9
155835	229533.I1RK95	pp	229533.I1S253
155836	229533.I1RK95	pp	229533.I1S2B8
155837	229533.I1RK95	pp	229533.I1RRM8
155838	229533.I1RK95	pp	229533.I1RRT2
155839	229533.I1RK95	pp	229533.I1S0J3
155840	229533.I1RK95	pp	229533.I1RZ39
155841	229533.I1RK95	pp	229533.I1RZ52
155842	229533.I1RK95	pp	229533.Q4IMD1
155843	229533.I1RK95	pp	229533.I1RVY0
155844	229533.I1RK95	pp	229533.I1RRT8
155845	229533.I1RK95	pp	229533.Q4IAS1
155846	229533.I1RK95	pp	229533.I1S5R1
155847	229533.I1RK95	pp	229533.I1RXC2
155848	229533.I1RK95	pp	229533.I1S268
155849	229533.I1RK95	pp	229533.I1RTD5
155850	229533.I1RK95	pp	229533.I1RVZ2
155851	229533.I1RK95	pp	229533.I1SAN0
155852	229533.I1RK95	pp	229533.I1RKJ6
155853	229533.I1RK95	pp	229533.I1RTA1
155854	229533.I1RK95	pp	229533.O74638
155855	229533.I1RK95	pp	229533.V6RAC9

155856	229533.I1RK95	pp	229533.I1S9Y1
155857	229533.I1RK95	pp	229533.I1RT40
155858	229533.I1RK95	pp	229533.I1RSZ3
155859	229533.I1RK95	pp	229533.V6R283
155860	229533.I1RK95	pp	229533.I1RV91
155861	229533.I1RK95	pp	229533.I1S0L4
155862	229533.I1RK95	pp	229533.I1RWS4
155863	229533.I1RK95	pp	229533.Q4HXF0
155864	229533.I1RK95	pp	229533.I1RRJ8
155865	229533.I1RK95	pp	229533.I1RYI5
155866	229533.I1RK95	pp	229533.I1S2H4
155867	229533.I1RK95	pp	229533.I1RQG4
155868	229533.I1RK95	pp	229533.I1RPP3
155869	229533.I1RK95	pp	229533.I1RS20
155870	229533.I1RK95	pp	229533.V6RUY9
155871	229533.I1RK95	pp	229533.I1RPV3
155872	229533.I1RK95	pp	229533.I1RL04
155873	229533.I1RK95	pp	229533.V6R3J1
155874	229533.I1RK95	pp	229533.I1S8P5
155875	229533.I1RK95	pp	229533.I1RSX0
155876	229533.I1RK95	pp	229533.I1RN67
155877	229533.I1RK95	pp	229533.I1RSX1
155878	229533.I1RK95	pp	229533.I1RMG6
155879	229533.I1RK95	pp	229533.I1RQ30
155880	229533.I1RK95	pp	229533.I1RS30
155881	229533.I1RK95	pp	229533.I1RQ65
155882	229533.I1RK95	pp	229533.I1S1U7
155883	229533.I1RK95	pp	229533.Q4I830
155884	229533.I1RK95	pp	229533.Q4HZ42
155885	229533.I1RK95	pp	229533.I1RZ20
155886	229533.I1RK95	pp	229533.I1RKL3
155887	229533.I1RK95	pp	229533.I1S274
155888	229533.I1RK95	pp	229533.V6RGY3
155889	229533.I1RK95	pp	229533.I1RS47
155890	229533.I1RK95	pp	229533.I1RXU9
155891	229533.I1RK95	pp	229533.I1RRQ3
155892	229533.I1RK95	pp	229533.I1S2E8
155893	229533.I1RK95	pp	229533.I1RRI2
155894	229533.I1RK95	pp	229533.I1RNC1
155895	229533.I1RK95	pp	229533.I1RQE7
155896	229533.I1RK95	pp	229533.I1RUG3
155897	229533.I1RK95	pp	229533.I1RZQ6
155898	229533.I1RK95	pp	229533.I1RSD8
155899	229533.I1RK95	pp	229533.V6R042
155900	229533.I1RK95	pp	229533.I1RXR1
155901	229533.I1RK95	pp	229533.I1RYW3
155902	229533.I1RK95	pp	229533.I1RSQ3
155903	229533.I1RK95	pp	229533.V6RMW6
155904	229533.I1RK95	pp	229533.I1RSS4
155905	229533.I1RK95	pp	229533.I1RT03
155906	229533.I1RK95	pp	229533.I1RXU0
155907	229533.I1RK95	pp	229533.I1RVX0
155908	229533.I1RK95	pp	229533.I1RTE9
155909	229533.I1RK95	pp	229533.I1RSI6
155910	229533.I1RK95	pp	229533.I1S1C7
155911	229533.I1RK95	pp	229533.I1RRV1
155912	229533.I1RK95	pp	229533.I1RSY2
155913	229533.I1RK95	pp	229533.I1RRN2
155914	229533.I1RK95	pp	229533.I1S2C0
155915	229533.I1RK95	pp	229533.I1S1X9
155916	229533.I1RK95	pp	229533.I1RMN6
155917	229533.I1RK95	pp	229533.I1RKC6
155918	229533.I1RK95	pp	229533.I1RSG8
155919	229533.I1RK95	pp	229533.I1RPM7
155920	229533.I1RK95	pp	229533.I1S1J6
155921	229533.I1RK95	pp	229533.V6RRH2
155922	229533.I1RK95	pp	229533.I1S855
155923	229533.I1RK95	pp	229533.I1RSU2
155924	229533.I1RK95	pp	229533.I1RKB6
155925	229533.I1RK95	pp	229533.I1RR25
155926	229533.I1RK95	pp	229533.V6RGN5
155927	229533.I1RK95	pp	229533.I1RM25
155928	229533.I1RK95	pp	229533.I1RT36

155929 229533.I1RK95 pp 229533.I1S5R6
155930 229533.I1RK95 pp 229533.I1RUG4
155931 229533.I1RK95 pp 229533.I1RNU6
155932 229533.I1RK95 pp 229533.I1RSD4
155933 229533.I1RK95 pp 229533.I1S1W3
155934 229533.I1RK95 pp 229533.Q4I624
155935 229533.I1RK95 pp 229533.I1RM96
155936 229533.I1RK95 pp 229533.I1S0N5
155937 229533.I1RK95 pp 229533.I1S1W9
155938 229533.I1RK95 pp 229533.I1S0J1
155939 229533.I1RK95 pp 229533.I1S4T3
155940 229533.I1RK95 pp 229533.Q4HZ68
155941 229533.I1RK95 pp 229533.I1RUP9
155942 229533.I1RK95 pp 229533.I1S296
155943 229533.I1RK95 pp 229533.V6R057
155944 229533.I1RK95 pp 229533.I1RTA0
155945 229533.I1RK95 pp 229533.I1S060
155946 229533.I1RK95 pp 229533.I1RWE8
155947 229533.I1RK95 pp 229533.I1RRR7
155948 229533.I1RK95 pp 229533.I1RWD3
155949 229533.I1RK95 pp 229533.Q4I327
155950 229533.I1RK95 pp 229533.I1RRN6
155951 229533.I1RK95 pp 229533.Q4I7K4
155952 229533.A0A1C3YJW1 pp 229533.I1RF85
155953 229533.A0A1C3YJW1 pp 229533.I1RIH8
155954 229533.A0A1C3YJW1 pp 229533.I1RDB1
155955 229533.A0A1C3YJW1 pp 229533.I1R9M7
155956 229533.A0A1C3YJW1 pp 229533.I1RZ63
155957 229533.A0A1C3YJW1 pp 229533.I1RC22
155958 229533.A0A1C3YJW1 pp 229533.I1RWJ6
155959 229533.A0A1C3YJW1 pp 229533.I1RK75
155960 229533.A0A1C3YJW1 pp 229533.I1RB75
155961 229533.A0A1C3YJW1 pp 229533.I1RB41
155962 229533.A0A1C3YJW1 pp 229533.I1S0J7
155963 229533.A0A1C3YJW1 pp 229533.Q4IJM4
155964 229533.A0A1C3YJW1 pp 229533.I1RRR6
155965 229533.A0A1C3YJW1 pp 229533.I1RRN5
155966 229533.A0A1C3YJW1 pp 229533.A0A1C3YK57
155967 229533.A0A1C3YJW1 pp 229533.I1RJS7
155968 229533.A0A1C3YJW1 pp 229533.I1RUQ3
155969 229533.A0A1C3YJW1 pp 229533.A0A1C3YLX2
155970 229533.A0A1C3YJW1 pp 229533.I1RQA3
155971 229533.A0A1C3YJW1 pp 229533.Q4HYR2
155972 229533.A0A1C3YJW1 pp 229533.I1RUN8
155973 229533.A0A1C3YJW1 pp 229533.I1S1W4
155974 229533.A0A1C3YJW1 pp 229533.I1RYX6
155975 229533.A0A1C3YJW1 pp 229533.I1RJE9
155976 229533.A0A1C3YJW1 pp 229533.I1RM49
155977 229533.A0A1C3YJW1 pp 229533.I1RWT6
155978 229533.A0A1C3YJW1 pp 229533.I1RC17
155979 229533.A0A1C3YJW1 pp 229533.I1S423
155980 229533.A0A1C3YJW1 pp 229533.Q4HVQ9
155981 229533.A0A1C3YJW1 pp 229533.I1RXJ0
155982 229533.A0A1C3YJW1 pp 229533.I1RB74
155983 229533.A0A1C3YJW1 pp 229533.I1RY44
155984 229533.A0A1C3YJW1 pp 229533.A0A1C3YN83
155985 229533.A0A1C3YJW1 pp 229533.I1RC34
155986 229533.A0A1C3YJW1 pp 229533.I1RCU2
155987 229533.A0A1C3YJW1 pp 229533.I1SAF7
155988 229533.A0A1C3YJW1 pp 229533.I1RZ51
155989 229533.A0A1C3YJW1 pp 229533.I1RT12
155990 229533.A0A1C3YJW1 pp 229533.I1RAM7
155991 229533.A0A1C3YJW1 pp 229533.I1S098
155992 229533.A0A1C3YJW1 pp 229533.I1S386
155993 229533.A0A1C3YJW1 pp 229533.I1S2E9
155994 229533.I1RFY6 pp 229533.I1S9Y1
155995 229533.I1RFY6 pp 229533.I1S5R1
155996 229533.I1RFY6 pp 229533.I1S253
155997 229533.I1RFY6 pp 229533.I1RUG3
155998 229533.I1RFY6 pp 229533.I1RQE7
155999 229533.I1RFY6 pp 229533.I1RW61
156000 229533.I1RFY6 pp 229533.I1RRI1
156001 229533.I1RFY6 pp 229533.Q4IER0

156002 229533.I1RFY6 pp 229533.I1RMC2
156003 229533.I1RFY6 pp 229533.I1RT33
156004 229533.I1RFY6 pp 229533.I1RYJ6
156005 229533.I1RFY6 pp 229533.I1RQ33
156006 229533.I1RFY6 pp 229533.I1RR72
156007 229533.I1RFY6 pp 229533.I1RT05
156008 229533.I1RFY6 pp 229533.V6RCZ8
156009 229533.I1RFY6 pp 229533.I1S5B0
156010 229533.I1RFY6 pp 229533.I1RZB2
156011 229533.I1RFY6 pp 229533.I1RJU3
156012 229533.I1RFY6 pp 229533.I1S0F1
156013 229533.I1RFY6 pp 229533.I1RR24
156014 229533.I1S8R8 pp 229533.V6RDP1
156015 229533.A0A1C3YN95 pp 229533.I1RUV5
156016 229533.A0A1C3YN95 pp 229533.I1RGU8
156017 229533.A0A1C3YN95 pp 229533.I1S1J0
156018 229533.A0A1C3YN95 pp 229533.I1RHQ4
156019 229533.I1RQM4 pp 229533.I1S368
156020 229533.I1RQM4 pp 229533.I1RWN0
156021 229533.I1RQM4 pp 229533.I1RW69
156022 229533.I1RQM4 pp 229533.V6RK96
156023 229533.I1RQM4 pp 229533.I1RZM4
156024 229533.I1RQM4 pp 229533.V6RHD0
156025 229533.I1RQM4 pp 229533.V6RDH3
156026 229533.I1RQM4 pp 229533.I1S3G4
156027 229533.I1RQM4 pp 229533.I1RUP5
156028 229533.I1RQM4 pp 229533.V6RXR0
156029 229533.I1RQM4 pp 229533.I1RTP2
156030 229533.I1RQM4 pp 229533.I1S1W8
156031 229533.I1RQM4 pp 229533.V6QU65
156032 229533.A0A098DIZ7 pp 229533.I1S2L1
156033 229533.A0A098DIZ7 pp 229533.A0A1C3YLQ4
156034 229533.A0A098DIZ7 pp 229533.A0A1C3YJ07
156035 229533.A0A098DIZ7 pp 229533.I1RWM4
156036 229533.A0A098DIZ7 pp 229533.I1S368
156037 229533.A0A098DIZ7 pp 229533.A0A1C3YIX3
156038 229533.A0A098DIZ7 pp 229533.A0A1C3YMF6
156039 229533.A0A098DIZ7 pp 229533.I1RWN0
156040 229533.A0A098DIZ7 pp 229533.I1RTP2
156041 229533.A0A098DIZ7 pp 229533.Q4I8Q4
156042 229533.A0A098DIZ7 pp 229533.A0A1C3YK95
156043 229533.A0A098DIZ7 pp 229533.I1RPG8
156044 229533.A0A098DIZ7 pp 229533.I1REI4
156045 229533.A0A098DIZ7 pp 229533.I1RV85
156046 229533.A0A098DIZ7 pp 229533.A0A098DXB1
156047 229533.A0A098DIZ7 pp 229533.Q4ICM0
156048 229533.A0A098DIZ7 pp 229533.I1S360
156049 229533.A0A098DIZ7 pp 229533.I1RDZ5
156050 229533.A0A098DIZ7 pp 229533.I1RQM4
156051 229533.A0A098DIZ7 pp 229533.A0A0E0S5J3
156052 229533.A0A098DIZ7 pp 229533.A0A1C3YJ69
156053 229533.A0A098DIZ7 pp 229533.I1RMU7
156054 229533.A0A098DIZ7 pp 229533.I1R9P1
156055 229533.A0A098DIZ7 pp 229533.A0A1C3YJJ7
156056 229533.A0A098DIZ7 pp 229533.I1REB5
156057 229533.A0A098DIZ7 pp 229533.I1RE88
156058 229533.A0A098DIZ7 pp 229533.A0A0E0SIE3
156059 229533.A0A0E0S5J3 pp 229533.I1RWM4
156060 229533.A0A0E0S5J3 pp 229533.I1REI4
156061 229533.A0A0E0S5J3 pp 229533.I1RTI3
156062 229533.A0A0E0S5J3 pp 229533.Q4I8Q4
156063 229533.A0A0E0S5J3 pp 229533.I1RTP2
156064 229533.A0A0E0S5J3 pp 229533.I1RDZ5
156065 229533.A0A0E0S5J3 pp 229533.I1RV85
156066 229533.A0A0E0S5J3 pp 229533.I1REB5
156067 229533.A0A0E0S5J3 pp 229533.I1RE88
156068 229533.A0A0E0S5J3 pp 229533.A0A1C3YJJ7
156069 229533.A0A0E0S5J3 pp 229533.I1RMU7
156070 229533.I1RD51 pp 229533.I1RD52
156071 229533.I1RD51 pp 229533.Q4HTT2
156072 229533.I1RFT4 pp 229533.I1RT00
156073 229533.I1RE88 pp 229533.I1RTP2
156074 229533.I1RE88 pp 229533.I1RWN0

156075 229533.I1RE88 pp 229533.I1RQM4
156076 229533.I1RE88 pp 229533.Q4ICM0
156077 229533.I1RE88 pp 229533.I1S360
156078 229533.I1RE88 pp 229533.I1RWM4
156079 229533.I1RE88 pp 229533.I1S368
156080 229533.I1RE88 pp 229533.I1REB5
156081 229533.I1RE88 pp 229533.I1RV85
156082 229533.I1RE88 pp 229533.I1REI4
156083 229533.I1RE88 pp 229533.I1S2L1
156084 229533.I1RE88 pp 229533.I1RMU7
156085 229533.I1RE88 pp 229533.Q4I8Q4
156086 229533.I1RE88 pp 229533.I1RPG8
156087 229533.A0A1C3YKR8 pp 229533.Q4IJU0
156088 229533.A0A1C3YKR8 pp 229533.I1RUG4
156089 229533.A0A1C3YKR8 pp 229533.I1REN3
156090 229533.A0A1C3YKR8 pp 229533.I1RAI9
156091 229533.A0A1C3YKR8 pp 229533.I1RAE0
156092 229533.A0A1C3YKR8 pp 229533.I1RKF3
156093 229533.A0A1C3YKR8 pp 229533.I1RR25
156094 229533.A0A1C3YKR8 pp 229533.I1RCB7
156095 229533.A0A1C3YKR8 pp 229533.I1RXU0
156096 229533.A0A1C3YKR8 pp 229533.Q4I5I4
156097 229533.A0A1C3YKR8 pp 229533.V6QUE6
156098 229533.A0A1C3YKR8 pp 229533.I1RG11
156099 229533.A0A1C3YKR8 pp 229533.Q4IPC8
156100 229533.A0A1C3YKR8 pp 229533.I1RUG0
156101 229533.A0A1C3YKR8 pp 229533.I1RD38
156102 229533.A0A1C3YKR8 pp 229533.I1RK79
156103 229533.A0A1C3YKR8 pp 229533.I1RMQ7
156104 229533.A0A1C3YKR8 pp 229533.Q4HYB8
156105 229533.A0A1C3YKR8 pp 229533.I1RC24
156106 229533.A0A1C3YKR8 pp 229533.I1RN07
156107 229533.A0A1C3YKR8 pp 229533.I1RZB5
156108 229533.A0A1C3YKR8 pp 229533.I1S5Z2
156109 229533.A0A1C3YKR8 pp 229533.Q4IB50
156110 229533.A0A1C3YKR8 pp 229533.I1RD70
156111 229533.A0A1C3YKR8 pp 229533.I1S276
156112 229533.A0A1C3YKR8 pp 229533.I1RW69
156113 229533.A0A1C3YKR8 pp 229533.I1RE54
156114 229533.A0A1C3YKR8 pp 229533.I1RT99
156115 229533.A0A1C3YKR8 pp 229533.I1RC16
156116 229533.A0A1C3YKR8 pp 229533.A0A1C3YKX5
156117 229533.A0A1C3YKR8 pp 229533.Q4I099
156118 229533.A0A1C3YKR8 pp 229533.A0A1C3YN55
156119 229533.A0A1C3YKR8 pp 229533.Q4I5U9
156120 229533.A0A1C3YKR8 pp 229533.I1S0R5
156121 229533.A0A1C3YKR8 pp 229533.I1RRG3
156122 229533.A0A1C3YKR8 pp 229533.Q4HYQ4
156123 229533.A0A1C3YKR8 pp 229533.I1RA84
156124 229533.A0A1C3YKR8 pp 229533.V6QWX8
156125 229533.A0A1C3YKR8 pp 229533.I1RBJ1
156126 229533.A0A1C3YKR8 pp 229533.I1RQ88
156127 229533.A0A1C3YKR8 pp 229533.I1RTC2
156128 229533.A0A1C3YKR8 pp 229533.I1RSW9
156129 229533.A0A1C3YKR8 pp 229533.I1RSZ3
156130 229533.A0A1C3YKR8 pp 229533.Q4I5W5
156131 229533.A0A1C3YKR8 pp 229533.I1RBC7
156132 229533.A0A1C3YIR8 pp 229533.I1RI82
156133 229533.A0A1C3YIR8 pp 229533.I1RJI3
156134 229533.A0A1C3YIR8 pp 229533.I1RE14
156135 229533.A0A1C3YIR8 pp 229533.I1S7M3
156136 229533.A0A1C3YIR8 pp 229533.A0A1C3YJW9
156137 229533.A0A1C3YIR8 pp 229533.Q4HYI0
156138 229533.A0A1C3YIR8 pp 229533.Q4HTT2
156139 229533.A0A1C3YIR8 pp 229533.I1RXF0
156140 229533.A0A1C3YIR8 pp 229533.I1RZM8
156141 229533.A0A1C3YIR8 pp 229533.A0A1C3YJZ9
156142 229533.A0A1C3YIR8 pp 229533.V6RCZ8
156143 229533.A0A1C3YIR8 pp 229533.I1RQ31
156144 229533.A0A1C3YIR8 pp 229533.I1RR51
156145 229533.A0A1C3YIR8 pp 229533.Q4I5I4
156146 229533.A0A1C3YIR8 pp 229533.I1S6K9
156147 229533.A0A1C3YIR8 pp 229533.I1S9U1

156148 229533.A0A1C3YIR8 pp 229533.I1RXC4
156149 229533.A0A1C3YIR8 pp 229533.I1RS18
156150 229533.A0A1C3YIR8 pp 229533.I1S504
156151 229533.A0A1C3YIR8 pp 229533.I1RVZ4
156152 229533.A0A1C3YIR8 pp 229533.I1RB27
156153 229533.A0A1C3YIR8 pp 229533.I1RM65
156154 229533.A0A1C3YIR8 pp 229533.A0A1C3YJR3
156155 229533.A0A1C3YIR8 pp 229533.V6QUE6
156156 229533.A0A1C3YIR8 pp 229533.I1S0F0
156157 229533.A0A1C3YIR8 pp 229533.I1RN07
156158 229533.A0A1C3YIR8 pp 229533.I1RAR2
156159 229533.A0A1C3YIR8 pp 229533.I1RW69
156160 229533.A0A1C3YIR8 pp 229533.I1RYY5
156161 229533.A0A1C3YIR8 pp 229533.I1RC15
156162 229533.A0A1C3YIR8 pp 229533.I1RRV4
156163 229533.A0A1C3YIR8 pp 229533.I1RYI7
156164 229533.A0A1C3YIR8 pp 229533.I1S9J6
156165 229533.A0A1C3YIR8 pp 229533.A0A1C3YKV3
156166 229533.A0A1C3YIR8 pp 229533.I1RQ81
156167 229533.A0A1C3YIR8 pp 229533.I1RY03
156168 229533.A0A1C3YIR8 pp 229533.I1RA94
156169 229533.A0A1C3YIR8 pp 229533.I1RXE5
156170 229533.A0A1C3YIR8 pp 229533.I1S5B0
156171 229533.A0A1C3YIR8 pp 229533.I1RUY5
156172 229533.A0A1C3YIR8 pp 229533.A0A1C3YKF1
156173 229533.A0A1C3YIR8 pp 229533.Q4ICA8
156174 229533.A0A1C3YIR8 pp 229533.I1RJL2
156175 229533.A0A1C3YIR8 pp 229533.I1RYS4
156176 229533.A0A1C3YIR8 pp 229533.Q4I5R3
156177 229533.A0A1C3YIR8 pp 229533.I1RJ17
156178 229533.A0A1C3YIR8 pp 229533.I1RCG6
156179 229533.A0A1C3YIR8 pp 229533.I1RPK5
156180 229533.A0A1C3YIR8 pp 229533.I1S887
156181 229533.A0A1C3YIR8 pp 229533.I1RSI1
156182 229533.A0A1C3YIR8 pp 229533.I1RQ93
156183 229533.A0A1C3YIR8 pp 229533.A0A1C3YK68
156184 229533.A0A1C3YIR8 pp 229533.I1RU02
156185 229533.A0A1C3YIR8 pp 229533.I1RMQ7
156186 229533.A0A1C3YIR8 pp 229533.I1RDC9
156187 229533.A0A1C3YIR8 pp 229533.I1RT01
156188 229533.A0A1C3YIR8 pp 229533.A0A1C3YK34
156189 229533.A0A1C3YIR8 pp 229533.I1RCM5
156190 229533.A0A1C3YIR8 pp 229533.I1RZH4
156191 229533.A0A1C3YIR8 pp 229533.I1RZK5
156192 229533.A0A1C3YIR8 pp 229533.I1S419
156193 229533.A0A1C3YIR8 pp 229533.I1RRT0
156194 229533.A0A1C3YIR8 pp 229533.I1RZB5
156195 229533.A0A1C3YIR8 pp 229533.I1RT99
156196 229533.A0A1C3YIR8 pp 229533.I1S0A0
156197 229533.A0A1C3YIR8 pp 229533.I1RAZ3
156198 229533.A0A1C3YIR8 pp 229533.I1RC16
156199 229533.A0A1C3YIR8 pp 229533.I1RT35
156200 229533.A0A1C3YIR8 pp 229533.I1RWB0
156201 229533.A0A1C3YIR8 pp 229533.Q4IER8
156202 229533.A0A1C3YIR8 pp 229533.I1RWT6
156203 229533.A0A1C3YIR8 pp 229533.I1S1J7
156204 229533.A0A1C3YIR8 pp 229533.I1RF71
156205 229533.A0A1C3YIR8 pp 229533.I1RZU9
156206 229533.A0A1C3YIR8 pp 229533.I1RA36
156207 229533.A0A1C3YIR8 pp 229533.I1RZL5
156208 229533.A0A1C3YIR8 pp 229533.A0A1C3YKB0
156209 229533.A0A1C3YIR8 pp 229533.I1RK15
156210 229533.A0A1C3YIR8 pp 229533.I1RDZ6
156211 229533.A0A1C3YIR8 pp 229533.I1RQ19
156212 229533.A0A1C3YIR8 pp 229533.I1S5Z2
156213 229533.A0A1C3YIR8 pp 229533.I1RJY2
156214 229533.A0A1C3YIR8 pp 229533.I1RRY9
156215 229533.A0A1C3YIR8 pp 229533.I1S255
156216 229533.A0A1C3YIR8 pp 229533.I1RA18
156217 229533.A0A1C3YIR8 pp 229533.I1RPR9
156218 229533.A0A1C3YIR8 pp 229533.A0A1C3YKY6
156219 229533.A0A1C3YIR8 pp 229533.I1RCW8
156220 229533.A0A1C3YIR8 pp 229533.I1REK4

156221 229533.A0A1C3YIR8 pp 229533.I1RGR8
156222 229533.A0A1C3YIR8 pp 229533.V6QV38
156223 229533.A0A1C3YIR8 pp 229533.Q4IJM4
156224 229533.A0A1C3YIR8 pp 229533.A0A1C3YKR8
156225 229533.A0A1C3YIR8 pp 229533.I1RBL7
156226 229533.A0A1C3YIR8 pp 229533.I1RXD7
156227 229533.A0A1C3YIR8 pp 229533.I1RLP1
156228 229533.A0A1C3YIR8 pp 229533.I1RN65
156229 229533.A0A1C3YIR8 pp 229533.V6RBN2
156230 229533.A0A1C3YIR8 pp 229533.I1RE00
156231 229533.A0A1C3YIR8 pp 229533.V6RUT5
156232 229533.A0A1C3YIR8 pp 229533.I1RZZ1
156233 229533.A0A1C3YIR8 pp 229533.I1S4Q4
156234 229533.A0A1C3YIR8 pp 229533.I1RAT7
156235 229533.A0A1C3YIR8 pp 229533.I1RPV5
156236 229533.A0A1C3YIR8 pp 229533.I1REL7
156237 229533.A0A1C3YIR8 pp 229533.Q4IJK2
156238 229533.A0A1C3YIR8 pp 229533.I1S7M2
156239 229533.A0A1C3YIR8 pp 229533.I1S0R7
156240 229533.A0A1C3YIR8 pp 229533.Q4IJU0
156241 229533.A0A1C3YIR8 pp 229533.I1RFQ5
156242 229533.A0A1C3YIR8 pp 229533.I1RQA3
156243 229533.A0A1C3YIR8 pp 229533.I1RML9
156244 229533.A0A1C3YIR8 pp 229533.A0A1C3YJN7
156245 229533.A0A1C3YIR8 pp 229533.I1RE31
156246 229533.A0A1C3YIR8 pp 229533.I1RN17
156247 229533.A0A1C3YIR8 pp 229533.I1RP96
156248 229533.A0A1C3YIR8 pp 229533.I1RK95
156249 229533.A0A1C3YIR8 pp 229533.I1REK8
156250 229533.A0A1C3YIR8 pp 229533.I1RP86
156251 229533.A0A1C3YIR8 pp 229533.I1RA48
156252 229533.A0A1C3YIR8 pp 229533.I1RCR5
156253 229533.A0A1C3YIR8 pp 229533.I1RQZ2
156254 229533.A0A1C3YIR8 pp 229533.I1REN3
156255 229533.A0A1C3YIR8 pp 229533.I1RAE0
156256 229533.A0A1C3YIR8 pp 229533.I1RMU8
156257 229533.A0A1C3YIR8 pp 229533.Q4IQX3
156258 229533.A0A1C3YIR8 pp 229533.I1RX46
156259 229533.A0A1C3YIR8 pp 229533.I1RDU0
156260 229533.A0A1C3YIR8 pp 229533.A0A1C3YKH8
156261 229533.A0A1C3YIR8 pp 229533.A0A1C3YJT5
156262 229533.A0A1C3YIR8 pp 229533.I1RRG3
156263 229533.A0A1C3YIR8 pp 229533.Q4HYQ4
156264 229533.A0A1C3YIR8 pp 229533.I1RP21
156265 229533.A0A1C3YIR8 pp 229533.A0A1C3YL54
156266 229533.A0A1C3YIR8 pp 229533.A0A1C3YM63
156267 229533.A0A1C3YIR8 pp 229533.I1S0T2
156268 229533.A0A1C3YIR8 pp 229533.I1RBD5
156269 229533.A0A1C3YIR8 pp 229533.A0A1C3YJJ2
156270 229533.A0A1C3YIR8 pp 229533.A0A1C3YK88
156271 229533.A0A1C3YIR8 pp 229533.I1RLV1
156272 229533.A0A1C3YIR8 pp 229533.I1S201
156273 229533.A0A1C3YIR8 pp 229533.I1RKQ5
156274 229533.A0A1C3YIR8 pp 229533.I1RRX5
156275 229533.A0A1C3YIR8 pp 229533.I1RFC0
156276 229533.A0A1C3YIR8 pp 229533.I1RRW3
156277 229533.A0A1C3YIR8 pp 229533.I1RA85
156278 229533.A0A1C3YIR8 pp 229533.I1RAV6
156279 229533.A0A1C3YIR8 pp 229533.A0A1C3YJ68
156280 229533.A0A1C3YIR8 pp 229533.A0A1C3YIW9
156281 229533.A0A1C3YIR8 pp 229533.I1RSQ7
156282 229533.A0A1C3YIR8 pp 229533.I1RT33
156283 229533.A0A1C3YIR8 pp 229533.I1RKB1
156284 229533.A0A1C3YIR8 pp 229533.I1RBW4
156285 229533.A0A1C3YIR8 pp 229533.I1RD52
156286 229533.A0A1C3YIR8 pp 229533.V6QWX8
156287 229533.A0A1C3YIR8 pp 229533.I1RA46
156288 229533.A0A1C3YIR8 pp 229533.V6RTJ8
156289 229533.A0A1C3YIR8 pp 229533.A0A1C3YN26
156290 229533.A0A1C3YIR8 pp 229533.I1RPN4
156291 229533.A0A1C3YIR8 pp 229533.I1RDD3
156292 229533.A0A1C3YIR8 pp 229533.I1R9Y4
156293 229533.A0A1C3YIR8 pp 229533.I1RTC2

156294 229533.A0A1C3YIR8 pp 229533.I1RAX6
156295 229533.A0A1C3YIR8 pp 229533.I1RM36
156296 229533.A0A1C3YIR8 pp 229533.I1RW91
156297 229533.A0A1C3YIR8 pp 229533.I1RW71
156298 229533.A0A1C3YIR8 pp 229533.I1S0X6
156299 229533.A0A1C3YIR8 pp 229533.I1S4S4
156300 229533.A0A1C3YIR8 pp 229533.I1RBT1
156301 229533.A0A1C3YIR8 pp 229533.A0A1C3YK13
156302 229533.A0A1C3YIR8 pp 229533.I1S4S7
156303 229533.A0A1C3YIR8 pp 229533.I1RNH1
156304 229533.A0A1C3YIR8 pp 229533.I1RMU7
156305 229533.A0A1C3YIR8 pp 229533.I1RW90
156306 229533.A0A1C3YIR8 pp 229533.A0A1C3YK11
156307 229533.A0A1C3YIR8 pp 229533.I1RB50
156308 229533.A0A1C3YIR8 pp 229533.I1RWL3
156309 229533.A0A1C3YIR8 pp 229533.I1RNL0
156310 229533.A0A1C3YIR8 pp 229533.I1RR24
156311 229533.A0A1C3YIR8 pp 229533.I1RUG0
156312 229533.A0A1C3YIR8 pp 229533.I1RZR6
156313 229533.A0A1C3YIR8 pp 229533.I1RJU3
156314 229533.A0A1C3YIR8 pp 229533.I1RPW8
156315 229533.A0A1C3YIR8 pp 229533.I1S182
156316 229533.A0A1C3YIR8 pp 229533.I1RC98
156317 229533.A0A1C3YIR8 pp 229533.I1RTF4
156318 229533.A0A1C3YIR8 pp 229533.I1RD22
156319 229533.A0A1C3YIR8 pp 229533.I1RCQ6
156320 229533.A0A1C3YIR8 pp 229533.I1RM67
156321 229533.A0A1C3YIR8 pp 229533.I1RRN2
156322 229533.A0A1C3YIR8 pp 229533.I1RMN6
156323 229533.A0A1C3YIR8 pp 229533.I1RCU3
156324 229533.A0A1C3YIR8 pp 229533.I1S0C6
156325 229533.A0A1C3YIR8 pp 229533.I1RSU2
156326 229533.A0A1C3YIR8 pp 229533.I1RCS1
156327 229533.A0A1C3YIR8 pp 229533.I1RDU9
156328 229533.A0A1C3YIR8 pp 229533.I1RS66
156329 229533.A0A1C3YIR8 pp 229533.Q4IPN9
156330 229533.A0A1C3YIR8 pp 229533.I1RHL7
156331 229533.A0A1C3YIR8 pp 229533.I1RNQ6
156332 229533.A0A1C3YIR8 pp 229533.I1S7J8
156333 229533.A0A1C3YIR8 pp 229533.I1RB71
156334 229533.A0A1C3YIR8 pp 229533.I1S1W8
156335 229533.A0A1C3YIR8 pp 229533.I1RQ42
156336 229533.A0A1C3YIR8 pp 229533.I1RNP4
156337 229533.A0A1C3YIR8 pp 229533.I1RG11
156338 229533.A0A1C3YIR8 pp 229533.I1RVJ1
156339 229533.A0A1C3YIR8 pp 229533.A0A1C3YLR6
156340 229533.A0A1C3YIR8 pp 229533.I1RCR8
156341 229533.A0A1C3YIR8 pp 229533.I1RW25
156342 229533.A0A1C3YIR8 pp 229533.A0A1C3YIZ3
156343 229533.A0A1C3YIR8 pp 229533.I1RUG3
156344 229533.A0A1C3YIR8 pp 229533.Q4IPC8
156345 229533.A0A1C3YIR8 pp 229533.I1RNC1
156346 229533.A0A1C3YIR8 pp 229533.V6RGN5
156347 229533.A0A1C3YIR8 pp 229533.A0A1C3YLQ8
156348 229533.A0A1C3YIR8 pp 229533.I1RMV0
156349 229533.A0A1C3YIR8 pp 229533.I1RNB8
156350 229533.A0A1C3YIR8 pp 229533.I1RQZ3
156351 229533.A0A1C3YIR8 pp 229533.V6RMW6
156352 229533.A0A1C3YIR8 pp 229533.I1RPU8
156353 229533.A0A1C3YIR8 pp 229533.I1RRF7
156354 229533.A0A1C3YIR8 pp 229533.I1RZ42
156355 229533.A0A1C3YIR8 pp 229533.I1S5R6
156356 229533.A0A1C3YIR8 pp 229533.V6R042
156357 229533.A0A1C3YIR8 pp 229533.I1RE54
156358 229533.A0A1C3YIR8 pp 229533.I1S0J1
156359 229533.A0A1C3YIR8 pp 229533.I1RCB7
156360 229533.A0A1C3YIR8 pp 229533.I1RA55
156361 229533.A0A1C3YIR8 pp 229533.I1RPK0
156362 229533.A0A1C3YIR8 pp 229533.V6RLM7
156363 229533.A0A1C3YIR8 pp 229533.I1RSQ3
156364 229533.A0A1C3YIR8 pp 229533.I1RC72
156365 229533.A0A1C3YIR8 pp 229533.I1RSD4
156366 229533.A0A1C3YIR8 pp 229533.A0A1C3YJ12

156367	229533.A0A1C3YIR8	pp	229533.I1RXU0
156368	229533.A0A1C3YIR8	pp	229533.I1S7W5
156369	229533.A0A1C3YIR8	pp	229533.Q4I7S1
156370	229533.A0A1C3YIR8	pp	229533.I1RJW8
156371	229533.A0A1C3YIR8	pp	229533.I1RTA0
156372	229533.A0A1C3YIR8	pp	229533.A0A1C3YL34
156373	229533.A0A1C3YIR8	pp	229533.I1RSA4
156374	229533.A0A1C3YIR8	pp	229533.I1RSI6
156375	229533.A0A1C3YIR8	pp	229533.A0A1C3YLC1
156376	229533.A0A1C3YIR8	pp	229533.A0A1C3YLB6
156377	229533.A0A1C3YIR8	pp	229533.A0A1C3YLS9
156378	229533.A0A1C3YIR8	pp	229533.I1S1W3
156379	229533.A0A1C3YIR8	pp	229533.Q4IQT8
156380	229533.A0A1C3YIR8	pp	229533.I1S3N1
156381	229533.A0A1C3YIR8	pp	229533.Q4I5U9
156382	229533.A0A1C3YIR8	pp	229533.I1RY44
156383	229533.A0A1C3YIR8	pp	229533.Q4HYB8
156384	229533.A0A1C3YIR8	pp	229533.I1RE01
156385	229533.A0A1C3YIR8	pp	229533.I1R9X7
156386	229533.A0A1C3YIR8	pp	229533.I1S8U9
156387	229533.A0A1C3YIR8	pp	229533.I1RAW0
156388	229533.A0A1C3YIR8	pp	229533.I1RM96
156389	229533.A0A1C3YIR8	pp	229533.I1S9Z4
156390	229533.A0A1C3YIR8	pp	229533.I1RFP4
156391	229533.A0A1C3YIR8	pp	229533.I1RZU4
156392	229533.A0A1C3YIR8	pp	229533.A0A1C3YK33
156393	229533.A0A1C3YIR8	pp	229533.I1S276
156394	229533.A0A1C3YIR8	pp	229533.I1RYW3
156395	229533.A0A1C3YIR8	pp	229533.I1S1C7
156396	229533.A0A1C3YIR8	pp	229533.I1RWF3
156397	229533.A0A1C3YIR8	pp	229533.I1S1W9
156398	229533.A0A1C3YIR8	pp	229533.I1RWN6
156399	229533.A0A1C3YIR8	pp	229533.I1RXU9
156400	229533.A0A1C3YIR8	pp	229533.A0A1C3YN55
156401	229533.A0A1C3YIR8	pp	229533.I1RD38
156402	229533.A0A1C3YIR8	pp	229533.A0A1C3YKY0
156403	229533.A0A1C3YIR8	pp	229533.I1RWN5
156404	229533.A0A1C3YIR8	pp	229533.I1RK92
156405	229533.A0A1C3YIR8	pp	229533.I1RKR8
156406	229533.A0A1C3YIR8	pp	229533.A0A1C3YKP5
156407	229533.A0A1C3YIR8	pp	229533.I1RCW6
156408	229533.A0A1C3YIR8	pp	229533.I1RZQ6
156409	229533.A0A1C3YIR8	pp	229533.I1RK79
156410	229533.A0A1C3YIR8	pp	229533.I1RR61
156411	229533.A0A1C3YIR8	pp	229533.I1S5N3
156412	229533.A0A1C3YIR8	pp	229533.I1S2E8
156413	229533.A0A1C3YIR8	pp	229533.I1S2C0
156414	229533.A0A1C3YIR8	pp	229533.I1RRN6
156415	229533.A0A1C3YIR8	pp	229533.I1RQE7
156416	229533.A0A1C3YIR8	pp	229533.I1RCH5
156417	229533.A0A1C3YIR8	pp	229533.I1RBJ1
156418	229533.A0A1C3YIR8	pp	229533.I1RJY3
156419	229533.A0A1C3YIR8	pp	229533.I1RRI2
156420	229533.A0A1C3YIR8	pp	229533.I1S2C4
156421	229533.A0A1C3YIR8	pp	229533.I1S8R3
156422	229533.A0A1C3YIR8	pp	229533.I1RMV1
156423	229533.A0A1C3YIR8	pp	229533.I1S0E8
156424	229533.A0A1C3YIR8	pp	229533.I1RW23
156425	229533.A0A1C3YIR8	pp	229533.I1RKC6
156426	229533.A0A1C3YIR8	pp	229533.I1RTH6
156427	229533.A0A1C3YIR8	pp	229533.I1RFL4
156428	229533.A0A1C3YIR8	pp	229533.V6R0U9
156429	229533.A0A1C3YIR8	pp	229533.I1RSD8
156430	229533.A0A1C3YIR8	pp	229533.I1RSG8
156431	229533.A0A1C3YIR8	pp	229533.I1RA70
156432	229533.A0A1C3YIR8	pp	229533.I1RB92
156433	229533.A0A1C3YIR8	pp	229533.V6RRH2
156434	229533.A0A1C3YIR8	pp	229533.I1RG90
156435	229533.A0A1C3YIR8	pp	229533.I1RPM7
156436	229533.A0A1C3YIR8	pp	229533.I1RK71
156437	229533.A0A1C3YIR8	pp	229533.I1RJV3
156438	229533.A0A1C3YIR8	pp	229533.Q4HWE2
156439	229533.A0A1C3YIR8	pp	229533.I1RS41

229533.A0A1C3YIR8 pp 229533.I1RR25
229533.A0A1C3YIR8 pp 229533.I1S8V0
229533.A0A1C3YIR8 pp 229533.I1RXR1
229533.A0A1C3YIR8 pp 229533.A0A1C3YMM2
229533.A0A1C3YIR8 pp 229533.I1RSS4
229533.A0A1C3YIR8 pp 229533.V6RUY9
229533.A0A1C3YIR8 pp 229533.I1RGI5
229533.A0A1C3YIR8 pp 229533.Q4I624
229533.A0A1C3YIR8 pp 229533.I1RSY2
229533.A0A1C3YIR8 pp 229533.I1RUG4
229533.A0A1C3YIR8 pp 229533.I1R9C7
229533.A0A1C3YIR8 pp 229533.I1RS06
229533.A0A1C3YIR8 pp 229533.I1RT04
229533.A0A1C3YIR8 pp 229533.I1RAI9
229533.A0A1C3YIR8 pp 229533.I1RWT1
229533.A0A1C3YIR8 pp 229533.I1RVX0
229533.A0A1C3YIR8 pp 229533.I1S0N5
229533.A0A1C3YIR8 pp 229533.A0A1C3YL55
229533.A0A1C3YIR8 pp 229533.I1RT03
229533.A0A1C3YIR8 pp 229533.I1RTD5
229533.A0A1C3YIR8 pp 229533.V6R2A1
229533.A0A1C3YIR8 pp 229533.I1RQ88
229533.A0A1C3YIR8 pp 229533.Q4HXF0
229533.A0A1C3YIR8 pp 229533.A0A1C3YMK1
229533.A0A1C3YIR8 pp 229533.I1RSZ3
229533.A0A1C3YIR8 pp 229533.I1RML0
229533.A0A1C3YIR8 pp 229533.I1R9R7
229533.A0A1C3YIR8 pp 229533.I1RHQ5
229533.A0A1C3YIR8 pp 229533.I1S0B1
229533.A0A1C3YIR8 pp 229533.I1RW80
229533.A0A1C3YIR8 pp 229533.V6R3J1
229533.A0A1C3YIR8 pp 229533.I1RT40
229533.A0A1C3YIR8 pp 229533.I1RTA1
229533.A0A1C3YIR8 pp 229533.I1RV91
229533.A0A1C3YIR8 pp 229533.A0A1C3YJI6
229533.A0A1C3YIR8 pp 229533.I1RZM2
229533.A0A1C3YIR8 pp 229533.I1S2E9
229533.A0A1C3YIR8 pp 229533.I1RFL3
229533.A0A1C3YIR8 pp 229533.A0A1C3YKM6
229533.A0A1C3YIR8 pp 229533.I1RSX0
229533.A0A1C3YIR8 pp 229533.I1RHL8
229533.A0A1C3YIR8 pp 229533.I1RFW1
229533.A0A1C3YIR8 pp 229533.I1RJL4
229533.A0A1C3YIR8 pp 229533.A0A1C3YM67
229533.A0A1C3YIR8 pp 229533.I1RQS5
229533.A0A1C3YIR8 pp 229533.I1RXF2
229533.A0A1C3YIR8 pp 229533.V6R283
229533.A0A1C3YIR8 pp 229533.Q4I5W5
229533.A0A1C3YIR8 pp 229533.I1RFL7
229533.A0A1C3YIR8 pp 229533.Q4IJ11
229533.A0A1C3YIR8 pp 229533.A0A1C3YLP8
229533.A0A1C3YIR8 pp 229533.I1RRM8
229533.A0A1C3YIR8 pp 229533.I1RNG4
229533.A0A1C3YIR8 pp 229533.I1RPP3
229533.A0A1C3YIR8 pp 229533.I1RS43
229533.A0A1C3YIR8 pp 229533.Q4IMD1
229533.A0A1C3YIR8 pp 229533.I1S503
229533.A0A1C3YIR8 pp 229533.I1S8P5
229533.A0A1C3YIR8 pp 229533.I1R9N1
229533.A0A1C3YIR8 pp 229533.Q4IPA4
229533.A0A1C3YIR8 pp 229533.I1RQ65
229533.A0A1C3YIR8 pp 229533.I1RYI5
229533.A0A1C3YIR8 pp 229533.I1RAB5
229533.A0A1C3YIR8 pp 229533.A0A1C3YLK8
229533.A0A1C3YIR8 pp 229533.I1S4M5
229533.A0A1C3YIR8 pp 229533.A0A1C3YIU5
229533.A0A1C3YIR8 pp 229533.I1RTH2
229533.A0A1C3YIR8 pp 229533.A0A1C3YL15
229533.A0A1C3YIR8 pp 229533.I1RJV4
229533.A0A1C3YIR8 pp 229533.I1RFT1
229533.A0A1C3YIR8 pp 229533.I1RC31
229533.A0A1C3YIR8 pp 229533.I1RC05
229533.A0A1C3YIR8 pp 229533.A0A1C3YN63

229533.A0A1C3YIR8	pp	229533.I1RGA4
229533.A0A1C3YIR8	pp	229533.I1RBC7
229533.A0A1C3YIR8	pp	229533.I1RVZ2
229533.A0A1C3YIR8	pp	229533.I1RX63
229533.A0A1C3YIR8	pp	229533.I1RS47
229533.A0A1C3YIR8	pp	229533.I1S214
229533.A0A1C3YIR8	pp	229533.I1RZ29
229533.A0A1C3YIR8	pp	229533.I1S2P0
229533.A0A1C3YIR8	pp	229533.I1RRT8
229533.A0A1C3YIR8	pp	229533.I1SAA0
229533.A0A1C3YIR8	pp	229533.I1RE30
229533.A0A1C3YIR8	pp	229533.I1RSQ4
229533.A0A1C3YIR8	pp	229533.I1RC66
229533.A0A1C3YIR8	pp	229533.I1RJL3
229533.A0A1C3YIR8	pp	229533.I1S9Y1
229533.A0A1C3YIR8	pp	229533.I1S3N0
229533.A0A1C3YIR8	pp	229533.I1RD36
229533.A0A1C3YIR8	pp	229533.I1RZZ9
229533.A0A1C3YIR8	pp	229533.I1RS30
229533.A0A1C3YIR8	pp	229533.V6R3X5
229533.A0A1C3YIR8	pp	229533.I1RWI5
229533.A0A1C3YIR8	pp	229533.A0A1C3YJK3
229533.A0A1C3YIR8	pp	229533.A0A1C3YJW4
229533.A0A1C3YIR8	pp	229533.I1S0L4
229533.A0A1C3YIR8	pp	229533.I1RN67
229533.A0A1C3YIR8	pp	229533.I1RR72
229533.A0A1C3YIR8	pp	229533.I1S2H4
229533.A0A1C3YIR8	pp	229533.I1RJD6
229533.A0A1C3YIR8	pp	229533.I1RKX6
229533.A0A1C3YIR8	pp	229533.A0A1C3YJD4
229533.A0A1C3YIR8	pp	229533.I1RRI6
229533.A0A1C3YIR8	pp	229533.I1S253
229533.A0A1C3YIR8	pp	229533.I1RC29
229533.A0A1C3YIR8	pp	229533.I1RLP9
229533.A0A1C3YIR8	pp	229533.A0A1C3YKX5
229533.A0A1C3YIR8	pp	229533.I1S3G4
229533.A0A1C3YIR8	pp	229533.Q4IB50
229533.A0A1C3YIR8	pp	229533.I1RC24
229533.A0A1C3YIR8	pp	229533.Q4HTT1
229533.A0A1C3YIR8	pp	229533.A0A1C3YN33
229533.A0A1C3YIR8	pp	229533.I1R9Q0
229533.A0A1C3YIR8	pp	229533.I1RE76
229533.A0A1C3YIR8	pp	229533.I1RC00
229533.A0A1C3YIR8	pp	229533.I1RFS3
229533.A0A1C3YIR8	pp	229533.I1RZ39
229533.A0A1C3YIR8	pp	229533.I1S0J3
229533.A0A1C3YIR8	pp	229533.I1RBY4
229533.A0A1C3YIR8	pp	229533.I1RL04
229533.A0A1C3YIR8	pp	229533.I1RRM5
229533.A0A1C3YIR8	pp	229533.A0A1C3YKM9
229533.A0A1C3YIR8	pp	229533.I1S6Y8
229533.A0A1C3YIR8	pp	229533.I1RLR0
229533.A0A1C3YIR8	pp	229533.I1RA11
229533.A0A1C3YIR8	pp	229533.I1RPV3
229533.A0A1C3YIR8	pp	229533.I1RXC2
229533.A0A1C3YIR8	pp	229533.A0A1C3YLW6
229533.A0A1C3YIR8	pp	229533.I1S2B8
229533.A0A1C3YIR8	pp	229533.O74638
229533.A0A1C3YIR8	pp	229533.I1RVL8
229533.A0A1C3YIR8	pp	229533.I1RVY0
229533.A0A1C3YIR8	pp	229533.I1S5R1
229533.A0A1C3YIR8	pp	229533.I1RA84
229533.A0A1C3YIR8	pp	229533.I1RLV3
229533.A0A1C3YIR8	pp	229533.I1RSZ7
229533.A0A1C3YIR8	pp	229533.I1S0R5
229533.A0A1C3YIR8	pp	229533.I1RBR5
229533.A0A1C3YIR8	pp	229533.Q4I099
229533.A0A1C3YIR8	pp	229533.I1R9M0
229533.A0A1C3YIR8	pp	229533.I1RN04
229533.A0A1C3YIR8	pp	229533.I1RYE2
229533.A0A1C3YIR8	pp	229533.I1RWI0
229533.A0A1C3YIR8	pp	229533.I1RM42
229533.A0A1C3YIR8	pp	229533.A0A1C3YJZ2

229533.A0A1C3YIR8 pp 229533.A0A1C3YMA1
229533.A0A1C3YIR8 pp 229533.A0A1C3YMY3
229533.A0A1C3YIR8 pp 229533.I1SAN0
229533.A0A0E0SD93 pp 229533.I1RC95
229533.A0A0E0SD93 pp 229533.I1RKK0
229533.A0A0E0SD93 pp 229533.I1RC17
229533.A0A0E0SD93 pp 229533.I1RA43
229533.A0A0E0SD93 pp 229533.I1RAM7
229533.A0A0E0SD93 pp 229533.I1R9Y9
229533.A0A0E0SD93 pp 229533.V6RKY7
229533.A0A0E0SD93 pp 229533.A0A1C3YJN3
229533.A0A0E0SD93 pp 229533.V6RVG3
229533.A0A0E0SD93 pp 229533.I1S1W4
229533.A0A0E0SD93 pp 229533.I1RC22
229533.A0A0E0SD93 pp 229533.I1RN08
229533.A0A0E0SD93 pp 229533.I1RMM9
229533.A0A0E0SD93 pp 229533.I1RRY3
229533.A0A0E0SD93 pp 229533.I1S0G0
229533.A0A0E0SD93 pp 229533.I1RNY9
229533.A0A0E0SD93 pp 229533.A0A1C3YM29
229533.A0A0E0SD93 pp 229533.I1RB75
229533.A0A0E0SD93 pp 229533.I1RC97
229533.A0A0E0SD93 pp 229533.I1RDB1
229533.A0A0E0SD93 pp 229533.I1RZ63
229533.A0A0E0SD93 pp 229533.A0A1C3YI28
229533.A0A0E0SD93 pp 229533.I1S0N1
229533.A0A0E0SD93 pp 229533.I1S439
229533.A0A0E0SD93 pp 229533.I1RPX0
229533.A0A0E0SD93 pp 229533.I1S0M2
229533.A0A0E0SD93 pp 229533.I1RAN2
229533.A0A0E0SD93 pp 229533.A0A1C3YKF2
229533.A0A0E0SD93 pp 229533.I1RNP7
229533.A0A0E0SD93 pp 229533.I1RQ26
229533.A0A0E0SD93 pp 229533.I1RJM7
229533.A0A0E0SD93 pp 229533.I1RD18
229533.A0A0E0SD93 pp 229533.A0A1C3YJ81
229533.A0A0E0SD93 pp 229533.I1RUN8
229533.A0A0E0SD93 pp 229533.I1RJH1
229533.A0A0E0SD93 pp 229533.A0A1C3YIL0
229533.A0A0E0SD93 pp 229533.I1RYH9
229533.A0A0E0SD93 pp 229533.I1RB74
229533.I1RGD2 pp 229533.I1RWD1
229533.I1RGD2 pp 229533.I1RXF4
229533.I1RGD2 pp 229533.I1RRL4
229533.I1RSW5 pp 229533.I1RT36
229533.I1RSW5 pp 229533.I1RW37
229533.I1RSW5 pp 229533.I1RZQ6
229533.I1RSW5 pp 229533.I1RWQ0
229533.I1RSW5 pp 229533.V6RV57
229533.I1RSW5 pp 229533.I1RZD9
229533.I1RSW5 pp 229533.I1S2A8
229533.I1RSW5 pp 229533.P0C431
229533.I1RSW5 pp 229533.I1S050
229533.I1RSW5 pp 229533.I1S095
229533.I1RSW5 pp 229533.I1S0S6
229533.I1RBC7 pp 229533.I1RPW9
229533.I1RBC7 pp 229533.I1RWL3
229533.I1RBC7 pp 229533.I1RS66
229533.I1RBC7 pp 229533.I1RR24
229533.I1RBC7 pp 229533.I1S4S4
229533.I1RBC7 pp 229533.I1RFQ7
229533.I1RBC7 pp 229533.I1RM67
229533.I1RBC7 pp 229533.I1RL23
229533.I1RBC7 pp 229533.I1RCL4
229533.I1RBC7 pp 229533.I1RT80
229533.I1RBC7 pp 229533.I1RNV6
229533.I1RBC7 pp 229533.I1RPU0
229533.I1RBC7 pp 229533.Q4HVW2
229533.I1RBC7 pp 229533.Q4IFI0
229533.I1RBC7 pp 229533.Q4IJH1
229533.I1RBC7 pp 229533.I1RDA0
229533.I1RBC7 pp 229533.I1RTF4
229533.I1RBC7 pp 229533.V6QYF0

156659	229533.I1RBC7	pp	229533.I1RW90
156660	229533.I1RBC7	pp	229533.I1RMU7
156661	229533.I1RBC7	pp	229533.I1RG51
156662	229533.I1RBC7	pp	229533.I1RUG0
156663	229533.I1RBC7	pp	229533.I1S1W8
156664	229533.I1RBC7	pp	229533.I1RPW8
156665	229533.I1RBC7	pp	229533.I1RRH5
156666	229533.I1RBC7	pp	229533.I1RJU3
156667	229533.I1RBC7	pp	229533.V6QWX8
156668	229533.I1RBC7	pp	229533.I1RD52
156669	229533.I1RBC7	pp	229533.I1RBT1
156670	229533.I1RBC7	pp	229533.I1RQM6
156671	229533.I1RBC7	pp	229533.I1RS19
156672	229533.I1RBC7	pp	229533.I1RCS1
156673	229533.I1RBC7	pp	229533.I1RZR6
156674	229533.I1RBC7	pp	229533.I1RT33
156675	229533.I1RBC7	pp	229533.I1RNM7
156676	229533.I1RBC7	pp	229533.I1RW91
156677	229533.I1RBC7	pp	229533.I1RNH1
156678	229533.I1RBC7	pp	229533.I1RT05
156679	229533.I1RBC7	pp	229533.I1RKB1
156680	229533.I1RBC7	pp	229533.I1RQ42
156681	229533.I1RBC7	pp	229533.I1RRY9
156682	229533.I1RBC7	pp	229533.I1RZ99
156683	229533.I1RBC7	pp	229533.I1RSR3
156684	229533.I1RBC7	pp	229533.V6RTJ8
156685	229533.I1RBC7	pp	229533.I1RX46
156686	229533.I1RBC7	pp	229533.I1RQJ5
156687	229533.I1RBC7	pp	229533.I1RFC0
156688	229533.I1RBC7	pp	229533.I1RJY2
156689	229533.I1RBC7	pp	229533.I1S0T2
156690	229533.I1RBC7	pp	229533.I1RRX5
156691	229533.I1RBC7	pp	229533.V6QV38
156692	229533.I1RBC7	pp	229533.I1RBL7
156693	229533.I1RBC7	pp	229533.I1RBW4
156694	229533.I1RBC7	pp	229533.I1RSQ7
156695	229533.I1RBC7	pp	229533.I1RRY1
156696	229533.I1RBC7	pp	229533.I1RKQ5
156697	229533.I1RBC7	pp	229533.I1RYS0
156698	229533.I1RBC7	pp	229533.I1RTC2
156699	229533.I1RBC7	pp	229533.I1RXD7
156700	229533.I1RBC7	pp	229533.I1RW71
156701	229533.I1RBC7	pp	229533.I1RML9
156702	229533.I1RBC7	pp	229533.I1S4S7
156703	229533.I1RBC7	pp	229533.I1S5Z2
156704	229533.I1RBC7	pp	229533.I1RP96
156705	229533.I1RBC7	pp	229533.I1RU02
156706	229533.I1RBC7	pp	229533.I1RQA2
156707	229533.I1RBC7	pp	229533.I1RPN4
156708	229533.I1RBC7	pp	229533.I1RBD2
156709	229533.I1RBC7	pp	229533.I1RSI4
156710	229533.I1RBC7	pp	229533.Q4HYQ4
156711	229533.I1RBC7	pp	229533.Q4INZ9
156712	229533.I1RBC7	pp	229533.I1REL7
156713	229533.I1RBC7	pp	229533.I1RCM5
156714	229533.I1RBC7	pp	229533.I1RZK5
156715	229533.I1RBC7	pp	229533.I1RRG3
156716	229533.I1RBC7	pp	229533.I1RJL2
156717	229533.I1RBC7	pp	229533.I1RPU4
156718	229533.I1RBC7	pp	229533.Q4IJU0
156719	229533.I1RBC7	pp	229533.Q4IER8
156720	229533.I1RBC7	pp	229533.I1RDG0
156721	229533.I1RBC7	pp	229533.Q4I5I4
156722	229533.I1RBC7	pp	229533.I1RN99
156723	229533.I1RBC7	pp	229533.I1RK95
156724	229533.I1RBC7	pp	229533.I1RZB5
156725	229533.I1RBC7	pp	229533.I1RBE7
156726	229533.I1RBC7	pp	229533.I1RDU0
156727	229533.I1RBC7	pp	229533.I1RN17
156728	229533.I1RBC7	pp	229533.I1RVZ4
156729	229533.I1RBC7	pp	229533.Q4IBS2
156730	229533.I1RBC7	pp	229533.I1RF08
156731	229533.I1RBC7	pp	229533.V6QUE6

156732	229533.I1RBC7	pp	229533.V6RBN2
156733	229533.I1RBC7	pp	229533.I1RCR5
156734	229533.I1RBC7	pp	229533.I1REK8
156735	229533.I1RBC7	pp	229533.I1REN3
156736	229533.I1RBC7	pp	229533.I1RE00
156737	229533.I1RBC7	pp	229533.I1S4Q4
156738	229533.I1RBC7	pp	229533.Q4IJK2
156739	229533.I1RBC7	pp	229533.I1RRV4
156740	229533.I1RBC7	pp	229533.I1RGC2
156741	229533.I1RBC7	pp	229533.I1RSI0
156742	229533.I1RBC7	pp	229533.I1S2U2
156743	229533.I1RBC7	pp	229533.I1RR51
156744	229533.I1RBC7	pp	229533.I1RFJ9
156745	229533.I1RBC7	pp	229533.I1RVZ0
156746	229533.I1RBC7	pp	229533.I1S044
156747	229533.I1RBC7	pp	229533.I1S5B0
156748	229533.I1RBC7	pp	229533.Q4HYI0
156749	229533.I1RBC7	pp	229533.I1S255
156750	229533.I1RBC7	pp	229533.I1RBE4
156751	229533.I1RBC7	pp	229533.V6RCZ8
156752	229533.I1RBC7	pp	229533.V6R227
156753	229533.I1RBC7	pp	229533.I1S6K9
156754	229533.I1RBC7	pp	229533.I1RUY5
156755	229533.I1RBC7	pp	229533.I1RRT0
156756	229533.I1RBC7	pp	229533.I1RMQ4
156757	229533.I1RBC7	pp	229533.I1S0A0
156758	229533.I1RBC7	pp	229533.Q4HTT2
156759	229533.I1RBC7	pp	229533.I1RZF6
156760	229533.I1RBC7	pp	229533.I1RE14
156761	229533.I1RBC7	pp	229533.I1RJI3
156762	229533.I1RBC7	pp	229533.I1RCW8
156763	229533.I1RBC7	pp	229533.I1RX14
156764	229533.I1RBC7	pp	229533.I1S9U1
156765	229533.I1RBC7	pp	229533.I1RXC4
156766	229533.I1RBC7	pp	229533.I1RXE5
156767	229533.I1RBC7	pp	229533.I1S021
156768	229533.I1RBC7	pp	229533.I1RDC9
156769	229533.I1RBC7	pp	229533.I1RQ31
156770	229533.I1RBC7	pp	229533.I1RNH6
156771	229533.I1RBC7	pp	229533.I1RC16
156772	229533.I1RBC7	pp	229533.I1RCG6
156773	229533.I1RBC7	pp	229533.I1RG56
156774	229533.I1RBC7	pp	229533.I1RMQ7
156775	229533.I1RBC7	pp	229533.I1RT35
156776	229533.I1RBC7	pp	229533.I1S419
156777	229533.I1RBC7	pp	229533.I1RXF0
156778	229533.I1RBC7	pp	229533.I1RT01
156779	229533.I1RBC7	pp	229533.I1S0L4
156780	229533.I1RBC7	pp	229533.I1RZM8
156781	229533.I1RBC7	pp	229533.I1RT99
156782	229533.I1RBC7	pp	229533.I1RBR5
156783	229533.I1RBC7	pp	229533.I1RGF6
156784	229533.I1RBC7	pp	229533.I1S2H4
156785	229533.I1RBC7	pp	229533.I1RVY0
156786	229533.I1RBC7	pp	229533.I1S2B8
156787	229533.I1RBC7	pp	229533.I1RS18
156788	229533.I1RBC7	pp	229533.I1RC15
156789	229533.I1RBC7	pp	229533.I1S0R5
156790	229533.I1RBC7	pp	229533.I1RS20
156791	229533.I1RBC7	pp	229533.Q4IJ56
156792	229533.I1RBC7	pp	229533.I1RJZ7
156793	229533.I1RBC7	pp	229533.I1S3G4
156794	229533.I1RBC7	pp	229533.I1RD64
156795	229533.I1RBC7	pp	229533.I1RZ39
156796	229533.I1RBC7	pp	229533.I1RJ17
156797	229533.I1RBC7	pp	229533.I1RXC2
156798	229533.I1RBC7	pp	229533.I1RR72
156799	229533.I1RBC7	pp	229533.I1RN04
156800	229533.I1RBC7	pp	229533.I1RC24
156801	229533.I1RBC7	pp	229533.O74638
156802	229533.I1RBC7	pp	229533.I1RZS6
156803	229533.I1RBC7	pp	229533.I1RVN1
156804	229533.I1RBC7	pp	229533.I1RC66

156805	229533.I1RBC7	pp	229533.I1RX24
156806	229533.I1RBC7	pp	229533.Q4HTT1
156807	229533.I1RBC7	pp	229533.I1S5R1
156808	229533.I1RBC7	pp	229533.I1RRT8
156809	229533.I1RBC7	pp	229533.I1RZZ9
156810	229533.I1RBC7	pp	229533.I1RSQ4
156811	229533.I1RBC7	pp	229533.I1RBG9
156812	229533.I1RBC7	pp	229533.I1RWI5
156813	229533.I1RBC7	pp	229533.I1RC29
156814	229533.I1RBC7	pp	229533.I1RKF9
156815	229533.I1RBC7	pp	229533.I1S9Y1
156816	229533.I1RBC7	pp	229533.Q4IF76
156817	229533.I1RBC7	pp	229533.I1RKJ6
156818	229533.I1RBC7	pp	229533.I1RFS3
156819	229533.I1RBC7	pp	229533.I1RPV3
156820	229533.I1RBC7	pp	229533.I1RVW0
156821	229533.I1RBC7	pp	229533.I1RSZ7
156822	229533.I1RBC7	pp	229533.I1S6Y8
156823	229533.I1RBC7	pp	229533.I1S253
156824	229533.I1RBC7	pp	229533.I1SAN0
156825	229533.I1RBC7	pp	229533.Q4I099
156826	229533.I1RBC7	pp	229533.Q4HXF0
156827	229533.I1RBC7	pp	229533.I1RVL8
156828	229533.I1RBC7	pp	229533.V6RAC9
156829	229533.I1RBC7	pp	229533.I1RC82
156830	229533.I1RBC7	pp	229533.I1RBY4
156831	229533.I1RBC7	pp	229533.I1RSZ3
156832	229533.I1RBC7	pp	229533.I1RJL3
156833	229533.I1RBC7	pp	229533.I1RTD5
156834	229533.I1RBC7	pp	229533.I1RGA4
156835	229533.I1RBC7	pp	229533.I1RFN1
156836	229533.I1RBC7	pp	229533.I1S8P5
156837	229533.I1RBC7	pp	229533.Q4I5W5
156838	229533.I1RBC7	pp	229533.I1RQ88
156839	229533.I1RBC7	pp	229533.I1RQS5
156840	229533.I1RBC7	pp	229533.I1RV91
156841	229533.I1RBC7	pp	229533.I1RYI5
156842	229533.I1RBC7	pp	229533.I1RPP3
156843	229533.I1RBC7	pp	229533.Q4IMD1
156844	229533.I1RBC7	pp	229533.I1RFL3
156845	229533.I1RBC7	pp	229533.I1RFL7
156846	229533.I1RBC7	pp	229533.I1RYK0
156847	229533.I1RBC7	pp	229533.I1RT40
156848	229533.I1RBC7	pp	229533.I1RQ65
156849	229533.I1RBC7	pp	229533.V6R3J1
156850	229533.I1RBC7	pp	229533.I1RW80
156851	229533.I1RBC7	pp	229533.I1S1F3
156852	229533.I1RBC7	pp	229533.I1RRJ8
156853	229533.I1RBC7	pp	229533.I1RS47
156854	229533.I1RBC7	pp	229533.I1RKL3
156855	229533.I1RBC7	pp	229533.Q4I830
156856	229533.I1RBC7	pp	229533.I1RFT1
156857	229533.I1RBC7	pp	229533.I1RTA1
156858	229533.I1RBC7	pp	229533.I1RSX0
156859	229533.I1RBC7	pp	229533.I1RTH2
156860	229533.I1RBC7	pp	229533.I1RJL4
156861	229533.I1RBC7	pp	229533.Q4IJ11
156862	229533.I1RBC7	pp	229533.Q4HZ42
156863	229533.I1RBC7	pp	229533.I1RCP7
156864	229533.I1RBC7	pp	229533.I1S214
156865	229533.I1RBC7	pp	229533.I1RKB2
156866	229533.I1RBC7	pp	229533.I1RZM2
156867	229533.I1RBC7	pp	229533.I1RFW1
156868	229533.I1RBC7	pp	229533.I1RRQ3
156869	229533.I1RBC7	pp	229533.I1RC05
156870	229533.I1RBC7	pp	229533.I1RE30
156871	229533.I1RBC7	pp	229533.I1RVZ2
156872	229533.I1RBC7	pp	229533.I1RC03
156873	229533.I1RBC7	pp	229533.I1RRP3
156874	229533.I1RBC7	pp	229533.Q4IN00
156875	229533.I1RBC7	pp	229533.I1S058
156876	229533.I1RBC7	pp	229533.V6R283
156877	229533.I1RBC7	pp	229533.I1RN67

156878	229533.I1RBC7	pp	229533.I1RYW3
156879	229533.I1RBC7	pp	229533.I1RNG4
156880	229533.I1RBC7	pp	229533.I1S276
156881	229533.I1RBC7	pp	229533.I1RS30
156882	229533.I1RBC7	pp	229533.I1RS43
156883	229533.I1RBC7	pp	229533.I1S1C7
156884	229533.I1RBC7	pp	229533.I1S274
156885	229533.I1RBC7	pp	229533.I1RZQ6
156886	229533.I1RBC7	pp	229533.I1S1X9
156887	229533.I1RBC7	pp	229533.I1RCL3
156888	229533.I1RBC7	pp	229533.I1RD38
156889	229533.I1RBC7	pp	229533.I1RVX0
156890	229533.I1RBC7	pp	229533.I1RT03
156891	229533.I1RBC7	pp	229533.I1RCW6
156892	229533.I1RBC7	pp	229533.I1RK79
156893	229533.I1RBC7	pp	229533.I1RPM7
156894	229533.I1RBC7	pp	229533.I1RSS4
156895	229533.I1RBC7	pp	229533.I1RR25
156896	229533.I1RBC7	pp	229533.I1RJV3
156897	229533.I1RBC7	pp	229533.I1RK71
156898	229533.I1RBC7	pp	229533.V6R057
156899	229533.I1RBC7	pp	229533.I1RCH5
156900	229533.I1RBC7	pp	229533.I1RR61
156901	229533.I1RBC7	pp	229533.I1RDJ8
156902	229533.I1RBC7	pp	229533.I1RJY3
156903	229533.I1RBC7	pp	229533.I1S296
156904	229533.I1RBC7	pp	229533.I1S2E8
156905	229533.I1RBC7	pp	229533.I1RJM5
156906	229533.I1RBC7	pp	229533.I1RQE7
156907	229533.I1RBC7	pp	229533.I1S2C4
156908	229533.I1RBC7	pp	229533.I1S2C0
156909	229533.I1RBC7	pp	229533.I1RW23
156910	229533.I1RBC7	pp	229533.Q4HZ68
156911	229533.I1RBC7	pp	229533.I1RFL4
156912	229533.I1RBC7	pp	229533.I1RTE9
156913	229533.I1RBC7	pp	229533.Q4I624
156914	229533.I1RBC7	pp	229533.I1RUG4
156915	229533.I1RBC7	pp	229533.I1RSD8
156916	229533.I1RBC7	pp	229533.I1RMV1
156917	229533.I1RBC7	pp	229533.I1RSG8
156918	229533.I1RBC7	pp	229533.V6RRH2
156919	229533.I1RBC7	pp	229533.I1RRI2
156920	229533.I1RBC7	pp	229533.I1RD22
156921	229533.I1RBC7	pp	229533.I1S0E8
156922	229533.I1RBC7	pp	229533.Q4I5U9
156923	229533.I1RBC7	pp	229533.Q4IPC8
156924	229533.I1RBC7	pp	229533.I1RM96
156925	229533.I1RBC7	pp	229533.I1RSU2
156926	229533.I1RBC7	pp	229533.I1RMN6
156927	229533.I1RBC7	pp	229533.I1RC27
156928	229533.I1RBC7	pp	229533.I1RXU0
156929	229533.I1RBC7	pp	229533.I1RNQ6
156930	229533.I1RBC7	pp	229533.I1RPU8
156931	229533.I1RBC7	pp	229533.I1S5R6
156932	229533.I1RBC7	pp	229533.I1RJW8
156933	229533.I1RBC7	pp	229533.I1RC54
156934	229533.I1RBC7	pp	229533.I1RRF7
156935	229533.I1RBC7	pp	229533.V6RMW6
156936	229533.I1RBC7	pp	229533.I1RWF3
156937	229533.I1RBC7	pp	229533.I1S4T3
156938	229533.I1RBC7	pp	229533.V6R042
156939	229533.I1RBC7	pp	229533.I1RRN2
156940	229533.I1RBC7	pp	229533.Q4HZB8
156941	229533.I1RBC7	pp	229533.I1S1W9
156942	229533.I1RBC7	pp	229533.I1RCB7
156943	229533.I1RBC7	pp	229533.I1RSI6
156944	229533.I1RBC7	pp	229533.I1RNB8
156945	229533.I1RBC7	pp	229533.I1RSQ3
156946	229533.I1RBC7	pp	229533.I1RC72
156947	229533.I1RBC7	pp	229533.I1RNU6
156948	229533.I1RBC7	pp	229533.I1RSD4
156949	229533.I1RBC7	pp	229533.I1S0J1
156950	229533.I1RBC7	pp	229533.I1RUG3

156951 229533.I1RBC7 pp 229533.I1RCR8
156952 229533.I1RBC7 pp 229533.Q4HYB8
156953 229533.I1RBC7 pp 229533.I1RNC1
156954 229533.I1RBC7 pp 229533.Q4IQT8
156955 229533.I1RBC7 pp 229533.I1RRN6
156956 229533.I1RBC7 pp 229533.I1RTA0
156957 229533.I1RBC7 pp 229533.I1RBJ1
156958 229533.I1RBC7 pp 229533.I1RE01
156959 229533.I1RBZ9 pp 229533.I1RCL8
156960 229533.I1RBZ9 pp 229533.I1RT36
156961 229533.I1RBZ9 pp 229533.I1RZE4
156962 229533.I1RBZ9 pp 229533.I1RRW0
156963 229533.I1RBZ9 pp 229533.I1S0M2
156964 229533.I1RBZ9 pp 229533.I1RQB4
156965 229533.I1RBZ9 pp 229533.V6RKY7
156966 229533.I1RBZ9 pp 229533.I1RRP7
156967 229533.I1RBZ9 pp 229533.I1S1W4
156968 229533.I1RBZ9 pp 229533.I1RQ72
156969 229533.I1RBZ9 pp 229533.I1RRY3
156970 229533.I1RBZ9 pp 229533.I1RDZ4
156971 229533.I1RBZ9 pp 229533.V6RVG3
156972 229533.I1RBZ9 pp 229533.I1RY44
156973 229533.I1RBZ9 pp 229533.I1S439
156974 229533.I1RBZ9 pp 229533.I1RC97
156975 229533.I1RBZ9 pp 229533.I1S2E9
156976 229533.I1RBZ9 pp 229533.I1RMP0
156977 229533.I1RBZ9 pp 229533.I1RX38
156978 229533.I1RBZ9 pp 229533.I1RNF4
156979 229533.I1RBZ9 pp 229533.I1S3G4
156980 229533.I1RBZ9 pp 229533.I1RN65
156981 229533.I1RBZ9 pp 229533.I1RC17
156982 229533.I1RBZ9 pp 229533.I1RPD6
156983 229533.I1RBZ9 pp 229533.I1RQA3
156984 229533.I1RBZ9 pp 229533.I1RNP7
156985 229533.I1RBZ9 pp 229533.I1RC00
156986 229533.I1RBZ9 pp 229533.I1S0Z7
156987 229533.I1RBZ9 pp 229533.I1RKK0
156988 229533.I1RBZ9 pp 229533.I1RST9
156989 229533.I1RBZ9 pp 229533.I1RLU1
156990 229533.I1RBZ9 pp 229533.I1RMM9
156991 229533.I1RBZ9 pp 229533.I1RC95
156992 229533.I1RBZ9 pp 229533.I1RJ88
156993 229533.I1RBZ9 pp 229533.I1RC71
156994 229533.I1RBZ9 pp 229533.I1RWT6
156995 229533.I1RBZ9 pp 229533.I1RUN8
156996 229533.I1RBZ9 pp 229533.I1RM09
156997 229533.I1RBZ9 pp 229533.I1S267
156998 229533.I1RBZ9 pp 229533.I1RUQ5
156999 229533.I1RBZ9 pp 229533.I1RN08
157000 229533.I1RBZ9 pp 229533.I1RZH4
157001 229533.I1RBZ9 pp 229533.I1RC22
157002 229533.I1RBZ9 pp 229533.I1RD18
157003 229533.I1RBZ9 pp 229533.I1RQ26
157004 229533.I1RBZ9 pp 229533.I1RPK5
157005 229533.I1RBZ9 pp 229533.I1RX70
157006 229533.I1RBZ9 pp 229533.I1RDB1
157007 229533.I1RBZ9 pp 229533.I1RV66
157008 229533.I1RBZ9 pp 229533.I1RNC0
157009 229533.I1RBZ9 pp 229533.I1RWJ6
157010 229533.I1RBZ9 pp 229533.I1S1W8
157011 229533.I1RBZ9 pp 229533.I1S224
157012 229533.I1RBZ9 pp 229533.I1RYH9
157013 229533.A0A1C3YJU0 pp 229533.I1RTJ2
157014 229533.A0A1C3YJU0 pp 229533.V6RK96
157015 229533.A0A1C3YJU0 pp 229533.I1RW69
157016 229533.A0A1C3YJU0 pp 229533.I1RST9
157017 229533.A0A1C3YJU0 pp 229533.I1RZK8
157018 229533.A0A1C3YJU0 pp 229533.I1RUY5
157019 229533.A0A1C3YJU0 pp 229533.V6QU63
157020 229533.A0A1C3YJU0 pp 229533.V6RDH3
157021 229533.A0A1C3YJU0 pp 229533.I1RX59
157022 229533.A0A1C3YJU0 pp 229533.I1RA13
157023 229533.A0A1C3YJU0 pp 229533.I1RA50

157024 229533.A0A1C3YJU0 pp 229533.I1S8F8
157025 229533.A0A1C3YJU0 pp 229533.I1RDU0
157026 229533.A0A1C3YJU0 pp 229533.V6QU65
157027 229533.A0A1C3YJU0 pp 229533.I1RNG5
157028 229533.A0A1C3YJU0 pp 229533.I1RCT7
157029 229533.A0A1C3YJU0 pp 229533.I1RCL0
157030 229533.A0A1C3YJU0 pp 229533.I1RCU7
157031 229533.A0A1C3YJU0 pp 229533.I1RBE7
157032 229533.A0A1C3YJU0 pp 229533.I1RC74
157033 229533.A0A1C3YJU0 pp 229533.I1S6H3
157034 229533.A0A1C3YJU0 pp 229533.V6RS91
157035 229533.A0A1C3YJU0 pp 229533.I1S241
157036 229533.A0A1C3YJU0 pp 229533.I1RNT2
157037 229533.A0A1C3YJU0 pp 229533.I1RNM7
157038 229533.A0A1C3YJU0 pp 229533.I1RTG4
157039 229533.A0A1C3YJU0 pp 229533.I1RGK9
157040 229533.A0A1C3YJU0 pp 229533.I1S094
157041 229533.A0A1C3YJU0 pp 229533.I1RZM4
157042 229533.A0A1C3YJU0 pp 229533.I1RQM4
157043 229533.A0A1C3YJU0 pp 229533.I1RRL5
157044 229533.A0A1C3YJU0 pp 229533.I1S1W8
157045 229533.A0A1C3YJU0 pp 229533.I1RY49
157046 229533.A0A1C3YJU0 pp 229533.I1RZ38
157047 229533.A0A1C3YJU0 pp 229533.I1RQE8
157048 229533.A0A1C3YJU0 pp 229533.I1RDN3
157049 229533.A0A1C3YJU0 pp 229533.I1RKB2
157050 229533.A0A1C3YJU0 pp 229533.I1RLA2
157051 229533.A0A1C3YJU0 pp 229533.I1S334
157052 229533.A0A1C3YJU0 pp 229533.V6RW66
157053 229533.A0A1C3YJU0 pp 229533.I1S0A5
157054 229533.A0A1C3YJU0 pp 229533.I1RW11
157055 229533.A0A1C3YJU0 pp 229533.I1RPV8
157056 229533.A0A1C3YJU0 pp 229533.I1RNG4
157057 229533.A0A1C3YJU0 pp 229533.I1RFK3
157058 229533.A0A1C3YJU0 pp 229533.V6RHD0
157059 229533.A0A1C3YJU0 pp 229533.I1RZ56
157060 229533.A0A1C3YJU0 pp 229533.I1S279
157061 229533.A0A1C3YJU0 pp 229533.I1RJS1
157062 229533.A0A1C3YJU0 pp 229533.I1RAH3
157063 229533.A0A1C3YJU0 pp 229533.Q4I1N3
157064 229533.A0A1C3YJU0 pp 229533.I1S3H8
157065 229533.A0A1C3YJU0 pp 229533.I1RBY9
157066 229533.A0A1C3YJU0 pp 229533.A0A1C3YKN4
157067 229533.A0A1C3YJU0 pp 229533.I1S3G4
157068 229533.A0A1C3YJU0 pp 229533.I1RE58
157069 229533.A0A1C3YJU0 pp 229533.I1RZ25
157070 229533.A0A1C3YJU0 pp 229533.V6RK40
157071 229533.A0A1C3YJU0 pp 229533.I1RGZ3
157072 229533.A0A1C3YJU0 pp 229533.V6RXR0
157073 229533.I1RTP2 pp 229533.I1S2L1
157074 229533.I1RTP2 pp 229533.I1S2B0
157075 229533.I1RTP2 pp 229533.I1RWN0
157076 229533.I1RTP2 pp 229533.I1RY34
157077 229533.I1RTP2 pp 229533.Q4I8Q4
157078 229533.I1RTP2 pp 229533.I1S8X0
157079 229533.I1RTP2 pp 229533.I1RW82
157080 229533.I1RTP2 pp 229533.I1S360
157081 229533.I1RTP2 pp 229533.Q4ICM0
157082 229533.I1RTP2 pp 229533.I1S368
157083 229533.I1RTP2 pp 229533.I1RV85
157084 229533.I1RF42 pp 229533.V6RXR0
157085 229533.I1RF42 pp 229533.I1RYM5
157086 229533.I1RF42 pp 229533.I1RHU0
157087 229533.I1RF42 pp 229533.V6QU65
157088 229533.I1RF42 pp 229533.V6RHD0
157089 229533.I1RVU9 pp 229533.I1RW37
157090 229533.I1RVU9 pp 229533.I1RZQ6
157091 229533.I1RRC5 pp 229533.I1RRE0
157092 229533.I1RBE0 pp 229533.I1RW71
157093 229533.I1RBE0 pp 229533.I1RJZ7
157094 229533.I1RFV6 pp 229533.I1RP18
157095 229533.I1RFV6 pp 229533.I1RS07
157096 229533.I1RFV6 pp 229533.I1RZV4

157097	229533.I1RFV6	pp	229533.I1RZD9
157098	229533.I1RFV6	pp	229533.I1S7L6
157099	229533.I1RFV6	pp	229533.I1RRG8
157100	229533.I1RFV6	pp	229533.I1S095
157101	229533.I1RFV6	pp	229533.I1SAP6
157102	229533.I1RFV6	pp	229533.V6RB81
157103	229533.I1RFV6	pp	229533.I1RZK3
157104	229533.I1RV85	pp	229533.Q4I8Q4
157105	229533.I1RV85	pp	229533.I1RWM4
157106	229533.I1RBD8	pp	229533.I1RQK2
157107	229533.I1RBD8	pp	229533.I1RPR4
157108	229533.I1RHP8	pp	229533.V6RDP1
157109	229533.I1RHP8	pp	229533.I1RMD3
157110	229533.I1RHP8	pp	229533.I1RST8
157111	229533.I1RPR4	pp	229533.Q4I2W2
157112	229533.I1RPR4	pp	229533.I1RZH5
157113	229533.I1RPR4	pp	229533.I1S0B1
157114	229533.I1RPR4	pp	229533.I1RPX5
157115	229533.I1RPR4	pp	229533.I1RQK2
157116	229533.I1RPR4	pp	229533.I1RPU8
157117	229533.I1RE53	pp	229533.I1RXY7
157118	229533.I1RE53	pp	229533.I1RS84
157119	229533.I1RE53	pp	229533.I1RWF4
157120	229533.I1RE53	pp	229533.I1REP3
157121	229533.I1RE53	pp	229533.I1RY11
157122	229533.I1RE53	pp	229533.I1RZR5
157123	229533.I1RE53	pp	229533.V6R382
157124	229533.I1RE53	pp	229533.I1RHJ2
157125	229533.I1RE53	pp	229533.V6RN69
157126	229533.I1RE53	pp	229533.I1RZJ8
157127	229533.I1RE53	pp	229533.I1SAT2
157128	229533.I1RE53	pp	229533.I1RFK3
157129	229533.I1RE53	pp	229533.I1S0V7
157130	229533.I1RE53	pp	229533.I1RSU2
157131	229533.I1RE53	pp	229533.I1RLT3
157132	229533.I1RE53	pp	229533.I1RMJ3
157133	229533.I1RE53	pp	229533.I1RM55
157134	229533.I1RE53	pp	229533.I1RPU8
157135	229533.I1RE53	pp	229533.I1RH83
157136	229533.I1RE53	pp	229533.I1RN84
157137	229533.I1RE53	pp	229533.I1S8Y1
157138	229533.I1RE53	pp	229533.I1RK02
157139	229533.I1RE53	pp	229533.I1RSS7
157140	229533.I1RE53	pp	229533.I1RT75
157141	229533.I1RE53	pp	229533.I1S2C4
157142	229533.I1RE53	pp	229533.I1RIB6
157143	229533.I1RE53	pp	229533.I1RWT0
157144	229533.I1RE53	pp	229533.I1RP69
157145	229533.I1RE53	pp	229533.I1RTA9
157146	229533.I1RE53	pp	229533.I1RZJ3
157147	229533.I1RE53	pp	229533.Q4HXF0
157148	229533.I1RE53	pp	229533.I1RR68
157149	229533.I1RE53	pp	229533.I1RSX4
157150	229533.I1RE53	pp	229533.I1RS40
157151	229533.I1RE53	pp	229533.I1RZQ6
157152	229533.I1RE53	pp	229533.I1S0C2
157153	229533.I1RE53	pp	229533.I1S0H5
157154	229533.I1RE53	pp	229533.I1S770
157155	229533.I1RE53	pp	229533.I1RZ20
157156	229533.I1RE53	pp	229533.I1RM54
157157	229533.I1RE53	pp	229533.I1RZ52
157158	229533.I1RE53	pp	229533.I1RNG4
157159	229533.I1RE53	pp	229533.I1RPY0
157160	229533.I1RE53	pp	229533.I1RSK5
157161	229533.I1RE53	pp	229533.I1S4D0
157162	229533.I1RE53	pp	229533.I1RG14
157163	229533.I1RE53	pp	229533.I1RFY0
157164	229533.I1RE53	pp	229533.I1RKA7
157165	229533.I1RE53	pp	229533.I1S2E2
157166	229533.I1RE53	pp	229533.I1S3G4
157167	229533.I1RE53	pp	229533.O74638
157168	229533.I1RE53	pp	229533.I1RVR0
157169	229533.I1RE53	pp	229533.I1RZT0

157170	229533.I1RE53	pp	229533.I1S267
157171	229533.I1RE53	pp	229533.I1S8X1
157172	229533.I1RE53	pp	229533.I1RT96
157173	229533.I1RE53	pp	229533.I1S3A5
157174	229533.I1RE53	pp	229533.V6RY56
157175	229533.I1RE53	pp	229533.V6RQW4
157176	229533.I1RE53	pp	229533.I1RM30
157177	229533.I1RE53	pp	229533.V6RK96
157178	229533.I1RE53	pp	229533.I1RIY0
157179	229533.I1RE53	pp	229533.I1S659
157180	229533.I1RE53	pp	229533.I1RJT5
157181	229533.I1RE53	pp	229533.Q4HVU7
157182	229533.I1RE53	pp	229533.I1RUL3
157183	229533.I1RE53	pp	229533.I1RM26
157184	229533.I1RE53	pp	229533.I1S0N3
157185	229533.I1RE53	pp	229533.I1S3K6
157186	229533.I1RE53	pp	229533.Q4I5G1
157187	229533.I1RE53	pp	229533.I1S0V6
157188	229533.I1RE53	pp	229533.I1S2A1
157189	229533.I1RE53	pp	229533.I1RIB5
157190	229533.I1RE53	pp	229533.I1RIJ2
157191	229533.I1RE53	pp	229533.I1RRS6
157192	229533.I1RE53	pp	229533.I1S1P8
157193	229533.I1RE53	pp	229533.I1S0K2
157194	229533.I1RE53	pp	229533.I1RYD3
157195	229533.I1RE53	pp	229533.I1RT09
157196	229533.I1RE53	pp	229533.V6RBN2
157197	229533.I1RE53	pp	229533.I1RPN1
157198	229533.I1RE53	pp	229533.I1RYX6
157199	229533.I1RE53	pp	229533.I1S9D4
157200	229533.I1RE53	pp	229533.I1RYA9
157201	229533.I1RE53	pp	229533.I1S084
157202	229533.I1RE53	pp	229533.I1RJR9
157203	229533.I1RE53	pp	229533.I1S029
157204	229533.I1RE53	pp	229533.I1RW01
157205	229533.I1RE53	pp	229533.V6R7V8
157206	229533.I1RE53	pp	229533.I1RZM4
157207	229533.I1RE53	pp	229533.I1S5D0
157208	229533.I1RE53	pp	229533.I1RYU5
157209	229533.I1RE53	pp	229533.V6RS91
157210	229533.I1RE53	pp	229533.I1RVX9
157211	229533.I1RE53	pp	229533.I1RP86
157212	229533.I1RE53	pp	229533.I1RW54
157213	229533.I1RE53	pp	229533.I1RYU6
157214	229533.I1RE53	pp	229533.I1S1W8
157215	229533.I1RE53	pp	229533.I1RFK7
157216	229533.I1RE53	pp	229533.V6QUG0
157217	229533.I1RE53	pp	229533.I1RY21
157218	229533.I1RE53	pp	229533.I1RNM7
157219	229533.I1RE53	pp	229533.I1S3K8
157220	229533.I1RE53	pp	229533.I1RQE8
157221	229533.I1RE53	pp	229533.I1S4S1
157222	229533.I1RE53	pp	229533.I1RMU7
157223	229533.I1RP18	pp	229533.I1RVN1
157224	229533.I1RT76	pp	229533.I1RZ29
157225	229533.I1RTI3	pp	229533.Q4I8Q4
157226	229533.I1RTI3	pp	229533.V6RDP1
157227	229533.I1RTI3	pp	229533.I1RV85
157228	229533.I1RXH6	pp	229533.I1S278
157229	229533.I1RXH6	pp	229533.I1S439
157230	229533.I1RXH6	pp	229533.V6RVG3
157231	229533.I1RXH6	pp	229533.Q4IEA7
157232	229533.I1RXH6	pp	229533.I1S1W4
157233	229533.I1RXH6	pp	229533.I1RZC3
157234	229533.I1RXH6	pp	229533.V6RKY7
157235	229533.I1RXH6	pp	229533.I1RYL5
157236	229533.I1RXH6	pp	229533.I1RZE4
157237	229533.I1RXH6	pp	229533.I1RY32
157238	229533.I1RXH6	pp	229533.I1S0X7
157239	229533.I1RXH6	pp	229533.I1S248
157240	229533.I1RXH6	pp	229533.I1S247
157241	229533.I1RXH6	pp	229533.I1S286
157242	229533.I1RXH6	pp	229533.I1S7X9

157243	229533.I1S027	pp	229533.I1S2B8
157244	229533.I1S673	pp	229533.V6RHD0
157245	229533.I1S673	pp	229533.I1S9T6
157246	229533.I1S673	pp	229533.V6QU65
157247	229533.I1S673	pp	229533.V6R4J6
157248	229533.I1S673	pp	229533.I1S694
157249	229533.I1S673	pp	229533.V6RK96
157250	229533.I1S673	pp	229533.V6RXR0
157251	229533.I1S673	pp	229533.I1S6A2
157252	229533.I1RNG4	pp	229533.I1RSI2
157253	229533.I1RNG4	pp	229533.I1S6Y8
157254	229533.I1RNG4	pp	229533.I1RR72
157255	229533.I1RNG4	pp	229533.I1S1C3
157256	229533.I1RNG4	pp	229533.I1S2J4
157257	229533.I1RNG4	pp	229533.I1RZ86
157258	229533.I1RNG4	pp	229533.I1S4Z7
157259	229533.I1RNG4	pp	229533.I1S277
157260	229533.I1RNG4	pp	229533.I1RVZ3
157261	229533.I1RNG4	pp	229533.I1S3X7
157262	229533.I1RNG4	pp	229533.I1S6D1
157263	229533.I1RNG4	pp	229533.I1S6P5
157264	229533.I1RNG4	pp	229533.Q4IMJ1
157265	229533.I1RNG4	pp	229533.V6RK40
157266	229533.I1RNG4	pp	229533.I1S7U6
157267	229533.I1RNG4	pp	229533.I1RUS5
157268	229533.I1RNG4	pp	229533.V6RXR0
157269	229533.I1RNG4	pp	229533.I1RVW0
157270	229533.I1RNG4	pp	229533.Q4IPI4
157271	229533.I1RNG4	pp	229533.I1RYB8
157272	229533.I1RNG4	pp	229533.I1RW43
157273	229533.I1RNG4	pp	229533.V6R5D2
157274	229533.I1RNG4	pp	229533.I1RRT8
157275	229533.I1RNG4	pp	229533.I1RZ29
157276	229533.I1RNG4	pp	229533.I1S0Y7
157277	229533.I1RNG4	pp	229533.I1RQG7
157278	229533.I1RNG4	pp	229533.I1RZ52
157279	229533.I1RNG4	pp	229533.I1RUE7
157280	229533.I1RNG4	pp	229533.I1S4Z8
157281	229533.I1RNG4	pp	229533.O74638
157282	229533.I1RNG4	pp	229533.I1RZT0
157283	229533.I1RNG4	pp	229533.I1RQ38
157284	229533.I1RNG4	pp	229533.I1S1L0
157285	229533.I1RNG4	pp	229533.I1RRJ6
157286	229533.I1RNG4	pp	229533.V6QZ48
157287	229533.I1RNG4	pp	229533.I1RY72
157288	229533.I1RNG4	pp	229533.I1S253
157289	229533.I1RNG4	pp	229533.I1S2V2
157290	229533.I1RNG4	pp	229533.I1RP28
157291	229533.I1RNG4	pp	229533.I1RWK9
157292	229533.I1RNG4	pp	229533.I1S2A6
157293	229533.I1RNG4	pp	229533.I1RSK5
157294	229533.I1RNG4	pp	229533.I1RYA8
157295	229533.I1RNG4	pp	229533.I1S4B6
157296	229533.I1RNG4	pp	229533.I1SAR2
157297	229533.I1RNG4	pp	229533.I1RZ39
157298	229533.I1RNG4	pp	229533.V6RHD0
157299	229533.I1RNG4	pp	229533.I1S9C6
157300	229533.I1RNG4	pp	229533.I1RZ83
157301	229533.I1RNG4	pp	229533.I1RVU7
157302	229533.I1RNG4	pp	229533.V6QV31
157303	229533.I1RNG4	pp	229533.I1RRF5
157304	229533.I1RNG4	pp	229533.I1S3H8
157305	229533.I1RNG4	pp	229533.I1RVR7
157306	229533.I1RNG4	pp	229533.I1RQG4
157307	229533.I1RNG4	pp	229533.I1SAH9
157308	229533.I1RNG4	pp	229533.I1S3G4
157309	229533.I1RNG4	pp	229533.V6RZD4
157310	229533.I1RNG4	pp	229533.I1S4B7
157311	229533.I1RNG4	pp	229533.I1RYW9
157312	229533.I1RNG4	pp	229533.I1RSQ9
157313	229533.I1RNG4	pp	229533.I1RZA7
157314	229533.I1RNG4	pp	229533.I1S2Q4
157315	229533.I1RNG4	pp	229533.V6R2Z2

157316	229533.I1RNG4	pp	229533.I1RR06
157317	229533.I1RNG4	pp	229533.I1RY60
157318	229533.I1RNG4	pp	229533.I1S673
157319	229533.I1RNG4	pp	229533.Q4HVQ9
157320	229533.I1RNG4	pp	229533.I1S5L5
157321	229533.I1RNG4	pp	229533.I1RZ20
157322	229533.I1RNG4	pp	229533.I1RYC5
157323	229533.I1RNG4	pp	229533.I1RS47
157324	229533.I1RNG4	pp	229533.I1S6M5
157325	229533.I1RNG4	pp	229533.I1S299
157326	229533.I1RNG4	pp	229533.I1RQM7
157327	229533.I1RNG4	pp	229533.I1S3K7
157328	229533.I1RNG4	pp	229533.I1RPD6
157329	229533.I1RNG4	pp	229533.I1RP27
157330	229533.I1RNG4	pp	229533.I1RY62
157331	229533.I1RNG4	pp	229533.I1RXF8
157332	229533.I1RNG4	pp	229533.I1RTD5
157333	229533.I1RNG4	pp	229533.I1RNW6
157334	229533.I1RNG4	pp	229533.I1S0Q5
157335	229533.I1RNG4	pp	229533.I1RWF5
157336	229533.I1RNG4	pp	229533.V6RIE5
157337	229533.I1RNG4	pp	229533.I1RYE9
157338	229533.I1RNG4	pp	229533.I1RW00
157339	229533.I1RNG4	pp	229533.I1RNG7
157340	229533.I1RNG4	pp	229533.I1S9T5
157341	229533.I1RNG4	pp	229533.I1RZN1
157342	229533.I1RNG4	pp	229533.I1RT75
157343	229533.I1RNG4	pp	229533.I1RP53
157344	229533.I1RNG4	pp	229533.Q4HXF0
157345	229533.I1RNG4	pp	229533.I1RS06
157346	229533.I1RNG4	pp	229533.V6RUY9
157347	229533.I1RNG4	pp	229533.I1RQE7
157348	229533.I1RNG4	pp	229533.I1S8R2
157349	229533.I1RNG4	pp	229533.I1RQW4
157350	229533.I1RNG4	pp	229533.V6RF62
157351	229533.I1RNG4	pp	229533.I1RP69
157352	229533.I1RNG4	pp	229533.I1S2T6
157353	229533.I1RNG4	pp	229533.I1RZV5
157354	229533.I1RNG4	pp	229533.I1RP97
157355	229533.I1RNG4	pp	229533.I1RSX4
157356	229533.I1RNG4	pp	229533.V6R3J1
157357	229533.I1RNG4	pp	229533.I1RY74
157358	229533.I1RNG4	pp	229533.I1RVE8
157359	229533.I1RNG4	pp	229533.I1RWW7
157360	229533.I1RNG4	pp	229533.I1RY58
157361	229533.I1RNG4	pp	229533.I1RNW2
157362	229533.I1RNG4	pp	229533.V6QVH8
157363	229533.I1RNG4	pp	229533.I1S332
157364	229533.I1RNG4	pp	229533.I1RSS7
157365	229533.I1RNG4	pp	229533.I1S6G9
157366	229533.I1RNG4	pp	229533.I1RP12
157367	229533.I1RNG4	pp	229533.V6RUB1
157368	229533.I1RNG4	pp	229533.I1RXW3
157369	229533.I1RNG4	pp	229533.I1S149
157370	229533.I1RNG4	pp	229533.I1S0H7
157371	229533.I1RNG4	pp	229533.I1S0I3
157372	229533.I1RNG4	pp	229533.I1S8P6
157373	229533.I1RNG4	pp	229533.I1S684
157374	229533.I1RNG4	pp	229533.I1RTY9
157375	229533.I1RNG4	pp	229533.I1RZ70
157376	229533.I1RNG4	pp	229533.I1RNU7
157377	229533.I1RNG4	pp	229533.I1S8H5
157378	229533.I1RNG4	pp	229533.I1RSX9
157379	229533.I1RNG4	pp	229533.I1SAJ3
157380	229533.I1RNG4	pp	229533.I1RNV4
157381	229533.I1RNG4	pp	229533.I1RS77
157382	229533.I1RNG4	pp	229533.I1RVW8
157383	229533.I1RNG4	pp	229533.I1S123
157384	229533.I1RNG4	pp	229533.I1RPA1
157385	229533.I1RNG4	pp	229533.I1RSP7
157386	229533.I1RNG4	pp	229533.I1RRI2
157387	229533.I1RNG4	pp	229533.I1RUE3
157388	229533.I1RNG4	pp	229533.I1S7W5

157389	229533.I1RNG4	pp	229533.I1RYQ0
157390	229533.I1RNG4	pp	229533.I1RZQ6
157391	229533.I1RNG4	pp	229533.V6R382
157392	229533.I1RNG4	pp	229533.I1S1T5
157393	229533.I1RNG4	pp	229533.I1RW21
157394	229533.I1RNG4	pp	229533.I1RUP2
157395	229533.I1RNG4	pp	229533.I1S1B0
157396	229533.I1RNG4	pp	229533.I1S9Z4
157397	229533.I1RNG4	pp	229533.I1S5C5
157398	229533.I1RNG4	pp	229533.V6RSW9
157399	229533.I1RNG4	pp	229533.V6RDZ4
157400	229533.I1RNG4	pp	229533.V6RSF3
157401	229533.I1RNG4	pp	229533.V6QX21
157402	229533.I1RNG4	pp	229533.I1RWZ3
157403	229533.I1RNG4	pp	229533.I1S302
157404	229533.I1RNG4	pp	229533.I1RQW1
157405	229533.I1RNG4	pp	229533.I1S0X1
157406	229533.I1RNG4	pp	229533.Q4I665
157407	229533.I1RNG4	pp	229533.I1RYV2
157408	229533.I1RNG4	pp	229533.I1RTA3
157409	229533.I1RNG4	pp	229533.V6R756
157410	229533.I1RNG4	pp	229533.I1RQ30
157411	229533.I1RNG4	pp	229533.I1RPK0
157412	229533.I1RNG4	pp	229533.I1RZQ1
157413	229533.I1RNG4	pp	229533.V6RLM7
157414	229533.I1RNG4	pp	229533.I1RR42
157415	229533.I1RNG4	pp	229533.I1RRF0
157416	229533.I1RNG4	pp	229533.I1SAV3
157417	229533.I1RNG4	pp	229533.I1S8H4
157418	229533.I1RNG4	pp	229533.I1S380
157419	229533.I1RNG4	pp	229533.I1RZ95
157420	229533.I1RNG4	pp	229533.I1RWH4
157421	229533.I1RNG4	pp	229533.I1S685
157422	229533.I1RNG4	pp	229533.I1S1W9
157423	229533.I1RNG4	pp	229533.I1S599
157424	229533.I1RNG4	pp	229533.I1RR05
157425	229533.I1RNG4	pp	229533.I1S282
157426	229533.I1RNG4	pp	229533.I1S147
157427	229533.I1RNG4	pp	229533.I1RTU5
157428	229533.I1RNG4	pp	229533.I1S6S6
157429	229533.I1RNG4	pp	229533.I1RNL4
157430	229533.I1RNG4	pp	229533.I1S256
157431	229533.I1RNG4	pp	229533.I1RUP5
157432	229533.I1RNG4	pp	229533.I1RSB8
157433	229533.I1RNG4	pp	229533.I1RSU2
157434	229533.I1RNG4	pp	229533.I1RYS3
157435	229533.I1RNG4	pp	229533.I1S5R6
157436	229533.I1RNG4	pp	229533.I1RVZ7
157437	229533.I1RNG4	pp	229533.V6RAG6
157438	229533.I1RNG4	pp	229533.V6R9L8
157439	229533.I1RNG4	pp	229533.I1RQX9
157440	229533.I1RNG4	pp	229533.I1RQ21
157441	229533.I1RNG4	pp	229533.V6RN69
157442	229533.I1RNG4	pp	229533.I1RST4
157443	229533.I1RNG4	pp	229533.I1S111
157444	229533.I1RNG4	pp	229533.I1S0B7
157445	229533.I1RNG4	pp	229533.I1RTD1
157446	229533.I1RNG4	pp	229533.I1RUQ7
157447	229533.I1RNG4	pp	229533.I1S1Q0
157448	229533.I1RNG4	pp	229533.I1RNM7
157449	229533.I1RNG4	pp	229533.V6RRM0
157450	229533.I1RNG4	pp	229533.I1SAI4
157451	229533.I1RNG4	pp	229533.I1RPZ4
157452	229533.I1RNG4	pp	229533.I1RX18
157453	229533.I1RNG4	pp	229533.I1RS90
157454	229533.I1RNG4	pp	229533.I1S7L6
157455	229533.I1RNG4	pp	229533.V6RPQ8
157456	229533.I1RNG4	pp	229533.I1RVD9
157457	229533.I1RNG4	pp	229533.I1RPN8
157458	229533.I1RNG4	pp	229533.I1RPA4
157459	229533.I1RNG4	pp	229533.I1S686
157460	229533.I1RNG4	pp	229533.I1RYU5
157461	229533.I1RNG4	pp	229533.I1RTG4

157462 229533.I1RNG4 pp 229533.I1S7Q8
157463 229533.I1RNG4 pp 229533.I1RT41
157464 229533.I1RNG4 pp 229533.I1RS87
157465 229533.I1RNG4 pp 229533.I1RYZ2
157466 229533.I1RNG4 pp 229533.I1S334
157467 229533.I1RNG4 pp 229533.I1S1W8
157468 229533.I1RNG4 pp 229533.V6R0W1
157469 229533.I1RNG4 pp 229533.I1S270
157470 229533.I1RNG4 pp 229533.I1S1P9
157471 229533.I1RNG4 pp 229533.I1SAF0
157472 229533.I1RNG4 pp 229533.I1RRL5
157473 229533.I1RNG4 pp 229533.I1S029
157474 229533.I1RNG4 pp 229533.I1S724
157475 229533.I1RNG4 pp 229533.I1S8Z2
157476 229533.I1RNG4 pp 229533.I1RPW2
157477 229533.I1RNG4 pp 229533.I1RRG1
157478 229533.I1RNG4 pp 229533.I1RY49
157479 229533.I1RNG4 pp 229533.I1S670
157480 229533.I1RNG4 pp 229533.I1RPI4
157481 229533.I1RNG4 pp 229533.I1S8E0
157482 229533.I1RNG4 pp 229533.I1S3K8
157483 229533.I1RNG4 pp 229533.V6RW66
157484 229533.I1RNG4 pp 229533.V6QWD8
157485 229533.I1RNG4 pp 229533.I1RVB2
157486 229533.I1RNG4 pp 229533.V6R1D2
157487 229533.I1RNG4 pp 229533.I1RQ60
157488 229533.I1RNG4 pp 229533.I1S0R1
157489 229533.I1RNG4 pp 229533.I1RQE8
157490 229533.I1RNG4 pp 229533.I1S4S5
157491 229533.I1RNG4 pp 229533.I1RZM4
157492 229533.I1RNG4 pp 229533.I1RYD3
157493 229533.I1RNG4 pp 229533.I1RZB7
157494 229533.I1RNG4 pp 229533.I1RUW0
157495 229533.I1RNG4 pp 229533.I1RYQ4
157496 229533.I1RNG4 pp 229533.I1RRX5
157497 229533.I1RNG4 pp 229533.I1RSQ2
157498 229533.I1RNG4 pp 229533.I1S2P2
157499 229533.I1RNG4 pp 229533.I1RNW1
157500 229533.I1RNG4 pp 229533.I1RSI4
157501 229533.I1RNG4 pp 229533.I1S241
157502 229533.I1RNG4 pp 229533.I1RYJ7
157503 229533.I1RNG4 pp 229533.I1RT77
157504 229533.I1RNG4 pp 229533.I1RTZ9
157505 229533.I1RNG4 pp 229533.I1RXB1
157506 229533.I1RNG4 pp 229533.V6RH92
157507 229533.I1RNG4 pp 229533.I1S4S7
157508 229533.I1RNG4 pp 229533.I1RPE7
157509 229533.I1RNG4 pp 229533.I1RYK1
157510 229533.I1RNG4 pp 229533.I1S9D4
157511 229533.I1RNG4 pp 229533.I1RSC1
157512 229533.I1RNG4 pp 229533.I1S0K1
157513 229533.I1RNG4 pp 229533.V6QVH2
157514 229533.I1RNG4 pp 229533.I1RUV4
157515 229533.I1RNG4 pp 229533.I1RQH1
157516 229533.I1RNG4 pp 229533.I1RQE1
157517 229533.I1RNG4 pp 229533.I1RY96
157518 229533.I1RNG4 pp 229533.I1RYX6
157519 229533.I1RNG4 pp 229533.I1S1B8
157520 229533.I1RNG4 pp 229533.I1RWC4
157521 229533.I1RNG4 pp 229533.I1RNZ6
157522 229533.I1RNG4 pp 229533.I1RYM6
157523 229533.I1RNG4 pp 229533.I1RQQ8
157524 229533.I1RNG4 pp 229533.I1RQ14
157525 229533.I1RNG4 pp 229533.I1RRY1
157526 229533.I1RNG4 pp 229533.I1RNQ1
157527 229533.I1RNG4 pp 229533.I1S338
157528 229533.I1RNG4 pp 229533.I1S3A5
157529 229533.I1RNG4 pp 229533.I1S0T2
157530 229533.I1RNG4 pp 229533.I1RXY4
157531 229533.I1RNG4 pp 229533.V6RS91
157532 229533.I1RNG4 pp 229533.I1S2C8
157533 229533.I1RNG4 pp 229533.I1RVX9
157534 229533.I1RNG4 pp 229533.I1RX59

157535 229533.I1RNG4 pp 229533.I1RTY0
157536 229533.I1RNG4 pp 229533.V6QU99
157537 229533.I1RNG4 pp 229533.I1RPI5
157538 229533.I1RNG4 pp 229533.I1S3E3
157539 229533.I1RNG4 pp 229533.V6RAM6
157540 229533.I1RNG4 pp 229533.I1RQL4
157541 229533.I1RNG4 pp 229533.V6RJ29
157542 229533.I1RNG4 pp 229533.I1S308
157543 229533.I1RNG4 pp 229533.I1RQX2
157544 229533.I1RNG4 pp 229533.I1RUQ3
157545 229533.I1RNG4 pp 229533.V6RQW4
157546 229533.I1RNG4 pp 229533.I1S4G3
157547 229533.I1RNG4 pp 229533.I1RTU0
157548 229533.I1RNG4 pp 229533.I1RT65
157549 229533.I1RNG4 pp 229533.I1RNG5
157550 229533.I1RNG4 pp 229533.V6RKK5
157551 229533.I1RNG4 pp 229533.I1RPT0
157552 229533.I1RNG4 pp 229533.I1RXV9
157553 229533.I1RNG4 pp 229533.V6RDH3
157554 229533.I1RNG4 pp 229533.I1RNH9
157555 229533.I1RNG4 pp 229533.V6QU65
157556 229533.I1RNG4 pp 229533.I1SAJ7
157557 229533.I1RNG4 pp 229533.I1RYM5
157558 229533.I1RNG4 pp 229533.I1RQA5
157559 229533.I1RNG4 pp 229533.I1S2M7
157560 229533.I1RNG4 pp 229533.V6RY56
157561 229533.I1RNG4 pp 229533.Q4IQC1
157562 229533.I1RNG4 pp 229533.I1RWD7
157563 229533.I1RNG4 pp 229533.V6RGB5
157564 229533.I1RNG4 pp 229533.V6RHZ8
157565 229533.I1RNG4 pp 229533.I1S4Q4
157566 229533.I1RNG4 pp 229533.I1RVY2
157567 229533.I1RNG4 pp 229533.V6R3V4
157568 229533.I1RNG4 pp 229533.I1S2K0
157569 229533.I1RNG4 pp 229533.I1RYL5
157570 229533.I1RNG4 pp 229533.I1RQE0
157571 229533.I1RNG4 pp 229533.I1S0N3
157572 229533.I1RNG4 pp 229533.I1RZK8
157573 229533.I1RNG4 pp 229533.I1RW69
157574 229533.I1RNG4 pp 229533.I1S1L9
157575 229533.I1RNG4 pp 229533.I1RVB7
157576 229533.I1RNG4 pp 229533.V6RPN0
157577 229533.I1RNG4 pp 229533.I1RU76
157578 229533.I1RNG4 pp 229533.I1S183
157579 229533.I1RNG4 pp 229533.I1S1J7
157580 229533.I1RNG4 pp 229533.I1RYA2
157581 229533.I1RNG4 pp 229533.I1RPW1
157582 229533.I1RNG4 pp 229533.I1RWB0
157583 229533.I1RNG4 pp 229533.I1S3D8
157584 229533.I1RNG4 pp 229533.I1S8H9
157585 229533.I1RNG4 pp 229533.I1RRY9
157586 229533.I1RNG4 pp 229533.I1RP66
157587 229533.I1RNG4 pp 229533.I1RW87
157588 229533.I1RNG4 pp 229533.I1RUY4
157589 229533.I1RNG4 pp 229533.I1RV44
157590 229533.I1RNG4 pp 229533.I1RWD2
157591 229533.I1RNG4 pp 229533.I1S5J9
157592 229533.I1RNG4 pp 229533.I1RPR9
157593 229533.I1RNG4 pp 229533.I1RXX0
157594 229533.I1RNG4 pp 229533.I1RPL2
157595 229533.I1RNG4 pp 229533.I1RVA9
157596 229533.I1RNG4 pp 229533.I1S0Z3
157597 229533.I1RNG4 pp 229533.V6RK96
157598 229533.I1RNG4 pp 229533.I1S0T1
157599 229533.I1RNG4 pp 229533.V6RAN2
157600 229533.I1RNG4 pp 229533.V6QU63
157601 229533.I1RNG4 pp 229533.I1RS07
157602 229533.I1RNG4 pp 229533.I1S419
157603 229533.I1RNG4 pp 229533.I1RVC7
157604 229533.I1RNG4 pp 229533.I1RVP5
157605 229533.I1RNG4 pp 229533.I1S0A0
157606 229533.I1RNG4 pp 229533.I1S4D0
157607 229533.I1RNG4 pp 229533.I1RTJ2

157608	229533.I1RNG4	pp	229533.I1S2E2
157609	229533.I1RNG4	pp	229533.I1RVR0
157610	229533.I1RNG4	pp	229533.I1S0Q4
157611	229533.I1RNG4	pp	229533.I1S2V7
157612	229533.I1RNG4	pp	229533.I1RSX2
157613	229533.I1RNG4	pp	229533.I1S6A8
157614	229533.I1RNG4	pp	229533.I1RXF0
157615	229533.I1RNG4	pp	229533.I1S019
157616	229533.I1RNG4	pp	229533.V6RPS5
157617	229533.I1RNG4	pp	229533.I1RNY6
157618	229533.I1RNG4	pp	229533.I1RQQ6
157619	229533.I1RNG4	pp	229533.I1S3K6
157620	229533.I1RNG4	pp	229533.I1RUI6
157621	229533.I1RNG4	pp	229533.I1RSI8
157622	229533.I1RNG4	pp	229533.I1S5R5
157623	229533.I1RNG4	pp	229533.I1RVQ9
157624	229533.I1RNG4	pp	229533.I1RRF9
157625	229533.I1RNG4	pp	229533.I1RYI7
157626	229533.I1RNG4	pp	229533.I1S235
157627	229533.I1RNG4	pp	229533.I1RPR1
157628	229533.I1RNG4	pp	229533.V6QUE6
157629	229533.I1RNG4	pp	229533.I1RST9
157630	229533.I1RNG4	pp	229533.I1RPM6
157631	229533.I1RNG4	pp	229533.I1RR51
157632	229533.I1RNG4	pp	229533.I1RXH2
157633	229533.I1RNG4	pp	229533.I1RUY5
157634	229533.I1RG11	pp	229533.I1S999
157635	229533.I1RG11	pp	229533.I1RNH1
157636	229533.I1RG11	pp	229533.I1RP96
157637	229533.I1RG11	pp	229533.I1S4S7
157638	229533.I1RG11	pp	229533.I1RML9
157639	229533.I1RG11	pp	229533.I1RTC2
157640	229533.I1RG11	pp	229533.V6RTJ8
157641	229533.I1RG11	pp	229533.I1RW71
157642	229533.I1RG11	pp	229533.I1RXD7
157643	229533.I1RG11	pp	229533.I1RKB1
157644	229533.I1RG11	pp	229533.I1RLV1
157645	229533.I1RG11	pp	229533.I1RPN4
157646	229533.I1RG11	pp	229533.I1S0T2
157647	229533.I1RG11	pp	229533.I1RGR8
157648	229533.I1RG11	pp	229533.I1RKQ5
157649	229533.I1RG11	pp	229533.Q4INZ9
157650	229533.I1RG11	pp	229533.I1RSQ7
157651	229533.I1RG11	pp	229533.I1RQJ5
157652	229533.I1RG11	pp	229533.I1RP21
157653	229533.I1RG11	pp	229533.Q4IQX3
157654	229533.I1RG11	pp	229533.I1RT33
157655	229533.I1RG11	pp	229533.I1RRX5
157656	229533.I1RG11	pp	229533.I1RSI4
157657	229533.I1RG11	pp	229533.I1RX46
157658	229533.I1RG11	pp	229533.I1RPW8
157659	229533.I1RG11	pp	229533.Q4HYQ4
157660	229533.I1RG11	pp	229533.I1RMU8
157661	229533.I1RG11	pp	229533.I1RRY1
157662	229533.I1RG11	pp	229533.I1RRG3
157663	229533.I1RG11	pp	229533.I1S182
157664	229533.I1RG11	pp	229533.I1S4S4
157665	229533.I1RG11	pp	229533.Q5I2J3
157666	229533.I1RG11	pp	229533.I1RWL3
157667	229533.I1RG11	pp	229533.I1RR24
157668	229533.I1RG11	pp	229533.I1RZR6
157669	229533.I1RG11	pp	229533.I1S8Z2
157670	229533.I1RG11	pp	229533.Q4HZS8
157671	229533.I1RG11	pp	229533.I1RS66
157672	229533.I1RG11	pp	229533.I1RQM6
157673	229533.I1RG11	pp	229533.V6QWX8
157674	229533.I1RG11	pp	229533.I1RT80
157675	229533.I1RG11	pp	229533.I1RUR0
157676	229533.I1RG11	pp	229533.I1RQ42
157677	229533.I1RG11	pp	229533.I1RKK0
157678	229533.I1RG11	pp	229533.I1RNP4
157679	229533.I1RG11	pp	229533.I1RPU4
157680	229533.I1RG11	pp	229533.I1RJU3

157681 229533.I1RG11 pp 229533.I1RUY5
157682 229533.I1RG11 pp 229533.I1RHL7
157683 229533.I1RG11 pp 229533.I1RN91
157684 229533.I1RG11 pp 229533.I1RUG0
157685 229533.I1RG11 pp 229533.I1S044
157686 229533.I1RG11 pp 229533.I1RMU7
157687 229533.I1RG11 pp 229533.I1RRS8
157688 229533.I1RG11 pp 229533.I1RW90
157689 229533.I1RG11 pp 229533.I1RVJ1
157690 229533.I1RG11 pp 229533.I1RRV4
157691 229533.I1RG11 pp 229533.I1S5B0
157692 229533.I1RG11 pp 229533.I1RW54
157693 229533.I1RG11 pp 229533.I1RW91
157694 229533.I1RG11 pp 229533.I1S1W8
157695 229533.I1RG11 pp 229533.Q4HTT2
157696 229533.I1RG11 pp 229533.V6QUE6
157697 229533.I1RG11 pp 229533.Q4I5I4
157698 229533.I1RG11 pp 229533.I1RR51
157699 229533.I1RG11 pp 229533.I1RGC2
157700 229533.I1RG11 pp 229533.I1RQ31
157701 229533.I1RG11 pp 229533.Q4IPN9
157702 229533.I1RG11 pp 229533.I1RVZ4
157703 229533.I1RG11 pp 229533.I1RNM7
157704 229533.I1RG11 pp 229533.I1RXF0
157705 229533.I1RG11 pp 229533.V6RCZ8
157706 229533.I1RG11 pp 229533.I1RZM8
157707 229533.I1RG11 pp 229533.I1RRY9
157708 229533.I1RG11 pp 229533.I1RJI3
157709 229533.I1RG11 pp 229533.I1RJY2
157710 229533.I1RG11 pp 229533.Q4HYI0
157711 229533.I1RG11 pp 229533.I1S6K9
157712 229533.I1RG11 pp 229533.I1RZL5
157713 229533.I1RG11 pp 229533.I1RZF6
157714 229533.I1RG11 pp 229533.I1RZU9
157715 229533.I1RG11 pp 229533.I1RNH6
157716 229533.I1RG11 pp 229533.I1RQA2
157717 229533.I1RG11 pp 229533.I1S9U1
157718 229533.I1RG11 pp 229533.I1RSI1
157719 229533.I1RG11 pp 229533.V6R227
157720 229533.I1RG11 pp 229533.I1S255
157721 229533.I1RG11 pp 229533.I1RS18
157722 229533.I1RG11 pp 229533.I1S5Z2
157723 229533.I1RG11 pp 229533.I1RXE5
157724 229533.I1RG11 pp 229533.I1RZH4
157725 229533.I1RG11 pp 229533.I1RXC4
157726 229533.I1RG11 pp 229533.I1RWT6
157727 229533.I1RG11 pp 229533.I1RRV5
157728 229533.I1RG11 pp 229533.Q4IER8
157729 229533.I1RG11 pp 229533.I1RRT0
157730 229533.I1RG11 pp 229533.I1RZB5
157731 229533.I1RG11 pp 229533.I1RT35
157732 229533.I1RG11 pp 229533.I1S0P4
157733 229533.I1RG11 pp 229533.I1RMQ7
157734 229533.I1RG11 pp 229533.I1RMQ4
157735 229533.I1RG11 pp 229533.I1RZK5
157736 229533.I1RG11 pp 229533.I1RT99
157737 229533.I1RG11 pp 229533.I1RJ17
157738 229533.I1RG11 pp 229533.I1RT01
157739 229533.I1RG11 pp 229533.I1RU02
157740 229533.I1RG11 pp 229533.I1RYS4
157741 229533.I1RG11 pp 229533.I1RPK5
157742 229533.I1RG11 pp 229533.I1RQA5
157743 229533.I1RG11 pp 229533.V6QV38
157744 229533.I1RG11 pp 229533.I1S419
157745 229533.I1RG11 pp 229533.I1RSP1
157746 229533.I1RG11 pp 229533.I1RM65
157747 229533.I1RG11 pp 229533.Q4I5W5
157748 229533.I1RG11 pp 229533.I1RJL2
157749 229533.I1RG11 pp 229533.I1RN07
157750 229533.I1RG11 pp 229533.I1RN65
157751 229533.I1RG11 pp 229533.I1RYY5
157752 229533.I1RG11 pp 229533.I1RRM8
157753 229533.I1RG11 pp 229533.I1RN17

157754	229533.I1RG11	pp	229533.I1RQZ2
157755	229533.I1RG11	pp	229533.I1RQS5
157756	229533.I1RG11	pp	229533.Q4IMD1
157757	229533.I1RG11	pp	229533.I1RVZ0
157758	229533.I1RG11	pp	229533.I1RK95
157759	229533.I1RG11	pp	229533.I1RN99
157760	229533.I1RG11	pp	229533.I1RSX0
157761	229533.I1RG11	pp	229533.V6R3J1
157762	229533.I1RG11	pp	229533.I1RQA3
157763	229533.I1RG11	pp	229533.Q4IJU0
157764	229533.I1RG11	pp	229533.I1S4Q4
157765	229533.I1RG11	pp	229533.I1RW80
157766	229533.I1RG11	pp	229533.Q4IJM4
157767	229533.I1RG11	pp	229533.Q4IJK2
157768	229533.I1RG11	pp	229533.I1RSI0
157769	229533.I1RG11	pp	229533.Q4HXF0
157770	229533.I1RG11	pp	229533.V6RBN2
157771	229533.I1RG11	pp	229533.I1RHQ5
157772	229533.I1RG11	pp	229533.I1RWT1
157773	229533.I1RG11	pp	229533.I1RGA4
157774	229533.I1RG11	pp	229533.I1RQ88
157775	229533.I1RG11	pp	229533.I1RTD5
157776	229533.I1RG11	pp	229533.I1RR95
157777	229533.I1RG11	pp	229533.Q4IJ11
157778	229533.I1RG11	pp	229533.I1RTH2
157779	229533.I1RG11	pp	229533.I1RMT6
157780	229533.I1RG11	pp	229533.I1RX63
157781	229533.I1RG11	pp	229533.I1S2E9
157782	229533.I1RG11	pp	229533.I1RV91
157783	229533.I1RG11	pp	229533.I1RYI5
157784	229533.I1RG11	pp	229533.I1RS47
157785	229533.I1RG11	pp	229533.I1RT40
157786	229533.I1RG11	pp	229533.I1S1F3
157787	229533.I1RG11	pp	229533.I1RYK0
157788	229533.I1RG11	pp	229533.Q4IPA4
157789	229533.I1RG11	pp	229533.I1RPP3
157790	229533.I1RG11	pp	229533.I1RSZ3
157791	229533.I1RG11	pp	229533.V6RAC9
157792	229533.I1RG11	pp	229533.I1S8P5
157793	229533.I1RG11	pp	229533.I1RRJ8
157794	229533.I1RG11	pp	229533.I1RS30
157795	229533.I1RG11	pp	229533.I1RN67
157796	229533.I1RG11	pp	229533.I1RRV6
157797	229533.I1RG11	pp	229533.I1S9Y1
157798	229533.I1RG11	pp	229533.I1RS43
157799	229533.I1RG11	pp	229533.I1RKJ6
157800	229533.I1RG11	pp	229533.I1RRP3
157801	229533.I1RG11	pp	229533.I1RND9
157802	229533.I1RG11	pp	229533.I1RRT8
157803	229533.I1RG11	pp	229533.V6R283
157804	229533.I1RG11	pp	229533.I1S5R1
157805	229533.I1RG11	pp	229533.I1RHL8
157806	229533.I1RG11	pp	229533.I1S3N0
157807	229533.I1RG11	pp	229533.I1RNG4
157808	229533.I1RG11	pp	229533.I1RTA1
157809	229533.I1RG11	pp	229533.I1RZM2
157810	229533.I1RG11	pp	229533.O74638
157811	229533.I1RG11	pp	229533.I1RXC2
157812	229533.I1RG11	pp	229533.I1RKB2
157813	229533.I1RG11	pp	229533.I1RJL3
157814	229533.I1RG11	pp	229533.I1RZ39
157815	229533.I1RG11	pp	229533.I1RRQ3
157816	229533.I1RG11	pp	229533.I1RVZ2
157817	229533.I1RG11	pp	229533.I1RG13
157818	229533.I1RG11	pp	229533.I1RUM5
157819	229533.I1RG11	pp	229533.I1S0J3
157820	229533.I1RG11	pp	229533.I1S4M5
157821	229533.I1RG11	pp	229533.I1S3G4
157822	229533.I1RG11	pp	229533.I1S253
157823	229533.I1RG11	pp	229533.Q4IB50
157824	229533.I1RG11	pp	229533.I1S2H4
157825	229533.I1RG11	pp	229533.I1RN04
157826	229533.I1RG11	pp	229533.Q4I099

157827	229533.I1RG11	pp	229533.I1RWI5
157828	229533.I1RG11	pp	229533.I1RSZ7
157829	229533.I1RG11	pp	229533.I1S0L4
157830	229533.I1RG11	pp	229533.I1RVL8
157831	229533.I1RG11	pp	229533.I1RSQ4
157832	229533.I1RG11	pp	229533.I1RZZ9
157833	229533.I1RG11	pp	229533.Q4I662
157834	229533.I1RG11	pp	229533.I1RVN1
157835	229533.I1RG11	pp	229533.I1RVW0
157836	229533.I1RG11	pp	229533.I1SAN0
157837	229533.I1RG11	pp	229533.I1RM42
157838	229533.I1RG11	pp	229533.I1S6Y8
157839	229533.I1RG11	pp	229533.I1RPV3
157840	229533.I1RG11	pp	229533.I1RWI0
157841	229533.I1RG11	pp	229533.I1S5R6
157842	229533.I1RG11	pp	229533.I1S0R5
157843	229533.I1RG11	pp	229533.I1RJZ7
157844	229533.I1RG11	pp	229533.I1S3N1
157845	229533.I1RG11	pp	229533.I1RLR0
157846	229533.I1RG11	pp	229533.Q4IPC8
157847	229533.I1RG11	pp	229533.I1RRF7
157848	229533.I1RG11	pp	229533.I1RVY0
157849	229533.I1RG11	pp	229533.Q4I7S1
157850	229533.I1RG11	pp	229533.I1S2B8
157851	229533.I1RG11	pp	229533.I1RXU0
157852	229533.I1RG11	pp	229533.I1RNQ6
157853	229533.I1RG11	pp	229533.V6R042
157854	229533.I1RG11	pp	229533.I1S0C6
157855	229533.I1RG11	pp	229533.I1RSU2
157856	229533.I1RG11	pp	229533.Q4HTT1
157857	229533.I1RG11	pp	229533.I1RMN6
157858	229533.I1RG11	pp	229533.I1RRN2
157859	229533.I1RG11	pp	229533.I1RR72
157860	229533.I1RG11	pp	229533.V6RMW6
157861	229533.I1RG11	pp	229533.I1RSQ3
157862	229533.I1RG11	pp	229533.I1RPU8
157863	229533.I1RG11	pp	229533.I1RZ86
157864	229533.I1RG11	pp	229533.I1RJY3
157865	229533.I1RG11	pp	229533.I1S2C0
157866	229533.I1RG11	pp	229533.I1RWF3
157867	229533.I1RG11	pp	229533.I1S1W9
157868	229533.I1RG11	pp	229533.I1RNC1
157869	229533.I1RG11	pp	229533.Q4HZB8
157870	229533.I1RG11	pp	229533.I1RRN6
157871	229533.I1RG11	pp	229533.I1RZQ6
157872	229533.I1RG11	pp	229533.I1RUG3
157873	229533.I1RG11	pp	229533.Q4I5U9
157874	229533.I1RG11	pp	229533.I1RK79
157875	229533.I1RG11	pp	229533.I1RM96
157876	229533.I1RG11	pp	229533.I1RQ21
157877	229533.I1RG11	pp	229533.I1RY44
157878	229533.I1RG11	pp	229533.I1RSA4
157879	229533.I1RG11	pp	229533.I1S1C7
157880	229533.I1RG11	pp	229533.I1S0J1
157881	229533.I1RG11	pp	229533.I1RJW8
157882	229533.I1RG11	pp	229533.I1RK92
157883	229533.I1RG11	pp	229533.I1RYW3
157884	229533.I1RG11	pp	229533.I1RWX5
157885	229533.I1RG11	pp	229533.I1S274
157886	229533.I1RG11	pp	229533.I1RZU4
157887	229533.I1RG11	pp	229533.I1RRI2
157888	229533.I1RG11	pp	229533.I1S276
157889	229533.I1RG11	pp	229533.Q4IN00
157890	229533.I1RG11	pp	229533.I1RQZ3
157891	229533.I1RG11	pp	229533.I1RZ42
157892	229533.I1RG11	pp	229533.I1RUG4
157893	229533.I1RG11	pp	229533.I1RS41
157894	229533.I1RG11	pp	229533.Q4HYB8
157895	229533.I1RG11	pp	229533.I1RJV3
157896	229533.I1RG11	pp	229533.I1RNB8
157897	229533.I1RG11	pp	229533.Q4IQT8
157898	229533.I1RG11	pp	229533.I1RPM7
157899	229533.I1RG11	pp	229533.Q4I624

157900	229533.I1RG11	pp	229533.I1RR25
157901	229533.I1RG11	pp	229533.I1RK71
157902	229533.I1RG11	pp	229533.I1RMU1
157903	229533.I1RG11	pp	229533.I1RSG8
157904	229533.I1RG11	pp	229533.I1RG90
157905	229533.I1RG11	pp	229533.V6RRH2
157906	229533.I1RG11	pp	229533.I1RSD4
157907	229533.I1RG11	pp	229533.I1RTA0
157908	229533.I1RG11	pp	229533.V6R0U9
157909	229533.I1RG11	pp	229533.I1S2C4
157910	229533.I1RG11	pp	229533.I1RS06
157911	229533.I1RG11	pp	229533.I1RVX0
157912	229533.I1RG11	pp	229533.I1RT03
157913	229533.I1RG11	pp	229533.I1RW23
157914	229533.I1RG11	pp	229533.I1RSD8
157915	229533.I1RG11	pp	229533.I1RSS4
157916	229533.I1RG11	pp	229533.I1RMV1
157917	229533.I1RG11	pp	229533.I1S0E8
157918	229533.I1RG11	pp	229533.I1RR61
157919	229533.I1RG11	pp	229533.I1S2E8
157920	229533.I1RG11	pp	229533.I1RQE7
157921	229533.I1RJH1	pp	229533.I1RZE4
157922	229533.I1RJH1	pp	229533.I1RRY3
157923	229533.I1RJH1	pp	229533.Q4IJM4
157924	229533.I1RJH1	pp	229533.I1S238
157925	229533.I1RJH1	pp	229533.I1RMM9
157926	229533.I1RJH1	pp	229533.I1RUQ5
157927	229533.I1RJH1	pp	229533.I1RN08
157928	229533.I1RJH1	pp	229533.I1RYY5
157929	229533.I1RJH1	pp	229533.V6RVG3
157930	229533.I1RJH1	pp	229533.V6RKY7
157931	229533.I1RJH1	pp	229533.I1S1W4
157932	229533.I1RJH1	pp	229533.I1RWA3
157933	229533.I1RJH1	pp	229533.V6RDP1
157934	229533.I1RJH1	pp	229533.I1RST8
157935	229533.I1RJH1	pp	229533.I1RKK0
157936	229533.I1RJH1	pp	229533.I1S439
157937	229533.I1RJH1	pp	229533.I1RUN8
157938	229533.I1RJH1	pp	229533.Q4IMN9
157939	229533.I1RJH1	pp	229533.I1S0M2
157940	229533.I1RJH1	pp	229533.I1RX70
157941	229533.I1RJH1	pp	229533.I1RNP7
157942	229533.I1RJH1	pp	229533.I1S246
157943	229533.I1RJH1	pp	229533.I1RYH9
157944	229533.I1RJH1	pp	229533.I1S2G7
157945	229533.I1RJH1	pp	229533.I1RMA1
157946	229533.I1RJH1	pp	229533.I1RQ26
157947	229533.I1RPU8	pp	229533.V6RQW4
157948	229533.I1RPU8	pp	229533.V6RY56
157949	229533.I1RPU8	pp	229533.I1S216
157950	229533.I1RPU8	pp	229533.I1S4Q4
157951	229533.I1RPU8	pp	229533.I1S0K2
157952	229533.I1RPU8	pp	229533.V6R057
157953	229533.I1RPU8	pp	229533.I1RRP7
157954	229533.I1RPU8	pp	229533.I1RS41
157955	229533.I1RPU8	pp	229533.V6RBN2
157956	229533.I1RPU8	pp	229533.I1RVX0
157957	229533.I1RPU8	pp	229533.I1S2M8
157958	229533.I1RPU8	pp	229533.I1RUE2
157959	229533.I1RPU8	pp	229533.I1S132
157960	229533.I1RPU8	pp	229533.I1RXV9
157961	229533.I1RPU8	pp	229533.I1RSL5
157962	229533.I1RPU8	pp	229533.I1RZQ6
157963	229533.I1RPU8	pp	229533.I1RTJ7
157964	229533.I1RPU8	pp	229533.I1RYR6
157965	229533.I1RPU8	pp	229533.I1S2C4
157966	229533.I1RPU8	pp	229533.I1RYE6
157967	229533.I1RPU8	pp	229533.I1RS17
157968	229533.I1RPU8	pp	229533.I1RTH6
157969	229533.I1RPU8	pp	229533.I1S3B7
157970	229533.I1RPU8	pp	229533.V6QUE6
157971	229533.I1RPU8	pp	229533.I1S3K6
157972	229533.I1RPU8	pp	229533.I1RYU5

157973 229533.I1RPU8 pp 229533.I1RRV4
157974 229533.I1RPU8 pp 229533.I1S2C5
157975 229533.I1RPU8 pp 229533.I1RW54
157976 229533.I1RPU8 pp 229533.I1RVR0
157977 229533.I1RPU8 pp 229533.I1S6H1
157978 229533.I1RPU8 pp 229533.I1RS18
157979 229533.I1RPU8 pp 229533.I1RV18
157980 229533.I1RPU8 pp 229533.I1S8G4
157981 229533.I1RPU8 pp 229533.O74638
157982 229533.I1RPU8 pp 229533.I1S4D0
157983 229533.I1RPU8 pp 229533.I1RZT0
157984 229533.I1RPU8 pp 229533.I1S2G9
157985 229533.I1RPU8 pp 229533.I1S076
157986 229533.I1RPU8 pp 229533.I1SAN9
157987 229533.I1RPU8 pp 229533.I1S3T7
157988 229533.I1RPU8 pp 229533.I1RX22
157989 229533.I1RPU8 pp 229533.I1RWL3
157990 229533.I1RPU8 pp 229533.I1S029
157991 229533.I1RPU8 pp 229533.I1RQS9
157992 229533.I1RPU8 pp 229533.I1S2H4
157993 229533.I1RPU8 pp 229533.I1S3K8
157994 229533.I1RPU8 pp 229533.V6R1K4
157995 229533.I1RPU8 pp 229533.I1SAI0
157996 229533.I1RPU8 pp 229533.I1RSQ4
157997 229533.I1RPU8 pp 229533.I1S1F3
157998 229533.I1RPU8 pp 229533.I1RXR5
157999 229533.I1RPU8 pp 229533.I1S1P8
158000 229533.I1RPU8 pp 229533.I1RT40
158001 229533.I1RPU8 pp 229533.I1SAJ0
158002 229533.I1RPU8 pp 229533.I1RR68
158003 229533.I1RPU8 pp 229533.I1RTA9
158004 229533.I1RPU8 pp 229533.Q4HXF0
158005 229533.I1RPU8 pp 229533.I1RRX5
158006 229533.I1RPU8 pp 229533.I1S5G3
158007 229533.I1RPU8 pp 229533.I1S2W1
158008 229533.I1RPU8 pp 229533.I1RZ20
158009 229533.I1RPU8 pp 229533.I1RZH6
158010 229533.I1RPU8 pp 229533.I1RXW3
158011 229533.I1RPU8 pp 229533.I1RQ14
158012 229533.I1RPU8 pp 229533.I1S084
158013 229533.A0A098D2Y5 pp 229533.Q4I8C9
158014 229533.A0A098D2Y5 pp 229533.I1RPK0
158015 229533.A0A098D2Y5 pp 229533.A0A0E0SNH8
158016 229533.A0A098D2Y5 pp 229533.A0A1C3YII1
158017 229533.A0A098D2Y5 pp 229533.I1RGL3
158018 229533.A0A098D2Y5 pp 229533.I1RN36
158019 229533.A0A098D2Y5 pp 229533.I1RRZ6
158020 229533.A0A098D2Y5 pp 229533.A0A098DLY3
158021 229533.A0A098D2Y5 pp 229533.I1RWQ0
158022 229533.A0A098D2Y5 pp 229533.I1RSW5
158023 229533.A0A098D2Y5 pp 229533.I1RQM0
158024 229533.A0A098D2Y5 pp 229533.I1RCI5
158025 229533.A0A098D2Y5 pp 229533.I1RZQ6
158026 229533.A0A098D2Y5 pp 229533.I1RVU9
158027 229533.A0A098D2Y5 pp 229533.I1RZD9
158028 229533.A0A098D2Y5 pp 229533.I1RPK5
158029 229533.A0A098D2Y5 pp 229533.I1RPM8
158030 229533.A0A098D2Y5 pp 229533.I1RJS2
158031 229533.A0A098D2Y5 pp 229533.V6RV57
158032 229533.A0A098D2Y5 pp 229533.P0C431
158033 229533.A0A098D2Y5 pp 229533.I1RXG6
158034 229533.A0A098D2Y5 pp 229533.A0A0E0S6X1
158035 229533.A0A098D2Y5 pp 229533.I1RYZ7
158036 229533.A0A098D2Y5 pp 229533.A0A0E0SNY0
158037 229533.A0A098D2Y5 pp 229533.A0A0E0RQT8
158038 229533.A0A098D2Y5 pp 229533.Q4IJ79
158039 229533.A0A098D2Y5 pp 229533.I1RP97
158040 229533.A0A098D2Y5 pp 229533.I1RBE4
158041 229533.A0A098D2Y5 pp 229533.I1RC46
158042 229533.A0A098D2Y5 pp 229533.I1RRB5
158043 229533.A0A098D2Y5 pp 229533.I1RTG7
158044 229533.A0A098D2Y5 pp 229533.V6RQW4
158045 229533.A0A098D2Y5 pp 229533.I1S0Z3

158046 229533.A0A098D2Y5 pp 229533.I1RKE9
158047 229533.A0A098D2Y5 pp 229533.I1RJZ7
158048 229533.A0A098D2Y5 pp 229533.I1RE49
158049 229533.A0A098D2Y5 pp 229533.A0A1C3YL10
158050 229533.A0A098D2Y5 pp 229533.I1RFH9
158051 229533.A0A098D2Y5 pp 229533.A0A1C3YJF1
158052 229533.A0A098D2Y5 pp 229533.I1RT41
158053 229533.A0A098D2Y5 pp 229533.I1RN81
158054 229533.A0A098D2Y5 pp 229533.I1RZX9
158055 229533.A0A098D2Y5 pp 229533.A0A1C3YIJ4
158056 229533.A0A098D2Y5 pp 229533.I1RNF9
158057 229533.A0A098D2Y5 pp 229533.I1RS10
158058 229533.A0A098D2Y5 pp 229533.A0A1C3YMD9
158059 229533.A0A098D2Y5 pp 229533.I1S095
158060 229533.A0A098D2Y5 pp 229533.I1RPI2
158061 229533.A0A098D2Y5 pp 229533.I1RZV4
158062 229533.A0A098D2Y5 pp 229533.I1RWJ6
158063 229533.A0A098D2Y5 pp 229533.I1RMU7
158064 229533.A0A098D2Y5 pp 229533.I1RW71
158065 229533.A0A098D2Y5 pp 229533.A0A1C3YL54
158066 229533.A0A0E0RU18 pp 229533.A0A1C3YMD9
158067 229533.A0A0E0RU18 pp 229533.I1RRY9
158068 229533.A0A0E0RU18 pp 229533.I1RFV6
158069 229533.A0A0E0RU18 pp 229533.I1S7L6
158070 229533.A0A0E0RU18 pp 229533.I1RZD9
158071 229533.A0A0E0RU18 pp 229533.I1RZV4
158072 229533.A0A0E0RU18 pp 229533.I1S095
158073 229533.I1RIY3 pp 229533.P0C431
158074 229533.I1RIY3 pp 229533.I1RPK0
158075 229533.I1RIY3 pp 229533.I1RZQ6
158076 229533.I1RIY3 pp 229533.I1RRR6
158077 229533.I1RIY3 pp 229533.I1RWB5
158078 229533.I1RIY3 pp 229533.V6RDP1
158079 229533.I1RIY3 pp 229533.I1RST8
158080 229533.I1RH63 pp 229533.I1RNG4
158081 229533.I1S091 pp 229533.I1SAP6
158082 229533.I1S091 pp 229533.Q4IJM4
158083 229533.I1S091 pp 229533.I1S2G7
158084 229533.I1S2D2 pp 229533.V6RH92
158085 229533.A0A1C3YLH6 pp 229533.A0A1C3YLH7
158086 229533.A0A1C3YLH6 pp 229533.I1RZV4
158087 229533.A0A1C3YLH6 pp 229533.I1S095
158088 229533.A0A1C3YLH6 pp 229533.I1S254
158089 229533.A0A1C3YLH6 pp 229533.I1RTQ9
158090 229533.A0A1C3YLH6 pp 229533.I1RWL9
158091 229533.A0A1C3YLH6 pp 229533.I1RSV4
158092 229533.A0A1C3YLH6 pp 229533.I1RA34
158093 229533.A0A1C3YLH6 pp 229533.I1RN81
158094 229533.A0A1C3YLH6 pp 229533.I1RDK3
158095 229533.A0A1C3YLH6 pp 229533.I1RH68
158096 229533.A0A1C3YLH6 pp 229533.I1RTB7
158097 229533.A0A1C3YLH6 pp 229533.I1RMR5
158098 229533.A0A1C3YLH6 pp 229533.I1RT41
158099 229533.A0A1C3YLH6 pp 229533.I1RD69
158100 229533.A0A1C3YLH6 pp 229533.I1S0Z2
158101 229533.A0A1C3YLH6 pp 229533.I1RX77
158102 229533.A0A1C3YLH6 pp 229533.I1S2C1
158103 229533.A0A1C3YLH6 pp 229533.I1S0S6
158104 229533.A0A1C3YLH6 pp 229533.I1RB98
158105 229533.A0A1C3YLH6 pp 229533.I1S0V5
158106 229533.A0A1C3YLH6 pp 229533.I1S1V3
158107 229533.A0A1C3YLH6 pp 229533.I1RMU8
158108 229533.A0A1C3YLH6 pp 229533.I1RMU7
158109 229533.A0A1C3YLH6 pp 229533.I1RK32
158110 229533.A0A1C3YLH6 pp 229533.I1RY98
158111 229533.A0A1C3YLH6 pp 229533.I1RB15
158112 229533.A0A1C3YLH6 pp 229533.I1REA6
158113 229533.A0A1C3YLH6 pp 229533.V6QUU6
158114 229533.A0A1C3YLH6 pp 229533.I1RZM1
158115 229533.A0A1C3YLH6 pp 229533.I1RXB5
158116 229533.A0A1C3YLH6 pp 229533.I1RIE2
158117 229533.A0A1C3YLH6 pp 229533.V6REE9
158118 229533.A0A1C3YLH6 pp 229533.I1S0Z3

158119 229533.A0A1C3YLH6 pp 229533.I1RNP1
158120 229533.A0A1C3YLH6 pp 229533.I1RBS4
158121 229533.A0A1C3YLH6 pp 229533.I1RPK9
158122 229533.A0A1C3YLH6 pp 229533.P0C431
158123 229533.A0A1C3YLH6 pp 229533.A0A1C3YN54
158124 229533.A0A1C3YLH6 pp 229533.I1S207
158125 229533.A0A1C3YLH6 pp 229533.I1RHI1
158126 229533.A0A1C3YLH6 pp 229533.I1R991
158127 229533.A0A1C3YLH6 pp 229533.A0A1C3YMD9
158128 229533.A0A1C3YLH6 pp 229533.I1RWA9
158129 229533.A0A1C3YLH6 pp 229533.V6RR76
158130 229533.A0A1C3YLH6 pp 229533.A0A1C3YM95
158131 229533.A0A1C3YLH6 pp 229533.I1RIG4
158132 229533.A0A1C3YLH6 pp 229533.I1RUP6
158133 229533.A0A1C3YLH6 pp 229533.A0A1C3YM82
158134 229533.A0A1C3YLH6 pp 229533.I1RCN6
158135 229533.A0A1C3YLH6 pp 229533.I1RWA5
158136 229533.A0A1C3YLH6 pp 229533.I1RB49
158137 229533.A0A1C3YLH6 pp 229533.I1RE19
158138 229533.A0A1C3YLH6 pp 229533.I1RTG9
158139 229533.A0A1C3YLH6 pp 229533.I1RQN9
158140 229533.A0A1C3YLH6 pp 229533.I1S549
158141 229533.A0A1C3YLH6 pp 229533.I1S7X9
158142 229533.A0A1C3YLH6 pp 229533.I1RNG6
158143 229533.A0A1C3YLH6 pp 229533.I1RRT8
158144 229533.A0A1C3YLH6 pp 229533.I1RBI5
158145 229533.A0A1C3YLH6 pp 229533.I1RD45
158146 229533.A0A1C3YLH6 pp 229533.I1RAN3
158147 229533.A0A1C3YLH6 pp 229533.V6R476
158148 229533.A0A1C3YLH6 pp 229533.I1RCD4
158149 229533.A0A1C3YLH6 pp 229533.A0A1C3YLL6
158150 229533.A0A1C3YLH6 pp 229533.I1RZD9
158151 229533.A0A1C3YLH6 pp 229533.I1S5S8
158152 229533.A0A1C3YLH6 pp 229533.I1RM82
158153 229533.A0A1C3YLH6 pp 229533.I1RC46
158154 229533.A0A1C3YLH6 pp 229533.I1RZ60
158155 229533.A0A1C3YLH6 pp 229533.I1RSB0
158156 229533.A0A1C3YLH6 pp 229533.I1RKR7
158157 229533.A0A1C3YLH6 pp 229533.Q4IJ79
158158 229533.A0A1C3YLH6 pp 229533.I1RBD6
158159 229533.A0A1C3YLH6 pp 229533.I1RTE6
158160 229533.A0A1C3YLH6 pp 229533.Q4I665
158161 229533.A0A1C3YLH6 pp 229533.I1RJS9
158162 229533.A0A1C3YLH6 pp 229533.I1RD10
158163 229533.A0A1C3YLH6 pp 229533.I1RTA3
158164 229533.A0A1C3YLH6 pp 229533.I1RZQ6
158165 229533.A0A1C3YLH6 pp 229533.Q4I2W2
158166 229533.A0A1C3YLH6 pp 229533.I1S9U6
158167 229533.A0A1C3YLH6 pp 229533.V6RDZ4
158168 229533.A0A1C3YLH6 pp 229533.I1RB26
158169 229533.I1RMY1 pp 229533.I1RSX9
158170 229533.I1RMY1 pp 229533.I1RY16
158171 229533.I1RMY1 pp 229533.I1RZA8
158172 229533.I1RMY1 pp 229533.I1S673
158173 229533.I1RMY1 pp 229533.I1RZM4
158174 229533.I1RMY1 pp 229533.I1S9T6
158175 229533.I1RMY1 pp 229533.V6RHD0
158176 229533.I1RMY1 pp 229533.I1RVY1
158177 229533.I1RMY1 pp 229533.I1RN84
158178 229533.I1RMY1 pp 229533.I1RNG4
158179 229533.I1RMY1 pp 229533.I1S6A2
158180 229533.I1RMY1 pp 229533.I1RQE8
158181 229533.I1RMY1 pp 229533.I1RW10
158182 229533.I1RMY1 pp 229533.I1RVR2
158183 229533.I1RMY1 pp 229533.I1S0Y7
158184 229533.I1RMY1 pp 229533.I1S694
158185 229533.I1RMY1 pp 229533.I1RW69
158186 229533.I1RMY1 pp 229533.V6RXR0
158187 229533.I1RMY1 pp 229533.V6RK96
158188 229533.I1RMY1 pp 229533.V6QU65
158189 229533.I1RMY1 pp 229533.I1RR29
158190 229533.I1RMY1 pp 229533.I1RSK5
158191 229533.I1RMY1 pp 229533.V6R4J6

158192	229533.A0A098D0Z4	pp	229533.A0A1C3YKZ8
158193	229533.A0A098D0Z4	pp	229533.I1RRG3
158194	229533.A0A098D0Z4	pp	229533.Q4I8Q4
158195	229533.A0A098D0Z4	pp	229533.I1RST9
158196	229533.A0A098D0Z4	pp	229533.V6QUE6
158197	229533.A0A098D0Z4	pp	229533.I1RMU7
158198	229533.A0A098D0Z4	pp	229533.I1RCV1
158199	229533.A0A098D0Z4	pp	229533.A0A098DGB7
158200	229533.A0A098D0Z4	pp	229533.I1RM26
158201	229533.A0A098D0Z4	pp	229533.I1S029
158202	229533.A0A098D0Z4	pp	229533.V6RY56
158203	229533.A0A098D0Z4	pp	229533.I1S4D0
158204	229533.A0A098D0Z4	pp	229533.A0A0E0RQT8
158205	229533.A0A098D0Z4	pp	229533.I1RVX9
158206	229533.A0A098D0Z4	pp	229533.A0A1C3YJR3
158207	229533.A0A098D0Z4	pp	229533.I1RJT5
158208	229533.A0A098D0Z4	pp	229533.I1S9Y1
158209	229533.A0A098D0Z4	pp	229533.I1REI4
158210	229533.A0A098D0Z4	pp	229533.I1RK95
158211	229533.A0A098D0Z4	pp	229533.I1S0E4
158212	229533.A0A098D0Z4	pp	229533.A0A098DIZ7
158213	229533.A0A098D0Z4	pp	229533.A0A0E0S5J3
158214	229533.A0A098D0Z4	pp	229533.I1RE88
158215	229533.A0A098D0Z4	pp	229533.A0A1C3YIR8
158216	229533.A0A098D0Z4	pp	229533.I1RSW5
158217	229533.A0A098D0Z4	pp	229533.I1RBC7
158218	229533.A0A098D0Z4	pp	229533.I1RTP2
158219	229533.A0A098D0Z4	pp	229533.I1RVU9
158220	229533.A0A098D0Z4	pp	229533.I1RV85
158221	229533.A0A098D0Z4	pp	229533.I1RE53
158222	229533.A0A098D0Z4	pp	229533.I1RTI3
158223	229533.A0A098D0Z4	pp	229533.I1RNG4
158224	229533.A0A098D0Z4	pp	229533.I1RG11
158225	229533.A0A098D0Z4	pp	229533.I1RPU8
158226	229533.A0A098D0Z4	pp	229533.A0A098D2Y5
158227	229533.A0A098D0Z4	pp	229533.A0A1C3YLH6
158228	229533.I1RRG8	pp	229533.I1S095
158229	229533.I1RRG8	pp	229533.I1S7L6
158230	229533.I1RRG8	pp	229533.I1RS07
158231	229533.I1RRG8	pp	229533.I1RZV4
158232	229533.I1RRG8	pp	229533.I1RZD9
158233	229533.I1RRG8	pp	229533.I1RRY9
158234	229533.I1REN4	pp	229533.I1SAT1
158235	229533.I1REN4	pp	229533.I1RW72
158236	229533.I1REN4	pp	229533.I1RW38
158237	229533.I1REN4	pp	229533.I1RXU1
158238	229533.I1REN4	pp	229533.V6R9X9
158239	229533.I1REN4	pp	229533.V6RR76
158240	229533.I1REN4	pp	229533.I1RWQ1
158241	229533.I1REN4	pp	229533.V6RKE4
158242	229533.I1REN4	pp	229533.I1S0S6
158243	229533.I1REN4	pp	229533.I1RY44
158244	229533.I1REN4	pp	229533.I1RQN9
158245	229533.I1REN4	pp	229533.I1RTB7
158246	229533.I1REN4	pp	229533.I1RYI3
158247	229533.I1REN4	pp	229533.I1RH68
158248	229533.I1REN4	pp	229533.I1RPE1
158249	229533.I1REN4	pp	229533.I1RNS3
158250	229533.I1REN4	pp	229533.I1RWT6
158251	229533.I1REN4	pp	229533.I1RIG4
158252	229533.I1REN4	pp	229533.I1RM09
158253	229533.I1REN4	pp	229533.I1RWF5
158254	229533.I1REN4	pp	229533.I1RW76
158255	229533.I1REN4	pp	229533.I1S7X9
158256	229533.I1REN4	pp	229533.I1RX09
158257	229533.I1REN4	pp	229533.I1RPK5
158258	229533.I1REN4	pp	229533.I1RQA3
158259	229533.I1REN4	pp	229533.I1RG99
158260	229533.I1REN4	pp	229533.I1RZ91
158261	229533.I1REN4	pp	229533.V6QW25
158262	229533.I1REN4	pp	229533.P0C431
158263	229533.I1REN4	pp	229533.I1RSP4
158264	229533.I1REN4	pp	229533.I1RS38

158265	229533.I1REN4	pp	229533.I1RZA4
158266	229533.I1REN4	pp	229533.I1S635
158267	229533.I1REN4	pp	229533.I1S2E9
158268	229533.I1REN4	pp	229533.I1RX50
158269	229533.I1REN4	pp	229533.Q4ICG5
158270	229533.I1REN4	pp	229533.I1RW32
158271	229533.I1REN4	pp	229533.I1RJT1
158272	229533.I1REN4	pp	229533.I1RTD0
158273	229533.I1REN4	pp	229533.V6RQ48
158274	229533.I1REN4	pp	229533.I1RN81
158275	229533.I1REN4	pp	229533.I1RTF0
158276	229533.A0A1C3YJA1	pp	229533.I1RYM5
158277	229533.A0A1C3YJA1	pp	229533.V6RXR0
158278	229533.A0A1C3YJA1	pp	229533.V6QU65
158279	229533.A0A1C3YJA1	pp	229533.I1RHU0
158280	229533.A0A1C3YJA1	pp	229533.V6RHD0
158281	229533.A0A1C3YJA1	pp	229533.I1RF42
158282	229533.I1RWI6	pp	229533.I1S635
158283	229533.I1RWI6	pp	229533.I1S034
158284	229533.I1RWI6	pp	229533.V6R476
158285	229533.I1RWI6	pp	229533.I1S8E0
158286	229533.I1RWI6	pp	229533.V6QUU6
158287	229533.I1RWI6	pp	229533.I1RX68
158288	229533.I1RWI6	pp	229533.I1RZL3
158289	229533.I1RWI6	pp	229533.I1RX09
158290	229533.I1RWI6	pp	229533.I1S559
158291	229533.I1RWI6	pp	229533.Q4INI2
158292	229533.I1RWI6	pp	229533.V6RR76
158293	229533.I1RWI6	pp	229533.I1RXS3
158294	229533.I1RWI6	pp	229533.I1S254
158295	229533.I1RWI6	pp	229533.I1S270
158296	229533.I1RWI6	pp	229533.I1S0Z3
158297	229533.I1RWI6	pp	229533.I1RYN9
158298	229533.I1RWI6	pp	229533.Q4HZE0
158299	229533.I1RWI6	pp	229533.I1S0X0
158300	229533.I1RWI6	pp	229533.Q4IPI4
158301	229533.I1RWI6	pp	229533.I1RX69
158302	229533.I1RWI6	pp	229533.I1RZ91
158303	229533.I1RWI6	pp	229533.I1RZR2
158304	229533.I1RWI6	pp	229533.V6QW25
158305	229533.I1RWI6	pp	229533.I1S0Z7
158306	229533.I1RWI6	pp	229533.Q4I963
158307	229533.A0A0E0SBU8	pp	229533.I1RSI0
158308	229533.A0A0E0SBU8	pp	229533.I1REK8
158309	229533.A0A0E0SBU8	pp	229533.Q4IJK2
158310	229533.A0A0E0SBU8	pp	229533.I1RRY9
158311	229533.A0A0E0SBU8	pp	229533.I1RB92
158312	229533.A0A0E0SBU8	pp	229533.I1RJV3
158313	229533.A0A0E0SBU8	pp	229533.I1RBT1
158314	229533.A0A0E0SBU8	pp	229533.A0A1C3YHX6
158315	229533.A0A0E0SBU8	pp	229533.V6RG06
158316	229533.A0A0E0SBU8	pp	229533.I1RAW0
158317	229533.A0A0E0SBU8	pp	229533.I1RJU3
158318	229533.A0A0E0SBU8	pp	229533.I1S1C7
158319	229533.A0A0E0SBU8	pp	229533.A0A1C3YIP5
158320	229533.A0A0E0SBU8	pp	229533.I1RD52
158321	229533.A0A0E0SBU8	pp	229533.I1RA70
158322	229533.A0A0E0SBU8	pp	229533.I1S1W9
158323	229533.A0A0E0SBU8	pp	229533.I1S4S7
158324	229533.A0A0E0SBU8	pp	229533.A0A1C3YKB0
158325	229533.A0A0E0SBU8	pp	229533.I1RXC4
158326	229533.A0A0E0SBU8	pp	229533.I1RW71
158327	229533.A0A0E0SBU8	pp	229533.I1RNK6
158328	229533.A0A0E0SBU8	pp	229533.A0A1C3YK34
158329	229533.A0A0E0SBU8	pp	229533.A0A1C3YKV3
158330	229533.A0A0E0SBU8	pp	229533.I1RSQ7
158331	229533.A0A0E0SBU8	pp	229533.I1RQ81
158332	229533.A0A0E0SBU8	pp	229533.I1RS18
158333	229533.A0A0E0SBU8	pp	229533.I1RE01
158334	229533.A0A0E0SBU8	pp	229533.I1RX46
158335	229533.A0A0E0SBU8	pp	229533.I1RTA0
158336	229533.A0A0E0SBU8	pp	229533.I1S0J1
158337	229533.A0A0E0SBU8	pp	229533.I1RD22

158338 229533.A0A0E0SBU8 pp 229533.I1S5R6
158339 229533.A0A0E0SBU8 pp 229533.I1RZM8
158340 229533.A0A0E0SBU8 pp 229533.I1RR24
158341 229533.A0A0E0SBU8 pp 229533.I1RBY4
158342 229533.A0A0E0SBU8 pp 229533.V6RTJ8
158343 229533.A0A0E0SBU8 pp 229533.A0A0E0SFN0
158344 229533.A0A0E0SBU8 pp 229533.I1RSZ7
158345 229533.A0A0E0SBU8 pp 229533.I1S5R1
158346 229533.A0A0E0SBU8 pp 229533.I1RNC1
158347 229533.A0A0E0SBU8 pp 229533.I1RXD7
158348 229533.A0A0E0SBU8 pp 229533.I1RUG3
158349 229533.A0A0E0SBU8 pp 229533.I1RFL4
158350 229533.A0A0E0SBU8 pp 229533.I1RCR8
158351 229533.A0A0E0SBU8 pp 229533.I1RXC2
158352 229533.A0A0E0SBU8 pp 229533.I1RCH5
158353 229533.A0A0E0SBU8 pp 229533.I1S2H4
158354 229533.A0A0E0SBU8 pp 229533.I1RRV1
158355 229533.A0A0E0SBU8 pp 229533.I1RQE7
158356 229533.A0A0E0SBU8 pp 229533.I1RSQ3
158357 229533.A0A0E0SBU8 pp 229533.I1RZZ9
158358 229533.A0A0E0SBU8 pp 229533.I1RT03
158359 229533.A0A0E0SBU8 pp 229533.I1RZ39
158360 229533.A0A0E0SBU8 pp 229533.V6R042
158361 229533.A0A0E0SBU8 pp 229533.I1RVX0
158362 229533.A0A0E0SBU8 pp 229533.V6RMW6
158363 229533.A0A0E0SBU8 pp 229533.A0A1C3YKM9
158364 229533.A0A0E0SBU8 pp 229533.I1RN67
158365 229533.A0A0E0SBU8 pp 229533.I1R9M0
158366 229533.A0A0E0SBU8 pp 229533.I1RJZ7
158367 229533.A0A0E0SBU8 pp 229533.I1RTA1
158368 229533.A0A0E0SBU8 pp 229533.I1RVZ2
158369 229533.A0A0E0SBU8 pp 229533.I1S2B8
158370 229533.A0A0E0SBU8 pp 229533.A0A1C3YLK8
158371 229533.A0A0E0SBU8 pp 229533.I1RYI5
158372 229533.A0A0E0SBU8 pp 229533.I1RPP3
158373 229533.A0A0E0SBU8 pp 229533.I1RGA4
158374 229533.A0A0E0SBU8 pp 229533.A0A1C3YJI6
158375 229533.A0A0E0SBU8 pp 229533.I1RT40
158376 229533.A0A0E0SBU8 pp 229533.I1S8P5
158377 229533.A0A0E0SBU8 pp 229533.I1RTD5
158378 229533.I1RE69 pp 229533.I1RWS7
158379 229533.I1RE69 pp 229533.I1S4T3
158380 229533.A0A1C3YJG7 pp 229533.I1RQS1
158381 229533.A0A1C3YJG7 pp 229533.I1RPE1
158382 229533.A0A1C3YJG7 pp 229533.I1S7X9
158383 229533.A0A1C3YJG7 pp 229533.I1RW83
158384 229533.A0A1C3YJG7 pp 229533.I1RCN6
158385 229533.A0A1C3YJG7 pp 229533.A0A1C3YKJ1
158386 229533.A0A1C3YJG7 pp 229533.I1RW72
158387 229533.A0A1C3YJG7 pp 229533.A0A1C3YM82
158388 229533.A0A1C3YJG7 pp 229533.I1RFL7
158389 229533.A0A1C3YJG7 pp 229533.A0A1C3YK57
158390 229533.A0A1C3YJG7 pp 229533.I1RML3
158391 229533.A0A1C3YJG7 pp 229533.I1RC18
158392 229533.A0A1C3YJG7 pp 229533.I1RV91
158393 229533.A0A1C3YJG7 pp 229533.I1RRN0
158394 229533.A0A1C3YJG7 pp 229533.A0A1C3YME0
158395 229533.A0A1C3YJG7 pp 229533.A0A1C3YJP1
158396 229533.A0A1C3YJG7 pp 229533.I1S074
158397 229533.A0A1C3YJG7 pp 229533.I1RS47
158398 229533.A0A1C3YJG7 pp 229533.I1RC71
158399 229533.A0A1C3YJG7 pp 229533.I1S549
158400 229533.A0A1C3YJG7 pp 229533.I1RD33
158401 229533.A0A1C3YJG7 pp 229533.I1RNN1
158402 229533.A0A1C3YJG7 pp 229533.I1RUC4
158403 229533.A0A1C3YJG7 pp 229533.I1S0J7
158404 229533.A0A1C3YJG7 pp 229533.I1RJU5
158405 229533.A0A1C3YJG7 pp 229533.I1RJP3
158406 229533.A0A1C3YJG7 pp 229533.I1S0P7
158407 229533.A0A1C3YJG7 pp 229533.I1S2E9
158408 229533.A0A1C3YJG7 pp 229533.I1RZ63
158409 229533.A0A1C3YJG7 pp 229533.I1S594
158410 229533.A0A1C3YJG7 pp 229533.I1RMU3

158411 229533.A0A1C3YJG7 pp 229533.I1RRZ3
158412 229533.A0A1C3YJG7 pp 229533.I1RSU6
158413 229533.A0A1C3YJG7 pp 229533.I1RAD4
158414 229533.A0A1C3YJG7 pp 229533.I1RNP7
158415 229533.A0A1C3YJG7 pp 229533.I1S1X7
158416 229533.A0A1C3YJG7 pp 229533.I1RX50
158417 229533.A0A1C3YJG7 pp 229533.I1RNZ8
158418 229533.A0A1C3YJG7 pp 229533.I1RSF2
158419 229533.A0A1C3YJG7 pp 229533.I1RP25
158420 229533.A0A1C3YJG7 pp 229533.A0A1C3YMQ5
158421 229533.A0A1C3YJG7 pp 229533.I1RNG8
158422 229533.A0A1C3YJG7 pp 229533.I1RGM4
158423 229533.A0A1C3YJG7 pp 229533.A0A1C3YK08
158424 229533.A0A1C3YJG7 pp 229533.A0A1C3YK90
158425 229533.A0A1C3YJG7 pp 229533.I1RMA4
158426 229533.A0A1C3YJG7 pp 229533.Q4HZB8
158427 229533.A0A1C3YJG7 pp 229533.I1RY44
158428 229533.A0A1C3YJG7 pp 229533.I1RZ95
158429 229533.A0A1C3YJG7 pp 229533.I1S4N8
158430 229533.A0A1C3YJG7 pp 229533.I1RW37
158431 229533.A0A1C3YJG7 pp 229533.I1RPS0
158432 229533.A0A1C3YJG7 pp 229533.I1RYF2
158433 229533.A0A1C3YJG7 pp 229533.Q4IN00
158434 229533.A0A1C3YJG7 pp 229533.I1S2N3
158435 229533.A0A1C3YJG7 pp 229533.I1RLS9
158436 229533.A0A1C3YJG7 pp 229533.I1RBU1
158437 229533.A0A1C3YJG7 pp 229533.I1RT36
158438 229533.A0A1C3YJG7 pp 229533.I1RJU7
158439 229533.A0A1C3YJG7 pp 229533.I1RM49
158440 229533.A0A1C3YJG7 pp 229533.I1RPP7
158441 229533.A0A1C3YJG7 pp 229533.I1RYZ9
158442 229533.A0A1C3YJG7 pp 229533.I1RMV6
158443 229533.A0A1C3YJG7 pp 229533.I1S834
158444 229533.A0A1C3YJG7 pp 229533.I1RNS3
158445 229533.A0A1C3YJG7 pp 229533.A0A1C3YLX3
158446 229533.A0A1C3YJG7 pp 229533.I1RCT1
158447 229533.A0A1C3YJG7 pp 229533.I1RC63
158448 229533.A0A1C3YJG7 pp 229533.A0A1C3YJI0
158449 229533.A0A1C3YJG7 pp 229533.I1REK7
158450 229533.A0A1C3YJG7 pp 229533.I1RTG0
158451 229533.A0A1C3YJG7 pp 229533.I1RAK3
158452 229533.A0A1C3YJG7 pp 229533.I1RRN5
158453 229533.A0A1C3YJG7 pp 229533.Q4IB89
158454 229533.A0A1C3YJG7 pp 229533.I1RW88
158455 229533.A0A1C3YJG7 pp 229533.I1S749
158456 229533.A0A1C3YJG7 pp 229533.I1S4N7
158457 229533.A0A1C3YJG7 pp 229533.V6QV09
158458 229533.A0A1C3YJG7 pp 229533.I1RK75
158459 229533.A0A1C3YJG7 pp 229533.I1RB41
158460 229533.A0A1C3YJG7 pp 229533.I1S7K6
158461 229533.A0A1C3YJG7 pp 229533.I1RXM0
158462 229533.A0A1C3YJG7 pp 229533.I1RXV4
158463 229533.A0A1C3YJG7 pp 229533.I1RCM2
158464 229533.A0A1C3YJG7 pp 229533.I1RM41
158465 229533.A0A1C3YJG7 pp 229533.I1RZ12
158466 229533.A0A1C3YJG7 pp 229533.I1RPK9
158467 229533.A0A1C3YJG7 pp 229533.Q4INZ9
158468 229533.A0A1C3YJG7 pp 229533.I1RVH2
158469 229533.A0A1C3YJG7 pp 229533.I1S1W5
158470 229533.A0A1C3YJG7 pp 229533.I1RKA1
158471 229533.A0A1C3YJG7 pp 229533.I1RQ41
158472 229533.A0A1C3YJG7 pp 229533.A0A1C3YKR7
158473 229533.A0A1C3YJG7 pp 229533.I1RS57
158474 229533.A0A1C3YJG7 pp 229533.I1RL24
158475 229533.A0A1C3YJG7 pp 229533.I1REA6
158476 229533.A0A1C3YJG7 pp 229533.E6PBP1
158477 229533.A0A1C3YJG7 pp 229533.I1RU80
158478 229533.A0A1C3YJG7 pp 229533.I1RWT6
158479 229533.A0A1C3YJG7 pp 229533.I1RY98
158480 229533.A0A1C3YJG7 pp 229533.I1RC91
158481 229533.A0A1C3YJG7 pp 229533.I1S039
158482 229533.A0A1C3YJG7 pp 229533.I1SA77
158483 229533.A0A1C3YJG7 pp 229533.I1RQ70

158484	229533.A0A1C3YJG7	pp	229533.I1RC22
158485	229533.A0A1C3YJG7	pp	229533.I1S9X9
158486	229533.A0A1C3YJG7	pp	229533.I1RYL5
158487	229533.A0A1C3YJG7	pp	229533.I1S1V3
158488	229533.A0A1C3YJG7	pp	229533.I1RJT1
158489	229533.A0A1C3YJG7	pp	229533.I1RSG0
158490	229533.A0A1C3YJG7	pp	229533.I1RFS8
158491	229533.A0A1C3YJG7	pp	229533.I1RJI1
158492	229533.A0A1C3YJG7	pp	229533.I1RMK1
158493	229533.A0A1C3YJG7	pp	229533.I1RTF0
158494	229533.A0A1C3YJG7	pp	229533.I1S278
158495	229533.A0A1C3YJG7	pp	229533.I1RSP4
158496	229533.A0A1C3YJG7	pp	229533.A0A1C3YN89
158497	229533.A0A1C3YJG7	pp	229533.I1RBH5
158498	229533.A0A1C3YJG7	pp	229533.I1RME5
158499	229533.A0A1C3YJG7	pp	229533.I1RG72
158500	229533.A0A1C3YJG7	pp	229533.A0A1C3YJH8
158501	229533.A0A1C3YJG7	pp	229533.I1RC17
158502	229533.A0A1C3YJG7	pp	229533.I1S275
158503	229533.A0A1C3YJG7	pp	229533.I1S0Z7
158504	229533.A0A1C3YJG7	pp	229533.I1RQA3
158505	229533.A0A1C3YJG7	pp	229533.A0A1C3YKU6
158506	229533.A0A1C3YJG7	pp	229533.I1RYZ0
158507	229533.A0A1C3YJG7	pp	229533.I1RFE0
158508	229533.A0A1C3YJG7	pp	229533.I1RCK2
158509	229533.A0A1C3YJG7	pp	229533.A0A1C3YLM3
158510	229533.A0A1C3YM00	pp	229533.I1RNB8
158511	229533.A0A1C3YM00	pp	229533.I1RG90
158512	229533.A0A1C3YM00	pp	229533.I1RT35
158513	229533.A0A1C3YJU5	pp	229533.I1RQE8
158514	229533.A0A1C3YJU5	pp	229533.I1RSK5
158515	229533.A0A1C3YJU5	pp	229533.I1S694
158516	229533.A0A1C3YJU5	pp	229533.I1RF59
158517	229533.A0A1C3YJU5	pp	229533.I1RSX9
158518	229533.A0A1C3YJU5	pp	229533.A0A1C3YLI1
158519	229533.A0A1C3YJU5	pp	229533.V6R4J6
158520	229533.A0A1C3YJU5	pp	229533.V6QU65
158521	229533.A0A1C3YJU5	pp	229533.I1RC48
158522	229533.A0A1C3YJU5	pp	229533.I1RHA8
158523	229533.A0A1C3YJU5	pp	229533.A0A1C3YJW4
158524	229533.A0A1C3YJU5	pp	229533.A0A1C3YKN4
158525	229533.A0A1C3YJU5	pp	229533.I1RI11
158526	229533.A0A1C3YJU5	pp	229533.I1RVY1
158527	229533.A0A1C3YJU5	pp	229533.I1S673
158528	229533.A0A1C3YJU5	pp	229533.I1RVR2
158529	229533.A0A1C3YJU5	pp	229533.I1RN84
158530	229533.A0A1C3YJU5	pp	229533.I1RNG4
158531	229533.A0A1C3YJU5	pp	229533.I1RMY1
158532	229533.A0A1C3YJU5	pp	229533.I1RW10
158533	229533.A0A1C3YJU5	pp	229533.V6RXR0
158534	229533.A0A1C3YJU5	pp	229533.V6RHD0
158535	229533.A0A1C3YJU5	pp	229533.I1S0Y7
158536	229533.A0A1C3YJU5	pp	229533.I1S6A2
158537	229533.A0A1C3YJU5	pp	229533.I1RW69
158538	229533.A0A1C3YJU5	pp	229533.A0A1C3YLE2
158539	229533.A0A1C3YJU5	pp	229533.V6RK96
158540	229533.A0A1C3YJU5	pp	229533.I1RZA8
158541	229533.A0A1C3YJU5	pp	229533.A0A1C3YNB3
158542	229533.A0A1C3YJU5	pp	229533.I1RA90
158543	229533.A0A1C3YJU5	pp	229533.I1S9T6
158544	229533.A0A1C3YJU5	pp	229533.I1RAY9
158545	229533.A0A1C3YJU5	pp	229533.I1RY16
158546	229533.A0A1C3YJU5	pp	229533.I1RZM4
158547	229533.A0A1C3YJU5	pp	229533.I1RR29
158548	229533.A0A1C3YJU5	pp	229533.I1RMJ3
158549	229533.A0A1C3YJU5	pp	229533.I1RH32
158550	229533.A0A098D0Y5	pp	229533.I1RE15
158551	229533.A0A098D0Y5	pp	229533.I1REN4
158552	229533.A0A098D0Y5	pp	229533.I1RPK5
158553	229533.A0A098D0Y5	pp	229533.I1RWI6
158554	229533.A0A098D0Y5	pp	229533.A0A1C3YJG7
158555	229533.A0A098D0Y5	pp	229533.A0A1C3YK51
158556	229533.I1RL76	pp	229533.V6QWD8

158557	229533.I1RL76	pp	229533.I1RSQ9
158558	229533.I1RL76	pp	229533.I1S4L8
158559	229533.A0A098E2N1	pp	229533.I1RS07
158560	229533.A0A098E2N1	pp	229533.A0A1C3YMS7
158561	229533.A0A098E2N1	pp	229533.I1S095
158562	229533.A0A098E2N1	pp	229533.I1RZV4
158563	229533.A0A098E2N1	pp	229533.V6RG22
158564	229533.A0A098E2N1	pp	229533.I1RFV6
158565	229533.A0A098E2N1	pp	229533.I1RP18
158566	229533.A0A098E2N1	pp	229533.I1RT76
158567	229533.A0A098E2N1	pp	229533.A0A1C3YKL8
158568	229533.A0A098E2N1	pp	229533.A0A1C3YM36
158569	229533.A0A098E2N1	pp	229533.I1RPE5
158570	229533.A0A098E2N1	pp	229533.I1S7L6
158571	229533.A0A098E2N1	pp	229533.A0A0E0RW18
158572	229533.A0A098E2N1	pp	229533.I1RCE6
158573	229533.A0A098E2N1	pp	229533.A0A0E0RU18
158574	229533.A0A098E2N1	pp	229533.I1RZD9
158575	229533.A0A098E2N1	pp	229533.I1RRG8
158576	229533.A0A098E2N1	pp	229533.Q00909
158577	229533.A0A098E2N1	pp	229533.I1R9Y2
158578	229533.A0A098E2N1	pp	229533.A0A1C3YMD9
158579	229533.A0A098E2N1	pp	229533.I1RTG9
158580	229533.A0A098E2N1	pp	229533.I1RY40
158581	229533.A0A098E2N1	pp	229533.A0A1C3YLH7
158582	229533.A0A098E2N1	pp	229533.A0A1C3YKR7
158583	229533.A0A098E2N1	pp	229533.I1RBR4
158584	229533.A0A098E2N1	pp	229533.V6RPN0
158585	229533.A0A098E2N1	pp	229533.A0A1C3YJ14
158586	229533.A0A098E2N1	pp	229533.I1RGC4
158587	229533.I1RXY7	pp	229533.I1RZ70
158588	229533.I1RXY7	pp	229533.I1S7X9
158589	229533.I1RXY7	pp	229533.I1S8R4
158590	229533.I1RXY7	pp	229533.I1S064
158591	229533.I1RXY7	pp	229533.V6R382
158592	229533.I1RXY7	pp	229533.V6RM02
158593	229533.I1RXY7	pp	229533.I1RYK0
158594	229533.I1RXY7	pp	229533.I1RYP2
158595	229533.I1RXY7	pp	229533.I1S0I3
158596	229533.I1RXY7	pp	229533.I1RY57
158597	229533.I1RXY7	pp	229533.V6QV31
158598	229533.I1RXY7	pp	229533.V6RHD0
158599	229533.I1RXY7	pp	229533.I1S0Y7
158600	229533.I1RXY7	pp	229533.I1S4B7
158601	229533.I1RXY7	pp	229533.I1S3G4
158602	229533.I1RXY7	pp	229533.Q4HXF0
158603	229533.I1RXY7	pp	229533.V6RK96
158604	229533.I1RXY7	pp	229533.I1RZ29
158605	229533.I1RXY7	pp	229533.I1S1F5
158606	229533.I1RXY7	pp	229533.I1S9C6
158607	229533.I1RXY7	pp	229533.I1RZU2
158608	229533.I1RXY7	pp	229533.I1RZK8
158609	229533.I1RXY7	pp	229533.I1S0A0
158610	229533.I1RXY7	pp	229533.V6RXR0
158611	229533.I1RXY7	pp	229533.I1S4B6
158612	229533.I1RXY7	pp	229533.I1RZM4
158613	229533.I1RXY7	pp	229533.I1S0K0
158614	229533.I1RXY7	pp	229533.I1S4S4
158615	229533.I1RXY7	pp	229533.I1S0N3
158616	229533.I1RXY7	pp	229533.I1S4L8
158617	229533.I1RXY7	pp	229533.I1S241
158618	229533.I1RXY7	pp	229533.V6RDH3
158619	229533.I1RXY7	pp	229533.Q4HZQ1
158620	229533.I1RXY7	pp	229533.I1RYQ4
158621	229533.I1RXY7	pp	229533.I1S8C1
158622	229533.I1RXY7	pp	229533.I1S3L2
158623	229533.I1RXY7	pp	229533.V6QU65
158624	229533.I1RXY7	pp	229533.I1RZE7
158625	229533.I1RXY7	pp	229533.I1S2C1
158626	229533.I1RXY7	pp	229533.I1RY49
158627	229533.I1RXY7	pp	229533.I1RYH6
158628	229533.I1RXY7	pp	229533.I1S767
158629	229533.I1RXY7	pp	229533.I1RYX6

158630 229533.I1RXY7 pp 229533.I1RYM6
158631 229533.I1RXY7 pp 229533.V6QWD8
158632 229533.I1RXY7 pp 229533.I1S1W8
158633 229533.I1RJD9 pp 229533.I1S303
158634 229533.I1RJD9 pp 229533.I1RVC7
158635 229533.I1RJD9 pp 229533.I1S2I7
158636 229533.I1RJD9 pp 229533.I1S069
158637 229533.I1RJD9 pp 229533.I1RY66
158638 229533.I1RJD9 pp 229533.I1RKP1
158639 229533.I1RJD9 pp 229533.I1RR60
158640 229533.I1RJD9 pp 229533.I1S3M2
158641 229533.I1RJD9 pp 229533.I1RJZ2
158642 229533.A0A0E0SQG6 pp 229533.I1RJD9
158643 229533.I1RP31 pp 229533.I1RQ31
158644 229533.I1RP31 pp 229533.I1S253
158645 229533.I1RP31 pp 229533.I1RT80
158646 229533.I1RP31 pp 229533.I1S021
158647 229533.I1RP31 pp 229533.I1RQA2
158648 229533.I1RP31 pp 229533.I1S4L8
158649 229533.I1RP31 pp 229533.I1RQM6
158650 229533.A0A098E2M3 pp 229533.I1RD21
158651 229533.A0A098E2M3 pp 229533.I1S1F5
158652 229533.A0A098E2M3 pp 229533.I1RYZ9
158653 229533.A0A098E2M3 pp 229533.I1RAU6
158654 229533.A0A098E2M3 pp 229533.I1RC17
158655 229533.A0A098E2M3 pp 229533.I1RWM6
158656 229533.A0A098E2M3 pp 229533.I1REE1
158657 229533.A0A098E2M3 pp 229533.I1S4L8
158658 229533.A0A098E2M3 pp 229533.I1S224
158659 229533.A0A098E2M3 pp 229533.I1RME5
158660 229533.A0A098E2M3 pp 229533.I1RRX6
158661 229533.A0A098E2M3 pp 229533.I1RKA1
158662 229533.A0A098E2M3 pp 229533.I1RQS4
158663 229533.A0A098E2M3 pp 229533.V6QV09
158664 229533.A0A098E2M3 pp 229533.I1RDV9
158665 229533.A0A098E2M3 pp 229533.I1RL24
158666 229533.A0A098E2M3 pp 229533.I1S039
158667 229533.A0A098E2M3 pp 229533.A0A1C3YJT5
158668 229533.A0A098E2M3 pp 229533.V6RKK5
158669 229533.A0A098E2M3 pp 229533.V6RDH3
158670 229533.A0A098E2M3 pp 229533.A0A0E0SC50
158671 229533.A0A098E2M3 pp 229533.A0A1C3YJH8
158672 229533.A0A098E2M3 pp 229533.I1RJK7
158673 229533.A0A098E2M3 pp 229533.I1S1W5
158674 229533.A0A098E2M3 pp 229533.I1RB75
158675 229533.A0A098E2M3 pp 229533.I1RD18
158676 229533.A0A098E2M3 pp 229533.I1RC22
158677 229533.A0A098E2M3 pp 229533.I1RAN0
158678 229533.A0A098E2M3 pp 229533.I1RT45
158679 229533.A0A098E2M3 pp 229533.A0A1C3YJW1
158680 229533.A0A098E2M3 pp 229533.A0A0E0RVZ1
158681 229533.A0A098E2M3 pp 229533.A0A1C3YJG7
158682 229533.A0A098E2M3 pp 229533.I1RJU6
158683 229533.A0A098E2M3 pp 229533.I1S2G7
158684 229533.A0A098E2M3 pp 229533.I1RVN2
158685 229533.A0A098E2M3 pp 229533.I1RRW0
158686 229533.A0A098E2M3 pp 229533.Q4IJM4
158687 229533.A0A098E2M3 pp 229533.A0A0E0RMV2
158688 229533.A0A098E2M3 pp 229533.I1RE79
158689 229533.A0A098E2M3 pp 229533.A0A1C3YM91
158690 229533.A0A098E2M3 pp 229533.I1RB74
158691 229533.A0A098E2M3 pp 229533.I1RFL7
158692 229533.A0A098E2M3 pp 229533.I1RYY5
158693 229533.A0A098E2M3 pp 229533.A0A1C3YI39
158694 229533.A0A098E2M3 pp 229533.I1RKL3
158695 229533.A0A098E2M3 pp 229533.I1RBF3
158696 229533.A0A098E2M3 pp 229533.A0A0E0S1U8
158697 229533.A0A098E2M3 pp 229533.I1RGL3
158698 229533.A0A098E2M3 pp 229533.I1S004
158699 229533.A0A098E2M3 pp 229533.I1RB41
158700 229533.A0A098E2M3 pp 229533.I1RBG9
158701 229533.A0A098E2M3 pp 229533.I1RV91
158702 229533.A0A098E2M3 pp 229533.A0A0E0S9G3

158703 229533.A0A098E2M3 pp 229533.A0A1C3YM29
158704 229533.A0A098E2M3 pp 229533.A0A1C3YK57
158705 229533.A0A098E2M3 pp 229533.I1RK75
158706 229533.A0A098E2M3 pp 229533.I1RY58
158707 229533.A0A098E2M3 pp 229533.A0A0E0SQG9
158708 229533.A0A098E2M3 pp 229533.I1RPK4
158709 229533.A0A098E2M3 pp 229533.I1S098
158710 229533.A0A098E2M3 pp 229533.I1RC14
158711 229533.A0A098E2M3 pp 229533.I1RFL4
158712 229533.A0A098E2M3 pp 229533.I1RZ63
158713 229533.A0A098E2M3 pp 229533.I1RRR6
158714 229533.A0A098E2M3 pp 229533.I1RX50
158715 229533.A0A098E2M3 pp 229533.I1RRN5
158716 229533.A0A098E2M3 pp 229533.I1RRZ6
158717 229533.A0A098E2M3 pp 229533.I1RMU9
158718 229533.A0A098E2M3 pp 229533.I1S1W4
158719 229533.A0A098E2M3 pp 229533.A0A1C3YIN6
158720 229533.A0A098E2M3 pp 229533.I1RC08
158721 229533.A0A098E2M3 pp 229533.I1RZT1
158722 229533.A0A098E2M3 pp 229533.A0A1C3YJC3
158723 229533.A0A098E2M3 pp 229533.I1RW03
158724 229533.I1RE79 pp 229533.V6QV31
158725 229533.I1RE79 pp 229533.I1RKP1
158726 229533.I1RE79 pp 229533.I1S5R1
158727 229533.I1RE79 pp 229533.I1RH91
158728 229533.I1RE79 pp 229533.I1RRI1
158729 229533.I1RE79 pp 229533.I1RFI8
158730 229533.I1RE79 pp 229533.I1RZ63
158731 229533.I1RE79 pp 229533.I1RTI7
158732 229533.I1RE79 pp 229533.I1RKD3
158733 229533.I1RE79 pp 229533.I1RR72
158734 229533.I1RE79 pp 229533.I1S253
158735 229533.I1RE79 pp 229533.I1RF33
158736 229533.I1RE79 pp 229533.I1RSA5
158737 229533.I1RE79 pp 229533.Q4HXF0
158738 229533.I1RE79 pp 229533.I1S0J7
158739 229533.I1RE79 pp 229533.I1RMJ3
158740 229533.I1RE79 pp 229533.I1RKG2
158741 229533.I1RE79 pp 229533.I1RYP2
158742 229533.I1RE79 pp 229533.I1RFI9
158743 229533.I1RE79 pp 229533.Q4IPH4
158744 229533.I1RE79 pp 229533.I1RQC0
158745 229533.I1RE79 pp 229533.I1RQE7
158746 229533.I1RE79 pp 229533.I1RMU9
158747 229533.I1RE79 pp 229533.I1RW26
158748 229533.I1RE79 pp 229533.V6RBG5
158749 229533.I1RE79 pp 229533.I1RR45
158750 229533.I1RE79 pp 229533.I1RJU6
158751 229533.I1RE79 pp 229533.I1RK75
158752 229533.I1RE79 pp 229533.Q4HXF6
158753 229533.I1RE79 pp 229533.I1RUQ7
158754 229533.I1RE79 pp 229533.I1RGY6
158755 229533.I1RE79 pp 229533.I1REW3
158756 229533.I1RE79 pp 229533.I1RIU0
158757 229533.I1RE79 pp 229533.I1RFK3
158758 229533.I1RE79 pp 229533.I1RGL3
158759 229533.I1RE79 pp 229533.Q4I1N3
158760 229533.I1RE79 pp 229533.V6RDH3
158761 229533.I1RE79 pp 229533.I1RN84
158762 229533.I1RE79 pp 229533.I1RUG3
158763 229533.I1RE79 pp 229533.I1RQ33
158764 229533.I1RE79 pp 229533.I1RMW4
158765 229533.I1RE79 pp 229533.I1S0S5
158766 229533.I1RE79 pp 229533.I1S064
158767 229533.I1RE79 pp 229533.I1RKG3
158768 229533.I1RE79 pp 229533.I1RZK5
158769 229533.I1RE79 pp 229533.I1REK8
158770 229533.I1RE79 pp 229533.I1RRM3
158771 229533.I1RE79 pp 229533.I1RJU3
158772 229533.I1RE79 pp 229533.I1RRZ6
158773 229533.I1RE79 pp 229533.I1RYM5
158774 229533.I1RE79 pp 229533.V6RKK5
158775 229533.I1RE79 pp 229533.I1RSC5

158776	229533.I1RE79	pp	229533.I1RMM8
158777	229533.I1RE79	pp	229533.I1RYU3
158778	229533.I1RE79	pp	229533.V6QT89
158779	229533.I1RE79	pp	229533.I1RN52
158780	229533.I1RE79	pp	229533.I1RSF1
158781	229533.I1RE79	pp	229533.I1RFY0
158782	229533.I1RE79	pp	229533.I1RX59
158783	229533.I1RE79	pp	229533.I1RGK9
158784	229533.I1RE79	pp	229533.I1RPM6
158785	229533.I1RE79	pp	229533.I1S4L8
158786	229533.I1RE79	pp	229533.I1RM49
158787	229533.I1RE79	pp	229533.I1S5B0
158788	229533.I1RE79	pp	229533.V6RCZ8
158789	229533.I1RE79	pp	229533.I1RRK5
158790	229533.I1RE79	pp	229533.I1RTJ2
158791	229533.I1RE79	pp	229533.I1RZK8
158792	229533.I1RE79	pp	229533.I1S1F5
158793	229533.I1RZK3	pp	229533.I1S830
158794	229533.I1RZK3	pp	229533.I1S213
158795	229533.I1RZK3	pp	229533.I1S5B5
158796	229533.I1RZK3	pp	229533.I1RZK4
158797	229533.I1RZK3	pp	229533.Q4HYZ2
158798	229533.I1RZK3	pp	229533.I1RZK6
158799	229533.I1RZK3	pp	229533.Q4ILI9
158800	229533.I1RZK3	pp	229533.I1S2B6
158801	229533.I1RZK3	pp	229533.Q4IN63
158802	229533.I1RZK3	pp	229533.I1S287
158803	229533.I1RZK3	pp	229533.I1SAM5
158804	229533.I1RZK3	pp	229533.I1S1U6
158805	229533.I1RZK3	pp	229533.I1S1V4
158806	229533.A0A1C3YK57	pp	229533.Q4I4A4
158807	229533.A0A1C3YK57	pp	229533.I1RKA1
158808	229533.A0A1C3YK57	pp	229533.I1S039
158809	229533.A0A1C3YK57	pp	229533.I1S1F5
158810	229533.A0A1C3YK57	pp	229533.I1RD21
158811	229533.A0A1C3YK57	pp	229533.I1S1W5
158812	229533.A0A1C3YK57	pp	229533.I1RC91
158813	229533.A0A1C3YK57	pp	229533.I1RC17
158814	229533.A0A1C3YK57	pp	229533.I1RC20
158815	229533.A0A1C3YK57	pp	229533.I1RC14
158816	229533.A0A1C3YK57	pp	229533.I1S4L8
158817	229533.A0A1C3YK57	pp	229533.I1RV91
158818	229533.A0A1C3YK57	pp	229533.V6RKK5
158819	229533.A0A1C3YK57	pp	229533.I1RAN0
158820	229533.A0A1C3YK57	pp	229533.I1RX50
158821	229533.A0A1C3YK57	pp	229533.I1RYY5
158822	229533.A0A1C3YK57	pp	229533.I1RT45
158823	229533.A0A1C3YK57	pp	229533.Q4IPI4
158824	229533.A0A1C3YK57	pp	229533.I1RB75
158825	229533.A0A1C3YK57	pp	229533.Q4IJM4
158826	229533.A0A1C3YK57	pp	229533.I1S2G7
158827	229533.A0A1C3YK57	pp	229533.I1RBF3
158828	229533.A0A1C3YK57	pp	229533.I1RJZ7
158829	229533.A0A1C3YK57	pp	229533.I1RJU6
158830	229533.A0A1C3YK57	pp	229533.I1RVN2
158831	229533.A0A1C3YK57	pp	229533.I1RFL7
158832	229533.A0A1C3YK57	pp	229533.I1RB74
158833	229533.A0A1C3YK57	pp	229533.I1S004
158834	229533.A0A1C3YK57	pp	229533.I1RQG4
158835	229533.A0A1C3YK57	pp	229533.I1RC08
158836	229533.A0A1C3YK57	pp	229533.I1S098
158837	229533.A0A1C3YK57	pp	229533.Q4I7L0
158838	229533.A0A1C3YK57	pp	229533.I1RE79
158839	229533.A0A1C3YK57	pp	229533.I1RGL3
158840	229533.A0A1C3YK57	pp	229533.I1RPK4
158841	229533.A0A1C3YK57	pp	229533.A0A1C3YM29
158842	229533.A0A1C3YK57	pp	229533.I1RYN4
158843	229533.A0A1C3YK57	pp	229533.I1RRR6
158844	229533.A0A1C3YK57	pp	229533.I1RRZ6
158845	229533.A0A1C3YK57	pp	229533.I1RY58
158846	229533.A0A1C3YK57	pp	229533.I1S1W4
158847	229533.A0A1C3YK57	pp	229533.I1RZT1
158848	229533.A0A1C3YK57	pp	229533.I1RMU9

158849 229533.A0A1C3YK57 pp 229533.I1RW03
158850 229533.A0A1C3YI52 pp 229533.I1RIF2
158851 229533.A0A1C3YHP8 pp 229533.I1RNC0
158852 229533.A0A1C3YHP8 pp 229533.I1RN89
158853 229533.A0A1C3YHP8 pp 229533.I1RL24
158854 229533.A0A1C3YHP8 pp 229533.A0A1C3YL96
158855 229533.A0A1C3YHP8 pp 229533.I1S224
158856 229533.A0A1C3YHP8 pp 229533.I1RD18
158857 229533.A0A1C3YHP8 pp 229533.I1RRX6
158858 229533.A0A1C3YHP8 pp 229533.I1S1W5
158859 229533.A0A1C3YHP8 pp 229533.A0A1C3YJT5
158860 229533.A0A1C3YHP8 pp 229533.I1RTG0
158861 229533.A0A1C3YHP8 pp 229533.I1S039
158862 229533.A0A1C3YHP8 pp 229533.I1RQ13
158863 229533.A0A1C3YHP8 pp 229533.I1RIH8
158864 229533.A0A1C3YHP8 pp 229533.I1RC91
158865 229533.A0A1C3YHP8 pp 229533.I1RLX7
158866 229533.A0A1C3YHP8 pp 229533.I1RNV6
158867 229533.A0A1C3YHP8 pp 229533.I1RYZ9
158868 229533.A0A1C3YHP8 pp 229533.I1RWJ6
158869 229533.A0A1C3YHP8 pp 229533.I1RM49
158870 229533.A0A1C3YHP8 pp 229533.A0A1C3YJW1
158871 229533.A0A1C3YHP8 pp 229533.I1RYY5
158872 229533.A0A1C3YHP8 pp 229533.I1RDV9
158873 229533.A0A1C3YHP8 pp 229533.I1RQ92
158874 229533.A0A1C3YHP8 pp 229533.V6RKK5
158875 229533.A0A1C3YHP8 pp 229533.I1RWT6
158876 229533.A0A1C3YHP8 pp 229533.I1RKA1
158877 229533.A0A1C3YHP8 pp 229533.A0A1C3YK66
158878 229533.A0A1C3YHP8 pp 229533.I1RT62
158879 229533.A0A1C3YHP8 pp 229533.I1RJK7
158880 229533.A0A1C3YHP8 pp 229533.I1RD21
158881 229533.A0A1C3YHP8 pp 229533.I1S1F5
158882 229533.A0A1C3YHP8 pp 229533.I1RT45
158883 229533.A0A1C3YHP8 pp 229533.I1RB75
158884 229533.A0A1C3YHP8 pp 229533.I1RZ12
158885 229533.A0A1C3YHP8 pp 229533.I1RQS4
158886 229533.A0A1C3YHP8 pp 229533.V6RG22
158887 229533.A0A1C3YHP8 pp 229533.I1RTC2
158888 229533.A0A1C3YHP8 pp 229533.I1RPK5
158889 229533.A0A1C3YHP8 pp 229533.A0A1C3YMB6
158890 229533.A0A1C3YHP8 pp 229533.A0A1C3YJF1
158891 229533.A0A1C3YHP8 pp 229533.Q4IJM4
158892 229533.A0A1C3YHP8 pp 229533.I1RZ51
158893 229533.A0A1C3YHP8 pp 229533.A0A1C3YKY3
158894 229533.A0A1C3YHP8 pp 229533.A0A1C3YLX2
158895 229533.A0A1C3YHP8 pp 229533.I1RQA3
158896 229533.A0A1C3YHP8 pp 229533.I1RMQ7
158897 229533.A0A1C3YHP8 pp 229533.I1RAU6
158898 229533.A0A1C3YHP8 pp 229533.V6QV09
158899 229533.A0A1C3YHP8 pp 229533.I1RPU4
158900 229533.A0A1C3YHP8 pp 229533.A0A1C3YK37
158901 229533.A0A1C3YHP8 pp 229533.I1RC17
158902 229533.A0A1C3YHP8 pp 229533.I1RC95
158903 229533.A0A1C3YHP8 pp 229533.I1RHB0
158904 229533.A0A1C3YHP8 pp 229533.I1RK75
158905 229533.A0A1C3YHP8 pp 229533.I1RX50
158906 229533.A0A1C3YHP8 pp 229533.I1S1J6
158907 229533.A0A1C3YHP8 pp 229533.A0A1C3YIW6
158908 229533.A0A1C3YHP8 pp 229533.I1RME5
158909 229533.A0A1C3YHP8 pp 229533.I1S0J7
158910 229533.A0A1C3YHP8 pp 229533.I1S4L8
158911 229533.A0A1C3YHP8 pp 229533.A0A1C3YL67
158912 229533.A0A1C3YHP8 pp 229533.I1RPW0
158913 229533.A0A1C3YHP8 pp 229533.I1RBF3
158914 229533.A0A1C3YHP8 pp 229533.A0A1C3YK57
158915 229533.A0A1C3YHP8 pp 229533.I1RY58
158916 229533.A0A1C3YHP8 pp 229533.I1REE1
158917 229533.A0A1C3YHP8 pp 229533.I1RPC2
158918 229533.A0A1C3YHP8 pp 229533.A0A1C3YI39
158919 229533.A0A1C3YHP8 pp 229533.I1R9N1
158920 229533.A0A1C3YHP8 pp 229533.I1RRP7
158921 229533.A0A1C3YHP8 pp 229533.I1RBJ0

229533.A0A1C3YHP8	pp	229533.I1RZT1
229533.A0A1C3YHP8	pp	229533.I1RJU6
229533.A0A1C3YHP8	pp	229533.I1RB74
229533.A0A1C3YHP8	pp	229533.I1RFL7
229533.A0A1C3YHP8	pp	229533.Q4I7L0
229533.A0A1C3YHP8	pp	229533.A0A1C3YKT6
229533.A0A1C3YHP8	pp	229533.I1RJH8
229533.A0A1C3YHP8	pp	229533.I1RPK4
229533.A0A1C3YHP8	pp	229533.I1RTN0
229533.A0A1C3YHP8	pp	229533.I1RGC6
229533.A0A1C3YHP8	pp	229533.I1RRT2
229533.A0A1C3YHP8	pp	229533.I1S4R1
229533.A0A1C3YHP8	pp	229533.I1RUF8
229533.A0A1C3YHP8	pp	229533.I1RMU9
229533.A0A1C3YHP8	pp	229533.A0A1C3YIN6
229533.A0A1C3YHP8	pp	229533.I1RX38
229533.A0A1C3YHP8	pp	229533.I1RZK9
229533.A0A1C3YHP8	pp	229533.I1S098
229533.A0A1C3YHP8	pp	229533.I1REX8
229533.A0A1C3YHP8	pp	229533.I1S2E9
229533.A0A1C3YHP8	pp	229533.I1RZ63
229533.A0A1C3YHP8	pp	229533.A0A1C3YIN1
229533.A0A1C3YHP8	pp	229533.I1RC14
229533.A0A1C3YHP8	pp	229533.I1RRZ6
229533.A0A1C3YHP8	pp	229533.I1RGD1
229533.A0A1C3YHP8	pp	229533.I1S1W4
229533.A0A1C3YHP8	pp	229533.I1RY44
229533.A0A1C3YHP8	pp	229533.I1RBV5
229533.A0A1C3YHP8	pp	229533.A0A1C3YKJ6
229533.A0A1C3YHP8	pp	229533.I1RV91
229533.A0A1C3YHP8	pp	229533.A0A1C3YJ67
229533.A0A1C3YHP8	pp	229533.I1RGL3
229533.A0A1C3YHP8	pp	229533.I1R9Y7
229533.A0A1C3YHP8	pp	229533.I1S2G7
229533.A0A1C3YHP8	pp	229533.A0A1C3YM29
229533.A0A1C3YHP8	pp	229533.A0A1C3YJG7
229533.A0A1C3YHP8	pp	229533.I1RVN2
229533.A0A1C3YHP8	pp	229533.A0A1C3YK43
229533.A0A1C3YHP8	pp	229533.I1S9S6
229533.A0A1C3YHP8	pp	229533.V6RGI1
229533.A0A1C3YHP8	pp	229533.I1SAF7
229533.A0A1C3YHP8	pp	229533.I1RC08
229533.A0A1C3YHP8	pp	229533.I1RQD1
229533.A0A1C3YHP8	pp	229533.A0A1C3YJC3
229533.A0A1C3YHP8	pp	229533.A0A1C3YM91
229533.A0A1C3YHP8	pp	229533.I1RRW0
229533.A0A1C3YHP8	pp	229533.I1RMA4
229533.A0A1C3YHP8	pp	229533.I1RE79
229533.A0A0E0RP23	pp	229533.A0A1C3YLS7
229533.A0A0E0RP23	pp	229533.I1S7T4
229533.A0A0E0RP23	pp	229533.I1RZP8
229533.A0A0E0RP23	pp	229533.A0A1C3YKU7
229533.A0A0E0RP23	pp	229533.I1RZ60
229533.A0A0E0RP23	pp	229533.I1S2B6
229533.A0A0E0RP23	pp	229533.I1RQD6
229533.A0A0E0RP23	pp	229533.A0A1C3YIE7
229533.A0A0E0RP23	pp	229533.I1RKI7
229533.A0A0E0RP23	pp	229533.I1RN82
229533.A0A0E0RP23	pp	229533.I1RW76
229533.A0A0E0RP23	pp	229533.A0A1C3YJW5
229533.A0A0E0RP23	pp	229533.A0A1C3YN27
229533.A0A0E0RP23	pp	229533.I1RE18
229533.A0A0E0RP23	pp	229533.I1RX87
229533.A0A0E0RP23	pp	229533.A0A0E0RR64
229533.A0A0E0RP23	pp	229533.I1S0V5
229533.A0A0E0RP23	pp	229533.I1RPP5
229533.A0A0E0RP23	pp	229533.I1SAM5
229533.A0A0E0RP23	pp	229533.I1RZS5
229533.A0A0E0RP23	pp	229533.I1RMN3
229533.A0A0E0RP23	pp	229533.I1S1V4
229533.A0A0E0RP23	pp	229533.I1RXF5
229533.A0A0E0RP23	pp	229533.I1S0Y1
229533.A0A0E0RP23	pp	229533.I1RA25

158995	229533.A0A0E0RP23	pp	229533.I1RYP0
158996	229533.A0A0E0RP23	pp	229533.I1S278
158997	229533.A0A0E0RP23	pp	229533.I1RCN6
158998	229533.A0A0E0RP23	pp	229533.I1RBT0
158999	229533.A0A0E0RP23	pp	229533.Q4ILI9
159000	229533.A0A0E0RP23	pp	229533.A0A0E0SJS1
159001	229533.A0A0E0RP23	pp	229533.I1RCV3
159002	229533.A0A0E0RP23	pp	229533.I1RQP7
159003	229533.A0A0E0RP23	pp	229533.I1RT93
159004	229533.A0A0E0RP23	pp	229533.A0A1C3YLE3
159005	229533.I1RK75	pp	229533.I1RQ92
159006	229533.I1RK75	pp	229533.I1RM49
159007	229533.I1RK75	pp	229533.I1RYZ9
159008	229533.I1RK75	pp	229533.I1RT62
159009	229533.I1RK75	pp	229533.I1RKA1
159010	229533.I1RK75	pp	229533.V6QV09
159011	229533.I1RK75	pp	229533.I1RN89
159012	229533.I1RK75	pp	229533.I1RZ12
159013	229533.I1RK75	pp	229533.I1RQ13
159014	229533.I1RK75	pp	229533.I1RYY5
159015	229533.I1RK75	pp	229533.I1S039
159016	229533.I1RK75	pp	229533.I1S1W5
159017	229533.I1RK75	pp	229533.V6RKK5
159018	229533.I1RK75	pp	229533.I1RME5
159019	229533.I1RK75	pp	229533.I1RWJ6
159020	229533.I1RK75	pp	229533.I1RL24
159021	229533.I1RK75	pp	229533.Q4IJM4
159022	229533.I1RK75	pp	229533.I1RT45
159023	229533.I1RK75	pp	229533.Q4I7L0
159024	229533.I1RK75	pp	229533.I1RWT6
159025	229533.I1RK75	pp	229533.I1S4L8
159026	229533.I1RK75	pp	229533.I1RPK4
159027	229533.I1RK75	pp	229533.I1RUF8
159028	229533.I1RK75	pp	229533.I1RQA3
159029	229533.I1RK75	pp	229533.I1RZ63
159030	229533.I1RK75	pp	229533.I1S1F5
159031	229533.I1RK75	pp	229533.I1S4R1
159032	229533.I1RK75	pp	229533.V6RG22
159033	229533.I1RK75	pp	229533.I1S098
159034	229533.I1RK75	pp	229533.I1RMQ7
159035	229533.I1RK75	pp	229533.I1RX50
159036	229533.I1RK75	pp	229533.I1RV91
159037	229533.I1RK75	pp	229533.I1RRZ6
159038	229533.I1RK75	pp	229533.I1S2G7
159039	229533.I1RK75	pp	229533.I1RPC2
159040	229533.I1RK75	pp	229533.I1S9S6
159041	229533.I1RK75	pp	229533.I1RVN2
159042	229533.I1RK75	pp	229533.V6RGI1
159043	229533.I1RK75	pp	229533.I1RZT1
159044	229533.I1RK75	pp	229533.I1S1W4
159045	229533.I1RK75	pp	229533.I1RY58
159046	229533.I1RK75	pp	229533.I1RMA4
159047	229533.I1RK75	pp	229533.I1RMU9
159048	229533.I1RBT0	pp	229533.I1RY40
159049	229533.I1RBT0	pp	229533.I1RZK3
159050	229533.I1RBT0	pp	229533.I1RWZ9
159051	229533.I1RBT0	pp	229533.I1RFW0
159052	229533.I1RBT0	pp	229533.I1RKI1
159053	229533.I1RBT0	pp	229533.Q4I785
159054	229533.I1RBT0	pp	229533.I1RQD6
159055	229533.I1RBT0	pp	229533.I1RW76
159056	229533.I1RBT0	pp	229533.I1S287
159057	229533.I1RBT0	pp	229533.I1RN70
159058	229533.I1RBT0	pp	229533.I1RNK8
159059	229533.I1RBT0	pp	229533.I1RSH3
159060	229533.I1RBT0	pp	229533.I1RZS5
159061	229533.I1RBT0	pp	229533.I1RX87
159062	229533.I1RBT0	pp	229533.I1RP92
159063	229533.I1RBT0	pp	229533.I1RCQ0
159064	229533.I1RBT0	pp	229533.I1RNM1
159065	229533.I1RBT0	pp	229533.I1S1V4
159066	229533.I1RBT0	pp	229533.I1S0V5
159067	229533.I1RBT0	pp	229533.I1RR74

159068 229533.I1RBT0 pp 229533.I1RQF7
159069 229533.I1RBT0 pp 229533.I1SAM5
159070 229533.I1RBT0 pp 229533.I1RGC1
159071 229533.I1RBT0 pp 229533.I1RYP0
159072 229533.I1RBT0 pp 229533.I1RCN6
159073 229533.I1RBT0 pp 229533.I1RNH7
159074 229533.I1RBT0 pp 229533.I1RXF5
159075 229533.I1RBT0 pp 229533.I1S278
159076 229533.I1RBT0 pp 229533.I1REU2
159077 229533.I1RBT0 pp 229533.I1RTG0
159078 229533.I1RBT0 pp 229533.I1RCV3
159079 229533.I1RBT0 pp 229533.I1RQP7
159080 229533.I1RBT0 pp 229533.I1RFK0
159081 229533.I1RBT0 pp 229533.I1RNM3
159082 229533.I1RBT0 pp 229533.V6RXW9
159083 229533.I1RBT0 pp 229533.V6QY25
159084 229533.I1RBT0 pp 229533.I1RNG2
159085 229533.I1RBT0 pp 229533.I1S2B6
159086 229533.I1RBT0 pp 229533.I1RPG2
159087 229533.I1RBT0 pp 229533.I1RE18
159088 229533.I1RBT0 pp 229533.I1RMN3
159089 229533.I1RBT0 pp 229533.I1S0S8
159090 229533.I1RBT0 pp 229533.I1S1U6
159091 229533.Q4HWB3 pp 229533.Q4HZI0
159092 229533.Q4HWB3 pp 229533.Q4I5M4
159093 229533.Q4HWB3 pp 229533.Q4IQ72
159094 229533.I1RM37 pp 229533.I1RWR7
159095 229533.I1RM37 pp 229533.I1RZN1
159096 229533.I1RM37 pp 229533.I1RPR9
159097 229533.I1RM37 pp 229533.I1RPE0
159098 229533.I1RM37 pp 229533.V6R3J1
159099 229533.I1RM37 pp 229533.I1RS50
159100 229533.I1RM37 pp 229533.I1RR24
159101 229533.I1RM37 pp 229533.I1S5R1
159102 229533.I1RM37 pp 229533.I1RXD7
159103 229533.I1RM37 pp 229533.I1S821
159104 229533.I1RM37 pp 229533.I1RRM3
159105 229533.I1RM37 pp 229533.I1RXC2
159106 229533.I1RM37 pp 229533.V6R9Z0
159107 229533.I1RM37 pp 229533.I1RVX9
159108 229533.I1RM37 pp 229533.V6RCZ8
159109 229533.I1RM37 pp 229533.I1RUL3
159110 229533.I1RM37 pp 229533.I1RVQ3
159111 229533.I1RM37 pp 229533.I1S4S7
159112 229533.I1RM37 pp 229533.I1RT40
159113 229533.I1RM37 pp 229533.I1RXC4
159114 229533.I1RM37 pp 229533.I1RZM8
159115 229533.I1RM37 pp 229533.V6RM66
159116 229533.I1RM37 pp 229533.I1RSZ7
159117 229533.I1RM37 pp 229533.I1RNB2
159118 229533.I1RM37 pp 229533.I1RQ85
159119 229533.I1RM37 pp 229533.I1RRI1
159120 229533.I1RM37 pp 229533.I1RQE7
159121 229533.I1RM37 pp 229533.I1RQ33
159122 229533.I1RM37 pp 229533.I1RXD6
159123 229533.I1RM37 pp 229533.I1RVZ2
159124 229533.I1RM37 pp 229533.I1RPU2
159125 229533.I1RM37 pp 229533.I1S1W9
159126 229533.I1RM37 pp 229533.I1RPN1
159127 229533.I1RM37 pp 229533.I1S0W4
159128 229533.I1RD09 pp 229533.I1RPW8
159129 229533.I1RD09 pp 229533.I1S182
159130 229533.I1RD09 pp 229533.I1RW80
159131 229533.I1RD09 pp 229533.I1RQ42
159132 229533.I1RD09 pp 229533.I1RN04
159133 229533.I1RD09 pp 229533.I1RQE0
159134 229533.I1RD09 pp 229533.I1RW91
159135 229533.A0A1C3YJK5 pp 229533.I1RPN1
159136 229533.A0A1C3YJK5 pp 229533.I1RM49
159137 229533.A0A1C3YJK5 pp 229533.I1RD21
159138 229533.A0A1C3YJK5 pp 229533.A0A1C3YNA1
159139 229533.A0A1C3YJK5 pp 229533.Q4HZQ1
159140 229533.A0A1C3YJK5 pp 229533.V6QUG0

159141 229533.A0A1C3YJK5 pp 229533.I1RN51
159142 229533.A0A1C3YJK5 pp 229533.I1RA94
159143 229533.A0A1C3YJK5 pp 229533.I1RKA7
159144 229533.A0A1C3YJK5 pp 229533.V6R7V8
159145 229533.A0A1C3YJK5 pp 229533.I1RGK9
159146 229533.A0A1C3YJK5 pp 229533.I1S4L8
159147 229533.A0A1C3YJK5 pp 229533.I1RTJ2
159148 229533.A0A1C3YJK5 pp 229533.I1RBE4
159149 229533.A0A1C3YJK5 pp 229533.I1S4S1
159150 229533.A0A1C3YJK5 pp 229533.I1RMQ6
159151 229533.A0A1C3YJK5 pp 229533.I1RKZ4
159152 229533.A0A1C3YJK5 pp 229533.I1RCN8
159153 229533.A0A1C3YJK5 pp 229533.A0A1C3YJQ6
159154 229533.A0A1C3YJK5 pp 229533.I1S1F5
159155 229533.A0A1C3YJK5 pp 229533.I1RT45
159156 229533.A0A1C3YJK5 pp 229533.I1S2A1
159157 229533.A0A1C3YJK5 pp 229533.I1RT09
159158 229533.A0A1C3YJK5 pp 229533.I1RFY0
159159 229533.A0A1C3YJK5 pp 229533.Q4HVU7
159160 229533.A0A1C3YJK5 pp 229533.I1RYU0
159161 229533.A0A1C3YJK5 pp 229533.I1RUQ3
159162 229533.A0A1C3YJK5 pp 229533.I1RC08
159163 229533.A0A1C3YJK5 pp 229533.I1RAE8
159164 229533.A0A1C3YJK5 pp 229533.I1RFI9
159165 229533.A0A1C3YJK5 pp 229533.V6RN69
159166 229533.A0A1C3YJK5 pp 229533.I1RBB3
159167 229533.A0A1C3YJK5 pp 229533.I1RYG3
159168 229533.A0A1C3YJK5 pp 229533.I1R9Y8
159169 229533.A0A1C3YJK5 pp 229533.I1RUL3
159170 229533.A0A1C3YJK5 pp 229533.I1RE79
159171 229533.A0A1C3YJK5 pp 229533.I1RZD3
159172 229533.A0A1C3YJK5 pp 229533.I1RPL3
159173 229533.A0A1C3YJK5 pp 229533.I1RMW4
159174 229533.A0A1C3YJK5 pp 229533.I1RPY0
159175 229533.A0A1C3YJK5 pp 229533.A0A1C3YLM7
159176 229533.A0A1C3YJK5 pp 229533.Q4IN52
159177 229533.A0A1C3YJK5 pp 229533.I1RW26
159178 229533.A0A1C3YJK5 pp 229533.A0A1C3YNB1
159179 229533.A0A1C3YJK5 pp 229533.I1RE53
159180 229533.A0A1C3YJK5 pp 229533.I1S0C2
159181 229533.A0A1C3YJK5 pp 229533.I1RRY3
159182 229533.A0A1C3YJK5 pp 229533.I1RP31
159183 229533.A0A1C3YJK5 pp 229533.Q4IEV5
159184 229533.A0A1C3YJK5 pp 229533.I1RMK9
159185 229533.A0A1C3YJK5 pp 229533.I1RQ21
159186 229533.A0A1C3YJK5 pp 229533.I1RBT8
159187 229533.A0A1C3YJK5 pp 229533.A0A1C3YLB6
159188 229533.A0A1C3YJK5 pp 229533.V6RM66
159189 229533.A0A1C3YJK5 pp 229533.I1RVT8
159190 229533.I1RC08 pp 229533.I1RF33
159191 229533.I1RC08 pp 229533.I1S0J7
159192 229533.I1RC08 pp 229533.I1RGY6
159193 229533.I1RC08 pp 229533.I1RXY7
159194 229533.I1RC08 pp 229533.I1RZ63
159195 229533.I1RC08 pp 229533.I1RP31
159196 229533.I1RC08 pp 229533.I1RFI9
159197 229533.I1RC08 pp 229533.I1RYG3
159198 229533.I1RC08 pp 229533.I1RE79
159199 229533.I1RC08 pp 229533.I1RK75
159200 229533.I1RC08 pp 229533.I1RL76
159201 229533.I1RC08 pp 229533.I1RD21
159202 229533.I1RC08 pp 229533.I1RMU9
159203 229533.I1RC08 pp 229533.V6QUG0
159204 229533.I1RC08 pp 229533.I1RM49
159205 229533.I1RC08 pp 229533.I1RW06
159206 229533.I1RC08 pp 229533.V6R7V8
159207 229533.I1RC08 pp 229533.I1S4S1
159208 229533.I1RC08 pp 229533.I1RGK9
159209 229533.I1RC08 pp 229533.I1RFY0
159210 229533.I1RC08 pp 229533.I1S1F5
159211 229533.I1RC08 pp 229533.I1RTJ2
159212 229533.I1RC08 pp 229533.I1S2A1
159213 229533.I1SAM5 pp 229533.Q4I6S5

229533.I1SAM5 pp 229533.Q4IN63
229533.I1RFI9 pp 229533.I1RTJ2
229533.I1RFI9 pp 229533.I1S1F5
229533.I1RFI9 pp 229533.I1RFY0
229533.I1RFI9 pp 229533.I1RI02
229533.I1RFI9 pp 229533.V6QUG0
229533.I1RFI9 pp 229533.I1S4L8
229533.I1RFI9 pp 229533.I1S569
229533.I1RFI9 pp 229533.V6R7V8
229533.I1RFI9 pp 229533.I1S4S1
229533.I1RFI9 pp 229533.I1RP31
229533.I1RFI9 pp 229533.I1RGK9
229533.I1RFI9 pp 229533.I1RPN1
229533.I1RFI9 pp 229533.I1RYG3
229533.I1RFI9 pp 229533.I1S2A1
229533.I1RFI9 pp 229533.I1RPY0
229533.I1RFI9 pp 229533.I1RYU0
229533.I1RFI9 pp 229533.I1RM49
229533.I1RFI9 pp 229533.I1RUL3
229533.A0A0E0S3U6 pp 229533.I1RCF0
229533.A0A0E0S3U6 pp 229533.I1RYP0
229533.A0A0E0S3U6 pp 229533.I1RBS3
229533.A0A0E0S3U6 pp 229533.I1RSH3
229533.A0A0E0S3U6 pp 229533.I1S287
229533.A0A0E0S3U6 pp 229533.I1RLL8
229533.A0A0E0S3U6 pp 229533.I1S0B4
229533.A0A0E0S3U6 pp 229533.I1RBT0
229533.A0A0E0S3U6 pp 229533.I1S1U6
229533.A0A0E0S3U6 pp 229533.I1RFD5
229533.A0A0E0S3U6 pp 229533.I1S0V5
229533.A0A0E0S3U6 pp 229533.I1RCV3
229533.A0A0E0S3U6 pp 229533.I1RAP3
229533.A0A0E0S3U6 pp 229533.I1RBN0
229533.A0A0E0S3U6 pp 229533.I1RZ17
229533.A0A0E0S3U6 pp 229533.A0A0E0SP22
229533.A0A0E0S3U6 pp 229533.I1RE44
229533.A0A0E0S3U6 pp 229533.I1RMA0
229533.A0A0E0S3U6 pp 229533.I1RMN3
229533.A0A0E0S3U6 pp 229533.I1RFP5
229533.A0A0E0S3U6 pp 229533.I1RSW4
229533.A0A0E0S3U6 pp 229533.I1RQP7
229533.A0A0E0S3U6 pp 229533.I1RQD6
229533.A0A0E0S3U6 pp 229533.I1RRF6
229533.A0A0E0S3U6 pp 229533.I1S278
229533.A0A0E0S3U6 pp 229533.I1RD96
229533.A0A0E0S3U6 pp 229533.Q4ILH3
229533.A0A0E0S3U6 pp 229533.I1RX87
229533.A0A0E0S3U6 pp 229533.I1RTF2
229533.A0A0E0S3U6 pp 229533.A0A1C3YKT4
229533.I1RC80 pp 229533.I1RT16
229533.I1RC80 pp 229533.I1RYN7
229533.I1RC80 pp 229533.I1RE51
229533.I1RC80 pp 229533.I1S1V2
229533.I1RC80 pp 229533.I1RPU1
229533.I1RYG3 pp 229533.I1S4L8
229533.I1RYG3 pp 229533.I1S021
229533.I1RYG3 pp 229533.I1S253
229533.I1RC90 pp 229533.I1RT16
229533.I1RC90 pp 229533.I1RYN7
229533.I1RC90 pp 229533.I1RE51
229533.I1RC90 pp 229533.I1RPU1
229533.I1RC90 pp 229533.I1S1V2
229533.I1RC90 pp 229533.I1S0N5
229533.I1RC90 pp 229533.Q4HTT1
229533.I1RMU9 pp 229533.I1S1W8
229533.I1RMU9 pp 229533.I1RYP2
229533.I1RMU9 pp 229533.I1RNG4
229533.I1RMU9 pp 229533.I1RTJ2
229533.I1RMU9 pp 229533.I1RYD3
229533.I1RMU9 pp 229533.I1RZ39
229533.I1RMU9 pp 229533.I1RRZ6
229533.I1RMU9 pp 229533.I1S3G4
229533.I1RMU9 pp 229533.I1RMV0

159287 229533.I1RMU9 pp 229533.I1RZ63
159288 229533.I1RMU9 pp 229533.Q4I665
159289 229533.I1RMU9 pp 229533.I1S0J7
159290 229533.I1RMU9 pp 229533.I1RQP5
159291 229533.I1RMU9 pp 229533.V6RDZ4
159292 229533.I1RMU9 pp 229533.V6RKK5
159293 229533.I1RMU9 pp 229533.V6QUV7
159294 229533.I1RSH3 pp 229533.I1RTF2
159295 229533.I1RSH3 pp 229533.Q4HY20
159296 229533.I1RSH3 pp 229533.I1S278
159297 229533.I1RSH3 pp 229533.I1S1U6
159298 229533.I1RSH3 pp 229533.I1RZ89
159299 229533.I1RSH3 pp 229533.I1RWZ9
159300 229533.I1RSH3 pp 229533.I1S2B6
159301 229533.I1RSH3 pp 229533.I1S287
159302 229533.I1RSH3 pp 229533.V6QY25
159303 229533.I1RSH3 pp 229533.I1RY40
159304 229533.I1RSH3 pp 229533.V6QXP4
159305 229533.I1RSH3 pp 229533.I1S0V5
159306 229533.I1RSH3 pp 229533.I1RYP0
159307 229533.I1RSH3 pp 229533.I1RX87
159308 229533.I1RSH3 pp 229533.I1RSW4
159309 229533.I1RCQ1 pp 229533.V6RVJ3
159310 229533.I1RCQ1 pp 229533.I1RSX9
159311 229533.I1RCQ1 pp 229533.I1RKF0
159312 229533.I1RCQ1 pp 229533.I1RMW4
159313 229533.I1RCQ1 pp 229533.Q4HVU7
159314 229533.A0A1C3YI88 pp 229533.V6R3J1
159315 229533.A0A1C3YI88 pp 229533.I1S7M8
159316 229533.A0A1C3YI88 pp 229533.I1RMJ3
159317 229533.A0A1C3YI88 pp 229533.V6QWB1
159318 229533.A0A1C3YI88 pp 229533.I1RWS4
159319 229533.A0A1C3YI88 pp 229533.I1S746
159320 229533.A0A1C3YI88 pp 229533.I1RGR7
159321 229533.A0A1C3YI88 pp 229533.I1S693
159322 229533.A0A1C3YI88 pp 229533.I1S2P8
159323 229533.A0A1C3YI88 pp 229533.I1RTG7
159324 229533.A0A1C3YI88 pp 229533.I1RVT1
159325 229533.A0A1C3YI88 pp 229533.I1RE13
159326 229533.A0A1C3YI88 pp 229533.I1RJ85
159327 229533.A0A1C3YI88 pp 229533.I1S8Q2
159328 229533.A0A1C3YI88 pp 229533.I1RYX7
159329 229533.A0A1C3YI88 pp 229533.A0A1C3YMG4
159330 229533.A0A1C3YI88 pp 229533.I1RGK9
159331 229533.A0A1C3YI88 pp 229533.I1RMU9
159332 229533.A0A1C3YI88 pp 229533.I1RCW1
159333 229533.A0A1C3YI88 pp 229533.I1S2Z8
159334 229533.A0A1C3YI88 pp 229533.A0A1C3YKF3
159335 229533.A0A1C3YI88 pp 229533.Q4IMJ1
159336 229533.A0A1C3YI88 pp 229533.I1RXV9
159337 229533.A0A1C3YI88 pp 229533.I1RSI0
159338 229533.A0A1C3YI88 pp 229533.I1RBA4
159339 229533.A0A1C3YI88 pp 229533.I1RIU1
159340 229533.A0A1C3YI88 pp 229533.V6RKK5
159341 229533.A0A1C3YI88 pp 229533.I1RW06
159342 229533.A0A1C3YI88 pp 229533.I1RJU4
159343 229533.A0A1C3YI88 pp 229533.I1RYJ6
159344 229533.A0A1C3YI88 pp 229533.I1S250
159345 229533.A0A1C3YI88 pp 229533.I1RTJ2
159346 229533.A0A1C3YI88 pp 229533.I1RMX0
159347 229533.A0A1C3YI88 pp 229533.I1S072
159348 229533.A0A1C3YI88 pp 229533.I1RI91
159349 229533.A0A1C3YI88 pp 229533.I1RZP0
159350 229533.A0A1C3YI88 pp 229533.I1RVV7
159351 229533.A0A1C3YI88 pp 229533.I1RBP0
159352 229533.A0A1C3YI88 pp 229533.I1RG29
159353 229533.A0A1C3YI88 pp 229533.I1RC08
159354 229533.A0A1C3YI48 pp 229533.I1RW91
159355 229533.A0A1C3YI48 pp 229533.I1RRF7
159356 229533.A0A1C3YI48 pp 229533.I1RD09
159357 229533.A0A1C3YI48 pp 229533.I1RW80
159358 229533.A0A1C3YI48 pp 229533.I1RZ42
159359 229533.A0A1C3YI48 pp 229533.I1RR61

159360 229533.A0A1C3YI48 pp 229533.I1RN04
159361 229533.A0A1C3YI48 pp 229533.I1RPW8
159362 229533.A0A1C3YI48 pp 229533.I1RQ42
159363 229533.A0A1C3YI48 pp 229533.I1RCG6
159364 229533.A0A1C3YI48 pp 229533.I1RKF3
159365 229533.A0A1C3YI48 pp 229533.I1S182
159366 229533.I1RCN6 pp 229533.V6R476
159367 229533.I1RCN6 pp 229533.V6QUU6
159368 229533.I1RCN6 pp 229533.I1S1V4
159369 229533.I1RCN6 pp 229533.I1RZS5
159370 229533.I1RCN6 pp 229533.I1RZ60
159371 229533.I1RCN6 pp 229533.I1RX87
159372 229533.I1RCN6 pp 229533.I1RVS1
159373 229533.I1RCN6 pp 229533.I1RCQ0
159374 229533.I1RCN6 pp 229533.I1RXU1
159375 229533.I1RCN6 pp 229533.I1RMF2
159376 229533.I1RCN6 pp 229533.I1RNP1
159377 229533.I1RCN6 pp 229533.I1RE19
159378 229533.I1RCN6 pp 229533.I1S0V5
159379 229533.I1RCN6 pp 229533.I1RN81
159380 229533.I1RCN6 pp 229533.I1RTQ9
159381 229533.I1RCN6 pp 229533.I1RQD6
159382 229533.I1RCN6 pp 229533.I1RJH8
159383 229533.I1RCN6 pp 229533.I1S4Y4
159384 229533.I1RCN6 pp 229533.I1RDK3
159385 229533.I1RCN6 pp 229533.I1RQ70
159386 229533.I1RCN6 pp 229533.I1S9X9
159387 229533.I1RCN6 pp 229533.I1RX84
159388 229533.I1RCN6 pp 229533.I1S039
159389 229533.I1RCN6 pp 229533.I1RQF7
159390 229533.I1RCN6 pp 229533.I1RPG2
159391 229533.I1RCN6 pp 229533.I1RKI7
159392 229533.I1RCN6 pp 229533.I1RW76
159393 229533.I1RCN6 pp 229533.I1S0Y1
159394 229533.I1RCN6 pp 229533.I1RNH7
159395 229533.I1RCN6 pp 229533.I1RMV6
159396 229533.I1RCN6 pp 229533.I1RE18
159397 229533.I1RCN6 pp 229533.I1RN82
159398 229533.I1RCN6 pp 229533.I1RKT9
159399 229533.I1RCN6 pp 229533.I1S0X0
159400 229533.I1RCN6 pp 229533.I1S254
159401 229533.I1RCN6 pp 229533.I1S2B6
159402 229533.I1RCN6 pp 229533.Q4ILI9
159403 229533.I1RCN6 pp 229533.I1RNG2
159404 229533.I1RCN6 pp 229533.I1SAM5
159405 229533.I1RCN6 pp 229533.Q4HYZ2
159406 229533.I1RCN6 pp 229533.I1RYW8
159407 229533.I1RCN6 pp 229533.I1RZK3
159408 229533.I1RCN6 pp 229533.I1RMJ1
159409 229533.I1RCN6 pp 229533.I1S278
159410 229533.I1RCN6 pp 229533.V6QW25
159411 229533.I1RCN6 pp 229533.I1RZB4
159412 229533.I1RCN6 pp 229533.I1RQP7
159413 229533.I1RCN6 pp 229533.I1RTC1
159414 229533.I1RCN6 pp 229533.I1RMP2
159415 229533.I1RCN6 pp 229533.I1S830
159416 229533.I1RCN6 pp 229533.I1RMN3
159417 229533.I1RCN6 pp 229533.I1RXF5
159418 229533.I1RCN6 pp 229533.V6RR76
159419 229533.I1RCN6 pp 229533.I1RRU9
159420 229533.I1RF33 pp 229533.I1RFY0
159421 229533.I1RF33 pp 229533.I1RTJ2
159422 229533.I1RF33 pp 229533.I1S1F5
159423 229533.I1RF33 pp 229533.V6QUG0
159424 229533.I1RF33 pp 229533.I1S4L8
159425 229533.I1RF33 pp 229533.V6R7V8
159426 229533.I1RF33 pp 229533.I1RPY0
159427 229533.I1RF33 pp 229533.I1S2A1
159428 229533.I1RF33 pp 229533.I1RGK9
159429 229533.I1RF33 pp 229533.I1RYU0
159430 229533.I1RF33 pp 229533.I1RP31
159431 229533.I1RF33 pp 229533.I1S4S1
159432 229533.I1RF33 pp 229533.I1RYG3

159433 229533.I1RF33 pp 229533.I1RM49
159434 229533.I1RF33 pp 229533.I1RUL3
159435 229533.I1RF33 pp 229533.I1RPN1
159436 229533.A0A098DHE1 pp 229533.A0A1C3YLA2
159437 229533.A0A098DHE1 pp 229533.A0A1C3YHP3
159438 229533.A0A098DHE1 pp 229533.A0A1C3YLC6
159439 229533.A0A098DHE1 pp 229533.I1RC80
159440 229533.A0A098DHE1 pp 229533.I1RC90
159441 229533.A0A098DHE1 pp 229533.I1RT16
159442 229533.A0A098DHE1 pp 229533.I1RE51
159443 229533.A0A098DHE1 pp 229533.I1RYN7
159444 229533.A0A098DHE1 pp 229533.A0A098DZN5
159445 229533.A0A098DHE1 pp 229533.I1S1V2
159446 229533.A0A098DRI1 pp 229533.I1RBS3
159447 229533.A0A098DRI1 pp 229533.I1RAX8
159448 229533.A0A098DRI1 pp 229533.I1RX54
159449 229533.A0A098DRI1 pp 229533.I1S287
159450 229533.A0A098DRI1 pp 229533.I1RQC4
159451 229533.A0A098DRI1 pp 229533.A0A0E0SP22
159452 229533.A0A098DRI1 pp 229533.I1RE44
159453 229533.A0A098DRI1 pp 229533.I1RMN3
159454 229533.A0A098DRI1 pp 229533.I1RRC8
159455 229533.A0A098DRI1 pp 229533.I1S1U6
159456 229533.A0A098DRI1 pp 229533.I1RQN8
159457 229533.A0A098DRI1 pp 229533.A0A0E0RTT2
159458 229533.A0A098DRI1 pp 229533.I1RRC9
159459 229533.A0A098DRI1 pp 229533.I1RT00
159460 229533.A0A098DRI1 pp 229533.I1RUU3
159461 229533.A0A098DRI1 pp 229533.I1S1Z9
159462 229533.A0A098DRI1 pp 229533.I1RD43
159463 229533.A0A098DRI1 pp 229533.I1S2B6
159464 229533.A0A098DRI1 pp 229533.I1RFP5
159465 229533.A0A098DRI1 pp 229533.I1RBC0
159466 229533.A0A098DRI1 pp 229533.I1RX87
159467 229533.A0A098DRI1 pp 229533.A0A1C3YNC1
159468 229533.A0A098DRI1 pp 229533.I1RDD0
159469 229533.A0A098DRI1 pp 229533.A0A1C3YI75
159470 229533.A0A098DRI1 pp 229533.I1RCV3
159471 229533.A0A098DRI1 pp 229533.I1RLP4
159472 229533.A0A098DRI1 pp 229533.I1RSW4
159473 229533.A0A098DRI1 pp 229533.V6QXP4
159474 229533.A0A098DRI1 pp 229533.Q4HY20
159475 229533.A0A098DRI1 pp 229533.I1RTF2
159476 229533.A0A098DRI1 pp 229533.I1RY40
159477 229533.A0A098DRI1 pp 229533.I1RWU9
159478 229533.A0A098DRI1 pp 229533.I1RMM1
159479 229533.A0A098DRI1 pp 229533.I1RWZ9
159480 229533.A0A098DRI1 pp 229533.I1RXF0
159481 229533.A0A098DRI1 pp 229533.I1RQP7
159482 229533.A0A098DRI1 pp 229533.A0A1C3YJG7
159483 229533.A0A098DRI1 pp 229533.A0A1C3YMI5
159484 229533.I1RZ63 pp 229533.V6RKY7
159485 229533.I1RZ63 pp 229533.I1S0N4
159486 229533.I1RZ63 pp 229533.I1S1W4
159487 229533.I1RZ63 pp 229533.I1S0S8
159488 229533.I1RZ63 pp 229533.I1RZT1
159489 229533.I1RZ63 pp 229533.Q4HZB8
159490 229533.I1RZ63 pp 229533.I1RZE4
159491 229533.I1RZ63 pp 229533.I1S1F5
159492 229533.I1RZ63 pp 229533.V6RGI1
159493 229533.I1RZ63 pp 229533.I1S2G7
159494 229533.I1RZ63 pp 229533.I1S0M2
159495 229533.I1RZ63 pp 229533.I1S4L8
159496 229533.I1RZ63 pp 229533.Q4HXT6
159497 229533.I1RZ63 pp 229533.Q4IJM4
159498 229533.I1RZ63 pp 229533.I1S098
159499 229533.I1RZ63 pp 229533.I1S0R9
159500 229533.I1RZ63 pp 229533.I1S2E9
159501 229533.I1RZ63 pp 229533.I1S0N8
159502 229533.I1RZ63 pp 229533.V6QV09
159503 229533.I1RZ63 pp 229533.V6RKK5
159504 229533.I1RZ63 pp 229533.V6RDH3
159505 229533.I1RZ63 pp 229533.I1S0J7

229533.I1RZ63 pp 229533.I1S224
229533.I1RZ63 pp 229533.I1S1W5
229533.I1RZ63 pp 229533.Q4IPI4
229533.I1RZ63 pp 229533.I1S283
229533.I1RZ63 pp 229533.I1S039
229533.I1RL83 pp 229533.I1S0V1
229533.I1RGY6 pp 229533.I1S2A1
229533.I1RGY6 pp 229533.I1S4L8
229533.I1RGY6 pp 229533.I1RPN1
229533.I1RGY6 pp 229533.I1RM49
229533.I1RGY6 pp 229533.I1S4S1
229533.I1RGY6 pp 229533.I1RTJ2
229533.I1RGY6 pp 229533.I1S1F5
229533.I1RGY6 pp 229533.V6QUG0
229533.I1RGY6 pp 229533.I1RUL3
229533.I1RGY6 pp 229533.I1RYU0
229533.I1RGY6 pp 229533.V6R7V8
229533.I1RGY6 pp 229533.I1RP31
229533.I1RGY6 pp 229533.I1RYG3
229533.I1RGY6 pp 229533.I1RPY0
229533.I1S2A1 pp 229533.V6RLM7
229533.I1S2A1 pp 229533.I1S5R6
229533.I1S2A1 pp 229533.V6R7V8
229533.I1S2A1 pp 229533.I1S4L8
229533.I1S2A1 pp 229533.Q4HVU7
229533.I1S2A1 pp 229533.V6QWB1
229533.I1S2A1 pp 229533.V6QUG0
229533.I1S2A1 pp 229533.V6RN69
229533.I1S2A1 pp 229533.I1S4S1
229533.I1RNH7 pp 229533.I1RX87
229533.I1RNH7 pp 229533.I1RQH7
229533.I1RNH7 pp 229533.I1S5B5
229533.I1RNH7 pp 229533.I1S1V4
229533.I1RNH7 pp 229533.I1RQF9
229533.I1RNH7 pp 229533.I1RZK3
229533.I1RNH7 pp 229533.I1RQF7
229533.I1RNH7 pp 229533.Q4IB50
229533.I1RNH7 pp 229533.I1S2B6
229533.I1RNH7 pp 229533.I1RP87
229533.I1RNH7 pp 229533.I1SAM5
229533.I1RNH7 pp 229533.I1RPC6
229533.I1RNH7 pp 229533.I1RWZ9
229533.I1RNH7 pp 229533.I1RQP7
229533.I1RNH7 pp 229533.I1RPG2
229533.I1RNH7 pp 229533.I1RW76
229533.I1RNH7 pp 229533.I1RYP0
229533.A0A1C3YLA6 pp 229533.I1RMM8
229533.A0A1C3YLA6 pp 229533.I1RQE8
229533.A0A1C3YLA6 pp 229533.I1RBT1
229533.A0A1C3YLA6 pp 229533.A0A1C3YN26
229533.A0A1C3YLA6 pp 229533.I1RC11
229533.A0A1C3YLA6 pp 229533.I1RR24
229533.A0A1C3YLA6 pp 229533.I1RW06
229533.A0A1C3YLA6 pp 229533.I1RYJ7
229533.A0A1C3YLA6 pp 229533.A0A1C3YLR6
229533.A0A1C3YLA6 pp 229533.I1S1W8
229533.A0A1C3YLA6 pp 229533.I1RP86
229533.A0A1C3YLA6 pp 229533.I1RAX6
229533.A0A1C3YLA6 pp 229533.I1RYU5
229533.A0A1C3YLA6 pp 229533.I1RX46
229533.A0A1C3YLA6 pp 229533.I1RFK7
229533.A0A1C3YLA6 pp 229533.A0A1C3YLE9
229533.A0A1C3YLA6 pp 229533.I1RRX5
229533.A0A1C3YLA6 pp 229533.I1RVJ6
229533.A0A1C3YLA6 pp 229533.I1RBE4
229533.A0A1C3YLA6 pp 229533.I1RZM4
229533.A0A1C3YLA6 pp 229533.A0A1C3YN25
229533.A0A1C3YLA6 pp 229533.V6RCZ8
229533.A0A1C3YLA6 pp 229533.I1RTG7
229533.A0A1C3YLA6 pp 229533.I1RXB1
229533.A0A1C3YLA6 pp 229533.I1RD52
229533.A0A1C3YLA6 pp 229533.I1RZM8
229533.A0A1C3YLA6 pp 229533.I1RYX6

159579 229533.A0A1C3YLA6 pp 229533.I1RW43
159580 229533.A0A1C3YLA6 pp 229533.V6R3X5
159581 229533.A0A1C3YLA6 pp 229533.I1RXF0
159582 229533.A0A1C3YLA6 pp 229533.I1RA94
159583 229533.A0A1C3YLA6 pp 229533.I1RSQ4
159584 229533.A0A1C3YLA6 pp 229533.I1S5B0
159585 229533.A0A1C3YLA6 pp 229533.I1S130
159586 229533.A0A1C3YLA6 pp 229533.I1RFU8
159587 229533.A0A1C3YLA6 pp 229533.I1RZ29
159588 229533.A0A1C3YLA6 pp 229533.I1RRV4
159589 229533.A0A1C3YLA6 pp 229533.I1RCM5
159590 229533.A0A1C3YLA6 pp 229533.I1RZK5
159591 229533.A0A1C3YLA6 pp 229533.I1S8V3
159592 229533.A0A1C3YLA6 pp 229533.I1S2H4
159593 229533.A0A1C3YLA6 pp 229533.I1RYI7
159594 229533.A0A1C3YLA6 pp 229533.I1RY03
159595 229533.A0A1C3YLA6 pp 229533.I1RBL7
159596 229533.A0A1C3YLA6 pp 229533.I1RFM6
159597 229533.A0A1C3YLA6 pp 229533.I1S3K6
159598 229533.A0A1C3YLA6 pp 229533.I1RXV9
159599 229533.A0A1C3YLA6 pp 229533.I1S3G4
159600 229533.A0A1C3YLA6 pp 229533.O74638
159601 229533.A0A1C3YLA6 pp 229533.I1RVR0
159602 229533.A0A1C3YLA6 pp 229533.I1RCR5
159603 229533.A0A1C3YLA6 pp 229533.I1RS18
159604 229533.A0A1C3YLA6 pp 229533.I1S0A0
159605 229533.A0A1C3YLA6 pp 229533.I1RVY2
159606 229533.A0A1C3YLA6 pp 229533.I1RRT8
159607 229533.A0A1C3YLA6 pp 229533.I1RC29
159608 229533.A0A1C3YLA6 pp 229533.I1RBE7
159609 229533.A0A1C3YLA6 pp 229533.I1RBF3
159610 229533.A0A1C3YLA6 pp 229533.I1RZ20
159611 229533.A0A1C3YLA6 pp 229533.V6RK96
159612 229533.A0A1C3YLA6 pp 229533.I1RFS3
159613 229533.A0A1C3YLA6 pp 229533.I1RN67
159614 229533.A0A1C3YLA6 pp 229533.I1RM30
159615 229533.A0A1C3YLA6 pp 229533.I1RN88
159616 229533.A0A1C3YLA6 pp 229533.I1RA90
159617 229533.A0A1C3YLA6 pp 229533.I1RNG4
159618 229533.A0A1C3YLA6 pp 229533.I1RC31
159619 229533.A0A1C3YLA6 pp 229533.I1RY62
159620 229533.A0A1C3YLA6 pp 229533.I1RTI7
159621 229533.A0A1C3YLA6 pp 229533.I1R9Y8
159622 229533.A0A1C3YLA6 pp 229533.Q4HXF0
159623 229533.A0A1C3YLA6 pp 229533.I1RB92
159624 229533.A0A1C3YLA6 pp 229533.I1RPD6
159625 229533.A0A1C3YLA6 pp 229533.V6QVH8
159626 229533.A0A1C3YLA6 pp 229533.I1RP97
159627 229533.A0A1C3YLA6 pp 229533.I1RBS0
159628 229533.A0A1C3YLA6 pp 229533.V6RSF3
159629 229533.A0A1C3YLA6 pp 229533.I1RCP7
159630 229533.A0A1C3YLA6 pp 229533.I1RBC7
159631 229533.A0A1C3YLA6 pp 229533.I1RQE7
159632 229533.A0A1C3YLA6 pp 229533.I1RCH5
159633 229533.A0A1C3YLA6 pp 229533.I1RJW6
159634 229533.A0A1C3YLA6 pp 229533.I1RAU7
159635 229533.A0A1C3YLA6 pp 229533.I1RYW3
159636 229533.A0A1C3YLA6 pp 229533.I1RRI2
159637 229533.A0A1C3YLA6 pp 229533.I1RXW3
159638 229533.A0A1C3YLA6 pp 229533.I1RP69
159639 229533.A0A1C3YLA6 pp 229533.I1RFT1
159640 229533.A0A1C3YLA6 pp 229533.I1S256
159641 229533.A0A1C3YLA6 pp 229533.I1RG11
159642 229533.A0A1C3YLA6 pp 229533.I1RKB6
159643 229533.A0A1C3YLA6 pp 229533.I1RPP3
159644 229533.A0A1C3YLA6 pp 229533.I1RYK0
159645 229533.A0A1C3YLA6 pp 229533.I1RVX0
159646 229533.A0A1C3YLA6 pp 229533.I1RCB4
159647 229533.A0A1C3YLA6 pp 229533.I1RIH0
159648 229533.A0A1C3YLA6 pp 229533.I1RN63
159649 229533.A0A1C3YLA6 pp 229533.I1RPK0
159650 229533.A0A1C3YLA6 pp 229533.V6RLM7
159651 229533.A0A1C3YLA6 pp 229533.I1RE01

159652 229533.A0A1C3YLA6 pp 229533.V6QT89
159653 229533.A0A1C3YLA6 pp 229533.I1S0J1
159654 229533.A0A1C3YLA6 pp 229533.I1S8V4
159655 229533.A0A1C3YLA6 pp 229533.I1RSL5
159656 229533.I1S1F5 pp 229533.I1S4L8
159657 229533.I1S1F5 pp 229533.V6RKK5
159658 229533.I1S1F5 pp 229533.I1S8Z2
159659 229533.I1RAH7 pp 229533.I1S2B6
159660 229533.I1RAH7 pp 229533.I1RPG2
159661 229533.I1RAH7 pp 229533.I1RUN2
159662 229533.I1RAH7 pp 229533.I1RE18
159663 229533.I1RAH7 pp 229533.I1RCV3
159664 229533.I1RAH7 pp 229533.I1RKI1
159665 229533.I1RAH7 pp 229533.I1RWZ9
159666 229533.I1RAH7 pp 229533.I1RQD6
159667 229533.I1RAH7 pp 229533.I1RZK3
159668 229533.I1RAH7 pp 229533.I1RCL9
159669 229533.I1RAH7 pp 229533.I1RX87
159670 229533.I1RAH7 pp 229533.I1RRC3
159671 229533.I1RAH7 pp 229533.I1RQP7
159672 229533.I1RAH7 pp 229533.I1S0S8
159673 229533.I1RAH7 pp 229533.I1RQH7
159674 229533.I1RAH7 pp 229533.I1RND7
159675 229533.I1RAH7 pp 229533.Q4IN63
159676 229533.I1RAH7 pp 229533.I1RMJ1
159677 229533.I1RAH7 pp 229533.I1S1V4
159678 229533.I1RAH7 pp 229533.Q4ILI9
159679 229533.I1RAH7 pp 229533.I1RUN1
159680 229533.I1RAH7 pp 229533.I1RBF2
159681 229533.I1RAH7 pp 229533.I1RJZ6
159682 229533.I1RAH7 pp 229533.I1RQF7
159683 229533.I1RAH7 pp 229533.I1SAM5
159684 229533.I1RAH7 pp 229533.I1RW76
159685 229533.I1RAH7 pp 229533.I1S287
159686 229533.I1RAH7 pp 229533.I1RCF0
159687 229533.I1RAH7 pp 229533.I1RBT0
159688 229533.I1RAH7 pp 229533.I1RCH2
159689 229533.I1RAH7 pp 229533.I1S0V5
159690 229533.I1RAH7 pp 229533.I1RMN3
159691 229533.I1RAH7 pp 229533.I1RAH8
159692 229533.I1RAH7 pp 229533.I1RFK0
159693 229533.I1RAH7 pp 229533.I1RCN6
159694 229533.I1RAH7 pp 229533.I1RFV4
159695 229533.I1RAH7 pp 229533.I1RQF9
159696 229533.I1RAH7 pp 229533.I1RCQ0
159697 229533.I1RAH7 pp 229533.I1RZJ9
159698 229533.I1RAH7 pp 229533.I1RYP0
159699 229533.I1RAH7 pp 229533.I1RNH7
159700 229533.A0A1C3YKE0 pp 229533.I1S2A1
159701 229533.A0A1C3YKE0 pp 229533.I1RYU0
159702 229533.A0A1C3YKE0 pp 229533.I1S2K0
159703 229533.A0A1C3YKE0 pp 229533.I1RUL3
159704 229533.A0A1C3YKE0 pp 229533.I1RPN1
159705 229533.A0A1C3YKE0 pp 229533.I1RM49
159706 229533.A0A1C3YKE0 pp 229533.I1RD21
159707 229533.A0A1C3YKE0 pp 229533.I1S2J3
159708 229533.A0A1C3YKE0 pp 229533.I1SAJ8
159709 229533.A0A1C3YKE0 pp 229533.A0A1C3YNA1
159710 229533.A0A1C3YKE0 pp 229533.V6QUG0
159711 229533.A0A1C3YKE0 pp 229533.I1S1F5
159712 229533.A0A1C3YKE0 pp 229533.I1S2J8
159713 229533.A0A1C3YKE0 pp 229533.I1RFY0
159714 229533.A0A1C3YKE0 pp 229533.I1RGK9
159715 229533.A0A1C3YKE0 pp 229533.V6R7V8
159716 229533.A0A1C3YKE0 pp 229533.I1RTJ2
159717 229533.A0A1C3YKE0 pp 229533.I1S4L8
159718 229533.A0A1C3YKE0 pp 229533.I1S4S1
159719 229533.A0A1C3YKE0 pp 229533.I1RYG3
159720 229533.A0A1C3YKE0 pp 229533.I1RP31
159721 229533.A0A1C3YKE0 pp 229533.I1RPY0
159722 229533.A0A1C3YKE0 pp 229533.I1SAJ7
159723 229533.A0A1C3YKE0 pp 229533.A0A1C3YLM7
159724 229533.A0A1C3YKE0 pp 229533.I1S2J6

229533.A0A1C3YKE0	pp	229533.I1RC08
229533.A0A1C3YKE0	pp	229533.A0A1C3YLB6
229533.A0A1C3YKE0	pp	229533.I1RE79
229533.A0A1C3YKE0	pp	229533.I1RBT8
229533.A0A1C3YKE0	pp	229533.A0A1C3YKS5
229533.A0A1C3YKE0	pp	229533.I1S2J5
229533.I1RZ79	pp	229533.I1S1C7
229533.I1RZ79	pp	229533.V6RTJ8
229533.I1RZ79	pp	229533.I1S5R6
229533.I1RZ79	pp	229533.Q4IJK2
229533.I1RZ79	pp	229533.I1RZM8
229533.I1RZ79	pp	229533.I1S4S7
229533.I1RZ79	pp	229533.I1RZZ9
229533.I1RZ79	pp	229533.V6R042
229533.I1RZ79	pp	229533.I1S2H4
229533.I1RZ79	pp	229533.I1S5R1
229533.I1RZ79	pp	229533.I1S8P5
229533.I1RZ79	pp	229533.I1S0J1
229533.I1RZ79	pp	229533.I1S2B8
229533.I1RZ79	pp	229533.V6RMW6
229533.I1RZ79	pp	229533.I1S1W9
229533.I1RW26	pp	229533.Q4IJU0
229533.I1RW26	pp	229533.I1RY63
229533.I1RW26	pp	229533.I1S0K2
229533.I1RW26	pp	229533.V6RQW4
229533.I1RW26	pp	229533.V6QWZ7
229533.I1RW26	pp	229533.Q4IEK8
229533.I1RW26	pp	229533.V6RKK5
229533.I1RW26	pp	229533.Q4IJ56
229533.I1RW26	pp	229533.V6R227
229533.I1RW26	pp	229533.I1RXF0
229533.I1RW26	pp	229533.V6QYF0
229533.I1RW26	pp	229533.Q4IFI0
229533.I1RW26	pp	229533.V6QUE6
229533.I1RW26	pp	229533.Q4I1B1
229533.I1RW26	pp	229533.I1RYJ6
229533.I1RW26	pp	229533.I1S0F1
229533.I1RW26	pp	229533.Q4IPI1
229533.I1RW26	pp	229533.Q4IN63
229533.I1RW26	pp	229533.Q4IJH1
229533.I1RW26	pp	229533.I1RW71
229533.I1RW26	pp	229533.I1RY21
229533.I1RW26	pp	229533.Q4HYB8
229533.I1RW26	pp	229533.I1RY92
229533.I1RW26	pp	229533.Q4I327
229533.I1RW26	pp	229533.I1S558
229533.I1RW26	pp	229533.I1S253
229533.I1RW26	pp	229533.I1S0P0
229533.I1RW26	pp	229533.Q4I5M4
229533.I1RW26	pp	229533.I1S289
229533.I1RW26	pp	229533.I1RWJ4
229533.I1RW26	pp	229533.V6RGY3
229533.I1RW26	pp	229533.I1S0J1
229533.I1RW26	pp	229533.Q4I662
229533.I1RW26	pp	229533.Q4I8X0
229533.I1RW26	pp	229533.Q4HZ68
229533.I1RW26	pp	229533.Q4IMD1
229533.I1RW26	pp	229533.I1S4T3
229533.I1RW26	pp	229533.V6RLW4
229533.I1RW26	pp	229533.Q4IQW0
229533.I1RW26	pp	229533.I1RZS6
229533.I1RW26	pp	229533.Q4IEV5
229533.I1RW26	pp	229533.Q4IR18
229533.I1RW26	pp	229533.I1RWS4
229533.I1RW26	pp	229533.Q4IB65
229533.I1RW26	pp	229533.Q4HXF0
229533.I1RTJ2	pp	229533.I1S4L8
229533.I1RTJ2	pp	229533.I1S3V2
229533.I1RTJ2	pp	229533.I1SAE8
229533.I1RTJ2	pp	229533.Q4I1N3
229533.I1RTJ2	pp	229533.I1S0H2
229533.I1RTJ2	pp	229533.V6RKK5
229533.I1RTJ2	pp	229533.Q4I061

159798 229533.I1RTJ2 pp 229533.I1S1F5
159799 229533.I1RTJ2 pp 229533.Q4HZ68
159800 229533.I1RTJ2 pp 229533.I1RZK8
159801 229533.I1RTJ2 pp 229533.I1RX59
159802 229533.I1RTJ2 pp 229533.V6RDH3
159803 229533.I1RTJ2 pp 229533.I1RWT0
159804 229533.I1RTJ2 pp 229533.I1RTP5
159805 229533.I1RTJ2 pp 229533.I1RXB6
159806 229533.I1RTJ2 pp 229533.V6RH92
159807 229533.I1RTJ2 pp 229533.I1RW11
159808 229533.I1RTJ2 pp 229533.I1RUQ7
159809 229533.I1RTJ2 pp 229533.I1RY21
159810 229533.I1RTJ2 pp 229533.I1RZH8
159811 229533.I1RTJ2 pp 229533.Q4IAS1
159812 229533.I1RTJ2 pp 229533.I1RYD3
159813 229533.I1RTJ2 pp 229533.V6QV31
159814 229533.I1RTJ2 pp 229533.V6RS91
159815 229533.I1RTJ2 pp 229533.I1RYQ4
159816 229533.I1RTJ2 pp 229533.I1RYQ1
159817 229533.I1RTJ2 pp 229533.I1RZP0
159818 229533.I1RPW2 pp 229533.I1S9Z4
159819 229533.I1RPW2 pp 229533.V6RLM7
159820 229533.I1RPW2 pp 229533.I1S0A0
159821 229533.I1RPW2 pp 229533.I1RS18
159822 229533.I1RPW2 pp 229533.V6RQW4
159823 229533.I1RPW2 pp 229533.I1SAJ3
159824 229533.I1RPW2 pp 229533.I1S2H3
159825 229533.I1RPW2 pp 229533.I1RTU0
159826 229533.I1RPW2 pp 229533.I1RQE0
159827 229533.I1RPW2 pp 229533.I1RU76
159828 229533.I1RPW2 pp 229533.I1RUV4
159829 229533.I1RPW2 pp 229533.I1RY74
159830 229533.I1RPW2 pp 229533.V6RUB1
159831 229533.I1RPW2 pp 229533.I1RZA7
159832 229533.I1RPW2 pp 229533.I1RVX9
159833 229533.I1RPW2 pp 229533.I1S3G4
159834 229533.I1RPW2 pp 229533.I1S5L5
159835 229533.I1RPW2 pp 229533.I1S4F4
159836 229533.I1RPW2 pp 229533.I1S2H4
159837 229533.I1RPW2 pp 229533.I1RZ20
159838 229533.I1RPW2 pp 229533.I1S1W8
159839 229533.I1RPW2 pp 229533.I1S3X7
159840 229533.I1RPW2 pp 229533.I1RVZ3
159841 229533.I1RPW2 pp 229533.I1RYU5
159842 229533.I1RPW2 pp 229533.I1S6P5
159843 229533.A0A098D6G8 pp 229533.I1RBE4
159844 229533.A0A098D6G8 pp 229533.I1S4L8
159845 229533.A0A098D6G8 pp 229533.I1RC08
159846 229533.A0A098D6G8 pp 229533.I1RWP4
159847 229533.A0A098D6G8 pp 229533.I1RK03
159848 229533.I1RAF6 pp 229533.I1RE44
159849 229533.I1RAF6 pp 229533.I1RMN3
159850 229533.I1RAF6 pp 229533.I1RTF2
159851 229533.I1RAF6 pp 229533.I1S1U6
159852 229533.I1RAF6 pp 229533.I1S278
159853 229533.I1RAF6 pp 229533.I1RFP5
159854 229533.I1RAF6 pp 229533.I1RCV3
159855 229533.I1RAF6 pp 229533.I1RQP7
159856 229533.I1RAF6 pp 229533.I1RX87
159857 229533.I1RAF6 pp 229533.I1RBT0
159858 229533.I1RAF6 pp 229533.I1RSW4
159859 229533.I1RAF6 pp 229533.I1RQD6
159860 229533.I1RAF6 pp 229533.I1RYP0
159861 229533.I1RAF6 pp 229533.I1RCF0
159862 229533.I1RAF6 pp 229533.I1S0V5
159863 229533.I1RAF6 pp 229533.I1RBS3
159864 229533.I1RAF6 pp 229533.I1RSH3
159865 229533.I1RAF6 pp 229533.I1S287
159866 229533.I1RE63 pp 229533.I1RM26
159867 229533.I1RE63 pp 229533.I1RF38
159868 229533.I1RE63 pp 229533.V6RQW4
159869 229533.I1RE63 pp 229533.I1RW69
159870 229533.I1RE63 pp 229533.I1S4Q4

159871	229533.I1RE63	pp	229533.I1RJT5
159872	229533.I1RE63	pp	229533.I1S0A0
159873	229533.I1RE63	pp	229533.I1RQE0
159874	229533.I1RE63	pp	229533.I1RF53
159875	229533.I1RE63	pp	229533.I1S2C4
159876	229533.I1RE63	pp	229533.I1RPD6
159877	229533.I1RE63	pp	229533.I1RU76
159878	229533.I1RE63	pp	229533.I1RNG4
159879	229533.I1RE63	pp	229533.I1RZ20
159880	229533.I1RE63	pp	229533.I1S1W8
159881	229533.I1RE63	pp	229533.I1S6P5
159882	229533.I1RE63	pp	229533.I1S5L5
159883	229533.I1RE63	pp	229533.I1RZA7
159884	229533.I1RE63	pp	229533.I1RYU5
159885	229533.I1RE63	pp	229533.I1RVX9
159886	229533.I1RE63	pp	229533.I1RNG7
159887	229533.I1RE63	pp	229533.I1S3G4
159888	229533.I1RE63	pp	229533.V6RUB1
159889	229533.I1RE63	pp	229533.I1RPW2
159890	229533.I1RE63	pp	229533.I1RW43
159891	229533.I1RE63	pp	229533.I1RVZ3
159892	229533.I1RGM9	pp	229533.I1RQ42
159893	229533.I1RGM9	pp	229533.I1RW91
159894	229533.I1RGM9	pp	229533.I1RN04
159895	229533.I1RGM9	pp	229533.I1RW80
159896	229533.I1RGM9	pp	229533.I1S182
159897	229533.I1RGM9	pp	229533.I1RPW8
159898	229533.I1RGM9	pp	229533.I1RZ42
159899	229533.I1RGM9	pp	229533.I1RQE0
159900	229533.I1RD21	pp	229533.I1RM49
159901	229533.I1RD21	pp	229533.V6RUB1
159902	229533.I1RD21	pp	229533.I1RGY6
159903	229533.I1RD21	pp	229533.I1RK80
159904	229533.I1RD21	pp	229533.V6RS91
159905	229533.I1RD21	pp	229533.I1RW11
159906	229533.I1RD21	pp	229533.I1RT45
159907	229533.I1RD21	pp	229533.I1RW26
159908	229533.I1RD21	pp	229533.I1RZA7
159909	229533.I1RD21	pp	229533.I1RF33
159910	229533.I1RD21	pp	229533.I1RY74
159911	229533.I1RD21	pp	229533.V6RKK5
159912	229533.I1RD21	pp	229533.I1S8Z2
159913	229533.I1RD21	pp	229533.I1RFK6
159914	229533.I1RD21	pp	229533.I1RE58
159915	229533.I1RD21	pp	229533.I1RZ63
159916	229533.I1RD21	pp	229533.I1RX59
159917	229533.I1RD21	pp	229533.I1S0J7
159918	229533.I1RD21	pp	229533.V6QV31
159919	229533.I1RD21	pp	229533.I1RN51
159920	229533.I1RD21	pp	229533.I1S6P5
159921	229533.I1RD21	pp	229533.I1RGK9
159922	229533.I1RD21	pp	229533.I1RFI9
159923	229533.I1RD21	pp	229533.I1RD22
159924	229533.I1RD21	pp	229533.I1S147
159925	229533.I1RD21	pp	229533.I1RTJ2
159926	229533.I1RD21	pp	229533.I1RFK3
159927	229533.I1RD21	pp	229533.I1RE79
159928	229533.I1RD21	pp	229533.I1RXY7
159929	229533.I1RD21	pp	229533.I1RZK8
159930	229533.I1RD21	pp	229533.I1S4L8
159931	229533.I1RD21	pp	229533.I1RK75
159932	229533.I1RD21	pp	229533.V6RDH3
159933	229533.I1RT45	pp	229533.V6RLW4
159934	229533.I1RT45	pp	229533.I1S0J7
159935	229533.I1RT45	pp	229533.I1RW26
159936	229533.I1RT45	pp	229533.I1S253
159937	229533.I1RT45	pp	229533.I1RZ63
159938	229533.I1RT45	pp	229533.V6RKY8
159939	229533.I1RT45	pp	229533.Q4I5M4
159940	229533.I1RT45	pp	229533.I1RTD7
159941	229533.I1RM49	pp	229533.I1S039
159942	229533.I1RM49	pp	229533.I1S098
159943	229533.I1RM49	pp	229533.I1RV91

159944 229533.I1RM49 pp 229533.V6R7V8
159945 229533.I1RM49 pp 229533.I1RNM7
159946 229533.I1RM49 pp 229533.I1S503
159947 229533.I1RM49 pp 229533.I1S2G7
159948 229533.I1RM49 pp 229533.I1RMJ3
159949 229533.I1RM49 pp 229533.I1RW06
159950 229533.I1RM49 pp 229533.I1RW26
159951 229533.I1RM49 pp 229533.I1RVN2
159952 229533.I1RM49 pp 229533.I1RZ63
159953 229533.I1RM49 pp 229533.I1S253
159954 229533.I1RM49 pp 229533.I1RN51
159955 229533.I1RM49 pp 229533.I1S272
159956 229533.I1RM49 pp 229533.I1RTJ2
159957 229533.I1RM49 pp 229533.I1RPK4
159958 229533.I1RM49 pp 229533.I1S4L8
159959 229533.I1RM49 pp 229533.I1RX50
159960 229533.I1RM49 pp 229533.I1S1W4
159961 229533.I1RM49 pp 229533.I1S1F5
159962 229533.I1RM49 pp 229533.I1RTD7
159963 229533.I1RM49 pp 229533.I1RRZ6
159964 229533.I1RM49 pp 229533.I1S504
159965 229533.I1RM49 pp 229533.V6RLW4
159966 229533.I1RM49 pp 229533.Q4IRI9
159967 229533.I1RM49 pp 229533.I1RSY2
159968 229533.I1RM49 pp 229533.I1RMU9
159969 229533.I1RM49 pp 229533.I1RY58
159970 229533.I1RM49 pp 229533.I1RMN5
159971 229533.I1RM49 pp 229533.I1S0J7
159972 229533.I1RM49 pp 229533.Q4IJM4
159973 229533.I1RM49 pp 229533.I1RT62
159974 229533.I1RM49 pp 229533.I1RWT6
159975 229533.I1RM49 pp 229533.I1RYY5
159976 229533.I1RM49 pp 229533.I1RNF5
159977 229533.I1RM49 pp 229533.I1S2A1
159978 229533.I1RM49 pp 229533.I1RY63
159979 229533.I1RM49 pp 229533.I1S1W5
159980 229533.I1RM49 pp 229533.V6RKK5
159981 229533.I1RM49 pp 229533.I1RT45
159982 229533.I1RM49 pp 229533.I1RQA3
159983 229533.I1RNL0 pp 229533.I1RYW3
159984 229533.I1RNL0 pp 229533.I1RWX5
159985 229533.I1RNL0 pp 229533.V6QUE6
159986 229533.I1RNL0 pp 229533.I1RW71
159987 229533.I1RNL0 pp 229533.I1RRT8
159988 229533.I1RUF7 pp 229533.I1S2B8
159989 229533.I1RUF7 pp 229533.I1S5R1
159990 229533.I1RUF7 pp 229533.I1RZ39
159991 229533.I1RUF7 pp 229533.I1RXC2
159992 229533.I1RUF7 pp 229533.I1RXD7
159993 229533.I1RUF7 pp 229533.I1RYI5
159994 229533.I1RUF7 pp 229533.I1S821
159995 229533.I1RUF7 pp 229533.I1RXD6
159996 229533.I1RUF7 pp 229533.I1RZM8
159997 229533.I1RUF7 pp 229533.I1S4S7
159998 229533.I1RUF7 pp 229533.I1S0W4
159999 229533.I1RUF7 pp 229533.I1S5R6
160000 229533.I1RUF7 pp 229533.I1RZK5
160001 229533.I1RUF7 pp 229533.I1RX95
160002 229533.I1RUF7 pp 229533.I1RWR7
160003 229533.I1RUF7 pp 229533.I1RVZ2
160004 229533.I1RUF7 pp 229533.I1RZV3
160005 229533.I1RUF7 pp 229533.I1RXC4
160006 229533.I1RUF7 pp 229533.I1RVX0
160007 229533.I1RUF7 pp 229533.I1S1W9
160008 229533.I1RUF7 pp 229533.Q4IJK2
160009 229533.I1RCQ0 pp 229533.I1RW76
160010 229533.I1RCQ0 pp 229533.I1RFV4
160011 229533.I1RCQ0 pp 229533.I1RQF7
160012 229533.I1RCQ0 pp 229533.I1RRC3
160013 229533.I1RCQ0 pp 229533.I1S1V4
160014 229533.I1RCQ0 pp 229533.I1RN04
160015 229533.I1RCQ0 pp 229533.I1RYP0
160016 229533.I1RCQ0 pp 229533.I1RZK3

160017	229533.I1RCQ0	pp	229533.I1RNH7
160018	229533.I1RCQ0	pp	229533.I1RX87
160019	229533.I1RCQ0	pp	229533.I1RQP7
160020	229533.I1RCQ0	pp	229533.I1RQH7
160021	229533.I1RCQ0	pp	229533.Q4HYZ2
160022	229533.I1RCQ0	pp	229533.I1RQD6
160023	229533.I1RCQ0	pp	229533.I1RCV3
160024	229533.I1RCQ0	pp	229533.I1RYW8
160025	229533.I1RCQ0	pp	229533.I1SAM5
160026	229533.I1RCQ0	pp	229533.I1RUN1
160027	229533.I1RCQ0	pp	229533.I1S830
160028	229533.I1RCQ0	pp	229533.I1RFK0
160029	229533.I1RCQ0	pp	229533.I1RMJ1
160030	229533.I1RCQ0	pp	229533.I1RMU8
160031	229533.I1RCQ0	pp	229533.I1RND7
160032	229533.I1RCQ0	pp	229533.I1S287
160033	229533.I1RCQ0	pp	229533.V6RGN5
160034	229533.I1RCQ0	pp	229533.Q4ILI9
160035	229533.I1RCQ0	pp	229533.I1S0S8
160036	229533.I1RCQ0	pp	229533.I1RM71
160037	229533.I1RCQ0	pp	229533.I1RE18
160038	229533.I1RCQ0	pp	229533.I1RK73
160039	229533.I1RCQ0	pp	229533.I1RUN2
160040	229533.I1RCQ0	pp	229533.I1RPG2
160041	229533.I1RCQ0	pp	229533.I1RS34
160042	229533.I1RCQ0	pp	229533.I1S2B6
160043	229533.I1RH23	pp	229533.I1RSX9
160044	229533.I1RH23	pp	229533.I1RND4
160045	229533.I1RH23	pp	229533.I1RNG4
160046	229533.I1RH23	pp	229533.I1RUP5
160047	229533.I1RH23	pp	229533.I1RRG1
160048	229533.I1RH23	pp	229533.I1RJS0
160049	229533.I1RXX8	pp	229533.I1S3G4
160050	229533.I1RXX8	pp	229533.I1S0T2
160051	229533.I1RFY0	pp	229533.I1RWK9
160052	229533.I1RFY0	pp	229533.I1S2V2
160053	229533.I1RFY0	pp	229533.I1S253
160054	229533.I1RFY0	pp	229533.I1RPY0
160055	229533.I1RFY0	pp	229533.Q4I7T5
160056	229533.I1RFY0	pp	229533.I1RW26
160057	229533.I1RFY0	pp	229533.I1RGZ3
160058	229533.I1RFY0	pp	229533.I1RVT8
160059	229533.I1RFY0	pp	229533.I1RR72
160060	229533.I1RFY0	pp	229533.I1RQ21
160061	229533.I1RFY0	pp	229533.I1RRZ6
160062	229533.I1RFY0	pp	229533.I1RGY6
160063	229533.I1RFY0	pp	229533.I1S4S1
160064	229533.I1RFY0	pp	229533.I1RGL3
160065	229533.I1RFY0	pp	229533.I1S0C2
160066	229533.I1RFY0	pp	229533.Q4I1N3
160067	229533.I1RFY0	pp	229533.I1S1W5
160068	229533.I1RFY0	pp	229533.I1S6Y3
160069	229533.I1RFY0	pp	229533.I1RVX8
160070	229533.I1RFY0	pp	229533.I1RPN8
160071	229533.I1RFY0	pp	229533.I1RVS1
160072	229533.I1RFY0	pp	229533.I1RUQ7
160073	229533.I1RFY0	pp	229533.I1RY21
160074	229533.I1RFY0	pp	229533.I1RUQ3
160075	229533.I1RFY0	pp	229533.I1RKK8
160076	229533.I1RFY0	pp	229533.I1S4S4
160077	229533.I1RFY0	pp	229533.I1S2A1
160078	229533.I1RFY0	pp	229533.V6R7V8
160079	229533.I1RFY0	pp	229533.V6RN69
160080	229533.I1RFY0	pp	229533.I1S0I8
160081	229533.I1RFY0	pp	229533.I1RNM7
160082	229533.I1RFY0	pp	229533.I1RT09
160083	229533.I1RFY0	pp	229533.Q4I5G1
160084	229533.I1RFY0	pp	229533.V6QUG0
160085	229533.I1RFY0	pp	229533.I1RWH4
160086	229533.I1RFY0	pp	229533.I1RM03
160087	229533.I1RFY0	pp	229533.I1RM49
160088	229533.I1RFY0	pp	229533.I1RKZ4
160089	229533.I1RFY0	pp	229533.I1S235

160090 229533.I1RFY0 pp 229533.I1RPN1
160091 229533.I1RFY0 pp 229533.I1S4L8
160092 229533.I1RFY0 pp 229533.I1RUL3
160093 229533.I1RFY0 pp 229533.I1RMW4
160094 229533.I1RFY0 pp 229533.I1RQA5
160095 229533.I1RFY0 pp 229533.Q4HVU7
160096 229533.I1RFY0 pp 229533.I1RP66
160097 229533.I1RFY0 pp 229533.I1RYL5
160098 229533.I1RFY0 pp 229533.I1RQR6
160099 229533.I1RFY0 pp 229533.I1RW87
160100 229533.I1RNR2 pp 229533.I1S0R3
160101 229533.I1RNR2 pp 229533.I1RWJ4
160102 229533.I1RNR2 pp 229533.I1SAN9
160103 229533.I1RNR2 pp 229533.Q4I1Y5
160104 229533.I1RNR2 pp 229533.I1RPW4
160105 229533.I1RNR2 pp 229533.I1S0R4
160106 229533.I1RNR2 pp 229533.I1RRV1
160107 229533.I1RNR2 pp 229533.I1S0N4
160108 229533.I1RNR2 pp 229533.I1S558
160109 229533.I1RNR2 pp 229533.I1RQ81
160110 229533.I1RNR2 pp 229533.I1RW71
160111 229533.I1RJP3 pp 229533.Q4HY20
160112 229533.I1RJP3 pp 229533.I1RTF2
160113 229533.I1RJP3 pp 229533.I1RRC9
160114 229533.I1RJP3 pp 229533.I1RY40
160115 229533.I1RJP3 pp 229533.I1RT00
160116 229533.I1RJP3 pp 229533.I1RMM1
160117 229533.I1RJP3 pp 229533.I1RQN8
160118 229533.I1RJP3 pp 229533.I1RSW4
160119 229533.I1RJP3 pp 229533.I1RRC8
160120 229533.I1RJP3 pp 229533.V6QXP4
160121 229533.I1RJP3 pp 229533.I1RX87
160122 229533.A0A0E0SC40 pp 229533.A0A1C3YK94
160123 229533.A0A0E0SC40 pp 229533.I1S209
160124 229533.A0A0E0SC40 pp 229533.A0A1C3YL10
160125 229533.A0A0E0SC40 pp 229533.I1S5X9
160126 229533.A0A0E0SC40 pp 229533.Q4IBU4
160127 229533.A0A0E0SC40 pp 229533.I1RZF6
160128 229533.A0A0E0SC40 pp 229533.V6RJP8
160129 229533.A0A0E0SC40 pp 229533.I1RCZ8
160130 229533.A0A0E0SC40 pp 229533.I1RKI2
160131 229533.A0A0E0SC40 pp 229533.I1RMP0
160132 229533.A0A0E0SC40 pp 229533.I1S8V3
160133 229533.A0A0E0SC40 pp 229533.I1RQ69
160134 229533.A0A0E0SC40 pp 229533.I1RJT1
160135 229533.A0A0E0SC40 pp 229533.A0A1C3YK56
160136 229533.A0A0E0SC40 pp 229533.I1RY21
160137 229533.A0A0E0SC40 pp 229533.I1RJR8
160138 229533.A0A0E0SC40 pp 229533.I1RXX8
160139 229533.I1RU81 pp 229533.I1RVB6
160140 229533.I1RU81 pp 229533.I1RW91
160141 229533.I1RGK9 pp 229533.I1RKB2
160142 229533.I1RGK9 pp 229533.V6RS91
160143 229533.I1RGK9 pp 229533.I1RYD3
160144 229533.I1RGK9 pp 229533.I1RMH4
160145 229533.I1RGK9 pp 229533.I1RGY6
160146 229533.I1RGK9 pp 229533.V6RH92
160147 229533.I1RGK9 pp 229533.I1RW11
160148 229533.I1RGK9 pp 229533.I1RZP0
160149 229533.I1RGK9 pp 229533.I1RNG4
160150 229533.I1RGK9 pp 229533.I1RQ65
160151 229533.I1RGK9 pp 229533.I1RY21
160152 229533.I1RGK9 pp 229533.Q4IAS1
160153 229533.I1RGK9 pp 229533.V6QV31
160154 229533.I1RGK9 pp 229533.I1RTJ2
160155 229533.I1RGK9 pp 229533.I1RQ48
160156 229533.I1RGK9 pp 229533.I1RZK8
160157 229533.I1RGK9 pp 229533.I1RZH8
160158 229533.I1RGK9 pp 229533.I1S4L8
160159 229533.I1RGK9 pp 229533.I1RYQ1
160160 229533.I1RGK9 pp 229533.I1RM49
160161 229533.I1RGK9 pp 229533.I1RUQ7
160162 229533.I1RGK9 pp 229533.I1RMK7

160163 229533.I1RGK9 pp 229533.I1RXB6
160164 229533.I1RGK9 pp 229533.I1RHK2
160165 229533.I1RGK9 pp 229533.I1S1F5
160166 229533.I1RGK9 pp 229533.I1SAE8
160167 229533.I1RGK9 pp 229533.Q4HZ68
160168 229533.I1RGK9 pp 229533.I1S3V2
160169 229533.I1RGK9 pp 229533.Q4I1N3
160170 229533.I1RGK9 pp 229533.I1S0H2
160171 229533.I1RGK9 pp 229533.I1RX59
160172 229533.I1RGK9 pp 229533.I1RRZ6
160173 229533.I1RGK9 pp 229533.V6RDH3
160174 229533.I1RGK9 pp 229533.I1RWT0
160175 229533.I1RGK9 pp 229533.I1RGL3
160176 229533.I1RGK9 pp 229533.I1RS40
160177 229533.I1RGK9 pp 229533.Q4I061
160178 229533.I1RGK9 pp 229533.V6RKK5
160179 229533.I1RGK9 pp 229533.I1RMU9
160180 229533.A0A1C3YLV0 pp 229533.I1R9W4
160181 229533.A0A1C3YLV0 pp 229533.I1RNG4
160182 229533.A0A1C3YLV0 pp 229533.I1RZ20
160183 229533.A0A1C3YLV0 pp 229533.I1RPD6
160184 229533.A0A1C3YLV0 pp 229533.I1RA94
160185 229533.A0A1C3YLV0 pp 229533.I1RXI0
160186 229533.A0A1C3YLV0 pp 229533.I1RE63
160187 229533.A0A1C3YLV0 pp 229533.I1S4F4
160188 229533.A0A1C3YLV0 pp 229533.I1S6P6
160189 229533.A0A1C3YLV0 pp 229533.I1RCB4
160190 229533.A0A1C3YLV0 pp 229533.I1REV2
160191 229533.A0A1C3YLV0 pp 229533.A0A1C3YN25
160192 229533.A0A1C3YLV0 pp 229533.I1RW43
160193 229533.A0A1C3YLV0 pp 229533.I1S064
160194 229533.A0A1C3YLV0 pp 229533.I1S0A0
160195 229533.A0A1C3YLV0 pp 229533.I1RJT5
160196 229533.A0A1C3YLV0 pp 229533.I1RN80
160197 229533.A0A1C3YLV0 pp 229533.I1RVX9
160198 229533.A0A1C3YLV0 pp 229533.I1RF40
160199 229533.A0A1C3YLV0 pp 229533.I1S2H3
160200 229533.A0A1C3YLV0 pp 229533.I1RR12
160201 229533.A0A1C3YLV0 pp 229533.V6R9Z3
160202 229533.A0A1C3YLV0 pp 229533.I1RU76
160203 229533.A0A1C3YLV0 pp 229533.I1RY49
160204 229533.A0A1C3YLV0 pp 229533.I1RPW2
160205 229533.A0A1C3YLV0 pp 229533.V6RQW4
160206 229533.A0A1C3YLV0 pp 229533.I1RM26
160207 229533.A0A1C3YLV0 pp 229533.I1RYU5
160208 229533.A0A1C3YLV0 pp 229533.I1RBE7
160209 229533.A0A1C3YLV0 pp 229533.I1RQE0
160210 229533.I1RXG2 pp 229533.I1S1Z9
160211 229533.V6R7V8 pp 229533.V6RN69
160212 229533.V6R7V8 pp 229533.V6RKK5
160213 229533.V6R7V8 pp 229533.V6RLM7
160214 229533.A0A0E0SBW3 pp 229533.I1RJU9
160215 229533.A0A0E0SBW3 pp 229533.I1RLM3
160216 229533.A0A0E0SBW3 pp 229533.A0A0E0SP71
160217 229533.A0A098DLG3 pp 229533.A0A098DWB1
160218 229533.A0A098DLG3 pp 229533.I1S1U6
160219 229533.A0A098DLG3 pp 229533.I1RBT0
160220 229533.A0A098DLG3 pp 229533.A0A0E0SP22
160221 229533.A0A098DLG3 pp 229533.I1RMN3
160222 229533.A0A098DLG3 pp 229533.I1RE44
160223 229533.A0A098DLG3 pp 229533.I1RFP5
160224 229533.A0A098DLG3 pp 229533.A0A0E0RR64
160225 229533.A0A098DLG3 pp 229533.I1RCF0
160226 229533.A0A098DLG3 pp 229533.I1RCV3
160227 229533.A0A098DLG3 pp 229533.A0A098DUU3
160228 229533.A0A098DLG3 pp 229533.I1RT58
160229 229533.A0A098DLG3 pp 229533.I1RQP7
160230 229533.A0A098DLG3 pp 229533.I1RYP0
160231 229533.A0A098DLG3 pp 229533.A0A1C3YKJ7
160232 229533.A0A098DLG3 pp 229533.I1RSW4
160233 229533.A0A098DLG3 pp 229533.I1S278
160234 229533.A0A098DLG3 pp 229533.I1RQD6
160235 229533.A0A098DLG3 pp 229533.I1RBS3

160236 229533.A0A098DLG3 pp 229533.I1RY59
160237 229533.A0A098DLG3 pp 229533.A0A1C3YMH5
160238 229533.A0A098DLG3 pp 229533.I1RTF2
160239 229533.A0A098DLG3 pp 229533.I1RX87
160240 229533.A0A098DLG3 pp 229533.A0A1C3YKT4
160241 229533.A0A098DLG3 pp 229533.I1RSH3
160242 229533.A0A098DLG3 pp 229533.I1RJS4
160243 229533.A0A098DLG3 pp 229533.I1RJL5
160244 229533.A0A098DLG3 pp 229533.I1S0V5
160245 229533.A0A098DLG3 pp 229533.I1S287
160246 229533.I1RZ29 pp 229533.I1S3N0
160247 229533.I1RZ29 pp 229533.I1S8P5
160248 229533.I1RZ29 pp 229533.V6RUB1
160249 229533.I1RZ29 pp 229533.Q4HZI0
160250 229533.I1RZ29 pp 229533.I1RZ52
160251 229533.I1RZ29 pp 229533.I1RZA7
160252 229533.I1RZ29 pp 229533.I1RZN1
160253 229533.I1RZ29 pp 229533.I1RZ39
160254 229533.I1RZ29 pp 229533.I1S3X8
160255 229533.I1RZ29 pp 229533.I1S4P6
160256 229533.I1RZ29 pp 229533.I1S216
160257 229533.I1RZ29 pp 229533.V6RXR0
160258 229533.I1RZ29 pp 229533.Q4HVQ9
160259 229533.I1RZ29 pp 229533.I1S2F2
160260 229533.I1RZ29 pp 229533.I1S064
160261 229533.I1RZ29 pp 229533.V6RLM7
160262 229533.I1RZ29 pp 229533.I1S6P5
160263 229533.I1RZ29 pp 229533.Q4I1Y5
160264 229533.I1RZ29 pp 229533.V6RRM0
160265 229533.I1RZ29 pp 229533.I1S3G4
160266 229533.I1RZ29 pp 229533.V6RSF3
160267 229533.I1RZ29 pp 229533.I1S0I3
160268 229533.I1RZ29 pp 229533.I1S169
160269 229533.I1RZ29 pp 229533.I1SAJ3
160270 229533.I1RZ29 pp 229533.V6QWD8
160271 229533.I1RZ29 pp 229533.I1S2V3
160272 229533.I1RZ29 pp 229533.I1RZT0
160273 229533.I1RZ29 pp 229533.I1RZQ1
160274 229533.I1RZ29 pp 229533.I1S5C5
160275 229533.I1RZ29 pp 229533.I1S1W8
160276 229533.I1RZ29 pp 229533.V6RN31
160277 229533.I1RZ29 pp 229533.I1S3K8
160278 229533.I1RZ29 pp 229533.I1SAK7
160279 229533.I1RZ29 pp 229533.V6RQW4
160280 229533.I1RZ29 pp 229533.Q4I5G1
160281 229533.I1RZ29 pp 229533.I1S183
160282 229533.I1RZ29 pp 229533.I1S0A0
160283 229533.I1RZ29 pp 229533.V6QUE6
160284 229533.A0A0E0RMV2 pp 229533.I1RM49
160285 229533.A0A0E0RMV2 pp 229533.I1S1W5
160286 229533.A0A0E0RMV2 pp 229533.A0A0E0SC50
160287 229533.A0A0E0RMV2 pp 229533.A0A0E0S1U8
160288 229533.A0A0E0RMV2 pp 229533.I1RFS8
160289 229533.A0A0E0RMV2 pp 229533.I1RMV6
160290 229533.A0A0E0RMV2 pp 229533.I1S0P7
160291 229533.A0A0E0RMV2 pp 229533.I1RZ63
160292 229533.A0A0E0RMV2 pp 229533.I1RNG8
160293 229533.A0A0E0RMV2 pp 229533.I1S4N8
160294 229533.A0A0E0RMV2 pp 229533.I1S9X9
160295 229533.A0A0E0RMV2 pp 229533.I1S278
160296 229533.A0A0E0RMV2 pp 229533.A0A1C3YME0
160297 229533.A0A0E0RMV2 pp 229533.A0A1C3YLM3
160298 229533.A0A0E0RMV2 pp 229533.I1RKA1
160299 229533.A0A0E0RMV2 pp 229533.I1RX50
160300 229533.A0A0E0RMV2 pp 229533.I1RXV4
160301 229533.A0A0E0RMV2 pp 229533.A0A1C3YJG7
160302 229533.A0A0E0RMV2 pp 229533.I1S0J7
160303 229533.A0A0E0RMV2 pp 229533.I1RK75
160304 229533.A0A0E0RMV2 pp 229533.I1S039
160305 229533.A0A0E0RMV2 pp 229533.A0A1C3YK57
160306 229533.A0A0E0RMV2 pp 229533.I1S4N7
160307 229533.A0A0E0RMV2 pp 229533.A0A1C3YHP8
160308 229533.A0A0E0RMV2 pp 229533.I1S1X7

160309	229533.V6QUG0	pp	229533.V6RLM7
160310	229533.V6QUG0	pp	229533.V6RN69
160311	229533.V6QUG0	pp	229533.V6QWB1
160312	229533.V6QUG0	pp	229533.V6R7V8
160313	229533.I1RNG2	pp	229533.I1RYP0
160314	229533.I1RNG2	pp	229533.Q4HY20
160315	229533.I1RNG2	pp	229533.I1RX95
160316	229533.I1RNG2	pp	229533.V6RXW9
160317	229533.I1RNG2	pp	229533.I1RXT7
160318	229533.I1RNG2	pp	229533.I1SAM5
160319	229533.I1RNG2	pp	229533.I1S051
160320	229533.I1RNG2	pp	229533.I1S278
160321	229533.I1RNG2	pp	229533.I1RQD6
160322	229533.I1RNG2	pp	229533.I1S2B6
160323	229533.I1RNG2	pp	229533.I1RPY3
160324	229533.I1RNG2	pp	229533.I1S287
160325	229533.I1RNG2	pp	229533.I1S1V4
160326	229533.I1RNG2	pp	229533.I1RW76
160327	229533.I1RNG2	pp	229533.I1RXF5
160328	229533.I1RNG2	pp	229533.I1RQP7
160329	229533.I1RNG2	pp	229533.I1RX87
160330	229533.I1RNG2	pp	229533.I1RUN9
160331	229533.I1RNG2	pp	229533.I1RQF9
160332	229533.I1RNG2	pp	229533.I1S0V5
160333	229533.I1RGN1	pp	229533.I1RMP5
160334	229533.I1RGN1	pp	229533.I1RU37
160335	229533.I1RD81	pp	229533.I1RM26
160336	229533.I1RD81	pp	229533.V6R9Z3
160337	229533.I1RD81	pp	229533.I1S3K8
160338	229533.I1RD81	pp	229533.I1S2D5
160339	229533.I1RD81	pp	229533.I1RYU5
160340	229533.I1RD81	pp	229533.I1RPW2
160341	229533.I1RD81	pp	229533.V6RQW4
160342	229533.I1RD81	pp	229533.I1RU76
160343	229533.I1RD81	pp	229533.I1RQE0
160344	229533.I1RD81	pp	229533.I1RY49
160345	229533.I1RD81	pp	229533.I1RN80
160346	229533.I1RD81	pp	229533.I1RJT5
160347	229533.I1RD81	pp	229533.I1S0A0
160348	229533.I1RD81	pp	229533.I1S4F4
160349	229533.I1RD81	pp	229533.I1RR12
160350	229533.I1RD81	pp	229533.I1S6P6
160351	229533.I1RD81	pp	229533.I1RVX9
160352	229533.I1RD81	pp	229533.I1RNG4
160353	229533.I1RD81	pp	229533.I1S2H3
160354	229533.I1RD81	pp	229533.I1RM69
160355	229533.I1RD81	pp	229533.I1RPD6
160356	229533.I1RD81	pp	229533.I1RZ20
160357	229533.I1RD81	pp	229533.I1RW43
160358	229533.I1RD81	pp	229533.Q4I8S2
160359	229533.I1RD81	pp	229533.I1RZT0
160360	229533.I1RD81	pp	229533.I1RE63
160361	229533.I1RD81	pp	229533.I1REV2
160362	229533.I1RD81	pp	229533.I1RE58
160363	229533.I1RD81	pp	229533.I1RI56
160364	229533.I1RD81	pp	229533.I1RF40
160365	229533.I1RD81	pp	229533.I1S064
160366	229533.I1RD81	pp	229533.I1RSX9
160367	229533.I1S4S1	pp	229533.V6R7V8
160368	229533.I1S4S1	pp	229533.Q4HVU7
160369	229533.I1S4S1	pp	229533.V6QWB1
160370	229533.I1S4S1	pp	229533.V6RN69
160371	229533.I1S4S1	pp	229533.I1S5R6
160372	229533.I1S4S1	pp	229533.V6RLM7
160373	229533.I1RFP7	pp	229533.I1RX17
160374	229533.I1RFP7	pp	229533.I1S7X9
160375	229533.I1S2B6	pp	229533.V6RKY7
160376	229533.I1S2B6	pp	229533.I1S830
160377	229533.I1S2B6	pp	229533.Q4IN63
160378	229533.I1S2B6	pp	229533.I1SA32
160379	229533.I1S2B6	pp	229533.I1S5B5
160380	229533.I1S2B6	pp	229533.V6RXW9
160381	229533.I1S2B6	pp	229533.V6QXP4

229533.I1S2B6 pp 229533.I1SAM5
229533.I1RAE7 pp 229533.I1RU37
229533.I1RAE7 pp 229533.I1RMP5
229533.A0A098D0U1 pp 229533.I1RL76
229533.A0A098D0U1 pp 229533.I1RXY7
229533.A0A098D0U1 pp 229533.I1RP31
229533.A0A098D0U1 pp 229533.A0A098E2M3
229533.A0A098D0U1 pp 229533.I1RE79
229533.A0A098D0U1 pp 229533.A0A1C3YK57
229533.A0A098D0U1 pp 229533.A0A1C3YHP8
229533.A0A098D0U1 pp 229533.I1RK75
229533.A0A098D0U1 pp 229533.A0A1C3YJK5
229533.A0A098D0U1 pp 229533.I1RC08
229533.A0A098D0U1 pp 229533.I1RFI9
229533.A0A098D0U1 pp 229533.I1RYG3
229533.A0A098D0U1 pp 229533.I1RMU9
229533.A0A098D0U1 pp 229533.I1S0J7
229533.A0A098D0U1 pp 229533.A0A1C3YI88
229533.A0A098D0U1 pp 229533.I1RF33
229533.A0A098D0U1 pp 229533.I1RZ63
229533.A0A098D0U1 pp 229533.A0A098D5D4
229533.A0A098D0U1 pp 229533.I1RGY6
229533.A0A098D0U1 pp 229533.I1S2A1
229533.A0A098D0U1 pp 229533.I1S1F5
229533.A0A098D0U1 pp 229533.A0A1C3YKE0
229533.A0A098D0U1 pp 229533.I1RTJ2
229533.A0A098D0U1 pp 229533.A0A098D6G8
229533.A0A098D0U1 pp 229533.I1RD21
229533.A0A098D0U1 pp 229533.I1RM49
229533.A0A098D0U1 pp 229533.I1RFY0
229533.A0A098D0U1 pp 229533.I1RGK9
229533.A0A098D0U1 pp 229533.V6R7V8
229533.A0A098D0U1 pp 229533.I1RW06
229533.A0A098D0U1 pp 229533.V6QUG0
229533.A0A098D0U1 pp 229533.I1S4S1
229533.I1RPS7 pp 229533.I1S0C6
229533.I1RPS7 pp 229533.I1RQ42
229533.I1RPS7 pp 229533.I1S0K9
229533.I1RPS7 pp 229533.I1S182
229533.I1RPS7 pp 229533.I1RR61
229533.I1RPS7 pp 229533.I1RW80
229533.I1RPS7 pp 229533.I1RW91
229533.I1RPS7 pp 229533.I1RPW8
229533.A0A0E0SC15 pp 229533.I1RGN1
229533.A0A0E0SC15 pp 229533.I1RJS2
229533.A0A0E0SC15 pp 229533.I1RAE7
229533.I1RCK4 pp 229533.I1S246
229533.I1RCK4 pp 229533.V6R3J1
229533.I1RCK4 pp 229533.I1RVA7
229533.I1RCK4 pp 229533.Q4IBK5
229533.I1RCK4 pp 229533.I1RYT7
229533.I1RCK4 pp 229533.Q4IPA4
229533.I1RCK4 pp 229533.I1RFZ2
229533.I1RCK4 pp 229533.I1S214
229533.I1RCK4 pp 229533.Q4IAS1
229533.I1RCK4 pp 229533.Q4I5R9
229533.I1RCK4 pp 229533.Q4HYC1
229533.I1RCK4 pp 229533.I1RMY3
229533.I1RCK4 pp 229533.I1RY40
229533.I1RCK4 pp 229533.I1RJS4
229533.I1RCK4 pp 229533.I1RCU7
229533.I1RCK4 pp 229533.I1RQC5
229533.I1RCK4 pp 229533.I1S2F3
229533.I1RCK4 pp 229533.I1RS94
229533.I1RCK4 pp 229533.I1RQ80
229533.I1RCK4 pp 229533.I1S1V5
229533.I1RCK4 pp 229533.I1RK77
229533.I1RCK4 pp 229533.Q4I7X1
229533.I1RCK4 pp 229533.Q4IPN9
229533.I1RCK4 pp 229533.I1RNT2
229533.I1RCK4 pp 229533.Q4IPB3
229533.I1RCK4 pp 229533.I1RCQ6
229533.I1RCK4 pp 229533.Q4I7S1

229533.I1RCK4 pp 229533.I1RNB8
229533.I1RCK4 pp 229533.I1RYL5
229533.I1RCK4 pp 229533.I1S0H1
229533.I1RCK4 pp 229533.I1RE46
229533.I1RCK4 pp 229533.Q4IE79
229533.I1RCK4 pp 229533.I1RQ49
229533.I1RCK4 pp 229533.I1RNE4
229533.I1RCK4 pp 229533.I1RDY1
229533.I1RCK4 pp 229533.I1RSL8
229533.I1RCK4 pp 229533.I1S044
229533.I1RCK4 pp 229533.I1RZT1
229533.I1RCK4 pp 229533.I1RDY5
229533.I1RCK4 pp 229533.I1RRE0
229533.I1RCK4 pp 229533.I1RQ21
229533.I1RCK4 pp 229533.Q4IPB8
229533.I1RCK4 pp 229533.I1RM32
229533.I1RCK4 pp 229533.I1S471
229533.I1RCK4 pp 229533.V6QV38
229533.I1RCK4 pp 229533.I1RTE5
229533.I1RCK4 pp 229533.I1RYJ8
229533.I1RCK4 pp 229533.I1RGC2
229533.I1RCK4 pp 229533.I1RD70
229533.I1RCK4 pp 229533.I1RZV9
229533.I1RCK4 pp 229533.I1RT35
229533.I1RCK4 pp 229533.Q4IAA0
229533.I1RCK4 pp 229533.I1S0P4
229533.I1RCK4 pp 229533.I1RDG0
229533.I1RCK4 pp 229533.I1RJS5
229533.I1RCK4 pp 229533.I1RCK5
229533.I1RCK4 pp 229533.V6REZ4
229533.I1RCK4 pp 229533.Q4IPH4
229533.I1RCK4 pp 229533.Q4I1Y1
229533.I1RCK4 pp 229533.I1RNG5
229533.I1RCK4 pp 229533.I1S5B5
229533.I1RCK4 pp 229533.Q4HXF6
229533.I1RCK4 pp 229533.I1RIR9
229533.I1RCK4 pp 229533.I1RCM6
229533.A0A1C3YIV7 pp 229533.I1RJF1
229533.A0A1C3YIV7 pp 229533.A0A1C3YIX7
229533.A0A1C3YIV7 pp 229533.I1S3G4
229533.A0A1C3YIV7 pp 229533.I1RDJ7
229533.A0A1C3YIV7 pp 229533.A0A1C3YJW4
229533.A0A1C3YIV7 pp 229533.I1RXH3
229533.A0A1C3YIV7 pp 229533.I1RQX9
229533.A0A1C3YIV7 pp 229533.I1R989
229533.A0A1C3YIV7 pp 229533.I1RW43
229533.A0A1C3YIV7 pp 229533.I1RXG6
229533.A0A1C3YIV7 pp 229533.I1RNG7
229533.A0A1C3YIV7 pp 229533.I1RMM8
229533.A0A1C3YIV7 pp 229533.I1S084
229533.A0A1C3YIV7 pp 229533.A0A1C3YK49
229533.A0A1C3YIV7 pp 229533.A0A1C3YN25
229533.A0A1C3YIV7 pp 229533.I1RPW2
229533.A0A1C3YIV7 pp 229533.I1RZT0
229533.A0A1C3YIV7 pp 229533.I1RVX9
229533.A0A1C3YIV7 pp 229533.I1RZ29
229533.A0A1C3YIV7 pp 229533.V6RUB1
229533.A0A1C3YIV7 pp 229533.I1S5L5
229533.A0A1C3YIV7 pp 229533.I1S3K8
229533.A0A1C3YIV7 pp 229533.I1RI56
229533.A0A1C3YIV7 pp 229533.I1RM69
229533.A0A1C3YIV7 pp 229533.A0A1C3YJ36
229533.A0A1C3YIV7 pp 229533.I1S1W8
229533.A0A1C3YIV7 pp 229533.I1S6P5
229533.A0A1C3YIV7 pp 229533.I1RZ20
229533.A0A1C3YIV7 pp 229533.I1RE63
229533.A0A1C3YIV7 pp 229533.A0A1C3YLV0
229533.A0A1C3YIV7 pp 229533.I1RYU5
229533.A0A1C3YIV7 pp 229533.I1S1P8
229533.A0A1C3YIV7 pp 229533.I1RYY2
229533.A0A1C3YIV7 pp 229533.I1S2H3
229533.A0A1C3YIV7 pp 229533.I1S4F4
229533.A0A1C3YIV7 pp 229533.I1RNG4

160528 229533.A0A1C3YIV7 pp 229533.I1RF53
160529 229533.A0A1C3YIV7 pp 229533.I1RCB4
160530 229533.A0A1C3YIV7 pp 229533.I1RC83
160531 229533.A0A1C3YIV7 pp 229533.I1RPD6
160532 229533.A0A1C3YIV7 pp 229533.I1RM68
160533 229533.A0A1C3YIV7 pp 229533.A0A1C3YKV2
160534 229533.A0A1C3YIV7 pp 229533.I1RAR1
160535 229533.A0A1C3YIV7 pp 229533.A0A1C3YK58
160536 229533.A0A1C3YIV7 pp 229533.I1R9B8
160537 229533.A0A1C3YIV7 pp 229533.I1RF38
160538 229533.A0A1C3YIV7 pp 229533.I1RQE0
160539 229533.A0A1C3YIV7 pp 229533.I1RZA7
160540 229533.A0A1C3YIV7 pp 229533.I1RM26
160541 229533.A0A1C3YIV7 pp 229533.I1RBE7
160542 229533.A0A1C3YIV7 pp 229533.I1RV23
160543 229533.A0A1C3YIV7 pp 229533.I1RD81
160544 229533.A0A1C3YIV7 pp 229533.I1RD80
160545 229533.I1RQ96 pp 229533.I1S2B9
160546 229533.I1RQ96 pp 229533.I1RS90
160547 229533.I1RQ96 pp 229533.I1RYX5
160548 229533.I1RNB8 pp 229533.I1RPM4
160549 229533.I1RNB8 pp 229533.I1RX81
160550 229533.I1RNB8 pp 229533.I1RX24
160551 229533.I1RNB8 pp 229533.I1S524
160552 229533.I1RNB8 pp 229533.I1RPY4
160553 229533.I1RNB8 pp 229533.Q4IJH1
160554 229533.I1RNB8 pp 229533.I1RZG7
160555 229533.I1RNB8 pp 229533.I1RUG0
160556 229533.I1RNB8 pp 229533.Q4I099
160557 229533.I1RNB8 pp 229533.I1RU69
160558 229533.I1RNB8 pp 229533.I1RTS2
160559 229533.I1RNB8 pp 229533.I1RWL3
160560 229533.I1RNB8 pp 229533.V6QWX8
160561 229533.I1RNB8 pp 229533.I1RW25
160562 229533.I1RNB8 pp 229533.Q4IF76
160563 229533.I1RNB8 pp 229533.Q4IMN9
160564 229533.I1RNB8 pp 229533.I1RZZ9
160565 229533.I1RNB8 pp 229533.I1RVJ1
160566 229533.I1RNB8 pp 229533.Q4IR09
160567 229533.I1RNB8 pp 229533.Q4IPA4
160568 229533.I1RNB8 pp 229533.I1RS20
160569 229533.I1RNB8 pp 229533.I1S0R5
160570 229533.I1RNB8 pp 229533.I1RRT8
160571 229533.I1RNB8 pp 229533.I1RS03
160572 229533.I1RNB8 pp 229533.I1RQK2
160573 229533.I1RNB8 pp 229533.I1RRW3
160574 229533.I1RNB8 pp 229533.I1RS66
160575 229533.I1RNB8 pp 229533.I1S0B6
160576 229533.I1RNB8 pp 229533.I1RWI0
160577 229533.I1RNB8 pp 229533.I1RW90
160578 229533.I1RNB8 pp 229533.I1S9L6
160579 229533.I1RNB8 pp 229533.Q4IPN9
160580 229533.I1RNB8 pp 229533.I1S2A8
160581 229533.I1RNB8 pp 229533.Q4I5W5
160582 229533.I1RNB8 pp 229533.I1RVQ0
160583 229533.I1RNB8 pp 229533.I1S214
160584 229533.I1RNB8 pp 229533.I1RYI9
160585 229533.I1RNB8 pp 229533.I1RY40
160586 229533.I1RNB8 pp 229533.I1RS94
160587 229533.I1RNB8 pp 229533.I1S9L5
160588 229533.I1RNB8 pp 229533.I1S5S2
160589 229533.I1RNB8 pp 229533.Q4I7T5
160590 229533.I1RNB8 pp 229533.I1S8U8
160591 229533.I1RNB8 pp 229533.I1S4M5
160592 229533.I1RNB8 pp 229533.I1RS43
160593 229533.I1RNB8 pp 229533.I1RUM5
160594 229533.I1RNB8 pp 229533.I1RS72
160595 229533.I1RNB8 pp 229533.I1RND9
160596 229533.I1RNB8 pp 229533.I1RS75
160597 229533.I1RNB8 pp 229533.V6REZ4
160598 229533.I1RNB8 pp 229533.I1RXF2
160599 229533.I1RNB8 pp 229533.Q4I785
160600 229533.I1RNB8 pp 229533.I1RZ59

160601	229533.I1RNB8	pp	229533.I1RPY7
160602	229533.I1RNB8	pp	229533.I1S7M2
160603	229533.I1RNB8	pp	229533.Q4HYQ4
160604	229533.I1RNB8	pp	229533.I1S0R7
160605	229533.I1RNB8	pp	229533.I1S0B1
160606	229533.I1RNB8	pp	229533.I1S246
160607	229533.I1RNB8	pp	229533.I1RYB1
160608	229533.I1RNB8	pp	229533.I1RWK3
160609	229533.I1RNB8	pp	229533.I1S0L8
160610	229533.I1RNB8	pp	229533.I1RT04
160611	229533.I1RNB8	pp	229533.I1RYJ9
160612	229533.I1RNB8	pp	229533.I1S6F1
160613	229533.I1RNB8	pp	229533.I1S0V4
160614	229533.I1RNB8	pp	229533.I1RXU7
160615	229533.I1RNB8	pp	229533.I1S0Z9
160616	229533.I1RNB8	pp	229533.Q4IQW0
160617	229533.I1RNB8	pp	229533.I1RR25
160618	229533.I1RNB8	pp	229533.I1S0N7
160619	229533.I1RNB8	pp	229533.Q4IF44
160620	229533.I1RNB8	pp	229533.I1RYT7
160621	229533.I1RNB8	pp	229533.I1RQR3
160622	229533.I1RNB8	pp	229533.I1RQZ2
160623	229533.I1RNB8	pp	229533.I1S8V0
160624	229533.I1RNB8	pp	229533.Q4HWE2
160625	229533.I1RNB8	pp	229533.I1RS28
160626	229533.I1RNB8	pp	229533.I1RSD4
160627	229533.I1RNB8	pp	229533.I1S9Y0
160628	229533.I1RNB8	pp	229533.I1RS06
160629	229533.I1RNB8	pp	229533.I1RT03
160630	229533.I1RNB8	pp	229533.I1RSL8
160631	229533.I1RNB8	pp	229533.I1RQP8
160632	229533.I1RNB8	pp	229533.I1S1X9
160633	229533.I1RNB8	pp	229533.I1RRI2
160634	229533.I1RNB8	pp	229533.I1RNY8
160635	229533.I1RNB8	pp	229533.Q4I7F9
160636	229533.I1RNB8	pp	229533.I1RZS7
160637	229533.I1RNB8	pp	229533.I1RPS0
160638	229533.I1RNB8	pp	229533.I1RQ49
160639	229533.I1RNB8	pp	229533.I1RZZ1
160640	229533.I1RNB8	pp	229533.I1RQE7
160641	229533.I1RNB8	pp	229533.I1RX14
160642	229533.I1RNB8	pp	229533.I1S9M9
160643	229533.I1RNB8	pp	229533.I1S1C7
160644	229533.I1RNB8	pp	229533.I1RNE4
160645	229533.I1RNB8	pp	229533.I1RRT0
160646	229533.I1RNB8	pp	229533.I1RYW3
160647	229533.I1RNB8	pp	229533.Q4HYB8
160648	229533.I1RNB8	pp	229533.I1RZU4
160649	229533.I1RNB8	pp	229533.I1RZL5
160650	229533.I1RNB8	pp	229533.I1RZH4
160651	229533.I1RNB8	pp	229533.V6RY33
160652	229533.I1RNB8	pp	229533.I1RYW2
160653	229533.I1RNB8	pp	229533.Q4IR08
160654	229533.I1RNB8	pp	229533.I1RSH4
160655	229533.I1RNB8	pp	229533.I1RWN6
160656	229533.I1RNB8	pp	229533.I1RTE5
160657	229533.I1RNB8	pp	229533.V6RIS2
160658	229533.I1RNB8	pp	229533.I1RND5
160659	229533.I1RNB8	pp	229533.I1RW99
160660	229533.I1RNB8	pp	229533.I1S5Z2
160661	229533.I1RNB8	pp	229533.I1RSA4
160662	229533.I1RNB8	pp	229533.I1RPA0
160663	229533.I1RNB8	pp	229533.Q4IB70
160664	229533.I1RNB8	pp	229533.I1RT99
160665	229533.I1RNB8	pp	229533.I1S471
160666	229533.I1RNB8	pp	229533.I1RWN5
160667	229533.I1RNB8	pp	229533.Q4IP34
160668	229533.I1RNB8	pp	229533.I1RT35
160669	229533.I1RNB8	pp	229533.I1RYJ8
160670	229533.I1RNB8	pp	229533.I1RWX8
160671	229533.I1RNB8	pp	229533.I1RVZ4
160672	229533.I1RNB8	pp	229533.I1RZV9
160673	229533.I1RNB8	pp	229533.I1S5B5

160674 229533.I1RNB8 pp 229533.Q4I5I4
160675 229533.I1RNB8 pp 229533.I1S0P4
160676 229533.I1RNB8 pp 229533.I1RQD8
160677 229533.I1RNB8 pp 229533.I1S2C4
160678 229533.I1RNB8 pp 229533.V6QXG1
160679 229533.I1RNB8 pp 229533.I1RRU9
160680 229533.I1RNB8 pp 229533.I1RWD3
160681 229533.I1RNB8 pp 229533.I1RZB5
160682 229533.I1RNB8 pp 229533.I1RZK5
160683 229533.I1RNB8 pp 229533.I1RZ11
160684 229533.I1RNB8 pp 229533.I1RT47
160685 229533.I1RNB8 pp 229533.I1RTH6
160686 229533.I1RNB8 pp 229533.Q4I7K4
160687 229533.I1RNB8 pp 229533.V6QUE6
160688 229533.I1RNB8 pp 229533.Q4IAA0
160689 229533.I1RNB8 pp 229533.I1RSQ3
160690 229533.I1RNB8 pp 229533.I1RPP4
160691 229533.I1RNB8 pp 229533.I1S8U9
160692 229533.I1RNB8 pp 229533.I1S7M3
160693 229533.I1RNB8 pp 229533.I1RSH9
160694 229533.I1RNB8 pp 229533.I1RX47
160695 229533.I1RNB8 pp 229533.I1RS17
160696 229533.I1RNB8 pp 229533.V6R042
160697 229533.I1RNB8 pp 229533.I1RSA8
160698 229533.I1RNB8 pp 229533.Q4I7X1
160699 229533.I1RDI6 pp 229533.I1RPT0
160700 229533.I1RDI6 pp 229533.I1RUY6
160701 229533.I1RDI6 pp 229533.I1RNU7
160702 229533.A0A098DIB7 pp 229533.A0A1C3YN62
160703 229533.A0A098DIB7 pp 229533.A0A1C3YII4
160704 229533.A0A098DIB7 pp 229533.A0A1C3YL16
160705 229533.I1RTH2 pp 229533.Q4HTT2
160706 229533.I1RTH2 pp 229533.Q4HTT1
160707 229533.I1RTH2 pp 229533.I1RZR6
160708 229533.I1RTH2 pp 229533.V6QUE6
160709 229533.I1RTH2 pp 229533.Q4I5I4
160710 229533.I1RTH2 pp 229533.Q4I5W5
160711 229533.I1RTH2 pp 229533.Q4IAA0
160712 229533.I1RTH2 pp 229533.Q4IJ11
160713 229533.I1RTH2 pp 229533.I1RU02
160714 229533.I1RTH2 pp 229533.Q4HYQ4
160715 229533.I1RTH2 pp 229533.Q4HYB8
160716 229533.I1RTH2 pp 229533.I1RZB5
160717 229533.I1RTH2 pp 229533.Q4IER8
160718 229533.I1RTH2 pp 229533.Q4IJU0
160719 229533.A0A1C3YJ23 pp 229533.I1RQC3
160720 229533.A0A1C3YJ23 pp 229533.I1RMM1
160721 229533.A0A1C3YJ23 pp 229533.I1RY40
160722 229533.A0A1C3YJ23 pp 229533.V6QXP4
160723 229533.A0A1C3YJ23 pp 229533.I1RTF2
160724 229533.A0A1C3YJ23 pp 229533.I1RQN8
160725 229533.A0A1C3YJ23 pp 229533.I1RRC9
160726 229533.A0A1C3YJ23 pp 229533.I1RRC8
160727 229533.A0A1C3YJ23 pp 229533.I1RMN3
160728 229533.A0A1C3YJ23 pp 229533.I1RQC4
160729 229533.A0A1C3YJ23 pp 229533.I1RE44
160730 229533.I1RU76 pp 229533.I1S1W8
160731 229533.I1RU76 pp 229533.I1RZ29
160732 229533.I1RU76 pp 229533.I1S5L5
160733 229533.I1RU76 pp 229533.I1RYU5
160734 229533.I1RU76 pp 229533.I1RVX9
160735 229533.I1RU76 pp 229533.I1RW43
160736 229533.I1RU76 pp 229533.I1RZ20
160737 229533.I1RU76 pp 229533.I1S3G4
160738 229533.A0A0E0SQ84 pp 229533.I1RNG4
160739 229533.Q4IAA0 pp 229533.V6RIS2
160740 229533.Q4IAA0 pp 229533.V6QXG1
160741 229533.Q4IAA0 pp 229533.V6RY33
160742 229533.Q4IAA0 pp 229533.Q4IJH1
160743 229533.Q4IAA0 pp 229533.Q4IPB8
160744 229533.Q4IAA0 pp 229533.Q4IB70
160745 229533.Q4IAA0 pp 229533.V6RDH3
160746 229533.Q4IAA0 pp 229533.V6RGN5

160747 229533.Q4IAA0 pp 229533.Q4IFI0
160748 229533.Q4IAA0 pp 229533.Q4IBS2
160749 229533.Q4IAA0 pp 229533.V6REZ4
160750 229533.Q4IAA0 pp 229533.Q4IRI9
160751 229533.Q4IAA0 pp 229533.Q4IQW0
160752 229533.Q4IAA0 pp 229533.V6QYF0
160753 229533.Q4IAA0 pp 229533.V6RNM4
160754 229533.Q4IAA0 pp 229533.V6R227
160755 229533.Q4IAA0 pp 229533.Q4IF76
160756 229533.Q4IAA0 pp 229533.Q4IPB3
160757 229533.Q4IAA0 pp 229533.Q4IBK5
160758 229533.Q4IAA0 pp 229533.Q4IPI1
160759 229533.Q4IAA0 pp 229533.Q4IEK8
160760 229533.Q4IAA0 pp 229533.Q4IAS1
160761 229533.Q4IAA0 pp 229533.Q4IJ56
160762 229533.I1RC83 pp 229533.I1RF40
160763 229533.I1RC83 pp 229533.I1S2H3
160764 229533.I1RC83 pp 229533.I1RSX9
160765 229533.I1RC83 pp 229533.I1S064
160766 229533.I1RC83 pp 229533.I1RW43
160767 229533.I1RC83 pp 229533.I1RCB4
160768 229533.I1RC83 pp 229533.I1RN80
160769 229533.I1RC83 pp 229533.I1S6P6
160770 229533.I1RC83 pp 229533.I1RNG4
160771 229533.I1RC83 pp 229533.I1REV2
160772 229533.I1RC83 pp 229533.I1RPD6
160773 229533.I1RC83 pp 229533.I1RY49
160774 229533.I1RC83 pp 229533.I1RU76
160775 229533.I1RC83 pp 229533.I1RPW2
160776 229533.I1RC83 pp 229533.I1RE63
160777 229533.I1RC83 pp 229533.I1RYU5
160778 229533.I1RC83 pp 229533.V6RQW4
160779 229533.I1RC83 pp 229533.I1RZ20
160780 229533.I1RC83 pp 229533.I1RVX9
160781 229533.I1RC83 pp 229533.I1S4F4
160782 229533.I1RC83 pp 229533.I1RM26
160783 229533.I1RC83 pp 229533.I1RR12
160784 229533.I1RC83 pp 229533.I1RQE0
160785 229533.I1RC83 pp 229533.V6R9Z3
160786 229533.I1RC83 pp 229533.I1RJT5
160787 229533.I1RC83 pp 229533.I1S0A0
160788 229533.I1RHQ9 pp 229533.I1RQE0
160789 229533.I1RHQ9 pp 229533.I1S182
160790 229533.I1RHQ9 pp 229533.I1RPW8
160791 229533.I1RHQ9 pp 229533.I1RW91
160792 229533.I1RHQ9 pp 229533.I1RW80
160793 229533.I1RHQ9 pp 229533.I1RQ42
160794 229533.I1RHQ9 pp 229533.I1RN04
160795 229533.I1RHQ9 pp 229533.I1RZ42
160796 229533.I1RC01 pp 229533.I1S333
160797 229533.I1RC01 pp 229533.I1RS37
160798 229533.I1RC01 pp 229533.I1RWM0
160799 229533.I1RC01 pp 229533.I1RCI2
160800 229533.I1RFP5 pp 229533.I1RJP3
160801 229533.I1RFP5 pp 229533.I1RQC3
160802 229533.I1RFP5 pp 229533.I1RX87
160803 229533.I1RFP5 pp 229533.I1RYP0
160804 229533.I1RFP5 pp 229533.I1RY40
160805 229533.I1RFP5 pp 229533.V6QXP4
160806 229533.I1RFP5 pp 229533.I1RMM1
160807 229533.I1RFP5 pp 229533.Q4HY20
160808 229533.I1RFP5 pp 229533.I1RSH3
160809 229533.I1RFP5 pp 229533.I1RQN8
160810 229533.I1RFP5 pp 229533.I1S0V5
160811 229533.I1RFP5 pp 229533.I1RTF2
160812 229533.I1RFP5 pp 229533.I1RRC9
160813 229533.I1RFP5 pp 229533.I1RMN3
160814 229533.I1RFP5 pp 229533.I1S1U6
160815 229533.I1RFP5 pp 229533.I1RQC4
160816 229533.I1RFP5 pp 229533.I1RRC8
160817 229533.I1RFP5 pp 229533.I1S2B6
160818 229533.I1RZE1 pp 229533.I1S8U9
160819 229533.I1RZE1 pp 229533.I1RZL5

160820 229533.I1RZE1 pp 229533.I1S2A8
160821 229533.I1RZE1 pp 229533.I1S8V0
160822 229533.I1RJU1 pp 229533.I1RP97
160823 229533.I1RJU1 pp 229533.I1RMR5
160824 229533.I1RJU1 pp 229533.I1RYB7
160825 229533.I1RJU1 pp 229533.V6RR76
160826 229533.I1RJU1 pp 229533.I1RN81
160827 229533.I1RSW4 pp 229533.I1RX87
160828 229533.I1RSW4 pp 229533.I1RY40
160829 229533.I1RSW4 pp 229533.V6QXP4
160830 229533.I1RSW4 pp 229533.I1S0V5
160831 229533.I1RSW4 pp 229533.I1RYP0
160832 229533.I1RSW4 pp 229533.I1RTG0
160833 229533.I1RSW4 pp 229533.I1RVN2
160834 229533.I1RSW4 pp 229533.Q4HZK7
160835 229533.I1RSW4 pp 229533.I1S2B6
160836 229533.I1RSW4 pp 229533.Q4HY20
160837 229533.I1RSW4 pp 229533.I1RTF2
160838 229533.A0A1C3YN86 pp 229533.Q4IPI1
160839 229533.A0A1C3YN86 pp 229533.I1REF2
160840 229533.A0A1C3YN86 pp 229533.I1RYJ6
160841 229533.A0A1C3YN86 pp 229533.I1RJV7
160842 229533.A0A1C3YN86 pp 229533.I1REK4
160843 229533.A0A1C3YN86 pp 229533.I1RB27
160844 229533.A0A1C3YN86 pp 229533.I1RP86
160845 229533.A0A1C3YN86 pp 229533.I1RE66
160846 229533.A0A1C3YN86 pp 229533.I1RMD5
160847 229533.A0A1C3YN86 pp 229533.I1RFY0
160848 229533.A0A1C3YN86 pp 229533.I1RXF0
160849 229533.A0A1C3YN86 pp 229533.I1S0P0
160850 229533.A0A1C3YN86 pp 229533.V6QWZ7
160851 229533.A0A1C3YN86 pp 229533.V6RQW4
160852 229533.A0A1C3YN86 pp 229533.I1RN51
160853 229533.A0A1C3YN86 pp 229533.I1RW06
160854 229533.A0A1C3YN86 pp 229533.I1RT33
160855 229533.A0A1C3YN86 pp 229533.I1RM67
160856 229533.A0A1C3YN86 pp 229533.V6RKK5
160857 229533.A0A1C3YN86 pp 229533.I1RY21
160858 229533.A0A1C3YN86 pp 229533.I1RMK7
160859 229533.A0A1C3YN86 pp 229533.I1RE65
160860 229533.A0A1C3YN86 pp 229533.I1RAH6
160861 229533.A0A1C3YN86 pp 229533.I1S0F1
160862 229533.A0A1C3YN86 pp 229533.I1RT45
160863 229533.A0A1C3YN86 pp 229533.I1S0K2
160864 229533.A0A1C3YN86 pp 229533.Q4I327
160865 229533.A0A1C3YN86 pp 229533.I1RE79
160866 229533.A0A1C3YN86 pp 229533.I1RBT1
160867 229533.A0A1C3YN86 pp 229533.I1RY92
160868 229533.A0A1C3YN86 pp 229533.I1RT05
160869 229533.A0A1C3YN86 pp 229533.I1S558
160870 229533.A0A1C3YN86 pp 229533.V6RLW4
160871 229533.A0A1C3YN86 pp 229533.I1RTD7
160872 229533.A0A1C3YN86 pp 229533.I1RA52
160873 229533.A0A1C3YN86 pp 229533.I1RPJ3
160874 229533.A0A1C3YN86 pp 229533.I1RWJ4
160875 229533.A0A1C3YN86 pp 229533.I1RFL4
160876 229533.A0A1C3YN86 pp 229533.I1RSS4
160877 229533.A0A1C3YN86 pp 229533.I1RR72
160878 229533.A0A1C3YN86 pp 229533.I1RL04
160879 229533.A0A1C3YN86 pp 229533.I1RKF9
160880 229533.A0A1C3YN86 pp 229533.I1RSC0
160881 229533.A0A1C3YN86 pp 229533.I1RP97
160882 229533.A0A1C3YN86 pp 229533.I1RK80
160883 229533.A0A1C3YN86 pp 229533.I1RC31
160884 229533.A0A1C3YN86 pp 229533.I1RVZ2
160885 229533.A0A1C3YN86 pp 229533.I1S253
160886 229533.A0A1C3YN86 pp 229533.I1RWS4
160887 229533.A0A1C3YI81 pp 229533.I1RRI2
160888 229533.A0A1C3YI81 pp 229533.I1RN17
160889 229533.A0A1C3YI81 pp 229533.A0A1C3YL25
160890 229533.A0A1C3YI81 pp 229533.I1RUG4
160891 229533.A0A1C3YI81 pp 229533.I1S8V0
160892 229533.A0A1C3YI81 pp 229533.A0A1C3YJW7

160893 229533.A0A1C3YI81 pp 229533.V6REZ4
160894 229533.A0A1C3YI81 pp 229533.A0A1C3YJ51
160895 229533.A0A1C3YI81 pp 229533.I1S0B1
160896 229533.A0A1C3YI81 pp 229533.I1RG90
160897 229533.A0A1C3YI81 pp 229533.A0A1C3YMF9
160898 229533.A0A1C3YI81 pp 229533.I1RW99
160899 229533.A0A1C3YI81 pp 229533.I1RQZ2
160900 229533.A0A1C3YI81 pp 229533.I1RA38
160901 229533.A0A1C3YI81 pp 229533.Q4IPC8
160902 229533.A0A1C3YI81 pp 229533.A0A1C3YL99
160903 229533.A0A1C3YI81 pp 229533.I1RQE7
160904 229533.A0A1C3YI81 pp 229533.Q4I5I4
160905 229533.A0A1C3YI81 pp 229533.I1S0N7
160906 229533.A0A1C3YI81 pp 229533.Q4I7X1
160907 229533.A0A1C3YI81 pp 229533.A0A1C3YKM6
160908 229533.A0A1C3YI81 pp 229533.Q4I5W5
160909 229533.A0A1C3YI81 pp 229533.A0A1C3YJ12
160910 229533.A0A1C3YI81 pp 229533.I1S8U9
160911 229533.A0A1C3YI81 pp 229533.I1RN49
160912 229533.A0A1C3YI81 pp 229533.Q4IAS1
160913 229533.A0A1C3YI81 pp 229533.I1RSA4
160914 229533.A0A1C3YI81 pp 229533.A0A1C3YN55
160915 229533.A0A1C3YI81 pp 229533.I1RBV3
160916 229533.A0A1C3YI81 pp 229533.I1RT35
160917 229533.A0A1C3YI81 pp 229533.I1RQ88
160918 229533.A0A1C3YI81 pp 229533.I1S1V5
160919 229533.A0A1C3YI81 pp 229533.A0A1C3YII4
160920 229533.A0A1C3YI81 pp 229533.I1RBD6
160921 229533.A0A1C3YI81 pp 229533.I1RNB8
160922 229533.A0A1C3YI81 pp 229533.Q4IF44
160923 229533.A0A1C3YI81 pp 229533.A0A1C3YM64
160924 229533.A0A1C3YI81 pp 229533.I1RB50
160925 229533.A0A1C3YI81 pp 229533.I1RN28
160926 229533.A0A1C3YI81 pp 229533.I1RZL5
160927 229533.A0A1C3YI81 pp 229533.I1RA36
160928 229533.A0A1C3YI81 pp 229533.I1RHI1
160929 229533.A0A1C3YI81 pp 229533.I1R9Q0
160930 229533.A0A1C3YI81 pp 229533.Q4IPA4
160931 229533.A0A1C3YI81 pp 229533.I1RNT2
160932 229533.A0A1C3YI81 pp 229533.I1RE45
160933 229533.A0A1C3YI81 pp 229533.I1RFU3
160934 229533.A0A1C3YI81 pp 229533.I1RQK2
160935 229533.A0A1C3YI81 pp 229533.I1S0G0
160936 229533.A0A1C3YI81 pp 229533.I1S5Z2
160937 229533.A0A1C3YI81 pp 229533.I1S382
160938 229533.A0A1C3YI81 pp 229533.I1RW25
160939 229533.A0A1C3YI81 pp 229533.I1RD52
160940 229533.A0A1C3YI81 pp 229533.I1S214
160941 229533.A0A1C3YI81 pp 229533.I1RDD3
160942 229533.A0A1C3YI81 pp 229533.I1RA46
160943 229533.A0A1C3YI81 pp 229533.I1RDG8
160944 229533.A0A1C3YI81 pp 229533.I1RS03
160945 229533.A0A1C3YI81 pp 229533.A0A1C3YJV9
160946 229533.A0A1C3YI81 pp 229533.I1RAW7
160947 229533.A0A1C3YI81 pp 229533.I1RB53
160948 229533.A0A1C3YI81 pp 229533.A0A1C3YK68
160949 229533.A0A1C3YI81 pp 229533.I1S246
160950 229533.A0A1C3YI81 pp 229533.I1S9U2
160951 229533.A0A1C3YI81 pp 229533.Q4IPN9
160952 229533.A0A1C3YI81 pp 229533.I1RK70
160953 229533.A0A1C3YI81 pp 229533.I1RCQ6
160954 229533.A0A1C3YI81 pp 229533.I1R976
160955 229533.A0A1C3YI81 pp 229533.Q4IR09
160956 229533.A0A1C3YI81 pp 229533.I1S951
160957 229533.A0A1C3YI81 pp 229533.I1RZP2
160958 229533.A0A1C3YI81 pp 229533.I1RRT8
160959 229533.A0A0E0SQ29 pp 229533.I1RWL9
160960 229533.A0A0E0SQ29 pp 229533.I1RC01
160961 229533.A0A0E0SQ29 pp 229533.I1RJU1
160962 229533.I1RQN8 pp 229533.I1RRC8
160963 229533.I1RQN8 pp 229533.I1RS34
160964 229533.I1RQN8 pp 229533.I1S0V5
160965 229533.I1RQN8 pp 229533.I1RRC9

160966	229533.I1RQN8	pp	229533.I1S1U6
160967	229533.I1RQN8	pp	229533.I1S287
160968	229533.I1RQN8	pp	229533.I1RSH3
160969	229533.I1RQN8	pp	229533.I1RRC6
160970	229533.I1RQN8	pp	229533.I1RTF2
160971	229533.I1RQN8	pp	229533.I1RUN9
160972	229533.I1RQN8	pp	229533.I1RQP7
160973	229533.I1RQN8	pp	229533.I1RXF0
160974	229533.I1RQN8	pp	229533.V6QXP4
160975	229533.I1RQN8	pp	229533.I1S0L3
160976	229533.I1RQN8	pp	229533.I1S278
160977	229533.I1RQN8	pp	229533.Q4HY20
160978	229533.I1RQN8	pp	229533.I1RWZ9
160979	229533.I1RQN8	pp	229533.I1RY40
160980	229533.I1RQN8	pp	229533.I1RX87
160981	229533.I1RQN8	pp	229533.I1RVA8
160982	229533.I1RQN8	pp	229533.V6QUV7
160983	229533.I1RQN8	pp	229533.I1RSW4
160984	229533.I1RQN8	pp	229533.I1RYP0
160985	229533.I1RQN8	pp	229533.I1RZ94
160986	229533.I1RF53	pp	229533.I1S4F4
160987	229533.I1RF53	pp	229533.I1RVX9
160988	229533.I1RF53	pp	229533.I1S064
160989	229533.I1RF53	pp	229533.I1RPD6
160990	229533.I1RF53	pp	229533.I1RJT5
160991	229533.I1RF53	pp	229533.I1RPW2
160992	229533.I1RF53	pp	229533.I1S0A0
160993	229533.I1RF53	pp	229533.I1RNG4
160994	229533.I1RF53	pp	229533.I1RY49
160995	229533.I1RF53	pp	229533.I1RZ20
160996	229533.I1RF53	pp	229533.I1RM26
160997	229533.I1RF53	pp	229533.I1RR12
160998	229533.I1RF53	pp	229533.I1RW43
160999	229533.I1RF53	pp	229533.I1RN80
161000	229533.I1RF53	pp	229533.I1RYU5
161001	229533.I1RF53	pp	229533.I1RU76
161002	229533.I1RF53	pp	229533.I1RQE0
161003	229533.I1RF53	pp	229533.I1S2H3
161004	229533.I1RZS7	pp	229533.Q4IAS1
161005	229533.I1RZS7	pp	229533.I1S503
161006	229533.I1RZS7	pp	229533.V6QW22
161007	229533.I1RZS7	pp	229533.V6RTJ8
161008	229533.I1RZS7	pp	229533.I1S0N7
161009	229533.I1RZS7	pp	229533.Q4HW67
161010	229533.I1RZS7	pp	229533.I1S0L3
161011	229533.I1RZS7	pp	229533.Q4HZ42
161012	229533.I1RZS7	pp	229533.I1RZT6
161013	229533.I1RZS7	pp	229533.I1S246
161014	229533.I1RZS7	pp	229533.I1S4S4
161015	229533.I1RZS7	pp	229533.I1S5B0
161016	229533.I1RZS7	pp	229533.V6R042
161017	229533.I1RZS7	pp	229533.I1RZZ9
161018	229533.I1RZS7	pp	229533.V6RMW6
161019	229533.I1RZS7	pp	229533.Q4IAA0
161020	229533.I1RZS7	pp	229533.I1S1C7
161021	229533.I1RZS7	pp	229533.I1S289
161022	229533.I1RZS7	pp	229533.V6RIV9
161023	229533.I1RZS7	pp	229533.I1S471
161024	229533.I1RZS7	pp	229533.I1RZW8
161025	229533.I1RZS7	pp	229533.Q4HWE2
161026	229533.I1RZS7	pp	229533.I1S504
161027	229533.I1RCJ5	pp	229533.I1RRX1
161028	229533.I1RCJ5	pp	229533.I1RZQ6
161029	229533.I1RCJ5	pp	229533.I1RZD9
161030	229533.I1RCJ5	pp	229533.I1RP88
161031	229533.I1RCJ5	pp	229533.I1RS64
161032	229533.I1RCJ5	pp	229533.I1S0E9
161033	229533.I1RCJ5	pp	229533.I1RN54
161034	229533.I1RCJ5	pp	229533.I1RWQ0
161035	229533.I1RCJ5	pp	229533.I1S095
161036	229533.I1RCJ5	pp	229533.I1RZV4
161037	229533.I1RCJ5	pp	229533.I1RD84
161038	229533.I1RCJ5	pp	229533.I1RSW5

161039 229533.I1RCJ5 pp 229533.I1RT19
161040 229533.I1RCJ5 pp 229533.I1RXB5
161041 229533.I1RCJ5 pp 229533.I1RD69
161042 229533.I1RCJ5 pp 229533.I1S077
161043 229533.A0A1C3YKU7 pp 229533.I1RQD6
161044 229533.A0A1C3YKU7 pp 229533.I1S6S4
161045 229533.A0A1C3YKU7 pp 229533.I1RAS9
161046 229533.A0A1C3YKU7 pp 229533.I1RX87
161047 229533.A0A1C3YKU7 pp 229533.I1S2B6
161048 229533.A0A1C3YKU7 pp 229533.I1RNG2
161049 229533.A0A1C3YKU7 pp 229533.I1RQH7
161050 229533.A0A1C3YKU7 pp 229533.I1RPG2
161051 229533.A0A1C3YKU7 pp 229533.I1RE03
161052 229533.A0A1C3YKU7 pp 229533.I1S1V4
161053 229533.A0A1C3YKU7 pp 229533.I1RE18
161054 229533.A0A1C3YKU7 pp 229533.I1RZS5
161055 229533.A0A1C3YKU7 pp 229533.I1RPU5
161056 229533.A0A1C3YKU7 pp 229533.I1RQP5
161057 229533.A0A1C3YKU7 pp 229533.I1RMF2
161058 229533.A0A1C3YKU7 pp 229533.I1RND7
161059 229533.A0A1C3YKU7 pp 229533.I1RBF2
161060 229533.A0A1C3YKU7 pp 229533.I1RFS7
161061 229533.A0A1C3YKU7 pp 229533.I1RJZ6
161062 229533.A0A1C3YKU7 pp 229533.I1RCH2
161063 229533.A0A1C3YKU7 pp 229533.I1RQF7
161064 229533.A0A1C3YKU7 pp 229533.I1RFV4
161065 229533.A0A1C3YKU7 pp 229533.I1RMN3
161066 229533.A0A1C3YKU7 pp 229533.A0A1C3YN81
161067 229533.A0A1C3YKU7 pp 229533.I1RAH7
161068 229533.A0A1C3YKU7 pp 229533.I1RCL9
161069 229533.A0A1C3YKU7 pp 229533.I1RZ60
161070 229533.A0A1C3YKU7 pp 229533.I1RW76
161071 229533.A0A1C3YKU7 pp 229533.I1RXF5
161072 229533.A0A1C3YKU7 pp 229533.I1RWU9
161073 229533.A0A1C3YKU7 pp 229533.I1RW58
161074 229533.A0A1C3YKU7 pp 229533.I1S287
161075 229533.A0A1C3YKU7 pp 229533.Q4IN63
161076 229533.A0A1C3YKU7 pp 229533.I1RQP7
161077 229533.A0A1C3YKU7 pp 229533.I1S278
161078 229533.A0A1C3YKU7 pp 229533.I1RCQ0
161079 229533.A0A1C3YKU7 pp 229533.I1S0V5
161080 229533.A0A1C3YKU7 pp 229533.I1S830
161081 229533.A0A1C3YKU7 pp 229533.I1RNA3
161082 229533.A0A1C3YKU7 pp 229533.I1SAM5
161083 229533.A0A1C3YKU7 pp 229533.I1RBT0
161084 229533.A0A1C3YKU7 pp 229533.I1RFK0
161085 229533.A0A1C3YKU7 pp 229533.I1RCV3
161086 229533.A0A1C3YKU7 pp 229533.I1RYS8
161087 229533.A0A1C3YKU7 pp 229533.A0A1C3YL89
161088 229533.A0A1C3YKU7 pp 229533.I1RK33
161089 229533.A0A1C3YKU7 pp 229533.I1RNH7
161090 229533.A0A1C3YKU7 pp 229533.I1RCN6
161091 229533.A0A1C3YKU7 pp 229533.I1RZK3
161092 229533.A0A1C3YKU7 pp 229533.Q4I6S5
161093 229533.I1S8U9 pp 229533.Q4IR09
161094 229533.I1S8U9 pp 229533.Q4I7X1
161095 229533.I1S8U9 pp 229533.Q4IPN9
161096 229533.I1S8U9 pp 229533.V6QUE6
161097 229533.I1S8U9 pp 229533.V6RIS2
161098 229533.I1S8U9 pp 229533.Q4IF44
161099 229533.I1S8U9 pp 229533.Q4IPC8
161100 229533.I1S8U9 pp 229533.Q4IPA4
161101 229533.I1RA99 pp 229533.I1REL1
161102 229533.I1RA99 pp 229533.I1RX47
161103 229533.I1RA99 pp 229533.I1RGZ5
161104 229533.I1RA99 pp 229533.I1RWU2
161105 229533.I1RA99 pp 229533.I1RG11
161106 229533.I1RA99 pp 229533.I1S471
161107 229533.I1RA99 pp 229533.I1RCS2
161108 229533.I1RA99 pp 229533.I1RNR6
161109 229533.I1RA99 pp 229533.Q4IAA0
161110 229533.I1RA99 pp 229533.I1S246
161111 229533.I1RA99 pp 229533.I1RJI0

229533.I1RA99 pp 229533.I1RN60
229533.I1RA99 pp 229533.I1RPW8
229533.I1RA99 pp 229533.I1RWL9
229533.I1RA99 pp 229533.I1RNB8
229533.I1RA99 pp 229533.I1RKK6
229533.I1RA99 pp 229533.I1RK72
229533.I1RA99 pp 229533.I1RCG6
229533.I1RA99 pp 229533.I1RAN3
229533.I1RA99 pp 229533.I1RAI1
229533.I1RA99 pp 229533.I1RAC1
229533.I1RA99 pp 229533.I1RBX0
229533.I1RA99 pp 229533.I1RG57
229533.I1RA99 pp 229533.I1RZG7
229533.I1RA99 pp 229533.I1RT35
229533.I1RA99 pp 229533.I1RSJ0
229533.I1RA99 pp 229533.I1RQ42
229533.I1RA99 pp 229533.I1S5Z2
229533.I1RA99 pp 229533.I1S9M9
229533.I1RA99 pp 229533.I1RS03
229533.I1RA99 pp 229533.I1RCH7
229533.I1RA99 pp 229533.I1RDX6
229533.I1RA99 pp 229533.I1RW80
229533.I1RA99 pp 229533.I1S0Z9
229533.I1RPG2 pp 229533.Q4ILI9
229533.I1RPG2 pp 229533.I1RW76
229533.I1RPG2 pp 229533.I1S287
229533.I1RPG2 pp 229533.I1RYP0
229533.I1RPG2 pp 229533.I1SAM5
229533.I1RPG2 pp 229533.I1RQP7
229533.I1RPG2 pp 229533.I1S5B5
229533.I1RPG2 pp 229533.I1RZK3
229533.I1RPG2 pp 229533.I1RX87
229533.I1RPG2 pp 229533.I1RQH7
229533.I1RPG2 pp 229533.I1S2B6
229533.I1RPG2 pp 229533.I1RQF7
229533.I1RPG2 pp 229533.I1S1U6
229533.I1RPG2 pp 229533.I1S1V4
229533.A0A098E2A4 pp 229533.I1RR24
229533.A0A098E2A4 pp 229533.A0A0E0RSD7
229533.A0A098E2A4 pp 229533.I1RYJ7
229533.A0A098E2A4 pp 229533.I1RW06
229533.A0A098E2A4 pp 229533.A0A1C3YLR6
229533.A0A098E2A4 pp 229533.I1RC11
229533.A0A098E2A4 pp 229533.A0A0E0RUI4
229533.A0A098E2A4 pp 229533.I1RMM8
229533.A0A098E2A4 pp 229533.I1RQE8
229533.A0A098E2A4 pp 229533.I1S3K8
229533.A0A098E2A4 pp 229533.A0A1C3YN26
229533.A0A098E2A4 pp 229533.A0A1C3YHX8
229533.A0A098E2A4 pp 229533.I1RBT1
229533.A0A098E2A4 pp 229533.I1RYU5
229533.A0A098E2A4 pp 229533.I1RAX6
229533.A0A098E2A4 pp 229533.I1RX46
229533.A0A098E2A4 pp 229533.I1RYX6
229533.A0A098E2A4 pp 229533.I1S1P8
229533.A0A098E2A4 pp 229533.I1RD52
229533.A0A098E2A4 pp 229533.I1S1W8
229533.A0A098E2A4 pp 229533.I1S130
229533.A0A098E2A4 pp 229533.I1RP86
229533.A0A098E2A4 pp 229533.I1RQX9
229533.A0A098E2A4 pp 229533.I1RTU1
229533.A0A098E2A4 pp 229533.I1RRX5
229533.A0A098E2A4 pp 229533.I1RFK7
229533.A0A098E2A4 pp 229533.I1RZM4
229533.A0A098E2A4 pp 229533.A0A1C3YJL7
229533.A0A098E2A4 pp 229533.A0A0E0S6V8
229533.A0A098E2A4 pp 229533.I1RBE7
229533.A0A098E2A4 pp 229533.I1S3K6
229533.A0A098E2A4 pp 229533.I1RVY2
229533.A0A098E2A4 pp 229533.A0A1C3YK13
229533.A0A098E2A4 pp 229533.A0A1C3YLE9
229533.A0A098E2A4 pp 229533.I1RXB1
229533.A0A098E2A4 pp 229533.I1RRV4

161185 229533.A0A098E2A4 pp 229533.I1S084
161186 229533.A0A098E2A4 pp 229533.A0A098E320
161187 229533.A0A098E2A4 pp 229533.I1RCR5
161188 229533.A0A098E2A4 pp 229533.I1RK47
161189 229533.A0A098E2A4 pp 229533.I1RYI7
161190 229533.A0A098E2A4 pp 229533.I1RXV9
161191 229533.A0A098E2A4 pp 229533.I1RBL7
161192 229533.A0A098E2A4 pp 229533.I1RFM6
161193 229533.A0A098E2A4 pp 229533.V6RCZ8
161194 229533.A0A098E2A4 pp 229533.I1RCM5
161195 229533.A0A098E2A4 pp 229533.I1RBE4
161196 229533.A0A098E2A4 pp 229533.A0A1C3YJW4
161197 229533.A0A098E2A4 pp 229533.I1RZK5
161198 229533.A0A098E2A4 pp 229533.A0A1C3YIV7
161199 229533.A0A098E2A4 pp 229533.A0A098E4W0
161200 229533.A0A098E2A4 pp 229533.I1RFU8
161201 229533.A0A098E2A4 pp 229533.A0A0E0S6X1
161202 229533.A0A098E2A4 pp 229533.I1S3G4
161203 229533.A0A098E2A4 pp 229533.I1RVJ6
161204 229533.A0A098E2A4 pp 229533.A0A1C3YK58
161205 229533.A0A098E2A4 pp 229533.I1S2H4
161206 229533.A0A098E2A4 pp 229533.I1RY03
161207 229533.A0A098E2A4 pp 229533.I1RXF0
161208 229533.A0A098E2A4 pp 229533.A0A1C3YKB0
161209 229533.A0A098E2A4 pp 229533.A0A1C3YJR3
161210 229533.A0A098E2A4 pp 229533.I1RA90
161211 229533.A0A098E2A4 pp 229533.A0A1C3YJZ0
161212 229533.A0A098E2A4 pp 229533.I1RRT8
161213 229533.A0A098E2A4 pp 229533.O74638
161214 229533.A0A098E2A4 pp 229533.I1RZM8
161215 229533.A0A098E2A4 pp 229533.I1RTI7
161216 229533.A0A098E2A4 pp 229533.I1RZT0
161217 229533.A0A098E2A4 pp 229533.I1R9Y8
161218 229533.A0A098E2A4 pp 229533.I1RS18
161219 229533.A0A098E2A4 pp 229533.I1RVR0
161220 229533.A0A098E2A4 pp 229533.A0A1C3YKV3
161221 229533.A0A098E2A4 pp 229533.V6RK96
161222 229533.A0A098E2A4 pp 229533.V6R3X5
161223 229533.A0A098E2A4 pp 229533.I1RW43
161224 229533.A0A098E2A4 pp 229533.I1RZ52
161225 229533.A0A098E2A4 pp 229533.A0A1C3YN25
161226 229533.A0A098E2A4 pp 229533.I1RTG7
161227 229533.A0A098E2A4 pp 229533.I1RM30
161228 229533.A0A098E2A4 pp 229533.Q4IMJ1
161229 229533.A0A098E2A4 pp 229533.I1S0A0
161230 229533.A0A098E2A4 pp 229533.A0A1C3YK49
161231 229533.A0A098E2A4 pp 229533.I1S8V3
161232 229533.A0A098E2A4 pp 229533.I1RBF3
161233 229533.A0A098E2A4 pp 229533.I1RC29
161234 229533.A0A098E2A4 pp 229533.I1RZ29
161235 229533.A0A098E2A4 pp 229533.I1S5B0
161236 229533.A0A098E2A4 pp 229533.I1S6P5
161237 229533.A0A098E2A4 pp 229533.I1RA94
161238 229533.A0A098E2A4 pp 229533.I1RN63
161239 229533.A0A098E2A4 pp 229533.A0A1C3YI20
161240 229533.A0A098E2A4 pp 229533.I1RSQ4
161241 229533.A0A098E2A4 pp 229533.I1RN88
161242 229533.A0A098E2A4 pp 229533.I1RFS3
161243 229533.A0A098E2A4 pp 229533.I1RZ20
161244 229533.A0A098E2A4 pp 229533.A0A1C3YL82
161245 229533.A0A098E2A4 pp 229533.A0A0E0SC46
161246 229533.A0A098E2A4 pp 229533.Q4I1T9
161247 229533.A0A098E2A4 pp 229533.I1RDJ7
161248 229533.A0A098E2A4 pp 229533.I1RBS0
161249 229533.A0A098E2A4 pp 229533.I1RB92
161250 229533.A0A098E2A4 pp 229533.I1RC31
161251 229533.A0A098E2A4 pp 229533.I1RY62
161252 229533.A0A098E2A4 pp 229533.I1RBC7
161253 229533.A0A098E2A4 pp 229533.I1RXW3
161254 229533.A0A098E2A4 pp 229533.I1RIH0
161255 229533.A0A098E2A4 pp 229533.V6RUB1
161256 229533.A0A098E2A4 pp 229533.A0A1C3YKV2
161257 229533.A0A098E2A4 pp 229533.I1RVX0

161258 229533.A0A098E2A4 pp 229533.I1RPD6
161259 229533.A0A098E2A4 pp 229533.I1RCH5
161260 229533.A0A098E2A4 pp 229533.I1RN67
161261 229533.A0A098E2A4 pp 229533.I1RNG4
161262 229533.A0A098E2A4 pp 229533.Q4HVQ9
161263 229533.A0A098E2A4 pp 229533.V6QVH8
161264 229533.A0A098E2A4 pp 229533.I1RJW6
161265 229533.A0A098E2A4 pp 229533.I1RYK0
161266 229533.A0A098E2A4 pp 229533.I1RQE7
161267 229533.A0A098E2A4 pp 229533.I1RPP3
161268 229533.A0A098E2A4 pp 229533.I1RCP7
161269 229533.A0A098E2A4 pp 229533.I1RKB6
161270 229533.A0A098E2A4 pp 229533.A0A0E0SMR8
161271 229533.A0A098E2A4 pp 229533.I1RP97
161272 229533.A0A098E2A4 pp 229533.Q4HXF0
161273 229533.A0A098E2A4 pp 229533.I1RFT1
161274 229533.A0A098E2A4 pp 229533.I1RZA7
161275 229533.A0A098E2A4 pp 229533.V6RSF3
161276 229533.A0A098E2A4 pp 229533.I1RRI2
161277 229533.A0A098E2A4 pp 229533.I1RPK0
161278 229533.A0A098E2A4 pp 229533.V6RLM7
161279 229533.A0A098E2A4 pp 229533.A0A0E0SMT9
161280 229533.A0A098E2A4 pp 229533.I1RAU7
161281 229533.A0A098E2A4 pp 229533.I1RAR1
161282 229533.A0A098E2A4 pp 229533.I1RYW3
161283 229533.A0A098E2A4 pp 229533.I1R9B8
161284 229533.A0A098E2A4 pp 229533.I1RP69
161285 229533.A0A098E2A4 pp 229533.V6RN31
161286 229533.A0A098E2A4 pp 229533.I1RSL5
161287 229533.A0A098E2A4 pp 229533.I1RD80
161288 229533.A0A098E2A4 pp 229533.I1S0J1
161289 229533.A0A098E2A4 pp 229533.I1S8V4
161290 229533.A0A098E2A4 pp 229533.I1RE01
161291 229533.A0A098E2A4 pp 229533.I1RG11
161292 229533.A0A098E2A4 pp 229533.I1RV23
161293 229533.A0A098E2A4 pp 229533.V6QT89
161294 229533.A0A098E2A4 pp 229533.I1RCB4
161295 229533.A0A098E2A4 pp 229533.I1S256
161296 229533.A0A098E2A4 pp 229533.A0A0E0SI42
161297 229533.A0A098E2A4 pp 229533.A0A1C3YI05
161298 229533.A0A098E2A4 pp 229533.Q4HTS9
161299 229533.I1RDY5 pp 229533.I1RYB9
161300 229533.I1RDY5 pp 229533.I1RNE4
161301 229533.I1RDY5 pp 229533.I1RSP1
161302 229533.I1RDY5 pp 229533.I1RJS0
161303 229533.I1RDY5 pp 229533.I1RK83
161304 229533.I1RDY5 pp 229533.I1SA48
161305 229533.I1RDY5 pp 229533.I1RSL6
161306 229533.I1RDY5 pp 229533.I1RYT7
161307 229533.I1RDY5 pp 229533.I1RYJ8
161308 229533.I1RDY5 pp 229533.I1RWH5
161309 229533.I1RDY5 pp 229533.I1RRE0
161310 229533.I1RDY5 pp 229533.I1RJS5
161311 229533.I1RDY5 pp 229533.I1RZ99
161312 229533.I1RDY5 pp 229533.I1RZE1
161313 229533.I1RDY5 pp 229533.I1RW24
161314 229533.I1RDY5 pp 229533.I1RSL8
161315 229533.I1RDY5 pp 229533.I1RDZ4
161316 229533.I1RDY5 pp 229533.I1RWB5
161317 229533.I1RDY5 pp 229533.I1S6E5
161318 229533.I1RDY5 pp 229533.I1RK95
161319 229533.I1RDY5 pp 229533.I1RW71
161320 229533.I1RDY5 pp 229533.I1RM96
161321 229533.I1RDY5 pp 229533.I1RYP7
161322 229533.I1RDY5 pp 229533.I1RRQ3
161323 229533.I1RDY5 pp 229533.I1RNT2
161324 229533.I1RDY5 pp 229533.I1SA47
161325 229533.I1RDY5 pp 229533.I1S2A8
161326 229533.I1RDY5 pp 229533.V6REZ4
161327 229533.I1RDY5 pp 229533.I1RTE5
161328 229533.I1RDY5 pp 229533.I1RFW3
161329 229533.I1RDY5 pp 229533.I1RG51
161330 229533.I1RDY5 pp 229533.I1RND6

161331 229533.I1RDY5 pp 229533.I1REX4
161332 229533.I1RDY5 pp 229533.I1S8E2
161333 229533.I1RDY5 pp 229533.I1RJS4
161334 229533.I1RDY5 pp 229533.I1RYT5
161335 229533.I1RDY5 pp 229533.I1RK77
161336 229533.I1RRS4 pp 229533.I1S0C6
161337 229533.A0A1C3YKD5 pp 229533.I1RSE6
161338 229533.A0A1C3YKD5 pp 229533.I1RDE9
161339 229533.A0A1C3YKD5 pp 229533.I1RJK8
161340 229533.A0A1C3YKD5 pp 229533.I1RXB5
161341 229533.A0A1C3YKD5 pp 229533.I1RYD8
161342 229533.A0A1C3YKD5 pp 229533.I1RDE8
161343 229533.A0A1C3YKD5 pp 229533.I1RBX2
161344 229533.I1RMQ5 pp 229533.I1RZ95
161345 229533.I1RMQ5 pp 229533.I1S9U6
161346 229533.I1RMQ5 pp 229533.I1RWH4
161347 229533.I1RMQ5 pp 229533.I1RN84
161348 229533.I1RMQ5 pp 229533.I1RRV1
161349 229533.I1RMQ5 pp 229533.I1RQ21
161350 229533.I1RMQ5 pp 229533.I1RPK0
161351 229533.I1RMQ5 pp 229533.I1RPN8
161352 229533.I1RMQ5 pp 229533.I1S0N4
161353 229533.I1RMQ5 pp 229533.I1S8V4
161354 229533.I1RMQ5 pp 229533.I1RYW3
161355 229533.I1RMQ5 pp 229533.I1RSL8
161356 229533.I1RMQ5 pp 229533.V6QX21
161357 229533.I1RMQ5 pp 229533.I1RZE4
161358 229533.I1RMQ5 pp 229533.I1RTA0
161359 229533.I1RMQ5 pp 229533.I1S2E8
161360 229533.I1RMQ5 pp 229533.I1S238
161361 229533.I1RMQ5 pp 229533.Q4HZI0
161362 229533.I1RMQ5 pp 229533.I1RZZ9
161363 229533.I1RMQ5 pp 229533.I1RQF2
161364 229533.I1RMQ5 pp 229533.V6RRH2
161365 229533.I1RMQ5 pp 229533.I1S5S8
161366 229533.I1RMQ5 pp 229533.I1S8G4
161367 229533.I1RMQ5 pp 229533.I1RRT8
161368 229533.I1RMQ5 pp 229533.I1RSH0
161369 229533.I1RMQ5 pp 229533.I1S247
161370 229533.I1RMQ5 pp 229533.Q4HXF0
161371 229533.I1RMQ5 pp 229533.O74638
161372 229533.I1RMQ5 pp 229533.I1S8P5
161373 229533.I1RMQ5 pp 229533.I1RP27
161374 229533.I1RMQ5 pp 229533.I1RQG4
161375 229533.I1RMQ5 pp 229533.I1RR95
161376 229533.I1RMQ5 pp 229533.I1RRM9
161377 229533.I1RMQ5 pp 229533.I1RSV2
161378 229533.I1RMQ5 pp 229533.I1S9Y1
161379 229533.I1RMQ5 pp 229533.V6QUE6
161380 229533.I1RMQ5 pp 229533.I1RP28
161381 229533.I1RMQ5 pp 229533.I1S2V2
161382 229533.I1RMQ5 pp 229533.I1RX22
161383 229533.I1RMQ5 pp 229533.I1RWK9
161384 229533.I1RMQ5 pp 229533.I1S209
161385 229533.I1RMQ5 pp 229533.I1RNG4
161386 229533.I1RMQ5 pp 229533.I1S5B0
161387 229533.I1RMQ5 pp 229533.I1S1V5
161388 229533.I1RMQ5 pp 229533.I1RN67
161389 229533.I1RMQ5 pp 229533.I1RZH4
161390 229533.I1RMQ5 pp 229533.I1RNF4
161391 229533.I1RMQ5 pp 229533.I1RS20
161392 229533.I1RMQ5 pp 229533.I1S2G9
161393 229533.I1RMQ5 pp 229533.I1RS47
161394 229533.I1RMQ5 pp 229533.I1RPL2
161395 229533.I1RMQ5 pp 229533.I1S8V3
161396 229533.I1RMQ5 pp 229533.Q4IAA0
161397 229533.I1RMQ5 pp 229533.I1S4Z7
161398 229533.I1RMQ5 pp 229533.I1RN88
161399 229533.I1RMQ5 pp 229533.I1RQG7
161400 229533.I1RMQ5 pp 229533.I1RQ81
161401 229533.I1RMQ5 pp 229533.V6RBN2
161402 229533.I1RMQ5 pp 229533.I1RN65
161403 229533.I1RMQ5 pp 229533.I1S0Z3

161404 229533.I1RMQ5 pp 229533.I1RP66
161405 229533.I1RMQ5 pp 229533.I1RSI0
161406 229533.I1RMQ5 pp 229533.I1RQQ6
161407 229533.I1RMQ5 pp 229533.I1S235
161408 229533.I1RMQ5 pp 229533.I1RZM1
161409 229533.I1RMQ5 pp 229533.I1RWD7
161410 229533.I1RMQ5 pp 229533.I1RYL5
161411 229533.I1RMQ5 pp 229533.I1S439
161412 229533.I1RMQ5 pp 229533.Q4ICA8
161413 229533.I1RMQ5 pp 229533.I1S050
161414 229533.I1RMQ5 pp 229533.I1RQA5
161415 229533.I1RMQ5 pp 229533.I1RXT0
161416 229533.I1RMQ5 pp 229533.I1RSP1
161417 229533.I1RMQ5 pp 229533.V6RXW9
161418 229533.I1RMQ5 pp 229533.I1RNY7
161419 229533.I1RMQ5 pp 229533.I1RW87
161420 229533.I1RMQ5 pp 229533.I1RQQ8
161421 229533.I1RMQ5 pp 229533.V6RTJ8
161422 229533.I1RMQ5 pp 229533.I1RRT6
161423 229533.I1RMQ5 pp 229533.I1RP86
161424 229533.I1RMQ5 pp 229533.I1RQH1
161425 229533.I1RMQ5 pp 229533.I1RZZ1
161426 229533.I1RMQ5 pp 229533.I1RW71
161427 229533.I1RMQ5 pp 229533.I1RQ26
161428 229533.I1RMQ5 pp 229533.Q5I2J3
161429 229533.I1RMQ5 pp 229533.I1RPN4
161430 229533.I1RMQ5 pp 229533.V6R1K4
161431 229533.I1RMQ5 pp 229533.Q4HZS8
161432 229533.I1RMQ5 pp 229533.I1S270
161433 229533.I1RMQ5 pp 229533.I1RQS9
161434 229533.I1RMQ5 pp 229533.I1RZ02
161435 229533.I1RMQ5 pp 229533.I1RT41
161436 229533.I1RMQ5 pp 229533.I1RYH9
161437 229533.I1RE18 pp 229533.I1RMN3
161438 229533.I1RE18 pp 229533.I1RQF7
161439 229533.I1RE18 pp 229533.I1RQD6
161440 229533.I1RE18 pp 229533.I1RFV4
161441 229533.I1RE18 pp 229533.I1RND7
161442 229533.I1RE18 pp 229533.I1RXF5
161443 229533.I1RE18 pp 229533.I1RJZ6
161444 229533.I1RE18 pp 229533.I1RMF2
161445 229533.I1RE18 pp 229533.I1S278
161446 229533.I1RE18 pp 229533.I1S0V5
161447 229533.I1RE18 pp 229533.I1RXT2
161448 229533.I1RE18 pp 229533.I1RQP7
161449 229533.I1RE18 pp 229533.V6QUV7
161450 229533.I1RE18 pp 229533.I1RJS5
161451 229533.I1RE18 pp 229533.I1RZ60
161452 229533.I1RE18 pp 229533.Q4IN63
161453 229533.I1RE18 pp 229533.I1S287
161454 229533.I1RE18 pp 229533.I1RMF9
161455 229533.I1RE18 pp 229533.I1S5B5
161456 229533.I1RE18 pp 229533.I1RW76
161457 229533.I1RE18 pp 229533.I1RFK0
161458 229533.I1RE18 pp 229533.I1RQH7
161459 229533.I1RE18 pp 229533.I1RNG2
161460 229533.I1RE18 pp 229533.I1S2B6
161461 229533.I1RE18 pp 229533.I1RX87
161462 229533.I1RE18 pp 229533.I1RZS5
161463 229533.I1RE18 pp 229533.I1RZK3
161464 229533.I1RE18 pp 229533.I1SAM5
161465 229533.I1RE18 pp 229533.I1RPG2
161466 229533.I1RE18 pp 229533.I1RJS4
161467 229533.I1RE18 pp 229533.I1S1V4
161468 229533.I1RE18 pp 229533.I1RNH7
161469 229533.I1RE18 pp 229533.I1RYP0
161470 229533.A0A098DHB5 pp 229533.I1RR68
161471 229533.A0A098DHB5 pp 229533.I1RVX9
161472 229533.A0A098DHB5 pp 229533.I1RNG7
161473 229533.A0A098DHB5 pp 229533.A0A1C3YLA6
161474 229533.A0A098DHB5 pp 229533.I1S4F4
161475 229533.A0A098DHB5 pp 229533.I1RW26
161476 229533.A0A098DHB5 pp 229533.I1RPW2

```
229533.A0A098DHB5   pp  229533.I1RNG4
229533.A0A098DHB5   pp  229533.I1RE63
229533.A0A098DHB5   pp  229533.I1RZ20
229533.A0A098DHB5   pp  229533.I1S5L5
229533.A0A098DHB5   pp  229533.I1RW43
229533.A0A098DHB5   pp  229533.I1S3G4
229533.A0A098DHB5   pp  229533.A0A0E0SMT9
229533.A0A098DHB5   pp  229533.A0A1C3YLV0
229533.A0A098DHB5   pp  229533.I1RYU5
229533.A0A098DHB5   pp  229533.I1RZ29
229533.A0A098DHB5   pp  229533.I1RD81
229533.A0A098DHB5   pp  229533.I1REV2
229533.A0A098DHB5   pp  229533.I1S1W8
229533.A0A098DHB5   pp  229533.I1RF38
229533.A0A098DHB5   pp  229533.A0A1C3YIV7
229533.A0A098DHB5   pp  229533.I1RCB4
229533.A0A098DHB5   pp  229533.I1RRV4
229533.A0A098DHB5   pp  229533.I1RU76
229533.A0A098DHB5   pp  229533.I1RC83
229533.A0A098DHB5   pp  229533.I1RN80
229533.A0A098DHB5   pp  229533.A0A1C3YN86
229533.A0A098DHB5   pp  229533.I1RF53
229533.A0A098DHB5   pp  229533.I1S2H3
229533.A0A098DHB5   pp  229533.V6RQW4
229533.A0A098DHB5   pp  229533.I1RM26
229533.A0A098DHB5   pp  229533.A0A098E2A4
229533.A0A098DHB5   pp  229533.A0A098DKG5
229533.A0A098DHB5   pp  229533.I1RBE7
229533.A0A098DHB5   pp  229533.I1RQE0
229533.I1RNK6   pp  229533.I1RW71
229533.I1RNK6   pp  229533.I1RRX5
229533.I1RNK6   pp  229533.V6R3J1
229533.I1RQ49   pp  229533.I1S0P4
229533.I1RQ49   pp  229533.I1RTE5
229533.I1RQ49   pp  229533.I1RT35
229533.I1RQ49   pp  229533.I1S7W5
229533.I1RQ49   pp  229533.I1RSL8
229533.I1RQ49   pp  229533.Q4IPB8
229533.I1RQ49   pp  229533.Q4HXF6
229533.I1RQ49   pp  229533.Q4IAA0
229533.I1RQ49   pp  229533.I1RYM3
229533.I1RQ49   pp  229533.I1S2B9
229533.I1RQ49   pp  229533.I1RWG1
229533.I1RQ49   pp  229533.I1RZV9
229533.I1RQ49   pp  229533.I1S471
229533.I1RQ49   pp  229533.I1RQ80
229533.I1RQ49   pp  229533.Q4IE79
229533.I1RQ49   pp  229533.I1S9G7
229533.I1RQ49   pp  229533.I1S2F3
229533.I1RQ49   pp  229533.Q4I7X1
229533.I1RQ49   pp  229533.I1S246
229533.I1RQ49   pp  229533.Q4IBK5
229533.I1RQ49   pp  229533.I1S5B5
229533.I1RQ49   pp  229533.I1RZT1
229533.I1RQ49   pp  229533.I1RYT7
229533.I1RQ49   pp  229533.Q4IPH4
229533.I1RQ49   pp  229533.Q4I1Y1
229533.I1RQ49   pp  229533.I1RRT2
229533.I1RQ49   pp  229533.V6REZ4
229533.I1RQ49   pp  229533.Q4I5R9
229533.I1RQ49   pp  229533.I1RQC5
229533.I1RQ49   pp  229533.I1RS94
229533.I1RQ49   pp  229533.I1S9V9
229533.I1RQ49   pp  229533.I1S0L8
229533.I1RQ49   pp  229533.I1RY40
229533.I1RQ49   pp  229533.I1S0X5
229533.I1RQ49   pp  229533.I1RSM2
229533.I1RQ49   pp  229533.Q4IPB3
229533.I1RUY1   pp  229533.I1RZ42
229533.I1RUY1   pp  229533.I1S182
229533.I1RUY1   pp  229533.I1RW80
229533.I1RUY1   pp  229533.I1RW91
229533.A0A098DH83   pp  229533.I1RNG4
```

161550 229533.I1RQD6 pp 229533.I1RSH3
161551 229533.I1RQD6 pp 229533.I1S1U6
161552 229533.I1RQD6 pp 229533.I1RZ60
161553 229533.I1RQD6 pp 229533.I1RSI3
161554 229533.I1RQD6 pp 229533.I1RW76
161555 229533.I1RQD6 pp 229533.I1RQN8
161556 229533.I1RQD6 pp 229533.I1RUN9
161557 229533.I1RQD6 pp 229533.I1S287
161558 229533.I1RQD6 pp 229533.I1RTF2
161559 229533.I1RQD6 pp 229533.V6QXP4
161560 229533.I1RQD6 pp 229533.I1RX87
161561 229533.I1RQD6 pp 229533.I1S830
161562 229533.I1RQD6 pp 229533.I1RZS5
161563 229533.I1RQD6 pp 229533.I1RY40
161564 229533.I1RQD6 pp 229533.I1RWZ9
161565 229533.I1RQD6 pp 229533.I1S1V4
161566 229533.I1RQD6 pp 229533.I1RSQ2
161567 229533.I1RQD6 pp 229533.I1RZK3
161568 229533.I1RQD6 pp 229533.I1RXF5
161569 229533.I1RQD6 pp 229533.I1RQF7
161570 229533.I1RQD6 pp 229533.I1S051
161571 229533.I1RQD6 pp 229533.I1RQP7
161572 229533.I1RQD6 pp 229533.I1S278
161573 229533.I1RQD6 pp 229533.I1RXT7
161574 229533.I1RQD6 pp 229533.I1RXH6
161575 229533.I1RQD6 pp 229533.I1RX95
161576 229533.I1RQD6 pp 229533.I1SAM5
161577 229533.I1RQD6 pp 229533.I1RYP0
161578 229533.I1RQD6 pp 229533.I1RYB8
161579 229533.I1RQD6 pp 229533.V6RXW9
161580 229533.I1RQD6 pp 229533.I1S2B6
161581 229533.I1RQD6 pp 229533.I1RUP5
161582 229533.I1RQD6 pp 229533.I1RQF9
161583 229533.I1RQD6 pp 229533.I1RZK4
161584 229533.I1RQD6 pp 229533.I1S0V5
161585 229533.I1RQD6 pp 229533.I1S2K7
161586 229533.I1RQD6 pp 229533.I1S296
161587 229533.I1RSL8 pp 229533.I1RTG5
161588 229533.I1RSL8 pp 229533.I1RZL9
161589 229533.I1RSL8 pp 229533.I1S2C0
161590 229533.I1RSL8 pp 229533.I1S2A8
161591 229533.I1RSL8 pp 229533.I1RZE1
161592 229533.I1RSL8 pp 229533.I1S0N5
161593 229533.I1RSL8 pp 229533.I1S490
161594 229533.I1RSL8 pp 229533.I1RTD3
161595 229533.I1RSL8 pp 229533.I1S8E2
161596 229533.I1RSL8 pp 229533.I1RYB9
161597 229533.I1RSL8 pp 229533.I1S2F2
161598 229533.I1RSL8 pp 229533.I1RVA7
161599 229533.I1RSL8 pp 229533.I1RW36
161600 229533.I1RSL8 pp 229533.I1RYT7
161601 229533.I1RSL8 pp 229533.I1RWT7
161602 229533.I1RSL8 pp 229533.I1RW24
161603 229533.I1RSL8 pp 229533.I1S604
161604 229533.I1RSL8 pp 229533.I1S5B5
161605 229533.I1RSL8 pp 229533.I1RTE5
161606 229533.I1RSL8 pp 229533.I1S209
161607 229533.I1RSL8 pp 229533.I1RWG5
161608 229533.I1RSL8 pp 229533.I1RZM2
161609 229533.I1RSL8 pp 229533.I1RYE4
161610 229533.I1RSL8 pp 229533.I1RSP1
161611 229533.I1RSL8 pp 229533.I1RYJ8
161612 229533.I1RSL8 pp 229533.V6RXE7
161613 229533.I1RSL8 pp 229533.Q4ICA8
161614 229533.I1RSL8 pp 229533.I1S0D4
161615 229533.I1RSL8 pp 229533.I1RT35
161616 229533.I1RSL8 pp 229533.I1S6S3
161617 229533.I1RSL8 pp 229533.I1RZJ9
161618 229533.I1RSL8 pp 229533.Q5I2J3
161619 229533.I1RSL8 pp 229533.Q4HY71
161620 229533.A0A098DQN8 pp 229533.A0A1C3YKL8
161621 229533.A0A098DQN8 pp 229533.I1RZJ3
161622 229533.A0A098DQN8 pp 229533.I1RY11

229533.A0A098DQN8	pp	229533.I1RWA4
229533.A0A098DQN8	pp	229533.I1RZR5
229533.A0A098DQN8	pp	229533.I1RA51
229533.A0A098DQN8	pp	229533.I1RT96
229533.Q4I7X1	pp	229533.Q4IAS1
229533.Q4I7X1	pp	229533.Q4IMD1
229533.Q4I7X1	pp	229533.Q4IR09
229533.Q4I7X1	pp	229533.V6R227
229533.Q4I7X1	pp	229533.Q4IR18
229533.Q4I7X1	pp	229533.Q4IPA4
229533.Q4I7X1	pp	229533.Q4IPN9
229533.Q4I7X1	pp	229533.Q4IF44
229533.Q4I7X1	pp	229533.V6REZ4
229533.Q4I7X1	pp	229533.V6QV38
229533.Q4I7X1	pp	229533.Q4IPI4
229533.Q4I7X1	pp	229533.Q4IPC8
229533.I1RR74	pp	229533.I1RT65
229533.I1RR74	pp	229533.I1RYM4
229533.I1RR74	pp	229533.I1RYP0
229533.I1RR74	pp	229533.I1S0S8
229533.I1RR74	pp	229533.I1RWD5
229533.I1RR74	pp	229533.V6QWP4
229533.I1RR74	pp	229533.I1S0Z3
229533.I1RR74	pp	229533.I1RX87
229533.I1RR74	pp	229533.I1RRF0
229533.I1RR74	pp	229533.Q4I2D7
229533.I1RR74	pp	229533.Q4ICM0
229533.I1RR74	pp	229533.I1RTC7
229533.I1RR74	pp	229533.I1RST4
229533.I1RR74	pp	229533.I1RUJ0
229533.I1RR74	pp	229533.I1S522
229533.I1RR74	pp	229533.I1RZA5
229533.I1RR74	pp	229533.I1S270
229533.I1RR74	pp	229533.Q4INI2
229533.I1RR74	pp	229533.Q4IPI4
229533.I1RR74	pp	229533.I1RT41
229533.A0A1C3YL32	pp	229533.I1S0M8
229533.A0A1C3YL32	pp	229533.I1RB00
229533.I1RTE5	pp	229533.Q4IE79
229533.I1RTE5	pp	229533.I1S285
229533.I1RTE5	pp	229533.I1RYT7
229533.I1RTE5	pp	229533.I1RY40
229533.I1RTE5	pp	229533.I1S2F3
229533.I1RTE5	pp	229533.I1S0P4
229533.I1RTE5	pp	229533.Q4IPN9
229533.I1RTE5	pp	229533.I1S246
229533.I1RTE5	pp	229533.Q4IAA0
229533.I1RTE5	pp	229533.I1RYJ8
229533.I1RTE5	pp	229533.I1RZV9
229533.I1RTE5	pp	229533.I1RZT1
229533.I1RTE5	pp	229533.V6REZ4
229533.I1RTE5	pp	229533.I1S2A8
229533.I1RTE5	pp	229533.I1S5B5
229533.I1RRK9	pp	229533.I1RS29
229533.I1RRK9	pp	229533.I1S1F3
229533.I1RRK9	pp	229533.I1RT96
229533.I1RRK9	pp	229533.I1S0K2
229533.I1RRK9	pp	229533.I1RY21
229533.I1RRK9	pp	229533.V6RJP8
229533.I1RRK9	pp	229533.I1RY92
229533.I1RRK9	pp	229533.I1RYT8
229533.I1RRK9	pp	229533.I1RZJ3
229533.I1RRK9	pp	229533.I1RS05
229533.I1RRK9	pp	229533.I1S1K6
229533.I1RRK9	pp	229533.I1S5X9
229533.I1RRK9	pp	229533.I1S0R8
229533.I1RRK9	pp	229533.I1RW01
229533.I1RRK9	pp	229533.I1S0Q7
229533.I1RRK9	pp	229533.I1RWA3
229533.I1RRK9	pp	229533.I1S0P0
229533.I1RRK9	pp	229533.I1RZR5
229533.I1RRK9	pp	229533.I1S464
229533.I1RRK9	pp	229533.I1RXD9

229533.I1RRK9	pp	229533.I1RY11
229533.I1RGW2	pp	229533.I1S5S8
229533.I1RGW2	pp	229533.I1S9U6
229533.I1RGW2	pp	229533.I1RNH4
229533.I1RGW2	pp	229533.I1RRM1
229533.I1RGW2	pp	229533.I1RPQ2
229533.I1RGW2	pp	229533.I1RJT5
229533.I1RX47	pp	229533.Q4I7F9
229533.I1RX47	pp	229533.I1RZQ5
229533.I1RX47	pp	229533.I1S8V0
229533.I1RX47	pp	229533.Q4IPH4
229533.I1RX47	pp	229533.I1S9M9
229533.I1RX47	pp	229533.I1RZG7
229533.I1RX47	pp	229533.I1S0Z9
229533.I1RX47	pp	229533.I1RYJ9
229533.I1RX47	pp	229533.V6QVD5
229533.I1RX47	pp	229533.I1RYB1
229533.I1RX47	pp	229533.I1S0B1
229533.I1RX47	pp	229533.I1RX81
229533.I1RX47	pp	229533.I1RYW3
229533.I1RX47	pp	229533.I1S1X9
229533.I1RX47	pp	229533.Q4I1Y1
229533.I1RX47	pp	229533.Q4IJH1
229533.I1RX47	pp	229533.Q4HXF6
229533.I1RX47	pp	229533.I1S5Z2
229533.I1RX47	pp	229533.Q4IAA0
229533.I1RX47	pp	229533.Q4IPB3
229533.I1RX47	pp	229533.Q4IF76
229533.I1RX47	pp	229533.I1S9L0
229533.I1RX47	pp	229533.I1S9L5
229533.I1RX47	pp	229533.I1S4M5
229533.I1RX47	pp	229533.Q4IRI9
229533.I1RX47	pp	229533.I1RZL5
229533.I1RX47	pp	229533.I1S0B6
229533.I1RX47	pp	229533.I1S246
229533.I1RX47	pp	229533.Q4IBK5
229533.I1RX47	pp	229533.I1RXF2
229533.I1RX47	pp	229533.Q4IB70
229533.I1RX47	pp	229533.I1S7M2
229533.I1RX47	pp	229533.I1S2Y5
229533.I1RX47	pp	229533.I1S9L6
229533.I1RX47	pp	229533.Q4IPB8
229533.I1RX47	pp	229533.Q4IE79
229533.I1RX47	pp	229533.I1S8U9
229533.I1RX47	pp	229533.I1S7M3
229533.I1RX47	pp	229533.I1S471
229533.I1RX47	pp	229533.I1S0N7
229533.I1RX47	pp	229533.Q4IP34
229533.I1RX47	pp	229533.V6QXG1
229533.I1RX47	pp	229533.I1RX51
229533.I1RX47	pp	229533.I1S1C6
229533.I1RX47	pp	229533.Q4I5R9
229533.I1S2B7	pp	229533.I1S4P4
229533.I1RCB2	pp	229533.I1RRC6
229533.I1RCB2	pp	229533.I1RQ41
229533.I1RCB2	pp	229533.I1RGS6
229533.I1RCB2	pp	229533.I1RXF0
229533.I1RCB2	pp	229533.I1RYL5
229533.I1RCB2	pp	229533.I1REK9
229533.I1RCB2	pp	229533.I1RD39
229533.I1RCB2	pp	229533.I1RX25
229533.I1RCB2	pp	229533.I1RNG2
229533.I1RA51	pp	229533.I1RPY0
229533.I1RA51	pp	229533.I1RRK9
229533.I1RA51	pp	229533.I1RXT2
229533.I1RA51	pp	229533.I1S1K6
229533.I1RA51	pp	229533.I1S8E9
229533.I1RA51	pp	229533.I1RW01
229533.I1RA51	pp	229533.I1RL16
229533.I1RA51	pp	229533.I1RT96
229533.I1RA51	pp	229533.I1RJU9
229533.I1RA51	pp	229533.I1RE53
229533.I1RA51	pp	229533.I1RZJ3

229533.I1RA51	pp	229533.I1RY11
229533.I1RA51	pp	229533.I1RVX8
229533.I1RA51	pp	229533.I1RZR5
229533.I1RA51	pp	229533.I1RLM3
229533.I1RA51	pp	229533.I1RXX1
229533.I1RYP7	pp	229533.V6REZ4
229533.I1RYP7	pp	229533.V6QUV7
229533.I1RYP7	pp	229533.I1S2H4
229533.I1RYP7	pp	229533.Q4IRI9
229533.I1RYP7	pp	229533.I1S0P4
229533.I1RYP7	pp	229533.Q4INS6
229533.I1RCT0	pp	229533.I1RYX5
229533.I1RCT0	pp	229533.I1S2B9
229533.I1RCT0	pp	229533.I1RS90
229533.I1RQF7	pp	229533.I1RZ87
229533.I1RQF7	pp	229533.I1RQP7
229533.I1RQF7	pp	229533.I1S1U6
229533.I1RQF7	pp	229533.Q4ILI9
229533.I1RQF7	pp	229533.I1RUN2
229533.I1RQF7	pp	229533.I1RR61
229533.I1RQF7	pp	229533.I1S2B6
229533.I1RQF7	pp	229533.Q4IQT8
229533.I1RQF7	pp	229533.I1S5B5
229533.I1RQF7	pp	229533.I1RUN1
229533.I1RQF7	pp	229533.I1SAM5
229533.I1RQF7	pp	229533.I1S0V5
229533.I1RQF7	pp	229533.V6RLW2
229533.I1RQF7	pp	229533.Q4I2W2
229533.I1RQF7	pp	229533.I1RQF9
229533.I1RQF7	pp	229533.I1RS34
229533.I1RQF7	pp	229533.I1RQJ5
229533.I1RQF7	pp	229533.I1RRC3
229533.I1RQF7	pp	229533.I1S287
229533.I1RQF7	pp	229533.I1S0N5
229533.I1RQF7	pp	229533.Q4HYZ2
229533.I1RQF7	pp	229533.I1RR74
229533.I1RQF7	pp	229533.I1RW76
229533.I1RQF7	pp	229533.I1S4Q7
229533.I1RQF7	pp	229533.I1RX87
229533.I1RQF7	pp	229533.I1RYP0
229533.I1RQF7	pp	229533.I1RWZ9
229533.I1RQF7	pp	229533.I1RQH7
229533.I1RQF7	pp	229533.I1RY40
229533.I1RQF7	pp	229533.I1RZK3
229533.I1RQF7	pp	229533.I1S1V4
229533.I1S6Y3	pp	229533.Q4IJU0
229533.I1RYJ8	pp	229533.I1S2A8
229533.I1RYJ8	pp	229533.I1RZZ3
229533.I1RYJ8	pp	229533.I1RYT7
229533.I1RYJ8	pp	229533.I1S0Y1
229533.I1RYJ8	pp	229533.I1S219
229533.I1RYJ8	pp	229533.I1RZE1
229533.I1RYJ8	pp	229533.I1S0P4
229533.I1RYJ8	pp	229533.I1S491
229533.I1RYJ8	pp	229533.V6REZ4
229533.I1RYJ8	pp	229533.I1S044
229533.I1RYJ8	pp	229533.I1S5B5
229533.I1RYJ8	pp	229533.I1RZV9
229533.I1RYJ8	pp	229533.V6QYF0
229533.I1RYJ8	pp	229533.Q4ICA8
229533.I1RIX1	pp	229533.I1RW57
229533.I1RIX1	pp	229533.I1S2A8
229533.I1RIX1	pp	229533.I1S092
229533.I1RIX1	pp	229533.I1RSZ1
229533.I1RIX1	pp	229533.V6RDH3
229533.I1RIX1	pp	229533.Q4IAA0
229533.I1RDY2	pp	229533.I1RWU2
229533.I1RDY2	pp	229533.I1S246
229533.I1RDY2	pp	229533.I1RKD2
229533.I1RDY2	pp	229533.I1RPW8
229533.I1RDY2	pp	229533.I1RNB8
229533.I1RDY2	pp	229533.I1RW80
229533.I1RDY2	pp	229533.Q4IAA0

161842 229533.I1RDY2 pp 229533.I1RZG7
161843 229533.I1RDY2 pp 229533.I1RS03
161844 229533.I1RDY2 pp 229533.I1RWU3
161845 229533.I1RDY2 pp 229533.I1RT35
161846 229533.I1RDY2 pp 229533.I1RQ42
161847 229533.I1RDY2 pp 229533.I1S0H1
161848 229533.I1RDY2 pp 229533.I1RX47
161849 229533.I1RDY2 pp 229533.I1S471
161850 229533.I1RDY2 pp 229533.I1RN60
161851 229533.I1RDY2 pp 229533.I1RG11
161852 229533.I1RDY2 pp 229533.I1RK72
161853 229533.I1RDY2 pp 229533.I1RG57
161854 229533.I1RDY2 pp 229533.I1S5Z2
161855 229533.I1RDY2 pp 229533.I1S0Z9
161856 229533.I1RDY2 pp 229533.I1S9M9
161857 229533.A0A1C3YKL8 pp 229533.Q4I5G1
161858 229533.A0A1C3YKL8 pp 229533.I1RXX1
161859 229533.A0A1C3YKL8 pp 229533.Q00909
161860 229533.A0A1C3YKL8 pp 229533.I1RIY0
161861 229533.A0A1C3YKL8 pp 229533.I1RCV1
161862 229533.A0A1C3YKL8 pp 229533.I1RCE6
161863 229533.A0A1C3YKL8 pp 229533.I1RRK9
161864 229533.A0A1C3YKL8 pp 229533.I1RW69
161865 229533.A0A1C3YKL8 pp 229533.V6RPN0
161866 229533.A0A1C3YKL8 pp 229533.I1S1W8
161867 229533.A0A1C3YKL8 pp 229533.I1RXB6
161868 229533.A0A1C3YKL8 pp 229533.I1RA51
161869 229533.A0A1C3YKL8 pp 229533.I1R9Y2
161870 229533.A0A1C3YKL8 pp 229533.I1RT96
161871 229533.A0A1C3YKL8 pp 229533.V6RH92
161872 229533.A0A1C3YKL8 pp 229533.V6RG22
161873 229533.A0A1C3YKL8 pp 229533.I1S5R5
161874 229533.A0A1C3YKL8 pp 229533.I1S130
161875 229533.A0A1C3YKL8 pp 229533.I1RD42
161876 229533.A0A1C3YKL8 pp 229533.I1RZ92
161877 229533.A0A1C3YKL8 pp 229533.I1RBT7
161878 229533.A0A1C3YKL8 pp 229533.I1RZ52
161879 229533.A0A1C3YKL8 pp 229533.I1RL16
161880 229533.A0A1C3YKL8 pp 229533.I1RVX8
161881 229533.A0A1C3YKL8 pp 229533.I1RPY0
161882 229533.A0A1C3YKL8 pp 229533.I1RW01
161883 229533.A0A1C3YKL8 pp 229533.A0A1C3YMK0
161884 229533.A0A1C3YKL8 pp 229533.I1RDU5
161885 229533.A0A1C3YKL8 pp 229533.I1RY35
161886 229533.A0A1C3YKL8 pp 229533.I1S3G4
161887 229533.A0A1C3YKL8 pp 229533.I1RZJ3
161888 229533.A0A1C3YKL8 pp 229533.I1RE53
161889 229533.A0A1C3YKL8 pp 229533.I1RZR5
161890 229533.A0A1C3YKL8 pp 229533.I1RZD0
161891 229533.A0A1C3YKL8 pp 229533.A0A1C3YM61
161892 229533.A0A1C3YKL8 pp 229533.I1S1K6
161893 229533.A0A1C3YKL8 pp 229533.I1RY11
161894 229533.A0A1C3YKL8 pp 229533.I1RCG7
161895 229533.A0A1C3YKL8 pp 229533.I1RXV3
161896 229533.A0A1C3YKL8 pp 229533.I1RPK0
161897 229533.A0A1C3YKL8 pp 229533.A0A1C3YMS7
161898 229533.A0A1C3YKL8 pp 229533.I1RQM9
161899 229533.I1S1V4 pp 229533.Q4IN63
161900 229533.I1S1V4 pp 229533.I1S6S4
161901 229533.I1S1V4 pp 229533.I1S2B6
161902 229533.I1S1V4 pp 229533.I1S830
161903 229533.I1S1V4 pp 229533.I1S278
161904 229533.I1S1V4 pp 229533.Q4I6S5
161905 229533.I1S1V4 pp 229533.I1SAM5
161906 229533.I1RRI2 pp 229533.Q4IBS2
161907 229533.I1RRI2 pp 229533.I1S5B0
161908 229533.I1RRI2 pp 229533.I1RW99
161909 229533.I1RRI2 pp 229533.I1RWT6
161910 229533.I1RRI2 pp 229533.I1RZM8
161911 229533.I1RRI2 pp 229533.I1RZF6
161912 229533.I1RRI2 pp 229533.I1S021
161913 229533.I1RRI2 pp 229533.V6R227
161914 229533.I1RRI2 pp 229533.I1RXF0

229533.I1RRI2 pp 229533.I1RYB9
229533.I1RRI2 pp 229533.Q4I283
229533.I1RRI2 pp 229533.I1RXC4
229533.I1RRI2 pp 229533.I1S6K9
229533.I1RRI2 pp 229533.I1RVJ6
229533.I1RRI2 pp 229533.V6RCZ8
229533.I1RRI2 pp 229533.I1RRV4
229533.I1RRI2 pp 229533.I1RX14
229533.I1RRI2 pp 229533.I1SA32
229533.I1RRI2 pp 229533.V6QUE6
229533.I1RRI2 pp 229533.I1RSR3
229533.I1RRI2 pp 229533.Q4IJ56
229533.I1RRI2 pp 229533.V6RCG5
229533.I1RRI2 pp 229533.I1RSR8
229533.I1RRI2 pp 229533.I1S419
229533.I1RRI2 pp 229533.I1RRY9
229533.I1RRI2 pp 229533.I1RT35
229533.I1RRI2 pp 229533.I1S0M9
229533.I1RRI2 pp 229533.I1RZM1
229533.I1RRI2 pp 229533.I1RY03
229533.I1RRI2 pp 229533.I1RS18
229533.I1RRI2 pp 229533.Q4ICA8
229533.I1RRI2 pp 229533.Q4I8P5
229533.I1RRI2 pp 229533.I1RTD9
229533.I1RRI2 pp 229533.I1RZB5
229533.I1RRI2 pp 229533.Q4IAA0
229533.I1RRI2 pp 229533.I1RZK5
229533.I1RRI2 pp 229533.Q4IEK8
229533.I1RRI2 pp 229533.I1S887
229533.I1RRI2 pp 229533.Q4IPI1
229533.I1RRI2 pp 229533.I1S504
229533.I1RRI2 pp 229533.I1S1J7
229533.I1RRI2 pp 229533.I1RZE5
229533.I1RRI2 pp 229533.Q4I2J8
229533.I1RRI2 pp 229533.I1S2U2
229533.I1RRI2 pp 229533.I1S0T2
229533.I1RRI2 pp 229533.Q4I5Z5
229533.I1RRI2 pp 229533.I1RVZ0
229533.I1RRI2 pp 229533.V6RTJ8
229533.I1RRI2 pp 229533.I1RX46
229533.I1RRI2 pp 229533.I1RSI0
229533.I1RRI2 pp 229533.Q4IJK2
229533.I1RRI2 pp 229533.I1RZK6
229533.I1RRI2 pp 229533.I1S7A8
229533.I1RRI2 pp 229533.I1S4Q4
229533.I1RRI2 pp 229533.I1RVQ0
229533.I1RRI2 pp 229533.I1RZZ1
229533.I1RRI2 pp 229533.Q4INZ9
229533.I1RRI2 pp 229533.I1S6F0
229533.I1RRI2 pp 229533.I1RSC6
229533.I1RRI2 pp 229533.I1S0R7
229533.I1RRI2 pp 229533.I1RY63
229533.I1RRI2 pp 229533.I1RT98
229533.I1RRI2 pp 229533.I1S0L3
229533.I1RRI2 pp 229533.I1RT33
229533.I1RRI2 pp 229533.I1RUQ2
229533.I1RRI2 pp 229533.I1S0D4
229533.I1RRI2 pp 229533.Q4I5R9
229533.I1RRI2 pp 229533.V6RUT5
229533.I1RRI2 pp 229533.I1S5R6
229533.I1RRI2 pp 229533.I1RXD7
229533.I1RRI2 pp 229533.Q4IJT5
229533.I1RRI2 pp 229533.I1RWQ8
229533.I1RRI2 pp 229533.V6R1K4
229533.I1RRI2 pp 229533.V6RGN5
229533.I1RRI2 pp 229533.I1RWD4
229533.I1RRI2 pp 229533.I1S0N4
229533.I1RRI2 pp 229533.I1S4S7
229533.I1RRI2 pp 229533.I1RZJ5
229533.I1RRI2 pp 229533.I1S9U2
229533.I1RRI2 pp 229533.V6QW22
229533.I1RRI2 pp 229533.I1RW71
229533.I1RRI2 pp 229533.I1RT05

161988	229533.I1RRI2	pp	229533.I1S0X6
161989	229533.I1RRI2	pp	229533.I1RSQ7
161990	229533.I1RRI2	pp	229533.I1RTF4
161991	229533.I1RRI2	pp	229533.Q4IN63
161992	229533.I1RRI2	pp	229533.I1RZ99
161993	229533.I1RRI2	pp	229533.I1RUG3
161994	229533.I1RRI2	pp	229533.I1RRX5
161995	229533.I1RRI2	pp	229533.Q4I327
161996	229533.I1RRI2	pp	229533.Q4IJH1
161997	229533.I1RRI2	pp	229533.I1RSI6
161998	229533.I1RRI2	pp	229533.I1RWJ8
161999	229533.I1RRI2	pp	229533.I1RT80
162000	229533.I1RRI2	pp	229533.I1S6U8
162001	229533.I1RRI2	pp	229533.I1RRV1
162002	229533.I1RRI2	pp	229533.I1S4S4
162003	229533.I1RRI2	pp	229533.V6RXB3
162004	229533.I1RRI2	pp	229533.Q4IQK7
162005	229533.I1RRI2	pp	229533.V6RGY0
162006	229533.I1RRI2	pp	229533.Q4HVW2
162007	229533.I1RRI2	pp	229533.V6QYF0
162008	229533.I1RRI2	pp	229533.I1RRQ6
162009	229533.I1RRI2	pp	229533.I1RSQ3
162010	229533.I1RRI2	pp	229533.Q4IFI0
162011	229533.I1RRI2	pp	229533.I1RW25
162012	229533.I1RRI2	pp	229533.I1SA33
162013	229533.I1RRI2	pp	229533.I1RSK1
162014	229533.I1RRI2	pp	229533.I1RX25
162015	229533.I1RRI2	pp	229533.V6R042
162016	229533.I1RRI2	pp	229533.V6RMW6
162017	229533.I1RRI2	pp	229533.I1S256
162018	229533.I1RRI2	pp	229533.I1RSU2
162019	229533.I1RRI2	pp	229533.I1S274
162020	229533.I1RRI2	pp	229533.I1RRN2
162021	229533.I1RRI2	pp	229533.Q4I624
162022	229533.I1RRI2	pp	229533.I1RXU9
162023	229533.I1RRI2	pp	229533.I1S016
162024	229533.I1RRI2	pp	229533.V6RGY3
162025	229533.I1RRI2	pp	229533.Q4HZ68
162026	229533.I1RRI2	pp	229533.I1RWN5
162027	229533.I1RRI2	pp	229533.I1S1U7
162028	229533.I1RRI2	pp	229533.V6RIV9
162029	229533.I1RRI2	pp	229533.V6R057
162030	229533.I1RRI2	pp	229533.I1S1W9
162031	229533.I1RRI2	pp	229533.I1S296
162032	229533.I1RRI2	pp	229533.I1S1C7
162033	229533.I1RRI2	pp	229533.I1S4T3
162034	229533.I1RRI2	pp	229533.I1RWN6
162035	229533.I1RRI2	pp	229533.I1RTD7
162036	229533.I1RRI2	pp	229533.I1RS64
162037	229533.I1RRI2	pp	229533.V6QVH8
162038	229533.I1RRI2	pp	229533.I1RSD8
162039	229533.I1RRI2	pp	229533.I1RYW3
162040	229533.I1RRI2	pp	229533.I1RSS4
162041	229533.I1RRI2	pp	229533.I1S1W3
162042	229533.I1RRI2	pp	229533.I1S2E8
162043	229533.I1RRI2	pp	229533.I1RY44
162044	229533.I1RRI2	pp	229533.I1RZW8
162045	229533.I1RRI2	pp	229533.I1S8V4
162046	229533.I1RRI2	pp	229533.I1RXR1
162047	229533.I1RRI2	pp	229533.I1RWX5
162048	229533.I1RRI2	pp	229533.I1RT03
162049	229533.I1RRI2	pp	229533.I1RRR7
162050	229533.I1RRI2	pp	229533.I1RRN5
162051	229533.I1RRI2	pp	229533.I1RTH6
162052	229533.I1RRI2	pp	229533.I1RVX0
162053	229533.I1RRI2	pp	229533.I1S1X9
162054	229533.I1RRI2	pp	229533.I1S8R3
162055	229533.I1RRI2	pp	229533.I1RTA0
162056	229533.I1RRI2	pp	229533.Q4HZK7
162057	229533.I1RRI2	pp	229533.I1RSY2
162058	229533.I1RRI2	pp	229533.I1S060
162059	229533.I1RRI2	pp	229533.I1RTE9
162060	229533.I1RRI2	pp	229533.I1RW46

162061	229533.I1RRI2	pp	229533.Q4IAS1
162062	229533.I1RRI2	pp	229533.I1S0J1
162063	229533.I1RRI2	pp	229533.I1S503
162064	229533.I1RRI2	pp	229533.I1S1J6
162065	229533.I1RRI2	pp	229533.I1RS17
162066	229533.I1RRI2	pp	229533.I1S2C0
162067	229533.I1RRI2	pp	229533.V6RLM7
162068	229533.I1RRI2	pp	229533.I1RRZ0
162069	229533.I1RRI2	pp	229533.V6RRH2
162070	229533.I1RRI2	pp	229533.V6R3J1
162071	229533.I1RRI2	pp	229533.I1RSX0
162072	229533.I1RRI2	pp	229533.I1RRT2
162073	229533.I1RRI2	pp	229533.I1S5R1
162074	229533.I1RRI2	pp	229533.Q4I8X0
162075	229533.I1RRI2	pp	229533.Q4HZ42
162076	229533.I1RRI2	pp	229533.O74638
162077	229533.I1RRI2	pp	229533.I1RXC2
162078	229533.I1RRI2	pp	229533.I1RS47
162079	229533.I1RRI2	pp	229533.I1S1B6
162080	229533.I1RRI2	pp	229533.Q4IEV5
162081	229533.I1RRI2	pp	229533.I1RS20
162082	229533.I1RRI2	pp	229533.I1RZB2
162083	229533.I1RRI2	pp	229533.V6R283
162084	229533.I1RRI2	pp	229533.Q4IQW0
162085	229533.I1RRI2	pp	229533.I1S0L4
162086	229533.I1RRI2	pp	229533.I1RT40
162087	229533.I1RRI2	pp	229533.I1S2H4
162088	229533.I1RRI2	pp	229533.I1RWK3
162089	229533.I1RRI2	pp	229533.I1RV91
162090	229533.I1RRI2	pp	229533.I1RXF7
162091	229533.I1RRI2	pp	229533.I1RTD5
162092	229533.I1RRI2	pp	229533.Q4IF76
162093	229533.I1RRI2	pp	229533.I1S2M6
162094	229533.I1RRI2	pp	229533.I1RVZ2
162095	229533.I1RRI2	pp	229533.I1RWI5
162096	229533.I1RRI2	pp	229533.I1RZZ9
162097	229533.I1RRI2	pp	229533.I1RTA1
162098	229533.I1RRI2	pp	229533.Q4IR18
162099	229533.I1RRI2	pp	229533.Q4HXF0
162100	229533.I1RRI2	pp	229533.I1RSQ4
162101	229533.I1RRI2	pp	229533.I1RT04
162102	229533.I1RRI2	pp	229533.Q4I830
162103	229533.I1RRI2	pp	229533.I1RZ29
162104	229533.I1RRI2	pp	229533.V6RUY9
162105	229533.I1RRI2	pp	229533.I1S8V3
162106	229533.I1RRI2	pp	229533.I1RSX1
162107	229533.I1RRI2	pp	229533.I1RRJ8
162108	229533.I1RRI2	pp	229533.I1RTG7
162109	229533.I1RRI2	pp	229533.I1S8P5
162110	229533.I1RRI2	pp	229533.I1S2E9
162111	229533.I1RRI2	pp	229533.I1RYI5
162112	229533.I1RRI2	pp	229533.I1RZ39
162113	229533.I1RRI2	pp	229533.I1S0F6
162114	229533.I1RRI2	pp	229533.I1S8E2
162115	229533.I1RRI2	pp	229533.V6R3X5
162116	229533.I1RRI2	pp	229533.I1RS30
162117	229533.I1RRI2	pp	229533.I1S9Y1
162118	229533.I1RRI2	pp	229533.I1RRP3
162119	229533.I1RRI2	pp	229533.I1RWF7
162120	229533.I1RRI2	pp	229533.I1S2B8
162121	229533.I1RRI2	pp	229533.I1RY62
162122	229533.I1RRI2	pp	229533.I1S253
162123	229533.I1RRI2	pp	229533.I1RRQ3
162124	229533.I1RRI2	pp	229533.I1RZV6
162125	229533.I1RRI2	pp	229533.I1RRM5
162126	229533.I1RRI2	pp	229533.I1RSZ7
162127	229533.I1RRI2	pp	229533.I1RW61
162128	229533.I1RRI2	pp	229533.Q4I662
162129	229533.I1RRI2	pp	229533.I1S6Y8
162130	229533.I1RRI2	pp	229533.I1RYT5
162131	229533.I1RRI2	pp	229533.I1RZS6
162132	229533.I1RRI2	pp	229533.I1RX24
162133	229533.I1RRH3	pp	229533.I1RY50

162134 229533.I1RRH3 pp 229533.I1S4P4
162135 229533.I1RRH3 pp 229533.I1S2B7
162136 229533.I1RRH3 pp 229533.I1S9L0
162137 229533.I1RRH3 pp 229533.I1S044
162138 229533.I1RRH3 pp 229533.I1RXB5
162139 229533.I1RRH3 pp 229533.I1RYW2
162140 229533.I1RRH3 pp 229533.I1S1C6
162141 229533.I1RZS5 pp 229533.I1S0V5
162142 229533.I1RZS5 pp 229533.I1S7T4
162143 229533.I1RZS5 pp 229533.I1S1V4
162144 229533.I1RZS5 pp 229533.I1S0Y1
162145 229533.I1RZS5 pp 229533.I1SAM5
162146 229533.I1RZS5 pp 229533.Q4ILI9
162147 229533.I1RZS5 pp 229533.I1S278
162148 229533.I1RZS5 pp 229533.I1S2B6
162149 229533.I1RW01 pp 229533.I1RY11
162150 229533.I1RW01 pp 229533.I1RZR5
162151 229533.I1RW01 pp 229533.I1RZJ3
162152 229533.I1RW01 pp 229533.V6RM66
162153 229533.I1RW01 pp 229533.I1S1K6
162154 229533.I1RW01 pp 229533.I1RWN3
162155 229533.I1RW01 pp 229533.V6RLW2
162156 229533.I1RW01 pp 229533.I1RZT4
162157 229533.I1S0P4 pp 229533.Q4IAS1
162158 229533.I1S0P4 pp 229533.V6R3J1
162159 229533.I1S0P4 pp 229533.I1S1V7
162160 229533.I1S0P4 pp 229533.I1S471
162161 229533.I1S0P4 pp 229533.I1S0X5
162162 229533.I1S0P4 pp 229533.I1S214
162163 229533.I1S0P4 pp 229533.Q4IPA4
162164 229533.I1S0P4 pp 229533.Q4HVW2
162165 229533.I1S0P4 pp 229533.I1S246
162166 229533.I1S0P4 pp 229533.V6REZ4
162167 229533.I1S0P4 pp 229533.I1S2A8
162168 229533.I1S0P4 pp 229533.Q4HY71
162169 229533.I1S0P4 pp 229533.V6R0U9
162170 229533.I1S0P4 pp 229533.Q4HZ68
162171 229533.I1S0P4 pp 229533.Q4I5I4
162172 229533.I1S0P4 pp 229533.Q4IPN9
162173 229533.I1S0P4 pp 229533.Q4IMN9
162174 229533.I1S0P4 pp 229533.Q4IAA0
162175 229533.I1S0P4 pp 229533.I1S5Z2
162176 229533.I1S0P4 pp 229533.Q4I7X1
162177 229533.I1S0P4 pp 229533.I1S5B5
162178 229533.A0A0E0SDU6 pp 229533.A0A0E0SKH0
162179 229533.A0A0E0SDU6 pp 229533.I1RE18
162180 229533.A0A0E0SDU6 pp 229533.I1RPG2
162181 229533.A0A0E0SDU6 pp 229533.I1RQH7
162182 229533.A0A0E0SDU6 pp 229533.A0A0E0SGH2
162183 229533.A0A0E0SDU6 pp 229533.I1RMU9
162184 229533.A0A0E0SDU6 pp 229533.I1RBS4
162185 229533.A0A0E0SDU6 pp 229533.I1RJS5
162186 229533.A0A0E0SDU6 pp 229533.I1RBF2
162187 229533.A0A0E0SDU6 pp 229533.I1RND7
162188 229533.A0A0E0SDU6 pp 229533.I1S1V4
162189 229533.A0A0E0SDU6 pp 229533.I1RJS4
162190 229533.A0A0E0SDU6 pp 229533.I1RYW8
162191 229533.A0A0E0SDU6 pp 229533.I1SAM5
162192 229533.A0A0E0SDU6 pp 229533.I1RJZ6
162193 229533.A0A0E0SDU6 pp 229533.A0A1C3YIH8
162194 229533.A0A0E0SDU6 pp 229533.I1RNA3
162195 229533.A0A0E0SDU6 pp 229533.I1RMF2
162196 229533.A0A0E0SDU6 pp 229533.I1RNH7
162197 229533.A0A0E0SDU6 pp 229533.I1RMJ1
162198 229533.A0A0E0SDU6 pp 229533.I1RCN6
162199 229533.A0A0E0SDU6 pp 229533.I1RFK0
162200 229533.A0A0E0SDU6 pp 229533.I1RQF7
162201 229533.A0A0E0SDU6 pp 229533.I1RZ41
162202 229533.A0A0E0SDU6 pp 229533.A0A1C3YID3
162203 229533.A0A0E0SDU6 pp 229533.A0A1C3YIE3
162204 229533.A0A0E0SDU6 pp 229533.I1RAH7
162205 229533.A0A0E0SDU6 pp 229533.I1RZK3
162206 229533.A0A0E0SDU6 pp 229533.I1RFV4

162207 229533.A0A0E0SDU6 pp 229533.I1RCL9
162208 229533.A0A0E0SDU6 pp 229533.I1RG99
162209 229533.A0A0E0SDU6 pp 229533.I1RCH2
162210 229533.A0A0E0SDU6 pp 229533.I1S5B5
162211 229533.A0A0E0SDU6 pp 229533.I1S287
162212 229533.A0A0E0SDU6 pp 229533.I1RA98
162213 229533.A0A0E0SDU6 pp 229533.A0A1C3YKU7
162214 229533.A0A0E0SDU6 pp 229533.I1RW58
162215 229533.A0A0E0SDU6 pp 229533.A0A1C3YL89
162216 229533.A0A0E0SDU6 pp 229533.I1S2B6
162217 229533.A0A0E0SDU6 pp 229533.I1RW76
162218 229533.A0A0E0SDU6 pp 229533.Q4ILI9
162219 229533.A0A0E0SDU6 pp 229533.I1RQF9
162220 229533.A0A0E0SDU6 pp 229533.I1S830
162221 229533.A0A0E0SDU6 pp 229533.Q4IN63
162222 229533.A0A0E0SDU6 pp 229533.I1RIG4
162223 229533.A0A0E0SDU6 pp 229533.A0A1C3YKT4
162224 229533.A0A0E0SDU6 pp 229533.I1RCQ0
162225 229533.I1RT35 pp 229533.Q4HWE2
162226 229533.I1RT35 pp 229533.V6REZ4
162227 229533.I1RT35 pp 229533.Q4I7F9
162228 229533.I1RT35 pp 229533.I1S1X9
162229 229533.I1RT35 pp 229533.I1RW23
162230 229533.I1RT35 pp 229533.I1S9M9
162231 229533.I1RT35 pp 229533.I1S8V0
162232 229533.I1RT35 pp 229533.I1S0B1
162233 229533.I1RT35 pp 229533.I1RZ59
162234 229533.I1RT35 pp 229533.I1RZU4
162235 229533.I1RT35 pp 229533.Q4IJU0
162236 229533.I1RT35 pp 229533.Q4I5U9
162237 229533.I1RT35 pp 229533.I1RT99
162238 229533.I1RT35 pp 229533.I1RYJ9
162239 229533.I1RT35 pp 229533.V6QXG1
162240 229533.I1RT35 pp 229533.I1S0P4
162241 229533.I1RT35 pp 229533.I1RWN6
162242 229533.I1RT35 pp 229533.I1S276
162243 229533.I1RT35 pp 229533.I1S5B5
162244 229533.I1RT35 pp 229533.I1S6F1
162245 229533.I1RT35 pp 229533.I1RWX8
162246 229533.I1RT35 pp 229533.I1S2C4
162247 229533.I1RT35 pp 229533.I1RZB5
162248 229533.I1RT35 pp 229533.Q4I7X1
162249 229533.I1RT35 pp 229533.I1S5Z2
162250 229533.I1RT35 pp 229533.I1RZW8
162251 229533.I1RT35 pp 229533.I1RZV9
162252 229533.I1RT35 pp 229533.I1RTH6
162253 229533.I1RT35 pp 229533.Q4I7K4
162254 229533.I1RT35 pp 229533.I1S8U9
162255 229533.I1RT35 pp 229533.I1RWD3
162256 229533.I1RT35 pp 229533.I1S7M3
162257 229533.I1RT35 pp 229533.I1RX47
162258 229533.I1RT35 pp 229533.Q4IAA0
162259 229533.I1RT35 pp 229533.I1RZL5
162260 229533.I1RT35 pp 229533.Q4IP34
162261 229533.I1RT35 pp 229533.I1RZS7
162262 229533.I1RT35 pp 229533.I1RX81
162263 229533.I1RT35 pp 229533.I1RX14
162264 229533.I1RT35 pp 229533.I1RX24
162265 229533.I1RT35 pp 229533.Q4IPC8
162266 229533.I1RT35 pp 229533.Q4HYB8
162267 229533.I1RT35 pp 229533.V6RIS2
162268 229533.I1RT35 pp 229533.I1RYU4
162269 229533.I1RT35 pp 229533.I1S524
162270 229533.I1RT35 pp 229533.I1S471
162271 229533.I1RT35 pp 229533.I1RYJ8
162272 229533.I1RT35 pp 229533.I1RZG7
162273 229533.I1RT35 pp 229533.I1RTE5
162274 229533.I1RT35 pp 229533.I1S9L6
162275 229533.I1RT35 pp 229533.I1RZ11
162276 229533.I1RT35 pp 229533.I1RTS2
162277 229533.I1RT35 pp 229533.Q4I099
162278 229533.I1RT35 pp 229533.I1RVZ4
162279 229533.I1RT35 pp 229533.Q4I5I4

162280 229533.I1RT35 pp 229533.I1S0X5
162281 229533.I1RT35 pp 229533.I1S214
162282 229533.I1RT35 pp 229533.I1RVJ1
162283 229533.I1RT35 pp 229533.V6QUE6
162284 229533.I1RT35 pp 229533.I1S8U8
162285 229533.I1RT35 pp 229533.I1S0R5
162286 229533.I1RT35 pp 229533.I1S5S2
162287 229533.I1RT35 pp 229533.I1S7M2
162288 229533.I1RT35 pp 229533.I1RT47
162289 229533.I1RT35 pp 229533.I1RW90
162290 229533.I1RT35 pp 229533.Q4IJ11
162291 229533.I1RT35 pp 229533.I1S2A8
162292 229533.I1RT35 pp 229533.Q4IPN9
162293 229533.I1RT35 pp 229533.I1RWL3
162294 229533.I1RT35 pp 229533.I1RUG0
162295 229533.I1RT35 pp 229533.V6QWX8
162296 229533.I1RT35 pp 229533.Q4IR09
162297 229533.I1RT35 pp 229533.I1RXF2
162298 229533.I1RT35 pp 229533.I1S9L5
162299 229533.I1RT35 pp 229533.I1S0B6
162300 229533.I1RT35 pp 229533.I1RXU7
162301 229533.I1RT35 pp 229533.Q4IF44
162302 229533.I1RT35 pp 229533.I1RYI9
162303 229533.I1RT35 pp 229533.Q4IQW0
162304 229533.I1RT35 pp 229533.I1RYX9
162305 229533.I1RT35 pp 229533.Q4IAS1
162306 229533.I1RT35 pp 229533.I1RUM5
162307 229533.I1RT35 pp 229533.V6RTJ8
162308 229533.I1RT35 pp 229533.I1RTC2
162309 229533.I1RT35 pp 229533.I1S2Y5
162310 229533.I1RT35 pp 229533.I1RYT7
162311 229533.I1RT35 pp 229533.I1S9Y0
162312 229533.I1RT35 pp 229533.Q4HYQ4
162313 229533.I1RT35 pp 229533.I1S0N7
162314 229533.I1RT35 pp 229533.Q4I5W5
162315 229533.I1RT35 pp 229533.I1S246
162316 229533.I1RCZ1 pp 229533.I1REC0
162317 229533.I1RDE0 pp 229533.I1RJV8
162318 229533.I1RDE0 pp 229533.I1RRM8
162319 229533.I1RDE0 pp 229533.I1RKK2
162320 229533.I1RDE0 pp 229533.I1RMD3
162321 229533.I1RDE0 pp 229533.V6RY56
162322 229533.A0A098D8F4 pp 229533.A0A0E0SP71
162323 229533.A0A0E0RV13 pp 229533.I1RIX1
162324 229533.A0A0E0RV13 pp 229533.I1RE19
162325 229533.A0A0E0RV13 pp 229533.I1RCI5
162326 229533.A0A0E0RV13 pp 229533.I1S285
162327 229533.A0A0E0RV13 pp 229533.I1RMP2
162328 229533.A0A0E0RV13 pp 229533.I1RTA3
162329 229533.A0A0E0RV13 pp 229533.A0A1C3YMF9
162330 229533.A0A0E0RV13 pp 229533.I1RCZ1
162331 229533.I1S471 pp 229533.Q4HVW2
162332 229533.I1S471 pp 229533.I1S472
162333 229533.I1S471 pp 229533.Q4IPH4
162334 229533.I1S471 pp 229533.Q4IJH1
162335 229533.I1S471 pp 229533.Q4I662
162336 229533.I1S471 pp 229533.Q4IFI0
162337 229533.I1S471 pp 229533.I1S5S2
162338 229533.I1S471 pp 229533.I1S4M5
162339 229533.I1S471 pp 229533.Q4HZ68
162340 229533.I1S471 pp 229533.Q4HW67
162341 229533.I1S471 pp 229533.Q4IF76
162342 229533.I1S471 pp 229533.V6REZ4
162343 229533.I1S471 pp 229533.I1S5B5
162344 229533.I1S471 pp 229533.I1S854
162345 229533.I1S471 pp 229533.I1S6F0
162346 229533.I1S471 pp 229533.Q4I830
162347 229533.I1S471 pp 229533.I1S7M2
162348 229533.I1S471 pp 229533.I1S855
162349 229533.I1S471 pp 229533.Q4I7F9
162350 229533.I1S471 pp 229533.Q4I1Y1
162351 229533.I1S471 pp 229533.Q4HZ42
162352 229533.I1S471 pp 229533.Q4IAS1

162353 229533.I1S471 pp 229533.I1S9M9
162354 229533.I1S471 pp 229533.Q4I7K4
162355 229533.I1S471 pp 229533.Q4HXF6
162356 229533.I1S471 pp 229533.V6QXG1
162357 229533.I1S471 pp 229533.Q4IB70
162358 229533.I1S471 pp 229533.Q4IPB8
162359 229533.I1S471 pp 229533.Q4IBS2
162360 229533.I1S471 pp 229533.I1S6F1
162361 229533.I1S471 pp 229533.Q4IJ56
162362 229533.I1S471 pp 229533.I1S7M3
162363 229533.I1S471 pp 229533.Q4IAA0
162364 229533.I1S471 pp 229533.Q4IP34
162365 229533.I1S471 pp 229533.Q4IEK8
162366 229533.I1RMY6 pp 229533.I1RX39
162367 229533.I1RT96 pp 229533.V6R3J1
162368 229533.I1RT96 pp 229533.I1S8H1
162369 229533.I1RT96 pp 229533.I1S2I9
162370 229533.I1RT96 pp 229533.I1RW01
162371 229533.I1RT96 pp 229533.I1S1F3
162372 229533.I1RT96 pp 229533.I1RZJ3
162373 229533.I1RT96 pp 229533.I1RY11
162374 229533.I1RT96 pp 229533.V6RLW2
162375 229533.I1RT96 pp 229533.Q4HYQ4
162376 229533.I1RT96 pp 229533.I1RZR5
162377 229533.A0A098DMM9 pp 229533.I1RSH4
162378 229533.A0A098DMM9 pp 229533.A0A1C3YM79
162379 229533.A0A098DMM9 pp 229533.I1RSA4
162380 229533.A0A098DMM9 pp 229533.I1RWX8
162381 229533.A0A098DMM9 pp 229533.I1RT35
162382 229533.A0A098DMM9 pp 229533.V6QXG1
162383 229533.A0A098DMM9 pp 229533.A0A1C3YJ42
162384 229533.A0A098DMM9 pp 229533.A0A1C3YJW0
162385 229533.A0A098DMM9 pp 229533.V6RMW6
162386 229533.A0A098DMM9 pp 229533.I1S8U9
162387 229533.A0A098DMM9 pp 229533.Q4IAA0
162388 229533.A0A098DMM9 pp 229533.I1S7M3
162389 229533.A0A098DMM9 pp 229533.I1RX47
162390 229533.A0A098DMM9 pp 229533.I1RN28
162391 229533.A0A098DMM9 pp 229533.I1RM25
162392 229533.A0A098DMM9 pp 229533.I1RSA8
162393 229533.A0A098DMM9 pp 229533.Q4IP34
162394 229533.A0A098DMM9 pp 229533.I1RSH9
162395 229533.A0A098DMM9 pp 229533.I1RNB8
162396 229533.A0A098DMM9 pp 229533.I1S471
162397 229533.A0A098DMM9 pp 229533.A0A1C3YHK3
162398 229533.A0A098DMM9 pp 229533.I1RSD4
162399 229533.A0A098DMM9 pp 229533.I1RK72
162400 229533.A0A098DMM9 pp 229533.I1RRL0
162401 229533.A0A098DMM9 pp 229533.V6RY33
162402 229533.A0A098DMM9 pp 229533.I1RQD8
162403 229533.A0A098DMM9 pp 229533.I1RT47
162404 229533.A0A098DMM9 pp 229533.I1RN09
162405 229533.A0A098DMM9 pp 229533.I1S1X9
162406 229533.A0A098DMM9 pp 229533.I1RG57
162407 229533.A0A098DMM9 pp 229533.I1RQZ2
162408 229533.A0A098DMM9 pp 229533.I1RDX6
162409 229533.A0A098DMM9 pp 229533.A0A0E0S8Y8
162410 229533.A0A098DMM9 pp 229533.I1RK90
162411 229533.A0A098DMM9 pp 229533.A0A1C3YHW3
162412 229533.A0A098DMM9 pp 229533.I1REF5
162413 229533.A0A098DMM9 pp 229533.I1RBZ8
162414 229533.A0A098DMM9 pp 229533.A0A1C3YHW5
162415 229533.A0A098DMM9 pp 229533.A0A1C3YKM6
162416 229533.A0A098DMM9 pp 229533.I1S0N7
162417 229533.A0A098DMM9 pp 229533.Q4I7F9
162418 229533.A0A098DMM9 pp 229533.I1RZL5
162419 229533.A0A098DMM9 pp 229533.I1RPA0
162420 229533.A0A098DMM9 pp 229533.I1RK92
162421 229533.A0A098DMM9 pp 229533.A0A1C3YJV9
162422 229533.A0A098DMM9 pp 229533.I1RE31
162423 229533.A0A098DMM9 pp 229533.I1S9M9
162424 229533.A0A098DMM9 pp 229533.I1S5Z2
162425 229533.A0A098DMM9 pp 229533.I1S8V0

162426 229533.A0A098DMM9 pp 229533.I1S2Z9
162427 229533.A0A098DMM9 pp 229533.I1RMH5
162428 229533.A0A098DMM9 pp 229533.I1RC84
162429 229533.A0A098DMM9 pp 229533.I1RCA7
162430 229533.A0A098DMM9 pp 229533.I1RX24
162431 229533.A0A098DMM9 pp 229533.A0A0E0S1T0
162432 229533.A0A098DMM9 pp 229533.A0A1C3YL25
162433 229533.A0A098DMM9 pp 229533.I1S0B1
162434 229533.A0A098DMM9 pp 229533.I1RK19
162435 229533.A0A098DMM9 pp 229533.I1RX81
162436 229533.A0A098DMM9 pp 229533.I1RK96
162437 229533.A0A098DMM9 pp 229533.V6QVD5
162438 229533.A0A098DMM9 pp 229533.I1S4M5
162439 229533.A0A098DMM9 pp 229533.I1RA46
162440 229533.A0A098DMM9 pp 229533.I1RYB1
162441 229533.A0A098DMM9 pp 229533.I1R9N6
162442 229533.A0A098DMM9 pp 229533.I1RAI1
162443 229533.A0A098DMM9 pp 229533.I1RDD3
162444 229533.A0A098DMM9 pp 229533.I1RCQ6
162445 229533.A0A098DMM9 pp 229533.I1RCS2
162446 229533.A0A098DMM9 pp 229533.I1RG90
162447 229533.A0A098DMM9 pp 229533.I1RYJ9
162448 229533.A0A098DMM9 pp 229533.I1RBX0
162449 229533.A0A098DMM9 pp 229533.A0A0E0RZU7
162450 229533.A0A098DMM9 pp 229533.I1RZG7
162451 229533.A0A098DMM9 pp 229533.I1S246
162452 229533.A0A098DMM9 pp 229533.A0A1C3YNC3
162453 229533.A0A098DMM9 pp 229533.I1RTS2
162454 229533.A0A098DMM9 pp 229533.I1RRU8
162455 229533.A0A098DMM9 pp 229533.I1RQK2
162456 229533.A0A098DMM9 pp 229533.I1S0Z9
162457 229533.A0A098DMM9 pp 229533.I1RCD6
162458 229533.A0A098DMM9 pp 229533.I1RD56
162459 229533.A0A098DMM9 pp 229533.I1RE45
162460 229533.A0A098DMM9 pp 229533.I1RCH7
162461 229533.A0A098DMM9 pp 229533.I1RAC1
162462 229533.A0A098DMM9 pp 229533.I1RLU5
162463 229533.A0A098DMM9 pp 229533.A0A1C3YHQ4
162464 229533.A0A098DMM9 pp 229533.A0A0E0SC46
162465 229533.A0A098DMM9 pp 229533.I1S0B6
162466 229533.A0A098DMM9 pp 229533.A0A098E0D1
162467 229533.A0A098DMM9 pp 229533.I1RCI4
162468 229533.A0A098DMM9 pp 229533.I1RE76
162469 229533.A0A098DMM9 pp 229533.I1RRW3
162470 229533.A0A098DMM9 pp 229533.A0A1C3YML2
162471 229533.A0A098DMM9 pp 229533.I1RMV5
162472 229533.A0A098DMM9 pp 229533.I1RG13
162473 229533.A0A098DMM9 pp 229533.A0A1C3YL11
162474 229533.A0A098DMM9 pp 229533.I1RS03
162475 229533.A0A098DMM9 pp 229533.I1RXF2
162476 229533.A0A098DMM9 pp 229533.A0A1C3YM64
162477 229533.A0A098DMM9 pp 229533.I1RB50
162478 229533.A0A098DMM9 pp 229533.I1S7M2
162479 229533.A0A098DMM9 pp 229533.I1RN60
162480 229533.A0A098DMM9 pp 229533.A0A0E0SQI9
162481 229533.I1RFQ9 pp 229533.I1RPW8
162482 229533.I1RFQ9 pp 229533.I1RN04
162483 229533.I1RFQ9 pp 229533.I1RW91
162484 229533.I1RFQ9 pp 229533.I1RRF7
162485 229533.I1RC18 pp 229533.I1RJT1
162486 229533.I1RC18 pp 229533.V6REE9
162487 229533.I1RC18 pp 229533.I1RN46
162488 229533.I1RC18 pp 229533.I1S439
162489 229533.I1RC18 pp 229533.I1RXD7
162490 229533.I1RC18 pp 229533.I1S217
162491 229533.I1RC18 pp 229533.I1RFI5
162492 229533.I1RC18 pp 229533.V6RJP8
162493 229533.I1RC18 pp 229533.Q4IJU0
162494 229533.I1RC18 pp 229533.I1RC95
162495 229533.I1RC18 pp 229533.I1RZK6
162496 229533.I1RC18 pp 229533.Q4IJW4
162497 229533.I1RC18 pp 229533.I1RN80
162498 229533.I1RC18 pp 229533.I1RGU0

162499	229533.I1RC18	pp	229533.I1RGB1
162500	229533.I1RC18	pp	229533.V6QV09
162501	229533.I1RC18	pp	229533.V6RTJ8
162502	229533.I1RC18	pp	229533.Q4I6B0
162503	229533.I1RC18	pp	229533.I1S1W5
162504	229533.I1RC18	pp	229533.I1RQA5
162505	229533.I1RC18	pp	229533.I1RUN2
162506	229533.I1RC18	pp	229533.I1RKB6
162507	229533.I1RC18	pp	229533.I1RJR8
162508	229533.I1RC18	pp	229533.I1S2C1
162509	229533.I1RC18	pp	229533.I1RRS8
162510	229533.I1RC18	pp	229533.I1RG57
162511	229533.I1RC18	pp	229533.I1RYT8
162512	229533.I1RC18	pp	229533.I1RY21
162513	229533.I1RC18	pp	229533.Q4I2W2
162514	229533.I1RC18	pp	229533.I1S0R9
162515	229533.I1RC18	pp	229533.I1RQ21
162516	229533.I1RC18	pp	229533.I1S020
162517	229533.I1RC18	pp	229533.I1RPA4
162518	229533.I1RC18	pp	229533.I1S039
162519	229533.I1RC18	pp	229533.I1S4P6
162520	229533.I1RC18	pp	229533.I1S5C4
162521	229533.I1RC18	pp	229533.I1RDK3
162522	229533.I1RC18	pp	229533.I1RTE6
162523	229533.I1RC18	pp	229533.I1RNJ5
162524	229533.I1RC18	pp	229533.Q4IBU4
162525	229533.I1RC18	pp	229533.I1S834
162526	229533.I1RC18	pp	229533.I1RS50
162527	229533.I1RC18	pp	229533.V6R3J1
162528	229533.I1RC18	pp	229533.I1RFS5
162529	229533.I1RC18	pp	229533.I1S5X9
162530	229533.I1RC18	pp	229533.Q4IPZ1
162531	229533.I1RC18	pp	229533.I1RSY2
162532	229533.I1RC18	pp	229533.I1S8A6
162533	229533.I1RC18	pp	229533.I1RZV5
162534	229533.I1RC18	pp	229533.V6RVG3
162535	229533.I1RC18	pp	229533.I1RJT3
162536	229533.I1RC18	pp	229533.V6QUV7
162537	229533.I1RC18	pp	229533.I1RTE0
162538	229533.I1RC18	pp	229533.I1RZH5
162539	229533.I1RC18	pp	229533.I1RCH5
162540	229533.I1RC18	pp	229533.I1REV2
162541	229533.I1RC18	pp	229533.I1RLZ4
162542	229533.I1RC18	pp	229533.I1RCD4
162543	229533.I1RC18	pp	229533.V6RB81
162544	229533.I1RC18	pp	229533.I1RGB0
162545	229533.I1RC18	pp	229533.Q4I099
162546	229533.I1RC18	pp	229533.I1RTN3
162547	229533.I1RC18	pp	229533.I1RRZ3
162548	229533.I1RC18	pp	229533.I1RY57
162549	229533.I1RC18	pp	229533.Q4I7T5
162550	229533.I1RC18	pp	229533.I1RQG4
162551	229533.I1RC18	pp	229533.I1RT38
162552	229533.I1RC18	pp	229533.I1RSQ4
162553	229533.I1RC18	pp	229533.I1S277
162554	229533.I1RC18	pp	229533.I1REV8
162555	229533.I1RC18	pp	229533.I1RKR9
162556	229533.I1RC18	pp	229533.I1S0J7
162557	229533.I1RMU1	pp	229533.Q4IAA0
162558	229533.I1RMU1	pp	229533.I1RT35
162559	229533.I1RMU1	pp	229533.I1S0Z9
162560	229533.I1RMU1	pp	229533.I1S471
162561	229533.I1RMU1	pp	229533.I1RNB8
162562	229533.I1RMU1	pp	229533.I1RQ42
162563	229533.I1RMU1	pp	229533.I1RX47
162564	229533.I1RMU1	pp	229533.I1RS03
162565	229533.I1RMU1	pp	229533.I1S5Z2
162566	229533.I1RMU1	pp	229533.I1S9M9
162567	229533.I1RMU1	pp	229533.I1RPW8
162568	229533.I1RMU1	pp	229533.I1RZG7
162569	229533.I1RMU1	pp	229533.I1RN60
162570	229533.I1RMU1	pp	229533.I1S246
162571	229533.I1RMU1	pp	229533.I1RWU2

162572	229533.I1RMU1	pp	229533.I1RW80
162573	229533.I1RU42	pp	229533.I1RW80
162574	229533.I1RU42	pp	229533.I1RW91
162575	229533.I1RU42	pp	229533.I1S182
162576	229533.I1RBZ1	pp	229533.I1RR61
162577	229533.I1RBZ1	pp	229533.I1RP20
162578	229533.I1RBZ1	pp	229533.I1RFN2
162579	229533.I1RBZ1	pp	229533.I1RQB7
162580	229533.I1RBZ1	pp	229533.I1RDA8
162581	229533.I1RBZ1	pp	229533.I1RJQ3
162582	229533.I1RBZ1	pp	229533.I1RRH5
162583	229533.I1RBZ1	pp	229533.I1S8W6
162584	229533.A0A1C3YI09	pp	229533.I1RX39
162585	229533.A0A1C3YI09	pp	229533.A0A1C3YIW9
162586	229533.A0A1C3YI09	pp	229533.A0A1C3YLB0
162587	229533.A0A1C3YJQ3	pp	229533.A0A1C3YLP6
162588	229533.A0A1C3YJQ3	pp	229533.I1RSS7
162589	229533.I1RB43	pp	229533.I1RDY5
162590	229533.I1RB43	pp	229533.I1S5B0
162591	229533.I1RB43	pp	229533.I1RYJ8
162592	229533.I1RB43	pp	229533.I1RZE1
162593	229533.I1RB43	pp	229533.I1RRI2
162594	229533.I1RB43	pp	229533.I1RSL8
162595	229533.I1RB43	pp	229533.I1RFW3
162596	229533.I1RB43	pp	229533.I1RC00
162597	229533.I1RB43	pp	229533.I1REX4
162598	229533.I1RBQ0	pp	229533.I1RDA8
162599	229533.I1RBQ0	pp	229533.I1RFH2
162600	229533.I1RBQ0	pp	229533.I1RMN2
162601	229533.I1RBQ0	pp	229533.I1RRH5
162602	229533.I1RBQ0	pp	229533.I1RQF7
162603	229533.I1RBQ0	pp	229533.I1S4Q7
162604	229533.I1RBQ0	pp	229533.I1RKD2
162605	229533.I1RVX8	pp	229533.I1S969
162606	229533.I1RVX8	pp	229533.I1RZ38
162607	229533.I1RVX8	pp	229533.I1S8D2
162608	229533.I1RVX8	pp	229533.I1S3V2
162609	229533.I1RVX8	pp	229533.I1S8D3
162610	229533.I1RVX8	pp	229533.V6QVV9
162611	229533.I1RVX8	pp	229533.I1RY11
162612	229533.I1RVX8	pp	229533.I1RYU6
162613	229533.I1RVX8	pp	229533.I1RW55
162614	229533.I1RVX8	pp	229533.I1RZX3
162615	229533.I1RVX8	pp	229533.I1RY81
162616	229533.I1RVX8	pp	229533.I1S968
162617	229533.I1RVX8	pp	229533.I1S8E9
162618	229533.I1RVX8	pp	229533.V6RWB3
162619	229533.I1RVX8	pp	229533.I1RYP5
162620	229533.I1RVX8	pp	229533.I1S0K2
162621	229533.I1RVX8	pp	229533.I1RZ52
162622	229533.I1RVX8	pp	229533.I1RZJ3
162623	229533.I1RVX8	pp	229533.I1RZ15
162624	229533.I1RL74	pp	229533.I1S402
162625	229533.I1RL74	pp	229533.I1S388
162626	229533.I1RL74	pp	229533.I1RXN9
162627	229533.I1RL74	pp	229533.I1RW06
162628	229533.I1RRE0	pp	229533.V6R223
162629	229533.I1RRE0	pp	229533.I1RWE8
162630	229533.I1RRE0	pp	229533.Q4IQW0
162631	229533.I1RRE0	pp	229533.Q4I5R9
162632	229533.I1RRE0	pp	229533.I1RT35
162633	229533.I1RRE0	pp	229533.I1S0P4
162634	229533.I1RRE0	pp	229533.I1RW24
162635	229533.I1RRE0	pp	229533.I1S4S8
162636	229533.I1RRE0	pp	229533.I1RZE1
162637	229533.I1RRE0	pp	229533.I1RSL8
162638	229533.I1RRE0	pp	229533.I1RW71
162639	229533.I1RRE0	pp	229533.I1RSB7
162640	229533.I1RRE0	pp	229533.I1RSP1
162641	229533.I1RRE0	pp	229533.I1RZV9
162642	229533.I1RRE0	pp	229533.I1RYJ8
162643	229533.I1RRE0	pp	229533.I1S2A8
162644	229533.I1RRE0	pp	229533.I1RTE9

162645 229533.A0A098DMK7 pp 229533.A0A1C3YID4
162646 229533.A0A098DMK7 pp 229533.A0A0E0S2K0
162647 229533.A0A098DMK7 pp 229533.I1RB03
162648 229533.A0A098DMK7 pp 229533.A0A1C3YLB0
162649 229533.A0A098DMK7 pp 229533.I1SAK2
162650 229533.A0A098DMK7 pp 229533.I1RB77
162651 229533.A0A098DMK7 pp 229533.A0A1C3YIW9
162652 229533.A0A098DMK7 pp 229533.I1RX39
162653 229533.I1RJ35 pp 229533.I1RS90
162654 229533.I1RJ35 pp 229533.I1RYX5
162655 229533.I1RJ35 pp 229533.I1S2B9
162656 229533.A0A1C3YJ68 pp 229533.I1RW71
162657 229533.A0A1C3YJ68 pp 229533.I1S273
162658 229533.A0A1C3YJ68 pp 229533.A0A1C3YLU7
162659 229533.A0A1C3YJ68 pp 229533.V6QUE6
162660 229533.A0A1C3YJ68 pp 229533.I1RSY1
162661 229533.A0A1C3YJ68 pp 229533.I1RAB6
162662 229533.A0A1C3YJ68 pp 229533.A0A1C3YNB1
162663 229533.A0A1C3YJ68 pp 229533.I1RBS0
162664 229533.A0A1C3YJ68 pp 229533.I1RYW3
162665 229533.A0A1C3YJ68 pp 229533.I1RMJ3
162666 229533.A0A1C3YJ68 pp 229533.I1RRT8
162667 229533.A0A1C3YJ68 pp 229533.A0A1C3YLL1
162668 229533.A0A1C3YJ68 pp 229533.I1RWX5
162669 229533.I1RSR3 pp 229533.I1S060
162670 229533.I1RSR3 pp 229533.Q4HYB8
162671 229533.I1RSR3 pp 229533.I1S1J6
162672 229533.I1RSR3 pp 229533.I1SA33
162673 229533.I1RSR3 pp 229533.I1RW46
162674 229533.I1RSR3 pp 229533.I1S0J1
162675 229533.I1RSR3 pp 229533.V6RGN5
162676 229533.I1RSR3 pp 229533.V6RGY3
162677 229533.I1RSR3 pp 229533.I1S274
162678 229533.I1RSR3 pp 229533.V6R057
162679 229533.I1RSR3 pp 229533.I1S296
162680 229533.I1RSR3 pp 229533.Q4HZ68
162681 229533.I1RSR3 pp 229533.I1RUG3
162682 229533.I1RSR3 pp 229533.I1S2E8
162683 229533.I1RSR3 pp 229533.Q4I327
162684 229533.I1RSR3 pp 229533.I1S1X9
162685 229533.I1RSR3 pp 229533.I1RZW8
162686 229533.I1RSR3 pp 229533.I1S4T3
162687 229533.I1RSR3 pp 229533.I1S2C0
162688 229533.I1RSR3 pp 229533.I1RSS4
162689 229533.I1RSR3 pp 229533.I1S8R3
162690 229533.I1RSR3 pp 229533.Q4IAS1
162691 229533.I1RSR3 pp 229533.V6RIV9
162692 229533.I1RSR3 pp 229533.I1S1B6
162693 229533.I1RSR3 pp 229533.I1RTE9
162694 229533.I1RSR3 pp 229533.Q4HXF0
162695 229533.I1RSR3 pp 229533.I1S0F6
162696 229533.I1RSR3 pp 229533.I1RZM8
162697 229533.I1RSR3 pp 229533.I1RW99
162698 229533.I1RSR3 pp 229533.Q4HZ42
162699 229533.I1RSR3 pp 229533.Q4IQW0
162700 229533.I1RSR3 pp 229533.V6RCZ8
162701 229533.I1RSR3 pp 229533.I1RT40
162702 229533.I1RSR3 pp 229533.I1S021
162703 229533.I1RSR3 pp 229533.Q4IR18
162704 229533.I1RSR3 pp 229533.I1RWK3
162705 229533.I1RSR3 pp 229533.I1RXF7
162706 229533.I1RSR3 pp 229533.V6RCG5
162707 229533.I1RSR3 pp 229533.V6R283
162708 229533.I1RSR3 pp 229533.Q4IEK8
162709 229533.I1RSR3 pp 229533.I1RT35
162710 229533.I1RSR3 pp 229533.Q4IF76
162711 229533.I1RSR3 pp 229533.Q4I830
162712 229533.I1RSR3 pp 229533.I1S2A8
162713 229533.I1RSR3 pp 229533.I1S2M6
162714 229533.I1RSR3 pp 229533.I1RZF6
162715 229533.I1RSR3 pp 229533.I1RSZ7
162716 229533.I1RSR3 pp 229533.I1S2H4
162717 229533.I1RSR3 pp 229533.I1RYT5

162718	229533.I1RSR3	pp	229533.I1S5R1
162719	229533.I1RSR3	pp	229533.V6R227
162720	229533.I1RSR3	pp	229533.I1RW61
162721	229533.I1RSR3	pp	229533.I1S9U1
162722	229533.I1RSR3	pp	229533.Q4I662
162723	229533.I1RSR3	pp	229533.I1RW41
162724	229533.I1RSR3	pp	229533.I1RX24
162725	229533.I1RSR3	pp	229533.I1RX14
162726	229533.I1RSR3	pp	229533.I1S0L4
162727	229533.I1RSR3	pp	229533.I1RZS6
162728	229533.I1RSR3	pp	229533.I1RZZ9
162729	229533.I1RSR3	pp	229533.I1SA32
162730	229533.I1RSR3	pp	229533.I1S0M9
162731	229533.I1RSR3	pp	229533.I1S0R7
162732	229533.I1RSR3	pp	229533.Q4I8P5
162733	229533.I1RSR3	pp	229533.Q4IJ56
162734	229533.I1RSR3	pp	229533.Q4IBS2
162735	229533.I1RSR3	pp	229533.I1S5B0
162736	229533.I1RSR3	pp	229533.I1RWD4
162737	229533.I1RSR3	pp	229533.I1RUQ2
162738	229533.I1RSR3	pp	229533.I1RTD9
162739	229533.I1RSR3	pp	229533.I1S6F0
162740	229533.I1RSR3	pp	229533.I1RVQ0
162741	229533.I1RSR3	pp	229533.I1RSR8
162742	229533.I1RSR3	pp	229533.I1S9U2
162743	229533.I1RSR3	pp	229533.I1RVZ0
162744	229533.I1RSR3	pp	229533.I1RT05
162745	229533.I1RSR3	pp	229533.I1RT33
162746	229533.I1RSR3	pp	229533.I1S0L3
162747	229533.I1RSR3	pp	229533.I1S0F1
162748	229533.I1RSR3	pp	229533.I1RT80
162749	229533.I1RSR3	pp	229533.I1S2L2
162750	229533.I1RSR3	pp	229533.I1S0Z9
162751	229533.I1RSR3	pp	229533.I1RW71
162752	229533.I1RSR3	pp	229533.I1RTF4
162753	229533.I1RSR3	pp	229533.I1RZE5
162754	229533.I1RSR3	pp	229533.I1RZ99
162755	229533.I1RSR3	pp	229533.I1S2U2
162756	229533.I1RSR3	pp	229533.Q4HVW2
162757	229533.I1RSR3	pp	229533.I1RY63
162758	229533.I1RSR3	pp	229533.Q4IJH1
162759	229533.I1RSR3	pp	229533.I1S4S4
162760	229533.I1RSR3	pp	229533.Q4I5R9
162761	229533.I1RSR3	pp	229533.V6QW22
162762	229533.I1RSR3	pp	229533.Q4IFI0
162763	229533.I1RSR3	pp	229533.I1RWJ8
162764	229533.I1RSR3	pp	229533.V6QYF0
162765	229533.I1RSR3	pp	229533.Q4IPI1
162766	229533.I1RSR3	pp	229533.I1RW25
162767	229533.I1RYN2	pp	229533.I1S212
162768	229533.I1RYN2	pp	229533.I1S0R8
162769	229533.I1RYN2	pp	229533.I1RZ43
162770	229533.I1RYN2	pp	229533.I1S0Q7
162771	229533.I1RYN2	pp	229533.I1S8F8
162772	229533.I1RYN2	pp	229533.I1S0K2
162773	229533.I1RZL5	pp	229533.I1S0B6
162774	229533.I1RZL5	pp	229533.I1S0N7
162775	229533.I1RZL5	pp	229533.I1S0B1
162776	229533.I1RZL5	pp	229533.Q4IPA4
162777	229533.I1RZL5	pp	229533.Q4IF44
162778	229533.I1RZL5	pp	229533.I1S246
162779	229533.I1RZL5	pp	229533.I1S471
162780	229533.I1RZL5	pp	229533.I1S7M2
162781	229533.I1RZL5	pp	229533.Q4IR09
162782	229533.I1RZL5	pp	229533.I1S1U7
162783	229533.I1RZL5	pp	229533.I1S5Z2
162784	229533.I1RZL5	pp	229533.I1S7M3
162785	229533.I1RZL5	pp	229533.Q4IB70
162786	229533.I1RTQ5	pp	229533.I1RX39
162787	229533.I1S0R9	pp	229533.I1S217
162788	229533.I1S0R9	pp	229533.I1S439
162789	229533.I1S0R9	pp	229533.I1S2C1
162790	229533.I1S0R9	pp	229533.V6RTJ8

162791 229533.I1S0R9 pp 229533.Q4IJU0
162792 229533.I1S0R9 pp 229533.I1S8A6
162793 229533.I1S0R9 pp 229533.V6REE9
162794 229533.I1S0R9 pp 229533.V6QV09
162795 229533.I1S0R9 pp 229533.Q4I6B0
162796 229533.I1S0R9 pp 229533.I1S277
162797 229533.I1S0R9 pp 229533.I1S267
162798 229533.I1S0R9 pp 229533.V6RB81
162799 229533.I1S0R9 pp 229533.I1S4P6
162800 229533.I1S0R9 pp 229533.V6QUV7
162801 229533.I1S0R9 pp 229533.I1S5C4
162802 229533.I1S0R9 pp 229533.Q4I099
162803 229533.I1S0R9 pp 229533.V6RVG3
162804 229533.I1S0R9 pp 229533.I1S834
162805 229533.I1S0R9 pp 229533.Q4IBU4
162806 229533.I1S0R9 pp 229533.V6R3J1
162807 229533.Q4IF44 pp 229533.Q4IPA4
162808 229533.Q4IF44 pp 229533.Q4IPN9
162809 229533.Q4IF44 pp 229533.V6REZ4
162810 229533.Q4IF44 pp 229533.Q4IPC8
162811 229533.Q4IF44 pp 229533.Q4IR09
162812 229533.I1RDC3 pp 229533.I1RX39
162813 229533.I1RDC3 pp 229533.I1RKB7
162814 229533.I1RDC3 pp 229533.I1RG62
162815 229533.I1RDC3 pp 229533.I1S0R8
162816 229533.I1RDC3 pp 229533.I1RZ43
162817 229533.Q4I5U9 pp 229533.Q4IJU0
162818 229533.Q4I5U9 pp 229533.Q4I7N9
162819 229533.Q4I5U9 pp 229533.Q4IPC8
162820 229533.Q4I5U9 pp 229533.V6QWX8
162821 229533.Q4I5U9 pp 229533.V6QUE6
162822 229533.Q4I5U9 pp 229533.Q4I5W5
162823 229533.Q4I5U9 pp 229533.Q4IJ11
162824 229533.Q4I5U9 pp 229533.Q4IB50
162825 229533.A0A098DVI8 pp 229533.I1RC61
162826 229533.A0A098DVI8 pp 229533.I1S4D0
162827 229533.A0A098DVI8 pp 229533.I1S241
162828 229533.A0A098DVI8 pp 229533.A0A1C3YM35
162829 229533.A0A098DVI8 pp 229533.A0A1C3YHQ3
162830 229533.A0A098DVI8 pp 229533.I1RRX5
162831 229533.A0A098DVI8 pp 229533.I1RZM4
162832 229533.A0A098DVI8 pp 229533.I1RUI6
162833 229533.A0A098DVI8 pp 229533.I1RHZ5
162834 229533.A0A098DVI8 pp 229533.I1RJS0
162835 229533.A0A098DVI8 pp 229533.I1RST9
162836 229533.A0A098DVI8 pp 229533.I1RRG3
162837 229533.A0A098DVI8 pp 229533.I1RXB1
162838 229533.A0A098DVI8 pp 229533.I1RL49
162839 229533.A0A098DVI8 pp 229533.I1RMM8
162840 229533.A0A098DVI8 pp 229533.I1RYM6
162841 229533.A0A098DVI8 pp 229533.I1RXV9
162842 229533.A0A098DVI8 pp 229533.I1RQE8
162843 229533.A0A098DVI8 pp 229533.I1R9Y4
162844 229533.A0A098DVI8 pp 229533.I1RWL4
162845 229533.A0A098DVI8 pp 229533.I1RC39
162846 229533.A0A098DVI8 pp 229533.I1S8C1
162847 229533.A0A098DVI8 pp 229533.I1RT09
162848 229533.A0A098DVI8 pp 229533.I1S3L2
162849 229533.A0A098DVI8 pp 229533.I1RXS0
162850 229533.A0A098DVI8 pp 229533.I1RAG9
162851 229533.A0A098DVI8 pp 229533.A0A1C3YKZ8
162852 229533.A0A098DVI8 pp 229533.I1RYJ7
162853 229533.A0A098DVI8 pp 229533.A0A0E0S6V8
162854 229533.A0A098DVI8 pp 229533.I1RDE6
162855 229533.A0A098DVI8 pp 229533.I1RFB6
162856 229533.A0A098DVI8 pp 229533.A0A1C3YJJ2
162857 229533.A0A098DVI8 pp 229533.A0A1C3YKR5
162858 229533.A0A098DVI8 pp 229533.I1S0K0
162859 229533.A0A098DVI8 pp 229533.I1RJT5
162860 229533.A0A098DVI8 pp 229533.I1RTG4
162861 229533.A0A098DVI8 pp 229533.I1RQP2
162862 229533.A0A098DVI8 pp 229533.I1RVY2
162863 229533.A0A098DVI8 pp 229533.V6RY56

162864 229533.A0A098DVI8 pp 229533.V6RK96
162865 229533.A0A098DVI8 pp 229533.I1RA90
162866 229533.A0A098DVI8 pp 229533.I1RFD4
162867 229533.A0A098DVI8 pp 229533.I1REV6
162868 229533.A0A098DVI8 pp 229533.I1RUY6
162869 229533.A0A098DVI8 pp 229533.A0A1C3YLP6
162870 229533.A0A098DVI8 pp 229533.I1RY12
162871 229533.A0A098DVI8 pp 229533.A0A1C3YLI1
162872 229533.A0A098DVI8 pp 229533.I1RJY7
162873 229533.A0A098DVI8 pp 229533.I1RC48
162874 229533.A0A098DVI8 pp 229533.I1RCV1
162875 229533.A0A098DVI8 pp 229533.I1S0N3
162876 229533.A0A098DVI8 pp 229533.I1R982
162877 229533.A0A098DVI8 pp 229533.I1RFM6
162878 229533.A0A098DVI8 pp 229533.I1RWF5
162879 229533.A0A098DVI8 pp 229533.I1S4B7
162880 229533.A0A098DVI8 pp 229533.I1RWL8
162881 229533.A0A098DVI8 pp 229533.I1RMJ3
162882 229533.A0A098DVI8 pp 229533.I1RSK5
162883 229533.A0A098DVI8 pp 229533.I1S4B6
162884 229533.A0A098DVI8 pp 229533.V6RIE5
162885 229533.A0A098DVI8 pp 229533.I1S9C6
162886 229533.A0A098DVI8 pp 229533.I1RKB2
162887 229533.A0A098DVI8 pp 229533.A0A1C3YKN4
162888 229533.A0A098DVI8 pp 229533.I1RXW3
162889 229533.A0A098DVI8 pp 229533.V6R382
162890 229533.A0A098DVI8 pp 229533.I1RNB4
162891 229533.A0A098DVI8 pp 229533.A0A1C3YKV2
162892 229533.A0A098DVI8 pp 229533.I1RSS7
162893 229533.A0A098DVI8 pp 229533.I1RY62
162894 229533.A0A098DVI8 pp 229533.I1RBF3
162895 229533.A0A098DVI8 pp 229533.I1RJ14
162896 229533.A0A098DVI8 pp 229533.I1RD80
162897 229533.A0A098DVI8 pp 229533.A0A1C3YLE2
162898 229533.A0A098DVI8 pp 229533.I1RVR2
162899 229533.A0A098DVI8 pp 229533.V6RN69
162900 229533.A0A098DVI8 pp 229533.I1RN88
162901 229533.A0A098DVI8 pp 229533.I1RV23
162902 229533.A0A098DVI8 pp 229533.I1S256
162903 229533.A0A098DVI8 pp 229533.I1RB38
162904 229533.A0A098DVI8 pp 229533.A0A1C3YLB6
162905 229533.A0A098DVI8 pp 229533.I1RSL5
162906 229533.A0A098DVI8 pp 229533.I1RGC3
162907 229533.A0A098DVI8 pp 229533.I1RXY7
162908 229533.A0A098DVI8 pp 229533.I1RUP5
162909 229533.A0A098DVI8 pp 229533.I1S8R4
162910 229533.A0A098DVI8 pp 229533.I1RN84
162911 229533.A0A098DVI8 pp 229533.A0A1C3YI05
162912 229533.A0A098DVI8 pp 229533.I1RPZ5
162913 229533.A0A098DVI8 pp 229533.V6RM02
162914 229533.A0A098DVI8 pp 229533.I1RZ70
162915 229533.A0A098DVI8 pp 229533.I1RSX9
162916 229533.A0A098DVI8 pp 229533.I1RUR8
162917 229533.A0A098DVI8 pp 229533.V6RSF3
162918 229533.A0A098DVI8 pp 229533.I1RFF3
162919 229533.A0A098DVI8 pp 229533.A0A1C3YHU3
162920 229533.A0A098DVI8 pp 229533.A0A0E0SBC4
162921 229533.A0A098DVI8 pp 229533.I1RJV3
162922 229533.I1RBZ8 pp 229533.I1RX51
162923 229533.I1RBZ8 pp 229533.Q4IAA0
162924 229533.I1RBZ8 pp 229533.I1RQC5
162925 229533.I1RBZ8 pp 229533.I1RCA7
162926 229533.I1RBZ8 pp 229533.I1RQK2
162927 229533.I1RBZ8 pp 229533.I1RD56
162928 229533.I1RBZ8 pp 229533.Q4IRI9
162929 229533.I1RBZ8 pp 229533.Q4IPB8
162930 229533.I1RBZ8 pp 229533.I1RRW3
162931 229533.I1RBZ8 pp 229533.I1RKK4
162932 229533.I1RBZ8 pp 229533.I1RK19
162933 229533.I1RBZ8 pp 229533.I1RM25
162934 229533.I1RBZ8 pp 229533.I1RLU5
162935 229533.I1RBZ8 pp 229533.I1RFG0
162936 229533.I1RBZ8 pp 229533.I1RNB8

162937	229533.I1RBZ8	pp	229533.I1RFQ1
162938	229533.I1RBZ8	pp	229533.I1RG13
162939	229533.I1RBZ8	pp	229533.I1RS28
162940	229533.I1RBZ8	pp	229533.I1RS75
162941	229533.I1RBZ8	pp	229533.I1RSD4
162942	229533.I1RBZ8	pp	229533.I1RMY3
162943	229533.I1RBZ8	pp	229533.I1RW78
162944	229533.I1RBZ8	pp	229533.I1RSA4
162945	229533.I1RBZ8	pp	229533.I1S7M3
162946	229533.I1RBZ8	pp	229533.I1RRL0
162947	229533.I1RBZ8	pp	229533.I1RT35
162948	229533.I1RBZ8	pp	229533.I1RND5
162949	229533.I1RBZ8	pp	229533.I1RX47
162950	229533.I1RBZ8	pp	229533.I1RCS2
162951	229533.I1RBZ8	pp	229533.I1RSA8
162952	229533.I1RBZ8	pp	229533.I1RWX8
162953	229533.I1RBZ8	pp	229533.Q4IE79
162954	229533.I1RBZ8	pp	229533.I1RZS7
162955	229533.I1RBZ8	pp	229533.V6QXG1
162956	229533.I1RBZ8	pp	229533.I1RK72
162957	229533.I1RBZ8	pp	229533.I1RFQ0
162958	229533.I1RBZ8	pp	229533.Q4IBK5
162959	229533.I1RBZ8	pp	229533.V6REZ4
162960	229533.I1RBZ8	pp	229533.I1RSW2
162961	229533.I1RBZ8	pp	229533.I1S0P4
162962	229533.I1RBZ8	pp	229533.I1RZL5
162963	229533.I1RBZ8	pp	229533.V6RIS2
162964	229533.I1RBZ8	pp	229533.I1S246
162965	229533.I1RBZ8	pp	229533.Q4IP34
162966	229533.I1RBZ8	pp	229533.I1RPA0
162967	229533.I1RBZ8	pp	229533.I1RZV9
162968	229533.I1RBZ8	pp	229533.I1RIR9
162969	229533.I1RBZ8	pp	229533.I1RCI4
162970	229533.I1RBZ8	pp	229533.I1RPY7
162971	229533.I1RBZ8	pp	229533.I1S471
162972	229533.I1RBZ8	pp	229533.I1RYB1
162973	229533.I1RBZ8	pp	229533.I1S056
162974	229533.I1RBZ8	pp	229533.I1S5Z2
162975	229533.I1RBZ8	pp	229533.Q4IB70
162976	229533.I1RBZ8	pp	229533.I1RMV5
162977	229533.I1RBZ8	pp	229533.I1RG90
162978	229533.I1RBZ8	pp	229533.I1RZ11
162979	229533.I1RBZ8	pp	229533.I1RCN5
162980	229533.I1RBZ8	pp	229533.I1S6F1
162981	229533.I1RBZ8	pp	229533.I1S0N7
162982	229533.I1RBZ8	pp	229533.I1RMH5
162983	229533.I1RBZ8	pp	229533.Q4HXF6
162984	229533.I1RBZ8	pp	229533.I1S0Z9
162985	229533.I1RBZ8	pp	229533.I1RC84
162986	229533.I1RBZ8	pp	229533.I1RT47
162987	229533.I1RBZ8	pp	229533.Q4I1Y1
162988	229533.I1RBZ8	pp	229533.I1RG17
162989	229533.I1RBZ8	pp	229533.I1RG57
162990	229533.I1RBZ8	pp	229533.I1RN09
162991	229533.I1RBZ8	pp	229533.I1RX81
162992	229533.I1RBZ8	pp	229533.I1RGC2
162993	229533.I1RBZ8	pp	229533.I1RYI9
162994	229533.I1RBZ8	pp	229533.Q4IJH1
162995	229533.I1RBZ8	pp	229533.I1RQZ2
162996	229533.I1RBZ8	pp	229533.I1RQR3
162997	229533.I1RBZ8	pp	229533.I1RT91
162998	229533.I1RBZ8	pp	229533.I1RNT2
162999	229533.I1RBZ8	pp	229533.I1S7M2
163000	229533.I1RBZ8	pp	229533.I1RXF2
163001	229533.I1RBZ8	pp	229533.I1RE71
163002	229533.I1RBZ8	pp	229533.I1RN59
163003	229533.I1RBZ8	pp	229533.Q4IPB3
163004	229533.I1RBZ8	pp	229533.I1RPY4
163005	229533.I1RBZ8	pp	229533.I1RK90
163006	229533.I1RBZ8	pp	229533.I1RU69
163007	229533.I1RBZ8	pp	229533.I1RCQ6
163008	229533.I1RBZ8	pp	229533.I1RYU4
163009	229533.I1RBZ8	pp	229533.Q4IF76

163010 229533.I1RBZ8 pp 229533.I1RMW7
163011 229533.I1RBZ8 pp 229533.I1RTS2
163012 229533.I1RBZ8 pp 229533.I1RE31
163013 229533.I1RBZ8 pp 229533.I1RZG7
163014 229533.I1RBZ8 pp 229533.I1RZQ5
163015 229533.I1RBZ8 pp 229533.I1S9L0
163016 229533.I1RBZ8 pp 229533.I1RST1
163017 229533.I1S0Z1 pp 229533.I1S208
163018 229533.I1S0Z1 pp 229533.I1S8W6
163019 229533.I1S0Z1 pp 229533.I1S969
163020 229533.A0A1C3YIG7 pp 229533.I1RW71
163021 229533.A0A1C3YIG7 pp 229533.I1S273
163022 229533.A0A1C3YIG7 pp 229533.V6QUE6
163023 229533.A0A1C3YIG7 pp 229533.I1RAB6
163024 229533.A0A1C3YIG7 pp 229533.A0A1C3YLU7
163025 229533.A0A1C3YIG7 pp 229533.A0A1C3YLL1
163026 229533.A0A1C3YIG7 pp 229533.I1RBS0
163027 229533.A0A1C3YIG7 pp 229533.I1RRT8
163028 229533.A0A1C3YIG7 pp 229533.I1RMJ3
163029 229533.A0A1C3YIG7 pp 229533.A0A1C3YIR8
163030 229533.A0A1C3YIG7 pp 229533.I1RWX5
163031 229533.A0A1C3YIG7 pp 229533.I1RSY1
163032 229533.A0A1C3YIG7 pp 229533.I1RYW3
163033 229533.A0A1C3YIG7 pp 229533.A0A1C3YNB1
163034 229533.I1S1F3 pp 229533.I1S8R2
163035 229533.I1S1F3 pp 229533.I1S7W5
163036 229533.I1S1F3 pp 229533.I1S266
163037 229533.I1S1F3 pp 229533.I1S5X9
163038 229533.I1S1F3 pp 229533.I1S464
163039 229533.I1S1F3 pp 229533.I1S299
163040 229533.I1S1F3 pp 229533.V6RLM7
163041 229533.I1S1F3 pp 229533.I1S3V2
163042 229533.I1S1F3 pp 229533.I1S6Y3
163043 229533.I1S1F3 pp 229533.Q4I5G1
163044 229533.I1S1F3 pp 229533.O74638
163045 229533.I1S1F3 pp 229533.V6R9Z3
163046 229533.I1S1F3 pp 229533.I1S5U0
163047 229533.I1S1F3 pp 229533.V6RAC9
163048 229533.I1S1F3 pp 229533.I1S6P6
163049 229533.I1S1F3 pp 229533.I1S8S9
163050 229533.I1S1F3 pp 229533.V6RJP8
163051 229533.I1S1F3 pp 229533.I1S212
163052 229533.I1S1F3 pp 229533.V6R3J1
163053 229533.I1S1F3 pp 229533.I1S8F8
163054 229533.I1S1F3 pp 229533.V6RLW2
163055 229533.I1RY80 pp 229533.I1RYQ1
163056 229533.I1RY80 pp 229533.Q4HVU7
163057 229533.I1RY80 pp 229533.Q4HZQ1
163058 229533.I1RY80 pp 229533.I1S2A1
163059 229533.I1RY80 pp 229533.I1SA55
163060 229533.I1RY80 pp 229533.I1S0C2
163061 229533.I1RER8 pp 229533.I1RY49
163062 229533.I1RER8 pp 229533.I1RW43
163063 229533.I1RER8 pp 229533.I1RM26
163064 229533.I1RER8 pp 229533.V6RRM0
163065 229533.I1RER8 pp 229533.I1RVX9
163066 229533.I1RWG8 pp 229533.V6RGU1
163067 229533.I1RWG8 pp 229533.I1S0C6
163068 229533.I1RWG8 pp 229533.I1S9H8
163069 229533.I1RWG8 pp 229533.I1S182
163070 229533.I1RWG8 pp 229533.I1S5Z5
163071 229533.V6RAM6 pp 229533.V6RPS5
163072 229533.V6RAM6 pp 229533.V6RY56
163073 229533.V6RAM6 pp 229533.V6RBG5
163074 229533.V6RAM6 pp 229533.V6REA4
163075 229533.V6RAM6 pp 229533.V6RM09
163076 229533.I1RJS5 pp 229533.I1RZV9
163077 229533.I1RJS5 pp 229533.I1RRE0
163078 229533.I1RJS5 pp 229533.I1S0P4
163079 229533.I1RJS5 pp 229533.Q4IAA0
163080 229533.I1RJS5 pp 229533.I1RSP1
163081 229533.I1RJS5 pp 229533.I1RJZ6
163082 229533.I1RJS5 pp 229533.I1S2B6

163083	229533.I1RJS5	pp	229533.I1RTE5
163084	229533.I1RJS5	pp	229533.Q4I7X1
163085	229533.I1RJS5	pp	229533.I1RT35
163086	229533.I1RJS5	pp	229533.I1RQF7
163087	229533.I1RJS5	pp	229533.V6REZ4
163088	229533.I1RJS5	pp	229533.I1RYJ8
163089	229533.I1RJS5	pp	229533.Q4IPH4
163090	229533.I1RJS5	pp	229533.I1RNT2
163091	229533.I1RJS5	pp	229533.I1RNE4
163092	229533.I1RJS5	pp	229533.I1RQ80
163093	229533.I1RJS5	pp	229533.I1RTF4
163094	229533.I1RJS5	pp	229533.I1RY40
163095	229533.I1RJS5	pp	229533.I1RQH7
163096	229533.I1RJS5	pp	229533.I1S246
163097	229533.I1RJS5	pp	229533.I1RPG2
163098	229533.I1RJS5	pp	229533.I1RZT1
163099	229533.I1RJS5	pp	229533.I1RLU5
163100	229533.I1RJS5	pp	229533.Q4I5R9
163101	229533.I1RJS5	pp	229533.V6QUV7
163102	229533.I1RJS5	pp	229533.Q4HXF6
163103	229533.I1RJS5	pp	229533.I1S2F3
163104	229533.I1RJS5	pp	229533.I1RQ49
163105	229533.I1RJS5	pp	229533.I1RSL8
163106	229533.I1RJS5	pp	229533.I1S2A8
163107	229533.I1RJS5	pp	229533.I1RNB8
163108	229533.I1RJS5	pp	229533.Q4IN63
163109	229533.I1RJS5	pp	229533.I1RNH7
163110	229533.I1RJS5	pp	229533.I1RUP8
163111	229533.I1RJS5	pp	229533.I1RND7
163112	229533.I1RJS5	pp	229533.I1RW24
163113	229533.I1RJS5	pp	229533.I1SAM5
163114	229533.I1RJS5	pp	229533.I1RXT9
163115	229533.I1RJS5	pp	229533.I1RS94
163116	229533.I1RJS5	pp	229533.I1RMF2
163117	229533.I1RJS5	pp	229533.I1S471
163118	229533.I1RJS5	pp	229533.I1RZK3
163119	229533.I1RJS5	pp	229533.I1RMY3
163120	229533.I1RJS5	pp	229533.I1RYT7
163121	229533.I1RJS5	pp	229533.I1RVA7
163122	229533.I1RJS5	pp	229533.V6R3J1
163123	229533.I1RDU9	pp	229533.V6QUE6
163124	229533.I1RDU9	pp	229533.I1RWX5
163125	229533.I1RDU9	pp	229533.I1RYW3
163126	229533.I1RDU9	pp	229533.I1RMJ3
163127	229533.I1RDU9	pp	229533.I1S273
163128	229533.I1RDU9	pp	229533.I1RRT8
163129	229533.I1RDU9	pp	229533.I1RSY1
163130	229533.I1RDU9	pp	229533.I1RW71
163131	229533.I1RBM4	pp	229533.I1RX39
163132	229533.I1RBM4	pp	229533.I1SAK2
163133	229533.I1RAN0	pp	229533.I1S5B5
163134	229533.I1RAN0	pp	229533.I1S2B6
163135	229533.I1RAN0	pp	229533.I1RNE4
163136	229533.I1RAN0	pp	229533.I1RDY1
163137	229533.I1RAN0	pp	229533.I1RZT1
163138	229533.I1RAN0	pp	229533.I1RRE0
163139	229533.I1RAN0	pp	229533.I1RZV9
163140	229533.I1RAN0	pp	229533.I1RD70
163141	229533.I1RAN0	pp	229533.I1RJS5
163142	229533.I1RAN0	pp	229533.I1RRR6
163143	229533.I1RAN0	pp	229533.I1RJK7
163144	229533.I1RAN0	pp	229533.I1RDV9
163145	229533.I1RAN0	pp	229533.V6REZ4
163146	229533.I1RAN0	pp	229533.I1RIR9
163147	229533.I1RAN0	pp	229533.I1REE1
163148	229533.I1RAN0	pp	229533.I1RQS4
163149	229533.I1RAN0	pp	229533.I1RRW0
163150	229533.I1RAN0	pp	229533.I1S0Z9
163151	229533.I1RAN0	pp	229533.I1RMA4
163152	229533.I1RAN0	pp	229533.I1REL7
163153	229533.I1RAN0	pp	229533.Q4IE79
163154	229533.I1RAN0	pp	229533.I1RME5
163155	229533.I1RAN0	pp	229533.I1RYJ8

163156 229533.I1RAN0 pp 229533.I1S224
163157 229533.I1RAN0 pp 229533.I1RRX6
163158 229533.I1RAN0 pp 229533.I1RVA7
163159 229533.I1RAN0 pp 229533.I1RCK4
163160 229533.I1RAN0 pp 229533.I1RXT9
163161 229533.I1RAN0 pp 229533.I1RSL8
163162 229533.I1RAN0 pp 229533.V6R3J1
163163 229533.I1RAN0 pp 229533.I1RQ49
163164 229533.I1RAN0 pp 229533.I1RYT7
163165 229533.I1RAN0 pp 229533.I1RZ12
163166 229533.I1RAN0 pp 229533.I1RTE5
163167 229533.I1RAN0 pp 229533.I1RW24
163168 229533.I1RAN0 pp 229533.I1S0P4
163169 229533.I1RAN0 pp 229533.I1RAW7
163170 229533.I1RAN0 pp 229533.I1RQ80
163171 229533.I1RAN0 pp 229533.I1RNT2
163172 229533.I1RAN0 pp 229533.I1S2A8
163173 229533.I1RAN0 pp 229533.I1RD18
163174 229533.I1RAN0 pp 229533.I1RTG0
163175 229533.I1RAN0 pp 229533.I1RC00
163176 229533.I1RAN0 pp 229533.I1RFZ2
163177 229533.I1RAN0 pp 229533.I1RCU7
163178 229533.I1RAN0 pp 229533.I1RJS4
163179 229533.I1RAN0 pp 229533.I1RMY3
163180 229533.I1RAN0 pp 229533.I1S1V5
163181 229533.I1RAN0 pp 229533.I1RC05
163182 229533.A0A1C3YL13 pp 229533.I1S273
163183 229533.A0A1C3YL13 pp 229533.A0A1C3YLL1
163184 229533.A0A1C3YL13 pp 229533.I1RMJ3
163185 229533.A0A1C3YL13 pp 229533.I1RSY1
163186 229533.A0A1C3YL13 pp 229533.A0A1C3YNB1
163187 229533.A0A1C3YL13 pp 229533.I1RAB6
163188 229533.I1RC39 pp 229533.I1RGI4
163189 229533.I1RC39 pp 229533.V6R756
163190 229533.I1RC39 pp 229533.I1RV23
163191 229533.I1RC39 pp 229533.I1RLT3
163192 229533.I1RC39 pp 229533.I1RJS1
163193 229533.I1RC39 pp 229533.I1RZ56
163194 229533.I1RC39 pp 229533.V6RM02
163195 229533.I1RC39 pp 229533.I1RF40
163196 229533.I1RC39 pp 229533.I1S6P6
163197 229533.I1RC39 pp 229533.I1RPV8
163198 229533.I1RC39 pp 229533.I1RVR2
163199 229533.I1RC39 pp 229533.I1RD80
163200 229533.I1RC39 pp 229533.V6RCJ5
163201 229533.I1RC39 pp 229533.I1RDJ7
163202 229533.I1RC39 pp 229533.I1RGZ3
163203 229533.I1RC39 pp 229533.I1S0H5
163204 229533.I1RC39 pp 229533.I1S6P5
163205 229533.I1RC39 pp 229533.V6R5D2
163206 229533.I1RC39 pp 229533.I1S024
163207 229533.I1RC39 pp 229533.I1RZA7
163208 229533.I1RC39 pp 229533.V6RY56
163209 229533.I1RC39 pp 229533.I1RNG7
163210 229533.I1RC39 pp 229533.I1RKB2
163211 229533.I1RC39 pp 229533.V6R2Z2
163212 229533.I1RC39 pp 229533.I1RLZ6
163213 229533.I1RC39 pp 229533.V6RUB1
163214 229533.I1RC39 pp 229533.I1RNG4
163215 229533.I1RC39 pp 229533.V6RGB5
163216 229533.I1RC39 pp 229533.I1S0A5
163217 229533.I1RC39 pp 229533.I1RRM1
163218 229533.I1RC39 pp 229533.I1RCV1
163219 229533.I1RC39 pp 229533.V6R9Z3
163220 229533.I1RC39 pp 229533.I1RC48
163221 229533.I1RC39 pp 229533.I1RNH4
163222 229533.I1RC39 pp 229533.I1RST9
163223 229533.I1RC39 pp 229533.I1RW55
163224 229533.I1RC39 pp 229533.I1RJA8
163225 229533.I1RC39 pp 229533.I1RJT5
163226 229533.I1RC39 pp 229533.I1S4D0
163227 229533.I1RC39 pp 229533.I1RDU0
163228 229533.I1RC39 pp 229533.I1RUY5

163229 229533.I1RC39 pp 229533.I1S0A0
163230 229533.I1RC39 pp 229533.I1RMD3
163231 229533.I1RC39 pp 229533.I1RF38
163232 229533.I1RC39 pp 229533.I1SAI4
163233 229533.I1RC39 pp 229533.V6R0W1
163234 229533.I1RC39 pp 229533.I1RNM7
163235 229533.I1RC39 pp 229533.I1RQX9
163236 229533.I1RC39 pp 229533.I1S094
163237 229533.I1RC39 pp 229533.I1S273
163238 229533.I1RC39 pp 229533.I1S6H3
163239 229533.I1RC39 pp 229533.I1RDN3
163240 229533.I1RC39 pp 229533.V6RAM6
163241 229533.I1RC39 pp 229533.V6QU99
163242 229533.I1RC39 pp 229533.V6R1K4
163243 229533.I1RC39 pp 229533.I1RH36
163244 229533.I1RC39 pp 229533.I1RQU9
163245 229533.I1RC39 pp 229533.I1RZY1
163246 229533.I1RC39 pp 229533.I1RHT5
163247 229533.I1RTL0 pp 229533.I1RX39
163248 229533.I1RTL0 pp 229533.I1RZ43
163249 229533.I1RTL0 pp 229533.I1S0R8
163250 229533.I1S5B5 pp 229533.I1SAM5
163251 229533.I1S5B5 pp 229533.V6REZ4
163252 229533.I1S5B5 pp 229533.Q4I7X1
163253 229533.I1S5B5 pp 229533.I1S5B6
163254 229533.I1S5B5 pp 229533.V6R3J1
163255 229533.I1S5B5 pp 229533.Q4IAA0
163256 229533.I1S5B5 pp 229533.Q4IN63
163257 229533.I1RK24 pp 229533.I1S464
163258 229533.I1RK24 pp 229533.I1RMK7
163259 229533.I1RK24 pp 229533.I1RS05
163260 229533.I1RK24 pp 229533.I1RXD9
163261 229533.I1RK24 pp 229533.V6RJP8
163262 229533.I1RK24 pp 229533.I1RYT8
163263 229533.I1RK24 pp 229533.I1RWA3
163264 229533.I1RK24 pp 229533.I1RMJ3
163265 229533.I1RK24 pp 229533.I1S273
163266 229533.I1RK24 pp 229533.I1S7J8
163267 229533.I1RK24 pp 229533.I1RM09
163268 229533.I1RK24 pp 229533.I1S8P5
163269 229533.I1RK24 pp 229533.I1S0K2
163270 229533.I1RK24 pp 229533.I1RY21
163271 229533.I1RK24 pp 229533.I1S5C6
163272 229533.I1RK24 pp 229533.I1RTA0
163273 229533.I1RK24 pp 229533.I1S5X9
163274 229533.I1RK24 pp 229533.I1S8S9
163275 229533.I1RK24 pp 229533.I1RSY1
163276 229533.I1RK24 pp 229533.I1RS29
163277 229533.I1RJF5 pp 229533.I1RX39
163278 229533.V6QU99 pp 229533.V6REA4
163279 229533.V6QU99 pp 229533.V6RBG5
163280 229533.V6QU99 pp 229533.V6R556
163281 229533.V6QU99 pp 229533.V6R0H4
163282 229533.V6QU99 pp 229533.V6RM09
163283 229533.V6QU99 pp 229533.V6RPS5
163284 229533.V6QU99 pp 229533.V6RY56
163285 229533.I1RSM2 pp 229533.I1S0L8
163286 229533.I1RSM2 pp 229533.I1RXU7
163287 229533.I1RSM2 pp 229533.Q4INZ9
163288 229533.I1RSM2 pp 229533.Q4HZB8
163289 229533.I1RSM2 pp 229533.Q4IAS1
163290 229533.I1RSM2 pp 229533.Q4IAA0
163291 229533.I1RSM2 pp 229533.I1RW71
163292 229533.I1RSM2 pp 229533.I1RY40
163293 229533.I1RSM2 pp 229533.Q4HW67
163294 229533.I1RSM2 pp 229533.I1RT35
163295 229533.I1RSM2 pp 229533.I1S6F0
163296 229533.I1RSM2 pp 229533.I1S246
163297 229533.I1RSM2 pp 229533.Q4HVW2
163298 229533.I1RSM2 pp 229533.I1S0P4
163299 229533.I1RSM2 pp 229533.Q4IEK8
163300 229533.I1RSM2 pp 229533.I1RZ94
163301 229533.I1RSM2 pp 229533.Q4HZ68

163302 229533.I1RSM2 pp 229533.Q4IN00
163303 229533.A0A1C3YLM6 pp 229533.I1S0K2
163304 229533.A0A1C3YLM6 pp 229533.I1S0R8
163305 229533.A0A1C3YLM6 pp 229533.I1RAV5
163306 229533.A0A1C3YLM6 pp 229533.I1RZ43
163307 229533.A0A1C3YLM6 pp 229533.I1RPP7
163308 229533.A0A1C3YLM6 pp 229533.I1S8F8
163309 229533.A0A1C3YLM6 pp 229533.A0A1C3YN89
163310 229533.A0A1C3YLM6 pp 229533.I1S0Q7
163311 229533.A0A1C3YLM6 pp 229533.I1S8S9
163312 229533.A0A1C3YLM6 pp 229533.I1RAM9
163313 229533.A0A1C3YLM6 pp 229533.I1RQJ3
163314 229533.A0A1C3YLM6 pp 229533.I1RFJ6
163315 229533.A0A1C3YLM6 pp 229533.I1RP02
163316 229533.A0A1C3YLM6 pp 229533.I1RFS5
163317 229533.A0A1C3YLM6 pp 229533.I1S212
163318 229533.A0A1C3YLM6 pp 229533.I1RNM7
163319 229533.A0A1C3YLM6 pp 229533.I1RMK7
163320 229533.A0A1C3YLM6 pp 229533.I1RY21
163321 229533.A0A1C3YLM6 pp 229533.A0A1C3YMQ5
163322 229533.A0A1C3YLM6 pp 229533.I1RSF2
163323 229533.A0A1C3YLM6 pp 229533.I1S0P0
163324 229533.A0A1C3YLM6 pp 229533.I1RG62
163325 229533.A0A1C3YLM6 pp 229533.I1RX39
163326 229533.A0A1C3YLM6 pp 229533.I1RBE6
163327 229533.A0A1C3YLM6 pp 229533.I1RKB7
163328 229533.A0A1C3YLM6 pp 229533.I1RMQ6
163329 229533.A0A1C3YLM6 pp 229533.A0A1C3YMC1
163330 229533.A0A1C3YLM6 pp 229533.I1RY92
163331 229533.A0A1C3YII4 pp 229533.I1S8U9
163332 229533.A0A1C3YII4 pp 229533.I1RNQ6
163333 229533.A0A1C3YII4 pp 229533.A0A1C3YJ12
163334 229533.A0A1C3YII4 pp 229533.I1RG11
163335 229533.A0A1C3YII4 pp 229533.Q4IPC8
163336 229533.A0A1C3YII4 pp 229533.Q4HWE2
163337 229533.A0A1C3YII4 pp 229533.I1RDG8
163338 229533.A0A1C3YII4 pp 229533.I1RQK2
163339 229533.A0A1C3YII4 pp 229533.A0A1C3YMF9
163340 229533.A0A1C3YII4 pp 229533.A0A1C3YJW7
163341 229533.A0A1C3YII4 pp 229533.A0A1C3YM64
163342 229533.A0A1C3YII4 pp 229533.Q4IR09
163343 229533.A0A1C3YII4 pp 229533.A0A1C3YL15
163344 229533.A0A1C3YII4 pp 229533.I1RR25
163345 229533.A0A1C3YII4 pp 229533.I1S214
163346 229533.A0A1C3YII4 pp 229533.Q4IF44
163347 229533.A0A1C3YII4 pp 229533.I1RN28
163348 229533.A0A1C3YII4 pp 229533.I1S8V0
163349 229533.A0A1C3YII4 pp 229533.I1S0P4
163350 229533.A0A1C3YII4 pp 229533.A0A1C3YM29
163351 229533.A0A1C3YII4 pp 229533.I1RT35
163352 229533.A0A1C3YII4 pp 229533.I1RPS0
163353 229533.A0A1C3YII4 pp 229533.I1S0N7
163354 229533.A0A1C3YII4 pp 229533.A0A1C3YKM6
163355 229533.A0A1C3YII4 pp 229533.Q4I7X1
163356 229533.A0A1C3YII4 pp 229533.I1RYW2
163357 229533.A0A1C3YII4 pp 229533.I1RNE4
163358 229533.A0A1C3YII4 pp 229533.I1RE14
163359 229533.A0A1C3YII4 pp 229533.I1S382
163360 229533.A0A1C3YII4 pp 229533.I1RDD3
163361 229533.A0A1C3YII4 pp 229533.I1RCK4
163362 229533.A0A1C3YII4 pp 229533.I1RA46
163363 229533.A0A1C3YII4 pp 229533.Q4I5I4
163364 229533.A0A1C3YII4 pp 229533.I1RS03
163365 229533.A0A1C3YII4 pp 229533.I1RE45
163366 229533.A0A1C3YII4 pp 229533.I1S246
163367 229533.A0A1C3YII4 pp 229533.I1R9Q0
163368 229533.A0A1C3YII4 pp 229533.I1S951
163369 229533.A0A1C3YII4 pp 229533.A0A1C3YIR8
163370 229533.A0A1C3YII4 pp 229533.I1RQZ2
163371 229533.A0A1C3YII4 pp 229533.I1RA38
163372 229533.A0A1C3YII4 pp 229533.I1RAW7
163373 229533.A0A1C3YII4 pp 229533.I1RWB3
163374 229533.A0A1C3YII4 pp 229533.A0A1C3YL25

163375 229533.A0A1C3YII4 pp 229533.I1RYB1
163376 229533.A0A1C3YII4 pp 229533.I1RY40
163377 229533.A0A1C3YII4 pp 229533.I1S1V5
163378 229533.A0A1C3YII4 pp 229533.A0A1C3YL53
163379 229533.A0A1C3YII4 pp 229533.I1RZP2
163380 229533.A0A1C3YII4 pp 229533.I1RNB8
163381 229533.A0A1C3YII4 pp 229533.I1RN49
163382 229533.A0A1C3YII4 pp 229533.I1RSD4
163383 229533.A0A1C3YII4 pp 229533.I1RZL5
163384 229533.A0A1C3YII4 pp 229533.I1S5Z2
163385 229533.A0A1C3YII4 pp 229533.Q4IPN9
163386 229533.A0A1C3YII4 pp 229533.I1R976
163387 229533.A0A1C3YII4 pp 229533.A0A1C3YMB5
163388 229533.A0A1C3YII4 pp 229533.I1RFU3
163389 229533.A0A1C3YII4 pp 229533.A0A1C3YJV9
163390 229533.A0A1C3YII4 pp 229533.I1RZV9
163391 229533.I1RC36 pp 229533.Q4I073
163392 229533.I1RC36 pp 229533.Q4I0K3
163393 229533.I1RC36 pp 229533.I1S252
163394 229533.I1RC36 pp 229533.I1S5B1
163395 229533.I1RC36 pp 229533.I1REU0
163396 229533.I1RC36 pp 229533.I1RGJ2
163397 229533.I1RC36 pp 229533.I1RLR7
163398 229533.A0A1C3YKQ4 pp 229533.A0A1C3YLB0
163399 229533.A0A1C3YKQ4 pp 229533.I1RX39
163400 229533.A0A0E0RUW5 pp 229533.I1RB74
163401 229533.A0A0E0RUW5 pp 229533.V6RKY7
163402 229533.A0A0E0RUW5 pp 229533.I1RC95
163403 229533.A0A0E0RUW5 pp 229533.Q4IJM4
163404 229533.A0A0E0RUW5 pp 229533.A0A1C3YK51
163405 229533.A0A0E0RUW5 pp 229533.I1S439
163406 229533.A0A0E0RUW5 pp 229533.I1RB75
163407 229533.A0A0E0RUW5 pp 229533.I1RYH9
163408 229533.A0A0E0RUW5 pp 229533.A0A1C3YM11
163409 229533.A0A0E0RUW5 pp 229533.I1RUQ5
163410 229533.I1RAC1 pp 229533.I1RM25
163411 229533.I1RAC1 pp 229533.I1RNB8
163412 229533.I1RAC1 pp 229533.I1RFQ1
163413 229533.I1RAC1 pp 229533.I1RSD4
163414 229533.I1RAC1 pp 229533.I1RS28
163415 229533.I1RAC1 pp 229533.I1S060
163416 229533.I1RAC1 pp 229533.I1RRL0
163417 229533.I1RAC1 pp 229533.Q4IPB8
163418 229533.I1RAC1 pp 229533.V6RY33
163419 229533.I1RAC1 pp 229533.I1RBH8
163420 229533.I1RAC1 pp 229533.I1RM96
163421 229533.I1RAC1 pp 229533.I1RSA4
163422 229533.I1RAC1 pp 229533.I1RND5
163423 229533.I1RAC1 pp 229533.V6QXG1
163424 229533.I1RAC1 pp 229533.I1RX51
163425 229533.I1RAC1 pp 229533.I1S471
163426 229533.I1RAC1 pp 229533.I1RX47
163427 229533.I1RAC1 pp 229533.I1S056
163428 229533.I1RAC1 pp 229533.I1RMN6
163429 229533.I1RAC1 pp 229533.I1S8E2
163430 229533.I1RAC1 pp 229533.I1RT47
163431 229533.I1RAC1 pp 229533.Q4HXF6
163432 229533.I1RAC1 pp 229533.I1RU69
163433 229533.I1RAC1 pp 229533.Q4IF76
163434 229533.I1RAC1 pp 229533.I1RPL3
163435 229533.I1RAC1 pp 229533.I1S0B1
163436 229533.I1RAC1 pp 229533.I1S2E8
163437 229533.I1RAC1 pp 229533.I1RZQ5
163438 229533.I1RAC1 pp 229533.I1RST1
163439 229533.I1RAC1 pp 229533.V6QVD5
163440 229533.I1RAC1 pp 229533.I1RKJ6
163441 229533.I1RAC1 pp 229533.I1RAU7
163442 229533.I1RAC1 pp 229533.I1RG90
163443 229533.I1RAC1 pp 229533.Q4IAA0
163444 229533.I1RAC1 pp 229533.I1RAF3
163445 229533.I1RAC1 pp 229533.I1RME9
163446 229533.I1RAC1 pp 229533.I1RFG0
163447 229533.I1RAC1 pp 229533.I1RYJ9

229533.I1RAC1	pp	229533.I1RG57
229533.I1RAC1	pp	229533.I1RMU1
229533.I1RAC1	pp	229533.Q4IRI9
229533.I1RAC1	pp	229533.I1RK90
229533.I1RAC1	pp	229533.I1RND9
229533.I1RAC1	pp	229533.I1RXF2
229533.I1RAC1	pp	229533.I1S0M9
229533.I1RAC1	pp	229533.I1RBZ8
229533.I1RAC1	pp	229533.I1RN28
229533.I1RAC1	pp	229533.I1RDX6
229533.I1RAC1	pp	229533.I1RK92
229533.I1RAC1	pp	229533.I1RBV3
229533.I1RAC1	pp	229533.I1RCN5
229533.I1RAC1	pp	229533.I1RCM3
229533.I1RAC1	pp	229533.I1RPY5
229533.I1RAC1	pp	229533.I1RC84
229533.I1RAC1	pp	229533.I1RAF8
229533.I1RAC1	pp	229533.I1RK72
229533.I1RAC1	pp	229533.I1RT17
229533.I1RAC1	pp	229533.I1S0N7
229533.I1RAC1	pp	229533.I1RCA7
229533.I1RAC1	pp	229533.I1RZF6
229533.I1RAC1	pp	229533.I1RWU2
229533.I1RAC1	pp	229533.I1RUM5
229533.I1RAC1	pp	229533.I1RLV9
229533.I1RAC1	pp	229533.I1RBG9
229533.I1RAC1	pp	229533.I1RT35
229533.I1RAC1	pp	229533.I1RK96
229533.I1RAC1	pp	229533.I1RKL3
229533.I1RAC1	pp	229533.I1RJF9
229533.I1RAC1	pp	229533.I1RWX8
229533.I1RAC1	pp	229533.I1RPY4
229533.I1RAC1	pp	229533.I1RK23
229533.I1RAC1	pp	229533.I1RFS3
229533.I1RAC1	pp	229533.I1RAI1
229533.I1RAC1	pp	229533.I1S4M5
229533.I1RAC1	pp	229533.I1RCS2
229533.I1RAC1	pp	229533.Q4IBK5
229533.I1RAC1	pp	229533.I1S7M3
229533.I1RAC1	pp	229533.Q4I662
229533.I1RAC1	pp	229533.I1RNH6
229533.I1RAC1	pp	229533.I1RSW2
229533.I1RAC1	pp	229533.I1RBX0
229533.I1RAC1	pp	229533.I1RQ65
229533.I1RAC1	pp	229533.Q4IE79
229533.I1RAC1	pp	229533.I1RE45
229533.I1RAC1	pp	229533.I1RQK2
229533.I1RAC1	pp	229533.I1RSA8
229533.I1RAC1	pp	229533.I1RMV5
229533.I1RAC1	pp	229533.Q4I7F9
229533.I1RAC1	pp	229533.I1RS03
229533.I1RAC1	pp	229533.Q4IP34
229533.I1RAC1	pp	229533.I1S9M9
229533.I1RAC1	pp	229533.I1RS83
229533.I1RAC1	pp	229533.I1RR73
229533.I1RAC1	pp	229533.I1RRW3
229533.I1RAC1	pp	229533.I1RFP9
229533.I1RAC1	pp	229533.I1RPY7
229533.I1RAC1	pp	229533.I1RQD8
229533.I1RAC1	pp	229533.I1RLU5
229533.I1RAC1	pp	229533.I1RRU9
229533.I1RAC1	pp	229533.I1S0Z9
229533.I1RAC1	pp	229533.I1RQC5
229533.I1RAC1	pp	229533.I1RGC2
229533.I1RAC1	pp	229533.Q4I1Y1
229533.I1RAC1	pp	229533.I1RDG0
229533.I1RAC1	pp	229533.I1S7M2
229533.I1RAC1	pp	229533.I1RPZ3
229533.I1RAC1	pp	229533.I1RQZ2
229533.I1RAC1	pp	229533.I1RSR8
229533.I1RAC1	pp	229533.I1RDY2
229533.I1RAC1	pp	229533.Q4IPI1
229533.I1RAC1	pp	229533.I1RZK6

163521	229533.I1RAC1	pp	229533.I1S9L0
163522	229533.I1RAC1	pp	229533.I1S1C6
163523	229533.I1RAC1	pp	229533.I1RN59
163524	229533.I1RAC1	pp	229533.I1RZL5
163525	229533.I1RAC1	pp	229533.I1S0B6
163526	229533.I1RAC1	pp	229533.I1RX81
163527	229533.I1RAC1	pp	229533.I1RAE1
163528	229533.I1RAC1	pp	229533.Q4IPB3
163529	229533.I1RAC1	pp	229533.I1RBL7
163530	229533.I1RAC1	pp	229533.I1RPA0
163531	229533.I1RAC1	pp	229533.I1RPU0
163532	229533.I1RAC1	pp	229533.Q4IB70
163533	229533.I1RAC1	pp	229533.I1RZG7
163534	229533.I1RAC1	pp	229533.I1RCQ6
163535	229533.I1RAC1	pp	229533.I1RCH7
163536	229533.I1RAC1	pp	229533.I1RAN5
163537	229533.I1RAC1	pp	229533.I1RS75
163538	229533.I1RAC1	pp	229533.I1RQJ5
163539	229533.I1RAC1	pp	229533.Q4ICG5
163540	229533.I1RAC1	pp	229533.I1RCI3
163541	229533.I1RAC1	pp	229533.I1S2Y5
163542	229533.I1RAC1	pp	229533.I1RCI4
163543	229533.I1RAC1	pp	229533.I1S246
163544	229533.I1RAC1	pp	229533.I1RB50
163545	229533.I1RAC1	pp	229533.I1RN60
163546	229533.I1RAC1	pp	229533.Q4IJH1
163547	229533.I1RAC1	pp	229533.I1RD56
163548	229533.I1RAC1	pp	229533.I1RN91
163549	229533.I1RRB2	pp	229533.I1S9F9
163550	229533.I1S273	pp	229533.I1S5N3
163551	229533.I1S273	pp	229533.V6RY56
163552	229533.I1S273	pp	229533.I1S2P0
163553	229533.I1S273	pp	229533.I1S4D0
163554	229533.I1S273	pp	229533.I1S7J8
163555	229533.I1RE24	pp	229533.I1RZG7
163556	229533.I1RE24	pp	229533.I1S7M3
163557	229533.I1RE24	pp	229533.I1S7M2
163558	229533.I1RE24	pp	229533.I1RX81
163559	229533.I1RE24	pp	229533.I1S273
163560	229533.I1RE24	pp	229533.I1RG57
163561	229533.I1RE24	pp	229533.I1RND5
163562	229533.I1RE24	pp	229533.I1RK90
163563	229533.I1RE24	pp	229533.I1RSY1
163564	229533.I1RE24	pp	229533.I1RG90
163565	229533.I1RE24	pp	229533.I1RK92
163566	229533.I1RE24	pp	229533.I1RSA4
163567	229533.I1RE24	pp	229533.V6QXG1
163568	229533.I1RE24	pp	229533.I1S7X9
163569	229533.I1RE24	pp	229533.I1RS28
163570	229533.I1RE24	pp	229533.I1RMJ3
163571	229533.I1RE24	pp	229533.I1S4M5
163572	229533.I1RE24	pp	229533.I1RSD4
163573	229533.I1RE24	pp	229533.I1RXF2
163574	229533.I1RWL9	pp	229533.Q4IBU4
163575	229533.I1RWL9	pp	229533.I1RZQ6
163576	229533.I1RWL9	pp	229533.I1RX09
163577	229533.I1RWL9	pp	229533.Q4IJ79
163578	229533.I1RWL9	pp	229533.I1RYD3
163579	229533.I1SA55	pp	229533.Q4HZQ1
163580	229533.I1RNE4	pp	229533.I1RQ21
163581	229533.I1RNE4	pp	229533.Q4I283
163582	229533.I1RNE4	pp	229533.I1S0P4
163583	229533.I1RNE4	pp	229533.I1S5B5
163584	229533.I1RNE4	pp	229533.I1RTE5
163585	229533.I1RNE4	pp	229533.I1RYL5
163586	229533.I1RNE4	pp	229533.I1RNG5
163587	229533.I1RNE4	pp	229533.I1RZT1
163588	229533.I1RNE4	pp	229533.I1RT35
163589	229533.I1RNE4	pp	229533.I1RQQ5
163590	229533.I1RNE4	pp	229533.I1RZV9
163591	229533.I1RNE4	pp	229533.V6REZ4
163592	229533.I1RNE4	pp	229533.I1RYW7
163593	229533.I1RNE4	pp	229533.I1S044

163594 229533.I1RNE4 pp 229533.I1S283
163595 229533.I1RNE4 pp 229533.I1RW71
163596 229533.I1RNE4 pp 229533.I1S279
163597 229533.I1RNE4 pp 229533.I1S208
163598 229533.I1RNE4 pp 229533.I1RYJ8
163599 229533.I1RNE4 pp 229533.I1RSI4
163600 229533.I1RNE4 pp 229533.Q4IAA0
163601 229533.I1RNE4 pp 229533.I1RPU4
163602 229533.I1RNE4 pp 229533.I1RQ49
163603 229533.I1RNE4 pp 229533.I1RSL8
163604 229533.I1RNE4 pp 229533.I1S2F3
163605 229533.I1RNE4 pp 229533.Q4IPN9
163606 229533.I1RNE4 pp 229533.I1RY40
163607 229533.I1RNE4 pp 229533.I1RUG1
163608 229533.I1RNE4 pp 229533.Q4IPA4
163609 229533.I1RNE4 pp 229533.I1RQK2
163610 229533.I1RNE4 pp 229533.I1RNT2
163611 229533.I1RNE4 pp 229533.I1RYT7
163612 229533.I1RNE4 pp 229533.I1S214
163613 229533.I1RNE4 pp 229533.I1RS94
163614 229533.I1RNE4 pp 229533.Q4I5R9
163615 229533.I1RNE4 pp 229533.I1S0N7
163616 229533.I1RJU4 pp 229533.I1RKK0
163617 229533.I1RJU4 pp 229533.I1RPM6
163618 229533.I1RJU4 pp 229533.V6QVH8
163619 229533.I1RJU4 pp 229533.I1RZM1
163620 229533.I1RJU4 pp 229533.I1RWM4
163621 229533.I1RJU4 pp 229533.V6RY56
163622 229533.I1RJU4 pp 229533.I1S3F3
163623 229533.I1RJU4 pp 229533.Q4IMJ1
163624 229533.I1RJU4 pp 229533.I1S7M8
163625 229533.I1RJU4 pp 229533.I1RTI7
163626 229533.I1RJU4 pp 229533.I1S2E2
163627 229533.I1RJU4 pp 229533.I1RZP0
163628 229533.I1RJU4 pp 229533.I1RMD1
163629 229533.I1RJU4 pp 229533.I1S095
163630 229533.A0A098E413 pp 229533.I1SAK2
163631 229533.A0A098E413 pp 229533.A0A1C3YIW9
163632 229533.A0A098E413 pp 229533.I1RX39
163633 229533.A0A098E413 pp 229533.A0A1C3YID4
163634 229533.A0A098E413 pp 229533.A0A1C3YLB0
163635 229533.I1RCH9 pp 229533.I1S8P5
163636 229533.I1RCH9 pp 229533.I1S464
163637 229533.I1RCH9 pp 229533.I1RS05
163638 229533.I1RCH9 pp 229533.I1RMJ3
163639 229533.I1RCH9 pp 229533.I1RGI5
163640 229533.I1RCH9 pp 229533.I1S273
163641 229533.I1RCH9 pp 229533.I1RYT8
163642 229533.I1RCH9 pp 229533.I1RXD9
163643 229533.I1RCH9 pp 229533.I1RWA3
163644 229533.I1RCH9 pp 229533.I1S5C6
163645 229533.I1RCH9 pp 229533.V6RJP8
163646 229533.I1RCH9 pp 229533.I1RY21
163647 229533.I1RCH9 pp 229533.I1RMK7
163648 229533.I1RCH9 pp 229533.I1S8S9
163649 229533.I1RCH9 pp 229533.I1RTA0
163650 229533.I1RCH9 pp 229533.I1RFJ6
163651 229533.I1RCH9 pp 229533.I1RSY1
163652 229533.I1RCH9 pp 229533.I1S7J8
163653 229533.I1RCH9 pp 229533.I1S0K2
163654 229533.I1RCH9 pp 229533.I1S5X9
163655 229533.I1RCH9 pp 229533.I1RS29
163656 229533.A0A0E0SBS3 pp 229533.I1RWL9
163657 229533.A0A0E0SBS3 pp 229533.V6RLW4
163658 229533.A0A0E0SBS3 pp 229533.I1RS79
163659 229533.I1RHT5 pp 229533.V6RCC0
163660 229533.I1RHT5 pp 229533.I1RRS6
163661 229533.I1RHT5 pp 229533.I1S5D0
163662 229533.I1RHT5 pp 229533.I1RW54
163663 229533.I1RHT5 pp 229533.I1S969
163664 229533.I1RHT5 pp 229533.I1RMA2
163665 229533.I1RHT5 pp 229533.I1S6H3
163666 229533.I1RHT5 pp 229533.I1S048

163667 229533.I1RHT5 pp 229533.I1RWL3
163668 229533.I1RHT5 pp 229533.I1RQV7
163669 229533.I1RHT5 pp 229533.I1RLA2
163670 229533.I1RHT5 pp 229533.I1RWM4
163671 229533.I1RHT5 pp 229533.V6R5T7
163672 229533.I1RHT5 pp 229533.I1RYU6
163673 229533.I1RHT5 pp 229533.I1RQV8
163674 229533.I1RHT5 pp 229533.I1RPM6
163675 229533.I1RHT5 pp 229533.I1S094
163676 229533.I1RHT5 pp 229533.I1RW55
163677 229533.I1RHT5 pp 229533.I1RYP5
163678 229533.I1RHT5 pp 229533.I1RST9
163679 229533.I1RHT5 pp 229533.I1RIB5
163680 229533.I1RHT5 pp 229533.V6RHX2
163681 229533.I1RHT5 pp 229533.I1S8D3
163682 229533.I1RHT5 pp 229533.I1RZ25
163683 229533.I1RHT5 pp 229533.V6RWB3
163684 229533.I1RHT5 pp 229533.I1S8H9
163685 229533.I1RHT5 pp 229533.I1RY12
163686 229533.I1RHT5 pp 229533.I1RQJ7
163687 229533.I1RHT5 pp 229533.I1S267
163688 229533.I1RHT5 pp 229533.I1S659
163689 229533.I1RHT5 pp 229533.I1S968
163690 229533.I1RHT5 pp 229533.I1S0A5
163691 229533.I1RHT5 pp 229533.I1RIM7
163692 229533.I1RHT5 pp 229533.I1RRR9
163693 229533.I1RHT5 pp 229533.I1RY81
163694 229533.I1RHT5 pp 229533.I1RWF5
163695 229533.I1RHT5 pp 229533.I1RW82
163696 229533.I1RHT5 pp 229533.I1RMK9
163697 229533.I1RHT5 pp 229533.I1SAT2
163698 229533.I1RHT5 pp 229533.I1RN84
163699 229533.I1RHT5 pp 229533.I1RSA5
163700 229533.I1RHT5 pp 229533.V6RCJ5
163701 229533.I1RHT5 pp 229533.I1RK55
163702 229533.I1RHT5 pp 229533.I1RIJ6
163703 229533.I1RHT5 pp 229533.I1RZ56
163704 229533.I1RHT5 pp 229533.I1RPV8
163705 229533.I1RHT5 pp 229533.I1RZ89
163706 229533.I1RHT5 pp 229533.I1S2C4
163707 229533.I1RHT5 pp 229533.I1RVX8
163708 229533.I1RHT5 pp 229533.I1RJS1
163709 229533.I1RHT5 pp 229533.I1RHU8
163710 229533.I1RHT5 pp 229533.I1S6V0
163711 229533.I1S7M8 pp 229533.Q4I8P5
163712 229533.I1S7M8 pp 229533.I1SA32
163713 229533.I1S7M8 pp 229533.V6RGI1
163714 229533.I1S7M8 pp 229533.Q4IBU4
163715 229533.I1S7M8 pp 229533.I1S9D4
163716 229533.I1S7M8 pp 229533.I1SA33
163717 229533.I1S7M8 pp 229533.I1S7M9
163718 229533.I1RDY1 pp 229533.I1RY59
163719 229533.I1RDY1 pp 229533.I1RQA8
163720 229533.I1RDY1 pp 229533.I1RS94
163721 229533.I1RDY1 pp 229533.I1S0C7
163722 229533.I1RDY1 pp 229533.I1RMY3
163723 229533.I1RDY1 pp 229533.I1RY40
163724 229533.I1RDY1 pp 229533.I1RLU5
163725 229533.I1RDY1 pp 229533.I1S1Y5
163726 229533.I1RDY1 pp 229533.I1RDY5
163727 229533.I1RDY1 pp 229533.I1RYT7
163728 229533.I1RDY1 pp 229533.I1S8W6
163729 229533.I1RDY1 pp 229533.I1RRH5
163730 229533.I1RDY1 pp 229533.I1RQ80
163731 229533.I1RDY1 pp 229533.I1RSL8
163732 229533.I1RDY1 pp 229533.I1RJS4
163733 229533.I1RDY1 pp 229533.I1S2F3
163734 229533.I1RDY1 pp 229533.I1S246
163735 229533.I1RDY1 pp 229533.I1RQ49
163736 229533.I1RDY1 pp 229533.Q4IAA0
163737 229533.I1RDY1 pp 229533.I1RNT2
163738 229533.I1RDY1 pp 229533.I1RYJ8
163739 229533.I1RDY1 pp 229533.I1RNB8

163740 229533.I1RDY1 pp 229533.I1RZV9
163741 229533.I1RDY1 pp 229533.I1S2A8
163742 229533.I1RDY1 pp 229533.I1RNB7
163743 229533.I1RDY1 pp 229533.Q4IE79
163744 229533.I1RDY1 pp 229533.I1RQB7
163745 229533.I1RDY1 pp 229533.I1RMH6
163746 229533.I1RDY1 pp 229533.I1S0P4
163747 229533.I1RDY1 pp 229533.I1RY14
163748 229533.I1RDY1 pp 229533.Q4IPN9
163749 229533.I1RDY1 pp 229533.I1RJS5
163750 229533.I1RDY1 pp 229533.I1RT35
163751 229533.I1RDY1 pp 229533.I1RZT1
163752 229533.I1RDY1 pp 229533.I1RWC6
163753 229533.I1RDY1 pp 229533.I1RJL5
163754 229533.I1RDY1 pp 229533.I1S5B5
163755 229533.I1RDY1 pp 229533.I1RIR9
163756 229533.I1RDY1 pp 229533.V6REZ4
163757 229533.I1RDY1 pp 229533.I1RNE4
163758 229533.I1REZ6 pp 229533.I1RIB9
163759 229533.I1REZ6 pp 229533.I1SAS4
163760 229533.I1REZ6 pp 229533.I1RLN1
163761 229533.I1REZ6 pp 229533.I1RZ43
163762 229533.I1REZ6 pp 229533.I1RKB7
163763 229533.I1REZ6 pp 229533.I1S0R8
163764 229533.I1REZ6 pp 229533.I1S1J0
163765 229533.I1REZ6 pp 229533.I1RX39
163766 229533.I1REZ6 pp 229533.I1RPV5
163767 229533.I1REZ6 pp 229533.I1RG62
163768 229533.I1REZ6 pp 229533.I1RUV5
163769 229533.I1REZ6 pp 229533.I1RHQ4
163770 229533.I1S7J8 pp 229533.V6QUE6
163771 229533.A0A0E0RUV3 pp 229533.I1RJU4
163772 229533.A0A0E0RUV3 pp 229533.I1S7M8
163773 229533.A0A0E0RUV3 pp 229533.I1RSL5
163774 229533.A0A1C3YKG7 pp 229533.I1RW16
163775 229533.A0A1C3YKG7 pp 229533.I1RPK0
163776 229533.A0A1C3YKG7 pp 229533.I1RML0
163777 229533.A0A1C3YKG7 pp 229533.I1RNB8
163778 229533.A0A1C3YKG7 pp 229533.V6R0U9
163779 229533.A0A1C3YKG7 pp 229533.V6R223
163780 229533.A0A1C3YKG7 pp 229533.I1RDY5
163781 229533.A0A1C3YKG7 pp 229533.I1RSL8
163782 229533.A0A1C3YKG7 pp 229533.I1RZE1
163783 229533.A0A1C3YKG7 pp 229533.I1RTE6
163784 229533.A0A1C3YKG7 pp 229533.I1RD70
163785 229533.A0A1C3YKG7 pp 229533.I1RJS5
163786 229533.A0A1C3YKG7 pp 229533.A0A1C3YL15
163787 229533.A0A1C3YKG7 pp 229533.I1RQN9
163788 229533.A0A1C3YKG7 pp 229533.I1RXF8
163789 229533.A0A1C3YKG7 pp 229533.I1S1V5
163790 229533.A0A1C3YKG7 pp 229533.I1RKJ8
163791 229533.A0A1C3YKG7 pp 229533.I1RC00
163792 229533.A0A1C3YKG7 pp 229533.I1RFW3
163793 229533.A0A1C3YKG7 pp 229533.I1RCM6
163794 229533.A0A1C3YKG7 pp 229533.A0A1C3YMK1
163795 229533.A0A1C3YKG7 pp 229533.A0A1C3YLF5
163796 229533.A0A1C3YKG7 pp 229533.I1RBL4
163797 229533.A0A1C3YKG7 pp 229533.I1RRE0
163798 229533.A0A1C3YKG7 pp 229533.V6REE9
163799 229533.A0A1C3YKG7 pp 229533.I1RIX1
163800 229533.A0A1C3YKG7 pp 229533.I1S2A8
163801 229533.A0A1C3YKG7 pp 229533.I1RMH4
163802 229533.A0A1C3YKG7 pp 229533.I1RCD4
163803 229533.A0A1C3YKG7 pp 229533.I1RJS4
163804 229533.A0A1C3YKG7 pp 229533.I1S5B5
163805 229533.A0A1C3YKG7 pp 229533.I1RDY1
163806 229533.A0A1C3YKG7 pp 229533.I1RT35
163807 229533.A0A1C3YKG7 pp 229533.I1RAN0
163808 229533.A0A1C3YKG7 pp 229533.I1S0P4
163809 229533.A0A1C3YKG7 pp 229533.I1S0S6
163810 229533.A0A1C3YKG7 pp 229533.I1RLX6
163811 229533.A0A1C3YKG7 pp 229533.I1RSP1
163812 229533.A0A1C3YKG7 pp 229533.I1RTB7

229533.A0A1C3YKG7 pp 229533.I1RTE5
229533.A0A1C3YKG7 pp 229533.I1RN31
229533.A0A1C3YKG7 pp 229533.I1RH68
229533.A0A1C3YKG7 pp 229533.I1RAW7
229533.A0A1C3YKG7 pp 229533.I1RYJ8
229533.A0A1C3YKG7 pp 229533.I1RQ80
229533.A0A1C3YKG7 pp 229533.I1REX4
229533.A0A1C3YKG7 pp 229533.I1RJX2
229533.A0A1C3YKG7 pp 229533.A0A1C3YLD3
229533.A0A1C3YKG7 pp 229533.I1RKJ7
229533.A0A1C3YKG7 pp 229533.P0C431
229533.A0A1C3YKG7 pp 229533.I1RK32
229533.A0A1C3YKG7 pp 229533.I1S0Z2
229533.A0A1C3YI01 pp 229533.I1RVX9
229533.A0A1C3YI01 pp 229533.I1S724
229533.A0A1C3YI01 pp 229533.I1RW43
229533.A0A1C3YI01 pp 229533.I1RY49
229533.A0A1C3YI01 pp 229533.V6RRM0
229533.A0A1C3YI01 pp 229533.I1RM26
229533.A0A1C3YN65 pp 229533.I1RE19
229533.A0A1C3YN65 pp 229533.I1RCI5
229533.A0A1C3YN65 pp 229533.I1RIX1
229533.A0A1C3YN65 pp 229533.I1RMP2
229533.A0A1C3YN65 pp 229533.I1RTA3
229533.I1RQX9 pp 229533.I1RTJ7
229533.I1RQX9 pp 229533.I1RYU0
229533.I1RQX9 pp 229533.I1RXH3
229533.I1RQX9 pp 229533.I1RVV7
229533.I1RQX9 pp 229533.V6RY56
229533.I1RQX9 pp 229533.I1S4D0
229533.I1RQX9 pp 229533.I1SAI4
229533.I1RQX9 pp 229533.I1RXV3
229533.I1RQX9 pp 229533.I1RV18
229533.I1RQX9 pp 229533.I1RTU5
229533.I1RQX9 pp 229533.V6RAM6
229533.I1RQX9 pp 229533.I1S0T1
229533.I1RQX9 pp 229533.I1S3K8
229533.I1RQX9 pp 229533.I1RZT0
229533.I1RQX9 pp 229533.I1S084
229533.I1RQX9 pp 229533.V6QU99
229533.I1RQX9 pp 229533.I1S6P5
229533.I1RQX9 pp 229533.V6R0W1
229533.I1RQX9 pp 229533.I1RY96
229533.I1RQX9 pp 229533.V6RUB1
229533.I1RQX9 pp 229533.V6R5D2
229533.I1RQX9 pp 229533.I1S2C4
229533.I1RQX9 pp 229533.I1S1P8
229533.I1RQX9 pp 229533.V6R756
229533.I1RQX9 pp 229533.V6R2Z2
229533.I1RQX9 pp 229533.I1SAJ0
229533.I1RQX9 pp 229533.I1RW15
229533.I1RQX9 pp 229533.I1RZA7
229533.I1RQX9 pp 229533.I1S6H1
229533.I1RQX9 pp 229533.I1RV23
229533.I1RQX9 pp 229533.I1RZ20
229533.A0A1C3YLQ8 pp 229533.A0A1C3YNB1
229533.A0A1C3YLQ8 pp 229533.I1RSY1
229533.A0A1C3YLQ8 pp 229533.I1RYW3
229533.A0A1C3YLQ8 pp 229533.I1RBS0
229533.A0A1C3YLQ8 pp 229533.I1RWX5
229533.A0A1C3YLQ8 pp 229533.I1RRT8
229533.A0A1C3YLQ8 pp 229533.V6QUE6
229533.A0A1C3YLQ8 pp 229533.A0A1C3YLU7
229533.A0A1C3YLQ8 pp 229533.I1RMJ3
229533.A0A1C3YLQ8 pp 229533.I1RAB6
229533.A0A1C3YLQ8 pp 229533.I1S273
229533.A0A1C3YLQ8 pp 229533.I1RW71
229533.I1S5Z2 pp 229533.I1S8V0
229533.I1S5Z2 pp 229533.Q4IPC8
229533.I1S5Z2 pp 229533.V6QUE6
229533.I1S5Z2 pp 229533.Q4I5I4
229533.I1S5Z2 pp 229533.I1SA47
229533.I1S5Z2 pp 229533.Q4IPA4

163886 229533.I1S5Z2 pp 229533.Q4HWE2
163887 229533.I1S5Z2 pp 229533.Q4I5U9
163888 229533.I1S5Z2 pp 229533.I1S8U9
163889 229533.I1S5Z2 pp 229533.Q4I7X1
163890 229533.I1S5Z2 pp 229533.I1S9M9
163891 229533.I1S5Z2 pp 229533.Q4I5W5
163892 229533.I1S5Z2 pp 229533.I1S5Z3
163893 229533.I1S5Z2 pp 229533.Q4IF44
163894 229533.I1S5Z2 pp 229533.Q4HYQ4
163895 229533.I1S5Z2 pp 229533.Q4IAA0
163896 229533.I1S5Z2 pp 229533.Q4IR09
163897 229533.I1S5Z2 pp 229533.Q4IPN9
163898 229533.I1S5Z2 pp 229533.V6QWX8
163899 229533.I1RYH7 pp 229533.V6R3J1
163900 229533.I1RYH7 pp 229533.V6R8C4
163901 229533.I1RYH7 pp 229533.I1S8F0
163902 229533.I1SAI4 pp 229533.V6RBG5
163903 229533.I1SAI4 pp 229533.V6R0H4
163904 229533.I1SAI4 pp 229533.V6RPS5
163905 229533.I1SAI4 pp 229533.V6R556
163906 229533.I1SAI4 pp 229533.V6RY56
163907 229533.I1SAI4 pp 229533.I1SAJ0
163908 229533.I1SAI4 pp 229533.V6RM09
163909 229533.I1SAI4 pp 229533.V6REA4
163910 229533.I1SAI4 pp 229533.V6QTX7
163911 229533.A0A1C3YL93 pp 229533.I1RE19
163912 229533.A0A1C3YL93 pp 229533.I1RMA1
163913 229533.A0A1C3YL93 pp 229533.I1RSR2
163914 229533.A0A1C3YL93 pp 229533.Q4IMN9
163915 229533.A0A1C3YL93 pp 229533.I1RNF3
163916 229533.A0A1C3YL93 pp 229533.I1RPE3
163917 229533.A0A1C3YL93 pp 229533.I1S246
163918 229533.A0A1C3YL93 pp 229533.I1RJH1
163919 229533.A0A1C3YL93 pp 229533.I1RNL7
163920 229533.A0A1C3YL93 pp 229533.A0A1C3YM61
163921 229533.A0A1C3YL93 pp 229533.I1RMY3
163922 229533.A0A1C3YL93 pp 229533.I1RE46
163923 229533.A0A1C3YL93 pp 229533.I1RN60
163924 229533.A0A1C3YL93 pp 229533.V6REZ4
163925 229533.A0A1C3YL93 pp 229533.I1S0P4
163926 229533.A0A1C3YL93 pp 229533.I1RTA3
163927 229533.A0A1C3YL93 pp 229533.I1RMP2
163928 229533.A0A1C3YL93 pp 229533.I1RRF9
163929 229533.A0A1C3YL93 pp 229533.I1S2C0
163930 229533.I1S1V6 pp 229533.I1SAK2
163931 229533.I1RJX3 pp 229533.I1RMJ3
163932 229533.I1RJX3 pp 229533.I1RSY1
163933 229533.I1RJX3 pp 229533.I1S7J8
163934 229533.I1RJX3 pp 229533.I1S273
163935 229533.I1RZV9 pp 229533.Q4IPN9
163936 229533.I1RZV9 pp 229533.I1S2F3
163937 229533.I1RZV9 pp 229533.Q4HYC1
163938 229533.I1RZV9 pp 229533.Q4I7L0
163939 229533.I1RZV9 pp 229533.I1S214
163940 229533.I1RZV9 pp 229533.Q4I061
163941 229533.I1RZV9 pp 229533.I1S5B5
163942 229533.I1RZV9 pp 229533.V6REZ4
163943 229533.I1RZV9 pp 229533.Q4IPA4
163944 229533.I1RZV9 pp 229533.I1S246
163945 229533.I1RZV9 pp 229533.I1S471
163946 229533.I1RZV9 pp 229533.Q4I7X1
163947 229533.I1RZV9 pp 229533.I1S0P4
163948 229533.I1RZV9 pp 229533.Q4I877
163949 229533.I1RZV9 pp 229533.I1S044
163950 229533.I1RZV9 pp 229533.Q4IE79
163951 229533.I1RZV9 pp 229533.Q4IAA0
163952 229533.I1RLR7 pp 229533.I1RNC0
163953 229533.I1RLR7 pp 229533.Q4IB63
163954 229533.I1RLR7 pp 229533.V6QY25
163955 229533.I1RL71 pp 229533.I1RZ43
163956 229533.I1RL71 pp 229533.I1S0R8
163957 229533.I1RL71 pp 229533.I1RX39
163958 229533.I1RN49 pp 229533.Q4IPN9

163959 229533.I1RN49 pp 229533.Q4IR09
163960 229533.I1RN49 pp 229533.Q4IJP1
163961 229533.I1RN49 pp 229533.I1S214
163962 229533.I1RN49 pp 229533.Q4IPA4
163963 229533.I1RN49 pp 229533.I1S7W2
163964 229533.I1RN49 pp 229533.I1RWI0
163965 229533.I1RN49 pp 229533.Q4IB50
163966 229533.I1RN49 pp 229533.I1RZ02
163967 229533.I1RN49 pp 229533.Q4IF44
163968 229533.I1RN49 pp 229533.I1RQZ2
163969 229533.I1RN49 pp 229533.Q4I7N9
163970 229533.I1RN49 pp 229533.I1S8U9
163971 229533.I1RN49 pp 229533.I1RQ19
163972 229533.I1RN49 pp 229533.I1RZP2
163973 229533.I1RN49 pp 229533.I1S8V0
163974 229533.I1RN49 pp 229533.Q4I7X1
163975 229533.I1RN49 pp 229533.I1RYV3
163976 229533.I1RN49 pp 229533.I1S5Z2
163977 229533.I1RN49 pp 229533.Q4IPC8
163978 229533.I1RN49 pp 229533.I1RZH4
163979 229533.I1RN49 pp 229533.I1RSD1
163980 229533.I1RN49 pp 229533.I1RZL5
163981 229533.I1RN49 pp 229533.I1S4Z5
163982 229533.I1RN49 pp 229533.Q4I5R3
163983 229533.A0A0E0SBH8 pp 229533.I1RTQ9
163984 229533.A0A0E0SBH8 pp 229533.A0A1C3YN54
163985 229533.I1RPP1 pp 229533.I1RX39
163986 229533.V6R0W1 pp 229533.V6R556
163987 229533.V6R0W1 pp 229533.V6RY56
163988 229533.V6R0W1 pp 229533.V6RPS5
163989 229533.V6R0W1 pp 229533.V6RBG5
163990 229533.V6R0W1 pp 229533.V6RM09
163991 229533.V6R0W1 pp 229533.V6REA4
163992 229533.I1S9L5 pp 229533.Q4IRI9
163993 229533.I1S9L5 pp 229533.V6RY33
163994 229533.I1S9L5 pp 229533.V6QXG1
163995 229533.I1S9L5 pp 229533.V6RIS2
163996 229533.I1S9L5 pp 229533.Q4IB70
163997 229533.A0A1C3YJV9 pp 229533.Q4IF44
163998 229533.A0A1C3YJV9 pp 229533.A0A1C3YL99
163999 229533.A0A1C3YJV9 pp 229533.I1RYU6
164000 229533.A0A1C3YJV9 pp 229533.I1RS03
164001 229533.A0A1C3YJV9 pp 229533.I1RA84
164002 229533.A0A1C3YJV9 pp 229533.I1RMY3
164003 229533.A0A1C3YJV9 pp 229533.I1RFU3
164004 229533.A0A1C3YJV9 pp 229533.I1S382
164005 229533.A0A1C3YJV9 pp 229533.I1RQK2
164006 229533.A0A1C3YJV9 pp 229533.I1S214
164007 229533.A0A1C3YJV9 pp 229533.I1RE45
164008 229533.A0A1C3YJV9 pp 229533.Q4IPA4
164009 229533.A0A1C3YJV9 pp 229533.I1RYV4
164010 229533.A0A1C3YJV9 pp 229533.I1R976
164011 229533.A0A1C3YJV9 pp 229533.I1S246
164012 229533.A0A1C3YJV9 pp 229533.I1RAW7
164013 229533.A0A1C3YJV9 pp 229533.I1RCZ3
164014 229533.A0A1C3YJV9 pp 229533.I1RDD3
164015 229533.A0A1C3YJV9 pp 229533.I1RA46
164016 229533.A0A1C3YJV9 pp 229533.I1S1V5
164017 229533.A0A1C3YJV9 pp 229533.Q4IPN9
164018 229533.A0A1C3YJV9 pp 229533.I1RCU7
164019 229533.A0A1C3YJV9 pp 229533.I1RCK4
164020 229533.A0A1C3YJV9 pp 229533.I1RZP2
164021 229533.A0A1C3YJV9 pp 229533.I1RN49
164022 229533.A0A1C3YJV9 pp 229533.I1S0N7
164023 229533.A0A1C3YJV9 pp 229533.A0A1C3YKM6
164024 229533.A0A1C3YJV9 pp 229533.I1S951
164025 229533.A0A1C3YJV9 pp 229533.Q4IPC8
164026 229533.A0A1C3YJV9 pp 229533.I1S8U9
164027 229533.A0A1C3YJV9 pp 229533.Q4I7X1
164028 229533.A0A1C3YJV9 pp 229533.Q4IR09
164029 229533.A0A1C3YJV9 pp 229533.I1RZL5
164030 229533.A0A1C3YJV9 pp 229533.I1RHI1
164031 229533.A0A1C3YJV9 pp 229533.I1RNE4

164032 229533.A0A1C3YJV9 pp 229533.I1RT35
164033 229533.A0A1C3YJV9 pp 229533.I1RNT2
164034 229533.A0A1C3YJV9 pp 229533.I1R9Q0
164035 229533.A0A1C3YJV9 pp 229533.A0A1C3YM64
164036 229533.A0A1C3YJV9 pp 229533.I1RDG8
164037 229533.A0A1C3YJV9 pp 229533.I1RXE5
164038 229533.A0A1C3YJV9 pp 229533.I1RFI3
164039 229533.A0A1C3YJV9 pp 229533.I1RBJ1
164040 229533.A0A1C3YJV9 pp 229533.I1RN28
164041 229533.A0A1C3YJV9 pp 229533.I1RNB8
164042 229533.A0A1C3YJV9 pp 229533.I1RIR9
164043 229533.A0A1C3YJV9 pp 229533.A0A1C3YJW7
164044 229533.A0A1C3YJV9 pp 229533.I1RN17
164045 229533.A0A1C3YJV9 pp 229533.V6REZ4
164046 229533.A0A1C3YJV9 pp 229533.I1RBD6
164047 229533.A0A1C3YJV9 pp 229533.I1S5Z2
164048 229533.A0A1C3YJV9 pp 229533.A0A1C3YL25
164049 229533.A0A1C3YJV9 pp 229533.I1RZV9
164050 229533.A0A1C3YJV9 pp 229533.I1SA47
164051 229533.A0A1C3YJV9 pp 229533.I1S8V0
164052 229533.A0A1C3YJV9 pp 229533.I1RQZ2
164053 229533.A0A1C3YJV9 pp 229533.A0A1C3YMF9
164054 229533.A0A1C3YJV9 pp 229533.I1RA38
164055 229533.A0A1C3YI11 pp 229533.I1S5X9
164056 229533.A0A1C3YI11 pp 229533.I1RS29
164057 229533.A0A1C3YI11 pp 229533.A0A1C3YNB1
164058 229533.A0A1C3YI11 pp 229533.I1RSY1
164059 229533.A0A1C3YI11 pp 229533.I1S8S9
164060 229533.A0A1C3YI11 pp 229533.I1RFJ6
164061 229533.A0A1C3YI11 pp 229533.I1RWA3
164062 229533.A0A1C3YI11 pp 229533.A0A1C3YMM2
164063 229533.A0A1C3YI11 pp 229533.A0A1C3YN33
164064 229533.A0A1C3YI11 pp 229533.I1RAB6
164065 229533.A0A1C3YI11 pp 229533.I1RTA0
164066 229533.A0A1C3YI11 pp 229533.I1RYT8
164067 229533.A0A1C3YI11 pp 229533.V6RJP8
164068 229533.A0A1C3YI11 pp 229533.I1S273
164069 229533.A0A1C3YI11 pp 229533.I1S0K2
164070 229533.A0A1C3YI11 pp 229533.I1RA52
164071 229533.A0A1C3YI11 pp 229533.I1S5C6
164072 229533.A0A1C3YI11 pp 229533.I1S7J8
164073 229533.A0A1C3YI11 pp 229533.I1RY21
164074 229533.A0A1C3YI11 pp 229533.A0A1C3YL46
164075 229533.A0A1C3YI11 pp 229533.I1RMK7
164076 229533.A0A1C3YI11 pp 229533.I1RMJ3
164077 229533.A0A1C3YI11 pp 229533.I1S8P5
164078 229533.A0A1C3YI11 pp 229533.I1S464
164079 229533.A0A1C3YI11 pp 229533.I1RXD9
164080 229533.A0A1C3YI11 pp 229533.A0A1C3YLL1
164081 229533.A0A1C3YI11 pp 229533.I1RS05
164082 229533.A0A1C3YI11 pp 229533.I1RGI5
164083 229533.I1RHX8 pp 229533.I1RX39
164084 229533.I1RW55 pp 229533.I1RZ89
164085 229533.I1RW55 pp 229533.I1RZ25
164086 229533.I1RW55 pp 229533.I1RZ56
164087 229533.I1RW55 pp 229533.I1S2C4
164088 229533.I1RW55 pp 229533.I1RWF5
164089 229533.I1RW55 pp 229533.I1RW82
164090 229533.I1RW55 pp 229533.I1S6V0
164091 229533.I1RW55 pp 229533.I1S968
164092 229533.I1RW55 pp 229533.I1SAT2
164093 229533.I1RW55 pp 229533.I1S659
164094 229533.I1RW55 pp 229533.V6RHX2
164095 229533.I1RW55 pp 229533.I1S0A5
164096 229533.I1RW55 pp 229533.I1S267
164097 229533.I1RW55 pp 229533.V6RCJ5
164098 229533.I1RW55 pp 229533.I1RY81
164099 229533.I1RW55 pp 229533.I1S2F7
164100 229533.I1RW55 pp 229533.V6RCC0
164101 229533.I1RW55 pp 229533.I1S5D0
164102 229533.I1RW55 pp 229533.V6RWB3
164103 229533.I1RW55 pp 229533.I1RWM4
164104 229533.I1RW55 pp 229533.I1RY12

229533.I1RW55	pp	229533.V6R5T7
229533.I1RW55	pp	229533.I1S8D3
229533.I1RW55	pp	229533.I1S969
229533.I1RW55	pp	229533.I1RWL3
229533.I1RW55	pp	229533.I1S048
229533.I1RW55	pp	229533.I1RYU6
229533.I1RW55	pp	229533.I1S6H3
229533.I1RW55	pp	229533.I1S094
229533.I1RW55	pp	229533.I1RYP5
229533.I1RB53	pp	229533.I1RS20
229533.I1RB53	pp	229533.I1RPU3
229533.I1RB53	pp	229533.I1RZZ9
229533.I1RB53	pp	229533.I1RR73
229533.I1RB53	pp	229533.I1RGA4
229533.I1RB53	pp	229533.I1RBY4
229533.I1RB53	pp	229533.I1RRT8
229533.I1RB53	pp	229533.I1RGY5
229533.I1RB53	pp	229533.I1RN63
229533.I1RB53	pp	229533.I1S2B8
229533.I1RB53	pp	229533.I1RJZ7
229533.I1RB53	pp	229533.I1RWK9
229533.I1RB53	pp	229533.Q4HW67
229533.I1RB53	pp	229533.I1S0X5
229533.I1RB53	pp	229533.I1S2V2
229533.I1RB53	pp	229533.I1RCJ7
229533.I1RB53	pp	229533.I1RPP3
229533.I1RB53	pp	229533.I1RT40
229533.I1RB53	pp	229533.I1RSV2
229533.I1RB53	pp	229533.I1RJV3
229533.I1RB53	pp	229533.I1RN67
229533.I1RB53	pp	229533.I1S2E9
229533.I1RB53	pp	229533.I1S855
229533.I1RB53	pp	229533.I1S2E8
229533.I1RB53	pp	229533.I1RNG4
229533.I1RB53	pp	229533.Q4IEV5
229533.I1RB53	pp	229533.I1RT04
229533.I1RB53	pp	229533.I1RQE7
229533.I1RB53	pp	229533.I1S0N7
229533.I1RB53	pp	229533.Q4IAS1
229533.I1RB53	pp	229533.I1RFL4
229533.I1RB53	pp	229533.I1RQK2
229533.I1RB53	pp	229533.I1SAE3
229533.I1RB53	pp	229533.I1RVX0
229533.I1RB53	pp	229533.I1S503
229533.I1RB53	pp	229533.Q4HZ68
229533.I1RB53	pp	229533.I1RT03
229533.I1RB53	pp	229533.Q4HZ42
229533.I1RB53	pp	229533.I1RE71
229533.I1RB53	pp	229533.I1RBS6
229533.I1RB53	pp	229533.I1RP27
229533.I1RB53	pp	229533.I1RZW8
229533.I1RB53	pp	229533.I1RCI8
229533.I1RB53	pp	229533.I1S0N4
229533.I1RB53	pp	229533.I1RH64
229533.I1RB53	pp	229533.I1RNC1
229533.I1RB53	pp	229533.I1RPN8
229533.I1RB53	pp	229533.I1S0J1
229533.I1RB53	pp	229533.I1RYW3
229533.I1RB53	pp	229533.I1RMQ5
229533.I1RB53	pp	229533.I1RCR8
229533.I1RB53	pp	229533.I1REF5
229533.I1RB53	pp	229533.I1S1C7
229533.I1RB53	pp	229533.V6R042
229533.I1RB53	pp	229533.I1RPS0
229533.I1RB53	pp	229533.I1RZS7
229533.I1RB53	pp	229533.Q4HYB8
229533.I1RB53	pp	229533.V6RMW6
229533.I1RB53	pp	229533.I1RSQ3
229533.I1RB53	pp	229533.I1RBD6
229533.I1RB53	pp	229533.I1S2C0
229533.I1RB53	pp	229533.I1RD22
229533.I1RB53	pp	229533.I1RTH6
229533.I1RB53	pp	229533.Q4I7K4

164178 229533.I1RB53 pp 229533.I1RWD3
164179 229533.I1RB53 pp 229533.I1S854
164180 229533.I1RB53 pp 229533.I1RWH4
164181 229533.I1RB53 pp 229533.I1RNB8
164182 229533.I1RB53 pp 229533.I1RFQ7
164183 229533.I1RB53 pp 229533.I1S4S4
164184 229533.I1RB53 pp 229533.I1RY44
164185 229533.I1RB53 pp 229533.I1RK70
164186 229533.I1RB53 pp 229533.V6RTJ8
164187 229533.I1RB53 pp 229533.V6RIV9
164188 229533.I1RB53 pp 229533.I1RM03
164189 229533.I1RB53 pp 229533.I1RN61
164190 229533.I1RB53 pp 229533.I1RW25
164191 229533.I1RB53 pp 229533.I1RCY0
164192 229533.I1RB53 pp 229533.I1RZ02
164193 229533.I1RB53 pp 229533.I1S0S0
164194 229533.I1RB53 pp 229533.I1RC64
164195 229533.I1RB53 pp 229533.I1RP08
164196 229533.I1RB53 pp 229533.I1RSQ7
164197 229533.I1RB53 pp 229533.I1RXU7
164198 229533.I1RB53 pp 229533.I1RBC9
164199 229533.I1RB53 pp 229533.I1RDI8
164200 229533.I1RB53 pp 229533.I1S6F0
164201 229533.I1RB53 pp 229533.I1RMT4
164202 229533.I1RB53 pp 229533.I1RNI4
164203 229533.I1RB53 pp 229533.I1RSM2
164204 229533.I1RB53 pp 229533.Q4HVW2
164205 229533.I1RB53 pp 229533.I1S0R7
164206 229533.I1RB53 pp 229533.I1RVQ0
164207 229533.I1RB53 pp 229533.I1RQC9
164208 229533.I1RB53 pp 229533.I1RLU5
164209 229533.I1RB53 pp 229533.I1RX46
164210 229533.I1RB53 pp 229533.I1S0D4
164211 229533.I1RB53 pp 229533.I1RW87
164212 229533.I1RB53 pp 229533.I1RYL5
164213 229533.I1RB53 pp 229533.I1RZZ1
164214 229533.I1RB53 pp 229533.I1RHI1
164215 229533.I1RB53 pp 229533.I1S0C1
164216 229533.I1RB53 pp 229533.I1RBC4
164217 229533.I1RB53 pp 229533.I1RCG8
164218 229533.I1RB53 pp 229533.I1RRU9
164219 229533.I1RB53 pp 229533.I1RW99
164220 229533.I1RB53 pp 229533.I1RKK8
164221 229533.I1RB53 pp 229533.I1RN99
164222 229533.I1RB53 pp 229533.Q4IJK2
164223 229533.I1RB53 pp 229533.I1RZH4
164224 229533.I1RB53 pp 229533.I1RN65
164225 229533.I1RB53 pp 229533.I1RE00
164226 229533.I1RB53 pp 229533.Q4IAA0
164227 229533.I1RB53 pp 229533.I1S6F1
164228 229533.I1RB53 pp 229533.I1RP66
164229 229533.I1RB53 pp 229533.I1RMC0
164230 229533.I1RB53 pp 229533.I1RWD7
164231 229533.I1RB53 pp 229533.I1RQ81
164232 229533.I1RB53 pp 229533.Q4IEK8
164233 229533.I1RB53 pp 229533.I1RT35
164234 229533.I1RB53 pp 229533.I1RN58
164235 229533.I1RB53 pp 229533.I1S504
164236 229533.I1RB53 pp 229533.I1RBB7
164237 229533.I1RNN0 pp 229533.I1RNN1
164238 229533.A0A098DPE0 pp 229533.A0A1C3YIW9
164239 229533.A0A098DPE0 pp 229533.I1RX39
164240 229533.A0A098DPE0 pp 229533.A0A1C3YLB0
164241 229533.I1RR22 pp 229533.I1S323
164242 229533.I1RR22 pp 229533.I1RRT2
164243 229533.I1RND9 pp 229533.I1RQ42
164244 229533.I1RND9 pp 229533.I1RWU2
164245 229533.I1RND9 pp 229533.I1RS03
164246 229533.I1RND9 pp 229533.I1S4M5
164247 229533.I1RND9 pp 229533.I1S246
164248 229533.I1RND9 pp 229533.I1RX81
164249 229533.I1RND9 pp 229533.I1RS75
164250 229533.I1RND9 pp 229533.I1RT35

164251	229533.I1RND9	pp	229533.I1RSA4
164252	229533.I1RND9	pp	229533.I1RPY4
164253	229533.I1RND9	pp	229533.Q4IPB3
164254	229533.I1RND9	pp	229533.I1RW80
164255	229533.I1RND9	pp	229533.I1RZG7
164256	229533.I1RND9	pp	229533.I1RWX8
164257	229533.I1RND9	pp	229533.I1S0Z9
164258	229533.I1RND9	pp	229533.I1RST1
164259	229533.I1RND9	pp	229533.I1S9M9
164260	229533.I1RND9	pp	229533.V6QXG1
164261	229533.I1RND9	pp	229533.I1RX51
164262	229533.I1RND9	pp	229533.I1RPA0
164263	229533.I1RND9	pp	229533.I1S5Z2
164264	229533.I1RND9	pp	229533.Q4IAA0
164265	229533.I1RND9	pp	229533.I1RPW8
164266	229533.I1RND9	pp	229533.I1RSD4
164267	229533.I1RND9	pp	229533.I1RS28
164268	229533.I1RND9	pp	229533.Q4IB70
164269	229533.I1RND9	pp	229533.I1RT47
164270	229533.I1RND9	pp	229533.I1S471
164271	229533.I1RND9	pp	229533.I1RX47
164272	229533.I1S2P0	pp	229533.V6QUE6
164273	229533.I1RRM1	pp	229533.V6QVH2
164274	229533.I1RRM1	pp	229533.I1S0A5
164275	229533.I1RRM1	pp	229533.V6R2Z2
164276	229533.I1RRM1	pp	229533.I1SAI4
164277	229533.I1RRM1	pp	229533.I1RV23
164278	229533.I1RRM1	pp	229533.I1S224
164279	229533.I1RRM1	pp	229533.V6RAM6
164280	229533.I1RRM1	pp	229533.V6R5D2
164281	229533.I1RRM1	pp	229533.V6RZD4
164282	229533.I1RRM1	pp	229533.I1RZV5
164283	229533.I1RRM1	pp	229533.I1RY49
164284	229533.I1RRM1	pp	229533.V6RRM0
164285	229533.I1RRM1	pp	229533.I1RVX9
164286	229533.I1RRM1	pp	229533.V6R0W1
164287	229533.I1RRM1	pp	229533.I1S4S5
164288	229533.I1RRM1	pp	229533.V6QU99
164289	229533.I1RRM1	pp	229533.I1RW43
164290	229533.I1RRM1	pp	229533.V6R3V4
164291	229533.I1RRM1	pp	229533.I1S150
164292	229533.I1RRM1	pp	229533.V6RF62
164293	229533.I1RRM1	pp	229533.V6R398
164294	229533.I1RRM1	pp	229533.V6R756
164295	229533.I1RRM1	pp	229533.I1S8H9
164296	229533.I1RRM1	pp	229533.I1RWD2
164297	229533.A0A0E0RT42	pp	229533.I1RW43
164298	229533.A0A0E0RT42	pp	229533.I1RY49
164299	229533.A0A0E0RT42	pp	229533.I1RVX9
164300	229533.A0A0E0RT42	pp	229533.I1RM26
164301	229533.A0A0E0RT42	pp	229533.V6RRM0
164302	229533.A0A098DW31	pp	229533.A0A1C3YLB0
164303	229533.A0A098DW31	pp	229533.A0A1C3YIW9
164304	229533.A0A098DW31	pp	229533.I1RX39
164305	229533.I1RNM9	pp	229533.I1RNN1
164306	229533.I1RR36	pp	229533.I1S323
164307	229533.I1RR36	pp	229533.I1RRT2
164308	229533.I1RJF9	pp	229533.I1RZQ5
164309	229533.I1RJF9	pp	229533.I1RPY4
164310	229533.I1RJF9	pp	229533.I1RZG7
164311	229533.I1RJF9	pp	229533.I1RX24
164312	229533.I1RJF9	pp	229533.Q4IPB3
164313	229533.I1RJF9	pp	229533.I1S1X9
164314	229533.I1RJF9	pp	229533.I1S0B1
164315	229533.I1RJF9	pp	229533.Q4IE79
164316	229533.I1RJF9	pp	229533.I1RMV5
164317	229533.I1RJF9	pp	229533.I1S7M3
164318	229533.I1RJF9	pp	229533.I1RX47
164319	229533.I1RJF9	pp	229533.I1RXF2
164320	229533.I1RJF9	pp	229533.I1S7M2
164321	229533.I1RJF9	pp	229533.I1RK90
164322	229533.I1RJF9	pp	229533.I1RLU0
164323	229533.I1RJF9	pp	229533.I1S056

164324 229533.I1RJF9 pp 229533.I1S1C6
164325 229533.I1RJF9 pp 229533.I1RN59
164326 229533.I1RJF9 pp 229533.I1RYW3
164327 229533.I1RJF9 pp 229533.I1RRL0
164328 229533.I1RJF9 pp 229533.Q4I1Y1
164329 229533.I1RJF9 pp 229533.Q4IBK5
164330 229533.I1RJF9 pp 229533.I1RQC5
164331 229533.I1RJF9 pp 229533.I1RT70
164332 229533.I1RJF9 pp 229533.I1RK92
164333 229533.I1RJF9 pp 229533.V6QXG1
164334 229533.I1RJF9 pp 229533.I1S2Y5
164335 229533.I1RJF9 pp 229533.I1RPY5
164336 229533.I1RJF9 pp 229533.I1RS28
164337 229533.I1RJF9 pp 229533.I1RND5
164338 229533.I1RJF9 pp 229533.I1S4M5
164339 229533.I1RJF9 pp 229533.I1RME9
164340 229533.I1RJF9 pp 229533.I1RSA4
164341 229533.I1RJF9 pp 229533.Q4IPB8
164342 229533.I1S5N3 pp 229533.V6QUE6
164343 229533.I1RYT7 pp 229533.I1S5B5
164344 229533.I1RYT7 pp 229533.I1S0P4
164345 229533.I1RYT7 pp 229533.Q4IAA0
164346 229533.I1RYT7 pp 229533.Q4I7X1
164347 229533.I1RYT7 pp 229533.I1RZV9
164348 229533.I1RYT7 pp 229533.Q4IPN9
164349 229533.I1RYT7 pp 229533.I1S471
164350 229533.I1RYT7 pp 229533.V6REZ4
164351 229533.I1RYT7 pp 229533.I1S2F3
164352 229533.I1RYT7 pp 229533.I1S246
164353 229533.I1RYT7 pp 229533.I1RZT1
164354 229533.I1RYT7 pp 229533.I1S2A8
164355 229533.I1RYT7 pp 229533.I1S214
164356 229533.I1RYT7 pp 229533.I1S0N7
164357 229533.I1RF38 pp 229533.I1RNV4
164358 229533.I1RF38 pp 229533.I1RM26
164359 229533.I1RF38 pp 229533.I1RU76
164360 229533.I1RF38 pp 229533.V6RHZ8
164361 229533.I1RF38 pp 229533.V6RGB5
164362 229533.I1RF38 pp 229533.V6R9Z3
164363 229533.I1RF38 pp 229533.I1S2H3
164364 229533.I1RF38 pp 229533.I1RN80
164365 229533.I1RF38 pp 229533.I1RJA8
164366 229533.I1RF38 pp 229533.I1RQE0
164367 229533.I1RF38 pp 229533.I1RGI2
164368 229533.I1RF38 pp 229533.I1RF40
164369 229533.I1RF38 pp 229533.I1RPH3
164370 229533.I1RF38 pp 229533.I1S0A0
164371 229533.I1RF38 pp 229533.I1S064
164372 229533.I1RF38 pp 229533.I1RW43
164373 229533.I1RF38 pp 229533.I1RVX9
164374 229533.I1RF38 pp 229533.I1RY49
164375 229533.I1RF38 pp 229533.I1RPW2
164376 229533.I1RF38 pp 229533.I1RH36
164377 229533.I1RF38 pp 229533.I1S4F4
164378 229533.I1RF38 pp 229533.I1RNG7
164379 229533.I1RF38 pp 229533.I1RNG4
164380 229533.I1RF38 pp 229533.I1RJT5
164381 229533.I1RF38 pp 229533.I1S6P6
164382 229533.I1RF38 pp 229533.I1RYU5
164383 229533.I1RF38 pp 229533.I1RQ70
164384 229533.I1RF38 pp 229533.I1RZ20
164385 229533.I1RF38 pp 229533.I1RR12
164386 229533.I1RF38 pp 229533.I1RPD6
164387 229533.I1RI81 pp 229533.I1RZP1
164388 229533.I1RYE8 pp 229533.Q4IL82
164389 229533.A0A1C3YLZ2 pp 229533.I1RZ25
164390 229533.I1RY40 pp 229533.I1S0N7
164391 229533.I1RY40 pp 229533.Q4I5R9
164392 229533.I1RY40 pp 229533.I1RYP0
164393 229533.I1RY40 pp 229533.I1S287
164394 229533.I1RY40 pp 229533.I1S0L8
164395 229533.I1RY40 pp 229533.I1S4N5
164396 229533.I1RY40 pp 229533.I1S284

229533.I1RY40 pp 229533.I1S2F3
229533.I1RY40 pp 229533.V6QXP4
229533.I1RY40 pp 229533.I1RYT7
229533.I1RY40 pp 229533.I1RZ94
229533.I1RY40 pp 229533.I1S0V5
229533.I1RY40 pp 229533.Q4IPA4
229533.I1RY40 pp 229533.V6QYF0
229533.I1RY40 pp 229533.I1RYZ7
229533.I1RY40 pp 229533.I1S0X5
229533.I1RY40 pp 229533.I1S9V9
229533.I1RY40 pp 229533.Q4I7X1
229533.I1RY40 pp 229533.I1S278
229533.I1RY40 pp 229533.I1RZM1
229533.I1RY40 pp 229533.I1S193
229533.I1RY40 pp 229533.I1RYX5
229533.I1RY40 pp 229533.V6QW25
229533.I1RY40 pp 229533.I1S471
229533.I1RY40 pp 229533.I1S1Y9
229533.I1RY40 pp 229533.I1S2B9
229533.I1RY40 pp 229533.I1RYM3
229533.I1RY40 pp 229533.I1S0P4
229533.I1RY40 pp 229533.I1S0N4
229533.I1RY40 pp 229533.I1S5B5
229533.I1RY40 pp 229533.I1RZT1
229533.I1RY40 pp 229533.I1S9G7
229533.I1RY40 pp 229533.Q4HZB8
229533.I1RY40 pp 229533.Q4IAA0
229533.I1RY40 pp 229533.I1S2B6
229533.I1RY40 pp 229533.I1RYL5
229533.I1RY40 pp 229533.V6RXW9
229533.I1RY40 pp 229533.Q4HY20
229533.I1RY40 pp 229533.I1RZV9
229533.I1RY40 pp 229533.I1S1U6
229533.I1RY40 pp 229533.V6R0U9
229533.I1RY40 pp 229533.V6QUV7
229533.I1RY40 pp 229533.V6REZ4
229533.I1RKX6 pp 229533.I1RYW3
229533.I1RKX6 pp 229533.I1RMJ3
229533.I1RKX6 pp 229533.I1RSY1
229533.I1RKX6 pp 229533.I1RWX5
229533.I1RKX6 pp 229533.I1RW71
229533.I1RKX6 pp 229533.V6QUE6
229533.I1RKX6 pp 229533.I1RRT8
229533.I1RKX6 pp 229533.I1S273
229533.A0A1C3YIP2 pp 229533.I1RDJ7
229533.A0A1C3YIP2 pp 229533.V6RBG5
229533.A0A1C3YIP2 pp 229533.I1RZ38
229533.A0A1C3YIP2 pp 229533.I1S3H8
229533.A0A1C3YIP2 pp 229533.I1RF24
229533.A0A1C3YIP2 pp 229533.I1RKB2
229533.A0A1C3YIP2 pp 229533.I1SAJ0
229533.A0A1C3YIP2 pp 229533.I1RNG4
229533.A0A1C3YIP2 pp 229533.I1RGZ3
229533.A0A1C3YIP2 pp 229533.A0A1C3YKN4
229533.A0A1C3YIP2 pp 229533.I1RI19
229533.A0A1C3YIP2 pp 229533.V6R0H4
229533.A0A1C3YIP2 pp 229533.I1RMT6
229533.A0A1C3YIP2 pp 229533.I1RAC0
229533.A0A1C3YIP2 pp 229533.A0A1C3YKV2
229533.A0A1C3YIP2 pp 229533.I1S0A5
229533.A0A1C3YIP2 pp 229533.V6R556
229533.A0A1C3YIP2 pp 229533.I1RJS1
229533.A0A1C3YIP2 pp 229533.I1RZ26
229533.A0A1C3YIP2 pp 229533.A0A1C3YJL9
229533.A0A1C3YIP2 pp 229533.I1S149
229533.A0A1C3YIP2 pp 229533.A0A1C3YJM7
229533.A0A1C3YIP2 pp 229533.I1RZ25
229533.A0A1C3YIP2 pp 229533.I1RTY9
229533.A0A1C3YIP2 pp 229533.I1RPV8
229533.A0A1C3YIP2 pp 229533.I1RF85
229533.A0A1C3YIP2 pp 229533.I1RD80
229533.A0A1C3YIP2 pp 229533.I1R9X3
229533.A0A1C3YIP2 pp 229533.I1RHT3

229533.A0A1C3YIP2 pp 229533.I1R9B8
229533.A0A1C3YIP2 pp 229533.I1RV23
229533.A0A1C3YIP2 pp 229533.I1RTU5
229533.A0A1C3YIP2 pp 229533.I1RQX9
229533.A0A1C3YIP2 pp 229533.I1S094
229533.A0A1C3YIP2 pp 229533.I1RJG4
229533.A0A1C3YIP2 pp 229533.I1RZ56
229533.A0A1C3YIP2 pp 229533.I1RHL9
229533.A0A1C3YIP2 pp 229533.I1RNM7
229533.A0A1C3YIP2 pp 229533.I1RVI8
229533.A0A1C3YIP2 pp 229533.I1RJE7
229533.A0A1C3YIP2 pp 229533.V6QTX7
229533.A0A1C3YIP2 pp 229533.I1S6H1
229533.A0A1C3YIP2 pp 229533.I1RRL5
229533.A0A1C3YIP2 pp 229533.I1RDN3
229533.A0A1C3YIP2 pp 229533.I1S334
229533.A0A1C3YIP2 pp 229533.V6REA4
229533.A0A1C3YIP2 pp 229533.I1RTT0
229533.A0A1C3YIP2 pp 229533.I1RY96
229533.A0A1C3YIP2 pp 229533.A0A1C3YKZ8
229533.A0A1C3YIP2 pp 229533.I1RLA2
229533.A0A1C3YIP2 pp 229533.I1RV06
229533.A0A1C3YIP2 pp 229533.I1REE6
229533.A0A1C3YIP2 pp 229533.I1R9F2
229533.A0A1C3YIP2 pp 229533.I1RC39
229533.A0A1C3YIP2 pp 229533.I1RF16
229533.A0A1C3YIP2 pp 229533.I1RMS5
229533.A0A1C3YIP2 pp 229533.V6RM09
229533.A0A1C3YIP2 pp 229533.I1S6H3
229533.A0A1C3YIP2 pp 229533.I1RCU2
229533.A0A1C3YIP2 pp 229533.I1S0H2
229533.A0A1C3YIP2 pp 229533.I1RDU0
229533.A0A1C3YIP2 pp 229533.I1RNH4
229533.A0A1C3YIP2 pp 229533.I1S269
229533.A0A1C3YIP2 pp 229533.A0A1C3YKH5
229533.A0A1C3YIP2 pp 229533.I1R9C0
229533.A0A1C3YIP2 pp 229533.I1RA13
229533.A0A1C3YIP2 pp 229533.I1RTJ7
229533.A0A1C3YIP2 pp 229533.I1S4D0
229533.A0A1C3YIP2 pp 229533.V6RPS5
229533.A0A1C3YIP2 pp 229533.I1RRM1
229533.A0A1C3YIP2 pp 229533.I1RK03
229533.A0A1C3YIP2 pp 229533.I1RJK0
229533.A0A1C3YIP2 pp 229533.I1RJT5
229533.A0A1C3YIP2 pp 229533.V6RY56
229533.A0A1C3YIP2 pp 229533.I1S405
229533.A0A1C3YIP2 pp 229533.I1RE90
229533.A0A1C3YIP2 pp 229533.I1RCV1
229533.A0A1C3YIP2 pp 229533.I1RST9
229533.A0A1C3YIP2 pp 229533.I1S0T1
229533.A0A1C3YIP2 pp 229533.I1RUY5
229533.A0A1C3YIP2 pp 229533.I1RIK8
229533.A0A1C3YIP2 pp 229533.I1S8F8
229533.A0A1C3YIP2 pp 229533.I1RDV8
229533.A0A1C3YIP2 pp 229533.I1RIW5
229533.A0A1C3YIP2 pp 229533.I1RV18
229533.A0A1C3YIP2 pp 229533.I1RAG3
229533.I1S1D4 pp 229533.V6RRM0
229533.I1RXP4 pp 229533.I1SAK2
229533.I1RLP9 pp 229533.I1RMJ3
229533.I1RLP9 pp 229533.I1RYW3
229533.I1RLP9 pp 229533.I1RWX5
229533.I1RLP9 pp 229533.I1RSY1
229533.I1RLP9 pp 229533.I1RRT8
229533.I1RLP9 pp 229533.V6QUE6
229533.I1RLP9 pp 229533.I1S273
229533.I1RLP9 pp 229533.I1RW71
229533.I1RQZ2 pp 229533.I1S5Z2
229533.I1RQZ2 pp 229533.Q4IB70
229533.I1RQZ2 pp 229533.I1RRT0
229533.I1RQZ2 pp 229533.I1RZB5
229533.I1RQZ2 pp 229533.I1RT35
229533.I1RQZ2 pp 229533.I1RSA4

164543 229533.I1RQZ2 pp 229533.I1RRW3
164544 229533.I1RQZ2 pp 229533.I1S246
164545 229533.I1RQZ2 pp 229533.I1RSH9
164546 229533.I1RQZ2 pp 229533.I1S7M3
164547 229533.I1RQZ2 pp 229533.I1RZE1
164548 229533.I1RQZ2 pp 229533.I1RRG3
164549 229533.I1RQZ2 pp 229533.I1S7M2
164550 229533.I1RQZ2 pp 229533.I1RSD4
164551 229533.I1RQZ2 pp 229533.I1RZG7
164552 229533.I1RQZ2 pp 229533.I1S471
164553 229533.I1RQZ2 pp 229533.I1RXF2
164554 229533.I1RQZ2 pp 229533.I1S0N7
164555 229533.I1RQZ2 pp 229533.Q4IF44
164556 229533.I1RQZ2 pp 229533.I1RX47
164557 229533.I1RQZ2 pp 229533.Q4IR09
164558 229533.I1RQZ2 pp 229533.I1RS03
164559 229533.I1RQZ2 pp 229533.I1S0B6
164560 229533.I1RQZ2 pp 229533.I1RYJ9
164561 229533.I1RQZ2 pp 229533.I1RS66
164562 229533.I1RQZ2 pp 229533.I1RR25
164563 229533.I1RQZ2 pp 229533.I1S1U7
164564 229533.I1RQZ2 pp 229533.I1RTS2
164565 229533.I1RQZ2 pp 229533.I1S0B1
164566 229533.I1RQZ2 pp 229533.Q4IPA4
164567 229533.I1S1G0 pp 229533.I1S1G1
164568 229533.I1S0N7 pp 229533.Q4HWE2
164569 229533.I1S0N7 pp 229533.I1S5Z2
164570 229533.I1S0N7 pp 229533.Q4I7X1
164571 229533.I1S0N7 pp 229533.I1S8U9
164572 229533.I1S0N7 pp 229533.I1S8V0
164573 229533.I1S0N7 pp 229533.I1S0N8
164574 229533.I1S0N7 pp 229533.I1S471
164575 229533.I1S0N7 pp 229533.V6REZ4
164576 229533.I1S0N7 pp 229533.I1S6F1
164577 229533.I1S0N7 pp 229533.I1SA47
164578 229533.I1S0N7 pp 229533.I1S0P4
164579 229533.I1S0N7 pp 229533.Q4IAA0
164580 229533.I1S0N7 pp 229533.Q4IPA4
164581 229533.I1S0N7 pp 229533.I1S4Y4
164582 229533.I1S0N7 pp 229533.I1S214
164583 229533.I1S0N7 pp 229533.Q4IR09
164584 229533.I1S0N7 pp 229533.Q4IF44
164585 229533.I1S0N7 pp 229533.Q4IPN9
164586 229533.I1S0N7 pp 229533.I1S246
164587 229533.I1RME9 pp 229533.I1RZG7
164588 229533.I1RME9 pp 229533.I1RSA8
164589 229533.I1RME9 pp 229533.Q4IRI9
164590 229533.I1RME9 pp 229533.I1RT35
164591 229533.I1RME9 pp 229533.I1RWX8
164592 229533.I1RME9 pp 229533.I1S7M3
164593 229533.I1RME9 pp 229533.I1RSD4
164594 229533.I1RME9 pp 229533.Q4IPB3
164595 229533.I1RME9 pp 229533.Q4IB70
164596 229533.I1RME9 pp 229533.I1RX81
164597 229533.I1RME9 pp 229533.I1S0Z9
164598 229533.I1RME9 pp 229533.I1RNB8
164599 229533.I1RME9 pp 229533.I1RS28
164600 229533.I1RME9 pp 229533.I1RPA0
164601 229533.I1RME9 pp 229533.I1RN09
164602 229533.I1RME9 pp 229533.I1S9M0
164603 229533.I1RME9 pp 229533.I1RS75
164604 229533.I1RME9 pp 229533.I1RN59
164605 229533.I1RME9 pp 229533.Q4I1Y1
164606 229533.I1RME9 pp 229533.I1RX51
164607 229533.I1RME9 pp 229533.I1S056
164608 229533.I1RME9 pp 229533.I1RT47
164609 229533.I1RME9 pp 229533.I1S1C6
164610 229533.I1RME9 pp 229533.I1RX47
164611 229533.I1RME9 pp 229533.I1RZQ5
164612 229533.I1RME9 pp 229533.I1S7M2
164613 229533.I1RME9 pp 229533.V6QXG1
164614 229533.I1RME9 pp 229533.I1RND5
164615 229533.I1RME9 pp 229533.I1S1X9

229533.I1RME9	pp	229533.I1RSA4
229533.I1RME9	pp	229533.I1RND9
229533.I1RME9	pp	229533.I1RRL0
229533.I1RME9	pp	229533.I1RPY4
229533.I1RME9	pp	229533.I1RMV5
229533.I1RME9	pp	229533.I1RXF2
229533.I1RME9	pp	229533.I1RST1
229533.I1RME9	pp	229533.I1RX24
229533.I1RME9	pp	229533.I1S4M5
229533.I1RJ04	pp	229533.I1RMJ3
229533.I1RJ04	pp	229533.I1S273
229533.I1RJ04	pp	229533.I1S7J8
229533.I1RJ04	pp	229533.I1RSY1
229533.I1RCG8	pp	229533.Q4HY71
229533.I1RCG8	pp	229533.I1S0N7
229533.I1RCG8	pp	229533.I1RJZ7
229533.I1RCG8	pp	229533.I1RN60
229533.I1RCG8	pp	229533.I1RS94
229533.I1RCG8	pp	229533.Q4IQK7
229533.I1RCG8	pp	229533.I1RDY5
229533.I1RCG8	pp	229533.I1RJI3
229533.I1RCG8	pp	229533.I1RNB8
229533.I1RCG8	pp	229533.I1S0P4
229533.I1RCG8	pp	229533.I1RMY3
229533.I1RCG8	pp	229533.I1S1U7
229533.I1RCG8	pp	229533.I1SA47
229533.I1RCG8	pp	229533.I1S5Z2
229533.I1RCG8	pp	229533.I1SA48
229533.I1RCG8	pp	229533.I1S246
229533.I1RR96	pp	229533.Q4ICM0
229533.I1RMY3	pp	229533.V6REZ4
229533.I1RMY3	pp	229533.V6R3J1
229533.I1RMY3	pp	229533.I1RYT7
229533.I1RMY3	pp	229533.I1RZT1
229533.I1RMY3	pp	229533.I1RS94
229533.I1RMY3	pp	229533.I1RYJ9
229533.I1RMY3	pp	229533.I1RS41
229533.I1RMY3	pp	229533.I1S214
229533.I1RMY3	pp	229533.I1RZV9
229533.I1RMY3	pp	229533.I1RSV2
229533.I1RMY3	pp	229533.I1RNG5
229533.I1RMY3	pp	229533.I1RQ49
229533.I1RMY3	pp	229533.I1RNB8
229533.I1RMY3	pp	229533.I1RNE4
229533.I1RMY3	pp	229533.I1S5B5
229533.I1RMY3	pp	229533.Q4HYB8
229533.I1RMY3	pp	229533.I1S471
229533.I1RMY3	pp	229533.I1RYL5
229533.I1RMY3	pp	229533.I1RWQ8
229533.I1RMY3	pp	229533.I1S0P4
229533.I1RMY3	pp	229533.I1S279
229533.I1RMY3	pp	229533.I1RTE5
229533.I1RMY3	pp	229533.I1RT35
229533.I1RMY3	pp	229533.I1RNT2
229533.I1RMY3	pp	229533.I1S246
229533.I1RMY3	pp	229533.Q4IAA0
229533.I1RMY3	pp	229533.I1RY40
229533.I1RMY3	pp	229533.Q4I7X1
229533.I1RMY3	pp	229533.Q4IP34
229533.A0A098DC40	pp	229533.I1RHQ4
229533.A0A098DC40	pp	229533.A0A0E0RPV7
229533.A0A098DC40	pp	229533.A0A1C3YLB0
229533.A0A098DC40	pp	229533.I1RZ43
229533.A0A098DC40	pp	229533.I1S0R8
229533.A0A098DC40	pp	229533.I1RPV5
229533.A0A098DC40	pp	229533.I1REL7
229533.A0A098DC40	pp	229533.A0A1C3YIW9
229533.A0A098DC40	pp	229533.I1RX39
229533.A0A098DC40	pp	229533.I1RUV5
229533.A0A098DC40	pp	229533.I1RKB7
229533.A0A098DC40	pp	229533.I1S1J0
229533.A0A098DC40	pp	229533.I1RG62
229533.I1RNC2	pp	229533.I1RZ86

164689 229533.I1RNC2 pp 229533.I1RRL9
164690 229533.I1RNC2 pp 229533.I1S9X9
164691 229533.I1RNC2 pp 229533.I1RXF0
164692 229533.I1RNC2 pp 229533.I1RT28
164693 229533.I1RNC2 pp 229533.I1RZP1
164694 229533.I1RNH4 pp 229533.I1S150
164695 229533.I1RNH4 pp 229533.V6RF62
164696 229533.I1RNH4 pp 229533.V6R398
164697 229533.I1RNH4 pp 229533.I1RWD2
164698 229533.I1RNH4 pp 229533.I1S8H9
164699 229533.I1RNH4 pp 229533.I1RRM1
164700 229533.I1RNH4 pp 229533.V6R5D2
164701 229533.I1RNH4 pp 229533.V6RRM0
164702 229533.I1RNH4 pp 229533.V6R756
164703 229533.I1RNH4 pp 229533.V6R3V4
164704 229533.I1RNH4 pp 229533.I1RV23
164705 229533.I1RNH4 pp 229533.I1S224
164706 229533.I1RNH4 pp 229533.I1SAI4
164707 229533.I1RNH4 pp 229533.I1RW43
164708 229533.I1RNH4 pp 229533.I1RY49
164709 229533.I1RNH4 pp 229533.V6R0W1
164710 229533.I1RNH4 pp 229533.V6QVH2
164711 229533.I1RNH4 pp 229533.I1RVX9
164712 229533.I1RNH4 pp 229533.V6QU99
164713 229533.I1RNH4 pp 229533.I1S4S5
164714 229533.I1RNH4 pp 229533.I1S0A5
164715 229533.I1RNH4 pp 229533.V6RZD4
164716 229533.I1RNH4 pp 229533.V6RAM6
164717 229533.I1RNH4 pp 229533.I1RZV5
164718 229533.I1RNH4 pp 229533.V6R2Z2
164719 229533.A0A1C3YL95 pp 229533.I1RJ49
164720 229533.A0A1C3YL95 pp 229533.I1RFP1
164721 229533.A0A1C3YL95 pp 229533.I1RYH1
164722 229533.A0A1C3YL95 pp 229533.I1RNE9
164723 229533.A0A1C3YL95 pp 229533.I1RJS0
164724 229533.A0A1C3YL95 pp 229533.I1S0Y2
164725 229533.A0A1C3YL95 pp 229533.I1S7H2
164726 229533.A0A1C3YL95 pp 229533.I1S5S8
164727 229533.A0A1C3YL95 pp 229533.I1RUP5
164728 229533.A0A1C3YL95 pp 229533.I1RKL3
164729 229533.I1S1C6 pp 229533.V6QXG1
164730 229533.I1S1C6 pp 229533.Q4IRI9
164731 229533.I1S1C6 pp 229533.Q4I1Y1
164732 229533.I1S1C6 pp 229533.Q4I7F9
164733 229533.I1S1C6 pp 229533.I1S4M5
164734 229533.I1S1C6 pp 229533.I1S9Y0
164735 229533.I1S1C6 pp 229533.I1S7M2
164736 229533.I1S1C6 pp 229533.I1S2Y5
164737 229533.I1S1C6 pp 229533.I1S1X9
164738 229533.I1S1C6 pp 229533.I1S7M3
164739 229533.I1S214 pp 229533.Q4IAA0
164740 229533.I1S214 pp 229533.I1S246
164741 229533.I1S214 pp 229533.Q4I7X1
164742 229533.I1S214 pp 229533.I1S788
164743 229533.I1S214 pp 229533.Q4I5I4
164744 229533.I1S214 pp 229533.Q4IR09
164745 229533.I1S214 pp 229533.I1S5Z2
164746 229533.I1S214 pp 229533.Q4IAS1
164747 229533.I1S214 pp 229533.I1S8V0
164748 229533.I1S214 pp 229533.Q4IF44
164749 229533.I1S214 pp 229533.Q4IPC8
164750 229533.I1S214 pp 229533.Q4IPN9
164751 229533.I1S214 pp 229533.I1S8U9
164752 229533.I1S214 pp 229533.Q4IPA4
164753 229533.I1S214 pp 229533.V6REZ4
164754 229533.A0A0E0SPT6 pp 229533.I1RM26
164755 229533.A0A0E0SPT6 pp 229533.I1RVX9
164756 229533.I1S2K2 pp 229533.I1S2N3
164757 229533.A0A1C3YJQ4 pp 229533.A0A1C3YL95
164758 229533.A0A1C3YKM6 pp 229533.I1RCB7
164759 229533.A0A1C3YKM6 pp 229533.I1RX47
164760 229533.A0A1C3YKM6 pp 229533.I1RT47
164761 229533.A0A1C3YKM6 pp 229533.I1RG11

229533.A0A1C3YKM6 pp 229533.V6RIS2
229533.A0A1C3YKM6 pp 229533.I1RSD4
229533.A0A1C3YKM6 pp 229533.I1RFQ1
229533.A0A1C3YKM6 pp 229533.I1RZE1
229533.A0A1C3YKM6 pp 229533.I1RUN9
229533.A0A1C3YKM6 pp 229533.I1RSH9
229533.A0A1C3YKM6 pp 229533.I1RNB8
229533.A0A1C3YKM6 pp 229533.I1RQR3
229533.A0A1C3YKM6 pp 229533.I1RS06
229533.A0A1C3YKM6 pp 229533.I1S471
229533.A0A1C3YKM6 pp 229533.I1RSA4
229533.A0A1C3YKM6 pp 229533.I1RCS2
229533.A0A1C3YKM6 pp 229533.I1RCA7
229533.A0A1C3YKM6 pp 229533.V6RY33
229533.A0A1C3YKM6 pp 229533.I1S0B1
229533.A0A1C3YKM6 pp 229533.I1RYJ9
229533.A0A1C3YKM6 pp 229533.I1S4M5
229533.A0A1C3YKM6 pp 229533.I1RBH8
229533.A0A1C3YKM6 pp 229533.I1RK79
229533.A0A1C3YKM6 pp 229533.I1RG57
229533.A0A1C3YKM6 pp 229533.I1S464
229533.A0A1C3YKM6 pp 229533.I1RG90
229533.A0A1C3YKM6 pp 229533.I1RR25
229533.A0A1C3YKM6 pp 229533.I1RRQ4
229533.A0A1C3YKM6 pp 229533.I1RBZ8
229533.A0A1C3YKM6 pp 229533.I1RC84
229533.A0A1C3YKM6 pp 229533.I1S214
229533.A0A1C3YKM6 pp 229533.I1RK92
229533.A0A1C3YKM6 pp 229533.Q4IF44
229533.A0A1C3YKM6 pp 229533.I1RBV3
229533.A0A1C3YKM6 pp 229533.I1RJL2
229533.A0A1C3YKM6 pp 229533.Q4IPA4
229533.A0A1C3YKM6 pp 229533.A0A1C3YNC3
229533.A0A1C3YKM6 pp 229533.I1RZB5
229533.A0A1C3YKM6 pp 229533.I1RXF2
229533.A0A1C3YKM6 pp 229533.I1RQK2
229533.A0A1C3YKM6 pp 229533.I1S0N7
229533.A0A1C3YKM6 pp 229533.I1RC24
229533.A0A1C3YKM6 pp 229533.I1S5Z2
229533.A0A1C3YKM6 pp 229533.I1S524
229533.A0A1C3YKM6 pp 229533.I1RS03
229533.A0A1C3YKM6 pp 229533.Q4IB70
229533.A0A1C3YKM6 pp 229533.I1RMV5
229533.A0A1C3YKM6 pp 229533.I1RE45
229533.A0A1C3YKM6 pp 229533.I1RTS2
229533.A0A1C3YKM6 pp 229533.I1S7M3
229533.A0A1C3YKM6 pp 229533.Q4I5I4
229533.A0A1C3YKM6 pp 229533.I1RMH5
229533.A0A1C3YKM6 pp 229533.I1RA36
229533.A0A1C3YKM6 pp 229533.I1RK96
229533.A0A1C3YKM6 pp 229533.I1RZL5
229533.A0A1C3YKM6 pp 229533.I1RQD8
229533.A0A1C3YKM6 pp 229533.Q4IP34
229533.A0A1C3YKM6 pp 229533.I1S7M2
229533.A0A1C3YKM6 pp 229533.I1RPA0
229533.A0A1C3YKM6 pp 229533.I1RN49
229533.A0A1C3YKM6 pp 229533.Q4I7F9
229533.A0A1C3YKM6 pp 229533.A0A1C3YML3
229533.A0A1C3YKM6 pp 229533.I1RRT0
229533.A0A1C3YKM6 pp 229533.I1RRG3
229533.A0A1C3YKM6 pp 229533.I1RZZ1
229533.A0A1C3YKM6 pp 229533.I1RN28
229533.A0A1C3YKM6 pp 229533.I1RYD3
229533.A0A1C3YKM6 pp 229533.I1RE31
229533.A0A1C3YKM6 pp 229533.I1RT35
229533.A0A1C3YKM6 pp 229533.I1RQZ2
229533.A0A1C3YKM6 pp 229533.I1S246
229533.A0A1C3YKM6 pp 229533.I1RWX8
229533.A0A1C3YKM6 pp 229533.A0A1C3YL25
229533.A0A1C3YKM6 pp 229533.I1RPY7
229533.A0A1C3YKM6 pp 229533.A0A1C3YM64
229533.A0A1C3YKM6 pp 229533.I1RBW4
229533.A0A1C3YKM6 pp 229533.I1RK19

229533.A0A1C3YKM6 pp 229533.I1RB50
229533.A0A1C3YKM6 pp 229533.I1RN60
229533.A0A1C3YKM6 pp 229533.A0A1C3YL53
229533.A0A1C3YKM6 pp 229533.I1RRW3
229533.A0A1C3YKM6 pp 229533.I1RAE1
229533.A0A1C3YKM6 pp 229533.I1RLU5
229533.A0A1C3YKM6 pp 229533.I1RS66
229533.A0A1C3YKM6 pp 229533.I1S0B6
229533.A0A1C3YKM6 pp 229533.I1RMW7
229533.A0A1C3YKM6 pp 229533.A0A1C3YL11
229533.A0A1C3YKM6 pp 229533.Q4IR09
229533.A0A1C3YKM6 pp 229533.I1RZG7
229533.A0A1C3YKM6 pp 229533.I1RCQ6
229533.A0A1C3YKM6 pp 229533.I1RX81
229533.A0A1C3YKM6 pp 229533.I1RCD6
229533.A0A1C3YKM6 pp 229533.I1RCI3
229533.A0A1C3YLE5 pp 229533.I1RRV1
229533.A0A1C3YLE5 pp 229533.I1RNB8
229533.A0A1C3YLE5 pp 229533.I1S246
229533.A0A1C3YLE5 pp 229533.I1RG90
229533.A0A1C3YLE5 pp 229533.I1RT35
229533.I1S3R9 pp 229533.V6RSM7
229533.A0A098DNZ7 pp 229533.A0A1C3YLB0
229533.A0A098DNZ7 pp 229533.A0A1C3YIW9
229533.A0A098DNZ7 pp 229533.I1RX39
229533.A0A098DNZ7 pp 229533.Q4I9B7
229533.A0A1C3YKV2 pp 229533.I1SAI4
229533.A0A1C3YKV2 pp 229533.I1S273
229533.A0A1C3YKV2 pp 229533.I1RQX9
229533.A0A1C3YKV2 pp 229533.I1RD80
229533.A0A1C3YKV2 pp 229533.I1RRM1
229533.A0A1C3YKV2 pp 229533.I1RZH8
229533.A0A1C3YKV2 pp 229533.I1RZY1
229533.A0A1C3YKV2 pp 229533.I1RCV1
229533.A0A1C3YKV2 pp 229533.I1S084
229533.A0A1C3YKV2 pp 229533.I1RDJ7
229533.A0A1C3YKV2 pp 229533.I1RK03
229533.A0A1C3YKV2 pp 229533.V6QU99
229533.A0A1C3YKV2 pp 229533.V6RY56
229533.A0A1C3YKV2 pp 229533.I1RZT0
229533.A0A1C3YKV2 pp 229533.V6R5D2
229533.A0A1C3YKV2 pp 229533.V6RAM6
229533.A0A1C3YKV2 pp 229533.A0A1C3YKZ8
229533.A0A1C3YKV2 pp 229533.I1RNH4
229533.A0A1C3YKV2 pp 229533.I1S0H5
229533.A0A1C3YKV2 pp 229533.I1S4D0
229533.A0A1C3YKV2 pp 229533.V6R1K4
229533.A0A1C3YKV2 pp 229533.I1S6P5
229533.A0A1C3YKV2 pp 229533.I1RC48
229533.A0A1C3YKV2 pp 229533.I1RC39
229533.A0A1C3YKV2 pp 229533.I1RVR2
229533.A0A1C3YKV2 pp 229533.I1S1P8
229533.A0A1C3YKV2 pp 229533.I1SAE8
229533.A0A1C3YKV2 pp 229533.V6RUB1
229533.A0A1C3YKV2 pp 229533.I1RX43
229533.A0A1C3YKV2 pp 229533.I1RMD3
229533.A0A1C3YKV2 pp 229533.I1S0A0
229533.A0A1C3YKV2 pp 229533.I1RW76
229533.A0A1C3YKV2 pp 229533.I1RLT3
229533.A0A1C3YKV2 pp 229533.I1RJT5
229533.A0A1C3YKV2 pp 229533.I1RZ60
229533.A0A1C3YKV2 pp 229533.I1RQF6
229533.A0A1C3YKV2 pp 229533.V6R756
229533.A0A1C3YKV2 pp 229533.A0A1C3YN30
229533.A0A1C3YKV2 pp 229533.V6RGY3
229533.A0A1C3YKV2 pp 229533.I1S024
229533.A0A1C3YKV2 pp 229533.A0A1C3YLE2
229533.A0A1C3YKV2 pp 229533.V6R0W1
229533.A0A1C3YKV2 pp 229533.I1RZ20
229533.A0A1C3YKV2 pp 229533.I1R9B8
229533.A0A1C3YKV2 pp 229533.I1RZA7
229533.A0A1C3YKV2 pp 229533.I1RAR1
229533.A0A1C3YKV2 pp 229533.A0A1C3YNB1

164908 229533.A0A1C3YKV2 pp 229533.I1S3K8
164909 229533.A0A1C3YKV2 pp 229533.V6RM02
164910 229533.A0A1C3YKV2 pp 229533.I1RV23
164911 229533.A0A1C3YKV2 pp 229533.V6R2Z2
164912 229533.I1S9L6 pp 229533.V6QXG1
164913 229533.I1S9L6 pp 229533.V6RY33
164914 229533.I1S9L6 pp 229533.Q4IRI9
164915 229533.I1S9L6 pp 229533.Q4IB70
164916 229533.I1S9L6 pp 229533.V6RIS2
164917 229533.I1RN91 pp 229533.I1RZG7
164918 229533.I1RN91 pp 229533.I1S246
164919 229533.I1RN91 pp 229533.I1RS03
164920 229533.I1RN91 pp 229533.Q4IAA0
164921 229533.I1RN91 pp 229533.I1RWU2
164922 229533.I1RN91 pp 229533.I1RQ42
164923 229533.I1RN91 pp 229533.I1S9M9
164924 229533.I1RN91 pp 229533.I1RPW8
164925 229533.I1RN91 pp 229533.I1S471
164926 229533.I1RN91 pp 229533.I1RW80
164927 229533.I1RN91 pp 229533.I1RNB8
164928 229533.I1RN91 pp 229533.I1S5Z2
164929 229533.I1RN91 pp 229533.I1S0Z9
164930 229533.I1RN91 pp 229533.I1RT35
164931 229533.I1RN91 pp 229533.I1RX47
164932 229533.I1RTX4 pp 229533.I1S7L6
164933 229533.A0A1C3YKC2 pp 229533.V6RRM0
164934 229533.A0A1C3YKC2 pp 229533.I1RM26
164935 229533.A0A1C3YKC2 pp 229533.I1S2I5
164936 229533.A0A1C3YKC2 pp 229533.I1S2I4
164937 229533.A0A1C3YKC2 pp 229533.I1RY49
164938 229533.A0A1C3YKC2 pp 229533.I1RVX9
164939 229533.A0A1C3YKC2 pp 229533.I1RW43
164940 229533.A0A098DS77 pp 229533.I1RXM0
164941 229533.A0A098DS77 pp 229533.E6PBP1
164942 229533.A0A098DS77 pp 229533.I1S2K2
164943 229533.A0A098DS77 pp 229533.I1S2N3
164944 229533.A0A098DS77 pp 229533.I1RIJ1
164945 229533.I1RF87 pp 229533.I1RX39
164946 229533.I1RFL6 pp 229533.I1RJH8
164947 229533.I1RFL6 pp 229533.I1RMG3
164948 229533.I1RFL6 pp 229533.I1RZA5
164949 229533.I1RFL6 pp 229533.I1RX25
164950 229533.I1RFL6 pp 229533.I1RQ82
164951 229533.I1RFL6 pp 229533.I1RQ23
164952 229533.I1RFL6 pp 229533.I1RNL7
164953 229533.I1RFL6 pp 229533.I1RTF5
164954 229533.I1RFL6 pp 229533.I1RJH7
164955 229533.I1RFL6 pp 229533.I1RZJ5
164956 229533.I1RFL6 pp 229533.I1S9G7
164957 229533.I1RFL6 pp 229533.I1RRR5
164958 229533.I1RFL6 pp 229533.I1RQ41
164959 229533.I1RFL6 pp 229533.Q4HY20
164960 229533.I1RFL6 pp 229533.I1S0Z6
164961 229533.I1RPB1 pp 229533.I1RT87
164962 229533.I1RPB1 pp 229533.I1RPB2
164963 229533.I1RPB1 pp 229533.I1S2J3
164964 229533.I1RAE1 pp 229533.I1S5S2
164965 229533.I1RAE1 pp 229533.I1RXF2
164966 229533.I1RAE1 pp 229533.I1RME6
164967 229533.I1RAE1 pp 229533.I1S7M2
164968 229533.I1RAE1 pp 229533.I1RZ11
164969 229533.I1RAE1 pp 229533.I1RBV3
164970 229533.I1RAE1 pp 229533.I1RQD8
164971 229533.I1RAE1 pp 229533.I1RBU6
164972 229533.I1RAE1 pp 229533.I1RT47
164973 229533.I1RAE1 pp 229533.I1S4Y4
164974 229533.I1RAE1 pp 229533.I1RYI9
164975 229533.I1RAE1 pp 229533.I1S193
164976 229533.I1RAE1 pp 229533.I1RQR3
164977 229533.I1RAE1 pp 229533.I1RK96
164978 229533.I1RAE1 pp 229533.I1RX81
164979 229533.I1RAE1 pp 229533.Q4I7F9
164980 229533.I1RAE1 pp 229533.I1RCA7

164981 229533.I1RAE1 pp 229533.I1RCQ6
164982 229533.I1RAE1 pp 229533.I1RDJ8
164983 229533.I1RAE1 pp 229533.I1S4M5
164984 229533.I1RAE1 pp 229533.I1RZZ1
164985 229533.I1RAE1 pp 229533.I1S524
164986 229533.I1RAE1 pp 229533.I1RAW7
164987 229533.I1RAE1 pp 229533.Q4I948
164988 229533.I1RAE1 pp 229533.I1RS75
164989 229533.I1RAE1 pp 229533.I1RCI3
164990 229533.I1RAE1 pp 229533.I1RSD8
164991 229533.I1RAE1 pp 229533.I1RMV5
164992 229533.I1RAE1 pp 229533.I1RB50
164993 229533.I1RAE1 pp 229533.Q4IPH4
164994 229533.I1RAE1 pp 229533.I1S0B6
164995 229533.I1RAE1 pp 229533.I1RMW7
164996 229533.I1RAE1 pp 229533.I1RRQ4
164997 229533.I1RAE1 pp 229533.I1RSD4
164998 229533.I1RAE1 pp 229533.I1RFQ1
164999 229533.I1RAE1 pp 229533.I1S0B1
165000 229533.I1RAE1 pp 229533.I1S0Z9
165001 229533.I1RAE1 pp 229533.I1RK72
165002 229533.I1RAE1 pp 229533.I1RG57
165003 229533.I1RAE1 pp 229533.I1RG90
165004 229533.I1RAE1 pp 229533.V6RY33
165005 229533.I1RAE1 pp 229533.I1RBH8
165006 229533.I1RAE1 pp 229533.I1RSR3
165007 229533.I1RAE1 pp 229533.I1RAN0
165008 229533.I1RAE1 pp 229533.I1RSA4
165009 229533.I1RAE1 pp 229533.I1RPA0
165010 229533.I1RAE1 pp 229533.I1RK92
165011 229533.I1RAE1 pp 229533.I1RWX8
165012 229533.I1RAE1 pp 229533.I1RZV9
165013 229533.I1RAE1 pp 229533.I1S7M3
165014 229533.I1RAE1 pp 229533.I1RSA8
165015 229533.I1RAE1 pp 229533.I1RC84
165016 229533.I1RAE1 pp 229533.I1S0N4
165017 229533.I1RAE1 pp 229533.V6RIS2
165018 229533.I1RAE1 pp 229533.I1RS83
165019 229533.A0A1C3YKF4 pp 229533.I1RY40
165020 229533.A0A1C3YKF4 pp 229533.I1RW71
165021 229533.A0A1C3YKF4 pp 229533.I1RCU7
165022 229533.A0A1C3YKF4 pp 229533.Q4IAA0
165023 229533.A0A1C3YKF4 pp 229533.I1RZV9
165024 229533.A0A1C3YKF4 pp 229533.I1RC00
165025 229533.A0A1C3YKF4 pp 229533.I1RJZ7
165026 229533.A0A1C3YKF4 pp 229533.I1RNT2
165027 229533.A0A1C3YKF4 pp 229533.A0A1C3YM64
165028 229533.A0A1C3YKF4 pp 229533.I1RCK4
165029 229533.A0A1C3YKF4 pp 229533.A0A1C3YMK1
165030 229533.A0A1C3YKF4 pp 229533.I1RYT7
165031 229533.A0A1C3YKF4 pp 229533.I1RS94
165032 229533.A0A1C3YKF4 pp 229533.I1S279
165033 229533.A0A1C3YKF4 pp 229533.I1RZT1
165034 229533.A0A1C3YKF4 pp 229533.I1S0P4
165035 229533.A0A1C3YKF4 pp 229533.I1RMY3
165036 229533.A0A1C3YKF4 pp 229533.V6REZ4
165037 229533.A0A1C3YKF4 pp 229533.I1RBZ8
165038 229533.I1RIY8 pp 229533.I1RWA6
165039 229533.I1RIY8 pp 229533.I1RW98
165040 229533.I1RIY8 pp 229533.I1RY33
165041 229533.I1RIY8 pp 229533.I1S6X3
165042 229533.I1RIY8 pp 229533.I1S2I4
165043 229533.I1RIY8 pp 229533.I1RWG0
165044 229533.A0A098DWS1 pp 229533.A0A1C3YIW9
165045 229533.A0A098DWS1 pp 229533.I1RX39
165046 229533.A0A098DWS1 pp 229533.A0A1C3YLB0
165047 229533.I1RIR9 pp 229533.Q4HYB8
165048 229533.I1RIR9 pp 229533.I1RY40
165049 229533.I1RIR9 pp 229533.I1S246
165050 229533.I1RIR9 pp 229533.I1RQ49
165051 229533.I1RIR9 pp 229533.I1S5B5
165052 229533.I1RIR9 pp 229533.I1RZV9
165053 229533.I1RIR9 pp 229533.I1RNB8

165054 229533.I1RIR9 pp 229533.I1RNT2
165055 229533.I1RIR9 pp 229533.I1RS94
165056 229533.I1RIR9 pp 229533.I1RT35
165057 229533.I1RIR9 pp 229533.I1S0P4
165058 229533.I1RIR9 pp 229533.I1S214
165059 229533.I1RIR9 pp 229533.I1RLX4
165060 229533.I1RIR9 pp 229533.I1S471
165061 229533.I1RIR9 pp 229533.I1RTE5
165062 229533.I1RIR9 pp 229533.I1S2L3
165063 229533.I1RIR9 pp 229533.Q4IP34
165064 229533.I1RIR9 pp 229533.I1RYJ9
165065 229533.I1RIR9 pp 229533.Q4IAA0
165066 229533.I1RIR9 pp 229533.V6REZ4
165067 229533.I1RIR9 pp 229533.I1RYT7
165068 229533.I1RIR9 pp 229533.I1RSV2
165069 229533.I1RIR9 pp 229533.I1RJS4
165070 229533.I1RIR9 pp 229533.I1RJS5
165071 229533.I1RIR9 pp 229533.I1RZT1
165072 229533.I1RIR9 pp 229533.I1RNE4
165073 229533.V6R9Z3 pp 229533.V6RZD4
165074 229533.V6R9Z3 pp 229533.V6RF62
165075 229533.A0A098DS24 pp 229533.Q4IMM0
165076 229533.A0A098DS24 pp 229533.A0A1C3YM21
165077 229533.A0A098DS24 pp 229533.Q4IBX0
165078 229533.I1S8F0 pp 229533.Q4I7T5
165079 229533.I1S8F0 pp 229533.I1S8Y0
165080 229533.I1S8F0 pp 229533.I1S8Y1
165081 229533.I1S8F0 pp 229533.Q4I375
165082 229533.I1S8F0 pp 229533.V6RCG5
165083 229533.I1S8F0 pp 229533.Q4I6B0
165084 229533.I1S8F0 pp 229533.Q4I2W2
165085 229533.I1S8F0 pp 229533.Q4IJK2
165086 229533.I1S8F0 pp 229533.Q4IK03
165087 229533.I1S8F0 pp 229533.Q4IJW4
165088 229533.I1S8F0 pp 229533.Q4IB65
165089 229533.I1S8F0 pp 229533.Q4HZ95
165090 229533.I1S8F0 pp 229533.Q4I099
165091 229533.I1S8F0 pp 229533.Q4IPZ1
165092 229533.I1RDD3 pp 229533.I1RZE1
165093 229533.I1RDD3 pp 229533.I1RZP2
165094 229533.I1RDD3 pp 229533.I1RSD4
165095 229533.I1RDD3 pp 229533.I1RNB8
165096 229533.I1RDD3 pp 229533.I1RQK2
165097 229533.I1RDD3 pp 229533.Q4IPA4
165098 229533.I1RDD3 pp 229533.I1RX47
165099 229533.I1RDD3 pp 229533.I1RSA4
165100 229533.I1RDD3 pp 229533.I1RK79
165101 229533.I1RDD3 pp 229533.I1S214
165102 229533.I1RDD3 pp 229533.Q4IF44
165103 229533.I1RDD3 pp 229533.I1RXF2
165104 229533.I1RDD3 pp 229533.Q4IPC8
165105 229533.I1RDD3 pp 229533.V6RIS2
165106 229533.I1RDD3 pp 229533.I1RRG3
165107 229533.I1RDD3 pp 229533.I1RK71
165108 229533.I1RDD3 pp 229533.I1S2E8
165109 229533.I1RDD3 pp 229533.I1S0N7
165110 229533.I1RDD3 pp 229533.I1S246
165111 229533.I1RDD3 pp 229533.I1S0B1
165112 229533.I1RDD3 pp 229533.I1RRW3
165113 229533.I1RDD3 pp 229533.I1RS03
165114 229533.I1RDD3 pp 229533.I1RLU5
165115 229533.I1RDD3 pp 229533.I1RE45
165116 229533.I1RDD3 pp 229533.I1S7M2
165117 229533.I1RDD3 pp 229533.I1RG90
165118 229533.I1RDD3 pp 229533.I1RN28
165119 229533.I1RDD3 pp 229533.I1RT35
165120 229533.I1RDD3 pp 229533.I1S7M3
165121 229533.I1RDD3 pp 229533.I1RN49
165122 229533.I1RDD3 pp 229533.Q4IPN9
165123 229533.I1RDD3 pp 229533.I1RZG7
165124 229533.I1RDD3 pp 229533.I1S0B6
165125 229533.I1RDD3 pp 229533.Q4I7X1
165126 229533.I1RDD3 pp 229533.I1RQD8

165127	229533.I1RDD3	pp	229533.V6QUE6
165128	229533.I1RDD3	pp	229533.I1RZZ1
165129	229533.I1RDD3	pp	229533.Q4IR09
165130	229533.I1RDD3	pp	229533.I1RPY7
165131	229533.I1RDD3	pp	229533.I1RRT0
165132	229533.I1RDD3	pp	229533.I1S5Z2
165133	229533.I1RDD3	pp	229533.I1RJL2
165134	229533.I1RDD3	pp	229533.I1RZB5
165135	229533.I1RDD3	pp	229533.Q4IB70
165136	229533.I1RKQ6	pp	229533.I1RX39
165137	229533.I1RKQ6	pp	229533.I1RZ43
165138	229533.I1RKQ6	pp	229533.I1S0R8
165139	229533.I1RPZ3	pp	229533.Q4IE79
165140	229533.I1RPZ3	pp	229533.I1RX47
165141	229533.I1RPZ3	pp	229533.I1RU69
165142	229533.I1RPZ3	pp	229533.I1RST1
165143	229533.I1RPZ3	pp	229533.Q4IF76
165144	229533.I1RPZ3	pp	229533.Q4IAA0
165145	229533.I1RPZ3	pp	229533.I1S056
165146	229533.I1RPZ3	pp	229533.I1RT17
165147	229533.I1RPZ3	pp	229533.I1RS28
165148	229533.I1RPZ3	pp	229533.I1RSA4
165149	229533.I1RPZ3	pp	229533.Q4IRI9
165150	229533.I1RPZ3	pp	229533.I1RRL0
165151	229533.I1RPZ3	pp	229533.V6QXG1
165152	229533.I1RPZ3	pp	229533.I1RZG7
165153	229533.I1RPZ3	pp	229533.Q4I1Y1
165154	229533.I1RPZ3	pp	229533.I1RQC5
165155	229533.I1RPZ3	pp	229533.Q4IBK5
165156	229533.I1RPZ3	pp	229533.I1RZQ5
165157	229533.I1RPZ3	pp	229533.Q4IJH1
165158	229533.I1RPZ3	pp	229533.Q4IPB8
165159	229533.I1RPZ3	pp	229533.I1S246
165160	229533.I1S2E7	pp	229533.I1SAK2
165161	229533.I1RWW5	pp	229533.I1RZG7
165162	229533.I1RWW5	pp	229533.I1RWX4
165163	229533.I1RN60	pp	229533.I1S246
165164	229533.I1RN60	pp	229533.I1RQ92
165165	229533.I1RN60	pp	229533.I1RRW3
165166	229533.I1RN60	pp	229533.I1RQK2
165167	229533.I1RN60	pp	229533.I1S0N7
165168	229533.I1RN60	pp	229533.I1RND9
165169	229533.I1RN60	pp	229533.I1RUM5
165170	229533.I1RN60	pp	229533.I1RX70
165171	229533.I1RN60	pp	229533.I1RS03
165172	229533.I1RN60	pp	229533.Q4IAA0
165173	229533.I1RN60	pp	229533.I1RNB8
165174	229533.I1RN60	pp	229533.I1RN91
165175	229533.I1RN60	pp	229533.I1S0P4
165176	229533.I1RN60	pp	229533.I1RT35
165177	229533.I1RN60	pp	229533.I1S9M9
165178	229533.I1RN60	pp	229533.I1RQZ2
165179	229533.I1RN60	pp	229533.I1S471
165180	229533.I1RN60	pp	229533.I1RZL5
165181	229533.I1RN60	pp	229533.I1RQP5
165182	229533.I1RN60	pp	229533.I1RPY7
165183	229533.I1RN60	pp	229533.I1S5Z2
165184	229533.I1S212	pp	229533.I1S5X9
165185	229533.I1S212	pp	229533.I1S8S9
165186	229533.I1S212	pp	229533.V6RJP8
165187	229533.I1S212	pp	229533.I1S277
165188	229533.I1S212	pp	229533.Q4HTT2
165189	229533.I1S212	pp	229533.V6R3J1
165190	229533.I1S212	pp	229533.I1S464
165191	229533.I1RA46	pp	229533.I1S0N7
165192	229533.I1RA46	pp	229533.I1S214
165193	229533.I1RA46	pp	229533.I1RC24
165194	229533.I1RA46	pp	229533.I1RQK2
165195	229533.I1RA46	pp	229533.I1RBH8
165196	229533.I1RA46	pp	229533.I1RSA4
165197	229533.I1RA46	pp	229533.Q4IF44
165198	229533.I1RA46	pp	229533.Q4IB65
165199	229533.I1RA46	pp	229533.I1RXF2

165200 229533.I1RA46 pp 229533.I1RBV3
165201 229533.I1RA46 pp 229533.I1RZE1
165202 229533.I1RA46 pp 229533.I1RSD4
165203 229533.I1RA46 pp 229533.I1RCS2
165204 229533.I1RA46 pp 229533.Q4IPA4
165205 229533.I1RA46 pp 229533.I1RNB8
165206 229533.I1RA46 pp 229533.I1RS03
165207 229533.I1RA46 pp 229533.I1RZP2
165208 229533.I1RA46 pp 229533.I1RE45
165209 229533.I1RA46 pp 229533.I1RBD6
165210 229533.I1RA46 pp 229533.I1RT47
165211 229533.I1RA46 pp 229533.V6RIS2
165212 229533.I1RA46 pp 229533.I1S4M5
165213 229533.I1RA46 pp 229533.I1RN49
165214 229533.I1RA46 pp 229533.I1SA47
165215 229533.I1RA46 pp 229533.Q4IPC8
165216 229533.I1RA46 pp 229533.I1S4Y4
165217 229533.I1RA46 pp 229533.I1RX47
165218 229533.I1RA46 pp 229533.I1S7M2
165219 229533.I1RA46 pp 229533.I1RK71
165220 229533.I1RA46 pp 229533.I1S2E8
165221 229533.I1RA46 pp 229533.I1RZG7
165222 229533.I1RA46 pp 229533.I1RG90
165223 229533.I1RA46 pp 229533.I1S0B1
165224 229533.I1RA46 pp 229533.I1RK92
165225 229533.I1RA46 pp 229533.I1RBZ8
165226 229533.I1RA46 pp 229533.I1RCQ6
165227 229533.I1RA46 pp 229533.I1RK79
165228 229533.I1RA46 pp 229533.I1RX81
165229 229533.I1RA46 pp 229533.I1RB50
165230 229533.I1RA46 pp 229533.I1RT35
165231 229533.I1RA46 pp 229533.Q4IPN9
165232 229533.I1RA46 pp 229533.I1RLU5
165233 229533.I1RA46 pp 229533.I1RRT0
165234 229533.I1RA46 pp 229533.I1RRW3
165235 229533.I1RA46 pp 229533.I1RCL4
165236 229533.I1RA46 pp 229533.I1RN28
165237 229533.I1RA46 pp 229533.I1S0B6
165238 229533.I1RA46 pp 229533.I1S246
165239 229533.I1RA46 pp 229533.I1RAR2
165240 229533.I1RA46 pp 229533.Q4IR09
165241 229533.I1RA46 pp 229533.V6QUE6
165242 229533.I1RA46 pp 229533.I1RRG3
165243 229533.I1RA46 pp 229533.Q4IR08
165244 229533.I1RA46 pp 229533.I1RQD8
165245 229533.I1RA46 pp 229533.I1RN17
165246 229533.I1RA46 pp 229533.I1RCG8
165247 229533.I1RA46 pp 229533.I1RE31
165248 229533.I1RA46 pp 229533.I1S7M3
165249 229533.I1RA46 pp 229533.I1RZZ1
165250 229533.I1RA46 pp 229533.I1S5Z2
165251 229533.I1RA46 pp 229533.Q4I7X1
165252 229533.I1RA46 pp 229533.I1RE14
165253 229533.I1RA46 pp 229533.I1SA48
165254 229533.I1RA46 pp 229533.I1RPY7
165255 229533.I1RA46 pp 229533.I1RPA0
165256 229533.I1RA46 pp 229533.I1RZB5
165257 229533.I1RA46 pp 229533.I1RBD2
165258 229533.I1RA46 pp 229533.I1RJL2
165259 229533.I1RHG0 pp 229533.I1SAK2
165260 229533.I1RHG0 pp 229533.I1RX39
165261 229533.I1RIU4 pp 229533.I1RJ10
165262 229533.I1RIU4 pp 229533.I1RIY8
165263 229533.A0A098E525 pp 229533.I1RRL5
165264 229533.A0A098E525 pp 229533.I1RHL9
165265 229533.A0A098E525 pp 229533.I1RDN3
165266 229533.A0A098E525 pp 229533.I1S334
165267 229533.A0A098E525 pp 229533.I1RVI8
165268 229533.A0A098E525 pp 229533.I1RJE7
165269 229533.A0A098E525 pp 229533.I1S094
165270 229533.A0A098E525 pp 229533.I1RNM7
165271 229533.A0A098E525 pp 229533.I1RJG4
165272 229533.A0A098E525 pp 229533.I1RV06

165273	229533.A0A098E525	pp	229533.I1RTT0
165274	229533.A0A098E525	pp	229533.V6QTX7
165275	229533.A0A098E525	pp	229533.I1RQX9
165276	229533.A0A098E525	pp	229533.I1RF16
165277	229533.A0A098E525	pp	229533.A0A1C3YKZ8
165278	229533.A0A098E525	pp	229533.I1RY96
165279	229533.A0A098E525	pp	229533.I1RLA2
165280	229533.A0A098E525	pp	229533.I1RMS5
165281	229533.A0A098E525	pp	229533.I1S6H3
165282	229533.A0A098E525	pp	229533.A0A1C3YKH5
165283	229533.A0A098E525	pp	229533.V6RM09
165284	229533.A0A098E525	pp	229533.I1RDU0
165285	229533.A0A098E525	pp	229533.I1RA13
165286	229533.A0A098E525	pp	229533.I1RCU2
165287	229533.A0A098E525	pp	229533.A0A0E0RTH0
165288	229533.A0A098E525	pp	229533.V6REA4
165289	229533.A0A098E525	pp	229533.I1S269
165290	229533.A0A098E525	pp	229533.I1RNH4
165291	229533.A0A098E525	pp	229533.I1RTJ7
165292	229533.A0A098E525	pp	229533.I1RC39
165293	229533.A0A098E525	pp	229533.I1R9C0
165294	229533.A0A098E525	pp	229533.I1RJK0
165295	229533.A0A098E525	pp	229533.I1RK03
165296	229533.A0A098E525	pp	229533.I1RJT5
165297	229533.A0A098E525	pp	229533.I1S8F8
165298	229533.A0A098E525	pp	229533.I1R9F2
165299	229533.A0A098E525	pp	229533.I1REE6
165300	229533.A0A098E525	pp	229533.I1RCV1
165301	229533.A0A098E525	pp	229533.V6RY56
165302	229533.A0A098E525	pp	229533.I1RRM1
165303	229533.A0A098E525	pp	229533.A0A0E0RQ82
165304	229533.A0A098E525	pp	229533.I1RE90
165305	229533.A0A098E525	pp	229533.I1S0T1
165306	229533.A0A098E525	pp	229533.I1RDV8
165307	229533.A0A098E525	pp	229533.I1S0H2
165308	229533.A0A098E525	pp	229533.I1S405
165309	229533.A0A098E525	pp	229533.I1S4D0
165310	229533.A0A098E525	pp	229533.I1RST9
165311	229533.A0A098E525	pp	229533.I1RDJ7
165312	229533.A0A098E525	pp	229533.I1RIW5
165313	229533.A0A098E525	pp	229533.V6RPS5
165314	229533.A0A098E525	pp	229533.I1SAJ0
165315	229533.A0A098E525	pp	229533.I1RAG3
165316	229533.A0A098E525	pp	229533.V6R0H4
165317	229533.A0A098E525	pp	229533.I1RV18
165318	229533.A0A098E525	pp	229533.I1RNG4
165319	229533.A0A098E525	pp	229533.I1RIK8
165320	229533.A0A098E525	pp	229533.I1S0A5
165321	229533.A0A098E525	pp	229533.I1RKB2
165322	229533.A0A098E525	pp	229533.I1RUY5
165323	229533.A0A098E525	pp	229533.I1RZ38
165324	229533.A0A098E525	pp	229533.A0A1C3YKV2
165325	229533.A0A098E525	pp	229533.A0A0E0RTW2
165326	229533.A0A098E525	pp	229533.A0A1C3YKN4
165327	229533.A0A098E525	pp	229533.I1RAC0
165328	229533.A0A098E525	pp	229533.I1RGZ3
165329	229533.A0A098E525	pp	229533.A0A1C3YHG7
165330	229533.A0A098E525	pp	229533.I1S3H8
165331	229533.A0A098E525	pp	229533.I1RMT6
165332	229533.A0A098E525	pp	229533.I1RI19
165333	229533.A0A098E525	pp	229533.V6RBG5
165334	229533.A0A098E525	pp	229533.A0A0E0S2K9
165335	229533.A0A098E525	pp	229533.V6R556
165336	229533.A0A098E525	pp	229533.I1RZ25
165337	229533.A0A098E525	pp	229533.I1RF24
165338	229533.A0A098E525	pp	229533.A0A1C3YJL9
165339	229533.A0A098E525	pp	229533.A0A1C3YJM7
165340	229533.A0A098E525	pp	229533.I1RZ26
165341	229533.A0A098E525	pp	229533.I1R9B8
165342	229533.A0A098E525	pp	229533.I1S149
165343	229533.A0A098E525	pp	229533.I1RJS1
165344	229533.A0A098E525	pp	229533.I1RF85
165345	229533.A0A098E525	pp	229533.A0A1C3YHU3

229533.A0A098E525	pp	229533.I1RTY9
229533.A0A098E525	pp	229533.I1R9X3
229533.A0A098E525	pp	229533.I1RHT3
229533.A0A098E525	pp	229533.I1RD80
229533.A0A098E525	pp	229533.I1RPV8
229533.A0A098E525	pp	229533.I1RZ56
229533.A0A098E525	pp	229533.I1RTU5
229533.A0A098E525	pp	229533.I1S6H1
229533.A0A098E525	pp	229533.I1RV23
229533.A0A1C3YM64	pp	229533.I1S8U9
229533.A0A1C3YM64	pp	229533.Q4I7X1
229533.A0A1C3YM64	pp	229533.I1RJV2
229533.A0A1C3YM64	pp	229533.I1RAC1
229533.A0A1C3YM64	pp	229533.I1RNE4
229533.A0A1C3YM64	pp	229533.I1RK92
229533.A0A1C3YM64	pp	229533.I1RE71
229533.A0A1C3YM64	pp	229533.I1RMQ5
229533.A0A1C3YM64	pp	229533.I1S471
229533.A0A1C3YM64	pp	229533.I1RZT1
229533.A0A1C3YM64	pp	229533.I1RNB8
229533.A0A1C3YM64	pp	229533.I1RZL5
229533.A0A1C3YM64	pp	229533.I1RN28
229533.A0A1C3YM64	pp	229533.I1RBZ8
229533.A0A1C3YM64	pp	229533.Q4IAA0
229533.A0A1C3YM64	pp	229533.I1RYW2
229533.A0A1C3YM64	pp	229533.I1RZS7
229533.A0A1C3YM64	pp	229533.Q4IB70
229533.A0A1C3YM64	pp	229533.I1RZH4
229533.A0A1C3YM64	pp	229533.I1S5Z2
229533.A0A1C3YM64	pp	229533.I1S0P4
229533.A0A1C3YM64	pp	229533.I1RZV9
229533.A0A1C3YM64	pp	229533.I1RT35
229533.A0A1C3YM64	pp	229533.I1RB53
229533.A0A1C3YM64	pp	229533.I1RQZ2
229533.A0A1C3YM64	pp	229533.I1S6F1
229533.A0A1C3YM64	pp	229533.I1RZZ1
229533.A0A1C3YM64	pp	229533.I1RYJ9
229533.A0A1C3YM64	pp	229533.V6REZ4
229533.A0A1C3YM64	pp	229533.I1RPY7
229533.A0A1C3YM64	pp	229533.I1S8V0
229533.A0A1C3YM64	pp	229533.I1RYB1
229533.A0A1C3YM64	pp	229533.I1RY40
229533.A0A1C3YM64	pp	229533.I1RYT7
229533.A0A1C3YM64	pp	229533.Q4IF44
229533.A0A1C3YM64	pp	229533.I1S214
229533.A0A1C3YM64	pp	229533.I1RN60
229533.A0A1C3YM64	pp	229533.I1RA46
229533.A0A1C3YM64	pp	229533.Q4IR09
229533.A0A1C3YM64	pp	229533.I1S4M5
229533.A0A1C3YM64	pp	229533.I1RDD3
229533.A0A1C3YM64	pp	229533.Q4IPN9
229533.A0A1C3YM64	pp	229533.I1RCU7
229533.A0A1C3YM64	pp	229533.I1S246
229533.A0A1C3YM64	pp	229533.I1RQ80
229533.A0A1C3YM64	pp	229533.I1RLU5
229533.A0A1C3YM64	pp	229533.I1RRW3
229533.A0A1C3YM64	pp	229533.Q4IPA4
229533.A0A1C3YM64	pp	229533.I1RB59
229533.A0A1C3YM64	pp	229533.I1RYU4
229533.A0A1C3YM64	pp	229533.I1RBX0
229533.A0A1C3YM64	pp	229533.I1RCH7
229533.A0A1C3YM64	pp	229533.I1RLS1
229533.A0A1C3YM64	pp	229533.I1RE45
229533.A0A1C3YM64	pp	229533.I1RFQ0
229533.A0A1C3YM64	pp	229533.I1RS03
229533.A0A1C3YJW3	pp	229533.I1RW43
229533.A0A1C3YJW3	pp	229533.I1RVX9
229533.A0A1C3YJW3	pp	229533.I1RY49
229533.A0A1C3YJW3	pp	229533.V6RRM0
229533.A0A1C3YJW3	pp	229533.I1RM26
229533.A0A1C3YLX6	pp	229533.I1RNG4
229533.A0A1C3YLX6	pp	229533.I1RD89
229533.A0A098DI50	pp	229533.A0A0E0RPV7

229533.A0A098DI50	pp	229533.I1S0R8
229533.A0A098DI50	pp	229533.A0A1C3YLB0
229533.A0A098DI50	pp	229533.I1RZ43
229533.A0A098DI50	pp	229533.I1RX39
229533.A0A098DI50	pp	229533.A0A1C3YIW9
229533.A0A098DI50	pp	229533.I1RG62
229533.A0A098DI50	pp	229533.I1RKB7
229533.I1RNG7	pp	229533.I1RPW2
229533.I1RNG7	pp	229533.I1RY49
229533.I1RNG7	pp	229533.V6R9Z3
229533.I1RNG7	pp	229533.I1RYU5
229533.I1RNG7	pp	229533.I1S4F4
229533.I1RNG7	pp	229533.I1RZ20
229533.I1RNG7	pp	229533.I1RPD6
229533.I1RNG7	pp	229533.V6RGB5
229533.I1RNG7	pp	229533.I1RQE0
229533.I1RNG7	pp	229533.I1RR12
229533.I1RNG7	pp	229533.I1S6P6
229533.I1RNG7	pp	229533.I1RU76
229533.I1RNG7	pp	229533.I1RW43
229533.I1RNG7	pp	229533.I1S064
229533.I1RNG7	pp	229533.I1RVX9
229533.I1RNG7	pp	229533.I1S2H3
229533.I1RNG7	pp	229533.I1S0A0
229533.I1RGW6	pp	229533.I1RIU4
229533.I1RGW6	pp	229533.I1RY33
229533.I1RGW6	pp	229533.I1RW98
229533.I1RMD1	pp	229533.I1RMG6
229533.I1RMD1	pp	229533.I1RQ30
229533.I1RMD1	pp	229533.I1RYW6
229533.I1S2Y5	pp	229533.I1S9L6
229533.I1S2Y5	pp	229533.I1S9L5
229533.I1S2Y5	pp	229533.I1S4M5
229533.I1S2Y5	pp	229533.Q4IRI9
229533.I1S2Y5	pp	229533.Q4IAA0
229533.I1S2Y5	pp	229533.Q4IBK5
229533.I1S2Y5	pp	229533.Q4I5R9
229533.I1S2Y5	pp	229533.Q4IPB3
229533.I1S2Y5	pp	229533.I1S471
229533.I1S2Y5	pp	229533.I1S7M3
229533.I1S2Y5	pp	229533.Q4IE79
229533.I1S2Y5	pp	229533.Q4HXF6
229533.I1S2Y5	pp	229533.V6QXG1
229533.I1S2Y5	pp	229533.Q4IB70
229533.I1S2Y5	pp	229533.I1S7M2
229533.I1S2Y5	pp	229533.Q4IPH4
229533.I1S2Y5	pp	229533.I1S8F8
229533.I1S2Y5	pp	229533.Q4I1Y1
229533.I1S2Y5	pp	229533.Q4IPB8
229533.I1S0Q7	pp	229533.I1S8S9
229533.I1S0Q7	pp	229533.I1S5U0
229533.I1S0Q7	pp	229533.I1S212
229533.I1S0Q7	pp	229533.Q4HYR2
229533.I1S0Q7	pp	229533.I1S266
229533.I1S0Q7	pp	229533.Q4I5G1
229533.I1S0Q7	pp	229533.I1S0R8
229533.I1S0Q7	pp	229533.I1S0U0
229533.I1S0Q7	pp	229533.V6R3J1
229533.I1S0Q7	pp	229533.I1S0R1
229533.I1S0Q7	pp	229533.I1S5X9
229533.I1S0Q7	pp	229533.I1S5C6
229533.I1S0Q7	pp	229533.Q4HZB8
229533.I1S0Q7	pp	229533.I1S8F0
229533.I1S0Q7	pp	229533.V6RJP8
229533.I1S0Q7	pp	229533.I1S464
229533.I1S0Q7	pp	229533.I1S8F8
229533.I1S0Q7	pp	229533.I1S458
229533.I1S0Q7	pp	229533.I1S1F3
229533.I1S0Q7	pp	229533.I1S0T7
229533.A0A098E320	pp	229533.A0A0E0S8J8
229533.A0A098E320	pp	229533.A0A0E0SBC4
229533.A0A098E320	pp	229533.I1RYA0
229533.A0A098E320	pp	229533.I1RAR1

165492	229533.A0A098E320	pp	229533.I1R9B8
165493	229533.A0A098E320	pp	229533.A0A0E0SA26
165494	229533.A0A098E320	pp	229533.V6RRM0
165495	229533.A0A098E320	pp	229533.I1S024
165496	229533.A0A098E320	pp	229533.V6RUB1
165497	229533.A0A098E320	pp	229533.I1RV23
165498	229533.A0A098E320	pp	229533.I1S0V7
165499	229533.A0A098E320	pp	229533.I1RWW7
165500	229533.A0A098E320	pp	229533.I1RZA7
165501	229533.A0A098E320	pp	229533.I1S147
165502	229533.A0A098E320	pp	229533.A0A1C3YJP4
165503	229533.A0A098E320	pp	229533.A0A1C3YJ47
165504	229533.A0A098E320	pp	229533.I1RA62
165505	229533.A0A098E320	pp	229533.I1RKB2
165506	229533.A0A098E320	pp	229533.I1RL81
165507	229533.A0A098E320	pp	229533.V6RLM7
165508	229533.A0A098E320	pp	229533.I1RPD6
165509	229533.A0A098E320	pp	229533.A0A1C3YKV2
165510	229533.A0A098E320	pp	229533.I1RB38
165511	229533.A0A098E320	pp	229533.I1RZ20
165512	229533.A0A098E320	pp	229533.I1RNB4
165513	229533.A0A098E320	pp	229533.I1RE63
165514	229533.A0A098E320	pp	229533.A0A1C3YK49
165515	229533.A0A098E320	pp	229533.I1RD80
165516	229533.A0A098E320	pp	229533.I1RYC8
165517	229533.A0A098E320	pp	229533.I1RYR3
165518	229533.A0A098E320	pp	229533.I1RZT0
165519	229533.A0A098E320	pp	229533.I1RYW9
165520	229533.A0A098E320	pp	229533.V6RN31
165521	229533.A0A098E320	pp	229533.I1RZ29
165522	229533.A0A098E320	pp	229533.I1RF85
165523	229533.A0A098E320	pp	229533.A0A1C3YKW4
165524	229533.A0A098E320	pp	229533.I1S3N0
165525	229533.A0A098E320	pp	229533.I1S3G4
165526	229533.A0A098E320	pp	229533.I1RNG4
165527	229533.A0A098E320	pp	229533.I1RKU0
165528	229533.A0A098E320	pp	229533.I1RE58
165529	229533.A0A098E320	pp	229533.I1RDU6
165530	229533.A0A098E320	pp	229533.A0A1C3YJ22
165531	229533.A0A098E320	pp	229533.I1RJT5
165532	229533.A0A098E320	pp	229533.I1SAE8
165533	229533.A0A098E320	pp	229533.I1RZY1
165534	229533.A0A098E320	pp	229533.I1RNW1
165535	229533.A0A098E320	pp	229533.V6QV31
165536	229533.A0A098E320	pp	229533.I1RD21
165537	229533.A0A098E320	pp	229533.I1RDJ7
165538	229533.A0A098E320	pp	229533.I1REA5
165539	229533.A0A098E320	pp	229533.I1S8F8
165540	229533.A0A098E320	pp	229533.I1RCU2
165541	229533.A0A098E320	pp	229533.I1RIL0
165542	229533.A0A098E320	pp	229533.I1S6P5
165543	229533.A0A098E320	pp	229533.I1RR12
165544	229533.A0A098E320	pp	229533.I1RCT7
165545	229533.A0A098E320	pp	229533.I1S8S9
165546	229533.A0A098E320	pp	229533.I1S1P8
165547	229533.A0A098E320	pp	229533.I1S3X8
165548	229533.A0A098E320	pp	229533.A0A1C3YLW6
165549	229533.A0A098E320	pp	229533.I1RC39
165550	229533.A0A098E320	pp	229533.I1RFI4
165551	229533.A0A098E320	pp	229533.I1RHT4
165552	229533.A0A098E320	pp	229533.A0A1C3YKZ8
165553	229533.A0A098E320	pp	229533.I1RJ03
165554	229533.A0A098E320	pp	229533.I1RG33
165555	229533.A0A098E320	pp	229533.I1RVZ3
165556	229533.A0A098E320	pp	229533.A0A1C3YHG7
165557	229533.A0A098E320	pp	229533.A0A1C3YM86
165558	229533.A0A098E320	pp	229533.A0A1C3YIV7
165559	229533.A0A098E320	pp	229533.A0A0E0RU85
165560	229533.A0A098E320	pp	229533.I1RP86
165561	229533.A0A098E320	pp	229533.I1RH30
165562	229533.A0A098E320	pp	229533.I1RAG9
165563	229533.A0A098E320	pp	229533.I1RY49
165564	229533.A0A098E320	pp	229533.I1S2V3

165565 229533.A0A098E320 pp 229533.I1RPR1
165566 229533.A0A098E320 pp 229533.A0A1C3YK58
165567 229533.A0A098E320 pp 229533.A0A1C3YIJ9
165568 229533.A0A098E320 pp 229533.I1RJ72
165569 229533.A0A098E320 pp 229533.I1RPW2
165570 229533.A0A098E320 pp 229533.I1RNY6
165571 229533.A0A098E320 pp 229533.V6QWD8
165572 229533.A0A098E320 pp 229533.I1RGU6
165573 229533.A0A098E320 pp 229533.I1RA94
165574 229533.A0A098E320 pp 229533.I1RIA1
165575 229533.A0A098E320 pp 229533.I1S084
165576 229533.A0A098E320 pp 229533.I1RPM6
165577 229533.A0A098E320 pp 229533.I1RZH8
165578 229533.A0A098E320 pp 229533.I1S1W8
165579 229533.A0A098E320 pp 229533.A0A1C3YIX7
165580 229533.A0A098E320 pp 229533.I1RXE6
165581 229533.A0A098E320 pp 229533.I1RNM7
165582 229533.A0A098E320 pp 229533.I1RLQ5
165583 229533.A0A098E320 pp 229533.I1RQX9
165584 229533.A0A098E320 pp 229533.I1RM66
165585 229533.A0A098E320 pp 229533.I1RC74
165586 229533.A0A098E320 pp 229533.I1S3K8
165587 229533.A0A098E320 pp 229533.I1RNP4
165588 229533.A0A098E320 pp 229533.I1RMU5
165589 229533.A0A1C3YL25 pp 229533.Q4IPB3
165590 229533.A0A1C3YL25 pp 229533.Q4IPN9
165591 229533.A0A1C3YL25 pp 229533.I1RCH7
165592 229533.A0A1C3YL25 pp 229533.I1RFQ0
165593 229533.A0A1C3YL25 pp 229533.I1S7M3
165594 229533.A0A1C3YL25 pp 229533.I1RSA8
165595 229533.A0A1C3YL25 pp 229533.Q4I7X1
165596 229533.A0A1C3YL25 pp 229533.Q4IE79
165597 229533.A0A1C3YL25 pp 229533.I1RK23
165598 229533.A0A1C3YL25 pp 229533.I1RSM2
165599 229533.A0A1C3YL25 pp 229533.I1RCK4
165600 229533.A0A1C3YL25 pp 229533.I1RBU6
165601 229533.A0A1C3YL25 pp 229533.I1RZ11
165602 229533.A0A1C3YL25 pp 229533.I1RRU9
165603 229533.A0A1C3YL25 pp 229533.I1RPZ3
165604 229533.A0A1C3YL25 pp 229533.I1S7M2
165605 229533.A0A1C3YL25 pp 229533.I1RGC2
165606 229533.A0A1C3YL25 pp 229533.I1RAU6
165607 229533.A0A1C3YL25 pp 229533.I1RDD3
165608 229533.A0A1C3YL25 pp 229533.I1S246
165609 229533.A0A1C3YL25 pp 229533.I1RS83
165610 229533.A0A1C3YL25 pp 229533.I1S044
165611 229533.A0A1C3YL25 pp 229533.I1RLU5
165612 229533.A0A1C3YL25 pp 229533.I1RA46
165613 229533.A0A1C3YL25 pp 229533.I1RRW3
165614 229533.A0A1C3YL25 pp 229533.Q4IRI9
165615 229533.A0A1C3YL25 pp 229533.I1RD56
165616 229533.A0A1C3YL25 pp 229533.Q4IAA0
165617 229533.A0A1C3YL25 pp 229533.I1RNT2
165618 229533.A0A1C3YL25 pp 229533.A0A1C3YM64
165619 229533.A0A1C3YL25 pp 229533.I1RB50
165620 229533.A0A1C3YL25 pp 229533.I1RN28
165621 229533.A0A1C3YL25 pp 229533.I1RN91
165622 229533.A0A1C3YL25 pp 229533.Q4IJ56
165623 229533.A0A1C3YL25 pp 229533.Q4IJH1
165624 229533.A0A1C3YL25 pp 229533.I1RN60
165625 229533.A0A1C3YL25 pp 229533.I1RCU7
165626 229533.A0A1C3YL25 pp 229533.I1S0P4
165627 229533.A0A1C3YL25 pp 229533.I1S0B6
165628 229533.A0A1C3YL25 pp 229533.I1RT35
165629 229533.A0A1C3YL25 pp 229533.I1R9X2
165630 229533.A0A1C3YL25 pp 229533.I1RTE5
165631 229533.A0A1C3YL25 pp 229533.I1RWU2
165632 229533.A0A1C3YL25 pp 229533.I1RDY2
165633 229533.A0A1C3YL25 pp 229533.I1RDY1
165634 229533.A0A1C3YL25 pp 229533.I1S5B5
165635 229533.A0A1C3YL25 pp 229533.I1RUM5
165636 229533.A0A1C3YL25 pp 229533.Q4IR09
165637 229533.A0A1C3YL25 pp 229533.I1RCQ6

165638	229533.A0A1C3YL25	pp	229533.I1REX4
165639	229533.A0A1C3YL25	pp	229533.I1RMY3
165640	229533.A0A1C3YL25	pp	229533.I1RAC1
165641	229533.A0A1C3YL25	pp	229533.Q4I1Y1
165642	229533.A0A1C3YL25	pp	229533.I1S214
165643	229533.A0A1C3YL25	pp	229533.A0A1C3YLE5
165644	229533.A0A1C3YL25	pp	229533.I1RFG0
165645	229533.A0A1C3YL25	pp	229533.I1S056
165646	229533.A0A1C3YL25	pp	229533.I1RS94
165647	229533.A0A1C3YL25	pp	229533.I1S5Z2
165648	229533.A0A1C3YL25	pp	229533.Q4IB70
165649	229533.A0A1C3YL25	pp	229533.I1S471
165650	229533.A0A1C3YL25	pp	229533.I1RA99
165651	229533.A0A1C3YL25	pp	229533.I1RND9
165652	229533.A0A1C3YL25	pp	229533.I1RQ49
165653	229533.A0A1C3YL25	pp	229533.I1RZV9
165654	229533.A0A1C3YL25	pp	229533.I1RAF3
165655	229533.A0A1C3YL25	pp	229533.Q4IPA4
165656	229533.A0A1C3YL25	pp	229533.I1RMH5
165657	229533.A0A1C3YL25	pp	229533.I1RA36
165658	229533.A0A1C3YL25	pp	229533.I1RQK2
165659	229533.A0A1C3YL25	pp	229533.I1RZS7
165660	229533.A0A1C3YL25	pp	229533.Q4I7K4
165661	229533.A0A1C3YL25	pp	229533.I1RZL5
165662	229533.A0A1C3YL25	pp	229533.I1S9M9
165663	229533.A0A1C3YL25	pp	229533.I1RU69
165664	229533.A0A1C3YL25	pp	229533.I1RWD3
165665	229533.A0A1C3YL25	pp	229533.Q4IF76
165666	229533.A0A1C3YL25	pp	229533.I1RM25
165667	229533.A0A1C3YL25	pp	229533.I1RNB8
165668	229533.A0A1C3YL25	pp	229533.I1RCG8
165669	229533.A0A1C3YL25	pp	229533.I1RDG0
165670	229533.A0A1C3YL25	pp	229533.I1RQZ2
165671	229533.A0A1C3YL25	pp	229533.I1RJS4
165672	229533.A0A1C3YL25	pp	229533.I1RPY7
165673	229533.A0A1C3YL25	pp	229533.I1RSA4
165674	229533.A0A1C3YL25	pp	229533.I1RAI1
165675	229533.A0A1C3YL25	pp	229533.I1RST1
165676	229533.A0A1C3YL25	pp	229533.V6RY33
165677	229533.A0A1C3YL25	pp	229533.A0A1C3YM79
165678	229533.A0A1C3YL25	pp	229533.Q4IPB8
165679	229533.A0A1C3YL25	pp	229533.I1RBH8
165680	229533.A0A1C3YL25	pp	229533.I1RE00
165681	229533.A0A1C3YL25	pp	229533.I1RYB1
165682	229533.A0A1C3YL25	pp	229533.I1RYU4
165683	229533.A0A1C3YL25	pp	229533.I1S0Z9
165684	229533.A0A1C3YL25	pp	229533.I1S6F1
165685	229533.A0A1C3YL25	pp	229533.I1RG57
165686	229533.A0A1C3YL25	pp	229533.I1RBV3
165687	229533.A0A1C3YL25	pp	229533.I1RMU1
165688	229533.A0A1C3YL25	pp	229533.A0A1C3YMK1
165689	229533.A0A1C3YL25	pp	229533.I1RBX0
165690	229533.A0A1C3YL25	pp	229533.A0A1C3YL93
165691	229533.A0A1C3YL25	pp	229533.Q4IF44
165692	229533.A0A1C3YL25	pp	229533.I1RJS5
165693	229533.A0A1C3YL25	pp	229533.I1RTG3
165694	229533.A0A1C3YL25	pp	229533.I1RBZ8
165695	229533.A0A1C3YL25	pp	229533.I1RCN5
165696	229533.A0A1C3YL25	pp	229533.I1RPL3
165697	229533.A0A1C3YL25	pp	229533.I1RXF2
165698	229533.A0A1C3YL25	pp	229533.I1RDX6
165699	229533.A0A1C3YL25	pp	229533.I1RS03
165700	229533.A0A1C3YL25	pp	229533.I1RE45
165701	229533.A0A1C3YL25	pp	229533.I1RYT7
165702	229533.A0A1C3YL25	pp	229533.I1S0X5
165703	229533.A0A1C3YL25	pp	229533.Q4HXF6
165704	229533.A0A1C3YL25	pp	229533.I1RQR3
165705	229533.A0A1C3YL25	pp	229533.I1S0N7
165706	229533.A0A1C3YL25	pp	229533.Q4IBK5
165707	229533.A0A1C3YL25	pp	229533.I1RX47
165708	229533.A0A1C3YL25	pp	229533.I1S0B1
165709	229533.A0A1C3YL25	pp	229533.I1RYJ9
165710	229533.A0A1C3YL25	pp	229533.I1RG90

165711 229533.A0A1C3YL25 pp 229533.I1S8U9
165712 229533.A0A1C3YL25 pp 229533.I1RCS2
165713 229533.A0A1C3YL25 pp 229533.I1RN09
165714 229533.A0A1C3YL25 pp 229533.I1S8V0
165715 229533.A0A1C3YL25 pp 229533.V6REZ4
165716 229533.A0A1C3YL25 pp 229533.I1RIR9
165717 229533.A0A1C3YL25 pp 229533.V6RIS2
165718 229533.I1RJW0 pp 229533.I1SAK2
165719 229533.I1RJW0 pp 229533.I1RX39
165720 229533.A0A1C3YL82 pp 229533.I1RYK0
165721 229533.A0A1C3YL82 pp 229533.I1RSS7
165722 229533.A0A1C3YL82 pp 229533.I1RKB2
165723 229533.A0A1C3YL82 pp 229533.V6RZD4
165724 229533.A0A1C3YL82 pp 229533.I1RLZ4
165725 229533.A0A1C3YL82 pp 229533.I1RZ20
165726 229533.A0A1C3YL82 pp 229533.I1R9W4
165727 229533.A0A1C3YL82 pp 229533.I1RPD6
165728 229533.A0A1C3YL82 pp 229533.I1RC31
165729 229533.A0A1C3YL82 pp 229533.Q4HXF0
165730 229533.A0A1C3YL82 pp 229533.I1RZV5
165731 229533.A0A1C3YL82 pp 229533.I1RR68
165732 229533.A0A1C3YL82 pp 229533.I1RZB2
165733 229533.A0A1C3YL82 pp 229533.I1RNB4
165734 229533.A0A1C3YL82 pp 229533.I1RNG4
165735 229533.A0A1C3YL82 pp 229533.I1RE53
165736 229533.A0A1C3YL82 pp 229533.I1RNG7
165737 229533.A0A1C3YL82 pp 229533.I1RJH8
165738 229533.A0A1C3YL82 pp 229533.I1RZT0
165739 229533.A0A1C3YL82 pp 229533.A0A1C3YLA6
165740 229533.A0A1C3YL82 pp 229533.I1S3G4
165741 229533.A0A1C3YL82 pp 229533.I1RCB4
165742 229533.A0A1C3YL82 pp 229533.I1RYP6
165743 229533.A0A1C3YL82 pp 229533.I1RJH7
165744 229533.A0A1C3YL82 pp 229533.V6R382
165745 229533.A0A1C3YL82 pp 229533.O74638
165746 229533.A0A1C3YL82 pp 229533.I1RZ39
165747 229533.A0A1C3YL82 pp 229533.I1RXY7
165748 229533.A0A1C3YL82 pp 229533.I1S2C4
165749 229533.A0A1C3YL82 pp 229533.I1RW43
165750 229533.A0A1C3YL82 pp 229533.I1RVR2
165751 229533.A0A1C3YL82 pp 229533.I1RA52
165752 229533.A0A1C3YL82 pp 229533.V6R5D2
165753 229533.A0A1C3YL82 pp 229533.I1RZ29
165754 229533.A0A1C3YL82 pp 229533.A0A1C3YMD8
165755 229533.A0A1C3YL82 pp 229533.V6RN31
165756 229533.A0A1C3YL82 pp 229533.I1RZV8
165757 229533.A0A1C3YL82 pp 229533.I1RCG5
165758 229533.A0A1C3YL82 pp 229533.I1RKB6
165759 229533.A0A1C3YL82 pp 229533.A0A1C3YLI5
165760 229533.A0A1C3YL82 pp 229533.I1RPU8
165761 229533.A0A1C3YL82 pp 229533.V6QVH2
165762 229533.A0A1C3YL82 pp 229533.I1S0I3
165763 229533.A0A1C3YL82 pp 229533.I1RAN1
165764 229533.A0A1C3YL82 pp 229533.I1RYU5
165765 229533.A0A1C3YL82 pp 229533.I1S4S5
165766 229533.A0A1C3YL82 pp 229533.I1RGT3
165767 229533.A0A1C3YL82 pp 229533.I1S1W8
165768 229533.A0A1C3YL82 pp 229533.I1RS77
165769 229533.A0A1C3YL82 pp 229533.I1RSI4
165770 229533.A0A1C3YL82 pp 229533.I1R9Y2
165771 229533.A0A1C3YL82 pp 229533.I1RDT5
165772 229533.A0A1C3YL82 pp 229533.I1RH36
165773 229533.A0A1C3YL82 pp 229533.I1RFK7
165774 229533.A0A1C3YL82 pp 229533.I1RJC5
165775 229533.A0A1C3YL82 pp 229533.I1RIJ2
165776 229533.A0A1C3YL82 pp 229533.I1RP69
165777 229533.A0A1C3YL82 pp 229533.V6RF62
165778 229533.A0A1C3YL82 pp 229533.A0A1C3YLR6
165779 229533.A0A1C3YL82 pp 229533.I1RYX6
165780 229533.A0A1C3YL82 pp 229533.I1S3K8
165781 229533.A0A1C3YL82 pp 229533.I1RC39
165782 229533.A0A1C3YL82 pp 229533.I1RBE4
165783 229533.A0A1C3YL82 pp 229533.I1RAG9

229533.A0A1C3YL82	pp	229533.I1RP86
229533.A0A1C3YL82	pp	229533.I1RVX9
229533.A0A1C3YL82	pp	229533.I1RH67
229533.A0A1C3YL82	pp	229533.I1RY49
229533.A0A1C3YL82	pp	229533.I1RA94
229533.A0A1C3YL82	pp	229533.I1S3K6
229533.A0A1C3YL82	pp	229533.I1RAR3
229533.A0A1C3YL82	pp	229533.I1RAR9
229533.A0A1C3YL82	pp	229533.I1S0A0
229533.A0A1C3YL82	pp	229533.I1RAG5
229533.A0A1C3YL82	pp	229533.I1RPW2
229533.A0A1C3YL82	pp	229533.I1RF38
229533.A0A1C3YL82	pp	229533.I1RVR0
229533.A0A1C3YL82	pp	229533.I1RA90
229533.A0A1C3YL82	pp	229533.I1RMM8
229533.A0A1C3YL82	pp	229533.I1RW69
229533.A0A1C3YL82	pp	229533.V6R3V4
229533.A0A1C3YL82	pp	229533.A0A1C3YN26
229533.A0A1C3YL82	pp	229533.I1S8H9
229533.A0A1C3YL82	pp	229533.I1RWL3
229533.A0A1C3YL82	pp	229533.I1RCV1
229533.A0A1C3YL82	pp	229533.A0A1C3YLP6
229533.A0A1C3YL82	pp	229533.I1RPW1
229533.A0A1C3YL82	pp	229533.I1RQE0
229533.A0A1C3YL82	pp	229533.I1RM30
229533.A0A1C3YL82	pp	229533.I1RBE7
229533.A0A1C3YL82	pp	229533.I1RJT5
229533.A0A1C3YL82	pp	229533.I1S2C8
229533.A0A1C3YL82	pp	229533.I1RJA8
229533.A0A1C3YL82	pp	229533.V6RQW4
229533.A0A1C3YL82	pp	229533.V6RGB5
229533.A0A1C3YL82	pp	229533.I1RM26
229533.A0A098E365	pp	229533.I1RYT7
229533.A0A098E365	pp	229533.A0A1C3YJ51
229533.A0A098E365	pp	229533.V6REZ4
229533.A0A098E365	pp	229533.I1RJZ7
229533.A0A098E365	pp	229533.I1RZT1
229533.A0A098E365	pp	229533.I1RC00
229533.A0A098E365	pp	229533.I1RBZ8
229533.A0A098E365	pp	229533.Q4IAA0
229533.A0A098E365	pp	229533.I1RS94
229533.A0A098E365	pp	229533.I1S0P4
229533.A0A098E365	pp	229533.I1RMY3
229533.A0A098E365	pp	229533.A0A1C3YMK1
229533.A0A098E365	pp	229533.I1RCK4
229533.A0A098E365	pp	229533.I1RZV9
229533.A0A098E365	pp	229533.I1RW71
229533.A0A098E365	pp	229533.I1RY40
229533.A0A098E365	pp	229533.I1RNT2
229533.A0A098E365	pp	229533.A0A1C3YM64
229533.A0A098E365	pp	229533.I1RCU7
229533.I1RBU6	pp	229533.V6RIS2
229533.I1RBU6	pp	229533.I1RS83
229533.I1RBU6	pp	229533.I1RN09
229533.I1RBU6	pp	229533.I1RG17
229533.I1RBU6	pp	229533.I1RZ11
229533.I1RBU6	pp	229533.I1RC95
229533.I1RBU6	pp	229533.I1RQD8
229533.I1RBU6	pp	229533.I1RSA8
229533.I1RBU6	pp	229533.V6RY33
229533.I1RBU6	pp	229533.I1RK19
229533.I1RBU6	pp	229533.I1RCA7
229533.I1RBU6	pp	229533.I1RKK4
229533.I1RBU6	pp	229533.I1RCS2
229533.I1RBU6	pp	229533.I1RWX8
229533.I1RBU6	pp	229533.I1S246
229533.I1RBU6	pp	229533.I1RSA4
229533.I1RBU6	pp	229533.Q4IJH1
229533.I1RBU6	pp	229533.I1RS75
229533.I1RBU6	pp	229533.I1RSD4
229533.I1RBU6	pp	229533.Q4IF76
229533.I1RBU6	pp	229533.I1RBZ8
229533.I1RBU6	pp	229533.I1RFQ1

165857	229533.I1RBU6	pp	229533.Q4IPH4
165858	229533.I1RBU6	pp	229533.I1RYJ9
165859	229533.I1RBU6	pp	229533.I1RCI3
165860	229533.I1RBU6	pp	229533.I1RMW7
165861	229533.I1RBU6	pp	229533.I1RMH5
165862	229533.I1RBU6	pp	229533.I1RMV5
165863	229533.I1RBU6	pp	229533.I1REX2
165864	229533.I1RBU6	pp	229533.I1RG90
165865	229533.I1RBU6	pp	229533.Q4IB70
165866	229533.I1RBU6	pp	229533.V6QVM5
165867	229533.I1RBU6	pp	229533.I1RQR3
165868	229533.I1RBU6	pp	229533.I1RYB1
165869	229533.I1RBU6	pp	229533.I1RZZ1
165870	229533.I1RBU6	pp	229533.I1RK96
165871	229533.I1RBU6	pp	229533.I1RCQ6
165872	229533.I1RBU6	pp	229533.I1RTS2
165873	229533.I1RBU6	pp	229533.I1RFG0
165874	229533.I1RBU6	pp	229533.I1S524
165875	229533.I1RBU6	pp	229533.I1RPY4
165876	229533.I1RS06	pp	229533.V6RMW6
165877	229533.I1RS06	pp	229533.I1S0B1
165878	229533.I1RS06	pp	229533.I1S2B8
165879	229533.I1RS06	pp	229533.I1RSZ7
165880	229533.I1RS06	pp	229533.I1S1W9
165881	229533.I1RS06	pp	229533.I1RWI5
165882	229533.I1RS06	pp	229533.Q4HW67
165883	229533.I1RS06	pp	229533.I1RZ39
165884	229533.I1RS06	pp	229533.I1RSQ4
165885	229533.I1RS06	pp	229533.I1S3G4
165886	229533.I1RS06	pp	229533.I1RYI5
165887	229533.I1RS06	pp	229533.I1RSA4
165888	229533.I1RS06	pp	229533.I1RXF2
165889	229533.I1RS06	pp	229533.I1RT35
165890	229533.I1RS06	pp	229533.V6QUE6
165891	229533.I1RS06	pp	229533.I1S7M3
165892	229533.I1RS06	pp	229533.I1S7M2
165893	229533.I1RS06	pp	229533.I1S4S7
165894	229533.I1RS06	pp	229533.I1S1W8
165895	229533.I1RZA7	pp	229533.I1S3N0
165896	229533.I1RZA7	pp	229533.I1S024
165897	229533.I1RZA7	pp	229533.I1RZH8
165898	229533.I1RZA7	pp	229533.I1S3X8
165899	229533.I1RZA7	pp	229533.V6RUB1
165900	229533.I1RZA7	pp	229533.V6RV57
165901	229533.I1RZA7	pp	229533.I1S202
165902	229533.I1RZA7	pp	229533.I1S6P5
165903	229533.I1RZA7	pp	229533.I1S0V7
165904	229533.I1RZA7	pp	229533.I1S147
165905	229533.I1RZA7	pp	229533.V6RLM7
165906	229533.I1RZA7	pp	229533.I1RZY1
165907	229533.I1RZA7	pp	229533.V6RRM0
165908	229533.I1RZA7	pp	229533.I1S3G4
165909	229533.I1RZA7	pp	229533.I1S084
165910	229533.I1RZA7	pp	229533.V6QV31
165911	229533.I1RZA7	pp	229533.I1S2V3
165912	229533.I1RZA7	pp	229533.I1S1W8
165913	229533.I1RZA7	pp	229533.V6QWD8
165914	229533.I1RZA7	pp	229533.I1RZT0
165915	229533.I1RZA7	pp	229533.V6RN31
165916	229533.I1RZA7	pp	229533.I1S1P8
165917	229533.I1RZA7	pp	229533.I1S3K8
165918	229533.I1RZA7	pp	229533.I1S8S9
165919	229533.I1RZA7	pp	229533.I1S8F8
165920	229533.I1RZA7	pp	229533.I1SAE8
165921	229533.I1S8V0	pp	229533.Q4I7X1
165922	229533.I1S8V0	pp	229533.Q4IR09
165923	229533.I1S8V0	pp	229533.Q4IF44
165924	229533.I1S8V0	pp	229533.Q4IPC8
165925	229533.I1S8V0	pp	229533.Q4IPA4
165926	229533.I1S8V0	pp	229533.Q4IPN9
165927	229533.I1S8V0	pp	229533.V6QUE6
165928	229533.I1S8V0	pp	229533.V6RIS2
165929	229533.I1RIF9	pp	229533.I1SAK2

165930 229533.I1RIF9 pp 229533.I1RX39
165931 229533.I1RIF9 pp 229533.I1S2L0
165932 229533.I1RIF9 pp 229533.I1S3S2
165933 229533.A0A098DS79 pp 229533.V6RY56
165934 229533.A0A098DS79 pp 229533.I1RZY1
165935 229533.A0A098DS79 pp 229533.A0A1C3YJ68
165936 229533.A0A098DS79 pp 229533.I1RC39
165937 229533.A0A098DS79 pp 229533.A0A1C3YKZ8
165938 229533.A0A098DS79 pp 229533.A0A1C3YIX7
165939 229533.A0A098DS79 pp 229533.I1S4D0
165940 229533.A0A098DS79 pp 229533.A0A1C3YIG7
165941 229533.A0A098DS79 pp 229533.I1RDU9
165942 229533.A0A098DS79 pp 229533.A0A1C3YL13
165943 229533.A0A098DS79 pp 229533.A0A1C3YK58
165944 229533.A0A098DS79 pp 229533.I1RK24
165945 229533.A0A098DS79 pp 229533.I1RJT5
165946 229533.A0A098DS79 pp 229533.I1RSY1
165947 229533.A0A098DS79 pp 229533.I1S273
165948 229533.A0A098DS79 pp 229533.I1S024
165949 229533.A0A098DS79 pp 229533.I1RE24
165950 229533.A0A098DS79 pp 229533.I1RCH9
165951 229533.A0A098DS79 pp 229533.I1S7J8
165952 229533.A0A098DS79 pp 229533.I1RCV1
165953 229533.A0A098DS79 pp 229533.A0A1C3YLQ8
165954 229533.A0A098DS79 pp 229533.A0A1C3YLL1
165955 229533.A0A098DS79 pp 229533.I1RMJ3
165956 229533.A0A098DS79 pp 229533.I1RJX3
165957 229533.A0A098DS79 pp 229533.I1RAB6
165958 229533.A0A098DS79 pp 229533.I1RV23
165959 229533.A0A098DS79 pp 229533.A0A1C3YI11
165960 229533.A0A098DS79 pp 229533.A0A1C3YKV2
165961 229533.A0A098DS79 pp 229533.I1RD80
165962 229533.A0A098DS79 pp 229533.I1S2P0
165963 229533.A0A098DS79 pp 229533.I1S5N3
165964 229533.A0A098DS79 pp 229533.I1RKX6
165965 229533.A0A098DS79 pp 229533.I1RLP9
165966 229533.A0A098DS79 pp 229533.I1RLT3
165967 229533.A0A098DS79 pp 229533.I1RVZ3
165968 229533.A0A098DS79 pp 229533.I1RJ04
165969 229533.A0A098DS79 pp 229533.A0A1C3YNB1
165970 229533.A0A098DS79 pp 229533.I1S0H5
165971 229533.I1S2A8 pp 229533.Q4HXF6
165972 229533.I1S2A8 pp 229533.P0C431
165973 229533.I1S2A8 pp 229533.Q4HY71
165974 229533.I1S2A8 pp 229533.V6REE9
165975 229533.I1S2A8 pp 229533.I1S5B5
165976 229533.I1RQB5 pp 229533.I1S9L5
165977 229533.I1RQB5 pp 229533.Q4I7F9
165978 229533.I1RQB5 pp 229533.I1RS83
165979 229533.I1RQB5 pp 229533.I1RRQ4
165980 229533.I1RQB5 pp 229533.V6RY33
165981 229533.I1RQB5 pp 229533.I1RQP8
165982 229533.I1RQB5 pp 229533.I1RQD8
165983 229533.I1RQB5 pp 229533.I1S9L6
165984 229533.I1RQB5 pp 229533.I1S1C6
165985 229533.I1RQB5 pp 229533.I1RSA4
165986 229533.I1RDJ7 pp 229533.I1S2C4
165987 229533.I1RDJ7 pp 229533.I1RHT3
165988 229533.I1RDJ7 pp 229533.I1RTU5
165989 229533.I1RDJ7 pp 229533.I1RX43
165990 229533.I1RDJ7 pp 229533.I1S0T1
165991 229533.I1RDJ7 pp 229533.V6R756
165992 229533.I1RDJ7 pp 229533.I1RT74
165993 229533.I1RDJ7 pp 229533.I1S6H1
165994 229533.I1RDJ7 pp 229533.V6R2Z2
165995 229533.I1RDJ7 pp 229533.I1SAJ0
165996 229533.I1RDJ7 pp 229533.I1RV23
165997 229533.I1RDJ7 pp 229533.I1RJK0
165998 229533.I1RDJ7 pp 229533.I1RZA7
165999 229533.I1RDJ7 pp 229533.I1RJT5
166000 229533.I1RDJ7 pp 229533.I1S4D0
166001 229533.I1RDJ7 pp 229533.I1RNG4
166002 229533.I1RDJ7 pp 229533.I1RVV7

229533.I1RDJ7 pp 229533.I1RYY2
229533.I1RDJ7 pp 229533.I1RV18
229533.I1RDJ7 pp 229533.V6QU99
229533.I1RDJ7 pp 229533.I1S084
229533.I1RDJ7 pp 229533.I1RZ20
229533.I1RDJ7 pp 229533.V6RUB1
229533.I1RDJ7 pp 229533.I1RTJ7
229533.I1RDJ7 pp 229533.V6RY56
229533.I1RDJ7 pp 229533.I1RP53
229533.I1RDJ7 pp 229533.I1RYU0
229533.I1RDJ7 pp 229533.I1SAI4
229533.I1RDJ7 pp 229533.V6R5D2
229533.I1RDJ7 pp 229533.I1S6P5
229533.I1RDJ7 pp 229533.I1S3K8
229533.I1RDJ7 pp 229533.I1RZT0
229533.I1RDJ7 pp 229533.I1RY96
229533.I1RDJ7 pp 229533.I1RJE7
229533.I1RDJ7 pp 229533.V6R0W1
229533.I1RDJ7 pp 229533.I1S1P8
229533.I1RDJ7 pp 229533.I1RXH3
229533.I1RDJ7 pp 229533.V6RAM6
229533.I1RDJ7 pp 229533.I1RXV3
229533.I1RRC7 pp 229533.I1RRC8
229533.I1RTX2 pp 229533.I1RZ25
229533.I1RWS8 pp 229533.I1S4T3
229533.I1RUM5 pp 229533.I1S471
229533.I1RUM5 pp 229533.Q4IAA0
229533.I1RUM5 pp 229533.I1S9M9
229533.I1RUM5 pp 229533.I1S0Z9
229533.I1RUM5 pp 229533.I1RX47
229533.I1RUM5 pp 229533.I1RWU2
229533.I1RUM5 pp 229533.I1S5Z2
229533.I1RUM5 pp 229533.I1RZG7
229533.I1RUM5 pp 229533.I1RW80
229533.I1RUM5 pp 229533.I1S246
229533.A0A1C3YM43 pp 229533.I1RB50
229533.A0A1C3YM43 pp 229533.I1RW25
229533.A0A1C3YM43 pp 229533.I1R9M0
229533.A0A1C3YM43 pp 229533.I1RZZ1
229533.A0A1C3YM43 pp 229533.I1RR72
229533.A0A1C3YM43 pp 229533.Q4IJH1
229533.A0A1C3YM43 pp 229533.I1S2E8
229533.A0A1C3YM43 pp 229533.I1RBG9
229533.A0A1C3YM43 pp 229533.I1RT05
229533.A0A1C3YM43 pp 229533.I1RQM6
229533.A0A1C3YM43 pp 229533.I1RMQ0
229533.A0A1C3YM43 pp 229533.I1RN18
229533.A0A1C3YM43 pp 229533.Q4I948
229533.A0A1C3YM43 pp 229533.V6QYF0
229533.A0A1C3YM43 pp 229533.I1RQ65
229533.A0A1C3YM43 pp 229533.I1RMR1
229533.A0A1C3YM43 pp 229533.I1RDX0
229533.A0A1C3YM43 pp 229533.I1S0L3
229533.A0A1C3YM43 pp 229533.I1S8F0
229533.A0A1C3YM43 pp 229533.I1RA36
229533.A0A1C3YM43 pp 229533.V6R8C4
229533.A0A1C3YM43 pp 229533.I1RSR3
229533.A0A1C3YM43 pp 229533.I1RT33
229533.A0A1C3YM43 pp 229533.Q4IPI1
229533.A0A1C3YM43 pp 229533.I1RTE9
229533.A0A1C3YM43 pp 229533.A0A1C3YN63
229533.A0A1C3YM43 pp 229533.I1RRZ0
229533.A0A1C3YM43 pp 229533.I1RJL2
229533.A0A1C3YM43 pp 229533.I1RS30
229533.A0A1C3YM43 pp 229533.I1S2C0
229533.A0A1C3YM43 pp 229533.I1RYI9
229533.A0A1C3YM43 pp 229533.I1RJM7
229533.A0A1C3YM43 pp 229533.I1RA70
229533.A0A1C3YM43 pp 229533.I1RKQ5
229533.A0A1C3YM43 pp 229533.I1RZB5
229533.A0A1C3YM43 pp 229533.I1R9X7
229533.A0A1C3YM43 pp 229533.I1RT04
229533.A0A1C3YM43 pp 229533.I1RS17

166076 229533.A0A1C3YM43 pp 229533.I1RQ81
166077 229533.A0A1C3YM43 pp 229533.I1RQJ4
166078 229533.A0A1C3YM43 pp 229533.I1RRI2
166079 229533.A0A1C3YM43 pp 229533.I1S0R7
166080 229533.A0A1C3YM43 pp 229533.I1RC72
166081 229533.A0A1C3YM43 pp 229533.I1RE00
166082 229533.A0A1C3YM43 pp 229533.I1RTD7
166083 229533.A0A1C3YM43 pp 229533.I1S0J1
166084 229533.A0A1C3YM43 pp 229533.I1RYH7
166085 229533.A0A1C3YM43 pp 229533.V6RGN5
166086 229533.A0A1C3YM43 pp 229533.I1RBC1
166087 229533.A0A1C3YM43 pp 229533.I1RSR8
166088 229533.A0A1C3YM43 pp 229533.I1RM96
166089 229533.A0A1C3YM43 pp 229533.I1RKC6
166090 229533.A0A1C3YM43 pp 229533.I1RSQ3
166091 229533.A0A1C3YM43 pp 229533.I1RC17
166092 229533.A0A1C3YM43 pp 229533.I1RLU6
166093 229533.A0A1C3YM43 pp 229533.I1RSS4
166094 229533.A0A1C3YM43 pp 229533.V6RCG5
166095 229533.A0A1C3YM43 pp 229533.Q4ICA8
166096 229533.A0A1C3YM43 pp 229533.I1RZK6
166097 229533.A0A1C3YM43 pp 229533.I1RSI6
166098 229533.A0A1C3YM43 pp 229533.I1RQ31
166099 229533.I1RLT7 pp 229533.I1RRQ3
166100 229533.I1RLT7 pp 229533.I1RPN6
166101 229533.Q4HY71 pp 229533.Q4ICA8
166102 229533.I1RV23 pp 229533.I1RZT0
166103 229533.I1RV23 pp 229533.V6RM02
166104 229533.I1RV23 pp 229533.I1S6P5
166105 229533.I1RV23 pp 229533.I1S0H5
166106 229533.I1RV23 pp 229533.V6R5D2
166107 229533.I1RV23 pp 229533.I1S3K8
166108 229533.I1RV23 pp 229533.I1RVR2
166109 229533.I1RV23 pp 229533.I1RX43
166110 229533.I1RV23 pp 229533.V6R756
166111 229533.I1RV23 pp 229533.I1S273
166112 229533.I1RV23 pp 229533.I1RZA7
166113 229533.I1RV23 pp 229533.V6R2Z2
166114 229533.I1RV23 pp 229533.V6RUB1
166115 229533.I1RV23 pp 229533.I1RW76
166116 229533.I1RV23 pp 229533.I1RZY1
166117 229533.I1RV23 pp 229533.I1SAI4
166118 229533.I1RV23 pp 229533.V6RY56
166119 229533.I1RV23 pp 229533.I1RZH8
166120 229533.I1RV23 pp 229533.I1SAE8
166121 229533.I1RV23 pp 229533.I1S4D0
166122 229533.I1RV23 pp 229533.I1RZ60
166123 229533.I1RV23 pp 229533.I1S024
166124 229533.I1RV23 pp 229533.I1S084
166125 229533.I1RV23 pp 229533.V6QU99
166126 229533.I1RV23 pp 229533.I1S1P8
166127 229533.I1RV23 pp 229533.V6RAM6
166128 229533.I1RV23 pp 229533.I1RZ20
166129 229533.I1RV23 pp 229533.V6R0W1
166130 229533.I1RV23 pp 229533.I1S0A0
166131 229533.I1RV23 pp 229533.V6R1K4
166132 229533.I1RBE6 pp 229533.I1S0R8
166133 229533.I1RBE6 pp 229533.I1RQE7
166134 229533.I1RBE6 pp 229533.I1S5X9
166135 229533.I1RBE6 pp 229533.I1RCH5
166136 229533.I1RBE6 pp 229533.I1RYA0
166137 229533.I1RBE6 pp 229533.I1RNJ5
166138 229533.I1RBE6 pp 229533.Q4IPZ1
166139 229533.I1RBE6 pp 229533.I1RBT6
166140 229533.I1RBE6 pp 229533.Q4HZ95
166141 229533.I1RBE6 pp 229533.I1RZD3
166142 229533.I1RBE6 pp 229533.I1RFJ6
166143 229533.I1RBE6 pp 229533.I1S2C4
166144 229533.I1RBE6 pp 229533.I1RWT0
166145 229533.I1RBE6 pp 229533.I1RMI1
166146 229533.I1RBE6 pp 229533.I1S0R9
166147 229533.I1RBE6 pp 229533.I1RPK0
166148 229533.I1RBE6 pp 229533.I1RC54

166149 229533.I1RBE6 pp 229533.V6RRM0
166150 229533.I1RBE6 pp 229533.I1RKF4
166151 229533.I1RBE6 pp 229533.Q4I375
166152 229533.I1RBE6 pp 229533.I1RYC8
166153 229533.I1RBE6 pp 229533.I1RF85
166154 229533.I1RBE6 pp 229533.I1S0Q7
166155 229533.I1RBE6 pp 229533.I1RSU1
166156 229533.I1RBE6 pp 229533.I1RE65
166157 229533.I1RBE6 pp 229533.I1RSQ4
166158 229533.I1RBE6 pp 229533.I1RFS5
166159 229533.I1RBE6 pp 229533.I1RGB0
166160 229533.I1RBE6 pp 229533.I1RN84
166161 229533.I1RBE6 pp 229533.V6R1X4
166162 229533.I1RBE6 pp 229533.I1RRI1
166163 229533.I1RBE6 pp 229533.I1RZ86
166164 229533.I1RBE6 pp 229533.I1RPY0
166165 229533.I1RBE6 pp 229533.I1RPY8
166166 229533.I1RBE6 pp 229533.I1RKD3
166167 229533.I1RBE6 pp 229533.Q4I7L0
166168 229533.I1RBE6 pp 229533.I1RC29
166169 229533.I1RBE6 pp 229533.I1RY57
166170 229533.I1RBE6 pp 229533.I1RXD9
166171 229533.I1RBE6 pp 229533.I1RC18
166172 229533.I1RBE6 pp 229533.I1RWA3
166173 229533.I1RBE6 pp 229533.I1RX39
166174 229533.I1RBE6 pp 229533.Q4I099
166175 229533.I1RBE6 pp 229533.I1RN88
166176 229533.I1RBE6 pp 229533.Q4I7T5
166177 229533.I1RBE6 pp 229533.I1S464
166178 229533.I1RBE6 pp 229533.V6R3J1
166179 229533.I1RBE6 pp 229533.I1RZN1
166180 229533.I1RBE6 pp 229533.V6RM66
166181 229533.I1RBE6 pp 229533.I1RY72
166182 229533.I1RBE6 pp 229533.I1RKB2
166183 229533.I1RBE6 pp 229533.I1RGC3
166184 229533.I1RBE6 pp 229533.Q4HVQ9
166185 229533.I1RBE6 pp 229533.I1RFI4
166186 229533.I1RBE6 pp 229533.I1RUR8
166187 229533.I1RBE6 pp 229533.I1RC34
166188 229533.I1RBE6 pp 229533.I1RY92
166189 229533.I1RBE6 pp 229533.I1S058
166190 229533.I1RBE6 pp 229533.I1S1F3
166191 229533.I1RBE6 pp 229533.I1RKF5
166192 229533.I1RBE6 pp 229533.I1RUQ3
166193 229533.I1RBE6 pp 229533.I1RT38
166194 229533.I1RBE6 pp 229533.I1RYW9
166195 229533.I1RBE6 pp 229533.I1S0K2
166196 229533.I1RBE6 pp 229533.I1RG33
166197 229533.I1RBE6 pp 229533.I1RM26
166198 229533.I1RBE6 pp 229533.I1RS29
166199 229533.I1RBE6 pp 229533.I1RL81
166200 229533.I1RBE6 pp 229533.I1RPP5
166201 229533.I1RBE6 pp 229533.I1RQA5
166202 229533.I1RBE6 pp 229533.I1RYE5
166203 229533.I1RBE6 pp 229533.I1RJY7
166204 229533.I1RBE6 pp 229533.I1RZ43
166205 229533.I1RBE6 pp 229533.I1RCM5
166206 229533.I1RBE6 pp 229533.I1S8S9
166207 229533.I1RBE6 pp 229533.V6RJP8
166208 229533.I1RBE6 pp 229533.I1RUL3
166209 229533.I1RBE6 pp 229533.I1S8F8
166210 229533.I1RBE6 pp 229533.I1RRS8
166211 229533.I1RBE6 pp 229533.I1RNM7
166212 229533.I1RBE6 pp 229533.I1RI27
166213 229533.I1RBE6 pp 229533.I1RNY6
166214 229533.I1RBE6 pp 229533.I1RPR9
166215 229533.I1RBE6 pp 229533.I1RYN2
166216 229533.I1RBE6 pp 229533.I1RGB1
166217 229533.I1RBE6 pp 229533.I1RQS2
166218 229533.I1RBE6 pp 229533.I1RP02
166219 229533.I1RBE6 pp 229533.I1RXE6
166220 229533.I1RBE6 pp 229533.I1S212
166221 229533.I1RBE6 pp 229533.I1RYT8

166222 229533.I1RBE6 pp 229533.I1RFY0
166223 229533.I1RBE6 pp 229533.I1RQR6
166224 229533.I1RBE6 pp 229533.I1RG62
166225 229533.I1RBE6 pp 229533.I1RVX9
166226 229533.I1RBE6 pp 229533.I1RMK7
166227 229533.I1RBE6 pp 229533.I1S2C1
166228 229533.I1RBE6 pp 229533.I1RGU0
166229 229533.I1RBE6 pp 229533.I1RX17
166230 229533.I1RBE6 pp 229533.I1RKB7
166231 229533.I1RBE6 pp 229533.I1RC49
166232 229533.I1RBE6 pp 229533.I1RPN1
166233 229533.I1RBE6 pp 229533.I1RMQ6
166234 229533.I1RBE6 pp 229533.I1RFV2
166235 229533.I1RBE6 pp 229533.I1RCU2
166236 229533.I1RBE6 pp 229533.I1RE66
166237 229533.I1RBE6 pp 229533.I1S5C6
166238 229533.I1RBE6 pp 229533.I1RRX5
166239 229533.I1RBE6 pp 229533.I1RY21
166240 229533.I1RBE6 pp 229533.I1S8C1
166241 229533.I1RBE6 pp 229533.I1RS05
166242 229533.I1RBE6 pp 229533.I1RP86
166243 229533.I1RBE6 pp 229533.I1S0P0
166244 229533.I1RW24 pp 229533.Q4I963
166245 229533.I1RW24 pp 229533.I1S224
166246 229533.I1RW24 pp 229533.I1RYJ8
166247 229533.I1RW24 pp 229533.I1RZZ1
166248 229533.I1RW24 pp 229533.Q4INI2
166249 229533.I1RW24 pp 229533.I1RYT7
166250 229533.I1RW24 pp 229533.Q4INS6
166251 229533.I1RW24 pp 229533.I1S0P4
166252 229533.I1RW24 pp 229533.I1RWI8
166253 229533.I1RW24 pp 229533.I1RZE1
166254 229533.I1RW24 pp 229533.I1RY44
166255 229533.I1RW24 pp 229533.I1RZ94
166256 229533.I1RW24 pp 229533.V6REZ4
166257 229533.I1RW24 pp 229533.I1RY29
166258 229533.I1RW24 pp 229533.I1S2E9
166259 229533.I1RW24 pp 229533.I1S2H4
166260 229533.I1RW24 pp 229533.I1S265
166261 229533.I1RW24 pp 229533.I1S2A8
166262 229533.I1RB46 pp 229533.I1RBE7
166263 229533.A0A1C3YII3 pp 229533.I1RY59
166264 229533.A0A1C3YII3 pp 229533.I1RW53
166265 229533.A0A1C3YII3 pp 229533.I1RBI1
166266 229533.A0A1C3YII3 pp 229533.I1RYR9
166267 229533.A0A1C3YII3 pp 229533.A0A1C3YN65
166268 229533.A0A1C3YII3 pp 229533.A0A1C3YKR1
166269 229533.V6R2Z2 pp 229533.V6RM09
166270 229533.V6R2Z2 pp 229533.V6RPS5
166271 229533.V6R2Z2 pp 229533.V6REA4
166272 229533.V6R2Z2 pp 229533.V6RY56
166273 229533.V6R2Z2 pp 229533.V6RBG5
166274 229533.V6R2Z2 pp 229533.V6R556
166275 229533.I1RYJ9 pp 229533.Q4IJH1
166276 229533.I1RYJ9 pp 229533.Q4I1Y1
166277 229533.I1RYJ9 pp 229533.I1S5Z2
166278 229533.I1RYJ9 pp 229533.I1RYU4
166279 229533.I1RYJ9 pp 229533.Q4IB70
166280 229533.I1RYJ9 pp 229533.I1RZG7
166281 229533.I1RYJ9 pp 229533.I1RZV9
166282 229533.I1RYJ9 pp 229533.Q4IPB3
166283 229533.I1RYJ9 pp 229533.Q4HXF6
166284 229533.I1RYJ9 pp 229533.I1S0Z9
166285 229533.I1RYJ9 pp 229533.I1S9L0
166286 229533.I1RYJ9 pp 229533.I1S0P4
166287 229533.I1RYJ9 pp 229533.Q4IPB8
166288 229533.I1RYJ9 pp 229533.I1S7M3
166289 229533.I1RYJ9 pp 229533.V6REZ4
166290 229533.I1RYJ9 pp 229533.I1S6F1
166291 229533.I1RYJ9 pp 229533.Q4IE79
166292 229533.I1RYJ9 pp 229533.I1RZL5
166293 229533.I1RYJ9 pp 229533.I1S246
166294 229533.I1RYJ9 pp 229533.I1RZS7

166295 229533.I1RYJ9 pp 229533.I1RZ11
166296 229533.I1RYJ9 pp 229533.Q4IBK5
166297 229533.I1RYJ9 pp 229533.Q4IF76
166298 229533.I1RYJ9 pp 229533.I1S7M2
166299 229533.I1RYJ9 pp 229533.Q4IAA0
166300 229533.I1RYJ9 pp 229533.I1S471
166301 229533.I1RYJ9 pp 229533.Q4IRI9
166302 229533.I1RYJ9 pp 229533.I1S0N7
166303 229533.I1RYJ9 pp 229533.V6RIS2
166304 229533.I1RYJ9 pp 229533.I1S056
166305 229533.A0A1C3YI42 pp 229533.I1RY59
166306 229533.A0A1C3YI42 pp 229533.I1RJS4
166307 229533.A0A1C3YI42 pp 229533.I1RT58
166308 229533.I1RWS6 pp 229533.I1RWS7
166309 229533.A0A1C3YKB7 pp 229533.I1RMT6
166310 229533.A0A1C3YKB7 pp 229533.I1S6H1
166311 229533.A0A1C3YKB7 pp 229533.I1RV23
166312 229533.A0A1C3YKB7 pp 229533.I1RHT3
166313 229533.A0A1C3YKB7 pp 229533.V6R556
166314 229533.A0A1C3YKB7 pp 229533.I1R9X3
166315 229533.A0A1C3YKB7 pp 229533.I1S0A5
166316 229533.A0A1C3YKB7 pp 229533.A0A1C3YKV2
166317 229533.A0A1C3YKB7 pp 229533.I1RPV8
166318 229533.A0A1C3YKB7 pp 229533.I1RTU5
166319 229533.A0A1C3YKB7 pp 229533.I1RNG4
166320 229533.A0A1C3YKB7 pp 229533.I1RJS1
166321 229533.A0A1C3YKB7 pp 229533.I1RF24
166322 229533.A0A1C3YKB7 pp 229533.I1RZ56
166323 229533.A0A1C3YKB7 pp 229533.I1RZ26
166324 229533.A0A1C3YKB7 pp 229533.I1RD80
166325 229533.A0A1C3YKB7 pp 229533.I1S3H8
166326 229533.A0A1C3YKB7 pp 229533.V6RBG5
166327 229533.A0A1C3YKB7 pp 229533.I1RF85
166328 229533.A0A1C3YKB7 pp 229533.I1RST9
166329 229533.A0A1C3YKB7 pp 229533.I1S149
166330 229533.A0A1C3YKB7 pp 229533.A0A1C3YKN4
166331 229533.A0A1C3YKB7 pp 229533.I1RTY9
166332 229533.A0A1C3YKB7 pp 229533.I1R9B8
166333 229533.A0A1C3YKB7 pp 229533.I1RKB2
166334 229533.A0A1C3YKB7 pp 229533.I1RZ38
166335 229533.A0A1C3YKB7 pp 229533.I1S405
166336 229533.A0A1C3YKB7 pp 229533.I1RIK8
166337 229533.A0A1C3YKB7 pp 229533.I1RZ25
166338 229533.A0A1C3YKB7 pp 229533.V6R0H4
166339 229533.A0A1C3YKB7 pp 229533.I1RUY5
166340 229533.A0A1C3YKB7 pp 229533.I1RV18
166341 229533.A0A1C3YKB7 pp 229533.I1SAJ0
166342 229533.A0A1C3YKB7 pp 229533.I1RDV8
166343 229533.A0A1C3YKB7 pp 229533.I1RAC0
166344 229533.A0A1C3YKB7 pp 229533.I1RAG3
166345 229533.A0A1C3YKB7 pp 229533.I1S8F8
166346 229533.A0A1C3YKB7 pp 229533.I1RDJ7
166347 229533.A0A1C3YKB7 pp 229533.I1RIW5
166348 229533.A0A1C3YKB7 pp 229533.I1S0H2
166349 229533.A0A1C3YKB7 pp 229533.I1RI19
166350 229533.A0A1C3YKB7 pp 229533.V6RPS5
166351 229533.A0A1C3YKB7 pp 229533.I1RGZ3
166352 229533.A0A1C3YKB7 pp 229533.I1RJT5
166353 229533.A0A1C3YKB7 pp 229533.I1S4D0
166354 229533.A0A1C3YKB7 pp 229533.I1RRM1
166355 229533.A0A1C3YKB7 pp 229533.I1RJK0
166356 229533.A0A1C3YKB7 pp 229533.V6RY56
166357 229533.A0A1C3YKB7 pp 229533.I1RCV1
166358 229533.A0A1C3YKB7 pp 229533.I1S0T1
166359 229533.A0A1C3YKB7 pp 229533.I1RE90
166360 229533.A0A1C3YKB7 pp 229533.I1R9C0
166361 229533.A0A1C3YKB7 pp 229533.I1RTJ7
166362 229533.A0A1C3YKB7 pp 229533.I1RNH4
166363 229533.A0A1C3YKB7 pp 229533.I1RK03
166364 229533.A0A1C3YKB7 pp 229533.A0A1C3YKZ8
166365 229533.A0A1C3YKB7 pp 229533.I1RA13
166366 229533.A0A1C3YKB7 pp 229533.I1RY96
166367 229533.A0A1C3YKB7 pp 229533.I1RCU2

166368	229533.A0A1C3YKB7	pp	229533.I1S269
166369	229533.A0A1C3YKB7	pp	229533.I1RC39
166370	229533.A0A1C3YKB7	pp	229533.A0A1C3YKH5
166371	229533.A0A1C3YKB7	pp	229533.I1RDU0
166372	229533.A0A1C3YKB7	pp	229533.V6REA4
166373	229533.A0A1C3YKB7	pp	229533.I1REE6
166374	229533.A0A1C3YKB7	pp	229533.I1R9F2
166375	229533.A0A1C3YKB7	pp	229533.I1RMS5
166376	229533.A0A1C3YKB7	pp	229533.V6RM09
166377	229533.A0A1C3YKB7	pp	229533.I1RLA2
166378	229533.A0A1C3YKB7	pp	229533.I1RF16
166379	229533.A0A1C3YKB7	pp	229533.I1RTT0
166380	229533.A0A1C3YKB7	pp	229533.I1RV06
166381	229533.A0A1C3YKB7	pp	229533.V6QTX7
166382	229533.A0A1C3YKB7	pp	229533.I1RDN3
166383	229533.A0A1C3YKB7	pp	229533.I1S6H3
166384	229533.A0A1C3YKB7	pp	229533.I1S094
166385	229533.A0A1C3YKB7	pp	229533.I1S334
166386	229533.A0A1C3YKB7	pp	229533.I1RJG4
166387	229533.A0A1C3YKB7	pp	229533.I1RJE7
166388	229533.A0A1C3YKB7	pp	229533.I1RNM7
166389	229533.A0A1C3YKB7	pp	229533.I1RQX9
166390	229533.A0A1C3YKB7	pp	229533.I1RVI8
166391	229533.A0A1C3YKB7	pp	229533.I1RHL9
166392	229533.A0A1C3YKB7	pp	229533.I1RRL5
166393	229533.A0A1C3YJ51	pp	229533.A0A1C3YJQ6
166394	229533.A0A1C3YJ51	pp	229533.Q4I7X1
166395	229533.A0A1C3YJ51	pp	229533.I1RCK4
166396	229533.A0A1C3YJ51	pp	229533.I1RYJ8
166397	229533.A0A1C3YJ51	pp	229533.I1RQ49
166398	229533.A0A1C3YJ51	pp	229533.A0A1C3YJI7
166399	229533.A0A1C3YJ51	pp	229533.I1RNB8
166400	229533.A0A1C3YJ51	pp	229533.Q4IAA0
166401	229533.A0A1C3YJ51	pp	229533.I1S0P4
166402	229533.A0A1C3YJ51	pp	229533.I1RT35
166403	229533.A0A1C3YJ51	pp	229533.I1RQB7
166404	229533.A0A1C3YJ51	pp	229533.I1RTE5
166405	229533.A0A1C3YJ51	pp	229533.I1S2F3
166406	229533.A0A1C3YJ51	pp	229533.I1RB17
166407	229533.A0A1C3YJ51	pp	229533.I1RDY1
166408	229533.A0A1C3YJ51	pp	229533.A0A1C3YK58
166409	229533.A0A1C3YJ51	pp	229533.I1RYL5
166410	229533.A0A1C3YJ51	pp	229533.I1RNE4
166411	229533.A0A1C3YJ51	pp	229533.I1RY40
166412	229533.A0A1C3YJ51	pp	229533.I1RYJ9
166413	229533.A0A1C3YJ51	pp	229533.I1RS94
166414	229533.A0A1C3YJ51	pp	229533.V6REZ4
166415	229533.A0A1C3YJ51	pp	229533.I1S5B5
166416	229533.A0A1C3YJ51	pp	229533.I1RAN0
166417	229533.A0A1C3YJ51	pp	229533.A0A1C3YKG7
166418	229533.A0A1C3YJ51	pp	229533.I1RFG0
166419	229533.A0A1C3YJ51	pp	229533.I1RIR9
166420	229533.A0A1C3YJ51	pp	229533.I1S214
166421	229533.A0A1C3YJ51	pp	229533.I1RMY3
166422	229533.A0A1C3YJ51	pp	229533.I1RJS5
166423	229533.A0A1C3YJ51	pp	229533.A0A1C3YL25
166424	229533.A0A1C3YJ51	pp	229533.A0A1C3YMK1
166425	229533.A0A1C3YJ51	pp	229533.A0A1C3YL93
166426	229533.A0A1C3YJ51	pp	229533.I1RZV9
166427	229533.A0A1C3YJ51	pp	229533.I1RYT7
166428	229533.A0A1C3YJ51	pp	229533.I1RRH5
166429	229533.A0A1C3YJ51	pp	229533.I1RZT1
166430	229533.A0A1C3YJ51	pp	229533.Q4HY71
166431	229533.A0A1C3YJ51	pp	229533.I1RBZ8
166432	229533.A0A1C3YJ51	pp	229533.Q4IF76
166433	229533.A0A1C3YJ51	pp	229533.I1RKD2
166434	229533.A0A1C3YJ51	pp	229533.A0A1C3YKF4
166435	229533.A0A1C3YJ51	pp	229533.I1S246
166436	229533.A0A1C3YJ51	pp	229533.I1RNT2
166437	229533.A0A1C3YJ51	pp	229533.I1RDA8
166438	229533.A0A1C3YJ51	pp	229533.I1RNG5
166439	229533.A0A1C3YJ51	pp	229533.I1RCU7
166440	229533.A0A1C3YJ51	pp	229533.Q4IJH1

166441 229533.A0A1C3YJ51 pp 229533.I1RC00
166442 229533.A0A1C3YJ51 pp 229533.I1RNB7
166443 229533.A0A1C3YJ51 pp 229533.I1R9X2
166444 229533.A0A1C3YJ51 pp 229533.I1S2A8
166445 229533.A0A1C3YJ51 pp 229533.Q4IPN9
166446 229533.A0A1C3YJ51 pp 229533.I1RJS4
166447 229533.A0A1C3YJ51 pp 229533.I1RBZ1
166448 229533.V6R5D2 pp 229533.V6RM09
166449 229533.V6R5D2 pp 229533.V6RY56
166450 229533.V6R5D2 pp 229533.V6REA4
166451 229533.V6R5D2 pp 229533.V6RK96
166452 229533.V6R5D2 pp 229533.V6RPS5
166453 229533.V6R5D2 pp 229533.V6RBG5
166454 229533.A0A0E0S9G3 pp 229533.Q4IE79
166455 229533.A0A0E0S9G3 pp 229533.I1RRE0
166456 229533.A0A0E0S9G3 pp 229533.I1REE1
166457 229533.A0A0E0S9G3 pp 229533.I1RRR6
166458 229533.A0A0E0S9G3 pp 229533.I1RMA4
166459 229533.A0A0E0S9G3 pp 229533.A0A1C3YJ51
166460 229533.A0A0E0S9G3 pp 229533.I1RQS4
166461 229533.A0A0E0S9G3 pp 229533.I1REL7
166462 229533.A0A0E0S9G3 pp 229533.I1RRW0
166463 229533.A0A0E0S9G3 pp 229533.I1S0Z9
166464 229533.A0A0E0S9G3 pp 229533.I1RME5
166465 229533.A0A0E0S9G3 pp 229533.V6REZ4
166466 229533.A0A0E0S9G3 pp 229533.A0A1C3YM91
166467 229533.A0A0E0S9G3 pp 229533.I1RXT9
166468 229533.A0A0E0S9G3 pp 229533.I1RJK7
166469 229533.A0A0E0S9G3 pp 229533.I1RCK4
166470 229533.A0A0E0S9G3 pp 229533.I1RIR9
166471 229533.A0A0E0S9G3 pp 229533.I1RDV9
166472 229533.A0A0E0S9G3 pp 229533.I1RYJ8
166473 229533.A0A0E0S9G3 pp 229533.A0A1C3YIE7
166474 229533.A0A0E0S9G3 pp 229533.A0A0E0SQG9
166475 229533.A0A0E0S9G3 pp 229533.I1RSL8
166476 229533.A0A0E0S9G3 pp 229533.I1RQ49
166477 229533.A0A0E0S9G3 pp 229533.I1RVA7
166478 229533.A0A0E0S9G3 pp 229533.I1R9X7
166479 229533.A0A0E0S9G3 pp 229533.I1RYT7
166480 229533.A0A0E0S9G3 pp 229533.I1S0P4
166481 229533.A0A0E0S9G3 pp 229533.V6R3J1
166482 229533.A0A0E0S9G3 pp 229533.I1RNT2
166483 229533.A0A0E0S9G3 pp 229533.I1RTE5
166484 229533.A0A0E0S9G3 pp 229533.I1S2A8
166485 229533.A0A0E0S9G3 pp 229533.I1RNE4
166486 229533.A0A0E0S9G3 pp 229533.I1RJS4
166487 229533.A0A0E0S9G3 pp 229533.I1RTG0
166488 229533.A0A0E0S9G3 pp 229533.I1RDY1
166489 229533.A0A0E0S9G3 pp 229533.A0A0E0SLZ3
166490 229533.A0A0E0S9G3 pp 229533.I1RAW7
166491 229533.A0A0E0S9G3 pp 229533.I1RQ80
166492 229533.A0A0E0S9G3 pp 229533.I1S1V5
166493 229533.A0A0E0S9G3 pp 229533.I1S2B6
166494 229533.A0A0E0S9G3 pp 229533.I1RCU7
166495 229533.A0A0E0S9G3 pp 229533.I1RAE1
166496 229533.A0A0E0S9G3 pp 229533.I1RC00
166497 229533.A0A0E0S9G3 pp 229533.I1RZ12
166498 229533.A0A0E0S9G3 pp 229533.I1S5B5
166499 229533.A0A0E0S9G3 pp 229533.I1RW24
166500 229533.A0A0E0S9G3 pp 229533.I1RD18
166501 229533.A0A0E0S9G3 pp 229533.A0A1C3YKG7
166502 229533.A0A0E0S9G3 pp 229533.A0A1C3YI39
166503 229533.A0A0E0S9G3 pp 229533.I1RJS5
166504 229533.A0A0E0S9G3 pp 229533.I1RZV9
166505 229533.A0A0E0S9G3 pp 229533.A0A1C3YK57
166506 229533.A0A0E0S9G3 pp 229533.I1S224
166507 229533.A0A0E0S9G3 pp 229533.A0A1C3YIP6
166508 229533.A0A0E0S9G3 pp 229533.I1RC05
166509 229533.A0A0E0S9G3 pp 229533.I1RMY3
166510 229533.A0A0E0S9G3 pp 229533.I1RZT1
166511 229533.A0A0E0S9G3 pp 229533.I1RFZ2
166512 229533.A0A0E0S9G3 pp 229533.I1RRX6
166513 229533.A0A0E0S9G3 pp 229533.I1RD70

166514 229533.I1RPY7 pp 229533.I1S4M5
166515 229533.I1RPY7 pp 229533.Q4I7F9
166516 229533.I1RPY7 pp 229533.I1S9M9
166517 229533.I1RPY7 pp 229533.I1S246
166518 229533.I1RPY7 pp 229533.I1S8V0
166519 229533.I1RPY7 pp 229533.I1S0B6
166520 229533.I1RPY7 pp 229533.I1RX24
166521 229533.I1RPY7 pp 229533.I1RYJ9
166522 229533.I1RPY7 pp 229533.I1RZG7
166523 229533.I1RPY7 pp 229533.I1RX81
166524 229533.I1RPY7 pp 229533.I1RQK2
166525 229533.I1RPY7 pp 229533.I1RRW3
166526 229533.I1RPY7 pp 229533.I1S0B1
166527 229533.I1RPY7 pp 229533.I1S0Z9
166528 229533.I1RPY7 pp 229533.I1RTS2
166529 229533.I1RPY7 pp 229533.I1RYB1
166530 229533.I1RPY7 pp 229533.I1S0N7
166531 229533.I1RPY7 pp 229533.I1RS03
166532 229533.I1RPY7 pp 229533.I1RZL5
166533 229533.I1RPY7 pp 229533.I1S8U9
166534 229533.I1RPY7 pp 229533.I1S5Z2
166535 229533.I1RPY7 pp 229533.V6QVD5
166536 229533.I1RPY7 pp 229533.I1RX47
166537 229533.I1RPY7 pp 229533.I1S1X9
166538 229533.I1RPY7 pp 229533.Q4IP34
166539 229533.I1RPY7 pp 229533.I1S471
166540 229533.I1RPY7 pp 229533.I1RQZ2
166541 229533.I1RPY7 pp 229533.I1RT47
166542 229533.I1RPY7 pp 229533.I1RSA4
166543 229533.I1RPY7 pp 229533.V6QXG1
166544 229533.I1RPY7 pp 229533.I1RSH9
166545 229533.I1RPY7 pp 229533.I1RQD8
166546 229533.I1RPY7 pp 229533.I1RSD4
166547 229533.I1RPY7 pp 229533.I1RRL0
166548 229533.I1RPY7 pp 229533.Q4IAA0
166549 229533.I1RPY7 pp 229533.I1RT35
166550 229533.I1RPY7 pp 229533.I1RWX8
166551 229533.A0A1C3YK32 pp 229533.I1RIA9
166552 229533.A0A1C3YK32 pp 229533.I1S166
166553 229533.A0A1C3YK32 pp 229533.I1RDN2
166554 229533.A0A1C3YLX1 pp 229533.I1RNG4
166555 229533.I1RNB5 pp 229533.I1RVX9
166556 229533.I1RNB5 pp 229533.V6RRM0
166557 229533.I1RNB5 pp 229533.I1RW43
166558 229533.I1RNB5 pp 229533.I1RY49
166559 229533.A0A098DS22 pp 229533.I1RK79
166560 229533.A0A098DS22 pp 229533.I1S276
166561 229533.A0A098DS22 pp 229533.I1RK71
166562 229533.A0A098DS22 pp 229533.I1RPM7
166563 229533.A0A098DS22 pp 229533.Q4HYB8
166564 229533.A0A098DS22 pp 229533.I1RWF3
166565 229533.A0A098DS22 pp 229533.A0A1C3YN55
166566 229533.A0A098DS22 pp 229533.I1RCB7
166567 229533.A0A098DS22 pp 229533.I1RXU0
166568 229533.A0A098DS22 pp 229533.I1RG11
166569 229533.A0A098DS22 pp 229533.Q4IPC8
166570 229533.A0A098DS22 pp 229533.A0A0E0RN05
166571 229533.A0A098DS22 pp 229533.I1RC15
166572 229533.A0A098DS22 pp 229533.I1RVL8
166573 229533.A0A098DS22 pp 229533.I1RRT0
166574 229533.A0A098DS22 pp 229533.A0A1C3YI23
166575 229533.A0A098DS22 pp 229533.I1RVY0
166576 229533.A0A098DS22 pp 229533.I1RR25
166577 229533.A0A098DS22 pp 229533.I1RE14
166578 229533.A0A098DS22 pp 229533.I1RE30
166579 229533.A0A098DS22 pp 229533.I1RC24
166580 229533.A0A098DS22 pp 229533.A0A1C3YIR8
166581 229533.A0A098DS22 pp 229533.V6QUE6
166582 229533.A0A098DS22 pp 229533.I1RAR2
166583 229533.A0A098DS22 pp 229533.I1RSZ3
166584 229533.A0A098DS22 pp 229533.Q4I5W5
166585 229533.A0A098DS22 pp 229533.Q4I5I4
166586 229533.A0A098DS22 pp 229533.I1RZB5

166587	229533.A0A098DS22	pp	229533.I1RUG0
166588	229533.A0A098DS22	pp	229533.I1RJL2
166589	229533.A0A098DS22	pp	229533.I1RTC2
166590	229533.A0A098DS22	pp	229533.I1RRG3
166591	229533.A0A098DS22	pp	229533.V6QWX8
166592	229533.A0A098DS22	pp	229533.I1RKB1
166593	229533.A0A1C3YIE7	pp	229533.I1RCU2
166594	229533.A0A1C3YIE7	pp	229533.I1S2Y5
166595	229533.A0A1C3YIE7	pp	229533.I1RQ80
166596	229533.A0A1C3YIE7	pp	229533.I1RLU5
166597	229533.A0A1C3YIE7	pp	229533.I1RAW7
166598	229533.A0A1C3YIE7	pp	229533.I1RD56
166599	229533.A0A1C3YIE7	pp	229533.I1RD52
166600	229533.A0A1C3YIE7	pp	229533.I1RPZ3
166601	229533.A0A1C3YIE7	pp	229533.I1RY40
166602	229533.A0A1C3YIE7	pp	229533.I1RVQ0
166603	229533.A0A1C3YIE7	pp	229533.A0A1C3YIP6
166604	229533.A0A1C3YIE7	pp	229533.I1RQC9
166605	229533.A0A1C3YIE7	pp	229533.I1RX46
166606	229533.A0A1C3YIE7	pp	229533.I1RPF1
166607	229533.A0A1C3YIE7	pp	229533.I1RSQ7
166608	229533.A0A1C3YIE7	pp	229533.I1RMR5
166609	229533.A0A1C3YIE7	pp	229533.I1S7M2
166610	229533.A0A1C3YIE7	pp	229533.Q4INZ9
166611	229533.A0A1C3YIE7	pp	229533.I1S246
166612	229533.A0A1C3YIE7	pp	229533.I1RCQ6
166613	229533.A0A1C3YIE7	pp	229533.I1RZG7
166614	229533.A0A1C3YIE7	pp	229533.I1RFY6
166615	229533.A0A1C3YIE7	pp	229533.I1RYJ6
166616	229533.A0A1C3YIE7	pp	229533.I1RW25
166617	229533.A0A1C3YIE7	pp	229533.I1RAX6
166618	229533.A0A1C3YIE7	pp	229533.I1R9N6
166619	229533.A0A1C3YIE7	pp	229533.I1RCI4
166620	229533.A0A1C3YIE7	pp	229533.Q4IJH1
166621	229533.A0A1C3YIE7	pp	229533.I1RJU3
166622	229533.A0A1C3YIE7	pp	229533.A0A1C3YKV3
166623	229533.A0A1C3YIE7	pp	229533.Q4IRI9
166624	229533.A0A1C3YIE7	pp	229533.Q4IAA0
166625	229533.A0A1C3YIE7	pp	229533.I1S7T4
166626	229533.A0A1C3YIE7	pp	229533.A0A1C3YLE3
166627	229533.A0A1C3YIE7	pp	229533.I1RDC9
166628	229533.A0A1C3YIE7	pp	229533.I1RB50
166629	229533.A0A1C3YIE7	pp	229533.I1RPY5
166630	229533.A0A1C3YIE7	pp	229533.I1RT05
166631	229533.A0A1C3YIE7	pp	229533.I1RN59
166632	229533.A0A1C3YIE7	pp	229533.I1RN03
166633	229533.A0A1C3YIE7	pp	229533.Q4IE79
166634	229533.A0A1C3YIE7	pp	229533.I1RTE5
166635	229533.A0A1C3YIE7	pp	229533.I1S7M3
166636	229533.A0A1C3YIE7	pp	229533.I1RNT2
166637	229533.A0A1C3YIE7	pp	229533.I1RT93
166638	229533.A0A1C3YIE7	pp	229533.I1RPU4
166639	229533.A0A1C3YIE7	pp	229533.A0A1C3YN26
166640	229533.A0A1C3YIE7	pp	229533.I1RZS5
166641	229533.A0A1C3YIE7	pp	229533.I1RR24
166642	229533.A0A1C3YIE7	pp	229533.A0A1C3YKB0
166643	229533.A0A1C3YIE7	pp	229533.I1S0P4
166644	229533.A0A1C3YIE7	pp	229533.I1RT35
166645	229533.A0A1C3YIE7	pp	229533.I1S5B0
166646	229533.A0A1C3YIE7	pp	229533.I1RRK8
166647	229533.A0A1C3YIE7	pp	229533.I1RZM1
166648	229533.A0A1C3YIE7	pp	229533.I1RCU7
166649	229533.A0A1C3YIE7	pp	229533.A0A1C3YK11
166650	229533.A0A1C3YIE7	pp	229533.I1RCK4
166651	229533.A0A1C3YIE7	pp	229533.I1RYL5
166652	229533.A0A1C3YIE7	pp	229533.V6RCZ8
166653	229533.A0A1C3YIE7	pp	229533.I1RXX1
166654	229533.A0A1C3YIE7	pp	229533.A0A1C3YLU7
166655	229533.A0A1C3YIE7	pp	229533.I1RRU9
166656	229533.A0A1C3YIE7	pp	229533.I1RA36
166657	229533.A0A1C3YIE7	pp	229533.I1RCM6
166658	229533.A0A1C3YIE7	pp	229533.I1RDG0
166659	229533.A0A1C3YIE7	pp	229533.A0A1C3YKN5

166660	229533.A0A1C3YIE7	pp	229533.I1RFP9
166661	229533.A0A1C3YIE7	pp	229533.I1RGB2
166662	229533.A0A1C3YIE7	pp	229533.I1RRY9
166663	229533.A0A1C3YIE7	pp	229533.I1RUI2
166664	229533.A0A1C3YIE7	pp	229533.I1REK8
166665	229533.A0A1C3YIE7	pp	229533.I1S9L0
166666	229533.A0A1C3YIE7	pp	229533.A0A1C3YL25
166667	229533.A0A1C3YIE7	pp	229533.Q4I2J8
166668	229533.A0A1C3YIE7	pp	229533.I1RAF3
166669	229533.A0A1C3YIE7	pp	229533.I1RPP5
166670	229533.A0A1C3YIE7	pp	229533.I1RFZ2
166671	229533.A0A1C3YIE7	pp	229533.A0A1C3YIJ3
166672	229533.A0A1C3YIE7	pp	229533.I1RBU8
166673	229533.A0A1C3YIE7	pp	229533.I1RE00
166674	229533.A0A1C3YIE7	pp	229533.I1RJB9
166675	229533.A0A1C3YIE7	pp	229533.I1RS94
166676	229533.A0A1C3YIE7	pp	229533.I1RFU8
166677	229533.A0A1C3YIE7	pp	229533.I1RZV9
166678	229533.A0A1C3YIE7	pp	229533.I1RN67
166679	229533.A0A1C3YIE7	pp	229533.I1RAN0
166680	229533.A0A1C3YIE7	pp	229533.I1RWU2
166681	229533.A0A1C3YIE7	pp	229533.I1RAY2
166682	229533.A0A1C3YIE7	pp	229533.I1RQA5
166683	229533.A0A1C3YIE7	pp	229533.I1RMY3
166684	229533.A0A1C3YIE7	pp	229533.I1RAC1
166685	229533.A0A1C3YIE7	pp	229533.I1RPP3
166686	229533.A0A1C3YIE7	pp	229533.I1RCM5
166687	229533.A0A1C3YIE7	pp	229533.I1RBS0
166688	229533.A0A1C3YIE7	pp	229533.I1RYK0
166689	229533.A0A1C3YIE7	pp	229533.Q4I1Y1
166690	229533.A0A1C3YIE7	pp	229533.I1RFG0
166691	229533.A0A1C3YIE7	pp	229533.Q4IBK5
166692	229533.A0A1C3YIE7	pp	229533.I1RZK5
166693	229533.A0A1C3YIE7	pp	229533.I1RT04
166694	229533.A0A1C3YIE7	pp	229533.A0A1C3YJ21
166695	229533.A0A1C3YIE7	pp	229533.I1RKG2
166696	229533.A0A1C3YIE7	pp	229533.I1RWS4
166697	229533.A0A1C3YIE7	pp	229533.I1RDY1
166698	229533.A0A1C3YIE7	pp	229533.I1RXF2
166699	229533.A0A1C3YIE7	pp	229533.I1RCS2
166700	229533.A0A1C3YIE7	pp	229533.A0A1C3YJI6
166701	229533.A0A1C3YIE7	pp	229533.I1RBS6
166702	229533.A0A1C3YIE7	pp	229533.I1RT17
166703	229533.A0A1C3YIE7	pp	229533.I1RBV3
166704	229533.A0A1C3YIE7	pp	229533.I1S1V5
166705	229533.A0A1C3YIE7	pp	229533.I1RL82
166706	229533.A0A1C3YIE7	pp	229533.V6R3J1
166707	229533.A0A1C3YIE7	pp	229533.A0A1C3YMK1
166708	229533.A0A1C3YIE7	pp	229533.I1RYT7
166709	229533.A0A1C3YIE7	pp	229533.A0A1C3YIS7
166710	229533.A0A1C3YIE7	pp	229533.I1RRT8
166711	229533.A0A1C3YIE7	pp	229533.I1RVY0
166712	229533.A0A1C3YIE7	pp	229533.I1S5R1
166713	229533.A0A1C3YIE7	pp	229533.I1RC46
166714	229533.A0A1C3YIE7	pp	229533.I1RK96
166715	229533.A0A1C3YIE7	pp	229533.I1RE76
166716	229533.A0A1C3YIE7	pp	229533.I1RCN2
166717	229533.A0A1C3YIE7	pp	229533.I1RKD3
166718	229533.A0A1C3YIE7	pp	229533.I1RNB8
166719	229533.A0A1C3YIE7	pp	229533.I1RTG7
166720	229533.A0A1C3YIE7	pp	229533.I1RKG3
166721	229533.A0A1C3YIE7	pp	229533.I1RM25
166722	229533.A0A1C3YIE7	pp	229533.I1RR73
166723	229533.A0A1C3YIE7	pp	229533.I1RW61
166724	229533.A0A1C3YIE7	pp	229533.I1RPM4
166725	229533.A0A1C3YIE7	pp	229533.V6RLM7
166726	229533.A0A1C3YIE7	pp	229533.Q4IF76
166727	229533.A0A1C3YIE7	pp	229533.I1RPY4
166728	229533.A0A1C3YIE7	pp	229533.I1RU69
166729	229533.A0A1C3YIE7	pp	229533.I1RPK0
166730	229533.A0A1C3YIE7	pp	229533.I1RHM4
166731	229533.A0A1C3YIE7	pp	229533.I1REC6
166732	229533.A0A1C3YIE7	pp	229533.A0A1C3YJK3

166733 229533.A0A1C3YIE7 pp 229533.I1RC00
166734 229533.A0A1C3YIE7 pp 229533.A0A1C3YMF9
166735 229533.A0A1C3YIE7 pp 229533.V6RE46
166736 229533.A0A1C3YIE7 pp 229533.I1RKP1
166737 229533.A0A1C3YIE7 pp 229533.I1RPP4
166738 229533.A0A1C3YIE7 pp 229533.A0A1C3YLB6
166739 229533.A0A1C3YIE7 pp 229533.I1RRL0
166740 229533.A0A1C3YIE7 pp 229533.I1RCU1
166741 229533.A0A1C3YIE7 pp 229533.I1RST1
166742 229533.A0A1C3YIE7 pp 229533.Q4HXF6
166743 229533.A0A1C3YIE7 pp 229533.I1RFQ1
166744 229533.A0A1C3YIE7 pp 229533.I1RRI1
166745 229533.A0A1C3YIE7 pp 229533.I1RS28
166746 229533.A0A1C3YIE7 pp 229533.I1RQ49
166747 229533.A0A1C3YIE7 pp 229533.Q4IPB8
166748 229533.A0A1C3YIE7 pp 229533.A0A1C3YLZ6
166749 229533.A0A1C3YIE7 pp 229533.I1RB92
166750 229533.A0A1C3YIE7 pp 229533.I1RFL4
166751 229533.A0A1C3YIE7 pp 229533.V6QXG1
166752 229533.A0A1C3YIE7 pp 229533.I1RND5
166753 229533.A0A1C3YIE7 pp 229533.I1RQE7
166754 229533.A0A1C3YIE7 pp 229533.I1RSF1
166755 229533.A0A1C3YIE7 pp 229533.I1RQ21
166756 229533.A0A1C3YIE7 pp 229533.I1RPL3
166757 229533.A0A1C3YIE7 pp 229533.I1RCH5
166758 229533.A0A1C3YIE7 pp 229533.I1RSA4
166759 229533.A0A1C3YIE7 pp 229533.I1RIR9
166760 229533.A0A1C3YIE7 pp 229533.I1RRM3
166761 229533.A0A1C3YIE7 pp 229533.I1S4P6
166762 229533.A0A1C3YIE7 pp 229533.Q4HZB8
166763 229533.A0A1C3YIE7 pp 229533.V6REZ4
166764 229533.A0A1C3YIE7 pp 229533.I1RQ33
166765 229533.A0A1C3YIE7 pp 229533.V6R042
166766 229533.A0A1C3YIE7 pp 229533.I1RE79
166767 229533.A0A1C3YIE7 pp 229533.I1RAU7
166768 229533.A0A1C3YIE7 pp 229533.I1RX51
166769 229533.A0A1C3YIE7 pp 229533.Q4IPH4
166770 229533.A0A1C3YIE7 pp 229533.I1RNC1
166771 229533.A0A1C3YIE7 pp 229533.I1RZP8
166772 229533.A0A1C3YIE7 pp 229533.I1RK90
166773 229533.A0A1C3YIE7 pp 229533.I1RX47
166774 229533.A0A1C3YIE7 pp 229533.I1RYJ9
166775 229533.A0A1C3YIE7 pp 229533.I1S056
166776 229533.A0A1C3YIE7 pp 229533.I1RNE4
166777 229533.A0A1C3YIE7 pp 229533.A0A1C3YJ51
166778 229533.A0A1C3YIE7 pp 229533.Q4IN00
166779 229533.A0A1C3YIE7 pp 229533.I1RG90
166780 229533.A0A1C3YIE7 pp 229533.I1S0B1
166781 229533.A0A1C3YIE7 pp 229533.I1S471
166782 229533.A0A1C3YIE7 pp 229533.I1RXT9
166783 229533.A0A1C3YIE7 pp 229533.I1RTH6
166784 229533.A0A1C3YIE7 pp 229533.I1RD70
166785 229533.A0A1C3YIE7 pp 229533.I1RWX5
166786 229533.A0A1C3YIE7 pp 229533.I1S247
166787 229533.A0A1C3YIE7 pp 229533.I1RF85
166788 229533.A0A1C3YIE7 pp 229533.I1RTA3
166789 229533.A0A1C3YIE7 pp 229533.I1RZT1
166790 229533.A0A1C3YIE7 pp 229533.I1RA70
166791 229533.A0A1C3YIE7 pp 229533.I1RG57
166792 229533.A0A1C3YIE7 pp 229533.I1REC7
166793 229533.A0A1C3YIE7 pp 229533.I1RBZ8
166794 229533.A0A1C3YIE7 pp 229533.I1RJS5
166795 229533.A0A1C3YIE7 pp 229533.I1RCN5
166796 229533.A0A1C3YIE7 pp 229533.I1RJV3
166797 229533.A0A1C3YIE7 pp 229533.V6RDZ4
166798 229533.I1RG17 pp 229533.I1RYI9
166799 229533.I1RG17 pp 229533.I1RSA8
166800 229533.I1RG17 pp 229533.Q4IP34
166801 229533.I1RG17 pp 229533.I1RX81
166802 229533.I1RG17 pp 229533.I1S056
166803 229533.I1RG17 pp 229533.I1RPY4
166804 229533.I1RG17 pp 229533.I1RZG7
166805 229533.I1RG17 pp 229533.I1RZ11

229533.I1RG17	pp	229533.I1RMV5
229533.I1RG17	pp	229533.I1RTS2
229533.I1RG17	pp	229533.I1RWW5
229533.I1RG17	pp	229533.I1RT47
229533.I1RG17	pp	229533.I1RPY5
229533.I1RG17	pp	229533.I1RQR3
229533.I1RG17	pp	229533.I1RKK4
229533.I1RG17	pp	229533.I1RZQ5
229533.I1RG17	pp	229533.I1RSD4
229533.I1RG17	pp	229533.I1RK19
229533.I1RG17	pp	229533.I1S2Y5
229533.I1RG17	pp	229533.I1RYB1
229533.I1RG17	pp	229533.I1RYJ9
229533.I1RG17	pp	229533.I1RK72
229533.I1RG17	pp	229533.I1RZD0
229533.I1RG17	pp	229533.I1RSA4
229533.I1RG17	pp	229533.I1RG57
229533.I1RG17	pp	229533.I1RND5
229533.I1RG17	pp	229533.I1S8F8
229533.I1RG17	pp	229533.I1RPA0
229533.I1RG17	pp	229533.V6QXG1
229533.I1RG17	pp	229533.I1RZT1
229533.I1RG17	pp	229533.I1RMH5
229533.I1RI16	pp	229533.I1S1Q6
229533.I1RI16	pp	229533.I1RLN6
229533.I1RI16	pp	229533.I1RJA2
229533.I1RI16	pp	229533.I1RII8
229533.A0A1C3YLF5	pp	229533.I1RDA0
229533.A0A1C3YLF5	pp	229533.I1RMK3
229533.A0A1C3YLF5	pp	229533.I1RJU3
229533.A0A1C3YLF5	pp	229533.Q4IJH1
229533.A0A1C3YLF5	pp	229533.A0A1C3YN30
229533.A0A1C3YLF5	pp	229533.I1RTF4
229533.A0A1C3YLF5	pp	229533.I1RT80
229533.A0A1C3YLF5	pp	229533.I1RA11
229533.A0A1C3YLF5	pp	229533.I1RNV6
229533.A0A1C3YLF5	pp	229533.I1RM67
229533.A0A1C3YLF5	pp	229533.I1S5R1
229533.A0A1C3YLF5	pp	229533.I1RPU0
229533.A0A1C3YLF5	pp	229533.I1RRT8
229533.A0A1C3YLF5	pp	229533.I1RKJ6
229533.A0A1C3YLF5	pp	229533.I1RWJ8
229533.A0A1C3YLF5	pp	229533.I1S9U2
229533.A0A1C3YLF5	pp	229533.I1S4S4
229533.A0A1C3YLF5	pp	229533.I1S0L4
229533.A0A1C3YLF5	pp	229533.I1RW41
229533.A0A1C3YLF5	pp	229533.I1RJZ7
229533.A0A1C3YLF5	pp	229533.I1RD64
229533.A0A1C3YLF5	pp	229533.I1RQS8
229533.A0A1C3YLF5	pp	229533.I1RRM5
229533.A0A1C3YLF5	pp	229533.I1RL23
229533.A0A1C3YLF5	pp	229533.I1S2H4
229533.A0A1C3YLF5	pp	229533.I1RR24
229533.A0A1C3YLF5	pp	229533.I1RC00
229533.A0A1C3YLF5	pp	229533.I1RGF6
229533.A0A1C3YLF5	pp	229533.I1RQM6
229533.A0A1C3YLF5	pp	229533.I1RFH8
229533.A0A1C3YLF5	pp	229533.I1R9G0
229533.A0A1C3YLF5	pp	229533.I1RFS3
229533.A0A1C3YLF5	pp	229533.I1REF2
229533.A0A1C3YLF5	pp	229533.I1RAF8
229533.A0A1C3YLF5	pp	229533.I1RT40
229533.A0A1C3YLF5	pp	229533.Q4IF76
229533.A0A1C3YLF5	pp	229533.I1RC66
229533.A0A1C3YLF5	pp	229533.Q4I662
229533.A0A1C3YLF5	pp	229533.I1RBT1
229533.A0A1C3YLF5	pp	229533.I1RV91
229533.A0A1C3YLF5	pp	229533.I1RZZ9
229533.A0A1C3YLF5	pp	229533.I1RXD7
229533.A0A1C3YLF5	pp	229533.I1S6Y8
229533.A0A1C3YLF5	pp	229533.I1RPM4
229533.A0A1C3YLF5	pp	229533.I1RUQ2
229533.A0A1C3YLF5	pp	229533.I1S2A8

166879	229533.A0A1C3YLF5	pp	229533.I1RG51
166880	229533.A0A1C3YLF5	pp	229533.Q4HXF0
166881	229533.A0A1C3YLF5	pp	229533.I1S6U8
166882	229533.A0A1C3YLF5	pp	229533.I1RW61
166883	229533.A0A1C3YLF5	pp	229533.I1RCS1
166884	229533.A0A1C3YLF5	pp	229533.I1S0R7
166885	229533.A0A1C3YLF5	pp	229533.I1RT04
166886	229533.A0A1C3YLF5	pp	229533.I1RN63
166887	229533.A0A1C3YLF5	pp	229533.I1RYT5
166888	229533.A0A1C3YLF5	pp	229533.V6QW22
166889	229533.A0A1C3YLF5	pp	229533.I1RRJ8
166890	229533.A0A1C3YLF5	pp	229533.I1RJL4
166891	229533.A0A1C3YLF5	pp	229533.I1RZS6
166892	229533.A0A1C3YLF5	pp	229533.I1RM06
166893	229533.A0A1C3YLF5	pp	229533.Q4IPI1
166894	229533.A0A1C3YLF5	pp	229533.I1RN61
166895	229533.A0A1C3YLF5	pp	229533.I1RSQ7
166896	229533.A0A1C3YLF5	pp	229533.I1S0T2
166897	229533.A0A1C3YLF5	pp	229533.A0A1C3YMW0
166898	229533.A0A1C3YLF5	pp	229533.Q4IQK7
166899	229533.A0A1C3YLF5	pp	229533.I1RE22
166900	229533.A0A1C3YLF5	pp	229533.I1RQJ5
166901	229533.A0A1C3YLF5	pp	229533.I1RRT2
166902	229533.A0A1C3YLF5	pp	229533.I1RNA0
166903	229533.A0A1C3YLF5	pp	229533.I1RZ99
166904	229533.A0A1C3YLF5	pp	229533.I1RJW6
166905	229533.A0A1C3YLF5	pp	229533.I1RBG9
166906	229533.A0A1C3YLF5	pp	229533.I1RBB8
166907	229533.A0A1C3YLF5	pp	229533.A0A1C3YLK8
166908	229533.A0A1C3YLF5	pp	229533.I1RWF7
166909	229533.A0A1C3YLF5	pp	229533.I1RKQ5
166910	229533.A0A1C3YLF5	pp	229533.V6QYF0
166911	229533.A0A1C3YLF5	pp	229533.I1RR00
166912	229533.A0A1C3YLF5	pp	229533.I1RRZ0
166913	229533.A0A1C3YLF5	pp	229533.I1RFT1
166914	229533.A0A1C3YLF5	pp	229533.I1RLV9
166915	229533.A0A1C3YLF5	pp	229533.Q4IAS1
166916	229533.A0A1C3YLF5	pp	229533.I1RX24
166917	229533.A0A1C3YLF5	pp	229533.I1RSX1
166918	229533.A0A1C3YLF5	pp	229533.I1RW25
166919	229533.A0A1C3YLF5	pp	229533.I1RPK2
166920	229533.A0A1C3YLF5	pp	229533.Q4I830
166921	229533.A0A1C3YLF5	pp	229533.I1RAC2
166922	229533.A0A1C3YLF5	pp	229533.I1RPU3
166923	229533.A0A1C3YLF5	pp	229533.I1RJV4
166924	229533.A0A1C3YLF5	pp	229533.I1RKL3
166925	229533.A0A1C3YLF5	pp	229533.I1RBY4
166926	229533.A0A1C3YLF5	pp	229533.I1RBC9
166927	229533.A0A1C3YLF5	pp	229533.I1RW71
166928	229533.A0A1C3YLF5	pp	229533.Q4IQW0
166929	229533.A0A1C3YLF5	pp	229533.I1RJ74
166930	229533.A0A1C3YLF5	pp	229533.I1RBC7
166931	229533.A0A1C3YLF5	pp	229533.Q4IFI0
166932	229533.A0A1C3YLF5	pp	229533.I1RFN1
166933	229533.A0A1C3YLF5	pp	229533.I1RNH1
166934	229533.A0A1C3YLF5	pp	229533.I1S1J6
166935	229533.A0A1C3YLF5	pp	229533.I1RVQ0
166936	229533.A0A1C3YLF5	pp	229533.I1S6F0
166937	229533.A0A1C3YLF5	pp	229533.I1RC82
166938	229533.A0A1C3YLF5	pp	229533.I1RKC6
166939	229533.A0A1C3YLF5	pp	229533.I1S1B6
166940	229533.A0A1C3YLF5	pp	229533.I1RAZ8
166941	229533.A0A1C3YLF5	pp	229533.I1RBS6
166942	229533.A0A1C3YLF5	pp	229533.I1RNK2
166943	229533.A0A1C3YLF5	pp	229533.I1RRQ3
166944	229533.A0A1C3YLF5	pp	229533.A0A1C3YN63
166945	229533.A0A1C3YLF5	pp	229533.I1S2M6
166946	229533.A0A1C3YLF5	pp	229533.I1RFW5
166947	229533.A0A1C3YLF5	pp	229533.I1RVZ0
166948	229533.A0A1C3YLF5	pp	229533.V6R283
166949	229533.A0A1C3YLF5	pp	229533.I1S0F6
166950	229533.A0A1C3YLF5	pp	229533.I1RN67
166951	229533.A0A1C3YLF5	pp	229533.I1RQ72

166952	229533.A0A1C3YLF5	pp	229533.I1S0L3
166953	229533.A0A1C3YLF5	pp	229533.I1RC03
166954	229533.A0A1C3YLF5	pp	229533.I1RXF7
166955	229533.A0A1C3YLF5	pp	229533.Q4I624
166956	229533.A0A1C3YLF5	pp	229533.I1RD17
166957	229533.A0A1C3YLF5	pp	229533.I1RKA0
166958	229533.A0A1C3YLF5	pp	229533.V6R1K4
166959	229533.A0A1C3YLF5	pp	229533.I1REK8
166960	229533.A0A1C3YLF5	pp	229533.I1RWK3
166961	229533.A0A1C3YLF5	pp	229533.Q4HZ68
166962	229533.A0A1C3YLF5	pp	229533.I1RQ65
166963	229533.A0A1C3YLF5	pp	229533.I1S8R3
166964	229533.A0A1C3YLF5	pp	229533.I1RNF4
166965	229533.A0A1C3YLF5	pp	229533.I1S296
166966	229533.A0A1C3YLF5	pp	229533.I1RCG4
166967	229533.A0A1C3YLF5	pp	229533.Q4HZ42
166968	229533.A0A1C3YLF5	pp	229533.I1RSG8
166969	229533.A0A1C3YLF5	pp	229533.I1RF08
166970	229533.A0A1C3YLF5	pp	229533.I1RT33
166971	229533.A0A1C3YLF5	pp	229533.I1RJV3
166972	229533.A0A1C3YLF5	pp	229533.V6R057
166973	229533.A0A1C3YLF5	pp	229533.I1RD52
166974	229533.A0A1C3YLF5	pp	229533.A0A1C3YLS4
166975	229533.A0A1C3YLF5	pp	229533.I1RCL3
166976	229533.A0A1C3YLF5	pp	229533.I1RQA2
166977	229533.A0A1C3YLF5	pp	229533.V6RGY3
166978	229533.A0A1C3YLF5	pp	229533.I1RNH6
166979	229533.A0A1C3YLF5	pp	229533.I1RS47
166980	229533.A0A1C3YLF5	pp	229533.I1RZW8
166981	229533.A0A1C3YLF5	pp	229533.I1RMN7
166982	229533.A0A1C3YLF5	pp	229533.I1S021
166983	229533.A0A1C3YLF5	pp	229533.I1RRI2
166984	229533.A0A1C3YLF5	pp	229533.I1RSR3
166985	229533.A0A1C3YLF5	pp	229533.Q4I327
166986	229533.A0A1C3YLF5	pp	229533.I1RRE0
166987	229533.A0A1C3YLF5	pp	229533.I1RNY2
166988	229533.A0A1C3YLF5	pp	229533.I1RBL7
166989	229533.A0A1C3YLF5	pp	229533.I1RYW3
166990	229533.A0A1C3YLF5	pp	229533.I1S2E8
166991	229533.A0A1C3YLF5	pp	229533.I1RI82
166992	229533.A0A1C3YLF5	pp	229533.I1RTE9
166993	229533.A0A1C3YLF5	pp	229533.I1RZM8
166994	229533.A0A1C3YLF5	pp	229533.I1RQE7
166995	229533.A0A1C3YLF5	pp	229533.I1RJZ6
166996	229533.A0A1C3YLF5	pp	229533.I1RDJ8
166997	229533.A0A1C3YLF5	pp	229533.I1RZZ1
166998	229533.A0A1C3YLF5	pp	229533.I1RCH5
166999	229533.A0A1C3YLF5	pp	229533.I1S2C0
167000	229533.A0A1C3YLF5	pp	229533.I1RW46
167001	229533.A0A1C3YLF5	pp	229533.I1RS18
167002	229533.A0A1C3YLF5	pp	229533.Q4IN63
167003	229533.A0A1C3YLF5	pp	229533.I1S2U2
167004	229533.A0A1C3YLF5	pp	229533.I1RMC0
167005	229533.A0A1C3YLF5	pp	229533.I1R9X7
167006	229533.A0A1C3YLF5	pp	229533.I1RFL4
167007	229533.A0A1C3YLF5	pp	229533.I1RB27
167008	229533.A0A1C3YLF5	pp	229533.I1RUG3
167009	229533.A0A1C3YLF5	pp	229533.I1RSD8
167010	229533.A0A1C3YLF5	pp	229533.Q4I8P5
167011	229533.A0A1C3YLF5	pp	229533.I1RQ31
167012	229533.A0A1C3YLF5	pp	229533.I1RN18
167013	229533.A0A1C3YLF5	pp	229533.I1RK95
167014	229533.A0A1C3YLF5	pp	229533.I1RB92
167015	229533.A0A1C3YLF5	pp	229533.I1RNU6
167016	229533.A0A1C3YLF5	pp	229533.I1RS17
167017	229533.A0A1C3YLF5	pp	229533.Q4IJ56
167018	229533.A0A1C3YLF5	pp	229533.V6RCG5
167019	229533.A0A1C3YLF5	pp	229533.I1RDG0
167020	229533.A0A1C3YLF5	pp	229533.I1RR51
167021	229533.A0A1C3YLF5	pp	229533.I1RSI6
167022	229533.A0A1C3YLF5	pp	229533.I1RN99
167023	229533.A0A1C3YLF5	pp	229533.I1RSC6
167024	229533.A0A1C3YLF5	pp	229533.I1RM96

167025 229533.A0A1C3YLF5 pp 229533.V6RCZ8
167026 229533.A0A1C3YLF5 pp 229533.I1RSS4
167027 229533.A0A1C3YLF5 pp 229533.I1RA48
167028 229533.A0A1C3YLF5 pp 229533.A0A1C3YLS9
167029 229533.A0A1C3YLF5 pp 229533.I1R9V7
167030 229533.A0A1C3YLF5 pp 229533.Q4IEK8
167031 229533.A0A1C3YLF5 pp 229533.I1S1X9
167032 229533.A0A1C3YLF5 pp 229533.Q4IBS2
167033 229533.A0A1C3YLF5 pp 229533.I1RSI0
167034 229533.A0A1C3YLF5 pp 229533.I1RE00
167035 229533.A0A1C3YLF5 pp 229533.I1RC33
167036 229533.A0A1C3YLF5 pp 229533.I1S419
167037 229533.A0A1C3YLF5 pp 229533.I1RG56
167038 229533.A0A1C3YLF5 pp 229533.I1SA33
167039 229533.A0A1C3YLF5 pp 229533.I1RFJ9
167040 229533.A0A1C3YLF5 pp 229533.I1RMN6
167041 229533.A0A1C3YLF5 pp 229533.I1RPY1
167042 229533.A0A1C3YLF5 pp 229533.I1RF19
167043 229533.A0A1C3YLF5 pp 229533.V6RIV9
167044 229533.A0A1C3YLF5 pp 229533.I1RZE5
167045 229533.A0A1C3YLF5 pp 229533.I1RS64
167046 229533.A0A1C3YLF5 pp 229533.V6QUE6
167047 229533.A0A1C3YLF5 pp 229533.I1RW99
167048 229533.A0A1C3YLF5 pp 229533.V6R227
167049 229533.A0A1C3YLF5 pp 229533.I1RRR7
167050 229533.A0A1C3YLF5 pp 229533.V6RGN5
167051 229533.A0A1C3YLF5 pp 229533.I1RZF6
167052 229533.A0A1C3YLF5 pp 229533.I1S5B0
167053 229533.A0A1C3YLF5 pp 229533.I1S0J1
167054 229533.A0A1C3YLF5 pp 229533.I1RC27
167055 229533.A0A1C3YLF5 pp 229533.I1RC72
167056 229533.A0A1C3YLF5 pp 229533.I1SA32
167057 229533.A0A1C3YLF5 pp 229533.I1RE01
167058 229533.I1S6P5 pp 229533.V6RN31
167059 229533.I1S6P5 pp 229533.V6RV57
167060 229533.I1S6P5 pp 229533.I1S8F8
167061 229533.I1S6P5 pp 229533.V6QV31
167062 229533.I1S6P5 pp 229533.V6RUB1
167063 229533.I1S6P5 pp 229533.V6QWD8
167064 229533.I1S6P5 pp 229533.V6RRM0
167065 229533.I1S6P5 pp 229533.V6RLM7
167066 229533.I1S6P5 pp 229533.I1SAE8
167067 229533.I1RNT2 pp 229533.I1S214
167068 229533.I1RNT2 pp 229533.I1RQC5
167069 229533.I1RNT2 pp 229533.I1RY40
167070 229533.I1RNT2 pp 229533.Q4HYQ4
167071 229533.I1RNT2 pp 229533.Q4IPN9
167072 229533.I1RNT2 pp 229533.I1RS94
167073 229533.I1RNT2 pp 229533.I1RYT7
167074 229533.I1RNT2 pp 229533.I1S2F3
167075 229533.I1RNT2 pp 229533.I1RSI4
167076 229533.I1RNT2 pp 229533.V6R3J1
167077 229533.I1RNT2 pp 229533.Q4IPB3
167078 229533.I1RNT2 pp 229533.I1S246
167079 229533.I1RNT2 pp 229533.I1S2A8
167080 229533.I1RNT2 pp 229533.Q4IBK5
167081 229533.I1RNT2 pp 229533.I1RXF2
167082 229533.I1RNT2 pp 229533.Q4I283
167083 229533.I1RNT2 pp 229533.Q4I5R9
167084 229533.I1RNT2 pp 229533.I1RT17
167085 229533.I1RNT2 pp 229533.Q4IPH4
167086 229533.I1RNT2 pp 229533.I1RZT1
167087 229533.I1RNT2 pp 229533.Q4IPB8
167088 229533.I1RNT2 pp 229533.I1RQQ5
167089 229533.I1RNT2 pp 229533.V6REZ4
167090 229533.I1RNT2 pp 229533.I1RZV9
167091 229533.I1RNT2 pp 229533.I1RTE5
167092 229533.I1RNT2 pp 229533.I1RYJ9
167093 229533.I1RNT2 pp 229533.I1RT35
167094 229533.I1RNT2 pp 229533.I1S5B5
167095 229533.I1RNT2 pp 229533.I1S0P4
167096 229533.I1RNT2 pp 229533.Q4I1Y1
167097 229533.I1RNT2 pp 229533.Q4I7X1

167098	229533.I1RNT2	pp	229533.Q4IAA0
167099	229533.I1RNT2	pp	229533.Q4HXF6
167100	229533.I1RNT2	pp	229533.I1RW16
167101	229533.I1RNT2	pp	229533.I1RSP1
167102	229533.I1RNT2	pp	229533.Q4IE79
167103	229533.I1RNT2	pp	229533.I1RQ49
167104	229533.I1RNT2	pp	229533.I1RYL5
167105	229533.I1RNT2	pp	229533.I1RYJ8
167106	229533.I1RNT2	pp	229533.I1S044
167107	229533.I1RQX6	pp	229533.I1RS41
167108	229533.I1RQX6	pp	229533.I1S7S9
167109	229533.I1REX2	pp	229533.I1RQR3
167110	229533.I1REX2	pp	229533.I1RSD4
167111	229533.I1REX2	pp	229533.I1RK96
167112	229533.I1REX2	pp	229533.I1S9L6
167113	229533.I1REX2	pp	229533.I1RX81
167114	229533.I1REX2	pp	229533.V6RY33
167115	229533.I1REX2	pp	229533.I1RL04
167116	229533.I1REX2	pp	229533.I1RZZ1
167117	229533.I1REX2	pp	229533.I1S7S4
167118	229533.I1REX2	pp	229533.I1RG90
167119	229533.I1REX2	pp	229533.Q4IPH4
167120	229533.I1REX2	pp	229533.I1RWX8
167121	229533.I1REX2	pp	229533.I1RMV5
167122	229533.I1REX2	pp	229533.I1RFG0
167123	229533.I1REX2	pp	229533.I1RYB1
167124	229533.I1REX2	pp	229533.I1RSA4
167125	229533.I1REX2	pp	229533.Q4IJH1
167126	229533.I1REX2	pp	229533.I1S5S2
167127	229533.I1REX2	pp	229533.I1S0B1
167128	229533.I1REX2	pp	229533.Q4IF76
167129	229533.I1REX2	pp	229533.I1RSA8
167130	229533.I1REX2	pp	229533.V6RIS2
167131	229533.I1REX2	pp	229533.I1RS83
167132	229533.I1REX2	pp	229533.I1RMW7
167133	229533.I1REX2	pp	229533.I1RK92
167134	229533.I1REX2	pp	229533.I1RYI9
167135	229533.I1REX2	pp	229533.I1RQP8
167136	229533.I1REX2	pp	229533.I1RG17
167137	229533.I1REX2	pp	229533.I1RPA0
167138	229533.I1REX2	pp	229533.I1S9L5
167139	229533.I1REX2	pp	229533.I1S4M5
167140	229533.I1REX2	pp	229533.I1RT47
167141	229533.I1REX2	pp	229533.I1RQD8
167142	229533.I1REX2	pp	229533.I1RZ11
167143	229533.I1REX2	pp	229533.I1RN09
167144	229533.I1S6P6	pp	229533.V6R9L8
167145	229533.I1S6P6	pp	229533.V6RZD4
167146	229533.I1S6P6	pp	229533.I1S8H9
167147	229533.I1S6P6	pp	229533.V6QVH2
167148	229533.I1S6P6	pp	229533.V6R3V4
167149	229533.I1S6P6	pp	229533.V6RF62
167150	229533.I1S3L0	pp	229533.I1S6L6
167151	229533.I1RFA9	pp	229533.I1RJA2
167152	229533.I1RFA9	pp	229533.I1RLN6
167153	229533.I1RFA9	pp	229533.I1RI16
167154	229533.I1RFA9	pp	229533.I1S1Q6
167155	229533.I1RFA9	pp	229533.I1RII8
167156	229533.A0A098D2W6	pp	229533.I1RY40
167157	229533.A0A098D2W6	pp	229533.I1RS94
167158	229533.A0A098D2W6	pp	229533.I1RFG0
167159	229533.A0A098D2W6	pp	229533.I1S2F3
167160	229533.A0A098D2W6	pp	229533.I1S214
167161	229533.A0A098D2W6	pp	229533.A0A1C3YI39
167162	229533.A0A098D2W6	pp	229533.Q4IPN9
167163	229533.A0A098D2W6	pp	229533.I1RMY3
167164	229533.A0A098D2W6	pp	229533.I1RYT7
167165	229533.A0A098D2W6	pp	229533.A0A098DU58
167166	229533.A0A098D2W6	pp	229533.I1RJS4
167167	229533.A0A098D2W6	pp	229533.I1S246
167168	229533.A0A098D2W6	pp	229533.I1RCU7
167169	229533.A0A098D2W6	pp	229533.I1RC00
167170	229533.A0A098D2W6	pp	229533.I1RIR9

167171 229533.A0A098D2W6 pp 229533.A0A098E365
167172 229533.A0A098D2W6 pp 229533.I1R9X2
167173 229533.A0A098D2W6 pp 229533.A0A1C3YL25
167174 229533.A0A098D2W6 pp 229533.A0A1C3YMK1
167175 229533.A0A098D2W6 pp 229533.A0A1C3YIE7
167176 229533.A0A098D2W6 pp 229533.Q4HY71
167177 229533.A0A098D2W6 pp 229533.A0A1C3YKF4
167178 229533.A0A098D2W6 pp 229533.Q4IF76
167179 229533.A0A098D2W6 pp 229533.I1RNT2
167180 229533.A0A098D2W6 pp 229533.Q4IJH1
167181 229533.A0A098D2W6 pp 229533.A0A1C3YI81
167182 229533.A0A098D2W6 pp 229533.I1RQ49
167183 229533.A0A098D2W6 pp 229533.I1RNB8
167184 229533.A0A098D2W6 pp 229533.Q4IAA0
167185 229533.A0A098D2W6 pp 229533.I1RNG5
167186 229533.A0A098D2W6 pp 229533.A0A1C3YKG7
167187 229533.A0A098D2W6 pp 229533.I1RJS5
167188 229533.A0A098D2W6 pp 229533.V6QUE6
167189 229533.A0A098D2W6 pp 229533.I1RNB7
167190 229533.A0A098D2W6 pp 229533.I1S2A8
167191 229533.A0A098D2W6 pp 229533.I1RZV9
167192 229533.A0A098D2W6 pp 229533.I1RT35
167193 229533.A0A098D2W6 pp 229533.I1RCK4
167194 229533.A0A098D2W6 pp 229533.I1RYJ8
167195 229533.A0A098D2W6 pp 229533.I1RZT1
167196 229533.A0A098D2W6 pp 229533.A0A1C3YK58
167197 229533.A0A098D2W6 pp 229533.Q4I7X1
167198 229533.A0A098D2W6 pp 229533.I1S0P4
167199 229533.A0A098D2W6 pp 229533.I1RBZ8
167200 229533.A0A098D2W6 pp 229533.I1RDY1
167201 229533.A0A098D2W6 pp 229533.A0A1C3YKJ3
167202 229533.A0A098D2W6 pp 229533.I1RTE5
167203 229533.A0A098D2W6 pp 229533.A0A0E0S9G3
167204 229533.A0A098D2W6 pp 229533.I1RNE4
167205 229533.A0A098D2W6 pp 229533.I1RYJ9
167206 229533.A0A098D2W6 pp 229533.V6REZ4
167207 229533.A0A098D2W6 pp 229533.I1RYL5
167208 229533.A0A098D2W6 pp 229533.I1S5B5
167209 229533.A0A098D2W6 pp 229533.I1RAN0
167210 229533.I1RF60 pp 229533.I1RF63
167211 229533.I1RF60 pp 229533.I1RF62
167212 229533.I1RF60 pp 229533.I1RF61
167213 229533.I1RF60 pp 229533.I1RWI5
167214 229533.I1RF60 pp 229533.I1RQ29
167215 229533.I1RF60 pp 229533.V6RN69
167216 229533.I1RII8 pp 229533.I1RLN6
167217 229533.I1RII8 pp 229533.I1RIJ2
167218 229533.I1RII8 pp 229533.I1S3T9
167219 229533.I1RII8 pp 229533.I1S1Q6
167220 229533.I1RXJ2 pp 229533.I1RXN6
167221 229533.I1S2C1 pp 229533.I1S7X9
167222 229533.I1S2C1 pp 229533.I1S8A6
167223 229533.I1S2C1 pp 229533.V6RVG3
167224 229533.I1S2C1 pp 229533.I1S439
167225 229533.I1S2C1 pp 229533.V6RR76
167226 229533.I1S2C1 pp 229533.V6RLH0
167227 229533.I1R9X2 pp 229533.Q4IN00
167228 229533.I1R9X2 pp 229533.I1RYW3
167229 229533.I1R9X2 pp 229533.I1RPS0
167230 229533.I1R9X2 pp 229533.I1REF5
167231 229533.I1R9X2 pp 229533.I1RJZ7
167232 229533.I1R9X2 pp 229533.I1RCD4
167233 229533.I1R9X2 pp 229533.Q4HZB8
167234 229533.I1R9X2 pp 229533.I1RAU7
167235 229533.I1R9X2 pp 229533.I1RTG9
167236 229533.I1R9X2 pp 229533.Q4HZ68
167237 229533.I1R9X2 pp 229533.I1RWD3
167238 229533.I1R9X2 pp 229533.Q4I7K4
167239 229533.I1R9X2 pp 229533.I1RS20
167240 229533.I1R9X2 pp 229533.I1RM82
167241 229533.I1R9X2 pp 229533.I1RAS2
167242 229533.I1R9X2 pp 229533.I1RNB8
167243 229533.I1R9X2 pp 229533.I1RJS4

167244	229533.I1R9X2	pp	229533.I1S855
167245	229533.I1R9X2	pp	229533.I1RN48
167246	229533.I1R9X2	pp	229533.I1RJS5
167247	229533.I1R9X2	pp	229533.I1RCM3
167248	229533.I1R9X2	pp	229533.I1RQ49
167249	229533.I1R9X2	pp	229533.I1S2A8
167250	229533.I1R9X2	pp	229533.I1S289
167251	229533.I1R9X2	pp	229533.I1RC72
167252	229533.I1R9X2	pp	229533.I1RFT6
167253	229533.I1R9X2	pp	229533.I1RN63
167254	229533.I1R9X2	pp	229533.Q4HW67
167255	229533.I1R9X2	pp	229533.I1RZS7
167256	229533.I1R9X2	pp	229533.I1RWI5
167257	229533.I1R9X2	pp	229533.I1RP27
167258	229533.I1R9X2	pp	229533.Q4HZ42
167259	229533.I1R9X2	pp	229533.I1S0N4
167260	229533.I1R9X2	pp	229533.I1RCI8
167261	229533.I1R9X2	pp	229533.I1S0L8
167262	229533.I1R9X2	pp	229533.I1RE87
167263	229533.I1R9X2	pp	229533.I1RMY3
167264	229533.I1R9X2	pp	229533.I1RRV1
167265	229533.I1R9X2	pp	229533.I1RN67
167266	229533.I1R9X2	pp	229533.V6REE9
167267	229533.I1R9X2	pp	229533.I1RB53
167268	229533.I1R9X2	pp	229533.Q4IAS1
167269	229533.I1R9X2	pp	229533.I1RSV2
167270	229533.I1R9X2	pp	229533.I1RK70
167271	229533.I1R9X2	pp	229533.I1RWD7
167272	229533.I1R9X2	pp	229533.Q4IEK8
167273	229533.I1R9X2	pp	229533.I1RXX1
167274	229533.I1R9X2	pp	229533.I1RZM1
167275	229533.I1R9X2	pp	229533.I1S9U2
167276	229533.I1R9X2	pp	229533.I1RPA7
167277	229533.I1R9X2	pp	229533.I1RT35
167278	229533.I1R9X2	pp	229533.I1S0P4
167279	229533.I1R9X2	pp	229533.I1RFQ7
167280	229533.I1R9X2	pp	229533.I1RN72
167281	229533.I1R9X2	pp	229533.I1RNE7
167282	229533.I1R9X2	pp	229533.Q4HVW2
167283	229533.I1R9X2	pp	229533.Q4IAA0
167284	229533.I1R9X2	pp	229533.I1RQ81
167285	229533.I1R9X2	pp	229533.I1RNI4
167286	229533.I1R9X2	pp	229533.Q4IBS2
167287	229533.I1R9X2	pp	229533.I1RRU9
167288	229533.I1R9X2	pp	229533.I1S6F0
167289	229533.I1R9X2	pp	229533.I1RMC0
167290	229533.I1R9X2	pp	229533.I1RN58
167291	229533.I1R9X2	pp	229533.I1RY40
167292	229533.I1R9X2	pp	229533.I1RN61
167293	229533.I1R9X2	pp	229533.Q4IPX8
167294	229533.I1R9X2	pp	229533.I1RAV4
167295	229533.I1R9X2	pp	229533.I1RW71
167296	229533.I1R9X2	pp	229533.I1RME6
167297	229533.I1R9X2	pp	229533.I1S246
167298	229533.I1R9X2	pp	229533.I1RKF8
167299	229533.I1R9X2	pp	229533.I1S0R7
167300	229533.I1R9X2	pp	229533.I1RXU7
167301	229533.I1R9X2	pp	229533.Q4INZ9
167302	229533.I1R9X2	pp	229533.I1S854
167303	229533.I1RD80	pp	229533.V6R1K4
167304	229533.I1RD80	pp	229533.I1S1P8
167305	229533.I1RD80	pp	229533.I1RNH4
167306	229533.I1RD80	pp	229533.I1RPQ2
167307	229533.I1RD80	pp	229533.I1S4S5
167308	229533.I1RD80	pp	229533.I1RZF7
167309	229533.I1RD80	pp	229533.I1RZH8
167310	229533.I1RD80	pp	229533.I1RDJ4
167311	229533.I1RD80	pp	229533.I1RZY1
167312	229533.I1RD80	pp	229533.I1RRM1
167313	229533.I1RD80	pp	229533.V6R0W1
167314	229533.I1RD80	pp	229533.V6QU99
167315	229533.I1RD80	pp	229533.I1S084
167316	229533.I1RD80	pp	229533.V6R3V4

167317 229533.I1RD80 pp 229533.I1RKB5
167318 229533.I1RD80 pp 229533.I1RZ20
167319 229533.I1RD80 pp 229533.I1RUM7
167320 229533.I1RD80 pp 229533.I1RMD3
167321 229533.I1RD80 pp 229533.V6R2Z2
167322 229533.I1RD80 pp 229533.V6RAM6
167323 229533.I1RD80 pp 229533.I1RZA7
167324 229533.I1RD80 pp 229533.I1RXX0
167325 229533.I1RD80 pp 229533.I1RYI6
167326 229533.I1RD80 pp 229533.I1SAE8
167327 229533.I1RD80 pp 229533.I1S273
167328 229533.I1RD80 pp 229533.V6RUB1
167329 229533.I1RD80 pp 229533.I1RQX9
167330 229533.I1RD80 pp 229533.I1S3K8
167331 229533.I1RD80 pp 229533.V6RIE5
167332 229533.I1RD80 pp 229533.I1RJT5
167333 229533.I1RD80 pp 229533.I1S4D0
167334 229533.I1RD80 pp 229533.I1S0A0
167335 229533.I1RD80 pp 229533.I1SAI4
167336 229533.I1RD80 pp 229533.I1RK03
167337 229533.I1RD80 pp 229533.I1RZ78
167338 229533.I1RD80 pp 229533.I1S5S8
167339 229533.I1RD80 pp 229533.I1RVR2
167340 229533.I1RD80 pp 229533.I1S024
167341 229533.I1RD80 pp 229533.V6RF62
167342 229533.I1RD80 pp 229533.V6RY56
167343 229533.I1RD80 pp 229533.I1S0H5
167344 229533.I1RD80 pp 229533.I1RGU7
167345 229533.I1RD80 pp 229533.V6R5D2
167346 229533.I1RD80 pp 229533.I1S6P5
167347 229533.I1RD80 pp 229533.I1RZT0
167348 229533.I1RD80 pp 229533.I1RDJ7
167349 229533.I1RD80 pp 229533.I1RPS2
167350 229533.I1RD80 pp 229533.I1RGV6
167351 229533.I1RD80 pp 229533.I1RX43
167352 229533.I1RD80 pp 229533.I1RV23
167353 229533.I1RD80 pp 229533.V6RM02
167354 229533.I1RD80 pp 229533.I1S9U6
167355 229533.I1RD80 pp 229533.I1RVF8
167356 229533.I1RD80 pp 229533.I1RLT3
167357 229533.I1RD80 pp 229533.V6QUV7
167358 229533.I1RD80 pp 229533.V6R756
167359 229533.I1RD80 pp 229533.I1S444
167360 229533.I1RTZ1 pp 229533.I1RWM4
167361 229533.I1RJS4 pp 229533.I1RPG2
167362 229533.I1RJS4 pp 229533.I1RQ49
167363 229533.I1RJS4 pp 229533.I1RNE4
167364 229533.I1RJS4 pp 229533.V6REZ4
167365 229533.I1RJS4 pp 229533.I1RSL8
167366 229533.I1RJS4 pp 229533.I1RXT9
167367 229533.I1RJS4 pp 229533.I1RND7
167368 229533.I1RJS4 pp 229533.I1RNB8
167369 229533.I1RJS4 pp 229533.I1RUP8
167370 229533.I1RJS4 pp 229533.I1RZT1
167371 229533.I1RJS4 pp 229533.I1S471
167372 229533.I1RJS4 pp 229533.I1SAN0
167373 229533.I1RJS4 pp 229533.Q4IN63
167374 229533.I1RJS4 pp 229533.I1S2A8
167375 229533.I1RJS4 pp 229533.I1RZV9
167376 229533.I1RJS4 pp 229533.I1RRE0
167377 229533.I1RJS4 pp 229533.I1RMF2
167378 229533.I1RJS4 pp 229533.I1S1Y5
167379 229533.I1RJS4 pp 229533.I1RZ27
167380 229533.I1RJS4 pp 229533.I1RWC6
167381 229533.I1RJS4 pp 229533.I1RKK6
167382 229533.I1RJS4 pp 229533.I1RVA7
167383 229533.I1RJS4 pp 229533.I1RS94
167384 229533.I1RJS4 pp 229533.I1S2B6
167385 229533.I1RJS4 pp 229533.I1RYT7
167386 229533.I1RJS4 pp 229533.I1RSP1
167387 229533.I1RJS4 pp 229533.I1RMY3
167388 229533.I1RJS4 pp 229533.I1RT35
167389 229533.I1RJS4 pp 229533.I1RZK3

167390 229533.I1RJS4 pp 229533.I1RY59
167391 229533.I1RJS4 pp 229533.V6R3J1
167392 229533.I1RJS4 pp 229533.I1RNH7
167393 229533.I1RJS4 pp 229533.I1SAM5
167394 229533.I1RJS4 pp 229533.I1RTE5
167395 229533.I1RJS4 pp 229533.I1S0P4
167396 229533.I1RJS4 pp 229533.I1RW24
167397 229533.I1RJS4 pp 229533.I1RJZ6
167398 229533.I1RJS4 pp 229533.Q4I7X1
167399 229533.I1RJS4 pp 229533.Q4IAA0
167400 229533.I1RJS4 pp 229533.I1RYJ8
167401 229533.I1RJS4 pp 229533.I1RQH7
167402 229533.I1RJS4 pp 229533.I1RNT2
167403 229533.I1RJS4 pp 229533.I1RQF7
167404 229533.I1RJS4 pp 229533.I1RY40
167405 229533.I1RJS4 pp 229533.I1RTF4
167406 229533.I1RJS4 pp 229533.I1S2F3
167407 229533.I1RJS4 pp 229533.I1RLU5
167408 229533.I1RJS4 pp 229533.I1S246
167409 229533.I1RJS4 pp 229533.I1RKK4
167410 229533.I1RJS4 pp 229533.I1RQ80
167411 229533.I1RJG6 pp 229533.I1S1C6
167412 229533.I1RJG6 pp 229533.I1S7M3
167413 229533.I1RJG6 pp 229533.I1S056
167414 229533.I1RJG6 pp 229533.I1RX47
167415 229533.I1RJG6 pp 229533.I1RN59
167416 229533.I1RJG6 pp 229533.I1RXF2
167417 229533.I1RJG6 pp 229533.I1S7M2
167418 229533.I1RJG6 pp 229533.I1RSA4
167419 229533.I1RJG6 pp 229533.I1RK90
167420 229533.I1RJG6 pp 229533.I1RQC5
167421 229533.I1RJG6 pp 229533.I1RYW3
167422 229533.I1RJG6 pp 229533.I1RND5
167423 229533.I1RJG6 pp 229533.V6QXG1
167424 229533.I1RJG6 pp 229533.I1S0B1
167425 229533.I1RJG6 pp 229533.Q4I1Y1
167426 229533.I1RJG6 pp 229533.I1S2Y5
167427 229533.I1RJG6 pp 229533.I1RMV5
167428 229533.I1RJG6 pp 229533.I1RS28
167429 229533.I1RJG6 pp 229533.I1RPY4
167430 229533.I1RJG6 pp 229533.I1RPY5
167431 229533.I1RJG6 pp 229533.Q4IBK5
167432 229533.I1RJG6 pp 229533.I1RZG7
167433 229533.I1RJG6 pp 229533.Q4IPB3
167434 229533.I1RJG6 pp 229533.I1RZQ5
167435 229533.I1RJG6 pp 229533.I1RRL0
167436 229533.I1RJG6 pp 229533.I1RK92
167437 229533.I1RJG6 pp 229533.I1S4M5
167438 229533.I1RJG6 pp 229533.I1S1X9
167439 229533.I1RJG6 pp 229533.I1RX24
167440 229533.A0A098DS16 pp 229533.I1S7X9
167441 229533.A0A098DS16 pp 229533.I1S2C1
167442 229533.A0A098DS16 pp 229533.I1RYH6
167443 229533.A0A1C3YHU3 pp 229533.V6R2Z2
167444 229533.A0A1C3YHU3 pp 229533.I1S299
167445 229533.A0A1C3YHU3 pp 229533.I1S4F4
167446 229533.A0A1C3YHU3 pp 229533.I1RFB0
167447 229533.A0A1C3YHU3 pp 229533.I1RZ38
167448 229533.A0A1C3YHU3 pp 229533.I1RKB2
167449 229533.A0A1C3YHU3 pp 229533.I1RMJ3
167450 229533.A0A1C3YHU3 pp 229533.I1S7X9
167451 229533.A0A1C3YHU3 pp 229533.I1RNF4
167452 229533.A0A1C3YHU3 pp 229533.I1RN88
167453 229533.A0A1C3YHU3 pp 229533.I1RL81
167454 229533.A0A1C3YHU3 pp 229533.I1RY62
167455 229533.A0A1C3YHU3 pp 229533.I1RC31
167456 229533.A0A1C3YHU3 pp 229533.I1S0A5
167457 229533.A0A1C3YHU3 pp 229533.A0A1C3YMQ5
167458 229533.A0A1C3YHU3 pp 229533.I1RK55
167459 229533.A0A1C3YHU3 pp 229533.I1RGC3
167460 229533.A0A1C3YHU3 pp 229533.I1RA52
167461 229533.A0A1C3YHU3 pp 229533.V6R5D2
167462 229533.A0A1C3YHU3 pp 229533.A0A1C3YJ56

167463	229533.A0A1C3YHU3	pp	229533.I1RF24
167464	229533.A0A1C3YHU3	pp	229533.A0A1C3YLE2
167465	229533.A0A1C3YHU3	pp	229533.I1RVR2
167466	229533.A0A1C3YHU3	pp	229533.V6RUY9
167467	229533.A0A1C3YHU3	pp	229533.I1RBF3
167468	229533.A0A1C3YHU3	pp	229533.I1RUR8
167469	229533.A0A1C3YHU3	pp	229533.I1RBY9
167470	229533.A0A1C3YHU3	pp	229533.I1S968
167471	229533.A0A1C3YHU3	pp	229533.I1RSK5
167472	229533.A0A1C3YHU3	pp	229533.I1RHJ0
167473	229533.A0A1C3YHU3	pp	229533.I1RH91
167474	229533.A0A1C3YHU3	pp	229533.A0A1C3YI20
167475	229533.A0A1C3YHU3	pp	229533.V6RM02
167476	229533.A0A1C3YHU3	pp	229533.I1RMT6
167477	229533.A0A1C3YHU3	pp	229533.I1S3T7
167478	229533.A0A1C3YHU3	pp	229533.A0A1C3YKB7
167479	229533.A0A1C3YHU3	pp	229533.I1S8R2
167480	229533.A0A1C3YHU3	pp	229533.I1RE52
167481	229533.A0A1C3YHU3	pp	229533.I1RWF5
167482	229533.A0A1C3YHU3	pp	229533.I1RYA0
167483	229533.A0A1C3YHU3	pp	229533.I1RSS7
167484	229533.A0A1C3YHU3	pp	229533.I1RXY7
167485	229533.A0A1C3YHU3	pp	229533.I1RGX1
167486	229533.A0A1C3YHU3	pp	229533.I1S210
167487	229533.A0A1C3YHU3	pp	229533.I1RMK7
167488	229533.A0A1C3YHU3	pp	229533.I1RQ72
167489	229533.A0A1C3YHU3	pp	229533.A0A1C3YM29
167490	229533.A0A1C3YHU3	pp	229533.I1RQP2
167491	229533.A0A1C3YHU3	pp	229533.I1RYC8
167492	229533.A0A1C3YHU3	pp	229533.I1RMH2
167493	229533.A0A1C3YHU3	pp	229533.I1RN84
167494	229533.A0A1C3YHU3	pp	229533.I1S8R4
167495	229533.A0A1C3YHU3	pp	229533.I1RMF1
167496	229533.A0A1C3YHU3	pp	229533.I1RW54
167497	229533.A0A1C3YHU3	pp	229533.I1S2C4
167498	229533.A0A1C3YHU3	pp	229533.I1SAI4
167499	229533.A0A1C3YHU3	pp	229533.I1RNV4
167500	229533.A0A1C3YHU3	pp	229533.I1RNM7
167501	229533.A0A1C3YHU3	pp	229533.I1RKZ7
167502	229533.A0A1C3YHU3	pp	229533.V6R756
167503	229533.A0A1C3YHU3	pp	229533.A0A1C3YK33
167504	229533.A0A1C3YHU3	pp	229533.V6RRM0
167505	229533.A0A1C3YHU3	pp	229533.I1RN81
167506	229533.A0A1C3YHU3	pp	229533.I1RYP5
167507	229533.A0A1C3YHU3	pp	229533.I1S2H3
167508	229533.A0A1C3YHU3	pp	229533.I1RF85
167509	229533.A0A1C3YHU3	pp	229533.I1RPA6
167510	229533.A0A1C3YHU3	pp	229533.I1S0V7
167511	229533.A0A1C3YHU3	pp	229533.A0A1C3YMK0
167512	229533.A0A1C3YHU3	pp	229533.I1S256
167513	229533.A0A1C3YHU3	pp	229533.I1RYH6
167514	229533.A0A1C3YHU3	pp	229533.V6R382
167515	229533.A0A1C3YHU3	pp	229533.I1RDN3
167516	229533.A0A1C3YHU3	pp	229533.A0A1C3YJ04
167517	229533.A0A1C3YHU3	pp	229533.A0A1C3YJV7
167518	229533.A0A1C3YHU3	pp	229533.I1RY21
167519	229533.A0A1C3YHU3	pp	229533.V6R0W1
167520	229533.A0A1C3YHU3	pp	229533.I1RDE6
167521	229533.A0A1C3YHU3	pp	229533.I1R9F2
167522	229533.A0A1C3YHU3	pp	229533.I1S2C1
167523	229533.A0A1C3YHU3	pp	229533.I1RGU3
167524	229533.A0A1C3YHU3	pp	229533.I1RRX5
167525	229533.A0A1C3YHU3	pp	229533.I1S0K0
167526	229533.A0A1C3YHU3	pp	229533.I1RZM4
167527	229533.A0A1C3YHU3	pp	229533.I1RQE8
167528	229533.A0A1C3YHU3	pp	229533.I1RNH4
167529	229533.A0A1C3YHU3	pp	229533.I1RYQ4
167530	229533.A0A1C3YHU3	pp	229533.I1RJN7
167531	229533.A0A1C3YHU3	pp	229533.I1R9B6
167532	229533.A0A1C3YHU3	pp	229533.I1RHT5
167533	229533.A0A1C3YHU3	pp	229533.I1S2W9
167534	229533.A0A1C3YHU3	pp	229533.A0A1C3YIR5
167535	229533.A0A1C3YHU3	pp	229533.I1RCU2

167536 229533.A0A1C3YHU3 pp 229533.I1RQ60
167537 229533.A0A1C3YHU3 pp 229533.I1R9Y4
167538 229533.A0A1C3YHU3 pp 229533.I1S2F7
167539 229533.A0A1C3YHU3 pp 229533.I1RWL3
167540 229533.A0A1C3YHU3 pp 229533.I1RA13
167541 229533.A0A1C3YHU3 pp 229533.I1S3L2
167542 229533.A0A1C3YHU3 pp 229533.I1RFS6
167543 229533.A0A1C3YHU3 pp 229533.I1RC39
167544 229533.A0A1C3YHU3 pp 229533.I1S0K2
167545 229533.A0A1C3YHU3 pp 229533.I1S8C1
167546 229533.A0A1C3YHU3 pp 229533.I1RG33
167547 229533.A0A1C3YHU3 pp 229533.A0A1C3YKZ8
167548 229533.A0A1C3YHU3 pp 229533.A0A1C3YLC2
167549 229533.A0A1C3YHU3 pp 229533.I1RUY4
167550 229533.A0A1C3YHU3 pp 229533.I1RXS0
167551 229533.A0A1C3YHU3 pp 229533.A0A1C3YLI1
167552 229533.A0A1C3YHU3 pp 229533.V6RR76
167553 229533.A0A1C3YHU3 pp 229533.I1RJY7
167554 229533.A0A1C3YHU3 pp 229533.V6RAM6
167555 229533.A0A1C3YHU3 pp 229533.A0A1C3YIP2
167556 229533.A0A1C3YHU3 pp 229533.I1RIM7
167557 229533.A0A1C3YHU3 pp 229533.I1RXZ4
167558 229533.A0A1C3YHU3 pp 229533.A0A1C3YKR5
167559 229533.A0A1C3YHU3 pp 229533.A0A1C3YLB3
167560 229533.A0A1C3YHU3 pp 229533.V6RK96
167561 229533.A0A1C3YHU3 pp 229533.I1RAQ4
167562 229533.A0A1C3YHU3 pp 229533.A0A1C3YL54
167563 229533.A0A1C3YHU3 pp 229533.V6QU99
167564 229533.A0A1C3YHU3 pp 229533.I1RJT5
167565 229533.A0A1C3YHU3 pp 229533.I1S0V6
167566 229533.A0A1C3YHU3 pp 229533.I1RA90
167567 229533.A0A1C3YHU3 pp 229533.I1RRM1
167568 229533.A0A1C3YHU3 pp 229533.I1RDQ2
167569 229533.A0A1C3YHU3 pp 229533.I1RDV8
167570 229533.A0A1C3YHU3 pp 229533.A0A1C3YLP6
167571 229533.A0A1C3YHU3 pp 229533.I1S8F8
167572 229533.A0A1C3YHU3 pp 229533.A0A1C3YKY6
167573 229533.A0A1C3YHU3 pp 229533.A0A1C3YKX1
167574 229533.A0A1C3YHU3 pp 229533.I1RY12
167575 229533.A0A1C3YHU3 pp 229533.I1RC48
167576 229533.A0A1C3YHU3 pp 229533.I1RPR1
167577 229533.A0A1C3YHU3 pp 229533.V6RAN2
167578 229533.A0A1C3YHU3 pp 229533.A0A1C3YIJ9
167579 229533.A0A1C3YHU3 pp 229533.I1RW55
167580 229533.A0A1C3YHU3 pp 229533.I1RXE6
167581 229533.A0A1C3YHU3 pp 229533.I1RAG3
167582 229533.A0A1C3YHU3 pp 229533.I1RHZ5
167583 229533.A0A1C3YHU3 pp 229533.I1REE2
167584 229533.A0A1C3YHU3 pp 229533.I1RST9
167585 229533.I1RCU7 pp 229533.I1RYJ8
167586 229533.I1RCU7 pp 229533.Q4I7X1
167587 229533.I1RCU7 pp 229533.I1RGC2
167588 229533.I1RCU7 pp 229533.I1S044
167589 229533.I1RCU7 pp 229533.I1RDY5
167590 229533.I1RCU7 pp 229533.I1S471
167591 229533.I1RCU7 pp 229533.I1RNB8
167592 229533.I1RCU7 pp 229533.I1RQ49
167593 229533.I1RCU7 pp 229533.I1S0P4
167594 229533.I1RCU7 pp 229533.I1RW16
167595 229533.I1RCU7 pp 229533.Q4IAA0
167596 229533.I1RCU7 pp 229533.I1RT35
167597 229533.I1RCU7 pp 229533.Q4I283
167598 229533.I1RCU7 pp 229533.I1RTE5
167599 229533.I1RCU7 pp 229533.I1RQ21
167600 229533.I1RCU7 pp 229533.I1RZK4
167601 229533.I1RCU7 pp 229533.I1RNE4
167602 229533.I1RCU7 pp 229533.Q4IE79
167603 229533.I1RCU7 pp 229533.I1RSI1
167604 229533.I1RCU7 pp 229533.I1RDY1
167605 229533.I1RCU7 pp 229533.I1RQQ5
167606 229533.I1RCU7 pp 229533.Q4IPB8
167607 229533.I1RCU7 pp 229533.I1RNG5
167608 229533.I1RCU7 pp 229533.I1RYL5

167609	229533.I1RCU7	pp	229533.I1S0X1
167610	229533.I1RCU7	pp	229533.I1RSC0
167611	229533.I1RCU7	pp	229533.Q4I5R9
167612	229533.I1RCU7	pp	229533.Q4HXF6
167613	229533.I1RCU7	pp	229533.I1RDG0
167614	229533.I1RCU7	pp	229533.I1RVA7
167615	229533.I1RCU7	pp	229533.I1RG90
167616	229533.I1RCU7	pp	229533.Q4I1Y1
167617	229533.I1RCU7	pp	229533.I1S5B5
167618	229533.I1RCU7	pp	229533.V6R0U9
167619	229533.I1RCU7	pp	229533.I1RYT7
167620	229533.I1RCU7	pp	229533.I1RJS5
167621	229533.I1RCU7	pp	229533.V6REZ4
167622	229533.I1RCU7	pp	229533.V6R3J1
167623	229533.I1RCU7	pp	229533.Q4IPH4
167624	229533.I1RCU7	pp	229533.I1RIR9
167625	229533.I1RCU7	pp	229533.I1RZV9
167626	229533.I1RCU7	pp	229533.I1S0N7
167627	229533.I1RCU7	pp	229533.I1RIF7
167628	229533.I1RCU7	pp	229533.I1RUE3
167629	229533.I1RCU7	pp	229533.I1S246
167630	229533.I1RCU7	pp	229533.Q4IBK5
167631	229533.I1RCU7	pp	229533.I1RP12
167632	229533.I1RCU7	pp	229533.I1RTY9
167633	229533.I1RCU7	pp	229533.I1S2F3
167634	229533.I1RCU7	pp	229533.I1RZT1
167635	229533.I1RCU7	pp	229533.I1RQP1
167636	229533.I1RCU7	pp	229533.I1RW71
167637	229533.I1RCU7	pp	229533.I1S0K1
167638	229533.I1RCU7	pp	229533.I1RQW1
167639	229533.I1RCU7	pp	229533.Q4I099
167640	229533.I1RCU7	pp	229533.I1RMY3
167641	229533.I1RCU7	pp	229533.I1RY40
167642	229533.I1RCU7	pp	229533.I1RW24
167643	229533.I1RCU7	pp	229533.I1RQK2
167644	229533.I1RCU7	pp	229533.Q4IPB3
167645	229533.I1RCU7	pp	229533.I1RK77
167646	229533.I1RCU7	pp	229533.I1RNT2
167647	229533.I1RCU7	pp	229533.I1RGK3
167648	229533.I1RCU7	pp	229533.I1RS94
167649	229533.I1RCU7	pp	229533.I1S214
167650	229533.I1RCU7	pp	229533.I1RQC5
167651	229533.I1RCU7	pp	229533.I1S2A8
167652	229533.I1RCU7	pp	229533.I1RJS4
167653	229533.I1RCU7	pp	229533.I1RX38
167654	229533.I1RCU7	pp	229533.Q4IPN9
167655	229533.I1RCU7	pp	229533.I1RJZ7
167656	229533.I1RCU7	pp	229533.I1RCU8
167657	229533.I1RCU7	pp	229533.I1RN31
167658	229533.I1RCU7	pp	229533.I1SAH9
167659	229533.I1RX51	pp	229533.I1S0B1
167660	229533.I1RX51	pp	229533.I1RYB1
167661	229533.I1RX51	pp	229533.Q4IB70
167662	229533.I1RX51	pp	229533.Q4IPH4
167663	229533.I1RX51	pp	229533.I1S0Z9
167664	229533.I1RX51	pp	229533.Q4HXF6
167665	229533.I1RX51	pp	229533.I1S1X9
167666	229533.I1RX51	pp	229533.Q4I1Y1
167667	229533.I1RX51	pp	229533.Q4IJH1
167668	229533.I1RX51	pp	229533.Q4IF76
167669	229533.I1RX51	pp	229533.I1S9L6
167670	229533.I1RX51	pp	229533.Q4IPB3
167671	229533.I1RX51	pp	229533.I1RZQ5
167672	229533.I1RX51	pp	229533.I1RX81
167673	229533.I1RX51	pp	229533.Q4IPB8
167674	229533.I1RX51	pp	229533.Q4IRI9
167675	229533.I1RX51	pp	229533.Q4IAA0
167676	229533.I1RX51	pp	229533.I1RZG7
167677	229533.I1RX51	pp	229533.V6QXG1
167678	229533.I1RX51	pp	229533.I1S9L5
167679	229533.I1RX51	pp	229533.Q4I5R9
167680	229533.I1RX51	pp	229533.I1RXF2
167681	229533.I1RX51	pp	229533.I1S7M2

229533.I1RX51	pp	229533.I1S4M5
229533.I1RX51	pp	229533.I1S2Y5
229533.I1RX51	pp	229533.Q4IBK5
229533.I1RX51	pp	229533.I1S7M3
229533.I1RX51	pp	229533.Q4IE79
229533.V6R756	pp	229533.V6RM09
229533.V6R756	pp	229533.V6RPS5
229533.V6R756	pp	229533.V6RY56
229533.V6R756	pp	229533.V6REA4
229533.V6R756	pp	229533.V6RBG5
229533.I1RBD1	pp	229533.I1RI16
229533.I1RBD1	pp	229533.I1S1Q6
229533.I1RBD1	pp	229533.I1RFA9
229533.I1RBD1	pp	229533.I1RII8
229533.I1RBD1	pp	229533.I1RDI8
229533.I1RBD1	pp	229533.I1RJA2
229533.I1RBD1	pp	229533.I1RLN6
229533.I1S246	pp	229533.I1S8V0
229533.I1S246	pp	229533.Q4IRI9
229533.I1S246	pp	229533.Q4IPB8
229533.I1S246	pp	229533.Q4IAA0
229533.I1S246	pp	229533.V6REZ4
229533.I1S246	pp	229533.V6RY33
229533.I1S246	pp	229533.Q4HXF6
229533.I1S246	pp	229533.I1S6F1
229533.I1S246	pp	229533.Q4IJ56
229533.I1S246	pp	229533.V6RIS2
229533.I1S246	pp	229533.Q4IF44
229533.I1S246	pp	229533.Q4I7K4
229533.I1S246	pp	229533.Q4IE79
229533.I1S246	pp	229533.Q4I7X1
229533.I1S246	pp	229533.I1S7M3
229533.I1S246	pp	229533.I1S9M9
229533.I1S246	pp	229533.I1S471
229533.I1S246	pp	229533.Q4IPB3
229533.I1S246	pp	229533.Q4IF76
229533.I1S246	pp	229533.Q4IPN9
229533.I1S246	pp	229533.Q4IB70
229533.I1S246	pp	229533.I1S8U9
229533.I1S246	pp	229533.I1S5Z2
229533.I1S246	pp	229533.Q4I1Y1
229533.I1S246	pp	229533.Q4IR09
229533.I1S246	pp	229533.Q4IBK5
229533.I1S246	pp	229533.I1S5B5
229533.I1S246	pp	229533.Q4IPA4
229533.I1S246	pp	229533.Q4IJH1
229533.I1S246	pp	229533.I1S7M2
229533.I1RVY4	pp	229533.I1RY98
229533.I1RVY4	pp	229533.Q4IN63
229533.I1RVY4	pp	229533.V6RQR4
229533.I1RLT3	pp	229533.V6RY56
229533.I1RLT3	pp	229533.I1S0H5
229533.I1RLT3	pp	229533.I1RZY1
229533.I1RLT3	pp	229533.I1S273
229533.I1RLT3	pp	229533.I1S024
229533.I1RLT3	pp	229533.I1S4D0
229533.I1RLT3	pp	229533.I1RV23
229533.I1RLT3	pp	229533.I1RXX0
229533.I1RLT3	pp	229533.I1RSX2
229533.I1RZP2	pp	229533.Q4IF44
229533.I1RZP2	pp	229533.Q4IPA4
229533.I1RZP2	pp	229533.I1S8U9
229533.I1RZP2	pp	229533.Q4IPN9
229533.I1RZP2	pp	229533.Q4I7X1
229533.I1RZP2	pp	229533.I1S5Z2
229533.I1RZP2	pp	229533.Q4IR09
229533.I1RZP2	pp	229533.Q4IPC8
229533.I1RZP2	pp	229533.I1S8V0
229533.I1RZP2	pp	229533.I1S214
229533.I1R9B8	pp	229533.I1S3K8
229533.I1R9B8	pp	229533.I1SAI4
229533.I1R9B8	pp	229533.V6RAM6
229533.I1R9B8	pp	229533.V6QU99

167755 229533.I1R9B8 pp 229533.I1RNJ7
167756 229533.I1R9B8 pp 229533.I1S084
167757 229533.I1R9B8 pp 229533.I1S1P8
167758 229533.I1R9B8 pp 229533.I1RC39
167759 229533.I1R9B8 pp 229533.I1RCV1
167760 229533.I1R9B8 pp 229533.I1RY96
167761 229533.I1R9B8 pp 229533.I1RYU0
167762 229533.I1R9B8 pp 229533.V6RY56
167763 229533.I1R9B8 pp 229533.I1RTJ7
167764 229533.I1R9B8 pp 229533.V6R0W1
167765 229533.I1R9B8 pp 229533.I1S0T1
167766 229533.I1R9B8 pp 229533.I1S4D0
167767 229533.I1R9B8 pp 229533.I1RJE7
167768 229533.I1R9B8 pp 229533.I1RJK0
167769 229533.I1R9B8 pp 229533.I1RJT5
167770 229533.I1R9B8 pp 229533.I1RVV7
167771 229533.I1R9B8 pp 229533.I1RXH3
167772 229533.I1R9B8 pp 229533.I1RV18
167773 229533.I1R9B8 pp 229533.I1RZT0
167774 229533.I1R9B8 pp 229533.I1S2C4
167775 229533.I1R9B8 pp 229533.V6R756
167776 229533.I1R9B8 pp 229533.I1RXV3
167777 229533.I1R9B8 pp 229533.I1S6P5
167778 229533.I1R9B8 pp 229533.I1RX43
167779 229533.I1R9B8 pp 229533.I1RAR1
167780 229533.I1R9B8 pp 229533.V6R5D2
167781 229533.I1R9B8 pp 229533.I1S6H1
167782 229533.I1R9B8 pp 229533.V6RUB1
167783 229533.I1R9B8 pp 229533.V6R2Z2
167784 229533.I1R9B8 pp 229533.I1RV23
167785 229533.I1R9B8 pp 229533.I1RYY2
167786 229533.I1R9B8 pp 229533.I1RZA7
167787 229533.I1R9B8 pp 229533.I1RTU5
167788 229533.I1R9B8 pp 229533.I1RD80
167789 229533.I1R9B8 pp 229533.I1RHT3
167790 229533.I1R9B8 pp 229533.I1SAJ0
167791 229533.I1R9B8 pp 229533.I1RNG4
167792 229533.I1R9B8 pp 229533.I1RZ20
167793 229533.A0A1C3YLD8 pp 229533.I1RRM5
167794 229533.A0A1C3YLD8 pp 229533.I1RBY4
167795 229533.A0A1C3YLD8 pp 229533.I1S2B8
167796 229533.A0A1C3YLD8 pp 229533.I1RW25
167797 229533.A0A1C3YLD8 pp 229533.I1RJU3
167798 229533.A0A1C3YLD8 pp 229533.I1RBC9
167799 229533.A0A1C3YLD8 pp 229533.I1RR73
167800 229533.A0A1C3YLD8 pp 229533.I1RZZ9
167801 229533.A0A1C3YLD8 pp 229533.I1RPP3
167802 229533.A0A1C3YLD8 pp 229533.I1RSQ7
167803 229533.A0A1C3YLD8 pp 229533.I1RBT1
167804 229533.A0A1C3YLD8 pp 229533.V6QW22
167805 229533.A0A1C3YLD8 pp 229533.I1S0L4
167806 229533.A0A1C3YLD8 pp 229533.I1RNE7
167807 229533.A0A1C3YLD8 pp 229533.I1RWK3
167808 229533.A0A1C3YLD8 pp 229533.I1RD52
167809 229533.A0A1C3YLD8 pp 229533.I1RS17
167810 229533.A0A1C3YLD8 pp 229533.I1RZM8
167811 229533.A0A1C3YLD8 pp 229533.I1S0L3
167812 229533.A0A1C3YLD8 pp 229533.I1RPP4
167813 229533.A0A1C3YLD8 pp 229533.I1RN67
167814 229533.A0A1C3YLD8 pp 229533.I1S504
167815 229533.A0A1C3YLD8 pp 229533.I1RE01
167816 229533.A0A1C3YLD8 pp 229533.I1RIQ1
167817 229533.A0A1C3YLD8 pp 229533.I1RAU5
167818 229533.A0A1C3YLD8 pp 229533.I1RD22
167819 229533.A0A1C3YLD8 pp 229533.I1RVQ0
167820 229533.A0A1C3YLD8 pp 229533.V6RIV9
167821 229533.A0A1C3YLD8 pp 229533.A0A1C3YME6
167822 229533.A0A1C3YLD8 pp 229533.I1RRN5
167823 229533.A0A1C3YLD8 pp 229533.I1RQC9
167824 229533.A0A1C3YLD8 pp 229533.I1RN18
167825 229533.A0A1C3YLD8 pp 229533.I1RB92
167826 229533.A0A1C3YLD8 pp 229533.Q4HZB8
167827 229533.A0A1C3YLD8 pp 229533.I1RT04

167828 229533.A0A1C3YLD8 pp 229533.I1RNQ5
167829 229533.A0A1C3YLD8 pp 229533.I1RBS6
167830 229533.A0A1C3YLD8 pp 229533.I1RZM1
167831 229533.A0A1C3YLD8 pp 229533.I1RFL4
167832 229533.A0A1C3YLD8 pp 229533.V6RTJ8
167833 229533.A0A1C3YLD8 pp 229533.Q4IJK2
167834 229533.A0A1C3YLD8 pp 229533.I1RQE7
167835 229533.A0A1C3YLD8 pp 229533.I1RJV4
167836 229533.A0A1C3YLD8 pp 229533.I1REK8
167837 229533.A0A1C3YLD8 pp 229533.V6RMW6
167838 229533.A0A1C3YLD8 pp 229533.I1RD17
167839 229533.A0A1C3YLD8 pp 229533.I1S503
167840 229533.A0A1C3YLD8 pp 229533.V6RCG5
167841 229533.A0A1C3YLD8 pp 229533.I1RAU7
167842 229533.A0A1C3YLD8 pp 229533.I1RJW6
167843 229533.A0A1C3YLD8 pp 229533.V6R042
167844 229533.A0A1C3YLD8 pp 229533.I1RA70
167845 229533.A0A1C3YLD8 pp 229533.I1RFU8
167846 229533.A0A1C3YLD8 pp 229533.I1RCG4
167847 229533.A0A1C3YLD8 pp 229533.I1RX46
167848 229533.A0A1C3YLD8 pp 229533.I1RQF9
167849 229533.A0A1C3YLD8 pp 229533.I1RTH6
167850 229533.A0A1C3YLD8 pp 229533.I1RSQ3
167851 229533.A0A1C3YLD8 pp 229533.I1RUG3
167852 229533.A0A1C3YLD8 pp 229533.I1RZW8
167853 229533.A0A1C3YLD8 pp 229533.I1RQJ4
167854 229533.A0A1C3YLD8 pp 229533.V6QUW4
167855 229533.A0A1C3YLD8 pp 229533.I1RWN6
167856 229533.A0A1C3YLD8 pp 229533.I1RNC1
167857 229533.A0A1C3YLD8 pp 229533.I1RRY9
167858 229533.A0A1C3YLD8 pp 229533.I1RPM4
167859 229533.A0A1C3YLD8 pp 229533.I1RAW0
167860 229533.A0A1C3YLD8 pp 229533.I1RJV3
167861 229533.A0A098DRZ7 pp 229533.I1RS05
167862 229533.A0A098DRZ7 pp 229533.I1RYT8
167863 229533.A0A098DRZ7 pp 229533.V6RJP8
167864 229533.A0A098DRZ7 pp 229533.I1RS29
167865 229533.A0A098DRZ7 pp 229533.I1RXD9
167866 229533.A0A098DRZ7 pp 229533.I1RA52
167867 229533.A0A098DRZ7 pp 229533.A0A1C3YM56
167868 229533.A0A098DRZ7 pp 229533.I1S5X9
167869 229533.I1RF40 pp 229533.V6R3V4
167870 229533.I1RF40 pp 229533.I1S8H9
167871 229533.I1RF40 pp 229533.I1RN80
167872 229533.I1RF40 pp 229533.I1S7B8
167873 229533.I1RF40 pp 229533.I1RJA8
167874 229533.I1RF40 pp 229533.I1RVX9
167875 229533.I1RF40 pp 229533.V6R9L8
167876 229533.I1RF40 pp 229533.V6QVH2
167877 229533.I1RF40 pp 229533.I1RH67
167878 229533.I1RF40 pp 229533.I1S2H3
167879 229533.I1RF40 pp 229533.I1S4S5
167880 229533.I1RF40 pp 229533.V6RGB5
167881 229533.I1RF40 pp 229533.I1RH36
167882 229533.I1RF40 pp 229533.I1RM26
167883 229533.I1RF40 pp 229533.V6RF62
167884 229533.I1RF40 pp 229533.I1RJC5
167885 229533.I1RF40 pp 229533.I1RF85
167886 229533.I1RF40 pp 229533.I1RMJ3
167887 229533.I1RF40 pp 229533.I1S4F4
167888 229533.I1RF40 pp 229533.I1RNG7
167889 229533.I1RF40 pp 229533.I1S1F3
167890 229533.I1RF40 pp 229533.V6RZD4
167891 229533.A0A098DEG3 pp 229533.I1RC24
167892 229533.A0A098DEG3 pp 229533.A0A1C3YIR8
167893 229533.A0A098DEG3 pp 229533.I1RZP2
167894 229533.A0A098DEG3 pp 229533.A0A1C3YI62
167895 229533.A0A098DEG3 pp 229533.I1RE45
167896 229533.A0A098DEG3 pp 229533.I1S0N7
167897 229533.A0A098DEG3 pp 229533.I1RS03
167898 229533.A0A098DEG3 pp 229533.I1RCS2
167899 229533.A0A098DEG3 pp 229533.I1RXF2
167900 229533.A0A098DEG3 pp 229533.A0A1C3YJW7

167901	229533.A0A098DEG3	pp	229533.I1RG90
167902	229533.A0A098DEG3	pp	229533.I1RQK2
167903	229533.A0A098DEG3	pp	229533.Q4IF44
167904	229533.A0A098DEG3	pp	229533.A0A1C3YNC3
167905	229533.A0A098DEG3	pp	229533.Q4IPA4
167906	229533.A0A098DEG3	pp	229533.I1S214
167907	229533.A0A098DEG3	pp	229533.I1S0B1
167908	229533.A0A098DEG3	pp	229533.I1RX47
167909	229533.A0A098DEG3	pp	229533.V6RIS2
167910	229533.A0A098DEG3	pp	229533.I1RBV3
167911	229533.A0A098DEG3	pp	229533.Q4IPC8
167912	229533.A0A098DEG3	pp	229533.I1RK79
167913	229533.A0A098DEG3	pp	229533.Q4IR09
167914	229533.A0A098DEG3	pp	229533.I1S2E8
167915	229533.A0A098DEG3	pp	229533.I1RCL4
167916	229533.A0A098DEG3	pp	229533.I1RRG3
167917	229533.A0A098DEG3	pp	229533.I1RNB8
167918	229533.A0A098DEG3	pp	229533.I1S0B6
167919	229533.A0A098DEG3	pp	229533.I1RZE1
167920	229533.A0A098DEG3	pp	229533.A0A1C3YI81
167921	229533.A0A098DEG3	pp	229533.I1RRW3
167922	229533.A0A098DEG3	pp	229533.Q4IPN9
167923	229533.A0A098DEG3	pp	229533.I1RLU5
167924	229533.A0A098DEG3	pp	229533.I1RZB5
167925	229533.A0A098DEG3	pp	229533.I1RSD4
167926	229533.A0A098DEG3	pp	229533.I1RBH8
167927	229533.A0A098DEG3	pp	229533.I1RZG7
167928	229533.A0A098DEG3	pp	229533.I1RSA4
167929	229533.A0A098DEG3	pp	229533.I1RCQ6
167930	229533.A0A098DEG3	pp	229533.A0A1C3YM64
167931	229533.A0A098DEG3	pp	229533.I1RB50
167932	229533.A0A098DEG3	pp	229533.I1RJL2
167933	229533.A0A098DEG3	pp	229533.I1RA36
167934	229533.A0A098DEG3	pp	229533.A0A1C3YJV9
167935	229533.A0A098DEG3	pp	229533.I1S7M2
167936	229533.A0A098DEG3	pp	229533.I1S5Z2
167937	229533.A0A098DEG3	pp	229533.A0A1C3YII4
167938	229533.A0A098DEG3	pp	229533.I1RZZ1
167939	229533.A0A098DEG3	pp	229533.I1RN49
167940	229533.A0A098DEG3	pp	229533.A0A0E0RTC6
167941	229533.A0A098DEG3	pp	229533.A0A1C3YL25
167942	229533.A0A098DEG3	pp	229533.I1RPY7
167943	229533.A0A098DEG3	pp	229533.I1S246
167944	229533.A0A098DEG3	pp	229533.Q4I7X1
167945	229533.A0A098DEG3	pp	229533.V6QUE6
167946	229533.A0A098DEG3	pp	229533.A0A1C3YI23
167947	229533.A0A098DEG3	pp	229533.I1S7M3
167948	229533.A0A098DEG3	pp	229533.I1RQD8
167949	229533.A0A098DEG3	pp	229533.I1RN28
167950	229533.A0A098DEG3	pp	229533.I1RRT0
167951	229533.A0A098DEG3	pp	229533.I1RT35
167952	229533.A0A098DEG3	pp	229533.A0A098DMM9
167953	229533.A0A098DEG3	pp	229533.A0A098E4T8
167954	229533.I1RPY5	pp	229533.I1RQP8
167955	229533.I1RPY5	pp	229533.I1S0Z9
167956	229533.I1RPY5	pp	229533.Q4I1Y1
167957	229533.I1RPY5	pp	229533.Q4IPH4
167958	229533.I1RPY5	pp	229533.I1RST1
167959	229533.I1RPY5	pp	229533.I1S0B1
167960	229533.I1RPY5	pp	229533.I1RZG7
167961	229533.I1RPY5	pp	229533.I1RZQ5
167962	229533.I1RPY5	pp	229533.I1RU69
167963	229533.I1RPY5	pp	229533.Q4IPB3
167964	229533.I1RPY5	pp	229533.I1RQR3
167965	229533.I1RPY5	pp	229533.Q4HXF6
167966	229533.I1RPY5	pp	229533.I1RX24
167967	229533.I1RPY5	pp	229533.I1S1X9
167968	229533.I1RPY5	pp	229533.I1RT47
167969	229533.I1RPY5	pp	229533.I1RT17
167970	229533.I1RPY5	pp	229533.I1RX81
167971	229533.I1RPY5	pp	229533.I1RQD8
167972	229533.I1RPY5	pp	229533.Q4I5R9
167973	229533.I1RPY5	pp	229533.I1RYI9

167974 229533.I1RPY5 pp 229533.I1RXF2
167975 229533.I1RPY5 pp 229533.I1RZ11
167976 229533.I1RPY5 pp 229533.I1S1C6
167977 229533.I1RPY5 pp 229533.I1S471
167978 229533.I1RPY5 pp 229533.I1S7M2
167979 229533.I1RPY5 pp 229533.Q4IE79
167980 229533.I1RPY5 pp 229533.I1RSA8
167981 229533.I1RPY5 pp 229533.Q4IPB8
167982 229533.I1RPY5 pp 229533.I1S9L6
167983 229533.I1RPY5 pp 229533.I1RQC5
167984 229533.I1RPY5 pp 229533.I1RX47
167985 229533.I1RPY5 pp 229533.I1RRL0
167986 229533.I1RPY5 pp 229533.Q4IBK5
167987 229533.I1RPY5 pp 229533.I1RS75
167988 229533.I1RPY5 pp 229533.I1RSD4
167989 229533.I1RPY5 pp 229533.I1RX51
167990 229533.I1RPY5 pp 229533.I1RS28
167991 229533.I1RPY5 pp 229533.I1S7M3
167992 229533.I1RPY5 pp 229533.I1RWU2
167993 229533.I1RPY5 pp 229533.Q4IRI9
167994 229533.I1RPY5 pp 229533.I1S4M5
167995 229533.I1RPY5 pp 229533.I1RT35
167996 229533.I1RPY5 pp 229533.I1S9L5
167997 229533.I1RPY5 pp 229533.V6QXG1
167998 229533.I1RPY5 pp 229533.Q4IAA0
167999 229533.I1RPY5 pp 229533.I1RWX8
168000 229533.I1RPY5 pp 229533.I1S8F8
168001 229533.I1RPY5 pp 229533.I1RYW3
168002 229533.I1RPY5 pp 229533.Q4IB70
168003 229533.I1RPY5 pp 229533.I1RSA4
168004 229533.I1S2Y4 pp 229533.I1S984
168005 229533.I1S2Y4 pp 229533.I1S574
168006 229533.A0A1C3YNB1 pp 229533.I1S024
168007 229533.A0A1C3YNB1 pp 229533.V6RY56
168008 229533.A0A1C3YNB1 pp 229533.I1RSY1
168009 229533.A0A1C3YNB1 pp 229533.I1RJ04
168010 229533.A0A1C3YNB1 pp 229533.I1S0H5
168011 229533.A0A1C3YNB1 pp 229533.I1RAB6
168012 229533.A0A1C3YNB1 pp 229533.I1RLT3
168013 229533.A0A1C3YNB1 pp 229533.I1RVZ3
168014 229533.A0A1C3YNB1 pp 229533.I1RCV1
168015 229533.A0A1C3YNB1 pp 229533.I1S273
168016 229533.A0A1C3YNB1 pp 229533.I1RJX3
168017 229533.A0A1C3YNB1 pp 229533.I1S5N3
168018 229533.A0A1C3YNB1 pp 229533.I1RCH9
168019 229533.A0A1C3YNB1 pp 229533.I1RDU9
168020 229533.A0A1C3YNB1 pp 229533.I1RLP9
168021 229533.A0A1C3YNB1 pp 229533.I1S2P0
168022 229533.A0A1C3YNB1 pp 229533.I1RKX6
168023 229533.A0A1C3YNB1 pp 229533.I1RE24
168024 229533.A0A1C3YNB1 pp 229533.I1RD80
168025 229533.A0A1C3YNB1 pp 229533.I1RK24
168026 229533.A0A1C3YNB1 pp 229533.I1RJT5
168027 229533.A0A1C3YNB1 pp 229533.I1RC39
168028 229533.A0A1C3YNB1 pp 229533.I1RMJ3
168029 229533.A0A1C3YNB1 pp 229533.I1RV23
168030 229533.A0A1C3YNB1 pp 229533.V6RM66
168031 229533.A0A1C3YNB1 pp 229533.I1S7J8
168032 229533.A0A1C3YNB1 pp 229533.I1S4D0
168033 229533.A0A1C3YNB1 pp 229533.I1RZY1
168034 229533.Q4IPN9 pp 229533.V6REZ4
168035 229533.Q4IPN9 pp 229533.Q4IR09
168036 229533.I1RGX0 pp 229533.I1S574
168037 229533.I1RGX0 pp 229533.I1S2Y4
168038 229533.I1RGX0 pp 229533.I1S984
168039 229533.I1RGX0 pp 229533.I1RNA1
168040 229533.V6RM02 pp 229533.V6RSF3
168041 229533.V6RM02 pp 229533.V6RY56
168042 229533.V6RM02 pp 229533.V6RN69
168043 229533.I1RRW3 pp 229533.I1S8U9
168044 229533.I1RRW3 pp 229533.I1RX47
168045 229533.I1RRW3 pp 229533.I1S7M3
168046 229533.I1RRW3 pp 229533.I1S5Z2

168047 229533.I1RRW3 pp 229533.V6RIS2
168048 229533.I1RRW3 pp 229533.I1RZL5
168049 229533.I1RRW3 pp 229533.I1RT47
168050 229533.I1RRW3 pp 229533.I1S2E8
168051 229533.I1RRW3 pp 229533.I1S471
168052 229533.I1RRW3 pp 229533.Q4IAA0
168053 229533.I1RRW3 pp 229533.I1S9M9
168054 229533.I1RRW3 pp 229533.I1RYJ9
168055 229533.I1RRW3 pp 229533.I1RSD4
168056 229533.I1RRW3 pp 229533.I1S8V0
168057 229533.I1RRW3 pp 229533.I1RWX8
168058 229533.I1RRW3 pp 229533.I1RXF2
168059 229533.I1RRW3 pp 229533.I1RT35
168060 229533.I1RRW3 pp 229533.I1S0N7
168061 229533.I1RRW3 pp 229533.I1S7M2
168062 229533.I1RRW3 pp 229533.I1RX81
168063 229533.I1RRW3 pp 229533.I1S4M5
168064 229533.I1RRW3 pp 229533.I1RVT9
168065 229533.I1RRW3 pp 229533.I1S0B6
168066 229533.I1RRW3 pp 229533.I1S246
168067 229533.I1RRW3 pp 229533.I1RZG7
168068 229533.I1RRW3 pp 229533.I1RS03
168069 229533.A0A098DH82 pp 229533.I1RZY1
168070 229533.A0A098DH82 pp 229533.A0A098DVI8
168071 229533.A0A098DH82 pp 229533.V6RAM6
168072 229533.A0A098DH82 pp 229533.A0A1C3YKZ8
168073 229533.A0A098DH82 pp 229533.I1RC39
168074 229533.A0A098DH82 pp 229533.V6R1K4
168075 229533.A0A098DH82 pp 229533.V6QU99
168076 229533.A0A098DH82 pp 229533.A0A1C3YN30
168077 229533.A0A098DH82 pp 229533.I1RH36
168078 229533.A0A098DH82 pp 229533.I1RDN3
168079 229533.A0A098DH82 pp 229533.I1S273
168080 229533.A0A098DH82 pp 229533.I1RQU9
168081 229533.A0A098DH82 pp 229533.I1RHT5
168082 229533.A0A098DH82 pp 229533.I1S6H3
168083 229533.A0A098DH82 pp 229533.I1RST9
168084 229533.A0A098DH82 pp 229533.I1RQX9
168085 229533.A0A098DH82 pp 229533.I1SAI4
168086 229533.A0A098DH82 pp 229533.I1S094
168087 229533.A0A098DH82 pp 229533.I1RNM7
168088 229533.A0A098DH82 pp 229533.I1RUY5
168089 229533.A0A098DH82 pp 229533.I1S4D0
168090 229533.A0A098DH82 pp 229533.V6R0W1
168091 229533.A0A098DH82 pp 229533.I1RW55
168092 229533.A0A098DH82 pp 229533.I1RMD3
168093 229533.A0A098DH82 pp 229533.I1RRM1
168094 229533.A0A098DH82 pp 229533.I1RF38
168095 229533.A0A098DH82 pp 229533.A0A1C3YIP2
168096 229533.A0A098DH82 pp 229533.I1RJT5
168097 229533.A0A098DH82 pp 229533.A0A1C3YK58
168098 229533.A0A098DH82 pp 229533.I1RC48
168099 229533.A0A098DH82 pp 229533.I1RCV1
168100 229533.A0A098DH82 pp 229533.I1S024
168101 229533.A0A098DH82 pp 229533.I1S0A0
168102 229533.A0A098DH82 pp 229533.I1RLZ6
168103 229533.A0A098DH82 pp 229533.I1RNH4
168104 229533.A0A098DH82 pp 229533.I1S0A5
168105 229533.A0A098DH82 pp 229533.V6RGB5
168106 229533.A0A098DH82 pp 229533.A0A1C3YKV2
168107 229533.A0A098DH82 pp 229533.A0A1C3YIR5
168108 229533.A0A098DH82 pp 229533.I1RDU0
168109 229533.A0A098DH82 pp 229533.I1RJA8
168110 229533.A0A098DH82 pp 229533.V6R9Z3
168111 229533.A0A098DH82 pp 229533.I1RA13
168112 229533.A0A098DH82 pp 229533.V6RUB1
168113 229533.A0A098DH82 pp 229533.I1RNG4
168114 229533.A0A098DH82 pp 229533.A0A1C3YKN4
168115 229533.A0A098DH82 pp 229533.V6RY56
168116 229533.A0A098DH82 pp 229533.I1RKB2
168117 229533.A0A098DH82 pp 229533.A0A098E525
168118 229533.A0A098DH82 pp 229533.I1RNG7
168119 229533.A0A098DH82 pp 229533.A0A098E320

168120 229533.A0A098DH82 pp 229533.A0A1C3YL82
168121 229533.A0A098DH82 pp 229533.I1RZA7
168122 229533.A0A098DH82 pp 229533.A0A098DS79
168123 229533.A0A098DH82 pp 229533.I1RDJ7
168124 229533.A0A098DH82 pp 229533.A0A1C3YLE2
168125 229533.A0A098DH82 pp 229533.I1RV23
168126 229533.A0A098DH82 pp 229533.I1RGI4
168127 229533.A0A098DH82 pp 229533.V6R2Z2
168128 229533.A0A098DH82 pp 229533.A0A1C3YKB7
168129 229533.A0A098DH82 pp 229533.V6R5D2
168130 229533.A0A098DH82 pp 229533.V6RCJ5
168131 229533.A0A098DH82 pp 229533.I1RVR2
168132 229533.A0A098DH82 pp 229533.I1S0H5
168133 229533.A0A098DH82 pp 229533.I1RPV8
168134 229533.A0A098DH82 pp 229533.I1RZ56
168135 229533.A0A098DH82 pp 229533.I1S6P5
168136 229533.A0A098DH82 pp 229533.I1S6P6
168137 229533.A0A098DH82 pp 229533.I1RGZ3
168138 229533.A0A098DH82 pp 229533.I1RD80
168139 229533.A0A098DH82 pp 229533.I1RJS1
168140 229533.A0A098DH82 pp 229533.A0A1C3YHU3
168141 229533.A0A098DH82 pp 229533.V6R756
168142 229533.A0A098DH82 pp 229533.I1RLT3
168143 229533.A0A098DH82 pp 229533.I1R9B8
168144 229533.A0A098DH82 pp 229533.I1RF40
168145 229533.A0A098DH82 pp 229533.A0A1C3YNB1
168146 229533.A0A098DH82 pp 229533.V6RM02
168147 229533.I1RLU5 pp 229533.Q4I7F9
168148 229533.I1RLU5 pp 229533.I1S9M9
168149 229533.I1RLU5 pp 229533.I1RZQ5
168150 229533.I1RLU5 pp 229533.I1RPL3
168151 229533.I1RLU5 pp 229533.I1RQZ2
168152 229533.I1RLU5 pp 229533.Q4HXF6
168153 229533.I1RLU5 pp 229533.I1S855
168154 229533.I1RLU5 pp 229533.V6QVD5
168155 229533.I1RLU5 pp 229533.I1RPY7
168156 229533.I1RLU5 pp 229533.I1RYJ9
168157 229533.I1RLU5 pp 229533.I1S0B1
168158 229533.I1RLU5 pp 229533.Q4IB70
168159 229533.I1RLU5 pp 229533.I1RZL5
168160 229533.I1RLU5 pp 229533.I1RZV9
168161 229533.I1RLU5 pp 229533.I1RQ49
168162 229533.I1RLU5 pp 229533.I1S8V0
168163 229533.I1RLU5 pp 229533.I1S6F1
168164 229533.I1RLU5 pp 229533.V6REZ4
168165 229533.I1RLU5 pp 229533.I1RZS7
168166 229533.I1RLU5 pp 229533.I1RS28
168167 229533.I1RLU5 pp 229533.I1RRL0
168168 229533.I1RLU5 pp 229533.Q4HZ68
168169 229533.I1RLU5 pp 229533.I1S5B5
168170 229533.I1RLU5 pp 229533.I1RPS0
168171 229533.I1RLU5 pp 229533.Q4I7K4
168172 229533.I1RLU5 pp 229533.I1S0C1
168173 229533.I1RLU5 pp 229533.I1RWD3
168174 229533.I1RLU5 pp 229533.Q4IRI9
168175 229533.I1RLU5 pp 229533.Q4IAA0
168176 229533.I1RLU5 pp 229533.Q4I1Y1
168177 229533.I1RLU5 pp 229533.Q4IJ56
168178 229533.I1RLU5 pp 229533.I1RNB8
168179 229533.I1RLU5 pp 229533.I1S8U9
168180 229533.I1RLU5 pp 229533.I1S246
168181 229533.I1RLU5 pp 229533.Q4IBS2
168182 229533.I1RLU5 pp 229533.I1RZB5
168183 229533.I1RLU5 pp 229533.Q4HW67
168184 229533.I1RLU5 pp 229533.I1RRU9
168185 229533.I1RLU5 pp 229533.Q4IP34
168186 229533.I1RLU5 pp 229533.I1RSA4
168187 229533.I1RLU5 pp 229533.I1RND5
168188 229533.I1RLU5 pp 229533.I1S471
168189 229533.I1RLU5 pp 229533.I1S4M5
168190 229533.I1RLU5 pp 229533.Q4IEK8
168191 229533.I1RLU5 pp 229533.V6QXG1
168192 229533.I1RLU5 pp 229533.Q4I830

168193 229533.I1RLU5 pp 229533.I1S6F0
168194 229533.I1RLU5 pp 229533.I1S0P4
168195 229533.I1RLU5 pp 229533.Q4I662
168196 229533.I1RLU5 pp 229533.I1RT35
168197 229533.I1RLU5 pp 229533.I1RQC5
168198 229533.I1RLU5 pp 229533.I1RYU4
168199 229533.I1RLU5 pp 229533.I1RWX8
168200 229533.I1RLU5 pp 229533.I1RPY4
168201 229533.I1RLU5 pp 229533.Q4HZ42
168202 229533.I1RLU5 pp 229533.I1RZG7
168203 229533.I1RLU5 pp 229533.I1RS94
168204 229533.I1RLU5 pp 229533.I1RRW3
168205 229533.I1RLU5 pp 229533.I1RS03
168206 229533.I1RLU5 pp 229533.I1RTE5
168207 229533.I1RLU5 pp 229533.Q4IPB8
168208 229533.I1RLU5 pp 229533.Q4IQ72
168209 229533.I1RLU5 pp 229533.I1RMV5
168210 229533.I1RLU5 pp 229533.I1RX47
168211 229533.I1RLU5 pp 229533.Q4IFI0
168212 229533.I1RLU5 pp 229533.I1RN59
168213 229533.I1RLU5 pp 229533.I1RQK2
168214 229533.I1RLU5 pp 229533.I1S7M3
168215 229533.I1RLU5 pp 229533.I1S7M2
168216 229533.I1RLU5 pp 229533.I1RXF8
168217 229533.I1RLU5 pp 229533.I1RT17
168218 229533.I1RLU5 pp 229533.Q4IF76
168219 229533.I1RLU5 pp 229533.I1RMC0
168220 229533.I1RLU5 pp 229533.I1RYT7
168221 229533.I1RLU5 pp 229533.Q4HVW2
168222 229533.I1RLU5 pp 229533.I1RXF2
168223 229533.I1RLU5 pp 229533.I1S0N7
168224 229533.I1RLU5 pp 229533.I1RN60
168225 229533.I1RLU5 pp 229533.I1RTF4
168226 229533.I1RLU5 pp 229533.I1S854
168227 229533.I1RLU5 pp 229533.Q4IJH1
168228 229533.I1RLU5 pp 229533.I1RPZ3
168229 229533.I1RLU5 pp 229533.I1RST1
168230 229533.I1RLU5 pp 229533.Q4IAS1
168231 229533.I1RBX0 pp 229533.I1S9M9
168232 229533.I1RBX0 pp 229533.I1S5Z2
168233 229533.I1RBX0 pp 229533.I1RCA9
168234 229533.I1RBX0 pp 229533.I1RRF0
168235 229533.I1RBX0 pp 229533.I1RN91
168236 229533.I1RBX0 pp 229533.I1RMU1
168237 229533.I1RBX0 pp 229533.I1RQP5
168238 229533.I1RBX0 pp 229533.I1RN60
168239 229533.I1RBX0 pp 229533.I1RDY2
168240 229533.I1RBX0 pp 229533.I1RRW3
168241 229533.I1RBX0 pp 229533.I1RLU5
168242 229533.I1RBX0 pp 229533.I1RQK2
168243 229533.I1RBX0 pp 229533.I1S214
168244 229533.I1RBX0 pp 229533.I1RCH7
168245 229533.I1RBX0 pp 229533.I1RPY7
168246 229533.I1RBX0 pp 229533.I1RNB8
168247 229533.I1RBX0 pp 229533.I1S0P4
168248 229533.I1RBX0 pp 229533.I1S246
168249 229533.I1RBX0 pp 229533.I1RND9
168250 229533.I1RBX0 pp 229533.I1RS03
168251 229533.I1RBX0 pp 229533.I1S471
168252 229533.I1RBX0 pp 229533.I1RT35
168253 229533.I1RBX0 pp 229533.I1RDX6
168254 229533.I1RBX0 pp 229533.Q4IAA0
168255 229533.I1RBX0 pp 229533.I1S0N7
168256 229533.I1RBX0 pp 229533.I1RPF5
168257 229533.I1RBX0 pp 229533.I1RUM5
168258 229533.I1RSR2 pp 229533.I1S1X2
168259 229533.I1RSR2 pp 229533.I1RZY6
168260 229533.I1RSR2 pp 229533.I1RWT1
168261 229533.I1RSR2 pp 229533.I1RYR9
168262 229533.I1RSR2 pp 229533.I1RY38
168263 229533.I1RR83 pp 229533.I1RZ17
168264 229533.I1RS47 pp 229533.V6QUE6
168265 229533.I1RS47 pp 229533.Q4IBS2

168266 229533.I1RS47 pp 229533.I1RZM8
168267 229533.I1RS47 pp 229533.I1RW99
168268 229533.I1RS47 pp 229533.I1S5B0
168269 229533.I1RS47 pp 229533.V6RCZ8
168270 229533.I1RS47 pp 229533.V6RCG5
168271 229533.I1RS47 pp 229533.I1RXF0
168272 229533.I1RS47 pp 229533.I1RZM1
168273 229533.I1RS47 pp 229533.Q4IEK8
168274 229533.I1RS47 pp 229533.I1RZF6
168275 229533.I1RS47 pp 229533.Q4ICA8
168276 229533.I1RS47 pp 229533.V6R227
168277 229533.I1RS47 pp 229533.V6RBN2
168278 229533.I1RS47 pp 229533.I1S504
168279 229533.I1RS47 pp 229533.I1RY03
168280 229533.I1RS47 pp 229533.I1S887
168281 229533.I1RS47 pp 229533.I1RZK5
168282 229533.I1RS47 pp 229533.I1S2U2
168283 229533.I1RS47 pp 229533.I1RZB5
168284 229533.I1RS47 pp 229533.I1SA32
168285 229533.I1RS47 pp 229533.Q4I948
168286 229533.I1RS47 pp 229533.Q4IAA0
168287 229533.I1RS47 pp 229533.Q4IJK2
168288 229533.I1RS47 pp 229533.Q4I8P5
168289 229533.I1RS47 pp 229533.I1RZE5
168290 229533.I1RS47 pp 229533.I1RVZ0
168291 229533.I1RS47 pp 229533.I1RXB6
168292 229533.I1RS47 pp 229533.I1RSI0
168293 229533.I1RS47 pp 229533.Q4IPI1
168294 229533.I1RS47 pp 229533.I1RX46
168295 229533.I1RS47 pp 229533.Q4I5Z5
168296 229533.I1RS47 pp 229533.V6RTJ8
168297 229533.I1RS47 pp 229533.Q4IJ56
168298 229533.I1RS47 pp 229533.I1RZK6
168299 229533.I1RS47 pp 229533.I1RWD7
168300 229533.I1RS47 pp 229533.I1S1J7
168301 229533.I1RS47 pp 229533.V6RUT5
168302 229533.I1RS47 pp 229533.I1RSR3
168303 229533.I1RS47 pp 229533.Q4INZ9
168304 229533.I1RS47 pp 229533.Q4IQK7
168305 229533.I1RS47 pp 229533.I1RZZ1
168306 229533.I1RS47 pp 229533.V6QYF0
168307 229533.I1RS47 pp 229533.I1RUQ2
168308 229533.I1RS47 pp 229533.I1S0R7
168309 229533.I1RS47 pp 229533.I1SA33
168310 229533.I1RS47 pp 229533.I1S6F0
168311 229533.I1RS47 pp 229533.I1RXD7
168312 229533.I1RS47 pp 229533.I1RWD4
168313 229533.I1RS47 pp 229533.I1RVQ0
168314 229533.I1RS47 pp 229533.I1RT05
168315 229533.I1RS47 pp 229533.V6RGN5
168316 229533.I1RS47 pp 229533.I1S9U2
168317 229533.I1RS47 pp 229533.I1S0L3
168318 229533.I1RS47 pp 229533.I1S0N4
168319 229533.I1RS47 pp 229533.V6R1K4
168320 229533.I1RS47 pp 229533.I1S5R6
168321 229533.I1RS47 pp 229533.Q4HVW2
168322 229533.I1RS47 pp 229533.I1RT33
168323 229533.I1RS47 pp 229533.Q4IJH1
168324 229533.I1RS47 pp 229533.I1RTF4
168325 229533.I1RS47 pp 229533.Q4I327
168326 229533.I1RS47 pp 229533.V6QW22
168327 229533.I1RS47 pp 229533.I1RZ99
168328 229533.I1RS47 pp 229533.I1RSQ7
168329 229533.I1RS47 pp 229533.I1RUG3
168330 229533.I1RS47 pp 229533.I1RXH6
168331 229533.I1RS47 pp 229533.I1RT80
168332 229533.I1RS47 pp 229533.I1S4S7
168333 229533.I1RS47 pp 229533.I1RW71
168334 229533.I1RS47 pp 229533.I1RW25
168335 229533.I1RS47 pp 229533.Q4IN63
168336 229533.I1RS47 pp 229533.I1RWJ8
168337 229533.I1RS47 pp 229533.I1RSQ3
168338 229533.I1RS47 pp 229533.I1S558

168339 229533.I1RS47 pp 229533.V6R042
168340 229533.I1RS47 pp 229533.Q4IFI0
168341 229533.I1RS47 pp 229533.V6RMW6
168342 229533.I1RS47 pp 229533.I1RY44
168343 229533.I1RS47 pp 229533.I1RSI6
168344 229533.I1RS47 pp 229533.I1RWN6
168345 229533.I1RS47 pp 229533.I1S1W3
168346 229533.I1RS47 pp 229533.I1RWN5
168347 229533.I1RS47 pp 229533.V6RGY3
168348 229533.I1RS47 pp 229533.I1S1J6
168349 229533.I1RS47 pp 229533.I1S1U7
168350 229533.I1RS47 pp 229533.V6RIV9
168351 229533.I1RS47 pp 229533.Q4HZ68
168352 229533.I1RS47 pp 229533.I1S296
168353 229533.I1RS47 pp 229533.I1S274
168354 229533.I1RS47 pp 229533.V6R057
168355 229533.I1RS47 pp 229533.I1S4T3
168356 229533.I1RS47 pp 229533.I1RXU9
168357 229533.I1RS47 pp 229533.Q4I624
168358 229533.I1RS47 pp 229533.I1S2K7
168359 229533.I1RS47 pp 229533.I1RS64
168360 229533.I1RS47 pp 229533.I1S0J1
168361 229533.I1RS47 pp 229533.I1RZW8
168362 229533.I1RS47 pp 229533.I1S1C7
168363 229533.I1RS47 pp 229533.I1S060
168364 229533.I1RS47 pp 229533.I1S2E8
168365 229533.I1RS47 pp 229533.I1RT03
168366 229533.I1RS47 pp 229533.I1RYW3
168367 229533.I1RS47 pp 229533.I1RSD8
168368 229533.I1RS47 pp 229533.I1RTE9
168369 229533.I1RS47 pp 229533.I1RW46
168370 229533.I1RS47 pp 229533.Q4IQW0
168371 229533.I1RS47 pp 229533.I1S2C0
168372 229533.I1RS47 pp 229533.I1RVX0
168373 229533.I1RS47 pp 229533.I1RTH6
168374 229533.I1RS47 pp 229533.I1S1B6
168375 229533.I1RS47 pp 229533.I1S1X9
168376 229533.I1RS47 pp 229533.I1S8R3
168377 229533.I1RS47 pp 229533.I1RSS4
168378 229533.I1RS47 pp 229533.I1RV91
168379 229533.I1RS47 pp 229533.I1RXR1
168380 229533.I1RS47 pp 229533.I1RTD5
168381 229533.I1RS47 pp 229533.Q4IAS1
168382 229533.I1RS47 pp 229533.Q4HZ42
168383 229533.I1RS47 pp 229533.I1RT40
168384 229533.I1RS47 pp 229533.I1S2E9
168385 229533.I1RS47 pp 229533.I1S2M6
168386 229533.I1RS47 pp 229533.I1RWK3
168387 229533.I1RS47 pp 229533.Q4HXF0
168388 229533.I1RS47 pp 229533.I1S503
168389 229533.I1RS47 pp 229533.V6R283
168390 229533.I1RS47 pp 229533.I1RXF7
168391 229533.I1RS47 pp 229533.I1RWI5
168392 229533.I1RS47 pp 229533.I1S0F6
168393 229533.I1RS47 pp 229533.I1RT04
168394 229533.I1RS47 pp 229533.I1RW26
168395 229533.I1RS47 pp 229533.I1RSQ4
168396 229533.I1RS47 pp 229533.I1RZZ9
168397 229533.I1RS47 pp 229533.Q4IF76
168398 229533.I1RS47 pp 229533.Q4I830
168399 229533.I1RS47 pp 229533.Q4IEV5
168400 229533.I1RS47 pp 229533.I1RSV2
168401 229533.I1RS47 pp 229533.I1RSX1
168402 229533.I1RS47 pp 229533.I1RZS6
168403 229533.I1RS47 pp 229533.V6R3X5
168404 229533.I1RS47 pp 229533.Q4I662
168405 229533.I1RS47 pp 229533.O74638
168406 229533.I1RS47 pp 229533.I1S2A8
168407 229533.I1RS47 pp 229533.I1S5R1
168408 229533.I1RS47 pp 229533.I1RZ39
168409 229533.I1RS47 pp 229533.I1RX24
168410 229533.I1RS47 pp 229533.I1RW41
168411 229533.I1RS47 pp 229533.Q4IMJ1

168412 229533.I1RS47 pp 229533.I1S0L4
168413 229533.I1RS47 pp 229533.I1S2B8
168414 229533.I1RS47 pp 229533.I1S2H4
168415 229533.I1RS47 pp 229533.I1S0J7
168416 229533.I1RS47 pp 229533.I1RWJ4
168417 229533.I1RS47 pp 229533.I1RVZ3
168418 229533.I1RJV2 pp 229533.I1RQB5
168419 229533.I1RJV2 pp 229533.I1S9L6
168420 229533.I1RJV2 pp 229533.I1RME6
168421 229533.I1RJV2 pp 229533.V6RY33
168422 229533.I1RJV2 pp 229533.Q4ICA8
168423 229533.I1RJV2 pp 229533.I1RZJ1
168424 229533.I1RJV2 pp 229533.I1S9L5
168425 229533.I1RJV2 pp 229533.I1RRL0
168426 229533.I1RJV2 pp 229533.I1RQP8
168427 229533.I1RJV2 pp 229533.I1RYW3
168428 229533.I1RJV2 pp 229533.I1RS83
168429 229533.I1RJV2 pp 229533.Q4IJM4
168430 229533.I1RJV2 pp 229533.Q4I7F9
168431 229533.I1RJV2 pp 229533.Q4I948
168432 229533.A0A098D862 pp 229533.I1RX38
168433 229533.A0A098D862 pp 229533.A0A1C3YKJ6
168434 229533.A0A098D862 pp 229533.I1RPW0
168435 229533.A0A098D862 pp 229533.I1RQ88
168436 229533.A0A098D862 pp 229533.I1RAI9
168437 229533.A0A098D862 pp 229533.I1RBV5
168438 229533.A0A098D862 pp 229533.A0A0E0SNZ9
168439 229533.A0A098D862 pp 229533.A0A1C3YK37
168440 229533.A0A098D862 pp 229533.I1RPU4
168441 229533.I1RAI1 pp 229533.I1RZL5
168442 229533.I1RAI1 pp 229533.I1S5Z2
168443 229533.I1RAI1 pp 229533.I1RNB8
168444 229533.I1RAI1 pp 229533.I1RT35
168445 229533.I1RAI1 pp 229533.Q4IAA0
168446 229533.I1RAI1 pp 229533.I1S0P4
168447 229533.I1RAI1 pp 229533.I1RUM5
168448 229533.I1RAI1 pp 229533.I1S246
168449 229533.I1RAI1 pp 229533.V6RKY7
168450 229533.I1RAI1 pp 229533.I1RKF0
168451 229533.I1RAI1 pp 229533.I1RLU5
168452 229533.I1RAI1 pp 229533.I1RX47
168453 229533.I1RAI1 pp 229533.I1RQZ2
168454 229533.I1RAI1 pp 229533.I1S9M9
168455 229533.I1RAI1 pp 229533.I1RND9
168456 229533.I1RAI1 pp 229533.I1S471
168457 229533.I1RAI1 pp 229533.I1RDX6
168458 229533.I1RAI1 pp 229533.I1S0N7
168459 229533.I1RAI1 pp 229533.I1RBX0
168460 229533.I1RAI1 pp 229533.I1RCH7
168461 229533.I1RAI1 pp 229533.I1RPY7
168462 229533.I1RAI1 pp 229533.I1RMU1
168463 229533.I1RAI1 pp 229533.I1RFQ0
168464 229533.I1RAI1 pp 229533.I1RS03
168465 229533.I1RAI1 pp 229533.I1RDY2
168466 229533.I1RAI1 pp 229533.I1RQP5
168467 229533.I1RAI1 pp 229533.I1RN91
168468 229533.I1RAI1 pp 229533.I1RN60
168469 229533.V6RJP8 pp 229533.V6RPN0
168470 229533.I1RLC1 pp 229533.I1RY49
168471 229533.I1RLC1 pp 229533.I1RW43
168472 229533.I1RLC1 pp 229533.I1RQV7
168473 229533.I1RLC1 pp 229533.V6RRM0
168474 229533.I1RLC1 pp 229533.I1RM26
168475 229533.I1RLC1 pp 229533.I1RVX9
168476 229533.I1RNW6 pp 229533.I1RVX9
168477 229533.I1RNW6 pp 229533.I1S3G4
168478 229533.I1RNW6 pp 229533.I1S1W8
168479 229533.I1S0X5 pp 229533.Q4HZ68
168480 229533.I1S0X5 pp 229533.Q4IAA0
168481 229533.I1S0X5 pp 229533.Q4IAS1
168482 229533.I1S0X5 pp 229533.Q4HZB8
168483 229533.I1S0X5 pp 229533.Q4HVW2
168484 229533.I1S0X5 pp 229533.Q4IEK8

168485 229533.I1S0X5 pp 229533.Q4IN00
168486 229533.I1S0X5 pp 229533.I1S0X6
168487 229533.I1S0X5 pp 229533.I1S246
168488 229533.I1S0X5 pp 229533.I1S6F0
168489 229533.I1S0X5 pp 229533.Q4HW67
168490 229533.I1S0X5 pp 229533.Q4INZ9
168491 229533.I1R9N8 pp 229533.I1RKF5
168492 229533.I1R9N8 pp 229533.I1RWA3
168493 229533.I1R9N8 pp 229533.I1S464
168494 229533.I1R9N8 pp 229533.I1RS05
168495 229533.I1R9N8 pp 229533.I1RE40
168496 229533.I1R9N8 pp 229533.I1RY92
168497 229533.I1R9N8 pp 229533.I1S0I9
168498 229533.I1R9N8 pp 229533.I1S0P0
168499 229533.I1R9N8 pp 229533.I1RFJ6
168500 229533.I1R9N8 pp 229533.I1RYT8
168501 229533.I1R9N8 pp 229533.I1RE66
168502 229533.I1R9N8 pp 229533.I1RY21
168503 229533.I1R9N8 pp 229533.I1RSC0
168504 229533.I1R9N8 pp 229533.I1RA52
168505 229533.I1R9N8 pp 229533.I1RKF4
168506 229533.I1R9N8 pp 229533.V6RJP8
168507 229533.I1R9N8 pp 229533.I1S5C6
168508 229533.I1R9N8 pp 229533.I1RQR6
168509 229533.I1R9N8 pp 229533.I1RA68
168510 229533.I1R9N8 pp 229533.I1S8S9
168511 229533.I1R9N8 pp 229533.I1S0K2
168512 229533.I1R9N8 pp 229533.I1RS29
168513 229533.I1R9N8 pp 229533.I1RYQ1
168514 229533.I1R9N8 pp 229533.I1S5X9
168515 229533.I1R9N8 pp 229533.I1RXD9
168516 229533.I1R9N8 pp 229533.I1S458
168517 229533.I1R9N8 pp 229533.I1S0R1
168518 229533.I1R9N8 pp 229533.I1RE65
168519 229533.I1R9N8 pp 229533.I1RE39
168520 229533.I1R9N8 pp 229533.I1RMK7
168521 229533.I1R9N8 pp 229533.I1RP02
168522 229533.I1R9N8 pp 229533.I1RQJ3
168523 229533.I1RS50 pp 229533.Q4IK03
168524 229533.I1RS50 pp 229533.I1RSR8
168525 229533.I1RS50 pp 229533.I1S0C2
168526 229533.I1RS50 pp 229533.I1S9G7
168527 229533.I1RS50 pp 229533.I1S7L6
168528 229533.I1RS50 pp 229533.I1RY72
168529 229533.I1RS50 pp 229533.I1S0R9
168530 229533.I1RS50 pp 229533.Q4IB65
168531 229533.I1RS50 pp 229533.I1RX00
168532 229533.I1RS50 pp 229533.V6RM66
168533 229533.I1RS50 pp 229533.Q4IPZ1
168534 229533.I1RS50 pp 229533.V6RSM7
168535 229533.I1RS50 pp 229533.I1RZI8
168536 229533.I1RS50 pp 229533.I1RYH7
168537 229533.I1RS50 pp 229533.V6QWZ7
168538 229533.I1RQK2 pp 229533.I1RYU4
168539 229533.I1RQK2 pp 229533.I1S5Z2
168540 229533.I1RQK2 pp 229533.I1RYW2
168541 229533.I1RQK2 pp 229533.I1RYT7
168542 229533.I1RQK2 pp 229533.Q4IPN9
168543 229533.I1RQK2 pp 229533.I1RZL5
168544 229533.I1RQK2 pp 229533.I1RS03
168545 229533.I1RQK2 pp 229533.I1S0B1
168546 229533.I1RQK2 pp 229533.Q4IR09
168547 229533.I1RQK2 pp 229533.I1RQZ2
168548 229533.I1RQK2 pp 229533.I1RZS7
168549 229533.I1RQK2 pp 229533.I1S246
168550 229533.I1RQK2 pp 229533.I1RZT1
168551 229533.I1RQK2 pp 229533.I1RYB1
168552 229533.I1RQK2 pp 229533.Q4IAA0
168553 229533.I1RQK2 pp 229533.I1S6F1
168554 229533.I1RQK2 pp 229533.I1S214
168555 229533.I1RQK2 pp 229533.I1RYJ9
168556 229533.I1RQK2 pp 229533.I1RRW3
168557 229533.I1RQK2 pp 229533.I1RT35

168558	229533.I1RQK2	pp	229533.I1RY40
168559	229533.I1RQK2	pp	229533.V6REZ4
168560	229533.I1RQK2	pp	229533.I1S8V0
168561	229533.I1RQK2	pp	229533.Q4IF44
168562	229533.I1RQK2	pp	229533.Q4I7X1
168563	229533.I1RQK2	pp	229533.I1S0P4
168564	229533.I1RQK2	pp	229533.I1S471
168565	229533.I1RQK2	pp	229533.I1S8U9
168566	229533.I1RP02	pp	229533.I1RS05
168567	229533.I1RP02	pp	229533.I1RXD9
168568	229533.I1RP02	pp	229533.I1S1F3
168569	229533.I1RP02	pp	229533.I1RSX0
168570	229533.I1RP02	pp	229533.I1RSC0
168571	229533.I1RP02	pp	229533.I1RWG0
168572	229533.I1RP02	pp	229533.V6R3J1
168573	229533.I1RP02	pp	229533.I1S1N7
168574	229533.I1RP02	pp	229533.I1RYC7
168575	229533.I1RP02	pp	229533.I1RX39
168576	229533.I1RP02	pp	229533.I1RXZ3
168577	229533.I1RP02	pp	229533.I1S0R1
168578	229533.I1RP02	pp	229533.I1S0T7
168579	229533.I1RP02	pp	229533.I1S458
168580	229533.I1RP02	pp	229533.I1RPV3
168581	229533.I1RP02	pp	229533.I1S464
168582	229533.I1RP02	pp	229533.I1RRX5
168583	229533.I1RP02	pp	229533.I1S0U0
168584	229533.I1RP02	pp	229533.I1RWA3
168585	229533.I1RP02	pp	229533.I1RY92
168586	229533.I1RP02	pp	229533.I1RY21
168587	229533.I1RP02	pp	229533.I1RRK9
168588	229533.I1RP02	pp	229533.I1S5C6
168589	229533.I1RP02	pp	229533.I1S0Q7
168590	229533.I1RP02	pp	229533.I1RYT8
168591	229533.I1RP02	pp	229533.I1S0P0
168592	229533.I1RP02	pp	229533.I1RYX5
168593	229533.I1RP02	pp	229533.I1RR65
168594	229533.I1RP02	pp	229533.I1S5R5
168595	229533.I1RP02	pp	229533.I1RQJ3
168596	229533.I1RP02	pp	229533.I1S212
168597	229533.I1RP02	pp	229533.I1S8F8
168598	229533.I1RP02	pp	229533.I1S6K9
168599	229533.I1RP02	pp	229533.I1RZ68
168600	229533.I1RP02	pp	229533.I1RPR9
168601	229533.I1RP02	pp	229533.I1RU95
168602	229533.I1RP02	pp	229533.I1RQR6
168603	229533.I1RP02	pp	229533.I1RRN2
168604	229533.I1RP02	pp	229533.I1S0R8
168605	229533.I1RP02	pp	229533.I1RW13
168606	229533.I1RP02	pp	229533.I1RXE6
168607	229533.I1RP02	pp	229533.I1RYC8
168608	229533.I1RP02	pp	229533.V6RJP8
168609	229533.I1RP02	pp	229533.I1S0K2
168610	229533.I1RP02	pp	229533.I1S8S9
168611	229533.I1RP02	pp	229533.I1S5X9
168612	229533.I1RP02	pp	229533.I1RS29
168613	229533.I1RP02	pp	229533.I1S266
168614	229533.I1RP02	pp	229533.I1RYA0
168615	229533.I1RP02	pp	229533.I1RP38
168616	229533.I1RP02	pp	229533.I1RYM5
168617	229533.I1RP02	pp	229533.I1S5U0
168618	229533.I1S9K9	pp	229533.I1S9L0
168619	229533.I1RCH7	pp	229533.Q4I7L0
168620	229533.I1RCH7	pp	229533.I1RDY2
168621	229533.I1RCH7	pp	229533.I1S5Z2
168622	229533.I1RCH7	pp	229533.Q4HVW2
168623	229533.I1RCH7	pp	229533.I1RQP5
168624	229533.I1RCH7	pp	229533.I1RN91
168625	229533.I1RCH7	pp	229533.I1RMU1
168626	229533.I1RCH7	pp	229533.I1RN60
168627	229533.I1RCH7	pp	229533.I1RNK2
168628	229533.I1RCH7	pp	229533.I1RMY3
168629	229533.I1RCH7	pp	229533.Q4IEK8
168630	229533.I1RCH7	pp	229533.I1RND9

168631	229533.I1RCH7	pp	229533.I1RT35
168632	229533.I1RCH7	pp	229533.Q4HZ68
168633	229533.I1RCH7	pp	229533.I1RS03
168634	229533.I1RCH7	pp	229533.I1RUM5
168635	229533.I1RCH7	pp	229533.I1RNB8
168636	229533.I1RCH7	pp	229533.I1RRR7
168637	229533.I1RCH7	pp	229533.I1S0P4
168638	229533.I1RCH7	pp	229533.I1S0N7
168639	229533.I1RCH7	pp	229533.I1RIR9
168640	229533.I1RCH7	pp	229533.I1RLU5
168641	229533.I1RCH7	pp	229533.I1S9M9
168642	229533.I1RCH7	pp	229533.I1RQK2
168643	229533.I1RCH7	pp	229533.I1RRW3
168644	229533.I1RCH7	pp	229533.I1RQ13
168645	229533.I1RCH7	pp	229533.Q4IAS1
168646	229533.I1RCH7	pp	229533.I1RCS2
168647	229533.I1RCH7	pp	229533.I1S193
168648	229533.I1RCH7	pp	229533.Q4IAA0
168649	229533.I1RCH7	pp	229533.I1S246
168650	229533.I1RCH7	pp	229533.I1S471
168651	229533.I1RCH7	pp	229533.I1RDX6
168652	229533.I1RCH7	pp	229533.Q4I7X1
168653	229533.I1RYT8	pp	229533.I1S325
168654	229533.I1RYT8	pp	229533.I1S458
168655	229533.I1RYT8	pp	229533.I1S3T7
168656	229533.I1RYT8	pp	229533.I1S5C6
168657	229533.I1RYT8	pp	229533.I1S0R1
168658	229533.I1RYT8	pp	229533.I1S8Z2
168659	229533.I1RYT8	pp	229533.I1S1N7
168660	229533.I1RYT8	pp	229533.I1S1F3
168661	229533.I1RYT8	pp	229533.I1S0U0
168662	229533.I1RYT8	pp	229533.Q4I099
168663	229533.I1RYT8	pp	229533.I1S0T7
168664	229533.I1RYT8	pp	229533.Q4IQX3
168665	229533.I1RYT8	pp	229533.I1S8S9
168666	229533.I1RYT8	pp	229533.I1S0P0
168667	229533.I1RYT8	pp	229533.I1S0K2
168668	229533.I1RYT8	pp	229533.Q4IJW4
168669	229533.I1RYT8	pp	229533.I1S464
168670	229533.I1RYT8	pp	229533.I1S0I9
168671	229533.I1RYT8	pp	229533.I1S5U0
168672	229533.I1RYT8	pp	229533.I1S5X9
168673	229533.I1RYT8	pp	229533.I1S212
168674	229533.I1RYT8	pp	229533.I1RZ43
168675	229533.I1RYT8	pp	229533.I1S0R8
168676	229533.I1RYT8	pp	229533.I1S5R5
168677	229533.I1RYT8	pp	229533.I1RZM8
168678	229533.I1RYT8	pp	229533.I1S0Q7
168679	229533.I1RYT8	pp	229533.I1S266
168680	229533.I1RYT8	pp	229533.I1RZ68
168681	229533.I1RYT8	pp	229533.I1S8F8
168682	229533.I1RYT8	pp	229533.V6RPN0
168683	229533.I1RYT8	pp	229533.V6RJP8
168684	229533.A0A098E2E9	pp	229533.I1RFJ1
168685	229533.A0A098E2E9	pp	229533.I1RS50
168686	229533.A0A098E2E9	pp	229533.I1S9K9
168687	229533.A0A098E2E9	pp	229533.V6RM66
168688	229533.I1RL15	pp	229533.I1RPB1
168689	229533.I1RL15	pp	229533.I1S2J3
168690	229533.I1RL15	pp	229533.I1S1K2
168691	229533.Q4IPA4	pp	229533.Q4IPC8
168692	229533.Q4IPA4	pp	229533.Q4IPN9
168693	229533.Q4IPA4	pp	229533.Q4IR09
168694	229533.I1RMM5	pp	229533.I1S9L0
168695	229533.I1RMM5	pp	229533.I1RMW7
168696	229533.I1RMM5	pp	229533.I1RSD4
168697	229533.I1RMM5	pp	229533.I1S7M3
168698	229533.I1RMM5	pp	229533.I1RPA0
168699	229533.I1RMM5	pp	229533.Q4HW67
168700	229533.I1RMM5	pp	229533.I1RS83
168701	229533.I1RMM5	pp	229533.V6RY33
168702	229533.I1RMM5	pp	229533.I1S524
168703	229533.I1RMM5	pp	229533.V6RIS2

168704 229533.I1RMM5 pp 229533.I1RSA4
168705 229533.I1RMM5 pp 229533.I1S4Y4
168706 229533.I1RMM5 pp 229533.I1RRQ4
168707 229533.I1RMM5 pp 229533.I1RND5
168708 229533.I1RMM5 pp 229533.I1RQD8
168709 229533.I1RMM5 pp 229533.I1RWX8
168710 229533.I1RMM5 pp 229533.I1RT47
168711 229533.I1RMM5 pp 229533.I1S0B1
168712 229533.I1RMM5 pp 229533.I1S5S2
168713 229533.I1RMM5 pp 229533.I1RXF2
168714 229533.I1RMM5 pp 229533.I1S7M2
168715 229533.I1RA52 pp 229533.I1S464
168716 229533.I1RA52 pp 229533.I1S299
168717 229533.I1RA52 pp 229533.I1RPP3
168718 229533.I1RA52 pp 229533.I1RYI5
168719 229533.I1RA52 pp 229533.I1RL81
168720 229533.I1RA52 pp 229533.I1S7X9
168721 229533.I1RA52 pp 229533.I1RB25
168722 229533.I1RA52 pp 229533.I1S1F3
168723 229533.I1RA52 pp 229533.I1RX96
168724 229533.I1RA52 pp 229533.I1RKB2
168725 229533.I1RA52 pp 229533.I1RW26
168726 229533.I1RA52 pp 229533.I1RGA4
168727 229533.I1RA52 pp 229533.I1RBE6
168728 229533.I1RA52 pp 229533.I1RBC7
168729 229533.I1RA52 pp 229533.I1RX39
168730 229533.I1RA52 pp 229533.I1RN42
168731 229533.I1RA52 pp 229533.I1RE53
168732 229533.I1RA52 pp 229533.I1RN67
168733 229533.I1RA52 pp 229533.I1RYF9
168734 229533.I1RA52 pp 229533.I1RYQ1
168735 229533.I1RA52 pp 229533.I1RWA3
168736 229533.I1RA52 pp 229533.I1RRM8
168737 229533.I1RA52 pp 229533.Q4HXF0
168738 229533.I1RA52 pp 229533.I1RBF3
168739 229533.I1RA52 pp 229533.I1RVR2
168740 229533.I1RA52 pp 229533.I1RZ60
168741 229533.I1RA52 pp 229533.I1RL04
168742 229533.I1RA52 pp 229533.I1RN88
168743 229533.I1RA52 pp 229533.I1RC34
168744 229533.I1RA52 pp 229533.I1RK39
168745 229533.I1RA52 pp 229533.I1RE40
168746 229533.I1RA52 pp 229533.Q4HVQ9
168747 229533.I1RA52 pp 229533.I1RXD9
168748 229533.I1RA52 pp 229533.I1S3G4
168749 229533.I1RA52 pp 229533.I1RSC0
168750 229533.I1RA52 pp 229533.I1RFI4
168751 229533.I1RA52 pp 229533.V6R3J1
168752 229533.I1RA52 pp 229533.I1S3T7
168753 229533.I1RA52 pp 229533.I1RSF2
168754 229533.I1RA52 pp 229533.I1RAW3
168755 229533.I1RA52 pp 229533.I1RR65
168756 229533.I1RA52 pp 229533.I1RYX5
168757 229533.I1RA52 pp 229533.O74638
168758 229533.I1RA52 pp 229533.I1S2B8
168759 229533.I1RA52 pp 229533.I1RYC8
168760 229533.I1RA52 pp 229533.I1RY92
168761 229533.I1RA52 pp 229533.I1RUR8
168762 229533.I1RA52 pp 229533.I1RB64
168763 229533.I1RA52 pp 229533.I1RKF4
168764 229533.I1RA52 pp 229533.I1RAP2
168765 229533.I1RA52 pp 229533.I1RSE9
168766 229533.I1RA52 pp 229533.I1RZ39
168767 229533.I1RA52 pp 229533.I1RPY0
168768 229533.I1RA52 pp 229533.I1RVX0
168769 229533.I1RA52 pp 229533.V6RLM7
168770 229533.I1RA52 pp 229533.I1RMV0
168771 229533.I1RA52 pp 229533.I1S458
168772 229533.I1RA52 pp 229533.I1RE39
168773 229533.I1RA52 pp 229533.I1RSQ4
168774 229533.I1RA52 pp 229533.I1RB38
168775 229533.I1RA52 pp 229533.I1RYA0
168776 229533.I1RA52 pp 229533.I1RVT8

168777 229533.I1RA52 pp 229533.I1RR78
168778 229533.I1RA52 pp 229533.I1RFS5
168779 229533.I1RA52 pp 229533.I1RVN1
168780 229533.I1RA52 pp 229533.I1S5X9
168781 229533.I1RA52 pp 229533.I1S0R8
168782 229533.I1RA52 pp 229533.I1S3V2
168783 229533.I1RA52 pp 229533.I1S7W5
168784 229533.I1RA52 pp 229533.Q4HZ34
168785 229533.I1RA52 pp 229533.V6RRM0
168786 229533.I1RA52 pp 229533.I1S0V7
168787 229533.I1RA52 pp 229533.I1S8R2
168788 229533.I1RA52 pp 229533.I1RCH5
168789 229533.I1RA52 pp 229533.I1RI95
168790 229533.I1RA52 pp 229533.Q4I174
168791 229533.I1RA52 pp 229533.V6RXB3
168792 229533.I1RA52 pp 229533.I1RF85
168793 229533.I1RA52 pp 229533.I1RU95
168794 229533.I1RA52 pp 229533.I1RGI8
168795 229533.I1RA52 pp 229533.V6RMW6
168796 229533.I1RA52 pp 229533.I1RE65
168797 229533.I1RA52 pp 229533.I1RGM0
168798 229533.I1RA52 pp 229533.I1RBS5
168799 229533.I1RA52 pp 229533.I1RG11
168800 229533.I1RA52 pp 229533.I1RBP6
168801 229533.I1RA52 pp 229533.I1RWG0
168802 229533.I1RA52 pp 229533.I1RQJ3
168803 229533.I1RA52 pp 229533.I1RVX8
168804 229533.I1RA52 pp 229533.I1RFJ6
168805 229533.I1RA52 pp 229533.I1S5U0
168806 229533.I1RA52 pp 229533.I1S0Q7
168807 229533.I1RA52 pp 229533.I1RYQ3
168808 229533.I1RA52 pp 229533.I1RJB3
168809 229533.I1RA52 pp 229533.I1RBT6
168810 229533.I1RA52 pp 229533.I1S0P0
168811 229533.I1RA52 pp 229533.I1RZJ3
168812 229533.I1RA52 pp 229533.I1RLN1
168813 229533.I1RA52 pp 229533.I1RE66
168814 229533.I1RA52 pp 229533.I1RPP7
168815 229533.I1RA52 pp 229533.I1S8C1
168816 229533.I1RA52 pp 229533.I1S266
168817 229533.I1RA52 pp 229533.I1RG62
168818 229533.I1RA52 pp 229533.I1S4S7
168819 229533.I1RA52 pp 229533.I1RYT8
168820 229533.I1RA52 pp 229533.V6RTJ8
168821 229533.I1RA52 pp 229533.I1RRX5
168822 229533.I1RA52 pp 229533.I1RRK9
168823 229533.I1RA52 pp 229533.I1S0T7
168824 229533.I1RA52 pp 229533.I1RNF7
168825 229533.I1RA52 pp 229533.Q4I5R9
168826 229533.I1RA52 pp 229533.I1S1W8
168827 229533.I1RA52 pp 229533.I1RAI4
168828 229533.I1RA52 pp 229533.I1RCU2
168829 229533.I1RA52 pp 229533.Q4IQX3
168830 229533.I1RA52 pp 229533.I1RYC7
168831 229533.I1RA52 pp 229533.I1RGJ4
168832 229533.I1RA52 pp 229533.I1RW54
168833 229533.I1RA52 pp 229533.I1RLH3
168834 229533.I1RA52 pp 229533.I1RS05
168835 229533.I1RA52 pp 229533.I1RBB1
168836 229533.I1RA52 pp 229533.I1RDH5
168837 229533.I1RA52 pp 229533.I1RMQ6
168838 229533.I1RA52 pp 229533.I1S325
168839 229533.I1RA52 pp 229533.I1RLP1
168840 229533.I1RA52 pp 229533.I1RMK7
168841 229533.I1RA52 pp 229533.I1REJ4
168842 229533.I1RA52 pp 229533.I1RKB7
168843 229533.I1RA52 pp 229533.I1RNM7
168844 229533.I1RA52 pp 229533.I1RMK4
168845 229533.I1RA52 pp 229533.I1REF0
168846 229533.I1RA52 pp 229533.I1S0U0
168847 229533.I1RA52 pp 229533.I1S1N7
168848 229533.I1RA52 pp 229533.I1S287
168849 229533.I1RA52 pp 229533.I1RZW7

168850 229533.I1RA52 pp 229533.I1S0R1
168851 229533.I1RA52 pp 229533.I1RF63
168852 229533.I1RA52 pp 229533.I1RIQ3
168853 229533.I1RA52 pp 229533.I1RA68
168854 229533.I1RA52 pp 229533.I1RC98
168855 229533.I1RA52 pp 229533.V6RJP8
168856 229533.I1RA52 pp 229533.I1RYI6
168857 229533.I1RA52 pp 229533.I1RNE7
168858 229533.I1RA52 pp 229533.I1RP02
168859 229533.I1RA52 pp 229533.I1RY21
168860 229533.I1RA52 pp 229533.I1REU9
168861 229533.I1RA52 pp 229533.I1S5C6
168862 229533.I1RA52 pp 229533.I1RM67
168863 229533.I1RA52 pp 229533.I1RW13
168864 229533.I1RA52 pp 229533.V6R227
168865 229533.I1RA52 pp 229533.I1RXE6
168866 229533.I1RA52 pp 229533.I1REE2
168867 229533.I1RA52 pp 229533.I1S8F8
168868 229533.I1RA52 pp 229533.I1RYM5
168869 229533.I1RA52 pp 229533.I1RQR6
168870 229533.I1RA52 pp 229533.Q4I5G1
168871 229533.I1RA52 pp 229533.I1RJY7
168872 229533.I1RA52 pp 229533.I1RCH9
168873 229533.I1RA52 pp 229533.I1RK24
168874 229533.I1RA52 pp 229533.I1S5R5
168875 229533.I1RA52 pp 229533.V6RPN0
168876 229533.I1RA52 pp 229533.I1RPR1
168877 229533.I1RA52 pp 229533.I1RWB0
168878 229533.I1RA52 pp 229533.I1RKF5
168879 229533.I1RA52 pp 229533.I1RAV5
168880 229533.I1RA52 pp 229533.I1RZ14
168881 229533.I1RA52 pp 229533.I1RUQ3
168882 229533.I1RA52 pp 229533.I1RZM8
168883 229533.I1RA52 pp 229533.I1S212
168884 229533.I1RA52 pp 229533.I1RP38
168885 229533.I1RA52 pp 229533.I1RVZ9
168886 229533.I1RA52 pp 229533.I1RXF0
168887 229533.I1RA52 pp 229533.I1RS29
168888 229533.I1RA52 pp 229533.I1S0K2
168889 229533.I1RA52 pp 229533.I1RAZ3
168890 229533.I1RA52 pp 229533.I1RQA5
168891 229533.I1RA52 pp 229533.I1RNY6
168892 229533.I1RA52 pp 229533.I1RG33
168893 229533.I1RA52 pp 229533.I1RAM9
168894 229533.I1RA52 pp 229533.I1S0V6
168895 229533.I1RA52 pp 229533.I1RZ68
168896 229533.I1RA52 pp 229533.I1S8S9
168897 229533.I1RA52 pp 229533.I1RPR9
168898 229533.I1RA52 pp 229533.I1S128
168899 229533.A0A098E2B1 pp 229533.I1RLM3
168900 229533.A0A098E2B1 pp 229533.I1RJU9
168901 229533.A0A098E2B1 pp 229533.A0A0E0SP71
168902 229533.I1RMK7 pp 229533.I1S0K2
168903 229533.I1RMK7 pp 229533.I1RUQ3
168904 229533.I1RMK7 pp 229533.I1RS29
168905 229533.I1RMK7 pp 229533.Q4I5G1
168906 229533.I1RMK7 pp 229533.I1S8F8
168907 229533.I1RMK7 pp 229533.I1S4Q4
168908 229533.I1RMK7 pp 229533.I1RZ68
168909 229533.I1RMK7 pp 229533.I1S8S9
168910 229533.I1RMK7 pp 229533.V6RJP8
168911 229533.I1RMK7 pp 229533.I1RVV7
168912 229533.I1RMK7 pp 229533.I1RP38
168913 229533.I1RMK7 pp 229533.I1S212
168914 229533.I1RMK7 pp 229533.I1RTJ2
168915 229533.I1RMK7 pp 229533.I1RMW4
168916 229533.I1RMK7 pp 229533.I1RXE6
168917 229533.I1RMK7 pp 229533.I1RY21
168918 229533.I1RMK7 pp 229533.I1RNY6
168919 229533.I1RMK7 pp 229533.I1RW13
168920 229533.I1RMK7 pp 229533.I1RPR1
168921 229533.I1RMK7 pp 229533.I1S0R1
168922 229533.I1RMK7 pp 229533.I1S5R5

168923 229533.I1RMK7 pp 229533.I1S5C6
168924 229533.I1RMK7 pp 229533.I1S0U0
168925 229533.I1RMK7 pp 229533.I1RQR6
168926 229533.I1RMK7 pp 229533.I1RP02
168927 229533.I1RMK7 pp 229533.I1RYC7
168928 229533.I1RMK7 pp 229533.I1S0T7
168929 229533.I1RMK7 pp 229533.I1RNM7
168930 229533.I1RMK7 pp 229533.I1S0P0
168931 229533.I1RMK7 pp 229533.I1RRX5
168932 229533.I1RMK7 pp 229533.I1RRK9
168933 229533.I1RMK7 pp 229533.I1S0R8
168934 229533.I1RMK7 pp 229533.I1RNF7
168935 229533.I1RMK7 pp 229533.I1S8C1
168936 229533.I1RMK7 pp 229533.I1RYT8
168937 229533.I1RMK7 pp 229533.I1RYN2
168938 229533.I1RMK7 pp 229533.I1RWG0
168939 229533.I1RMK7 pp 229533.I1S8R2
168940 229533.I1RMK7 pp 229533.I1RYQ3
168941 229533.I1RMK7 pp 229533.I1RMQ6
168942 229533.I1RMK7 pp 229533.I1S5X9
168943 229533.I1RMK7 pp 229533.I1RS05
168944 229533.I1RMK7 pp 229533.I1RYA0
168945 229533.I1RMK7 pp 229533.I1RQJ3
168946 229533.I1RMK7 pp 229533.I1S0Q7
168947 229533.I1RMK7 pp 229533.I1S5U0
168948 229533.I1RMK7 pp 229533.I1RN42
168949 229533.I1RMK7 pp 229533.V6RXB3
168950 229533.I1RMK7 pp 229533.I1S266
168951 229533.I1RMK7 pp 229533.V6R1X4
168952 229533.I1RMK7 pp 229533.I1RW26
168953 229533.I1RMK7 pp 229533.I1RVT8
168954 229533.I1RMK7 pp 229533.I1RT88
168955 229533.I1RMK7 pp 229533.I1RU95
168956 229533.I1RMK7 pp 229533.Q4HZ34
168957 229533.I1RMK7 pp 229533.I1S1F3
168958 229533.I1RMK7 pp 229533.I1RN84
168959 229533.I1RMK7 pp 229533.I1RWF5
168960 229533.I1RMK7 pp 229533.I1RYC8
168961 229533.I1RMK7 pp 229533.I1RXD9
168962 229533.I1RMK7 pp 229533.Q4I174
168963 229533.I1RMK7 pp 229533.I1RUR8
168964 229533.I1RMK7 pp 229533.V6R3J1
168965 229533.I1RMK7 pp 229533.I1S3L8
168966 229533.I1RMK7 pp 229533.V6RRM0
168967 229533.I1RMK7 pp 229533.I1RY92
168968 229533.I1RMK7 pp 229533.I1S458
168969 229533.I1RMK7 pp 229533.I1RPY0
168970 229533.I1RMK7 pp 229533.Q4HVQ9
168971 229533.I1RMK7 pp 229533.I1RWA3
168972 229533.I1RMK7 pp 229533.I1S299
168973 229533.I1RMK7 pp 229533.I1S3T7
168974 229533.I1RMK7 pp 229533.I1RSC0
168975 229533.I1RMK7 pp 229533.I1RZ90
168976 229533.I1RMK7 pp 229533.I1S464
168977 229533.I1RMK7 pp 229533.I1RX39
168978 229533.I1RMK7 pp 229533.I1RYQ1
168979 229533.A0A1C3YIZ4 pp 229533.V6RM09
168980 229533.A0A1C3YIZ4 pp 229533.V6REA4
168981 229533.A0A1C3YIZ4 pp 229533.V6RN16
168982 229533.A0A1C3YIZ4 pp 229533.I1RY21
168983 229533.A0A1C3YIZ4 pp 229533.I1RZ12
168984 229533.A0A1C3YIZ4 pp 229533.I1S5C6
168985 229533.A0A1C3YIZ4 pp 229533.V6QTX7
168986 229533.A0A1C3YIZ4 pp 229533.I1RYT8
168987 229533.A0A1C3YIZ4 pp 229533.I1RME5
168988 229533.A0A1C3YIZ4 pp 229533.A0A1C3YJC4
168989 229533.A0A1C3YIZ4 pp 229533.A0A1C3YJZ0
168990 229533.A0A1C3YIZ4 pp 229533.V6R5T7
168991 229533.A0A1C3YIZ4 pp 229533.V6RJP8
168992 229533.A0A1C3YIZ4 pp 229533.I1RY16
168993 229533.A0A1C3YIZ4 pp 229533.I1RFN8
168994 229533.A0A1C3YIZ4 pp 229533.I1S8S9
168995 229533.A0A1C3YIZ4 pp 229533.I1RMK7

168996 229533.A0A1C3YIZ4 pp 229533.I1RS05
168997 229533.A0A1C3YIZ4 pp 229533.I1RRQ7
168998 229533.A0A1C3YIZ4 pp 229533.I1S0P0
168999 229533.A0A1C3YIZ4 pp 229533.I1RJL1
169000 229533.A0A1C3YIZ4 pp 229533.I1RAM9
169001 229533.A0A1C3YIZ4 pp 229533.I1RQR6
169002 229533.A0A1C3YIZ4 pp 229533.I1RS29
169003 229533.A0A1C3YIZ4 pp 229533.I1RAV5
169004 229533.A0A1C3YIZ4 pp 229533.I1RE66
169005 229533.A0A1C3YIZ4 pp 229533.I1S8D3
169006 229533.A0A1C3YIZ4 pp 229533.V6RWB3
169007 229533.A0A1C3YIZ4 pp 229533.I1RAJ0
169008 229533.A0A1C3YIZ4 pp 229533.I1RP02
169009 229533.A0A1C3YIZ4 pp 229533.I1S0K2
169010 229533.A0A1C3YIZ4 pp 229533.I1RE40
169011 229533.A0A1C3YIZ4 pp 229533.I1RXD9
169012 229533.A0A1C3YIZ4 pp 229533.V6RDP1
169013 229533.A0A1C3YIZ4 pp 229533.I1RZA8
169014 229533.A0A1C3YIZ4 pp 229533.I1R9N8
169015 229533.A0A1C3YIZ4 pp 229533.I1RKF5
169016 229533.A0A1C3YIZ4 pp 229533.A0A1C3YLR9
169017 229533.A0A1C3YIZ4 pp 229533.I1RCT9
169018 229533.A0A1C3YIZ4 pp 229533.I1RNG4
169019 229533.A0A1C3YIZ4 pp 229533.V6R556
169020 229533.A0A1C3YIZ4 pp 229533.I1RE39
169021 229533.A0A1C3YIZ4 pp 229533.I1RE65
169022 229533.A0A1C3YIZ4 pp 229533.I1RMA4
169023 229533.A0A1C3YIZ4 pp 229533.I1RW82
169024 229533.A0A1C3YIZ4 pp 229533.A0A1C3YJQ2
169025 229533.A0A1C3YIZ4 pp 229533.I1S464
169026 229533.A0A1C3YIZ4 pp 229533.I1S0Q7
169027 229533.A0A1C3YIZ4 pp 229533.I1RI11
169028 229533.A0A1C3YIZ4 pp 229533.I1S458
169029 229533.A0A1C3YIZ4 pp 229533.I1RQJ3
169030 229533.A0A1C3YIZ4 pp 229533.I1RY92
169031 229533.A0A1C3YIZ4 pp 229533.I1RKF4
169032 229533.A0A1C3YIZ4 pp 229533.A0A1C3YJU7
169033 229533.A0A1C3YIZ4 pp 229533.A0A1C3YLB6
169034 229533.A0A1C3YIZ4 pp 229533.I1RFJ6
169035 229533.A0A1C3YIZ4 pp 229533.I1S694
169036 229533.A0A1C3YIZ4 pp 229533.I1RA52
169037 229533.A0A1C3YIZ4 pp 229533.I1RTP2
169038 229533.A0A1C3YIZ4 pp 229533.A0A1C3YM56
169039 229533.A0A1C3YIZ4 pp 229533.I1RWA3
169040 229533.A0A1C3YIZ4 pp 229533.I1S5X9
169041 229533.A0A1C3YIZ4 pp 229533.I1RHU8
169042 229533.I1RNW2 pp 229533.I1RVX9
169043 229533.I1RNW2 pp 229533.I1S3G4
169044 229533.I1RNW2 pp 229533.I1S1W8
169045 229533.I1RNW2 pp 229533.I1S019
169046 229533.I1RNW2 pp 229533.I1S686
169047 229533.I1RS94 pp 229533.Q4HY71
169048 229533.I1RS94 pp 229533.Q4I7F9
169049 229533.I1RS94 pp 229533.V6REZ4
169050 229533.I1RS94 pp 229533.Q4IJH1
169051 229533.I1RS94 pp 229533.Q4IF76
169052 229533.I1RS94 pp 229533.I1RXZ4
169053 229533.I1RS94 pp 229533.I1S5B5
169054 229533.I1RS94 pp 229533.I1RZV9
169055 229533.I1RS94 pp 229533.Q4I662
169056 229533.I1RS94 pp 229533.I1S246
169057 229533.I1RS94 pp 229533.I1RY40
169058 229533.I1RS94 pp 229533.I1S0P4
169059 229533.I1RS94 pp 229533.I1RYT7
169060 229533.I1RS94 pp 229533.I1RTE5
169061 229533.I1RS94 pp 229533.Q4IAA0
169062 229533.I1RS94 pp 229533.I1RT35
169063 229533.I1RS94 pp 229533.I1RWD3
169064 229533.I1RS94 pp 229533.Q4I7K4
169065 229533.I1RS94 pp 229533.Q4IAS1
169066 229533.I1RS94 pp 229533.I1S471
169067 229533.I1RRU2 pp 229533.I1RWI5
169068 229533.I1RTS4 pp 229533.I1RZ25

169069 229533.I1RFP4 pp 229533.I1RSI1
169070 229533.I1RFP4 pp 229533.I1RSD4
169071 229533.I1RFP4 pp 229533.I1S9J6
169072 229533.I1RFP4 pp 229533.I1S0J3
169073 229533.I1RFP4 pp 229533.Q4IJH1
169074 229533.I1RFP4 pp 229533.Q4IJT0
169075 229533.I1RFP4 pp 229533.Q4IF76
169076 229533.I1RFP4 pp 229533.I1RFQ5
169077 229533.I1RFP4 pp 229533.I1S0N5
169078 229533.I1RFP4 pp 229533.I1RX38
169079 229533.I1RFP4 pp 229533.I1RVY0
169080 229533.I1RFP4 pp 229533.I1RM36
169081 229533.I1RFP4 pp 229533.Q4HTT2
169082 229533.I1RFP4 pp 229533.I1RKQ5
169083 229533.I1RFP4 pp 229533.V6QUE6
169084 229533.I1RFP4 pp 229533.I1RNH1
169085 229533.I1RFP4 pp 229533.Q4IMD1
169086 229533.I1RFP4 pp 229533.I1RRG3
169087 229533.I1RWA3 pp 229533.I1RWG0
169088 229533.I1RWA3 pp 229533.I1RYQ3
169089 229533.I1RWA3 pp 229533.I1S1F3
169090 229533.I1RWA3 pp 229533.I1S0P0
169091 229533.I1RWA3 pp 229533.I1RYC7
169092 229533.I1RWA3 pp 229533.I1RYQ1
169093 229533.I1RWA3 pp 229533.Q4IJW4
169094 229533.I1RWA3 pp 229533.I1RYI5
169095 229533.I1RWA3 pp 229533.I1S5C6
169096 229533.I1RWA3 pp 229533.I1S0T7
169097 229533.I1RWA3 pp 229533.I1S464
169098 229533.I1RWA3 pp 229533.I1RX39
169099 229533.I1RWA3 pp 229533.I1RXD9
169100 229533.I1RWA3 pp 229533.I1S8F0
169101 229533.I1RWA3 pp 229533.I1RY21
169102 229533.I1RWA3 pp 229533.I1RXZ3
169103 229533.I1RWA3 pp 229533.I1RYT8
169104 229533.I1RWA3 pp 229533.I1S0R1
169105 229533.I1RWA3 pp 229533.V6RJP8
169106 229533.I1RWA3 pp 229533.I1S8F8
169107 229533.I1RWA3 pp 229533.I1RXE6
169108 229533.I1RWA3 pp 229533.I1S0U0
169109 229533.I1RWA3 pp 229533.I1RYX5
169110 229533.I1RWA3 pp 229533.I1RYI6
169111 229533.I1RWA3 pp 229533.I1RZ68
169112 229533.I1RWA3 pp 229533.I1S458
169113 229533.I1RWA3 pp 229533.V6RPN0
169114 229533.I1RWA3 pp 229533.I1S1N7
169115 229533.I1RWA3 pp 229533.I1S0Q7
169116 229533.I1RWA3 pp 229533.I1RY92
169117 229533.I1RWA3 pp 229533.I1S5R5
169118 229533.I1RWA3 pp 229533.I1RYM5
169119 229533.I1RWA3 pp 229533.I1S5U0
169120 229533.I1RWA3 pp 229533.I1S266
169121 229533.I1RWA3 pp 229533.I1S0K2
169122 229533.I1RWA3 pp 229533.I1S0R8
169123 229533.I1RWA3 pp 229533.I1S8S9
169124 229533.I1RWA3 pp 229533.I1S212
169125 229533.I1RWA3 pp 229533.I1S5X9
169126 229533.I1RWA3 pp 229533.I1RZ43
169127 229533.I1RSQ9 pp 229533.I1S3G4
169128 229533.I1RSQ9 pp 229533.I1RYQ4
169129 229533.I1RSQ9 pp 229533.I1RY49
169130 229533.I1RSQ9 pp 229533.I1RZT0
169131 229533.I1RSQ9 pp 229533.I1S1W8
169132 229533.I1RSQ9 pp 229533.I1S3K8
169133 229533.A0A1C3YM61 pp 229533.I1R9U0
169134 229533.A0A1C3YM61 pp 229533.I1RKI1
169135 229533.A0A1C3YM61 pp 229533.I1RDE7
169136 229533.A0A1C3YM61 pp 229533.I1RB35
169137 229533.A0A1C3YM61 pp 229533.I1RC35
169138 229533.A0A1C3YM61 pp 229533.I1RNN1
169139 229533.A0A1C3YM61 pp 229533.I1RCX1
169140 229533.A0A1C3YM61 pp 229533.I1RKD3
169141 229533.A0A1C3YM61 pp 229533.I1RDE9

169142 229533.A0A1C3YM61 pp 229533.I1RXB8
169143 229533.A0A1C3YM61 pp 229533.V6RVJ3
169144 229533.A0A1C3YM61 pp 229533.I1RS94
169145 229533.A0A1C3YM61 pp 229533.I1S0S4
169146 229533.A0A1C3YM61 pp 229533.I1SAD2
169147 229533.A0A1C3YM61 pp 229533.Q4IPI4
169148 229533.A0A1C3YM61 pp 229533.I1S8X0
169149 229533.A0A1C3YM61 pp 229533.I1RY35
169150 229533.A0A1C3YM61 pp 229533.I1S2E9
169151 229533.A0A1C3YM61 pp 229533.I1RFL7
169152 229533.A0A1C3YM61 pp 229533.I1RVL8
169153 229533.A0A1C3YM61 pp 229533.I1RZP7
169154 229533.A0A1C3YM61 pp 229533.I1RR95
169155 229533.A0A1C3YM61 pp 229533.I1RMA1
169156 229533.A0A1C3YM61 pp 229533.I1RSZ3
169157 229533.A0A1C3YM61 pp 229533.I1S7X9
169158 229533.A0A1C3YM61 pp 229533.I1RIU0
169159 229533.A0A1C3YM61 pp 229533.I1RPF5
169160 229533.A0A1C3YM61 pp 229533.I1RPX7
169161 229533.A0A1C3YM61 pp 229533.I1RTE0
169162 229533.A0A1C3YM61 pp 229533.I1RRF0
169163 229533.A0A1C3YM61 pp 229533.I1RQL9
169164 229533.A0A1C3YM61 pp 229533.I1RUU5
169165 229533.A0A1C3YM61 pp 229533.I1RD02
169166 229533.A0A1C3YM61 pp 229533.I1S276
169167 229533.A0A1C3YM61 pp 229533.I1RUF8
169168 229533.A0A1C3YM61 pp 229533.I1S5L1
169169 229533.A0A1C3YM61 pp 229533.A0A1C3YMN5
169170 229533.A0A1C3YM61 pp 229533.I1RZT1
169171 229533.A0A1C3YM61 pp 229533.I1R9N1
169172 229533.A0A1C3YM61 pp 229533.I1RNG4
169173 229533.A0A1C3YM61 pp 229533.I1RD70
169174 229533.A0A1C3YM61 pp 229533.I1RQC0
169175 229533.A0A1C3YM61 pp 229533.I1RPG1
169176 229533.A0A1C3YM61 pp 229533.I1RJM9
169177 229533.A0A1C3YM61 pp 229533.I1RPM7
169178 229533.A0A1C3YM61 pp 229533.I1RND7
169179 229533.A0A1C3YM61 pp 229533.I1RTA3
169180 229533.A0A1C3YM61 pp 229533.I1RXU0
169181 229533.A0A1C3YM61 pp 229533.I1RRN5
169182 229533.A0A1C3YM61 pp 229533.I1RTG5
169183 229533.A0A1C3YM61 pp 229533.A0A1C3YM91
169184 229533.A0A1C3YM61 pp 229533.I1RDH0
169185 229533.A0A1C3YM61 pp 229533.I1RRW0
169186 229533.A0A1C3YM61 pp 229533.I1RMK8
169187 229533.A0A1C3YM61 pp 229533.I1RY58
169188 229533.A0A1C3YM61 pp 229533.I1RST4
169189 229533.A0A1C3YM61 pp 229533.Q4I650
169190 229533.A0A1C3YM61 pp 229533.I1S4P4
169191 229533.A0A1C3YM61 pp 229533.I1RY44
169192 229533.A0A1C3YM61 pp 229533.I1R9U3
169193 229533.A0A1C3YM61 pp 229533.I1RJM4
169194 229533.A0A1C3YM61 pp 229533.I1RPK0
169195 229533.A0A1C3YM61 pp 229533.V6QX58
169196 229533.A0A1C3YM61 pp 229533.A0A1C3YN55
169197 229533.A0A1C3YM61 pp 229533.A0A1C3YMC9
169198 229533.A0A1C3YM61 pp 229533.I1RPE5
169199 229533.A0A1C3YM61 pp 229533.I1RHA9
169200 229533.A0A1C3YM61 pp 229533.A0A1C3YN58
169201 229533.A0A1C3YM61 pp 229533.I1RT41
169202 229533.A0A1C3YM61 pp 229533.I1S064
169203 229533.A0A1C3YM61 pp 229533.I1S224
169204 229533.A0A1C3YM61 pp 229533.I1RQ70
169205 229533.A0A1C3YM61 pp 229533.I1RZL9
169206 229533.A0A1C3YM61 pp 229533.I1RW46
169207 229533.A0A1C3YM61 pp 229533.I1RZ94
169208 229533.A0A1C3YM61 pp 229533.I1RWJ8
169209 229533.A0A1C3YM61 pp 229533.Q4IMN9
169210 229533.A0A1C3YM61 pp 229533.I1RI75
169211 229533.A0A1C3YM61 pp 229533.Q4HZS8
169212 229533.A0A1C3YM61 pp 229533.I1RYU3
169213 229533.A0A1C3YM61 pp 229533.I1RAT7
169214 229533.A0A1C3YM61 pp 229533.I1S5U9

229533.A0A1C3YM61 pp 229533.I1RNF3
229533.A0A1C3YM61 pp 229533.I1RL88
229533.A0A1C3YM61 pp 229533.I1RZ92
229533.A0A1C3YM61 pp 229533.I1RKB1
229533.A0A1C3YM61 pp 229533.I1RTQ9
229533.A0A1C3YM61 pp 229533.I1RB22
229533.A0A1C3YM61 pp 229533.I1RZU6
229533.A0A1C3YM61 pp 229533.I1RT65
229533.A0A1C3YM61 pp 229533.I1RYY5
229533.A0A1C3YM61 pp 229533.I1RX84
229533.A0A1C3YM61 pp 229533.I1RAQ4
229533.A0A1C3YM61 pp 229533.I1RU50
229533.A0A1C3YM61 pp 229533.A0A1C3YN54
229533.A0A1C3YM61 pp 229533.I1S130
229533.A0A1C3YM61 pp 229533.Q5I2J3
229533.A0A1C3YM61 pp 229533.I1RTC2
229533.A0A1C3YM61 pp 229533.Q4HYI0
229533.A0A1C3YM61 pp 229533.I1RJH1
229533.A0A1C3YM61 pp 229533.I1RJK7
229533.A0A1C3YM61 pp 229533.I1RHU0
229533.A0A1C3YM61 pp 229533.I1RUK7
229533.A0A1C3YM61 pp 229533.I1REG5
229533.A0A1C3YM61 pp 229533.I1RWT6
229533.A0A1C3YM61 pp 229533.I1RMS2
229533.A0A1C3YM61 pp 229533.I1REN3
229533.A0A1C3YM61 pp 229533.Q4IBX0
229533.A0A1C3YM61 pp 229533.I1RQS4
229533.A0A1C3YM61 pp 229533.I1RU70
229533.A0A1C3YM61 pp 229533.I1RC43
229533.A0A1C3YM61 pp 229533.V6RG22
229533.A0A1C3YM61 pp 229533.I1S0Z3
229533.A0A1C3YM61 pp 229533.Q4IJM4
229533.A0A1C3YM61 pp 229533.I1RN43
229533.A0A1C3YM61 pp 229533.I1RMQ7
229533.A0A1C3YM61 pp 229533.Q4ICA8
229533.A0A1C3YM61 pp 229533.I1RZP6
229533.A0A1C3YM61 pp 229533.I1RQA3
229533.A0A1C3YM61 pp 229533.I1RCN8
229533.A0A1C3YM61 pp 229533.I1RA72
229533.A0A1C3YM61 pp 229533.I1RB27
229533.A0A098D0Q6 pp 229533.I1RCK4
229533.A0A098D0Q6 pp 229533.I1RNB8
229533.A0A098D0Q6 pp 229533.A0A098DIB7
229533.A0A098D0Q6 pp 229533.Q4IAA0
229533.A0A098D0Q6 pp 229533.I1RZE1
229533.A0A098D0Q6 pp 229533.A0A1C3YI81
229533.A0A098D0Q6 pp 229533.I1RZS7
229533.A0A098D0Q6 pp 229533.I1S8U9
229533.A0A098D0Q6 pp 229533.I1RA99
229533.A0A098D0Q6 pp 229533.I1RDY5
229533.A0A098D0Q6 pp 229533.I1RMQ5
229533.A0A098D0Q6 pp 229533.I1RQ49
229533.A0A098D0Q6 pp 229533.I1RSL8
229533.A0A098D0Q6 pp 229533.Q4I7X1
229533.A0A098D0Q6 pp 229533.I1RTE5
229533.A0A098D0Q6 pp 229533.I1RX47
229533.A0A098D0Q6 pp 229533.I1RYP7
229533.A0A098D0Q6 pp 229533.I1RYJ8
229533.A0A098D0Q6 pp 229533.I1RDY2
229533.A0A098D0Q6 pp 229533.I1RRI2
229533.A0A098D0Q6 pp 229533.I1S0P4
229533.A0A098D0Q6 pp 229533.I1RT35
229533.A0A098D0Q6 pp 229533.I1S471
229533.A0A098D0Q6 pp 229533.A0A098DMM9
229533.A0A098D0Q6 pp 229533.I1RMU1
229533.A0A098D0Q6 pp 229533.I1RB43
229533.A0A098D0Q6 pp 229533.I1RRE0
229533.A0A098D0Q6 pp 229533.I1RSR3
229533.A0A098D0Q6 pp 229533.I1RZL5
229533.A0A098D0Q6 pp 229533.Q4IF44
229533.A0A098D0Q6 pp 229533.I1RBZ8
229533.A0A098D0Q6 pp 229533.I1RJS5
229533.A0A098D0Q6 pp 229533.I1RAN0

169288 229533.A0A098D0Q6 pp 229533.I1S5B5
169289 229533.A0A098D0Q6 pp 229533.I1RSM2
169290 229533.A0A098D0Q6 pp 229533.A0A1C3YII4
169291 229533.A0A098D0Q6 pp 229533.I1RAC1
169292 229533.A0A098D0Q6 pp 229533.I1RNE4
169293 229533.A0A098D0Q6 pp 229533.I1RDY1
169294 229533.A0A098D0Q6 pp 229533.A0A1C3YKG7
169295 229533.A0A098D0Q6 pp 229533.I1S5Z2
169296 229533.A0A098D0Q6 pp 229533.A0A1C3YL93
169297 229533.A0A098D0Q6 pp 229533.I1RZV9
169298 229533.A0A098D0Q6 pp 229533.I1RN49
169299 229533.A0A098D0Q6 pp 229533.A0A1C3YJV9
169300 229533.A0A098D0Q6 pp 229533.I1RB53
169301 229533.A0A098D0Q6 pp 229533.I1RND9
169302 229533.A0A098D0Q6 pp 229533.I1RYT7
169303 229533.A0A098D0Q6 pp 229533.I1RY40
169304 229533.A0A098D0Q6 pp 229533.I1RQZ2
169305 229533.A0A098D0Q6 pp 229533.I1S0N7
169306 229533.A0A098D0Q6 pp 229533.I1RCG8
169307 229533.A0A098D0Q6 pp 229533.I1RMY3
169308 229533.A0A098D0Q6 pp 229533.I1S214
169309 229533.A0A098D0Q6 pp 229533.A0A1C3YKM6
169310 229533.A0A098D0Q6 pp 229533.I1RN91
169311 229533.A0A098D0Q6 pp 229533.A0A1C3YKF4
169312 229533.A0A098D0Q6 pp 229533.I1RIR9
169313 229533.A0A098D0Q6 pp 229533.I1RDD3
169314 229533.A0A098D0Q6 pp 229533.V6REZ4
169315 229533.A0A098D0Q6 pp 229533.I1RN60
169316 229533.A0A098D0Q6 pp 229533.I1RA46
169317 229533.A0A098D0Q6 pp 229533.A0A1C3YM64
169318 229533.A0A098D0Q6 pp 229533.I1RMD1
169319 229533.A0A098D0Q6 pp 229533.A0A1C3YL25
169320 229533.A0A098D0Q6 pp 229533.A0A098E365
169321 229533.A0A098D0Q6 pp 229533.I1S8V0
169322 229533.A0A098D0Q6 pp 229533.I1S2A8
169323 229533.A0A098D0Q6 pp 229533.I1RUM5
169324 229533.A0A098D0Q6 pp 229533.Q4HY71
169325 229533.A0A098D0Q6 pp 229533.I1RW24
169326 229533.A0A098D0Q6 pp 229533.I1RYJ9
169327 229533.A0A098D0Q6 pp 229533.A0A1C3YJ51
169328 229533.A0A098D0Q6 pp 229533.A0A0E0S9G3
169329 229533.A0A098D0Q6 pp 229533.I1RPY7
169330 229533.A0A098D0Q6 pp 229533.A0A1C3YIE7
169331 229533.A0A098D0Q6 pp 229533.A0A1C3YLF5
169332 229533.A0A098D0Q6 pp 229533.I1RNT2
169333 229533.A0A098D0Q6 pp 229533.A0A098D2W6
169334 229533.A0A098D0Q6 pp 229533.I1R9X2
169335 229533.A0A098D0Q6 pp 229533.I1RJS4
169336 229533.A0A098D0Q6 pp 229533.I1RCU7
169337 229533.A0A098D0Q6 pp 229533.I1S246
169338 229533.A0A098D0Q6 pp 229533.I1RZP2
169339 229533.A0A098D0Q6 pp 229533.A0A1C3YLD8
169340 229533.A0A098D0Q6 pp 229533.A0A098DEG3
169341 229533.A0A098D0Q6 pp 229533.Q4IPN9
169342 229533.A0A098D0Q6 pp 229533.I1RRW3
169343 229533.A0A098D0Q6 pp 229533.I1RLU5
169344 229533.A0A098D0Q6 pp 229533.I1RBX0
169345 229533.A0A098D0Q6 pp 229533.I1RSR2
169346 229533.A0A098D0Q6 pp 229533.I1RS47
169347 229533.A0A098D0Q6 pp 229533.I1RAI1
169348 229533.A0A098D0Q6 pp 229533.I1S0X5
169349 229533.A0A098D0Q6 pp 229533.I1RQK2
169350 229533.A0A098D0Q6 pp 229533.I1RCH7
169351 229533.A0A098D0Q6 pp 229533.Q4IPA4
169352 229533.A0A098D0Q6 pp 229533.I1RPE3
169353 229533.A0A098D0Q6 pp 229533.Q4IMN9
169354 229533.A0A098D0Q6 pp 229533.I1RS94
169355 229533.A0A098D0Q6 pp 229533.A0A1C3YM61
169356 229533.I1S0R1 pp 229533.I1S5C6
169357 229533.I1S0R1 pp 229533.I1S0R2
169358 229533.I1S0R1 pp 229533.I1S694
169359 229533.I1S0R1 pp 229533.V6RJP8
169360 229533.I1S0R1 pp 229533.I1S8S9

169361 229533.I1S0R1 pp 229533.I1S458
169362 229533.I1S0R1 pp 229533.I1S5X9
169363 229533.I1S0R1 pp 229533.I1S464
169364 229533.I1RYD3 pp 229533.I1S0G6
169365 229533.I1RYD3 pp 229533.I1RZ52
169366 229533.I1RYD3 pp 229533.V6RY56
169367 229533.I1RYD3 pp 229533.O74638
169368 229533.I1RYD3 pp 229533.I1RZ20
169369 229533.I1RYD3 pp 229533.I1RYM0
169370 229533.I1RYD3 pp 229533.I1S326
169371 229533.I1RYD3 pp 229533.I1RYM5
169372 229533.I1RYD3 pp 229533.I1S2E2
169373 229533.I1RYD3 pp 229533.I1RYU5
169374 229533.I1RYD3 pp 229533.I1S029
169375 229533.I1RYD3 pp 229533.V6RH92
169376 229533.I1RYD3 pp 229533.I1S363
169377 229533.I1RYD3 pp 229533.V6RQW4
169378 229533.I1RYD3 pp 229533.I1S2Z4
169379 229533.I1RYD3 pp 229533.I1S4D0
169380 229533.I1RYD3 pp 229533.I1S9D4
169381 229533.A0A1C3YL46 pp 229533.I1RMQ6
169382 229533.A0A1C3YL46 pp 229533.I1S464
169383 229533.A0A1C3YL46 pp 229533.I1RS05
169384 229533.A0A1C3YL46 pp 229533.I1RY92
169385 229533.A0A1C3YL46 pp 229533.I1RPY0
169386 229533.A0A1C3YL46 pp 229533.I1S0R8
169387 229533.A0A1C3YL46 pp 229533.I1S0K2
169388 229533.A0A1C3YL46 pp 229533.I1S128
169389 229533.A0A1C3YL46 pp 229533.I1RP02
169390 229533.A0A1C3YL46 pp 229533.I1S5X9
169391 229533.A0A1C3YL46 pp 229533.I1RNF7
169392 229533.A0A1C3YL46 pp 229533.I1RBT6
169393 229533.A0A1C3YL46 pp 229533.I1RXD9
169394 229533.A0A1C3YL46 pp 229533.I1RE66
169395 229533.A0A1C3YL46 pp 229533.I1S0P0
169396 229533.A0A1C3YL46 pp 229533.I1RS29
169397 229533.A0A1C3YL46 pp 229533.I1RFJ6
169398 229533.A0A1C3YL46 pp 229533.I1S8S9
169399 229533.A0A1C3YL46 pp 229533.I1RB38
169400 229533.A0A1C3YL46 pp 229533.I1RCH9
169401 229533.A0A1C3YL46 pp 229533.I1S8F8
169402 229533.A0A1C3YL46 pp 229533.A0A1C3YLB6
169403 229533.A0A1C3YL46 pp 229533.I1S0Q7
169404 229533.A0A1C3YL46 pp 229533.I1RAM9
169405 229533.A0A1C3YL46 pp 229533.I1S5C6
169406 229533.A0A1C3YL46 pp 229533.I1RYM5
169407 229533.A0A1C3YL46 pp 229533.I1RKF4
169408 229533.A0A1C3YL46 pp 229533.I1RY21
169409 229533.A0A1C3YL46 pp 229533.I1RMK7
169410 229533.A0A1C3YL46 pp 229533.I1RYQ3
169411 229533.A0A1C3YL46 pp 229533.A0A1C3YM56
169412 229533.A0A1C3YL46 pp 229533.I1RE39
169413 229533.A0A1C3YL46 pp 229533.I1RG62
169414 229533.A0A1C3YL46 pp 229533.I1RKF5
169415 229533.A0A1C3YL46 pp 229533.I1RK24
169416 229533.A0A1C3YL46 pp 229533.I1RE65
169417 229533.A0A1C3YL46 pp 229533.I1RYT8
169418 229533.A0A1C3YL46 pp 229533.I1RA52
169419 229533.A0A1C3YL46 pp 229533.I1RE40
169420 229533.A0A1C3YL46 pp 229533.I1RBE6
169421 229533.A0A1C3YL46 pp 229533.I1RWA3
169422 229533.A0A1C3YL46 pp 229533.V6RJP8
169423 229533.I1RQP8 pp 229533.I1S524
169424 229533.I1RQP8 pp 229533.I1S5S2
169425 229533.I1RQP8 pp 229533.I1RZG7
169426 229533.I1RQP8 pp 229533.I1S2Y5
169427 229533.I1RQP8 pp 229533.I1RXF2
169428 229533.I1RQP8 pp 229533.I1RX81
169429 229533.I1RQP8 pp 229533.I1S7M2
169430 229533.I1RQP8 pp 229533.I1RT47
169431 229533.I1RQP8 pp 229533.I1RZ11
169432 229533.I1RQP8 pp 229533.I1RS75
169433 229533.I1RQP8 pp 229533.V6RIS2

169434 229533.I1RQP8 pp 229533.I1RS83
169435 229533.I1RQP8 pp 229533.I1S4M5
169436 229533.I1RQP8 pp 229533.I1RZQ5
169437 229533.I1RQP8 pp 229533.I1RW71
169438 229533.I1RQP8 pp 229533.I1RWU2
169439 229533.I1RQP8 pp 229533.I1RWQ8
169440 229533.I1RQP8 pp 229533.I1S7M3
169441 229533.I1RQP8 pp 229533.I1RX47
169442 229533.I1RQP8 pp 229533.I1RS28
169443 229533.I1RQP8 pp 229533.I1RSD4
169444 229533.I1RQP8 pp 229533.I1RST1
169445 229533.I1RQP8 pp 229533.I1RX51
169446 229533.I1RQP8 pp 229533.I1RSA4
169447 229533.I1RQP8 pp 229533.I1RSA8
169448 229533.I1RQP8 pp 229533.Q4IRI9
169449 229533.I1RQP8 pp 229533.I1RRL0
169450 229533.I1RQP8 pp 229533.V6QXG1
169451 229533.I1RQP8 pp 229533.I1RWX8
169452 229533.I1RQP8 pp 229533.I1RT35
169453 229533.I1RQP8 pp 229533.V6RY33
169454 229533.I1RQP8 pp 229533.I1RYW3
169455 229533.I1RQP8 pp 229533.Q4IB70
169456 229533.I1RQP8 pp 229533.I1S0B1
169457 229533.I1S1E2 pp 229533.I1S1E4
169458 229533.I1S1E2 pp 229533.I1S1E3
169459 229533.A0A098D5D4 pp 229533.I1S2H3
169460 229533.A0A098D5D4 pp 229533.A0A1C3YJ17
169461 229533.A0A098D5D4 pp 229533.I1RYA0
169462 229533.A0A098D5D4 pp 229533.I1RKF2
169463 229533.A0A098D5D4 pp 229533.I1RGL3
169464 229533.A0A098D5D4 pp 229533.A0A098E3X8
169465 229533.A0A098D5D4 pp 229533.I1RZJ3
169466 229533.A0A098D5D4 pp 229533.A0A1C3YHV5
169467 229533.A0A098D5D4 pp 229533.Q4I1N3
169468 229533.A0A098D5D4 pp 229533.I1RY74
169469 229533.A0A098D5D4 pp 229533.I1RS77
169470 229533.A0A098D5D4 pp 229533.A0A098DKG5
169471 229533.A0A098D5D4 pp 229533.A0A098DCQ7
169472 229533.A0A098D5D4 pp 229533.I1RGC6
169473 229533.A0A098D5D4 pp 229533.I1RMU9
169474 229533.A0A098D5D4 pp 229533.I1S5H2
169475 229533.A0A098D5D4 pp 229533.V6QZS3
169476 229533.A0A098D5D4 pp 229533.A0A1C3YHU7
169477 229533.A0A098D5D4 pp 229533.A0A1C3YHP8
169478 229533.A0A098D5D4 pp 229533.I1R9U7
169479 229533.A0A098D5D4 pp 229533.I1RBA4
169480 229533.A0A098D5D4 pp 229533.Q4I1Y5
169481 229533.A0A098D5D4 pp 229533.I1RQ30
169482 229533.A0A098D5D4 pp 229533.A0A1C3YKG0
169483 229533.A0A098D5D4 pp 229533.I1RK75
169484 229533.A0A098D5D4 pp 229533.I1RYC8
169485 229533.A0A098D5D4 pp 229533.A0A1C3YLT9
169486 229533.A0A098D5D4 pp 229533.I1RC08
169487 229533.A0A098D5D4 pp 229533.I1RCB4
169488 229533.A0A098D5D4 pp 229533.I1RE79
169489 229533.A0A098D5D4 pp 229533.I1S147
169490 229533.A0A098D5D4 pp 229533.I1RFX5
169491 229533.A0A098D5D4 pp 229533.I1RFK3
169492 229533.A0A098D5D4 pp 229533.I1RWF4
169493 229533.A0A098D5D4 pp 229533.I1RRZ6
169494 229533.A0A098D5D4 pp 229533.I1RN84
169495 229533.A0A098D5D4 pp 229533.I1RG46
169496 229533.A0A098D5D4 pp 229533.I1RA65
169497 229533.A0A098D5D4 pp 229533.I1RP70
169498 229533.A0A098D5D4 pp 229533.I1RLW5
169499 229533.A0A098D5D4 pp 229533.I1S0G6
169500 229533.A0A098D5D4 pp 229533.A0A1C3YLB6
169501 229533.A0A098D5D4 pp 229533.A0A0E0SH57
169502 229533.A0A098D5D4 pp 229533.V6RLM7
169503 229533.A0A098D5D4 pp 229533.I1RQ21
169504 229533.A0A098D5D4 pp 229533.I1RSF1
169505 229533.A0A098D5D4 pp 229533.I1S490
169506 229533.A0A098D5D4 pp 229533.A0A1C3YJF9

169507 229533.A0A098D5D4 pp 229533.I1RFI4
169508 229533.A0A098D5D4 pp 229533.I1RMH4
169509 229533.A0A098D5D4 pp 229533.I1RE52
169510 229533.A0A098D5D4 pp 229533.I1RKP5
169511 229533.A0A098D5D4 pp 229533.I1RFI8
169512 229533.A0A098D5D4 pp 229533.I1RTI7
169513 229533.A0A098D5D4 pp 229533.I1RPY0
169514 229533.A0A098D5D4 pp 229533.A0A1C3YIS7
169515 229533.A0A098D5D4 pp 229533.A0A098E3T0
169516 229533.A0A098D5D4 pp 229533.I1RZ63
169517 229533.A0A098D5D4 pp 229533.A0A1C3YK57
169518 229533.A0A098D5D4 pp 229533.I1RB58
169519 229533.A0A098D5D4 pp 229533.I1RZ52
169520 229533.A0A098D5D4 pp 229533.V6RVJ3
169521 229533.A0A098D5D4 pp 229533.A0A098DJ03
169522 229533.A0A098D5D4 pp 229533.A0A1C3YKK9
169523 229533.A0A098D5D4 pp 229533.A0A1C3YHI3
169524 229533.A0A098D5D4 pp 229533.I1S3L8
169525 229533.A0A098D5D4 pp 229533.A0A098DAD8
169526 229533.A0A098D5D4 pp 229533.O74638
169527 229533.A0A098D5D4 pp 229533.I1RBF3
169528 229533.A0A098D5D4 pp 229533.A0A1C3YLL1
169529 229533.A0A098D5D4 pp 229533.I1REV2
169530 229533.A0A098D5D4 pp 229533.I1RGJ3
169531 229533.A0A098D5D4 pp 229533.I1S0J7
169532 229533.A0A098D5D4 pp 229533.I1RKP1
169533 229533.A0A098D5D4 pp 229533.I1RG58
169534 229533.A0A098D5D4 pp 229533.I1R9K3
169535 229533.A0A098D5D4 pp 229533.V6QV31
169536 229533.A0A098D5D4 pp 229533.I1RZU2
169537 229533.A0A098D5D4 pp 229533.I1RG71
169538 229533.A0A098D5D4 pp 229533.Q4HXF0
169539 229533.A0A098D5D4 pp 229533.I1RMJ3
169540 229533.A0A098D5D4 pp 229533.I1RSV2
169541 229533.A0A098D5D4 pp 229533.A0A1C3YM01
169542 229533.A0A098D5D4 pp 229533.A0A1C3YLE2
169543 229533.A0A098D5D4 pp 229533.A0A1C3YN70
169544 229533.A0A098D5D4 pp 229533.A0A098DZL0
169545 229533.A0A098D5D4 pp 229533.A0A1C3YJ21
169546 229533.A0A098D5D4 pp 229533.I1RZ25
169547 229533.A0A098D5D4 pp 229533.I1S326
169548 229533.A0A098D5D4 pp 229533.I1RAP1
169549 229533.A0A098D5D4 pp 229533.I1RZ90
169550 229533.A0A098D5D4 pp 229533.A0A098E2M3
169551 229533.A0A098D5D4 pp 229533.A0A1C3YMC1
169552 229533.A0A098D5D4 pp 229533.A0A098DE31
169553 229533.A0A098D5D4 pp 229533.A0A1C3YLU9
169554 229533.A0A098D5D4 pp 229533.A0A098DH99
169555 229533.A0A098D5D4 pp 229533.I1RWF9
169556 229533.A0A098D5D4 pp 229533.A0A098DF57
169557 229533.A0A098D5D4 pp 229533.A0A1C3YJP4
169558 229533.A0A098D5D4 pp 229533.I1RMG6
169559 229533.A0A098D5D4 pp 229533.A0A0E0SQJ6
169560 229533.A0A098D5D4 pp 229533.I1RE53
169561 229533.A0A098D5D4 pp 229533.I1RNT1
169562 229533.A0A098D5D4 pp 229533.I1RMG7
169563 229533.A0A098D5D4 pp 229533.I1RFB0
169564 229533.A0A098D5D4 pp 229533.I1S4F4
169565 229533.A0A098D5D4 pp 229533.I1RZD0
169566 229533.A0A098D5D4 pp 229533.I1RCL0
169567 229533.A0A098D5D4 pp 229533.I1RNG4
169568 229533.A0A098D5D4 pp 229533.A0A1C3YM61
169569 229533.A0A098D5D4 pp 229533.A0A1C3YI88
169570 229533.A0A098D5D4 pp 229533.V6RGB5
169571 229533.A0A098D5D4 pp 229533.I1R9E0
169572 229533.A0A098D5D4 pp 229533.I1RAM9
169573 229533.A0A098D5D4 pp 229533.I1RLI4
169574 229533.A0A098D5D4 pp 229533.I1RW11
169575 229533.A0A098D5D4 pp 229533.I1RZ20
169576 229533.A0A098D5D4 pp 229533.V6RQW4
169577 229533.A0A098D5D4 pp 229533.I1RL81
169578 229533.A0A098D5D4 pp 229533.V6RDH3
169579 229533.A0A098D5D4 pp 229533.I1RY81

169580	229533.A0A098D5D4	pp	229533.A0A1C3YL98
169581	229533.A0A098D5D4	pp	229533.I1RQ48
169582	229533.A0A098D5D4	pp	229533.I1RK03
169583	229533.A0A098D5D4	pp	229533.I1RBE7
169584	229533.A0A098D5D4	pp	229533.I1RT88
169585	229533.A0A098D5D4	pp	229533.I1RQE0
169586	229533.A0A098D5D4	pp	229533.I1S2Z4
169587	229533.A0A098D5D4	pp	229533.I1RBU8
169588	229533.A0A098D5D4	pp	229533.I1RN80
169589	229533.A0A098D5D4	pp	229533.A0A1C3YKF3
169590	229533.A0A098D5D4	pp	229533.I1RSR6
169591	229533.A0A098D5D4	pp	229533.V6RY56
169592	229533.A0A098D5D4	pp	229533.I1RIY0
169593	229533.A0A098D5D4	pp	229533.I1S2C8
169594	229533.A0A098D5D4	pp	229533.A0A098DTS1
169595	229533.A0A098D5D4	pp	229533.A0A098DGA1
169596	229533.A0A098D5D4	pp	229533.I1RG33
169597	229533.A0A098D5D4	pp	229533.I1RCV1
169598	229533.A0A098D5D4	pp	229533.I1RUL3
169599	229533.A0A098D5D4	pp	229533.I1RLR2
169600	229533.A0A098D5D4	pp	229533.A0A0E0SMY1
169601	229533.A0A098D5D4	pp	229533.A0A098D6Y5
169602	229533.A0A098D5D4	pp	229533.A0A1C3YJH4
169603	229533.A0A098D5D4	pp	229533.A0A1C3YLC2
169604	229533.A0A098D5D4	pp	229533.V6RKK5
169605	229533.A0A098D5D4	pp	229533.A0A0E0SFT7
169606	229533.A0A098D5D4	pp	229533.I1RJT5
169607	229533.A0A098D5D4	pp	229533.I1S0K2
169608	229533.A0A098D5D4	pp	229533.I1RYM5
169609	229533.A0A098D5D4	pp	229533.I1RGG6
169610	229533.A0A098D5D4	pp	229533.I1RK47
169611	229533.A0A098D5D4	pp	229533.V6QVL9
169612	229533.A0A098D5D4	pp	229533.I1S4D0
169613	229533.A0A098D5D4	pp	229533.I1S1F5
169614	229533.A0A098D5D4	pp	229533.I1RJA8
169615	229533.A0A098D5D4	pp	229533.I1RMB6
169616	229533.A0A098D5D4	pp	229533.I1RVW6
169617	229533.A0A098D5D4	pp	229533.I1S267
169618	229533.A0A098D5D4	pp	229533.I1RIE8
169619	229533.A0A098D5D4	pp	229533.I1RB11
169620	229533.A0A098D5D4	pp	229533.I1RXB6
169621	229533.A0A098D5D4	pp	229533.I1RC48
169622	229533.A0A098D5D4	pp	229533.I1S235
169623	229533.A0A098D5D4	pp	229533.I1RVV7
169624	229533.A0A098D5D4	pp	229533.I1RST9
169625	229533.A0A098D5D4	pp	229533.I1S2E2
169626	229533.A0A098D5D4	pp	229533.I1RTJ2
169627	229533.A0A098D5D4	pp	229533.I1S8F8
169628	229533.A0A098D5D4	pp	229533.A0A1C3YK94
169629	229533.A0A098D5D4	pp	229533.I1RE90
169630	229533.A0A098D5D4	pp	229533.I1S4L8
169631	229533.A0A098D5D4	pp	229533.V6RH92
169632	229533.A0A098D5D4	pp	229533.A0A0E0SN30
169633	229533.A0A098D5D4	pp	229533.I1RLA2
169634	229533.A0A098D5D4	pp	229533.I1RCN8
169635	229533.A0A098D5D4	pp	229533.I1RHM1
169636	229533.A0A098D5D4	pp	229533.I1RWG5
169637	229533.A0A098D5D4	pp	229533.I1S049
169638	229533.A0A098D5D4	pp	229533.I1RDZ7
169639	229533.A0A098D5D4	pp	229533.I1RMA2
169640	229533.A0A098D5D4	pp	229533.I1RZE7
169641	229533.A0A098D5D4	pp	229533.I1RYM0
169642	229533.A0A098D5D4	pp	229533.I1RE48
169643	229533.A0A098D5D4	pp	229533.I1RYV4
169644	229533.A0A098D5D4	pp	229533.I1S029
169645	229533.A0A098D5D4	pp	229533.I1S363
169646	229533.A0A098D5D4	pp	229533.I1RWY2
169647	229533.A0A098D5D4	pp	229533.I1RY21
169648	229533.A0A098D5D4	pp	229533.I1RAN8
169649	229533.A0A098D5D4	pp	229533.A0A098DPB8
169650	229533.A0A098D5D4	pp	229533.I1RQ76
169651	229533.A0A098D5D4	pp	229533.I1RTT0
169652	229533.A0A098D5D4	pp	229533.I1RGK9

169653 229533.A0A098D5D4 pp 229533.I1REI0
169654 229533.A0A098D5D4 pp 229533.I1RC11
169655 229533.A0A098D5D4 pp 229533.A0A1C3YLR6
169656 229533.A0A098D5D4 pp 229533.I1RHK2
169657 229533.A0A098D5D4 pp 229533.V6R6H5
169658 229533.A0A098D5D4 pp 229533.V6RS91
169659 229533.A0A098D5D4 pp 229533.I1RYU5
169660 229533.A0A098D5D4 pp 229533.I1RDU5
169661 229533.A0A098D5D4 pp 229533.A0A098DVZ4
169662 229533.A0A098D5D4 pp 229533.I1RM49
169663 229533.A0A098D5D4 pp 229533.I1RQL4
169664 229533.A0A098D5D4 pp 229533.A0A1C3YI71
169665 229533.A0A098D5D4 pp 229533.I1RZP0
169666 229533.A0A098D5D4 pp 229533.A0A0E0RU85
169667 229533.A0A098D5D4 pp 229533.Q4HZQ1
169668 229533.A0A098D5D4 pp 229533.I1RH36
169669 229533.A0A098D5D4 pp 229533.A0A0E0RNI4
169670 229533.A0A098D5D4 pp 229533.I1S9D4
169671 229533.A0A098D5D4 pp 229533.I1RD42
169672 229533.A0A098D5D4 pp 229533.I1RYD3
169673 229533.A0A098D5D4 pp 229533.I1RD21
169674 229533.A0A098D5D4 pp 229533.I1RT62
169675 229533.A0A098D5D4 pp 229533.A0A1C3YKZ8
169676 229533.A0A098D5D4 pp 229533.I1S130
169677 229533.A0A098D5D4 pp 229533.A0A0E0SQ99
169678 229533.A0A098D5D4 pp 229533.I1RPN1
169679 229533.A0A098D5D4 pp 229533.I1RBT7
169680 229533.A0A098D5D4 pp 229533.I1S6D7
169681 229533.A0A098D5D4 pp 229533.I1S767
169682 229533.A0A098D5D4 pp 229533.A0A0E0RX50
169683 229533.I1S5C6 pp 229533.I1S5X9
169684 229533.I1S5C6 pp 229533.I1S8S9
169685 229533.I1S5C6 pp 229533.V6RJP8
169686 229533.A0A098D0P1 pp 229533.I1RYD3
169687 229533.A0A098D0P1 pp 229533.A0A098D5D4
169688 229533.I1RTK1 pp 229533.I1RTK5
169689 229533.I1RTK1 pp 229533.I1S0K2
169690 229533.I1RTK1 pp 229533.Q4I5G1
169691 229533.I1RRQ4 pp 229533.I1RZZ1
169692 229533.I1RRQ4 pp 229533.Q4I7F9
169693 229533.I1RRQ4 pp 229533.I1RT47
169694 229533.I1RRQ4 pp 229533.I1RSS4
169695 229533.I1RRQ4 pp 229533.I1RSA4
169696 229533.I1RRQ4 pp 229533.V6RIS2
169697 229533.I1RRQ4 pp 229533.I1RS83
169698 229533.I1RRQ4 pp 229533.I1RYW3
169699 229533.I1RRQ4 pp 229533.V6RY33
169700 229533.I1RRQ4 pp 229533.I1RWX8
169701 229533.I1RRQ4 pp 229533.I1S0B1
169702 229533.I1RRQ4 pp 229533.I1RSD4
169703 229533.I1RRQ4 pp 229533.I1RX70
169704 229533.I1RRQ4 pp 229533.I1S0Z9
169705 229533.I1RRQ4 pp 229533.I1S0B6
169706 229533.I1RRQ4 pp 229533.I1RUQ6
169707 229533.I1RRQ4 pp 229533.I1RX81
169708 229533.I1RRQ4 pp 229533.I1S524
169709 229533.I1RRQ4 pp 229533.I1S5S2
169710 229533.I1RRQ4 pp 229533.I1S4Y4
169711 229533.I1RRQ4 pp 229533.I1S4M5
169712 229533.I1RY92 pp 229533.I1RYT8
169713 229533.I1RY92 pp 229533.I1RYI6
169714 229533.I1RY92 pp 229533.I1S0R1
169715 229533.I1RY92 pp 229533.I1S0U0
169716 229533.I1RY92 pp 229533.I1S1W8
169717 229533.I1RY92 pp 229533.I1S5C6
169718 229533.I1RY92 pp 229533.I1S0T7
169719 229533.I1RY92 pp 229533.I1RYC7
169720 229533.I1RY92 pp 229533.I1S8F8
169721 229533.I1RY92 pp 229533.V6RPN0
169722 229533.I1RY92 pp 229533.I1S1N7
169723 229533.I1RY92 pp 229533.I1S0P0
169724 229533.I1RY92 pp 229533.Q4I5G1
169725 229533.I1RY92 pp 229533.I1S4S7

```
229533.I1RY92   pp  229533.V6RXW9
229533.I1RY92   pp  229533.I1S0K2
229533.I1RY92   pp  229533.I1S0V6
229533.I1RY92   pp  229533.I1RYX5
229533.I1RY92   pp  229533.I1S8S9
229533.I1RY92   pp  229533.V6RJP8
229533.I1RY92   pp  229533.Q4I5R9
229533.I1RY92   pp  229533.I1RZM8
229533.I1RY92   pp  229533.I1S5R5
229533.I1RY92   pp  229533.I1S212
229533.I1RY92   pp  229533.I1RZ39
229533.I1RY92   pp  229533.I1RZ68
229533.I1RY92   pp  229533.I1S458
229533.I1RY92   pp  229533.I1S5X9
229533.I1RY92   pp  229533.I1RYI5
229533.I1RY92   pp  229533.I1S464
229533.I1RY92   pp  229533.I1RYA0
229533.I1RY92   pp  229533.I1S3G4
229533.I1RY92   pp  229533.I1S266
229533.I1RY92   pp  229533.I1S7W5
229533.I1RY92   pp  229533.I1S5U0
229533.I1RY92   pp  229533.I1S1F3
229533.I1RY92   pp  229533.I1S7X9
229533.I1RY92   pp  229533.I1S0R8
229533.I1RY92   pp  229533.I1S0Q7
229533.I1RY92   pp  229533.I1S0V7
229533.I1RY92   pp  229533.I1S6Y3
229533.I1RY92   pp  229533.V6RLM7
229533.I1RY92   pp  229533.I1RYC8
229533.I1S1E3   pp  229533.I1S1E4
229533.A0A1C3YIN0   pp  229533.I1S635
229533.A0A1C3YIN0   pp  229533.I1RU69
229533.A0A1C3YIN0   pp  229533.I1RTF0
229533.A0A1C3YIN0   pp  229533.I1RPH7
229533.A0A1C3YIN0   pp  229533.I1REN4
229533.A0A1C3YIN0   pp  229533.I1RNR3
229533.A0A1C3YIN0   pp  229533.I1SAT1
229533.A0A1C3YIN0   pp  229533.V6RQ48
229533.A0A1C3YIN0   pp  229533.I1S9Z3
229533.A0A1C3YIN0   pp  229533.I1RNK3
229533.A0A1C3YIN0   pp  229533.I1RE20
229533.A0A1C3YIN0   pp  229533.I1RE15
229533.A0A1C3YIN0   pp  229533.I1RX24
229533.A0A1C3YIN0   pp  229533.A0A1C3YJH8
229533.A0A1C3YIN0   pp  229533.V6RKE4
229533.A0A1C3YIN0   pp  229533.I1RSP4
229533.A0A1C3YIN0   pp  229533.I1RTD0
229533.A0A1C3YIN0   pp  229533.I1REF9
229533.A0A1C3YIN0   pp  229533.I1RPW9
229533.A0A1C3YIN0   pp  229533.A0A1C3YK51
229533.A0A1C3YIN0   pp  229533.I1RH68
229533.A0A1C3YIN0   pp  229533.Q4ICG5
229533.A0A1C3YIN0   pp  229533.I1RTB7
229533.A0A1C3YIN0   pp  229533.I1S2E9
229533.A0A1C3YIN0   pp  229533.I1RW76
229533.A0A1C3YIN0   pp  229533.I1RD56
229533.A0A1C3YIN0   pp  229533.I1RXU1
229533.A0A1C3YIN0   pp  229533.I1RV91
229533.A0A1C3YIN0   pp  229533.I1RLP1
229533.A0A1C3YIN0   pp  229533.I1RQN9
229533.A0A1C3YIN0   pp  229533.I1S0S6
229533.A0A1C3YIN0   pp  229533.I1RW32
229533.A0A1C3YIN0   pp  229533.I1RSL9
229533.A0A1C3YIN0   pp  229533.I1RS38
229533.A0A1C3YIN0   pp  229533.I1RWA5
229533.A0A1C3YIN0   pp  229533.I1RGA4
229533.A0A1C3YIN0   pp  229533.I1RLX5
229533.A0A1C3YIN0   pp  229533.I1RQA3
229533.A0A1C3YIN0   pp  229533.I1RAC1
229533.A0A1C3YIN0   pp  229533.I1RZA4
229533.A0A1C3YIN0   pp  229533.I1S7X9
229533.A0A1C3YIN0   pp  229533.I1RW84
229533.A0A1C3YIN0   pp  229533.A0A1C3YJ29
```

169799 229533.A0A1C3YIN0 pp 229533.I1RCN5
169800 229533.A0A1C3YIN0 pp 229533.I1S1X9
169801 229533.A0A1C3YIN0 pp 229533.I1RWB0
169802 229533.A0A1C3YIN0 pp 229533.I1RZ91
169803 229533.A0A1C3YIN0 pp 229533.I1RRN5
169804 229533.A0A1C3YIN0 pp 229533.V6R042
169805 229533.A0A1C3YIN0 pp 229533.I1RYB1
169806 229533.A0A1C3YIN0 pp 229533.I1RNW8
169807 229533.A0A1C3YIN0 pp 229533.I1RXF5
169808 229533.A0A1C3YIN0 pp 229533.I1S0Z9
169809 229533.A0A1C3YIN0 pp 229533.I1RB41
169810 229533.A0A1C3YIN0 pp 229533.P0C431
169811 229533.A0A1C3YIN0 pp 229533.I1RCK2
169812 229533.A0A1C3YIN0 pp 229533.I1RC17
169813 229533.A0A1C3YIN0 pp 229533.V6R9X9
169814 229533.A0A1C3YIN0 pp 229533.I1S7W5
169815 229533.A0A1C3YIN0 pp 229533.I1RX09
169816 229533.A0A1C3YIN0 pp 229533.I1RWA9
169817 229533.A0A1C3YIN0 pp 229533.I1RPK5
169818 229533.A0A1C3YIN0 pp 229533.I1RM25
169819 229533.A0A1C3YIN0 pp 229533.I1RY44
169820 229533.A0A1C3YIN0 pp 229533.I1RWT6
169821 229533.A0A1C3YIN0 pp 229533.I1RLT6
169822 229533.A0A1C3YJP7 pp 229533.I1S1E2
169823 229533.A0A1C3YJP7 pp 229533.I1S1E4
169824 229533.A0A1C3YJP7 pp 229533.I1S1E3
169825 229533.I1RPL3 pp 229533.Q4IE79
169826 229533.I1RPL3 pp 229533.I1RTD9
169827 229533.I1RPL3 pp 229533.Q4IF76
169828 229533.I1RPL3 pp 229533.Q4IPB8
169829 229533.I1RPL3 pp 229533.I1RX47
169830 229533.I1RPL3 pp 229533.I1RU69
169831 229533.I1RPL3 pp 229533.I1RQC5
169832 229533.I1RPL3 pp 229533.V6QXG1
169833 229533.I1RPL3 pp 229533.I1RSW2
169834 229533.I1RPL3 pp 229533.I1RSA4
169835 229533.I1RPL3 pp 229533.I1S0B1
169836 229533.I1RPL3 pp 229533.I1RY50
169837 229533.I1RPL3 pp 229533.Q4IAA0
169838 229533.I1RPL3 pp 229533.I1S4M5
169839 229533.I1RPL3 pp 229533.I1S056
169840 229533.I1RPL3 pp 229533.Q4IRI9
169841 229533.I1RPL3 pp 229533.I1RT17
169842 229533.I1RPL3 pp 229533.I1RST1
169843 229533.I1RPL3 pp 229533.I1RRL0
169844 229533.I1RPL3 pp 229533.I1RZQ5
169845 229533.I1RPL3 pp 229533.I1RPZ3
169846 229533.I1RPL3 pp 229533.I1RZG7
169847 229533.I1RPL3 pp 229533.I1S246
169848 229533.I1RPL3 pp 229533.Q4IJH1
169849 229533.I1RPL3 pp 229533.I1RPY8
169850 229533.I1RPL3 pp 229533.Q4IBK5
169851 229533.I1RPL3 pp 229533.Q4I1Y1
169852 229533.I1RPL3 pp 229533.I1RS28
169853 229533.I1RPL3 pp 229533.I1RPY4
169854 229533.Q4ICG5 pp 229533.V6RQ48
169855 229533.Q4ICG5 pp 229533.V6RKE4
169856 229533.Q4ICG5 pp 229533.V6R042
169857 229533.A0A0E0SAR4 pp 229533.I1RBB1
169858 229533.A0A0E0SAR4 pp 229533.I1RDH5
169859 229533.A0A0E0SAR4 pp 229533.I1RMK7
169860 229533.A0A0E0SAR4 pp 229533.I1RAI4
169861 229533.A0A0E0SAR4 pp 229533.I1S1W8
169862 229533.A0A0E0SAR4 pp 229533.I1RNA0
169863 229533.A0A0E0SAR4 pp 229533.I1RW54
169864 229533.A0A0E0SAR4 pp 229533.I1RMK3
169865 229533.A0A0E0SAR4 pp 229533.I1RNM7
169866 229533.A0A0E0SAR4 pp 229533.I1RX17
169867 229533.A0A0E0SAR4 pp 229533.I1S0U0
169868 229533.A0A0E0SAR4 pp 229533.A0A1C3YLR6
169869 229533.A0A0E0SAR4 pp 229533.I1RMQ6
169870 229533.A0A0E0SAR4 pp 229533.I1RKB7
169871 229533.A0A0E0SAR4 pp 229533.I1RW25

169872	229533.A0A0E0SAR4	pp	229533.A0A1C3YK11
169873	229533.A0A0E0SAR4	pp	229533.I1RZT4
169874	229533.A0A0E0SAR4	pp	229533.I1RTF4
169875	229533.A0A0E0SAR4	pp	229533.I1RNF7
169876	229533.A0A0E0SAR4	pp	229533.I1R9Y4
169877	229533.A0A0E0SAR4	pp	229533.I1RG62
169878	229533.A0A0E0SAR4	pp	229533.I1RBT1
169879	229533.A0A0E0SAR4	pp	229533.I1REF0
169880	229533.A0A0E0SAR4	pp	229533.V6RLW2
169881	229533.A0A0E0SAR4	pp	229533.I1RYT8
169882	229533.A0A0E0SAR4	pp	229533.I1RRX5
169883	229533.A0A0E0SAR4	pp	229533.I1S1N7
169884	229533.A0A0E0SAR4	pp	229533.I1RWN3
169885	229533.A0A0E0SAR4	pp	229533.I1S9U2
169886	229533.A0A0E0SAR4	pp	229533.I1RTH7
169887	229533.A0A0E0SAR4	pp	229533.I1RRK9
169888	229533.A0A0E0SAR4	pp	229533.I1S4S7
169889	229533.A0A0E0SAR4	pp	229533.I1RZW7
169890	229533.A0A0E0SAR4	pp	229533.I1RLP1
169891	229533.A0A0E0SAR4	pp	229533.I1RR24
169892	229533.A0A0E0SAR4	pp	229533.I1S0T7
169893	229533.A0A0E0SAR4	pp	229533.I1RS05
169894	229533.A0A0E0SAR4	pp	229533.A0A1C3YLM6
169895	229533.A0A0E0SAR4	pp	229533.I1RCU2
169896	229533.A0A0E0SAR4	pp	229533.I1RGJ4
169897	229533.A0A0E0SAR4	pp	229533.I1S0R1
169898	229533.A0A0E0SAR4	pp	229533.I1S287
169899	229533.A0A0E0SAR4	pp	229533.I1RM67
169900	229533.A0A0E0SAR4	pp	229533.I1RWG0
169901	229533.A0A0E0SAR4	pp	229533.I1RYQ3
169902	229533.A0A0E0SAR4	pp	229533.I1RY21
169903	229533.A0A0E0SAR4	pp	229533.I1RYU6
169904	229533.A0A0E0SAR4	pp	229533.I1S5C6
169905	229533.A0A0E0SAR4	pp	229533.I1RMK4
169906	229533.A0A0E0SAR4	pp	229533.I1RT98
169907	229533.A0A0E0SAR4	pp	229533.A0A1C3YIW9
169908	229533.A0A0E0SAR4	pp	229533.I1RJB3
169909	229533.A0A0E0SAR4	pp	229533.I1RA49
169910	229533.A0A0E0SAR4	pp	229533.I1S0P0
169911	229533.A0A0E0SAR4	pp	229533.I1RKF5
169912	229533.A0A0E0SAR4	pp	229533.V6RTJ8
169913	229533.A0A0E0SAR4	pp	229533.I1RUQ3
169914	229533.A0A0E0SAR4	pp	229533.I1RE66
169915	229533.A0A0E0SAR4	pp	229533.I1RJY7
169916	229533.A0A0E0SAR4	pp	229533.I1RYC7
169917	229533.A0A0E0SAR4	pp	229533.Q4I5Z5
169918	229533.A0A0E0SAR4	pp	229533.Q4I5R9
169919	229533.A0A0E0SAR4	pp	229533.I1R9V7
169920	229533.A0A0E0SAR4	pp	229533.Q4I5G1
169921	229533.A0A0E0SAR4	pp	229533.I1S0K2
169922	229533.A0A0E0SAR4	pp	229533.I1RPR1
169923	229533.A0A0E0SAR4	pp	229533.A0A1C3YJW9
169924	229533.A0A0E0SAR4	pp	229533.I1RG33
169925	229533.A0A0E0SAR4	pp	229533.A0A1C3YKY4
169926	229533.A0A0E0SAR4	pp	229533.A0A1C3YK68
169927	229533.A0A0E0SAR4	pp	229533.I1RBU8
169928	229533.A0A0E0SAR4	pp	229533.A0A1C3YN89
169929	229533.A0A0E0SAR4	pp	229533.I1RS29
169930	229533.A0A0E0SAR4	pp	229533.A0A1C3YLB0
169931	229533.A0A0E0SAR4	pp	229533.I1RP38
169932	229533.A0A0E0SAR4	pp	229533.I1RAZ3
169933	229533.A0A0E0SAR4	pp	229533.I1RAV5
169934	229533.A0A0E0SAR4	pp	229533.V6RCZ8
169935	229533.A0A0E0SAR4	pp	229533.I1RK24
169936	229533.A0A0E0SAR4	pp	229533.A0A1C3YIF3
169937	229533.A0A0E0SAR4	pp	229533.A0A1C3YIK4
169938	229533.A0A0E0SAR4	pp	229533.V6RPN0
169939	229533.A0A0E0SAR4	pp	229533.A0A1C3YLB3
169940	229533.A0A0E0SAR4	pp	229533.I1RAM9
169941	229533.A0A0E0SAR4	pp	229533.I1S8S9
169942	229533.A0A0E0SAR4	pp	229533.I1RBE4
169943	229533.A0A0E0SAR4	pp	229533.A0A1C3YHX6
169944	229533.A0A0E0SAR4	pp	229533.A0A1C3YMC4

169945 229533.A0A0E0SAR4 pp 229533.I1RPR9
169946 229533.A0A0E0SAR4 pp 229533.A0A1C3YKV3
169947 229533.A0A0E0SAR4 pp 229533.I1RT96
169948 229533.A0A0E0SAR4 pp 229533.I1RNH6
169949 229533.A0A0E0SAR4 pp 229533.I1RA68
169950 229533.A0A0E0SAR4 pp 229533.A0A1C3YK34
169951 229533.A0A0E0SAR4 pp 229533.A0A1C3YN86
169952 229533.A0A0E0SAR4 pp 229533.I1S212
169953 229533.A0A0E0SAR4 pp 229533.I1RZ68
169954 229533.A0A0E0SAR4 pp 229533.A0A1C3YKY6
169955 229533.A0A0E0SAR4 pp 229533.I1RWB0
169956 229533.A0A0E0SAR4 pp 229533.I1RZM8
169957 229533.A0A0E0SAR4 pp 229533.A0A1C3YIJ9
169958 229533.A0A0E0SAR4 pp 229533.A0A1C3YKB0
169959 229533.A0A0E0SAR4 pp 229533.V6RJP8
169960 229533.A0A0E0SAR4 pp 229533.I1RPY0
169961 229533.A0A0E0SAR4 pp 229533.I1RNY6
169962 229533.A0A0E0SAR4 pp 229533.A0A1C3YHJ9
169963 229533.A0A0E0SAR4 pp 229533.I1RWA3
169964 229533.A0A0E0SAR4 pp 229533.I1RCH9
169965 229533.A0A0E0SAR4 pp 229533.I1RGK5
169966 229533.A0A0E0SAR4 pp 229533.A0A1C3YJN3
169967 229533.A0A0E0SAR4 pp 229533.I1R9N8
169968 229533.A0A0E0SAR4 pp 229533.A0A1C3YL46
169969 229533.A0A0E0SAR4 pp 229533.I1RN88
169970 229533.A0A0E0SAR4 pp 229533.I1S8F8
169971 229533.A0A0E0SAR4 pp 229533.I1RW13
169972 229533.A0A0E0SAR4 pp 229533.I1S5R5
169973 229533.A0A0E0SAR4 pp 229533.I1S5B0
169974 229533.A0A0E0SAR4 pp 229533.I1RSF2
169975 229533.A0A0E0SAR4 pp 229533.A0A1C3YK94
169976 229533.A0A0E0SAR4 pp 229533.I1R9Y8
169977 229533.A0A0E0SAR4 pp 229533.A0A1C3YMQ5
169978 229533.A0A0E0SAR4 pp 229533.A0A1C3YI20
169979 229533.A0A0E0SAR4 pp 229533.I1RVN1
169980 229533.A0A0E0SAR4 pp 229533.I1RXE6
169981 229533.A0A0E0SAR4 pp 229533.I1S458
169982 229533.A0A0E0SAR4 pp 229533.I1RGC3
169983 229533.A0A0E0SAR4 pp 229533.I1RP02
169984 229533.A0A0E0SAR4 pp 229533.I1RAW3
169985 229533.A0A0E0SAR4 pp 229533.A0A1C3YI30
169986 229533.A0A0E0SAR4 pp 229533.I1RYX5
169987 229533.A0A0E0SAR4 pp 229533.I1RSQ4
169988 229533.A0A0E0SAR4 pp 229533.I1REE2
169989 229533.A0A0E0SAR4 pp 229533.A0A1C3YJK3
169990 229533.A0A0E0SAR4 pp 229533.I1S3T7
169991 229533.A0A0E0SAR4 pp 229533.I1RFI4
169992 229533.A0A0E0SAR4 pp 229533.I1RQR6
169993 229533.A0A0E0SAR4 pp 229533.A0A1C3YHG7
169994 229533.A0A0E0SAR4 pp 229533.I1RA52
169995 229533.A0A0E0SAR4 pp 229533.I1RFY0
169996 229533.A0A0E0SAR4 pp 229533.A0A1C3YIZ4
169997 229533.A0A0E0SAR4 pp 229533.A0A1C3YKT4
169998 229533.A0A0E0SAR4 pp 229533.I1S2B8
169999 229533.A0A0E0SAR4 pp 229533.I1RKP1
170000 229533.A0A0E0SAR4 pp 229533.I1RBF3
170001 229533.A0A0E0SAR4 pp 229533.A0A0E0SPS2
170002 229533.A0A0E0SAR4 pp 229533.A0A1C3YJD4
170003 229533.A0A0E0SAR4 pp 229533.I1S3G4
170004 229533.A0A0E0SAR4 pp 229533.I1R9M0
170005 229533.A0A0E0SAR4 pp 229533.A0A1C3YJI5
170006 229533.A0A0E0SAR4 pp 229533.I1RK39
170007 229533.A0A0E0SAR4 pp 229533.I1RY92
170008 229533.A0A0E0SAR4 pp 229533.A0A1C3YM60
170009 229533.A0A0E0SAR4 pp 229533.I1RA11
170010 229533.A0A0E0SAR4 pp 229533.O74638
170011 229533.A0A0E0SAR4 pp 229533.I1RE40
170012 229533.A0A0E0SAR4 pp 229533.I1RB64
170013 229533.A0A0E0SAR4 pp 229533.A0A1C3YJ21
170014 229533.A0A0E0SAR4 pp 229533.A0A1C3YKM9
170015 229533.A0A0E0SAR4 pp 229533.I1RZ39
170016 229533.A0A0E0SAR4 pp 229533.I1RKI7
170017 229533.A0A0E0SAR4 pp 229533.I1RNW4

170018	229533.A0A0E0SAR4	pp	229533.I1RRJ8
170019	229533.A0A0E0SAR4	pp	229533.I1S299
170020	229533.A0A0E0SAR4	pp	229533.I1RWF5
170021	229533.A0A0E0SAR4	pp	229533.I1S464
170022	229533.A0A0E0SAR4	pp	229533.I1RPP3
170023	229533.A0A0E0SAR4	pp	229533.I1RXD9
170024	229533.A0A0E0SAR4	pp	229533.I1RYI5
170025	229533.A0A0E0SAR4	pp	229533.I1RKB2
170026	229533.A0A0E0SAR4	pp	229533.I1RYQ1
170027	229533.A0A0E0SAR4	pp	229533.I1RMJ3
170028	229533.A0A0E0SAR4	pp	229533.I1RE39
170029	229533.A0A0E0SAR4	pp	229533.I1RGA4
170030	229533.A0A0E0SAR4	pp	229533.I1R995
170031	229533.A0A0E0SAR4	pp	229533.I1RFN1
170032	229533.A0A0E0SAR4	pp	229533.I1RVX0
170033	229533.A0A0E0SAR4	pp	229533.I1RX96
170034	229533.A0A0E0SAR4	pp	229533.I1RBE6
170035	229533.A0A0E0SAR4	pp	229533.I1S0R8
170036	229533.A0A0E0SAR4	pp	229533.I1S7X9
170037	229533.A0A0E0SAR4	pp	229533.I1RX39
170038	229533.A0A0E0SAR4	pp	229533.I1S1F3
170039	229533.A0A0E0SAR4	pp	229533.V6R3J1
170040	229533.A0A0E0SAR4	pp	229533.I1S8R2
170041	229533.A0A0E0SAR4	pp	229533.I1RL81
170042	229533.A0A0E0SAR4	pp	229533.I1RTK5
170043	229533.A0A0E0SAR4	pp	229533.A0A1C3YI11
170044	229533.A0A0E0SAR4	pp	229533.I1RC18
170045	229533.A0A0E0SAR4	pp	229533.A0A1C3YMC1
170046	229533.A0A0E0SAR4	pp	229533.I1RCH5
170047	229533.A0A0E0SAR4	pp	229533.I1RYA0
170048	229533.A0A0E0SAR4	pp	229533.I1S5X9
170049	229533.A0A0E0SAR4	pp	229533.I1RW26
170050	229533.A0A0E0SAR4	pp	229533.I1RN42
170051	229533.A0A0E0SAR4	pp	229533.A0A1C3YLK8
170052	229533.A0A0E0SAR4	pp	229533.A0A1C3YLE2
170053	229533.A0A0E0SAR4	pp	229533.I1RC34
170054	229533.A0A0E0SAR4	pp	229533.Q4HXF0
170055	229533.A0A0E0SAR4	pp	229533.Q4HVQ9
170056	229533.A0A0E0SAR4	pp	229533.A0A1C3YM56
170057	229533.A0A0E0SAR4	pp	229533.I1RJT3
170058	229533.A0A0E0SAR4	pp	229533.I1RSC0
170059	229533.A0A0E0SAR4	pp	229533.I1S7W5
170060	229533.A0A0E0SAR4	pp	229533.I1RBT6
170061	229533.A0A0E0SAR4	pp	229533.I1S266
170062	229533.A0A0E0SAR4	pp	229533.A0A1C3YLB6
170063	229533.A0A0E0SAR4	pp	229533.I1RB38
170064	229533.A0A0E0SAR4	pp	229533.A0A0E0SK74
170065	229533.A0A0E0SAR4	pp	229533.I1RB92
170066	229533.A0A0E0SAR4	pp	229533.A0A1C3YJU7
170067	229533.A0A0E0SAR4	pp	229533.I1RLN1
170068	229533.A0A0E0SAR4	pp	229533.I1RVT8
170069	229533.A0A0E0SAR4	pp	229533.A0A1C3YMS7
170070	229533.A0A0E0SAR4	pp	229533.I1S5U0
170071	229533.A0A0E0SAR4	pp	229533.I1RE01
170072	229533.A0A0E0SAR4	pp	229533.I1RTE9
170073	229533.A0A0E0SAR4	pp	229533.I1RWT0
170074	229533.A0A0E0SAR4	pp	229533.I1RN84
170075	229533.A0A0E0SAR4	pp	229533.I1RPP7
170076	229533.A0A0E0SAR4	pp	229533.A0A0E0SJN2
170077	229533.A0A0E0SAR4	pp	229533.I1RSF1
170078	229533.A0A0E0SAR4	pp	229533.I1RGK4
170079	229533.A0A0E0SAR4	pp	229533.Q4I327
170080	229533.A0A0E0SAR4	pp	229533.I1RU26
170081	229533.A0A0E0SAR4	pp	229533.I1RE79
170082	229533.A0A0E0SAR4	pp	229533.I1RYC8
170083	229533.A0A0E0SAR4	pp	229533.I1S0R9
170084	229533.A0A0E0SAR4	pp	229533.I1RKF4
170085	229533.A0A0E0SAR4	pp	229533.I1RCG5
170086	229533.A0A0E0SAR4	pp	229533.V6RMW6
170087	229533.A0A0E0SAR4	pp	229533.I1RG11
170088	229533.A0A0E0SAR4	pp	229533.I1RF85
170089	229533.A0A0E0SAR4	pp	229533.V6RRM0
170090	229533.A0A0E0SAR4	pp	229533.I1RU95

170091 229533.A0A0E0SAR4 pp 229533.I1RI95
170092 229533.A0A0E0SAR4 pp 229533.Q4I174
170093 229533.A0A0E0SAR4 pp 229533.I1RSQ3
170094 229533.A0A0E0SAR4 pp 229533.I1RBS5
170095 229533.A0A0E0SAR4 pp 229533.V6R042
170096 229533.A0A0E0SAR4 pp 229533.I1RGI8
170097 229533.A0A0E0SAR4 pp 229533.I1RFJ6
170098 229533.A0A0E0SAR4 pp 229533.I1RQJ3
170099 229533.A0A0E0SAR4 pp 229533.I1RE65
170100 229533.A0A0E0SAR4 pp 229533.I1RFS5
170101 229533.A0A0E0SAR4 pp 229533.I1RKB6
170102 229533.A0A0E0SAR4 pp 229533.Q4HZ34
170103 229533.A0A0E0SAR4 pp 229533.I1S0Q7
170104 229533.A0A0E0SAR4 pp 229533.I1RMV0
170105 229533.A0A0E0SAR4 pp 229533.A0A1C3YJQ2
170106 229533.A0A0E0SAR4 pp 229533.I1RE52
170107 229533.I1RL07 pp 229533.I1S1C8
170108 229533.I1RL07 pp 229533.Q4I0J6
170109 229533.A0A1C3YK51 pp 229533.I1RWT6
170110 229533.A0A1C3YK51 pp 229533.V6RXW9
170111 229533.A0A1C3YK51 pp 229533.I1RRI4
170112 229533.A0A1C3YK51 pp 229533.V6QW25
170113 229533.A0A1C3YK51 pp 229533.I1RWQ1
170114 229533.A0A1C3YK51 pp 229533.I1RUM7
170115 229533.A0A1C3YK51 pp 229533.I1RLT6
170116 229533.A0A1C3YK51 pp 229533.I1RIG4
170117 229533.A0A1C3YK51 pp 229533.I1S0Z3
170118 229533.A0A1C3YK51 pp 229533.I1S0X0
170119 229533.A0A1C3YK51 pp 229533.I1RPL2
170120 229533.A0A1C3YK51 pp 229533.V6RR76
170121 229533.A0A1C3YK51 pp 229533.I1S278
170122 229533.A0A1C3YK51 pp 229533.I1RBC3
170123 229533.A0A1C3YK51 pp 229533.I1RM09
170124 229533.A0A1C3YK51 pp 229533.I1RA72
170125 229533.A0A1C3YK51 pp 229533.I1RQC4
170126 229533.A0A1C3YK51 pp 229533.I1RWA9
170127 229533.A0A1C3YK51 pp 229533.A0A1C3YMC0
170128 229533.A0A1C3YK51 pp 229533.I1RBY8
170129 229533.A0A1C3YK51 pp 229533.I1RCK2
170130 229533.A0A1C3YK51 pp 229533.A0A1C3YME3
170131 229533.A0A1C3YK51 pp 229533.I1REI8
170132 229533.A0A1C3YK51 pp 229533.I1RBR0
170133 229533.A0A1C3YK51 pp 229533.I1RD63
170134 229533.A0A1C3YK51 pp 229533.I1RX09
170135 229533.A0A1C3YK51 pp 229533.I1RQA3
170136 229533.A0A1C3YK51 pp 229533.I1RPK5
170137 229533.A0A1C3YK51 pp 229533.I1RWA5
170138 229533.A0A1C3YK51 pp 229533.I1RPU4
170139 229533.A0A1C3YK51 pp 229533.I1RAU6
170140 229533.A0A1C3YK51 pp 229533.A0A1C3YN54
170141 229533.A0A1C3YK51 pp 229533.I1RAL2
170142 229533.A0A1C3YK51 pp 229533.I1RJT1
170143 229533.A0A1C3YK51 pp 229533.I1RS38
170144 229533.A0A1C3YK51 pp 229533.I1RBC4
170145 229533.A0A1C3YK51 pp 229533.I1RTB7
170146 229533.A0A1C3YK51 pp 229533.I1RWI6
170147 229533.A0A1C3YK51 pp 229533.I1RH68
170148 229533.A0A1C3YK51 pp 229533.I1RYB7
170149 229533.A0A1C3YK51 pp 229533.I1S0N8
170150 229533.A0A1C3YK51 pp 229533.A0A1C3YKU6
170151 229533.A0A1C3YK51 pp 229533.I1RYI3
170152 229533.A0A1C3YK51 pp 229533.I1RTF2
170153 229533.A0A1C3YK51 pp 229533.I1RJS0
170154 229533.A0A1C3YK51 pp 229533.I1RKA0
170155 229533.A0A1C3YK51 pp 229533.I1RJY4
170156 229533.A0A1C3YK51 pp 229533.V6R9X9
170157 229533.A0A1C3YK51 pp 229533.Q4I963
170158 229533.A0A1C3YK51 pp 229533.Q4HTT2
170159 229533.A0A1C3YK51 pp 229533.P0C431
170160 229533.A0A1C3YK51 pp 229533.I1S9Z3
170161 229533.A0A1C3YK51 pp 229533.I1S2B6
170162 229533.A0A1C3YK51 pp 229533.Q5I2J3
170163 229533.A0A1C3YK51 pp 229533.I1RE18

170164 229533.A0A1C3YK51 pp 229533.I1RFU6
170165 229533.A0A1C3YK51 pp 229533.I1RQJ7
170166 229533.A0A1C3YK51 pp 229533.I1RSJ1
170167 229533.A0A1C3YK51 pp 229533.V6RUT5
170168 229533.A0A1C3YK51 pp 229533.I1SAT1
170169 229533.A0A1C3YK51 pp 229533.I1RR94
170170 229533.A0A1C3YK51 pp 229533.I1RWZ9
170171 229533.A0A1C3YK51 pp 229533.I1RBS4
170172 229533.A0A1C3YK51 pp 229533.I1RM11
170173 229533.A0A1C3YK51 pp 229533.I1S1Z9
170174 229533.A0A1C3YK51 pp 229533.I1S034
170175 229533.A0A1C3YK51 pp 229533.Q4ICG5
170176 229533.A0A1C3YK51 pp 229533.A0A1C3YKT9
170177 229533.A0A1C3YK51 pp 229533.I1RSQ2
170178 229533.A0A1C3YK51 pp 229533.I1RC49
170179 229533.A0A1C3YK51 pp 229533.A0A1C3YL89
170180 229533.A0A1C3YK51 pp 229533.I1RW38
170181 229533.A0A1C3YK51 pp 229533.I1REK1
170182 229533.A0A1C3YK51 pp 229533.I1RTD0
170183 229533.A0A1C3YK51 pp 229533.I1RC58
170184 229533.A0A1C3YK51 pp 229533.I1RTF0
170185 229533.A0A1C3YK51 pp 229533.I1RQA9
170186 229533.A0A1C3YK51 pp 229533.I1RT41
170187 229533.A0A1C3YK51 pp 229533.I1S9Q3
170188 229533.A0A1C3YK51 pp 229533.I1S254
170189 229533.A0A1C3YK51 pp 229533.I1S635
170190 229533.A0A1C3YK51 pp 229533.A0A1C3YLN8
170191 229533.A0A1C3YK51 pp 229533.I1RYY6
170192 229533.A0A1C3YK51 pp 229533.V6QT89
170193 229533.A0A1C3YK51 pp 229533.I1RX67
170194 229533.A0A1C3YK51 pp 229533.I1RHQ0
170195 229533.A0A1C3YK51 pp 229533.I1RCK9
170196 229533.A0A1C3YK51 pp 229533.I1S0S6
170197 229533.A0A1C3YK51 pp 229533.I1RN81
170198 229533.A0A1C3YK51 pp 229533.I1RUP5
170199 229533.A0A1C3YK51 pp 229533.I1RTQ9
170200 229533.A0A1C3YK51 pp 229533.I1RQK5
170201 229533.A0A1C3YK51 pp 229533.I1S9B2
170202 229533.A0A1C3YK51 pp 229533.I1RQ21
170203 229533.A0A1C3YK51 pp 229533.I1RA34
170204 229533.A0A1C3YK51 pp 229533.I1RNS3
170205 229533.A0A1C3YK51 pp 229533.I1RCU4
170206 229533.A0A1C3YK51 pp 229533.I1RX87
170207 229533.A0A1C3YK51 pp 229533.I1RS64
170208 229533.A0A1C3YK51 pp 229533.I1RXT2
170209 229533.A0A1C3YK51 pp 229533.I1RPI2
170210 229533.A0A1C3YK51 pp 229533.I1RY44
170211 229533.A0A1C3YK51 pp 229533.I1RPS0
170212 229533.A0A1C3YK51 pp 229533.V6QUU6
170213 229533.A0A1C3YK51 pp 229533.I1RS90
170214 229533.A0A1C3YK51 pp 229533.I1RAZ1
170215 229533.A0A1C3YK51 pp 229533.I1S0R9
170216 229533.A0A1C3YK51 pp 229533.I1RG11
170217 229533.A0A1C3YK51 pp 229533.I1REC4
170218 229533.A0A1C3YK51 pp 229533.I1RUN9
170219 229533.A0A1C3YK51 pp 229533.I1S1V4
170220 229533.A0A1C3YK51 pp 229533.I1RYW3
170221 229533.A0A1C3YK51 pp 229533.I1RAH5
170222 229533.A0A1C3YK51 pp 229533.I1RSP4
170223 229533.A0A1C3YK51 pp 229533.I1RW32
170224 229533.A0A1C3YK51 pp 229533.I1RWH4
170225 229533.A0A1C3YK51 pp 229533.I1RCJ9
170226 229533.A0A1C3YK51 pp 229533.I1RE20
170227 229533.A0A1C3YK51 pp 229533.I1RKT9
170228 229533.A0A1C3YK51 pp 229533.A0A1C3YLT9
170229 229533.A0A1C3YK51 pp 229533.I1RPU5
170230 229533.A0A1C3YK51 pp 229533.I1RXF5
170231 229533.A0A1C3YK51 pp 229533.V6R042
170232 229533.A0A1C3YK51 pp 229533.I1S757
170233 229533.A0A1C3YK51 pp 229533.A0A1C3YMZ1
170234 229533.A0A1C3YK51 pp 229533.I1REF9
170235 229533.A0A1C3YK51 pp 229533.I1REK3
170236 229533.A0A1C3YK51 pp 229533.V6RKE4

170237 229533.A0A1C3YK51 pp 229533.I1RZ91
170238 229533.A0A1C3YK51 pp 229533.I1RP87
170239 229533.A0A1C3YK51 pp 229533.I1RB41
170240 229533.A0A1C3YK51 pp 229533.I1RMM1
170241 229533.A0A1C3YK51 pp 229533.I1RE44
170242 229533.A0A1C3YK51 pp 229533.A0A1C3YLL6
170243 229533.A0A1C3YK51 pp 229533.A0A1C3YM29
170244 229533.A0A1C3YK51 pp 229533.I1S2E9
170245 229533.A0A1C3YK51 pp 229533.I1RPS2
170246 229533.A0A1C3YK51 pp 229533.I1RC18
170247 229533.A0A1C3YK51 pp 229533.I1RPU7
170248 229533.A0A1C3YK51 pp 229533.I1RW72
170249 229533.A0A1C3YK51 pp 229533.I1RD10
170250 229533.A0A1C3YK51 pp 229533.I1RCT2
170251 229533.A0A1C3YK51 pp 229533.I1RLX5
170252 229533.A0A1C3YK51 pp 229533.I1RNA3
170253 229533.A0A1C3YK51 pp 229533.A0A1C3YM95
170254 229533.A0A1C3YK51 pp 229533.I1RG99
170255 229533.A0A1C3YK51 pp 229533.I1RAQ3
170256 229533.A0A1C3YK51 pp 229533.I1RW76
170257 229533.A0A1C3YK51 pp 229533.I1RW93
170258 229533.A0A1C3YK51 pp 229533.I1RN70
170259 229533.A0A1C3YK51 pp 229533.I1S8X0
170260 229533.A0A1C3YK51 pp 229533.Q4IPI4
170261 229533.A0A1C3YK51 pp 229533.A0A1C3YM82
170262 229533.A0A1C3YK51 pp 229533.I1RTE0
170263 229533.A0A1C3YK51 pp 229533.I1S7X9
170264 229533.A0A1C3YK51 pp 229533.I1RFV4
170265 229533.A0A1C3YK51 pp 229533.V6QUV7
170266 229533.A0A1C3YK51 pp 229533.I1RGC1
170267 229533.A0A1C3YK51 pp 229533.I1RXF7
170268 229533.A0A1C3YK51 pp 229533.I1RYB8
170269 229533.A0A1C3YK51 pp 229533.I1RKF9
170270 229533.A0A1C3YK51 pp 229533.I1RJS5
170271 229533.A0A1C3YK51 pp 229533.I1RQC3
170272 229533.A0A1C3YK51 pp 229533.I1RNR2
170273 229533.A0A1C3YK51 pp 229533.I1RW84
170274 229533.A0A1C3YK51 pp 229533.I1RZA4
170275 229533.A0A1C3YK51 pp 229533.I1RSZ1
170276 229533.A0A1C3YK51 pp 229533.A0A1C3YK90
170277 229533.A0A1C3YK51 pp 229533.I1RGA4
170278 229533.A0A1C3YK51 pp 229533.I1RBC7
170279 229533.A0A1C3YK51 pp 229533.A0A1C3YKU7
170280 229533.A0A1C3YK51 pp 229533.I1RPM4
170281 229533.A0A1C3YK51 pp 229533.I1RE15
170282 229533.A0A1C3YK51 pp 229533.I1S1B6
170283 229533.A0A1C3YK51 pp 229533.I1RAS9
170284 229533.A0A1C3YK51 pp 229533.I1RX76
170285 229533.A0A1C3YK51 pp 229533.I1RAU2
170286 229533.A0A1C3YK51 pp 229533.I1RPX7
170287 229533.A0A1C3YK51 pp 229533.I1RPE1
170288 229533.A0A1C3YK51 pp 229533.I1RW41
170289 229533.A0A1C3YK51 pp 229533.I1RQ38
170290 229533.A0A1C3YK51 pp 229533.I1RRH0
170291 229533.A0A1C3YK51 pp 229533.I1RYU1
170292 229533.A0A1C3YK51 pp 229533.V6R476
170293 229533.A0A1C3YK51 pp 229533.I1RQN9
170294 229533.A0A1C3YK51 pp 229533.I1RXU1
170295 229533.A0A1C3YK51 pp 229533.I1RNK3
170296 229533.A0A1C3YK51 pp 229533.V6RQ48
170297 229533.A0A1C3YK51 pp 229533.I1RNR3
170298 229533.A0A1C3YK51 pp 229533.I1S2A8
170299 229533.A0A1C3YK51 pp 229533.I1REN4
170300 229533.A0A1C3YK51 pp 229533.I1RFD1
170301 229533.A0A1C3YK51 pp 229533.I1RKR7
170302 229533.A0A1C3YK51 pp 229533.I1RZ63
170303 229533.I1S458 pp 229533.I1S464
170304 229533.I1S458 pp 229533.V6RJP8
170305 229533.I1S458 pp 229533.I1S8S9
170306 229533.I1S458 pp 229533.I1S5X9
170307 229533.I1S458 pp 229533.I1S5C6
170308 229533.I1RKZ9 pp 229533.I1RMR8
170309 229533.I1RTD0 pp 229533.V6RQ48

170310 229533.I1RTD0 pp 229533.I1SAT1
170311 229533.I1RTD0 pp 229533.I1RX09
170312 229533.I1RTD0 pp 229533.I1S9Z3
170313 229533.I1RTD0 pp 229533.I1RWQ1
170314 229533.I1RTD0 pp 229533.Q4ICG5
170315 229533.I1RTD0 pp 229533.I1S830
170316 229533.I1RTD0 pp 229533.V6R9X9
170317 229533.I1RTD0 pp 229533.I1RXF5
170318 229533.I1RTD0 pp 229533.I1RZ91
170319 229533.I1RTD0 pp 229533.I1S2W9
170320 229533.I1RTD0 pp 229533.V6QW25
170321 229533.I1RTD0 pp 229533.I1S635
170322 229533.I1RTD0 pp 229533.V6RKE4
170323 229533.I1RTD0 pp 229533.I1RW93
170324 229533.I1RTD0 pp 229533.I1RW84
170325 229533.I1RTD0 pp 229533.I1RYU1
170326 229533.I1RTD0 pp 229533.I1RZA4
170327 229533.I1RTD0 pp 229533.I1RW76
170328 229533.I1RTD0 pp 229533.I1RW32
170329 229533.I1RTD0 pp 229533.I1RXU1
170330 229533.I1RWP9 pp 229533.V6RQ48
170331 229533.I1RWP9 pp 229533.I1RZA4
170332 229533.I1RWP9 pp 229533.V6RKE4
170333 229533.I1S464 pp 229533.V6RJP8
170334 229533.I1S464 pp 229533.I1S8F0
170335 229533.I1S464 pp 229533.I1S5C6
170336 229533.I1S464 pp 229533.I1S5U0
170337 229533.I1S464 pp 229533.I1S5R5
170338 229533.I1S464 pp 229533.I1S8S9
170339 229533.I1S464 pp 229533.Q4IPZ1
170340 229533.I1S464 pp 229533.I1S5X9
170341 229533.I1S464 pp 229533.I1S8F8
170342 229533.I1RE15 pp 229533.I1RW76
170343 229533.I1RE15 pp 229533.I1S2E9
170344 229533.I1RE15 pp 229533.I1RPE1
170345 229533.I1RE15 pp 229533.I1RQN9
170346 229533.I1RE15 pp 229533.I1RV91
170347 229533.I1RE15 pp 229533.I1RYB8
170348 229533.I1RE15 pp 229533.I1RXU1
170349 229533.I1RE15 pp 229533.I1RW72
170350 229533.I1RE15 pp 229533.I1RYU1
170351 229533.I1RE15 pp 229533.I1RSZ1
170352 229533.I1RE15 pp 229533.I1S7X9
170353 229533.I1RE15 pp 229533.V6R042
170354 229533.I1RE15 pp 229533.I1RXF5
170355 229533.I1RE15 pp 229533.I1RNS3
170356 229533.I1RE15 pp 229533.I1REF9
170357 229533.I1RE15 pp 229533.V6RKE4
170358 229533.I1RE15 pp 229533.I1S0S6
170359 229533.I1RE15 pp 229533.I1RZ91
170360 229533.I1RE15 pp 229533.I1REK1
170361 229533.I1RE15 pp 229533.I1RTB7
170362 229533.I1RE15 pp 229533.I1RWH4
170363 229533.I1RE15 pp 229533.I1RH68
170364 229533.I1RE15 pp 229533.V6RQ48
170365 229533.I1RE15 pp 229533.I1RW32
170366 229533.I1RE15 pp 229533.I1RY44
170367 229533.I1RE15 pp 229533.I1REN4
170368 229533.I1RE15 pp 229533.I1RNR3
170369 229533.I1RE15 pp 229533.I1RNK3
170370 229533.I1RE15 pp 229533.I1SAT1
170371 229533.I1RE15 pp 229533.I1RSQ2
170372 229533.I1RE15 pp 229533.I1RTF0
170373 229533.I1RE15 pp 229533.I1RQ38
170374 229533.I1RE15 pp 229533.I1RW84
170375 229533.I1RE15 pp 229533.I1RZA4
170376 229533.I1RE15 pp 229533.I1S635
170377 229533.I1RE15 pp 229533.Q4ICG5
170378 229533.I1RE15 pp 229533.I1RLX5
170379 229533.I1RE15 pp 229533.I1RW93
170380 229533.I1RE15 pp 229533.I1REC4
170381 229533.I1RE15 pp 229533.I1RKT9
170382 229533.I1RE15 pp 229533.I1RT41

170383 229533.I1RE15 pp 229533.I1REK3
170384 229533.I1RE15 pp 229533.I1RE20
170385 229533.I1RE15 pp 229533.P0C431
170386 229533.I1RE15 pp 229533.I1RSP4
170387 229533.I1RE15 pp 229533.V6QW25
170388 229533.I1RE15 pp 229533.I1RTD0
170389 229533.I1RE15 pp 229533.I1S0X0
170390 229533.I1RE15 pp 229533.I1S278
170391 229533.I1RE15 pp 229533.I1RM09
170392 229533.I1RE15 pp 229533.I1RWI6
170393 229533.I1RE15 pp 229533.V6R9X9
170394 229533.I1RE15 pp 229533.I1RIG4
170395 229533.I1RE15 pp 229533.I1RX09
170396 229533.I1RE15 pp 229533.I1RPK5
170397 229533.I1RE15 pp 229533.I1RS38
170398 229533.I1RE15 pp 229533.I1RWT6
170399 229533.I1RE15 pp 229533.I1RLT6
170400 229533.I1RE15 pp 229533.I1REI8
170401 229533.I1RE15 pp 229533.I1RQA3
170402 229533.A0A1C3YIM4 pp 229533.I1RLU5
170403 229533.A0A1C3YIM4 pp 229533.I1RSA4
170404 229533.A0A1C3YIM4 pp 229533.I1RCI4
170405 229533.A0A1C3YIM4 pp 229533.I1RND5
170406 229533.A0A1C3YIM4 pp 229533.I1RQC5
170407 229533.A0A1C3YIM4 pp 229533.I1RAH7
170408 229533.A0A1C3YIM4 pp 229533.I1RG90
170409 229533.A0A1C3YIM4 pp 229533.V6QXG1
170410 229533.A0A1C3YIM4 pp 229533.I1RMV5
170411 229533.A0A1C3YIM4 pp 229533.Q4IAA0
170412 229533.A0A1C3YIM4 pp 229533.I1RPY4
170413 229533.A0A1C3YIM4 pp 229533.I1RCQ6
170414 229533.A0A1C3YIM4 pp 229533.I1RFQ1
170415 229533.A0A1C3YIM4 pp 229533.I1RPL3
170416 229533.A0A1C3YIM4 pp 229533.I1RZQ5
170417 229533.A0A1C3YIM4 pp 229533.I1RS28
170418 229533.A0A1C3YIM4 pp 229533.I1RZG7
170419 229533.A0A1C3YIM4 pp 229533.I1RRL0
170420 229533.A0A1C3YIM4 pp 229533.I1RA36
170421 229533.A0A1C3YIM4 pp 229533.I1RCS2
170422 229533.A0A1C3YIM4 pp 229533.I1RPZ3
170423 229533.A0A1C3YIM4 pp 229533.I1RST1
170424 229533.A0A1C3YIM4 pp 229533.I1RG57
170425 229533.A0A1C3YIM4 pp 229533.I1RB50
170426 229533.A0A1C3YIM4 pp 229533.Q4I1Y1
170427 229533.A0A1C3YIM4 pp 229533.I1RX08
170428 229533.A0A1C3YIM4 pp 229533.I1RN59
170429 229533.A0A1C3YIM4 pp 229533.I1RX47
170430 229533.A0A1C3YIM4 pp 229533.I1RK90
170431 229533.A0A1C3YIM4 pp 229533.Q4IE79
170432 229533.I1RYQ3 pp 229533.I1S464
170433 229533.I1RYQ3 pp 229533.I1S5C6
170434 229533.I1RYQ3 pp 229533.I1RYT8
170435 229533.I1RYQ3 pp 229533.V6RJP8
170436 229533.I1RYQ3 pp 229533.I1S8S9
170437 229533.I1RYQ3 pp 229533.I1S0K2
170438 229533.I1RYQ3 pp 229533.I1S5X9
170439 229533.I1RXU1 pp 229533.I1S2E9
170440 229533.I1RXU1 pp 229533.V6RKE4
170441 229533.I1RXU1 pp 229533.Q4ICG5
170442 229533.I1RXU1 pp 229533.I1S635
170443 229533.I1RXU1 pp 229533.I1S0X0
170444 229533.I1RXU1 pp 229533.V6RQ48
170445 229533.I1RXU1 pp 229533.I1SAT1
170446 229533.I1RXU1 pp 229533.I1RYI3
170447 229533.I1RXU1 pp 229533.I1RZA4
170448 229533.I1RXU1 pp 229533.I1RZ91
170449 229533.I1RXU1 pp 229533.I1RYP1
170450 229533.I1RXU1 pp 229533.I1RY44
170451 229533.I1RXU1 pp 229533.V6QW25
170452 229533.I1RXU1 pp 229533.I1S2W9
170453 229533.I1RXU1 pp 229533.V6R9X9
170454 229533.I1RJD6 pp 229533.I1RUP5
170455 229533.I1RJD6 pp 229533.I1RYW3

170456 229533.I1RJD6 pp 229533.I1RWX5
170457 229533.I1RJD6 pp 229533.I1RJS0
170458 229533.I1RJD6 pp 229533.I1RW71
170459 229533.I1RJD6 pp 229533.I1RRT8
170460 229533.I1RJD6 pp 229533.V6QUE6
170461 229533.I1RJD6 pp 229533.I1RPI4
170462 229533.I1RJD6 pp 229533.I1RTE9
170463 229533.I1RXD9 pp 229533.I1S464
170464 229533.I1RXD9 pp 229533.I1S0P0
170465 229533.I1RXD9 pp 229533.I1S0T7
170466 229533.I1RXD9 pp 229533.Q4I7T5
170467 229533.I1RXD9 pp 229533.I1RYQ3
170468 229533.I1RXD9 pp 229533.I1RYQ1
170469 229533.I1RXD9 pp 229533.I1S1F3
170470 229533.I1RXD9 pp 229533.I1S458
170471 229533.I1RXD9 pp 229533.I1RXF1
170472 229533.I1RXD9 pp 229533.I1RYC7
170473 229533.I1RXD9 pp 229533.I1RY92
170474 229533.I1RXD9 pp 229533.I1S8F8
170475 229533.I1RXD9 pp 229533.I1S8F0
170476 229533.I1RXD9 pp 229533.I1S325
170477 229533.I1RXD9 pp 229533.I1RYT8
170478 229533.I1RXD9 pp 229533.I1RY21
170479 229533.I1RXD9 pp 229533.I1S0U0
170480 229533.I1RXD9 pp 229533.V6RJP8
170481 229533.I1RXD9 pp 229533.I1S5C6
170482 229533.I1RXD9 pp 229533.I1S0R1
170483 229533.I1RXD9 pp 229533.I1S0R8
170484 229533.I1RXD9 pp 229533.I1RY11
170485 229533.I1RXD9 pp 229533.I1RZ68
170486 229533.I1RXD9 pp 229533.I1S0K2
170487 229533.I1RXD9 pp 229533.I1S0Q7
170488 229533.I1RXD9 pp 229533.I1S8S9
170489 229533.I1RXD9 pp 229533.I1S266
170490 229533.I1RXD9 pp 229533.I1RZ43
170491 229533.I1RXD9 pp 229533.I1RXE6
170492 229533.I1RXD9 pp 229533.I1S5X9
170493 229533.I1RXD9 pp 229533.I1RYM5
170494 229533.I1RXD9 pp 229533.I1S5U0
170495 229533.I1RXD9 pp 229533.I1S212
170496 229533.I1RQR3 pp 229533.V6RIS2
170497 229533.I1RQR3 pp 229533.I1RS83
170498 229533.I1RQR3 pp 229533.I1RSA8
170499 229533.I1RQR3 pp 229533.I1RT47
170500 229533.I1RQR3 pp 229533.I1RZ11
170501 229533.I1RQR3 pp 229533.I1RS28
170502 229533.I1RQR3 pp 229533.V6RY33
170503 229533.I1RQR3 pp 229533.I1RSD4
170504 229533.I1RQR3 pp 229533.Q4IPB8
170505 229533.I1RQR3 pp 229533.I1RRL0
170506 229533.I1RQR3 pp 229533.Q4IQT8
170507 229533.I1RQR3 pp 229533.Q4IPH4
170508 229533.I1RQR3 pp 229533.I1RSA4
170509 229533.I1RQR3 pp 229533.I1RWX8
170510 229533.I1RQR3 pp 229533.I1S2Y5
170511 229533.I1RQR3 pp 229533.V6QXG1
170512 229533.I1RQR3 pp 229533.I1S0N5
170513 229533.I1RQR3 pp 229533.I1RS75
170514 229533.I1RQR3 pp 229533.I1RRQ4
170515 229533.I1RQR3 pp 229533.I1S246
170516 229533.I1RQR3 pp 229533.I1RT35
170517 229533.I1RQR3 pp 229533.I1RYJ9
170518 229533.I1RQR3 pp 229533.I1RYB1
170519 229533.I1RQR3 pp 229533.I1S9J6
170520 229533.I1RQR3 pp 229533.Q4IB70
170521 229533.I1RQR3 pp 229533.I1S5S2
170522 229533.I1RQR3 pp 229533.Q4IBK5
170523 229533.I1RQR3 pp 229533.I1RT91
170524 229533.I1RQR3 pp 229533.I1RRW3
170525 229533.I1RQR3 pp 229533.V6RLW2
170526 229533.I1RQR3 pp 229533.Q4IJH1
170527 229533.I1RQR3 pp 229533.I1RYI9
170528 229533.I1RQR3 pp 229533.Q4IF76

170529	229533.I1RQR3	pp	229533.I1RZZ1
170530	229533.I1RQR3	pp	229533.I1RR61
170531	229533.I1RQR3	pp	229533.I1S4M5
170532	229533.I1RQR3	pp	229533.I1RZK6
170533	229533.I1RQR3	pp	229533.I1RX81
170534	229533.I1RQR3	pp	229533.I1S283
170535	229533.I1RQR3	pp	229533.I1S2G9
170536	229533.I1RQR3	pp	229533.I1RZG7
170537	229533.I1RQR3	pp	229533.I1S524
170538	229533.I1RT91	pp	229533.I1S9Y1
170539	229533.I1RT91	pp	229533.I1RZ11
170540	229533.I1RT91	pp	229533.V6RQR4
170541	229533.I1RT91	pp	229533.I1RWX8
170542	229533.I1RT91	pp	229533.I1RZG7
170543	229533.I1RT91	pp	229533.I1RYI9
170544	229533.I1RT91	pp	229533.Q4IBK5
170545	229533.I1RT91	pp	229533.V6QXG1
170546	229533.I1RT91	pp	229533.I1RX81
170547	229533.I1S0P0	pp	229533.I1S3G4
170548	229533.I1S0P0	pp	229533.I1S0R8
170549	229533.I1S0P0	pp	229533.I1S1F3
170550	229533.I1S0P0	pp	229533.I1S5X9
170551	229533.I1S0P0	pp	229533.I1S5U0
170552	229533.I1S0P0	pp	229533.I1S7X9
170553	229533.I1S0P0	pp	229533.I1S458
170554	229533.I1S0P0	pp	229533.I1S464
170555	229533.I1S0P0	pp	229533.I1S7W5
170556	229533.I1S0P0	pp	229533.I1S0V7
170557	229533.I1S0P0	pp	229533.I1S1N7
170558	229533.I1S0P0	pp	229533.I1S266
170559	229533.I1S0P0	pp	229533.I1S0Q7
170560	229533.I1S0P0	pp	229533.I1S6Y3
170561	229533.I1S0P0	pp	229533.Q4I5G1
170562	229533.I1S0P0	pp	229533.I1S5C6
170563	229533.I1S0P0	pp	229533.V6RLM7
170564	229533.I1S0P0	pp	229533.I1S0R1
170565	229533.I1S0P0	pp	229533.I1S4S7
170566	229533.I1S0P0	pp	229533.I1S1W8
170567	229533.I1S0P0	pp	229533.I1S8S9
170568	229533.I1S0P0	pp	229533.I1S0U0
170569	229533.I1S0P0	pp	229533.I1S0T7
170570	229533.I1S0P0	pp	229533.I1S0V6
170571	229533.I1S0P0	pp	229533.I1S212
170572	229533.I1S0P0	pp	229533.V6RJP8
170573	229533.I1S0P0	pp	229533.V6RPN0
170574	229533.I1S0P0	pp	229533.Q4I5R9
170575	229533.I1S0P0	pp	229533.V6RXW9
170576	229533.I1S0P0	pp	229533.I1S5R5
170577	229533.I1S0P0	pp	229533.I1S8F8
170578	229533.I1RZA4	pp	229533.Q4ICG5
170579	229533.I1RZA4	pp	229533.V6R9X9
170580	229533.I1RZA4	pp	229533.I1SAT1
170581	229533.I1RZA4	pp	229533.I1S635
170582	229533.I1RRI6	pp	229533.I1RYW3
170583	229533.I1RRI6	pp	229533.V6QUE6
170584	229533.I1RRI6	pp	229533.I1RRT8
170585	229533.I1RRI6	pp	229533.I1RWX5
170586	229533.I1RRI6	pp	229533.I1RW71
170587	229533.A0A1C3YM56	pp	229533.I1RXD9
170588	229533.A0A1C3YM56	pp	229533.I1S5C6
170589	229533.A0A1C3YM56	pp	229533.I1S5X9
170590	229533.A0A1C3YM56	pp	229533.I1RSC0
170591	229533.A0A1C3YM56	pp	229533.I1RY21
170592	229533.A0A1C3YM56	pp	229533.I1RS29
170593	229533.A0A1C3YM56	pp	229533.I1S8S9
170594	229533.A0A1C3YM56	pp	229533.I1RKF4
170595	229533.A0A1C3YM56	pp	229533.A0A1C3YMZ4
170596	229533.A0A1C3YM56	pp	229533.I1S458
170597	229533.A0A1C3YM56	pp	229533.I1RE66
170598	229533.A0A1C3YM56	pp	229533.I1RFJ6
170599	229533.A0A1C3YM56	pp	229533.I1RYT8
170600	229533.A0A1C3YM56	pp	229533.I1RWA3
170601	229533.A0A1C3YM56	pp	229533.I1S0R1

170602 229533.A0A1C3YM56 pp 229533.I1S0K2
170603 229533.A0A1C3YM56 pp 229533.I1S0R8
170604 229533.A0A1C3YM56 pp 229533.I1RQR6
170605 229533.A0A1C3YM56 pp 229533.I1RS05
170606 229533.A0A1C3YM56 pp 229533.I1S0Q7
170607 229533.A0A1C3YM56 pp 229533.A0A1C3YMC4
170608 229533.A0A1C3YM56 pp 229533.V6RJP8
170609 229533.A0A1C3YM56 pp 229533.I1RMK7
170610 229533.A0A1C3YM56 pp 229533.I1RE39
170611 229533.A0A1C3YM56 pp 229533.I1RKF5
170612 229533.A0A1C3YM56 pp 229533.I1S464
170613 229533.A0A1C3YM56 pp 229533.I1REU9
170614 229533.A0A1C3YM56 pp 229533.I1RA52
170615 229533.A0A1C3YM56 pp 229533.I1RBP6
170616 229533.A0A1C3YM56 pp 229533.I1S0I9
170617 229533.A0A1C3YM56 pp 229533.I1R9N8
170618 229533.A0A1C3YM56 pp 229533.I1S325
170619 229533.A0A1C3YM56 pp 229533.I1RP02
170620 229533.A0A1C3YM56 pp 229533.I1RLH3
170621 229533.A0A1C3YM56 pp 229533.I1RIQ3
170622 229533.A0A1C3YM56 pp 229533.I1RE65
170623 229533.A0A1C3YM56 pp 229533.I1RE40
170624 229533.I1RIL9 pp 229533.I1RIM7
170625 229533.I1RIL9 pp 229533.I1RJU9
170626 229533.I1RIL9 pp 229533.I1RLM3
170627 229533.I1RIL9 pp 229533.I1RXN6
170628 229533.I1RIL9 pp 229533.I1RXJ0
170629 229533.I1RQX3 pp 229533.I1S7W2
170630 229533.I1RQX3 pp 229533.I1RRH0
170631 229533.I1RQX3 pp 229533.I1S270
170632 229533.I1RQX3 pp 229533.Q4INI2
170633 229533.I1RQX3 pp 229533.I1S034
170634 229533.I1RQX3 pp 229533.I1RZL3
170635 229533.I1RQX3 pp 229533.V6R1X4
170636 229533.I1RQX3 pp 229533.I1RRH6
170637 229533.I1RQX3 pp 229533.I1S1X5
170638 229533.I1RQX3 pp 229533.I1RWI6
170639 229533.A0A1C3YK19 pp 229533.I1RG90
170640 229533.A0A1C3YK19 pp 229533.I1RSW2
170641 229533.A0A1C3YK19 pp 229533.I1RMV5
170642 229533.A0A1C3YK19 pp 229533.I1RU69
170643 229533.A0A1C3YK19 pp 229533.I1RN59
170644 229533.A0A1C3YK19 pp 229533.I1RK90
170645 229533.A0A1C3YK19 pp 229533.Q4IF76
170646 229533.A0A1C3YK19 pp 229533.I1RB50
170647 229533.A0A1C3YK19 pp 229533.I1RG57
170648 229533.A0A1C3YK19 pp 229533.Q4I1Y1
170649 229533.A0A1C3YK19 pp 229533.I1RAC1
170650 229533.A0A1C3YK19 pp 229533.Q4IJH1
170651 229533.A0A1C3YK19 pp 229533.Q4IB70
170652 229533.A0A1C3YK19 pp 229533.I1RCN5
170653 229533.A0A1C3YK19 pp 229533.I1RB86
170654 229533.A0A1C3YK19 pp 229533.I1RA36
170655 229533.A0A1C3YK19 pp 229533.I1RST1
170656 229533.A0A1C3YK19 pp 229533.I1S2Y5
170657 229533.A0A1C3YK19 pp 229533.I1S9L0
170658 229533.A0A1C3YK19 pp 229533.I1RFQ1
170659 229533.A0A1C3YK19 pp 229533.I1RS28
170660 229533.A0A1C3YK19 pp 229533.I1RPY4
170661 229533.A0A1C3YK19 pp 229533.I1RCQ6
170662 229533.A0A1C3YK19 pp 229533.I1RPY5
170663 229533.A0A1C3YK19 pp 229533.I1RRL0
170664 229533.A0A1C3YK19 pp 229533.I1RPL3
170665 229533.A0A1C3YK19 pp 229533.I1RCS2
170666 229533.A0A1C3YK19 pp 229533.I1RZG7
170667 229533.A0A1C3YK19 pp 229533.I1RZQ5
170668 229533.A0A1C3YK19 pp 229533.I1RCA7
170669 229533.A0A1C3YK19 pp 229533.Q4IRI9
170670 229533.A0A1C3YK19 pp 229533.I1RCI4
170671 229533.A0A1C3YK19 pp 229533.Q4IAA0
170672 229533.A0A1C3YK19 pp 229533.I1RD56
170673 229533.A0A1C3YK19 pp 229533.I1RLU5
170674 229533.A0A1C3YK19 pp 229533.I1RBH8

170675 229533.A0A1C3YK19 pp 229533.I1RM25
170676 229533.A0A1C3YK19 pp 229533.I1RND5
170677 229533.A0A1C3YK19 pp 229533.I1RSA4
170678 229533.A0A1C3YK19 pp 229533.Q4IPB8
170679 229533.A0A1C3YK19 pp 229533.I1RX47
170680 229533.A0A1C3YK19 pp 229533.V6QXG1
170681 229533.A0A1C3YK19 pp 229533.I1RFG0
170682 229533.A0A1C3YK19 pp 229533.I1RK23
170683 229533.A0A1C3YK19 pp 229533.I1RPZ3
170684 229533.A0A1C3YK19 pp 229533.I1RPY8
170685 229533.A0A1C3YK19 pp 229533.I1S056
170686 229533.I1RWH8 pp 229533.I1RXG6
170687 229533.I1RWH8 pp 229533.Q4I8X0
170688 229533.I1RWH8 pp 229533.I1RXF8
170689 229533.I1RWH8 pp 229533.I1RY63
170690 229533.I1RWH8 pp 229533.I1S1Y7
170691 229533.I1S5X9 pp 229533.Q4ICG5
170692 229533.I1S5X9 pp 229533.Q4IJW4
170693 229533.I1S5X9 pp 229533.V6RJP8
170694 229533.I1S5X9 pp 229533.I1S8S9
170695 229533.I1S5X9 pp 229533.Q4IPZ1
170696 229533.I1S5X9 pp 229533.I1S8Z2
170697 229533.I1S5X9 pp 229533.Q4I6B0
170698 229533.I1RCJ9 pp 229533.I1RZD6
170699 229533.I1RCJ9 pp 229533.I1RZV1
170700 229533.I1RCJ9 pp 229533.I1RRH6
170701 229533.I1RCJ9 pp 229533.I1RWI6
170702 229533.I1RCJ9 pp 229533.I1S0Z3
170703 229533.I1RCJ9 pp 229533.I1RE49
170704 229533.I1RCJ9 pp 229533.I1RKR9
170705 229533.I1RCJ9 pp 229533.I1RZ91
170706 229533.I1RCJ9 pp 229533.V6R1X4
170707 229533.I1RCJ9 pp 229533.I1S7W2
170708 229533.I1RCJ9 pp 229533.I1S270
170709 229533.I1RCJ9 pp 229533.I1RT41
170710 229533.I1RCJ9 pp 229533.V6RGY0
170711 229533.I1RCJ9 pp 229533.Q4INI2
170712 229533.I1RCJ9 pp 229533.I1REK3
170713 229533.I1RCJ9 pp 229533.I1S0X0
170714 229533.I1RCJ9 pp 229533.I1RNK3
170715 229533.I1RCJ9 pp 229533.I1RSD7
170716 229533.I1RCJ9 pp 229533.I1RZL3
170717 229533.I1RCJ9 pp 229533.I1RCT2
170718 229533.I1RCJ9 pp 229533.I1RRH0
170719 229533.I1RCJ9 pp 229533.I1RQP5
170720 229533.I1RCJ9 pp 229533.I1S8E0
170721 229533.I1RCJ9 pp 229533.Q4I963
170722 229533.I1RCJ9 pp 229533.I1S1X5
170723 229533.I1RCJ9 pp 229533.I1S034
170724 229533.I1RCJ9 pp 229533.I1S559
170725 229533.I1RJM9 pp 229533.I1RNV6
170726 229533.I1RJM9 pp 229533.I1RTA6
170727 229533.I1RJM9 pp 229533.I1RNG8
170728 229533.I1RJM9 pp 229533.I1RZD9
170729 229533.I1RJM9 pp 229533.I1RY40
170730 229533.I1RJM9 pp 229533.I1RYZ7
170731 229533.I1RJM9 pp 229533.I1RLX6
170732 229533.I1RJM9 pp 229533.I1S0L8
170733 229533.I1RJM9 pp 229533.I1RJS2
170734 229533.I1RJM9 pp 229533.I1S050
170735 229533.I1RJM9 pp 229533.I1RNF9
170736 229533.I1RJM9 pp 229533.I1RZM1
170737 229533.I1RJM9 pp 229533.Q4I5U9
170738 229533.I1RS05 pp 229533.I1S458
170739 229533.I1RS05 pp 229533.I1RZ39
170740 229533.I1RS05 pp 229533.I1S325
170741 229533.I1RS05 pp 229533.I1RWA3
170742 229533.I1RS05 pp 229533.I1RY92
170743 229533.I1RS05 pp 229533.I1S0U0
170744 229533.I1RS05 pp 229533.I1RY21
170745 229533.I1RS05 pp 229533.I1RYT8
170746 229533.I1RS05 pp 229533.I1S5C6
170747 229533.I1RS05 pp 229533.I1S0R1

170748	229533.I1RS05	pp	229533.V6QUG0
170749	229533.I1RS05	pp	229533.I1S0P0
170750	229533.I1RS05	pp	229533.I1RYC7
170751	229533.I1RS05	pp	229533.I1RX39
170752	229533.I1RS05	pp	229533.I1RYQ1
170753	229533.I1RS05	pp	229533.I1RYQ3
170754	229533.I1RS05	pp	229533.I1S0T7
170755	229533.I1RS05	pp	229533.I1S5X9
170756	229533.I1RS05	pp	229533.V6RM66
170757	229533.I1RS05	pp	229533.I1S1F3
170758	229533.I1RS05	pp	229533.I1S4S7
170759	229533.I1RS05	pp	229533.I1RWG0
170760	229533.I1RS05	pp	229533.I1RXD9
170761	229533.I1RS05	pp	229533.I1S0K2
170762	229533.I1RS05	pp	229533.I1RS29
170763	229533.I1RS05	pp	229533.I1RSC0
170764	229533.I1RS05	pp	229533.I1S464
170765	229533.I1RS05	pp	229533.I1S266
170766	229533.I1RS05	pp	229533.I1S8S9
170767	229533.I1RS05	pp	229533.I1S0R8
170768	229533.I1RS05	pp	229533.I1S8F0
170769	229533.I1RS05	pp	229533.I1RYM5
170770	229533.I1RS05	pp	229533.I1S5U0
170771	229533.I1RS05	pp	229533.I1S8F8
170772	229533.I1RS05	pp	229533.I1RWA4
170773	229533.I1RS05	pp	229533.I1RW13
170774	229533.I1RS05	pp	229533.I1RXE6
170775	229533.I1RS05	pp	229533.V6RJP8
170776	229533.I1RS05	pp	229533.I1RWR7
170777	229533.I1RS05	pp	229533.I1RU95
170778	229533.I1RS05	pp	229533.I1S212
170779	229533.I1RS05	pp	229533.I1S0Q7
170780	229533.I1RS05	pp	229533.I1RZ68
170781	229533.I1RS05	pp	229533.I1RVT8
170782	229533.I1RS05	pp	229533.V6QWP4
170783	229533.I1RM09	pp	229533.Q4IB89
170784	229533.I1RM09	pp	229533.I1RTN6
170785	229533.I1RM09	pp	229533.I1S0D1
170786	229533.I1RM09	pp	229533.I1RPP7
170787	229533.I1RM09	pp	229533.I1RWE1
170788	229533.I1RM09	pp	229533.I1S1W4
170789	229533.I1RM09	pp	229533.I1RXF5
170790	229533.I1RM09	pp	229533.I1RRY3
170791	229533.I1RM09	pp	229533.I1S0M2
170792	229533.I1RM09	pp	229533.V6RVG3
170793	229533.I1RM09	pp	229533.I1RWP9
170794	229533.I1RM09	pp	229533.I1RT36
170795	229533.I1RM09	pp	229533.I1RTG2
170796	229533.I1RM09	pp	229533.I1S7X9
170797	229533.I1RM09	pp	229533.I1S2E9
170798	229533.I1RM09	pp	229533.I1RY44
170799	229533.I1RM09	pp	229533.I1RNK3
170800	229533.I1RM09	pp	229533.I1S2I4
170801	229533.I1RM09	pp	229533.I1RTG9
170802	229533.I1RM09	pp	229533.I1RN66
170803	229533.I1RM09	pp	229533.I1RV91
170804	229533.I1RM09	pp	229533.I1RPU7
170805	229533.I1RM09	pp	229533.I1RMP0
170806	229533.I1RM09	pp	229533.I1RSF2
170807	229533.I1RM09	pp	229533.I1RQN9
170808	229533.I1RM09	pp	229533.I1RMM9
170809	229533.I1RM09	pp	229533.I1RN08
170810	229533.I1RM09	pp	229533.I1RQA3
170811	229533.I1RM09	pp	229533.I1S0N8
170812	229533.I1RM09	pp	229533.I1S1B1
170813	229533.I1RM09	pp	229533.P0C431
170814	229533.I1RM09	pp	229533.I1RWT6
170815	229533.I1RM09	pp	229533.I1S1V3
170816	229533.I1RM09	pp	229533.I1RRK8
170817	229533.I1RM09	pp	229533.I1S439
170818	229533.I1RM09	pp	229533.I1RMW7
170819	229533.I1RM09	pp	229533.I1RQA9
170820	229533.I1RM09	pp	229533.I1RQ26

170821 229533.I1RM09 pp 229533.I1S0S6
170822 229533.I1RM09 pp 229533.I1RPK5
170823 229533.I1RM09 pp 229533.I1RTB7
170824 229533.I1RM09 pp 229533.I1RYH9
170825 229533.I1RM09 pp 229533.I1RM10
170826 229533.I1RKF5 pp 229533.V6RJP8
170827 229533.I1RKF5 pp 229533.I1RPY8
170828 229533.I1RKF5 pp 229533.I1RQR6
170829 229533.I1RKF5 pp 229533.I1RP02
170830 229533.I1RKF5 pp 229533.I1RS29
170831 229533.I1RKF5 pp 229533.I1S5X9
170832 229533.I1RKF5 pp 229533.I1RQJ3
170833 229533.I1RKF5 pp 229533.I1RY92
170834 229533.I1RKF5 pp 229533.I1S0Q7
170835 229533.I1RKF5 pp 229533.I1S458
170836 229533.I1RKF5 pp 229533.I1RPR9
170837 229533.I1RKF5 pp 229533.I1S0K2
170838 229533.I1RKF5 pp 229533.I1RS05
170839 229533.I1RKF5 pp 229533.I1S8S9
170840 229533.I1RKF5 pp 229533.I1S1F3
170841 229533.I1RKF5 pp 229533.I1RYT8
170842 229533.I1RKF5 pp 229533.I1RXD9
170843 229533.I1RKF5 pp 229533.I1RMK7
170844 229533.I1RKF5 pp 229533.I1RWA3
170845 229533.I1RKF5 pp 229533.I1S0P0
170846 229533.I1RKF5 pp 229533.I1S464
170847 229533.I1RKF5 pp 229533.I1S0R1
170848 229533.I1RKF5 pp 229533.I1S5C6
170849 229533.I1RKF5 pp 229533.I1RY21
170850 229533.A0A1C3YLE9 pp 229533.Q4HXF0
170851 229533.A0A1C3YLE9 pp 229533.I1RPD6
170852 229533.A0A1C3YLE9 pp 229533.I1RMN6
170853 229533.A0A1C3YLE9 pp 229533.I1RA06
170854 229533.A0A1C3YLE9 pp 229533.I1RQ31
170855 229533.A0A1C3YLE9 pp 229533.I1RS47
170856 229533.A0A1C3YLE9 pp 229533.V6R283
170857 229533.A0A1C3YLE9 pp 229533.I1RRT2
170858 229533.A0A1C3YLE9 pp 229533.I1RXU0
170859 229533.A0A1C3YLE9 pp 229533.I1RR95
170860 229533.A0A1C3YLE9 pp 229533.V6RXB3
170861 229533.A0A1C3YLE9 pp 229533.I1RC82
170862 229533.A0A1C3YLE9 pp 229533.I1RRJ8
170863 229533.A0A1C3YLE9 pp 229533.I1RW71
170864 229533.A0A1C3YLE9 pp 229533.I1RM67
170865 229533.A0A1C3YLE9 pp 229533.I1RXG6
170866 229533.A0A1C3YLE9 pp 229533.I1S1W3
170867 229533.A0A1C3YLE9 pp 229533.I1RUQ2
170868 229533.A0A1C3YLE9 pp 229533.I1RRT8
170869 229533.A0A1C3YLE9 pp 229533.Q4IEK8
170870 229533.A0A1C3YLE9 pp 229533.I1RMK3
170871 229533.A0A1C3YLE9 pp 229533.I1S1J7
170872 229533.A0A1C3YLE9 pp 229533.Q4IJH1
170873 229533.A0A1C3YLE9 pp 229533.I1RC33
170874 229533.A0A1C3YLE9 pp 229533.I1RG56
170875 229533.A0A1C3YLE9 pp 229533.I1RIE2
170876 229533.A0A1C3YLE9 pp 229533.V6R227
170877 229533.A0A1C3YLE9 pp 229533.I1RRI2
170878 229533.A0A1C3YLE9 pp 229533.Q4IMJ1
170879 229533.A0A1C3YLE9 pp 229533.I1RSL5
170880 229533.A0A1C3YLE9 pp 229533.I1RE76
170881 229533.A0A1C3YLE9 pp 229533.I1S207
170882 229533.A0A1C3YLE9 pp 229533.I1RJZ7
170883 229533.A0A1C3YLE9 pp 229533.I1RPJ3
170884 229533.A0A1C3YLE9 pp 229533.I1RG51
170885 229533.A0A1C3YLE9 pp 229533.A0A1C3YMA1
170886 229533.A0A1C3YLE9 pp 229533.I1RC72
170887 229533.A0A1C3YLE9 pp 229533.I1RHJ2
170888 229533.A0A1C3YLE9 pp 229533.I1RE00
170889 229533.A0A1C3YLE9 pp 229533.I1S2C0
170890 229533.A0A1C3YLE9 pp 229533.V6R7V8
170891 229533.A0A1C3YLE9 pp 229533.I1RF19
170892 229533.A0A1C3YLE9 pp 229533.I1RZK6
170893 229533.A0A1C3YLE9 pp 229533.I1RAZ8

229533.A0A1C3YLE9	pp	229533.I1S2A1
229533.I1RE20	pp	229533.I1S2W9
229533.I1RE20	pp	229533.I1RFK0
229533.I1RE20	pp	229533.I1RX67
229533.I1RE20	pp	229533.I1REI8
229533.I1RE20	pp	229533.I1RX09
229533.I1RE20	pp	229533.I1RN82
229533.I1RE20	pp	229533.I1RPK5
229533.I1RE20	pp	229533.I1RWQ1
229533.I1RE20	pp	229533.I1S1Z9
229533.I1RE20	pp	229533.Q4ICG5
229533.I1RE20	pp	229533.I1RTD0
229533.I1RE20	pp	229533.I1RRZ9
229533.I1RE20	pp	229533.I1RLT6
229533.I1RE20	pp	229533.I1RS38
229533.I1RE20	pp	229533.I1RKE9
229533.I1RE20	pp	229533.I1S635
229533.I1RE20	pp	229533.I1RNK8
229533.I1RE20	pp	229533.I1RIG4
229533.I1RE20	pp	229533.I1RM09
229533.I1RE20	pp	229533.I1RW38
229533.I1RE20	pp	229533.I1REK1
229533.I1RE20	pp	229533.I1RSQ2
229533.I1RE20	pp	229533.I1S9Z3
229533.I1RE20	pp	229533.I1RU42
229533.I1RE20	pp	229533.V6QW25
229533.I1RE20	pp	229533.I1S9X9
229533.I1RE20	pp	229533.I1SAT1
229533.I1RE20	pp	229533.V6R9X9
229533.I1RE20	pp	229533.I1RTF0
229533.I1RE20	pp	229533.I1REC4
229533.I1RE20	pp	229533.I1RWZ9
229533.I1RE20	pp	229533.I1RTQ9
229533.I1RE20	pp	229533.I1RSP4
229533.I1RE20	pp	229533.I1RM11
229533.I1RE20	pp	229533.I1RYB8
229533.I1RE20	pp	229533.I1RJT0
229533.I1RE20	pp	229533.I1S5Y0
229533.I1RE20	pp	229533.I1RMV0
229533.I1RE20	pp	229533.I1RNW8
229533.I1RE20	pp	229533.I1RKR9
229533.I1RE20	pp	229533.I1RGC1
229533.I1RE20	pp	229533.I1RW32
229533.I1RE20	pp	229533.I1RQK5
229533.I1RE20	pp	229533.I1RXF5
229533.I1RE20	pp	229533.I1RNR3
229533.I1RE20	pp	229533.I1RZ91
229533.I1RE20	pp	229533.I1RZA4
229533.I1RE20	pp	229533.V6RQ48
229533.I1RE20	pp	229533.I1REN4
229533.I1RE20	pp	229533.I1RW84
229533.I1RE20	pp	229533.I1RE57
229533.I1RE20	pp	229533.I1RQN8
229533.I1RE20	pp	229533.I1REF9
229533.I1RE20	pp	229533.V6RKE4
229533.I1RE20	pp	229533.I1RKR7
229533.I1RE20	pp	229533.I1RG99
229533.I1RE20	pp	229533.I1RQ38
229533.I1RE20	pp	229533.I1RW93
229533.I1RE20	pp	229533.I1RW76
229533.I1RE20	pp	229533.I1RN70
229533.I1RE20	pp	229533.I1RNK3
229533.I1RE20	pp	229533.I1RXU1
229533.I1RE20	pp	229533.I1RYU1
229533.I1RFJ6	pp	229533.I1S266
229533.I1RFJ6	pp	229533.I1RVT8
229533.I1RFJ6	pp	229533.I1RYI6
229533.I1RFJ6	pp	229533.I1S5U0
229533.I1RFJ6	pp	229533.Q4IJM4
229533.I1RFJ6	pp	229533.I1RKF5
229533.I1RFJ6	pp	229533.V6RJP8
229533.I1RFJ6	pp	229533.I1RP38
229533.I1RFJ6	pp	229533.I1RYA0

170967 229533.I1RFJ6 pp 229533.I1RWT0
170968 229533.I1RFJ6 pp 229533.I1S5X9
170969 229533.I1RFJ6 pp 229533.I1RWA4
170970 229533.I1RFJ6 pp 229533.I1RS29
170971 229533.I1RFJ6 pp 229533.I1S8S9
170972 229533.I1RFJ6 pp 229533.I1S0K2
170973 229533.I1RFJ6 pp 229533.I1RU95
170974 229533.I1RFJ6 pp 229533.I1RYC8
170975 229533.I1RFJ6 pp 229533.I1S0R8
170976 229533.I1RFJ6 pp 229533.I1RLN1
170977 229533.I1RFJ6 pp 229533.I1RKF4
170978 229533.I1RFJ6 pp 229533.I1RG33
170979 229533.I1RFJ6 pp 229533.I1RGI8
170980 229533.I1RFJ6 pp 229533.I1S8F8
170981 229533.I1RFJ6 pp 229533.I1RI95
170982 229533.I1RFJ6 pp 229533.I1RPY8
170983 229533.I1RFJ6 pp 229533.I1S5R5
170984 229533.I1RFJ6 pp 229533.I1RP02
170985 229533.I1RFJ6 pp 229533.I1S3V2
170986 229533.I1RFJ6 pp 229533.I1S212
170987 229533.I1RFJ6 pp 229533.I1RK24
170988 229533.I1RFJ6 pp 229533.I1RQJ3
170989 229533.I1RFJ6 pp 229533.I1RW13
170990 229533.I1RFJ6 pp 229533.I1S0Q7
170991 229533.I1RFJ6 pp 229533.I1RUR8
170992 229533.I1RFJ6 pp 229533.I1RZ68
170993 229533.I1RFJ6 pp 229533.I1RXE6
170994 229533.I1RFJ6 pp 229533.I1RRK9
170995 229533.I1RFJ6 pp 229533.I1RQR6
170996 229533.I1RFJ6 pp 229533.I1RNF7
170997 229533.I1RFJ6 pp 229533.I1RY92
170998 229533.I1RFJ6 pp 229533.I1RGJ4
170999 229533.I1RFJ6 pp 229533.I1RL81
171000 229533.I1RFJ6 pp 229533.I1RFS5
171001 229533.I1RFJ6 pp 229533.I1RPY0
171002 229533.I1RFJ6 pp 229533.I1S1F3
171003 229533.I1RFJ6 pp 229533.I1RKD3
171004 229533.I1RFJ6 pp 229533.I1RJU3
171005 229533.I1RFJ6 pp 229533.I1RTM8
171006 229533.I1RFJ6 pp 229533.I1RKB7
171007 229533.I1RFJ6 pp 229533.I1RWA3
171008 229533.I1RFJ6 pp 229533.I1RWG0
171009 229533.I1RFJ6 pp 229533.I1RMK7
171010 229533.I1RFJ6 pp 229533.I1RMQ6
171011 229533.I1RFJ6 pp 229533.I1RNM7
171012 229533.I1RFJ6 pp 229533.I1S458
171013 229533.I1RFJ6 pp 229533.I1RN42
171014 229533.I1RFJ6 pp 229533.I1S0U0
171015 229533.I1RFJ6 pp 229533.I1RYQ3
171016 229533.I1RFJ6 pp 229533.I1RJB3
171017 229533.I1RFJ6 pp 229533.I1RGC3
171018 229533.I1RFJ6 pp 229533.I1S1N7
171019 229533.I1RFJ6 pp 229533.I1RYT8
171020 229533.I1RFJ6 pp 229533.I1S0R1
171021 229533.I1RFJ6 pp 229533.I1RK39
171022 229533.I1RFJ6 pp 229533.I1S0P0
171023 229533.I1RFJ6 pp 229533.I1RKP1
171024 229533.I1RFJ6 pp 229533.I1RNE7
171025 229533.I1RFJ6 pp 229533.I1RS05
171026 229533.I1RFJ6 pp 229533.I1RG62
171027 229533.I1RFJ6 pp 229533.I1RXD9
171028 229533.I1RFJ6 pp 229533.I1RY21
171029 229533.I1RFJ6 pp 229533.I1RX39
171030 229533.I1RFJ6 pp 229533.I1RYC7
171031 229533.I1RFJ6 pp 229533.I1S5C6
171032 229533.I1RFJ6 pp 229533.I1RYQ1
171033 229533.I1RFJ6 pp 229533.I1RRX5
171034 229533.I1RFJ6 pp 229533.I1S464
171035 229533.I1RFJ6 pp 229533.I1RSC0
171036 229533.I1RFJ6 pp 229533.I1S0T7
171037 229533.I1S077 pp 229533.Q4IB89
171038 229533.I1S077 pp 229533.V6RDZ4
171039 229533.I1S077 pp 229533.Q4I7L0

171040	229533.I1S842	pp	229533.Q4ILI9
171041	229533.I1RKF4	pp	229533.I1RWA3
171042	229533.I1RKF4	pp	229533.I1S458
171043	229533.I1RKF4	pp	229533.I1RYT8
171044	229533.I1RKF4	pp	229533.I1RMK7
171045	229533.I1RKF4	pp	229533.I1S464
171046	229533.I1RKF4	pp	229533.I1RY92
171047	229533.I1RKF4	pp	229533.I1RY21
171048	229533.I1RKF4	pp	229533.I1S0P0
171049	229533.I1RKF4	pp	229533.I1S5C6
171050	229533.I1RKF4	pp	229533.I1S0Q7
171051	229533.I1RKF4	pp	229533.I1RXD9
171052	229533.I1RKF4	pp	229533.I1S1F3
171053	229533.I1RKF4	pp	229533.I1RQR6
171054	229533.I1RKF4	pp	229533.I1S0R1
171055	229533.I1RKF4	pp	229533.I1S8S9
171056	229533.I1RKF4	pp	229533.I1RS05
171057	229533.I1RKF4	pp	229533.I1RP02
171058	229533.I1RKF4	pp	229533.I1S5X9
171059	229533.I1RKF4	pp	229533.I1RPR9
171060	229533.I1RKF4	pp	229533.I1RS29
171061	229533.I1RKF4	pp	229533.I1S0K2
171062	229533.I1RKF4	pp	229533.V6RJP8
171063	229533.I1RKF4	pp	229533.I1RQJ3
171064	229533.I1RKF4	pp	229533.I1RPY8
171065	229533.I1RQP5	pp	229533.I1RXF5
171066	229533.I1RQP5	pp	229533.Q4HZB8
171067	229533.I1RQP5	pp	229533.I1RZV1
171068	229533.I1RQP5	pp	229533.Q4IPI4
171069	229533.I1RQP5	pp	229533.I1RS90
171070	229533.I1RQP5	pp	229533.I1RSZ6
171071	229533.I1RQP5	pp	229533.I1RST4
171072	229533.I1RQP5	pp	229533.I1RQX3
171073	229533.I1RQP5	pp	229533.I1RRF9
171074	229533.I1RQP5	pp	229533.I1S7W2
171075	229533.I1RQP5	pp	229533.I1RUN2
171076	229533.I1RQP5	pp	229533.I1RTE0
171077	229533.I1RQP5	pp	229533.I1RRF0
171078	229533.I1RQP5	pp	229533.I1RZL3
171079	229533.I1RQP5	pp	229533.Q4IEV5
171080	229533.I1RQP5	pp	229533.I1S9M9
171081	229533.I1RQP5	pp	229533.I1S267
171082	229533.I1RQP5	pp	229533.I1S128
171083	229533.I1RQP5	pp	229533.V6R476
171084	229533.I1RQP5	pp	229533.I1RWY1
171085	229533.I1RQP5	pp	229533.I1RRC3
171086	229533.I1RQP5	pp	229533.I1RSD7
171087	229533.I1RQP5	pp	229533.Q4I963
171088	229533.I1RQP5	pp	229533.V6RR76
171089	229533.I1RQP5	pp	229533.I1RQP7
171090	229533.I1RQP5	pp	229533.I1RT65
171091	229533.I1RQP5	pp	229533.I1S0Z3
171092	229533.I1RQP5	pp	229533.Q4INI2
171093	229533.I1RQP5	pp	229533.I1S0Z7
171094	229533.I1RQP5	pp	229533.I1S270
171095	229533.I1RQP5	pp	229533.I1S5Z2
171096	229533.I1RQP5	pp	229533.I1S0X0
171097	229533.I1RQP5	pp	229533.I1S8E0
171098	229533.I1RQP5	pp	229533.I1RT41
171099	229533.I1RQP5	pp	229533.I1RWI6
171100	229533.I1RQP5	pp	229533.I1RSI4
171101	229533.I1RQP5	pp	229533.I1RVU1
171102	229533.I1RQP5	pp	229533.I1S254
171103	229533.I1RQP5	pp	229533.I1S1V4
171104	229533.I1RQP5	pp	229533.V6QUU6
171105	229533.I1RPK5	pp	229533.I1RW72
171106	229533.I1RPK5	pp	229533.Q4I7T5
171107	229533.I1RPK5	pp	229533.I1S7X9
171108	229533.I1RPK5	pp	229533.I1RXU1
171109	229533.I1RPK5	pp	229533.I1S2V2
171110	229533.I1RPK5	pp	229533.I1RSB0
171111	229533.I1RPK5	pp	229533.I1RW76
171112	229533.I1RPK5	pp	229533.I1RQ88

171113 229533.I1RPK5 pp 229533.I1RZD9
171114 229533.I1RPK5 pp 229533.I1RWK9
171115 229533.I1RPK5 pp 229533.I1RTM8
171116 229533.I1RPK5 pp 229533.I1RX21
171117 229533.I1RPK5 pp 229533.I1RQ32
171118 229533.I1RPK5 pp 229533.I1RX38
171119 229533.I1RPK5 pp 229533.I1RWH4
171120 229533.I1RPK5 pp 229533.I1RTG9
171121 229533.I1RPK5 pp 229533.I1RR07
171122 229533.I1RPK5 pp 229533.I1RTG2
171123 229533.I1RPK5 pp 229533.Q4HYB8
171124 229533.I1RPK5 pp 229533.I1RZS8
171125 229533.I1RPK5 pp 229533.I1RT94
171126 229533.I1RPK5 pp 229533.I1RPV0
171127 229533.I1RPK5 pp 229533.I1RPN8
171128 229533.I1RPK5 pp 229533.I1S0D1
171129 229533.I1RPK5 pp 229533.I1RQ21
171130 229533.I1RPK5 pp 229533.I1RW32
171131 229533.I1RPK5 pp 229533.I1RWB4
171132 229533.I1RPK5 pp 229533.I1RRC6
171133 229533.I1RPK5 pp 229533.I1S1C7
171134 229533.I1RPK5 pp 229533.I1S0Z8
171135 229533.I1RPK5 pp 229533.I1S2C4
171136 229533.I1RPK5 pp 229533.I1RW23
171137 229533.I1RPK5 pp 229533.I1RYD5
171138 229533.I1RPK5 pp 229533.I1RRT6
171139 229533.I1RPK5 pp 229533.I1RZD3
171140 229533.I1RPK5 pp 229533.I1RPW0
171141 229533.I1RPK5 pp 229533.I1RT03
171142 229533.I1RPK5 pp 229533.I1RR25
171143 229533.I1RPK5 pp 229533.I1RT62
171144 229533.I1RPK5 pp 229533.I1RX84
171145 229533.I1RPK5 pp 229533.I1S095
171146 229533.I1RPK5 pp 229533.I1RWC2
171147 229533.I1RPK5 pp 229533.I1RZV4
171148 229533.I1RPK5 pp 229533.I1RSP4
171149 229533.I1RPK5 pp 229533.I1RTF0
171150 229533.I1RPK5 pp 229533.I1RTD0
171151 229533.I1RPK5 pp 229533.I1RWL3
171152 229533.I1RPK5 pp 229533.I1RQ70
171153 229533.I1RPK5 pp 229533.I1RTQ9
171154 229533.I1RPK5 pp 229533.I1S1D0
171155 229533.I1RPK5 pp 229533.I1S235
171156 229533.I1RPK5 pp 229533.Q4I5I4
171157 229533.I1RPK5 pp 229533.V6QW25
171158 229533.I1RPK5 pp 229533.Q4ICA8
171159 229533.I1RPK5 pp 229533.I1RWI6
171160 229533.I1RPK5 pp 229533.I1RT11
171161 229533.I1RPK5 pp 229533.I1RT01
171162 229533.I1RPK5 pp 229533.I1RPU4
171163 229533.I1RPK5 pp 229533.I1RRT0
171164 229533.I1RPK5 pp 229533.I1RWQ1
171165 229533.I1RPK5 pp 229533.I1RWT6
171166 229533.I1RPK5 pp 229533.I1RW87
171167 229533.I1RPK5 pp 229533.I1RZB5
171168 229533.I1RPK5 pp 229533.I1RQA3
171169 229533.I1RPK5 pp 229533.I1RYL5
171170 229533.I1RPK5 pp 229533.I1RYV3
171171 229533.I1RPK5 pp 229533.I1S2W9
171172 229533.A0A1C3YMG7 pp 229533.V6QWZ7
171173 229533.A0A1C3YMG7 pp 229533.I1RZT1
171174 229533.A0A1C3YMG7 pp 229533.V6RRH2
171175 229533.A0A1C3YMG7 pp 229533.Q4I8X0
171176 229533.A0A1C3YMG7 pp 229533.I1RW68
171177 229533.A0A1C3YMG7 pp 229533.I1RCW1
171178 229533.A0A1C3YMG7 pp 229533.I1RYJ6
171179 229533.A0A1C3YMG7 pp 229533.I1RRP3
171180 229533.A0A1C3YMG7 pp 229533.I1RWS4
171181 229533.I1RNR3 pp 229533.V6RQ48
171182 229533.I1RNR3 pp 229533.I1RW32
171183 229533.I1RNR3 pp 229533.I1SAT1
171184 229533.I1RNR3 pp 229533.Q4ICG5
171185 229533.I1RNR3 pp 229533.I1RTF0

171186 229533.I1RNR3 pp 229533.I1RWA5
171187 229533.I1RNR3 pp 229533.V6RKE4
171188 229533.I1RNR3 pp 229533.I1RTD0
171189 229533.I1RNR3 pp 229533.I1RSP4
171190 229533.I1RNR3 pp 229533.I1RWA9
171191 229533.I1RNR3 pp 229533.I1RSL9
171192 229533.I1RNR3 pp 229533.I1RXU1
171193 229533.I1RNR3 pp 229533.I1RZA4
171194 229533.I1RNR3 pp 229533.I1RW76
171195 229533.I1RNR3 pp 229533.I1S635
171196 229533.I1RNR3 pp 229533.I1RS38
171197 229533.I1RNR3 pp 229533.I1RZ91
171198 229533.A0A1C3YJU7 pp 229533.V6RJP8
171199 229533.A0A1C3YJU7 pp 229533.I1RI95
171200 229533.A0A1C3YJU7 pp 229533.I1RC17
171201 229533.A0A1C3YJU7 pp 229533.I1R9N8
171202 229533.A0A1C3YJU7 pp 229533.I1RE65
171203 229533.A0A1C3YJU7 pp 229533.I1RW13
171204 229533.A0A1C3YJU7 pp 229533.I1RYC8
171205 229533.A0A1C3YJU7 pp 229533.A0A1C3YK94
171206 229533.A0A1C3YJU7 pp 229533.I1RXE6
171207 229533.A0A1C3YJU7 pp 229533.I1RQR6
171208 229533.A0A1C3YJU7 pp 229533.I1RKF4
171209 229533.A0A1C3YJU7 pp 229533.I1RAV5
171210 229533.A0A1C3YJU7 pp 229533.I1RLN1
171211 229533.A0A1C3YJU7 pp 229533.I1S8F8
171212 229533.A0A1C3YJU7 pp 229533.I1RFS5
171213 229533.A0A1C3YJU7 pp 229533.I1S266
171214 229533.A0A1C3YJU7 pp 229533.I1RRN2
171215 229533.A0A1C3YJU7 pp 229533.I1S6K9
171216 229533.A0A1C3YJU7 pp 229533.A0A1C3YLB6
171217 229533.A0A1C3YJU7 pp 229533.I1RA68
171218 229533.A0A1C3YJU7 pp 229533.I1RB38
171219 229533.A0A1C3YJU7 pp 229533.I1RKF5
171220 229533.A0A1C3YJU7 pp 229533.A0A1C3YKY6
171221 229533.A0A1C3YJU7 pp 229533.I1S5R5
171222 229533.A0A1C3YJU7 pp 229533.I1RE39
171223 229533.A0A1C3YJU7 pp 229533.I1S5U0
171224 229533.A0A1C3YJU7 pp 229533.I1RYA0
171225 229533.A0A1C3YJU7 pp 229533.I1RP38
171226 229533.A0A1C3YJU7 pp 229533.A0A1C3YLB0
171227 229533.A0A1C3YJU7 pp 229533.I1RQJ3
171228 229533.A0A1C3YJU7 pp 229533.I1RS29
171229 229533.A0A1C3YJU7 pp 229533.I1RAM9
171230 229533.A0A1C3YJU7 pp 229533.I1S0Q7
171231 229533.A0A1C3YJU7 pp 229533.I1S8S9
171232 229533.A0A1C3YJU7 pp 229533.I1S5X9
171233 229533.A0A1C3YJU7 pp 229533.I1S0K2
171234 229533.A0A1C3YJU7 pp 229533.I1RL81
171235 229533.A0A1C3YJU7 pp 229533.A0A1C3YMC4
171236 229533.A0A1C3YJU7 pp 229533.I1S1F3
171237 229533.A0A1C3YJU7 pp 229533.I1RWG0
171238 229533.A0A1C3YJU7 pp 229533.I1S0R8
171239 229533.A0A1C3YJU7 pp 229533.I1RZ68
171240 229533.A0A1C3YJU7 pp 229533.I1RG33
171241 229533.A0A1C3YJU7 pp 229533.I1RBT6
171242 229533.A0A1C3YJU7 pp 229533.I1RU95
171243 229533.A0A1C3YJU7 pp 229533.A0A1C3YM56
171244 229533.A0A1C3YJU7 pp 229533.I1RGI8
171245 229533.A0A1C3YJU7 pp 229533.I1RGJ4
171246 229533.A0A1C3YJU7 pp 229533.I1RYM5
171247 229533.A0A1C3YJU7 pp 229533.I1RYC7
171248 229533.A0A1C3YJU7 pp 229533.I1RN42
171249 229533.A0A1C3YJU7 pp 229533.I1S0P0
171250 229533.A0A1C3YJU7 pp 229533.I1RJB3
171251 229533.A0A1C3YJU7 pp 229533.I1RPR9
171252 229533.A0A1C3YJU7 pp 229533.I1RE66
171253 229533.A0A1C3YJU7 pp 229533.I1RBE6
171254 229533.A0A1C3YJU7 pp 229533.I1S464
171255 229533.A0A1C3YJU7 pp 229533.I1S212
171256 229533.A0A1C3YJU7 pp 229533.I1RX39
171257 229533.A0A1C3YJU7 pp 229533.I1RRX5
171258 229533.A0A1C3YJU7 pp 229533.I1RFJ6

171259	229533.A0A1C3YJU7	pp	229533.I1RA49
171260	229533.A0A1C3YJU7	pp	229533.A0A1C3YLM6
171261	229533.A0A1C3YJU7	pp	229533.I1S0T7
171262	229533.A0A1C3YJU7	pp	229533.I1RYQ1
171263	229533.A0A1C3YJU7	pp	229533.I1RFI4
171264	229533.A0A1C3YJU7	pp	229533.I1RA52
171265	229533.A0A1C3YJU7	pp	229533.I1RYX5
171266	229533.A0A1C3YJU7	pp	229533.I1S0R1
171267	229533.A0A1C3YJU7	pp	229533.I1RSC0
171268	229533.A0A1C3YJU7	pp	229533.I1RAW3
171269	229533.A0A1C3YJU7	pp	229533.I1RR65
171270	229533.A0A1C3YJU7	pp	229533.I1RSX0
171271	229533.A0A1C3YJU7	pp	229533.I1RYT8
171272	229533.A0A1C3YJU7	pp	229533.I1RY21
171273	229533.A0A1C3YJU7	pp	229533.V6R3J1
171274	229533.A0A1C3YJU7	pp	229533.I1RG62
171275	229533.A0A1C3YJU7	pp	229533.I1S5C6
171276	229533.A0A1C3YJU7	pp	229533.A0A1C3YMC1
171277	229533.A0A1C3YJU7	pp	229533.I1RRK9
171278	229533.A0A1C3YJU7	pp	229533.I1RAI4
171279	229533.A0A1C3YJU7	pp	229533.I1RY92
171280	229533.A0A1C3YJU7	pp	229533.I1RK39
171281	229533.A0A1C3YJU7	pp	229533.A0A1C3YM60
171282	229533.A0A1C3YJU7	pp	229533.I1RWA3
171283	229533.A0A1C3YJU7	pp	229533.I1RS05
171284	229533.A0A1C3YJU7	pp	229533.I1RB64
171285	229533.A0A1C3YJU7	pp	229533.I1RBB1
171286	229533.A0A1C3YJU7	pp	229533.I1RE40
171287	229533.A0A1C3YJU7	pp	229533.I1RKB7
171288	229533.A0A1C3YJU7	pp	229533.I1RMQ6
171289	229533.A0A1C3YJU7	pp	229533.I1RMK7
171290	229533.A0A1C3YJU7	pp	229533.I1RXD9
171291	229533.A0A1C3YJU7	pp	229533.I1RNM7
171292	229533.A0A1C3YJU7	pp	229533.I1S458
171293	229533.A0A1C3YJU7	pp	229533.I1R9Y4
171294	229533.A0A1C3YJU7	pp	229533.I1S0U0
171295	229533.A0A1C3YJU7	pp	229533.I1RXZ3
171296	229533.A0A1C3YJU7	pp	229533.A0A1C3YL46
171297	229533.A0A1C3YJU7	pp	229533.I1RPV3
171298	229533.A0A1C3YJU7	pp	229533.I1REF0
171299	229533.A0A1C3YJU7	pp	229533.I1S1N7
171300	229533.A0A098D0H2	pp	229533.A0A1C3YIN0
171301	229533.A0A098D0H2	pp	229533.Q4ICG5
171302	229533.A0A098D0H2	pp	229533.A0A1C3YK51
171303	229533.A0A098D0H2	pp	229533.I1RTD0
171304	229533.A0A098D0H2	pp	229533.I1RWP9
171305	229533.A0A098D0H2	pp	229533.I1RE15
171306	229533.A0A098D0H2	pp	229533.I1RXU1
171307	229533.A0A098D0H2	pp	229533.V6RKE4
171308	229533.A0A098D0H2	pp	229533.I1RZA4
171309	229533.A0A098D0H2	pp	229533.I1RQX3
171310	229533.A0A098D0H2	pp	229533.I1RCJ9
171311	229533.A0A098D0H2	pp	229533.I1RM09
171312	229533.A0A098D0H2	pp	229533.I1RE20
171313	229533.A0A098D0H2	pp	229533.V6RQ48
171314	229533.A0A098D0H2	pp	229533.I1RPK5
171315	229533.A0A098D0H2	pp	229533.I1RNR3
171316	229533.A0A1C3YM33	pp	229533.I1RPY8
171317	229533.A0A1C3YM33	pp	229533.I1RRS3
171318	229533.A0A1C3YM33	pp	229533.I1RB38
171319	229533.A0A1C3YM33	pp	229533.Q4IPZ1
171320	229533.A0A1C3YM33	pp	229533.I1RB86
171321	229533.A0A1C3YM33	pp	229533.Q4IB65
171322	229533.A0A1C3YM33	pp	229533.I1RCM1
171323	229533.A0A1C3YM33	pp	229533.I1RCH8
171324	229533.I1RS29	pp	229533.I1S1F3
171325	229533.I1RS29	pp	229533.I1S0T7
171326	229533.I1RS29	pp	229533.I1RWG0
171327	229533.I1RS29	pp	229533.I1RYQ3
171328	229533.I1RS29	pp	229533.I1RYI5
171329	229533.I1RS29	pp	229533.I1S464
171330	229533.I1RS29	pp	229533.I1S0R1
171331	229533.I1RS29	pp	229533.I1S8F0

171332	229533.I1RS29	pp	229533.I1RSC0
171333	229533.I1RS29	pp	229533.I1RYC7
171334	229533.I1RS29	pp	229533.I1S458
171335	229533.I1RS29	pp	229533.I1RYE5
171336	229533.I1RS29	pp	229533.I1RX39
171337	229533.I1RS29	pp	229533.I1S0P0
171338	229533.I1RS29	pp	229533.I1RY92
171339	229533.I1RS29	pp	229533.I1RUI0
171340	229533.I1RS29	pp	229533.I1S1N7
171341	229533.I1RS29	pp	229533.I1RXD9
171342	229533.I1RS29	pp	229533.I1S0U0
171343	229533.I1RS29	pp	229533.I1RYQ1
171344	229533.I1RS29	pp	229533.I1RWA3
171345	229533.I1RS29	pp	229533.I1S325
171346	229533.I1RS29	pp	229533.I1S5C6
171347	229533.I1RS29	pp	229533.I1RYT8
171348	229533.I1RS29	pp	229533.I1RYC8
171349	229533.I1RS29	pp	229533.I1RY21
171350	229533.I1RS29	pp	229533.I1S8F8
171351	229533.I1RS29	pp	229533.V6RJP8
171352	229533.I1RS29	pp	229533.I1S266
171353	229533.I1RS29	pp	229533.I1RW13
171354	229533.I1RS29	pp	229533.I1RYM5
171355	229533.I1RS29	pp	229533.V6RPN0
171356	229533.I1RS29	pp	229533.I1RXE6
171357	229533.I1RS29	pp	229533.I1RYA0
171358	229533.I1RS29	pp	229533.I1RZ68
171359	229533.I1RS29	pp	229533.I1RWA4
171360	229533.I1RS29	pp	229533.I1S0Q7
171361	229533.I1RS29	pp	229533.I1S5R5
171362	229533.I1RS29	pp	229533.I1S0R8
171363	229533.I1RS29	pp	229533.I1S212
171364	229533.I1RS29	pp	229533.I1S5X9
171365	229533.I1RS29	pp	229533.I1S8S9
171366	229533.I1RS29	pp	229533.I1S0K2
171367	229533.I1RS29	pp	229533.I1RU95
171368	229533.I1RS29	pp	229533.I1S5U0
171369	229533.I1RWT6	pp	229533.Q4I5R3
171370	229533.I1RWT6	pp	229533.Q4I8P5
171371	229533.I1RWT6	pp	229533.Q4HTT2
171372	229533.I1RWT6	pp	229533.I1RZM1
171373	229533.I1RWT6	pp	229533.I1RX09
171374	229533.I1RWT6	pp	229533.I1SA32
171375	229533.I1RWT6	pp	229533.I1S267
171376	229533.I1RWT6	pp	229533.Q4IER8
171377	229533.I1RWT6	pp	229533.I1S5B0
171378	229533.I1RWT6	pp	229533.I1RY03
171379	229533.I1RWT6	pp	229533.I1RYZ0
171380	229533.I1RWT6	pp	229533.I1RZM8
171381	229533.I1RWT6	pp	229533.I1RZB5
171382	229533.I1RWT6	pp	229533.I1RYV3
171383	229533.I1RWT6	pp	229533.I1RXZ4
171384	229533.I1RWT6	pp	229533.Q4IJM4
171385	229533.I1RWT6	pp	229533.Q4ICG5
171386	229533.I1RWT6	pp	229533.V6QW22
171387	229533.I1RWT6	pp	229533.V6QV09
171388	229533.I1RWT6	pp	229533.I1RZ12
171389	229533.I1RWT6	pp	229533.I1S1J7
171390	229533.I1RWT6	pp	229533.Q4IJP1
171391	229533.I1RWT6	pp	229533.I1S1W5
171392	229533.I1RWT6	pp	229533.I1S635
171393	229533.I1RWT6	pp	229533.I1RZ51
171394	229533.I1RWT6	pp	229533.I1RYZ9
171395	229533.I1RWT6	pp	229533.I1RYH9
171396	229533.I1RWT6	pp	229533.I1RY44
171397	229533.I1RWT6	pp	229533.I1RZ91
171398	229533.I1RWT6	pp	229533.I1S0X6
171399	229533.I1RWT6	pp	229533.I1RX70
171400	229533.I1RWT6	pp	229533.I1RXU0
171401	229533.I1RWT6	pp	229533.I1RXF5
171402	229533.I1RWT6	pp	229533.I1S016
171403	229533.I1RWT6	pp	229533.I1RZC3
171404	229533.I1RWT6	pp	229533.V6RMW6

171405 229533.I1RWT6 pp 229533.V6RIV9
171406 229533.I1RWT6 pp 229533.I1S0E8
171407 229533.I1RWT6 pp 229533.I1RWX5
171408 229533.I1RWT6 pp 229533.I1RZW8
171409 229533.I1RWT6 pp 229533.I1RZX3
171410 229533.I1RWT6 pp 229533.I1S276
171411 229533.I1RWT6 pp 229533.I1S0J1
171412 229533.I1RWT6 pp 229533.I1RYW3
171413 229533.I1RWT6 pp 229533.I1SAF7
171414 229533.I1RWT6 pp 229533.Q4IB89
171415 229533.I1RWT6 pp 229533.I1S0A6
171416 229533.I1RWT6 pp 229533.I1RZE4
171417 229533.I1RWT6 pp 229533.I1S3H8
171418 229533.I1RWT6 pp 229533.I1S0X7
171419 229533.I1RWT6 pp 229533.I1S0F6
171420 229533.I1RWT6 pp 229533.I1RZP8
171421 229533.I1RWT6 pp 229533.I1S8R3
171422 229533.I1RWT6 pp 229533.I1RY58
171423 229533.I1RWT6 pp 229533.I1S2G7
171424 229533.I1RWT6 pp 229533.I1S5R1
171425 229533.I1RWT6 pp 229533.I1S098
171426 229533.I1RWT6 pp 229533.I1RY32
171427 229533.I1RWT6 pp 229533.I1S004
171428 229533.I1RWT6 pp 229533.I1S1V5
171429 229533.I1RWT6 pp 229533.I1RXF0
171430 229533.I1RWT6 pp 229533.I1S2B8
171431 229533.I1RWT6 pp 229533.I1S7X9
171432 229533.I1RWT6 pp 229533.I1S0J7
171433 229533.I1RWT6 pp 229533.Q4IJK2
171434 229533.I1RWT6 pp 229533.I1S2H4
171435 229533.I1RWT6 pp 229533.V6RCG5
171436 229533.I1RWT6 pp 229533.I1S0L4
171437 229533.I1RWT6 pp 229533.V6RLW4
171438 229533.I1RWT6 pp 229533.I1S604
171439 229533.I1RWT6 pp 229533.V6RCZ8
171440 229533.I1RWT6 pp 229533.V6QV38
171441 229533.I1RWT6 pp 229533.I1S0J3
171442 229533.I1RWT6 pp 229533.V6R3X5
171443 229533.I1RWT6 pp 229533.I1S0N8
171444 229533.I1RWT6 pp 229533.I1S439
171445 229533.I1RWT6 pp 229533.I1RZK5
171446 229533.I1RWT6 pp 229533.Q4IR87
171447 229533.I1RWT6 pp 229533.I1S504
171448 229533.I1RWT6 pp 229533.I1RYY5
171449 229533.I1RWT6 pp 229533.I1SAT1
171450 229533.I1RWT6 pp 229533.Q4I7N9
171451 229533.I1RWT6 pp 229533.I1RXD7
171452 229533.I1RWT6 pp 229533.Q4IEA7
171453 229533.I1RWT6 pp 229533.I1RZZ1
171454 229533.I1RWT6 pp 229533.I1RYI3
171455 229533.I1RWT6 pp 229533.I1S1W4
171456 229533.I1RWT6 pp 229533.I1S0F5
171457 229533.I1RWT6 pp 229533.I1RXH6
171458 229533.I1RWT6 pp 229533.I1S0R7
171459 229533.I1RWT6 pp 229533.I1S0L3
171460 229533.I1RWT6 pp 229533.I1S0D1
171461 229533.I1RWT6 pp 229533.V6RTJ8
171462 229533.I1RWT6 pp 229533.I1S2K7
171463 229533.I1RWT6 pp 229533.I1S224
171464 229533.I1RWT6 pp 229533.I1RX46
171465 229533.I1RWT6 pp 229533.V6RVG3
171466 229533.I1RWT6 pp 229533.I1S0N4
171467 229533.I1RWT6 pp 229533.V6RKY7
171468 229533.I1RWT6 pp 229533.I1RX25
171469 229533.I1RWT6 pp 229533.I1SA33
171470 229533.I1RWT6 pp 229533.I1S334
171471 229533.I1RWT6 pp 229533.I1S0M2
171472 229533.I1RWT6 pp 229533.V6R042
171473 229533.I1RWT6 pp 229533.I1RZT1
171474 229533.I1RWT6 pp 229533.I1RZ94
171475 229533.I1RWT6 pp 229533.V6RGI1
171476 229533.I1RWT6 pp 229533.I1RZZ9
171477 229533.I1RWT6 pp 229533.I1S286

171478 229533.I1RWT6 pp 229533.I1S9S6
171479 229533.I1RWT6 pp 229533.I1S1C7
171480 229533.I1RWT6 pp 229533.I1S757
171481 229533.I1RWT6 pp 229533.I1RZ39
171482 229533.I1RWT6 pp 229533.I1RZP1
171483 229533.I1RWT6 pp 229533.I1RXU1
171484 229533.I1RWT6 pp 229533.I1RZK9
171485 229533.I1RWT6 pp 229533.I1RZ63
171486 229533.I1RWT6 pp 229533.I1RX64
171487 229533.I1RWT6 pp 229533.I1S549
171488 229533.I1RWT6 pp 229533.I1RY29
171489 229533.I1RWT6 pp 229533.I1S2E9
171490 229533.I1RWT6 pp 229533.I1S503
171491 229533.I1RWT6 pp 229533.I1S0M6
171492 229533.I1RWT6 pp 229533.I1RX38
171493 229533.I1RWT6 pp 229533.Q4HTT1
171494 229533.I1RWT6 pp 229533.I1S074
171495 229533.I1RWT6 pp 229533.Q4I7L0
171496 229533.A0A1C3YJQ2 pp 229533.I1S0T7
171497 229533.A0A1C3YJQ2 pp 229533.A0A1C3YLB6
171498 229533.A0A1C3YJQ2 pp 229533.I1RGJ4
171499 229533.A0A1C3YJQ2 pp 229533.A0A1C3YJU7
171500 229533.A0A1C3YJQ2 pp 229533.A0A1C3YMS7
171501 229533.A0A1C3YJQ2 pp 229533.I1RWG0
171502 229533.A0A1C3YJQ2 pp 229533.I1RA49
171503 229533.A0A1C3YJQ2 pp 229533.I1RW13
171504 229533.A0A1C3YJQ2 pp 229533.I1RYQ1
171505 229533.A0A1C3YJQ2 pp 229533.I1R9N8
171506 229533.A0A1C3YJQ2 pp 229533.I1S1F3
171507 229533.A0A1C3YJQ2 pp 229533.I1RXE6
171508 229533.A0A1C3YJQ2 pp 229533.I1RI95
171509 229533.A0A1C3YJQ2 pp 229533.I1RP02
171510 229533.A0A1C3YJQ2 pp 229533.V6RJP8
171511 229533.A0A1C3YJQ2 pp 229533.I1RYC7
171512 229533.A0A1C3YJQ2 pp 229533.I1RE65
171513 229533.A0A1C3YJQ2 pp 229533.I1RJB3
171514 229533.A0A1C3YJQ2 pp 229533.I1RE66
171515 229533.A0A1C3YJQ2 pp 229533.I1RBE6
171516 229533.A0A1C3YJQ2 pp 229533.I1RX39
171517 229533.A0A1C3YJQ2 pp 229533.I1S0P0
171518 229533.A0A1C3YJQ2 pp 229533.I1RKF4
171519 229533.A0A1C3YJQ2 pp 229533.I1RN42
171520 229533.A0A1C3YJQ2 pp 229533.I1RAW3
171521 229533.A0A1C3YJQ2 pp 229533.A0A1C3YM56
171522 229533.A0A1C3YJQ2 pp 229533.I1S8F0
171523 229533.A0A1C3YJQ2 pp 229533.I1RBT6
171524 229533.A0A1C3YJQ2 pp 229533.I1RAV5
171525 229533.A0A1C3YJQ2 pp 229533.I1RA52
171526 229533.A0A1C3YJQ2 pp 229533.I1S266
171527 229533.A0A1C3YJQ2 pp 229533.I1RRX5
171528 229533.A0A1C3YJQ2 pp 229533.I1RQR6
171529 229533.A0A1C3YJQ2 pp 229533.V6RPN0
171530 229533.A0A1C3YJQ2 pp 229533.I1S464
171531 229533.A0A1C3YJQ2 pp 229533.I1RG62
171532 229533.A0A1C3YJQ2 pp 229533.I1RYT8
171533 229533.A0A1C3YJQ2 pp 229533.I1RFS5
171534 229533.A0A1C3YJQ2 pp 229533.I1RSC0
171535 229533.A0A1C3YJQ2 pp 229533.I1RA68
171536 229533.A0A1C3YJQ2 pp 229533.I1S0R1
171537 229533.A0A1C3YJQ2 pp 229533.I1RLN1
171538 229533.A0A1C3YJQ2 pp 229533.I1RQJ3
171539 229533.A0A1C3YJQ2 pp 229533.I1RFJ6
171540 229533.A0A1C3YJQ2 pp 229533.I1RRK9
171541 229533.A0A1C3YJQ2 pp 229533.I1R9M0
171542 229533.A0A1C3YJQ2 pp 229533.I1S5R5
171543 229533.A0A1C3YJQ2 pp 229533.I1RKF5
171544 229533.A0A1C3YJQ2 pp 229533.I1S212
171545 229533.A0A1C3YJQ2 pp 229533.I1RBB1
171546 229533.A0A1C3YJQ2 pp 229533.I1S0Q7
171547 229533.A0A1C3YJQ2 pp 229533.I1S5C6
171548 229533.A0A1C3YJQ2 pp 229533.A0A1C3YM60
171549 229533.A0A1C3YJQ2 pp 229533.I1RMK7
171550 229533.A0A1C3YJQ2 pp 229533.I1RE39

171551 229533.A0A1C3YJQ2 pp 229533.I1RK39
171552 229533.A0A1C3YJQ2 pp 229533.I1RAI4
171553 229533.A0A1C3YJQ2 pp 229533.I1RY21
171554 229533.A0A1C3YJQ2 pp 229533.I1RE40
171555 229533.A0A1C3YJQ2 pp 229533.I1RWA3
171556 229533.A0A1C3YJQ2 pp 229533.I1S0K2
171557 229533.A0A1C3YJQ2 pp 229533.I1S0R8
171558 229533.A0A1C3YJQ2 pp 229533.I1RB64
171559 229533.A0A1C3YJQ2 pp 229533.A0A1C3YLB0
171560 229533.A0A1C3YJQ2 pp 229533.I1RS05
171561 229533.A0A1C3YJQ2 pp 229533.I1S5U0
171562 229533.A0A1C3YJQ2 pp 229533.I1RJU3
171563 229533.A0A1C3YJQ2 pp 229533.A0A1C3YMC4
171564 229533.A0A1C3YJQ2 pp 229533.I1RP38
171565 229533.A0A1C3YJQ2 pp 229533.I1RY92
171566 229533.A0A1C3YJQ2 pp 229533.I1RMQ6
171567 229533.A0A1C3YJQ2 pp 229533.I1S0B7
171568 229533.A0A1C3YJQ2 pp 229533.I1S0U0
171569 229533.A0A1C3YJQ2 pp 229533.I1RNM7
171570 229533.A0A1C3YJQ2 pp 229533.I1RZ68
171571 229533.A0A1C3YJQ2 pp 229533.I1S5X9
171572 229533.A0A1C3YJQ2 pp 229533.I1R9Y4
171573 229533.A0A1C3YJQ2 pp 229533.I1RKB7
171574 229533.A0A1C3YJQ2 pp 229533.I1RXD9
171575 229533.A0A1C3YJQ2 pp 229533.I1S458
171576 229533.A0A1C3YJQ2 pp 229533.I1S1N7
171577 229533.A0A1C3YJQ2 pp 229533.I1RAM9
171578 229533.A0A1C3YJQ2 pp 229533.I1RS29
171579 229533.A0A1C3YJQ2 pp 229533.I1RGI8
171580 229533.A0A1C3YJQ2 pp 229533.I1REF0
171581 229533.A0A1C3YJQ2 pp 229533.I1RU95
171582 229533.A0A1C3YJQ2 pp 229533.I1S8S9
171583 229533.I1RBR0 pp 229533.I1RT65
171584 229533.I1RBR0 pp 229533.I1RZR2
171585 229533.I1RBR0 pp 229533.I1RQP5
171586 229533.I1RBR0 pp 229533.I1RQX3
171587 229533.I1RBR0 pp 229533.Q4I963
171588 229533.I1RBR0 pp 229533.I1RQM8
171589 229533.I1RBR0 pp 229533.I1RKI1
171590 229533.I1RBR0 pp 229533.I1S8E0
171591 229533.I1RBR0 pp 229533.I1RZL3
171592 229533.I1RBR0 pp 229533.I1RSD7
171593 229533.I1RBR0 pp 229533.I1RCT2
171594 229533.I1RBR0 pp 229533.I1RRH0
171595 229533.I1RBR0 pp 229533.I1REK3
171596 229533.I1RBR0 pp 229533.I1RCJ9
171597 229533.I1RBR0 pp 229533.I1RN81
171598 229533.I1RBR0 pp 229533.I1S0X0
171599 229533.I1RBR0 pp 229533.I1S270
171600 229533.I1RBR0 pp 229533.V6QW25
171601 229533.I1RBR0 pp 229533.I1RE15
171602 229533.I1RBR0 pp 229533.I1RKR9
171603 229533.I1RBR0 pp 229533.I1RT41
171604 229533.I1RBR0 pp 229533.I1RST4
171605 229533.I1RBR0 pp 229533.I1RZ91
171606 229533.I1RBR0 pp 229533.I1RJB5
171607 229533.I1RBR0 pp 229533.I1RWI6
171608 229533.I1RBR0 pp 229533.I1S559
171609 229533.I1RBR0 pp 229533.I1RN49
171610 229533.I1RBR0 pp 229533.I1S034
171611 229533.I1RBR0 pp 229533.I1RX09
171612 229533.I1RBR0 pp 229533.I1S0Z3
171613 229533.A0A1C3YLB5 pp 229533.Q4ILI9
171614 229533.A0A1C3YLB5 pp 229533.I1RTQ9
171615 229533.A0A1C3YLB5 pp 229533.A0A1C3YN54
171616 229533.I1RQA3 pp 229533.I1RZ91
171617 229533.I1RQA3 pp 229533.I1RQB4
171618 229533.I1RQA3 pp 229533.I1RUG3
171619 229533.I1RQA3 pp 229533.I1RXU0
171620 229533.I1RQA3 pp 229533.V6RIV9
171621 229533.I1RQA3 pp 229533.I1RY44
171622 229533.I1RQA3 pp 229533.I1RXF5
171623 229533.I1RQA3 pp 229533.I1RSQ3

171624 229533.I1RQA3 pp 229533.I1RRE5
171625 229533.I1RQA3 pp 229533.I1RT03
171626 229533.I1RQA3 pp 229533.I1S016
171627 229533.I1RQA3 pp 229533.I1RZC3
171628 229533.I1RQA3 pp 229533.I1RRN5
171629 229533.I1RQA3 pp 229533.V6RMW6
171630 229533.I1RQA3 pp 229533.I1RW84
171631 229533.I1RQA3 pp 229533.I1S0J1
171632 229533.I1RQA3 pp 229533.I1RSR9
171633 229533.I1RQA3 pp 229533.I1RRV1
171634 229533.I1RQA3 pp 229533.Q4IB89
171635 229533.I1RQA3 pp 229533.I1RWN6
171636 229533.I1RQA3 pp 229533.I1RS17
171637 229533.I1RQA3 pp 229533.I1RSX8
171638 229533.I1RQA3 pp 229533.I1RZX3
171639 229533.I1RQA3 pp 229533.I1SAF7
171640 229533.I1RQA3 pp 229533.I1RQD1
171641 229533.I1RQA3 pp 229533.I1S0A6
171642 229533.I1RQA3 pp 229533.I1S276
171643 229533.I1RQA3 pp 229533.I1RZE4
171644 229533.I1RQA3 pp 229533.I1RSI3
171645 229533.I1RQA3 pp 229533.I1RWT5
171646 229533.I1RQA3 pp 229533.I1RQE7
171647 229533.I1RQA3 pp 229533.I1RWX5
171648 229533.I1RQA3 pp 229533.I1S3H8
171649 229533.I1RQA3 pp 229533.I1RRI2
171650 229533.I1RQA3 pp 229533.I1S0F6
171651 229533.I1RQA3 pp 229533.I1S0E8
171652 229533.I1RQA3 pp 229533.I1RZW8
171653 229533.I1RQA3 pp 229533.I1RY58
171654 229533.I1RQA3 pp 229533.I1RW32
171655 229533.I1RQA3 pp 229533.I1S2G7
171656 229533.I1RQA3 pp 229533.I1RYW3
171657 229533.I1RQA3 pp 229533.I1RY32
171658 229533.I1RQA3 pp 229533.I1RWK3
171659 229533.I1RQA3 pp 229533.I1RZP8
171660 229533.I1RQA3 pp 229533.I1RW83
171661 229533.I1RQA3 pp 229533.I1RT28
171662 229533.I1RQA3 pp 229533.I1S098
171663 229533.I1RQA3 pp 229533.I1S7X9
171664 229533.I1RQA3 pp 229533.I1S0J7
171665 229533.I1RQA3 pp 229533.I1RRB5
171666 229533.I1RQA3 pp 229533.I1S8R3
171667 229533.I1RQA3 pp 229533.I1RUF8
171668 229533.I1RQA3 pp 229533.I1RTB2
171669 229533.I1RQA3 pp 229533.I1RSV2
171670 229533.I1RQA3 pp 229533.V6RLW4
171671 229533.I1RQA3 pp 229533.I1RSZ3
171672 229533.I1RQA3 pp 229533.I1RT40
171673 229533.I1RQA3 pp 229533.I1S2B8
171674 229533.I1RQA3 pp 229533.I1S004
171675 229533.I1RQA3 pp 229533.I1RRM5
171676 229533.I1RQA3 pp 229533.I1RZM1
171677 229533.I1RQA3 pp 229533.I1S5R1
171678 229533.I1RQA3 pp 229533.I1RUN8
171679 229533.I1RQA3 pp 229533.I1S1V5
171680 229533.I1RQA3 pp 229533.I1SA32
171681 229533.I1RQA3 pp 229533.I1RSZ1
171682 229533.I1RQA3 pp 229533.I1RVL8
171683 229533.I1RQA3 pp 229533.I1S0X7
171684 229533.I1RQA3 pp 229533.I1RX09
171685 229533.I1RQA3 pp 229533.I1S0L4
171686 229533.I1RQA3 pp 229533.I1RRY9
171687 229533.I1RQA3 pp 229533.I1S2H4
171688 229533.I1RQA3 pp 229533.I1RS18
171689 229533.I1RQA3 pp 229533.I1RU69
171690 229533.I1RQA3 pp 229533.Q4I5R3
171691 229533.I1RQA3 pp 229533.I1RUQ5
171692 229533.I1RQA3 pp 229533.V6R3X5
171693 229533.I1RQA3 pp 229533.I1RS38
171694 229533.I1RQA3 pp 229533.Q4I8P5
171695 229533.I1RQA3 pp 229533.I1RY03
171696 229533.I1RQA3 pp 229533.I1RU02

171697 229533.I1RQA3 pp 229533.I1S267
171698 229533.I1RQA3 pp 229533.I1S0J3
171699 229533.I1RQA3 pp 229533.Q4IER8
171700 229533.I1RQA3 pp 229533.I1RU88
171701 229533.I1RQA3 pp 229533.I1RZM8
171702 229533.I1RQA3 pp 229533.I1S5B0
171703 229533.I1RQA3 pp 229533.Q4HTT2
171704 229533.I1RQA3 pp 229533.I1RZB5
171705 229533.I1RQA3 pp 229533.I1RW99
171706 229533.I1RQA3 pp 229533.I1RQS4
171707 229533.I1RQA3 pp 229533.I1RTG0
171708 229533.I1RQA3 pp 229533.I1RXZ4
171709 229533.I1RQA3 pp 229533.I1RVQ0
171710 229533.I1RQA3 pp 229533.I1RW38
171711 229533.I1RQA3 pp 229533.V6QW22
171712 229533.I1RQA3 pp 229533.Q4IJP1
171713 229533.I1RQA3 pp 229533.I1RYZ0
171714 229533.I1RQA3 pp 229533.I1RSQ7
171715 229533.I1RQA3 pp 229533.I1RZ12
171716 229533.I1RQA3 pp 229533.I1RYV3
171717 229533.I1RQA3 pp 229533.Q4IJM4
171718 229533.I1RQA3 pp 229533.I1RZ51
171719 229533.I1RQA3 pp 229533.I1S1J7
171720 229533.I1RQA3 pp 229533.I1RUQ6
171721 229533.I1RQA3 pp 229533.V6QV09
171722 229533.I1RQA3 pp 229533.Q4ICG5
171723 229533.I1RQA3 pp 229533.I1RYH9
171724 229533.I1RQA3 pp 229533.I1RYZ9
171725 229533.I1RQA3 pp 229533.I1RRT1
171726 229533.I1RQA3 pp 229533.I1S635
171727 229533.I1RQA3 pp 229533.I1RRX6
171728 229533.I1RQA3 pp 229533.I1RWJ8
171729 229533.I1RQA3 pp 229533.I1RRL5
171730 229533.I1RQA3 pp 229533.I1SA33
171731 229533.I1RQA3 pp 229533.I1S0X6
171732 229533.I1RQA3 pp 229533.I1RW25
171733 229533.I1RQA3 pp 229533.I1RX70
171734 229533.I1RQA3 pp 229533.I1S1W5
171735 229533.I1RQA3 pp 229533.V6RVG3
171736 229533.I1RQA3 pp 229533.I1S0N4
171737 229533.I1RQA3 pp 229533.I1RWJ6
171738 229533.I1RQA3 pp 229533.I1RVX0
171739 229533.I1RQA3 pp 229533.I1RVN2
171740 229533.I1RQA3 pp 229533.I1RT36
171741 229533.I1RQA3 pp 229533.I1RTN0
171742 229533.I1RQA3 pp 229533.I1RWN5
171743 229533.I1RQA3 pp 229533.V6RKY7
171744 229533.I1RQA3 pp 229533.V6RGI1
171745 229533.I1RQA3 pp 229533.I1RSK1
171746 229533.I1RQA3 pp 229533.I1RS41
171747 229533.I1RQA3 pp 229533.I1RRY3
171748 229533.I1RQA3 pp 229533.I1S9S6
171749 229533.I1RQA3 pp 229533.I1S286
171750 229533.I1RQA3 pp 229533.I1S1W4
171751 229533.I1RQA3 pp 229533.I1S0M2
171752 229533.I1RQA3 pp 229533.I1S2K7
171753 229533.I1RQA3 pp 229533.I1RRW0
171754 229533.I1RQA3 pp 229533.I1RZT1
171755 229533.I1RQA3 pp 229533.I1RW46
171756 229533.I1RQA3 pp 229533.I1RRP7
171757 229533.I1RQA3 pp 229533.I1RSD1
171758 229533.I1RQA3 pp 229533.V6R042
171759 229533.I1RQA3 pp 229533.I1S0D1
171760 229533.I1RQA3 pp 229533.I1S1C7
171761 229533.I1RQA3 pp 229533.I1RR25
171762 229533.I1RQA3 pp 229533.I1RSH9
171763 229533.I1RQA3 pp 229533.I1S757
171764 229533.I1RQA3 pp 229533.I1RZK9
171765 229533.I1RQA3 pp 229533.I1RRY4
171766 229533.I1RQA3 pp 229533.I1RTH6
171767 229533.I1RQA3 pp 229533.I1S0M6
171768 229533.I1RQA3 pp 229533.I1RX64
171769 229533.I1RQA3 pp 229533.I1RTD5

171770	229533.I1RQA3	pp	229533.I1S549
171771	229533.I1RQA3	pp	229533.I1RW76
171772	229533.I1RQA3	pp	229533.I1RXU1
171773	229533.I1RQA3	pp	229533.I1RZP1
171774	229533.I1RQA3	pp	229533.I1RY29
171775	229533.I1RQA3	pp	229533.Q4HTT1
171776	229533.I1RQA3	pp	229533.I1RV91
171777	229533.I1RQA3	pp	229533.I1S604
171778	229533.I1RQA3	pp	229533.I1RRT8
171779	229533.I1RQA3	pp	229533.I1S503
171780	229533.I1RQA3	pp	229533.Q4I7L0
171781	229533.I1RQA3	pp	229533.I1S2E9
171782	229533.I1RQA3	pp	229533.I1RX38
171783	229533.I1RQA3	pp	229533.I1RXF0
171784	229533.I1RQA3	pp	229533.I1S074
171785	229533.I1RQA3	pp	229533.I1RWI5
171786	229533.I1RQA3	pp	229533.I1RWI8
171787	229533.I1RQA3	pp	229533.V6RCG5
171788	229533.I1RQA3	pp	229533.I1RZZ9
171789	229533.I1RQA3	pp	229533.I1RT04
171790	229533.I1RQA3	pp	229533.I1RZ63
171791	229533.I1RQA3	pp	229533.I1S504
171792	229533.I1RQA3	pp	229533.V6RCZ8
171793	229533.I1RQA3	pp	229533.I1RR73
171794	229533.I1RQA3	pp	229533.I1RSI0
171795	229533.I1RQA3	pp	229533.Q4IEA7
171796	229533.I1RQA3	pp	229533.I1RS07
171797	229533.I1RQA3	pp	229533.Q4IR87
171798	229533.I1RQA3	pp	229533.I1RSS1
171799	229533.I1RQA3	pp	229533.I1RRT0
171800	229533.I1RQA3	pp	229533.I1RZ39
171801	229533.I1RQA3	pp	229533.V6QV38
171802	229533.I1RQA3	pp	229533.I1RZZ1
171803	229533.I1RQA3	pp	229533.I1S439
171804	229533.I1RQA3	pp	229533.I1RRU9
171805	229533.I1RQA3	pp	229533.Q4I7N9
171806	229533.I1RQA3	pp	229533.I1RZK5
171807	229533.I1RQA3	pp	229533.I1RST9
171808	229533.I1RQA3	pp	229533.I1RYY5
171809	229533.I1RQA3	pp	229533.I1RW71
171810	229533.I1RQA3	pp	229533.I1S0N8
171811	229533.I1RQA3	pp	229533.Q4IJK2
171812	229533.I1RQA3	pp	229533.I1SAT1
171813	229533.I1RQA3	pp	229533.I1RTC2
171814	229533.I1RQA3	pp	229533.V6RTJ8
171815	229533.I1RQA3	pp	229533.I1RT62
171816	229533.I1RQA3	pp	229533.I1RX46
171817	229533.I1RQA3	pp	229533.I1RYI3
171818	229533.I1RQA3	pp	229533.I1RXD7
171819	229533.I1RQA3	pp	229533.I1S0F5
171820	229533.I1RQA3	pp	229533.I1S0L3
171821	229533.I1RQA3	pp	229533.I1RQC9
171822	229533.I1RQA3	pp	229533.I1S224
171823	229533.I1RQA3	pp	229533.I1RSI4
171824	229533.I1RQA3	pp	229533.I1S0R7
171825	229533.I1RQA3	pp	229533.I1RX25
171826	229533.I1RQA3	pp	229533.I1RXH6
171827	229533.I1RQA3	pp	229533.I1RRG3
171828	229533.I1RQA3	pp	229533.I1RSX7
171829	229533.I1RQA3	pp	229533.I1S334
171830	229533.I1RQA3	pp	229533.I1RV66
171831	229533.I1RQA3	pp	229533.I1RR94
171832	229533.I1RQA3	pp	229533.I1RZ94
171833	229533.I1RQA3	pp	229533.I1RQA9
171834	229533.I1RQA3	pp	229533.I1RR24
171835	229533.I1S8S9	pp	229533.Q4HZ34
171836	229533.I1S8S9	pp	229533.Q4I174
171837	229533.I1S8S9	pp	229533.V6RUB1
171838	229533.I1S8S9	pp	229533.V6QUG0
171839	229533.I1S8S9	pp	229533.Q4HVQ9
171840	229533.I1S8S9	pp	229533.V6R1X4
171841	229533.I1S8S9	pp	229533.V6R3J1
171842	229533.I1S8S9	pp	229533.V6RJP8

171843 229533.I1S7W2 pp 229533.Q4IPI4
171844 229533.I1S7W2 pp 229533.I1S8X0
171845 229533.I1S7W2 pp 229533.Q4I2D7
171846 229533.I1S7W2 pp 229533.I1S8E0
171847 229533.I1S7W2 pp 229533.V6QUU6
171848 229533.I1S7W2 pp 229533.Q4INI2
171849 229533.I1S7W2 pp 229533.Q4I963
171850 229533.I1S7W2 pp 229533.V6RB81
171851 229533.I1S7W2 pp 229533.V6RR76
171852 229533.A0A098D0D9 pp 229533.A0A1C3YM33
171853 229533.A0A098D0D9 pp 229533.I1RWT6
171854 229533.A0A098D0D9 pp 229533.I1RQA3
171855 229533.A0A098D828 pp 229533.V6RJP8
171856 229533.A0A098D828 pp 229533.I1R9N8
171857 229533.A0A098D828 pp 229533.I1RP02
171858 229533.A0A098D828 pp 229533.I1RYT8
171859 229533.A0A098D828 pp 229533.A0A0E0RPV7
171860 229533.A0A098D828 pp 229533.I1RA52
171861 229533.A0A098D828 pp 229533.I1RMK7
171862 229533.A0A098D828 pp 229533.A0A1C3YIZ4
171863 229533.A0A098D828 pp 229533.I1RWA3
171864 229533.A0A098D828 pp 229533.I1S0R1
171865 229533.A0A098D828 pp 229533.A0A1C3YL46
171866 229533.A0A098D828 pp 229533.I1S5C6
171867 229533.A0A098D828 pp 229533.I1RY92
171868 229533.A0A098D828 pp 229533.I1RY21
171869 229533.A0A098D828 pp 229533.A0A0E0SAR4
171870 229533.A0A098D828 pp 229533.I1S458
171871 229533.A0A098D828 pp 229533.V6QWX8
171872 229533.A0A098D828 pp 229533.I1S464
171873 229533.A0A098D828 pp 229533.I1RYQ3
171874 229533.A0A098D828 pp 229533.I1RXD9
171875 229533.A0A098D828 pp 229533.I1S0P0
171876 229533.A0A098D828 pp 229533.A0A1C3YM56
171877 229533.A0A098D828 pp 229533.I1S5X9
171878 229533.A0A098D828 pp 229533.I1RS05
171879 229533.A0A098D828 pp 229533.I1RKF5
171880 229533.A0A098D828 pp 229533.I1RFJ6
171881 229533.A0A098D828 pp 229533.I1RKF4
171882 229533.A0A098D828 pp 229533.I1S0K2
171883 229533.A0A098D828 pp 229533.A0A1C3YLB6
171884 229533.A0A098D828 pp 229533.A0A1C3YJU7
171885 229533.A0A098D828 pp 229533.I1RE65
171886 229533.A0A098D828 pp 229533.I1RS29
171887 229533.A0A098D828 pp 229533.A0A1C3YJQ2
171888 229533.A0A098D828 pp 229533.I1S8S9
171889 229533.I1RNC0 pp 229533.I1RV91
171890 229533.I1RNC0 pp 229533.I1RZ63
171891 229533.I1RNC0 pp 229533.I1S2E9
171892 229533.I1RNC0 pp 229533.I1RQ88
171893 229533.I1RNC0 pp 229533.V6RKY7
171894 229533.I1RNC0 pp 229533.V6RVG3
171895 229533.I1RNC0 pp 229533.I1S0J7
171896 229533.I1RNC0 pp 229533.I1RY44
171897 229533.I1RNC0 pp 229533.I1RQM0
171898 229533.I1RNC0 pp 229533.I1S1W4
171899 229533.I1RNC0 pp 229533.Q4HYB8
171900 229533.I1RNC0 pp 229533.I1S1D3
171901 229533.I1RNC0 pp 229533.I1RUN8
171902 229533.I1RNC0 pp 229533.I1RNP7
171903 229533.I1RNC0 pp 229533.I1S1V5
171904 229533.I1RNC0 pp 229533.I1RYF8
171905 229533.I1RNC0 pp 229533.I1RPL5
171906 229533.I1RNC0 pp 229533.I1S0D1
171907 229533.I1RNC0 pp 229533.I1RZE4
171908 229533.I1RNC0 pp 229533.I1RWT6
171909 229533.I1RNC0 pp 229533.I1RX38
171910 229533.I1RNC0 pp 229533.I1S0M2
171911 229533.I1RNC0 pp 229533.I1RZH4
171912 229533.I1RNC0 pp 229533.I1RW16
171913 229533.I1RNC0 pp 229533.I1RPU4
171914 229533.I1RNC0 pp 229533.I1RPW0
171915 229533.I1RNC0 pp 229533.I1RRW0

171916 229533.I1RNC0 pp 229533.I1RRP7
171917 229533.I1RNC0 pp 229533.I1RRY3
171918 229533.I1RNC0 pp 229533.I1RQ80
171919 229533.I1RNC0 pp 229533.Q4HTT2
171920 229533.I1RNC0 pp 229533.I1RQ26
171921 229533.I1RNC0 pp 229533.I1RQS4
171922 229533.I1RNC0 pp 229533.I1RSP7
171923 229533.I1RNC0 pp 229533.I1RYH9
171924 229533.I1RNC0 pp 229533.Q4I5Z5
171925 229533.I1RNC0 pp 229533.I1S224
171926 229533.I1RNC0 pp 229533.I1S439
171927 229533.I1RNC0 pp 229533.I1RYM2
171928 229533.I1RNC0 pp 229533.I1S6Y1
171929 229533.I1RNC0 pp 229533.I1RQA3
171930 229533.I1RXY4 pp 229533.I1S3G4
171931 229533.I1RXY4 pp 229533.I1S1W8
171932 229533.I1RYV3 pp 229533.Q4IAK7
171933 229533.I1RYV3 pp 229533.I1S625
171934 229533.I1RYV3 pp 229533.V6R3J1
171935 229533.I1RYV3 pp 229533.I1SAG3
171936 229533.I1RYV3 pp 229533.Q4IMD1
171937 229533.I1RYV3 pp 229533.I1S0E2
171938 229533.I1RYV3 pp 229533.I1SAA0
171939 229533.I1RYV3 pp 229533.Q4IB50
171940 229533.I1RYV3 pp 229533.Q4HTT2
171941 229533.I1RYV3 pp 229533.I1S2E9
171942 229533.I1RYV3 pp 229533.Q4IJP1
171943 229533.I1RYV3 pp 229533.Q4I5R3
171944 229533.I1RYV3 pp 229533.I1S7H9
171945 229533.I1RYV3 pp 229533.Q4HTT1
171946 229533.I1RYV3 pp 229533.I1RYX3
171947 229533.I1RYV3 pp 229533.I1S566
171948 229533.I1RYV3 pp 229533.I1S259
171949 229533.I1RYV3 pp 229533.I1S6Y1
171950 229533.I1RYV3 pp 229533.Q4IER8
171951 229533.I1RYV3 pp 229533.I1S4Z5
171952 229533.I1RYV3 pp 229533.I1S193
171953 229533.I1RYV3 pp 229533.I1S1U7
171954 229533.I1RYV3 pp 229533.I1S926
171955 229533.I1RYV3 pp 229533.Q4IN63
171956 229533.I1RYV3 pp 229533.I1S567
171957 229533.I1RYV3 pp 229533.I1S1X2
171958 229533.I1RYV3 pp 229533.I1S0L6
171959 229533.I1RYV3 pp 229533.I1SA28
171960 229533.I1RYV3 pp 229533.I1S8J7
171961 229533.I1RYV3 pp 229533.I1S3A2
171962 229533.A0A1C3YKX2 pp 229533.I1RTQ9
171963 229533.A0A1C3YKX2 pp 229533.I1RKR7
171964 229533.A0A1C3YKX2 pp 229533.Q4ILI9
171965 229533.A0A1C3YKX2 pp 229533.I1RVN2
171966 229533.A0A1C3YKX2 pp 229533.A0A1C3YN54
171967 229533.I1RZD6 pp 229533.I1S270
171968 229533.I1RZD6 pp 229533.I1S8E0
171969 229533.I1RZD6 pp 229533.I1S7W2
171970 229533.I1RZD6 pp 229533.Q4I963
171971 229533.I1RZD6 pp 229533.I1RZV1
171972 229533.I1RWT2 pp 229533.I1S1Y7
171973 229533.I1RWT2 pp 229533.I1S0F0
171974 229533.I1RWT2 pp 229533.I1RZQ6
171975 229533.I1RWT2 pp 229533.I1S6Y1
171976 229533.I1RWT2 pp 229533.Q4IER8
171977 229533.I1RWT2 pp 229533.Q4IMD1
171978 229533.I1RWT2 pp 229533.I1RZM1
171979 229533.I1RXC4 pp 229533.V6R042
171980 229533.I1RXC4 pp 229533.I1RZV3
171981 229533.I1RXC4 pp 229533.V6RMW6
171982 229533.I1RXC4 pp 229533.I1S5R6
171983 229533.I1RXC4 pp 229533.I1RZ79
171984 229533.I1RXC4 pp 229533.I1S437
171985 229533.I1RXC4 pp 229533.I1RYW3
171986 229533.I1RXC4 pp 229533.Q4I624
171987 229533.I1RXC4 pp 229533.I1S0J1
171988 229533.I1RXC4 pp 229533.I1S1C7

171989	229533.I1RXC4	pp	229533.I1S1W9
171990	229533.I1RXC4	pp	229533.I1RYI5
171991	229533.I1RXC4	pp	229533.I1S0L4
171992	229533.I1RXC4	pp	229533.Q4IPZ1
171993	229533.I1RXC4	pp	229533.I1S0W4
171994	229533.I1RXC4	pp	229533.I1RYR0
171995	229533.I1RXC4	pp	229533.I1RXF8
171996	229533.I1RXC4	pp	229533.I1S1B2
171997	229533.I1RXC4	pp	229533.I1S5R1
171998	229533.I1RXC4	pp	229533.I1RZB2
171999	229533.I1RXC4	pp	229533.I1S2H4
172000	229533.I1RXC4	pp	229533.I1S5B0
172001	229533.I1RXC4	pp	229533.V6QUE6
172002	229533.I1RXC4	pp	229533.I1S2B8
172003	229533.I1RXC4	pp	229533.I1S821
172004	229533.I1RXC4	pp	229533.I1S277
172005	229533.I1RXC4	pp	229533.I1S9Y1
172006	229533.I1RXC4	pp	229533.I1S6K9
172007	229533.I1RXC4	pp	229533.I1S1J7
172008	229533.I1RXC4	pp	229533.I1RZ39
172009	229533.I1RXC4	pp	229533.Q4IJK2
172010	229533.I1RXC4	pp	229533.I1RZZ9
172011	229533.I1RXC4	pp	229533.I1RZK5
172012	229533.I1RXC4	pp	229533.I1RXF0
172013	229533.I1RXC4	pp	229533.I1RY86
172014	229533.I1RXC4	pp	229533.V6RCZ8
172015	229533.I1RXC4	pp	229533.I1RYS0
172016	229533.I1RXC4	pp	229533.I1RZM8
172017	229533.I1RXC4	pp	229533.V6RTJ8
172018	229533.I1RXC4	pp	229533.I1RXD7
172019	229533.I1RXC4	pp	229533.I1S438
172020	229533.I1RXC4	pp	229533.I1S4S7
172021	229533.I1RXC4	pp	229533.I1RXD6
172022	229533.I1RXC4	pp	229533.I1S4S4
172023	229533.I1RVJ6	pp	229533.I1S1W3
172024	229533.I1RVJ6	pp	229533.I1S0R7
172025	229533.I1RVJ6	pp	229533.I1RZ52
172026	229533.I1RVJ6	pp	229533.I1S0A0
172027	229533.I1RVJ6	pp	229533.V6QVM5
172028	229533.I1RVJ6	pp	229533.Q4IMJ1
172029	229533.I1RVJ6	pp	229533.I1RW06
172030	229533.I1RVJ6	pp	229533.V6RGN5
172031	229533.Q4HTT2	pp	229533.Q5I2J3
172032	229533.Q4HTT2	pp	229533.Q4IPI1
172033	229533.Q4HTT2	pp	229533.Q4HZS8
172034	229533.Q4HTT2	pp	229533.Q4HYQ4
172035	229533.Q4HTT2	pp	229533.Q4IJP1
172036	229533.Q4HTT2	pp	229533.V6QWX8
172037	229533.Q4HTT2	pp	229533.Q4I948
172038	229533.Q4HTT2	pp	229533.V6RPS5
172039	229533.Q4HTT2	pp	229533.Q4IQX3
172040	229533.Q4HTT2	pp	229533.Q4IR08
172041	229533.Q4HTT2	pp	229533.Q4I5I4
172042	229533.Q4HTT2	pp	229533.Q4ICA8
172043	229533.Q4HTT2	pp	229533.Q4IJU0
172044	229533.Q4HTT2	pp	229533.Q4I5R3
172045	229533.Q4HTT2	pp	229533.V6QUE6
172046	229533.Q4HTT2	pp	229533.Q4I7N9
172047	229533.Q4HTT2	pp	229533.Q4IER8
172048	229533.Q4HTT2	pp	229533.Q4IEV4
172049	229533.Q4HTT2	pp	229533.Q4IAK7
172050	229533.Q4HTT2	pp	229533.Q4IL82
172051	229533.Q4HTT2	pp	229533.V6QV38
172052	229533.Q4HTT2	pp	229533.Q4IPI4
172053	229533.Q4HTT2	pp	229533.Q4I5V3
172054	229533.Q4HTT2	pp	229533.Q4I7L0
172055	229533.Q4HTT2	pp	229533.Q4IJ11
172056	229533.Q4HTT2	pp	229533.V6R3J1
172057	229533.Q4HTT2	pp	229533.Q4IMD1
172058	229533.Q4HTT2	pp	229533.V6R0U9
172059	229533.Q4HTT2	pp	229533.Q4HYB8
172060	229533.Q4HTT2	pp	229533.Q4I5W5
172061	229533.Q4HTT2	pp	229533.Q4IB89

172062 229533.Q4HTT2 pp 229533.Q4IB50
172063 229533.Q4I0M9 pp 229533.V6RDZ4
172064 229533.Q4I0M9 pp 229533.Q4ICI6
172065 229533.Q4I0M9 pp 229533.Q4INW6
172066 229533.Q4I0M9 pp 229533.Q4ICG8
172067 229533.Q4I7L0 pp 229533.Q4IQX3
172068 229533.Q4I7L0 pp 229533.Q4IJM4
172069 229533.Q4I7L0 pp 229533.Q4IA56
172070 229533.Q4I7L0 pp 229533.Q4IR08
172071 229533.Q4I7L0 pp 229533.Q4IMD1
172072 229533.Q4I7L0 pp 229533.Q4ICA8
172073 229533.Q4I7L0 pp 229533.Q4IER8
172074 229533.Q4I7L0 pp 229533.V6R3J1
172075 229533.Q4I7L0 pp 229533.Q4IPI4
172076 229533.Q4I7L0 pp 229533.Q4IEV4
172077 229533.Q4I7L0 pp 229533.Q4IBR4
172078 229533.Q4I7L0 pp 229533.Q4IB50
172079 229533.I1S8E0 pp 229533.V6R382
172080 229533.I1S8E0 pp 229533.Q4HXF0
172081 229533.I1S8E0 pp 229533.Q4I963
172082 229533.I1S8E0 pp 229533.Q4INI2
172083 229533.I1RZH6 pp 229533.V6QWZ7
172084 229533.I1RZH6 pp 229533.I1S2A1
172085 229533.I1RZH6 pp 229533.Q4IER0
172086 229533.A0A098D1L9 pp 229533.I1RCW8
172087 229533.A0A098D1L9 pp 229533.I1RMK8
172088 229533.A0A098D1L9 pp 229533.I1RU02
172089 229533.A0A098D1L9 pp 229533.Q4IER8
172090 229533.A0A098D1L9 pp 229533.A0A0E0SQV5
172091 229533.A0A098D1L9 pp 229533.I1RAX3
172092 229533.A0A098D1L9 pp 229533.I1S0F0
172093 229533.A0A098D1L9 pp 229533.I1RPL5
172094 229533.A0A098D1L9 pp 229533.A0A098DUZ1
172095 229533.A0A098D1L9 pp 229533.I1S1Y7
172096 229533.A0A098D1L9 pp 229533.I1S6Y1
172097 229533.A0A098D1L9 pp 229533.Q4IMD1
172098 229533.A0A098D1L9 pp 229533.I1RDA5
172099 229533.A0A098D1L9 pp 229533.I1RVH7
172100 229533.A0A098D1L9 pp 229533.I1RFW1
172101 229533.A0A098D1L9 pp 229533.A0A1C3YIS1
172102 229533.I1RG14 pp 229533.I1RM67
172103 229533.I1RG14 pp 229533.I1RS18
172104 229533.I1RG14 pp 229533.I1S4S4
172105 229533.I1RG14 pp 229533.V6RCZ8
172106 229533.I1RG14 pp 229533.I1RXC4
172107 229533.I1RG14 pp 229533.I1RW71
172108 229533.I1RG14 pp 229533.I1S4S7
172109 229533.I1RG14 pp 229533.I1S3S3
172110 229533.I1RG14 pp 229533.I1RXD7
172111 229533.I1RG14 pp 229533.I1RTD3
172112 229533.I1RG14 pp 229533.I1RPN4
172113 229533.I1RG14 pp 229533.I1RSQ7
172114 229533.I1RG14 pp 229533.I1RXF0
172115 229533.I1RG14 pp 229533.I1RZM8
172116 229533.I1RG14 pp 229533.V6RTJ8
172117 229533.I1RG14 pp 229533.I1RT77
172118 229533.I1RG14 pp 229533.I1RM09
172119 229533.I1RG14 pp 229533.I1RZ39
172120 229533.I1RG14 pp 229533.I1RX46
172121 229533.I1RG14 pp 229533.I1RWI5
172122 229533.I1RG14 pp 229533.I1S1W8
172123 229533.I1RG14 pp 229533.I1RRV4
172124 229533.I1RG14 pp 229533.I1S5B0
172125 229533.I1RG14 pp 229533.I1RJU3
172126 229533.I1RG14 pp 229533.I1RR24
172127 229533.I1RG14 pp 229533.I1RZZ9
172128 229533.I1RG14 pp 229533.I1RSQ4
172129 229533.I1RG14 pp 229533.I1RN67
172130 229533.I1RG14 pp 229533.I1RGA6
172131 229533.I1RG14 pp 229533.Q4HXF0
172132 229533.I1RG14 pp 229533.I1RR68
172133 229533.I1RG14 pp 229533.Q4IJK2
172134 229533.I1RG14 pp 229533.I1RQ82

172135 229533.I1RG14 pp 229533.I1S2H4
172136 229533.I1RG14 pp 229533.I1S3G4
172137 229533.I1RG14 pp 229533.I1S4Q4
172138 229533.I1RG14 pp 229533.I1RT75
172139 229533.I1RG14 pp 229533.I1RZK5
172140 229533.I1RG14 pp 229533.I1RT40
172141 229533.I1RG14 pp 229533.I1S0L4
172142 229533.I1RG14 pp 229533.I1RTD5
172143 229533.I1RG14 pp 229533.I1S9G7
172144 229533.I1RG14 pp 229533.I1S5R1
172145 229533.I1RG14 pp 229533.I1RGA4
172146 229533.I1RG14 pp 229533.I1RJH8
172147 229533.I1RG14 pp 229533.O74638
172148 229533.I1RG14 pp 229533.I1RXC2
172149 229533.I1RG14 pp 229533.I1RRY9
172150 229533.I1RG14 pp 229533.I1RYI5
172151 229533.I1RG14 pp 229533.I1RSZ7
172152 229533.I1RG14 pp 229533.I1RZB2
172153 229533.I1RG14 pp 229533.I1RPP3
172154 229533.I1RG14 pp 229533.I1RJH7
172155 229533.I1RG14 pp 229533.I1RKB2
172156 229533.I1RG14 pp 229533.V6RLM7
172157 229533.I1RG14 pp 229533.I1RL04
172158 229533.I1RG14 pp 229533.I1S2B8
172159 229533.I1RG14 pp 229533.I1S0J1
172160 229533.I1RG14 pp 229533.I1RJZ7
172161 229533.I1RG14 pp 229533.I1S1W9
172162 229533.I1RG14 pp 229533.I1RTA1
172163 229533.I1RG14 pp 229533.I1S5R6
172164 229533.I1RG14 pp 229533.I1RVZ2
172165 229533.I1RG14 pp 229533.I1RRV1
172166 229533.I1RG14 pp 229533.I1RVX0
172167 229533.I1RG14 pp 229533.I1RSQ3
172168 229533.I1RG14 pp 229533.V6R042
172169 229533.I1RG14 pp 229533.I1RIH0
172170 229533.I1RG14 pp 229533.I1RT03
172171 229533.I1RG14 pp 229533.V6QVH8
172172 229533.I1RG14 pp 229533.V6RMW6
172173 229533.I1RG14 pp 229533.I1RNC1
172174 229533.I1RG14 pp 229533.I1RQE7
172175 229533.I1RG14 pp 229533.I1RJV3
172176 229533.I1RG14 pp 229533.I1RUG3
172177 229533.I1RG14 pp 229533.V6RRH2
172178 229533.I1RG14 pp 229533.Q4I624
172179 229533.I1RG14 pp 229533.I1S1C7
172180 229533.I1S019 pp 229533.I1S3G4
172181 229533.I1S019 pp 229533.I1S686
172182 229533.I1S019 pp 229533.I1S1W8
172183 229533.I1RBJ0 pp 229533.Q4HTT2
172184 229533.I1RBJ0 pp 229533.I1S193
172185 229533.I1RBJ0 pp 229533.Q4I7X1
172186 229533.I1RBJ0 pp 229533.I1S567
172187 229533.I1RBJ0 pp 229533.I1RPK5
172188 229533.I1RBJ0 pp 229533.I1RC17
172189 229533.I1RBJ0 pp 229533.Q4I5R3
172190 229533.I1RBJ0 pp 229533.I1RKL0
172191 229533.I1RBJ0 pp 229533.I1RMQ7
172192 229533.I1RBJ0 pp 229533.I1RSD1
172193 229533.I1RBJ0 pp 229533.I1RJ34
172194 229533.I1RBJ0 pp 229533.I1RMV6
172195 229533.I1RBJ0 pp 229533.I1S4Z5
172196 229533.I1RBJ0 pp 229533.I1RDH3
172197 229533.I1RBJ0 pp 229533.Q4IN63
172198 229533.I1RBJ0 pp 229533.I1RQ91
172199 229533.I1RBJ0 pp 229533.I1S259
172200 229533.I1RBJ0 pp 229533.I1RQ93
172201 229533.I1RBJ0 pp 229533.I1RVB5
172202 229533.I1RBJ0 pp 229533.I1RU02
172203 229533.I1RBJ0 pp 229533.I1RPL5
172204 229533.I1RBJ0 pp 229533.I1SA28
172205 229533.I1RBJ0 pp 229533.Q4IER8
172206 229533.I1RBJ0 pp 229533.I1RRN5
172207 229533.I1RBJ0 pp 229533.I1RY44

172208	229533.I1RBJ0	pp	229533.I1RGC6
172209	229533.I1RBJ0	pp	229533.I1REZ5
172210	229533.I1RBJ0	pp	229533.I1S1X2
172211	229533.I1RBJ0	pp	229533.I1S1U7
172212	229533.I1RBJ0	pp	229533.I1S6Y1
172213	229533.I1RBJ0	pp	229533.I1RWT6
172214	229533.I1RBJ0	pp	229533.I1RME4
172215	229533.I1RBJ0	pp	229533.I1RYX3
172216	229533.I1RBJ0	pp	229533.I1S926
172217	229533.I1RBJ0	pp	229533.I1RS62
172218	229533.I1RBJ0	pp	229533.I1RQ19
172219	229533.I1RBJ0	pp	229533.I1S8J7
172220	229533.I1RBJ0	pp	229533.I1RCW8
172221	229533.I1RBJ0	pp	229533.I1RYV3
172222	229533.I1RBJ0	pp	229533.I1RLJ8
172223	229533.I1RBJ0	pp	229533.I1S0L6
172224	229533.I1RBJ0	pp	229533.V6R3J1
172225	229533.I1RBJ0	pp	229533.Q4I7N9
172226	229533.I1RBJ0	pp	229533.I1RJZ6
172227	229533.I1RBJ0	pp	229533.I1S625
172228	229533.I1RBJ0	pp	229533.I1RCS3
172229	229533.I1RBJ0	pp	229533.I1RUF8
172230	229533.I1RBJ0	pp	229533.I1RV91
172231	229533.I1RBJ0	pp	229533.I1S2E9
172232	229533.I1RBJ0	pp	229533.I1S3A2
172233	229533.I1RBJ0	pp	229533.I1SAG3
172234	229533.I1RBJ0	pp	229533.I1RQA3
172235	229533.I1RBJ0	pp	229533.I1RIV7
172236	229533.I1RBJ0	pp	229533.I1RVV5
172237	229533.I1RBJ0	pp	229533.Q4IMD1
172238	229533.I1RBJ0	pp	229533.I1RHP9
172239	229533.I1RBJ0	pp	229533.I1RVH7
172240	229533.I1RBJ0	pp	229533.I1RWT1
172241	229533.I1RBJ0	pp	229533.I1RN49
172242	229533.I1RBJ0	pp	229533.I1RQ54
172243	229533.I1RBJ0	pp	229533.I1RT52
172244	229533.I1RBJ0	pp	229533.I1RI51
172245	229533.I1RBJ0	pp	229533.I1RG34
172246	229533.I1RBJ0	pp	229533.I1RMY7
172247	229533.I1RBJ0	pp	229533.I1RFW1
172248	229533.I1RBJ0	pp	229533.I1S0E2
172249	229533.I1RBJ0	pp	229533.I1RDV4
172250	229533.I1RBJ0	pp	229533.I1RQ13
172251	229533.I1RBJ0	pp	229533.I1RCM0
172252	229533.I1RBJ0	pp	229533.Q4HTT1
172253	229533.I1RBJ0	pp	229533.I1RES8
172254	229533.I1RBJ0	pp	229533.Q4IB50
172255	229533.I1RBJ0	pp	229533.I1S566
172256	229533.I1RBJ0	pp	229533.I1RVY0
172257	229533.I1RBJ0	pp	229533.I1RJX2
172258	229533.I1RBJ0	pp	229533.I1RT62
172259	229533.I1RBJ0	pp	229533.I1RK36
172260	229533.I1RBJ0	pp	229533.I1SAA0
172261	229533.I1RBJ0	pp	229533.I1RKJ7
172262	229533.I1RBJ0	pp	229533.I1RDA5
172263	229533.I1RBJ0	pp	229533.I1S7H9
172264	229533.I1RBJ0	pp	229533.Q4I7L0
172265	229533.I1RBJ0	pp	229533.I1RC32
172266	229533.I1RS19	pp	229533.I1RW71
172267	229533.I1RS19	pp	229533.V6QWZ7
172268	229533.I1RS19	pp	229533.V6RGY0
172269	229533.I1RS19	pp	229533.Q4I8X0
172270	229533.I1RS19	pp	229533.I1RWJ4
172271	229533.I1RS19	pp	229533.I1RZ94
172272	229533.I1RS19	pp	229533.I1RY63
172273	229533.I1RS19	pp	229533.I1RTE9
172274	229533.I1RS19	pp	229533.I1S558
172275	229533.I1RS19	pp	229533.I1RTD7
172276	229533.I1RF82	pp	229533.I1RZJ9
172277	229533.I1RF82	pp	229533.I1RPN4
172278	229533.I1RF82	pp	229533.I1RMQ4
172279	229533.I1RF82	pp	229533.V6RTJ8
172280	229533.I1RF82	pp	229533.I1RGS5

172281 229533.I1RF82 pp 229533.I1RRV4
172282 229533.I1RF82 pp 229533.I1SAK7
172283 229533.I1RF82 pp 229533.Q4I5R9
172284 229533.I1RF82 pp 229533.I1RTD3
172285 229533.I1RF82 pp 229533.I1RK15
172286 229533.I1RF82 pp 229533.I1S1W8
172287 229533.I1RF82 pp 229533.I1RG14
172288 229533.I1RF82 pp 229533.I1RM35
172289 229533.I1RF82 pp 229533.I1S4Q4
172290 229533.I1RF82 pp 229533.I1RGB3
172291 229533.I1RF82 pp 229533.I1RWE8
172292 229533.I1RF82 pp 229533.I1S0B7
172293 229533.I1RF82 pp 229533.I1RSL8
172294 229533.I1RF82 pp 229533.I1RJU3
172295 229533.I1RF82 pp 229533.I1RYJ7
172296 229533.I1RF82 pp 229533.V6RBN2
172297 229533.I1RF82 pp 229533.V6R057
172298 229533.I1RF82 pp 229533.I1S216
172299 229533.I1RF82 pp 229533.I1RQS9
172300 229533.I1RF82 pp 229533.V6RRH2
172301 229533.I1RF82 pp 229533.V6R1K4
172302 229533.I1RF82 pp 229533.I1RN67
172303 229533.I1RF82 pp 229533.I1RMI8
172304 229533.I1RF82 pp 229533.O74638
172305 229533.I1RF82 pp 229533.V6R2A1
172306 229533.I1RF82 pp 229533.I1RSQ4
172307 229533.I1RF82 pp 229533.I1RX22
172308 229533.I1RF82 pp 229533.I1RYB2
172309 229533.I1RF82 pp 229533.I1RI34
172310 229533.I1RF82 pp 229533.I1S2E8
172311 229533.I1RF82 pp 229533.I1S8G4
172312 229533.I1RF82 pp 229533.V6RXR0
172313 229533.I1RF82 pp 229533.I1RRP7
172314 229533.I1RF82 pp 229533.I1RL04
172315 229533.I1RF82 pp 229533.I1RZ29
172316 229533.I1RF82 pp 229533.I1S3G4
172317 229533.A0A1C3YL20 pp 229533.I1RNG4
172318 229533.A0A1C3YL20 pp 229533.I1RPD6
172319 229533.A0A1C3YL20 pp 229533.I1S064
172320 229533.A0A1C3YL20 pp 229533.I1S3H8
172321 229533.A0A1C3YL20 pp 229533.I1RRZ6
172322 229533.A0A1C3YL20 pp 229533.I1RT81
172323 229533.A0A1C3YL20 pp 229533.I1S0K7
172324 229533.A0A1C3YL20 pp 229533.V6R382
172325 229533.A0A1C3YL20 pp 229533.I1RW00
172326 229533.A0A1C3YL20 pp 229533.I1RD16
172327 229533.A0A1C3YL20 pp 229533.I1RAY9
172328 229533.A0A1C3YL20 pp 229533.I1R9W4
172329 229533.A0A1C3YL20 pp 229533.I1RGL3
172330 229533.A0A1C3YL20 pp 229533.I1RP97
172331 229533.A0A1C3YL20 pp 229533.I1RMW3
172332 229533.A0A1C3YL20 pp 229533.I1S7W2
172333 229533.A0A1C3YL20 pp 229533.I1RWF9
172334 229533.A0A1C3YL20 pp 229533.I1RPQ7
172335 229533.A0A1C3YL20 pp 229533.Q4HXF0
172336 229533.A0A1C3YL20 pp 229533.I1S0Y7
172337 229533.A0A1C3YL20 pp 229533.I1RKR9
172338 229533.A0A1C3YL20 pp 229533.A0A1C3YL56
172339 229533.A0A1C3YL20 pp 229533.I1RSS7
172340 229533.A0A1C3YL20 pp 229533.I1RA90
172341 229533.A0A1C3YL20 pp 229533.I1RZL3
172342 229533.A0A1C3YL20 pp 229533.I1RWI6
172343 229533.A0A1C3YL20 pp 229533.I1RA94
172344 229533.A0A1C3YL20 pp 229533.I1RWA5
172345 229533.A0A1C3YL20 pp 229533.I1RC61
172346 229533.A0A1C3YL20 pp 229533.I1RJY7
172347 229533.A0A1C3YL20 pp 229533.I1RIX0
172348 229533.A0A1C3YL20 pp 229533.Q4I963
172349 229533.A0A1C3YL20 pp 229533.A0A1C3YLP6
172350 229533.A0A1C3YL20 pp 229533.I1RQG9
172351 229533.A0A1C3YL20 pp 229533.I1RVX9
172352 229533.A0A1C3YL20 pp 229533.I1RM26
172353 229533.A0A1C3YL20 pp 229533.I1RYH0

229533.A0A1C3YL20 pp 229533.I1RBE7
229533.A0A1C3YL20 pp 229533.I1RQP5
229533.A0A1C3YL20 pp 229533.I1RN81
229533.A0A1C3YL20 pp 229533.A0A1C3YLR6
229533.A0A1C3YL20 pp 229533.I1RRL5
229533.A0A1C3YL20 pp 229533.I1S8C1
229533.A0A1C3YL20 pp 229533.I1RBJ8
229533.A0A1C3YL20 pp 229533.I1S334
229533.A0A1C3YL20 pp 229533.I1RMM8
229533.A0A1C3YL20 pp 229533.I1RSJ2
229533.A0A1C3YL20 pp 229533.V6QUU6
229533.A0A1C3YJ34 pp 229533.I1RVH7
229533.A0A1C3YJ34 pp 229533.I1RFW1
229533.A0A1C3YJ34 pp 229533.Q4IMD1
229533.A0A1C3YJ34 pp 229533.A0A1C3YN58
229533.A0A1C3YJ34 pp 229533.I1RDA5
229533.A0A1C3YJ34 pp 229533.I1RCW8
229533.A0A1C3YJ34 pp 229533.I1RAX3
229533.A0A1C3YJ34 pp 229533.I1RMK8
229533.A0A1C3YJ34 pp 229533.I1S1Y7
229533.A0A1C3YJ34 pp 229533.I1RU02
229533.A0A1C3YJ34 pp 229533.I1S0F0
229533.A0A1C3YJ34 pp 229533.I1RPL5
229533.A0A1C3YJ34 pp 229533.Q4IER8
229533.A0A1C3YJ34 pp 229533.I1S6Y1
229533.I1RTD3 pp 229533.I1RZJ9
229533.I1RTD3 pp 229533.I1RWE8
229533.I1RTD3 pp 229533.I1RX22
229533.I1RTD3 pp 229533.V6R1K4
229533.I1RTD3 pp 229533.V6R2A1
229533.I1RTD3 pp 229533.V6RTJ8
229533.I1RTD3 pp 229533.I1S2E8
229533.I1RTD3 pp 229533.I1S8G4
229533.I1RTD3 pp 229533.V6R223
229533.I1RTD3 pp 229533.V6R057
229533.I1RTD3 pp 229533.O74638
229533.I1RTD3 pp 229533.Q4I5R9
229533.I1RTD3 pp 229533.I1S3G4
229533.I1RTD3 pp 229533.I1S1W8
229533.I1RTD3 pp 229533.I1S216
229533.I1RTD3 pp 229533.I1S0B7
229533.I1RTD3 pp 229533.I1S7R5
229533.I1RTD3 pp 229533.V6RRH2
229533.I1RTD3 pp 229533.V6RBN2
229533.I1RTD3 pp 229533.I1S111
229533.I1RTD3 pp 229533.I1RVV7
229533.I1RTD3 pp 229533.V6RJ21
229533.I1RTD3 pp 229533.I1RUE2
229533.I1RTD3 pp 229533.I1S4Q4
229533.I1R9F4 pp 229533.I1RXN6
229533.I1R9F4 pp 229533.I1RGI5
229533.I1R9F4 pp 229533.V6RDP1
229533.I1R9F4 pp 229533.I1RLM3
229533.I1R9F4 pp 229533.I1RJU9
229533.I1S4Z5 pp 229533.I1S6Y1
229533.I1S4Z5 pp 229533.Q4IER8
229533.I1S4Z5 pp 229533.Q4I5R3
229533.I1S4Z5 pp 229533.Q4IPI4
229533.I1S4Z5 pp 229533.I1S567
229533.I1S4Z5 pp 229533.I1S926
229533.I1S4Z5 pp 229533.I1S566
229533.I1S4Z5 pp 229533.I1S7H9
229533.I1S4Z5 pp 229533.I1S625
229533.I1S4Z5 pp 229533.Q4HTT2
229533.I1S4Z5 pp 229533.Q4IB50
229533.A0A098D0B5 pp 229533.I1RNC0
229533.A0A098D0B5 pp 229533.I1RYV3
229533.A0A098D0B5 pp 229533.I1RWT2
229533.A0A098D0B5 pp 229533.Q4HTT2
229533.A0A098D0B5 pp 229533.Q4I7L0
229533.A0A098D0B5 pp 229533.A0A098D1L9
229533.A0A098D0B5 pp 229533.I1RBJ0
229533.A0A098D0B5 pp 229533.A0A1C3YJ34

172427 229533.A0A098D0B5 pp 229533.I1S4Z5
172428 229533.A0A098DH36 pp 229533.A0A1C3YIF4
172429 229533.A0A098DH36 pp 229533.I1RQP5
172430 229533.A0A098DH36 pp 229533.I1RT65
172431 229533.A0A098DH36 pp 229533.I1S0Z3
172432 229533.A0A098DH36 pp 229533.I1RWI6
172433 229533.A0A098DH36 pp 229533.I1RT41
172434 229533.A0A098DH36 pp 229533.A0A1C3YK51
172435 229533.A0A098DH36 pp 229533.I1S270
172436 229533.A0A098DH36 pp 229533.I1RBR0
172437 229533.A0A098DH36 pp 229533.I1S7W2
172438 229533.A0A098DH36 pp 229533.A0A098DRX7
172439 229533.A0A098DH36 pp 229533.I1RTE0
172440 229533.A0A098DH36 pp 229533.I1RKR9
172441 229533.A0A098DH36 pp 229533.A0A1C3YJZ4
172442 229533.A0A098DH36 pp 229533.I1RZD6
172443 229533.A0A098DH36 pp 229533.Q4INI2
172444 229533.A0A098DH36 pp 229533.A0A098DZN5
172445 229533.A0A098DH36 pp 229533.Q4IPI4
172446 229533.A0A098DH36 pp 229533.I1S8E0
172447 229533.A0A098DH36 pp 229533.I1RZV1
172448 229533.A0A098DH36 pp 229533.I1RE49
172449 229533.A0A098DH36 pp 229533.I1RST4
172450 229533.A0A098DH36 pp 229533.A0A1C3YL20
172451 229533.A0A098DH36 pp 229533.I1RZL3
172452 229533.A0A098DH36 pp 229533.I1RKI1
172453 229533.A0A098DH36 pp 229533.A0A0E0SMG8
172454 229533.I1REX4 pp 229533.I1RFS3
172455 229533.I1REX4 pp 229533.I1RKJ6
172456 229533.I1REX4 pp 229533.I1RX24
172457 229533.I1REX4 pp 229533.I1RFW3
172458 229533.I1REX4 pp 229533.I1S9Y1
172459 229533.I1REX4 pp 229533.O74638
172460 229533.I1REX4 pp 229533.I1RYT7
172461 229533.I1REX4 pp 229533.I1S2A8
172462 229533.I1REX4 pp 229533.I1RS20
172463 229533.I1REX4 pp 229533.Q4HXF0
172464 229533.I1REX4 pp 229533.I1RP27
172465 229533.I1REX4 pp 229533.V6REZ4
172466 229533.I1REX4 pp 229533.I1RRQ3
172467 229533.I1REX4 pp 229533.I1RZ20
172468 229533.I1REX4 pp 229533.I1RS47
172469 229533.I1REX4 pp 229533.I1RNF4
172470 229533.I1REX4 pp 229533.I1RQ72
172471 229533.I1REX4 pp 229533.I1RSD8
172472 229533.I1REX4 pp 229533.V6R283
172473 229533.I1REX4 pp 229533.I1RRR7
172474 229533.I1REX4 pp 229533.I1S2E8
172475 229533.I1REX4 pp 229533.I1RFW1
172476 229533.I1REX4 pp 229533.I1S1X9
172477 229533.I1REX4 pp 229533.I1RZE1
172478 229533.I1REX4 pp 229533.I1RSV2
172479 229533.I1REX4 pp 229533.I1RT80
172480 229533.I1REX4 pp 229533.I1RRI2
172481 229533.I1REX4 pp 229533.I1RMN6
172482 229533.I1REX4 pp 229533.I1RSL8
172483 229533.I1REX4 pp 229533.I1RNH1
172484 229533.I1REX4 pp 229533.I1S246
172485 229533.I1REX4 pp 229533.I1RW71
172486 229533.I1REX4 pp 229533.Q4HZK7
172487 229533.I1REX4 pp 229533.I1RG51
172488 229533.I1REX4 pp 229533.V6RGN5
172489 229533.I1REX4 pp 229533.I1S020
172490 229533.I1REX4 pp 229533.I1RQJ5
172491 229533.I1REX4 pp 229533.I1RK95
172492 229533.I1REX4 pp 229533.I1RQA2
172493 229533.I1REX4 pp 229533.I1RRE0
172494 229533.I1REX4 pp 229533.Q4IJH1
172495 229533.I1REX4 pp 229533.I1RSP1
172496 229533.I1REX4 pp 229533.Q4IER8
172497 229533.I1REX4 pp 229533.I1S0P4
172498 229533.I1REX4 pp 229533.I1RU02
172499 229533.I1REX4 pp 229533.I1RG56

172500	229533.I1REX4	pp	229533.V6R227
172501	229533.I1REX4	pp	229533.Q4IJ56
172502	229533.I1REX4	pp	229533.Q4IEK8
172503	229533.I1REX4	pp	229533.I1RYJ8
172504	229533.I1REX4	pp	229533.I1RRV4
172505	229533.I1REX4	pp	229533.Q4I6A4
172506	229533.I1REX4	pp	229533.Q4IR08
172507	229533.I1REX4	pp	229533.I1RQ31
172508	229533.I1RAN5	pp	229533.I1RT05
172509	229533.I1RAN5	pp	229533.Q4IF76
172510	229533.I1RAN5	pp	229533.I1RU69
172511	229533.I1RAN5	pp	229533.I1S9U2
172512	229533.I1RAN5	pp	229533.I1RPW9
172513	229533.I1RAN5	pp	229533.I1S0F1
172514	229533.I1RAN5	pp	229533.I1RFS3
172515	229533.I1RAN5	pp	229533.I1S503
172516	229533.I1RAN5	pp	229533.Q4I662
172517	229533.I1RAN5	pp	229533.I1RNT2
172518	229533.I1RAN5	pp	229533.I1RS19
172519	229533.I1RAN5	pp	229533.Q4I5R9
172520	229533.I1RAN5	pp	229533.I1S8E2
172521	229533.I1RAN5	pp	229533.I1RG51
172522	229533.I1RAN5	pp	229533.Q4IAS1
172523	229533.I1RAN5	pp	229533.V6QYF0
172524	229533.I1RAN5	pp	229533.I1RT80
172525	229533.I1RAN5	pp	229533.I1RLV9
172526	229533.I1RAN5	pp	229533.I1RKF9
172527	229533.I1RAN5	pp	229533.I1RZ39
172528	229533.I1RAN5	pp	229533.I1RBG9
172529	229533.I1RAN5	pp	229533.I1RX24
172530	229533.I1RAN5	pp	229533.Q4IJH1
172531	229533.I1RAN5	pp	229533.Q4IPI1
172532	229533.I1RAN5	pp	229533.I1RJL4
172533	229533.I1RAN5	pp	229533.I1RX46
172534	229533.I1RAN5	pp	229533.I1RND6
172535	229533.I1RAN5	pp	229533.I1RZS6
172536	229533.I1RAN5	pp	229533.I1RM06
172537	229533.I1RAN5	pp	229533.I1RAX6
172538	229533.I1RAN5	pp	229533.V6R3X5
172539	229533.I1RAN5	pp	229533.I1RGA4
172540	229533.I1RAN5	pp	229533.I1RC82
172541	229533.I1RAN5	pp	229533.I1RFN1
172542	229533.I1RAN5	pp	229533.I1RR73
172543	229533.I1RAN5	pp	229533.I1S2B8
172544	229533.I1RAN5	pp	229533.Q4IFI0
172545	229533.I1RAN5	pp	229533.I1RBC7
172546	229533.I1RAN5	pp	229533.Q4HVW2
172547	229533.I1RAN5	pp	229533.I1RSL9
172548	229533.I1RAN5	pp	229533.I1RC03
172549	229533.I1RAN5	pp	229533.I1RJZ7
172550	229533.I1RAN5	pp	229533.I1RW25
172551	229533.I1RAN5	pp	229533.I1RBN1
172552	229533.I1RAN5	pp	229533.V6RG79
172553	229533.I1RAN5	pp	229533.I1RDA0
172554	229533.I1RAN5	pp	229533.V6R283
172555	229533.I1RAN5	pp	229533.I1RKJ6
172556	229533.I1RAN5	pp	229533.I1RPU0
172557	229533.I1RAN5	pp	229533.I1S8I8
172558	229533.I1RAN5	pp	229533.I1S9Y1
172559	229533.I1RAN5	pp	229533.I1RWH5
172560	229533.I1RAN5	pp	229533.I1RFW5
172561	229533.I1RAN5	pp	229533.I1RR00
172562	229533.I1RAN5	pp	229533.I1RZV6
172563	229533.I1RAN5	pp	229533.I1RZJ6
172564	229533.I1RAN5	pp	229533.I1RT98
172565	229533.I1RAN5	pp	229533.I1RW71
172566	229533.I1RAN5	pp	229533.I1RKL3
172567	229533.I1RAN5	pp	229533.I1RM67
172568	229533.I1RAN5	pp	229533.I1RTD5
172569	229533.I1RAN5	pp	229533.I1RPG6
172570	229533.I1RAN5	pp	229533.Q4I830
172571	229533.I1RAN5	pp	229533.Q4IR18
172572	229533.I1RAN5	pp	229533.I1RD64

172573	229533.I1RAN5	pp	229533.I1RRQ3
172574	229533.I1RAN5	pp	229533.I1S0R7
172575	229533.I1RAN5	pp	229533.I1RSX1
172576	229533.I1RAN5	pp	229533.I1RFG0
172577	229533.I1RAN5	pp	229533.I1RT04
172578	229533.I1RAN5	pp	229533.I1S6F0
172579	229533.I1RAN5	pp	229533.I1RSQ7
172580	229533.I1RAN5	pp	229533.I1RPP3
172581	229533.I1RAN5	pp	229533.I1RQJ5
172582	229533.I1RAN5	pp	229533.Q4HZ42
172583	229533.I1RAN5	pp	229533.I1RXD7
172584	229533.I1RAN5	pp	229533.I1RQ31
172585	229533.I1RAN5	pp	229533.I1RD56
172586	229533.I1RAN5	pp	229533.I1RSR3
172587	229533.I1RAN5	pp	229533.I1RMC0
172588	229533.I1RAN5	pp	229533.I1RRH5
172589	229533.I1RAN5	pp	229533.I1RAW0
172590	229533.I1RAN5	pp	229533.I1RQ65
172591	229533.I1RAN5	pp	229533.I1RRY9
172592	229533.I1RAN5	pp	229533.I1RYB9
172593	229533.I1RAN5	pp	229533.I1RWN6
172594	229533.I1RAN5	pp	229533.I1RSD8
172595	229533.I1RAN5	pp	229533.I1RY03
172596	229533.I1RAN5	pp	229533.I1S021
172597	229533.I1RAN5	pp	229533.I1RSR8
172598	229533.I1RAN5	pp	229533.Q4I327
172599	229533.I1RAN5	pp	229533.I1RNH6
172600	229533.I1RAN5	pp	229533.I1RJZ6
172601	229533.I1RAN5	pp	229533.I1S2E8
172602	229533.I1RAN5	pp	229533.I1S1C7
172603	229533.I1RAN5	pp	229533.I1RZZ1
172604	229533.I1RAN5	pp	229533.I1RZK6
172605	229533.I1RAN5	pp	229533.V6RMW6
172606	229533.I1RAN5	pp	229533.V6R042
172607	229533.I1RAN5	pp	229533.I1RWX5
172608	229533.I1RAN5	pp	229533.Q4HWE2
172609	229533.I1RAN5	pp	229533.I1RTH6
172610	229533.I1RAN5	pp	229533.I1RSS4
172611	229533.I1RAN5	pp	229533.I1RFU8
172612	229533.I1RAN5	pp	229533.I1S1X9
172613	229533.I1RAN5	pp	229533.I1RGC2
172614	229533.I1RAN5	pp	229533.I1S2C0
172615	229533.I1RAN5	pp	229533.I1RMN6
172616	229533.I1RAN5	pp	229533.I1RAN6
172617	229533.I1RAN5	pp	229533.I1RK83
172618	229533.I1RAN5	pp	229533.I1RY63
172619	229533.I1RAN5	pp	229533.I1RAU5
172620	229533.I1RAN5	pp	229533.Q4IBS2
172621	229533.I1RAN5	pp	229533.I1RTE9
172622	229533.I1RAN5	pp	229533.I1RT03
172623	229533.I1RAN5	pp	229533.I1RD22
172624	229533.I1RAN5	pp	229533.I1RSG2
172625	229533.I1RAN5	pp	229533.I1RVX0
172626	229533.I1RAN5	pp	229533.I1RG11
172627	229533.I1RAN5	pp	229533.I1RC27
172628	229533.I1RAN5	pp	229533.I1RZK5
172629	229533.I1RAN5	pp	229533.I1S044
172630	229533.I1RAN5	pp	229533.I1RK95
172631	229533.I1RAN5	pp	229533.I1RDG0
172632	229533.I1RAN5	pp	229533.I1RQA2
172633	229533.I1RAN5	pp	229533.I1RN99
172634	229533.I1RAN5	pp	229533.I1RCN5
172635	229533.I1RAN5	pp	229533.V6RGN5
172636	229533.I1RAN5	pp	229533.I1RC81
172637	229533.I1RAN5	pp	229533.I1RX14
172638	229533.I1RAN5	pp	229533.I1RLP2
172639	229533.I1RAN5	pp	229533.I1S0Z9
172640	229533.I1RAN5	pp	229533.I1RKC6
172641	229533.I1RAN5	pp	229533.I1S060
172642	229533.I1RAN5	pp	229533.I1RJV3
172643	229533.I1RAN5	pp	229533.I1RAZ8
172644	229533.I1RAN5	pp	229533.I1RVZ0
172645	229533.I1RAN5	pp	229533.I1RBZ3

172646 229533.I1RAN5 pp 229533.I1RCL3
172647 229533.I1RAN5 pp 229533.I1RC72
172648 229533.I1RAN5 pp 229533.I1RNK2
172649 229533.I1RAN5 pp 229533.I1RFJ9
172650 229533.I1RAN5 pp 229533.I1S274
172651 229533.I1RAN5 pp 229533.I1S0J1
172652 229533.I1RAN5 pp 229533.I1RWB5
172653 229533.I1RAN5 pp 229533.I1RE00
172654 229533.I1RAN5 pp 229533.I1RYB1
172655 229533.I1RAN5 pp 229533.I1RBL7
172656 229533.I1RAN5 pp 229533.I1RF08
172657 229533.I1RAN5 pp 229533.I1RM25
172658 229533.I1RAN5 pp 229533.I1S4T3
172659 229533.I1RAN5 pp 229533.I1RNU6
172660 229533.I1RAN5 pp 229533.I1S0M9
172661 229533.I1RAN5 pp 229533.Q4HZ68
172662 229533.I1RAN5 pp 229533.I1RAU7
172663 229533.I1RAN5 pp 229533.I1RWN5
172664 229533.I1RAN5 pp 229533.Q4IN63
172665 229533.I1RAN5 pp 229533.Q4IJ56
172666 229533.I1RAN5 pp 229533.I1RB27
172667 229533.I1RAN5 pp 229533.Q4IEK8
172668 229533.I1RAN5 pp 229533.I1RNQ5
172669 229533.I1RAN5 pp 229533.Q4IAA0
172670 229533.I1RAN5 pp 229533.I1RM96
172671 229533.I1RAN5 pp 229533.I1RCR8
172672 229533.I1RAN5 pp 229533.I1RDK7
172673 229533.I1RAN5 pp 229533.I1RG56
172674 229533.I1RAN5 pp 229533.I1RZF6
172675 229533.I1RAN5 pp 229533.I1S504
172676 229533.I1RAN5 pp 229533.V6R227
172677 229533.I1RAN5 pp 229533.I1RRR7
172678 229533.A0A1C3YIX3 pp 229533.I1RFY0
172679 229533.A0A1C3YIX3 pp 229533.I1S368
172680 229533.A0A1C3YIX3 pp 229533.I1S034
172681 229533.A0A1C3YIX3 pp 229533.A0A1C3YJ07
172682 229533.A0A1C3YIX3 pp 229533.I1RQM4
172683 229533.A0A1C3YIX3 pp 229533.I1RDZ5
172684 229533.A0A1C3YIX3 pp 229533.I1RED2
172685 229533.A0A1C3YIX3 pp 229533.I1REI2
172686 229533.A0A1C3YIX3 pp 229533.I1S1Z9
172687 229533.A0A1C3YIX3 pp 229533.I1RMR5
172688 229533.A0A1C3YIX3 pp 229533.I1S0G1
172689 229533.A0A1C3YIX3 pp 229533.A0A1C3YLQ0
172690 229533.A0A1C3YIX3 pp 229533.I1REC4
172691 229533.A0A1C3YIX3 pp 229533.Q4I1H6
172692 229533.A0A1C3YIX3 pp 229533.I1RRC4
172693 229533.A0A1C3YIX3 pp 229533.I1RK49
172694 229533.A0A1C3YIX3 pp 229533.I1RYY6
172695 229533.A0A1C3YIX3 pp 229533.I1RW69
172696 229533.A0A1C3YIX3 pp 229533.I1RFU6
172697 229533.A0A1C3YIX3 pp 229533.I1RCN6
172698 229533.A0A1C3YIX3 pp 229533.I1RE88
172699 229533.A0A1C3YIX3 pp 229533.I1S5R2
172700 229533.A0A1C3YIX3 pp 229533.I1REB5
172701 229533.A0A1C3YIX3 pp 229533.I1S8F9
172702 229533.A0A1C3YIX3 pp 229533.A0A1C3YJJ7
172703 229533.A0A1C3YIX3 pp 229533.I1S360
172704 229533.A0A1C3YIX3 pp 229533.I1RWN0
172705 229533.A0A1C3YIX3 pp 229533.A0A1C3YMF6
172706 229533.A0A1C3YIX3 pp 229533.A0A1C3YLQ4
172707 229533.A0A1C3YIX3 pp 229533.I1S2B0
172708 229533.A0A1C3YIX3 pp 229533.I1S2L1
172709 229533.A0A1C3YIX3 pp 229533.I1RTP2
172710 229533.I1RAN6 pp 229533.I1RQR6
172711 229533.I1RAN6 pp 229533.Q4I6B0
172712 229533.I1RAN6 pp 229533.Q4I375
172713 229533.I1RAN6 pp 229533.I1RQ21
172714 229533.I1RAN6 pp 229533.Q4I7T5
172715 229533.I1RAN6 pp 229533.I1RT38
172716 229533.I1RAN6 pp 229533.Q4ILH3
172717 229533.A0A098D053 pp 229533.I1RAN5
172718 229533.A0A098D053 pp 229533.I1RAN6

172719	229533.A0A098DR54	pp	229533.I1RCL4
172720	229533.A0A098DR54	pp	229533.A0A0E0RSD7
172721	229533.A0A098DR54	pp	229533.I1RPA7
172722	229533.A0A098DR54	pp	229533.A0A1C3YM63
172723	229533.A0A098DR54	pp	229533.I1RRX5
172724	229533.A0A098DR54	pp	229533.I1RSQ7
172725	229533.A0A098DR54	pp	229533.I1RW71
172726	229533.A0A098DR54	pp	229533.A0A1C3YK13
172727	229533.A0A098DR54	pp	229533.I1S0B7
172728	229533.A0A098DR54	pp	229533.V6RBN2
172729	229533.A0A098DR54	pp	229533.I1RK95
172730	229533.A0A098DR54	pp	229533.I1RPN4
172731	229533.A0A098DR54	pp	229533.A0A1C3YIP5
172732	229533.A0A098DR54	pp	229533.A0A1C3YKH8
172733	229533.A0A098DR54	pp	229533.A0A098DY09
172734	229533.A0A098DR54	pp	229533.I1S4Q4
172735	229533.A0A098DR54	pp	229533.I1S1W8
172736	229533.A0A098DR54	pp	229533.V6R1K4
172737	229533.A0A098DR54	pp	229533.I1RFX8
172738	229533.A0A098DR54	pp	229533.I1RB39
172739	229533.A0A098DR54	pp	229533.I1REL7
172740	229533.A0A098DR54	pp	229533.I1RM35
172741	229533.A0A098DR54	pp	229533.I1RYX6
172742	229533.A0A098DR54	pp	229533.I1REK8
172743	229533.A0A098DR54	pp	229533.A0A1C3YLR6
172744	229533.A0A098DR54	pp	229533.A0A1C3YN58
172745	229533.A0A098DR54	pp	229533.I1RME6
172746	229533.A0A098DR54	pp	229533.A0A1C3YKS6
172747	229533.A0A098DR54	pp	229533.I1RCR5
172748	229533.A0A098DR54	pp	229533.I1RQS9
172749	229533.A0A098DR54	pp	229533.I1REK4
172750	229533.A0A098DR54	pp	229533.A0A1C3YN30
172751	229533.A0A098DR54	pp	229533.I1RJU3
172752	229533.A0A098DR54	pp	229533.A0A1C3YHS4
172753	229533.A0A098DR54	pp	229533.A0A1C3YHX6
172754	229533.A0A098DR54	pp	229533.A0A1C3YK68
172755	229533.A0A098DR54	pp	229533.I1RXD7
172756	229533.A0A098DR54	pp	229533.A0A0E0SFN0
172757	229533.A0A098DR54	pp	229533.I1RAZ3
172758	229533.A0A098DR54	pp	229533.I1RN72
172759	229533.A0A098DR54	pp	229533.I1RM67
172760	229533.A0A098DR54	pp	229533.I1S3S3
172761	229533.A0A098DR54	pp	229533.I1RS18
172762	229533.A0A098DR54	pp	229533.A0A098E4W0
172763	229533.A0A098DR54	pp	229533.I1RRY9
172764	229533.A0A098DR54	pp	229533.Q4I061
172765	229533.A0A098DR54	pp	229533.I1RX46
172766	229533.A0A098DR54	pp	229533.A0A1C3YJJ2
172767	229533.A0A098DR54	pp	229533.I1RBL7
172768	229533.A0A098DR54	pp	229533.I1RBI1
172769	229533.A0A098DR54	pp	229533.A0A1C3YKV3
172770	229533.A0A098DR54	pp	229533.I1RF82
172771	229533.A0A098DR54	pp	229533.I1RG14
172772	229533.A0A098DR54	pp	229533.I1RK15
172773	229533.A0A098DR54	pp	229533.A0A098DVZ4
172774	229533.A0A098DR54	pp	229533.A0A1C3YJR3
172775	229533.A0A098DR54	pp	229533.I1RXF0
172776	229533.A0A098DR54	pp	229533.I1RZM8
172777	229533.A0A098DR54	pp	229533.I1RMQ4
172778	229533.A0A098DR54	pp	229533.A0A0E0S134
172779	229533.A0A098DR54	pp	229533.A0A0E0RSG8
172780	229533.A0A098DR54	pp	229533.A0A1C3YK34
172781	229533.A0A098DR54	pp	229533.A0A1C3YIK4
172782	229533.A0A098DR54	pp	229533.V6RTJ8
172783	229533.A0A098DR54	pp	229533.I1S4Z8
172784	229533.A0A098DR54	pp	229533.I1RC05
172785	229533.A0A098DR54	pp	229533.A0A1C3YK75
172786	229533.A0A098DR54	pp	229533.A0A1C3YJ29
172787	229533.A0A098DR54	pp	229533.I1RN67
172788	229533.A0A098DR54	pp	229533.A0A1C3YLU7
172789	229533.A0A098DR54	pp	229533.I1RTD3
172790	229533.A0A098DR54	pp	229533.I1RGA6
172791	229533.A0A098DR54	pp	229533.A0A1C3YI20

172792	229533.A0A098DR54	pp	229533.I1RSQ4
172793	229533.A0A098DR54	pp	229533.I1RTD5
172794	229533.A0A098DR54	pp	229533.A0A1C3YJI6
172795	229533.A0A098DR54	pp	229533.I1RZZ9
172796	229533.A0A098DR54	pp	229533.V6RCZ8
172797	229533.A0A098DR54	pp	229533.I1RT40
172798	229533.A0A098DR54	pp	229533.A0A1C3YL55
172799	229533.A0A098DR54	pp	229533.V6QUE6
172800	229533.A0A098DR54	pp	229533.A0A1C3YIR8
172801	229533.A0A098DR54	pp	229533.A0A1C3YLK8
172802	229533.A0A098DR54	pp	229533.I1S8G4
172803	229533.A0A098DR54	pp	229533.I1RQM7
172804	229533.A0A098DR54	pp	229533.I1RB73
172805	229533.A0A098DR54	pp	229533.O74638
172806	229533.A0A098DR54	pp	229533.I1RRV4
172807	229533.A0A098DR54	pp	229533.I1S5B0
172808	229533.A0A098DR54	pp	229533.I1RZB2
172809	229533.A0A098DR54	pp	229533.I1RGA4
172810	229533.A0A098DR54	pp	229533.A0A1C3YIS9
172811	229533.A0A098DR54	pp	229533.I1RFS3
172812	229533.A0A098DR54	pp	229533.I1S5R1
172813	229533.A0A098DR54	pp	229533.I1RBC7
172814	229533.A0A098DR54	pp	229533.I1S9Y1
172815	229533.A0A098DR54	pp	229533.I1RKY2
172816	229533.A0A098DR54	pp	229533.I1RSL9
172817	229533.A0A098DR54	pp	229533.A0A1C3YJH9
172818	229533.A0A098DR54	pp	229533.I1RCN2
172819	229533.A0A098DR54	pp	229533.I1RCH5
172820	229533.A0A098DR54	pp	229533.I1RBY4
172821	229533.A0A098DR54	pp	229533.A0A1C3YKU8
172822	229533.A0A098DR54	pp	229533.I1S2B8
172823	229533.A0A098DR54	pp	229533.A0A0E0SC46
172824	229533.A0A098DR54	pp	229533.A0A1C3YJK3
172825	229533.A0A098DR54	pp	229533.I1S2H4
172826	229533.A0A098DR54	pp	229533.I1S2E8
172827	229533.A0A098DR54	pp	229533.I1RFL4
172828	229533.A0A098DR54	pp	229533.I1S3G4
172829	229533.A0A098DR54	pp	229533.I1RJZ7
172830	229533.A0A098DR54	pp	229533.I1RI34
172831	229533.A0A098DR54	pp	229533.V6R057
172832	229533.A0A098DR54	pp	229533.I1RTA1
172833	229533.A0A098DR54	pp	229533.I1RVX0
172834	229533.A0A098DR54	pp	229533.I1RKB2
172835	229533.A0A098DR54	pp	229533.I1RPP3
172836	229533.A0A098DR54	pp	229533.I1RWX5
172837	229533.A0A098DR54	pp	229533.V6R2A1
172838	229533.A0A098DR54	pp	229533.I1RYW3
172839	229533.A0A098DR54	pp	229533.A0A1C3YIH5
172840	229533.A0A098DR54	pp	229533.I1RCD8
172841	229533.A0A098DR54	pp	229533.A0A098E0A5
172842	229533.A0A098DR54	pp	229533.I1RFT1
172843	229533.A0A098DR54	pp	229533.I1RX22
172844	229533.A0A098DR54	pp	229533.I1RCW6
172845	229533.A0A098DR54	pp	229533.I1RB44
172846	229533.A0A098DR54	pp	229533.V6RRH2
172847	229533.A0A098DR54	pp	229533.A0A0E0S8Z0
172848	229533.A0A098DR54	pp	229533.I1RPD6
172849	229533.A0A098DR54	pp	229533.I1RA70
172850	229533.A0A098DR54	pp	229533.I1RJV3
172851	229533.A0A098DR54	pp	229533.I1RKB6
172852	229533.A0A098DR54	pp	229533.I1S216
172853	229533.A0A098DR54	pp	229533.I1RRP7
172854	229533.A0A098DR54	pp	229533.I1RAW0
172855	229533.A0A098DR54	pp	229533.I1S770
172856	229533.A0A098DR54	pp	229533.A0A1C3YKY0
172857	229533.A0A098DR54	pp	229533.I1S0J1
172858	229533.A0A098DR54	pp	229533.I1RE01
172859	229533.A0A098DR54	pp	229533.I1RPK0
172860	229533.A0A098DR54	pp	229533.I1RS17
172861	229533.A0A098DR54	pp	229533.I1RXY7
172862	229533.A0A098DR54	pp	229533.I1RVZ7
172863	229533.A0A098DR54	pp	229533.I1RMI8
172864	229533.A0A098DR54	pp	229533.I1RWE8

172865	229533.A0A098DR54	pp	229533.I1RUG3
172866	229533.A0A098DR54	pp	229533.I1RPU8
172867	229533.A0A098DR54	pp	229533.V6RMW6
172868	229533.A0A098DR54	pp	229533.V6R042
172869	229533.A0A098DR54	pp	229533.I1SAN9
172870	229533.A0A098DR54	pp	229533.I1RD22
172871	229533.A0A098DR54	pp	229533.I1RG11
172872	229533.A0A1C3YKM2	pp	229533.I1RPU4
172873	229533.A0A1C3YKM2	pp	229533.V6RN44
172874	229533.A0A1C3YKM2	pp	229533.I1RBS7
172875	229533.A0A1C3YKM2	pp	229533.I1S9X9
172876	229533.A0A1C3YKM2	pp	229533.A0A1C3YKR7
172877	229533.A0A1C3YKM2	pp	229533.I1S0J7
172878	229533.A0A1C3YKM2	pp	229533.I1S1W5
172879	229533.A0A1C3YKM2	pp	229533.I1RY72
172880	229533.I1RGI4	pp	229533.I1RNM7
172881	229533.I1RGI4	pp	229533.I1S1W8
172882	229533.I1RGI4	pp	229533.I1S360
172883	229533.I1RGI4	pp	229533.I1RWM4
172884	229533.I1RGI4	pp	229533.I1S3G4
172885	229533.I1RGI4	pp	229533.I1RGI5
172886	229533.I1RGI4	pp	229533.I1RPM5
172887	229533.I1RGI4	pp	229533.I1RVR7
172888	229533.I1RGI4	pp	229533.I1RPM6
172889	229533.I1RGI4	pp	229533.I1S0H5
172890	229533.I1RGI4	pp	229533.I1RZV5
172891	229533.I1RGI4	pp	229533.I1RLZ4
172892	229533.I1RGI4	pp	229533.I1RXE6
172893	229533.I1RGI4	pp	229533.I1RYX6
172894	229533.I1RGI4	pp	229533.V6RRM0
172895	229533.I1RGI4	pp	229533.I1RQU9
172896	229533.I1RGI4	pp	229533.I1S2L1
172897	229533.I1RGI4	pp	229533.I1RLZ6
172898	229533.I1RGI4	pp	229533.I1S8F8
172899	229533.I1RFN8	pp	229533.I1RNG8
172900	229533.I1RFN8	pp	229533.I1S0P7
172901	229533.I1RFN8	pp	229533.I1S4N8
172902	229533.I1RFN8	pp	229533.I1RRC7
172903	229533.I1RFN8	pp	229533.I1RXT2
172904	229533.I1RFN8	pp	229533.I1RRG7
172905	229533.I1RFN8	pp	229533.I1RR07
172906	229533.I1RFN8	pp	229533.I1RW37
172907	229533.I1RFN8	pp	229533.I1S4N7
172908	229533.I1RFN8	pp	229533.Q4I1B1
172909	229533.I1RFN8	pp	229533.I1RKA1
172910	229533.I1RFN8	pp	229533.I1RW82
172911	229533.I1RFN8	pp	229533.I1S8Q3
172912	229533.I1RFN8	pp	229533.I1RWF4
172913	229533.I1RFN8	pp	229533.Q4I6S5
172914	229533.I1RFN8	pp	229533.V6QX21
172915	229533.I1RFN8	pp	229533.I1RTP2
172916	229533.I1RFN8	pp	229533.I1RFS8
172917	229533.I1RFN8	pp	229533.I1S1X7
172918	229533.I1RFN8	pp	229533.I1RQM9
172919	229533.I1RFN8	pp	229533.I1S9X9
172920	229533.I1RFN8	pp	229533.I1S1W5
172921	229533.I1RFN8	pp	229533.I1S039
172922	229533.I1RFN8	pp	229533.I1RMF2
172923	229533.I1RFN8	pp	229533.I1S4Z7
172924	229533.I1RFN8	pp	229533.I1RHU8
172925	229533.I1RFN8	pp	229533.I1S0J7
172926	229533.I1RFN8	pp	229533.I1RX50
172927	229533.I1RMT3	pp	229533.I1RTQ9
172928	229533.I1RMT3	pp	229533.Q4ILI9
172929	229533.V6R9L8	pp	229533.V6R9Z3
172930	229533.I1RQ82	pp	229533.I1RWC9
172931	229533.I1RQ82	pp	229533.I1RVX1
172932	229533.I1RQ82	pp	229533.I1RTF5
172933	229533.I1RQ82	pp	229533.I1S9G7
172934	229533.I1RQ82	pp	229533.I1S1W8
172935	229533.I1RQ82	pp	229533.V6RM66
172936	229533.I1RQ82	pp	229533.I1S5R5
172937	229533.I1RQ82	pp	229533.I1RRR5

229533.I1RQ82 pp 229533.I1RRS8
229533.I1RQ82 pp 229533.I1S2B9
229533.I1RQ82 pp 229533.I1RZS4
229533.I1RQ82 pp 229533.I1RY40
229533.I1RQ82 pp 229533.I1RSQ4
229533.I1RQ82 pp 229533.I1S9V9
229533.I1RQ82 pp 229533.I1RS50
229533.I1RQ82 pp 229533.I1RY72
229533.I1RQ82 pp 229533.I1S3G4
229533.Q4I1B1 pp 229533.Q4IA62
229533.Q4I1B1 pp 229533.Q4IMZ7
229533.Q4I1B1 pp 229533.Q4I8B6
229533.Q4I1B1 pp 229533.Q4IN63
229533.I1S0B0 pp 229533.I1S2B9
229533.I1S0B0 pp 229533.I1S0I2
229533.I1S0B0 pp 229533.I1S7W5
229533.A0A1C3YI30 pp 229533.I1SAJ3
229533.A0A1C3YI30 pp 229533.I1RRI2
229533.A0A1C3YI30 pp 229533.I1RB44
229533.A0A1C3YI30 pp 229533.I1RYW3
229533.A0A1C3YI30 pp 229533.I1S437
229533.A0A1C3YI30 pp 229533.I1S1C7
229533.A0A1C3YI30 pp 229533.I1RKJ8
229533.A0A1C3YI30 pp 229533.Q4IPZ1
229533.A0A1C3YI30 pp 229533.I1RA70
229533.A0A1C3YI30 pp 229533.I1RCD8
229533.A0A1C3YI30 pp 229533.I1RML0
229533.A0A1C3YI30 pp 229533.I1RBR1
229533.A0A1C3YI30 pp 229533.V6RRH2
229533.A0A1C3YI30 pp 229533.I1RQ33
229533.A0A1C3YI30 pp 229533.Q4I624
229533.A0A1C3YI30 pp 229533.I1S0R8
229533.A0A1C3YI30 pp 229533.I1RQE7
229533.A0A1C3YI30 pp 229533.I1RCH5
229533.A0A1C3YI30 pp 229533.I1RFL4
229533.A0A1C3YI30 pp 229533.I1RB92
229533.A0A1C3YI30 pp 229533.I1S5X9
229533.A0A1C3YI30 pp 229533.I1RSS4
229533.A0A1C3YI30 pp 229533.I1RJV3
229533.A0A1C3YI30 pp 229533.I1RT03
229533.A0A1C3YI30 pp 229533.I1RE89
229533.A0A1C3YI30 pp 229533.I1RAW0
229533.A0A1C3YI30 pp 229533.A0A1C3YKY0
229533.A0A1C3YI30 pp 229533.I1RRN2
229533.A0A1C3YI30 pp 229533.I1RD22
229533.A0A1C3YI30 pp 229533.I1RVX0
229533.A0A1C3YI30 pp 229533.I1RSU2
229533.A0A1C3YI30 pp 229533.I1S0J1
229533.A0A1C3YI30 pp 229533.I1RG11
229533.A0A1C3YI30 pp 229533.I1RSY2
229533.A0A1C3YI30 pp 229533.A0A1C3YLB6
229533.A0A1C3YI30 pp 229533.I1RE01
229533.A0A1C3YI30 pp 229533.I1S5R6
229533.A0A1C3YI30 pp 229533.A0A1C3YJU7
229533.A0A1C3YI30 pp 229533.I1RNB8
229533.A0A1C3YI30 pp 229533.I1RPK0
229533.A0A1C3YI30 pp 229533.I1RY92
229533.A0A1C3YI30 pp 229533.I1RVF8
229533.A0A1C3YI30 pp 229533.I1RZ39
229533.A0A1C3YI30 pp 229533.I1RCR8
229533.A0A1C3YI30 pp 229533.A0A1C3YKM9
229533.A0A1C3YI30 pp 229533.I1RNC1
229533.A0A1C3YI30 pp 229533.I1RA11
229533.A0A1C3YI30 pp 229533.I1RE76
229533.A0A1C3YI30 pp 229533.I1S5R1
229533.A0A1C3YI30 pp 229533.I1RBY4
229533.A0A1C3YI30 pp 229533.I1RRT8
229533.A0A1C3YI30 pp 229533.I1RY57
229533.A0A1C3YI30 pp 229533.I1S2B8
229533.A0A1C3YI30 pp 229533.I1RUG3
229533.A0A1C3YI30 pp 229533.I1R9M0
229533.A0A1C3YI30 pp 229533.I1RSQ3
229533.A0A1C3YI30 pp 229533.O74638

173011	229533.A0A1C3YI30	pp	229533.I1S4Z8
173012	229533.A0A1C3YI30	pp	229533.A0A1C3YMA1
173013	229533.A0A1C3YI30	pp	229533.I1RXC2
173014	229533.A0A1C3YI30	pp	229533.V6R042
173015	229533.A0A1C3YI30	pp	229533.I1S277
173016	229533.A0A1C3YI30	pp	229533.I1RBS5
173017	229533.A0A1C3YI30	pp	229533.I1RRM3
173018	229533.A0A1C3YI30	pp	229533.I1S9Y1
173019	229533.A0A1C3YI30	pp	229533.I1RVW0
173020	229533.A0A1C3YI30	pp	229533.A0A1C3YJK3
173021	229533.A0A1C3YI30	pp	229533.I1RPU8
173022	229533.A0A1C3YI30	pp	229533.I1RL04
173023	229533.A0A1C3YI30	pp	229533.V6RMW6
173024	229533.A0A1C3YI30	pp	229533.I1RBW1
173025	229533.A0A1C3YI30	pp	229533.I1S0L4
173026	229533.A0A1C3YI30	pp	229533.I1RPV3
173027	229533.A0A1C3YI30	pp	229533.I1RRI1
173028	229533.A0A1C3YI30	pp	229533.I1RW61
173029	229533.A0A1C3YI30	pp	229533.A0A1C3YL34
173030	229533.A0A1C3YI30	pp	229533.I1RRV1
173031	229533.A0A1C3YI30	pp	229533.I1RPE0
173032	229533.A0A1C3YI30	pp	229533.I1RSZ7
173033	229533.A0A1C3YI30	pp	229533.I1S2H4
173034	229533.A0A1C3YI30	pp	229533.I1RZV3
173035	229533.A0A1C3YI30	pp	229533.I1RTN3
173036	229533.A0A1C3YI30	pp	229533.I1S1W9
173037	229533.A0A1C3YI30	pp	229533.I1RC66
173038	229533.A0A1C3YI30	pp	229533.I1RR72
173039	229533.A0A1C3YI30	pp	229533.A0A1C3YIR8
173040	229533.A0A1C3YI30	pp	229533.I1RXZ3
173041	229533.A0A1C3YI30	pp	229533.I1RGU7
173042	229533.A0A1C3YI30	pp	229533.I1RWI5
173043	229533.A0A1C3YI30	pp	229533.I1RSX0
173044	229533.A0A1C3YI30	pp	229533.I1RZZ9
173045	229533.A0A1C3YI30	pp	229533.I1RVZ7
173046	229533.A0A1C3YI30	pp	229533.I1RXW3
173047	229533.A0A1C3YI30	pp	229533.I1RPU2
173048	229533.A0A1C3YI30	pp	229533.A0A1C3YLK8
173049	229533.A0A1C3YI30	pp	229533.I1RSQ4
173050	229533.A0A1C3YI30	pp	229533.A0A1C3YM05
173051	229533.A0A1C3YI30	pp	229533.A0A1C3YKB9
173052	229533.A0A1C3YI30	pp	229533.V6QT89
173053	229533.A0A1C3YI30	pp	229533.I1S253
173054	229533.A0A1C3YI30	pp	229533.I1RZB2
173055	229533.A0A1C3YI30	pp	229533.I1RQM7
173056	229533.A0A1C3YI30	pp	229533.I1RCR5
173057	229533.A0A1C3YI30	pp	229533.I1RGA4
173058	229533.A0A1C3YI30	pp	229533.I1RBC7
173059	229533.A0A1C3YI30	pp	229533.I1RXF8
173060	229533.A0A1C3YI30	pp	229533.I1REK8
173061	229533.A0A1C3YI30	pp	229533.A0A1C3YIS9
173062	229533.A0A1C3YI30	pp	229533.Q4IJK2
173063	229533.A0A1C3YI30	pp	229533.I1RTD5
173064	229533.A0A1C3YI30	pp	229533.I1RPP3
173065	229533.A0A1C3YI30	pp	229533.I1RFT1
173066	229533.A0A1C3YI30	pp	229533.I1RT40
173067	229533.A0A1C3YI30	pp	229533.I1RK95
173068	229533.A0A1C3YI30	pp	229533.A0A1C3YJI6
173069	229533.A0A1C3YI30	pp	229533.I1RXV9
173070	229533.A0A1C3YI30	pp	229533.I1RWF9
173071	229533.A0A1C3YI30	pp	229533.I1RRV4
173072	229533.A0A1C3YI30	pp	229533.I1R9W4
173073	229533.A0A1C3YI30	pp	229533.I1RC18
173074	229533.A0A1C3YI30	pp	229533.I1RW26
173075	229533.A0A1C3YI30	pp	229533.I1S1J7
173076	229533.A0A1C3YI30	pp	229533.I1RN67
173077	229533.A0A1C3YI30	pp	229533.I1RVZ2
173078	229533.A0A1C3YI30	pp	229533.V6QUE6
173079	229533.A0A1C3YI30	pp	229533.I1S5B0
173080	229533.A0A1C3YI30	pp	229533.A0A1C3YN86
173081	229533.A0A1C3YI30	pp	229533.I1RP02
173082	229533.A0A1C3YI30	pp	229533.I1RTA1
173083	229533.A0A1C3YI30	pp	229533.I1RRY9

173084 229533.A0A1C3YI30 pp 229533.I1RJN9
173085 229533.A0A1C3YI30 pp 229533.I1RYR0
173086 229533.A0A1C3YI30 pp 229533.I1RQD2
173087 229533.A0A1C3YI30 pp 229533.I1RWR7
173088 229533.A0A1C3YI30 pp 229533.I1RYB9
173089 229533.A0A1C3YI30 pp 229533.I1RWS4
173090 229533.A0A1C3YI30 pp 229533.I1RNT9
173091 229533.A0A1C3YI30 pp 229533.I1REV8
173092 229533.A0A1C3YI30 pp 229533.I1RYI5
173093 229533.A0A1C3YI30 pp 229533.I1RXF0
173094 229533.A0A1C3YI30 pp 229533.I1RZM8
173095 229533.A0A1C3YI30 pp 229533.I1S8S9
173096 229533.A0A1C3YI30 pp 229533.A0A1C3YN57
173097 229533.A0A1C3YI30 pp 229533.I1RZK5
173098 229533.A0A1C3YI30 pp 229533.A0A1C3YKY4
173099 229533.A0A1C3YI30 pp 229533.I1S6K9
173100 229533.A0A1C3YI30 pp 229533.A0A1C3YJN3
173101 229533.A0A1C3YI30 pp 229533.I1RT35
173102 229533.A0A1C3YI30 pp 229533.I1RCN8
173103 229533.A0A1C3YI30 pp 229533.I1S4Q4
173104 229533.A0A1C3YI30 pp 229533.I1RQ85
173105 229533.A0A1C3YI30 pp 229533.I1RBE4
173106 229533.A0A1C3YI30 pp 229533.A0A1C3YL25
173107 229533.A0A1C3YI30 pp 229533.V6RCZ8
173108 229533.A0A1C3YI30 pp 229533.I1RJU3
173109 229533.A0A1C3YI30 pp 229533.I1RT42
173110 229533.A0A1C3YI30 pp 229533.I1RG14
173111 229533.A0A1C3YI30 pp 229533.A0A1C3YKB0
173112 229533.A0A1C3YI30 pp 229533.A0A1C3YJR3
173113 229533.A0A1C3YI30 pp 229533.V6RTJ8
173114 229533.A0A1C3YI30 pp 229533.V6RJP8
173115 229533.A0A1C3YI30 pp 229533.I1RBW0
173116 229533.A0A1C3YI30 pp 229533.I1RAX6
173117 229533.A0A1C3YI30 pp 229533.I1RYT8
173118 229533.A0A1C3YI30 pp 229533.I1RMK4
173119 229533.A0A1C3YI30 pp 229533.Q4IFI0
173120 229533.A0A1C3YI30 pp 229533.A0A1C3YK34
173121 229533.A0A1C3YI30 pp 229533.I1RXC4
173122 229533.A0A1C3YI30 pp 229533.I1RYJ6
173123 229533.A0A1C3YI30 pp 229533.I1RS18
173124 229533.A0A1C3YI30 pp 229533.A0A1C3YLR6
173125 229533.A0A1C3YI30 pp 229533.I1RXD7
173126 229533.A0A1C3YI30 pp 229533.I1RA35
173127 229533.A0A1C3YI30 pp 229533.I1S0B7
173128 229533.A0A1C3YI30 pp 229533.A0A1C3YIK4
173129 229533.A0A1C3YI30 pp 229533.A0A1C3YKV3
173130 229533.A0A1C3YI30 pp 229533.I1REK4
173131 229533.A0A1C3YI30 pp 229533.I1RM67
173132 229533.A0A1C3YI30 pp 229533.I1RXH3
173133 229533.A0A1C3YI30 pp 229533.I1RPA4
173134 229533.A0A1C3YI30 pp 229533.I1S0P0
173135 229533.A0A1C3YI30 pp 229533.I1S438
173136 229533.A0A1C3YI30 pp 229533.I1S4S4
173137 229533.A0A1C3YI30 pp 229533.I1RXD6
173138 229533.A0A1C3YI30 pp 229533.A0A1C3YIP5
173139 229533.A0A1C3YI30 pp 229533.I1RR24
173140 229533.A0A1C3YI30 pp 229533.I1RRY1
173141 229533.A0A1C3YI30 pp 229533.I1RY86
173142 229533.A0A1C3YI30 pp 229533.I1RX46
173143 229533.A0A1C3YI30 pp 229533.I1S0F1
173144 229533.A0A1C3YI30 pp 229533.I1RT33
173145 229533.A0A1C3YI30 pp 229533.I1R9Y4
173146 229533.A0A1C3YI30 pp 229533.I1RD52
173147 229533.A0A1C3YI30 pp 229533.I1RBT1
173148 229533.A0A1C3YI30 pp 229533.I1RRG3
173149 229533.A0A1C3YI30 pp 229533.I1RBR2
173150 229533.A0A1C3YI30 pp 229533.I1S246
173151 229533.A0A1C3YI30 pp 229533.I1RRX5
173152 229533.A0A1C3YI30 pp 229533.I1RSQ7
173153 229533.A0A1C3YI30 pp 229533.I1S4S7
173154 229533.A0A1C3YI30 pp 229533.A0A1C3YK13
173155 229533.I1RZV5 pp 229533.V6RLM7
173156 229533.I1RZV5 pp 229533.I1S0R9

173157	229533.I1RZV5	pp	229533.I1S2L1
173158	229533.I1RZV5	pp	229533.Q4IBU4
173159	229533.I1RZV5	pp	229533.I1S360
173160	229533.I1RZV5	pp	229533.I1S3T7
173161	229533.I1RZV5	pp	229533.I1SAB4
173162	229533.I1RZV5	pp	229533.I1S149
173163	229533.I1RZV5	pp	229533.I1S3G4
173164	229533.I1RZV5	pp	229533.I1S8F8
173165	229533.I1RZV5	pp	229533.I1S0I8
173166	229533.I1RZV5	pp	229533.I1S3K0
173167	229533.I1RZV5	pp	229533.I1S7M8
173168	229533.I1RZV5	pp	229533.I1S0A5
173169	229533.I1RZV5	pp	229533.I1S694
173170	229533.I1RZV5	pp	229533.V6RPS5
173171	229533.I1RZV5	pp	229533.I1S1W8
173172	229533.I1RZV5	pp	229533.I1S8C1
173173	229533.I1RZV5	pp	229533.I1S6H3
173174	229533.I1RZV5	pp	229533.I1S0H2
173175	229533.I1RZV5	pp	229533.I1S094
173176	229533.I1RZH4	pp	229533.V6QW22
173177	229533.I1RZH4	pp	229533.Q4HYZ2
173178	229533.I1RZH4	pp	229533.I1S0L3
173179	229533.I1RZH4	pp	229533.I1S0X6
173180	229533.I1RZH4	pp	229533.V6RTJ8
173181	229533.I1RZH4	pp	229533.V6QV09
173182	229533.I1RZH4	pp	229533.I1S0S6
173183	229533.I1RZH4	pp	229533.I1S224
173184	229533.I1RZH4	pp	229533.I1S439
173185	229533.I1RZH4	pp	229533.I1SAT1
173186	229533.I1RZH4	pp	229533.I1S6F1
173187	229533.I1RZH4	pp	229533.Q5I2J3
173188	229533.I1RZH4	pp	229533.Q4IQC1
173189	229533.I1RZH4	pp	229533.I1S0Z3
173190	229533.I1RZH4	pp	229533.P0C431
173191	229533.I1RZH4	pp	229533.V6REE9
173192	229533.I1RZH4	pp	229533.Q4I0M9
173193	229533.I1RZH4	pp	229533.V6RCG5
173194	229533.I1RZH4	pp	229533.Q4I5I4
173195	229533.I1RZH4	pp	229533.V6RVG3
173196	229533.I1RZH4	pp	229533.I1S4R1
173197	229533.I1RZH4	pp	229533.V6RKY7
173198	229533.I1RZH4	pp	229533.I1RZZ9
173199	229533.I1RZH4	pp	229533.V6RIV9
173200	229533.I1RZH4	pp	229533.I1S7X9
173201	229533.I1RZH4	pp	229533.V6R042
173202	229533.I1RZH4	pp	229533.I1S0L8
173203	229533.I1RZH4	pp	229533.I1S1V5
173204	229533.I1RZH4	pp	229533.I1S2E9
173205	229533.I1RZH4	pp	229533.Q4INW6
173206	229533.I1RZH4	pp	229533.I1S0M2
173207	229533.I1RZH4	pp	229533.V6RMW6
173208	229533.I1RZH4	pp	229533.Q4IER0
173209	229533.I1RZH4	pp	229533.Q4ICG8
173210	229533.I1RZH4	pp	229533.Q4IPC8
173211	229533.I1RZH4	pp	229533.I1RZS7
173212	229533.I1RZH4	pp	229533.V6RGI1
173213	229533.I1RZH4	pp	229533.I1S1W4
173214	229533.I1S4S4	pp	229533.I1S6K9
173215	229533.I1S4S4	pp	229533.V6RCZ8
173216	229533.I1S4S4	pp	229533.V6RMW6
173217	229533.I1S4S4	pp	229533.Q4HXF0
173218	229533.I1S4S4	pp	229533.V6R042
173219	229533.I1S4S4	pp	229533.I1S4Z8
173220	229533.I1S4S4	pp	229533.O74638
173221	229533.I1S4S4	pp	229533.V6R1K4
173222	229533.I1S4S4	pp	229533.V6QUE6
173223	229533.I1S4S4	pp	229533.I1S5R1
173224	229533.I1S4S4	pp	229533.I1S4S7
173225	229533.I1S4S4	pp	229533.V6R3J1
173226	229533.I1S4S4	pp	229533.V6R057
173227	229533.I1S4S4	pp	229533.Q4I624
173228	229533.I1S4S4	pp	229533.V6RPQ8
173229	229533.I1S4S4	pp	229533.V6RTJ8

229533.I1S4S4 pp 229533.I1S5R6
229533.I1S4S4 pp 229533.Q4IJK2
229533.I1S4S4 pp 229533.I1S7W5
229533.I1S4S4 pp 229533.V6RBN2
229533.I1S4S4 pp 229533.I1S5B0
229533.A0A1C3YMF6 pp 229533.I1RZT6
229533.A0A1C3YMF6 pp 229533.I1RTP2
229533.A0A1C3YMF6 pp 229533.I1RQM4
229533.A0A1C3YMF6 pp 229533.I1RDZ5
229533.A0A1C3YMF6 pp 229533.I1RZJ8
229533.A0A1C3YMF6 pp 229533.I1S8X0
229533.A0A1C3YMF6 pp 229533.I1RQ48
229533.A0A1C3YMF6 pp 229533.I1S360
229533.A0A1C3YMF6 pp 229533.I1RDQ0
229533.A0A1C3YMF6 pp 229533.I1RWN0
229533.A0A1C3YMF6 pp 229533.I1RDL0
229533.A0A1C3YMF6 pp 229533.I1RE88
229533.A0A1C3YMF6 pp 229533.I1REB5
229533.A0A1C3YMF6 pp 229533.I1S2L1
229533.A0A1C3YMF6 pp 229533.Q4IQK7
229533.A0A1C3YMF6 pp 229533.I1S368
229533.A0A1C3YMF6 pp 229533.I1S108
229533.A0A1C3YMF6 pp 229533.I1RB45
229533.A0A1C3YMF6 pp 229533.I1RK49
229533.A0A1C3YMF6 pp 229533.I1RKZ8
229533.A0A1C3YMF6 pp 229533.V6RXE7
229533.A0A1C3YMF6 pp 229533.I1RXJ5
229533.A0A1C3YMF6 pp 229533.I1RMG4
229533.I1RCK2 pp 229533.Q4ICG5
229533.I1RCK2 pp 229533.I1REK1
229533.I1RCK2 pp 229533.I1RFV5
229533.I1RCK2 pp 229533.I1RJT1
229533.I1RCK2 pp 229533.I1RE15
229533.I1RCK2 pp 229533.I1RWP9
229533.I1RCK2 pp 229533.I1RT41
229533.I1RCK2 pp 229533.V6RQ48
229533.I1RCK2 pp 229533.I1RNR3
229533.I1RCK2 pp 229533.I1RPK5
229533.I1RCK2 pp 229533.I1RS35
229533.I1RCK2 pp 229533.I1REN4
229533.I1RCK2 pp 229533.V6RKE4
229533.I1RCK2 pp 229533.I1RWI6
229533.I1RCK2 pp 229533.I1S1W5
229533.I1RCK2 pp 229533.I1S039
229533.I1RCK2 pp 229533.I1RRK8
229533.I1RCK2 pp 229533.I1S9T1
229533.I1RCK2 pp 229533.I1RE20
229533.I1RCK2 pp 229533.I1RTF0
229533.I1RCK2 pp 229533.I1RXU1
229533.I1RCK2 pp 229533.I1REC4
229533.I1RCK2 pp 229533.V6R9X9
229533.I1RCK2 pp 229533.I1RX54
229533.I1RCK2 pp 229533.I1S4H6
229533.I1RCK2 pp 229533.I1RPX9
229533.I1RCK2 pp 229533.I1REK3
229533.I1RCK2 pp 229533.Q4IBU4
229533.I1RCK2 pp 229533.I1RXF5
229533.I1RCK2 pp 229533.I1RZA4
229533.I1S3L3 pp 229533.I1S9J6
229533.I1S3L3 pp 229533.Q4IB50
229533.I1S3L3 pp 229533.Q4I7L0
229533.A0A1C3YK49 pp 229533.I1RZT0
229533.A0A1C3YK49 pp 229533.I1RZA7
229533.A0A1C3YK49 pp 229533.I1RDJ7
229533.A0A1C3YK49 pp 229533.I1RZ20
229533.A0A1C3YK49 pp 229533.I1S1P8
229533.A0A1C3YK49 pp 229533.I1S084
229533.A0A1C3YK49 pp 229533.A0A1C3YKV2
229533.A0A1C3YK49 pp 229533.V6RUB1
229533.A0A1C3YK49 pp 229533.I1RAR1
229533.A0A1C3YK49 pp 229533.I1R9B8
229533.A0A1C3YK49 pp 229533.I1RQE0
229533.A0A1C3YK49 pp 229533.I1S6P5

173303 229533.A0A1C3YK49 pp 229533.I1RQX9
173304 229533.A0A1C3YK49 pp 229533.I1RD80
173305 229533.A0A1C3YK49 pp 229533.I1S3K8
173306 229533.A0A1C3YK49 pp 229533.A0A1C3YK58
173307 229533.A0A1C3YK49 pp 229533.I1RV23
173308 229533.I1RRV4 pp 229533.I1RX46
173309 229533.I1RRV4 pp 229533.I1S1W8
173310 229533.I1RRV4 pp 229533.I1S0B7
173311 229533.I1RRV4 pp 229533.V6RTJ8
173312 229533.I1RRV4 pp 229533.I1RZM8
173313 229533.I1RRV4 pp 229533.I1RTD3
173314 229533.I1RRV4 pp 229533.I1RW71
173315 229533.I1RRV4 pp 229533.I1RXF0
173316 229533.I1RRV4 pp 229533.I1RRX5
173317 229533.I1RRV4 pp 229533.V6RCZ8
173318 229533.I1RRV4 pp 229533.I1RSQ7
173319 229533.I1RRV4 pp 229533.I1RT33
173320 229533.I1RRV4 pp 229533.V6QUE6
173321 229533.I1RRV4 pp 229533.Q4I5R9
173322 229533.I1RRV4 pp 229533.I1RYX6
173323 229533.I1RRV4 pp 229533.I1RXD7
173324 229533.I1RRV4 pp 229533.V6R1K4
173325 229533.I1RRV4 pp 229533.I1S3K6
173326 229533.I1RRV4 pp 229533.I1RYI7
173327 229533.I1RRV4 pp 229533.I1RVR0
173328 229533.I1RRV4 pp 229533.I1S835
173329 229533.I1RRV4 pp 229533.I1S5B0
173330 229533.I1RRV4 pp 229533.I1RS18
173331 229533.I1RRV4 pp 229533.I1S4S4
173332 229533.I1RRV4 pp 229533.I1S4Q4
173333 229533.I1RRV4 pp 229533.I1S0A0
173334 229533.I1RRV4 pp 229533.I1RSI0
173335 229533.I1RRV4 pp 229533.I1RX22
173336 229533.I1RRV4 pp 229533.Q4I1Y1
173337 229533.I1RRV4 pp 229533.I1S8G4
173338 229533.I1RRV4 pp 229533.I1RTD5
173339 229533.I1RRV4 pp 229533.I1RT40
173340 229533.I1RRV4 pp 229533.V6RBN2
173341 229533.I1RRV4 pp 229533.I1RRY9
173342 229533.I1RRV4 pp 229533.I1RZZ9
173343 229533.I1RRV4 pp 229533.I1S2B8
173344 229533.I1RRV4 pp 229533.Q4HXF0
173345 229533.I1RRV4 pp 229533.I1S1V7
173346 229533.I1RRV4 pp 229533.I1S8P5
173347 229533.I1RRV4 pp 229533.I1RSQ4
173348 229533.I1RRV4 pp 229533.I1RZ52
173349 229533.I1RRV4 pp 229533.I1S147
173350 229533.I1RRV4 pp 229533.I1S0Y7
173351 229533.I1RRV4 pp 229533.I1RTA0
173352 229533.I1RRV4 pp 229533.I1RZ39
173353 229533.I1RRV4 pp 229533.I1S0J1
173354 229533.I1RRV4 pp 229533.I1S4Z8
173355 229533.I1RRV4 pp 229533.I1S9Y1
173356 229533.I1RRV4 pp 229533.I1RS17
173357 229533.I1RRV4 pp 229533.O74638
173358 229533.I1RRV4 pp 229533.I1RUG3
173359 229533.I1RRV4 pp 229533.I1SAN9
173360 229533.I1RRV4 pp 229533.I1S5R1
173361 229533.I1RRV4 pp 229533.I1S2H4
173362 229533.I1RRV4 pp 229533.I1RZ95
173363 229533.I1RRV4 pp 229533.V6RLM7
173364 229533.I1RRV4 pp 229533.V6RMW6
173365 229533.I1RRV4 pp 229533.I1S3G4
173366 229533.I1RRV4 pp 229533.I1S1W9
173367 229533.I1RRV4 pp 229533.V6R042
173368 229533.I1RRV4 pp 229533.I1RWE8
173369 229533.I1RRV4 pp 229533.I1S1C7
173370 229533.I1RRV4 pp 229533.I1RVZ7
173371 229533.I1RRV4 pp 229533.Q4I5V2
173372 229533.I1RRV4 pp 229533.I1RSS4
173373 229533.I1RRV4 pp 229533.I1RYW3
173374 229533.I1RRV4 pp 229533.I1RY74
173375 229533.I1RRV4 pp 229533.I1RWX5

173376 229533.I1RRV4 pp 229533.I1RT03
173377 229533.I1RRV4 pp 229533.I1RVX0
173378 229533.I1RRV4 pp 229533.I1RZJ3
173379 229533.I1RRV4 pp 229533.V6R2A1
173380 229533.I1RRV4 pp 229533.I1S216
173381 229533.I1RRV4 pp 229533.V6R057
173382 229533.I1RRV4 pp 229533.V6RRH2
173383 229533.A0A098DRQ4 pp 229533.I1S9X9
173384 229533.A0A098DRQ4 pp 229533.I1RX50
173385 229533.A0A098DRQ4 pp 229533.A0A1C3YK51
173386 229533.A0A098DRQ4 pp 229533.I1RMR5
173387 229533.A0A098DRQ4 pp 229533.I1RCS1
173388 229533.A0A098DRQ4 pp 229533.I1RNM5
173389 229533.A0A098DRQ4 pp 229533.I1RXT2
173390 229533.A0A098DRQ4 pp 229533.I1RNG8
173391 229533.A0A098DRQ4 pp 229533.I1RG99
173392 229533.A0A098DRQ4 pp 229533.I1RKA1
173393 229533.A0A098DRQ4 pp 229533.I1RS43
173394 229533.A0A098DRQ4 pp 229533.I1S0G1
173395 229533.A0A098DRQ4 pp 229533.I1S1W5
173396 229533.A0A098DRQ4 pp 229533.I1S034
173397 229533.A0A098DRQ4 pp 229533.I1S5R2
173398 229533.A0A098DRQ4 pp 229533.A0A1C3YLQ0
173399 229533.A0A098DRQ4 pp 229533.I1RR07
173400 229533.A0A098DRQ4 pp 229533.I1S8F9
173401 229533.A0A098DRQ4 pp 229533.V6QW25
173402 229533.A0A098DRQ4 pp 229533.I1REC4
173403 229533.A0A098DRQ4 pp 229533.Q4HYZ2
173404 229533.A0A098DRQ4 pp 229533.I1RMK1
173405 229533.A0A098DRQ4 pp 229533.I1RC52
173406 229533.A0A098DRQ4 pp 229533.I1RYY6
173407 229533.A0A098DRQ4 pp 229533.Q4ILI9
173408 229533.A0A098DRQ4 pp 229533.I1RFU6
173409 229533.A0A098DRQ4 pp 229533.I1S8Q3
173410 229533.A0A098DRQ4 pp 229533.Q4I1H6
173411 229533.A0A098DRQ4 pp 229533.I1RFN8
173412 229533.A0A098DRQ4 pp 229533.Q4I1B1
173413 229533.A0A098DRQ4 pp 229533.V6QX21
173414 229533.A0A098DRQ4 pp 229533.I1RD63
173415 229533.I1RBX4 pp 229533.I1S573
173416 229533.I1S0H5 pp 229533.I1S2L1
173417 229533.I1S0H5 pp 229533.I1S4D0
173418 229533.I1S0H5 pp 229533.I1S273
173419 229533.I1S0H5 pp 229533.I1S360
173420 229533.I1S0H5 pp 229533.V6RY56
173421 229533.Q4HYC1 pp 229533.Q4ID21
173422 229533.Q4HYC1 pp 229533.V6QWB1
173423 229533.Q4HYC1 pp 229533.Q4I5V2
173424 229533.I1RAL7 pp 229533.I1S278
173425 229533.I1RAL7 pp 229533.I1RXT2
173426 229533.I1RAL7 pp 229533.I1S8Q3
173427 229533.I1RAL7 pp 229533.I1RMK9
173428 229533.I1RAL7 pp 229533.V6QX21
173429 229533.I1RAL7 pp 229533.I1RBR3
173430 229533.A0A098DY09 pp 229533.I1RX22
173431 229533.A0A098DY09 pp 229533.I1RC31
173432 229533.A0A098DY09 pp 229533.I1RC05
173433 229533.A0A098DY09 pp 229533.I1RN67
173434 229533.A0A098DY09 pp 229533.I1RNG4
173435 229533.A0A098DY09 pp 229533.I1RW26
173436 229533.A0A098DY09 pp 229533.I1RPP3
173437 229533.A0A098DY09 pp 229533.I1S8P5
173438 229533.A0A098DY09 pp 229533.A0A098E0A5
173439 229533.A0A098DY09 pp 229533.I1RTA1
173440 229533.A0A098DY09 pp 229533.I1RZH6
173441 229533.A0A098DY09 pp 229533.I1RVZ2
173442 229533.A0A098DY09 pp 229533.I1RWS4
173443 229533.A0A098DY09 pp 229533.I1RBW1
173444 229533.A0A098DY09 pp 229533.I1S2H4
173445 229533.A0A098DY09 pp 229533.I1RRT8
173446 229533.A0A098DY09 pp 229533.A0A1C3YKU8
173447 229533.A0A098DY09 pp 229533.A0A1C3YLA6
173448 229533.A0A098DY09 pp 229533.A0A1C3YIS9

173449	229533.A0A098DY09	pp	229533.I1RGA4
173450	229533.A0A098DY09	pp	229533.A0A1C3YHI3
173451	229533.A0A098DY09	pp	229533.I1S9Y1
173452	229533.A0A098DY09	pp	229533.I1S2B8
173453	229533.A0A098DY09	pp	229533.I1S253
173454	229533.A0A098DY09	pp	229533.I1RR72
173455	229533.A0A098DY09	pp	229533.I1RBF3
173456	229533.A0A098DY09	pp	229533.I1RSQ4
173457	229533.A0A098DY09	pp	229533.I1RP97
173458	229533.A0A098DY09	pp	229533.I1RL04
173459	229533.A0A098DY09	pp	229533.Q4HXF0
173460	229533.A0A098DY09	pp	229533.I1RZZ9
173461	229533.A0A098DY09	pp	229533.I1S5R6
173462	229533.A0A098DY09	pp	229533.A0A1C3YIR8
173463	229533.A0A098DY09	pp	229533.I1S8G4
173464	229533.A0A098DY09	pp	229533.I1RTD5
173465	229533.A0A098DY09	pp	229533.I1RSU2
173466	229533.A0A098DY09	pp	229533.A0A1C3YJI6
173467	229533.A0A098DY09	pp	229533.I1RQ75
173468	229533.A0A098DY09	pp	229533.I1RZ52
173469	229533.A0A098DY09	pp	229533.I1RPU8
173470	229533.A0A098DY09	pp	229533.A0A1C3YN83
173471	229533.A0A098DY09	pp	229533.V6RLM7
173472	229533.A0A098DY09	pp	229533.I1RB92
173473	229533.A0A098DY09	pp	229533.V6R2A1
173474	229533.A0A098DY09	pp	229533.V6RMW6
173475	229533.A0A098DY09	pp	229533.I1RSS4
173476	229533.A0A098DY09	pp	229533.I1RS17
173477	229533.A0A098DY09	pp	229533.A0A1C3YJJ0
173478	229533.A0A098DY09	pp	229533.V6R042
173479	229533.A0A098DY09	pp	229533.I1S064
173480	229533.A0A098DY09	pp	229533.I1RFL4
173481	229533.A0A098DY09	pp	229533.V6RTJ8
173482	229533.A0A098DY09	pp	229533.I1RRV1
173483	229533.A0A098DY09	pp	229533.I1RMF6
173484	229533.A0A098DY09	pp	229533.I1RTA0
173485	229533.A0A098DY09	pp	229533.I1RRP7
173486	229533.A0A098DY09	pp	229533.I1SAN9
173487	229533.A0A098DY09	pp	229533.A0A1C3YKY0
173488	229533.A0A098DY09	pp	229533.A0A1C3YJJ2
173489	229533.A0A098DY09	pp	229533.I1RNC1
173490	229533.A0A098DY09	pp	229533.I1RME6
173491	229533.A0A098DY09	pp	229533.Q4I5V2
173492	229533.A0A098DY09	pp	229533.I1RFV3
173493	229533.A0A098DY09	pp	229533.I1REK4
173494	229533.A0A098DY09	pp	229533.I1RP86
173495	229533.A0A098DY09	pp	229533.I1RMI8
173496	229533.A0A098DY09	pp	229533.I1RA70
173497	229533.A0A098DY09	pp	229533.A0A1C3YJL7
173498	229533.A0A098DY09	pp	229533.V6RS91
173499	229533.A0A098DY09	pp	229533.I1RRI2
173500	229533.A0A098DY09	pp	229533.I1RWX5
173501	229533.A0A098DY09	pp	229533.I1RWE8
173502	229533.A0A098DY09	pp	229533.A0A1C3YM63
173503	229533.A0A098DY09	pp	229533.A0A0E0RSD7
173504	229533.A0A098DY09	pp	229533.I1SAJ3
173505	229533.A0A098DY09	pp	229533.I1RYJ6
173506	229533.A0A098DY09	pp	229533.I1RR24
173507	229533.A0A098DY09	pp	229533.V6R1K4
173508	229533.A0A098DY09	pp	229533.I1RCW6
173509	229533.A0A098DY09	pp	229533.I1RM67
173510	229533.A0A098DY09	pp	229533.I1RB44
173511	229533.A0A098DY09	pp	229533.I1S216
173512	229533.A0A098DY09	pp	229533.A0A0E0RX50
173513	229533.A0A098DY09	pp	229533.I1RYX6
173514	229533.A0A098DY09	pp	229533.I1S4S4
173515	229533.A0A098DY09	pp	229533.A0A1C3YK11
173516	229533.A0A098DY09	pp	229533.I1RT33
173517	229533.A0A098DY09	pp	229533.V6R057
173518	229533.A0A098DY09	pp	229533.I1RCL4
173519	229533.A0A098DY09	pp	229533.A0A1C3YN30
173520	229533.A0A098DY09	pp	229533.A0A098E2A4
173521	229533.A0A098DY09	pp	229533.V6RRH2

173522	229533.A0A098DY09	pp	229533.I1RJU3
173523	229533.A0A098DY09	pp	229533.I1RPN4
173524	229533.A0A098DY09	pp	229533.I1S0F1
173525	229533.A0A098DY09	pp	229533.I1RRG3
173526	229533.A0A098DY09	pp	229533.I1RI34
173527	229533.A0A098DY09	pp	229533.A0A1C3YN26
173528	229533.A0A098DY09	pp	229533.A0A1C3YLU7
173529	229533.A0A098DY09	pp	229533.A0A1C3YKS6
173530	229533.A0A098DY09	pp	229533.I1RTD3
173531	229533.A0A098DY09	pp	229533.I1RAZ3
173532	229533.A0A098DY09	pp	229533.I1RQS9
173533	229533.A0A098DY09	pp	229533.A0A098E4W0
173534	229533.A0A098DY09	pp	229533.I1RTJ2
173535	229533.A0A098DY09	pp	229533.A0A1C3YHS4
173536	229533.A0A098DY09	pp	229533.I1RAQ4
173537	229533.A0A098DY09	pp	229533.I1RB39
173538	229533.A0A098DY09	pp	229533.I1RM35
173539	229533.A0A098DY09	pp	229533.A0A1C3YLR6
173540	229533.A0A098DY09	pp	229533.I1RBE7
173541	229533.A0A098DY09	pp	229533.I1RXF0
173542	229533.A0A098DY09	pp	229533.I1RPE5
173543	229533.A0A098DY09	pp	229533.I1RJL9
173544	229533.A0A098DY09	pp	229533.I1REK8
173545	229533.A0A098DY09	pp	229533.I1RBW0
173546	229533.A0A098DY09	pp	229533.I1S4Q4
173547	229533.A0A098DY09	pp	229533.I1RG14
173548	229533.A0A098DY09	pp	229533.I1RGK9
173549	229533.A0A098DY09	pp	229533.I1RK15
173550	229533.A0A098DY09	pp	229533.A0A1C3YJR3
173551	229533.A0A098DY09	pp	229533.I1RCL0
173552	229533.A0A098DY09	pp	229533.I1S0B7
173553	229533.A0A098DY09	pp	229533.I1REL7
173554	229533.A0A098DY09	pp	229533.I1RK95
173555	229533.A0A098DY09	pp	229533.A0A1C3YKH8
173556	229533.A0A098DY09	pp	229533.I1RF82
173557	229533.A0A098DY09	pp	229533.A0A1C3YIK4
173558	229533.A0A098DY09	pp	229533.I1RS18
173559	229533.A0A098DY09	pp	229533.I1S835
173560	229533.A0A098DY09	pp	229533.I1RBI1
173561	229533.A0A098DY09	pp	229533.V6RBN2
173562	229533.A0A098DY09	pp	229533.I1RA94
173563	229533.A0A098DY09	pp	229533.A0A1C3YI30
173564	229533.A0A098DY09	pp	229533.A0A1C3YL10
173565	229533.A0A098DY09	pp	229533.Q4IJK2
173566	229533.A0A098DY09	pp	229533.I1RMQ4
173567	229533.A0A098DY09	pp	229533.A0A0E0S134
173568	229533.A0A098DY09	pp	229533.A0A1C3YK34
173569	229533.A0A098DY09	pp	229533.I1RRY9
173570	229533.A0A098DY09	pp	229533.V6QUE6
173571	229533.A0A098DY09	pp	229533.I1RIY0
173572	229533.A0A098DY09	pp	229533.I1RRV4
173573	229533.A0A098DY09	pp	229533.A0A1C3YKF3
173574	229533.A0A098DY09	pp	229533.A0A1C3YN86
173575	229533.A0A098DY09	pp	229533.A0A1C3YK68
173576	229533.I1RXT2	pp	229533.Q4I1H6
173577	229533.I1RXT2	pp	229533.I1S1W5
173578	229533.I1RXT2	pp	229533.I1S0G1
173579	229533.I1RXT2	pp	229533.I1S039
173580	229533.I1RXT2	pp	229533.I1S0J7
173581	229533.I1RXT2	pp	229533.I1S9X9
173582	229533.I1RXT2	pp	229533.Q4I1B1
173583	229533.I1RXT2	pp	229533.I1S8Q3
173584	229533.I1RXT2	pp	229533.I1S8F9
173585	229533.I1RXT2	pp	229533.V6QX21
173586	229533.I1RXT2	pp	229533.I1S5R2
173587	229533.I1RXT2	pp	229533.I1S034
173588	229533.I1RXT2	pp	229533.I1RYY6
173589	229533.I1RXT2	pp	229533.I1S0P7
173590	229533.I1S3W0	pp	229533.Q4IB50
173591	229533.I1S3W0	pp	229533.I1S9J6
173592	229533.I1S3W0	pp	229533.Q4I7L0
173593	229533.A0A1C3YJ07	pp	229533.A0A1C3YLQ4
173594	229533.A0A1C3YJ07	pp	229533.I1REB5

173595 229533.A0A1C3YJ07 pp 229533.I1S2L1
173596 229533.A0A1C3YJ07 pp 229533.I1S360
173597 229533.A0A1C3YJ07 pp 229533.I1RCN6
173598 229533.A0A1C3YJ07 pp 229533.A0A1C3YMF6
173599 229533.A0A1C3YJ07 pp 229533.I1RWN0
173600 229533.A0A1C3YJ07 pp 229533.I1S368
173601 229533.A0A1C3YJ07 pp 229533.I1RE88
173602 229533.A0A1C3YJ07 pp 229533.A0A1C3YJJ7
173603 229533.A0A1C3YJ07 pp 229533.I1RTP2
173604 229533.A0A1C3YJ07 pp 229533.I1RFY0
173605 229533.A0A1C3YJ07 pp 229533.I1RW69
173606 229533.A0A1C3YJ07 pp 229533.I1S2B0
173607 229533.A0A1C3YJ07 pp 229533.I1RQM4
173608 229533.A0A1C3YJ07 pp 229533.I1RDZ5
173609 229533.A0A1C3YJ07 pp 229533.I1RED2
173610 229533.A0A1C3YJ07 pp 229533.I1RK49
173611 229533.A0A1C3YJ07 pp 229533.I1REI2
173612 229533.A0A1C3YJ07 pp 229533.I1RRC4
173613 229533.I1RNG8 pp 229533.I1RX50
173614 229533.I1RNG8 pp 229533.I1S0J7
173615 229533.I1RNG8 pp 229533.I1S4N8
173616 229533.I1RNG8 pp 229533.I1RPS0
173617 229533.I1RNG8 pp 229533.I1RZD9
173618 229533.I1RNG8 pp 229533.I1RQG7
173619 229533.I1RNG8 pp 229533.I1RXV4
173620 229533.I1RNG8 pp 229533.I1S549
173621 229533.I1RNG8 pp 229533.I1RSB0
173622 229533.I1RNG8 pp 229533.I1S4P4
173623 229533.I1RNG8 pp 229533.V6QX21
173624 229533.I1RNG8 pp 229533.I1RTQ9
173625 229533.I1RNG8 pp 229533.I1RW37
173626 229533.I1RNG8 pp 229533.I1RZG0
173627 229533.I1RNG8 pp 229533.I1RTG9
173628 229533.I1RNG8 pp 229533.I1S039
173629 229533.I1RNG8 pp 229533.I1RTA6
173630 229533.I1RNG8 pp 229533.I1S1X7
173631 229533.I1RNG8 pp 229533.I1S4N7
173632 229533.I1RNG8 pp 229533.I1RWJ1
173633 229533.I1RNG8 pp 229533.I1RPA7
173634 229533.I1RNG8 pp 229533.I1RUP2
173635 229533.I1RNG8 pp 229533.I1S8Q3
173636 229533.I1RNG8 pp 229533.I1RR07
173637 229533.I1RNG8 pp 229533.Q4HYC1
173638 229533.I1RNG8 pp 229533.I1RZP7
173639 229533.I1RNG8 pp 229533.I1S1W5
173640 229533.I1RNG8 pp 229533.Q4I1B1
173641 229533.I1RNG8 pp 229533.I1S0L8
173642 229533.I1RNG8 pp 229533.I1RZH4
173643 229533.I1RNG8 pp 229533.I1S0P7
173644 229533.I1RNG8 pp 229533.I1S9X9
173645 229533.I1RNG8 pp 229533.I1S050
173646 229533.I1RNG8 pp 229533.I1RYM3
173647 229533.I1RNG8 pp 229533.I1RWG1
173648 229533.I1RNG8 pp 229533.I1RZV4
173649 229533.I1RNG8 pp 229533.I1RZP6
173650 229533.I1RNG8 pp 229533.I1RQ01
173651 229533.I1RNG8 pp 229533.V6R6F6
173652 229533.I1RNG8 pp 229533.I1S095
173653 229533.I1RNG8 pp 229533.I1S287
173654 229533.I1RNG8 pp 229533.I1RXT2
173655 229533.I1RNG8 pp 229533.I1RP29
173656 229533.I1RZR5 pp 229533.I1S8H1
173657 229533.I1RZR5 pp 229533.Q4IJP1
173658 229533.I1RZR5 pp 229533.Q4INW6
173659 229533.I1RZR5 pp 229533.Q4I0M9
173660 229533.I1RZR5 pp 229533.Q4I5G1
173661 229533.I1RZR5 pp 229533.I1S2I9
173662 229533.I1RLZ6 pp 229533.I1S2L1
173663 229533.I1RLZ6 pp 229533.V6RRM0
173664 229533.I1RLZ6 pp 229533.I1S3G4
173665 229533.I1RLZ6 pp 229533.I1RVR7
173666 229533.I1RLZ6 pp 229533.I1RPM5
173667 229533.I1RLZ6 pp 229533.I1RPM6

173668 229533.I1RLZ6 pp 229533.I1S0H5
173669 229533.I1RLZ6 pp 229533.I1S360
173670 229533.I1RLZ6 pp 229533.I1RWM4
173671 229533.I1RLZ6 pp 229533.I1RXE6
173672 229533.I1RLZ6 pp 229533.I1RQU9
173673 229533.I1RLZ6 pp 229533.I1S8F8
173674 229533.I1RLZ6 pp 229533.I1RZV5
173675 229533.I1RLZ6 pp 229533.I1RYX6
173676 229533.I1RLZ6 pp 229533.I1S1W8
173677 229533.I1RLZ6 pp 229533.I1RNM7
173678 229533.I1RR07 pp 229533.I1S0Z5
173679 229533.I1RR07 pp 229533.I1S0P7
173680 229533.I1RR07 pp 229533.I1S1W5
173681 229533.I1RR07 pp 229533.I1RXT2
173682 229533.I1RR07 pp 229533.I1S9X9
173683 229533.I1RR07 pp 229533.Q4I1B1
173684 229533.I1RR07 pp 229533.I1S039
173685 229533.I1RR07 pp 229533.I1RZB8
173686 229533.I1RR07 pp 229533.I1RX50
173687 229533.I1RR07 pp 229533.I1RW37
173688 229533.I1RR07 pp 229533.I1S0J7
173689 229533.I1S4Q4 pp 229533.V6RCZ8
173690 229533.I1S4Q4 pp 229533.V6R042
173691 229533.I1S4Q4 pp 229533.I1S5B0
173692 229533.I1S4Q4 pp 229533.I1S5R6
173693 229533.I1S4Q4 pp 229533.V6R1K4
173694 229533.I1S4Q4 pp 229533.V6RBN2
173695 229533.I1S4Q4 pp 229533.I1SAN9
173696 229533.I1S4Q4 pp 229533.V6QUE6
173697 229533.I1S4Q4 pp 229533.V6R2A1
173698 229533.I1S4Q4 pp 229533.I1S4S4
173699 229533.I1S4Q4 pp 229533.V6RPQ8
173700 229533.I1S4Q4 pp 229533.I1S8G4
173701 229533.I1S4Q4 pp 229533.V6RTJ8
173702 229533.I1S4Q4 pp 229533.I1SAJ3
173703 229533.I1S4Q4 pp 229533.V6RRH2
173704 229533.I1S4Q4 pp 229533.I1S9Y1
173705 229533.I1S4Q4 pp 229533.I1S5R1
173706 229533.I1S4Q4 pp 229533.V6R057
173707 229533.I1S4Q4 pp 229533.O74638
173708 229533.I1S4Q4 pp 229533.Q4HXF0
173709 229533.I1RQU9 pp 229533.I1S1W8
173710 229533.I1RQU9 pp 229533.I1RYX6
173711 229533.I1RQU9 pp 229533.I1RXE6
173712 229533.I1RQU9 pp 229533.I1RWM4
173713 229533.I1RQU9 pp 229533.V6RRM0
173714 229533.I1RQU9 pp 229533.I1S8F8
173715 229533.I1RQU9 pp 229533.I1S360
173716 229533.I1RQU9 pp 229533.I1S2L1
173717 229533.I1RQU9 pp 229533.I1S3G4
173718 229533.I1RQU9 pp 229533.I1RVR7
173719 229533.I1RQU9 pp 229533.I1RZV5
173720 229533.I1RQU9 pp 229533.I1S0H5
173721 229533.I1RKA1 pp 229533.I1RZR6
173722 229533.I1RKA1 pp 229533.I1RXT2
173723 229533.I1RKA1 pp 229533.I1RM49
173724 229533.I1RKA1 pp 229533.I1RZJ9
173725 229533.I1RKA1 pp 229533.I1S0P7
173726 229533.I1RKA1 pp 229533.I1RNG8
173727 229533.I1RKA1 pp 229533.I1S0J7
173728 229533.I1RKA1 pp 229533.I1RT41
173729 229533.I1RKA1 pp 229533.I1RX50
173730 229533.I1RKA1 pp 229533.I1S1W5
173731 229533.I1RKA1 pp 229533.I1S1X7
173732 229533.I1RKA1 pp 229533.I1S039
173733 229533.I1RKA1 pp 229533.I1RZ63
173734 229533.I1RKA1 pp 229533.I1S9X9
173735 229533.I1RKA1 pp 229533.I1S4N7
173736 229533.I1RKA1 pp 229533.I1RW37
173737 229533.I1RKA1 pp 229533.I1S8Q3
173738 229533.I1RKA1 pp 229533.I1RS41
173739 229533.I1RKA1 pp 229533.I1S0L1
173740 229533.I1RKA1 pp 229533.I1RXV4

173741 229533.I1RKA1 pp 229533.I1S4N8
173742 229533.I1RKA1 pp 229533.I1RR07
173743 229533.I1RKA1 pp 229533.I1RMV6
173744 229533.I1RKA1 pp 229533.V6QX21
173745 229533.A0A0E0SIE3 pp 229533.I1REB5
173746 229533.A0A0E0SIE3 pp 229533.I1RE88
173747 229533.A0A0E0SIE3 pp 229533.I1S108
173748 229533.A0A0E0SIE3 pp 229533.A0A1C3YJJ7
173749 229533.A0A0E0SIE3 pp 229533.I1RZT6
173750 229533.A0A0E0SIE3 pp 229533.I1RDZ5
173751 229533.A0A0E0SIE3 pp 229533.I1RQM4
173752 229533.A0A0E0SIE3 pp 229533.I1S360
173753 229533.A0A0E0SIE3 pp 229533.I1RB45
173754 229533.A0A0E0SIE3 pp 229533.I1RXJ5
173755 229533.A0A0E0SIE3 pp 229533.I1RK49
173756 229533.A0A0E0SIE3 pp 229533.I1RTP2
173757 229533.A0A0E0SIE3 pp 229533.I1RMG4
173758 229533.A0A0E0SIE3 pp 229533.A0A1C3YIX3
173759 229533.A0A0E0SIE3 pp 229533.I1RWN0
173760 229533.A0A0E0SIE3 pp 229533.I1RDQ0
173761 229533.A0A0E0SIE3 pp 229533.A0A1C3YMF6
173762 229533.A0A0E0SIE3 pp 229533.A0A1C3YJ07
173763 229533.A0A0E0SIE3 pp 229533.A0A1C3YLQ4
173764 229533.A0A0E0SIE3 pp 229533.I1S2L1
173765 229533.A0A0E0SIE3 pp 229533.I1S368
173766 229533.I1RZR6 pp 229533.Q4HYQ4
173767 229533.I1RZR6 pp 229533.Q4IJ11
173768 229533.I1RZR6 pp 229533.Q4HTT1
173769 229533.I1RZR6 pp 229533.Q4I5W5
173770 229533.I1RZR6 pp 229533.I1S201
173771 229533.I1RZR6 pp 229533.Q4IB50
173772 229533.I1RZR6 pp 229533.I1S0C6
173773 229533.I1RZR6 pp 229533.Q4IER8
173774 229533.I1RZR6 pp 229533.Q4I5R3
173775 229533.I1RZR6 pp 229533.V6QUE6
173776 229533.I1RZR6 pp 229533.Q4I7N9
173777 229533.I1RZR6 pp 229533.Q4HTT2
173778 229533.I1RZR6 pp 229533.V6QWX8
173779 229533.I1RZR6 pp 229533.Q4IJU0
173780 229533.I1RZR6 pp 229533.Q4I5I4
173781 229533.I1RZR6 pp 229533.I1S289
173782 229533.I1RZR6 pp 229533.Q4HYB8
173783 229533.A0A1C3YKR7 pp 229533.V6QUV7
173784 229533.A0A1C3YKR7 pp 229533.I1RT76
173785 229533.A0A1C3YKR7 pp 229533.I1S444
173786 229533.A0A1C3YKR7 pp 229533.I1RHE5
173787 229533.A0A1C3YKR7 pp 229533.I1S2L3
173788 229533.A0A1C3YKR7 pp 229533.I1RJQ5
173789 229533.A0A1C3YKR7 pp 229533.I1RZ78
173790 229533.A0A1C3YKR7 pp 229533.I1RM09
173791 229533.A0A1C3YKR7 pp 229533.A0A1C3YM36
173792 229533.A0A1C3YKR7 pp 229533.A0A1C3YN73
173793 229533.A0A1C3YKR7 pp 229533.I1RQ01
173794 229533.A0A1C3YKR7 pp 229533.Q4HY20
173795 229533.A0A1C3YKR7 pp 229533.A0A1C3YML3
173796 229533.A0A1C3YKR7 pp 229533.I1S1W5
173797 229533.A0A1C3YKR7 pp 229533.I1RMM6
173798 229533.I1S3S3 pp 229533.V6R1K4
173799 229533.I1S3S3 pp 229533.V6RBN2
173800 229533.I1S3S3 pp 229533.Q4HXF0
173801 229533.I1S3S3 pp 229533.I1S4Q4
173802 229533.I1S3S3 pp 229533.O74638
173803 229533.I1S3S3 pp 229533.I1S8P5
173804 229533.I1S3S3 pp 229533.V6RTJ8
173805 229533.I1S3S3 pp 229533.V6R057
173806 229533.I1S3S3 pp 229533.V6RMW6
173807 229533.Q4I1T9 pp 229533.V6RN31
173808 229533.I1S1W5 pp 229533.V6R6F6
173809 229533.I1S1W5 pp 229533.I1S287
173810 229533.I1S1W5 pp 229533.Q4I1B1
173811 229533.I1S1W5 pp 229533.I1S278
173812 229533.I1S1W5 pp 229533.I1S8Q3
173813 229533.I1S1W5 pp 229533.V6QV09

173814	229533.I1S1W5	pp	229533.V6QX21
173815	229533.I1S1W5	pp	229533.Q4IBU4
173816	229533.I1S1W5	pp	229533.I1S9X9
173817	229533.I1S1W5	pp	229533.Q4IB89
173818	229533.I1S1W5	pp	229533.I1S1X7
173819	229533.I1S1W5	pp	229533.I1S2E9
173820	229533.I1S1W5	pp	229533.Q4I6S5
173821	229533.I1S1W5	pp	229533.I1S4N7
173822	229533.I1S1W5	pp	229533.I1S4N8
173823	229533.I1S1W5	pp	229533.I1S248
173824	229533.I1S1W5	pp	229533.I1S5R2
173825	229533.A0A098DH35	pp	229533.I1RWN0
173826	229533.A0A098DH35	pp	229533.A0A1C3YIX3
173827	229533.A0A098DH35	pp	229533.I1RGI5
173828	229533.A0A098DH35	pp	229533.I1RWM4
173829	229533.A0A098DH35	pp	229533.I1RGI4
173830	229533.A0A098DH35	pp	229533.V6R9L8
173831	229533.A0A098DH35	pp	229533.I1S368
173832	229533.A0A098DH35	pp	229533.I1RZV5
173833	229533.A0A098DH35	pp	229533.A0A1C3YMF6
173834	229533.A0A098DH35	pp	229533.A0A098DXB1
173835	229533.A0A098DH35	pp	229533.I1S0H5
173836	229533.A0A098DH35	pp	229533.A0A1C3YLQ4
173837	229533.A0A098DH35	pp	229533.I1RTP2
173838	229533.A0A098DH35	pp	229533.A0A1C3YJ07
173839	229533.A0A098DH35	pp	229533.I1RLZ6
173840	229533.A0A098DH35	pp	229533.I1RQU9
173841	229533.A0A098DH35	pp	229533.A0A0E0SIE3
173842	229533.A0A098DH35	pp	229533.I1REB5
173843	229533.A0A098DH35	pp	229533.I1RDZ5
173844	229533.A0A098DH35	pp	229533.I1RE88
173845	229533.A0A098DH35	pp	229533.A0A098DIZ7
173846	229533.A0A098DH35	pp	229533.A0A1C3YJJ7
173847	229533.A0A0E0SC50	pp	229533.I1S278
173848	229533.A0A0E0SC50	pp	229533.I1S4N8
173849	229533.A0A0E0SC50	pp	229533.I1RR07
173850	229533.A0A0E0SC50	pp	229533.I1RMV6
173851	229533.A0A0E0SC50	pp	229533.Q4I1B1
173852	229533.A0A0E0SC50	pp	229533.V6QX21
173853	229533.A0A0E0SC50	pp	229533.I1S4N7
173854	229533.A0A0E0SC50	pp	229533.I1RFN8
173855	229533.A0A0E0SC50	pp	229533.A0A1C3YHP8
173856	229533.A0A0E0SC50	pp	229533.A0A1C3YMK7
173857	229533.A0A0E0SC50	pp	229533.I1S8Q3
173858	229533.A0A0E0SC50	pp	229533.I1RXV4
173859	229533.A0A0E0SC50	pp	229533.I1RW37
173860	229533.A0A0E0SC50	pp	229533.I1RK75
173861	229533.A0A0E0SC50	pp	229533.A0A1C3YLM3
173862	229533.A0A0E0SC50	pp	229533.I1RJT1
173863	229533.A0A0E0SC50	pp	229533.I1RT45
173864	229533.A0A0E0SC50	pp	229533.I1S1X7
173865	229533.A0A0E0SC50	pp	229533.A0A1C3YME0
173866	229533.A0A0E0SC50	pp	229533.I1S1W5
173867	229533.A0A0E0SC50	pp	229533.I1S0P7
173868	229533.A0A0E0SC50	pp	229533.I1RNG8
173869	229533.A0A0E0SC50	pp	229533.A0A1C3YI39
173870	229533.A0A0E0SC50	pp	229533.I1S039
173871	229533.A0A0E0SC50	pp	229533.I1RXT2
173872	229533.A0A0E0SC50	pp	229533.A0A1C3YK57
173873	229533.A0A0E0SC50	pp	229533.I1S0J7
173874	229533.A0A0E0SC50	pp	229533.I1RM49
173875	229533.A0A0E0SC50	pp	229533.A0A1C3YM82
173876	229533.A0A0E0SC50	pp	229533.I1RX50
173877	229533.A0A0E0SC50	pp	229533.A0A1C3YJG7
173878	229533.A0A0E0SC50	pp	229533.I1RKA1
173879	229533.A0A0E0SC50	pp	229533.I1RX00
173880	229533.A0A0E0SC50	pp	229533.I1RSQ4
173881	229533.A0A0E0SC50	pp	229533.I1RFS8
173882	229533.A0A0E0SC50	pp	229533.I1RZ63
173883	229533.A0A0E0SC50	pp	229533.I1S9X9
173884	229533.A0A098E2S1	pp	229533.I1RPC6
173885	229533.A0A098E2S1	pp	229533.I1REK8
173886	229533.A0A098E2S1	pp	229533.A0A0E0S134

173887 229533.A0A098E2S1 pp 229533.A0A1C3YLB6
173888 229533.A0A098E2S1 pp 229533.A0A1C3YI30
173889 229533.A0A098E2S1 pp 229533.I1S5B0
173890 229533.A0A098E2S1 pp 229533.Q4IPH4
173891 229533.A0A098E2S1 pp 229533.V6RCZ8
173892 229533.A0A098E2S1 pp 229533.I1RQ33
173893 229533.A0A098E2S1 pp 229533.I1S0S5
173894 229533.A0A098E2S1 pp 229533.Q4HXF6
173895 229533.A0A098E2S1 pp 229533.I1RB92
173896 229533.A0A098E2S1 pp 229533.I1RUG3
173897 229533.A0A098E2S1 pp 229533.I1RQE7
173898 229533.A0A098E2S1 pp 229533.I1RFV3
173899 229533.A0A098E2S1 pp 229533.I1RSR3
173900 229533.A0A098E2S1 pp 229533.I1R9X4
173901 229533.A0A098E2S1 pp 229533.A0A1C3YKB0
173902 229533.A0A098E2S1 pp 229533.V6QYF0
173903 229533.A0A098E2S1 pp 229533.I1RG14
173904 229533.A0A098E2S1 pp 229533.A0A1C3YK36
173905 229533.A0A098E2S1 pp 229533.I1RPF1
173906 229533.A0A098E2S1 pp 229533.I1RJU3
173907 229533.A0A098E2S1 pp 229533.I1RW61
173908 229533.A0A098E2S1 pp 229533.I1RSQ4
173909 229533.A0A098E2S1 pp 229533.I1RL04
173910 229533.A0A098E2S1 pp 229533.I1RR24
173911 229533.A0A098E2S1 pp 229533.I1RT05
173912 229533.A0A098E2S1 pp 229533.I1RRI1
173913 229533.A0A098E2S1 pp 229533.I1S0F1
173914 229533.A0A098E2S1 pp 229533.A0A1C3YJK3
173915 229533.A0A098E2S1 pp 229533.I1RD52
173916 229533.A0A098E2S1 pp 229533.I1RWS4
173917 229533.A0A098E2S1 pp 229533.I1S5R1
173918 229533.A0A098E2S1 pp 229533.I1RFY6
173919 229533.A0A098E2S1 pp 229533.A0A1C3YM63
173920 229533.A0A098E2S1 pp 229533.I1RC82
173921 229533.A0A098E2S1 pp 229533.I1RM67
173922 229533.A0A098E2S1 pp 229533.I1RFT4
173923 229533.A0A098E2S1 pp 229533.I1RMM8
173924 229533.A0A098E2S1 pp 229533.I1RZB2
173925 229533.A0A098E2S1 pp 229533.A0A1C3YIE7
173926 229533.A0A098E2S1 pp 229533.I1R9W4
173927 229533.A0A098E2S1 pp 229533.A0A1C3YJI6
173928 229533.A0A098E2S1 pp 229533.I1RYJ6
173929 229533.I1S4N8 pp 229533.I1S9X9
173930 229533.I1S4N8 pp 229533.I1S549
173931 229533.A0A1C3YHS4 pp 229533.I1RG11
173932 229533.A0A1C3YHS4 pp 229533.I1RD22
173933 229533.A0A1C3YHS4 pp 229533.I1RRN2
173934 229533.A0A1C3YHS4 pp 229533.I1S256
173935 229533.A0A1C3YHS4 pp 229533.I1SAN9
173936 229533.A0A1C3YHS4 pp 229533.V6R042
173937 229533.A0A1C3YHS4 pp 229533.I1RMQ5
173938 229533.A0A1C3YHS4 pp 229533.I1RJV3
173939 229533.A0A1C3YHS4 pp 229533.I1RUG3
173940 229533.A0A1C3YHS4 pp 229533.I1RA70
173941 229533.A0A1C3YHS4 pp 229533.I1RNC1
173942 229533.A0A1C3YHS4 pp 229533.I1S2C4
173943 229533.A0A1C3YHS4 pp 229533.I1RWE8
173944 229533.A0A1C3YHS4 pp 229533.V6R057
173945 229533.A0A1C3YHS4 pp 229533.I1RYW3
173946 229533.A0A1C3YHS4 pp 229533.I1S216
173947 229533.A0A1C3YHS4 pp 229533.I1RCW6
173948 229533.A0A1C3YHS4 pp 229533.I1RMI8
173949 229533.A0A1C3YHS4 pp 229533.I1RB44
173950 229533.A0A1C3YHS4 pp 229533.I1RVZ7
173951 229533.A0A1C3YHS4 pp 229533.V6RRH2
173952 229533.A0A1C3YHS4 pp 229533.I1RRI2
173953 229533.A0A1C3YHS4 pp 229533.I1RN67
173954 229533.A0A1C3YHS4 pp 229533.I1RP69
173955 229533.A0A1C3YHS4 pp 229533.I1RC05
173956 229533.A0A1C3YHS4 pp 229533.I1S1W9
173957 229533.A0A1C3YHS4 pp 229533.I1RCD8
173958 229533.A0A1C3YHS4 pp 229533.I1RVX0
173959 229533.A0A1C3YHS4 pp 229533.I1RY62

173960	229533.A0A1C3YHS4	pp	229533.V6R2A1
173961	229533.A0A1C3YHS4	pp	229533.I1RS17
173962	229533.A0A1C3YHS4	pp	229533.I1RX22
173963	229533.A0A1C3YHS4	pp	229533.V6RLM7
173964	229533.A0A1C3YHS4	pp	229533.I1RP28
173965	229533.A0A1C3YHS4	pp	229533.I1RFL4
173966	229533.A0A1C3YHS4	pp	229533.I1RFT1
173967	229533.A0A1C3YHS4	pp	229533.I1RTA0
173968	229533.A0A1C3YHS4	pp	229533.I1S2E8
173969	229533.A0A1C3YHS4	pp	229533.I1RQG4
173970	229533.A0A1C3YHS4	pp	229533.I1RQE7
173971	229533.A0A1C3YHS4	pp	229533.I1RE01
173972	229533.A0A1C3YHS4	pp	229533.I1RPP3
173973	229533.A0A1C3YHS4	pp	229533.I1S0J1
173974	229533.A0A1C3YHS4	pp	229533.I1RCH5
173975	229533.A0A1C3YHS4	pp	229533.A0A1C3YKY0
173976	229533.A0A1C3YHS4	pp	229533.I1S274
173977	229533.A0A1C3YHS4	pp	229533.I1S8P5
173978	229533.A0A1C3YHS4	pp	229533.I1RAW0
173979	229533.A0A1C3YHS4	pp	229533.I1S9Y1
173980	229533.A0A1C3YHS4	pp	229533.I1RVZ2
173981	229533.A0A1C3YHS4	pp	229533.O74638
173982	229533.A0A1C3YHS4	pp	229533.A0A1C3YLK8
173983	229533.A0A1C3YHS4	pp	229533.I1RTA1
173984	229533.A0A1C3YHS4	pp	229533.I1RSX0
173985	229533.A0A1C3YHS4	pp	229533.I1RGA4
173986	229533.A0A1C3YHS4	pp	229533.I1RRT8
173987	229533.A0A1C3YHS4	pp	229533.A0A1C3YIS9
173988	229533.A0A1C3YHS4	pp	229533.I1RT75
173989	229533.A0A1C3YHS4	pp	229533.I1RBC7
173990	229533.A0A1C3YHS4	pp	229533.I1S5R1
173991	229533.A0A1C3YHS4	pp	229533.Q4HXF0
173992	229533.A0A1C3YHS4	pp	229533.I1RQM7
173993	229533.A0A1C3YHS4	pp	229533.I1RCJ7
173994	229533.A0A1C3YHS4	pp	229533.I1RW43
173995	229533.A0A1C3YHS4	pp	229533.I1RMQ4
173996	229533.A0A1C3YHS4	pp	229533.A0A1C3YI30
173997	229533.A0A1C3YHS4	pp	229533.A0A1C3YKM9
173998	229533.A0A1C3YHS4	pp	229533.I1RZ39
173999	229533.A0A1C3YHS4	pp	229533.I1RT40
174000	229533.A0A1C3YHS4	pp	229533.A0A1C3YJI6
174001	229533.A0A1C3YHS4	pp	229533.I1RTD5
174002	229533.A0A1C3YHS4	pp	229533.I1S5B0
174003	229533.A0A1C3YHS4	pp	229533.A0A1C3YMA1
174004	229533.A0A1C3YHS4	pp	229533.I1S4Z8
174005	229533.A0A1C3YHS4	pp	229533.A0A1C3YIR8
174006	229533.A0A1C3YHS4	pp	229533.I1S8G4
174007	229533.A0A1C3YHS4	pp	229533.V6QUE6
174008	229533.A0A1C3YHS4	pp	229533.I1RSQ4
174009	229533.A0A1C3YHS4	pp	229533.I1RRV4
174010	229533.A0A1C3YHS4	pp	229533.I1RJZ7
174011	229533.A0A1C3YHS4	pp	229533.A0A1C3YKU8
174012	229533.A0A1C3YHS4	pp	229533.I1RBY4
174013	229533.A0A1C3YHS4	pp	229533.I1RZZ9
174014	229533.A0A1C3YHS4	pp	229533.I1S3K6
174015	229533.A0A1C3YHS4	pp	229533.I1RWI5
174016	229533.A0A1C3YHS4	pp	229533.I1RE76
174017	229533.A0A1C3YHS4	pp	229533.I1RFT2
174018	229533.A0A1C3YHS4	pp	229533.I1RBF3
174019	229533.A0A1C3YHS4	pp	229533.I1RFS3
174020	229533.A0A1C3YHS4	pp	229533.I1RKY2
174021	229533.A0A1C3YHS4	pp	229533.A0A1C3YI20
174022	229533.A0A1C3YHS4	pp	229533.A0A1C3YJR3
174023	229533.A0A1C3YHS4	pp	229533.I1RG14
174024	229533.A0A1C3YHS4	pp	229533.I1RTD3
174025	229533.A0A1C3YHS4	pp	229533.I1RK15
174026	229533.A0A1C3YHS4	pp	229533.I1RPV3
174027	229533.A0A1C3YHS4	pp	229533.V6RCZ8
174028	229533.A0A1C3YHS4	pp	229533.I1RL04
174029	229533.A0A1C3YHS4	pp	229533.I1RBE4
174030	229533.A0A1C3YHS4	pp	229533.I1RN88
174031	229533.A0A1C3YHS4	pp	229533.I1S2H4
174032	229533.A0A1C3YHS4	pp	229533.A0A1C3YKB0

229533.A0A1C3YHS4 pp 229533.I1RF82
229533.A0A1C3YHS4 pp 229533.I1S6K9
229533.A0A1C3YHS4 pp 229533.A0A1C3YKV3
229533.A0A1C3YHS4 pp 229533.I1RXB6
229533.A0A1C3YHS4 pp 229533.I1RVR0
229533.A0A1C3YHS4 pp 229533.A0A1C3YJW4
229533.A0A1C3YHS4 pp 229533.I1RBL7
229533.A0A1C3YHS4 pp 229533.I1RZM8
229533.A0A1C3YHS4 pp 229533.I1RRY9
229533.A0A1C3YHS4 pp 229533.I1REK8
229533.A0A1C3YHS4 pp 229533.I1RS18
229533.A0A1C3YHS4 pp 229533.I1RCR5
229533.A0A1C3YHS4 pp 229533.I1S1J7
229533.A0A1C3YHS4 pp 229533.A0A1C3YIK4
229533.A0A1C3YHS4 pp 229533.I1RM35
229533.A0A1C3YHS4 pp 229533.A0A1C3YK34
229533.A0A1C3YHS4 pp 229533.I1RM30
229533.A0A1C3YHS4 pp 229533.I1RAZ3
229533.A0A1C3YHS4 pp 229533.I1REL7
229533.A0A1C3YHS4 pp 229533.A0A1C3YK68
229533.A0A1C3YHS4 pp 229533.I1RZK5
229533.A0A1C3YHS4 pp 229533.I1RRG3
229533.A0A1C3YHS4 pp 229533.I1S4Q4
229533.A0A1C3YHS4 pp 229533.I1RAX6
229533.A0A1C3YHS4 pp 229533.A0A1C3YHX6
229533.A0A1C3YHS4 pp 229533.A0A1C3YKH8
229533.A0A1C3YHS4 pp 229533.I1RD52
229533.A0A1C3YHS4 pp 229533.V6R1K4
229533.A0A1C3YHS4 pp 229533.I1RK95
229533.A0A1C3YHS4 pp 229533.A0A1C3YIP5
229533.A0A1C3YHS4 pp 229533.V6RTJ8
229533.A0A1C3YHS4 pp 229533.Q4IJK2
229533.A0A1C3YHS4 pp 229533.I1RX46
229533.A0A1C3YHS4 pp 229533.I1RPN4
229533.A0A1C3YHS4 pp 229533.V6RBN2
229533.A0A1C3YHS4 pp 229533.I1RW71
229533.A0A1C3YHS4 pp 229533.A0A1C3YJJ2
229533.A0A1C3YHS4 pp 229533.I1RP86
229533.A0A1C3YHS4 pp 229533.I1RXD7
229533.A0A1C3YHS4 pp 229533.I1S0B7
229533.A0A1C3YHS4 pp 229533.I1RME6
229533.A0A1C3YHS4 pp 229533.A0A1C3YK13
229533.A0A1C3YHS4 pp 229533.I1RSQ7
229533.A0A1C3YHS4 pp 229533.I1S8E9
229533.A0A1C3YHS4 pp 229533.I1RRX5
229533.A0A1C3YHS4 pp 229533.I1RC11
229533.A0A1C3YHS4 pp 229533.I1RCL4
229533.A0A1C3YHS4 pp 229533.I1RB39
229533.A0A1C3YHS4 pp 229533.A0A1C3YN30
229533.A0A1C3YHS4 pp 229533.A0A1C3YLR6
229533.A0A1C3YHS4 pp 229533.I1RFK7
229533.A0A1C3YHS4 pp 229533.I1RJU3
229533.A0A1C3YHS4 pp 229533.I1R9Y4
229533.A0A1C3YHS4 pp 229533.A0A1C3YKS6
229533.A0A1C3YHS4 pp 229533.I1RQS9
229533.A0A1C3YHS4 pp 229533.A0A1C3YHX8
229533.A0A098DH24 pp 229533.I1RI11
229533.A0A098DH24 pp 229533.I1RY16
229533.A0A098DH24 pp 229533.I1RZA8
229533.A0A098DH24 pp 229533.I1S694
229533.I1RW37 pp 229533.I1S0J7
229533.I1RW37 pp 229533.I1S0L8
229533.I1RW37 pp 229533.I1RX50
229533.I1RW37 pp 229533.I1S0E4
229533.I1RW37 pp 229533.Q4I1B1
229533.I1RW37 pp 229533.I1S039
229533.I1RW37 pp 229533.I1RZB8
229533.I1RW37 pp 229533.I1S1W5
229533.I1RW37 pp 229533.I1RXT2
229533.I1RW37 pp 229533.I1S9X9
229533.I1RW37 pp 229533.I1RZH4
229533.I1RW37 pp 229533.I1S0Z5
229533.I1RW37 pp 229533.I1S4N8

229533.I1RW37 pp 229533.I1RW38
229533.I1RW37 pp 229533.I1S4N7
229533.I1RW37 pp 229533.I1S0P7
229533.I1S694 pp 229533.V6RVE9
229533.I1S694 pp 229533.I1S6B5
229533.I1S694 pp 229533.V6QYX9
229533.I1S694 pp 229533.V6R0H4
229533.I1S694 pp 229533.V6R556
229533.I1S694 pp 229533.V6QXV1
229533.I1S694 pp 229533.V6RM09
229533.I1S694 pp 229533.I1S697
229533.I1S694 pp 229533.V6REA4
229533.I1S694 pp 229533.V6QTX7
229533.I1S694 pp 229533.I1S9K9
229533.I1S694 pp 229533.I1S6C7
229533.I1S694 pp 229533.V6QZF0
229533.A0A098DPY0 pp 229533.I1RX50
229533.A0A098DPY0 pp 229533.I1S0J7
229533.A0A098DPY0 pp 229533.I1S039
229533.A0A098DPY0 pp 229533.I1S1W5
229533.A0A098DPY0 pp 229533.A0A0E0SC50
229533.A0A098DPY0 pp 229533.I1RFS8
229533.A0A098DPY0 pp 229533.I1S9X9
229533.A0A098DPY0 pp 229533.I1RKA1
229533.A0A098DPY0 pp 229533.I1RXT2
229533.A0A098DPY0 pp 229533.I1S0P7
229533.A0A098DPY0 pp 229533.I1RNG8
229533.A0A098DPY0 pp 229533.A0A1C3YME0
229533.A0A098DPY0 pp 229533.I1RAL7
229533.A0A098DPY0 pp 229533.A0A098DRQ4
229533.A0A098DPY0 pp 229533.I1RMV6
229533.A0A098DPY0 pp 229533.V6QX21
229533.A0A098DPY0 pp 229533.I1RW37
229533.A0A098DPY0 pp 229533.I1S4N7
229533.A0A098DPY0 pp 229533.I1RFN8
229533.A0A098DPY0 pp 229533.A0A0E0SDV0
229533.A0A098DPY0 pp 229533.I1RR07
229533.I1S1K5 pp 229533.V6RDP1
229533.I1S4N7 pp 229533.I1S9X9
229533.I1S4N7 pp 229533.I1S549
229533.I1S4N7 pp 229533.Q4I6S5
229533.I1RI44 pp 229533.I1RX38
229533.I1RI44 pp 229533.I1S0C2
229533.I1RI11 pp 229533.I1RZV5
229533.I1RI11 pp 229533.V6RVE9
229533.I1RI11 pp 229533.I1RWF5
229533.I1RI11 pp 229533.I1RLZ4
229533.I1RI11 pp 229533.V6R556
229533.I1RI11 pp 229533.I1S4E0
229533.I1RI11 pp 229533.I1S673
229533.I1RI11 pp 229533.I1S6B5
229533.I1RI11 pp 229533.I1RZQ1
229533.I1RI11 pp 229533.I1RSA5
229533.I1RI11 pp 229533.I1RZ25
229533.I1RI11 pp 229533.I1RWY4
229533.I1RI11 pp 229533.V6R0H4
229533.I1RI11 pp 229533.I1S2W0
229533.I1RI11 pp 229533.I1RT26
229533.I1RI11 pp 229533.I1RUP5
229533.I1RI11 pp 229533.I1S6C7
229533.I1RI11 pp 229533.I1RSX9
229533.I1RI11 pp 229533.I1RXN7
229533.I1RI11 pp 229533.V6QYX9
229533.I1RI11 pp 229533.I1S9K9
229533.I1RI11 pp 229533.I1S0R1
229533.I1RI11 pp 229533.V6REA4
229533.I1RI11 pp 229533.I1RV06
229533.I1RI11 pp 229533.I1RLA2
229533.I1RI11 pp 229533.I1RIM5
229533.I1RI11 pp 229533.I1S269
229533.I1RI11 pp 229533.I1RWY5
229533.I1RI11 pp 229533.I1S405
229533.I1RI11 pp 229533.V6RM09

174179	229533.I1RI11	pp	229533.I1S048
174180	229533.I1RI11	pp	229533.I1RST9
174181	229533.I1RI11	pp	229533.V6QTX7
174182	229533.I1RI11	pp	229533.I1RJS0
174183	229533.I1RI11	pp	229533.V6QZF0
174184	229533.I1RI11	pp	229533.V6QXV1
174185	229533.I1RI11	pp	229533.I1S267
174186	229533.I1RI11	pp	229533.I1S697
174187	229533.I1RI11	pp	229533.I1S3B2
174188	229533.I1RI11	pp	229533.I1RVI8
174189	229533.I1RI11	pp	229533.I1S0H2
174190	229533.I1RI11	pp	229533.I1RMY1
174191	229533.I1RI11	pp	229533.I1S3K0
174192	229533.I1RI11	pp	229533.I1RY39
174193	229533.I1RI11	pp	229533.I1RYM5
174194	229533.I1S1W9	pp	229533.V6RBN2
174195	229533.I1S1W9	pp	229533.Q4IJK2
174196	229533.I1S1W9	pp	229533.Q4I624
174197	229533.I1S1W9	pp	229533.V6R042
174198	229533.I1S1W9	pp	229533.V6RRH2
174199	229533.I1S1W9	pp	229533.I1S821
174200	229533.I1S1W9	pp	229533.V6QUE6
174201	229533.I1S1W9	pp	229533.V6RCZ8
174202	229533.I1S1W9	pp	229533.V6R9Z0
174203	229533.I1S1W9	pp	229533.V6RMW6
174204	229533.I1S1W9	pp	229533.I1S6K9
174205	229533.I1S1W9	pp	229533.I1S9Y1
174206	229533.I1S1W9	pp	229533.I1S5B0
174207	229533.I1S1W9	pp	229533.I1S4S4
174208	229533.I1S1W9	pp	229533.I1S5R6
174209	229533.I1S1W9	pp	229533.I1S2B8
174210	229533.I1S1W9	pp	229533.I1S9I5
174211	229533.I1S1W9	pp	229533.I1S2H4
174212	229533.I1S1W9	pp	229533.I1S268
174213	229533.I1S1W9	pp	229533.I1S2R0
174214	229533.I1S1W9	pp	229533.Q4HVQ9
174215	229533.I1S1W9	pp	229533.I1S5R1
174216	229533.I1S1W9	pp	229533.I1S4S7
174217	229533.I1S1W9	pp	229533.V6RTJ8
174218	229533.I1RSP4	pp	229533.I1RZA4
174219	229533.I1RSP4	pp	229533.Q4IBU4
174220	229533.I1RSP4	pp	229533.I1S9T1
174221	229533.I1RSP4	pp	229533.I1RXF5
174222	229533.I1RSP4	pp	229533.V6R9X9
174223	229533.I1RSP4	pp	229533.I1RWI6
174224	229533.I1RSP4	pp	229533.I1RWP9
174225	229533.I1RSP4	pp	229533.V6RQ48
174226	229533.I1RSP4	pp	229533.I1RX54
174227	229533.I1RSP4	pp	229533.I1RT41
174228	229533.I1RSP4	pp	229533.I1S1W5
174229	229533.I1RSP4	pp	229533.I1RXU1
174230	229533.I1RSP4	pp	229533.I1S034
174231	229533.I1RSP4	pp	229533.V6RKE4
174232	229533.I1RSP4	pp	229533.I1S039
174233	229533.I1RSP4	pp	229533.Q4ICG5
174234	229533.I1RSP4	pp	229533.Q4I963
174235	229533.I1RZA8	pp	229533.V6QYX9
174236	229533.I1RZA8	pp	229533.I1S0R1
174237	229533.I1RZA8	pp	229533.I1S3B2
174238	229533.I1RZA8	pp	229533.I1S0H2
174239	229533.I1RZA8	pp	229533.I1S405
174240	229533.I1RZA8	pp	229533.V6QZF0
174241	229533.I1RZA8	pp	229533.I1S048
174242	229533.I1RZA8	pp	229533.V6REA4
174243	229533.I1RZA8	pp	229533.I1S9K9
174244	229533.I1RZA8	pp	229533.I1S2W0
174245	229533.I1RZA8	pp	229533.I1S267
174246	229533.I1RZA8	pp	229533.I1S269
174247	229533.I1RZA8	pp	229533.V6QTX7
174248	229533.I1RZA8	pp	229533.I1S697
174249	229533.I1RZA8	pp	229533.I1S3K0
174250	229533.I1RZA8	pp	229533.I1S6C7
174251	229533.I1RZA8	pp	229533.V6QXV1

174252 229533.I1RZA8 pp 229533.V6R556
174253 229533.I1RZA8 pp 229533.I1S673
174254 229533.I1RZA8 pp 229533.I1RZV5
174255 229533.I1RZA8 pp 229533.V6RM09
174256 229533.I1RZA8 pp 229533.I1RZQ1
174257 229533.I1RZA8 pp 229533.I1S4E0
174258 229533.I1RZA8 pp 229533.V6RVE9
174259 229533.I1RZA8 pp 229533.I1S6B5
174260 229533.I1RZA8 pp 229533.V6R0H4
174261 229533.I1RM80 pp 229533.I1RXN6
174262 229533.I1RM80 pp 229533.V6RDP1
174263 229533.I1RFS8 pp 229533.I1RMV6
174264 229533.I1RFS8 pp 229533.I1S4N7
174265 229533.I1RFS8 pp 229533.I1S4N8
174266 229533.I1RFS8 pp 229533.I1RX50
174267 229533.I1RFS8 pp 229533.I1S1W5
174268 229533.I1RFS8 pp 229533.I1S9X9
174269 229533.I1RFS8 pp 229533.I1S039
174270 229533.I1RFS8 pp 229533.I1S0P7
174271 229533.I1RFS8 pp 229533.I1S0J7
174272 229533.I1RFS8 pp 229533.I1S287
174273 229533.I1RFS8 pp 229533.I1RNG8
174274 229533.I1RFS8 pp 229533.I1RKA1
174275 229533.A0A1C3YJN8 pp 229533.I1RMR5
174276 229533.A0A1C3YJN8 pp 229533.I1RJS2
174277 229533.I1S9G7 pp 229533.I1S9V9
174278 229533.I1RRG1 pp 229533.V6RW66
174279 229533.I1RRG1 pp 229533.V6QU63
174280 229533.I1RRG1 pp 229533.I1RXH3
174281 229533.I1RRG1 pp 229533.I1RYZ2
174282 229533.I1RRG1 pp 229533.V6RJ29
174283 229533.I1RRG1 pp 229533.Q4INS6
174284 229533.I1RRG1 pp 229533.I1S0H2
174285 229533.I1RRG1 pp 229533.I1S405
174286 229533.I1RRG1 pp 229533.V6RLM7
174287 229533.I1RRG1 pp 229533.V6RAG6
174288 229533.I1RRG1 pp 229533.I1RYM5
174289 229533.I1RRG1 pp 229533.I1S6C7
174290 229533.I1RRG1 pp 229533.I1S3D8
174291 229533.I1RRG1 pp 229533.I1RUQ7
174292 229533.I1RRG1 pp 229533.I1S3K0
174293 229533.I1RRG1 pp 229533.V6RK40
174294 229533.I1RRG1 pp 229533.I1RXN7
174295 229533.I1S324 pp 229533.I1S325
174296 229533.I1S9X9 pp 229533.Q4I6S5
174297 229533.I1S9X9 pp 229533.Q4I1B1
174298 229533.I1S9X9 pp 229533.V6RG22
174299 229533.I1S9X9 pp 229533.V6QX21
174300 229533.I1RY16 pp 229533.I1RZQ1
174301 229533.I1RY16 pp 229533.I1S6B5
174302 229533.I1RY16 pp 229533.V6R0H4
174303 229533.I1RY16 pp 229533.I1RZ25
174304 229533.I1RY16 pp 229533.V6RVE9
174305 229533.I1RY16 pp 229533.I1S673
174306 229533.I1RY16 pp 229533.I1RZ38
174307 229533.I1RY16 pp 229533.I1RZV5
174308 229533.I1RY16 pp 229533.V6R556
174309 229533.I1RY16 pp 229533.I1S4E0
174310 229533.I1RY16 pp 229533.I1S405
174311 229533.I1RY16 pp 229533.I1S0H2
174312 229533.I1RY16 pp 229533.I1S3K0
174313 229533.I1RY16 pp 229533.V6RWB3
174314 229533.I1RY16 pp 229533.I1S3T6
174315 229533.I1RY16 pp 229533.I1S0A5
174316 229533.I1RY16 pp 229533.I1RY39
174317 229533.I1RY16 pp 229533.I1S8D3
174318 229533.I1RY16 pp 229533.I1S267
174319 229533.I1RY16 pp 229533.V6QYX9
174320 229533.I1RY16 pp 229533.I1RYM5
174321 229533.I1RY16 pp 229533.I1S2W0
174322 229533.I1RY16 pp 229533.I1S0R1
174323 229533.I1RY16 pp 229533.V6QTX7
174324 229533.I1RY16 pp 229533.I1S3B2

174325 229533.I1RY16 pp 229533.V6QXV1
174326 229533.I1RY16 pp 229533.I1S048
174327 229533.I1RY16 pp 229533.I1S269
174328 229533.I1RY16 pp 229533.I1S6C7
174329 229533.I1RY16 pp 229533.I1S697
174330 229533.I1RY16 pp 229533.I1S9K9
174331 229533.I1RY16 pp 229533.V6QZF0
174332 229533.I1RY16 pp 229533.I1S223
174333 229533.I1RY16 pp 229533.V6RM09
174334 229533.I1RY16 pp 229533.V6REA4
174335 229533.I1RTL2 pp 229533.I1RZ94
174336 229533.A0A098DGY5 pp 229533.I1S694
174337 229533.A0A098DGY5 pp 229533.I1SA62
174338 229533.A0A098DGY5 pp 229533.I1RI11
174339 229533.A0A098DGY5 pp 229533.I1RZA8
174340 229533.A0A098DGY5 pp 229533.I1RRG1
174341 229533.A0A098DGY5 pp 229533.I1RY16
174342 229533.I1RS35 pp 229533.I1RY32
174343 229533.I1RS35 pp 229533.I1S7A5
174344 229533.I1RS35 pp 229533.I1RZP1
174345 229533.I1RS35 pp 229533.I1RY40
174346 229533.I1RS35 pp 229533.I1RX80
174347 229533.I1RS35 pp 229533.I1RS69
174348 229533.I1RS35 pp 229533.I1S7M8
174349 229533.I1RS35 pp 229533.Q4IPX8
174350 229533.I1RS35 pp 229533.I1RZE7
174351 229533.I1RS35 pp 229533.I1RUR5
174352 229533.I1RS35 pp 229533.I1SAF7
174353 229533.I1RS35 pp 229533.I1RZC3
174354 229533.I1RS35 pp 229533.I1RZ89
174355 229533.I1RS35 pp 229533.Q4IBU4
174356 229533.I1RS35 pp 229533.I1RXF0
174357 229533.I1RS35 pp 229533.I1RSI3
174358 229533.I1RS35 pp 229533.I1RSG0
174359 229533.I1RS35 pp 229533.I1S0R2
174360 229533.I1RS41 pp 229533.Q4I7X1
174361 229533.I1RS41 pp 229533.Q4HTT2
174362 229533.I1RS41 pp 229533.I1RV79
174363 229533.I1RS41 pp 229533.Q4I5I4
174364 229533.I1RS41 pp 229533.I1RWT6
174365 229533.I1RS41 pp 229533.I1RZB5
174366 229533.I1RS41 pp 229533.I1S9T1
174367 229533.I1RS41 pp 229533.Q4IER8
174368 229533.I1RS41 pp 229533.Q4I5R3
174369 229533.I1RS41 pp 229533.I1RU02
174370 229533.I1RS41 pp 229533.I1S002
174371 229533.I1RS41 pp 229533.Q4HYQ4
174372 229533.I1RS41 pp 229533.I1S5Z2
174373 229533.I1RS41 pp 229533.I1RSI1
174374 229533.I1RS41 pp 229533.I1RVX1
174375 229533.I1RS41 pp 229533.I1RT01
174376 229533.I1RS41 pp 229533.I1RYC3
174377 229533.I1RS41 pp 229533.Q4I7N9
174378 229533.I1RS41 pp 229533.I1RZR6
174379 229533.I1RS41 pp 229533.Q4IJU0
174380 229533.I1RS41 pp 229533.Q4IF44
174381 229533.I1RS41 pp 229533.V6QWX8
174382 229533.I1RS41 pp 229533.I1S0V1
174383 229533.I1RS41 pp 229533.I1RV91
174384 229533.I1RS41 pp 229533.I1RV82
174385 229533.I1RS41 pp 229533.I1S1K9
174386 229533.I1RS41 pp 229533.Q4HYB8
174387 229533.I1RS41 pp 229533.Q4IJ11
174388 229533.I1RS41 pp 229533.I1RSH9
174389 229533.I1RS41 pp 229533.Q4IB50
174390 229533.I1RS41 pp 229533.I1RY44
174391 229533.I1RS41 pp 229533.I1S0J3
174392 229533.I1RS41 pp 229533.I1RTH2
174393 229533.I1RS41 pp 229533.I1RWE1
174394 229533.I1RS41 pp 229533.I1S2E9
174395 229533.I1RS41 pp 229533.Q4I5W5
174396 229533.I1RS41 pp 229533.I1RW23
174397 229533.I1RS41 pp 229533.Q4HTT1

174398 229533.I1RS41 pp 229533.Q4HY90
174399 229533.I1RS41 pp 229533.I1RX38
174400 229533.I1RS41 pp 229533.I1S2R6
174401 229533.A0A1C3YLE6 pp 229533.I1RNY7
174402 229533.A0A1C3YLE6 pp 229533.I1RMR7
174403 229533.A0A1C3YLE6 pp 229533.I1S1V1
174404 229533.A0A1C3YLE6 pp 229533.I1RCJ5
174405 229533.A0A1C3YLE6 pp 229533.I1RAU1
174406 229533.A0A1C3YLE6 pp 229533.I1RWG6
174407 229533.A0A1C3YLE6 pp 229533.I1RBW7
174408 229533.I1RMV6 pp 229533.I1S0P7
174409 229533.I1RMV6 pp 229533.I1RYU1
174410 229533.I1RMV6 pp 229533.I1RZP6
174411 229533.I1RMV6 pp 229533.I1S4N8
174412 229533.I1RMV6 pp 229533.I1RNG8
174413 229533.I1RMV6 pp 229533.I1RNA3
174414 229533.I1RMV6 pp 229533.I1S278
174415 229533.I1RMV6 pp 229533.I1S4N7
174416 229533.I1RMV6 pp 229533.I1RQG7
174417 229533.I1RMV6 pp 229533.I1RW37
174418 229533.I1RMV6 pp 229533.I1RYD3
174419 229533.I1RMV6 pp 229533.I1RUP2
174420 229533.I1RMV6 pp 229533.I1RX50
174421 229533.I1RMV6 pp 229533.I1RZP7
174422 229533.I1RMV6 pp 229533.I1RXV4
174423 229533.I1RMV6 pp 229533.I1S039
174424 229533.I1RMV6 pp 229533.I1S1X7
174425 229533.I1RMV6 pp 229533.I1S0J7
174426 229533.I1RMV6 pp 229533.I1S9X9
174427 229533.I1RMV6 pp 229533.I1RTA6
174428 229533.I1RMV6 pp 229533.I1S1W5
174429 229533.A0A1C3YIG9 pp 229533.I1RZH5
174430 229533.A0A1C3YIG9 pp 229533.A0A1C3YJI6
174431 229533.A0A1C3YIG9 pp 229533.I1RT40
174432 229533.A0A1C3YIG9 pp 229533.A0A1C3YL55
174433 229533.A0A1C3YIG9 pp 229533.I1RP97
174434 229533.A0A1C3YIG9 pp 229533.I1RC86
174435 229533.A0A1C3YIG9 pp 229533.I1RC18
174436 229533.A0A1C3YIG9 pp 229533.I1R9Y8
174437 229533.A0A1C3YIG9 pp 229533.Q4IB65
174438 229533.A0A1C3YIG9 pp 229533.I1RSZ7
174439 229533.A0A1C3YIG9 pp 229533.I1RL04
174440 229533.A0A1C3YIG9 pp 229533.I1RBC2
174441 229533.A0A1C3YIG9 pp 229533.I1RTA1
174442 229533.A0A1C3YIG9 pp 229533.I1RYI5
174443 229533.A0A1C3YIG9 pp 229533.A0A1C3YK08
174444 229533.A0A1C3YIG9 pp 229533.I1RY57
174445 229533.A0A1C3YIG9 pp 229533.I1RSQ4
174446 229533.A0A1C3YIG9 pp 229533.I1RQ21
174447 229533.A0A1C3YIG9 pp 229533.I1RAD4
174448 229533.A0A1C3YIG9 pp 229533.I1RT38
174449 229533.A0A1C3YIG9 pp 229533.I1RB38
174450 229533.A0A1C3YIG9 pp 229533.A0A1C3YLK8
174451 229533.A0A1C3YIG9 pp 229533.I1RZ39
174452 229533.A0A1C3YIG9 pp 229533.I1RKB6
174453 229533.A0A1C3YIG9 pp 229533.I1RFS5
174454 229533.A0A1C3YIG9 pp 229533.Q4IBU4
174455 229533.A0A1C3YIG9 pp 229533.I1RS50
174456 229533.A0A1C3YIG9 pp 229533.I1S0R9
174457 229533.A0A1C3YIG9 pp 229533.I1RZS4
174458 229533.A0A1C3YIG9 pp 229533.I1RZ95
174459 229533.A0A1C3YIG9 pp 229533.I1RTE9
174460 229533.A0A1C3YIG9 pp 229533.I1S7L6
174461 229533.A0A1C3YIG9 pp 229533.V6R1X4
174462 229533.A0A1C3YIG9 pp 229533.I1RD52
174463 229533.A0A1C3YIG9 pp 229533.I1S4S7
174464 229533.A0A1C3YIG9 pp 229533.A0A1C3YK13
174465 229533.A0A1C3YIG9 pp 229533.I1RRS8
174466 229533.A0A1C3YIG9 pp 229533.I1RPA4
174467 229533.A0A1C3YIG9 pp 229533.I1RX80
174468 229533.A0A1C3YIG9 pp 229533.Q4IPZ1
174469 229533.A0A1C3YIG9 pp 229533.I1RNJ5
174470 229533.A0A1C3YIG9 pp 229533.I1RCH5

229533.A0A1C3YIG9 pp 229533.I1RW54
229533.A0A1C3YIG9 pp 229533.I1REK8
229533.A0A1C3YIG9 pp 229533.I1S4S4
229533.A0A1C3YIG9 pp 229533.I1RR24
229533.A0A1C3YIG9 pp 229533.I1RCR5
229533.A0A1C3YIG9 pp 229533.I1RPP5
229533.A0A1C3YIG9 pp 229533.I1RJU3
229533.A0A1C3YIG9 pp 229533.A0A1C3YJ14
229533.A0A1C3YIG9 pp 229533.I1RY63
229533.A0A1C3YIG9 pp 229533.I1S2C1
229533.A0A1C3YIG9 pp 229533.I1RJT1
229533.A0A1C3YIG9 pp 229533.A0A1C3YJN3
229533.A0A1C3YIG9 pp 229533.A0A1C3YK36
229533.A0A1C3YIG9 pp 229533.I1RQA5
229533.A0A1C3YIG9 pp 229533.V6RJP8
229533.A0A1C3YIG9 pp 229533.A0A1C3YIK4
229533.A0A1C3YIG9 pp 229533.A0A1C3YK34
229533.A0A1C3YIG9 pp 229533.I1RQR6
229533.A0A1C3YL53 pp 229533.I1S9U2
229533.A0A1C3YL53 pp 229533.I1RQR3
229533.A0A1C3YL53 pp 229533.I1RYU4
229533.V6QX21 pp 229533.V6RKY8
229533.A0A098DGX5 pp 229533.I1S0K0
229533.A0A098DGX5 pp 229533.I1RHZ5
229533.A0A098DGX5 pp 229533.I1RQP2
229533.A0A098DGX5 pp 229533.I1RC48
229533.A0A098DGX5 pp 229533.I1S8R4
229533.A0A098DGX5 pp 229533.I1S3L2
229533.A0A098DGX5 pp 229533.I1RXS0
229533.I1S8Q3 pp 229533.I1S9X9
229533.I1S8Q3 pp 229533.Q4I1B1
229533.I1S8Q3 pp 229533.V6QX21
229533.I1S8Q3 pp 229533.Q4IB50
229533.I1S4S7 pp 229533.I1S8D3
229533.I1S4S7 pp 229533.I1S6V0
229533.I1S4S7 pp 229533.V6QUE6
229533.I1S4S7 pp 229533.V6R9Z0
229533.I1S4S7 pp 229533.I1S5X9
229533.I1S4S7 pp 229533.V6RWB3
229533.I1S4S7 pp 229533.I1S6K9
229533.I1S4S7 pp 229533.Q4I624
229533.I1S4S7 pp 229533.V6RCZ8
229533.I1S4S7 pp 229533.Q4IJK2
229533.I1S4S7 pp 229533.I1S887
229533.I1S4S7 pp 229533.V6RBN2
229533.I1S4S7 pp 229533.I1S5R6
229533.I1S4S7 pp 229533.Q4IPZ1
229533.I1S4S7 pp 229533.I1S821
229533.I1S4S7 pp 229533.V6R042
229533.I1S4S7 pp 229533.I1S9Y1
229533.I1S4S7 pp 229533.V6R5T7
229533.I1S4S7 pp 229533.I1S5B0
229533.I1S4S7 pp 229533.V6RMW6
229533.I1S4S7 pp 229533.V6RHX2
229533.I1S4S7 pp 229533.I1S7W5
229533.I1S4S7 pp 229533.V6RCJ5
229533.I1S4S7 pp 229533.V6RTJ8
229533.I1S4S7 pp 229533.I1S9Z4
229533.I1S4S7 pp 229533.I1S6Y8
229533.I1S4S7 pp 229533.I1S5R1
229533.I1S4S7 pp 229533.V6RUT5
229533.I1S4S7 pp 229533.V6RCC0
229533.I1S4S7 pp 229533.Q4IB65
229533.I1S4S7 pp 229533.I1S4Z8
229533.I1S4S7 pp 229533.Q4HVQ9
229533.I1S4S7 pp 229533.V6R3J1
229533.I1S4S7 pp 229533.I1S9I5
229533.A0A0E0SPG1 pp 229533.I1RD70
229533.A0A0E0SPG1 pp 229533.A0A1C3YIP6
229533.A0A0E0SPG1 pp 229533.I1S1C8
229533.A0A0E0SPG1 pp 229533.A0A1C3YK06
229533.I1S0L8 pp 229533.Q4HZB8
229533.I1S0L8 pp 229533.I1S282

174544	229533.I1S0L8	pp	229533.I1S9G7
174545	229533.I1S0L8	pp	229533.V6REE9
174546	229533.I1S0L8	pp	229533.I1S2D7
174547	229533.I1S0L8	pp	229533.I1S9V9
174548	229533.I1S0L8	pp	229533.I1S1Y9
174549	229533.I1S0L8	pp	229533.I1S7W5
174550	229533.I1S0L8	pp	229533.I1S2B9
174551	229533.I1S0L8	pp	229533.I1S0X5
174552	229533.I1RB39	pp	229533.I1REL7
174553	229533.I1RB39	pp	229533.I1RBI1
174554	229533.I1RB39	pp	229533.I1RMQ4
174555	229533.I1RB39	pp	229533.I1RRV4
174556	229533.I1RB39	pp	229533.I1S4Q4
174557	229533.I1RB39	pp	229533.I1RTD3
174558	229533.I1RB39	pp	229533.I1RMC0
174559	229533.I1RB39	pp	229533.V6RTJ8
174560	229533.I1RB39	pp	229533.I1REK8
174561	229533.I1RB39	pp	229533.I1RM35
174562	229533.I1RB39	pp	229533.I1RBE4
174563	229533.I1RB39	pp	229533.I1S0B7
174564	229533.I1RB39	pp	229533.V6RBN2
174565	229533.I1RB39	pp	229533.I1S1W8
174566	229533.I1RB39	pp	229533.V6RRH2
174567	229533.I1RB39	pp	229533.I1RF82
174568	229533.I1RB39	pp	229533.I1S8S9
174569	229533.I1RB39	pp	229533.V6R057
174570	229533.I1RB39	pp	229533.I1RCL4
174571	229533.I1RB39	pp	229533.I1RQS9
174572	229533.I1RB39	pp	229533.I1RG14
174573	229533.I1RB39	pp	229533.I1RJU3
174574	229533.I1RB39	pp	229533.I1S216
174575	229533.I1RB39	pp	229533.I1RWE8
174576	229533.I1RB39	pp	229533.I1RK15
174577	229533.I1RB39	pp	229533.I1S247
174578	229533.I1RB39	pp	229533.I1RPN4
174579	229533.I1RB39	pp	229533.I1RMI8
174580	229533.I1RB39	pp	229533.V6R1K4
174581	229533.I1RB39	pp	229533.I1RL04
174582	229533.I1RB39	pp	229533.V6R2A1
174583	229533.I1RB39	pp	229533.I1RR72
174584	229533.I1RB39	pp	229533.I1RI34
174585	229533.I1RB39	pp	229533.I1S8G4
174586	229533.I1RB39	pp	229533.O74638
174587	229533.I1RB39	pp	229533.I1RCW6
174588	229533.I1RB39	pp	229533.I1RRP7
174589	229533.I1RB39	pp	229533.I1RB44
174590	229533.I1RB39	pp	229533.I1RN67
174591	229533.I1RB39	pp	229533.I1RX22
174592	229533.I1RB39	pp	229533.I1S3G4
174593	229533.I1RB39	pp	229533.I1RC05
174594	229533.I1RB39	pp	229533.I1RZJ3
174595	229533.I1S039	pp	229533.I1S278
174596	229533.I1S039	pp	229533.I1S8Q3
174597	229533.I1S039	pp	229533.I1S4N8
174598	229533.I1S039	pp	229533.I1S4N7
174599	229533.I1S039	pp	229533.I1S0R9
174600	229533.I1S039	pp	229533.Q4I6S5
174601	229533.I1S039	pp	229533.Q4IBU4
174602	229533.I1S039	pp	229533.V6QX21
174603	229533.I1S039	pp	229533.I1S0Z3
174604	229533.I1S039	pp	229533.I1S1X7
174605	229533.I1S039	pp	229533.I1S9X9
174606	229533.I1S039	pp	229533.I1S1W5
174607	229533.I1S039	pp	229533.I1S0P7
174608	229533.I1S039	pp	229533.I1S0J7
174609	229533.A0A1C3YI45	pp	229533.I1RAL2
174610	229533.A0A1C3YI45	pp	229533.I1S082
174611	229533.A0A1C3YI45	pp	229533.I1RNY2
174612	229533.A0A1C3YI45	pp	229533.I1RW41
174613	229533.A0A1C3YI45	pp	229533.I1RCJ5
174614	229533.A0A1C3YI45	pp	229533.I1RMB3
174615	229533.A0A1C3YI45	pp	229533.I1S1V1
174616	229533.A0A1C3YI45	pp	229533.I1RDE9

174617 229533.A0A1C3YI45 pp 229533.I1R9Y4
174618 229533.A0A1C3YI45 pp 229533.A0A1C3YKD5
174619 229533.A0A1C3YI45 pp 229533.I1RWG6
174620 229533.A0A1C3YI45 pp 229533.I1RS64
174621 229533.A0A1C3YI45 pp 229533.I1S1B6
174622 229533.A0A1C3YI45 pp 229533.I1RCD4
174623 229533.A0A1C3YI45 pp 229533.I1RBW7
174624 229533.A0A1C3YI45 pp 229533.I1RZH4
174625 229533.A0A1C3YI45 pp 229533.A0A1C3YIA8
174626 229533.A0A1C3YI45 pp 229533.I1RNY7
174627 229533.A0A1C3YI45 pp 229533.A0A1C3YJP1
174628 229533.A0A1C3YI45 pp 229533.I1RKA0
174629 229533.A0A1C3YI45 pp 229533.I1S2B7
174630 229533.A0A1C3YI45 pp 229533.A0A1C3YIP6
174631 229533.A0A1C3YI45 pp 229533.I1RXF7
174632 229533.A0A1C3YI45 pp 229533.I1RBX2
174633 229533.A0A1C3YI45 pp 229533.A0A1C3YI80
174634 229533.A0A1C3YI45 pp 229533.I1RXB5
174635 229533.A0A1C3YI45 pp 229533.V6REE9
174636 229533.A0A1C3YI45 pp 229533.I1RRH3
174637 229533.I1RQ01 pp 229533.I1S1W5
174638 229533.I1RQ01 pp 229533.I1S0J7
174639 229533.I1RQ01 pp 229533.I1RX50
174640 229533.I1RQ01 pp 229533.I1RW37
174641 229533.I1RQ01 pp 229533.I1S9X9
174642 229533.I1RRS8 pp 229533.Q4IPZ1
174643 229533.I1RRS8 pp 229533.I1RTD5
174644 229533.I1RRS8 pp 229533.I1RSQ4
174645 229533.I1RRS8 pp 229533.I1RZ39
174646 229533.I1RRS8 pp 229533.I1RT40
174647 229533.I1RRS8 pp 229533.I1RZ95
174648 229533.I1RRS8 pp 229533.V6R3J1
174649 229533.I1RRS8 pp 229533.I1RXC2
174650 229533.I1RRS8 pp 229533.I1RS50
174651 229533.I1RRS8 pp 229533.I1S268
174652 229533.I1RRS8 pp 229533.I1S1W3
174653 229533.I1RRS8 pp 229533.I1S5R1
174654 229533.I1RRS8 pp 229533.I1S1C7
174655 229533.I1RRS8 pp 229533.I1RY57
174656 229533.I1RRS8 pp 229533.I1RSZ7
174657 229533.I1RRS8 pp 229533.I1S0R9
174658 229533.I1RRS8 pp 229533.I1RTA1
174659 229533.I1RRS8 pp 229533.I1RVZ2
174660 229533.I1RRS8 pp 229533.Q4I099
174661 229533.I1RRS8 pp 229533.I1RSY2
174662 229533.I1RRS8 pp 229533.I1RYI5
174663 229533.I1RRS8 pp 229533.I1RTE9
174664 229533.I1RRS8 pp 229533.I1RVX0
174665 229533.I1RRS8 pp 229533.Q4I7T5
174666 229533.I1RRS8 pp 229533.I1S2B8
174667 229533.I1RRS8 pp 229533.I1RY72
174668 229533.I1RRS8 pp 229533.V6RM66
174669 229533.I1RRS8 pp 229533.I1RRY9
174670 229533.I1RRS8 pp 229533.I1RZM8
174671 229533.I1RRS8 pp 229533.I1RT03
174672 229533.I1RRS8 pp 229533.I1RXC4
174673 229533.I1RRS8 pp 229533.V6RCZ8
174674 229533.I1RRS8 pp 229533.I1S2A1
174675 229533.I1RRS8 pp 229533.V6RUT5
174676 229533.I1RRS8 pp 229533.I1RY63
174677 229533.I1RRS8 pp 229533.I1RXD7
174678 229533.I1RRS8 pp 229533.I1RZK5
174679 229533.I1RRS8 pp 229533.V6RJP8
174680 229533.I1RRS8 pp 229533.I1S4S4
174681 229533.I1RRS8 pp 229533.I1S2C1
174682 229533.I1RRS8 pp 229533.I1RX46
174683 229533.I1RRS8 pp 229533.I1S4S7
174684 229533.I1RRS8 pp 229533.I1RVX9
174685 229533.I1RRS8 pp 229533.I1RW54
174686 229533.I1RAZ3 pp 229533.I1S0B7
174687 229533.I1RAZ3 pp 229533.I1S4S4
174688 229533.I1RAZ3 pp 229533.I1RCL4
174689 229533.I1RAZ3 pp 229533.Q4IPI1

174690 229533.I1RAZ3 pp 229533.I1RM67
174691 229533.I1RAZ3 pp 229533.I1RJU3
174692 229533.I1RAZ3 pp 229533.I1RQS9
174693 229533.I1RAZ3 pp 229533.I1RB39
174694 229533.I1RAZ3 pp 229533.I1RBT1
174695 229533.I1RAZ3 pp 229533.I1RX46
174696 229533.I1RAZ3 pp 229533.I1RYX6
174697 229533.I1RAZ3 pp 229533.I1RME6
174698 229533.I1RAZ3 pp 229533.I1RP86
174699 229533.I1RAZ3 pp 229533.I1RYU5
174700 229533.I1RAZ3 pp 229533.I1RBF3
174701 229533.I1RAZ3 pp 229533.I1RPN4
174702 229533.I1RAZ3 pp 229533.V6R1K4
174703 229533.I1RAZ3 pp 229533.I1RSZ7
174704 229533.I1RAZ3 pp 229533.I1RYJ6
174705 229533.I1RAZ3 pp 229533.V6RTJ8
174706 229533.I1RAZ3 pp 229533.I1RL04
174707 229533.I1RAZ3 pp 229533.I1RCR5
174708 229533.I1RAZ3 pp 229533.I1RKY2
174709 229533.I1RAZ3 pp 229533.I1RM35
174710 229533.I1RAZ3 pp 229533.I1REK8
174711 229533.I1RAZ3 pp 229533.I1RF82
174712 229533.I1RAZ3 pp 229533.I1RK15
174713 229533.I1RAZ3 pp 229533.I1S9Y1
174714 229533.I1RAZ3 pp 229533.I1REL7
174715 229533.I1RAZ3 pp 229533.I1S4Q4
174716 229533.I1RAZ3 pp 229533.I1RMQ4
174717 229533.I1RAZ3 pp 229533.I1RBI1
174718 229533.I1RAZ3 pp 229533.I1RK95
174719 229533.I1RAZ3 pp 229533.I1RYI7
174720 229533.I1RAZ3 pp 229533.I1S5R1
174721 229533.I1RAZ3 pp 229533.O74638
174722 229533.I1RAZ3 pp 229533.I1RRV4
174723 229533.I1RAZ3 pp 229533.V6RBN2
174724 229533.I1RAZ3 pp 229533.I1RDE7
174725 229533.I1RAZ3 pp 229533.V6QUE6
174726 229533.I1RAZ3 pp 229533.I1RQA5
174727 229533.I1RAZ3 pp 229533.I1S8G4
174728 229533.I1RAZ3 pp 229533.I1RX22
174729 229533.I1RAZ3 pp 229533.I1RTD3
174730 229533.I1RAZ3 pp 229533.I1RFT1
174731 229533.I1RAZ3 pp 229533.I1RYI5
174732 229533.I1RAZ3 pp 229533.I1RZZ9
174733 229533.I1RAZ3 pp 229533.I1S4Z8
174734 229533.I1RAZ3 pp 229533.I1RXF0
174735 229533.I1RAZ3 pp 229533.I1RSQ4
174736 229533.I1RAZ3 pp 229533.I1RWS4
174737 229533.I1RAZ3 pp 229533.I1RT03
174738 229533.I1RAZ3 pp 229533.I1RBC7
174739 229533.I1RAZ3 pp 229533.I1RZ20
174740 229533.I1RAZ3 pp 229533.I1RQM7
174741 229533.I1RAZ3 pp 229533.I1RN67
174742 229533.I1RAZ3 pp 229533.I1RGA4
174743 229533.I1RAZ3 pp 229533.I1RI34
174744 229533.I1RAZ3 pp 229533.Q4HXF0
174745 229533.I1RAZ3 pp 229533.I1RCH5
174746 229533.I1RAZ3 pp 229533.I1RPD6
174747 229533.I1RAZ3 pp 229533.I1RFL4
174748 229533.I1RAZ3 pp 229533.V6R057
174749 229533.I1RAZ3 pp 229533.I1RRP7
174750 229533.I1RAZ3 pp 229533.I1RC05
174751 229533.I1RAZ3 pp 229533.V6RLM7
174752 229533.I1RAZ3 pp 229533.I1RKB6
174753 229533.I1RAZ3 pp 229533.I1S216
174754 229533.I1RAZ3 pp 229533.I1RB44
174755 229533.I1RAZ3 pp 229533.I1RCW6
174756 229533.I1RAZ3 pp 229533.V6RRH2
174757 229533.I1RAZ3 pp 229533.I1S1C7
174758 229533.I1RAZ3 pp 229533.I1S0J1
174759 229533.I1RAZ3 pp 229533.I1RYW3
174760 229533.I1RAZ3 pp 229533.I1S064
174761 229533.I1RAZ3 pp 229533.V6R2A1
174762 229533.I1RAZ3 pp 229533.I1RCD8

229533.I1RAZ3 pp 229533.I1RG11
229533.I1RAZ3 pp 229533.I1RD22
229533.I1RAZ3 pp 229533.I1RCB4
229533.I1RAZ3 pp 229533.I1RWE8
229533.I1RAZ3 pp 229533.I1RVZ7
229533.I1RAZ3 pp 229533.I1RMI8
229533.I1RAZ3 pp 229533.V6R042
229533.I1RAZ3 pp 229533.I1SAN9
229533.I1RAZ3 pp 229533.I1RS17
229533.I1RAZ3 pp 229533.I1RCR8
229533.I1RAZ3 pp 229533.I1RUG3
229533.I1RAZ3 pp 229533.I1RPU8
229533.I1S3D7 pp 229533.V6QV38
229533.A0A1C3YM82 pp 229533.I1RWG1
229533.A0A1C3YM82 pp 229533.I1RKA1
229533.A0A1C3YM82 pp 229533.I1RLU5
229533.A0A1C3YM82 pp 229533.I1S1V3
229533.A0A1C3YM82 pp 229533.A0A1C3YN36
229533.A0A1C3YM82 pp 229533.I1S039
229533.A0A1C3YM82 pp 229533.I1R991
229533.A0A1C3YM82 pp 229533.I1RCK9
229533.A0A1C3YM82 pp 229533.I1S095
229533.A0A1C3YM82 pp 229533.I1RTA6
229533.A0A1C3YM82 pp 229533.I1RN72
229533.A0A1C3YM82 pp 229533.I1RZV4
229533.A0A1C3YM82 pp 229533.I1RW71
229533.A0A1C3YM82 pp 229533.I1RPA7
229533.A0A1C3YM82 pp 229533.I1S1W5
229533.A0A1C3YM82 pp 229533.I1RCY0
229533.A0A1C3YM82 pp 229533.I1RNJ2
229533.A0A1C3YM82 pp 229533.I1RAU9
229533.A0A1C3YM82 pp 229533.I1RKD7
229533.A0A1C3YM82 pp 229533.I1S788
229533.A0A1C3YM82 pp 229533.I1R9X2
229533.A0A1C3YM82 pp 229533.I1RQL4
229533.A0A1C3YM82 pp 229533.I1RT41
229533.A0A1C3YM82 pp 229533.I1RB15
229533.A0A1C3YM82 pp 229533.I1RDB1
229533.A0A1C3YM82 pp 229533.Q4INZ9
229533.A0A1C3YM82 pp 229533.I1RRT1
229533.A0A1C3YM82 pp 229533.I1RC49
229533.A0A1C3YM82 pp 229533.I1RCJ2
229533.A0A1C3YM82 pp 229533.I1RY40
229533.A0A1C3YM82 pp 229533.I1RNG8
229533.A0A1C3YM82 pp 229533.I1RSM2
229533.A0A1C3YM82 pp 229533.Q4IAA0
229533.A0A1C3YM82 pp 229533.I1RZR6
229533.A0A1C3YM82 pp 229533.I1S1Y9
229533.A0A1C3YM82 pp 229533.I1RYM3
229533.A0A1C3YM82 pp 229533.I1RME6
229533.A0A1C3YM82 pp 229533.V6REE9
229533.A0A1C3YM82 pp 229533.I1RB75
229533.A0A1C3YM82 pp 229533.I1S6F1
229533.A0A1C3YM82 pp 229533.I1RWD7
229533.A0A1C3YM82 pp 229533.I1RKJ2
229533.A0A1C3YM82 pp 229533.I1RJS0
229533.A0A1C3YM82 pp 229533.I1RC95
229533.A0A1C3YM82 pp 229533.I1RZP6
229533.A0A1C3YM82 pp 229533.I1RM09
229533.A0A1C3YM82 pp 229533.I1S7M3
229533.A0A1C3YM82 pp 229533.I1S2B6
229533.A0A1C3YM82 pp 229533.I1RQA3
229533.A0A1C3YM82 pp 229533.Q4I948
229533.A0A1C3YM82 pp 229533.I1RN65
229533.A0A1C3YM82 pp 229533.Q4ICA8
229533.A0A1C3YM82 pp 229533.Q4HTT2
229533.A0A1C3YM82 pp 229533.I1S2D7
229533.A0A1C3YM82 pp 229533.I1RBL8
229533.A0A1C3YM82 pp 229533.I1RCJ5
229533.A0A1C3YM82 pp 229533.I1RA38
229533.A0A1C3YM82 pp 229533.I1RB53
229533.A0A1C3YM82 pp 229533.P0C431
229533.A0A1C3YM82 pp 229533.I1S9G7

174836	229533.A0A1C3YM82	pp	229533.I1RWT6
174837	229533.A0A1C3YM82	pp	229533.I1RJU5
174838	229533.A0A1C3YM82	pp	229533.A0A1C3YMD9
174839	229533.A0A1C3YM82	pp	229533.I1RZH4
174840	229533.A0A1C3YM82	pp	229533.I1S0J7
174841	229533.A0A1C3YM82	pp	229533.I1RMQ7
174842	229533.A0A1C3YM82	pp	229533.A0A1C3YME9
174843	229533.A0A1C3YM82	pp	229533.I1REC6
174844	229533.A0A1C3YM82	pp	229533.I1RJZ7
174845	229533.A0A1C3YM82	pp	229533.I1RCD4
174846	229533.A0A1C3YM82	pp	229533.I1S0X5
174847	229533.A0A1C3YM82	pp	229533.I1RSB0
174848	229533.A0A1C3YM82	pp	229533.I1S183
174849	229533.A0A1C3YM82	pp	229533.I1RRJ6
174850	229533.A0A1C3YM82	pp	229533.I1RZG0
174851	229533.A0A1C3YM82	pp	229533.I1RQ81
174852	229533.A0A1C3YM82	pp	229533.I1RQG7
174853	229533.A0A1C3YM82	pp	229533.I1RZD9
174854	229533.A0A1C3YM82	pp	229533.I1REC7
174855	229533.A0A1C3YM82	pp	229533.I1RTG9
174856	229533.A0A1C3YM82	pp	229533.I1RS20
174857	229533.A0A1C3YM82	pp	229533.I1RMU9
174858	229533.A0A1C3YM82	pp	229533.I1RTN0
174859	229533.A0A1C3YM82	pp	229533.I1RA40
174860	229533.A0A1C3YM82	pp	229533.I1S9V9
174861	229533.A0A1C3YM82	pp	229533.I1RAE3
174862	229533.A0A1C3YM82	pp	229533.I1RC18
174863	229533.A0A1C3YM82	pp	229533.I1RSW5
174864	229533.A0A1C3YM82	pp	229533.I1RMJ8
174865	229533.A0A1C3YM82	pp	229533.I1S0L8
174866	229533.A0A1C3YM82	pp	229533.I1RZP7
174867	229533.A0A1C3YM82	pp	229533.I1RP88
174868	229533.A0A1C3YM82	pp	229533.I1RB40
174869	229533.A0A1C3YM82	pp	229533.I1RB74
174870	229533.A0A1C3YM82	pp	229533.I1RE71
174871	229533.A0A1C3YM82	pp	229533.I1RBA6
174872	229533.A0A1C3YM82	pp	229533.I1S2E9
174873	229533.A0A1C3YM82	pp	229533.I1RTE6
174874	229533.A0A1C3YM82	pp	229533.Q4IN00
174875	229533.A0A1C3YM82	pp	229533.I1RAE2
174876	229533.A0A1C3YM82	pp	229533.I1RUP5
174877	229533.A0A1C3YM82	pp	229533.I1RAU7
174878	229533.A0A1C3YM82	pp	229533.I1RMV6
174879	229533.A0A1C3YM82	pp	229533.I1RDJ8
174880	229533.A0A1C3YM82	pp	229533.I1RAE5
174881	229533.A0A1C3YM82	pp	229533.Q4HZB8
174882	229533.A0A1C3YM82	pp	229533.Q4IB89
174883	229533.A0A1C3YM82	pp	229533.I1RZS7
174884	229533.A0A1C3YM82	pp	229533.I1S4P6
174885	229533.A0A1C3YM82	pp	229533.I1RY44
174886	229533.A0A1C3YM82	pp	229533.I1RWB5
174887	229533.A0A1C3YM82	pp	229533.I1RNB8
174888	229533.A0A1C3YM82	pp	229533.I1RUP2
174889	229533.A0A1C3YM82	pp	229533.I1RMV0
174890	229533.A0A1C3YM82	pp	229533.I1S282
174891	229533.A0A1C3YM82	pp	229533.I1RW37
174892	229533.A0A1C3YM82	pp	229533.I1RAE4
174893	229533.A0A1C3YM82	pp	229533.I1S2B9
174894	229533.A0A1C3YM82	pp	229533.I1RRS7
174895	229533.A0A1C3YM82	pp	229533.I1RT36
174896	229533.A0A1C3YM82	pp	229533.I1RS90
174897	229533.A0A1C3YM82	pp	229533.I1RSC7
174898	229533.A0A1C3YK68	pp	229533.I1RME6
174899	229533.A0A1C3YK68	pp	229533.I1RYJ6
174900	229533.A0A1C3YK68	pp	229533.I1RP86
174901	229533.A0A1C3YK68	pp	229533.V6RTJ8
174902	229533.A0A1C3YK68	pp	229533.I1RJU3
174903	229533.A0A1C3YK68	pp	229533.I1RQS9
174904	229533.A0A1C3YK68	pp	229533.A0A1C3YN26
174905	229533.A0A1C3YK68	pp	229533.I1RX46
174906	229533.A0A1C3YK68	pp	229533.A0A1C3YKS6
174907	229533.A0A1C3YK68	pp	229533.I1RM67
174908	229533.A0A1C3YK68	pp	229533.I1RBT1

229533.A0A1C3YK68	pp	229533.I1RCL4
229533.A0A1C3YK68	pp	229533.I1S4S4
229533.A0A1C3YK68	pp	229533.I1RW71
229533.A0A1C3YK68	pp	229533.I1RPN4
229533.A0A1C3YK68	pp	229533.V6QUE6
229533.A0A1C3YK68	pp	229533.A0A1C3YM63
229533.A0A1C3YK68	pp	229533.I1RYU5
229533.A0A1C3YK68	pp	229533.I1RYX6
229533.A0A1C3YK68	pp	229533.I1RYI7
229533.A0A1C3YK68	pp	229533.I1S1W8
229533.A0A1C3YK68	pp	229533.I1RRV4
229533.A0A1C3YK68	pp	229533.V6R1K4
229533.A0A1C3YK68	pp	229533.I1S0B7
229533.A0A1C3YK68	pp	229533.A0A1C3YKV3
229533.A0A1C3YK68	pp	229533.I1RB39
229533.A0A1C3YK68	pp	229533.I1RMQ4
229533.A0A1C3YK68	pp	229533.A0A1C3YLR6
229533.A0A1C3YK68	pp	229533.I1RBI1
229533.A0A1C3YK68	pp	229533.I1RTD3
229533.A0A1C3YK68	pp	229533.A0A1C3YN30
229533.A0A1C3YK68	pp	229533.I1RK15
229533.A0A1C3YK68	pp	229533.A0A1C3YKB0
229533.A0A1C3YK68	pp	229533.I1RZP1
229533.A0A1C3YK68	pp	229533.I1RXF0
229533.A0A1C3YK68	pp	229533.I1RF82
229533.A0A1C3YK68	pp	229533.V6R283
229533.A0A1C3YK68	pp	229533.I1RQA5
229533.A0A1C3YK68	pp	229533.I1RA94
229533.A0A1C3YK68	pp	229533.A0A1C3YKH8
229533.A0A1C3YK68	pp	229533.I1RAZ3
229533.A0A1C3YK68	pp	229533.I1RWS4
229533.A0A1C3YK68	pp	229533.V6RBN2
229533.A0A1C3YK68	pp	229533.I1RAQ4
229533.A0A1C3YK68	pp	229533.I1RRE0
229533.A0A1C3YK68	pp	229533.I1RZ20
229533.A0A1C3YK68	pp	229533.I1RCR5
229533.A0A1C3YK68	pp	229533.I1REK8
229533.A0A1C3YK68	pp	229533.I1RBL7
229533.A0A1C3YK68	pp	229533.I1RPD6
229533.A0A1C3YK68	pp	229533.I1RM35
229533.A0A1C3YK68	pp	229533.I1RK95
229533.A0A1C3YK68	pp	229533.I1RC05
229533.A0A1C3YK68	pp	229533.I1S4Q4
229533.A0A1C3YK68	pp	229533.I1RX22
229533.A0A1C3YK68	pp	229533.I1RL04
229533.A0A1C3YK68	pp	229533.I1REL7
229533.A0A1C3YK68	pp	229533.I1RN67
229533.A0A1C3YK68	pp	229533.I1RKY2
229533.A0A1C3YK68	pp	229533.Q4HXF0
229533.A0A1C3YK68	pp	229533.I1RFS3
229533.A0A1C3YK68	pp	229533.I1RQM7
229533.A0A1C3YK68	pp	229533.A0A1C3YL55
229533.A0A1C3YK68	pp	229533.I1RDE7
229533.A0A1C3YK68	pp	229533.I1S8G4
229533.A0A1C3YK68	pp	229533.I1RA52
229533.A0A1C3YK68	pp	229533.I1S4Z8
229533.A0A1C3YK68	pp	229533.I1RZZ9
229533.A0A1C3YK68	pp	229533.A0A1C3YKU8
229533.A0A1C3YK68	pp	229533.I1RBC7
229533.A0A1C3YK68	pp	229533.I1RGA4
229533.A0A1C3YK68	pp	229533.I1RJZ7
229533.A0A1C3YK68	pp	229533.I1RSQ4
229533.A0A1C3YK68	pp	229533.I1S8P5
229533.A0A1C3YK68	pp	229533.A0A1C3YLK8
229533.A0A1C3YK68	pp	229533.I1RPU8
229533.A0A1C3YK68	pp	229533.I1RYI5
229533.A0A1C3YK68	pp	229533.V6R042
229533.A0A1C3YK68	pp	229533.I1S3G4
229533.A0A1C3YK68	pp	229533.I1RRM9
229533.A0A1C3YK68	pp	229533.O74638
229533.A0A1C3YK68	pp	229533.I1SAN9
229533.A0A1C3YK68	pp	229533.I1RFT1
229533.A0A1C3YK68	pp	229533.I1RTA0

174982 229533.A0A1C3YK68 pp 229533.I1RCR8
174983 229533.A0A1C3YK68 pp 229533.I1S5R1
174984 229533.A0A1C3YK68 pp 229533.I1RVZ7
174985 229533.A0A1C3YK68 pp 229533.I1RUG3
174986 229533.A0A1C3YK68 pp 229533.I1S9Y1
174987 229533.A0A1C3YK68 pp 229533.I1RNC1
174988 229533.A0A1C3YK68 pp 229533.I1RQ21
174989 229533.A0A1C3YK68 pp 229533.I1RWE8
174990 229533.A0A1C3YK68 pp 229533.I1RG11
174991 229533.A0A1C3YK68 pp 229533.V6R2A1
174992 229533.A0A1C3YK68 pp 229533.I1RBF3
174993 229533.A0A1C3YK68 pp 229533.I1RMI8
174994 229533.A0A1C3YK68 pp 229533.I1S0N4
174995 229533.A0A1C3YK68 pp 229533.I1RD22
174996 229533.A0A1C3YK68 pp 229533.I1RA70
174997 229533.A0A1C3YK68 pp 229533.I1RYW3
174998 229533.A0A1C3YK68 pp 229533.I1RCB4
174999 229533.A0A1C3YK68 pp 229533.I1RT03
175000 229533.A0A1C3YK68 pp 229533.I1S1C7
175001 229533.A0A1C3YK68 pp 229533.I1RS17
175002 229533.A0A1C3YK68 pp 229533.I1RCH5
175003 229533.A0A1C3YK68 pp 229533.I1S216
175004 229533.A0A1C3YK68 pp 229533.I1RFL4
175005 229533.A0A1C3YK68 pp 229533.I1RB44
175006 229533.A0A1C3YK68 pp 229533.I1RCW6
175007 229533.A0A1C3YK68 pp 229533.I1RAW0
175008 229533.A0A1C3YK68 pp 229533.V6RLM7
175009 229533.A0A1C3YK68 pp 229533.I1RRP7
175010 229533.A0A1C3YK68 pp 229533.V6R057
175011 229533.A0A1C3YK68 pp 229533.I1RCD8
175012 229533.A0A1C3YK68 pp 229533.I1S0J1
175013 229533.A0A1C3YK68 pp 229533.V6RRH2
175014 229533.A0A1C3YK68 pp 229533.A0A1C3YKY0
175015 229533.A0A1C3YK68 pp 229533.I1RI34
175016 229533.A0A1C3YK68 pp 229533.I1RKB6
175017 229533.A0A1C3YK68 pp 229533.I1S064
175018 229533.I1RPN4 pp 229533.I1S4Z8
175019 229533.I1RPN4 pp 229533.I1RZZ9
175020 229533.I1RPN4 pp 229533.I1S2B8
175021 229533.I1RPN4 pp 229533.I1RSQ4
175022 229533.I1RPN4 pp 229533.I1S8G4
175023 229533.I1RPN4 pp 229533.I1S2H4
175024 229533.I1RPN4 pp 229533.O74638
175025 229533.I1RPN4 pp 229533.I1RX22
175026 229533.I1RPN4 pp 229533.I1S3G4
175027 229533.I1RPN4 pp 229533.I1RTD5
175028 229533.I1RPN4 pp 229533.I1RPP3
175029 229533.I1RPN4 pp 229533.I1S8P5
175030 229533.I1RPN4 pp 229533.I1RT40
175031 229533.I1RPN4 pp 229533.I1S5R1
175032 229533.I1RPN4 pp 229533.I1S216
175033 229533.I1RPN4 pp 229533.V6R283
175034 229533.I1RPN4 pp 229533.I1RTA1
175035 229533.I1RPN4 pp 229533.I1RQM7
175036 229533.I1RPN4 pp 229533.V6R057
175037 229533.I1RPN4 pp 229533.I1S9Y1
175038 229533.I1RPN4 pp 229533.I1RZB2
175039 229533.I1RPN4 pp 229533.I1RWX5
175040 229533.I1RPN4 pp 229533.I1RVX0
175041 229533.I1RPN4 pp 229533.I1RYW3
175042 229533.I1RPN4 pp 229533.V6RRH2
175043 229533.I1RPN4 pp 229533.I1RPU8
175044 229533.I1RPN4 pp 229533.Q4I2W2
175045 229533.I1RPN4 pp 229533.V6RMW6
175046 229533.I1RPN4 pp 229533.I1RXY7
175047 229533.I1RPN4 pp 229533.V6R2A1
175048 229533.I1RPN4 pp 229533.I1RVZ7
175049 229533.I1RPN4 pp 229533.V6R042
175050 229533.I1RPN4 pp 229533.I1S2E8
175051 229533.I1RPN4 pp 229533.I1RWE8
175052 229533.I1RPN4 pp 229533.I1RRP7
175053 229533.I1RPN4 pp 229533.I1RRV1
175054 229533.I1RPN4 pp 229533.I1SAN9

175055 229533.I1RPN4 pp 229533.I1RQS9
175056 229533.I1RPN4 pp 229533.V6R1K4
175057 229533.I1RPN4 pp 229533.I1RUG3
175058 229533.I1RPN4 pp 229533.I1RS17
175059 229533.I1RPN4 pp 229533.I1S1W8
175060 229533.I1RPN4 pp 229533.I1RYX6
175061 229533.I1RPN4 pp 229533.I1S0J1
175062 229533.I1RPN4 pp 229533.I1S0B7
175063 229533.I1RPN4 pp 229533.I1RTA0
175064 229533.I1RPN4 pp 229533.I1RSQ7
175065 229533.I1RPN4 pp 229533.I1RS18
175066 229533.I1RPN4 pp 229533.I1RRX5
175067 229533.I1RPN4 pp 229533.I1S3S3
175068 229533.I1RPN4 pp 229533.I1S5B0
175069 229533.I1RPN4 pp 229533.I1RXD7
175070 229533.I1RPN4 pp 229533.V6RTJ8
175071 229533.I1RPN4 pp 229533.I1RW71
175072 229533.I1RPN4 pp 229533.I1S4Q4
175073 229533.I1RPN4 pp 229533.I1RX46
175074 229533.I1RPN4 pp 229533.I1S1Y9
175075 229533.I1RPN4 pp 229533.I1RRV4
175076 229533.I1RPN4 pp 229533.I1RSI0
175077 229533.I1RPN4 pp 229533.V6QUE6
175078 229533.I1RPN4 pp 229533.I1RTD3
175079 229533.I1RPN4 pp 229533.V6RBN2
175080 229533.I1RPN4 pp 229533.Q4I061
175081 229533.I1RPN4 pp 229533.V6RCZ8
175082 229533.I1RPN4 pp 229533.I1RRY9
175083 229533.I1RPN4 pp 229533.I1RXF0
175084 229533.I1RPN4 pp 229533.I1RSP1
175085 229533.I1RPN4 pp 229533.I1RZM8
175086 229533.I1S270 pp 229533.Q4HY90
175087 229533.I1S270 pp 229533.I1S7W2
175088 229533.I1S270 pp 229533.Q4INI2
175089 229533.I1S270 pp 229533.I1S8X0
175090 229533.I1S270 pp 229533.Q4I963
175091 229533.I1S270 pp 229533.V6R476
175092 229533.I1S270 pp 229533.Q4HZE0
175093 229533.I1S270 pp 229533.I1S8E0
175094 229533.I1S270 pp 229533.V6RR76
175095 229533.I1S270 pp 229533.I1S559
175096 229533.I1S270 pp 229533.V6QUU6
175097 229533.I1S270 pp 229533.Q4ICA8
175098 229533.I1S270 pp 229533.Q5I2J3
175099 229533.I1S270 pp 229533.Q4I2D7
175100 229533.A0A1C3YLS7 pp 229533.I1RX84
175101 229533.A0A1C3YLS7 pp 229533.I1RCE7
175102 229533.A0A1C3YLS7 pp 229533.Q4ILI9
175103 229533.A0A1C3YLS7 pp 229533.I1RZ60
175104 229533.A0A1C3YLS7 pp 229533.I1RZK3
175105 229533.A0A1C3YLS7 pp 229533.I1RA25
175106 229533.A0A1C3YLS7 pp 229533.I1S254
175107 229533.A0A1C3YLS7 pp 229533.A0A1C3YN54
175108 229533.A0A1C3YLS7 pp 229533.I1RQP5
175109 229533.A0A1C3YLS7 pp 229533.I1RCN6
175110 229533.A0A1C3YLS7 pp 229533.I1RTC1
175111 229533.A0A1C3YLS7 pp 229533.V6RR76
175112 229533.A0A1C3YLS7 pp 229533.I1RT41
175113 229533.A0A1C3YLS7 pp 229533.I1RD10
175114 229533.A0A1C3YLS7 pp 229533.Q4HYZ2
175115 229533.A0A1C3YLS7 pp 229533.I1RQF7
175116 229533.A0A1C3YLS7 pp 229533.V6R476
175117 229533.A0A1C3YLS7 pp 229533.I1RTG5
175118 229533.A0A1C3YLS7 pp 229533.I1RYW8
175119 229533.A0A1C3YLS7 pp 229533.I1RZS5
175120 229533.A0A1C3YLS7 pp 229533.I1RMJ1
175121 229533.A0A1C3YLS7 pp 229533.I1RTQ9
175122 229533.A0A1C3YLS7 pp 229533.V6QUU6
175123 229533.A0A1C3YLS7 pp 229533.I1RN81
175124 229533.A0A1C3YLS7 pp 229533.I1RQ70
175125 229533.A0A098DSB7 pp 229533.A0A1C3YJN3
175126 229533.A0A098DSB7 pp 229533.A0A1C3YJR3
175127 229533.A0A098DSB7 pp 229533.I1RB17

175128 229533.A0A098DSB7 pp 229533.I1RQ85
175129 229533.A0A098DSB7 pp 229533.V6QUE6
175130 229533.A0A098DSB7 pp 229533.I1RG14
175131 229533.A0A098DSB7 pp 229533.A0A1C3YKB0
175132 229533.A0A098DSB7 pp 229533.I1S1B2
175133 229533.A0A098DSB7 pp 229533.A0A1C3YI30
175134 229533.A0A098DSB7 pp 229533.A0A1C3YK34
175135 229533.A0A098DSB7 pp 229533.I1RQD2
175136 229533.A0A098DSB7 pp 229533.A0A1C3YKV3
175137 229533.A0A098DSB7 pp 229533.A0A1C3YJV6
175138 229533.A0A098DSB7 pp 229533.I1RS18
175139 229533.A0A098DSB7 pp 229533.A0A1C3YIK4
175140 229533.A0A098DSB7 pp 229533.A0A0E0RV05
175141 229533.A0A098DSB7 pp 229533.I1S5B0
175142 229533.A0A098DSB7 pp 229533.I1S1J7
175143 229533.A0A098DSB7 pp 229533.I1RXF0
175144 229533.A0A098DSB7 pp 229533.I1RYS0
175145 229533.A0A098DSB7 pp 229533.I1RZM8
175146 229533.A0A098DSB7 pp 229533.I1RBL7
175147 229533.A0A098DSB7 pp 229533.I1S821
175148 229533.A0A098DSB7 pp 229533.I1RWR7
175149 229533.A0A098DSB7 pp 229533.I1RRY9
175150 229533.A0A098DSB7 pp 229533.V6RCZ8
175151 229533.A0A098DSB7 pp 229533.I1RNT9
175152 229533.A0A098DSB7 pp 229533.I1RGC5
175153 229533.A0A098DSB7 pp 229533.I1RZK5
175154 229533.A0A098DSB7 pp 229533.A0A1C3YHX6
175155 229533.A0A098DSB7 pp 229533.I1RDY1
175156 229533.A0A098DSB7 pp 229533.I1RBU8
175157 229533.A0A098DSB7 pp 229533.I1S6K9
175158 229533.A0A098DSB7 pp 229533.A0A098E4W0
175159 229533.A0A098DSB7 pp 229533.I1RCR8
175160 229533.A0A098DSB7 pp 229533.I1REK8
175161 229533.A0A098DSB7 pp 229533.I1RCR5
175162 229533.A0A098DSB7 pp 229533.A0A0E0SFN0
175163 229533.A0A098DSB7 pp 229533.I1RXD7
175164 229533.A0A098DSB7 pp 229533.I1RNC1
175165 229533.A0A098DSB7 pp 229533.A0A0E0SKJ9
175166 229533.A0A098DSB7 pp 229533.A0A1C3YIP5
175167 229533.A0A098DSB7 pp 229533.I1RXD6
175168 229533.A0A098DSB7 pp 229533.Q4IJK2
175169 229533.A0A098DSB7 pp 229533.I1RUG3
175170 229533.A0A098DSB7 pp 229533.I1RK95
175171 229533.A0A098DSB7 pp 229533.I1RY86
175172 229533.A0A098DSB7 pp 229533.I1RVF8
175173 229533.A0A098DSB7 pp 229533.I1RRH5
175174 229533.A0A098DSB7 pp 229533.I1RD52
175175 229533.A0A098DSB7 pp 229533.I1RX46
175176 229533.A0A098DSB7 pp 229533.I1RAX6
175177 229533.A0A098DSB7 pp 229533.V6R042
175178 229533.A0A098DSB7 pp 229533.I1RBR2
175179 229533.A0A098DSB7 pp 229533.V6RTJ8
175180 229533.A0A098DSB7 pp 229533.A0A1C3YM63
175181 229533.A0A098DSB7 pp 229533.I1RX95
175182 229533.A0A098DSB7 pp 229533.I1RSQ3
175183 229533.A0A098DSB7 pp 229533.I1RRG3
175184 229533.A0A098DSB7 pp 229533.V6RMW6
175185 229533.A0A098DSB7 pp 229533.A0A0E0SBU8
175186 229533.A0A098DSB7 pp 229533.A0A1C3YK13
175187 229533.A0A098DSB7 pp 229533.I1S4S7
175188 229533.A0A098DSB7 pp 229533.A0A0E0S035
175189 229533.A0A098DSB7 pp 229533.I1RPY3
175190 229533.A0A098DSB7 pp 229533.I1RRM3
175191 229533.A0A098DSB7 pp 229533.I1RJU3
175192 229533.A0A098DSB7 pp 229533.I1RKJ5
175193 229533.A0A098DSB7 pp 229533.I1RW71
175194 229533.A0A098DSB7 pp 229533.I1RSQ7
175195 229533.A0A098DSB7 pp 229533.I1RAG7
175196 229533.A0A098DSB7 pp 229533.I1S4S4
175197 229533.A0A098DSB7 pp 229533.I1RG62
175198 229533.A0A098DSB7 pp 229533.I1RW25
175199 229533.A0A098DSB7 pp 229533.I1S1W9
175200 229533.A0A098DSB7 pp 229533.I1RRS8

175201	229533.A0A098DSB7	pp	229533.A0A0E0RSD7
175202	229533.A0A098DSB7	pp	229533.I1RR24
175203	229533.A0A098DSB7	pp	229533.I1RZV3
175204	229533.A0A098DSB7	pp	229533.I1R9Y4
175205	229533.A0A098DSB7	pp	229533.I1RPA4
175206	229533.A0A098DSB7	pp	229533.I1RM37
175207	229533.A0A098DSB7	pp	229533.I1S438
175208	229533.A0A098DSB7	pp	229533.I1RBT1
175209	229533.A0A098DSB7	pp	229533.A0A1C3YHX8
175210	229533.A0A098DSB7	pp	229533.I1RPU2
175211	229533.A0A098DSB7	pp	229533.I1RAW0
175212	229533.A0A098DSB7	pp	229533.I1S437
175213	229533.A0A098DSB7	pp	229533.A0A1C3YIK9
175214	229533.A0A098DSB7	pp	229533.I1RSF1
175215	229533.A0A098DSB7	pp	229533.I1RD22
175216	229533.A0A098DSB7	pp	229533.A0A1C3YLB6
175217	229533.A0A098DSB7	pp	229533.I1RYW3
175218	229533.A0A098DSB7	pp	229533.I1RRN2
175219	229533.A0A098DSB7	pp	229533.I1RE01
175220	229533.A0A098DSB7	pp	229533.I1S5R6
175221	229533.A0A098DSB7	pp	229533.I1S1C7
175222	229533.A0A098DSB7	pp	229533.I1S0J1
175223	229533.A0A098DSB7	pp	229533.I1RRI2
175224	229533.A0A098DSB7	pp	229533.I1RG11
175225	229533.A0A098DSB7	pp	229533.I1RPK0
175226	229533.A0A098DSB7	pp	229533.I1RB26
175227	229533.A0A098DSB7	pp	229533.I1RE89
175228	229533.A0A098DSB7	pp	229533.I1RVZ2
175229	229533.A0A098DSB7	pp	229533.I1RZ79
175230	229533.A0A098DSB7	pp	229533.I1RJV3
175231	229533.A0A098DSB7	pp	229533.I1RA70
175232	229533.A0A098DSB7	pp	229533.I1RJN9
175233	229533.A0A098DSB7	pp	229533.I1RB92
175234	229533.A0A098DSB7	pp	229533.I1RTA1
175235	229533.A0A098DSB7	pp	229533.I1RKJ8
175236	229533.A0A098DSB7	pp	229533.I1RBR1
175237	229533.A0A098DSB7	pp	229533.I1RUF7
175238	229533.A0A098DSB7	pp	229533.Q4IPZ1
175239	229533.A0A098DSB7	pp	229533.I1RFT1
175240	229533.A0A098DSB7	pp	229533.A0A1C3YLK8
175241	229533.A0A098DSB7	pp	229533.I1RYI5
175242	229533.A0A098DSB7	pp	229533.I1RC34
175243	229533.A0A098DSB7	pp	229533.I1RPP3
175244	229533.A0A098DSB7	pp	229533.I1RML0
175245	229533.A0A098DSB7	pp	229533.I1RSX0
175246	229533.A0A098DSB7	pp	229533.Q4I624
175247	229533.A0A098DSB7	pp	229533.I1RBC7
175248	229533.A0A098DSB7	pp	229533.I1S9Y1
175249	229533.A0A098DSB7	pp	229533.I1RGA4
175250	229533.A0A098DSB7	pp	229533.I1RA11
175251	229533.A0A098DSB7	pp	229533.I1S5R1
175252	229533.A0A098DSB7	pp	229533.I1RQ33
175253	229533.A0A098DSB7	pp	229533.I1RFL4
175254	229533.A0A098DSB7	pp	229533.I1RZB2
175255	229533.A0A098DSB7	pp	229533.I1RQE7
175256	229533.A0A098DSB7	pp	229533.I1RT40
175257	229533.A0A098DSB7	pp	229533.I1RRT8
175258	229533.A0A098DSB7	pp	229533.A0A1C3YJI6
175259	229533.A0A098DSB7	pp	229533.I1S2H4
175260	229533.A0A098DSB7	pp	229533.I1RXF8
175261	229533.A0A098DSB7	pp	229533.I1RCH5
175262	229533.A0A098DSB7	pp	229533.I1RVX0
175263	229533.A0A098DSB7	pp	229533.I1RPE0
175264	229533.A0A098DSB7	pp	229533.I1RTD5
175265	229533.A0A098DSB7	pp	229533.A0A1C3YJ21
175266	229533.A0A098DSB7	pp	229533.I1REC5
175267	229533.A0A098DSB7	pp	229533.I1RT03
175268	229533.A0A098DSB7	pp	229533.I1RDF7
175269	229533.A0A098DSB7	pp	229533.I1RSY2
175270	229533.A0A098DSB7	pp	229533.I1RKP1
175271	229533.A0A098DSB7	pp	229533.I1RN67
175272	229533.A0A098DSB7	pp	229533.I1S0L4
175273	229533.A0A098DSB7	pp	229533.I1RDA8

175274 229533.A0A098DSB7 pp 229533.A0A0E0SC46
175275 229533.A0A098DSB7 pp 229533.I1S0W4
175276 229533.A0A098DSB7 pp 229533.I1RRI1
175277 229533.A0A098DSB7 pp 229533.I1RE76
175278 229533.A0A098DSB7 pp 229533.I1RBY4
175279 229533.A0A098DSB7 pp 229533.I1RSA5
175280 229533.A0A098DSB7 pp 229533.I1S277
175281 229533.A0A098DSB7 pp 229533.A0A1C3YIR8
175282 229533.A0A098DSB7 pp 229533.I1RWI5
175283 229533.A0A098DSB7 pp 229533.I1RSQ4
175284 229533.A0A098DSB7 pp 229533.I1RYR0
175285 229533.A0A098DSB7 pp 229533.I1RZZ9
175286 229533.A0A098DSB7 pp 229533.A0A1C3YMA1
175287 229533.A0A098DSB7 pp 229533.I1RSZ7
175288 229533.A0A098DSB7 pp 229533.I1RFS3
175289 229533.A0A098DSB7 pp 229533.I1RL04
175290 229533.A0A098DSB7 pp 229533.I1RPV3
175291 229533.A0A098DSB7 pp 229533.I1RC66
175292 229533.A0A098DSB7 pp 229533.I1RZ39
175293 229533.A0A098DSB7 pp 229533.A0A1C3YKM9
175294 229533.A0A098DSB7 pp 229533.A0A1C3YIU2
175295 229533.A0A098DSB7 pp 229533.I1RGU7
175296 229533.A0A098DSB7 pp 229533.I1RJZ7
175297 229533.A0A098DSB7 pp 229533.I1S2B8
175298 229533.A0A098DSB7 pp 229533.I1RBZ1
175299 229533.A0A098DSB7 pp 229533.I1R9M0
175300 229533.I1S0P7 pp 229533.V6QX21
175301 229533.I1S0P7 pp 229533.I1S1D0
175302 229533.I1S0P7 pp 229533.Q4IB50
175303 229533.I1S0P7 pp 229533.I1S4N8
175304 229533.I1S0P7 pp 229533.I1S278
175305 229533.I1S0P7 pp 229533.I1S1W5
175306 229533.I1S0P7 pp 229533.I1S1X7
175307 229533.I1S0P7 pp 229533.I1S4N7
175308 229533.I1S0P7 pp 229533.I1S9X9
175309 229533.I1S0P7 pp 229533.I1S8Q3
175310 229533.I1RAR9 pp 229533.I1RPU8
175311 229533.I1RAR9 pp 229533.I1S2C4
175312 229533.I1RAR9 pp 229533.I1RZ29
175313 229533.I1RAR9 pp 229533.I1S1W8
175314 229533.I1RAR9 pp 229533.I1S3G4
175315 229533.I1RAR9 pp 229533.V6RRM0
175316 229533.I1RAR9 pp 229533.I1RM35
175317 229533.I1RAR9 pp 229533.I1S029
175318 229533.I1RAR9 pp 229533.V6QWD8
175319 229533.I1RAR9 pp 229533.O74638
175320 229533.I1RAR9 pp 229533.V6RY56
175321 229533.I1RAR9 pp 229533.I1RSQ9
175322 229533.I1RAR9 pp 229533.I1RCV1
175323 229533.I1RAR9 pp 229533.I1RE53
175324 229533.I1RAR9 pp 229533.I1RJT5
175325 229533.I1RAR9 pp 229533.I1RMM8
175326 229533.I1RAR9 pp 229533.I1RQ14
175327 229533.I1RAR9 pp 229533.I1RNG4
175328 229533.I1RAR9 pp 229533.I1S4D0
175329 229533.I1RX50 pp 229533.I1RXT2
175330 229533.I1RX50 pp 229533.I1S039
175331 229533.I1RX50 pp 229533.I1RXV4
175332 229533.I1RX50 pp 229533.I1S9X9
175333 229533.I1RX50 pp 229533.I1S1X7
175334 229533.I1RX50 pp 229533.I1S0J7
175335 229533.I1RX50 pp 229533.I1RZ63
175336 229533.I1RX50 pp 229533.V6QX21
175337 229533.I1RX50 pp 229533.I1S4N8
175338 229533.I1RX50 pp 229533.I1S278
175339 229533.I1RX50 pp 229533.I1S4N7
175340 229533.I1RX50 pp 229533.I1S1W5
175341 229533.I1RX50 pp 229533.I1S0P7
175342 229533.I1RM35 pp 229533.V6RRH2
175343 229533.I1RM35 pp 229533.I1RQE7
175344 229533.I1RM35 pp 229533.V6R057
175345 229533.I1RM35 pp 229533.I1S216
175346 229533.I1RM35 pp 229533.I1RSY2

229533.I1RM35	pp	229533.V6R2A1
229533.I1RM35	pp	229533.I1RVX0
229533.I1RM35	pp	229533.I1RVZ7
229533.I1RM35	pp	229533.I1S8V3
229533.I1RM35	pp	229533.I1S8V4
229533.I1RM35	pp	229533.I1RRP7
229533.I1RM35	pp	229533.I1RSQ4
229533.I1RM35	pp	229533.I1RMI8
229533.I1RM35	pp	229533.I1RZZ9
229533.I1RM35	pp	229533.I1RRV1
229533.I1RM35	pp	229533.I1S4Z8
229533.I1RM35	pp	229533.I1S8G4
229533.I1RM35	pp	229533.I1RSL8
229533.I1RM35	pp	229533.I1S0M6
229533.I1RM35	pp	229533.I1RWE8
229533.I1RM35	pp	229533.I1RTA0
229533.I1RM35	pp	229533.I1RX22
229533.I1RM35	pp	229533.O74638
229533.I1RM35	pp	229533.I1RNU6
229533.I1RM35	pp	229533.I1RN67
229533.I1RM35	pp	229533.I1RM82
229533.I1RM35	pp	229533.I1RWS4
229533.I1RM35	pp	229533.I1RVZ2
229533.I1RM35	pp	229533.I1RPP3
229533.I1RM35	pp	229533.I1RQM7
229533.I1RM35	pp	229533.I1RS17
229533.I1RM35	pp	229533.I1S8P5
229533.I1RM35	pp	229533.I1RTF4
229533.I1RM35	pp	229533.V6R1K4
229533.I1RM35	pp	229533.Q4HXF0
229533.I1RM35	pp	229533.I1RTD5
229533.I1RM35	pp	229533.I1RZJ9
229533.I1RM35	pp	229533.I1RRY9
229533.I1RM35	pp	229533.I1S4Q4
229533.I1RM35	pp	229533.I1RN61
229533.I1RM35	pp	229533.I1RRE0
229533.I1RM35	pp	229533.I1RPN4
229533.I1RM35	pp	229533.I1RN08
229533.I1RM35	pp	229533.Q4I2J8
229533.I1RM35	pp	229533.I1RX46
229533.I1RM35	pp	229533.I1RWD7
229533.I1RM35	pp	229533.I1S9U1
229533.I1RM35	pp	229533.I1RMQ4
229533.I1RM35	pp	229533.V6RBN2
229533.I1RM35	pp	229533.I1RRV4
229533.I1RM35	pp	229533.V6RTJ8
229533.I1RM35	pp	229533.Q4IAA0
229533.I1RM35	pp	229533.I1S0B7
229533.I1RM35	pp	229533.I1RMR1
229533.I1RM35	pp	229533.I1RTD3
229533.I1RM35	pp	229533.I1RYJ6
229533.I1RM35	pp	229533.V6QUE6
229533.I1RM35	pp	229533.I1S3S3
229533.I1RM35	pp	229533.I1RQS9
229533.A0A1C3YLU8	pp	229533.I1RW06
229533.A0A1C3YLU8	pp	229533.I1R9N6
229533.A0A098DGW9	pp	229533.I1RRJ8
229533.A0A098DGW9	pp	229533.I1RRV4
229533.A0A098DGW9	pp	229533.I1RM67
229533.A0A098DGW9	pp	229533.A0A1C3YN30
229533.A0A098DGW9	pp	229533.I1RQ31
229533.A0A098DGW9	pp	229533.I1RAZ3
229533.A0A098DGW9	pp	229533.A0A1C3YK68
229533.A0A098DGW9	pp	229533.A0A1C3YLS7
229533.A0A098DGW9	pp	229533.I1RAR9
229533.A0A098DGW9	pp	229533.I1RM35
229533.A0A098DDV7	pp	229533.I1RCJ2
229533.A0A098DDV7	pp	229533.A0A1C3YME0
229533.A0A098DDV7	pp	229533.I1RSM2
229533.A0A098DDV7	pp	229533.Q4INZ9
229533.A0A098DDV7	pp	229533.I1RME6
229533.A0A098DDV7	pp	229533.I1RLP1
229533.A0A098DDV7	pp	229533.I1RNG8

175420	229533.A0A098DDV7	pp	229533.I1R991
175421	229533.A0A098DDV7	pp	229533.I1RB40
175422	229533.A0A098DDV7	pp	229533.I1RSB0
175423	229533.A0A098DDV7	pp	229533.I1RAU9
175424	229533.A0A098DDV7	pp	229533.I1RKD7
175425	229533.A0A098DDV7	pp	229533.I1RQL4
175426	229533.A0A098DDV7	pp	229533.I1RBA6
175427	229533.A0A098DDV7	pp	229533.I1RZP7
175428	229533.A0A098DDV7	pp	229533.A0A1C3YM76
175429	229533.A0A098DDV7	pp	229533.I1RA40
175430	229533.A0A098DDV7	pp	229533.I1R9X2
175431	229533.A0A098DDV7	pp	229533.A0A0E0S0J3
175432	229533.A0A098DDV7	pp	229533.I1S0L8
175433	229533.A0A098DDV7	pp	229533.I1RTG9
175434	229533.A0A098DDV7	pp	229533.I1RPA7
175435	229533.A0A098DDV7	pp	229533.I1S095
175436	229533.A0A098DDV7	pp	229533.I1RNJ2
175437	229533.A0A098DDV7	pp	229533.I1RZV4
175438	229533.A0A098DDV7	pp	229533.A0A0E0SPS2
175439	229533.A0A098DDV7	pp	229533.I1REC6
175440	229533.A0A098DDV7	pp	229533.I1S2B9
175441	229533.A0A098DDV7	pp	229533.I1RN81
175442	229533.A0A098DDV7	pp	229533.A0A0E0SDV0
175443	229533.A0A098DDV7	pp	229533.A0A1C3YLH7
175444	229533.A0A098DDV7	pp	229533.A0A1C3YM82
175445	229533.A0A098DDV7	pp	229533.I1RW71
175446	229533.A0A098DDV7	pp	229533.I1RZD9
175447	229533.A0A098DDV7	pp	229533.I1S7W5
175448	229533.A0A098DDV7	pp	229533.I1S039
175449	229533.A0A098DDV7	pp	229533.I1RPN4
175450	229533.A0A098DDV7	pp	229533.I1RQ49
175451	229533.A0A098DDV7	pp	229533.A0A1C3YJC0
175452	229533.A0A098DDV7	pp	229533.A0A1C3YIH5
175453	229533.A0A098DDV7	pp	229533.A0A098DUK3
175454	229533.A0A098DDV7	pp	229533.I1RCD4
175455	229533.A0A098DDV7	pp	229533.A0A1C3YIZ3
175456	229533.A0A098DDV7	pp	229533.A0A0E0SFI7
175457	229533.A0A098DDV7	pp	229533.A0A1C3YJG7
175458	229533.A0A098DDV7	pp	229533.I1RY40
175459	229533.A0A098DDV7	pp	229533.I1RZH4
175460	229533.A0A098DDV7	pp	229533.A0A1C3YI04
175461	229533.A0A098DDV7	pp	229533.I1RWG1
175462	229533.A0A098DDV7	pp	229533.I1RJU5
175463	229533.A0A098DDV7	pp	229533.I1S0J7
175464	229533.A0A098DDV7	pp	229533.I1RUP2
175465	229533.A0A098DDV7	pp	229533.I1RJZ7
175466	229533.A0A098DDV7	pp	229533.I1RAE3
175467	229533.A0A098DDV7	pp	229533.I1RNN1
175468	229533.A0A098DDV7	pp	229533.I1RYM3
175469	229533.A0A098DDV7	pp	229533.I1RFT6
175470	229533.A0A098DDV7	pp	229533.I1S9V9
175471	229533.A0A098DDV7	pp	229533.I1RQG7
175472	229533.A0A098DDV7	pp	229533.I1S0X5
175473	229533.A0A098DDV7	pp	229533.V6RR76
175474	229533.A0A098DDV7	pp	229533.I1RM09
175475	229533.A0A098DDV7	pp	229533.I1RQ81
175476	229533.A0A098DDV7	pp	229533.I1RB15
175477	229533.A0A098DDV7	pp	229533.I1RTA6
175478	229533.A0A098DDV7	pp	229533.A0A1C3YIQ2
175479	229533.A0A098DDV7	pp	229533.I1RNB8
175480	229533.A0A098DDV7	pp	229533.I1RBL8
175481	229533.A0A098DDV7	pp	229533.I1RAE4
175482	229533.A0A098DDV7	pp	229533.I1RZP6
175483	229533.A0A098DDV7	pp	229533.I1RZG0
175484	229533.A0A098DDV7	pp	229533.I1S282
175485	229533.A0A098DDV7	pp	229533.A0A0E0SC50
175486	229533.A0A098DDV7	pp	229533.A0A1C3YM54
175487	229533.A0A098DDV7	pp	229533.I1RN72
175488	229533.A0A098DDV7	pp	229533.I1RWB0
175489	229533.A0A098DDV7	pp	229533.I1RJS0
175490	229533.A0A098DDV7	pp	229533.Q4HZB8
175491	229533.A0A098DDV7	pp	229533.I1RUP5
175492	229533.A0A098DDV7	pp	229533.I1RAE2

175493	229533.A0A098DDV7	pp	229533.A0A0E0SAK0
175494	229533.A0A098DDV7	pp	229533.I1REC7
175495	229533.A0A098DDV7	pp	229533.I1S9G7
175496	229533.A0A098DDV7	pp	229533.I1RW37
175497	229533.A0A098DDV7	pp	229533.A0A098DSP0
175498	229533.A0A098DDV7	pp	229533.A0A1C3YMD9
175499	229533.A0A098DDV7	pp	229533.A0A1C3YME9
175500	229533.A0A098DDV7	pp	229533.A0A1C3YLW8
175501	229533.A0A098DDV7	pp	229533.Q4IN00
175502	229533.A0A098DDV7	pp	229533.I1RKJ2
175503	229533.A0A098DDV7	pp	229533.A0A098DRS5
175504	229533.A0A098DDV7	pp	229533.A0A0E0RU69
175505	229533.A0A098DDV7	pp	229533.A0A0E0SKT2
175506	229533.A0A098DDV7	pp	229533.I1RAE5
175507	229533.A0A098DDV7	pp	229533.V6REE9
175508	229533.A0A098DDV7	pp	229533.I1RAU7
175509	229533.A0A098DDV7	pp	229533.I1RTE6
175510	229533.A0A098DDV7	pp	229533.I1RP88
175511	229533.A0A098DDV7	pp	229533.I1RQ82
175512	229533.A0A098DDV7	pp	229533.I1RPS0
175513	229533.A0A098DDV7	pp	229533.I1RMV6
175514	229533.A0A1C3YME0	pp	229533.Q4IBU4
175515	229533.A0A1C3YME0	pp	229533.I1S0K5
175516	229533.A0A1C3YME0	pp	229533.I1RD63
175517	229533.A0A1C3YME0	pp	229533.I1RLT6
175518	229533.A0A1C3YME0	pp	229533.I1RAZ1
175519	229533.A0A1C3YME0	pp	229533.I1RC22
175520	229533.A0A1C3YME0	pp	229533.I1RMG4
175521	229533.A0A1C3YME0	pp	229533.I1RW37
175522	229533.A0A1C3YME0	pp	229533.I1RM09
175523	229533.A0A1C3YME0	pp	229533.I1RNR9
175524	229533.A0A1C3YME0	pp	229533.I1S278
175525	229533.A0A1C3YME0	pp	229533.Q4ICM9
175526	229533.A0A1C3YME0	pp	229533.I1RMV6
175527	229533.A0A1C3YME0	pp	229533.I1S4N7
175528	229533.A0A1C3YME0	pp	229533.I1RM77
175529	229533.A0A1C3YME0	pp	229533.I1RC52
175530	229533.A0A1C3YME0	pp	229533.I1RW84
175531	229533.A0A1C3YME0	pp	229533.I1RK86
175532	229533.A0A1C3YME0	pp	229533.I1RDE8
175533	229533.A0A1C3YME0	pp	229533.I1RUP2
175534	229533.A0A1C3YME0	pp	229533.I1S4N8
175535	229533.A0A1C3YME0	pp	229533.I1RCN6
175536	229533.A0A1C3YME0	pp	229533.I1RG99
175537	229533.A0A1C3YME0	pp	229533.I1RA96
175538	229533.A0A1C3YME0	pp	229533.I1RNG8
175539	229533.A0A1C3YME0	pp	229533.I1RN66
175540	229533.A0A1C3YME0	pp	229533.I1RC58
175541	229533.A0A1C3YME0	pp	229533.I1RZP7
175542	229533.A0A1C3YME0	pp	229533.I1S0P7
175543	229533.A0A1C3YME0	pp	229533.I1RZP6
175544	229533.A0A1C3YME0	pp	229533.I1RB14
175545	229533.A0A1C3YME0	pp	229533.I1S368
175546	229533.A0A1C3YME0	pp	229533.I1RYU1
175547	229533.A0A1C3YME0	pp	229533.I1RNA3
175548	229533.A0A1C3YME0	pp	229533.Q4HYZ2
175549	229533.A0A1C3YME0	pp	229533.A0A1C3YN81
175550	229533.A0A1C3YME0	pp	229533.I1R9X6
175551	229533.A0A1C3YME0	pp	229533.I1RXV4
175552	229533.A0A1C3YME0	pp	229533.I1RWV4
175553	229533.A0A1C3YME0	pp	229533.I1RZN1
175554	229533.A0A1C3YME0	pp	229533.Q4I6S5
175555	229533.A0A1C3YME0	pp	229533.I1S9X9
175556	229533.A0A1C3YME0	pp	229533.I1RD02
175557	229533.A0A1C3YME0	pp	229533.I1R9N6
175558	229533.A0A1C3YME0	pp	229533.I1RNV6
175559	229533.A0A1C3YME0	pp	229533.I1RM62
175560	229533.A0A1C3YME0	pp	229533.I1RMF2
175561	229533.A0A1C3YME0	pp	229533.I1RK33
175562	229533.A0A1C3YME0	pp	229533.I1S039
175563	229533.A0A1C3YME0	pp	229533.I1RKA1
175564	229533.A0A1C3YME0	pp	229533.I1RFV2
175565	229533.A0A1C3YME0	pp	229533.I1RFS8

175566 229533.A0A1C3YME0 pp 229533.I1RJ78
175567 229533.A0A1C3YME0 pp 229533.I1RTA6
175568 229533.A0A1C3YME0 pp 229533.I1S1W5
175569 229533.A0A1C3YME0 pp 229533.I1S0J7
175570 229533.A0A1C3YME0 pp 229533.I1S1X7
175571 229533.A0A1C3YME0 pp 229533.I1RFV4
175572 229533.A0A1C3YME0 pp 229533.I1RX50
175573 229533.A0A1C3YME0 pp 229533.I1RB35
175574 229533.A0A1C3YME0 pp 229533.I1RPD3
175575 229533.A0A1C3YME0 pp 229533.I1RQ61
175576 229533.A0A1C3YME0 pp 229533.I1RQG7
175577 229533.I1RT77 pp 229533.V6RLM7
175578 229533.I1RT77 pp 229533.I1S268
175579 229533.I1RT77 pp 229533.I1S0T4
175580 229533.I1RKR9 pp 229533.I1RT41
175581 229533.I1RKR9 pp 229533.Q4HZS8
175582 229533.I1RKR9 pp 229533.I1RN81
175583 229533.I1RKR9 pp 229533.I1RR74
175584 229533.I1RKR9 pp 229533.V6QUU6
175585 229533.I1RKR9 pp 229533.Q4INI2
175586 229533.I1RKR9 pp 229533.I1S270
175587 229533.I1RKR9 pp 229533.I1RWA5
175588 229533.I1RKR9 pp 229533.I1S8E0
175589 229533.I1RKR9 pp 229533.I1RQM8
175590 229533.I1RKR9 pp 229533.Q4ICA8
175591 229533.I1RKR9 pp 229533.Q4I948
175592 229533.I1RKR9 pp 229533.I1S254
175593 229533.I1RKR9 pp 229533.I1S0Z3
175594 229533.I1RKR9 pp 229533.Q5I2J3
175595 229533.I1RKR9 pp 229533.I1RZH4
175596 229533.I1RKR9 pp 229533.I1RT65
175597 229533.I1RKR9 pp 229533.I1RX09
175598 229533.I1RKR9 pp 229533.I1S034
175599 229533.I1RKR9 pp 229533.V6REE9
175600 229533.I1RKR9 pp 229533.I1RWA9
175601 229533.I1RKR9 pp 229533.I1RQP5
175602 229533.I1RKR9 pp 229533.I1RW71
175603 229533.I1RKR9 pp 229533.I1RSD7
175604 229533.I1RKR9 pp 229533.Q4IER8
175605 229533.I1RKR9 pp 229533.Q4I963
175606 229533.I1RKR9 pp 229533.I1S0X0
175607 229533.I1RKR9 pp 229533.V6RR76
175608 229533.I1RKR9 pp 229533.I1RMG2
175609 229533.I1RKR9 pp 229533.I1S248
175610 229533.I1RKR9 pp 229533.I1RWI6
175611 229533.I1RKR9 pp 229533.I1RVY0
175612 229533.I1RKR9 pp 229533.I1S209
175613 229533.I1RKR9 pp 229533.I1RQP3
175614 229533.I1RKR9 pp 229533.V6R476
175615 229533.I1RKR9 pp 229533.I1S0J7
175616 229533.I1RKR9 pp 229533.I1S8X0
175617 229533.I1RKR9 pp 229533.I1S7W2
175618 229533.I1RKR9 pp 229533.I1RRM9
175619 229533.I1RKR9 pp 229533.I1RQD6
175620 229533.I1RKR9 pp 229533.I1RZL3
175621 229533.I1RKR9 pp 229533.I1RPX7
175622 229533.I1RKR9 pp 229533.I1RNG4
175623 229533.I1RKR9 pp 229533.I1RX68
175624 229533.I1RKR9 pp 229533.Q4HY90
175625 229533.I1RKR9 pp 229533.Q4HZE0
175626 229533.I1RKR9 pp 229533.I1RM37
175627 229533.I1RKR9 pp 229533.I1RTG5
175628 229533.I1RKR9 pp 229533.I1RXS3
175629 229533.I1RKR9 pp 229533.I1RUP5
175630 229533.I1RKR9 pp 229533.I1S0S8
175631 229533.I1RKR9 pp 229533.I1RR95
175632 229533.I1RKR9 pp 229533.I1RZD6
175633 229533.I1RKR9 pp 229533.I1S559
175634 229533.I1RKR9 pp 229533.I1RZL9
175635 229533.I1RKR9 pp 229533.I1RQP1
175636 229533.I1RKR9 pp 229533.I1RTE0
175637 229533.I1RKR9 pp 229533.I1RX69
175638 229533.I1RKR9 pp 229533.I1RQX3

175639	229533.I1RKR9	pp	229533.I1S0R9
175640	229533.I1RKR9	pp	229533.I1RPG1
175641	229533.I1RKR9	pp	229533.I1RST4
175642	229533.I1RKR9	pp	229533.I1RZV1
175643	229533.I1RKR9	pp	229533.I1S0N4
175644	229533.I1RKR9	pp	229533.I1RWH4
175645	229533.I1RKR9	pp	229533.I1RSC7
175646	229533.I1RKR9	pp	229533.I1RSZ6
175647	229533.I1RNV7	pp	229533.Q4IMD1
175648	229533.I1RNV7	pp	229533.I1RXE5
175649	229533.I1RNV7	pp	229533.I1RVY0
175650	229533.I1RNV7	pp	229533.I1RQB7
175651	229533.I1RNV7	pp	229533.I1RQ19
175652	229533.I1RNV7	pp	229533.I1RPX5
175653	229533.I1RNV7	pp	229533.Q4HTT2
175654	229533.I1RNV7	pp	229533.Q4IER8
175655	229533.I1RNV7	pp	229533.Q4I5R3
175656	229533.I1RNV7	pp	229533.I1S196
175657	229533.I1RNV7	pp	229533.I1RYV3
175658	229533.I1RNV7	pp	229533.I1RU02
175659	229533.I1RNV7	pp	229533.I1RRH5
175660	229533.I1RNV7	pp	229533.I1RWU3
175661	229533.I1RNV7	pp	229533.I1RUQ6
175662	229533.I1RNV7	pp	229533.V6QV38
175663	229533.I1REP3	pp	229533.Q4HXF0
175664	229533.I1REP3	pp	229533.O74638
175665	229533.I1REP3	pp	229533.I1RZ20
175666	229533.I1REP3	pp	229533.I1RQ48
175667	229533.I1REP3	pp	229533.I1RQ30
175668	229533.I1REP3	pp	229533.I1RMG6
175669	229533.I1REP3	pp	229533.I1RPY0
175670	229533.I1REP3	pp	229533.I1RTI7
175671	229533.I1REP3	pp	229533.I1RZ52
175672	229533.I1REP3	pp	229533.I1RWF4
175673	229533.I1REP3	pp	229533.V6RQW4
175674	229533.I1REP3	pp	229533.I1RKF2
175675	229533.I1REP3	pp	229533.I1S9D4
175676	229533.I1REP3	pp	229533.I1RYU5
175677	229533.I1REP3	pp	229533.I1RTJ2
175678	229533.I1REP3	pp	229533.I1RIY0
175679	229533.I1REP3	pp	229533.I1RMG7
175680	229533.I1REP3	pp	229533.I1S2E2
175681	229533.I1S0J7	pp	229533.I1S4N8
175682	229533.I1S0J7	pp	229533.I1S1W4
175683	229533.I1S0J7	pp	229533.V6RGI1
175684	229533.I1S0J7	pp	229533.I1S0P7
175685	229533.I1S0J7	pp	229533.V6QX21
175686	229533.I1S0J7	pp	229533.Q4IBU4
175687	229533.I1S0J7	pp	229533.I1S4N7
175688	229533.I1S0J7	pp	229533.I1S2E9
175689	229533.I1S0J7	pp	229533.I1S2G7
175690	229533.I1S0J7	pp	229533.I1S4L8
175691	229533.I1S0J7	pp	229533.I1S1X7
175692	229533.I1S0J7	pp	229533.I1S278
175693	229533.I1S0J7	pp	229533.I1S1J6
175694	229533.I1S0J7	pp	229533.I1S1W5
175695	229533.I1S0J7	pp	229533.V6RKK5
175696	229533.I1S0J7	pp	229533.I1S8Q3
175697	229533.I1S0J7	pp	229533.V6QV09
175698	229533.I1S0J7	pp	229533.I1S1F5
175699	229533.I1S0J7	pp	229533.I1S9X9
175700	229533.I1S0J7	pp	229533.Q4IJM4
175701	229533.I1S0J7	pp	229533.I1S224
175702	229533.A0A098D065	pp	229533.A0A1C3YKM2
175703	229533.A0A098D065	pp	229533.I1RFN8
175704	229533.A0A098D065	pp	229533.Q4I1B1
175705	229533.A0A098D065	pp	229533.I1RZH4
175706	229533.A0A098D065	pp	229533.I1RCK2
175707	229533.A0A098D065	pp	229533.A0A098DRQ4
175708	229533.A0A098D065	pp	229533.I1RAL7
175709	229533.A0A098D065	pp	229533.I1RXT2
175710	229533.A0A098D065	pp	229533.I1RNG8
175711	229533.A0A098D065	pp	229533.I1RR07

175712 229533.A0A098D065 pp 229533.I1RKA1
175713 229533.A0A098D065 pp 229533.I1RZR6
175714 229533.A0A098D065 pp 229533.A0A1C3YKR7
175715 229533.A0A098D065 pp 229533.I1S1W5
175716 229533.A0A098D065 pp 229533.A0A0E0SC50
175717 229533.A0A098D065 pp 229533.I1S4N8
175718 229533.A0A098D065 pp 229533.I1RW37
175719 229533.A0A098D065 pp 229533.A0A098DPY0
175720 229533.A0A098D065 pp 229533.I1S4N7
175721 229533.A0A098D065 pp 229533.I1RSP4
175722 229533.A0A098D065 pp 229533.I1RFS8
175723 229533.A0A098D065 pp 229533.I1S9X9
175724 229533.A0A098D065 pp 229533.I1RS35
175725 229533.A0A098D065 pp 229533.I1RS41
175726 229533.A0A098D065 pp 229533.I1RMV6
175727 229533.A0A098D065 pp 229533.V6QX21
175728 229533.A0A098D065 pp 229533.I1S8Q3
175729 229533.A0A098D065 pp 229533.I1S0L8
175730 229533.A0A098D065 pp 229533.I1S039
175731 229533.A0A098D065 pp 229533.I1RQ01
175732 229533.A0A098D065 pp 229533.A0A1C3YM82
175733 229533.A0A098D065 pp 229533.I1S270
175734 229533.A0A098D065 pp 229533.I1S0P7
175735 229533.A0A098D065 pp 229533.I1RX50
175736 229533.A0A098D065 pp 229533.A0A098DDV7
175737 229533.A0A098D065 pp 229533.A0A1C3YME0
175738 229533.A0A098D065 pp 229533.I1RKR9
175739 229533.A0A098D065 pp 229533.I1S0J7
175740 229533.I1RBK1 pp 229533.I1RE53
175741 229533.I1RBK1 pp 229533.I1RZ20
175742 229533.I1RBK1 pp 229533.I1RQ48
175743 229533.I1RBK1 pp 229533.I1RMG6
175744 229533.I1RBK1 pp 229533.I1S444
175745 229533.I1RBK1 pp 229533.I1RCB4
175746 229533.I1RBK1 pp 229533.O74638
175747 229533.I1RBK1 pp 229533.I1RWF4
175748 229533.I1RBK1 pp 229533.I1S8F8
175749 229533.I1RBK1 pp 229533.I1RZ52
175750 229533.I1RBK1 pp 229533.I1S2E2
175751 229533.I1RBK1 pp 229533.I1RQ30
175752 229533.I1RBK1 pp 229533.I1RIY0
175753 229533.I1RBK1 pp 229533.I1S9D4
175754 229533.I1RBK1 pp 229533.V6RQW4
175755 229533.I1RBK1 pp 229533.I1RTI7
175756 229533.I1RBK1 pp 229533.Q4ID21
175757 229533.I1RBK1 pp 229533.I1RMG7
175758 229533.I1RBK1 pp 229533.I1RYU5
175759 229533.I1RBK1 pp 229533.V6QYF0
175760 229533.I1S2V9 pp 229533.Q4ICM0
175761 229533.I1S8X1 pp 229533.I1S9D4
175762 229533.I1S8X1 pp 229533.V6RQW4
175763 229533.I1S8X1 pp 229533.O74638
175764 229533.I1S5R6 pp 229533.I1S9Y1
175765 229533.I1S5R6 pp 229533.O74638
175766 229533.I1S5R6 pp 229533.I1SAJ3
175767 229533.I1S5R6 pp 229533.Q4HVQ9
175768 229533.I1S5R6 pp 229533.I1S6Y8
175769 229533.I1S5R6 pp 229533.V6R3J1
175770 229533.I1S5R6 pp 229533.V6RTJ8
175771 229533.I1S5R6 pp 229533.V6R7V8
175772 229533.I1S5R6 pp 229533.I1S9Z4
175773 229533.I1S5R6 pp 229533.Q4HXF0
175774 229533.I1S5R6 pp 229533.Q4I624
175775 229533.I1S5R6 pp 229533.V6RLM7
175776 229533.I1S5R6 pp 229533.V6R042
175777 229533.I1S5R6 pp 229533.V6RBN2
175778 229533.I1S5R6 pp 229533.V6QUG0
175779 229533.I1S5R6 pp 229533.I1S9I5
175780 229533.I1S5R6 pp 229533.Q4IJK2
175781 229533.I1S5R6 pp 229533.Q4I5G1
175782 229533.I1S5R6 pp 229533.I1S6K9
175783 229533.I1S5R6 pp 229533.I1S821
175784 229533.I1S5R6 pp 229533.V6RMW6

175785	229533.I1S5R6	pp	229533.V6RCZ8
175786	229533.I1S5R6	pp	229533.V6QUE6
175787	229533.Q4HTS9	pp	229533.V6QVM5
175788	229533.Q4HTS9	pp	229533.V6QT89
175789	229533.Q4HTS9	pp	229533.Q4IMJ1
175790	229533.I1RIH7	pp	229533.I1RV62
175791	229533.I1RIH7	pp	229533.I1RJA4
175792	229533.I1RCW6	pp	229533.Q4IJK2
175793	229533.I1RCW6	pp	229533.I1RXF0
175794	229533.I1RCW6	pp	229533.V6RBN2
175795	229533.I1RCW6	pp	229533.I1RZM8
175796	229533.I1RCW6	pp	229533.I1RTD3
175797	229533.I1RCW6	pp	229533.I1RF82
175798	229533.I1RCW6	pp	229533.I1RX46
175799	229533.I1RCW6	pp	229533.V6RCZ8
175800	229533.I1RCW6	pp	229533.V6QUE6
175801	229533.I1RCW6	pp	229533.V6RTJ8
175802	229533.I1RCW6	pp	229533.I1RRV4
175803	229533.I1RCW6	pp	229533.I1S3S3
175804	229533.I1RCW6	pp	229533.I1RK15
175805	229533.I1RCW6	pp	229533.I1RMQ4
175806	229533.I1RCW6	pp	229533.I1RS18
175807	229533.I1RCW6	pp	229533.I1RJ17
175808	229533.I1RCW6	pp	229533.I1S5B0
175809	229533.I1RCW6	pp	229533.I1RME6
175810	229533.I1RCW6	pp	229533.I1RQS9
175811	229533.I1RCW6	pp	229533.I1RXD7
175812	229533.I1RCW6	pp	229533.I1S1J7
175813	229533.I1RCW6	pp	229533.I1S4S7
175814	229533.I1RCW6	pp	229533.I1RJU3
175815	229533.I1RCW6	pp	229533.I1RD52
175816	229533.I1RCW6	pp	229533.I1RRY9
175817	229533.I1RCW6	pp	229533.I1RPN4
175818	229533.I1RCW6	pp	229533.I1RD22
175819	229533.I1RCW6	pp	229533.I1S4Q4
175820	229533.I1RCW6	pp	229533.I1S0B7
175821	229533.I1RCW6	pp	229533.I1REK8
175822	229533.I1RCW6	pp	229533.I1RFC0
175823	229533.I1RCW6	pp	229533.I1RM35
175824	229533.I1RCW6	pp	229533.I1S5R6
175825	229533.I1RCW6	pp	229533.I1RRX5
175826	229533.I1RCW6	pp	229533.I1RVZ7
175827	229533.I1RCW6	pp	229533.I1RSQ7
175828	229533.I1RCW6	pp	229533.I1RG11
175829	229533.I1RCW6	pp	229533.I1RFL3
175830	229533.I1RCW6	pp	229533.I1RUG3
175831	229533.I1RCW6	pp	229533.I1RQM7
175832	229533.I1RCW6	pp	229533.I1RE01
175833	229533.I1RCW6	pp	229533.I1RT40
175834	229533.I1RCW6	pp	229533.I1RTD5
175835	229533.I1RCW6	pp	229533.I1RNC1
175836	229533.I1RCW6	pp	229533.I1S0J1
175837	229533.I1RCW6	pp	229533.Q4HXF0
175838	229533.I1RCW6	pp	229533.I1RPK0
175839	229533.I1RCW6	pp	229533.I1RRV1
175840	229533.I1RCW6	pp	229533.I1SAN9
175841	229533.I1RCW6	pp	229533.I1RJV3
175842	229533.I1RCW6	pp	229533.I1RNG4
175843	229533.I1RCW6	pp	229533.I1RYW3
175844	229533.I1RCW6	pp	229533.I1RN67
175845	229533.I1RCW6	pp	229533.I1RX22
175846	229533.I1RCW6	pp	229533.I1RMI8
175847	229533.I1RCW6	pp	229533.V6R042
175848	229533.I1RCW6	pp	229533.V6RRH2
175849	229533.I1RCW6	pp	229533.I1S058
175850	229533.I1RCW6	pp	229533.I1RTA1
175851	229533.I1RCW6	pp	229533.I1RWE8
175852	229533.I1RCW6	pp	229533.V6R057
175853	229533.I1RCW6	pp	229533.I1RFT1
175854	229533.I1RCW6	pp	229533.I1RE76
175855	229533.I1RCW6	pp	229533.I1S2B8
175856	229533.I1RCW6	pp	229533.I1RPP3
175857	229533.I1RCW6	pp	229533.Q4I624

175858	229533.I1RCW6	pp	229533.O74638
175859	229533.I1RCW6	pp	229533.I1RYI5
175860	229533.I1RCW6	pp	229533.I1S4Z8
175861	229533.I1RCW6	pp	229533.I1RSZ7
175862	229533.I1RCW6	pp	229533.I1RFL4
175863	229533.I1RCW6	pp	229533.I1S216
175864	229533.I1RCW6	pp	229533.I1S5R1
175865	229533.I1RCW6	pp	229533.I1RKY2
175866	229533.I1RCW6	pp	229533.I1RVX0
175867	229533.I1RCW6	pp	229533.I1RQE7
175868	229533.I1RCW6	pp	229533.I1RL04
175869	229533.I1RCW6	pp	229533.I1S2H4
175870	229533.I1RCW6	pp	229533.I1S0L4
175871	229533.I1RCW6	pp	229533.V6R2A1
175872	229533.I1RCW6	pp	229533.I1RSQ4
175873	229533.I1RCW6	pp	229533.I1RGA4
175874	229533.I1RCW6	pp	229533.I1RGA6
175875	229533.I1RCW6	pp	229533.I1RZZ9
175876	229533.I1RCW6	pp	229533.I1S8G4
175877	229533.V6QVH8	pp	229533.V6RLM7
175878	229533.V6QVH8	pp	229533.V6RG79
175879	229533.V6QVH8	pp	229533.V6RSF3
175880	229533.V6QVH8	pp	229533.V6RQW4
175881	229533.V6QVH8	pp	229533.V6RUT5
175882	229533.V6QVH8	pp	229533.V6QVM5
175883	229533.I1RAI5	pp	229533.Q4IBX0
175884	229533.I1RAI5	pp	229533.Q4IMM0
175885	229533.I1RL80	pp	229533.I1RV62
175886	229533.I1RDH9	pp	229533.I1RZR5
175887	229533.I1RDH9	pp	229533.I1RQE1
175888	229533.I1RDH9	pp	229533.I1RNG4
175889	229533.A0A098E219	pp	229533.I1RAI5
175890	229533.A0A098E219	pp	229533.V6RGU1
175891	229533.A0A0E0S6Y2	pp	229533.A0A1C3YI50
175892	229533.A0A0E0S6Y2	pp	229533.I1RHG5
175893	229533.A0A0E0S6Y2	pp	229533.I1RSY2
175894	229533.A0A0E0S6Y2	pp	229533.I1RC81
175895	229533.A0A0E0S6Y2	pp	229533.I1RA63
175896	229533.A0A0E0S6Y2	pp	229533.A0A1C3YN83
175897	229533.A0A0E0S6Y2	pp	229533.I1RFC0
175898	229533.A0A0E0S6Y2	pp	229533.I1RNJ2
175899	229533.A0A0E0S6Y2	pp	229533.I1RFL3
175900	229533.A0A0E0S6Y2	pp	229533.I1RK03
175901	229533.A0A0E0S6Y2	pp	229533.I1S6E5
175902	229533.A0A0E0S6Y2	pp	229533.A0A1C3YL10
175903	229533.A0A0E0S6Y2	pp	229533.A0A1C3YKY6
175904	229533.A0A0E0S6Y2	pp	229533.I1RD63
175905	229533.A0A0E0S6Y2	pp	229533.I1RJ17
175906	229533.A0A0E0S6Y2	pp	229533.A0A1C3YI30
175907	229533.A0A0E0S6Y2	pp	229533.I1RVP5
175908	229533.A0A0E0S6Y2	pp	229533.A0A1C3YN57
175909	229533.I1RHZ4	pp	229533.I1RUZ4
175910	229533.I1RHZ4	pp	229533.I1RJA4
175911	229533.I1RHZ4	pp	229533.I1RV62
175912	229533.A0A098CZX5	pp	229533.A0A1C3YLG7
175913	229533.A0A098CZX5	pp	229533.I1RIH7
175914	229533.A0A098CZX5	pp	229533.I1RL80
175915	229533.A0A098CZX5	pp	229533.I1S6E6
175916	229533.A0A098CZX5	pp	229533.I1RHZ4
175917	229533.I1RDD6	pp	229533.I1S0H1
175918	229533.I1RDD6	pp	229533.I1RNE7
175919	229533.I1RDD6	pp	229533.I1RN48
175920	229533.A0A098DVZ4	pp	229533.A0A1C3YKW4
175921	229533.A0A098DVZ4	pp	229533.I1RF85
175922	229533.A0A098DVZ4	pp	229533.I1RRF0
175923	229533.A0A098DVZ4	pp	229533.A0A1C3YK33
175924	229533.A0A098DVZ4	pp	229533.I1RKB6
175925	229533.A0A098DVZ4	pp	229533.I1RG11
175926	229533.A0A098DVZ4	pp	229533.V6RF62
175927	229533.A0A098DVZ4	pp	229533.A0A0E0SBC4
175928	229533.A0A098DVZ4	pp	229533.I1REV3
175929	229533.A0A098DVZ4	pp	229533.I1S2C4
175930	229533.A0A098DVZ4	pp	229533.I1RST4

175931 229533.A0A098DVZ4 pp 229533.I1RYW3
175932 229533.A0A098DVZ4 pp 229533.I1RWH4
175933 229533.A0A098DVZ4 pp 229533.I1RRI2
175934 229533.A0A098DVZ4 pp 229533.I1RBA4
175935 229533.A0A098DVZ4 pp 229533.I1RZ70
175936 229533.A0A098DVZ4 pp 229533.I1S0V7
175937 229533.A0A098DVZ4 pp 229533.I1RD22
175938 229533.A0A098DVZ4 pp 229533.I1RY74
175939 229533.A0A098DVZ4 pp 229533.I1RWX5
175940 229533.A0A098DVZ4 pp 229533.I1RRV1
175941 229533.A0A098DVZ4 pp 229533.I1SAJ3
175942 229533.A0A098DVZ4 pp 229533.I1RQE7
175943 229533.A0A098DVZ4 pp 229533.I1RE84
175944 229533.A0A098DVZ4 pp 229533.I1RS90
175945 229533.A0A098DVZ4 pp 229533.I1RBS5
175946 229533.A0A098DVZ4 pp 229533.V6RRM0
175947 229533.A0A098DVZ4 pp 229533.I1RFF3
175948 229533.A0A098DVZ4 pp 229533.I1RTE6
175949 229533.A0A098DVZ4 pp 229533.I1RM37
175950 229533.A0A098DVZ4 pp 229533.I1RE52
175951 229533.A0A098DVZ4 pp 229533.I1RUG3
175952 229533.A0A098DVZ4 pp 229533.I1RAK7
175953 229533.A0A098DVZ4 pp 229533.I1RH91
175954 229533.A0A098DVZ4 pp 229533.A0A098E375
175955 229533.A0A098DVZ4 pp 229533.I1RW43
175956 229533.A0A098DVZ4 pp 229533.I1RE79
175957 229533.A0A098DVZ4 pp 229533.A0A1C3YKM9
175958 229533.A0A098DVZ4 pp 229533.I1RSK5
175959 229533.A0A098DVZ4 pp 229533.Q4HYB8
175960 229533.A0A098DVZ4 pp 229533.V6QT89
175961 229533.A0A098DVZ4 pp 229533.I1RGX1
175962 229533.A0A098DVZ4 pp 229533.A0A1C3YLB6
175963 229533.A0A098DVZ4 pp 229533.I1S2A9
175964 229533.A0A098DVZ4 pp 229533.I1RTG7
175965 229533.A0A098DVZ4 pp 229533.I1RQ21
175966 229533.A0A098DVZ4 pp 229533.I1S4B6
175967 229533.A0A098DVZ4 pp 229533.A0A0E0SID1
175968 229533.A0A098DVZ4 pp 229533.I1RC00
175969 229533.A0A098DVZ4 pp 229533.I1S2H4
175970 229533.A0A098DVZ4 pp 229533.I1S5X3
175971 229533.A0A098DVZ4 pp 229533.I1RSQ4
175972 229533.A0A098DVZ4 pp 229533.I1S3G4
175973 229533.A0A098DVZ4 pp 229533.I1S0J7
175974 229533.A0A098DVZ4 pp 229533.I1RIQ7
175975 229533.A0A098DVZ4 pp 229533.I1S5R1
175976 229533.A0A098DVZ4 pp 229533.V6RUB1
175977 229533.A0A098DVZ4 pp 229533.I1RKI1
175978 229533.A0A098DVZ4 pp 229533.A0A1C3YI20
175979 229533.A0A098DVZ4 pp 229533.I1RZ29
175980 229533.A0A098DVZ4 pp 229533.I1RBL4
175981 229533.A0A098DVZ4 pp 229533.A0A1C3YHG7
175982 229533.A0A098DVZ4 pp 229533.V6RKY8
175983 229533.A0A098DVZ4 pp 229533.I1RKR9
175984 229533.A0A098DVZ4 pp 229533.I1RBY4
175985 229533.A0A098DVZ4 pp 229533.I1RSS7
175986 229533.A0A098DVZ4 pp 229533.V6RUY9
175987 229533.A0A098DVZ4 pp 229533.I1RA52
175988 229533.A0A098DVZ4 pp 229533.Q4IPI4
175989 229533.A0A098DVZ4 pp 229533.A0A1C3YMA1
175990 229533.A0A098DVZ4 pp 229533.I1RM54
175991 229533.A0A098DVZ4 pp 229533.I1R9Y8
175992 229533.A0A098DVZ4 pp 229533.A0A1C3YKU8
175993 229533.A0A098DVZ4 pp 229533.I1RT75
175994 229533.A0A098DVZ4 pp 229533.A0A1C3YI27
175995 229533.A0A098DVZ4 pp 229533.I1RC29
175996 229533.A0A098DVZ4 pp 229533.I1RZA7
175997 229533.A0A098DVZ4 pp 229533.I1RCD4
175998 229533.A0A098DVZ4 pp 229533.A0A0E0SQG9
175999 229533.A0A098DVZ4 pp 229533.I1RBF3
176000 229533.A0A098DVZ4 pp 229533.I1RT40
176001 229533.A0A098DVZ4 pp 229533.I1RPP3
176002 229533.A0A098DVZ4 pp 229533.A0A1C3YLL4
176003 229533.A0A098DVZ4 pp 229533.I1S4B7

176004	229533.A0A098DVZ4	pp	229533.I1RWF5
176005	229533.A0A098DVZ4	pp	229533.I1RU18
176006	229533.A0A098DVZ4	pp	229533.I1RLZ4
176007	229533.A0A098DVZ4	pp	229533.I1REC6
176008	229533.A0A098DVZ4	pp	229533.A0A1C3YKN4
176009	229533.A0A098DVZ4	pp	229533.I1RYK0
176010	229533.A0A098DVZ4	pp	229533.I1RN88
176011	229533.A0A098DVZ4	pp	229533.A0A098E2F0
176012	229533.A0A098DVZ4	pp	229533.I1RQG4
176013	229533.A0A098DVZ4	pp	229533.I1RZN1
176014	229533.A0A098DVZ4	pp	229533.I1RWS4
176015	229533.A0A098DVZ4	pp	229533.A0A1C3YLA6
176016	229533.A0A098DVZ4	pp	229533.I1RQ48
176017	229533.A0A098DVZ4	pp	229533.I1RN67
176018	229533.A0A098DVZ4	pp	229533.I1RA62
176019	229533.A0A098DVZ4	pp	229533.I1RR95
176020	229533.A0A098DVZ4	pp	229533.A0A1C3YLK5
176021	229533.A0A098DVZ4	pp	229533.I1RMJ3
176022	229533.A0A098DVZ4	pp	229533.A0A1C3YM61
176023	229533.A0A098DVZ4	pp	229533.I1RE19
176024	229533.A0A098DVZ4	pp	229533.A0A1C3YJ56
176025	229533.A0A098DVZ4	pp	229533.I1RMG6
176026	229533.A0A098DVZ4	pp	229533.I1RBS0
176027	229533.A0A098DVZ4	pp	229533.I1RKB2
176028	229533.A0A098DVZ4	pp	229533.I1RVU7
176029	229533.A0A098DVZ4	pp	229533.I1S097
176030	229533.A0A098DVZ4	pp	229533.I1RVZ2
176031	229533.A0A098DVZ4	pp	229533.A0A1C3YLC2
176032	229533.A0A098DVZ4	pp	229533.I1RPW1
176033	229533.A0A098DVZ4	pp	229533.I1S4D0
176034	229533.A0A098DVZ4	pp	229533.I1RYU0
176035	229533.A0A098DVZ4	pp	229533.I1RA90
176036	229533.A0A098DVZ4	pp	229533.A0A1C3YLP6
176037	229533.A0A098DVZ4	pp	229533.I1RBL7
176038	229533.A0A098DVZ4	pp	229533.I1RPR9
176039	229533.A0A098DVZ4	pp	229533.A0A1C3YJ93
176040	229533.A0A098DVZ4	pp	229533.I1RUY6
176041	229533.A0A098DVZ4	pp	229533.I1S1J7
176042	229533.A0A098DVZ4	pp	229533.I1S0N3
176043	229533.A0A098DVZ4	pp	229533.I1RRY9
176044	229533.A0A098DVZ4	pp	229533.I1S9U1
176045	229533.A0A098DVZ4	pp	229533.I1RUY4
176046	229533.A0A098DVZ4	pp	229533.A0A1C3YIJ3
176047	229533.A0A098DVZ4	pp	229533.A0A0E0RQ52
176048	229533.A0A098DVZ4	pp	229533.V6R3V4
176049	229533.A0A098DVZ4	pp	229533.I1S0V6
176050	229533.A0A098DVZ4	pp	229533.I1RWP4
176051	229533.A0A098DVZ4	pp	229533.I1RG66
176052	229533.A0A098DVZ4	pp	229533.I1RT65
176053	229533.A0A098DVZ4	pp	229533.V6REE9
176054	229533.A0A098DVZ4	pp	229533.I1RDU0
176055	229533.A0A098DVZ4	pp	229533.I1RUQ3
176056	229533.A0A098DVZ4	pp	229533.I1S8F8
176057	229533.A0A098DVZ4	pp	229533.A0A098E4T8
176058	229533.A0A098DVZ4	pp	229533.A0A1C3YJ26
176059	229533.A0A098DVZ4	pp	229533.I1RVZ9
176060	229533.A0A098DVZ4	pp	229533.I1RYL5
176061	229533.A0A098DVZ4	pp	229533.I1S0Z3
176062	229533.A0A098DVZ4	pp	229533.I1RAQ4
176063	229533.A0A098DVZ4	pp	229533.I1RQA3
176064	229533.A0A098DVZ4	pp	229533.Q4HYR2
176065	229533.A0A098DVZ4	pp	229533.I1RPL2
176066	229533.A0A098DVZ4	pp	229533.A0A0E0RVH6
176067	229533.A0A098DVZ4	pp	229533.A0A1C3YK94
176068	229533.A0A098DVZ4	pp	229533.I1RXE6
176069	229533.A0A098DVZ4	pp	229533.V6QUE6
176070	229533.A0A098DVZ4	pp	229533.I1RQE0
176071	229533.A0A098DVZ4	pp	229533.A0A098E320
176072	229533.A0A098DVZ4	pp	229533.I1RBE7
176073	229533.A0A098DVZ4	pp	229533.I1RFD4
176074	229533.A0A098DVZ4	pp	229533.A0A1C3YMT2
176075	229533.A0A098DVZ4	pp	229533.I1RYM5
176076	229533.A0A098DVZ4	pp	229533.I1S267

176077 229533.A0A098DVZ4 pp 229533.A0A1C3YKR5
176078 229533.A0A098DVZ4 pp 229533.A0A0E0SF53
176079 229533.A0A098DVZ4 pp 229533.I1RJY7
176080 229533.A0A098DVZ4 pp 229533.I1RHR3
176081 229533.A0A098DVZ4 pp 229533.I1RFV9
176082 229533.A0A098DVZ4 pp 229533.Q4HTT2
176083 229533.A0A098DVZ4 pp 229533.A0A1C3YLI1
176084 229533.A0A098DVZ4 pp 229533.I1RE00
176085 229533.A0A098DVZ4 pp 229533.V6RPN0
176086 229533.A0A098DVZ4 pp 229533.I1RS18
176087 229533.A0A098DVZ4 pp 229533.A0A1C3YIK4
176088 229533.A0A098DVZ4 pp 229533.A0A1C3YLR6
176089 229533.A0A098DVZ4 pp 229533.I1RC11
176090 229533.A0A098DVZ4 pp 229533.A0A1C3YK34
176091 229533.A0A098DVZ4 pp 229533.I1RW69
176092 229533.A0A098DVZ4 pp 229533.I1RJT5
176093 229533.A0A098DVZ4 pp 229533.A0A1C3YHC6
176094 229533.A0A098DVZ4 pp 229533.I1RCU4
176095 229533.A0A098DVZ4 pp 229533.I1RSB7
176096 229533.A0A098DVZ4 pp 229533.I1RJC5
176097 229533.A0A098DVZ4 pp 229533.I1RT41
176098 229533.A0A098DVZ4 pp 229533.I1S7Q8
176099 229533.A0A098DVZ4 pp 229533.A0A1C3YMC0
176100 229533.A0A098DVZ4 pp 229533.A0A1C3YMK2
176101 229533.A0A098DVZ4 pp 229533.I1RW54
176102 229533.A0A098DVZ4 pp 229533.I1S8Z2
176103 229533.A0A098DVZ4 pp 229533.I1S338
176104 229533.A0A098DVZ4 pp 229533.I1RM67
176105 229533.A0A098DVZ4 pp 229533.I1RMQ6
176106 229533.A0A098DVZ4 pp 229533.I1RKB7
176107 229533.A0A098DVZ4 pp 229533.I1RJU3
176108 229533.A0A098DVZ4 pp 229533.I1S270
176109 229533.A0A098DVZ4 pp 229533.I1RMK7
176110 229533.A0A098DVZ4 pp 229533.I1RY16
176111 229533.A0A098DVZ4 pp 229533.I1RTG4
176112 229533.A0A098DVZ4 pp 229533.A0A1C3YL54
176113 229533.A0A098DVZ4 pp 229533.A0A098DXS1
176114 229533.A0A098DVZ4 pp 229533.I1RH48
176115 229533.A0A098DVZ4 pp 229533.A0A1C3YN77
176116 229533.A0A098DVZ4 pp 229533.I1S039
176117 229533.A0A098DVZ4 pp 229533.I1R9Y4
176118 229533.A0A098DVZ4 pp 229533.I1RKB5
176119 229533.A0A098DVZ4 pp 229533.A0A1C3YLC3
176120 229533.A0A098DVZ4 pp 229533.A0A1C3YJ69
176121 229533.A0A098DVZ4 pp 229533.Q4HZS8
176122 229533.A0A098DVZ4 pp 229533.I1RRR5
176123 229533.A0A098DVZ4 pp 229533.I1RFV2
176124 229533.A0A098DVZ4 pp 229533.A0A1C3YJ33
176125 229533.A0A098DVZ4 pp 229533.I1RYD3
176126 229533.A0A098DVZ4 pp 229533.I1RR12
176127 229533.A0A098DVZ4 pp 229533.I1RCM8
176128 229533.A0A098DVZ4 pp 229533.I1RKI3
176129 229533.A0A098DVZ4 pp 229533.I1RM55
176130 229533.A0A098DVZ4 pp 229533.V6QUV7
176131 229533.A0A098DVZ4 pp 229533.V6R1K4
176132 229533.A0A098DVZ4 pp 229533.A0A1C3YKZ8
176133 229533.A0A098DVZ4 pp 229533.I1RAW0
176134 229533.A0A098DVZ4 pp 229533.A0A1C3YHU3
176135 229533.A0A098DVZ4 pp 229533.I1RVQ0
176136 229533.A0A098DVZ4 pp 229533.I1REC7
176137 229533.A0A098DVZ4 pp 229533.I1RA70
176138 229533.A0A098DVZ4 pp 229533.A0A0E0S8Z0
176139 229533.A0A098DVZ4 pp 229533.I1RCG5
176140 229533.A0A098DVZ4 pp 229533.A0A0E0RU55
176141 229533.A0A098DVZ4 pp 229533.I1RNU7
176142 229533.A0A098DVZ4 pp 229533.I1RHG5
176143 229533.A0A098DVZ4 pp 229533.I1S3V2
176144 229533.A0A098DVZ4 pp 229533.A0A0E0SI42
176145 229533.A0A098DVZ4 pp 229533.I1RAU7
176146 229533.A0A098DVZ4 pp 229533.A0A098E2A4
176147 229533.A0A098DVZ4 pp 229533.Q4I1N3
176148 229533.A0A098DVZ4 pp 229533.I1RWE1
176149 229533.A0A098DVZ4 pp 229533.I1RSU2

176150	229533.A0A098DVZ4	pp	229533.I1RXY7
176151	229533.A0A098DVZ4	pp	229533.I1RPZ5
176152	229533.A0A098DVZ4	pp	229533.A0A1C3YIB0
176153	229533.A0A098DVZ4	pp	229533.I1S6S6
176154	229533.A0A098DVZ4	pp	229533.I1RVX0
176155	229533.A0A098DVZ4	pp	229533.I1RNM3
176156	229533.A0A098DVZ4	pp	229533.I1RIH0
176157	229533.A0A098DVZ4	pp	229533.I1RAN1
176158	229533.A0A098DVZ4	pp	229533.A0A1C3YMK4
176159	229533.A0A098DVZ4	pp	229533.I1S147
176160	229533.A0A098DVZ4	pp	229533.A0A1C3YMK7
176161	229533.A0A098DVZ4	pp	229533.I1RSA4
176162	229533.A0A098DVZ4	pp	229533.I1RCH5
176163	229533.A0A098DVZ4	pp	229533.A0A1C3YMW5
176164	229533.A0A098DVZ4	pp	229533.I1RND4
176165	229533.A0A098DVZ4	pp	229533.V6QVH8
176166	229533.A0A098DVZ4	pp	229533.I1RZQ1
176167	229533.A0A098DVZ4	pp	229533.I1S8R2
176168	229533.A0A098DVZ4	pp	229533.I1RPK0
176169	229533.A0A098DVZ4	pp	229533.I1S0S5
176170	229533.A0A098DVZ4	pp	229533.V6RLM7
176171	229533.A0A098DVZ4	pp	229533.A0A0E0SC46
176172	229533.A0A098DVZ4	pp	229533.I1RMQ5
176173	229533.A0A098DVZ4	pp	229533.A0A0E0RU58
176174	229533.A0A098DVZ4	pp	229533.A0A1C3YIF8
176175	229533.A0A098DVZ4	pp	229533.A0A1C3YJ04
176176	229533.A0A098DVZ4	pp	229533.A0A1C3YMS7
176177	229533.A0A098DVZ4	pp	229533.I1RBW1
176178	229533.A0A098DVZ4	pp	229533.I1S9C6
176179	229533.A0A098DVZ4	pp	229533.V6R042
176180	229533.A0A098DVZ4	pp	229533.A0A1C3YI53
176181	229533.A0A098DVZ4	pp	229533.I1RRT8
176182	229533.A0A098DVZ4	pp	229533.I1RHJ2
176183	229533.A0A098DVZ4	pp	229533.I1RTA0
176184	229533.A0A098DVZ4	pp	229533.I1RQI4
176185	229533.A0A098DVZ4	pp	229533.A0A0E0SK74
176186	229533.A0A098DVZ4	pp	229533.A0A0E0SAG8
176187	229533.A0A098DVZ4	pp	229533.I1RZ39
176188	229533.A0A098DVZ4	pp	229533.I1RDE7
176189	229533.A0A098DVZ4	pp	229533.A0A1C3YJI5
176190	229533.A0A098DVZ4	pp	229533.I1RZZ9
176191	229533.A0A098DVZ4	pp	229533.A0A1C3YML7
176192	229533.A0A098DVZ4	pp	229533.A0A1C3YIR8
176193	229533.A0A098DVZ4	pp	229533.A0A0E0S648
176194	229533.A0A098DVZ4	pp	229533.I1RVZ3
176195	229533.A0A098DVZ4	pp	229533.A0A0E0SH57
176196	229533.A0A098DVZ4	pp	229533.I1R9N7
176197	229533.A0A098DVZ4	pp	229533.I1RGA4
176198	229533.A0A098DVZ4	pp	229533.I1RBC7
176199	229533.A0A098DVZ4	pp	229533.V6RV57
176200	229533.A0A098DVZ4	pp	229533.A0A1C3YKL7
176201	229533.A0A098DVZ4	pp	229533.I1RJV4
176202	229533.A0A098DVZ4	pp	229533.I1RTD5
176203	229533.A0A098DVZ4	pp	229533.A0A0E0SPV0
176204	229533.A0A098DVZ4	pp	229533.I1RBY9
176205	229533.A0A098DVZ4	pp	229533.V6R3J1
176206	229533.A0A098DVZ4	pp	229533.I1RFI4
176207	229533.A0A098DVZ4	pp	229533.I1S1F3
176208	229533.A0A098DVZ4	pp	229533.I1RGC3
176209	229533.A0A098DVZ4	pp	229533.I1RUR8
176210	229533.A0A098DVZ4	pp	229533.I1RNG4
176211	229533.A0A098DVZ4	pp	229533.I1RZV5
176212	229533.A0A098DVZ4	pp	229533.I1RP28
176213	229533.A0A098DVZ4	pp	229533.I1RVY0
176214	229533.A0A098DVZ4	pp	229533.I1RC31
176215	229533.A0A098DVZ4	pp	229533.I1RE76
176216	229533.A0A098DVZ4	pp	229533.I1RP97
176217	229533.A0A098DVZ4	pp	229533.I1RMP2
176218	229533.A0A098DVZ4	pp	229533.I1RR68
176219	229533.A0A098DVZ4	pp	229533.I1RGZ3
176220	229533.A0A098DVZ4	pp	229533.I1S6P5
176221	229533.A0A098DVZ4	pp	229533.I1RPD6
176222	229533.A0A098DVZ4	pp	229533.A0A1C3YJ36

229533.A0A098DVZ4 pp 229533.A0A1C3YIE7
229533.A0A098DVZ4 pp 229533.I1RT04
229533.A0A098DVZ4 pp 229533.I1S299
229533.A0A098DVZ4 pp 229533.A0A1C3YL55
229533.A0A098DVZ4 pp 229533.Q4HXF0
229533.A0A098DVZ4 pp 229533.I1RFS3
229533.A0A098DVZ4 pp 229533.I1RBX1
229533.A0A098DVZ4 pp 229533.I1RL04
229533.A0A098DVZ4 pp 229533.I1RFT1
229533.A0A098DVZ4 pp 229533.A0A1C3YJP4
229533.A0A098DVZ4 pp 229533.I1RXX1
229533.A0A098DVZ4 pp 229533.A0A1C3YKF3
229533.A0A098DVZ4 pp 229533.A0A0E0SMT9
229533.A0A098DVZ4 pp 229533.I1RKI7
229533.A0A098DVZ4 pp 229533.I1RUY5
229533.A0A098DVZ4 pp 229533.I1S8P5
229533.A0A098DVZ4 pp 229533.I1RA94
229533.A0A098DVZ4 pp 229533.I1RY12
229533.A0A098DVZ4 pp 229533.I1RX72
229533.A0A098DVZ4 pp 229533.A0A098E4W0
229533.A0A098DVZ4 pp 229533.I1RCV1
229533.A0A098DVZ4 pp 229533.I1RCM5
229533.A0A098DVZ4 pp 229533.I1RRV4
229533.A0A098DVZ4 pp 229533.I1RN63
229533.A0A098DVZ4 pp 229533.I1RRU9
229533.A0A098DVZ4 pp 229533.A0A1C3YM97
229533.A0A098DVZ4 pp 229533.A0A1C3YL82
229533.A0A098DVZ4 pp 229533.I1RQA5
229533.A0A098DVZ4 pp 229533.A0A0E0SMG8
229533.A0A098DVZ4 pp 229533.A0A1C3YIF4
229533.A0A098DVZ4 pp 229533.A0A1C3YHQ3
229533.A0A098DVZ4 pp 229533.I1RH82
229533.A0A098DVZ4 pp 229533.I1REK8
229533.A0A098DVZ4 pp 229533.A0A0E0RQ72
229533.A0A098DVZ4 pp 229533.I1RBE4
229533.A0A098DVZ4 pp 229533.I1RPR1
229533.A0A098DVZ4 pp 229533.A0A1C3YLU7
229533.A0A098DVZ4 pp 229533.P0C431
229533.A0A098DVZ4 pp 229533.I1RKE1
229533.A0A098DVZ4 pp 229533.I1RPT0
229533.A0A098DVZ4 pp 229533.I1RQP5
229533.A0A098DVZ4 pp 229533.I1RCR5
229533.A0A098DVZ4 pp 229533.A0A1C3YM86
229533.A0A098DVZ4 pp 229533.V6RY56
229533.A0A098DVZ4 pp 229533.I1RWT6
229533.A0A098DVZ4 pp 229533.I1S5R5
229533.A0A098DVZ4 pp 229533.A0A1C3YIJ9
229533.A0A098DVZ4 pp 229533.I1RMG7
229533.A0A098DVZ4 pp 229533.A0A1C3YK58
229533.A0A098DVZ4 pp 229533.I1RXF0
229533.A0A098DVZ4 pp 229533.A0A1C3YJZ0
229533.A0A098DVZ4 pp 229533.I1RFJ1
229533.A0A098DVZ4 pp 229533.A0A1C3YKV3
229533.A0A098DVZ4 pp 229533.I1RR44
229533.A0A098DVZ4 pp 229533.I1RBC4
229533.A0A098DVZ4 pp 229533.I1RP72
229533.A0A098DVZ4 pp 229533.I1RGB2
229533.A0A098DVZ4 pp 229533.I1RXB6
229533.A0A098DVZ4 pp 229533.A0A1C3YJA9
229533.A0A098DVZ4 pp 229533.I1RP73
229533.A0A098DVZ4 pp 229533.I1RVP5
229533.A0A098DVZ4 pp 229533.A0A1C3YJR3
229533.A0A098DVZ4 pp 229533.I1RWM4
229533.A0A098DVZ4 pp 229533.I1RG14
229533.A0A098DVZ4 pp 229533.I1RA72
229533.A0A098DVZ4 pp 229533.A0A1C3YK11
229533.A0A098DVZ4 pp 229533.I1RAG5
229533.A0A098DVZ4 pp 229533.I1RQ12
229533.A0A098DVZ4 pp 229533.I1RMD3
229533.A0A098DVZ4 pp 229533.I1RJX2
229533.A0A098DVZ4 pp 229533.A0A1C3YLD3
229533.A0A098DVZ4 pp 229533.I1RBW0
229533.A0A098DVZ4 pp 229533.I1S1W8

229533.A0A098DVZ4 pp 229533.I1S0A0
229533.A0A098DVZ4 pp 229533.I1RMM8
229533.A0A098DVZ4 pp 229533.I1RDC9
229533.A0A098DVZ4 pp 229533.I1RJN7
229533.A0A098DVZ4 pp 229533.I1RZ94
229533.A0A098DVZ4 pp 229533.I1RMQ4
229533.A0A098DVZ4 pp 229533.I1RA32
229533.A0A098DVZ4 pp 229533.A0A0E0SAR4
229533.A0A098DVZ4 pp 229533.V6RK96
229533.A0A098DVZ4 pp 229533.I1RQE8
229533.A0A098DVZ4 pp 229533.I1RNM7
229533.A0A098DVZ4 pp 229533.A0A0E0RSD7
229533.A0A098DVZ4 pp 229533.I1RY49
229533.A0A098DVZ4 pp 229533.I1RKJ7
229533.A0A098DVZ4 pp 229533.I1RAM6
229533.A0A098DVZ4 pp 229533.Q4IR09
229533.A0A098DVZ4 pp 229533.I1RP86
229533.A0A098DVZ4 pp 229533.A0A1C3YN26
229533.A0A098DVZ4 pp 229533.A0A098DZN5
229533.A0A098DVZ4 pp 229533.A0A1C3YN58
229533.A0A098DVZ4 pp 229533.A0A0E0RU85
229533.A0A098DVZ4 pp 229533.A0A1C3YJL7
229533.A0A098DVZ4 pp 229533.A0A098E0Z2
229533.A0A098DVZ4 pp 229533.I1RYJ6
229533.A0A098DVZ4 pp 229533.I1RQH1
229533.A0A098DVZ4 pp 229533.I1RVV3
229533.A0A098DVZ4 pp 229533.A0A1C3YI55
229533.A0A098DVZ4 pp 229533.V6RTJ8
229533.A0A098DVZ4 pp 229533.I1RHF1
229533.A0A098DVZ4 pp 229533.I1RD52
229533.A0A098DVZ4 pp 229533.I1RQ14
229533.A0A098DVZ4 pp 229533.I1RZ16
229533.A0A098DVZ4 pp 229533.I1RYM6
229533.A0A098DVZ4 pp 229533.I1RQL4
229533.A0A098DVZ4 pp 229533.I1RPI5
229533.A0A098DVZ4 pp 229533.I1RYX6
229533.A0A098DVZ4 pp 229533.Q5I2J3
229533.A0A098DVZ4 pp 229533.I1S8C1
229533.A0A098DVZ4 pp 229533.A0A1C3YK13
229533.A0A098DVZ4 pp 229533.I1R9Z6
229533.A0A098DVZ4 pp 229533.I1RPN4
229533.A0A098DVZ4 pp 229533.I1RYL8
229533.A0A098DVZ4 pp 229533.I1RRX5
229533.A0A098DVZ4 pp 229533.I1RZM4
229533.A0A098DVZ4 pp 229533.A0A0E0RNI4
229533.A0A098DVZ4 pp 229533.I1RCU2
229533.A0A098DVZ4 pp 229533.I1RZT6
229533.A0A098DVZ4 pp 229533.Q4IJT5
229533.A0A098DVZ4 pp 229533.I1S4S5
229533.A0A098DVZ4 pp 229533.A0A1C3YMK3
229533.I1RW06 pp 229533.I1S334
229533.I1RW06 pp 229533.I1RZC4
229533.I1RW06 pp 229533.I1S3F7
229533.I1RW06 pp 229533.V6QVM5
229533.I1RW06 pp 229533.I1S693
229533.I1RW06 pp 229533.I1S8Q2
229533.I1RW06 pp 229533.I1RZ52
229533.I1RW06 pp 229533.I1RZW7
229533.I1RW06 pp 229533.I1S887
229533.I1RW06 pp 229533.I1RYX7
229533.I1RW06 pp 229533.Q4IMJ1
229533.I1RW06 pp 229533.I1RYU0
229533.I1RW06 pp 229533.V6RUT5
229533.I1RW06 pp 229533.I1S2M0
229533.I1RW06 pp 229533.O74638
229533.I1RW06 pp 229533.I1S250
229533.I1RW06 pp 229533.I1S2Z8
229533.I1RW06 pp 229533.I1RZZ1
229533.I1RW06 pp 229533.I1RWP4
229533.I1RW06 pp 229533.I1RYJ6
229533.I1RW06 pp 229533.I1S072
229533.I1RW06 pp 229533.I1S2P8
229533.I1RW06 pp 229533.I1RWS4

176369 229533.I1RW06 pp 229533.Q4I7X1
176370 229533.I1RW06 pp 229533.I1RX96
176371 229533.I1RW06 pp 229533.I1S4L8
176372 229533.I1RW06 pp 229533.I1S3H8
176373 229533.I1RW06 pp 229533.I1S746
176374 229533.I1RW06 pp 229533.Q4HXF0
176375 229533.I1RW06 pp 229533.I1RW26
176376 229533.I1RW06 pp 229533.I1RZN1
176377 229533.I1RW06 pp 229533.I1RXU9
176378 229533.I1RW06 pp 229533.I1S2C0
176379 229533.I1RW06 pp 229533.I1RXG6
176380 229533.I1RW06 pp 229533.I1S2A9
176381 229533.I1RW06 pp 229533.V6RLM7
176382 229533.I1RW06 pp 229533.I1RXR1
176383 229533.I1RW06 pp 229533.I1S0H7
176384 229533.I1RW06 pp 229533.V6QVH8
176385 229533.I1RW06 pp 229533.V6QT89
176386 229533.I1RW06 pp 229533.I1RWK4
176387 229533.I1RW06 pp 229533.I1S5X3
176388 229533.I1RW06 pp 229533.I1S1W3
176389 229533.I1RW06 pp 229533.Q4HY90
176390 229533.I1RW06 pp 229533.I1RZQ1
176391 229533.I1RSM8 pp 229533.I1RVJ8
176392 229533.I1RSM8 pp 229533.I1S272
176393 229533.I1RSM8 pp 229533.I1S2B9
176394 229533.I1RSM8 pp 229533.V6RPQ8
176395 229533.I1RSM8 pp 229533.I1RST0
176396 229533.I1RSM8 pp 229533.I1RZM0
176397 229533.I1RSM8 pp 229533.I1RW80
176398 229533.I1RSM8 pp 229533.I1SAM9
176399 229533.I1RSM8 pp 229533.I1RZN1
176400 229533.I1RSM8 pp 229533.I1RSV4
176401 229533.I1RSM8 pp 229533.I1RW91
176402 229533.I1RIH0 pp 229533.I1RNG4
176403 229533.I1RIH0 pp 229533.I1RKI7
176404 229533.I1RIH0 pp 229533.Q4HXF0
176405 229533.I1RIH0 pp 229533.I1RZ52
176406 229533.I1RIH0 pp 229533.I1RMG6
176407 229533.I1RIH0 pp 229533.Q4IMJ1
176408 229533.I1RIH0 pp 229533.I1RPD6
176409 229533.I1RIH0 pp 229533.I1RTI7
176410 229533.I1RIH0 pp 229533.I1RSL5
176411 229533.I1RIH0 pp 229533.V6QVM5
176412 229533.I1RIH0 pp 229533.V6RG79
176413 229533.I1RIH0 pp 229533.I1S3G4
176414 229533.I1RIH0 pp 229533.I1S0H7
176415 229533.I1RIH0 pp 229533.V6RLM7
176416 229533.I1RIH0 pp 229533.I1S1W3
176417 229533.I1RIH0 pp 229533.I1RXW3
176418 229533.I1RIH0 pp 229533.I1RXU9
176419 229533.I1RIH0 pp 229533.O74638
176420 229533.I1RIH0 pp 229533.I1RM82
176421 229533.I1RIH0 pp 229533.I1RQ30
176422 229533.I1RIH0 pp 229533.I1RPK0
176423 229533.I1RIH0 pp 229533.V6RSF3
176424 229533.I1RIH0 pp 229533.I1RZQ1
176425 229533.I1RIH0 pp 229533.I1RTG7
176426 229533.I1RIH0 pp 229533.I1RXR1
176427 229533.I1RIH0 pp 229533.I1RRI2
176428 229533.I1RIH0 pp 229533.I1RYI7
176429 229533.I1RIH0 pp 229533.I1RVY2
176430 229533.I1RIH0 pp 229533.I1RW06
176431 229533.I1RIH0 pp 229533.I1RJT2
176432 229533.I1RIH0 pp 229533.I1RXB1
176433 229533.I1RIH0 pp 229533.I1RKY6
176434 229533.I1RIH0 pp 229533.I1S1W8
176435 229533.I1RIH0 pp 229533.I1RXV9
176436 229533.I1RIH0 pp 229533.I1RPI5
176437 229533.I1RIH0 pp 229533.I1RMG7
176438 229533.I1RIH0 pp 229533.I1S338
176439 229533.I1RIH0 pp 229533.I1RKI3
176440 229533.I1RIH0 pp 229533.I1RZZ1
176441 229533.I1RIH0 pp 229533.I1S0R7

176442	229533.I1RIH0	pp	229533.I1RIY0
176443	229533.I1RIH0	pp	229533.I1RPW1
176444	229533.I1RIH0	pp	229533.I1RYJ7
176445	229533.I1RIH0	pp	229533.I1S887
176446	229533.I1RIH0	pp	229533.V6RUT5
176447	229533.V6RLM7	pp	229533.V6RUY9
176448	229533.V6RLM7	pp	229533.V6RV57
176449	229533.V6RLM7	pp	229533.V6RPQ8
176450	229533.V6RLM7	pp	229533.V6RTJ8
176451	229533.V6RLM7	pp	229533.V6RY56
176452	229533.V6RLM7	pp	229533.V6RUB1
176453	229533.V6RLM7	pp	229533.V6RMW6
176454	229533.V6RLM7	pp	229533.V6RZD4
176455	229533.V6RLM7	pp	229533.V6RQW4
176456	229533.V6RLM7	pp	229533.V6RRM0
176457	229533.I1RIB6	pp	229533.I1RPY0
176458	229533.I1RIB6	pp	229533.O74638
176459	229533.I1RIB6	pp	229533.I1RKF2
176460	229533.I1RIB6	pp	229533.I1RZ52
176461	229533.I1RIB6	pp	229533.I1RMG6
176462	229533.I1RIB6	pp	229533.I1RTI7
176463	229533.I1RIB6	pp	229533.I1RZ20
176464	229533.I1RIB6	pp	229533.I1RWF4
176465	229533.I1RIB6	pp	229533.I1RQ48
176466	229533.I1RIB6	pp	229533.I1RQ30
176467	229533.I1RIB6	pp	229533.Q4HXF0
176468	229533.I1RIB6	pp	229533.I1S9D4
176469	229533.I1RIB6	pp	229533.I1RYU5
176470	229533.I1RIB6	pp	229533.I1RMG7
176471	229533.I1RIB6	pp	229533.I1S2E2
176472	229533.I1RIB6	pp	229533.V6RQW4
176473	229533.I1RIB6	pp	229533.I1RIY0
176474	229533.I1RFI5	pp	229533.I1RQP8
176475	229533.I1RFI5	pp	229533.I1RKG3
176476	229533.I1RFI5	pp	229533.I1S9L5
176477	229533.I1RFI5	pp	229533.I1S9L6
176478	229533.I1RFI5	pp	229533.I1S6Y3
176479	229533.I1RFI5	pp	229533.I1RWQ8
176480	229533.I1RFI5	pp	229533.I1RKG2
176481	229533.I1RFI5	pp	229533.I1RRF7
176482	229533.I1RFI5	pp	229533.I1RW80
176483	229533.I1RFI5	pp	229533.I1RNC1
176484	229533.I1RFI5	pp	229533.I1RX04
176485	229533.I1RFI5	pp	229533.I1RXU0
176486	229533.I1RYK0	pp	229533.O74638
176487	229533.I1RYK0	pp	229533.Q4HXF0
176488	229533.I1RYK0	pp	229533.V6RV57
176489	229533.I1RYK0	pp	229533.I1RZV5
176490	229533.I1RYK0	pp	229533.I1S2H4
176491	229533.I1RYK0	pp	229533.I1S256
176492	229533.I1RYK0	pp	229533.I1S0V7
176493	229533.I1RYK0	pp	229533.I1S147
176494	229533.I1RYK0	pp	229533.I1S065
176495	229533.I1RYK0	pp	229533.V6RUY9
176496	229533.I1RYK0	pp	229533.I1S5X3
176497	229533.I1RYK0	pp	229533.I1S2B8
176498	229533.I1RYK0	pp	229533.V6QT89
176499	229533.I1RYK0	pp	229533.V6RG79
176500	229533.I1RYK0	pp	229533.I1RYS3
176501	229533.I1RYK0	pp	229533.I1S2A9
176502	229533.I1RYK0	pp	229533.I1S5C5
176503	229533.I1RYK0	pp	229533.V6RRM0
176504	229533.I1RYK0	pp	229533.I1S274
176505	229533.I1RYK0	pp	229533.V6RMW6
176506	229533.I1RYK0	pp	229533.I1S2C4
176507	229533.I1RYK0	pp	229533.I1S8C1
176508	229533.I1RYK0	pp	229533.Q4IR09
176509	229533.I1RYK0	pp	229533.V6RLM7
176510	229533.I1RYK0	pp	229533.V6RN16
176511	229533.I1RYK0	pp	229533.I1RYL8
176512	229533.I1RYK0	pp	229533.I1RZ16
176513	229533.I1RYK0	pp	229533.I1S8F8
176514	229533.I1RYK0	pp	229533.Q4IJK2

176515 229533.I1RYK0 pp 229533.I1RYU6
176516 229533.I1RYK0 pp 229533.V6RAN2
176517 229533.I1RYK0 pp 229533.I1S8S9
176518 229533.I1RYK0 pp 229533.I1S0V6
176519 229533.I1RYK0 pp 229533.I1S0N3
176520 229533.I1RCK7 pp 229533.I1RCN2
176521 229533.I1RCK7 pp 229533.I1RVY0
176522 229533.I1RCK7 pp 229533.I1RGW0
176523 229533.I1RCK7 pp 229533.I1RMA5
176524 229533.I1RCK7 pp 229533.I1RS43
176525 229533.I1RCK7 pp 229533.I1RKR6
176526 229533.I1RCK7 pp 229533.V6QV38
176527 229533.I1RCK7 pp 229533.I1RQM2
176528 229533.I1RCK7 pp 229533.I1RJW8
176529 229533.I1RCK7 pp 229533.I1RYR9
176530 229533.I1RCK7 pp 229533.I1RXE5
176531 229533.I1RCK7 pp 229533.I1RDC9
176532 229533.A0A0E0RWW0 pp 229533.I1RD22
176533 229533.A0A0E0RWW0 pp 229533.V6QUE6
176534 229533.A0A0E0RWW0 pp 229533.I1S5B0
176535 229533.A0A0E0RWW0 pp 229533.I1RG11
176536 229533.A0A0E0RWW0 pp 229533.I1RYB9
176537 229533.A0A0E0RWW0 pp 229533.I1RNC1
176538 229533.A0A0E0RWW0 pp 229533.I1RUG3
176539 229533.A0A0E0RWW0 pp 229533.I1RXF0
176540 229533.A0A0E0RWW0 pp 229533.A0A1C3YN57
176541 229533.A0A0E0RWW0 pp 229533.A0A0E0SID1
176542 229533.A0A0E0RWW0 pp 229533.A0A1C3YJR3
176543 229533.A0A0E0RWW0 pp 229533.I1RJ17
176544 229533.A0A0E0RWW0 pp 229533.I1RSQ3
176545 229533.A0A0E0RWW0 pp 229533.I1S0J1
176546 229533.A0A0E0RWW0 pp 229533.I1RS18
176547 229533.A0A0E0RWW0 pp 229533.A0A1C3YJI6
176548 229533.A0A0E0RWW0 pp 229533.I1RT40
176549 229533.A0A0E0RWW0 pp 229533.V6R042
176550 229533.A0A0E0RWW0 pp 229533.I1RXD7
176551 229533.A0A0E0RWW0 pp 229533.A0A0E0SFN0
176552 229533.A0A0E0RWW0 pp 229533.Q4HXF0
176553 229533.A0A0E0RWW0 pp 229533.V6RCZ8
176554 229533.A0A0E0RWW0 pp 229533.A0A1C3YKV3
176555 229533.A0A0E0RWW0 pp 229533.I1RBZ3
176556 229533.A0A0E0RWW0 pp 229533.I1RK83
176557 229533.A0A0E0RWW0 pp 229533.I1RJV3
176558 229533.A0A0E0RWW0 pp 229533.I1RC05
176559 229533.A0A0E0RWW0 pp 229533.I1RA70
176560 229533.A0A0E0RWW0 pp 229533.I1RN67
176561 229533.A0A0E0RWW0 pp 229533.A0A1C3YIP5
176562 229533.A0A0E0RWW0 pp 229533.I1RD52
176563 229533.A0A0E0RWW0 pp 229533.I1RC81
176564 229533.A0A0E0RWW0 pp 229533.I1RAW0
176565 229533.A0A0E0RWW0 pp 229533.I1S6E5
176566 229533.A0A0E0RWW0 pp 229533.I1RBL7
176567 229533.A0A0E0RWW0 pp 229533.A0A0E0S6Y2
176568 229533.A0A0E0RWW0 pp 229533.I1RRI2
176569 229533.A0A0E0RWW0 pp 229533.I1RT33
176570 229533.A0A0E0RWW0 pp 229533.I1REL7
176571 229533.A0A0E0RWW0 pp 229533.A0A1C3YI50
176572 229533.A0A0E0RWW0 pp 229533.I1RPP3
176573 229533.A0A0E0RWW0 pp 229533.I1RCW6
176574 229533.A0A0E0RWW0 pp 229533.I1RCR5
176575 229533.A0A0E0RWW0 pp 229533.I1RYW3
176576 229533.A0A0E0RWW0 pp 229533.I1RCH5
176577 229533.A0A0E0RWW0 pp 229533.I1RSQ7
176578 229533.A0A0E0RWW0 pp 229533.A0A1C3YK13
176579 229533.A0A0E0RWW0 pp 229533.I1RQE7
176580 229533.A0A0E0RWW0 pp 229533.I1S2H4
176581 229533.A0A0E0RWW0 pp 229533.I1RFT1
176582 229533.A0A0E0RWW0 pp 229533.I1RFL4
176583 229533.A0A0E0RWW0 pp 229533.I1S5R1
176584 229533.A0A0E0RWW0 pp 229533.I1RRT8
176585 229533.A0A0E0RWW0 pp 229533.A0A1C3YI20
176586 229533.A0A0E0RWW0 pp 229533.I1RBT1
176587 229533.A0A0E0RWW0 pp 229533.I1RZZ9

229533.A0A0E0RWW0	pp	229533.Q4IJK2
229533.A0A0E0RWW0	pp	229533.I1RK95
229533.A0A0E0RWW0	pp	229533.I1RSQ4
229533.A0A0E0RWW0	pp	229533.O74638
229533.A0A0E0RWW0	pp	229533.I1RVX0
229533.A0A0E0RWW0	pp	229533.I1RM67
229533.A0A0E0RWW0	pp	229533.A0A1C3YIR8
229533.A0A0E0RWW0	pp	229533.I1RZ39
229533.A0A0E0RWW0	pp	229533.I1RSS4
229533.A0A0E0RWW0	pp	229533.A0A1C3YKM9
229533.A0A0E0RWW0	pp	229533.A0A1C3YHV8
229533.A0A0E0RWW0	pp	229533.I1RBY4
229533.A0A0E0RWW0	pp	229533.I1S9Y1
229533.A0A0E0RWW0	pp	229533.I1RX46
229533.A0A0E0RWW0	pp	229533.I1S4S4
229533.A0A0E0RWW0	pp	229533.I1R9M0
229533.A0A0E0RWW0	pp	229533.I1RBC7
229533.A0A0E0RWW0	pp	229533.I1RIY2
229533.A0A0E0RWW0	pp	229533.I1RGA4
229533.A0A0E0RWW0	pp	229533.I1R9Y2
229533.A0A0E0RWW0	pp	229533.A0A0E0SC46
229533.A0A0E0RWW0	pp	229533.I1RL04
229533.A0A0E0RWW0	pp	229533.I1RFS3
229533.A0A0E0RWW0	pp	229533.I1RSZ7
229533.A0A0E0RWW0	pp	229533.I1RCE6
229533.I1RND4	pp	229533.I1RWF5
229533.I1RND4	pp	229533.I1RQI4
229533.I1RND4	pp	229533.I1RZN1
229533.I1RND4	pp	229533.I1RYI5
229533.I1RND4	pp	229533.V6RAG6
229533.I1RND4	pp	229533.I1S5R6
229533.I1RND4	pp	229533.V6RLM7
229533.I1RND4	pp	229533.I1RYK0
229533.I1RND4	pp	229533.O74638
229533.I1RND4	pp	229533.I1RRJ3
229533.I1RND4	pp	229533.V6RK40
229533.I1RND4	pp	229533.I1RTG7
229533.I1RND4	pp	229533.I1RSZ7
229533.I1RND4	pp	229533.I1RPD6
229533.I1RND4	pp	229533.I1RW64
229533.I1RND4	pp	229533.I1S2A9
229533.I1RND4	pp	229533.I1S5X3
229533.I1RND4	pp	229533.I1RP72
229533.I1RND4	pp	229533.V6RAN2
229533.I1RND4	pp	229533.I1RR12
229533.I1RND4	pp	229533.I1RRX5
229533.I1RND4	pp	229533.I1RST9
229533.I1RND4	pp	229533.I1RY49
229533.I1RND4	pp	229533.I1S130
229533.I1RND4	pp	229533.I1RNU7
229533.I1RND4	pp	229533.I1RP73
229533.I1RND4	pp	229533.I1RRD0
229533.I1RND4	pp	229533.I1RUY6
229533.I1RND4	pp	229533.I1RR44
229533.I1RND4	pp	229533.I1RP86
229533.I1RND4	pp	229533.V6RW66
229533.I1RND4	pp	229533.V6RJ29
229533.I1RND4	pp	229533.I1RYZ2
229533.I1RND4	pp	229533.I1RPT0
229533.I1RND4	pp	229533.V6QU63
229533.I1RND4	pp	229533.I1RUY4
229533.I1RND4	pp	229533.I1RY12
229533.I1RND4	pp	229533.I1S3D8
229533.I1RGA6	pp	229533.V6RBN2
229533.I1RGA6	pp	229533.I1RPA4
229533.I1RGA6	pp	229533.I1RM35
229533.I1RGA6	pp	229533.I1S4Q4
229533.I1RGA6	pp	229533.I1RJU3
229533.I1RGA6	pp	229533.I1RPN4
229533.I1RGA6	pp	229533.V6R1K4
229533.I1RGA6	pp	229533.O74638
229533.I1RGA6	pp	229533.V6RTJ8
229533.I1RGA6	pp	229533.I1RVX0

176661 229533.I1RGA6 pp 229533.I1RPP3
176662 229533.I1RGA6 pp 229533.I1RS17
176663 229533.I1RGA6 pp 229533.V6R057
176664 229533.I1RGA6 pp 229533.I1S8P5
176665 229533.I1RGA6 pp 229533.I1RTA0
176666 229533.I1RGA6 pp 229533.I1RWE8
176667 229533.I1RGA6 pp 229533.I1RTA1
176668 229533.I1RGA6 pp 229533.Q4HXF0
176669 229533.I1RGA6 pp 229533.I1RN67
176670 229533.I1RGA6 pp 229533.I1RL04
176671 229533.I1RGA6 pp 229533.I1RZB2
176672 229533.I1RGA6 pp 229533.V6RMW6
176673 229533.I1RGA6 pp 229533.I1S2B8
176674 229533.I1RGA6 pp 229533.I1RWI5
176675 229533.I1RGA6 pp 229533.I1S277
176676 229533.I1RFV9 pp 229533.I1RNG4
176677 229533.I1RFV9 pp 229533.I1RPD6
176678 229533.I1RFV9 pp 229533.I1RUV4
176679 229533.I1RFV9 pp 229533.I1RXY7
176680 229533.I1RFV9 pp 229533.I1RJF2
176681 229533.I1RFV9 pp 229533.I1RPE7
176682 229533.I1RFV9 pp 229533.I1RXG6
176683 229533.I1RFV9 pp 229533.I1RMH9
176684 229533.I1RFV9 pp 229533.I1S5X3
176685 229533.I1RFV9 pp 229533.I1RHF1
176686 229533.I1RFV9 pp 229533.I1RP73
176687 229533.I1RFV9 pp 229533.I1RIJ5
176688 229533.I1RFV9 pp 229533.I1S2A9
176689 229533.I1RFV9 pp 229533.I1RVZ3
176690 229533.I1RFV9 pp 229533.I1RP72
176691 229533.I1RFV9 pp 229533.I1RW06
176692 229533.I1RFV9 pp 229533.I1RMJ4
176693 229533.I1RFV9 pp 229533.I1RHH5
176694 229533.I1RFV9 pp 229533.I1S9T8
176695 229533.I1RFV9 pp 229533.I1RTU0
176696 229533.I1RFV9 pp 229533.Q4IPH4
176697 229533.I1RFV9 pp 229533.I1S3X7
176698 229533.I1RFV9 pp 229533.I1S0K2
176699 229533.I1RFV9 pp 229533.I1S3G4
176700 229533.I1RFV9 pp 229533.I1S1W8
176701 229533.I1RFV9 pp 229533.I1RSK5
176702 229533.I1RFV9 pp 229533.I1RY74
176703 229533.I1RFV9 pp 229533.I1RK01
176704 229533.I1RFV9 pp 229533.V6QWD8
176705 229533.I1RS40 pp 229533.V6RQW4
176706 229533.I1RS40 pp 229533.I1RTJ2
176707 229533.I1RS40 pp 229533.I1S2E2
176708 229533.I1RS40 pp 229533.I1RYU5
176709 229533.I1RS40 pp 229533.I1RZ20
176710 229533.I1RS40 pp 229533.I1S9D4
176711 229533.I1RS40 pp 229533.I1RWF4
176712 229533.I1RS40 pp 229533.Q4HXF0
176713 229533.I1RS40 pp 229533.I1RYA0
176714 229533.I1RS40 pp 229533.I1RYC8
176715 229533.I1RS40 pp 229533.I1RTI7
176716 229533.I1RS40 pp 229533.O74638
176717 229533.I1RS40 pp 229533.I1RZ52
176718 229533.I1S9T8 pp 229533.Q4IMN9
176719 229533.I1S0K1 pp 229533.I1S5D0
176720 229533.I1S0K1 pp 229533.I1S6H3
176721 229533.I1S0K1 pp 229533.I1SAT2
176722 229533.I1S0K1 pp 229533.I1S267
176723 229533.I1S0K1 pp 229533.I1S8H5
176724 229533.I1S0K1 pp 229533.I1S659
176725 229533.I1S0K1 pp 229533.I1SAH9
176726 229533.I1S0K1 pp 229533.I1S1T5
176727 229533.V6RRH2 pp 229533.V6RTJ8
176728 229533.A0A1C3YJI4 pp 229533.I1RSK5
176729 229533.A0A1C3YJI4 pp 229533.I1RY49
176730 229533.A0A1C3YJI4 pp 229533.I1RW43
176731 229533.A0A1C3YJI4 pp 229533.A0A1C3YJW4
176732 229533.A0A1C3YJI4 pp 229533.I1S2A9
176733 229533.A0A1C3YJI4 pp 229533.I1S3G4

229533.A0A1C3YJI4 pp 229533.I1RAG5
229533.A0A1C3YJI4 pp 229533.I1RMR3
229533.A0A1C3YJI4 pp 229533.V6RLM7
229533.A0A1C3YJI4 pp 229533.I1RP73
229533.A0A1C3YJI4 pp 229533.I1RQ81
229533.A0A1C3YJI4 pp 229533.I1RC05
229533.A0A1C3YJI4 pp 229533.I1S5X3
229533.A0A1C3YJI4 pp 229533.I1RNG4
229533.A0A1C3YJI4 pp 229533.I1RHF1
229533.A0A1C3YJI4 pp 229533.A0A1C3YMK3
229533.A0A1C3YJI4 pp 229533.I1RXY7
229533.A0A1C3YJI4 pp 229533.A0A1C3YM63
229533.A0A1C3YJI4 pp 229533.I1RP72
229533.A0A1C3YJI4 pp 229533.I1RC61
229533.A0A1C3YJI4 pp 229533.I1RN61
229533.A0A1C3YJI4 pp 229533.A0A1C3YKZ4
229533.A0A1C3YJI4 pp 229533.I1RVF2
229533.A0A1C3YJI4 pp 229533.I1RFV9
229533.A0A1C3YJI4 pp 229533.I1R9Z6
229533.A0A1C3YJI4 pp 229533.I1RRV1
229533.A0A1C3YJI4 pp 229533.I1RC91
229533.A0A1C3YJI4 pp 229533.I1RK03
229533.A0A1C3YJI4 pp 229533.I1RA94
229533.A0A1C3YJI4 pp 229533.A0A1C3YLI1
229533.A0A1C3YJI4 pp 229533.I1RMM6
229533.A0A1C3YJI4 pp 229533.A0A1C3YMD6
229533.A0A1C3YJI4 pp 229533.I1RMF1
229533.A0A1C3YJI4 pp 229533.A0A1C3YLL4
229533.A0A1C3YJI4 pp 229533.I1S0I3
229533.A0A1C3YJI4 pp 229533.I1S4I8
229533.A0A1C3YJI4 pp 229533.I1RY74
229533.A0A1C3YJI4 pp 229533.V6QVH8
229533.A0A1C3YJI4 pp 229533.I1S1W8
229533.A0A1C3YJI4 pp 229533.I1RW06
229533.A0A1C3YJI4 pp 229533.I1S9T8
229533.A0A1C3YJI4 pp 229533.I1RAQ4
229533.A0A1C3YJI4 pp 229533.A0A1C3YML7
229533.A0A1C3YJI4 pp 229533.I1S4I7
229533.A0A1C3YJI4 pp 229533.I1RIH0
229533.A0A1C3YJI4 pp 229533.I1S000
229533.I1RH75 pp 229533.I1RHJ2
229533.I1RH75 pp 229533.I1RR61
229533.I1RH75 pp 229533.I1RW80
229533.I1RH75 pp 229533.I1RZN1
229533.I1RH75 pp 229533.I1S2B9
229533.I1RH75 pp 229533.I1RRN4
229533.I1RH75 pp 229533.I1RRC4
229533.I1RH75 pp 229533.I1RQQ0
229533.I1RH75 pp 229533.I1RRF7
229533.I1RH75 pp 229533.I1RQ42
229533.I1RH75 pp 229533.I1RN04
229533.I1RH75 pp 229533.I1RMG4
229533.I1RH75 pp 229533.I1RW91
229533.I1RH75 pp 229533.I1RKF3
229533.I1RH75 pp 229533.I1RTP5
229533.I1RH75 pp 229533.I1RYQ4
229533.I1RH75 pp 229533.I1RYX5
229533.I1RH75 pp 229533.I1RPW8
229533.I1RH75 pp 229533.I1RQM9
229533.I1RH75 pp 229533.I1RNJ7
229533.I1RH75 pp 229533.I1RTJ2
229533.I1RH75 pp 229533.I1S5Z5
229533.A0A098CZS6 pp 229533.A0A098DVZ4
229533.A0A098CZS6 pp 229533.I1RW06
229533.A0A098CZS6 pp 229533.V6RLM7
229533.A0A098CZS6 pp 229533.I1RYK0
229533.A0A098CZS6 pp 229533.I1RND4
229533.A0A098CZS6 pp 229533.I1RFV9
229533.A0A098CZS6 pp 229533.I1S9T8
229533.A0A098CZS6 pp 229533.A0A1C3YJI4
229533.I1RSQ4 pp 229533.I1S0B7
229533.I1RSQ4 pp 229533.I1RW54
229533.I1RSQ4 pp 229533.I1S8Z2

176807 229533.I1RSQ4 pp 229533.I1S1W8
176808 229533.I1RSQ4 pp 229533.I1S039
176809 229533.I1RSQ4 pp 229533.V6RTJ8
176810 229533.I1RSQ4 pp 229533.I1S4S4
176811 229533.I1RSQ4 pp 229533.I1S0T2
176812 229533.I1RSQ4 pp 229533.I1RX46
176813 229533.I1RSQ4 pp 229533.I1S2C1
176814 229533.I1RSQ4 pp 229533.V6RQW4
176815 229533.I1RSQ4 pp 229533.I1RY21
176816 229533.I1RSQ4 pp 229533.I1RUQ3
176817 229533.I1RSQ4 pp 229533.I1RT33
176818 229533.I1RSQ4 pp 229533.I1S4Q4
176819 229533.I1RSQ4 pp 229533.I1RXD7
176820 229533.I1RSQ4 pp 229533.V6R1K4
176821 229533.I1RSQ4 pp 229533.I1RYJ6
176822 229533.I1RSQ4 pp 229533.Q4IJK2
176823 229533.I1RSQ4 pp 229533.V6RUT5
176824 229533.I1RSQ4 pp 229533.I1S0P0
176825 229533.I1RSQ4 pp 229533.I1S0K2
176826 229533.I1RSQ4 pp 229533.I1RXD6
176827 229533.I1RSQ4 pp 229533.V6RS91
176828 229533.I1RSQ4 pp 229533.I1S4S7
176829 229533.I1RSQ4 pp 229533.I1RSQ7
176830 229533.I1RSQ4 pp 229533.I1RYU6
176831 229533.I1RSQ4 pp 229533.V6RBN2
176832 229533.I1RSQ4 pp 229533.I1S217
176833 229533.I1RSQ4 pp 229533.I1S887
176834 229533.I1RSQ4 pp 229533.I1S9G7
176835 229533.I1RSQ4 pp 229533.Q4I5G1
176836 229533.I1RSQ4 pp 229533.I1RWR7
176837 229533.I1RSQ4 pp 229533.I1S0V6
176838 229533.I1RSQ4 pp 229533.I1RY12
176839 229533.I1RSQ4 pp 229533.I1RZK5
176840 229533.I1RSQ4 pp 229533.I1RUY4
176841 229533.I1RSQ4 pp 229533.I1S835
176842 229533.I1RSQ4 pp 229533.I1RWB0
176843 229533.I1RSQ4 pp 229533.V6RJP8
176844 229533.I1RSQ4 pp 229533.I1S8S9
176845 229533.I1RSQ4 pp 229533.I1RUY5
176846 229533.I1RSQ4 pp 229533.I1S5B0
176847 229533.I1RSQ4 pp 229533.I1RWP4
176848 229533.I1RSQ4 pp 229533.I1RYI7
176849 229533.I1RSQ4 pp 229533.I1S1J7
176850 229533.I1RSQ4 pp 229533.V6QUE6
176851 229533.I1RSQ4 pp 229533.I1S3K6
176852 229533.I1RSQ4 pp 229533.I1S4Z8
176853 229533.I1RSQ4 pp 229533.I1S8F8
176854 229533.I1RSQ4 pp 229533.I1S2B8
176855 229533.I1RSQ4 pp 229533.I1S419
176856 229533.I1RSQ4 pp 229533.V6RCZ8
176857 229533.I1RSQ4 pp 229533.I1RUR8
176858 229533.I1RSQ4 pp 229533.I1RTD3
176859 229533.I1RSQ4 pp 229533.I1RVR0
176860 229533.I1RSQ4 pp 229533.I1RTN3
176861 229533.I1RSQ4 pp 229533.I1RXC4
176862 229533.I1RSQ4 pp 229533.I1S6K9
176863 229533.I1RSQ4 pp 229533.I1RSZ7
176864 229533.I1RSQ4 pp 229533.I1RZM8
176865 229533.I1RSQ4 pp 229533.I1S9Y1
176866 229533.I1RSQ4 pp 229533.I1RW61
176867 229533.I1RSQ4 pp 229533.Q4I099
176868 229533.I1RSQ4 pp 229533.I1RXF0
176869 229533.I1RSQ4 pp 229533.I1S6Y8
176870 229533.I1RSQ4 pp 229533.I1RXC2
176871 229533.I1RSQ4 pp 229533.I1S277
176872 229533.I1RSQ4 pp 229533.I1S2H4
176873 229533.I1RSQ4 pp 229533.I1RZ52
176874 229533.I1RSQ4 pp 229533.O74638
176875 229533.I1RSQ4 pp 229533.I1RY57
176876 229533.I1RSQ4 pp 229533.I1RSX0
176877 229533.I1RSQ4 pp 229533.I1RY92
176878 229533.I1RSQ4 pp 229533.I1S5R1
176879 229533.I1RSQ4 pp 229533.I1S3G4

176880	229533.I1RSQ4	pp	229533.Q4HXF0
176881	229533.I1RSQ4	pp	229533.I1S3T7
176882	229533.I1RSQ4	pp	229533.V6RUY9
176883	229533.I1RSQ4	pp	229533.I1S0L4
176884	229533.I1RSQ4	pp	229533.I1RW43
176885	229533.I1RSQ4	pp	229533.I1RYP2
176886	229533.I1RSQ4	pp	229533.I1RT40
176887	229533.I1RSQ4	pp	229533.I1RZ39
176888	229533.I1RSQ4	pp	229533.I1RZZ9
176889	229533.I1RSQ4	pp	229533.I1RXF8
176890	229533.I1RSQ4	pp	229533.I1RTD5
176891	229533.I1RSQ4	pp	229533.I1RWI5
176892	229533.I1RSQ4	pp	229533.I1S8P5
176893	229533.I1RSQ4	pp	229533.I1RYK0
176894	229533.I1RSQ4	pp	229533.I1RWF5
176895	229533.I1RSQ4	pp	229533.I1RT38
176896	229533.I1RSQ4	pp	229533.I1RYI5
176897	229533.I1RSQ4	pp	229533.I1RVN1
176898	229533.I1RSQ4	pp	229533.Q4I624
176899	229533.I1RSQ4	pp	229533.V6R057
176900	229533.I1RSQ4	pp	229533.I1S1F3
176901	229533.I1RSQ4	pp	229533.I1RYW9
176902	229533.I1RSQ4	pp	229533.V6RRH2
176903	229533.I1RSQ4	pp	229533.V6RG79
176904	229533.I1RSQ4	pp	229533.I1RVZ2
176905	229533.I1RSQ4	pp	229533.V6R949
176906	229533.I1RSQ4	pp	229533.I1RWS4
176907	229533.I1RSQ4	pp	229533.I1RY62
176908	229533.I1RSQ4	pp	229533.I1RZD3
176909	229533.I1RSQ4	pp	229533.I1RZN1
176910	229533.I1RSQ4	pp	229533.I1S1C7
176911	229533.I1RSQ4	pp	229533.I1RTA1
176912	229533.I1RSQ4	pp	229533.I1RXR1
176913	229533.I1RSQ4	pp	229533.I1RZB2
176914	229533.I1RSQ4	pp	229533.Q4I1N3
176915	229533.I1RSQ4	pp	229533.I1RYW3
176916	229533.I1RSQ4	pp	229533.I1RSS4
176917	229533.I1RSQ4	pp	229533.V6QT89
176918	229533.I1RSQ4	pp	229533.I1SAJ3
176919	229533.I1RSQ4	pp	229533.Q4HVQ9
176920	229533.I1RSQ4	pp	229533.V6R3J1
176921	229533.I1RSQ4	pp	229533.I1RT03
176922	229533.I1RSQ4	pp	229533.I1RVX0
176923	229533.I1RSQ4	pp	229533.I1RYA0
176924	229533.I1RSQ4	pp	229533.V6R2A1
176925	229533.I1RSQ4	pp	229533.I1S1W9
176926	229533.I1RSQ4	pp	229533.I1RYC8
176927	229533.I1RSQ4	pp	229533.I1RVZ7
176928	229533.I1RSQ4	pp	229533.I1S5R6
176929	229533.I1RSQ4	pp	229533.I1RXU9
176930	229533.I1RSQ4	pp	229533.V6RRM0
176931	229533.I1RSQ4	pp	229533.Q4I5V2
176932	229533.I1RSQ4	pp	229533.I1RSU2
176933	229533.I1RSQ4	pp	229533.I1S0R9
176934	229533.I1RSQ4	pp	229533.I1RZV3
176935	229533.I1RSQ4	pp	229533.I1S0V7
176936	229533.I1RSQ4	pp	229533.I1S147
176937	229533.I1RSQ4	pp	229533.I1S256
176938	229533.I1RSQ4	pp	229533.I1S1W3
176939	229533.I1RSQ4	pp	229533.I1RUP2
176940	229533.I1RSQ4	pp	229533.V6RLM7
176941	229533.I1RSQ4	pp	229533.I1RTA0
176942	229533.I1RSQ4	pp	229533.I1RZ95
176943	229533.I1RSQ4	pp	229533.V6R1X4
176944	229533.I1RSQ4	pp	229533.V6R042
176945	229533.I1RSQ4	pp	229533.V6RMW6
176946	229533.I1RSQ4	pp	229533.I1S0J1
176947	229533.I1RSQ4	pp	229533.I1RUG3
176948	229533.I1RSQ4	pp	229533.I1S7W5
176949	229533.A0A0E0SPA8	pp	229533.I1RNG4
176950	229533.A0A0E0SPA8	pp	229533.I1RPD6
176951	229533.A0A1C3YJ16	pp	229533.I1RQL4
176952	229533.A0A1C3YJ16	pp	229533.I1S8K7

176953	229533.A0A1C3YJ16	pp	229533.I1RF62
176954	229533.A0A1C3YJ16	pp	229533.I1S1J9
176955	229533.A0A1C3YJ16	pp	229533.I1RMP2
176956	229533.A0A1C3YJ16	pp	229533.I1RE19
176957	229533.A0A1C3YJ16	pp	229533.I1RZ28
176958	229533.A0A1C3YJ16	pp	229533.I1RMG9
176959	229533.A0A1C3YJ16	pp	229533.I1RF64
176960	229533.A0A1C3YJ16	pp	229533.I1RYV4
176961	229533.I1RVK4	pp	229533.I1RY11
176962	229533.I1RMH0	pp	229533.I1RQL4
176963	229533.I1RMH0	pp	229533.I1RYV4
176964	229533.I1RMH0	pp	229533.I1S1J9
176965	229533.I1RMH0	pp	229533.I1RMP2
176966	229533.I1RMH0	pp	229533.I1RZ28
176967	229533.I1RMH0	pp	229533.I1S8K7
176968	229533.A0A0E0RZF3	pp	229533.A0A1C3YLB6
176969	229533.A0A0E0RZF3	pp	229533.I1RE52
176970	229533.A0A0E0RZF3	pp	229533.I1RIV2
176971	229533.A0A0E0RZF3	pp	229533.I1RZ56
176972	229533.A0A0E0RZF3	pp	229533.I1RPV8
176973	229533.A0A0E0RZF3	pp	229533.I1RH83
176974	229533.A0A0E0RZF3	pp	229533.I1RHU8
176975	229533.A0A0E0RZF3	pp	229533.I1RKR8
176976	229533.A0A0E0RZF3	pp	229533.I1RJS1
176977	229533.A0A0E0RZF3	pp	229533.I1SAT2
176978	229533.A0A0E0RZF3	pp	229533.I1S8H5
176979	229533.A0A0E0RZF3	pp	229533.I1RF85
176980	229533.A0A0E0RZF3	pp	229533.I1S1T5
176981	229533.A0A0E0RZF3	pp	229533.I1RRF7
176982	229533.A0A0E0RZF3	pp	229533.I1RNG4
176983	229533.A0A0E0RZF3	pp	229533.I1RN04
176984	229533.A0A0E0RZF3	pp	229533.I1RW82
176985	229533.A0A0E0RZF3	pp	229533.I1RW80
176986	229533.A0A0E0RZF3	pp	229533.A0A1C3YHG7
176987	229533.A0A0E0RZF3	pp	229533.I1RF49
176988	229533.A0A0E0RZF3	pp	229533.I1S0A5
176989	229533.A0A0E0RZF3	pp	229533.A0A1C3YMP1
176990	229533.A0A0E0RZF3	pp	229533.I1RCG6
176991	229533.A0A0E0RZF3	pp	229533.I1RA13
176992	229533.A0A0E0RZF3	pp	229533.I1RST9
176993	229533.A0A0E0RZF3	pp	229533.I1S267
176994	229533.A0A0E0RZF3	pp	229533.I1RIB5
176995	229533.A0A0E0RZF3	pp	229533.I1S659
176996	229533.A0A0E0RZF3	pp	229533.I1RW91
176997	229533.A0A0E0RZF3	pp	229533.I1RCU2
176998	229533.A0A0E0RZF3	pp	229533.A0A1C3YHX6
176999	229533.A0A0E0RZF3	pp	229533.I1RRS6
177000	229533.A0A0E0RZF3	pp	229533.I1R9F2
177001	229533.A0A0E0RZF3	pp	229533.I1RPW8
177002	229533.A0A0E0RZF3	pp	229533.I1S094
177003	229533.A0A0E0RZF3	pp	229533.I1S5D0
177004	229533.A0A0E0RZF3	pp	229533.I1RCU7
177005	229533.A0A0E0RZF3	pp	229533.I1S6H3
177006	229533.A0A0E0RZF3	pp	229533.I1RQV7
177007	229533.A0A0E0RZF3	pp	229533.I1RDN3
177008	229533.A0A098CZG8	pp	229533.A0A1C3YJ16
177009	229533.A0A098CZG8	pp	229533.I1RMH0
177010	229533.I1RY65	pp	229533.I1SAS4
177011	229533.I1RY65	pp	229533.I1S1Q3
177012	229533.I1RY65	pp	229533.I1SAS3
177013	229533.I1RY65	pp	229533.V6RR38
177014	229533.I1RY65	pp	229533.I1RZZ3
177015	229533.I1RY65	pp	229533.V6RPN0
177016	229533.I1RPA6	pp	229533.I1RQB4
177017	229533.I1RPA6	pp	229533.I1RSI3
177018	229533.I1RPA6	pp	229533.V6RVG3
177019	229533.I1RPA6	pp	229533.V6QX58
177020	229533.I1RPA6	pp	229533.I1S1W4
177021	229533.I1RPA6	pp	229533.V6RR76
177022	229533.I1RPA6	pp	229533.I1S2B6
177023	229533.I1RPA6	pp	229533.I1RZK4
177024	229533.I1RPA6	pp	229533.I1RSS1
177025	229533.I1RPA6	pp	229533.I1S4I7

177026 229533.I1RPA6 pp 229533.I1S296
177027 229533.I1RPA6 pp 229533.I1S2K7
177028 229533.I1RPA6 pp 229533.I1RQ72
177029 229533.I1RPA6 pp 229533.I1S2D9
177030 229533.I1RPA6 pp 229533.I1RX20
177031 229533.I1RPA6 pp 229533.I1RV66
177032 229533.I1RPA6 pp 229533.I1RY57
177033 229533.I1RPA6 pp 229533.I1RQD4
177034 229533.I1RPA6 pp 229533.I1S2C1
177035 229533.I1RPA6 pp 229533.I1RPH1
177036 229533.I1RPA6 pp 229533.I1RX77
177037 229533.I1RPA6 pp 229533.I1S0X7
177038 229533.I1RPA6 pp 229533.I1RXH6
177039 229533.I1RPA6 pp 229533.I1S7X9
177040 229533.I1RPA6 pp 229533.I1RUN8
177041 229533.A0A0E0S8Z0 pp 229533.I1RY92
177042 229533.A0A0E0S8Z0 pp 229533.I1RB64
177043 229533.A0A0E0S8Z0 pp 229533.I1RZ39
177044 229533.A0A0E0S8Z0 pp 229533.I1RN42
177045 229533.A0A0E0S8Z0 pp 229533.I1RXD9
177046 229533.A0A0E0S8Z0 pp 229533.I1RN67
177047 229533.A0A0E0S8Z0 pp 229533.A0A1C3YKM9
177048 229533.A0A0E0S8Z0 pp 229533.I1S458
177049 229533.A0A0E0S8Z0 pp 229533.I1RWI5
177050 229533.A0A0E0S8Z0 pp 229533.I1RGA4
177051 229533.A0A0E0S8Z0 pp 229533.I1RBE6
177052 229533.A0A0E0S8Z0 pp 229533.I1RX39
177053 229533.A0A0E0S8Z0 pp 229533.I1RPD6
177054 229533.A0A0E0S8Z0 pp 229533.I1RYQ1
177055 229533.A0A0E0S8Z0 pp 229533.A0A1C3YI20
177056 229533.A0A0E0S8Z0 pp 229533.I1S3G4
177057 229533.A0A0E0S8Z0 pp 229533.I1S5U0
177058 229533.A0A0E0S8Z0 pp 229533.I1RNW4
177059 229533.A0A0E0S8Z0 pp 229533.I1RWT0
177060 229533.A0A0E0S8Z0 pp 229533.I1RSC0
177061 229533.A0A0E0S8Z0 pp 229533.I1RA11
177062 229533.A0A0E0S8Z0 pp 229533.I1RRT8
177063 229533.A0A0E0S8Z0 pp 229533.I1RPP7
177064 229533.A0A0E0S8Z0 pp 229533.O74638
177065 229533.A0A0E0S8Z0 pp 229533.V6R3J1
177066 229533.A0A0E0S8Z0 pp 229533.I1RK26
177067 229533.A0A0E0S8Z0 pp 229533.I1RU26
177068 229533.A0A0E0S8Z0 pp 229533.I1S464
177069 229533.A0A0E0S8Z0 pp 229533.I1RPY0
177070 229533.A0A0E0S8Z0 pp 229533.I1RRI2
177071 229533.A0A0E0S8Z0 pp 229533.I1RWA3
177072 229533.A0A0E0S8Z0 pp 229533.A0A1C3YN78
177073 229533.A0A0E0S8Z0 pp 229533.I1RYI5
177074 229533.A0A0E0S8Z0 pp 229533.I1RU95
177075 229533.A0A0E0S8Z0 pp 229533.I1RGI8
177076 229533.A0A0E0S8Z0 pp 229533.I1RPP3
177077 229533.A0A0E0S8Z0 pp 229533.A0A1C3YKY0
177078 229533.A0A0E0S8Z0 pp 229533.I1S7X9
177079 229533.A0A0E0S8Z0 pp 229533.I1RN21
177080 229533.A0A0E0S8Z0 pp 229533.I1RFJ6
177081 229533.A0A0E0S8Z0 pp 229533.A0A1C3YL46
177082 229533.A0A0E0S8Z0 pp 229533.I1RVX0
177083 229533.A0A0E0S8Z0 pp 229533.I1RA52
177084 229533.A0A0E0S8Z0 pp 229533.I1RX96
177085 229533.A0A0E0S8Z0 pp 229533.A0A1C3YMQ5
177086 229533.A0A0E0S8Z0 pp 229533.I1R995
177087 229533.A0A0E0S8Z0 pp 229533.I1RSF2
177088 229533.A0A0E0S8Z0 pp 229533.I1RB92
177089 229533.A0A0E0S8Z0 pp 229533.I1R9M0
177090 229533.A0A0E0S8Z0 pp 229533.A0A1C3YIZ4
177091 229533.A0A0E0S8Z0 pp 229533.I1RK39
177092 229533.A0A0E0S8Z0 pp 229533.I1RBT6
177093 229533.A0A0E0S8Z0 pp 229533.I1RKB2
177094 229533.A0A0E0S8Z0 pp 229533.I1RL81
177095 229533.A0A0E0S8Z0 pp 229533.I1S1F3
177096 229533.A0A0E0S8Z0 pp 229533.I1RYA0
177097 229533.A0A0E0S8Z0 pp 229533.I1RW26
177098 229533.A0A0E0S8Z0 pp 229533.A0A1C3YM56

177099	229533.A0A0E0S8Z0	pp	229533.I1RLN1
177100	229533.A0A0E0S8Z0	pp	229533.I1S266
177101	229533.A0A0E0S8Z0	pp	229533.A0A1C3YJQ2
177102	229533.A0A0E0S8Z0	pp	229533.I1RPY8
177103	229533.A0A0E0S8Z0	pp	229533.A0A1C3YJU7
177104	229533.A0A0E0S8Z0	pp	229533.I1S5X9
177105	229533.A0A0E0S8Z0	pp	229533.I1RYC8
177106	229533.A0A0E0S8Z0	pp	229533.I1RSQ3
177107	229533.A0A0E0S8Z0	pp	229533.I1S0R8
177108	229533.A0A0E0S8Z0	pp	229533.I1RQJ3
177109	229533.A0A0E0S8Z0	pp	229533.I1RKF4
177110	229533.A0A0E0S8Z0	pp	229533.I1RCH5
177111	229533.A0A0E0S8Z0	pp	229533.I1RGK4
177112	229533.A0A0E0S8Z0	pp	229533.A0A0E0SAR4
177113	229533.A0A0E0S8Z0	pp	229533.I1RKB7
177114	229533.A0A0E0S8Z0	pp	229533.I1S0Q7
177115	229533.A0A0E0S8Z0	pp	229533.I1RPN4
177116	229533.A0A0E0S8Z0	pp	229533.I1RE79
177117	229533.A0A0E0S8Z0	pp	229533.I1RMQ6
177118	229533.A0A0E0S8Z0	pp	229533.I1S4S7
177119	229533.A0A0E0S8Z0	pp	229533.I1R9Y4
177120	229533.A0A0E0S8Z0	pp	229533.I1REF0
177121	229533.A0A0E0S8Z0	pp	229533.I1RTH7
177122	229533.A0A0E0S8Z0	pp	229533.I1S0R1
177123	229533.A0A0E0S8Z0	pp	229533.I1RZW7
177124	229533.A0A0E0S8Z0	pp	229533.I1RI95
177125	229533.A0A0E0S8Z0	pp	229533.I1RE65
177126	229533.A0A0E0S8Z0	pp	229533.A0A0E0SGH7
177127	229533.A0A0E0S8Z0	pp	229533.V6RLW2
177128	229533.A0A0E0S8Z0	pp	229533.I1RG11
177129	229533.A0A0E0S8Z0	pp	229533.I1RFS5
177130	229533.A0A0E0S8Z0	pp	229533.I1RR24
177131	229533.A0A0E0S8Z0	pp	229533.I1RRX5
177132	229533.A0A0E0S8Z0	pp	229533.I1RFF8
177133	229533.A0A0E0S8Z0	pp	229533.I1S1A0
177134	229533.A0A0E0S8Z0	pp	229533.I1S0T7
177135	229533.A0A0E0S8Z0	pp	229533.A0A1C3YLB6
177136	229533.A0A0E0S8Z0	pp	229533.I1RB86
177137	229533.A0A0E0S8Z0	pp	229533.I1RCL4
177138	229533.A0A0E0S8Z0	pp	229533.I1RMK7
177139	229533.A0A0E0S8Z0	pp	229533.I1RDH5
177140	229533.A0A0E0S8Z0	pp	229533.I1RNM7
177141	229533.A0A0E0S8Z0	pp	229533.I1S5C6
177142	229533.A0A0E0S8Z0	pp	229533.I1RY21
177143	229533.A0A0E0S8Z0	pp	229533.I1RGJ4
177144	229533.A0A0E0S8Z0	pp	229533.I1RZT4
177145	229533.A0A0E0S8Z0	pp	229533.I1S1W8
177146	229533.A0A0E0S8Z0	pp	229533.I1S0U0
177147	229533.A0A0E0S8Z0	pp	229533.I1RYT8
177148	229533.A0A0E0S8Z0	pp	229533.I1RWG0
177149	229533.A0A0E0S8Z0	pp	229533.I1RWN3
177150	229533.A0A0E0S8Z0	pp	229533.A0A1C3YJ95
177151	229533.A0A0E0S8Z0	pp	229533.I1RW54
177152	229533.A0A0E0S8Z0	pp	229533.I1RJB3
177153	229533.A0A0E0S8Z0	pp	229533.I1RG62
177154	229533.A0A0E0S8Z0	pp	229533.I1RRK9
177155	229533.A0A0E0S8Z0	pp	229533.I1RE66
177156	229533.A0A0E0S8Z0	pp	229533.I1RS05
177157	229533.A0A0E0S8Z0	pp	229533.I1S0P0
177158	229533.A0A0E0S8Z0	pp	229533.A0A1C3YLB0
177159	229533.A0A0E0S8Z0	pp	229533.I1S287
177160	229533.A0A0E0S8Z0	pp	229533.I1RAZ3
177161	229533.A0A0E0S8Z0	pp	229533.I1RYC7
177162	229533.A0A0E0S8Z0	pp	229533.I1RP38
177163	229533.A0A0E0S8Z0	pp	229533.A0A1C3YK68
177164	229533.A0A0E0S8Z0	pp	229533.I1RAM9
177165	229533.A0A0E0S8Z0	pp	229533.Q4I5R9
177166	229533.A0A0E0S8Z0	pp	229533.I1S8S9
177167	229533.A0A0E0S8Z0	pp	229533.A0A1C3YIW9
177168	229533.A0A0E0S8Z0	pp	229533.I1RPR9
177169	229533.A0A0E0S8Z0	pp	229533.A0A1C3YNC6
177170	229533.A0A0E0S8Z0	pp	229533.I1S212
177171	229533.A0A0E0S8Z0	pp	229533.I1S3D8

177172	229533.A0A0E0S8Z0	pp	229533.A0A1C3YKY6
177173	229533.A0A0E0S8Z0	pp	229533.A0A1C3YN86
177174	229533.A0A0E0S8Z0	pp	229533.I1RJU8
177175	229533.A0A0E0S8Z0	pp	229533.I1RKF5
177176	229533.A0A0E0S8Z0	pp	229533.A0A1C3YL10
177177	229533.A0A0E0S8Z0	pp	229533.I1RS29
177178	229533.A0A0E0S8Z0	pp	229533.I1S0K2
177179	229533.A0A0E0S8Z0	pp	229533.I1RG33
177180	229533.A0A0E0S8Z0	pp	229533.Q4I5G1
177181	229533.A0A0E0S8Z0	pp	229533.I1S4Q4
177182	229533.A0A0E0S8Z0	pp	229533.I1RAV5
177183	229533.A0A0E0S8Z0	pp	229533.A0A1C3YN89
177184	229533.A0A0E0S8Z0	pp	229533.A0A1C3YHJ9
177185	229533.A0A0E0S8Z0	pp	229533.A0A1C3YIJ9
177186	229533.A0A0E0S8Z0	pp	229533.A0A1C3YMC4
177187	229533.A0A0E0S8Z0	pp	229533.I1RZ68
177188	229533.A0A0E0S8Z0	pp	229533.I1RP02
177189	229533.A0A0E0S8Z0	pp	229533.I1RW13
177190	229533.A0A0E0S8Z0	pp	229533.I1R9N8
177191	229533.A0A0E0S8Z0	pp	229533.I1RGK5
177192	229533.A0A0E0S8Z0	pp	229533.I1RXE6
177193	229533.A0A0E0S8Z0	pp	229533.I1RQR6
177194	229533.A0A0E0S8Z0	pp	229533.I1RRV4
177195	229533.A0A0E0S8Z0	pp	229533.A0A1C3YKT4
177196	229533.A0A0E0S8Z0	pp	229533.A0A1C3YKV3
177197	229533.A0A0E0S8Z0	pp	229533.A0A1C3YIK4
177198	229533.A0A0E0S8Z0	pp	229533.I1RT96
177199	229533.A0A0E0S8Z0	pp	229533.I1RMQ4
177200	229533.A0A0E0S8Z0	pp	229533.V6RJP8
177201	229533.A0A0E0S8Z0	pp	229533.I1RG14
177202	229533.A0A0E0S8Z0	pp	229533.I1S8F8
177203	229533.I1RJ17	pp	229533.I1S5B0
177204	229533.I1RJ17	pp	229533.I1RSQ3
177205	229533.I1RJ17	pp	229533.V6QUE6
177206	229533.I1RJ17	pp	229533.I1RYB9
177207	229533.I1RJ17	pp	229533.V6R042
177208	229533.I1RJ17	pp	229533.V6RCZ8
177209	229533.I1RJ17	pp	229533.I1RNC1
177210	229533.I1RJ17	pp	229533.I1RXF0
177211	229533.I1RJ17	pp	229533.I1RUG3
177212	229533.I1RJ17	pp	229533.I1RK95
177213	229533.I1RJ17	pp	229533.I1S6E5
177214	229533.I1RJ17	pp	229533.I1RVX0
177215	229533.I1RJ17	pp	229533.I1RS18
177216	229533.I1RJ17	pp	229533.I1S0J1
177217	229533.I1RJ17	pp	229533.I1RSS4
177218	229533.I1RJ17	pp	229533.I1RJV3
177219	229533.I1RJ17	pp	229533.Q4IJK2
177220	229533.I1RJ17	pp	229533.I1RRI2
177221	229533.I1RJ17	pp	229533.I1RK83
177222	229533.I1RJ17	pp	229533.I1S2H4
177223	229533.I1RJ17	pp	229533.I1RL04
177224	229533.I1RJ17	pp	229533.I1RSZ7
177225	229533.I1RJ17	pp	229533.O74638
177226	229533.I1RJ17	pp	229533.I1RYW3
177227	229533.I1RJ17	pp	229533.I1RQE7
177228	229533.I1RJ17	pp	229533.I1S5R1
177229	229533.I1RJ17	pp	229533.I1RT33
177230	229533.I1RJ17	pp	229533.I1RPP3
177231	229533.I1RJ17	pp	229533.I1RRT8
177232	229533.I1RJ17	pp	229533.I1RN67
177233	229533.I1RJ17	pp	229533.I1RSQ7
177234	229533.I1RJ17	pp	229533.I1S4S4
177235	229533.I1RJ17	pp	229533.I1RM67
177236	229533.I1RJ17	pp	229533.I1RX46
177237	229533.I1RJ17	pp	229533.I1S9Y1
177238	229533.I1RJ17	pp	229533.I1RZZ9
177239	229533.I1RJ17	pp	229533.I1RXD7
177240	229533.I1RJ17	pp	229533.I1RZ39
177241	229533.I1RJ17	pp	229533.I1RT40
177242	229533.I1RJ17	pp	229533.I1RSQ4
177243	229533.I1RJ17	pp	229533.Q4HXF0
177244	229533.A0A098CZ63	pp	229533.I1RPA6

177245 229533.A0A1C3YI86 pp 229533.I1RJU9
177246 229533.A0A1C3YI86 pp 229533.I1RLM3
177247 229533.V6RDP1 pp 229533.V6REA4
177248 229533.V6RDP1 pp 229533.V6RN16
177249 229533.V6RDP1 pp 229533.V6RVB4
177250 229533.V6RDP1 pp 229533.V6RKY7
177251 229533.V6RDP1 pp 229533.V6RVG3
177252 229533.V6RDP1 pp 229533.V6RM09
177253 229533.I1RIF7 pp 229533.I1RZ56
177254 229533.I1RIF7 pp 229533.I1RPV8
177255 229533.I1RIF7 pp 229533.I1RRF7
177256 229533.I1RIF7 pp 229533.I1RIV2
177257 229533.I1RIF7 pp 229533.I1SAT2
177258 229533.I1RIF7 pp 229533.I1RJS1
177259 229533.I1RIF7 pp 229533.I1S1T5
177260 229533.I1RIF7 pp 229533.I1RNG4
177261 229533.I1RIF7 pp 229533.I1RW80
177262 229533.I1RIF7 pp 229533.I1RKR8
177263 229533.I1RIF7 pp 229533.I1S8H5
177264 229533.I1RIF7 pp 229533.I1S0A5
177265 229533.I1RIF7 pp 229533.I1RST9
177266 229533.I1RIF7 pp 229533.I1RN04
177267 229533.I1RIF7 pp 229533.I1S267
177268 229533.I1RIF7 pp 229533.I1S659
177269 229533.I1RIF7 pp 229533.I1RPW8
177270 229533.I1RIF7 pp 229533.I1SAH9
177271 229533.I1RIF7 pp 229533.I1S5D0
177272 229533.I1RIF7 pp 229533.I1RQV7
177273 229533.I1RIF7 pp 229533.I1S094
177274 229533.I1RIF7 pp 229533.I1S0K1
177275 229533.I1RIF7 pp 229533.I1RRS6
177276 229533.I1RIF7 pp 229533.I1S6H3
177277 229533.I1RIF7 pp 229533.I1RW91
177278 229533.I1RWB5 pp 229533.I1RZV6
177279 229533.I1RWB5 pp 229533.I1S8E2
177280 229533.I1RWB5 pp 229533.I1S1X9
177281 229533.I1RWB5 pp 229533.I1RZZ1
177282 229533.I1RWB5 pp 229533.I1S2E8
177283 229533.I1RWB5 pp 229533.Q4IFI0
177284 229533.I1RWB5 pp 229533.I1RX24
177285 229533.I1RWB5 pp 229533.I1S7S9
177286 229533.I1RWB5 pp 229533.I1RZK6
177287 229533.I1RWB5 pp 229533.I1S044
177288 229533.I1RWB5 pp 229533.I1RXV1
177289 229533.I1RWB5 pp 229533.V6RGN5
177290 229533.I1RWB5 pp 229533.Q4I662
177291 229533.I1RWB5 pp 229533.I1RZS6
177292 229533.I1RWB5 pp 229533.V6R042
177293 229533.I1RWB5 pp 229533.V6RMW6
177294 229533.I1RWB5 pp 229533.I1S8R8
177295 229533.I1RWB5 pp 229533.Q4IPI1
177296 229533.I1RWB5 pp 229533.I1S2C0
177297 229533.I1RWB5 pp 229533.I1RX46
177298 229533.I1RWB5 pp 229533.Q4IN63
177299 229533.I1RWB5 pp 229533.I1RZQ6
177300 229533.I1RWB5 pp 229533.I1RWH5
177301 229533.I1RWB5 pp 229533.I1S6E5
177302 229533.I1RWB5 pp 229533.I1S0R7
177303 229533.I1RWB5 pp 229533.I1RYB9
177304 229533.I1RWB5 pp 229533.I1RYB1
177305 229533.I1RWB5 pp 229533.Q4I327
177306 229533.I1RWB5 pp 229533.I1S060
177307 229533.I1RWB5 pp 229533.I1S021
177308 229533.I1RWB5 pp 229533.I1S0M9
177309 229533.I1RWB5 pp 229533.I1RZF6
177310 229533.I1RWB5 pp 229533.I1S0J1
177311 229533.I1RQW1 pp 229533.I1S5D0
177312 229533.I1RQW1 pp 229533.I1RW80
177313 229533.I1RQW1 pp 229533.I1S0A5
177314 229533.I1RQW1 pp 229533.I1S8H5
177315 229533.I1RQW1 pp 229533.I1RZ56
177316 229533.I1RQW1 pp 229533.I1S3I4
177317 229533.I1RQW1 pp 229533.I1S094

229533.I1RQW1	pp	229533.I1SAT2
229533.I1RQW1	pp	229533.I1S6H3
229533.I1RQW1	pp	229533.I1RRF7
229533.I1RQW1	pp	229533.I1RW91
229533.I1RQW1	pp	229533.I1RRS6
229533.I1RQW1	pp	229533.I1S2N3
229533.I1RQW1	pp	229533.I1RST9
229533.I1RQW1	pp	229533.I1S267
229533.I1RQW1	pp	229533.I1S659
229533.I1RRR6	pp	229533.I1RSM3
229533.I1RRR6	pp	229533.I1RTF4
229533.I1RRR6	pp	229533.I1S8R8
229533.I1RRR6	pp	229533.I1RRT2
229533.I1RRR6	pp	229533.Q4IR18
229533.I1RRR6	pp	229533.V6R6F0
229533.I1RRR6	pp	229533.I1S004
229533.I1RRR6	pp	229533.V6R227
229533.I1RRR6	pp	229533.V6QVS3
229533.I1RRR6	pp	229533.I1RWD4
229533.I1RRR6	pp	229533.I1RXV1
229533.I1RRR6	pp	229533.V6RVB4
229533.I1RRR6	pp	229533.I1RZT1
229533.I1RRR6	pp	229533.I1RSL8
229533.I1RRR6	pp	229533.I1RW03
229533.I1RR68	pp	229533.I1S2C4
229533.I1RR68	pp	229533.I1S5R6
229533.I1RR68	pp	229533.V6RSF3
229533.I1RR68	pp	229533.I1S6V0
229533.I1RR68	pp	229533.I1S2H3
229533.I1RR68	pp	229533.I1S9Z4
229533.I1RR68	pp	229533.I1RSU2
229533.I1RR68	pp	229533.I1RRP7
229533.I1RR68	pp	229533.I1S147
229533.I1RR68	pp	229533.I1S8R2
229533.I1RR68	pp	229533.I1RZ20
229533.I1RR68	pp	229533.I1RSA5
229533.I1RR68	pp	229533.I1RYK0
229533.I1RR68	pp	229533.I1RW82
229533.I1RR68	pp	229533.Q4HXF0
229533.I1RR68	pp	229533.I1S299
229533.I1RR68	pp	229533.I1RTY9
229533.I1RR68	pp	229533.I1S4F4
229533.I1RR68	pp	229533.I1S968
229533.I1RR68	pp	229533.V6RCJ5
229533.I1RR68	pp	229533.I1RSF5
229533.I1RR68	pp	229533.I1RW43
229533.I1RR68	pp	229533.I1RWM4
229533.I1RR68	pp	229533.V6RHX2
229533.I1RR68	pp	229533.I1S3T7
229533.I1RR68	pp	229533.I1RST9
229533.I1RR68	pp	229533.I1S8D3
229533.I1RR68	pp	229533.I1S2Q4
229533.I1RR68	pp	229533.V6RWB3
229533.I1RR68	pp	229533.I1SAB4
229533.I1RR68	pp	229533.I1RVP5
229533.I1RR68	pp	229533.I1RY81
229533.I1RR68	pp	229533.I1S489
229533.I1RR68	pp	229533.V6RY56
229533.I1RR68	pp	229533.I1RTB8
229533.I1RR68	pp	229533.I1RVY2
229533.I1RR68	pp	229533.I1S3T6
229533.I1RR68	pp	229533.I1S1D0
229533.I1RR68	pp	229533.I1S969
229533.I1RR68	pp	229533.I1RXX1
229533.I1RR68	pp	229533.I1RYU5
229533.I1RR68	pp	229533.V6R5T7
229533.I1RR68	pp	229533.I1S223
229533.I1RR68	pp	229533.I1RW54
229533.I1RR68	pp	229533.I1RRX5
229533.I1RR68	pp	229533.I1RXB1
229533.I1RR68	pp	229533.I1RSW4
229533.I1RR68	pp	229533.I1RYP5
229533.I1RR68	pp	229533.I1RYX6

177391 229533.I1RR68 pp 229533.I1RVI8
177392 229533.I1RR68 pp 229533.I1S8C1
177393 229533.I1RR68 pp 229533.V6RCC0
177394 229533.I1RR68 pp 229533.I1RYJ7
177395 229533.I1RR68 pp 229533.I1RWL3
177396 229533.A0A1C3YN57 pp 229533.I1RSY2
177397 229533.A0A1C3YN57 pp 229533.A0A1C3YN83
177398 229533.A0A1C3YN57 pp 229533.I1RJ17
177399 229533.A0A1C3YN57 pp 229533.I1RD63
177400 229533.A0A1C3YN57 pp 229533.I1RDV8
177401 229533.A0A1C3YN57 pp 229533.I1RHG5
177402 229533.A0A1C3YN57 pp 229533.I1RN63
177403 229533.A0A1C3YN57 pp 229533.I1RVP5
177404 229533.A0A1C3YN57 pp 229533.I1RFL3
177405 229533.A0A1C3YN57 pp 229533.I1RAG3
177406 229533.A0A1C3YN57 pp 229533.I1RC81
177407 229533.A0A1C3YN57 pp 229533.I1RK03
177408 229533.A0A1C3YN57 pp 229533.I1RA63
177409 229533.A0A1C3YN57 pp 229533.I1RFC0
177410 229533.A0A1C3YN57 pp 229533.I1RNJ2
177411 229533.A0A1C3YN57 pp 229533.I1S6E5
177412 229533.A0A1C3YN57 pp 229533.I1RT05
177413 229533.I1RPK0 pp 229533.I1RRF9
177414 229533.I1RPK0 pp 229533.V6QUE6
177415 229533.I1RPK0 pp 229533.I1RYI7
177416 229533.I1RPK0 pp 229533.I1S6K9
177417 229533.I1RPK0 pp 229533.V6RCZ8
177418 229533.I1RPK0 pp 229533.I1RVJ6
177419 229533.I1RPK0 pp 229533.V6RCG5
177420 229533.I1RPK0 pp 229533.I1RQD2
177421 229533.I1RPK0 pp 229533.I1RUY5
177422 229533.I1RPK0 pp 229533.I1RZM8
177423 229533.I1RPK0 pp 229533.I1RXV1
177424 229533.I1RPK0 pp 229533.I1S5B0
177425 229533.I1RPK0 pp 229533.Q4IE79
177426 229533.I1RPK0 pp 229533.I1RXE6
177427 229533.I1RPK0 pp 229533.I1RYB9
177428 229533.I1RPK0 pp 229533.I1RXC4
177429 229533.I1RPK0 pp 229533.I1RRV4
177430 229533.I1RPK0 pp 229533.I1RVP5
177431 229533.I1RPK0 pp 229533.V6RK96
177432 229533.I1RPK0 pp 229533.I1S504
177433 229533.I1RPK0 pp 229533.V6RG22
177434 229533.I1RPK0 pp 229533.I1S0N3
177435 229533.I1RPK0 pp 229533.I1S887
177436 229533.I1RPK0 pp 229533.I1RZF5
177437 229533.I1RPK0 pp 229533.Q4I1Y1
177438 229533.I1RPK0 pp 229533.I1RZK5
177439 229533.I1RPK0 pp 229533.V6QVS3
177440 229533.I1RPK0 pp 229533.I1RYU0
177441 229533.I1RPK0 pp 229533.I1RRY9
177442 229533.I1RPK0 pp 229533.I1RWR7
177443 229533.I1RPK0 pp 229533.I1RY12
177444 229533.I1RPK0 pp 229533.I1RQ85
177445 229533.I1RPK0 pp 229533.I1S0V6
177446 229533.I1RPK0 pp 229533.I1RS18
177447 229533.I1RPK0 pp 229533.I1RXM6
177448 229533.I1RPK0 pp 229533.Q4IJK2
177449 229533.I1RPK0 pp 229533.I1S1J7
177450 229533.I1RPK0 pp 229533.I1RVY2
177451 229533.I1RPK0 pp 229533.I1RWH8
177452 229533.I1RPK0 pp 229533.I1S8R8
177453 229533.I1RPK0 pp 229533.I1RY86
177454 229533.I1RPK0 pp 229533.V6RS91
177455 229533.I1RPK0 pp 229533.V6RTJ8
177456 229533.I1RPK0 pp 229533.I1RVX9
177457 229533.I1RPK0 pp 229533.I1RT72
177458 229533.I1RPK0 pp 229533.I1S236
177459 229533.I1RPK0 pp 229533.Q4I5R9
177460 229533.I1RPK0 pp 229533.V6QW22
177461 229533.I1RPK0 pp 229533.I1RQC5
177462 229533.I1RPK0 pp 229533.I1RSQ7
177463 229533.I1RPK0 pp 229533.I1RS10

177464	229533.I1RPK0	pp	229533.I1RRX5
177465	229533.I1RPK0	pp	229533.I1S338
177466	229533.I1RPK0	pp	229533.Q5I2J3
177467	229533.I1RPK0	pp	229533.I1RZM4
177468	229533.I1RPK0	pp	229533.I1RX46
177469	229533.I1RPK0	pp	229533.I1RXD6
177470	229533.I1RPK0	pp	229533.I1RVQ0
177471	229533.I1RPK0	pp	229533.I1RXD7
177472	229533.I1RPK0	pp	229533.V6RUT5
177473	229533.I1RPK0	pp	229533.I1S8C1
177474	229533.I1RPK0	pp	229533.I1RXB1
177475	229533.I1RPK0	pp	229533.I1RW90
177476	229533.I1RPK0	pp	229533.I1RRG3
177477	229533.I1RPK0	pp	229533.I1RZU6
177478	229533.I1RPK0	pp	229533.I1RPW2
177479	229533.I1RPK0	pp	229533.I1RR24
177480	229533.I1RPK0	pp	229533.I1S4S7
177481	229533.I1RPK0	pp	229533.I1RPN4
177482	229533.I1RPK0	pp	229533.I1RWJ8
177483	229533.I1RPK0	pp	229533.I1S4S4
177484	229533.I1RPK0	pp	229533.V6QWX8
177485	229533.I1RPK0	pp	229533.I1RSU2
177486	229533.I1RPK0	pp	229533.I1RW06
177487	229533.I1RPK0	pp	229533.I1RYJ7
177488	229533.I1RPK0	pp	229533.I1RWH4
177489	229533.I1RPK0	pp	229533.I1S0V7
177490	229533.I1RPK0	pp	229533.I1RQE8
177491	229533.I1RPK0	pp	229533.Q4IPB8
177492	229533.I1RPK0	pp	229533.I1RW25
177493	229533.I1RPK0	pp	229533.I1RRZ6
177494	229533.I1RPK0	pp	229533.I1S147
177495	229533.I1RPK0	pp	229533.I1S438
177496	229533.I1RPK0	pp	229533.I1RVZ7
177497	229533.I1RPK0	pp	229533.I1S256
177498	229533.I1RPK0	pp	229533.I1RPU2
177499	229533.I1RPK0	pp	229533.Q4IPB3
177500	229533.I1RPK0	pp	229533.I1RRN2
177501	229533.I1RPK0	pp	229533.V6R042
177502	229533.I1RPK0	pp	229533.I1RTG6
177503	229533.I1RPK0	pp	229533.I1RQ21
177504	229533.I1RPK0	pp	229533.I1S5R6
177505	229533.I1RPK0	pp	229533.I1RSQ3
177506	229533.I1RPK0	pp	229533.I1RSL5
177507	229533.I1RPK0	pp	229533.V6RSF3
177508	229533.I1RPK0	pp	229533.Q4HZB8
177509	229533.I1RPK0	pp	229533.V6RIV9
177510	229533.I1RPK0	pp	229533.I1RRM3
177511	229533.I1RPK0	pp	229533.V6RMW6
177512	229533.I1RPK0	pp	229533.I1RWN5
177513	229533.I1RPK0	pp	229533.I1S2C0
177514	229533.I1RPK0	pp	229533.I1RUG3
177515	229533.I1RPK0	pp	229533.I1RVF8
177516	229533.I1RPK0	pp	229533.I1S0R9
177517	229533.I1RPK0	pp	229533.I1S437
177518	229533.I1RPK0	pp	229533.I1RW46
177519	229533.I1RPK0	pp	229533.I1S1W9
177520	229533.I1RPK0	pp	229533.I1RW78
177521	229533.I1RPK0	pp	229533.I1S1C7
177522	229533.I1RPK0	pp	229533.I1S1W3
177523	229533.I1RPK0	pp	229533.I1S0J1
177524	229533.I1RPK0	pp	229533.I1RZV3
177525	229533.I1RPK0	pp	229533.V6RLM7
177526	229533.I1RPK0	pp	229533.I1RYW3
177527	229533.I1RPK0	pp	229533.Q4ILH3
177528	229533.I1RPK0	pp	229533.I1RXU9
177529	229533.I1RPK0	pp	229533.I1RPP4
177530	229533.I1RPK0	pp	229533.I1RWX5
177531	229533.I1RPK0	pp	229533.I1RRI2
177532	229533.I1RPK0	pp	229533.I1RWN6
177533	229533.I1RPK0	pp	229533.V6R057
177534	229533.I1RPK0	pp	229533.I1S9Z4
177535	229533.I1RPK0	pp	229533.Q4I624
177536	229533.I1RPK0	pp	229533.I1RTA3

177537 229533.I1RPK0 pp 229533.I1RTA0
177538 229533.I1RPK0 pp 229533.I1RTH6
177539 229533.I1RPK0 pp 229533.I1RQ33
177540 229533.I1RPK0 pp 229533.I1RXR1
177541 229533.I1RPK0 pp 229533.I1RVX0
177542 229533.I1RPK0 pp 229533.I1RQM7
177543 229533.I1RPK0 pp 229533.I1S216
177544 229533.I1RPK0 pp 229533.I1RSX0
177545 229533.I1RPK0 pp 229533.I1RT03
177546 229533.I1RPK0 pp 229533.I1S503
177547 229533.I1RPK0 pp 229533.I1RSY2
177548 229533.I1RPK0 pp 229533.I1RR25
177549 229533.I1RPK0 pp 229533.Q4HXF6
177550 229533.I1RPK0 pp 229533.V6R3J1
177551 229533.I1RPK0 pp 229533.Q4IPH4
177552 229533.I1RPK0 pp 229533.V6R6F0
177553 229533.I1RPK0 pp 229533.I1S8P5
177554 229533.I1RPK0 pp 229533.I1RPP3
177555 229533.I1RPK0 pp 229533.Q4HXF0
177556 229533.I1RPK0 pp 229533.V6QVH8
177557 229533.I1RPK0 pp 229533.V6RG79
177558 229533.I1RPK0 pp 229533.I1RYR0
177559 229533.I1RPK0 pp 229533.I1RQE7
177560 229533.I1RPK0 pp 229533.I1RT75
177561 229533.I1RPK0 pp 229533.I1RX39
177562 229533.I1RPK0 pp 229533.I1RT04
177563 229533.I1RPK0 pp 229533.I1S237
177564 229533.I1RPK0 pp 229533.I1RRT8
177565 229533.I1RPK0 pp 229533.I1RWK3
177566 229533.I1RPK0 pp 229533.I1S5R1
177567 229533.I1RPK0 pp 229533.Q4IBK5
177568 229533.I1RPK0 pp 229533.I1RT40
177569 229533.I1RPK0 pp 229533.I1RXC2
177570 229533.I1RPK0 pp 229533.I1RXA9
177571 229533.I1RPK0 pp 229533.I1RVZ2
177572 229533.I1RPK0 pp 229533.I1RXF8
177573 229533.I1RPK0 pp 229533.I1RTA1
177574 229533.I1RPK0 pp 229533.O74638
177575 229533.I1RPK0 pp 229533.I1RVU7
177576 229533.I1RPK0 pp 229533.I1RWF5
177577 229533.I1RPK0 pp 229533.I1RTG7
177578 229533.I1RPK0 pp 229533.I1RTD5
177579 229533.I1RPK0 pp 229533.I1RZ39
177580 229533.I1RPK0 pp 229533.I1S2H4
177581 229533.I1RPK0 pp 229533.I1RR73
177582 229533.I1RPK0 pp 229533.I1RZ83
177583 229533.I1RPK0 pp 229533.I1S3T7
177584 229533.I1RPK0 pp 229533.I1S2A8
177585 229533.I1RPK0 pp 229533.I1RXB2
177586 229533.I1RPK0 pp 229533.I1RY62
177587 229533.I1RPK0 pp 229533.I1RYI5
177588 229533.I1RPK0 pp 229533.I1S0L4
177589 229533.I1RPK0 pp 229533.I1RSQ4
177590 229533.I1RPK0 pp 229533.I1RRI1
177591 229533.I1RPK0 pp 229533.I1RXV3
177592 229533.I1RPK0 pp 229533.I1RZ29
177593 229533.I1RPK0 pp 229533.I1RZZ9
177594 229533.I1RPK0 pp 229533.V6RVB4
177595 229533.I1RPK0 pp 229533.I1RZ52
177596 229533.I1RPK0 pp 229533.I1RT38
177597 229533.I1RPK0 pp 229533.I1RSZ7
177598 229533.I1RPK0 pp 229533.I1S2B8
177599 229533.I1RPK0 pp 229533.I1S4Z8
177600 229533.I1RPK0 pp 229533.I1RPV3
177601 229533.I1RPK0 pp 229533.I1RPM4
177602 229533.A0A1C3YJK3 pp 229533.Q4HXF6
177603 229533.A0A1C3YJK3 pp 229533.I1RB92
177604 229533.A0A1C3YJK3 pp 229533.I1RK95
177605 229533.A0A1C3YJK3 pp 229533.I1RSS4
177606 229533.A0A1C3YJK3 pp 229533.I1RQE7
177607 229533.A0A1C3YJK3 pp 229533.Q4IPH4
177608 229533.A0A1C3YJK3 pp 229533.A0A1C3YL10
177609 229533.A0A1C3YJK3 pp 229533.I1RQ31

177610 229533.A0A1C3YJK3 pp 229533.I1RUG3
177611 229533.A0A1C3YJK3 pp 229533.I1RFV3
177612 229533.A0A1C3YJK3 pp 229533.I1RQ33
177613 229533.A0A1C3YJK3 pp 229533.I1REK8
177614 229533.A0A1C3YJK3 pp 229533.I1S0T4
177615 229533.A0A1C3YJK3 pp 229533.A0A1C3YK68
177616 229533.A0A1C3YJK3 pp 229533.I1S5B0
177617 229533.A0A1C3YJK3 pp 229533.I1RAZ3
177618 229533.A0A1C3YJK3 pp 229533.I1RA94
177619 229533.A0A1C3YJK3 pp 229533.I1RCD8
177620 229533.A0A1C3YJK3 pp 229533.I1RAQ4
177621 229533.A0A1C3YJK3 pp 229533.I1RAH6
177622 229533.A0A1C3YJK3 pp 229533.I1S4Q4
177623 229533.A0A1C3YJK3 pp 229533.I1RB44
177624 229533.A0A1C3YJK3 pp 229533.Q4I5G1
177625 229533.A0A1C3YJK3 pp 229533.I1RSR3
177626 229533.A0A1C3YJK3 pp 229533.V6QUE6
177627 229533.A0A1C3YJK3 pp 229533.A0A1C3YN86
177628 229533.A0A1C3YJK3 pp 229533.A0A1C3YKY0
177629 229533.A0A1C3YJK3 pp 229533.I1RKB6
177630 229533.A0A1C3YJK3 pp 229533.I1RBS5
177631 229533.A0A1C3YJK3 pp 229533.V6RCZ8
177632 229533.A0A1C3YJK3 pp 229533.I1S9Z4
177633 229533.A0A1C3YJK3 pp 229533.I1S1W9
177634 229533.A0A1C3YJK3 pp 229533.I1RS18
177635 229533.A0A1C3YJK3 pp 229533.I1S253
177636 229533.A0A1C3YJK3 pp 229533.A0A1C3YK36
177637 229533.A0A1C3YJK3 pp 229533.V6RLM7
177638 229533.A0A1C3YJK3 pp 229533.A0A1C3YLB6
177639 229533.A0A1C3YJK3 pp 229533.A0A1C3YKB0
177640 229533.A0A1C3YJK3 pp 229533.I1RCM7
177641 229533.A0A1C3YJK3 pp 229533.V6QYF0
177642 229533.A0A1C3YJK3 pp 229533.I1RMM8
177643 229533.A0A1C3YJK3 pp 229533.I1RJU3
177644 229533.A0A1C3YJK3 pp 229533.I1RG14
177645 229533.A0A1C3YJK3 pp 229533.I1RDE7
177646 229533.A0A1C3YJK3 pp 229533.I1S0B7
177647 229533.A0A1C3YJK3 pp 229533.I1RPA4
177648 229533.A0A1C3YJK3 pp 229533.I1RVZ7
177649 229533.A0A1C3YJK3 pp 229533.I1S0F1
177650 229533.A0A1C3YJK3 pp 229533.I1RRY1
177651 229533.A0A1C3YJK3 pp 229533.I1RW61
177652 229533.A0A1C3YJK3 pp 229533.I1RSQ4
177653 229533.A0A1C3YJK3 pp 229533.I1RTK5
177654 229533.A0A1C3YJK3 pp 229533.A0A1C3YLK8
177655 229533.A0A1C3YJK3 pp 229533.I1RT05
177656 229533.A0A1C3YJK3 pp 229533.A0A1C3YLR6
177657 229533.A0A1C3YJK3 pp 229533.I1RA45
177658 229533.A0A1C3YJK3 pp 229533.I1S4Z8
177659 229533.A0A1C3YJK3 pp 229533.I1RRI1
177660 229533.A0A1C3YJK3 pp 229533.I1RPN4
177661 229533.A0A1C3YJK3 pp 229533.I1R9Y8
177662 229533.A0A1C3YJK3 pp 229533.I1RBW1
177663 229533.A0A1C3YJK3 pp 229533.I1RVW0
177664 229533.A0A1C3YJK3 pp 229533.I1RTA1
177665 229533.A0A1C3YJK3 pp 229533.I1RR24
177666 229533.A0A1C3YJK3 pp 229533.I1S2H4
177667 229533.A0A1C3YJK3 pp 229533.A0A1C3YJR2
177668 229533.A0A1C3YJK3 pp 229533.I1S277
177669 229533.A0A1C3YJK3 pp 229533.I1RWS4
177670 229533.A0A1C3YJK3 pp 229533.I1RP86
177671 229533.A0A1C3YJK3 pp 229533.I1RBW0
177672 229533.A0A1C3YJK3 pp 229533.O74638
177673 229533.A0A1C3YJK3 pp 229533.I1S0Z6
177674 229533.A0A1C3YJK3 pp 229533.I1RT75
177675 229533.A0A1C3YJK3 pp 229533.I1S5R1
177676 229533.A0A1C3YJK3 pp 229533.A0A1C3YL55
177677 229533.A0A1C3YJK3 pp 229533.Q4IBK5
177678 229533.A0A1C3YJK3 pp 229533.I1RM67
177679 229533.A0A1C3YJK3 pp 229533.I1RYJ6
177680 229533.A0A1C3YJK3 pp 229533.I1RFY6
177681 229533.A0A1C3YJK3 pp 229533.I1S9Y1
177682 229533.A0A1C3YJK3 pp 229533.I1RD52

177683 229533.A0A1C3YJK3 pp 229533.I1RT40
177684 229533.A0A1C3YJK3 pp 229533.I1RQM7
177685 229533.A0A1C3YJK3 pp 229533.I1RT33
177686 229533.A0A1C3YJK3 pp 229533.I1RPF1
177687 229533.A0A1C3YJK3 pp 229533.I1RR72
177688 229533.A0A1C3YJK3 pp 229533.I1RZB2
177689 229533.A0A1C3YJK3 pp 229533.I1RRW3
177690 229533.A0A1C3YJK3 pp 229533.I1RC82
177691 229533.A0A1C3YJK3 pp 229533.I1RN67
177692 229533.A0A1C3YJK3 pp 229533.I1RW26
177693 229533.I1RCH4 pp 229533.I1RFH9
177694 229533.I1RCH4 pp 229533.I1RKF3
177695 229533.I1RCH4 pp 229533.I1RQ42
177696 229533.I1RCH4 pp 229533.I1RRC4
177697 229533.I1RCH4 pp 229533.I1RW91
177698 229533.I1RCH4 pp 229533.I1RFV2
177699 229533.I1RCH4 pp 229533.I1RNJ7
177700 229533.I1RCH4 pp 229533.I1RMG4
177701 229533.I1RCH4 pp 229533.I1RRN4
177702 229533.I1RCH4 pp 229533.I1RPW8
177703 229533.I1RCH4 pp 229533.I1RCM8
177704 229533.I1RCH4 pp 229533.I1RHJ2
177705 229533.I1RCH4 pp 229533.I1RYX5
177706 229533.I1RCH4 pp 229533.I1RRF7
177707 229533.I1RCH4 pp 229533.I1S2B9
177708 229533.I1RCH4 pp 229533.I1RN04
177709 229533.I1RCH4 pp 229533.I1S5Z5
177710 229533.I1RCH4 pp 229533.I1RZN1
177711 229533.I1RCH4 pp 229533.I1RQM9
177712 229533.I1RCH4 pp 229533.I1RR61
177713 229533.I1RCH4 pp 229533.I1RW80
177714 229533.I1RAM7 pp 229533.I1RC22
177715 229533.I1RAM7 pp 229533.I1RMM9
177716 229533.I1RAM7 pp 229533.I1RUQ5
177717 229533.I1RAM7 pp 229533.I1RYY5
177718 229533.I1RAM7 pp 229533.I1RXX1
177719 229533.I1RAM7 pp 229533.I1RN08
177720 229533.I1RAM7 pp 229533.I1RFU8
177721 229533.I1RAM7 pp 229533.I1RXV1
177722 229533.I1RAM7 pp 229533.I1RRY9
177723 229533.I1RAM7 pp 229533.I1RCL7
177724 229533.I1RAM7 pp 229533.I1RZH4
177725 229533.I1RAM7 pp 229533.I1RB75
177726 229533.I1RAM7 pp 229533.I1S439
177727 229533.I1RAM7 pp 229533.I1RQ81
177728 229533.I1RAM7 pp 229533.I1RQA3
177729 229533.I1RAM7 pp 229533.I1RN65
177730 229533.I1RAM7 pp 229533.Q4ICA8
177731 229533.I1RAM7 pp 229533.V6RCG5
177732 229533.I1RAM7 pp 229533.I1RM09
177733 229533.I1RAM7 pp 229533.I1RWT6
177734 229533.I1RAM7 pp 229533.Q4IJM4
177735 229533.I1RAM7 pp 229533.I1RC95
177736 229533.I1RAM7 pp 229533.V6QVS3
177737 229533.I1RAM7 pp 229533.I1RKK0
177738 229533.I1RAM7 pp 229533.I1RYZ0
177739 229533.I1RAM7 pp 229533.I1RC17
177740 229533.I1RAM7 pp 229533.I1RPU4
177741 229533.I1RAM7 pp 229533.I1RMD3
177742 229533.I1RAM7 pp 229533.V6RTJ8
177743 229533.I1RAM7 pp 229533.I1RWJ6
177744 229533.I1RAM7 pp 229533.I1RBT1
177745 229533.I1RAM7 pp 229533.I1S224
177746 229533.I1RAM7 pp 229533.I1RYZ9
177747 229533.I1RAM7 pp 229533.I1RX70
177748 229533.I1RAM7 pp 229533.I1RXH6
177749 229533.I1RAM7 pp 229533.I1RQ26
177750 229533.I1RAM7 pp 229533.I1RJH1
177751 229533.I1RAM7 pp 229533.I1RYH9
177752 229533.I1RAM7 pp 229533.I1RDB1
177753 229533.I1RAM7 pp 229533.I1RNC0
177754 229533.I1RAM7 pp 229533.I1RV66
177755 229533.I1RAM7 pp 229533.V6QV09

177756 229533.I1RAM7 pp 229533.I1RD18
177757 229533.I1RAM7 pp 229533.I1RR24
177758 229533.I1RAM7 pp 229533.I1RN31
177759 229533.I1RAM7 pp 229533.I1RLX6
177760 229533.I1RAM7 pp 229533.I1RAW7
177761 229533.I1RAM7 pp 229533.I1RL24
177762 229533.I1RAM7 pp 229533.I1RW71
177763 229533.I1RAM7 pp 229533.I1RQC9
177764 229533.I1RAM7 pp 229533.I1RIY3
177765 229533.I1RAM7 pp 229533.I1RHP8
177766 229533.I1RAM7 pp 229533.I1S8R8
177767 229533.I1RAM7 pp 229533.I1RHQ8
177768 229533.I1RAM7 pp 229533.I1RBZ9
177769 229533.I1RAM7 pp 229533.I1RTH6
177770 229533.I1RAM7 pp 229533.I1RHJ1
177771 229533.I1RAM7 pp 229533.I1RRY3
177772 229533.I1RAM7 pp 229533.I1RZQ6
177773 229533.I1RAM7 pp 229533.I1RNY9
177774 229533.I1RAM7 pp 229533.I1RDZ4
177775 229533.I1RAM7 pp 229533.I1RT36
177776 229533.I1RAM7 pp 229533.I1RS41
177777 229533.I1RAM7 pp 229533.I1RRP7
177778 229533.I1RAM7 pp 229533.I1RFL4
177779 229533.I1RAM7 pp 229533.I1RB41
177780 229533.I1RAM7 pp 229533.I1RB92
177781 229533.I1RAM7 pp 229533.I1RAU7
177782 229533.I1RAM7 pp 229533.V6RGI1
177783 229533.I1RAM7 pp 229533.I1RZE4
177784 229533.I1RAM7 pp 229533.I1RQ72
177785 229533.I1RAM7 pp 229533.I1RS90
177786 229533.I1RAM7 pp 229533.I1RCL8
177787 229533.I1RAM7 pp 229533.I1RHJ2
177788 229533.I1RAM7 pp 229533.I1S0M2
177789 229533.I1RAM7 pp 229533.I1RQB4
177790 229533.I1RAM7 pp 229533.I1RTI3
177791 229533.I1RAM7 pp 229533.I1RPW0
177792 229533.I1RAM7 pp 229533.I1RE01
177793 229533.I1RAM7 pp 229533.I1RRW0
177794 229533.I1RAM7 pp 229533.I1S1D3
177795 229533.I1RAM7 pp 229533.I1S247
177796 229533.I1RAM7 pp 229533.V6R1X4
177797 229533.I1RAM7 pp 229533.I1RNY8
177798 229533.I1RAM7 pp 229533.I1RRN5
177799 229533.I1RAM7 pp 229533.I1S2K7
177800 229533.I1RAM7 pp 229533.I1RMQ5
177801 229533.I1RAM7 pp 229533.V6RMW6
177802 229533.I1RAM7 pp 229533.I1S1W4
177803 229533.I1RAM7 pp 229533.I1RZZ9
177804 229533.I1RAM7 pp 229533.I1RJW4
177805 229533.I1RAM7 pp 229533.I1S0D1
177806 229533.I1RAM7 pp 229533.I1RAN2
177807 229533.I1RAM7 pp 229533.I1RUN8
177808 229533.I1RAM7 pp 229533.I1RJ88
177809 229533.I1RAM7 pp 229533.I1RW16
177810 229533.I1RAM7 pp 229533.I1S0X7
177811 229533.I1RAM7 pp 229533.I1RR73
177812 229533.I1RAM7 pp 229533.I1RZ63
177813 229533.I1RAM7 pp 229533.V6RKY7
177814 229533.I1RAM7 pp 229533.V6RVB4
177815 229533.I1RAM7 pp 229533.I1RY44
177816 229533.I1RAM7 pp 229533.I1RMF5
177817 229533.I1RAM7 pp 229533.I1S549
177818 229533.I1RAM7 pp 229533.I1RAY4
177819 229533.I1RAM7 pp 229533.I1RVY0
177820 229533.I1RAM7 pp 229533.I1RJZ7
177821 229533.I1RAM7 pp 229533.I1S1V5
177822 229533.I1RAM7 pp 229533.I1RRM9
177823 229533.I1RAM7 pp 229533.I1RG99
177824 229533.I1RAM7 pp 229533.V6RVG3
177825 229533.I1RAM7 pp 229533.I1RNP7
177826 229533.I1RAM7 pp 229533.I1RPU3
177827 229533.I1RAM7 pp 229533.I1RBY4
177828 229533.I1RAM7 pp 229533.I1RBV5

177829	229533.I1RAM7	pp	229533.I1RQM0
177830	229533.I1RAM7	pp	229533.I1RLU1
177831	229533.I1RAM7	pp	229533.V6R6F0
177832	229533.I1RAM7	pp	229533.I1RX38
177833	229533.I1RAM7	pp	229533.I1RV91
177834	229533.I1RAM7	pp	229533.I1S2E9
177835	229533.I1RAM7	pp	229533.I1S3L1
177836	229533.I1RAM7	pp	229533.I1RCJ7
177837	229533.I1RAM7	pp	229533.I1RB74
177838	229533.I1RAM7	pp	229533.I1RPP3
177839	229533.I1RAM7	pp	229533.I1RFL7
177840	229533.I1RAM7	pp	229533.I1RQG4
177841	229533.I1RAM7	pp	229533.I1RN67
177842	229533.I1RAM7	pp	229533.I1RPD6
177843	229533.I1RAM7	pp	229533.I1RNF4
177844	229533.I1RAM7	pp	229533.I1RC97
177845	229533.I1RKY9	pp	229533.I1RWT6
177846	229533.I1RKY9	pp	229533.I1RQA3
177847	229533.I1R9X3	pp	229533.I1RF49
177848	229533.I1R9X3	pp	229533.I1RR68
177849	229533.I1R9X3	pp	229533.V6R5D2
177850	229533.I1R9X3	pp	229533.I1S0A5
177851	229533.I1R9X3	pp	229533.I1S2J4
177852	229533.I1R9X3	pp	229533.I1RN04
177853	229533.I1R9X3	pp	229533.I1RW80
177854	229533.I1R9X3	pp	229533.V6R2Z2
177855	229533.I1R9X3	pp	229533.I1RJS1
177856	229533.I1R9X3	pp	229533.I1RNG4
177857	229533.I1R9X3	pp	229533.I1S9Z4
177858	229533.I1R9X3	pp	229533.I1RH83
177859	229533.I1R9X3	pp	229533.I1S8H5
177860	229533.I1R9X3	pp	229533.V6R756
177861	229533.I1R9X3	pp	229533.I1RF85
177862	229533.I1R9X3	pp	229533.I1RTY9
177863	229533.I1R9X3	pp	229533.I1RIV2
177864	229533.I1R9X3	pp	229533.I1RHU8
177865	229533.I1R9X3	pp	229533.I1RE52
177866	229533.I1R9X3	pp	229533.I1SAT2
177867	229533.I1R9X3	pp	229533.I1RCU7
177868	229533.I1R9X3	pp	229533.I1S1T5
177869	229533.I1R9X3	pp	229533.I1RRF7
177870	229533.I1R9X3	pp	229533.I1S6H3
177871	229533.I1R9X3	pp	229533.I1S094
177872	229533.I1R9X3	pp	229533.I1RDN3
177873	229533.I1R9X3	pp	229533.I1SAI4
177874	229533.I1R9X3	pp	229533.I1RPV8
177875	229533.I1R9X3	pp	229533.I1RMW9
177876	229533.I1R9X3	pp	229533.I1RKR8
177877	229533.I1R9X3	pp	229533.I1RQV7
177878	229533.I1R9X3	pp	229533.I1RW91
177879	229533.I1R9X3	pp	229533.I1RZ56
177880	229533.I1R9X3	pp	229533.I1S5D0
177881	229533.I1R9X3	pp	229533.V6R0W1
177882	229533.I1R9X3	pp	229533.V6QU99
177883	229533.I1R9X3	pp	229533.V6RAM6
177884	229533.I1R9X3	pp	229533.I1RRS6
177885	229533.I1R9X3	pp	229533.I1RCU2
177886	229533.I1R9X3	pp	229533.I1RPW8
177887	229533.I1R9X3	pp	229533.I1RST9
177888	229533.I1R9X3	pp	229533.I1RIB5
177889	229533.I1R9X3	pp	229533.I1S489
177890	229533.I1R9X3	pp	229533.I1S659
177891	229533.I1R9X3	pp	229533.I1RA13
177892	229533.I1R9X3	pp	229533.I1RCG6
177893	229533.I1R9X3	pp	229533.I1S267
177894	229533.I1RHJ2	pp	229533.I1S3L1
177895	229533.I1RHJ2	pp	229533.I1RR68
177896	229533.I1RHJ2	pp	229533.V6R6F0
177897	229533.I1RHJ2	pp	229533.I1RMJ3
177898	229533.I1RHJ2	pp	229533.I1RKB2
177899	229533.I1RHJ2	pp	229533.I1RJW4
177900	229533.I1RHJ2	pp	229533.I1S8X0
177901	229533.I1RHJ2	pp	229533.V6RV57

177902 229533.I1RHJ2 pp 229533.V6RLM7
177903 229533.I1RHJ2 pp 229533.I1S9Z4
177904 229533.I1RHJ2 pp 229533.I1RK02
177905 229533.I1RHJ2 pp 229533.I1S1F3
177906 229533.I1RHJ2 pp 229533.I1RSM8
177907 229533.I1RHJ2 pp 229533.I1RYK0
177908 229533.I1RHJ2 pp 229533.I1RJT5
177909 229533.I1RHJ2 pp 229533.I1RL81
177910 229533.I1RHJ2 pp 229533.V6RVB4
177911 229533.I1RHJ2 pp 229533.I1RYC8
177912 229533.I1RHJ2 pp 229533.I1RN84
177913 229533.I1RHJ2 pp 229533.I1S8F8
177914 229533.I1RHJ2 pp 229533.I1RP86
177915 229533.I1RHJ2 pp 229533.I1RND4
177916 229533.I1RHJ2 pp 229533.I1RSA3
177917 229533.I1RHJ2 pp 229533.I1RMD3
177918 229533.I1RHJ2 pp 229533.I1RYA0
177919 229533.I1RHJ2 pp 229533.I1RPQ2
177920 229533.I1RHJ2 pp 229533.I1RTI3
177921 229533.I1RHJ2 pp 229533.I1S8R8
177922 229533.I1RHJ2 pp 229533.V6RAN2
177923 229533.I1RHJ2 pp 229533.V6QVS3
177924 229533.I1RHJ2 pp 229533.I1RIP6
177925 229533.I1RHJ2 pp 229533.I1RNM7
177926 229533.I1RHJ2 pp 229533.I1RST8
177927 229533.I1RHJ2 pp 229533.I1S5C5
177928 229533.I1RHJ2 pp 229533.I1RMK7
177929 229533.I1RHJ2 pp 229533.I1RXV1
177930 229533.I1RHJ2 pp 229533.I1RXE6
177931 229533.I1RHJ2 pp 229533.I1RTP2
177932 229533.I1RHJ2 pp 229533.V6RDP1
177933 229533.I1RHJ2 pp 229533.I1RIY3
177934 229533.I1RHJ2 pp 229533.I1RHP8
177935 229533.I1RHJ2 pp 229533.I1RQ14
177936 229533.I1RHJ2 pp 229533.I1RRC4
177937 229533.I1RHJ2 pp 229533.I1RMQ6
177938 229533.I1RHJ2 pp 229533.I1S4Q4
177939 229533.I1RHJ2 pp 229533.I1RJU8
177940 229533.A0A1C3YKY0 pp 229533.I1RS17
177941 229533.A0A1C3YKY0 pp 229533.I1RNG4
177942 229533.A0A1C3YKY0 pp 229533.I1S256
177943 229533.A0A1C3YKY0 pp 229533.I1RN67
177944 229533.A0A1C3YKY0 pp 229533.I1RD22
177945 229533.A0A1C3YKY0 pp 229533.I1RX22
177946 229533.A0A1C3YKY0 pp 229533.I1S5R6
177947 229533.A0A1C3YKY0 pp 229533.I1RY62
177948 229533.A0A1C3YKY0 pp 229533.I1RC27
177949 229533.A0A1C3YKY0 pp 229533.I1RC05
177950 229533.A0A1C3YKY0 pp 229533.I1RBF3
177951 229533.A0A1C3YKY0 pp 229533.V6RPQ8
177952 229533.A0A1C3YKY0 pp 229533.I1S9Y1
177953 229533.A0A1C3YKY0 pp 229533.I1RRT8
177954 229533.A0A1C3YKY0 pp 229533.I1RE63
177955 229533.A0A1C3YKY0 pp 229533.I1RYI5
177956 229533.A0A1C3YKY0 pp 229533.I1RG11
177957 229533.A0A1C3YKY0 pp 229533.I1S5R1
177958 229533.A0A1C3YKY0 pp 229533.I1RUG3
177959 229533.A0A1C3YKY0 pp 229533.I1RPP3
177960 229533.A0A1C3YKY0 pp 229533.O74638
177961 229533.A0A1C3YKY0 pp 229533.A0A1C3YLK8
177962 229533.A0A1C3YKY0 pp 229533.I1SAN9
177963 229533.A0A1C3YKY0 pp 229533.I1RGA4
177964 229533.A0A1C3YKY0 pp 229533.I1RBC7
177965 229533.A0A1C3YKY0 pp 229533.I1RT40
177966 229533.A0A1C3YKY0 pp 229533.I1S2H4
177967 229533.A0A1C3YKY0 pp 229533.I1RZZ9
177968 229533.A0A1C3YKY0 pp 229533.V6R042
177969 229533.A0A1C3YKY0 pp 229533.I1RTD5
177970 229533.A0A1C3YKY0 pp 229533.I1RPU8
177971 229533.A0A1C3YKY0 pp 229533.I1RSQ4
177972 229533.A0A1C3YKY0 pp 229533.Q4HXF0
177973 229533.A0A1C3YKY0 pp 229533.V6RRH2
177974 229533.A0A1C3YKY0 pp 229533.I1S8G4

229533.A0A1C3YKY0	pp	229533.I1RP69
229533.A0A1C3YKY0	pp	229533.I1RJZ7
229533.A0A1C3YKY0	pp	229533.I1RBY4
229533.A0A1C3YKY0	pp	229533.I1RE76
229533.A0A1C3YKY0	pp	229533.I1RGA6
229533.A0A1C3YKY0	pp	229533.A0A1C3YMA1
229533.A0A1C3YKY0	pp	229533.I1RZ39
229533.A0A1C3YKY0	pp	229533.V6R057
229533.A0A1C3YKY0	pp	229533.I1RN88
229533.A0A1C3YKY0	pp	229533.I1RMI8
229533.A0A1C3YKY0	pp	229533.I1RL04
229533.A0A1C3YKY0	pp	229533.I1S216
229533.A0A1C3YKY0	pp	229533.I1RWE8
229533.A0A1C3YKY0	pp	229533.I1RKY2
229533.A0A1C3YKY0	pp	229533.I1RFL4
229533.A0A1C3YKY0	pp	229533.I1RCH5
229533.A0A1C3YKY0	pp	229533.I1S0J1
229533.A0A1C3YKY0	pp	229533.I1RHJ2
229533.A0A1C3YKY0	pp	229533.I1RX46
229533.A0A1C3YKY0	pp	229533.V6RTJ8
229533.A0A1C3YKY0	pp	229533.I1RP86
229533.A0A1C3YKY0	pp	229533.I1S2E8
229533.A0A1C3YKY0	pp	229533.I1RJV3
229533.A0A1C3YKY0	pp	229533.V6R2A1
229533.A0A1C3YKY0	pp	229533.I1RB44
229533.A0A1C3YKY0	pp	229533.I1RAW0
229533.A0A1C3YKY0	pp	229533.I1RCW6
229533.A0A1C3YKY0	pp	229533.I1S3S3
229533.A0A1C3YKY0	pp	229533.I1RME6
229533.A0A1C3YKY0	pp	229533.I1RVX0
229533.A0A1C3YKY0	pp	229533.I1RRI2
229533.A0A1C3YKY0	pp	229533.I1RQS9
229533.A0A1C3YKY0	pp	229533.I1RXD7
229533.A0A1C3YKY0	pp	229533.I1SAJ3
229533.A0A1C3YKY0	pp	229533.A0A1C3YN26
229533.A0A1C3YKY0	pp	229533.A0A1C3YLR6
229533.A0A1C3YKY0	pp	229533.I1RMM8
229533.A0A1C3YKY0	pp	229533.I1RA70
229533.A0A1C3YKY0	pp	229533.V6R1K4
229533.A0A1C3YKY0	pp	229533.I1RB39
229533.A0A1C3YKY0	pp	229533.I1RJU3
229533.A0A1C3YKY0	pp	229533.I1RFK7
229533.A0A1C3YKY0	pp	229533.I1S0B7
229533.A0A1C3YKY0	pp	229533.A0A1C3YN30
229533.A0A1C3YKY0	pp	229533.A0A1C3YM63
229533.A0A1C3YKY0	pp	229533.I1RA06
229533.A0A1C3YKY0	pp	229533.I1RW71
229533.A0A1C3YKY0	pp	229533.I1RPN4
229533.A0A1C3YKY0	pp	229533.I1RMK7
229533.A0A1C3YKY0	pp	229533.I1RCZ3
229533.A0A1C3YKY0	pp	229533.I1RF82
229533.A0A1C3YKY0	pp	229533.I1RK81
229533.A0A1C3YKY0	pp	229533.I1RSQ7
229533.A0A1C3YKY0	pp	229533.I1S4S4
229533.A0A1C3YKY0	pp	229533.I1RK15
229533.A0A1C3YKY0	pp	229533.I1RG14
229533.A0A1C3YKY0	pp	229533.I1RCL4
229533.A0A1C3YKY0	pp	229533.I1RVX9
229533.A0A1C3YKY0	pp	229533.I1RMQ4
229533.A0A1C3YKY0	pp	229533.I1RM67
229533.A0A1C3YKY0	pp	229533.I1RXF0
229533.A0A1C3YKY0	pp	229533.I1RBI1
229533.A0A1C3YKY0	pp	229533.I1RM30
229533.A0A1C3YKY0	pp	229533.I1RC61
229533.A0A1C3YKY0	pp	229533.I1RQ81
229533.A0A1C3YKY0	pp	229533.V6RCZ8
229533.A0A1C3YKY0	pp	229533.I1RS18
229533.A0A1C3YKY0	pp	229533.I1RTD3
229533.A0A1C3YKY0	pp	229533.I1RM26
229533.A0A1C3YKY0	pp	229533.I1RVR0
229533.A0A1C3YKY0	pp	229533.I1REL7
229533.A0A1C3YKY0	pp	229533.I1RYI7
229533.A0A1C3YKY0	pp	229533.I1RRV4

178048	229533.A0A1C3YKY0	pp	229533.I1S4Q4
178049	229533.A0A1C3YKY0	pp	229533.I1RM35
178050	229533.A0A1C3YKY0	pp	229533.I1REK8
178051	229533.A0A1C3YKY0	pp	229533.V6RBN2
178052	229533.A0A1C3YKY0	pp	229533.V6QUE6
178053	229533.A0A1C3YKY0	pp	229533.I1S3K6
178054	229533.A0A1C3YKY0	pp	229533.I1RK95
178055	229533.A0A1C3YKY0	pp	229533.I1S5B0
178056	229533.A0A1C3YKY0	pp	229533.I1S1J7
178057	229533.A0A1C3YKY0	pp	229533.I1RA94
178058	229533.A0A1C3YKY0	pp	229533.I1RRY9
178059	229533.A0A1C3YKY0	pp	229533.I1RAZ3
178060	229533.A0A098D8H0	pp	229533.A0A1C3YKA6
178061	229533.A0A098D8H0	pp	229533.I1RY44
178062	229533.A0A098D8H0	pp	229533.V6RVG3
178063	229533.A0A098D8H0	pp	229533.I1RT36
178064	229533.A0A098D8H0	pp	229533.V6RGI1
178065	229533.A0A098D8H0	pp	229533.I1RAY4
178066	229533.A0A098D8H0	pp	229533.A0A1C3YII1
178067	229533.A0A098D8H0	pp	229533.V6RKY7
178068	229533.A0A098D8H0	pp	229533.I1RBM3
178069	229533.A0A098D8H0	pp	229533.I1RMQ5
178070	229533.A0A098D8H0	pp	229533.V6RMW6
178071	229533.A0A098D8H0	pp	229533.I1RW16
178072	229533.A0A098D8H0	pp	229533.I1RPP4
178073	229533.A0A098D8H0	pp	229533.V6R042
178074	229533.A0A098D8H0	pp	229533.A0A098DH49
178075	229533.A0A098D8H0	pp	229533.I1S0D1
178076	229533.A0A098D8H0	pp	229533.I1RRW0
178077	229533.A0A098D8H0	pp	229533.A0A1C3YI28
178078	229533.A0A098D8H0	pp	229533.I1RQB4
178079	229533.A0A098D8H0	pp	229533.I1R9Y9
178080	229533.A0A098D8H0	pp	229533.I1RNY8
178081	229533.A0A098D8H0	pp	229533.I1S0M2
178082	229533.A0A098D8H0	pp	229533.A0A1C3YIB0
178083	229533.A0A098D8H0	pp	229533.A0A0E0RS85
178084	229533.A0A098D8H0	pp	229533.I1S1W4
178085	229533.A0A098D8H0	pp	229533.I1S2K7
178086	229533.A0A098D8H0	pp	229533.I1RJW4
178087	229533.A0A098D8H0	pp	229533.A0A098DJL5
178088	229533.A0A098D8H0	pp	229533.I1RRY3
178089	229533.A0A098D8H0	pp	229533.I1RA43
178090	229533.A0A098D8H0	pp	229533.I1S1D3
178091	229533.A0A098D8H0	pp	229533.I1S2E9
178092	229533.A0A098D8H0	pp	229533.I1RBV5
178093	229533.A0A098D8H0	pp	229533.I1RHJ2
178094	229533.A0A098D8H0	pp	229533.I1RRP7
178095	229533.A0A098D8H0	pp	229533.A0A1C3YIN6
178096	229533.A0A098D8H0	pp	229533.I1RHJ1
178097	229533.A0A098D8H0	pp	229533.A0A098E4P3
178098	229533.A0A098D8H0	pp	229533.I1RT04
178099	229533.A0A098D8H0	pp	229533.A0A098DF98
178100	229533.A0A098D8H0	pp	229533.I1RQ72
178101	229533.A0A098D8H0	pp	229533.I1RAU7
178102	229533.A0A098D8H0	pp	229533.I1R9Y7
178103	229533.A0A098D8H0	pp	229533.V6R6F0
178104	229533.A0A098D8H0	pp	229533.I1RB41
178105	229533.A0A098D8H0	pp	229533.A0A1C3YKJ6
178106	229533.A0A098D8H0	pp	229533.I1RQG4
178107	229533.A0A098D8H0	pp	229533.I1RB92
178108	229533.A0A098D8H0	pp	229533.I1RV91
178109	229533.A0A098D8H0	pp	229533.A0A0E0RP92
178110	229533.A0A098D8H0	pp	229533.I1RDZ4
178111	229533.A0A098D8H0	pp	229533.I1RNY9
178112	229533.A0A098D8H0	pp	229533.I1RPW0
178113	229533.A0A098D8H0	pp	229533.I1RHQ8
178114	229533.A0A098D8H0	pp	229533.I1RRN5
178115	229533.A0A098D8H0	pp	229533.I1RZE4
178116	229533.A0A098D8H0	pp	229533.I1RB49
178117	229533.A0A098D8H0	pp	229533.I1RX38
178118	229533.A0A098D8H0	pp	229533.I1RLU1
178119	229533.A0A098D8H0	pp	229533.I1RNP7
178120	229533.A0A098D8H0	pp	229533.I1RRM9

229533.A0A098D8H0 pp 229533.I1RNF4
229533.A0A098D8H0 pp 229533.I1S1V5
229533.A0A098D8H0 pp 229533.I1RC97
229533.A0A098D8H0 pp 229533.I1RTI3
229533.A0A098D8H0 pp 229533.I1RP28
229533.A0A098D8H0 pp 229533.V6RVB4
229533.A0A098D8H0 pp 229533.I1RCL8
229533.A0A098D8H0 pp 229533.I1RPD6
229533.A0A098D8H0 pp 229533.I1RFL4
229533.A0A098D8H0 pp 229533.A0A0E0RTX3
229533.A0A098D8H0 pp 229533.I1S3L1
229533.A0A098D8H0 pp 229533.I1RZ63
229533.A0A098D8H0 pp 229533.I1RPP3
229533.A0A098D8H0 pp 229533.I1RB74
229533.A0A098D8H0 pp 229533.I1RAN2
229533.A0A098D8H0 pp 229533.I1RFW0
229533.A0A098D8H0 pp 229533.I1RMF5
229533.A0A098D8H0 pp 229533.I1RFL7
229533.A0A098D8H0 pp 229533.A0A1C3YI10
229533.A0A098D8H0 pp 229533.I1RAN3
229533.A0A098D8H0 pp 229533.A0A0E0RQT8
229533.A0A098D8H0 pp 229533.A0A1C3YIG3
229533.A0A098D8H0 pp 229533.I1RM09
229533.A0A098D8H0 pp 229533.I1RR73
229533.A0A098D8H0 pp 229533.A0A1C3YK37
229533.A0A098D8H0 pp 229533.I1RUN8
229533.A0A098D8H0 pp 229533.I1RZZ9
229533.A0A098D8H0 pp 229533.A0A0E0SNY0
229533.A0A098D8H0 pp 229533.A0A1C3YHL0
229533.A0A098D8H0 pp 229533.A0A1C3YJI8
229533.A0A098D8H0 pp 229533.I1RMD3
229533.A0A098D8H0 pp 229533.A0A1C3YN39
229533.A0A098D8H0 pp 229533.V6QVS3
229533.A0A098D8H0 pp 229533.I1RJ88
229533.A0A098D8H0 pp 229533.I1RXV1
229533.A0A098D8H0 pp 229533.I1RQM0
229533.A0A098D8H0 pp 229533.I1RKK0
229533.A0A098D8H0 pp 229533.I1RWT6
229533.A0A098D8H0 pp 229533.I1S0X7
229533.A0A098D8H0 pp 229533.Q4ICA8
229533.A0A098D8H0 pp 229533.I1RC17
229533.A0A098D8H0 pp 229533.I1RPU4
229533.A0A098D8H0 pp 229533.I1S549
229533.A0A098D8H0 pp 229533.I1RC95
229533.A0A098D8H0 pp 229533.A0A1C3YKV3
229533.A0A098D8H0 pp 229533.A0A1C3YJZ0
229533.A0A098D8H0 pp 229533.I1RYY5
229533.A0A098D8H0 pp 229533.I1RAM7
229533.A0A098D8H0 pp 229533.I1RIR0
229533.A0A098D8H0 pp 229533.I1RMB2
229533.A0A098D8H0 pp 229533.I1RZH4
229533.A0A098D8H0 pp 229533.A0A098DGP5
229533.A0A098D8H0 pp 229533.A0A0E0SJS1
229533.A0A098D8H0 pp 229533.I1RSS1
229533.A0A098D8H0 pp 229533.I1RCL7
229533.A0A098D8H0 pp 229533.A0A0E0RTV8
229533.A0A098D8H0 pp 229533.I1RUQ5
229533.A0A098D8H0 pp 229533.I1RC22
229533.A0A098D8H0 pp 229533.I1S439
229533.A0A098D8H0 pp 229533.I1RRY9
229533.A0A098D8H0 pp 229533.I1RB75
229533.A0A098D8H0 pp 229533.I1RMM9
229533.A0A098D8H0 pp 229533.A0A1C3YIJ3
229533.A0A098D8H0 pp 229533.Q4IR87
229533.A0A098D8H0 pp 229533.I1RQA3
229533.A0A098D8H0 pp 229533.I1S0Z7
229533.A0A098D8H0 pp 229533.I1RYZ9
229533.A0A098D8H0 pp 229533.A0A0E0RVZ1
229533.A0A098D8H0 pp 229533.I1RFU8
229533.A0A098D8H0 pp 229533.Q4IJM4
229533.A0A098D8H0 pp 229533.A0A1C3YJW1
229533.A0A098D8H0 pp 229533.I1RN08
229533.A0A098D8H0 pp 229533.I1S8R8

229533.A0A098D8H0	pp	229533.A0A1C3YJF1
229533.A0A098D8H0	pp	229533.I1RN65
229533.A0A098D8H0	pp	229533.I1RPA6
229533.A0A098D8H0	pp	229533.I1RN31
229533.A0A098D8H0	pp	229533.A0A0E0RUI4
229533.A0A098D8H0	pp	229533.A0A0E0SD93
229533.A0A098D8H0	pp	229533.I1RLX6
229533.A0A098D8H0	pp	229533.I1RNC0
229533.A0A098D8H0	pp	229533.I1RBZ9
229533.A0A098D8H0	pp	229533.A0A1C3YIL0
229533.A0A098D8H0	pp	229533.A0A098DPS3
229533.A0A098D8H0	pp	229533.I1RQC9
229533.A0A098D8H0	pp	229533.I1RYH9
229533.A0A098D8H0	pp	229533.A0A098DT74
229533.A0A098D8H0	pp	229533.I1RHP8
229533.A0A098D8H0	pp	229533.V6QV09
229533.A0A098D8H0	pp	229533.I1RXH6
229533.A0A098D8H0	pp	229533.A0A1C3YKW5
229533.A0A098D8H0	pp	229533.I1S224
229533.A0A098D8H0	pp	229533.I1RJH1
229533.A0A098D8H0	pp	229533.A0A0E0SAC4
229533.A0A098D8H0	pp	229533.I1RAW7
229533.A0A098D8H0	pp	229533.I1RIY3
229533.A0A098D8H0	pp	229533.A0A098DQ41
229533.A0A098D8H0	pp	229533.I1RQ26
229533.A0A098D8H0	pp	229533.I1RL24
229533.A0A098D8H0	pp	229533.I1RFZ3
229533.A0A098D8H0	pp	229533.I1RR24
229533.A0A098D8H0	pp	229533.I1RD18
229533.A0A098D8H0	pp	229533.A0A098E0E1
229533.A0A098D8H0	pp	229533.A0A1C3YKF2
229533.A0A098D8H0	pp	229533.A0A1C3YJV7
229533.A0A098D8H0	pp	229533.A0A1C3YJH8
229533.A0A098D8H0	pp	229533.I1RV66
229533.A0A098D8H0	pp	229533.A0A0E0SA11
229533.A0A098D8H0	pp	229533.I1RDB1
229533.A0A098D8H0	pp	229533.I1RX70
229533.A0A098D8H0	pp	229533.I1RAM6
229533.A0A098D8H0	pp	229533.I1RWJ6
229533.A0A098D8H0	pp	229533.I1RMC8
229533.I1RT75	pp	229533.V6RRH2
229533.I1RT75	pp	229533.I1S274
229533.I1RT75	pp	229533.I1S5C5
229533.I1RT75	pp	229533.I1S5H2
229533.I1RT75	pp	229533.Q4IN00
229533.I1RT75	pp	229533.I1S3G4
229533.I1RT75	pp	229533.V6QUV7
229533.I1RT75	pp	229533.I1RVX0
229533.I1RT75	pp	229533.I1S2H4
229533.I1RT75	pp	229533.V6RSF3
229533.I1RT75	pp	229533.Q4HZB8
229533.I1RT75	pp	229533.V6RLM7
229533.I1RT75	pp	229533.I1S253
229533.I1RT75	pp	229533.I1S1F3
229533.I1RT75	pp	229533.I1RW43
229533.I1RT75	pp	229533.V6RKY8
229533.I1RT75	pp	229533.I1RWS4
229533.I1RT75	pp	229533.I1RZ39
229533.I1RT75	pp	229533.I1RYP2
229533.I1RT75	pp	229533.V6QUE6
229533.I1RT75	pp	229533.I1RWH8
229533.I1RT75	pp	229533.I1S0Z6
229533.I1RT75	pp	229533.Q4IJK2
229533.I1RT75	pp	229533.I1S5B0
229533.I1RT75	pp	229533.Q4HXF0
229533.I1RT75	pp	229533.V6RCZ8
229533.I1RT75	pp	229533.I1RYJ6
229533.I1RT75	pp	229533.I1S1W8
229533.I1RT75	pp	229533.I1RYI9
229533.I1RT75	pp	229533.I1S6D7
229533.I1RT75	pp	229533.Q4INZ9
229533.I1RT75	pp	229533.I1RYJ7
229533.I1SAH9	pp	229533.I1SAT2

229533.A0A1C3YII1 pp 229533.I1RW16
229533.A0A1C3YII1 pp 229533.I1RCU9
229533.A0A1C3YII1 pp 229533.I1RUN9
229533.A0A1C3YII1 pp 229533.I1RCJ4
229533.A0A1C3YII1 pp 229533.I1RZP2
229533.A0A1C3YII1 pp 229533.I1RZC3
229533.A0A1C3YII1 pp 229533.I1RHJ1
229533.A0A1C3YII1 pp 229533.I1RB87
229533.A0A1C3YII1 pp 229533.V6RVB4
229533.A0A1C3YII1 pp 229533.V6R6F0
229533.A0A1C3YII1 pp 229533.I1RCV6
229533.A0A1C3YII1 pp 229533.I1RWJ1
229533.A0A1C3YII1 pp 229533.I1RM94
229533.A0A1C3YII1 pp 229533.I1RCV0
229533.A0A1C3YII1 pp 229533.I1REI3
229533.A0A1C3YII1 pp 229533.I1RW15
229533.A0A1C3YII1 pp 229533.A0A1C3YII3
229533.A0A1C3YII1 pp 229533.A0A1C3YN78
229533.A0A1C3YII1 pp 229533.I1RU98
229533.A0A1C3YII1 pp 229533.V6RDP1
229533.A0A1C3YII1 pp 229533.I1RFF2
229533.A0A1C3YII1 pp 229533.A0A1C3YJK9
229533.A0A1C3YII1 pp 229533.I1RPX7
229533.A0A1C3YII1 pp 229533.I1RC37
229533.A0A1C3YII1 pp 229533.I1S7X9
229533.A0A1C3YII1 pp 229533.I1RZH4
229533.A0A1C3YII1 pp 229533.I1S0R1
229533.A0A1C3YII1 pp 229533.I1RHV1
229533.A0A1C3YII1 pp 229533.A0A1C3YMX3
229533.A0A1C3YII1 pp 229533.I1RKT7
229533.A0A1C3YII1 pp 229533.I1RHC6
229533.A0A1C3YII1 pp 229533.I1RSE4
229533.A0A1C3YII1 pp 229533.I1RM23
229533.A0A1C3YII1 pp 229533.V6RUT5
229533.A0A1C3YII1 pp 229533.V6RJ21
229533.A0A1C3YII1 pp 229533.I1RK45
229533.A0A1C3YII1 pp 229533.I1RBN0
229533.A0A1C3YII1 pp 229533.I1RCY0
229533.A0A1C3YII1 pp 229533.I1RS10
229533.A0A1C3YII1 pp 229533.I1RN31
229533.A0A1C3YII1 pp 229533.I1RDX7
229533.A0A1C3YII1 pp 229533.I1S274
229533.A0A1C3YII1 pp 229533.A0A1C3YLB2
229533.A0A1C3YII1 pp 229533.I1RQN8
229533.A0A1C3YII1 pp 229533.I1RER6
229533.A0A1C3YII1 pp 229533.I1S1W3
229533.A0A1C3YII1 pp 229533.I1RZK9
229533.A0A1C3YII1 pp 229533.V6RLM7
229533.A0A1C3YII1 pp 229533.I1RBD6
229533.A0A1C3YII1 pp 229533.A0A1C3YJX9
229533.A0A1C3YII1 pp 229533.I1RT76
229533.A0A1C3YII1 pp 229533.I1S5C4
229533.A0A1C3YII1 pp 229533.I1RQ06
229533.A0A1C3YII1 pp 229533.A0A1C3YK43
229533.A0A1C3YII1 pp 229533.I1RTI7
229533.A0A1C3YII1 pp 229533.I1RHY5
229533.A0A1C3YII1 pp 229533.I1RJW4
229533.A0A1C3YII1 pp 229533.I1RHJ2
229533.A0A1C3YII1 pp 229533.V6QVS3
229533.A0A1C3YII1 pp 229533.I1S6H4
229533.A0A1C3YII1 pp 229533.I1RE49
229533.A0A1C3YII1 pp 229533.A0A1C3YIZ4
229533.A0A1C3YII1 pp 229533.I1RAJ9
229533.A0A1C3YII1 pp 229533.I1RNG4
229533.A0A1C3YII1 pp 229533.A0A1C3YL99
229533.A0A1C3YII1 pp 229533.I1RMD3
229533.A0A1C3YII1 pp 229533.I1RXV1
229533.A0A1C3YII1 pp 229533.I1S8X0
229533.A0A1C3YII1 pp 229533.A0A1C3YJZ0
229533.A0A1C3YII1 pp 229533.I1RHI1
229533.A0A1C3YII1 pp 229533.I1RC88
229533.A0A1C3YII1 pp 229533.I1S0P4
229533.A0A1C3YII1 pp 229533.I1RST8

178340 229533.A0A1C3YII1 pp 229533.A0A1C3YN48
178341 229533.A0A1C3YII1 pp 229533.I1RAM7
178342 229533.A0A1C3YII1 pp 229533.A0A1C3YN32
178343 229533.A0A1C3YII1 pp 229533.I1S111
178344 229533.A0A1C3YII1 pp 229533.I1RG76
178345 229533.A0A1C3YII1 pp 229533.I1RDB1
178346 229533.A0A1C3YII1 pp 229533.I1S8R8
178347 229533.A0A1C3YII1 pp 229533.I1RLY6
178348 229533.A0A1C3YII1 pp 229533.I1RWC2
178349 229533.A0A1C3YII1 pp 229533.I1RMT4
178350 229533.A0A1C3YII1 pp 229533.I1RZ02
178351 229533.A0A1C3YII1 pp 229533.I1RNS2
178352 229533.A0A1C3YII1 pp 229533.I1RKA9
178353 229533.A0A1C3YII1 pp 229533.I1RIY3
178354 229533.A0A1C3YII1 pp 229533.I1RCU8
178355 229533.A0A1C3YII1 pp 229533.I1RLX6
178356 229533.A0A1C3YII1 pp 229533.I1RR49
178357 229533.A0A1C3YII1 pp 229533.I1RZU7
178358 229533.A0A1C3YII1 pp 229533.I1RCI0
178359 229533.A0A1C3YII1 pp 229533.I1RZT6
178360 229533.I1S1W6 pp 229533.V6RR38
178361 229533.I1S1W6 pp 229533.V6RXE7
178362 229533.I1S1W6 pp 229533.V6RPN0
178363 229533.I1S1W6 pp 229533.I1S2M5
178364 229533.A0A1C3YHQ6 pp 229533.Q4IJH1
178365 229533.A0A1C3YHQ6 pp 229533.I1S5H3
178366 229533.A0A1C3YHQ6 pp 229533.I1RG07
178367 229533.A0A1C3YHQ6 pp 229533.I1RWX2
178368 229533.A0A1C3YHQ6 pp 229533.I1RD01
178369 229533.A0A1C3YHQ6 pp 229533.A0A1C3YLG6
178370 229533.A0A1C3YHQ6 pp 229533.I1RC73
178371 229533.A0A1C3YHQ6 pp 229533.Q4IAS1
178372 229533.A0A0E0SAR5 pp 229533.I1RSY2
178373 229533.A0A0E0SAR5 pp 229533.I1RMU9
178374 229533.A0A0E0SAR5 pp 229533.A0A1C3YLH6
178375 229533.A0A0E0SAR5 pp 229533.I1S277
178376 229533.A0A0E0SAR5 pp 229533.I1RVN1
178377 229533.A0A0E0SAR5 pp 229533.Q4HZB8
178378 229533.A0A0E0SAR5 pp 229533.I1RNG4
178379 229533.A0A0E0SAR5 pp 229533.A0A1C3YIK4
178380 229533.A0A0E0SAR5 pp 229533.I1RPA4
178381 229533.A0A0E0SAR5 pp 229533.I1RM67
178382 229533.A0A0E0SAR5 pp 229533.A0A0E0SLM3
178383 229533.I1RBB1 pp 229533.I1RCW6
178384 229533.I1RBB1 pp 229533.I1RN42
178385 229533.I1RBB1 pp 229533.I1RY11
178386 229533.I1RBB1 pp 229533.I1RE65
178387 229533.I1RBB1 pp 229533.I1RVT8
178388 229533.I1RBB1 pp 229533.I1RSC0
178389 229533.I1RBB1 pp 229533.I1S8Y1
178390 229533.I1RBB1 pp 229533.I1S464
178391 229533.I1RBB1 pp 229533.I1S5X9
178392 229533.I1RBB1 pp 229533.I1RFJ6
178393 229533.I1RBB1 pp 229533.I1RXD9
178394 229533.I1RBB1 pp 229533.I1RWA3
178395 229533.I1RBB1 pp 229533.I1S0P0
178396 229533.I1RBB1 pp 229533.I1RE66
178397 229533.I1RBB1 pp 229533.I1RL04
178398 229533.I1RBB1 pp 229533.I1RY92
178399 229533.I1RBB1 pp 229533.I1RYT8
178400 229533.I1RBB1 pp 229533.V6RJP8
178401 229533.I1RBB1 pp 229533.I1RS05
178402 229533.I1RBB1 pp 229533.I1RMK7
178403 229533.I1RBB1 pp 229533.I1RP02
178404 229533.I1RBB1 pp 229533.I1RS29
178405 229533.I1RBB1 pp 229533.I1RY21
178406 229533.I1RBB1 pp 229533.I1S5R5
178407 229533.I1RBB1 pp 229533.I1S8S9
178408 229533.I1RBB1 pp 229533.I1S0K2
178409 229533.A0A1C3YL55 pp 229533.I1RBC1
178410 229533.A0A1C3YL55 pp 229533.V6RQW4
178411 229533.A0A1C3YL55 pp 229533.I1RG77
178412 229533.A0A1C3YL55 pp 229533.I1RE00

178413	229533.A0A1C3YL55	pp	229533.I1RN55
178414	229533.A0A1C3YL55	pp	229533.I1RK95
178415	229533.A0A1C3YL55	pp	229533.V6RBN2
178416	229533.A0A1C3YL55	pp	229533.I1RWP4
178417	229533.A0A1C3YL55	pp	229533.I1RA12
178418	229533.A0A1C3YL55	pp	229533.I1RS18
178419	229533.A0A1C3YL55	pp	229533.I1RA94
178420	229533.A0A1C3YL55	pp	229533.I1RPI0
178421	229533.A0A1C3YL55	pp	229533.I1S835
178422	229533.A0A1C3YL55	pp	229533.I1RRY9
178423	229533.A0A1C3YL55	pp	229533.I1S987
178424	229533.A0A1C3YL55	pp	229533.I1REK8
178425	229533.A0A1C3YL55	pp	229533.I1S5B0
178426	229533.A0A1C3YL55	pp	229533.I1RY12
178427	229533.A0A1C3YL55	pp	229533.I1RAZ3
178428	229533.A0A1C3YL55	pp	229533.I1S209
178429	229533.A0A1C3YL55	pp	229533.V6QUE6
178430	229533.A0A1C3YL55	pp	229533.I1RBW0
178431	229533.A0A1C3YL55	pp	229533.I1S1W8
178432	229533.A0A1C3YL55	pp	229533.I1RAQ4
178433	229533.A0A1C3YL55	pp	229533.I1RG14
178434	229533.A0A1C3YL55	pp	229533.I1RRV4
178435	229533.A0A1C3YL55	pp	229533.I1RDE4
178436	229533.A0A1C3YL55	pp	229533.I1RJS0
178437	229533.A0A1C3YL55	pp	229533.I1RZ94
178438	229533.A0A1C3YL55	pp	229533.I1RDJ1
178439	229533.A0A1C3YL55	pp	229533.I1RYP7
178440	229533.A0A1C3YL55	pp	229533.I1RD01
178441	229533.A0A1C3YL55	pp	229533.I1RRT1
178442	229533.A0A1C3YL55	pp	229533.Q4INS6
178443	229533.A0A1C3YL55	pp	229533.A0A1C3YN36
178444	229533.A0A1C3YL55	pp	229533.A0A1C3YM63
178445	229533.A0A1C3YL55	pp	229533.V6RCZ8
178446	229533.A0A1C3YL55	pp	229533.I1RCQ4
178447	229533.A0A1C3YL55	pp	229533.I1RQ80
178448	229533.A0A1C3YL55	pp	229533.I1S788
178449	229533.A0A1C3YL55	pp	229533.I1RAL9
178450	229533.A0A1C3YL55	pp	229533.I1RR24
178451	229533.A0A1C3YL55	pp	229533.I1RQH8
178452	229533.A0A1C3YL55	pp	229533.I1S4S4
178453	229533.A0A1C3YL55	pp	229533.I1RZM8
178454	229533.A0A1C3YL55	pp	229533.I1RWM6
178455	229533.A0A1C3YL55	pp	229533.I1RXF0
178456	229533.A0A1C3YL55	pp	229533.I1RPQ1
178457	229533.A0A1C3YL55	pp	229533.I1RCB3
178458	229533.A0A1C3YL55	pp	229533.A0A1C3YLR6
178459	229533.A0A1C3YL55	pp	229533.I1RJU3
178460	229533.A0A1C3YL55	pp	229533.I1RUR0
178461	229533.A0A1C3YL55	pp	229533.V6RTJ8
178462	229533.A0A1C3YL55	pp	229533.I1RQS9
178463	229533.A0A1C3YL55	pp	229533.I1RSY2
178464	229533.A0A1C3YL55	pp	229533.A0A1C3YML3
178465	229533.A0A1C3YL55	pp	229533.I1RP99
178466	229533.A0A1C3YL55	pp	229533.I1RD52
178467	229533.A0A1C3YL55	pp	229533.I1RPN4
178468	229533.A0A1C3YL55	pp	229533.I1RRY1
178469	229533.A0A1C3YL55	pp	229533.I1S4S7
178470	229533.A0A1C3YL55	pp	229533.I1RVX0
178471	229533.A0A1C3YL55	pp	229533.I1RB92
178472	229533.A0A1C3YL55	pp	229533.I1S3S3
178473	229533.A0A1C3YL55	pp	229533.I1RP86
178474	229533.A0A1C3YL55	pp	229533.V6RLM7
178475	229533.A0A1C3YL55	pp	229533.I1RSJ1
178476	229533.A0A1C3YL55	pp	229533.I1RQE7
178477	229533.A0A1C3YL55	pp	229533.I1RA10
178478	229533.A0A1C3YL55	pp	229533.I1RCH5
178479	229533.A0A1C3YL55	pp	229533.I1RDJ8
178480	229533.A0A1C3YL55	pp	229533.I1RKI2
178481	229533.A0A1C3YL55	pp	229533.I1RZT1
178482	229533.A0A1C3YL55	pp	229533.A0A1C3YLB6
178483	229533.A0A1C3YL55	pp	229533.I1RUG4
178484	229533.A0A1C3YL55	pp	229533.Q4IB89
178485	229533.A0A1C3YL55	pp	229533.I1RE01

178486	229533.A0A1C3YL55	pp	229533.I1RAW0
178487	229533.A0A1C3YL55	pp	229533.I1RKB6
178488	229533.A0A1C3YL55	pp	229533.I1RBY4
178489	229533.A0A1C3YL55	pp	229533.I1RSR9
178490	229533.A0A1C3YL55	pp	229533.I1RN57
178491	229533.A0A1C3YL55	pp	229533.I1S064
178492	229533.A0A1C3YL55	pp	229533.I1RVR2
178493	229533.A0A1C3YL55	pp	229533.I1RC54
178494	229533.A0A1C3YL55	pp	229533.I1RG11
178495	229533.A0A1C3YL55	pp	229533.I1RZ86
178496	229533.A0A1C3YL55	pp	229533.I1S0D5
178497	229533.A0A1C3YL55	pp	229533.Q4I5V2
178498	229533.A0A1C3YL55	pp	229533.I1RNA2
178499	229533.A0A1C3YL55	pp	229533.I1RBC8
178500	229533.A0A1C3YL55	pp	229533.I1RCA5
178501	229533.A0A1C3YL55	pp	229533.I1RD22
178502	229533.A0A1C3YL55	pp	229533.I1RBN9
178503	229533.A0A1C3YL55	pp	229533.I1RX64
178504	229533.A0A1C3YL55	pp	229533.I1S2H4
178505	229533.A0A1C3YL55	pp	229533.I1RTN6
178506	229533.A0A1C3YL55	pp	229533.V6RMW6
178507	229533.A0A1C3YL55	pp	229533.I1RBS5
178508	229533.A0A1C3YL55	pp	229533.I1S3G4
178509	229533.A0A1C3YL55	pp	229533.I1RBW1
178510	229533.A0A1C3YL55	pp	229533.V6R042
178511	229533.A0A1C3YL55	pp	229533.I1RDC0
178512	229533.A0A1C3YL55	pp	229533.I1RSP7
178513	229533.A0A1C3YL55	pp	229533.I1RD19
178514	229533.A0A1C3YL55	pp	229533.I1RJQ5
178515	229533.A0A1C3YL55	pp	229533.I1RRI2
178516	229533.A0A1C3YL55	pp	229533.I1RYW3
178517	229533.A0A1C3YL55	pp	229533.I1RUG3
178518	229533.A0A1C3YL55	pp	229533.I1RQI6
178519	229533.A0A1C3YL55	pp	229533.I1RZ98
178520	229533.A0A1C3YL55	pp	229533.I1S9Y1
178521	229533.A0A1C3YL55	pp	229533.I1RSC7
178522	229533.A0A1C3YL55	pp	229533.I1RQJ4
178523	229533.A0A1C3YL55	pp	229533.O74638
178524	229533.A0A1C3YL55	pp	229533.I1RW03
178525	229533.A0A1C3YL55	pp	229533.I1RMI8
178526	229533.A0A1C3YL55	pp	229533.I1RVW0
178527	229533.A0A1C3YL55	pp	229533.I1S998
178528	229533.A0A1C3YL55	pp	229533.I1RHD2
178529	229533.A0A1C3YL55	pp	229533.I1S4P6
178530	229533.A0A1C3YL55	pp	229533.I1RRT8
178531	229533.A0A1C3YL55	pp	229533.I1RGA6
178532	229533.A0A1C3YL55	pp	229533.A0A1C3YN55
178533	229533.A0A1C3YL55	pp	229533.I1RNG4
178534	229533.A0A1C3YL55	pp	229533.I1S2B8
178535	229533.A0A1C3YL55	pp	229533.I1S5R1
178536	229533.A0A1C3YL55	pp	229533.I1S523
178537	229533.A0A1C3YL55	pp	229533.I1RY29
178538	229533.A0A1C3YL55	pp	229533.I1RN67
178539	229533.A0A1C3YL55	pp	229533.I1RZ39
178540	229533.A0A1C3YL55	pp	229533.I1RY62
178541	229533.A0A1C3YL55	pp	229533.I1RX22
178542	229533.A0A1C3YL55	pp	229533.I1RSN7
178543	229533.A0A1C3YL55	pp	229533.I1RTD5
178544	229533.A0A1C3YL55	pp	229533.I1RT40
178545	229533.A0A1C3YL55	pp	229533.I1S8G4
178546	229533.A0A1C3YL55	pp	229533.I1RF02
178547	229533.A0A1C3YL55	pp	229533.I1RW24
178548	229533.A0A1C3YL55	pp	229533.I1RFT1
178549	229533.A0A1C3YL55	pp	229533.I1RZZ9
178550	229533.A0A1C3YL55	pp	229533.I1RPP3
178551	229533.A0A1C3YL55	pp	229533.A0A1C3YMJ3
178552	229533.A0A1C3YL55	pp	229533.I1RSQ4
178553	229533.A0A1C3YL55	pp	229533.I1RTA1
178554	229533.A0A1C3YL55	pp	229533.I1RWI5
178555	229533.A0A1C3YL55	pp	229533.I1RGA4
178556	229533.A0A1C3YL55	pp	229533.A0A1C3YLQ7
178557	229533.A0A1C3YL55	pp	229533.I1RYQ1
178558	229533.A0A1C3YL55	pp	229533.A0A1C3YLK8

178559 229533.A0A1C3YL55 pp 229533.I1RJV8
178560 229533.A0A1C3YL55 pp 229533.I1R9W7
178561 229533.A0A1C3YL55 pp 229533.I1RWI8
178562 229533.I1RWX2 pp 229533.I1S5H3
178563 229533.I1RWX2 pp 229533.I1SA55
178564 229533.I1S2C2 pp 229533.V6RR38
178565 229533.I1S2C2 pp 229533.V6RPN0
178566 229533.I1S2C2 pp 229533.I1SAS4
178567 229533.I1S2C2 pp 229533.I1SAS3
178568 229533.I1RTT3 pp 229533.Q4I7L0
178569 229533.I1RTT3 pp 229533.I1RXB5
178570 229533.I1RTT3 pp 229533.V6RDZ4
178571 229533.I1RTT3 pp 229533.Q4IB89
178572 229533.I1RTT3 pp 229533.I1RZT1
178573 229533.I1RKY4 pp 229533.I1RZ25
178574 229533.I1RZQ6 pp 229533.V6QVS3
178575 229533.I1RZQ6 pp 229533.P0C431
178576 229533.I1RZQ6 pp 229533.I1S095
178577 229533.I1RZQ6 pp 229533.I1S8R8
178578 229533.I1RZQ6 pp 229533.I1S4D0
178579 229533.I1RZQ6 pp 229533.V6QUE6
178580 229533.I1RZQ6 pp 229533.I1RZV4
178581 229533.I1RZQ6 pp 229533.V6R6F0
178582 229533.I1RZQ6 pp 229533.I1RZW8
178583 229533.I1RZQ6 pp 229533.I1S9Y1
178584 229533.I1RZQ6 pp 229533.V6RY56
178585 229533.I1RZQ6 pp 229533.I1S029
178586 229533.I1RZQ6 pp 229533.V6RVB4
178587 229533.I1RZQ6 pp 229533.I1S0E4
178588 229533.I1RYI5 pp 229533.I1RZV3
178589 229533.I1RYI5 pp 229533.I1RYW3
178590 229533.I1RYI5 pp 229533.I1S437
178591 229533.I1RYI5 pp 229533.I1S1C7
178592 229533.I1RYI5 pp 229533.Q4IPZ1
178593 229533.I1RYI5 pp 229533.I1S0R8
178594 229533.I1RYI5 pp 229533.I1S0J1
178595 229533.I1RYI5 pp 229533.I1S0F8
178596 229533.I1RYI5 pp 229533.I1S7W5
178597 229533.I1RYI5 pp 229533.Q4I624
178598 229533.I1RYI5 pp 229533.I1RZ79
178599 229533.I1RYI5 pp 229533.I1S6V0
178600 229533.I1RYI5 pp 229533.I1S5R1
178601 229533.I1RYI5 pp 229533.I1S5R6
178602 229533.I1RYI5 pp 229533.I1S9Z4
178603 229533.I1RYI5 pp 229533.I1S9Y1
178604 229533.I1RYI5 pp 229533.I1S016
178605 229533.I1RYI5 pp 229533.I1S2H4
178606 229533.I1RYI5 pp 229533.I1S1W9
178607 229533.I1RYI5 pp 229533.Q4I375
178608 229533.I1RYI5 pp 229533.O74638
178609 229533.I1RYI5 pp 229533.I1RYP2
178610 229533.I1RYI5 pp 229533.I1S0L4
178611 229533.I1RYI5 pp 229533.V6RMW6
178612 229533.I1RYI5 pp 229533.I1S0F7
178613 229533.I1RYI5 pp 229533.V6R042
178614 229533.I1RYI5 pp 229533.I1RYR0
178615 229533.I1RYI5 pp 229533.Q4HVQ9
178616 229533.I1RYI5 pp 229533.I1S2B8
178617 229533.I1RYI5 pp 229533.I1S8F0
178618 229533.I1RYI5 pp 229533.I1RZZ9
178619 229533.I1RYI5 pp 229533.I1RZ39
178620 229533.I1RYI5 pp 229533.I1S268
178621 229533.I1RYI5 pp 229533.I1S4Z8
178622 229533.I1RYI5 pp 229533.V6RCJ5
178623 229533.I1RYI5 pp 229533.I1S277
178624 229533.I1RYI5 pp 229533.I1S0W4
178625 229533.I1RYI5 pp 229533.V6RBN2
178626 229533.I1RYI5 pp 229533.I1S5B0
178627 229533.I1RYI5 pp 229533.I1S217
178628 229533.I1RYI5 pp 229533.I1S8D3
178629 229533.I1RYI5 pp 229533.V6RWB3
178630 229533.I1RYI5 pp 229533.I1RZB2
178631 229533.I1RYI5 pp 229533.V6R9Z0

178632 229533.I1RYI5 pp 229533.I1RYS0
178633 229533.I1RYI5 pp 229533.I1S6K9
178634 229533.I1RYI5 pp 229533.I1RZM8
178635 229533.I1RYI5 pp 229533.I1S4Q4
178636 229533.I1RYI5 pp 229533.I1S821
178637 229533.I1RYI5 pp 229533.I1RYT8
178638 229533.I1RYI5 pp 229533.Q4IJK2
178639 229533.I1RYI5 pp 229533.V6RCZ8
178640 229533.I1RYI5 pp 229533.I1RZK5
178641 229533.I1RYI5 pp 229533.V6R5T7
178642 229533.I1RYI5 pp 229533.V6RCC0
178643 229533.I1RYI5 pp 229533.V6QUE6
178644 229533.I1RYI5 pp 229533.I1S1J7
178645 229533.I1RYI5 pp 229533.V6QWP4
178646 229533.I1RYI5 pp 229533.V6RHX2
178647 229533.I1RYI5 pp 229533.I1S1B2
178648 229533.I1RYI5 pp 229533.I1S438
178649 229533.I1RYI5 pp 229533.I1S0P0
178650 229533.I1RYI5 pp 229533.I1S9I5
178651 229533.I1RYI5 pp 229533.I1S0B7
178652 229533.I1RYI5 pp 229533.I1S4S7
178653 229533.I1RYI5 pp 229533.I1S2R0
178654 229533.I1RYI5 pp 229533.I1S4S4
178655 229533.I1RYI5 pp 229533.V6RTJ8
178656 229533.I1RI75 pp 229533.I1RQ80
178657 229533.I1RI75 pp 229533.I1RZU6
178658 229533.I1RI75 pp 229533.I1RW71
178659 229533.I1RI75 pp 229533.I1RRB0
178660 229533.I1RI75 pp 229533.Q5I2J3
178661 229533.I1RI75 pp 229533.I1RQ93
178662 229533.I1RI75 pp 229533.Q4IBX0
178663 229533.I1RI75 pp 229533.I1RTQ9
178664 229533.I1RI75 pp 229533.Q4HTT2
178665 229533.I1RI75 pp 229533.I1RYL5
178666 229533.I1RI75 pp 229533.I1RWD5
178667 229533.I1RI75 pp 229533.I1S4Z7
178668 229533.I1RI75 pp 229533.I1RPL2
178669 229533.I1RI75 pp 229533.Q4I0J6
178670 229533.I1RI75 pp 229533.I1RUK7
178671 229533.I1RI75 pp 229533.I1RRF9
178672 229533.I1RI75 pp 229533.Q4IPI4
178673 229533.I1RI75 pp 229533.I1RQG4
178674 229533.I1RI75 pp 229533.I1S1C8
178675 229533.I1RI75 pp 229533.Q4I7L0
178676 229533.I1RI75 pp 229533.I1RJZ7
178677 229533.I1RI75 pp 229533.I1RTG5
178678 229533.I1RI75 pp 229533.I1S8P5
178679 229533.I1RI75 pp 229533.I1SAA0
178680 229533.I1RI75 pp 229533.I1S7X9
178681 229533.I1RI75 pp 229533.I1RMP2
178682 229533.I1RI75 pp 229533.V6QX21
178683 229533.I1RI75 pp 229533.I1RYW3
178684 229533.I1RI75 pp 229533.I1RMA7
178685 229533.I1RI75 pp 229533.I1RMQ5
178686 229533.I1RI75 pp 229533.I1S074
178687 229533.I1RI75 pp 229533.I1RP28
178688 229533.I1RI75 pp 229533.I1RTA0
178689 229533.I1REJ4 pp 229533.I1S5H3
178690 229533.I1REJ4 pp 229533.I1RJ42
178691 229533.I1REJ4 pp 229533.I1RSE9
178692 229533.I1REJ4 pp 229533.I1RZ60
178693 229533.I1REJ4 pp 229533.I1RNG4
178694 229533.A0A098E0A5 pp 229533.A0A1C3YJK3
178695 229533.A0A098E0A5 pp 229533.I1S3G4
178696 229533.A0A098E0A5 pp 229533.I1S2H4
178697 229533.A0A098E0A5 pp 229533.I1S5R1
178698 229533.A0A098E0A5 pp 229533.O74638
178699 229533.A0A098E0A5 pp 229533.I1RJC7
178700 229533.A0A098E0A5 pp 229533.I1S9Y1
178701 229533.A0A098E0A5 pp 229533.I1RZT0
178702 229533.A0A098E0A5 pp 229533.I1S076
178703 229533.A0A098E0A5 pp 229533.A0A1C3YKM9
178704 229533.A0A098E0A5 pp 229533.I1RZ39

178705	229533.A0A098E0A5	pp	229533.I1RX00
178706	229533.A0A098E0A5	pp	229533.A0A1C3YI20
178707	229533.A0A098E0A5	pp	229533.I1RGA6
178708	229533.A0A098E0A5	pp	229533.I1RSQ4
178709	229533.A0A098E0A5	pp	229533.I1RP69
178710	229533.A0A098E0A5	pp	229533.I1RCD8
178711	229533.A0A098E0A5	pp	229533.V6RRH2
178712	229533.A0A098E0A5	pp	229533.I1S8G4
178713	229533.A0A098E0A5	pp	229533.A0A1C3YIR8
178714	229533.A0A098E0A5	pp	229533.I1RB92
178715	229533.A0A098E0A5	pp	229533.I1S4Z8
178716	229533.A0A098E0A5	pp	229533.I1RXR5
178717	229533.A0A098E0A5	pp	229533.A0A1C3YMA1
178718	229533.A0A098E0A5	pp	229533.I1S277
178719	229533.A0A098E0A5	pp	229533.I1RI34
178720	229533.A0A098E0A5	pp	229533.I1RSS4
178721	229533.A0A098E0A5	pp	229533.I1RZZ9
178722	229533.A0A098E0A5	pp	229533.V6R2A1
178723	229533.A0A098E0A5	pp	229533.I1RA52
178724	229533.A0A098E0A5	pp	229533.A0A1C3YKU8
178725	229533.A0A098E0A5	pp	229533.I1RX22
178726	229533.A0A098E0A5	pp	229533.I1RBY4
178727	229533.A0A098E0A5	pp	229533.I1RE76
178728	229533.A0A098E0A5	pp	229533.I1RVX0
178729	229533.A0A098E0A5	pp	229533.I1RW26
178730	229533.A0A098E0A5	pp	229533.I1RQE7
178731	229533.A0A098E0A5	pp	229533.I1RE53
178732	229533.A0A098E0A5	pp	229533.I1RY74
178733	229533.A0A098E0A5	pp	229533.I1RS43
178734	229533.A0A098E0A5	pp	229533.I1RN67
178735	229533.A0A098E0A5	pp	229533.I1RCH5
178736	229533.A0A098E0A5	pp	229533.I1RFL4
178737	229533.A0A098E0A5	pp	229533.I1S2B8
178738	229533.A0A098E0A5	pp	229533.I1RYI5
178739	229533.A0A098E0A5	pp	229533.I1RVR2
178740	229533.A0A098E0A5	pp	229533.I1RPP3
178741	229533.A0A098E0A5	pp	229533.I1R9G4
178742	229533.A0A098E0A5	pp	229533.I1S5R6
178743	229533.A0A098E0A5	pp	229533.A0A1C3YJH9
178744	229533.A0A098E0A5	pp	229533.I1RL04
178745	229533.A0A098E0A5	pp	229533.I1RVZ2
178746	229533.A0A098E0A5	pp	229533.I1RKY2
178747	229533.A0A098E0A5	pp	229533.I1RD22
178748	229533.A0A098E0A5	pp	229533.I1RCB4
178749	229533.A0A098E0A5	pp	229533.I1RFS3
178750	229533.A0A098E0A5	pp	229533.I1RTA1
178751	229533.A0A098E0A5	pp	229533.I1RZB2
178752	229533.A0A098E0A5	pp	229533.I1RQM7
178753	229533.A0A098E0A5	pp	229533.I1RSU2
178754	229533.A0A098E0A5	pp	229533.V6R042
178755	229533.A0A098E0A5	pp	229533.I1SAJ0
178756	229533.A0A098E0A5	pp	229533.V6RMW6
178757	229533.A0A098E0A5	pp	229533.A0A1C3YIS9
178758	229533.A0A098E0A5	pp	229533.I1RGA4
178759	229533.A0A098E0A5	pp	229533.I1RWS4
178760	229533.A0A098E0A5	pp	229533.I1RAW0
178761	229533.A0A098E0A5	pp	229533.A0A1C3YLK8
178762	229533.A0A098E0A5	pp	229533.I1RPU8
178763	229533.A0A098E0A5	pp	229533.I1RLV9
178764	229533.A0A098E0A5	pp	229533.I1RRP7
178765	229533.A0A098E0A5	pp	229533.Q4HXF0
178766	229533.A0A098E0A5	pp	229533.A0A1C3YKY0
178767	229533.A0A098E0A5	pp	229533.A0A1C3YL55
178768	229533.A0A098E0A5	pp	229533.I1RT75
178769	229533.A0A098E0A5	pp	229533.I1SAN9
178770	229533.A0A098E0A5	pp	229533.Q4I5V2
178771	229533.A0A098E0A5	pp	229533.I1RUG3
178772	229533.A0A098E0A5	pp	229533.I1RE89
178773	229533.A0A098E0A5	pp	229533.I1S6H1
178774	229533.A0A098E0A5	pp	229533.I1S2C4
178775	229533.A0A098E0A5	pp	229533.I1RTD5
178776	229533.A0A098E0A5	pp	229533.I1RVZ7
178777	229533.A0A098E0A5	pp	229533.I1RYR6

178778	229533.A0A098E0A5	pp	229533.I1RWE8
178779	229533.A0A098E0A5	pp	229533.I1RMI8
178780	229533.A0A098E0A5	pp	229533.I1RT40
178781	229533.A0A098E0A5	pp	229533.I1RFL3
178782	229533.A0A098E0A5	pp	229533.V6RN31
178783	229533.A0A098E0A5	pp	229533.I1S5G3
178784	229533.A0A098E0A5	pp	229533.I1RB44
178785	229533.A0A098E0A5	pp	229533.I1S2C5
178786	229533.A0A098E0A5	pp	229533.I1RJW8
178787	229533.A0A098E0A5	pp	229533.I1RZ20
178788	229533.A0A098E0A5	pp	229533.I1RCW6
178789	229533.A0A098E0A5	pp	229533.I1RC05
178790	229533.A0A098E0A5	pp	229533.V6R382
178791	229533.A0A098E0A5	pp	229533.I1S216
178792	229533.A0A098E0A5	pp	229533.I1RHT3
178793	229533.A0A098E0A5	pp	229533.A0A1C3YHT1
178794	229533.A0A098E0A5	pp	229533.I1RS17
178795	229533.A0A098E0A5	pp	229533.V6R057
178796	229533.A0A098E0A5	pp	229533.A0A1C3YMA8
178797	229533.A0A098E0A5	pp	229533.A0A0E0RSS5
178798	229533.A0A098E0A5	pp	229533.I1RPK0
178799	229533.A0A098E0A5	pp	229533.A0A1C3YK68
178800	229533.A0A098E0A5	pp	229533.I1S0J1
178801	229533.A0A098E0A5	pp	229533.I1RFK7
178802	229533.A0A098E0A5	pp	229533.I1RMW4
178803	229533.A0A098E0A5	pp	229533.I1RPA4
178804	229533.A0A098E0A5	pp	229533.A0A098E4W0
178805	229533.A0A098E0A5	pp	229533.I1RAZ3
178806	229533.A0A098E0A5	pp	229533.V6RTJ8
178807	229533.A0A098E0A5	pp	229533.A0A1C3YHS4
178808	229533.A0A098E0A5	pp	229533.I1RYE6
178809	229533.A0A098E0A5	pp	229533.A0A1C3YHX6
178810	229533.A0A098E0A5	pp	229533.I1RE01
178811	229533.A0A098E0A5	pp	229533.I1REK8
178812	229533.A0A098E0A5	pp	229533.A0A0E0RSD7
178813	229533.A0A098E0A5	pp	229533.A0A1C3YKJ8
178814	229533.A0A098E0A5	pp	229533.A0A1C3YJJ2
178815	229533.A0A098E0A5	pp	229533.I1RX46
178816	229533.A0A098E0A5	pp	229533.I1RR24
178817	229533.A0A098E0A5	pp	229533.I1RM67
178818	229533.A0A098E0A5	pp	229533.I1RM35
178819	229533.A0A098E0A5	pp	229533.I1RHD1
178820	229533.A0A098E0A5	pp	229533.I1RCL4
178821	229533.A0A098E0A5	pp	229533.I1RVX9
178822	229533.A0A098E0A5	pp	229533.I1S4Q4
178823	229533.A0A098E0A5	pp	229533.I1RME6
178824	229533.A0A098E0A5	pp	229533.I1S3S3
178825	229533.A0A098E0A5	pp	229533.V6RQW4
178826	229533.A0A098E0A5	pp	229533.I1S4S4
178827	229533.A0A098E0A5	pp	229533.I1RTJ7
178828	229533.A0A098E0A5	pp	229533.I1RP86
178829	229533.A0A098E0A5	pp	229533.I1RAR9
178830	229533.A0A098E0A5	pp	229533.I1RJU3
178831	229533.A0A098E0A5	pp	229533.I1RM26
178832	229533.A0A098E0A5	pp	229533.A0A1C3YN30
178833	229533.A0A098E0A5	pp	229533.I1RYJ6
178834	229533.A0A098E0A5	pp	229533.I1REL7
178835	229533.A0A098E0A5	pp	229533.I1RIJ2
178836	229533.A0A098E0A5	pp	229533.V6R1K4
178837	229533.A0A098E0A5	pp	229533.I1RBT1
178838	229533.A0A098E0A5	pp	229533.I1RQS9
178839	229533.A0A098E0A5	pp	229533.Q4I5G1
178840	229533.A0A098E0A5	pp	229533.A0A1C3YKS6
178841	229533.A0A098E0A5	pp	229533.A0A1C3YLR6
178842	229533.A0A098E0A5	pp	229533.I1RB39
178843	229533.A0A098E0A5	pp	229533.I1RK95
178844	229533.A0A098E0A5	pp	229533.I1S3K8
178845	229533.A0A098E0A5	pp	229533.I1RT33
178846	229533.A0A098E0A5	pp	229533.I1REX0
178847	229533.A0A098E0A5	pp	229533.A0A1C3YKH8
178848	229533.A0A098E0A5	pp	229533.I1RD52
178849	229533.A0A098E0A5	pp	229533.A0A1C3YIP5
178850	229533.A0A098E0A5	pp	229533.I1S7L6

229533.A0A098E0A5 pp 229533.V6RBN2
229533.A0A098E0A5 pp 229533.I1RA94
229533.A0A098E0A5 pp 229533.I1RYU5
229533.A0A098E0A5 pp 229533.I1S1W8
229533.A0A098E0A5 pp 229533.I1S0B7
229533.A0A098E0A5 pp 229533.I1RFC0
229533.A0A098E0A5 pp 229533.I1RPN4
229533.A0A098E0A5 pp 229533.A0A1C3YM63
229533.A0A098E0A5 pp 229533.I1RRY9
229533.A0A098E0A5 pp 229533.I1S835
229533.A0A098E0A5 pp 229533.I1RBL7
229533.A0A098E0A5 pp 229533.I1S1J7
229533.A0A098E0A5 pp 229533.I1S5B0
229533.A0A098E0A5 pp 229533.A0A1C3YJ14
229533.A0A098E0A5 pp 229533.A0A1C3YN86
229533.A0A098E0A5 pp 229533.I1RV18
229533.A0A098E0A5 pp 229533.A0A0E0RWW0
229533.A0A098E0A5 pp 229533.I1RTD3
229533.A0A098E0A5 pp 229533.I1S3K6
229533.A0A098E0A5 pp 229533.I1RXF0
229533.A0A098E0A5 pp 229533.I1RYI7
229533.A0A098E0A5 pp 229533.I1RZM8
229533.A0A098E0A5 pp 229533.I1RRV4
229533.A0A098E0A5 pp 229533.I1RK15
229533.A0A098E0A5 pp 229533.I1RG14
229533.A0A098E0A5 pp 229533.I1RF82
229533.A0A098E0A5 pp 229533.V6QUE6
229533.A0A098E0A5 pp 229533.I1RS18
229533.A0A098E0A5 pp 229533.I1RVR0
229533.A0A098E0A5 pp 229533.V6RCZ8
229533.A0A098E0A5 pp 229533.I1RBE4
229533.A0A098E0A5 pp 229533.I1RW69
229533.A0A098E0A5 pp 229533.A0A1C3YKV3
229533.A0A098E0A5 pp 229533.I1RJK0
229533.A0A098E0A5 pp 229533.I1RM30
229533.A0A098E0A5 pp 229533.I1RMQ4
229533.A0A098E0A5 pp 229533.I1RBI1
229533.A0A098E0A5 pp 229533.I1RJ17
229533.A0A098E0A5 pp 229533.A0A1C3YK34
229533.A0A098E0A5 pp 229533.A0A0E0RSG8
229533.A0A098E0A5 pp 229533.I1S0A0
229533.I1RCU9 pp 229533.V6RVG3
229533.I1RCU9 pp 229533.I1RTI3
229533.I1RCU9 pp 229533.I1RF85
229533.I1RCU9 pp 229533.V6RKY7
229533.I1RCU9 pp 229533.I1RZE4
229533.I1RCU9 pp 229533.I1RJW4
229533.I1RCU9 pp 229533.I1S0X7
229533.I1RCU9 pp 229533.I1RHJ1
229533.I1RCU9 pp 229533.I1RCV0
229533.I1RCU9 pp 229533.I1RHJ2
229533.I1RCU9 pp 229533.I1RW83
229533.I1RCU9 pp 229533.I1RUN8
229533.I1RCU9 pp 229533.V6R6F0
229533.I1RCU9 pp 229533.I1S3L1
229533.I1RCU9 pp 229533.I1S439
229533.I1RCU9 pp 229533.I1RNP7
229533.I1RCU9 pp 229533.Q4IJM4
229533.I1RCU9 pp 229533.V6RVB4
229533.I1RCU9 pp 229533.I1RXV1
229533.I1RCU9 pp 229533.I1RUQ5
229533.I1RCU9 pp 229533.V6QVS3
229533.I1RCU9 pp 229533.I1RMD3
229533.I1RCU9 pp 229533.I1RHP8
229533.I1RCU9 pp 229533.I1RKK0
229533.I1RCU9 pp 229533.I1RVS4
229533.I1RCU9 pp 229533.I1RJH1
229533.I1RCU9 pp 229533.I1RYH9
229533.I1RCU9 pp 229533.I1S037
229533.I1RCU9 pp 229533.Q4ICA8
229533.I1RCU9 pp 229533.I1S8R8
229533.I1RCU9 pp 229533.I1RIY3
229533.I1RCU9 pp 229533.I1RX70

178924 229533.I1RCU9 pp 229533.I1RWC2
178925 229533.I1RLC8 pp 229533.I1RLM3
178926 229533.A0A1C3YJI6 pp 229533.I1RBL7
178927 229533.A0A1C3YJI6 pp 229533.I1RSR3
178928 229533.A0A1C3YJI6 pp 229533.I1RGB1
178929 229533.A0A1C3YJI6 pp 229533.I1RPR9
178930 229533.A0A1C3YJI6 pp 229533.I1RYS0
178931 229533.A0A1C3YJI6 pp 229533.A0A1C3YKY6
178932 229533.A0A1C3YJI6 pp 229533.A0A1C3YN86
178933 229533.A0A1C3YJI6 pp 229533.I1RUQ3
178934 229533.A0A1C3YJI6 pp 229533.I1RK95
178935 229533.A0A1C3YJI6 pp 229533.I1S1J7
178936 229533.A0A1C3YJI6 pp 229533.I1RGB2
178937 229533.A0A1C3YJI6 pp 229533.V6RWB3
178938 229533.A0A1C3YJI6 pp 229533.V6RBN2
178939 229533.A0A1C3YJI6 pp 229533.V6R9Z0
178940 229533.A0A1C3YJI6 pp 229533.I1RS18
178941 229533.A0A1C3YJI6 pp 229533.A0A1C3YKV3
178942 229533.A0A1C3YJI6 pp 229533.I1RJ17
178943 229533.A0A1C3YJI6 pp 229533.Q4IJK2
178944 229533.A0A1C3YJI6 pp 229533.A0A1C3YLX0
178945 229533.A0A1C3YJI6 pp 229533.I1RM35
178946 229533.A0A1C3YJI6 pp 229533.A0A1C3YK34
178947 229533.A0A1C3YJI6 pp 229533.I1RXC4
178948 229533.A0A1C3YJI6 pp 229533.I1REK8
178949 229533.A0A1C3YJI6 pp 229533.I1RCR5
178950 229533.A0A1C3YJI6 pp 229533.I1RQ85
178951 229533.A0A1C3YJI6 pp 229533.A0A1C3YKB0
178952 229533.A0A1C3YJI6 pp 229533.I1S4Q4
178953 229533.A0A1C3YJI6 pp 229533.I1RG14
178954 229533.A0A1C3YJI6 pp 229533.A0A1C3YJN3
178955 229533.A0A1C3YJI6 pp 229533.A0A1C3YJR3
178956 229533.A0A1C3YJI6 pp 229533.I1S217
178957 229533.A0A1C3YJI6 pp 229533.A0A1C3YJZ0
178958 229533.A0A1C3YJI6 pp 229533.V6RHX2
178959 229533.A0A1C3YJI6 pp 229533.I1RZK5
178960 229533.A0A1C3YJI6 pp 229533.A0A1C3YK36
178961 229533.A0A1C3YJI6 pp 229533.I1S887
178962 229533.A0A1C3YJI6 pp 229533.I1S419
178963 229533.A0A1C3YJI6 pp 229533.I1RZM8
178964 229533.A0A1C3YJI6 pp 229533.I1RXF0
178965 229533.A0A1C3YJI6 pp 229533.I1RBU8
178966 229533.A0A1C3YJI6 pp 229533.I1RR51
178967 229533.A0A1C3YJI6 pp 229533.I1RNT9
178968 229533.A0A1C3YJI6 pp 229533.I1RWR7
178969 229533.A0A1C3YJI6 pp 229533.A0A1C3YJV6
178970 229533.A0A1C3YJI6 pp 229533.I1RGC5
178971 229533.A0A1C3YJI6 pp 229533.I1S5B0
178972 229533.A0A1C3YJI6 pp 229533.I1RQD2
178973 229533.A0A1C3YJI6 pp 229533.I1RRY9
178974 229533.A0A1C3YJI6 pp 229533.Q4IFI0
178975 229533.A0A1C3YJI6 pp 229533.V6RCZ8
178976 229533.A0A1C3YJI6 pp 229533.I1S8D3
178977 229533.A0A1C3YJI6 pp 229533.I1S6K9
178978 229533.A0A1C3YJI6 pp 229533.I1S1B2
178979 229533.A0A1C3YJI6 pp 229533.A0A1C3YJW9
178980 229533.A0A1C3YJI6 pp 229533.I1RRV3
178981 229533.A0A1C3YJI6 pp 229533.V6RCC0
178982 229533.A0A1C3YJI6 pp 229533.I1RKJ5
178983 229533.A0A1C3YJI6 pp 229533.I1S821
178984 229533.A0A1C3YJI6 pp 229533.I1RJU3
178985 229533.A0A1C3YJI6 pp 229533.I1RRV4
178986 229533.A0A1C3YJI6 pp 229533.V6QUE6
178987 229533.A0A1C3YJI6 pp 229533.I1RY86
178988 229533.A0A1C3YJI6 pp 229533.I1S0F1
178989 229533.A0A1C3YJI6 pp 229533.I1RA85
178990 229533.A0A1C3YJI6 pp 229533.I1RBT1
178991 229533.A0A1C3YJI6 pp 229533.I1RT33
178992 229533.A0A1C3YJI6 pp 229533.I1RT05
178993 229533.A0A1C3YJI6 pp 229533.I1RPF1
178994 229533.A0A1C3YJI6 pp 229533.I1RXD6
178995 229533.A0A1C3YJI6 pp 229533.I1RQV7
178996 229533.A0A1C3YJI6 pp 229533.I1S438

178997 229533.A0A1C3YJI6 pp 229533.I1RE17
178998 229533.A0A1C3YJI6 pp 229533.I1RTB3
178999 229533.A0A1C3YJI6 pp 229533.I1R9Y4
179000 229533.A0A1C3YJI6 pp 229533.I1RR24
179001 229533.A0A1C3YJI6 pp 229533.A0A1C3YK11
179002 229533.A0A1C3YJI6 pp 229533.V6QYF0
179003 229533.A0A1C3YJI6 pp 229533.I1S4S4
179004 229533.A0A1C3YJI6 pp 229533.V6RUT5
179005 229533.A0A1C3YJI6 pp 229533.I1RCS1
179006 229533.A0A1C3YJI6 pp 229533.I1RGU0
179007 229533.A0A1C3YJI6 pp 229533.I1RXD7
179008 229533.A0A1C3YJI6 pp 229533.I1RPA4
179009 229533.A0A1C3YJI6 pp 229533.I1REK4
179010 229533.A0A1C3YJI6 pp 229533.V6R5T7
179011 229533.A0A1C3YJI6 pp 229533.I1RRX5
179012 229533.A0A1C3YJI6 pp 229533.I1RFC0
179013 229533.A0A1C3YJI6 pp 229533.I1RPY3
179014 229533.A0A1C3YJI6 pp 229533.I1RRS8
179015 229533.A0A1C3YJI6 pp 229533.I1RSQ7
179016 229533.A0A1C3YJI6 pp 229533.I1RYJ6
179017 229533.A0A1C3YJI6 pp 229533.I1S9I5
179018 229533.A0A1C3YJI6 pp 229533.I1S4S7
179019 229533.A0A1C3YJI6 pp 229533.A0A1C3YK13
179020 229533.A0A1C3YJI6 pp 229533.I1RNB2
179021 229533.A0A1C3YJI6 pp 229533.I1RQV8
179022 229533.A0A1C3YJI6 pp 229533.I1RPN4
179023 229533.A0A1C3YJI6 pp 229533.I1RW71
179024 229533.A0A1C3YJI6 pp 229533.I1RRG3
179025 229533.A0A1C3YJI6 pp 229533.I1RX95
179026 229533.A0A1C3YJI6 pp 229533.I1RB05
179027 229533.A0A1C3YJI6 pp 229533.I1RFL4
179028 229533.A0A1C3YJI6 pp 229533.A0A1C3YLZ6
179029 229533.A0A1C3YJI6 pp 229533.Q4IPH4
179030 229533.A0A1C3YJI6 pp 229533.I1RQ33
179031 229533.A0A1C3YJI6 pp 229533.V6RTJ8
179032 229533.A0A1C3YJI6 pp 229533.I1RBR2
179033 229533.A0A1C3YJI6 pp 229533.I1S2R0
179034 229533.A0A1C3YJI6 pp 229533.Q4I624
179035 229533.A0A1C3YJI6 pp 229533.I1RAX6
179036 229533.A0A1C3YJI6 pp 229533.I1RBD5
179037 229533.A0A1C3YJI6 pp 229533.I1S0T2
179038 229533.A0A1C3YJI6 pp 229533.I1RFY6
179039 229533.A0A1C3YJI6 pp 229533.A0A1C3YM63
179040 229533.A0A1C3YJI6 pp 229533.I1RD52
179041 229533.A0A1C3YJI6 pp 229533.I1RE89
179042 229533.A0A1C3YJI6 pp 229533.I1RML0
179043 229533.A0A1C3YJI6 pp 229533.I1RVQ3
179044 229533.A0A1C3YJI6 pp 229533.I1RCD8
179045 229533.A0A1C3YJI6 pp 229533.V6RRH2
179046 229533.A0A1C3YJI6 pp 229533.I1RBR1
179047 229533.A0A1C3YJI6 pp 229533.I1RX46
179048 229533.A0A1C3YJI6 pp 229533.I1RCR1
179049 229533.A0A1C3YJI6 pp 229533.I1S6V0
179050 229533.A0A1C3YJI6 pp 229533.Q4I5R9
179051 229533.A0A1C3YJI6 pp 229533.I1RPK0
179052 229533.A0A1C3YJI6 pp 229533.I1RKG3
179053 229533.A0A1C3YJI6 pp 229533.I1S0J1
179054 229533.A0A1C3YJI6 pp 229533.I1RUF7
179055 229533.A0A1C3YJI6 pp 229533.I1RKJ8
179056 229533.A0A1C3YJI6 pp 229533.I1RB92
179057 229533.A0A1C3YJI6 pp 229533.I1RA70
179058 229533.A0A1C3YJI6 pp 229533.A0A1C3YLC1
179059 229533.A0A1C3YJI6 pp 229533.I1RE01
179060 229533.A0A1C3YJI6 pp 229533.A0A1C3YLB6
179061 229533.A0A1C3YJI6 pp 229533.I1RSF1
179062 229533.A0A1C3YJI6 pp 229533.I1RRI2
179063 229533.A0A1C3YJI6 pp 229533.I1S0F8
179064 229533.A0A1C3YJI6 pp 229533.Q4HXF6
179065 229533.A0A1C3YJI6 pp 229533.I1RZ79
179066 229533.A0A1C3YJI6 pp 229533.I1RVX0
179067 229533.A0A1C3YJI6 pp 229533.I1RVZ7
179068 229533.A0A1C3YJI6 pp 229533.I1RPU2
179069 229533.A0A1C3YJI6 pp 229533.I1S437

179070 229533.A0A1C3YJI6 pp 229533.I1RB44
179071 229533.A0A1C3YJI6 pp 229533.I1RT03
179072 229533.A0A1C3YJI6 pp 229533.A0A1C3YKB9
179073 229533.A0A1C3YJI6 pp 229533.I1RM37
179074 229533.A0A1C3YJI6 pp 229533.I1S1W3
179075 229533.A0A1C3YJI6 pp 229533.I1RXR1
179076 229533.A0A1C3YJI6 pp 229533.I1RYW3
179077 229533.A0A1C3YJI6 pp 229533.I1RSS4
179078 229533.A0A1C3YJI6 pp 229533.I1RCH5
179079 229533.A0A1C3YJI6 pp 229533.I1S1C7
179080 229533.A0A1C3YJI6 pp 229533.I1RQE7
179081 229533.A0A1C3YJI6 pp 229533.I1RZV3
179082 229533.A0A1C3YJI6 pp 229533.I1RAW0
179083 229533.A0A1C3YJI6 pp 229533.I1RNC1
179084 229533.A0A1C3YJI6 pp 229533.I1RXU9
179085 229533.A0A1C3YJI6 pp 229533.I1RUG3
179086 229533.A0A1C3YJI6 pp 229533.A0A1C3YKY0
179087 229533.A0A1C3YJI6 pp 229533.I1RE79
179088 229533.A0A1C3YJI6 pp 229533.I1RJV3
179089 229533.A0A1C3YJI6 pp 229533.I1S1W9
179090 229533.A0A1C3YJI6 pp 229533.I1RG11
179091 229533.A0A1C3YJI6 pp 229533.I1RCR8
179092 229533.A0A1C3YJI6 pp 229533.I1RAG7
179093 229533.A0A1C3YJI6 pp 229533.I1RZ39
179094 229533.A0A1C3YJI6 pp 229533.A0A1C3YKM9
179095 229533.A0A1C3YJI6 pp 229533.I1RB26
179096 229533.A0A1C3YJI6 pp 229533.I1S016
179097 229533.A0A1C3YJI6 pp 229533.I1RWQ8
179098 229533.A0A1C3YJI6 pp 229533.I1RC66
179099 229533.A0A1C3YJI6 pp 229533.V6R042
179100 229533.A0A1C3YJI6 pp 229533.I1RWI5
179101 229533.A0A1C3YJI6 pp 229533.I1S5R6
179102 229533.A0A1C3YJI6 pp 229533.I1RSQ3
179103 229533.A0A1C3YJI6 pp 229533.I1RSQ4
179104 229533.A0A1C3YJI6 pp 229533.I1RRN2
179105 229533.A0A1C3YJI6 pp 229533.V6RMW6
179106 229533.A0A1C3YJI6 pp 229533.I1RD22
179107 229533.A0A1C3YJI6 pp 229533.I1RZZ9
179108 229533.A0A1C3YJI6 pp 229533.I1S0L4
179109 229533.A0A1C3YJI6 pp 229533.I1RSU2
179110 229533.A0A1C3YJI6 pp 229533.I1RR72
179111 229533.A0A1C3YJI6 pp 229533.I1RRM3
179112 229533.A0A1C3YJI6 pp 229533.I1RRI1
179113 229533.A0A1C3YJI6 pp 229533.A0A1C3YLS9
179114 229533.A0A1C3YJI6 pp 229533.I1RGB0
179115 229533.A0A1C3YJI6 pp 229533.I1RRV1
179116 229533.A0A1C3YJI6 pp 229533.I1RTN3
179117 229533.A0A1C3YJI6 pp 229533.I1RKP1
179118 229533.A0A1C3YJI6 pp 229533.I1RKD3
179119 229533.A0A1C3YJI6 pp 229533.I1RKJ4
179120 229533.A0A1C3YJI6 pp 229533.A0A1C3YKS0
179121 229533.A0A1C3YJI6 pp 229533.I1RVF8
179122 229533.A0A1C3YJI6 pp 229533.I1REC5
179123 229533.A0A1C3YJI6 pp 229533.A0A1C3YJK3
179124 229533.A0A1C3YJI6 pp 229533.I1RGU7
179125 229533.A0A1C3YJI6 pp 229533.I1S2H4
179126 229533.A0A1C3YJI6 pp 229533.I1RN67
179127 229533.A0A1C3YJI6 pp 229533.I1RFS3
179128 229533.A0A1C3YJI6 pp 229533.I1RL04
179129 229533.A0A1C3YJI6 pp 229533.I1RPE0
179130 229533.A0A1C3YJI6 pp 229533.I1S0W4
179131 229533.A0A1C3YJI6 pp 229533.I1S5R1
179132 229533.A0A1C3YJI6 pp 229533.I1S6Y8
179133 229533.A0A1C3YJI6 pp 229533.I1RDF7
179134 229533.A0A1C3YJI6 pp 229533.I1RPV3
179135 229533.A0A1C3YJI6 pp 229533.I1RW26
179136 229533.A0A1C3YJI6 pp 229533.I1RRT8
179137 229533.A0A1C3YJI6 pp 229533.A0A1C3YK39
179138 229533.A0A1C3YJI6 pp 229533.I1RXC2
179139 229533.A0A1C3YJI6 pp 229533.I1RKG2
179140 229533.A0A1C3YJI6 pp 229533.I1RW61
179141 229533.A0A1C3YJI6 pp 229533.I1RA11
179142 229533.A0A1C3YJI6 pp 229533.Q4HXF0

179143 229533.A0A1C3YJI6 pp 229533.A0A1C3YM60
179144 229533.A0A1C3YJI6 pp 229533.I1RSZ7
179145 229533.A0A1C3YJI6 pp 229533.I1RXF8
179146 229533.A0A1C3YJI6 pp 229533.I1S4Z8
179147 229533.A0A1C3YJI6 pp 229533.I1S9Y1
179148 229533.A0A1C3YJI6 pp 229533.I1RJQ5
179149 229533.A0A1C3YJI6 pp 229533.A0A1C3YMA1
179150 229533.A0A1C3YJI6 pp 229533.I1RTD5
179151 229533.A0A1C3YJI6 pp 229533.I1RYI5
179152 229533.A0A1C3YJI6 pp 229533.I1S277
179153 229533.A0A1C3YJI6 pp 229533.I1RPH6
179154 229533.A0A1C3YJI6 pp 229533.I1RE76
179155 229533.A0A1C3YJI6 pp 229533.I1RPP3
179156 229533.A0A1C3YJI6 pp 229533.I1RYP2
179157 229533.A0A1C3YJI6 pp 229533.I1RFL3
179158 229533.A0A1C3YJI6 pp 229533.A0A1C3YKU8
179159 229533.A0A1C3YJI6 pp 229533.I1RBY4
179160 229533.A0A1C3YJI6 pp 229533.I1RT40
179161 229533.A0A1C3YJI6 pp 229533.I1R9M0
179162 229533.A0A1C3YJI6 pp 229533.I1RFT1
179163 229533.A0A1C3YJI6 pp 229533.I1RQM7
179164 229533.A0A1C3YJI6 pp 229533.I1RIJ6
179165 229533.A0A1C3YJI6 pp 229533.I1RJN9
179166 229533.A0A1C3YJI6 pp 229533.I1RJZ7
179167 229533.A0A1C3YJI6 pp 229533.I1RTA1
179168 229533.A0A1C3YJI6 pp 229533.I1RVZ2
179169 229533.A0A1C3YJI6 pp 229533.I1RZB2
179170 229533.A0A1C3YJI6 pp 229533.I1S2B8
179171 229533.A0A1C3YJI6 pp 229533.A0A1C3YM05
179172 229533.A0A1C3YJI6 pp 229533.I1S268
179173 229533.A0A1C3YJI6 pp 229533.I1RBC7
179174 229533.A0A1C3YJI6 pp 229533.I1RWS4
179175 229533.A0A1C3YJI6 pp 229533.I1REV8
179176 229533.A0A1C3YJI6 pp 229533.V6RCJ5
179177 229533.A0A1C3YJI6 pp 229533.I1RYR0
179178 229533.A0A1C3YJI6 pp 229533.I1RC82
179179 229533.A0A1C3YJI6 pp 229533.I1RSA5
179180 229533.A0A1C3YJI6 pp 229533.I1RGA4
179181 229533.A0A1C3YJI6 pp 229533.I1RSX0
179182 229533.A0A1C3YJI6 pp 229533.I1R9H9
179183 229533.A0A1C3YJI6 pp 229533.I1S253
179184 229533.A0A1C3YJI6 pp 229533.I1S0F7
179185 229533.A0A1C3YJI6 pp 229533.V6R3J1
179186 229533.A0A1C3YJI6 pp 229533.I1RS47
179187 229533.A0A1C3YJI6 pp 229533.Q4HVQ9
179188 229533.A0A1C3YJI6 pp 229533.I1RC34
179189 229533.A0A1C3YJI6 pp 229533.A0A1C3YLK8
179190 229533.A0A1C3YJI6 pp 229533.I1RRK3
179191 229533.A0A1C3YMJ5 pp 229533.I1RJU9
179192 229533.A0A1C3YMJ5 pp 229533.I1RLM3
179193 229533.I1RDB1 pp 229533.I1RF85
179194 229533.I1RDB1 pp 229533.I1RHJ1
179195 229533.I1RDB1 pp 229533.I1RRY3
179196 229533.I1RDB1 pp 229533.I1RHJ2
179197 229533.I1RDB1 pp 229533.I1S1D3
179198 229533.I1RDB1 pp 229533.I1RRP7
179199 229533.I1RDB1 pp 229533.I1RFL4
179200 229533.I1RDB1 pp 229533.I1RJS3
179201 229533.I1RDB1 pp 229533.I1RZE4
179202 229533.I1RDB1 pp 229533.I1RTI3
179203 229533.I1RDB1 pp 229533.I1S2K7
179204 229533.I1RDB1 pp 229533.I1RRN5
179205 229533.I1RDB1 pp 229533.I1RDZ4
179206 229533.I1RDB1 pp 229533.I1RJW4
179207 229533.I1RDB1 pp 229533.I1RNY9
179208 229533.I1RDB1 pp 229533.I1RNY8
179209 229533.I1RDB1 pp 229533.I1RPW0
179210 229533.I1RDB1 pp 229533.I1RQ72
179211 229533.I1RDB1 pp 229533.I1RHQ8
179212 229533.I1RDB1 pp 229533.V6RVG3
179213 229533.I1RDB1 pp 229533.I1RPS0
179214 229533.I1RDB1 pp 229533.I1S0D1
179215 229533.I1RDB1 pp 229533.I1RW16

179216	229533.I1RDB1	pp	229533.I1RTH6
179217	229533.I1RDB1	pp	229533.V6RKY7
179218	229533.I1RDB1	pp	229533.V6RGI1
179219	229533.I1RDB1	pp	229533.I1RY44
179220	229533.I1RDB1	pp	229533.I1RT36
179221	229533.I1RDB1	pp	229533.V6RVB4
179222	229533.I1RDB1	pp	229533.I1S1W4
179223	229533.I1RDB1	pp	229533.I1S0M2
179224	229533.I1RDB1	pp	229533.I1RJ88
179225	229533.I1RDB1	pp	229533.I1S1V5
179226	229533.I1RDB1	pp	229533.I1RQB4
179227	229533.I1RDB1	pp	229533.I1RUN8
179228	229533.I1RDB1	pp	229533.I1RRW0
179229	229533.I1RDB1	pp	229533.I1RNP7
179230	229533.I1RDB1	pp	229533.I1RMF5
179231	229533.I1RDB1	pp	229533.I1S549
179232	229533.I1RDB1	pp	229533.V6RMW6
179233	229533.I1RDB1	pp	229533.O74638
179234	229533.I1RDB1	pp	229533.I1RLU1
179235	229533.I1RDB1	pp	229533.I1S0X7
179236	229533.I1RDB1	pp	229533.I1RJZ7
179237	229533.I1RDB1	pp	229533.I1RQM0
179238	229533.I1RDB1	pp	229533.I1RPP3
179239	229533.I1RDB1	pp	229533.I1RZZ9
179240	229533.I1RDB1	pp	229533.I1RX38
179241	229533.I1RDB1	pp	229533.I1RPD6
179242	229533.I1RDB1	pp	229533.I1RN67
179243	229533.I1RDB1	pp	229533.I1RW83
179244	229533.I1RDB1	pp	229533.I1RR73
179245	229533.I1RDB1	pp	229533.I1RQG4
179246	229533.I1RDB1	pp	229533.I1RP28
179247	229533.I1RDB1	pp	229533.I1RNF4
179248	229533.I1RDB1	pp	229533.I1RQS4
179249	229533.I1RDB1	pp	229533.I1RJK7
179250	229533.I1RDB1	pp	229533.I1RFL7
179251	229533.I1RDB1	pp	229533.I1RV91
179252	229533.I1RDB1	pp	229533.I1S439
179253	229533.I1RDB1	pp	229533.V6R6F0
179254	229533.I1RDB1	pp	229533.I1S3L1
179255	229533.I1RDB1	pp	229533.I1S2E9
179256	229533.I1RDB1	pp	229533.I1RQA3
179257	229533.I1RDB1	pp	229533.I1RYY5
179258	229533.I1RDB1	pp	229533.I1RUQ5
179259	229533.I1RDB1	pp	229533.I1RN65
179260	229533.I1RDB1	pp	229533.I1RMM9
179261	229533.I1RDB1	pp	229533.Q4IJM4
179262	229533.I1RDB1	pp	229533.I1RZB5
179263	229533.I1RDB1	pp	229533.I1RPU4
179264	229533.I1RDB1	pp	229533.I1RRY9
179265	229533.I1RDB1	pp	229533.I1RKK0
179266	229533.I1RDB1	pp	229533.I1RZH4
179267	229533.I1RDB1	pp	229533.I1RN08
179268	229533.I1RDB1	pp	229533.I1S0Z7
179269	229533.I1RDB1	pp	229533.I1RZM1
179270	229533.I1RDB1	pp	229533.I1RFU8
179271	229533.I1RDB1	pp	229533.Q4ICA8
179272	229533.I1RDB1	pp	229533.I1RXV1
179273	229533.I1RDB1	pp	229533.I1RL24
179274	229533.I1RDB1	pp	229533.I1RNM7
179275	229533.I1RDB1	pp	229533.V6QVS3
179276	229533.I1RDB1	pp	229533.I1RNC0
179277	229533.I1RDB1	pp	229533.I1RIY3
179278	229533.I1RDB1	pp	229533.I1RWT6
179279	229533.I1RDB1	pp	229533.I1RMD3
179280	229533.I1RDB1	pp	229533.I1RN31
179281	229533.I1RDB1	pp	229533.I1RPA6
179282	229533.I1RDB1	pp	229533.I1RW25
179283	229533.I1RDB1	pp	229533.I1RM09
179284	229533.I1RDB1	pp	229533.I1S224
179285	229533.I1RDB1	pp	229533.I1RX70
179286	229533.I1RDB1	pp	229533.I1RWJ6
179287	229533.I1RDB1	pp	229533.I1RYH9
179288	229533.I1RDB1	pp	229533.I1RR24

229533.I1RDB1	pp	229533.I1RJX2
229533.I1RDB1	pp	229533.I1RQ26
229533.I1RDB1	pp	229533.I1RJH1
229533.I1RDB1	pp	229533.I1RV66
229533.I1RDB1	pp	229533.V6QV09
229533.I1RDB1	pp	229533.I1RXH6
229533.I1RDB1	pp	229533.I1RW71
229533.I1RDB1	pp	229533.I1RLX6
229533.I1RDB1	pp	229533.I1RYZ9
229533.I1RDB1	pp	229533.I1S8R8
229533.I1RDB1	pp	229533.I1RHP8
229533.I1RXC2	pp	229533.I1S0J1
229533.I1RXC2	pp	229533.I1RZ39
229533.I1RXC2	pp	229533.I1S1W9
229533.I1RXC2	pp	229533.V6R042
229533.I1RXC2	pp	229533.V6RMW6
229533.I1RXC2	pp	229533.I1S5R6
229533.I1RXC2	pp	229533.I1S5R1
229533.I1RXC2	pp	229533.I1S437
229533.I1RXC2	pp	229533.I1S2H4
229533.I1RXC2	pp	229533.I1S9Y1
229533.I1RXC2	pp	229533.I1RZV3
229533.I1RXC2	pp	229533.I1S2B8
229533.I1RXC2	pp	229533.I1RZ79
229533.I1RXC2	pp	229533.I1RYW3
229533.I1RXC2	pp	229533.I1S0L4
229533.I1RXC2	pp	229533.I1S1C7
229533.I1RXC2	pp	229533.Q4IPZ1
229533.I1RXC2	pp	229533.I1RXF0
229533.I1RXC2	pp	229533.Q4I624
229533.I1RXC2	pp	229533.I1RZB2
229533.I1RXC2	pp	229533.I1S277
229533.I1RXC2	pp	229533.I1RYR0
229533.I1RXC2	pp	229533.I1S0W4
229533.I1RXC2	pp	229533.I1RXF8
229533.I1RXC2	pp	229533.I1S821
229533.I1RXC2	pp	229533.I1RYI5
229533.I1RXC2	pp	229533.I1RZM8
229533.I1RXC2	pp	229533.V6RTJ8
229533.I1RXC2	pp	229533.I1S1B2
229533.I1RXC2	pp	229533.V6QUE6
229533.I1RXC2	pp	229533.I1RZZ9
229533.I1RXC2	pp	229533.I1S6K9
229533.I1RXC2	pp	229533.I1S1J7
229533.I1RXC2	pp	229533.I1RXD6
229533.I1RXC2	pp	229533.I1RYS0
229533.I1RXC2	pp	229533.V6RCZ8
229533.I1RXC2	pp	229533.I1S5B0
229533.I1RXC2	pp	229533.I1S4S7
229533.I1RXC2	pp	229533.I1RZK5
229533.I1RXC2	pp	229533.Q4IJK2
229533.I1RXC2	pp	229533.I1RXD7
229533.I1RXC2	pp	229533.I1RY86
229533.I1RXC2	pp	229533.I1S4S4
229533.I1RXC2	pp	229533.I1S438
229533.I1RC05	pp	229533.I1RRV1
229533.I1RC05	pp	229533.V6R042
229533.I1RC05	pp	229533.I1SAN9
229533.I1RC05	pp	229533.I1RYW3
229533.I1RC05	pp	229533.I1RUG3
229533.I1RC05	pp	229533.I1RNC1
229533.I1RC05	pp	229533.Q4HZ42
229533.I1RC05	pp	229533.Q4I624
229533.I1RC05	pp	229533.I1RCD8
229533.I1RC05	pp	229533.V6R057
229533.I1RC05	pp	229533.I1RWE8
229533.I1RC05	pp	229533.I1RX22
229533.I1RC05	pp	229533.I1RMI8
229533.I1RC05	pp	229533.I1RS47
229533.I1RC05	pp	229533.V6RRH2
229533.I1RC05	pp	229533.I1S0J1
229533.I1RC05	pp	229533.I1RVX0
229533.I1RC05	pp	229533.I1RE01

179362	229533.I1RC05	pp	229533.I1RNG4
179363	229533.I1RC05	pp	229533.I1RQG4
179364	229533.I1RC05	pp	229533.I1RG11
179365	229533.I1RC05	pp	229533.I1RN67
179366	229533.I1RC05	pp	229533.I1S5R6
179367	229533.I1RC05	pp	229533.I1RQE7
179368	229533.I1RC05	pp	229533.I1RFL4
179369	229533.I1RC05	pp	229533.I1RCH5
179370	229533.I1RC05	pp	229533.I1RYI5
179371	229533.I1RC05	pp	229533.I1RD22
179372	229533.I1RC05	pp	229533.I1RJV3
179373	229533.I1RC05	pp	229533.I1RPP3
179374	229533.I1RC05	pp	229533.Q4IJ56
179375	229533.I1RC05	pp	229533.I1RS18
179376	229533.I1RC05	pp	229533.Q4I5V2
179377	229533.I1RC05	pp	229533.I1RFT1
179378	229533.I1RC05	pp	229533.I1RTA1
179379	229533.I1RC05	pp	229533.I1RQM7
179380	229533.I1RC05	pp	229533.I1RMQ4
179381	229533.I1RC05	pp	229533.I1RCW6
179382	229533.I1RC05	pp	229533.I1RJ17
179383	229533.I1RC05	pp	229533.I1RE76
179384	229533.I1RC05	pp	229533.Q4HXF0
179385	229533.I1RC05	pp	229533.I1RGA4
179386	229533.I1RC05	pp	229533.I1RL04
179387	229533.I1RC05	pp	229533.I1RKY2
179388	229533.I1RC05	pp	229533.I1RRV4
179389	229533.I1RC05	pp	229533.I1S5B0
179390	229533.I1RC05	pp	229533.I1RSZ7
179391	229533.I1RC05	pp	229533.I1RTD5
179392	229533.I1RC05	pp	229533.I1S0L4
179393	229533.I1RC05	pp	229533.I1RT40
179394	229533.I1RC05	pp	229533.V6QUE6
179395	229533.I1RC05	pp	229533.I1RFL3
179396	229533.I1RC05	pp	229533.I1S4Z8
179397	229533.I1RC05	pp	229533.V6RCZ8
179398	229533.I1RC05	pp	229533.I1S1J7
179399	229533.I1RC05	pp	229533.I1S2H4
179400	229533.I1RC05	pp	229533.I1RZM8
179401	229533.I1RC05	pp	229533.I1RTD3
179402	229533.I1RC05	pp	229533.I1S5R1
179403	229533.I1RC05	pp	229533.V6R1K4
179404	229533.I1RC05	pp	229533.O74638
179405	229533.I1RC05	pp	229533.I1RXF0
179406	229533.I1RC05	pp	229533.I1RGA6
179407	229533.I1RC05	pp	229533.I1RD52
179408	229533.I1RC05	pp	229533.I1RC66
179409	229533.I1RC05	pp	229533.I1RRX5
179410	229533.I1RC05	pp	229533.I1RCR5
179411	229533.I1RC05	pp	229533.I1RFC0
179412	229533.I1RC05	pp	229533.I1RSQ7
179413	229533.I1RC05	pp	229533.I1REK8
179414	229533.I1RC05	pp	229533.I1RE00
179415	229533.I1RC05	pp	229533.I1RM35
179416	229533.I1RC05	pp	229533.I1RPN4
179417	229533.I1RC05	pp	229533.I1S8G4
179418	229533.I1RC05	pp	229533.I1S4Q4
179419	229533.I1RC05	pp	229533.I1S4S7
179420	229533.I1RC05	pp	229533.V6RTJ8
179421	229533.I1RC05	pp	229533.I1RZZ9
179422	229533.I1RC05	pp	229533.Q4IJK2
179423	229533.I1RC05	pp	229533.V6RBN2
179424	229533.I1RC05	pp	229533.I1RX46
179425	229533.I1RC05	pp	229533.I1RSQ4
179426	229533.I1RC05	pp	229533.Q4I948
179427	229533.I1RC05	pp	229533.I1RXD7
179428	229533.I1RC05	pp	229533.I1RME6
179429	229533.I1RC05	pp	229533.I1RF82
179430	229533.I1RC05	pp	229533.I1S3S3
179431	229533.I1RC05	pp	229533.I1RRY9
179432	229533.I1RC05	pp	229533.I1RJU3
179433	229533.I1RC05	pp	229533.I1S0B7
179434	229533.I1RC05	pp	229533.I1RCL4

229533.I1RC05	pp	229533.I1RQS9
229533.I1RCU8	pp	229533.V6R6F0
229533.I1RCU8	pp	229533.V6RVB4
229533.I1RCU8	pp	229533.I1RZ78
229533.I1RCU8	pp	229533.I1S3L1
229533.I1RCU8	pp	229533.I1RNP7
229533.I1RCU8	pp	229533.I1RW83
229533.I1RCU8	pp	229533.I1RUN8
229533.I1RCU8	pp	229533.I1S0X7
229533.I1RCU8	pp	229533.I1RZE4
229533.I1RCU8	pp	229533.V6RKY7
229533.I1RCU8	pp	229533.I1RTI3
229533.I1RCU8	pp	229533.I1RHJ1
229533.I1RCU8	pp	229533.I1RHJ2
229533.I1RCU8	pp	229533.V6RVG3
229533.I1RCU8	pp	229533.I1RF85
229533.I1RCU8	pp	229533.I1RIY3
229533.I1RCU8	pp	229533.I1RJW4
229533.I1RCU8	pp	229533.I1S8R8
229533.I1RCU8	pp	229533.I1RWC2
229533.I1RCU8	pp	229533.I1RYH9
229533.I1RCU8	pp	229533.Q4IJM4
229533.I1RCU8	pp	229533.I1S037
229533.I1RCU8	pp	229533.I1RX70
229533.I1RCU8	pp	229533.I1RJH1
229533.I1RCU8	pp	229533.I1S439
229533.I1RCU8	pp	229533.I1RMD3
229533.I1RCU8	pp	229533.I1RUQ5
229533.I1RCU8	pp	229533.I1RHP8
229533.I1RCU8	pp	229533.Q4ICA8
229533.I1RCU8	pp	229533.I1RVS4
229533.I1RCU8	pp	229533.V6QVS3
229533.I1RCU8	pp	229533.I1RXV1
229533.I1RCU8	pp	229533.I1RKK0
229533.A0A1C3YM25	pp	229533.I1RCC2
229533.A0A1C3YM25	pp	229533.V6RR38
229533.A0A1C3YM25	pp	229533.V6RPN0
229533.A0A1C3YM25	pp	229533.I1RZZ3
229533.A0A1C3YM25	pp	229533.I1RRQ1
229533.A0A1C3YM25	pp	229533.I1RT23
229533.A0A1C3YM25	pp	229533.I1RI22
229533.A0A1C3YM25	pp	229533.I1S1Q3
229533.A0A1C3YM25	pp	229533.I1RJG1
229533.A0A1C3YM25	pp	229533.I1RNT1
229533.A0A1C3YM25	pp	229533.I1RQ76
229533.A0A1C3YM25	pp	229533.I1RF79
229533.A0A1C3YM25	pp	229533.I1RQ48
229533.A0A1C3YM25	pp	229533.I1RR90
229533.A0A1C3YM25	pp	229533.I1RFM2
229533.A0A1C3YM25	pp	229533.I1RY35
229533.A0A1C3YM25	pp	229533.A0A1C3YMS7
229533.A0A1C3YM25	pp	229533.I1RCA0
229533.A0A1C3YM25	pp	229533.I1SAS4
229533.A0A1C3YM25	pp	229533.I1SAS3
229533.I1RTA1	pp	229533.V6RWB3
229533.I1RTA1	pp	229533.I1S217
229533.I1RTA1	pp	229533.I1RYS0
229533.I1RTA1	pp	229533.I1RZM8
229533.I1RTA1	pp	229533.V6RBN2
229533.I1RTA1	pp	229533.I1RXF0
229533.I1RTA1	pp	229533.Q4IJK2
229533.I1RTA1	pp	229533.V6R9Z0
229533.I1RTA1	pp	229533.I1S1J7
229533.I1RTA1	pp	229533.I1RUQ3
229533.I1RTA1	pp	229533.I1S6K9
229533.I1RTA1	pp	229533.I1RWR7
229533.I1RTA1	pp	229533.V6RCZ8
229533.I1RTA1	pp	229533.I1RXC4
229533.I1RTA1	pp	229533.I1RZK5
229533.I1RTA1	pp	229533.I1S1B2
229533.I1RTA1	pp	229533.I1S821
229533.I1RTA1	pp	229533.I1S8D3
229533.I1RTA1	pp	229533.V6RHX2

229533.I1RTA1 pp 229533.I1S9I5
229533.I1RTA1 pp 229533.I1S887
229533.I1RTA1 pp 229533.V6QUE6
229533.I1RTA1 pp 229533.I1RVX0
229533.I1RTA1 pp 229533.I1S835
229533.I1RTA1 pp 229533.V6RCC0
229533.I1RTA1 pp 229533.I1S5B0
229533.I1RTA1 pp 229533.V6R5T7
229533.I1RTA1 pp 229533.Q4I5V2
229533.I1RTA1 pp 229533.I1RXR1
229533.I1RTA1 pp 229533.I1RW54
229533.I1RTA1 pp 229533.I1RVQ3
229533.I1RTA1 pp 229533.I1RXU9
229533.I1RTA1 pp 229533.I1S4S4
229533.I1RTA1 pp 229533.I1S438
229533.I1RTA1 pp 229533.I1S0F1
229533.I1RTA1 pp 229533.I1S274
229533.I1RTA1 pp 229533.I1RY86
229533.I1RTA1 pp 229533.I1RXD6
229533.I1RTA1 pp 229533.I1RX95
229533.I1RTA1 pp 229533.I1RYW3
229533.I1RTA1 pp 229533.I1RW71
229533.I1RTA1 pp 229533.I1RZ79
229533.I1RTA1 pp 229533.I1RTB3
229533.I1RTA1 pp 229533.I1S4S7
229533.I1RTA1 pp 229533.V6RUT5
229533.I1RTA1 pp 229533.I1RX46
229533.I1RTA1 pp 229533.I1S1C7
229533.I1RTA1 pp 229533.I1S3S3
229533.I1RTA1 pp 229533.I1RXD7
229533.I1RTA1 pp 229533.I1S437
229533.I1RTA1 pp 229533.I1S0F8
229533.I1RTA1 pp 229533.I1S338
229533.I1RTA1 pp 229533.I1S6V0
229533.I1RTA1 pp 229533.V6RTJ8
229533.I1RTA1 pp 229533.V6RRH2
229533.I1RTA1 pp 229533.I1RVF8
229533.I1RTA1 pp 229533.I1RTH6
229533.I1RTA1 pp 229533.Q4I624
229533.I1RTA1 pp 229533.I1RUG3
229533.I1RTA1 pp 229533.I1RUF7
229533.I1RTA1 pp 229533.I1RZV3
229533.I1RTA1 pp 229533.I1S1W9
229533.I1RTA1 pp 229533.I1S1W3
229533.I1RTA1 pp 229533.I1RXC2
229533.I1RTA1 pp 229533.V6RMW6
229533.I1RTA1 pp 229533.I1RTN3
229533.I1RTA1 pp 229533.V6R042
229533.I1RTA1 pp 229533.I1S016
229533.I1RTA1 pp 229533.O74638
229533.I1RTA1 pp 229533.I1S5R1
229533.I1RTA1 pp 229533.I1RVZ7
229533.I1RTA1 pp 229533.I1S0J1
229533.I1RTA1 pp 229533.I1S9Y1
229533.I1RTA1 pp 229533.I1RZQ1
229533.I1RTA1 pp 229533.I1S0L4
229533.I1RTA1 pp 229533.I1S9Z4
229533.I1RTA1 pp 229533.I1S2B8
229533.I1RTA1 pp 229533.I1S2H4
229533.I1RTA1 pp 229533.I1S4Z8
229533.I1RTA1 pp 229533.Q4HVQ9
229533.I1RTA1 pp 229533.I1S277
229533.I1RTA1 pp 229533.I1S5R6
229533.I1RTA1 pp 229533.I1RWI5
229533.I1RTA1 pp 229533.I1RW61
229533.I1RTA1 pp 229533.I1RZZ9
229533.I1RTA1 pp 229533.I1S0W4
229533.I1RTA1 pp 229533.I1S268
229533.I1RTA1 pp 229533.I1S0F7
229533.I1RTA1 pp 229533.V6RCJ5
229533.I1RTA1 pp 229533.I1RTD5
229533.I1RTA1 pp 229533.I1RVZ2
229533.I1RTA1 pp 229533.I1RXF8

229533.I1RTA1 pp 229533.I1S253
229533.I1RTA1 pp 229533.I1RZ39
229533.I1RTA1 pp 229533.Q4HXF0
229533.I1RTA1 pp 229533.I1RYK0
229533.I1RTA1 pp 229533.I1RYI5
229533.I1RTA1 pp 229533.I1RYR0
229533.I1RTA1 pp 229533.I1RYP2
229533.A0A1C3YIQ5 pp 229533.A0A1C3YLU9
229533.A0A1C3YIQ5 pp 229533.O74638
229533.A0A1C3YIQ5 pp 229533.I1R9K3
229533.A0A1C3YIQ5 pp 229533.I1RE53
229533.A0A1C3YIQ5 pp 229533.A0A1C3YM01
229533.A0A1C3YIQ5 pp 229533.I1RMG6
229533.A0A1C3YIQ5 pp 229533.I1RQ48
229533.A0A1C3YIQ5 pp 229533.I1RTI7
229533.A0A1C3YIQ5 pp 229533.I1RIY0
229533.A0A1C3YIQ5 pp 229533.I1RQ30
229533.A0A1C3YIQ5 pp 229533.I1RZ20
229533.A0A1C3YIQ5 pp 229533.I1RZ52
229533.A0A1C3YIQ5 pp 229533.V6RQW4
229533.A0A1C3YIQ5 pp 229533.I1S9D4
229533.A0A1C3YIQ5 pp 229533.I1RYU5
229533.A0A1C3YIQ5 pp 229533.I1S2E2
229533.A0A1C3YIQ5 pp 229533.I1RCB4
229533.A0A1C3YIQ5 pp 229533.I1RMG7
229533.A0A1C3YIB4 pp 229533.I1RQV7
229533.A0A1C3YIB4 pp 229533.I1RCU7
229533.A0A1C3YIB4 pp 229533.I1S5D0
229533.A0A1C3YIB4 pp 229533.I1S6H3
229533.A0A1C3YIB4 pp 229533.I1S094
229533.A0A1C3YIB4 pp 229533.I1RW91
229533.A0A1C3YIB4 pp 229533.I1RPW8
229533.A0A1C3YIB4 pp 229533.I1RDN3
229533.A0A1C3YIB4 pp 229533.I1R9F2
229533.A0A1C3YIB4 pp 229533.I1RST9
229533.A0A1C3YIB4 pp 229533.I1S267
229533.A0A1C3YIB4 pp 229533.I1RCU2
229533.A0A1C3YIB4 pp 229533.I1RRS6
229533.A0A1C3YIB4 pp 229533.I1S659
229533.A0A1C3YIB4 pp 229533.I1RA13
229533.A0A1C3YIB4 pp 229533.I1S0A5
229533.A0A1C3YIB4 pp 229533.I1RIB5
229533.A0A1C3YIB4 pp 229533.I1RH83
229533.A0A1C3YIB4 pp 229533.I1RCG6
229533.A0A1C3YIB4 pp 229533.I1RNG4
229533.A0A1C3YIB4 pp 229533.I1RN04
229533.A0A1C3YIB4 pp 229533.I1RF85
229533.A0A1C3YIB4 pp 229533.I1RJS1
229533.A0A1C3YIB4 pp 229533.I1RPV8
229533.A0A1C3YIB4 pp 229533.I1RW80
229533.A0A1C3YIB4 pp 229533.I1RAP1
229533.A0A1C3YIB4 pp 229533.I1RZ56
229533.A0A1C3YIB4 pp 229533.A0A1C3YLB6
229533.A0A1C3YIB4 pp 229533.I1RE52
229533.A0A1C3YIB4 pp 229533.I1SAT2
229533.I1RST8 pp 229533.I1S439
229533.I1RST8 pp 229533.Q4IJU0
229533.I1RST8 pp 229533.I1RXV1
229533.I1RST8 pp 229533.Q4ICA8
229533.I1RST8 pp 229533.I1S0X0
229533.I1RST8 pp 229533.V6QVS3
229533.I1RST8 pp 229533.I1RUQ5
229533.I1RST8 pp 229533.I1RX70
229533.I1RST8 pp 229533.I1RYH9
229533.I1RST8 pp 229533.I1S8R8
229533.I1RST8 pp 229533.V6R6F0
229533.I1RST8 pp 229533.I1RTI3
229533.I1RST8 pp 229533.Q4HYB8
229533.I1RST8 pp 229533.V6R3J1
229533.I1RST8 pp 229533.V6RKY7
229533.I1RST8 pp 229533.I1RUN8
229533.I1RST8 pp 229533.V6RVG3
229533.I1RST8 pp 229533.I1RZE4

179654 229533.I1RST8 pp 229533.I1RW83
179655 229533.I1RST8 pp 229533.I1S3L1
179656 229533.I1RST8 pp 229533.I1S0X7
179657 229533.I1RST8 pp 229533.V6RVB4
179658 229533.A0A098CZE9 pp 229533.V6RDP1
179659 229533.A0A098CZE9 pp 229533.I1RWB5
179660 229533.A0A098CZE9 pp 229533.I1RRR6
179661 229533.A0A098CZE9 pp 229533.I1RPK0
179662 229533.A0A098CZE9 pp 229533.I1RAM7
179663 229533.A0A098CZE9 pp 229533.I1RHJ2
179664 229533.A0A098CZE9 pp 229533.A0A098D8H0
179665 229533.A0A098CZE9 pp 229533.A0A1C3YII1
179666 229533.A0A098CZE9 pp 229533.A0A1C3YHQ6
179667 229533.A0A098CZE9 pp 229533.I1S5H3
179668 229533.A0A098CZE9 pp 229533.I1RWX2
179669 229533.A0A098CZE9 pp 229533.I1RZQ6
179670 229533.A0A098CZE9 pp 229533.I1REJ4
179671 229533.A0A098CZE9 pp 229533.I1RCU9
179672 229533.A0A098CZE9 pp 229533.I1RDB1
179673 229533.A0A098CZE9 pp 229533.I1RCU8
179674 229533.A0A098CZE9 pp 229533.I1RST8
179675 229533.I1R9Y2 pp 229533.I1RPD6
179676 229533.I1R9Y2 pp 229533.I1RW43
179677 229533.I1R9Y2 pp 229533.Q4HXF0
179678 229533.I1R9Y2 pp 229533.I1RVN1
179679 229533.I1R9Y2 pp 229533.I1RY29
179680 229533.I1R9Y2 pp 229533.I1S1K6
179681 229533.I1R9Y2 pp 229533.I1S2H4
179682 229533.I1R9Y2 pp 229533.I1S3G4
179683 229533.I1R9Y2 pp 229533.O74638
179684 229533.I1R9Y2 pp 229533.I1RFL3
179685 229533.I1R9Y2 pp 229533.I1RED3
179686 229533.I1R9Y2 pp 229533.I1RSM8
179687 229533.I1R9Y2 pp 229533.I1RY35
179688 229533.I1R9Y2 pp 229533.I1RNM4
179689 229533.I1R9Y2 pp 229533.I1RSC7
179690 229533.I1R9Y2 pp 229533.I1RUF8
179691 229533.I1R9Y2 pp 229533.V6RPQ8
179692 229533.I1R9Y2 pp 229533.I1RWF9
179693 229533.I1R9Y2 pp 229533.I1RFV6
179694 229533.I1R9Y2 pp 229533.I1RPE5
179695 229533.I1R9Y2 pp 229533.I1RA70
179696 229533.I1R9Y2 pp 229533.I1RCH4
179697 229533.I1R9Y2 pp 229533.I1RFC0
179698 229533.I1R9Y2 pp 229533.I1RPQ2
179699 229533.I1R9Y2 pp 229533.I1S209
179700 229533.I1R9Y2 pp 229533.I1RL16
179701 229533.I1R9Y2 pp 229533.I1RD42
179702 229533.I1R9Y2 pp 229533.I1RBT7
179703 229533.I1R9Y2 pp 229533.I1RH75
179704 229533.I1R9Y2 pp 229533.V6RPN0
179705 229533.I1R9Y2 pp 229533.I1RDU5
179706 229533.I1R9Y2 pp 229533.I1RKI2
179707 229533.I1R9Y2 pp 229533.I1RPZ1
179708 229533.I1R9Y2 pp 229533.I1S130
179709 229533.I1R9Y2 pp 229533.I1S4S4
179710 229533.I1R9Y2 pp 229533.I1RS18
179711 229533.I1R9Y2 pp 229533.I1RZ92
179712 229533.I1R9Y2 pp 229533.I1S0A0
179713 229533.I1R9Y2 pp 229533.V6RG22
179714 229533.I1R9Y2 pp 229533.I1RCL4
179715 229533.I1R9Y2 pp 229533.V6RH92
179716 229533.I1R9Y2 pp 229533.I1RMQ4
179717 229533.I1R9Y2 pp 229533.I1RJ17
179718 229533.I1R9Y2 pp 229533.I1RM67
179719 229533.I1R9Y2 pp 229533.I1S1W8
179720 229533.I1R9Y2 pp 229533.I1RCE6
179721 229533.I1R9Y2 pp 229533.I1RJL9
179722 229533.I1R9Y2 pp 229533.I1RSV4
179723 229533.I1R9Y2 pp 229533.Q4I5G1
179724 229533.I1R9Y2 pp 229533.I1RXX1
179725 229533.I1R9Y2 pp 229533.Q00909
179726 229533.I1R9Y2 pp 229533.I1RED2

179727 229533.I1R9Y2 pp 229533.V6RBN2
179728 229533.A0A1C3YIQ4 pp 229533.I1RE65
179729 229533.A0A1C3YIQ4 pp 229533.I1RMW7
179730 229533.I1RZB2 pp 229533.I1S183
179731 229533.I1RZB2 pp 229533.I1S0L4
179732 229533.I1RZB2 pp 229533.V6RLM7
179733 229533.I1RZB2 pp 229533.I1S1W9
179734 229533.I1RZB2 pp 229533.I1S5B0
179735 229533.I1RZB2 pp 229533.V6RCZ8
179736 229533.I1RZB2 pp 229533.I1S0A0
179737 229533.I1RZB2 pp 229533.I1S5R1
179738 229533.I1RZB2 pp 229533.I1S338
179739 229533.I1RZB2 pp 229533.I1S2H4
179740 229533.I1RZB2 pp 229533.I1RZM8
179741 229533.I1RZB2 pp 229533.Q4HXF0
179742 229533.I1RZB2 pp 229533.I1S4S4
179743 229533.I1RZB2 pp 229533.Q4I624
179744 229533.I1RZB2 pp 229533.O74638
179745 229533.I1RZB2 pp 229533.Q4IFI0
179746 229533.I1RZB2 pp 229533.I1S4S7
179747 229533.I1RZB2 pp 229533.I1S0F1
179748 229533.I1RZB2 pp 229533.I1RZZ9
179749 229533.I1RCE6 pp 229533.I1RN23
179750 229533.I1RCE6 pp 229533.I1RPE5
179751 229533.I1RCE6 pp 229533.I1RY35
179752 229533.I1RCE6 pp 229533.I1RED3
179753 229533.I1RCE6 pp 229533.I1S1W8
179754 229533.I1RCE6 pp 229533.O74638
179755 229533.I1RCE6 pp 229533.I1RSV4
179756 229533.I1RCE6 pp 229533.V6RH92
179757 229533.I1RCE6 pp 229533.I1S2H4
179758 229533.I1RCE6 pp 229533.I1S3G4
179759 229533.I1RCE6 pp 229533.I1RVN1
179760 229533.I1RCE6 pp 229533.I1RW43
179761 229533.I1RCE6 pp 229533.I1S1K6
179762 229533.I1RCE6 pp 229533.I1RCL4
179763 229533.I1RCE6 pp 229533.I1S4S4
179764 229533.I1RCE6 pp 229533.I1RPD6
179765 229533.I1RCE6 pp 229533.I1RPZ1
179766 229533.I1RCE6 pp 229533.I1RM67
179767 229533.I1RCE6 pp 229533.I1RPQ2
179768 229533.I1RCE6 pp 229533.I1S130
179769 229533.I1RCE6 pp 229533.I1RWF9
179770 229533.I1RCE6 pp 229533.I1RH75
179771 229533.I1RCE6 pp 229533.I1RFC0
179772 229533.I1RCE6 pp 229533.V6RBN2
179773 229533.I1RCE6 pp 229533.I1RSM8
179774 229533.I1RCE6 pp 229533.I1RL16
179775 229533.I1RCE6 pp 229533.I1RD42
179776 229533.I1RCE6 pp 229533.Q00909
179777 229533.I1RCE6 pp 229533.Q4HXF0
179778 229533.I1RCE6 pp 229533.I1RMQ4
179779 229533.I1RCE6 pp 229533.V6RG22
179780 229533.I1RCE6 pp 229533.I1RXX1
179781 229533.I1RCE6 pp 229533.I1RFL3
179782 229533.I1RCE6 pp 229533.I1RDU5
179783 229533.I1RCE6 pp 229533.I1RZ92
179784 229533.I1RCE6 pp 229533.I1RJ17
179785 229533.I1RCE6 pp 229533.V6RPQ8
179786 229533.I1RCE6 pp 229533.V6RPN0
179787 229533.I1RCE6 pp 229533.I1RCH4
179788 229533.I1RCE6 pp 229533.I1RS18
179789 229533.I1RCE6 pp 229533.I1RED2
179790 229533.I1RCE6 pp 229533.Q4I5G1
179791 229533.I1R9B6 pp 229533.I1RVX9
179792 229533.I1R9B6 pp 229533.I1RZY1
179793 229533.I1R9B6 pp 229533.I1RTY0
179794 229533.I1R9B6 pp 229533.I1RQE1
179795 229533.I1R9B6 pp 229533.I1S1B8
179796 229533.I1R9B6 pp 229533.I1RMK4
179797 229533.I1R9B6 pp 229533.I1RYX6
179798 229533.I1R9B6 pp 229533.V6QWD8
179799 229533.I1R9B6 pp 229533.I1RAJ6

179800	229533.I1R9B6	pp	229533.I1RY49
179801	229533.I1R9B6	pp	229533.I1RAG9
179802	229533.I1R9B6	pp	229533.I1RY12
179803	229533.I1R9B6	pp	229533.I1S5P3
179804	229533.I1R9B6	pp	229533.I1RWF5
179805	229533.I1R9B6	pp	229533.V6QU65
179806	229533.I1R9B6	pp	229533.I1RSQ9
179807	229533.I1R9B6	pp	229533.I1RAR3
179808	229533.I1R9B6	pp	229533.I1RYQ4
179809	229533.I1R9B6	pp	229533.I1RNB4
179810	229533.I1R9B6	pp	229533.I1RM26
179811	229533.I1R9B6	pp	229533.I1RBE7
179812	229533.I1R9B6	pp	229533.I1S1W8
179813	229533.I1R9B6	pp	229533.I1RNG4
179814	229533.I1R9B6	pp	229533.I1S4G3
179815	229533.I1R9B6	pp	229533.I1RC74
179816	229533.I1R9B6	pp	229533.I1RZ83
179817	229533.I1R9B6	pp	229533.I1RX59
179818	229533.I1R9B6	pp	229533.I1RG66
179819	229533.I1R9B6	pp	229533.I1RA94
179820	229533.I1R9B6	pp	229533.I1RZK8
179821	229533.I1R9B6	pp	229533.V6RHD0
179822	229533.I1R9B6	pp	229533.I1RBY9
179823	229533.I1R9B6	pp	229533.I1S0I3
179824	229533.I1R9B6	pp	229533.I1S024
179825	229533.I1R9B6	pp	229533.I1S2Q4
179826	229533.I1R9B6	pp	229533.I1RY74
179827	229533.I1R9B6	pp	229533.I1RJJ7
179828	229533.I1R9B6	pp	229533.I1S3G4
179829	229533.I1R9B6	pp	229533.I1S147
179830	229533.I1R9B6	pp	229533.I1RGC3
179831	229533.I1R9B6	pp	229533.I1RH91
179832	229533.I1R9B6	pp	229533.I1RKZ7
179833	229533.I1R9B6	pp	229533.I1RAV0
179834	229533.I1R9B6	pp	229533.V6RXR0
179835	229533.I1R9B6	pp	229533.I1RXY7
179836	229533.I1R9B6	pp	229533.I1RWZ3
179837	229533.I1R9B6	pp	229533.I1RNV4
179838	229533.I1R9B6	pp	229533.I1RAH3
179839	229533.I1R9B6	pp	229533.I1RUR8
179840	229533.I1RRA5	pp	229533.V6RG22
179841	229533.I1RRA5	pp	229533.I1S130
179842	229533.I1RRA5	pp	229533.I1S2M5
179843	229533.I1RRA5	pp	229533.I1RZ92
179844	229533.A0A0E0SP71	pp	229533.I1RVK4
179845	229533.A0A0E0SP71	pp	229533.I1RY65
179846	229533.A0A0E0SP71	pp	229533.I1REL9
179847	229533.A0A0E0SP71	pp	229533.A0A1C3YI86
179848	229533.A0A0E0SP71	pp	229533.I1RWF4
179849	229533.A0A0E0SP71	pp	229533.I1S671
179850	229533.A0A0E0SP71	pp	229533.V6RPN0
179851	229533.A0A0E0SP71	pp	229533.I1RCH0
179852	229533.A0A0E0SP71	pp	229533.I1RUQ7
179853	229533.A0A0E0SP71	pp	229533.I1RIL9
179854	229533.A0A0E0SP71	pp	229533.I1RFK3
179855	229533.A0A0E0SP71	pp	229533.I1RJS0
179856	229533.A0A0E0SP71	pp	229533.V6RG22
179857	229533.A0A0E0SP71	pp	229533.A0A1C3YLB6
179858	229533.A0A0E0SP71	pp	229533.I1S2S8
179859	229533.A0A0E0SP71	pp	229533.A0A1C3YMS7
179860	229533.A0A0E0SP71	pp	229533.I1REP4
179861	229533.A0A0E0SP71	pp	229533.I1S1W6
179862	229533.A0A0E0SP71	pp	229533.I1S5R5
179863	229533.A0A0E0SP71	pp	229533.I1RGC4
179864	229533.A0A0E0SP71	pp	229533.I1RBR4
179865	229533.A0A0E0SP71	pp	229533.I1S2C2
179866	229533.A0A0E0SP71	pp	229533.I1RE80
179867	229533.A0A0E0SP71	pp	229533.I1S8M3
179868	229533.A0A0E0SP71	pp	229533.I1RT23
179869	229533.A0A0E0SP71	pp	229533.I1RLC8
179870	229533.A0A0E0SP71	pp	229533.A0A1C3YMJ5
179871	229533.A0A0E0SP71	pp	229533.A0A1C3YM25
179872	229533.A0A0E0SP71	pp	229533.A0A1C3YLL7

179873 229533.A0A0E0SP71 pp 229533.A0A1C3YKL8
179874 229533.A0A0E0SP71 pp 229533.I1RPW1
179875 229533.A0A0E0SP71 pp 229533.I1RV54
179876 229533.A0A0E0SP71 pp 229533.I1RZW1
179877 229533.A0A0E0SP71 pp 229533.I1RFM2
179878 229533.A0A0E0SP71 pp 229533.I1RRA5
179879 229533.A0A0E0SP71 pp 229533.I1RTR2
179880 229533.A0A0E0SP71 pp 229533.I1RZZ3
179881 229533.A0A0E0SP71 pp 229533.I1RNX0
179882 229533.A0A0E0SP71 pp 229533.I1RT57
179883 229533.A0A0E0SP71 pp 229533.I1SAS3
179884 229533.A0A0E0SP71 pp 229533.I1RXJ2
179885 229533.A0A0E0SP71 pp 229533.I1RXX1
179886 229533.A0A0E0SP71 pp 229533.I1RUP5
179887 229533.A0A0E0SP71 pp 229533.I1S167
179888 229533.A0A0E0SP71 pp 229533.I1SAS4
179889 229533.A0A0E0SP71 pp 229533.I1RI22
179890 229533.A0A0E0SP71 pp 229533.I1RCA0
179891 229533.A0A0E0SP71 pp 229533.I1RIX8
179892 229533.A0A0E0SP71 pp 229533.A0A1C3YMA3
179893 229533.A0A0E0SP71 pp 229533.I1RQM4
179894 229533.A0A0E0SP71 pp 229533.I1RQM2
179895 229533.A0A0E0SP71 pp 229533.I1RAG2
179896 229533.A0A0E0SP71 pp 229533.I1RVB6
179897 229533.A0A0E0SP71 pp 229533.I1RLZ7
179898 229533.A0A0E0SP71 pp 229533.I1SAV2
179899 229533.A0A0E0SP71 pp 229533.I1RRJ7
179900 229533.A0A0E0SP71 pp 229533.I1RL16
179901 229533.A0A0E0SP71 pp 229533.I1RSX9
179902 229533.A0A0E0SP71 pp 229533.I1R9F4
179903 229533.A0A0E0SP71 pp 229533.I1RNT1
179904 229533.A0A0E0SP71 pp 229533.V6RIE5
179905 229533.A0A0E0SP71 pp 229533.I1RY81
179906 229533.A0A0E0SP71 pp 229533.I1RTC4
179907 229533.A0A0E0SP71 pp 229533.I1RCR9
179908 229533.A0A0E0SP71 pp 229533.I1RXN6
179909 229533.A0A0E0SP71 pp 229533.I1RI23
179910 229533.A0A0E0SP71 pp 229533.I1RPF2
179911 229533.A0A0E0SP71 pp 229533.I1RU20
179912 229533.A0A0E0SP71 pp 229533.I1RUJ7
179913 229533.A0A0E0SP71 pp 229533.I1RCC2
179914 229533.A0A0E0SP71 pp 229533.I1RYW0
179915 229533.A0A0E0SP71 pp 229533.I1RPI2
179916 229533.A0A0E0SP71 pp 229533.A0A1C3YMN2
179917 229533.A0A0E0SP71 pp 229533.I1RM80
179918 229533.A0A0E0SP71 pp 229533.I1RIZ8
179919 229533.A0A0E0SP71 pp 229533.I1S350
179920 229533.A0A0E0SP71 pp 229533.I1RTW0
179921 229533.A0A0E0SP71 pp 229533.I1RPY2
179922 229533.A0A0E0SP71 pp 229533.I1SAF8
179923 229533.A0A0E0SP71 pp 229533.V6RR38
179924 229533.A0A0E0SP71 pp 229533.I1S2M5
179925 229533.A0A0E0SP71 pp 229533.I1RCG7
179926 229533.A0A0E0SP71 pp 229533.I1RSY4
179927 229533.A0A0E0SP71 pp 229533.I1RZ92
179928 229533.A0A0E0SP71 pp 229533.A0A1C3YKC3
179929 229533.A0A0E0SP71 pp 229533.I1RKG4
179930 229533.A0A0E0SP71 pp 229533.I1RVT4
179931 229533.A0A0E0SP71 pp 229533.I1RKA5
179932 229533.A0A0E0SP71 pp 229533.I1RQ48
179933 229533.A0A0E0SP71 pp 229533.I1RGK2
179934 229533.A0A0E0SP71 pp 229533.I1S1K6
179935 229533.A0A0E0SP71 pp 229533.A0A1C3YMV4
179936 229533.A0A0E0SP71 pp 229533.I1S130
179937 229533.A0A0E0SP71 pp 229533.I1RNK1
179938 229533.A0A0E0SP71 pp 229533.I1RKG6
179939 229533.A0A0E0SP71 pp 229533.I1S5K2
179940 229533.A0A0E0SP71 pp 229533.I1RZD0
179941 229533.A0A0E0SP71 pp 229533.I1RRQ1
179942 229533.A0A0E0SP71 pp 229533.I1RLD2
179943 229533.A0A0E0SP71 pp 229533.I1S1K5
179944 229533.A0A0E0SP71 pp 229533.I1R984
179945 229533.A0A0E0SP71 pp 229533.I1RDZ5

179946 229533.A0A0E0SP71 pp 229533.I1RHV5
179947 229533.A0A0E0SP71 pp 229533.I1RIB7
179948 229533.A0A0E0SP71 pp 229533.I1RJR2
179949 229533.A0A0E0SP71 pp 229533.I1RPE5
179950 229533.A0A0E0SP71 pp 229533.I1RXV8
179951 229533.A0A0E0SP71 pp 229533.I1RFP1
179952 229533.A0A0E0SP71 pp 229533.I1RJ25
179953 229533.A0A0E0SP71 pp 229533.I1S108
179954 229533.A0A0E0SP71 pp 229533.I1REM6
179955 229533.A0A0E0SP71 pp 229533.I1S1Q3
179956 229533.A0A0E0SP71 pp 229533.I1RIT5
179957 229533.A0A0E0SP71 pp 229533.I1RF79
179958 229533.A0A0E0SP71 pp 229533.I1RY34
179959 229533.A0A0E0SP71 pp 229533.I1RLC9
179960 229533.A0A0E0SP71 pp 229533.I1RDI7
179961 229533.A0A0E0SP71 pp 229533.I1RQ76
179962 229533.A0A0E0SP71 pp 229533.I1SAK7
179963 229533.A0A0E0SP71 pp 229533.I1RFA1
179964 229533.A0A0E0SP71 pp 229533.I1RA51
179965 229533.A0A0E0SP71 pp 229533.A0A1C3YMJ7
179966 229533.A0A0E0SP71 pp 229533.I1REM2
179967 229533.A0A0E0SP71 pp 229533.I1RMA2
179968 229533.A0A0E0SP71 pp 229533.I1RJG1
179969 229533.A0A0E0SP71 pp 229533.I1RR90
179970 229533.A0A0E0SP71 pp 229533.I1RY35
179971 229533.I1RVZ2 pp 229533.I1RZZ9
179972 229533.I1RVZ2 pp 229533.I1RWI5
179973 229533.I1RVZ2 pp 229533.I1RZ39
179974 229533.I1RVZ2 pp 229533.I1S0F1
179975 229533.I1RVZ2 pp 229533.V6RCJ5
179976 229533.I1RVZ2 pp 229533.I1RXC2
179977 229533.I1RVZ2 pp 229533.I1S5R1
179978 229533.I1RVZ2 pp 229533.I1S2H4
179979 229533.I1RVZ2 pp 229533.I1S3G4
179980 229533.I1RVZ2 pp 229533.I1S253
179981 229533.I1RVZ2 pp 229533.I1S4S4
179982 229533.I1RVZ2 pp 229533.I1S9Y1
179983 229533.I1RVZ2 pp 229533.I1RW61
179984 229533.I1RVZ2 pp 229533.V6R5T7
179985 229533.I1RVZ2 pp 229533.I1S0L4
179986 229533.I1RVZ2 pp 229533.I1S2B8
179987 229533.I1RVZ2 pp 229533.Q4HVQ9
179988 229533.I1RVZ2 pp 229533.V6RCC0
179989 229533.I1RVZ2 pp 229533.I1S8F0
179990 229533.I1RVZ2 pp 229533.I1S0B7
179991 229533.I1RVZ2 pp 229533.I1RX46
179992 229533.I1RVZ2 pp 229533.I1S9I5
179993 229533.I1RVZ2 pp 229533.I1S1W8
179994 229533.I1RVZ2 pp 229533.I1RZB2
179995 229533.I1RVZ2 pp 229533.I1S438
179996 229533.I1RVZ2 pp 229533.I1S277
179997 229533.I1RVZ2 pp 229533.I1S4Z8
179998 229533.I1RVZ2 pp 229533.I1S4S7
179999 229533.I1RVZ2 pp 229533.I1RW71
180000 229533.I1RVZ2 pp 229533.I1RXF8
180001 229533.I1RVZ2 pp 229533.V6RTJ8
180002 229533.I1RVZ2 pp 229533.Q4HXF0
180003 229533.I1RVZ2 pp 229533.I1RYI5
180004 229533.I1RVZ2 pp 229533.I1RYR0
180005 229533.I1RVZ2 pp 229533.I1RXD7
180006 229533.I1RVZ2 pp 229533.I1RX95
180007 229533.I1RVZ2 pp 229533.I1S0W4
180008 229533.I1RVZ2 pp 229533.I1S2R0
180009 229533.I1RVZ2 pp 229533.I1RW26
180010 229533.I1RVZ2 pp 229533.I1RYP2
180011 229533.I1RVZ2 pp 229533.I1RZK5
180012 229533.I1RVZ2 pp 229533.I1RXD6
180013 229533.I1RVZ2 pp 229533.I1RY86
180014 229533.I1RVZ2 pp 229533.I1S6V0
180015 229533.I1RVZ2 pp 229533.I1RYU0
180016 229533.I1RVZ2 pp 229533.I1S0F7
180017 229533.I1RVZ2 pp 229533.I1S821
180018 229533.I1RVZ2 pp 229533.I1S1J7

180019 229533.I1RVZ2 pp 229533.V6RCZ8
180020 229533.I1RVZ2 pp 229533.V6R9Z0
180021 229533.I1RVZ2 pp 229533.I1S6K9
180022 229533.I1RVZ2 pp 229533.I1S437
180023 229533.I1RVZ2 pp 229533.I1RYS0
180024 229533.I1RVZ2 pp 229533.I1RYW3
180025 229533.I1RVZ2 pp 229533.I1S1C7
180026 229533.I1RVZ2 pp 229533.I1RZ79
180027 229533.I1RVZ2 pp 229533.I1S274
180028 229533.I1RVZ2 pp 229533.V6R042
180029 229533.I1RVZ2 pp 229533.I1S0F8
180030 229533.I1RVZ2 pp 229533.V6RMW6
180031 229533.I1RVZ2 pp 229533.V6QUE6
180032 229533.I1RVZ2 pp 229533.Q4IJK2
180033 229533.I1RVZ2 pp 229533.I1RWR7
180034 229533.I1RVZ2 pp 229533.I1S1B2
180035 229533.I1RVZ2 pp 229533.I1S8D3
180036 229533.I1RVZ2 pp 229533.V6RWB3
180037 229533.I1RVZ2 pp 229533.I1RWQ8
180038 229533.I1RVZ2 pp 229533.V6RRH2
180039 229533.I1RVZ2 pp 229533.I1RZM8
180040 229533.I1RVZ2 pp 229533.I1S5R6
180041 229533.I1RVZ2 pp 229533.Q4I624
180042 229533.I1RVZ2 pp 229533.I1RXC4
180043 229533.I1RVZ2 pp 229533.I1RXF0
180044 229533.I1RVZ2 pp 229533.I1S5B0
180045 229533.I1RVZ2 pp 229533.I1S064
180046 229533.I1RVZ2 pp 229533.I1S0J1
180047 229533.I1RVZ2 pp 229533.I1RZV3
180048 229533.I1RVZ2 pp 229533.V6RHX2
180049 229533.I1RVZ2 pp 229533.I1S1W9
180050 229533.I1RVZ2 pp 229533.I1RWP4
180051 229533.I1RVZ2 pp 229533.I1RVZ7
180052 229533.A0A0E0RRM0 pp 229533.I1RPV8
180053 229533.A0A0E0RRM0 pp 229533.I1RN04
180054 229533.A0A0E0RRM0 pp 229533.A0A1C3YHG7
180055 229533.A0A0E0RRM0 pp 229533.I1S094
180056 229533.A0A0E0RRM0 pp 229533.I1S0A5
180057 229533.A0A0E0RRM0 pp 229533.I1RW91
180058 229533.A0A0E0RRM0 pp 229533.I1RW80
180059 229533.A0A0E0RRM0 pp 229533.I1RNG4
180060 229533.A0A0E0RRM0 pp 229533.I1RF85
180061 229533.A0A0E0RRM0 pp 229533.I1SAT2
180062 229533.A0A0E0RRM0 pp 229533.I1S6H3
180063 229533.A0A0E0RRM0 pp 229533.I1RJS1
180064 229533.A0A0E0RRM0 pp 229533.A0A0E0RSI1
180065 229533.A0A0E0RRM0 pp 229533.I1RCU7
180066 229533.A0A0E0RRM0 pp 229533.I1RQV7
180067 229533.A0A0E0RRM0 pp 229533.I1RE52
180068 229533.A0A0E0RRM0 pp 229533.A0A1C3YLB6
180069 229533.A0A0E0RRM0 pp 229533.I1RH83
180070 229533.A0A0E0RRM0 pp 229533.I1RZ56
180071 229533.A0A0E0RRM0 pp 229533.I1R9F2
180072 229533.A0A0E0RRM0 pp 229533.I1S5D0
180073 229533.A0A0E0RRM0 pp 229533.I1RRS6
180074 229533.A0A0E0RRM0 pp 229533.I1RA13
180075 229533.A0A0E0RRM0 pp 229533.I1S659
180076 229533.A0A0E0RRM0 pp 229533.I1RDN3
180077 229533.A0A0E0RRM0 pp 229533.I1RCU2
180078 229533.A0A0E0RRM0 pp 229533.A0A1C3YHX6
180079 229533.A0A0E0RRM0 pp 229533.I1RPW8
180080 229533.A0A0E0RRM0 pp 229533.I1RST9
180081 229533.A0A0E0RRM0 pp 229533.I1RIB5
180082 229533.A0A0E0RRM0 pp 229533.I1S267
180083 229533.A0A0E0RRM0 pp 229533.I1RCG6
180084 229533.I1S2M5 pp 229533.I1SAS3
180085 229533.I1S2M5 pp 229533.I1SAS4
180086 229533.I1S2M5 pp 229533.V6RG22
180087 229533.I1S2M5 pp 229533.V6RR38
180088 229533.I1S2M5 pp 229533.V6RPN0
180089 229533.I1S2M5 pp 229533.Q4IPH4
180090 229533.A0A1C3YJR2 pp 229533.I1S274
180091 229533.A0A1C3YJR2 pp 229533.I1S3T6

180092 229533.A0A1C3YJR2 pp 229533.I1RG14
180093 229533.A0A1C3YJR2 pp 229533.I1RSS4
180094 229533.A0A1C3YJR2 pp 229533.I1RX70
180095 229533.A0A1C3YJR2 pp 229533.A0A1C3YKM9
180096 229533.A0A1C3YJR2 pp 229533.I1RZ39
180097 229533.A0A1C3YJR2 pp 229533.I1RT82
180098 229533.A0A1C3YJR2 pp 229533.I1RWH8
180099 229533.A0A1C3YJR2 pp 229533.I1RTG6
180100 229533.A0A1C3YJR2 pp 229533.I1RM67
180101 229533.A0A1C3YJR2 pp 229533.I1RB27
180102 229533.A0A1C3YJR2 pp 229533.I1RR72
180103 229533.A0A1C3YJR2 pp 229533.I1RYH9
180104 229533.A0A1C3YJR2 pp 229533.I1RP97
180105 229533.A0A1C3YJR2 pp 229533.I1RSZ7
180106 229533.A0A1C3YJR2 pp 229533.I1RT75
180107 229533.A0A1C3YJR2 pp 229533.Q4HXF0
180108 229533.A0A1C3YJR2 pp 229533.I1REF2
180109 229533.A0A1C3YJR2 pp 229533.I1RYJ6
180110 229533.A0A1C3YJR2 pp 229533.I1S223
180111 229533.A0A1C3YJR2 pp 229533.I1RWS4
180112 229533.A0A1C3YJR2 pp 229533.I1RT33
180113 229533.A0A1C3YJR2 pp 229533.I1RFY6
180114 229533.A0A1C3YJR2 pp 229533.I1S1F3
180115 229533.A0A1C3YJR2 pp 229533.I1RNG4
180116 229533.I1RJK8 pp 229533.I1RXB5
180117 229533.I1RJK8 pp 229533.I1RKG4
180118 229533.I1RJK8 pp 229533.Q4I2W2
180119 229533.I1RJK8 pp 229533.I1RKR7
180120 229533.I1RJK8 pp 229533.I1RQ70
180121 229533.I1RJK8 pp 229533.I1RN81
180122 229533.I1RJK8 pp 229533.I1RRH3
180123 229533.I1RJK8 pp 229533.Q4IMN9
180124 229533.I1RJK8 pp 229533.I1RMF2
180125 229533.I1RJK8 pp 229533.I1RRH6
180126 229533.I1RJK8 pp 229533.I1RUK7
180127 229533.I1RJK8 pp 229533.I1RMK1
180128 229533.I1RVB6 pp 229533.I1S2M5
180129 229533.I1RVB6 pp 229533.I1S1S9
180130 229533.I1RVB6 pp 229533.I1RVC0
180131 229533.I1RVB6 pp 229533.I1S6X6
180132 229533.I1RNX0 pp 229533.I1S5K2
180133 229533.I1RNX0 pp 229533.I1S1Q3
180134 229533.I1RNX0 pp 229533.I1RQ76
180135 229533.I1RNX0 pp 229533.I1RY35
180136 229533.I1RNX0 pp 229533.I1RQ48
180137 229533.I1RNX0 pp 229533.I1RVE0
180138 229533.I1RNX0 pp 229533.I1SAA1
180139 229533.I1RNX0 pp 229533.I1RR90
180140 229533.I1RNX0 pp 229533.I1RS80
180141 229533.I1RNX0 pp 229533.I1RRQ1
180142 229533.I1RNX0 pp 229533.I1S341
180143 229533.I1RNX0 pp 229533.V6RPN0
180144 229533.I1RNX0 pp 229533.I1SAS4
180145 229533.I1RNX0 pp 229533.V6RR38
180146 229533.I1RNX0 pp 229533.I1RZZ3
180147 229533.I1RNX0 pp 229533.I1SAS3
180148 229533.I1RNX0 pp 229533.I1RT23
180149 229533.I1RNX0 pp 229533.I1SAS1
180150 229533.I1RJH7 pp 229533.I1S9V9
180151 229533.I1RJH7 pp 229533.I1RSQ4
180152 229533.I1RJH7 pp 229533.I1RNL7
180153 229533.I1RJH7 pp 229533.I1RTF5
180154 229533.I1RJH7 pp 229533.I1S2B9
180155 229533.I1RJH7 pp 229533.I1RNN7
180156 229533.I1RJH7 pp 229533.I1S3G4
180157 229533.I1RJH7 pp 229533.I1RZ60
180158 229533.I1RJH7 pp 229533.I1RJP2
180159 229533.I1RJH7 pp 229533.I1S4Q6
180160 229533.I1RJH7 pp 229533.I1RNF5
180161 229533.I1RJH7 pp 229533.I1RWC9
180162 229533.I1RJH7 pp 229533.I1RWC6
180163 229533.I1RJH7 pp 229533.I1RRR5
180164 229533.I1RJH7 pp 229533.I1S0A0

180165	229533.I1RJH7	pp	229533.I1S1W8
180166	229533.I1RJH7	pp	229533.I1RY40
180167	229533.I1RJH7	pp	229533.I1S2I7
180168	229533.I1RFL5	pp	229533.Q4I298
180169	229533.I1RFL5	pp	229533.I1RNG4
180170	229533.I1RIM6	pp	229533.I1S267
180171	229533.I1RIM6	pp	229533.I1RWF9
180172	229533.I1RIM6	pp	229533.I1RNM7
180173	229533.I1RIM6	pp	229533.I1RST9
180174	229533.I1RNV4	pp	229533.I1RY12
180175	229533.I1RNV4	pp	229533.I1RYX6
180176	229533.I1RNV4	pp	229533.I1S4G3
180177	229533.I1RNV4	pp	229533.I1RX59
180178	229533.I1RNV4	pp	229533.I1RQE1
180179	229533.I1RNV4	pp	229533.I1S1B8
180180	229533.I1RNV4	pp	229533.I1RTY0
180181	229533.I1RNV4	pp	229533.V6RHZ8
180182	229533.I1RNV4	pp	229533.I1RZK8
180183	229533.I1RNV4	pp	229533.V6QWD8
180184	229533.I1RNV4	pp	229533.I1RVX9
180185	229533.I1RNV4	pp	229533.V6QU65
180186	229533.I1RNV4	pp	229533.I1S1W8
180187	229533.I1RNV4	pp	229533.I1RZY1
180188	229533.I1RNV4	pp	229533.I1RSQ9
180189	229533.I1RNV4	pp	229533.I1RY49
180190	229533.I1RNV4	pp	229533.I1RWZ3
180191	229533.I1RNV4	pp	229533.I1RXY7
180192	229533.I1RNV4	pp	229533.I1RQ70
180193	229533.I1RNV4	pp	229533.I1S147
180194	229533.I1RNV4	pp	229533.I1RZ83
180195	229533.I1RNV4	pp	229533.I1RYQ4
180196	229533.I1RNV4	pp	229533.V6RXR0
180197	229533.I1RNV4	pp	229533.I1RUR8
180198	229533.I1RNV4	pp	229533.V6RHD0
180199	229533.I1RNV4	pp	229533.I1RPH3
180200	229533.I1RNV4	pp	229533.I1S0I3
180201	229533.I1RNV4	pp	229533.I1RY74
180202	229533.I1RNV4	pp	229533.I1S2Q4
180203	229533.I1RNV4	pp	229533.I1S024
180204	229533.I1RNV4	pp	229533.I1RWF5
180205	229533.I1RNV4	pp	229533.I1S3G4
180206	229533.I1RCE9	pp	229533.I1RNM7
180207	229533.I1RT57	pp	229533.I1RY11
180208	229533.I1S1N7	pp	229533.I1S464
180209	229533.I1S1N7	pp	229533.I1S8S9
180210	229533.I1S1N7	pp	229533.V6RJP8
180211	229533.I1S1N7	pp	229533.I1S5X9
180212	229533.I1S1N7	pp	229533.I1S5R5
180213	229533.I1REM2	pp	229533.I1RFA2
180214	229533.I1REM2	pp	229533.I1RU81
180215	229533.I1REM2	pp	229533.I1S1S9
180216	229533.I1REM2	pp	229533.I1S2M5
180217	229533.I1REM2	pp	229533.I1RQ48
180218	229533.I1REM2	pp	229533.I1RYA9
180219	229533.I1REM2	pp	229533.I1RNT1
180220	229533.I1REM2	pp	229533.I1REM6
180221	229533.I1REM2	pp	229533.V6RPN0
180222	229533.I1REM2	pp	229533.I1S2J5
180223	229533.I1REM2	pp	229533.I1RR90
180224	229533.I1REM2	pp	229533.I1RRQ1
180225	229533.I1REM2	pp	229533.V6RR38
180226	229533.I1REM2	pp	229533.I1RY35
180227	229533.I1REM2	pp	229533.I1RH44
180228	229533.I1REM2	pp	229533.I1RGV3
180229	229533.I1REM2	pp	229533.I1RQ76
180230	229533.I1REM2	pp	229533.I1REM3
180231	229533.I1REM2	pp	229533.I1RT23
180232	229533.I1REM2	pp	229533.I1RJG1
180233	229533.I1REM2	pp	229533.I1RI22
180234	229533.I1REM2	pp	229533.I1S1Q3
180235	229533.I1REM2	pp	229533.I1RVB6
180236	229533.I1REM2	pp	229533.I1RUW5
180237	229533.I1REM2	pp	229533.I1RZZ3

180238	229533.I1REM2	pp	229533.I1RF79
180239	229533.I1REM2	pp	229533.I1RF98
180240	229533.I1REM2	pp	229533.I1RFM2
180241	229533.I1REM2	pp	229533.I1SAS4
180242	229533.I1REM2	pp	229533.I1RIA9
180243	229533.I1REM2	pp	229533.I1SAS3
180244	229533.I1RJH8	pp	229533.I1S9V9
180245	229533.I1RJH8	pp	229533.I1RZ60
180246	229533.I1RJH8	pp	229533.I1S3G4
180247	229533.I1RJH8	pp	229533.I1S2I7
180248	229533.I1RJH8	pp	229533.I1RSQ4
180249	229533.I1RJH8	pp	229533.I1S0A0
180250	229533.I1RJH8	pp	229533.I1RY40
180251	229533.I1RJH8	pp	229533.I1S2B9
180252	229533.I1RJH8	pp	229533.I1RNF5
180253	229533.I1RJH8	pp	229533.I1RWC9
180254	229533.I1RJH8	pp	229533.I1S4Q6
180255	229533.I1RJH8	pp	229533.I1RJK7
180256	229533.I1RJH8	pp	229533.I1RTF5
180257	229533.I1RJH8	pp	229533.I1RNN7
180258	229533.I1RJH8	pp	229533.Q4I4A4
180259	229533.I1RJH8	pp	229533.I1RRR5
180260	229533.I1RJH8	pp	229533.I1RNL7
180261	229533.I1RJH8	pp	229533.Q4I1Y5
180262	229533.I1RJH8	pp	229533.I1S1W8
180263	229533.I1RJH8	pp	229533.I1RMJ1
180264	229533.A0A098DGU8	pp	229533.I1RC81
180265	229533.A0A098DGU8	pp	229533.I1RBZ3
180266	229533.A0A098DGU8	pp	229533.I1RBL7
180267	229533.A0A098DGU8	pp	229533.I1RAW0
180268	229533.A0A098DGU8	pp	229533.Q4IJK2
180269	229533.A0A098DGU8	pp	229533.I1RFL4
180270	229533.A0A098DGU8	pp	229533.I1RQE7
180271	229533.A0A098DGU8	pp	229533.I1RCH5
180272	229533.A0A098DGU8	pp	229533.I1RSS4
180273	229533.A0A098DGU8	pp	229533.I1RVX0
180274	229533.A0A098DGU8	pp	229533.I1RCW6
180275	229533.A0A098DGU8	pp	229533.I1RYW3
180276	229533.A0A098DGU8	pp	229533.I1RK95
180277	229533.A0A098DGU8	pp	229533.A0A0E0S6Y2
180278	229533.A0A098DGU8	pp	229533.I1REL7
180279	229533.A0A098DGU8	pp	229533.I1S6E5
180280	229533.A0A098DGU8	pp	229533.I1RRI2
180281	229533.A0A098DGU8	pp	229533.V6QUE6
180282	229533.A0A098DGU8	pp	229533.A0A098E4W0
180283	229533.A0A098DGU8	pp	229533.I1RA70
180284	229533.A0A098DGU8	pp	229533.I1RCR5
180285	229533.A0A098DGU8	pp	229533.I1RD22
180286	229533.A0A098DGU8	pp	229533.A0A1C3YI50
180287	229533.A0A098DGU8	pp	229533.I1RXF0
180288	229533.A0A098DGU8	pp	229533.A0A0E0RWW0
180289	229533.A0A098DGU8	pp	229533.I1RK83
180290	229533.A0A098DGU8	pp	229533.I1RG11
180291	229533.A0A098DGU8	pp	229533.I1RJV3
180292	229533.A0A098DGU8	pp	229533.I1RZ39
180293	229533.A0A098DGU8	pp	229533.I1RNC1
180294	229533.A0A098DGU8	pp	229533.I1S5B0
180295	229533.A0A098DGU8	pp	229533.I1RUG3
180296	229533.A0A098DGU8	pp	229533.I1RYB9
180297	229533.A0A098DGU8	pp	229533.A0A1C3YKM9
180298	229533.A0A098DGU8	pp	229533.I1S0J1
180299	229533.A0A098DGU8	pp	229533.I1S9Y1
180300	229533.A0A098DGU8	pp	229533.I1S4S4
180301	229533.A0A098DGU8	pp	229533.V6RCZ8
180302	229533.A0A098DGU8	pp	229533.I1RJ17
180303	229533.A0A098DGU8	pp	229533.V6R042
180304	229533.A0A098DGU8	pp	229533.A0A1C3YKV3
180305	229533.A0A098DGU8	pp	229533.I1RM67
180306	229533.A0A098DGU8	pp	229533.I1S5R1
180307	229533.A0A098DGU8	pp	229533.I1RRT8
180308	229533.A0A098DGU8	pp	229533.I1RSQ3
180309	229533.A0A098DGU8	pp	229533.A0A1C3YN57
180310	229533.A0A098DGU8	pp	229533.I1RS18

180311 229533.A0A098DGU8 pp 229533.I1RIY2
180312 229533.A0A098DGU8 pp 229533.A0A1C3YJR3
180313 229533.A0A098DGU8 pp 229533.A0A0E0SID1
180314 229533.A0A098DGU8 pp 229533.O74638
180315 229533.A0A098DGU8 pp 229533.A0A1C3YIR8
180316 229533.A0A098DGU8 pp 229533.I1RT40
180317 229533.A0A098DGU8 pp 229533.I1RBT1
180318 229533.A0A098DGU8 pp 229533.I1S2H4
180319 229533.A0A098DGU8 pp 229533.A0A1C3YJI6
180320 229533.A0A098DGU8 pp 229533.Q4HXF0
180321 229533.A0A098DGU8 pp 229533.I1RSQ4
180322 229533.A0A098DGU8 pp 229533.A0A098DPR9
180323 229533.A0A098DGU8 pp 229533.A0A0E0SFN0
180324 229533.A0A098DGU8 pp 229533.I1RXD7
180325 229533.A0A098DGU8 pp 229533.A0A0E0RSD7
180326 229533.A0A098DGU8 pp 229533.A0A0E0SC46
180327 229533.A0A098DGU8 pp 229533.I1RSZ7
180328 229533.A0A098DGU8 pp 229533.I1RZZ9
180329 229533.A0A098DGU8 pp 229533.A0A1C3YI20
180330 229533.A0A098DGU8 pp 229533.I1RFS3
180331 229533.A0A098DGU8 pp 229533.I1RX46
180332 229533.A0A098DGU8 pp 229533.I1RC05
180333 229533.A0A098DGU8 pp 229533.I1RBC7
180334 229533.A0A098DGU8 pp 229533.I1RL04
180335 229533.A0A098DGU8 pp 229533.I1R9Y2
180336 229533.A0A098DGU8 pp 229533.I1RT33
180337 229533.A0A098DGU8 pp 229533.I1RCE6
180338 229533.A0A098DGU8 pp 229533.I1RGA4
180339 229533.A0A098DGU8 pp 229533.I1RD52
180340 229533.A0A098DGU8 pp 229533.I1RFT1
180341 229533.A0A098DGU8 pp 229533.I1R9M0
180342 229533.A0A098DGU8 pp 229533.A0A1C3YHV8
180343 229533.A0A098DGU8 pp 229533.A0A098E0A5
180344 229533.A0A098DGU8 pp 229533.A0A1C3YK13
180345 229533.A0A098DGU8 pp 229533.I1RSQ7
180346 229533.A0A098DGU8 pp 229533.I1RBY4
180347 229533.A0A098DGU8 pp 229533.I1RPP3
180348 229533.A0A098DGU8 pp 229533.A0A1C3YIP5
180349 229533.A0A098DGU8 pp 229533.I1RN67
180350 229533.I1RYQ4 pp 229533.I1RZK8
180351 229533.I1RYQ4 pp 229533.I1S2Q4
180352 229533.I1RYQ4 pp 229533.V6QWD8
180353 229533.I1RYQ4 pp 229533.I1S1B8
180354 229533.I1RYQ4 pp 229533.I1RZY1
180355 229533.I1RYQ4 pp 229533.I1RYX6
180356 229533.I1RYQ4 pp 229533.V6QU65
180357 229533.I1RYQ4 pp 229533.I1S1W8
180358 229533.I1RYQ4 pp 229533.I1S4G3
180359 229533.I1RYQ4 pp 229533.I1S3G4
180360 229533.I1RYQ4 pp 229533.I1S099
180361 229533.I1RYQ4 pp 229533.I1RZ83
180362 229533.I1RYQ4 pp 229533.I1S147
180363 229533.I1RYQ4 pp 229533.V6RXR0
180364 229533.I1RYQ4 pp 229533.I1S024
180365 229533.I1RYQ4 pp 229533.V6R5D2
180366 229533.I1RYQ4 pp 229533.V6RHD0
180367 229533.I1RYQ4 pp 229533.V6R382
180368 229533.I1RYQ4 pp 229533.I1S0I3
180369 229533.A0A098CZ12 pp 229533.I1R9B6
180370 229533.A0A098CZ12 pp 229533.A0A0E0SP71
180371 229533.A0A098CZ12 pp 229533.I1S2M5
180372 229533.A0A098CZ12 pp 229533.I1RVB6
180373 229533.A0A098CZ12 pp 229533.I1RNV4
180374 229533.A0A098CZ12 pp 229533.I1REM2
180375 229533.A0A098CZ12 pp 229533.I1RYQ4
180376 229533.I1RRJ7 pp 229533.I1RWN8
180377 229533.I1RUE3 pp 229533.I1RZ56
180378 229533.I1RUE3 pp 229533.I1S8H5
180379 229533.I1RUE3 pp 229533.I1S1T5
180380 229533.I1RUE3 pp 229533.I1RW80
180381 229533.I1RUE3 pp 229533.I1S0A5
180382 229533.I1RUE3 pp 229533.I1SAT2
180383 229533.I1RUE3 pp 229533.I1S6H3

180384 229533.I1RUE3 pp 229533.I1S659
180385 229533.I1RUE3 pp 229533.I1S5D0
180386 229533.I1RUE3 pp 229533.I1RW91
180387 229533.I1RUE3 pp 229533.I1S267
180388 229533.I1RUE3 pp 229533.I1S094
180389 229533.A0A1C3YJH9 pp 229533.I1REK8
180390 229533.A0A1C3YJH9 pp 229533.I1REL7
180391 229533.A0A1C3YJH9 pp 229533.I1RM35
180392 229533.A0A1C3YJH9 pp 229533.I1S4Q4
180393 229533.A0A1C3YJH9 pp 229533.V6RBN2
180394 229533.A0A1C3YJH9 pp 229533.A0A1C3YJJ2
180395 229533.A0A1C3YJH9 pp 229533.V6RTJ8
180396 229533.A0A1C3YJH9 pp 229533.A0A1C3YJL7
180397 229533.A0A1C3YJH9 pp 229533.I1REK4
180398 229533.A0A1C3YJH9 pp 229533.I1RPN4
180399 229533.A0A1C3YJH9 pp 229533.V6R1K4
180400 229533.A0A1C3YJH9 pp 229533.I1RBE4
180401 229533.A0A1C3YJH9 pp 229533.I1RM67
180402 229533.A0A1C3YJH9 pp 229533.I1RJU3
180403 229533.A0A1C3YJH9 pp 229533.A0A1C3YN30
180404 229533.A0A1C3YJH9 pp 229533.I1RG14
180405 229533.A0A1C3YJH9 pp 229533.A0A1C3YK34
180406 229533.A0A1C3YJH9 pp 229533.V6R057
180407 229533.A0A1C3YJH9 pp 229533.I1RCW6
180408 229533.A0A1C3YJH9 pp 229533.A0A1C3YLR6
180409 229533.A0A1C3YJH9 pp 229533.I1RPA4
180410 229533.A0A1C3YJH9 pp 229533.V6RMW6
180411 229533.A0A1C3YJH9 pp 229533.I1RD22
180412 229533.A0A1C3YJH9 pp 229533.I1RVX0
180413 229533.A0A1C3YJH9 pp 229533.A0A1C3YL55
180414 229533.A0A1C3YJH9 pp 229533.I1RS17
180415 229533.A0A1C3YJH9 pp 229533.I1RWE8
180416 229533.A0A1C3YJH9 pp 229533.Q4HXF0
180417 229533.A0A1C3YJH9 pp 229533.I1RN67
180418 229533.A0A1C3YJH9 pp 229533.I1RTA0
180419 229533.A0A1C3YJH9 pp 229533.I1RC05
180420 229533.A0A1C3YJH9 pp 229533.A0A1C3YKY0
180421 229533.A0A1C3YJH9 pp 229533.I1S8P5
180422 229533.A0A1C3YJH9 pp 229533.I1RTA1
180423 229533.A0A1C3YJH9 pp 229533.I1RPP3
180424 229533.A0A1C3YJH9 pp 229533.O74638
180425 229533.A0A1C3YJH9 pp 229533.I1S277
180426 229533.A0A1C3YJH9 pp 229533.I1RL04
180427 229533.A0A1C3YJH9 pp 229533.I1S2B8
180428 229533.I1RL04 pp 229533.V6RTJ8
180429 229533.I1RL04 pp 229533.I1RX46
180430 229533.I1RL04 pp 229533.I1RYX6
180431 229533.I1RL04 pp 229533.I1RSQ7
180432 229533.I1RL04 pp 229533.I1RZM4
180433 229533.I1RL04 pp 229533.I1S3S3
180434 229533.I1RL04 pp 229533.I1RXD7
180435 229533.I1RL04 pp 229533.I1S4S7
180436 229533.I1RL04 pp 229533.I1RRS8
180437 229533.I1RL04 pp 229533.I1RQD2
180438 229533.I1RL04 pp 229533.I1S5B0
180439 229533.I1RL04 pp 229533.I1RPU4
180440 229533.I1RL04 pp 229533.I1S4S4
180441 229533.I1RL04 pp 229533.I1RPA4
180442 229533.I1RL04 pp 229533.I1RNC0
180443 229533.I1RL04 pp 229533.I1RXH3
180444 229533.I1RL04 pp 229533.I1RR24
180445 229533.I1RL04 pp 229533.I1S438
180446 229533.I1RL04 pp 229533.I1RZM8
180447 229533.I1RL04 pp 229533.I1RQE8
180448 229533.I1RL04 pp 229533.I1RXF0
180449 229533.I1RL04 pp 229533.I1RTD3
180450 229533.I1RL04 pp 229533.V6R7V8
180451 229533.I1RL04 pp 229533.I1RQ85
180452 229533.I1RL04 pp 229533.V6RK96
180453 229533.I1RL04 pp 229533.I1RMQ4
180454 229533.I1RL04 pp 229533.I1RRY9
180455 229533.I1RL04 pp 229533.I1RXC4
180456 229533.I1RL04 pp 229533.I1S6K9

180457 229533.I1RL04 pp 229533.I1RXX1
180458 229533.I1RL04 pp 229533.V6RCZ8
180459 229533.I1RL04 pp 229533.I1RWR7
180460 229533.I1RL04 pp 229533.I1S4Q4
180461 229533.I1RL04 pp 229533.I1RS18
180462 229533.I1RL04 pp 229533.I1RZK5
180463 229533.I1RL04 pp 229533.I1RY62
180464 229533.I1RL04 pp 229533.V6QUE6
180465 229533.I1RL04 pp 229533.I1RN67
180466 229533.I1RL04 pp 229533.I1RN42
180467 229533.I1RL04 pp 229533.V6RBN2
180468 229533.I1RL04 pp 229533.O74638
180469 229533.I1RL04 pp 229533.Q4IJK2
180470 229533.I1RL04 pp 229533.I1RRV4
180471 229533.I1RL04 pp 229533.I1RW26
180472 229533.I1RL04 pp 229533.I1RXC2
180473 229533.I1RL04 pp 229533.I1S5R1
180474 229533.I1RL04 pp 229533.I1RZ83
180475 229533.I1RL04 pp 229533.I1S2A1
180476 229533.I1RL04 pp 229533.I1RQM7
180477 229533.I1RL04 pp 229533.I1RTA1
180478 229533.I1RL04 pp 229533.I1RSX0
180479 229533.I1RL04 pp 229533.I1RRT8
180480 229533.I1RL04 pp 229533.I1RVZ2
180481 229533.I1RL04 pp 229533.I1RYR0
180482 229533.I1RL04 pp 229533.I1S9Y1
180483 229533.I1RL04 pp 229533.I1RZB2
180484 229533.I1RL04 pp 229533.I1RYI5
180485 229533.I1RL04 pp 229533.I1RPP3
180486 229533.I1RL04 pp 229533.I1RTD5
180487 229533.I1RL04 pp 229533.I1RXF8
180488 229533.I1RL04 pp 229533.I1S1V5
180489 229533.I1RL04 pp 229533.I1RPK0
180490 229533.I1RL04 pp 229533.I1RT40
180491 229533.I1RL04 pp 229533.I1RRI1
180492 229533.I1RL04 pp 229533.I1S2B8
180493 229533.I1RL04 pp 229533.I1S0L4
180494 229533.I1RL04 pp 229533.I1RRM7
180495 229533.I1RL04 pp 229533.I1RRN2
180496 229533.I1RL04 pp 229533.I1RPE0
180497 229533.I1RL04 pp 229533.I1RX38
180498 229533.I1RL04 pp 229533.I1S4Z8
180499 229533.I1RL04 pp 229533.I1S2H4
180500 229533.I1RL04 pp 229533.I1S5R6
180501 229533.I1RL04 pp 229533.I1RW64
180502 229533.I1RL04 pp 229533.I1S256
180503 229533.I1RL04 pp 229533.I1S277
180504 229533.I1RL04 pp 229533.I1RZZ9
180505 229533.I1RL04 pp 229533.I1RN88
180506 229533.I1RL04 pp 229533.V6RGI1
180507 229533.I1RL04 pp 229533.I1RSQ4
180508 229533.I1RL04 pp 229533.I1RPV3
180509 229533.I1RL04 pp 229533.I1RWI5
180510 229533.I1RL04 pp 229533.I1RUG3
180511 229533.I1RL04 pp 229533.I1RNC1
180512 229533.I1RL04 pp 229533.I1RXV3
180513 229533.I1RL04 pp 229533.I1RZ39
180514 229533.I1RL04 pp 229533.I1RRM3
180515 229533.I1RL04 pp 229533.I1RSZ7
180516 229533.I1RL04 pp 229533.V6RMW6
180517 229533.I1RL04 pp 229533.I1RZV3
180518 229533.I1RL04 pp 229533.I1RT03
180519 229533.I1RL04 pp 229533.I1RSQ3
180520 229533.I1RL04 pp 229533.I1RPW0
180521 229533.I1RL04 pp 229533.I1RVZ7
180522 229533.I1RL04 pp 229533.I1RVX0
180523 229533.I1RL04 pp 229533.I1S1W9
180524 229533.I1RL04 pp 229533.I1RRV1
180525 229533.I1RL04 pp 229533.I1RRP7
180526 229533.I1RL04 pp 229533.V6R042
180527 229533.I1RL04 pp 229533.I1RRI2
180528 229533.I1RL04 pp 229533.I1RPU2
180529 229533.I1RL04 pp 229533.I1RML0

180530 229533.I1RL04 pp 229533.I1S064
180531 229533.I1RL04 pp 229533.V6QT89
180532 229533.I1RL04 pp 229533.I1SAJ3
180533 229533.I1RL04 pp 229533.Q4I624
180534 229533.I1RL04 pp 229533.I1RQ33
180535 229533.I1RL04 pp 229533.I1S0J1
180536 229533.I1RL04 pp 229533.I1RQE7
180537 229533.I1RL04 pp 229533.I1S437
180538 229533.I1RL04 pp 229533.I1S1C7
180539 229533.I1RL39 pp 229533.Q4IEK8
180540 229533.I1RL39 pp 229533.I1RWC9
180541 229533.I1RL39 pp 229533.V6R0U9
180542 229533.I1RL39 pp 229533.Q4IAS1
180543 229533.I1RL39 pp 229533.Q4HVW2
180544 229533.I1S0X1 pp 229533.I1S8H5
180545 229533.I1S0X1 pp 229533.I1S5D0
180546 229533.I1S0X1 pp 229533.I1S659
180547 229533.I1S0X1 pp 229533.I1S1T5
180548 229533.I1S0X1 pp 229533.I1SAT2
180549 229533.I1S0X1 pp 229533.I1S267
180550 229533.I1S0X1 pp 229533.I1S2J4
180551 229533.I1S0X1 pp 229533.I1S6H3
180552 229533.I1RSZ7 pp 229533.I1S1C7
180553 229533.I1RSZ7 pp 229533.Q4IPZ1
180554 229533.I1RSZ7 pp 229533.Q4I624
180555 229533.I1RSZ7 pp 229533.I1RYW3
180556 229533.I1RSZ7 pp 229533.I1RUF7
180557 229533.I1RSZ7 pp 229533.I1S0F8
180558 229533.I1RSZ7 pp 229533.I1RVQ3
180559 229533.I1RSZ7 pp 229533.I1RVX0
180560 229533.I1RSZ7 pp 229533.I1RT03
180561 229533.I1RSZ7 pp 229533.I1S0L4
180562 229533.I1RSZ7 pp 229533.I1S437
180563 229533.I1RSZ7 pp 229533.I1RZ79
180564 229533.I1RSZ7 pp 229533.I1RWQ8
180565 229533.I1RSZ7 pp 229533.V6R042
180566 229533.I1RSZ7 pp 229533.I1S5R6
180567 229533.I1RSZ7 pp 229533.I1S2H4
180568 229533.I1RSZ7 pp 229533.I1S256
180569 229533.I1RSZ7 pp 229533.I1RVF8
180570 229533.I1RSZ7 pp 229533.I1S5R1
180571 229533.I1RSZ7 pp 229533.V6RMW6
180572 229533.I1RSZ7 pp 229533.I1RUG3
180573 229533.I1RSZ7 pp 229533.I1S2B8
180574 229533.I1RSZ7 pp 229533.O74638
180575 229533.I1RSZ7 pp 229533.I1RVZ7
180576 229533.I1RSZ7 pp 229533.I1RTN3
180577 229533.I1RSZ7 pp 229533.I1RZV3
180578 229533.I1RSZ7 pp 229533.I1RXC2
180579 229533.I1RSZ7 pp 229533.I1RW61
180580 229533.I1RSZ7 pp 229533.I1S268
180581 229533.I1RSZ7 pp 229533.I1RZB2
180582 229533.I1RSZ7 pp 229533.I1S9Y1
180583 229533.I1RSZ7 pp 229533.I1RZ39
180584 229533.I1RSZ7 pp 229533.I1S1W9
180585 229533.I1RSZ7 pp 229533.I1S016
180586 229533.I1RSZ7 pp 229533.Q4HVQ9
180587 229533.I1RSZ7 pp 229533.I1RWI5
180588 229533.I1RSZ7 pp 229533.I1S0J1
180589 229533.I1RSZ7 pp 229533.I1RZZ9
180590 229533.I1RSZ7 pp 229533.Q4HXF0
180591 229533.I1RSZ7 pp 229533.I1RT40
180592 229533.I1RSZ7 pp 229533.I1S4Z8
180593 229533.I1RSZ7 pp 229533.I1RXF8
180594 229533.I1RSZ7 pp 229533.I1RT75
180595 229533.I1RSZ7 pp 229533.I1S277
180596 229533.I1RSZ7 pp 229533.I1RY62
180597 229533.I1RSZ7 pp 229533.I1RTD5
180598 229533.I1RSZ7 pp 229533.I1S0F7
180599 229533.I1RSZ7 pp 229533.V6RBN2
180600 229533.I1RSZ7 pp 229533.I1RYS0
180601 229533.I1RSZ7 pp 229533.I1RYI5
180602 229533.I1RSZ7 pp 229533.Q4IJK2

229533.I1RSZ7 pp 229533.I1S0W4
229533.I1RSZ7 pp 229533.Q4I0K3
229533.I1RSZ7 pp 229533.I1S1J7
229533.I1RSZ7 pp 229533.I1S5B0
229533.I1RSZ7 pp 229533.I1S1B2
229533.I1RSZ7 pp 229533.V6R9Z0
229533.I1RSZ7 pp 229533.I1RVZ2
229533.I1RSZ7 pp 229533.V6QUE6
229533.I1RSZ7 pp 229533.I1RTA1
229533.I1RSZ7 pp 229533.I1RWS4
229533.I1RSZ7 pp 229533.I1S6K9
229533.I1RSZ7 pp 229533.I1RZK5
229533.I1RSZ7 pp 229533.I1RYR0
229533.I1RSZ7 pp 229533.V6RCZ8
229533.I1RSZ7 pp 229533.I1RWR7
229533.I1RSZ7 pp 229533.I1S821
229533.I1RSZ7 pp 229533.I1S217
229533.I1RSZ7 pp 229533.I1RXD7
229533.I1RSZ7 pp 229533.I1RUQ3
229533.I1RSZ7 pp 229533.I1RXF0
229533.I1RSZ7 pp 229533.I1RZM8
229533.I1RSZ7 pp 229533.I1S9I5
229533.I1RSZ7 pp 229533.I1RY86
229533.I1RSZ7 pp 229533.I1S438
229533.I1RSZ7 pp 229533.I1RYJ6
229533.I1RSZ7 pp 229533.V6RTJ8
229533.I1RSZ7 pp 229533.I1RT33
229533.I1RSZ7 pp 229533.V6QYF0
229533.I1RSZ7 pp 229533.I1RXD6
229533.I1RSZ7 pp 229533.I1RXC4
229533.I1RSZ7 pp 229533.I1S4S7
229533.I1RSZ7 pp 229533.I1RX46
229533.I1RSZ7 pp 229533.I1S4S4
229533.I1RSZ7 pp 229533.I1RW71
229533.I1RSZ7 pp 229533.I1RY49
229533.I1RSZ7 pp 229533.I1RVX9
229533.I1RSZ7 pp 229533.I1RTB3
229533.I1RSZ7 pp 229533.I1S0F1
229533.I1RSZ7 pp 229533.I1S2R0
229533.I1RSZ7 pp 229533.I1RX95
229533.I1RIX8 pp 229533.I1RJU9
229533.I1RIX8 pp 229533.I1RLM3
229533.A0A1C3YN60 pp 229533.I1RBC1
229533.A0A1C3YN60 pp 229533.I1RYR1
229533.A0A1C3YN60 pp 229533.I1RFJ2
229533.A0A1C3YN60 pp 229533.I1RMK1
229533.A0A1C3YN60 pp 229533.Q4INI2
229533.I1REG4 pp 229533.I1RJA7
229533.I1REG4 pp 229533.I1RMP5
229533.I1REG4 pp 229533.I1SA27
229533.I1REG4 pp 229533.I1RPT0
229533.I1REG4 pp 229533.I1RIN3
229533.I1S1B3 pp 229533.I1S338
229533.A0A0E0S035 pp 229533.A0A1C3YHX6
229533.A0A0E0S035 pp 229533.Q4IBS2
229533.A0A0E0S035 pp 229533.Q4IJ56
229533.A0A0E0S035 pp 229533.I1S2M8
229533.A0A0E0S035 pp 229533.Q4IJK2
229533.A0A0E0S035 pp 229533.I1RWR7
229533.A0A0E0S035 pp 229533.A0A1C3YL10
229533.A0A0E0S035 pp 229533.I1REK8
229533.A0A0E0S035 pp 229533.I1RQA2
229533.A0A0E0S035 pp 229533.V6RCZ8
229533.A0A0E0S035 pp 229533.A0A1C3YKV3
229533.A0A0E0S035 pp 229533.Q4IAA0
229533.A0A0E0S035 pp 229533.I1RVX0
229533.A0A0E0S035 pp 229533.A0A1C3YK34
229533.A0A0E0S035 pp 229533.I1RXC4
229533.A0A0E0S035 pp 229533.I1RQ85
229533.A0A0E0S035 pp 229533.I1RZM8
229533.A0A0E0S035 pp 229533.A0A1C3YI30
229533.A0A0E0S035 pp 229533.I1RQD2
229533.A0A0E0S035 pp 229533.I1RQE7

180676 229533.A0A0E0S035 pp 229533.A0A1C3YJV6
180677 229533.A0A0E0S035 pp 229533.I1S5B0
180678 229533.A0A0E0S035 pp 229533.A0A1C3YKB0
180679 229533.A0A0E0S035 pp 229533.I1RCH5
180680 229533.A0A0E0S035 pp 229533.A0A1C3YJN3
180681 229533.A0A0E0S035 pp 229533.I1RJU3
180682 229533.A0A0E0S035 pp 229533.I1RQ33
180683 229533.A0A0E0S035 pp 229533.I1S0F1
180684 229533.A0A0E0S035 pp 229533.I1S4S4
180685 229533.A0A0E0S035 pp 229533.I1S438
180686 229533.A0A0E0S035 pp 229533.V6R0U9
180687 229533.A0A0E0S035 pp 229533.I1RIV0
180688 229533.A0A0E0S035 pp 229533.I1RBR1
180689 229533.A0A0E0S035 pp 229533.I1S4S7
180690 229533.A0A0E0S035 pp 229533.A0A0E0SFN0
180691 229533.A0A0E0S035 pp 229533.I1RXD7
180692 229533.A0A0E0S035 pp 229533.I1RSQ3
180693 229533.A0A0E0S035 pp 229533.A0A1C3YHW3
180694 229533.A0A0E0S035 pp 229533.I1RBR2
180695 229533.A0A0E0S035 pp 229533.V6RTJ8
180696 229533.A0A0E0S035 pp 229533.I1RSQ7
180697 229533.A0A0E0S035 pp 229533.V6R042
180698 229533.A0A0E0S035 pp 229533.I1RD52
180699 229533.A0A0E0S035 pp 229533.I1RRM3
180700 229533.A0A0E0S035 pp 229533.I1RTE9
180701 229533.A0A0E0S035 pp 229533.I1S437
180702 229533.A0A0E0S035 pp 229533.V6RMW6
180703 229533.A0A0E0S035 pp 229533.I1S132
180704 229533.A0A0E0S035 pp 229533.A0A1C3YLB6
180705 229533.A0A0E0S035 pp 229533.I1RE01
180706 229533.A0A0E0S035 pp 229533.I1RNC1
180707 229533.A0A0E0S035 pp 229533.I1S0J1
180708 229533.A0A0E0S035 pp 229533.I1RAW0
180709 229533.A0A0E0S035 pp 229533.I1RUG3
180710 229533.A0A0E0S035 pp 229533.I1RJV3
180711 229533.A0A0E0S035 pp 229533.A0A0E0SKJ9
180712 229533.A0A0E0S035 pp 229533.Q4HWE2
180713 229533.A0A0E0S035 pp 229533.I1RPU2
180714 229533.A0A0E0S035 pp 229533.I1S1W9
180715 229533.A0A0E0S035 pp 229533.I1S5R6
180716 229533.A0A0E0S035 pp 229533.I1RD22
180717 229533.A0A0E0S035 pp 229533.I1RZV3
180718 229533.A0A0E0S035 pp 229533.I1RSZ7
180719 229533.A0A0E0S035 pp 229533.I1RL04
180720 229533.A0A0E0S035 pp 229533.I1RPK0
180721 229533.A0A0E0S035 pp 229533.Q4I662
180722 229533.A0A0E0S035 pp 229533.I1RKD4
180723 229533.A0A0E0S035 pp 229533.A0A1C3YKM9
180724 229533.A0A0E0S035 pp 229533.I1RPP3
180725 229533.A0A0E0S035 pp 229533.I1RZ39
180726 229533.A0A0E0S035 pp 229533.I1RSQ4
180727 229533.A0A0E0S035 pp 229533.I1S2B8
180728 229533.A0A0E0S035 pp 229533.I1RYR0
180729 229533.A0A0E0S035 pp 229533.I1R9M0
180730 229533.A0A0E0S035 pp 229533.I1RPE0
180731 229533.A0A0E0S035 pp 229533.I1RVZ2
180732 229533.A0A0E0S035 pp 229533.I1RRI1
180733 229533.A0A0E0S035 pp 229533.A0A1C3YJI6
180734 229533.A0A0E0S035 pp 229533.I1RGA4
180735 229533.A0A0E0S035 pp 229533.I1RTA1
180736 229533.A0A0E0S035 pp 229533.A0A1C3YLK8
180737 229533.A0A0E0S035 pp 229533.Q4IAS1
180738 229533.A0A0E0S035 pp 229533.I1S5R1
180739 229533.A0A0E0S035 pp 229533.I1RJN9
180740 229533.A0A0E0S035 pp 229533.A0A1C3YL15
180741 229533.A0A0E0S035 pp 229533.I1RDF7
180742 229533.A0A0E0S035 pp 229533.I1RXC2
180743 229533.A0A0E0S035 pp 229533.I1RYI5
180744 229533.A0A0E0S035 pp 229533.I1S0W4
180745 229533.A0A0E0S035 pp 229533.I1RNG4
180746 229533.I1RP12 pp 229533.I1S1T5
180747 229533.I1RP12 pp 229533.I1RW80
180748 229533.I1RP12 pp 229533.I1S8H5

180749 229533.I1RP12 pp 229533.I1RW91
180750 229533.I1RP12 pp 229533.I1S0A5
180751 229533.I1RP12 pp 229533.I1RZ56
180752 229533.I1RP12 pp 229533.I1SAT2
180753 229533.I1RP12 pp 229533.I1RPV8
180754 229533.I1RP12 pp 229533.I1S6H3
180755 229533.I1RP12 pp 229533.I1RRF7
180756 229533.I1RP12 pp 229533.I1S094
180757 229533.I1RP12 pp 229533.I1S5D0
180758 229533.I1RP12 pp 229533.I1RRS6
180759 229533.I1RP12 pp 229533.I1S659
180760 229533.I1RP12 pp 229533.I1RQV7
180761 229533.I1RP12 pp 229533.I1RPW8
180762 229533.I1RP12 pp 229533.I1RST9
180763 229533.I1RP12 pp 229533.I1S267
180764 229533.A0A098D7S6 pp 229533.A0A1C3YIK4
180765 229533.A0A098D7S6 pp 229533.I1RS18
180766 229533.A0A098D7S6 pp 229533.A0A1C3YKV3
180767 229533.A0A098D7S6 pp 229533.A0A0E0S134
180768 229533.A0A098D7S6 pp 229533.I1RXC4
180769 229533.A0A098D7S6 pp 229533.Q4I0M9
180770 229533.A0A098D7S6 pp 229533.A0A1C3YK34
180771 229533.A0A098D7S6 pp 229533.A0A1C3YKB0
180772 229533.A0A098D7S6 pp 229533.I1RG14
180773 229533.A0A098D7S6 pp 229533.I1RF82
180774 229533.A0A098D7S6 pp 229533.I1RTD3
180775 229533.A0A098D7S6 pp 229533.V6RCZ8
180776 229533.A0A098D7S6 pp 229533.I1RM09
180777 229533.A0A098D7S6 pp 229533.I1RXF0
180778 229533.A0A098D7S6 pp 229533.I1RZM8
180779 229533.A0A098D7S6 pp 229533.I1S5B0
180780 229533.A0A098D7S6 pp 229533.Q4IJK2
180781 229533.A0A098D7S6 pp 229533.A0A098DR54
180782 229533.A0A098D7S6 pp 229533.A0A098DR40
180783 229533.A0A098D7S6 pp 229533.I1RM67
180784 229533.A0A098D7S6 pp 229533.I1RA94
180785 229533.A0A098D7S6 pp 229533.I1RQ82
180786 229533.A0A098D7S6 pp 229533.A0A1C3YI30
180787 229533.A0A098D7S6 pp 229533.I1S4S4
180788 229533.A0A098D7S6 pp 229533.I1RRV4
180789 229533.A0A098D7S6 pp 229533.I1REK8
180790 229533.A0A098D7S6 pp 229533.A0A098DY09
180791 229533.A0A098D7S6 pp 229533.I1RD52
180792 229533.A0A098D7S6 pp 229533.A0A1C3YIP5
180793 229533.A0A098D7S6 pp 229533.I1RZR5
180794 229533.A0A098D7S6 pp 229533.I1S4Q4
180795 229533.A0A098D7S6 pp 229533.V6RLM7
180796 229533.A0A098D7S6 pp 229533.I1RXD7
180797 229533.A0A098D7S6 pp 229533.A0A0E0SFN0
180798 229533.A0A098D7S6 pp 229533.I1RZK5
180799 229533.A0A098D7S6 pp 229533.I1S3S3
180800 229533.A0A098D7S6 pp 229533.I1RCV1
180801 229533.A0A098D7S6 pp 229533.A0A098E4W0
180802 229533.A0A098D7S6 pp 229533.A0A098E2S1
180803 229533.A0A098D7S6 pp 229533.I1S0J1
180804 229533.A0A098D7S6 pp 229533.A0A1C3YHS4
180805 229533.A0A098D7S6 pp 229533.A0A1C3YJL7
180806 229533.A0A098D7S6 pp 229533.A0A1C3YHX6
180807 229533.A0A098D7S6 pp 229533.I1RE01
180808 229533.A0A098D7S6 pp 229533.I1RRY9
180809 229533.A0A098D7S6 pp 229533.I1S1W9
180810 229533.A0A098D7S6 pp 229533.V6RTJ8
180811 229533.A0A098D7S6 pp 229533.I1S9G7
180812 229533.A0A098D7S6 pp 229533.I1RAX6
180813 229533.A0A098D7S6 pp 229533.I1RSQ7
180814 229533.A0A098D7S6 pp 229533.I1RX46
180815 229533.A0A098D7S6 pp 229533.V6RMW6
180816 229533.A0A098D7S6 pp 229533.I1RW71
180817 229533.A0A098D7S6 pp 229533.I1RSQ3
180818 229533.A0A098D7S6 pp 229533.A0A1C3YIG9
180819 229533.A0A098D7S6 pp 229533.A0A098DVZ4
180820 229533.A0A098D7S6 pp 229533.I1S1W8
180821 229533.A0A098D7S6 pp 229533.I1S4S7

180822 229533.A0A098D7S6 pp 229533.A0A1C3YK13
180823 229533.A0A098D7S6 pp 229533.I1RB39
180824 229533.A0A098D7S6 pp 229533.A0A1C3YLR6
180825 229533.A0A098D7S6 pp 229533.I1RRS8
180826 229533.A0A098D7S6 pp 229533.V6R042
180827 229533.A0A098D7S6 pp 229533.I1RPN4
180828 229533.A0A098D7S6 pp 229533.A0A098DSB7
180829 229533.A0A098D7S6 pp 229533.I1RRV1
180830 229533.A0A098D7S6 pp 229533.I1RS37
180831 229533.A0A098D7S6 pp 229533.A0A1C3YM63
180832 229533.A0A098D7S6 pp 229533.I1RJU3
180833 229533.A0A098D7S6 pp 229533.I1RUG3
180834 229533.A0A098D7S6 pp 229533.I1RT77
180835 229533.A0A098D7S6 pp 229533.I1RNC1
180836 229533.A0A098D7S6 pp 229533.A0A1C3YN26
180837 229533.A0A098D7S6 pp 229533.A0A1C3YHX8
180838 229533.A0A098D7S6 pp 229533.I1RBT1
180839 229533.A0A098D7S6 pp 229533.I1RR24
180840 229533.A0A098D7S6 pp 229533.I1RCR8
180841 229533.A0A098D7S6 pp 229533.I1S5R6
180842 229533.A0A098D7S6 pp 229533.V6QVH8
180843 229533.A0A098D7S6 pp 229533.I1RDH9
180844 229533.A0A098D7S6 pp 229533.I1RD22
180845 229533.A0A098D7S6 pp 229533.I1RZ39
180846 229533.A0A098D7S6 pp 229533.I1RFL4
180847 229533.A0A098D7S6 pp 229533.A0A1C3YKM9
180848 229533.A0A098D7S6 pp 229533.A0A1C3YNA2
180849 229533.A0A098D7S6 pp 229533.I1RC66
180850 229533.A0A098D7S6 pp 229533.I1RIH0
180851 229533.A0A098D7S6 pp 229533.I1RB92
180852 229533.A0A098D7S6 pp 229533.I1RT03
180853 229533.A0A098D7S6 pp 229533.Q4I624
180854 229533.A0A098D7S6 pp 229533.I1RVX0
180855 229533.A0A098D7S6 pp 229533.I1RGA6
180856 229533.A0A098D7S6 pp 229533.A0A1C3YI20
180857 229533.A0A098D7S6 pp 229533.I1RZZ9
180858 229533.A0A098D7S6 pp 229533.V6RRH2
180859 229533.A0A098D7S6 pp 229533.I1RSQ4
180860 229533.A0A098D7S6 pp 229533.I1RWI5
180861 229533.A0A098D7S6 pp 229533.I1RCH5
180862 229533.A0A098D7S6 pp 229533.I1RQE7
180863 229533.A0A098D7S6 pp 229533.Q4INW6
180864 229533.A0A098D7S6 pp 229533.A0A0E0S8Z0
180865 229533.A0A098D7S6 pp 229533.I1RN67
180866 229533.A0A098D7S6 pp 229533.I1RA70
180867 229533.A0A098D7S6 pp 229533.I1S0L4
180868 229533.A0A098D7S6 pp 229533.I1RE53
180869 229533.A0A098D7S6 pp 229533.I1S1C7
180870 229533.A0A098D7S6 pp 229533.I1RR68
180871 229533.A0A098D7S6 pp 229533.A0A1C3YJK3
180872 229533.A0A098D7S6 pp 229533.I1S2H4
180873 229533.A0A098D7S6 pp 229533.I1RAW0
180874 229533.A0A098D7S6 pp 229533.A0A1C3YKY0
180875 229533.A0A098D7S6 pp 229533.I1S3G4
180876 229533.A0A098D7S6 pp 229533.I1RT75
180877 229533.A0A098D7S6 pp 229533.I1RJV3
180878 229533.A0A098D7S6 pp 229533.Q4HXF0
180879 229533.A0A098D7S6 pp 229533.I1RPP3
180880 229533.A0A098D7S6 pp 229533.O74638
180881 229533.A0A098D7S6 pp 229533.A0A1C3YL55
180882 229533.A0A098D7S6 pp 229533.I1RTD5
180883 229533.A0A098D7S6 pp 229533.I1RYI5
180884 229533.A0A098D7S6 pp 229533.A0A098E0A5
180885 229533.A0A098D7S6 pp 229533.A0A1C3YJI6
180886 229533.A0A098D7S6 pp 229533.I1RT40
180887 229533.A0A098D7S6 pp 229533.I1RXC2
180888 229533.A0A098D7S6 pp 229533.I1RTA1
180889 229533.A0A098D7S6 pp 229533.I1S5R1
180890 229533.A0A098D7S6 pp 229533.I1RKB2
180891 229533.A0A098D7S6 pp 229533.I1RE19
180892 229533.A0A098D7S6 pp 229533.I1RZB2
180893 229533.A0A098D7S6 pp 229533.A0A1C3YHI3
180894 229533.A0A098D7S6 pp 229533.I1RA11

229533.A0A098D7S6 pp 229533.I1RVZ2
229533.A0A098D7S6 pp 229533.A0A1C3YJR2
229533.A0A098D7S6 pp 229533.I1RJH7
229533.A0A098D7S6 pp 229533.A0A0E0SLZ3
229533.A0A098D7S6 pp 229533.I1S9Y1
229533.A0A098D7S6 pp 229533.I1RJH8
229533.A0A098D7S6 pp 229533.I1RGA4
229533.A0A098D7S6 pp 229533.I1RFZ2
229533.A0A098D7S6 pp 229533.A0A1C3YLK8
229533.A0A098D7S6 pp 229533.A0A1C3YJH9
229533.A0A098D7S6 pp 229533.I1RL04
229533.A0A098D7S6 pp 229533.I1RSZ7
229533.A0A098D7S6 pp 229533.I1R9Y8
229533.A0A098D7S6 pp 229533.I1RBY4
229533.A0A098D7S6 pp 229533.I1R9M0
229533.A0A098D7S6 pp 229533.I1S2B8
229533.A0A098D7S6 pp 229533.I1RJZ7
229533.I1RAG2 pp 229533.I1RY11
229533.I1RAG2 pp 229533.I1RP72
229533.A0A1C3YIR5 pp 229533.I1RDU0
229533.A0A1C3YIR5 pp 229533.I1RA13
229533.A0A1C3YIR5 pp 229533.A0A1C3YKH5
229533.A0A1C3YIR5 pp 229533.I1RNH4
229533.A0A1C3YIR5 pp 229533.V6RY56
229533.A0A1C3YIR5 pp 229533.I1RE90
229533.A0A1C3YIR5 pp 229533.I1RK03
229533.A0A1C3YIR5 pp 229533.I1S0T1
229533.A0A1C3YIR5 pp 229533.I1RJK0
229533.A0A1C3YIR5 pp 229533.I1RTJ7
229533.A0A1C3YIR5 pp 229533.I1RJT5
229533.A0A1C3YIR5 pp 229533.I1RCV1
229533.A0A1C3YIR5 pp 229533.V6RPS5
229533.A0A1C3YIR5 pp 229533.I1S0H2
229533.A0A1C3YIR5 pp 229533.I1RRM1
229533.A0A1C3YIR5 pp 229533.I1RAG3
229533.A0A1C3YIR5 pp 229533.I1RIW5
229533.A0A1C3YIR5 pp 229533.I1RQX9
229533.A0A1C3YIR5 pp 229533.I1S8F8
229533.A0A1C3YIR5 pp 229533.I1RJG4
229533.A0A1C3YIR5 pp 229533.I1RV18
229533.A0A1C3YIR5 pp 229533.I1RDV8
229533.A0A1C3YIR5 pp 229533.I1S334
229533.A0A1C3YIR5 pp 229533.I1RUY5
229533.A0A1C3YIR5 pp 229533.I1S4D0
229533.A0A1C3YIR5 pp 229533.I1RIK8
229533.A0A1C3YIR5 pp 229533.I1RDN3
229533.A0A1C3YIR5 pp 229533.V6QTX7
229533.A0A1C3YIR5 pp 229533.I1RV06
229533.A0A1C3YIR5 pp 229533.I1RTT0
229533.A0A1C3YIR5 pp 229533.I1S405
229533.A0A1C3YIR5 pp 229533.I1RVI8
229533.A0A1C3YIR5 pp 229533.I1RST9
229533.A0A1C3YIR5 pp 229533.I1RLA2
229533.A0A1C3YIR5 pp 229533.I1RJE7
229533.A0A1C3YIR5 pp 229533.I1RFC3
229533.A0A1C3YIR5 pp 229533.V6REA4
229533.A0A1C3YIR5 pp 229533.I1RRL5
229533.A0A1C3YIR5 pp 229533.I1S094
229533.A0A1C3YIR5 pp 229533.I1RHL9
229533.A0A1C3YIR5 pp 229533.I1S6H3
229533.A0A1C3YIR5 pp 229533.I1RNM7
229533.A0A1C3YIR5 pp 229533.I1S269
229533.A0A1C3YIR5 pp 229533.V6RM09
229533.A0A1C3YIR5 pp 229533.A0A1C3YJL9
229533.A0A1C3YIR5 pp 229533.I1R9B8
229533.A0A1C3YIR5 pp 229533.I1RY96
229533.A0A1C3YIR5 pp 229533.A0A1C3YKZ8
229533.A0A1C3YIR5 pp 229533.I1RC39
229533.A0A1C3YIR5 pp 229533.I1SAJ0
229533.A0A1C3YIR5 pp 229533.I1RTY9
229533.A0A1C3YIR5 pp 229533.I1RL35
229533.A0A1C3YIR5 pp 229533.I1RF16
229533.A0A1C3YIR5 pp 229533.I1RMS5

229533.A0A1C3YIR5 pp 229533.I1S149
229533.A0A1C3YIR5 pp 229533.A0A1C3YJM7
229533.A0A1C3YIR5 pp 229533.V6R0H4
229533.A0A1C3YIR5 pp 229533.I1R9F2
229533.A0A1C3YIR5 pp 229533.I1RZ38
229533.A0A1C3YIR5 pp 229533.I1REE6
229533.A0A1C3YIR5 pp 229533.I1RZ26
229533.A0A1C3YIR5 pp 229533.I1RJS1
229533.A0A1C3YIR5 pp 229533.I1S3G0
229533.A0A1C3YIR5 pp 229533.I1RKB2
229533.A0A1C3YIR5 pp 229533.I1RTU5
229533.A0A1C3YIR5 pp 229533.I1RPV8
229533.A0A1C3YIR5 pp 229533.A0A1C3YKN4
229533.A0A1C3YIR5 pp 229533.V6RBG5
229533.A0A1C3YIR5 pp 229533.I1R9X3
229533.A0A1C3YIR5 pp 229533.I1S3H8
229533.A0A1C3YIR5 pp 229533.I1RHT3
229533.A0A1C3YIR5 pp 229533.I1S478
229533.A0A1C3YIR5 pp 229533.I1RF24
229533.A0A1C3YIR5 pp 229533.I1RD80
229533.A0A1C3YIR5 pp 229533.I1RNG4
229533.A0A1C3YIR5 pp 229533.I1RZ56
229533.A0A1C3YIR5 pp 229533.I1S6H1
229533.A0A1C3YIR5 pp 229533.I1RFC2
229533.A0A1C3YIR5 pp 229533.I1RGZ3
229533.A0A1C3YIR5 pp 229533.A0A1C3YKV2
229533.A0A1C3YIR5 pp 229533.I1S0A5
229533.A0A1C3YIR5 pp 229533.V6R556
229533.A0A1C3YIR5 pp 229533.I1RV23
229533.A0A1C3YIR5 pp 229533.I1RI19
229533.A0A1C3YIR5 pp 229533.I1RMT6
229533.A0A1C3YIR5 pp 229533.I1RZ25
229533.A0A1C3YIR5 pp 229533.I1RDJ7
229533.A0A1C3YIR5 pp 229533.I1RAC0
229533.I1S3G0 pp 229533.I1S478
229533.I1RML0 pp 229533.Q4IJ56
229533.I1RML0 pp 229533.I1RQ85
229533.I1RML0 pp 229533.I1RWP4
229533.I1RML0 pp 229533.I1RXC4
229533.I1RML0 pp 229533.Q4IAA0
229533.I1RML0 pp 229533.I1RZM8
229533.I1RML0 pp 229533.V6RCZ8
229533.I1RML0 pp 229533.I1RQD2
229533.I1RML0 pp 229533.I1S5B0
229533.I1RML0 pp 229533.I1RPK0
229533.I1RML0 pp 229533.I1S4S4
229533.I1RML0 pp 229533.I1S2M8
229533.I1RML0 pp 229533.Q4IJK2
229533.I1RML0 pp 229533.I1S0J1
229533.I1RML0 pp 229533.Q4IBS2
229533.I1RML0 pp 229533.I1RWR7
229533.I1RML0 pp 229533.I1RQA2
229533.I1RML0 pp 229533.I1RPU2
229533.I1RML0 pp 229533.I1RSQ7
229533.I1RML0 pp 229533.I1RZS7
229533.I1RML0 pp 229533.I1S4S7
229533.I1RML0 pp 229533.Q4IPI1
229533.I1RML0 pp 229533.I1RZV3
229533.I1RML0 pp 229533.I1S1W9
229533.I1RML0 pp 229533.I1S438
229533.I1RML0 pp 229533.V6QYF0
229533.I1RML0 pp 229533.V6R042
229533.I1RML0 pp 229533.I1RS75
229533.I1RML0 pp 229533.I1RSQ3
229533.I1RML0 pp 229533.Q4IJH1
229533.I1RML0 pp 229533.I1RXD7
229533.I1RML0 pp 229533.V6RMW6
229533.I1RML0 pp 229533.V6RTJ8
229533.I1RML0 pp 229533.I1RYI5
229533.I1RML0 pp 229533.I1S5R6
229533.I1RML0 pp 229533.I1S0F1
229533.I1RML0 pp 229533.Q4I327
229533.I1RML0 pp 229533.I1RVX0

181041 229533.I1RML0 pp 229533.I1RRM3
181042 229533.I1RML0 pp 229533.I1RPP3
181043 229533.I1RML0 pp 229533.I1RNC1
181044 229533.I1RML0 pp 229533.I1RUG3
181045 229533.I1RML0 pp 229533.I1RTA1
181046 229533.I1RML0 pp 229533.Q4HWE2
181047 229533.I1RML0 pp 229533.I1RQ33
181048 229533.I1RML0 pp 229533.I1RVZ2
181049 229533.I1RML0 pp 229533.I1RQE7
181050 229533.I1RML0 pp 229533.Q4I662
181051 229533.I1RML0 pp 229533.Q4IAS1
181052 229533.I1RML0 pp 229533.V6R0U9
181053 229533.I1RML0 pp 229533.Q4IEV5
181054 229533.I1RML0 pp 229533.I1RYR0
181055 229533.I1RML0 pp 229533.I1RSZ7
181056 229533.I1RML0 pp 229533.I1RNG4
181057 229533.I1RML0 pp 229533.I1RTE9
181058 229533.I1RML0 pp 229533.I1S0W4
181059 229533.I1RML0 pp 229533.I1RWI0
181060 229533.I1RML0 pp 229533.I1S2B8
181061 229533.I1RML0 pp 229533.I1RXF8
181062 229533.I1RML0 pp 229533.I1S437
181063 229533.I1RML0 pp 229533.I1RZ39
181064 229533.I1RML0 pp 229533.I1S2A8
181065 229533.I1RML0 pp 229533.I1S132
181066 229533.I1RML0 pp 229533.Q4IF76
181067 229533.I1RML0 pp 229533.I1RSQ4
181068 229533.I1RML0 pp 229533.I1S5R1
181069 229533.I1RML0 pp 229533.I1RPE0
181070 229533.I1RML0 pp 229533.I1RRI1
181071 229533.I1RML0 pp 229533.I1RXC2
181072 229533.I1RBX2 pp 229533.Q4I2W2
181073 229533.I1RBX2 pp 229533.I1RD70
181074 229533.I1RBX2 pp 229533.I1RXB5
181075 229533.I1RBX2 pp 229533.I1RPW0
181076 229533.I1RBX2 pp 229533.I1RRF9
181077 229533.I1RBX2 pp 229533.I1RRH3
181078 229533.I1RBX2 pp 229533.I1RCM6
181079 229533.I1RBX2 pp 229533.I1RLX4
181080 229533.I1RBX2 pp 229533.I1RDE9
181081 229533.I1RBX2 pp 229533.I1RUK7
181082 229533.I1RBX2 pp 229533.I1RKR7
181083 229533.I1RBX2 pp 229533.I1RWG6
181084 229533.I1RBX2 pp 229533.I1RQ70
181085 229533.I1RBX2 pp 229533.I1S4P4
181086 229533.I1RBX2 pp 229533.I1RC35
181087 229533.I1RBX2 pp 229533.I1RFZ2
181088 229533.I1RBX2 pp 229533.I1S2B7
181089 229533.I1RBX2 pp 229533.I1RMF2
181090 229533.I1RBX2 pp 229533.I1RRT4
181091 229533.I1RBX2 pp 229533.I1RFS6
181092 229533.I1RBX2 pp 229533.I1RQ80
181093 229533.I1RED3 pp 229533.I1RRF7
181094 229533.I1RED3 pp 229533.I1RHJ2
181095 229533.I1RED3 pp 229533.I1S2B9
181096 229533.I1RED3 pp 229533.I1S5Z5
181097 229533.I1RED3 pp 229533.I1RZN1
181098 229533.I1RED3 pp 229533.I1RR61
181099 229533.I1RED3 pp 229533.I1RYX5
181100 229533.I1RED3 pp 229533.I1RW80
181101 229533.I1RED3 pp 229533.I1RRN4
181102 229533.I1RED3 pp 229533.I1RN04
181103 229533.I1RED3 pp 229533.I1RQM9
181104 229533.I1RED3 pp 229533.I1RRC4
181105 229533.I1RED3 pp 229533.I1RMG4
181106 229533.I1RED3 pp 229533.I1RKF3
181107 229533.I1RED3 pp 229533.I1RNJ7
181108 229533.I1RED3 pp 229533.I1RPW8
181109 229533.I1RED3 pp 229533.I1RFH9
181110 229533.I1RED3 pp 229533.I1RFV2
181111 229533.I1RED3 pp 229533.I1RQ42
181112 229533.I1RED3 pp 229533.I1RW91
181113 229533.I1RFC2 pp 229533.I1S478

229533.I1RFC2 pp 229533.I1RFC3
229533.I1RFC2 pp 229533.I1S3G0
229533.I1RFC2 pp 229533.I1RL35
229533.A0A098DTM7 pp 229533.Q4IEK8
229533.A0A098DTM7 pp 229533.Q4IAS1
229533.A0A098DTM7 pp 229533.I1RED5
229533.A0A098DTM7 pp 229533.I1RKJ8
229533.A0A098DTM7 pp 229533.Q4HVW2
229533.A0A098DTM7 pp 229533.I1RJ42
229533.A0A098DTM7 pp 229533.V6R0U9
229533.A0A098DTM7 pp 229533.I1RWC9
229533.I1RL35 pp 229533.I1RX39
229533.I1RL35 pp 229533.I1S3G0
229533.I1RL35 pp 229533.I1S478
229533.I1RTY9 pp 229533.I1S9Z4
229533.I1RTY9 pp 229533.V6R756
229533.I1RTY9 pp 229533.I1RZ56
229533.I1RTY9 pp 229533.I1S8H5
229533.I1RTY9 pp 229533.V6R5D2
229533.I1RTY9 pp 229533.I1S1T5
229533.I1RTY9 pp 229533.I1S2J4
229533.I1RTY9 pp 229533.I1RW80
229533.I1RTY9 pp 229533.V6R2Z2
229533.I1RTY9 pp 229533.I1SAT2
229533.I1RTY9 pp 229533.I1S0A5
229533.I1RTY9 pp 229533.I1S659
229533.I1RTY9 pp 229533.I1S267
229533.I1RTY9 pp 229533.I1S5D0
229533.I1RTY9 pp 229533.I1S094
229533.I1RTY9 pp 229533.I1RW91
229533.I1RTY9 pp 229533.V6R0W1
229533.I1RTY9 pp 229533.I1SAI4
229533.I1RTY9 pp 229533.I1S6H3
229533.I1RTY9 pp 229533.I1S489
229533.I1RTY9 pp 229533.V6QU99
229533.I1RTY9 pp 229533.V6RAM6
229533.A0A1C3YIL4 pp 229533.I1S478
229533.A0A1C3YIL4 pp 229533.I1S3G0
229533.A0A1C3YIL4 pp 229533.I1RFC2
229533.A0A1C3YIL4 pp 229533.V6R3U5
229533.A0A1C3YIL4 pp 229533.I1RFC3
229533.A0A1C3YIL4 pp 229533.A0A1C3YIR5
229533.A0A1C3YIL4 pp 229533.I1RAH8
229533.A0A1C3YIL4 pp 229533.I1RL35
229533.A0A0E0SG28 pp 229533.A0A1C3YID5
229533.A0A0E0SG28 pp 229533.A0A1C3YKJ7
229533.A0A0E0SG28 pp 229533.I1RDY1
229533.A0A0E0SG28 pp 229533.I1RRF6
229533.A0A0E0SG28 pp 229533.I1RRD2
229533.A0A0E0SG28 pp 229533.I1RUN4
229533.A0A0E0SG28 pp 229533.I1RTE5
229533.A0A0E0SG28 pp 229533.A0A1C3YMH5
229533.A0A0E0SG28 pp 229533.I1RJS4
229533.A0A0E0SG28 pp 229533.I1R9Z0
229533.A0A098D7N8 pp 229533.A0A1C3YIR5
229533.A0A098D7N8 pp 229533.I1S3G0
229533.A0A098D7N8 pp 229533.I1S478
229533.A0A098D7N8 pp 229533.A0A0E0RTV8
229533.A0A098D7N8 pp 229533.I1RFC2
229533.A0A098D7N8 pp 229533.I1RL35
229533.A0A098D7N8 pp 229533.I1RFC3
229533.A0A098D7N8 pp 229533.A0A1C3YIL4
229533.I1RCE0 pp 229533.I1RW71
229533.I1RCE0 pp 229533.I1RFU8
229533.I1RCE0 pp 229533.I1RWC3
229533.I1RCE0 pp 229533.I1RJZ7
229533.I1RCE0 pp 229533.I1RWS7
229533.I1RGK3 pp 229533.I1S6H3
229533.I1RGK3 pp 229533.I1S094
229533.I1RGK3 pp 229533.I1RW91
229533.I1RGK3 pp 229533.I1RRS6
229533.I1RGK3 pp 229533.I1RPW8
229533.I1RGK3 pp 229533.I1RQV7

181187 229533.I1RGK3 pp 229533.I1S5D0
181188 229533.I1RGK3 pp 229533.I1RST9
181189 229533.I1RGK3 pp 229533.I1S0A5
181190 229533.I1RGK3 pp 229533.I1RIB5
181191 229533.I1RGK3 pp 229533.I1S267
181192 229533.I1RGK3 pp 229533.I1RNG4
181193 229533.I1RGK3 pp 229533.I1RN04
181194 229533.I1RGK3 pp 229533.I1S659
181195 229533.I1RGK3 pp 229533.I1RW80
181196 229533.I1RGK3 pp 229533.I1S1T5
181197 229533.I1RGK3 pp 229533.I1SAT2
181198 229533.I1RGK3 pp 229533.I1S2J4
181199 229533.I1RGK3 pp 229533.I1RRF7
181200 229533.I1RGK3 pp 229533.I1RH83
181201 229533.I1RGK3 pp 229533.I1RZ56
181202 229533.I1RGK3 pp 229533.I1RJS1
181203 229533.I1RGK3 pp 229533.I1RPV8
181204 229533.I1RGK3 pp 229533.I1RHU8
181205 229533.I1RGK3 pp 229533.I1S8H5
181206 229533.I1RGK3 pp 229533.I1RIV2
181207 229533.I1RGK3 pp 229533.I1RKR8
181208 229533.A0A1C3YIP8 pp 229533.I1RK95
181209 229533.A0A1C3YIP8 pp 229533.I1RXU0
181210 229533.A0A1C3YIP8 pp 229533.I1RSG8
181211 229533.A0A1C3YIP8 pp 229533.I1RK79
181212 229533.A0A1C3YIP8 pp 229533.I1RTC2
181213 229533.A0A1C3YIP8 pp 229533.I1RRY9
181214 229533.A0A1C3YIP8 pp 229533.I1RTD5
181215 229533.A0A1C3YIP8 pp 229533.I1RVY0
181216 229533.A0A1C3YIP8 pp 229533.I1RUP7
181217 229533.Q4I1Y1 pp 229533.Q4IRI9
181218 229533.Q4I1Y1 pp 229533.Q4IAA0
181219 229533.Q4I1Y1 pp 229533.Q4IE79
181220 229533.Q4I1Y1 pp 229533.Q4IB70
181221 229533.Q4I1Y1 pp 229533.Q4IBK5
181222 229533.Q4I1Y1 pp 229533.Q4IPB8
181223 229533.Q4I1Y1 pp 229533.V6QXG1
181224 229533.Q4I1Y1 pp 229533.Q4IPB3
181225 229533.Q4I1Y1 pp 229533.Q4IF76
181226 229533.Q4I1Y1 pp 229533.Q4IJH1
181227 229533.A0A098D7M0 pp 229533.I1RC91
181228 229533.A0A098D7M0 pp 229533.A0A1C3YM29
181229 229533.A0A098D7M0 pp 229533.I1RAX3
181230 229533.A0A098D7M0 pp 229533.I1S020
181231 229533.I1RE89 pp 229533.I1RKD4
181232 229533.I1RE89 pp 229533.I1RSQ4
181233 229533.I1RE89 pp 229533.I1RSZ7
181234 229533.I1RE89 pp 229533.I1RZ39
181235 229533.I1RE89 pp 229533.I1RW61
181236 229533.I1RE89 pp 229533.I1RL04
181237 229533.I1RE89 pp 229533.I1RPV3
181238 229533.I1RE89 pp 229533.I1RSX0
181239 229533.I1RE89 pp 229533.Q4HVQ9
181240 229533.I1RE89 pp 229533.I1S5R1
181241 229533.I1RE89 pp 229533.I1S2B8
181242 229533.I1RE89 pp 229533.I1RXC2
181243 229533.I1RE89 pp 229533.I1RGU7
181244 229533.I1RE89 pp 229533.I1RGA4
181245 229533.I1RE89 pp 229533.I1S277
181246 229533.I1RE89 pp 229533.I1REC5
181247 229533.I1RE89 pp 229533.I1RZB2
181248 229533.I1RE89 pp 229533.I1RPE0
181249 229533.I1RE89 pp 229533.I1RYR0
181250 229533.I1RE89 pp 229533.I1S4Z8
181251 229533.I1RE89 pp 229533.I1RQM7
181252 229533.I1RE89 pp 229533.I1RT40
181253 229533.I1RE89 pp 229533.I1RKP1
181254 229533.I1RE89 pp 229533.I1RTA1
181255 229533.I1RE89 pp 229533.I1RVZ2
181256 229533.I1RE89 pp 229533.I1RRI1
181257 229533.I1RE89 pp 229533.I1RTD5
181258 229533.I1RE89 pp 229533.I1RJN9
181259 229533.I1RE89 pp 229533.I1RQE7

181260 229533.I1RE89 pp 229533.I1RT03
181261 229533.I1RE89 pp 229533.I1RYI5
181262 229533.I1RE89 pp 229533.V6R042
181263 229533.I1RE89 pp 229533.I1RPP3
181264 229533.I1RE89 pp 229533.I1RSQ3
181265 229533.I1RE89 pp 229533.I1RRM3
181266 229533.I1RE89 pp 229533.I1S0W4
181267 229533.I1RE89 pp 229533.I1RVX0
181268 229533.I1RE89 pp 229533.I1RNG4
181269 229533.I1RE89 pp 229533.I1RUF7
181270 229533.I1RE89 pp 229533.V6R0U9
181271 229533.I1RE89 pp 229533.I1RSA5
181272 229533.I1RE89 pp 229533.V6RMW6
181273 229533.I1RE89 pp 229533.I1S5R6
181274 229533.I1RE89 pp 229533.I1S437
181275 229533.I1RE89 pp 229533.I1RRN2
181276 229533.I1RE89 pp 229533.I1S1C7
181277 229533.I1RE89 pp 229533.I1RQ33
181278 229533.I1RE89 pp 229533.I1RPK0
181279 229533.I1RE89 pp 229533.I1RNC1
181280 229533.I1RE89 pp 229533.I1S132
181281 229533.I1RE89 pp 229533.I1RUG3
181282 229533.I1RE89 pp 229533.I1RZV3
181283 229533.I1RE89 pp 229533.I1S1W9
181284 229533.I1RE89 pp 229533.I1RJV3
181285 229533.I1RE89 pp 229533.I1RVQ3
181286 229533.I1RE89 pp 229533.I1RM37
181287 229533.I1RE89 pp 229533.I1RVF8
181288 229533.I1RE89 pp 229533.I1RPU2
181289 229533.I1RE89 pp 229533.I1S438
181290 229533.I1RE89 pp 229533.I1RVZ7
181291 229533.I1RE89 pp 229533.I1RKJ4
181292 229533.I1RE89 pp 229533.I1RSF1
181293 229533.I1RE89 pp 229533.I1RX46
181294 229533.I1RE89 pp 229533.I1S9I5
181295 229533.I1RE89 pp 229533.I1S0F1
181296 229533.I1RE89 pp 229533.Q4IFI0
181297 229533.I1RE89 pp 229533.I1RHK2
181298 229533.I1RE89 pp 229533.I1RJU3
181299 229533.I1RE89 pp 229533.V6RTJ8
181300 229533.I1RE89 pp 229533.I1RX95
181301 229533.I1RE89 pp 229533.I1RKJ5
181302 229533.I1RE89 pp 229533.I1RNB2
181303 229533.I1RE89 pp 229533.I1S4S4
181304 229533.I1RE89 pp 229533.I1RR24
181305 229533.I1RE89 pp 229533.I1S4S7
181306 229533.I1RE89 pp 229533.I1RXD7
181307 229533.I1RE89 pp 229533.I1RPY3
181308 229533.I1RE89 pp 229533.I1RIV0
181309 229533.I1RE89 pp 229533.I1RTB3
181310 229533.I1RE89 pp 229533.I1RZK5
181311 229533.I1RE89 pp 229533.I1RPA4
181312 229533.I1RE89 pp 229533.I1RSQ7
181313 229533.I1RE89 pp 229533.I1RXD6
181314 229533.I1RE89 pp 229533.I1REK8
181315 229533.I1RE89 pp 229533.I1RMW6
181316 229533.I1RE89 pp 229533.V6R9Z0
181317 229533.I1RE89 pp 229533.I1RRJ7
181318 229533.I1RE89 pp 229533.I1RZM8
181319 229533.I1RE89 pp 229533.I1RY86
181320 229533.I1RE89 pp 229533.I1RRY9
181321 229533.I1RE89 pp 229533.I1S821
181322 229533.I1RE89 pp 229533.I1RWR7
181323 229533.I1RE89 pp 229533.I1RNT9
181324 229533.I1RE89 pp 229533.I1RWN8
181325 229533.I1RE89 pp 229533.Q4IJK2
181326 229533.I1RE89 pp 229533.I1RGC5
181327 229533.I1RE89 pp 229533.I1RGB2
181328 229533.I1RE89 pp 229533.I1S6K9
181329 229533.I1RE89 pp 229533.V6RCZ8
181330 229533.I1RE89 pp 229533.V6RSM7
181331 229533.I1RE89 pp 229533.I1S5B0
181332 229533.I1RE89 pp 229533.I1S2M8

181333 229533.I1RE89 pp 229533.I1RK95
181334 229533.I1RE89 pp 229533.I1RQ85
181335 229533.I1RE89 pp 229533.I1RXC4
181336 229533.I1RE89 pp 229533.I1RUQ3
181337 229533.I1RE89 pp 229533.I1RRV3
181338 229533.I1RE89 pp 229533.I1S1B2
181339 229533.I1RE89 pp 229533.V6QUE6
181340 229533.I1RE89 pp 229533.I1RQD2
181341 229533.I1RU20 pp 229533.I1S8L1
181342 229533.I1RKJ8 pp 229533.I1RXC2
181343 229533.I1RKJ8 pp 229533.I1S5R1
181344 229533.I1RKJ8 pp 229533.I1RPE0
181345 229533.I1RKJ8 pp 229533.I1RRI1
181346 229533.I1RKJ8 pp 229533.I1RSQ4
181347 229533.I1RKJ8 pp 229533.I1RZ39
181348 229533.I1RKJ8 pp 229533.I1S5R6
181349 229533.I1RKJ8 pp 229533.I1RL04
181350 229533.I1RKJ8 pp 229533.I1S2B8
181351 229533.I1RKJ8 pp 229533.V6R042
181352 229533.I1RKJ8 pp 229533.Q4IAS1
181353 229533.I1RKJ8 pp 229533.I1RSQ3
181354 229533.I1RKJ8 pp 229533.I1S0W4
181355 229533.I1RKJ8 pp 229533.I1RSZ7
181356 229533.I1RKJ8 pp 229533.I1RNG4
181357 229533.I1RKJ8 pp 229533.I1RRM3
181358 229533.I1RKJ8 pp 229533.I1RYR0
181359 229533.I1RKJ8 pp 229533.I1RYI5
181360 229533.I1RKJ8 pp 229533.I1S437
181361 229533.I1RKJ8 pp 229533.V6RMW6
181362 229533.I1RKJ8 pp 229533.I1RPP3
181363 229533.I1RKJ8 pp 229533.I1S132
181364 229533.I1RKJ8 pp 229533.I1RUG3
181365 229533.I1RKJ8 pp 229533.I1RNC1
181366 229533.I1RKJ8 pp 229533.I1RQ33
181367 229533.I1RKJ8 pp 229533.I1RML0
181368 229533.I1RKJ8 pp 229533.I1RPU2
181369 229533.I1RKJ8 pp 229533.I1RVZ2
181370 229533.I1RKJ8 pp 229533.V6R0U9
181371 229533.I1RKJ8 pp 229533.I1RTA1
181372 229533.I1RKJ8 pp 229533.I1S1W9
181373 229533.I1RKJ8 pp 229533.I1S4S4
181374 229533.I1RKJ8 pp 229533.I1RZV3
181375 229533.I1RKJ8 pp 229533.I1S0F1
181376 229533.I1RKJ8 pp 229533.I1RQE7
181377 229533.I1RKJ8 pp 229533.I1RVX0
181378 229533.I1RKJ8 pp 229533.I1S5B0
181379 229533.I1RKJ8 pp 229533.I1RPK0
181380 229533.I1RKJ8 pp 229533.I1RLU5
181381 229533.I1RKJ8 pp 229533.I1S438
181382 229533.I1RKJ8 pp 229533.I1RXD7
181383 229533.I1RKJ8 pp 229533.V6RTJ8
181384 229533.I1RKJ8 pp 229533.I1RQD2
181385 229533.I1RKJ8 pp 229533.I1RSQ7
181386 229533.I1RKJ8 pp 229533.I1S4S7
181387 229533.I1RKJ8 pp 229533.V6RCZ8
181388 229533.I1RKJ8 pp 229533.I1RZM8
181389 229533.I1RKJ8 pp 229533.I1RWR7
181390 229533.I1RKJ8 pp 229533.I1S2M8
181391 229533.I1RKJ8 pp 229533.I1RL39
181392 229533.I1RKJ8 pp 229533.I1RQ85
181393 229533.I1RKJ8 pp 229533.I1RWH8
181394 229533.I1RKJ8 pp 229533.I1RXC4
181395 229533.I1RKJ8 pp 229533.Q4IJK2
181396 229533.A0A098DEU5 pp 229533.I1RPR1
181397 229533.A0A098DEU5 pp 229533.I1RIV2
181398 229533.A0A098DEU5 pp 229533.A0A1C3YIP3
181399 229533.A0A098DEU5 pp 229533.A0A098E2M9
181400 229533.A0A098DEU5 pp 229533.A0A098E3B9
181401 229533.A0A098DEU5 pp 229533.A0A098E0Z2
181402 229533.A0A098DEU5 pp 229533.I1RK69
181403 229533.A0A098DEU5 pp 229533.I1S5U5
181404 229533.A0A098DEU5 pp 229533.I1RVL7
181405 229533.A0A098DEU5 pp 229533.I1RHK0

229533.A0A098DEU5 pp 229533.A0A0E0SA95
229533.A0A098DEU5 pp 229533.I1S9Z4
229533.A0A098DEU5 pp 229533.I1RQ48
229533.A0A098DEU5 pp 229533.A0A1C3YJ35
229533.A0A098DEU5 pp 229533.I1RF49
229533.A0A098DEU5 pp 229533.I1S1L0
229533.A0A098DEU5 pp 229533.A0A1C3YKA2
229533.A0A098DEU5 pp 229533.I1RVM4
229533.I1RYZ0 pp 229533.I1S1V1
229533.I1RYZ0 pp 229533.V6RVG3
229533.I1RYZ0 pp 229533.I1S016
229533.I1RYZ0 pp 229533.I1SAA0
229533.I1RYZ0 pp 229533.I1S1W4
229533.I1RYZ0 pp 229533.I1S2E9
229533.I1RYZ0 pp 229533.I1S0M2
229533.I1RYZ0 pp 229533.I1RZE4
229533.I1RYZ0 pp 229533.V6RKY7
229533.I1RYZ0 pp 229533.I1S7X9
229533.I1RYZ0 pp 229533.I1S9T2
229533.I1RYZ0 pp 229533.I1S439
229533.I1RYZ0 pp 229533.I1S927
229533.I1S5E5 pp 229533.I1S9Z4
229533.I1S5E5 pp 229533.I1S5U5
229533.I1RM22 pp 229533.I1RT35
229533.I1RM22 pp 229533.V6R0U9
229533.I1RM22 pp 229533.I1RNB8
229533.I1S537 pp 229533.V6R382
229533.A0A098DC45 pp 229533.I1RVL7
229533.A0A098DC45 pp 229533.I1RHK0
229533.A0A098DC45 pp 229533.I1S9Z4
229533.A0A098DC45 pp 229533.A0A098E2M9
229533.A0A098DC45 pp 229533.I1RIV2
229533.A0A098DC45 pp 229533.A0A1C3YIP3
229533.A0A098DC45 pp 229533.A0A098E3B9
229533.A0A098DC45 pp 229533.I1RF49
229533.A0A098DC45 pp 229533.I1S1L0
229533.A0A098DC45 pp 229533.I1RPR1
229533.A0A098DC45 pp 229533.A0A1C3YJ35
229533.A0A098DC45 pp 229533.I1RVM4
229533.A0A098DC45 pp 229533.I1RQ48
229533.A0A098DC45 pp 229533.I1S5U5
229533.A0A098DC45 pp 229533.I1RK69
229533.A0A098DC45 pp 229533.A0A1C3YKA2
229533.A0A098DC45 pp 229533.A0A098E0Z2
229533.I1RIZ8 pp 229533.I1S1Q3
229533.I1RIZ8 pp 229533.I1RZZ3
229533.I1RIZ8 pp 229533.I1RRQ1
229533.I1RIZ8 pp 229533.I1SAS3
229533.I1RIZ8 pp 229533.I1RQ76
229533.I1RIZ8 pp 229533.I1RT23
229533.I1RIZ8 pp 229533.V6RR38
229533.I1RIZ8 pp 229533.I1RR90
229533.I1RIZ8 pp 229533.V6RPN0
229533.I1RIZ8 pp 229533.I1SAS4
229533.I1RIZ8 pp 229533.I1RQ48
229533.I1RIZ8 pp 229533.I1RJG1
229533.I1RIZ8 pp 229533.I1RY35
229533.I1RIZ8 pp 229533.I1RNT1
229533.I1RVT5 pp 229533.I1S9Z4
229533.I1RVT5 pp 229533.I1S5U5
229533.I1RXF8 pp 229533.I1S438
229533.I1RXF8 pp 229533.I1S0F1
229533.I1RXF8 pp 229533.I1S5B0
229533.I1RXF8 pp 229533.I1S2M8
229533.I1RXF8 pp 229533.Q4IJK2
229533.I1RXF8 pp 229533.I1S4S4
229533.I1RXF8 pp 229533.I1S4S7
229533.I1RXF8 pp 229533.I1S2M5
229533.I1RXF8 pp 229533.I1RZM8
229533.I1RXF8 pp 229533.V6RTJ8
229533.I1RXF8 pp 229533.V6RCZ8
229533.I1RXF8 pp 229533.Q4IAS1
229533.I1RXF8 pp 229533.I1S5R6

181479 229533.I1RXF8 pp 229533.I1S5R1
181480 229533.I1RXF8 pp 229533.I1RZ39
181481 229533.I1RXF8 pp 229533.I1S2B8
181482 229533.I1RXF8 pp 229533.V6R042
181483 229533.I1RXF8 pp 229533.I1RYI5
181484 229533.I1RXF8 pp 229533.V6RMW6
181485 229533.I1RXF8 pp 229533.I1S437
181486 229533.I1RXF8 pp 229533.I1S0W4
181487 229533.I1RXF8 pp 229533.I1S132
181488 229533.I1RXF8 pp 229533.I1RZV3
181489 229533.I1RXF8 pp 229533.I1S1W9
181490 229533.I1RXF8 pp 229533.V6R0U9
181491 229533.I1RXF8 pp 229533.I1RYR0
181492 229533.Q4IE79 pp 229533.V6QXG1
181493 229533.Q4IE79 pp 229533.Q4IN00
181494 229533.Q4IE79 pp 229533.Q4IJH1
181495 229533.Q4IE79 pp 229533.Q4IPB3
181496 229533.Q4IE79 pp 229533.Q4IPB8
181497 229533.Q4IE79 pp 229533.V6RDZ4
181498 229533.Q4IE79 pp 229533.Q4IRI9
181499 229533.Q4IE79 pp 229533.Q4IF76
181500 229533.Q4IE79 pp 229533.Q5I2J3
181501 229533.I1RD90 pp 229533.V6R0U9
181502 229533.I1RD90 pp 229533.I1RJ42
181503 229533.I1RD90 pp 229533.I1RKJ8
181504 229533.I1RD90 pp 229533.I1RWC9
181505 229533.A0A098DGU0 pp 229533.I1RRJ7
181506 229533.A0A098DGU0 pp 229533.I1S0P4
181507 229533.A0A098DGU0 pp 229533.I1RDG0
181508 229533.A0A098DGU0 pp 229533.I1RL39
181509 229533.A0A098DGU0 pp 229533.I1RA84
181510 229533.A0A098DGU0 pp 229533.I1REG4
181511 229533.A0A098DGU0 pp 229533.A0A0E0S035
181512 229533.A0A098DGU0 pp 229533.I1RZ13
181513 229533.A0A098DGU0 pp 229533.I1RWP4
181514 229533.A0A098DGU0 pp 229533.I1RRT0
181515 229533.A0A098DGU0 pp 229533.V6R0U9
181516 229533.A0A098DGU0 pp 229533.I1RGC2
181517 229533.A0A098DGU0 pp 229533.I1RML0
181518 229533.A0A098DGU0 pp 229533.I1S044
181519 229533.A0A098DGU0 pp 229533.Q4HTT2
181520 229533.A0A098DGU0 pp 229533.I1RC00
181521 229533.A0A098DGU0 pp 229533.A0A098DTM7
181522 229533.A0A098DGU0 pp 229533.A0A1C3YIR8
181523 229533.A0A098DGU0 pp 229533.I1RC24
181524 229533.A0A098DGU0 pp 229533.V6REZ4
181525 229533.A0A098DGU0 pp 229533.Q4HW67
181526 229533.A0A098DGU0 pp 229533.I1RR25
181527 229533.A0A098DGU0 pp 229533.I1RCU7
181528 229533.A0A098DGU0 pp 229533.A0A1C3YKG7
181529 229533.A0A098DGU0 pp 229533.Q4IMD1
181530 229533.A0A098DGU0 pp 229533.I1RY40
181531 229533.A0A098DGU0 pp 229533.I1RE89
181532 229533.A0A098DGU0 pp 229533.I1RYD9
181533 229533.A0A098DGU0 pp 229533.I1RKJ8
181534 229533.A0A098DGU0 pp 229533.I1S0V4
181535 229533.A0A098DGU0 pp 229533.I1RBJ1
181536 229533.A0A098DGU0 pp 229533.I1RM22
181537 229533.A0A098DGU0 pp 229533.A0A1C3YMK1
181538 229533.A0A098DGU0 pp 229533.I1RG11
181539 229533.A0A098DGU0 pp 229533.I1RXF8
181540 229533.A0A098DGU0 pp 229533.I1RD90
181541 229533.I1RTW0 pp 229533.I1RXN6
181542 229533.I1RTW0 pp 229533.V6RDP1
181543 229533.I1RDS5 pp 229533.I1S9Z4
181544 229533.I1RDS5 pp 229533.I1RFC3
181545 229533.I1RDS5 pp 229533.I1RIV2
181546 229533.I1RDS5 pp 229533.I1RPR1
181547 229533.I1RDS5 pp 229533.I1RHK0
181548 229533.I1RDS5 pp 229533.I1RQ48
181549 229533.I1RDS5 pp 229533.I1RVL7
181550 229533.I1RDS5 pp 229533.I1RK69
181551 229533.I1RDS5 pp 229533.I1S3K7

181552 229533.I1RDS5 pp 229533.I1S1L0
181553 229533.I1RDS5 pp 229533.I1S5U5
181554 229533.I1RDS5 pp 229533.I1RF49
181555 229533.I1RDS5 pp 229533.I1RVM4
181556 229533.I1RPY2 pp 229533.I1RR94
181557 229533.A0A1C3YIF3 pp 229533.I1S8S9
181558 229533.A0A1C3YIF3 pp 229533.I1S0C2
181559 229533.A0A1C3YIF3 pp 229533.I1RAE8
181560 229533.A0A1C3YIF3 pp 229533.I1S0K2
181561 229533.A0A1C3YIF3 pp 229533.Q4HVU7
181562 229533.A0A1C3YIF3 pp 229533.I1RN42
181563 229533.A0A1C3YIF3 pp 229533.I1RA90
181564 229533.A0A1C3YIF3 pp 229533.I1RY21
181565 229533.A0A1C3YIF3 pp 229533.I1RA52
181566 229533.A0A1C3YIF3 pp 229533.I1RMK7
181567 229533.I1RHS7 pp 229533.I1RI58
181568 229533.I1RHS7 pp 229533.I1RIV2
181569 229533.I1RHS7 pp 229533.I1S9Z4
181570 229533.I1RHS7 pp 229533.I1S1T5
181571 229533.I1RHS7 pp 229533.I1RVL7
181572 229533.I1RHS7 pp 229533.I1S1L0
181573 229533.I1RHS7 pp 229533.I1RPR1
181574 229533.I1RHS7 pp 229533.I1SAF3
181575 229533.I1RHS7 pp 229533.I1RVM4
181576 229533.I1RHS7 pp 229533.I1RK69
181577 229533.I1RHS7 pp 229533.I1S5U5
181578 229533.I1RHS7 pp 229533.I1RVD9
181579 229533.I1RHS7 pp 229533.I1S2J4
181580 229533.I1RHS7 pp 229533.I1S3K7
181581 229533.I1RHS7 pp 229533.I1RUA7
181582 229533.I1RHS7 pp 229533.I1RQ48
181583 229533.I1RNK9 pp 229533.I1RNM7
181584 229533.I1RNK9 pp 229533.I1S267
181585 229533.I1RNK9 pp 229533.I1RST9
181586 229533.I1RNK9 pp 229533.I1RWF9
181587 229533.I1RWV9 pp 229533.I1S2J4
181588 229533.I1RWV9 pp 229533.I1S3K7
181589 229533.I1RWV9 pp 229533.I1S1K2
181590 229533.I1RWV9 pp 229533.I1S1L0
181591 229533.I1RWV9 pp 229533.I1S9Z4
181592 229533.I1RWV9 pp 229533.I1S1T5
181593 229533.I1RWV9 pp 229533.I1RYI5
181594 229533.I1RWV9 pp 229533.I1S5U5
181595 229533.I1RWV9 pp 229533.I1S8H5
181596 229533.I1RRV9 pp 229533.I1S073
181597 229533.I1RRV9 pp 229533.I1S272
181598 229533.I1RLA7 pp 229533.I1RVB6
181599 229533.I1RLA7 pp 229533.I1RR33
181600 229533.I1RLA7 pp 229533.I1S8H5
181601 229533.I1RLA7 pp 229533.I1RVC0
181602 229533.I1RLA7 pp 229533.I1S9Z4
181603 229533.I1RLA7 pp 229533.I1RVL7
181604 229533.I1RLA7 pp 229533.I1RVB5
181605 229533.I1RLA7 pp 229533.I1RPR1
181606 229533.I1RLA7 pp 229533.I1RVM4
181607 229533.I1RLA7 pp 229533.I1S1L0
181608 229533.I1RLA7 pp 229533.I1S5U5
181609 229533.I1RLA7 pp 229533.I1RQ48
181610 229533.I1RVD8 pp 229533.I1S3K7
181611 229533.I1RVD8 pp 229533.I1RVD9
181612 229533.I1RVD8 pp 229533.I1RVL7
181613 229533.I1RVD8 pp 229533.I1S1L0
181614 229533.I1RVD8 pp 229533.I1S2J4
181615 229533.I1RVD8 pp 229533.I1RVM4
181616 229533.I1RVD8 pp 229533.I1S5U5
181617 229533.I1RVD8 pp 229533.I1S9Z4
181618 229533.I1RVD8 pp 229533.I1S8H5
181619 229533.A0A1C3YKC3 pp 229533.I1RY11
181620 229533.I1RPZ7 pp 229533.I1RVM4
181621 229533.I1RPZ7 pp 229533.I1S9Z4
181622 229533.I1RPZ7 pp 229533.I1S5U5
181623 229533.I1RPZ7 pp 229533.I1RVL7
181624 229533.I1RPZ7 pp 229533.I1RQ48

229533.A0A1C3YKU1	pp	229533.I1RRV9
229533.A0A1C3YKU1	pp	229533.I1RZ13
229533.A0A1C3YKU1	pp	229533.I1S073
229533.A0A1C3YKU1	pp	229533.I1RKK2
229533.A0A1C3YKU1	pp	229533.I1RK84
229533.V6RAN2	pp	229533.V6RN16
229533.I1RKG4	pp	229533.I1RWM7
229533.I1RKG4	pp	229533.V6RR76
229533.I1RKG4	pp	229533.I1S1B1
229533.I1RKG4	pp	229533.I1RLM3
229533.I1RKG4	pp	229533.I1RRB0
229533.I1RKG4	pp	229533.I1RTG5
229533.I1RKG4	pp	229533.Q4ILI9
229533.I1RKG4	pp	229533.I1RQP5
229533.I1RKG4	pp	229533.I1S8E0
229533.I1RKG4	pp	229533.I1S0Z3
229533.I1RKG4	pp	229533.I1RT41
229533.I1RKG4	pp	229533.I1RRT4
229533.I1RKG4	pp	229533.I1RN81
229533.I1REX6	pp	229533.I1RVM4
229533.I1REX6	pp	229533.I1S1L0
229533.I1REX6	pp	229533.I1RF49
229533.I1REX6	pp	229533.I1S5U5
229533.I1REX6	pp	229533.I1RQ48
229533.I1REX6	pp	229533.I1RK69
229533.I1REX6	pp	229533.I1RIV2
229533.I1REX6	pp	229533.I1RHK0
229533.I1REX6	pp	229533.I1RVL7
229533.I1REX6	pp	229533.I1RPR1
229533.I1REX6	pp	229533.I1S9Z4
229533.I1RVT4	pp	229533.V6RLM7
229533.I1RA68	pp	229533.I1S5X9
229533.I1RA68	pp	229533.I1RWA3
229533.I1RA68	pp	229533.I1RE39
229533.I1RA68	pp	229533.I1S458
229533.I1RA68	pp	229533.I1RFJ6
229533.I1RA68	pp	229533.I1S464
229533.I1RA68	pp	229533.I1RBQ9
229533.I1RA68	pp	229533.I1RE65
229533.I1RA68	pp	229533.I1RKF4
229533.I1RA68	pp	229533.I1RYQ1
229533.I1RA68	pp	229533.I1S0P0
229533.I1RA68	pp	229533.I1RQJ3
229533.I1RA68	pp	229533.I1RXD9
229533.I1RA68	pp	229533.I1RS05
229533.I1RA68	pp	229533.I1RYT8
229533.I1RA68	pp	229533.I1RP02
229533.I1RA68	pp	229533.I1RS29
229533.I1RA68	pp	229533.I1RMK7
229533.I1RA68	pp	229533.I1S5C6
229533.I1RA68	pp	229533.I1RKF5
229533.I1RA68	pp	229533.I1S0K2
229533.I1RA68	pp	229533.I1RY21
229533.I1RA68	pp	229533.I1S8S9
229533.I1RA68	pp	229533.V6RJP8
229533.I1RK84	pp	229533.I1RRV9
229533.I1RK84	pp	229533.I1RKK2
229533.I1RKA5	pp	229533.I1RP72
229533.I1RKA5	pp	229533.I1RY11
229533.I1RKK2	pp	229533.I1RRM8
229533.I1RKK2	pp	229533.I1S073
229533.I1RKK2	pp	229533.I1RMD3
229533.I1RKK2	pp	229533.I1RRV9
229533.I1RJE8	pp	229533.I1RVL7
229533.I1RJE8	pp	229533.I1S8H5
229533.I1RJE8	pp	229533.I1S1T5
229533.I1RJE8	pp	229533.I1S9Z4
229533.I1RJE8	pp	229533.I1RPR1
229533.I1RJE8	pp	229533.I1S2J4
229533.I1RJE8	pp	229533.I1RR05
229533.I1RJE8	pp	229533.I1RVM4
229533.I1RJE8	pp	229533.I1RQ48
229533.I1RJE8	pp	229533.I1S1L0

181698 229533.I1RJE8 pp 229533.I1S5U5
181699 229533.I1RJE8 pp 229533.I1RK69
181700 229533.A0A098E1Q7 pp 229533.I1RBF4
181701 229533.A0A098E1Q7 pp 229533.V6RAN2
181702 229533.A0A098E1Q7 pp 229533.A0A1C3YMK0
181703 229533.A0A098E1Q7 pp 229533.I1RJQ5
181704 229533.A0A098E1Q7 pp 229533.I1RCG7
181705 229533.A0A098E1Q7 pp 229533.A0A1C3YHZ7
181706 229533.A0A098E1Q7 pp 229533.I1RC31
181707 229533.A0A098DGT4 pp 229533.I1RPU8
181708 229533.A0A098DGT4 pp 229533.I1RCB4
181709 229533.A0A098DGT4 pp 229533.V6R057
181710 229533.A0A098DGT4 pp 229533.I1RRV9
181711 229533.A0A098DGT4 pp 229533.A0A098E0A5
181712 229533.A0A098DGT4 pp 229533.I1RZ20
181713 229533.A0A098DGT4 pp 229533.Q4HXF0
181714 229533.A0A098DGT4 pp 229533.I1RS17
181715 229533.A0A098DGT4 pp 229533.A0A1C3YKU1
181716 229533.A0A098DGT4 pp 229533.V6R1K4
181717 229533.A0A098DGT4 pp 229533.I1RK84
181718 229533.A0A098DGT4 pp 229533.I1RKK2
181719 229533.A0A098DGT4 pp 229533.I1RYU5
181720 229533.A0A098DGT4 pp 229533.A0A1C3YN30
181721 229533.I1RGK2 pp 229533.V6RPN0
181722 229533.I1RGK2 pp 229533.I1RJG1
181723 229533.I1RGK2 pp 229533.I1SAS4
181724 229533.I1RGK2 pp 229533.I1SAS3
181725 229533.I1RGK2 pp 229533.I1RY35
181726 229533.I1RGK2 pp 229533.I1S1Q3
181727 229533.I1RGK2 pp 229533.I1RR90
181728 229533.I1RGK2 pp 229533.I1RZZ3
181729 229533.I1RGK2 pp 229533.I1RQ48
181730 229533.I1RGK2 pp 229533.I1RNT1
181731 229533.I1RGK2 pp 229533.I1RQ76
181732 229533.I1RGK2 pp 229533.I1RT23
181733 229533.I1RGK2 pp 229533.I1RI22
181734 229533.I1RGK2 pp 229533.V6RR38
181735 229533.I1RGK2 pp 229533.I1RRQ1
181736 229533.I1S5B6 pp 229533.V6RBN2
181737 229533.A0A1C3YMV4 pp 229533.I1RY11
181738 229533.A0A1C3YMV4 pp 229533.I1RBY4
181739 229533.A0A098D7H6 pp 229533.A0A1C3YIH7
181740 229533.A0A098D7H6 pp 229533.I1RFL7
181741 229533.A0A098D7H6 pp 229533.A0A1C3YJG7
181742 229533.A0A098D7H6 pp 229533.I1REG5
181743 229533.I1RWE8 pp 229533.I1RX22
181744 229533.I1RWE8 pp 229533.V6R223
181745 229533.I1RWE8 pp 229533.I1RWS4
181746 229533.I1RWE8 pp 229533.I1S216
181747 229533.I1RWE8 pp 229533.I1S8P5
181748 229533.I1RWE8 pp 229533.Q4HXF0
181749 229533.I1RWE8 pp 229533.V6R057
181750 229533.I1RWE8 pp 229533.V6RRH2
181751 229533.I1RWE8 pp 229533.O74638
181752 229533.I1RWE8 pp 229533.I1S0M6
181753 229533.I1RWE8 pp 229533.V6R2A1
181754 229533.I1RWE8 pp 229533.I1S4Z8
181755 229533.I1RWE8 pp 229533.I1S8G4
181756 229533.I1RWE8 pp 229533.I1S7R5
181757 229533.I1RWE8 pp 229533.V6R1K4
181758 229533.I1RWE8 pp 229533.I1RZJ1
181759 229533.I1RWE8 pp 229533.I1S0B7
181760 229533.I1RWE8 pp 229533.I1S4Q4
181761 229533.I1RWE8 pp 229533.V6RTJ8
181762 229533.I1RWE8 pp 229533.V6RBN2
181763 229533.I1RWE8 pp 229533.I1S241
181764 229533.I1RWE8 pp 229533.I1S5S3
181765 229533.I1RWE8 pp 229533.I1S111
181766 229533.I1RWE8 pp 229533.I1RYJ6
181767 229533.I1RWE8 pp 229533.V6RJ21
181768 229533.I1RWE8 pp 229533.I1S3S3
181769 229533.I1RZT6 pp 229533.I1S5C5
181770 229533.I1RZT6 pp 229533.I1S272

181771	229533.I1RZT6	pp	229533.V6RXE7
181772	229533.I1RZT6	pp	229533.I1S0A0
181773	229533.I1RZT6	pp	229533.I1S0Y1
181774	229533.I1RNK1	pp	229533.I1RXX1
181775	229533.I1RNK1	pp	229533.I1S604
181776	229533.I1RNK1	pp	229533.Q4IR08
181777	229533.I1RNK1	pp	229533.I1RTE7
181778	229533.I1RNK1	pp	229533.I1S2M5
181779	229533.I1RNK1	pp	229533.I1RY25
181780	229533.I1RNK1	pp	229533.Q4I7L0
181781	229533.I1RNK1	pp	229533.Q4IBR4
181782	229533.I1RNK1	pp	229533.I1RW64
181783	229533.I1RA47	pp	229533.I1RNG4
181784	229533.I1RA47	pp	229533.Q4I298
181785	229533.I1RKG6	pp	229533.I1RLM3
181786	229533.I1RE86	pp	229533.I1RPD3
181787	229533.I1RE86	pp	229533.I1RGB2
181788	229533.I1RE86	pp	229533.I1RNG4
181789	229533.I1RE86	pp	229533.I1RLB5
181790	229533.I1RE86	pp	229533.I1RJ78
181791	229533.I1RE86	pp	229533.I1RPW1
181792	229533.I1RE86	pp	229533.I1RSI2
181793	229533.I1RE86	pp	229533.I1RYK0
181794	229533.I1RE86	pp	229533.I1SAJ3
181795	229533.I1RE86	pp	229533.I1RMG4
181796	229533.I1RE86	pp	229533.I1RRG3
181797	229533.I1RE86	pp	229533.I1RZT6
181798	229533.I1RE86	pp	229533.I1RFV2
181799	229533.I1RE86	pp	229533.I1RZ29
181800	229533.I1RE86	pp	229533.I1S5C5
181801	229533.I1RE86	pp	229533.I1RWV4
181802	229533.I1RE86	pp	229533.I1RP86
181803	229533.I1RE86	pp	229533.I1RZN1
181804	229533.I1RE86	pp	229533.I1RM77
181805	229533.I1RE86	pp	229533.I1S368
181806	229533.I1RE86	pp	229533.I1RT75
181807	229533.I1RE86	pp	229533.I1S183
181808	229533.I1RE86	pp	229533.I1RYS3
181809	229533.I1RE86	pp	229533.I1RVW7
181810	229533.I1RE86	pp	229533.V6RQW4
181811	229533.I1RE86	pp	229533.I1RNR9
181812	229533.I1RE86	pp	229533.I1RHJ2
181813	229533.I1RE86	pp	229533.I1RG95
181814	229533.I1RE86	pp	229533.I1RUY5
181815	229533.I1S5K2	pp	229533.V6RR38
181816	229533.I1S5K2	pp	229533.I1SAS4
181817	229533.I1S5K2	pp	229533.I1SAS3
181818	229533.I1S5K2	pp	229533.V6RPN0
181819	229533.I1RBZ6	pp	229533.I1RVC7
181820	229533.I1RBZ6	pp	229533.I1S2I7
181821	229533.I1RBZ6	pp	229533.I1RJD9
181822	229533.I1RBZ6	pp	229533.I1RI44
181823	229533.I1RBZ6	pp	229533.I1RR60
181824	229533.I1RBZ6	pp	229533.I1RNN7
181825	229533.I1RBZ6	pp	229533.I1S3M2
181826	229533.I1RBZ6	pp	229533.I1RBZ7
181827	229533.I1RBZ6	pp	229533.I1S069
181828	229533.I1RBZ6	pp	229533.I1RKP1
181829	229533.I1RSI2	pp	229533.V6RS91
181830	229533.I1RSI2	pp	229533.I1S5C5
181831	229533.I1RSI2	pp	229533.I1RYK0
181832	229533.I1RSI2	pp	229533.I1RZT6
181833	229533.I1RSI2	pp	229533.Q4I1N3
181834	229533.I1RSI2	pp	229533.I1S1W8
181835	229533.I1RSI2	pp	229533.I1S3G4
181836	229533.I1RSI2	pp	229533.I1RYS3
181837	229533.A0A1C3YKA3	pp	229533.I1RJQ5
181838	229533.I1RLD2	pp	229533.I1RLM3
181839	229533.A0A1C3YK76	pp	229533.I1RLB5
181840	229533.A0A1C3YK76	pp	229533.I1RMG4
181841	229533.A0A1C3YK76	pp	229533.I1RG95
181842	229533.A0A1C3YK76	pp	229533.I1S368
181843	229533.A0A1C3YK76	pp	229533.I1SAJ3

229533.A0A1C3YK76	pp	229533.I1RPW1
229533.A0A1C3YK76	pp	229533.I1RVW7
229533.A0A1C3YK76	pp	229533.I1RUY5
229533.A0A1C3YK76	pp	229533.I1RA96
229533.A0A1C3YK76	pp	229533.I1RDU0
229533.A0A1C3YK76	pp	229533.I1RGB2
229533.A0A1C3YK76	pp	229533.I1RZN1
229533.A0A1C3YK76	pp	229533.V6RQW4
229533.A0A1C3YK76	pp	229533.I1RBE7
229533.A0A1C3YK76	pp	229533.I1RYS3
229533.A0A1C3YK76	pp	229533.I1RYK0
229533.A0A1C3YK76	pp	229533.A0A1C3YLC3
229533.A0A1C3YK76	pp	229533.A0A1C3YN83
229533.A0A1C3YK76	pp	229533.I1S183
229533.A0A1C3YK76	pp	229533.I1RZ29
229533.A0A1C3YK76	pp	229533.I1RSI2
229533.A0A1C3YK76	pp	229533.I1RRG3
229533.A0A1C3YK76	pp	229533.I1RM77
229533.A0A1C3YK76	pp	229533.I1RNW0
229533.A0A1C3YK76	pp	229533.I1RZT6
229533.A0A1C3YK76	pp	229533.I1RFV2
229533.A0A1C3YK76	pp	229533.I1RNR9
229533.A0A1C3YK76	pp	229533.I1RPD3
229533.A0A1C3YK76	pp	229533.I1RC31
229533.A0A1C3YK76	pp	229533.I1RJ78
229533.A0A1C3YK76	pp	229533.I1RNG4
229533.A0A1C3YK76	pp	229533.I1RHJ2
229533.A0A1C3YK76	pp	229533.I1RT75
229533.A0A1C3YK76	pp	229533.I1RWV4
229533.A0A1C3YK76	pp	229533.I1RE86
229533.A0A1C3YK76	pp	229533.I1RP86
229533.I1S024	pp	229533.V6QV31
229533.I1S024	pp	229533.I1S0H5
229533.I1S024	pp	229533.I1S273
229533.I1S024	pp	229533.V6RY56
229533.I1S024	pp	229533.I1S4D0
229533.I1S024	pp	229533.I1SAE8
229533.I1S024	pp	229533.Q4IPN9
229533.I1S024	pp	229533.I1S6P5
229533.I1S024	pp	229533.V6RUB1
229533.I1RYS3	pp	229533.I1S3G4
229533.I1RYS3	pp	229533.Q4I1N3
229533.I1RYS3	pp	229533.I1RYU0
229533.I1RYS3	pp	229533.I1S5C5
229533.I1RYS3	pp	229533.Q4HWE2
229533.I1RYS3	pp	229533.I1S1W8
229533.I1RYS3	pp	229533.V6RS91
229533.I1RYS3	pp	229533.I1RZT6
229533.Q4IPB3	pp	229533.V6QXG1
229533.Q4IPB3	pp	229533.Q4IPB8
229533.Q4IPB3	pp	229533.Q4IRI9
229533.Q4IPB3	pp	229533.V6RY33

Supplementary File S3. Molecular docking model generation details

```
          AutoDock 4.2 Release 4.2.6
         (C) 1989-2012 The Scripps Research Institute
        AutoDock comes with ABSOLUTELY NO WARRANTY.
        AutoDock is free software, and you are welcome
        to redistribute it under certain conditions;
        for details type 'autodock4 -C'

                     main.cc  $Revision: 1.213 $

                   Compiled on Jul 18 2014 at 15:34:58


This file was created at:          1:51 32" p.m., 01/27/2025
                   on host:     "BAYSALO"
Current Working Directory = "D:\Autodock"

      ________________________________________________________________

                   SETTING UP DEFAULT PARAMETER LIBRARY
      ________________________________________________________________


Random number generator was seeded with values 11736, 1737975092.
Docking parameter file (DPF) used for this docking:     D:/Autodock/dock.dpf
DPF> autodock_parameter_version 4.2       # used by autodock to validate parameter set

    Autodock parameter version 4.2.
DPF> outlev 1                             # diagnostic output level

Output Level = 1  ADT-COMPATIBLE OUTPUT DURING DOCKING.
DPF> intelec                              # calculate internal electrostatics

Electrostatic energies will be calculated for all non-bonds between moving atoms.
DPF> seed pid time                        # seeds for random generator

Random number generator was seeded with values 11736, 1737975092.
DPF> ligand_types A C NA OA N HD          # atoms types in ligand

DPF> fld receptor.maps.fld                # grid_data_file

Opening Grid Map Dimensions file:       receptor.maps.fld
Grid Point Spacing =                0.436 Angstroms

Even Number of User-specified Grid Points = 40 x-points
                        40 y-points
                        40 z-points

Coordinates of Central Grid Point of Maps = (7.069, 4.409, 3.928)
Macromolecule file used to create Grid Maps =   receptor.pdbqt
Grid Parameter file used to create Grid Maps =  D:/Autodock/grid.gpf
Minimum coordinates in grid = (-1.651, -4.311, -4.792)
Maximum coordinates in grid = (15.789, 13.129, 12.648)

DPF> map receptor.A.map                   # atom-specific affinity map

DPF> map receptor.C.map                   # atom-specific affinity map

DPF> map receptor.NA.map                  # atom-specific affinity map

DPF> map receptor.OA.map                  # atom-specific affinity map

DPF> map receptor.N.map                   # atom-specific affinity map

DPF> map receptor.HD.map                  # atom-specific affinity map

DPF> elecmap receptor.e.map               # electrostatics map

DPF> desolvmap receptor.d.map             # desolvation map

DPF> move ligand1.pdbqt                   # small molecule

1,4-interactions will be _ignored_ in the non-bonded internal energy calculation.
```

```
Ligand PDBQT file = "ligand1.pdbqt"

INPUT LIGAND PDBQT FILE:
________________________

INPUT-LIGAND-PDBQT: REMARK  6 active torsions:
INPUT-LIGAND-PDBQT: REMARK  status: ('A' for Active; 'I' for Inactive)
INPUT-LIGAND-PDBQT: REMARK    1  A    between atoms: N_5  and  C_10
INPUT-LIGAND-PDBQT: REMARK    2  A    between atoms: N_6  and  C_17
INPUT-LIGAND-PDBQT: REMARK    3  A    between atoms: N_6  and  C_26
INPUT-LIGAND-PDBQT: REMARK    4  A    between atoms: C_15  and  C_17
INPUT-LIGAND-PDBQT: REMARK    5  A    between atoms: C_19  and  C_23
INPUT-LIGAND-PDBQT: REMARK    6  A    between atoms: C_26  and  C_27
INPUT-LIGAND-PDBQT: ROOT
INPUT-LIGAND-PDBQT: ATOM      1  O   UNL d   1       2.742   0.625   1.200  0.00  0.00    -0.312 OA
INPUT-LIGAND-PDBQT: ATOM      2  C   UNL d   1       3.932  -0.018   1.012  0.00  0.00     0.078 A
INPUT-LIGAND-PDBQT: ATOM      3  C   UNL d   1       4.925   0.896   0.688  0.00  0.00    -0.018 A
INPUT-LIGAND-PDBQT: ATOM      4  C   UNL d   1       4.283   2.174   0.650  0.00  0.00     0.006 A
INPUT-LIGAND-PDBQT: ATOM      5  C   UNL d   1       2.952   1.947   0.974  0.00  0.00     0.086 A
INPUT-LIGAND-PDBQT: ATOM      6  C   UNL d   1       6.360   0.625   0.425  0.00  0.00     0.052 C
INPUT-LIGAND-PDBQT: ATOM      7  C   UNL d   1       4.686   3.495   0.382  0.00  0.00     0.015 A
INPUT-LIGAND-PDBQT: ATOM      8  C   UNL d   1       1.982   2.943   1.056  0.00  0.00     0.038 A
INPUT-LIGAND-PDBQT: ATOM      9  C   UNL d   1       3.733   4.522   0.455  0.00  0.00     0.001 A
INPUT-LIGAND-PDBQT: ATOM     10  C   UNL d   1       2.404   4.249   0.787  0.00  0.00     0.003 A
INPUT-LIGAND-PDBQT: ENDROOT
INPUT-LIGAND-PDBQT: BRANCH   2  11
INPUT-LIGAND-PDBQT: ATOM     11  C   UNL d   1       3.912  -1.492   1.216  0.00  0.00     0.191 C
INPUT-LIGAND-PDBQT: BRANCH  11  12
INPUT-LIGAND-PDBQT: ATOM     12  N   UNL d   1       3.741  -2.196  -0.058  0.00  0.00    -0.347 N
INPUT-LIGAND-PDBQT: ATOM     13  C   UNL d   1       4.965  -2.591  -0.741  0.00  0.00     0.153 C
INPUT-LIGAND-PDBQT: BRANCH  12  14
INPUT-LIGAND-PDBQT: ATOM     14  C   UNL d   1       2.547  -2.351  -0.749  0.00  0.00     0.243 C
INPUT-LIGAND-PDBQT: ATOM     15  O   UNL d   1       2.492  -2.869  -1.864  0.00  0.00    -0.268 OA
INPUT-LIGAND-PDBQT: BRANCH  14  16
INPUT-LIGAND-PDBQT: ATOM     16  C   UNL d   1       1.287  -1.868  -0.112  0.00  0.00     0.075 C
INPUT-LIGAND-PDBQT: ATOM     17  C   UNL d   1       0.147  -1.884  -0.824  0.00  0.00     0.018 C
INPUT-LIGAND-PDBQT: BRANCH  17  18
INPUT-LIGAND-PDBQT: ATOM     18  C   UNL d   1      -1.151  -1.401  -0.342  0.00  0.00    -0.018 A
INPUT-LIGAND-PDBQT: ATOM     19  C   UNL d   1      -2.082  -0.902  -1.254  0.00  0.00     0.024 A
INPUT-LIGAND-PDBQT: ATOM     20  C   UNL d   1      -3.355  -0.440  -0.849  0.00  0.00    -0.011 A
INPUT-LIGAND-PDBQT: ATOM     21  C   UNL d   1      -4.232   0.087  -1.947  0.00  0.00     0.045 C
INPUT-LIGAND-PDBQT: ATOM     22  C   UNL d   1      -3.655  -0.552   0.504  0.00  0.00     0.136 A
INPUT-LIGAND-PDBQT: ATOM     23  C   UNL d   1      -5.323   1.069  -1.549  0.00  0.00     0.047 C
INPUT-LIGAND-PDBQT: ATOM     24  C   UNL d   1      -6.396   0.363  -0.751  0.00  0.00     0.275 C
INPUT-LIGAND-PDBQT: ATOM     25  C   UNL d   1      -6.047   0.212   0.721  0.00  0.00     0.254 C
INPUT-LIGAND-PDBQT: ATOM     26  O   UNL d   1      -6.941   0.465   1.540  0.00  0.00    -0.271 OA
INPUT-LIGAND-PDBQT: ATOM     27  N   UNL d   1      -4.842  -0.211   1.140  0.00
```

```
0.00    -0.309 N
INPUT-LIGAND-PDBQT: ATOM     28  H   UNL d   1      -4.761  -0.339   2.146  0.00  0.00     0.170 HD
INPUT-LIGAND-PDBQT: ATOM     29  N   UNL d   1      -2.775  -1.030   1.407  0.00  0.00    -0.242 NA
INPUT-LIGAND-PDBQT: ATOM     30  C   UNL d   1      -1.559  -1.446   0.978  0.00  0.00     0.130 A
INPUT-LIGAND-PDBQT: BRANCH  24  31
INPUT-LIGAND-PDBQT: ATOM     31  N   UNL d   1      -7.665   1.185  -0.838  0.00  0.00    -0.066 N
INPUT-LIGAND-PDBQT: ATOM     32  H   UNL d   1      -8.147   1.118  -1.740  0.00  0.00     0.275 HD
INPUT-LIGAND-PDBQT: ATOM     33  H   UNL d   1      -7.493   2.172  -0.607  0.00  0.00     0.275 HD
INPUT-LIGAND-PDBQT: ATOM     34  H   UNL d   1      -8.300   0.851  -0.092  0.00  0.00     0.275 HD
INPUT-LIGAND-PDBQT: ENDBRANCH  24  31
INPUT-LIGAND-PDBQT: ENDBRANCH  17  18
INPUT-LIGAND-PDBQT: ENDBRANCH  14  16
INPUT-LIGAND-PDBQT: ENDBRANCH  12  14
INPUT-LIGAND-PDBQT: ENDBRANCH  11  12
INPUT-LIGAND-PDBQT: ENDBRANCH   2  11
INPUT-LIGAND-PDBQT: TORSDOF 5
________________________________________________________________________________

Total charge on ligand                               =  +1.003 e
REMARK  6 active torsions:
REMARK  status: ('A' for Active; 'I' for Inactive)
REMARK    1  A    between atoms: N_5  and  C_10
REMARK    2  A    between atoms: N_6  and  C_17
REMARK    3  A    between atoms: N_6  and  C_26
REMARK    4  A    between atoms: C_15  and  C_17
REMARK    5  A    between atoms: C_19  and  C_23
REMARK    6  A    between atoms: C_26  and  C_27


Number of Rotatable Bonds in Small Molecule =   6 torsions
Number of atoms in ligand:  34

Number of non-hydrogen atoms in ligand:  30

Number of vibrational degrees of freedom of ligand:  96


Number of torsional degrees of freedom = 5
Estimated loss of torsional free energy upon binding = +1.4915 kcal/mol


DPF> about -0.813 0.218 0.087            # small molecule center

Small molecule center of rotation = (-0.813, +0.218, +0.087)

DPF> tran0 random                         # initial coordinates/A or random

Initial translation =           (13.438, 0.334, 0.375) Angstroms
DPF> quaternion0 random                   # initial orientation

Each run will begin with a new, random initial orientation.
Initial quaternion,  (x,y,z,w) =    ( 0.823, -0.418, -0.169, -0.346 ),
DPF> dihe0 random                         # initial dihedrals (relative) or random

DPF> torsdof 5                            # torsional degrees of freedom

Number of torsional degrees of freedom = 5
Free energy coefficient for torsional degrees of freedom = 0.2983, the factory default value.

Estimated loss of torsional free energy upon binding = +1.4915 kcal/mol

DPF> rmstol 2.0                           # cluster_tolerance/A

Maximum RMS tolerance for conformational cluster analysis = 2.00 Angstroms
DPF> extnrg 1000.0                        # external grid energy
```

```
External grid energy (beyond grid map walls) = 1000.00

DPF> e0max 0.0 10000                          # max initial energy; max number of retries

Using user-specified maximum number of retries for simanneal initialization, 10000 retries.
If the simanneal initial energy is greater than e0max, 0.000,
then a new, random initial state will be created.

DPF> ga_pop_size 150                          # number of individuals in population

A population of 150 individuals will be used
DPF> ga_num_evals 2500000                     # maximum number of energy evaluations

There will be at most 2500000 function evaluations used.
DPF> ga_num_generations 27000                 # maximum number of generations

The GA will run for at most 27000 generations.
DPF> ga_elitism 1                             # number of top individuals to survive to next generation

The 1 best will be preserved each GA generation.
DPF> ga_mutation_rate 0.02                    # rate of gene mutation

The mutation rate is 0.020000.
DPF> ga_crossover_rate 0.8                    # rate of crossover

The crossover rate is 0.800000.
DPF> ga_window_size 10                        #

The GA's selection window is 10 generations.
DPF> ga_cauchy_alpha 0.0                      # Alpha parameter of Cauchy distribution

The alpha parameter (for the Cauchy distribution) is being set to 0.000000.
DPF> ga_cauchy_beta 1.0                       # Beta parameter Cauchy distribution

The beta parameter (for the Cauchy distribution) is being set to 1.000000.
DPF> set_ga                                   # set the above parameters for GA or LGA

DPF> sw_max_its 300                           # iterations of Solis & Wets local search

Solis & Wets algorithms will perform at most 300 iterations.
DPF> sw_max_succ 4                            # consecutive successes before changing rho

Solis & Wets algorithms expand rho every 4 in a row successes.
DPF> sw_max_fail 4                            # consecutive failures before changing rho

Solis & Wets algorithms contract rho every 4 in a row failures.
DPF> sw_rho 1.0                               # size of local search space to sample

rho is set to 1.000000.
DPF> sw_lb_rho 0.01                           # lower bound on rho

rho will never get smaller than 0.010000.
DPF> ls_search_freq 0.06                      # probability of performing local search on individual

Local search will be performed with frequency 0.060000.
DPF> set_psw1                                 # set the above pseudo-Solis & Wets parameters

Creating a new Local Search object using the pseudo-Solis-Wets algorithm (pSW1) with the current settings.

DPF> unbound_model bound                      # state of unbound ligand

DPF> ga_run 15                                # do this many hybrid GA-LS runs

centering ligand on specified point: -0.813 0.218 0.087
Furthest true ligand atom from "about" center is 7.612 Angstroms (maxrad).
Number of requested GA dockings = 15 runs
Unbound model to be used is 'same as bound' [AutoDock 4.2 default].
```

```
    BEGINNING GENETIC ALGORITHM DOCKING 1 of 15
Run: 1 Seed: 169016444 1697134460 [ Run 1 of 15 GA/GALS ]
Beginning LAMARCKIAN GENETIC ALGORITHM (LGA), with a maximum of 2500000 energy evaluations.

Final-Value: -10.899


    FINAL GENETIC ALGORITHM DOCKED STATE
    _______________________________________________


Detailed state:  trans 4.050 3.776 5.341 quatxyzw 0.482002 -0.100026 -0.673719 0.551154 center -0.813 0.218 0.087 ntor 6 -1.0423 8.1215 19.4363 -152.6014 173.0394 -51.8373
State:    4.050   3.776   5.341   0.578 -0.120 -0.807 113.108     -1.04    8.12 19.44 -152.60  173.04  -51.84

DOCKED: MODEL        1
DOCKED: USER    Run = 1
DOCKED: USER    DPF = D:/Autodock/dock.dpf
DOCKED: USER
DOCKED: USER    Estimated Free Energy of Binding    =   -7.60 kcal/mol [=(1)+(2)+(3)-(4)]
DOCKED: USER    Estimated Inhibition Constant, Ki   =    2.69 uM (micromolar) [Temperature = 298.15 K]
DOCKED: USER
DOCKED: USER    (1) Final Intermolecular Energy     =   -9.09 kcal/mol
DOCKED: USER        vdW + Hbond + desolv Energy     =   -9.22 kcal/mol
DOCKED: USER        Electrostatic Energy            =   +0.13 kcal/mol
DOCKED: USER    (2) Final Total Internal Energy     =   -1.81 kcal/mol
DOCKED: USER    (3) Torsional Free Energy           =   +1.49 kcal/mol
DOCKED: USER    (4) Unbound System's Energy  [=(2)] =   -1.81 kcal/mol
DOCKED: USER
DOCKED: USER
DOCKED: USER    NEWDPF move ligand1.pdbqt
DOCKED: USER    NEWDPF about -0.813000 0.218000 0.087000
DOCKED: USER    NEWDPF tran0 4.050302 3.776373 5.340507
DOCKED: USER    NEWDPF quaternion0 0.482002 -0.100026 -0.673719 0.551154
DOCKED: USER    NEWDPF axisangle0 0.577661 -0.119877 -0.807427 113.107521
DOCKED: USER    NEWDPF quat0 0.577661 -0.119877 -0.807427 113.107521
DOCKED: USER    NEWDPF dihe0 -1.04 8.12 19.44 -152.60 173.04 -51.84
DOCKED: USER  keepresnum = 1
DOCKED: USER
DOCKED: REMARK  6 active torsions:
DOCKED: REMARK  status: ('A' for Active; 'I' for Inactive)
DOCKED: REMARK    1  A    between atoms: N_5  and  C_10
DOCKED: REMARK    2  A    between atoms: N_6  and  C_17
DOCKED: REMARK    3  A    between atoms: N_6  and  C_26
DOCKED: REMARK    4  A    between atoms: C_15  and  C_17
DOCKED: REMARK    5  A    between atoms: C_19  and  C_23
DOCKED: REMARK    6  A    between atoms: C_26  and  C_27
DOCKED: USER                              x       y       z     vdW  Elec       q    Type
DOCKED: USER                           _______ _______ _______ _____ _____    ______ ____
DOCKED: ROOT
DOCKED: ATOM      1  O   UNL d   1       3.365   6.663   3.052 -0.21 +0.13    -0.312 OA
DOCKED: ATOM      2  C   UNL d   1       4.092   7.547   2.306 -0.26 -0.02    +0.078 A
DOCKED: ATOM      3  C   UNL d   1       3.572   7.632   1.022 -0.23 +0.00    -0.018 A
DOCKED: ATOM      4  C   UNL d   1       2.473   6.716   0.983 -0.28 -0.00    +0.006 A
DOCKED: ATOM      5  C   UNL d   1       2.393   6.156   2.252 -0.27 -0.02    +0.086 A
DOCKED: ATOM      6  C   UNL d   1       4.044   8.485  -0.096 -0.20 -0.01    +0.052 C
DOCKED: ATOM      7  C   UNL d   1       1.539   6.306   0.015 -0.32 -0.00    +0.015 A
DOCKED: ATOM      8  C   UNL d   1       1.443   5.213   2.636 -0.35 -0.01    +0.038
```

```
A
DOCKED: ATOM      9  C   UNL d   1       0.569   5.356   0.370 -0.39 -0.00    +0.001
A
DOCKED: ATOM     10  C   UNL d   1       0.523   4.820   1.659 -0.54 -0.00    +0.003
A
DOCKED: ENDROOT
DOCKED: BRANCH   2  11
DOCKED: ATOM     11  C   UNL d   1       5.217   8.219   3.011 -0.32 -0.07    +0.191
C
DOCKED: BRANCH  11  12
DOCKED: ATOM     12  N   UNL d   1       5.979   7.260   3.816 -0.28 +0.13    -0.347
N
DOCKED: ATOM     13  C   UNL d   1       5.596   7.154   5.217 -0.33 -0.12    +0.153
C
DOCKED: BRANCH  12  14
DOCKED: ATOM     14  C   UNL d   1       7.131   6.596   3.418 -0.39 -0.06    +0.243
C
DOCKED: ATOM     15  O   UNL d   1       7.855   7.013   2.515 -0.37 +0.04    -0.268
OA
DOCKED: BRANCH  14  16
DOCKED: ATOM     16  C   UNL d   1       7.500   5.328   4.114 -0.15 -0.02    +0.075
C
DOCKED: ATOM     17  C   UNL d   1       8.726   4.811   3.924 -0.81 -0.00    +0.018
C
DOCKED: BRANCH  17  18
DOCKED: ATOM     18  C   UNL d   1       9.207   3.555   4.508 -0.63 -0.00    -0.018
A
DOCKED: ATOM     19  C   UNL d   1      10.293   2.898   3.929 +0.28 +0.00    +0.024
A
DOCKED: ATOM     20  C   UNL d   1      10.811   1.694   4.458 -0.19 -0.00    -0.011
A
DOCKED: ATOM     21  C   UNL d   1      11.967   1.101   3.707 -0.38 +0.00    +0.045
C
DOCKED: ATOM     22  C   UNL d   1      10.212   1.218   5.619 -0.40 +0.02    +0.136
A
DOCKED: ATOM     23  C   UNL d   1      12.200  -0.394   3.866 -0.26 +0.01    +0.047
C
DOCKED: ATOM     24  C   UNL d   1      12.684  -0.702   5.264 -0.13 +0.08    +0.275
C
DOCKED: ATOM     25  C   UNL d   1      11.557  -0.809   6.279 -0.25 +0.15    +0.254
C
DOCKED: ATOM     26  O   UNL d   1      11.607  -1.753   7.079 -0.64 -0.29    -0.271
OA
DOCKED: ATOM     27  N   UNL d   1      10.560   0.090   6.351 -0.26 -0.16    -0.309
N
DOCKED: ATOM     28  H   UNL d   1       9.910  -0.055   7.120 +0.16 +0.14    +0.170
HD
DOCKED: ATOM     29  N   UNL d   1       9.161   1.831   6.200 -0.41 -0.07    -0.242
NA
DOCKED: ATOM     30  C   UNL d   1       8.685   2.974   5.649 -0.47 +0.02    +0.130
A
DOCKED: BRANCH  24  31
DOCKED: ATOM     31  N   UNL d   1      13.398  -2.038   5.232 -0.07 -0.02    -0.066
N
DOCKED: ATOM     32  H   UNL d   1      14.322  -2.009   4.790 +0.04 +0.07    +0.275
HD
DOCKED: ATOM     33  H   UNL d   1      12.821  -2.764   4.787 +0.07 +0.08    +0.275
HD
DOCKED: ATOM     34  H   UNL d   1      13.503  -2.355   6.212 +0.07 +0.12    +0.275
HD
DOCKED: ENDBRANCH  24  31
DOCKED: ENDBRANCH  17  18
DOCKED: ENDBRANCH  14  16
DOCKED: ENDBRANCH  12  14
DOCKED: ENDBRANCH  11  12
DOCKED: ENDBRANCH   2  11
DOCKED: TORSDOF 5
DOCKED: TER
DOCKED: ENDMDL
________________________________________________________________________________


    BEGINNING GENETIC ALGORITHM DOCKING 2 of 15
```

```
Run: 2 Seed: 1857940070 572023952 [ Run 2 of 15 GA/GALS ]
Beginning LAMARCKIAN GENETIC ALGORITHM (LGA), with a maximum of 2500000 energy evaluations.

Final-Value: -11.760


    FINAL GENETIC ALGORITHM DOCKED STATE
    _______________________________________________


Detailed state:  trans 6.191 5.256 -0.537 quatxyzw -0.345352 -0.658944 -0.604073 0.285692 center -0.813 0.218 0.087 ntor 6 -50.2549 170.2963 69.4484 -41.8676 141.5424 -38.1584
State:    6.191   5.256  -0.537  -0.360 -0.688 -0.630 146.800    -50.25  170.30  69.45  -41.87  141.54  -38.16

DOCKED: MODEL        2
DOCKED: USER    Run = 2
DOCKED: USER    DPF = D:/Autodock/dock.dpf
DOCKED: USER
DOCKED: USER    Estimated Free Energy of Binding    =   -7.43 kcal/mol  [=(1)+(2)+(3)-(4)]
DOCKED: USER    Estimated Inhibition Constant, Ki   =    3.56 uM (micromolar)  [Temperature = 298.15 K]
DOCKED: USER
DOCKED: USER    (1) Final Intermolecular Energy     =   -8.92 kcal/mol
DOCKED: USER        vdW + Hbond + desolv Energy     =   -7.23 kcal/mol
DOCKED: USER        Electrostatic Energy            =   -1.70 kcal/mol
DOCKED: USER    (2) Final Total Internal Energy     =   -2.84 kcal/mol
DOCKED: USER    (3) Torsional Free Energy           =   +1.49 kcal/mol
DOCKED: USER    (4) Unbound System's Energy  [=(2)] =   -2.84 kcal/mol
DOCKED: USER
DOCKED: USER
DOCKED: USER    NEWDPF move ligand1.pdbqt
DOCKED: USER    NEWDPF about -0.813000 0.218000 0.087000
DOCKED: USER    NEWDPF tran0 6.190553 5.256177 -0.536762
DOCKED: USER    NEWDPF quaternion0 -0.345352 -0.658944 -0.604073 0.285692
DOCKED: USER    NEWDPF axisangle0 -0.360372 -0.687602 -0.630345 146.799611
DOCKED: USER    NEWDPF quat0 -0.360372 -0.687602 -0.630345 146.799611
DOCKED: USER    NEWDPF dihe0 -50.25 170.30 69.45 -41.87 141.54 -38.16
DOCKED: USER  keepresnum = 1
DOCKED: USER
DOCKED: REMARK  6 active torsions:
DOCKED: REMARK  status: ('A' for Active; 'I' for Inactive)
DOCKED: REMARK    1  A    between atoms: N_5  and  C_10
DOCKED: REMARK    2  A    between atoms: N_6  and  C_17
DOCKED: REMARK    3  A    between atoms: N_6  and  C_26
DOCKED: REMARK    4  A    between atoms: C_15  and  C_17
DOCKED: REMARK    5  A    between atoms: C_19  and  C_23
DOCKED: REMARK    6  A    between atoms: C_26  and  C_27
DOCKED: USER                              x       y       z     vdW  Elec       q    Type
DOCKED: USER                           _______ _______ _______ _____ _____    ______ ____
DOCKED: ROOT
DOCKED: ATOM      1  O   UNL d   1       4.992   8.781  -0.107 -0.12 +0.03    -0.312 OA
DOCKED: ATOM      2  C   UNL d   1       4.060   9.600  -0.677 -0.11 -0.01    +0.078 A
DOCKED: ATOM      3  C   UNL d   1       3.310  10.230   0.306 -0.06 +0.00    -0.018 A
DOCKED: ATOM      4  C   UNL d   1       3.804   9.733   1.554 -0.12 -0.00    +0.006 A
DOCKED: ATOM      5  C   UNL d   1       4.832   8.855   1.239 -0.20 -0.02    +0.086 A
DOCKED: ATOM      6  C   UNL d   1       2.213  11.212   0.124 -0.01 -0.01    +0.052 C
DOCKED: ATOM      7  C   UNL d   1       3.495   9.937   2.911 -0.13 -0.01    +0.015 A
DOCKED: ATOM      8  C   UNL d   1       5.587   8.159   2.181 -0.29 -0.01    +0.038 A
DOCKED: ATOM      9  C   UNL d   1       4.236   9.251   3.885 -0.28 -0.00    +0.001
```

```
A
DOCKED: ATOM     10  C   UNL d   1       5.265   8.377   3.524 -0.41 -0.00    +0.003 A
DOCKED: ENDROOT
DOCKED: BRANCH   2  11
DOCKED: ATOM     11  C   UNL d   1       4.072   9.659  -2.164 -0.11 -0.02    +0.191 C
DOCKED: BRANCH  11  12
DOCKED: ATOM     12  N   UNL d   1       3.881   8.327  -2.744 -0.10 +0.02    -0.347 N
DOCKED: ATOM     13  C   UNL d   1       4.889   7.895  -3.702 -0.21 -0.01    +0.153 C
DOCKED: BRANCH  12  14
DOCKED: ATOM     14  C   UNL d   1       2.749   7.534  -2.607 -0.16 -0.01    +0.243 C
DOCKED: ATOM     15  O   UNL d   1       2.215   6.976  -3.565 -0.14 +0.01    -0.268 OA
DOCKED: BRANCH  14  16
DOCKED: ATOM     16  C   UNL d   1       2.163   7.358  -1.247 -0.22 -0.01    +0.075 C
DOCKED: ATOM     17  C   UNL d   1       2.124   6.128  -0.706 -0.35 -0.00    +0.018 C
DOCKED: BRANCH  17  18
DOCKED: ATOM     18  C   UNL d   1       1.540   5.802   0.599 -0.35 +0.00    -0.018 A
DOCKED: ATOM     19  C   UNL d   1       1.702   6.686   1.666 -0.29 -0.01    +0.024 A
DOCKED: ATOM     20  C   UNL d   1       1.146   6.433   2.941 -0.35 +0.00    -0.011 A
DOCKED: ATOM     21  C   UNL d   1       1.429   7.470   3.988 -0.31 -0.04    +0.045 C
DOCKED: ATOM     22  C   UNL d   1       0.384   5.276   3.067 -0.36 -0.03    +0.136 A
DOCKED: ATOM     23  C   UNL d   1       1.357   7.023   5.440 -0.04 -0.07    +0.047 C
DOCKED: ATOM     24  C   UNL d   1      -0.075   6.727   5.826 -0.27 -0.29    +0.275 C
DOCKED: ATOM     25  C   UNL d   1      -0.533   5.337   5.414 -0.40 -0.11    +0.254 C
DOCKED: ATOM     26  O   UNL d   1      -1.175   4.686   6.250 -0.48 +0.10    -0.271 OA
DOCKED: ATOM     27  N   UNL d   1      -0.296   4.829   4.193 -0.26 +0.08    -0.309 N
DOCKED: ATOM     28  H   UNL d   1      -0.718   3.919   4.020 +0.12 -0.02    +0.170 HD
DOCKED: ATOM     29  N   UNL d   1       0.211   4.405   2.053 -0.35 +0.03    -0.242 NA
DOCKED: ATOM     30  C   UNL d   1       0.776   4.678   0.853 -0.34 -0.01    +0.130 A
DOCKED: BRANCH  24  31
DOCKED: ATOM     31  N   UNL d   1      -0.184   6.817   7.334 +0.19 +0.10    -0.066 N
DOCKED: ATOM     32  H   UNL d   1       0.527   7.411   7.771 -0.24 -0.73    +0.275 HD
DOCKED: ATOM     33  H   UNL d   1      -0.178   5.886   7.770 -0.14 -0.30    +0.275 HD
DOCKED: ATOM     34  H   UNL d   1      -1.121   7.199   7.552 -0.33 -0.38    +0.275 HD
DOCKED: ENDBRANCH  24  31
DOCKED: ENDBRANCH  17  18
DOCKED: ENDBRANCH  14  16
DOCKED: ENDBRANCH  12  14
DOCKED: ENDBRANCH  11  12
DOCKED: ENDBRANCH   2  11
DOCKED: TORSDOF 5
DOCKED: TER
DOCKED: ENDMDL
________________________________________________________________________________


    BEGINNING GENETIC ALGORITHM DOCKING 3 of 15
Run: 3 Seed: 636218694 697539420 [ Run 3 of 15 GA/GALS ]
Beginning LAMARCKIAN GENETIC ALGORITHM (LGA), with a maximum of 2500000 energy
```

```
evaluations.

Final-Value: -11.019


    FINAL GENETIC ALGORITHM DOCKED STATE
    _______________________________________________


Detailed state:  trans 4.472 3.226 4.249 quatxyzw 0.383192 -0.069490 -0.690216 0.609867 center -0.813 0.218 0.087 ntor 6 -4.0874 23.3326 0.0604 -166.6281 -168.8679 -65.8991
State:    4.472   3.226   4.249   0.484 -0.088 -0.871 104.840     -4.09   23.33   0.06 -166.63 -168.87  -65.90

DOCKED: MODEL        3
DOCKED: USER    Run = 3
DOCKED: USER    DPF = D:/Autodock/dock.dpf
DOCKED: USER
DOCKED: USER    Estimated Free Energy of Binding    =   -7.82 kcal/mol  [=(1)+(2)+(3)-(4)]
DOCKED: USER    Estimated Inhibition Constant, Ki   =    1.86 uM (micromolar)  [Temperature = 298.15 K]
DOCKED: USER
DOCKED: USER    (1) Final Intermolecular Energy     =   -9.31 kcal/mol
DOCKED: USER        vdW + Hbond + desolv Energy     =   -9.46 kcal/mol
DOCKED: USER        Electrostatic Energy            =   +0.15 kcal/mol
DOCKED: USER    (2) Final Total Internal Energy     =   -1.71 kcal/mol
DOCKED: USER    (3) Torsional Free Energy           =   +1.49 kcal/mol
DOCKED: USER    (4) Unbound System's Energy  [=(2)] =   -1.71 kcal/mol
DOCKED: USER
DOCKED: USER
DOCKED: USER    NEWDPF move ligand1.pdbqt
DOCKED: USER    NEWDPF about -0.813000 0.218000 0.087000
DOCKED: USER    NEWDPF tran0 4.472324 3.226055 4.249154
DOCKED: USER    NEWDPF quaternion0 0.383192 -0.069490 -0.690216 0.609867
DOCKED: USER    NEWDPF axisangle0 0.483520 -0.087685 -0.870930 104.840294
DOCKED: USER    NEWDPF quat0 0.483520 -0.087685 -0.870930 104.840294
DOCKED: USER    NEWDPF dihe0 -4.09 23.33 0.06 -166.63 -168.87 -65.90
DOCKED: USER  keepresnum = 1
DOCKED: USER
DOCKED: REMARK  6 active torsions:
DOCKED: REMARK  status: ('A' for Active; 'I' for Inactive)
DOCKED: REMARK    1  A    between atoms: N_5  and  C_10
DOCKED: REMARK    2  A    between atoms: N_6  and  C_17
DOCKED: REMARK    3  A    between atoms: N_6  and  C_26
DOCKED: REMARK    4  A    between atoms: C_15  and  C_17
DOCKED: REMARK    5  A    between atoms: C_19  and  C_23
DOCKED: REMARK    6  A    between atoms: C_26  and  C_27
DOCKED: USER                              x       y       z     vdW  Elec       q    Type
DOCKED: USER                           _______ _______ _______ _____ _____    ______ ____
DOCKED: ROOT
DOCKED: ATOM      1  O   UNL d   1       3.747   6.556   2.692 -0.20 +0.10    -0.312 OA
DOCKED: ATOM      2  C   UNL d   1       4.451   7.547   2.069 -0.26 -0.02    +0.078 A
DOCKED: ATOM      3  C   UNL d   1       3.814   7.923   0.894 -0.22 +0.00    -0.018 A
DOCKED: ATOM      4  C   UNL d   1       2.663   7.080   0.787 -0.26 -0.00    +0.006 A
DOCKED: ATOM      5  C   UNL d   1       2.672   6.269   1.914 -0.23 -0.02    +0.086 A
DOCKED: ATOM      6  C   UNL d   1       4.227   8.973  -0.069 -0.19 -0.01    +0.052 C
DOCKED: ATOM      7  C   UNL d   1       1.614   6.921  -0.138 -0.25 -0.00    +0.015 A
DOCKED: ATOM      8  C   UNL d   1       1.708   5.304   2.197 -0.27 -0.01    +0.038 A
DOCKED: ATOM      9  C   UNL d   1       0.627   5.958   0.117 -0.36 -0.00    +0.001 A
DOCKED: ATOM     10  C   UNL d   1       0.674   5.164   1.265 -0.49 -0.00    +0.003
```

```
A
DOCKED: ENDROOT
DOCKED: BRANCH   2  11
DOCKED: ATOM     11  C   UNL d   1       5.679   8.010   2.771 -0.34 -0.06    +0.191 C
DOCKED: BRANCH  11  12
DOCKED: ATOM     12  N   UNL d   1       6.276   6.924   3.554 -0.26 +0.11    -0.347 N
DOCKED: ATOM     13  C   UNL d   1       5.613   6.599   4.808 -0.47 -0.10    +0.153 C
DOCKED: BRANCH  12  14
DOCKED: ATOM     14  C   UNL d   1       7.500   6.317   3.303 -0.40 -0.05    +0.243 C
DOCKED: ATOM     15  O   UNL d   1       8.284   6.739   2.454 -0.43 +0.04    -0.268 OA
DOCKED: BRANCH  14  16
DOCKED: ATOM     16  C   UNL d   1       7.871   5.108   4.095 -0.42 -0.02    +0.075 C
DOCKED: ATOM     17  C   UNL d   1       8.985   4.431   3.770 -0.71 -0.00    +0.018 C
DOCKED: BRANCH  17  18
DOCKED: ATOM     18  C   UNL d   1       9.446   3.206   4.430 -0.52 -0.00    -0.018 A
DOCKED: ATOM     19  C   UNL d   1      10.560   2.530   3.929 -0.01 +0.00    +0.024 A
DOCKED: ATOM     20  C   UNL d   1      11.060   1.355   4.535 -0.29 -0.00    -0.011 A
DOCKED: ATOM     21  C   UNL d   1      12.250   0.735   3.863 -0.35 +0.01    +0.045 C
DOCKED: ATOM     22  C   UNL d   1      10.414   0.929   5.690 -0.41 +0.03    +0.136 A
DOCKED: ATOM     23  C   UNL d   1      12.482  -0.749   4.099 -0.23 +0.01    +0.047 C
DOCKED: ATOM     24  C   UNL d   1      12.907  -0.990   5.530 -0.11 +0.10    +0.275 C
DOCKED: ATOM     25  C   UNL d   1      11.739  -1.056   6.500 -0.23 +0.18    +0.254 C
DOCKED: ATOM     26  O   UNL d   1      11.759  -1.963   7.344 -0.65 -0.32    -0.271 OA
DOCKED: ATOM     27  N   UNL d   1      10.735  -0.162   6.488 -0.23 -0.19    -0.309 N
DOCKED: ATOM     28  H   UNL d   1      10.054  -0.275   7.235 +0.14 +0.16    +0.170 HD
DOCKED: ATOM     29  N   UNL d   1       9.336   1.562   6.197 -0.37 -0.07    -0.242 NA
DOCKED: ATOM     30  C   UNL d   1       8.879   2.675   5.574 -0.54 +0.02    +0.130 A
DOCKED: BRANCH  24  31
DOCKED: ATOM     31  N   UNL d   1      13.627  -2.321   5.590 -0.06 -0.02    -0.066 N
DOCKED: ATOM     32  H   UNL d   1      14.550  -2.319   5.144 +0.04 +0.07    +0.275 HD
DOCKED: ATOM     33  H   UNL d   1      13.053  -3.079   5.200 +0.06 +0.09    +0.275 HD
DOCKED: ATOM     34  H   UNL d   1      13.738  -2.566   6.589 +0.06 +0.12    +0.275 HD
DOCKED: ENDBRANCH  24  31
DOCKED: ENDBRANCH  17  18
DOCKED: ENDBRANCH  14  16
DOCKED: ENDBRANCH  12  14
DOCKED: ENDBRANCH  11  12
DOCKED: ENDBRANCH   2  11
DOCKED: TORSDOF 5
DOCKED: TER
DOCKED: ENDMDL
________________________________________________________________________________


    BEGINNING GENETIC ALGORITHM DOCKING 4 of 15
Run: 4 Seed: 318362053 1229107611 [ Run 4 of 15 GA/GALS ]
Beginning LAMARCKIAN GENETIC ALGORITHM (LGA), with a maximum of 2500000 energy evaluations.
```

```
Final-Value: -9.473


    FINAL GENETIC ALGORITHM DOCKED STATE
    _______________________________________________


Detailed state:  trans 10.345 2.970 2.831 quatxyzw -0.122820 0.188497 -0.531561 -0.816594 center -0.813 0.218 0.087 ntor 6 -1.5244 44.1995 90.4544 46.1260 155.3635 -171.2317
State:   10.345   2.970   2.831  -0.213  0.327 -0.921 -70.509     -1.52   44.20   90.45   46.13  155.36 -171.23

DOCKED: MODEL        4
DOCKED: USER    Run = 4
DOCKED: USER    DPF = D:/Autodock/dock.dpf
DOCKED: USER
DOCKED: USER    Estimated Free Energy of Binding    =   -5.81 kcal/mol [=(1)+(2)+(3)-(4)]
DOCKED: USER    Estimated Inhibition Constant, Ki   =   55.23 uM (micromolar) [Temperature = 298.15 K]
DOCKED: USER
DOCKED: USER    (1) Final Intermolecular Energy     =   -7.30 kcal/mol
DOCKED: USER        vdW + Hbond + desolv Energy     =   -7.06 kcal/mol
DOCKED: USER        Electrostatic Energy            =   -0.24 kcal/mol
DOCKED: USER    (2) Final Total Internal Energy     =   -2.17 kcal/mol
DOCKED: USER    (3) Torsional Free Energy           =   +1.49 kcal/mol
DOCKED: USER    (4) Unbound System's Energy  [=(2)] =   -2.17 kcal/mol
DOCKED: USER
DOCKED: USER
DOCKED: USER    NEWDPF move ligand1.pdbqt
DOCKED: USER    NEWDPF about -0.813000 0.218000 0.087000
DOCKED: USER    NEWDPF tran0 10.344927 2.970430 2.830932
DOCKED: USER    NEWDPF quaternion0 -0.122820 0.188497 -0.531561 -0.816594
DOCKED: USER    NEWDPF axisangle0 -0.212782 0.326564 -0.920912 -70.509357
DOCKED: USER    NEWDPF quat0 -0.212782 0.326564 -0.920912 -70.509357
DOCKED: USER    NEWDPF dihe0 -1.52 44.20 90.45 46.13 155.36 -171.23
DOCKED: USER  keepresnum = 1
DOCKED: USER
DOCKED: REMARK  6 active torsions:
DOCKED: REMARK  status: ('A' for Active; 'I' for Inactive)
DOCKED: REMARK    1  A    between atoms: N_5  and  C_10
DOCKED: REMARK    2  A    between atoms: N_6  and  C_17
DOCKED: REMARK    3  A    between atoms: N_6  and  C_26
DOCKED: REMARK    4  A    between atoms: C_15  and  C_17
DOCKED: REMARK    5  A    between atoms: C_19  and  C_23
DOCKED: REMARK    6  A    between atoms: C_26  and  C_27
DOCKED: USER                                 x       y       z     vdW  Elec       q    Type
DOCKED: USER                              _______ _______ _______ _____ _____    ______ ____
DOCKED: ROOT
DOCKED: ATOM      1  O   UNL d   1      12.461  -0.115   3.038 -0.16 -0.03    -0.312 OA
DOCKED: ATOM      2  C   UNL d   1      12.283  -1.464   2.916 -0.17 +0.01    +0.078 A
DOCKED: ATOM      3  C   UNL d   1      13.253  -2.002   2.082 -0.11 -0.00    -0.018 A
DOCKED: ATOM      4  C   UNL d   1      14.053  -0.898   1.649 -0.10 +0.00    +0.006 A
DOCKED: ATOM      5  C   UNL d   1      13.525   0.227   2.267 -0.18 +0.01    +0.086 A
DOCKED: ATOM      6  C   UNL d   1      13.437  -3.424   1.700 -0.06 +0.01    +0.052 C
DOCKED: ATOM      7  C   UNL d   1      15.168  -0.732   0.807 -0.06 +0.00    +0.015 A
DOCKED: ATOM      8  C   UNL d   1      14.026   1.518   2.114 -0.28 +0.00    +0.038 A
DOCKED: ATOM      9  C   UNL d   1      15.697   0.555   0.630 -0.12 +0.00    +0.001 A
DOCKED: ATOM     10  C   UNL d   1      15.135   1.660   1.273 -0.20 -0.00    +0.003 A
DOCKED: ENDROOT
```

```
DOCKED: BRANCH   2  11
DOCKED: ATOM     11  C   UNL d   1      11.154  -2.042   3.694 +0.01 +0.04    +0.191
C
DOCKED: BRANCH  11  12
DOCKED: ATOM     12  N   UNL d   1      11.487  -2.129   5.118 -0.08 -0.12    -0.347
N
DOCKED: ATOM     13  C   UNL d   1      11.638  -3.472   5.661 -0.16 +0.07    +0.153
C
DOCKED: BRANCH  12  14
DOCKED: ATOM     14  C   UNL d   1      11.488  -1.073   6.020 -0.24 +0.13    +0.243
C
DOCKED: ATOM     15  O   UNL d   1      12.359  -0.940   6.880 -0.64 -0.21    -0.268
OA
DOCKED: BRANCH  14  16
DOCKED: ATOM     16  C   UNL d   1      10.389  -0.067   5.940 -0.12 +0.03    +0.075
C
DOCKED: ATOM     17  C   UNL d   1      10.597   1.075   5.262 -0.45 +0.00    +0.018
C
DOCKED: BRANCH  17  18
DOCKED: ATOM     18  C   UNL d   1       9.625   2.166   5.138 -0.56 -0.00    -0.018
A
DOCKED: ATOM     19  C   UNL d   1       9.811   3.146   4.161 -0.15 +0.00    +0.024
A
DOCKED: ATOM     20  C   UNL d   1       8.898   4.210   3.984 -0.68 +0.00    -0.011
A
DOCKED: ATOM     21  C   UNL d   1       9.251   5.194   2.907 -0.64 -0.00    +0.045
C
DOCKED: ATOM     22  C   UNL d   1       7.774   4.208   4.803 -0.24 -0.03    +0.136
A
DOCKED: ATOM     23  C   UNL d   1       8.662   6.591   3.026 -0.37 -0.01    +0.047
C
DOCKED: ATOM     24  C   UNL d   1       7.169   6.551   2.786 -0.08 -0.05    +0.275
C
DOCKED: ATOM     25  C   UNL d   1       6.374   6.152   4.019 +0.10 -0.09    +0.254
C
DOCKED: ATOM     26  O   UNL d   1       5.356   6.810   4.269 -0.40 +0.15    -0.271
OA
DOCKED: ATOM     27  N   UNL d   1       6.719   5.111   4.797 +0.32 +0.15    -0.309
N
DOCKED: ATOM     28  H   UNL d   1       6.063   4.905   5.547 -0.35 -0.11    +0.170
HD
DOCKED: ATOM     29  N   UNL d   1       7.569   3.275   5.755 -0.56 +0.00    -0.242
NA
DOCKED: ATOM     30  C   UNL d   1       8.479   2.281   5.902 -0.35 +0.02    +0.130
A
DOCKED: BRANCH  24  31
DOCKED: ATOM     31  N   UNL d   1       6.720   7.940   2.383 -0.26 +0.02    -0.066
N
DOCKED: ATOM     32  H   UNL d   1       6.993   8.210   1.433 +0.20 -0.04    +0.275
HD
DOCKED: ATOM     33  H   UNL d   1       7.042   8.651   3.053 -0.07 -0.10    +0.275
HD
DOCKED: ATOM     34  H   UNL d   1       5.688   7.964   2.456 +0.15 -0.08    +0.275
HD
DOCKED: ENDBRANCH  24  31
DOCKED: ENDBRANCH  17  18
DOCKED: ENDBRANCH  14  16
DOCKED: ENDBRANCH  12  14
DOCKED: ENDBRANCH  11  12
DOCKED: ENDBRANCH   2  11
DOCKED: TORSDOF 5
DOCKED: TER
DOCKED: ENDMDL
________________________________________________________________________________


    BEGINNING GENETIC ALGORITHM DOCKING 5 of 15
Run: 5 Seed: 1113997574 2119540956 [ Run 5 of 15 GA/GALS ]
Beginning LAMARCKIAN GENETIC ALGORITHM (LGA), with a maximum of 2500000 energy
evaluations.

Final-Value: -11.658
```

```
     FINAL GENETIC ALGORITHM DOCKED STATE
     ________________________________________________


Detailed state:  trans 6.246 5.300 0.559 quatxyzw 0.343780 0.556360 0.601156 -0.459228 center -0.813 0.218 0.087 ntor 6 -113.9617 -166.4693 4.1453 65.0355 23.8996 2.1132
State:    6.246   5.300   0.559   0.387  0.626  0.677 -125.325   -113.96 -166.47 4.15   65.04   23.90    2.11

DOCKED: MODEL        5
DOCKED: USER    Run = 5
DOCKED: USER    DPF = D:/Autodock/dock.dpf
DOCKED: USER
DOCKED: USER    Estimated Free Energy of Binding    =   -7.49 kcal/mol [=(1)+(2)+(3)-(4)]
DOCKED: USER    Estimated Inhibition Constant, Ki   =    3.21 uM (micromolar) [Temperature = 298.15 K]
DOCKED: USER
DOCKED: USER    (1) Final Intermolecular Energy     =   -8.99 kcal/mol
DOCKED: USER        vdW + Hbond + desolv Energy     =   -7.46 kcal/mol
DOCKED: USER        Electrostatic Energy            =   -1.52 kcal/mol
DOCKED: USER    (2) Final Total Internal Energy     =   -2.67 kcal/mol
DOCKED: USER    (3) Torsional Free Energy           =   +1.49 kcal/mol
DOCKED: USER    (4) Unbound System's Energy  [=(2)] =   -2.67 kcal/mol
DOCKED: USER
DOCKED: USER
DOCKED: USER    NEWDPF move ligand1.pdbqt
DOCKED: USER    NEWDPF about -0.813000 0.218000 0.087000
DOCKED: USER    NEWDPF tran0 6.246223 5.299738 0.559296
DOCKED: USER    NEWDPF quaternion0 0.343780 0.556360 0.601156 -0.459228
DOCKED: USER    NEWDPF axisangle0 0.387001 0.626307 0.676735 -125.325366
DOCKED: USER    NEWDPF quat0 0.387001 0.626307 0.676735 -125.325366
DOCKED: USER    NEWDPF dihe0 -113.96 -166.47 4.15 65.04 23.90 2.11
DOCKED: USER  keepresnum = 1
DOCKED: USER
DOCKED: REMARK  6 active torsions:
DOCKED: REMARK  status: ('A' for Active; 'I' for Inactive)
DOCKED: REMARK    1  A    between atoms: N_5  and  C_10
DOCKED: REMARK    2  A    between atoms: N_6  and  C_17
DOCKED: REMARK    3  A    between atoms: N_6  and  C_26
DOCKED: REMARK    4  A    between atoms: C_15  and  C_17
DOCKED: REMARK    5  A    between atoms: C_19  and  C_23
DOCKED: REMARK    6  A    between atoms: C_26  and  C_27
DOCKED: USER                              x       y       z     vdW  Elec       q    Type
DOCKED: USER                           _______ _______ _______ _____ _____    ______ ____
DOCKED: ROOT
DOCKED: ATOM      1  O   UNL d   1       5.991   9.032   0.774 -0.08 +0.04    -0.312 OA
DOCKED: ATOM      2  C   UNL d   1       5.519  10.052  -0.003 -0.22 -0.01    +0.078 A
DOCKED: ATOM      3  C   UNL d   1       4.725  10.903   0.753 -0.12 +0.00    -0.018 A
DOCKED: ATOM      4  C   UNL d   1       4.693  10.342   2.069 -0.17 -0.00    +0.006 A
DOCKED: ATOM      5  C   UNL d   1       5.486   9.203   2.022 -0.24 -0.03    +0.086 A
DOCKED: ATOM      6  C   UNL d   1       4.038  12.140   0.308 -0.04 -0.01    +0.052 C
DOCKED: ATOM      7  C   UNL d   1       4.083  10.678   3.292 -0.15 -0.01    +0.015 A
DOCKED: ATOM      8  C   UNL d   1       5.724   8.366   3.110 -0.38 -0.01    +0.038 A
DOCKED: ATOM      9  C   UNL d   1       4.302   9.855   4.407 -0.26 -0.00    +0.001 A
DOCKED: ATOM     10  C   UNL d   1       5.110   8.718   4.315 -0.39 -0.00    +0.003 A
DOCKED: ENDROOT
DOCKED: BRANCH   2  11
DOCKED: ATOM     11  C   UNL d   1       5.965  10.045  -1.423 -0.25 -0.01    +0.191
```

```
C
DOCKED: BRANCH  11  12
DOCKED: ATOM     12  N   UNL d   1       4.943   9.451  -2.290 -0.11 +0.03    -0.347
N
DOCKED: ATOM     13  C   UNL d   1       4.356  10.336  -3.287 -0.08 -0.01    +0.153
C
DOCKED: BRANCH  12  14
DOCKED: ATOM     14  C   UNL d   1       4.363   8.201  -2.128 -0.18 -0.01    +0.243
C
DOCKED: ATOM     15  O   UNL d   1       4.435   7.323  -2.988 -0.18 +0.01    -0.268
OA
DOCKED: BRANCH  14  16
DOCKED: ATOM     16  C   UNL d   1       3.625   7.916  -0.863 -0.23 -0.01    +0.075
C
DOCKED: ATOM     17  C   UNL d   1       3.013   6.729  -0.717 -0.34 -0.00    +0.018
C
DOCKED: BRANCH  17  18
DOCKED: ATOM     18  C   UNL d   1       2.218   6.331   0.450 -0.30 +0.00    -0.018
A
DOCKED: ATOM     19  C   UNL d   1       2.315   7.067   1.632 -0.25 -0.01    +0.024
A
DOCKED: ATOM     20  C   UNL d   1       1.560   6.742   2.782 -0.31 +0.00    -0.011
A
DOCKED: ATOM     21  C   UNL d   1       1.794   7.616   3.979 -0.31 -0.04    +0.045
C
DOCKED: ATOM     22  C   UNL d   1       0.675   5.676   2.663 -0.37 -0.03    +0.136
A
DOCKED: ATOM     23  C   UNL d   1       1.471   7.023   5.342 -0.07 -0.07    +0.047
C
DOCKED: ATOM     24  C   UNL d   1      -0.023   6.857   5.499 -0.27 -0.28    +0.275
C
DOCKED: ATOM     25  C   UNL d   1      -0.556   5.584   4.860 -0.34 -0.10    +0.254
C
DOCKED: ATOM     26  O   UNL d   1      -1.373   4.925   5.517 -0.42 +0.08    -0.271
OA
DOCKED: ATOM     27  N   UNL d   1      -0.200   5.190   3.625 -0.18 +0.08    -0.309
N
DOCKED: ATOM     28  H   UNL d   1      -0.683   4.361   3.287 +0.12 -0.02    +0.170
HD
DOCKED: ATOM     29  N   UNL d   1       0.561   4.948   1.534 -0.38 +0.03    -0.242
NA
DOCKED: ATOM     30  C   UNL d   1       1.317   5.284   0.460 -0.29 -0.01    +0.130
A
DOCKED: BRANCH  24  31
DOCKED: ATOM     31  N   UNL d   1      -0.337   6.792   6.979 -0.40 +0.09    -0.066
N
DOCKED: ATOM     32  H   UNL d   1       0.345   6.260   7.529 -0.03 -0.36    +0.275
HD
DOCKED: ATOM     33  H   UNL d   1      -1.283   6.427   7.149 +0.03 -0.24    +0.275
HD
DOCKED: ATOM     34  H   UNL d   1      -0.367   7.765   7.330 -0.28 -0.62    +0.275
HD
DOCKED: ENDBRANCH  24  31
DOCKED: ENDBRANCH  17  18
DOCKED: ENDBRANCH  14  16
DOCKED: ENDBRANCH  12  14
DOCKED: ENDBRANCH  11  12
DOCKED: ENDBRANCH   2  11
DOCKED: TORSDOF 5
DOCKED: TER
DOCKED: ENDMDL
________________________________________________________________________________


        BEGINNING GENETIC ALGORITHM DOCKING 6 of 15
Run: 6 Seed: 199063283 18331409 [ Run 6 of 15 GA/GALS ]
Beginning LAMARCKIAN GENETIC ALGORITHM (LGA), with a maximum of 2500000 energy
evaluations.

Final-Value: -11.534


        FINAL GENETIC ALGORITHM DOCKED STATE
```

```
    _______________________________________________


Detailed state:  trans 6.425 5.687 -0.774 quatxyzw -0.462742 -0.434139 -0.690879 0.346525 center -0.813 0.218 0.087 ntor 6 142.5318 -180.0000 25.0639 64.9654 44.7409 -11.7169
State:    6.425   5.687  -0.774  -0.493 -0.463 -0.737 139.450    142.53 -180.00 25.06   64.97   44.74  -11.72

DOCKED: MODEL        6
DOCKED: USER    Run = 6
DOCKED: USER    DPF = D:/Autodock/dock.dpf
DOCKED: USER
DOCKED: USER    Estimated Free Energy of Binding    =   -7.26 kcal/mol [=(1)+(2)+(3)-(4)]
DOCKED: USER    Estimated Inhibition Constant, Ki   =    4.77 uM (micromolar) [Temperature = 298.15 K]
DOCKED: USER
DOCKED: USER    (1) Final Intermolecular Energy     =   -8.75 kcal/mol
DOCKED: USER        vdW + Hbond + desolv Energy     =   -7.50 kcal/mol
DOCKED: USER        Electrostatic Energy            =   -1.25 kcal/mol
DOCKED: USER    (2) Final Total Internal Energy     =   -2.78 kcal/mol
DOCKED: USER    (3) Torsional Free Energy           =   +1.49 kcal/mol
DOCKED: USER    (4) Unbound System's Energy  [=(2)] =   -2.78 kcal/mol
DOCKED: USER
DOCKED: USER
DOCKED: USER    NEWDPF move ligand1.pdbqt
DOCKED: USER    NEWDPF about -0.813000 0.218000 0.087000
DOCKED: USER    NEWDPF tran0 6.425226 5.687261 -0.774041
DOCKED: USER    NEWDPF quaternion0 -0.462742 -0.434139 -0.690879 0.346525
DOCKED: USER    NEWDPF axisangle0 -0.493307 -0.462815 -0.736513 139.450167
DOCKED: USER    NEWDPF quat0 -0.493307 -0.462815 -0.736513 139.450167
DOCKED: USER    NEWDPF dihe0 142.53 -180.00 25.06 64.97 44.74 -11.72
DOCKED: USER  keepresnum = 1
DOCKED: USER
DOCKED: REMARK  6 active torsions:
DOCKED: REMARK  status: ('A' for Active; 'I' for Inactive)
DOCKED: REMARK    1  A    between atoms: N_5  and  C_10
DOCKED: REMARK    2  A    between atoms: N_6  and  C_17
DOCKED: REMARK    3  A    between atoms: N_6  and  C_26
DOCKED: REMARK    4  A    between atoms: C_15  and  C_17
DOCKED: REMARK    5  A    between atoms: C_19  and  C_23
DOCKED: REMARK    6  A    between atoms: C_26  and  C_27
DOCKED: USER                                 x       y       z     vdW  Elec       q    Type
DOCKED: USER                              _______ _______ _______ _____ _____    ______ ____
DOCKED: ROOT
DOCKED: ATOM      1  O   UNL d   1       6.262   8.973   1.021 -0.06 +0.05    -0.312 OA
DOCKED: ATOM      2  C   UNL d   1       5.740  10.214   0.795 -0.21 -0.02    +0.078 A
DOCKED: ATOM      3  C   UNL d   1       5.036  10.648   1.910 -0.16 +0.01    -0.018 A
DOCKED: ATOM      4  C   UNL d   1       5.114   9.583   2.861 -0.26 -0.00    +0.006 A
DOCKED: ATOM      5  C   UNL d   1       5.878   8.588   2.265 -0.25 -0.03    +0.086 A
DOCKED: ATOM      6  C   UNL d   1       4.333  11.942   2.095 -0.07 -0.01    +0.052 C
DOCKED: ATOM      7  C   UNL d   1       4.627   9.357   4.162 -0.31 -0.01    +0.015 A
DOCKED: ATOM      8  C   UNL d   1       6.200   7.376   2.869 -0.39 -0.01    +0.038 A
DOCKED: ATOM      9  C   UNL d   1       4.932   8.145   4.799 -0.34 -0.00    +0.001 A
DOCKED: ATOM     10  C   UNL d   1       5.706   7.172   4.162 -0.55 -0.00    +0.003 A
DOCKED: ENDROOT
DOCKED: BRANCH   2  11
DOCKED: ATOM     11  C   UNL d   1       6.052  10.818  -0.529 -0.21 -0.02    +0.191 C
DOCKED: BRANCH  11  12
```

```
DOCKED: ATOM     12  N   UNL d   1       5.111  10.354  -1.552 -0.07 +0.03    -0.347 N
DOCKED: ATOM     13  C   UNL d   1       4.613  11.367  -2.472 -0.05 -0.01    +0.153 C
DOCKED: BRANCH  12  14
DOCKED: ATOM     14  C   UNL d   1       4.520   9.099  -1.606 -0.16 -0.02    +0.243 C
DOCKED: ATOM     15  O   UNL d   1       4.667   8.340  -2.564 -0.19 +0.01    -0.268 OA
DOCKED: BRANCH  14  16
DOCKED: ATOM     16  C   UNL d   1       3.675   8.659  -0.457 -0.17 -0.01    +0.075 C
DOCKED: ATOM     17  C   UNL d   1       3.403   7.351  -0.309 -0.29 -0.00    +0.018 C
DOCKED: BRANCH  17  18
DOCKED: ATOM     18  C   UNL d   1       2.554   6.784   0.744 -0.27 +0.00    -0.018 A
DOCKED: ATOM     19  C   UNL d   1       2.455   7.435   1.974 -0.22 -0.01    +0.024 A
DOCKED: ATOM     20  C   UNL d   1       1.639   6.945   3.018 -0.30 +0.00    -0.011 A
DOCKED: ATOM     21  C   UNL d   1       1.659   7.747   4.287 -0.30 -0.05    +0.045 C
DOCKED: ATOM     22  C   UNL d   1       0.897   5.801   2.743 -0.35 -0.04    +0.136 A
DOCKED: ATOM     23  C   UNL d   1       1.281   7.022   5.569 +0.03 -0.07    +0.047 C
DOCKED: ATOM     24  C   UNL d   1      -0.193   6.684   5.563 -0.29 -0.25    +0.275 C
DOCKED: ATOM     25  C   UNL d   1      -0.514   5.412   4.794 -0.34 -0.10    +0.254 C
DOCKED: ATOM     26  O   UNL d   1      -1.308   4.621   5.321 -0.37 +0.06    -0.271 OA
DOCKED: ATOM     27  N   UNL d   1      -0.002   5.152   3.579 -0.25 +0.08    -0.309 N
DOCKED: ATOM     28  H   UNL d   1      -0.353   4.304   3.141 +0.09 -0.02    +0.170 HD
DOCKED: ATOM     29  N   UNL d   1       0.974   5.152   1.563 -0.35 +0.03    -0.242 NA
DOCKED: ATOM     30  C   UNL d   1       1.782   5.647   0.595 -0.18 -0.01    +0.130 A
DOCKED: BRANCH  24  31
DOCKED: ATOM     31  N   UNL d   1      -0.634   6.474   6.997 -0.42 +0.07    -0.066 N
DOCKED: ATOM     32  H   UNL d   1      -1.321   5.724   7.119 +0.20 -0.17    +0.275 HD
DOCKED: ATOM     33  H   UNL d   1      -0.989   7.344   7.415 -0.35 -0.39    +0.275 HD
DOCKED: ATOM     34  H   UNL d   1       0.212   6.248   7.549 -0.07 -0.36    +0.275 HD
DOCKED: ENDBRANCH  24  31
DOCKED: ENDBRANCH  17  18
DOCKED: ENDBRANCH  14  16
DOCKED: ENDBRANCH  12  14
DOCKED: ENDBRANCH  11  12
DOCKED: ENDBRANCH   2  11
DOCKED: TORSDOF 5
DOCKED: TER
DOCKED: ENDMDL
________________________________________________________________________________


    BEGINNING GENETIC ALGORITHM DOCKING 7 of 15
Run: 7 Seed: 1194492545 619365546 [ Run 7 of 15 GA/GALS ]
Beginning LAMARCKIAN GENETIC ALGORITHM (LGA), with a maximum of 2500000 energy evaluations.

Final-Value: -10.816


    FINAL GENETIC ALGORITHM DOCKED STATE
    _______________________________________________
```

```
Detailed state:  trans 4.671 8.743 0.964 quatxyzw -0.216459 0.768794 0.559071 -0.222580 center -0.813 0.218 0.087 ntor 6 -40.2637 -98.1823 -84.8551 -75.7758 151.9959 -55.0117
State:    4.671   8.743   0.964  -0.222  0.789  0.573 -154.279    -40.26  -98.18  -84.86  -75.78  152.00  -55.01

DOCKED: MODEL        7
DOCKED: USER    Run = 7
DOCKED: USER    DPF = D:/Autodock/dock.dpf
DOCKED: USER
DOCKED: USER    Estimated Free Energy of Binding    =   -6.29 kcal/mol  [=(1)+(2)+(3)-(4)]
DOCKED: USER    Estimated Inhibition Constant, Ki   =   24.65 uM (micromolar)  [Temperature = 298.15 K]
DOCKED: USER
DOCKED: USER    (1) Final Intermolecular Energy     =   -7.78 kcal/mol
DOCKED: USER        vdW + Hbond + desolv Energy     =   -6.13 kcal/mol
DOCKED: USER        Electrostatic Energy            =   -1.65 kcal/mol
DOCKED: USER    (2) Final Total Internal Energy     =   -3.04 kcal/mol
DOCKED: USER    (3) Torsional Free Energy           =   +1.49 kcal/mol
DOCKED: USER    (4) Unbound System's Energy  [=(2)] =   -3.04 kcal/mol
DOCKED: USER
DOCKED: USER
DOCKED: USER    NEWDPF move ligand1.pdbqt
DOCKED: USER    NEWDPF about -0.813000 0.218000 0.087000
DOCKED: USER    NEWDPF tran0 4.671203 8.743212 0.964308
DOCKED: USER    NEWDPF quaternion0 -0.216459 0.768794 0.559071 -0.222580
DOCKED: USER    NEWDPF axisangle0 -0.222028 0.788576 0.573456 -154.278721
DOCKED: USER    NEWDPF quat0 -0.222028 0.788576 0.573456 -154.278721
DOCKED: USER    NEWDPF dihe0 -40.26 -98.18 -84.86 -75.78 152.00 -55.01
DOCKED: USER  keepresnum = 1
DOCKED: USER
DOCKED: REMARK  6 active torsions:
DOCKED: REMARK  status: ('A' for Active; 'I' for Inactive)
DOCKED: REMARK    1  A    between atoms: N_5  and  C_10
DOCKED: REMARK    2  A    between atoms: N_6  and  C_17
DOCKED: REMARK    3  A    between atoms: N_6  and  C_26
DOCKED: REMARK    4  A    between atoms: C_15  and  C_17
DOCKED: REMARK    5  A    between atoms: C_19  and  C_23
DOCKED: REMARK    6  A    between atoms: C_26  and  C_27
DOCKED: USER                              x       y       z     vdW  Elec       q    Type
DOCKED: USER                           _______ _______ _______ _____ _____    ______ ____
DOCKED: ROOT
DOCKED: ATOM      1  O   UNL d   1       1.676   9.623  -1.109 -0.02 +0.04    -0.312 OA
DOCKED: ATOM      2  C   UNL d   1       1.071   9.163  -2.243 -0.05 -0.01    +0.078 A
DOCKED: ATOM      3  C   UNL d   1      -0.295   9.027  -2.036 -0.06 +0.00    -0.018 A
DOCKED: ATOM      4  C   UNL d   1      -0.524   9.404  -0.675 -0.07 -0.00    +0.006 A
DOCKED: ATOM      5  C   UNL d   1       0.715   9.761  -0.160 -0.03 -0.01    +0.086 A
DOCKED: ATOM      6  C   UNL d   1      -1.322   8.579  -3.009 -0.08 -0.00    +0.052 C
DOCKED: ATOM      7  C   UNL d   1      -1.644   9.485   0.171 -0.09 -0.00    +0.015 A
DOCKED: ATOM      8  C   UNL d   1       0.927  10.201   1.144 -0.03 -0.01    +0.038 A
DOCKED: ATOM      9  C   UNL d   1      -1.465   9.924   1.492 -0.07 -0.00    +0.001 A
DOCKED: ATOM     10  C   UNL d   1      -0.200  10.276   1.968 -0.04 -0.00    +0.003 A
DOCKED: ENDROOT
DOCKED: BRANCH   2  11
DOCKED: ATOM     11  C   UNL d   1       1.965   8.945  -3.413 -0.06 -0.02    +0.191 C
DOCKED: BRANCH  11  12
DOCKED: ATOM     12  N   UNL d   1       3.232   8.337  -2.999 -0.07 +0.02    -0.347 N
```

```
DOCKED: ATOM     13  C   UNL d   1       4.427   9.145  -3.199 -0.18 -0.01    +0.153 C
DOCKED: BRANCH  12  14
DOCKED: ATOM     14  C   UNL d   1       3.414   7.015  -2.615 -0.21 -0.01    +0.243 C
DOCKED: ATOM     15  O   UNL d   1       3.849   6.158  -3.384 -0.12 +0.01    -0.268 OA
DOCKED: BRANCH  14  16
DOCKED: ATOM     16  C   UNL d   1       3.066   6.624  -1.217 -0.32 -0.00    +0.075 C
DOCKED: ATOM     17  C   UNL d   1       1.810   6.236  -0.941 -0.33 -0.00    +0.018 C
DOCKED: BRANCH  17  18
DOCKED: ATOM     18  C   UNL d   1       1.318   5.868   0.390 -0.38 +0.00    -0.018 A
DOCKED: ATOM     19  C   UNL d   1       1.499   6.747   1.459 -0.28 -0.01    +0.024 A
DOCKED: ATOM     20  C   UNL d   1       1.030   6.453   2.760 -0.35 +0.00    -0.011 A
DOCKED: ATOM     21  C   UNL d   1       1.324   7.490   3.804 -0.30 -0.03    +0.045 C
DOCKED: ATOM     22  C   UNL d   1       0.334   5.260   2.914 -0.35 -0.03    +0.136 A
DOCKED: ATOM     23  C   UNL d   1       1.358   7.024   5.251 -0.21 -0.06    +0.047 C
DOCKED: ATOM     24  C   UNL d   1      -0.034   6.656   5.714 -0.29 -0.27    +0.275 C
DOCKED: ATOM     25  C   UNL d   1      -0.448   5.250   5.310 -0.39 -0.11    +0.254 C
DOCKED: ATOM     26  O   UNL d   1      -1.009   4.559   6.172 -0.48 +0.10    -0.271 OA
DOCKED: ATOM     27  N   UNL d   1      -0.258   4.767   4.070 -0.24 +0.07    -0.309 N
DOCKED: ATOM     28  H   UNL d   1      -0.646   3.840   3.909 +0.13 -0.02    +0.170 HD
DOCKED: ATOM     29  N   UNL d   1       0.144   4.392   1.899 -0.35 +0.03    -0.242 NA
DOCKED: ATOM     30  C   UNL d   1       0.625   4.705   0.672 -0.29 -0.01    +0.130 A
DOCKED: BRANCH  24  31
DOCKED: ATOM     31  N   UNL d   1      -0.060   6.723   7.227 -0.10 +0.10    -0.066 N
DOCKED: ATOM     32  H   UNL d   1       0.532   7.460   7.624 -0.14 -0.76    +0.275 HD
DOCKED: ATOM     33  H   UNL d   1       0.181   5.817   7.650 +0.00 -0.29    +0.275 HD
DOCKED: ATOM     34  H   UNL d   1      -1.041   6.890   7.511 -0.29 -0.35    +0.275 HD
DOCKED: ENDBRANCH  24  31
DOCKED: ENDBRANCH  17  18
DOCKED: ENDBRANCH  14  16
DOCKED: ENDBRANCH  12  14
DOCKED: ENDBRANCH  11  12
DOCKED: ENDBRANCH   2  11
DOCKED: TORSDOF 5
DOCKED: TER
DOCKED: ENDMDL
________________________________________________________________________________


    BEGINNING GENETIC ALGORITHM DOCKING 8 of 15
Run: 8 Seed: 1721432613 27074660 [ Run 8 of 15 GA/GALS ]
Beginning LAMARCKIAN GENETIC ALGORITHM (LGA), with a maximum of 2500000 energy evaluations.

Final-Value: -11.573


    FINAL GENETIC ALGORITHM DOCKED STATE
    _______________________________________________


Detailed state:  trans 1.156 5.624 0.454 quatxyzw 0.686524 0.509852 0.182105
```

```
-0.485359 center -0.813 0.218 0.087 ntor 6 -48.6130 134.6579 102.4467 -46.5323 173.6317 -117.7786
State:    1.156   5.624   0.454   0.785  0.583  0.208 -121.928    -48.61  134.66  102.45  -46.53  173.63 -117.78

DOCKED: MODEL        8
DOCKED: USER    Run = 8
DOCKED: USER    DPF = D:/Autodock/dock.dpf
DOCKED: USER
DOCKED: USER    Estimated Free Energy of Binding    =   -7.50 kcal/mol [=(1)+(2)+(3)-(4)]
DOCKED: USER    Estimated Inhibition Constant, Ki   =    3.21 uM (micromolar) [Temperature = 298.15 K]
DOCKED: USER
DOCKED: USER    (1) Final Intermolecular Energy     =   -8.99 kcal/mol
DOCKED: USER        vdW + Hbond + desolv Energy     =   -7.27 kcal/mol
DOCKED: USER        Electrostatic Energy            =   -1.71 kcal/mol
DOCKED: USER    (2) Final Total Internal Energy     =   -2.59 kcal/mol
DOCKED: USER    (3) Torsional Free Energy           =   +1.49 kcal/mol
DOCKED: USER    (4) Unbound System's Energy  [=(2)] =   -2.59 kcal/mol
DOCKED: USER
DOCKED: USER
DOCKED: USER    NEWDPF move ligand1.pdbqt
DOCKED: USER    NEWDPF about -0.813000 0.218000 0.087000
DOCKED: USER    NEWDPF tran0 1.155532 5.624100 0.454476
DOCKED: USER    NEWDPF quaternion0 0.686524 0.509852 0.182105 -0.485359
DOCKED: USER    NEWDPF axisangle0 0.785214 0.583145 0.208283 -121.928045
DOCKED: USER    NEWDPF quat0 0.785214 0.583145 0.208283 -121.928045
DOCKED: USER    NEWDPF dihe0 -48.61 134.66 102.45 -46.53 173.63 -117.78
DOCKED: USER  keepresnum = 1
DOCKED: USER
DOCKED: REMARK  6 active torsions:
DOCKED: REMARK  status: ('A' for Active; 'I' for Inactive)
DOCKED: REMARK    1  A    between atoms: N_5  and  C_10
DOCKED: REMARK    2  A    between atoms: N_6  and  C_17
DOCKED: REMARK    3  A    between atoms: N_6  and  C_26
DOCKED: REMARK    4  A    between atoms: C_15  and  C_17
DOCKED: REMARK    5  A    between atoms: C_19  and  C_23
DOCKED: REMARK    6  A    between atoms: C_26  and  C_27
DOCKED: USER                              x       y       z     vdW  Elec       q    Type
DOCKED: USER                           _______ _______ _______ _____ _____    ______ ____
DOCKED: ROOT
DOCKED: ATOM      1  O   UNL d   1       3.669   8.203  -0.584 -0.11 +0.03    -0.312 OA
DOCKED: ATOM      2  C   UNL d   1       3.685   9.342  -1.336 -0.11 -0.01    +0.078 A
DOCKED: ATOM      3  C   UNL d   1       4.332  10.360  -0.651 -0.11 +0.00    -0.018 A
DOCKED: ATOM      4  C   UNL d   1       4.707   9.804   0.613 -0.18 -0.00    +0.006 A
DOCKED: ATOM      5  C   UNL d   1       4.279   8.483   0.596 -0.16 -0.01    +0.086 A
DOCKED: ATOM      6  C   UNL d   1       4.588  11.747  -1.112 -0.06 -0.01    +0.052 C
DOCKED: ATOM      7  C   UNL d   1       5.365  10.275   1.764 -0.18 -0.00    +0.015 A
DOCKED: ATOM      8  C   UNL d   1       4.460   7.585   1.644 -0.24 -0.01    +0.038 A
DOCKED: ATOM      9  C   UNL d   1       5.563   9.395   2.839 -0.24 -0.00    +0.001 A
DOCKED: ATOM     10  C   UNL d   1       5.117   8.072   2.778 -0.36 -0.00    +0.003 A
DOCKED: ENDROOT
DOCKED: BRANCH   2  11
DOCKED: ATOM     11  C   UNL d   1       3.057   9.240  -2.682 -0.08 -0.02    +0.191 C
DOCKED: BRANCH  11  12
DOCKED: ATOM     12  N   UNL d   1       3.006   7.846  -3.133 -0.08 +0.02    -0.347 N
DOCKED: ATOM     13  C   UNL d   1       4.116   7.401  -3.963 -0.25 -0.01    +0.153 C
```

```
DOCKED: BRANCH  12  14
DOCKED: ATOM     14  C   UNL d   1       1.925   6.985  -2.998 -0.17 -0.01    +0.243 C
DOCKED: ATOM     15  O   UNL d   1       1.458   6.359  -3.949 -0.16 +0.01    -0.268 OA
DOCKED: BRANCH  14  16
DOCKED: ATOM     16  C   UNL d   1       1.310   6.819  -1.649 -0.24 -0.00    +0.075 C
DOCKED: ATOM     17  C   UNL d   1       1.848   5.936  -0.790 -0.33 -0.00    +0.018 C
DOCKED: BRANCH  17  18
DOCKED: ATOM     18  C   UNL d   1       1.325   5.640   0.547 -0.39 +0.00    -0.018 A
DOCKED: ATOM     19  C   UNL d   1       1.502   6.564   1.577 -0.30 -0.01    +0.024 A
DOCKED: ATOM     20  C   UNL d   1       1.003   6.340   2.881 -0.36 +0.00    -0.011 A
DOCKED: ATOM     21  C   UNL d   1       1.296   7.420   3.881 -0.31 -0.03    +0.045 C
DOCKED: ATOM     22  C   UNL d   1       0.281   5.168   3.076 -0.33 -0.03    +0.136 A
DOCKED: ATOM     23  C   UNL d   1       1.293   7.022   5.349 -0.15 -0.07    +0.047 C
DOCKED: ATOM     24  C   UNL d   1      -0.114   6.700   5.800 -0.28 -0.28    +0.275 C
DOCKED: ATOM     25  C   UNL d   1      -0.546   5.284   5.454 -0.40 -0.11    +0.254 C
DOCKED: ATOM     26  O   UNL d   1      -1.137   4.645   6.335 -0.48 +0.10    -0.271 OA
DOCKED: ATOM     27  N   UNL d   1      -0.342   4.741   4.242 -0.26 +0.08    -0.309 N
DOCKED: ATOM     28  H   UNL d   1      -0.744   3.815   4.117 +0.13 -0.02    +0.170 HD
DOCKED: ATOM     29  N   UNL d   1       0.094   4.257   2.100 -0.22 +0.03    -0.242 NA
DOCKED: ATOM     30  C   UNL d   1       0.605   4.504   0.869 -0.37 -0.01    +0.130 A
DOCKED: BRANCH  24  31
DOCKED: ATOM     31  N   UNL d   1      -0.167   6.839   7.308 +0.17 +0.10    -0.066 N
DOCKED: ATOM     32  H   UNL d   1       0.526   7.487   7.694 -0.17 -0.77    +0.275 HD
DOCKED: ATOM     33  H   UNL d   1      -0.089   5.926   7.775 -0.15 -0.31    +0.275 HD
DOCKED: ATOM     34  H   UNL d   1      -1.115   7.175   7.552 -0.33 -0.38    +0.275 HD
DOCKED: ENDBRANCH  24  31
DOCKED: ENDBRANCH  17  18
DOCKED: ENDBRANCH  14  16
DOCKED: ENDBRANCH  12  14
DOCKED: ENDBRANCH  11  12
DOCKED: ENDBRANCH   2  11
DOCKED: TORSDOF 5
DOCKED: TER
DOCKED: ENDMDL
________________________________________________________________________________


    BEGINNING GENETIC ALGORITHM DOCKING 9 of 15
Run: 9 Seed: 197390380 893244483 [ Run 9 of 15 GA/GALS ]
Beginning LAMARCKIAN GENETIC ALGORITHM (LGA), with a maximum of 2500000 energy evaluations.

Final-Value: -10.340


    FINAL GENETIC ALGORITHM DOCKED STATE
    ______________________________________________


Detailed state:  trans 6.387 7.630 -3.501 quatxyzw 0.201183 0.876647 0.409328 0.153186 center -0.813 0.218 0.087 ntor 6 -3.5213 -54.6361 173.0281 79.6463 82.2290 -36.2414
```

```
State:    6.387   7.630  -3.501   0.204  0.887  0.414 162.377     -3.52  -54.64  173.03   79.65   82.23  -36.24

DOCKED: MODEL        9
DOCKED: USER    Run = 9
DOCKED: USER    DPF = D:/Autodock/dock.dpf
DOCKED: USER
DOCKED: USER    Estimated Free Energy of Binding    =   -6.34 kcal/mol  [=(1)+(2)+(3)-(4)]
DOCKED: USER    Estimated Inhibition Constant, Ki   =   22.39 uM (micromolar)  [Temperature = 298.15 K]
DOCKED: USER
DOCKED: USER    (1) Final Intermolecular Energy     =   -7.84 kcal/mol
DOCKED: USER        vdW + Hbond + desolv Energy     =   -6.24 kcal/mol
DOCKED: USER        Electrostatic Energy            =   -1.60 kcal/mol
DOCKED: USER    (2) Final Total Internal Energy     =   -2.50 kcal/mol
DOCKED: USER    (3) Torsional Free Energy           =   +1.49 kcal/mol
DOCKED: USER    (4) Unbound System's Energy  [=(2)] =   -2.50 kcal/mol
DOCKED: USER
DOCKED: USER
DOCKED: USER    NEWDPF move ligand1.pdbqt
DOCKED: USER    NEWDPF about -0.813000 0.218000 0.087000
DOCKED: USER    NEWDPF tran0 6.387047 7.629937 -3.500876
DOCKED: USER    NEWDPF quaternion0 0.201183 0.876647 0.409328 0.153186
DOCKED: USER    NEWDPF axisangle0 0.203586 0.887118 0.414216 162.376826
DOCKED: USER    NEWDPF quat0 0.203586 0.887118 0.414216 162.376826
DOCKED: USER    NEWDPF dihe0 -3.52 -54.64 173.03 79.65 82.23 -36.24
DOCKED: USER  keepresnum = 1
DOCKED: USER
DOCKED: REMARK  6 active torsions:
DOCKED: REMARK  status: ('A' for Active; 'I' for Inactive)
DOCKED: REMARK    1  A    between atoms: N_5  and  C_10
DOCKED: REMARK    2  A    between atoms: N_6  and  C_17
DOCKED: REMARK    3  A    between atoms: N_6  and  C_26
DOCKED: REMARK    4  A    between atoms: C_15  and  C_17
DOCKED: REMARK    5  A    between atoms: C_19  and  C_23
DOCKED: REMARK    6  A    between atoms: C_26  and  C_27
DOCKED: USER                              x       y       z     vdW  Elec       q    Type
DOCKED: USER                           _______ _______ _______ _____ _____    ______ ____
DOCKED: ROOT
DOCKED: ATOM      1  O   UNL d   1       3.366   9.543  -2.381 -0.04 +0.03    -0.312 OA
DOCKED: ATOM      2  C   UNL d   1       2.040   9.292  -2.171 -0.05 -0.01    +0.078 A
DOCKED: ATOM      3  C   UNL d   1       1.645   9.799  -0.941 -0.04 +0.00    -0.018 A
DOCKED: ATOM      4  C   UNL d   1       2.819  10.369  -0.358 -0.04 -0.00    +0.006 A
DOCKED: ATOM      5  C   UNL d   1       3.838  10.187  -1.283 -0.07 -0.01    +0.086 A
DOCKED: ATOM      6  C   UNL d   1       0.291   9.762  -0.335 -0.05 -0.01    +0.052 C
DOCKED: ATOM      7  C   UNL d   1       3.127  11.024   0.849 -0.03 -0.00    +0.015 A
DOCKED: ATOM      8  C   UNL d   1       5.152  10.612  -1.101 -0.15 -0.00    +0.038 A
DOCKED: ATOM      9  C   UNL d   1       4.442  11.463   1.065 -0.08 -0.00    +0.001 A
DOCKED: ATOM     10  C   UNL d   1       5.436  11.261   0.105 -0.15 -0.00    +0.003 A
DOCKED: ENDROOT
DOCKED: BRANCH   2  11
DOCKED: ATOM     11  C   UNL d   1       1.331   8.585  -3.273 -0.07 -0.01    +0.191 C
DOCKED: BRANCH  11  12
DOCKED: ATOM     12  N   UNL d   1       1.941   7.279  -3.536 -0.08 +0.02    -0.347 N
DOCKED: ATOM     13  C   UNL d   1       1.260   6.440  -4.513 -0.21 -0.00    +0.153 C
DOCKED: BRANCH  12  14
DOCKED: ATOM     14  C   UNL d   1       2.980   6.705  -2.816 -0.21 -0.01    +0.243
```

```
C
DOCKED: ATOM     15  O   UNL d   1       4.127   6.635  -3.255 -0.09 +0.01    -0.268
OA
DOCKED: BRANCH  14  16
DOCKED: ATOM     16  C   UNL d   1       2.687   6.151  -1.462 -0.27 -0.00    +0.075
C
DOCKED: ATOM     17  C   UNL d   1       1.525   6.465  -0.864 -0.32 -0.00    +0.018
C
DOCKED: BRANCH  17  18
DOCKED: ATOM     18  C   UNL d   1       1.126   6.029   0.478 -0.37 +0.00    -0.018
A
DOCKED: ATOM     19  C   UNL d   1       1.327   6.880   1.566 -0.27 -0.01    +0.024
A
DOCKED: ATOM     20  C   UNL d   1       0.946   6.521   2.879 -0.34 +0.00    -0.011
A
DOCKED: ATOM     21  C   UNL d   1       1.250   7.536   3.942 -0.31 -0.04    +0.045
C
DOCKED: ATOM     22  C   UNL d   1       0.314   5.292   3.028 -0.35 -0.03    +0.136
A
DOCKED: ATOM     23  C   UNL d   1       1.386   7.024   5.368 -0.10 -0.07    +0.047
C
DOCKED: ATOM     24  C   UNL d   1       0.041   6.575   5.892 -0.30 -0.28    +0.275
C
DOCKED: ATOM     25  C   UNL d   1      -0.330   5.165   5.461 -0.39 -0.11    +0.254
C
DOCKED: ATOM     26  O   UNL d   1      -0.810   4.420   6.327 -0.50 +0.10    -0.271
OA
DOCKED: ATOM     27  N   UNL d   1      -0.189   4.733   4.196 -0.24 +0.08    -0.309
N
DOCKED: ATOM     28  H   UNL d   1      -0.542   3.795   4.024 +0.14 -0.02    +0.170
HD
DOCKED: ATOM     29  N   UNL d   1       0.107   4.450   1.995 -0.34 +0.03    -0.242
NA
DOCKED: ATOM     30  C   UNL d   1       0.504   4.827   0.755 -0.35 -0.01    +0.130
A
DOCKED: BRANCH  24  31
DOCKED: ATOM     31  N   UNL d   1       0.097   6.590   7.406 -0.19 +0.10    -0.066
N
DOCKED: ATOM     32  H   UNL d   1       0.117   7.530   7.815 -0.33 -0.70    +0.275
HD
DOCKED: ATOM     33  H   UNL d   1       0.889   6.038   7.761 +0.09 -0.34    +0.275
HD
DOCKED: ATOM     34  H   UNL d   1      -0.742   6.092   7.750 -0.15 -0.30    +0.275
HD
DOCKED: ENDBRANCH  24  31
DOCKED: ENDBRANCH  17  18
DOCKED: ENDBRANCH  14  16
DOCKED: ENDBRANCH  12  14
DOCKED: ENDBRANCH  11  12
DOCKED: ENDBRANCH   2  11
DOCKED: TORSDOF 5
DOCKED: TER
DOCKED: ENDMDL
________________________________________________________________________________


    BEGINNING GENETIC ALGORITHM DOCKING 10 of 15
Run: 10 Seed: 1693290148 421878558 [ Run 10 of 15 GA/GALS ]
Beginning LAMARCKIAN GENETIC ALGORITHM (LGA), with a maximum of 2500000 energy
evaluations.

Final-Value: -11.371


    FINAL GENETIC ALGORITHM DOCKED STATE
    _______________________________________________


Detailed state:  trans 5.667 4.733 0.539 quatxyzw -0.289167 -0.694116 -0.512370
0.414804 center -0.813 0.218 0.087 ntor 6 -175.6151 169.4804 70.4719 61.6172 10.2196
37.2973
State:    5.667   4.733   0.539  -0.318 -0.763 -0.563 130.986   -175.62  169.48
70.47   61.62   10.22   37.30
```

```
DOCKED: MODEL       10
DOCKED: USER    Run = 10
DOCKED: USER    DPF = D:/Autodock/dock.dpf
DOCKED: USER
DOCKED: USER    Estimated Free Energy of Binding    =   -7.07 kcal/mol  [=(1)+(2)+(3)-(4)]
DOCKED: USER    Estimated Inhibition Constant, Ki   =    6.52 uM (micromolar)  [Temperature = 298.15 K]
DOCKED: USER
DOCKED: USER    (1) Final Intermolecular Energy     =   -8.57 kcal/mol
DOCKED: USER        vdW + Hbond + desolv Energy     =   -6.86 kcal/mol
DOCKED: USER        Electrostatic Energy            =   -1.71 kcal/mol
DOCKED: USER    (2) Final Total Internal Energy     =   -2.80 kcal/mol
DOCKED: USER    (3) Torsional Free Energy           =   +1.49 kcal/mol
DOCKED: USER    (4) Unbound System's Energy  [=(2)] =   -2.80 kcal/mol
DOCKED: USER
DOCKED: USER
DOCKED: USER    NEWDPF move ligand1.pdbqt
DOCKED: USER    NEWDPF about -0.813000 0.218000 0.087000
DOCKED: USER    NEWDPF tran0 5.666810 4.733308 0.539287
DOCKED: USER    NEWDPF quaternion0 -0.289167 -0.694116 -0.512370 0.414804
DOCKED: USER    NEWDPF axisangle0 -0.317797 -0.762840 -0.563099 130.986055
DOCKED: USER    NEWDPF quat0 -0.317797 -0.762840 -0.563099 130.986055
DOCKED: USER    NEWDPF dihe0 -175.62 169.48 70.47 61.62 10.22 37.30
DOCKED: USER  keepresnum = 1
DOCKED: USER
DOCKED: REMARK  6 active torsions:
DOCKED: REMARK  status: ('A' for Active; 'I' for Inactive)
DOCKED: REMARK    1  A    between atoms: N_5  and  C_10
DOCKED: REMARK    2  A    between atoms: N_6  and  C_17
DOCKED: REMARK    3  A    between atoms: N_6  and  C_26
DOCKED: REMARK    4  A    between atoms: C_15  and  C_17
DOCKED: REMARK    5  A    between atoms: C_19  and  C_23
DOCKED: REMARK    6  A    between atoms: C_26  and  C_27
DOCKED: USER                                 x       y       z     vdW  Elec       q    Type
DOCKED: USER                              _______ _______ _______ _____ _____    ______ ____
DOCKED: ROOT
DOCKED: ATOM      1  O   UNL d   1       4.891   8.321  -0.213 -0.03 +0.03    -0.312 OA
DOCKED: ATOM      2  C   UNL d   1       4.161   9.018  -1.133 -0.16 -0.01    +0.078 A
DOCKED: ATOM      3  C   UNL d   1       3.371   9.968  -0.498 -0.07 +0.00    -0.018 A
DOCKED: ATOM      4  C   UNL d   1       3.621   9.812   0.902 -0.09 -0.00    +0.006 A
DOCKED: ATOM      5  C   UNL d   1       4.560   8.795   1.015 -0.18 -0.02    +0.086 A
DOCKED: ATOM      6  C   UNL d   1       2.447  10.946  -1.123 -0.02 -0.01    +0.052 C
DOCKED: ATOM      7  C   UNL d   1       3.160  10.426   2.081 -0.07 -0.00    +0.015 A
DOCKED: ATOM      8  C   UNL d   1       5.082   8.339   2.223 -0.30 -0.01    +0.038 A
DOCKED: ATOM      9  C   UNL d   1       3.665   9.988   3.314 -0.17 -0.00    +0.001 A
DOCKED: ATOM     10  C   UNL d   1       4.610   8.963   3.383 -0.30 -0.00    +0.003 A
DOCKED: ENDROOT
DOCKED: BRANCH   2  11
DOCKED: ATOM     11  C   UNL d   1       4.383   8.645  -2.556 -0.19 -0.01    +0.191 C
DOCKED: BRANCH  11  12
DOCKED: ATOM     12  N   UNL d   1       3.109   8.482  -3.261 -0.05 +0.02    -0.347 N
DOCKED: ATOM     13  C   UNL d   1       2.714   9.581  -4.131 -0.06 -0.01    +0.153 C
DOCKED: BRANCH  12  14
DOCKED: ATOM     14  C   UNL d   1       2.175   7.480  -3.035 -0.14 -0.01    +0.243 C
DOCKED: ATOM     15  O   UNL d   1       1.780   6.732  -3.929 -0.15 +0.01    -0.268
```

```
OA
DOCKED: BRANCH  14  16
DOCKED: ATOM     16  C   UNL d   1       1.632   7.310  -1.656 -0.20 -0.01    +0.075
C
DOCKED: ATOM     17  C   UNL d   1       1.874   6.165  -0.995 -0.33 -0.00    +0.018
C
DOCKED: BRANCH  17  18
DOCKED: ATOM     18  C   UNL d   1       1.368   5.845   0.344 -0.37 +0.00    -0.018
A
DOCKED: ATOM     19  C   UNL d   1       1.326   6.841   1.320 -0.27 -0.00    +0.024
A
DOCKED: ATOM     20  C   UNL d   1       0.832   6.595   2.622 -0.33 +0.00    -0.011
A
DOCKED: ATOM     21  C   UNL d   1       0.875   7.764   3.562 -0.27 -0.03    +0.045
C
DOCKED: ATOM     22  C   UNL d   1       0.342   5.317   2.869 -0.35 -0.03    +0.136
A
DOCKED: ATOM     23  C   UNL d   1       0.895   7.452   5.051 -0.17 -0.06    +0.047
C
DOCKED: ATOM     24  C   UNL d   1      -0.439   6.889   5.485 -0.28 -0.24    +0.275
C
DOCKED: ATOM     25  C   UNL d   1      -0.578   5.399   5.214 -0.39 -0.11    +0.254
C
DOCKED: ATOM     26  O   UNL d   1      -1.067   4.706   6.116 -0.47 +0.10    -0.271
OA
DOCKED: ATOM     27  N   UNL d   1      -0.229   4.841   4.042 -0.25 +0.08    -0.309
N
DOCKED: ATOM     28  H   UNL d   1      -0.440   3.849   3.963 +0.14 -0.02    +0.170
HD
DOCKED: ATOM     29  N   UNL d   1       0.371   4.336   1.944 -0.35 +0.03    -0.242
NA
DOCKED: ATOM     30  C   UNL d   1       0.869   4.610   0.715 -0.25 -0.01    +0.130
A
DOCKED: BRANCH  24  31
DOCKED: ATOM     31  N   UNL d   1      -0.574   7.096   6.979 -0.31 +0.09    -0.066
N
DOCKED: ATOM     32  H   UNL d   1      -0.376   6.260   7.538 -0.15 -0.33    +0.275
HD
DOCKED: ATOM     33  H   UNL d   1      -1.499   7.470   7.227 -0.14 -0.30    +0.275
HD
DOCKED: ATOM     34  H   UNL d   1       0.089   7.844   7.247 -0.14 -0.84    +0.275
HD
DOCKED: ENDBRANCH  24  31
DOCKED: ENDBRANCH  17  18
DOCKED: ENDBRANCH  14  16
DOCKED: ENDBRANCH  12  14
DOCKED: ENDBRANCH  11  12
DOCKED: ENDBRANCH   2  11
DOCKED: TORSDOF 5
DOCKED: TER
DOCKED: ENDMDL
________________________________________________________________________________


    BEGINNING GENETIC ALGORITHM DOCKING 11 of 15
Run: 11 Seed: 148451833 1661404380 [ Run 11 of 15 GA/GALS ]
Beginning LAMARCKIAN GENETIC ALGORITHM (LGA), with a maximum of 2500000 energy
evaluations.

Final-Value: -12.015


    FINAL GENETIC ALGORITHM DOCKED STATE
    _______________________________________________


Detailed state:  trans 2.731 3.531 -0.709 quatxyzw -0.516214 -0.559986 -0.411898
0.500279 center -0.813 0.218 0.087 ntor 6 -34.3438 163.6345 151.1719 -65.3313
132.1275 -27.3305
State:    2.731   3.531  -0.709  -0.596 -0.647 -0.476 119.963    -34.34  163.63
151.17  -65.33  132.13  -27.33

DOCKED: MODEL       11
```

```
DOCKED: USER    Run = 11
DOCKED: USER    DPF = D:/Autodock/dock.dpf
DOCKED: USER
DOCKED: USER    Estimated Free Energy of Binding    =   -7.50 kcal/mol [=(1)+(2)+(3)-(4)]
DOCKED: USER    Estimated Inhibition Constant, Ki   =    3.16 uM (micromolar) [Temperature = 298.15 K]
DOCKED: USER
DOCKED: USER    (1) Final Intermolecular Energy     =   -9.00 kcal/mol
DOCKED: USER        vdW + Hbond + desolv Energy     =   -7.37 kcal/mol
DOCKED: USER        Electrostatic Energy            =   -1.63 kcal/mol
DOCKED: USER    (2) Final Total Internal Energy     =   -3.02 kcal/mol
DOCKED: USER    (3) Torsional Free Energy           =   +1.49 kcal/mol
DOCKED: USER    (4) Unbound System's Energy  [=(2)] =   -3.02 kcal/mol
DOCKED: USER
DOCKED: USER
DOCKED: USER    NEWDPF move ligand1.pdbqt
DOCKED: USER    NEWDPF about -0.813000 0.218000 0.087000
DOCKED: USER    NEWDPF tran0 2.731090 3.531301 -0.708772
DOCKED: USER    NEWDPF quaternion0 -0.516214 -0.559986 -0.411898 0.500279
DOCKED: USER    NEWDPF axisangle0 -0.596183 -0.646737 -0.475707 119.963095
DOCKED: USER    NEWDPF quat0 -0.596183 -0.646737 -0.475707 119.963095
DOCKED: USER    NEWDPF dihe0 -34.34 163.63 151.17 -65.33 132.13 -27.33
DOCKED: USER  keepresnum = 1
DOCKED: USER
DOCKED: REMARK  6 active torsions:
DOCKED: REMARK  status: ('A' for Active; 'I' for Inactive)
DOCKED: REMARK    1  A    between atoms: N_5  and  C_10
DOCKED: REMARK    2  A    between atoms: N_6  and  C_17
DOCKED: REMARK    3  A    between atoms: N_6  and  C_26
DOCKED: REMARK    4  A    between atoms: C_15  and  C_17
DOCKED: REMARK    5  A    between atoms: C_19  and  C_23
DOCKED: REMARK    6  A    between atoms: C_26  and  C_27
DOCKED: USER                              x       y       z     vdW  Elec       q    Type
DOCKED: USER                           _______ _______ _______ _____ _____    ______ ____
DOCKED: ROOT
DOCKED: ATOM      1  O   UNL d   1       4.015   7.042  -0.969 -0.20 +0.02    -0.312 OA
DOCKED: ATOM      2  C   UNL d   1       3.763   8.149  -1.728 -0.19 -0.01    +0.078 A
DOCKED: ATOM      3  C   UNL d   1       3.628   9.267  -0.917 -0.12 +0.00    -0.018 A
DOCKED: ATOM      4  C   UNL d   1       3.781   8.796   0.426 -0.16 -0.00    +0.006 A
DOCKED: ATOM      5  C   UNL d   1       4.018   7.432   0.331 -0.18 -0.01    +0.086 A
DOCKED: ATOM      6  C   UNL d   1       3.372  10.668  -1.334 -0.05 -0.01    +0.052 C
DOCKED: ATOM      7  C   UNL d   1       3.750   9.379   1.706 -0.13 -0.00    +0.015 A
DOCKED: ATOM      8  C   UNL d   1       4.232   6.594   1.423 -0.16 -0.01    +0.038 A
DOCKED: ATOM      9  C   UNL d   1       3.961   8.563   2.827 -0.24 -0.00    +0.001 A
DOCKED: ATOM     10  C   UNL d   1       4.198   7.193   2.686 -0.33 -0.00    +0.003 A
DOCKED: ENDROOT
DOCKED: BRANCH   2  11
DOCKED: ATOM     11  C   UNL d   1       3.718   7.930  -3.200 -0.20 -0.01    +0.191 C
DOCKED: BRANCH  11  12
DOCKED: ATOM     12  N   UNL d   1       2.547   7.134  -3.577 -0.10 +0.02    -0.347 N
DOCKED: ATOM     13  C   UNL d   1       2.781   6.064  -4.536 -0.24 -0.00    +0.153 C
DOCKED: BRANCH  12  14
DOCKED: ATOM     14  C   UNL d   1       1.228   7.421  -3.252 -0.12 -0.01    +0.243 C
DOCKED: ATOM     15  O   UNL d   1       0.410   7.802  -4.088 -0.06 +0.02    -0.268 OA
DOCKED: BRANCH  14  16
```

```
DOCKED: ATOM     16  C   UNL d   1       0.793   7.263  -1.833 -0.20 -0.01    +0.075 C
DOCKED: ATOM     17  C   UNL d   1       1.310   6.263  -1.099 -0.31 -0.00    +0.018 C
DOCKED: BRANCH  17  18
DOCKED: ATOM     18  C   UNL d   1       0.943   5.964   0.289 -0.37 +0.00    -0.018 A
DOCKED: ATOM     19  C   UNL d   1       0.841   7.004   1.214 -0.26 -0.00    +0.024 A
DOCKED: ATOM     20  C   UNL d   1       0.473   6.777   2.560 -0.30 +0.00    -0.011 A
DOCKED: ATOM     21  C   UNL d   1       0.431   7.997   3.434 -0.24 -0.03    +0.045 C
DOCKED: ATOM     22  C   UNL d   1       0.169   5.466   2.909 -0.30 -0.03    +0.136 A
DOCKED: ATOM     23  C   UNL d   1       0.593   7.780   4.930 -0.10 -0.06    +0.047 C
DOCKED: ATOM     24  C   UNL d   1      -0.625   7.083   5.493 -0.27 -0.24    +0.275 C
DOCKED: ATOM     25  C   UNL d   1      -0.590   5.574   5.309 -0.38 -0.12    +0.254 C
DOCKED: ATOM     26  O   UNL d   1      -0.922   4.881   6.281 -0.45 +0.11    -0.271 OA
DOCKED: ATOM     27  N   UNL d   1      -0.254   4.994   4.144 -0.25 +0.08    -0.309 N
DOCKED: ATOM     28  H   UNL d   1      -0.341   3.980   4.132 +0.11 -0.03    +0.170 HD
DOCKED: ATOM     29  N   UNL d   1       0.259   4.442   2.036 -0.36 +0.03    -0.242 NA
DOCKED: ATOM     30  C   UNL d   1       0.632   4.702   0.759 -0.33 -0.01    +0.130 A
DOCKED: BRANCH  24  31
DOCKED: ATOM     31  N   UNL d   1      -0.682   7.361   6.981 -0.23 +0.10    -0.066 N
DOCKED: ATOM     32  H   UNL d   1      -0.436   8.322   7.237 -0.27 -0.74    +0.275 HD
DOCKED: ATOM     33  H   UNL d   1      -0.101   6.699   7.512 -0.22 -0.42    +0.275 HD
DOCKED: ATOM     34  H   UNL d   1      -1.649   7.166   7.294 -0.13 -0.26    +0.275 HD
DOCKED: ENDBRANCH  24  31
DOCKED: ENDBRANCH  17  18
DOCKED: ENDBRANCH  14  16
DOCKED: ENDBRANCH  12  14
DOCKED: ENDBRANCH  11  12
DOCKED: ENDBRANCH   2  11
DOCKED: TORSDOF 5
DOCKED: TER
DOCKED: ENDMDL
________________________________________________________________________________


        BEGINNING GENETIC ALGORITHM DOCKING 12 of 15
Run: 12 Seed: 1866504810 1375020577 [ Run 12 of 15 GA/GALS ]
Beginning LAMARCKIAN GENETIC ALGORITHM (LGA), with a maximum of 2500000 energy evaluations.

Final-Value: -10.036


        FINAL GENETIC ALGORITHM DOCKED STATE
        _______________________________________________


Detailed state:  trans 6.616 4.910 -0.391 quatxyzw -0.025615 0.698664 0.697877 -0.155500 center -0.813 0.218 0.087 ntor 6 177.4309 -89.2833 173.7197 94.0712 43.5323 178.7420
State:    6.616   4.910  -0.391  -0.026  0.707  0.706 -162.108    177.43  -89.28 173.72   94.07   43.53  178.74

DOCKED: MODEL        12
DOCKED: USER    Run = 12
DOCKED: USER    DPF = D:/Autodock/dock.dpf
```

```
DOCKED: USER
DOCKED: USER    Estimated Free Energy of Binding    =   -5.82 kcal/mol  [=(1)+(2)+(3)-(4)]
DOCKED: USER    Estimated Inhibition Constant, Ki   =   54.45 uM (micromolar)  [Temperature = 298.15 K]
DOCKED: USER
DOCKED: USER    (1) Final Intermolecular Energy     =   -7.31 kcal/mol
DOCKED: USER        vdW + Hbond + desolv Energy     =   -6.09 kcal/mol
DOCKED: USER        Electrostatic Energy            =   -1.22 kcal/mol
DOCKED: USER    (2) Final Total Internal Energy     =   -2.73 kcal/mol
DOCKED: USER    (3) Torsional Free Energy           =   +1.49 kcal/mol
DOCKED: USER    (4) Unbound System's Energy  [=(2)] =   -2.73 kcal/mol
DOCKED: USER
DOCKED: USER
DOCKED: USER    NEWDPF move ligand1.pdbqt
DOCKED: USER    NEWDPF about -0.813000 0.218000 0.087000
DOCKED: USER    NEWDPF tran0 6.615514 4.910440 -0.391263
DOCKED: USER    NEWDPF quaternion0 -0.025615 0.698664 0.697877 -0.155500
DOCKED: USER    NEWDPF axisangle0 -0.025931 0.707267 0.706470 -162.108374
DOCKED: USER    NEWDPF quat0 -0.025931 0.707267 0.706470 -162.108374
DOCKED: USER    NEWDPF dihe0 177.43 -89.28 173.72 94.07 43.53 178.74
DOCKED: USER  keepresnum = 1
DOCKED: USER
DOCKED: REMARK  6 active torsions:
DOCKED: REMARK  status: ('A' for Active; 'I' for Inactive)
DOCKED: REMARK    1  A    between atoms: N_5  and  C_10
DOCKED: REMARK    2  A    between atoms: N_6  and  C_17
DOCKED: REMARK    3  A    between atoms: N_6  and  C_26
DOCKED: REMARK    4  A    between atoms: C_15  and  C_17
DOCKED: REMARK    5  A    between atoms: C_19  and  C_23
DOCKED: REMARK    6  A    between atoms: C_26  and  C_27
DOCKED: USER                              x       y       z     vdW  Elec       q    Type
DOCKED: USER                           _______ _______ _______ _____ _____    ______ ____
DOCKED: ROOT
DOCKED: ATOM      1  O   UNL d   1       3.336   6.659  -0.872 -0.21 +0.02    -0.312 OA
DOCKED: ATOM      2  C   UNL d   1       2.334   6.674  -1.799 -0.24 -0.00    +0.078 A
DOCKED: ATOM      3  C   UNL d   1       1.100   6.558  -1.174 -0.27 +0.00    -0.018 A
DOCKED: ATOM      4  C   UNL d   1       1.380   6.436   0.224 -0.32 -0.00    +0.006 A
DOCKED: ATOM      5  C   UNL d   1       2.761   6.507   0.348 -0.26 -0.01    +0.086 A
DOCKED: ATOM      6  C   UNL d   1      -0.243   6.553  -1.805 -0.24 -0.00    +0.052 C
DOCKED: ATOM      7  C   UNL d   1       0.615   6.278   1.393 -0.35 -0.00    +0.015 A
DOCKED: ATOM      8  C   UNL d   1       3.446   6.437   1.559 -0.19 -0.01    +0.038 A
DOCKED: ATOM      9  C   UNL d   1       1.274   6.202   2.629 -0.38 -0.00    +0.001 A
DOCKED: ATOM     10  C   UNL d   1       2.666   6.281   2.709 -0.29 -0.00    +0.003 A
DOCKED: ENDROOT
DOCKED: BRANCH   2  11
DOCKED: ATOM     11  C   UNL d   1       2.763   6.835  -3.215 -0.22 -0.01    +0.191 C
DOCKED: BRANCH  11  12
DOCKED: ATOM     12  N   UNL d   1       2.927   8.250  -3.557 -0.06 +0.02    -0.347 N
DOCKED: ATOM     13  C   UNL d   1       4.119   8.591  -4.321 -0.19 -0.01    +0.153 C
DOCKED: BRANCH  12  14
DOCKED: ATOM     14  C   UNL d   1       2.162   9.300  -3.067 -0.04 -0.02    +0.243 C
DOCKED: ATOM     15  O   UNL d   1       1.150   9.703  -3.640 -0.02 +0.02    -0.268 OA
DOCKED: BRANCH  14  16
DOCKED: ATOM     16  C   UNL d   1       2.589   9.963  -1.800 -0.05 -0.01    +0.075 C
```

```
DOCKED: ATOM     17  C   UNL d   1       3.523   9.371  -1.036 -0.13 -0.00    +0.018 C
DOCKED: BRANCH  17  18
DOCKED: ATOM     18  C   UNL d   1       4.005   9.885   0.250 -0.11 +0.00    -0.018 A
DOCKED: ATOM     19  C   UNL d   1       3.113  10.019   1.316 -0.07 -0.01    +0.024 A
DOCKED: ATOM     20  C   UNL d   1       3.524  10.491   2.583 -0.09 +0.00    -0.011 A
DOCKED: ATOM     21  C   UNL d   1       2.441  10.599   3.616 -0.13 -0.03    +0.045 C
DOCKED: ATOM     22  C   UNL d   1       4.878  10.772   2.732 -0.13 -0.05    +0.136 A
DOCKED: ATOM     23  C   UNL d   1       2.671  11.577   4.759 -0.18 -0.04    +0.047 C
DOCKED: ATOM     24  C   UNL d   1       3.770  11.071   5.666 -0.09 -0.31    +0.275 C
DOCKED: ATOM     25  C   UNL d   1       5.165  11.396   5.157 -0.19 -0.13    +0.254 C
DOCKED: ATOM     26  O   UNL d   1       5.982  11.821   5.985 -0.17 +0.08    -0.271 OA
DOCKED: ATOM     27  N   UNL d   1       5.533  11.205   3.878 -0.12 +0.12    -0.309 N
DOCKED: ATOM     28  H   UNL d   1       6.513  11.392   3.683 -0.33 -0.07    +0.170 HD
DOCKED: ATOM     29  N   UNL d   1       5.761  10.651   1.720 -0.09 +0.08    -0.242 NA
DOCKED: ATOM     30  C   UNL d   1       5.321  10.212   0.517 -0.18 -0.02    +0.130 A
DOCKED: BRANCH  24  31
DOCKED: ATOM     31  N   UNL d   1       3.610  11.737   7.016 -0.24 +0.07    -0.066 N
DOCKED: ATOM     32  H   UNL d   1       4.320  12.447   7.221 +0.06 -0.17    +0.275 HD
DOCKED: ATOM     33  H   UNL d   1       3.577  11.047   7.778 -0.33 -0.42    +0.275 HD
DOCKED: ATOM     34  H   UNL d   1       2.676  12.183   7.031 -0.25 -0.33    +0.275 HD
DOCKED: ENDBRANCH  24  31
DOCKED: ENDBRANCH  17  18
DOCKED: ENDBRANCH  14  16
DOCKED: ENDBRANCH  12  14
DOCKED: ENDBRANCH  11  12
DOCKED: ENDBRANCH   2  11
DOCKED: TORSDOF 5
DOCKED: TER
DOCKED: ENDMDL
________________________________________________________________________________


    BEGINNING GENETIC ALGORITHM DOCKING 13 of 15
Run: 13 Seed: 241337205 957980048 [ Run 13 of 15 GA/GALS ]
Beginning LAMARCKIAN GENETIC ALGORITHM (LGA), with a maximum of 2500000 energy evaluations.

Final-Value: -11.579


    FINAL GENETIC ALGORITHM DOCKED STATE
    _______________________________________________


Detailed state:  trans 7.478 6.522 -1.057 quatxyzw 0.196245 0.744103 0.627946 -0.116109 center -0.813 0.218 0.087 ntor 6 -51.5383 129.7776 113.3307 -51.0694 155.8137 -14.3487
State:    7.478   6.522  -1.057   0.198   0.749   0.632 -166.665    -51.54  129.78  113.33  -51.07  155.81  -14.35

DOCKED: MODEL       13
DOCKED: USER    Run = 13
DOCKED: USER    DPF = D:/Autodock/dock.dpf
DOCKED: USER
DOCKED: USER    Estimated Free Energy of Binding    =   -7.25 kcal/mol
```

```
[=(1)+(2)+(3)-(4)]
DOCKED: USER    Estimated Inhibition Constant, Ki   =    4.85 uM (micromolar) [Temperature = 298.15 K]
DOCKED: USER
DOCKED: USER    (1) Final Intermolecular Energy     =   -8.74 kcal/mol
DOCKED: USER        vdW + Hbond + desolv Energy     =   -7.14 kcal/mol
DOCKED: USER        Electrostatic Energy            =   -1.60 kcal/mol
DOCKED: USER    (2) Final Total Internal Energy     =   -2.84 kcal/mol
DOCKED: USER    (3) Torsional Free Energy           =   +1.49 kcal/mol
DOCKED: USER    (4) Unbound System's Energy  [=(2)] =   -2.84 kcal/mol
DOCKED: USER
DOCKED: USER
DOCKED: USER    NEWDPF move ligand1.pdbqt
DOCKED: USER    NEWDPF about -0.813000 0.218000 0.087000
DOCKED: USER    NEWDPF tran0 7.478362 6.522100 -1.056868
DOCKED: USER    NEWDPF quaternion0 0.196245 0.744103 0.627946 -0.116109
DOCKED: USER    NEWDPF axisangle0 0.197582 0.749170 0.632223 -166.664849
DOCKED: USER    NEWDPF quat0 0.197582 0.749170 0.632223 -166.664849
DOCKED: USER    NEWDPF dihe0 -51.54 129.78 113.33 -51.07 155.81 -14.35
DOCKED: USER  keepresnum = 1
DOCKED: USER
DOCKED: REMARK  6 active torsions:
DOCKED: REMARK  status: ('A' for Active; 'I' for Inactive)
DOCKED: REMARK    1  A    between atoms: N_5  and  C_10
DOCKED: REMARK    2  A    between atoms: N_6  and  C_17
DOCKED: REMARK    3  A    between atoms: N_6  and  C_26
DOCKED: REMARK    4  A    between atoms: C_15  and  C_17
DOCKED: REMARK    5  A    between atoms: C_19  and  C_23
DOCKED: REMARK    6  A    between atoms: C_26  and  C_27
DOCKED: USER                              x       y       z     vdW  Elec       q    Type
DOCKED: USER                           _______ _______ _______ _____ _____    ______ ____
DOCKED: ROOT
DOCKED: ATOM      1  O   UNL d   1       4.819   9.123  -0.601 -0.13 +0.03    -0.312 OA
DOCKED: ATOM      2  C   UNL d   1       3.580   9.390  -1.109 -0.10 -0.01    +0.078 A
DOCKED: ATOM      3  C   UNL d   1       2.688   9.660  -0.080 -0.06 +0.00    -0.018 A
DOCKED: ATOM      4  C   UNL d   1       3.434   9.517   1.132 -0.10 -0.00    +0.006 A
DOCKED: ATOM      5  C   UNL d   1       4.730   9.191   0.751 -0.18 -0.01    +0.086 A
DOCKED: ATOM      6  C   UNL d   1       1.252  10.018  -0.192 -0.04 -0.01    +0.052 C
DOCKED: ATOM      7  C   UNL d   1       3.154   9.632   2.506 -0.11 -0.01    +0.015 A
DOCKED: ATOM      8  C   UNL d   1       5.779   8.973   1.641 -0.22 -0.01    +0.038 A
DOCKED: ATOM      9  C   UNL d   1       4.189   9.418   3.428 -0.24 -0.00    +0.001 A
DOCKED: ATOM     10  C   UNL d   1       5.479   9.095   3.002 -0.31 -0.00    +0.003 A
DOCKED: ENDROOT
DOCKED: BRANCH   2  11
DOCKED: ATOM     11  C   UNL d   1       3.468   9.365  -2.593 -0.09 -0.02    +0.191 C
DOCKED: BRANCH  11  12
DOCKED: ATOM     12  N   UNL d   1       3.318   7.992  -3.085 -0.09 +0.02    -0.347 N
DOCKED: ATOM     13  C   UNL d   1       4.439   7.458  -3.844 -0.24 -0.01    +0.153 C
DOCKED: BRANCH  12  14
DOCKED: ATOM     14  C   UNL d   1       2.152   7.239  -3.053 -0.16 -0.01    +0.243 C
DOCKED: ATOM     15  O   UNL d   1       1.655   6.748  -4.066 -0.14 +0.01    -0.268 OA
DOCKED: BRANCH  14  16
DOCKED: ATOM     16  C   UNL d   1       1.481   7.021  -1.738 -0.22 -0.01    +0.075 C
DOCKED: ATOM     17  C   UNL d   1       2.032   6.169  -0.856 -0.35 -0.00    +0.018 C
```

```
DOCKED: BRANCH  17  18
DOCKED: ATOM     18  C   UNL d   1       1.460   5.827   0.450 -0.37 +0.00    -0.018 A
DOCKED: ATOM     19  C   UNL d   1       1.610   6.710   1.520 -0.28 -0.01    +0.024 A
DOCKED: ATOM     20  C   UNL d   1       1.065   6.441   2.797 -0.35 +0.00    -0.011 A
DOCKED: ATOM     21  C   UNL d   1       1.333   7.480   3.847 -0.31 -0.03    +0.045 C
DOCKED: ATOM     22  C   UNL d   1       0.327   5.270   2.923 -0.35 -0.03    +0.136 A
DOCKED: ATOM     23  C   UNL d   1       1.277   7.026   5.297 -0.20 -0.06    +0.047 C
DOCKED: ATOM     24  C   UNL d   1      -0.147   6.701   5.689 -0.29 -0.27    +0.275 C
DOCKED: ATOM     25  C   UNL d   1      -0.579   5.304   5.274 -0.39 -0.10    +0.254 C
DOCKED: ATOM     26  O   UNL d   1      -1.204   4.637   6.110 -0.48 +0.09    -0.271 OA
DOCKED: ATOM     27  N   UNL d   1      -0.339   4.805   4.050 -0.24 +0.07    -0.309 N
DOCKED: ATOM     28  H   UNL d   1      -0.744   3.888   3.876 +0.12 -0.02    +0.170 HD
DOCKED: ATOM     29  N   UNL d   1       0.166   4.399   1.906 -0.36 +0.03    -0.242 NA
DOCKED: ATOM     30  C   UNL d   1       0.720   4.687   0.704 -0.29 -0.01    +0.130 A
DOCKED: BRANCH  24  31
DOCKED: ATOM     31  N   UNL d   1      -0.250   6.783   7.198 -0.03 +0.10    -0.066 N
DOCKED: ATOM     32  H   UNL d   1       0.464   7.373   7.636 -0.17 -0.71    +0.275 HD
DOCKED: ATOM     33  H   UNL d   1      -0.244   5.849   7.628 -0.05 -0.28    +0.275 HD
DOCKED: ATOM     34  H   UNL d   1      -1.185   7.166   7.422 -0.32 -0.34    +0.275 HD
DOCKED: ENDBRANCH  24  31
DOCKED: ENDBRANCH  17  18
DOCKED: ENDBRANCH  14  16
DOCKED: ENDBRANCH  12  14
DOCKED: ENDBRANCH  11  12
DOCKED: ENDBRANCH   2  11
DOCKED: TORSDOF 5
DOCKED: TER
DOCKED: ENDMDL
________________________________________________________________________________


    BEGINNING GENETIC ALGORITHM DOCKING 14 of 15
Run: 14 Seed: 72767237 2020956876 [ Run 14 of 15 GA/GALS ]
Beginning LAMARCKIAN GENETIC ALGORITHM (LGA), with a maximum of 2500000 energy evaluations.

Final-Value: -9.533


    FINAL GENETIC ALGORITHM DOCKED STATE
    _______________________________________________


Detailed state:  trans 2.108 11.983 -1.243 quatxyzw 0.771625 -0.575243 -0.269577 0.031921 center -0.813 0.218 0.087 ntor 6 147.1494 178.7638 -164.6457 86.0161 26.3611 -28.0698
State:    2.108  11.983  -1.243   0.772 -0.576 -0.270 176.342    147.15  178.76  -164.65   86.02   26.36  -28.07

DOCKED: MODEL       14
DOCKED: USER    Run = 14
DOCKED: USER    DPF = D:/Autodock/dock.dpf
DOCKED: USER
DOCKED: USER    Estimated Free Energy of Binding    =   -5.38 kcal/mol [=(1)+(2)+(3)-(4)]
DOCKED: USER    Estimated Inhibition Constant, Ki   =  114.70 uM (micromolar)
```

```
[Temperature = 298.15 K]
DOCKED: USER
DOCKED: USER    (1) Final Intermolecular Energy     =   -6.87 kcal/mol
DOCKED: USER        vdW + Hbond + desolv Energy     =   -5.26 kcal/mol
DOCKED: USER        Electrostatic Energy            =   -1.60 kcal/mol
DOCKED: USER    (2) Final Total Internal Energy     =   -2.67 kcal/mol
DOCKED: USER    (3) Torsional Free Energy           =   +1.49 kcal/mol
DOCKED: USER    (4) Unbound System's Energy  [=(2)] =   -2.67 kcal/mol
DOCKED: USER
DOCKED: USER
DOCKED: USER    NEWDPF move ligand1.pdbqt
DOCKED: USER    NEWDPF about -0.813000 0.218000 0.087000
DOCKED: USER    NEWDPF tran0 2.108396 11.983153 -1.243157
DOCKED: USER    NEWDPF quaternion0 0.771625 -0.575243 -0.269577 0.031921
DOCKED: USER    NEWDPF axisangle0 0.772018 -0.575536 -0.269714 176.341557
DOCKED: USER    NEWDPF quat0 0.772018 -0.575536 -0.269714 176.341557
DOCKED: USER    NEWDPF dihe0 147.15 178.76 -164.65 86.02 26.36 -28.07
DOCKED: USER  keepresnum = 1
DOCKED: USER
DOCKED: REMARK  6 active torsions:
DOCKED: REMARK  status: ('A' for Active; 'I' for Inactive)
DOCKED: REMARK    1  A    between atoms: N_5  and  C_10
DOCKED: REMARK    2  A    between atoms: N_6  and  C_17
DOCKED: REMARK    3  A    between atoms: N_6  and  C_26
DOCKED: REMARK    4  A    between atoms: C_15  and  C_17
DOCKED: REMARK    5  A    between atoms: C_19  and  C_23
DOCKED: REMARK    6  A    between atoms: C_26  and  C_27
DOCKED: USER                              x       y       z     vdW  Elec       q    Type
DOCKED: USER                           _______ _______ _______ _____ _____    ______ ____
DOCKED: ROOT
DOCKED: ATOM      1  O   UNL d   1       2.003   9.152  -3.695 -0.03 +0.03    -0.312 OA
DOCKED: ATOM      2  C   UNL d   1       2.886   8.264  -4.242 -0.14 -0.00    +0.078 A
DOCKED: ATOM      3  C   UNL d   1       2.373   6.976  -4.177 -0.24 +0.00    -0.018 A
DOCKED: ATOM      4  C   UNL d   1       1.108   7.092  -3.520 -0.19 -0.00    +0.006 A
DOCKED: ATOM      5  C   UNL d   1       0.933   8.443  -3.253 -0.07 -0.01    +0.086 A
DOCKED: ATOM      6  C   UNL d   1       2.995   5.723  -4.673 -0.23 -0.00    +0.052 C
DOCKED: ATOM      7  C   UNL d   1       0.091   6.201  -3.129 -0.16 -0.00    +0.015 A
DOCKED: ATOM      8  C   UNL d   1      -0.186   8.982  -2.624 -0.05 -0.00    +0.038 A
DOCKED: ATOM      9  C   UNL d   1      -1.049   6.711  -2.492 -0.21 -0.00    +0.001 A
DOCKED: ATOM     10  C   UNL d   1      -1.185   8.079  -2.245 -0.13 -0.00    +0.003 A
DOCKED: ENDROOT
DOCKED: BRANCH   2  11
DOCKED: ATOM     11  C   UNL d   1       4.139   8.850  -4.791 -0.16 -0.01    +0.191 C
DOCKED: BRANCH  11  12
DOCKED: ATOM     12  N   UNL d   1       4.981   9.388  -3.719 -0.10 +0.03    -0.347 N
DOCKED: ATOM     13  C   UNL d   1       6.298   8.784  -3.575 -0.13 -0.02    +0.153 C
DOCKED: BRANCH  12  14
DOCKED: ATOM     14  C   UNL d   1       4.575  10.273  -2.730 -0.08 -0.02    +0.243 C
DOCKED: ATOM     15  O   UNL d   1       4.799  11.482  -2.793 -0.03 +0.03    -0.268 OA
DOCKED: BRANCH  14  16
DOCKED: ATOM     16  C   UNL d   1       3.840   9.734  -1.549 -0.11 -0.01    +0.075 C
DOCKED: ATOM     17  C   UNL d   1       3.745   8.402  -1.390 -0.22 -0.00    +0.018 C
DOCKED: BRANCH  17  18
DOCKED: ATOM     18  C   UNL d   1       3.019   7.735  -0.304 -0.20 +0.00    -0.018
```

```
A
DOCKED: ATOM     19  C   UNL d   1        2.909   8.363   0.937 -0.14 -0.00    +0.024 A
DOCKED: ATOM     20  C   UNL d   1        2.206   7.776   2.014 -0.19 +0.00    -0.011 A
DOCKED: ATOM     21  C   UNL d   1        2.198   8.566   3.290 -0.21 -0.03    +0.045 C
DOCKED: ATOM     22  C   UNL d   1        1.586   6.558   1.761 -0.25 -0.03    +0.136 A
DOCKED: ATOM     23  C   UNL d   1        1.969   7.794   4.580 -0.18 -0.06    +0.047 C
DOCKED: ATOM     24  C   UNL d   1        0.544   7.292   4.642 -0.22 -0.26    +0.275 C
DOCKED: ATOM     25  C   UNL d   1        0.331   5.997   3.873 -0.28 -0.11    +0.254 C
DOCKED: ATOM     26  O   UNL d   1       -0.341   5.118   4.429 -0.39 +0.08    -0.271 OA
DOCKED: ATOM     27  N   UNL d   1        0.808   5.805   2.631 -0.24 +0.08    -0.309 N
DOCKED: ATOM     28  H   UNL d   1        0.534   4.925   2.201 +0.08 -0.03    +0.170 HD
DOCKED: ATOM     29  N   UNL d   1        1.676   5.929   0.571 -0.22 +0.03    -0.242 NA
DOCKED: ATOM     30  C   UNL d   1        2.374   6.519  -0.429 -0.25 -0.01    +0.130 A
DOCKED: BRANCH  24  31
DOCKED: ATOM     31  N   UNL d   1        0.202   7.024   6.093 -0.31 +0.10    -0.066 N
DOCKED: ATOM     32  H   UNL d   1       -0.326   6.159   6.246 +0.23 -0.23    +0.275 HD
DOCKED: ATOM     33  H   UNL d   1       -0.297   7.817   6.516 -0.20 -0.56    +0.275 HD
DOCKED: ATOM     34  H   UNL d   1        1.094   6.970   6.614 -0.01 -0.57    +0.275 HD
DOCKED: ENDBRANCH  24  31
DOCKED: ENDBRANCH  17  18
DOCKED: ENDBRANCH  14  16
DOCKED: ENDBRANCH  12  14
DOCKED: ENDBRANCH  11  12
DOCKED: ENDBRANCH   2  11
DOCKED: TORSDOF 5
DOCKED: TER
DOCKED: ENDMDL
________________________________________________________________________________


    BEGINNING GENETIC ALGORITHM DOCKING 15 of 15
Run: 15 Seed: 1155453944 970604047 [ Run 15 of 15 GA/GALS ]
Beginning LAMARCKIAN GENETIC ALGORITHM (LGA), with a maximum of 2500000 energy evaluations.

Final-Value: -11.516


    FINAL GENETIC ALGORITHM DOCKED STATE
    _______________________________________________


Detailed state:  trans 3.754 4.146 -0.612 quatxyzw -0.461198 -0.650396 -0.459711 0.391085 center -0.813 0.218 0.087 ntor 6 154.7152 99.7520 165.6366 -74.1023 158.7061 -48.4158
State:    3.754   4.146  -0.612  -0.501 -0.707 -0.499 133.956    154.72   99.75  165.64  -74.10  158.71  -48.42

DOCKED: MODEL       15
DOCKED: USER    Run = 15
DOCKED: USER    DPF = D:/Autodock/dock.dpf
DOCKED: USER
DOCKED: USER    Estimated Free Energy of Binding    =   -7.16 kcal/mol [=(1)+(2)+(3)-(4)]
DOCKED: USER    Estimated Inhibition Constant, Ki   =    5.68 uM (micromolar) [Temperature = 298.15 K]
DOCKED: USER
```

```
DOCKED: USER    (1) Final Intermolecular Energy     =   -8.65 kcal/mol
DOCKED: USER        vdW + Hbond + desolv Energy     =   -6.98 kcal/mol
DOCKED: USER        Electrostatic Energy            =   -1.67 kcal/mol
DOCKED: USER    (2) Final Total Internal Energy     =   -2.87 kcal/mol
DOCKED: USER    (3) Torsional Free Energy           =   +1.49 kcal/mol
DOCKED: USER    (4) Unbound System's Energy  [=(2)] =   -2.87 kcal/mol
DOCKED: USER
DOCKED: USER
DOCKED: USER    NEWDPF move ligand1.pdbqt
DOCKED: USER    NEWDPF about -0.813000 0.218000 0.087000
DOCKED: USER    NEWDPF tran0 3.754086 4.146128 -0.611958
DOCKED: USER    NEWDPF quaternion0 -0.461198 -0.650396 -0.459711 0.391085
DOCKED: USER    NEWDPF axisangle0 -0.501109 -0.706680 -0.499493 133.955988
DOCKED: USER    NEWDPF quat0 -0.501109 -0.706680 -0.499493 133.955988
DOCKED: USER    NEWDPF dihe0 154.72 99.75 165.64 -74.10 158.71 -48.42
DOCKED: USER  keepresnum = 1
DOCKED: USER
DOCKED: REMARK  6 active torsions:
DOCKED: REMARK  status: ('A' for Active; 'I' for Inactive)
DOCKED: REMARK    1  A    between atoms: N_5  and  C_10
DOCKED: REMARK    2  A    between atoms: N_6  and  C_17
DOCKED: REMARK    3  A    between atoms: N_6  and  C_26
DOCKED: REMARK    4  A    between atoms: C_15  and  C_17
DOCKED: REMARK    5  A    between atoms: C_19  and  C_23
DOCKED: REMARK    6  A    between atoms: C_26  and  C_27
DOCKED: USER                              x       y       z     vdW  Elec       q    Type
DOCKED: USER                           _______ _______ _______ _____ _____    ______ ____
DOCKED: ROOT
DOCKED: ATOM      1  O   UNL d   1       3.935   7.883  -0.825 -0.15 +0.02    -0.312 OA
DOCKED: ATOM      2  C   UNL d   1       3.285   8.883  -1.491 -0.12 -0.01    +0.078 A
DOCKED: ATOM      3  C   UNL d   1       2.936   9.897  -0.611 -0.06 +0.00    -0.018 A
DOCKED: ATOM      4  C   UNL d   1       3.380   9.466   0.679 -0.10 -0.00    +0.006 A
DOCKED: ATOM      5  C   UNL d   1       3.985   8.232   0.486 -0.17 -0.01    +0.086 A
DOCKED: ATOM      6  C   UNL d   1       2.240  11.171  -0.921 -0.02 -0.01    +0.052 C
DOCKED: ATOM      7  C   UNL d   1       3.339   9.990   1.984 -0.09 -0.00    +0.015 A
DOCKED: ATOM      8  C   UNL d   1       4.562   7.472   1.501 -0.15 -0.01    +0.038 A
DOCKED: ATOM      9  C   UNL d   1       3.910   9.249   3.030 -0.20 -0.00    +0.001 A
DOCKED: ATOM     10  C   UNL d   1       4.511   8.011   2.790 -0.32 -0.00    +0.003 A
DOCKED: ENDROOT
DOCKED: BRANCH   2  11
DOCKED: ATOM     11  C   UNL d   1       3.127   8.688  -2.958 -0.11 -0.01    +0.191 C
DOCKED: BRANCH  11  12
DOCKED: ATOM     12  N   UNL d   1       2.629   7.342  -3.258 -0.10 +0.02    -0.347 N
DOCKED: ATOM     13  C   UNL d   1       3.582   6.429  -3.873 -0.26 -0.00    +0.153 C
DOCKED: BRANCH  12  14
DOCKED: ATOM     14  C   UNL d   1       1.306   6.927  -3.183 -0.15 -0.01    +0.243 C
DOCKED: ATOM     15  O   UNL d   1       0.588   6.828  -4.177 -0.11 +0.01    -0.268 OA
DOCKED: BRANCH  14  16
DOCKED: ATOM     16  C   UNL d   1       0.740   6.592  -1.843 -0.25 -0.00    +0.075 C
DOCKED: ATOM     17  C   UNL d   1       1.577   6.281  -0.839 -0.33 -0.00    +0.018 C
DOCKED: BRANCH  17  18
DOCKED: ATOM     18  C   UNL d   1       1.160   5.890   0.512 -0.38 +0.00    -0.018 A
DOCKED: ATOM     19  C   UNL d   1       1.364   6.769   1.576 -0.28 -0.01    +0.024
```

```
A
DOCKED: ATOM     20  C   UNL d   1        0.966   6.454   2.895 -0.35 +0.00    -0.011 A
DOCKED: ATOM     21  C   UNL d   1        1.276   7.495   3.931 -0.31 -0.04    +0.045 C
DOCKED: ATOM     22  C   UNL d   1        0.314   5.239   3.074 -0.35 -0.03    +0.136 A
DOCKED: ATOM     23  C   UNL d   1        1.391   7.023   5.372 -0.09 -0.07    +0.047 C
DOCKED: ATOM     24  C   UNL d   1        0.035   6.610   5.897 -0.29 -0.29    +0.275 C
DOCKED: ATOM     25  C   UNL d   1       -0.354   5.193   5.504 -0.40 -0.11    +0.254 C
DOCKED: ATOM     26  O   UNL d   1       -0.852   4.481   6.386 -0.50 +0.11    -0.271 OA
DOCKED: ATOM     27  N   UNL d   1       -0.208   4.723   4.253 -0.25 +0.08    -0.309 N
DOCKED: ATOM     28  H   UNL d   1       -0.574   3.785   4.105 +0.14 -0.02    +0.170 HD
DOCKED: ATOM     29  N   UNL d   1        0.104   4.371   2.064 -0.31 +0.03    -0.242 NA
DOCKED: ATOM     30  C   UNL d   1        0.517   4.706   0.818 -0.37 -0.01    +0.130 A
DOCKED: BRANCH  24  31
DOCKED: ATOM     31  N   UNL d   1        0.078   6.669   7.410 -0.07 +0.10    -0.066 N
DOCKED: ATOM     32  H   UNL d   1       -0.274   5.825   7.873 -0.18 -0.29    +0.275 HD
DOCKED: ATOM     33  H   UNL d   1       -0.416   7.496   7.770 -0.37 -0.58    +0.275 HD
DOCKED: ATOM     34  H   UNL d   1        1.065   6.816   7.685 +0.07 -0.53    +0.275 HD
DOCKED: ENDBRANCH  24  31
DOCKED: ENDBRANCH  17  18
DOCKED: ENDBRANCH  14  16
DOCKED: ENDBRANCH  12  14
DOCKED: ENDBRANCH  11  12
DOCKED: ENDBRANCH   2  11
DOCKED: TORSDOF 5
DOCKED: TER
DOCKED: ENDMDL
________________________________________________________________________________

DPF> analysis                             # perform a ranked cluster analysis


        CLUSTER ANALYSIS OF CONFORMATIONS
        _________________________________

Number of conformations = 15

RMSD cluster analysis will be performed using the ligand atoms only (34 / 34 total atoms).

Outputting structurally similar clusters, ranked in order of increasing energy.



________________________________________________________________________________

Number of distinct conformational clusters found = 6,  out of 15 runs,
Using an rmsd-tolerance of 2.0 A


    CLUSTERING HISTOGRAM
    ____________________

________________________________________________________________________________
     |            |     |            |     |
Clus | Lowest     | Run | Mean       | Num | Histogram
-ter | Binding    |     | Binding    | in  |
Rank | Energy     |     | Energy     | Clus|    5    10   15   20   25   30   35
_____|____________|_____|____________|_____|____:____|____:____|____:____|____:___
   1 |     -7.82  |   3 |     -7.71  |   2 |##
```

```
    2 |     -7.50 |  11 |     -7.22 |   9 |#########
    3 |     -6.29 |   7 |     -6.29 |   1 |#
    4 |     -5.82 |  12 |     -5.82 |   1 |#
    5 |     -5.81 |   4 |     -5.81 |   1 |#
    6 |     -5.38 |  14 |     -5.38 |   1 |#
_____|___________|_____|___________|_____|______________________________________


Number of multi-member conformational clusters found = 2, out of 15 runs.

    RMSD TABLE
    __________

_____________________________________________________________________
     |      |      |            |         |                 |
Rank | Sub- | Run  | Binding    | Cluster | Reference       | Grep
     | Rank |      | Energy     | RMSD    | RMSD            | Pattern
_____|______|______|____________|_________|_________________|___________
   1      1      3       -7.82      0.00      9.24           RANKING
   1      2      1       -7.60      0.48      9.12           RANKING
   2      1     11       -7.50      0.00      7.44           RANKING
   2      2      8       -7.50      1.02      7.52           RANKING
   2      3      5       -7.49      1.72      7.83           RANKING
   2      4      2       -7.43      1.18      7.60           RANKING
   2      5      6       -7.26      1.96      7.88           RANKING
   2      6     13       -7.25      1.10      7.54           RANKING
   2      7     15       -7.16      0.79      7.49           RANKING
   2      8     10       -7.07      0.93      7.66           RANKING
   2      9      9       -6.34      1.47      7.73           RANKING
   3      1      7       -6.29      0.00      7.66           RANKING
   4      1     12       -5.82      0.00     10.00           RANKING
   5      1      4       -5.81      0.00      9.22           RANKING
   6      1     14       -5.38      0.00      8.03           RANKING
_______________________________________________________________________



    INFORMATION ENTROPY ANALYSIS FOR THIS CLUSTERING
    ________________________________________________


Information entropy for this clustering = 0.48  (rmstol = 2.00 Angstrom)

_______________________________________________________________________


    STATISTICAL MECHANICAL ANALYSIS
    _______________________________


Partition function, Q =    15.18            at Temperature, T = 298.15 K
Free energy,        A ~ -1611.38 kcal/mol   at Temperature, T = 298.15 K
Internal energy,    U =    -6.92 kcal/mol   at Temperature, T = 298.15 K
Entropy,            S =     5.38 kcal/mol/K at Temperature, T = 298.15 K

_______________________________________________________________________


    LOWEST ENERGY DOCKED CONFORMATION from EACH CLUSTER
    ___________________________________________________



Keeping original residue number (specified in the input PDBQ file) for outputting.

MODEL        3
USER    Run = 3
USER    Cluster Rank = 1
USER    Number of conformations in this cluster = 2
USER
USER    RMSD from reference structure       = 9.245 A
USER
USER    Estimated Free Energy of Binding    =   -7.82 kcal/mol  [=(1)+(2)+(3)-(4)]
```

```
USER    Estimated Inhibition Constant, Ki   =    1.86 uM (micromolar)  [Temperature = 298.15 K]
USER
USER    (1) Final Intermolecular Energy     =   -9.31 kcal/mol
USER        vdW + Hbond + desolv Energy     =   -9.46 kcal/mol
USER        Electrostatic Energy            =   +0.15 kcal/mol
USER    (2) Final Total Internal Energy     =   -1.71 kcal/mol
USER    (3) Torsional Free Energy           =   +1.49 kcal/mol
USER    (4) Unbound System's Energy  [=(2)] =   -1.71 kcal/mol
USER
USER
USER
USER    DPF = D:/Autodock/dock.dpf
USER    NEWDPF move ligand1.pdbqt
USER    NEWDPF about    -0.813000 0.218000 0.087000
USER    NEWDPF tran0    4.472324 3.226055 4.249154
USER    NEWDPF axisangle0   0.483520 -0.087685 -0.870930 104.840294
USER    NEWDPF quaternion0  0.383192 -0.069490 -0.690216 0.609867
USER    NEWDPF dihe0    -4.09 23.33 0.06 -166.63 -168.87 -65.90
USER
USER                              x       y       z     vdW   Elec       q      RMS
ATOM      1  O   UNL d   1       3.747   6.556   2.692 -0.20 +0.10    -0.312     9.245
ATOM      2  C   UNL d   1       4.451   7.547   2.069 -0.26 -0.02    +0.078     9.245
ATOM      3  C   UNL d   1       3.814   7.923   0.894 -0.22 +0.00    -0.018     9.245
ATOM      4  C   UNL d   1       2.663   7.080   0.787 -0.26 -0.00    +0.006     9.245
ATOM      5  C   UNL d   1       2.672   6.269   1.914 -0.23 -0.02    +0.086     9.245
ATOM      6  C   UNL d   1       4.227   8.973  -0.069 -0.19 -0.01    +0.052     9.245
ATOM      7  C   UNL d   1       1.614   6.921  -0.138 -0.25 -0.00    +0.015     9.245
ATOM      8  C   UNL d   1       1.708   5.304   2.197 -0.27 -0.01    +0.038     9.245
ATOM      9  C   UNL d   1       0.627   5.958   0.117 -0.36 -0.00    +0.001     9.245
ATOM     10  C   UNL d   1       0.674   5.164   1.265 -0.49 -0.00    +0.003     9.245
ATOM     11  C   UNL d   1       5.679   8.010   2.771 -0.34 -0.06    +0.191     9.245
ATOM     12  N   UNL d   1       6.276   6.924   3.554 -0.26 +0.11    -0.347     9.245
ATOM     13  C   UNL d   1       5.613   6.599   4.808 -0.47 -0.10    +0.153     9.245
ATOM     14  C   UNL d   1       7.500   6.317   3.303 -0.40 -0.05    +0.243     9.245
ATOM     15  O   UNL d   1       8.284   6.739   2.454 -0.43 +0.04    -0.268     9.245
ATOM     16  C   UNL d   1       7.871   5.108   4.095 -0.42 -0.02    +0.075     9.245
ATOM     17  C   UNL d   1       8.985   4.431   3.770 -0.71 -0.00    +0.018     9.245
ATOM     18  C   UNL d   1       9.446   3.206   4.430 -0.52 -0.00    -0.018     9.245
ATOM     19  C   UNL d   1      10.560   2.530   3.929 -0.01 +0.00    +0.024     9.245
ATOM     20  C   UNL d   1      11.060   1.355   4.535 -0.29 -0.00    -0.011     9.245
ATOM     21  C   UNL d   1      12.250   0.735   3.863 -0.35 +0.01    +0.045     9.245
ATOM     22  C   UNL d   1      10.414   0.929   5.690 -0.41 +0.03    +0.136     9.245
ATOM     23  C   UNL d   1      12.482  -0.749   4.099 -0.23 +0.01    +0.047     9.245
ATOM     24  C   UNL d   1      12.907  -0.990   5.530 -0.11 +0.10    +0.275     9.245
ATOM     25  C   UNL d   1      11.739  -1.056   6.500 -0.23 +0.18    +0.254     9.245
ATOM     26  O   UNL d   1      11.759  -1.963   7.344 -0.65 -0.32    -0.271     9.245
ATOM     27  N   UNL d   1      10.735  -0.162   6.488 -0.23 -0.19    -0.309     9.245
ATOM     28  H   UNL d   1      10.054  -0.275   7.235 +0.14 +0.16    +0.170     9.245
ATOM     29  N   UNL d   1       9.336   1.562   6.197 -0.37 -0.07    -0.242     9.245
ATOM     30  C   UNL d   1       8.879   2.675   5.574 -0.54 +0.02    +0.130     9.245
ATOM     31  N   UNL d   1      13.627  -2.321   5.590 -0.06 -0.02    -0.066     9.245
ATOM     32  H   UNL d   1      14.550  -2.319   5.144 +0.04 +0.07    +0.275     9.245
ATOM     33  H   UNL d   1      13.053  -3.079   5.200 +0.06 +0.09    +0.275     9.245
ATOM     34  H   UNL d   1      13.738  -2.566   6.589 +0.06 +0.12    +0.275     9.245
TER
ENDMDL
MODEL       11
USER    Run = 11
USER    Cluster Rank = 2
USER    Number of conformations in this cluster = 9
USER
USER    RMSD from reference structure       = 7.444 A
USER
USER    Estimated Free Energy of Binding    =   -7.50 kcal/mol  [=(1)+(2)+(3)-(4)]
USER    Estimated Inhibition Constant, Ki   =    3.16 uM (micromolar)  [Temperature = 298.15 K]
USER
USER    (1) Final Intermolecular Energy     =   -9.00 kcal/mol
USER        vdW + Hbond + desolv Energy     =   -7.37 kcal/mol
USER        Electrostatic Energy            =   -1.63 kcal/mol
USER    (2) Final Total Internal Energy     =   -3.02 kcal/mol
USER    (3) Torsional Free Energy           =   +1.49 kcal/mol
```

```
USER    (4) Unbound System's Energy  [=(2)] =   -3.02 kcal/mol
USER
USER
USER
USER    DPF = D:/Autodock/dock.dpf
USER    NEWDPF move ligand1.pdbqt
USER    NEWDPF about    -0.813000 0.218000 0.087000
USER    NEWDPF tran0    2.731090 3.531301 -0.708772
USER    NEWDPF axisangle0   -0.596183 -0.646737 -0.475707 119.963095
USER    NEWDPF quaternion0  -0.516214 -0.559986 -0.411898 0.500279
USER    NEWDPF dihe0    -34.34 163.63 151.17 -65.33 132.13 -27.33
USER
USER                              x       y       z     vdW   Elec        q     RMS
ATOM      1  O   UNL d   1       4.015   7.042  -0.969 -0.20 +0.02    -0.312     7.444
ATOM      2  C   UNL d   1       3.763   8.149  -1.728 -0.19 -0.01    +0.078     7.444
ATOM      3  C   UNL d   1       3.628   9.267  -0.917 -0.12 +0.00    -0.018     7.444
ATOM      4  C   UNL d   1       3.781   8.796   0.426 -0.16 -0.00    +0.006     7.444
ATOM      5  C   UNL d   1       4.018   7.432   0.331 -0.18 -0.01    +0.086     7.444
ATOM      6  C   UNL d   1       3.372  10.668  -1.334 -0.05 -0.01    +0.052     7.444
ATOM      7  C   UNL d   1       3.750   9.379   1.706 -0.13 -0.00    +0.015     7.444
ATOM      8  C   UNL d   1       4.232   6.594   1.423 -0.16 -0.01    +0.038     7.444
ATOM      9  C   UNL d   1       3.961   8.563   2.827 -0.24 -0.00    +0.001     7.444
ATOM     10  C   UNL d   1       4.198   7.193   2.686 -0.33 -0.00    +0.003     7.444
ATOM     11  C   UNL d   1       3.718   7.930  -3.200 -0.20 -0.01    +0.191     7.444
ATOM     12  N   UNL d   1       2.547   7.134  -3.577 -0.10 +0.02    -0.347     7.444
ATOM     13  C   UNL d   1       2.781   6.064  -4.536 -0.24 -0.00    +0.153     7.444
ATOM     14  C   UNL d   1       1.228   7.421  -3.252 -0.12 -0.01    +0.243     7.444
ATOM     15  O   UNL d   1       0.410   7.802  -4.088 -0.06 +0.02    -0.268     7.444
ATOM     16  C   UNL d   1       0.793   7.263  -1.833 -0.20 -0.01    +0.075     7.444
ATOM     17  C   UNL d   1       1.310   6.263  -1.099 -0.31 -0.00    +0.018     7.444
ATOM     18  C   UNL d   1       0.943   5.964   0.289 -0.37 +0.00    -0.018     7.444
ATOM     19  C   UNL d   1       0.841   7.004   1.214 -0.26 -0.00    +0.024     7.444
ATOM     20  C   UNL d   1       0.473   6.777   2.560 -0.30 +0.00    -0.011     7.444
ATOM     21  C   UNL d   1       0.431   7.997   3.434 -0.24 -0.03    +0.045     7.444
ATOM     22  C   UNL d   1       0.169   5.466   2.909 -0.30 -0.03    +0.136     7.444
ATOM     23  C   UNL d   1       0.593   7.780   4.930 -0.10 -0.06    +0.047     7.444
ATOM     24  C   UNL d   1      -0.625   7.083   5.493 -0.27 -0.24    +0.275     7.444
ATOM     25  C   UNL d   1      -0.590   5.574   5.309 -0.38 -0.12    +0.254     7.444
ATOM     26  O   UNL d   1      -0.922   4.881   6.281 -0.45 +0.11    -0.271     7.444
ATOM     27  N   UNL d   1      -0.254   4.994   4.144 -0.25 +0.08    -0.309     7.444
ATOM     28  H   UNL d   1      -0.341   3.980   4.132 +0.11 -0.03    +0.170     7.444
ATOM     29  N   UNL d   1       0.259   4.442   2.036 -0.36 +0.03    -0.242     7.444
ATOM     30  C   UNL d   1       0.632   4.702   0.759 -0.33 -0.01    +0.130     7.444
ATOM     31  N   UNL d   1      -0.682   7.361   6.981 -0.23 +0.10    -0.066     7.444
ATOM     32  H   UNL d   1      -0.436   8.322   7.237 -0.27 -0.74    +0.275     7.444
ATOM     33  H   UNL d   1      -0.101   6.699   7.512 -0.22 -0.42    +0.275     7.444
ATOM     34  H   UNL d   1      -1.649   7.166   7.294 -0.13 -0.26    +0.275     7.444
TER
ENDMDL
MODEL        7
USER    Run = 7
USER    Cluster Rank = 3
USER    Number of conformations in this cluster = 1
USER
USER    RMSD from reference structure       = 7.661 A
USER
USER    Estimated Free Energy of Binding    =   -6.29 kcal/mol  [=(1)+(2)+(3)-(4)]
USER    Estimated Inhibition Constant, Ki   =   24.65 uM (micromolar)  [Temperature = 298.15 K]
USER
USER    (1) Final Intermolecular Energy     =   -7.78 kcal/mol
USER        vdW + Hbond + desolv Energy     =   -6.13 kcal/mol
USER        Electrostatic Energy            =   -1.65 kcal/mol
USER    (2) Final Total Internal Energy     =   -3.04 kcal/mol
USER    (3) Torsional Free Energy           =   +1.49 kcal/mol
USER    (4) Unbound System's Energy  [=(2)] =   -3.04 kcal/mol
USER
USER
USER
USER    DPF = D:/Autodock/dock.dpf
USER    NEWDPF move ligand1.pdbqt
USER    NEWDPF about    -0.813000 0.218000 0.087000
USER    NEWDPF tran0    4.671203 8.743212 0.964308
```

```
USER    NEWDPF axisangle0   -0.222028 0.788576 0.573456 -154.278721
USER    NEWDPF quaternion0  -0.216459 0.768794 0.559071 -0.222580
USER    NEWDPF dihe0    -40.26 -98.18 -84.86 -75.78 152.00 -55.01
USER
USER                              x       y       z     vdW   Elec        q      RMS
ATOM      1  O   UNL d   1       1.676   9.623  -1.109 -0.02 +0.04    -0.312     7.661
ATOM      2  C   UNL d   1       1.071   9.163  -2.243 -0.05 -0.01    +0.078     7.661
ATOM      3  C   UNL d   1      -0.295   9.027  -2.036 -0.06 +0.00    -0.018     7.661
ATOM      4  C   UNL d   1      -0.524   9.404  -0.675 -0.07 -0.00    +0.006     7.661
ATOM      5  C   UNL d   1       0.715   9.761  -0.160 -0.03 -0.01    +0.086     7.661
ATOM      6  C   UNL d   1      -1.322   8.579  -3.009 -0.08 -0.00    +0.052     7.661
ATOM      7  C   UNL d   1      -1.644   9.485   0.171 -0.09 -0.00    +0.015     7.661
ATOM      8  C   UNL d   1       0.927  10.201   1.144 -0.03 -0.01    +0.038     7.661
ATOM      9  C   UNL d   1      -1.465   9.924   1.492 -0.07 -0.00    +0.001     7.661
ATOM     10  C   UNL d   1      -0.200  10.276   1.968 -0.04 -0.00    +0.003     7.661
ATOM     11  C   UNL d   1       1.965   8.945  -3.413 -0.06 -0.02    +0.191     7.661
ATOM     12  N   UNL d   1       3.232   8.337  -2.999 -0.07 +0.02    -0.347     7.661
ATOM     13  C   UNL d   1       4.427   9.145  -3.199 -0.18 -0.01    +0.153     7.661
ATOM     14  C   UNL d   1       3.414   7.015  -2.615 -0.21 -0.01    +0.243     7.661
ATOM     15  O   UNL d   1       3.849   6.158  -3.384 -0.12 +0.01    -0.268     7.661
ATOM     16  C   UNL d   1       3.066   6.624  -1.217 -0.32 -0.00    +0.075     7.661
ATOM     17  C   UNL d   1       1.810   6.236  -0.941 -0.33 -0.00    +0.018     7.661
ATOM     18  C   UNL d   1       1.318   5.868   0.390 -0.38 +0.00    -0.018     7.661
ATOM     19  C   UNL d   1       1.499   6.747   1.459 -0.28 -0.01    +0.024     7.661
ATOM     20  C   UNL d   1       1.030   6.453   2.760 -0.35 +0.00    -0.011     7.661
ATOM     21  C   UNL d   1       1.324   7.490   3.804 -0.30 -0.03    +0.045     7.661
ATOM     22  C   UNL d   1       0.334   5.260   2.914 -0.35 -0.03    +0.136     7.661
ATOM     23  C   UNL d   1       1.358   7.024   5.251 -0.21 -0.06    +0.047     7.661
ATOM     24  C   UNL d   1      -0.034   6.656   5.714 -0.29 -0.27    +0.275     7.661
ATOM     25  C   UNL d   1      -0.448   5.250   5.310 -0.39 -0.11    +0.254     7.661
ATOM     26  O   UNL d   1      -1.009   4.559   6.172 -0.48 +0.10    -0.271     7.661
ATOM     27  N   UNL d   1      -0.258   4.767   4.070 -0.24 +0.07    -0.309     7.661
ATOM     28  H   UNL d   1      -0.646   3.840   3.909 +0.13 -0.02    +0.170     7.661
ATOM     29  N   UNL d   1       0.144   4.392   1.899 -0.35 +0.03    -0.242     7.661
ATOM     30  C   UNL d   1       0.625   4.705   0.672 -0.29 -0.01    +0.130     7.661
ATOM     31  N   UNL d   1      -0.060   6.723   7.227 -0.10 +0.10    -0.066     7.661
ATOM     32  H   UNL d   1       0.532   7.460   7.624 -0.14 -0.76    +0.275     7.661
ATOM     33  H   UNL d   1       0.181   5.817   7.650 +0.00 -0.29    +0.275     7.661
ATOM     34  H   UNL d   1      -1.041   6.890   7.511 -0.29 -0.35    +0.275     7.661
TER
ENDMDL
MODEL       12
USER    Run = 12
USER    Cluster Rank = 4
USER    Number of conformations in this cluster = 1
USER
USER    RMSD from reference structure       = 10.002 A
USER
USER    Estimated Free Energy of Binding    =   -5.82 kcal/mol  [=(1)+(2)+(3)-(4)]
USER    Estimated Inhibition Constant, Ki   =   54.45 uM (micromolar)  [Temperature = 
298.15 K]
USER
USER    (1) Final Intermolecular Energy     =   -7.31 kcal/mol
USER        vdW + Hbond + desolv Energy     =   -6.09 kcal/mol
USER        Electrostatic Energy            =   -1.22 kcal/mol
USER    (2) Final Total Internal Energy     =   -2.73 kcal/mol
USER    (3) Torsional Free Energy           =   +1.49 kcal/mol
USER    (4) Unbound System's Energy  [=(2)] =   -2.73 kcal/mol
USER
USER
USER
USER    DPF = D:/Autodock/dock.dpf
USER    NEWDPF move ligand1.pdbqt
USER    NEWDPF about    -0.813000 0.218000 0.087000
USER    NEWDPF tran0    6.615514 4.910440 -0.391263
USER    NEWDPF axisangle0   -0.025931 0.707267 0.706470 -162.108374
USER    NEWDPF quaternion0  -0.025615 0.698664 0.697877 -0.155500
USER    NEWDPF dihe0    177.43 -89.28 173.72 94.07 43.53 178.74
USER
USER                              x       y       z     vdW   Elec        q      RMS
ATOM      1  O   UNL d   1       3.336   6.659  -0.872 -0.21 +0.02    -0.312    10.002
ATOM      2  C   UNL d   1       2.334   6.674  -1.799 -0.24 -0.00    +0.078    10.002
ATOM      3  C   UNL d   1       1.100   6.558  -1.174 -0.27 +0.00    -0.018    10.002
```

```
ATOM      4  C   UNL d   1       1.380   6.436   0.224 -0.32 -0.00    +0.006    10.002
ATOM      5  C   UNL d   1       2.761   6.507   0.348 -0.26 -0.01    +0.086    10.002
ATOM      6  C   UNL d   1      -0.243   6.553  -1.805 -0.24 -0.00    +0.052    10.002
ATOM      7  C   UNL d   1       0.615   6.278   1.393 -0.35 -0.00    +0.015    10.002
ATOM      8  C   UNL d   1       3.446   6.437   1.559 -0.19 -0.01    +0.038    10.002
ATOM      9  C   UNL d   1       1.274   6.202   2.629 -0.38 -0.00    +0.001    10.002
ATOM     10  C   UNL d   1       2.666   6.281   2.709 -0.29 -0.00    +0.003    10.002
ATOM     11  C   UNL d   1       2.763   6.835  -3.215 -0.22 -0.01    +0.191    10.002
ATOM     12  N   UNL d   1       2.927   8.250  -3.557 -0.06 +0.02    -0.347    10.002
ATOM     13  C   UNL d   1       4.119   8.591  -4.321 -0.19 -0.01    +0.153    10.002
ATOM     14  C   UNL d   1       2.162   9.300  -3.067 -0.04 -0.02    +0.243    10.002
ATOM     15  O   UNL d   1       1.150   9.703  -3.640 -0.02 +0.02    -0.268    10.002
ATOM     16  C   UNL d   1       2.589   9.963  -1.800 -0.05 -0.01    +0.075    10.002
ATOM     17  C   UNL d   1       3.523   9.371  -1.036 -0.13 -0.00    +0.018    10.002
ATOM     18  C   UNL d   1       4.005   9.885   0.250 -0.11 +0.00    -0.018    10.002
ATOM     19  C   UNL d   1       3.113  10.019   1.316 -0.07 -0.01    +0.024    10.002
ATOM     20  C   UNL d   1       3.524  10.491   2.583 -0.09 +0.00    -0.011    10.002
ATOM     21  C   UNL d   1       2.441  10.599   3.616 -0.13 -0.03    +0.045    10.002
ATOM     22  C   UNL d   1       4.878  10.772   2.732 -0.13 -0.05    +0.136    10.002
ATOM     23  C   UNL d   1       2.671  11.577   4.759 -0.18 -0.04    +0.047    10.002
ATOM     24  C   UNL d   1       3.770  11.071   5.666 -0.09 -0.31    +0.275    10.002
ATOM     25  C   UNL d   1       5.165  11.396   5.157 -0.19 -0.13    +0.254    10.002
ATOM     26  O   UNL d   1       5.982  11.821   5.985 -0.17 +0.08    -0.271    10.002
ATOM     27  N   UNL d   1       5.533  11.205   3.878 -0.12 +0.12    -0.309    10.002
ATOM     28  H   UNL d   1       6.513  11.392   3.683 -0.33 -0.07    +0.170    10.002
ATOM     29  N   UNL d   1       5.761  10.651   1.720 -0.09 +0.08    -0.242    10.002
ATOM     30  C   UNL d   1       5.321  10.212   0.517 -0.18 -0.02    +0.130    10.002
ATOM     31  N   UNL d   1       3.610  11.737   7.016 -0.24 +0.07    -0.066    10.002
ATOM     32  H   UNL d   1       4.320  12.447   7.221 +0.06 -0.17    +0.275    10.002
ATOM     33  H   UNL d   1       3.577  11.047   7.778 -0.33 -0.42    +0.275    10.002
ATOM     34  H   UNL d   1       2.676  12.183   7.031 -0.25 -0.33    +0.275    10.002
TER
ENDMDL
MODEL        4
USER    Run = 4
USER    Cluster Rank = 5
USER    Number of conformations in this cluster = 1
USER  
USER    RMSD from reference structure       = 9.219 A
USER  
USER    Estimated Free Energy of Binding    =   -5.81 kcal/mol  [=(1)+(2)+(3)-(4)]
USER    Estimated Inhibition Constant, Ki   =   55.23 uM (micromolar)  [Temperature = 298.15 K]
USER    
USER    (1) Final Intermolecular Energy     =   -7.30 kcal/mol
USER        vdW + Hbond + desolv Energy     =   -7.06 kcal/mol
USER        Electrostatic Energy            =   -0.24 kcal/mol
USER    (2) Final Total Internal Energy     =   -2.17 kcal/mol
USER    (3) Torsional Free Energy           =   +1.49 kcal/mol
USER    (4) Unbound System's Energy  [=(2)] =   -2.17 kcal/mol
USER    
USER    
USER  
USER    DPF = D:/Autodock/dock.dpf
USER    NEWDPF move ligand1.pdbqt
USER    NEWDPF about    -0.813000 0.218000 0.087000
USER    NEWDPF tran0    10.344927 2.970430 2.830932
USER    NEWDPF axisangle0   -0.212782 0.326564 -0.920912 -70.509357
USER    NEWDPF quaternion0  -0.122820 0.188497 -0.531561 -0.816594
USER    NEWDPF dihe0    -1.52 44.20 90.45 46.13 155.36 -171.23
USER  
USER                              x       y       z    vdW   Elec        q     RMS 
ATOM      1  O   UNL d   1      12.461  -0.115   3.038 -0.16 -0.03    -0.312     9.219
ATOM      2  C   UNL d   1      12.283  -1.464   2.916 -0.17 +0.01    +0.078     9.219
ATOM      3  C   UNL d   1      13.253  -2.002   2.082 -0.11 -0.00    -0.018     9.219
ATOM      4  C   UNL d   1      14.053  -0.898   1.649 -0.10 +0.00    +0.006     9.219
ATOM      5  C   UNL d   1      13.525   0.227   2.267 -0.18 +0.01    +0.086     9.219
ATOM      6  C   UNL d   1      13.437  -3.424   1.700 -0.06 +0.01    +0.052     9.219
ATOM      7  C   UNL d   1      15.168  -0.732   0.807 -0.06 +0.00    +0.015     9.219
ATOM      8  C   UNL d   1      14.026   1.518   2.114 -0.28 +0.00    +0.038     9.219
ATOM      9  C   UNL d   1      15.697   0.555   0.630 -0.12 +0.00    +0.001     9.219
ATOM     10  C   UNL d   1      15.135   1.660   1.273 -0.20 -0.00    +0.003     9.219
ATOM     11  C   UNL d   1      11.154  -2.042   3.694 +0.01 +0.04    +0.191     9.219
```

```
ATOM     12  N   UNL d   1      11.487  -2.129   5.118 -0.08 -0.12    -0.347      9.219
ATOM     13  C   UNL d   1      11.638  -3.472   5.661 -0.16 +0.07    +0.153      9.219
ATOM     14  C   UNL d   1      11.488  -1.073   6.020 -0.24 +0.13    +0.243      9.219
ATOM     15  O   UNL d   1      12.359  -0.940   6.880 -0.64 -0.21    -0.268      9.219
ATOM     16  C   UNL d   1      10.389  -0.067   5.940 -0.12 +0.03    +0.075      9.219
ATOM     17  C   UNL d   1      10.597   1.075   5.262 -0.45 +0.00    +0.018      9.219
ATOM     18  C   UNL d   1       9.625   2.166   5.138 -0.56 -0.00    -0.018      9.219
ATOM     19  C   UNL d   1       9.811   3.146   4.161 -0.15 +0.00    +0.024      9.219
ATOM     20  C   UNL d   1       8.898   4.210   3.984 -0.68 +0.00    -0.011      9.219
ATOM     21  C   UNL d   1       9.251   5.194   2.907 -0.64 -0.00    +0.045      9.219
ATOM     22  C   UNL d   1       7.774   4.208   4.803 -0.24 -0.03    +0.136      9.219
ATOM     23  C   UNL d   1       8.662   6.591   3.026 -0.37 -0.01    +0.047      9.219
ATOM     24  C   UNL d   1       7.169   6.551   2.786 -0.08 -0.05    +0.275      9.219
ATOM     25  C   UNL d   1       6.374   6.152   4.019 +0.10 -0.09    +0.254      9.219
ATOM     26  O   UNL d   1       5.356   6.810   4.269 -0.40 +0.15    -0.271      9.219
ATOM     27  N   UNL d   1       6.719   5.111   4.797 +0.32 +0.15    -0.309      9.219
ATOM     28  H   UNL d   1       6.063   4.905   5.547 -0.35 -0.11    +0.170      9.219
ATOM     29  N   UNL d   1       7.569   3.275   5.755 -0.56 +0.00    -0.242      9.219
ATOM     30  C   UNL d   1       8.479   2.281   5.902 -0.35 +0.02    +0.130      9.219
ATOM     31  N   UNL d   1       6.720   7.940   2.383 -0.26 +0.02    -0.066      9.219
ATOM     32  H   UNL d   1       6.993   8.210   1.433 +0.20 -0.04    +0.275      9.219
ATOM     33  H   UNL d   1       7.042   8.651   3.053 -0.07 -0.10    +0.275      9.219
ATOM     34  H   UNL d   1       5.688   7.964   2.456 +0.15 -0.08    +0.275      9.219
TER
ENDMDL
MODEL       14
USER    Run = 14
USER    Cluster Rank = 6
USER    Number of conformations in this cluster = 1
USER
USER    RMSD from reference structure       = 8.033 A
USER
USER    Estimated Free Energy of Binding    =   -5.38 kcal/mol  [=(1)+(2)+(3)-(4)]
USER    Estimated Inhibition Constant, Ki   =  114.70 uM (micromolar)  [Temperature =
298.15 K]
USER
USER    (1) Final Intermolecular Energy     =   -6.87 kcal/mol
USER        vdW + Hbond + desolv Energy     =   -5.26 kcal/mol
USER        Electrostatic Energy            =   -1.60 kcal/mol
USER    (2) Final Total Internal Energy     =   -2.67 kcal/mol
USER    (3) Torsional Free Energy           =   +1.49 kcal/mol
USER    (4) Unbound System's Energy  [=(2)] =   -2.67 kcal/mol
USER
USER
USER
USER    DPF = D:/Autodock/dock.dpf
USER    NEWDPF move ligand1.pdbqt
USER    NEWDPF about    -0.813000 0.218000 0.087000
USER    NEWDPF tran0    2.108396 11.983153 -1.243157
USER    NEWDPF axisangle0   0.772018 -0.575536 -0.269714 176.341557
USER    NEWDPF quaternion0  0.771625 -0.575243 -0.269577 0.031921
USER    NEWDPF dihe0    147.15 178.76 -164.65 86.02 26.36 -28.07
USER
USER                              x       y       z    vdW   Elec        q     RMS
ATOM      1  O   UNL d   1       2.003   9.152  -3.695 -0.03 +0.03    -0.312      8.033
ATOM      2  C   UNL d   1       2.886   8.264  -4.242 -0.14 -0.00    +0.078      8.033
ATOM      3  C   UNL d   1       2.373   6.976  -4.177 -0.24 +0.00    -0.018      8.033
ATOM      4  C   UNL d   1       1.108   7.092  -3.520 -0.19 -0.00    +0.006      8.033
ATOM      5  C   UNL d   1       0.933   8.443  -3.253 -0.07 -0.01    +0.086      8.033
ATOM      6  C   UNL d   1       2.995   5.723  -4.673 -0.23 -0.00    +0.052      8.033
ATOM      7  C   UNL d   1       0.091   6.201  -3.129 -0.16 -0.00    +0.015      8.033
ATOM      8  C   UNL d   1      -0.186   8.982  -2.624 -0.05 -0.00    +0.038      8.033
ATOM      9  C   UNL d   1      -1.049   6.711  -2.492 -0.21 -0.00    +0.001      8.033
ATOM     10  C   UNL d   1      -1.185   8.079  -2.245 -0.13 -0.00    +0.003      8.033
ATOM     11  C   UNL d   1       4.139   8.850  -4.791 -0.16 -0.01    +0.191      8.033
ATOM     12  N   UNL d   1       4.981   9.388  -3.719 -0.10 +0.03    -0.347      8.033
ATOM     13  C   UNL d   1       6.298   8.784  -3.575 -0.13 -0.02    +0.153      8.033
ATOM     14  C   UNL d   1       4.575  10.273  -2.730 -0.08 -0.02    +0.243      8.033
ATOM     15  O   UNL d   1       4.799  11.482  -2.793 -0.03 +0.03    -0.268      8.033
ATOM     16  C   UNL d   1       3.840   9.734  -1.549 -0.11 -0.01    +0.075      8.033
ATOM     17  C   UNL d   1       3.745   8.402  -1.390 -0.22 -0.00    +0.018      8.033
ATOM     18  C   UNL d   1       3.019   7.735  -0.304 -0.20 +0.00    -0.018      8.033
ATOM     19  C   UNL d   1       2.909   8.363   0.937 -0.14 -0.00    +0.024      8.033
```

```
ATOM     20  C   UNL d   1       2.206   7.776   2.014 -0.19 +0.00    -0.011     8.033
ATOM     21  C   UNL d   1       2.198   8.566   3.290 -0.21 -0.03    +0.045     8.033
ATOM     22  C   UNL d   1       1.586   6.558   1.761 -0.25 -0.03    +0.136     8.033
ATOM     23  C   UNL d   1       1.969   7.794   4.580 -0.18 -0.06    +0.047     8.033
ATOM     24  C   UNL d   1       0.544   7.292   4.642 -0.22 -0.26    +0.275     8.033
ATOM     25  C   UNL d   1       0.331   5.997   3.873 -0.28 -0.11    +0.254     8.033
ATOM     26  O   UNL d   1      -0.341   5.118   4.429 -0.39 +0.08    -0.271     8.033
ATOM     27  N   UNL d   1       0.808   5.805   2.631 -0.24 +0.08    -0.309     8.033
ATOM     28  H   UNL d   1       0.534   4.925   2.201 +0.08 -0.03    +0.170     8.033
ATOM     29  N   UNL d   1       1.676   5.929   0.571 -0.22 +0.03    -0.242     8.033
ATOM     30  C   UNL d   1       2.374   6.519  -0.429 -0.25 -0.01    +0.130     8.033
ATOM     31  N   UNL d   1       0.202   7.024   6.093 -0.31 +0.10    -0.066     8.033
ATOM     32  H   UNL d   1      -0.326   6.159   6.246 +0.23 -0.23    +0.275     8.033
ATOM     33  H   UNL d   1      -0.297   7.817   6.516 -0.20 -0.56    +0.275     8.033
ATOM     34  H   UNL d   1       1.094   6.970   6.614 -0.01 -0.57    +0.275     8.033
TER
ENDMDL


AVSFLD: # AVS field file
AVSFLD: #
AVSFLD: # Created by AutoDock
AVSFLD: #
AVSFLD: ndim=2            # number of dimensions in the field
AVSFLD: nspace=1          # number of physical coordinates
AVSFLD: veclen=7          # vector size
AVSFLD: dim1=34           # atoms
AVSFLD: dim2=6            # conformations
AVSFLD: data=Real        # data type (byte,integer,Real,double)
AVSFLD: field=uniform    # field coordinate layout
AVSFLD: label= x y z vdW Elec q RMS
AVSFLD: variable 1 file = D:/Autodock/dock.dlg.pdb filetype = ascii offset = 5 stride = 12
AVSFLD: variable 2 file = D:/Autodock/dock.dlg.pdb filetype = ascii offset = 6 stride = 12
AVSFLD: variable 3 file = D:/Autodock/dock.dlg.pdb filetype = ascii offset = 7 stride = 12
AVSFLD: variable 4 file = D:/Autodock/dock.dlg.pdb filetype = ascii offset = 8 stride = 12
AVSFLD: variable 5 file = D:/Autodock/dock.dlg.pdb filetype = ascii offset = 9 stride = 12
AVSFLD: variable 6 file = D:/Autodock/dock.dlg.pdb filetype = ascii offset = 10 stride = 12
AVSFLD: variable 7 file = D:/Autodock/dock.dlg.pdb filetype = ascii offset = 11 stride = 12
AVSFLD: # end of file

>>> Closing the docking parameter file (DPF)...
This docking finished at:           1:57 40" p.m., 01/27/2025


________________________________________________________________________________

D:/Autodock/autodock4.exe: Successful Completion on "BAYSALO"

Real= 6m 08.18s,  CPU= 6m 04.36s,  System= 0.31s
________________________________________________________________________________
```

Search SWISS-MODEL Repository

A0A2H3GNL7 (A0A2H3GNL7_GIBZA) *Gibberella zeae (Wheat head blight fungus) (Fusarium graminearum)*

Supplementary file S4 : Structural and sequence information for uncharacterized protein A0A2H3GNL7 from Fusarium graminearum

Uncharacterized protein

347 aa; Sequence (Fasta)

50 100 150 200 250 300

A0A2H3GNL7

monomer 1-347

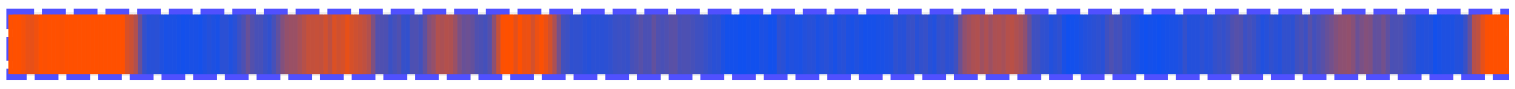

No models have been built for this target sequence.

Build Models

Available Structures

1 AlphaFold Model

	Model ID	Oligo-state	Avg pLDDT	Range	Trg-Mdl Seq Id (%)
◉	AF-A0A2H3GNL7-F1<br>*Download*	monomer	76.50<br>Compare ☐		100.0<br>*Structure Assessment*

Alignments

AF-A0A2H3GNL7-F1-model_v4

```
     Target MRSHAGPITWASVVWLVLGLFQLANCASVDNKLSHGRERI  40
AlphaFold.A MRSHAGPITWASVVWLVLGLFQLANCASVDNKLSHGRERI  40

     Target WLWEMYNIFVDIEGADKQDLILPQDKNAKFVYRRHYSLDK  80
AlphaFold.A WLWEMYNIFVDIEGADKQDLILPQDKNAKFVYRRHYSLDK  80

     Target NTVDGRLSYAEFMADLEAKLTPLDMDYSLQAPNSEGIVPG 120
AlphaFold.A NTVDGRLSYAEFMADLEAKLTPLDMDYSLQAPNSEGIVPG 120

     Target KDGQASGKPSVAEAVETLKLKGWAQTMKVDQVTSGKTKDY 160
AlphaFold.A KDGQASGKPSVAEAVETLKLKGWAQTMKVDQVTSGKTKDY 160

     Target GELVGRVDKKFQDYYDKLGSDPKWKRKLIETNQRTNKLTG 200
AlphaFold.A GELVGRVDKKFQDYYDKLGSDPKWKRKLIETNQRTNKLTG 200

     Target EIVALRQQEADDWVIKQLTQDADHNNPKVPDEDKKLGIGL 240
AlphaFold.A EIVALRQQEADDWVIKQLTQDADHNNPKVPDEDKKLGIGL 240

     Target SRDDLVIERVRSSIPGATTYERVDLVRTFLEHPNQAEFLE 280
AlphaFold.A SRDDLVIERVRSSIPGATTYERVDLVRTFLEHPNQAEFLE 280

     Target KLSTAGIKGGIQGLINWADMLGNERYANWGAGFTDANKSH 320
AlphaFold.A KLSTAGIKGGIQGLINWADMLGNERYANWGAGFTDANKSH 320

     Target HQAKSLWLAMHDSSTKRLGGLSPSCSR              347
AlphaFold.A HQAKSLWLAMHDSSTKRLGGLSPSCSR              347
```

AlphaFold AF-A0A2H3GNL7-F1-model-v4; Created: 2022-06-01;

Average Model Confidence (pLDDT) : 76.50 ; Local Confidence

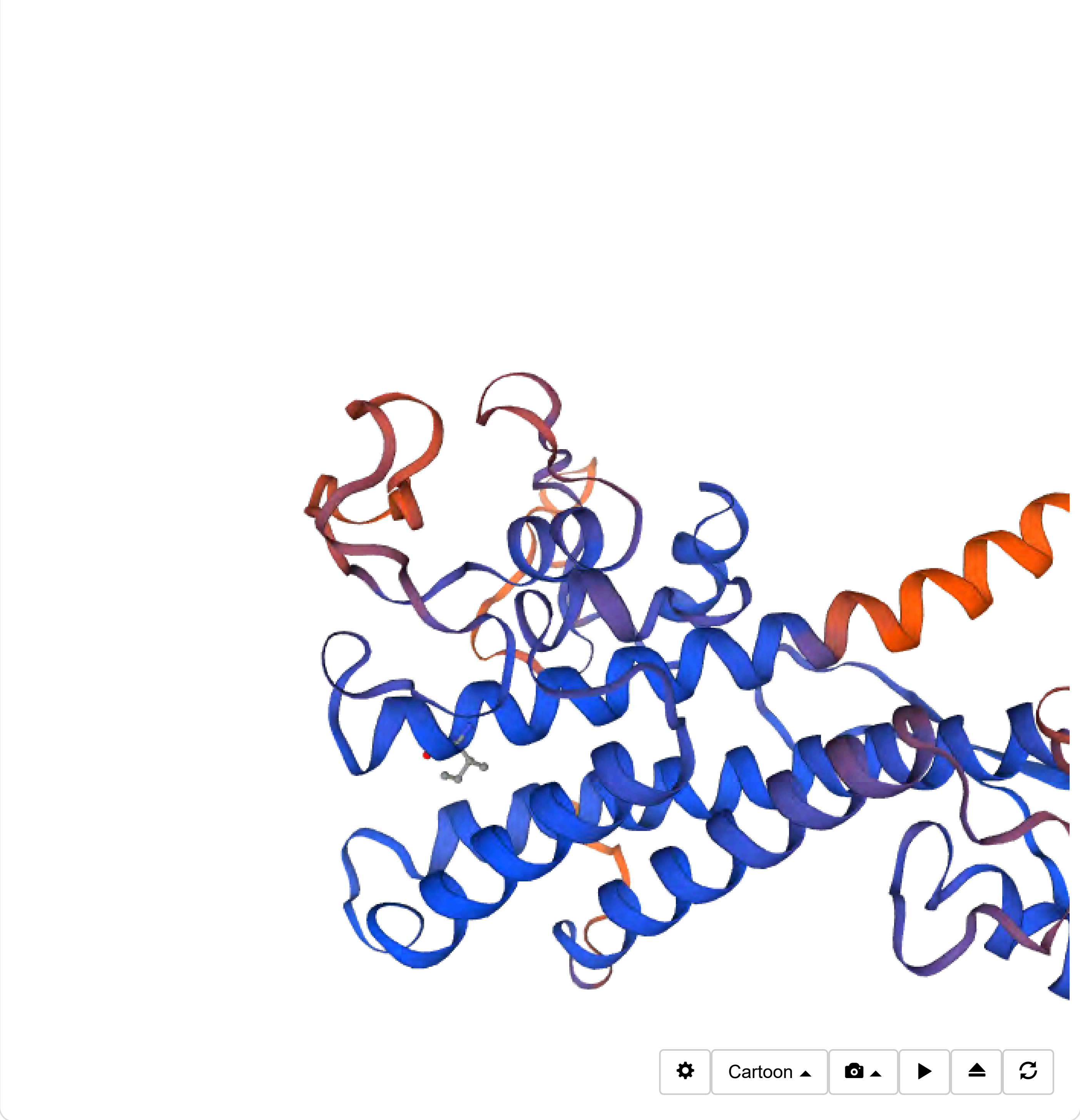
Cartoon
